# Patterns of PCR Amplification Artifacts of the Fungal Barcode Marker in a Hybrid Mushroom

**DOI:** 10.3389/fmicb.2019.02686

**Published:** 2019-11-19

**Authors:** Jun-Liang Zhou, Jianping Xu, An-Guo Jiao, Li Yang, Jie Chen, Philippe Callac, Yu Liu, Shou-Xian Wang

**Affiliations:** ^1^Institute of Plant and Environment Protection, Beijing Academy of Agriculture and Forestry Sciences, Beijing Engineering Research Center for Edible Mushroom, Beijing, China; ^2^International Exchange and Cooperation Department, Kunming University, Kunming, China; ^3^Department of Biology, McMaster University, Hamilton, ON, Canada; ^4^Laboratory for Conservation and Utilization of Bio-Resources and Key Laboratory for Microbial Resources of the Ministry of Education, Yunnan University, Kunming, China; ^5^Instituto de Ecología, Veracruz, Mexico; ^6^MycSA, INRA, Villenave d’Ornon, France

**Keywords:** polymerase chain reaction, ITS, mutation spectrum, chimera sequence, recombination

## Abstract

The polymerase chain reaction (PCR) is widely used in modern biology and medicine. However, PCR artifacts can complicate the interpretation of PCR-based results. The internal transcribed spacer (ITS) region of the ribosomal RNA gene cluster is the consensus fungal barcode marker and suspected PCR artifacts have been reported in many studies, especially for the analyses of environmental fungal samples. At present, the patterns of PCR artifacts in the whole fungal ITS region (ITS1+5.8S+ITS2) are not known. In this study, we analyzed the error rates of PCR at three template complexity levels using the divergent copies of ITS from the mushroom *Agaricus subrufescens*. Our results showed that PCR using the Phusion^®^ High-Fidelity DNA Polymerase has a per nucleotide error rate of about 4 × 10^–6^ per replication. Among the detected mutations, transitions were much more frequent than transversions, insertions, and deletions. When divergent alleles were mixed as templates in the same reaction, a significant proportion (∼30%) of recombinant molecules were detected. The *in vitro* mixed-template results were comparable to those obtained from using the genomic DNA of the original mushroom specimen as template. Our results indicate that caution should be in place when interpreting ITS sequences from individual fungal specimens, especially those containing divergent ITS copies. Similar results could also happen to PCR-based analyses of other multicopy DNA fragments as well as single-copy DNA sequences with divergent alleles in diploid organisms.

## Introduction

The polymerase chain reaction (PCR), an *in vitro* method for the enzymatic replication of specific DNA sequences, enables the amplification of large amounts of target DNA fragments from one or a few template molecules (Mullis et al., 1986; Mullis and Faloona, 1987; Saiki et al., 1988). As an easy and efficient way to amplify a target DNA fragment, this technique has been widely used in diverse fields of life sciences and medicine, including molecular biology, ecology, evolutionary biology, and applied fields such as the diagnosis of infectious diseases and DNA-based species identification (Zhou and Cui, 2017). However, during PCR, the changes in temperature during each cycle and the use of multiple cycles can damage components of the reaction, reducing the efficiency and accuracy of the process in later cycles. While the application of thermostable DNA polymerases isolated from thermophiles such as *Thermus aquaticus* (*Taq*) have significantly improved the efficiency of PCR, artifacts can be produced during the PCR procedure (Cline et al., 1996; Ishii and Fukui, 2001; Kanagawa, 2003; Kurata et al., 2004; Acinas et al., 2005; Sharifian, 2010; Schloss et al., 2011; Bjørnsgaard Aas et al., 2017). Indeed, measurable error rates of several thermostable DNA polymerases in *in vitro* PCR have been reported by using a *lacIOZ*α-based fidelity assay (Cline et al., 1996; Hogrefe and Borns, 2003; Biles and Connolly, 2004; Jozwiakowski and Connolly, 2009). Due to its common use for bacterial taxonomy and environmental microbial ecology research, the 16S rRNA gene has also been used as the template to estimate PCR error rate (Ishii and Fukui, 2001; Kurata et al., 2004; Acinas et al., 2005; Sharifian, 2010; Schloss et al., 2011). These studies have shown that template complexity can influence the frequencies of PCR artifacts (Farrelly et al., 1996; Suzuki and Giovannoni, 1996; Speksnijder et al., 2001; Sharifian, 2010; Schloss et al., 2011). However, these studies have targeted single or low-copy number genes (1–15 copies) within individual organisms (Stoddard et al., 2015). Recently, Bjørnsgaard Aas et al. (2017) used the high-copy number DNA fragment ITS2 to examine the rate of chimera formation of different fungal communities. However, artifacts of the short ITS2 region within fungal community samples may not be representative for the types of artifacts of the whole ITS region that contains ITS1, 5.8S, and ITS2. In addition, chimera is only one type of PCR artifact. Furthermore, PCR artifacts based on individual specimens may be different from those based on complex community samples. Indeed, the patterns of PCR artifacts based on intra-strain genetic variation of high copy number genes, especially those with potentially divergent intragenomic sequences, remain unknown.

The nuclear ribosomal internal transcribed spacer (ITS) region is the consensus DNA barcode marker for fungi (Schoch et al., 2012). The ITS region as well as its surrounding ribosomal genes 18S rRNA and 28S rRNA are tandemly repeated in all fungi and many other eukaryotes genomes (O’Brien et al., 2005; Kiss, 2012; Schoch et al., 2012). In most eukaryotes, the ITS DNA fragment can be detected at tens to hundreds or even to thousands of copies per cell (Longo et al., 2013; Diaz et al., 2017; Minamoto et al., 2017; Rebollar et al., 2017). Within many of these organisms, the different copies may have distinct sequence information (Smith et al., 2007; Nilsson et al., 2008; Kovács et al., 2011; Li et al., 2013; Weitemier et al., 2015). Though intragenomic variability may have limited impacts on species richness estimates (Lindner et al., 2013), such variations can impact PCR and DNA sequence-based identifications of strains, geographic populations, and species (Xu, 2016). For example, in a recent study of a cultivated mushroom – *Agaricus subrufescens* Peck – collected in Saint-Leon, Chen et al. (2016) found that there were three main types of ITS sequences (called types A, B, and C) within a single specimen. Types A and B sequences were similar to those amplified from *A. subrufescens* specimens collected from the Americas and Europe while type C sequence was close to those found in Oceanian and Asian specimens (Chen et al., 2016). These three types of ITS sequences differ from each other at 7–9 nucleotide positions, and the similarities among the three types are about 98.8%. The ITS sequences and other data suggested that the specimen was a natural hybrid. Interestingly, in their PCR-based amplification and sequencing analyses, 61 other minor types of ITS sequences were found and these minor sequences differed from the three main ITS sequence types either at a single or several positions (Chen et al., 2016). Among these 61 sequences, eight had unique nucleotides at specific positions, while other 53 types were consistent with results of recombination between two or three of the three main types. It was not known whether those minor types represent their true distributions within that natural specimen or they were PCR and sequencing artifacts (Chen et al., 2016). Putative chimera DNA sequences have also been reported in metagenomic studies of fungal diversity using ITS (e.g., O’Brien et al., 2005; Lindner et al., 2013) and in studies of single genes in hybrid fungi (e.g., Xu and Mitchell, 2003). At present, the extent of PCR artifacts for the whole fungal ITS region has not been experimentally quantified.

The objectives of this study are to use an *in vitro* system to investigate: (i) the mutation rate and mutational pattern of ITS sequences during PCR and (ii) the effect of ITS template complexity on mutation rate and chimera formation and detection. To accomplish these two objectives, we used the three types of cloned ITS sequences from the mushroom *A. subrufescens* mentioned above (Chen et al., 2016) as starting materials. These three sequences were PCR-amplified either individually, mixed in twos (three combinations), or mixed in three (one combination), for a total of seven treatments. To minimize mutations generated due to sequencing error that are relatively common in next generation sequencing platforms, the amplified fragments were first cloned into *Escherichia coli*. and single colonies were individually sequenced using the Sanger sequencing method. In total, over 5400 high-quality ITS sequences were obtained and analyzed. These sequences were then compared with those obtained using the original genomic DNA of the mushroom strain as templates, with the rest of the protocols being identical to the above-mentioned artificial treatments. The details of the materials and methods, results, and their relevance to ITS sequence heterogeneity within and between samples are described below.

## Materials and Methods

### Strains, DNA Extraction and PCR Amplification

*Agaricus subrufescens* strain CA487 is used in this study. Both the original fruiting body specimen and the mycelial culture from its tissue are available upon request at INRA MycSA Bordeaux. The strain has been deposited at the CIRM-CF in Marseilles where it will appear in the catalog in a few months^1^. Total genomic DNA was extracted from the dried specimen by using the FH plant DNA kit II (Demeter Biotech Co., Ltd., Beijing, China), following the manufacturer’s instructions (Zhou and Cui, 2017). DNA concentration was measured by Eppendorf BioPhotometer^®^ D30 (Eppendorf, Germany) and adjusted to 25 ng/μl.

**FIGURE 1:**
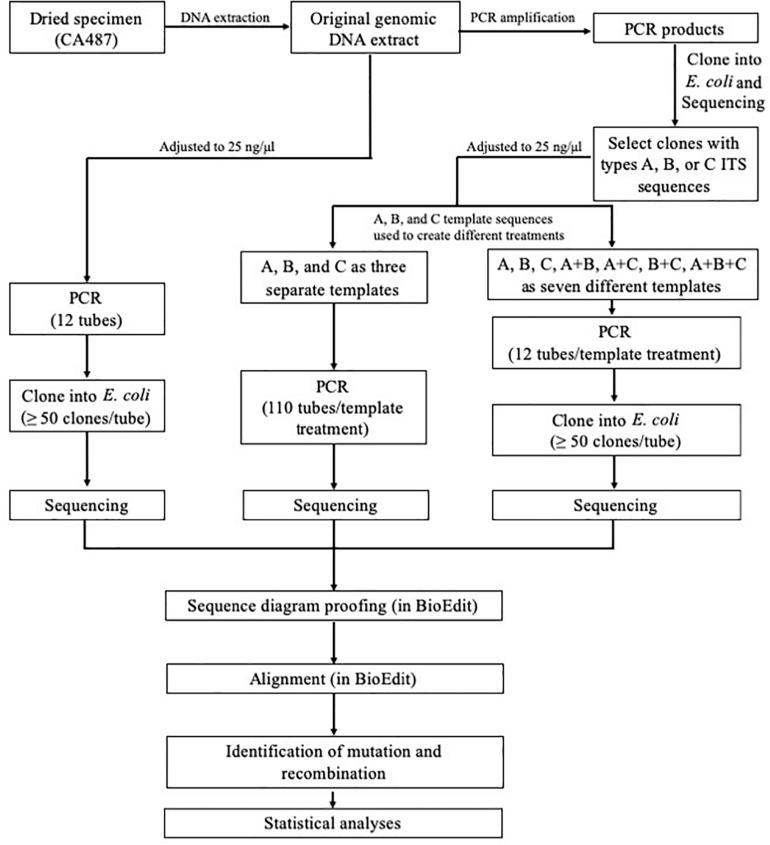
Experimental design for assessing *in vitro* PCR artifacts of the consensus fungal barcode marker ITS.

The overall experimental design is shown in Figure 1. Briefly, the three main types of ITS sequences (A, B, and C) previously identified by Chen et al. (2016) were isolated through cloning into *E. coli* followed by PCR amplification (primer pair ITS4 + ITS5) and sequencing (primer pair ITS4 + ITS5) for confirmation, using protocols described previously (Chen et al., 2016). The concentrations of the three confirmed ITS sequence types (purified ITS amplification fragments) were individually adjusted to 25 ng/μl. These diluted products were then mixed to create the following four combinations A+B, A+C, B+C, and A+B+C mixtures. For each combination, the concentrations of individual components were equal. In total, eight types of DNA templates, A, B, C, A+B, A+C, B+C, A+B+C, and PCR product based on the original genomic DNA extraction of strain CA487, were analyzed in this study.

For PCR amplification, two common primers, ITS4 and ITS5 (White et al., 1990), were used to amplify the ITS DNA fragment. The PCR protocol followed Chen et al. (2016) with the following modifications (Cline et al., 1996; Kobayashi et al., 1999) to reduce error rate: the pH of the reaction mixture was adjusted to 8.5–8.7 by Tris–HCl buffer and the final PCR reaction mixture of 50 μl for each of the eight treatments contained 15 μM each of the two primers, 50 ng of ITS DNA template, 100 μM each of dATP, dGTP, dCTP, and dTTP, 2 mM MgSO_4_, and 1 U Phusion^®^ High-Fidelity DNA Polymerase [New England Biolabs (Beijing) Ltd., Beijing, China]. One hundred and ten tubes of types A, B, and C, and twelve tubes for each of the other five treatments (A+B, A+C, B+C, A+B+C, and PCR product of the natural sample) were amplified (Figure 1). For each PCR reaction, the total template ITS DNA concentration was the same (i.e., 50 ng per tube). PCRs were performed on S1000^TM^ Thermal Cycler (Bio-Rad Laboratories, Hercules, CA, United States). The positions of the PCR tubes among treatment groups were randomized in the PCR machine to minimize edge and position effects on amplification efficiency that might occur for individual treatments.

To minimize PCR artifacts, we followed the PCR procedure described by Acinas et al. (2005) and Zhao et al. (2011) with the following parameters: (1) initial denaturation 95°C for 3 min, (2) denaturation at 94°C for 1 min, (3) annealing at 55°C for 1 min, (4) extension at 72°C for 1 min 30 s, (5) repeat for 30 cycles of steps 2–4, and (6) final extension at 72°C for 5 min. After amplification, the amount of output DNA concentrations were measured by Eppendorf BioPhotometer^®^ D30 (Eppendorf, Germany).

### Cloning and Sequencing

The PCR products from each tube were purified using the QIAquick PCR purification kit (Qiagen, Hilden, Germany), following the manufacturer’s instructions. After adding adenine at the 3′ ends of the purified PCR products, the products were then cloned into the pMD19-T vector (Takara, Tokyo, Japan), and transformed into *Escherichia coli* competent cells Top10 (TianGen Biotech., Co., Ltd., Beijing, China). Then, the bacteria were plated on an LB agar medium containing ampicillin, X-Gal, and IPTG (Seidman et al., 1985). After cultivating for 12 h, at least 50 white colonies of each LB plate were randomly chosen to further separately propagate in LB liquid medium in a shaker incubator.

All direct PCR products (for individual types A, B, and C only) and cloned PCR products (for all the eight treatments) were purified to remove excess primers and dNTP using PureLink PCR purification kits (Invitrogen, United States) and sequenced at SGM (SinoGenoMax Co., Ltd., Beijing, China). Sequencing of the direct PCR products used the primers ITS5/ITS4. Sequencing of the cloned ITS products used the M13F/M13R primer pair (Schoelkopf et al., 2005) flanking the ITS cloning site on the plasmid vector.

### Error Rate Calculation

In this study, the first-generation DNA sequencing (i.e., Sanger sequencing) technology was used to obtain sequences from our DNA fragments. The accuracy of this sequencing method is very high, ∼99.999% (Liu et al., 2012). Given the length of each ITS sequence (<650 bases), an error rate of <0.001% is too low to generate artifacts during sequencing reaction. So, we ignored this sequencing error in the present study.

In this study, the error rate (ER) for each treatment was calculated by using the equation of Keohavong and Thilly (1989) and Cline et al. (1996):

$$\text{ER}=\frac{m}{b\times d}$$

In this formula, *m* is the total number of artificial mutations occurred among all the sequences of each treatment, *b* is the number of total bases (i.e., product of the length of each sequence × the number of sequenced DNA fragments within each treatment), and *d* is the number of template doublings which is determined using the equation 2^d^ = (amount output DNA)/(amount of input DNA). The unit for error rate calculated based on this formula is the number of mutations per base per PCR replication.

### Chimera Determination and Comparison

Aside from mutations as characterized by base substitutions, deletions and insertions, recombination can also occur during PCR to generate chimera sequences. In this study, we define a chimera as a single DNA sequence read that contains unique nucleotides from two or more of the input parental sequences. That is, if a sequence contains unique signature nucleotides from two or more of the input ITS types, but without any new mutation, we define it as a recombinant (or chimera) type. To identify the chimera sequences, we compared all the non-parental sequences with those of the parental ones in each reaction by using the computer program BioEdit 7.2.5 (Hall, 1999). In this analysis, all the sequences of each reaction were aligned, and if the mutated nucleotides of the non-parental sequences matched all those of the parental sequences, these non-parental sequences are classified as chimeras. Aside from the direct visual comparisons, we also ran all ITS sequences from each of the eight treatments to identify the frequency and prevalence of chimeras using BioEdit 7.2.5 (Hall, 1999).

### Statistical Analyses

Several tests were applied to examine the statistical significance of the observed differences in PCR artifact among treatment groups. Specifically, the IBM SPSS Statistics V22.0 (IBM, Chicago, IL, United States) was used to perform the multiple *t*-tests with Bonferroni correction to derive the statistical significances of the observed difference among treatments. This same program was used to calculate Pearson correlation coefficients between template complexity and PCR artifact frequency.

## Results

In this study, using the Sanger sequencing method, we obtained a total of 5429 ITS sequences from eight treatments involving three types of template DNA from the mushroom *Agaricus subrufescens* CA487. All the chromatograms are available upon request and the detailed sequences are presented in the Supplementary Data Sheets 1–11, with data from each of the 11 treatments in a different Supplementary File. Among these 5429 ITS sequences, 188 (Table 1, 3.46%) contained 207 (Table 2) mutated nucleotide sites (including insertions and deletions) not found in the original type sequences. Specifically, among the 188 sequences, 171 contained only one variable site per sequence, 15 contained 2 variable sites per sequence, and 2 contained 3 variable sites per sequence. In addition, high percentages of *in vitro* recombinant sequences were detected from the five treatment groups containing two to three types of template ITS sequences. Below we describe the details of the recovered sequences.

**TABLE 1:** Mutation and recombination frequencies detected within each of the 11 PCR treatments.

**Treatment**	**Total**	**Identical sequences**			**Recombined sequences without mutated sites**				**Recombined sequences with mutated sites**	**Novel sequences without recombination**	**Total novel sequences**	**Accuracy rate (% ± SD)^1^**
							
		**A**	**B**	**C**	**1**	**2**	**3**	**>3**				
A (PCR)	110	110 (100%)	–	–	–	–	–	–	–	–	–	100 ± 0 a
B (PCR)	109	–	109 (100%)	–	–	–	–	–	–	–	–	100 ± 0 a
C (PCR)	110	–	–	110 (100%)	–	–	–	–	–	–	–	100 ± 0 a
A (Clone)	653	633 (96.94%)	–	–	–	–	–	–	–	20 (3.06%)	20 (3.06%)	96.94 ± 1.06 b
B (Clone)	641	–	618 (96.41%)	–	–	–	–	–	–	23 (3.59%)	23 (3.59%)	96.41 ± 1.47 b
C (Clone)	648	–	–	624 (96.30%)	–	–	–	–	–	24 (3.70%)	24 (3.70%)	96.30 ± 1.12 b
A+B	621	243 (39.13%)	190 (30.60%)	–	101 (16.27%)	43 (6.92%)	20 (3.22%)	3 (0.48%)	8 (1.29%)	13 (2.09%)	188 (30.27%)	69.51 ± 2.52 c
B+C	630	–	204 (32.38%)	221 (35.08%)	108 (17.14%)	50 (7.94%)	23 (3.65%)	3 (0.48%)	5 (0.79%)	16 (2.54%)	205 (32.54%)	67.33 ± 3.36 c
A+C	635	218 (34.33%)	–	231 (36.38%)	105 (16.54%)	44 (6.93%)	16 (2.52%)	1 (0.16%)	7 (1.10%)	13 (2.05%)	186 (29.29%)	69.91 ± 2.20 c
A+B+C	636	150 (23.58%)	122 (19.21%)	136 (21.38%)	112 (17.61%)	56 (8.80%)	28 (4.40%)	4 (0.63%)	11 (1.73%)	17 (2.67%)	228 (35.85%)	64.65 ± 3.38 c
Original DNA	636	169 (26.57%)	131 (20.60%)	138 (21.70%)	103 (16.20%)	47 (7.39%)	15 (2.36%)	2 (0.31%)	11 (1.73%)	20 (3.14%)	198 (31.13%)	68.07 ± 3.55 c
Total	5429	1523 (28.05%)	1374 (25.31%)	1460 (26.89%)	529 (16.75%)^2^	240 (7.60%)^2^	102 (3.23%)^2^	13 (0.41%)^2^	42 (1.33%)^2^	146 (3.69%)^3^	1072 (21.02%)^3^	

*^1^Treatments with different letters a, b, and c indicate that the accuracy rates are statistically significantly different from each other. ^2^The results were calculated using the equation *R* = *a*/*b*, where a is the total recombined sequence of each item and b is the total sequence number of the treatments A+B, A+C, B+C, A+B+C and original DNA, i.e., 42/3158. ^3^The results were calculated using the equation *R* = *x*/*y*, where *x* is the sequence number of each item and y is the total sequence numbers of the treatments A (Clone), B (Clone), C (Clone), A+B, A+C, B+C, A+B+C and original DNA, i.e., 1072/5100.*

**TABLE 2:** Summary mutations and error rate of each PCR treatment.

**Treatment type**	**Total no.**	**Transition mutations**				**Transversion mutations**							**Insertion**	**Deletion**	**Total No. variations**	**Error rate (×10^–6^ ± SD)^1^**
							
		**A to G**	**G to A**	**C to T**	**T to C**	**A to C**	**A to T**	**G to T**	**C to A**	**C to G**	**T to A**	**T to G**				
A (PCR)	110	–	–	–	–	–	–	–	–	–	–	–	–	–	0	0 a
B (PCR)	109	–	–	–	–	–	–	–	–	–	–	–	–	–	0	0 a
C (PCR)	110	–	–	–	–	–	–	–	–	-	–	–	–	–	0	0 a
A (Clone)	653	2	6	5	3	–	1	1	–	–	2	–	–	–	20	3.53 ± 0.22 b
B (Clone)	641	1	8	6	1	–	2	1	1	–	2	1	–	–	23	4.05 ± 0.31 bc
C (Clone)	648	2	7	11	1	–	–	1	–	–	1	1	–	–	24	3.96 ± 0.46 bc
A + B	621	–	7	12	1	–	1	–	1	–	2	–	–	–	24	4.5 ± 0.55 c
B + C	630	2	6	7	2	1	1	–	2	–	1	1	1 (G)	1 (T)	25	4.51 ± 0.41 c
A + C	635	2	7	8	2	1	–	1	1	–	1	–	–	–	23	4.2 ± 0.29 c
A + B + C	636	2	10	8	1	1	1	1	2	1	1	1	–	1 (T)	31	5.43 ± 0.33 d
Original DNA	636	3	9	11	3	1	2	3	2	–	1	1	–	1 (T)	37	6.47 ± 0.39 d
Total	5429	14	60	68	14	4	8	8	9	1	11	5	2	3	207	

*^1^Treatments with different letters a, b, c, and d indicate that the error rates are statistically significantly different from each other.*

### Mutations Recovered in Single-Template Treatments

As shown in Table 1, among the 329 directly sequenced PCR products of each single type A, B, and C, no mutated base was observed and all 329 sequence chromatographs were identical to either the A, B, or C type sequences respectively. This result is consistent with the overall high-fidelity nature of the PCR amplification and Sanger sequencing system. However, mutations have likely occurred but because those mutated sequences would be in minority, the mutated bases would not interfere with the dominant wild-type sequence in each reaction. After cloning, the individual amplified DNA fragments were separated and the potential mutations could be revealed by sequencing individual cloned DNA fragments. In total, 20, 23, and 24 of the cloned sequences with one or more mutations were obtained from among 653 type A, 641 type B and 648 type C clones, respectively. The above results indicated that between 3 and 4% of the PCR amplified sequences contained mutations, with an error rate of about 4 × 10^–6^ (range of 3.53–4.05 × 10^–6^). Among the three single template treatments, each of the variant sequences contained only one mutation each, and no insertion or deletion was found (Tables 1, 2).

### Mutations Recovered in Treatments Using Two or Three Templates

Similar proportions of sequences with mutated nucleotides were found in the treatments with two or more types of templates, with a frequency range of 3.15% (templates A+C combination) to 4.87% (template DNA from the original mushroom specimen) (Table 1). Interestingly, though the replication error rates were overall very similar among the treatments, there was evidence for increasing error rate as template complexity increased. Specifically, there was a high Pearson correlation coefficient (*R* = 0.9288, df = 7, *p* = 0.000855) between template complexity and PCR error rate among the eight treatments (Table 2) where ITS sequences were obtained based on cloned PCR products. In this study, the highest PCR error rate (∼6 × 10^–6^) was found when the genomic DNA of the original specimen was used as template.

Even though *A. subrufescens* strain CA487 was found to contain three types of ITS sequences, the exact copy number and complexity of ITS sequences within this strain are not known. Indeed, some of the variant sequences found when using the original specimen as template may represent true ITS sequences present within the specimen and not PCR artifacts. A direct comparison between PCR error rate of the artificially mixed sample A+B+C (5.43 × 10^–6^) with that of the original mushroom sample (5.99 × 10^–6^) suggests that about ∼10% [(5.99–5.43)/5.43 = 10.31%] of the minor variants in the original mushroom specimen may represent true variants and not PCR artifacts. However, the 10% difference in error rates between the artificially mixed template (A+B+C) and the template from the original mushroom was statistically not significant. Excluding template from the original mushroom sample from Pearson correlation test still indicated a statistically significant positive correlation between template complexity and PCR error rate (*R* = 0.9274, df = 6, *p* = 0.007715).

### Spectrum of Nucleotide Substitutions in *In vitro* PCR

The specific mutations detected in this study from PCR artifacts are summarized in Table 2. Because the ITS complexity in the original mushroom specimen is not known, we used mutations detected in the remaining seven treatments to identify the types of mutations accumulated during PCR. Overall, transitional mutations outnumbered transversion mutations by 3.4 folds (156 transition mutations vs. 46 transversion mutations). Of the transition mutations, the C → T (68 detected) and G → A (60 detected) were much more common than the T → C (14 detected) and the A → G (14 detected) mutations (Table 2). Among the transversion mutations, the most frequent was T → A, followed by C → A, A → T, G → T, T → G, A → C, and C → G. In two of the template treatments (B+C and A+B+C), one insertion and one deletion were detected in each treatment. In the mushroom specimen DNA as PCR template, a single deletion was found in one of the 636 colonies. Interestingly, all three deletions involved strings of the thymine nucleotide (Table 2). The results suggest that among the novel variants, the insertion/deletion events represent about 2.4% (5/207) of mutational events.

Table 3 shows the distribution of *in vitro* PCR mutations among the three regions of the sequenced DNA fragment: ITS1, 5.8S, and ITS2. Overall, though statistically insignificant, the ITS2 region had more mutations and an overall higher error rate than the other two regions (ITS1 and 5.8S) (Table 3). However, there are several differences among the different types of mutations. For example, among the transversion mutations, the T → A, G → T, and T → G mutations were the most frequent in the ITS1 region, while the C → A and C → G were the most common in the ITS2 region. Interestingly, there was no T → A, A → C, and C → G mutation in the 5.8S region. Moreover, aside from one thymine deletion in the ITS1 region, all other insertions and deletions were found in the ITS2 region.

**TABLE 3:** Relative mutation frequencies in different regions of the ITS marker.

**Treatment or variation type**	**Mutation ratio (%)**			**Error rate (×10^–6^)**		
		
	**ITS1**	**5.8S**	**ITS2**	**ITS1**	**5.8S**	**ITS2**
A	40	20	40	3.43	3.35	4.08
B	30.4	26.1	43.5	2.8	5.02	5.07
C	37.5	25	37.5	3.41	4.51	4.12
A+B	33.3	20.8	45.8	3.4	4.32	5.73
A+C	30.4	17.4	52.2	3.23	3.47	6.22
B+C	32	20	48	3.46	4.29	6.25
A+B+C	35.5	25.8	38.7	4.45	6.51	6.2
Original	29.7	24.3	45.9	4.14	7.01	8.03
G to A	25	16.7	58.3	0.76	1.06	2.25
A to G	35.7	14.3	50	0.25	0.21	0.45
T to C	28.6	28.6	42.8	0.2	0.42	0.39
C to T	29.4	22.1	48.5	1.01	1.59	2.12
T to A	66.7	–	33.3	0.4	–	0.26
A to C	50	–	50	0.1	–	0.13
C to A	33.3	22.2	44.4	0.15	0.21	0.26
C to G	–	–	100	–	–	0.06
G to T	57.1	14.3	28.6	0.2	0.11	0.13
T to G	60	20	20	0.15	0.11	0.06

To investigate the potential effect of base compositions on the differences in mutational spectrum among ITS1, 5.8S, and ITS2 regions, we calculated the base compositions in all the three regions. Table 4 shows the base compositions of the forward sense strand of the whole ITS region and its three different parts (ITS1, 5.8S and ITS2) in the three ITS types of *A. subrufescens* strain CA487. Overall, while some nucleotide compositional differences were observed among the three regions of ITS, the differences were statistically insignificant between the ITS1 and ITS2 regions (*P* > 0.5). In contrast, the 5.8S region had significantly higher percentages of A and C nucleotides and lower T nucleotides than those in the ITS1 and ITS2 regions (*P* < 0.05), likely due to the functional constraint of the 5.8S rRNA gene. However, the observed nucleotide compositional differences were not significantly correlated to the nucleotide mutational patterns observed in Table 3 (*P* > 0.2).

**TABLE 4:** Base compositions of one single strand of the whole ITS region and its three constituent parts (ITS1, 5.8S and ITS2) in the three ITS sequence types of *A. subrufescens* strain CA487.

	**Type A**				**Type B**				**Type C**			
			
	**Whole region**	**ITS1**	**5.8S**	**ITS2**	**Whole region**	**ITS1**	**5.8S**	**ITS2**	**Whole region**	**ITS1**	**5.8S**	**ITS2**
Total length	662	292	154	216	661	291	154	216	660	291	154	215
G	154	68	37	49	156	70	38	48	154	68	37	49
C	126	50	33	43	127	51	33	43	128	51	33	44
A	151	63	41	47	149	60	40	49	149	62	41	46
T	231	111	43	77	229	110	43	76	229	110	43	76
G content	23.3%	23.3%	24%	22.7%	23.6%	24.1%	24.7%	22.2%	23.3%	23.4%	24%	22.8%
C content	19%	17.1%	21.4%	19.9%	19.2%	17.5%	21.4%	19.9%	19.4%	17.5%	21.4%	20.5%
A content	22.8%	21.6%	26.6%	21.8%	22.5%	20.6%	26%	22.7%	22.6%	21.3%	26.6%	21.4%
T content	34.9%	38%	27.9%	35.6%	34.6%	37.8%	27.9%	35.2%	34.7%	37.8%	27.9%	35.3%
GC content	42.3%	40.4%	45.5%	42.6%	42.8%	41.6%	46.1%	42.1%	42.7%	40.9%	45.5%	43.3%
AT content	57.7%	59.6%	54.5%	57.4%	57.2%	58.4%	53.9%	57.9%	57.3%	59.1%	54.5%	56.7%

### Recombination During *In vitro* PCR

The summary results of our chimera detection are shown in Table 1. Among the five samples with two or three ITS templates each, the relative frequencies of recombined sequences ranged between 27.24 and 33.18%, with the highest percentage found in the A+B+C treatment group. However, these differences among template complexity treatments are statistically insignificant. Interestingly, though the template complexity is similar or potentially higher than the A+B+C treatment, the PCR products derived from using original mushroom DNA as template yielded the second lowest frequency of chimeric sequences (27.99%) among the five treatments.

**FIGURE 2:**
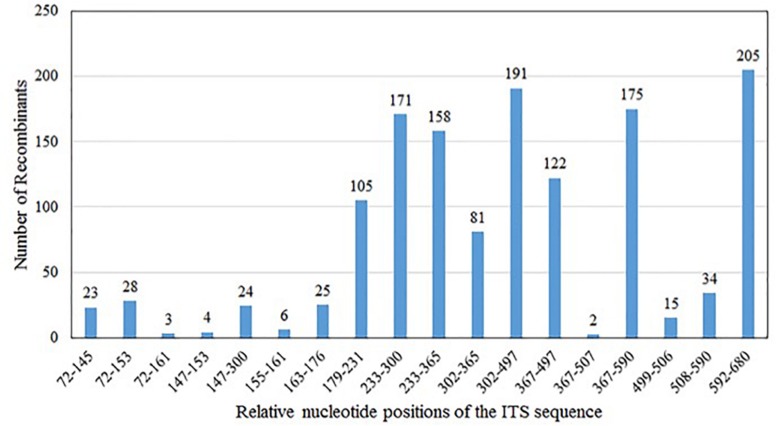
Distribution of *in vitro* PCR recombination events along the sequenced ITS DNA fragment. In the present study, base positions 1–32 are part of the 18S; positions 33–324 (type A), and 33–323 (types B and C) represent ITS1; positions 325–478 (type A) and 324–477 (types B and C) represent 5.8S; positions 479–694 (type A), 478–693 (type B), and 478–692 (type C) represent ITS2; and positions 695–731 (type A), 694–730 (type B), and 693–729 (type C) are part of the 26S. Because of the different lengths of types A, B, and C sequences, their aligned dataset was used to infer the regions where recombination occurred. Type A and C have the same 5.8S gene sequence, while type B has a different base at the position 366, adenine for type A and C, guanine for type B.

Our analyses showed that the majority (∼60%) of the chimeric sequences were likely generated due to one recombination event; about 27% were generated by double recombination events; about 11% due to triple recombination event; and the remaining ∼2% due to >3 recombination events (Table 1). Figure 2 summarizes the distributions of the likely recombination events across the ITS regions. Here, because the three types of ITS sequences differed slightly in their lengths, the recombination breakpoints (regions) were inferred based on their combined aligned sequences. However, due to the high sequence similarities and the long tracks of identical sequences among types A, B, and C sequences, we are unable to pinpoint the exact locations for most recombination events. Instead, only the range of nucleotide positions where each recombination event was identified.

## Discussion

In this study, we analyzed the *in vitro* PCR mutation spectrum and recombination frequencies in the fungal barcode marker ITS, using three divergent alleles found within the natural hybrid strain CA487 of the mushroom *A. subrufescens*. Using the high-fidelity amplification system, our study identified an *in vitro* PCR replication error rate of around 4 × 10^–6^ per base per PCR replication, generating about 4% of sequences with nucleotide substitutions and/or deletions/insertions. Significantly, we found that in PCR containing two or more ITS templates, about 30% of the PCR products were recombinants, with each containing signature nucleotides of two or more parental templates. Below we discuss the potential mechanisms and implications of our results.

Ideally, a perfect PCR means all the amplified sequences are exactly the same as the original template(s). However, in reality, artificial molecules are frequently produced (Cline et al., 1996; Sharifian, 2010; Kulibaba and Liashenko, 2016). These artifacts include those derived from mutation, recombination (Brakenhoff et al., 1991; Komatsoulis and Waterman, 1997; Haas et al., 2011), and heteroduplex formation when double-stranded DNA contain sites with non-complementary bases such as A–C and G–T pairs (L’Abbé et al., 1992; Thompson et al., 2002; Kulibaba and Liashenko, 2016). Previous studies have shown that primer sequence matching, template length, the type of DNA polymerase, annealing temperature, extension time, and the number of PCR cycles could all contribute to the generation of PCR artifacts (Suzuki and Giovannoni, 1996; Polz and Cavanaugh, 1998; Suzuki et al., 1998; Ishii and Fukui, 2001; Kanagawa, 2003; Sharifian, 2010). For example, previous studies using mixed 16S rDNA from environmental bacteria as templates showed that disproportionally high numbers of PCR artifacts were generated in later PCR cycles (Ishii and Fukui, 2001; Kanagawa, 2003). In our present study, we followed the recommended protocol for minimizing PCR artifacts by using the perfectly matched primers, the suggested annealing temperature, an extension time that’s about twice that needed to complete the synthesis of each molecule in each PCR cycle, and a limited number (30) of PCR cycles. In addition, Phusion^®^ High-Fidelity DNA Polymerase was chosen. According to the supplier of this enzyme, the New England Biolabs, this polymerase has a 50-fold and 6-fold higher fidelity than the commonly used *Taq* and *Pfu* DNA Polymerases, respectively. Thus, the artifacts observed here likely represent the intrinsic inaccuracies of the *in vitro* PCR system.

Our results showed that, in single-template PCRs, no artificial molecule was detected when the PCR product was sequenced directly. However, after cloning the PCR products, about 4% of the generated sequences contained one or more mutations. The seemingly conflicting results are understandable because the relatively low frequency of sequences with mutated bases would be overshadowed by the wildtype sequence in the chromatogram of direct PCR products. Our observed error rate of about 4 × 10^–6^ per nucleotide per replication is much lower than those observed in previous studies using the *Taq* DNA Polymerase [2.1 × 10^–4^ per nucleotide per replication as reported by Keohavong and Thilly (1989); 2.0 × 10^–5^ per nucleotide per replication by Lundberg et al. (1991); 2.6 × 10^–5^ per nucleotide per replication by Frey and Suppmann (1995); 8.0 × 10^–6^ per nucleotide per replication by Cline et al. (1996); 3.3 × 10^–5^ per nucleotide per replication by Acinas et al. (2005); and 1.0 × 10^–5^ per nucleotide per replication by Biles and Connolly (2004)]. This is because the Phusion^®^ High-Fidelity DNA Polymerase has the 3′–5′ exonuclease activity while *Taq* DNA polymerase is devoid of 3′–5′ exonuclease activity and cannot excise mis-incorporated bases produced during PCR amplification (Tindall and Kunkel, 1988). However, we would like to mention that the fidelity of Phusion^®^ High-Fidelity DNA Polymerase observed in our study was not 50 × higher than the highest reported so far for commonly used *Taq* Polymerases-based systems.

At present, we can’t exclude the possibility that mutation and artifacts can happen during plasmid cloning and replication within *E. coli*. However, we believe their effect is likely very small compared to the *in vitro* PCR. In this *E. coli* cloning system, each bacterial cell typically only takes in a single plasmid, thus, it’s highly unlikely that there was *in vivo* recombination. Secondly, the bacterial strain used for cloning has the complete set of functional DNA mutation repair system with an estimated mutation rate of <10^–10^ per base per replication. This rate is significantly lower than what we have estimated in the *in vitro* PCR system. We also note that the error rate in our PCR with the Phusion^®^ High-Fidelity DNA Polymerase is higher than that reported for *Pfu* DNA Polymerase (1.6 × 10^–6^ per nucleotide per replication for Lundberg et al., 1991; 1.3 × 10^–6^ per nucleotide per replication for Cline et al., 1996). The exact reason for the difference is not known but likely related to the different methods used in estimating error rate. In the studies by Lundberg et al. (1991) and Cline et al. (1996), they used the *lacIOZ*α-based fidelity assay, which depended on the counts of blue colonies and white colonies growing on agar plates. While efficient, such an assay likely under-estimated the error rate because certain mutations would have no phenotypic effects. Furthermore, their method could not ascertain the specific number of mutated nucleotides within an amplified sequence. In our analyses, all the nucleotides on the target fragment were sequenced by Sanger chain termination method, and all the mutations were counted.

With two or three types of templates in each PCR reaction, we detected chimera sequences at an average frequency of 31.8%. Among the recombinant sequences, ∼60% of the recombinants were likely produced from single cross-over events while the remaining 40% were from two or more recombination events. We should note that the lack of chimera sequences in the single template experiments was unlikely due to the lack of recombination in those reactions. Instead, *in vitro* recombination has likely occurred but due to the absence of markers to distinguish parental from recombinant sequences, we are unable to infer recombination events in those treatments. Indeed, the inferred recombination frequencies in the mixed template treatments were likely under-estimates of the true frequencies and that more polymorphic nucleotide sites (up to a certain degree) among the templates would likely lead to recovery of a higher frequency of chimera sequences.

There were 207 single-base mutations in the 5429 ITS sequences (Table 2). Among these mutations, most were transitions, while less than three percent were single-base insertions and deletions. Our observed mutational spectrum is similar to those observed by Cadwell and Joyce (1992); Collins and Jukes (1994) and Ebersberger et al. (2002), but different from that by Brodin et al. (2013), who estimated transition errors as 48-times more common than transversion errors. Transition mutations are more likely than transversions because of the similar structures between nucleotides involved in transitional mutations. Of all the four transition types, C → T and G → A mutations were much more frequent than T → C and A → G mutations. At present, the reason(s) for the significantly biased transitional mutation patterns observed here is not known.

The observed accuracy rate in this study for the original mushroom DNA sample is significantly higher than that reported in the study by Chen et al. (2016) for the same mushroom specimen. In Chen et al. (2016) study, among the 284 sequenced clones, 164 (57.8%) were of the A, B, and C types while the remaining 120 (42.2%) were of other types. In this study, among the 636 sequenced clones, 438 (68.9%) were of the A, B, and C types while the remaining 198 were of the other types. The reason for the observed 10% increase in accuracy rate in the current study was most likely due to the use of a high-fidelity DNA polymerase and a protocol that minimizes PCR artifacts.

## Conclusion

In conclusion, our study is the first report on the *in vitro* PCR mutational patterns of the whole ITS region (ITS1 + 5.8S + ITS2) of fungi. Our analyses revealed an overall nucleotide substitution rate of about 4 × 10^–6^ per nucleotide per replication using the Phusion^®^ High-Fidelity DNA Polymerase following the recommended protocols to minimize mutations. Our results revealed that such an *in vitro* PCR mutation rate has a negligible effect on the quality and accuracy of ITS sequences derived from direct sequencing of PCR products. However, if the amplified PCR products were cloned first before sequencing (such as in most metagenomic studies), PCR artifacts (at least 3% and likely higher depending on the PCR amplification system) would be generated and reported as part of the “sequence diversity” in each sample. A more significant concern is the generation of chimera sequences due to the *in vitro* recombination during PCR. Specifically, our analyses showed that over 27% of cloned ITS sequences were PCR recombinant artifacts which were revealed when the PCR reaction mixture contained two to more homologous sequences. In individual samples containing two or more divergent alleles within each sample (such as the ITS sequences in the hybrid mushroom *A. subrufescens* CA487 and all metagenomic studies), care should be taken to filter out such artifacts before sequence analyses and submission. Specifically, our results suggest that both minor frequency sequences containing one to a few nucleotide differences from the main sequence types and sequences containing signature nucleotides from two or more of the main sequence types (to ∼30% in overall frequency) in the sample should be treated as PCR artifacts and be excluded from analyses.

## Data Availability Statement

The raw data supporting the conclusions of this manuscript will be made available by the authors, without undue reservation, to any qualified researcher.

## Author Contributions

JX and S-XW conceived the study. J-LZ, A-GJ, and LY conducted the experiments. JC and PC contributed the samples and data interpretation. YL contributed the reagents. J-LZ and JX analyzed the data. J-LZ, S-XW, and JX drafted the manuscript. All authors contributed to finalizing the manuscript.

## Conflict of Interest

The authors declare that the research was conducted in the absence of any commercial or financial relationships that could be construed as a potential conflict of interest. The reviewer MF-O declared a shared affiliation, with no collaboration, with one of the authors, PC, to the handling Editor at time of review.

## Footnotes

1 https://www6.inra.fr/cirm_eng/The-CIRM-activities/Strain-and-DNA-catalogs
