# Supplementary material for: Patterns of PCR Amplification Artifacts of the Fungal Barcode Marker in a Hybrid Mushroom
Source: Front Microbiol. 2019 Nov 19;10:2686. doi: 10.3389/fmicb.2019.02686 (PMC6877668; doi:10.3389/fmicb.2019.02686)
Supplement: Supplementary file 1 [file Data_Sheet_1.PDF]

>A1_8
TTTCCGTAGGTGAACCTGCGGAAGGATCATTATTGAATTATGTTTCTAGATAGGTTGTAG
CTGGCTCTTTTAGAGCATGTGCACGCCTGTTTGGACTTCATTTTCATCCACCTGTGCACC
TATTGTAGTCTTTGGTTGGGTTAGGAGGAAGTGATCATTGTATCAGCATCTGCTGGGAGT
GAGGACTTGCATTGTGAAAGCTTTGCTGTCCTTGATGTGATCATGGAATCTTTTTCTCAC
TAGAGTCTATGTCACTCATTATACTCTGTCGAATGTCATTGAATGTCTTTACATGGGCTT
GTATGCCTATGAAAATTGTAATACAACTTTCAGCAACGGATCTCTTGGCTCTCGCATCGA
TGAAGAACGCAGCGAAATGCGATAAGTAATGTGAATTGCAGAATTCAGTGAATCATCGAA
TCTTTGAACGCATCTTGCGCTCCTTGGTATTCCGAGGAGCATGCCTGTTTGAGTGTCATT
AAATTCTCAACTCTCTTATACTTTTTTGTAAAAGAGAGCTTGGACTGTGGAGGCTTGCTG
GCCACTTTTTGGGGTCAGCTCCTCTGAAATGCATTAGCGGAACCGTTTGCGATCTGCCAC
AAGTGTGATAAGTTATCTACACTGGCGAGGGGATTGCTCTCTGTAATGTTCAGCTTCTAA
TTGTCTCTACTTTGTGAGACTACTTTTGAATGCTTGACCTCAAATCAGGTAGGACTACCC
GCTGAACTTAA
>A8_52
TTTCCGTAGGTGAACCTGCGGAAGGATCATTATTGAATTATGTTTCTAGATAGGTTGTAG
CTGGCTCTTTTAGAGCATGTGCACGCCTGTTTGGACTTCATTTTCATCCACCTGTGCACC
TATTGTAGTCTTTGGTTGGGTTAGGAGGAAGTGATCATTGTATCAGCATCTGCTGGGAGT
GAGGACTTGCATTGTGAAAGCTTTGCTGTCCTTGATGTGATCATGGAATCTTTTTCTCAC
TAGAGTCTATGTCACTCATTATACTCTGTCGAATGTCATTGAATGTCTTTACATGGGCTT
GTATGCCTATGAAAATTGTAATACAACTTTCAGCAACGGATCTCTTGGCTCTCGCATCGA
TGAAGAACGCAGCGAAATGCGATAAGTAATGTGAATTGCAGAATTCAGTGAATCATCGAA
TCTTTGAACGCATCTTGCGCTCCTTGGTATTCCGAGGAGCATGCCTGTTTGAGTGTCATT
AAATTCTCAACTCTCTTATACTTTTTTGTAAAAGAGAGCTTGGACTGTGGAGGCTTGCTG
GCCACTTTTTGGGGTCAGCTCCTCTGAAATGCATTAGCGGAACCGTTTGCGATCTGCCAC
AAGTGTGATAAGTTATCTACACTGGCGAGGGGATTGCTCTCTGTAATGTTCAGCTTCTAA
TTGTCTCTACTTTGTGAGACTACTTTTGAATGCTTGACCTCAAATCAGGTAGGACTACCC
GCTGAACTTAA
>A8_63
TTTCCGTAGGTGAACCTGCGGAAGGATCATTATTGAATTATGTTTCTAGATAGGTTGTAG
CTGGCTCTTTTAGAGCATGTGCACGCCTGTTTGGACTTCATTTTCATCCACCTGTGCACC
TATTGTAGTCTTTGGTTGGGTTAGGAGGAAGTGATCATTGTATCAGCATCTGCTGGGAGT
GAGGACTTGCATTGTGAAAGCTTTGCTGTCCTTGATGTGATCATGGAATCTTTTTCTCAC
TAGAGTCTATGTCACTCATTATACTCTGTCGAATGTCATTGAATGTCTTTACATGGGCTT
GTATGCCTATGAAAATTGTAATACAACTTTCAGCAACGGATCTCTTGGCTCTCGCATCGA
TGAAGAACGCAGCGAAATGCGATAAGTAATGTGAATTGCAGAATTCAGTGAATCATCGAA
TCTTTGAACGCATCTTGCGCTCCTTGGTATTCCGAGGAGCATGCCTGTTTGAGTGTCATT
AAATTCTCAACTCTCTTATACTTTTTTGTAAAAGAGAGCTTGGACTGTGGAGGCTTGCTG
GCCACTTTTTGGGGTCAGCTCCTCTGAAATGCATTAGCGGAACCGTTTGCGATCTGCCAC
AAGTGTGATAAGTTATCTACACTGGCGAGGGGATTGCTCTCTGTAATGTTCAGCTTCTAA
TTGTCTCTACTTTGTGAGACTACTTTTGAATGCTTGACCTCAAATCAGGTAGGACTACCC
GCTGAACTTAA
>A1_1
TTTCCGTAGGTGAACCTGCGGAAGGATCATTATTGAATTATGTTTCTAGATAGGTTGTAG
CTGGCTCTTTTAGAGCATGTGCACGCCTGTTTGGACTTCATTTTCATCCACCTGTGCACC
TATTGTAGTCTTTGGTTGGGTTAGGAGGAAGTGATCATTGTATCAGCATCTGCTGGGAGT
GAGGACTTGCATTGTGAAAGCTTTGCTGTCCTTGATGTGATCATGGAATCTTTTTCTCAC
TAGAGTCTATGTCACTCATTATACTCTGTCGAATGTCATTGAATGTCTTTACATGGGCTT
GTATGCCTATGAAAATTGTAATACAACTTTCAGCAACGGATCTCTTGGCTCTCGCATCGA
TGAAGAACGCAGCGAAATGCGATAAGTAATGTGAATTGCAGAATTCAGTGAATCATCGAA

TCTTTGAACGCATCTTGCGCTCCTTGGTATTCCGAGGAGCATGCCTGTTTGAGTGTCATT
AAATTCTCAACTCTCTTATACTTTTTTGTAAAAGAGAGCTTGGACTGTGGAGGCTTGCTG
GCCACTTTTTGGGGTCAGCTCCTCTGAAATGCATTAGCGGAACCGTTTGCGATCTGCCAC
AAGTGTGATAAGTTATCTACACTGGCGAGGGGATTGCTCTCTGTAATGTTCAGCTTCTAA
TTGTCTCTACTTTGTGAGACTACTTTTGAATGCTTGACCTCAAATCAGGTAGGACTACCC
GCTGAACTTAA
>A1_13
TTTCCGTAGGTGAACCTGCGGAAGGATCATTATTGAATTATGTTTCTAGATAGGTTGTAG
CTGGCTCTTTTAGAGCATGTGCACGCCTGTTTGGACTTCATTTTCATCCACCTGTGCACC
TATTGTAGTCTTTGGTTGGGTTAGGAGGAAGTGATCATTGTATCAGCATCTGCTGGGAGT
GAGGACTTGCATTGTGAAAGCTTTGCTGTCCTTGATGTGATCATGGAATCTTTTTCTCAC
TAGAGTCTATGTCACTCATTATACTCTGTCGAATGTCATTGAATGTCTTTACATGGGCTT
GTATGCCTATGAAAATTGTAATACAACTTTCAGCAACGGATCTCTTGGCTCTCGCATCGA
TGAAGAACGCAGCGAAATGCGATAAGTAATGTGAATTGCAGAATTCAGTGAATCATCGAA
TCTTTGAACGCATCTTGCGCTCCTTGGTATTCCGAGGAGCATGCCTGTTTGAGTGTCATT
AAATTCTCAACTCTCTTATACTTTTTTGTAAAAGAGAGCTTGGACTGTGGAGGCTTGCTG
GCCACTTTTTGGGGTCAGCTCCTCTGAAATGCATTAGCGGAACCGTTTGCGATCTGCCAC
AAGTGTGATAAGTTATCTACACTGGCGAGGGGATTGCTCTCTGTAATGTTCAGCTTCTAA
TTGTCTCTACTTTGTGAGACTACTTTTGAATGCTTGACCTCAAATCAGGTAGGACTACCC
GCTGAACTTAA
>A8_1
TTTCCGTAGGTGAACCTGCGGAAGGATCATTATTGAATTATGTTTCTAGATAGGTTGTAG
CTGGCTCTTTTAGAGCATGTGCACGCCTGTTTGGACTTCATTTTCATCCACCTGTGCACC
TATTGTAGTCTTTGGTTGGGTTAGGAGGAAGTGATCATTGTATCAGCATCTGCTGGGAGT
GAGGACTTGCATTGTGAAAGCTTTGCTGTCCTTGATGTGATCATGGAATCTTTTTCTCAC
TAGAGTCTATGTCACTCATTATACTCTGTCGAATGTCATTGAATGTCTTTACATGGGCTT
GTATGCCTATGAAAATTGTAATACAACTTTCAGCAACGGATCTCTTGGCTCTCGCATCGA
TGAAGAACGCAGCGAAATGCGATAAGTAATGTGAATTGCAGAATTCAGTGAATCATCGAA
TCTTTGAACGCATCTTGCGCTCCTTGGTATTCCGAGGAGCATGCCTGTTTGAGTGTCATT
AAATTCTCAACTCTCTTATACTTTTTTGTAAAAGAGAGCTTGGACTGTGGAGGCTTGCTG
GCCACTTTTTGGGGTCAGCTCCTCTGAAATGCATTAGCGGAACCGTTTGCGATCTGCCAC
AAGTGTGATAAGTTATCTACACTGGCGAGGGGATTGCTCTCTGTAATGTTCAGCTTCTAA
TTGTCTCTACTTTGTGAGACTACTTTTGAATGCTTGACCTCAAATCAGGTAGGACTACCC
GCTGAACTTAA
>A8_3
TTTCCGTAGGTGAACCTGCGGAAGGATCATTATTGAATTATGTTTCTAGATAGGTTGTAG
CTGGCTCTTTTAGAGCATGTGCACGCCTGTTTGGACTTCATTTTCATCCACCTGTGCACC
TATTGTAGTCTTTGGTTGGGTTAGGAGGAAGTGATCATTGTATCAGCATCTGCTGGGAGT
GAGGACTTGCATTGTGAAAGCTTTGCTGTCCTTGATGTGATCATGGAATCTTTTTCTCAC
TAGAGTCTATGTCACTCATTATACTCTGTCGAATGTCATTGAATGTCTTTACATGGGCTT
GTATGCCTATGAAAATTGTAATACAACTTTCAGCAACGGATCTCTTGGCTCTCGCATCGA
TGAAGAACGCAGCGAAATGCGATAAGTAATGTGAATTGCAGAATTCAGTGAATCATCGAA
TCTTTGAACGCATCTTGCGCTCCTTGGTATTCCGAGGAGCATGCCTGTTTGAGTGTCATT
AAATTCTCAACTCTCTTATACTTTTTTGTAAAAGAGAGCTTGGACTGTGGAGGCTTGCTG
GCCACTTTTTGGGGTCAGCTCCTCTGAAATGCATTAGCGGAACCGTTTGCGATCTGCCAC
AAGTGTGATAAGTTATCTACACTGGCGAGGGGATTGCTCTCTGTAATGTTCAGCTTCTAA
TTGTCTCTACTTTGTGAGACTACTTTTGAATGCTTGACCTCAAATCAGGTAGGACTACCC
GCTGAACTTAA
>A8_4
TTTCCGTAGGTGAACCTGCGGAAGGATCATTATTGAATTATGTTTCTAGATAGGTTGTAG

CTGGCTCTTTTAGAGCATGTGCACGCCTGTTTGGACTTCATTTTCATCCACCTGTGCACC
TATTGTAGTCTTTGGTTGGGTTAGGAGGAAGTGATCATTGTATCAGCATCTGCTGGGAGT
GAGGACTTGCATTGTGAAAGCTTTGCTGTCCTTGATGTGATCATGGAATCTTTTTCTCAC
TAGAGTCTATGTCACTCATTATACTCTGTCGAATGTCATTGAATGTCTTTACATGGGCTT
GTATGCCTATGAAAATTGTAATACAACTTTCAGCAACGGATCTCTTGGCTCTCGCATCGA
TGAAGAACGCAGCGAAATGCGATAAGTAATGTGAATTGCAGAATTCAGTGAATCATCGAA
TCTTTGAACGCATCTTGCGCTCCTTGGTATTCCGAGGAGCATGCCTGTTTGAGTGTCATT
AAATTCTCAACTCTCTTATACTTTTTTGTAAAAGAGAGCTTGGACTGTGGAGGCTTGCTG
GCCACTTTTTGGGGTCAGCTCCTCTGAAATGCATTAGCGGAACCGTTTGCGATCTGCCAC
AAGTGTGATAAGTTATCTACACTGGCGAGGGGATTGCTCTCTGTAATGTTCAGCTTCTAA
TTGTCTCTACTTTGTGAGACTACTTTTGAATGCTTGACCTCAAATCAGGTAGGACTACCC
GCTGAACTTAA
>A8_5
TTTCCGTAGGTGAACCTGCGGAAGGATCATTATTGAATTATGTTTCTAGATAGGTTGTAG
CTGGCTCTTTTAGAGCATGTGCACGCCTGTTTGGACTTCATTTTCATCCACCTGTGCACC
TATTGTAGTCTTTGGTTGGGTTAGGAGGAAGTGATCATTGTATCAGCATCTGCTGGGAGT
GAGGACTTGCATTGTGAAAGCTTTGCTGTCCTTGATGTGATCATGGAATCTTTTTCTCAC
TAGAGTCTATGTCACTCATTATACTCTGTCGAATGTCATTGAATGTCTTTACATGGGCTT
GTATGCCTATGAAAATTGTAATACAACTTTCAGCAACGGATCTCTTGGCTCTCGCATCGA
TGAAGAACGCAGCGAAATGCGATAAGTAATGTGAATTGCAGAATTCAGTGAATCATCGAA
TCTTTGAACGCATCTTGCGCTCCTTGGTATTCCGAGGAGCATGCCTGTTTGAGTGTCATT
AAATTCTCAACTCTCTTATACTTTTTTGTAAAAGAGAGCTTGGACTGTGGAGGCTTGCTG
GCCACTTTTTGGGGTCAGCTCCTCTGAAATGCATTAGCGGAACCGTTTGCGATCTGCCAC
AAGTGTGATAAGTTATCTACACTGGCGAGGGGATTGCTCTCTGTAATGTTCAGCTTCTAA
TTGTCTCTACTTTGTGAGACTACTTTTGAATGCTTGACCTCAAATCAGGTAGGACTACCC
GCTGAACTTAA
>A8_6
TTTCCGTAGGTGAACCTGCGGAAGGATCATTATTGAATTATGTTTCTAGATAGGTTGTAG
CTGGCTCTTTTAGAGCATGTGCACGCCTGTTTGGACTTCATTTTCATCCACCTGTGCACC
TATTGTAGTCTTTGGTTGGGTTAGGAGGAAGTGATCATTGTATCAGCATCTGCTGGGAGT
GAGGACTTGCATTGTGAAAGCTTTGCTGTCCTTGATGTGATCATGGAATCTTTTTCTCAC
TAGAGTCTATGTCACTCATTATACTCTGTCGAATGTCATTGAATGTCTTTACATGGGCTT
GTATGCCTATGAAAATTGTAATACAACTTTCAGCAACGGATCTCTTGGCTCTCGCATCGA
TGAAGAACGCAGCGAAATGCGATAAGTAATGTGAATTGCAGAATTCAGTGAATCATCGAA
TCTTTGAACGCATCTTGCGCTCCTTGGTATTCCGAGGAGCATGCCTGTTTGAGTGTCATT
AAATTCTCAACTCTCTTATACTTTTTTGTAAAAGAGAGCTTGGACTGTGGAGGCTTGCTG
GCCACTTTTTGGGGTCAGCTCCTCTGAAATGCATTAGCGGAACCGTTTGCGATCTGCCAC
AAGTGTGATAAGTTATCTACACTGGCGAGGGGATTGCTCTCTGTAATGTTCAGCTTCTAA
TTGTCTCTACTTTGTGAGACTACTTTTGAATGCTTGACCTCAAATCAGGTAGGACTACCC
GCTGAACTTAA
>A8_7
TTTCCGTAGGTGAACCTGCGGAAGGATCATTATTGAATTATGTTTCTAGATAGGTTGTAG
CTGGCTCTTTTAGAGCATGTGCACGCCTGTTTGGACTTCATTTTCATCCACCTGTGCACC
TATTGTAGTCTTTGGTTGGGTTAGGAGGAAGTGATCATTGTATCAGCATCTGCTGGGAGT
GAGGACTTGCATTGTGAAAGCTTTGCTGTCCTTGATGTGATCATGGAATCTTTTTCTCAC
TAGAGTCTATGTCACTCATTATACTCTGTCGAATGTCATTGAATGTCTTTACATGGGCTT
GTATGCCTATGAAAATTGTAATACAACTTTCAGCAACGGATCTCTTGGCTCTCGCATCGA
TGAAGAACGCAGCGAAATGCGATAAGTAATGTGAATTGCAGAATTCAGTGAATCATCGAA
TCTTTGAACGCATCTTGCGCTCCTTGGTATTCCGAGGAGCATGCCTGTTTGAGTGTCATT
AAATTCTCAACTCTCTTATACTTTTTTGTAAAAGAGAGCTTGGACTGTGGAGGCTTGCTG

GCCACTTTTTGGGGTCAGCTCCTCTGAAATGCATTAGCGGAACCGTTTGCGATCTGCCAC
AAGTGTGATAAGTTATCTACACTGGCGAGGGGATTGCTCTCTGTAATGTTCAGCTTCTAA
TTGTCTCTACTTTGTGAGACTACTTTTGAATGCTTGACCTCAAATCAGGTAGGACTACCC
GCTGAACTTAA
>A8_8
TTTCCGTAGGTGAACCTGCGGAAGGATCATTATTGAATTATGTTTCTAGATAGGTTGTAG
CTGGCTCTTTTAGAGCATGTGCACGCCTGTTTGGACTTCATTTTCATCCACCTGTGCACC
TATTGTAGTCTTTGGTTGGGTTAGGAGGAAGTGATCATTGTATCAGCATCTGCTGGGAGT
GAGGACTTGCATTGTGAAAGCTTTGCTGTCCTTGATGTGATCATGGAATCTTTTTCTCAC
TAGAGTCTATGTCACTCATTATACTCTGTCGAATGTCATTGAATGTCTTTACATGGGCTT
GTATGCCTATGAAAATTGTAATACAACTTTCAGCAACGGATCTCTTGGCTCTCGCATCGA
TGAAGAACGCAGCGAAATGCGATAAGTAATGTGAATTGCAGAATTCAGTGAATCATCGAA
TCTTTGAACGCATCTTGCGCTCCTTGGTATTCCGAGGAGCATGCCTGTTTGAGTGTCATT
AAATTCTCAACTCTCTTATACTTTTTGTAAAAGAGAGCTTGGACTGTGGAGGCTTGCTG
GCCACTTTTTGGGGTCAGCTCCTCTGAAATGCATTAGCGGAACCGTTTGCGATCTGCCAC
AAGTGTGATAAGTTATCTACACTGGCGAGGGGATTGCTCTCTGTAATGTTCAGCTTCTAA
TTGTCTCTACTTTGTGAGACTACTTTTGAATGCTTGACCTCAAATCAGGTAGGACTACCC
GCTGAACTTAA
>A8_53
TTTCCGTAGGTGAACCTGCGGAAGGATCATTATTGAATTATGTTTCTAGATAGGTTGTAG
CTGGCTCTTTTAGAGCATGTGCACGCCTGTTTGGACTTCATTTTCATCCACCTGTGCACC
TATTGTAGTCTTTGGTTGGGTTAGGAGGAAGTGATCATTGTATCAGCATCTGCTGGGAGT
GAGGACTTGCATTGTGAAAGCTTTGCTGTCCTTGATGTGATCATGGAATCTTTTTCTCAC
TAGAGTCTATGTCACTCATTATACTCTGTCGAATGTCATTGAATGTCTTTACATGGGCTT
GTATGCCTATGAAAATTGTAATACAACTTTCAGCAACGGATCTCTTGGCTCTCGCATCGA
TGAAGAACGCAGCGAAATGCGATAAGTAATGTGAATTGCAGAATTCAGTGAATCATCGAA
TCTTTGAACGCATCTTGCGCTCCTTGGTATTCCGAGGAGCATGCCTGTTTGAGTGTCATT
AAATTCTCAACTCTCTTATACTTTTTGTAAAAGAGAGCTTGGACTGTGGAGGCTTGCTG
GCCACTTTTTGGGGTCAGCTCCTCTGAAATGCATTAGCGGAACCGTTTGCGATCTGCCAC
AAGTGTGATAAGTTATCTACACTGGCGAGGGGATTGCTCTCTGTAATGTTCAGCTTCTAA
TTGTCTCTACTTTGTGAGACTACTTTTGAATGCTTGACCTCAAATCAGGTAGGACTACCC
GCTGAACTTAA
>A8_54
TTTCCGTAGGTGAACCTGCGGAAGGATCATTATTGAATTATGTTTCTAGATAGGTTGTAG
CTGGCTCTTTTAGAGCATGTGCACGCCTGTTTGGACTTCATTTTCATCCACCTGTGCACC
TATTGTAGTCTTTGGTTGGGTTAGGAGGAAGTGATCATTGTATCAGCATCTGCTGGGAGT
GAGGACTTGCATTGTGAAAGCTTTGCTGTCCTTGATGTGATCATGGAATCTTTTTCTCAC
TAGAGTCTATGTCACTCATTATACTCTGTCGAATGTCATTGAATGTCTTTACATGGGCTT
GTATGCCTATGAAAATTGTAATACAACTTTCAGCAACGGATCTCTTGGCTCTCGCATCGA
TGAAGAACGCAGCGAAATGCGATAAGTAATGTGAATTGCAGAATTCAGTGAATCATCGAA
TCTTTGAACGCATCTTGCGCTCCTTGGTATTCCGAGGAGCATGCCTGTTTGAGTGTCATT
AAATTCTCAACTCTCTTATACTTTTTGTAAAAGAGAGCTTGGACTGTGGAGGCTTGCTG
GCCACTTTTTGGGGTCAGCTCCTCTGAAATGCATTAGCGGAACCGTTTGCGATCTGCCAC
AAGTGTGATAAGTTATCTACACTGGCGAGGGGATTGCTCTCTGTAATGTTCAGCTTCTAA
TTGTCTCTACTTTGTGAGACTACTTTTGAATGCTTGACCTCAAATCAGGTAGGACTACCC
GCTGAACTTAA
>A8_55
TTTCCGTAGGTGAACCTGCGGAAGGATCATTATTGAATTATGTTTCTAGATAGGTTGTAG
CTGGCTCTTTTAGAGCATGTGCACGCCTGTTTGGACTTCATTTTCATCCACCTGTGCACC
TATTGTAGTCTTTGGTTGGGTTAGGAGGAAGTGATCATTGTATCAGCATCTGCTGGGAGT

GAGGACTTGCATTGTGAAAGCTTTGCTGTCCTTGATGTGATCATGGAATCTTTTTCTCAC
TAGAGTCTATGTCACTCATTATACTCTGTCGAATGTCATTGAATGTCTTTACATGGGCTT
GTATGCCTATGAAAATTGTAATACAACTTTCAGCAACGGATCTCTTGGCTCTCGCATCGA
TGAAGAACGCAGCGAAATGCGATAAGTAATGTGAATTGCAGAATTCAGTGAATCATCGAA
TCTTTGAACGCATCTTGCGCTCCTTGGTATTCCGAGGAGCATGCCTGTTTGAGTGTCATT
AAATTCTCAACTCTCTTATACTTTTTTGTAAAAGAGAGCTTGGACTGTGGAGGCTTGCTG
GCCACTTTTTGGGGTCAGCTCCTCTGAAATGCATTAGCGGAACCGTTTGCGATCTGCCAC
AAGTGTGATAAGTTATCTACACTGGCGAGGGGATTGCTCTCTGTAATGTTCAGCTTCTAA
TTGTCTCTACTTTGTGAGACTACTTTTGAATGCTTGACCTCAAATCAGGTAGGACTACCC
GCTGAACTTAA
>A8_56
TTTCCGTAGGTGAACCTGCGGAAGGATCATTATTGAATTATGTTTCTAGATAGGTTGTAG
CTGGCTCTTTTAGAGCATGTGCACGCCTGTTTGGACTTCATTTTCATCCACCTGTGCACC
TATTGTAGTCTTTGGTTGGGTTAGGAGGAAGTGATCATTGTATCAGCATCTGCTGGGAGT
GAGGACTTGCATTGTGAAAGCTTTGCTGTCCTTGATGTGATCATGGAATCTTTTTCTCAC
TAGAGTCTATGTCACTCATTATACTCTGTCGAATGTCATTGAATGTCTTTACATGGGCTT
GTATGCCTATGAAAATTGTAATACAACTTTCAGCAACGGATCTCTTGGCTCTCGCATCGA
TGAAGAACGCAGCGAAATGCGATAAGTAATGTGAATTGCAGAATTCAGTGAATCATCGAA
TCTTTGAACGCATCTTGCGCTCCTTGGTATTCCGAGGAGCATGCCTGTTTGAGTGTCATT
AAATTCTCAACTCTCTTATACTTTTTTGTAAAAGAGAGCTTGGACTGTGGAGGCTTGCTG
GCCACTTTTTGGGGTCAGCTCCTCTGAAATGCATTAGCGGAACCGTTTGCGATCTGCCAC
AAGTGTGATAAGTTATCTACACTGGCGAGGGGATTGCTCTCTGTAATGTTCAGCTTCTAA
TTGTCTCTACTTTGTGAGACTACTTTTGAATGCTTGACCTCAAATCAGGTAGGACTACCC
GCTGAACTTAA
>A8_57
TTTCCGTAGGTGAACCTGCGGAAGGATCATTATTGAATTATGTTTCTAGATAGGTTGTAG
CTGGCTCTTTTAGAGCATGTGCACGCCTGTTTGGACTTCATTTTCATCCACCTGTGCACC
TATTGTAGTCTTTGGTTGGGTTAGGAGGAAGTGATCATTGTATCAGCATCTGCTGGGAGT
GAGGACTTGCATTGTGAAAGCTTTGCTGTCCTTGATGTGATCATGGAATCTTTTTCTCAC
TAGAGTCTATGTCACTCATTATACTCTGTCGAATGTCATTGAATGTCTTTACATGGGCTT
GTATGCCTATGAAAATTGTAATACAACTTTCAGCAACGGATCTCTTGGCTCTCGCATCGA
TGAAGAACGCAGCGAAATGCGATAAGTAATGTGAATTGCAGAATTCAGTGAATCATCGAA
TCTTTGAACGCATCTTGCGCTCCTTGGTATTCCGAGGAGCATGCCTGTTTGAGTGTCATT
AAATTCTCAACTCTCTTATACTTTTTTGTAAAAGAGAGCTTGGACTGTGGAGGCTTGCTG
GCCACTTTTTGGGGTCAGCTCCTCTGAAATGCATTAGCGGAACCGTTTGCGATCTGCCAC
AAGTGTGATAAGTTATCTACACTGGCGAGGGGATTGCTCTCTGTAATGTTCAGCTTCTAA
TTGTCTCTACTTTGTGAGACTACTTTTGAATGCTTGACCTCAAATCAGGTAGGACTACCC
GCTGAACTTAA
>A8_58
TTTCCGTAGGTGAACCTGCGGAAGGATCATTATTGAATTATGTTTCTAGATAGGTTGTAG
CTGGCTCTTTTAGAGCATGTGCACGCCTGTTTGGACTTCATTTTCATCCACCTGTGCACC
TATTGTAGTCTTTGGTTGGGTTAGGAGGAAGTGATCATTGTATCAGCATCTGCTGGGAGT
GAGGACTTGCATTGTGAAAGCTTTGCTGTCCTTGATGTGATCATGGAATCTTTTTCTCAC
TAGAGTCTATGTCACTCATTATACTCTGTCGAATGTCATTGAATGTCTTTACATGGGCTT
GTATGCCTATGAAAATTGTAATACAACTTTCAGCAACGGATCTCTTGGCTCTCGCATCGA
TGAAGAACGCAGCGAAATGCGATAAGTAATGTGAATTGCAGAATTCAGTGAATCATCGAA
TCTTTGAACGCATCTTGCGCTCCTTGGTATTCCGAGGAGCATGCCTGTTTGAGTGTCATT
AAATTCTCAACTCTCTTATACTTTTTTGTAAAAGAGAGCTTGGACTGTGGAGGCTTGCTG
GCCACTTTTTGGGGTCAGCTCCTCTGAAATGCATTAGCGGAACCGTTTGCGATCTGCCAC
AAGTGTGATAAGTTATCTACACTGGCGAGGGGATTGCTCTCTGTAATGTTCAGCTTCTAA

TTGTCTCTACTTTGTGAGACTACTTTTGAATGCTTGACCTCAAATCAGGTAGGACTACCC
GCTGAACTTAA
>A1_2
TTTCCGTAGGTGAACCTGCGGAAGGATCATTATTGAATTATGTTTCTAGATAGGTTGTAG
CTGGCTCTTTTAGAGCATGTGCACGCCTGTTTGGACTTCATTTTCATCCACCTGTGCACC
TATTGTAGTCTTTGGTTGGGTTAGGAGGAAGTGATCATTGTATCAGCATCTGCTGGGAGT
GAGGACTTGCATTGTGAAAGCTTTGCTGTCCTTGATGTGATCATGGAATCTTTTTCTCAC
TAGAGTCTATGTCACTCATTATACTCTGTCGAATGTCATTGAATGTCTTTACATGGGCTT
GTATGCCTATGAAAATTGTAATACAACTTTCAGCAACGGATCTCTTGGCTCTCGCATCGA
TGAAGAACGCAGCGAAATGCGATAAGTAATGTGAATTGCAGAATTCAGTGAATCATCGAA
TCTTTGAACGCATCTTGCGCTCCTTGGTATTCCGAGGAGCATGCCTGTTTGAGTGTCATT
AAATTCTCAACTCTCTTATACTTTTTTGTAAAAGAGAGCTTGGACTGTGGAGGCTTGCTG
GCCACTTTTTGGGGTCAGCTCCTCTGAAATGCATTAGCGGAACCGTTTGCGATCTGCCAC
AAGTGTGATAAGTTATCTACACTGGCGAGGGGATTGCTCTCTGTAATGTTCAGCTTCTAA
TTGTCTCTACTTTGTGAGACTACTTTTGAATGCTTGACCTCAAATCAGGTAGGACTACCC
GCTGAACTTAA
>A1_3
TTTCCGTAGGTGAACCTGCGGAAGGATCATTATTGAATTATGTTTCTAGATAGGTTGTAG
CTGGCTCTTTTAGAGCATGTGCACGCCTGTTTGGACTTCATTTTCATCCACCTGTGCACC
TATTGTAGTCTTTGGTTGGGTTAGGAGGAAGTGATCATTGTATCAGCATCTGCTGGGAGT
GAGGACTTGCATTGTGAAAGCTTTGCTGTCCTTGATGTGATCATGGAATCTTTTTCTCAC
TAGAGTCTATGTCACTCATTATACTCTGTCGAATGTCATTGAATGTCTTTACATGGGCTT
GTATGCCTATGAAAATTGTAATACAACTTTCAGCAACGGATCTCTTGGCTCTCGCATCGA
TGAAGAACGCAGCGAAATGCGATAAGTAATGTGAATTGCAGAATTCAGTGAATCATCGAA
TCTTTGAACGCATCTTGCGCTCCTTGGTATTCCGAGGAGCATGCCTGTTTGAGTGTCATT
AAATTCTCAACTCTCTTATACTTTTTTGTAAAAGAGAGCTTGGACTGTGGAGGCTTGCTG
GCCACTTTTTGGGGTCAGCTCCTCTGAAATGCATTAGCGGAACCGTTTGCGATCTGCCAC
AAGTGTGATAAGTTATCTACACTGGCGAGGGGATTGCTCTCTGTAATGTTCAGCTTCTAA
TTGTCTCTACTTTGTGAGACTACTTTTGAATGCTTGACCTCAAATCAGGTAGGACTACCC
GCTGAACTTAA
>A1_5
TTTCCGTAGGTGAACCTGCGGAAGGATCATTATTGAATTATGTTTCTAGATAGGTTGTAG
CTGGCTCTTTTAGAGCATGTGCACGCCTGTTTGGACTTCATTTTCATCCACCTGTGCACC
TATTGTAGTCTTTGGTTGGGTTAGGAGGAAGTGATCATTGTATCAGCATCTGCTGGGAGT
GAGGACTTGCATTGTGAAAGCTTTGCTGTCCTTGATGTGATCATGGAATCTTTTTCTCAC
TAGAGTCTATGTCACTCATTATACTCTGTCGAATGTCATTGAATGTCTTTACATGGGCTT
GTATGCCTATGAAAATTGTAATACAACTTTCAGCAACGGATCTCTTGGCTCTCGCATCGA
TGAAGAACGCAGCGAAATGCGATAAGTAATGTGAATTGCAGAATTCAGTGAATCATCGAA
TCTTTGAACGCATCTTGCGCTCCTTGGTATTCCGAGGAGCATGCCTGTTTGAGTGTCATT
AAATTCTCAACTCTCTTATACTTTTTTGTAAAAGAGAGCTTGGACTGTGGAGGCTTGCTG
GCCACTTTTTGGGGTCAGCTCCTCTGAAATGCATTAGCGGAACCGTTTGCGATCTGCCAC
AAGTGTGATAAGTTATCTACACTGGCGAGGGGATTGCTCTCTGTAATGTTCAGCTTCTAA
TTGTCTCTACTTTGTGAGACTACTTTTGAATGCTTGACCTCAAATCAGGTAGGACTACCC
GCTGAACTTAA
>A1_7
TTTCCGTAGGTGAACCTGCGGAAGGATCATTATTGAATTATGTTTCTAGATAGGTTGTAG
CTGGCTCTTTTAGAGCATGTGCACGCCTGTTTGGACTTCATTTTCATCCACCTGTGCACC
TATTGTAGTCTTTGGTTGGGTTAGGAGGAAGTGATCATTGTATCAGCATCTGCTGGGAGT
GAGGACTTGCATTGTGAAAGCTTTGCTGTCCTTGATGTGATCATGGAATCTTTTTCTCAC
TAGAGTCTATGTCACTCATTATACTCTGTCGAATGTCATTGAATGTCTTTACATGGGCTT

GTATGCCTATGAAAATTGTAATACAACTTTCAGCAACGGATCTCTTGGCTCTCGCATCGA
TGAAGAACGCAGCGAAATGCGATAAGTAATGTGAATTGCAGAATTCAGTGAATCATCGAA
TCTTTGAACGCATCTTGCGCTCCTTGGTATTCCGAGGAGCATGCCTGTTTGAGTGTCATT
AAATTCTCAACTCTCTTATACTTTTTTGTAAAAGAGAGCTTGGACTGTGGAGGCTTGCTG
GCCACTTTTTGGGGTCAGCTCCTCTGAAATGCATTAGCGGAACCGTTTGCGATCTGCCAC
AAGTGTGATAAGTTATCTACACTGGCGAGGGGATTGCTCTCTGTAATGTTCAGCTTCTAA
TTGTCTCTACTTTGTGAGACTACTTTTGAATGCTTGACCTCAAATCAGGTAGGACTACCC
GCTGAACTTAA
>A1_9
TTTCCGTAGGTGAACCTGCGGAAGGATCATTATTGAATTATGTTTCTAGATAGGTTGTAG
CTGGCTCTTTTAGAGCATGTGCACGCCTGTTTGGACTTCATTTTCATCCACCTGTGCACC
TATTGTAGTCTTTGGTTGGGTTAGGAGGAAGTGATCATTGTATCAGCATCTGCTGGGAGT
GAGGACTTGCATTGTGAAAGCTTTGCTGTCCTTGATGTGATCATGGAATCTTTTTCTCAC
TAGAGTCTATGTCACTCATTATACTCTGTCGAATGTCATTGAATGTCTTTACATGGGCTT
GTATGCCTATGAAAATTGTAATACAACTTTCAGCAACGGATCTCTTGGCTCTCGCATCGA
TGAAGAACGCAGCGAAATGCGATAAGTAATGTGAATTGCAGAATTCAGTGAATCATCGAA
TCTTTGAACGCATCTTGCGCTCCTTGGTATTCCGAGGAGCATGCCTGTTTGAGTGTCATT
AAATTCTCAACTCTCTTATACTTTTTTGTAAAAGAGAGCTTGGACTGTGGAGGCTTGCTG
GCCACTTTTTGGGGTCAGCTCCTCTGAAATGCATTAGCGGAACCGTTTGCGATCTGCCAC
AAGTGTGATAAGTTATCTACACTGGCGAGGGGATTGCTCTCTGTAATGTTCAGCTTCTAA
TTGTCTCTACTTTGTGAGACTACTTTTGAATGCTTGACCTCAAATCAGGTAGGACTACCC
GCTGAACTTAA
>A1_10
TTTCCGTAGGTGAACCTGCGGAAGGATCATTATTGAATTATGTTTCTAGATAGGTTGTAG
CTGGCTCTTTTAGAGCATGTGCACGCCTGTTTGGACTTCATTTTCATCCACCTGTGCACC
TATTGTAGTCTTTGGTTGGGTTAGGAGGAAGTGATCATTGTATCAGCATCTGCTGGGAGT
GAGGACTTGCATTGTGAAAGCTTTGCTGTCCTTGATGTGATCATGGAATCTTTTTCTCAC
TAGAGTCTATGTCACTCATTATACTCTGTCGAATGTCATTGAATGTCTTTACATGGGCTT
GTATGCCTATGAAAATTGTAATACAACTTTCAGCAACGGATCTCTTGGCTCTCGCATCGA
TGAAGAACGCAGCGAAATGCGATAAGTAATGTGAATTGCAGAATTCAGTGAATCATCGAA
TCTTTGAACGCATCTTGCGCTCCTTGGTATTCCGAGGAGCATGCCTGTTTGAGTGTCATT
AAATTCTCAACTCTCTTATACTTTTTTGTAAAAGAGAGCTTGGACTGTGGAGGCTTGCTG
GCCACTTTTTGGGGTCAGCTCCTCTGAAATGCATTAGCGGAACCGTTTGCGATCTGCCAC
AAGTGTGATAAGTTATCTACACTGGCGAGGGGATTGCTCTCTGTAATGTTCAGCTTCTAA
TTGTCTCTACTTTGTGAGACTACTTTTGAATGCTTGACCTCAAATCAGGTAGGACTACCC
GCTGAACTTAA
>A1_11
TTTCCGTAGGTGAACCTGCGGAAGGATCATTATTGAATTATGTTTCTAGATAGGTTGTAG
CTGGCTCTTTTAGAGCATGTGCACGCCTGTTTGGACTTCATTTTCATCCACCTGTGCACC
TATTGTAGTCTTTGGTTGGGTTAGGAGGAAGTGATCATTGTATCAGCATCTGCTGGGAGT
GAGGACTTGCATTGTGAAAGCTTTGCTGTCCTTGATGTGATCATGGAATCTTTTTCTCAC
TAGAGTCTATGTCACTCATTATACTCTGTCGAATGTCATTGAATGTCTTTACATGGGCTT
GTATGCCTATGAAAATTGTAATACAACTTTCAGCAACGGATCTCTTGGCTCTCGCATCGA
TGAAGAACGCAGCGAAATGCGATAAGTAATGTGAATTGCAGAATTCAGTGAATCATCGAA
TCTTTGAACGCATCTTGCGCTCCTTGGTATTCCGAGGAGCATGCCTGTTTGAGTGTCATT
AAATTCTCAACTCTCTTATACTTTTTTGTAAAAGAGAGCTTGGACTGTGGAGGCTTGCTG
GCCACTTTTTGGGGTCAGCTCCTCTGAAATGCATTAGCGGAACCGTTTGCGATCTGCCAC
AAGTGTGATAAGTTATCTACACTGGCGAGGGGATTGCTCTCTGTAATGTTCAGCTTCTAA
TTGTCTCTACTTTGTGAGACTACTTTTGAATGCTTGACCTCAAATCAGGTAGGACTACCC
GCTGAACTTAA

>A8_59
TTTCCGTAGGTGAACCTGCGGAAGGATCATTATTGAATTATGTTTCTAGATAGGTTGTAG
CTGGCTCTTTTAGAGCATGTGCACGCCTGTTTGGACTTCATTTTCATCCACCTGTGCACC
TATTGTAGTCTTTGGTTGGGTTAGGAGGAAGTGATCATTGTATCAGCATCTGCTGGGAGT
GAGGACTTGCATTGTGAAAGCTTTGCTGTCCTTGATGTGATCATGGAATCTTTTTCTCAC
TAGAGTCTATGTCACTCATTATACTCTGTCGAATGTCATTGAATGTCTTTACATGGGCTT
GTATGCCTATGAAAATTGTAATACAACTTTCAGCAACGGATCTCTTGGCTCTCGCATCGA
TGAAGAACGCAGCGAAATGCGATAAGTAATGTGAATTGCAGAATTCAGTGAATCATCGAA
TCTTTGAACGCATCTTGCGCTCCTTGGTATTCCGAGGAGCATGCCTGTTTGAGTGTCATT
AAATTCTCAACTCTCTTATACTTTTTTGTAAAAGAGAGCTTGGACTGTGGAGGCTTGCTG
GCCACTTTTTGGGGTCAGCTCCTCTGAAATGCATTAGCGGAACCGTTTGCGATCTGCCAC
AAGTGTGATAAGTTATCTACACTGGCGAGGGGATTGCTCTCTGTAATGTTCAGCTTCTAA
TTGTCTCTACTTTGTGAGACTACTTTTGAATGCTTGACCTCAAATCAGGTAGGACTACCC
GCTGAACTTAA
>A8_60
TTTCCGTAGGTGAACCTGCGGAAGGATCATTATTGAATTATGTTTCTAGATAGGTTGTAG
CTGGCTCTTTTAGAGCATGTGCACGCCTGTTTGGACTTCATTTTCATCCACCTGTGCACC
TATTGTAGTCTTTGGTTGGGTTAGGAGGAAGTGATCATTGTATCAGCATCTGCTGGGAGT
GAGGACTTGCATTGTGAAAGCTTTGCTGTCCTTGATGTGATCATGGAATCTTTTTCTCAC
TAGAGTCTATGTCACTCATTATACTCTGTCGAATGTCATTGAATGTCTTTACATGGGCTT
GTATGCCTATGAAAATTGTAATACAACTTTCAGCAACGGATCTCTTGGCTCTCGCATCGA
TGAAGAACGCAGCGAAATGCGATAAGTAATGTGAATTGCAGAATTCAGTGAATCATCGAA
TCTTTGAACGCATCTTGCGCTCCTTGGTATTCCGAGGAGCATGCCTGTTTGAGTGTCATT
AAATTCTCAACTCTCTTATACTTTTTTGTAAAAGAGAGCTTGGACTGTGGAGGCTTGCTG
GCCACTTTTTGGGGTCAGCTCCTCTGAAATGCATTAGCGGAACCGTTTGCGATCTGCCAC
AAGTGTGATAAGTTATCTACACTGGCGAGGGGATTGCTCTCTGTAATGTTCAGCTTCTAA
TTGTCTCTACTTTGTGAGACTACTTTTGAATGCTTGACCTCAAATCAGGTAGGACTACCC
GCTGAACTTAA
>A8_61
TTTCCGTAGGTGAACCTGCGGAAGGATCATTATTGAATTATGTTTCTAGATAGGTTGTAG
CTGGCTCTTTTAGAGCATGTGCACGCCTGTTTGGACTTCATTTTCATCCACCTGTGCACC
TATTGTAGTCTTTGGTTGGGTTAGGAGGAAGTGATCATTGTATCAGCATCTGCTGGGAGT
GAGGACTTGCATTGTGAAAGCTTTGCTGTCCTTGATGTGATCATGGAATCTTTTTCTCAC
TAGAGTCTATGTCACTCATTATACTCTGTCGAATGTCATTGAATGTCTTTACATGGGCTT
GTATGCCTATGAAAATTGTAATACAACTTTCAGCAACGGATCTCTTGGCTCTCGCATCGA
TGAAGAACGCAGCGAAATGCGATAAGTAATGTGAATTGCAGAATTCAGTGAATCATCGAA
TCTTTGAACGCATCTTGCGCTCCTTGGTATTCCGAGGAGCATGCCTGTTTGAGTGTCATT
AAATTCTCAACTCTCTTATACTTTTTTGTAAAAGAGAGCTTGGACTGTGGAGGCTTGCTG
GCCACTTTTTGGGGTCAGCTCCTCTGAAATGCATTAGCGGAACCGTTTGCGATCTGCCAC
AAGTGTGATAAGTTATCTACACTGGCGAGGGGATTGCTCTCTGTAATGTTCAGCTTCTAA
TTGTCTCTACTTTGTGAGACTACTTTTGAATGCTTGACCTCAAATCAGGTAGGACTACCC
GCTGAACTTAA
>A8_62
TTTCCGTAGGTGAACCTGCGGAAGGATCATTATTGAATTATGTTTCTAGATAGGTTGTAG
CTGGCTCTTTTAGAGCATGTGCACGCCTGTTTGGACTTCATTTTCATCCACCTGTGCACC
TATTGTAGTCTTTGGTTGGGTTAGGAGGAAGTGATCATTGTATCAGCATCTGCTGGGAGT
GAGGACTTGCATTGTGAAAGCTTTGCTGTCCTTGATGTGATCATGGAATCTTTTTCTCAC
TAGAGTCTATGTCACTCATTATACTCTGTCGAATGTCATTGAATGTCTTTACATGGGCTT
GTATGCCTATGAAAATTGTAATACAACTTTCAGCAACGGATCTCTTGGCTCTCGCATCGA
TGAAGAACGCAGCGAAATGCGATAAGTAATGTGAATTGCAGAATTCAGTGAATCATCGAA

TCTTTGAACGCATCTTGCGCTCCTTGGTATTCCGAGGAGCATGCCTGTTTGAGTGTCATT
AAATTCTCAACTCTCTTATACTTTTTTGTAAAAGAGAGCTTGGACTGTGGAGGCTTGCTG
GCCACTTTTTGGGGTCAGCTCCTCTGAAATGCATTAGCGGAACCGTTTGCGATCTGCCAC
AAGTGTGATAAGTTATCTACACTGGCGAGGGGATTGCTCTCTGTAATGTTCAGCTTCTAA
TTGTCTCTACTTTGTGAGACTACTTTTGAATGCTTGACCTCAAATCAGGTAGGACTACCC
GCTGAACTTAA
>A8_9
TTTCCGTAGGTGAACCTGCGGAAGGATCATTATTGAATTATGTTTCTAGATAGGTTGTAG
CTGGCTCTTTTAGAGCATGTGCACGCCTGTTTGGACTTCATTTTCATCCACCTGTGCACC
TATTGTAGTCTTTGGTTGGGTTAGGAGGAAGTGATCATTGTATCAGCATCTGCTGGGAGT
GAGGACTTGCATTGTGAAAGCTTTGCTGTCCTTGATGTGATCATGGAATCTTTTTCTCAC
TAGAGTCTATGTCACTCATTATACTCTGTCGAATGTCATTGAATGTCTTTACATGGGCTT
GTATGCCTATGAAAATTGTAATACAACTTTCAGCAACGGATCTCTTGGCTCTCGCATCGA
TGAAGAACGCAGCGAAATGCGATAAGTAATGTGAATTGCAGAATTCAGTGAATCATCGAA
TCTTTGAACGCATCTTGCGCTCCTTGGTATTCCGAGGAGCATGCCTGTTTGAGTGTCATT
AAATTCTCAACTCTCTTATACTTTTTTGTAAAAGAGAGCTTGGACTGTGGAGGCTTGCTG
GCCACTTTTTGGGGTCAGCTCCTCTGAAATGCATTAGCGGAACCGTTTGCGATCTGCCAC
AAGTGTGATAAGTTATCTACACTGGCGAGGGGATTGCTCTCTGTAATGTTCAGCTTCTAA
TTGTCTCTACTTTGTGAGACTACTTTTGAATGCTTGACCTCAAATCAGGTAGGACTACCC
GCTGAACTTAA
>A8_10
TTTCCGTAGGTGAACCTGCGGAAGGATCATTATTGAATTATGTTTCTAGATAGGTTGTAG
CTGGCTCTTTTAGAGCATGTGCACGCCTGTTTGGACTTCATTTTCATCCACCTGTGCACC
TATTGTAGTCTTTGGTTGGGTTAGGAGGAAGTGATCATTGTATCAGCATCTGCTGGGAGT
GAGGACTTGCATTGTGAAAGCTTTGCTGTCCTTGATGTGATCATGGAATCTTTTTCTCAC
TAGAGTCTATGTCACTCATTATACTCTGTCGAATGTCATTGAATGTCTTTACATGGGCTT
GTATGCCTATGAAAATTGTAATACAACTTTCAGCAACGGATCTCTTGGCTCTCGCATCGA
TGAAGAACGCAGCGAAATGCGATAAGTAATGTGAATTGCAGAATTCAGTGAATCATCGAA
TCTTTGAACGCATCTTGCGCTCCTTGGTATTCCGAGGAGCATGCCTGTTTGAGTGTCATT
AAATTCTCAACTCTCTTATACTTTTTTGTAAAAGAGAGCTTGGACTGTGGAGGCTTGCTG
GCCACTTTTTGGGGTCAGCTCCTCTGAAATGCATTAGCGGAACCGTTTGCGATCTGCCAC
AAGTGTGATAAGTTATCTACACTGGCGAGGGGATTGCTCTCTGTAATGTTCAGCTTCTAA
TTGTCTCTACTTTGTGAGACTACTTTTGAATGCTTGACCTCAAATCAGGTAGGACTACCC
GCTGAACTTAA
>A8_11
TTTCCGTAGGTGAACCTGCGGAAGGATCATTATTGAATTATGTTTCTAGATAGGTTGTAG
CTGGCTCTTTTAGAGCATGTGCACGCCTGTTTGGACTTCATTTTCATCCACCTGTGCACC
TATTGTAGTCTTTGGTTGGGTTAGGAGGAAGTGATCATTGTATCAGCATCTGCTGGGAGT
GAGGACTTGCATTGTGAAAGCTTTGCTGTCCTTGATGTGATCATGGAATCTTTTTCTCAC
TAGAGTCTATGTCACTCATTATACTCTGTCGAATGTCATTGAATGTCTTTACATGGGCTT
GTATGCCTATGAAAATTGTAATACAACTTTCAGCAACGGATCTCTTGGCTCTCGCATCGA
TGAAGAACGCAGCGAAATGCGATAAGTAATGTGAATTGCAGAATTCAGTGAATCATCGAA
TCTTTGAACGCATCTTGCGCTCCTTGGTATTCCGAGGAGCATGCCTGTTTGAGTGTCATT
AAATTCTCAACTCTCTTATACTTTTTTGTAAAAGAGAGCTTGGACTGTGGAGGCTTGCTG
GCCACTTTTTGGGGTCAGCTCCTCTGAAATGCATTAGCGGAACCGTTTGCGATCTGCCAC
AAGTGTGATAAGTTATCTACACTGGCGAGGGGATTGCTCTCTGTAATGTTCAGCTTCTAA
TTGTCTCTACTTTGTGAGACTACTTTTGAATGCTTGACCTCAAATCAGGTAGGACTACCC
GCTGAACTTAA
>A8_12
TTTCCGTAGGTGAACCTGCGGAAGGATCATTATTGAATTATGTTTCTAGATAGGTTGTAG

CTGGCTCTTTTAGAGCATGTGCACGCCTGTTTGGACTTCATTTTCATCCACCTGTGCACC
TATTGTAGTCTTTGGTTGGGTTAGGAGGAAGTGATCATTGTATCAGCATCTGCTGGGAGT
GAGGACTTGCATTGTGAAAGCTTTGCTGTCCTTGATGTGATCATGGAATCTTTTTCTCAC
TAGAGTCTATGTCACTCATTATACTCTGTCGAATGTCATTGAATGTCTTTACATGGGCTT
GTATGCCTATGAAAATTGTAATACAACTTTCAGCAACGGATCTCTTGGCTCTCGCATCGA
TGAAGAACGCAGCGAAATGCGATAAGTAATGTGAATTGCAGAATTCAGTGAATCATCGAA
TCTTTGAACGCATCTTGCGCTCCTTGGTATTCCGAGGAGCATGCCTGTTTGAGTGTCATT
AAATTCTCAACTCTCTTATACTTTTTTGTAAAAGAGAGCTTGGACTGTGGAGGCTTGCTG
GCCACTTTTTGGGGTCAGCTCCTCTGAAATGCATTAGCGGAACCGTTTGCGATCTGCCAC
AAGTGTGATAAGTTATCTACACTGGCGAGGGGATTGCTCTCTGTAATGTTCAGCTTCTAA
TTGTCTCTACTTTGTGAGACTACTTTTGAATGCTTGACCTCAAATCAGGTAGGACTACCC
GCTGAACTTAA
>A8_13
TTTCCGTAGGTGAACCTGCGGAAGGATCATTATTGAATTATGTTTCTAGATAGGTTGTAG
CTGGCTCTTTTAGAGCATGTGCACGCCTGTTTGGACTTCATTTTCATCCACCTGTGCACC
TATTGTAGTCTTTGGTTGGGTTAGGAGGAAGTGATCATTGTATCAGCATCTGCTGGGAGT
GAGGACTTGCATTGTGAAAGCTTTGCTGTCCTTGATGTGATCATGGAATCTTTTTCTCAC
TAGAGTCTATGTCACTCATTATACTCTGTCGAATGTCATTGAATGTCTTTACATGGGCTT
GTATGCCTATGAAAATTGTAATACAACTTTCAGCAACGGATCTCTTGGCTCTCGCATCGA
TGAAGAACGCAGCGAAATGCGATAAGTAATGTGAATTGCAGAATTCAGTGAATCATCGAA
TCTTTGAACGCATCTTGCGCTCCTTGGTATTCCGAGGAGCATGCCTGTTTGAGTGTCATT
AAATTCTCAACTCTCTTATACTTTTTTGTAAAAGAGAGCTTGGACTGTGGAGGCTTGCTG
GCCACTTTTTGGGGTCAGCTCCTCTGAAATGCATTAGCGGAACCGTTTGCGATCTGCCAC
AAGTGTGATAAGTTATCTACACTGGCGAGGGGATTGCTCTCTGTAATGTTCAGCTTCTAA
TTGTCTCTACTTTGTGAGACTACTTTTGAATGCTTGACCTCAAATCAGGTAGGACTACCC
GCTGAACTTAA
>A8_14
TTTCCGTAGGTGAACCTGCGGAAGGATCATTATTGAATTATGTTTCTAGATAGGTTGTAG
CTGGCTCTTTTAGAGCATGTGCACGCCTGTTTGGACTTCATTTTCATCCACCTGTGCACC
TATTGTAGTCTTTGGTTGGGTTAGGAGGAAGTGATCATTGTATCAGCATCTGCTGGGAGT
GAGGACTTGCATTGTGAAAGCTTTGCTGTCCTTGATGTGATCATGGAATCTTTTTCTCAC
TAGAGTCTATGTCACTCATTATACTCTGTCGAATGTCATTGAATGTCTTTACATGGGCTT
GTATGCCTATGAAAATTGTAATACAACTTTCAGCAACGGATCTCTTGGCTCTCGCATCGA
TGAAGAACGCAGCGAAATGCGATAAGTAATGTGAATTGCAGAATTCAGTGAATCATCGAA
TCTTTGAACGCATCTTGCGCTCCTTGGTATTCCGAGGAGCATGCCTGTTTGAGTGTCATT
AAATTCTCAACTCTCTTATACTTTTTTGTAAAAGAGAGCTTGGACTGTGGAGGCTTGCTG
GCCACTTTTTGGGGTCAGCTCCTCTGAAATGCATTAGCGGAACCGTTTGCGATCTGCCAC
AAGTGTGATAAGTTATCTACACTGGCGAGGGGATTGCTCTCTGTAATGTTCAGCTTCTAA
TTGTCTCTACTTTGTGAGACTACTTTTGAATGCTTGACCTCAAATCAGGTAGGACTACCC
GCTGAACTTAA
>A8_15
TTTCCGTAGGTGAACCTGCGGAAGGATCATTATTGAATTATGTTTCTAGATAGGTTGTAG
CTGGCTCTTTTAGAGCATGTGCACGCCTGTTTGGACTTCATTTTCATCCACCTGTGCACC
TATTGTAGTCTTTGGTTGGGTTAGGAGGAAGTGATCATTGTATCAGCATCTGCTGGGAGT
GAGGACTTGCATTGTGAAAGCTTTGCTGTCCTTGATGTGATCATGGAATCTTTTTCTCAC
TAGAGTCTATGTCACTCATTATACTCTGTCGAATGTCATTGAATGTCTTTACATGGGCTT
GTATGCCTATGAAAATTGTAATACAACTTTCAGCAACGGATCTCTTGGCTCTCGCATCGA
TGAAGAACGCAGCGAAATGCGATAAGTAATGTGAATTGCAGAATTCAGTGAATCATCGAA
TCTTTGAACGCATCTTGCGCTCCTTGGTATTCCGAGGAGCATGCCTGTTTGAGTGTCATT
AAATTCTCAACTCTCTTATACTTTTTTGTAAAAGAGAGCTTGGACTGTGGAGGCTTGCTG

GCCACTTTTTGGGGTCAGCTCCTCTGAAATGCATTAGCGGAACCGTTTGCGATCTGCCAC
AAGTGTGATAAGTTATCTACACTGGCGAGGGGATTGCTCTCTGTAATGTTCAGCTTCTAA
TTGTCTCTACTTTGTGAGACTACTTTTGAATGCTTGACCTCAAATCAGGTAGGACTACCC
GCTGAACTTAA
>A8_16
TTTCCGTAGGTGAACCTGCGGAAGGATCATTATTGAATTATGTTTCTAGATAGGTTGTAG
CTGGCTCTTTTAGAGCATGTGCACGCCTGTTTGGACTTCATTTTCATCCACCTGTGCACC
TATTGTAGTCTTTGGTTGGGTTAGGAGGAAGTGATCATTGTATCAGCATCTGCTGGGAGT
GAGGACTTGCATTGTGAAAGCTTTGCTGTCCTTGATGTGATCATGGAATCTTTTTCTCAC
TAGAGTCTATGTCACTCATTATACTCTGTCGAATGTCATTGAATGTCTTTACATGGGCTT
GTATGCCTATGAAAATTGTAATACAACTTTCAGCAACGGATCTCTTGGCTCTCGCATCGA
TGAAGAACGCAGCGAAATGCGATAAGTAATGTGAATTGCAGAATTCAGTGAATCATCGAA
TCTTTGAACGCATCTTGCGCTCCTTGGTATTCCGAGGAGCATGCCTGTTTGAGTGTCATT
AAATTCTCAACTCTCTTATACTTTTTTGTAAAAGAGAGCTTGGACTGTGGAGGCTTGCTG
GCCACTTTTTGGGGTCAGCTCCTCTGAAATGCATTAGCGGAACCGTTTGCGATCTGCCAC
AAGTGTGATAAGTTATCTACACTGGCGAGGGGATTGCTCTCTGTAATGTTCAGCTTCTAA
TTGTCTCTACTTTGTGAGACTACTTTTGAATGCTTGACCTCAAATCAGGTAGGACTACCC
GCTGAACTTAA
>A8_17
TTTCCGTAGGTGAACCTGCGGAAGGATCATTATTGAATTATGTTTCTAGATAGGTTGTAG
CTGGCTCTTTTAGAGCATGTGCACGCCTGTTTGGACTTCATTTTCATCCACCTGTGCACC
TATTGTAGTCTTTGGTTGGGTTAGGAGGAAGTGATCATTGTATCAGCATCTGCTGGGAGT
GAGGACTTGCATTGTGAAAGCTTTGCTGTCCTTGATGTGATCATGGAATCTTTTTCTCAC
TAGAGTCTATGTCACTCATTATACTCTGTCGAATGTCATTGAATGTCTTTACATGGGCTT
GTATGCCTATGAAAATTGTAATACAACTTTCAGCAACGGATCTCTTGGCTCTCGCATCGA
TGAAGAACGCAGCGAAATGCGATAAGTAATGTGAATTGCAGAATTCAGTGAATCATCGAA
TCTTTGAACGCATCTTGCGCTCCTTGGTATTCCGAGGAGCATGCCTGTTTGAGTGTCATT
AAATTCTCAACTCTCTTATACTTTTTTGTAAAAGAGAGCTTGGACTGTGGAGGCTTGCTG
GCCACTTTTTGGGGTCAGCTCCTCTGAAATGCATTAGCGGAACCGTTTGCGATCTGCCAC
AAGTGTGATAAGTTATCTACACTGGCGAGGGGATTGCTCTCTGTAATGTTCAGCTTCTAA
TTGTCTCTACTTTGTGAGACTACTTTTGAATGCTTGACCTCAAATCAGGTAGGACTACCC
GCTGAACTTAA
>A8_18
TTTCCGTAGGTGAACCTGCGGAAGGATCATTATTGAATTATGTTTCTAGATAGGTTGTAG
CTGGCTCTTTTAGAGCATGTGCACGCCTGTTTGGACTTCATTTTCATCCACCTGTGCACC
TATTGTAGTCTTTGGTTGGGTTAGGAGGAAGTGATCATTGTATCAGCATCTGCTGGGAGT
GAGGACTTGCATTGTGAAAGCTTTGCTGTCCTTGATGTGATCATGGAATCTTTTTCTCAC
TAGAGTCTATGTCACTCATTATACTCTGTCGAATGTCATTGAATGTCTTTACATGGGCTT
GTATGCCTATGAAAATTGTAATACAACTTTCAGCAACGGATCTCTTGGCTCTCGCATCGA
TGAAGAACGCAGCGAAATGCGATAAGTAATGTGAATTGCAGAATTCAGTGAATCATCGAA
TCTTTGAACGCATCTTGCGCTCCTTGGTATTCCGAGGAGCATGCCTGTTTGAGTGTCATT
AAATTCTCAACTCTCTTATACTTTTTTGTAAAAGAGAGCTTGGACTGTGGAGGCTTGCTG
GCCACTTTTTGGGGTCAGCTCCTCTGAAATGCATTAGCGGAACCGTTTGCGATCTGCCAC
AAGTGTGATAAGTTATCTACACTGGCGAGGGGATTGCTCTCTGTAATGTTCAGCTTCTAA
TTGTCTCTACTTTGTGAGACTACTTTTGAATGCTTGACCTCAAATCAGGTAGGACTACCC
GCTGAACTTAA
>A8_20
TTTCCGTAGGTGAACCTGCGGAAGGATCATTATTGAATTATGTTTCTAGATAGGTTGTAG
CTGGCTCTTTTAGAGCATGTGCACGCCTGTTTGGACTTCATTTTCATCCACCTGTGCACC
TATTGTAGTCTTTGGTTGGGTTAGGAGGAAGTGATCATTGTATCAGCATCTGCTGGGAGT

GAGGACTTGCATTGTGAAAGCTTTGCTGTCCTTGATGTGATCATGGAATCTTTTTCTCAC
TAGAGTCTATGTCACTCATTATACTCTGTCGAATGTCATTGAATGTCTTTACATGGGCTT
GTATGCCTATGAAAATTGTAATACAACTTTCAGCAACGGATCTCTTGGCTCTCGCATCGA
TGAAGAACGCAGCGAAATGCGATAAGTAATGTGAATTGCAGAATTCAGTGAATCATCGAA
TCTTTGAACGCATCTTGCGCTCCTTGGTATTCCGAGGAGCATGCCTGTTTGAGTGTCATT
AAATTCTCAACTCTCTTATACTTTTTTGTAAAAGAGAGCTTGGACTGTGGAGGCTTGCTG
GCCACTTTTTGGGGTCAGCTCCTCTGAAATGCATTAGCGGAACCGTTTGCGATCTGCCAC
AAGTGTGATAAGTTATCTACACTGGCGAGGGGATTGCTCTCTGTAATGTTCAGCTTCTAA
TTGTCTCTACTTTGTGAGACTACTTTTGAATGCTTGACCTCAAATCAGGTAGGACTACCC
GCTGAACTTAA
>A8_21
TTTCCGTAGGTGAACCTGCGGAAGGATCATTATTGAATTATGTTTCTAGATAGGTTGTAG
CTGGCTCTTTTAGAGCATGTGCACGCCTGTTTGGACTTCATTTTCATCCACCTGTGCACC
TATTGTAGTCTTTGGTTGGGTTAGGAGGAAGTGATCATTGTATCAGCATCTGCTGGGAGT
GAGGACTTGCATTGTGAAAGCTTTGCTGTCCTTGATGTGATCATGGAATCTTTTTCTCAC
TAGAGTCTATGTCACTCATTATACTCTGTCGAATGTCATTGAATGTCTTTACATGGGCTT
GTATGCCTATGAAAATTGTAATACAACTTTCAGCAACGGATCTCTTGGCTCTCGCATCGA
TGAAGAACGCAGCGAAATGCGATAAGTAATGTGAATTGCAGAATTCAGTGAATCATCGAA
TCTTTGAACGCATCTTGCGCTCCTTGGTATTCCGAGGAGCATGCCTGTTTGAGTGTCATT
AAATTCTCAACTCTCTTATACTTTTTTGTAAAAGAGAGCTTGGACTGTGGAGGCTTGCTG
GCCACTTTTTGGGGTCAGCTCCTCTGAAATGCATTAGCGGAACCGTTTGCGATCTGCCAC
AAGTGTGATAAGTTATCTACACTGGCGAGGGGATTGCTCTCTGTAATGTTCAGCTTCTAA
TTGTCTCTACTTTGTGAGACTACTTTTGAATGCTTGACCTCAAATCAGGTAGGACTACCC
GCTGAACTTAA
>A8_22
TTTCCGTAGGTGAACCTGCGGAAGGATCATTATTGAATTATGTTTCTAGATAGGTTGTAG
CTGGCTCTTTTAGAGCATGTGCACGCCTGTTTGGACTTCATTTTCATCCACCTGTGCACC
TATTGTAGTCTTTGGTTGGGTTAGGAGGAAGTGATCATTGTATCAGCATCTGCTGGGAGT
GAGGACTTGCATTGTGAAAGCTTTGCTGTCCTTGATGTGATCATGGAATCTTTTTCTCAC
TAGAGTCTATGTCACTCATTATACTCTGTCGAATGTCATTGAATGTCTTTACATGGGCTT
GTATGCCTATGAAAATTGTAATACAACTTTCAGCAACGGATCTCTTGGCTCTCGCATCGA
TGAAGAACGCAGCGAAATGCGATAAGTAATGTGAATTGCAGAATTCAGTGAATCATCGAA
TCTTTGAACGCATCTTGCGCTCCTTGGTATTCCGAGGAGCATGCCTGTTTGAGTGTCATT
AAATTCTCAACTCTCTTATACTTTTTTGTAAAAGAGAGCTTGGACTGTGGAGGCTTGCTG
GCCACTTTTTGGGGTCAGCTCCTCTGAAATGCATTAGCGGAACCGTTTGCGATCTGCCAC
AAGTGTGATAAGTTATCTACACTGGCGAGGGGATTGCTCTCTGTAATGTTCAGCTTCTAA
TTGTCTCTACTTTGTGAGACTACTTTTGAATGCTTGACCTCAAATCAGGTAGGACTACCC
GCTGAACTTAA
>A8_23
TTTCCGTAGGTGAACCTGCGGAAGGATCATTATTGAATTATGTTTCTAGATAGGTTGTAG
CTGGCTCTTTTAGAGCATGTGCACGCCTGTTTGGACTTCATTTTCATCCACCTGTGCACC
TATTGTAGTCTTTGGTTGGGTTAGGAGGAAGTGATCATTGTATCAGCATCTGCTGGGAGT
GAGGACTTGCATTGTGAAAGCTTTGCTGTCCTTGATGTGATCATGGAATCTTTTTCTCAC
TAGAGTCTATGTCACTCATTATACTCTGTCGAATGTCATTGAATGTCTTTACATGGGCTT
GTATGCCTATGAAAATTGTAATACAACTTTCAGCAACGGATCTCTTGGCTCTCGCATCGA
TGAAGAACGCAGCGAAATGCGATAAGTAATGTGAATTGCAGAATTCAGTGAATCATCGAA
TCTTTGAACGCATCTTGCGCTCCTTGGTATTCCGAGGAGCATGCCTGTTTGAGTGTCATT
AAATTCTCAACTCTCTTATACTTTTTTGTAAAAGAGAGCTTGGACTGTGGAGGCTTGCTG
GCCACTTTTTGGGGTCAGCTCCTCTGAAATGCATTAGCGGAACCGTTTGCGATCTGCCAC
AAGTGTGATAAGTTATCTACACTGGCGAGGGGATTGCTCTCTGTAATGTTCAGCTTCTAA

TTGTCTCTACTTTGTGAGACTACTTTTGAATGCTTGACCTCAAATCAGGTAGGACTACCC
GCTGAACTTAA
>A8_25
TTTCCGTAGGTGAACCTGCGGAAGGATCATTATTGAATTATGTTTCTAGATAGGTTGTAG
CTGGCTCTTTTAGAGCATGTGCACGCCTGTTTGGACTTCATTTTCATCCACCTGTGCACC
TATTGTAGTCTTTGGTTGGGTTAGGAGGAAGTGATCATTGTATCAGCATCTGCTGGGAGT
GAGGACTTGCATTGTGAAAGCTTTGCTGTCCTTGATGTGATCATGGAATCTTTTTCTCAC
TAGAGTCTATGTCACTCATTATACTCTGTCGAATGTCATTGAATGTCTTTACATGGGCTT
GTATGCCTATGAAAATTGTAATACAACTTTCAGCAACGGATCTCTTGGCTCTCGCATCGA
TGAAGAACGCAGCGAAATGCGATAAGTAATGTGAATTGCAGAATTCAGTGAATCATCGAA
TCTTTGAACGCATCTTGCGCTCCTTGGTATTCCGAGGAGCATGCCTGTTTGAGTGTCATT
AAATTCTCAACTCTCTTATACTTTTTTGTAAAAGAGAGCTTGGACTGTGGAGGCTTGCTG
GCCACTTTTTGGGGTCAGCTCCTCTGAAATGCATTAGCGGAACCGTTTGCGATCTGCCAC
AAGTGTGATAAGTTATCTACACTGGCGAGGGGATTGCTCTCTGTAATGTTCAGCTTCTAA
TTGTCTCTACTTTGTGAGACTACTTTTGAATGCTTGACCTCAAATCAGGTAGGACTACCC
GCTGAACTTAA
>A8_26
TTTCCGTAGGTGAACCTGCGGAAGGATCATTATTGAATTATGTTTCTAGATAGGTTGTAG
CTGGCTCTTTTAGAGCATGTGCACGCCTGTTTGGACTTCATTTTCATCCACCTGTGCACC
TATTGTAGTCTTTGGTTGGGTTAGGAGGAAGTGATCATTGTATCAGCATCTGCTGGGAGT
GAGGACTTGCATTGTGAAAGCTTTGCTGTCCTTGATGTGATCATGGAATCTTTTTCTCAC
TAGAGTCTATGTCACTCATTATACTCTGTCGAATGTCATTGAATGTCTTTACATGGGCTT
GTATGCCTATGAAAATTGTAATACAACTTTCAGCAACGGATCTCTTGGCTCTCGCATCGA
TGAAGAACGCAGCGAAATGCGATAAGTAATGTGAATTGCAGAATTCAGTGAATCATCGAA
TCTTTGAACGCATCTTGCGCTCCTTGGTATTCCGAGGAGCATGCCTGTTTGAGTGTCATT
AAATTCTCAACTCTCTTATACTTTTTTGTAAAAGAGAGCTTGGACTGTGGAGGCTTGCTG
GCCACTTTTTGGGGTCAGCTCCTCTGAAATGCATTAGCGGAACCGTTTGCGATCTGCCAC
AAGTGTGATAAGTTATCTACACTGGCGAGGGGATTGCTCTCTGTAATGTTCAGCTTCTAA
TTGTCTCTACTTTGTGAGACTACTTTTGAATGCTTGACCTCAAATCAGGTAGGACTACCC
GCTGAACTTAA
>A8_28
TTTCCGTAGGTGAACCTGCGGAAGGATCATTATTGAATTATGTTTCTAGATAGGTTGTAG
CTGGCTCTTTTAGAGCATGTGCACGCCTGTTTGGACTTCATTTTCATCCACCTGTGCACC
TATTGTAGTCTTTGGTTGGGTTAGGAGGAAGTGATCATTGTATCAGCATCTGCTGGGAGT
GAGGACTTGCATTGTGAAAGCTTTGCTGTCCTTGATGTGATCATGGAATCTTTTTCTCAC
TAGAGTCTATGTCACTCATTATACTCTGTCGAATGTCATTGAATGTCTTTACATGGGCTT
GTATGCCTATGAAAATTGTAATACAACTTTCAGCAACGGATCTCTTGGCTCTCGCATCGA
TGAAGAACGCAGCGAAATGCGATAAGTAATGTGAATTGCAGAATTCAGTGAATCATCGAA
TCTTTGAACGCATCTTGCGCTCCTTGGTATTCCGAGGAGCATGCCTGTTTGAGTGTCATT
AAATTCTCAACTCTCTTATACTTTTTTGTAAAAGAGAGCTTGGACTGTGGAGGCTTGCTG
GCCACTTTTTGGGGTCAGCTCCTCTGAAATGCATTAGCGGAACCGTTTGCGATCTGCCAC
AAGTGTGATAAGTTATCTACACTGGCGAGGGGATTGCTCTCTGTAATGTTCAGCTTCTAA
TTGTCTCTACTTTGTGAGACTACTTTTGAATGCTTGACCTCAAATCAGGTAGGACTACCC
GCTGAACTTAA
>A8_30
TTTCCGTAGGTGAACCTGCGGAAGGATCATTATTGAATTATGTTTCTAGATAGGTTGTAG
CTGGCTCTTTTAGAGCATGTGCACGCCTGTTTGGACTTCATTTTCATCCACCTGTGCACC
TATTGTAGTCTTTGGTTGGGTTAGGAGGAAGTGATCATTGTATCAGCATCTGCTGGGAGT
GAGGACTTGCATTGTGAAAGCTTTGCTGTCCTTGATGTGATCATGGAATCTTTTTCTCAC
TAGAGTCTATGTCACTCATTATACTCTGTCGAATGTCATTGAATGTCTTTACATGGGCTT

GTATGCCTATGAAAATTGTAATACAACTTTCAGCAACGGATCTCTTGGCTCTCGCATCGA
TGAAGAACGCAGCGAAATGCGATAAGTAATGTGAATTGCAGAATTCAGTGAATCATCGAA
TCTTTGAACGCATCTTGCGCTCCTTGGTATTCCGAGGAGCATGCCTGTTTGAGTGTCATT
AAATTCTCAACTCTCTTATACTTTTTTGTAAAAGAGAGCTTGGACTGTGGAGGCTTGCTG
GCCACTTTTTGGGGTCAGCTCCTCTGAAATGCATTAGCGGAACCGTTTGCGATCTGCCAC
AAGTGTGATAAGTTATCTACACTGGCGAGGGGATTGCTCTCTGTAATGTTCAGCTTCTAA
TTGTCTCTACTTTGTGAGACTACTTTTGAATGCTTGACCTCAAATCAGGTAGGACTACCC
GCTGAACTTAA
>A8_31
TTTCCGTAGGTGAACCTGCGGAAGGATCATTATTGAATTATGTTTCTAGATAGGTTGTAG
CTGGCTCTTTTAGAGCATGTGCACGCCTGTTTGGACTTCATTTTCATCCACCTGTGCACC
TATTGTAGTCTTTGGTTGGGTTAGGAGGAAGTGATCATTGTATCAGCATCTGCTGGGAGT
GAGGACTTGCATTGTGAAAGCTTTGCTGTCCTTGATGTGATCATGGAATCTTTTTCTCAC
TAGAGTCTATGTCACTCATTATACTCTGTCGAATGTCATTGAATGTCTTTACATGGGCTT
GTATGCCTATGAAAATTGTAATACAACTTTCAGCAACGGATCTCTTGGCTCTCGCATCGA
TGAAGAACGCAGCGAAATGCGATAAGTAATGTGAATTGCAGAATTCAGTGAATCATCGAA
TCTTTGAACGCATCTTGCGCTCCTTGGTATTCCGAGGAGCATGCCTGTTTGAGTGTCATT
AAATTCTCAACTCTCTTATACTTTTTTGTAAAAGAGAGCTTGGACTGTGGAGGCTTGCTG
GCCACTTTTTGGGGTCAGCTCCTCTGAAATGCATTAGCGGAACCGTTTGCGATCTGCCAC
AAGTGTGATAAGTTATCTACACTGGCGAGGGGATTGCTCTCTGTAATGTTCAGCTTCTAA
TTGTCTCTACTTTGTGAGACTACTTTTGAATGCTTGACCTCAAATCAGGTAGGACTACCC
GCTGAACTTAA
>A8_33
TTTCCGTAGGTGAACCTGCGGAAGGATCATTATTGAATTATGTTTCTAGATAGGTTGTAG
CTGGCTCTTTTAGAGCATGTGCACGCCTGTTTGGACTTCATTTTCATCCACCTGTGCACC
TATTGTAGTCTTTGGTTGGGTTAGGAGGAAGTGATCATTGTATCAGCATCTGCTGGGAGT
GAGGACTTGCATTGTGAAAGCTTTGCTGTCCTTGATGTGATCATGGAATCTTTTTCTCAC
TAGAGTCTATGTCACTCATTATACTCTGTCGAATGTCATTGAATGTCTTTACATGGGCTT
GTATGCCTATGAAAATTGTAATACAACTTTCAGCAACGGATCTCTTGGCTCTCGCATCGA
TGAAGAACGCAGCGAAATGCGATAAGTAATGTGAATTGCAGAATTCAGTGAATCATCGAA
TCTTTGAACGCATCTTGCGCTCCTTGGTATTCCGAGGAGCATGCCTGTTTGAGTGTCATT
AAATTCTCAACTCTCTTATACTTTTTTGTAAAAGAGAGCTTGGACTGTGGAGGCTTGCTG
GCCACTTTTTGGGGTCAGCTCCTCTGAAATGCATTAGCGGAACCGTTTGCGATCTGCCAC
AAGTGTGATAAGTTATCTACACTGGCGAGGGGATTGCTCTCTGTAATGTTCAGCTTCTAA
TTGTCTCTACTTTGTGAGACTACTTTTGAATGCTTGACCTCAAATCAGGTAGGACTACCC
GCTGAACTTAA
>A8_34
TTTCCGTAGGTGAACCTGCGGAAGGATCATTATTGAATTATGTTTCTAGATAGGTTGTAG
CTGGCTCTTTTAGAGCATGTGCACGCCTGTTTGGACTTCATTTTCATCCACCTGTGCACC
TATTGTAGTCTTTGGTTGGGTTAGGAGGAAGTGATCATTGTATCAGCATCTGCTGGGAGT
GAGGACTTGCATTGTGAAAGCTTTGCTGTCCTTGATGTGATCATGGAATCTTTTTCTCAC
TAGAGTCTATGTCACTCATTATACTCTGTCGAATGTCATTGAATGTCTTTACATGGGCTT
GTATGCCTATGAAAATTGTAATACAACTTTCAGCAACGGATCTCTTGGCTCTCGCATCGA
TGAAGAACGCAGCGAAATGCGATAAGTAATGTGAATTGCAGAATTCAGTGAATCATCGAA
TCTTTGAACGCATCTTGCGCTCCTTGGTATTCCGAGGAGCATGCCTGTTTGAGTGTCATT
AAATTCTCAACTCTCTTATACTTTTTTGTAAAAGAGAGCTTGGACTGTGGAGGCTTGCTG
GCCACTTTTTGGGGTCAGCTCCTCTGAAATGCATTAGCGGAACCGTTTGCGATCTGCCAC
AAGTGTGATAAGTTATCTACACTGGCGAGGGGATTGCTCTCTGTAATGTTCAGCTTCTAA
TTGTCTCTACTTTGTGAGACTACTTTTGAATGCTTGACCTCAAATCAGGTAGGACTACCC
GCTGAACTTAA

>A8_35
TTTCCGTAGGTGAACCTGCGGAAGGATCATTATTGAATTATGTTTCTAGATAGGTTGTAG
CTGGCTCTTTTAGAGCATGTGCACGCCTGTTTGGACTTCATTTTCATCCACCTGTGCACC
TATTGTAGTCTTTGGTTGGGTTAGGAGGAAGTGATCATTGTATCAGCATCTGCTGGGAGT
GAGGACTTGCATTGTGAAAGCTTTGCTGTCCTTGATGTGATCATGGAATCTTTTTCTCAC
TAGAGTCTATGTCACTCATTATACTCTGTCGAATGTCATTGAATGTCTTTACATGGGCTT
GTATGCCTATGAAAATTGTAATACAACTTTCAGCAACGGATCTCTTGGCTCTCGCATCGA
TGAAGAACGCAGCGAAATGCGATAAGTAATGTGAATTGCAGAATTCAGTGAATCATCGAA
TCTTTGAACGCATCTTGCGCTCCTTGGTATTCCGAGGAGCATGCCTGTTTGAGTGTCATT
AAATTCTCAACTCTCTTATACTTTTTTGTAAAAGAGAGCTTGGACTGTGGAGGCTTGCTG
GCCACTTTTTGGGGTCAGCTCCTCTGAAATGCATTAGCGGAACCGTTTGCGATCTGCCAC
AAGTGTGATAAGTTATCTACACTGGCGAGGGGATTGCTCTCTGTAATGTTCAGCTTCTAA
TTGTCTCTACTTTGTGAGACTACTTTTGAATGCTTGACCTCAAATCAGGTAGGACTACCC
GCTGAACTTAA
>A8_37
TTTCCGTAGGTGAACCTGCGGAAGGATCATTATTGAATTATGTTTCTAGATAGGTTGTAG
CTGGCTCTTTTAGAGCATGTGCACGCCTGTTTGGACTTCATTTTCATCCACCTGTGCACC
TATTGTAGTCTTTGGTTGGGTTAGGAGGAAGTGATCATTGTATCAGCATCTGCTGGGAGT
GAGGACTTGCATTGTGAAAGCTTTGCTGTCCTTGATGTGATCATGGAATCTTTTTCTCAC
TAGAGTCTATGTCACTCATTATACTCTGTCGAATGTCATTGAATGTCTTTACATGGGCTT
GTATGCCTATGAAAATTGTAATACAACTTTCAGCAACGGATCTCTTGGCTCTCGCATCGA
TGAAGAACGCAGCGAAATGCGATAAGTAATGTGAATTGCAGAATTCAGTGAATCATCGAA
TCTTTGAACGCATCTTGCGCTCCTTGGTATTCCGAGGAGCATGCCTGTTTGAGTGTCATT
AAATTCTCAACTCTCTTATACTTTTTTGTAAAAGAGAGCTTGGACTGTGGAGGCTTGCTG
GCCACTTTTTGGGGTCAGCTCCTCTGAAATGCATTAGCGGAACCGTTTGCGATCTGCCAC
AAGTGTGATAAGTTATCTACACTGGCGAGGGGATTGCTCTCTGTAATGTTCAGCTTCTAA
TTGTCTCTACTTTGTGAGACTACTTTTGAATGCTTGACCTCAAATCAGGTAGGACTACCC
GCTGAACTTAA
>A8_38
TTTCCGTAGGTGAACCTGCGGAAGGATCATTATTGAATTATGTTTCTAGATAGGTTGTAG
CTGGCTCTTTTAGAGCATGTGCACGCCTGTTTGGACTTCATTTTCATCCACCTGTGCACC
TATTGTAGTCTTTGGTTGGGTTAGGAGGAAGTGATCATTGTATCAGCATCTGCTGGGAGT
GAGGACTTGCATTGTGAAAGCTTTGCTGTCCTTGATGTGATCATGGAATCTTTTTCTCAC
TAGAGTCTATGTCACTCATTATACTCTGTCGAATGTCATTGAATGTCTTTACATGGGCTT
GTATGCCTATGAAAATTGTAATACAACTTTCAGCAACGGATCTCTTGGCTCTCGCATCGA
TGAAGAACGCAGCGAAATGCGATAAGTAATGTGAATTGCAGAATTCAGTGAATCATCGAA
TCTTTGAACGCATCTTGCGCTCCTTGGTATTCCGAGGAGCATGCCTGTTTGAGTGTCATT
AAATTCTCAACTCTCTTATACTTTTTTGTAAAAGAGAGCTTGGACTGTGGAGGCTTGCTG
GCCACTTTTTGGGGTCAGCTCCTCTGAAATGCATTAGCGGAACCGTTTGCGATCTGCCAC
AAGTGTGATAAGTTATCTACACTGGCGAGGGGATTGCTCTCTGTAATGTTCAGCTTCTAA
TTGTCTCTACTTTGTGAGACTACTTTTGAATGCTTGACCTCAAATCAGGTAGGACTACCC
GCTGAACTTAA
>A8_39
TTTCCGTAGGTGAACCTGCGGAAGGATCATTATTGAATTATGTTTCTAGATAGGTTGTAG
CTGGCTCTTTTAGAGCATGTGCACGCCTGTTTGGACTTCATTTTCATCCACCTGTGCACC
TATTGTAGTCTTTGGTTGGGTTAGGAGGAAGTGATCATTGTATCAGCATCTGCTGGGAGT
GAGGACTTGCATTGTGAAAGCTTTGCTGTCCTTGATGTGATCATGGAATCTTTTTCTCAC
TAGAGTCTATGTCACTCATTATACTCTGTCGAATGTCATTGAATGTCTTTACATGGGCTT
GTATGCCTATGAAAATTGTAATACAACTTTCAGCAACGGATCTCTTGGCTCTCGCATCGA
TGAAGAACGCAGCGAAATGCGATAAGTAATGTGAATTGCAGAATTCAGTGAATCATCGAA

TCTTTGAACGCATCTTGCGCTCCTTGGTATTCCGAGGAGCATGCCTGTTTGAGTGTCATT
AAATTCTCAACTCTCTTATACTTTTTTGTAAAAGAGAGCTTGGACTGTGGAGGCTTGCTG
GCCACTTTTTGGGGTCAGCTCCTCTGAAATGCATTAGCGGAACCGTTTGCGATCTGCCAC
AAGTGTGATAAGTTATCTACACTGGCGAGGGGATTGCTCTCTGTAATGTTCAGCTTCTAA
TTGTCTCTACTTTGTGAGACTACTTTTGAATGCTTGACCTCAAATCAGGTAGGACTACCC
GCTGAACTTAA
>A8_40
TTTCCGTAGGTGAACCTGCGGAAGGATCATTATTGAATTATGTTTCTAGATAGGTTGTAG
CTGGCTCTTTTAGAGCATGTGCACGCCTGTTTGGACTTCATTTTCATCCACCTGTGCACC
TATTGTAGTCTTTGGTTGGGTTAGGAGGAAGTGATCATTGTATCAGCATCTGCTGGGAGT
GAGGACTTGCATTGTGAAAGCTTTGCTGTCCTTGATGTGATCATGGAATCTTTTTCTCAC
TAGAGTCTATGTCACTCATTATACTCTGTCGAATGTCATTGAATGTCTTTACATGGGCTT
GTATGCCTATGAAAATTGTAATACAACTTTCAGCAACGGATCTCTTGGCTCTCGCATCGA
TGAAGAACGCAGCGAAATGCGATAAGTAATGTGAATTGCAGAATTCAGTGAATCATCGAA
TCTTTGAACGCATCTTGCGCTCCTTGGTATTCCGAGGAGCATGCCTGTTTGAGTGTCATT
AAATTCTCAACTCTCTTATACTTTTTTGTAAAAGAGAGCTTGGACTGTGGAGGCTTGCTG
GCCACTTTTTGGGGTCAGCTCCTCTGAAATGCATTAGCGGAACCGTTTGCGATCTGCCAC
AAGTGTGATAAGTTATCTACACTGGCGAGGGGATTGCTCTCTGTAATGTTCAGCTTCTAA
TTGTCTCTACTTTGTGAGACTACTTTTGAATGCTTGACCTCAAATCAGGTAGGACTACCC
GCTGAACTTAA
>A8_41
TTTCCGTAGGTGAACCTGCGGAAGGATCATTATTGAATTATGTTTCTAGATAGGTTGTAG
CTGGCTCTTTTAGAGCATGTGCACGCCTGTTTGGACTTCATTTTCATCCACCTGTGCACC
TATTGTAGTCTTTGGTTGGGTTAGGAGGAAGTGATCATTGTATCAGCATCTGCTGGGAGT
GAGGACTTGCATTGTGAAAGCTTTGCTGTCCTTGATGTGATCATGGAATCTTTTTCTCAC
TAGAGTCTATGTCACTCATTATACTCTGTCGAATGTCATTGAATGTCTTTACATGGGCTT
GTATGCCTATGAAAATTGTAATACAACTTTCAGCAACGGATCTCTTGGCTCTCGCATCGA
TGAAGAACGCAGCGAAATGCGATAAGTAATGTGAATTGCAGAATTCAGTGAATCATCGAA
TCTTTGAACGCATCTTGCGCTCCTTGGTATTCCGAGGAGCATGCCTGTTTGAGTGTCATT
AAATTCTCAACTCTCTTATACTTTTTTGTAAAAGAGAGCTTGGACTGTGGAGGCTTGCTG
GCCACTTTTTGGGGTCAGCTCCTCTGAAATGCATTAGCGGAACCGTTTGCGATCTGCCAC
AAGTGTGATAAGTTATCTACACTGGCGAGGGGATTGCTCTCTGTAATGTTCAGCTTCTAA
TTGTCTCTACTTTGTGAGACTACTTTTGAATGCTTGACCTCAAATCAGGTAGGACTACCC
GCTGAACTTAA
>A8_42
TTTCCGTAGGTGAACCTGCGGAAGGATCATTATTGAATTATGTTTCTAGATAGGTTGTAG
CTGGCTCTTTTAGAGCATGTGCACGCCTGTTTGGACTTCATTTTCATCCACCTGTGCACC
TATTGTAGTCTTTGGTTGGGTTAGGAGGAAGTGATCATTGTATCAGCATCTGCTGGGAGT
GAGGACTTGCATTGTGAAAGCTTTGCTGTCCTTGATGTGATCATGGAATCTTTTTCTCAC
TAGAGTCTATGTCACTCATTATACTCTGTCGAATGTCATTGAATGTCTTTACATGGGCTT
GTATGCCTATGAAAATTGTAATACAACTTTCAGCAACGGATCTCTTGGCTCTCGCATCGA
TGAAGAACGCAGCGAAATGCGATAAGTAATGTGAATTGCAGAATTCAGTGAATCATCGAA
TCTTTGAACGCATCTTGCGCTCCTTGGTATTCCGAGGAGCATGCCTGTTTGAGTGTCATT
AAATTCTCAACTCTCTTATACTTTTTTGTAAAAGAGAGCTTGGACTGTGGAGGCTTGCTG
GCCACTTTTTGGGGTCAGCTCCTCTGAAATGCATTAGCGGAACCGTTTGCGATCTGCCAC
AAGTGTGATAAGTTATCTACACTGGCGAGGGGATTGCTCTCTGTAATGTTCAGCTTCTAA
TTGTCTCTACTTTGTGAGACTACTTTTGAATGCTTGACCTCAAATCAGGTAGGACTACCC
GCTGAACTTAA
>A8_43
TTTCCGTAGGTGAACCTGCGGAAGGATCATTATTGAATTATGTTTCTAGATAGGTTGTAG

CTGGCTCTTTTAGAGCATGTGCACGCCTGTTTGGACTTCATTTTCATCCACCTGTGCACC
TATTGTAGTCTTTGGTTGGGTTAGGAGGAAGTGATCATTGTATCAGCATCTGCTGGGAGT
GAGGACTTGCATTGTGAAAGCTTTGCTGTCCTTGATGTGATCATGGAATCTTTTTCTCAC
TAGAGTCTATGTCACTCATTATACTCTGTCGAATGTCATTGAATGTCTTTACATGGGCTT
GTATGCCTATGAAAATTGTAATACAACTTTCAGCAACGGATCTCTTGGCTCTCGCATCGA
TGAAGAACGCAGCGAAATGCGATAAGTAATGTGAATTGCAGAATTCAGTGAATCATCGAA
TCTTTGAACGCATCTTGCGCTCCTTGGTATTCCGAGGAGCATGCCTGTTTGAGTGTCATT
AAATTCTCAACTCTCTTATACTTTTTTGTAAAAGAGAGCTTGGACTGTGGAGGCTTGCTG
GCCACTTTTTGGGGTCAGCTCCTCTGAAATGCATTAGCGGAACCGTTTGCGATCTGCCAC
AAGTGTGATAAGTTATCTACACTGGCGAGGGGATTGCTCTCTGTAATGTTCAGCTTCTAA
TTGTCTCTACTTTGTGAGACTACTTTTGAATGCTTGACCTCAAATCAGGTAGGACTACCC
GCTGAACTTAA
>A1_23
TTTCCGTAGGTGAACCTGCGGAAGGATCATTATTGAATTATGTTTCTAGATAGGTTGTAG
CTGGCTCTTTTAGAGCATGTGCACGCCTGTTTGGACTTCATTTTCATCCACCTGTGCACC
TATTGTAGTCTTTGGTTGGGTTAGGAGGAAGTGATCATTGTATCAGCATCTGCTGGGAGT
GAGGACTTGCATTGTGAAAGCTTTGCTGTCCTTGATGTGATCATGGAATCTTTTTCTCAC
TAGAGTCTATGTCACTCATTATACTCTGTCGAATGTCATTGAATGTCTTTACATGGGCTT
GTATGCCTATGAAAATTGTAATACAACTTTCAGCAACGGATCTCTTGGCTCTCGCATCGA
TGAAGAACGCAGCGAAATGCGATAAGTAATGTGAATTGCAGAATTCAGTGAATCATCGAA
TCTTTGAACGCATCTTGCGCTCCTTGGTATTCCGAGGAGCATGCCTGTTTGAGTGTCATT
AAATTCTCAACTCTCTTATACTTTTTTGTAAAAGAGAGCTTGGACTGTGGAGGCTTGCTG
GCCACTTTTTGGGGTCAGCTCCTCTGAAATGCATTAGCGGAACCGTTTGCGATCTGCCAC
AAGTGTGATAAGTTATCTACACTGGCGAGGGGATTGCTCTCTGTAATGTTCAGCTTCTAA
TTGTCTCTACTTTGTGAGACTACTTTTGAATGCTTGACCTCAAATCAGGTAGGACTACCC
GCTGAACTTAA
>A1_24
TTTCCGTAGGTGAACCTGCGGAAGGATCATTATTGAATTATGTTTCTAGATAGGTTGTAG
CTGGCTCTTTTAGAGCATGTGCACGCCTGTTTGGACTTCATTTTCATCCACCTGTGCACC
TATTGTAGTCTTTGGTTGGGTTAGGAGGAAGTGATCATTGTATCAGCATCTGCTGGGAGT
GAGGACTTGCATTGTGAAAGCTTTGCTGTCCTTGATGTGATCATGGAATCTTTTTCTCAC
TAGAGTCTATGTCACTCATTATACTCTGTCGAATGTCATTGAATGTCTTTACATGGGCTT
GTATGCCTATGAAAATTGTAATACAACTTTCAGCAACGGATCTCTTGGCTCTCGCATCGA
TGAAGAACGCAGCGAAATGCGATAAGTAATGTGAATTGCAGAATTCAGTGAATCATCGAA
TCTTTGAACGCATCTTGCGCTCCTTGGTATTCCGAGGAGCATGCCTGTTTGAGTGTCATT
AAATTCTCAACTCTCTTATACTTTTTTGTAAAAGAGAGCTTGGACTGTGGAGGCTTGCTG
GCCACTTTTTGGGGTCAGCTCCTCTGAAATGCATTAGCGGAACCGTTTGCGATCTGCCAC
AAGTGTGATAAGTTATCTACACTGGCGAGGGGATTGCTCTCTGTAATGTTCAGCTTCTAA
TTGTCTCTACTTTGTGAGACTACTTTTGAATGCTTGACCTCAAATCAGGTAGGACTACCC
GCTGAACTTAA
>A1_25
TTTCCGTAGGTGAACCTGCGGAAGGATCATTATTGAATTATGTTTCTAGATAGGTTGTAG
CTGGCTCTTTTAGAGCATGTGCACGCCTGTTTGGACTTCATTTTCATCCACCTGTGCACC
TATTGTAGTCTTTGGTTGGGTTAGGAGGAAGTGATCATTGTATCAGCATCTGCTGGGAGT
GAGGACTTGCATTGTGAAAGCTTTGCTGTCCTTGATGTGATCATGGAATCTTTTTCTCAC
TAGAGTCTATGTCACTCATTATACTCTGTCGAATGTCATTGAATGTCTTTACATGGGCTT
GTATGCCTATGAAAATTGTAATACAACTTTCAGCAACGGATCTCTTGGCTCTCGCATCGA
TGAAGAACGCAGCGAAATGCGATAAGTAATGTGAATTGCAGAATTCAGTGAATCATCGAA
TCTTTGAACGCATCTTGCGCTCCTTGGTATTCCGAGGAGCATGCCTGTTTGAGTGTCATT
AAATTCTCAACTCTCTTATACTTTTTTGTAAAAGAGAGCTTGGACTGTGGAGGCTTGCTG

GCCACTTTTTGGGGTCAGCTCCTCTGAAATGCATTAGCGGAACCGTTTGCGATCTGCCAC
AAGTGTGATAAGTTATCTACACTGGCGAGGGGATTGCTCTCTGTAATGTTCAGCTTCTAA
TTGTCTCTACTTTGTGAGACTACTTTTGAATGCTTGACCTCAAATCAGGTAGGACTACCC
GCTGAACTTAA
>A1_26
TTTCCGTAGGTGAACCTGCGGAAGGATCATTATTGAATTATGTTTCTAGATAGGTTGTAG
CTGGCTCTTTTAGAGCATGTGCACGCCTGTTTGGACTTCATTTTCATCCACCTGTGCACC
TATTGTAGTCTTTGGTTGGGTTAGGAGGAAGTGATCATTGTATCAGCATCTGCTGGGAGT
GAGGACTTGCATTGTGAAAGCTTTGCTGTCCTTGATGTGATCATGGAATCTTTTTCTCAC
TAGAGTCTATGTCACTCATTATACTCTGTCGAATGTCATTGAATGTCTTTACATGGGCTT
GTATGCCTATGAAAATTGTAATACAACTTTCAGCAACGGATCTCTTGGCTCTCGCATCGA
TGAAGAACGCAGCGAAATGCGATAAGTAATGTGAATTGCAGAATTCAGTGAATCATCGAA
TCTTTGAACGCATCTTGCGCTCCTTGGTATTCCGAGGAGCATGCCTGTTTGAGTGTCATT
AAATTCTCAACTCTCTTATACTTTTTTGTAAAAGAGAGCTTGGACTGTGGAGGCTTGCTG
GCCACTTTTTGGGGTCAGCTCCTCTGAAATGCATTAGCGGAACCGTTTGCGATCTGCCAC
AAGTGTGATAAGTTATCTACACTGGCGAGGGGATTGCTCTCTGTAATGTTCAGCTTCTAA
TTGTCTCTACTTTGTGAGACTACTTTTGAATGCTTGACCTCAAATCAGGTAGGACTACCC
GCTGAACTTAA
>A1_27
TTTCCGTAGGTGAACCTGCGGAAGGATCATTATTGAATTATGTTTCTAGATAGGTTGTAG
CTGGCTCTTTTAGAGCATGTGCACGCCTGTTTGGACTTCATTTTCATCCACCTGTGCACC
TATTGTAGTCTTTGGTTGGGTTAGGAGGAAGTGATCATTGTATCAGCATCTGCTGGGAGT
GAGGACTTGCATTGTGAAAGCTTTGCTGTCCTTGATGTGATCATGGAATCTTTTTCTCAC
TAGAGTCTATGTCACTCATTATACTCTGTCGAATGTCATTGAATGTCTTTACATGGGCTT
GTATGCCTATGAAAATTGTAATACAACTTTCAGCAACGGATCTCTTGGCTCTCGCATCGA
TGAAGAACGCAGCGAAATGCGATAAGTAATGTGAATTGCAGAATTCAGTGAATCATCGAA
TCTTTGAACGCATCTTGCGCTCCTTGGTATTCCGAGGAGCATGCCTGTTTGAGTGTCATT
AAATTCTCAACTCTCTTATACTTTTTTGTAAAAGAGAGCTTGGACTGTGGAGGCTTGCTG
GCCACTTTTTGGGGTCAGCTCCTCTGAAATGCATTAGCGGAACCGTTTGCGATCTGCCAC
AAGTGTGATAAGTTATCTACACTGGCGAGGGGATTGCTCTCTGTAATGTTCAGCTTCTAA
TTGTCTCTACTTTGTGAGACTACTTTTGAATGCTTGACCTCAAATCAGGTAGGACTACCC
GCTGAACTTAA
>A1_28
TTTCCGTAGGTGAACCTGCGGAAGGATCATTATTGAATTATGTTTCTAGATAGGTTGTAG
CTGGCTCTTTTAGAGCATGTGCACGCCTGTTTGGACTTCATTTTCATCCACCTGTGCACC
TATTGTAGTCTTTGGTTGGGTTAGGAGGAAGTGATCATTGTATCAGCATCTGCTGGGAGT
GAGGACTTGCATTGTGAAAGCTTTGCTGTCCTTGATGTGATCATGGAATCTTTTTCTCAC
TAGAGTCTATGTCACTCATTATACTCTGTCGAATGTCATTGAATGTCTTTACATGGGCTT
GTATGCCTATGAAAATTGTAATACAACTTTCAGCAACGGATCTCTTGGCTCTCGCATCGA
TGAAGAACGCAGCGAAATGCGATAAGTAATGTGAATTGCAGAATTCAGTGAATCATCGAA
TCTTTGAACGCATCTTGCGCTCCTTGGTATTCCGAGGAGCATGCCTGTTTGAGTGTCATT
AAATTCTCAACTCTCTTATACTTTTTTGTAAAAGAGAGCTTGGACTGTGGAGGCTTGCTG
GCCACTTTTTGGGGTCAGCTCCTCTGAAATGCATTAGCGGAACCGTTTGCGATCTGCCAC
AAGTGTGATAAGTTATCTACACTGGCGAGGGGATTGCTCTCTGTAATGTTCAGCTTCTAA
TTGTCTCTACTTTGTGAGACTACTTTTGAATGCTTGACCTCAAATCAGGTAGGACTACCC
GCTGAACTTAA
>A1_29
TTTCCGTAGGTGAACCTGCGGAAGGATCATTATTGAATTATGTTTCTAGATAGGTTGTAG
CTGGCTCTTTTAGAGCATGTGCACGCCTGTTTGGACTTCATTTTCATCCACCTGTGCACC
TATTGTAGTCTTTGGTTGGGTTAGGAGGAAGTGATCATTGTATCAGCATCTGCTGGGAGT

GAGGACTTGCATTGTGAAAGCTTTGCTGTCCTTGATGTGATCATGGAATCTTTTTCTCAC
TAGAGTCTATGTCACTCATTATACTCTGTCGAATGTCATTGAATGTCTTTACATGGGCTT
GTATGCCTATGAAAATTGTAATACAACTTTCAGCAACGGATCTCTTGGCTCTCGCATCGA
TGAAGAACGCAGCGAAATGCGATAAGTAATGTGAATTGCAGAATTCAGTGAATCATCGAA
TCTTTGAACGCATCTTGCGCTCCTTGGTATTCCGAGGAGCATGCCTGTTTGAGTGTCATT
AAATTCTCAACTCTCTTATACTTTTTTGTAAAAGAGAGCTTGGACTGTGGAGGCTTGCTG
GCCACTTTTTGGGGTCAGCTCCTCTGAAATGCATTAGCGGAACCGTTTGCGATCTGCCAC
AAGTGTGATAAGTTATCTACACTGGCGAGGGGATTGCTCTCTGTAATGTTCAGCTTCTAA
TTGTCTCTACTTTGTGAGACTACTTTTGAATGCTTGACCTCAAATCAGGTAGGACTACCC
GCTGAACTTAA
>A8_44
TTTCCGTAGGTGAACCTGCGGAAGGATCATTATTGAATTATGTTTCTAGATAGGTTGTAG
CTGGCTCTTTTAGAGCATGTGCACGCCTGTTTGGACTTCATTTTCATCCACCTGTGCACC
TATTGTAGTCTTTGGTTGGGTTAGGAGGAAGTGATCATTGTATCAGCATCTGCTGGGAGT
GAGGACTTGCATTGTGAAAGCTTTGCTGTCCTTGATGTGATCATGGAATCTTTTTCTCAC
TAGAGTCTATGTCACTCATTATACTCTGTCGAATGTCATTGAATGTCTTTACATGGGCTT
GTATGCCTATGAAAATTGTAATACAACTTTCAGCAACGGATCTCTTGGCTCTCGCATCGA
TGAAGAACGCAGCGAAATGCGATAAGTAATGTGAATTGCAGAATTCAGTGAATCATCGAA
TCTTTGAACGCATCTTGCGCTCCTTGGTATTCCGAGGAGCATGCCTGTTTGAGTGTCATT
AAATTCTCAACTCTCTTATACTTTTTTGTAAAAGAGAGCTTGGACTGTGGAGGCTTGCTG
GCCACTTTTTGGGGTCAGCTCCTCTGAAATGCATTAGCGGAACCGTTTGCGATCTGCCAC
AAGTGTGATAAGTTATCTACACTGGCGAGGGGATTGCTCTCTGTAATGTTCAGCTTCTAA
TTGTCTCTACTTTGTGAGACTACTTTTGAATGCTTGACCTCAAATCAGGTAGGACTACCC
GCTGAACTTAA
>A8_45
TTTCCGTAGGTGAACCTGCGGAAGGATCATTATTGAATTATGTTTCTAGATAGGTTGTAG
CTGGCTCTTTTAGAGCATGTGCACGCCTGTTTGGACTTCATTTTCATCCACCTGTGCACC
TATTGTAGTCTTTGGTTGGGTTAGGAGGAAGTGATCATTGTATCAGCATCTGCTGGGAGT
GAGGACTTGCATTGTGAAAGCTTTGCTGTCCTTGATGTGATCATGGAATCTTTTTCTCAC
TAGAGTCTATGTCACTCATTATACTCTGTCGAATGTCATTGAATGTCTTTACATGGGCTT
GTATGCCTATGAAAATTGTAATACAACTTTCAGCAACGGATCTCTTGGCTCTCGCATCGA
TGAAGAACGCAGCGAAATGCGATAAGTAATGTGAATTGCAGAATTCAGTGAATCATCGAA
TCTTTGAACGCATCTTGCGCTCCTTGGTATTCCGAGGAGCATGCCTGTTTGAGTGTCATT
AAATTCTCAACTCTCTTATACTTTTTTGTAAAAGAGAGCTTGGACTGTGGAGGCTTGCTG
GCCACTTTTTGGGGTCAGCTCCTCTGAAATGCATTAGCGGAACCGTTTGCGATCTGCCAC
AAGTGTGATAAGTTATCTACACTGGCGAGGGGATTGCTCTCTGTAATGTTCAGCTTCTAA
TTGTCTCTACTTTGTGAGACTACTTTTGAATGCTTGACCTCAAATCAGGTAGGACTACCC
GCTGAACTTAA
>A8_47
TTTCCGTAGGTGAACCTGCGGAAGGATCATTATTGAATTATGTTTCTAGATAGGTTGTAG
CTGGCTCTTTTAGAGCATGTGCACGCCTGTTTGGACTTCATTTTCATCCACCTGTGCACC
TATTGTAGTCTTTGGTTGGGTTAGGAGGAAGTGATCATTGTATCAGCATCTGCTGGGAGT
GAGGACTTGCATTGTGAAAGCTTTGCTGTCCTTGATGTGATCATGGAATCTTTTTCTCAC
TAGAGTCTATGTCACTCATTATACTCTGTCGAATGTCATTGAATGTCTTTACATGGGCTT
GTATGCCTATGAAAATTGTAATACAACTTTCAGCAACGGATCTCTTGGCTCTCGCATCGA
TGAAGAACGCAGCGAAATGCGATAAGTAATGTGAATTGCAGAATTCAGTGAATCATCGAA
TCTTTGAACGCATCTTGCGCTCCTTGGTATTCCGAGGAGCATGCCTGTTTGAGTGTCATT
AAATTCTCAACTCTCTTATACTTTTTTGTAAAAGAGAGCTTGGACTGTGGAGGCTTGCTG
GCCACTTTTTGGGGTCAGCTCCTCTGAAATGCATTAGCGGAACCGTTTGCGATCTGCCAC
AAGTGTGATAAGTTATCTACACTGGCGAGGGGATTGCTCTCTGTAATGTTCAGCTTCTAA

TTGTCTCTACTTTGTGAGACTACTTTTGAATGCTTGACCTCAAATCAGGTAGGACTACCC
GCTGAACTTAA
>A8_48
TTTCCGTAGGTGAACCTGCGGAAGGATCATTATTGAATTATGTTTCTAGATAGGTTGTAG
CTGGCTCTTTTAGAGCATGTGCACGCCTGTTTGGACTTCATTTTCATCCACCTGTGCACC
TATTGTAGTCTTTGGTTGGGTTAGGAGGAAGTGATCATTGTATCAGCATCTGCTGGGAGT
GAGGACTTGCATTGTGAAAGCTTTGCTGTCCTTGATGTGATCATGGAATCTTTTTCTCAC
TAGAGTCTATGTCACTCATTATACTCTGTCGAATGTCATTGAATGTCTTTACATGGGCTT
GTATGCCTATGAAAATTGTAATACAACTTTCAGCAACGGATCTCTTGGCTCTCGCATCGA
TGAAGAACGCAGCGAAATGCGATAAGTAATGTGAATTGCAGAATTCAGTGAATCATCGAA
TCTTTGAACGCATCTTGCGCTCCTTGGTATTCCGAGGAGCATGCCTGTTTGAGTGTCATT
AAATTCTCAACTCTCTTATACTTTTTTGTAAAAGAGAGCTTGGACTGTGGAGGCTTGCTG
GCCACTTTTTGGGGTCAGCTCCTCTGAAATGCATTAGCGGAACCGTTTGCGATCTGCCAC
AAGTGTGATAAGTTATCTACACTGGCGAGGGGATTGCTCTCTGTAATGTTCAGCTTCTAA
TTGTCTCTACTTTGTGAGACTACTTTTGAATGCTTGACCTCAAATCAGGTAGGACTACCC
GCTGAACTTAA
>A8_49
TTTCCGTAGGTGAACCTGCGGAAGGATCATTATTGAATTATGTTTCTAGATAGGTTGTAG
CTGGCTCTTTTAGAGCATGTGCACGCCTGTTTGGACTTCATTTTCATCCACCTGTGCACC
TATTGTAGTCTTTGGTTGGGTTAGGAGGAAGTGATCATTGTATCAGCATCTGCTGGGAGT
GAGGACTTGCATTGTGAAAGCTTTGCTGTCCTTGATGTGATCATGGAATCTTTTTCTCAC
TAGAGTCTATGTCACTCATTATACTCTGTCGAATGTCATTGAATGTCTTTACATGGGCTT
GTATGCCTATGAAAATTGTAATACAACTTTCAGCAACGGATCTCTTGGCTCTCGCATCGA
TGAAGAACGCAGCGAAATGCGATAAGTAATGTGAATTGCAGAATTCAGTGAATCATCGAA
TCTTTGAACGCATCTTGCGCTCCTTGGTATTCCGAGGAGCATGCCTGTTTGAGTGTCATT
AAATTCTCAACTCTCTTATACTTTTTTGTAAAAGAGAGCTTGGACTGTGGAGGCTTGCTG
GCCACTTTTTGGGGTCAGCTCCTCTGAAATGCATTAGCGGAACCGTTTGCGATCTGCCAC
AAGTGTGATAAGTTATCTACACTGGCGAGGGGATTGCTCTCTGTAATGTTCAGCTTCTAA
TTGTCTCTACTTTGTGAGACTACTTTTGAATGCTTGACCTCAAATCAGGTAGGACTACCC
GCTGAACTTAA
>A8_50
TTTCCGTAGGTGAACCTGCGGAAGGATCATTATTGAATTATGTTTCTAGATAGGTTGTAG
CTGGCTCTTTTAGAGCATGTGCACGCCTGTTTGGACTTCATTTTCATCCACCTGTGCACC
TATTGTAGTCTTTGGTTGGGTTAGGAGGAAGTGATCATTGTATCAGCATCTGCTGGGAGT
GAGGACTTGCATTGTGAAAGCTTTGCTGTCCTTGATGTGATCATGGAATCTTTTTCTCAC
TAGAGTCTATGTCACTCATTATACTCTGTCGAATGTCATTGAATGTCTTTACATGGGCTT
GTATGCCTATGAAAATTGTAATACAACTTTCAGCAACGGATCTCTTGGCTCTCGCATCGA
TGAAGAACGCAGCGAAATGCGATAAGTAATGTGAATTGCAGAATTCAGTGAATCATCGAA
TCTTTGAACGCATCTTGCGCTCCTTGGTATTCCGAGGAGCATGCCTGTTTGAGTGTCATT
AAATTCTCAACTCTCTTATACTTTTTTGTAAAAGAGAGCTTGGACTGTGGAGGCTTGCTG
GCCACTTTTTGGGGTCAGCTCCTCTGAAATGCATTAGCGGAACCGTTTGCGATCTGCCAC
AAGTGTGATAAGTTATCTACACTGGCGAGGGGATTGCTCTCTGTAATGTTCAGCTTCTAA
TTGTCTCTACTTTGTGAGACTACTTTTGAATGCTTGACCTCAAATCAGGTAGGACTACCC
GCTGAACTTAA
>A8_51
TTTCCGTAGGTGAACCTGCGGAAGGATCATTATTGAATTATGTTTCTAGATAGGTTGTAG
CTGGCTCTTTTAGAGCATGTGCACGCCTGTTTGGACTTCATTTTCATCCACCTGTGCACC
TATTGTAGTCTTTGGTTGGGTTAGGAGGAAGTGATCATTGTATCAGCATCTGCTGGGAGT
GAGGACTTGCATTGTGAAAGCTTTGCTGTCCTTGATGTGATCATGGAATCTTTTTCTCAC
TAGAGTCTATGTCACTCATTATACTCTGTCGAATGTCATTGAATGTCTTTACATGGGCTT

GTATGCCTATGAAAATTGTAATACAACTTTCAGCAACGGATCTCTTGGCTCTCGCATCGA
TGAAGAACGCAGCGAAATGCGATAAGTAATGTGAATTGCAGAATTCAGTGAATCATCGAA
TCTTTGAACGCATCTTGCGCTCCTTGGTATTCCGAGGAGCATGCCTGTTTGAGTGTCATT
AAATTCTCAACTCTCTTATACTTTTTTGTAAAAGAGAGCTTGGACTGTGGAGGCTTGCTG
GCCACTTTTTGGGGTCAGCTCCTCTGAAATGCATTAGCGGAACCGTTTGCGATCTGCCAC
AAGTGTGATAAGTTATCTACACTGGCGAGGGGATTGCTCTCTGTAATGTTCAGCTTCTAA
TTGTCTCTACTTTGTGAGACTACTTTTGAATGCTTGACCTCAAATCAGGTAGGACTACCC
GCTGAACTTAA
>A1_16
TTTCCGTAGGTGAACCTGCGGAAGGATCATTATTGAATTATGTTTCTAGATAGGTTGTAG
CTGGCTCTTTTAGAGCATGTGCACGCCTGTTTGGACTTCATTTTCATCCACCTGTGCACC
TATTGTAGTCTTTGGTTGGGTTAGGAGGAAGTGATCATTGTATCAGCATCTGCTGGGAGT
GAGGACTTGCATTGTGAAAGCTTTGCTGTCCTTGATGTGATCATGGAATCTTTTTCTCAC
TAGAGTCTATGTCACTCATTATACTCTGTCGAATGTCATTGAATGTCTTTACATGGGCTT
GTATGCCTATGAAAATTGTAATACAACTTTCAGCAACGGATCTCTTGGCTCTCGCATCGA
TGAAGAACGCAGCGAAATGCGATAAGTAATGTGAATTGCAGAATTCAGTGAATCATCGAA
TCTTTGAACGCATCTTGCGCTCCTTGGTATTCCGAGGAGCATGCCTGTTTGAGTGTCATT
AAATTCTCAACTCTCTTATACTTTTTTGTAAAAGAGAGCTTGGACTGTGGAGGCTTGCTG
GCCACTTTTTGGGGTCAGCTCCTCTGAAATGCATTAGCGGAACCGTTTGCGATCTGCCAC
AAGTGTGATAAGTTATCTACACTGGCGAGGGGATTGCTCTCTGTAATGTTCAGCTTCTAA
TTGTCTCTACTTTGTGAGACTACTTTTGAATGCTTGACCTCAAATCAGGTAGGACTACCC
GCTGAACTTAA
>A1_17
TTTCCGTAGGTGAACCTGCGGAAGGATCATTATTGAATTATGTTTCTAGATAGGTTGTAG
CTGGCTCTTTTAGAGCATGTGCACGCCTGTTTGGACTTCATTTTCATCCACCTGTGCACC
TATTGTAGTCTTTGGTTGGGTTAGGAGGAAGTGATCATTGTATCAGCATCTGCTGGGAGT
GAGGACTTGCATTGTGAAAGCTTTGCTGTCCTTGATGTGATCATGGAATCTTTTTCTCAC
TAGAGTCTATGTCACTCATTATACTCTGTCGAATGTCATTGAATGTCTTTACATGGGCTT
GTATGCCTATGAAAATTGTAATACAACTTTCAGCAACGGATCTCTTGGCTCTCGCATCGA
TGAAGAACGCAGCGAAATGCGATAAGTAATGTGAATTGCAGAATTCAGTGAATCATCGAA
TCTTTGAACGCATCTTGCGCTCCTTGGTATTCCGAGGAGCATGCCTGTTTGAGTGTCATT
AAATTCTCAACTCTCTTATACTTTTTTGTAAAAGAGAGCTTGGACTGTGGAGGCTTGCTG
GCCACTTTTTGGGGTCAGCTCCTCTGAAATGCATTAGCGGAACCGTTTGCGATCTGCCAC
AAGTGTGATAAGTTATCTACACTGGCGAGGGGATTGCTCTCTGTAATGTTCAGCTTCTAA
TTGTCTCTACTTTGTGAGACTACTTTTGAATGCTTGACCTCAAATCAGGTAGGACTACCC
GCTGAACTTAA
>A1_18
TTTCCGTAGGTGAACCTGCGGAAGGATCATTATTGAATTATGTTTCTAGATAGGTTGTAG
CTGGCTCTTTTAGAGCATGTGCACGCCTGTTTGGACTTCATTTTCATCCACCTGTGCACC
TATTGTAGTCTTTGGTTGGGTTAGGAGGAAGTGATCATTGTATCAGCATCTGCTGGGAGT
GAGGACTTGCATTGTGAAAGCTTTGCTGTCCTTGATGTGATCATGGAATCTTTTTCTCAC
TAGAGTCTATGTCACTCATTATACTCTGTCGAATGTCATTGAATGTCTTTACATGGGCTT
GTATGCCTATGAAAATTGTAATACAACTTTCAGCAACGGATCTCTTGGCTCTCGCATCGA
TGAAGAACGCAGCGAAATGCGATAAGTAATGTGAATTGCAGAATTCAGTGAATCATCGAA
TCTTTGAACGCATCTTGCGCTCCTTGGTATTCCGAGGAGCATGCCTGTTTGAGTGTCATT
AAATTCTCAACTCTCTTATACTTTTTTGTAAAAGAGAGCTTGGACTGTGGAGGCTTGCTG
GCCACTTTTTGGGGTCAGCTCCTCTGAAATGCATTAGCGGAACCGTTTGCGATCTGCCAC
AAGTGTGATAAGTTATCTACACTGGCGAGGGGATTGCTCTCTGTAATGTTCAGCTTCTAA
TTGTCTCTACTTTGTGAGACTACTTTTGAATGCTTGACCTCAAATCAGGTAGGACTACCC
GCTGAACTTAA

>A1_19
TTTCCGTAGGTGAACCTGCGGAAGGATCATTATTGAATTATGTTTCTAGATAGGTTGTAG
CTGGCTCTTTTAGAGCATGTGCACGCCTGTTTGGACTTCATTTTCATCCACCTGTGCACC
TATTGTAGTCTTTGGTTGGGTTAGGAGGAAGTGATCATTGTATCAGCATCTGCTGGGAGT
GAGGACTTGCATTGTGAAAGCTTTGCTGTCCTTGATGTGATCATGGAATCTTTTTCTCAC
TAGAGTCTATGTCACTCATTATACTCTGTCGAATGTCATTGAATGTCTTTACATGGGCTT
GTATGCCTATGAAAATTGTAATACAACTTTCAGCAACGGATCTCTTGGCTCTCGCATCGA
TGAAGAACGCAGCGAAATGCGATAAGTAATGTGAATTGCAGAATTCAGTGAATCATCGAA
TCTTTGAACGCATCTTGCGCTCCTTGGTATTCCGAGGAGCATGCCTGTTTGAGTGTCATT
AAATTCTCAACTCTCTTATACTTTTTTGTAAAAGAGAGCTTGGACTGTGGAGGCTTGCTG
GCCACTTTTTGGGGTCAGCTCCTCTGAAATGCATTAGCGGAACCGTTTGCGATCTGCCAC
AAGTGTGATAAGTTATCTACACTGGCGAGGGGATTGCTCTCTGTAATGTTCAGCTTCTAA
TTGTCTCTACTTTGTGAGACTACTTTTGAATGCTTGACCTCAAATCAGGTAGGACTACCC
GCTGAACTTAA
>A1_20
TTTCCGTAGGTGAACCTGCGGAAGGATCATTATTGAATTATGTTTCTAGATAGGTTGTAG
CTGGCTCTTTTAGAGCATGTGCACGCCTGTTTGGACTTCATTTTCATCCACCTGTGCACC
TATTGTAGTCTTTGGTTGGGTTAGGAGGAAGTGATCATTGTATCAGCATCTGCTGGGAGT
GAGGACTTGCATTGTGAAAGCTTTGCTGTCCTTGATGTGATCATGGAATCTTTTTCTCAC
TAGAGTCTATGTCACTCATTATACTCTGTCGAATGTCATTGAATGTCTTTACATGGGCTT
GTATGCCTATGAAAATTGTAATACAACTTTCAGCAACGGATCTCTTGGCTCTCGCATCGA
TGAAGAACGCAGCGAAATGCGATAAGTAATGTGAATTGCAGAATTCAGTGAATCATCGAA
TCTTTGAACGCATCTTGCGCTCCTTGGTATTCCGAGGAGCATGCCTGTTTGAGTGTCATT
AAATTCTCAACTCTCTTATACTTTTTTGTAAAAGAGAGCTTGGACTGTGGAGGCTTGCTG
GCCACTTTTTGGGGTCAGCTCCTCTGAAATGCATTAGCGGAACCGTTTGCGATCTGCCAC
AAGTGTGATAAGTTATCTACACTGGCGAGGGGATTGCTCTCTGTAATGTTCAGCTTCTAA
TTGTCTCTACTTTGTGAGACTACTTTTGAATGCTTGACCTCAAATCAGGTAGGACTACCC
GCTGAACTTAA
>A1_21
TTTCCGTAGGTGAACCTGCGGAAGGATCATTATTGAATTATGTTTCTAGATAGGTTGTAG
CTGGCTCTTTTAGAGCATGTGCACGCCTGTTTGGACTTCATTTTCATCCACCTGTGCACC
TATTGTAGTCTTTGGTTGGGTTAGGAGGAAGTGATCATTGTATCAGCATCTGCTGGGAGT
GAGGACTTGCATTGTGAAAGCTTTGCTGTCCTTGATGTGATCATGGAATCTTTTTCTCAC
TAGAGTCTATGTCACTCATTATACTCTGTCGAATGTCATTGAATGTCTTTACATGGGCTT
GTATGCCTATGAAAATTGTAATACAACTTTCAGCAACGGATCTCTTGGCTCTCGCATCGA
TGAAGAACGCAGCGAAATGCGATAAGTAATGTGAATTGCAGAATTCAGTGAATCATCGAA
TCTTTGAACGCATCTTGCGCTCCTTGGTATTCCGAGGAGCATGCCTGTTTGAGTGTCATT
AAATTCTCAACTCTCTTATACTTTTTTGTAAAAGAGAGCTTGGACTGTGGAGGCTTGCTG
GCCACTTTTTGGGGTCAGCTCCTCTGAAATGCATTAGCGGAACCGTTTGCGATCTGCCAC
AAGTGTGATAAGTTATCTACACTGGCGAGGGGATTGCTCTCTGTAATGTTCAGCTTCTAA
TTGTCTCTACTTTGTGAGACTACTTTTGAATGCTTGACCTCAAATCAGGTAGGACTACCC
GCTGAACTTAA
>A1_22
TTTCCGTAGGTGAACCTGCGGAAGGATCATTATTGAATTATGTTTCTAGATAGGTTGTAG
CTGGCTCTTTTAGAGCATGTGCACGCCTGTTTGGACTTCATTTTCATCCACCTGTGCACC
TATTGTAGTCTTTGGTTGGGTTAGGAGGAAGTGATCATTGTATCAGCATCTGCTGGGAGT
GAGGACTTGCATTGTGAAAGCTTTGCTGTCCTTGATGTGATCATGGAATCTTTTTCTCAC
TAGAGTCTATGTCACTCATTATACTCTGTCGAATGTCATTGAATGTCTTTACATGGGCTT
GTATGCCTATGAAAATTGTAATACAACTTTCAGCAACGGATCTCTTGGCTCTCGCATCGA
TGAAGAACGCAGCGAAATGCGATAAGTAATGTGAATTGCAGAATTCAGTGAATCATCGAA

TCTTTGAACGCATCTTGCGCTCCTTGGTATTCCGAGGAGCATGCCTGTTTGAGTGTCATT
AAATTCTCAACTCTCTTATACTTTTTTGTAAAAGAGAGCTTGGACTGTGGAGGCTTGCTG
GCCACTTTTTGGGGTCAGCTCCTCTGAAATGCATTAGCGGAACCGTTTGCGATCTGCCAC
AAGTGTGATAAGTTATCTACACTGGCGAGGGGATTGCTCTCTGTAATGTTCAGCTTCTAA
TTGTCTCTACTTTGTGAGACTACTTTTGAATGCTTGACCTCAAATCAGGTAGGACTACCC
GCTGAACTTAA
>A1_30
TTTCCGTAGGTGAACCTGCGGAAGGATCATTATTGAATTATGTTTCTAGATAGGTTGTAG
CTGGCTCTTTTAGAGCATGTGCACGCCTGTTTGGACTTCATTTTCATCCACCTGTGCACC
TATTGTAGTCTTTGGTTGGGTTAGGAGGAAGTGATCATTGTATCAGCATCTGCTGGGAGT
GAGGACTTGCATTGTGAAAGCTTTGCTGTCCTTGATGTGATCATGGAATCTTTTTCTCAC
TAGAGTCTATGTCACTCATTATACTCTGTCGAATGTCATTGAATGTCTTTACATGGGCTT
GTATGCCTATGAAAATTGTAATACAACTTTCAGCAACGGATCTCTTGGCTCTCGCATCGA
TGAAGAACGCAGCGAAATGCGATAAGTAATGTGAATTGCAGAATTCAGTGAATCATCGAA
TCTTTGAACGCATCTTGCGCTCCTTGGTATTCCGAGGAGCATGCCTGTTTGAGTGTCATT
AAATTCTCAACTCTCTTATACTTTTTTGTAAAAGAGAGCTTGGACTGTGGAGGCTTGCTG
GCCACTTTTTGGGGTCAGCTCCTCTGAAATGCATTAGCGGAACCGTTTGCGATCTGCCAC
AAGTGTGATAAGTTATCTACACTGGCGAGGGGATTGCTCTCTGTAATGTTCAGCTTCTAA
TTGTCTCTACTTTGTGAGACTACTTTTGAATGCTTGACCTCAAATCAGGTAGGACTACCC
GCTGAACTTAA
>A1_33
TTTCCGTAGGTGAACCTGCGGAAGGATCATTATTGAATTATGTTTCTAGATAGGTTGTAG
CTGGCTCTTTTAGAGCATGTGCACGCCTGTTTGGACTTCATTTTCATCCACCTGTGCACC
TATTGTAGTCTTTGGTTGGGTTAGGAGGAAGTGATCATTGTATCAGCATCTGCTGGGAGT
GAGGACTTGCATTGTGAAAGCTTTGCTGTCCTTGATGTGATCATGGAATCTTTTTCTCAC
TAGAGTCTATGTCACTCATTATACTCTGTCGAATGTCATTGAATGTCTTTACATGGGCTT
GTATGCCTATGAAAATTGTAATACAACTTTCAGCAACGGATCTCTTGGCTCTCGCATCGA
TGAAGAACGCAGCGAAATGCGATAAGTAATGTGAATTGCAGAATTCAGTGAATCATCGAA
TCTTTGAACGCATCTTGCGCTCCTTGGTATTCCGAGGAGCATGCCTGTTTGAGTGTCATT
AAATTCTCAACTCTCTTATACTTTTTTGTAAAAGAGAGCTTGGACTGTGGAGGCTTGCTG
GCCACTTTTTGGGGTCAGCTCCTCTGAAATGCATTAGCGGAACCGTTTGCGATCTGCCAC
AAGTGTGATAAGTTATCTACACTGGCGAGGGGATTGCTCTCTGTAATGTTCAGCTTCTAA
TTGTCTCTACTTTGTGAGACTACTTTTGAATGCTTGACCTCAAATCAGGTAGGACTACCC
GCTGAACTTAA
>A1_34
TTTCCGTAGGTGAACCTGCGGAAGGATCATTATTGAATTATGTTTCTAGATAGGTTGTAG
CTGGCTCTTTTAGAGCATGTGCACGCCTGTTTGGACTTCATTTTCATCCACCTGTGCACC
TATTGTAGTCTTTGGTTGGGTTAGGAGGAAGTGATCATTGTATCAGCATCTGCTGGGAGT
GAGGACTTGCATTGTGAAAGCTTTGCTGTCCTTGATGTGATCATGGAATCTTTTTCTCAC
TAGAGTCTATGTCACTCATTATACTCTGTCGAATGTCATTGAATGTCTTTACATGGGCTT
GTATGCCTATGAAAATTGTAATACAACTTTCAGCAACGGATCTCTTGGCTCTCGCATCGA
TGAAGAACGCAGCGAAATGCGATAAGTAATGTGAATTGCAGAATTCAGTGAATCATCGAA
TCTTTGAACGCATCTTGCGCTCCTTGGTATTCCGAGGAGCATGCCTGTTTGAGTGTCATT
AAATTCTCAACTCTCTTATACTTTTTTGTAAAAGAGAGCTTGGACTGTGGAGGCTTGCTG
GCCACTTTTTGGGGTCAGCTCCTCTGAAATGCATTAGCGGAACCGTTTGCGATCTGCCAC
AAGTGTGATAAGTTATCTACACTGGCGAGGGGATTGCTCTCTGTAATGTTCAGCTTCTAA
TTGTCTCTACTTTGTGAGACTACTTTTGAATGCTTGACCTCAAATCAGGTAGGACTACCC
GCTGAACTTAA
>A1_35
TTTCCGTAGGTGAACCTGCGGAAGGATCATTATTGAATTATGTTTCTAGATAGGTTGTAG

CTGGCTCTTTTAGAGCATGTGCACGCCTGTTTGGACTTCATTTTCATCCACCTGTGCACC
TATTGTAGTCTTTGGTTGGGTTAGGAGGAAGTGATCATTGTATCAGCATCTGCTGGGAGT
GAGGACTTGCATTGTGAAAGCTTTGCTGTCCTTGATGTGATCATGGAATCTTTTTCTCAC
TAGAGTCTATGTCACTCATTATACTCTGTCGAATGTCATTGAATGTCTTTACATGGGCTT
GTATGCCTATGAAAATTGTAATACAACTTTCAGCAACGGATCTCTTGGCTCTCGCATCGA
TGAAGAACGCAGCGAAATGCGATAAGTAATGTGAATTGCAGAATTCAGTGAATCATCGAA
TCTTTGAACGCATCTTGCGCTCCTTGGTATTCCGAGGAGCATGCCTGTTTGAGTGTCATT
AAATTCTCAACTCTCTTATACTTTTTTGTAAAAGAGAGCTTGGACTGTGGAGGCTTGCTG
GCCACTTTTTGGGGTCAGCTCCTCTGAAATGCATTAGCGGAACCGTTTGCGATCTGCCAC
AAGTGTGATAAGTTATCTACACTGGCGAGGGGATTGCTCTCTGTAATGTTCAGCTTCTAA
TTGTCTCTACTTTGTGAGACTACTTTTGAATGCTTGACCTCAAATCAGGTAGGACTACCC
GCTGAACTTAA
>A1_36
TTTCCGTAGGTGAACCTGCGGAAGGATCATTATTGAATTATGTTTCTAGATAGGTTGTAG
CTGGCTCTTTTAGAGCATGTGCACGCCTGTTTGGACTTCATTTTCATCCACCTGTGCACC
TATTGTAGTCTTTGGTTGGGTTAGGAGGAAGTGATCATTGTATCAGCATCTGCTGGGAGT
GAGGACTTGCATTGTGAAAGCTTTGCTGTCCTTGATGTGATCATGGAATCTTTTTCTCAC
TAGAGTCTATGTCACTCATTATACTCTGTCGAATGTCATTGAATGTCTTTACATGGGCTT
GTATGCCTATGAAAATTGTAATACAACTTTCAGCAACGGATCTCTTGGCTCTCGCATCGA
TGAAGAACGCAGCGAAATGCGATAAGTAATGTGAATTGCAGAATTCAGTGAATCATCGAA
TCTTTGAACGCATCTTGCGCTCCTTGGTATTCCGAGGAGCATGCCTGTTTGAGTGTCATT
AAATTCTCAACTCTCTTATACTTTTTTGTAAAAGAGAGCTTGGACTGTGGAGGCTTGCTG
GCCACTTTTTGGGGTCAGCTCCTCTGAAATGCATTAGCGGAACCGTTTGCGATCTGCCAC
AAGTGTGATAAGTTATCTACACTGGCGAGGGGATTGCTCTCTGTAATGTTCAGCTTCTAA
TTGTCTCTACTTTGTGAGACTACTTTTGAATGCTTGACCTCAAATCAGGTAGGACTACCC
GCTGAACTTAA
>A1_49
TTTCCGTAGGTGAACCTGCGGAAGGATCATTATTGAATTATGTTTCTAGATAGGTTGTAG
CTGGCTCTTTTAGAGCATGTGCACGCCTGTTTGGACTTCATTTTCATCCACCTGTGCACC
TATTGTAGTCTTTGGTTGGGTTAGGAGGAAGTGATCATTGTATCAGCATCTGCTGGGAGT
GAGGACTTGCATTGTGAAAGCTTTGCTGTCCTTGATGTGATCATGGAATCTTTTTCTCAC
TAGAGTCTATGTCACTCATTATACTCTGTCGAATGTCATTGAATGTCTTTACATGGGCTT
GTATGCCTATGAAAATTGTAATACAACTTTCAGCAACGGATCTCTTGGCTCTCGCATCGA
TGAAGAACGCAGCGAAATGCGATAAGTAATGTGAATTGCAGAATTCAGTGAATCATCGAA
TCTTTGAACGCATCTTGCGCTCCTTGGTATTCCGAGGAGCATGCCTGTTTGAGTGTCATT
AAATTCTCAACTCTCTTATACTTTTTTGTAAAAGAGAGCTTGGACTGTGGAGGCTTGCTG
GCCACTTTTTGGGGTCAGCTCCTCTGAAATGCATTAGCGGAACCGTTTGCGATCTGCCAC
AAGTGTGATAAGTTATCTACACTGGCGAGGGGATTGCTCTCTGTAATGTTCAGCTTCTAA
TTGTCTCTACTTTGTGAGACTACTTTTGAATGCTTGACCTCAAATCAGGTAGGACTACCC
GCTGAACTTAA
>A1_50
TTTCCGTAGGTGAACCTGCGGAAGGATCATTATTGAATTATGTTTCTAGATAGGTTGTAG
CTGGCTCTTTTAGAGCATGTGCACGCCTGTTTGGACTTCATTTTCATCCACCTGTGCACC
TATTGTAGTCTTTGGTTGGGTTAGGAGGAAGTGATCATTGTATCAGCATCTGCTGGGAGT
GAGGACTTGCATTGTGAAAGCTTTGCTGTCCTTGATGTGATCATGGAATCTTTTTCTCAC
TAGAGTCTATGTCACTCATTATACTCTGTCGAATGTCATTGAATGTCTTTACATGGGCTT
GTATGCCTATGAAAATTGTAATACAACTTTCAGCAACGGATCTCTTGGCTCTCGCATCGA
TGAAGAACGCAGCGAAATGCGATAAGTAATGTGAATTGCAGAATTCAGTGAATCATCGAA
TCTTTGAACGCATCTTGCGCTCCTTGGTATTCCGAGGAGCATGCCTGTTTGAGTGTCATT
AAATTCTCAACTCTCTTATACTTTTTTGTAAAAGAGAGCTTGGACTGTGGAGGCTTGCTG

GCCACTTTTTGGGGTCAGCTCCTCTGAAATGCATTAGCGGAACCGTTTGCGATCTGCCAC
AAGTGTGATAAGTTATCTACACTGGCGAGGGGATTGCTCTCTGTAATGTTCAGCTTCTAA
TTGTCTCTACTTTGTGAGACTACTTTTGAATGCTTGACCTCAAATCAGGTAGGACTACCC
GCTGAACTTAA
>A1-51
TTTCCGTAGGTGAACCTGCGGAAGGATCATTATTGAATTATGTTTCTAGATAGGTTGTAG
CTGGCTCTTTTAGAGCATGTGCACGCCTGTTTGGACTTCATTTTCATCCACCTGTGCACC
TATTGTAGTCTTTGGTTGGGTTAGGAGGAAGTGATCATTGTATCAGCATCTGCTGGGAGT
GAGGACTTGCATTGTGAAAGCTTTGCTGTCCTTGATGTGATCATGGAATCTTTTTCTCAC
TAGAGTCTATGTCACTCATTATACTCTGTCGAATGTCATTGAATGTCTTTACATGGGCTT
GTATGCCTATGAAAATTGTAATACAACTTTCAGCAACGGATCTCTTGGCTCTCGCATCGA
TGAAGAACGCAGCGAAATGCGATAAGTAATGTGAATTGCAGAATTCAGTGAATCATCGAA
TCTTTGAACGCATCTTGCGCTCCTTGGTATTCCGAGGAGCATGCCTGTTTGAGTGTCATT
AAATTCTCAACTCTCTTATACTTTTTTGTAAAAGAGAGCTTGGACTGTGGAGGCTTGCTG
GCCACTTTTTGGGGTCAGCTCCTCTGAAATGCATTAGCGGAACCGTTTGCGATCTGCCAC
AAGTGTGATAAGTTATCTACACTGGCGAGGGGATTGCTCTCTGTAATGTTCAGCTTCTAA
TTGTCTCTACTTTGTGAGACTACTTTTGAATGCTTGACCTCAAATCAGGTAGGACTACCC
GCTGAACTTAA
>A1-52
TTTCCGTAGGTGAACCTGCGGAAGGATCATTATTGAATTATGTTTCTAGATAGGTTGTAG
CTGGCTCTTTTAGAGCATGTGCACGCCTGTTTGGACTTCATTTTCATCCACCTGTGCACC
TATTGTAGTCTTTGGTTGGGTTAGGAGGAAGTGATCATTGTATCAGCATCTGCTGGGAGT
GAGGACTTGCATTGTGAAAGCTTTGCTGTCCTTGATGTGATCATGGAATCTTTTTCTCAC
TAGAGTCTATGTCACTCATTATACTCTGTCGAATGTCATTGAATGTCTTTACATGGGCTT
GTATGCCTATGAAAATTGTAATACAACTTTCAGCAACGGATCTCTTGGCTCTCGCATCGA
TGAAGAACGCAGCGAAATGCGATAAGTAATGTGAATTGCAGAATTCAGTGAATCATCGAA
TCTTTGAACGCATCTTGCGCTCCTTGGTATTCCGAGGAGCATGCCTGTTTGAGTGTCATT
AAATTCTCAACTCTCTTATACTTTTTTGTAAAAGAGAGCTTGGACTGTGGAGGCTTGCTG
GCCACTTTTTGGGGTCAGCTCCTCTGAAATGCATTAGCGGAACCGTTTGCGATCTGCCAC
AAGTGTGATAAGTTATCTACACTGGCGAGGGGATTGCTCTCTGTAATGTTCAGCTTCTAA
TTGTCTCTACTTTGTGAGACTACTTTTGAATGCTTGACCTCAAATCAGGTAGGACTACCC
GCTGAACTTAA
>A1-53
TTTCCGTAGGTGAACCTGCGGAAGGATCATTATTGAATTATGTTTCTAGATAGGTTGTAG
CTGGCTCTTTTAGAGCATGTGCACGCCTGTTTGGACTTCATTTTCATCCACCTGTGCACC
TATTGTAGTCTTTGGTTGGGTTAGGAGGAAGTGATCATTGTATCAGCATCTGCTGGGAGT
GAGGACTTGCATTGTGAAAGCTTTGCTGTCCTTGATGTGATCATGGAATCTTTTTCTCAC
TAGAGTCTATGTCACTCATTATACTCTGTCGAATGTCATTGAATGTCTTTACATGGGCTT
GTATGCCTATGAAAATTGTAATACAACTTTCAGCAACGGATCTCTTGGCTCTCGCATCGA
TGAAGAACGCAGCGAAATGCGATAAGTAATGTGAATTGCAGAATTCAGTGAATCATCGAA
TCTTTGAACGCATCTTGCGCTCCTTGGTATTCCGAGGAGCATGCCTGTTTGAGTGTCATT
AAATTCTCAACTCTCTTATACTTTTTTGTAAAAGAGAGCTTGGACTGTGGAGGCTTGCTG
GCCACTTTTTGGGGTCAGCTCCTCTGAAATGCATTAGCGGAACCGTTTGCGATCTGCCAC
AAGTGTGATAAGTTATCTACACTGGCGAGGGGATTGCTCTCTGTAATGTTCAGCTTCTAA
TTGTCTCTACTTTGTGAGACTACTTTTGAATGCTTGACCTCAAATCAGGTAGGACTACCC
GCTGAACTTAA
>A1-54
TTTCCGTAGGTGAACCTGCGGAAGGATCATTATTGAATTATGTTTCTAGATAGGTTGTAG
CTGGCTCTTTTAGAGCATGTGCACGCCTGTTTGGACTTCATTTTCATCCACCTGTGCACC
TATTGTAGTCTTTGGTTGGGTTAGGAGGAAGTGATCATTGTATCAGCATCTGCTGGGAGT

GAGGACTTGCATTGTGAAAGCTTTGCTGTCCTTGATGTGATCATGGAATCTTTTTCTCAC
TAGAGTCTATGTCACTCATTATACTCTGTCGAATGTCATTGAATGTCTTTACATGGGCTT
GTATGCCTATGAAAATTGTAATACAACTTTCAGCAACGGATCTCTTGGCTCTCGCATCGA
TGAAGAACGCAGCGAAATGCGATAAGTAATGTGAATTGCAGAATTCAGTGAATCATCGAA
TCTTTGAACGCATCTTGCGCTCCTTGGTATTCCGAGGAGCATGCCTGTTTGAGTGTCATT
AAATTCTCAACTCTCTTATACTTTTTTGTAAAAGAGAGCTTGGACTGTGGAGGCTTGCTG
GCCACTTTTTGGGGTCAGCTCCTCTGAAATGCATTAGCGGAACCGTTTGCGATCTGCCAC
AAGTGTGATAAGTTATCTACACTGGCGAGGGGATTGCTCTCTGTAATGTTCAGCTTCTAA
TTGTCTCTACTTTGTGAGACTACTTTTGAATGCTTGACCTCAAATCAGGTAGGACTACCC
GCTGAACTTAA
>A1–55
TTTCCGTAGGTGAACCTGCGGAAGGATCATTATTGAATTATGTTTCTAGATAGGTTGTAG
CTGGCTCTTTTAGAGCATGTGCACGCCTGTTTGGACTTCATTTTCATCCACCTGTGCACC
TATTGTAGTCTTTGGTTGGGTTAGGAGGAAGTGATCATTGTATCAGCATCTGCTGGGAGT
GAGGACTTGCATTGTGAAAGCTTTGCTGTCCTTGATGTGATCATGGAATCTTTTTCTCAC
TAGAGTCTATGTCACTCATTATACTCTGTCGAATGTCATTGAATGTCTTTACATGGGCTT
GTATGCCTATGAAAATTGTAATACAACTTTCAGCAACGGATCTCTTGGCTCTCGCATCGA
TGAAGAACGCAGCGAAATGCGATAAGTAATGTGAATTGCAGAATTCAGTGAATCATCGAA
TCTTTGAACGCATCTTGCGCTCCTTGGTATTCCGAGGAGCATGCCTGTTTGAGTGTCATT
AAATTCTCAACTCTCTTATACTTTTTTGTAAAAGAGAGCTTGGACTGTGGAGGCTTGCTG
GCCACTTTTTGGGGTCAGCTCCTCTGAAATGCATTAGCGGAACCGTTTGCGATCTGCCAC
AAGTGTGATAAGTTATCTACACTGGCGAGGGGATTGCTCTCTGTAATGTTCAGCTTCTAA
TTGTCTCTACTTTGTGAGACTACTTTTGAATGCTTGACCTCAAATCAGGTAGGACTACCC
GCTGAACTTAA
>A1–56
TTTCCGTAGGTGAACCTGCGGAAGGATCATTATTGAATTATGTTTCTAGATAGGTTGTAG
CTGGCTCTTTTAGAGCATGTGCACGCCTGTTTGGACTTCATTTTCATCCACCTGTGCACC
TATTGTAGTCTTTGGTTGGGTTAGGAGGAAGTGATCATTGTATCAGCATCTGCTGGGAGT
GAGGACTTGCATTGTGAAAGCTTTGCTGTCCTTGATGTGATCATGGAATCTTTTTCTCAC
TAGAGTCTATGTCACTCATTATACTCTGTCGAATGTCATTGAATGTCTTTACATGGGCTT
GTATGCCTATGAAAATTGTAATACAACTTTCAGCAACGGATCTCTTGGCTCTCGCATCGA
TGAAGAACGCAGCGAAATGCGATAAGTAATGTGAATTGCAGAATTCAGTGAATCATCGAA
TCTTTGAACGCATCTTGCGCTCCTTGGTATTCCGAGGAGCATGCCTGTTTGAGTGTCATT
AAATTCTCAACTCTCTTATACTTTTTTGTAAAAGAGAGCTTGGACTGTGGAGGCTTGCTG
GCCACTTTTTGGGGTCAGCTCCTCTGAAATGCATTAGCGGAACCGTTTGCGATCTGCCAC
AAGTGTGATAAGTTATCTACACTGGCGAGGGGATTGCTCTCTGTAATGTTCAGCTTCTAA
TTGTCTCTACTTTGTGAGACTACTTTTGAATGCTTGACCTCAAATCAGGTAGGACTACCC
GCTGAACTTAA
>A1–57
TTTCCGTAGGTGAACCTGCGGAAGGATCATTATTGAATTATGTTTCTAGATAGGTTGTAG
CTGGCTCTTTTAGAGCATGTGCACGCCTGTTTGGACTTCATTTTCATCCACCTGTGCACC
TATTGTAGTCTTTGGTTGGGTTAGGAGGAAGTGATCATTGTATCAGCATCTGCTGGGAGT
GAGGACTTGCATTGTGAAAGCTTTGCTGTCCTTGATGTGATCATGGAATCTTTTTCTCAC
TAGAGTCTATGTCACTCATTATACTCTGTCGAATGTCATTGAATGTCTTTACATGGGCTT
GTATGCCTATGAAAATTGTAATACAACTTTCAGCAACGGATCTCTTGGCTCTCGCATCGA
TGAAGAACGCAGCGAAATGCGATAAGTAATGTGAATTGCAGAATTCAGTGAATCATCGAA
TCTTTGAACGCATCTTGCGCTCCTTGGTATTCCGAGGAGCATGCCTGTTTGAGTGTCATT
AAATTCTCAACTCTCTTATACTTTTTTGTAAAAGAGAGCTTGGACTGTGGAGGCTTGCTG
GCCACTTTTTGGGGTCAGCTCCTCTGAAATGCATTAGCGGAACCGTTTGCGATCTGCCAC
AAGTGTGATAAGTTATCTACACTGGCGAGGGGATTGCTCTCTGTAATGTTCAGCTTCTAA

TTGTCTCTACTTTGTGAGACTACTTTTGAATGCTTGACCTCAAATCAGGTAGGACTACCC
GCTGAACTTAA
>A1-58
TTTCCGTAGGTGAACCTGCGGAAGGATCATTATTGAATTATGTTTCTAGATAGGTTGTAG
CTGGCTCTTTTAGAGCATGTGCACGCCTGTTTGGACTTCATTTTCATCCACCTGTGCACC
TATTGTAGTCTTTGGTTGGGTTAGGAGGAAGTGATCATTGTATCAGCATCTGCTGGGAGT
GAGGACTTGCATTGTGAAAGCTTTGCTGTCCTTGATGTGATCATGGAATCTTTTTCTCAC
TAGAGTCTATGTCACTCATTATACTCTGTCGAATGTCATTGAATGTCTTTACATGGGCTT
GTATGCCTATGAAAATTGTAATACAACTTTCAGCAACGGATCTCTTGGCTCTCGCATCGA
TGAAGAACGCAGCGAAATGCGATAAGTAATGTGAATTGCAGAATTCAGTGAATCATCGAA
TCTTTGAACGCATCTTGCGCTCCTTGGTATTCCGAGGAGCATGCCTGTTTGAGTGTCATT
AAATTCTCAACTCTCTTATACTTTTTTGTAAAAGAGAGCTTGGACTGTGGAGGCTTGCTG
GCCACTTTTTGGGGTCAGCTCCTCTGAAATGCATTAGCGGAACCGTTTGCGATCTGCCAC
AAGTGTGATAAGTTATCTACACTGGCGAGGGGATTGCTCTCTGTAATGTTCAGCTTCTAA
TTGTCTCTACTTTGTGAGACTACTTTTGAATGCTTGACCTCAAATCAGGTAGGACTACCC
GCTGAACTTAA
>A1-59
TTTCCGTAGGTGAACCTGCGGAAGGATCATTATTGAATTATGTTTCTAGATAGGTTGTAG
CTGGCTCTTTTAGAGCATGTGCACGCCTGTTTGGACTTCATTTTCATCCACCTGTGCACC
TATTGTAGTCTTTGGTTGGGTTAGGAGGAAGTGATCATTGTATCAGCATCTGCTGGGAGT
GAGGACTTGCATTGTGAAAGCTTTGCTGTCCTTGATGTGATCATGGAATCTTTTTCTCAC
TAGAGTCTATGTCACTCATTATACTCTGTCGAATGTCATTGAATGTCTTTACATGGGCTT
GTATGCCTATGAAAATTGTAATACAACTTTCAGCAACGGATCTCTTGGCTCTCGCATCGA
TGAAGAACGCAGCGAAATGCGATAAGTAATGTGAATTGCAGAATTCAGTGAATCATCGAA
TCTTTGAACGCATCTTGCGCTCCTTGGTATTCCGAGGAGCATGCCTGTTTGAGTGTCATT
AAATTCTCAACTCTCTTATACTTTTTTGTAAAAGAGAGCTTGGACTGTGGAGGCTTGCTG
GCCACTTTTTGGGGTCAGCTCCTCTGAAATGCATTAGCGGAACCGTTTGCGATCTGCCAC
AAGTGTGATAAGTTATCTACACTGGCGAGGGGATTGCTCTCTGTAATGTTCAGCTTCTAA
TTGTCTCTACTTTGTGAGACTACTTTTGAATGCTTGACCTCAAATCAGGTAGGACTACCC
GCTGAACTTAA
>A1-60
TTTCCGTAGGTGAACCTGCGGAAGGATCATTATTGAATTATGTTTCTAGATAGGTTGTAG
CTGGCTCTTTTAGAGCATGTGCACGCCTGTTTGGACTTCATTTTCATCCACCTGTGCACC
TATTGTAGTCTTTGGTTGGGTTAGGAGGAAGTGATCATTGTATCAGCATCTGCTGGGAGT
GAGGACTTGCATTGTGAAAGCTTTGCTGTCCTTGATGTGATCATGGAATCTTTTTCTCAC
TAGAGTCTATGTCACTCATTATACTCTGTCGAATGTCATTGAATGTCTTTACATGGGCTT
GTATGCCTATGAAAATTGTAATACAACTTTCAGCAACGGATCTCTTGGCTCTCGCATCGA
TGAAGAACGCAGCGAAATGCGATAAGTAATGTGAATTGCAGAATTCAGTGAATCATCGAA
TCTTTGAACGCATCTTGCGCTCCTTGGTATTCCGAGGAGCATGCCTGTTTGAGTGTCATT
AAATTCTCAACTCTCTTATACTTTTTTGTAAAAGAGAGCTTGGACTGTGGAGGCTTGCTG
GCCACTTTTTGGGGTCAGCTCCTCTGAAATGCATTAGCGGAACCGTTTGCGATCTGCCAC
AAGTGTGATAAGTTATCTACACTGGCGAGGGGATTGCTCTCTGTAATGTTCAGCTTCTAA
TTGTCTCTACTTTGTGAGACTACTTTTGAATGCTTGACCTCAAATCAGGTAGGACTACCC
GCTGAACTTAA
>A1-61
TTTCCGTAGGTGAACCTGCGGAAGGATCATTATTGAATTATGTTTCTAGATAGGTTGTAG
CTGGCTCTTTTAGAGCATGTGCACGCCTGTTTGGACTTCATTTTCATCCACCTGTGCACC
TATTGTAGTCTTTGGTTGGGTTAGGAGGAAGTGATCATTGTATCAGCATCTGCTGGGAGT
GAGGACTTGCATTGTGAAAGCTTTGCTGTCCTTGATGTGATCATGGAATCTTTTTCTCAC
TAGAGTCTATGTCACTCATTATACTCTGTCGAATGTCATTGAATGTCTTTACATGGGCTT

GTATGCCTATGAAAATTGTAATACAACTTTCAGCAACGGATCTCTTGGCTCTCGCATCGA
TGAAGAACGCAGCGAAATGCGATAAGTAATGTGAATTGCAGAATTCAGTGAATCATCGAA
TCTTTGAACGCATCTTGCGCTCCTTGGTATTCCGAGGAGCATGCCTGTTTGAGTGTCATT
AAATTCTCAACTCTCTTATACTTTTTTGTAAAAGAGAGCTTGGACTGTGGAGGCTTGCTG
GCCACTTTTTGGGGTCAGCTCCTCTGAAATGCATTAGCGGAACCGTTTGCGATCTGCCAC
AAGTGTGATAAGTTATCTACACTGGCGAGGGGATTGCTCTCTGTAATGTTCAGCTTCTAA
TTGTCTCTACTTTGTGAGACTACTTTTGAATGCTTGACCTCAAATCAGGTAGGACTACCC
GCTGAACTTAA
>A1-69
TTTCCGTAGGTGAACCTGCGGAAGGATCATTATTGAATTATGTTTCTAGATAGGTTGTAG
CTGGCTCTTTTAGAGCATGTGCACGCCTGTTTGGACTTCATTTTCATCCACCTGTGCACC
TATTGTAGTCTTTGGTTGGGTTAGGAGGAAGTGATCATTGTATCAGCATCTGCTGGGAGT
GAGGACTTGCATTGTGAAAGCTTTGCTGTCCTTGATGTGATCATGGAATCTTTTTCTCAC
TAGAGTCTATGTCACTCATTATACTCTGTCGAATGTCATTGAATGTCTTTACATGGGCTT
GTATGCCTATGAAAATTGTAATACAACTTTCAGCAACGGATCTCTTGGCTCTCGCATCGA
TGAAGAACGCAGCGAAATGCGATAAGTAATGTGAATTGCAGAATTCAGTGAATCATCGAA
TCTTTGAACGCATCTTGCGCTCCTTGGTATTCCGAGGAGCATGCCTGTTTGAGTGTCATT
AAATTCTCAACTCTCTTATACTTTTTTGTAAAAGAGAGCTTGGACTGTGGAGGCTTGCTG
GCCACTTTTTGGGGTCAGCTCCTCTGAAATGCATTAGCGGAACCGTTTGCGATCTGCCAC
AAGTGTGATAAGTTATCTACACTGGCGAGGGGATTGCTCTCTGTAATGTTCAGCTTCTAA
TTGTCTCTACTTTGTGAGACTACTTTTGAATGCTTGACCTCAAATCAGGTAGGACTACCC
GCTGAACTTAA
>A1-70
TTTCCGTAGGTGAACCTGCGGAAGGATCATTATTGAATTATGTTTCTAGATAGGTTGTAG
CTGGCTCTTTTAGAGCATGTGCACGCCTGTTTGGACTTCATTTTCATCCACCTGTGCACC
TATTGTAGTCTTTGGTTGGGTTAGGAGGAAGTGATCATTGTATCAGCATCTGCTGGGAGT
GAGGACTTGCATTGTGAAAGCTTTGCTGTCCTTGATGTGATCATGGAATCTTTTTCTCAC
TAGAGTCTATGTCACTCATTATACTCTGTCGAATGTCATTGAATGTCTTTACATGGGCTT
GTATGCCTATGAAAATTGTAATACAACTTTCAGCAACGGATCTCTTGGCTCTCGCATCGA
TGAAGAACGCAGCGAAATGCGATAAGTAATGTGAATTGCAGAATTCAGTGAATCATCGAA
TCTTTGAACGCATCTTGCGCTCCTTGGTATTCCGAGGAGCATGCCTGTTTGAGTGTCATT
AAATTCTCAACTCTCTTATACTTTTTTGTAAAAGAGAGCTTGGACTGTGGAGGCTTGCTG
GCCACTTTTTGGGGTCAGCTCCTCTGAAATGCATTAGCGGAACCGTTTGCGATCTGCCAC
AAGTGTGATAAGTTATCTACACTGGCGAGGGGATTGCTCTCTGTAATGTTCAGCTTCTAA
TTGTCTCTACTTTGTGAGACTACTTTTGAATGCTTGACCTCAAATCAGGTAGGACTACCC
GCTGAACTTAA
>A2_1
TTTCCGTAGGTGAACCTGCGGAAGGATCATTATTGAATTATGTTTCTAGATAGGTTGTAG
CTGGCTCTTTTAGAGCATGTGCACGCCTGTTTGGACTTCATTTTCATCCACCTGTGCACC
TATTGTAGTCTTTGGTTGGGTTAGGAGGAAGTGATCATTGTATCAGCATCTGCTGGGAGT
GAGGACTTGCATTGTGAAAGCTTTGCTGTCCTTGATGTGATCATGGAATCTTTTTCTCAC
TAGAGTCTATGTCACTCATTATACTCTGTCGAATGTCATTGAATGTCTTTACATGGGCTT
GTATGCCTATGAAAATTGTAATACAACTTTCAGCAACGGATCTCTTGGCTCTCGCATCGA
TGAAGAACGCAGCGAAATGCGATAAGTAATGTGAATTGCAGAATTCAGTGAATCATCGAA
TCTTTGAACGCATCTTGCGCTCCTTGGTATTCCGAGGAGCATGCCTGTTTGAGTGTCATT
AAATTCTCAACTCTCTTATACTTTTTTGTAAAAGAGAGCTTGGACTGTGGAGGCTTGCTG
GCCACTTTTTGGGGTCAGCTCCTCTGAAATGCATTAGCGGAACCGTTTGCGATCTGCCAC
AAGTGTGATAAGTTATCTACACTGGCGAGGGGATTGCTCTCTGTAATGTTCAGCTTCTAA
TTGTCTCTACTTTGTGAGACTACTTTTGAATGCTTGACCTCAAATCAGGTAGGACTACCC
GCTGAACTTAA

>A2_2
TTTCCGTAGGTGAACCTGCGGAAGGATCATTATTGAATTATGTTTCTAGATAGGTTGTAG
CTGGCTCTTTTAGAGCATGTGCACGCCTGTTTGGACTTCATTTTCATCCACCTGTGCACC
TATTGTAGTCTTTGGTTGGGTTAGGAGGAAGTGATCATTGTATCAGCATCTGCTGGGAGT
GAGGACTTGCATTGTGAAAGCTTTGCTGTCCTTGATGTGATCATGGAATCTTTTTCTCAC
TAGAGTCTATGTCACTCATTATACTCTGTCGAATGTCATTGAATGTCTTTACATGGGCTT
GTATGCCTATGAAAATTGTAATACAACTTTCAGCAACGGATCTCTTGGCTCTCGCATCGA
TGAAGAACGCAGCGAAATGCGATAAGTAATGTGAATTGCAGAATTCAGTGAATCATCGAA
TCTTTGAACGCATCTTGCGCTCCTTGGTATTCCGAGGAGCATGCCTGTTTGAGTGTCATT
AAATTCTCAACTCTCTTATACTTTTTTGTAAAAGAGAGCTTGGACTGTGGAGGCTTGCTG
GCCACTTTTTGGGGTCAGCTCCTCTGAAATGCATTAGCGGAACCGTTTGCGATCTGCCAC
AAGTGTGATAAGTTATCTACACTGGCGAGGGGATTGCTCTCTGTAATGTTCAGCTTCTAA
TTGTCTCTACTTTGTGAGACTACTTTTGAATGCTTGACCTCAAATCAGGTAGGACTACCC
GCTGAACTTAA
>A2_3
TTTCCGTAGGTGAACCTGCGGAAGGATCATTATTGAATTATGTTTCTAGATAGGTTGTAG
CTGGCTCTTTTAGAGCATGTGCACGCCTGTTTGGACTTCATTTTCATCCACCTGTGCACC
TATTGTAGTCTTTGGTTGGGTTAGGAGGAAGTGATCATTGTATCAGCATCTGCTGGGAGT
GAGGACTTGCATTGTGAAAGCTTTGCTGTCCTTGATGTGATCATGGAATCTTTTTCTCAC
TAGAGTCTATGTCACTCATTATACTCTGTCGAATGTCATTGAATGTCTTTACATGGGCTT
GTATGCCTATGAAAATTGTAATACAACTTTCAGCAACGGATCTCTTGGCTCTCGCATCGA
TGAAGAACGCAGCGAAATGCGATAAGTAATGTGAATTGCAGAATTCAGTGAATCATCGAA
TCTTTGAACGCATCTTGCGCTCCTTGGTATTCCGAGGAGCATGCCTGTTTGAGTGTCATT
AAATTCTCAACTCTCTTATACTTTTTTGTAAAAGAGAGCTTGGACTGTGGAGGCTTGCTG
GCCACTTTTTGGGGTCAGCTCCTCTGAAATGCATTAGCGGAACCGTTTGCGATCTGCCAC
AAGTGTGATAAGTTATCTACACTGGCGAGGGGATTGCTCTCTGTAATGTTCAGCTTCTAA
TTGTCTCTACTTTGTGAGACTACTTTTGAATGCTTGACCTCAAATCAGGTAGGACTACCC
GCTGAACTTAA
>A1_37
TTTCCGTAGGTGAACCTGCGGAAGGATCATTATTGAATTATGTTTCTAGATAGGTTGTAG
CTGGCTCTTTTAGAGCATGTGCACGCCTGTTTGGACTTCATTTTCATCCACCTGTGCACC
TATTGTAGTCTTTGGTTGGGTTAGGAGGAAGTGATCATTGTATCAGCATCTGCTGGGAGT
GAGGACTTGCATTGTGAAAGCTTTGCTGTCCTTGATGTGATCATGGAATCTTTTTCTCAC
TAGAGTCTATGTCACTCATTATACTCTGTCGAATGTCATTGAATGTCTTTACATGGGCTT
GTATGCCTATGAAAATTGTAATACAACTTTCAGCAACGGATCTCTTGGCTCTCGCATCGA
TGAAGAACGCAGCGAAATGCGATAAGTAATGTGAATTGCAGAATTCAGTGAATCATCGAA
TCTTTGAACGCATCTTGCGCTCCTTGGTATTCCGAGGAGCATGCCTGTTTGAGTGTCATT
AAATTCTCAACTCTCTTATACTTTTTTGTAAAAGAGAGCTTGGACTGTGGAGGCTTGCTG
GCCACTTTTTGGGGTCAGCTCCTCTGAAATGCATTAGCGGAACCGTTTGCGATCTGCCAC
AAGTGTGATAAGTTATCTACACTGGCGAGGGGATTGCTCTCTGTAATGTTCAGCTTCTAA
TTGTCTCTACTTTGTGAGACTACTTTTGAATGCTTGACCTCAAATCAGGTAGGACTACCC
GCTGAACTTAA
>A1_38
TTTCCGTAGGTGAACCTGCGGAAGGATCATTATTGAATTATGTTTCTAGATAGGTTGTAG
CTGGCTCTTTTAGAGCATGTGCACGCCTGTTTGGACTTCATTTTCATCCACCTGTGCACC
TATTGTAGTCTTTGGTTGGGTTAGGAGGAAGTGATCATTGTATCAGCATCTGCTGGGAGT
GAGGACTTGCATTGTGAAAGCTTTGCTGTCCTTGATGTGATCATGGAATCTTTTTCTCAC
TAGAGTCTATGTCACTCATTATACTCTGTCGAATGTCATTGAATGTCTTTACATGGGCTT
GTATGCCTATGAAAATTGTAATACAACTTTCAGCAACGGATCTCTTGGCTCTCGCATCGA
TGAAGAACGCAGCGAAATGCGATAAGTAATGTGAATTGCAGAATTCAGTGAATCATCGAA

TCTTTGAACGCATCTTGCGCTCCTTGGTATTCCGAGGAGCATGCCTGTTTGAGTGTCATT
AAATTCTCAACTCTCTTATACTTTTTTGTAAAAGAGAGCTTGGACTGTGGAGGCTTGCTG
GCCACTTTTTGGGGTCAGCTCCTCTGAAATGCATTAGCGGAACCGTTTGCGATCTGCCAC
AAGTGTGATAAGTTATCTACACTGGCGAGGGGATTGCTCTCTGTAATGTTCAGCTTCTAA
TTGTCTCTACTTTGTGAGACTACTTTTGAATGCTTGACCTCAAATCAGGTAGGACTACCC
GCTGAACTTAA
>A1_39
TTTCCGTAGGTGAACCTGCGGAAGGATCATTATTGAATTATGTTTCTAGATAGGTTGTAG
CTGGCTCTTTTAGAGCATGTGCACGCCTGTTTGGACTTCATTTTCATCCACCTGTGCACC
TATTGTAGTCTTTGGTTGGGTTAGGAGGAAGTGATCATTGTATCAGCATCTGCTGGGAGT
GAGGACTTGCATTGTGAAAGCTTTGCTGTCCTTGATGTGATCATGGAATCTTTTTCTCAC
TAGAGTCTATGTCACTCATTATACTCTGTCGAATGTCATTGAATGTCTTTACATGGGCTT
GTATGCCTATGAAAATTGTAATACAACTTTCAGCAACGGATCTCTTGGCTCTCGCATCGA
TGAAGAACGCAGCGAAATGCGATAAGTAATGTGAATTGCAGAATTCAGTGAATCATCGAA
TCTTTGAACGCATCTTGCGCTCCTTGGTATTCCGAGGAGCATGCCTGTTTGAGTGTCATT
AAATTCTCAACTCTCTTATACTTTTTTGTAAAAGAGAGCTTGGACTGTGGAGGCTTGCTG
GCCACTTTTTGGGGTCAGCTCCTCTGAAATGCATTAGCGGAACCGTTTGCGATCTGCCAC
AAGTGTGATAAGTTATCTACACTGGCGAGGGGATTGCTCTCTGTAATGTTCAGCTTCTAA
TTGTCTCTACTTTGTGAGACTACTTTTGAATGCTTGACCTCAAATCAGGTAGGACTACCC
GCTGAACTTAA
>A1_48
TTTCCGTAGGTGAACCTGCGGAAGGATCATTATTGAATTATGTTTCTAGATAGGTTGTAG
CTGGCTCTTTTAGAGCATGTGCACGCCTGTTTGGACTTCATTTTCATCCACCTGTGCACC
TATTGTAGTCTTTGGTTGGGTTAGGAGGAAGTGATCATTGTATCAGCATCTGCTGGGAGT
GAGGACTTGCATTGTGAAAGCTTTGCTGTCCTTGATGTGATCATGGAATCTTTTTCTCAC
TAGAGTCTATGTCACTCATTATACTCTGTCGAATGTCATTGAATGTCTTTACATGGGCTT
GTATGCCTATGAAAATTGTAATACAACTTTCAGCAACGGATCTCTTGGCTCTCGCATCGA
TGAAGAACGCAGCGAAATGCGATAAGTAATGTGAATTGCAGAATTCAGTGAATCATCGAA
TCTTTGAACGCATCTTGCGCTCCTTGGTATTCCGAGGAGCATGCCTGTTTGAGTGTCATT
AAATTCTCAACTCTCTTATACTTTTTTGTAAAAGAGAGCTTGGACTGTGGAGGCTTGCTG
GCCACTTTTTGGGGTCAGCTCCTCTGAAATGCATTAGCGGAACCGTTTGCGATCTGCCAC
AAGTGTGATAAGTTATCTACACTGGCGAGGGGATTGCTCTCTGTAATGTTCAGCTTCTAA
TTGTCTCTACTTTGTGAGACTACTTTTGAATGCTTGACCTCAAATCAGGTAGGACTACCC
GCTGAACTTAA
>A2_4
TTTCCGTAGGTGAACCTGCGGAAGGATCATTATTGAATTATGTTTCTAGATAGGTTGTAG
CTGGCTCTTTTAGAGCATGTGCACGCCTGTTTGGACTTCATTTTCATCCACCTGTGCACC
TATTGTAGTCTTTGGTTGGGTTAGGAGGAAGTGATCATTGTATCAGCATCTGCTGGGAGT
GAGGACTTGCATTGTGAAAGCTTTGCTGTCCTTGATGTGATCATGGAATCTTTTTCTCAC
TAGAGTCTATGTCACTCATTATACTCTGTCGAATGTCATTGAATGTCTTTACATGGGCTT
GTATGCCTATGAAAATTGTAATACAACTTTCAGCAACGGATCTCTTGGCTCTCGCATCGA
TGAAGAACGCAGCGAAATGCGATAAGTAATGTGAATTGCAGAATTCAGTGAATCATCGAA
TCTTTGAACGCATCTTGCGCTCCTTGGTATTCCGAGGAGCATGCCTGTTTGAGTGTCATT
AAATTCTCAACTCTCTTATACTTTTTTGTAAAAGAGAGCTTGGACTGTGGAGGCTTGCTG
GCCACTTTTTGGGGTCAGCTCCTCTGAAATGCATTAGCGGAACCGTTTGCGATCTGCCAC
AAGTGTGATAAGTTATCTACACTGGCGAGGGGATTGCTCTCTGTAATGTTCAGCTTCTAA
TTGTCTCTACTTTGTGAGACTACTTTTGAATGCTTGACCTCAAATCAGGTAGGACTACCC
GCTGAACTTAA
>A2_5
TTTCCGTAGGTGAACCTGCGGAAGGATCATTATTGAATTATGTTTCTAGATAGGTTGTAG

CTGGCTCTTTTAGAGCATGTGCACGCCTGTTTGGACTTCATTTTCATCCACCTGTGCACC
TATTGTAGTCTTTGGTTGGGTTAGGAGGAAGTGATCATTGTATCAGCATCTGCTGGGAGT
GAGGACTTGCATTGTGAAAGCTTTGCTGTCCTTGATGTGATCATGGAATCTTTTTCTCAC
TAGAGTCTATGTCACTCATTATACTCTGTCGAATGTCATTGAATGTCTTTACATGGGCTT
GTATGCCTATGAAAATTGTAATACAACTTTCAGCAACGGATCTCTTGGCTCTCGCATCGA
TGAAGAACGCAGCGAAATGCGATAAGTAATGTGAATTGCAGAATTCAGTGAATCATCGAA
TCTTTGAACGCATCTTGCGCTCCTTGGTATTCCGAGGAGCATGCCTGTTTGAGTGTCATT
AAATTCTCAACTCTCTTATACTTTTTTGTAAAAGAGAGCTTGGACTGTGGAGGCTTGCTG
GCCACTTTTTGGGGTCAGCTCCTCTGAAATGCATTAGCGGAACCGTTTGCGATCTGCCAC
AAGTGTGATAAGTTATCTACACTGGCGAGGGGATTGCTCTCTGTAATGTTCAGCTTCTAA
TTGTCTCTACTTTGTGAGACTACTTTTGAATGCTTGACCTCAAATCAGGTAGGACTACCC
GCTGAACTTAA
>A2_6
TTTCCGTAGGTGAACCTGCGGAAGGATCATTATTGAATTATGTTTCTAGATAGGTTGTAG
CTGGCTCTTTTAGAGCATGTGCACGCCTGTTTGGACTTCATTTTCATCCACCTGTGCACC
TATTGTAGTCTTTGGTTGGGTTAGGAGGAAGTGATCATTGTATCAGCATCTGCTGGGAGT
GAGGACTTGCATTGTGAAAGCTTTGCTGTCCTTGATGTGATCATGGAATCTTTTTCTCAC
TAGAGTCTATGTCACTCATTATACTCTGTCGAATGTCATTGAATGTCTTTACATGGGCTT
GTATGCCTATGAAAATTGTAATACAACTTTCAGCAACGGATCTCTTGGCTCTCGCATCGA
TGAAGAACGCAGCGAAATGCGATAAGTAATGTGAATTGCAGAATTCAGTGAATCATCGAA
TCTTTGAACGCATCTTGCGCTCCTTGGTATTCCGAGGAGCATGCCTGTTTGAGTGTCATT
AAATTCTCAACTCTCTTATACTTTTTTGTAAAAGAGAGCTTGGACTGTGGAGGCTTGCTG
GCCACTTTTTGGGGTCAGCTCCTCTGAAATGCATTAGCGGAACCGTTTGCGATCTGCCAC
AAGTGTGATAAGTTATCTACACTGGCGAGGGGATTGCTCTCTGTAATGTTCAGCTTCTAA
TTGTCTCTACTTTGTGAGACTACTTTTGAATGCTTGACCTCAAATCAGGTAGGACTACCC
GCTGAACTTAA
>A2_7
TTTCCGTAGGTGAACCTGCGGAAGGATCATTATTGAATTATGTTTCTAGATAGGTTGTAG
CTGGCTCTTTTAGAGCATGTGCACGCCTGTTTGGACTTCATTTTCATCCACCTGTGCACC
TATTGTAGTCTTTGGTTGGGTTAGGAGGAAGTGATCATTGTATCAGCATCTGCTGGGAGT
GAGGACTTGCATTGTGAAAGCTTTGCTGTCCTTGATGTGATCATGGAATCTTTTTCTCAC
TAGAGTCTATGTCACTCATTATACTCTGTCGAATGTCATTGAATGTCTTTACATGGGCTT
GTATGCCTATGAAAATTGTAATACAACTTTCAGCAACGGATCTCTTGGCTCTCGCATCGA
TGAAGAACGCAGCGAAATGCGATAAGTAATGTGAATTGCAGAATTCAGTGAATCATCGAA
TCTTTGAACGCATCTTGCGCTCCTTGGTATTCCGAGGAGCATGCCTGTTTGAGTGTCATT
AAATTCTCAACTCTCTTATACTTTTTTGTAAAAGAGAGCTTGGACTGTGGAGGCTTGCTG
GCCACTTTTTGGGGTCAGCTCCTCTGAAATGCATTAGCGGAACCGTTTGCGATCTGCCAC
AAGTGTGATAAGTTATCTACACTGGCGAGGGGATTGCTCTCTGTAATGTTCAGCTTCTAA
TTGTCTCTACTTTGTGAGACTACTTTTGAATGCTTGACCTCAAATCAGGTAGGACTACCC
GCTGAACTTAA
>A2_8
TTTCCGTAGGTGAACCTGCGGAAGGATCATTATTGAATTATGTTTCTAGATAGGTTGTAG
CTGGCTCTTTTAGAGCATGTGCACGCCTGTTTGGACTTCATTTTCATCCACCTGTGCACC
TATTGTAGTCTTTGGTTGGGTTAGGAGGAAGTGATCATTGTATCAGCATCTGCTGGGAGT
GAGGACTTGCATTGTGAAAGCTTTGCTGTCCTTGATGTGATCATGGAATCTTTTTCTCAC
TAGAGTCTATGTCACTCATTATACTCTGTCGAATGTCATTGAATGTCTTTACATGGGCTT
GTATGCCTATGAAAATTGTAATACAACTTTCAGCAACGGATCTCTTGGCTCTCGCATCGA
TGAAGAACGCAGCGAAATGCGATAAGTAATGTGAATTGCAGAATTCAGTGAATCATCGAA
TCTTTGAACGCATCTTGCGCTCCTTGGTATTCCGAGGAGCATGCCTGTTTGAGTGTCATT
AAATTCTCAACTCTCTTATACTTTTTTGTAAAAGAGAGCTTGGACTGTGGAGGCTTGCTG

GCCACTTTTTGGGGTCAGCTCCTCTGAAATGCATTAGCGGAACCGTTTGCGATCTGCCAC
AAGTGTGATAAGTTATCTACACTGGCGAGGGGATTGCTCTCTGTAATGTTCAGCTTCTAA
TTGTCTCTACTTTGTGAGACTACTTTTGAATGCTTGACCTCAAATCAGGTAGGACTACCC
GCTGAACTTAA
>A1-62
TTTCCGTAGGTGAACCTGCGGAAGGATCATTATTGAATTATGTTTCTAGATAGGTTGTAG
CTGGCTCTTTTAGAGCATGTGCACGCCTGTTTGGACTTCATTTTCATCCACCTGTGCACC
TATTGTAGTCTTTGGTTGGGTTAGGAGGAAGTGATCATTGTATCAGCATCTGCTGGGAGT
GAGGACTTGCATTGTGAAAGCTTTGCTGTCCTTGATGTGATCATGGAATCTTTTTCTCAC
TAGAGTCTATGTCACTCATTATACTCTGTCGAATGTCATTGAATGTCTTTACATGGGCTT
GTATGCCTATGAAAATTGTAATACAACTTTCAGCAACGGATCTCTTGGCTCTCGCATCGA
TGAAGAACGCAGCGAAATGCGATAAGTAATGTGAATTGCAGAATTCAGTGAATCATCGAA
TCTTTGAACGCATCTTGCGCTCCTTGGTATTCCGAGGAGCATGCCTGTTTGAGTGTCATT
AAATTCTCAACTCTCTTATACTTTTTTGTAAAAGAGAGCTTGGACTGTGGAGGCTTGCTG
GCCACTTTTTGGGGTCAGCTCCTCTGAAATGCATTAGCGGAACCGTTTGCGATCTGCCAC
AAGTGTGATAAGTTATCTACACTGGCGAGGGGATTGCTCTCTGTAATGTTCAGCTTCTAA
TTGTCTCTACTTTGTGAGACTACTTTTGAATGCTTGACCTCAAATCAGGTAGGACTACCC
GCTGAACTTAA
>A3_8
TTTCCGTAGGTGAACCTGCGGAAGGATCATTATTGAATTATGTTTCTAGATAGGTTGTAG
CTGGCTCTTTTAGAGCATGTGCACGCCTGTTTGGACTTCATTTTCATCCACCTGTGCACC
TATTGTAGTCTTTGGTTGGGTTAGGAGGAAGTGATCATTGTATCAGCATCTGCTGGGAGT
GAGGACTTGCATTGTGAAAGCTTTGCTGTCCTTGATGTGATCATGGAATCTTTTTCTCAC
TAGAGTCTATGTCACTCATTATACTCTGTCGAATGTCATTGAATGTCTTTACATGGGCTT
GTATGCCTATGAAAATTGTAATACAACTTTCAGCAACGGATCTCTTGGCTCTCGCATCGA
TGAAGAACGCAGCGAAATGCGATAAGTAATGTGAATTGCAGAATTCAGTGAATCATCGAA
TCTTTGAACGCATCTTGCGCTCCTTGGTATTCCGAGGAGCATGCCTGTTTGAGTGTCATT
AAATTCTCAACTCTCTTATACTTTTTTGTAAAAGAGAGCTTGGACTGTGGAGGCTTGCTG
GCCACTTTTTGGGGTCAGCTCCTCTGAAATGCATTAGCGGAACCGTTTGCGATCTGCCAC
AAGTGTGATAAGTTATCTACACTGGCGAGGGGATTGCTCTCTGTAATGTTCAGCTTCTAA
TTGTCTCTACTTTGTGAGACTACTTTTGAATGCTTGACCTCAAATCAGGTAGGACTACCC
GCTGAACTTAA
>A3_9
TTTCCGTAGGTGAACCTGCGGAAGGATCATTATTGAATTATGTTTCTAGATAGGTTGTAG
CTGGCTCTTTTAGAGCATGTGCACGCCTGTTTGGACTTCATTTTCATCCACCTGTGCACC
TATTGTAGTCTTTGGTTGGGTTAGGAGGAAGTGATCATTGTATCAGCATCTGCTGGGAGT
GAGGACTTGCATTGTGAAAGCTTTGCTGTCCTTGATGTGATCATGGAATCTTTTTCTCAC
TAGAGTCTATGTCACTCATTATACTCTGTCGAATGTCATTGAATGTCTTTACATGGGCTT
GTATGCCTATGAAAATTGTAATACAACTTTCAGCAACGGATCTCTTGGCTCTCGCATCGA
TGAAGAACGCAGCGAAATGCGATAAGTAATGTGAATTGCAGAATTCAGTGAATCATCGAA
TCTTTGAACGCATCTTGCGCTCCTTGGTATTCCGAGGAGCATGCCTGTTTGAGTGTCATT
AAATTCTCAACTCTCTTATACTTTTTTGTAAAAGAGAGCTTGGACTGTGGAGGCTTGCTG
GCCACTTTTTGGGGTCAGCTCCTCTGAAATGCATTAGCGGAACCGTTTGCGATCTGCCAC
AAGTGTGATAAGTTATCTACACTGGCGAGGGGATTGCTCTCTGTAATGTTCAGCTTCTAA
TTGTCTCTACTTTGTGAGACTACTTTTGAATGCTTGACCTCAAATCAGGTAGGACTACCC
GCTGAACTTAA
>A3_10
TTTCCGTAGGTGAACCTGCGGAAGGATCATTATTGAATTATGTTTCTAGATAGGTTGTAG
CTGGCTCTTTTAGAGCATGTGCACGCCTGTTTGGACTTCATTTTCATCCACCTGTGCACC
TATTGTAGTCTTTGGTTGGGTTAGGAGGAAGTGATCATTGTATCAGCATCTGCTGGGAGT

GAGGACTTGCATTGTGAAAGCTTTGCTGTCCTTGATGTGATCATGGAATCTTTTTCTCAC
TAGAGTCTATGTCACTCATTATACTCTGTCGAATGTCATTGAATGTCTTTACATGGGCTT
GTATGCCTATGAAAATTGTAATACAACTTTCAGCAACGGATCTCTTGGCTCTCGCATCGA
TGAAGAACGCAGCGAAATGCGATAAGTAATGTGAATTGCAGAATTCAGTGAATCATCGAA
TCTTTGAACGCATCTTGCGCTCCTTGGTATTCCGAGGAGCATGCCTGTTTGAGTGTCATT
AAATTCTCAACTCTCTTATACTTTTTTGTAAAAGAGAGCTTGGACTGTGGAGGCTTGCTG
GCCACTTTTTGGGGTCAGCTCCTCTGAAATGCATTAGCGGAACCGTTTGCGATCTGCCAC
AAGTGTGATAAGTTATCTACACTGGCGAGGGGATTGCTCTCTGTAATGTTCAGCTTCTAA
TTGTCTCTACTTTGTGAGACTACTTTTGAATGCTTGACCTCAAATCAGGTAGGACTACCC
GCTGAACTTAA
>A3_11
TTTCCGTAGGTGAACCTGCGGAAGGATCATTATTGAATTATGTTTCTAGATAGGTTGTAG
CTGGCTCTTTTAGAGCATGTGCACGCCTGTTTGGACTTCATTTTCATCCACCTGTGCACC
TATTGTAGTCTTTGGTTGGGTTAGGAGGAAGTGATCATTGTATCAGCATCTGCTGGGAGT
GAGGACTTGCATTGTGAAAGCTTTGCTGTCCTTGATGTGATCATGGAATCTTTTTCTCAC
TAGAGTCTATGTCACTCATTATACTCTGTCGAATGTCATTGAATGTCTTTACATGGGCTT
GTATGCCTATGAAAATTGTAATACAACTTTCAGCAACGGATCTCTTGGCTCTCGCATCGA
TGAAGAACGCAGCGAAATGCGATAAGTAATGTGAATTGCAGAATTCAGTGAATCATCGAA
TCTTTGAACGCATCTTGCGCTCCTTGGTATTCCGAGGAGCATGCCTGTTTGAGTGTCATT
AAATTCTCAACTCTCTTATACTTTTTTGTAAAAGAGAGCTTGGACTGTGGAGGCTTGCTG
GCCACTTTTTGGGGTCAGCTCCTCTGAAATGCATTAGCGGAACCGTTTGCGATCTGCCAC
AAGTGTGATAAGTTATCTACACTGGCGAGGGGATTGCTCTCTGTAATGTTCAGCTTCTAA
TTGTCTCTACTTTGTGAGACTACTTTTGAATGCTTGACCTCAAATCAGGTAGGACTACCC
GCTGAACTTAA
>A3_12
TTTCCGTAGGTGAACCTGCGGAAGGATCATTATTGAATTATGTTTCTAGATAGGTTGTAG
CTGGCTCTTTTAGAGCATGTGCACGCCTGTTTGGACTTCATTTTCATCCACCTGTGCACC
TATTGTAGTCTTTGGTTGGGTTAGGAGGAAGTGATCATTGTATCAGCATCTGCTGGGAGT
GAGGACTTGCATTGTGAAAGCTTTGCTGTCCTTGATGTGATCATGGAATCTTTTTCTCAC
TAGAGTCTATGTCACTCATTATACTCTGTCGAATGTCATTGAATGTCTTTACATGGGCTT
GTATGCCTATGAAAATTGTAATACAACTTTCAGCAACGGATCTCTTGGCTCTCGCATCGA
TGAAGAACGCAGCGAAATGCGATAAGTAATGTGAATTGCAGAATTCAGTGAATCATCGAA
TCTTTGAACGCATCTTGCGCTCCTTGGTATTCCGAGGAGCATGCCTGTTTGAGTGTCATT
AAATTCTCAACTCTCTTATACTTTTTTGTAAAAGAGAGCTTGGACTGTGGAGGCTTGCTG
GCCACTTTTTGGGGTCAGCTCCTCTGAAATGCATTAGCGGAACCGTTTGCGATCTGCCAC
AAGTGTGATAAGTTATCTACACTGGCGAGGGGATTGCTCTCTGTAATGTTCAGCTTCTAA
TTGTCTCTACTTTGTGAGACTACTTTTGAATGCTTGACCTCAAATCAGGTAGGACTACCC
GCTGAACTTAA
>A3_13
TTTCCGTAGGTGAACCTGCGGAAGGATCATTATTGAATTATGTTTCTAGATAGGTTGTAG
CTGGCTCTTTTAGAGCATGTGCACGCCTGTTTGGACTTCATTTTCATCCACCTGTGCACC
TATTGTAGTCTTTGGTTGGGTTAGGAGGAAGTGATCATTGTATCAGCATCTGCTGGGAGT
GAGGACTTGCATTGTGAAAGCTTTGCTGTCCTTGATGTGATCATGGAATCTTTTTCTCAC
TAGAGTCTATGTCACTCATTATACTCTGTCGAATGTCATTGAATGTCTTTACATGGGCTT
GTATGCCTATGAAAATTGTAATACAACTTTCAGCAACGGATCTCTTGGCTCTCGCATCGA
TGAAGAACGCAGCGAAATGCGATAAGTAATGTGAATTGCAGAATTCAGTGAATCATCGAA
TCTTTGAACGCATCTTGCGCTCCTTGGTATTCCGAGGAGCATGCCTGTTTGAGTGTCATT
AAATTCTCAACTCTCTTATACTTTTTTGTAAAAGAGAGCTTGGACTGTGGAGGCTTGCTG
GCCACTTTTTGGGGTCAGCTCCTCTGAAATGCATTAGCGGAACCGTTTGCGATCTGCCAC
AAGTGTGATAAGTTATCTACACTGGCGAGGGGATTGCTCTCTGTAATGTTCAGCTTCTAA

TTGTCTCTACTTTGTGAGACTACTTTTGAATGCTTGACCTCAAATCAGGTAGGACTACCC
GCTGAACTTAA
>A1–63
TTTCCGTAGGTGAACCTGCGGAAGGATCATTATTGAATTATGTTTCTAGATAGGTTGTAG
CTGGCTCTTTTAGAGCATGTGCACGCCTGTTTGGACTTCATTTTCATCCACCTGTGCACC
TATTGTAGTCTTTGGTTGGGTTAGGAGGAAGTGATCATTGTATCAGCATCTGCTGGGAGT
GAGGACTTGCATTGTGAAAGCTTTGCTGTCCTTGATGTGATCATGGAATCTTTTTCTCAC
TAGAGTCTATGTCACTCATTATACTCTGTCGAATGTCATTGAATGTCTTTACATGGGCTT
GTATGCCTATGAAAATTGTAATACAACTTTCAGCAACGGATCTCTTGGCTCTCGCATCGA
TGAAGAACGCAGCGAAATGCGATAAGTAATGTGAATTGCAGAATTCAGTGAATCATCGAA
TCTTTGAACGCATCTTGCGCTCCTTGGTATTCCGAGGAGCATGCCTGTTTGAGTGTCATT
AAATTCTCAACTCTCTTATACTTTTTTGTAAAAGAGAGCTTGGACTGTGGAGGCTTGCTG
GCCACTTTTTGGGGTCAGCTCCTCTGAAATGCATTAGCGGAACCGTTTGCGATCTGCCAC
AAGTGTGATAAGTTATCTACACTGGCGAGGGGATTGCTCTCTGTAATGTTCAGCTTCTAA
TTGTCTCTACTTTGTGAGACTACTTTTGAATGCTTGACCTCAAATCAGGTAGGACTACCC
GCTGAACTTAA
>A1–64
TTTCCGTAGGTGAACCTGCGGAAGGATCATTATTGAATTATGTTTCTAGATAGGTTGTAG
CTGGCTCTTTTAGAGCATGTGCACGCCTGTTTGGACTTCATTTTCATCCACCTGTGCACC
TATTGTAGTCTTTGGTTGGGTTAGGAGGAAGTGATCATTGTATCAGCATCTGCTGGGAGT
GAGGACTTGCATTGTGAAAGCTTTGCTGTCCTTGATGTGATCATGGAATCTTTTTCTCAC
TAGAGTCTATGTCACTCATTATACTCTGTCGAATGTCATTGAATGTCTTTACATGGGCTT
GTATGCCTATGAAAATTGTAATACAACTTTCAGCAACGGATCTCTTGGCTCTCGCATCGA
TGAAGAACGCAGCGAAATGCGATAAGTAATGTGAATTGCAGAATTCAGTGAATCATCGAA
TCTTTGAACGCATCTTGCGCTCCTTGGTATTCCGAGGAGCATGCCTGTTTGAGTGTCATT
AAATTCTCAACTCTCTTATACTTTTTTGTAAAAGAGAGCTTGGACTGTGGAGGCTTGCTG
GCCACTTTTTGGGGTCAGCTCCTCTGAAATGCATTAGCGGAACCGTTTGCGATCTGCCAC
AAGTGTGATAAGTTATCTACACTGGCGAGGGGATTGCTCTCTGTAATGTTCAGCTTCTAA
TTGTCTCTACTTTGTGAGACTACTTTTGAATGCTTGACCTCAAATCAGGTAGGACTACCC
GCTGAACTTAA
>A1–65
TTTCCGTAGGTGAACCTGCGGAAGGATCATTATTGAATTATGTTTCTAGATAGGTTGTAG
CTGGCTCTTTTAGAGCATGTGCACGCCTGTTTGGACTTCATTTTCATCCACCTGTGCACC
TATTGTAGTCTTTGGTTGGGTTAGGAGGAAGTGATCATTGTATCAGCATCTGCTGGGAGT
GAGGACTTGCATTGTGAAAGCTTTGCTGTCCTTGATGTGATCATGGAATCTTTTTCTCAC
TAGAGTCTATGTCACTCATTATACTCTGTCGAATGTCATTGAATGTCTTTACATGGGCTT
GTATGCCTATGAAAATTGTAATACAACTTTCAGCAACGGATCTCTTGGCTCTCGCATCGA
TGAAGAACGCAGCGAAATGCGATAAGTAATGTGAATTGCAGAATTCAGTGAATCATCGAA
TCTTTGAACGCATCTTGCGCTCCTTGGTATTCCGAGGAGCATGCCTGTTTGAGTGTCATT
AAATTCTCAACTCTCTTATACTTTTTTGTAAAAGAGAGCTTGGACTGTGGAGGCTTGCTG
GCCACTTTTTGGGGTCAGCTCCTCTGAAATGCATTAGCGGAACCGTTTGCGATCTGCCAC
AAGTGTGATAAGTTATCTACACTGGCGAGGGGATTGCTCTCTGTAATGTTCAGCTTCTAA
TTGTCTCTACTTTGTGAGACTACTTTTGAATGCTTGACCTCAAATCAGGTAGGACTACCC
GCTGAACTTAA
>A1–66
TTTCCGTAGGTGAACCTGCGGAAGGATCATTATTGAATTATGTTTCTAGATAGGTTGTAG
CTGGCTCTTTTAGAGCATGTGCACGCCTGTTTGGACTTCATTTTCATCCACCTGTGCACC
TATTGTAGTCTTTGGTTGGGTTAGGAGGAAGTGATCATTGTATCAGCATCTGCTGGGAGT
GAGGACTTGCATTGTGAAAGCTTTGCTGTCCTTGATGTGATCATGGAATCTTTTTCTCAC
TAGAGTCTATGTCACTCATTATACTCTGTCGAATGTCATTGAATGTCTTTACATGGGCTT

GTATGCCTATGAAAATTGTAATACAACTTTCAGCAACGGATCTCTTGGCTCTCGCATCGA
TGAAGAACGCAGCGAAATGCGATAAGTAATGTGAATTGCAGAATTCAGTGAATCATCGAA
TCTTTGAACGCATCTTGCGCTCCTTGGTATTCCGAGGAGCATGCCTGTTTGAGTGTCATT
AAATTCTCAACTCTCTTATACTTTTTTGTAAAAGAGAGCTTGGACTGTGGAGGCTTGCTG
GCCACTTTTTGGGGTCAGCTCCTCTGAAATGCATTAGCGGAACCGTTTGCGATCTGCCAC
AAGTGTGATAAGTTATCTACACTGGCGAGGGGATTGCTCTCTGTAATGTTCAGCTTCTAA
TTGTCTCTACTTTGTGAGACTACTTTTGAATGCTTGACCTCAAATCAGGTAGGACTACCC
GCTGAACTTAA
>A1-67
TTTCCGTAGGTGAACCTGCGGAAGGATCATTATTGAATTATGTTTCTAGATAGGTTGTAG
CTGGCTCTTTTAGAGCATGTGCACGCCTGTTTGGACTTCATTTTCATCCACCTGTGCACC
TATTGTAGTCTTTGGTTGGGTTAGGAGGAAGTGATCATTGTATCAGCATCTGCTGGGAGT
GAGGACTTGCATTGTGAAAGCTTTGCTGTCCTTGATGTGATCATGGAATCTTTTTCTCAC
TAGAGTCTATGTCACTCATTATACTCTGTCGAATGTCATTGAATGTCTTTACATGGGCTT
GTATGCCTATGAAAATTGTAATACAACTTTCAGCAACGGATCTCTTGGCTCTCGCATCGA
TGAAGAACGCAGCGAAATGCGATAAGTAATGTGAATTGCAGAATTCAGTGAATCATCGAA
TCTTTGAACGCATCTTGCGCTCCTTGGTATTCCGAGGAGCATGCCTGTTTGAGTGTCATT
AAATTCTCAACTCTCTTATACTTTTTTGTAAAAGAGAGCTTGGACTGTGGAGGCTTGCTG
GCCACTTTTTGGGGTCAGCTCCTCTGAAATGCATTAGCGGAACCGTTTGCGATCTGCCAC
AAGTGTGATAAGTTATCTACACTGGCGAGGGGATTGCTCTCTGTAATGTTCAGCTTCTAA
TTGTCTCTACTTTGTGAGACTACTTTTGAATGCTTGACCTCAAATCAGGTAGGACTACCC
GCTGAACTTAA
>A1-68
TTTCCGTAGGTGAACCTGCGGAAGGATCATTATTGAATTATGTTTCTAGATAGGTTGTAG
CTGGCTCTTTTAGAGCATGTGCACGCCTGTTTGGACTTCATTTTCATCCACCTGTGCACC
TATTGTAGTCTTTGGTTGGGTTAGGAGGAAGTGATCATTGTATCAGCATCTGCTGGGAGT
GAGGACTTGCATTGTGAAAGCTTTGCTGTCCTTGATGTGATCATGGAATCTTTTTCTCAC
TAGAGTCTATGTCACTCATTATACTCTGTCGAATGTCATTGAATGTCTTTACATGGGCTT
GTATGCCTATGAAAATTGTAATACAACTTTCAGCAACGGATCTCTTGGCTCTCGCATCGA
TGAAGAACGCAGCGAAATGCGATAAGTAATGTGAATTGCAGAATTCAGTGAATCATCGAA
TCTTTGAACGCATCTTGCGCTCCTTGGTATTCCGAGGAGCATGCCTGTTTGAGTGTCATT
AAATTCTCAACTCTCTTATACTTTTTTGTAAAAGAGAGCTTGGACTGTGGAGGCTTGCTG
GCCACTTTTTGGGGTCAGCTCCTCTGAAATGCATTAGCGGAACCGTTTGCGATCTGCCAC
AAGTGTGATAAGTTATCTACACTGGCGAGGGGATTGCTCTCTGTAATGTTCAGCTTCTAA
TTGTCTCTACTTTGTGAGACTACTTTTGAATGCTTGACCTCAAATCAGGTAGGACTACCC
GCTGAACTTAA
>A2_9
TTTCCGTAGGTGAACCTGCGGAAGGATCATTATTGAATTATGTTTCTAGATAGGTTGTAG
CTGGCTCTTTTAGAGCATGTGCACGCCTGTTTGGACTTCATTTTCATCCACCTGTGCACC
TATTGTAGTCTTTGGTTGGGTTAGGAGGAAGTGATCATTGTATCAGCATCTGCTGGGAGT
GAGGACTTGCATTGTGAAAGCTTTGCTGTCCTTGATGTGATCATGGAATCTTTTTCTCAC
TAGAGTCTATGTCACTCATTATACTCTGTCGAATGTCATTGAATGTCTTTACATGGGCTT
GTATGCCTATGAAAATTGTAATACAACTTTCAGCAACGGATCTCTTGGCTCTCGCATCGA
TGAAGAACGCAGCGAAATGCGATAAGTAATGTGAATTGCAGAATTCAGTGAATCATCGAA
TCTTTGAACGCATCTTGCGCTCCTTGGTATTCCGAGGAGCATGCCTGTTTGAGTGTCATT
AAATTCTCAACTCTCTTATACTTTTTTGTAAAAGAGAGCTTGGACTGTGGAGGCTTGCTG
GCCACTTTTTGGGGTCAGCTCCTCTGAAATGCATTAGCGGAACCGTTTGCGATCTGCCAC
AAGTGTGATAAGTTATCTACACTGGCGAGGGGATTGCTCTCTGTAATGTTCAGCTTCTAA
TTGTCTCTACTTTGTGAGACTACTTTTGAATGCTTGACCTCAAATCAGGTAGGACTACCC
GCTGAACTTAA

>A2_10
TTTCCGTAGGTGAACCTGCGGAAGGATCATTATTGAATTATGTTTCTAGATAGGTTGTAG
CTGGCTCTTTTAGAGCATGTGCACGCCTGTTTGGACTTCATTTTCATCCACCTGTGCACC
TATTGTAGTCTTTGGTTGGGTTAGGAGGAAGTGATCATTGTATCAGCATCTGCTGGGAGT
GAGGACTTGCATTGTGAAAGCTTTGCTGTCCTTGATGTGATCATGGAATCTTTTTCTCAC
TAGAGTCTATGTCACTCATTATACTCTGTCGAATGTCATTGAATGTCTTTACATGGGCTT
GTATGCCTATGAAAATTGTAATACAACTTTCAGCAACGGATCTCTTGGCTCTCGCATCGA
TGAAGAACGCAGCGAAATGCGATAAGTAATGTGAATTGCAGAATTCAGTGAATCATCGAA
TCTTTGAACGCATCTTGCGCTCCTTGGTATTCCGAGGAGCATGCCTGTTTGAGTGTCATT
AAATTCTCAACTCTCTTATACTTTTTGTAAAAGAGAGCTTGGACTGTGGAGGCTTGCTG
GCCACTTTTTGGGGTCAGCTCCTCTGAAATGCATTAGCGGAACCGTTTGCGATCTGCCAC
AAGTGTGATAAGTTATCTACACTGGCGAGGGGATTGCTCTCTGTAATGTTCAGCTTCTAA
TTGTCTCTACTTTGTGAGACTACTTTTGAATGCTTGACCTCAAATCAGGTAGGACTACCC
GCTGAACTTAA
>A2_11
TTTCCGTAGGTGAACCTGCGGAAGGATCATTATTGAATTATGTTTCTAGATAGGTTGTAG
CTGGCTCTTTTAGAGCATGTGCACGCCTGTTTGGACTTCATTTTCATCCACCTGTGCACC
TATTGTAGTCTTTGGTTGGGTTAGGAGGAAGTGATCATTGTATCAGCATCTGCTGGGAGT
GAGGACTTGCATTGTGAAAGCTTTGCTGTCCTTGATGTGATCATGGAATCTTTTTCTCAC
TAGAGTCTATGTCACTCATTATACTCTGTCGAATGTCATTGAATGTCTTTACATGGGCTT
GTATGCCTATGAAAATTGTAATACAACTTTCAGCAACGGATCTCTTGGCTCTCGCATCGA
TGAAGAACGCAGCGAAATGCGATAAGTAATGTGAATTGCAGAATTCAGTGAATCATCGAA
TCTTTGAACGCATCTTGCGCTCCTTGGTATTCCGAGGAGCATGCCTGTTTGAGTGTCATT
AAATTCTCAACTCTCTTATACTTTTTGTAAAAGAGAGCTTGGACTGTGGAGGCTTGCTG
GCCACTTTTTGGGGTCAGCTCCTCTGAAATGCATTAGCGGAACCGTTTGCGATCTGCCAC
AAGTGTGATAAGTTATCTACACTGGCGAGGGGATTGCTCTCTGTAATGTTCAGCTTCTAA
TTGTCTCTACTTTGTGAGACTACTTTTGAATGCTTGACCTCAAATCAGGTAGGACTACCC
GCTGAACTTAA
>A2_12
TTTCCGTAGGTGAACCTGCGGAAGGATCATTATTGAATTATGTTTCTAGATAGGTTGTAG
CTGGCTCTTTTAGAGCATGTGCACGCCTGTTTGGACTTCATTTTCATCCACCTGTGCACC
TATTGTAGTCTTTGGTTGGGTTAGGAGGAAGTGATCATTGTATCAGCATCTGCTGGGAGT
GAGGACTTGCATTGTGAAAGCTTTGCTGTCCTTGATGTGATCATGGAATCTTTTTCTCAC
TAGAGTCTATGTCACTCATTATACTCTGTCGAATGTCATTGAATGTCTTTACATGGGCTT
GTATGCCTATGAAAATTGTAATACAACTTTCAGCAACGGATCTCTTGGCTCTCGCATCGA
TGAAGAACGCAGCGAAATGCGATAAGTAATGTGAATTGCAGAATTCAGTGAATCATCGAA
TCTTTGAACGCATCTTGCGCTCCTTGGTATTCCGAGGAGCATGCCTGTTTGAGTGTCATT
AAATTCTCAACTCTCTTATACTTTTTGTAAAAGAGAGCTTGGACTGTGGAGGCTTGCTG
GCCACTTTTTGGGGTCAGCTCCTCTGAAATGCATTAGCGGAACCGTTTGCGATCTGCCAC
AAGTGTGATAAGTTATCTACACTGGCGAGGGGATTGCTCTCTGTAATGTTCAGCTTCTAA
TTGTCTCTACTTTGTGAGACTACTTTTGAATGCTTGACCTCAAATCAGGTAGGACTACCC
GCTGAACTTAA
>A2_13
TTTCCGTAGGTGAACCTGCGGAAGGATCATTATTGAATTATGTTTCTAGATAGGTTGTAG
CTGGCTCTTTTAGAGCATGTGCACGCCTGTTTGGACTTCATTTTCATCCACCTGTGCACC
TATTGTAGTCTTTGGTTGGGTTAGGAGGAAGTGATCATTGTATCAGCATCTGCTGGGAGT
GAGGACTTGCATTGTGAAAGCTTTGCTGTCCTTGATGTGATCATGGAATCTTTTTCTCAC
TAGAGTCTATGTCACTCATTATACTCTGTCGAATGTCATTGAATGTCTTTACATGGGCTT
GTATGCCTATGAAAATTGTAATACAACTTTCAGCAACGGATCTCTTGGCTCTCGCATCGA
TGAAGAACGCAGCGAAATGCGATAAGTAATGTGAATTGCAGAATTCAGTGAATCATCGAA

TCTTTGAACGCATCTTGCGCTCCTTGGTATTCCGAGGAGCATGCCTGTTTGAGTGTCATT
AAATTCTCAACTCTCTTATACTTTTTTGTAAAAGAGAGCTTGGACTGTGGAGGCTTGCTG
GCCACTTTTTGGGGTCAGCTCCTCTGAAATGCATTAGCGGAACCGTTTGCGATCTGCCAC
AAGTGTGATAAGTTATCTACACTGGCGAGGGGATTGCTCTCTGTAATGTTCAGCTTCTAA
TTGTCTCTACTTTGTGAGACTACTTTTGAATGCTTGACCTCAAATCAGGTAGGACTACCC
GCTGAACTTAA
>A2_14
TTTCCGTAGGTGAACCTGCGGAAGGATCATTATTGAATTATGTTTCTAGATAGGTTGTAG
CTGGCTCTTTTAGAGCATGTGCACGCCTGTTTGGACTTCATTTTCATCCACCTGTGCACC
TATTGTAGTCTTTGGTTGGGTTAGGAGGAAGTGATCATTGTATCAGCATCTGCTGGGAGT
GAGGACTTGCATTGTGAAAGCTTTGCTGTCCTTGATGTGATCATGGAATCTTTTTCTCAC
TAGAGTCTATGTCACTCATTATACTCTGTCGAATGTCATTGAATGTCTTTACATGGGCTT
GTATGCCTATGAAAATTGTAATACAACTTTCAGCAACGGATCTCTTGGCTCTCGCATCGA
TGAAGAACGCAGCGAAATGCGATAAGTAATGTGAATTGCAGAATTCAGTGAATCATCGAA
TCTTTGAACGCATCTTGCGCTCCTTGGTATTCCGAGGAGCATGCCTGTTTGAGTGTCATT
AAATTCTCAACTCTCTTATACTTTTTTGTAAAAGAGAGCTTGGACTGTGGAGGCTTGCTG
GCCACTTTTTGGGGTCAGCTCCTCTGAAATGCATTAGCGGAACCGTTTGCGATCTGCCAC
AAGTGTGATAAGTTATCTACACTGGCGAGGGGATTGCTCTCTGTAATGTTCAGCTTCTAA
TTGTCTCTACTTTGTGAGACTACTTTTGAATGCTTGACCTCAAATCAGGTAGGACTACCC
GCTGAACTTAA
>A1_40
TTTCCGTAGGTGAACCTGCGGAAGGATCATTATTGAATTATGTTTCTAGATAGGTTGTAG
CTGGCTCTTTTAGAGCATGTGCACGCCTGTTTGGACTTCATTTTCATCCACCTGTGCACC
TATTGTAGTCTTTGGTTGGGTTAGGAGGAAGTGATCATTGTATCAGCATCTGCTGGGAGT
GAGGACTTGCATTGTGAAAGCTTTGCTGTCCTTGATGTGATCATGGAATCTTTTTCTCAC
TAGAGTCTATGTCACTCATTATACTCTGTCGAATGTCATTGAATGTCTTTACATGGGCTT
GTATGCCTATGAAAATTGTAATACAACTTTCAGCAACGGATCTCTTGGCTCTCGCATCGA
TGAAGAACGCAGCGAAATGCGATAAGTAATGTGAATTGCAGAATTCAGTGAATCATCGAA
TCTTTGAACGCATCTTGCGCTCCTTGGTATTCCGAGGAGCATGCCTGTTTGAGTGTCATT
AAATTCTCAACTCTCTTATACTTTTTTGTAAAAGAGAGCTTGGACTGTGGAGGCTTGCTG
GCCACTTTTTGGGGTCAGCTCCTCTGAAATGCATTAGCGGAACCGTTTGCGATCTGCCAC
AAGTGTGATAAGTTATCTACACTGGCGAGGGGATTGCTCTCTGTAATGTTCAGCTTCTAA
TTGTCTCTACTTTGTGAGACTACTTTTGAATGCTTGACCTCAAATCAGGTAGGACTACCC
GCTGAACTTAA
>A1_41
TTTCCGTAGGTGAACCTGCGGAAGGATCATTATTGAATTATGTTTCTAGATAGGTTGTAG
CTGGCTCTTTTAGAGCATGTGCACGCCTGTTTGGACTTCATTTTCATCCACCTGTGCACC
TATTGTAGTCTTTGGTTGGGTTAGGAGGAAGTGATCATTGTATCAGCATCTGCTGGGAGT
GAGGACTTGCATTGTGAAAGCTTTGCTGTCCTTGATGTGATCATGGAATCTTTTTCTCAC
TAGAGTCTATGTCACTCATTATACTCTGTCGAATGTCATTGAATGTCTTTACATGGGCTT
GTATGCCTATGAAAATTGTAATACAACTTTCAGCAACGGATCTCTTGGCTCTCGCATCGA
TGAAGAACGCAGCGAAATGCGATAAGTAATGTGAATTGCAGAATTCAGTGAATCATCGAA
TCTTTGAACGCATCTTGCGCTCCTTGGTATTCCGAGGAGCATGCCTGTTTGAGTGTCATT
AAATTCTCAACTCTCTTATACTTTTTTGTAAAAGAGAGCTTGGACTGTGGAGGCTTGCTG
GCCACTTTTTGGGGTCAGCTCCTCTGAAATGCATTAGCGGAACCGTTTGCGATCTGCCAC
AAGTGTGATAAGTTATCTACACTGGCGAGGGGATTGCTCTCTGTAATGTTCAGCTTCTAA
TTGTCTCTACTTTGTGAGACTACTTTTGAATGCTTGACCTCAAATCAGGTAGGACTACCC
GCTGAACTTAA
>A1_42
TTTCCGTAGGTGAACCTGCGGAAGGATCATTATTGAATTATGTTTCTAGATAGGTTGTAG

CTGGCTCTTTTAGAGCATGTGCACGCCTGTTTGGACTTCATTTTCATCCACCTGTGCACC
TATTGTAGTCTTTGGTTGGGTTAGGAGGAAGTGATCATTGTATCAGCATCTGCTGGGAGT
GAGGACTTGCATTGTGAAAGCTTTGCTGTCCTTGATGTGATCATGGAATCTTTTTCTCAC
TAGAGTCTATGTCACTCATTATACTCTGTCGAATGTCATTGAATGTCTTTACATGGGCTT
GTATGCCTATGAAAATTGTAATACAACTTTCAGCAACGGATCTCTTGGCTCTCGCATCGA
TGAAGAACGCAGCGAAATGCGATAAGTAATGTGAATTGCAGAATTCAGTGAATCATCGAA
TCTTTGAACGCATCTTGCGCTCCTTGGTATTCCGAGGAGCATGCCTGTTTGAGTGTCATT
AAATTCTCAACTCTCTTATACTTTTTTGTAAAAGAGAGCTTGGACTGTGGAGGCTTGCTG
GCCACTTTTTGGGGTCAGCTCCTCTGAAATGCATTAGCGGAACCGTTTGCGATCTGCCAC
AAGTGTGATAAGTTATCTACACTGGCGAGGGGATTGCTCTCTGTAATGTTCAGCTTCTAA
TTGTCTCTACTTTGTGAGACTACTTTTGAATGCTTGACCTCAAATCAGGTAGGACTACCC
GCTGAACTTAA
>A1_43
TTTCCGTAGGTGAACCTGCGGAAGGATCATTATTGAATTATGTTTCTAGATAGGTTGTAG
CTGGCTCTTTTAGAGCATGTGCACGCCTGTTTGGACTTCATTTTCATCCACCTGTGCACC
TATTGTAGTCTTTGGTTGGGTTAGGAGGAAGTGATCATTGTATCAGCATCTGCTGGGAGT
GAGGACTTGCATTGTGAAAGCTTTGCTGTCCTTGATGTGATCATGGAATCTTTTTCTCAC
TAGAGTCTATGTCACTCATTATACTCTGTCGAATGTCATTGAATGTCTTTACATGGGCTT
GTATGCCTATGAAAATTGTAATACAACTTTCAGCAACGGATCTCTTGGCTCTCGCATCGA
TGAAGAACGCAGCGAAATGCGATAAGTAATGTGAATTGCAGAATTCAGTGAATCATCGAA
TCTTTGAACGCATCTTGCGCTCCTTGGTATTCCGAGGAGCATGCCTGTTTGAGTGTCATT
AAATTCTCAACTCTCTTATACTTTTTTGTAAAAGAGAGCTTGGACTGTGGAGGCTTGCTG
GCCACTTTTTGGGGTCAGCTCCTCTGAAATGCATTAGCGGAACCGTTTGCGATCTGCCAC
AAGTGTGATAAGTTATCTACACTGGCGAGGGGATTGCTCTCTGTAATGTTCAGCTTCTAA
TTGTCTCTACTTTGTGAGACTACTTTTGAATGCTTGACCTCAAATCAGGTAGGACTACCC
GCTGAACTTAA
>A1_44
TTTCCGTAGGTGAACCTGCGGAAGGATCATTATTGAATTATGTTTCTAGATAGGTTGTAG
CTGGCTCTTTTAGAGCATGTGCACGCCTGTTTGGACTTCATTTTCATCCACCTGTGCACC
TATTGTAGTCTTTGGTTGGGTTAGGAGGAAGTGATCATTGTATCAGCATCTGCTGGGAGT
GAGGACTTGCATTGTGAAAGCTTTGCTGTCCTTGATGTGATCATGGAATCTTTTTCTCAC
TAGAGTCTATGTCACTCATTATACTCTGTCGAATGTCATTGAATGTCTTTACATGGGCTT
GTATGCCTATGAAAATTGTAATACAACTTTCAGCAACGGATCTCTTGGCTCTCGCATCGA
TGAAGAACGCAGCGAAATGCGATAAGTAATGTGAATTGCAGAATTCAGTGAATCATCGAA
TCTTTGAACGCATCTTGCGCTCCTTGGTATTCCGAGGAGCATGCCTGTTTGAGTGTCATT
AAATTCTCAACTCTCTTATACTTTTTTGTAAAAGAGAGCTTGGACTGTGGAGGCTTGCTG
GCCACTTTTTGGGGTCAGCTCCTCTGAAATGCATTAGCGGAACCGTTTGCGATCTGCCAC
AAGTGTGATAAGTTATCTACACTGGCGAGGGGATTGCTCTCTGTAATGTTCAGCTTCTAA
TTGTCTCTACTTTGTGAGACTACTTTTGAATGCTTGACCTCAAATCAGGTAGGACTACCC
GCTGAACTTAA
>A1_45
TTTCCGTAGGTGAACCTGCGGAAGGATCATTATTGAATTATGTTTCTAGATAGGTTGTAG
CTGGCTCTTTTAGAGCATGTGCACGCCTGTTTGGACTTCATTTTCATCCACCTGTGCACC
TATTGTAGTCTTTGGTTGGGTTAGGAGGAAGTGATCATTGTATCAGCATCTGCTGGGAGT
GAGGACTTGCATTGTGAAAGCTTTGCTGTCCTTGATGTGATCATGGAATCTTTTTCTCAC
TAGAGTCTATGTCACTCATTATACTCTGTCGAATGTCATTGAATGTCTTTACATGGGCTT
GTATGCCTATGAAAATTGTAATACAACTTTCAGCAACGGATCTCTTGGCTCTCGCATCGA
TGAAGAACGCAGCGAAATGCGATAAGTAATGTGAATTGCAGAATTCAGTGAATCATCGAA
TCTTTGAACGCATCTTGCGCTCCTTGGTATTCCGAGGAGCATGCCTGTTTGAGTGTCATT
AAATTCTCAACTCTCTTATACTTTTTTGTAAAAGAGAGCTTGGACTGTGGAGGCTTGCTG

GCCACTTTTTGGGGTCAGCTCCTCTGAAATGCATTAGCGGAACCGTTTGCGATCTGCCAC
AAGTGTGATAAGTTATCTACACTGGCGAGGGGATTGCTCTCTGTAATGTTCAGCTTCTAA
TTGTCTCTACTTTGTGAGACTACTTTTGAATGCTTGACCTCAAATCAGGTAGGACTACCC
GCTGAACTTAA
>A1_46
TTTCCGTAGGTGAACCTGCGGAAGGATCATTATTGAATTATGTTTCTAGATAGGTTGTAG
CTGGCTCTTTTAGAGCATGTGCACGCCTGTTTGGACTTCATTTTCATCCACCTGTGCACC
TATTGTAGTCTTTGGTTGGGTTAGGAGGAAGTGATCATTGTATCAGCATCTGCTGGGAGT
GAGGACTTGCATTGTGAAAGCTTTGCTGTCCTTGATGTGATCATGGAATCTTTTTCTCAC
TAGAGTCTATGTCACTCATTATACTCTGTCGAATGTCATTGAATGTCTTTACATGGGCTT
GTATGCCTATGAAAATTGTAATACAACTTTCAGCAACGGATCTCTTGGCTCTCGCATCGA
TGAAGAACGCAGCGAAATGCGATAAGTAATGTGAATTGCAGAATTCAGTGAATCATCGAA
TCTTTGAACGCATCTTGCGCTCCTTGGTATTCCGAGGAGCATGCCTGTTTGAGTGTCATT
AAATTCTCAACTCTCTTATACTTTTTTGTAAAAGAGAGCTTGGACTGTGGAGGCTTGCTG
GCCACTTTTTGGGGTCAGCTCCTCTGAAATGCATTAGCGGAACCGTTTGCGATCTGCCAC
AAGTGTGATAAGTTATCTACACTGGCGAGGGGATTGCTCTCTGTAATGTTCAGCTTCTAA
TTGTCTCTACTTTGTGAGACTACTTTTGAATGCTTGACCTCAAATCAGGTAGGACTACCC
GCTGAACTTAA
>A1_47
TTTCCGTAGGTGAACCTGCGGAAGGATCATTATTGAATTATGTTTCTAGATAGGTTGTAG
CTGGCTCTTTTAGAGCATGTGCACGCCTGTTTGGACTTCATTTTCATCCACCTGTGCACC
TATTGTAGTCTTTGGTTGGGTTAGGAGGAAGTGATCATTGTATCAGCATCTGCTGGGAGT
GAGGACTTGCATTGTGAAAGCTTTGCTGTCCTTGATGTGATCATGGAATCTTTTTCTCAC
TAGAGTCTATGTCACTCATTATACTCTGTCGAATGTCATTGAATGTCTTTACATGGGCTT
GTATGCCTATGAAAATTGTAATACAACTTTCAGCAACGGATCTCTTGGCTCTCGCATCGA
TGAAGAACGCAGCGAAATGCGATAAGTAATGTGAATTGCAGAATTCAGTGAATCATCGAA
TCTTTGAACGCATCTTGCGCTCCTTGGTATTCCGAGGAGCATGCCTGTTTGAGTGTCATT
AAATTCTCAACTCTCTTATACTTTTTTGTAAAAGAGAGCTTGGACTGTGGAGGCTTGCTG
GCCACTTTTTGGGGTCAGCTCCTCTGAAATGCATTAGCGGAACCGTTTGCGATCTGCCAC
AAGTGTGATAAGTTATCTACACTGGCGAGGGGATTGCTCTCTGTAATGTTCAGCTTCTAA
TTGTCTCTACTTTGTGAGACTACTTTTGAATGCTTGACCTCAAATCAGGTAGGACTACCC
GCTGAACTTAA
>A2_15
TTTCCGTAGGTGAACCTGCGGAAGGATCATTATTGAATTATGTTTCTAGATAGGTTGTAG
CTGGCTCTTTTAGAGCATGTGCACGCCTGTTTGGACTTCATTTTCATCCACCTGTGCACC
TATTGTAGTCTTTGGTTGGGTTAGGAGGAAGTGATCATTGTATCAGCATCTGCTGGGAGT
GAGGACTTGCATTGTGAAAGCTTTGCTGTCCTTGATGTGATCATGGAATCTTTTTCTCAC
TAGAGTCTATGTCACTCATTATACTCTGTCGAATGTCATTGAATGTCTTTACATGGGCTT
GTATGCCTATGAAAATTGTAATACAACTTTCAGCAACGGATCTCTTGGCTCTCGCATCGA
TGAAGAACGCAGCGAAATGCGATAAGTAATGTGAATTGCAGAATTCAGTGAATCATCGAA
TCTTTGAACGCATCTTGCGCTCCTTGGTATTCCGAGGAGCATGCCTGTTTGAGTGTCATT
AAATTCTCAACTCTCTTATACTTTTTTGTAAAAGAGAGCTTGGACTGTGGAGGCTTGCTG
GCCACTTTTTGGGGTCAGCTCCTCTGAAATGCATTAGCGGAACCGTTTGCGATCTGCCAC
AAGTGTGATAAGTTATCTACACTGGCGAGGGGATTGCTCTCTGTAATGTTCAGCTTCTAA
TTGTCTCTACTTTGTGAGACTACTTTTGAATGCTTGACCTCAAATCAGGTAGGACTACCC
GCTGAACTTAA
>A2_16
TTTCCGTAGGTGAACCTGCGGAAGGATCATTATTGAATTATGTTTCTAGATAGGTTGTAG
CTGGCTCTTTTAGAGCATGTGCACGCCTGTTTGGACTTCATTTTCATCCACCTGTGCACC
TATTGTAGTCTTTGGTTGGGTTAGGAGGAAGTGATCATTGTATCAGCATCTGCTGGGAGT

GAGGACTTGCATTGTGAAAGCTTTGCTGTCCTTGATGTGATCATGGAATCTTTTTCTCAC
TAGAGTCTATGTCACTCATTATACTCTGTCGAATGTCATTGAATGTCTTTACATGGGCTT
GTATGCCTATGAAAATTGTAATACAACTTTCAGCAACGGATCTCTTGGCTCTCGCATCGA
TGAAGAACGCAGCGAAATGCGATAAGTAATGTGAATTGCAGAATTCAGTGAATCATCGAA
TCTTTGAACGCATCTTGCGCTCCTTGGTATTCCGAGGAGCATGCCTGTTTGAGTGTCATT
AAATTCTCAACTCTCTTATACTTTTTTGTAAAAGAGAGCTTGGACTGTGGAGGCTTGCTG
GCCACTTTTTGGGGTCAGCTCCTCTGAAATGCATTAGCGGAACCGTTTGCGATCTGCCAC
AAGTGTGATAAGTTATCTACACTGGCGAGGGGATTGCTCTCTGTAATGTTCAGCTTCTAA
TTGTCTCTACTTTGTGAGACTACTTTTGAATGCTTGACCTCAAATCAGGTAGGACTACCC
GCTGAACTTAA
>A3_5
TTTCCGTAGGTGAACCTGCGGAAGGATCATTATTGAATTATGTTTCTAGATAGGTTGTAG
CTGGCTCTTTTAGAGCATGTGCACGCCTGTTTGGACTTCATTTTCATCCACCTGTGCACC
TATTGTAGTCTTTGGTTGGGTTAGGAGGAAGTGATCATTGTATCAGCATCTGCTGGGAGT
GAGGACTTGCATTGTGAAAGCTTTGCTGTCCTTGATGTGATCATGGAATCTTTTTCTCAC
TAGAGTCTATGTCACTCATTATACTCTGTCGAATGTCATTGAATGTCTTTACATGGGCTT
GTATGCCTATGAAAATTGTAATACAACTTTCAGCAACGGATCTCTTGGCTCTCGCATCGA
TGAAGAACGCAGCGAAATGCGATAAGTAATGTGAATTGCAGAATTCAGTGAATCATCGAA
TCTTTGAACGCATCTTGCGCTCCTTGGTATTCCGAGGAGCATGCCTGTTTGAGTGTCATT
AAATTCTCAACTCTCTTATACTTTTTTGTAAAAGAGAGCTTGGACTGTGGAGGCTTGCTG
GCCACTTTTTGGGGTCAGCTCCTCTGAAATGCATTAGCGGAACCGTTTGCGATCTGCCAC
AAGTGTGATAAGTTATCTACACTGGCGAGGGGATTGCTCTCTGTAATGTTCAGCTTCTAA
TTGTCTCTACTTTGTGAGACTACTTTTGAATGCTTGACCTCAAATCAGGTAGGACTACCC
GCTGAACTTAA
>A3_6
TTTCCGTAGGTGAACCTGCGGAAGGATCATTATTGAATTATGTTTCTAGATAGGTTGTAG
CTGGCTCTTTTAGAGCATGTGCACGCCTGTTTGGACTTCATTTTCATCCACCTGTGCACC
TATTGTAGTCTTTGGTTGGGTTAGGAGGAAGTGATCATTGTATCAGCATCTGCTGGGAGT
GAGGACTTGCATTGTGAAAGCTTTGCTGTCCTTGATGTGATCATGGAATCTTTTTCTCAC
TAGAGTCTATGTCACTCATTATACTCTGTCGAATGTCATTGAATGTCTTTACATGGGCTT
GTATGCCTATGAAAATTGTAATACAACTTTCAGCAACGGATCTCTTGGCTCTCGCATCGA
TGAAGAACGCAGCGAAATGCGATAAGTAATGTGAATTGCAGAATTCAGTGAATCATCGAA
TCTTTGAACGCATCTTGCGCTCCTTGGTATTCCGAGGAGCATGCCTGTTTGAGTGTCATT
AAATTCTCAACTCTCTTATACTTTTTTGTAAAAGAGAGCTTGGACTGTGGAGGCTTGCTG
GCCACTTTTTGGGGTCAGCTCCTCTGAAATGCATTAGCGGAACCGTTTGCGATCTGCCAC
AAGTGTGATAAGTTATCTACACTGGCGAGGGGATTGCTCTCTGTAATGTTCAGCTTCTAA
TTGTCTCTACTTTGTGAGACTACTTTTGAATGCTTGACCTCAAATCAGGTAGGACTACCC
GCTGAACTTAA
>A3_7
TTTCCGTAGGTGAACCTGCGGAAGGATCATTATTGAATTATGTTTCTAGATAGGTTGTAG
CTGGCTCTTTTAGAGCATGTGCACGCCTGTTTGGACTTCATTTTCATCCACCTGTGCACC
TATTGTAGTCTTTGGTTGGGTTAGGAGGAAGTGATCATTGTATCAGCATCTGCTGGGAGT
GAGGACTTGCATTGTGAAAGCTTTGCTGTCCTTGATGTGATCATGGAATCTTTTTCTCAC
TAGAGTCTATGTCACTCATTATACTCTGTCGAATGTCATTGAATGTCTTTACATGGGCTT
GTATGCCTATGAAAATTGTAATACAACTTTCAGCAACGGATCTCTTGGCTCTCGCATCGA
TGAAGAACGCAGCGAAATGCGATAAGTAATGTGAATTGCAGAATTCAGTGAATCATCGAA
TCTTTGAACGCATCTTGCGCTCCTTGGTATTCCGAGGAGCATGCCTGTTTGAGTGTCATT
AAATTCTCAACTCTCTTATACTTTTTTGTAAAAGAGAGCTTGGACTGTGGAGGCTTGCTG
GCCACTTTTTGGGGTCAGCTCCTCTGAAATGCATTAGCGGAACCGTTTGCGATCTGCCAC
AAGTGTGATAAGTTATCTACACTGGCGAGGGGATTGCTCTCTGTAATGTTCAGCTTCTAA

TTGTCTCTACTTTGTGAGACTACTTTTGAATGCTTGACCTCAAATCAGGTAGGACTACCC
GCTGAACTTAA
>A3_14
TTTCCGTAGGTGAACCTGCGGAAGGATCATTATTGAATTATGTTTCTAGATAGGTTGTAG
CTGGCTCTTTTAGAGCATGTGCACGCCTGTTTGGACTTCATTTTCATCCACCTGTGCACC
TATTGTAGTCTTTGGTTGGGTTAGGAGGAAGTGATCATTGTATCAGCATCTGCTGGGAGT
GAGGACTTGCATTGTGAAAGCTTTGCTGTCCTTGATGTGATCATGGAATCTTTTTCTCAC
TAGAGTCTATGTCACTCATTATACTCTGTCGAATGTCATTGAATGTCTTTACATGGGCTT
GTATGCCTATGAAAATTGTAATACAACTTTCAGCAACGGATCTCTTGGCTCTCGCATCGA
TGAAGAACGCAGCGAAATGCGATAAGTAATGTGAATTGCAGAATTCAGTGAATCATCGAA
TCTTTGAACGCATCTTGCGCTCCTTGGTATTCCGAGGAGCATGCCTGTTTGAGTGTCATT
AAATTCTCAACTCTCTTATACTTTTTTGTAAAAGAGAGCTTGGACTGTGGAGGCTTGCTG
GCCACTTTTTGGGGTCAGCTCCTCTGAAATGCATTAGCGGAACCGTTTGCGATCTGCCAC
AAGTGTGATAAGTTATCTACACTGGCGAGGGGATTGCTCTCTGTAATGTTCAGCTTCTAA
TTGTCTCTACTTTGTGAGACTACTTTTGAATGCTTGACCTCAAATCAGGTAGGACTACCC
GCTGAACTTAA
>A3_15
TTTCCGTAGGTGAACCTGCGGAAGGATCATTATTGAATTATGTTTCTAGATAGGTTGTAG
CTGGCTCTTTTAGAGCATGTGCACGCCTGTTTGGACTTCATTTTCATCCACCTGTGCACC
TATTGTAGTCTTTGGTTGGGTTAGGAGGAAGTGATCATTGTATCAGCATCTGCTGGGAGT
GAGGACTTGCATTGTGAAAGCTTTGCTGTCCTTGATGTGATCATGGAATCTTTTTCTCAC
TAGAGTCTATGTCACTCATTATACTCTGTCGAATGTCATTGAATGTCTTTACATGGGCTT
GTATGCCTATGAAAATTGTAATACAACTTTCAGCAACGGATCTCTTGGCTCTCGCATCGA
TGAAGAACGCAGCGAAATGCGATAAGTAATGTGAATTGCAGAATTCAGTGAATCATCGAA
TCTTTGAACGCATCTTGCGCTCCTTGGTATTCCGAGGAGCATGCCTGTTTGAGTGTCATT
AAATTCTCAACTCTCTTATACTTTTTTGTAAAAGAGAGCTTGGACTGTGGAGGCTTGCTG
GCCACTTTTTGGGGTCAGCTCCTCTGAAATGCATTAGCGGAACCGTTTGCGATCTGCCAC
AAGTGTGATAAGTTATCTACACTGGCGAGGGGATTGCTCTCTGTAATGTTCAGCTTCTAA
TTGTCTCTACTTTGTGAGACTACTTTTGAATGCTTGACCTCAAATCAGGTAGGACTACCC
GCTGAACTTAA
>A2_17
TTTCCGTAGGTGAACCTGCGGAAGGATCATTATTGAATTATGTTTCTAGATAGGTTGTAG
CTGGCTCTTTTAGAGCATGTGCACGCCTGTTTGGACTTCATTTTCATCCACCTGTGCACC
TATTGTAGTCTTTGGTTGGGTTAGGAGGAAGTGATCATTGTATCAGCATCTGCTGGGAGT
GAGGACTTGCATTGTGAAAGCTTTGCTGTCCTTGATGTGATCATGGAATCTTTTTCTCAC
TAGAGTCTATGTCACTCATTATACTCTGTCGAATGTCATTGAATGTCTTTACATGGGCTT
GTATGCCTATGAAAATTGTAATACAACTTTCAGCAACGGATCTCTTGGCTCTCGCATCGA
TGAAGAACGCAGCGAAATGCGATAAGTAATGTGAATTGCAGAATTCAGTGAATCATCGAA
TCTTTGAACGCATCTTGCGCTCCTTGGTATTCCGAGGAGCATGCCTGTTTGAGTGTCATT
AAATTCTCAACTCTCTTATACTTTTTTGTAAAAGAGAGCTTGGACTGTGGAGGCTTGCTG
GCCACTTTTTGGGGTCAGCTCCTCTGAAATGCATTAGCGGAACCGTTTGCGATCTGCCAC
AAGTGTGATAAGTTATCTACACTGGCGAGGGGATTGCTCTCTGTAATGTTCAGCTTCTAA
TTGTCTCTACTTTGTGAGACTACTTTTGAATGCTTGACCTCAAATCAGGTAGGACTACCC
GCTGAACTTAA
>A2−62
TTTCCGTAGGTGAACCTGCGGAAGGATCATTATTGAATTATGTTTCTAGATAGGTTGTAG
CTGGCTCTTTTAGAGCATGTGCACGCCTGTTTGGACTTCATTTTCATCCACCTGTGCACC
TATTGTAGTCTTTGGTTGGGTTAGGAGGAAGTGATCATTGTATCAGCATCTGCTGGGAGT
GAGGACTTGCATTGTGAAAGCTTTGCTGTCCTTGATGTGATCATGGAATCTTTTTCTCAC
TAGAGTCTATGTCACTCATTATACTCTGTCGAATGTCATTGAATGTCTTTACATGGGCTT

GTATGCCTATGAAAATTGTAATACAACTTTCAGCAACGGATCTCTTGGCTCTCGCATCGA
TGAAGAACGCAGCGAAATGCGATAAGTAATGTGAATTGCAGAATTCAGTGAATCATCGAA
TCTTTGAACGCATCTTGCGCTCCTTGGTATTCCGAGGAGCATGCCTGTTTGAGTGTCATT
AAATTCTCAACTCTCTTATACTTTTTTGTAAAAGAGAGCTTGGACTGTGGAGGCTTGCTG
GCCACTTTTTGGGGTCAGCTCCTCTGAAATGCATTAGCGGAACCGTTTGCGATCTGCCAC
AAGTGTGATAAGTTATCTACACTGGCGAGGGGATTGCTCTCTGTAATGTTCAGCTTCTAA
TTGTCTCTACTTTGTGAGACTACTTTTGAATGCTTGACCTCAAATCAGGTAGGACTACCC
GCTGAACTTAA
>A2-64
TTTCCGTAGGTGAACCTGCGGAAGGATCATTATTGAATTATGTTTCTAGATAGGTTGTAG
CTGGCTCTTTTAGAGCATGTGCACGCCTGTTTGGACTTCATTTTCATCCACCTGTGCACC
TATTGTAGTCTTTGGTTGGGTTAGGAGGAAGTGATCATTGTATCAGCATCTGCTGGGAGT
GAGGACTTGCATTGTGAAAGCTTTGCTGTCCTTGATGTGATCATGGAATCTTTTTCTCAC
TAGAGTCTATGTCACTCATTATACTCTGTCGAATGTCATTGAATGTCTTTACATGGGCTT
GTATGCCTATGAAAATTGTAATACAACTTTCAGCAACGGATCTCTTGGCTCTCGCATCGA
TGAAGAACGCAGCGAAATGCGATAAGTAATGTGAATTGCAGAATTCAGTGAATCATCGAA
TCTTTGAACGCATCTTGCGCTCCTTGGTATTCCGAGGAGCATGCCTGTTTGAGTGTCATT
AAATTCTCAACTCTCTTATACTTTTTTGTAAAAGAGAGCTTGGACTGTGGAGGCTTGCTG
GCCACTTTTTGGGGTCAGCTCCTCTGAAATGCATTAGCGGAACCGTTTGCGATCTGCCAC
AAGTGTGATAAGTTATCTACACTGGCGAGGGGATTGCTCTCTGTAATGTTCAGCTTCTAA
TTGTCTCTACTTTGTGAGACTACTTTTGAATGCTTGACCTCAAATCAGGTAGGACTACCC
GCTGAACTTAA
>A2-65
TTTCCGTAGGTGAACCTGCGGAAGGATCATTATTGAATTATGTTTCTAGATAGGTTGTAG
CTGGCTCTTTTAGAGCATGTGCACGCCTGTTTGGACTTCATTTTCATCCACCTGTGCACC
TATTGTAGTCTTTGGTTGGGTTAGGAGGAAGTGATCATTGTATCAGCATCTGCTGGGAGT
GAGGACTTGCATTGTGAAAGCTTTGCTGTCCTTGATGTGATCATGGAATCTTTTTCTCAC
TAGAGTCTATGTCACTCATTATACTCTGTCGAATGTCATTGAATGTCTTTACATGGGCTT
GTATGCCTATGAAAATTGTAATACAACTTTCAGCAACGGATCTCTTGGCTCTCGCATCGA
TGAAGAACGCAGCGAAATGCGATAAGTAATGTGAATTGCAGAATTCAGTGAATCATCGAA
TCTTTGAACGCATCTTGCGCTCCTTGGTATTCCGAGGAGCATGCCTGTTTGAGTGTCATT
AAATTCTCAACTCTCTTATACTTTTTTGTAAAAGAGAGCTTGGACTGTGGAGGCTTGCTG
GCCACTTTTTGGGGTCAGCTCCTCTGAAATGCATTAGCGGAACCGTTTGCGATCTGCCAC
AAGTGTGATAAGTTATCTACACTGGCGAGGGGATTGCTCTCTGTAATGTTCAGCTTCTAA
TTGTCTCTACTTTGTGAGACTACTTTTGAATGCTTGACCTCAAATCAGGTAGGACTACCC
GCTGAACTTAA
>A2-66
TTTCCGTAGGTGAACCTGCGGAAGGATCATTATTGAATTATGTTTCTAGATAGGTTGTAG
CTGGCTCTTTTAGAGCATGTGCACGCCTGTTTGGACTTCATTTTCATCCACCTGTGCACC
TATTGTAGTCTTTGGTTGGGTTAGGAGGAAGTGATCATTGTATCAGCATCTGCTGGGAGT
GAGGACTTGCATTGTGAAAGCTTTGCTGTCCTTGATGTGATCATGGAATCTTTTTCTCAC
TAGAGTCTATGTCACTCATTATACTCTGTCGAATGTCATTGAATGTCTTTACATGGGCTT
GTATGCCTATGAAAATTGTAATACAACTTTCAGCAACGGATCTCTTGGCTCTCGCATCGA
TGAAGAACGCAGCGAAATGCGATAAGTAATGTGAATTGCAGAATTCAGTGAATCATCGAA
TCTTTGAACGCATCTTGCGCTCCTTGGTATTCCGAGGAGCATGCCTGTTTGAGTGTCATT
AAATTCTCAACTCTCTTATACTTTTTTGTAAAAGAGAGCTTGGACTGTGGAGGCTTGCTG
GCCACTTTTTGGGGTCAGCTCCTCTGAAATGCATTAGCGGAACCGTTTGCGATCTGCCAC
AAGTGTGATAAGTTATCTACACTGGCGAGGGGATTGCTCTCTGTAATGTTCAGCTTCTAA
TTGTCTCTACTTTGTGAGACTACTTTTGAATGCTTGACCTCAAATCAGGTAGGACTACCC
GCTGAACTTAA

>A2–70
TTTCCGTAGGTGAACCTGCGGAAGGATCATTATTGAATTATGTTTCTAGATAGGTTGTAG
CTGGCTCTTTTAGAGCATGTGCACGCCTGTTTGGACTTCATTTTCATCCACCTGTGCACC
TATTGTAGTCTTTGGTTGGGTTAGGAGGAAGTGATCATTGTATCAGCATCTGCTGGGAGT
GAGGACTTGCATTGTGAAAGCTTTGCTGTCCTTGATGTGATCATGGAATCTTTTTCTCAC
TAGAGTCTATGTCACTCATTATACTCTGTCGAATGTCATTGAATGTCTTTACATGGGCTT
GTATGCCTATGAAAATTGTAATACAACTTTCAGCAACGGATCTCTTGGCTCTCGCATCGA
TGAAGAACGCAGCGAAATGCGATAAGTAATGTGAATTGCAGAATTCAGTGAATCATCGAA
TCTTTGAACGCATCTTGCGCTCCTTGGTATTCCGAGGAGCATGCCTGTTTGAGTGTCATT
AAATTCTCAACTCTCTTATACTTTTTTGTAAAAGAGAGCTTGGACTGTGGAGGCTTGCTG
GCCACTTTTTGGGGTCAGCTCCTCTGAAATGCATTAGCGGAACCGTTTGCGATCTGCCAC
AAGTGTGATAAGTTATCTACACTGGCGAGGGGATTGCTCTCTGTAATGTTCAGCTTCTAA
TTGTCTCTACTTTGTGAGACTACTTTTGAATGCTTGACCTCAAATCAGGTAGGACTACCC
GCTGAACTTAA
>A3_1
TTTCCGTAGGTGAACCTGCGGAAGGATCATTATTGAATTATGTTTCTAGATAGGTTGTAG
CTGGCTCTTTTAGAGCATGTGCACGCCTGTTTGGACTTCATTTTCATCCACCTGTGCACC
TATTGTAGTCTTTGGTTGGGTTAGGAGGAAGTGATCATTGTATCAGCATCTGCTGGGAGT
GAGGACTTGCATTGTGAAAGCTTTGCTGTCCTTGATGTGATCATGGAATCTTTTTCTCAC
TAGAGTCTATGTCACTCATTATACTCTGTCGAATGTCATTGAATGTCTTTACATGGGCTT
GTATGCCTATGAAAATTGTAATACAACTTTCAGCAACGGATCTCTTGGCTCTCGCATCGA
TGAAGAACGCAGCGAAATGCGATAAGTAATGTGAATTGCAGAATTCAGTGAATCATCGAA
TCTTTGAACGCATCTTGCGCTCCTTGGTATTCCGAGGAGCATGCCTGTTTGAGTGTCATT
AAATTCTCAACTCTCTTATACTTTTTTGTAAAAGAGAGCTTGGACTGTGGAGGCTTGCTG
GCCACTTTTTGGGGTCAGCTCCTCTGAAATGCATTAGCGGAACCGTTTGCGATCTGCCAC
AAGTGTGATAAGTTATCTACACTGGCGAGGGGATTGCTCTCTGTAATGTTCAGCTTCTAA
TTGTCTCTACTTTGTGAGACTACTTTTGAATGCTTGACCTCAAATCAGGTAGGACTACCC
GCTGAACTTAA
>A3_2
TTTCCGTAGGTGAACCTGCGGAAGGATCATTATTGAATTATGTTTCTAGATAGGTTGTAG
CTGGCTCTTTTAGAGCATGTGCACGCCTGTTTGGACTTCATTTTCATCCACCTGTGCACC
TATTGTAGTCTTTGGTTGGGTTAGGAGGAAGTGATCATTGTATCAGCATCTGCTGGGAGT
GAGGACTTGCATTGTGAAAGCTTTGCTGTCCTTGATGTGATCATGGAATCTTTTTCTCAC
TAGAGTCTATGTCACTCATTATACTCTGTCGAATGTCATTGAATGTCTTTACATGGGCTT
GTATGCCTATGAAAATTGTAATACAACTTTCAGCAACGGATCTCTTGGCTCTCGCATCGA
TGAAGAACGCAGCGAAATGCGATAAGTAATGTGAATTGCAGAATTCAGTGAATCATCGAA
TCTTTGAACGCATCTTGCGCTCCTTGGTATTCCGAGGAGCATGCCTGTTTGAGTGTCATT
AAATTCTCAACTCTCTTATACTTTTTTGTAAAAGAGAGCTTGGACTGTGGAGGCTTGCTG
GCCACTTTTTGGGGTCAGCTCCTCTGAAATGCATTAGCGGAACCGTTTGCGATCTGCCAC
AAGTGTGATAAGTTATCTACACTGGCGAGGGGATTGCTCTCTGTAATGTTCAGCTTCTAA
TTGTCTCTACTTTGTGAGACTACTTTTGAATGCTTGACCTCAAATCAGGTAGGACTACCC
GCTGAACTTAA
>A3_3
TTTCCGTAGGTGAACCTGCGGAAGGATCATTATTGAATTATGTTTCTAGATAGGTTGTAG
CTGGCTCTTTTAGAGCATGTGCACGCCTGTTTGGACTTCATTTTCATCCACCTGTGCACC
TATTGTAGTCTTTGGTTGGGTTAGGAGGAAGTGATCATTGTATCAGCATCTGCTGGGAGT
GAGGACTTGCATTGTGAAAGCTTTGCTGTCCTTGATGTGATCATGGAATCTTTTTCTCAC
TAGAGTCTATGTCACTCATTATACTCTGTCGAATGTCATTGAATGTCTTTACATGGGCTT
GTATGCCTATGAAAATTGTAATACAACTTTCAGCAACGGATCTCTTGGCTCTCGCATCGA
TGAAGAACGCAGCGAAATGCGATAAGTAATGTGAATTGCAGAATTCAGTGAATCATCGAA

TCTTTGAACGCATCTTGCGCTCCTTGGTATTCCGAGGAGCATGCCTGTTTGAGTGTCATT
AAATTCTCAACTCTCTTATACTTTTTTGTAAAAGAGAGCTTGGACTGTGGAGGCTTGCTG
GCCACTTTTTGGGGTCAGCTCCTCTGAAATGCATTAGCGGAACCGTTTGCGATCTGCCAC
AAGTGTGATAAGTTATCTACACTGGCGAGGGGATTGCTCTCTGTAATGTTCAGCTTCTAA
TTGTCTCTACTTTGTGAGACTACTTTTGAATGCTTGACCTCAAATCAGGTAGGACTACCC
GCTGAACTTAA
>A3_4
TTTCCGTAGGTGAACCTGCGGAAGGATCATTATTGAATTATGTTTCTAGATAGGTTGTAG
CTGGCTCTTTTAGAGCATGTGCACGCCTGTTTGGACTTCATTTTCATCCACCTGTGCACC
TATTGTAGTCTTTGGTTGGGTTAGGAGGAAGTGATCATTGTATCAGCATCTGCTGGGAGT
GAGGACTTGCATTGTGAAAGCTTTGCTGTCCTTGATGTGATCATGGAATCTTTTTCTCAC
TAGAGTCTATGTCACTCATTATACTCTGTCGAATGTCATTGAATGTCTTTACATGGGCTT
GTATGCCTATGAAAATTGTAATACAACTTTCAGCAACGGATCTCTTGGCTCTCGCATCGA
TGAAGAACGCAGCGAAATGCGATAAGTAATGTGAATTGCAGAATTCAGTGAATCATCGAA
TCTTTGAACGCATCTTGCGCTCCTTGGTATTCCGAGGAGCATGCCTGTTTGAGTGTCATT
AAATTCTCAACTCTCTTATACTTTTTTGTAAAAGAGAGCTTGGACTGTGGAGGCTTGCTG
GCCACTTTTTGGGGTCAGCTCCTCTGAAATGCATTAGCGGAACCGTTTGCGATCTGCCAC
AAGTGTGATAAGTTATCTACACTGGCGAGGGGATTGCTCTCTGTAATGTTCAGCTTCTAA
TTGTCTCTACTTTGTGAGACTACTTTTGAATGCTTGACCTCAAATCAGGTAGGACTACCC
GCTGAACTTAA
>A3_16
TTTCCGTAGGTGAACCTGCGGAAGGATCATTATTGAATTATGTTTCTAGATAGGTTGTAG
CTGGCTCTTTTAGAGCATGTGCACGCCTGTTTGGACTTCATTTTCATCCACCTGTGCACC
TATTGTAGTCTTTGGTTGGGTTAGGAGGAAGTGATCATTGTATCAGCATCTGCTGGGAGT
GAGGACTTGCATTGTGAAAGCTTTGCTGTCCTTGATGTGATCATGGAATCTTTTTCTCAC
TAGAGTCTATGTCACTCATTATACTCTGTCGAATGTCATTGAATGTCTTTACATGGGCTT
GTATGCCTATGAAAATTGTAATACAACTTTCAGCAACGGATCTCTTGGCTCTCGCATCGA
TGAAGAACGCAGCGAAATGCGATAAGTAATGTGAATTGCAGAATTCAGTGAATCATCGAA
TCTTTGAACGCATCTTGCGCTCCTTGGTATTCCGAGGAGCATGCCTGTTTGAGTGTCATT
AAATTCTCAACTCTCTTATACTTTTTTGTAAAAGAGAGCTTGGACTGTGGAGGCTTGCTG
GCCACTTTTTGGGGTCAGCTCCTCTGAAATGCATTAGCGGAACCGTTTGCGATCTGCCAC
AAGTGTGATAAGTTATCTACACTGGCGAGGGGATTGCTCTCTGTAATGTTCAGCTTCTAA
TTGTCTCTACTTTGTGAGACTACTTTTGAATGCTTGACCTCAAATCAGGTAGGACTACCC
GCTGAACTTAA
>A3_17
TTTCCGTAGGTGAACCTGCGGAAGGATCATTATTGAATTATGTTTCTAGATAGGTTGTAG
CTGGCTCTTTTAGAGCATGTGCACGCCTGTTTGGACTTCATTTTCATCCACCTGTGCACC
TATTGTAGTCTTTGGTTGGGTTAGGAGGAAGTGATCATTGTATCAGCATCTGCTGGGAGT
GAGGACTTGCATTGTGAAAGCTTTGCTGTCCTTGATGTGATCATGGAATCTTTTTCTCAC
TAGAGTCTATGTCACTCATTATACTCTGTCGAATGTCATTGAATGTCTTTACATGGGCTT
GTATGCCTATGAAAATTGTAATACAACTTTCAGCAACGGATCTCTTGGCTCTCGCATCGA
TGAAGAACGCAGCGAAATGCGATAAGTAATGTGAATTGCAGAATTCAGTGAATCATCGAA
TCTTTGAACGCATCTTGCGCTCCTTGGTATTCCGAGGAGCATGCCTGTTTGAGTGTCATT
AAATTCTCAACTCTCTTATACTTTTTTGTAAAAGAGAGCTTGGACTGTGGAGGCTTGCTG
GCCACTTTTTGGGGTCAGCTCCTCTGAAATGCATTAGCGGAACCGTTTGCGATCTGCCAC
AAGTGTGATAAGTTATCTACACTGGCGAGGGGATTGCTCTCTGTAATGTTCAGCTTCTAA
TTGTCTCTACTTTGTGAGACTACTTTTGAATGCTTGACCTCAAATCAGGTAGGACTACCC
GCTGAACTTAA
>A2_18
TTTCCGTAGGTGAACCTGCGGAAGGATCATTATTGAATTATGTTTCTAGATAGGTTGTAG

CTGGCTCTTTTAGAGCATGTGCACGCCTGTTTGGACTTCATTTTCATCCACCTGTGCACC
TATTGTAGTCTTTGGTTGGGTTAGGAGGAAGTGATCATTGTATCAGCATCTGCTGGGAGT
GAGGACTTGCATTGTGAAAGCTTTGCTGTCCTTGATGTGATCATGGAATCTTTTTCTCAC
TAGAGTCTATGTCACTCATTATACTCTGTCGAATGTCATTGAATGTCTTTACATGGGCTT
GTATGCCTATGAAAATTGTAATACAACTTTCAGCAACGGATCTCTTGGCTCTCGCATCGA
TGAAGAACGCAGCGAAATGCGATAAGTAATGTGAATTGCAGAATTCAGTGAATCATCGAA
TCTTTGAACGCATCTTGCGCTCCTTGGTATTCCGAGGAGCATGCCTGTTTGAGTGTCATT
AAATTCTCAACTCTCTTATACTTTTTTGTAAAAGAGAGCTTGGACTGTGGAGGCTTGCTG
GCCACTTTTTGGGGTCAGCTCCTCTGAAATGCATTAGCGGAACCGTTTGCGATCTGCCAC
AAGTGTGATAAGTTATCTACACTGGCGAGGGGATTGCTCTCTGTAATGTTCAGCTTCTAA
TTGTCTCTACTTTGTGAGACTACTTTTGAATGCTTGACCTCAAATCAGGTAGGACTACCC
GCTGAACTTAA
>A2_19
TTTCCGTAGGTGAACCTGCGGAAGGATCATTATTGAATTATGTTTCTAGATAGGTTGTAG
CTGGCTCTTTTAGAGCATGTGCACGCCTGTTTGGACTTCATTTTCATCCACCTGTGCACC
TATTGTAGTCTTTGGTTGGGTTAGGAGGAAGTGATCATTGTATCAGCATCTGCTGGGAGT
GAGGACTTGCATTGTGAAAGCTTTGCTGTCCTTGATGTGATCATGGAATCTTTTTCTCAC
TAGAGTCTATGTCACTCATTATACTCTGTCGAATGTCATTGAATGTCTTTACATGGGCTT
GTATGCCTATGAAAATTGTAATACAACTTTCAGCAACGGATCTCTTGGCTCTCGCATCGA
TGAAGAACGCAGCGAAATGCGATAAGTAATGTGAATTGCAGAATTCAGTGAATCATCGAA
TCTTTGAACGCATCTTGCGCTCCTTGGTATTCCGAGGAGCATGCCTGTTTGAGTGTCATT
AAATTCTCAACTCTCTTATACTTTTTTGTAAAAGAGAGCTTGGACTGTGGAGGCTTGCTG
GCCACTTTTTGGGGTCAGCTCCTCTGAAATGCATTAGCGGAACCGTTTGCGATCTGCCAC
AAGTGTGATAAGTTATCTACACTGGCGAGGGGATTGCTCTCTGTAATGTTCAGCTTCTAA
TTGTCTCTACTTTGTGAGACTACTTTTGAATGCTTGACCTCAAATCAGGTAGGACTACCC
GCTGAACTTAA
>A2_20
TTTCCGTAGGTGAACCTGCGGAAGGATCATTATTGAATTATGTTTCTAGATAGGTTGTAG
CTGGCTCTTTTAGAGCATGTGCACGCCTGTTTGGACTTCATTTTCATCCACCTGTGCACC
TATTGTAGTCTTTGGTTGGGTTAGGAGGAAGTGATCATTGTATCAGCATCTGCTGGGAGT
GAGGACTTGCATTGTGAAAGCTTTGCTGTCCTTGATGTGATCATGGAATCTTTTTCTCAC
TAGAGTCTATGTCACTCATTATACTCTGTCGAATGTCATTGAATGTCTTTACATGGGCTT
GTATGCCTATGAAAATTGTAATACAACTTTCAGCAACGGATCTCTTGGCTCTCGCATCGA
TGAAGAACGCAGCGAAATGCGATAAGTAATGTGAATTGCAGAATTCAGTGAATCATCGAA
TCTTTGAACGCATCTTGCGCTCCTTGGTATTCCGAGGAGCATGCCTGTTTGAGTGTCATT
AAATTCTCAACTCTCTTATACTTTTTTGTAAAAGAGAGCTTGGACTGTGGAGGCTTGCTG
GCCACTTTTTGGGGTCAGCTCCTCTGAAATGCATTAGCGGAACCGTTTGCGATCTGCCAC
AAGTGTGATAAGTTATCTACACTGGCGAGGGGATTGCTCTCTGTAATGTTCAGCTTCTAA
TTGTCTCTACTTTGTGAGACTACTTTTGAATGCTTGACCTCAAATCAGGTAGGACTACCC
GCTGAACTTAA
>A2_21
TTTCCGTAGGTGAACCTGCGGAAGGATCATTATTGAATTATGTTTCTAGATAGGTTGTAG
CTGGCTCTTTTAGAGCATGTGCACGCCTGTTTGGACTTCATTTTCATCCACCTGTGCACC
TATTGTAGTCTTTGGTTGGGTTAGGAGGAAGTGATCATTGTATCAGCATCTGCTGGGAGT
GAGGACTTGCATTGTGAAAGCTTTGCTGTCCTTGATGTGATCATGGAATCTTTTTCTCAC
TAGAGTCTATGTCACTCATTATACTCTGTCGAATGTCATTGAATGTCTTTACATGGGCTT
GTATGCCTATGAAAATTGTAATACAACTTTCAGCAACGGATCTCTTGGCTCTCGCATCGA
TGAAGAACGCAGCGAAATGCGATAAGTAATGTGAATTGCAGAATTCAGTGAATCATCGAA
TCTTTGAACGCATCTTGCGCTCCTTGGTATTCCGAGGAGCATGCCTGTTTGAGTGTCATT
AAATTCTCAACTCTCTTATACTTTTTTGTAAAAGAGAGCTTGGACTGTGGAGGCTTGCTG

GCCACTTTTTGGGGTCAGCTCCTCTGAAATGCATTAGCGGAACCGTTTGCGATCTGCCAC
AAGTGTGATAAGTTATCTACACTGGCGAGGGGATTGCTCTCTGTAATGTTCAGCTTCTAA
TTGTCTCTACTTTGTGAGACTACTTTTGAATGCTTGACCTCAAATCAGGTAGGACTACCC
GCTGAACTTAA
>A2_22
TTTCCGTAGGTGAACCTGCGGAAGGATCATTATTGAATTATGTTTCTAGATAGGTTGTAG
CTGGCTCTTTTAGAGCATGTGCACGCCTGTTTGGACTTCATTTTCATCCACCTGTGCACC
TATTGTAGTCTTTGGTTGGGTTAGGAGGAAGTGATCATTGTATCAGCATCTGCTGGGAGT
GAGGACTTGCATTGTGAAAGCTTTGCTGTCCTTGATGTGATCATGGAATCTTTTTCTCAC
TAGAGTCTATGTCACTCATTATACTCTGTCGAATGTCATTGAATGTCTTTACATGGGCTT
GTATGCCTATGAAAATTGTAATACAACTTTCAGCAACGGATCTCTTGGCTCTCGCATCGA
TGAAGAACGCAGCGAAATGCGATAAGTAATGTGAATTGCAGAATTCAGTGAATCATCGAA
TCTTTGAACGCATCTTGCGCTCCTTGGTATTCCGAGGAGCATGCCTGTTTGAGTGTCATT
AAATTCTCAACTCTCTTATACTTTTTTGTAAAAGAGAGCTTGGACTGTGGAGGCTTGCTG
GCCACTTTTTGGGGTCAGCTCCTCTGAAATGCATTAGCGGAACCGTTTGCGATCTGCCAC
AAGTGTGATAAGTTATCTACACTGGCGAGGGGATTGCTCTCTGTAATGTTCAGCTTCTAA
TTGTCTCTACTTTGTGAGACTACTTTTGAATGCTTGACCTCAAATCAGGTAGGACTACCC
GCTGAACTTAA
>A2_23
TTTCCGTAGGTGAACCTGCGGAAGGATCATTATTGAATTATGTTTCTAGATAGGTTGTAG
CTGGCTCTTTTAGAGCATGTGCACGCCTGTTTGGACTTCATTTTCATCCACCTGTGCACC
TATTGTAGTCTTTGGTTGGGTTAGGAGGAAGTGATCATTGTATCAGCATCTGCTGGGAGT
GAGGACTTGCATTGTGAAAGCTTTGCTGTCCTTGATGTGATCATGGAATCTTTTTCTCAC
TAGAGTCTATGTCACTCATTATACTCTGTCGAATGTCATTGAATGTCTTTACATGGGCTT
GTATGCCTATGAAAATTGTAATACAACTTTCAGCAACGGATCTCTTGGCTCTCGCATCGA
TGAAGAACGCAGCGAAATGCGATAAGTAATGTGAATTGCAGAATTCAGTGAATCATCGAA
TCTTTGAACGCATCTTGCGCTCCTTGGTATTCCGAGGAGCATGCCTGTTTGAGTGTCATT
AAATTCTCAACTCTCTTATACTTTTTTGTAAAAGAGAGCTTGGACTGTGGAGGCTTGCTG
GCCACTTTTTGGGGTCAGCTCCTCTGAAATGCATTAGCGGAACCGTTTGCGATCTGCCAC
AAGTGTGATAAGTTATCTACACTGGCGAGGGGATTGCTCTCTGTAATGTTCAGCTTCTAA
TTGTCTCTACTTTGTGAGACTACTTTTGAATGCTTGACCTCAAATCAGGTAGGACTACCC
GCTGAACTTAA
>A2_24
TTTCCGTAGGTGAACCTGCGGAAGGATCATTATTGAATTATGTTTCTAGATAGGTTGTAG
CTGGCTCTTTTAGAGCATGTGCACGCCTGTTTGGACTTCATTTTCATCCACCTGTGCACC
TATTGTAGTCTTTGGTTGGGTTAGGAGGAAGTGATCATTGTATCAGCATCTGCTGGGAGT
GAGGACTTGCATTGTGAAAGCTTTGCTGTCCTTGATGTGATCATGGAATCTTTTTCTCAC
TAGAGTCTATGTCACTCATTATACTCTGTCGAATGTCATTGAATGTCTTTACATGGGCTT
GTATGCCTATGAAAATTGTAATACAACTTTCAGCAACGGATCTCTTGGCTCTCGCATCGA
TGAAGAACGCAGCGAAATGCGATAAGTAATGTGAATTGCAGAATTCAGTGAATCATCGAA
TCTTTGAACGCATCTTGCGCTCCTTGGTATTCCGAGGAGCATGCCTGTTTGAGTGTCATT
AAATTCTCAACTCTCTTATACTTTTTTGTAAAAGAGAGCTTGGACTGTGGAGGCTTGCTG
GCCACTTTTTGGGGTCAGCTCCTCTGAAATGCATTAGCGGAACCGTTTGCGATCTGCCAC
AAGTGTGATAAGTTATCTACACTGGCGAGGGGATTGCTCTCTGTAATGTTCAGCTTCTAA
TTGTCTCTACTTTGTGAGACTACTTTTGAATGCTTGACCTCAAATCAGGTAGGACTACCC
GCTGAACTTAA
>A2_25
TTTCCGTAGGTGAACCTGCGGAAGGATCATTATTGAATTATGTTTCTAGATAGGTTGTAG
CTGGCTCTTTTAGAGCATGTGCACGCCTGTTTGGACTTCATTTTCATCCACCTGTGCACC
TATTGTAGTCTTTGGTTGGGTTAGGAGGAAGTGATCATTGTATCAGCATCTGCTGGGAGT

GAGGACTTGCATTGTGAAAGCTTTGCTGTCCTTGATGTGATCATGGAATCTTTTTCTCAC
TAGAGTCTATGTCACTCATTATACTCTGTCGAATGTCATTGAATGTCTTTACATGGGCTT
GTATGCCTATGAAAATTGTAATACAACTTTCAGCAACGGATCTCTTGGCTCTCGCATCGA
TGAAGAACGCAGCGAAATGCGATAAGTAATGTGAATTGCAGAATTCAGTGAATCATCGAA
TCTTTGAACGCATCTTGCGCTCCTTGGTATTCCGAGGAGCATGCCTGTTTGAGTGTCATT
AAATTCTCAACTCTCTTATACTTTTTTGTAAAAGAGAGCTTGGACTGTGGAGGCTTGCTG
GCCACTTTTTGGGGTCAGCTCCTCTGAAATGCATTAGCGGAACCGTTTGCGATCTGCCAC
AAGTGTGATAAGTTATCTACACTGGCGAGGGGATTGCTCTCTGTAATGTTCAGCTTCTAA
TTGTCTCTACTTTGTGAGACTACTTTTGAATGCTTGACCTCAAATCAGGTAGGACTACCC
GCTGAACTTAA
>A2_26
TTTCCGTAGGTGAACCTGCGGAAGGATCATTATTGAATTATGTTTCTAGATAGGTTGTAG
CTGGCTCTTTTAGAGCATGTGCACGCCTGTTTGGACTTCATTTTCATCCACCTGTGCACC
TATTGTAGTCTTTGGTTGGGTTAGGAGGAAGTGATCATTGTATCAGCATCTGCTGGGAGT
GAGGACTTGCATTGTGAAAGCTTTGCTGTCCTTGATGTGATCATGGAATCTTTTTCTCAC
TAGAGTCTATGTCACTCATTATACTCTGTCGAATGTCATTGAATGTCTTTACATGGGCTT
GTATGCCTATGAAAATTGTAATACAACTTTCAGCAACGGATCTCTTGGCTCTCGCATCGA
TGAAGAACGCAGCGAAATGCGATAAGTAATGTGAATTGCAGAATTCAGTGAATCATCGAA
TCTTTGAACGCATCTTGCGCTCCTTGGTATTCCGAGGAGCATGCCTGTTTGAGTGTCATT
AAATTCTCAACTCTCTTATACTTTTTTGTAAAAGAGAGCTTGGACTGTGGAGGCTTGCTG
GCCACTTTTTGGGGTCAGCTCCTCTGAAATGCATTAGCGGAACCGTTTGCGATCTGCCAC
AAGTGTGATAAGTTATCTACACTGGCGAGGGGATTGCTCTCTGTAATGTTCAGCTTCTAA
TTGTCTCTACTTTGTGAGACTACTTTTGAATGCTTGACCTCAAATCAGGTAGGACTACCC
GCTGAACTTAA
>A2_28
TTTCCGTAGGTGAACCTGCGGAAGGATCATTATTGAATTATGTTTCTAGATAGGTTGTAG
CTGGCTCTTTTAGAGCATGTGCACGCCTGTTTGGACTTCATTTTCATCCACCTGTGCACC
TATTGTAGTCTTTGGTTGGGTTAGGAGGAAGTGATCATTGTATCAGCATCTGCTGGGAGT
GAGGACTTGCATTGTGAAAGCTTTGCTGTCCTTGATGTGATCATGGAATCTTTTTCTCAC
TAGAGTCTATGTCACTCATTATACTCTGTCGAATGTCATTGAATGTCTTTACATGGGCTT
GTATGCCTATGAAAATTGTAATACAACTTTCAGCAACGGATCTCTTGGCTCTCGCATCGA
TGAAGAACGCAGCGAAATGCGATAAGTAATGTGAATTGCAGAATTCAGTGAATCATCGAA
TCTTTGAACGCATCTTGCGCTCCTTGGTATTCCGAGGAGCATGCCTGTTTGAGTGTCATT
AAATTCTCAACTCTCTTATACTTTTTTGTAAAAGAGAGCTTGGACTGTGGAGGCTTGCTG
GCCACTTTTTGGGGTCAGCTCCTCTGAAATGCATTAGCGGAACCGTTTGCGATCTGCCAC
AAGTGTGATAAGTTATCTACACTGGCGAGGGGATTGCTCTCTGTAATGTTCAGCTTCTAA
TTGTCTCTACTTTGTGAGACTACTTTTGAATGCTTGACCTCAAATCAGGTAGGACTACCC
GCTGAACTTAA
>A2_29
TTTCCGTAGGTGAACCTGCGGAAGGATCATTATTGAATTATGTTTCTAGATAGGTTGTAG
CTGGCTCTTTTAGAGCATGTGCACGCCTGTTTGGACTTCATTTTCATCCACCTGTGCACC
TATTGTAGTCTTTGGTTGGGTTAGGAGGAAGTGATCATTGTATCAGCATCTGCTGGGAGT
GAGGACTTGCATTGTGAAAGCTTTGCTGTCCTTGATGTGATCATGGAATCTTTTTCTCAC
TAGAGTCTATGTCACTCATTATACTCTGTCGAATGTCATTGAATGTCTTTACATGGGCTT
GTATGCCTATGAAAATTGTAATACAACTTTCAGCAACGGATCTCTTGGCTCTCGCATCGA
TGAAGAACGCAGCGAAATGCGATAAGTAATGTGAATTGCAGAATTCAGTGAATCATCGAA
TCTTTGAACGCATCTTGCGCTCCTTGGTATTCCGAGGAGCATGCCTGTTTGAGTGTCATT
AAATTCTCAACTCTCTTATACTTTTTTGTAAAAGAGAGCTTGGACTGTGGAGGCTTGCTG
GCCACTTTTTGGGGTCAGCTCCTCTGAAATGCATTAGCGGAACCGTTTGCGATCTGCCAC
AAGTGTGATAAGTTATCTACACTGGCGAGGGGATTGCTCTCTGTAATGTTCAGCTTCTAA

TTGTCTCTACTTTGTGAGACTACTTTTGAATGCTTGACCTCAAATCAGGTAGGACTACCC
GCTGAACTTAA
>A2_30
TTTCCGTAGGTGAACCTGCGGAAGGATCATTATTGAATTATGTTTCTAGATAGGTTGTAG
CTGGCTCTTTTAGAGCATGTGCACGCCTGTTTGGACTTCATTTTCATCCACCTGTGCACC
TATTGTAGTCTTTGGTTGGGTTAGGAGGAAGTGATCATTGTATCAGCATCTGCTGGGAGT
GAGGACTTGCATTGTGAAAGCTTTGCTGTCCTTGATGTGATCATGGAATCTTTTTCTCAC
TAGAGTCTATGTCACTCATTATACTCTGTCGAATGTCATTGAATGTCTTTACATGGGCTT
GTATGCCTATGAAAATTGTAATACAACTTTCAGCAACGGATCTCTTGGCTCTCGCATCGA
TGAAGAACGCAGCGAAATGCGATAAGTAATGTGAATTGCAGAATTCAGTGAATCATCGAA
TCTTTGAACGCATCTTGCGCTCCTTGGTATTCCGAGGAGCATGCCTGTTTGAGTGTCATT
AAATTCTCAACTCTCTTATACTTTTTTGTAAAAGAGAGCTTGGACTGTGGAGGCTTGCTG
GCCACTTTTTGGGGTCAGCTCCTCTGAAATGCATTAGCGGAACCGTTTGCGATCTGCCAC
AAGTGTGATAAGTTATCTACACTGGCGAGGGGATTGCTCTCTGTAATGTTCAGCTTCTAA
TTGTCTCTACTTTGTGAGACTACTTTTGAATGCTTGACCTCAAATCAGGTAGGACTACCC
GCTGAACTTAA
>A2_32
TTTCCGTAGGTGAACCTGCGGAAGGATCATTATTGAATTATGTTTCTAGATAGGTTGTAG
CTGGCTCTTTTAGAGCATGTGCACGCCTGTTTGGACTTCATTTTCATCCACCTGTGCACC
TATTGTAGTCTTTGGTTGGGTTAGGAGGAAGTGATCATTGTATCAGCATCTGCTGGGAGT
GAGGACTTGCATTGTGAAAGCTTTGCTGTCCTTGATGTGATCATGGAATCTTTTTCTCAC
TAGAGTCTATGTCACTCATTATACTCTGTCGAATGTCATTGAATGTCTTTACATGGGCTT
GTATGCCTATGAAAATTGTAATACAACTTTCAGCAACGGATCTCTTGGCTCTCGCATCGA
TGAAGAACGCAGCGAAATGCGATAAGTAATGTGAATTGCAGAATTCAGTGAATCATCGAA
TCTTTGAACGCATCTTGCGCTCCTTGGTATTCCGAGGAGCATGCCTGTTTGAGTGTCATT
AAATTCTCAACTCTCTTATACTTTTTTGTAAAAGAGAGCTTGGACTGTGGAGGCTTGCTG
GCCACTTTTTGGGGTCAGCTCCTCTGAAATGCATTAGCGGAACCGTTTGCGATCTGCCAC
AAGTGTGATAAGTTATCTACACTGGCGAGGGGATTGCTCTCTGTAATGTTCAGCTTCTAA
TTGTCTCTACTTTGTGAGACTACTTTTGAATGCTTGACCTCAAATCAGGTAGGACTACCC
GCTGAACTTAA
>A2_33
TTTCCGTAGGTGAACCTGCGGAAGGATCATTATTGAATTATGTTTCTAGATAGGTTGTAG
CTGGCTCTTTTAGAGCATGTGCACGCCTGTTTGGACTTCATTTTCATCCACCTGTGCACC
TATTGTAGTCTTTGGTTGGGTTAGGAGGAAGTGATCATTGTATCAGCATCTGCTGGGAGT
GAGGACTTGCATTGTGAAAGCTTTGCTGTCCTTGATGTGATCATGGAATCTTTTTCTCAC
TAGAGTCTATGTCACTCATTATACTCTGTCGAATGTCATTGAATGTCTTTACATGGGCTT
GTATGCCTATGAAAATTGTAATACAACTTTCAGCAACGGATCTCTTGGCTCTCGCATCGA
TGAAGAACGCAGCGAAATGCGATAAGTAATGTGAATTGCAGAATTCAGTGAATCATCGAA
TCTTTGAACGCATCTTGCGCTCCTTGGTATTCCGAGGAGCATGCCTGTTTGAGTGTCATT
AAATTCTCAACTCTCTTATACTTTTTTGTAAAAGAGAGCTTGGACTGTGGAGGCTTGCTG
GCCACTTTTTGGGGTCAGCTCCTCTGAAATGCATTAGCGGAACCGTTTGCGATCTGCCAC
AAGTGTGATAAGTTATCTACACTGGCGAGGGGATTGCTCTCTGTAATGTTCAGCTTCTAA
TTGTCTCTACTTTGTGAGACTACTTTTGAATGCTTGACCTCAAATCAGGTAGGACTACCC
GCTGAACTTAA
>A2_34
TTTCCGTAGGTGAACCTGCGGAAGGATCATTATTGAATTATGTTTCTAGATAGGTTGTAG
CTGGCTCTTTTAGAGCATGTGCACGCCTGTTTGGACTTCATTTTCATCCACCTGTGCACC
TATTGTAGTCTTTGGTTGGGTTAGGAGGAAGTGATCATTGTATCAGCATCTGCTGGGAGT
GAGGACTTGCATTGTGAAAGCTTTGCTGTCCTTGATGTGATCATGGAATCTTTTTCTCAC
TAGAGTCTATGTCACTCATTATACTCTGTCGAATGTCATTGAATGTCTTTACATGGGCTT

GTATGCCTATGAAAATTGTAATACAACTTTCAGCAACGGATCTCTTGGCTCTCGCATCGA
TGAAGAACGCAGCGAAATGCGATAAGTAATGTGAATTGCAGAATTCAGTGAATCATCGAA
TCTTTGAACGCATCTTGCGCTCCTTGGTATTCCGAGGAGCATGCCTGTTTGAGTGTCATT
AAATTCTCAACTCTCTTATACTTTTTTGTAAAAGAGAGCTTGGACTGTGGAGGCTTGCTG
GCCACTTTTTGGGGTCAGCTCCTCTGAAATGCATTAGCGGAACCGTTTGCGATCTGCCAC
AAGTGTGATAAGTTATCTACACTGGCGAGGGGATTGCTCTCTGTAATGTTCAGCTTCTAA
TTGTCTCTACTTTGTGAGACTACTTTTGAATGCTTGACCTCAAATCAGGTAGGACTACCC
GCTGAACTTAA
>A2_35
TTTCCGTAGGTGAACCTGCGGAAGGATCATTATTGAATTATGTTTCTAGATAGGTTGTAG
CTGGCTCTTTTAGAGCATGTGCACGCCTGTTTGGACTTCATTTTCATCCACCTGTGCACC
TATTGTAGTCTTTGGTTGGGTTAGGAGGAAGTGATCATTGTATCAGCATCTGCTGGGAGT
GAGGACTTGCATTGTGAAAGCTTTGCTGTCCTTGATGTGATCATGGAATCTTTTTCTCAC
TAGAGTCTATGTCACTCATTATACTCTGTCGAATGTCATTGAATGTCTTTACATGGGCTT
GTATGCCTATGAAAATTGTAATACAACTTTCAGCAACGGATCTCTTGGCTCTCGCATCGA
TGAAGAACGCAGCGAAATGCGATAAGTAATGTGAATTGCAGAATTCAGTGAATCATCGAA
TCTTTGAACGCATCTTGCGCTCCTTGGTATTCCGAGGAGCATGCCTGTTTGAGTGTCATT
AAATTCTCAACTCTCTTATACTTTTTTGTAAAAGAGAGCTTGGACTGTGGAGGCTTGCTG
GCCACTTTTTGGGGTCAGCTCCTCTGAAATGCATTAGCGGAACCGTTTGCGATCTGCCAC
AAGTGTGATAAGTTATCTACACTGGCGAGGGGATTGCTCTCTGTAATGTTCAGCTTCTAA
TTGTCTCTACTTTGTGAGACTACTTTTGAATGCTTGACCTCAAATCAGGTAGGACTACCC
GCTGAACTTAA
>A2_38
TTTCCGTAGGTGAACCTGCGGAAGGATCATTATTGAATTATGTTTCTAGATAGGTTGTAG
CTGGCTCTTTTAGAGCATGTGCACGCCTGTTTGGACTTCATTTTCATCCACCTGTGCACC
TATTGTAGTCTTTGGTTGGGTTAGGAGGAAGTGATCATTGTATCAGCATCTGCTGGGAGT
GAGGACTTGCATTGTGAAAGCTTTGCTGTCCTTGATGTGATCATGGAATCTTTTTCTCAC
TAGAGTCTATGTCACTCATTATACTCTGTCGAATGTCATTGAATGTCTTTACATGGGCTT
GTATGCCTATGAAAATTGTAATACAACTTTCAGCAACGGATCTCTTGGCTCTCGCATCGA
TGAAGAACGCAGCGAAATGCGATAAGTAATGTGAATTGCAGAATTCAGTGAATCATCGAA
TCTTTGAACGCATCTTGCGCTCCTTGGTATTCCGAGGAGCATGCCTGTTTGAGTGTCATT
AAATTCTCAACTCTCTTATACTTTTTTGTAAAAGAGAGCTTGGACTGTGGAGGCTTGCTG
GCCACTTTTTGGGGTCAGCTCCTCTGAAATGCATTAGCGGAACCGTTTGCGATCTGCCAC
AAGTGTGATAAGTTATCTACACTGGCGAGGGGATTGCTCTCTGTAATGTTCAGCTTCTAA
TTGTCTCTACTTTGTGAGACTACTTTTGAATGCTTGACCTCAAATCAGGTAGGACTACCC
GCTGAACTTAA
>A2_39
TTTCCGTAGGTGAACCTGCGGAAGGATCATTATTGAATTATGTTTCTAGATAGGTTGTAG
CTGGCTCTTTTAGAGCATGTGCACGCCTGTTTGGACTTCATTTTCATCCACCTGTGCACC
TATTGTAGTCTTTGGTTGGGTTAGGAGGAAGTGATCATTGTATCAGCATCTGCTGGGAGT
GAGGACTTGCATTGTGAAAGCTTTGCTGTCCTTGATGTGATCATGGAATCTTTTTCTCAC
TAGAGTCTATGTCACTCATTATACTCTGTCGAATGTCATTGAATGTCTTTACATGGGCTT
GTATGCCTATGAAAATTGTAATACAACTTTCAGCAACGGATCTCTTGGCTCTCGCATCGA
TGAAGAACGCAGCGAAATGCGATAAGTAATGTGAATTGCAGAATTCAGTGAATCATCGAA
TCTTTGAACGCATCTTGCGCTCCTTGGTATTCCGAGGAGCATGCCTGTTTGAGTGTCATT
AAATTCTCAACTCTCTTATACTTTTTTGTAAAAGAGAGCTTGGACTGTGGAGGCTTGCTG
GCCACTTTTTGGGGTCAGCTCCTCTGAAATGCATTAGCGGAACCGTTTGCGATCTGCCAC
AAGTGTGATAAGTTATCTACACTGGCGAGGGGATTGCTCTCTGTAATGTTCAGCTTCTAA
TTGTCTCTACTTTGTGAGACTACTTTTGAATGCTTGACCTCAAATCAGGTAGGACTACCC
GCTGAACTTAA

>A2_40
TTTCCGTAGGTGAACCTGCGGAAGGATCATTATTGAATTATGTTTCTAGATAGGTTGTAG
CTGGCTCTTTTAGAGCATGTGCACGCCTGTTTGGACTTCATTTTCATCCACCTGTGCACC
TATTGTAGTCTTTGGTTGGGTTAGGAGGAAGTGATCATTGTATCAGCATCTGCTGGGAGT
GAGGACTTGCATTGTGAAAGCTTTGCTGTCCTTGATGTGATCATGGAATCTTTTTCTCAC
TAGAGTCTATGTCACTCATTATACTCTGTCGAATGTCATTGAATGTCTTTACATGGGCTT
GTATGCCTATGAAAATTGTAATACAACTTTCAGCAACGGATCTCTTGGCTCTCGCATCGA
TGAAGAACGCAGCGAAATGCGATAAGTAATGTGAATTGCAGAATTCAGTGAATCATCGAA
TCTTTGAACGCATCTTGCGCTCCTTGGTATTCCGAGGAGCATGCCTGTTTGAGTGTCATT
AAATTCTCAACTCTCTTATACTTTTTTGTAAAAGAGAGCTTGGACTGTGGAGGCTTGCTG
GCCACTTTTTGGGGTCAGCTCCTCTGAAATGCATTAGCGGAACCGTTTGCGATCTGCCAC
AAGTGTGATAAGTTATCTACACTGGCGAGGGGATTGCTCTCTGTAATGTTCAGCTTCTAA
TTGTCTCTACTTTGTGAGACTACTTTTGAATGCTTGACCTCAAATCAGGTAGGACTACCC
GCTGAACTTAA
>A2_42
TTTCCGTAGGTGAACCTGCGGAAGGATCATTATTGAATTATGTTTCTAGATAGGTTGTAG
CTGGCTCTTTTAGAGCATGTGCACGCCTGTTTGGACTTCATTTTCATCCACCTGTGCACC
TATTGTAGTCTTTGGTTGGGTTAGGAGGAAGTGATCATTGTATCAGCATCTGCTGGGAGT
GAGGACTTGCATTGTGAAAGCTTTGCTGTCCTTGATGTGATCATGGAATCTTTTTCTCAC
TAGAGTCTATGTCACTCATTATACTCTGTCGAATGTCATTGAATGTCTTTACATGGGCTT
GTATGCCTATGAAAATTGTAATACAACTTTCAGCAACGGATCTCTTGGCTCTCGCATCGA
TGAAGAACGCAGCGAAATGCGATAAGTAATGTGAATTGCAGAATTCAGTGAATCATCGAA
TCTTTGAACGCATCTTGCGCTCCTTGGTATTCCGAGGAGCATGCCTGTTTGAGTGTCATT
AAATTCTCAACTCTCTTATACTTTTTTGTAAAAGAGAGCTTGGACTGTGGAGGCTTGCTG
GCCACTTTTTGGGGTCAGCTCCTCTGAAATGCATTAGCGGAACCGTTTGCGATCTGCCAC
AAGTGTGATAAGTTATCTACACTGGCGAGGGGATTGCTCTCTGTAATGTTCAGCTTCTAA
TTGTCTCTACTTTGTGAGACTACTTTTGAATGCTTGACCTCAAATCAGGTAGGACTACCC
GCTGAACTTAA
>A2_43
TTTCCGTAGGTGAACCTGCGGAAGGATCATTATTGAATTATGTTTCTAGATAGGTTGTAG
CTGGCTCTTTTAGAGCATGTGCACGCCTGTTTGGACTTCATTTTCATCCACCTGTGCACC
TATTGTAGTCTTTGGTTGGGTTAGGAGGAAGTGATCATTGTATCAGCATCTGCTGGGAGT
GAGGACTTGCATTGTGAAAGCTTTGCTGTCCTTGATGTGATCATGGAATCTTTTTCTCAC
TAGAGTCTATGTCACTCATTATACTCTGTCGAATGTCATTGAATGTCTTTACATGGGCTT
GTATGCCTATGAAAATTGTAATACAACTTTCAGCAACGGATCTCTTGGCTCTCGCATCGA
TGAAGAACGCAGCGAAATGCGATAAGTAATGTGAATTGCAGAATTCAGTGAATCATCGAA
TCTTTGAACGCATCTTGCGCTCCTTGGTATTCCGAGGAGCATGCCTGTTTGAGTGTCATT
AAATTCTCAACTCTCTTATACTTTTTTGTAAAAGAGAGCTTGGACTGTGGAGGCTTGCTG
GCCACTTTTTGGGGTCAGCTCCTCTGAAATGCATTAGCGGAACCGTTTGCGATCTGCCAC
AAGTGTGATAAGTTATCTACACTGGCGAGGGGATTGCTCTCTGTAATGTTCAGCTTCTAA
TTGTCTCTACTTTGTGAGACTACTTTTGAATGCTTGACCTCAAATCAGGTAGGACTACCC
GCTGAACTTAA
>A2_44
TTTCCGTAGGTGAACCTGCGGAAGGATCATTATTGAATTATGTTTCTAGATAGGTTGTAG
CTGGCTCTTTTAGAGCATGTGCACGCCTGTTTGGACTTCATTTTCATCCACCTGTGCACC
TATTGTAGTCTTTGGTTGGGTTAGGAGGAAGTGATCATTGTATCAGCATCTGCTGGGAGT
GAGGACTTGCATTGTGAAAGCTTTGCTGTCCTTGATGTGATCATGGAATCTTTTTCTCAC
TAGAGTCTATGTCACTCATTATACTCTGTCGAATGTCATTGAATGTCTTTACATGGGCTT
GTATGCCTATGAAAATTGTAATACAACTTTCAGCAACGGATCTCTTGGCTCTCGCATCGA
TGAAGAACGCAGCGAAATGCGATAAGTAATGTGAATTGCAGAATTCAGTGAATCATCGAA

TCTTTGAACGCATCTTGCGCTCCTTGGTATTCCGAGGAGCATGCCTGTTTGAGTGTCATT
AAATTCTCAACTCTCTTATACTTTTTTGTAAAAGAGAGCTTGGACTGTGGAGGCTTGCTG
GCCACTTTTTGGGGTCAGCTCCTCTGAAATGCATTAGCGGAACCGTTTGCGATCTGCCAC
AAGTGTGATAAGTTATCTACACTGGCGAGGGGATTGCTCTCTGTAATGTTCAGCTTCTAA
TTGTCTCTACTTTGTGAGACTACTTTTGAATGCTTGACCTCAAATCAGGTAGGACTACCC
GCTGAACTTAA
>A2_46
TTTCCGTAGGTGAACCTGCGGAAGGATCATTATTGAATTATGTTTCTAGATAGGTTGTAG
CTGGCTCTTTTAGAGCATGTGCACGCCTGTTTGGACTTCATTTTCATCCACCTGTGCACC
TATTGTAGTCTTTGGTTGGGTTAGGAGGAAGTGATCATTGTATCAGCATCTGCTGGGAGT
GAGGACTTGCATTGTGAAAGCTTTGCTGTCCTTGATGTGATCATGGAATCTTTTTCTCAC
TAGAGTCTATGTCACTCATTATACTCTGTCGAATGTCATTGAATGTCTTTACATGGGCTT
GTATGCCTATGAAAATTGTAATACAACTTTCAGCAACGGATCTCTTGGCTCTCGCATCGA
TGAAGAACGCAGCGAAATGCGATAAGTAATGTGAATTGCAGAATTCAGTGAATCATCGAA
TCTTTGAACGCATCTTGCGCTCCTTGGTATTCCGAGGAGCATGCCTGTTTGAGTGTCATT
AAATTCTCAACTCTCTTATACTTTTTTGTAAAAGAGAGCTTGGACTGTGGAGGCTTGCTG
GCCACTTTTTGGGGTCAGCTCCTCTGAAATGCATTAGCGGAACCGTTTGCGATCTGCCAC
AAGTGTGATAAGTTATCTACACTGGCGAGGGGATTGCTCTCTGTAATGTTCAGCTTCTAA
TTGTCTCTACTTTGTGAGACTACTTTTGAATGCTTGACCTCAAATCAGGTAGGACTACCC
GCTGAACTTAA
>A2_47
TTTCCGTAGGTGAACCTGCGGAAGGATCATTATTGAATTATGTTTCTAGATAGGTTGTAG
CTGGCTCTTTTAGAGCATGTGCACGCCTGTTTGGACTTCATTTTCATCCACCTGTGCACC
TATTGTAGTCTTTGGTTGGGTTAGGAGGAAGTGATCATTGTATCAGCATCTGCTGGGAGT
GAGGACTTGCATTGTGAAAGCTTTGCTGTCCTTGATGTGATCATGGAATCTTTTTCTCAC
TAGAGTCTATGTCACTCATTATACTCTGTCGAATGTCATTGAATGTCTTTACATGGGCTT
GTATGCCTATGAAAATTGTAATACAACTTTCAGCAACGGATCTCTTGGCTCTCGCATCGA
TGAAGAACGCAGCGAAATGCGATAAGTAATGTGAATTGCAGAATTCAGTGAATCATCGAA
TCTTTGAACGCATCTTGCGCTCCTTGGTATTCCGAGGAGCATGCCTGTTTGAGTGTCATT
AAATTCTCAACTCTCTTATACTTTTTTGTAAAAGAGAGCTTGGACTGTGGAGGCTTGCTG
GCCACTTTTTGGGGTCAGCTCCTCTGAAATGCATTAGCGGAACCGTTTGCGATCTGCCAC
AAGTGTGATAAGTTATCTACACTGGCGAGGGGATTGCTCTCTGTAATGTTCAGCTTCTAA
TTGTCTCTACTTTGTGAGACTACTTTTGAATGCTTGACCTCAAATCAGGTAGGACTACCC
GCTGAACTTAA
>A2_48
TTTCCGTAGGTGAACCTGCGGAAGGATCATTATTGAATTATGTTTCTAGATAGGTTGTAG
CTGGCTCTTTTAGAGCATGTGCACGCCTGTTTGGACTTCATTTTCATCCACCTGTGCACC
TATTGTAGTCTTTGGTTGGGTTAGGAGGAAGTGATCATTGTATCAGCATCTGCTGGGAGT
GAGGACTTGCATTGTGAAAGCTTTGCTGTCCTTGATGTGATCATGGAATCTTTTTCTCAC
TAGAGTCTATGTCACTCATTATACTCTGTCGAATGTCATTGAATGTCTTTACATGGGCTT
GTATGCCTATGAAAATTGTAATACAACTTTCAGCAACGGATCTCTTGGCTCTCGCATCGA
TGAAGAACGCAGCGAAATGCGATAAGTAATGTGAATTGCAGAATTCAGTGAATCATCGAA
TCTTTGAACGCATCTTGCGCTCCTTGGTATTCCGAGGAGCATGCCTGTTTGAGTGTCATT
AAATTCTCAACTCTCTTATACTTTTTTGTAAAAGAGAGCTTGGACTGTGGAGGCTTGCTG
GCCACTTTTTGGGGTCAGCTCCTCTGAAATGCATTAGCGGAACCGTTTGCGATCTGCCAC
AAGTGTGATAAGTTATCTACACTGGCGAGGGGATTGCTCTCTGTAATGTTCAGCTTCTAA
TTGTCTCTACTTTGTGAGACTACTTTTGAATGCTTGACCTCAAATCAGGTAGGACTACCC
GCTGAACTTAA
>A2_49
TTTCCGTAGGTGAACCTGCGGAAGGATCATTATTGAATTATGTTTCTAGATAGGTTGTAG

CTGGCTCTTTTAGAGCATGTGCACGCCTGTTTGGACTTCATTTTCATCCACCTGTGCACC
TATTGTAGTCTTTGGTTGGGTTAGGAGGAAGTGATCATTGTATCAGCATCTGCTGGGAGT
GAGGACTTGCATTGTGAAAGCTTTGCTGTCCTTGATGTGATCATGGAATCTTTTTCTCAC
TAGAGTCTATGTCACTCATTATACTCTGTCGAATGTCATTGAATGTCTTTACATGGGCTT
GTATGCCTATGAAAATTGTAATACAACTTTCAGCAACGGATCTCTTGGCTCTCGCATCGA
TGAAGAACGCAGCGAAATGCGATAAGTAATGTGAATTGCAGAATTCAGTGAATCATCGAA
TCTTTGAACGCATCTTGCGCTCCTTGGTATTCCGAGGAGCATGCCTGTTTGAGTGTCATT
AAATTCTCAACTCTCTTATACTTTTTTGTAAAAGAGAGCTTGGACTGTGGAGGCTTGCTG
GCCACTTTTTGGGGTCAGCTCCTCTGAAATGCATTAGCGGAACCGTTTGCGATCTGCCAC
AAGTGTGATAAGTTATCTACACTGGCGAGGGGATTGCTCTCTGTAATGTTCAGCTTCTAA
TTGTCTCTACTTTGTGAGACTACTTTTGAATGCTTGACCTCAAATCAGGTAGGACTACCC
GCTGAACTTAA
>A2_50
TTTCCGTAGGTGAACCTGCGGAAGGATCATTATTGAATTATGTTTCTAGATAGGTTGTAG
CTGGCTCTTTTAGAGCATGTGCACGCCTGTTTGGACTTCATTTTCATCCACCTGTGCACC
TATTGTAGTCTTTGGTTGGGTTAGGAGGAAGTGATCATTGTATCAGCATCTGCTGGGAGT
GAGGACTTGCATTGTGAAAGCTTTGCTGTCCTTGATGTGATCATGGAATCTTTTTCTCAC
TAGAGTCTATGTCACTCATTATACTCTGTCGAATGTCATTGAATGTCTTTACATGGGCTT
GTATGCCTATGAAAATTGTAATACAACTTTCAGCAACGGATCTCTTGGCTCTCGCATCGA
TGAAGAACGCAGCGAAATGCGATAAGTAATGTGAATTGCAGAATTCAGTGAATCATCGAA
TCTTTGAACGCATCTTGCGCTCCTTGGTATTCCGAGGAGCATGCCTGTTTGAGTGTCATT
AAATTCTCAACTCTCTTATACTTTTTTGTAAAAGAGAGCTTGGACTGTGGAGGCTTGCTG
GCCACTTTTTGGGGTCAGCTCCTCTGAAATGCATTAGCGGAACCGTTTGCGATCTGCCAC
AAGTGTGATAAGTTATCTACACTGGCGAGGGGATTGCTCTCTGTAATGTTCAGCTTCTAA
TTGTCTCTACTTTGTGAGACTACTTTTGAATGCTTGACCTCAAATCAGGTAGGACTACCC
GCTGAACTTAA
>A2_51
TTTCCGTAGGTGAACCTGCGGAAGGATCATTATTGAATTATGTTTCTAGATAGGTTGTAG
CTGGCTCTTTTAGAGCATGTGCACGCCTGTTTGGACTTCATTTTCATCCACCTGTGCACC
TATTGTAGTCTTTGGTTGGGTTAGGAGGAAGTGATCATTGTATCAGCATCTGCTGGGAGT
GAGGACTTGCATTGTGAAAGCTTTGCTGTCCTTGATGTGATCATGGAATCTTTTTCTCAC
TAGAGTCTATGTCACTCATTATACTCTGTCGAATGTCATTGAATGTCTTTACATGGGCTT
GTATGCCTATGAAAATTGTAATACAACTTTCAGCAACGGATCTCTTGGCTCTCGCATCGA
TGAAGAACGCAGCGAAATGCGATAAGTAATGTGAATTGCAGAATTCAGTGAATCATCGAA
TCTTTGAACGCATCTTGCGCTCCTTGGTATTCCGAGGAGCATGCCTGTTTGAGTGTCATT
AAATTCTCAACTCTCTTATACTTTTTTGTAAAAGAGAGCTTGGACTGTGGAGGCTTGCTG
GCCACTTTTTGGGGTCAGCTCCTCTGAAATGCATTAGCGGAACCGTTTGCGATCTGCCAC
AAGTGTGATAAGTTATCTACACTGGCGAGGGGATTGCTCTCTGTAATGTTCAGCTTCTAA
TTGTCTCTACTTTGTGAGACTACTTTTGAATGCTTGACCTCAAATCAGGTAGGACTACCC
GCTGAACTTAA
>A2_52
TTTCCGTAGGTGAACCTGCGGAAGGATCATTATTGAATTATGTTTCTAGATAGGTTGTAG
CTGGCTCTTTTAGAGCATGTGCACGCCTGTTTGGACTTCATTTTCATCCACCTGTGCACC
TATTGTAGTCTTTGGTTGGGTTAGGAGGAAGTGATCATTGTATCAGCATCTGCTGGGAGT
GAGGACTTGCATTGTGAAAGCTTTGCTGTCCTTGATGTGATCATGGAATCTTTTTCTCAC
TAGAGTCTATGTCACTCATTATACTCTGTCGAATGTCATTGAATGTCTTTACATGGGCTT
GTATGCCTATGAAAATTGTAATACAACTTTCAGCAACGGATCTCTTGGCTCTCGCATCGA
TGAAGAACGCAGCGAAATGCGATAAGTAATGTGAATTGCAGAATTCAGTGAATCATCGAA
TCTTTGAACGCATCTTGCGCTCCTTGGTATTCCGAGGAGCATGCCTGTTTGAGTGTCATT
AAATTCTCAACTCTCTTATACTTTTTTGTAAAAGAGAGCTTGGACTGTGGAGGCTTGCTG

GCCACTTTTTGGGGTCAGCTCCTCTGAAATGCATTAGCGGAACCGTTTGCGATCTGCCAC
AAGTGTGATAAGTTATCTACACTGGCGAGGGGATTGCTCTCTGTAATGTTCAGCTTCTAA
TTGTCTCTACTTTGTGAGACTACTTTTGAATGCTTGACCTCAAATCAGGTAGGACTACCC
GCTGAACTTAA
>A2_54
TTTCCGTAGGTGAACCTGCGGAAGGATCATTATTGAATTATGTTTCTAGATAGGTTGTAG
CTGGCTCTTTTAGAGCATGTGCACGCCTGTTTGGACTTCATTTTCATCCACCTGTGCACC
TATTGTAGTCTTTGGTTGGGTTAGGAGGAAGTGATCATTGTATCAGCATCTGCTGGGAGT
GAGGACTTGCATTGTGAAAGCTTTGCTGTCCTTGATGTGATCATGGAATCTTTTTCTCAC
TAGAGTCTATGTCACTCATTATACTCTGTCGAATGTCATTGAATGTCTTTACATGGGCTT
GTATGCCTATGAAAATTGTAATACAACTTTCAGCAACGGATCTCTTGGCTCTCGCATCGA
TGAAGAACGCAGCGAAATGCGATAAGTAATGTGAATTGCAGAATTCAGTGAATCATCGAA
TCTTTGAACGCATCTTGCGCTCCTTGGTATTCCGAGGAGCATGCCTGTTTGAGTGTCATT
AAATTCTCAACTCTCTTATACTTTTTTGTAAAAGAGAGCTTGGACTGTGGAGGCTTGCTG
GCCACTTTTTGGGGTCAGCTCCTCTGAAATGCATTAGCGGAACCGTTTGCGATCTGCCAC
AAGTGTGATAAGTTATCTACACTGGCGAGGGGATTGCTCTCTGTAATGTTCAGCTTCTAA
TTGTCTCTACTTTGTGAGACTACTTTTGAATGCTTGACCTCAAATCAGGTAGGACTACCC
GCTGAACTTAA
>A2-55
TTTCCGTAGGTGAACCTGCGGAAGGATCATTATTGAATTATGTTTCTAGATAGGTTGTAG
CTGGCTCTTTTAGAGCATGTGCACGCCTGTTTGGACTTCATTTTCATCCACCTGTGCACC
TATTGTAGTCTTTGGTTGGGTTAGGAGGAAGTGATCATTGTATCAGCATCTGCTGGGAGT
GAGGACTTGCATTGTGAAAGCTTTGCTGTCCTTGATGTGATCATGGAATCTTTTTCTCAC
TAGAGTCTATGTCACTCATTATACTCTGTCGAATGTCATTGAATGTCTTTACATGGGCTT
GTATGCCTATGAAAATTGTAATACAACTTTCAGCAACGGATCTCTTGGCTCTCGCATCGA
TGAAGAACGCAGCGAAATGCGATAAGTAATGTGAATTGCAGAATTCAGTGAATCATCGAA
TCTTTGAACGCATCTTGCGCTCCTTGGTATTCCGAGGAGCATGCCTGTTTGAGTGTCATT
AAATTCTCAACTCTCTTATACTTTTTTGTAAAAGAGAGCTTGGACTGTGGAGGCTTGCTG
GCCACTTTTTGGGGTCAGCTCCTCTGAAATGCATTAGCGGAACCGTTTGCGATCTGCCAC
AAGTGTGATAAGTTATCTACACTGGCGAGGGGATTGCTCTCTGTAATGTTCAGCTTCTAA
TTGTCTCTACTTTGTGAGACTACTTTTGAATGCTTGACCTCAAATCAGGTAGGACTACCC
GCTGAACTTAA
>A2-57
TTTCCGTAGGTGAACCTGCGGAAGGATCATTATTGAATTATGTTTCTAGATAGGTTGTAG
CTGGCTCTTTTAGAGCATGTGCACGCCTGTTTGGACTTCATTTTCATCCACCTGTGCACC
TATTGTAGTCTTTGGTTGGGTTAGGAGGAAGTGATCATTGTATCAGCATCTGCTGGGAGT
GAGGACTTGCATTGTGAAAGCTTTGCTGTCCTTGATGTGATCATGGAATCTTTTTCTCAC
TAGAGTCTATGTCACTCATTATACTCTGTCGAATGTCATTGAATGTCTTTACATGGGCTT
GTATGCCTATGAAAATTGTAATACAACTTTCAGCAACGGATCTCTTGGCTCTCGCATCGA
TGAAGAACGCAGCGAAATGCGATAAGTAATGTGAATTGCAGAATTCAGTGAATCATCGAA
TCTTTGAACGCATCTTGCGCTCCTTGGTATTCCGAGGAGCATGCCTGTTTGAGTGTCATT
AAATTCTCAACTCTCTTATACTTTTTTGTAAAAGAGAGCTTGGACTGTGGAGGCTTGCTG
GCCACTTTTTGGGGTCAGCTCCTCTGAAATGCATTAGCGGAACCGTTTGCGATCTGCCAC
AAGTGTGATAAGTTATCTACACTGGCGAGGGGATTGCTCTCTGTAATGTTCAGCTTCTAA
TTGTCTCTACTTTGTGAGACTACTTTTGAATGCTTGACCTCAAATCAGGTAGGACTACCC
GCTGAACTTAA
>A2-59
TTTCCGTAGGTGAACCTGCGGAAGGATCATTATTGAATTATGTTTCTAGATAGGTTGTAG
CTGGCTCTTTTAGAGCATGTGCACGCCTGTTTGGACTTCATTTTCATCCACCTGTGCACC
TATTGTAGTCTTTGGTTGGGTTAGGAGGAAGTGATCATTGTATCAGCATCTGCTGGGAGT

GAGGACTTGCATTGTGAAAGCTTTGCTGTCCTTGATGTGATCATGGAATCTTTTTCTCAC
TAGAGTCTATGTCACTCATTATACTCTGTCGAATGTCATTGAATGTCTTTACATGGGCTT
GTATGCCTATGAAAATTGTAATACAACTTTCAGCAACGGATCTCTTGGCTCTCGCATCGA
TGAAGAACGCAGCGAAATGCGATAAGTAATGTGAATTGCAGAATTCAGTGAATCATCGAA
TCTTTGAACGCATCTTGCGCTCCTTGGTATTCCGAGGAGCATGCCTGTTTGAGTGTCATT
AAATTCTCAACTCTCTTATACTTTTTTGTAAAAGAGAGCTTGGACTGTGGAGGCTTGCTG
GCCACTTTTTGGGGTCAGCTCCTCTGAAATGCATTAGCGGAACCGTTTGCGATCTGCCAC
AAGTGTGATAAGTTATCTACACTGGCGAGGGGATTGCTCTCTGTAATGTTCAGCTTCTAA
TTGTCTCTACTTTGTGAGACTACTTTTGAATGCTTGACCTCAAATCAGGTAGGACTACCC
GCTGAACTTAA
>A2-60
TTTCCGTAGGTGAACCTGCGGAAGGATCATTATTGAATTATGTTTCTAGATAGGTTGTAG
CTGGCTCTTTTAGAGCATGTGCACGCCTGTTTGGACTTCATTTTCATCCACCTGTGCACC
TATTGTAGTCTTTGGTTGGGTTAGGAGGAAGTGATCATTGTATCAGCATCTGCTGGGAGT
GAGGACTTGCATTGTGAAAGCTTTGCTGTCCTTGATGTGATCATGGAATCTTTTTCTCAC
TAGAGTCTATGTCACTCATTATACTCTGTCGAATGTCATTGAATGTCTTTACATGGGCTT
GTATGCCTATGAAAATTGTAATACAACTTTCAGCAACGGATCTCTTGGCTCTCGCATCGA
TGAAGAACGCAGCGAAATGCGATAAGTAATGTGAATTGCAGAATTCAGTGAATCATCGAA
TCTTTGAACGCATCTTGCGCTCCTTGGTATTCCGAGGAGCATGCCTGTTTGAGTGTCATT
AAATTCTCAACTCTCTTATACTTTTTTGTAAAAGAGAGCTTGGACTGTGGAGGCTTGCTG
GCCACTTTTTGGGGTCAGCTCCTCTGAAATGCATTAGCGGAACCGTTTGCGATCTGCCAC
AAGTGTGATAAGTTATCTACACTGGCGAGGGGATTGCTCTCTGTAATGTTCAGCTTCTAA
TTGTCTCTACTTTGTGAGACTACTTTTGAATGCTTGACCTCAAATCAGGTAGGACTACCC
GCTGAACTTAA
>A2-61
TTTCCGTAGGTGAACCTGCGGAAGGATCATTATTGAATTATGTTTCTAGATAGGTTGTAG
CTGGCTCTTTTAGAGCATGTGCACGCCTGTTTGGACTTCATTTTCATCCACCTGTGCACC
TATTGTAGTCTTTGGTTGGGTTAGGAGGAAGTGATCATTGTATCAGCATCTGCTGGGAGT
GAGGACTTGCATTGTGAAAGCTTTGCTGTCCTTGATGTGATCATGGAATCTTTTTCTCAC
TAGAGTCTATGTCACTCATTATACTCTGTCGAATGTCATTGAATGTCTTTACATGGGCTT
GTATGCCTATGAAAATTGTAATACAACTTTCAGCAACGGATCTCTTGGCTCTCGCATCGA
TGAAGAACGCAGCGAAATGCGATAAGTAATGTGAATTGCAGAATTCAGTGAATCATCGAA
TCTTTGAACGCATCTTGCGCTCCTTGGTATTCCGAGGAGCATGCCTGTTTGAGTGTCATT
AAATTCTCAACTCTCTTATACTTTTTTGTAAAAGAGAGCTTGGACTGTGGAGGCTTGCTG
GCCACTTTTTGGGGTCAGCTCCTCTGAAATGCATTAGCGGAACCGTTTGCGATCTGCCAC
AAGTGTGATAAGTTATCTACACTGGCGAGGGGATTGCTCTCTGTAATGTTCAGCTTCTAA
TTGTCTCTACTTTGTGAGACTACTTTTGAATGCTTGACCTCAAATCAGGTAGGACTACCC
GCTGAACTTAA
>A3_18
TTTCCGTAGGTGAACCTGCGGAAGGATCATTATTGAATTATGTTTCTAGATAGGTTGTAG
CTGGCTCTTTTAGAGCATGTGCACGCCTGTTTGGACTTCATTTTCATCCACCTGTGCACC
TATTGTAGTCTTTGGTTGGGTTAGGAGGAAGTGATCATTGTATCAGCATCTGCTGGGAGT
GAGGACTTGCATTGTGAAAGCTTTGCTGTCCTTGATGTGATCATGGAATCTTTTTCTCAC
TAGAGTCTATGTCACTCATTATACTCTGTCGAATGTCATTGAATGTCTTTACATGGGCTT
GTATGCCTATGAAAATTGTAATACAACTTTCAGCAACGGATCTCTTGGCTCTCGCATCGA
TGAAGAACGCAGCGAAATGCGATAAGTAATGTGAATTGCAGAATTCAGTGAATCATCGAA
TCTTTGAACGCATCTTGCGCTCCTTGGTATTCCGAGGAGCATGCCTGTTTGAGTGTCATT
AAATTCTCAACTCTCTTATACTTTTTTGTAAAAGAGAGCTTGGACTGTGGAGGCTTGCTG
GCCACTTTTTGGGGTCAGCTCCTCTGAAATGCATTAGCGGAACCGTTTGCGATCTGCCAC
AAGTGTGATAAGTTATCTACACTGGCGAGGGGATTGCTCTCTGTAATGTTCAGCTTCTAA

TTGTCTCTACTTTGTGAGACTACTTTTGAATGCTTGACCTCAAATCAGGTAGGACTACCC
GCTGAACTTAA
>A3_19
TTTCCGTAGGTGAACCTGCGGAAGGATCATTATTGAATTATGTTTCTAGATAGGTTGTAG
CTGGCTCTTTTAGAGCATGTGCACGCCTGTTTGGACTTCATTTTCATCCACCTGTGCACC
TATTGTAGTCTTTGGTTGGGTTAGGAGGAAGTGATCATTGTATCAGCATCTGCTGGGAGT
GAGGACTTGCATTGTGAAAGCTTTGCTGTCCTTGATGTGATCATGGAATCTTTTTCTCAC
TAGAGTCTATGTCACTCATTATACTCTGTCGAATGTCATTGAATGTCTTTACATGGGCTT
GTATGCCTATGAAAATTGTAATACAACTTTCAGCAACGGATCTCTTGGCTCTCGCATCGA
TGAAGAACGCAGCGAAATGCGATAAGTAATGTGAATTGCAGAATTCAGTGAATCATCGAA
TCTTTGAACGCATCTTGCGCTCCTTGGTATTCCGAGGAGCATGCCTGTTTGAGTGTCATT
AAATTCTCAACTCTCTTATACTTTTTTGTAAAAGAGAGCTTGGACTGTGGAGGCTTGCTG
GCCACTTTTTGGGGTCAGCTCCTCTGAAATGCATTAGCGGAACCGTTTGCGATCTGCCAC
AAGTGTGATAAGTTATCTACACTGGCGAGGGGATTGCTCTCTGTAATGTTCAGCTTCTAA
TTGTCTCTACTTTGTGAGACTACTTTTGAATGCTTGACCTCAAATCAGGTAGGACTACCC
GCTGAACTTAA
>A3_20
TTTCCGTAGGTGAACCTGCGGAAGGATCATTATTGAATTATGTTTCTAGATAGGTTGTAG
CTGGCTCTTTTAGAGCATGTGCACGCCTGTTTGGACTTCATTTTCATCCACCTGTGCACC
TATTGTAGTCTTTGGTTGGGTTAGGAGGAAGTGATCATTGTATCAGCATCTGCTGGGAGT
GAGGACTTGCATTGTGAAAGCTTTGCTGTCCTTGATGTGATCATGGAATCTTTTTCTCAC
TAGAGTCTATGTCACTCATTATACTCTGTCGAATGTCATTGAATGTCTTTACATGGGCTT
GTATGCCTATGAAAATTGTAATACAACTTTCAGCAACGGATCTCTTGGCTCTCGCATCGA
TGAAGAACGCAGCGAAATGCGATAAGTAATGTGAATTGCAGAATTCAGTGAATCATCGAA
TCTTTGAACGCATCTTGCGCTCCTTGGTATTCCGAGGAGCATGCCTGTTTGAGTGTCATT
AAATTCTCAACTCTCTTATACTTTTTTGTAAAAGAGAGCTTGGACTGTGGAGGCTTGCTG
GCCACTTTTTGGGGTCAGCTCCTCTGAAATGCATTAGCGGAACCGTTTGCGATCTGCCAC
AAGTGTGATAAGTTATCTACACTGGCGAGGGGATTGCTCTCTGTAATGTTCAGCTTCTAA
TTGTCTCTACTTTGTGAGACTACTTTTGAATGCTTGACCTCAAATCAGGTAGGACTACCC
GCTGAACTTAA
>A3_21
TTTCCGTAGGTGAACCTGCGGAAGGATCATTATTGAATTATGTTTCTAGATAGGTTGTAG
CTGGCTCTTTTAGAGCATGTGCACGCCTGTTTGGACTTCATTTTCATCCACCTGTGCACC
TATTGTAGTCTTTGGTTGGGTTAGGAGGAAGTGATCATTGTATCAGCATCTGCTGGGAGT
GAGGACTTGCATTGTGAAAGCTTTGCTGTCCTTGATGTGATCATGGAATCTTTTTCTCAC
TAGAGTCTATGTCACTCATTATACTCTGTCGAATGTCATTGAATGTCTTTACATGGGCTT
GTATGCCTATGAAAATTGTAATACAACTTTCAGCAACGGATCTCTTGGCTCTCGCATCGA
TGAAGAACGCAGCGAAATGCGATAAGTAATGTGAATTGCAGAATTCAGTGAATCATCGAA
TCTTTGAACGCATCTTGCGCTCCTTGGTATTCCGAGGAGCATGCCTGTTTGAGTGTCATT
AAATTCTCAACTCTCTTATACTTTTTTGTAAAAGAGAGCTTGGACTGTGGAGGCTTGCTG
GCCACTTTTTGGGGTCAGCTCCTCTGAAATGCATTAGCGGAACCGTTTGCGATCTGCCAC
AAGTGTGATAAGTTATCTACACTGGCGAGGGGATTGCTCTCTGTAATGTTCAGCTTCTAA
TTGTCTCTACTTTGTGAGACTACTTTTGAATGCTTGACCTCAAATCAGGTAGGACTACCC
GCTGAACTTAA
>A3_22
TTTCCGTAGGTGAACCTGCGGAAGGATCATTATTGAATTATGTTTCTAGATAGGTTGTAG
CTGGCTCTTTTAGAGCATGTGCACGCCTGTTTGGACTTCATTTTCATCCACCTGTGCACC
TATTGTAGTCTTTGGTTGGGTTAGGAGGAAGTGATCATTGTATCAGCATCTGCTGGGAGT
GAGGACTTGCATTGTGAAAGCTTTGCTGTCCTTGATGTGATCATGGAATCTTTTTCTCAC
TAGAGTCTATGTCACTCATTATACTCTGTCGAATGTCATTGAATGTCTTTACATGGGCTT

GTATGCCTATGAAAATTGTAATACAACTTTCAGCAACGGATCTCTTGGCTCTCGCATCGA
TGAAGAACGCAGCGAAATGCGATAAGTAATGTGAATTGCAGAATTCAGTGAATCATCGAA
TCTTTGAACGCATCTTGCGCTCCTTGGTATTCCGAGGAGCATGCCTGTTTGAGTGTCATT
AAATTCTCAACTCTCTTATACTTTTTTGTAAAAGAGAGCTTGGACTGTGGAGGCTTGCTG
GCCACTTTTTGGGGTCAGCTCCTCTGAAATGCATTAGCGGAACCGTTTGCGATCTGCCAC
AAGTGTGATAAGTTATCTACACTGGCGAGGGGATTGCTCTCTGTAATGTTCAGCTTCTAA
TTGTCTCTACTTTGTGAGACTACTTTTGAATGCTTGACCTCAAATCAGGTAGGACTACCC
GCTGAACTTAA
>A3_23
TTTCCGTAGGTGAACCTGCGGAAGGATCATTATTGAATTATGTTTCTAGATAGGTTGTAG
CTGGCTCTTTTAGAGCATGTGCACGCCTGTTTGGACTTCATTTTCATCCACCTGTGCACC
TATTGTAGTCTTTGGTTGGGTTAGGAGGAAGTGATCATTGTATCAGCATCTGCTGGGAGT
GAGGACTTGCATTGTGAAAGCTTTGCTGTCCTTGATGTGATCATGGAATCTTTTTCTCAC
TAGAGTCTATGTCACTCATTATACTCTGTCGAATGTCATTGAATGTCTTTACATGGGCTT
GTATGCCTATGAAAATTGTAATACAACTTTCAGCAACGGATCTCTTGGCTCTCGCATCGA
TGAAGAACGCAGCGAAATGCGATAAGTAATGTGAATTGCAGAATTCAGTGAATCATCGAA
TCTTTGAACGCATCTTGCGCTCCTTGGTATTCCGAGGAGCATGCCTGTTTGAGTGTCATT
AAATTCTCAACTCTCTTATACTTTTTTGTAAAAGAGAGCTTGGACTGTGGAGGCTTGCTG
GCCACTTTTTGGGGTCAGCTCCTCTGAAATGCATTAGCGGAACCGTTTGCGATCTGCCAC
AAGTGTGATAAGTTATCTACACTGGCGAGGGGATTGCTCTCTGTAATGTTCAGCTTCTAA
TTGTCTCTACTTTGTGAGACTACTTTTGAATGCTTGACCTCAAATCAGGTAGGACTACCC
GCTGAACTTAA
>A3_24
TTTCCGTAGGTGAACCTGCGGAAGGATCATTATTGAATTATGTTTCTAGATAGGTTGTAG
CTGGCTCTTTTAGAGCATGTGCACGCCTGTTTGGACTTCATTTTCATCCACCTGTGCACC
TATTGTAGTCTTTGGTTGGGTTAGGAGGAAGTGATCATTGTATCAGCATCTGCTGGGAGT
GAGGACTTGCATTGTGAAAGCTTTGCTGTCCTTGATGTGATCATGGAATCTTTTTCTCAC
TAGAGTCTATGTCACTCATTATACTCTGTCGAATGTCATTGAATGTCTTTACATGGGCTT
GTATGCCTATGAAAATTGTAATACAACTTTCAGCAACGGATCTCTTGGCTCTCGCATCGA
TGAAGAACGCAGCGAAATGCGATAAGTAATGTGAATTGCAGAATTCAGTGAATCATCGAA
TCTTTGAACGCATCTTGCGCTCCTTGGTATTCCGAGGAGCATGCCTGTTTGAGTGTCATT
AAATTCTCAACTCTCTTATACTTTTTTGTAAAAGAGAGCTTGGACTGTGGAGGCTTGCTG
GCCACTTTTTGGGGTCAGCTCCTCTGAAATGCATTAGCGGAACCGTTTGCGATCTGCCAC
AAGTGTGATAAGTTATCTACACTGGCGAGGGGATTGCTCTCTGTAATGTTCAGCTTCTAA
TTGTCTCTACTTTGTGAGACTACTTTTGAATGCTTGACCTCAAATCAGGTAGGACTACCC
GCTGAACTTAA
>A3_25
TTTCCGTAGGTGAACCTGCGGAAGGATCATTATTGAATTATGTTTCTAGATAGGTTGTAG
CTGGCTCTTTTAGAGCATGTGCACGCCTGTTTGGACTTCATTTTCATCCACCTGTGCACC
TATTGTAGTCTTTGGTTGGGTTAGGAGGAAGTGATCATTGTATCAGCATCTGCTGGGAGT
GAGGACTTGCATTGTGAAAGCTTTGCTGTCCTTGATGTGATCATGGAATCTTTTTCTCAC
TAGAGTCTATGTCACTCATTATACTCTGTCGAATGTCATTGAATGTCTTTACATGGGCTT
GTATGCCTATGAAAATTGTAATACAACTTTCAGCAACGGATCTCTTGGCTCTCGCATCGA
TGAAGAACGCAGCGAAATGCGATAAGTAATGTGAATTGCAGAATTCAGTGAATCATCGAA
TCTTTGAACGCATCTTGCGCTCCTTGGTATTCCGAGGAGCATGCCTGTTTGAGTGTCATT
AAATTCTCAACTCTCTTATACTTTTTTGTAAAAGAGAGCTTGGACTGTGGAGGCTTGCTG
GCCACTTTTTGGGGTCAGCTCCTCTGAAATGCATTAGCGGAACCGTTTGCGATCTGCCAC
AAGTGTGATAAGTTATCTACACTGGCGAGGGGATTGCTCTCTGTAATGTTCAGCTTCTAA
TTGTCTCTACTTTGTGAGACTACTTTTGAATGCTTGACCTCAAATCAGGTAGGACTACCC
GCTGAACTTAA

>A3_26
TTTCCGTAGGTGAACCTGCGGAAGGATCATTATTGAATTATGTTTCTAGATAGGTTGTAG
CTGGCTCTTTTAGAGCATGTGCACGCCTGTTTGGACTTCATTTTCATCCACCTGTGCACC
TATTGTAGTCTTTGGTTGGGTTAGGAGGAAGTGATCATTGTATCAGCATCTGCTGGGAGT
GAGGACTTGCATTGTGAAAGCTTTGCTGTCCTTGATGTGATCATGGAATCTTTTTCTCAC
TAGAGTCTATGTCACTCATTATACTCTGTCGAATGTCATTGAATGTCTTTACATGGGCTT
GTATGCCTATGAAAATTGTAATACAACTTTCAGCAACGGATCTCTTGGCTCTCGCATCGA
TGAAGAACGCAGCGAAATGCGATAAGTAATGTGAATTGCAGAATTCAGTGAATCATCGAA
TCTTTGAACGCATCTTGCGCTCCTTGGTATTCCGAGGAGCATGCCTGTTTGAGTGTCATT
AAATTCTCAACTCTCTTATACTTTTTTGTAAAAGAGAGCTTGGACTGTGGAGGCTTGCTG
GCCACTTTTTGGGGTCAGCTCCTCTGAAATGCATTAGCGGAACCGTTTGCGATCTGCCAC
AAGTGTGATAAGTTATCTACACTGGCGAGGGGATTGCTCTCTGTAATGTTCAGCTTCTAA
TTGTCTCTACTTTGTGAGACTACTTTTGAATGCTTGACCTCAAATCAGGTAGGACTACCC
GCTGAACTTAA
>A3_27
TTTCCGTAGGTGAACCTGCGGAAGGATCATTATTGAATTATGTTTCTAGATAGGTTGTAG
CTGGCTCTTTTAGAGCATGTGCACGCCTGTTTGGACTTCATTTTCATCCACCTGTGCACC
TATTGTAGTCTTTGGTTGGGTTAGGAGGAAGTGATCATTGTATCAGCATCTGCTGGGAGT
GAGGACTTGCATTGTGAAAGCTTTGCTGTCCTTGATGTGATCATGGAATCTTTTTCTCAC
TAGAGTCTATGTCACTCATTATACTCTGTCGAATGTCATTGAATGTCTTTACATGGGCTT
GTATGCCTATGAAAATTGTAATACAACTTTCAGCAACGGATCTCTTGGCTCTCGCATCGA
TGAAGAACGCAGCGAAATGCGATAAGTAATGTGAATTGCAGAATTCAGTGAATCATCGAA
TCTTTGAACGCATCTTGCGCTCCTTGGTATTCCGAGGAGCATGCCTGTTTGAGTGTCATT
AAATTCTCAACTCTCTTATACTTTTTTGTAAAAGAGAGCTTGGACTGTGGAGGCTTGCTG
GCCACTTTTTGGGGTCAGCTCCTCTGAAATGCATTAGCGGAACCGTTTGCGATCTGCCAC
AAGTGTGATAAGTTATCTACACTGGCGAGGGGATTGCTCTCTGTAATGTTCAGCTTCTAA
TTGTCTCTACTTTGTGAGACTACTTTTGAATGCTTGACCTCAAATCAGGTAGGACTACCC
GCTGAACTTAA
>A3_28
TTTCCGTAGGTGAACCTGCGGAAGGATCATTATTGAATTATGTTTCTAGATAGGTTGTAG
CTGGCTCTTTTAGAGCATGTGCACGCCTGTTTGGACTTCATTTTCATCCACCTGTGCACC
TATTGTAGTCTTTGGTTGGGTTAGGAGGAAGTGATCATTGTATCAGCATCTGCTGGGAGT
GAGGACTTGCATTGTGAAAGCTTTGCTGTCCTTGATGTGATCATGGAATCTTTTTCTCAC
TAGAGTCTATGTCACTCATTATACTCTGTCGAATGTCATTGAATGTCTTTACATGGGCTT
GTATGCCTATGAAAATTGTAATACAACTTTCAGCAACGGATCTCTTGGCTCTCGCATCGA
TGAAGAACGCAGCGAAATGCGATAAGTAATGTGAATTGCAGAATTCAGTGAATCATCGAA
TCTTTGAACGCATCTTGCGCTCCTTGGTATTCCGAGGAGCATGCCTGTTTGAGTGTCATT
AAATTCTCAACTCTCTTATACTTTTTTGTAAAAGAGAGCTTGGACTGTGGAGGCTTGCTG
GCCACTTTTTGGGGTCAGCTCCTCTGAAATGCATTAGCGGAACCGTTTGCGATCTGCCAC
AAGTGTGATAAGTTATCTACACTGGCGAGGGGATTGCTCTCTGTAATGTTCAGCTTCTAA
TTGTCTCTACTTTGTGAGACTACTTTTGAATGCTTGACCTCAAATCAGGTAGGACTACCC
GCTGAACTTAA
>A3_29
TTTCCGTAGGTGAACCTGCGGAAGGATCATTATTGAATTATGTTTCTAGATAGGTTGTAG
CTGGCTCTTTTAGAGCATGTGCACGCCTGTTTGGACTTCATTTTCATCCACCTGTGCACC
TATTGTAGTCTTTGGTTGGGTTAGGAGGAAGTGATCATTGTATCAGCATCTGCTGGGAGT
GAGGACTTGCATTGTGAAAGCTTTGCTGTCCTTGATGTGATCATGGAATCTTTTTCTCAC
TAGAGTCTATGTCACTCATTATACTCTGTCGAATGTCATTGAATGTCTTTACATGGGCTT
GTATGCCTATGAAAATTGTAATACAACTTTCAGCAACGGATCTCTTGGCTCTCGCATCGA
TGAAGAACGCAGCGAAATGCGATAAGTAATGTGAATTGCAGAATTCAGTGAATCATCGAA

TCTTTGAACGCATCTTGCGCTCCTTGGTATTCCGAGGAGCATGCCTGTTTGAGTGTCATT
AAATTCTCAACTCTCTTATACTTTTTTGTAAAAGAGAGCTTGGACTGTGGAGGCTTGCTG
GCCACTTTTTGGGGTCAGCTCCTCTGAAATGCATTAGCGGAACCGTTTGCGATCTGCCAC
AAGTGTGATAAGTTATCTACACTGGCGAGGGGATTGCTCTCTGTAATGTTCAGCTTCTAA
TTGTCTCTACTTTGTGAGACTACTTTTGAATGCTTGACCTCAAATCAGGTAGGACTACCC
GCTGAACTTAA
>A3_30
TTTCCGTAGGTGAACCTGCGGAAGGATCATTATTGAATTATGTTTCTAGATAGGTTGTAG
CTGGCTCTTTTAGAGCATGTGCACGCCTGTTTGGACTTCATTTTCATCCACCTGTGCACC
TATTGTAGTCTTTGGTTGGGTTAGGAGGAAGTGATCATTGTATCAGCATCTGCTGGGAGT
GAGGACTTGCATTGTGAAAGCTTTGCTGTCCTTGATGTGATCATGGAATCTTTTTCTCAC
TAGAGTCTATGTCACTCATTATACTCTGTCGAATGTCATTGAATGTCTTTACATGGGCTT
GTATGCCTATGAAAATTGTAATACAACTTTCAGCAACGGATCTCTTGGCTCTCGCATCGA
TGAAGAACGCAGCGAAATGCGATAAGTAATGTGAATTGCAGAATTCAGTGAATCATCGAA
TCTTTGAACGCATCTTGCGCTCCTTGGTATTCCGAGGAGCATGCCTGTTTGAGTGTCATT
AAATTCTCAACTCTCTTATACTTTTTTGTAAAAGAGAGCTTGGACTGTGGAGGCTTGCTG
GCCACTTTTTGGGGTCAGCTCCTCTGAAATGCATTAGCGGAACCGTTTGCGATCTGCCAC
AAGTGTGATAAGTTATCTACACTGGCGAGGGGATTGCTCTCTGTAATGTTCAGCTTCTAA
TTGTCTCTACTTTGTGAGACTACTTTTGAATGCTTGACCTCAAATCAGGTAGGACTACCC
GCTGAACTTAA
>A3_31
TTTCCGTAGGTGAACCTGCGGAAGGATCATTATTGAATTATGTTTCTAGATAGGTTGTAG
CTGGCTCTTTTAGAGCATGTGCACGCCTGTTTGGACTTCATTTTCATCCACCTGTGCACC
TATTGTAGTCTTTGGTTGGGTTAGGAGGAAGTGATCATTGTATCAGCATCTGCTGGGAGT
GAGGACTTGCATTGTGAAAGCTTTGCTGTCCTTGATGTGATCATGGAATCTTTTTCTCAC
TAGAGTCTATGTCACTCATTATACTCTGTCGAATGTCATTGAATGTCTTTACATGGGCTT
GTATGCCTATGAAAATTGTAATACAACTTTCAGCAACGGATCTCTTGGCTCTCGCATCGA
TGAAGAACGCAGCGAAATGCGATAAGTAATGTGAATTGCAGAATTCAGTGAATCATCGAA
TCTTTGAACGCATCTTGCGCTCCTTGGTATTCCGAGGAGCATGCCTGTTTGAGTGTCATT
AAATTCTCAACTCTCTTATACTTTTTTGTAAAAGAGAGCTTGGACTGTGGAGGCTTGCTG
GCCACTTTTTGGGGTCAGCTCCTCTGAAATGCATTAGCGGAACCGTTTGCGATCTGCCAC
AAGTGTGATAAGTTATCTACACTGGCGAGGGGATTGCTCTCTGTAATGTTCAGCTTCTAA
TTGTCTCTACTTTGTGAGACTACTTTTGAATGCTTGACCTCAAATCAGGTAGGACTACCC
GCTGAACTTAA
>A3_32
TTTCCGTAGGTGAACCTGCGGAAGGATCATTATTGAATTATGTTTCTAGATAGGTTGTAG
CTGGCTCTTTTAGAGCATGTGCACGCCTGTTTGGACTTCATTTTCATCCACCTGTGCACC
TATTGTAGTCTTTGGTTGGGTTAGGAGGAAGTGATCATTGTATCAGCATCTGCTGGGAGT
GAGGACTTGCATTGTGAAAGCTTTGCTGTCCTTGATGTGATCATGGAATCTTTTTCTCAC
TAGAGTCTATGTCACTCATTATACTCTGTCGAATGTCATTGAATGTCTTTACATGGGCTT
GTATGCCTATGAAAATTGTAATACAACTTTCAGCAACGGATCTCTTGGCTCTCGCATCGA
TGAAGAACGCAGCGAAATGCGATAAGTAATGTGAATTGCAGAATTCAGTGAATCATCGAA
TCTTTGAACGCATCTTGCGCTCCTTGGTATTCCGAGGAGCATGCCTGTTTGAGTGTCATT
AAATTCTCAACTCTCTTATACTTTTTTGTAAAAGAGAGCTTGGACTGTGGAGGCTTGCTG
GCCACTTTTTGGGGTCAGCTCCTCTGAAATGCATTAGCGGAACCGTTTGCGATCTGCCAC
AAGTGTGATAAGTTATCTACACTGGCGAGGGGATTGCTCTCTGTAATGTTCAGCTTCTAA
TTGTCTCTACTTTGTGAGACTACTTTTGAATGCTTGACCTCAAATCAGGTAGGACTACCC
GCTGAACTTAA
>A3_33
TTTCCGTAGGTGAACCTGCGGAAGGATCATTATTGAATTATGTTTCTAGATAGGTTGTAG

CTGGCTCTTTTAGAGCATGTGCACGCCTGTTTGGACTTCATTTTCATCCACCTGTGCACC
TATTGTAGTCTTTGGTTGGGTTAGGAGGAAGTGATCATTGTATCAGCATCTGCTGGGAGT
GAGGACTTGCATTGTGAAAGCTTTGCTGTCCTTGATGTGATCATGGAATCTTTTTCTCAC
TAGAGTCTATGTCACTCATTATACTCTGTCGAATGTCATTGAATGTCTTTACATGGGCTT
GTATGCCTATGAAAATTGTAATACAACTTTCAGCAACGGATCTCTTGGCTCTCGCATCGA
TGAAGAACGCAGCGAAATGCGATAAGTAATGTGAATTGCAGAATTCAGTGAATCATCGAA
TCTTTGAACGCATCTTGCGCTCCTTGGTATTCCGAGGAGCATGCCTGTTTGAGTGTCATT
AAATTCTCAACTCTCTTATACTTTTTTGTAAAAGAGAGCTTGGACTGTGGAGGCTTGCTG
GCCACTTTTTGGGGTCAGCTCCTCTGAAATGCATTAGCGGAACCGTTTGCGATCTGCCAC
AAGTGTGATAAGTTATCTACACTGGCGAGGGGATTGCTCTCTGTAATGTTCAGCTTCTAA
TTGTCTCTACTTTGTGAGACTACTTTTGAATGCTTGACCTCAAATCAGGTAGGACTACCC
GCTGAACTTAA
>A3_34
TTTCCGTAGGTGAACCTGCGGAAGGATCATTATTGAATTATGTTTCTAGATAGGTTGTAG
CTGGCTCTTTTAGAGCATGTGCACGCCTGTTTGGACTTCATTTTCATCCACCTGTGCACC
TATTGTAGTCTTTGGTTGGGTTAGGAGGAAGTGATCATTGTATCAGCATCTGCTGGGAGT
GAGGACTTGCATTGTGAAAGCTTTGCTGTCCTTGATGTGATCATGGAATCTTTTTCTCAC
TAGAGTCTATGTCACTCATTATACTCTGTCGAATGTCATTGAATGTCTTTACATGGGCTT
GTATGCCTATGAAAATTGTAATACAACTTTCAGCAACGGATCTCTTGGCTCTCGCATCGA
TGAAGAACGCAGCGAAATGCGATAAGTAATGTGAATTGCAGAATTCAGTGAATCATCGAA
TCTTTGAACGCATCTTGCGCTCCTTGGTATTCCGAGGAGCATGCCTGTTTGAGTGTCATT
AAATTCTCAACTCTCTTATACTTTTTTGTAAAAGAGAGCTTGGACTGTGGAGGCTTGCTG
GCCACTTTTTGGGGTCAGCTCCTCTGAAATGCATTAGCGGAACCGTTTGCGATCTGCCAC
AAGTGTGATAAGTTATCTACACTGGCGAGGGGATTGCTCTCTGTAATGTTCAGCTTCTAA
TTGTCTCTACTTTGTGAGACTACTTTTGAATGCTTGACCTCAAATCAGGTAGGACTACCC
GCTGAACTTAA
>A3_35
TTTCCGTAGGTGAACCTGCGGAAGGATCATTATTGAATTATGTTTCTAGATAGGTTGTAG
CTGGCTCTTTTAGAGCATGTGCACGCCTGTTTGGACTTCATTTTCATCCACCTGTGCACC
TATTGTAGTCTTTGGTTGGGTTAGGAGGAAGTGATCATTGTATCAGCATCTGCTGGGAGT
GAGGACTTGCATTGTGAAAGCTTTGCTGTCCTTGATGTGATCATGGAATCTTTTTCTCAC
TAGAGTCTATGTCACTCATTATACTCTGTCGAATGTCATTGAATGTCTTTACATGGGCTT
GTATGCCTATGAAAATTGTAATACAACTTTCAGCAACGGATCTCTTGGCTCTCGCATCGA
TGAAGAACGCAGCGAAATGCGATAAGTAATGTGAATTGCAGAATTCAGTGAATCATCGAA
TCTTTGAACGCATCTTGCGCTCCTTGGTATTCCGAGGAGCATGCCTGTTTGAGTGTCATT
AAATTCTCAACTCTCTTATACTTTTTTGTAAAAGAGAGCTTGGACTGTGGAGGCTTGCTG
GCCACTTTTTGGGGTCAGCTCCTCTGAAATGCATTAGCGGAACCGTTTGCGATCTGCCAC
AAGTGTGATAAGTTATCTACACTGGCGAGGGGATTGCTCTCTGTAATGTTCAGCTTCTAA
TTGTCTCTACTTTGTGAGACTACTTTTGAATGCTTGACCTCAAATCAGGTAGGACTACCC
GCTGAACTTAA
>A3_36
TTTCCGTAGGTGAACCTGCGGAAGGATCATTATTGAATTATGTTTCTAGATAGGTTGTAG
CTGGCTCTTTTAGAGCATGTGCACGCCTGTTTGGACTTCATTTTCATCCACCTGTGCACC
TATTGTAGTCTTTGGTTGGGTTAGGAGGAAGTGATCATTGTATCAGCATCTGCTGGGAGT
GAGGACTTGCATTGTGAAAGCTTTGCTGTCCTTGATGTGATCATGGAATCTTTTTCTCAC
TAGAGTCTATGTCACTCATTATACTCTGTCGAATGTCATTGAATGTCTTTACATGGGCTT
GTATGCCTATGAAAATTGTAATACAACTTTCAGCAACGGATCTCTTGGCTCTCGCATCGA
TGAAGAACGCAGCGAAATGCGATAAGTAATGTGAATTGCAGAATTCAGTGAATCATCGAA
TCTTTGAACGCATCTTGCGCTCCTTGGTATTCCGAGGAGCATGCCTGTTTGAGTGTCATT
AAATTCTCAACTCTCTTATACTTTTTTGTAAAAGAGAGCTTGGACTGTGGAGGCTTGCTG

GCCACTTTTTGGGGTCAGCTCCTCTGAAATGCATTAGCGGAACCGTTTGCGATCTGCCAC
AAGTGTGATAAGTTATCTACACTGGCGAGGGGATTGCTCTCTGTAATGTTCAGCTTCTAA
TTGTCTCTACTTTGTGAGACTACTTTTGAATGCTTGACCTCAAATCAGGTAGGACTACCC
GCTGAACTTAA
>A3_37
TTTCCGTAGGTGAACCTGCGGAAGGATCATTATTGAATTATGTTTCTAGATAGGTTGTAG
CTGGCTCTTTTAGAGCATGTGCACGCCTGTTTGGACTTCATTTTCATCCACCTGTGCACC
TATTGTAGTCTTTGGTTGGGTTAGGAGGAAGTGATCATTGTATCAGCATCTGCTGGGAGT
GAGGACTTGCATTGTGAAAGCTTTGCTGTCCTTGATGTGATCATGGAATCTTTTTCTCAC
TAGAGTCTATGTCACTCATTATACTCTGTCGAATGTCATTGAATGTCTTTACATGGGCTT
GTATGCCTATGAAAATTGTAATACAACTTTCAGCAACGGATCTCTTGGCTCTCGCATCGA
TGAAGAACGCAGCGAAATGCGATAAGTAATGTGAATTGCAGAATTCAGTGAATCATCGAA
TCTTTGAACGCATCTTGCGCTCCTTGGTATTCCGAGGAGCATGCCTGTTTGAGTGTCATT
AAATTCTCAACTCTCTTATACTTTTTTGTAAAAGAGAGCTTGGACTGTGGAGGCTTGCTG
GCCACTTTTTGGGGTCAGCTCCTCTGAAATGCATTAGCGGAACCGTTTGCGATCTGCCAC
AAGTGTGATAAGTTATCTACACTGGCGAGGGGATTGCTCTCTGTAATGTTCAGCTTCTAA
TTGTCTCTACTTTGTGAGACTACTTTTGAATGCTTGACCTCAAATCAGGTAGGACTACCC
GCTGAACTTAA
>A3_38
TTTCCGTAGGTGAACCTGCGGAAGGATCATTATTGAATTATGTTTCTAGATAGGTTGTAG
CTGGCTCTTTTAGAGCATGTGCACGCCTGTTTGGACTTCATTTTCATCCACCTGTGCACC
TATTGTAGTCTTTGGTTGGGTTAGGAGGAAGTGATCATTGTATCAGCATCTGCTGGGAGT
GAGGACTTGCATTGTGAAAGCTTTGCTGTCCTTGATGTGATCATGGAATCTTTTTCTCAC
TAGAGTCTATGTCACTCATTATACTCTGTCGAATGTCATTGAATGTCTTTACATGGGCTT
GTATGCCTATGAAAATTGTAATACAACTTTCAGCAACGGATCTCTTGGCTCTCGCATCGA
TGAAGAACGCAGCGAAATGCGATAAGTAATGTGAATTGCAGAATTCAGTGAATCATCGAA
TCTTTGAACGCATCTTGCGCTCCTTGGTATTCCGAGGAGCATGCCTGTTTGAGTGTCATT
AAATTCTCAACTCTCTTATACTTTTTTGTAAAAGAGAGCTTGGACTGTGGAGGCTTGCTG
GCCACTTTTTGGGGTCAGCTCCTCTGAAATGCATTAGCGGAACCGTTTGCGATCTGCCAC
AAGTGTGATAAGTTATCTACACTGGCGAGGGGATTGCTCTCTGTAATGTTCAGCTTCTAA
TTGTCTCTACTTTGTGAGACTACTTTTGAATGCTTGACCTCAAATCAGGTAGGACTACCC
GCTGAACTTAA
>A3_39
TTTCCGTAGGTGAACCTGCGGAAGGATCATTATTGAATTATGTTTCTAGATAGGTTGTAG
CTGGCTCTTTTAGAGCATGTGCACGCCTGTTTGGACTTCATTTTCATCCACCTGTGCACC
TATTGTAGTCTTTGGTTGGGTTAGGAGGAAGTGATCATTGTATCAGCATCTGCTGGGAGT
GAGGACTTGCATTGTGAAAGCTTTGCTGTCCTTGATGTGATCATGGAATCTTTTTCTCAC
TAGAGTCTATGTCACTCATTATACTCTGTCGAATGTCATTGAATGTCTTTACATGGGCTT
GTATGCCTATGAAAATTGTAATACAACTTTCAGCAACGGATCTCTTGGCTCTCGCATCGA
TGAAGAACGCAGCGAAATGCGATAAGTAATGTGAATTGCAGAATTCAGTGAATCATCGAA
TCTTTGAACGCATCTTGCGCTCCTTGGTATTCCGAGGAGCATGCCTGTTTGAGTGTCATT
AAATTCTCAACTCTCTTATACTTTTTTGTAAAAGAGAGCTTGGACTGTGGAGGCTTGCTG
GCCACTTTTTGGGGTCAGCTCCTCTGAAATGCATTAGCGGAACCGTTTGCGATCTGCCAC
AAGTGTGATAAGTTATCTACACTGGCGAGGGGATTGCTCTCTGTAATGTTCAGCTTCTAA
TTGTCTCTACTTTGTGAGACTACTTTTGAATGCTTGACCTCAAATCAGGTAGGACTACCC
GCTGAACTTAA
>A3_40
TTTCCGTAGGTGAACCTGCGGAAGGATCATTATTGAATTATGTTTCTAGATAGGTTGTAG
CTGGCTCTTTTAGAGCATGTGCACGCCTGTTTGGACTTCATTTTCATCCACCTGTGCACC
TATTGTAGTCTTTGGTTGGGTTAGGAGGAAGTGATCATTGTATCAGCATCTGCTGGGAGT

GAGGACTTGCATTGTGAAAGCTTTGCTGTCCTTGATGTGATCATGGAATCTTTTTCTCAC
TAGAGTCTATGTCACTCATTATACTCTGTCGAATGTCATTGAATGTCTTTACATGGGCTT
GTATGCCTATGAAAATTGTAATACAACTTTCAGCAACGGATCTCTTGGCTCTCGCATCGA
TGAAGAACGCAGCGAAATGCGATAAGTAATGTGAATTGCAGAATTCAGTGAATCATCGAA
TCTTTGAACGCATCTTGCGCTCCTTGGTATTCCGAGGAGCATGCCTGTTTGAGTGTCATT
AAATTCTCAACTCTCTTATACTTTTTTGTAAAAGAGAGCTTGGACTGTGGAGGCTTGCTG
GCCACTTTTTGGGGTCAGCTCCTCTGAAATGCATTAGCGGAACCGTTTGCGATCTGCCAC
AAGTGTGATAAGTTATCTACACTGGCGAGGGGATTGCTCTCTGTAATGTTCAGCTTCTAA
TTGTCTCTACTTTGTGAGACTACTTTTGAATGCTTGACCTCAAATCAGGTAGGACTACCC
GCTGAACTTAA
>A3_41
TTTCCGTAGGTGAACCTGCGGAAGGATCATTATTGAATTATGTTTCTAGATAGGTTGTAG
CTGGCTCTTTTAGAGCATGTGCACGCCTGTTTGGACTTCATTTTCATCCACCTGTGCACC
TATTGTAGTCTTTGGTTGGGTTAGGAGGAAGTGATCATTGTATCAGCATCTGCTGGGAGT
GAGGACTTGCATTGTGAAAGCTTTGCTGTCCTTGATGTGATCATGGAATCTTTTTCTCAC
TAGAGTCTATGTCACTCATTATACTCTGTCGAATGTCATTGAATGTCTTTACATGGGCTT
GTATGCCTATGAAAATTGTAATACAACTTTCAGCAACGGATCTCTTGGCTCTCGCATCGA
TGAAGAACGCAGCGAAATGCGATAAGTAATGTGAATTGCAGAATTCAGTGAATCATCGAA
TCTTTGAACGCATCTTGCGCTCCTTGGTATTCCGAGGAGCATGCCTGTTTGAGTGTCATT
AAATTCTCAACTCTCTTATACTTTTTTGTAAAAGAGAGCTTGGACTGTGGAGGCTTGCTG
GCCACTTTTTGGGGTCAGCTCCTCTGAAATGCATTAGCGGAACCGTTTGCGATCTGCCAC
AAGTGTGATAAGTTATCTACACTGGCGAGGGGATTGCTCTCTGTAATGTTCAGCTTCTAA
TTGTCTCTACTTTGTGAGACTACTTTTGAATGCTTGACCTCAAATCAGGTAGGACTACCC
GCTGAACTTAA
>A3_42
TTTCCGTAGGTGAACCTGCGGAAGGATCATTATTGAATTATGTTTCTAGATAGGTTGTAG
CTGGCTCTTTTAGAGCATGTGCACGCCTGTTTGGACTTCATTTTCATCCACCTGTGCACC
TATTGTAGTCTTTGGTTGGGTTAGGAGGAAGTGATCATTGTATCAGCATCTGCTGGGAGT
GAGGACTTGCATTGTGAAAGCTTTGCTGTCCTTGATGTGATCATGGAATCTTTTTCTCAC
TAGAGTCTATGTCACTCATTATACTCTGTCGAATGTCATTGAATGTCTTTACATGGGCTT
GTATGCCTATGAAAATTGTAATACAACTTTCAGCAACGGATCTCTTGGCTCTCGCATCGA
TGAAGAACGCAGCGAAATGCGATAAGTAATGTGAATTGCAGAATTCAGTGAATCATCGAA
TCTTTGAACGCATCTTGCGCTCCTTGGTATTCCGAGGAGCATGCCTGTTTGAGTGTCATT
AAATTCTCAACTCTCTTATACTTTTTTGTAAAAGAGAGCTTGGACTGTGGAGGCTTGCTG
GCCACTTTTTGGGGTCAGCTCCTCTGAAATGCATTAGCGGAACCGTTTGCGATCTGCCAC
AAGTGTGATAAGTTATCTACACTGGCGAGGGGATTGCTCTCTGTAATGTTCAGCTTCTAA
TTGTCTCTACTTTGTGAGACTACTTTTGAATGCTTGACCTCAAATCAGGTAGGACTACCC
GCTGAACTTAA
>A3_43
TTTCCGTAGGTGAACCTGCGGAAGGATCATTATTGAATTATGTTTCTAGATAGGTTGTAG
CTGGCTCTTTTAGAGCATGTGCACGCCTGTTTGGACTTCATTTTCATCCACCTGTGCACC
TATTGTAGTCTTTGGTTGGGTTAGGAGGAAGTGATCATTGTATCAGCATCTGCTGGGAGT
GAGGACTTGCATTGTGAAAGCTTTGCTGTCCTTGATGTGATCATGGAATCTTTTTCTCAC
TAGAGTCTATGTCACTCATTATACTCTGTCGAATGTCATTGAATGTCTTTACATGGGCTT
GTATGCCTATGAAAATTGTAATACAACTTTCAGCAACGGATCTCTTGGCTCTCGCATCGA
TGAAGAACGCAGCGAAATGCGATAAGTAATGTGAATTGCAGAATTCAGTGAATCATCGAA
TCTTTGAACGCATCTTGCGCTCCTTGGTATTCCGAGGAGCATGCCTGTTTGAGTGTCATT
AAATTCTCAACTCTCTTATACTTTTTTGTAAAAGAGAGCTTGGACTGTGGAGGCTTGCTG
GCCACTTTTTGGGGTCAGCTCCTCTGAAATGCATTAGCGGAACCGTTTGCGATCTGCCAC
AAGTGTGATAAGTTATCTACACTGGCGAGGGGATTGCTCTCTGTAATGTTCAGCTTCTAA

TTGTCTCTACTTTGTGAGACTACTTTTGAATGCTTGACCTCAAATCAGGTAGGACTACCC
GCTGAACTTAA
>A3_44
TTTCCGTAGGTGAACCTGCGGAAGGATCATTATTGAATTATGTTTCTAGATAGGTTGTAG
CTGGCTCTTTTAGAGCATGTGCACGCCTGTTTGGACTTCATTTTCATCCACCTGTGCACC
TATTGTAGTCTTTGGTTGGGTTAGGAGGAAGTGATCATTGTATCAGCATCTGCTGGGAGT
GAGGACTTGCATTGTGAAAGCTTTGCTGTCCTTGATGTGATCATGGAATCTTTTTCTCAC
TAGAGTCTATGTCACTCATTATACTCTGTCGAATGTCATTGAATGTCTTTACATGGGCTT
GTATGCCTATGAAAATTGTAATACAACTTTCAGCAACGGATCTCTTGGCTCTCGCATCGA
TGAAGAACGCAGCGAAATGCGATAAGTAATGTGAATTGCAGAATTCAGTGAATCATCGAA
TCTTTGAACGCATCTTGCGCTCCTTGGTATTCCGAGGAGCATGCCTGTTTGAGTGTCATT
AAATTCTCAACTCTCTTATACTTTTTTGTAAAAGAGAGCTTGGACTGTGGAGGCTTGCTG
GCCACTTTTTGGGGTCAGCTCCTCTGAAATGCATTAGCGGAACCGTTTGCGATCTGCCAC
AAGTGTGATAAGTTATCTACACTGGCGAGGGGATTGCTCTCTGTAATGTTCAGCTTCTAA
TTGTCTCTACTTTGTGAGACTACTTTTGAATGCTTGACCTCAAATCAGGTAGGACTACCC
GCTGAACTTAA
>A3_45
TTTCCGTAGGTGAACCTGCGGAAGGATCATTATTGAATTATGTTTCTAGATAGGTTGTAG
CTGGCTCTTTTAGAGCATGTGCACGCCTGTTTGGACTTCATTTTCATCCACCTGTGCACC
TATTGTAGTCTTTGGTTGGGTTAGGAGGAAGTGATCATTGTATCAGCATCTGCTGGGAGT
GAGGACTTGCATTGTGAAAGCTTTGCTGTCCTTGATGTGATCATGGAATCTTTTTCTCAC
TAGAGTCTATGTCACTCATTATACTCTGTCGAATGTCATTGAATGTCTTTACATGGGCTT
GTATGCCTATGAAAATTGTAATACAACTTTCAGCAACGGATCTCTTGGCTCTCGCATCGA
TGAAGAACGCAGCGAAATGCGATAAGTAATGTGAATTGCAGAATTCAGTGAATCATCGAA
TCTTTGAACGCATCTTGCGCTCCTTGGTATTCCGAGGAGCATGCCTGTTTGAGTGTCATT
AAATTCTCAACTCTCTTATACTTTTTTGTAAAAGAGAGCTTGGACTGTGGAGGCTTGCTG
GCCACTTTTTGGGGTCAGCTCCTCTGAAATGCATTAGCGGAACCGTTTGCGATCTGCCAC
AAGTGTGATAAGTTATCTACACTGGCGAGGGGATTGCTCTCTGTAATGTTCAGCTTCTAA
TTGTCTCTACTTTGTGAGACTACTTTTGAATGCTTGACCTCAAATCAGGTAGGACTACCC
GCTGAACTTAA
>A3_46
TTTCCGTAGGTGAACCTGCGGAAGGATCATTATTGAATTATGTTTCTAGATAGGTTGTAG
CTGGCTCTTTTAGAGCATGTGCACGCCTGTTTGGACTTCATTTTCATCCACCTGTGCACC
TATTGTAGTCTTTGGTTGGGTTAGGAGGAAGTGATCATTGTATCAGCATCTGCTGGGAGT
GAGGACTTGCATTGTGAAAGCTTTGCTGTCCTTGATGTGATCATGGAATCTTTTTCTCAC
TAGAGTCTATGTCACTCATTATACTCTGTCGAATGTCATTGAATGTCTTTACATGGGCTT
GTATGCCTATGAAAATTGTAATACAACTTTCAGCAACGGATCTCTTGGCTCTCGCATCGA
TGAAGAACGCAGCGAAATGCGATAAGTAATGTGAATTGCAGAATTCAGTGAATCATCGAA
TCTTTGAACGCATCTTGCGCTCCTTGGTATTCCGAGGAGCATGCCTGTTTGAGTGTCATT
AAATTCTCAACTCTCTTATACTTTTTTGTAAAAGAGAGCTTGGACTGTGGAGGCTTGCTG
GCCACTTTTTGGGGTCAGCTCCTCTGAAATGCATTAGCGGAACCGTTTGCGATCTGCCAC
AAGTGTGATAAGTTATCTACACTGGCGAGGGGATTGCTCTCTGTAATGTTCAGCTTCTAA
TTGTCTCTACTTTGTGAGACTACTTTTGAATGCTTGACCTCAAATCAGGTAGGACTACCC
GCTGAACTTAA
>A3_47
TTTCCGTAGGTGAACCTGCGGAAGGATCATTATTGAATTATGTTTCTAGATAGGTTGTAG
CTGGCTCTTTTAGAGCATGTGCACGCCTGTTTGGACTTCATTTTCATCCACCTGTGCACC
TATTGTAGTCTTTGGTTGGGTTAGGAGGAAGTGATCATTGTATCAGCATCTGCTGGGAGT
GAGGACTTGCATTGTGAAAGCTTTGCTGTCCTTGATGTGATCATGGAATCTTTTTCTCAC
TAGAGTCTATGTCACTCATTATACTCTGTCGAATGTCATTGAATGTCTTTACATGGGCTT

GTATGCCTATGAAAATTGTAATACAACTTTCAGCAACGGATCTCTTGGCTCTCGCATCGA
TGAAGAACGCAGCGAAATGCGATAAGTAATGTGAATTGCAGAATTCAGTGAATCATCGAA
TCTTTGAACGCATCTTGCGCTCCTTGGTATTCCGAGGAGCATGCCTGTTTGAGTGTCATT
AAATTCTCAACTCTCTTATACTTTTTTGTAAAAGAGAGCTTGGACTGTGGAGGCTTGCTG
GCCACTTTTTGGGGTCAGCTCCTCTGAAATGCATTAGCGGAACCGTTTGCGATCTGCCAC
AAGTGTGATAAGTTATCTACACTGGCGAGGGGATTGCTCTCTGTAATGTTCAGCTTCTAA
TTGTCTCTACTTTGTGAGACTACTTTTGAATGCTTGACCTCAAATCAGGTAGGACTACCC
GCTGAACTTAA
>A3_48
TTTCCGTAGGTGAACCTGCGGAAGGATCATTATTGAATTATGTTTCTAGATAGGTTGTAG
CTGGCTCTTTTAGAGCATGTGCACGCCTGTTTGGACTTCATTTTCATCCACCTGTGCACC
TATTGTAGTCTTTGGTTGGGTTAGGAGGAAGTGATCATTGTATCAGCATCTGCTGGGAGT
GAGGACTTGCATTGTGAAAGCTTTGCTGTCCTTGATGTGATCATGGAATCTTTTTCTCAC
TAGAGTCTATGTCACTCATTATACTCTGTCGAATGTCATTGAATGTCTTTACATGGGCTT
GTATGCCTATGAAAATTGTAATACAACTTTCAGCAACGGATCTCTTGGCTCTCGCATCGA
TGAAGAACGCAGCGAAATGCGATAAGTAATGTGAATTGCAGAATTCAGTGAATCATCGAA
TCTTTGAACGCATCTTGCGCTCCTTGGTATTCCGAGGAGCATGCCTGTTTGAGTGTCATT
AAATTCTCAACTCTCTTATACTTTTTTGTAAAAGAGAGCTTGGACTGTGGAGGCTTGCTG
GCCACTTTTTGGGGTCAGCTCCTCTGAAATGCATTAGCGGAACCGTTTGCGATCTGCCAC
AAGTGTGATAAGTTATCTACACTGGCGAGGGGATTGCTCTCTGTAATGTTCAGCTTCTAA
TTGTCTCTACTTTGTGAGACTACTTTTGAATGCTTGACCTCAAATCAGGTAGGACTACCC
GCTGAACTTAA
>A3_49
TTTCCGTAGGTGAACCTGCGGAAGGATCATTATTGAATTATGTTTCTAGATAGGTTGTAG
CTGGCTCTTTTAGAGCATGTGCACGCCTGTTTGGACTTCATTTTCATCCACCTGTGCACC
TATTGTAGTCTTTGGTTGGGTTAGGAGGAAGTGATCATTGTATCAGCATCTGCTGGGAGT
GAGGACTTGCATTGTGAAAGCTTTGCTGTCCTTGATGTGATCATGGAATCTTTTTCTCAC
TAGAGTCTATGTCACTCATTATACTCTGTCGAATGTCATTGAATGTCTTTACATGGGCTT
GTATGCCTATGAAAATTGTAATACAACTTTCAGCAACGGATCTCTTGGCTCTCGCATCGA
TGAAGAACGCAGCGAAATGCGATAAGTAATGTGAATTGCAGAATTCAGTGAATCATCGAA
TCTTTGAACGCATCTTGCGCTCCTTGGTATTCCGAGGAGCATGCCTGTTTGAGTGTCATT
AAATTCTCAACTCTCTTATACTTTTTTGTAAAAGAGAGCTTGGACTGTGGAGGCTTGCTG
GCCACTTTTTGGGGTCAGCTCCTCTGAAATGCATTAGCGGAACCGTTTGCGATCTGCCAC
AAGTGTGATAAGTTATCTACACTGGCGAGGGGATTGCTCTCTGTAATGTTCAGCTTCTAA
TTGTCTCTACTTTGTGAGACTACTTTTGAATGCTTGACCTCAAATCAGGTAGGACTACCC
GCTGAACTTAA
>A3_50
TTTCCGTAGGTGAACCTGCGGAAGGATCATTATTGAATTATGTTTCTAGATAGGTTGTAG
CTGGCTCTTTTAGAGCATGTGCACGCCTGTTTGGACTTCATTTTCATCCACCTGTGCACC
TATTGTAGTCTTTGGTTGGGTTAGGAGGAAGTGATCATTGTATCAGCATCTGCTGGGAGT
GAGGACTTGCATTGTGAAAGCTTTGCTGTCCTTGATGTGATCATGGAATCTTTTTCTCAC
TAGAGTCTATGTCACTCATTATACTCTGTCGAATGTCATTGAATGTCTTTACATGGGCTT
GTATGCCTATGAAAATTGTAATACAACTTTCAGCAACGGATCTCTTGGCTCTCGCATCGA
TGAAGAACGCAGCGAAATGCGATAAGTAATGTGAATTGCAGAATTCAGTGAATCATCGAA
TCTTTGAACGCATCTTGCGCTCCTTGGTATTCCGAGGAGCATGCCTGTTTGAGTGTCATT
AAATTCTCAACTCTCTTATACTTTTTTGTAAAAGAGAGCTTGGACTGTGGAGGCTTGCTG
GCCACTTTTTGGGGTCAGCTCCTCTGAAATGCATTAGCGGAACCGTTTGCGATCTGCCAC
AAGTGTGATAAGTTATCTACACTGGCGAGGGGATTGCTCTCTGTAATGTTCAGCTTCTAA
TTGTCTCTACTTTGTGAGACTACTTTTGAATGCTTGACCTCAAATCAGGTAGGACTACCC
GCTGAACTTAA

>A3-61
TTTCCGTAGGTGAACCTGCGGAAGGATCATTATTGAATTATGTTTCTAGATAGGTTGTAG
CTGGCTCTTTTAGAGCATGTGCACGCCTGTTTGGACTTCATTTTCATCCACCTGTGCACC
TATTGTAGTCTTTGGTTGGGTTAGGAGGAAGTGATCATTGTATCAGCATCTGCTGGGAGT
GAGGACTTGCATTGTGAAAGCTTTGCTGTCCTTGATGTGATCATGGAATCTTTTTCTCAC
TAGAGTCTATGTCACTCATTATACTCTGTCGAATGTCATTGAATGTCTTTACATGGGCTT
GTATGCCTATGAAAATTGTAATACAACTTTCAGCAACGGATCTCTTGGCTCTCGCATCGA
TGAAGAACGCAGCGAAATGCGATAAGTAATGTGAATTGCAGAATTCAGTGAATCATCGAA
TCTTTGAACGCATCTTGCGCTCCTTGGTATTCCGAGGAGCATGCCTGTTTGAGTGTCATT
AAATTCTCAACTCTCTTATACTTTTTTGTAAAAGAGAGCTTGGACTGTGGAGGCTTGCTG
GCCACTTTTTGGGGTCAGCTCCTCTGAAATGCATTAGCGGAACCGTTTGCGATCTGCCAC
AAGTGTGATAAGTTATCTACACTGGCGAGGGGATTGCTCTCTGTAATGTTCAGCTTCTAA
TTGTCTCTACTTTGTGAGACTACTTTTGAATGCTTGACCTCAAATCAGGTAGGACTACCC
GCTGAACTTAA
>A3-62
TTTCCGTAGGTGAACCTGCGGAAGGATCATTATTGAATTATGTTTCTAGATAGGTTGTAG
CTGGCTCTTTTAGAGCATGTGCACGCCTGTTTGGACTTCATTTTCATCCACCTGTGCACC
TATTGTAGTCTTTGGTTGGGTTAGGAGGAAGTGATCATTGTATCAGCATCTGCTGGGAGT
GAGGACTTGCATTGTGAAAGCTTTGCTGTCCTTGATGTGATCATGGAATCTTTTTCTCAC
TAGAGTCTATGTCACTCATTATACTCTGTCGAATGTCATTGAATGTCTTTACATGGGCTT
GTATGCCTATGAAAATTGTAATACAACTTTCAGCAACGGATCTCTTGGCTCTCGCATCGA
TGAAGAACGCAGCGAAATGCGATAAGTAATGTGAATTGCAGAATTCAGTGAATCATCGAA
TCTTTGAACGCATCTTGCGCTCCTTGGTATTCCGAGGAGCATGCCTGTTTGAGTGTCATT
AAATTCTCAACTCTCTTATACTTTTTTGTAAAAGAGAGCTTGGACTGTGGAGGCTTGCTG
GCCACTTTTTGGGGTCAGCTCCTCTGAAATGCATTAGCGGAACCGTTTGCGATCTGCCAC
AAGTGTGATAAGTTATCTACACTGGCGAGGGGATTGCTCTCTGTAATGTTCAGCTTCTAA
TTGTCTCTACTTTGTGAGACTACTTTTGAATGCTTGACCTCAAATCAGGTAGGACTACCC
GCTGAACTTAA
>A3-63
TTTCCGTAGGTGAACCTGCGGAAGGATCATTATTGAATTATGTTTCTAGATAGGTTGTAG
CTGGCTCTTTTAGAGCATGTGCACGCCTGTTTGGACTTCATTTTCATCCACCTGTGCACC
TATTGTAGTCTTTGGTTGGGTTAGGAGGAAGTGATCATTGTATCAGCATCTGCTGGGAGT
GAGGACTTGCATTGTGAAAGCTTTGCTGTCCTTGATGTGATCATGGAATCTTTTTCTCAC
TAGAGTCTATGTCACTCATTATACTCTGTCGAATGTCATTGAATGTCTTTACATGGGCTT
GTATGCCTATGAAAATTGTAATACAACTTTCAGCAACGGATCTCTTGGCTCTCGCATCGA
TGAAGAACGCAGCGAAATGCGATAAGTAATGTGAATTGCAGAATTCAGTGAATCATCGAA
TCTTTGAACGCATCTTGCGCTCCTTGGTATTCCGAGGAGCATGCCTGTTTGAGTGTCATT
AAATTCTCAACTCTCTTATACTTTTTTGTAAAAGAGAGCTTGGACTGTGGAGGCTTGCTG
GCCACTTTTTGGGGTCAGCTCCTCTGAAATGCATTAGCGGAACCGTTTGCGATCTGCCAC
AAGTGTGATAAGTTATCTACACTGGCGAGGGGATTGCTCTCTGTAATGTTCAGCTTCTAA
TTGTCTCTACTTTGTGAGACTACTTTTGAATGCTTGACCTCAAATCAGGTAGGACTACCC
GCTGAACTTAA
>A3-65
TTTCCGTAGGTGAACCTGCGGAAGGATCATTATTGAATTATGTTTCTAGATAGGTTGTAG
CTGGCTCTTTTAGAGCATGTGCACGCCTGTTTGGACTTCATTTTCATCCACCTGTGCACC
TATTGTAGTCTTTGGTTGGGTTAGGAGGAAGTGATCATTGTATCAGCATCTGCTGGGAGT
GAGGACTTGCATTGTGAAAGCTTTGCTGTCCTTGATGTGATCATGGAATCTTTTTCTCAC
TAGAGTCTATGTCACTCATTATACTCTGTCGAATGTCATTGAATGTCTTTACATGGGCTT
GTATGCCTATGAAAATTGTAATACAACTTTCAGCAACGGATCTCTTGGCTCTCGCATCGA
TGAAGAACGCAGCGAAATGCGATAAGTAATGTGAATTGCAGAATTCAGTGAATCATCGAA

TCTTTGAACGCATCTTGCGCTCCTTGGTATTCCGAGGAGCATGCCTGTTTGAGTGTCATT
AAATTCTCAACTCTCTTATACTTTTTTGTAAAAGAGAGCTTGGACTGTGGAGGCTTGCTG
GCCACTTTTTGGGGTCAGCTCCTCTGAAATGCATTAGCGGAACCGTTTGCGATCTGCCAC
AAGTGTGATAAGTTATCTACACTGGCGAGGGGATTGCTCTCTGTAATGTTCAGCTTCTAA
TTGTCTCTACTTTGTGAGACTACTTTTGAATGCTTGACCTCAAATCAGGTAGGACTACCC
GCTGAACTTAA
>A3-66
TTTCCGTAGGTGAACCTGCGGAAGGATCATTATTGAATTATGTTTCTAGATAGGTTGTAG
CTGGCTCTTTTAGAGCATGTGCACGCCTGTTTGGACTTCATTTTCATCCACCTGTGCACC
TATTGTAGTCTTTGGTTGGGTTAGGAGGAAGTGATCATTGTATCAGCATCTGCTGGGAGT
GAGGACTTGCATTGTGAAAGCTTTGCTGTCCTTGATGTGATCATGGAATCTTTTTCTCAC
TAGAGTCTATGTCACTCATTATACTCTGTCGAATGTCATTGAATGTCTTTACATGGGCTT
GTATGCCTATGAAAATTGTAATACAACTTTCAGCAACGGATCTCTTGGCTCTCGCATCGA
TGAAGAACGCAGCGAAATGCGATAAGTAATGTGAATTGCAGAATTCAGTGAATCATCGAA
TCTTTGAACGCATCTTGCGCTCCTTGGTATTCCGAGGAGCATGCCTGTTTGAGTGTCATT
AAATTCTCAACTCTCTTATACTTTTTTGTAAAAGAGAGCTTGGACTGTGGAGGCTTGCTG
GCCACTTTTTGGGGTCAGCTCCTCTGAAATGCATTAGCGGAACCGTTTGCGATCTGCCAC
AAGTGTGATAAGTTATCTACACTGGCGAGGGGATTGCTCTCTGTAATGTTCAGCTTCTAA
TTGTCTCTACTTTGTGAGACTACTTTTGAATGCTTGACCTCAAATCAGGTAGGACTACCC
GCTGAACTTAA
>A3-68
TTTCCGTAGGTGAACCTGCGGAAGGATCATTATTGAATTATGTTTCTAGATAGGTTGTAG
CTGGCTCTTTTAGAGCATGTGCACGCCTGTTTGGACTTCATTTTCATCCACCTGTGCACC
TATTGTAGTCTTTGGTTGGGTTAGGAGGAAGTGATCATTGTATCAGCATCTGCTGGGAGT
GAGGACTTGCATTGTGAAAGCTTTGCTGTCCTTGATGTGATCATGGAATCTTTTTCTCAC
TAGAGTCTATGTCACTCATTATACTCTGTCGAATGTCATTGAATGTCTTTACATGGGCTT
GTATGCCTATGAAAATTGTAATACAACTTTCAGCAACGGATCTCTTGGCTCTCGCATCGA
TGAAGAACGCAGCGAAATGCGATAAGTAATGTGAATTGCAGAATTCAGTGAATCATCGAA
TCTTTGAACGCATCTTGCGCTCCTTGGTATTCCGAGGAGCATGCCTGTTTGAGTGTCATT
AAATTCTCAACTCTCTTATACTTTTTTGTAAAAGAGAGCTTGGACTGTGGAGGCTTGCTG
GCCACTTTTTGGGGTCAGCTCCTCTGAAATGCATTAGCGGAACCGTTTGCGATCTGCCAC
AAGTGTGATAAGTTATCTACACTGGCGAGGGGATTGCTCTCTGTAATGTTCAGCTTCTAA
TTGTCTCTACTTTGTGAGACTACTTTTGAATGCTTGACCTCAAATCAGGTAGGACTACCC
GCTGAACTTAA
>A3-69
TTTCCGTAGGTGAACCTGCGGAAGGATCATTATTGAATTATGTTTCTAGATAGGTTGTAG
CTGGCTCTTTTAGAGCATGTGCACGCCTGTTTGGACTTCATTTTCATCCACCTGTGCACC
TATTGTAGTCTTTGGTTGGGTTAGGAGGAAGTGATCATTGTATCAGCATCTGCTGGGAGT
GAGGACTTGCATTGTGAAAGCTTTGCTGTCCTTGATGTGATCATGGAATCTTTTTCTCAC
TAGAGTCTATGTCACTCATTATACTCTGTCGAATGTCATTGAATGTCTTTACATGGGCTT
GTATGCCTATGAAAATTGTAATACAACTTTCAGCAACGGATCTCTTGGCTCTCGCATCGA
TGAAGAACGCAGCGAAATGCGATAAGTAATGTGAATTGCAGAATTCAGTGAATCATCGAA
TCTTTGAACGCATCTTGCGCTCCTTGGTATTCCGAGGAGCATGCCTGTTTGAGTGTCATT
AAATTCTCAACTCTCTTATACTTTTTTGTAAAAGAGAGCTTGGACTGTGGAGGCTTGCTG
GCCACTTTTTGGGGTCAGCTCCTCTGAAATGCATTAGCGGAACCGTTTGCGATCTGCCAC
AAGTGTGATAAGTTATCTACACTGGCGAGGGGATTGCTCTCTGTAATGTTCAGCTTCTAA
TTGTCTCTACTTTGTGAGACTACTTTTGAATGCTTGACCTCAAATCAGGTAGGACTACCC
GCTGAACTTAA
>A3-70
TTTCCGTAGGTGAACCTGCGGAAGGATCATTATTGAATTATGTTTCTAGATAGGTTGTAG

CTGGCTCTTTTAGAGCATGTGCACGCCTGTTTGGACTTCATTTTCATCCACCTGTGCACC
TATTGTAGTCTTTGGTTGGGTTAGGAGGAAGTGATCATTGTATCAGCATCTGCTGGGAGT
GAGGACTTGCATTGTGAAAGCTTTGCTGTCCTTGATGTGATCATGGAATCTTTTTCTCAC
TAGAGTCTATGTCACTCATTATACTCTGTCGAATGTCATTGAATGTCTTTACATGGGCTT
GTATGCCTATGAAAATTGTAATACAACTTTCAGCAACGGATCTCTTGGCTCTCGCATCGA
TGAAGAACGCAGCGAAATGCGATAAGTAATGTGAATTGCAGAATTCAGTGAATCATCGAA
TCTTTGAACGCATCTTGCGCTCCTTGGTATTCCGAGGAGCATGCCTGTTTGAGTGTCATT
AAATTCTCAACTCTCTTATACTTTTTTGTAAAAGAGAGCTTGGACTGTGGAGGCTTGCTG
GCCACTTTTTGGGGTCAGCTCCTCTGAAATGCATTAGCGGAACCGTTTGCGATCTGCCAC
AAGTGTGATAAGTTATCTACACTGGCGAGGGGATTGCTCTCTGTAATGTTCAGCTTCTAA
TTGTCTCTACTTTGTGAGACTACTTTTGAATGCTTGACCTCAAATCAGGTAGGACTACCC
GCTGAACTTAA
>A4_1
TTTCCGTAGGTGAACCTGCGGAAGGATCATTATTGAATTATGTTTCTAGATAGGTTGTAG
CTGGCTCTTTTAGAGCATGTGCACGCCTGTTTGGACTTCATTTTCATCCACCTGTGCACC
TATTGTAGTCTTTGGTTGGGTTAGGAGGAAGTGATCATTGTATCAGCATCTGCTGGGAGT
GAGGACTTGCATTGTGAAAGCTTTGCTGTCCTTGATGTGATCATGGAATCTTTTTCTCAC
TAGAGTCTATGTCACTCATTATACTCTGTCGAATGTCATTGAATGTCTTTACATGGGCTT
GTATGCCTATGAAAATTGTAATACAACTTTCAGCAACGGATCTCTTGGCTCTCGCATCGA
TGAAGAACGCAGCGAAATGCGATAAGTAATGTGAATTGCAGAATTCAGTGAATCATCGAA
TCTTTGAACGCATCTTGCGCTCCTTGGTATTCCGAGGAGCATGCCTGTTTGAGTGTCATT
AAATTCTCAACTCTCTTATACTTTTTTGTAAAAGAGAGCTTGGACTGTGGAGGCTTGCTG
GCCACTTTTTGGGGTCAGCTCCTCTGAAATGCATTAGCGGAACCGTTTGCGATCTGCCAC
AAGTGTGATAAGTTATCTACACTGGCGAGGGGATTGCTCTCTGTAATGTTCAGCTTCTAA
TTGTCTCTACTTTGTGAGACTACTTTTGAATGCTTGACCTCAAATCAGGTAGGACTACCC
GCTGAACTTAA
>A4_2
TTTCCGTAGGTGAACCTGCGGAAGGATCATTATTGAATTATGTTTCTAGATAGGTTGTAG
CTGGCTCTTTTAGAGCATGTGCACGCCTGTTTGGACTTCATTTTCATCCACCTGTGCACC
TATTGTAGTCTTTGGTTGGGTTAGGAGGAAGTGATCATTGTATCAGCATCTGCTGGGAGT
GAGGACTTGCATTGTGAAAGCTTTGCTGTCCTTGATGTGATCATGGAATCTTTTTCTCAC
TAGAGTCTATGTCACTCATTATACTCTGTCGAATGTCATTGAATGTCTTTACATGGGCTT
GTATGCCTATGAAAATTGTAATACAACTTTCAGCAACGGATCTCTTGGCTCTCGCATCGA
TGAAGAACGCAGCGAAATGCGATAAGTAATGTGAATTGCAGAATTCAGTGAATCATCGAA
TCTTTGAACGCATCTTGCGCTCCTTGGTATTCCGAGGAGCATGCCTGTTTGAGTGTCATT
AAATTCTCAACTCTCTTATACTTTTTTGTAAAAGAGAGCTTGGACTGTGGAGGCTTGCTG
GCCACTTTTTGGGGTCAGCTCCTCTGAAATGCATTAGCGGAACCGTTTGCGATCTGCCAC
AAGTGTGATAAGTTATCTACACTGGCGAGGGGATTGCTCTCTGTAATGTTCAGCTTCTAA
TTGTCTCTACTTTGTGAGACTACTTTTGAATGCTTGACCTCAAATCAGGTAGGACTACCC
GCTGAACTTAA
>A4_3
TTTCCGTAGGTGAACCTGCGGAAGGATCATTATTGAATTATGTTTCTAGATAGGTTGTAG
CTGGCTCTTTTAGAGCATGTGCACGCCTGTTTGGACTTCATTTTCATCCACCTGTGCACC
TATTGTAGTCTTTGGTTGGGTTAGGAGGAAGTGATCATTGTATCAGCATCTGCTGGGAGT
GAGGACTTGCATTGTGAAAGCTTTGCTGTCCTTGATGTGATCATGGAATCTTTTTCTCAC
TAGAGTCTATGTCACTCATTATACTCTGTCGAATGTCATTGAATGTCTTTACATGGGCTT
GTATGCCTATGAAAATTGTAATACAACTTTCAGCAACGGATCTCTTGGCTCTCGCATCGA
TGAAGAACGCAGCGAAATGCGATAAGTAATGTGAATTGCAGAATTCAGTGAATCATCGAA
TCTTTGAACGCATCTTGCGCTCCTTGGTATTCCGAGGAGCATGCCTGTTTGAGTGTCATT
AAATTCTCAACTCTCTTATACTTTTTTGTAAAAGAGAGCTTGGACTGTGGAGGCTTGCTG

GCCACTTTTTGGGGTCAGCTCCTCTGAAATGCATTAGCGGAACCGTTTGCGATCTGCCAC
AAGTGTGATAAGTTATCTACACTGGCGAGGGGATTGCTCTCTGTAATGTTCAGCTTCTAA
TTGTCTCTACTTTGTGAGACTACTTTTGAATGCTTGACCTCAAATCAGGTAGGACTACCC
GCTGAACTTAA
>A4_4
TTTCCGTAGGTGAACCTGCGGAAGGATCATTATTGAATTATGTTTCTAGATAGGTTGTAG
CTGGCTCTTTTAGAGCATGTGCACGCCTGTTTGGACTTCATTTTCATCCACCTGTGCACC
TATTGTAGTCTTTGGTTGGGTTAGGAGGAAGTGATCATTGTATCAGCATCTGCTGGGAGT
GAGGACTTGCATTGTGAAAGCTTTGCTGTCCTTGATGTGATCATGGAATCTTTTTCTCAC
TAGAGTCTATGTCACTCATTATACTCTGTCGAATGTCATTGAATGTCTTTACATGGGCTT
GTATGCCTATGAAAATTGTAATACAACTTTCAGCAACGGATCTCTTGGCTCTCGCATCGA
TGAAGAACGCAGCGAAATGCGATAAGTAATGTGAATTGCAGAATTCAGTGAATCATCGAA
TCTTTGAACGCATCTTGCGCTCCTTGGTATTCCGAGGAGCATGCCTGTTTGAGTGTCATT
AAATTCTCAACTCTCTTATACTTTTTTGTAAAAGAGAGCTTGGACTGTGGAGGCTTGCTG
GCCACTTTTTGGGGTCAGCTCCTCTGAAATGCATTAGCGGAACCGTTTGCGATCTGCCAC
AAGTGTGATAAGTTATCTACACTGGCGAGGGGATTGCTCTCTGTAATGTTCAGCTTCTAA
TTGTCTCTACTTTGTGAGACTACTTTTGAATGCTTGACCTCAAATCAGGTAGGACTACCC
GCTGAACTTAA
>A4_5
TTTCCGTAGGTGAACCTGCGGAAGGATCATTATTGAATTATGTTTCTAGATAGGTTGTAG
CTGGCTCTTTTAGAGCATGTGCACGCCTGTTTGGACTTCATTTTCATCCACCTGTGCACC
TATTGTAGTCTTTGGTTGGGTTAGGAGGAAGTGATCATTGTATCAGCATCTGCTGGGAGT
GAGGACTTGCATTGTGAAAGCTTTGCTGTCCTTGATGTGATCATGGAATCTTTTTCTCAC
TAGAGTCTATGTCACTCATTATACTCTGTCGAATGTCATTGAATGTCTTTACATGGGCTT
GTATGCCTATGAAAATTGTAATACAACTTTCAGCAACGGATCTCTTGGCTCTCGCATCGA
TGAAGAACGCAGCGAAATGCGATAAGTAATGTGAATTGCAGAATTCAGTGAATCATCGAA
TCTTTGAACGCATCTTGCGCTCCTTGGTATTCCGAGGAGCATGCCTGTTTGAGTGTCATT
AAATTCTCAACTCTCTTATACTTTTTTGTAAAAGAGAGCTTGGACTGTGGAGGCTTGCTG
GCCACTTTTTGGGGTCAGCTCCTCTGAAATGCATTAGCGGAACCGTTTGCGATCTGCCAC
AAGTGTGATAAGTTATCTACACTGGCGAGGGGATTGCTCTCTGTAATGTTCAGCTTCTAA
TTGTCTCTACTTTGTGAGACTACTTTTGAATGCTTGACCTCAAATCAGGTAGGACTACCC
GCTGAACTTAA
>A4_6
TTTCCGTAGGTGAACCTGCGGAAGGATCATTATTGAATTATGTTTCTAGATAGGTTGTAG
CTGGCTCTTTTAGAGCATGTGCACGCCTGTTTGGACTTCATTTTCATCCACCTGTGCACC
TATTGTAGTCTTTGGTTGGGTTAGGAGGAAGTGATCATTGTATCAGCATCTGCTGGGAGT
GAGGACTTGCATTGTGAAAGCTTTGCTGTCCTTGATGTGATCATGGAATCTTTTTCTCAC
TAGAGTCTATGTCACTCATTATACTCTGTCGAATGTCATTGAATGTCTTTACATGGGCTT
GTATGCCTATGAAAATTGTAATACAACTTTCAGCAACGGATCTCTTGGCTCTCGCATCGA
TGAAGAACGCAGCGAAATGCGATAAGTAATGTGAATTGCAGAATTCAGTGAATCATCGAA
TCTTTGAACGCATCTTGCGCTCCTTGGTATTCCGAGGAGCATGCCTGTTTGAGTGTCATT
AAATTCTCAACTCTCTTATACTTTTTTGTAAAAGAGAGCTTGGACTGTGGAGGCTTGCTG
GCCACTTTTTGGGGTCAGCTCCTCTGAAATGCATTAGCGGAACCGTTTGCGATCTGCCAC
AAGTGTGATAAGTTATCTACACTGGCGAGGGGATTGCTCTCTGTAATGTTCAGCTTCTAA
TTGTCTCTACTTTGTGAGACTACTTTTGAATGCTTGACCTCAAATCAGGTAGGACTACCC
GCTGAACTTAA
>A4_7
TTTCCGTAGGTGAACCTGCGGAAGGATCATTATTGAATTATGTTTCTAGATAGGTTGTAG
CTGGCTCTTTTAGAGCATGTGCACGCCTGTTTGGACTTCATTTTCATCCACCTGTGCACC
TATTGTAGTCTTTGGTTGGGTTAGGAGGAAGTGATCATTGTATCAGCATCTGCTGGGAGT

GAGGACTTGCATTGTGAAAGCTTTGCTGTCCTTGATGTGATCATGGAATCTTTTTCTCAC
TAGAGTCTATGTCACTCATTATACTCTGTCGAATGTCATTGAATGTCTTTACATGGGCTT
GTATGCCTATGAAAATTGTAATACAACTTTCAGCAACGGATCTCTTGGCTCTCGCATCGA
TGAAGAACGCAGCGAAATGCGATAAGTAATGTGAATTGCAGAATTCAGTGAATCATCGAA
TCTTTGAACGCATCTTGCGCTCCTTGGTATTCCGAGGAGCATGCCTGTTTGAGTGTCATT
AAATTCTCAACTCTCTTATACTTTTTTGTAAAAGAGAGCTTGGACTGTGGAGGCTTGCTG
GCCACTTTTTGGGGTCAGCTCCTCTGAAATGCATTAGCGGAACCGTTTGCGATCTGCCAC
AAGTGTGATAAGTTATCTACACTGGCGAGGGGATTGCTCTCTGTAATGTTCAGCTTCTAA
TTGTCTCTACTTTGTGAGACTACTTTTGAATGCTTGACCTCAAATCAGGTAGGACTACCC
GCTGAACTTAA
>A4_8
TTTCCGTAGGTGAACCTGCGGAAGGATCATTATTGAATTATGTTTCTAGATAGGTTGTAG
CTGGCTCTTTTAGAGCATGTGCACGCCTGTTTGGACTTCATTTTCATCCACCTGTGCACC
TATTGTAGTCTTTGGTTGGGTTAGGAGGAAGTGATCATTGTATCAGCATCTGCTGGGAGT
GAGGACTTGCATTGTGAAAGCTTTGCTGTCCTTGATGTGATCATGGAATCTTTTTCTCAC
TAGAGTCTATGTCACTCATTATACTCTGTCGAATGTCATTGAATGTCTTTACATGGGCTT
GTATGCCTATGAAAATTGTAATACAACTTTCAGCAACGGATCTCTTGGCTCTCGCATCGA
TGAAGAACGCAGCGAAATGCGATAAGTAATGTGAATTGCAGAATTCAGTGAATCATCGAA
TCTTTGAACGCATCTTGCGCTCCTTGGTATTCCGAGGAGCATGCCTGTTTGAGTGTCATT
AAATTCTCAACTCTCTTATACTTTTTTGTAAAAGAGAGCTTGGACTGTGGAGGCTTGCTG
GCCACTTTTTGGGGTCAGCTCCTCTGAAATGCATTAGCGGAACCGTTTGCGATCTGCCAC
AAGTGTGATAAGTTATCTACACTGGCGAGGGGATTGCTCTCTGTAATGTTCAGCTTCTAA
TTGTCTCTACTTTGTGAGACTACTTTTGAATGCTTGACCTCAAATCAGGTAGGACTACCC
GCTGAACTTAA
>A4_9
TTTCCGTAGGTGAACCTGCGGAAGGATCATTATTGAATTATGTTTCTAGATAGGTTGTAG
CTGGCTCTTTTAGAGCATGTGCACGCCTGTTTGGACTTCATTTTCATCCACCTGTGCACC
TATTGTAGTCTTTGGTTGGGTTAGGAGGAAGTGATCATTGTATCAGCATCTGCTGGGAGT
GAGGACTTGCATTGTGAAAGCTTTGCTGTCCTTGATGTGATCATGGAATCTTTTTCTCAC
TAGAGTCTATGTCACTCATTATACTCTGTCGAATGTCATTGAATGTCTTTACATGGGCTT
GTATGCCTATGAAAATTGTAATACAACTTTCAGCAACGGATCTCTTGGCTCTCGCATCGA
TGAAGAACGCAGCGAAATGCGATAAGTAATGTGAATTGCAGAATTCAGTGAATCATCGAA
TCTTTGAACGCATCTTGCGCTCCTTGGTATTCCGAGGAGCATGCCTGTTTGAGTGTCATT
AAATTCTCAACTCTCTTATACTTTTTTGTAAAAGAGAGCTTGGACTGTGGAGGCTTGCTG
GCCACTTTTTGGGGTCAGCTCCTCTGAAATGCATTAGCGGAACCGTTTGCGATCTGCCAC
AAGTGTGATAAGTTATCTACACTGGCGAGGGGATTGCTCTCTGTAATGTTCAGCTTCTAA
TTGTCTCTACTTTGTGAGACTACTTTTGAATGCTTGACCTCAAATCAGGTAGGACTACCC
GCTGAACTTAA
>A4_10
TTTCCGTAGGTGAACCTGCGGAAGGATCATTATTGAATTATGTTTCTAGATAGGTTGTAG
CTGGCTCTTTTAGAGCATGTGCACGCCTGTTTGGACTTCATTTTCATCCACCTGTGCACC
TATTGTAGTCTTTGGTTGGGTTAGGAGGAAGTGATCATTGTATCAGCATCTGCTGGGAGT
GAGGACTTGCATTGTGAAAGCTTTGCTGTCCTTGATGTGATCATGGAATCTTTTTCTCAC
TAGAGTCTATGTCACTCATTATACTCTGTCGAATGTCATTGAATGTCTTTACATGGGCTT
GTATGCCTATGAAAATTGTAATACAACTTTCAGCAACGGATCTCTTGGCTCTCGCATCGA
TGAAGAACGCAGCGAAATGCGATAAGTAATGTGAATTGCAGAATTCAGTGAATCATCGAA
TCTTTGAACGCATCTTGCGCTCCTTGGTATTCCGAGGAGCATGCCTGTTTGAGTGTCATT
AAATTCTCAACTCTCTTATACTTTTTTGTAAAAGAGAGCTTGGACTGTGGAGGCTTGCTG
GCCACTTTTTGGGGTCAGCTCCTCTGAAATGCATTAGCGGAACCGTTTGCGATCTGCCAC
AAGTGTGATAAGTTATCTACACTGGCGAGGGGATTGCTCTCTGTAATGTTCAGCTTCTAA

TTGTCTCTACTTTGTGAGACTACTTTTGAATGCTTGACCTCAAATCAGGTAGGACTACCC
GCTGAACTTAA
>A4_11
TTTCCGTAGGTGAACCTGCGGAAGGATCATTATTGAATTATGTTTCTAGATAGGTTGTAG
CTGGCTCTTTTAGAGCATGTGCACGCCTGTTTGGACTTCATTTTCATCCACCTGTGCACC
TATTGTAGTCTTTGGTTGGGTTAGGAGGAAGTGATCATTGTATCAGCATCTGCTGGGAGT
GAGGACTTGCATTGTGAAAGCTTTGCTGTCCTTGATGTGATCATGGAATCTTTTTCTCAC
TAGAGTCTATGTCACTCATTATACTCTGTCGAATGTCATTGAATGTCTTTACATGGGCTT
GTATGCCTATGAAAATTGTAATACAACTTTCAGCAACGGATCTCTTGGCTCTCGCATCGA
TGAAGAACGCAGCGAAATGCGATAAGTAATGTGAATTGCAGAATTCAGTGAATCATCGAA
TCTTTGAACGCATCTTGCGCTCCTTGGTATTCCGAGGAGCATGCCTGTTTGAGTGTCATT
AAATTCTCAACTCTCTTATACTTTTTTGTAAAAGAGAGCTTGGACTGTGGAGGCTTGCTG
GCCACTTTTTGGGGTCAGCTCCTCTGAAATGCATTAGCGGAACCGTTTGCGATCTGCCAC
AAGTGTGATAAGTTATCTACACTGGCGAGGGGATTGCTCTCTGTAATGTTCAGCTTCTAA
TTGTCTCTACTTTGTGAGACTACTTTTGAATGCTTGACCTCAAATCAGGTAGGACTACCC
GCTGAACTTAA
>A4_12
TTTCCGTAGGTGAACCTGCGGAAGGATCATTATTGAATTATGTTTCTAGATAGGTTGTAG
CTGGCTCTTTTAGAGCATGTGCACGCCTGTTTGGACTTCATTTTCATCCACCTGTGCACC
TATTGTAGTCTTTGGTTGGGTTAGGAGGAAGTGATCATTGTATCAGCATCTGCTGGGAGT
GAGGACTTGCATTGTGAAAGCTTTGCTGTCCTTGATGTGATCATGGAATCTTTTTCTCAC
TAGAGTCTATGTCACTCATTATACTCTGTCGAATGTCATTGAATGTCTTTACATGGGCTT
GTATGCCTATGAAAATTGTAATACAACTTTCAGCAACGGATCTCTTGGCTCTCGCATCGA
TGAAGAACGCAGCGAAATGCGATAAGTAATGTGAATTGCAGAATTCAGTGAATCATCGAA
TCTTTGAACGCATCTTGCGCTCCTTGGTATTCCGAGGAGCATGCCTGTTTGAGTGTCATT
AAATTCTCAACTCTCTTATACTTTTTTGTAAAAGAGAGCTTGGACTGTGGAGGCTTGCTG
GCCACTTTTTGGGGTCAGCTCCTCTGAAATGCATTAGCGGAACCGTTTGCGATCTGCCAC
AAGTGTGATAAGTTATCTACACTGGCGAGGGGATTGCTCTCTGTAATGTTCAGCTTCTAA
TTGTCTCTACTTTGTGAGACTACTTTTGAATGCTTGACCTCAAATCAGGTAGGACTACCC
GCTGAACTTAA
>A4_13
TTTCCGTAGGTGAACCTGCGGAAGGATCATTATTGAATTATGTTTCTAGATAGGTTGTAG
CTGGCTCTTTTAGAGCATGTGCACGCCTGTTTGGACTTCATTTTCATCCACCTGTGCACC
TATTGTAGTCTTTGGTTGGGTTAGGAGGAAGTGATCATTGTATCAGCATCTGCTGGGAGT
GAGGACTTGCATTGTGAAAGCTTTGCTGTCCTTGATGTGATCATGGAATCTTTTTCTCAC
TAGAGTCTATGTCACTCATTATACTCTGTCGAATGTCATTGAATGTCTTTACATGGGCTT
GTATGCCTATGAAAATTGTAATACAACTTTCAGCAACGGATCTCTTGGCTCTCGCATCGA
TGAAGAACGCAGCGAAATGCGATAAGTAATGTGAATTGCAGAATTCAGTGAATCATCGAA
TCTTTGAACGCATCTTGCGCTCCTTGGTATTCCGAGGAGCATGCCTGTTTGAGTGTCATT
AAATTCTCAACTCTCTTATACTTTTTTGTAAAAGAGAGCTTGGACTGTGGAGGCTTGCTG
GCCACTTTTTGGGGTCAGCTCCTCTGAAATGCATTAGCGGAACCGTTTGCGATCTGCCAC
AAGTGTGATAAGTTATCTACACTGGCGAGGGGATTGCTCTCTGTAATGTTCAGCTTCTAA
TTGTCTCTACTTTGTGAGACTACTTTTGAATGCTTGACCTCAAATCAGGTAGGACTACCC
GCTGAACTTAA
>A4_14
TTTCCGTAGGTGAACCTGCGGAAGGATCATTATTGAATTATGTTTCTAGATAGGTTGTAG
CTGGCTCTTTTAGAGCATGTGCACGCCTGTTTGGACTTCATTTTCATCCACCTGTGCACC
TATTGTAGTCTTTGGTTGGGTTAGGAGGAAGTGATCATTGTATCAGCATCTGCTGGGAGT
GAGGACTTGCATTGTGAAAGCTTTGCTGTCCTTGATGTGATCATGGAATCTTTTTCTCAC
TAGAGTCTATGTCACTCATTATACTCTGTCGAATGTCATTGAATGTCTTTACATGGGCTT

GTATGCCTATGAAAATTGTAATACAACTTTCAGCAACGGATCTCTTGGCTCTCGCATCGA
TGAAGAACGCAGCGAAATGCGATAAGTAATGTGAATTGCAGAATTCAGTGAATCATCGAA
TCTTTGAACGCATCTTGCGCTCCTTGGTATTCCGAGGAGCATGCCTGTTTGAGTGTCATT
AAATTCTCAACTCTCTTATACTTTTTTGTAAAAGAGAGCTTGGACTGTGGAGGCTTGCTG
GCCACTTTTTGGGGTCAGCTCCTCTGAAATGCATTAGCGGAACCGTTTGCGATCTGCCAC
AAGTGTGATAAGTTATCTACACTGGCGAGGGGATTGCTCTCTGTAATGTTCAGCTTCTAA
TTGTCTCTACTTTGTGAGACTACTTTTGAATGCTTGACCTCAAATCAGGTAGGACTACCC
GCTGAACTTAA
>A4_15
TTTCCGTAGGTGAACCTGCGGAAGGATCATTATTGAATTATGTTTCTAGATAGGTTGTAG
CTGGCTCTTTTAGAGCATGTGCACGCCTGTTTGGACTTCATTTTCATCCACCTGTGCACC
TATTGTAGTCTTTGGTTGGGTTAGGAGGAAGTGATCATTGTATCAGCATCTGCTGGGAGT
GAGGACTTGCATTGTGAAAGCTTTGCTGTCCTTGATGTGATCATGGAATCTTTTTCTCAC
TAGAGTCTATGTCACTCATTATACTCTGTCGAATGTCATTGAATGTCTTTACATGGGCTT
GTATGCCTATGAAAATTGTAATACAACTTTCAGCAACGGATCTCTTGGCTCTCGCATCGA
TGAAGAACGCAGCGAAATGCGATAAGTAATGTGAATTGCAGAATTCAGTGAATCATCGAA
TCTTTGAACGCATCTTGCGCTCCTTGGTATTCCGAGGAGCATGCCTGTTTGAGTGTCATT
AAATTCTCAACTCTCTTATACTTTTTTGTAAAAGAGAGCTTGGACTGTGGAGGCTTGCTG
GCCACTTTTTGGGGTCAGCTCCTCTGAAATGCATTAGCGGAACCGTTTGCGATCTGCCAC
AAGTGTGATAAGTTATCTACACTGGCGAGGGGATTGCTCTCTGTAATGTTCAGCTTCTAA
TTGTCTCTACTTTGTGAGACTACTTTTGAATGCTTGACCTCAAATCAGGTAGGACTACCC
GCTGAACTTAA
>A4_16
TTTCCGTAGGTGAACCTGCGGAAGGATCATTATTGAATTATGTTTCTAGATAGGTTGTAG
CTGGCTCTTTTAGAGCATGTGCACGCCTGTTTGGACTTCATTTTCATCCACCTGTGCACC
TATTGTAGTCTTTGGTTGGGTTAGGAGGAAGTGATCATTGTATCAGCATCTGCTGGGAGT
GAGGACTTGCATTGTGAAAGCTTTGCTGTCCTTGATGTGATCATGGAATCTTTTTCTCAC
TAGAGTCTATGTCACTCATTATACTCTGTCGAATGTCATTGAATGTCTTTACATGGGCTT
GTATGCCTATGAAAATTGTAATACAACTTTCAGCAACGGATCTCTTGGCTCTCGCATCGA
TGAAGAACGCAGCGAAATGCGATAAGTAATGTGAATTGCAGAATTCAGTGAATCATCGAA
TCTTTGAACGCATCTTGCGCTCCTTGGTATTCCGAGGAGCATGCCTGTTTGAGTGTCATT
AAATTCTCAACTCTCTTATACTTTTTTGTAAAAGAGAGCTTGGACTGTGGAGGCTTGCTG
GCCACTTTTTGGGGTCAGCTCCTCTGAAATGCATTAGCGGAACCGTTTGCGATCTGCCAC
AAGTGTGATAAGTTATCTACACTGGCGAGGGGATTGCTCTCTGTAATGTTCAGCTTCTAA
TTGTCTCTACTTTGTGAGACTACTTTTGAATGCTTGACCTCAAATCAGGTAGGACTACCC
GCTGAACTTAA
>A4_17
TTTCCGTAGGTGAACCTGCGGAAGGATCATTATTGAATTATGTTTCTAGATAGGTTGTAG
CTGGCTCTTTTAGAGCATGTGCACGCCTGTTTGGACTTCATTTTCATCCACCTGTGCACC
TATTGTAGTCTTTGGTTGGGTTAGGAGGAAGTGATCATTGTATCAGCATCTGCTGGGAGT
GAGGACTTGCATTGTGAAAGCTTTGCTGTCCTTGATGTGATCATGGAATCTTTTTCTCAC
TAGAGTCTATGTCACTCATTATACTCTGTCGAATGTCATTGAATGTCTTTACATGGGCTT
GTATGCCTATGAAAATTGTAATACAACTTTCAGCAACGGATCTCTTGGCTCTCGCATCGA
TGAAGAACGCAGCGAAATGCGATAAGTAATGTGAATTGCAGAATTCAGTGAATCATCGAA
TCTTTGAACGCATCTTGCGCTCCTTGGTATTCCGAGGAGCATGCCTGTTTGAGTGTCATT
AAATTCTCAACTCTCTTATACTTTTTTGTAAAAGAGAGCTTGGACTGTGGAGGCTTGCTG
GCCACTTTTTGGGGTCAGCTCCTCTGAAATGCATTAGCGGAACCGTTTGCGATCTGCCAC
AAGTGTGATAAGTTATCTACACTGGCGAGGGGATTGCTCTCTGTAATGTTCAGCTTCTAA
TTGTCTCTACTTTGTGAGACTACTTTTGAATGCTTGACCTCAAATCAGGTAGGACTACCC
GCTGAACTTAA

>A4_18
TTTCCGTAGGTGAACCTGCGGAAGGATCATTATTGAATTATGTTTCTAGATAGGTTGTAG
CTGGCTCTTTTAGAGCATGTGCACGCCTGTTTGGACTTCATTTTCATCCACCTGTGCACC
TATTGTAGTCTTTGGTTGGGTTAGGAGGAAGTGATCATTGTATCAGCATCTGCTGGGAGT
GAGGACTTGCATTGTGAAAGCTTTGCTGTCCTTGATGTGATCATGGAATCTTTTTCTCAC
TAGAGTCTATGTCACTCATTATACTCTGTCGAATGTCATTGAATGTCTTTACATGGGCTT
GTATGCCTATGAAAATTGTAATACAACTTTCAGCAACGGATCTCTTGGCTCTCGCATCGA
TGAAGAACGCAGCGAAATGCGATAAGTAATGTGAATTGCAGAATTCAGTGAATCATCGAA
TCTTTGAACGCATCTTGCGCTCCTTGGTATTCCGAGGAGCATGCCTGTTTGAGTGTCATT
AAATTCTCAACTCTCTTATACTTTTTGTAAAAGAGAGCTTGGACTGTGGAGGCTTGCTG
GCCACTTTTTGGGGTCAGCTCCTCTGAAATGCATTAGCGGAACCGTTTGCGATCTGCCAC
AAGTGTGATAAGTTATCTACACTGGCGAGGGGATTGCTCTCTGTAATGTTCAGCTTCTAA
TTGTCTCTACTTTGTGAGACTACTTTTGAATGCTTGACCTCAAATCAGGTAGGACTACCC
GCTGAACTTAA
>A4_19
TTTCCGTAGGTGAACCTGCGGAAGGATCATTATTGAATTATGTTTCTAGATAGGTTGTAG
CTGGCTCTTTTAGAGCATGTGCACGCCTGTTTGGACTTCATTTTCATCCACCTGTGCACC
TATTGTAGTCTTTGGTTGGGTTAGGAGGAAGTGATCATTGTATCAGCATCTGCTGGGAGT
GAGGACTTGCATTGTGAAAGCTTTGCTGTCCTTGATGTGATCATGGAATCTTTTTCTCAC
TAGAGTCTATGTCACTCATTATACTCTGTCGAATGTCATTGAATGTCTTTACATGGGCTT
GTATGCCTATGAAAATTGTAATACAACTTTCAGCAACGGATCTCTTGGCTCTCGCATCGA
TGAAGAACGCAGCGAAATGCGATAAGTAATGTGAATTGCAGAATTCAGTGAATCATCGAA
TCTTTGAACGCATCTTGCGCTCCTTGGTATTCCGAGGAGCATGCCTGTTTGAGTGTCATT
AAATTCTCAACTCTCTTATACTTTTTGTAAAAGAGAGCTTGGACTGTGGAGGCTTGCTG
GCCACTTTTTGGGGTCAGCTCCTCTGAAATGCATTAGCGGAACCGTTTGCGATCTGCCAC
AAGTGTGATAAGTTATCTACACTGGCGAGGGGATTGCTCTCTGTAATGTTCAGCTTCTAA
TTGTCTCTACTTTGTGAGACTACTTTTGAATGCTTGACCTCAAATCAGGTAGGACTACCC
GCTGAACTTAA
>A4_20
TTTCCGTAGGTGAACCTGCGGAAGGATCATTATTGAATTATGTTTCTAGATAGGTTGTAG
CTGGCTCTTTTAGAGCATGTGCACGCCTGTTTGGACTTCATTTTCATCCACCTGTGCACC
TATTGTAGTCTTTGGTTGGGTTAGGAGGAAGTGATCATTGTATCAGCATCTGCTGGGAGT
GAGGACTTGCATTGTGAAAGCTTTGCTGTCCTTGATGTGATCATGGAATCTTTTTCTCAC
TAGAGTCTATGTCACTCATTATACTCTGTCGAATGTCATTGAATGTCTTTACATGGGCTT
GTATGCCTATGAAAATTGTAATACAACTTTCAGCAACGGATCTCTTGGCTCTCGCATCGA
TGAAGAACGCAGCGAAATGCGATAAGTAATGTGAATTGCAGAATTCAGTGAATCATCGAA
TCTTTGAACGCATCTTGCGCTCCTTGGTATTCCGAGGAGCATGCCTGTTTGAGTGTCATT
AAATTCTCAACTCTCTTATACTTTTTGTAAAAGAGAGCTTGGACTGTGGAGGCTTGCTG
GCCACTTTTTGGGGTCAGCTCCTCTGAAATGCATTAGCGGAACCGTTTGCGATCTGCCAC
AAGTGTGATAAGTTATCTACACTGGCGAGGGGATTGCTCTCTGTAATGTTCAGCTTCTAA
TTGTCTCTACTTTGTGAGACTACTTTTGAATGCTTGACCTCAAATCAGGTAGGACTACCC
GCTGAACTTAA
>A4_21
TTTCCGTAGGTGAACCTGCGGAAGGATCATTATTGAATTATGTTTCTAGATAGGTTGTAG
CTGGCTCTTTTAGAGCATGTGCACGCCTGTTTGGACTTCATTTTCATCCACCTGTGCACC
TATTGTAGTCTTTGGTTGGGTTAGGAGGAAGTGATCATTGTATCAGCATCTGCTGGGAGT
GAGGACTTGCATTGTGAAAGCTTTGCTGTCCTTGATGTGATCATGGAATCTTTTTCTCAC
TAGAGTCTATGTCACTCATTATACTCTGTCGAATGTCATTGAATGTCTTTACATGGGCTT
GTATGCCTATGAAAATTGTAATACAACTTTCAGCAACGGATCTCTTGGCTCTCGCATCGA
TGAAGAACGCAGCGAAATGCGATAAGTAATGTGAATTGCAGAATTCAGTGAATCATCGAA

TCTTTGAACGCATCTTGCGCTCCTTGGTATTCCGAGGAGCATGCCTGTTTGAGTGTCATT
AAATTCTCAACTCTCTTATACTTTTTTGTAAAAGAGAGCTTGGACTGTGGAGGCTTGCTG
GCCACTTTTTGGGGTCAGCTCCTCTGAAATGCATTAGCGGAACCGTTTGCGATCTGCCAC
AAGTGTGATAAGTTATCTACACTGGCGAGGGGATTGCTCTCTGTAATGTTCAGCTTCTAA
TTGTCTCTACTTTGTGAGACTACTTTTGAATGCTTGACCTCAAATCAGGTAGGACTACCC
GCTGAACTTAA
>A4_22
TTTCCGTAGGTGAACCTGCGGAAGGATCATTATTGAATTATGTTTCTAGATAGGTTGTAG
CTGGCTCTTTTAGAGCATGTGCACGCCTGTTTGGACTTCATTTTCATCCACCTGTGCACC
TATTGTAGTCTTTGGTTGGGTTAGGAGGAAGTGATCATTGTATCAGCATCTGCTGGGAGT
GAGGACTTGCATTGTGAAAGCTTTGCTGTCCTTGATGTGATCATGGAATCTTTTTCTCAC
TAGAGTCTATGTCACTCATTATACTCTGTCGAATGTCATTGAATGTCTTTACATGGGCTT
GTATGCCTATGAAAATTGTAATACAACTTTCAGCAACGGATCTCTTGGCTCTCGCATCGA
TGAAGAACGCAGCGAAATGCGATAAGTAATGTGAATTGCAGAATTCAGTGAATCATCGAA
TCTTTGAACGCATCTTGCGCTCCTTGGTATTCCGAGGAGCATGCCTGTTTGAGTGTCATT
AAATTCTCAACTCTCTTATACTTTTTTGTAAAAGAGAGCTTGGACTGTGGAGGCTTGCTG
GCCACTTTTTGGGGTCAGCTCCTCTGAAATGCATTAGCGGAACCGTTTGCGATCTGCCAC
AAGTGTGATAAGTTATCTACACTGGCGAGGGGATTGCTCTCTGTAATGTTCAGCTTCTAA
TTGTCTCTACTTTGTGAGACTACTTTTGAATGCTTGACCTCAAATCAGGTAGGACTACCC
GCTGAACTTAA
>A4_23
TTTCCGTAGGTGAACCTGCGGAAGGATCATTATTGAATTATGTTTCTAGATAGGTTGTAG
CTGGCTCTTTTAGAGCATGTGCACGCCTGTTTGGACTTCATTTTCATCCACCTGTGCACC
TATTGTAGTCTTTGGTTGGGTTAGGAGGAAGTGATCATTGTATCAGCATCTGCTGGGAGT
GAGGACTTGCATTGTGAAAGCTTTGCTGTCCTTGATGTGATCATGGAATCTTTTTCTCAC
TAGAGTCTATGTCACTCATTATACTCTGTCGAATGTCATTGAATGTCTTTACATGGGCTT
GTATGCCTATGAAAATTGTAATACAACTTTCAGCAACGGATCTCTTGGCTCTCGCATCGA
TGAAGAACGCAGCGAAATGCGATAAGTAATGTGAATTGCAGAATTCAGTGAATCATCGAA
TCTTTGAACGCATCTTGCGCTCCTTGGTATTCCGAGGAGCATGCCTGTTTGAGTGTCATT
AAATTCTCAACTCTCTTATACTTTTTTGTAAAAGAGAGCTTGGACTGTGGAGGCTTGCTG
GCCACTTTTTGGGGTCAGCTCCTCTGAAATGCATTAGCGGAACCGTTTGCGATCTGCCAC
AAGTGTGATAAGTTATCTACACTGGCGAGGGGATTGCTCTCTGTAATGTTCAGCTTCTAA
TTGTCTCTACTTTGTGAGACTACTTTTGAATGCTTGACCTCAAATCAGGTAGGACTACCC
GCTGAACTTAA
>A4_25
TTTCCGTAGGTGAACCTGCGGAAGGATCATTATTGAATTATGTTTCTAGATAGGTTGTAG
CTGGCTCTTTTAGAGCATGTGCACGCCTGTTTGGACTTCATTTTCATCCACCTGTGCACC
TATTGTAGTCTTTGGTTGGGTTAGGAGGAAGTGATCATTGTATCAGCATCTGCTGGGAGT
GAGGACTTGCATTGTGAAAGCTTTGCTGTCCTTGATGTGATCATGGAATCTTTTTCTCAC
TAGAGTCTATGTCACTCATTATACTCTGTCGAATGTCATTGAATGTCTTTACATGGGCTT
GTATGCCTATGAAAATTGTAATACAACTTTCAGCAACGGATCTCTTGGCTCTCGCATCGA
TGAAGAACGCAGCGAAATGCGATAAGTAATGTGAATTGCAGAATTCAGTGAATCATCGAA
TCTTTGAACGCATCTTGCGCTCCTTGGTATTCCGAGGAGCATGCCTGTTTGAGTGTCATT
AAATTCTCAACTCTCTTATACTTTTTTGTAAAAGAGAGCTTGGACTGTGGAGGCTTGCTG
GCCACTTTTTGGGGTCAGCTCCTCTGAAATGCATTAGCGGAACCGTTTGCGATCTGCCAC
AAGTGTGATAAGTTATCTACACTGGCGAGGGGATTGCTCTCTGTAATGTTCAGCTTCTAA
TTGTCTCTACTTTGTGAGACTACTTTTGAATGCTTGACCTCAAATCAGGTAGGACTACCC
GCTGAACTTAA
>A4_26
TTTCCGTAGGTGAACCTGCGGAAGGATCATTATTGAATTATGTTTCTAGATAGGTTGTAG

CTGGCTCTTTTAGAGCATGTGCACGCCTGTTTGGACTTCATTTTCATCCACCTGTGCACC
TATTGTAGTCTTTGGTTGGGTTAGGAGGAAGTGATCATTGTATCAGCATCTGCTGGGAGT
GAGGACTTGCATTGTGAAAGCTTTGCTGTCCTTGATGTGATCATGGAATCTTTTTCTCAC
TAGAGTCTATGTCACTCATTATACTCTGTCGAATGTCATTGAATGTCTTTACATGGGCTT
GTATGCCTATGAAAATTGTAATACAACTTTCAGCAACGGATCTCTTGGCTCTCGCATCGA
TGAAGAACGCAGCGAAATGCGATAAGTAATGTGAATTGCAGAATTCAGTGAATCATCGAA
TCTTTGAACGCATCTTGCGCTCCTTGGTATTCCGAGGAGCATGCCTGTTTGAGTGTCATT
AAATTCTCAACTCTCTTATACTTTTTTGTAAAAGAGAGCTTGGACTGTGGAGGCTTGCTG
GCCACTTTTTGGGGTCAGCTCCTCTGAAATGCATTAGCGGAACCGTTTGCGATCTGCCAC
AAGTGTGATAAGTTATCTACACTGGCGAGGGGATTGCTCTCTGTAATGTTCAGCTTCTAA
TTGTCTCTACTTTGTGAGACTACTTTTGAATGCTTGACCTCAAATCAGGTAGGACTACCC
GCTGAACTTAA
>A4_27
TTTCCGTAGGTGAACCTGCGGAAGGATCATTATTGAATTATGTTTCTAGATAGGTTGTAG
CTGGCTCTTTTAGAGCATGTGCACGCCTGTTTGGACTTCATTTTCATCCACCTGTGCACC
TATTGTAGTCTTTGGTTGGGTTAGGAGGAAGTGATCATTGTATCAGCATCTGCTGGGAGT
GAGGACTTGCATTGTGAAAGCTTTGCTGTCCTTGATGTGATCATGGAATCTTTTTCTCAC
TAGAGTCTATGTCACTCATTATACTCTGTCGAATGTCATTGAATGTCTTTACATGGGCTT
GTATGCCTATGAAAATTGTAATACAACTTTCAGCAACGGATCTCTTGGCTCTCGCATCGA
TGAAGAACGCAGCGAAATGCGATAAGTAATGTGAATTGCAGAATTCAGTGAATCATCGAA
TCTTTGAACGCATCTTGCGCTCCTTGGTATTCCGAGGAGCATGCCTGTTTGAGTGTCATT
AAATTCTCAACTCTCTTATACTTTTTTGTAAAAGAGAGCTTGGACTGTGGAGGCTTGCTG
GCCACTTTTTGGGGTCAGCTCCTCTGAAATGCATTAGCGGAACCGTTTGCGATCTGCCAC
AAGTGTGATAAGTTATCTACACTGGCGAGGGGATTGCTCTCTGTAATGTTCAGCTTCTAA
TTGTCTCTACTTTGTGAGACTACTTTTGAATGCTTGACCTCAAATCAGGTAGGACTACCC
GCTGAACTTAA
>A4_28
TTTCCGTAGGTGAACCTGCGGAAGGATCATTATTGAATTATGTTTCTAGATAGGTTGTAG
CTGGCTCTTTTAGAGCATGTGCACGCCTGTTTGGACTTCATTTTCATCCACCTGTGCACC
TATTGTAGTCTTTGGTTGGGTTAGGAGGAAGTGATCATTGTATCAGCATCTGCTGGGAGT
GAGGACTTGCATTGTGAAAGCTTTGCTGTCCTTGATGTGATCATGGAATCTTTTTCTCAC
TAGAGTCTATGTCACTCATTATACTCTGTCGAATGTCATTGAATGTCTTTACATGGGCTT
GTATGCCTATGAAAATTGTAATACAACTTTCAGCAACGGATCTCTTGGCTCTCGCATCGA
TGAAGAACGCAGCGAAATGCGATAAGTAATGTGAATTGCAGAATTCAGTGAATCATCGAA
TCTTTGAACGCATCTTGCGCTCCTTGGTATTCCGAGGAGCATGCCTGTTTGAGTGTCATT
AAATTCTCAACTCTCTTATACTTTTTTGTAAAAGAGAGCTTGGACTGTGGAGGCTTGCTG
GCCACTTTTTGGGGTCAGCTCCTCTGAAATGCATTAGCGGAACCGTTTGCGATCTGCCAC
AAGTGTGATAAGTTATCTACACTGGCGAGGGGATTGCTCTCTGTAATGTTCAGCTTCTAA
TTGTCTCTACTTTGTGAGACTACTTTTGAATGCTTGACCTCAAATCAGGTAGGACTACCC
GCTGAACTTAA
>A4_29
TTTCCGTAGGTGAACCTGCGGAAGGATCATTATTGAATTATGTTTCTAGATAGGTTGTAG
CTGGCTCTTTTAGAGCATGTGCACGCCTGTTTGGACTTCATTTTCATCCACCTGTGCACC
TATTGTAGTCTTTGGTTGGGTTAGGAGGAAGTGATCATTGTATCAGCATCTGCTGGGAGT
GAGGACTTGCATTGTGAAAGCTTTGCTGTCCTTGATGTGATCATGGAATCTTTTTCTCAC
TAGAGTCTATGTCACTCATTATACTCTGTCGAATGTCATTGAATGTCTTTACATGGGCTT
GTATGCCTATGAAAATTGTAATACAACTTTCAGCAACGGATCTCTTGGCTCTCGCATCGA
TGAAGAACGCAGCGAAATGCGATAAGTAATGTGAATTGCAGAATTCAGTGAATCATCGAA
TCTTTGAACGCATCTTGCGCTCCTTGGTATTCCGAGGAGCATGCCTGTTTGAGTGTCATT
AAATTCTCAACTCTCTTATACTTTTTTGTAAAAGAGAGCTTGGACTGTGGAGGCTTGCTG

GCCACTTTTTGGGGTCAGCTCCTCTGAAATGCATTAGCGGAACCGTTTGCGATCTGCCAC
AAGTGTGATAAGTTATCTACACTGGCGAGGGGATTGCTCTCTGTAATGTTCAGCTTCTAA
TTGTCTCTACTTTGTGAGACTACTTTTGAATGCTTGACCTCAAATCAGGTAGGACTACCC
GCTGAACTTAA
>A4_30
TTTCCGTAGGTGAACCTGCGGAAGGATCATTATTGAATTATGTTTCTAGATAGGTTGTAG
CTGGCTCTTTTAGAGCATGTGCACGCCTGTTTGGACTTCATTTTCATCCACCTGTGCACC
TATTGTAGTCTTTGGTTGGGTTAGGAGGAAGTGATCATTGTATCAGCATCTGCTGGGAGT
GAGGACTTGCATTGTGAAAGCTTTGCTGTCCTTGATGTGATCATGGAATCTTTTTCTCAC
TAGAGTCTATGTCACTCATTATACTCTGTCGAATGTCATTGAATGTCTTTACATGGGCTT
GTATGCCTATGAAAATTGTAATACAACTTTCAGCAACGGATCTCTTGGCTCTCGCATCGA
TGAAGAACGCAGCGAAATGCGATAAGTAATGTGAATTGCAGAATTCAGTGAATCATCGAA
TCTTTGAACGCATCTTGCGCTCCTTGGTATTCCGAGGAGCATGCCTGTTTGAGTGTCATT
AAATTCTCAACTCTCTTATACTTTTTTGTAAAAGAGAGCTTGGACTGTGGAGGCTTGCTG
GCCACTTTTTGGGGTCAGCTCCTCTGAAATGCATTAGCGGAACCGTTTGCGATCTGCCAC
AAGTGTGATAAGTTATCTACACTGGCGAGGGGATTGCTCTCTGTAATGTTCAGCTTCTAA
TTGTCTCTACTTTGTGAGACTACTTTTGAATGCTTGACCTCAAATCAGGTAGGACTACCC
GCTGAACTTAA
>A4_32
TTTCCGTAGGTGAACCTGCGGAAGGATCATTATTGAATTATGTTTCTAGATAGGTTGTAG
CTGGCTCTTTTAGAGCATGTGCACGCCTGTTTGGACTTCATTTTCATCCACCTGTGCACC
TATTGTAGTCTTTGGTTGGGTTAGGAGGAAGTGATCATTGTATCAGCATCTGCTGGGAGT
GAGGACTTGCATTGTGAAAGCTTTGCTGTCCTTGATGTGATCATGGAATCTTTTTCTCAC
TAGAGTCTATGTCACTCATTATACTCTGTCGAATGTCATTGAATGTCTTTACATGGGCTT
GTATGCCTATGAAAATTGTAATACAACTTTCAGCAACGGATCTCTTGGCTCTCGCATCGA
TGAAGAACGCAGCGAAATGCGATAAGTAATGTGAATTGCAGAATTCAGTGAATCATCGAA
TCTTTGAACGCATCTTGCGCTCCTTGGTATTCCGAGGAGCATGCCTGTTTGAGTGTCATT
AAATTCTCAACTCTCTTATACTTTTTTGTAAAAGAGAGCTTGGACTGTGGAGGCTTGCTG
GCCACTTTTTGGGGTCAGCTCCTCTGAAATGCATTAGCGGAACCGTTTGCGATCTGCCAC
AAGTGTGATAAGTTATCTACACTGGCGAGGGGATTGCTCTCTGTAATGTTCAGCTTCTAA
TTGTCTCTACTTTGTGAGACTACTTTTGAATGCTTGACCTCAAATCAGGTAGGACTACCC
GCTGAACTTAA
>A4_33
TTTCCGTAGGTGAACCTGCGGAAGGATCATTATTGAATTATGTTTCTAGATAGGTTGTAG
CTGGCTCTTTTAGAGCATGTGCACGCCTGTTTGGACTTCATTTTCATCCACCTGTGCACC
TATTGTAGTCTTTGGTTGGGTTAGGAGGAAGTGATCATTGTATCAGCATCTGCTGGGAGT
GAGGACTTGCATTGTGAAAGCTTTGCTGTCCTTGATGTGATCATGGAATCTTTTTCTCAC
TAGAGTCTATGTCACTCATTATACTCTGTCGAATGTCATTGAATGTCTTTACATGGGCTT
GTATGCCTATGAAAATTGTAATACAACTTTCAGCAACGGATCTCTTGGCTCTCGCATCGA
TGAAGAACGCAGCGAAATGCGATAAGTAATGTGAATTGCAGAATTCAGTGAATCATCGAA
TCTTTGAACGCATCTTGCGCTCCTTGGTATTCCGAGGAGCATGCCTGTTTGAGTGTCATT
AAATTCTCAACTCTCTTATACTTTTTTGTAAAAGAGAGCTTGGACTGTGGAGGCTTGCTG
GCCACTTTTTGGGGTCAGCTCCTCTGAAATGCATTAGCGGAACCGTTTGCGATCTGCCAC
AAGTGTGATAAGTTATCTACACTGGCGAGGGGATTGCTCTCTGTAATGTTCAGCTTCTAA
TTGTCTCTACTTTGTGAGACTACTTTTGAATGCTTGACCTCAAATCAGGTAGGACTACCC
GCTGAACTTAA
>A4_34
TTTCCGTAGGTGAACCTGCGGAAGGATCATTATTGAATTATGTTTCTAGATAGGTTGTAG
CTGGCTCTTTTAGAGCATGTGCACGCCTGTTTGGACTTCATTTTCATCCACCTGTGCACC
TATTGTAGTCTTTGGTTGGGTTAGGAGGAAGTGATCATTGTATCAGCATCTGCTGGGAGT

GAGGACTTGCATTGTGAAAGCTTTGCTGTCCTTGATGTGATCATGGAATCTTTTTCTCAC
TAGAGTCTATGTCACTCATTATACTCTGTCGAATGTCATTGAATGTCTTTACATGGGCTT
GTATGCCTATGAAAATTGTAATACAACTTTCAGCAACGGATCTCTTGGCTCTCGCATCGA
TGAAGAACGCAGCGAAATGCGATAAGTAATGTGAATTGCAGAATTCAGTGAATCATCGAA
TCTTTGAACGCATCTTGCGCTCCTTGGTATTCCGAGGAGCATGCCTGTTTGAGTGTCATT
AAATTCTCAACTCTCTTATACTTTTTTGTAAAAGAGAGCTTGGACTGTGGAGGCTTGCTG
GCCACTTTTTGGGGTCAGCTCCTCTGAAATGCATTAGCGGAACCGTTTGCGATCTGCCAC
AAGTGTGATAAGTTATCTACACTGGCGAGGGGATTGCTCTCTGTAATGTTCAGCTTCTAA
TTGTCTCTACTTTGTGAGACTACTTTTGAATGCTTGACCTCAAATCAGGTAGGACTACCC
GCTGAACTTAA
>A4_35
TTTCCGTAGGTGAACCTGCGGAAGGATCATTATTGAATTATGTTTCTAGATAGGTTGTAG
CTGGCTCTTTTAGAGCATGTGCACGCCTGTTTGGACTTCATTTTCATCCACCTGTGCACC
TATTGTAGTCTTTGGTTGGGTTAGGAGGAAGTGATCATTGTATCAGCATCTGCTGGGAGT
GAGGACTTGCATTGTGAAAGCTTTGCTGTCCTTGATGTGATCATGGAATCTTTTTCTCAC
TAGAGTCTATGTCACTCATTATACTCTGTCGAATGTCATTGAATGTCTTTACATGGGCTT
GTATGCCTATGAAAATTGTAATACAACTTTCAGCAACGGATCTCTTGGCTCTCGCATCGA
TGAAGAACGCAGCGAAATGCGATAAGTAATGTGAATTGCAGAATTCAGTGAATCATCGAA
TCTTTGAACGCATCTTGCGCTCCTTGGTATTCCGAGGAGCATGCCTGTTTGAGTGTCATT
AAATTCTCAACTCTCTTATACTTTTTTGTAAAAGAGAGCTTGGACTGTGGAGGCTTGCTG
GCCACTTTTTGGGGTCAGCTCCTCTGAAATGCATTAGCGGAACCGTTTGCGATCTGCCAC
AAGTGTGATAAGTTATCTACACTGGCGAGGGGATTGCTCTCTGTAATGTTCAGCTTCTAA
TTGTCTCTACTTTGTGAGACTACTTTTGAATGCTTGACCTCAAATCAGGTAGGACTACCC
GCTGAACTTAA
>A4_36
TTTCCGTAGGTGAACCTGCGGAAGGATCATTATTGAATTATGTTTCTAGATAGGTTGTAG
CTGGCTCTTTTAGAGCATGTGCACGCCTGTTTGGACTTCATTTTCATCCACCTGTGCACC
TATTGTAGTCTTTGGTTGGGTTAGGAGGAAGTGATCATTGTATCAGCATCTGCTGGGAGT
GAGGACTTGCATTGTGAAAGCTTTGCTGTCCTTGATGTGATCATGGAATCTTTTTCTCAC
TAGAGTCTATGTCACTCATTATACTCTGTCGAATGTCATTGAATGTCTTTACATGGGCTT
GTATGCCTATGAAAATTGTAATACAACTTTCAGCAACGGATCTCTTGGCTCTCGCATCGA
TGAAGAACGCAGCGAAATGCGATAAGTAATGTGAATTGCAGAATTCAGTGAATCATCGAA
TCTTTGAACGCATCTTGCGCTCCTTGGTATTCCGAGGAGCATGCCTGTTTGAGTGTCATT
AAATTCTCAACTCTCTTATACTTTTTTGTAAAAGAGAGCTTGGACTGTGGAGGCTTGCTG
GCCACTTTTTGGGGTCAGCTCCTCTGAAATGCATTAGCGGAACCGTTTGCGATCTGCCAC
AAGTGTGATAAGTTATCTACACTGGCGAGGGGATTGCTCTCTGTAATGTTCAGCTTCTAA
TTGTCTCTACTTTGTGAGACTACTTTTGAATGCTTGACCTCAAATCAGGTAGGACTACCC
GCTGAACTTAA
>A4_37
TTTCCGTAGGTGAACCTGCGGAAGGATCATTATTGAATTATGTTTCTAGATAGGTTGTAG
CTGGCTCTTTTAGAGCATGTGCACGCCTGTTTGGACTTCATTTTCATCCACCTGTGCACC
TATTGTAGTCTTTGGTTGGGTTAGGAGGAAGTGATCATTGTATCAGCATCTGCTGGGAGT
GAGGACTTGCATTGTGAAAGCTTTGCTGTCCTTGATGTGATCATGGAATCTTTTTCTCAC
TAGAGTCTATGTCACTCATTATACTCTGTCGAATGTCATTGAATGTCTTTACATGGGCTT
GTATGCCTATGAAAATTGTAATACAACTTTCAGCAACGGATCTCTTGGCTCTCGCATCGA
TGAAGAACGCAGCGAAATGCGATAAGTAATGTGAATTGCAGAATTCAGTGAATCATCGAA
TCTTTGAACGCATCTTGCGCTCCTTGGTATTCCGAGGAGCATGCCTGTTTGAGTGTCATT
AAATTCTCAACTCTCTTATACTTTTTTGTAAAAGAGAGCTTGGACTGTGGAGGCTTGCTG
GCCACTTTTTGGGGTCAGCTCCTCTGAAATGCATTAGCGGAACCGTTTGCGATCTGCCAC
AAGTGTGATAAGTTATCTACACTGGCGAGGGGATTGCTCTCTGTAATGTTCAGCTTCTAA

TTGTCTCTACTTTGTGAGACTACTTTTGAATGCTTGACCTCAAATCAGGTAGGACTACCC
GCTGAACTTAA
>A4_38
TTTCCGTAGGTGAACCTGCGGAAGGATCATTATTGAATTATGTTTCTAGATAGGTTGTAG
CTGGCTCTTTTAGAGCATGTGCACGCCTGTTTGGACTTCATTTTCATCCACCTGTGCACC
TATTGTAGTCTTTGGTTGGGTTAGGAGGAAGTGATCATTGTATCAGCATCTGCTGGGAGT
GAGGACTTGCATTGTGAAAGCTTTGCTGTCCTTGATGTGATCATGGAATCTTTTTCTCAC
TAGAGTCTATGTCACTCATTATACTCTGTCGAATGTCATTGAATGTCTTTACATGGGCTT
GTATGCCTATGAAAATTGTAATACAACTTTCAGCAACGGATCTCTTGGCTCTCGCATCGA
TGAAGAACGCAGCGAAATGCGATAAGTAATGTGAATTGCAGAATTCAGTGAATCATCGAA
TCTTTGAACGCATCTTGCGCTCCTTGGTATTCCGAGGAGCATGCCTGTTTGAGTGTCATT
AAATTCTCAACTCTCTTATACTTTTTGTAAAAGAGAGCTTGGACTGTGGAGGCTTGCTG
GCCACTTTTTGGGGTCAGCTCCTCTGAAATGCATTAGCGGAACCGTTTGCGATCTGCCAC
AAGTGTGATAAGTTATCTACACTGGCGAGGGGATTGCTCTCTGTAATGTTCAGCTTCTAA
TTGTCTCTACTTTGTGAGACTACTTTTGAATGCTTGACCTCAAATCAGGTAGGACTACCC
GCTGAACTTAA
>A4_39
TTTCCGTAGGTGAACCTGCGGAAGGATCATTATTGAATTATGTTTCTAGATAGGTTGTAG
CTGGCTCTTTTAGAGCATGTGCACGCCTGTTTGGACTTCATTTTCATCCACCTGTGCACC
TATTGTAGTCTTTGGTTGGGTTAGGAGGAAGTGATCATTGTATCAGCATCTGCTGGGAGT
GAGGACTTGCATTGTGAAAGCTTTGCTGTCCTTGATGTGATCATGGAATCTTTTTCTCAC
TAGAGTCTATGTCACTCATTATACTCTGTCGAATGTCATTGAATGTCTTTACATGGGCTT
GTATGCCTATGAAAATTGTAATACAACTTTCAGCAACGGATCTCTTGGCTCTCGCATCGA
TGAAGAACGCAGCGAAATGCGATAAGTAATGTGAATTGCAGAATTCAGTGAATCATCGAA
TCTTTGAACGCATCTTGCGCTCCTTGGTATTCCGAGGAGCATGCCTGTTTGAGTGTCATT
AAATTCTCAACTCTCTTATACTTTTTGTAAAAGAGAGCTTGGACTGTGGAGGCTTGCTG
GCCACTTTTTGGGGTCAGCTCCTCTGAAATGCATTAGCGGAACCGTTTGCGATCTGCCAC
AAGTGTGATAAGTTATCTACACTGGCGAGGGGATTGCTCTCTGTAATGTTCAGCTTCTAA
TTGTCTCTACTTTGTGAGACTACTTTTGAATGCTTGACCTCAAATCAGGTAGGACTACCC
GCTGAACTTAA
>A4_40
TTTCCGTAGGTGAACCTGCGGAAGGATCATTATTGAATTATGTTTCTAGATAGGTTGTAG
CTGGCTCTTTTAGAGCATGTGCACGCCTGTTTGGACTTCATTTTCATCCACCTGTGCACC
TATTGTAGTCTTTGGTTGGGTTAGGAGGAAGTGATCATTGTATCAGCATCTGCTGGGAGT
GAGGACTTGCATTGTGAAAGCTTTGCTGTCCTTGATGTGATCATGGAATCTTTTTCTCAC
TAGAGTCTATGTCACTCATTATACTCTGTCGAATGTCATTGAATGTCTTTACATGGGCTT
GTATGCCTATGAAAATTGTAATACAACTTTCAGCAACGGATCTCTTGGCTCTCGCATCGA
TGAAGAACGCAGCGAAATGCGATAAGTAATGTGAATTGCAGAATTCAGTGAATCATCGAA
TCTTTGAACGCATCTTGCGCTCCTTGGTATTCCGAGGAGCATGCCTGTTTGAGTGTCATT
AAATTCTCAACTCTCTTATACTTTTTGTAAAAGAGAGCTTGGACTGTGGAGGCTTGCTG
GCCACTTTTTGGGGTCAGCTCCTCTGAAATGCATTAGCGGAACCGTTTGCGATCTGCCAC
AAGTGTGATAAGTTATCTACACTGGCGAGGGGATTGCTCTCTGTAATGTTCAGCTTCTAA
TTGTCTCTACTTTGTGAGACTACTTTTGAATGCTTGACCTCAAATCAGGTAGGACTACCC
GCTGAACTTAA
>A4_41
TTTCCGTAGGTGAACCTGCGGAAGGATCATTATTGAATTATGTTTCTAGATAGGTTGTAG
CTGGCTCTTTTAGAGCATGTGCACGCCTGTTTGGACTTCATTTTCATCCACCTGTGCACC
TATTGTAGTCTTTGGTTGGGTTAGGAGGAAGTGATCATTGTATCAGCATCTGCTGGGAGT
GAGGACTTGCATTGTGAAAGCTTTGCTGTCCTTGATGTGATCATGGAATCTTTTTCTCAC
TAGAGTCTATGTCACTCATTATACTCTGTCGAATGTCATTGAATGTCTTTACATGGGCTT

GTATGCCTATGAAAATTGTAATACAACTTTCAGCAACGGATCTCTTGGCTCTCGCATCGA
TGAAGAACGCAGCGAAATGCGATAAGTAATGTGAATTGCAGAATTCAGTGAATCATCGAA
TCTTTGAACGCATCTTGCGCTCCTTGGTATTCCGAGGAGCATGCCTGTTTGAGTGTCATT
AAATTCTCAACTCTCTTATACTTTTTTGTAAAAGAGAGCTTGGACTGTGGAGGCTTGCTG
GCCACTTTTTGGGGTCAGCTCCTCTGAAATGCATTAGCGGAACCGTTTGCGATCTGCCAC
AAGTGTGATAAGTTATCTACACTGGCGAGGGGATTGCTCTCTGTAATGTTCAGCTTCTAA
TTGTCTCTACTTTGTGAGACTACTTTTGAATGCTTGACCTCAAATCAGGTAGGACTACCC
GCTGAACTTAA
>A4_42
TTTCCGTAGGTGAACCTGCGGAAGGATCATTATTGAATTATGTTTCTAGATAGGTTGTAG
CTGGCTCTTTTAGAGCATGTGCACGCCTGTTTGGACTTCATTTTCATCCACCTGTGCACC
TATTGTAGTCTTTGGTTGGGTTAGGAGGAAGTGATCATTGTATCAGCATCTGCTGGGAGT
GAGGACTTGCATTGTGAAAGCTTTGCTGTCCTTGATGTGATCATGGAATCTTTTTCTCAC
TAGAGTCTATGTCACTCATTATACTCTGTCGAATGTCATTGAATGTCTTTACATGGGCTT
GTATGCCTATGAAAATTGTAATACAACTTTCAGCAACGGATCTCTTGGCTCTCGCATCGA
TGAAGAACGCAGCGAAATGCGATAAGTAATGTGAATTGCAGAATTCAGTGAATCATCGAA
TCTTTGAACGCATCTTGCGCTCCTTGGTATTCCGAGGAGCATGCCTGTTTGAGTGTCATT
AAATTCTCAACTCTCTTATACTTTTTTGTAAAAGAGAGCTTGGACTGTGGAGGCTTGCTG
GCCACTTTTTGGGGTCAGCTCCTCTGAAATGCATTAGCGGAACCGTTTGCGATCTGCCAC
AAGTGTGATAAGTTATCTACACTGGCGAGGGGATTGCTCTCTGTAATGTTCAGCTTCTAA
TTGTCTCTACTTTGTGAGACTACTTTTGAATGCTTGACCTCAAATCAGGTAGGACTACCC
GCTGAACTTAA
>A4_43
TTTCCGTAGGTGAACCTGCGGAAGGATCATTATTGAATTATGTTTCTAGATAGGTTGTAG
CTGGCTCTTTTAGAGCATGTGCACGCCTGTTTGGACTTCATTTTCATCCACCTGTGCACC
TATTGTAGTCTTTGGTTGGGTTAGGAGGAAGTGATCATTGTATCAGCATCTGCTGGGAGT
GAGGACTTGCATTGTGAAAGCTTTGCTGTCCTTGATGTGATCATGGAATCTTTTTCTCAC
TAGAGTCTATGTCACTCATTATACTCTGTCGAATGTCATTGAATGTCTTTACATGGGCTT
GTATGCCTATGAAAATTGTAATACAACTTTCAGCAACGGATCTCTTGGCTCTCGCATCGA
TGAAGAACGCAGCGAAATGCGATAAGTAATGTGAATTGCAGAATTCAGTGAATCATCGAA
TCTTTGAACGCATCTTGCGCTCCTTGGTATTCCGAGGAGCATGCCTGTTTGAGTGTCATT
AAATTCTCAACTCTCTTATACTTTTTTGTAAAAGAGAGCTTGGACTGTGGAGGCTTGCTG
GCCACTTTTTGGGGTCAGCTCCTCTGAAATGCATTAGCGGAACCGTTTGCGATCTGCCAC
AAGTGTGATAAGTTATCTACACTGGCGAGGGGATTGCTCTCTGTAATGTTCAGCTTCTAA
TTGTCTCTACTTTGTGAGACTACTTTTGAATGCTTGACCTCAAATCAGGTAGGACTACCC
GCTGAACTTAA
>A4_44
TTTCCGTAGGTGAACCTGCGGAAGGATCATTATTGAATTATGTTTCTAGATAGGTTGTAG
CTGGCTCTTTTAGAGCATGTGCACGCCTGTTTGGACTTCATTTTCATCCACCTGTGCACC
TATTGTAGTCTTTGGTTGGGTTAGGAGGAAGTGATCATTGTATCAGCATCTGCTGGGAGT
GAGGACTTGCATTGTGAAAGCTTTGCTGTCCTTGATGTGATCATGGAATCTTTTTCTCAC
TAGAGTCTATGTCACTCATTATACTCTGTCGAATGTCATTGAATGTCTTTACATGGGCTT
GTATGCCTATGAAAATTGTAATACAACTTTCAGCAACGGATCTCTTGGCTCTCGCATCGA
TGAAGAACGCAGCGAAATGCGATAAGTAATGTGAATTGCAGAATTCAGTGAATCATCGAA
TCTTTGAACGCATCTTGCGCTCCTTGGTATTCCGAGGAGCATGCCTGTTTGAGTGTCATT
AAATTCTCAACTCTCTTATACTTTTTTGTAAAAGAGAGCTTGGACTGTGGAGGCTTGCTG
GCCACTTTTTGGGGTCAGCTCCTCTGAAATGCATTAGCGGAACCGTTTGCGATCTGCCAC
AAGTGTGATAAGTTATCTACACTGGCGAGGGGATTGCTCTCTGTAATGTTCAGCTTCTAA
TTGTCTCTACTTTGTGAGACTACTTTTGAATGCTTGACCTCAAATCAGGTAGGACTACCC
GCTGAACTTAA

>A4_45
TTTCCGTAGGTGAACCTGCGGAAGGATCATTATTGAATTATGTTTCTAGATAGGTTGTAG
CTGGCTCTTTTAGAGCATGTGCACGCCTGTTTGGACTTCATTTTCATCCACCTGTGCACC
TATTGTAGTCTTTGGTTGGGTTAGGAGGAAGTGATCATTGTATCAGCATCTGCTGGGAGT
GAGGACTTGCATTGTGAAAGCTTTGCTGTCCTTGATGTGATCATGGAATCTTTTTCTCAC
TAGAGTCTATGTCACTCATTATACTCTGTCGAATGTCATTGAATGTCTTTACATGGGCTT
GTATGCCTATGAAAATTGTAATACAACTTTCAGCAACGGATCTCTTGGCTCTCGCATCGA
TGAAGAACGCAGCGAAATGCGATAAGTAATGTGAATTGCAGAATTCAGTGAATCATCGAA
TCTTTGAACGCATCTTGCGCTCCTTGGTATTCCGAGGAGCATGCCTGTTTGAGTGTCATT
AAATTCTCAACTCTCTTATACTTTTTTGTAAAAGAGAGCTTGGACTGTGGAGGCTTGCTG
GCCACTTTTTGGGGTCAGCTCCTCTGAAATGCATTAGCGGAACCGTTTGCGATCTGCCAC
AAGTGTGATAAGTTATCTACACTGGCGAGGGGATTGCTCTCTGTAATGTTCAGCTTCTAA
TTGTCTCTACTTTGTGAGACTACTTTTGAATGCTTGACCTCAAATCAGGTAGGACTACCC
GCTGAACTTAA
>A4_46
TTTCCGTAGGTGAACCTGCGGAAGGATCATTATTGAATTATGTTTCTAGATAGGTTGTAG
CTGGCTCTTTTAGAGCATGTGCACGCCTGTTTGGACTTCATTTTCATCCACCTGTGCACC
TATTGTAGTCTTTGGTTGGGTTAGGAGGAAGTGATCATTGTATCAGCATCTGCTGGGAGT
GAGGACTTGCATTGTGAAAGCTTTGCTGTCCTTGATGTGATCATGGAATCTTTTTCTCAC
TAGAGTCTATGTCACTCATTATACTCTGTCGAATGTCATTGAATGTCTTTACATGGGCTT
GTATGCCTATGAAAATTGTAATACAACTTTCAGCAACGGATCTCTTGGCTCTCGCATCGA
TGAAGAACGCAGCGAAATGCGATAAGTAATGTGAATTGCAGAATTCAGTGAATCATCGAA
TCTTTGAACGCATCTTGCGCTCCTTGGTATTCCGAGGAGCATGCCTGTTTGAGTGTCATT
AAATTCTCAACTCTCTTATACTTTTTTGTAAAAGAGAGCTTGGACTGTGGAGGCTTGCTG
GCCACTTTTTGGGGTCAGCTCCTCTGAAATGCATTAGCGGAACCGTTTGCGATCTGCCAC
AAGTGTGATAAGTTATCTACACTGGCGAGGGGATTGCTCTCTGTAATGTTCAGCTTCTAA
TTGTCTCTACTTTGTGAGACTACTTTTGAATGCTTGACCTCAAATCAGGTAGGACTACCC
GCTGAACTTAA
>A4_47
TTTCCGTAGGTGAACCTGCGGAAGGATCATTATTGAATTATGTTTCTAGATAGGTTGTAG
CTGGCTCTTTTAGAGCATGTGCACGCCTGTTTGGACTTCATTTTCATCCACCTGTGCACC
TATTGTAGTCTTTGGTTGGGTTAGGAGGAAGTGATCATTGTATCAGCATCTGCTGGGAGT
GAGGACTTGCATTGTGAAAGCTTTGCTGTCCTTGATGTGATCATGGAATCTTTTTCTCAC
TAGAGTCTATGTCACTCATTATACTCTGTCGAATGTCATTGAATGTCTTTACATGGGCTT
GTATGCCTATGAAAATTGTAATACAACTTTCAGCAACGGATCTCTTGGCTCTCGCATCGA
TGAAGAACGCAGCGAAATGCGATAAGTAATGTGAATTGCAGAATTCAGTGAATCATCGAA
TCTTTGAACGCATCTTGCGCTCCTTGGTATTCCGAGGAGCATGCCTGTTTGAGTGTCATT
AAATTCTCAACTCTCTTATACTTTTTTGTAAAAGAGAGCTTGGACTGTGGAGGCTTGCTG
GCCACTTTTTGGGGTCAGCTCCTCTGAAATGCATTAGCGGAACCGTTTGCGATCTGCCAC
AAGTGTGATAAGTTATCTACACTGGCGAGGGGATTGCTCTCTGTAATGTTCAGCTTCTAA
TTGTCTCTACTTTGTGAGACTACTTTTGAATGCTTGACCTCAAATCAGGTAGGACTACCC
GCTGAACTTAA
>A4_48
TTTCCGTAGGTGAACCTGCGGAAGGATCATTATTGAATTATGTTTCTAGATAGGTTGTAG
CTGGCTCTTTTAGAGCATGTGCACGCCTGTTTGGACTTCATTTTCATCCACCTGTGCACC
TATTGTAGTCTTTGGTTGGGTTAGGAGGAAGTGATCATTGTATCAGCATCTGCTGGGAGT
GAGGACTTGCATTGTGAAAGCTTTGCTGTCCTTGATGTGATCATGGAATCTTTTTCTCAC
TAGAGTCTATGTCACTCATTATACTCTGTCGAATGTCATTGAATGTCTTTACATGGGCTT
GTATGCCTATGAAAATTGTAATACAACTTTCAGCAACGGATCTCTTGGCTCTCGCATCGA
TGAAGAACGCAGCGAAATGCGATAAGTAATGTGAATTGCAGAATTCAGTGAATCATCGAA

TCTTTGAACGCATCTTGCGCTCCTTGGTATTCCGAGGAGCATGCCTGTTTGAGTGTCATT
AAATTCTCAACTCTCTTATACTTTTTTGTAAAAGAGAGCTTGGACTGTGGAGGCTTGCTG
GCCACTTTTTGGGGTCAGCTCCTCTGAAATGCATTAGCGGAACCGTTTGCGATCTGCCAC
AAGTGTGATAAGTTATCTACACTGGCGAGGGGATTGCTCTCTGTAATGTTCAGCTTCTAA
TTGTCTCTACTTTGTGAGACTACTTTTGAATGCTTGACCTCAAATCAGGTAGGACTACCC
GCTGAACTTAA
>A4_49
TTTCCGTAGGTGAACCTGCGGAAGGATCATTATTGAATTATGTTTCTAGATAGGTTGTAG
CTGGCTCTTTTAGAGCATGTGCACGCCTGTTTGGACTTCATTTTCATCCACCTGTGCACC
TATTGTAGTCTTTGGTTGGGTTAGGAGGAAGTGATCATTGTATCAGCATCTGCTGGGAGT
GAGGACTTGCATTGTGAAAGCTTTGCTGTCCTTGATGTGATCATGGAATCTTTTTCTCAC
TAGAGTCTATGTCACTCATTATACTCTGTCGAATGTCATTGAATGTCTTTACATGGGCTT
GTATGCCTATGAAAATTGTAATACAACTTTCAGCAACGGATCTCTTGGCTCTCGCATCGA
TGAAGAACGCAGCGAAATGCGATAAGTAATGTGAATTGCAGAATTCAGTGAATCATCGAA
TCTTTGAACGCATCTTGCGCTCCTTGGTATTCCGAGGAGCATGCCTGTTTGAGTGTCATT
AAATTCTCAACTCTCTTATACTTTTTTGTAAAAGAGAGCTTGGACTGTGGAGGCTTGCTG
GCCACTTTTTGGGGTCAGCTCCTCTGAAATGCATTAGCGGAACCGTTTGCGATCTGCCAC
AAGTGTGATAAGTTATCTACACTGGCGAGGGGATTGCTCTCTGTAATGTTCAGCTTCTAA
TTGTCTCTACTTTGTGAGACTACTTTTGAATGCTTGACCTCAAATCAGGTAGGACTACCC
GCTGAACTTAA
>A4_50
TTTCCGTAGGTGAACCTGCGGAAGGATCATTATTGAATTATGTTTCTAGATAGGTTGTAG
CTGGCTCTTTTAGAGCATGTGCACGCCTGTTTGGACTTCATTTTCATCCACCTGTGCACC
TATTGTAGTCTTTGGTTGGGTTAGGAGGAAGTGATCATTGTATCAGCATCTGCTGGGAGT
GAGGACTTGCATTGTGAAAGCTTTGCTGTCCTTGATGTGATCATGGAATCTTTTTCTCAC
TAGAGTCTATGTCACTCATTATACTCTGTCGAATGTCATTGAATGTCTTTACATGGGCTT
GTATGCCTATGAAAATTGTAATACAACTTTCAGCAACGGATCTCTTGGCTCTCGCATCGA
TGAAGAACGCAGCGAAATGCGATAAGTAATGTGAATTGCAGAATTCAGTGAATCATCGAA
TCTTTGAACGCATCTTGCGCTCCTTGGTATTCCGAGGAGCATGCCTGTTTGAGTGTCATT
AAATTCTCAACTCTCTTATACTTTTTTGTAAAAGAGAGCTTGGACTGTGGAGGCTTGCTG
GCCACTTTTTGGGGTCAGCTCCTCTGAAATGCATTAGCGGAACCGTTTGCGATCTGCCAC
AAGTGTGATAAGTTATCTACACTGGCGAGGGGATTGCTCTCTGTAATGTTCAGCTTCTAA
TTGTCTCTACTTTGTGAGACTACTTTTGAATGCTTGACCTCAAATCAGGTAGGACTACCC
GCTGAACTTAA
>A4-59
TTTCCGTAGGTGAACCTGCGGAAGGATCATTATTGAATTATGTTTCTAGATAGGTTGTAG
CTGGCTCTTTTAGAGCATGTGCACGCCTGTTTGGACTTCATTTTCATCCACCTGTGCACC
TATTGTAGTCTTTGGTTGGGTTAGGAGGAAGTGATCATTGTATCAGCATCTGCTGGGAGT
GAGGACTTGCATTGTGAAAGCTTTGCTGTCCTTGATGTGATCATGGAATCTTTTTCTCAC
TAGAGTCTATGTCACTCATTATACTCTGTCGAATGTCATTGAATGTCTTTACATGGGCTT
GTATGCCTATGAAAATTGTAATACAACTTTCAGCAACGGATCTCTTGGCTCTCGCATCGA
TGAAGAACGCAGCGAAATGCGATAAGTAATGTGAATTGCAGAATTCAGTGAATCATCGAA
TCTTTGAACGCATCTTGCGCTCCTTGGTATTCCGAGGAGCATGCCTGTTTGAGTGTCATT
AAATTCTCAACTCTCTTATACTTTTTTGTAAAAGAGAGCTTGGACTGTGGAGGCTTGCTG
GCCACTTTTTGGGGTCAGCTCCTCTGAAATGCATTAGCGGAACCGTTTGCGATCTGCCAC
AAGTGTGATAAGTTATCTACACTGGCGAGGGGATTGCTCTCTGTAATGTTCAGCTTCTAA
TTGTCTCTACTTTGTGAGACTACTTTTGAATGCTTGACCTCAAATCAGGTAGGACTACCC
GCTGAACTTAA
>A4-60
TTTCCGTAGGTGAACCTGCGGAAGGATCATTATTGAATTATGTTTCTAGATAGGTTGTAG

CTGGCTCTTTTAGAGCATGTGCACGCCTGTTTGGACTTCATTTTCATCCACCTGTGCACC
TATTGTAGTCTTTGGTTGGGTTAGGAGGAAGTGATCATTGTATCAGCATCTGCTGGGAGT
GAGGACTTGCATTGTGAAAGCTTTGCTGTCCTTGATGTGATCATGGAATCTTTTTCTCAC
TAGAGTCTATGTCACTCATTATACTCTGTCGAATGTCATTGAATGTCTTTACATGGGCTT
GTATGCCTATGAAAATTGTAATACAACTTTCAGCAACGGATCTCTTGGCTCTCGCATCGA
TGAAGAACGCAGCGAAATGCGATAAGTAATGTGAATTGCAGAATTCAGTGAATCATCGAA
TCTTTGAACGCATCTTGCGCTCCTTGGTATTCCGAGGAGCATGCCTGTTTGAGTGTCATT
AAATTCTCAACTCTCTTATACTTTTTTGTAAAAGAGAGCTTGGACTGTGGAGGCTTGCTG
GCCACTTTTTGGGGTCAGCTCCTCTGAAATGCATTAGCGGAACCGTTTGCGATCTGCCAC
AAGTGTGATAAGTTATCTACACTGGCGAGGGGATTGCTCTCTGTAATGTTCAGCTTCTAA
TTGTCTCTACTTTGTGAGACTACTTTTGAATGCTTGACCTCAAATCAGGTAGGACTACCC
GCTGAACTTAA
>A4-61
TTTCCGTAGGTGAACCTGCGGAAGGATCATTATTGAATTATGTTTCTAGATAGGTTGTAG
CTGGCTCTTTTAGAGCATGTGCACGCCTGTTTGGACTTCATTTTCATCCACCTGTGCACC
TATTGTAGTCTTTGGTTGGGTTAGGAGGAAGTGATCATTGTATCAGCATCTGCTGGGAGT
GAGGACTTGCATTGTGAAAGCTTTGCTGTCCTTGATGTGATCATGGAATCTTTTTCTCAC
TAGAGTCTATGTCACTCATTATACTCTGTCGAATGTCATTGAATGTCTTTACATGGGCTT
GTATGCCTATGAAAATTGTAATACAACTTTCAGCAACGGATCTCTTGGCTCTCGCATCGA
TGAAGAACGCAGCGAAATGCGATAAGTAATGTGAATTGCAGAATTCAGTGAATCATCGAA
TCTTTGAACGCATCTTGCGCTCCTTGGTATTCCGAGGAGCATGCCTGTTTGAGTGTCATT
AAATTCTCAACTCTCTTATACTTTTTTGTAAAAGAGAGCTTGGACTGTGGAGGCTTGCTG
GCCACTTTTTGGGGTCAGCTCCTCTGAAATGCATTAGCGGAACCGTTTGCGATCTGCCAC
AAGTGTGATAAGTTATCTACACTGGCGAGGGGATTGCTCTCTGTAATGTTCAGCTTCTAA
TTGTCTCTACTTTGTGAGACTACTTTTGAATGCTTGACCTCAAATCAGGTAGGACTACCC
GCTGAACTTAA
>A4-62
TTTCCGTAGGTGAACCTGCGGAAGGATCATTATTGAATTATGTTTCTAGATAGGTTGTAG
CTGGCTCTTTTAGAGCATGTGCACGCCTGTTTGGACTTCATTTTCATCCACCTGTGCACC
TATTGTAGTCTTTGGTTGGGTTAGGAGGAAGTGATCATTGTATCAGCATCTGCTGGGAGT
GAGGACTTGCATTGTGAAAGCTTTGCTGTCCTTGATGTGATCATGGAATCTTTTTCTCAC
TAGAGTCTATGTCACTCATTATACTCTGTCGAATGTCATTGAATGTCTTTACATGGGCTT
GTATGCCTATGAAAATTGTAATACAACTTTCAGCAACGGATCTCTTGGCTCTCGCATCGA
TGAAGAACGCAGCGAAATGCGATAAGTAATGTGAATTGCAGAATTCAGTGAATCATCGAA
TCTTTGAACGCATCTTGCGCTCCTTGGTATTCCGAGGAGCATGCCTGTTTGAGTGTCATT
AAATTCTCAACTCTCTTATACTTTTTTGTAAAAGAGAGCTTGGACTGTGGAGGCTTGCTG
GCCACTTTTTGGGGTCAGCTCCTCTGAAATGCATTAGCGGAACCGTTTGCGATCTGCCAC
AAGTGTGATAAGTTATCTACACTGGCGAGGGGATTGCTCTCTGTAATGTTCAGCTTCTAA
TTGTCTCTACTTTGTGAGACTACTTTTGAATGCTTGACCTCAAATCAGGTAGGACTACCC
GCTGAACTTAA
>A4-63
TTTCCGTAGGTGAACCTGCGGAAGGATCATTATTGAATTATGTTTCTAGATAGGTTGTAG
CTGGCTCTTTTAGAGCATGTGCACGCCTGTTTGGACTTCATTTTCATCCACCTGTGCACC
TATTGTAGTCTTTGGTTGGGTTAGGAGGAAGTGATCATTGTATCAGCATCTGCTGGGAGT
GAGGACTTGCATTGTGAAAGCTTTGCTGTCCTTGATGTGATCATGGAATCTTTTTCTCAC
TAGAGTCTATGTCACTCATTATACTCTGTCGAATGTCATTGAATGTCTTTACATGGGCTT
GTATGCCTATGAAAATTGTAATACAACTTTCAGCAACGGATCTCTTGGCTCTCGCATCGA
TGAAGAACGCAGCGAAATGCGATAAGTAATGTGAATTGCAGAATTCAGTGAATCATCGAA
TCTTTGAACGCATCTTGCGCTCCTTGGTATTCCGAGGAGCATGCCTGTTTGAGTGTCATT
AAATTCTCAACTCTCTTATACTTTTTTGTAAAAGAGAGCTTGGACTGTGGAGGCTTGCTG

GCCACTTTTTGGGGTCAGCTCCTCTGAAATGCATTAGCGGAACCGTTTGCGATCTGCCAC
AAGTGTGATAAGTTATCTACACTGGCGAGGGGATTGCTCTCTGTAATGTTCAGCTTCTAA
TTGTCTCTACTTTGTGAGACTACTTTTGAATGCTTGACCTCAAATCAGGTAGGACTACCC
GCTGAACTTAA
>A4-64
TTTCCGTAGGTGAACCTGCGGAAGGATCATTATTGAATTATGTTTCTAGATAGGTTGTAG
CTGGCTCTTTTAGAGCATGTGCACGCCTGTTTGGACTTCATTTTCATCCACCTGTGCACC
TATTGTAGTCTTTGGTTGGGTTAGGAGGAAGTGATCATTGTATCAGCATCTGCTGGGAGT
GAGGACTTGCATTGTGAAAGCTTTGCTGTCCTTGATGTGATCATGGAATCTTTTTCTCAC
TAGAGTCTATGTCACTCATTATACTCTGTCGAATGTCATTGAATGTCTTTACATGGGCTT
GTATGCCTATGAAAATTGTAATACAACTTTCAGCAACGGATCTCTTGGCTCTCGCATCGA
TGAAGAACGCAGCGAAATGCGATAAGTAATGTGAATTGCAGAATTCAGTGAATCATCGAA
TCTTTGAACGCATCTTGCGCTCCTTGGTATTCCGAGGAGCATGCCTGTTTGAGTGTCATT
AAATTCTCAACTCTCTTATACTTTTTTGTAAAAGAGAGCTTGGACTGTGGAGGCTTGCTG
GCCACTTTTTGGGGTCAGCTCCTCTGAAATGCATTAGCGGAACCGTTTGCGATCTGCCAC
AAGTGTGATAAGTTATCTACACTGGCGAGGGGATTGCTCTCTGTAATGTTCAGCTTCTAA
TTGTCTCTACTTTGTGAGACTACTTTTGAATGCTTGACCTCAAATCAGGTAGGACTACCC
GCTGAACTTAA
>A4-65
TTTCCGTAGGTGAACCTGCGGAAGGATCATTATTGAATTATGTTTCTAGATAGGTTGTAG
CTGGCTCTTTTAGAGCATGTGCACGCCTGTTTGGACTTCATTTTCATCCACCTGTGCACC
TATTGTAGTCTTTGGTTGGGTTAGGAGGAAGTGATCATTGTATCAGCATCTGCTGGGAGT
GAGGACTTGCATTGTGAAAGCTTTGCTGTCCTTGATGTGATCATGGAATCTTTTTCTCAC
TAGAGTCTATGTCACTCATTATACTCTGTCGAATGTCATTGAATGTCTTTACATGGGCTT
GTATGCCTATGAAAATTGTAATACAACTTTCAGCAACGGATCTCTTGGCTCTCGCATCGA
TGAAGAACGCAGCGAAATGCGATAAGTAATGTGAATTGCAGAATTCAGTGAATCATCGAA
TCTTTGAACGCATCTTGCGCTCCTTGGTATTCCGAGGAGCATGCCTGTTTGAGTGTCATT
AAATTCTCAACTCTCTTATACTTTTTTGTAAAAGAGAGCTTGGACTGTGGAGGCTTGCTG
GCCACTTTTTGGGGTCAGCTCCTCTGAAATGCATTAGCGGAACCGTTTGCGATCTGCCAC
AAGTGTGATAAGTTATCTACACTGGCGAGGGGATTGCTCTCTGTAATGTTCAGCTTCTAA
TTGTCTCTACTTTGTGAGACTACTTTTGAATGCTTGACCTCAAATCAGGTAGGACTACCC
GCTGAACTTAA
>A4-66
TTTCCGTAGGTGAACCTGCGGAAGGATCATTATTGAATTATGTTTCTAGATAGGTTGTAG
CTGGCTCTTTTAGAGCATGTGCACGCCTGTTTGGACTTCATTTTCATCCACCTGTGCACC
TATTGTAGTCTTTGGTTGGGTTAGGAGGAAGTGATCATTGTATCAGCATCTGCTGGGAGT
GAGGACTTGCATTGTGAAAGCTTTGCTGTCCTTGATGTGATCATGGAATCTTTTTCTCAC
TAGAGTCTATGTCACTCATTATACTCTGTCGAATGTCATTGAATGTCTTTACATGGGCTT
GTATGCCTATGAAAATTGTAATACAACTTTCAGCAACGGATCTCTTGGCTCTCGCATCGA
TGAAGAACGCAGCGAAATGCGATAAGTAATGTGAATTGCAGAATTCAGTGAATCATCGAA
TCTTTGAACGCATCTTGCGCTCCTTGGTATTCCGAGGAGCATGCCTGTTTGAGTGTCATT
AAATTCTCAACTCTCTTATACTTTTTTGTAAAAGAGAGCTTGGACTGTGGAGGCTTGCTG
GCCACTTTTTGGGGTCAGCTCCTCTGAAATGCATTAGCGGAACCGTTTGCGATCTGCCAC
AAGTGTGATAAGTTATCTACACTGGCGAGGGGATTGCTCTCTGTAATGTTCAGCTTCTAA
TTGTCTCTACTTTGTGAGACTACTTTTGAATGCTTGACCTCAAATCAGGTAGGACTACCC
GCTGAACTTAA
>A4-69
TTTCCGTAGGTGAACCTGCGGAAGGATCATTATTGAATTATGTTTCTAGATAGGTTGTAG
CTGGCTCTTTTAGAGCATGTGCACGCCTGTTTGGACTTCATTTTCATCCACCTGTGCACC
TATTGTAGTCTTTGGTTGGGTTAGGAGGAAGTGATCATTGTATCAGCATCTGCTGGGAGT

GAGGACTTGCATTGTGAAAGCTTTGCTGTCCTTGATGTGATCATGGAATCTTTTTCTCAC
TAGAGTCTATGTCACTCATTATACTCTGTCGAATGTCATTGAATGTCTTTACATGGGCTT
GTATGCCTATGAAAATTGTAATACAACTTTCAGCAACGGATCTCTTGGCTCTCGCATCGA
TGAAGAACGCAGCGAAATGCGATAAGTAATGTGAATTGCAGAATTCAGTGAATCATCGAA
TCTTTGAACGCATCTTGCGCTCCTTGGTATTCCGAGGAGCATGCCTGTTTGAGTGTCATT
AAATTCTCAACTCTCTTATACTTTTTTGTAAAAGAGAGCTTGGACTGTGGAGGCTTGCTG
GCCACTTTTTGGGGTCAGCTCCTCTGAAATGCATTAGCGGAACCGTTTGCGATCTGCCAC
AAGTGTGATAAGTTATCTACACTGGCGAGGGGATTGCTCTCTGTAATGTTCAGCTTCTAA
TTGTCTCTACTTTGTGAGACTACTTTTGAATGCTTGACCTCAAATCAGGTAGGACTACCC
GCTGAACTTAA
>A4-70
TTTCCGTAGGTGAACCTGCGGAAGGATCATTATTGAATTATGTTTCTAGATAGGTTGTAG
CTGGCTCTTTTAGAGCATGTGCACGCCTGTTTGGACTTCATTTTCATCCACCTGTGCACC
TATTGTAGTCTTTGGTTGGGTTAGGAGGAAGTGATCATTGTATCAGCATCTGCTGGGAGT
GAGGACTTGCATTGTGAAAGCTTTGCTGTCCTTGATGTGATCATGGAATCTTTTTCTCAC
TAGAGTCTATGTCACTCATTATACTCTGTCGAATGTCATTGAATGTCTTTACATGGGCTT
GTATGCCTATGAAAATTGTAATACAACTTTCAGCAACGGATCTCTTGGCTCTCGCATCGA
TGAAGAACGCAGCGAAATGCGATAAGTAATGTGAATTGCAGAATTCAGTGAATCATCGAA
TCTTTGAACGCATCTTGCGCTCCTTGGTATTCCGAGGAGCATGCCTGTTTGAGTGTCATT
AAATTCTCAACTCTCTTATACTTTTTTGTAAAAGAGAGCTTGGACTGTGGAGGCTTGCTG
GCCACTTTTTGGGGTCAGCTCCTCTGAAATGCATTAGCGGAACCGTTTGCGATCTGCCAC
AAGTGTGATAAGTTATCTACACTGGCGAGGGGATTGCTCTCTGTAATGTTCAGCTTCTAA
TTGTCTCTACTTTGTGAGACTACTTTTGAATGCTTGACCTCAAATCAGGTAGGACTACCC
GCTGAACTTAA
>A5_1
TTTCCGTAGGTGAACCTGCGGAAGGATCATTATTGAATTATGTTTCTAGATAGGTTGTAG
CTGGCTCTTTTAGAGCATGTGCACGCCTGTTTGGACTTCATTTTCATCCACCTGTGCACC
TATTGTAGTCTTTGGTTGGGTTAGGAGGAAGTGATCATTGTATCAGCATCTGCTGGGAGT
GAGGACTTGCATTGTGAAAGCTTTGCTGTCCTTGATGTGATCATGGAATCTTTTTCTCAC
TAGAGTCTATGTCACTCATTATACTCTGTCGAATGTCATTGAATGTCTTTACATGGGCTT
GTATGCCTATGAAAATTGTAATACAACTTTCAGCAACGGATCTCTTGGCTCTCGCATCGA
TGAAGAACGCAGCGAAATGCGATAAGTAATGTGAATTGCAGAATTCAGTGAATCATCGAA
TCTTTGAACGCATCTTGCGCTCCTTGGTATTCCGAGGAGCATGCCTGTTTGAGTGTCATT
AAATTCTCAACTCTCTTATACTTTTTTGTAAAAGAGAGCTTGGACTGTGGAGGCTTGCTG
GCCACTTTTTGGGGTCAGCTCCTCTGAAATGCATTAGCGGAACCGTTTGCGATCTGCCAC
AAGTGTGATAAGTTATCTACACTGGCGAGGGGATTGCTCTCTGTAATGTTCAGCTTCTAA
TTGTCTCTACTTTGTGAGACTACTTTTGAATGCTTGACCTCAAATCAGGTAGGACTACCC
GCTGAACTTAA
>A5_2
TTTCCGTAGGTGAACCTGCGGAAGGATCATTATTGAATTATGTTTCTAGATAGGTTGTAG
CTGGCTCTTTTAGAGCATGTGCACGCCTGTTTGGACTTCATTTTCATCCACCTGTGCACC
TATTGTAGTCTTTGGTTGGGTTAGGAGGAAGTGATCATTGTATCAGCATCTGCTGGGAGT
GAGGACTTGCATTGTGAAAGCTTTGCTGTCCTTGATGTGATCATGGAATCTTTTTCTCAC
TAGAGTCTATGTCACTCATTATACTCTGTCGAATGTCATTGAATGTCTTTACATGGGCTT
GTATGCCTATGAAAATTGTAATACAACTTTCAGCAACGGATCTCTTGGCTCTCGCATCGA
TGAAGAACGCAGCGAAATGCGATAAGTAATGTGAATTGCAGAATTCAGTGAATCATCGAA
TCTTTGAACGCATCTTGCGCTCCTTGGTATTCCGAGGAGCATGCCTGTTTGAGTGTCATT
AAATTCTCAACTCTCTTATACTTTTTTGTAAAAGAGAGCTTGGACTGTGGAGGCTTGCTG
GCCACTTTTTGGGGTCAGCTCCTCTGAAATGCATTAGCGGAACCGTTTGCGATCTGCCAC
AAGTGTGATAAGTTATCTACACTGGCGAGGGGATTGCTCTCTGTAATGTTCAGCTTCTAA

TTGTCTCTACTTTGTGAGACTACTTTTGAATGCTTGACCTCAAATCAGGTAGGACTACCC
GCTGAACTTAA
>A5_3
TTTCCGTAGGTGAACCTGCGGAAGGATCATTATTGAATTATGTTTCTAGATAGGTTGTAG
CTGGCTCTTTTAGAGCATGTGCACGCCTGTTTGGACTTCATTTTCATCCACCTGTGCACC
TATTGTAGTCTTTGGTTGGGTTAGGAGGAAGTGATCATTGTATCAGCATCTGCTGGGAGT
GAGGACTTGCATTGTGAAAGCTTTGCTGTCCTTGATGTGATCATGGAATCTTTTTCTCAC
TAGAGTCTATGTCACTCATTATACTCTGTCGAATGTCATTGAATGTCTTTACATGGGCTT
GTATGCCTATGAAAATTGTAATACAACTTTCAGCAACGGATCTCTTGGCTCTCGCATCGA
TGAAGAACGCAGCGAAATGCGATAAGTAATGTGAATTGCAGAATTCAGTGAATCATCGAA
TCTTTGAACGCATCTTGCGCTCCTTGGTATTCCGAGGAGCATGCCTGTTTGAGTGTCATT
AAATTCTCAACTCTCTTATACTTTTTTGTAAAAGAGAGCTTGGACTGTGGAGGCTTGCTG
GCCACTTTTTGGGGTCAGCTCCTCTGAAATGCATTAGCGGAACCGTTTGCGATCTGCCAC
AAGTGTGATAAGTTATCTACACTGGCGAGGGGATTGCTCTCTGTAATGTTCAGCTTCTAA
TTGTCTCTACTTTGTGAGACTACTTTTGAATGCTTGACCTCAAATCAGGTAGGACTACCC
GCTGAACTTAA
>A5_4
TTTCCGTAGGTGAACCTGCGGAAGGATCATTATTGAATTATGTTTCTAGATAGGTTGTAG
CTGGCTCTTTTAGAGCATGTGCACGCCTGTTTGGACTTCATTTTCATCCACCTGTGCACC
TATTGTAGTCTTTGGTTGGGTTAGGAGGAAGTGATCATTGTATCAGCATCTGCTGGGAGT
GAGGACTTGCATTGTGAAAGCTTTGCTGTCCTTGATGTGATCATGGAATCTTTTTCTCAC
TAGAGTCTATGTCACTCATTATACTCTGTCGAATGTCATTGAATGTCTTTACATGGGCTT
GTATGCCTATGAAAATTGTAATACAACTTTCAGCAACGGATCTCTTGGCTCTCGCATCGA
TGAAGAACGCAGCGAAATGCGATAAGTAATGTGAATTGCAGAATTCAGTGAATCATCGAA
TCTTTGAACGCATCTTGCGCTCCTTGGTATTCCGAGGAGCATGCCTGTTTGAGTGTCATT
AAATTCTCAACTCTCTTATACTTTTTTGTAAAAGAGAGCTTGGACTGTGGAGGCTTGCTG
GCCACTTTTTGGGGTCAGCTCCTCTGAAATGCATTAGCGGAACCGTTTGCGATCTGCCAC
AAGTGTGATAAGTTATCTACACTGGCGAGGGGATTGCTCTCTGTAATGTTCAGCTTCTAA
TTGTCTCTACTTTGTGAGACTACTTTTGAATGCTTGACCTCAAATCAGGTAGGACTACCC
GCTGAACTTAA
>A5_5
TTTCCGTAGGTGAACCTGCGGAAGGATCATTATTGAATTATGTTTCTAGATAGGTTGTAG
CTGGCTCTTTTAGAGCATGTGCACGCCTGTTTGGACTTCATTTTCATCCACCTGTGCACC
TATTGTAGTCTTTGGTTGGGTTAGGAGGAAGTGATCATTGTATCAGCATCTGCTGGGAGT
GAGGACTTGCATTGTGAAAGCTTTGCTGTCCTTGATGTGATCATGGAATCTTTTTCTCAC
TAGAGTCTATGTCACTCATTATACTCTGTCGAATGTCATTGAATGTCTTTACATGGGCTT
GTATGCCTATGAAAATTGTAATACAACTTTCAGCAACGGATCTCTTGGCTCTCGCATCGA
TGAAGAACGCAGCGAAATGCGATAAGTAATGTGAATTGCAGAATTCAGTGAATCATCGAA
TCTTTGAACGCATCTTGCGCTCCTTGGTATTCCGAGGAGCATGCCTGTTTGAGTGTCATT
AAATTCTCAACTCTCTTATACTTTTTTGTAAAAGAGAGCTTGGACTGTGGAGGCTTGCTG
GCCACTTTTTGGGGTCAGCTCCTCTGAAATGCATTAGCGGAACCGTTTGCGATCTGCCAC
AAGTGTGATAAGTTATCTACACTGGCGAGGGGATTGCTCTCTGTAATGTTCAGCTTCTAA
TTGTCTCTACTTTGTGAGACTACTTTTGAATGCTTGACCTCAAATCAGGTAGGACTACCC
GCTGAACTTAA
>A5_6
TTTCCGTAGGTGAACCTGCGGAAGGATCATTATTGAATTATGTTTCTAGATAGGTTGTAG
CTGGCTCTTTTAGAGCATGTGCACGCCTGTTTGGACTTCATTTTCATCCACCTGTGCACC
TATTGTAGTCTTTGGTTGGGTTAGGAGGAAGTGATCATTGTATCAGCATCTGCTGGGAGT
GAGGACTTGCATTGTGAAAGCTTTGCTGTCCTTGATGTGATCATGGAATCTTTTTCTCAC
TAGAGTCTATGTCACTCATTATACTCTGTCGAATGTCATTGAATGTCTTTACATGGGCTT

GTATGCCTATGAAAATTGTAATACAACTTTCAGCAACGGATCTCTTGGCTCTCGCATCGA
TGAAGAACGCAGCGAAATGCGATAAGTAATGTGAATTGCAGAATTCAGTGAATCATCGAA
TCTTTGAACGCATCTTGCGCTCCTTGGTATTCCGAGGAGCATGCCTGTTTGAGTGTCATT
AAATTCTCAACTCTCTTATACTTTTTTGTAAAAGAGAGCTTGGACTGTGGAGGCTTGCTG
GCCACTTTTTGGGGTCAGCTCCTCTGAAATGCATTAGCGGAACCGTTTGCGATCTGCCAC
AAGTGTGATAAGTTATCTACACTGGCGAGGGGATTGCTCTCTGTAATGTTCAGCTTCTAA
TTGTCTCTACTTTGTGAGACTACTTTTGAATGCTTGACCTCAAATCAGGTAGGACTACCC
GCTGAACTTAA
>A5_7
TTTCCGTAGGTGAACCTGCGGAAGGATCATTATTGAATTATGTTTCTAGATAGGTTGTAG
CTGGCTCTTTTAGAGCATGTGCACGCCTGTTTGGACTTCATTTTCATCCACCTGTGCACC
TATTGTAGTCTTTGGTTGGGTTAGGAGGAAGTGATCATTGTATCAGCATCTGCTGGGAGT
GAGGACTTGCATTGTGAAAGCTTTGCTGTCCTTGATGTGATCATGGAATCTTTTTCTCAC
TAGAGTCTATGTCACTCATTATACTCTGTCGAATGTCATTGAATGTCTTTACATGGGCTT
GTATGCCTATGAAAATTGTAATACAACTTTCAGCAACGGATCTCTTGGCTCTCGCATCGA
TGAAGAACGCAGCGAAATGCGATAAGTAATGTGAATTGCAGAATTCAGTGAATCATCGAA
TCTTTGAACGCATCTTGCGCTCCTTGGTATTCCGAGGAGCATGCCTGTTTGAGTGTCATT
AAATTCTCAACTCTCTTATACTTTTTTGTAAAAGAGAGCTTGGACTGTGGAGGCTTGCTG
GCCACTTTTTGGGGTCAGCTCCTCTGAAATGCATTAGCGGAACCGTTTGCGATCTGCCAC
AAGTGTGATAAGTTATCTACACTGGCGAGGGGATTGCTCTCTGTAATGTTCAGCTTCTAA
TTGTCTCTACTTTGTGAGACTACTTTTGAATGCTTGACCTCAAATCAGGTAGGACTACCC
GCTGAACTTAA
>A5_8
TTTCCGTAGGTGAACCTGCGGAAGGATCATTATTGAATTATGTTTCTAGATAGGTTGTAG
CTGGCTCTTTTAGAGCATGTGCACGCCTGTTTGGACTTCATTTTCATCCACCTGTGCACC
TATTGTAGTCTTTGGTTGGGTTAGGAGGAAGTGATCATTGTATCAGCATCTGCTGGGAGT
GAGGACTTGCATTGTGAAAGCTTTGCTGTCCTTGATGTGATCATGGAATCTTTTTCTCAC
TAGAGTCTATGTCACTCATTATACTCTGTCGAATGTCATTGAATGTCTTTACATGGGCTT
GTATGCCTATGAAAATTGTAATACAACTTTCAGCAACGGATCTCTTGGCTCTCGCATCGA
TGAAGAACGCAGCGAAATGCGATAAGTAATGTGAATTGCAGAATTCAGTGAATCATCGAA
TCTTTGAACGCATCTTGCGCTCCTTGGTATTCCGAGGAGCATGCCTGTTTGAGTGTCATT
AAATTCTCAACTCTCTTATACTTTTTTGTAAAAGAGAGCTTGGACTGTGGAGGCTTGCTG
GCCACTTTTTGGGGTCAGCTCCTCTGAAATGCATTAGCGGAACCGTTTGCGATCTGCCAC
AAGTGTGATAAGTTATCTACACTGGCGAGGGGATTGCTCTCTGTAATGTTCAGCTTCTAA
TTGTCTCTACTTTGTGAGACTACTTTTGAATGCTTGACCTCAAATCAGGTAGGACTACCC
GCTGAACTTAA
>A5_9
TTTCCGTAGGTGAACCTGCGGAAGGATCATTATTGAATTATGTTTCTAGATAGGTTGTAG
CTGGCTCTTTTAGAGCATGTGCACGCCTGTTTGGACTTCATTTTCATCCACCTGTGCACC
TATTGTAGTCTTTGGTTGGGTTAGGAGGAAGTGATCATTGTATCAGCATCTGCTGGGAGT
GAGGACTTGCATTGTGAAAGCTTTGCTGTCCTTGATGTGATCATGGAATCTTTTTCTCAC
TAGAGTCTATGTCACTCATTATACTCTGTCGAATGTCATTGAATGTCTTTACATGGGCTT
GTATGCCTATGAAAATTGTAATACAACTTTCAGCAACGGATCTCTTGGCTCTCGCATCGA
TGAAGAACGCAGCGAAATGCGATAAGTAATGTGAATTGCAGAATTCAGTGAATCATCGAA
TCTTTGAACGCATCTTGCGCTCCTTGGTATTCCGAGGAGCATGCCTGTTTGAGTGTCATT
AAATTCTCAACTCTCTTATACTTTTTTGTAAAAGAGAGCTTGGACTGTGGAGGCTTGCTG
GCCACTTTTTGGGGTCAGCTCCTCTGAAATGCATTAGCGGAACCGTTTGCGATCTGCCAC
AAGTGTGATAAGTTATCTACACTGGCGAGGGGATTGCTCTCTGTAATGTTCAGCTTCTAA
TTGTCTCTACTTTGTGAGACTACTTTTGAATGCTTGACCTCAAATCAGGTAGGACTACCC
GCTGAACTTAA

>A5_10
TTTCCGTAGGTGAACCTGCGGAAGGATCATTATTGAATTATGTTTCTAGATAGGTTGTAG
CTGGCTCTTTTAGAGCATGTGCACGCCTGTTTGGACTTCATTTTCATCCACCTGTGCACC
TATTGTAGTCTTTGGTTGGGTTAGGAGGAAGTGATCATTGTATCAGCATCTGCTGGGAGT
GAGGACTTGCATTGTGAAAGCTTTGCTGTCCTTGATGTGATCATGGAATCTTTTTCTCAC
TAGAGTCTATGTCACTCATTATACTCTGTCGAATGTCATTGAATGTCTTTACATGGGCTT
GTATGCCTATGAAAATTGTAATACAACTTTCAGCAACGGATCTCTTGGCTCTCGCATCGA
TGAAGAACGCAGCGAAATGCGATAAGTAATGTGAATTGCAGAATTCAGTGAATCATCGAA
TCTTTGAACGCATCTTGCGCTCCTTGGTATTCCGAGGAGCATGCCTGTTTGAGTGTCATT
AAATTCTCAACTCTCTTATACTTTTTTGTAAAAGAGAGCTTGGACTGTGGAGGCTTGCTG
GCCACTTTTTGGGGTCAGCTCCTCTGAAATGCATTAGCGGAACCGTTTGCGATCTGCCAC
AAGTGTGATAAGTTATCTACACTGGCGAGGGGATTGCTCTCTGTAATGTTCAGCTTCTAA
TTGTCTCTACTTTGTGAGACTACTTTTGAATGCTTGACCTCAAATCAGGTAGGACTACCC
GCTGAACTTAA
>A5_11
TTTCCGTAGGTGAACCTGCGGAAGGATCATTATTGAATTATGTTTCTAGATAGGTTGTAG
CTGGCTCTTTTAGAGCATGTGCACGCCTGTTTGGACTTCATTTTCATCCACCTGTGCACC
TATTGTAGTCTTTGGTTGGGTTAGGAGGAAGTGATCATTGTATCAGCATCTGCTGGGAGT
GAGGACTTGCATTGTGAAAGCTTTGCTGTCCTTGATGTGATCATGGAATCTTTTTCTCAC
TAGAGTCTATGTCACTCATTATACTCTGTCGAATGTCATTGAATGTCTTTACATGGGCTT
GTATGCCTATGAAAATTGTAATACAACTTTCAGCAACGGATCTCTTGGCTCTCGCATCGA
TGAAGAACGCAGCGAAATGCGATAAGTAATGTGAATTGCAGAATTCAGTGAATCATCGAA
TCTTTGAACGCATCTTGCGCTCCTTGGTATTCCGAGGAGCATGCCTGTTTGAGTGTCATT
AAATTCTCAACTCTCTTATACTTTTTTGTAAAAGAGAGCTTGGACTGTGGAGGCTTGCTG
GCCACTTTTTGGGGTCAGCTCCTCTGAAATGCATTAGCGGAACCGTTTGCGATCTGCCAC
AAGTGTGATAAGTTATCTACACTGGCGAGGGGATTGCTCTCTGTAATGTTCAGCTTCTAA
TTGTCTCTACTTTGTGAGACTACTTTTGAATGCTTGACCTCAAATCAGGTAGGACTACCC
GCTGAACTTAA
>A5_12
TTTCCGTAGGTGAACCTGCGGAAGGATCATTATTGAATTATGTTTCTAGATAGGTTGTAG
CTGGCTCTTTTAGAGCATGTGCACGCCTGTTTGGACTTCATTTTCATCCACCTGTGCACC
TATTGTAGTCTTTGGTTGGGTTAGGAGGAAGTGATCATTGTATCAGCATCTGCTGGGAGT
GAGGACTTGCATTGTGAAAGCTTTGCTGTCCTTGATGTGATCATGGAATCTTTTTCTCAC
TAGAGTCTATGTCACTCATTATACTCTGTCGAATGTCATTGAATGTCTTTACATGGGCTT
GTATGCCTATGAAAATTGTAATACAACTTTCAGCAACGGATCTCTTGGCTCTCGCATCGA
TGAAGAACGCAGCGAAATGCGATAAGTAATGTGAATTGCAGAATTCAGTGAATCATCGAA
TCTTTGAACGCATCTTGCGCTCCTTGGTATTCCGAGGAGCATGCCTGTTTGAGTGTCATT
AAATTCTCAACTCTCTTATACTTTTTTGTAAAAGAGAGCTTGGACTGTGGAGGCTTGCTG
GCCACTTTTTGGGGTCAGCTCCTCTGAAATGCATTAGCGGAACCGTTTGCGATCTGCCAC
AAGTGTGATAAGTTATCTACACTGGCGAGGGGATTGCTCTCTGTAATGTTCAGCTTCTAA
TTGTCTCTACTTTGTGAGACTACTTTTGAATGCTTGACCTCAAATCAGGTAGGACTACCC
GCTGAACTTAA
>A5_14
TTTCCGTAGGTGAACCTGCGGAAGGATCATTATTGAATTATGTTTCTAGATAGGTTGTAG
CTGGCTCTTTTAGAGCATGTGCACGCCTGTTTGGACTTCATTTTCATCCACCTGTGCACC
TATTGTAGTCTTTGGTTGGGTTAGGAGGAAGTGATCATTGTATCAGCATCTGCTGGGAGT
GAGGACTTGCATTGTGAAAGCTTTGCTGTCCTTGATGTGATCATGGAATCTTTTTCTCAC
TAGAGTCTATGTCACTCATTATACTCTGTCGAATGTCATTGAATGTCTTTACATGGGCTT
GTATGCCTATGAAAATTGTAATACAACTTTCAGCAACGGATCTCTTGGCTCTCGCATCGA
TGAAGAACGCAGCGAAATGCGATAAGTAATGTGAATTGCAGAATTCAGTGAATCATCGAA

TCTTTGAACGCATCTTGCGCTCCTTGGTATTCCGAGGAGCATGCCTGTTTGAGTGTCATT
AAATTCTCAACTCTCTTATACTTTTTTGTAAAAGAGAGCTTGGACTGTGGAGGCTTGCTG
GCCACTTTTTGGGGTCAGCTCCTCTGAAATGCATTAGCGGAACCGTTTGCGATCTGCCAC
AAGTGTGATAAGTTATCTACACTGGCGAGGGGATTGCTCTCTGTAATGTTCAGCTTCTAA
TTGTCTCTACTTTGTGAGACTACTTTTGAATGCTTGACCTCAAATCAGGTAGGACTACCC
GCTGAACTTAA
>A5_15
TTTCCGTAGGTGAACCTGCGGAAGGATCATTATTGAATTATGTTTCTAGATAGGTTGTAG
CTGGCTCTTTTAGAGCATGTGCACGCCTGTTTGGACTTCATTTTCATCCACCTGTGCACC
TATTGTAGTCTTTGGTTGGGTTAGGAGGAAGTGATCATTGTATCAGCATCTGCTGGGAGT
GAGGACTTGCATTGTGAAAGCTTTGCTGTCCTTGATGTGATCATGGAATCTTTTTCTCAC
TAGAGTCTATGTCACTCATTATACTCTGTCGAATGTCATTGAATGTCTTTACATGGGCTT
GTATGCCTATGAAAATTGTAATACAACTTTCAGCAACGGATCTCTTGGCTCTCGCATCGA
TGAAGAACGCAGCGAAATGCGATAAGTAATGTGAATTGCAGAATTCAGTGAATCATCGAA
TCTTTGAACGCATCTTGCGCTCCTTGGTATTCCGAGGAGCATGCCTGTTTGAGTGTCATT
AAATTCTCAACTCTCTTATACTTTTTTGTAAAAGAGAGCTTGGACTGTGGAGGCTTGCTG
GCCACTTTTTGGGGTCAGCTCCTCTGAAATGCATTAGCGGAACCGTTTGCGATCTGCCAC
AAGTGTGATAAGTTATCTACACTGGCGAGGGGATTGCTCTCTGTAATGTTCAGCTTCTAA
TTGTCTCTACTTTGTGAGACTACTTTTGAATGCTTGACCTCAAATCAGGTAGGACTACCC
GCTGAACTTAA
>A5_16
TTTCCGTAGGTGAACCTGCGGAAGGATCATTATTGAATTATGTTTCTAGATAGGTTGTAG
CTGGCTCTTTTAGAGCATGTGCACGCCTGTTTGGACTTCATTTTCATCCACCTGTGCACC
TATTGTAGTCTTTGGTTGGGTTAGGAGGAAGTGATCATTGTATCAGCATCTGCTGGGAGT
GAGGACTTGCATTGTGAAAGCTTTGCTGTCCTTGATGTGATCATGGAATCTTTTTCTCAC
TAGAGTCTATGTCACTCATTATACTCTGTCGAATGTCATTGAATGTCTTTACATGGGCTT
GTATGCCTATGAAAATTGTAATACAACTTTCAGCAACGGATCTCTTGGCTCTCGCATCGA
TGAAGAACGCAGCGAAATGCGATAAGTAATGTGAATTGCAGAATTCAGTGAATCATCGAA
TCTTTGAACGCATCTTGCGCTCCTTGGTATTCCGAGGAGCATGCCTGTTTGAGTGTCATT
AAATTCTCAACTCTCTTATACTTTTTTGTAAAAGAGAGCTTGGACTGTGGAGGCTTGCTG
GCCACTTTTTGGGGTCAGCTCCTCTGAAATGCATTAGCGGAACCGTTTGCGATCTGCCAC
AAGTGTGATAAGTTATCTACACTGGCGAGGGGATTGCTCTCTGTAATGTTCAGCTTCTAA
TTGTCTCTACTTTGTGAGACTACTTTTGAATGCTTGACCTCAAATCAGGTAGGACTACCC
GCTGAACTTAA
>A5_17
TTTCCGTAGGTGAACCTGCGGAAGGATCATTATTGAATTATGTTTCTAGATAGGTTGTAG
CTGGCTCTTTTAGAGCATGTGCACGCCTGTTTGGACTTCATTTTCATCCACCTGTGCACC
TATTGTAGTCTTTGGTTGGGTTAGGAGGAAGTGATCATTGTATCAGCATCTGCTGGGAGT
GAGGACTTGCATTGTGAAAGCTTTGCTGTCCTTGATGTGATCATGGAATCTTTTTCTCAC
TAGAGTCTATGTCACTCATTATACTCTGTCGAATGTCATTGAATGTCTTTACATGGGCTT
GTATGCCTATGAAAATTGTAATACAACTTTCAGCAACGGATCTCTTGGCTCTCGCATCGA
TGAAGAACGCAGCGAAATGCGATAAGTAATGTGAATTGCAGAATTCAGTGAATCATCGAA
TCTTTGAACGCATCTTGCGCTCCTTGGTATTCCGAGGAGCATGCCTGTTTGAGTGTCATT
AAATTCTCAACTCTCTTATACTTTTTTGTAAAAGAGAGCTTGGACTGTGGAGGCTTGCTG
GCCACTTTTTGGGGTCAGCTCCTCTGAAATGCATTAGCGGAACCGTTTGCGATCTGCCAC
AAGTGTGATAAGTTATCTACACTGGCGAGGGGATTGCTCTCTGTAATGTTCAGCTTCTAA
TTGTCTCTACTTTGTGAGACTACTTTTGAATGCTTGACCTCAAATCAGGTAGGACTACCC
GCTGAACTTAA
>A5_18
TTTCCGTAGGTGAACCTGCGGAAGGATCATTATTGAATTATGTTTCTAGATAGGTTGTAG

CTGGCTCTTTTAGAGCATGTGCACGCCTGTTTGGACTTCATTTTCATCCACCTGTGCACC
TATTGTAGTCTTTGGTTGGGTTAGGAGGAAGTGATCATTGTATCAGCATCTGCTGGGAGT
GAGGACTTGCATTGTGAAAGCTTTGCTGTCCTTGATGTGATCATGGAATCTTTTTCTCAC
TAGAGTCTATGTCACTCATTATACTCTGTCGAATGTCATTGAATGTCTTTACATGGGCTT
GTATGCCTATGAAAATTGTAATACAACTTTCAGCAACGGATCTCTTGGCTCTCGCATCGA
TGAAGAACGCAGCGAAATGCGATAAGTAATGTGAATTGCAGAATTCAGTGAATCATCGAA
TCTTTGAACGCATCTTGCGCTCCTTGGTATTCCGAGGAGCATGCCTGTTTGAGTGTCATT
AAATTCTCAACTCTCTTATACTTTTTTGTAAAAGAGAGCTTGGACTGTGGAGGCTTGCTG
GCCACTTTTTGGGGTCAGCTCCTCTGAAATGCATTAGCGGAACCGTTTGCGATCTGCCAC
AAGTGTGATAAGTTATCTACACTGGCGAGGGGATTGCTCTCTGTAATGTTCAGCTTCTAA
TTGTCTCTACTTTGTGAGACTACTTTTGAATGCTTGACCTCAAATCAGGTAGGACTACCC
GCTGAACTTAA
>A5_19
TTTCCGTAGGTGAACCTGCGGAAGGATCATTATTGAATTATGTTTCTAGATAGGTTGTAG
CTGGCTCTTTTAGAGCATGTGCACGCCTGTTTGGACTTCATTTTCATCCACCTGTGCACC
TATTGTAGTCTTTGGTTGGGTTAGGAGGAAGTGATCATTGTATCAGCATCTGCTGGGAGT
GAGGACTTGCATTGTGAAAGCTTTGCTGTCCTTGATGTGATCATGGAATCTTTTTCTCAC
TAGAGTCTATGTCACTCATTATACTCTGTCGAATGTCATTGAATGTCTTTACATGGGCTT
GTATGCCTATGAAAATTGTAATACAACTTTCAGCAACGGATCTCTTGGCTCTCGCATCGA
TGAAGAACGCAGCGAAATGCGATAAGTAATGTGAATTGCAGAATTCAGTGAATCATCGAA
TCTTTGAACGCATCTTGCGCTCCTTGGTATTCCGAGGAGCATGCCTGTTTGAGTGTCATT
AAATTCTCAACTCTCTTATACTTTTTTGTAAAAGAGAGCTTGGACTGTGGAGGCTTGCTG
GCCACTTTTTGGGGTCAGCTCCTCTGAAATGCATTAGCGGAACCGTTTGCGATCTGCCAC
AAGTGTGATAAGTTATCTACACTGGCGAGGGGATTGCTCTCTGTAATGTTCAGCTTCTAA
TTGTCTCTACTTTGTGAGACTACTTTTGAATGCTTGACCTCAAATCAGGTAGGACTACCC
GCTGAACTTAA
>A5_20
TTTCCGTAGGTGAACCTGCGGAAGGATCATTATTGAATTATGTTTCTAGATAGGTTGTAG
CTGGCTCTTTTAGAGCATGTGCACGCCTGTTTGGACTTCATTTTCATCCACCTGTGCACC
TATTGTAGTCTTTGGTTGGGTTAGGAGGAAGTGATCATTGTATCAGCATCTGCTGGGAGT
GAGGACTTGCATTGTGAAAGCTTTGCTGTCCTTGATGTGATCATGGAATCTTTTTCTCAC
TAGAGTCTATGTCACTCATTATACTCTGTCGAATGTCATTGAATGTCTTTACATGGGCTT
GTATGCCTATGAAAATTGTAATACAACTTTCAGCAACGGATCTCTTGGCTCTCGCATCGA
TGAAGAACGCAGCGAAATGCGATAAGTAATGTGAATTGCAGAATTCAGTGAATCATCGAA
TCTTTGAACGCATCTTGCGCTCCTTGGTATTCCGAGGAGCATGCCTGTTTGAGTGTCATT
AAATTCTCAACTCTCTTATACTTTTTTGTAAAAGAGAGCTTGGACTGTGGAGGCTTGCTG
GCCACTTTTTGGGGTCAGCTCCTCTGAAATGCATTAGCGGAACCGTTTGCGATCTGCCAC
AAGTGTGATAAGTTATCTACACTGGCGAGGGGATTGCTCTCTGTAATGTTCAGCTTCTAA
TTGTCTCTACTTTGTGAGACTACTTTTGAATGCTTGACCTCAAATCAGGTAGGACTACCC
GCTGAACTTAA
>A5_50
TTTCCGTAGGTGAACCTGCGGAAGGATCATTATTGAATTATGTTTCTAGATAGGTTGTAG
CTGGCTCTTTTAGAGCATGTGCACGCCTGTTTGGACTTCATTTTCATCCACCTGTGCACC
TATTGTAGTCTTTGGTTGGGTTAGGAGGAAGTGATCATTGTATCAGCATCTGCTGGGAGT
GAGGACTTGCATTGTGAAAGCTTTGCTGTCCTTGATGTGATCATGGAATCTTTTTCTCAC
TAGAGTCTATGTCACTCATTATACTCTGTCGAATGTCATTGAATGTCTTTACATGGGCTT
GTATGCCTATGAAAATTGTAATACAACTTTCAGCAACGGATCTCTTGGCTCTCGCATCGA
TGAAGAACGCAGCGAAATGCGATAAGTAATGTGAATTGCAGAATTCAGTGAATCATCGAA
TCTTTGAACGCATCTTGCGCTCCTTGGTATTCCGAGGAGCATGCCTGTTTGAGTGTCATT
AAATTCTCAACTCTCTTATACTTTTTTGTAAAAGAGAGCTTGGACTGTGGAGGCTTGCTG

GCCACTTTTTGGGGTCAGCTCCTCTGAAATGCATTAGCGGAACCGTTTGCGATCTGCCAC
AAGTGTGATAAGTTATCTACACTGGCGAGGGGATTGCTCTCTGTAATGTTCAGCTTCTAA
TTGTCTCTACTTTGTGAGACTACTTTTGAATGCTTGACCTCAAATCAGGTAGGACTACCC
GCTGAACTTAA
>A5-65
TTTCCGTAGGTGAACCTGCGGAAGGATCATTATTGAATTATGTTTCTAGATAGGTTGTAG
CTGGCTCTTTTAGAGCATGTGCACGCCTGTTTGGACTTCATTTTCATCCACCTGTGCACC
TATTGTAGTCTTTGGTTGGGTTAGGAGGAAGTGATCATTGTATCAGCATCTGCTGGGAGT
GAGGACTTGCATTGTGAAAGCTTTGCTGTCCTTGATGTGATCATGGAATCTTTTTCTCAC
TAGAGTCTATGTCACTCATTATACTCTGTCGAATGTCATTGAATGTCTTTACATGGGCTT
GTATGCCTATGAAAATTGTAATACAACTTTCAGCAACGGATCTCTTGGCTCTCGCATCGA
TGAAGAACGCAGCGAAATGCGATAAGTAATGTGAATTGCAGAATTCAGTGAATCATCGAA
TCTTTGAACGCATCTTGCGCTCCTTGGTATTCCGAGGAGCATGCCTGTTTGAGTGTCATT
AAATTCTCAACTCTCTTATACTTTTTTGTAAAAGAGAGCTTGGACTGTGGAGGCTTGCTG
GCCACTTTTTGGGGTCAGCTCCTCTGAAATGCATTAGCGGAACCGTTTGCGATCTGCCAC
AAGTGTGATAAGTTATCTACACTGGCGAGGGGATTGCTCTCTGTAATGTTCAGCTTCTAA
TTGTCTCTACTTTGTGAGACTACTTTTGAATGCTTGACCTCAAATCAGGTAGGACTACCC
GCTGAACTTAA
>A5-66
TTTCCGTAGGTGAACCTGCGGAAGGATCATTATTGAATTATGTTTCTAGATAGGTTGTAG
CTGGCTCTTTTAGAGCATGTGCACGCCTGTTTGGACTTCATTTTCATCCACCTGTGCACC
TATTGTAGTCTTTGGTTGGGTTAGGAGGAAGTGATCATTGTATCAGCATCTGCTGGGAGT
GAGGACTTGCATTGTGAAAGCTTTGCTGTCCTTGATGTGATCATGGAATCTTTTTCTCAC
TAGAGTCTATGTCACTCATTATACTCTGTCGAATGTCATTGAATGTCTTTACATGGGCTT
GTATGCCTATGAAAATTGTAATACAACTTTCAGCAACGGATCTCTTGGCTCTCGCATCGA
TGAAGAACGCAGCGAAATGCGATAAGTAATGTGAATTGCAGAATTCAGTGAATCATCGAA
TCTTTGAACGCATCTTGCGCTCCTTGGTATTCCGAGGAGCATGCCTGTTTGAGTGTCATT
AAATTCTCAACTCTCTTATACTTTTTTGTAAAAGAGAGCTTGGACTGTGGAGGCTTGCTG
GCCACTTTTTGGGGTCAGCTCCTCTGAAATGCATTAGCGGAACCGTTTGCGATCTGCCAC
AAGTGTGATAAGTTATCTACACTGGCGAGGGGATTGCTCTCTGTAATGTTCAGCTTCTAA
TTGTCTCTACTTTGTGAGACTACTTTTGAATGCTTGACCTCAAATCAGGTAGGACTACCC
GCTGAACTTAA
>A5-67
TTTCCGTAGGTGAACCTGCGGAAGGATCATTATTGAATTATGTTTCTAGATAGGTTGTAG
CTGGCTCTTTTAGAGCATGTGCACGCCTGTTTGGACTTCATTTTCATCCACCTGTGCACC
TATTGTAGTCTTTGGTTGGGTTAGGAGGAAGTGATCATTGTATCAGCATCTGCTGGGAGT
GAGGACTTGCATTGTGAAAGCTTTGCTGTCCTTGATGTGATCATGGAATCTTTTTCTCAC
TAGAGTCTATGTCACTCATTATACTCTGTCGAATGTCATTGAATGTCTTTACATGGGCTT
GTATGCCTATGAAAATTGTAATACAACTTTCAGCAACGGATCTCTTGGCTCTCGCATCGA
TGAAGAACGCAGCGAAATGCGATAAGTAATGTGAATTGCAGAATTCAGTGAATCATCGAA
TCTTTGAACGCATCTTGCGCTCCTTGGTATTCCGAGGAGCATGCCTGTTTGAGTGTCATT
AAATTCTCAACTCTCTTATACTTTTTTGTAAAAGAGAGCTTGGACTGTGGAGGCTTGCTG
GCCACTTTTTGGGGTCAGCTCCTCTGAAATGCATTAGCGGAACCGTTTGCGATCTGCCAC
AAGTGTGATAAGTTATCTACACTGGCGAGGGGATTGCTCTCTGTAATGTTCAGCTTCTAA
TTGTCTCTACTTTGTGAGACTACTTTTGAATGCTTGACCTCAAATCAGGTAGGACTACCC
GCTGAACTTAA
>A5-69
TTTCCGTAGGTGAACCTGCGGAAGGATCATTATTGAATTATGTTTCTAGATAGGTTGTAG
CTGGCTCTTTTAGAGCATGTGCACGCCTGTTTGGACTTCATTTTCATCCACCTGTGCACC
TATTGTAGTCTTTGGTTGGGTTAGGAGGAAGTGATCATTGTATCAGCATCTGCTGGGAGT

GAGGACTTGCATTGTGAAAGCTTTGCTGTCCTTGATGTGATCATGGAATCTTTTTCTCAC
TAGAGTCTATGTCACTCATTATACTCTGTCGAATGTCATTGAATGTCTTTACATGGGCTT
GTATGCCTATGAAAATTGTAATACAACTTTCAGCAACGGATCTCTTGGCTCTCGCATCGA
TGAAGAACGCAGCGAAATGCGATAAGTAATGTGAATTGCAGAATTCAGTGAATCATCGAA
TCTTTGAACGCATCTTGCGCTCCTTGGTATTCCGAGGAGCATGCCTGTTTGAGTGTCATT
AAATTCTCAACTCTCTTATACTTTTTTGTAAAAGAGAGCTTGGACTGTGGAGGCTTGCTG
GCCACTTTTTGGGGTCAGCTCCTCTGAAATGCATTAGCGGAACCGTTTGCGATCTGCCAC
AAGTGTGATAAGTTATCTACACTGGCGAGGGGATTGCTCTCTGTAATGTTCAGCTTCTAA
TTGTCTCTACTTTGTGAGACTACTTTTGAATGCTTGACCTCAAATCAGGTAGGACTACCC
GCTGAACTTAA
>A6_1
TTTCCGTAGGTGAACCTGCGGAAGGATCATTATTGAATTATGTTTCTAGATAGGTTGTAG
CTGGCTCTTTTAGAGCATGTGCACGCCTGTTTGGACTTCATTTTCATCCACCTGTGCACC
TATTGTAGTCTTTGGTTGGGTTAGGAGGAAGTGATCATTGTATCAGCATCTGCTGGGAGT
GAGGACTTGCATTGTGAAAGCTTTGCTGTCCTTGATGTGATCATGGAATCTTTTTCTCAC
TAGAGTCTATGTCACTCATTATACTCTGTCGAATGTCATTGAATGTCTTTACATGGGCTT
GTATGCCTATGAAAATTGTAATACAACTTTCAGCAACGGATCTCTTGGCTCTCGCATCGA
TGAAGAACGCAGCGAAATGCGATAAGTAATGTGAATTGCAGAATTCAGTGAATCATCGAA
TCTTTGAACGCATCTTGCGCTCCTTGGTATTCCGAGGAGCATGCCTGTTTGAGTGTCATT
AAATTCTCAACTCTCTTATACTTTTTTGTAAAAGAGAGCTTGGACTGTGGAGGCTTGCTG
GCCACTTTTTGGGGTCAGCTCCTCTGAAATGCATTAGCGGAACCGTTTGCGATCTGCCAC
AAGTGTGATAAGTTATCTACACTGGCGAGGGGATTGCTCTCTGTAATGTTCAGCTTCTAA
TTGTCTCTACTTTGTGAGACTACTTTTGAATGCTTGACCTCAAATCAGGTAGGACTACCC
GCTGAACTTAA
>A6_2
TTTCCGTAGGTGAACCTGCGGAAGGATCATTATTGAATTATGTTTCTAGATAGGTTGTAG
CTGGCTCTTTTAGAGCATGTGCACGCCTGTTTGGACTTCATTTTCATCCACCTGTGCACC
TATTGTAGTCTTTGGTTGGGTTAGGAGGAAGTGATCATTGTATCAGCATCTGCTGGGAGT
GAGGACTTGCATTGTGAAAGCTTTGCTGTCCTTGATGTGATCATGGAATCTTTTTCTCAC
TAGAGTCTATGTCACTCATTATACTCTGTCGAATGTCATTGAATGTCTTTACATGGGCTT
GTATGCCTATGAAAATTGTAATACAACTTTCAGCAACGGATCTCTTGGCTCTCGCATCGA
TGAAGAACGCAGCGAAATGCGATAAGTAATGTGAATTGCAGAATTCAGTGAATCATCGAA
TCTTTGAACGCATCTTGCGCTCCTTGGTATTCCGAGGAGCATGCCTGTTTGAGTGTCATT
AAATTCTCAACTCTCTTATACTTTTTTGTAAAAGAGAGCTTGGACTGTGGAGGCTTGCTG
GCCACTTTTTGGGGTCAGCTCCTCTGAAATGCATTAGCGGAACCGTTTGCGATCTGCCAC
AAGTGTGATAAGTTATCTACACTGGCGAGGGGATTGCTCTCTGTAATGTTCAGCTTCTAA
TTGTCTCTACTTTGTGAGACTACTTTTGAATGCTTGACCTCAAATCAGGTAGGACTACCC
GCTGAACTTAA
>A6_3
TTTCCGTAGGTGAACCTGCGGAAGGATCATTATTGAATTATGTTTCTAGATAGGTTGTAG
CTGGCTCTTTTAGAGCATGTGCACGCCTGTTTGGACTTCATTTTCATCCACCTGTGCACC
TATTGTAGTCTTTGGTTGGGTTAGGAGGAAGTGATCATTGTATCAGCATCTGCTGGGAGT
GAGGACTTGCATTGTGAAAGCTTTGCTGTCCTTGATGTGATCATGGAATCTTTTTCTCAC
TAGAGTCTATGTCACTCATTATACTCTGTCGAATGTCATTGAATGTCTTTACATGGGCTT
GTATGCCTATGAAAATTGTAATACAACTTTCAGCAACGGATCTCTTGGCTCTCGCATCGA
TGAAGAACGCAGCGAAATGCGATAAGTAATGTGAATTGCAGAATTCAGTGAATCATCGAA
TCTTTGAACGCATCTTGCGCTCCTTGGTATTCCGAGGAGCATGCCTGTTTGAGTGTCATT
AAATTCTCAACTCTCTTATACTTTTTTGTAAAAGAGAGCTTGGACTGTGGAGGCTTGCTG
GCCACTTTTTGGGGTCAGCTCCTCTGAAATGCATTAGCGGAACCGTTTGCGATCTGCCAC
AAGTGTGATAAGTTATCTACACTGGCGAGGGGATTGCTCTCTGTAATGTTCAGCTTCTAA

TTGTCTCTACTTTGTGAGACTACTTTTGAATGCTTGACCTCAAATCAGGTAGGACTACCC
GCTGAACTTAA
>A6_4
TTTCCGTAGGTGAACCTGCGGAAGGATCATTATTGAATTATGTTTCTAGATAGGTTGTAG
CTGGCTCTTTTAGAGCATGTGCACGCCTGTTTGGACTTCATTTTCATCCACCTGTGCACC
TATTGTAGTCTTTGGTTGGGTTAGGAGGAAGTGATCATTGTATCAGCATCTGCTGGGAGT
GAGGACTTGCATTGTGAAAGCTTTGCTGTCCTTGATGTGATCATGGAATCTTTTTCTCAC
TAGAGTCTATGTCACTCATTATACTCTGTCGAATGTCATTGAATGTCTTTACATGGGCTT
GTATGCCTATGAAAATTGTAATACAACTTTCAGCAACGGATCTCTTGGCTCTCGCATCGA
TGAAGAACGCAGCGAAATGCGATAAGTAATGTGAATTGCAGAATTCAGTGAATCATCGAA
TCTTTGAACGCATCTTGCGCTCCTTGGTATTCCGAGGAGCATGCCTGTTTGAGTGTCATT
AAATTCTCAACTCTCTTATACTTTTTTGTAAAAGAGAGCTTGGACTGTGGAGGCTTGCTG
GCCACTTTTTGGGGTCAGCTCCTCTGAAATGCATTAGCGGAACCGTTTGCGATCTGCCAC
AAGTGTGATAAGTTATCTACACTGGCGAGGGGATTGCTCTCTGTAATGTTCAGCTTCTAA
TTGTCTCTACTTTGTGAGACTACTTTTGAATGCTTGACCTCAAATCAGGTAGGACTACCC
GCTGAACTTAA
>A6_5
TTTCCGTAGGTGAACCTGCGGAAGGATCATTATTGAATTATGTTTCTAGATAGGTTGTAG
CTGGCTCTTTTAGAGCATGTGCACGCCTGTTTGGACTTCATTTTCATCCACCTGTGCACC
TATTGTAGTCTTTGGTTGGGTTAGGAGGAAGTGATCATTGTATCAGCATCTGCTGGGAGT
GAGGACTTGCATTGTGAAAGCTTTGCTGTCCTTGATGTGATCATGGAATCTTTTTCTCAC
TAGAGTCTATGTCACTCATTATACTCTGTCGAATGTCATTGAATGTCTTTACATGGGCTT
GTATGCCTATGAAAATTGTAATACAACTTTCAGCAACGGATCTCTTGGCTCTCGCATCGA
TGAAGAACGCAGCGAAATGCGATAAGTAATGTGAATTGCAGAATTCAGTGAATCATCGAA
TCTTTGAACGCATCTTGCGCTCCTTGGTATTCCGAGGAGCATGCCTGTTTGAGTGTCATT
AAATTCTCAACTCTCTTATACTTTTTTGTAAAAGAGAGCTTGGACTGTGGAGGCTTGCTG
GCCACTTTTTGGGGTCAGCTCCTCTGAAATGCATTAGCGGAACCGTTTGCGATCTGCCAC
AAGTGTGATAAGTTATCTACACTGGCGAGGGGATTGCTCTCTGTAATGTTCAGCTTCTAA
TTGTCTCTACTTTGTGAGACTACTTTTGAATGCTTGACCTCAAATCAGGTAGGACTACCC
GCTGAACTTAA
>A6_6
TTTCCGTAGGTGAACCTGCGGAAGGATCATTATTGAATTATGTTTCTAGATAGGTTGTAG
CTGGCTCTTTTAGAGCATGTGCACGCCTGTTTGGACTTCATTTTCATCCACCTGTGCACC
TATTGTAGTCTTTGGTTGGGTTAGGAGGAAGTGATCATTGTATCAGCATCTGCTGGGAGT
GAGGACTTGCATTGTGAAAGCTTTGCTGTCCTTGATGTGATCATGGAATCTTTTTCTCAC
TAGAGTCTATGTCACTCATTATACTCTGTCGAATGTCATTGAATGTCTTTACATGGGCTT
GTATGCCTATGAAAATTGTAATACAACTTTCAGCAACGGATCTCTTGGCTCTCGCATCGA
TGAAGAACGCAGCGAAATGCGATAAGTAATGTGAATTGCAGAATTCAGTGAATCATCGAA
TCTTTGAACGCATCTTGCGCTCCTTGGTATTCCGAGGAGCATGCCTGTTTGAGTGTCATT
AAATTCTCAACTCTCTTATACTTTTTTGTAAAAGAGAGCTTGGACTGTGGAGGCTTGCTG
GCCACTTTTTGGGGTCAGCTCCTCTGAAATGCATTAGCGGAACCGTTTGCGATCTGCCAC
AAGTGTGATAAGTTATCTACACTGGCGAGGGGATTGCTCTCTGTAATGTTCAGCTTCTAA
TTGTCTCTACTTTGTGAGACTACTTTTGAATGCTTGACCTCAAATCAGGTAGGACTACCC
GCTGAACTTAA
>A6_7
TTTCCGTAGGTGAACCTGCGGAAGGATCATTATTGAATTATGTTTCTAGATAGGTTGTAG
CTGGCTCTTTTAGAGCATGTGCACGCCTGTTTGGACTTCATTTTCATCCACCTGTGCACC
TATTGTAGTCTTTGGTTGGGTTAGGAGGAAGTGATCATTGTATCAGCATCTGCTGGGAGT
GAGGACTTGCATTGTGAAAGCTTTGCTGTCCTTGATGTGATCATGGAATCTTTTTCTCAC
TAGAGTCTATGTCACTCATTATACTCTGTCGAATGTCATTGAATGTCTTTACATGGGCTT

GTATGCCTATGAAAATTGTAATACAACTTTCAGCAACGGATCTCTTGGCTCTCGCATCGA
TGAAGAACGCAGCGAAATGCGATAAGTAATGTGAATTGCAGAATTCAGTGAATCATCGAA
TCTTTGAACGCATCTTGCGCTCCTTGGTATTCCGAGGAGCATGCCTGTTTGAGTGTCATT
AAATTCTCAACTCTCTTATACTTTTTTGTAAAAGAGAGCTTGGACTGTGGAGGCTTGCTG
GCCACTTTTTGGGGTCAGCTCCTCTGAAATGCATTAGCGGAACCGTTTGCGATCTGCCAC
AAGTGTGATAAGTTATCTACACTGGCGAGGGGATTGCTCTCTGTAATGTTCAGCTTCTAA
TTGTCTCTACTTTGTGAGACTACTTTTGAATGCTTGACCTCAAATCAGGTAGGACTACCC
GCTGAACTTAA
>A6_8
TTTCCGTAGGTGAACCTGCGGAAGGATCATTATTGAATTATGTTTCTAGATAGGTTGTAG
CTGGCTCTTTTAGAGCATGTGCACGCCTGTTTGGACTTCATTTTCATCCACCTGTGCACC
TATTGTAGTCTTTGGTTGGGTTAGGAGGAAGTGATCATTGTATCAGCATCTGCTGGGAGT
GAGGACTTGCATTGTGAAAGCTTTGCTGTCCTTGATGTGATCATGGAATCTTTTTCTCAC
TAGAGTCTATGTCACTCATTATACTCTGTCGAATGTCATTGAATGTCTTTACATGGGCTT
GTATGCCTATGAAAATTGTAATACAACTTTCAGCAACGGATCTCTTGGCTCTCGCATCGA
TGAAGAACGCAGCGAAATGCGATAAGTAATGTGAATTGCAGAATTCAGTGAATCATCGAA
TCTTTGAACGCATCTTGCGCTCCTTGGTATTCCGAGGAGCATGCCTGTTTGAGTGTCATT
AAATTCTCAACTCTCTTATACTTTTTTGTAAAAGAGAGCTTGGACTGTGGAGGCTTGCTG
GCCACTTTTTGGGGTCAGCTCCTCTGAAATGCATTAGCGGAACCGTTTGCGATCTGCCAC
AAGTGTGATAAGTTATCTACACTGGCGAGGGGATTGCTCTCTGTAATGTTCAGCTTCTAA
TTGTCTCTACTTTGTGAGACTACTTTTGAATGCTTGACCTCAAATCAGGTAGGACTACCC
GCTGAACTTAA
>A6_9
TTTCCGTAGGTGAACCTGCGGAAGGATCATTATTGAATTATGTTTCTAGATAGGTTGTAG
CTGGCTCTTTTAGAGCATGTGCACGCCTGTTTGGACTTCATTTTCATCCACCTGTGCACC
TATTGTAGTCTTTGGTTGGGTTAGGAGGAAGTGATCATTGTATCAGCATCTGCTGGGAGT
GAGGACTTGCATTGTGAAAGCTTTGCTGTCCTTGATGTGATCATGGAATCTTTTTCTCAC
TAGAGTCTATGTCACTCATTATACTCTGTCGAATGTCATTGAATGTCTTTACATGGGCTT
GTATGCCTATGAAAATTGTAATACAACTTTCAGCAACGGATCTCTTGGCTCTCGCATCGA
TGAAGAACGCAGCGAAATGCGATAAGTAATGTGAATTGCAGAATTCAGTGAATCATCGAA
TCTTTGAACGCATCTTGCGCTCCTTGGTATTCCGAGGAGCATGCCTGTTTGAGTGTCATT
AAATTCTCAACTCTCTTATACTTTTTTGTAAAAGAGAGCTTGGACTGTGGAGGCTTGCTG
GCCACTTTTTGGGGTCAGCTCCTCTGAAATGCATTAGCGGAACCGTTTGCGATCTGCCAC
AAGTGTGATAAGTTATCTACACTGGCGAGGGGATTGCTCTCTGTAATGTTCAGCTTCTAA
TTGTCTCTACTTTGTGAGACTACTTTTGAATGCTTGACCTCAAATCAGGTAGGACTACCC
GCTGAACTTAA
>A6_10
TTTCCGTAGGTGAACCTGCGGAAGGATCATTATTGAATTATGTTTCTAGATAGGTTGTAG
CTGGCTCTTTTAGAGCATGTGCACGCCTGTTTGGACTTCATTTTCATCCACCTGTGCACC
TATTGTAGTCTTTGGTTGGGTTAGGAGGAAGTGATCATTGTATCAGCATCTGCTGGGAGT
GAGGACTTGCATTGTGAAAGCTTTGCTGTCCTTGATGTGATCATGGAATCTTTTTCTCAC
TAGAGTCTATGTCACTCATTATACTCTGTCGAATGTCATTGAATGTCTTTACATGGGCTT
GTATGCCTATGAAAATTGTAATACAACTTTCAGCAACGGATCTCTTGGCTCTCGCATCGA
TGAAGAACGCAGCGAAATGCGATAAGTAATGTGAATTGCAGAATTCAGTGAATCATCGAA
TCTTTGAACGCATCTTGCGCTCCTTGGTATTCCGAGGAGCATGCCTGTTTGAGTGTCATT
AAATTCTCAACTCTCTTATACTTTTTTGTAAAAGAGAGCTTGGACTGTGGAGGCTTGCTG
GCCACTTTTTGGGGTCAGCTCCTCTGAAATGCATTAGCGGAACCGTTTGCGATCTGCCAC
AAGTGTGATAAGTTATCTACACTGGCGAGGGGATTGCTCTCTGTAATGTTCAGCTTCTAA
TTGTCTCTACTTTGTGAGACTACTTTTGAATGCTTGACCTCAAATCAGGTAGGACTACCC
GCTGAACTTAA

>A6_11
TTTCCGTAGGTGAACCTGCGGAAGGATCATTATTGAATTATGTTTCTAGATAGGTTGTAG
CTGGCTCTTTTAGAGCATGTGCACGCCTGTTTGGACTTCATTTTCATCCACCTGTGCACC
TATTGTAGTCTTTGGTTGGGTTAGGAGGAAGTGATCATTGTATCAGCATCTGCTGGGAGT
GAGGACTTGCATTGTGAAAGCTTTGCTGTCCTTGATGTGATCATGGAATCTTTTTCTCAC
TAGAGTCTATGTCACTCATTATACTCTGTCGAATGTCATTGAATGTCTTTACATGGGCTT
GTATGCCTATGAAAATTGTAATACAACTTTCAGCAACGGATCTCTTGGCTCTCGCATCGA
TGAAGAACGCAGCGAAATGCGATAAGTAATGTGAATTGCAGAATTCAGTGAATCATCGAA
TCTTTGAACGCATCTTGCGCTCCTTGGTATTCCGAGGAGCATGCCTGTTTGAGTGTCATT
AAATTCTCAACTCTCTTATACTTTTTGTAAAAGAGAGCTTGGACTGTGGAGGCTTGCTG
GCCACTTTTTGGGGTCAGCTCCTCTGAAATGCATTAGCGGAACCGTTTGCGATCTGCCAC
AAGTGTGATAAGTTATCTACACTGGCGAGGGGATTGCTCTCTGTAATGTTCAGCTTCTAA
TTGTCTCTACTTTGTGAGACTACTTTTGAATGCTTGACCTCAAATCAGGTAGGACTACCC
GCTGAACTTAA
>A6_13
TTTCCGTAGGTGAACCTGCGGAAGGATCATTATTGAATTATGTTTCTAGATAGGTTGTAG
CTGGCTCTTTTAGAGCATGTGCACGCCTGTTTGGACTTCATTTTCATCCACCTGTGCACC
TATTGTAGTCTTTGGTTGGGTTAGGAGGAAGTGATCATTGTATCAGCATCTGCTGGGAGT
GAGGACTTGCATTGTGAAAGCTTTGCTGTCCTTGATGTGATCATGGAATCTTTTTCTCAC
TAGAGTCTATGTCACTCATTATACTCTGTCGAATGTCATTGAATGTCTTTACATGGGCTT
GTATGCCTATGAAAATTGTAATACAACTTTCAGCAACGGATCTCTTGGCTCTCGCATCGA
TGAAGAACGCAGCGAAATGCGATAAGTAATGTGAATTGCAGAATTCAGTGAATCATCGAA
TCTTTGAACGCATCTTGCGCTCCTTGGTATTCCGAGGAGCATGCCTGTTTGAGTGTCATT
AAATTCTCAACTCTCTTATACTTTTTGTAAAAGAGAGCTTGGACTGTGGAGGCTTGCTG
GCCACTTTTTGGGGTCAGCTCCTCTGAAATGCATTAGCGGAACCGTTTGCGATCTGCCAC
AAGTGTGATAAGTTATCTACACTGGCGAGGGGATTGCTCTCTGTAATGTTCAGCTTCTAA
TTGTCTCTACTTTGTGAGACTACTTTTGAATGCTTGACCTCAAATCAGGTAGGACTACCC
GCTGAACTTAA
>A6_15
TTTCCGTAGGTGAACCTGCGGAAGGATCATTATTGAATTATGTTTCTAGATAGGTTGTAG
CTGGCTCTTTTAGAGCATGTGCACGCCTGTTTGGACTTCATTTTCATCCACCTGTGCACC
TATTGTAGTCTTTGGTTGGGTTAGGAGGAAGTGATCATTGTATCAGCATCTGCTGGGAGT
GAGGACTTGCATTGTGAAAGCTTTGCTGTCCTTGATGTGATCATGGAATCTTTTTCTCAC
TAGAGTCTATGTCACTCATTATACTCTGTCGAATGTCATTGAATGTCTTTACATGGGCTT
GTATGCCTATGAAAATTGTAATACAACTTTCAGCAACGGATCTCTTGGCTCTCGCATCGA
TGAAGAACGCAGCGAAATGCGATAAGTAATGTGAATTGCAGAATTCAGTGAATCATCGAA
TCTTTGAACGCATCTTGCGCTCCTTGGTATTCCGAGGAGCATGCCTGTTTGAGTGTCATT
AAATTCTCAACTCTCTTATACTTTTTGTAAAAGAGAGCTTGGACTGTGGAGGCTTGCTG
GCCACTTTTTGGGGTCAGCTCCTCTGAAATGCATTAGCGGAACCGTTTGCGATCTGCCAC
AAGTGTGATAAGTTATCTACACTGGCGAGGGGATTGCTCTCTGTAATGTTCAGCTTCTAA
TTGTCTCTACTTTGTGAGACTACTTTTGAATGCTTGACCTCAAATCAGGTAGGACTACCC
GCTGAACTTAA
>A6_19
TTTCCGTAGGTGAACCTGCGGAAGGATCATTATTGAATTATGTTTCTAGATAGGTTGTAG
CTGGCTCTTTTAGAGCATGTGCACGCCTGTTTGGACTTCATTTTCATCCACCTGTGCACC
TATTGTAGTCTTTGGTTGGGTTAGGAGGAAGTGATCATTGTATCAGCATCTGCTGGGAGT
GAGGACTTGCATTGTGAAAGCTTTGCTGTCCTTGATGTGATCATGGAATCTTTTTCTCAC
TAGAGTCTATGTCACTCATTATACTCTGTCGAATGTCATTGAATGTCTTTACATGGGCTT
GTATGCCTATGAAAATTGTAATACAACTTTCAGCAACGGATCTCTTGGCTCTCGCATCGA
TGAAGAACGCAGCGAAATGCGATAAGTAATGTGAATTGCAGAATTCAGTGAATCATCGAA

TCTTTGAACGCATCTTGCGCTCCTTGGTATTCCGAGGAGCATGCCTGTTTGAGTGTCATT
AAATTCTCAACTCTCTTATACTTTTTTGTAAAAGAGAGCTTGGACTGTGGAGGCTTGCTG
GCCACTTTTTGGGGTCAGCTCCTCTGAAATGCATTAGCGGAACCGTTTGCGATCTGCCAC
AAGTGTGATAAGTTATCTACACTGGCGAGGGGATTGCTCTCTGTAATGTTCAGCTTCTAA
TTGTCTCTACTTTGTGAGACTACTTTTGAATGCTTGACCTCAAATCAGGTAGGACTACCC
GCTGAACTTAA
>A6_20
TTTCCGTAGGTGAACCTGCGGAAGGATCATTATTGAATTATGTTTCTAGATAGGTTGTAG
CTGGCTCTTTTAGAGCATGTGCACGCCTGTTTGGACTTCATTTTCATCCACCTGTGCACC
TATTGTAGTCTTTGGTTGGGTTAGGAGGAAGTGATCATTGTATCAGCATCTGCTGGGAGT
GAGGACTTGCATTGTGAAAGCTTTGCTGTCCTTGATGTGATCATGGAATCTTTTTCTCAC
TAGAGTCTATGTCACTCATTATACTCTGTCGAATGTCATTGAATGTCTTTACATGGGCTT
GTATGCCTATGAAAATTGTAATACAACTTTCAGCAACGGATCTCTTGGCTCTCGCATCGA
TGAAGAACGCAGCGAAATGCGATAAGTAATGTGAATTGCAGAATTCAGTGAATCATCGAA
TCTTTGAACGCATCTTGCGCTCCTTGGTATTCCGAGGAGCATGCCTGTTTGAGTGTCATT
AAATTCTCAACTCTCTTATACTTTTTTGTAAAAGAGAGCTTGGACTGTGGAGGCTTGCTG
GCCACTTTTTGGGGTCAGCTCCTCTGAAATGCATTAGCGGAACCGTTTGCGATCTGCCAC
AAGTGTGATAAGTTATCTACACTGGCGAGGGGATTGCTCTCTGTAATGTTCAGCTTCTAA
TTGTCTCTACTTTGTGAGACTACTTTTGAATGCTTGACCTCAAATCAGGTAGGACTACCC
GCTGAACTTAA
>A6_24
TTTCCGTAGGTGAACCTGCGGAAGGATCATTATTGAATTATGTTTCTAGATAGGTTGTAG
CTGGCTCTTTTAGAGCATGTGCACGCCTGTTTGGACTTCATTTTCATCCACCTGTGCACC
TATTGTAGTCTTTGGTTGGGTTAGGAGGAAGTGATCATTGTATCAGCATCTGCTGGGAGT
GAGGACTTGCATTGTGAAAGCTTTGCTGTCCTTGATGTGATCATGGAATCTTTTTCTCAC
TAGAGTCTATGTCACTCATTATACTCTGTCGAATGTCATTGAATGTCTTTACATGGGCTT
GTATGCCTATGAAAATTGTAATACAACTTTCAGCAACGGATCTCTTGGCTCTCGCATCGA
TGAAGAACGCAGCGAAATGCGATAAGTAATGTGAATTGCAGAATTCAGTGAATCATCGAA
TCTTTGAACGCATCTTGCGCTCCTTGGTATTCCGAGGAGCATGCCTGTTTGAGTGTCATT
AAATTCTCAACTCTCTTATACTTTTTTGTAAAAGAGAGCTTGGACTGTGGAGGCTTGCTG
GCCACTTTTTGGGGTCAGCTCCTCTGAAATGCATTAGCGGAACCGTTTGCGATCTGCCAC
AAGTGTGATAAGTTATCTACACTGGCGAGGGGATTGCTCTCTGTAATGTTCAGCTTCTAA
TTGTCTCTACTTTGTGAGACTACTTTTGAATGCTTGACCTCAAATCAGGTAGGACTACCC
GCTGAACTTAA
>A6_31
TTTCCGTAGGTGAACCTGCGGAAGGATCATTATTGAATTATGTTTCTAGATAGGTTGTAG
CTGGCTCTTTTAGAGCATGTGCACGCCTGTTTGGACTTCATTTTCATCCACCTGTGCACC
TATTGTAGTCTTTGGTTGGGTTAGGAGGAAGTGATCATTGTATCAGCATCTGCTGGGAGT
GAGGACTTGCATTGTGAAAGCTTTGCTGTCCTTGATGTGATCATGGAATCTTTTTCTCAC
TAGAGTCTATGTCACTCATTATACTCTGTCGAATGTCATTGAATGTCTTTACATGGGCTT
GTATGCCTATGAAAATTGTAATACAACTTTCAGCAACGGATCTCTTGGCTCTCGCATCGA
TGAAGAACGCAGCGAAATGCGATAAGTAATGTGAATTGCAGAATTCAGTGAATCATCGAA
TCTTTGAACGCATCTTGCGCTCCTTGGTATTCCGAGGAGCATGCCTGTTTGAGTGTCATT
AAATTCTCAACTCTCTTATACTTTTTTGTAAAAGAGAGCTTGGACTGTGGAGGCTTGCTG
GCCACTTTTTGGGGTCAGCTCCTCTGAAATGCATTAGCGGAACCGTTTGCGATCTGCCAC
AAGTGTGATAAGTTATCTACACTGGCGAGGGGATTGCTCTCTGTAATGTTCAGCTTCTAA
TTGTCTCTACTTTGTGAGACTACTTTTGAATGCTTGACCTCAAATCAGGTAGGACTACCC
GCTGAACTTAA
>A6_56
TTTCCGTAGGTGAACCTGCGGAAGGATCATTATTGAATTATGTTTCTAGATAGGTTGTAG

CTGGCTCTTTTAGAGCATGTGCACGCCTGTTTGGACTTCATTTTCATCCACCTGTGCACC
TATTGTAGTCTTTGGTTGGGTTAGGAGGAAGTGATCATTGTATCAGCATCTGCTGGGAGT
GAGGACTTGCATTGTGAAAGCTTTGCTGTCCTTGATGTGATCATGGAATCTTTTTCTCAC
TAGAGTCTATGTCACTCATTATACTCTGTCGAATGTCATTGAATGTCTTTACATGGGCTT
GTATGCCTATGAAAATTGTAATACAACTTTCAGCAACGGATCTCTTGGCTCTCGCATCGA
TGAAGAACGCAGCGAAATGCGATAAGTAATGTGAATTGCAGAATTCAGTGAATCATCGAA
TCTTTGAACGCATCTTGCGCTCCTTGGTATTCCGAGGAGCATGCCTGTTTGAGTGTCATT
AAATTCTCAACTCTCTTATACTTTTTTGTAAAAGAGAGCTTGGACTGTGGAGGCTTGCTG
GCCACTTTTTGGGGTCAGCTCCTCTGAAATGCATTAGCGGAACCGTTTGCGATCTGCCAC
AAGTGTGATAAGTTATCTACACTGGCGAGGGGATTGCTCTCTGTAATGTTCAGCTTCTAA
TTGTCTCTACTTTGTGAGACTACTTTTGAATGCTTGACCTCAAATCAGGTAGGACTACCC
GCTGAACTTAA
>A5_21
TTTCCGTAGGTGAACCTGCGGAAGGATCATTATTGAATTATGTTTCTAGATAGGTTGTAG
CTGGCTCTTTTAGAGCATGTGCACGCCTGTTTGGACTTCATTTTCATCCACCTGTGCACC
TATTGTAGTCTTTGGTTGGGTTAGGAGGAAGTGATCATTGTATCAGCATCTGCTGGGAGT
GAGGACTTGCATTGTGAAAGCTTTGCTGTCCTTGATGTGATCATGGAATCTTTTTCTCAC
TAGAGTCTATGTCACTCATTATACTCTGTCGAATGTCATTGAATGTCTTTACATGGGCTT
GTATGCCTATGAAAATTGTAATACAACTTTCAGCAACGGATCTCTTGGCTCTCGCATCGA
TGAAGAACGCAGCGAAATGCGATAAGTAATGTGAATTGCAGAATTCAGTGAATCATCGAA
TCTTTGAACGCATCTTGCGCTCCTTGGTATTCCGAGGAGCATGCCTGTTTGAGTGTCATT
AAATTCTCAACTCTCTTATACTTTTTTGTAAAAGAGAGCTTGGACTGTGGAGGCTTGCTG
GCCACTTTTTGGGGTCAGCTCCTCTGAAATGCATTAGCGGAACCGTTTGCGATCTGCCAC
AAGTGTGATAAGTTATCTACACTGGCGAGGGGATTGCTCTCTGTAATGTTCAGCTTCTAA
TTGTCTCTACTTTGTGAGACTACTTTTGAATGCTTGACCTCAAATCAGGTAGGACTACCC
GCTGAACTTAA
>A5_22
TTTCCGTAGGTGAACCTGCGGAAGGATCATTATTGAATTATGTTTCTAGATAGGTTGTAG
CTGGCTCTTTTAGAGCATGTGCACGCCTGTTTGGACTTCATTTTCATCCACCTGTGCACC
TATTGTAGTCTTTGGTTGGGTTAGGAGGAAGTGATCATTGTATCAGCATCTGCTGGGAGT
GAGGACTTGCATTGTGAAAGCTTTGCTGTCCTTGATGTGATCATGGAATCTTTTTCTCAC
TAGAGTCTATGTCACTCATTATACTCTGTCGAATGTCATTGAATGTCTTTACATGGGCTT
GTATGCCTATGAAAATTGTAATACAACTTTCAGCAACGGATCTCTTGGCTCTCGCATCGA
TGAAGAACGCAGCGAAATGCGATAAGTAATGTGAATTGCAGAATTCAGTGAATCATCGAA
TCTTTGAACGCATCTTGCGCTCCTTGGTATTCCGAGGAGCATGCCTGTTTGAGTGTCATT
AAATTCTCAACTCTCTTATACTTTTTTGTAAAAGAGAGCTTGGACTGTGGAGGCTTGCTG
GCCACTTTTTGGGGTCAGCTCCTCTGAAATGCATTAGCGGAACCGTTTGCGATCTGCCAC
AAGTGTGATAAGTTATCTACACTGGCGAGGGGATTGCTCTCTGTAATGTTCAGCTTCTAA
TTGTCTCTACTTTGTGAGACTACTTTTGAATGCTTGACCTCAAATCAGGTAGGACTACCC
GCTGAACTTAA
>A5_23
TTTCCGTAGGTGAACCTGCGGAAGGATCATTATTGAATTATGTTTCTAGATAGGTTGTAG
CTGGCTCTTTTAGAGCATGTGCACGCCTGTTTGGACTTCATTTTCATCCACCTGTGCACC
TATTGTAGTCTTTGGTTGGGTTAGGAGGAAGTGATCATTGTATCAGCATCTGCTGGGAGT
GAGGACTTGCATTGTGAAAGCTTTGCTGTCCTTGATGTGATCATGGAATCTTTTTCTCAC
TAGAGTCTATGTCACTCATTATACTCTGTCGAATGTCATTGAATGTCTTTACATGGGCTT
GTATGCCTATGAAAATTGTAATACAACTTTCAGCAACGGATCTCTTGGCTCTCGCATCGA
TGAAGAACGCAGCGAAATGCGATAAGTAATGTGAATTGCAGAATTCAGTGAATCATCGAA
TCTTTGAACGCATCTTGCGCTCCTTGGTATTCCGAGGAGCATGCCTGTTTGAGTGTCATT
AAATTCTCAACTCTCTTATACTTTTTTGTAAAAGAGAGCTTGGACTGTGGAGGCTTGCTG

GCCACTTTTTGGGGTCAGCTCCTCTGAAATGCATTAGCGGAACCGTTTGCGATCTGCCAC
AAGTGTGATAAGTTATCTACACTGGCGAGGGGATTGCTCTCTGTAATGTTCAGCTTCTAA
TTGTCTCTACTTTGTGAGACTACTTTTGAATGCTTGACCTCAAATCAGGTAGGACTACCC
GCTGAACTTAA
>A5_25
TTTCCGTAGGTGAACCTGCGGAAGGATCATTATTGAATTATGTTTCTAGATAGGTTGTAG
CTGGCTCTTTTAGAGCATGTGCACGCCTGTTTGGACTTCATTTTCATCCACCTGTGCACC
TATTGTAGTCTTTGGTTGGGTTAGGAGGAAGTGATCATTGTATCAGCATCTGCTGGGAGT
GAGGACTTGCATTGTGAAAGCTTTGCTGTCCTTGATGTGATCATGGAATCTTTTTCTCAC
TAGAGTCTATGTCACTCATTATACTCTGTCGAATGTCATTGAATGTCTTTACATGGGCTT
GTATGCCTATGAAAATTGTAATACAACTTTCAGCAACGGATCTCTTGGCTCTCGCATCGA
TGAAGAACGCAGCGAAATGCGATAAGTAATGTGAATTGCAGAATTCAGTGAATCATCGAA
TCTTTGAACGCATCTTGCGCTCCTTGGTATTCCGAGGAGCATGCCTGTTTGAGTGTCATT
AAATTCTCAACTCTCTTATACTTTTTTGTAAAAGAGAGCTTGGACTGTGGAGGCTTGCTG
GCCACTTTTTGGGGTCAGCTCCTCTGAAATGCATTAGCGGAACCGTTTGCGATCTGCCAC
AAGTGTGATAAGTTATCTACACTGGCGAGGGGATTGCTCTCTGTAATGTTCAGCTTCTAA
TTGTCTCTACTTTGTGAGACTACTTTTGAATGCTTGACCTCAAATCAGGTAGGACTACCC
GCTGAACTTAA
>A5_26
TTTCCGTAGGTGAACCTGCGGAAGGATCATTATTGAATTATGTTTCTAGATAGGTTGTAG
CTGGCTCTTTTAGAGCATGTGCACGCCTGTTTGGACTTCATTTTCATCCACCTGTGCACC
TATTGTAGTCTTTGGTTGGGTTAGGAGGAAGTGATCATTGTATCAGCATCTGCTGGGAGT
GAGGACTTGCATTGTGAAAGCTTTGCTGTCCTTGATGTGATCATGGAATCTTTTTCTCAC
TAGAGTCTATGTCACTCATTATACTCTGTCGAATGTCATTGAATGTCTTTACATGGGCTT
GTATGCCTATGAAAATTGTAATACAACTTTCAGCAACGGATCTCTTGGCTCTCGCATCGA
TGAAGAACGCAGCGAAATGCGATAAGTAATGTGAATTGCAGAATTCAGTGAATCATCGAA
TCTTTGAACGCATCTTGCGCTCCTTGGTATTCCGAGGAGCATGCCTGTTTGAGTGTCATT
AAATTCTCAACTCTCTTATACTTTTTTGTAAAAGAGAGCTTGGACTGTGGAGGCTTGCTG
GCCACTTTTTGGGGTCAGCTCCTCTGAAATGCATTAGCGGAACCGTTTGCGATCTGCCAC
AAGTGTGATAAGTTATCTACACTGGCGAGGGGATTGCTCTCTGTAATGTTCAGCTTCTAA
TTGTCTCTACTTTGTGAGACTACTTTTGAATGCTTGACCTCAAATCAGGTAGGACTACCC
GCTGAACTTAA
>A5_27
TTTCCGTAGGTGAACCTGCGGAAGGATCATTATTGAATTATGTTTCTAGATAGGTTGTAG
CTGGCTCTTTTAGAGCATGTGCACGCCTGTTTGGACTTCATTTTCATCCACCTGTGCACC
TATTGTAGTCTTTGGTTGGGTTAGGAGGAAGTGATCATTGTATCAGCATCTGCTGGGAGT
GAGGACTTGCATTGTGAAAGCTTTGCTGTCCTTGATGTGATCATGGAATCTTTTTCTCAC
TAGAGTCTATGTCACTCATTATACTCTGTCGAATGTCATTGAATGTCTTTACATGGGCTT
GTATGCCTATGAAAATTGTAATACAACTTTCAGCAACGGATCTCTTGGCTCTCGCATCGA
TGAAGAACGCAGCGAAATGCGATAAGTAATGTGAATTGCAGAATTCAGTGAATCATCGAA
TCTTTGAACGCATCTTGCGCTCCTTGGTATTCCGAGGAGCATGCCTGTTTGAGTGTCATT
AAATTCTCAACTCTCTTATACTTTTTTGTAAAAGAGAGCTTGGACTGTGGAGGCTTGCTG
GCCACTTTTTGGGGTCAGCTCCTCTGAAATGCATTAGCGGAACCGTTTGCGATCTGCCAC
AAGTGTGATAAGTTATCTACACTGGCGAGGGGATTGCTCTCTGTAATGTTCAGCTTCTAA
TTGTCTCTACTTTGTGAGACTACTTTTGAATGCTTGACCTCAAATCAGGTAGGACTACCC
GCTGAACTTAA
>A5_28
TTTCCGTAGGTGAACCTGCGGAAGGATCATTATTGAATTATGTTTCTAGATAGGTTGTAG
CTGGCTCTTTTAGAGCATGTGCACGCCTGTTTGGACTTCATTTTCATCCACCTGTGCACC
TATTGTAGTCTTTGGTTGGGTTAGGAGGAAGTGATCATTGTATCAGCATCTGCTGGGAGT

GAGGACTTGCATTGTGAAAGCTTTGCTGTCCTTGATGTGATCATGGAATCTTTTTCTCAC
TAGAGTCTATGTCACTCATTATACTCTGTCGAATGTCATTGAATGTCTTTACATGGGCTT
GTATGCCTATGAAAATTGTAATACAACTTTCAGCAACGGATCTCTTGGCTCTCGCATCGA
TGAAGAACGCAGCGAAATGCGATAAGTAATGTGAATTGCAGAATTCAGTGAATCATCGAA
TCTTTGAACGCATCTTGCGCTCCTTGGTATTCCGAGGAGCATGCCTGTTTGAGTGTCATT
AAATTCTCAACTCTCTTATACTTTTTTGTAAAAGAGAGCTTGGACTGTGGAGGCTTGCTG
GCCACTTTTTGGGGTCAGCTCCTCTGAAATGCATTAGCGGAACCGTTTGCGATCTGCCAC
AAGTGTGATAAGTTATCTACACTGGCGAGGGGATTGCTCTCTGTAATGTTCAGCTTCTAA
TTGTCTCTACTTTGTGAGACTACTTTTGAATGCTTGACCTCAAATCAGGTAGGACTACCC
GCTGAACTTAA
>A5_29
TTTCCGTAGGTGAACCTGCGGAAGGATCATTATTGAATTATGTTTCTAGATAGGTTGTAG
CTGGCTCTTTTAGAGCATGTGCACGCCTGTTTGGACTTCATTTTCATCCACCTGTGCACC
TATTGTAGTCTTTGGTTGGGTTAGGAGGAAGTGATCATTGTATCAGCATCTGCTGGGAGT
GAGGACTTGCATTGTGAAAGCTTTGCTGTCCTTGATGTGATCATGGAATCTTTTTCTCAC
TAGAGTCTATGTCACTCATTATACTCTGTCGAATGTCATTGAATGTCTTTACATGGGCTT
GTATGCCTATGAAAATTGTAATACAACTTTCAGCAACGGATCTCTTGGCTCTCGCATCGA
TGAAGAACGCAGCGAAATGCGATAAGTAATGTGAATTGCAGAATTCAGTGAATCATCGAA
TCTTTGAACGCATCTTGCGCTCCTTGGTATTCCGAGGAGCATGCCTGTTTGAGTGTCATT
AAATTCTCAACTCTCTTATACTTTTTTGTAAAAGAGAGCTTGGACTGTGGAGGCTTGCTG
GCCACTTTTTGGGGTCAGCTCCTCTGAAATGCATTAGCGGAACCGTTTGCGATCTGCCAC
AAGTGTGATAAGTTATCTACACTGGCGAGGGGATTGCTCTCTGTAATGTTCAGCTTCTAA
TTGTCTCTACTTTGTGAGACTACTTTTGAATGCTTGACCTCAAATCAGGTAGGACTACCC
GCTGAACTTAA
>A5_30
TTTCCGTAGGTGAACCTGCGGAAGGATCATTATTGAATTATGTTTCTAGATAGGTTGTAG
CTGGCTCTTTTAGAGCATGTGCACGCCTGTTTGGACTTCATTTTCATCCACCTGTGCACC
TATTGTAGTCTTTGGTTGGGTTAGGAGGAAGTGATCATTGTATCAGCATCTGCTGGGAGT
GAGGACTTGCATTGTGAAAGCTTTGCTGTCCTTGATGTGATCATGGAATCTTTTTCTCAC
TAGAGTCTATGTCACTCATTATACTCTGTCGAATGTCATTGAATGTCTTTACATGGGCTT
GTATGCCTATGAAAATTGTAATACAACTTTCAGCAACGGATCTCTTGGCTCTCGCATCGA
TGAAGAACGCAGCGAAATGCGATAAGTAATGTGAATTGCAGAATTCAGTGAATCATCGAA
TCTTTGAACGCATCTTGCGCTCCTTGGTATTCCGAGGAGCATGCCTGTTTGAGTGTCATT
AAATTCTCAACTCTCTTATACTTTTTTGTAAAAGAGAGCTTGGACTGTGGAGGCTTGCTG
GCCACTTTTTGGGGTCAGCTCCTCTGAAATGCATTAGCGGAACCGTTTGCGATCTGCCAC
AAGTGTGATAAGTTATCTACACTGGCGAGGGGATTGCTCTCTGTAATGTTCAGCTTCTAA
TTGTCTCTACTTTGTGAGACTACTTTTGAATGCTTGACCTCAAATCAGGTAGGACTACCC
GCTGAACTTAA
>A5_31
TTTCCGTAGGTGAACCTGCGGAAGGATCATTATTGAATTATGTTTCTAGATAGGTTGTAG
CTGGCTCTTTTAGAGCATGTGCACGCCTGTTTGGACTTCATTTTCATCCACCTGTGCACC
TATTGTAGTCTTTGGTTGGGTTAGGAGGAAGTGATCATTGTATCAGCATCTGCTGGGAGT
GAGGACTTGCATTGTGAAAGCTTTGCTGTCCTTGATGTGATCATGGAATCTTTTTCTCAC
TAGAGTCTATGTCACTCATTATACTCTGTCGAATGTCATTGAATGTCTTTACATGGGCTT
GTATGCCTATGAAAATTGTAATACAACTTTCAGCAACGGATCTCTTGGCTCTCGCATCGA
TGAAGAACGCAGCGAAATGCGATAAGTAATGTGAATTGCAGAATTCAGTGAATCATCGAA
TCTTTGAACGCATCTTGCGCTCCTTGGTATTCCGAGGAGCATGCCTGTTTGAGTGTCATT
AAATTCTCAACTCTCTTATACTTTTTTGTAAAAGAGAGCTTGGACTGTGGAGGCTTGCTG
GCCACTTTTTGGGGTCAGCTCCTCTGAAATGCATTAGCGGAACCGTTTGCGATCTGCCAC
AAGTGTGATAAGTTATCTACACTGGCGAGGGGATTGCTCTCTGTAATGTTCAGCTTCTAA

TTGTCTCTACTTTGTGAGACTACTTTTGAATGCTTGACCTCAAATCAGGTAGGACTACCC
GCTGAACTTAA
>A5_32
TTTCCGTAGGTGAACCTGCGGAAGGATCATTATTGAATTATGTTTCTAGATAGGTTGTAG
CTGGCTCTTTTAGAGCATGTGCACGCCTGTTTGGACTTCATTTTCATCCACCTGTGCACC
TATTGTAGTCTTTGGTTGGGTTAGGAGGAAGTGATCATTGTATCAGCATCTGCTGGGAGT
GAGGACTTGCATTGTGAAAGCTTTGCTGTCCTTGATGTGATCATGGAATCTTTTTCTCAC
TAGAGTCTATGTCACTCATTATACTCTGTCGAATGTCATTGAATGTCTTTACATGGGCTT
GTATGCCTATGAAAATTGTAATACAACTTTCAGCAACGGATCTCTTGGCTCTCGCATCGA
TGAAGAACGCAGCGAAATGCGATAAGTAATGTGAATTGCAGAATTCAGTGAATCATCGAA
TCTTTGAACGCATCTTGCGCTCCTTGGTATTCCGAGGAGCATGCCTGTTTGAGTGTCATT
AAATTCTCAACTCTCTTATACTTTTTTGTAAAAGAGAGCTTGGACTGTGGAGGCTTGCTG
GCCACTTTTTGGGGTCAGCTCCTCTGAAATGCATTAGCGGAACCGTTTGCGATCTGCCAC
AAGTGTGATAAGTTATCTACACTGGCGAGGGGATTGCTCTCTGTAATGTTCAGCTTCTAA
TTGTCTCTACTTTGTGAGACTACTTTTGAATGCTTGACCTCAAATCAGGTAGGACTACCC
GCTGAACTTAA
>A5_34
TTTCCGTAGGTGAACCTGCGGAAGGATCATTATTGAATTATGTTTCTAGATAGGTTGTAG
CTGGCTCTTTTAGAGCATGTGCACGCCTGTTTGGACTTCATTTTCATCCACCTGTGCACC
TATTGTAGTCTTTGGTTGGGTTAGGAGGAAGTGATCATTGTATCAGCATCTGCTGGGAGT
GAGGACTTGCATTGTGAAAGCTTTGCTGTCCTTGATGTGATCATGGAATCTTTTTCTCAC
TAGAGTCTATGTCACTCATTATACTCTGTCGAATGTCATTGAATGTCTTTACATGGGCTT
GTATGCCTATGAAAATTGTAATACAACTTTCAGCAACGGATCTCTTGGCTCTCGCATCGA
TGAAGAACGCAGCGAAATGCGATAAGTAATGTGAATTGCAGAATTCAGTGAATCATCGAA
TCTTTGAACGCATCTTGCGCTCCTTGGTATTCCGAGGAGCATGCCTGTTTGAGTGTCATT
AAATTCTCAACTCTCTTATACTTTTTTGTAAAAGAGAGCTTGGACTGTGGAGGCTTGCTG
GCCACTTTTTGGGGTCAGCTCCTCTGAAATGCATTAGCGGAACCGTTTGCGATCTGCCAC
AAGTGTGATAAGTTATCTACACTGGCGAGGGGATTGCTCTCTGTAATGTTCAGCTTCTAA
TTGTCTCTACTTTGTGAGACTACTTTTGAATGCTTGACCTCAAATCAGGTAGGACTACCC
GCTGAACTTAA
>A5_36
TTTCCGTAGGTGAACCTGCGGAAGGATCATTATTGAATTATGTTTCTAGATAGGTTGTAG
CTGGCTCTTTTAGAGCATGTGCACGCCTGTTTGGACTTCATTTTCATCCACCTGTGCACC
TATTGTAGTCTTTGGTTGGGTTAGGAGGAAGTGATCATTGTATCAGCATCTGCTGGGAGT
GAGGACTTGCATTGTGAAAGCTTTGCTGTCCTTGATGTGATCATGGAATCTTTTTCTCAC
TAGAGTCTATGTCACTCATTATACTCTGTCGAATGTCATTGAATGTCTTTACATGGGCTT
GTATGCCTATGAAAATTGTAATACAACTTTCAGCAACGGATCTCTTGGCTCTCGCATCGA
TGAAGAACGCAGCGAAATGCGATAAGTAATGTGAATTGCAGAATTCAGTGAATCATCGAA
TCTTTGAACGCATCTTGCGCTCCTTGGTATTCCGAGGAGCATGCCTGTTTGAGTGTCATT
AAATTCTCAACTCTCTTATACTTTTTTGTAAAAGAGAGCTTGGACTGTGGAGGCTTGCTG
GCCACTTTTTGGGGTCAGCTCCTCTGAAATGCATTAGCGGAACCGTTTGCGATCTGCCAC
AAGTGTGATAAGTTATCTACACTGGCGAGGGGATTGCTCTCTGTAATGTTCAGCTTCTAA
TTGTCTCTACTTTGTGAGACTACTTTTGAATGCTTGACCTCAAATCAGGTAGGACTACCC
GCTGAACTTAA
>A5_37
TTTCCGTAGGTGAACCTGCGGAAGGATCATTATTGAATTATGTTTCTAGATAGGTTGTAG
CTGGCTCTTTTAGAGCATGTGCACGCCTGTTTGGACTTCATTTTCATCCACCTGTGCACC
TATTGTAGTCTTTGGTTGGGTTAGGAGGAAGTGATCATTGTATCAGCATCTGCTGGGAGT
GAGGACTTGCATTGTGAAAGCTTTGCTGTCCTTGATGTGATCATGGAATCTTTTTCTCAC
TAGAGTCTATGTCACTCATTATACTCTGTCGAATGTCATTGAATGTCTTTACATGGGCTT

GTATGCCTATGAAAATTGTAATACAACTTTCAGCAACGGATCTCTTGGCTCTCGCATCGA
TGAAGAACGCAGCGAAATGCGATAAGTAATGTGAATTGCAGAATTCAGTGAATCATCGAA
TCTTTGAACGCATCTTGCGCTCCTTGGTATTCCGAGGAGCATGCCTGTTTGAGTGTCATT
AAATTCTCAACTCTCTTATACTTTTTTGTAAAAGAGAGCTTGGACTGTGGAGGCTTGCTG
GCCACTTTTTGGGGTCAGCTCCTCTGAAATGCATTAGCGGAACCGTTTGCGATCTGCCAC
AAGTGTGATAAGTTATCTACACTGGCGAGGGGATTGCTCTCTGTAATGTTCAGCTTCTAA
TTGTCTCTACTTTGTGAGACTACTTTTGAATGCTTGACCTCAAATCAGGTAGGACTACCC
GCTGAACTTAA
>A5_38
TTTCCGTAGGTGAACCTGCGGAAGGATCATTATTGAATTATGTTTCTAGATAGGTTGTAG
CTGGCTCTTTTAGAGCATGTGCACGCCTGTTTGGACTTCATTTTCATCCACCTGTGCACC
TATTGTAGTCTTTGGTTGGGTTAGGAGGAAGTGATCATTGTATCAGCATCTGCTGGGAGT
GAGGACTTGCATTGTGAAAGCTTTGCTGTCCTTGATGTGATCATGGAATCTTTTTCTCAC
TAGAGTCTATGTCACTCATTATACTCTGTCGAATGTCATTGAATGTCTTTACATGGGCTT
GTATGCCTATGAAAATTGTAATACAACTTTCAGCAACGGATCTCTTGGCTCTCGCATCGA
TGAAGAACGCAGCGAAATGCGATAAGTAATGTGAATTGCAGAATTCAGTGAATCATCGAA
TCTTTGAACGCATCTTGCGCTCCTTGGTATTCCGAGGAGCATGCCTGTTTGAGTGTCATT
AAATTCTCAACTCTCTTATACTTTTTTGTAAAAGAGAGCTTGGACTGTGGAGGCTTGCTG
GCCACTTTTTGGGGTCAGCTCCTCTGAAATGCATTAGCGGAACCGTTTGCGATCTGCCAC
AAGTGTGATAAGTTATCTACACTGGCGAGGGGATTGCTCTCTGTAATGTTCAGCTTCTAA
TTGTCTCTACTTTGTGAGACTACTTTTGAATGCTTGACCTCAAATCAGGTAGGACTACCC
GCTGAACTTAA
>A5_39
TTTCCGTAGGTGAACCTGCGGAAGGATCATTATTGAATTATGTTTCTAGATAGGTTGTAG
CTGGCTCTTTTAGAGCATGTGCACGCCTGTTTGGACTTCATTTTCATCCACCTGTGCACC
TATTGTAGTCTTTGGTTGGGTTAGGAGGAAGTGATCATTGTATCAGCATCTGCTGGGAGT
GAGGACTTGCATTGTGAAAGCTTTGCTGTCCTTGATGTGATCATGGAATCTTTTTCTCAC
TAGAGTCTATGTCACTCATTATACTCTGTCGAATGTCATTGAATGTCTTTACATGGGCTT
GTATGCCTATGAAAATTGTAATACAACTTTCAGCAACGGATCTCTTGGCTCTCGCATCGA
TGAAGAACGCAGCGAAATGCGATAAGTAATGTGAATTGCAGAATTCAGTGAATCATCGAA
TCTTTGAACGCATCTTGCGCTCCTTGGTATTCCGAGGAGCATGCCTGTTTGAGTGTCATT
AAATTCTCAACTCTCTTATACTTTTTTGTAAAAGAGAGCTTGGACTGTGGAGGCTTGCTG
GCCACTTTTTGGGGTCAGCTCCTCTGAAATGCATTAGCGGAACCGTTTGCGATCTGCCAC
AAGTGTGATAAGTTATCTACACTGGCGAGGGGATTGCTCTCTGTAATGTTCAGCTTCTAA
TTGTCTCTACTTTGTGAGACTACTTTTGAATGCTTGACCTCAAATCAGGTAGGACTACCC
GCTGAACTTAA
>A5_40
TTTCCGTAGGTGAACCTGCGGAAGGATCATTATTGAATTATGTTTCTAGATAGGTTGTAG
CTGGCTCTTTTAGAGCATGTGCACGCCTGTTTGGACTTCATTTTCATCCACCTGTGCACC
TATTGTAGTCTTTGGTTGGGTTAGGAGGAAGTGATCATTGTATCAGCATCTGCTGGGAGT
GAGGACTTGCATTGTGAAAGCTTTGCTGTCCTTGATGTGATCATGGAATCTTTTTCTCAC
TAGAGTCTATGTCACTCATTATACTCTGTCGAATGTCATTGAATGTCTTTACATGGGCTT
GTATGCCTATGAAAATTGTAATACAACTTTCAGCAACGGATCTCTTGGCTCTCGCATCGA
TGAAGAACGCAGCGAAATGCGATAAGTAATGTGAATTGCAGAATTCAGTGAATCATCGAA
TCTTTGAACGCATCTTGCGCTCCTTGGTATTCCGAGGAGCATGCCTGTTTGAGTGTCATT
AAATTCTCAACTCTCTTATACTTTTTTGTAAAAGAGAGCTTGGACTGTGGAGGCTTGCTG
GCCACTTTTTGGGGTCAGCTCCTCTGAAATGCATTAGCGGAACCGTTTGCGATCTGCCAC
AAGTGTGATAAGTTATCTACACTGGCGAGGGGATTGCTCTCTGTAATGTTCAGCTTCTAA
TTGTCTCTACTTTGTGAGACTACTTTTGAATGCTTGACCTCAAATCAGGTAGGACTACCC
GCTGAACTTAA

>A5_41
TTTCCGTAGGTGAACCTGCGGAAGGATCATTATTGAATTATGTTTCTAGATAGGTTGTAG
CTGGCTCTTTTAGAGCATGTGCACGCCTGTTTGGACTTCATTTTCATCCACCTGTGCACC
TATTGTAGTCTTTGGTTGGGTTAGGAGGAAGTGATCATTGTATCAGCATCTGCTGGGAGT
GAGGACTTGCATTGTGAAAGCTTTGCTGTCCTTGATGTGATCATGGAATCTTTTTCTCAC
TAGAGTCTATGTCACTCATTATACTCTGTCGAATGTCATTGAATGTCTTTACATGGGCTT
GTATGCCTATGAAAATTGTAATACAACTTTCAGCAACGGATCTCTTGGCTCTCGCATCGA
TGAAGAACGCAGCGAAATGCGATAAGTAATGTGAATTGCAGAATTCAGTGAATCATCGAA
TCTTTGAACGCATCTTGCGCTCCTTGGTATTCCGAGGAGCATGCCTGTTTGAGTGTCATT
AAATTCTCAACTCTCTTATACTTTTTTGTAAAAGAGAGCTTGGACTGTGGAGGCTTGCTG
GCCACTTTTTGGGGTCAGCTCCTCTGAAATGCATTAGCGGAACCGTTTGCGATCTGCCAC
AAGTGTGATAAGTTATCTACACTGGCGAGGGGATTGCTCTCTGTAATGTTCAGCTTCTAA
TTGTCTCTACTTTGTGAGACTACTTTTGAATGCTTGACCTCAAATCAGGTAGGACTACCC
GCTGAACTTAA
>A5_42
TTTCCGTAGGTGAACCTGCGGAAGGATCATTATTGAATTATGTTTCTAGATAGGTTGTAG
CTGGCTCTTTTAGAGCATGTGCACGCCTGTTTGGACTTCATTTTCATCCACCTGTGCACC
TATTGTAGTCTTTGGTTGGGTTAGGAGGAAGTGATCATTGTATCAGCATCTGCTGGGAGT
GAGGACTTGCATTGTGAAAGCTTTGCTGTCCTTGATGTGATCATGGAATCTTTTTCTCAC
TAGAGTCTATGTCACTCATTATACTCTGTCGAATGTCATTGAATGTCTTTACATGGGCTT
GTATGCCTATGAAAATTGTAATACAACTTTCAGCAACGGATCTCTTGGCTCTCGCATCGA
TGAAGAACGCAGCGAAATGCGATAAGTAATGTGAATTGCAGAATTCAGTGAATCATCGAA
TCTTTGAACGCATCTTGCGCTCCTTGGTATTCCGAGGAGCATGCCTGTTTGAGTGTCATT
AAATTCTCAACTCTCTTATACTTTTTTGTAAAAGAGAGCTTGGACTGTGGAGGCTTGCTG
GCCACTTTTTGGGGTCAGCTCCTCTGAAATGCATTAGCGGAACCGTTTGCGATCTGCCAC
AAGTGTGATAAGTTATCTACACTGGCGAGGGGATTGCTCTCTGTAATGTTCAGCTTCTAA
TTGTCTCTACTTTGTGAGACTACTTTTGAATGCTTGACCTCAAATCAGGTAGGACTACCC
GCTGAACTTAA
>A5_43
TTTCCGTAGGTGAACCTGCGGAAGGATCATTATTGAATTATGTTTCTAGATAGGTTGTAG
CTGGCTCTTTTAGAGCATGTGCACGCCTGTTTGGACTTCATTTTCATCCACCTGTGCACC
TATTGTAGTCTTTGGTTGGGTTAGGAGGAAGTGATCATTGTATCAGCATCTGCTGGGAGT
GAGGACTTGCATTGTGAAAGCTTTGCTGTCCTTGATGTGATCATGGAATCTTTTTCTCAC
TAGAGTCTATGTCACTCATTATACTCTGTCGAATGTCATTGAATGTCTTTACATGGGCTT
GTATGCCTATGAAAATTGTAATACAACTTTCAGCAACGGATCTCTTGGCTCTCGCATCGA
TGAAGAACGCAGCGAAATGCGATAAGTAATGTGAATTGCAGAATTCAGTGAATCATCGAA
TCTTTGAACGCATCTTGCGCTCCTTGGTATTCCGAGGAGCATGCCTGTTTGAGTGTCATT
AAATTCTCAACTCTCTTATACTTTTTTGTAAAAGAGAGCTTGGACTGTGGAGGCTTGCTG
GCCACTTTTTGGGGTCAGCTCCTCTGAAATGCATTAGCGGAACCGTTTGCGATCTGCCAC
AAGTGTGATAAGTTATCTACACTGGCGAGGGGATTGCTCTCTGTAATGTTCAGCTTCTAA
TTGTCTCTACTTTGTGAGACTACTTTTGAATGCTTGACCTCAAATCAGGTAGGACTACCC
GCTGAACTTAA
>A5_45
TTTCCGTAGGTGAACCTGCGGAAGGATCATTATTGAATTATGTTTCTAGATAGGTTGTAG
CTGGCTCTTTTAGAGCATGTGCACGCCTGTTTGGACTTCATTTTCATCCACCTGTGCACC
TATTGTAGTCTTTGGTTGGGTTAGGAGGAAGTGATCATTGTATCAGCATCTGCTGGGAGT
GAGGACTTGCATTGTGAAAGCTTTGCTGTCCTTGATGTGATCATGGAATCTTTTTCTCAC
TAGAGTCTATGTCACTCATTATACTCTGTCGAATGTCATTGAATGTCTTTACATGGGCTT
GTATGCCTATGAAAATTGTAATACAACTTTCAGCAACGGATCTCTTGGCTCTCGCATCGA
TGAAGAACGCAGCGAAATGCGATAAGTAATGTGAATTGCAGAATTCAGTGAATCATCGAA

TCTTTGAACGCATCTTGCGCTCCTTGGTATTCCGAGGAGCATGCCTGTTTGAGTGTCATT
AAATTCTCAACTCTCTTATACTTTTTTGTAAAAGAGAGCTTGGACTGTGGAGGCTTGCTG
GCCACTTTTTGGGGTCAGCTCCTCTGAAATGCATTAGCGGAACCGTTTGCGATCTGCCAC
AAGTGTGATAAGTTATCTACACTGGCGAGGGGATTGCTCTCTGTAATGTTCAGCTTCTAA
TTGTCTCTACTTTGTGAGACTACTTTTGAATGCTTGACCTCAAATCAGGTAGGACTACCC
GCTGAACTTAA
>A5_46
TTTCCGTAGGTGAACCTGCGGAAGGATCATTATTGAATTATGTTTCTAGATAGGTTGTAG
CTGGCTCTTTTAGAGCATGTGCACGCCTGTTTGGACTTCATTTTCATCCACCTGTGCACC
TATTGTAGTCTTTGGTTGGGTTAGGAGGAAGTGATCATTGTATCAGCATCTGCTGGGAGT
GAGGACTTGCATTGTGAAAGCTTTGCTGTCCTTGATGTGATCATGGAATCTTTTTCTCAC
TAGAGTCTATGTCACTCATTATACTCTGTCGAATGTCATTGAATGTCTTTACATGGGCTT
GTATGCCTATGAAAATTGTAATACAACTTTCAGCAACGGATCTCTTGGCTCTCGCATCGA
TGAAGAACGCAGCGAAATGCGATAAGTAATGTGAATTGCAGAATTCAGTGAATCATCGAA
TCTTTGAACGCATCTTGCGCTCCTTGGTATTCCGAGGAGCATGCCTGTTTGAGTGTCATT
AAATTCTCAACTCTCTTATACTTTTTTGTAAAAGAGAGCTTGGACTGTGGAGGCTTGCTG
GCCACTTTTTGGGGTCAGCTCCTCTGAAATGCATTAGCGGAACCGTTTGCGATCTGCCAC
AAGTGTGATAAGTTATCTACACTGGCGAGGGGATTGCTCTCTGTAATGTTCAGCTTCTAA
TTGTCTCTACTTTGTGAGACTACTTTTGAATGCTTGACCTCAAATCAGGTAGGACTACCC
GCTGAACTTAA
>A5_47
TTTCCGTAGGTGAACCTGCGGAAGGATCATTATTGAATTATGTTTCTAGATAGGTTGTAG
CTGGCTCTTTTAGAGCATGTGCACGCCTGTTTGGACTTCATTTTCATCCACCTGTGCACC
TATTGTAGTCTTTGGTTGGGTTAGGAGGAAGTGATCATTGTATCAGCATCTGCTGGGAGT
GAGGACTTGCATTGTGAAAGCTTTGCTGTCCTTGATGTGATCATGGAATCTTTTTCTCAC
TAGAGTCTATGTCACTCATTATACTCTGTCGAATGTCATTGAATGTCTTTACATGGGCTT
GTATGCCTATGAAAATTGTAATACAACTTTCAGCAACGGATCTCTTGGCTCTCGCATCGA
TGAAGAACGCAGCGAAATGCGATAAGTAATGTGAATTGCAGAATTCAGTGAATCATCGAA
TCTTTGAACGCATCTTGCGCTCCTTGGTATTCCGAGGAGCATGCCTGTTTGAGTGTCATT
AAATTCTCAACTCTCTTATACTTTTTTGTAAAAGAGAGCTTGGACTGTGGAGGCTTGCTG
GCCACTTTTTGGGGTCAGCTCCTCTGAAATGCATTAGCGGAACCGTTTGCGATCTGCCAC
AAGTGTGATAAGTTATCTACACTGGCGAGGGGATTGCTCTCTGTAATGTTCAGCTTCTAA
TTGTCTCTACTTTGTGAGACTACTTTTGAATGCTTGACCTCAAATCAGGTAGGACTACCC
GCTGAACTTAA
>A5_48
TTTCCGTAGGTGAACCTGCGGAAGGATCATTATTGAATTATGTTTCTAGATAGGTTGTAG
CTGGCTCTTTTAGAGCATGTGCACGCCTGTTTGGACTTCATTTTCATCCACCTGTGCACC
TATTGTAGTCTTTGGTTGGGTTAGGAGGAAGTGATCATTGTATCAGCATCTGCTGGGAGT
GAGGACTTGCATTGTGAAAGCTTTGCTGTCCTTGATGTGATCATGGAATCTTTTTCTCAC
TAGAGTCTATGTCACTCATTATACTCTGTCGAATGTCATTGAATGTCTTTACATGGGCTT
GTATGCCTATGAAAATTGTAATACAACTTTCAGCAACGGATCTCTTGGCTCTCGCATCGA
TGAAGAACGCAGCGAAATGCGATAAGTAATGTGAATTGCAGAATTCAGTGAATCATCGAA
TCTTTGAACGCATCTTGCGCTCCTTGGTATTCCGAGGAGCATGCCTGTTTGAGTGTCATT
AAATTCTCAACTCTCTTATACTTTTTTGTAAAAGAGAGCTTGGACTGTGGAGGCTTGCTG
GCCACTTTTTGGGGTCAGCTCCTCTGAAATGCATTAGCGGAACCGTTTGCGATCTGCCAC
AAGTGTGATAAGTTATCTACACTGGCGAGGGGATTGCTCTCTGTAATGTTCAGCTTCTAA
TTGTCTCTACTTTGTGAGACTACTTTTGAATGCTTGACCTCAAATCAGGTAGGACTACCC
GCTGAACTTAA
>A5_49
TTTCCGTAGGTGAACCTGCGGAAGGATCATTATTGAATTATGTTTCTAGATAGGTTGTAG

CTGGCTCTTTTAGAGCATGTGCACGCCTGTTTGGACTTCATTTTCATCCACCTGTGCACC
TATTGTAGTCTTTGGTTGGGTTAGGAGGAAGTGATCATTGTATCAGCATCTGCTGGGAGT
GAGGACTTGCATTGTGAAAGCTTTGCTGTCCTTGATGTGATCATGGAATCTTTTTCTCAC
TAGAGTCTATGTCACTCATTATACTCTGTCGAATGTCATTGAATGTCTTTACATGGGCTT
GTATGCCTATGAAAATTGTAATACAACTTTCAGCAACGGATCTCTTGGCTCTCGCATCGA
TGAAGAACGCAGCGAAATGCGATAAGTAATGTGAATTGCAGAATTCAGTGAATCATCGAA
TCTTTGAACGCATCTTGCGCTCCTTGGTATTCCGAGGAGCATGCCTGTTTGAGTGTCATT
AAATTCTCAACTCTCTTATACTTTTTTGTAAAAGAGAGCTTGGACTGTGGAGGCTTGCTG
GCCACTTTTTGGGGTCAGCTCCTCTGAAATGCATTAGCGGAACCGTTTGCGATCTGCCAC
AAGTGTGATAAGTTATCTACACTGGCGAGGGGATTGCTCTCTGTAATGTTCAGCTTCTAA
TTGTCTCTACTTTGTGAGACTACTTTTGAATGCTTGACCTCAAATCAGGTAGGACTACCC
GCTGAACTTAA
>A6_57
TTTCCGTAGGTGAACCTGCGGAAGGATCATTATTGAATTATGTTTCTAGATAGGTTGTAG
CTGGCTCTTTTAGAGCATGTGCACGCCTGTTTGGACTTCATTTTCATCCACCTGTGCACC
TATTGTAGTCTTTGGTTGGGTTAGGAGGAAGTGATCATTGTATCAGCATCTGCTGGGAGT
GAGGACTTGCATTGTGAAAGCTTTGCTGTCCTTGATGTGATCATGGAATCTTTTTCTCAC
TAGAGTCTATGTCACTCATTATACTCTGTCGAATGTCATTGAATGTCTTTACATGGGCTT
GTATGCCTATGAAAATTGTAATACAACTTTCAGCAACGGATCTCTTGGCTCTCGCATCGA
TGAAGAACGCAGCGAAATGCGATAAGTAATGTGAATTGCAGAATTCAGTGAATCATCGAA
TCTTTGAACGCATCTTGCGCTCCTTGGTATTCCGAGGAGCATGCCTGTTTGAGTGTCATT
AAATTCTCAACTCTCTTATACTTTTTTGTAAAAGAGAGCTTGGACTGTGGAGGCTTGCTG
GCCACTTTTTGGGGTCAGCTCCTCTGAAATGCATTAGCGGAACCGTTTGCGATCTGCCAC
AAGTGTGATAAGTTATCTACACTGGCGAGGGGATTGCTCTCTGTAATGTTCAGCTTCTAA
TTGTCTCTACTTTGTGAGACTACTTTTGAATGCTTGACCTCAAATCAGGTAGGACTACCC
GCTGAACTTAA
>A6_58
TTTCCGTAGGTGAACCTGCGGAAGGATCATTATTGAATTATGTTTCTAGATAGGTTGTAG
CTGGCTCTTTTAGAGCATGTGCACGCCTGTTTGGACTTCATTTTCATCCACCTGTGCACC
TATTGTAGTCTTTGGTTGGGTTAGGAGGAAGTGATCATTGTATCAGCATCTGCTGGGAGT
GAGGACTTGCATTGTGAAAGCTTTGCTGTCCTTGATGTGATCATGGAATCTTTTTCTCAC
TAGAGTCTATGTCACTCATTATACTCTGTCGAATGTCATTGAATGTCTTTACATGGGCTT
GTATGCCTATGAAAATTGTAATACAACTTTCAGCAACGGATCTCTTGGCTCTCGCATCGA
TGAAGAACGCAGCGAAATGCGATAAGTAATGTGAATTGCAGAATTCAGTGAATCATCGAA
TCTTTGAACGCATCTTGCGCTCCTTGGTATTCCGAGGAGCATGCCTGTTTGAGTGTCATT
AAATTCTCAACTCTCTTATACTTTTTTGTAAAAGAGAGCTTGGACTGTGGAGGCTTGCTG
GCCACTTTTTGGGGTCAGCTCCTCTGAAATGCATTAGCGGAACCGTTTGCGATCTGCCAC
AAGTGTGATAAGTTATCTACACTGGCGAGGGGATTGCTCTCTGTAATGTTCAGCTTCTAA
TTGTCTCTACTTTGTGAGACTACTTTTGAATGCTTGACCTCAAATCAGGTAGGACTACCC
GCTGAACTTAA
>A6_60
TTTCCGTAGGTGAACCTGCGGAAGGATCATTATTGAATTATGTTTCTAGATAGGTTGTAG
CTGGCTCTTTTAGAGCATGTGCACGCCTGTTTGGACTTCATTTTCATCCACCTGTGCACC
TATTGTAGTCTTTGGTTGGGTTAGGAGGAAGTGATCATTGTATCAGCATCTGCTGGGAGT
GAGGACTTGCATTGTGAAAGCTTTGCTGTCCTTGATGTGATCATGGAATCTTTTTCTCAC
TAGAGTCTATGTCACTCATTATACTCTGTCGAATGTCATTGAATGTCTTTACATGGGCTT
GTATGCCTATGAAAATTGTAATACAACTTTCAGCAACGGATCTCTTGGCTCTCGCATCGA
TGAAGAACGCAGCGAAATGCGATAAGTAATGTGAATTGCAGAATTCAGTGAATCATCGAA
TCTTTGAACGCATCTTGCGCTCCTTGGTATTCCGAGGAGCATGCCTGTTTGAGTGTCATT
AAATTCTCAACTCTCTTATACTTTTTTGTAAAAGAGAGCTTGGACTGTGGAGGCTTGCTG

GCCACTTTTTGGGGTCAGCTCCTCTGAAATGCATTAGCGGAACCGTTTGCGATCTGCCAC
AAGTGTGATAAGTTATCTACACTGGCGAGGGGATTGCTCTCTGTAATGTTCAGCTTCTAA
TTGTCTCTACTTTGTGAGACTACTTTTGAATGCTTGACCTCAAATCAGGTAGGACTACCC
GCTGAACTTAA
>A6_61
TTTCCGTAGGTGAACCTGCGGAAGGATCATTATTGAATTATGTTTCTAGATAGGTTGTAG
CTGGCTCTTTTAGAGCATGTGCACGCCTGTTTGGACTTCATTTTCATCCACCTGTGCACC
TATTGTAGTCTTTGGTTGGGTTAGGAGGAAGTGATCATTGTATCAGCATCTGCTGGGAGT
GAGGACTTGCATTGTGAAAGCTTTGCTGTCCTTGATGTGATCATGGAATCTTTTTCTCAC
TAGAGTCTATGTCACTCATTATACTCTGTCGAATGTCATTGAATGTCTTTACATGGGCTT
GTATGCCTATGAAAATTGTAATACAACTTTCAGCAACGGATCTCTTGGCTCTCGCATCGA
TGAAGAACGCAGCGAAATGCGATAAGTAATGTGAATTGCAGAATTCAGTGAATCATCGAA
TCTTTGAACGCATCTTGCGCTCCTTGGTATTCCGAGGAGCATGCCTGTTTGAGTGTCATT
AAATTCTCAACTCTCTTATACTTTTTTGTAAAAGAGAGCTTGGACTGTGGAGGCTTGCTG
GCCACTTTTTGGGGTCAGCTCCTCTGAAATGCATTAGCGGAACCGTTTGCGATCTGCCAC
AAGTGTGATAAGTTATCTACACTGGCGAGGGGATTGCTCTCTGTAATGTTCAGCTTCTAA
TTGTCTCTACTTTGTGAGACTACTTTTGAATGCTTGACCTCAAATCAGGTAGGACTACCC
GCTGAACTTAA
>A6_62
TTTCCGTAGGTGAACCTGCGGAAGGATCATTATTGAATTATGTTTCTAGATAGGTTGTAG
CTGGCTCTTTTAGAGCATGTGCACGCCTGTTTGGACTTCATTTTCATCCACCTGTGCACC
TATTGTAGTCTTTGGTTGGGTTAGGAGGAAGTGATCATTGTATCAGCATCTGCTGGGAGT
GAGGACTTGCATTGTGAAAGCTTTGCTGTCCTTGATGTGATCATGGAATCTTTTTCTCAC
TAGAGTCTATGTCACTCATTATACTCTGTCGAATGTCATTGAATGTCTTTACATGGGCTT
GTATGCCTATGAAAATTGTAATACAACTTTCAGCAACGGATCTCTTGGCTCTCGCATCGA
TGAAGAACGCAGCGAAATGCGATAAGTAATGTGAATTGCAGAATTCAGTGAATCATCGAA
TCTTTGAACGCATCTTGCGCTCCTTGGTATTCCGAGGAGCATGCCTGTTTGAGTGTCATT
AAATTCTCAACTCTCTTATACTTTTTTGTAAAAGAGAGCTTGGACTGTGGAGGCTTGCTG
GCCACTTTTTGGGGTCAGCTCCTCTGAAATGCATTAGCGGAACCGTTTGCGATCTGCCAC
AAGTGTGATAAGTTATCTACACTGGCGAGGGGATTGCTCTCTGTAATGTTCAGCTTCTAA
TTGTCTCTACTTTGTGAGACTACTTTTGAATGCTTGACCTCAAATCAGGTAGGACTACCC
GCTGAACTTAA
>A6_64
TTTCCGTAGGTGAACCTGCGGAAGGATCATTATTGAATTATGTTTCTAGATAGGTTGTAG
CTGGCTCTTTTAGAGCATGTGCACGCCTGTTTGGACTTCATTTTCATCCACCTGTGCACC
TATTGTAGTCTTTGGTTGGGTTAGGAGGAAGTGATCATTGTATCAGCATCTGCTGGGAGT
GAGGACTTGCATTGTGAAAGCTTTGCTGTCCTTGATGTGATCATGGAATCTTTTTCTCAC
TAGAGTCTATGTCACTCATTATACTCTGTCGAATGTCATTGAATGTCTTTACATGGGCTT
GTATGCCTATGAAAATTGTAATACAACTTTCAGCAACGGATCTCTTGGCTCTCGCATCGA
TGAAGAACGCAGCGAAATGCGATAAGTAATGTGAATTGCAGAATTCAGTGAATCATCGAA
TCTTTGAACGCATCTTGCGCTCCTTGGTATTCCGAGGAGCATGCCTGTTTGAGTGTCATT
AAATTCTCAACTCTCTTATACTTTTTTGTAAAAGAGAGCTTGGACTGTGGAGGCTTGCTG
GCCACTTTTTGGGGTCAGCTCCTCTGAAATGCATTAGCGGAACCGTTTGCGATCTGCCAC
AAGTGTGATAAGTTATCTACACTGGCGAGGGGATTGCTCTCTGTAATGTTCAGCTTCTAA
TTGTCTCTACTTTGTGAGACTACTTTTGAATGCTTGACCTCAAATCAGGTAGGACTACCC
GCTGAACTTAA
>A7_42
TTTCCGTAGGTGAACCTGCGGAAGGATCATTATTGAATTATGTTTCTAGATAGGTTGTAG
CTGGCTCTTTTAGAGCATGTGCACGCCTGTTTGGACTTCATTTTCATCCACCTGTGCACC
TATTGTAGTCTTTGGTTGGGTTAGGAGGAAGTGATCATTGTATCAGCATCTGCTGGGAGT

GAGGACTTGCATTGTGAAAGCTTTGCTGTCCTTGATGTGATCATGGAATCTTTTTCTCAC
TAGAGTCTATGTCACTCATTATACTCTGTCGAATGTCATTGAATGTCTTTACATGGGCTT
GTATGCCTATGAAAATTGTAATACAACTTTCAGCAACGGATCTCTTGGCTCTCGCATCGA
TGAAGAACGCAGCGAAATGCGATAAGTAATGTGAATTGCAGAATTCAGTGAATCATCGAA
TCTTTGAACGCATCTTGCGCTCCTTGGTATTCCGAGGAGCATGCCTGTTTGAGTGTCATT
AAATTCTCAACTCTCTTATACTTTTTTGTAAAAGAGAGCTTGGACTGTGGAGGCTTGCTG
GCCACTTTTTGGGGTCAGCTCCTCTGAAATGCATTAGCGGAACCGTTTGCGATCTGCCAC
AAGTGTGATAAGTTATCTACACTGGCGAGGGGATTGCTCTCTGTAATGTTCAGCTTCTAA
TTGTCTCTACTTTGTGAGACTACTTTTGAATGCTTGACCTCAAATCAGGTAGGACTACCC
GCTGAACTTAA
>A7_43
TTTCCGTAGGTGAACCTGCGGAAGGATCATTATTGAATTATGTTTCTAGATAGGTTGTAG
CTGGCTCTTTTAGAGCATGTGCACGCCTGTTTGGACTTCATTTTCATCCACCTGTGCACC
TATTGTAGTCTTTGGTTGGGTTAGGAGGAAGTGATCATTGTATCAGCATCTGCTGGGAGT
GAGGACTTGCATTGTGAAAGCTTTGCTGTCCTTGATGTGATCATGGAATCTTTTTCTCAC
TAGAGTCTATGTCACTCATTATACTCTGTCGAATGTCATTGAATGTCTTTACATGGGCTT
GTATGCCTATGAAAATTGTAATACAACTTTCAGCAACGGATCTCTTGGCTCTCGCATCGA
TGAAGAACGCAGCGAAATGCGATAAGTAATGTGAATTGCAGAATTCAGTGAATCATCGAA
TCTTTGAACGCATCTTGCGCTCCTTGGTATTCCGAGGAGCATGCCTGTTTGAGTGTCATT
AAATTCTCAACTCTCTTATACTTTTTTGTAAAAGAGAGCTTGGACTGTGGAGGCTTGCTG
GCCACTTTTTGGGGTCAGCTCCTCTGAAATGCATTAGCGGAACCGTTTGCGATCTGCCAC
AAGTGTGATAAGTTATCTACACTGGCGAGGGGATTGCTCTCTGTAATGTTCAGCTTCTAA
TTGTCTCTACTTTGTGAGACTACTTTTGAATGCTTGACCTCAAATCAGGTAGGACTACCC
GCTGAACTTAA
>A7_44
TTTCCGTAGGTGAACCTGCGGAAGGATCATTATTGAATTATGTTTCTAGATAGGTTGTAG
CTGGCTCTTTTAGAGCATGTGCACGCCTGTTTGGACTTCATTTTCATCCACCTGTGCACC
TATTGTAGTCTTTGGTTGGGTTAGGAGGAAGTGATCATTGTATCAGCATCTGCTGGGAGT
GAGGACTTGCATTGTGAAAGCTTTGCTGTCCTTGATGTGATCATGGAATCTTTTTCTCAC
TAGAGTCTATGTCACTCATTATACTCTGTCGAATGTCATTGAATGTCTTTACATGGGCTT
GTATGCCTATGAAAATTGTAATACAACTTTCAGCAACGGATCTCTTGGCTCTCGCATCGA
TGAAGAACGCAGCGAAATGCGATAAGTAATGTGAATTGCAGAATTCAGTGAATCATCGAA
TCTTTGAACGCATCTTGCGCTCCTTGGTATTCCGAGGAGCATGCCTGTTTGAGTGTCATT
AAATTCTCAACTCTCTTATACTTTTTTGTAAAAGAGAGCTTGGACTGTGGAGGCTTGCTG
GCCACTTTTTGGGGTCAGCTCCTCTGAAATGCATTAGCGGAACCGTTTGCGATCTGCCAC
AAGTGTGATAAGTTATCTACACTGGCGAGGGGATTGCTCTCTGTAATGTTCAGCTTCTAA
TTGTCTCTACTTTGTGAGACTACTTTTGAATGCTTGACCTCAAATCAGGTAGGACTACCC
GCTGAACTTAA
>A7_47
TTTCCGTAGGTGAACCTGCGGAAGGATCATTATTGAATTATGTTTCTAGATAGGTTGTAG
CTGGCTCTTTTAGAGCATGTGCACGCCTGTTTGGACTTCATTTTCATCCACCTGTGCACC
TATTGTAGTCTTTGGTTGGGTTAGGAGGAAGTGATCATTGTATCAGCATCTGCTGGGAGT
GAGGACTTGCATTGTGAAAGCTTTGCTGTCCTTGATGTGATCATGGAATCTTTTTCTCAC
TAGAGTCTATGTCACTCATTATACTCTGTCGAATGTCATTGAATGTCTTTACATGGGCTT
GTATGCCTATGAAAATTGTAATACAACTTTCAGCAACGGATCTCTTGGCTCTCGCATCGA
TGAAGAACGCAGCGAAATGCGATAAGTAATGTGAATTGCAGAATTCAGTGAATCATCGAA
TCTTTGAACGCATCTTGCGCTCCTTGGTATTCCGAGGAGCATGCCTGTTTGAGTGTCATT
AAATTCTCAACTCTCTTATACTTTTTTGTAAAAGAGAGCTTGGACTGTGGAGGCTTGCTG
GCCACTTTTTGGGGTCAGCTCCTCTGAAATGCATTAGCGGAACCGTTTGCGATCTGCCAC
AAGTGTGATAAGTTATCTACACTGGCGAGGGGATTGCTCTCTGTAATGTTCAGCTTCTAA

TTGTCTCTACTTTGTGAGACTACTTTTGAATGCTTGACCTCAAATCAGGTAGGACTACCC
GCTGAACTTAA
>A7_48
TTTCCGTAGGTGAACCTGCGGAAGGATCATTATTGAATTATGTTTCTAGATAGGTTGTAG
CTGGCTCTTTTAGAGCATGTGCACGCCTGTTTGGACTTCATTTTCATCCACCTGTGCACC
TATTGTAGTCTTTGGTTGGGTTAGGAGGAAGTGATCATTGTATCAGCATCTGCTGGGAGT
GAGGACTTGCATTGTGAAAGCTTTGCTGTCCTTGATGTGATCATGGAATCTTTTTCTCAC
TAGAGTCTATGTCACTCATTATACTCTGTCGAATGTCATTGAATGTCTTTACATGGGCTT
GTATGCCTATGAAAATTGTAATACAACTTTCAGCAACGGATCTCTTGGCTCTCGCATCGA
TGAAGAACGCAGCGAAATGCGATAAGTAATGTGAATTGCAGAATTCAGTGAATCATCGAA
TCTTTGAACGCATCTTGCGCTCCTTGGTATTCCGAGGAGCATGCCTGTTTGAGTGTCATT
AAATTCTCAACTCTCTTATACTTTTTTGTAAAAGAGAGCTTGGACTGTGGAGGCTTGCTG
GCCACTTTTTGGGGTCAGCTCCTCTGAAATGCATTAGCGGAACCGTTTGCGATCTGCCAC
AAGTGTGATAAGTTATCTACACTGGCGAGGGGATTGCTCTCTGTAATGTTCAGCTTCTAA
TTGTCTCTACTTTGTGAGACTACTTTTGAATGCTTGACCTCAAATCAGGTAGGACTACCC
GCTGAACTTAA
>A7_49
TTTCCGTAGGTGAACCTGCGGAAGGATCATTATTGAATTATGTTTCTAGATAGGTTGTAG
CTGGCTCTTTTAGAGCATGTGCACGCCTGTTTGGACTTCATTTTCATCCACCTGTGCACC
TATTGTAGTCTTTGGTTGGGTTAGGAGGAAGTGATCATTGTATCAGCATCTGCTGGGAGT
GAGGACTTGCATTGTGAAAGCTTTGCTGTCCTTGATGTGATCATGGAATCTTTTTCTCAC
TAGAGTCTATGTCACTCATTATACTCTGTCGAATGTCATTGAATGTCTTTACATGGGCTT
GTATGCCTATGAAAATTGTAATACAACTTTCAGCAACGGATCTCTTGGCTCTCGCATCGA
TGAAGAACGCAGCGAAATGCGATAAGTAATGTGAATTGCAGAATTCAGTGAATCATCGAA
TCTTTGAACGCATCTTGCGCTCCTTGGTATTCCGAGGAGCATGCCTGTTTGAGTGTCATT
AAATTCTCAACTCTCTTATACTTTTTTGTAAAAGAGAGCTTGGACTGTGGAGGCTTGCTG
GCCACTTTTTGGGGTCAGCTCCTCTGAAATGCATTAGCGGAACCGTTTGCGATCTGCCAC
AAGTGTGATAAGTTATCTACACTGGCGAGGGGATTGCTCTCTGTAATGTTCAGCTTCTAA
TTGTCTCTACTTTGTGAGACTACTTTTGAATGCTTGACCTCAAATCAGGTAGGACTACCC
GCTGAACTTAA
>A7_52
TTTCCGTAGGTGAACCTGCGGAAGGATCATTATTGAATTATGTTTCTAGATAGGTTGTAG
CTGGCTCTTTTAGAGCATGTGCACGCCTGTTTGGACTTCATTTTCATCCACCTGTGCACC
TATTGTAGTCTTTGGTTGGGTTAGGAGGAAGTGATCATTGTATCAGCATCTGCTGGGAGT
GAGGACTTGCATTGTGAAAGCTTTGCTGTCCTTGATGTGATCATGGAATCTTTTTCTCAC
TAGAGTCTATGTCACTCATTATACTCTGTCGAATGTCATTGAATGTCTTTACATGGGCTT
GTATGCCTATGAAAATTGTAATACAACTTTCAGCAACGGATCTCTTGGCTCTCGCATCGA
TGAAGAACGCAGCGAAATGCGATAAGTAATGTGAATTGCAGAATTCAGTGAATCATCGAA
TCTTTGAACGCATCTTGCGCTCCTTGGTATTCCGAGGAGCATGCCTGTTTGAGTGTCATT
AAATTCTCAACTCTCTTATACTTTTTTGTAAAAGAGAGCTTGGACTGTGGAGGCTTGCTG
GCCACTTTTTGGGGTCAGCTCCTCTGAAATGCATTAGCGGAACCGTTTGCGATCTGCCAC
AAGTGTGATAAGTTATCTACACTGGCGAGGGGATTGCTCTCTGTAATGTTCAGCTTCTAA
TTGTCTCTACTTTGTGAGACTACTTTTGAATGCTTGACCTCAAATCAGGTAGGACTACCC
GCTGAACTTAA
>A7_54
TTTCCGTAGGTGAACCTGCGGAAGGATCATTATTGAATTATGTTTCTAGATAGGTTGTAG
CTGGCTCTTTTAGAGCATGTGCACGCCTGTTTGGACTTCATTTTCATCCACCTGTGCACC
TATTGTAGTCTTTGGTTGGGTTAGGAGGAAGTGATCATTGTATCAGCATCTGCTGGGAGT
GAGGACTTGCATTGTGAAAGCTTTGCTGTCCTTGATGTGATCATGGAATCTTTTTCTCAC
TAGAGTCTATGTCACTCATTATACTCTGTCGAATGTCATTGAATGTCTTTACATGGGCTT

GTATGCCTATGAAAATTGTAATACAACTTTCAGCAACGGATCTCTTGGCTCTCGCATCGA
TGAAGAACGCAGCGAAATGCGATAAGTAATGTGAATTGCAGAATTCAGTGAATCATCGAA
TCTTTGAACGCATCTTGCGCTCCTTGGTATTCCGAGGAGCATGCCTGTTTGAGTGTCATT
AAATTCTCAACTCTCTTATACTTTTTTGTAAAAGAGAGCTTGGACTGTGGAGGCTTGCTG
GCCACTTTTTGGGGTCAGCTCCTCTGAAATGCATTAGCGGAACCGTTTGCGATCTGCCAC
AAGTGTGATAAGTTATCTACACTGGCGAGGGGATTGCTCTCTGTAATGTTCAGCTTCTAA
TTGTCTCTACTTTGTGAGACTACTTTTGAATGCTTGACCTCAAATCAGGTAGGACTACCC
GCTGAACTTAA
>A7_56
TTTCCGTAGGTGAACCTGCGGAAGGATCATTATTGAATTATGTTTCTAGATAGGTTGTAG
CTGGCTCTTTTAGAGCATGTGCACGCCTGTTTGGACTTCATTTTCATCCACCTGTGCACC
TATTGTAGTCTTTGGTTGGGTTAGGAGGAAGTGATCATTGTATCAGCATCTGCTGGGAGT
GAGGACTTGCATTGTGAAAGCTTTGCTGTCCTTGATGTGATCATGGAATCTTTTTCTCAC
TAGAGTCTATGTCACTCATTATACTCTGTCGAATGTCATTGAATGTCTTTACATGGGCTT
GTATGCCTATGAAAATTGTAATACAACTTTCAGCAACGGATCTCTTGGCTCTCGCATCGA
TGAAGAACGCAGCGAAATGCGATAAGTAATGTGAATTGCAGAATTCAGTGAATCATCGAA
TCTTTGAACGCATCTTGCGCTCCTTGGTATTCCGAGGAGCATGCCTGTTTGAGTGTCATT
AAATTCTCAACTCTCTTATACTTTTTTGTAAAAGAGAGCTTGGACTGTGGAGGCTTGCTG
GCCACTTTTTGGGGTCAGCTCCTCTGAAATGCATTAGCGGAACCGTTTGCGATCTGCCAC
AAGTGTGATAAGTTATCTACACTGGCGAGGGGATTGCTCTCTGTAATGTTCAGCTTCTAA
TTGTCTCTACTTTGTGAGACTACTTTTGAATGCTTGACCTCAAATCAGGTAGGACTACCC
GCTGAACTTAA
>A6_35
TTTCCGTAGGTGAACCTGCGGAAGGATCATTATTGAATTATGTTTCTAGATAGGTTGTAG
CTGGCTCTTTTAGAGCATGTGCACGCCTGTTTGGACTTCATTTTCATCCACCTGTGCACC
TATTGTAGTCTTTGGTTGGGTTAGGAGGAAGTGATCATTGTATCAGCATCTGCTGGGAGT
GAGGACTTGCATTGTGAAAGCTTTGCTGTCCTTGATGTGATCATGGAATCTTTTTCTCAC
TAGAGTCTATGTCACTCATTATACTCTGTCGAATGTCATTGAATGTCTTTACATGGGCTT
GTATGCCTATGAAAATTGTAATACAACTTTCAGCAACGGATCTCTTGGCTCTCGCATCGA
TGAAGAACGCAGCGAAATGCGATAAGTAATGTGAATTGCAGAATTCAGTGAATCATCGAA
TCTTTGAACGCATCTTGCGCTCCTTGGTATTCCGAGGAGCATGCCTGTTTGAGTGTCATT
AAATTCTCAACTCTCTTATACTTTTTTGTAAAAGAGAGCTTGGACTGTGGAGGCTTGCTG
GCCACTTTTTGGGGTCAGCTCCTCTGAAATGCATTAGCGGAACCGTTTGCGATCTGCCAC
AAGTGTGATAAGTTATCTACACTGGCGAGGGGATTGCTCTCTGTAATGTTCAGCTTCTAA
TTGTCTCTACTTTGTGAGACTACTTTTGAATGCTTGACCTCAAATCAGGTAGGACTACCC
GCTGAACTTAA
>A6_36
TTTCCGTAGGTGAACCTGCGGAAGGATCATTATTGAATTATGTTTCTAGATAGGTTGTAG
CTGGCTCTTTTAGAGCATGTGCACGCCTGTTTGGACTTCATTTTCATCCACCTGTGCACC
TATTGTAGTCTTTGGTTGGGTTAGGAGGAAGTGATCATTGTATCAGCATCTGCTGGGAGT
GAGGACTTGCATTGTGAAAGCTTTGCTGTCCTTGATGTGATCATGGAATCTTTTTCTCAC
TAGAGTCTATGTCACTCATTATACTCTGTCGAATGTCATTGAATGTCTTTACATGGGCTT
GTATGCCTATGAAAATTGTAATACAACTTTCAGCAACGGATCTCTTGGCTCTCGCATCGA
TGAAGAACGCAGCGAAATGCGATAAGTAATGTGAATTGCAGAATTCAGTGAATCATCGAA
TCTTTGAACGCATCTTGCGCTCCTTGGTATTCCGAGGAGCATGCCTGTTTGAGTGTCATT
AAATTCTCAACTCTCTTATACTTTTTTGTAAAAGAGAGCTTGGACTGTGGAGGCTTGCTG
GCCACTTTTTGGGGTCAGCTCCTCTGAAATGCATTAGCGGAACCGTTTGCGATCTGCCAC
AAGTGTGATAAGTTATCTACACTGGCGAGGGGATTGCTCTCTGTAATGTTCAGCTTCTAA
TTGTCTCTACTTTGTGAGACTACTTTTGAATGCTTGACCTCAAATCAGGTAGGACTACCC
GCTGAACTTAA

>A6_37
TTTCCGTAGGTGAACCTGCGGAAGGATCATTATTGAATTATGTTTCTAGATAGGTTGTAG
CTGGCTCTTTTAGAGCATGTGCACGCCTGTTTGGACTTCATTTTCATCCACCTGTGCACC
TATTGTAGTCTTTGGTTGGGTTAGGAGGAAGTGATCATTGTATCAGCATCTGCTGGGAGT
GAGGACTTGCATTGTGAAAGCTTTGCTGTCCTTGATGTGATCATGGAATCTTTTTCTCAC
TAGAGTCTATGTCACTCATTATACTCTGTCGAATGTCATTGAATGTCTTTACATGGGCTT
GTATGCCTATGAAAATTGTAATACAACTTTCAGCAACGGATCTCTTGGCTCTCGCATCGA
TGAAGAACGCAGCGAAATGCGATAAGTAATGTGAATTGCAGAATTCAGTGAATCATCGAA
TCTTTGAACGCATCTTGCGCTCCTTGGTATTCCGAGGAGCATGCCTGTTTGAGTGTCATT
AAATTCTCAACTCTCTTATACTTTTTTGTAAAAGAGAGCTTGGACTGTGGAGGCTTGCTG
GCCACTTTTTGGGGTCAGCTCCTCTGAAATGCATTAGCGGAACCGTTTGCGATCTGCCAC
AAGTGTGATAAGTTATCTACACTGGCGAGGGGATTGCTCTCTGTAATGTTCAGCTTCTAA
TTGTCTCTACTTTGTGAGACTACTTTTGAATGCTTGACCTCAAATCAGGTAGGACTACCC
GCTGAACTTAA
>A6_38
TTTCCGTAGGTGAACCTGCGGAAGGATCATTATTGAATTATGTTTCTAGATAGGTTGTAG
CTGGCTCTTTTAGAGCATGTGCACGCCTGTTTGGACTTCATTTTCATCCACCTGTGCACC
TATTGTAGTCTTTGGTTGGGTTAGGAGGAAGTGATCATTGTATCAGCATCTGCTGGGAGT
GAGGACTTGCATTGTGAAAGCTTTGCTGTCCTTGATGTGATCATGGAATCTTTTTCTCAC
TAGAGTCTATGTCACTCATTATACTCTGTCGAATGTCATTGAATGTCTTTACATGGGCTT
GTATGCCTATGAAAATTGTAATACAACTTTCAGCAACGGATCTCTTGGCTCTCGCATCGA
TGAAGAACGCAGCGAAATGCGATAAGTAATGTGAATTGCAGAATTCAGTGAATCATCGAA
TCTTTGAACGCATCTTGCGCTCCTTGGTATTCCGAGGAGCATGCCTGTTTGAGTGTCATT
AAATTCTCAACTCTCTTATACTTTTTTGTAAAAGAGAGCTTGGACTGTGGAGGCTTGCTG
GCCACTTTTTGGGGTCAGCTCCTCTGAAATGCATTAGCGGAACCGTTTGCGATCTGCCAC
AAGTGTGATAAGTTATCTACACTGGCGAGGGGATTGCTCTCTGTAATGTTCAGCTTCTAA
TTGTCTCTACTTTGTGAGACTACTTTTGAATGCTTGACCTCAAATCAGGTAGGACTACCC
GCTGAACTTAA
>A6_39
TTTCCGTAGGTGAACCTGCGGAAGGATCATTATTGAATTATGTTTCTAGATAGGTTGTAG
CTGGCTCTTTTAGAGCATGTGCACGCCTGTTTGGACTTCATTTTCATCCACCTGTGCACC
TATTGTAGTCTTTGGTTGGGTTAGGAGGAAGTGATCATTGTATCAGCATCTGCTGGGAGT
GAGGACTTGCATTGTGAAAGCTTTGCTGTCCTTGATGTGATCATGGAATCTTTTTCTCAC
TAGAGTCTATGTCACTCATTATACTCTGTCGAATGTCATTGAATGTCTTTACATGGGCTT
GTATGCCTATGAAAATTGTAATACAACTTTCAGCAACGGATCTCTTGGCTCTCGCATCGA
TGAAGAACGCAGCGAAATGCGATAAGTAATGTGAATTGCAGAATTCAGTGAATCATCGAA
TCTTTGAACGCATCTTGCGCTCCTTGGTATTCCGAGGAGCATGCCTGTTTGAGTGTCATT
AAATTCTCAACTCTCTTATACTTTTTTGTAAAAGAGAGCTTGGACTGTGGAGGCTTGCTG
GCCACTTTTTGGGGTCAGCTCCTCTGAAATGCATTAGCGGAACCGTTTGCGATCTGCCAC
AAGTGTGATAAGTTATCTACACTGGCGAGGGGATTGCTCTCTGTAATGTTCAGCTTCTAA
TTGTCTCTACTTTGTGAGACTACTTTTGAATGCTTGACCTCAAATCAGGTAGGACTACCC
GCTGAACTTAA
>A6_42
TTTCCGTAGGTGAACCTGCGGAAGGATCATTATTGAATTATGTTTCTAGATAGGTTGTAG
CTGGCTCTTTTAGAGCATGTGCACGCCTGTTTGGACTTCATTTTCATCCACCTGTGCACC
TATTGTAGTCTTTGGTTGGGTTAGGAGGAAGTGATCATTGTATCAGCATCTGCTGGGAGT
GAGGACTTGCATTGTGAAAGCTTTGCTGTCCTTGATGTGATCATGGAATCTTTTTCTCAC
TAGAGTCTATGTCACTCATTATACTCTGTCGAATGTCATTGAATGTCTTTACATGGGCTT
GTATGCCTATGAAAATTGTAATACAACTTTCAGCAACGGATCTCTTGGCTCTCGCATCGA
TGAAGAACGCAGCGAAATGCGATAAGTAATGTGAATTGCAGAATTCAGTGAATCATCGAA

TCTTTGAACGCATCTTGCGCTCCTTGGTATTCCGAGGAGCATGCCTGTTTGAGTGTCATT
AAATTCTCAACTCTCTTATACTTTTTTGTAAAAGAGAGCTTGGACTGTGGAGGCTTGCTG
GCCACTTTTTGGGGTCAGCTCCTCTGAAATGCATTAGCGGAACCGTTTGCGATCTGCCAC
AAGTGTGATAAGTTATCTACACTGGCGAGGGGATTGCTCTCTGTAATGTTCAGCTTCTAA
TTGTCTCTACTTTGTGAGACTACTTTTGAATGCTTGACCTCAAATCAGGTAGGACTACCC
GCTGAACTTAA
>A6_43
TTTCCGTAGGTGAACCTGCGGAAGGATCATTATTGAATTATGTTTCTAGATAGGTTGTAG
CTGGCTCTTTTAGAGCATGTGCACGCCTGTTTGGACTTCATTTTCATCCACCTGTGCACC
TATTGTAGTCTTTGGTTGGGTTAGGAGGAAGTGATCATTGTATCAGCATCTGCTGGGAGT
GAGGACTTGCATTGTGAAAGCTTTGCTGTCCTTGATGTGATCATGGAATCTTTTTCTCAC
TAGAGTCTATGTCACTCATTATACTCTGTCGAATGTCATTGAATGTCTTTACATGGGCTT
GTATGCCTATGAAAATTGTAATACAACTTTCAGCAACGGATCTCTTGGCTCTCGCATCGA
TGAAGAACGCAGCGAAATGCGATAAGTAATGTGAATTGCAGAATTCAGTGAATCATCGAA
TCTTTGAACGCATCTTGCGCTCCTTGGTATTCCGAGGAGCATGCCTGTTTGAGTGTCATT
AAATTCTCAACTCTCTTATACTTTTTTGTAAAAGAGAGCTTGGACTGTGGAGGCTTGCTG
GCCACTTTTTGGGGTCAGCTCCTCTGAAATGCATTAGCGGAACCGTTTGCGATCTGCCAC
AAGTGTGATAAGTTATCTACACTGGCGAGGGGATTGCTCTCTGTAATGTTCAGCTTCTAA
TTGTCTCTACTTTGTGAGACTACTTTTGAATGCTTGACCTCAAATCAGGTAGGACTACCC
GCTGAACTTAA
>A6_44
TTTCCGTAGGTGAACCTGCGGAAGGATCATTATTGAATTATGTTTCTAGATAGGTTGTAG
CTGGCTCTTTTAGAGCATGTGCACGCCTGTTTGGACTTCATTTTCATCCACCTGTGCACC
TATTGTAGTCTTTGGTTGGGTTAGGAGGAAGTGATCATTGTATCAGCATCTGCTGGGAGT
GAGGACTTGCATTGTGAAAGCTTTGCTGTCCTTGATGTGATCATGGAATCTTTTTCTCAC
TAGAGTCTATGTCACTCATTATACTCTGTCGAATGTCATTGAATGTCTTTACATGGGCTT
GTATGCCTATGAAAATTGTAATACAACTTTCAGCAACGGATCTCTTGGCTCTCGCATCGA
TGAAGAACGCAGCGAAATGCGATAAGTAATGTGAATTGCAGAATTCAGTGAATCATCGAA
TCTTTGAACGCATCTTGCGCTCCTTGGTATTCCGAGGAGCATGCCTGTTTGAGTGTCATT
AAATTCTCAACTCTCTTATACTTTTTTGTAAAAGAGAGCTTGGACTGTGGAGGCTTGCTG
GCCACTTTTTGGGGTCAGCTCCTCTGAAATGCATTAGCGGAACCGTTTGCGATCTGCCAC
AAGTGTGATAAGTTATCTACACTGGCGAGGGGATTGCTCTCTGTAATGTTCAGCTTCTAA
TTGTCTCTACTTTGTGAGACTACTTTTGAATGCTTGACCTCAAATCAGGTAGGACTACCC
GCTGAACTTAA
>A6_46
TTTCCGTAGGTGAACCTGCGGAAGGATCATTATTGAATTATGTTTCTAGATAGGTTGTAG
CTGGCTCTTTTAGAGCATGTGCACGCCTGTTTGGACTTCATTTTCATCCACCTGTGCACC
TATTGTAGTCTTTGGTTGGGTTAGGAGGAAGTGATCATTGTATCAGCATCTGCTGGGAGT
GAGGACTTGCATTGTGAAAGCTTTGCTGTCCTTGATGTGATCATGGAATCTTTTTCTCAC
TAGAGTCTATGTCACTCATTATACTCTGTCGAATGTCATTGAATGTCTTTACATGGGCTT
GTATGCCTATGAAAATTGTAATACAACTTTCAGCAACGGATCTCTTGGCTCTCGCATCGA
TGAAGAACGCAGCGAAATGCGATAAGTAATGTGAATTGCAGAATTCAGTGAATCATCGAA
TCTTTGAACGCATCTTGCGCTCCTTGGTATTCCGAGGAGCATGCCTGTTTGAGTGTCATT
AAATTCTCAACTCTCTTATACTTTTTTGTAAAAGAGAGCTTGGACTGTGGAGGCTTGCTG
GCCACTTTTTGGGGTCAGCTCCTCTGAAATGCATTAGCGGAACCGTTTGCGATCTGCCAC
AAGTGTGATAAGTTATCTACACTGGCGAGGGGATTGCTCTCTGTAATGTTCAGCTTCTAA
TTGTCTCTACTTTGTGAGACTACTTTTGAATGCTTGACCTCAAATCAGGTAGGACTACCC
GCTGAACTTAA
>A6_47
TTTCCGTAGGTGAACCTGCGGAAGGATCATTATTGAATTATGTTTCTAGATAGGTTGTAG

CTGGCTCTTTTAGAGCATGTGCACGCCTGTTTGGACTTCATTTTCATCCACCTGTGCACC
TATTGTAGTCTTTGGTTGGGTTAGGAGGAAGTGATCATTGTATCAGCATCTGCTGGGAGT
GAGGACTTGCATTGTGAAAGCTTTGCTGTCCTTGATGTGATCATGGAATCTTTTTCTCAC
TAGAGTCTATGTCACTCATTATACTCTGTCGAATGTCATTGAATGTCTTTACATGGGCTT
GTATGCCTATGAAAATTGTAATACAACTTTCAGCAACGGATCTCTTGGCTCTCGCATCGA
TGAAGAACGCAGCGAAATGCGATAAGTAATGTGAATTGCAGAATTCAGTGAATCATCGAA
TCTTTGAACGCATCTTGCGCTCCTTGGTATTCCGAGGAGCATGCCTGTTTGAGTGTCATT
AAATTCTCAACTCTCTTATACTTTTTTGTAAAAGAGAGCTTGGACTGTGGAGGCTTGCTG
GCCACTTTTTGGGGTCAGCTCCTCTGAAATGCATTAGCGGAACCGTTTGCGATCTGCCAC
AAGTGTGATAAGTTATCTACACTGGCGAGGGGATTGCTCTCTGTAATGTTCAGCTTCTAA
TTGTCTCTACTTTGTGAGACTACTTTTGAATGCTTGACCTCAAATCAGGTAGGACTACCC
GCTGAACTTAA
>A6_48
TTTCCGTAGGTGAACCTGCGGAAGGATCATTATTGAATTATGTTTCTAGATAGGTTGTAG
CTGGCTCTTTTAGAGCATGTGCACGCCTGTTTGGACTTCATTTTCATCCACCTGTGCACC
TATTGTAGTCTTTGGTTGGGTTAGGAGGAAGTGATCATTGTATCAGCATCTGCTGGGAGT
GAGGACTTGCATTGTGAAAGCTTTGCTGTCCTTGATGTGATCATGGAATCTTTTTCTCAC
TAGAGTCTATGTCACTCATTATACTCTGTCGAATGTCATTGAATGTCTTTACATGGGCTT
GTATGCCTATGAAAATTGTAATACAACTTTCAGCAACGGATCTCTTGGCTCTCGCATCGA
TGAAGAACGCAGCGAAATGCGATAAGTAATGTGAATTGCAGAATTCAGTGAATCATCGAA
TCTTTGAACGCATCTTGCGCTCCTTGGTATTCCGAGGAGCATGCCTGTTTGAGTGTCATT
AAATTCTCAACTCTCTTATACTTTTTTGTAAAAGAGAGCTTGGACTGTGGAGGCTTGCTG
GCCACTTTTTGGGGTCAGCTCCTCTGAAATGCATTAGCGGAACCGTTTGCGATCTGCCAC
AAGTGTGATAAGTTATCTACACTGGCGAGGGGATTGCTCTCTGTAATGTTCAGCTTCTAA
TTGTCTCTACTTTGTGAGACTACTTTTGAATGCTTGACCTCAAATCAGGTAGGACTACCC
GCTGAACTTAA
>A6_50
TTTCCGTAGGTGAACCTGCGGAAGGATCATTATTGAATTATGTTTCTAGATAGGTTGTAG
CTGGCTCTTTTAGAGCATGTGCACGCCTGTTTGGACTTCATTTTCATCCACCTGTGCACC
TATTGTAGTCTTTGGTTGGGTTAGGAGGAAGTGATCATTGTATCAGCATCTGCTGGGAGT
GAGGACTTGCATTGTGAAAGCTTTGCTGTCCTTGATGTGATCATGGAATCTTTTTCTCAC
TAGAGTCTATGTCACTCATTATACTCTGTCGAATGTCATTGAATGTCTTTACATGGGCTT
GTATGCCTATGAAAATTGTAATACAACTTTCAGCAACGGATCTCTTGGCTCTCGCATCGA
TGAAGAACGCAGCGAAATGCGATAAGTAATGTGAATTGCAGAATTCAGTGAATCATCGAA
TCTTTGAACGCATCTTGCGCTCCTTGGTATTCCGAGGAGCATGCCTGTTTGAGTGTCATT
AAATTCTCAACTCTCTTATACTTTTTTGTAAAAGAGAGCTTGGACTGTGGAGGCTTGCTG
GCCACTTTTTGGGGTCAGCTCCTCTGAAATGCATTAGCGGAACCGTTTGCGATCTGCCAC
AAGTGTGATAAGTTATCTACACTGGCGAGGGGATTGCTCTCTGTAATGTTCAGCTTCTAA
TTGTCTCTACTTTGTGAGACTACTTTTGAATGCTTGACCTCAAATCAGGTAGGACTACCC
GCTGAACTTAA
>A6_54
TTTCCGTAGGTGAACCTGCGGAAGGATCATTATTGAATTATGTTTCTAGATAGGTTGTAG
CTGGCTCTTTTAGAGCATGTGCACGCCTGTTTGGACTTCATTTTCATCCACCTGTGCACC
TATTGTAGTCTTTGGTTGGGTTAGGAGGAAGTGATCATTGTATCAGCATCTGCTGGGAGT
GAGGACTTGCATTGTGAAAGCTTTGCTGTCCTTGATGTGATCATGGAATCTTTTTCTCAC
TAGAGTCTATGTCACTCATTATACTCTGTCGAATGTCATTGAATGTCTTTACATGGGCTT
GTATGCCTATGAAAATTGTAATACAACTTTCAGCAACGGATCTCTTGGCTCTCGCATCGA
TGAAGAACGCAGCGAAATGCGATAAGTAATGTGAATTGCAGAATTCAGTGAATCATCGAA
TCTTTGAACGCATCTTGCGCTCCTTGGTATTCCGAGGAGCATGCCTGTTTGAGTGTCATT
AAATTCTCAACTCTCTTATACTTTTTTGTAAAAGAGAGCTTGGACTGTGGAGGCTTGCTG

GCCACTTTTTGGGGTCAGCTCCTCTGAAATGCATTAGCGGAACCGTTTGCGATCTGCCAC
AAGTGTGATAAGTTATCTACACTGGCGAGGGGATTGCTCTCTGTAATGTTCAGCTTCTAA
TTGTCTCTACTTTGTGAGACTACTTTTGAATGCTTGACCTCAAATCAGGTAGGACTACCC
GCTGAACTTAA
>A6_55
TTTCCGTAGGTGAACCTGCGGAAGGATCATTATTGAATTATGTTTCTAGATAGGTTGTAG
CTGGCTCTTTTAGAGCATGTGCACGCCTGTTTGGACTTCATTTTCATCCACCTGTGCACC
TATTGTAGTCTTTGGTTGGGTTAGGAGGAAGTGATCATTGTATCAGCATCTGCTGGGAGT
GAGGACTTGCATTGTGAAAGCTTTGCTGTCCTTGATGTGATCATGGAATCTTTTTCTCAC
TAGAGTCTATGTCACTCATTATACTCTGTCGAATGTCATTGAATGTCTTTACATGGGCTT
GTATGCCTATGAAAATTGTAATACAACTTTCAGCAACGGATCTCTTGGCTCTCGCATCGA
TGAAGAACGCAGCGAAATGCGATAAGTAATGTGAATTGCAGAATTCAGTGAATCATCGAA
TCTTTGAACGCATCTTGCGCTCCTTGGTATTCCGAGGAGCATGCCTGTTTGAGTGTCATT
AAATTCTCAACTCTCTTATACTTTTTTGTAAAAGAGAGCTTGGACTGTGGAGGCTTGCTG
GCCACTTTTTGGGGTCAGCTCCTCTGAAATGCATTAGCGGAACCGTTTGCGATCTGCCAC
AAGTGTGATAAGTTATCTACACTGGCGAGGGGATTGCTCTCTGTAATGTTCAGCTTCTAA
TTGTCTCTACTTTGTGAGACTACTTTTGAATGCTTGACCTCAAATCAGGTAGGACTACCC
GCTGAACTTAA
>A7_57
TTTCCGTAGGTGAACCTGCGGAAGGATCATTATTGAATTATGTTTCTAGATAGGTTGTAG
CTGGCTCTTTTAGAGCATGTGCACGCCTGTTTGGACTTCATTTTCATCCACCTGTGCACC
TATTGTAGTCTTTGGTTGGGTTAGGAGGAAGTGATCATTGTATCAGCATCTGCTGGGAGT
GAGGACTTGCATTGTGAAAGCTTTGCTGTCCTTGATGTGATCATGGAATCTTTTTCTCAC
TAGAGTCTATGTCACTCATTATACTCTGTCGAATGTCATTGAATGTCTTTACATGGGCTT
GTATGCCTATGAAAATTGTAATACAACTTTCAGCAACGGATCTCTTGGCTCTCGCATCGA
TGAAGAACGCAGCGAAATGCGATAAGTAATGTGAATTGCAGAATTCAGTGAATCATCGAA
TCTTTGAACGCATCTTGCGCTCCTTGGTATTCCGAGGAGCATGCCTGTTTGAGTGTCATT
AAATTCTCAACTCTCTTATACTTTTTTGTAAAAGAGAGCTTGGACTGTGGAGGCTTGCTG
GCCACTTTTTGGGGTCAGCTCCTCTGAAATGCATTAGCGGAACCGTTTGCGATCTGCCAC
AAGTGTGATAAGTTATCTACACTGGCGAGGGGATTGCTCTCTGTAATGTTCAGCTTCTAA
TTGTCTCTACTTTGTGAGACTACTTTTGAATGCTTGACCTCAAATCAGGTAGGACTACCC
GCTGAACTTAA
>A7_58
TTTCCGTAGGTGAACCTGCGGAAGGATCATTATTGAATTATGTTTCTAGATAGGTTGTAG
CTGGCTCTTTTAGAGCATGTGCACGCCTGTTTGGACTTCATTTTCATCCACCTGTGCACC
TATTGTAGTCTTTGGTTGGGTTAGGAGGAAGTGATCATTGTATCAGCATCTGCTGGGAGT
GAGGACTTGCATTGTGAAAGCTTTGCTGTCCTTGATGTGATCATGGAATCTTTTTCTCAC
TAGAGTCTATGTCACTCATTATACTCTGTCGAATGTCATTGAATGTCTTTACATGGGCTT
GTATGCCTATGAAAATTGTAATACAACTTTCAGCAACGGATCTCTTGGCTCTCGCATCGA
TGAAGAACGCAGCGAAATGCGATAAGTAATGTGAATTGCAGAATTCAGTGAATCATCGAA
TCTTTGAACGCATCTTGCGCTCCTTGGTATTCCGAGGAGCATGCCTGTTTGAGTGTCATT
AAATTCTCAACTCTCTTATACTTTTTTGTAAAAGAGAGCTTGGACTGTGGAGGCTTGCTG
GCCACTTTTTGGGGTCAGCTCCTCTGAAATGCATTAGCGGAACCGTTTGCGATCTGCCAC
AAGTGTGATAAGTTATCTACACTGGCGAGGGGATTGCTCTCTGTAATGTTCAGCTTCTAA
TTGTCTCTACTTTGTGAGACTACTTTTGAATGCTTGACCTCAAATCAGGTAGGACTACCC
GCTGAACTTAA
>A7_60
TTTCCGTAGGTGAACCTGCGGAAGGATCATTATTGAATTATGTTTCTAGATAGGTTGTAG
CTGGCTCTTTTAGAGCATGTGCACGCCTGTTTGGACTTCATTTTCATCCACCTGTGCACC
TATTGTAGTCTTTGGTTGGGTTAGGAGGAAGTGATCATTGTATCAGCATCTGCTGGGAGT

GAGGACTTGCATTGTGAAAGCTTTGCTGTCCTTGATGTGATCATGGAATCTTTTTCTCAC
TAGAGTCTATGTCACTCATTATACTCTGTCGAATGTCATTGAATGTCTTTACATGGGCTT
GTATGCCTATGAAAATTGTAATACAACTTTCAGCAACGGATCTCTTGGCTCTCGCATCGA
TGAAGAACGCAGCGAAATGCGATAAGTAATGTGAATTGCAGAATTCAGTGAATCATCGAA
TCTTTGAACGCATCTTGCGCTCCTTGGTATTCCGAGGAGCATGCCTGTTTGAGTGTCATT
AAATTCTCAACTCTCTTATACTTTTTTGTAAAAGAGAGCTTGGACTGTGGAGGCTTGCTG
GCCACTTTTTGGGGTCAGCTCCTCTGAAATGCATTAGCGGAACCGTTTGCGATCTGCCAC
AAGTGTGATAAGTTATCTACACTGGCGAGGGGATTGCTCTCTGTAATGTTCAGCTTCTAA
TTGTCTCTACTTTGTGAGACTACTTTTGAATGCTTGACCTCAAATCAGGTAGGACTACCC
GCTGAACTTAA
>A7_62
TTTCCGTAGGTGAACCTGCGGAAGGATCATTATTGAATTATGTTTCTAGATAGGTTGTAG
CTGGCTCTTTTAGAGCATGTGCACGCCTGTTTGGACTTCATTTTCATCCACCTGTGCACC
TATTGTAGTCTTTGGTTGGGTTAGGAGGAAGTGATCATTGTATCAGCATCTGCTGGGAGT
GAGGACTTGCATTGTGAAAGCTTTGCTGTCCTTGATGTGATCATGGAATCTTTTTCTCAC
TAGAGTCTATGTCACTCATTATACTCTGTCGAATGTCATTGAATGTCTTTACATGGGCTT
GTATGCCTATGAAAATTGTAATACAACTTTCAGCAACGGATCTCTTGGCTCTCGCATCGA
TGAAGAACGCAGCGAAATGCGATAAGTAATGTGAATTGCAGAATTCAGTGAATCATCGAA
TCTTTGAACGCATCTTGCGCTCCTTGGTATTCCGAGGAGCATGCCTGTTTGAGTGTCATT
AAATTCTCAACTCTCTTATACTTTTTTGTAAAAGAGAGCTTGGACTGTGGAGGCTTGCTG
GCCACTTTTTGGGGTCAGCTCCTCTGAAATGCATTAGCGGAACCGTTTGCGATCTGCCAC
AAGTGTGATAAGTTATCTACACTGGCGAGGGGATTGCTCTCTGTAATGTTCAGCTTCTAA
TTGTCTCTACTTTGTGAGACTACTTTTGAATGCTTGACCTCAAATCAGGTAGGACTACCC
GCTGAACTTAA
>A7_63
TTTCCGTAGGTGAACCTGCGGAAGGATCATTATTGAATTATGTTTCTAGATAGGTTGTAG
CTGGCTCTTTTAGAGCATGTGCACGCCTGTTTGGACTTCATTTTCATCCACCTGTGCACC
TATTGTAGTCTTTGGTTGGGTTAGGAGGAAGTGATCATTGTATCAGCATCTGCTGGGAGT
GAGGACTTGCATTGTGAAAGCTTTGCTGTCCTTGATGTGATCATGGAATCTTTTTCTCAC
TAGAGTCTATGTCACTCATTATACTCTGTCGAATGTCATTGAATGTCTTTACATGGGCTT
GTATGCCTATGAAAATTGTAATACAACTTTCAGCAACGGATCTCTTGGCTCTCGCATCGA
TGAAGAACGCAGCGAAATGCGATAAGTAATGTGAATTGCAGAATTCAGTGAATCATCGAA
TCTTTGAACGCATCTTGCGCTCCTTGGTATTCCGAGGAGCATGCCTGTTTGAGTGTCATT
AAATTCTCAACTCTCTTATACTTTTTTGTAAAAGAGAGCTTGGACTGTGGAGGCTTGCTG
GCCACTTTTTGGGGTCAGCTCCTCTGAAATGCATTAGCGGAACCGTTTGCGATCTGCCAC
AAGTGTGATAAGTTATCTACACTGGCGAGGGGATTGCTCTCTGTAATGTTCAGCTTCTAA
TTGTCTCTACTTTGTGAGACTACTTTTGAATGCTTGACCTCAAATCAGGTAGGACTACCC
GCTGAACTTAA
>A6_65
TTTCCGTAGGTGAACCTGCGGAAGGATCATTATTGAATTATGTTTCTAGATAGGTTGTAG
CTGGCTCTTTTAGAGCATGTGCACGCCTGTTTGGACTTCATTTTCATCCACCTGTGCACC
TATTGTAGTCTTTGGTTGGGTTAGGAGGAAGTGATCATTGTATCAGCATCTGCTGGGAGT
GAGGACTTGCATTGTGAAAGCTTTGCTGTCCTTGATGTGATCATGGAATCTTTTTCTCAC
TAGAGTCTATGTCACTCATTATACTCTGTCGAATGTCATTGAATGTCTTTACATGGGCTT
GTATGCCTATGAAAATTGTAATACAACTTTCAGCAACGGATCTCTTGGCTCTCGCATCGA
TGAAGAACGCAGCGAAATGCGATAAGTAATGTGAATTGCAGAATTCAGTGAATCATCGAA
TCTTTGAACGCATCTTGCGCTCCTTGGTATTCCGAGGAGCATGCCTGTTTGAGTGTCATT
AAATTCTCAACTCTCTTATACTTTTTTGTAAAAGAGAGCTTGGACTGTGGAGGCTTGCTG
GCCACTTTTTGGGGTCAGCTCCTCTGAAATGCATTAGCGGAACCGTTTGCGATCTGCCAC
AAGTGTGATAAGTTATCTACACTGGCGAGGGGATTGCTCTCTGTAATGTTCAGCTTCTAA

TTGTCTCTACTTTGTGAGACTACTTTTGAATGCTTGACCTCAAATCAGGTAGGACTACCC
GCTGAACTTAA
>A6_66
TTTCCGTAGGTGAACCTGCGGAAGGATCATTATTGAATTATGTTTCTAGATAGGTTGTAG
CTGGCTCTTTTAGAGCATGTGCACGCCTGTTTGGACTTCATTTTCATCCACCTGTGCACC
TATTGTAGTCTTTGGTTGGGTTAGGAGGAAGTGATCATTGTATCAGCATCTGCTGGGAGT
GAGGACTTGCATTGTGAAAGCTTTGCTGTCCTTGATGTGATCATGGAATCTTTTTCTCAC
TAGAGTCTATGTCACTCATTATACTCTGTCGAATGTCATTGAATGTCTTTACATGGGCTT
GTATGCCTATGAAAATTGTAATACAACTTTCAGCAACGGATCTCTTGGCTCTCGCATCGA
TGAAGAACGCAGCGAAATGCGATAAGTAATGTGAATTGCAGAATTCAGTGAATCATCGAA
TCTTTGAACGCATCTTGCGCTCCTTGGTATTCCGAGGAGCATGCCTGTTTGAGTGTCATT
AAATTCTCAACTCTCTTATACTTTTTTGTAAAAGAGAGCTTGGACTGTGGAGGCTTGCTG
GCCACTTTTTGGGGTCAGCTCCTCTGAAATGCATTAGCGGAACCGTTTGCGATCTGCCAC
AAGTGTGATAAGTTATCTACACTGGCGAGGGGATTGCTCTCTGTAATGTTCAGCTTCTAA
TTGTCTCTACTTTGTGAGACTACTTTTGAATGCTTGACCTCAAATCAGGTAGGACTACCC
GCTGAACTTAA
>A6_67
TTTCCGTAGGTGAACCTGCGGAAGGATCATTATTGAATTATGTTTCTAGATAGGTTGTAG
CTGGCTCTTTTAGAGCATGTGCACGCCTGTTTGGACTTCATTTTCATCCACCTGTGCACC
TATTGTAGTCTTTGGTTGGGTTAGGAGGAAGTGATCATTGTATCAGCATCTGCTGGGAGT
GAGGACTTGCATTGTGAAAGCTTTGCTGTCCTTGATGTGATCATGGAATCTTTTTCTCAC
TAGAGTCTATGTCACTCATTATACTCTGTCGAATGTCATTGAATGTCTTTACATGGGCTT
GTATGCCTATGAAAATTGTAATACAACTTTCAGCAACGGATCTCTTGGCTCTCGCATCGA
TGAAGAACGCAGCGAAATGCGATAAGTAATGTGAATTGCAGAATTCAGTGAATCATCGAA
TCTTTGAACGCATCTTGCGCTCCTTGGTATTCCGAGGAGCATGCCTGTTTGAGTGTCATT
AAATTCTCAACTCTCTTATACTTTTTTGTAAAAGAGAGCTTGGACTGTGGAGGCTTGCTG
GCCACTTTTTGGGGTCAGCTCCTCTGAAATGCATTAGCGGAACCGTTTGCGATCTGCCAC
AAGTGTGATAAGTTATCTACACTGGCGAGGGGATTGCTCTCTGTAATGTTCAGCTTCTAA
TTGTCTCTACTTTGTGAGACTACTTTTGAATGCTTGACCTCAAATCAGGTAGGACTACCC
GCTGAACTTAA
>A6_68
TTTCCGTAGGTGAACCTGCGGAAGGATCATTATTGAATTATGTTTCTAGATAGGTTGTAG
CTGGCTCTTTTAGAGCATGTGCACGCCTGTTTGGACTTCATTTTCATCCACCTGTGCACC
TATTGTAGTCTTTGGTTGGGTTAGGAGGAAGTGATCATTGTATCAGCATCTGCTGGGAGT
GAGGACTTGCATTGTGAAAGCTTTGCTGTCCTTGATGTGATCATGGAATCTTTTTCTCAC
TAGAGTCTATGTCACTCATTATACTCTGTCGAATGTCATTGAATGTCTTTACATGGGCTT
GTATGCCTATGAAAATTGTAATACAACTTTCAGCAACGGATCTCTTGGCTCTCGCATCGA
TGAAGAACGCAGCGAAATGCGATAAGTAATGTGAATTGCAGAATTCAGTGAATCATCGAA
TCTTTGAACGCATCTTGCGCTCCTTGGTATTCCGAGGAGCATGCCTGTTTGAGTGTCATT
AAATTCTCAACTCTCTTATACTTTTTTGTAAAAGAGAGCTTGGACTGTGGAGGCTTGCTG
GCCACTTTTTGGGGTCAGCTCCTCTGAAATGCATTAGCGGAACCGTTTGCGATCTGCCAC
AAGTGTGATAAGTTATCTACACTGGCGAGGGGATTGCTCTCTGTAATGTTCAGCTTCTAA
TTGTCTCTACTTTGTGAGACTACTTTTGAATGCTTGACCTCAAATCAGGTAGGACTACCC
GCTGAACTTAA
>A6_69
TTTCCGTAGGTGAACCTGCGGAAGGATCATTATTGAATTATGTTTCTAGATAGGTTGTAG
CTGGCTCTTTTAGAGCATGTGCACGCCTGTTTGGACTTCATTTTCATCCACCTGTGCACC
TATTGTAGTCTTTGGTTGGGTTAGGAGGAAGTGATCATTGTATCAGCATCTGCTGGGAGT
GAGGACTTGCATTGTGAAAGCTTTGCTGTCCTTGATGTGATCATGGAATCTTTTTCTCAC
TAGAGTCTATGTCACTCATTATACTCTGTCGAATGTCATTGAATGTCTTTACATGGGCTT

GTATGCCTATGAAAATTGTAATACAACTTTCAGCAACGGATCTCTTGGCTCTCGCATCGA
TGAAGAACGCAGCGAAATGCGATAAGTAATGTGAATTGCAGAATTCAGTGAATCATCGAA
TCTTTGAACGCATCTTGCGCTCCTTGGTATTCCGAGGAGCATGCCTGTTTGAGTGTCATT
AAATTCTCAACTCTCTTATACTTTTTTGTAAAAGAGAGCTTGGACTGTGGAGGCTTGCTG
GCCACTTTTTGGGGTCAGCTCCTCTGAAATGCATTAGCGGAACCGTTTGCGATCTGCCAC
AAGTGTGATAAGTTATCTACACTGGCGAGGGGATTGCTCTCTGTAATGTTCAGCTTCTAA
TTGTCTCTACTTTGTGAGACTACTTTTGAATGCTTGACCTCAAATCAGGTAGGACTACCC
GCTGAACTTAA
>A7_5
TTTCCGTAGGTGAACCTGCGGAAGGATCATTATTGAATTATGTTTCTAGATAGGTTGTAG
CTGGCTCTTTTAGAGCATGTGCACGCCTGTTTGGACTTCATTTTCATCCACCTGTGCACC
TATTGTAGTCTTTGGTTGGGTTAGGAGGAAGTGATCATTGTATCAGCATCTGCTGGGAGT
GAGGACTTGCATTGTGAAAGCTTTGCTGTCCTTGATGTGATCATGGAATCTTTTTCTCAC
TAGAGTCTATGTCACTCATTATACTCTGTCGAATGTCATTGAATGTCTTTACATGGGCTT
GTATGCCTATGAAAATTGTAATACAACTTTCAGCAACGGATCTCTTGGCTCTCGCATCGA
TGAAGAACGCAGCGAAATGCGATAAGTAATGTGAATTGCAGAATTCAGTGAATCATCGAA
TCTTTGAACGCATCTTGCGCTCCTTGGTATTCCGAGGAGCATGCCTGTTTGAGTGTCATT
AAATTCTCAACTCTCTTATACTTTTTTGTAAAAGAGAGCTTGGACTGTGGAGGCTTGCTG
GCCACTTTTTGGGGTCAGCTCCTCTGAAATGCATTAGCGGAACCGTTTGCGATCTGCCAC
AAGTGTGATAAGTTATCTACACTGGCGAGGGGATTGCTCTCTGTAATGTTCAGCTTCTAA
TTGTCTCTACTTTGTGAGACTACTTTTGAATGCTTGACCTCAAATCAGGTAGGACTACCC
GCTGAACTTAA
>A7_6
TTTCCGTAGGTGAACCTGCGGAAGGATCATTATTGAATTATGTTTCTAGATAGGTTGTAG
CTGGCTCTTTTAGAGCATGTGCACGCCTGTTTGGACTTCATTTTCATCCACCTGTGCACC
TATTGTAGTCTTTGGTTGGGTTAGGAGGAAGTGATCATTGTATCAGCATCTGCTGGGAGT
GAGGACTTGCATTGTGAAAGCTTTGCTGTCCTTGATGTGATCATGGAATCTTTTTCTCAC
TAGAGTCTATGTCACTCATTATACTCTGTCGAATGTCATTGAATGTCTTTACATGGGCTT
GTATGCCTATGAAAATTGTAATACAACTTTCAGCAACGGATCTCTTGGCTCTCGCATCGA
TGAAGAACGCAGCGAAATGCGATAAGTAATGTGAATTGCAGAATTCAGTGAATCATCGAA
TCTTTGAACGCATCTTGCGCTCCTTGGTATTCCGAGGAGCATGCCTGTTTGAGTGTCATT
AAATTCTCAACTCTCTTATACTTTTTTGTAAAAGAGAGCTTGGACTGTGGAGGCTTGCTG
GCCACTTTTTGGGGTCAGCTCCTCTGAAATGCATTAGCGGAACCGTTTGCGATCTGCCAC
AAGTGTGATAAGTTATCTACACTGGCGAGGGGATTGCTCTCTGTAATGTTCAGCTTCTAA
TTGTCTCTACTTTGTGAGACTACTTTTGAATGCTTGACCTCAAATCAGGTAGGACTACCC
GCTGAACTTAA
>A7_7
TTTCCGTAGGTGAACCTGCGGAAGGATCATTATTGAATTATGTTTCTAGATAGGTTGTAG
CTGGCTCTTTTAGAGCATGTGCACGCCTGTTTGGACTTCATTTTCATCCACCTGTGCACC
TATTGTAGTCTTTGGTTGGGTTAGGAGGAAGTGATCATTGTATCAGCATCTGCTGGGAGT
GAGGACTTGCATTGTGAAAGCTTTGCTGTCCTTGATGTGATCATGGAATCTTTTTCTCAC
TAGAGTCTATGTCACTCATTATACTCTGTCGAATGTCATTGAATGTCTTTACATGGGCTT
GTATGCCTATGAAAATTGTAATACAACTTTCAGCAACGGATCTCTTGGCTCTCGCATCGA
TGAAGAACGCAGCGAAATGCGATAAGTAATGTGAATTGCAGAATTCAGTGAATCATCGAA
TCTTTGAACGCATCTTGCGCTCCTTGGTATTCCGAGGAGCATGCCTGTTTGAGTGTCATT
AAATTCTCAACTCTCTTATACTTTTTTGTAAAAGAGAGCTTGGACTGTGGAGGCTTGCTG
GCCACTTTTTGGGGTCAGCTCCTCTGAAATGCATTAGCGGAACCGTTTGCGATCTGCCAC
AAGTGTGATAAGTTATCTACACTGGCGAGGGGATTGCTCTCTGTAATGTTCAGCTTCTAA
TTGTCTCTACTTTGTGAGACTACTTTTGAATGCTTGACCTCAAATCAGGTAGGACTACCC
GCTGAACTTAA

>A7_8
TTTCCGTAGGTGAACCTGCGGAAGGATCATTATTGAATTATGTTTCTAGATAGGTTGTAG
CTGGCTCTTTTAGAGCATGTGCACGCCTGTTTGGACTTCATTTTCATCCACCTGTGCACC
TATTGTAGTCTTTGGTTGGGTTAGGAGGAAGTGATCATTGTATCAGCATCTGCTGGGAGT
GAGGACTTGCATTGTGAAAGCTTTGCTGTCCTTGATGTGATCATGGAATCTTTTTCTCAC
TAGAGTCTATGTCACTCATTATACTCTGTCGAATGTCATTGAATGTCTTTACATGGGCTT
GTATGCCTATGAAAATTGTAATACAACTTTCAGCAACGGATCTCTTGGCTCTCGCATCGA
TGAAGAACGCAGCGAAATGCGATAAGTAATGTGAATTGCAGAATTCAGTGAATCATCGAA
TCTTTGAACGCATCTTGCGCTCCTTGGTATTCCGAGGAGCATGCCTGTTTGAGTGTCATT
AAATTCTCAACTCTCTTATACTTTTTTGTAAAAGAGAGCTTGGACTGTGGAGGCTTGCTG
GCCACTTTTTGGGGTCAGCTCCTCTGAAATGCATTAGCGGAACCGTTTGCGATCTGCCAC
AAGTGTGATAAGTTATCTACACTGGCGAGGGGATTGCTCTCTGTAATGTTCAGCTTCTAA
TTGTCTCTACTTTGTGAGACTACTTTTGAATGCTTGACCTCAAATCAGGTAGGACTACCC
GCTGAACTTAA
>A7_9
TTTCCGTAGGTGAACCTGCGGAAGGATCATTATTGAATTATGTTTCTAGATAGGTTGTAG
CTGGCTCTTTTAGAGCATGTGCACGCCTGTTTGGACTTCATTTTCATCCACCTGTGCACC
TATTGTAGTCTTTGGTTGGGTTAGGAGGAAGTGATCATTGTATCAGCATCTGCTGGGAGT
GAGGACTTGCATTGTGAAAGCTTTGCTGTCCTTGATGTGATCATGGAATCTTTTTCTCAC
TAGAGTCTATGTCACTCATTATACTCTGTCGAATGTCATTGAATGTCTTTACATGGGCTT
GTATGCCTATGAAAATTGTAATACAACTTTCAGCAACGGATCTCTTGGCTCTCGCATCGA
TGAAGAACGCAGCGAAATGCGATAAGTAATGTGAATTGCAGAATTCAGTGAATCATCGAA
TCTTTGAACGCATCTTGCGCTCCTTGGTATTCCGAGGAGCATGCCTGTTTGAGTGTCATT
AAATTCTCAACTCTCTTATACTTTTTTGTAAAAGAGAGCTTGGACTGTGGAGGCTTGCTG
GCCACTTTTTGGGGTCAGCTCCTCTGAAATGCATTAGCGGAACCGTTTGCGATCTGCCAC
AAGTGTGATAAGTTATCTACACTGGCGAGGGGATTGCTCTCTGTAATGTTCAGCTTCTAA
TTGTCTCTACTTTGTGAGACTACTTTTGAATGCTTGACCTCAAATCAGGTAGGACTACCC
GCTGAACTTAA
>A7_11
TTTCCGTAGGTGAACCTGCGGAAGGATCATTATTGAATTATGTTTCTAGATAGGTTGTAG
CTGGCTCTTTTAGAGCATGTGCACGCCTGTTTGGACTTCATTTTCATCCACCTGTGCACC
TATTGTAGTCTTTGGTTGGGTTAGGAGGAAGTGATCATTGTATCAGCATCTGCTGGGAGT
GAGGACTTGCATTGTGAAAGCTTTGCTGTCCTTGATGTGATCATGGAATCTTTTTCTCAC
TAGAGTCTATGTCACTCATTATACTCTGTCGAATGTCATTGAATGTCTTTACATGGGCTT
GTATGCCTATGAAAATTGTAATACAACTTTCAGCAACGGATCTCTTGGCTCTCGCATCGA
TGAAGAACGCAGCGAAATGCGATAAGTAATGTGAATTGCAGAATTCAGTGAATCATCGAA
TCTTTGAACGCATCTTGCGCTCCTTGGTATTCCGAGGAGCATGCCTGTTTGAGTGTCATT
AAATTCTCAACTCTCTTATACTTTTTTGTAAAAGAGAGCTTGGACTGTGGAGGCTTGCTG
GCCACTTTTTGGGGTCAGCTCCTCTGAAATGCATTAGCGGAACCGTTTGCGATCTGCCAC
AAGTGTGATAAGTTATCTACACTGGCGAGGGGATTGCTCTCTGTAATGTTCAGCTTCTAA
TTGTCTCTACTTTGTGAGACTACTTTTGAATGCTTGACCTCAAATCAGGTAGGACTACCC
GCTGAACTTAA
>A7_12
TTTCCGTAGGTGAACCTGCGGAAGGATCATTATTGAATTATGTTTCTAGATAGGTTGTAG
CTGGCTCTTTTAGAGCATGTGCACGCCTGTTTGGACTTCATTTTCATCCACCTGTGCACC
TATTGTAGTCTTTGGTTGGGTTAGGAGGAAGTGATCATTGTATCAGCATCTGCTGGGAGT
GAGGACTTGCATTGTGAAAGCTTTGCTGTCCTTGATGTGATCATGGAATCTTTTTCTCAC
TAGAGTCTATGTCACTCATTATACTCTGTCGAATGTCATTGAATGTCTTTACATGGGCTT
GTATGCCTATGAAAATTGTAATACAACTTTCAGCAACGGATCTCTTGGCTCTCGCATCGA
TGAAGAACGCAGCGAAATGCGATAAGTAATGTGAATTGCAGAATTCAGTGAATCATCGAA

TCTTTGAACGCATCTTGCGCTCCTTGGTATTCCGAGGAGCATGCCTGTTTGAGTGTCATT
AAATTCTCAACTCTCTTATACTTTTTTGTAAAAGAGAGCTTGGACTGTGGAGGCTTGCTG
GCCACTTTTTGGGGTCAGCTCCTCTGAAATGCATTAGCGGAACCGTTTGCGATCTGCCAC
AAGTGTGATAAGTTATCTACACTGGCGAGGGGATTGCTCTCTGTAATGTTCAGCTTCTAA
TTGTCTCTACTTTGTGAGACTACTTTTGAATGCTTGACCTCAAATCAGGTAGGACTACCC
GCTGAACTTAA
>A7_14
TTTCCGTAGGTGAACCTGCGGAAGGATCATTATTGAATTATGTTTCTAGATAGGTTGTAG
CTGGCTCTTTTAGAGCATGTGCACGCCTGTTTGGACTTCATTTTCATCCACCTGTGCACC
TATTGTAGTCTTTGGTTGGGTTAGGAGGAAGTGATCATTGTATCAGCATCTGCTGGGAGT
GAGGACTTGCATTGTGAAAGCTTTGCTGTCCTTGATGTGATCATGGAATCTTTTTCTCAC
TAGAGTCTATGTCACTCATTATACTCTGTCGAATGTCATTGAATGTCTTTACATGGGCTT
GTATGCCTATGAAAATTGTAATACAACTTTCAGCAACGGATCTCTTGGCTCTCGCATCGA
TGAAGAACGCAGCGAAATGCGATAAGTAATGTGAATTGCAGAATTCAGTGAATCATCGAA
TCTTTGAACGCATCTTGCGCTCCTTGGTATTCCGAGGAGCATGCCTGTTTGAGTGTCATT
AAATTCTCAACTCTCTTATACTTTTTTGTAAAAGAGAGCTTGGACTGTGGAGGCTTGCTG
GCCACTTTTTGGGGTCAGCTCCTCTGAAATGCATTAGCGGAACCGTTTGCGATCTGCCAC
AAGTGTGATAAGTTATCTACACTGGCGAGGGGATTGCTCTCTGTAATGTTCAGCTTCTAA
TTGTCTCTACTTTGTGAGACTACTTTTGAATGCTTGACCTCAAATCAGGTAGGACTACCC
GCTGAACTTAA
>A7_15
TTTCCGTAGGTGAACCTGCGGAAGGATCATTATTGAATTATGTTTCTAGATAGGTTGTAG
CTGGCTCTTTTAGAGCATGTGCACGCCTGTTTGGACTTCATTTTCATCCACCTGTGCACC
TATTGTAGTCTTTGGTTGGGTTAGGAGGAAGTGATCATTGTATCAGCATCTGCTGGGAGT
GAGGACTTGCATTGTGAAAGCTTTGCTGTCCTTGATGTGATCATGGAATCTTTTTCTCAC
TAGAGTCTATGTCACTCATTATACTCTGTCGAATGTCATTGAATGTCTTTACATGGGCTT
GTATGCCTATGAAAATTGTAATACAACTTTCAGCAACGGATCTCTTGGCTCTCGCATCGA
TGAAGAACGCAGCGAAATGCGATAAGTAATGTGAATTGCAGAATTCAGTGAATCATCGAA
TCTTTGAACGCATCTTGCGCTCCTTGGTATTCCGAGGAGCATGCCTGTTTGAGTGTCATT
AAATTCTCAACTCTCTTATACTTTTTTGTAAAAGAGAGCTTGGACTGTGGAGGCTTGCTG
GCCACTTTTTGGGGTCAGCTCCTCTGAAATGCATTAGCGGAACCGTTTGCGATCTGCCAC
AAGTGTGATAAGTTATCTACACTGGCGAGGGGATTGCTCTCTGTAATGTTCAGCTTCTAA
TTGTCTCTACTTTGTGAGACTACTTTTGAATGCTTGACCTCAAATCAGGTAGGACTACCC
GCTGAACTTAA
>A7_19
TTTCCGTAGGTGAACCTGCGGAAGGATCATTATTGAATTATGTTTCTAGATAGGTTGTAG
CTGGCTCTTTTAGAGCATGTGCACGCCTGTTTGGACTTCATTTTCATCCACCTGTGCACC
TATTGTAGTCTTTGGTTGGGTTAGGAGGAAGTGATCATTGTATCAGCATCTGCTGGGAGT
GAGGACTTGCATTGTGAAAGCTTTGCTGTCCTTGATGTGATCATGGAATCTTTTTCTCAC
TAGAGTCTATGTCACTCATTATACTCTGTCGAATGTCATTGAATGTCTTTACATGGGCTT
GTATGCCTATGAAAATTGTAATACAACTTTCAGCAACGGATCTCTTGGCTCTCGCATCGA
TGAAGAACGCAGCGAAATGCGATAAGTAATGTGAATTGCAGAATTCAGTGAATCATCGAA
TCTTTGAACGCATCTTGCGCTCCTTGGTATTCCGAGGAGCATGCCTGTTTGAGTGTCATT
AAATTCTCAACTCTCTTATACTTTTTTGTAAAAGAGAGCTTGGACTGTGGAGGCTTGCTG
GCCACTTTTTGGGGTCAGCTCCTCTGAAATGCATTAGCGGAACCGTTTGCGATCTGCCAC
AAGTGTGATAAGTTATCTACACTGGCGAGGGGATTGCTCTCTGTAATGTTCAGCTTCTAA
TTGTCTCTACTTTGTGAGACTACTTTTGAATGCTTGACCTCAAATCAGGTAGGACTACCC
GCTGAACTTAA
>A7_23
TTTCCGTAGGTGAACCTGCGGAAGGATCATTATTGAATTATGTTTCTAGATAGGTTGTAG

CTGGCTCTTTTAGAGCATGTGCACGCCTGTTTGGACTTCATTTTCATCCACCTGTGCACC
TATTGTAGTCTTTGGTTGGGTTAGGAGGAAGTGATCATTGTATCAGCATCTGCTGGGAGT
GAGGACTTGCATTGTGAAAGCTTTGCTGTCCTTGATGTGATCATGGAATCTTTTTCTCAC
TAGAGTCTATGTCACTCATTATACTCTGTCGAATGTCATTGAATGTCTTTACATGGGCTT
GTATGCCTATGAAAATTGTAATACAACTTTCAGCAACGGATCTCTTGGCTCTCGCATCGA
TGAAGAACGCAGCGAAATGCGATAAGTAATGTGAATTGCAGAATTCAGTGAATCATCGAA
TCTTTGAACGCATCTTGCGCTCCTTGGTATTCCGAGGAGCATGCCTGTTTGAGTGTCATT
AAATTCTCAACTCTCTTATACTTTTTTGTAAAAGAGAGCTTGGACTGTGGAGGCTTGCTG
GCCACTTTTTGGGGTCAGCTCCTCTGAAATGCATTAGCGGAACCGTTTGCGATCTGCCAC
AAGTGTGATAAGTTATCTACACTGGCGAGGGGATTGCTCTCTGTAATGTTCAGCTTCTAA
TTGTCTCTACTTTGTGAGACTACTTTTGAATGCTTGACCTCAAATCAGGTAGGACTACCC
GCTGAACTTAA
>A7_24
TTTCCGTAGGTGAACCTGCGGAAGGATCATTATTGAATTATGTTTCTAGATAGGTTGTAG
CTGGCTCTTTTAGAGCATGTGCACGCCTGTTTGGACTTCATTTTCATCCACCTGTGCACC
TATTGTAGTCTTTGGTTGGGTTAGGAGGAAGTGATCATTGTATCAGCATCTGCTGGGAGT
GAGGACTTGCATTGTGAAAGCTTTGCTGTCCTTGATGTGATCATGGAATCTTTTTCTCAC
TAGAGTCTATGTCACTCATTATACTCTGTCGAATGTCATTGAATGTCTTTACATGGGCTT
GTATGCCTATGAAAATTGTAATACAACTTTCAGCAACGGATCTCTTGGCTCTCGCATCGA
TGAAGAACGCAGCGAAATGCGATAAGTAATGTGAATTGCAGAATTCAGTGAATCATCGAA
TCTTTGAACGCATCTTGCGCTCCTTGGTATTCCGAGGAGCATGCCTGTTTGAGTGTCATT
AAATTCTCAACTCTCTTATACTTTTTTGTAAAAGAGAGCTTGGACTGTGGAGGCTTGCTG
GCCACTTTTTGGGGTCAGCTCCTCTGAAATGCATTAGCGGAACCGTTTGCGATCTGCCAC
AAGTGTGATAAGTTATCTACACTGGCGAGGGGATTGCTCTCTGTAATGTTCAGCTTCTAA
TTGTCTCTACTTTGTGAGACTACTTTTGAATGCTTGACCTCAAATCAGGTAGGACTACCC
GCTGAACTTAA
>A7_26
TTTCCGTAGGTGAACCTGCGGAAGGATCATTATTGAATTATGTTTCTAGATAGGTTGTAG
CTGGCTCTTTTAGAGCATGTGCACGCCTGTTTGGACTTCATTTTCATCCACCTGTGCACC
TATTGTAGTCTTTGGTTGGGTTAGGAGGAAGTGATCATTGTATCAGCATCTGCTGGGAGT
GAGGACTTGCATTGTGAAAGCTTTGCTGTCCTTGATGTGATCATGGAATCTTTTTCTCAC
TAGAGTCTATGTCACTCATTATACTCTGTCGAATGTCATTGAATGTCTTTACATGGGCTT
GTATGCCTATGAAAATTGTAATACAACTTTCAGCAACGGATCTCTTGGCTCTCGCATCGA
TGAAGAACGCAGCGAAATGCGATAAGTAATGTGAATTGCAGAATTCAGTGAATCATCGAA
TCTTTGAACGCATCTTGCGCTCCTTGGTATTCCGAGGAGCATGCCTGTTTGAGTGTCATT
AAATTCTCAACTCTCTTATACTTTTTTGTAAAAGAGAGCTTGGACTGTGGAGGCTTGCTG
GCCACTTTTTGGGGTCAGCTCCTCTGAAATGCATTAGCGGAACCGTTTGCGATCTGCCAC
AAGTGTGATAAGTTATCTACACTGGCGAGGGGATTGCTCTCTGTAATGTTCAGCTTCTAA
TTGTCTCTACTTTGTGAGACTACTTTTGAATGCTTGACCTCAAATCAGGTAGGACTACCC
GCTGAACTTAA
>A7_27
TTTCCGTAGGTGAACCTGCGGAAGGATCATTATTGAATTATGTTTCTAGATAGGTTGTAG
CTGGCTCTTTTAGAGCATGTGCACGCCTGTTTGGACTTCATTTTCATCCACCTGTGCACC
TATTGTAGTCTTTGGTTGGGTTAGGAGGAAGTGATCATTGTATCAGCATCTGCTGGGAGT
GAGGACTTGCATTGTGAAAGCTTTGCTGTCCTTGATGTGATCATGGAATCTTTTTCTCAC
TAGAGTCTATGTCACTCATTATACTCTGTCGAATGTCATTGAATGTCTTTACATGGGCTT
GTATGCCTATGAAAATTGTAATACAACTTTCAGCAACGGATCTCTTGGCTCTCGCATCGA
TGAAGAACGCAGCGAAATGCGATAAGTAATGTGAATTGCAGAATTCAGTGAATCATCGAA
TCTTTGAACGCATCTTGCGCTCCTTGGTATTCCGAGGAGCATGCCTGTTTGAGTGTCATT
AAATTCTCAACTCTCTTATACTTTTTTGTAAAAGAGAGCTTGGACTGTGGAGGCTTGCTG

GCCACTTTTTGGGGTCAGCTCCTCTGAAATGCATTAGCGGAACCGTTTGCGATCTGCCAC
AAGTGTGATAAGTTATCTACACTGGCGAGGGGATTGCTCTCTGTAATGTTCAGCTTCTAA
TTGTCTCTACTTTGTGAGACTACTTTTGAATGCTTGACCTCAAATCAGGTAGGACTACCC
GCTGAACTTAA
>A7_28
TTTCCGTAGGTGAACCTGCGGAAGGATCATTATTGAATTATGTTTCTAGATAGGTTGTAG
CTGGCTCTTTTAGAGCATGTGCACGCCTGTTTGGACTTCATTTTCATCCACCTGTGCACC
TATTGTAGTCTTTGGTTGGGTTAGGAGGAAGTGATCATTGTATCAGCATCTGCTGGGAGT
GAGGACTTGCATTGTGAAAGCTTTGCTGTCCTTGATGTGATCATGGAATCTTTTTCTCAC
TAGAGTCTATGTCACTCATTATACTCTGTCGAATGTCATTGAATGTCTTTACATGGGCTT
GTATGCCTATGAAAATTGTAATACAACTTTCAGCAACGGATCTCTTGGCTCTCGCATCGA
TGAAGAACGCAGCGAAATGCGATAAGTAATGTGAATTGCAGAATTCAGTGAATCATCGAA
TCTTTGAACGCATCTTGCGCTCCTTGGTATTCCGAGGAGCATGCCTGTTTGAGTGTCATT
AAATTCTCAACTCTCTTATACTTTTTTGTAAAAGAGAGCTTGGACTGTGGAGGCTTGCTG
GCCACTTTTTGGGGTCAGCTCCTCTGAAATGCATTAGCGGAACCGTTTGCGATCTGCCAC
AAGTGTGATAAGTTATCTACACTGGCGAGGGGATTGCTCTCTGTAATGTTCAGCTTCTAA
TTGTCTCTACTTTGTGAGACTACTTTTGAATGCTTGACCTCAAATCAGGTAGGACTACCC
GCTGAACTTAA
>A7_30
TTTCCGTAGGTGAACCTGCGGAAGGATCATTATTGAATTATGTTTCTAGATAGGTTGTAG
CTGGCTCTTTTAGAGCATGTGCACGCCTGTTTGGACTTCATTTTCATCCACCTGTGCACC
TATTGTAGTCTTTGGTTGGGTTAGGAGGAAGTGATCATTGTATCAGCATCTGCTGGGAGT
GAGGACTTGCATTGTGAAAGCTTTGCTGTCCTTGATGTGATCATGGAATCTTTTTCTCAC
TAGAGTCTATGTCACTCATTATACTCTGTCGAATGTCATTGAATGTCTTTACATGGGCTT
GTATGCCTATGAAAATTGTAATACAACTTTCAGCAACGGATCTCTTGGCTCTCGCATCGA
TGAAGAACGCAGCGAAATGCGATAAGTAATGTGAATTGCAGAATTCAGTGAATCATCGAA
TCTTTGAACGCATCTTGCGCTCCTTGGTATTCCGAGGAGCATGCCTGTTTGAGTGTCATT
AAATTCTCAACTCTCTTATACTTTTTTGTAAAAGAGAGCTTGGACTGTGGAGGCTTGCTG
GCCACTTTTTGGGGTCAGCTCCTCTGAAATGCATTAGCGGAACCGTTTGCGATCTGCCAC
AAGTGTGATAAGTTATCTACACTGGCGAGGGGATTGCTCTCTGTAATGTTCAGCTTCTAA
TTGTCTCTACTTTGTGAGACTACTTTTGAATGCTTGACCTCAAATCAGGTAGGACTACCC
GCTGAACTTAA
>A7_32
TTTCCGTAGGTGAACCTGCGGAAGGATCATTATTGAATTATGTTTCTAGATAGGTTGTAG
CTGGCTCTTTTAGAGCATGTGCACGCCTGTTTGGACTTCATTTTCATCCACCTGTGCACC
TATTGTAGTCTTTGGTTGGGTTAGGAGGAAGTGATCATTGTATCAGCATCTGCTGGGAGT
GAGGACTTGCATTGTGAAAGCTTTGCTGTCCTTGATGTGATCATGGAATCTTTTTCTCAC
TAGAGTCTATGTCACTCATTATACTCTGTCGAATGTCATTGAATGTCTTTACATGGGCTT
GTATGCCTATGAAAATTGTAATACAACTTTCAGCAACGGATCTCTTGGCTCTCGCATCGA
TGAAGAACGCAGCGAAATGCGATAAGTAATGTGAATTGCAGAATTCAGTGAATCATCGAA
TCTTTGAACGCATCTTGCGCTCCTTGGTATTCCGAGGAGCATGCCTGTTTGAGTGTCATT
AAATTCTCAACTCTCTTATACTTTTTTGTAAAAGAGAGCTTGGACTGTGGAGGCTTGCTG
GCCACTTTTTGGGGTCAGCTCCTCTGAAATGCATTAGCGGAACCGTTTGCGATCTGCCAC
AAGTGTGATAAGTTATCTACACTGGCGAGGGGATTGCTCTCTGTAATGTTCAGCTTCTAA
TTGTCTCTACTTTGTGAGACTACTTTTGAATGCTTGACCTCAAATCAGGTAGGACTACCC
GCTGAACTTAA
>A7_33
TTTCCGTAGGTGAACCTGCGGAAGGATCATTATTGAATTATGTTTCTAGATAGGTTGTAG
CTGGCTCTTTTAGAGCATGTGCACGCCTGTTTGGACTTCATTTTCATCCACCTGTGCACC
TATTGTAGTCTTTGGTTGGGTTAGGAGGAAGTGATCATTGTATCAGCATCTGCTGGGAGT

GAGGACTTGCATTGTGAAAGCTTTGCTGTCCTTGATGTGATCATGGAATCTTTTTCTCAC
TAGAGTCTATGTCACTCATTATACTCTGTCGAATGTCATTGAATGTCTTTACATGGGCTT
GTATGCCTATGAAAATTGTAATACAACTTTCAGCAACGGATCTCTTGGCTCTCGCATCGA
TGAAGAACGCAGCGAAATGCGATAAGTAATGTGAATTGCAGAATTCAGTGAATCATCGAA
TCTTTGAACGCATCTTGCGCTCCTTGGTATTCCGAGGAGCATGCCTGTTTGAGTGTCATT
AAATTCTCAACTCTCTTATACTTTTTTGTAAAAGAGAGCTTGGACTGTGGAGGCTTGCTG
GCCACTTTTTGGGGTCAGCTCCTCTGAAATGCATTAGCGGAACCGTTTGCGATCTGCCAC
AAGTGTGATAAGTTATCTACACTGGCGAGGGGATTGCTCTCTGTAATGTTCAGCTTCTAA
TTGTCTCTACTTTGTGAGACTACTTTTGAATGCTTGACCTCAAATCAGGTAGGACTACCC
GCTGAACTTAA
>A7_37
TTTCCGTAGGTGAACCTGCGGAAGGATCATTATTGAATTATGTTTCTAGATAGGTTGTAG
CTGGCTCTTTTAGAGCATGTGCACGCCTGTTTGGACTTCATTTTCATCCACCTGTGCACC
TATTGTAGTCTTTGGTTGGGTTAGGAGGAAGTGATCATTGTATCAGCATCTGCTGGGAGT
GAGGACTTGCATTGTGAAAGCTTTGCTGTCCTTGATGTGATCATGGAATCTTTTTCTCAC
TAGAGTCTATGTCACTCATTATACTCTGTCGAATGTCATTGAATGTCTTTACATGGGCTT
GTATGCCTATGAAAATTGTAATACAACTTTCAGCAACGGATCTCTTGGCTCTCGCATCGA
TGAAGAACGCAGCGAAATGCGATAAGTAATGTGAATTGCAGAATTCAGTGAATCATCGAA
TCTTTGAACGCATCTTGCGCTCCTTGGTATTCCGAGGAGCATGCCTGTTTGAGTGTCATT
AAATTCTCAACTCTCTTATACTTTTTTGTAAAAGAGAGCTTGGACTGTGGAGGCTTGCTG
GCCACTTTTTGGGGTCAGCTCCTCTGAAATGCATTAGCGGAACCGTTTGCGATCTGCCAC
AAGTGTGATAAGTTATCTACACTGGCGAGGGGATTGCTCTCTGTAATGTTCAGCTTCTAA
TTGTCTCTACTTTGTGAGACTACTTTTGAATGCTTGACCTCAAATCAGGTAGGACTACCC
GCTGAACTTAA
>A7_38
TTTCCGTAGGTGAACCTGCGGAAGGATCATTATTGAATTATGTTTCTAGATAGGTTGTAG
CTGGCTCTTTTAGAGCATGTGCACGCCTGTTTGGACTTCATTTTCATCCACCTGTGCACC
TATTGTAGTCTTTGGTTGGGTTAGGAGGAAGTGATCATTGTATCAGCATCTGCTGGGAGT
GAGGACTTGCATTGTGAAAGCTTTGCTGTCCTTGATGTGATCATGGAATCTTTTTCTCAC
TAGAGTCTATGTCACTCATTATACTCTGTCGAATGTCATTGAATGTCTTTACATGGGCTT
GTATGCCTATGAAAATTGTAATACAACTTTCAGCAACGGATCTCTTGGCTCTCGCATCGA
TGAAGAACGCAGCGAAATGCGATAAGTAATGTGAATTGCAGAATTCAGTGAATCATCGAA
TCTTTGAACGCATCTTGCGCTCCTTGGTATTCCGAGGAGCATGCCTGTTTGAGTGTCATT
AAATTCTCAACTCTCTTATACTTTTTTGTAAAAGAGAGCTTGGACTGTGGAGGCTTGCTG
GCCACTTTTTGGGGTCAGCTCCTCTGAAATGCATTAGCGGAACCGTTTGCGATCTGCCAC
AAGTGTGATAAGTTATCTACACTGGCGAGGGGATTGCTCTCTGTAATGTTCAGCTTCTAA
TTGTCTCTACTTTGTGAGACTACTTTTGAATGCTTGACCTCAAATCAGGTAGGACTACCC
GCTGAACTTAA
>A7_39
TTTCCGTAGGTGAACCTGCGGAAGGATCATTATTGAATTATGTTTCTAGATAGGTTGTAG
CTGGCTCTTTTAGAGCATGTGCACGCCTGTTTGGACTTCATTTTCATCCACCTGTGCACC
TATTGTAGTCTTTGGTTGGGTTAGGAGGAAGTGATCATTGTATCAGCATCTGCTGGGAGT
GAGGACTTGCATTGTGAAAGCTTTGCTGTCCTTGATGTGATCATGGAATCTTTTTCTCAC
TAGAGTCTATGTCACTCATTATACTCTGTCGAATGTCATTGAATGTCTTTACATGGGCTT
GTATGCCTATGAAAATTGTAATACAACTTTCAGCAACGGATCTCTTGGCTCTCGCATCGA
TGAAGAACGCAGCGAAATGCGATAAGTAATGTGAATTGCAGAATTCAGTGAATCATCGAA
TCTTTGAACGCATCTTGCGCTCCTTGGTATTCCGAGGAGCATGCCTGTTTGAGTGTCATT
AAATTCTCAACTCTCTTATACTTTTTTGTAAAAGAGAGCTTGGACTGTGGAGGCTTGCTG
GCCACTTTTTGGGGTCAGCTCCTCTGAAATGCATTAGCGGAACCGTTTGCGATCTGCCAC
AAGTGTGATAAGTTATCTACACTGGCGAGGGGATTGCTCTCTGTAATGTTCAGCTTCTAA

TTGTCTCTACTTTGTGAGACTACTTTTGAATGCTTGACCTCAAATCAGGTAGGACTACCC
GCTGAACTTAA
>A7_65
TTTCCGTAGGTGAACCTGCGGAAGGATCATTATTGAATTATGTTTCTAGATAGGTTGTAG
CTGGCTCTTTTAGAGCATGTGCACGCCTGTTTGGACTTCATTTTCATCCACCTGTGCACC
TATTGTAGTCTTTGGTTGGGTTAGGAGGAAGTGATCATTGTATCAGCATCTGCTGGGAGT
GAGGACTTGCATTGTGAAAGCTTTGCTGTCCTTGATGTGATCATGGAATCTTTTTCTCAC
TAGAGTCTATGTCACTCATTATACTCTGTCGAATGTCATTGAATGTCTTTACATGGGCTT
GTATGCCTATGAAAATTGTAATACAACTTTCAGCAACGGATCTCTTGGCTCTCGCATCGA
TGAAGAACGCAGCGAAATGCGATAAGTAATGTGAATTGCAGAATTCAGTGAATCATCGAA
TCTTTGAACGCATCTTGCGCTCCTTGGTATTCCGAGGAGCATGCCTGTTTGAGTGTCATT
AAATTCTCAACTCTCTTATACTTTTTTGTAAAAGAGAGCTTGGACTGTGGAGGCTTGCTG
GCCACTTTTTGGGGTCAGCTCCTCTGAAATGCATTAGCGGAACCGTTTGCGATCTGCCAC
AAGTGTGATAAGTTATCTACACTGGCGAGGGGATTGCTCTCTGTAATGTTCAGCTTCTAA
TTGTCTCTACTTTGTGAGACTACTTTTGAATGCTTGACCTCAAATCAGGTAGGACTACCC
GCTGAACTTAA
>A7_66
TTTCCGTAGGTGAACCTGCGGAAGGATCATTATTGAATTATGTTTCTAGATAGGTTGTAG
CTGGCTCTTTTAGAGCATGTGCACGCCTGTTTGGACTTCATTTTCATCCACCTGTGCACC
TATTGTAGTCTTTGGTTGGGTTAGGAGGAAGTGATCATTGTATCAGCATCTGCTGGGAGT
GAGGACTTGCATTGTGAAAGCTTTGCTGTCCTTGATGTGATCATGGAATCTTTTTCTCAC
TAGAGTCTATGTCACTCATTATACTCTGTCGAATGTCATTGAATGTCTTTACATGGGCTT
GTATGCCTATGAAAATTGTAATACAACTTTCAGCAACGGATCTCTTGGCTCTCGCATCGA
TGAAGAACGCAGCGAAATGCGATAAGTAATGTGAATTGCAGAATTCAGTGAATCATCGAA
TCTTTGAACGCATCTTGCGCTCCTTGGTATTCCGAGGAGCATGCCTGTTTGAGTGTCATT
AAATTCTCAACTCTCTTATACTTTTTTGTAAAAGAGAGCTTGGACTGTGGAGGCTTGCTG
GCCACTTTTTGGGGTCAGCTCCTCTGAAATGCATTAGCGGAACCGTTTGCGATCTGCCAC
AAGTGTGATAAGTTATCTACACTGGCGAGGGGATTGCTCTCTGTAATGTTCAGCTTCTAA
TTGTCTCTACTTTGTGAGACTACTTTTGAATGCTTGACCTCAAATCAGGTAGGACTACCC
GCTGAACTTAA
>A9_18
TTTCCGTAGGTGAACCTGCGGAAGGATCATTATTGAATTATGTTTCTAGATAGGTTGTAG
CTGGCTCTTTTAGAGCATGTGCACGCCTGTTTGGACTTCATTTTCATCCACCTGTGCACC
TATTGTAGTCTTTGGTTGGGTTAGGAGGAAGTGATCATTGTATCAGCATCTGCTGGGAGT
GAGGACTTGCATTGTGAAAGCTTTGCTGTCCTTGATGTGATCATGGAATCTTTTTCTCAC
TAGAGTCTATGTCACTCATTATACTCTGTCGAATGTCATTGAATGTCTTTACATGGGCTT
GTATGCCTATGAAAATTGTAATACAACTTTCAGCAACGGATCTCTTGGCTCTCGCATCGA
TGAAGAACGCAGCGAAATGCGATAAGTAATGTGAATTGCAGAATTCAGTGAATCATCGAA
TCTTTGAACGCATCTTGCGCTCCTTGGTATTCCGAGGAGCATGCCTGTTTGAGTGTCATT
AAATTCTCAACTCTCTTATACTTTTTTGTAAAAGAGAGCTTGGACTGTGGAGGCTTGCTG
GCCACTTTTTGGGGTCAGCTCCTCTGAAATGCATTAGCGGAACCGTTTGCGATCTGCCAC
AAGTGTGATAAGTTATCTACACTGGCGAGGGGATTGCTCTCTGTAATGTTCAGCTTCTAA
TTGTCTCTACTTTGTGAGACTACTTTTGAATGCTTGACCTCAAATCAGGTAGGACTACCC
GCTGAACTTAA
>A9_19
TTTCCGTAGGTGAACCTGCGGAAGGATCATTATTGAATTATGTTTCTAGATAGGTTGTAG
CTGGCTCTTTTAGAGCATGTGCACGCCTGTTTGGACTTCATTTTCATCCACCTGTGCACC
TATTGTAGTCTTTGGTTGGGTTAGGAGGAAGTGATCATTGTATCAGCATCTGCTGGGAGT
GAGGACTTGCATTGTGAAAGCTTTGCTGTCCTTGATGTGATCATGGAATCTTTTTCTCAC
TAGAGTCTATGTCACTCATTATACTCTGTCGAATGTCATTGAATGTCTTTACATGGGCTT

GTATGCCTATGAAAATTGTAATACAACTTTCAGCAACGGATCTCTTGGCTCTCGCATCGA
TGAAGAACGCAGCGAAATGCGATAAGTAATGTGAATTGCAGAATTCAGTGAATCATCGAA
TCTTTGAACGCATCTTGCGCTCCTTGGTATTCCGAGGAGCATGCCTGTTTGAGTGTCATT
AAATTCTCAACTCTCTTATACTTTTTTGTAAAAGAGAGCTTGGACTGTGGAGGCTTGCTG
GCCACTTTTTGGGGTCAGCTCCTCTGAAATGCATTAGCGGAACCGTTTGCGATCTGCCAC
AAGTGTGATAAGTTATCTACACTGGCGAGGGGATTGCTCTCTGTAATGTTCAGCTTCTAA
TTGTCTCTACTTTGTGAGACTACTTTTGAATGCTTGACCTCAAATCAGGTAGGACTACCC
GCTGAACTTAA
>A9_21
TTTCCGTAGGTGAACCTGCGGAAGGATCATTATTGAATTATGTTTCTAGATAGGTTGTAG
CTGGCTCTTTTAGAGCATGTGCACGCCTGTTTGGACTTCATTTTCATCCACCTGTGCACC
TATTGTAGTCTTTGGTTGGGTTAGGAGGAAGTGATCATTGTATCAGCATCTGCTGGGAGT
GAGGACTTGCATTGTGAAAGCTTTGCTGTCCTTGATGTGATCATGGAATCTTTTTCTCAC
TAGAGTCTATGTCACTCATTATACTCTGTCGAATGTCATTGAATGTCTTTACATGGGCTT
GTATGCCTATGAAAATTGTAATACAACTTTCAGCAACGGATCTCTTGGCTCTCGCATCGA
TGAAGAACGCAGCGAAATGCGATAAGTAATGTGAATTGCAGAATTCAGTGAATCATCGAA
TCTTTGAACGCATCTTGCGCTCCTTGGTATTCCGAGGAGCATGCCTGTTTGAGTGTCATT
AAATTCTCAACTCTCTTATACTTTTTTGTAAAAGAGAGCTTGGACTGTGGAGGCTTGCTG
GCCACTTTTTGGGGTCAGCTCCTCTGAAATGCATTAGCGGAACCGTTTGCGATCTGCCAC
AAGTGTGATAAGTTATCTACACTGGCGAGGGGATTGCTCTCTGTAATGTTCAGCTTCTAA
TTGTCTCTACTTTGTGAGACTACTTTTGAATGCTTGACCTCAAATCAGGTAGGACTACCC
GCTGAACTTAA
>A9_22
TTTCCGTAGGTGAACCTGCGGAAGGATCATTATTGAATTATGTTTCTAGATAGGTTGTAG
CTGGCTCTTTTAGAGCATGTGCACGCCTGTTTGGACTTCATTTTCATCCACCTGTGCACC
TATTGTAGTCTTTGGTTGGGTTAGGAGGAAGTGATCATTGTATCAGCATCTGCTGGGAGT
GAGGACTTGCATTGTGAAAGCTTTGCTGTCCTTGATGTGATCATGGAATCTTTTTCTCAC
TAGAGTCTATGTCACTCATTATACTCTGTCGAATGTCATTGAATGTCTTTACATGGGCTT
GTATGCCTATGAAAATTGTAATACAACTTTCAGCAACGGATCTCTTGGCTCTCGCATCGA
TGAAGAACGCAGCGAAATGCGATAAGTAATGTGAATTGCAGAATTCAGTGAATCATCGAA
TCTTTGAACGCATCTTGCGCTCCTTGGTATTCCGAGGAGCATGCCTGTTTGAGTGTCATT
AAATTCTCAACTCTCTTATACTTTTTTGTAAAAGAGAGCTTGGACTGTGGAGGCTTGCTG
GCCACTTTTTGGGGTCAGCTCCTCTGAAATGCATTAGCGGAACCGTTTGCGATCTGCCAC
AAGTGTGATAAGTTATCTACACTGGCGAGGGGATTGCTCTCTGTAATGTTCAGCTTCTAA
TTGTCTCTACTTTGTGAGACTACTTTTGAATGCTTGACCTCAAATCAGGTAGGACTACCC
GCTGAACTTAA
>A9_23
TTTCCGTAGGTGAACCTGCGGAAGGATCATTATTGAATTATGTTTCTAGATAGGTTGTAG
CTGGCTCTTTTAGAGCATGTGCACGCCTGTTTGGACTTCATTTTCATCCACCTGTGCACC
TATTGTAGTCTTTGGTTGGGTTAGGAGGAAGTGATCATTGTATCAGCATCTGCTGGGAGT
GAGGACTTGCATTGTGAAAGCTTTGCTGTCCTTGATGTGATCATGGAATCTTTTTCTCAC
TAGAGTCTATGTCACTCATTATACTCTGTCGAATGTCATTGAATGTCTTTACATGGGCTT
GTATGCCTATGAAAATTGTAATACAACTTTCAGCAACGGATCTCTTGGCTCTCGCATCGA
TGAAGAACGCAGCGAAATGCGATAAGTAATGTGAATTGCAGAATTCAGTGAATCATCGAA
TCTTTGAACGCATCTTGCGCTCCTTGGTATTCCGAGGAGCATGCCTGTTTGAGTGTCATT
AAATTCTCAACTCTCTTATACTTTTTTGTAAAAGAGAGCTTGGACTGTGGAGGCTTGCTG
GCCACTTTTTGGGGTCAGCTCCTCTGAAATGCATTAGCGGAACCGTTTGCGATCTGCCAC
AAGTGTGATAAGTTATCTACACTGGCGAGGGGATTGCTCTCTGTAATGTTCAGCTTCTAA
TTGTCTCTACTTTGTGAGACTACTTTTGAATGCTTGACCTCAAATCAGGTAGGACTACCC
GCTGAACTTAA

>A9_24
TTTCCGTAGGTGAACCTGCGGAAGGATCATTATTGAATTATGTTTCTAGATAGGTTGTAG
CTGGCTCTTTTAGAGCATGTGCACGCCTGTTTGGACTTCATTTTCATCCACCTGTGCACC
TATTGTAGTCTTTGGTTGGGTTAGGAGGAAGTGATCATTGTATCAGCATCTGCTGGGAGT
GAGGACTTGCATTGTGAAAGCTTTGCTGTCCTTGATGTGATCATGGAATCTTTTTCTCAC
TAGAGTCTATGTCACTCATTATACTCTGTCGAATGTCATTGAATGTCTTTACATGGGCTT
GTATGCCTATGAAAATTGTAATACAACTTTCAGCAACGGATCTCTTGGCTCTCGCATCGA
TGAAGAACGCAGCGAAATGCGATAAGTAATGTGAATTGCAGAATTCAGTGAATCATCGAA
TCTTTGAACGCATCTTGCGCTCCTTGGTATTCCGAGGAGCATGCCTGTTTGAGTGTCATT
AAATTCTCAACTCTCTTATACTTTTTTGTAAAAGAGAGCTTGGACTGTGGAGGCTTGCTG
GCCACTTTTTGGGGTCAGCTCCTCTGAAATGCATTAGCGGAACCGTTTGCGATCTGCCAC
AAGTGTGATAAGTTATCTACACTGGCGAGGGGATTGCTCTCTGTAATGTTCAGCTTCTAA
TTGTCTCTACTTTGTGAGACTACTTTTGAATGCTTGACCTCAAATCAGGTAGGACTACCC
GCTGAACTTAA
>A9_25
TTTCCGTAGGTGAACCTGCGGAAGGATCATTATTGAATTATGTTTCTAGATAGGTTGTAG
CTGGCTCTTTTAGAGCATGTGCACGCCTGTTTGGACTTCATTTTCATCCACCTGTGCACC
TATTGTAGTCTTTGGTTGGGTTAGGAGGAAGTGATCATTGTATCAGCATCTGCTGGGAGT
GAGGACTTGCATTGTGAAAGCTTTGCTGTCCTTGATGTGATCATGGAATCTTTTTCTCAC
TAGAGTCTATGTCACTCATTATACTCTGTCGAATGTCATTGAATGTCTTTACATGGGCTT
GTATGCCTATGAAAATTGTAATACAACTTTCAGCAACGGATCTCTTGGCTCTCGCATCGA
TGAAGAACGCAGCGAAATGCGATAAGTAATGTGAATTGCAGAATTCAGTGAATCATCGAA
TCTTTGAACGCATCTTGCGCTCCTTGGTATTCCGAGGAGCATGCCTGTTTGAGTGTCATT
AAATTCTCAACTCTCTTATACTTTTTTGTAAAAGAGAGCTTGGACTGTGGAGGCTTGCTG
GCCACTTTTTGGGGTCAGCTCCTCTGAAATGCATTAGCGGAACCGTTTGCGATCTGCCAC
AAGTGTGATAAGTTATCTACACTGGCGAGGGGATTGCTCTCTGTAATGTTCAGCTTCTAA
TTGTCTCTACTTTGTGAGACTACTTTTGAATGCTTGACCTCAAATCAGGTAGGACTACCC
GCTGAACTTAA
>A9_27
TTTCCGTAGGTGAACCTGCGGAAGGATCATTATTGAATTATGTTTCTAGATAGGTTGTAG
CTGGCTCTTTTAGAGCATGTGCACGCCTGTTTGGACTTCATTTTCATCCACCTGTGCACC
TATTGTAGTCTTTGGTTGGGTTAGGAGGAAGTGATCATTGTATCAGCATCTGCTGGGAGT
GAGGACTTGCATTGTGAAAGCTTTGCTGTCCTTGATGTGATCATGGAATCTTTTTCTCAC
TAGAGTCTATGTCACTCATTATACTCTGTCGAATGTCATTGAATGTCTTTACATGGGCTT
GTATGCCTATGAAAATTGTAATACAACTTTCAGCAACGGATCTCTTGGCTCTCGCATCGA
TGAAGAACGCAGCGAAATGCGATAAGTAATGTGAATTGCAGAATTCAGTGAATCATCGAA
TCTTTGAACGCATCTTGCGCTCCTTGGTATTCCGAGGAGCATGCCTGTTTGAGTGTCATT
AAATTCTCAACTCTCTTATACTTTTTTGTAAAAGAGAGCTTGGACTGTGGAGGCTTGCTG
GCCACTTTTTGGGGTCAGCTCCTCTGAAATGCATTAGCGGAACCGTTTGCGATCTGCCAC
AAGTGTGATAAGTTATCTACACTGGCGAGGGGATTGCTCTCTGTAATGTTCAGCTTCTAA
TTGTCTCTACTTTGTGAGACTACTTTTGAATGCTTGACCTCAAATCAGGTAGGACTACCC
GCTGAACTTAA
>A9_28
TTTCCGTAGGTGAACCTGCGGAAGGATCATTATTGAATTATGTTTCTAGATAGGTTGTAG
CTGGCTCTTTTAGAGCATGTGCACGCCTGTTTGGACTTCATTTTCATCCACCTGTGCACC
TATTGTAGTCTTTGGTTGGGTTAGGAGGAAGTGATCATTGTATCAGCATCTGCTGGGAGT
GAGGACTTGCATTGTGAAAGCTTTGCTGTCCTTGATGTGATCATGGAATCTTTTTCTCAC
TAGAGTCTATGTCACTCATTATACTCTGTCGAATGTCATTGAATGTCTTTACATGGGCTT
GTATGCCTATGAAAATTGTAATACAACTTTCAGCAACGGATCTCTTGGCTCTCGCATCGA
TGAAGAACGCAGCGAAATGCGATAAGTAATGTGAATTGCAGAATTCAGTGAATCATCGAA

TCTTTGAACGCATCTTGCGCTCCTTGGTATTCCGAGGAGCATGCCTGTTTGAGTGTCATT
AAATTCTCAACTCTCTTATACTTTTTTGTAAAAGAGAGCTTGGACTGTGGAGGCTTGCTG
GCCACTTTTTGGGGTCAGCTCCTCTGAAATGCATTAGCGGAACCGTTTGCGATCTGCCAC
AAGTGTGATAAGTTATCTACACTGGCGAGGGGATTGCTCTCTGTAATGTTCAGCTTCTAA
TTGTCTCTACTTTGTGAGACTACTTTTGAATGCTTGACCTCAAATCAGGTAGGACTACCC
GCTGAACTTAA
>A9_30
TTTCCGTAGGTGAACCTGCGGAAGGATCATTATTGAATTATGTTTCTAGATAGGTTGTAG
CTGGCTCTTTTAGAGCATGTGCACGCCTGTTTGGACTTCATTTTCATCCACCTGTGCACC
TATTGTAGTCTTTGGTTGGGTTAGGAGGAAGTGATCATTGTATCAGCATCTGCTGGGAGT
GAGGACTTGCATTGTGAAAGCTTTGCTGTCCTTGATGTGATCATGGAATCTTTTTCTCAC
TAGAGTCTATGTCACTCATTATACTCTGTCGAATGTCATTGAATGTCTTTACATGGGCTT
GTATGCCTATGAAAATTGTAATACAACTTTCAGCAACGGATCTCTTGGCTCTCGCATCGA
TGAAGAACGCAGCGAAATGCGATAAGTAATGTGAATTGCAGAATTCAGTGAATCATCGAA
TCTTTGAACGCATCTTGCGCTCCTTGGTATTCCGAGGAGCATGCCTGTTTGAGTGTCATT
AAATTCTCAACTCTCTTATACTTTTTTGTAAAAGAGAGCTTGGACTGTGGAGGCTTGCTG
GCCACTTTTTGGGGTCAGCTCCTCTGAAATGCATTAGCGGAACCGTTTGCGATCTGCCAC
AAGTGTGATAAGTTATCTACACTGGCGAGGGGATTGCTCTCTGTAATGTTCAGCTTCTAA
TTGTCTCTACTTTGTGAGACTACTTTTGAATGCTTGACCTCAAATCAGGTAGGACTACCC
GCTGAACTTAA
>A9_31
TTTCCGTAGGTGAACCTGCGGAAGGATCATTATTGAATTATGTTTCTAGATAGGTTGTAG
CTGGCTCTTTTAGAGCATGTGCACGCCTGTTTGGACTTCATTTTCATCCACCTGTGCACC
TATTGTAGTCTTTGGTTGGGTTAGGAGGAAGTGATCATTGTATCAGCATCTGCTGGGAGT
GAGGACTTGCATTGTGAAAGCTTTGCTGTCCTTGATGTGATCATGGAATCTTTTTCTCAC
TAGAGTCTATGTCACTCATTATACTCTGTCGAATGTCATTGAATGTCTTTACATGGGCTT
GTATGCCTATGAAAATTGTAATACAACTTTCAGCAACGGATCTCTTGGCTCTCGCATCGA
TGAAGAACGCAGCGAAATGCGATAAGTAATGTGAATTGCAGAATTCAGTGAATCATCGAA
TCTTTGAACGCATCTTGCGCTCCTTGGTATTCCGAGGAGCATGCCTGTTTGAGTGTCATT
AAATTCTCAACTCTCTTATACTTTTTTGTAAAAGAGAGCTTGGACTGTGGAGGCTTGCTG
GCCACTTTTTGGGGTCAGCTCCTCTGAAATGCATTAGCGGAACCGTTTGCGATCTGCCAC
AAGTGTGATAAGTTATCTACACTGGCGAGGGGATTGCTCTCTGTAATGTTCAGCTTCTAA
TTGTCTCTACTTTGTGAGACTACTTTTGAATGCTTGACCTCAAATCAGGTAGGACTACCC
GCTGAACTTAA
>A9_32
TTTCCGTAGGTGAACCTGCGGAAGGATCATTATTGAATTATGTTTCTAGATAGGTTGTAG
CTGGCTCTTTTAGAGCATGTGCACGCCTGTTTGGACTTCATTTTCATCCACCTGTGCACC
TATTGTAGTCTTTGGTTGGGTTAGGAGGAAGTGATCATTGTATCAGCATCTGCTGGGAGT
GAGGACTTGCATTGTGAAAGCTTTGCTGTCCTTGATGTGATCATGGAATCTTTTTCTCAC
TAGAGTCTATGTCACTCATTATACTCTGTCGAATGTCATTGAATGTCTTTACATGGGCTT
GTATGCCTATGAAAATTGTAATACAACTTTCAGCAACGGATCTCTTGGCTCTCGCATCGA
TGAAGAACGCAGCGAAATGCGATAAGTAATGTGAATTGCAGAATTCAGTGAATCATCGAA
TCTTTGAACGCATCTTGCGCTCCTTGGTATTCCGAGGAGCATGCCTGTTTGAGTGTCATT
AAATTCTCAACTCTCTTATACTTTTTTGTAAAAGAGAGCTTGGACTGTGGAGGCTTGCTG
GCCACTTTTTGGGGTCAGCTCCTCTGAAATGCATTAGCGGAACCGTTTGCGATCTGCCAC
AAGTGTGATAAGTTATCTACACTGGCGAGGGGATTGCTCTCTGTAATGTTCAGCTTCTAA
TTGTCTCTACTTTGTGAGACTACTTTTGAATGCTTGACCTCAAATCAGGTAGGACTACCC
GCTGAACTTAA
>A7_67
TTTCCGTAGGTGAACCTGCGGAAGGATCATTATTGAATTATGTTTCTAGATAGGTTGTAG

CTGGCTCTTTTAGAGCATGTGCACGCCTGTTTGGACTTCATTTTCATCCACCTGTGCACC
TATTGTAGTCTTTGGTTGGGTTAGGAGGAAGTGATCATTGTATCAGCATCTGCTGGGAGT
GAGGACTTGCATTGTGAAAGCTTTGCTGTCCTTGATGTGATCATGGAATCTTTTTCTCAC
TAGAGTCTATGTCACTCATTATACTCTGTCGAATGTCATTGAATGTCTTTACATGGGCTT
GTATGCCTATGAAAATTGTAATACAACTTTCAGCAACGGATCTCTTGGCTCTCGCATCGA
TGAAGAACGCAGCGAAATGCGATAAGTAATGTGAATTGCAGAATTCAGTGAATCATCGAA
TCTTTGAACGCATCTTGCGCTCCTTGGTATTCCGAGGAGCATGCCTGTTTGAGTGTCATT
AAATTCTCAACTCTCTTATACTTTTTTGTAAAAGAGAGCTTGGACTGTGGAGGCTTGCTG
GCCACTTTTTGGGGTCAGCTCCTCTGAAATGCATTAGCGGAACCGTTTGCGATCTGCCAC
AAGTGTGATAAGTTATCTACACTGGCGAGGGGATTGCTCTCTGTAATGTTCAGCTTCTAA
TTGTCTCTACTTTGTGAGACTACTTTTGAATGCTTGACCTCAAATCAGGTAGGACTACCC
GCTGAACTTAA
>A7_70
TTTCCGTAGGTGAACCTGCGGAAGGATCATTATTGAATTATGTTTCTAGATAGGTTGTAG
CTGGCTCTTTTAGAGCATGTGCACGCCTGTTTGGACTTCATTTTCATCCACCTGTGCACC
TATTGTAGTCTTTGGTTGGGTTAGGAGGAAGTGATCATTGTATCAGCATCTGCTGGGAGT
GAGGACTTGCATTGTGAAAGCTTTGCTGTCCTTGATGTGATCATGGAATCTTTTTCTCAC
TAGAGTCTATGTCACTCATTATACTCTGTCGAATGTCATTGAATGTCTTTACATGGGCTT
GTATGCCTATGAAAATTGTAATACAACTTTCAGCAACGGATCTCTTGGCTCTCGCATCGA
TGAAGAACGCAGCGAAATGCGATAAGTAATGTGAATTGCAGAATTCAGTGAATCATCGAA
TCTTTGAACGCATCTTGCGCTCCTTGGTATTCCGAGGAGCATGCCTGTTTGAGTGTCATT
AAATTCTCAACTCTCTTATACTTTTTTGTAAAAGAGAGCTTGGACTGTGGAGGCTTGCTG
GCCACTTTTTGGGGTCAGCTCCTCTGAAATGCATTAGCGGAACCGTTTGCGATCTGCCAC
AAGTGTGATAAGTTATCTACACTGGCGAGGGGATTGCTCTCTGTAATGTTCAGCTTCTAA
TTGTCTCTACTTTGTGAGACTACTTTTGAATGCTTGACCTCAAATCAGGTAGGACTACCC
GCTGAACTTAA
>A7_71
TTTCCGTAGGTGAACCTGCGGAAGGATCATTATTGAATTATGTTTCTAGATAGGTTGTAG
CTGGCTCTTTTAGAGCATGTGCACGCCTGTTTGGACTTCATTTTCATCCACCTGTGCACC
TATTGTAGTCTTTGGTTGGGTTAGGAGGAAGTGATCATTGTATCAGCATCTGCTGGGAGT
GAGGACTTGCATTGTGAAAGCTTTGCTGTCCTTGATGTGATCATGGAATCTTTTTCTCAC
TAGAGTCTATGTCACTCATTATACTCTGTCGAATGTCATTGAATGTCTTTACATGGGCTT
GTATGCCTATGAAAATTGTAATACAACTTTCAGCAACGGATCTCTTGGCTCTCGCATCGA
TGAAGAACGCAGCGAAATGCGATAAGTAATGTGAATTGCAGAATTCAGTGAATCATCGAA
TCTTTGAACGCATCTTGCGCTCCTTGGTATTCCGAGGAGCATGCCTGTTTGAGTGTCATT
AAATTCTCAACTCTCTTATACTTTTTTGTAAAAGAGAGCTTGGACTGTGGAGGCTTGCTG
GCCACTTTTTGGGGTCAGCTCCTCTGAAATGCATTAGCGGAACCGTTTGCGATCTGCCAC
AAGTGTGATAAGTTATCTACACTGGCGAGGGGATTGCTCTCTGTAATGTTCAGCTTCTAA
TTGTCTCTACTTTGTGAGACTACTTTTGAATGCTTGACCTCAAATCAGGTAGGACTACCC
GCTGAACTTAA
>A7_72
TTTCCGTAGGTGAACCTGCGGAAGGATCATTATTGAATTATGTTTCTAGATAGGTTGTAG
CTGGCTCTTTTAGAGCATGTGCACGCCTGTTTGGACTTCATTTTCATCCACCTGTGCACC
TATTGTAGTCTTTGGTTGGGTTAGGAGGAAGTGATCATTGTATCAGCATCTGCTGGGAGT
GAGGACTTGCATTGTGAAAGCTTTGCTGTCCTTGATGTGATCATGGAATCTTTTTCTCAC
TAGAGTCTATGTCACTCATTATACTCTGTCGAATGTCATTGAATGTCTTTACATGGGCTT
GTATGCCTATGAAAATTGTAATACAACTTTCAGCAACGGATCTCTTGGCTCTCGCATCGA
TGAAGAACGCAGCGAAATGCGATAAGTAATGTGAATTGCAGAATTCAGTGAATCATCGAA
TCTTTGAACGCATCTTGCGCTCCTTGGTATTCCGAGGAGCATGCCTGTTTGAGTGTCATT
AAATTCTCAACTCTCTTATACTTTTTTGTAAAAGAGAGCTTGGACTGTGGAGGCTTGCTG

GCCACTTTTTGGGGTCAGCTCCTCTGAAATGCATTAGCGGAACCGTTTGCGATCTGCCAC
AAGTGTGATAAGTTATCTACACTGGCGAGGGGATTGCTCTCTGTAATGTTCAGCTTCTAA
TTGTCTCTACTTTGTGAGACTACTTTTGAATGCTTGACCTCAAATCAGGTAGGACTACCC
GCTGAACTTAA
>A7_73
TTTCCGTAGGTGAACCTGCGGAAGGATCATTATTGAATTATGTTTCTAGATAGGTTGTAG
CTGGCTCTTTTAGAGCATGTGCACGCCTGTTTGGACTTCATTTTCATCCACCTGTGCACC
TATTGTAGTCTTTGGTTGGGTTAGGAGGAAGTGATCATTGTATCAGCATCTGCTGGGAGT
GAGGACTTGCATTGTGAAAGCTTTGCTGTCCTTGATGTGATCATGGAATCTTTTTCTCAC
TAGAGTCTATGTCACTCATTATACTCTGTCGAATGTCATTGAATGTCTTTACATGGGCTT
GTATGCCTATGAAAATTGTAATACAACTTTCAGCAACGGATCTCTTGGCTCTCGCATCGA
TGAAGAACGCAGCGAAATGCGATAAGTAATGTGAATTGCAGAATTCAGTGAATCATCGAA
TCTTTGAACGCATCTTGCGCTCCTTGGTATTCCGAGGAGCATGCCTGTTTGAGTGTCATT
AAATTCTCAACTCTCTTATACTTTTTTGTAAAAGAGAGCTTGGACTGTGGAGGCTTGCTG
GCCACTTTTTGGGGTCAGCTCCTCTGAAATGCATTAGCGGAACCGTTTGCGATCTGCCAC
AAGTGTGATAAGTTATCTACACTGGCGAGGGGATTGCTCTCTGTAATGTTCAGCTTCTAA
TTGTCTCTACTTTGTGAGACTACTTTTGAATGCTTGACCTCAAATCAGGTAGGACTACCC
GCTGAACTTAA
>A7_75
TTTCCGTAGGTGAACCTGCGGAAGGATCATTATTGAATTATGTTTCTAGATAGGTTGTAG
CTGGCTCTTTTAGAGCATGTGCACGCCTGTTTGGACTTCATTTTCATCCACCTGTGCACC
TATTGTAGTCTTTGGTTGGGTTAGGAGGAAGTGATCATTGTATCAGCATCTGCTGGGAGT
GAGGACTTGCATTGTGAAAGCTTTGCTGTCCTTGATGTGATCATGGAATCTTTTTCTCAC
TAGAGTCTATGTCACTCATTATACTCTGTCGAATGTCATTGAATGTCTTTACATGGGCTT
GTATGCCTATGAAAATTGTAATACAACTTTCAGCAACGGATCTCTTGGCTCTCGCATCGA
TGAAGAACGCAGCGAAATGCGATAAGTAATGTGAATTGCAGAATTCAGTGAATCATCGAA
TCTTTGAACGCATCTTGCGCTCCTTGGTATTCCGAGGAGCATGCCTGTTTGAGTGTCATT
AAATTCTCAACTCTCTTATACTTTTTTGTAAAAGAGAGCTTGGACTGTGGAGGCTTGCTG
GCCACTTTTTGGGGTCAGCTCCTCTGAAATGCATTAGCGGAACCGTTTGCGATCTGCCAC
AAGTGTGATAAGTTATCTACACTGGCGAGGGGATTGCTCTCTGTAATGTTCAGCTTCTAA
TTGTCTCTACTTTGTGAGACTACTTTTGAATGCTTGACCTCAAATCAGGTAGGACTACCC
GCTGAACTTAA
>A9_1
TTTCCGTAGGTGAACCTGCGGAAGGATCATTATTGAATTATGTTTCTAGATAGGTTGTAG
CTGGCTCTTTTAGAGCATGTGCACGCCTGTTTGGACTTCATTTTCATCCACCTGTGCACC
TATTGTAGTCTTTGGTTGGGTTAGGAGGAAGTGATCATTGTATCAGCATCTGCTGGGAGT
GAGGACTTGCATTGTGAAAGCTTTGCTGTCCTTGATGTGATCATGGAATCTTTTTCTCAC
TAGAGTCTATGTCACTCATTATACTCTGTCGAATGTCATTGAATGTCTTTACATGGGCTT
GTATGCCTATGAAAATTGTAATACAACTTTCAGCAACGGATCTCTTGGCTCTCGCATCGA
TGAAGAACGCAGCGAAATGCGATAAGTAATGTGAATTGCAGAATTCAGTGAATCATCGAA
TCTTTGAACGCATCTTGCGCTCCTTGGTATTCCGAGGAGCATGCCTGTTTGAGTGTCATT
AAATTCTCAACTCTCTTATACTTTTTTGTAAAAGAGAGCTTGGACTGTGGAGGCTTGCTG
GCCACTTTTTGGGGTCAGCTCCTCTGAAATGCATTAGCGGAACCGTTTGCGATCTGCCAC
AAGTGTGATAAGTTATCTACACTGGCGAGGGGATTGCTCTCTGTAATGTTCAGCTTCTAA
TTGTCTCTACTTTGTGAGACTACTTTTGAATGCTTGACCTCAAATCAGGTAGGACTACCC
GCTGAACTTAA
>A9_3
TTTCCGTAGGTGAACCTGCGGAAGGATCATTATTGAATTATGTTTCTAGATAGGTTGTAG
CTGGCTCTTTTAGAGCATGTGCACGCCTGTTTGGACTTCATTTTCATCCACCTGTGCACC
TATTGTAGTCTTTGGTTGGGTTAGGAGGAAGTGATCATTGTATCAGCATCTGCTGGGAGT

GAGGACTTGCATTGTGAAAGCTTTGCTGTCCTTGATGTGATCATGGAATCTTTTTCTCAC
TAGAGTCTATGTCACTCATTATACTCTGTCGAATGTCATTGAATGTCTTTACATGGGCTT
GTATGCCTATGAAAATTGTAATACAACTTTCAGCAACGGATCTCTTGGCTCTCGCATCGA
TGAAGAACGCAGCGAAATGCGATAAGTAATGTGAATTGCAGAATTCAGTGAATCATCGAA
TCTTTGAACGCATCTTGCGCTCCTTGGTATTCCGAGGAGCATGCCTGTTTGAGTGTCATT
AAATTCTCAACTCTCTTATACTTTTTTGTAAAAGAGAGCTTGGACTGTGGAGGCTTGCTG
GCCACTTTTTGGGGTCAGCTCCTCTGAAATGCATTAGCGGAACCGTTTGCGATCTGCCAC
AAGTGTGATAAGTTATCTACACTGGCGAGGGGATTGCTCTCTGTAATGTTCAGCTTCTAA
TTGTCTCTACTTTGTGAGACTACTTTTGAATGCTTGACCTCAAATCAGGTAGGACTACCC
GCTGAACTTAA
>A9_4
TTTCCGTAGGTGAACCTGCGGAAGGATCATTATTGAATTATGTTTCTAGATAGGTTGTAG
CTGGCTCTTTTAGAGCATGTGCACGCCTGTTTGGACTTCATTTTCATCCACCTGTGCACC
TATTGTAGTCTTTGGTTGGGTTAGGAGGAAGTGATCATTGTATCAGCATCTGCTGGGAGT
GAGGACTTGCATTGTGAAAGCTTTGCTGTCCTTGATGTGATCATGGAATCTTTTTCTCAC
TAGAGTCTATGTCACTCATTATACTCTGTCGAATGTCATTGAATGTCTTTACATGGGCTT
GTATGCCTATGAAAATTGTAATACAACTTTCAGCAACGGATCTCTTGGCTCTCGCATCGA
TGAAGAACGCAGCGAAATGCGATAAGTAATGTGAATTGCAGAATTCAGTGAATCATCGAA
TCTTTGAACGCATCTTGCGCTCCTTGGTATTCCGAGGAGCATGCCTGTTTGAGTGTCATT
AAATTCTCAACTCTCTTATACTTTTTTGTAAAAGAGAGCTTGGACTGTGGAGGCTTGCTG
GCCACTTTTTGGGGTCAGCTCCTCTGAAATGCATTAGCGGAACCGTTTGCGATCTGCCAC
AAGTGTGATAAGTTATCTACACTGGCGAGGGGATTGCTCTCTGTAATGTTCAGCTTCTAA
TTGTCTCTACTTTGTGAGACTACTTTTGAATGCTTGACCTCAAATCAGGTAGGACTACCC
GCTGAACTTAA
>A9_5
TTTCCGTAGGTGAACCTGCGGAAGGATCATTATTGAATTATGTTTCTAGATAGGTTGTAG
CTGGCTCTTTTAGAGCATGTGCACGCCTGTTTGGACTTCATTTTCATCCACCTGTGCACC
TATTGTAGTCTTTGGTTGGGTTAGGAGGAAGTGATCATTGTATCAGCATCTGCTGGGAGT
GAGGACTTGCATTGTGAAAGCTTTGCTGTCCTTGATGTGATCATGGAATCTTTTTCTCAC
TAGAGTCTATGTCACTCATTATACTCTGTCGAATGTCATTGAATGTCTTTACATGGGCTT
GTATGCCTATGAAAATTGTAATACAACTTTCAGCAACGGATCTCTTGGCTCTCGCATCGA
TGAAGAACGCAGCGAAATGCGATAAGTAATGTGAATTGCAGAATTCAGTGAATCATCGAA
TCTTTGAACGCATCTTGCGCTCCTTGGTATTCCGAGGAGCATGCCTGTTTGAGTGTCATT
AAATTCTCAACTCTCTTATACTTTTTTGTAAAAGAGAGCTTGGACTGTGGAGGCTTGCTG
GCCACTTTTTGGGGTCAGCTCCTCTGAAATGCATTAGCGGAACCGTTTGCGATCTGCCAC
AAGTGTGATAAGTTATCTACACTGGCGAGGGGATTGCTCTCTGTAATGTTCAGCTTCTAA
TTGTCTCTACTTTGTGAGACTACTTTTGAATGCTTGACCTCAAATCAGGTAGGACTACCC
GCTGAACTTAA
>A9_6
TTTCCGTAGGTGAACCTGCGGAAGGATCATTATTGAATTATGTTTCTAGATAGGTTGTAG
CTGGCTCTTTTAGAGCATGTGCACGCCTGTTTGGACTTCATTTTCATCCACCTGTGCACC
TATTGTAGTCTTTGGTTGGGTTAGGAGGAAGTGATCATTGTATCAGCATCTGCTGGGAGT
GAGGACTTGCATTGTGAAAGCTTTGCTGTCCTTGATGTGATCATGGAATCTTTTTCTCAC
TAGAGTCTATGTCACTCATTATACTCTGTCGAATGTCATTGAATGTCTTTACATGGGCTT
GTATGCCTATGAAAATTGTAATACAACTTTCAGCAACGGATCTCTTGGCTCTCGCATCGA
TGAAGAACGCAGCGAAATGCGATAAGTAATGTGAATTGCAGAATTCAGTGAATCATCGAA
TCTTTGAACGCATCTTGCGCTCCTTGGTATTCCGAGGAGCATGCCTGTTTGAGTGTCATT
AAATTCTCAACTCTCTTATACTTTTTTGTAAAAGAGAGCTTGGACTGTGGAGGCTTGCTG
GCCACTTTTTGGGGTCAGCTCCTCTGAAATGCATTAGCGGAACCGTTTGCGATCTGCCAC
AAGTGTGATAAGTTATCTACACTGGCGAGGGGATTGCTCTCTGTAATGTTCAGCTTCTAA

TTGTCTCTACTTTGTGAGACTACTTTTGAATGCTTGACCTCAAATCAGGTAGGACTACCC
GCTGAACTTAA
>A9_8
TTTCCGTAGGTGAACCTGCGGAAGGATCATTATTGAATTATGTTTCTAGATAGGTTGTAG
CTGGCTCTTTTAGAGCATGTGCACGCCTGTTTGGACTTCATTTTCATCCACCTGTGCACC
TATTGTAGTCTTTGGTTGGGTTAGGAGGAAGTGATCATTGTATCAGCATCTGCTGGGAGT
GAGGACTTGCATTGTGAAAGCTTTGCTGTCCTTGATGTGATCATGGAATCTTTTTCTCAC
TAGAGTCTATGTCACTCATTATACTCTGTCGAATGTCATTGAATGTCTTTACATGGGCTT
GTATGCCTATGAAAATTGTAATACAACTTTCAGCAACGGATCTCTTGGCTCTCGCATCGA
TGAAGAACGCAGCGAAATGCGATAAGTAATGTGAATTGCAGAATTCAGTGAATCATCGAA
TCTTTGAACGCATCTTGCGCTCCTTGGTATTCCGAGGAGCATGCCTGTTTGAGTGTCATT
AAATTCTCAACTCTCTTATACTTTTTTGTAAAAGAGAGCTTGGACTGTGGAGGCTTGCTG
GCCACTTTTTGGGGTCAGCTCCTCTGAAATGCATTAGCGGAACCGTTTGCGATCTGCCAC
AAGTGTGATAAGTTATCTACACTGGCGAGGGGATTGCTCTCTGTAATGTTCAGCTTCTAA
TTGTCTCTACTTTGTGAGACTACTTTTGAATGCTTGACCTCAAATCAGGTAGGACTACCC
GCTGAACTTAA
>A9_9
TTTCCGTAGGTGAACCTGCGGAAGGATCATTATTGAATTATGTTTCTAGATAGGTTGTAG
CTGGCTCTTTTAGAGCATGTGCACGCCTGTTTGGACTTCATTTTCATCCACCTGTGCACC
TATTGTAGTCTTTGGTTGGGTTAGGAGGAAGTGATCATTGTATCAGCATCTGCTGGGAGT
GAGGACTTGCATTGTGAAAGCTTTGCTGTCCTTGATGTGATCATGGAATCTTTTTCTCAC
TAGAGTCTATGTCACTCATTATACTCTGTCGAATGTCATTGAATGTCTTTACATGGGCTT
GTATGCCTATGAAAATTGTAATACAACTTTCAGCAACGGATCTCTTGGCTCTCGCATCGA
TGAAGAACGCAGCGAAATGCGATAAGTAATGTGAATTGCAGAATTCAGTGAATCATCGAA
TCTTTGAACGCATCTTGCGCTCCTTGGTATTCCGAGGAGCATGCCTGTTTGAGTGTCATT
AAATTCTCAACTCTCTTATACTTTTTTGTAAAAGAGAGCTTGGACTGTGGAGGCTTGCTG
GCCACTTTTTGGGGTCAGCTCCTCTGAAATGCATTAGCGGAACCGTTTGCGATCTGCCAC
AAGTGTGATAAGTTATCTACACTGGCGAGGGGATTGCTCTCTGTAATGTTCAGCTTCTAA
TTGTCTCTACTTTGTGAGACTACTTTTGAATGCTTGACCTCAAATCAGGTAGGACTACCC
GCTGAACTTAA
>A9_10
TTTCCGTAGGTGAACCTGCGGAAGGATCATTATTGAATTATGTTTCTAGATAGGTTGTAG
CTGGCTCTTTTAGAGCATGTGCACGCCTGTTTGGACTTCATTTTCATCCACCTGTGCACC
TATTGTAGTCTTTGGTTGGGTTAGGAGGAAGTGATCATTGTATCAGCATCTGCTGGGAGT
GAGGACTTGCATTGTGAAAGCTTTGCTGTCCTTGATGTGATCATGGAATCTTTTTCTCAC
TAGAGTCTATGTCACTCATTATACTCTGTCGAATGTCATTGAATGTCTTTACATGGGCTT
GTATGCCTATGAAAATTGTAATACAACTTTCAGCAACGGATCTCTTGGCTCTCGCATCGA
TGAAGAACGCAGCGAAATGCGATAAGTAATGTGAATTGCAGAATTCAGTGAATCATCGAA
TCTTTGAACGCATCTTGCGCTCCTTGGTATTCCGAGGAGCATGCCTGTTTGAGTGTCATT
AAATTCTCAACTCTCTTATACTTTTTTGTAAAAGAGAGCTTGGACTGTGGAGGCTTGCTG
GCCACTTTTTGGGGTCAGCTCCTCTGAAATGCATTAGCGGAACCGTTTGCGATCTGCCAC
AAGTGTGATAAGTTATCTACACTGGCGAGGGGATTGCTCTCTGTAATGTTCAGCTTCTAA
TTGTCTCTACTTTGTGAGACTACTTTTGAATGCTTGACCTCAAATCAGGTAGGACTACCC
GCTGAACTTAA
>A9_11
TTTCCGTAGGTGAACCTGCGGAAGGATCATTATTGAATTATGTTTCTAGATAGGTTGTAG
CTGGCTCTTTTAGAGCATGTGCACGCCTGTTTGGACTTCATTTTCATCCACCTGTGCACC
TATTGTAGTCTTTGGTTGGGTTAGGAGGAAGTGATCATTGTATCAGCATCTGCTGGGAGT
GAGGACTTGCATTGTGAAAGCTTTGCTGTCCTTGATGTGATCATGGAATCTTTTTCTCAC
TAGAGTCTATGTCACTCATTATACTCTGTCGAATGTCATTGAATGTCTTTACATGGGCTT

GTATGCCTATGAAAATTGTAATACAACTTTCAGCAACGGATCTCTTGGCTCTCGCATCGA
TGAAGAACGCAGCGAAATGCGATAAGTAATGTGAATTGCAGAATTCAGTGAATCATCGAA
TCTTTGAACGCATCTTGCGCTCCTTGGTATTCCGAGGAGCATGCCTGTTTGAGTGTCATT
AAATTCTCAACTCTCTTATACTTTTTTGTAAAAGAGAGCTTGGACTGTGGAGGCTTGCTG
GCCACTTTTTGGGGTCAGCTCCTCTGAAATGCATTAGCGGAACCGTTTGCGATCTGCCAC
AAGTGTGATAAGTTATCTACACTGGCGAGGGGATTGCTCTCTGTAATGTTCAGCTTCTAA
TTGTCTCTACTTTGTGAGACTACTTTTGAATGCTTGACCTCAAATCAGGTAGGACTACCC
GCTGAACTTAA
>A9_13
TTTCCGTAGGTGAACCTGCGGAAGGATCATTATTGAATTATGTTTCTAGATAGGTTGTAG
CTGGCTCTTTTAGAGCATGTGCACGCCTGTTTGGACTTCATTTTCATCCACCTGTGCACC
TATTGTAGTCTTTGGTTGGGTTAGGAGGAAGTGATCATTGTATCAGCATCTGCTGGGAGT
GAGGACTTGCATTGTGAAAGCTTTGCTGTCCTTGATGTGATCATGGAATCTTTTTCTCAC
TAGAGTCTATGTCACTCATTATACTCTGTCGAATGTCATTGAATGTCTTTACATGGGCTT
GTATGCCTATGAAAATTGTAATACAACTTTCAGCAACGGATCTCTTGGCTCTCGCATCGA
TGAAGAACGCAGCGAAATGCGATAAGTAATGTGAATTGCAGAATTCAGTGAATCATCGAA
TCTTTGAACGCATCTTGCGCTCCTTGGTATTCCGAGGAGCATGCCTGTTTGAGTGTCATT
AAATTCTCAACTCTCTTATACTTTTTTGTAAAAGAGAGCTTGGACTGTGGAGGCTTGCTG
GCCACTTTTTGGGGTCAGCTCCTCTGAAATGCATTAGCGGAACCGTTTGCGATCTGCCAC
AAGTGTGATAAGTTATCTACACTGGCGAGGGGATTGCTCTCTGTAATGTTCAGCTTCTAA
TTGTCTCTACTTTGTGAGACTACTTTTGAATGCTTGACCTCAAATCAGGTAGGACTACCC
GCTGAACTTAA
>A9_14
TTTCCGTAGGTGAACCTGCGGAAGGATCATTATTGAATTATGTTTCTAGATAGGTTGTAG
CTGGCTCTTTTAGAGCATGTGCACGCCTGTTTGGACTTCATTTTCATCCACCTGTGCACC
TATTGTAGTCTTTGGTTGGGTTAGGAGGAAGTGATCATTGTATCAGCATCTGCTGGGAGT
GAGGACTTGCATTGTGAAAGCTTTGCTGTCCTTGATGTGATCATGGAATCTTTTTCTCAC
TAGAGTCTATGTCACTCATTATACTCTGTCGAATGTCATTGAATGTCTTTACATGGGCTT
GTATGCCTATGAAAATTGTAATACAACTTTCAGCAACGGATCTCTTGGCTCTCGCATCGA
TGAAGAACGCAGCGAAATGCGATAAGTAATGTGAATTGCAGAATTCAGTGAATCATCGAA
TCTTTGAACGCATCTTGCGCTCCTTGGTATTCCGAGGAGCATGCCTGTTTGAGTGTCATT
AAATTCTCAACTCTCTTATACTTTTTTGTAAAAGAGAGCTTGGACTGTGGAGGCTTGCTG
GCCACTTTTTGGGGTCAGCTCCTCTGAAATGCATTAGCGGAACCGTTTGCGATCTGCCAC
AAGTGTGATAAGTTATCTACACTGGCGAGGGGATTGCTCTCTGTAATGTTCAGCTTCTAA
TTGTCTCTACTTTGTGAGACTACTTTTGAATGCTTGACCTCAAATCAGGTAGGACTACCC
GCTGAACTTAA
>A9_15
TTTCCGTAGGTGAACCTGCGGAAGGATCATTATTGAATTATGTTTCTAGATAGGTTGTAG
CTGGCTCTTTTAGAGCATGTGCACGCCTGTTTGGACTTCATTTTCATCCACCTGTGCACC
TATTGTAGTCTTTGGTTGGGTTAGGAGGAAGTGATCATTGTATCAGCATCTGCTGGGAGT
GAGGACTTGCATTGTGAAAGCTTTGCTGTCCTTGATGTGATCATGGAATCTTTTTCTCAC
TAGAGTCTATGTCACTCATTATACTCTGTCGAATGTCATTGAATGTCTTTACATGGGCTT
GTATGCCTATGAAAATTGTAATACAACTTTCAGCAACGGATCTCTTGGCTCTCGCATCGA
TGAAGAACGCAGCGAAATGCGATAAGTAATGTGAATTGCAGAATTCAGTGAATCATCGAA
TCTTTGAACGCATCTTGCGCTCCTTGGTATTCCGAGGAGCATGCCTGTTTGAGTGTCATT
AAATTCTCAACTCTCTTATACTTTTTTGTAAAAGAGAGCTTGGACTGTGGAGGCTTGCTG
GCCACTTTTTGGGGTCAGCTCCTCTGAAATGCATTAGCGGAACCGTTTGCGATCTGCCAC
AAGTGTGATAAGTTATCTACACTGGCGAGGGGATTGCTCTCTGTAATGTTCAGCTTCTAA
TTGTCTCTACTTTGTGAGACTACTTTTGAATGCTTGACCTCAAATCAGGTAGGACTACCC
GCTGAACTTAA

>A9_16
TTTCCGTAGGTGAACCTGCGGAAGGATCATTATTGAATTATGTTTCTAGATAGGTTGTAG
CTGGCTCTTTTAGAGCATGTGCACGCCTGTTTGGACTTCATTTTCATCCACCTGTGCACC
TATTGTAGTCTTTGGTTGGGTTAGGAGGAAGTGATCATTGTATCAGCATCTGCTGGGAGT
GAGGACTTGCATTGTGAAAGCTTTGCTGTCCTTGATGTGATCATGGAATCTTTTTCTCAC
TAGAGTCTATGTCACTCATTATACTCTGTCGAATGTCATTGAATGTCTTTACATGGGCTT
GTATGCCTATGAAAATTGTAATACAACTTTCAGCAACGGATCTCTTGGCTCTCGCATCGA
TGAAGAACGCAGCGAAATGCGATAAGTAATGTGAATTGCAGAATTCAGTGAATCATCGAA
TCTTTGAACGCATCTTGCGCTCCTTGGTATTCCGAGGAGCATGCCTGTTTGAGTGTCATT
AAATTCTCAACTCTCTTATACTTTTTTGTAAAAGAGAGCTTGGACTGTGGAGGCTTGCTG
GCCACTTTTTGGGGTCAGCTCCTCTGAAATGCATTAGCGGAACCGTTTGCGATCTGCCAC
AAGTGTGATAAGTTATCTACACTGGCGAGGGGATTGCTCTCTGTAATGTTCAGCTTCTAA
TTGTCTCTACTTTGTGAGACTACTTTTGAATGCTTGACCTCAAATCAGGTAGGACTACCC
GCTGAACTTAA
>A9_17
TTTCCGTAGGTGAACCTGCGGAAGGATCATTATTGAATTATGTTTCTAGATAGGTTGTAG
CTGGCTCTTTTAGAGCATGTGCACGCCTGTTTGGACTTCATTTTCATCCACCTGTGCACC
TATTGTAGTCTTTGGTTGGGTTAGGAGGAAGTGATCATTGTATCAGCATCTGCTGGGAGT
GAGGACTTGCATTGTGAAAGCTTTGCTGTCCTTGATGTGATCATGGAATCTTTTTCTCAC
TAGAGTCTATGTCACTCATTATACTCTGTCGAATGTCATTGAATGTCTTTACATGGGCTT
GTATGCCTATGAAAATTGTAATACAACTTTCAGCAACGGATCTCTTGGCTCTCGCATCGA
TGAAGAACGCAGCGAAATGCGATAAGTAATGTGAATTGCAGAATTCAGTGAATCATCGAA
TCTTTGAACGCATCTTGCGCTCCTTGGTATTCCGAGGAGCATGCCTGTTTGAGTGTCATT
AAATTCTCAACTCTCTTATACTTTTTTGTAAAAGAGAGCTTGGACTGTGGAGGCTTGCTG
GCCACTTTTTGGGGTCAGCTCCTCTGAAATGCATTAGCGGAACCGTTTGCGATCTGCCAC
AAGTGTGATAAGTTATCTACACTGGCGAGGGGATTGCTCTCTGTAATGTTCAGCTTCTAA
TTGTCTCTACTTTGTGAGACTACTTTTGAATGCTTGACCTCAAATCAGGTAGGACTACCC
GCTGAACTTAA
>A9_34
TTTCCGTAGGTGAACCTGCGGAAGGATCATTATTGAATTATGTTTCTAGATAGGTTGTAG
CTGGCTCTTTTAGAGCATGTGCACGCCTGTTTGGACTTCATTTTCATCCACCTGTGCACC
TATTGTAGTCTTTGGTTGGGTTAGGAGGAAGTGATCATTGTATCAGCATCTGCTGGGAGT
GAGGACTTGCATTGTGAAAGCTTTGCTGTCCTTGATGTGATCATGGAATCTTTTTCTCAC
TAGAGTCTATGTCACTCATTATACTCTGTCGAATGTCATTGAATGTCTTTACATGGGCTT
GTATGCCTATGAAAATTGTAATACAACTTTCAGCAACGGATCTCTTGGCTCTCGCATCGA
TGAAGAACGCAGCGAAATGCGATAAGTAATGTGAATTGCAGAATTCAGTGAATCATCGAA
TCTTTGAACGCATCTTGCGCTCCTTGGTATTCCGAGGAGCATGCCTGTTTGAGTGTCATT
AAATTCTCAACTCTCTTATACTTTTTTGTAAAAGAGAGCTTGGACTGTGGAGGCTTGCTG
GCCACTTTTTGGGGTCAGCTCCTCTGAAATGCATTAGCGGAACCGTTTGCGATCTGCCAC
AAGTGTGATAAGTTATCTACACTGGCGAGGGGATTGCTCTCTGTAATGTTCAGCTTCTAA
TTGTCTCTACTTTGTGAGACTACTTTTGAATGCTTGACCTCAAATCAGGTAGGACTACCC
GCTGAACTTAA
>A10_2
TTTCCGTAGGTGAACCTGCGGAAGGATCATTATTGAATTATGTTTCTAGATAGGTTGTAG
CTGGCTCTTTTAGAGCATGTGCACGCCTGTTTGGACTTCATTTTCATCCACCTGTGCACC
TATTGTAGTCTTTGGTTGGGTTAGGAGGAAGTGATCATTGTATCAGCATCTGCTGGGAGT
GAGGACTTGCATTGTGAAAGCTTTGCTGTCCTTGATGTGATCATGGAATCTTTTTCTCAC
TAGAGTCTATGTCACTCATTATACTCTGTCGAATGTCATTGAATGTCTTTACATGGGCTT
GTATGCCTATGAAAATTGTAATACAACTTTCAGCAACGGATCTCTTGGCTCTCGCATCGA
TGAAGAACGCAGCGAAATGCGATAAGTAATGTGAATTGCAGAATTCAGTGAATCATCGAA

TCTTTGAACGCATCTTGCGCTCCTTGGTATTCCGAGGAGCATGCCTGTTTGAGTGTCATT
AAATTCTCAACTCTCTTATACTTTTTTGTAAAAGAGAGCTTGGACTGTGGAGGCTTGCTG
GCCACTTTTTGGGGTCAGCTCCTCTGAAATGCATTAGCGGAACCGTTTGCGATCTGCCAC
AAGTGTGATAAGTTATCTACACTGGCGAGGGGATTGCTCTCTGTAATGTTCAGCTTCTAA
TTGTCTCTACTTTGTGAGACTACTTTTGAATGCTTGACCTCAAATCAGGTAGGACTACCC
GCTGAACTTAA
>A10_5
TTTCCGTAGGTGAACCTGCGGAAGGATCATTATTGAATTATGTTTCTAGATAGGTTGTAG
CTGGCTCTTTTAGAGCATGTGCACGCCTGTTTGGACTTCATTTTCATCCACCTGTGCACC
TATTGTAGTCTTTGGTTGGGTTAGGAGGAAGTGATCATTGTATCAGCATCTGCTGGGAGT
GAGGACTTGCATTGTGAAAGCTTTGCTGTCCTTGATGTGATCATGGAATCTTTTTCTCAC
TAGAGTCTATGTCACTCATTATACTCTGTCGAATGTCATTGAATGTCTTTACATGGGCTT
GTATGCCTATGAAAATTGTAATACAACTTTCAGCAACGGATCTCTTGGCTCTCGCATCGA
TGAAGAACGCAGCGAAATGCGATAAGTAATGTGAATTGCAGAATTCAGTGAATCATCGAA
TCTTTGAACGCATCTTGCGCTCCTTGGTATTCCGAGGAGCATGCCTGTTTGAGTGTCATT
AAATTCTCAACTCTCTTATACTTTTTTGTAAAAGAGAGCTTGGACTGTGGAGGCTTGCTG
GCCACTTTTTGGGGTCAGCTCCTCTGAAATGCATTAGCGGAACCGTTTGCGATCTGCCAC
AAGTGTGATAAGTTATCTACACTGGCGAGGGGATTGCTCTCTGTAATGTTCAGCTTCTAA
TTGTCTCTACTTTGTGAGACTACTTTTGAATGCTTGACCTCAAATCAGGTAGGACTACCC
GCTGAACTTAA
>A10_7
TTTCCGTAGGTGAACCTGCGGAAGGATCATTATTGAATTATGTTTCTAGATAGGTTGTAG
CTGGCTCTTTTAGAGCATGTGCACGCCTGTTTGGACTTCATTTTCATCCACCTGTGCACC
TATTGTAGTCTTTGGTTGGGTTAGGAGGAAGTGATCATTGTATCAGCATCTGCTGGGAGT
GAGGACTTGCATTGTGAAAGCTTTGCTGTCCTTGATGTGATCATGGAATCTTTTTCTCAC
TAGAGTCTATGTCACTCATTATACTCTGTCGAATGTCATTGAATGTCTTTACATGGGCTT
GTATGCCTATGAAAATTGTAATACAACTTTCAGCAACGGATCTCTTGGCTCTCGCATCGA
TGAAGAACGCAGCGAAATGCGATAAGTAATGTGAATTGCAGAATTCAGTGAATCATCGAA
TCTTTGAACGCATCTTGCGCTCCTTGGTATTCCGAGGAGCATGCCTGTTTGAGTGTCATT
AAATTCTCAACTCTCTTATACTTTTTTGTAAAAGAGAGCTTGGACTGTGGAGGCTTGCTG
GCCACTTTTTGGGGTCAGCTCCTCTGAAATGCATTAGCGGAACCGTTTGCGATCTGCCAC
AAGTGTGATAAGTTATCTACACTGGCGAGGGGATTGCTCTCTGTAATGTTCAGCTTCTAA
TTGTCTCTACTTTGTGAGACTACTTTTGAATGCTTGACCTCAAATCAGGTAGGACTACCC
GCTGAACTTAA
>A10_8
TTTCCGTAGGTGAACCTGCGGAAGGATCATTATTGAATTATGTTTCTAGATAGGTTGTAG
CTGGCTCTTTTAGAGCATGTGCACGCCTGTTTGGACTTCATTTTCATCCACCTGTGCACC
TATTGTAGTCTTTGGTTGGGTTAGGAGGAAGTGATCATTGTATCAGCATCTGCTGGGAGT
GAGGACTTGCATTGTGAAAGCTTTGCTGTCCTTGATGTGATCATGGAATCTTTTTCTCAC
TAGAGTCTATGTCACTCATTATACTCTGTCGAATGTCATTGAATGTCTTTACATGGGCTT
GTATGCCTATGAAAATTGTAATACAACTTTCAGCAACGGATCTCTTGGCTCTCGCATCGA
TGAAGAACGCAGCGAAATGCGATAAGTAATGTGAATTGCAGAATTCAGTGAATCATCGAA
TCTTTGAACGCATCTTGCGCTCCTTGGTATTCCGAGGAGCATGCCTGTTTGAGTGTCATT
AAATTCTCAACTCTCTTATACTTTTTTGTAAAAGAGAGCTTGGACTGTGGAGGCTTGCTG
GCCACTTTTTGGGGTCAGCTCCTCTGAAATGCATTAGCGGAACCGTTTGCGATCTGCCAC
AAGTGTGATAAGTTATCTACACTGGCGAGGGGATTGCTCTCTGTAATGTTCAGCTTCTAA
TTGTCTCTACTTTGTGAGACTACTTTTGAATGCTTGACCTCAAATCAGGTAGGACTACCC
GCTGAACTTAA
>A10_10
TTTCCGTAGGTGAACCTGCGGAAGGATCATTATTGAATTATGTTTCTAGATAGGTTGTAG

CTGGCTCTTTTAGAGCATGTGCACGCCTGTTTGGACTTCATTTTCATCCACCTGTGCACC
TATTGTAGTCTTTGGTTGGGTTAGGAGGAAGTGATCATTGTATCAGCATCTGCTGGGAGT
GAGGACTTGCATTGTGAAAGCTTTGCTGTCCTTGATGTGATCATGGAATCTTTTTCTCAC
TAGAGTCTATGTCACTCATTATACTCTGTCGAATGTCATTGAATGTCTTTACATGGGCTT
GTATGCCTATGAAAATTGTAATACAACTTTCAGCAACGGATCTCTTGGCTCTCGCATCGA
TGAAGAACGCAGCGAAATGCGATAAGTAATGTGAATTGCAGAATTCAGTGAATCATCGAA
TCTTTGAACGCATCTTGCGCTCCTTGGTATTCCGAGGAGCATGCCTGTTTGAGTGTCATT
AAATTCTCAACTCTCTTATACTTTTTTGTAAAAGAGAGCTTGGACTGTGGAGGCTTGCTG
GCCACTTTTTGGGGTCAGCTCCTCTGAAATGCATTAGCGGAACCGTTTGCGATCTGCCAC
AAGTGTGATAAGTTATCTACACTGGCGAGGGGATTGCTCTCTGTAATGTTCAGCTTCTAA
TTGTCTCTACTTTGTGAGACTACTTTTGAATGCTTGACCTCAAATCAGGTAGGACTACCC
GCTGAACTTAA
>A10_11
TTTCCGTAGGTGAACCTGCGGAAGGATCATTATTGAATTATGTTTCTAGATAGGTTGTAG
CTGGCTCTTTTAGAGCATGTGCACGCCTGTTTGGACTTCATTTTCATCCACCTGTGCACC
TATTGTAGTCTTTGGTTGGGTTAGGAGGAAGTGATCATTGTATCAGCATCTGCTGGGAGT
GAGGACTTGCATTGTGAAAGCTTTGCTGTCCTTGATGTGATCATGGAATCTTTTTCTCAC
TAGAGTCTATGTCACTCATTATACTCTGTCGAATGTCATTGAATGTCTTTACATGGGCTT
GTATGCCTATGAAAATTGTAATACAACTTTCAGCAACGGATCTCTTGGCTCTCGCATCGA
TGAAGAACGCAGCGAAATGCGATAAGTAATGTGAATTGCAGAATTCAGTGAATCATCGAA
TCTTTGAACGCATCTTGCGCTCCTTGGTATTCCGAGGAGCATGCCTGTTTGAGTGTCATT
AAATTCTCAACTCTCTTATACTTTTTTGTAAAAGAGAGCTTGGACTGTGGAGGCTTGCTG
GCCACTTTTTGGGGTCAGCTCCTCTGAAATGCATTAGCGGAACCGTTTGCGATCTGCCAC
AAGTGTGATAAGTTATCTACACTGGCGAGGGGATTGCTCTCTGTAATGTTCAGCTTCTAA
TTGTCTCTACTTTGTGAGACTACTTTTGAATGCTTGACCTCAAATCAGGTAGGACTACCC
GCTGAACTTAA
>A10_12
TTTCCGTAGGTGAACCTGCGGAAGGATCATTATTGAATTATGTTTCTAGATAGGTTGTAG
CTGGCTCTTTTAGAGCATGTGCACGCCTGTTTGGACTTCATTTTCATCCACCTGTGCACC
TATTGTAGTCTTTGGTTGGGTTAGGAGGAAGTGATCATTGTATCAGCATCTGCTGGGAGT
GAGGACTTGCATTGTGAAAGCTTTGCTGTCCTTGATGTGATCATGGAATCTTTTTCTCAC
TAGAGTCTATGTCACTCATTATACTCTGTCGAATGTCATTGAATGTCTTTACATGGGCTT
GTATGCCTATGAAAATTGTAATACAACTTTCAGCAACGGATCTCTTGGCTCTCGCATCGA
TGAAGAACGCAGCGAAATGCGATAAGTAATGTGAATTGCAGAATTCAGTGAATCATCGAA
TCTTTGAACGCATCTTGCGCTCCTTGGTATTCCGAGGAGCATGCCTGTTTGAGTGTCATT
AAATTCTCAACTCTCTTATACTTTTTTGTAAAAGAGAGCTTGGACTGTGGAGGCTTGCTG
GCCACTTTTTGGGGTCAGCTCCTCTGAAATGCATTAGCGGAACCGTTTGCGATCTGCCAC
AAGTGTGATAAGTTATCTACACTGGCGAGGGGATTGCTCTCTGTAATGTTCAGCTTCTAA
TTGTCTCTACTTTGTGAGACTACTTTTGAATGCTTGACCTCAAATCAGGTAGGACTACCC
GCTGAACTTAA
>A10_15
TTTCCGTAGGTGAACCTGCGGAAGGATCATTATTGAATTATGTTTCTAGATAGGTTGTAG
CTGGCTCTTTTAGAGCATGTGCACGCCTGTTTGGACTTCATTTTCATCCACCTGTGCACC
TATTGTAGTCTTTGGTTGGGTTAGGAGGAAGTGATCATTGTATCAGCATCTGCTGGGAGT
GAGGACTTGCATTGTGAAAGCTTTGCTGTCCTTGATGTGATCATGGAATCTTTTTCTCAC
TAGAGTCTATGTCACTCATTATACTCTGTCGAATGTCATTGAATGTCTTTACATGGGCTT
GTATGCCTATGAAAATTGTAATACAACTTTCAGCAACGGATCTCTTGGCTCTCGCATCGA
TGAAGAACGCAGCGAAATGCGATAAGTAATGTGAATTGCAGAATTCAGTGAATCATCGAA
TCTTTGAACGCATCTTGCGCTCCTTGGTATTCCGAGGAGCATGCCTGTTTGAGTGTCATT
AAATTCTCAACTCTCTTATACTTTTTTGTAAAAGAGAGCTTGGACTGTGGAGGCTTGCTG

GCCACTTTTTGGGGTCAGCTCCTCTGAAATGCATTAGCGGAACCGTTTGCGATCTGCCAC
AAGTGTGATAAGTTATCTACACTGGCGAGGGGATTGCTCTCTGTAATGTTCAGCTTCTAA
TTGTCTCTACTTTGTGAGACTACTTTTGAATGCTTGACCTCAAATCAGGTAGGACTACCC
GCTGAACTTAA
>A10_16
TTTCCGTAGGTGAACCTGCGGAAGGATCATTATTGAATTATGTTTCTAGATAGGTTGTAG
CTGGCTCTTTTAGAGCATGTGCACGCCTGTTTGGACTTCATTTTCATCCACCTGTGCACC
TATTGTAGTCTTTGGTTGGGTTAGGAGGAAGTGATCATTGTATCAGCATCTGCTGGGAGT
GAGGACTTGCATTGTGAAAGCTTTGCTGTCCTTGATGTGATCATGGAATCTTTTTCTCAC
TAGAGTCTATGTCACTCATTATACTCTGTCGAATGTCATTGAATGTCTTTACATGGGCTT
GTATGCCTATGAAAATTGTAATACAACTTTCAGCAACGGATCTCTTGGCTCTCGCATCGA
TGAAGAACGCAGCGAAATGCGATAAGTAATGTGAATTGCAGAATTCAGTGAATCATCGAA
TCTTTGAACGCATCTTGCGCTCCTTGGTATTCCGAGGAGCATGCCTGTTTGAGTGTCATT
AAATTCTCAACTCTCTTATACTTTTTTGTAAAAGAGAGCTTGGACTGTGGAGGCTTGCTG
GCCACTTTTTGGGGTCAGCTCCTCTGAAATGCATTAGCGGAACCGTTTGCGATCTGCCAC
AAGTGTGATAAGTTATCTACACTGGCGAGGGGATTGCTCTCTGTAATGTTCAGCTTCTAA
TTGTCTCTACTTTGTGAGACTACTTTTGAATGCTTGACCTCAAATCAGGTAGGACTACCC
GCTGAACTTAA
>A9_36
TTTCCGTAGGTGAACCTGCGGAAGGATCATTATTGAATTATGTTTCTAGATAGGTTGTAG
CTGGCTCTTTTAGAGCATGTGCACGCCTGTTTGGACTTCATTTTCATCCACCTGTGCACC
TATTGTAGTCTTTGGTTGGGTTAGGAGGAAGTGATCATTGTATCAGCATCTGCTGGGAGT
GAGGACTTGCATTGTGAAAGCTTTGCTGTCCTTGATGTGATCATGGAATCTTTTTCTCAC
TAGAGTCTATGTCACTCATTATACTCTGTCGAATGTCATTGAATGTCTTTACATGGGCTT
GTATGCCTATGAAAATTGTAATACAACTTTCAGCAACGGATCTCTTGGCTCTCGCATCGA
TGAAGAACGCAGCGAAATGCGATAAGTAATGTGAATTGCAGAATTCAGTGAATCATCGAA
TCTTTGAACGCATCTTGCGCTCCTTGGTATTCCGAGGAGCATGCCTGTTTGAGTGTCATT
AAATTCTCAACTCTCTTATACTTTTTTGTAAAAGAGAGCTTGGACTGTGGAGGCTTGCTG
GCCACTTTTTGGGGTCAGCTCCTCTGAAATGCATTAGCGGAACCGTTTGCGATCTGCCAC
AAGTGTGATAAGTTATCTACACTGGCGAGGGGATTGCTCTCTGTAATGTTCAGCTTCTAA
TTGTCTCTACTTTGTGAGACTACTTTTGAATGCTTGACCTCAAATCAGGTAGGACTACCC
GCTGAACTTAA
>A9_38
TTTCCGTAGGTGAACCTGCGGAAGGATCATTATTGAATTATGTTTCTAGATAGGTTGTAG
CTGGCTCTTTTAGAGCATGTGCACGCCTGTTTGGACTTCATTTTCATCCACCTGTGCACC
TATTGTAGTCTTTGGTTGGGTTAGGAGGAAGTGATCATTGTATCAGCATCTGCTGGGAGT
GAGGACTTGCATTGTGAAAGCTTTGCTGTCCTTGATGTGATCATGGAATCTTTTTCTCAC
TAGAGTCTATGTCACTCATTATACTCTGTCGAATGTCATTGAATGTCTTTACATGGGCTT
GTATGCCTATGAAAATTGTAATACAACTTTCAGCAACGGATCTCTTGGCTCTCGCATCGA
TGAAGAACGCAGCGAAATGCGATAAGTAATGTGAATTGCAGAATTCAGTGAATCATCGAA
TCTTTGAACGCATCTTGCGCTCCTTGGTATTCCGAGGAGCATGCCTGTTTGAGTGTCATT
AAATTCTCAACTCTCTTATACTTTTTTGTAAAAGAGAGCTTGGACTGTGGAGGCTTGCTG
GCCACTTTTTGGGGTCAGCTCCTCTGAAATGCATTAGCGGAACCGTTTGCGATCTGCCAC
AAGTGTGATAAGTTATCTACACTGGCGAGGGGATTGCTCTCTGTAATGTTCAGCTTCTAA
TTGTCTCTACTTTGTGAGACTACTTTTGAATGCTTGACCTCAAATCAGGTAGGACTACCC
GCTGAACTTAA
>A9_39
TTTCCGTAGGTGAACCTGCGGAAGGATCATTATTGAATTATGTTTCTAGATAGGTTGTAG
CTGGCTCTTTTAGAGCATGTGCACGCCTGTTTGGACTTCATTTTCATCCACCTGTGCACC
TATTGTAGTCTTTGGTTGGGTTAGGAGGAAGTGATCATTGTATCAGCATCTGCTGGGAGT

GAGGACTTGCATTGTGAAAGCTTTGCTGTCCTTGATGTGATCATGGAATCTTTTTCTCAC
TAGAGTCTATGTCACTCATTATACTCTGTCGAATGTCATTGAATGTCTTTACATGGGCTT
GTATGCCTATGAAAATTGTAATACAACTTTCAGCAACGGATCTCTTGGCTCTCGCATCGA
TGAAGAACGCAGCGAAATGCGATAAGTAATGTGAATTGCAGAATTCAGTGAATCATCGAA
TCTTTGAACGCATCTTGCGCTCCTTGGTATTCCGAGGAGCATGCCTGTTTGAGTGTCATT
AAATTCTCAACTCTCTTATACTTTTTTGTAAAAGAGAGCTTGGACTGTGGAGGCTTGCTG
GCCACTTTTTGGGGTCAGCTCCTCTGAAATGCATTAGCGGAACCGTTTGCGATCTGCCAC
AAGTGTGATAAGTTATCTACACTGGCGAGGGGATTGCTCTCTGTAATGTTCAGCTTCTAA
TTGTCTCTACTTTGTGAGACTACTTTTGAATGCTTGACCTCAAATCAGGTAGGACTACCC
GCTGAACTTAA
>A9_40
TTTCCGTAGGTGAACCTGCGGAAGGATCATTATTGAATTATGTTTCTAGATAGGTTGTAG
CTGGCTCTTTTAGAGCATGTGCACGCCTGTTTGGACTTCATTTTCATCCACCTGTGCACC
TATTGTAGTCTTTGGTTGGGTTAGGAGGAAGTGATCATTGTATCAGCATCTGCTGGGAGT
GAGGACTTGCATTGTGAAAGCTTTGCTGTCCTTGATGTGATCATGGAATCTTTTTCTCAC
TAGAGTCTATGTCACTCATTATACTCTGTCGAATGTCATTGAATGTCTTTACATGGGCTT
GTATGCCTATGAAAATTGTAATACAACTTTCAGCAACGGATCTCTTGGCTCTCGCATCGA
TGAAGAACGCAGCGAAATGCGATAAGTAATGTGAATTGCAGAATTCAGTGAATCATCGAA
TCTTTGAACGCATCTTGCGCTCCTTGGTATTCCGAGGAGCATGCCTGTTTGAGTGTCATT
AAATTCTCAACTCTCTTATACTTTTTTGTAAAAGAGAGCTTGGACTGTGGAGGCTTGCTG
GCCACTTTTTGGGGTCAGCTCCTCTGAAATGCATTAGCGGAACCGTTTGCGATCTGCCAC
AAGTGTGATAAGTTATCTACACTGGCGAGGGGATTGCTCTCTGTAATGTTCAGCTTCTAA
TTGTCTCTACTTTGTGAGACTACTTTTGAATGCTTGACCTCAAATCAGGTAGGACTACCC
GCTGAACTTAA
>A9_41
TTTCCGTAGGTGAACCTGCGGAAGGATCATTATTGAATTATGTTTCTAGATAGGTTGTAG
CTGGCTCTTTTAGAGCATGTGCACGCCTGTTTGGACTTCATTTTCATCCACCTGTGCACC
TATTGTAGTCTTTGGTTGGGTTAGGAGGAAGTGATCATTGTATCAGCATCTGCTGGGAGT
GAGGACTTGCATTGTGAAAGCTTTGCTGTCCTTGATGTGATCATGGAATCTTTTTCTCAC
TAGAGTCTATGTCACTCATTATACTCTGTCGAATGTCATTGAATGTCTTTACATGGGCTT
GTATGCCTATGAAAATTGTAATACAACTTTCAGCAACGGATCTCTTGGCTCTCGCATCGA
TGAAGAACGCAGCGAAATGCGATAAGTAATGTGAATTGCAGAATTCAGTGAATCATCGAA
TCTTTGAACGCATCTTGCGCTCCTTGGTATTCCGAGGAGCATGCCTGTTTGAGTGTCATT
AAATTCTCAACTCTCTTATACTTTTTTGTAAAAGAGAGCTTGGACTGTGGAGGCTTGCTG
GCCACTTTTTGGGGTCAGCTCCTCTGAAATGCATTAGCGGAACCGTTTGCGATCTGCCAC
AAGTGTGATAAGTTATCTACACTGGCGAGGGGATTGCTCTCTGTAATGTTCAGCTTCTAA
TTGTCTCTACTTTGTGAGACTACTTTTGAATGCTTGACCTCAAATCAGGTAGGACTACCC
GCTGAACTTAA
>A9_44
TTTCCGTAGGTGAACCTGCGGAAGGATCATTATTGAATTATGTTTCTAGATAGGTTGTAG
CTGGCTCTTTTAGAGCATGTGCACGCCTGTTTGGACTTCATTTTCATCCACCTGTGCACC
TATTGTAGTCTTTGGTTGGGTTAGGAGGAAGTGATCATTGTATCAGCATCTGCTGGGAGT
GAGGACTTGCATTGTGAAAGCTTTGCTGTCCTTGATGTGATCATGGAATCTTTTTCTCAC
TAGAGTCTATGTCACTCATTATACTCTGTCGAATGTCATTGAATGTCTTTACATGGGCTT
GTATGCCTATGAAAATTGTAATACAACTTTCAGCAACGGATCTCTTGGCTCTCGCATCGA
TGAAGAACGCAGCGAAATGCGATAAGTAATGTGAATTGCAGAATTCAGTGAATCATCGAA
TCTTTGAACGCATCTTGCGCTCCTTGGTATTCCGAGGAGCATGCCTGTTTGAGTGTCATT
AAATTCTCAACTCTCTTATACTTTTTTGTAAAAGAGAGCTTGGACTGTGGAGGCTTGCTG
GCCACTTTTTGGGGTCAGCTCCTCTGAAATGCATTAGCGGAACCGTTTGCGATCTGCCAC
AAGTGTGATAAGTTATCTACACTGGCGAGGGGATTGCTCTCTGTAATGTTCAGCTTCTAA

TTGTCTCTACTTTGTGAGACTACTTTTGAATGCTTGACCTCAAATCAGGTAGGACTACCC
GCTGAACTTAA
>A9_46
TTTCCGTAGGTGAACCTGCGGAAGGATCATTATTGAATTATGTTTCTAGATAGGTTGTAG
CTGGCTCTTTTAGAGCATGTGCACGCCTGTTTGGACTTCATTTTCATCCACCTGTGCACC
TATTGTAGTCTTTGGTTGGGTTAGGAGGAAGTGATCATTGTATCAGCATCTGCTGGGAGT
GAGGACTTGCATTGTGAAAGCTTTGCTGTCCTTGATGTGATCATGGAATCTTTTTCTCAC
TAGAGTCTATGTCACTCATTATACTCTGTCGAATGTCATTGAATGTCTTTACATGGGCTT
GTATGCCTATGAAAATTGTAATACAACTTTCAGCAACGGATCTCTTGGCTCTCGCATCGA
TGAAGAACGCAGCGAAATGCGATAAGTAATGTGAATTGCAGAATTCAGTGAATCATCGAA
TCTTTGAACGCATCTTGCGCTCCTTGGTATTCCGAGGAGCATGCCTGTTTGAGTGTCATT
AAATTCTCAACTCTCTTATACTTTTTTGTAAAAGAGAGCTTGGACTGTGGAGGCTTGCTG
GCCACTTTTTGGGGTCAGCTCCTCTGAAATGCATTAGCGGAACCGTTTGCGATCTGCCAC
AAGTGTGATAAGTTATCTACACTGGCGAGGGGATTGCTCTCTGTAATGTTCAGCTTCTAA
TTGTCTCTACTTTGTGAGACTACTTTTGAATGCTTGACCTCAAATCAGGTAGGACTACCC
GCTGAACTTAA
>A9_47
TTTCCGTAGGTGAACCTGCGGAAGGATCATTATTGAATTATGTTTCTAGATAGGTTGTAG
CTGGCTCTTTTAGAGCATGTGCACGCCTGTTTGGACTTCATTTTCATCCACCTGTGCACC
TATTGTAGTCTTTGGTTGGGTTAGGAGGAAGTGATCATTGTATCAGCATCTGCTGGGAGT
GAGGACTTGCATTGTGAAAGCTTTGCTGTCCTTGATGTGATCATGGAATCTTTTTCTCAC
TAGAGTCTATGTCACTCATTATACTCTGTCGAATGTCATTGAATGTCTTTACATGGGCTT
GTATGCCTATGAAAATTGTAATACAACTTTCAGCAACGGATCTCTTGGCTCTCGCATCGA
TGAAGAACGCAGCGAAATGCGATAAGTAATGTGAATTGCAGAATTCAGTGAATCATCGAA
TCTTTGAACGCATCTTGCGCTCCTTGGTATTCCGAGGAGCATGCCTGTTTGAGTGTCATT
AAATTCTCAACTCTCTTATACTTTTTTGTAAAAGAGAGCTTGGACTGTGGAGGCTTGCTG
GCCACTTTTTGGGGTCAGCTCCTCTGAAATGCATTAGCGGAACCGTTTGCGATCTGCCAC
AAGTGTGATAAGTTATCTACACTGGCGAGGGGATTGCTCTCTGTAATGTTCAGCTTCTAA
TTGTCTCTACTTTGTGAGACTACTTTTGAATGCTTGACCTCAAATCAGGTAGGACTACCC
GCTGAACTTAA
>A9_51
TTTCCGTAGGTGAACCTGCGGAAGGATCATTATTGAATTATGTTTCTAGATAGGTTGTAG
CTGGCTCTTTTAGAGCATGTGCACGCCTGTTTGGACTTCATTTTCATCCACCTGTGCACC
TATTGTAGTCTTTGGTTGGGTTAGGAGGAAGTGATCATTGTATCAGCATCTGCTGGGAGT
GAGGACTTGCATTGTGAAAGCTTTGCTGTCCTTGATGTGATCATGGAATCTTTTTCTCAC
TAGAGTCTATGTCACTCATTATACTCTGTCGAATGTCATTGAATGTCTTTACATGGGCTT
GTATGCCTATGAAAATTGTAATACAACTTTCAGCAACGGATCTCTTGGCTCTCGCATCGA
TGAAGAACGCAGCGAAATGCGATAAGTAATGTGAATTGCAGAATTCAGTGAATCATCGAA
TCTTTGAACGCATCTTGCGCTCCTTGGTATTCCGAGGAGCATGCCTGTTTGAGTGTCATT
AAATTCTCAACTCTCTTATACTTTTTTGTAAAAGAGAGCTTGGACTGTGGAGGCTTGCTG
GCCACTTTTTGGGGTCAGCTCCTCTGAAATGCATTAGCGGAACCGTTTGCGATCTGCCAC
AAGTGTGATAAGTTATCTACACTGGCGAGGGGATTGCTCTCTGTAATGTTCAGCTTCTAA
TTGTCTCTACTTTGTGAGACTACTTTTGAATGCTTGACCTCAAATCAGGTAGGACTACCC
GCTGAACTTAA
>A10_30
TTTCCGTAGGTGAACCTGCGGAAGGATCATTATTGAATTATGTTTCTAGATAGGTTGTAG
CTGGCTCTTTTAGAGCATGTGCACGCCTGTTTGGACTTCATTTTCATCCACCTGTGCACC
TATTGTAGTCTTTGGTTGGGTTAGGAGGAAGTGATCATTGTATCAGCATCTGCTGGGAGT
GAGGACTTGCATTGTGAAAGCTTTGCTGTCCTTGATGTGATCATGGAATCTTTTTCTCAC
TAGAGTCTATGTCACTCATTATACTCTGTCGAATGTCATTGAATGTCTTTACATGGGCTT

GTATGCCTATGAAAATTGTAATACAACTTTCAGCAACGGATCTCTTGGCTCTCGCATCGA
TGAAGAACGCAGCGAAATGCGATAAGTAATGTGAATTGCAGAATTCAGTGAATCATCGAA
TCTTTGAACGCATCTTGCGCTCCTTGGTATTCCGAGGAGCATGCCTGTTTGAGTGTCATT
AAATTCTCAACTCTCTTATACTTTTTTGTAAAAGAGAGCTTGGACTGTGGAGGCTTGCTG
GCCACTTTTTGGGGTCAGCTCCTCTGAAATGCATTAGCGGAACCGTTTGCGATCTGCCAC
AAGTGTGATAAGTTATCTACACTGGCGAGGGGATTGCTCTCTGTAATGTTCAGCTTCTAA
TTGTCTCTACTTTGTGAGACTACTTTTGAATGCTTGACCTCAAATCAGGTAGGACTACCC
GCTGAACTTAA
>A10_31
TTTCCGTAGGTGAACCTGCGGAAGGATCATTATTGAATTATGTTTCTAGATAGGTTGTAG
CTGGCTCTTTTAGAGCATGTGCACGCCTGTTTGGACTTCATTTTCATCCACCTGTGCACC
TATTGTAGTCTTTGGTTGGGTTAGGAGGAAGTGATCATTGTATCAGCATCTGCTGGGAGT
GAGGACTTGCATTGTGAAAGCTTTGCTGTCCTTGATGTGATCATGGAATCTTTTTCTCAC
TAGAGTCTATGTCACTCATTATACTCTGTCGAATGTCATTGAATGTCTTTACATGGGCTT
GTATGCCTATGAAAATTGTAATACAACTTTCAGCAACGGATCTCTTGGCTCTCGCATCGA
TGAAGAACGCAGCGAAATGCGATAAGTAATGTGAATTGCAGAATTCAGTGAATCATCGAA
TCTTTGAACGCATCTTGCGCTCCTTGGTATTCCGAGGAGCATGCCTGTTTGAGTGTCATT
AAATTCTCAACTCTCTTATACTTTTTTGTAAAAGAGAGCTTGGACTGTGGAGGCTTGCTG
GCCACTTTTTGGGGTCAGCTCCTCTGAAATGCATTAGCGGAACCGTTTGCGATCTGCCAC
AAGTGTGATAAGTTATCTACACTGGCGAGGGGATTGCTCTCTGTAATGTTCAGCTTCTAA
TTGTCTCTACTTTGTGAGACTACTTTTGAATGCTTGACCTCAAATCAGGTAGGACTACCC
GCTGAACTTAA
>A10_33
TTTCCGTAGGTGAACCTGCGGAAGGATCATTATTGAATTATGTTTCTAGATAGGTTGTAG
CTGGCTCTTTTAGAGCATGTGCACGCCTGTTTGGACTTCATTTTCATCCACCTGTGCACC
TATTGTAGTCTTTGGTTGGGTTAGGAGGAAGTGATCATTGTATCAGCATCTGCTGGGAGT
GAGGACTTGCATTGTGAAAGCTTTGCTGTCCTTGATGTGATCATGGAATCTTTTTCTCAC
TAGAGTCTATGTCACTCATTATACTCTGTCGAATGTCATTGAATGTCTTTACATGGGCTT
GTATGCCTATGAAAATTGTAATACAACTTTCAGCAACGGATCTCTTGGCTCTCGCATCGA
TGAAGAACGCAGCGAAATGCGATAAGTAATGTGAATTGCAGAATTCAGTGAATCATCGAA
TCTTTGAACGCATCTTGCGCTCCTTGGTATTCCGAGGAGCATGCCTGTTTGAGTGTCATT
AAATTCTCAACTCTCTTATACTTTTTTGTAAAAGAGAGCTTGGACTGTGGAGGCTTGCTG
GCCACTTTTTGGGGTCAGCTCCTCTGAAATGCATTAGCGGAACCGTTTGCGATCTGCCAC
AAGTGTGATAAGTTATCTACACTGGCGAGGGGATTGCTCTCTGTAATGTTCAGCTTCTAA
TTGTCTCTACTTTGTGAGACTACTTTTGAATGCTTGACCTCAAATCAGGTAGGACTACCC
GCTGAACTTAA
>A10_34
TTTCCGTAGGTGAACCTGCGGAAGGATCATTATTGAATTATGTTTCTAGATAGGTTGTAG
CTGGCTCTTTTAGAGCATGTGCACGCCTGTTTGGACTTCATTTTCATCCACCTGTGCACC
TATTGTAGTCTTTGGTTGGGTTAGGAGGAAGTGATCATTGTATCAGCATCTGCTGGGAGT
GAGGACTTGCATTGTGAAAGCTTTGCTGTCCTTGATGTGATCATGGAATCTTTTTCTCAC
TAGAGTCTATGTCACTCATTATACTCTGTCGAATGTCATTGAATGTCTTTACATGGGCTT
GTATGCCTATGAAAATTGTAATACAACTTTCAGCAACGGATCTCTTGGCTCTCGCATCGA
TGAAGAACGCAGCGAAATGCGATAAGTAATGTGAATTGCAGAATTCAGTGAATCATCGAA
TCTTTGAACGCATCTTGCGCTCCTTGGTATTCCGAGGAGCATGCCTGTTTGAGTGTCATT
AAATTCTCAACTCTCTTATACTTTTTTGTAAAAGAGAGCTTGGACTGTGGAGGCTTGCTG
GCCACTTTTTGGGGTCAGCTCCTCTGAAATGCATTAGCGGAACCGTTTGCGATCTGCCAC
AAGTGTGATAAGTTATCTACACTGGCGAGGGGATTGCTCTCTGTAATGTTCAGCTTCTAA
TTGTCTCTACTTTGTGAGACTACTTTTGAATGCTTGACCTCAAATCAGGTAGGACTACCC
GCTGAACTTAA

>A10_35
TTTCCGTAGGTGAACCTGCGGAAGGATCATTATTGAATTATGTTTCTAGATAGGTTGTAG
CTGGCTCTTTTAGAGCATGTGCACGCCTGTTTGGACTTCATTTTCATCCACCTGTGCACC
TATTGTAGTCTTTGGTTGGGTTAGGAGGAAGTGATCATTGTATCAGCATCTGCTGGGAGT
GAGGACTTGCATTGTGAAAGCTTTGCTGTCCTTGATGTGATCATGGAATCTTTTTCTCAC
TAGAGTCTATGTCACTCATTATACTCTGTCGAATGTCATTGAATGTCTTTACATGGGCTT
GTATGCCTATGAAAATTGTAATACAACTTTCAGCAACGGATCTCTTGGCTCTCGCATCGA
TGAAGAACGCAGCGAAATGCGATAAGTAATGTGAATTGCAGAATTCAGTGAATCATCGAA
TCTTTGAACGCATCTTGCGCTCCTTGGTATTCCGAGGAGCATGCCTGTTTGAGTGTCATT
AAATTCTCAACTCTCTTATACTTTTTTGTAAAAGAGAGCTTGGACTGTGGAGGCTTGCTG
GCCACTTTTTGGGGTCAGCTCCTCTGAAATGCATTAGCGGAACCGTTTGCGATCTGCCAC
AAGTGTGATAAGTTATCTACACTGGCGAGGGGATTGCTCTCTGTAATGTTCAGCTTCTAA
TTGTCTCTACTTTGTGAGACTACTTTTGAATGCTTGACCTCAAATCAGGTAGGACTACCC
GCTGAACTTAA
>A10_36
TTTCCGTAGGTGAACCTGCGGAAGGATCATTATTGAATTATGTTTCTAGATAGGTTGTAG
CTGGCTCTTTTAGAGCATGTGCACGCCTGTTTGGACTTCATTTTCATCCACCTGTGCACC
TATTGTAGTCTTTGGTTGGGTTAGGAGGAAGTGATCATTGTATCAGCATCTGCTGGGAGT
GAGGACTTGCATTGTGAAAGCTTTGCTGTCCTTGATGTGATCATGGAATCTTTTTCTCAC
TAGAGTCTATGTCACTCATTATACTCTGTCGAATGTCATTGAATGTCTTTACATGGGCTT
GTATGCCTATGAAAATTGTAATACAACTTTCAGCAACGGATCTCTTGGCTCTCGCATCGA
TGAAGAACGCAGCGAAATGCGATAAGTAATGTGAATTGCAGAATTCAGTGAATCATCGAA
TCTTTGAACGCATCTTGCGCTCCTTGGTATTCCGAGGAGCATGCCTGTTTGAGTGTCATT
AAATTCTCAACTCTCTTATACTTTTTTGTAAAAGAGAGCTTGGACTGTGGAGGCTTGCTG
GCCACTTTTTGGGGTCAGCTCCTCTGAAATGCATTAGCGGAACCGTTTGCGATCTGCCAC
AAGTGTGATAAGTTATCTACACTGGCGAGGGGATTGCTCTCTGTAATGTTCAGCTTCTAA
TTGTCTCTACTTTGTGAGACTACTTTTGAATGCTTGACCTCAAATCAGGTAGGACTACCC
GCTGAACTTAA
>A10_37
TTTCCGTAGGTGAACCTGCGGAAGGATCATTATTGAATTATGTTTCTAGATAGGTTGTAG
CTGGCTCTTTTAGAGCATGTGCACGCCTGTTTGGACTTCATTTTCATCCACCTGTGCACC
TATTGTAGTCTTTGGTTGGGTTAGGAGGAAGTGATCATTGTATCAGCATCTGCTGGGAGT
GAGGACTTGCATTGTGAAAGCTTTGCTGTCCTTGATGTGATCATGGAATCTTTTTCTCAC
TAGAGTCTATGTCACTCATTATACTCTGTCGAATGTCATTGAATGTCTTTACATGGGCTT
GTATGCCTATGAAAATTGTAATACAACTTTCAGCAACGGATCTCTTGGCTCTCGCATCGA
TGAAGAACGCAGCGAAATGCGATAAGTAATGTGAATTGCAGAATTCAGTGAATCATCGAA
TCTTTGAACGCATCTTGCGCTCCTTGGTATTCCGAGGAGCATGCCTGTTTGAGTGTCATT
AAATTCTCAACTCTCTTATACTTTTTTGTAAAAGAGAGCTTGGACTGTGGAGGCTTGCTG
GCCACTTTTTGGGGTCAGCTCCTCTGAAATGCATTAGCGGAACCGTTTGCGATCTGCCAC
AAGTGTGATAAGTTATCTACACTGGCGAGGGGATTGCTCTCTGTAATGTTCAGCTTCTAA
TTGTCTCTACTTTGTGAGACTACTTTTGAATGCTTGACCTCAAATCAGGTAGGACTACCC
GCTGAACTTAA
>A10_39
TTTCCGTAGGTGAACCTGCGGAAGGATCATTATTGAATTATGTTTCTAGATAGGTTGTAG
CTGGCTCTTTTAGAGCATGTGCACGCCTGTTTGGACTTCATTTTCATCCACCTGTGCACC
TATTGTAGTCTTTGGTTGGGTTAGGAGGAAGTGATCATTGTATCAGCATCTGCTGGGAGT
GAGGACTTGCATTGTGAAAGCTTTGCTGTCCTTGATGTGATCATGGAATCTTTTTCTCAC
TAGAGTCTATGTCACTCATTATACTCTGTCGAATGTCATTGAATGTCTTTACATGGGCTT
GTATGCCTATGAAAATTGTAATACAACTTTCAGCAACGGATCTCTTGGCTCTCGCATCGA
TGAAGAACGCAGCGAAATGCGATAAGTAATGTGAATTGCAGAATTCAGTGAATCATCGAA

TCTTTGAACGCATCTTGCGCTCCTTGGTATTCCGAGGAGCATGCCTGTTTGAGTGTCATT
AAATTCTCAACTCTCTTATACTTTTTTGTAAAAGAGAGCTTGGACTGTGGAGGCTTGCTG
GCCACTTTTTGGGGTCAGCTCCTCTGAAATGCATTAGCGGAACCGTTTGCGATCTGCCAC
AAGTGTGATAAGTTATCTACACTGGCGAGGGGATTGCTCTCTGTAATGTTCAGCTTCTAA
TTGTCTCTACTTTGTGAGACTACTTTTGAATGCTTGACCTCAAATCAGGTAGGACTACCC
GCTGAACTTAA
>A9_54
TTTCCGTAGGTGAACCTGCGGAAGGATCATTATTGAATTATGTTTCTAGATAGGTTGTAG
CTGGCTCTTTTAGAGCATGTGCACGCCTGTTTGGACTTCATTTTCATCCACCTGTGCACC
TATTGTAGTCTTTGGTTGGGTTAGGAGGAAGTGATCATTGTATCAGCATCTGCTGGGAGT
GAGGACTTGCATTGTGAAAGCTTTGCTGTCCTTGATGTGATCATGGAATCTTTTTCTCAC
TAGAGTCTATGTCACTCATTATACTCTGTCGAATGTCATTGAATGTCTTTACATGGGCTT
GTATGCCTATGAAAATTGTAATACAACTTTCAGCAACGGATCTCTTGGCTCTCGCATCGA
TGAAGAACGCAGCGAAATGCGATAAGTAATGTGAATTGCAGAATTCAGTGAATCATCGAA
TCTTTGAACGCATCTTGCGCTCCTTGGTATTCCGAGGAGCATGCCTGTTTGAGTGTCATT
AAATTCTCAACTCTCTTATACTTTTTTGTAAAAGAGAGCTTGGACTGTGGAGGCTTGCTG
GCCACTTTTTGGGGTCAGCTCCTCTGAAATGCATTAGCGGAACCGTTTGCGATCTGCCAC
AAGTGTGATAAGTTATCTACACTGGCGAGGGGATTGCTCTCTGTAATGTTCAGCTTCTAA
TTGTCTCTACTTTGTGAGACTACTTTTGAATGCTTGACCTCAAATCAGGTAGGACTACCC
GCTGAACTTAA
>A9_57
TTTCCGTAGGTGAACCTGCGGAAGGATCATTATTGAATTATGTTTCTAGATAGGTTGTAG
CTGGCTCTTTTAGAGCATGTGCACGCCTGTTTGGACTTCATTTTCATCCACCTGTGCACC
TATTGTAGTCTTTGGTTGGGTTAGGAGGAAGTGATCATTGTATCAGCATCTGCTGGGAGT
GAGGACTTGCATTGTGAAAGCTTTGCTGTCCTTGATGTGATCATGGAATCTTTTTCTCAC
TAGAGTCTATGTCACTCATTATACTCTGTCGAATGTCATTGAATGTCTTTACATGGGCTT
GTATGCCTATGAAAATTGTAATACAACTTTCAGCAACGGATCTCTTGGCTCTCGCATCGA
TGAAGAACGCAGCGAAATGCGATAAGTAATGTGAATTGCAGAATTCAGTGAATCATCGAA
TCTTTGAACGCATCTTGCGCTCCTTGGTATTCCGAGGAGCATGCCTGTTTGAGTGTCATT
AAATTCTCAACTCTCTTATACTTTTTTGTAAAAGAGAGCTTGGACTGTGGAGGCTTGCTG
GCCACTTTTTGGGGTCAGCTCCTCTGAAATGCATTAGCGGAACCGTTTGCGATCTGCCAC
AAGTGTGATAAGTTATCTACACTGGCGAGGGGATTGCTCTCTGTAATGTTCAGCTTCTAA
TTGTCTCTACTTTGTGAGACTACTTTTGAATGCTTGACCTCAAATCAGGTAGGACTACCC
GCTGAACTTAA
>A9_58
TTTCCGTAGGTGAACCTGCGGAAGGATCATTATTGAATTATGTTTCTAGATAGGTTGTAG
CTGGCTCTTTTAGAGCATGTGCACGCCTGTTTGGACTTCATTTTCATCCACCTGTGCACC
TATTGTAGTCTTTGGTTGGGTTAGGAGGAAGTGATCATTGTATCAGCATCTGCTGGGAGT
GAGGACTTGCATTGTGAAAGCTTTGCTGTCCTTGATGTGATCATGGAATCTTTTTCTCAC
TAGAGTCTATGTCACTCATTATACTCTGTCGAATGTCATTGAATGTCTTTACATGGGCTT
GTATGCCTATGAAAATTGTAATACAACTTTCAGCAACGGATCTCTTGGCTCTCGCATCGA
TGAAGAACGCAGCGAAATGCGATAAGTAATGTGAATTGCAGAATTCAGTGAATCATCGAA
TCTTTGAACGCATCTTGCGCTCCTTGGTATTCCGAGGAGCATGCCTGTTTGAGTGTCATT
AAATTCTCAACTCTCTTATACTTTTTTGTAAAAGAGAGCTTGGACTGTGGAGGCTTGCTG
GCCACTTTTTGGGGTCAGCTCCTCTGAAATGCATTAGCGGAACCGTTTGCGATCTGCCAC
AAGTGTGATAAGTTATCTACACTGGCGAGGGGATTGCTCTCTGTAATGTTCAGCTTCTAA
TTGTCTCTACTTTGTGAGACTACTTTTGAATGCTTGACCTCAAATCAGGTAGGACTACCC
GCTGAACTTAA
>A9_59
TTTCCGTAGGTGAACCTGCGGAAGGATCATTATTGAATTATGTTTCTAGATAGGTTGTAG

CTGGCTCTTTTAGAGCATGTGCACGCCTGTTTGGACTTCATTTTCATCCACCTGTGCACC
TATTGTAGTCTTTGGTTGGGTTAGGAGGAAGTGATCATTGTATCAGCATCTGCTGGGAGT
GAGGACTTGCATTGTGAAAGCTTTGCTGTCCTTGATGTGATCATGGAATCTTTTTCTCAC
TAGAGTCTATGTCACTCATTATACTCTGTCGAATGTCATTGAATGTCTTTACATGGGCTT
GTATGCCTATGAAAATTGTAATACAACTTTCAGCAACGGATCTCTTGGCTCTCGCATCGA
TGAAGAACGCAGCGAAATGCGATAAGTAATGTGAATTGCAGAATTCAGTGAATCATCGAA
TCTTTGAACGCATCTTGCGCTCCTTGGTATTCCGAGGAGCATGCCTGTTTGAGTGTCATT
AAATTCTCAACTCTCTTATACTTTTTTGTAAAAGAGAGCTTGGACTGTGGAGGCTTGCTG
GCCACTTTTTGGGGTCAGCTCCTCTGAAATGCATTAGCGGAACCGTTTGCGATCTGCCAC
AAGTGTGATAAGTTATCTACACTGGCGAGGGGATTGCTCTCTGTAATGTTCAGCTTCTAA
TTGTCTCTACTTTGTGAGACTACTTTTGAATGCTTGACCTCAAATCAGGTAGGACTACCC
GCTGAACTTAA
>A9_60
TTTCCGTAGGTGAACCTGCGGAAGGATCATTATTGAATTATGTTTCTAGATAGGTTGTAG
CTGGCTCTTTTAGAGCATGTGCACGCCTGTTTGGACTTCATTTTCATCCACCTGTGCACC
TATTGTAGTCTTTGGTTGGGTTAGGAGGAAGTGATCATTGTATCAGCATCTGCTGGGAGT
GAGGACTTGCATTGTGAAAGCTTTGCTGTCCTTGATGTGATCATGGAATCTTTTTCTCAC
TAGAGTCTATGTCACTCATTATACTCTGTCGAATGTCATTGAATGTCTTTACATGGGCTT
GTATGCCTATGAAAATTGTAATACAACTTTCAGCAACGGATCTCTTGGCTCTCGCATCGA
TGAAGAACGCAGCGAAATGCGATAAGTAATGTGAATTGCAGAATTCAGTGAATCATCGAA
TCTTTGAACGCATCTTGCGCTCCTTGGTATTCCGAGGAGCATGCCTGTTTGAGTGTCATT
AAATTCTCAACTCTCTTATACTTTTTTGTAAAAGAGAGCTTGGACTGTGGAGGCTTGCTG
GCCACTTTTTGGGGTCAGCTCCTCTGAAATGCATTAGCGGAACCGTTTGCGATCTGCCAC
AAGTGTGATAAGTTATCTACACTGGCGAGGGGATTGCTCTCTGTAATGTTCAGCTTCTAA
TTGTCTCTACTTTGTGAGACTACTTTTGAATGCTTGACCTCAAATCAGGTAGGACTACCC
GCTGAACTTAA
>A9_61
TTTCCGTAGGTGAACCTGCGGAAGGATCATTATTGAATTATGTTTCTAGATAGGTTGTAG
CTGGCTCTTTTAGAGCATGTGCACGCCTGTTTGGACTTCATTTTCATCCACCTGTGCACC
TATTGTAGTCTTTGGTTGGGTTAGGAGGAAGTGATCATTGTATCAGCATCTGCTGGGAGT
GAGGACTTGCATTGTGAAAGCTTTGCTGTCCTTGATGTGATCATGGAATCTTTTTCTCAC
TAGAGTCTATGTCACTCATTATACTCTGTCGAATGTCATTGAATGTCTTTACATGGGCTT
GTATGCCTATGAAAATTGTAATACAACTTTCAGCAACGGATCTCTTGGCTCTCGCATCGA
TGAAGAACGCAGCGAAATGCGATAAGTAATGTGAATTGCAGAATTCAGTGAATCATCGAA
TCTTTGAACGCATCTTGCGCTCCTTGGTATTCCGAGGAGCATGCCTGTTTGAGTGTCATT
AAATTCTCAACTCTCTTATACTTTTTTGTAAAAGAGAGCTTGGACTGTGGAGGCTTGCTG
GCCACTTTTTGGGGTCAGCTCCTCTGAAATGCATTAGCGGAACCGTTTGCGATCTGCCAC
AAGTGTGATAAGTTATCTACACTGGCGAGGGGATTGCTCTCTGTAATGTTCAGCTTCTAA
TTGTCTCTACTTTGTGAGACTACTTTTGAATGCTTGACCTCAAATCAGGTAGGACTACCC
GCTGAACTTAA
>A9_65
TTTCCGTAGGTGAACCTGCGGAAGGATCATTATTGAATTATGTTTCTAGATAGGTTGTAG
CTGGCTCTTTTAGAGCATGTGCACGCCTGTTTGGACTTCATTTTCATCCACCTGTGCACC
TATTGTAGTCTTTGGTTGGGTTAGGAGGAAGTGATCATTGTATCAGCATCTGCTGGGAGT
GAGGACTTGCATTGTGAAAGCTTTGCTGTCCTTGATGTGATCATGGAATCTTTTTCTCAC
TAGAGTCTATGTCACTCATTATACTCTGTCGAATGTCATTGAATGTCTTTACATGGGCTT
GTATGCCTATGAAAATTGTAATACAACTTTCAGCAACGGATCTCTTGGCTCTCGCATCGA
TGAAGAACGCAGCGAAATGCGATAAGTAATGTGAATTGCAGAATTCAGTGAATCATCGAA
TCTTTGAACGCATCTTGCGCTCCTTGGTATTCCGAGGAGCATGCCTGTTTGAGTGTCATT
AAATTCTCAACTCTCTTATACTTTTTTGTAAAAGAGAGCTTGGACTGTGGAGGCTTGCTG

GCCACTTTTTGGGGTCAGCTCCTCTGAAATGCATTAGCGGAACCGTTTGCGATCTGCCAC
AAGTGTGATAAGTTATCTACACTGGCGAGGGGATTGCTCTCTGTAATGTTCAGCTTCTAA
TTGTCTCTACTTTGTGAGACTACTTTTGAATGCTTGACCTCAAATCAGGTAGGACTACCC
GCTGAACTTAA
>A9_66
TTTCCGTAGGTGAACCTGCGGAAGGATCATTATTGAATTATGTTTCTAGATAGGTTGTAG
CTGGCTCTTTTAGAGCATGTGCACGCCTGTTTGGACTTCATTTTCATCCACCTGTGCACC
TATTGTAGTCTTTGGTTGGGTTAGGAGGAAGTGATCATTGTATCAGCATCTGCTGGGAGT
GAGGACTTGCATTGTGAAAGCTTTGCTGTCCTTGATGTGATCATGGAATCTTTTTCTCAC
TAGAGTCTATGTCACTCATTATACTCTGTCGAATGTCATTGAATGTCTTTACATGGGCTT
GTATGCCTATGAAAATTGTAATACAACTTTCAGCAACGGATCTCTTGGCTCTCGCATCGA
TGAAGAACGCAGCGAAATGCGATAAGTAATGTGAATTGCAGAATTCAGTGAATCATCGAA
TCTTTGAACGCATCTTGCGCTCCTTGGTATTCCGAGGAGCATGCCTGTTTGAGTGTCATT
AAATTCTCAACTCTCTTATACTTTTTTGTAAAAGAGAGCTTGGACTGTGGAGGCTTGCTG
GCCACTTTTTGGGGTCAGCTCCTCTGAAATGCATTAGCGGAACCGTTTGCGATCTGCCAC
AAGTGTGATAAGTTATCTACACTGGCGAGGGGATTGCTCTCTGTAATGTTCAGCTTCTAA
TTGTCTCTACTTTGTGAGACTACTTTTGAATGCTTGACCTCAAATCAGGTAGGACTACCC
GCTGAACTTAA
>A9_67
TTTCCGTAGGTGAACCTGCGGAAGGATCATTATTGAATTATGTTTCTAGATAGGTTGTAG
CTGGCTCTTTTAGAGCATGTGCACGCCTGTTTGGACTTCATTTTCATCCACCTGTGCACC
TATTGTAGTCTTTGGTTGGGTTAGGAGGAAGTGATCATTGTATCAGCATCTGCTGGGAGT
GAGGACTTGCATTGTGAAAGCTTTGCTGTCCTTGATGTGATCATGGAATCTTTTTCTCAC
TAGAGTCTATGTCACTCATTATACTCTGTCGAATGTCATTGAATGTCTTTACATGGGCTT
GTATGCCTATGAAAATTGTAATACAACTTTCAGCAACGGATCTCTTGGCTCTCGCATCGA
TGAAGAACGCAGCGAAATGCGATAAGTAATGTGAATTGCAGAATTCAGTGAATCATCGAA
TCTTTGAACGCATCTTGCGCTCCTTGGTATTCCGAGGAGCATGCCTGTTTGAGTGTCATT
AAATTCTCAACTCTCTTATACTTTTTTGTAAAAGAGAGCTTGGACTGTGGAGGCTTGCTG
GCCACTTTTTGGGGTCAGCTCCTCTGAAATGCATTAGCGGAACCGTTTGCGATCTGCCAC
AAGTGTGATAAGTTATCTACACTGGCGAGGGGATTGCTCTCTGTAATGTTCAGCTTCTAA
TTGTCTCTACTTTGTGAGACTACTTTTGAATGCTTGACCTCAAATCAGGTAGGACTACCC
GCTGAACTTAA
>A10_1
TTTCCGTAGGTGAACCTGCGGAAGGATCATTATTGAATTATGTTTCTAGATAGGTTGTAG
CTGGCTCTTTTAGAGCATGTGCACGCCTGTTTGGACTTCATTTTCATCCACCTGTGCACC
TATTGTAGTCTTTGGTTGGGTTAGGAGGAAGTGATCATTGTATCAGCATCTGCTGGGAGT
GAGGACTTGCATTGTGAAAGCTTTGCTGTCCTTGATGTGATCATGGAATCTTTTTCTCAC
TAGAGTCTATGTCACTCATTATACTCTGTCGAATGTCATTGAATGTCTTTACATGGGCTT
GTATGCCTATGAAAATTGTAATACAACTTTCAGCAACGGATCTCTTGGCTCTCGCATCGA
TGAAGAACGCAGCGAAATGCGATAAGTAATGTGAATTGCAGAATTCAGTGAATCATCGAA
TCTTTGAACGCATCTTGCGCTCCTTGGTATTCCGAGGAGCATGCCTGTTTGAGTGTCATT
AAATTCTCAACTCTCTTATACTTTTTTGTAAAAGAGAGCTTGGACTGTGGAGGCTTGCTG
GCCACTTTTTGGGGTCAGCTCCTCTGAAATGCATTAGCGGAACCGTTTGCGATCTGCCAC
AAGTGTGATAAGTTATCTACACTGGCGAGGGGATTGCTCTCTGTAATGTTCAGCTTCTAA
TTGTCTCTACTTTGTGAGACTACTTTTGAATGCTTGACCTCAAATCAGGTAGGACTACCC
GCTGAACTTAA
>A10_18
TTTCCGTAGGTGAACCTGCGGAAGGATCATTATTGAATTATGTTTCTAGATAGGTTGTAG
CTGGCTCTTTTAGAGCATGTGCACGCCTGTTTGGACTTCATTTTCATCCACCTGTGCACC
TATTGTAGTCTTTGGTTGGGTTAGGAGGAAGTGATCATTGTATCAGCATCTGCTGGGAGT

GAGGACTTGCATTGTGAAAGCTTTGCTGTCCTTGATGTGATCATGGAATCTTTTTCTCAC
TAGAGTCTATGTCACTCATTATACTCTGTCGAATGTCATTGAATGTCTTTACATGGGCTT
GTATGCCTATGAAAATTGTAATACAACTTTCAGCAACGGATCTCTTGGCTCTCGCATCGA
TGAAGAACGCAGCGAAATGCGATAAGTAATGTGAATTGCAGAATTCAGTGAATCATCGAA
TCTTTGAACGCATCTTGCGCTCCTTGGTATTCCGAGGAGCATGCCTGTTTGAGTGTCATT
AAATTCTCAACTCTCTTATACTTTTTTGTAAAAGAGAGCTTGGACTGTGGAGGCTTGCTG
GCCACTTTTTGGGGTCAGCTCCTCTGAAATGCATTAGCGGAACCGTTTGCGATCTGCCAC
AAGTGTGATAAGTTATCTACACTGGCGAGGGGATTGCTCTCTGTAATGTTCAGCTTCTAA
TTGTCTCTACTTTGTGAGACTACTTTTGAATGCTTGACCTCAAATCAGGTAGGACTACCC
GCTGAACTTAA
>A10_19
TTTCCGTAGGTGAACCTGCGGAAGGATCATTATTGAATTATGTTTCTAGATAGGTTGTAG
CTGGCTCTTTTAGAGCATGTGCACGCCTGTTTGGACTTCATTTTCATCCACCTGTGCACC
TATTGTAGTCTTTGGTTGGGTTAGGAGGAAGTGATCATTGTATCAGCATCTGCTGGGAGT
GAGGACTTGCATTGTGAAAGCTTTGCTGTCCTTGATGTGATCATGGAATCTTTTTCTCAC
TAGAGTCTATGTCACTCATTATACTCTGTCGAATGTCATTGAATGTCTTTACATGGGCTT
GTATGCCTATGAAAATTGTAATACAACTTTCAGCAACGGATCTCTTGGCTCTCGCATCGA
TGAAGAACGCAGCGAAATGCGATAAGTAATGTGAATTGCAGAATTCAGTGAATCATCGAA
TCTTTGAACGCATCTTGCGCTCCTTGGTATTCCGAGGAGCATGCCTGTTTGAGTGTCATT
AAATTCTCAACTCTCTTATACTTTTTTGTAAAAGAGAGCTTGGACTGTGGAGGCTTGCTG
GCCACTTTTTGGGGTCAGCTCCTCTGAAATGCATTAGCGGAACCGTTTGCGATCTGCCAC
AAGTGTGATAAGTTATCTACACTGGCGAGGGGATTGCTCTCTGTAATGTTCAGCTTCTAA
TTGTCTCTACTTTGTGAGACTACTTTTGAATGCTTGACCTCAAATCAGGTAGGACTACCC
GCTGAACTTAA
>A10_20
TTTCCGTAGGTGAACCTGCGGAAGGATCATTATTGAATTATGTTTCTAGATAGGTTGTAG
CTGGCTCTTTTAGAGCATGTGCACGCCTGTTTGGACTTCATTTTCATCCACCTGTGCACC
TATTGTAGTCTTTGGTTGGGTTAGGAGGAAGTGATCATTGTATCAGCATCTGCTGGGAGT
GAGGACTTGCATTGTGAAAGCTTTGCTGTCCTTGATGTGATCATGGAATCTTTTTCTCAC
TAGAGTCTATGTCACTCATTATACTCTGTCGAATGTCATTGAATGTCTTTACATGGGCTT
GTATGCCTATGAAAATTGTAATACAACTTTCAGCAACGGATCTCTTGGCTCTCGCATCGA
TGAAGAACGCAGCGAAATGCGATAAGTAATGTGAATTGCAGAATTCAGTGAATCATCGAA
TCTTTGAACGCATCTTGCGCTCCTTGGTATTCCGAGGAGCATGCCTGTTTGAGTGTCATT
AAATTCTCAACTCTCTTATACTTTTTTGTAAAAGAGAGCTTGGACTGTGGAGGCTTGCTG
GCCACTTTTTGGGGTCAGCTCCTCTGAAATGCATTAGCGGAACCGTTTGCGATCTGCCAC
AAGTGTGATAAGTTATCTACACTGGCGAGGGGATTGCTCTCTGTAATGTTCAGCTTCTAA
TTGTCTCTACTTTGTGAGACTACTTTTGAATGCTTGACCTCAAATCAGGTAGGACTACCC
GCTGAACTTAA
>A10_21
TTTCCGTAGGTGAACCTGCGGAAGGATCATTATTGAATTATGTTTCTAGATAGGTTGTAG
CTGGCTCTTTTAGAGCATGTGCACGCCTGTTTGGACTTCATTTTCATCCACCTGTGCACC
TATTGTAGTCTTTGGTTGGGTTAGGAGGAAGTGATCATTGTATCAGCATCTGCTGGGAGT
GAGGACTTGCATTGTGAAAGCTTTGCTGTCCTTGATGTGATCATGGAATCTTTTTCTCAC
TAGAGTCTATGTCACTCATTATACTCTGTCGAATGTCATTGAATGTCTTTACATGGGCTT
GTATGCCTATGAAAATTGTAATACAACTTTCAGCAACGGATCTCTTGGCTCTCGCATCGA
TGAAGAACGCAGCGAAATGCGATAAGTAATGTGAATTGCAGAATTCAGTGAATCATCGAA
TCTTTGAACGCATCTTGCGCTCCTTGGTATTCCGAGGAGCATGCCTGTTTGAGTGTCATT
AAATTCTCAACTCTCTTATACTTTTTTGTAAAAGAGAGCTTGGACTGTGGAGGCTTGCTG
GCCACTTTTTGGGGTCAGCTCCTCTGAAATGCATTAGCGGAACCGTTTGCGATCTGCCAC
AAGTGTGATAAGTTATCTACACTGGCGAGGGGATTGCTCTCTGTAATGTTCAGCTTCTAA

TTGTCTCTACTTTGTGAGACTACTTTTGAATGCTTGACCTCAAATCAGGTAGGACTACCC
GCTGAACTTAA
>A11_30
TTTCCGTAGGTGAACCTGCGGAAGGATCATTATTGAATTATGTTTCTAGATAGGTTGTAG
CTGGCTCTTTTAGAGCATGTGCACGCCTGTTTGGACTTCATTTTCATCCACCTGTGCACC
TATTGTAGTCTTTGGTTGGGTTAGGAGGAAGTGATCATTGTATCAGCATCTGCTGGGAGT
GAGGACTTGCATTGTGAAAGCTTTGCTGTCCTTGATGTGATCATGGAATCTTTTTCTCAC
TAGAGTCTATGTCACTCATTATACTCTGTCGAATGTCATTGAATGTCTTTACATGGGCTT
GTATGCCTATGAAAATTGTAATACAACTTTCAGCAACGGATCTCTTGGCTCTCGCATCGA
TGAAGAACGCAGCGAAATGCGATAAGTAATGTGAATTGCAGAATTCAGTGAATCATCGAA
TCTTTGAACGCATCTTGCGCTCCTTGGTATTCCGAGGAGCATGCCTGTTTGAGTGTCATT
AAATTCTCAACTCTCTTATACTTTTTTGTAAAAGAGAGCTTGGACTGTGGAGGCTTGCTG
GCCACTTTTTGGGGTCAGCTCCTCTGAAATGCATTAGCGGAACCGTTTGCGATCTGCCAC
AAGTGTGATAAGTTATCTACACTGGCGAGGGGATTGCTCTCTGTAATGTTCAGCTTCTAA
TTGTCTCTACTTTGTGAGACTACTTTTGAATGCTTGACCTCAAATCAGGTAGGACTACCC
GCTGAACTTAA
>A11_31
TTTCCGTAGGTGAACCTGCGGAAGGATCATTATTGAATTATGTTTCTAGATAGGTTGTAG
CTGGCTCTTTTAGAGCATGTGCACGCCTGTTTGGACTTCATTTTCATCCACCTGTGCACC
TATTGTAGTCTTTGGTTGGGTTAGGAGGAAGTGATCATTGTATCAGCATCTGCTGGGAGT
GAGGACTTGCATTGTGAAAGCTTTGCTGTCCTTGATGTGATCATGGAATCTTTTTCTCAC
TAGAGTCTATGTCACTCATTATACTCTGTCGAATGTCATTGAATGTCTTTACATGGGCTT
GTATGCCTATGAAAATTGTAATACAACTTTCAGCAACGGATCTCTTGGCTCTCGCATCGA
TGAAGAACGCAGCGAAATGCGATAAGTAATGTGAATTGCAGAATTCAGTGAATCATCGAA
TCTTTGAACGCATCTTGCGCTCCTTGGTATTCCGAGGAGCATGCCTGTTTGAGTGTCATT
AAATTCTCAACTCTCTTATACTTTTTTGTAAAAGAGAGCTTGGACTGTGGAGGCTTGCTG
GCCACTTTTTGGGGTCAGCTCCTCTGAAATGCATTAGCGGAACCGTTTGCGATCTGCCAC
AAGTGTGATAAGTTATCTACACTGGCGAGGGGATTGCTCTCTGTAATGTTCAGCTTCTAA
TTGTCTCTACTTTGTGAGACTACTTTTGAATGCTTGACCTCAAATCAGGTAGGACTACCC
GCTGAACTTAA
>A11_32
TTTCCGTAGGTGAACCTGCGGAAGGATCATTATTGAATTATGTTTCTAGATAGGTTGTAG
CTGGCTCTTTTAGAGCATGTGCACGCCTGTTTGGACTTCATTTTCATCCACCTGTGCACC
TATTGTAGTCTTTGGTTGGGTTAGGAGGAAGTGATCATTGTATCAGCATCTGCTGGGAGT
GAGGACTTGCATTGTGAAAGCTTTGCTGTCCTTGATGTGATCATGGAATCTTTTTCTCAC
TAGAGTCTATGTCACTCATTATACTCTGTCGAATGTCATTGAATGTCTTTACATGGGCTT
GTATGCCTATGAAAATTGTAATACAACTTTCAGCAACGGATCTCTTGGCTCTCGCATCGA
TGAAGAACGCAGCGAAATGCGATAAGTAATGTGAATTGCAGAATTCAGTGAATCATCGAA
TCTTTGAACGCATCTTGCGCTCCTTGGTATTCCGAGGAGCATGCCTGTTTGAGTGTCATT
AAATTCTCAACTCTCTTATACTTTTTTGTAAAAGAGAGCTTGGACTGTGGAGGCTTGCTG
GCCACTTTTTGGGGTCAGCTCCTCTGAAATGCATTAGCGGAACCGTTTGCGATCTGCCAC
AAGTGTGATAAGTTATCTACACTGGCGAGGGGATTGCTCTCTGTAATGTTCAGCTTCTAA
TTGTCTCTACTTTGTGAGACTACTTTTGAATGCTTGACCTCAAATCAGGTAGGACTACCC
GCTGAACTTAA
>A11_33
TTTCCGTAGGTGAACCTGCGGAAGGATCATTATTGAATTATGTTTCTAGATAGGTTGTAG
CTGGCTCTTTTAGAGCATGTGCACGCCTGTTTGGACTTCATTTTCATCCACCTGTGCACC
TATTGTAGTCTTTGGTTGGGTTAGGAGGAAGTGATCATTGTATCAGCATCTGCTGGGAGT
GAGGACTTGCATTGTGAAAGCTTTGCTGTCCTTGATGTGATCATGGAATCTTTTTCTCAC
TAGAGTCTATGTCACTCATTATACTCTGTCGAATGTCATTGAATGTCTTTACATGGGCTT

GTATGCCTATGAAAATTGTAATACAACTTTCAGCAACGGATCTCTTGGCTCTCGCATCGA
TGAAGAACGCAGCGAAATGCGATAAGTAATGTGAATTGCAGAATTCAGTGAATCATCGAA
TCTTTGAACGCATCTTGCGCTCCTTGGTATTCCGAGGAGCATGCCTGTTTGAGTGTCATT
AAATTCTCAACTCTCTTATACTTTTTTGTAAAAGAGAGCTTGGACTGTGGAGGCTTGCTG
GCCACTTTTTGGGGTCAGCTCCTCTGAAATGCATTAGCGGAACCGTTTGCGATCTGCCAC
AAGTGTGATAAGTTATCTACACTGGCGAGGGGATTGCTCTCTGTAATGTTCAGCTTCTAA
TTGTCTCTACTTTGTGAGACTACTTTTGAATGCTTGACCTCAAATCAGGTAGGACTACCC
GCTGAACTTAA
>A11_35
TTTCCGTAGGTGAACCTGCGGAAGGATCATTATTGAATTATGTTTCTAGATAGGTTGTAG
CTGGCTCTTTTAGAGCATGTGCACGCCTGTTTGGACTTCATTTTCATCCACCTGTGCACC
TATTGTAGTCTTTGGTTGGGTTAGGAGGAAGTGATCATTGTATCAGCATCTGCTGGGAGT
GAGGACTTGCATTGTGAAAGCTTTGCTGTCCTTGATGTGATCATGGAATCTTTTTCTCAC
TAGAGTCTATGTCACTCATTATACTCTGTCGAATGTCATTGAATGTCTTTACATGGGCTT
GTATGCCTATGAAAATTGTAATACAACTTTCAGCAACGGATCTCTTGGCTCTCGCATCGA
TGAAGAACGCAGCGAAATGCGATAAGTAATGTGAATTGCAGAATTCAGTGAATCATCGAA
TCTTTGAACGCATCTTGCGCTCCTTGGTATTCCGAGGAGCATGCCTGTTTGAGTGTCATT
AAATTCTCAACTCTCTTATACTTTTTTGTAAAAGAGAGCTTGGACTGTGGAGGCTTGCTG
GCCACTTTTTGGGGTCAGCTCCTCTGAAATGCATTAGCGGAACCGTTTGCGATCTGCCAC
AAGTGTGATAAGTTATCTACACTGGCGAGGGGATTGCTCTCTGTAATGTTCAGCTTCTAA
TTGTCTCTACTTTGTGAGACTACTTTTGAATGCTTGACCTCAAATCAGGTAGGACTACCC
GCTGAACTTAA
>A11_36
TTTCCGTAGGTGAACCTGCGGAAGGATCATTATTGAATTATGTTTCTAGATAGGTTGTAG
CTGGCTCTTTTAGAGCATGTGCACGCCTGTTTGGACTTCATTTTCATCCACCTGTGCACC
TATTGTAGTCTTTGGTTGGGTTAGGAGGAAGTGATCATTGTATCAGCATCTGCTGGGAGT
GAGGACTTGCATTGTGAAAGCTTTGCTGTCCTTGATGTGATCATGGAATCTTTTTCTCAC
TAGAGTCTATGTCACTCATTATACTCTGTCGAATGTCATTGAATGTCTTTACATGGGCTT
GTATGCCTATGAAAATTGTAATACAACTTTCAGCAACGGATCTCTTGGCTCTCGCATCGA
TGAAGAACGCAGCGAAATGCGATAAGTAATGTGAATTGCAGAATTCAGTGAATCATCGAA
TCTTTGAACGCATCTTGCGCTCCTTGGTATTCCGAGGAGCATGCCTGTTTGAGTGTCATT
AAATTCTCAACTCTCTTATACTTTTTTGTAAAAGAGAGCTTGGACTGTGGAGGCTTGCTG
GCCACTTTTTGGGGTCAGCTCCTCTGAAATGCATTAGCGGAACCGTTTGCGATCTGCCAC
AAGTGTGATAAGTTATCTACACTGGCGAGGGGATTGCTCTCTGTAATGTTCAGCTTCTAA
TTGTCTCTACTTTGTGAGACTACTTTTGAATGCTTGACCTCAAATCAGGTAGGACTACCC
GCTGAACTTAA
>A11_37
TTTCCGTAGGTGAACCTGCGGAAGGATCATTATTGAATTATGTTTCTAGATAGGTTGTAG
CTGGCTCTTTTAGAGCATGTGCACGCCTGTTTGGACTTCATTTTCATCCACCTGTGCACC
TATTGTAGTCTTTGGTTGGGTTAGGAGGAAGTGATCATTGTATCAGCATCTGCTGGGAGT
GAGGACTTGCATTGTGAAAGCTTTGCTGTCCTTGATGTGATCATGGAATCTTTTTCTCAC
TAGAGTCTATGTCACTCATTATACTCTGTCGAATGTCATTGAATGTCTTTACATGGGCTT
GTATGCCTATGAAAATTGTAATACAACTTTCAGCAACGGATCTCTTGGCTCTCGCATCGA
TGAAGAACGCAGCGAAATGCGATAAGTAATGTGAATTGCAGAATTCAGTGAATCATCGAA
TCTTTGAACGCATCTTGCGCTCCTTGGTATTCCGAGGAGCATGCCTGTTTGAGTGTCATT
AAATTCTCAACTCTCTTATACTTTTTTGTAAAAGAGAGCTTGGACTGTGGAGGCTTGCTG
GCCACTTTTTGGGGTCAGCTCCTCTGAAATGCATTAGCGGAACCGTTTGCGATCTGCCAC
AAGTGTGATAAGTTATCTACACTGGCGAGGGGATTGCTCTCTGTAATGTTCAGCTTCTAA
TTGTCTCTACTTTGTGAGACTACTTTTGAATGCTTGACCTCAAATCAGGTAGGACTACCC
GCTGAACTTAA

>A11_38
TTTCCGTAGGTGAACCTGCGGAAGGATCATTATTGAATTATGTTTCTAGATAGGTTGTAG
CTGGCTCTTTTAGAGCATGTGCACGCCTGTTTGGACTTCATTTTCATCCACCTGTGCACC
TATTGTAGTCTTTGGTTGGGTTAGGAGGAAGTGATCATTGTATCAGCATCTGCTGGGAGT
GAGGACTTGCATTGTGAAAGCTTTGCTGTCCTTGATGTGATCATGGAATCTTTTTCTCAC
TAGAGTCTATGTCACTCATTATACTCTGTCGAATGTCATTGAATGTCTTTACATGGGCTT
GTATGCCTATGAAAATTGTAATACAACTTTCAGCAACGGATCTCTTGGCTCTCGCATCGA
TGAAGAACGCAGCGAAATGCGATAAGTAATGTGAATTGCAGAATTCAGTGAATCATCGAA
TCTTTGAACGCATCTTGCGCTCCTTGGTATTCCGAGGAGCATGCCTGTTTGAGTGTCATT
AAATTCTCAACTCTCTTATACTTTTTTGTAAAAGAGAGCTTGGACTGTGGAGGCTTGCTG
GCCACTTTTTGGGGTCAGCTCCTCTGAAATGCATTAGCGGAACCGTTTGCGATCTGCCAC
AAGTGTGATAAGTTATCTACACTGGCGAGGGGATTGCTCTCTGTAATGTTCAGCTTCTAA
TTGTCTCTACTTTGTGAGACTACTTTTGAATGCTTGACCTCAAATCAGGTAGGACTACCC
GCTGAACTTAA
>A11_39
TTTCCGTAGGTGAACCTGCGGAAGGATCATTATTGAATTATGTTTCTAGATAGGTTGTAG
CTGGCTCTTTTAGAGCATGTGCACGCCTGTTTGGACTTCATTTTCATCCACCTGTGCACC
TATTGTAGTCTTTGGTTGGGTTAGGAGGAAGTGATCATTGTATCAGCATCTGCTGGGAGT
GAGGACTTGCATTGTGAAAGCTTTGCTGTCCTTGATGTGATCATGGAATCTTTTTCTCAC
TAGAGTCTATGTCACTCATTATACTCTGTCGAATGTCATTGAATGTCTTTACATGGGCTT
GTATGCCTATGAAAATTGTAATACAACTTTCAGCAACGGATCTCTTGGCTCTCGCATCGA
TGAAGAACGCAGCGAAATGCGATAAGTAATGTGAATTGCAGAATTCAGTGAATCATCGAA
TCTTTGAACGCATCTTGCGCTCCTTGGTATTCCGAGGAGCATGCCTGTTTGAGTGTCATT
AAATTCTCAACTCTCTTATACTTTTTTGTAAAAGAGAGCTTGGACTGTGGAGGCTTGCTG
GCCACTTTTTGGGGTCAGCTCCTCTGAAATGCATTAGCGGAACCGTTTGCGATCTGCCAC
AAGTGTGATAAGTTATCTACACTGGCGAGGGGATTGCTCTCTGTAATGTTCAGCTTCTAA
TTGTCTCTACTTTGTGAGACTACTTTTGAATGCTTGACCTCAAATCAGGTAGGACTACCC
GCTGAACTTAA
>A11_40
TTTCCGTAGGTGAACCTGCGGAAGGATCATTATTGAATTATGTTTCTAGATAGGTTGTAG
CTGGCTCTTTTAGAGCATGTGCACGCCTGTTTGGACTTCATTTTCATCCACCTGTGCACC
TATTGTAGTCTTTGGTTGGGTTAGGAGGAAGTGATCATTGTATCAGCATCTGCTGGGAGT
GAGGACTTGCATTGTGAAAGCTTTGCTGTCCTTGATGTGATCATGGAATCTTTTTCTCAC
TAGAGTCTATGTCACTCATTATACTCTGTCGAATGTCATTGAATGTCTTTACATGGGCTT
GTATGCCTATGAAAATTGTAATACAACTTTCAGCAACGGATCTCTTGGCTCTCGCATCGA
TGAAGAACGCAGCGAAATGCGATAAGTAATGTGAATTGCAGAATTCAGTGAATCATCGAA
TCTTTGAACGCATCTTGCGCTCCTTGGTATTCCGAGGAGCATGCCTGTTTGAGTGTCATT
AAATTCTCAACTCTCTTATACTTTTTTGTAAAAGAGAGCTTGGACTGTGGAGGCTTGCTG
GCCACTTTTTGGGGTCAGCTCCTCTGAAATGCATTAGCGGAACCGTTTGCGATCTGCCAC
AAGTGTGATAAGTTATCTACACTGGCGAGGGGATTGCTCTCTGTAATGTTCAGCTTCTAA
TTGTCTCTACTTTGTGAGACTACTTTTGAATGCTTGACCTCAAATCAGGTAGGACTACCC
GCTGAACTTAA
>A10_25
TTTCCGTAGGTGAACCTGCGGAAGGATCATTATTGAATTATGTTTCTAGATAGGTTGTAG
CTGGCTCTTTTAGAGCATGTGCACGCCTGTTTGGACTTCATTTTCATCCACCTGTGCACC
TATTGTAGTCTTTGGTTGGGTTAGGAGGAAGTGATCATTGTATCAGCATCTGCTGGGAGT
GAGGACTTGCATTGTGAAAGCTTTGCTGTCCTTGATGTGATCATGGAATCTTTTTCTCAC
TAGAGTCTATGTCACTCATTATACTCTGTCGAATGTCATTGAATGTCTTTACATGGGCTT
GTATGCCTATGAAAATTGTAATACAACTTTCAGCAACGGATCTCTTGGCTCTCGCATCGA
TGAAGAACGCAGCGAAATGCGATAAGTAATGTGAATTGCAGAATTCAGTGAATCATCGAA

TCTTTGAACGCATCTTGCGCTCCTTGGTATTCCGAGGAGCATGCCTGTTTGAGTGTCATT
AAATTCTCAACTCTCTTATACTTTTTTGTAAAAGAGAGCTTGGACTGTGGAGGCTTGCTG
GCCACTTTTTGGGGTCAGCTCCTCTGAAATGCATTAGCGGAACCGTTTGCGATCTGCCAC
AAGTGTGATAAGTTATCTACACTGGCGAGGGGATTGCTCTCTGTAATGTTCAGCTTCTAA
TTGTCTCTACTTTGTGAGACTACTTTTGAATGCTTGACCTCAAATCAGGTAGGACTACCC
GCTGAACTTAA
>A10_26
TTTCCGTAGGTGAACCTGCGGAAGGATCATTATTGAATTATGTTTCTAGATAGGTTGTAG
CTGGCTCTTTTAGAGCATGTGCACGCCTGTTTGGACTTCATTTTCATCCACCTGTGCACC
TATTGTAGTCTTTGGTTGGGTTAGGAGGAAGTGATCATTGTATCAGCATCTGCTGGGAGT
GAGGACTTGCATTGTGAAAGCTTTGCTGTCCTTGATGTGATCATGGAATCTTTTTCTCAC
TAGAGTCTATGTCACTCATTATACTCTGTCGAATGTCATTGAATGTCTTTACATGGGCTT
GTATGCCTATGAAAATTGTAATACAACTTTCAGCAACGGATCTCTTGGCTCTCGCATCGA
TGAAGAACGCAGCGAAATGCGATAAGTAATGTGAATTGCAGAATTCAGTGAATCATCGAA
TCTTTGAACGCATCTTGCGCTCCTTGGTATTCCGAGGAGCATGCCTGTTTGAGTGTCATT
AAATTCTCAACTCTCTTATACTTTTTTGTAAAAGAGAGCTTGGACTGTGGAGGCTTGCTG
GCCACTTTTTGGGGTCAGCTCCTCTGAAATGCATTAGCGGAACCGTTTGCGATCTGCCAC
AAGTGTGATAAGTTATCTACACTGGCGAGGGGATTGCTCTCTGTAATGTTCAGCTTCTAA
TTGTCTCTACTTTGTGAGACTACTTTTGAATGCTTGACCTCAAATCAGGTAGGACTACCC
GCTGAACTTAA
>A10_28
TTTCCGTAGGTGAACCTGCGGAAGGATCATTATTGAATTATGTTTCTAGATAGGTTGTAG
CTGGCTCTTTTAGAGCATGTGCACGCCTGTTTGGACTTCATTTTCATCCACCTGTGCACC
TATTGTAGTCTTTGGTTGGGTTAGGAGGAAGTGATCATTGTATCAGCATCTGCTGGGAGT
GAGGACTTGCATTGTGAAAGCTTTGCTGTCCTTGATGTGATCATGGAATCTTTTTCTCAC
TAGAGTCTATGTCACTCATTATACTCTGTCGAATGTCATTGAATGTCTTTACATGGGCTT
GTATGCCTATGAAAATTGTAATACAACTTTCAGCAACGGATCTCTTGGCTCTCGCATCGA
TGAAGAACGCAGCGAAATGCGATAAGTAATGTGAATTGCAGAATTCAGTGAATCATCGAA
TCTTTGAACGCATCTTGCGCTCCTTGGTATTCCGAGGAGCATGCCTGTTTGAGTGTCATT
AAATTCTCAACTCTCTTATACTTTTTTGTAAAAGAGAGCTTGGACTGTGGAGGCTTGCTG
GCCACTTTTTGGGGTCAGCTCCTCTGAAATGCATTAGCGGAACCGTTTGCGATCTGCCAC
AAGTGTGATAAGTTATCTACACTGGCGAGGGGATTGCTCTCTGTAATGTTCAGCTTCTAA
TTGTCTCTACTTTGTGAGACTACTTTTGAATGCTTGACCTCAAATCAGGTAGGACTACCC
GCTGAACTTAA
>A10_29
TTTCCGTAGGTGAACCTGCGGAAGGATCATTATTGAATTATGTTTCTAGATAGGTTGTAG
CTGGCTCTTTTAGAGCATGTGCACGCCTGTTTGGACTTCATTTTCATCCACCTGTGCACC
TATTGTAGTCTTTGGTTGGGTTAGGAGGAAGTGATCATTGTATCAGCATCTGCTGGGAGT
GAGGACTTGCATTGTGAAAGCTTTGCTGTCCTTGATGTGATCATGGAATCTTTTTCTCAC
TAGAGTCTATGTCACTCATTATACTCTGTCGAATGTCATTGAATGTCTTTACATGGGCTT
GTATGCCTATGAAAATTGTAATACAACTTTCAGCAACGGATCTCTTGGCTCTCGCATCGA
TGAAGAACGCAGCGAAATGCGATAAGTAATGTGAATTGCAGAATTCAGTGAATCATCGAA
TCTTTGAACGCATCTTGCGCTCCTTGGTATTCCGAGGAGCATGCCTGTTTGAGTGTCATT
AAATTCTCAACTCTCTTATACTTTTTTGTAAAAGAGAGCTTGGACTGTGGAGGCTTGCTG
GCCACTTTTTGGGGTCAGCTCCTCTGAAATGCATTAGCGGAACCGTTTGCGATCTGCCAC
AAGTGTGATAAGTTATCTACACTGGCGAGGGGATTGCTCTCTGTAATGTTCAGCTTCTAA
TTGTCTCTACTTTGTGAGACTACTTTTGAATGCTTGACCTCAAATCAGGTAGGACTACCC
GCTGAACTTAA
>A10_40
TTTCCGTAGGTGAACCTGCGGAAGGATCATTATTGAATTATGTTTCTAGATAGGTTGTAG

CTGGCTCTTTTAGAGCATGTGCACGCCTGTTTGGACTTCATTTTCATCCACCTGTGCACC
TATTGTAGTCTTTGGTTGGGTTAGGAGGAAGTGATCATTGTATCAGCATCTGCTGGGAGT
GAGGACTTGCATTGTGAAAGCTTTGCTGTCCTTGATGTGATCATGGAATCTTTTTCTCAC
TAGAGTCTATGTCACTCATTATACTCTGTCGAATGTCATTGAATGTCTTTACATGGGCTT
GTATGCCTATGAAAATTGTAATACAACTTTCAGCAACGGATCTCTTGGCTCTCGCATCGA
TGAAGAACGCAGCGAAATGCGATAAGTAATGTGAATTGCAGAATTCAGTGAATCATCGAA
TCTTTGAACGCATCTTGCGCTCCTTGGTATTCCGAGGAGCATGCCTGTTTGAGTGTCATT
AAATTCTCAACTCTCTTATACTTTTTTGTAAAAGAGAGCTTGGACTGTGGAGGCTTGCTG
GCCACTTTTTGGGGTCAGCTCCTCTGAAATGCATTAGCGGAACCGTTTGCGATCTGCCAC
AAGTGTGATAAGTTATCTACACTGGCGAGGGGATTGCTCTCTGTAATGTTCAGCTTCTAA
TTGTCTCTACTTTGTGAGACTACTTTTGAATGCTTGACCTCAAATCAGGTAGGACTACCC
GCTGAACTTAA
>A10_41
TTTCCGTAGGTGAACCTGCGGAAGGATCATTATTGAATTATGTTTCTAGATAGGTTGTAG
CTGGCTCTTTTAGAGCATGTGCACGCCTGTTTGGACTTCATTTTCATCCACCTGTGCACC
TATTGTAGTCTTTGGTTGGGTTAGGAGGAAGTGATCATTGTATCAGCATCTGCTGGGAGT
GAGGACTTGCATTGTGAAAGCTTTGCTGTCCTTGATGTGATCATGGAATCTTTTTCTCAC
TAGAGTCTATGTCACTCATTATACTCTGTCGAATGTCATTGAATGTCTTTACATGGGCTT
GTATGCCTATGAAAATTGTAATACAACTTTCAGCAACGGATCTCTTGGCTCTCGCATCGA
TGAAGAACGCAGCGAAATGCGATAAGTAATGTGAATTGCAGAATTCAGTGAATCATCGAA
TCTTTGAACGCATCTTGCGCTCCTTGGTATTCCGAGGAGCATGCCTGTTTGAGTGTCATT
AAATTCTCAACTCTCTTATACTTTTTTGTAAAAGAGAGCTTGGACTGTGGAGGCTTGCTG
GCCACTTTTTGGGGTCAGCTCCTCTGAAATGCATTAGCGGAACCGTTTGCGATCTGCCAC
AAGTGTGATAAGTTATCTACACTGGCGAGGGGATTGCTCTCTGTAATGTTCAGCTTCTAA
TTGTCTCTACTTTGTGAGACTACTTTTGAATGCTTGACCTCAAATCAGGTAGGACTACCC
GCTGAACTTAA
>A10_42
TTTCCGTAGGTGAACCTGCGGAAGGATCATTATTGAATTATGTTTCTAGATAGGTTGTAG
CTGGCTCTTTTAGAGCATGTGCACGCCTGTTTGGACTTCATTTTCATCCACCTGTGCACC
TATTGTAGTCTTTGGTTGGGTTAGGAGGAAGTGATCATTGTATCAGCATCTGCTGGGAGT
GAGGACTTGCATTGTGAAAGCTTTGCTGTCCTTGATGTGATCATGGAATCTTTTTCTCAC
TAGAGTCTATGTCACTCATTATACTCTGTCGAATGTCATTGAATGTCTTTACATGGGCTT
GTATGCCTATGAAAATTGTAATACAACTTTCAGCAACGGATCTCTTGGCTCTCGCATCGA
TGAAGAACGCAGCGAAATGCGATAAGTAATGTGAATTGCAGAATTCAGTGAATCATCGAA
TCTTTGAACGCATCTTGCGCTCCTTGGTATTCCGAGGAGCATGCCTGTTTGAGTGTCATT
AAATTCTCAACTCTCTTATACTTTTTTGTAAAAGAGAGCTTGGACTGTGGAGGCTTGCTG
GCCACTTTTTGGGGTCAGCTCCTCTGAAATGCATTAGCGGAACCGTTTGCGATCTGCCAC
AAGTGTGATAAGTTATCTACACTGGCGAGGGGATTGCTCTCTGTAATGTTCAGCTTCTAA
TTGTCTCTACTTTGTGAGACTACTTTTGAATGCTTGACCTCAAATCAGGTAGGACTACCC
GCTGAACTTAA
>A10_43
TTTCCGTAGGTGAACCTGCGGAAGGATCATTATTGAATTATGTTTCTAGATAGGTTGTAG
CTGGCTCTTTTAGAGCATGTGCACGCCTGTTTGGACTTCATTTTCATCCACCTGTGCACC
TATTGTAGTCTTTGGTTGGGTTAGGAGGAAGTGATCATTGTATCAGCATCTGCTGGGAGT
GAGGACTTGCATTGTGAAAGCTTTGCTGTCCTTGATGTGATCATGGAATCTTTTTCTCAC
TAGAGTCTATGTCACTCATTATACTCTGTCGAATGTCATTGAATGTCTTTACATGGGCTT
GTATGCCTATGAAAATTGTAATACAACTTTCAGCAACGGATCTCTTGGCTCTCGCATCGA
TGAAGAACGCAGCGAAATGCGATAAGTAATGTGAATTGCAGAATTCAGTGAATCATCGAA
TCTTTGAACGCATCTTGCGCTCCTTGGTATTCCGAGGAGCATGCCTGTTTGAGTGTCATT
AAATTCTCAACTCTCTTATACTTTTTTGTAAAAGAGAGCTTGGACTGTGGAGGCTTGCTG

GCCACTTTTTGGGGTCAGCTCCTCTGAAATGCATTAGCGGAACCGTTTGCGATCTGCCAC
AAGTGTGATAAGTTATCTACACTGGCGAGGGGATTGCTCTCTGTAATGTTCAGCTTCTAA
TTGTCTCTACTTTGTGAGACTACTTTTGAATGCTTGACCTCAAATCAGGTAGGACTACCC
GCTGAACTTAA
>A10_44
TTTCCGTAGGTGAACCTGCGGAAGGATCATTATTGAATTATGTTTCTAGATAGGTTGTAG
CTGGCTCTTTTAGAGCATGTGCACGCCTGTTTGGACTTCATTTTCATCCACCTGTGCACC
TATTGTAGTCTTTGGTTGGGTTAGGAGGAAGTGATCATTGTATCAGCATCTGCTGGGAGT
GAGGACTTGCATTGTGAAAGCTTTGCTGTCCTTGATGTGATCATGGAATCTTTTTCTCAC
TAGAGTCTATGTCACTCATTATACTCTGTCGAATGTCATTGAATGTCTTTACATGGGCTT
GTATGCCTATGAAAATTGTAATACAACTTTCAGCAACGGATCTCTTGGCTCTCGCATCGA
TGAAGAACGCAGCGAAATGCGATAAGTAATGTGAATTGCAGAATTCAGTGAATCATCGAA
TCTTTGAACGCATCTTGCGCTCCTTGGTATTCCGAGGAGCATGCCTGTTTGAGTGTCATT
AAATTCTCAACTCTCTTATACTTTTTTGTAAAAGAGAGCTTGGACTGTGGAGGCTTGCTG
GCCACTTTTTGGGGTCAGCTCCTCTGAAATGCATTAGCGGAACCGTTTGCGATCTGCCAC
AAGTGTGATAAGTTATCTACACTGGCGAGGGGATTGCTCTCTGTAATGTTCAGCTTCTAA
TTGTCTCTACTTTGTGAGACTACTTTTGAATGCTTGACCTCAAATCAGGTAGGACTACCC
GCTGAACTTAA
>A10_45
TTTCCGTAGGTGAACCTGCGGAAGGATCATTATTGAATTATGTTTCTAGATAGGTTGTAG
CTGGCTCTTTTAGAGCATGTGCACGCCTGTTTGGACTTCATTTTCATCCACCTGTGCACC
TATTGTAGTCTTTGGTTGGGTTAGGAGGAAGTGATCATTGTATCAGCATCTGCTGGGAGT
GAGGACTTGCATTGTGAAAGCTTTGCTGTCCTTGATGTGATCATGGAATCTTTTTCTCAC
TAGAGTCTATGTCACTCATTATACTCTGTCGAATGTCATTGAATGTCTTTACATGGGCTT
GTATGCCTATGAAAATTGTAATACAACTTTCAGCAACGGATCTCTTGGCTCTCGCATCGA
TGAAGAACGCAGCGAAATGCGATAAGTAATGTGAATTGCAGAATTCAGTGAATCATCGAA
TCTTTGAACGCATCTTGCGCTCCTTGGTATTCCGAGGAGCATGCCTGTTTGAGTGTCATT
AAATTCTCAACTCTCTTATACTTTTTTGTAAAAGAGAGCTTGGACTGTGGAGGCTTGCTG
GCCACTTTTTGGGGTCAGCTCCTCTGAAATGCATTAGCGGAACCGTTTGCGATCTGCCAC
AAGTGTGATAAGTTATCTACACTGGCGAGGGGATTGCTCTCTGTAATGTTCAGCTTCTAA
TTGTCTCTACTTTGTGAGACTACTTTTGAATGCTTGACCTCAAATCAGGTAGGACTACCC
GCTGAACTTAA
>A10_47
TTTCCGTAGGTGAACCTGCGGAAGGATCATTATTGAATTATGTTTCTAGATAGGTTGTAG
CTGGCTCTTTTAGAGCATGTGCACGCCTGTTTGGACTTCATTTTCATCCACCTGTGCACC
TATTGTAGTCTTTGGTTGGGTTAGGAGGAAGTGATCATTGTATCAGCATCTGCTGGGAGT
GAGGACTTGCATTGTGAAAGCTTTGCTGTCCTTGATGTGATCATGGAATCTTTTTCTCAC
TAGAGTCTATGTCACTCATTATACTCTGTCGAATGTCATTGAATGTCTTTACATGGGCTT
GTATGCCTATGAAAATTGTAATACAACTTTCAGCAACGGATCTCTTGGCTCTCGCATCGA
TGAAGAACGCAGCGAAATGCGATAAGTAATGTGAATTGCAGAATTCAGTGAATCATCGAA
TCTTTGAACGCATCTTGCGCTCCTTGGTATTCCGAGGAGCATGCCTGTTTGAGTGTCATT
AAATTCTCAACTCTCTTATACTTTTTTGTAAAAGAGAGCTTGGACTGTGGAGGCTTGCTG
GCCACTTTTTGGGGTCAGCTCCTCTGAAATGCATTAGCGGAACCGTTTGCGATCTGCCAC
AAGTGTGATAAGTTATCTACACTGGCGAGGGGATTGCTCTCTGTAATGTTCAGCTTCTAA
TTGTCTCTACTTTGTGAGACTACTTTTGAATGCTTGACCTCAAATCAGGTAGGACTACCC
GCTGAACTTAA
>A10_48
TTTCCGTAGGTGAACCTGCGGAAGGATCATTATTGAATTATGTTTCTAGATAGGTTGTAG
CTGGCTCTTTTAGAGCATGTGCACGCCTGTTTGGACTTCATTTTCATCCACCTGTGCACC
TATTGTAGTCTTTGGTTGGGTTAGGAGGAAGTGATCATTGTATCAGCATCTGCTGGGAGT

GAGGACTTGCATTGTGAAAGCTTTGCTGTCCTTGATGTGATCATGGAATCTTTTTCTCAC
TAGAGTCTATGTCACTCATTATACTCTGTCGAATGTCATTGAATGTCTTTACATGGGCTT
GTATGCCTATGAAAATTGTAATACAACTTTCAGCAACGGATCTCTTGGCTCTCGCATCGA
TGAAGAACGCAGCGAAATGCGATAAGTAATGTGAATTGCAGAATTCAGTGAATCATCGAA
TCTTTGAACGCATCTTGCGCTCCTTGGTATTCCGAGGAGCATGCCTGTTTGAGTGTCATT
AAATTCTCAACTCTCTTATACTTTTTTGTAAAAGAGAGCTTGGACTGTGGAGGCTTGCTG
GCCACTTTTTGGGGTCAGCTCCTCTGAAATGCATTAGCGGAACCGTTTGCGATCTGCCAC
AAGTGTGATAAGTTATCTACACTGGCGAGGGGATTGCTCTCTGTAATGTTCAGCTTCTAA
TTGTCTCTACTTTGTGAGACTACTTTTGAATGCTTGACCTCAAATCAGGTAGGACTACCC
GCTGAACTTAA
>A10_49
TTTCCGTAGGTGAACCTGCGGAAGGATCATTATTGAATTATGTTTCTAGATAGGTTGTAG
CTGGCTCTTTTAGAGCATGTGCACGCCTGTTTGGACTTCATTTTCATCCACCTGTGCACC
TATTGTAGTCTTTGGTTGGGTTAGGAGGAAGTGATCATTGTATCAGCATCTGCTGGGAGT
GAGGACTTGCATTGTGAAAGCTTTGCTGTCCTTGATGTGATCATGGAATCTTTTTCTCAC
TAGAGTCTATGTCACTCATTATACTCTGTCGAATGTCATTGAATGTCTTTACATGGGCTT
GTATGCCTATGAAAATTGTAATACAACTTTCAGCAACGGATCTCTTGGCTCTCGCATCGA
TGAAGAACGCAGCGAAATGCGATAAGTAATGTGAATTGCAGAATTCAGTGAATCATCGAA
TCTTTGAACGCATCTTGCGCTCCTTGGTATTCCGAGGAGCATGCCTGTTTGAGTGTCATT
AAATTCTCAACTCTCTTATACTTTTTTGTAAAAGAGAGCTTGGACTGTGGAGGCTTGCTG
GCCACTTTTTGGGGTCAGCTCCTCTGAAATGCATTAGCGGAACCGTTTGCGATCTGCCAC
AAGTGTGATAAGTTATCTACACTGGCGAGGGGATTGCTCTCTGTAATGTTCAGCTTCTAA
TTGTCTCTACTTTGTGAGACTACTTTTGAATGCTTGACCTCAAATCAGGTAGGACTACCC
GCTGAACTTAA
>A11_28
TTTCCGTAGGTGAACCTGCGGAAGGATCATTATTGAATTATGTTTCTAGATAGGTTGTAG
CTGGCTCTTTTAGAGCATGTGCACGCCTGTTTGGACTTCATTTTCATCCACCTGTGCACC
TATTGTAGTCTTTGGTTGGGTTAGGAGGAAGTGATCATTGTATCAGCATCTGCTGGGAGT
GAGGACTTGCATTGTGAAAGCTTTGCTGTCCTTGATGTGATCATGGAATCTTTTTCTCAC
TAGAGTCTATGTCACTCATTATACTCTGTCGAATGTCATTGAATGTCTTTACATGGGCTT
GTATGCCTATGAAAATTGTAATACAACTTTCAGCAACGGATCTCTTGGCTCTCGCATCGA
TGAAGAACGCAGCGAAATGCGATAAGTAATGTGAATTGCAGAATTCAGTGAATCATCGAA
TCTTTGAACGCATCTTGCGCTCCTTGGTATTCCGAGGAGCATGCCTGTTTGAGTGTCATT
AAATTCTCAACTCTCTTATACTTTTTTGTAAAAGAGAGCTTGGACTGTGGAGGCTTGCTG
GCCACTTTTTGGGGTCAGCTCCTCTGAAATGCATTAGCGGAACCGTTTGCGATCTGCCAC
AAGTGTGATAAGTTATCTACACTGGCGAGGGGATTGCTCTCTGTAATGTTCAGCTTCTAA
TTGTCTCTACTTTGTGAGACTACTTTTGAATGCTTGACCTCAAATCAGGTAGGACTACCC
GCTGAACTTAA
>A11_41
TTTCCGTAGGTGAACCTGCGGAAGGATCATTATTGAATTATGTTTCTAGATAGGTTGTAG
CTGGCTCTTTTAGAGCATGTGCACGCCTGTTTGGACTTCATTTTCATCCACCTGTGCACC
TATTGTAGTCTTTGGTTGGGTTAGGAGGAAGTGATCATTGTATCAGCATCTGCTGGGAGT
GAGGACTTGCATTGTGAAAGCTTTGCTGTCCTTGATGTGATCATGGAATCTTTTTCTCAC
TAGAGTCTATGTCACTCATTATACTCTGTCGAATGTCATTGAATGTCTTTACATGGGCTT
GTATGCCTATGAAAATTGTAATACAACTTTCAGCAACGGATCTCTTGGCTCTCGCATCGA
TGAAGAACGCAGCGAAATGCGATAAGTAATGTGAATTGCAGAATTCAGTGAATCATCGAA
TCTTTGAACGCATCTTGCGCTCCTTGGTATTCCGAGGAGCATGCCTGTTTGAGTGTCATT
AAATTCTCAACTCTCTTATACTTTTTTGTAAAAGAGAGCTTGGACTGTGGAGGCTTGCTG
GCCACTTTTTGGGGTCAGCTCCTCTGAAATGCATTAGCGGAACCGTTTGCGATCTGCCAC
AAGTGTGATAAGTTATCTACACTGGCGAGGGGATTGCTCTCTGTAATGTTCAGCTTCTAA

TTGTCTCTACTTTGTGAGACTACTTTTGAATGCTTGACCTCAAATCAGGTAGGACTACCC
GCTGAACTTAA
>A11_44
TTTCCGTAGGTGAACCTGCGGAAGGATCATTATTGAATTATGTTTCTAGATAGGTTGTAG
CTGGCTCTTTTAGAGCATGTGCACGCCTGTTTGGACTTCATTTTCATCCACCTGTGCACC
TATTGTAGTCTTTGGTTGGGTTAGGAGGAAGTGATCATTGTATCAGCATCTGCTGGGAGT
GAGGACTTGCATTGTGAAAGCTTTGCTGTCCTTGATGTGATCATGGAATCTTTTTCTCAC
TAGAGTCTATGTCACTCATTATACTCTGTCGAATGTCATTGAATGTCTTTACATGGGCTT
GTATGCCTATGAAAATTGTAATACAACTTTCAGCAACGGATCTCTTGGCTCTCGCATCGA
TGAAGAACGCAGCGAAATGCGATAAGTAATGTGAATTGCAGAATTCAGTGAATCATCGAA
TCTTTGAACGCATCTTGCGCTCCTTGGTATTCCGAGGAGCATGCCTGTTTGAGTGTCATT
AAATTCTCAACTCTCTTATACTTTTTTGTAAAAGAGAGCTTGGACTGTGGAGGCTTGCTG
GCCACTTTTTGGGGTCAGCTCCTCTGAAATGCATTAGCGGAACCGTTTGCGATCTGCCAC
AAGTGTGATAAGTTATCTACACTGGCGAGGGGATTGCTCTCTGTAATGTTCAGCTTCTAA
TTGTCTCTACTTTGTGAGACTACTTTTGAATGCTTGACCTCAAATCAGGTAGGACTACCC
GCTGAACTTAA
>A11_46
TTTCCGTAGGTGAACCTGCGGAAGGATCATTATTGAATTATGTTTCTAGATAGGTTGTAG
CTGGCTCTTTTAGAGCATGTGCACGCCTGTTTGGACTTCATTTTCATCCACCTGTGCACC
TATTGTAGTCTTTGGTTGGGTTAGGAGGAAGTGATCATTGTATCAGCATCTGCTGGGAGT
GAGGACTTGCATTGTGAAAGCTTTGCTGTCCTTGATGTGATCATGGAATCTTTTTCTCAC
TAGAGTCTATGTCACTCATTATACTCTGTCGAATGTCATTGAATGTCTTTACATGGGCTT
GTATGCCTATGAAAATTGTAATACAACTTTCAGCAACGGATCTCTTGGCTCTCGCATCGA
TGAAGAACGCAGCGAAATGCGATAAGTAATGTGAATTGCAGAATTCAGTGAATCATCGAA
TCTTTGAACGCATCTTGCGCTCCTTGGTATTCCGAGGAGCATGCCTGTTTGAGTGTCATT
AAATTCTCAACTCTCTTATACTTTTTTGTAAAAGAGAGCTTGGACTGTGGAGGCTTGCTG
GCCACTTTTTGGGGTCAGCTCCTCTGAAATGCATTAGCGGAACCGTTTGCGATCTGCCAC
AAGTGTGATAAGTTATCTACACTGGCGAGGGGATTGCTCTCTGTAATGTTCAGCTTCTAA
TTGTCTCTACTTTGTGAGACTACTTTTGAATGCTTGACCTCAAATCAGGTAGGACTACCC
GCTGAACTTAA
>A11_47
TTTCCGTAGGTGAACCTGCGGAAGGATCATTATTGAATTATGTTTCTAGATAGGTTGTAG
CTGGCTCTTTTAGAGCATGTGCACGCCTGTTTGGACTTCATTTTCATCCACCTGTGCACC
TATTGTAGTCTTTGGTTGGGTTAGGAGGAAGTGATCATTGTATCAGCATCTGCTGGGAGT
GAGGACTTGCATTGTGAAAGCTTTGCTGTCCTTGATGTGATCATGGAATCTTTTTCTCAC
TAGAGTCTATGTCACTCATTATACTCTGTCGAATGTCATTGAATGTCTTTACATGGGCTT
GTATGCCTATGAAAATTGTAATACAACTTTCAGCAACGGATCTCTTGGCTCTCGCATCGA
TGAAGAACGCAGCGAAATGCGATAAGTAATGTGAATTGCAGAATTCAGTGAATCATCGAA
TCTTTGAACGCATCTTGCGCTCCTTGGTATTCCGAGGAGCATGCCTGTTTGAGTGTCATT
AAATTCTCAACTCTCTTATACTTTTTTGTAAAAGAGAGCTTGGACTGTGGAGGCTTGCTG
GCCACTTTTTGGGGTCAGCTCCTCTGAAATGCATTAGCGGAACCGTTTGCGATCTGCCAC
AAGTGTGATAAGTTATCTACACTGGCGAGGGGATTGCTCTCTGTAATGTTCAGCTTCTAA
TTGTCTCTACTTTGTGAGACTACTTTTGAATGCTTGACCTCAAATCAGGTAGGACTACCC
GCTGAACTTAA
>A11_48
TTTCCGTAGGTGAACCTGCGGAAGGATCATTATTGAATTATGTTTCTAGATAGGTTGTAG
CTGGCTCTTTTAGAGCATGTGCACGCCTGTTTGGACTTCATTTTCATCCACCTGTGCACC
TATTGTAGTCTTTGGTTGGGTTAGGAGGAAGTGATCATTGTATCAGCATCTGCTGGGAGT
GAGGACTTGCATTGTGAAAGCTTTGCTGTCCTTGATGTGATCATGGAATCTTTTTCTCAC
TAGAGTCTATGTCACTCATTATACTCTGTCGAATGTCATTGAATGTCTTTACATGGGCTT

GTATGCCTATGAAAATTGTAATACAACTTTCAGCAACGGATCTCTTGGCTCTCGCATCGA
TGAAGAACGCAGCGAAATGCGATAAGTAATGTGAATTGCAGAATTCAGTGAATCATCGAA
TCTTTGAACGCATCTTGCGCTCCTTGGTATTCCGAGGAGCATGCCTGTTTGAGTGTCATT
AAATTCTCAACTCTCTTATACTTTTTTGTAAAAGAGAGCTTGGACTGTGGAGGCTTGCTG
GCCACTTTTTGGGGTCAGCTCCTCTGAAATGCATTAGCGGAACCGTTTGCGATCTGCCAC
AAGTGTGATAAGTTATCTACACTGGCGAGGGGATTGCTCTCTGTAATGTTCAGCTTCTAA
TTGTCTCTACTTTGTGAGACTACTTTTGAATGCTTGACCTCAAATCAGGTAGGACTACCC
GCTGAACTTAA
>A11_49
TTTCCGTAGGTGAACCTGCGGAAGGATCATTATTGAATTATGTTTCTAGATAGGTTGTAG
CTGGCTCTTTTAGAGCATGTGCACGCCTGTTTGGACTTCATTTTCATCCACCTGTGCACC
TATTGTAGTCTTTGGTTGGGTTAGGAGGAAGTGATCATTGTATCAGCATCTGCTGGGAGT
GAGGACTTGCATTGTGAAAGCTTTGCTGTCCTTGATGTGATCATGGAATCTTTTTCTCAC
TAGAGTCTATGTCACTCATTATACTCTGTCGAATGTCATTGAATGTCTTTACATGGGCTT
GTATGCCTATGAAAATTGTAATACAACTTTCAGCAACGGATCTCTTGGCTCTCGCATCGA
TGAAGAACGCAGCGAAATGCGATAAGTAATGTGAATTGCAGAATTCAGTGAATCATCGAA
TCTTTGAACGCATCTTGCGCTCCTTGGTATTCCGAGGAGCATGCCTGTTTGAGTGTCATT
AAATTCTCAACTCTCTTATACTTTTTTGTAAAAGAGAGCTTGGACTGTGGAGGCTTGCTG
GCCACTTTTTGGGGTCAGCTCCTCTGAAATGCATTAGCGGAACCGTTTGCGATCTGCCAC
AAGTGTGATAAGTTATCTACACTGGCGAGGGGATTGCTCTCTGTAATGTTCAGCTTCTAA
TTGTCTCTACTTTGTGAGACTACTTTTGAATGCTTGACCTCAAATCAGGTAGGACTACCC
GCTGAACTTAA
>A11_54
TTTCCGTAGGTGAACCTGCGGAAGGATCATTATTGAATTATGTTTCTAGATAGGTTGTAG
CTGGCTCTTTTAGAGCATGTGCACGCCTGTTTGGACTTCATTTTCATCCACCTGTGCACC
TATTGTAGTCTTTGGTTGGGTTAGGAGGAAGTGATCATTGTATCAGCATCTGCTGGGAGT
GAGGACTTGCATTGTGAAAGCTTTGCTGTCCTTGATGTGATCATGGAATCTTTTTCTCAC
TAGAGTCTATGTCACTCATTATACTCTGTCGAATGTCATTGAATGTCTTTACATGGGCTT
GTATGCCTATGAAAATTGTAATACAACTTTCAGCAACGGATCTCTTGGCTCTCGCATCGA
TGAAGAACGCAGCGAAATGCGATAAGTAATGTGAATTGCAGAATTCAGTGAATCATCGAA
TCTTTGAACGCATCTTGCGCTCCTTGGTATTCCGAGGAGCATGCCTGTTTGAGTGTCATT
AAATTCTCAACTCTCTTATACTTTTTTGTAAAAGAGAGCTTGGACTGTGGAGGCTTGCTG
GCCACTTTTTGGGGTCAGCTCCTCTGAAATGCATTAGCGGAACCGTTTGCGATCTGCCAC
AAGTGTGATAAGTTATCTACACTGGCGAGGGGATTGCTCTCTGTAATGTTCAGCTTCTAA
TTGTCTCTACTTTGTGAGACTACTTTTGAATGCTTGACCTCAAATCAGGTAGGACTACCC
GCTGAACTTAA
>A11_55
TTTCCGTAGGTGAACCTGCGGAAGGATCATTATTGAATTATGTTTCTAGATAGGTTGTAG
CTGGCTCTTTTAGAGCATGTGCACGCCTGTTTGGACTTCATTTTCATCCACCTGTGCACC
TATTGTAGTCTTTGGTTGGGTTAGGAGGAAGTGATCATTGTATCAGCATCTGCTGGGAGT
GAGGACTTGCATTGTGAAAGCTTTGCTGTCCTTGATGTGATCATGGAATCTTTTTCTCAC
TAGAGTCTATGTCACTCATTATACTCTGTCGAATGTCATTGAATGTCTTTACATGGGCTT
GTATGCCTATGAAAATTGTAATACAACTTTCAGCAACGGATCTCTTGGCTCTCGCATCGA
TGAAGAACGCAGCGAAATGCGATAAGTAATGTGAATTGCAGAATTCAGTGAATCATCGAA
TCTTTGAACGCATCTTGCGCTCCTTGGTATTCCGAGGAGCATGCCTGTTTGAGTGTCATT
AAATTCTCAACTCTCTTATACTTTTTTGTAAAAGAGAGCTTGGACTGTGGAGGCTTGCTG
GCCACTTTTTGGGGTCAGCTCCTCTGAAATGCATTAGCGGAACCGTTTGCGATCTGCCAC
AAGTGTGATAAGTTATCTACACTGGCGAGGGGATTGCTCTCTGTAATGTTCAGCTTCTAA
TTGTCTCTACTTTGTGAGACTACTTTTGAATGCTTGACCTCAAATCAGGTAGGACTACCC
GCTGAACTTAA

>A11_56
TTTCCGTAGGTGAACCTGCGGAAGGATCATTATTGAATTATGTTTCTAGATAGGTTGTAG
CTGGCTCTTTTAGAGCATGTGCACGCCTGTTTGGACTTCATTTTCATCCACCTGTGCACC
TATTGTAGTCTTTGGTTGGGTTAGGAGGAAGTGATCATTGTATCAGCATCTGCTGGGAGT
GAGGACTTGCATTGTGAAAGCTTTGCTGTCCTTGATGTGATCATGGAATCTTTTTCTCAC
TAGAGTCTATGTCACTCATTATACTCTGTCGAATGTCATTGAATGTCTTTACATGGGCTT
GTATGCCTATGAAAATTGTAATACAACTTTCAGCAACGGATCTCTTGGCTCTCGCATCGA
TGAAGAACGCAGCGAAATGCGATAAGTAATGTGAATTGCAGAATTCAGTGAATCATCGAA
TCTTTGAACGCATCTTGCGCTCCTTGGTATTCCGAGGAGCATGCCTGTTTGAGTGTCATT
AAATTCTCAACTCTCTTATACTTTTTGTAAAAGAGAGCTTGGACTGTGGAGGCTTGCTG
GCCACTTTTTGGGGTCAGCTCCTCTGAAATGCATTAGCGGAACCGTTTGCGATCTGCCAC
AAGTGTGATAAGTTATCTACACTGGCGAGGGGATTGCTCTCTGTAATGTTCAGCTTCTAA
TTGTCTCTACTTTGTGAGACTACTTTTGAATGCTTGACCTCAAATCAGGTAGGACTACCC
GCTGAACTTAA
>A11_57
TTTCCGTAGGTGAACCTGCGGAAGGATCATTATTGAATTATGTTTCTAGATAGGTTGTAG
CTGGCTCTTTTAGAGCATGTGCACGCCTGTTTGGACTTCATTTTCATCCACCTGTGCACC
TATTGTAGTCTTTGGTTGGGTTAGGAGGAAGTGATCATTGTATCAGCATCTGCTGGGAGT
GAGGACTTGCATTGTGAAAGCTTTGCTGTCCTTGATGTGATCATGGAATCTTTTTCTCAC
TAGAGTCTATGTCACTCATTATACTCTGTCGAATGTCATTGAATGTCTTTACATGGGCTT
GTATGCCTATGAAAATTGTAATACAACTTTCAGCAACGGATCTCTTGGCTCTCGCATCGA
TGAAGAACGCAGCGAAATGCGATAAGTAATGTGAATTGCAGAATTCAGTGAATCATCGAA
TCTTTGAACGCATCTTGCGCTCCTTGGTATTCCGAGGAGCATGCCTGTTTGAGTGTCATT
AAATTCTCAACTCTCTTATACTTTTTGTAAAAGAGAGCTTGGACTGTGGAGGCTTGCTG
GCCACTTTTTGGGGTCAGCTCCTCTGAAATGCATTAGCGGAACCGTTTGCGATCTGCCAC
AAGTGTGATAAGTTATCTACACTGGCGAGGGGATTGCTCTCTGTAATGTTCAGCTTCTAA
TTGTCTCTACTTTGTGAGACTACTTTTGAATGCTTGACCTCAAATCAGGTAGGACTACCC
GCTGAACTTAA
>A10_50
TTTCCGTAGGTGAACCTGCGGAAGGATCATTATTGAATTATGTTTCTAGATAGGTTGTAG
CTGGCTCTTTTAGAGCATGTGCACGCCTGTTTGGACTTCATTTTCATCCACCTGTGCACC
TATTGTAGTCTTTGGTTGGGTTAGGAGGAAGTGATCATTGTATCAGCATCTGCTGGGAGT
GAGGACTTGCATTGTGAAAGCTTTGCTGTCCTTGATGTGATCATGGAATCTTTTTCTCAC
TAGAGTCTATGTCACTCATTATACTCTGTCGAATGTCATTGAATGTCTTTACATGGGCTT
GTATGCCTATGAAAATTGTAATACAACTTTCAGCAACGGATCTCTTGGCTCTCGCATCGA
TGAAGAACGCAGCGAAATGCGATAAGTAATGTGAATTGCAGAATTCAGTGAATCATCGAA
TCTTTGAACGCATCTTGCGCTCCTTGGTATTCCGAGGAGCATGCCTGTTTGAGTGTCATT
AAATTCTCAACTCTCTTATACTTTTTGTAAAAGAGAGCTTGGACTGTGGAGGCTTGCTG
GCCACTTTTTGGGGTCAGCTCCTCTGAAATGCATTAGCGGAACCGTTTGCGATCTGCCAC
AAGTGTGATAAGTTATCTACACTGGCGAGGGGATTGCTCTCTGTAATGTTCAGCTTCTAA
TTGTCTCTACTTTGTGAGACTACTTTTGAATGCTTGACCTCAAATCAGGTAGGACTACCC
GCTGAACTTAA
>A10_51
TTTCCGTAGGTGAACCTGCGGAAGGATCATTATTGAATTATGTTTCTAGATAGGTTGTAG
CTGGCTCTTTTAGAGCATGTGCACGCCTGTTTGGACTTCATTTTCATCCACCTGTGCACC
TATTGTAGTCTTTGGTTGGGTTAGGAGGAAGTGATCATTGTATCAGCATCTGCTGGGAGT
GAGGACTTGCATTGTGAAAGCTTTGCTGTCCTTGATGTGATCATGGAATCTTTTTCTCAC
TAGAGTCTATGTCACTCATTATACTCTGTCGAATGTCATTGAATGTCTTTACATGGGCTT
GTATGCCTATGAAAATTGTAATACAACTTTCAGCAACGGATCTCTTGGCTCTCGCATCGA
TGAAGAACGCAGCGAAATGCGATAAGTAATGTGAATTGCAGAATTCAGTGAATCATCGAA

TCTTTGAACGCATCTTGCGCTCCTTGGTATTCCGAGGAGCATGCCTGTTTGAGTGTCATT
AAATTCTCAACTCTCTTATACTTTTTTGTAAAAGAGAGCTTGGACTGTGGAGGCTTGCTG
GCCACTTTTTGGGGTCAGCTCCTCTGAAATGCATTAGCGGAACCGTTTGCGATCTGCCAC
AAGTGTGATAAGTTATCTACACTGGCGAGGGGATTGCTCTCTGTAATGTTCAGCTTCTAA
TTGTCTCTACTTTGTGAGACTACTTTTGAATGCTTGACCTCAAATCAGGTAGGACTACCC
GCTGAACTTAA
>A10_52
TTTCCGTAGGTGAACCTGCGGAAGGATCATTATTGAATTATGTTTCTAGATAGGTTGTAG
CTGGCTCTTTTAGAGCATGTGCACGCCTGTTTGGACTTCATTTTCATCCACCTGTGCACC
TATTGTAGTCTTTGGTTGGGTTAGGAGGAAGTGATCATTGTATCAGCATCTGCTGGGAGT
GAGGACTTGCATTGTGAAAGCTTTGCTGTCCTTGATGTGATCATGGAATCTTTTTCTCAC
TAGAGTCTATGTCACTCATTATACTCTGTCGAATGTCATTGAATGTCTTTACATGGGCTT
GTATGCCTATGAAAATTGTAATACAACTTTCAGCAACGGATCTCTTGGCTCTCGCATCGA
TGAAGAACGCAGCGAAATGCGATAAGTAATGTGAATTGCAGAATTCAGTGAATCATCGAA
TCTTTGAACGCATCTTGCGCTCCTTGGTATTCCGAGGAGCATGCCTGTTTGAGTGTCATT
AAATTCTCAACTCTCTTATACTTTTTTGTAAAAGAGAGCTTGGACTGTGGAGGCTTGCTG
GCCACTTTTTGGGGTCAGCTCCTCTGAAATGCATTAGCGGAACCGTTTGCGATCTGCCAC
AAGTGTGATAAGTTATCTACACTGGCGAGGGGATTGCTCTCTGTAATGTTCAGCTTCTAA
TTGTCTCTACTTTGTGAGACTACTTTTGAATGCTTGACCTCAAATCAGGTAGGACTACCC
GCTGAACTTAA
>A10_53
TTTCCGTAGGTGAACCTGCGGAAGGATCATTATTGAATTATGTTTCTAGATAGGTTGTAG
CTGGCTCTTTTAGAGCATGTGCACGCCTGTTTGGACTTCATTTTCATCCACCTGTGCACC
TATTGTAGTCTTTGGTTGGGTTAGGAGGAAGTGATCATTGTATCAGCATCTGCTGGGAGT
GAGGACTTGCATTGTGAAAGCTTTGCTGTCCTTGATGTGATCATGGAATCTTTTTCTCAC
TAGAGTCTATGTCACTCATTATACTCTGTCGAATGTCATTGAATGTCTTTACATGGGCTT
GTATGCCTATGAAAATTGTAATACAACTTTCAGCAACGGATCTCTTGGCTCTCGCATCGA
TGAAGAACGCAGCGAAATGCGATAAGTAATGTGAATTGCAGAATTCAGTGAATCATCGAA
TCTTTGAACGCATCTTGCGCTCCTTGGTATTCCGAGGAGCATGCCTGTTTGAGTGTCATT
AAATTCTCAACTCTCTTATACTTTTTTGTAAAAGAGAGCTTGGACTGTGGAGGCTTGCTG
GCCACTTTTTGGGGTCAGCTCCTCTGAAATGCATTAGCGGAACCGTTTGCGATCTGCCAC
AAGTGTGATAAGTTATCTACACTGGCGAGGGGATTGCTCTCTGTAATGTTCAGCTTCTAA
TTGTCTCTACTTTGTGAGACTACTTTTGAATGCTTGACCTCAAATCAGGTAGGACTACCC
GCTGAACTTAA
>A10_55
TTTCCGTAGGTGAACCTGCGGAAGGATCATTATTGAATTATGTTTCTAGATAGGTTGTAG
CTGGCTCTTTTAGAGCATGTGCACGCCTGTTTGGACTTCATTTTCATCCACCTGTGCACC
TATTGTAGTCTTTGGTTGGGTTAGGAGGAAGTGATCATTGTATCAGCATCTGCTGGGAGT
GAGGACTTGCATTGTGAAAGCTTTGCTGTCCTTGATGTGATCATGGAATCTTTTTCTCAC
TAGAGTCTATGTCACTCATTATACTCTGTCGAATGTCATTGAATGTCTTTACATGGGCTT
GTATGCCTATGAAAATTGTAATACAACTTTCAGCAACGGATCTCTTGGCTCTCGCATCGA
TGAAGAACGCAGCGAAATGCGATAAGTAATGTGAATTGCAGAATTCAGTGAATCATCGAA
TCTTTGAACGCATCTTGCGCTCCTTGGTATTCCGAGGAGCATGCCTGTTTGAGTGTCATT
AAATTCTCAACTCTCTTATACTTTTTTGTAAAAGAGAGCTTGGACTGTGGAGGCTTGCTG
GCCACTTTTTGGGGTCAGCTCCTCTGAAATGCATTAGCGGAACCGTTTGCGATCTGCCAC
AAGTGTGATAAGTTATCTACACTGGCGAGGGGATTGCTCTCTGTAATGTTCAGCTTCTAA
TTGTCTCTACTTTGTGAGACTACTTTTGAATGCTTGACCTCAAATCAGGTAGGACTACCC
GCTGAACTTAA
>A10_56
TTTCCGTAGGTGAACCTGCGGAAGGATCATTATTGAATTATGTTTCTAGATAGGTTGTAG

CTGGCTCTTTTAGAGCATGTGCACGCCTGTTTGGACTTCATTTTCATCCACCTGTGCACC
TATTGTAGTCTTTGGTTGGGTTAGGAGGAAGTGATCATTGTATCAGCATCTGCTGGGAGT
GAGGACTTGCATTGTGAAAGCTTTGCTGTCCTTGATGTGATCATGGAATCTTTTTCTCAC
TAGAGTCTATGTCACTCATTATACTCTGTCGAATGTCATTGAATGTCTTTACATGGGCTT
GTATGCCTATGAAAATTGTAATACAACTTTCAGCAACGGATCTCTTGGCTCTCGCATCGA
TGAAGAACGCAGCGAAATGCGATAAGTAATGTGAATTGCAGAATTCAGTGAATCATCGAA
TCTTTGAACGCATCTTGCGCTCCTTGGTATTCCGAGGAGCATGCCTGTTTGAGTGTCATT
AAATTCTCAACTCTCTTATACTTTTTTGTAAAAGAGAGCTTGGACTGTGGAGGCTTGCTG
GCCACTTTTTGGGGTCAGCTCCTCTGAAATGCATTAGCGGAACCGTTTGCGATCTGCCAC
AAGTGTGATAAGTTATCTACACTGGCGAGGGGATTGCTCTCTGTAATGTTCAGCTTCTAA
TTGTCTCTACTTTGTGAGACTACTTTTGAATGCTTGACCTCAAATCAGGTAGGACTACCC
GCTGAACTTAA
>A10_57
TTTCCGTAGGTGAACCTGCGGAAGGATCATTATTGAATTATGTTTCTAGATAGGTTGTAG
CTGGCTCTTTTAGAGCATGTGCACGCCTGTTTGGACTTCATTTTCATCCACCTGTGCACC
TATTGTAGTCTTTGGTTGGGTTAGGAGGAAGTGATCATTGTATCAGCATCTGCTGGGAGT
GAGGACTTGCATTGTGAAAGCTTTGCTGTCCTTGATGTGATCATGGAATCTTTTTCTCAC
TAGAGTCTATGTCACTCATTATACTCTGTCGAATGTCATTGAATGTCTTTACATGGGCTT
GTATGCCTATGAAAATTGTAATACAACTTTCAGCAACGGATCTCTTGGCTCTCGCATCGA
TGAAGAACGCAGCGAAATGCGATAAGTAATGTGAATTGCAGAATTCAGTGAATCATCGAA
TCTTTGAACGCATCTTGCGCTCCTTGGTATTCCGAGGAGCATGCCTGTTTGAGTGTCATT
AAATTCTCAACTCTCTTATACTTTTTTGTAAAAGAGAGCTTGGACTGTGGAGGCTTGCTG
GCCACTTTTTGGGGTCAGCTCCTCTGAAATGCATTAGCGGAACCGTTTGCGATCTGCCAC
AAGTGTGATAAGTTATCTACACTGGCGAGGGGATTGCTCTCTGTAATGTTCAGCTTCTAA
TTGTCTCTACTTTGTGAGACTACTTTTGAATGCTTGACCTCAAATCAGGTAGGACTACCC
GCTGAACTTAA
>A10_62
TTTCCGTAGGTGAACCTGCGGAAGGATCATTATTGAATTATGTTTCTAGATAGGTTGTAG
CTGGCTCTTTTAGAGCATGTGCACGCCTGTTTGGACTTCATTTTCATCCACCTGTGCACC
TATTGTAGTCTTTGGTTGGGTTAGGAGGAAGTGATCATTGTATCAGCATCTGCTGGGAGT
GAGGACTTGCATTGTGAAAGCTTTGCTGTCCTTGATGTGATCATGGAATCTTTTTCTCAC
TAGAGTCTATGTCACTCATTATACTCTGTCGAATGTCATTGAATGTCTTTACATGGGCTT
GTATGCCTATGAAAATTGTAATACAACTTTCAGCAACGGATCTCTTGGCTCTCGCATCGA
TGAAGAACGCAGCGAAATGCGATAAGTAATGTGAATTGCAGAATTCAGTGAATCATCGAA
TCTTTGAACGCATCTTGCGCTCCTTGGTATTCCGAGGAGCATGCCTGTTTGAGTGTCATT
AAATTCTCAACTCTCTTATACTTTTTTGTAAAAGAGAGCTTGGACTGTGGAGGCTTGCTG
GCCACTTTTTGGGGTCAGCTCCTCTGAAATGCATTAGCGGAACCGTTTGCGATCTGCCAC
AAGTGTGATAAGTTATCTACACTGGCGAGGGGATTGCTCTCTGTAATGTTCAGCTTCTAA
TTGTCTCTACTTTGTGAGACTACTTTTGAATGCTTGACCTCAAATCAGGTAGGACTACCC
GCTGAACTTAA
>A10_63
TTTCCGTAGGTGAACCTGCGGAAGGATCATTATTGAATTATGTTTCTAGATAGGTTGTAG
CTGGCTCTTTTAGAGCATGTGCACGCCTGTTTGGACTTCATTTTCATCCACCTGTGCACC
TATTGTAGTCTTTGGTTGGGTTAGGAGGAAGTGATCATTGTATCAGCATCTGCTGGGAGT
GAGGACTTGCATTGTGAAAGCTTTGCTGTCCTTGATGTGATCATGGAATCTTTTTCTCAC
TAGAGTCTATGTCACTCATTATACTCTGTCGAATGTCATTGAATGTCTTTACATGGGCTT
GTATGCCTATGAAAATTGTAATACAACTTTCAGCAACGGATCTCTTGGCTCTCGCATCGA
TGAAGAACGCAGCGAAATGCGATAAGTAATGTGAATTGCAGAATTCAGTGAATCATCGAA
TCTTTGAACGCATCTTGCGCTCCTTGGTATTCCGAGGAGCATGCCTGTTTGAGTGTCATT
AAATTCTCAACTCTCTTATACTTTTTTGTAAAAGAGAGCTTGGACTGTGGAGGCTTGCTG

GCCACTTTTTGGGGTCAGCTCCTCTGAAATGCATTAGCGGAACCGTTTGCGATCTGCCAC
AAGTGTGATAAGTTATCTACACTGGCGAGGGGATTGCTCTCTGTAATGTTCAGCTTCTAA
TTGTCTCTACTTTGTGAGACTACTTTTGAATGCTTGACCTCAAATCAGGTAGGACTACCC
GCTGAACTTAA
>A10_64
TTTCCGTAGGTGAACCTGCGGAAGGATCATTATTGAATTATGTTTCTAGATAGGTTGTAG
CTGGCTCTTTTAGAGCATGTGCACGCCTGTTTGGACTTCATTTTCATCCACCTGTGCACC
TATTGTAGTCTTTGGTTGGGTTAGGAGGAAGTGATCATTGTATCAGCATCTGCTGGGAGT
GAGGACTTGCATTGTGAAAGCTTTGCTGTCCTTGATGTGATCATGGAATCTTTTTCTCAC
TAGAGTCTATGTCACTCATTATACTCTGTCGAATGTCATTGAATGTCTTTACATGGGCTT
GTATGCCTATGAAAATTGTAATACAACTTTCAGCAACGGATCTCTTGGCTCTCGCATCGA
TGAAGAACGCAGCGAAATGCGATAAGTAATGTGAATTGCAGAATTCAGTGAATCATCGAA
TCTTTGAACGCATCTTGCGCTCCTTGGTATTCCGAGGAGCATGCCTGTTTGAGTGTCATT
AAATTCTCAACTCTCTTATACTTTTTTGTAAAAGAGAGCTTGGACTGTGGAGGCTTGCTG
GCCACTTTTTGGGGTCAGCTCCTCTGAAATGCATTAGCGGAACCGTTTGCGATCTGCCAC
AAGTGTGATAAGTTATCTACACTGGCGAGGGGATTGCTCTCTGTAATGTTCAGCTTCTAA
TTGTCTCTACTTTGTGAGACTACTTTTGAATGCTTGACCTCAAATCAGGTAGGACTACCC
GCTGAACTTAA
>A10_66
TTTCCGTAGGTGAACCTGCGGAAGGATCATTATTGAATTATGTTTCTAGATAGGTTGTAG
CTGGCTCTTTTAGAGCATGTGCACGCCTGTTTGGACTTCATTTTCATCCACCTGTGCACC
TATTGTAGTCTTTGGTTGGGTTAGGAGGAAGTGATCATTGTATCAGCATCTGCTGGGAGT
GAGGACTTGCATTGTGAAAGCTTTGCTGTCCTTGATGTGATCATGGAATCTTTTTCTCAC
TAGAGTCTATGTCACTCATTATACTCTGTCGAATGTCATTGAATGTCTTTACATGGGCTT
GTATGCCTATGAAAATTGTAATACAACTTTCAGCAACGGATCTCTTGGCTCTCGCATCGA
TGAAGAACGCAGCGAAATGCGATAAGTAATGTGAATTGCAGAATTCAGTGAATCATCGAA
TCTTTGAACGCATCTTGCGCTCCTTGGTATTCCGAGGAGCATGCCTGTTTGAGTGTCATT
AAATTCTCAACTCTCTTATACTTTTTTGTAAAAGAGAGCTTGGACTGTGGAGGCTTGCTG
GCCACTTTTTGGGGTCAGCTCCTCTGAAATGCATTAGCGGAACCGTTTGCGATCTGCCAC
AAGTGTGATAAGTTATCTACACTGGCGAGGGGATTGCTCTCTGTAATGTTCAGCTTCTAA
TTGTCTCTACTTTGTGAGACTACTTTTGAATGCTTGACCTCAAATCAGGTAGGACTACCC
GCTGAACTTAA
>A11_2
TTTCCGTAGGTGAACCTGCGGAAGGATCATTATTGAATTATGTTTCTAGATAGGTTGTAG
CTGGCTCTTTTAGAGCATGTGCACGCCTGTTTGGACTTCATTTTCATCCACCTGTGCACC
TATTGTAGTCTTTGGTTGGGTTAGGAGGAAGTGATCATTGTATCAGCATCTGCTGGGAGT
GAGGACTTGCATTGTGAAAGCTTTGCTGTCCTTGATGTGATCATGGAATCTTTTTCTCAC
TAGAGTCTATGTCACTCATTATACTCTGTCGAATGTCATTGAATGTCTTTACATGGGCTT
GTATGCCTATGAAAATTGTAATACAACTTTCAGCAACGGATCTCTTGGCTCTCGCATCGA
TGAAGAACGCAGCGAAATGCGATAAGTAATGTGAATTGCAGAATTCAGTGAATCATCGAA
TCTTTGAACGCATCTTGCGCTCCTTGGTATTCCGAGGAGCATGCCTGTTTGAGTGTCATT
AAATTCTCAACTCTCTTATACTTTTTTGTAAAAGAGAGCTTGGACTGTGGAGGCTTGCTG
GCCACTTTTTGGGGTCAGCTCCTCTGAAATGCATTAGCGGAACCGTTTGCGATCTGCCAC
AAGTGTGATAAGTTATCTACACTGGCGAGGGGATTGCTCTCTGTAATGTTCAGCTTCTAA
TTGTCTCTACTTTGTGAGACTACTTTTGAATGCTTGACCTCAAATCAGGTAGGACTACCC
GCTGAACTTAA
>A11_3
TTTCCGTAGGTGAACCTGCGGAAGGATCATTATTGAATTATGTTTCTAGATAGGTTGTAG
CTGGCTCTTTTAGAGCATGTGCACGCCTGTTTGGACTTCATTTTCATCCACCTGTGCACC
TATTGTAGTCTTTGGTTGGGTTAGGAGGAAGTGATCATTGTATCAGCATCTGCTGGGAGT

GAGGACTTGCATTGTGAAAGCTTTGCTGTCCTTGATGTGATCATGGAATCTTTTTCTCAC
TAGAGTCTATGTCACTCATTATACTCTGTCGAATGTCATTGAATGTCTTTACATGGGCTT
GTATGCCTATGAAAATTGTAATACAACTTTCAGCAACGGATCTCTTGGCTCTCGCATCGA
TGAAGAACGCAGCGAAATGCGATAAGTAATGTGAATTGCAGAATTCAGTGAATCATCGAA
TCTTTGAACGCATCTTGCGCTCCTTGGTATTCCGAGGAGCATGCCTGTTTGAGTGTCATT
AAATTCTCAACTCTCTTATACTTTTTTGTAAAAGAGAGCTTGGACTGTGGAGGCTTGCTG
GCCACTTTTTGGGGTCAGCTCCTCTGAAATGCATTAGCGGAACCGTTTGCGATCTGCCAC
AAGTGTGATAAGTTATCTACACTGGCGAGGGGATTGCTCTCTGTAATGTTCAGCTTCTAA
TTGTCTCTACTTTGTGAGACTACTTTTGAATGCTTGACCTCAAATCAGGTAGGACTACCC
GCTGAACTTAA
>A11_6
TTTCCGTAGGTGAACCTGCGGAAGGATCATTATTGAATTATGTTTCTAGATAGGTTGTAG
CTGGCTCTTTTAGAGCATGTGCACGCCTGTTTGGACTTCATTTTCATCCACCTGTGCACC
TATTGTAGTCTTTGGTTGGGTTAGGAGGAAGTGATCATTGTATCAGCATCTGCTGGGAGT
GAGGACTTGCATTGTGAAAGCTTTGCTGTCCTTGATGTGATCATGGAATCTTTTTCTCAC
TAGAGTCTATGTCACTCATTATACTCTGTCGAATGTCATTGAATGTCTTTACATGGGCTT
GTATGCCTATGAAAATTGTAATACAACTTTCAGCAACGGATCTCTTGGCTCTCGCATCGA
TGAAGAACGCAGCGAAATGCGATAAGTAATGTGAATTGCAGAATTCAGTGAATCATCGAA
TCTTTGAACGCATCTTGCGCTCCTTGGTATTCCGAGGAGCATGCCTGTTTGAGTGTCATT
AAATTCTCAACTCTCTTATACTTTTTTGTAAAAGAGAGCTTGGACTGTGGAGGCTTGCTG
GCCACTTTTTGGGGTCAGCTCCTCTGAAATGCATTAGCGGAACCGTTTGCGATCTGCCAC
AAGTGTGATAAGTTATCTACACTGGCGAGGGGATTGCTCTCTGTAATGTTCAGCTTCTAA
TTGTCTCTACTTTGTGAGACTACTTTTGAATGCTTGACCTCAAATCAGGTAGGACTACCC
GCTGAACTTAA
>A11_7
TTTCCGTAGGTGAACCTGCGGAAGGATCATTATTGAATTATGTTTCTAGATAGGTTGTAG
CTGGCTCTTTTAGAGCATGTGCACGCCTGTTTGGACTTCATTTTCATCCACCTGTGCACC
TATTGTAGTCTTTGGTTGGGTTAGGAGGAAGTGATCATTGTATCAGCATCTGCTGGGAGT
GAGGACTTGCATTGTGAAAGCTTTGCTGTCCTTGATGTGATCATGGAATCTTTTTCTCAC
TAGAGTCTATGTCACTCATTATACTCTGTCGAATGTCATTGAATGTCTTTACATGGGCTT
GTATGCCTATGAAAATTGTAATACAACTTTCAGCAACGGATCTCTTGGCTCTCGCATCGA
TGAAGAACGCAGCGAAATGCGATAAGTAATGTGAATTGCAGAATTCAGTGAATCATCGAA
TCTTTGAACGCATCTTGCGCTCCTTGGTATTCCGAGGAGCATGCCTGTTTGAGTGTCATT
AAATTCTCAACTCTCTTATACTTTTTTGTAAAAGAGAGCTTGGACTGTGGAGGCTTGCTG
GCCACTTTTTGGGGTCAGCTCCTCTGAAATGCATTAGCGGAACCGTTTGCGATCTGCCAC
AAGTGTGATAAGTTATCTACACTGGCGAGGGGATTGCTCTCTGTAATGTTCAGCTTCTAA
TTGTCTCTACTTTGTGAGACTACTTTTGAATGCTTGACCTCAAATCAGGTAGGACTACCC
GCTGAACTTAA
>A11_12
TTTCCGTAGGTGAACCTGCGGAAGGATCATTATTGAATTATGTTTCTAGATAGGTTGTAG
CTGGCTCTTTTAGAGCATGTGCACGCCTGTTTGGACTTCATTTTCATCCACCTGTGCACC
TATTGTAGTCTTTGGTTGGGTTAGGAGGAAGTGATCATTGTATCAGCATCTGCTGGGAGT
GAGGACTTGCATTGTGAAAGCTTTGCTGTCCTTGATGTGATCATGGAATCTTTTTCTCAC
TAGAGTCTATGTCACTCATTATACTCTGTCGAATGTCATTGAATGTCTTTACATGGGCTT
GTATGCCTATGAAAATTGTAATACAACTTTCAGCAACGGATCTCTTGGCTCTCGCATCGA
TGAAGAACGCAGCGAAATGCGATAAGTAATGTGAATTGCAGAATTCAGTGAATCATCGAA
TCTTTGAACGCATCTTGCGCTCCTTGGTATTCCGAGGAGCATGCCTGTTTGAGTGTCATT
AAATTCTCAACTCTCTTATACTTTTTTGTAAAAGAGAGCTTGGACTGTGGAGGCTTGCTG
GCCACTTTTTGGGGTCAGCTCCTCTGAAATGCATTAGCGGAACCGTTTGCGATCTGCCAC
AAGTGTGATAAGTTATCTACACTGGCGAGGGGATTGCTCTCTGTAATGTTCAGCTTCTAA

TTGTCTCTACTTTGTGAGACTACTTTTGAATGCTTGACCTCAAATCAGGTAGGACTACCC
GCTGAACTTAA
>A11_19
TTTCCGTAGGTGAACCTGCGGAAGGATCATTATTGAATTATGTTTCTAGATAGGTTGTAG
CTGGCTCTTTTAGAGCATGTGCACGCCTGTTTGGACTTCATTTTCATCCACCTGTGCACC
TATTGTAGTCTTTGGTTGGGTTAGGAGGAAGTGATCATTGTATCAGCATCTGCTGGGAGT
GAGGACTTGCATTGTGAAAGCTTTGCTGTCCTTGATGTGATCATGGAATCTTTTTCTCAC
TAGAGTCTATGTCACTCATTATACTCTGTCGAATGTCATTGAATGTCTTTACATGGGCTT
GTATGCCTATGAAAATTGTAATACAACTTTCAGCAACGGATCTCTTGGCTCTCGCATCGA
TGAAGAACGCAGCGAAATGCGATAAGTAATGTGAATTGCAGAATTCAGTGAATCATCGAA
TCTTTGAACGCATCTTGCGCTCCTTGGTATTCCGAGGAGCATGCCTGTTTGAGTGTCATT
AAATTCTCAACTCTCTTATACTTTTTTGTAAAAGAGAGCTTGGACTGTGGAGGCTTGCTG
GCCACTTTTTGGGGTCAGCTCCTCTGAAATGCATTAGCGGAACCGTTTGCGATCTGCCAC
AAGTGTGATAAGTTATCTACACTGGCGAGGGGATTGCTCTCTGTAATGTTCAGCTTCTAA
TTGTCTCTACTTTGTGAGACTACTTTTGAATGCTTGACCTCAAATCAGGTAGGACTACCC
GCTGAACTTAA
>A11_21
TTTCCGTAGGTGAACCTGCGGAAGGATCATTATTGAATTATGTTTCTAGATAGGTTGTAG
CTGGCTCTTTTAGAGCATGTGCACGCCTGTTTGGACTTCATTTTCATCCACCTGTGCACC
TATTGTAGTCTTTGGTTGGGTTAGGAGGAAGTGATCATTGTATCAGCATCTGCTGGGAGT
GAGGACTTGCATTGTGAAAGCTTTGCTGTCCTTGATGTGATCATGGAATCTTTTTCTCAC
TAGAGTCTATGTCACTCATTATACTCTGTCGAATGTCATTGAATGTCTTTACATGGGCTT
GTATGCCTATGAAAATTGTAATACAACTTTCAGCAACGGATCTCTTGGCTCTCGCATCGA
TGAAGAACGCAGCGAAATGCGATAAGTAATGTGAATTGCAGAATTCAGTGAATCATCGAA
TCTTTGAACGCATCTTGCGCTCCTTGGTATTCCGAGGAGCATGCCTGTTTGAGTGTCATT
AAATTCTCAACTCTCTTATACTTTTTTGTAAAAGAGAGCTTGGACTGTGGAGGCTTGCTG
GCCACTTTTTGGGGTCAGCTCCTCTGAAATGCATTAGCGGAACCGTTTGCGATCTGCCAC
AAGTGTGATAAGTTATCTACACTGGCGAGGGGATTGCTCTCTGTAATGTTCAGCTTCTAA
TTGTCTCTACTTTGTGAGACTACTTTTGAATGCTTGACCTCAAATCAGGTAGGACTACCC
GCTGAACTTAA
>A11_22
TTTCCGTAGGTGAACCTGCGGAAGGATCATTATTGAATTATGTTTCTAGATAGGTTGTAG
CTGGCTCTTTTAGAGCATGTGCACGCCTGTTTGGACTTCATTTTCATCCACCTGTGCACC
TATTGTAGTCTTTGGTTGGGTTAGGAGGAAGTGATCATTGTATCAGCATCTGCTGGGAGT
GAGGACTTGCATTGTGAAAGCTTTGCTGTCCTTGATGTGATCATGGAATCTTTTTCTCAC
TAGAGTCTATGTCACTCATTATACTCTGTCGAATGTCATTGAATGTCTTTACATGGGCTT
GTATGCCTATGAAAATTGTAATACAACTTTCAGCAACGGATCTCTTGGCTCTCGCATCGA
TGAAGAACGCAGCGAAATGCGATAAGTAATGTGAATTGCAGAATTCAGTGAATCATCGAA
TCTTTGAACGCATCTTGCGCTCCTTGGTATTCCGAGGAGCATGCCTGTTTGAGTGTCATT
AAATTCTCAACTCTCTTATACTTTTTTGTAAAAGAGAGCTTGGACTGTGGAGGCTTGCTG
GCCACTTTTTGGGGTCAGCTCCTCTGAAATGCATTAGCGGAACCGTTTGCGATCTGCCAC
AAGTGTGATAAGTTATCTACACTGGCGAGGGGATTGCTCTCTGTAATGTTCAGCTTCTAA
TTGTCTCTACTTTGTGAGACTACTTTTGAATGCTTGACCTCAAATCAGGTAGGACTACCC
GCTGAACTTAA
>A11_23
TTTCCGTAGGTGAACCTGCGGAAGGATCATTATTGAATTATGTTTCTAGATAGGTTGTAG
CTGGCTCTTTTAGAGCATGTGCACGCCTGTTTGGACTTCATTTTCATCCACCTGTGCACC
TATTGTAGTCTTTGGTTGGGTTAGGAGGAAGTGATCATTGTATCAGCATCTGCTGGGAGT
GAGGACTTGCATTGTGAAAGCTTTGCTGTCCTTGATGTGATCATGGAATCTTTTTCTCAC
TAGAGTCTATGTCACTCATTATACTCTGTCGAATGTCATTGAATGTCTTTACATGGGCTT

GTATGCCTATGAAAATTGTAATACAACTTTCAGCAACGGATCTCTTGGCTCTCGCATCGA
TGAAGAACGCAGCGAAATGCGATAAGTAATGTGAATTGCAGAATTCAGTGAATCATCGAA
TCTTTGAACGCATCTTGCGCTCCTTGGTATTCCGAGGAGCATGCCTGTTTGAGTGTCATT
AAATTCTCAACTCTCTTATACTTTTTTGTAAAAGAGAGCTTGGACTGTGGAGGCTTGCTG
GCCACTTTTTGGGGTCAGCTCCTCTGAAATGCATTAGCGGAACCGTTTGCGATCTGCCAC
AAGTGTGATAAGTTATCTACACTGGCGAGGGGATTGCTCTCTGTAATGTTCAGCTTCTAA
TTGTCTCTACTTTGTGAGACTACTTTTGAATGCTTGACCTCAAATCAGGTAGGACTACCC
GCTGAACTTAA
>A11_25
TTTCCGTAGGTGAACCTGCGGAAGGATCATTATTGAATTATGTTTCTAGATAGGTTGTAG
CTGGCTCTTTTAGAGCATGTGCACGCCTGTTTGGACTTCATTTTCATCCACCTGTGCACC
TATTGTAGTCTTTGGTTGGGTTAGGAGGAAGTGATCATTGTATCAGCATCTGCTGGGAGT
GAGGACTTGCATTGTGAAAGCTTTGCTGTCCTTGATGTGATCATGGAATCTTTTTCTCAC
TAGAGTCTATGTCACTCATTATACTCTGTCGAATGTCATTGAATGTCTTTACATGGGCTT
GTATGCCTATGAAAATTGTAATACAACTTTCAGCAACGGATCTCTTGGCTCTCGCATCGA
TGAAGAACGCAGCGAAATGCGATAAGTAATGTGAATTGCAGAATTCAGTGAATCATCGAA
TCTTTGAACGCATCTTGCGCTCCTTGGTATTCCGAGGAGCATGCCTGTTTGAGTGTCATT
AAATTCTCAACTCTCTTATACTTTTTTGTAAAAGAGAGCTTGGACTGTGGAGGCTTGCTG
GCCACTTTTTGGGGTCAGCTCCTCTGAAATGCATTAGCGGAACCGTTTGCGATCTGCCAC
AAGTGTGATAAGTTATCTACACTGGCGAGGGGATTGCTCTCTGTAATGTTCAGCTTCTAA
TTGTCTCTACTTTGTGAGACTACTTTTGAATGCTTGACCTCAAATCAGGTAGGACTACCC
GCTGAACTTAA
>A11_26
TTTCCGTAGGTGAACCTGCGGAAGGATCATTATTGAATTATGTTTCTAGATAGGTTGTAG
CTGGCTCTTTTAGAGCATGTGCACGCCTGTTTGGACTTCATTTTCATCCACCTGTGCACC
TATTGTAGTCTTTGGTTGGGTTAGGAGGAAGTGATCATTGTATCAGCATCTGCTGGGAGT
GAGGACTTGCATTGTGAAAGCTTTGCTGTCCTTGATGTGATCATGGAATCTTTTTCTCAC
TAGAGTCTATGTCACTCATTATACTCTGTCGAATGTCATTGAATGTCTTTACATGGGCTT
GTATGCCTATGAAAATTGTAATACAACTTTCAGCAACGGATCTCTTGGCTCTCGCATCGA
TGAAGAACGCAGCGAAATGCGATAAGTAATGTGAATTGCAGAATTCAGTGAATCATCGAA
TCTTTGAACGCATCTTGCGCTCCTTGGTATTCCGAGGAGCATGCCTGTTTGAGTGTCATT
AAATTCTCAACTCTCTTATACTTTTTTGTAAAAGAGAGCTTGGACTGTGGAGGCTTGCTG
GCCACTTTTTGGGGTCAGCTCCTCTGAAATGCATTAGCGGAACCGTTTGCGATCTGCCAC
AAGTGTGATAAGTTATCTACACTGGCGAGGGGATTGCTCTCTGTAATGTTCAGCTTCTAA
TTGTCTCTACTTTGTGAGACTACTTTTGAATGCTTGACCTCAAATCAGGTAGGACTACCC
GCTGAACTTAA
>A11_27
TTTCCGTAGGTGAACCTGCGGAAGGATCATTATTGAATTATGTTTCTAGATAGGTTGTAG
CTGGCTCTTTTAGAGCATGTGCACGCCTGTTTGGACTTCATTTTCATCCACCTGTGCACC
TATTGTAGTCTTTGGTTGGGTTAGGAGGAAGTGATCATTGTATCAGCATCTGCTGGGAGT
GAGGACTTGCATTGTGAAAGCTTTGCTGTCCTTGATGTGATCATGGAATCTTTTTCTCAC
TAGAGTCTATGTCACTCATTATACTCTGTCGAATGTCATTGAATGTCTTTACATGGGCTT
GTATGCCTATGAAAATTGTAATACAACTTTCAGCAACGGATCTCTTGGCTCTCGCATCGA
TGAAGAACGCAGCGAAATGCGATAAGTAATGTGAATTGCAGAATTCAGTGAATCATCGAA
TCTTTGAACGCATCTTGCGCTCCTTGGTATTCCGAGGAGCATGCCTGTTTGAGTGTCATT
AAATTCTCAACTCTCTTATACTTTTTTGTAAAAGAGAGCTTGGACTGTGGAGGCTTGCTG
GCCACTTTTTGGGGTCAGCTCCTCTGAAATGCATTAGCGGAACCGTTTGCGATCTGCCAC
AAGTGTGATAAGTTATCTACACTGGCGAGGGGATTGCTCTCTGTAATGTTCAGCTTCTAA
TTGTCTCTACTTTGTGAGACTACTTTTGAATGCTTGACCTCAAATCAGGTAGGACTACCC
GCTGAACTTAA

>A11_60
TTTCCGTAGGTGAACCTGCGGAAGGATCATTATTGAATTATGTTTCTAGATAGGTTGTAG
CTGGCTCTTTTAGAGCATGTGCACGCCTGTTTGGACTTCATTTTCATCCACCTGTGCACC
TATTGTAGTCTTTGGTTGGGTTAGGAGGAAGTGATCATTGTATCAGCATCTGCTGGGAGT
GAGGACTTGCATTGTGAAAGCTTTGCTGTCCTTGATGTGATCATGGAATCTTTTTCTCAC
TAGAGTCTATGTCACTCATTATACTCTGTCGAATGTCATTGAATGTCTTTACATGGGCTT
GTATGCCTATGAAAATTGTAATACAACTTTCAGCAACGGATCTCTTGGCTCTCGCATCGA
TGAAGAACGCAGCGAAATGCGATAAGTAATGTGAATTGCAGAATTCAGTGAATCATCGAA
TCTTTGAACGCATCTTGCGCTCCTTGGTATTCCGAGGAGCATGCCTGTTTGAGTGTCATT
AAATTCTCAACTCTCTTATACTTTTTTGTAAAAGAGAGCTTGGACTGTGGAGGCTTGCTG
GCCACTTTTTGGGGTCAGCTCCTCTGAAATGCATTAGCGGAACCGTTTGCGATCTGCCAC
AAGTGTGATAAGTTATCTACACTGGCGAGGGGATTGCTCTCTGTAATGTTCAGCTTCTAA
TTGTCTCTACTTTGTGAGACTACTTTTGAATGCTTGACCTCAAATCAGGTAGGACTACCC
GCTGAACTTAA
>A12_16
TTTCCGTAGGTGAACCTGCGGAAGGATCATTATTGAATTATGTTTCTAGATAGGTTGTAG
CTGGCTCTTTTAGAGCATGTGCACGCCTGTTTGGACTTCATTTTCATCCACCTGTGCACC
TATTGTAGTCTTTGGTTGGGTTAGGAGGAAGTGATCATTGTATCAGCATCTGCTGGGAGT
GAGGACTTGCATTGTGAAAGCTTTGCTGTCCTTGATGTGATCATGGAATCTTTTTCTCAC
TAGAGTCTATGTCACTCATTATACTCTGTCGAATGTCATTGAATGTCTTTACATGGGCTT
GTATGCCTATGAAAATTGTAATACAACTTTCAGCAACGGATCTCTTGGCTCTCGCATCGA
TGAAGAACGCAGCGAAATGCGATAAGTAATGTGAATTGCAGAATTCAGTGAATCATCGAA
TCTTTGAACGCATCTTGCGCTCCTTGGTATTCCGAGGAGCATGCCTGTTTGAGTGTCATT
AAATTCTCAACTCTCTTATACTTTTTTGTAAAAGAGAGCTTGGACTGTGGAGGCTTGCTG
GCCACTTTTTGGGGTCAGCTCCTCTGAAATGCATTAGCGGAACCGTTTGCGATCTGCCAC
AAGTGTGATAAGTTATCTACACTGGCGAGGGGATTGCTCTCTGTAATGTTCAGCTTCTAA
TTGTCTCTACTTTGTGAGACTACTTTTGAATGCTTGACCTCAAATCAGGTAGGACTACCC
GCTGAACTTAA
>A12_17
TTTCCGTAGGTGAACCTGCGGAAGGATCATTATTGAATTATGTTTCTAGATAGGTTGTAG
CTGGCTCTTTTAGAGCATGTGCACGCCTGTTTGGACTTCATTTTCATCCACCTGTGCACC
TATTGTAGTCTTTGGTTGGGTTAGGAGGAAGTGATCATTGTATCAGCATCTGCTGGGAGT
GAGGACTTGCATTGTGAAAGCTTTGCTGTCCTTGATGTGATCATGGAATCTTTTTCTCAC
TAGAGTCTATGTCACTCATTATACTCTGTCGAATGTCATTGAATGTCTTTACATGGGCTT
GTATGCCTATGAAAATTGTAATACAACTTTCAGCAACGGATCTCTTGGCTCTCGCATCGA
TGAAGAACGCAGCGAAATGCGATAAGTAATGTGAATTGCAGAATTCAGTGAATCATCGAA
TCTTTGAACGCATCTTGCGCTCCTTGGTATTCCGAGGAGCATGCCTGTTTGAGTGTCATT
AAATTCTCAACTCTCTTATACTTTTTTGTAAAAGAGAGCTTGGACTGTGGAGGCTTGCTG
GCCACTTTTTGGGGTCAGCTCCTCTGAAATGCATTAGCGGAACCGTTTGCGATCTGCCAC
AAGTGTGATAAGTTATCTACACTGGCGAGGGGATTGCTCTCTGTAATGTTCAGCTTCTAA
TTGTCTCTACTTTGTGAGACTACTTTTGAATGCTTGACCTCAAATCAGGTAGGACTACCC
GCTGAACTTAA
>A12_19
TTTCCGTAGGTGAACCTGCGGAAGGATCATTATTGAATTATGTTTCTAGATAGGTTGTAG
CTGGCTCTTTTAGAGCATGTGCACGCCTGTTTGGACTTCATTTTCATCCACCTGTGCACC
TATTGTAGTCTTTGGTTGGGTTAGGAGGAAGTGATCATTGTATCAGCATCTGCTGGGAGT
GAGGACTTGCATTGTGAAAGCTTTGCTGTCCTTGATGTGATCATGGAATCTTTTTCTCAC
TAGAGTCTATGTCACTCATTATACTCTGTCGAATGTCATTGAATGTCTTTACATGGGCTT
GTATGCCTATGAAAATTGTAATACAACTTTCAGCAACGGATCTCTTGGCTCTCGCATCGA
TGAAGAACGCAGCGAAATGCGATAAGTAATGTGAATTGCAGAATTCAGTGAATCATCGAA

TCTTTGAACGCATCTTGCGCTCCTTGGTATTCCGAGGAGCATGCCTGTTTGAGTGTCATT
AAATTCTCAACTCTCTTATACTTTTTTGTAAAAGAGAGCTTGGACTGTGGAGGCTTGCTG
GCCACTTTTTGGGGTCAGCTCCTCTGAAATGCATTAGCGGAACCGTTTGCGATCTGCCAC
AAGTGTGATAAGTTATCTACACTGGCGAGGGGATTGCTCTCTGTAATGTTCAGCTTCTAA
TTGTCTCTACTTTGTGAGACTACTTTTGAATGCTTGACCTCAAATCAGGTAGGACTACCC
GCTGAACTTAA
>A12_20
TTTCCGTAGGTGAACCTGCGGAAGGATCATTATTGAATTATGTTTCTAGATAGGTTGTAG
CTGGCTCTTTTAGAGCATGTGCACGCCTGTTTGGACTTCATTTTCATCCACCTGTGCACC
TATTGTAGTCTTTGGTTGGGTTAGGAGGAAGTGATCATTGTATCAGCATCTGCTGGGAGT
GAGGACTTGCATTGTGAAAGCTTTGCTGTCCTTGATGTGATCATGGAATCTTTTTCTCAC
TAGAGTCTATGTCACTCATTATACTCTGTCGAATGTCATTGAATGTCTTTACATGGGCTT
GTATGCCTATGAAAATTGTAATACAACTTTCAGCAACGGATCTCTTGGCTCTCGCATCGA
TGAAGAACGCAGCGAAATGCGATAAGTAATGTGAATTGCAGAATTCAGTGAATCATCGAA
TCTTTGAACGCATCTTGCGCTCCTTGGTATTCCGAGGAGCATGCCTGTTTGAGTGTCATT
AAATTCTCAACTCTCTTATACTTTTTTGTAAAAGAGAGCTTGGACTGTGGAGGCTTGCTG
GCCACTTTTTGGGGTCAGCTCCTCTGAAATGCATTAGCGGAACCGTTTGCGATCTGCCAC
AAGTGTGATAAGTTATCTACACTGGCGAGGGGATTGCTCTCTGTAATGTTCAGCTTCTAA
TTGTCTCTACTTTGTGAGACTACTTTTGAATGCTTGACCTCAAATCAGGTAGGACTACCC
GCTGAACTTAA
>A12_21
TTTCCGTAGGTGAACCTGCGGAAGGATCATTATTGAATTATGTTTCTAGATAGGTTGTAG
CTGGCTCTTTTAGAGCATGTGCACGCCTGTTTGGACTTCATTTTCATCCACCTGTGCACC
TATTGTAGTCTTTGGTTGGGTTAGGAGGAAGTGATCATTGTATCAGCATCTGCTGGGAGT
GAGGACTTGCATTGTGAAAGCTTTGCTGTCCTTGATGTGATCATGGAATCTTTTTCTCAC
TAGAGTCTATGTCACTCATTATACTCTGTCGAATGTCATTGAATGTCTTTACATGGGCTT
GTATGCCTATGAAAATTGTAATACAACTTTCAGCAACGGATCTCTTGGCTCTCGCATCGA
TGAAGAACGCAGCGAAATGCGATAAGTAATGTGAATTGCAGAATTCAGTGAATCATCGAA
TCTTTGAACGCATCTTGCGCTCCTTGGTATTCCGAGGAGCATGCCTGTTTGAGTGTCATT
AAATTCTCAACTCTCTTATACTTTTTTGTAAAAGAGAGCTTGGACTGTGGAGGCTTGCTG
GCCACTTTTTGGGGTCAGCTCCTCTGAAATGCATTAGCGGAACCGTTTGCGATCTGCCAC
AAGTGTGATAAGTTATCTACACTGGCGAGGGGATTGCTCTCTGTAATGTTCAGCTTCTAA
TTGTCTCTACTTTGTGAGACTACTTTTGAATGCTTGACCTCAAATCAGGTAGGACTACCC
GCTGAACTTAA
>A12_22
TTTCCGTAGGTGAACCTGCGGAAGGATCATTATTGAATTATGTTTCTAGATAGGTTGTAG
CTGGCTCTTTTAGAGCATGTGCACGCCTGTTTGGACTTCATTTTCATCCACCTGTGCACC
TATTGTAGTCTTTGGTTGGGTTAGGAGGAAGTGATCATTGTATCAGCATCTGCTGGGAGT
GAGGACTTGCATTGTGAAAGCTTTGCTGTCCTTGATGTGATCATGGAATCTTTTTCTCAC
TAGAGTCTATGTCACTCATTATACTCTGTCGAATGTCATTGAATGTCTTTACATGGGCTT
GTATGCCTATGAAAATTGTAATACAACTTTCAGCAACGGATCTCTTGGCTCTCGCATCGA
TGAAGAACGCAGCGAAATGCGATAAGTAATGTGAATTGCAGAATTCAGTGAATCATCGAA
TCTTTGAACGCATCTTGCGCTCCTTGGTATTCCGAGGAGCATGCCTGTTTGAGTGTCATT
AAATTCTCAACTCTCTTATACTTTTTTGTAAAAGAGAGCTTGGACTGTGGAGGCTTGCTG
GCCACTTTTTGGGGTCAGCTCCTCTGAAATGCATTAGCGGAACCGTTTGCGATCTGCCAC
AAGTGTGATAAGTTATCTACACTGGCGAGGGGATTGCTCTCTGTAATGTTCAGCTTCTAA
TTGTCTCTACTTTGTGAGACTACTTTTGAATGCTTGACCTCAAATCAGGTAGGACTACCC
GCTGAACTTAA
>A12_23
TTTCCGTAGGTGAACCTGCGGAAGGATCATTATTGAATTATGTTTCTAGATAGGTTGTAG

CTGGCTCTTTTAGAGCATGTGCACGCCTGTTTGGACTTCATTTTCATCCACCTGTGCACC
TATTGTAGTCTTTGGTTGGGTTAGGAGGAAGTGATCATTGTATCAGCATCTGCTGGGAGT
GAGGACTTGCATTGTGAAAGCTTTGCTGTCCTTGATGTGATCATGGAATCTTTTTCTCAC
TAGAGTCTATGTCACTCATTATACTCTGTCGAATGTCATTGAATGTCTTTACATGGGCTT
GTATGCCTATGAAAATTGTAATACAACTTTCAGCAACGGATCTCTTGGCTCTCGCATCGA
TGAAGAACGCAGCGAAATGCGATAAGTAATGTGAATTGCAGAATTCAGTGAATCATCGAA
TCTTTGAACGCATCTTGCGCTCCTTGGTATTCCGAGGAGCATGCCTGTTTGAGTGTCATT
AAATTCTCAACTCTCTTATACTTTTTTGTAAAAGAGAGCTTGGACTGTGGAGGCTTGCTG
GCCACTTTTTGGGGTCAGCTCCTCTGAAATGCATTAGCGGAACCGTTTGCGATCTGCCAC
AAGTGTGATAAGTTATCTACACTGGCGAGGGGATTGCTCTCTGTAATGTTCAGCTTCTAA
TTGTCTCTACTTTGTGAGACTACTTTTGAATGCTTGACCTCAAATCAGGTAGGACTACCC
GCTGAACTTAA
>A12_26
TTTCCGTAGGTGAACCTGCGGAAGGATCATTATTGAATTATGTTTCTAGATAGGTTGTAG
CTGGCTCTTTTAGAGCATGTGCACGCCTGTTTGGACTTCATTTTCATCCACCTGTGCACC
TATTGTAGTCTTTGGTTGGGTTAGGAGGAAGTGATCATTGTATCAGCATCTGCTGGGAGT
GAGGACTTGCATTGTGAAAGCTTTGCTGTCCTTGATGTGATCATGGAATCTTTTTCTCAC
TAGAGTCTATGTCACTCATTATACTCTGTCGAATGTCATTGAATGTCTTTACATGGGCTT
GTATGCCTATGAAAATTGTAATACAACTTTCAGCAACGGATCTCTTGGCTCTCGCATCGA
TGAAGAACGCAGCGAAATGCGATAAGTAATGTGAATTGCAGAATTCAGTGAATCATCGAA
TCTTTGAACGCATCTTGCGCTCCTTGGTATTCCGAGGAGCATGCCTGTTTGAGTGTCATT
AAATTCTCAACTCTCTTATACTTTTTTGTAAAAGAGAGCTTGGACTGTGGAGGCTTGCTG
GCCACTTTTTGGGGTCAGCTCCTCTGAAATGCATTAGCGGAACCGTTTGCGATCTGCCAC
AAGTGTGATAAGTTATCTACACTGGCGAGGGGATTGCTCTCTGTAATGTTCAGCTTCTAA
TTGTCTCTACTTTGTGAGACTACTTTTGAATGCTTGACCTCAAATCAGGTAGGACTACCC
GCTGAACTTAA
>A12_27
TTTCCGTAGGTGAACCTGCGGAAGGATCATTATTGAATTATGTTTCTAGATAGGTTGTAG
CTGGCTCTTTTAGAGCATGTGCACGCCTGTTTGGACTTCATTTTCATCCACCTGTGCACC
TATTGTAGTCTTTGGTTGGGTTAGGAGGAAGTGATCATTGTATCAGCATCTGCTGGGAGT
GAGGACTTGCATTGTGAAAGCTTTGCTGTCCTTGATGTGATCATGGAATCTTTTTCTCAC
TAGAGTCTATGTCACTCATTATACTCTGTCGAATGTCATTGAATGTCTTTACATGGGCTT
GTATGCCTATGAAAATTGTAATACAACTTTCAGCAACGGATCTCTTGGCTCTCGCATCGA
TGAAGAACGCAGCGAAATGCGATAAGTAATGTGAATTGCAGAATTCAGTGAATCATCGAA
TCTTTGAACGCATCTTGCGCTCCTTGGTATTCCGAGGAGCATGCCTGTTTGAGTGTCATT
AAATTCTCAACTCTCTTATACTTTTTTGTAAAAGAGAGCTTGGACTGTGGAGGCTTGCTG
GCCACTTTTTGGGGTCAGCTCCTCTGAAATGCATTAGCGGAACCGTTTGCGATCTGCCAC
AAGTGTGATAAGTTATCTACACTGGCGAGGGGATTGCTCTCTGTAATGTTCAGCTTCTAA
TTGTCTCTACTTTGTGAGACTACTTTTGAATGCTTGACCTCAAATCAGGTAGGACTACCC
GCTGAACTTAA
>A12_30
TTTCCGTAGGTGAACCTGCGGAAGGATCATTATTGAATTATGTTTCTAGATAGGTTGTAG
CTGGCTCTTTTAGAGCATGTGCACGCCTGTTTGGACTTCATTTTCATCCACCTGTGCACC
TATTGTAGTCTTTGGTTGGGTTAGGAGGAAGTGATCATTGTATCAGCATCTGCTGGGAGT
GAGGACTTGCATTGTGAAAGCTTTGCTGTCCTTGATGTGATCATGGAATCTTTTTCTCAC
TAGAGTCTATGTCACTCATTATACTCTGTCGAATGTCATTGAATGTCTTTACATGGGCTT
GTATGCCTATGAAAATTGTAATACAACTTTCAGCAACGGATCTCTTGGCTCTCGCATCGA
TGAAGAACGCAGCGAAATGCGATAAGTAATGTGAATTGCAGAATTCAGTGAATCATCGAA
TCTTTGAACGCATCTTGCGCTCCTTGGTATTCCGAGGAGCATGCCTGTTTGAGTGTCATT
AAATTCTCAACTCTCTTATACTTTTTTGTAAAAGAGAGCTTGGACTGTGGAGGCTTGCTG

GCCACTTTTTGGGGTCAGCTCCTCTGAAATGCATTAGCGGAACCGTTTGCGATCTGCCAC
AAGTGTGATAAGTTATCTACACTGGCGAGGGGATTGCTCTCTGTAATGTTCAGCTTCTAA
TTGTCTCTACTTTGTGAGACTACTTTTGAATGCTTGACCTCAAATCAGGTAGGACTACCC
GCTGAACTTAA
>A12_36
TTTCCGTAGGTGAACCTGCGGAAGGATCATTATTGAATTATGTTTCTAGATAGGTTGTAG
CTGGCTCTTTTAGAGCATGTGCACGCCTGTTTGGACTTCATTTTCATCCACCTGTGCACC
TATTGTAGTCTTTGGTTGGGTTAGGAGGAAGTGATCATTGTATCAGCATCTGCTGGGAGT
GAGGACTTGCATTGTGAAAGCTTTGCTGTCCTTGATGTGATCATGGAATCTTTTTCTCAC
TAGAGTCTATGTCACTCATTATACTCTGTCGAATGTCATTGAATGTCTTTACATGGGCTT
GTATGCCTATGAAAATTGTAATACAACTTTCAGCAACGGATCTCTTGGCTCTCGCATCGA
TGAAGAACGCAGCGAAATGCGATAAGTAATGTGAATTGCAGAATTCAGTGAATCATCGAA
TCTTTGAACGCATCTTGCGCTCCTTGGTATTCCGAGGAGCATGCCTGTTTGAGTGTCATT
AAATTCTCAACTCTCTTATACTTTTTTGTAAAAGAGAGCTTGGACTGTGGAGGCTTGCTG
GCCACTTTTTGGGGTCAGCTCCTCTGAAATGCATTAGCGGAACCGTTTGCGATCTGCCAC
AAGTGTGATAAGTTATCTACACTGGCGAGGGGATTGCTCTCTGTAATGTTCAGCTTCTAA
TTGTCTCTACTTTGTGAGACTACTTTTGAATGCTTGACCTCAAATCAGGTAGGACTACCC
GCTGAACTTAA
>A12_40
TTTCCGTAGGTGAACCTGCGGAAGGATCATTATTGAATTATGTTTCTAGATAGGTTGTAG
CTGGCTCTTTTAGAGCATGTGCACGCCTGTTTGGACTTCATTTTCATCCACCTGTGCACC
TATTGTAGTCTTTGGTTGGGTTAGGAGGAAGTGATCATTGTATCAGCATCTGCTGGGAGT
GAGGACTTGCATTGTGAAAGCTTTGCTGTCCTTGATGTGATCATGGAATCTTTTTCTCAC
TAGAGTCTATGTCACTCATTATACTCTGTCGAATGTCATTGAATGTCTTTACATGGGCTT
GTATGCCTATGAAAATTGTAATACAACTTTCAGCAACGGATCTCTTGGCTCTCGCATCGA
TGAAGAACGCAGCGAAATGCGATAAGTAATGTGAATTGCAGAATTCAGTGAATCATCGAA
TCTTTGAACGCATCTTGCGCTCCTTGGTATTCCGAGGAGCATGCCTGTTTGAGTGTCATT
AAATTCTCAACTCTCTTATACTTTTTTGTAAAAGAGAGCTTGGACTGTGGAGGCTTGCTG
GCCACTTTTTGGGGTCAGCTCCTCTGAAATGCATTAGCGGAACCGTTTGCGATCTGCCAC
AAGTGTGATAAGTTATCTACACTGGCGAGGGGATTGCTCTCTGTAATGTTCAGCTTCTAA
TTGTCTCTACTTTGTGAGACTACTTTTGAATGCTTGACCTCAAATCAGGTAGGACTACCC
GCTGAACTTAA
>A12_42
TTTCCGTAGGTGAACCTGCGGAAGGATCATTATTGAATTATGTTTCTAGATAGGTTGTAG
CTGGCTCTTTTAGAGCATGTGCACGCCTGTTTGGACTTCATTTTCATCCACCTGTGCACC
TATTGTAGTCTTTGGTTGGGTTAGGAGGAAGTGATCATTGTATCAGCATCTGCTGGGAGT
GAGGACTTGCATTGTGAAAGCTTTGCTGTCCTTGATGTGATCATGGAATCTTTTTCTCAC
TAGAGTCTATGTCACTCATTATACTCTGTCGAATGTCATTGAATGTCTTTACATGGGCTT
GTATGCCTATGAAAATTGTAATACAACTTTCAGCAACGGATCTCTTGGCTCTCGCATCGA
TGAAGAACGCAGCGAAATGCGATAAGTAATGTGAATTGCAGAATTCAGTGAATCATCGAA
TCTTTGAACGCATCTTGCGCTCCTTGGTATTCCGAGGAGCATGCCTGTTTGAGTGTCATT
AAATTCTCAACTCTCTTATACTTTTTTGTAAAAGAGAGCTTGGACTGTGGAGGCTTGCTG
GCCACTTTTTGGGGTCAGCTCCTCTGAAATGCATTAGCGGAACCGTTTGCGATCTGCCAC
AAGTGTGATAAGTTATCTACACTGGCGAGGGGATTGCTCTCTGTAATGTTCAGCTTCTAA
TTGTCTCTACTTTGTGAGACTACTTTTGAATGCTTGACCTCAAATCAGGTAGGACTACCC
GCTGAACTTAA
>A12_43
TTTCCGTAGGTGAACCTGCGGAAGGATCATTATTGAATTATGTTTCTAGATAGGTTGTAG
CTGGCTCTTTTAGAGCATGTGCACGCCTGTTTGGACTTCATTTTCATCCACCTGTGCACC
TATTGTAGTCTTTGGTTGGGTTAGGAGGAAGTGATCATTGTATCAGCATCTGCTGGGAGT

GAGGACTTGCATTGTGAAAGCTTTGCTGTCCTTGATGTGATCATGGAATCTTTTTCTCAC
TAGAGTCTATGTCACTCATTATACTCTGTCGAATGTCATTGAATGTCTTTACATGGGCTT
GTATGCCTATGAAAATTGTAATACAACTTTCAGCAACGGATCTCTTGGCTCTCGCATCGA
TGAAGAACGCAGCGAAATGCGATAAGTAATGTGAATTGCAGAATTCAGTGAATCATCGAA
TCTTTGAACGCATCTTGCGCTCCTTGGTATTCCGAGGAGCATGCCTGTTTGAGTGTCATT
AAATTCTCAACTCTCTTATACTTTTTTGTAAAAGAGAGCTTGGACTGTGGAGGCTTGCTG
GCCACTTTTTGGGGTCAGCTCCTCTGAAATGCATTAGCGGAACCGTTTGCGATCTGCCAC
AAGTGTGATAAGTTATCTACACTGGCGAGGGGATTGCTCTCTGTAATGTTCAGCTTCTAA
TTGTCTCTACTTTGTGAGACTACTTTTGAATGCTTGACCTCAAATCAGGTAGGACTACCC
GCTGAACTTAA
>A12_45
TTTCCGTAGGTGAACCTGCGGAAGGATCATTATTGAATTATGTTTCTAGATAGGTTGTAG
CTGGCTCTTTTAGAGCATGTGCACGCCTGTTTGGACTTCATTTTCATCCACCTGTGCACC
TATTGTAGTCTTTGGTTGGGTTAGGAGGAAGTGATCATTGTATCAGCATCTGCTGGGAGT
GAGGACTTGCATTGTGAAAGCTTTGCTGTCCTTGATGTGATCATGGAATCTTTTTCTCAC
TAGAGTCTATGTCACTCATTATACTCTGTCGAATGTCATTGAATGTCTTTACATGGGCTT
GTATGCCTATGAAAATTGTAATACAACTTTCAGCAACGGATCTCTTGGCTCTCGCATCGA
TGAAGAACGCAGCGAAATGCGATAAGTAATGTGAATTGCAGAATTCAGTGAATCATCGAA
TCTTTGAACGCATCTTGCGCTCCTTGGTATTCCGAGGAGCATGCCTGTTTGAGTGTCATT
AAATTCTCAACTCTCTTATACTTTTTTGTAAAAGAGAGCTTGGACTGTGGAGGCTTGCTG
GCCACTTTTTGGGGTCAGCTCCTCTGAAATGCATTAGCGGAACCGTTTGCGATCTGCCAC
AAGTGTGATAAGTTATCTACACTGGCGAGGGGATTGCTCTCTGTAATGTTCAGCTTCTAA
TTGTCTCTACTTTGTGAGACTACTTTTGAATGCTTGACCTCAAATCAGGTAGGACTACCC
GCTGAACTTAA
>A11_61
TTTCCGTAGGTGAACCTGCGGAAGGATCATTATTGAATTATGTTTCTAGATAGGTTGTAG
CTGGCTCTTTTAGAGCATGTGCACGCCTGTTTGGACTTCATTTTCATCCACCTGTGCACC
TATTGTAGTCTTTGGTTGGGTTAGGAGGAAGTGATCATTGTATCAGCATCTGCTGGGAGT
GAGGACTTGCATTGTGAAAGCTTTGCTGTCCTTGATGTGATCATGGAATCTTTTTCTCAC
TAGAGTCTATGTCACTCATTATACTCTGTCGAATGTCATTGAATGTCTTTACATGGGCTT
GTATGCCTATGAAAATTGTAATACAACTTTCAGCAACGGATCTCTTGGCTCTCGCATCGA
TGAAGAACGCAGCGAAATGCGATAAGTAATGTGAATTGCAGAATTCAGTGAATCATCGAA
TCTTTGAACGCATCTTGCGCTCCTTGGTATTCCGAGGAGCATGCCTGTTTGAGTGTCATT
AAATTCTCAACTCTCTTATACTTTTTTGTAAAAGAGAGCTTGGACTGTGGAGGCTTGCTG
GCCACTTTTTGGGGTCAGCTCCTCTGAAATGCATTAGCGGAACCGTTTGCGATCTGCCAC
AAGTGTGATAAGTTATCTACACTGGCGAGGGGATTGCTCTCTGTAATGTTCAGCTTCTAA
TTGTCTCTACTTTGTGAGACTACTTTTGAATGCTTGACCTCAAATCAGGTAGGACTACCC
GCTGAACTTAA
>A11_62
TTTCCGTAGGTGAACCTGCGGAAGGATCATTATTGAATTATGTTTCTAGATAGGTTGTAG
CTGGCTCTTTTAGAGCATGTGCACGCCTGTTTGGACTTCATTTTCATCCACCTGTGCACC
TATTGTAGTCTTTGGTTGGGTTAGGAGGAAGTGATCATTGTATCAGCATCTGCTGGGAGT
GAGGACTTGCATTGTGAAAGCTTTGCTGTCCTTGATGTGATCATGGAATCTTTTTCTCAC
TAGAGTCTATGTCACTCATTATACTCTGTCGAATGTCATTGAATGTCTTTACATGGGCTT
GTATGCCTATGAAAATTGTAATACAACTTTCAGCAACGGATCTCTTGGCTCTCGCATCGA
TGAAGAACGCAGCGAAATGCGATAAGTAATGTGAATTGCAGAATTCAGTGAATCATCGAA
TCTTTGAACGCATCTTGCGCTCCTTGGTATTCCGAGGAGCATGCCTGTTTGAGTGTCATT
AAATTCTCAACTCTCTTATACTTTTTTGTAAAAGAGAGCTTGGACTGTGGAGGCTTGCTG
GCCACTTTTTGGGGTCAGCTCCTCTGAAATGCATTAGCGGAACCGTTTGCGATCTGCCAC
AAGTGTGATAAGTTATCTACACTGGCGAGGGGATTGCTCTCTGTAATGTTCAGCTTCTAA

TTGTCTCTACTTTGTGAGACTACTTTTGAATGCTTGACCTCAAATCAGGTAGGACTACCC
GCTGAACTTAA
>A12_2
TTTCCGTAGGTGAACCTGCGGAAGGATCATTATTGAATTATGTTTCTAGATAGGTTGTAG
CTGGCTCTTTTAGAGCATGTGCACGCCTGTTTGGACTTCATTTTCATCCACCTGTGCACC
TATTGTAGTCTTTGGTTGGGTTAGGAGGAAGTGATCATTGTATCAGCATCTGCTGGGAGT
GAGGACTTGCATTGTGAAAGCTTTGCTGTCCTTGATGTGATCATGGAATCTTTTTCTCAC
TAGAGTCTATGTCACTCATTATACTCTGTCGAATGTCATTGAATGTCTTTACATGGGCTT
GTATGCCTATGAAAATTGTAATACAACTTTCAGCAACGGATCTCTTGGCTCTCGCATCGA
TGAAGAACGCAGCGAAATGCGATAAGTAATGTGAATTGCAGAATTCAGTGAATCATCGAA
TCTTTGAACGCATCTTGCGCTCCTTGGTATTCCGAGGAGCATGCCTGTTTGAGTGTCATT
AAATTCTCAACTCTCTTATACTTTTTGTAAAAGAGAGCTTGGACTGTGGAGGCTTGCTG
GCCACTTTTTGGGGTCAGCTCCTCTGAAATGCATTAGCGGAACCGTTTGCGATCTGCCAC
AAGTGTGATAAGTTATCTACACTGGCGAGGGGATTGCTCTCTGTAATGTTCAGCTTCTAA
TTGTCTCTACTTTGTGAGACTACTTTTGAATGCTTGACCTCAAATCAGGTAGGACTACCC
GCTGAACTTAA
>A12_3
TTTCCGTAGGTGAACCTGCGGAAGGATCATTATTGAATTATGTTTCTAGATAGGTTGTAG
CTGGCTCTTTTAGAGCATGTGCACGCCTGTTTGGACTTCATTTTCATCCACCTGTGCACC
TATTGTAGTCTTTGGTTGGGTTAGGAGGAAGTGATCATTGTATCAGCATCTGCTGGGAGT
GAGGACTTGCATTGTGAAAGCTTTGCTGTCCTTGATGTGATCATGGAATCTTTTTCTCAC
TAGAGTCTATGTCACTCATTATACTCTGTCGAATGTCATTGAATGTCTTTACATGGGCTT
GTATGCCTATGAAAATTGTAATACAACTTTCAGCAACGGATCTCTTGGCTCTCGCATCGA
TGAAGAACGCAGCGAAATGCGATAAGTAATGTGAATTGCAGAATTCAGTGAATCATCGAA
TCTTTGAACGCATCTTGCGCTCCTTGGTATTCCGAGGAGCATGCCTGTTTGAGTGTCATT
AAATTCTCAACTCTCTTATACTTTTTGTAAAAGAGAGCTTGGACTGTGGAGGCTTGCTG
GCCACTTTTTGGGGTCAGCTCCTCTGAAATGCATTAGCGGAACCGTTTGCGATCTGCCAC
AAGTGTGATAAGTTATCTACACTGGCGAGGGGATTGCTCTCTGTAATGTTCAGCTTCTAA
TTGTCTCTACTTTGTGAGACTACTTTTGAATGCTTGACCTCAAATCAGGTAGGACTACCC
GCTGAACTTAA
>A12_4
TTTCCGTAGGTGAACCTGCGGAAGGATCATTATTGAATTATGTTTCTAGATAGGTTGTAG
CTGGCTCTTTTAGAGCATGTGCACGCCTGTTTGGACTTCATTTTCATCCACCTGTGCACC
TATTGTAGTCTTTGGTTGGGTTAGGAGGAAGTGATCATTGTATCAGCATCTGCTGGGAGT
GAGGACTTGCATTGTGAAAGCTTTGCTGTCCTTGATGTGATCATGGAATCTTTTTCTCAC
TAGAGTCTATGTCACTCATTATACTCTGTCGAATGTCATTGAATGTCTTTACATGGGCTT
GTATGCCTATGAAAATTGTAATACAACTTTCAGCAACGGATCTCTTGGCTCTCGCATCGA
TGAAGAACGCAGCGAAATGCGATAAGTAATGTGAATTGCAGAATTCAGTGAATCATCGAA
TCTTTGAACGCATCTTGCGCTCCTTGGTATTCCGAGGAGCATGCCTGTTTGAGTGTCATT
AAATTCTCAACTCTCTTATACTTTTTGTAAAAGAGAGCTTGGACTGTGGAGGCTTGCTG
GCCACTTTTTGGGGTCAGCTCCTCTGAAATGCATTAGCGGAACCGTTTGCGATCTGCCAC
AAGTGTGATAAGTTATCTACACTGGCGAGGGGATTGCTCTCTGTAATGTTCAGCTTCTAA
TTGTCTCTACTTTGTGAGACTACTTTTGAATGCTTGACCTCAAATCAGGTAGGACTACCC
GCTGAACTTAA
>A12_7
TTTCCGTAGGTGAACCTGCGGAAGGATCATTATTGAATTATGTTTCTAGATAGGTTGTAG
CTGGCTCTTTTAGAGCATGTGCACGCCTGTTTGGACTTCATTTTCATCCACCTGTGCACC
TATTGTAGTCTTTGGTTGGGTTAGGAGGAAGTGATCATTGTATCAGCATCTGCTGGGAGT
GAGGACTTGCATTGTGAAAGCTTTGCTGTCCTTGATGTGATCATGGAATCTTTTTCTCAC
TAGAGTCTATGTCACTCATTATACTCTGTCGAATGTCATTGAATGTCTTTACATGGGCTT

GTATGCCTATGAAAATTGTAATACAACTTTCAGCAACGGATCTCTTGGCTCTCGCATCGA
TGAAGAACGCAGCGAAATGCGATAAGTAATGTGAATTGCAGAATTCAGTGAATCATCGAA
TCTTTGAACGCATCTTGCGCTCCTTGGTATTCCGAGGAGCATGCCTGTTTGAGTGTCATT
AAATTCTCAACTCTCTTATACTTTTTTGTAAAAGAGAGCTTGGACTGTGGAGGCTTGCTG
GCCACTTTTTGGGGTCAGCTCCTCTGAAATGCATTAGCGGAACCGTTTGCGATCTGCCAC
AAGTGTGATAAGTTATCTACACTGGCGAGGGGATTGCTCTCTGTAATGTTCAGCTTCTAA
TTGTCTCTACTTTGTGAGACTACTTTTGAATGCTTGACCTCAAATCAGGTAGGACTACCC
GCTGAACTTAA
>A12_8
TTTCCGTAGGTGAACCTGCGGAAGGATCATTATTGAATTATGTTTCTAGATAGGTTGTAG
CTGGCTCTTTTAGAGCATGTGCACGCCTGTTTGGACTTCATTTTCATCCACCTGTGCACC
TATTGTAGTCTTTGGTTGGGTTAGGAGGAAGTGATCATTGTATCAGCATCTGCTGGGAGT
GAGGACTTGCATTGTGAAAGCTTTGCTGTCCTTGATGTGATCATGGAATCTTTTTCTCAC
TAGAGTCTATGTCACTCATTATACTCTGTCGAATGTCATTGAATGTCTTTACATGGGCTT
GTATGCCTATGAAAATTGTAATACAACTTTCAGCAACGGATCTCTTGGCTCTCGCATCGA
TGAAGAACGCAGCGAAATGCGATAAGTAATGTGAATTGCAGAATTCAGTGAATCATCGAA
TCTTTGAACGCATCTTGCGCTCCTTGGTATTCCGAGGAGCATGCCTGTTTGAGTGTCATT
AAATTCTCAACTCTCTTATACTTTTTTGTAAAAGAGAGCTTGGACTGTGGAGGCTTGCTG
GCCACTTTTTGGGGTCAGCTCCTCTGAAATGCATTAGCGGAACCGTTTGCGATCTGCCAC
AAGTGTGATAAGTTATCTACACTGGCGAGGGGATTGCTCTCTGTAATGTTCAGCTTCTAA
TTGTCTCTACTTTGTGAGACTACTTTTGAATGCTTGACCTCAAATCAGGTAGGACTACCC
GCTGAACTTAA
>A12_10
TTTCCGTAGGTGAACCTGCGGAAGGATCATTATTGAATTATGTTTCTAGATAGGTTGTAG
CTGGCTCTTTTAGAGCATGTGCACGCCTGTTTGGACTTCATTTTCATCCACCTGTGCACC
TATTGTAGTCTTTGGTTGGGTTAGGAGGAAGTGATCATTGTATCAGCATCTGCTGGGAGT
GAGGACTTGCATTGTGAAAGCTTTGCTGTCCTTGATGTGATCATGGAATCTTTTTCTCAC
TAGAGTCTATGTCACTCATTATACTCTGTCGAATGTCATTGAATGTCTTTACATGGGCTT
GTATGCCTATGAAAATTGTAATACAACTTTCAGCAACGGATCTCTTGGCTCTCGCATCGA
TGAAGAACGCAGCGAAATGCGATAAGTAATGTGAATTGCAGAATTCAGTGAATCATCGAA
TCTTTGAACGCATCTTGCGCTCCTTGGTATTCCGAGGAGCATGCCTGTTTGAGTGTCATT
AAATTCTCAACTCTCTTATACTTTTTTGTAAAAGAGAGCTTGGACTGTGGAGGCTTGCTG
GCCACTTTTTGGGGTCAGCTCCTCTGAAATGCATTAGCGGAACCGTTTGCGATCTGCCAC
AAGTGTGATAAGTTATCTACACTGGCGAGGGGATTGCTCTCTGTAATGTTCAGCTTCTAA
TTGTCTCTACTTTGTGAGACTACTTTTGAATGCTTGACCTCAAATCAGGTAGGACTACCC
GCTGAACTTAA
>A12_13
TTTCCGTAGGTGAACCTGCGGAAGGATCATTATTGAATTATGTTTCTAGATAGGTTGTAG
CTGGCTCTTTTAGAGCATGTGCACGCCTGTTTGGACTTCATTTTCATCCACCTGTGCACC
TATTGTAGTCTTTGGTTGGGTTAGGAGGAAGTGATCATTGTATCAGCATCTGCTGGGAGT
GAGGACTTGCATTGTGAAAGCTTTGCTGTCCTTGATGTGATCATGGAATCTTTTTCTCAC
TAGAGTCTATGTCACTCATTATACTCTGTCGAATGTCATTGAATGTCTTTACATGGGCTT
GTATGCCTATGAAAATTGTAATACAACTTTCAGCAACGGATCTCTTGGCTCTCGCATCGA
TGAAGAACGCAGCGAAATGCGATAAGTAATGTGAATTGCAGAATTCAGTGAATCATCGAA
TCTTTGAACGCATCTTGCGCTCCTTGGTATTCCGAGGAGCATGCCTGTTTGAGTGTCATT
AAATTCTCAACTCTCTTATACTTTTTTGTAAAAGAGAGCTTGGACTGTGGAGGCTTGCTG
GCCACTTTTTGGGGTCAGCTCCTCTGAAATGCATTAGCGGAACCGTTTGCGATCTGCCAC
AAGTGTGATAAGTTATCTACACTGGCGAGGGGATTGCTCTCTGTAATGTTCAGCTTCTAA
TTGTCTCTACTTTGTGAGACTACTTTTGAATGCTTGACCTCAAATCAGGTAGGACTACCC
GCTGAACTTAA

>A12_14
TTTCCGTAGGTGAACCTGCGGAAGGATCATTATTGAATTATGTTTCTAGATAGGTTGTAG
CTGGCTCTTTTAGAGCATGTGCACGCCTGTTTGGACTTCATTTTCATCCACCTGTGCACC
TATTGTAGTCTTTGGTTGGGTTAGGAGGAAGTGATCATTGTATCAGCATCTGCTGGGAGT
GAGGACTTGCATTGTGAAAGCTTTGCTGTCCTTGATGTGATCATGGAATCTTTTTCTCAC
TAGAGTCTATGTCACTCATTATACTCTGTCGAATGTCATTGAATGTCTTTACATGGGCTT
GTATGCCTATGAAAATTGTAATACAACTTTCAGCAACGGATCTCTTGGCTCTCGCATCGA
TGAAGAACGCAGCGAAATGCGATAAGTAATGTGAATTGCAGAATTCAGTGAATCATCGAA
TCTTTGAACGCATCTTGCGCTCCTTGGTATTCCGAGGAGCATGCCTGTTTGAGTGTCATT
AAATTCTCAACTCTCTTATACTTTTTGTAAAAGAGAGCTTGGACTGTGGAGGCTTGCTG
GCCACTTTTTGGGGTCAGCTCCTCTGAAATGCATTAGCGGAACCGTTTGCGATCTGCCAC
AAGTGTGATAAGTTATCTACACTGGCGAGGGGATTGCTCTCTGTAATGTTCAGCTTCTAA
TTGTCTCTACTTTGTGAGACTACTTTTGAATGCTTGACCTCAAATCAGGTAGGACTACCC
GCTGAACTTAA
>A11_64
TTTCCGTAGGTGAACCTGCGGAAGGATCATTATTGAATTATGTTTCTAGATAGGTTGTAG
CTGGCTCTTTTAGAGCATGTGCACGCCTGTTTGGACTTCATTTTCATCCACCTGTGCACC
TATTGTAGTCTTTGGTTGGGTTAGGAGGAAGTGATCATTGTATCAGCATCTGCTGGGAGT
GAGGACTTGCATTGTGAAAGCTTTGCTGTCCTTGATGTGATCATGGAATCTTTTTCTCAC
TAGAGTCTATGTCACTCATTATACTCTGTCGAATGTCATTGAATGTCTTTACATGGGCTT
GTATGCCTATGAAAATTGTAATACAACTTTCAGCAACGGATCTCTTGGCTCTCGCATCGA
TGAAGAACGCAGCGAAATGCGATAAGTAATGTGAATTGCAGAATTCAGTGAATCATCGAA
TCTTTGAACGCATCTTGCGCTCCTTGGTATTCCGAGGAGCATGCCTGTTTGAGTGTCATT
AAATTCTCAACTCTCTTATACTTTTTGTAAAAGAGAGCTTGGACTGTGGAGGCTTGCTG
GCCACTTTTTGGGGTCAGCTCCTCTGAAATGCATTAGCGGAACCGTTTGCGATCTGCCAC
AAGTGTGATAAGTTATCTACACTGGCGAGGGGATTGCTCTCTGTAATGTTCAGCTTCTAA
TTGTCTCTACTTTGTGAGACTACTTTTGAATGCTTGACCTCAAATCAGGTAGGACTACCC
GCTGAACTTAA
>A11_65
TTTCCGTAGGTGAACCTGCGGAAGGATCATTATTGAATTATGTTTCTAGATAGGTTGTAG
CTGGCTCTTTTAGAGCATGTGCACGCCTGTTTGGACTTCATTTTCATCCACCTGTGCACC
TATTGTAGTCTTTGGTTGGGTTAGGAGGAAGTGATCATTGTATCAGCATCTGCTGGGAGT
GAGGACTTGCATTGTGAAAGCTTTGCTGTCCTTGATGTGATCATGGAATCTTTTTCTCAC
TAGAGTCTATGTCACTCATTATACTCTGTCGAATGTCATTGAATGTCTTTACATGGGCTT
GTATGCCTATGAAAATTGTAATACAACTTTCAGCAACGGATCTCTTGGCTCTCGCATCGA
TGAAGAACGCAGCGAAATGCGATAAGTAATGTGAATTGCAGAATTCAGTGAATCATCGAA
TCTTTGAACGCATCTTGCGCTCCTTGGTATTCCGAGGAGCATGCCTGTTTGAGTGTCATT
AAATTCTCAACTCTCTTATACTTTTTGTAAAAGAGAGCTTGGACTGTGGAGGCTTGCTG
GCCACTTTTTGGGGTCAGCTCCTCTGAAATGCATTAGCGGAACCGTTTGCGATCTGCCAC
AAGTGTGATAAGTTATCTACACTGGCGAGGGGATTGCTCTCTGTAATGTTCAGCTTCTAA
TTGTCTCTACTTTGTGAGACTACTTTTGAATGCTTGACCTCAAATCAGGTAGGACTACCC
GCTGAACTTAA
>A11_66
TTTCCGTAGGTGAACCTGCGGAAGGATCATTATTGAATTATGTTTCTAGATAGGTTGTAG
CTGGCTCTTTTAGAGCATGTGCACGCCTGTTTGGACTTCATTTTCATCCACCTGTGCACC
TATTGTAGTCTTTGGTTGGGTTAGGAGGAAGTGATCATTGTATCAGCATCTGCTGGGAGT
GAGGACTTGCATTGTGAAAGCTTTGCTGTCCTTGATGTGATCATGGAATCTTTTTCTCAC
TAGAGTCTATGTCACTCATTATACTCTGTCGAATGTCATTGAATGTCTTTACATGGGCTT
GTATGCCTATGAAAATTGTAATACAACTTTCAGCAACGGATCTCTTGGCTCTCGCATCGA
TGAAGAACGCAGCGAAATGCGATAAGTAATGTGAATTGCAGAATTCAGTGAATCATCGAA

TCTTTGAACGCATCTTGCGCTCCTTGGTATTCCGAGGAGCATGCCTGTTTGAGTGTCATT
AAATTCTCAACTCTCTTATACTTTTTTGTAAAAGAGAGCTTGGACTGTGGAGGCTTGCTG
GCCACTTTTTGGGGTCAGCTCCTCTGAAATGCATTAGCGGAACCGTTTGCGATCTGCCAC
AAGTGTGATAAGTTATCTACACTGGCGAGGGGATTGCTCTCTGTAATGTTCAGCTTCTAA
TTGTCTCTACTTTGTGAGACTACTTTTGAATGCTTGACCTCAAATCAGGTAGGACTACCC
GCTGAACTTAA
>A11_67
TTTCCGTAGGTGAACCTGCGGAAGGATCATTATTGAATTATGTTTCTAGATAGGTTGTAG
CTGGCTCTTTTAGAGCATGTGCACGCCTGTTTGGACTTCATTTTCATCCACCTGTGCACC
TATTGTAGTCTTTGGTTGGGTTAGGAGGAAGTGATCATTGTATCAGCATCTGCTGGGAGT
GAGGACTTGCATTGTGAAAGCTTTGCTGTCCTTGATGTGATCATGGAATCTTTTTCTCAC
TAGAGTCTATGTCACTCATTATACTCTGTCGAATGTCATTGAATGTCTTTACATGGGCTT
GTATGCCTATGAAAATTGTAATACAACTTTCAGCAACGGATCTCTTGGCTCTCGCATCGA
TGAAGAACGCAGCGAAATGCGATAAGTAATGTGAATTGCAGAATTCAGTGAATCATCGAA
TCTTTGAACGCATCTTGCGCTCCTTGGTATTCCGAGGAGCATGCCTGTTTGAGTGTCATT
AAATTCTCAACTCTCTTATACTTTTTTGTAAAAGAGAGCTTGGACTGTGGAGGCTTGCTG
GCCACTTTTTGGGGTCAGCTCCTCTGAAATGCATTAGCGGAACCGTTTGCGATCTGCCAC
AAGTGTGATAAGTTATCTACACTGGCGAGGGGATTGCTCTCTGTAATGTTCAGCTTCTAA
TTGTCTCTACTTTGTGAGACTACTTTTGAATGCTTGACCTCAAATCAGGTAGGACTACCC
GCTGAACTTAA
>A11_68
TTTCCGTAGGTGAACCTGCGGAAGGATCATTATTGAATTATGTTTCTAGATAGGTTGTAG
CTGGCTCTTTTAGAGCATGTGCACGCCTGTTTGGACTTCATTTTCATCCACCTGTGCACC
TATTGTAGTCTTTGGTTGGGTTAGGAGGAAGTGATCATTGTATCAGCATCTGCTGGGAGT
GAGGACTTGCATTGTGAAAGCTTTGCTGTCCTTGATGTGATCATGGAATCTTTTTCTCAC
TAGAGTCTATGTCACTCATTATACTCTGTCGAATGTCATTGAATGTCTTTACATGGGCTT
GTATGCCTATGAAAATTGTAATACAACTTTCAGCAACGGATCTCTTGGCTCTCGCATCGA
TGAAGAACGCAGCGAAATGCGATAAGTAATGTGAATTGCAGAATTCAGTGAATCATCGAA
TCTTTGAACGCATCTTGCGCTCCTTGGTATTCCGAGGAGCATGCCTGTTTGAGTGTCATT
AAATTCTCAACTCTCTTATACTTTTTTGTAAAAGAGAGCTTGGACTGTGGAGGCTTGCTG
GCCACTTTTTGGGGTCAGCTCCTCTGAAATGCATTAGCGGAACCGTTTGCGATCTGCCAC
AAGTGTGATAAGTTATCTACACTGGCGAGGGGATTGCTCTCTGTAATGTTCAGCTTCTAA
TTGTCTCTACTTTGTGAGACTACTTTTGAATGCTTGACCTCAAATCAGGTAGGACTACCC
GCTGAACTTAA
>A11_69
TTTCCGTAGGTGAACCTGCGGAAGGATCATTATTGAATTATGTTTCTAGATAGGTTGTAG
CTGGCTCTTTTAGAGCATGTGCACGCCTGTTTGGACTTCATTTTCATCCACCTGTGCACC
TATTGTAGTCTTTGGTTGGGTTAGGAGGAAGTGATCATTGTATCAGCATCTGCTGGGAGT
GAGGACTTGCATTGTGAAAGCTTTGCTGTCCTTGATGTGATCATGGAATCTTTTTCTCAC
TAGAGTCTATGTCACTCATTATACTCTGTCGAATGTCATTGAATGTCTTTACATGGGCTT
GTATGCCTATGAAAATTGTAATACAACTTTCAGCAACGGATCTCTTGGCTCTCGCATCGA
TGAAGAACGCAGCGAAATGCGATAAGTAATGTGAATTGCAGAATTCAGTGAATCATCGAA
TCTTTGAACGCATCTTGCGCTCCTTGGTATTCCGAGGAGCATGCCTGTTTGAGTGTCATT
AAATTCTCAACTCTCTTATACTTTTTTGTAAAAGAGAGCTTGGACTGTGGAGGCTTGCTG
GCCACTTTTTGGGGTCAGCTCCTCTGAAATGCATTAGCGGAACCGTTTGCGATCTGCCAC
AAGTGTGATAAGTTATCTACACTGGCGAGGGGATTGCTCTCTGTAATGTTCAGCTTCTAA
TTGTCTCTACTTTGTGAGACTACTTTTGAATGCTTGACCTCAAATCAGGTAGGACTACCC
GCTGAACTTAA
>A11_71
TTTCCGTAGGTGAACCTGCGGAAGGATCATTATTGAATTATGTTTCTAGATAGGTTGTAG

CTGGCTCTTTTAGAGCATGTGCACGCCTGTTTGGACTTCATTTTCATCCACCTGTGCACC
TATTGTAGTCTTTGGTTGGGTTAGGAGGAAGTGATCATTGTATCAGCATCTGCTGGGAGT
GAGGACTTGCATTGTGAAAGCTTTGCTGTCCTTGATGTGATCATGGAATCTTTTTCTCAC
TAGAGTCTATGTCACTCATTATACTCTGTCGAATGTCATTGAATGTCTTTACATGGGCTT
GTATGCCTATGAAAATTGTAATACAACTTTCAGCAACGGATCTCTTGGCTCTCGCATCGA
TGAAGAACGCAGCGAAATGCGATAAGTAATGTGAATTGCAGAATTCAGTGAATCATCGAA
TCTTTGAACGCATCTTGCGCTCCTTGGTATTCCGAGGAGCATGCCTGTTTGAGTGTCATT
AAATTCTCAACTCTCTTATACTTTTTTGTAAAAGAGAGCTTGGACTGTGGAGGCTTGCTG
GCCACTTTTTGGGGTCAGCTCCTCTGAAATGCATTAGCGGAACCGTTTGCGATCTGCCAC
AAGTGTGATAAGTTATCTACACTGGCGAGGGGATTGCTCTCTGTAATGTTCAGCTTCTAA
TTGTCTCTACTTTGTGAGACTACTTTTGAATGCTTGACCTCAAATCAGGTAGGACTACCC
GCTGAACTTAA
>A11_72
TTTCCGTAGGTGAACCTGCGGAAGGATCATTATTGAATTATGTTTCTAGATAGGTTGTAG
CTGGCTCTTTTAGAGCATGTGCACGCCTGTTTGGACTTCATTTTCATCCACCTGTGCACC
TATTGTAGTCTTTGGTTGGGTTAGGAGGAAGTGATCATTGTATCAGCATCTGCTGGGAGT
GAGGACTTGCATTGTGAAAGCTTTGCTGTCCTTGATGTGATCATGGAATCTTTTTCTCAC
TAGAGTCTATGTCACTCATTATACTCTGTCGAATGTCATTGAATGTCTTTACATGGGCTT
GTATGCCTATGAAAATTGTAATACAACTTTCAGCAACGGATCTCTTGGCTCTCGCATCGA
TGAAGAACGCAGCGAAATGCGATAAGTAATGTGAATTGCAGAATTCAGTGAATCATCGAA
TCTTTGAACGCATCTTGCGCTCCTTGGTATTCCGAGGAGCATGCCTGTTTGAGTGTCATT
AAATTCTCAACTCTCTTATACTTTTTTGTAAAAGAGAGCTTGGACTGTGGAGGCTTGCTG
GCCACTTTTTGGGGTCAGCTCCTCTGAAATGCATTAGCGGAACCGTTTGCGATCTGCCAC
AAGTGTGATAAGTTATCTACACTGGCGAGGGGATTGCTCTCTGTAATGTTCAGCTTCTAA
TTGTCTCTACTTTGTGAGACTACTTTTGAATGCTTGACCTCAAATCAGGTAGGACTACCC
GCTGAACTTAA
>A11_73
TTTCCGTAGGTGAACCTGCGGAAGGATCATTATTGAATTATGTTTCTAGATAGGTTGTAG
CTGGCTCTTTTAGAGCATGTGCACGCCTGTTTGGACTTCATTTTCATCCACCTGTGCACC
TATTGTAGTCTTTGGTTGGGTTAGGAGGAAGTGATCATTGTATCAGCATCTGCTGGGAGT
GAGGACTTGCATTGTGAAAGCTTTGCTGTCCTTGATGTGATCATGGAATCTTTTTCTCAC
TAGAGTCTATGTCACTCATTATACTCTGTCGAATGTCATTGAATGTCTTTACATGGGCTT
GTATGCCTATGAAAATTGTAATACAACTTTCAGCAACGGATCTCTTGGCTCTCGCATCGA
TGAAGAACGCAGCGAAATGCGATAAGTAATGTGAATTGCAGAATTCAGTGAATCATCGAA
TCTTTGAACGCATCTTGCGCTCCTTGGTATTCCGAGGAGCATGCCTGTTTGAGTGTCATT
AAATTCTCAACTCTCTTATACTTTTTTGTAAAAGAGAGCTTGGACTGTGGAGGCTTGCTG
GCCACTTTTTGGGGTCAGCTCCTCTGAAATGCATTAGCGGAACCGTTTGCGATCTGCCAC
AAGTGTGATAAGTTATCTACACTGGCGAGGGGATTGCTCTCTGTAATGTTCAGCTTCTAA
TTGTCTCTACTTTGTGAGACTACTTTTGAATGCTTGACCTCAAATCAGGTAGGACTACCC
GCTGAACTTAA
>A11_74
TTTCCGTAGGTGAACCTGCGGAAGGATCATTATTGAATTATGTTTCTAGATAGGTTGTAG
CTGGCTCTTTTAGAGCATGTGCACGCCTGTTTGGACTTCATTTTCATCCACCTGTGCACC
TATTGTAGTCTTTGGTTGGGTTAGGAGGAAGTGATCATTGTATCAGCATCTGCTGGGAGT
GAGGACTTGCATTGTGAAAGCTTTGCTGTCCTTGATGTGATCATGGAATCTTTTTCTCAC
TAGAGTCTATGTCACTCATTATACTCTGTCGAATGTCATTGAATGTCTTTACATGGGCTT
GTATGCCTATGAAAATTGTAATACAACTTTCAGCAACGGATCTCTTGGCTCTCGCATCGA
TGAAGAACGCAGCGAAATGCGATAAGTAATGTGAATTGCAGAATTCAGTGAATCATCGAA
TCTTTGAACGCATCTTGCGCTCCTTGGTATTCCGAGGAGCATGCCTGTTTGAGTGTCATT
AAATTCTCAACTCTCTTATACTTTTTTGTAAAAGAGAGCTTGGACTGTGGAGGCTTGCTG

GCCACTTTTTGGGGTCAGCTCCTCTGAAATGCATTAGCGGAACCGTTTGCGATCTGCCAC
AAGTGTGATAAGTTATCTACACTGGCGAGGGGATTGCTCTCTGTAATGTTCAGCTTCTAA
TTGTCTCTACTTTGTGAGACTACTTTTGAATGCTTGACCTCAAATCAGGTAGGACTACCC
GCTGAACTTAA
>A12_46
TTTCCGTAGGTGAACCTGCGGAAGGATCATTATTGAATTATGTTTCTAGATAGGTTGTAG
CTGGCTCTTTTAGAGCATGTGCACGCCTGTTTGGACTTCATTTTCATCCACCTGTGCACC
TATTGTAGTCTTTGGTTGGGTTAGGAGGAAGTGATCATTGTATCAGCATCTGCTGGGAGT
GAGGACTTGCATTGTGAAAGCTTTGCTGTCCTTGATGTGATCATGGAATCTTTTTCTCAC
TAGAGTCTATGTCACTCATTATACTCTGTCGAATGTCATTGAATGTCTTTACATGGGCTT
GTATGCCTATGAAAATTGTAATACAACTTTCAGCAACGGATCTCTTGGCTCTCGCATCGA
TGAAGAACGCAGCGAAATGCGATAAGTAATGTGAATTGCAGAATTCAGTGAATCATCGAA
TCTTTGAACGCATCTTGCGCTCCTTGGTATTCCGAGGAGCATGCCTGTTTGAGTGTCATT
AAATTCTCAACTCTCTTATACTTTTTTGTAAAAGAGAGCTTGGACTGTGGAGGCTTGCTG
GCCACTTTTTGGGGTCAGCTCCTCTGAAATGCATTAGCGGAACCGTTTGCGATCTGCCAC
AAGTGTGATAAGTTATCTACACTGGCGAGGGGATTGCTCTCTGTAATGTTCAGCTTCTAA
TTGTCTCTACTTTGTGAGACTACTTTTGAATGCTTGACCTCAAATCAGGTAGGACTACCC
GCTGAACTTAA
>A12_47
TTTCCGTAGGTGAACCTGCGGAAGGATCATTATTGAATTATGTTTCTAGATAGGTTGTAG
CTGGCTCTTTTAGAGCATGTGCACGCCTGTTTGGACTTCATTTTCATCCACCTGTGCACC
TATTGTAGTCTTTGGTTGGGTTAGGAGGAAGTGATCATTGTATCAGCATCTGCTGGGAGT
GAGGACTTGCATTGTGAAAGCTTTGCTGTCCTTGATGTGATCATGGAATCTTTTTCTCAC
TAGAGTCTATGTCACTCATTATACTCTGTCGAATGTCATTGAATGTCTTTACATGGGCTT
GTATGCCTATGAAAATTGTAATACAACTTTCAGCAACGGATCTCTTGGCTCTCGCATCGA
TGAAGAACGCAGCGAAATGCGATAAGTAATGTGAATTGCAGAATTCAGTGAATCATCGAA
TCTTTGAACGCATCTTGCGCTCCTTGGTATTCCGAGGAGCATGCCTGTTTGAGTGTCATT
AAATTCTCAACTCTCTTATACTTTTTTGTAAAAGAGAGCTTGGACTGTGGAGGCTTGCTG
GCCACTTTTTGGGGTCAGCTCCTCTGAAATGCATTAGCGGAACCGTTTGCGATCTGCCAC
AAGTGTGATAAGTTATCTACACTGGCGAGGGGATTGCTCTCTGTAATGTTCAGCTTCTAA
TTGTCTCTACTTTGTGAGACTACTTTTGAATGCTTGACCTCAAATCAGGTAGGACTACCC
GCTGAACTTAA
>A12_51
TTTCCGTAGGTGAACCTGCGGAAGGATCATTATTGAATTATGTTTCTAGATAGGTTGTAG
CTGGCTCTTTTAGAGCATGTGCACGCCTGTTTGGACTTCATTTTCATCCACCTGTGCACC
TATTGTAGTCTTTGGTTGGGTTAGGAGGAAGTGATCATTGTATCAGCATCTGCTGGGAGT
GAGGACTTGCATTGTGAAAGCTTTGCTGTCCTTGATGTGATCATGGAATCTTTTTCTCAC
TAGAGTCTATGTCACTCATTATACTCTGTCGAATGTCATTGAATGTCTTTACATGGGCTT
GTATGCCTATGAAAATTGTAATACAACTTTCAGCAACGGATCTCTTGGCTCTCGCATCGA
TGAAGAACGCAGCGAAATGCGATAAGTAATGTGAATTGCAGAATTCAGTGAATCATCGAA
TCTTTGAACGCATCTTGCGCTCCTTGGTATTCCGAGGAGCATGCCTGTTTGAGTGTCATT
AAATTCTCAACTCTCTTATACTTTTTTGTAAAAGAGAGCTTGGACTGTGGAGGCTTGCTG
GCCACTTTTTGGGGTCAGCTCCTCTGAAATGCATTAGCGGAACCGTTTGCGATCTGCCAC
AAGTGTGATAAGTTATCTACACTGGCGAGGGGATTGCTCTCTGTAATGTTCAGCTTCTAA
TTGTCTCTACTTTGTGAGACTACTTTTGAATGCTTGACCTCAAATCAGGTAGGACTACCC
GCTGAACTTAA
>A12_58
TTTCCGTAGGTGAACCTGCGGAAGGATCATTATTGAATTATGTTTCTAGATAGGTTGTAG
CTGGCTCTTTTAGAGCATGTGCACGCCTGTTTGGACTTCATTTTCATCCACCTGTGCACC
TATTGTAGTCTTTGGTTGGGTTAGGAGGAAGTGATCATTGTATCAGCATCTGCTGGGAGT

GAGGACTTGCATTGTGAAAGCTTTGCTGTCCTTGATGTGATCATGGAATCTTTTTCTCAC
TAGAGTCTATGTCACTCATTATACTCTGTCGAATGTCATTGAATGTCTTTACATGGGCTT
GTATGCCTATGAAAATTGTAATACAACTTTCAGCAACGGATCTCTTGGCTCTCGCATCGA
TGAAGAACGCAGCGAAATGCGATAAGTAATGTGAATTGCAGAATTCAGTGAATCATCGAA
TCTTTGAACGCATCTTGCGCTCCTTGGTATTCCGAGGAGCATGCCTGTTTGAGTGTCATT
AAATTCTCAACTCTCTTATACTTTTTTGTAAAAGAGAGCTTGGACTGTGGAGGCTTGCTG
GCCACTTTTTGGGGTCAGCTCCTCTGAAATGCATTAGCGGAACCGTTTGCGATCTGCCAC
AAGTGTGATAAGTTATCTACACTGGCGAGGGGATTGCTCTCTGTAATGTTCAGCTTCTAA
TTGTCTCTACTTTGTGAGACTACTTTTGAATGCTTGACCTCAAATCAGGTAGGACTACCC
GCTGAACTTAA
>A6_51
TTTCCGTAGGTGAACCTGCGGAAGGATCATTATTGAATTATGTTTCTAGATAGGTTGTAG
CTGGCTCTTTTAGAGCATGTGCACGCCTGTTTGGACTTCATTTTCATCCACCTGTGCACC
TATTGTAGTCTTTGGTTGGGTTAGGAGGAAGTGATCATTGTATCAGCATCTGCTGGGAGT
GAGGACTTGCATTGTGAAAGCTTTGCTGTCCTTGATGTGATCATGGAATCTTTTTCTCAC
TAGAGTCTATGTCACTCATTATACTCTGTCGAATGTCATTGAATGTCTTTACATGGGCTT
GTATGCCTATGAAAATTGTAATACAACTTTCAGCAACGGATCTCTTGGCTCTCGCATCGA
TGAAGAACGCAGCGAAATGCGATAAGTAATGTGAATTGCAGAATTCAGTGAATCATCGAA
TCTTTGAACGCATCTTGCGCTCCTTGGTATTCCGAGGAGCATGCCTGTTTGAGTGTCATT
AAATTCTCAACTCTCTTATACTTTTTTGTAAAAGAGAGCTTGGACTGTGGAGGCTTGCTG
GCCACTTTTTGGGGTCAGCTCCTCTGAAATGCATTAGCGGAACCGTTTGCGATCTGCCAC
AAGTGTGATAAGTTATCTACACTGGCGAGGGGATTGCTCTCTGTAATGTTCAGCTTCTAA
TTGTCTCTACTTTGTGAGACTACTTTTGAATGCTTGACCTCAAATCAGGTAGGACTACCC
GCTGAACTTAA
>A7_64
TTTCCGTAGGTGAACCTGCGGAAGGATCATTATTGAATTATGTTTCTAGATAGGTTGTAG
CTGGCTCTTTTAGAGCATGTGCACGCCTGTTTGGACTTCATTTTCATCCACCTGTGCACC
TATTGTAGTCTTTGGTTGGGTTAGGAGGAAGTGATCATTGTATCAGCATCTGCTGGGAGT
GAGGACTTGCATTGTGAAAGCTTTGCTGTCCTTGATGTGATCATGGAATCTTTTTCTCAC
TAGAGTCTATGTCACTCATTATACTCTGTCGAATGTCATTGAATGTCTTTACATGGGCTT
GTATGCCTATGAAAATTGTAATACAACTTTCAGCAACGGATCTCTTGGCTCTCGCATCGA
TGAAGAACGCAGCGAAATGCGATAAGTAATGTGAATTGCAGAATTCAGTGAATCATCGAA
TCTTTGAACGCATCTTGCGCTCCTTGGTATTCCGAGGAGCATGCCTGTTTGAGTGTCATT
AAATTCTCAACTCTCTTATACTTTTTTGTAAAAGAGAGCTTGGACTGTGGAGGCTTGCTG
GCCACTTTTTGGGGTCAGCTCCTCTGAAATGCATTAGCGGAACCGTTTGCGATCTGCCAC
AAGTGTGATAAGTTATCTACACTGGCGAGGGGATTGCTCTCTGTAATGTTCAGCTTCTAA
TTGTCTCTACTTTGTGAGACTACTTTTGAATGCTTGACCTCAAATCAGGTAGGACTACCC
GCTGAACTTAA
>A9_29
TTTCCGTAGGTGAACCTGCGGAAGGATCATTATTGAATTATGTTTCTAGATAGGTTGTAG
CTGGCTCTTTTAGAGCATGTGCACGCCTGTTTGGACTTCATTTTCATCCACCTGTGCACC
TATTGTAGTCTTTGGTTGGGTTAGGAGGAAGTGATCATTGTATCAGCATCTGCTGGGAGT
GAGGACTTGCATTGTGAAAGCTTTGCTGTCCTTGATGTGATCATGGAATCTTTTTCTCAC
TAGAGTCTATGTCACTCATTATACTCTGTCGAATGTCATTGAATGTCTTTACATGGGCTT
GTATGCCTATGAAAATTGTAATACAACTTTCAGCAACGGATCTCTTGGCTCTCGCATCGA
TGAAGAACGCAGCGAAATGCGATAAGTAATGTGAATTGCAGAATTCAGTGAATCATCGAA
TCTTTGAACGCATCTTGCGCTCCTTGGTATTCCGAGGAGCATGCCTGTTTGAGTGTCATT
AAATTCTCAACTCTCTTATACTTTTTTGTAAAAGAGAGCTTGGACTGTGGAGGCTTGCTG
GCCACTTTTTGGGGTCAGCTCCTCTGAAATGCATTAGCGGAACCGTTTGCGATCTGCCAC
AAGTGTGATAAGTTATCTACACTGGCGAGGGGATTGCTCTCTGTAATGTTCAGCTTCTAA

TTGTCTCTACTTTGTGAGACTACTTTTGAATGCTTGACCTCAAATCAGGTAGGACTACCC
GCTGAACTTAA
>A1_32
TTTCCGTAGGTGAACCTGCGGAAGGATCATTATTGAATTATGTTTCTAGATAGGTTGTAG
CTGGCTCTTTTAGAGCATGTGCACGCCTGTTTGGACTTCATTTTCATCCACCTGTGCACC
TATTGTAGTCTTTGGTTGGGTTAGGAGGAAGTGATCATTGTATCAGCATCTGCTGGGAGT
GAGGACTTGCATTGTGAAAGCTTTGCTGTCCTTGATGTGATCATGGAATCTTTTTCTCAC
TAGAGTCTATGTCACTCATTATACTCTGTCGAATGTCATTGAATGTCTTTACATGGGCTT
GTATGCCTATGAAAATTGTAATACAACTTTCAGCAACGGATCTCTTGGCTCTCGCATCGA
TGAAGAACGCAGCGAAATGCGATAAGTAATGTGAATTGCAGAATTCAGTGAATCATCGAA
TCTTTGAACGCATCTTGCGCTCCTTGGTATTCCGAGGAGCATGCCTGTTTGAGTGTCATT
AAATTCTCAACTCTCTTATACTTTTTTGTAAAAGAGAGCTTGGACTGTGGAGGCTTGCTG
GCCACTTTTTGGGGTCAGCTCCTCTGAAATGCATTAGCGGAACCGTTTGCGATCTGCCAC
AAGTGTGATAAGTTATCTACACTGGCGAGGGGATTGCTCTCTGTAATGTTCAGCTTCTAA
TTGTCTCTACTTTGTGAGACTACTTTTGAATGCTTGACCTCAAATCAGGTAGGACTACCC
GCTGAACTTAA
>A2_31
TTTCCGTAGGTGAACCTGCGGAAGGATCATTATTGAATTATGTTTCTAGATAGGTTGTAG
CTGGCTCTTTTAGAGCATGTGCACGCCTGTTTGGACTTCATTTTCATCCACCTGTGCACC
TATTGTAGTCTTTGGTTGGGTTAGGAGGAAGTGATCATTGTATCAGCATCTGCTGGGAGT
GAGGACTTGCATTGTGAAAGCTTTGCTGTCCTTGATGTGATCATGGAATCTTTTTCTCAC
TAGAGTCTATGTCACTCATTATACTCTGTCGAATGTCATTGAATGTCTTTACATGGGCTT
GTATGCCTATGAAAATTGTAATACAACTTTCAGCAACGGATCTCTTGGCTCTCGCATCGA
TGAAGAACGCAGCGAAATGCGATAAGTAATGTGAATTGCAGAATTCAGTGAATCATCGAA
TCTTTGAACGCATCTTGCGCTCCTTGGTATTCCGAGGAGCATGCCTGTTTGAGTGTCATT
AAATTCTCAACTCTCTTATACTTTTTTGTAAAAGAGAGCTTGGACTGTGGAGGCTTGCTG
GCCGCTTTTTGGGGTCAGCTCCTCTGAAATGCATTAGCGGAACCGTTTGCGATCTGCCAC
AAGTGTGATAAGTTATCTACACTGGCGAGGGGATTGCTCTCTGTAATGTTCAGCTTCTAA
TTGTCTCTACTTTGTGAGACTACTTTTGAATGCTTGACCTCAAATCAGGTAGGACTACCC
GCTGAACTTAA
>A3-67
TTTCCGTAGGTGAACCTGCGGAAGGATCATTATTGAATTATGTTTCTAGATAGGTTGTAG
CTGGCTCTTTTAGAGCATGTGCACGCCTGTTTGGACTTCATTTTCATCCACCTGTGCACC
TATTGTAGTCTTTGGTTGGGTTAGGAGGAAGTGATCATTGTATCAGCATCTGCTGGGAGT
GAGGACTTGCATTGTGAAAGCTTTGCTGTCCTTGATGTGATCATGGAATCTTTTTCTCAC
TAGAGTCTATGTCACTCATTATACTCTGTCGAATGTCATTGAATGTCTTTACATGGGCTT
GTATGCCTATGAAAATTGTAATACAACTTTCAGCAACGGATCTCTTGGCTCTCGCATCGA
TGAAGAACGCAGCGAAATGCGATAAGTAATGTGAATTGCAGAATTCAGTGAATCATCGAA
TCTTTGAACGCATCTTGCGCTCCTTGGTATTCCGAGGAGCATGCCTGTTTGAGTGTCATT
AAATTCTCAACTCTCTTATACTTTTTTGTAAAAGAGAGCTTGGACTGTGGAGGCTTGCTG
GCCACTTTTTGGGGTCAGCTCCTCTGAAATGCATTAGCGGAACCGTTTGCGATCTGCCAC
AAGTGTGATAAGTTATCTACACTGGCGAGGGGATTGCTCTCTGTAATGTTCAGCTTCTAA
TTGTCTCTACTTTGTGAGACTACTTTTGAATGCTTGACCTCAAATCAGGTAGGACTACCC
GCTGAACTTAA
>A4_24
TTTCCGTAGGTGAACCTGCGGAAGGATCATTATTGAATTATGTTTCTAGATAGGTTGTAG
CTGGCTCTTTTAGAGCATGTGCACGCCTGTTTGGACTTCATTTTCATCCACCTGTGCACC
TATTGTAGTCTTTGGTTGGGTTAGGAGGAAGTGATCATTGTATCAGCATCTGCTGGGAGT
GAGGACTTGCATTGTGAAAGCTTTGCTGTCCTTGATGTGATCATGGAATCTTTTTCTCAC
TAGAGTCTATGTCACTCATTATACTCTGTCGAATGTCATTGAATGTCTTTACATGGGCTT

GTATGCCTATGAAAATTGTAATACAACTTTCAGCAACGGATCTCTTGGCTCTCGCATCGA
TGAAGAACGCAGCGAAATGCGATAAGTAATGTGAATTGCAGAATTCAGTGAATCATCGAA
TCTTTGAACGCATCTTGCGCTCCTTGGTATTCCGAGGAGCATGCCTGTTTGAGTGTCATT
AAATTCTCAACTCTCTTATACTTTTTTGTAAAAGAGAGCTTGGACTGTGGAGGCTTGCTG
GCCACTTTTTGGGGTCAGCTCCTCTGAAATGCATTAGCGGAACCGTTTGCGATCTGCCAC
AAGTGTGATAAGTTATCTACACTGGCGAGGGGATTGCTCTCTGTAATGTTCAGCTTCTAA
TTGTCTCTACTTTGTGAGACTACTTTTGAATGCTTGACCTCAAATCAGGTAGGACTACCC
GCTGAACTTAA
>A5_33
TTTCCGTAGGTGAACCTGCGGAAGGATCATTATTGAATTATGTTTCTAGATAGGTTGTAG
CTGGCTCTTTTAGAGCATGTGCACGCCTGTTTGGACTTCATTTTCATCCACCTGTGCACC
TATTGTAGTCTTTGGTTGGGTTAGGAGGAAGTGATCATTGTATCAGCATCTGCTGGGAGT
GAGGACTTGCATTGTGAAAGCTTTGCTGTCCTTGATGTGATCATGGAATCTTTTTCTCAC
TAGAGTCTATGTCACTCATTATACTCTGTCGAATGTCATTGAATGTCTTTACATGGGCTT
GTATGCCTATGAAAATTGTAATACAACTTTCAGCAACGGATCTCTTGGCTCTCGCATCGA
TGAAGAACGCAGCGAAATGCGATAAGTAATGTGAATTGCAGAATTCAGTGAATCATCGAA
TCTTTGAACGCATCTTGCGCTCCTTGGTATTCCGAGGAGCATGCCTGTTTGAGTGTCATT
AAATTCTCAACTCTCTTATACTTTTTTGTAAAAGAGAGCTTGGACTGTGGAGGCTTGCTG
ACCACTTTTTGGGGTCAGCTCCTCTGAAATGCATTAGCGGAACCGTTTGCGATCTGCCAC
AAGTGTGATAAGTTATCTACACTGGCGAGGGGATTGCTCTCTGTAATGTTCAGCTTCTAA
TTGTCTCTACTTTGTGAGACTACTTTTGAATGCTTGACCTCAAATCAGGTAGGACTACCC
GCTGAACTTAA
>A7_13
TTTCCGTAGGTGAACCTGCGGAAGGATCATTATTGAATTATGTTTCTAGATAGGTTGTAG
CTGGCTCTTTTAGAGCATGTGCACGCCTGTTTGGACTTCATTTTCATCCACCTGTGCACC
TATTGTAGTCTTTGGTTGGGTTAGGAGGAAGTGATCATTGTATCAGCATCTGCTGGGAGT
GAGGACTTGCATTGTGAAAGCTTTGCTGTCCTTGATGTGATCATGGAATCTTTTTCTCAC
TAGAGTCTATGTCACTCATTATACTCTGTCGAATGTCATTGAATGTCTTTACATGGGCTT
GTATGCCTATGAAAATTGTAATACAACTTTCAGCAACGGATCTCTTGGCTCTCGCATCGA
TGAAGAACGCAGCGAAATGCGATAAGTAATGTGAATTGCAGAATTCAGTGAATCATCGAA
TCTTTGAACGCATCTTGCGCTCCTTGGTATTCCGAGGAGCATGCCTGTTTGAGTGTCATT
AAATTCTCAACTCTCTTATACTTTTTTGTAAAAGAGAGCTTGGACTGTGGAGGCTTGCTG
GCCACTTTTTGGGGTCAGCTCCTCTGAAATGCATTAGCGGAACCGTTTGCGATCTGCCAC
AAGTGTGATAAGTTATCTACACTGGCGAGGGGATTGCTCTCTGTAATGTTCAGCTTCTAA
TTGTCTCTACTTTGTGAGACTACTTTTGAATGCTTGACCTCAAATCAGGTAGGACTACCC
GCTGAACTTAA
>A7_74
TTTCCGTAGGTGAACCTGCGGAAGGATCATTATTGAATTATGTTTCTAGATAGGTTGTAG
CTGGCTCTTTTAGAGCATGTGCACGCCTGTTTGGACTTCATTTTCATCCACCTGTGCACC
TATTGTAGTCTTTGGTTGGGTTAGGAGGAAGTGATCATTGTATCAGCATCTGCTGGGAGT
GAGGACTTGCATTGTGAAAGCTTTGCTGTCCTTGATGTGATCATGGAATCTTTTTCTCAC
TAGAGTCTATGTCACTCATTATACTCTGTCGAATGTCATTGAATGTCTTTACATGGGCTT
GTATGCCTATGAAAATTGTAATACAACTTTCAGCAACGGATCTCTTGGCTCTCGCATCGA
TGAAGAACGCAGCGAAATGCGATAAGTAATGTGAATTGCAGAATTCAGTGAATCATCGAA
TCTTTGAACGCATCTTGCGCTCCTTGGTATTCCGAGGAGCATGCCTGTTTGAGTGTCATT
AAATTCTCAACTCTCTTATACTTTTTTGTAAAAGAGAGCTTGGACTGTGGAGGCTTGCTG
GCCACTTTTTGGGGTCAGCTCCTCTGAAATGCATTAGCGGAACCGTTTGCGATCTGCCAC
AAGTGTGATAAGTTATCTACACTGGCGAGGGGATTGCTCTCTGTAATGTTCAGCTTCTAA
TTGTCTCTACTTTGTGAGACTACTTTTGAATGCTTGACCTCAAATCAGGTAGGACTACCC
GCTGAACTTAA

>A6_12
TTTCCGTAGGTGAACCTGCGGAAGGATCATTATTGAATTATGTTTCTAGATAGGTTGTAG
CTGGCTCTTTTAGAGCATGTGCACGCCTGTTTGGACTTCATTTTCATCCACCTATGCACC
TATTGTAGTCTTTGGTTGGGTTAGGAGGAAGTGATCATTGTATCAGCATCTGCTGGGAGT
GAGGACTTGCATTGTGAAAGCTTTGCTGTCCTTGATGTGATCATGGAATCTTTTTCTCAC
TAGAGTCTATGTCACTCATTATACTCTGTCGAATGTCATTGAATGTCTTTACATGGGCTT
GTATGCCTATGAAAATTGTAATACAACTTTCAGCAACGGATCTCTTGGCTCTCGCATCGA
TGAAGAACGCAGCGAAATGCGATAAGTAATGTGAATTGCAGAATTCAGTGAATCATCGAA
TCTTTGAACGCATCTTGCGCTCCTTGGTATTCCGAGGAGCATGCCTGTTTGAGTGTCATT
AAATTCTCAACTCTCTTATACTTTTTTGTAAAAGAGAGCTTGGACTGTGGAGGCTTGCTG
GCCACTTTTTGGGGTCAGCTCCTCTGAAATGCATTAGCGGAACCGTTTGCGATCTGCCAC
AAGTGTGATAAGTTATCTACACTGGCGAGGGGATTGCTCTCTGTAATGTTCAGCTTCTAA
TTGTCTCTACTTTGTGAGACTACTTTTGAATGCTTGACCTCAAATCAGGTAGGACTACCC
GCTGAACTTAA
>A6_52
TTTCCGTAGGTGAACCTGCGGAAGGATCATTATTGAATTATGTTTCTAGATAGGTTGTAG
CTGGCTCTTTTAGAGCATGTGCACGCCTGTTTGGACTTCATTTTCATCCACCTGTGCACC
TATTGTAGTCTTTGGTTGGGTTAGGAGGAAGTGATCATTGTATCAGCATCTGCTGGGAGT
GAGGACTTGCATTGTGAAAGCTTTGCTGTCCTTGATGTGATCATGGAATCTTTTTCTCAC
TAGAGTCTATGTCACTCATTATACTCTGTCGAATGTCATTGAATGTCTTTACATGGGCTT
GTATGCCTATGAAAATTGTAATACAACTTTCAGCAACGGATCTCTTGGCTCTCGCATCGA
TGAAGAACGCAGCGAAATGCGATAAGTAATGTGAATTGCAGAATTCAGTGAATCATCGAA
TCTTTGAACGCATCTTGCGCTCCTTGGTATTCCGAGGAGCATGCCTGTTTGAGTGTCATT
AAATTCTCAACTCTCTTATACTTTTTTGTAAAAGAGAGCTTGGACTGTGGAGGCTTGCTG
GCCACTTTTTGGGGTCAGCTCCTCTGAAATGCATTAGCGGAACCGTTTGTGATCTGCCAC
AAGTGTGATAAGTTATCTACACTGGCGAGGGGATTGCTCTCTGTAATGTTCAGCTTCTAA
TTGTCTCTACTTTGTGAGACTACTTTTGAATGCTTGACCTCAAATCAGGTAGGACTACCC
GCTGAACTTAA
>A12_59
TTTCCGTAGGTGAACCTGCGGAAGGATCATTATTGAATTATGTTTCTAGATAGGTTGTAG
CTGGCTCTTTTAGAGCATGTGCACGCCTGTTTGGACTTCATTTTCATCCACCTGTGCACC
TATTGTAGTCTTTGGTTGGGTTAGGAGGAAGTGATCATTGTATCAGCATCTGCTGGGAGT
GAGGACTTGCATTGTGAAAGCTTTGCTGTCCTTGATGTGATCATGGAATCTTTTTCTCAC
TAGAGTCTATGTCACTCATTATACTCTGTCGAATGTCATTGAATGTCTTTACATGGGCTT
GTATGCCTATGAAAATTGTAATACAACTTTCAGCAACGGATCTCTTGGCTCTCGCATCGA
TGAAGAACGCAGCGAAATGCGATAAGTAATGTGAATTGCAGAATTCAGTGAATCATCGAA
TCTTTGAACGCATCTTGCGCTCCTTGGTATTCCGAGGAGCATGCCTGTTTGAGTGTCATT
AAATTCTCAACTCTCTTATACTTTTTTGTAAAAGAGAGCTTGGACTGTGGAGGCTTGCTG
GCCACTTTTTGGGGTCAGCTCCTCTGAAATGCATTAGCGGAACCGTTTGCGATCTGCCAC
AAGTGTGATAAGTTATCTACACTGGCGAGGGGATTGCTCTCTGTAATGTTCAGCTTCTAA
TTGTCTCTACTTTGTGAGACTACTTTTGAATGCTTGACCTCAAATCAGGTAGGACTACCC
GCTGAACTTAA
>A12_63
TTTCCGTAGGTGAACCTGCGGAAGGATCATTATTGAATTATGTTTCTAGATAGGTTGTAG
CTGGCTCTTTTAGAGCATGTGCACGCCTGTTTGGACTTCATTTTCATCCACCTGTGCACC
TATTGTAGTCTTTGGTTGGGTTAGGAGGAAGTGATCATTGTATCAGCATCTGCTGGGAGT
GAGGACTTGCATTGTGAAAGCTTTGCTGTCCTTGATGTGATCATGGAATCTTTTTCTCAC
TAGAGTCTATGTCACTCATTATACTCTGTCGAATGTCATTGAATGTCTTTACATGGGCTT
GTATGCCTATGAAAATTGTAATACAACTTTCAGCAACGGATCTCTTGGCTCTCGCATCGA
TGAAGAACGCAGCGAAATGCGATAAGTAATGTGAATTGCAGAATTCAGTGAATCATCGAA

TCTTTGAACGCATCTTGCGCTCCTTGGTATTCCGAGGAGCATGCCTGTTTGAGTGTCATT
AAATTCTCAACTCTCTTATACTTTTTTGTAAAAGAGAGCTTGGACTGTGGAGGCTTGCTG
GCCACTTTTTGGGGTCAGCTCCTCTGAAATGCATTAGCGGAACCGTTTGCGATCTGCCAC
AAGTGTGATAAGTTATCTACACTGGCGAGGGGATTGCTCTCTGTAATGTTCAGCTTCTAA
TTGTCTCTACTTTGTGAGACTACTTTTGAATGCTTGACCTCAAATCAGGTAGGACTACCC
GCTGAACTTAA
>A12_65
TTTCCGTAGGTGAACCTGCGGAAGGATCATTATTGAATTATGTTTCTAGATAGGTTGTAG
CTGGCTCTTTTAGAGCATGTGCACGCCTGTTTGGACTTCATTTTCATCCACCTGTGCACC
TATTGTAGTCTTTGGTTGGGTTAGGAGGAAGTGATCATTGTATCAGCATCTGCTGGGAGT
GAGGACTTGCATTGTGAAAGCTTTGCTGTCCTTGATGTGATCATGGAATCTTTTTCTCAC
TAGAGTCTATGTCACTCATTATACTCTGTCGAATGTCATTGAATGTCTTTACATGGGCTT
GTATGCCTATGAAAATTGTAATACAACTTTCAGCAACGGATCTCTTGGCTCTCGCATCGA
TGAAGAACGCAGCGAAATGCGATAAGTAATGTGAATTGCAGAATTCAGTGAATCATCGAA
TCTTTGAACGCATCTTGCGCTCCTTGGTATTCCGAGGAGCATGCCTGTTTGAGTGTCATT
AAATTCTCAACTCTCTTATACTTTTTTGTAAAAGAGAGCTTGGACTGTGGAGGCTTGCTG
GCCACTTTTTGGGGTCAGCTCCTCTGAAATGCATTAGCGGAACCGTTTGCGATCTGCCAC
AAGTGTGATAAGTTATCTACACTGGCGAGGGGATTGCTCTCTGTAATGTTCAGCTTCTAA
TTGTCTCTACTTTGTGAGACTACTTTTGAATGCTTGACCTCAAATCAGGTAGGACTACCC
GCTGAACTTAA
>A12_66
TTTCCGTAGGTGAACCTGCGGAAGGATCATTATTGAATTATGTTTCTAGATAGGTTGTAG
CTGGCTCTTTTAGAGCATGTGCACGCCTGTTTGGACTTCATTTTCATCCACCTGTGCACC
TATTGTAGTCTTTGGTTGGGTTAGGAGGAAGTGATCATTGTATCAGCATCTGCTGGGAGT
GAGGACTTGCATTGTGAAAGCTTTGCTGTCCTTGATGTGATCATGGAATCTTTTTCTCAC
TAGAGTCTATGTCACTCATTATACTCTGTCGAATGTCATTGAATGTCTTTACATGGGCTT
GTATGCCTATGAAAATTGTAATACAACTTTCAGCAACGGATCTCTTGGCTCTCGCATCGA
TGAAGAACGCAGCGAAATGCGATAAGTAATGTGAATTGCAGAATTCAGTGAATCATCGAA
TCTTTGAACGCATCTTGCGCTCCTTGGTATTCCGAGGAGCATGCCTGTTTGAGTGTCATT
AAATTCTCAACTCTCTTATACTTTTTTGTAAAAGAGAGCTTGGACTGTGGAGGCTTGCTG
GCCACTTTTTGGGGTCAGCTCCTCTGAAATGCATTAGCGGAACCGTTTGCGATCTGCCAC
AAGTGTGATAAGTTATCTACACTGGCGAGGGGATTGCTCTCTGTAATGTTCAGCTTCTAA
TTGTCTCTACTTTGTGAGACTACTTTTGAATGCTTGACCTCAAATCAGGTAGGACTACCC
GCTGAACTTAA
>A12_67
TTTCCGTAGGTGAACCTGCGGAAGGATCATTATTGAATTATGTTTCTAGATAGGTTGTAG
CTGGCTCTTTTAGAGCATGTGCACGCCTGTTTGGACTTCATTTTCATCCACCTGTGCACC
TATTGTAGTCTTTGGTTGGGTTAGGAGGAAGTGATCATTGTATCAGCATCTGCTGGGAGT
GAGGACTTGCATTGTGAAAGCTTTGCTGTCCTTGATGTGATCATGGAATCTTTTTCTCAC
TAGAGTCTATGTCACTCATTATACTCTGTCGAATGTCATTGAATGTCTTTACATGGGCTT
GTATGCCTATGAAAATTGTAATACAACTTTCAGCAACGGATCTCTTGGCTCTCGCATCGA
TGAAGAACGCAGCGAAATGCGATAAGTAATGTGAATTGCAGAATTCAGTGAATCATCGAA
TCTTTGAACGCATCTTGCGCTCCTTGGTATTCCGAGGAGCATGCCTGTTTGAGTGTCATT
AAATTCTCAACTCTCTTATACTTTTTTGTAAAAGAGAGCTTGGACTGTGGAGGCTTGCTG
GCCACTTTTTGGGGTCAGCTCCTCTGAAATGCATTAGCGGAACCGTTTGCGATCTGCCAC
AAGTGTGATAAGTTATCTACACTGGCGAGGGGATTGCTCTCTGTAATGTTCAGCTTCTAA
TTGTCTCTACTTTGTGAGACTACTTTTGAATGCTTGACCTCAAATCAGGTAGGACTACCC
GCTGAACTTAA
>A7_35
TTTCCGTAGGTGAACCTGCGGAAGGATCATTATTGAATTATGTTTCTAGATAGGTTGTAG

CTGGCTCTTTTAGAGCATGTGCACGCCTGTTTGGACTTCATTTTCATCCACCTGTGCACC
TATTGTAGTCTTTGGTTGGGTTAGGAGGAAGTGATCATTGTATCAGCATCTGCTGGGAGT
GAGGACTTGCATTGTGAAAGCTTTGCTGTCCTTGATGTGATCATGGAATCTTTTTCTCAC
TAGAGTCTATGTCACTCATTATACTCTGTCGAATGTCATTGAATGTCTTTACATGGGTTT
GTATGCCTATGAAAATTGTAATACAACTTTCAGCAACGGATCTCTTGGCTCTCGCATCGA
TGAAGAACGCAGCGAAATGCGATAAGTAATGTGAATTGCAGAATTCAGTGAATCATCGAA
TCTTTGAACGCATCTTGCGCTCCTTGGTATTCCGAGGAGCATGCCTGTTTGAGTGTCATT
AAATTCTCAACTCTCTTATACTTTTTTGTAAAAGAGAGCTTGGACTGTGGAGGCTTGCTG
GCCACTTTTTGGGGTCAGCTCCTCTGAAATGCATTAGCGGAACCGTTTGCGATCTGCCAC
AAGTGTGATAAGTTATCTACACTGGCGAGGGGATTGCTCTCTGTAATGTTCAGCTTCTAA
TTGTCTCTACTTTGTGAGACTACTTTTGAATGCTTGACCTCAAATCAGGTAGGACTACCC
GCTGAACTTAA
>A7_40
TTTCCGTAGGTGAACCTGCGGAAGGATCATTATTGAATTATGTTTCTAGATAGGTTGTAG
CTGGCTCTTTTAGAGCATGTGCACGCCTGTTTGGACTTCATTTTCATCCACCTGTGCACC
TATTGTAGTCTTTGGTTGGGTTAGGAGGAAGTGATCATTGTATCAGCATCTGCTGGGAGT
GAGGACTTGCATTGTGAAAGCTTTGCTGTCCTTGATGTGATCATGGAATCTTTTTCTCAC
TAGAGTCTATGTCACTCATTATACTCTGTCGAATGTCATTGAATGTCTTTACATGGGCTT
GTATGCCTATGAAAATTGTAATACAACTTTCAGCAACGGATCTCTTGGCTCTCGCATCGA
TGAAGAACGCAGCGAAATGCGATAAGTAATGTGAATTGCAGAATTCAGTGAATCATCGAA
TCTTTGAACGCATCTTGCGCTCCTTGGTATTCCGAGGAGCATGCCTGTTTGAGTGTCATT
AAATTCTCAACTCTCTTATACTTTTTTGTAAAAGAGAGCTTGGACTGTGGAGGCTTGCTG
GCCACTTTTTGGGGTCAGCTCCTCTGAAATGCATTAGCGGAACCGTTTGCGATCTGCCAC
AAGTGTGATAAGTTATCTACACTGGCGAGGGGATTGCTCTCTGTAATGTTCAGCTTCTAA
TTGTCTCTACTTTGTGAGACTACTTTTGAATGCTTGACCTCAAATCAGGTAGGACTACCC
GCTGAACTTAA
>A7_41
TTTCCGTAGGTGAACCTGCGGAAGGATCATTATTGAATTATGTTTCTAGATAGGTTGTAG
CTGGCTCTTTTAGAGCATGTGCACGCCTGTTTGGACTTCATTTTCATCCACCTGTGCACC
TATTGTAGTCTTTGGTTGGGTTAGGAGGAAGTGATCATTGTATCAGCATCTGCTGGGAGT
GAGGACTTGCATTGTGAAAGCTTTGCTGTCCTTGATGTGATCATGGAATCTTTTTCTCAC
TAGAGTCTATGTCACTCATTATACTCTGTCGAATGTCATTGAATGTCTTTACATGGGCTT
GTATGCCTATGAAAATTGTAATACAACTTTCAGCAACGGATCTCTTGGCTCTCGCATCGA
TGAAGAACGCAGCGAAATGCGATAAGTAATGTGAATTGCAGAATTCAGTGAATCATCGAA
TCTTTGAACGCATCTTGCGCTCCTTGGTATTCCGAGGAGCATGCCTGTTTGAGTGTCATT
AAATTCTCAACTCTCTTATACTTTTTTGTAAAAGAGAGCTTGGACTGTGGAGGCTTGCTG
GCCACTTTTTGGGGTCAGCTCCTCTGAAATGCATTAGCGGAACCGTTTGCGATCTGCCAC
AAGTGTGATAAGTTATCTACACTGGCGAGGGGATTGCTCTCTGTAATGTTCAGCTTCTAA
TTGTCCCTACTTTGTGAGACTACTTTTGAATGCTTGACCTCAAATCAGGTAGGACTACCC
GCTGAACTTAA
>A9_64
TTTCCGTAGGTGAACCTGCGGAAGGATCATTATTGAATTATGTTTCTAGATAGGTTGTAG
CTGGCTCTTTTAGAGCATGTGCACGCCTGTTTGGACTTCATTTTCATCCACCTGTGCACC
TATTGTAGTCTTTGGTTGGGTTAGGAGGAAGTGATCATTGTATCAACATCTGCTGGGAGT
GAGGACTTGCATTGTGAAAGCTTTGCTGTCCTTGATGTGATCATGGAATCTTTTTCTCAC
TAGAGTCTATGTCACTCATTATACTCTGTCGAATGTCATTGAATGTCTTTACATGGGCTT
GTATGCCTATGAAAATTGTAATACAACTTTCAGCAACGGATCTCTTGGCTCTCGCATCGA
TGAAGAACGCAGCGAAATGCGATAAGTAATGTGAATTGCAGAATTCAGTGAATCATCGAA
TCTTTGAACGCATCTTGCGCTCCTTGGTATTCCGAGGAGCATGCCTGTTTGAGTGTCATT
AAATTCTCAACTCTCTTATACTTTTTTGTAAAAGAGAGCTTGGACTGTGGAGGCTTGCTG

GCCACTTTTTGGGGTCAGCTCCTCTGAAATGCATTAGCGGAACCGTTTGCGATCTGCCAC
AAGTGTGATAAGTTATCTACACTGGCGAGGGGATTGCTCTCTGTAATGTTCAGCTTCTAA
TTGTCTCTACTTTGTGAGACTACTTTTGAATGCTTGACCTCAAATCAGGTAGGACTACCC
GCTGAACTTAA
>A10_38
TTTCCGTAGGTGAACCTGCGGAAGGATCATTATTGAATTATGTTTCTAGATAGGTTGTAG
CTGGCTCTTTTAGAGCATGTGCACGCCTGTTTGGACTTCATTTTCATCCACCTGTGCACC
TATTGTAGTCTTTGGTTGGGTTAGGAGGAAGTGATCATTGTATCAGCATCTGCTGGGAGT
GAGGACTTGCATTGTGAAAGCTTTGCTGTCCTTGATGTGATCATGGAATCTTTTTCTCAC
TAGAGTCTATGTCACTCATTATACTCTGTCGAATGTCATTGAATGTCTTTACATGGGCTT
GTATGCCTATGAAAATTGTAATACAACTTTCAGCAACGGATCTCTTGGCTCTCGCATCGA
TGAAGAACGCAGCGAAATGCGATAAGTAATGTGAATTGCAGAATTCAGTGAATCATCGAA
TCTTTGAACGCATCTTGCGCTCCTTGGTATTCCGAGGAGCATGCCTGTTTGAGTGTCATT
AAATTCTCAACTCTCTTATACTTTTTTGTAAAAGAGAGCTTGGACTGTGGAGGCTTGCTG
GCCACTTTTTGGGGTCAGCTCCTCTGAAATGCATTAGCGGAACCGTTTGCGATCTGCCAC
AAGTGTGATAAGTTATCTACACTGGCGAGGGGATTGCTCTCTGTAATGTTCAGCTTCTAA
TTGTCTCTACTTTGTGAGACTACTTTTGAATGCTTGACCTCAAATCAGGTAGGACTACCC
GCTGAACTTAA
>A12_52
TTTCCGTAGGTGAACCTGCGGAAGGATCATTATTGAATTATGTTTCTAGATAGGTTGTAG
CTGGCTCTTTTAGAGCATGTGCACGCCTGTTTGGACTTCATTTTCATCCACCTGTGCACC
TATTGTAGTCTTTGGTTGGGTTAGGAGGAAGTGATCATTGTATCAGCATCTGCTGGGAGT
GAGGACTTGCATTGTGAAAGCTTTGCTGTCCTTGATGTGATCATGGAATCTTTTTCTCAC
TAGAGTCTATGTCACTCATTATACTCTGTCGAATGTCATTGAATGTCTTTACATGGGCTT
GTATGCCTATGAAAATTGTAATACAACTTTCAGCAACGGATCTCTTGGCTCTCGCATCGA
TGAAGAACGCAGCGAAATGCGATAAGTAATGTGAATTGCAGAATTCAGTGAATCATCGAA
TCTTTGAACGCATCTTGCGCTCCTTGGTATTCCGAGGAGCATGCCTGTTTGAGTGTCATT
AAATTCTCAACTCTCTTATACTTTTTTGTAAAAGAGAGCTTGGACTGTGGAGGCTTGCTG
GCCACTTTTTGGGGTCAGCTCCTCTGAAATGCATTAGCGGAACCGTTTGCGATCTGCCAC
AAGTGTGATAAGTTATCTACACTGGCGAGGGGATTGCTCTCTGTAATGTTCAGCTTCTAA
TTGTCTCTACTTTGTGAGACTACTTTTGAATGCTTGACCTCAAATCAGGTAGGACTACCC
GCTGAACTTAA
>A12_53
TTTCCGTAGGTGAACCTGCGGAAGGATCATTATTGAATTATGTTTCTAGATAGGTTGTAG
CTGGCTCTTTTAGAGCATGTGCACGCCTGTTTGGACTTCATTTTCATCCACCTGTGCACC
TATTGTAGTCTTTGGTTGGGTTAGGAGGAAGTGATCATTGTATCAGCATCTGCTGGGAGT
GAGGACTTGCATTGTGAAAGCTTTGCTGTCCTTGATGTGATCATGGAATCTTTTTCTCAC
TAGAGTCTATGTCACTCATTATACTCTGTCGAATGTCATTGAATGTCTTTACATGGGCTT
GTATGCCTATGAAAATTGTAATACAACTTTCAGCAACGGATCTCTTGGCTCTCGCATCGA
TGAAGAACGCAGCGAAATGCGATAAGTAATGTGAATTGCAGAATTCAGTGAATCATCGAA
TCTTTGAACGCATCTTGCGCTCCTTGGTATTCCGAGGAGCATGCCTGTTTGAGTGTCATT
AAATTCTCAACTCTCTTATACTTTTTTGTAAAAGAGAGCTTGGACTGTGGAGGCTTGCTG
GCCACTTTTTGGGGTCAGCTCCTCTGAAATGCATTAGCGGAACCGTTTGCGATCTGCCAC
AAGTGTGATAAGTTATCTACACTGGCGAGGGGATTGCTCTCTGTAATGTTCAGCTTCTAA
TTGTCTCTACTTTGTGAGACTACTTTTGAATGCTTGACCTCAAATCAGGTAGGACTACCC
GCTGAACTTAA
>A12_55
TTTCCGTAGGTGAACCTGCGGAAGGATCATTATTGAATTATGTTTCTAGATAGGTTGTAG
CTGGCTCTTTTAGAGCATGTGCACGCCTGTTTGGACTTCATTTTCATCCACCTGTGCACC
TATTGTAGTCTTTGGTTGGGTTAGGAGGAAGTGATCATTGTATCAGCATCTGCTGGGAGT

GAGGACTTGCATTGTGAAAGCTTTGCTGTCCTTGATGTGATCATGGAATCTTTTTCTCAC
TAGAGTCTATGTCACTCATTATACTCTGTCGAATGTCATTGAATGTCTTTACATGGGCTT
GTATGCCTATGAAAATTGTAATACAACTTTCAGCAACGGATCTCTTGGCTCTCGCATCGA
TGAAGAACGCAGCGAAATGCGATAAGTAATGTGAATTGCAGAATTCAGTGAATCATCGAA
TCTTTGAACGCATCTTGCGCTCCTTGGTATTCCGAGGAGCATGCCTGTTTGAGTGTCATT
AAATTCTCAACTCTCTTATACTTTTTTGTAAAAGAGAGCTTGGACTGTGGAGGCTTGCTG
GCCACTTTTTGGGGTCAGCTCCTCTGAAATGCATTAGCGGAACCGTTTGCGATCTGCCAC
AAGTGTGATAAGTTATCTACACTGGCGAGGGGATTGCTCTCTGTAATGTTCAGCTTCTAA
TTGTCTCTACTTTGTGAGACTACTTTTGAATGCTTGACCTCAAATCAGGTAGGACTACCC
GCTGAACTTAA
>A12_56
TTTCCGTAGGTGAACCTGCGGAAGGATCATTATTGAATTATGTTTCTAGATAGGTTGTAG
CTGGCTCTTTTAGAGCATGTGCACGCCTGTTTGGACTTCATTTTCATCCACCTGTGCACC
TATTGTAGTCTTTGGTTGGGTTAGGAGGAAGTGATCATTGTATCAGCATCTGCTGGGAGT
GAGGACTTGCATTGTGAAAGCTTTGCTGTCCTTGATGTGATCATGGAATCTTTTTCTCAC
TAGAGTCTATGTCACTCATTATACTCTGTCGAATGTCATTGAATGTCTTTACATGGGCTT
GTATGCCTATGAAAATTGTAATACAACTTTCAGCAACGGATCTCTTGGCTCTCGCATCGA
TGAAGAACGCAGCGAAATGCGATAAGTAATGTGAATTGCAGAATTCAGTGAATCATCGAA
TCTTTGAACGCATCTTGCGCTCCTTGGTATTCCGAGGAGCATGCCTGTTTGAGTGTCATT
AAATTCTCAACTCTCTTATACTTTTTTGTAAAAGAGAGCTTGGACTGTGGAGGCTTGCTG
GCCACTTTTTGGGGTCAGCTCCTCTGAAATGCATTAGCGGAACCGTTTGCGATCTGCCAC
AAGTGTGATAAGTTATCTACACTGGCGAGGGGATTGCTCTCTGTAATGTTCAGCTTCTAA
TTGTCTCTACTTTGTGAGACTACTTTTGAATGCTTGACCTCAAATCAGGTAGGACTACCC
GCTGAACTTAA
>A12_57
TTTCCGTAGGTGAACCTGCGGAAGGATCATTATTGAATTATGTTTCTAGATAGGTTGTAG
CTGGCTCTTTTAGAGCATGTGCACGCCTGTTTGGACTTCATTTTCATCCACCTGTGCACC
TATTGTAGTCTTTGGTTGGGTTAGGAGGAAGTGATCATTGTATCAGCATCTGCTGGGAGT
GAGGACTTGCATTGTGAAAGCTTTGCTGTCCTTGATGTGATCATGGAATCTTTTTCTCAC
TAGAGTCTATGTCACTCATTATACTCTGTCGAATGTCATTGAATGTCTTTACATGGGCTT
GTATGCCTATGAAAATTGTAATACAACTTTCAGCAACGGATCTCTTGGCTCTCGCATCGA
TGAAGAACGCAGCGAAATGCGATAAGTAATGTGAATTGCAGAATTCAGTGAATCATCGAA
TCTTTGAACGCATCTTGCGCTCCTTGGTATTCCGAGGAGCATGCCTGTTTGAGTGTCATT
AAATTCTCAACTCTCTTATACTTTTTTGTAAAAGAGAGCTTGGACTGTGGAGGCTTGCTG
GCCACTTTTTGGGGTCAGCTCCTCTGAAATGCATTAGCGGAACCGTTTGCGATCTGCCAC
AAGTGTGATAAGTTATCTACACTGGCGAGGGGATTGCTCTCTGTAATGTTCAGCTTCTAA
TTGTCTCTACTTTGTGAGACTACTTTTGAATGCTTGACCTCAAATCAGGTAGGACTACCC
GCTGAACTTAA
>A10_54
TTTCCGTAGGTGAACCTGCGGAAGGATCATTATTGAATTATGTTTCTAGATAGGTTGTAG
CTGGCTCTTTTAGAGCATGTGCACGCCTGTTTGGACTTCATTTTCATCCACCTGTGCACC
TATTGTAGTCTTTGGTTGGGTTAGGAGGAAGTGATCATTGTATCAGCATCTGCTGGGAGT
GAGGACTTGCATTGTGAAAGCTTTGCTGTCCTTGATGTGATCATGGAATCTTTTTCTCAC
TAGAGTCTATGTCACTCATTATACTCTGTCGAATGTCATTGAATGTCTTTACATGGGCTT
GTATGTCTATGAAAATTGTAATACAACTTTCAGCAACGGATCTCTTGGCTCTCGCATCGA
TGAAGAACGCAGCGAAATGCGATAAGTAATGTGAATTGCAGAATTCAGTGAATCATCGAA
TCTTTGAACGCATCTTGCGCTCCTTGGTATTCCGAGGAGCATGCCTGTTTGAGTGTCATT
AAATTCTCAACTCTCTTATACTTTTTTGTAAAAGAGAGCTTGGACTGTGGAGGCTTGCTG
GCCACTTTTTGGGGTCAGCTCCTCTGAAATGCATTAGCGGAACCGTTTGCGATCTGCCAC
AAGTGTGATAAGTTATCTACACTGGCGAGGGGATTGCTCTCTGTAATGTTCAGCTTCTAA

TTGTCTCTACTTTGTGAGACTACTTTTGAATGCTTGACCTCAAATCAGGTAGGACTACCC
GCTGAACTTAA
>A10_65
TTTCCGTAGGTGAACCTGCGGAAGGATCATTATTGAATTATGTTTCTAGATAGGTTGTAG
CTGGCTCTTTTAGAGCATGTGCACGCCTGTTTGGACTTCATTTTCATCCACCTGTGCACC
TATTGTAGTCTTTGGTTGGGTTAGGAGGAAGTGATCATTGTATCAGCATCTGCTGGGAGT
GAGGACTTGCATTGTGAAAGCTTTGCTGTCCTTGATGTGATCATGGAATCTTTTTCTCAC
TAGAGTCTATGTCACTCATTATACTCTGTCGAATGTCATTGAATGTCTTTACATGGGCTT
GTATGCCTATGAAAATTGTAATACAACTTTCAGCAACGGATCTCTTGGCTCTCGCATCGA
TGAAGAACGCAGCGAAATGCGATAAGTAATGTGAATTGCAGAATTCAGTGAATCATCGAA
TCTTTGAACGCATCTTGCGCTCCTTGGTATTCCGAGGAGCATGCCTGTTTGAGTGTCATT
AAATTCTCAACTCTCTTATACTTTTTTGTAAAAGAGAGCTTGGACTGTGGAGGCTTGCTG
GCCACTTTTTGGGGTCAGCTCCTCTGAAATGCATTAGCGGAACCGTTTGCGATCTGTCAC
AAGTGTGATAAGTTATCTACACTGGCGAGGGGATTGCTCTCTGTAATGTTCAGCTTCTAA
TTGTCTCTACTTTGTGAGACTACTTTTGAATGCTTGACCTCAAATCAGGTAGGACTACCC
GCTGAACTTAA
>A11_51
TTTCCGTAGGTGAACCTGCGGAAGGATCATTATTGAATTATGTTTCTAGATAGGTTGTAG
CTGGCTCTTTTAGAGCATGTGCACGCCTGTTTGGACTTCATTTTCATCCACCTGTGCACC
TATTGTAGTCTTTGGTTGGGTTAGGAGGAAGTGATCATTGTATCAGCATCTGCTGGGAGT
GAGGACTTGCATTGTGAAAGCTTTGCTGTCCTTGATGTGATCATGGAATCTTTTTCTCAC
TAGAGTCTATGTCACTCATTATACTCTGTCGAATGTCATTGAATGTCTTTACATGGGCTT
GTATGCCTATGAAAATTGTAATACAACTTTCAGCAACGGATCTCTTGGCTCTCGCATCGA
TGAAGAACGCAGCGAAATGCGATAAGTAATGTGAATTGCAGAATTCAGTGAATCATCGAA
TCTTTGAACGCATCTTGTGCTCCTTGGTATTCCGAGGAGCATGCCTGTTTGAGTGTCATT
AAATTCTCAACTCTCTTATACTTTTTTGTAAAAGAGAGCTTGGACTGTGGAGGCTTGCTG
GCCACTTTTTGGGGTCAGCTCCTCTGAAATGCATTAGCGGAACCGTTTGCGATCTGCCAC
AAGTGTGATAAGTTATCTACACTGGCGAGGGGATTGCTCTCTGTAATGTTCAGCTTCTAA
TTGTCTCTACTTTGTGAGACTACTTTTGAATGCTTGACCTCAAATCAGGTAGGACTACCC
GCTGAACTTAA
>A11_63
TTTCCGTAGGTGAACCTGCGGAAGGATCATTATTGAATTATGTTTCTAGATAGGTTGTAG
CTGGCTCTTTTAGAGCATGTGCACGCCTGTTTGGACTTCATTTTCATCCACCTGTGCACC
TATTGTAGTCTTTGGTTGGGTTAGGAGGAAGTGATCATTGTATCAGCATCTGCTGGGAGT
GAGGACTTGCATTGTGAAAGCTTTGCTGTCCTTGATGTGATCATGGAATCTTTTTCTCAC
TAGAGTCTATGTCACTCATTATACTCTGTCGAATGTCATTGAATGTCTTTACATGGGCTT
GTATGCCTACGAAAATTGTAATACAACTTTCAGCAACGGATCTCTTGGCTCTCGCATCGA
TGAAGAACGCAGCGAAATGCGATAAGTAATGTGAATTGCAGAATTCAGTGAATCATCGAA
TCTTTGAACGCATCTTGCGCTCCTTGGTATTCCGAGGAGCATGCCTGTTTGAGTGTCATT
AAATTCTCAACTCTCTTATACTTTTTTGTAAAAGAGAGCTTGGACTGTGGAGGCTTGCTG
GCCACTTTTTGGGGTCAGCTCCTCTGAAATGCATTAGCGGAACCGTTTGCGATCTGCCAC
AAGTGTGATAAGTTATCTACACTGGCGAGGGGATTGCTCTCTGTAATGTTCAGCTTCTAA
TTGTCTCTACTTTGTGAGACTACTTTTGAATGCTTGACCTCAAATCAGGTAGGACTACCC
GCTGAACTTAA
>A11_70
TTTCCGTAGGTGAACCTGCGGAAGGATCATTATTGAATTATGTTTCTAGATAGGTTGTAG
CTGGCTCTTTTAGAGCATGTGCACGCCTGTTTGGACTTCATTTTCATCCACCTGTGCACC
TATTGTAGTCTTTGGTTGGGTTAGGAGGAAGTGATCATTGTATCAGCATCTGCTGGGAGT
GAGGACTTGCATTGTGAAAGCTTTGCTGTCCTTGATGTGATCATGGAATCTTTTTCTCAC
TAGAGTCTATGTCACTCATTATACTCTGTCGAATGTCATTGAATGTCTTTACATGGGCTT

GTATGCCTATGAAAATTGTAATACAACTTTCAGCAACGGATCTCTTGGCTCTCGCATCGA
TGAAGAACGCAGCGAAATGCGATAAGTAATGTGAATTGCAGAATTCAGTGAATCATCGAA
TCTTTGAACGCATCTTGCGCTCCTTGGTATTCCGAGGAGCATGCCTGTTTGAGTGTCATT
AAATTCTCAACTCTCTTATACTTTTTTGTAAAAGAGAACTTGGACTGTGGAGGCTTGCTG
GCCACTTTTTGGGGTCAGCTCCTCTGAAATGCATTAGCGGAACCGTTTGCGATCTGCCAC
AAGTGTGATAAGTTATCTACACTGGCGAGGGGATTGCTCTCTGTAATGTTCAGCTTCTAA
TTGTCTCTACTTTGTGAGACTACTTTTGAATGCTTGACCTCAAATCAGGTAGGACTACCC
GCTGAACTTAA
>A12_68
TTTCCGTAGGTGAACCTGCGGAAGGATCATTATTGAATTATGTTTCTAGATAGGTTGTAG
CTGGCTCTTTTAGAGCATGTGCACGCCTGTTTGGACTTCATTTTCATCCACCTGTGCACC
TATTGTAGTCTTTGGTTGGGTTAGGAGGAAGTGATCATTGTATCAGCATCTGCTGGGAGT
GAGGACTTGCATTGTGAAAGCTTTGCTGTCCTTGATGTGATCATGGAATCTTTTTCTCAC
TAGAGTCTATGTCACTCATTATACTCTGTCGAATGTCATTGAATGTCTTTACATGGGCTT
GTATGCCTATGAAAATTGTAATACAACTTTCAGCAACGGATCTCTTGGCTCTCGCATCGA
TGAAGAACGCAGCGAAATGCGATAAGTAATGTGAATTGCAGAATTCAGTGAATCATCGAA
TCTTTGAACGCATCTTGCGCTCCTTGGTATTCCGAGGAGCATGCCTGTTTGAGTGTCATT
AAATTCTCAACTCTCTTATACTTTTTTGTAAAAGAGAGCTTGGACTGTGGAGGCTTGCTG
GCCACTTTTTGGGGTCAGCTCCTCTGAAATGCATTAGCGGAACCGTTTGCGATCTGCCAC
AAGTGTGATAAGTTATCTACACTGGCGAGGGGATTGCTCTCTGTAATGTTCAGCTTCTAA
TTGTCTCTACTTTGTGAGACTACTTTTGAATGCTTGACCTCAAATCAGGTAGGACTACCC
GCTGAACTTAA
>A12_69
TTTCCGTAGGTGAACCTGCGGAAGGATCATTATTGAATTATGTTTCTAGATAGGTTGTAG
CTGGCTCTTTTAGAGCATGTGCACGCCTGTTTGGACTTCATTTTCATCCACCTGTGCACC
TATTGTAGTCTTTGGTTGGGTTAGGAGGAAGTGATCATTGTATCAGCATCTGCTGGGAGT
GAGGACTTGCATTGTGAAAGCTTTGCTGTCCTTGATGTGATCATGGAATCTTTTTCTCAC
TAGAGTCTATGTCACTCATTATACTCTGTCGAATGTCATTGAATGTCTTTACATGGGCTT
GTATGCCTATGAAAATTGTAATACAACTTTCAGCAACGGATCTCTTGGCTCTCGCATCGA
TGAAGAACGCAGCGAAATGCGATAAGTAATGTGAATTGCAGAATTCAGTGAATCATCGAA
TCTTTGAACGCATCTTGCGCTCCTTGGTATTCCGAGGAGCATGCCTGTTTGAGTGTCATT
AAATTCTCAACTCTCTTATACTTTTTTGTAAAAGAGAGCTTGGACTGTGGAGGCTTGCTG
GCCACTTTTTGGGGTCAGCTCCTCTGAAATGCATTAGCGGAACCGTTTGCGATCTGCCAC
AAGTGTGATAAGTTATCTACACTGGCGAGGGGATTGCTCTCTGTAATGTTCAGCTTCTAA
TTGTCTCTACTTTGTGAGACTACTTTTGAATGCTTGACCTCAAATCAGGTAGGACTACCC
GCTGAACTTAA
>A12_71
TTTCCGTAGGTGAACCTGCGGAAGGATCATTATTGAATTATGTTTCTAGATAGGTTGTAG
CTGGCTCTTTTAGAGCATGTGCACGCCTGTTTGGACTTCATTTTCATCCACCTGTGCACC
TATTGTAGTCTTTGGTTGGGTTAGGAGGAAGTGATCATTGTATCAGCATCTGCTGGGAGT
GAGGACTTGCATTGTGAAAGCTTTGCTGTCCTTGATGTGATCATGGAATCTTTTTCTCAC
TAGAGTCTATGTCACTCATTATACTCTGTCGAATGTCATTGAATGTCTTTACATGGGCTT
GTATGCCTATGAAAATTGTAATACAACTTTCAGCAACGGATCTCTTGGCTCTCGCATCGA
TGAAGAACGCAGCGAAATGCGATAAGTAATGTGAATTGCAGAATTCAGTGAATCATCGAA
TCTTTGAACGCATCTTGCGCTCCTTGGTATTCCGAGGAGCATGCCTGTTTGAGTGTCATT
AAATTCTCAACTCTCTTATACTTTTTTGTAAAAGAGAGCTTGGACTGTGGAGGCTTGCTG
GCCACTTTTTGGGGTCAGCTCCTCTGAAATGCATTAGCGGAACCGTTTGCGATCTGCCAC
AAGTGTGATAAGTTATCTACACTGGCGAGGGGATTGCTCTCTGTAATGTTCAGCTTCTAA
TTGTCTCTACTTTGTGAGACTACTTTTGAATGCTTGACCTCAAATCAGGTAGGACTACCC
GCTGAACTTAA

>A12_72
TTTCCGTAGGTGAACCTGCGGAAGGATCATTATTGAATTATGTTTCTAGATAGGTTGTAG
CTGGCTCTTTTAGAGCATGTGCACGCCTGTTTGGACTTCATTTTCATCCACCTGTGCACC
TATTGTAGTCTTTGGTTGGGTTAGGAGGAAGTGATCATTGTATCAGCATCTGCTGGGAGT
GAGGACTTGCATTGTGAAAGCTTTGCTGTCCTTGATGTGATCATGGAATCTTTTTCTCAC
TAGAGTCTATGTCACTCATTATACTCTGTCGAATGTCATTGAATGTCTTTACATGGGCTT
GTATGCCTATGAAAATTGTAATACAACTTTCAGCAACGGATCTCTTGGCTCTCGCATCGA
TGAAGAACGCAGCGAAATGCGATAAGTAATGTGAATTGCAGAATTCAGTGAATCATCGAA
TCTTTGAACGCATCTTGCGCTCCTTGGTATTCCGAGGAGCATGCCTGTTTGAGTGTCATT
AAATTCTCAACTCTCTTATACTTTTTTGTAAAAGAGAGCTTGGACTGTGGAGGCTTGCTG
GCCACTTTTTGGGGTCAGCTCCTCTGAAATGCATTAGCGGAACCGTTTGCGATCTGCCAC
AAGTGTGATAAGTTATCTACACTGGCGAGGGGATTGCTCTCTGTAATGTTCAGCTTCTAA
TTGTCTCTACTTTGTGAGACTACTTTTGAATGCTTGACCTCAAATCAGGTAGGACTACCC
GCTGAACTTAA
>A12_73
TTTCCGTAGGTGAACCTGCGGAAGGATCATTATTGAATTATGTTTCTAGATAGGTTGTAG
CTGGCTCTTTTAGAGCATGTGCACGCCTGTTTGGACTTCATTTTCATCCACCTGTGCACC
TATTGTAGTCTTTGGTTGGGTTAGGAGGAAGTGATCATTGTATCAGCATCTGCTGGGAGT
GAGGACTTGCATTGTGAAAGCTTTGCTGTCCTTGATGTGATCATGGAATCTTTTTCTCAC
TAGAGTCTATGTCACTCATTATACTCTGTCGAATGTCATTGAATGTCTTTACATGGGCTT
GTATGCCTATGAAAATTGTAATACAACTTTCAGCAACGGATCTCTTGGCTCTCGCATCGA
TGAAGAACGCAGCGAAATGCGATAAGTAATGTGAATTGCAGAATTCAGTGAATCATCGAA
TCTTTGAACGCATCTTGCGCTCCTTGGTATTCCGAGGAGCATGCCTGTTTGAGTGTCATT
AAATTCTCAACTCTCTTATACTTTTTTGTAAAAGAGAGCTTGGACTGTGGAGGCTTGCTG
GCCACTTTTTGGGGTCAGCTCCTCTGAAATGCATTAGCGGAACCGTTTGCGATCTGCCAC
AAGTGTGATAAGTTATCTACACTGGCGAGGGGATTGCTCTCTGTAATGTTCAGCTTCTAA
TTGTCTCTACTTTGTGAGACTACTTTTGAATGCTTGACCTCAAATCAGGTAGGACTACCC
GCTGAACTTAA
>A12_75
TTTCCGTAGGTGAACCTGCGGAAGGATCATTATTGAATTATGTTTCTAGATAGGTTGTAG
CTGGCTCTTTTAGAGCATGTGCACGCCTGTTTGGACTTCATTTTCATCCACCTGTGCACC
TATTGTAGTCTTTGGTTGGGTTAGGAGGAAGTGATCATTGTATCAGCATCTGCTGGGAGT
GAGGACTTGCATTGTGAAAGCTTTGCTGTCCTTGATGTGATCATGGAATCTTTTTCTCAC
TAGAGTCTATGTCACTCATTATACTCTGTCGAATGTCATTGAATGTCTTTACATGGGCTT
GTATGCCTATGAAAATTGTAATACAACTTTCAGCAACGGATCTCTTGGCTCTCGCATCGA
TGAAGAACGCAGCGAAATGCGATAAGTAATGTGAATTGCAGAATTCAGTGAATCATCGAA
TCTTTGAACGCATCTTGCGCTCCTTGGTATTCCGAGGAGCATGCCTGTTTGAGTGTCATT
AAATTCTCAACTCTCTTATACTTTTTTGTAAAAGAGAGCTTGGACTGTGGAGGCTTGCTG
GCCACTTTTTGGGGTCAGCTCCTCTGAAATGCATTAGCGGAACCGTTTGCGATCTGCCAC
AAGTGTGATAAGTTATCTACACTGGCGAGGGGATTGCTCTCTGTAATGTTCAGCTTCTAA
TTGTCTCTACTTTGTGAGACTACTTTTGAATGCTTGACCTCAAATCAGGTAGGACTACCC
GCTGAACTTAA
>A12_76
TTTCCGTAGGTGAACCTGCGGAAGGATCATTATTGAATTATGTTTCTAGATAGGTTGTAG
CTGGCTCTTTTAGAGCATGTGCACGCCTGTTTGGACTTCATTTTCATCCACCTGTGCACC
TATTGTAGTCTTTGGTTGGGTTAGGAGGAAGTGATCATTGTATCAGCATCTGCTGGGAGT
GAGGACTTGCATTGTGAAAGCTTTGCTGTCCTTGATGTGATCATGGAATCTTTTTCTCAC
TAGAGTCTATGTCACTCATTATACTCTGTCGAATGTCATTGAATGTCTTTACATGGGCTT
GTATGCCTATGAAAATTGTAATACAACTTTCAGCAACGGATCTCTTGGCTCTCGCATCGA
TGAAGAACGCAGCGAAATGCGATAAGTAATGTGAATTGCAGAATTCAGTGAATCATCGAA

TCTTTGAACGCATCTTGCGCTCCTTGGTATTCCGAGGAGCATGCCTGTTTGAGTGTCATT
AAATTCTCAACTCTCTTATACTTTTTTGTAAAAGAGAGCTTGGACTGTGGAGGCTTGCTG
GCCACTTTTTGGGGTCAGCTCCTCTGAAATGCATTAGCGGAACCGTTTGCGATCTGCCAC
AAGTGTGATAAGTTATCTACACTGGCGAGGGGATTGCTCTCTGTAATGTTCAGCTTCTAA
TTGTCTCTACTTTGTGAGACTACTTTTGAATGCTTGACCTCAAATCAGGTAGGACTACCC
GCTGAACTTAA
>A12_77
TTTCCGTAGGTGAACCTGCGGAAGGATCATTATTGAATTATGTTTCTAGATAGGTTGTAG
CTGGCTCTTTTAGAGCATGTGCACGCCTGTTTGGACTTCATTTTCATCCACCTGTGCACC
TATTGTAGTCTTTGGTTGGGTTAGGAGGAAGTGATCATTGTATCAGCATCTGCTGGGAGT
GAGGACTTGCATTGTGAAAGCTTTGCTGTCCTTGATGTGATCATGGAATCTTTTTCTCAC
TAGAGTCTATGTCACTCATTATACTCTGTCGAATGTCATTGAATGTCTTTACATGGGCTT
GTATGCCTATGAAAATTGTAATACAACTTTCAGCAACGGATCTCTTGGCTCTCGCATCGA
TGAAGAACGCAGCGAAATGCGATAAGTAATGTGAATTGCAGAATTCAGTGAATCATCGAA
TCTTTGAACGCATCTTGCGCTCCTTGGTATTCCGAGGAGCATGCCTGTTTGAGTGTCATT
AAATTCTCAACTCTCTTATACTTTTTTGTAAAAGAGAGCTTGGACTGTGGAGGCTTGCTG
GCCACTTTTTGGGGTCAGCTCCTCTGAAATGCATTAGCGGAACCGTTTGCGATCTGCCAC
AAGTGTGATAAGTTATCTACACTGGCGAGGGGATTGCTCTCTGTAATGTTCAGCTTCTAA
TTGTCTCTACTTTGTGAGACTACTTTTGAATGCTTGACCTCAAATCAGGTAGGACTACCC
GCTGAACTTAA
>A12_6
TTTCCGTAGGTGAACCTGCGGAAGGATCATTATTGAATTATGTTTCTAGATAGGTTGTAG
CTGGCTCTTTTAGAGCATGTGCACGCCTGTTTGGACTTCATTTTCATCCACCTGTGCACC
TATTGTAGTCTTTGGTTGGGTTAGGAGGAAGTGATCATTGTATCAGCATCTGCTGGGAGT
GAGGACTTGCATTGTGAAAGCTTTGCTGTCCTTGATGTGATCATGGAATCTTTTTCTCAC
TAGAGTCTATGTCACTCATTATACTCTGTCGAATGTCATTGAATGTCTTTACATGGGCTT
GTATGCCTATGAAAATTGTAATACAACTTTCAGCAACGGATCTCTTGGCTCTCGCATCGA
TGAAGAACGCAGCGAAATGCGATAAGTAATGTGAATTGCAGAATTCAGTGAATCATCGAA
TCTTTGAACGCATCTTGCGCTCCTTGGTATTCCGAGGAGCATGCCTGTTTGAGTGTCATT
AAATTCTCAACTCTCTTATACTTTTTTGTAAAAGAGAGCTTGGACTGTGGAGGCTTGCTG
GCCACTTTTTGGGGTCAGCTCCTCTGAAATGCATTAGCGGAACCGTTTGCGATCTGCCAC
AAGTGTGATAAGTTATCTACACTGGCGAGGGGATTGCTCTCTGTAATGTTCAGCTTCTAA
TTGTCTCTACTTTGTGAGACTACTTTTGAATGCTTGACCTCAAATCAGGTAGGACTACCC
ACTGAACTTAA
>A12_38
TTTCCGTAGGTGAACCTGCGGAAGGATCATTATTGAATTATGTTTCTAGATAGGTTGTAG
CTGGCTCTTTTAGAGCATGTGCACGCCTGTTTGGACTTCATTTTCATCCACCTGTGCACC
TATTGTAGTCTTTGGTTGGGTTAGGAGGAAGTGATCATTGTATCAGCATCTGCTGGGAGT
GAGGACTTGCATTGTGAAAGCTTTGCTGTCCTTGATGTGATCATGGAATCTTTTTCTCAC
TAGAGTCTATGTCACTCATTATACTCTGTCGAATGTCATTGAATGTCTTTACATGGGCTT
GTATGCCTATGAAAATTGTAATACAACTTTCAGCAACGGATCTCTTGGCTCTCGCATCGA
TGAAGAACGCAGCGAAATGCGATAAGTAATGTGAATTGCAGAATTCAGTGAATCATCGAA
TCTTTGAACGCATCTTGCGCTCCTTGGTATTCCGAGGAGCATGCCTGTTTGAGTGTCATT
AAATTCTCAACTCTCTTATACTTTTTTGTAAAAGAGAGCTTGGACTGTGGAGGCTTGCTG
GCCACTTTTTGGGGTCAGCTCCTCTGAAATGCATTAGCGGAACCGTTTGCGATCTGCCAC
AAGTGTGATAAGTTATCTACACTGGCGAGGGGATTGCTCTCTGTAATGTTCAGCTTCTAA
TTGTCTCTACTTTGTGAGACTACTTTTGAATGCTTGACCTCAAATCAGGTAGGACTACCC
GCTGAACTTAA
>A2_41
TTTCCGTAGGTGAACCTGCGGAAGGATCATTATTGAATTATGTTTCTAGATAGGTTGTAG

CTGGCTCTTTTAGAGCATGTGCACGCCTGTTTGGACTTCATTTTCATCCACCTGTGCACC
TATTGTAGTCTTTGGTTGGGTTAGGAGGAAGTGATCATTGTATCAGCATCTGCTGGGAGT
GAGGACTTGCATTGTGAAAGCTTTGCTGTCCTTGATGTGATCATGGAATCTTTTTCTCAC
TAGAGTCTATGTCACTCATTATACTCTGTCGAATGTCATTGAATGTCTTTACATGGGCTT
GTATGCCTATGAAAATTGTAATACAACTTTCAGCAACGGATCTCTTGGCTCTCGCATCGA
TGAAGAACGCAGCGAAATGCGATAAGTAATGTGAATTGCAGAATTCAGTGAATCATCGAA
TCTTTGAACGCATCTTGCGCTCCTTGGTATTCCGAGGAGCATGCCTGTTTGAGTGTCATT
AAATTCTCAACTCTCTTATACTTTTTTGTAAAAGAGAGCTTGGACTGTGGAGGCTTGCTG
GCCACTTTTTGGGGTCAGCTCCTCTGAAATGCATTAGCGGAACCGTTTGCGATCTGCCAC
AAGTGTGATAAGTTATCTACACTGGCGAGGGGATTGCTCTCTGTAATGTTCAGCTTCTAA
TTGTCTCTACTTTGTGAGACTACTTTTGAATGCTTGACCTCAAATCAGGTAGGACTACCC
GCTGAACTTAA
>A9_37
TTTCCGTAGGTGAACCTGCGGAAGGATCATTATTGAATTATGTTTCTAGATAGGTTGTAG
CTGGCTCTTTTAGAGCATGTGCACGCCTGTTTGGACTTCATTTTCATCCACCTGTGCACC
TATTGTAGTCTTTGGTTGGGTTAGGAGGAAGTGATCATTGTATCAGCATCTGCTGGGAGT
GAGGACTTGCATTGTGAAAGCTTTGCTGTCCTTGATGTGATCATGGAATCTTTTTCTCAC
TAGAGTCTATGTCACTCATTATACTCTGTCGAATGTCATTGAATGTCTTTACATGGGCTT
GTATGCCTATGAAAATTGTAATACAACTTTCAGCAACGGATCTCTTGGCTCTCGCATCGA
TGAAGAACGCAGCGAAATGCGATAAGTAATGTGAATTGCAGAATTCAGTGAATCATCGAA
TCTTTGAACGCATCTTGCGCTCCTTGGTATTCCGAGGAGCATGCCTGTTTGAGTGTCATT
AAATTCTCAACTCTCTTATACTTTTTTGTAAAAGAGAGCTTGGACTGTGGAGGCTTGCTG
GCCACTTTTTGGGGTCAGCTCCTCTGAAATGCATTAGCGGAACCGTTTGCGATCTGCCAC
AAGTGTGATAAGTTATCTACACTGGCGAGGGGATTGCTCTCTGTAATGTTCAGCTTCTAA
TTGTCTCTACTTTGTGAGACTACTTTTGAATGCTTGACCTCAAATCAGGTAGGACTACCC
GCTGAACTTAA
>A11_20
TTTCCGTAGGTGAACCTGCGGAAGGATCATTATTGAATTATGTTTCTAGATAGGTTGTAG
CTGGCTCTTTTAGAGCATGTGCACGCCTGTTTGGACTTCATTTTCATCCACCTGTGCACC
TATTGTAGTCTTTGGTTGGGTTAGGAGGAAGTGATCATTGTATCAGCATCTGCTGGGAGT
GAGGACTTGCATTGTGAAAGCTTTGCTGTCCTTGATGTGATCATGGAATCTTTTTCTCAC
TAGAGTCTATGTCACTCATTATACTCTGTCGAATGTCATTGAATGTCTTTACATGGGCTT
GTATGCCTATGAAAATTGTAATACAACTTTCAGCAACGGATCTCTTGGCTCTCGCATCGA
TGAAGAACGCAGCGAAATGCGATAAGTAATGTGAATTGCAGAATTCAGTGAATCATCGAA
TCTTTGAACGCATCTTGCGCTCCCTGGTATTCCGAGGAGCATGCCTGTTTGAGTGTCATT
AAATTCTCAACTCTCTTATACTTTTTTGTAAAAGAGAGCTTGGACTGTGGAGGCTTGCTG
GCCACTTTTTGGGGTCAGCTCCTCTGAAATGCATTAGCGGAACCGTTTGCGATCTGCCAC
AAGTGTGATAAGTTATCTACACTGGCGAGGGGATTGCTCTCTGTAATGTTCAGCTTCTAA
TTGTCTCTACTTTGTGAGACTACTTTTGAATGCTTGACCTCAAATCAGGTAGGACTACCC
GCTGAACTTAA
>A5_24
TTTCCGTAGGTGAACCTGCGGAAGGATCATTATTGAATTATGTTTCTAGATAGGTTGTAG
CTGGCTCTTTTAGAGCATGTGCACGCCTGTTTGGACTTCATTTTCATCCACCTGTGCACC
TATTGTAGTCTTTGGTTGGGTTAGGAGGAAGTGATCATTGTATCAGCATCTGCTGGGAGT
GAGGACTTGCATTGTGAAAGCTTTGCTGTCCTTGATGTGATCATGGAATCTTTTTCTCAC
TAGAGTCTATGTCACTCATTATACTCTGTCGAATGTCATTGAATGTCTTTACATGGGCTT
GTATGCCTATGAAAATTGTAATACAACTTTCAGCAACGGATCTCTTGGCTCTCGCATCGA
TGAAGAACGCAGCGAAATGCGATAAGTAATGTGAATTGCAGAATTCAGTGAATCATCGAA
TCTTTGAACGCATCTTGCGCTCCTTGGTATTCCGAGGAGCATGCCTGTTTGAGTGTCATT
AAATTCTCAACTCTCTTATACTTTTTTGTAAAAGAGAGCTTGGACTGTGGAGGCTTGCTG

GCCACTTTTTGGGGTCAGCTCCTCTGAAATGCATTAGCGGAACCGTTTGCGATCTGCCAC
AAGTGTGATAAGTTATCTACACTGGCGAGGGGATTGCTCTCTGTAATGTTCAGCTTCTAA
TTGTCTCTACTTTGTGAGACTACTTTTGAATGCTTGACCTCAAATCAGGTAGGACTACCC
GCTGAACTTAA
>A6_32
TTTCCGTAGGTGAACCTGCGGAAGGATCATTATTGAATTATGTTTCTAGATAGGTTGTAG
CTGGCTCTTTTAGAGCATGTGCACGCCTGTTTGGACTTCATTTTCATCCACCTGTGCACC
TATTGTAGTCTTTGGTTGGGTTAGGAGGAAGTGATCATTGTATCAGCATCTGCTGGGAGT
GAGGACTTGCATTGTGAAAGCTTTGCTGTCCTTGATGTGATCATGGAATCTTTTTCTCAC
TAGAGTCTATGTCACTCATTATACTCTGTCGAATGTCATTGAATGTCTTTACATGGGCTT
GTATGCCTATGAAAATTGTAATACAACTTTCAGCAACGGATCTCTTGGCTCTCGCATCGA
TGAAGAACGCAGCGAAATGCGATAAGTAATGTGAATTGCAGAATTCAGTGAATCATCGAA
TCTTTGAACGCATCTTGCGCTCCTTGGTATTCCGAGGAGCATGCCTGTTTGAGTGTCATT
AAATTCTCAACTCTCTTATACTTTTTTGTAAAAGAGAGCTTGGACTGTGGAGGCTTGCTG
GCCACTTTTTGGGGTCAGCTCCTCTGAAATGCATTAGCGGAACCGTTTGCAATCTGCCAC
AAGTGTGATAAGTTATCTACACTGGCGAGGGGATTGCTCTCTGTAATGTTCAGCTTCTAA
TTGTCTCTACTTTGTGAGACTACTTTTGAATGCTTGACCTCAAATCAGGTAGGACTACCC
GCTGAACTTAA
>A1_12
TTTCCGTAGGTGAACCTGCGGAAGGATCATTATTGAATTATGTTTCTAGATAGGTTGTAG
CTGGCTCTTTTAGAGCATGTGCACGCCTGTTTGGACTTCATTTTCATCCACCTGTGCACC
TATTGTAGTCATTGGTTGGGTTAGGAGGAAGTGATCATTGTATCAGCATCTGCTGGGAGT
GAGGACTTGCATTGTGAAAGCTTTGCTGTCCTTGATGTGATCATGGAATCTTTTTCTCAC
TAGAGTCTATGTCACTCATTATACTCTGTCGAATGTCATTGAATGTCTTTACATGGGCTT
GTATGCCTATGAAAATTGTAATACAACTTTCAGCAACGGATCTCTTGGCTCTCGCATCGA
TGAAGAACGCAGCGAAATGCGATAAGTAATGTGAATTGCAGAATTCAGTGAATCATCGAA
TCTTTGAACGCATCTTGCGCTCCTTGGTATTCCGAGGAGCATGCCTGTTTGAGTGTCATT
AAATTCTCAACTCTCTTATACTTTTTTGTAAAAGAGAGCTTGGACTGTGGAGGCTTGCTG
GCCACTTTTTGGGGTCAGCTCCTCTGAAATGCATTAGCGGAACCGTTTGCGATCTGCCAC
AAGTGTGATAAGTTATCTACACTGGCGAGGGGATTGCTCTCTGTAATGTTCAGCTTCTAA
TTGTCTCTACTTTGTGAGACTACTTTTGAATGCTTGACCTCAAATCAGGTAGGACTACCC
GCTGAACTTAA
>A2_45
TTTCCGTAGGTGAACCTGCGGAAGGATCATTATTGAATTATGTTTCTAGATAGGTTGTAG
CTGGCTCTTTTAGAGCATGTGCACGCCTGTTTGGACTTCATTTTCATCCACCTGTGCACC
TATTGTAGTCTTTGGTTGGGTTAGGAGGAAGTGATCATTGTATCAGCATCTGCTGGGAGT
GAGGACTTGCATTGTGAAAGCTTTGCTGTCCTTGATGTGATCATGGAATCTTTTTCTCAC
TAGAGTCTATGTCACTCATTATACTCTGTCGAATGTCATTGAATGTCTTTACATGGGCTT
GTATGCCTATGAAAATTGTAATACAACTTTCAGCAACGGATCTCTTGGCTCTCGCATCGA
TGAAGAACGCAGCGAAATGCGATAAGTAATGTGAATTGCAGAATTCAGTGAATCATCGAA
TCTTTGAACGCATCTTGCGCTCCTTGGTATTCCGAGGAGCATGCCTGTTTGAGTGTCATT
AAATTCTCAACTCTCTTATACTTTTTTGTAAAAGAGAGCTTGGACTGTGGAGGCTTGCTG
GCCACTTTTTGGGGTCAGCTCCTCTGAAATGCATTAGCGGAACCGTTTGCGATCTGCCAC
AAGTGTGATAAGTTATCTACACTGGCGAGGGGATTGCTCTCTGTAATGTTCAGCTTCTAA
TTGTCTCTACTTTGTGATACTACTTTTGAATGCTTGACCTCAAATCAGGTAGGACTACCC
GCTGAACTTAA
>A3-64
TTTCCGTAGGTGAACCTGCGGAAGGATCATTATTGAATTATGTTTCTAGATAGGTTGTAG
CTGGCTCTTTTAGAGCATGTGCACGCCTGTTTGGACTTCATTTTCATCCACCTGTGCACC
TATTGTAGTCTTTGGTTGGGTTAGGAGGAAGTGATCATTGTATCAGCATCTGCTGGGAGT

GAGGACTTGCATTGTGAAAGCTTTGCTGTCCTTGATGTGATCATGGAATCTTTTTCTCAC
TAGAGTCTATGTCACTCATTATACTCTGTCGAATGTCATTGAATGTCTTTACATGGGCTT
GTATGCCTATGAAAATTGTAATACAACTTTCAGCAACGGATCTCTTGGCTCTCGCATCGA
TGAAGAACGCAGCGAAATGCGATAAGTAATGTGAATTGCAGAATTCAGTGAATCATCGAA
TCTTTGAACGCATCTTGCGCTCCTTGGTATTCCGAGGAGCATGCCTGTTTGAGTGTCATT
AAATTCTCAACTCTCTTATACTTTTTTGTAAAAGAGAGCTTGGACTGTGGAGGCTTGCTG
GCCACTTTTTGGGGTCAGCTCCTCTGAAATGCATTAGCGGAACCGTTTGCGATCTGCCAC
AAGTGTGATAAGTTATCTACACTGGCGAGGGGATTGCTCTCTGTAATGTTCAGCATCTAA
TTGTCTCTACTTTGTGAGACTACTTTTGAATGCTTGACCTCAAATCAGGTAGGACTACCC
GCTGAACTTAA
>A4_31
TTTCCGTAGGTGAACCTGCGGAAGGATCATTATTGAATTATGTTTCTAGATAGGTTGTAG
CTGGCTCTTTTAGAGCATGTGCACGCCTGTTTGGACTTCATTTTCATCCACCTGTGCACC
TATTGTAGTCTTTGGTTGGGTTAGGAGGAAGTGATCATTGTATCAGCATCTGCTGGGAGT
GAGGACTTGCATTGTGAAAGCTTTGCTGTCCTTGATGTGATCATGGAATCTTTTTCTCAC
TAGAGTCTATGTCACTCATTATACTCTGTCGAATGTCATTGAATGTCTTTACATGGGCTT
GTATGCCTATGAAAATTGTAATACAACTTTCAGCAACGGATCTCTTGGCTCTCGCATCGA
TGAAGAACGCAGCGAAATGCGATAAGTAATGTGAATTGCAGAATTCAGTGAATCATCGAA
TCTTTGAACGCATCTTGCGCTCCTTGGTATTCCGAGGAGCATGCCTGTTTGAGTGTCATT
AAATTCTCAACTCTCTTATACTTTTTTGTAAAAGAGAGCTTGGACTGTGGAGGCTTGCTG
GCCACTTTTTGGGGTCAGCTCCTCTGAAATGCATTAGCGGAACCGTTTGCGATCTGCCAC
AAGTGTGATAAGTTATCTACACTGGCGAGGGGATTGCTCTCTGTAATGTTCAGCTTCTAA
TTGTCTCTACTTTGTGAGACTACTTTTGAATGCTTGACCTCAAATCAGGTAGGACTACCC
GCTGAACTTAA
>A6_45
TTTCCGTAGGTGAACCTGCGGAAGGATCATTATTGAATTATGTTTCTAGATAGGTTGTAG
CTGGCTCTTTTAGAGCATGTGCACGCCTGTTTGGACTTCATTTTCATCCACCTGTGCACC
TATTGTAGTCTTTGGTTGGGTTAGGAGGAAGTGATCATTGTATCAGCATCTGCTGGGAGT
GAGGACTTGCATTGTGAAAGCTTTGCTGTCCTTGATGTGATCATGGAATCTTTTTCTCAC
TAGAGTCTATGTCACTCATTATACTCTGTCGAATGTCATTGAATGTCTTTACATGGGCTT
GTTTGCCTATGAAAATTGTAATACAACTTTCAGCAACGGATCTCTTGGCTCTCGCATCGA
TGAAGAACGCAGCGAAATGCGATAAGTAATGTGAATTGCAGAATTCAGTGAATCATCGAA
TCTTTGAACGCATCTTGCGCTCCTTGGTATTCCGAGGAGCATGCCTGTTTGAGTGTCATT
AAATTCTCAACTCTCTTATACTTTTTTGTAAAAGAGAGCTTGGACTGTGGAGGCTTGCTG
GCCACTTTTTGGGGTCAGCTCCTCTGAAATGCATTAGCGGAACCGTTTGCGATCTGCCAC
AAGTGTGATAAGTTATCTACACTGGCGAGGGGATTGCTCTCTGTAATGTTCAGCTTCTAA
TTGTCTCTACTTTGTGAGACTACTTTTGAATGCTTGACCTCAAATCAGGTAGGACTACCC
GCTGAACTTAA
>A9_7
TTTCCGTAGGTGAACCTGCGGAAGGATCATTATTGAATTATGTTTCTAGATAGGTTGTAG
CTGGCTCTTTTAGAGCATGTGCACGCCTGTTTGGACTTCATTTTCATCCACCTGTGCACC
TATTGTAGTCTTTGGTTGGGTTAGGAGGAAGTGATCATTGTATCAGCATCTGCTGGGAGT
GAGGACTTGCATTGTGAAAGCTTTGCTGTCCTTGATGTGATCATGGAATCTTTTTCTCAC
TAGAGTCTATGTCACTCATTATACTCTGTCGAATGTCATTGAATGTCTTTACATGGGCTT
GTATGCCTATGAAAATTGTAATACAACTTTCAGCAACGGATCTCTTGGCTCTCGCATCGA
TGAAGAACGCAGCGAAATGCGATAAGTAATGTGAATTGCAGAATTCAGTGAATCATCGAA
TCTTTGAACGCATCTTGCGCTCCTTGGTATTCCGAGGAGCATGCCTGTTTGAGTGTCATT
AAATTCTCAACTCTCTTATACTTTTTTGTAAAAGAGAGCTTGGACTGTGGAGGCTTGCTG
GCCACTTTTTGGGGTCAGCTCCTCTGAAATGCATTAGCGGAACCGTTTGCGATCTGCCAC
AAGTGTGATAAGTTATCTACACTGGCGAGGGGATTGCTCTCTGTAATGTTCAGCTTCTAA

TTGTCTCTACTTTGTGAGACTACTTTTGAATGCTTGACCTCAAATCAGGTAGGACTACCC
GCTGAACTTAA
>A1_6
TTTCCGTAGGTGAACCTGCGGAAGGATCATTATTGAATTATGTTTCTAGATAGGTTGTAG
CTGGCTCTTTTAGAGCATGTGCACGCCTGTTTGGACTTCATTTTCATCCACCTGTGCACC
TATTGTAGTCTTTGGTTGGGTTAGGAGGAAGTGATCATTGTATCAGCATCTGCTGGGAGT
GAGGACTTGCATTGTGAAAGCTTTGCTGTCCTTGATGTGATCATGGAATCTTTTTCTCAC
TAGAGTCTATGTCACTCATTATACTCTGTCGAATGTCATTGAATGTCTTTACATGGGCTT
GTATGCCTATGAAAATTGTAATACAACTTTCAGCAACGGATCTCTTGGCTCTCGCATCGA
TGAAGAACGCAGCGAAATGCGATAAGTAATGTGAATTGCAGAATTCAGTGAATCATCGAA
TCTTTGAACGCATCTTGCGCTCCTTGGTATTCCGAGGAGCATGCCTGTTTGAGTGTCATT
AAATTCTCAACTCTCTTATACTTTTTTGTAAAAGAGAGCTTGGACTGTGGAGGCTTGCTG
GCCACTTTTTGGGGTCAGCTCCTCTGAAATGCATTAGCGGAACCGTTTGCGATCTGCCAC
AAGTGTGATAAGTTATCTACACTGGCGAGGGGATTGCTCTCTGTAATGTTCAGCTTCTAA
TTGTCTCTACTTTGTGAGACTACTTTTGAATGCTTGACCTCAAATCAGGTAGGACTACCC
GCTGAACTTAA
>A2–67
TTTCCGTAGGTGAACCTGCGGAAGGATCATTATTGAATTATGTTTCTAGATAGGTTGTAG
CTGGCTCTTTTAGAGCATGTGCACGCCTGTTTGGACTTCATTTTCATCCACCTGTGCACC
TATTGTAGTCTTTGGTTGGGTTAGGAGGAAGTGATCATTGTATCAGCATCTGCTGGGAGT
GAGGACTTGCATTGTGAAAGCTTTGCTGTCCTTGATGTGATCATGGAATCTTTTTCTCAC
TAGAGTCTATGTCACTCATTATACTCTGTCGAATGTCATTGAATGTCTTTACATGGGCTT
GTATGCCTATGAAAATTGTAATACAACTTTCAGCAACGGATCTCTTGGCTCTCGCATCGA
TGAAGGACGCAGCGAAATGCGATAAGTAATGTGAATTGCAGAATTCAGTGAATCATCGAA
TCTTTGAACGCATCTTGCGCTCCTTGGTATTCCGAGGAGCATGCCTGTTTGAGTGTCATT
AAATTCTCAACTCTCTTATACTTTTTTGTAAAAGAGAGCTTGGACTGTGGAGGCTTGCTG
GCCACTTTTTGGGGTCAGCTCCTCTGAAATGCATTAGCGGAACCGTTTGCGATCTGCCAC
AAGTGTGATAAGTTATCTACACTGGCGAGGGGATTGCTCTCTGTAATGTTCAGCTTCTAA
TTGTCTCTACTTTGTGAGACTACTTTTGAATGCTTGACCTCAAATCAGGTAGGACTACCC
GCTGAACTTAA
>A9_43
TTTCCGTAGGTGAACCTGCGGAAGGATCATTATTGAATTATGTTTCTAGATAGGTTGTAG
CTGGCTCTTTTAGAGCATGTGCACGCCTGTTTGGACTTCATTTTCATCCACCTGTGCACC
TATTGTAGTCTTTGGTTGGGTTAGGAGGAAGTGATCATTGTATCAGCATCTGCTGGGAGT
GAGGACTTGCATTGTGAAAGCTTTGCTGTCCTTGATGTGATCATGGAATCTTTTTCTCAC
TAGAGTCTATGTCACTCATTATACTCTGTCGAATGTCATTGAATGTCTTTACATGGGCTT
GTATGCCTATGAAAATTGTAATACAACTTTCAGCAACGGATCTCTTGGCTCTCGCATCGA
TGAAGAACGCAGCGAAATGCGATAAGTAATGTGAATTGCAGAATTCAGTGAATCATCGAA
TCTTTGAACGCATCTTGCGCTCCTTGGTATTCCGAGGAGCATGCCTGTTTGAGTGTCATT
AAATTCTCAACTCTCTTATACTTTTTTGTAAAAGAGAGCTTGGACTGTGGAGGCTTGCTG
GCCACTTTTTGGGGTCAGCTCCTCTGAAATGCATTAGCGGAACCGTTTGCGATCTGCCAC
AAGTGTGATAAGTTATCTACACTGGCGAGGGGATTGCTCTCTGTAATGTTCAGCTTCTAA
TTGTCTCTACTTTGTGAGACTACTTTTGAATGCTTGACCTCAAATCAGGTAGGACTACCC
GCTGAACTTAA
>A8_2
TTTCCGTAGGTGAACCTGCGGAAGGATCATTATTGAATTATGTTTCTAGATAGGTTGTAG
CTGGCTCTTTTAGAGCATGTGCACGCCTGTTTGGACTTCATTTTCATCCACCTGTGCACC
TATTGTAGTCTTTGGTTGGGTTAGGAGGAAGTGATCATTGTATCAGCATCTGCTGGGAGT
GAGGACTTGCATTGTGAAAGCTTTGCTGTCCTTGATGTGATCATGGAATCTTTTTCTCAC
TAGAGTCTATGTCACTCATTATACTCTGTCGAATGTCATTGAATGTCTTTACATGGGCTT

GTATGCCTATGAAAATTGTAATACAACTTTCAGCAACGGATCTCTTGGCTCTCGCATCGA
TGAAGAACGCAGCGAAATGCGATAAGTAATGTGAATTGCAGAATTCAGTGAATCATCGAA
TCTTTGAACGCATCTTGCGCTCCTTGGTATTCCGAGGAGCATGCCTGTTTGAGTGTCATT
AAATTCTCAACTCTCTTATACTTTTTTGTAAAAGAGAGCTTGGACTGTGGAGGCTTGCTG
GCCACTTTTTGGGGTCAGCTCCTCTGAAATGCATTAGCGGAACCGTTTGCGATCTGCCAC
AAGTGTGATAAGTTATCTACACTGGCGAGGGGATTGCTCTCTGTAATGTTCAGCTTCTAA
TTGTCTCTACTTTGTGAGACTACTTTTGAATGCTTGACCTCAAATCAGGTAGGACTACCC
GCTGAACTTAA
>A6_14
TTTCCGTAGGTGAACCTGCGGAAGGATCATTATTGAATTATGTTTCTAGATAGGTTGTAG
CTGGCTCTTTTAGAGCATGTGCACGCCTGTTTGGACTTCATTTTCATCCACCTGTGCACC
TATTGTAGTCTTTGGTTGGGTTAGGAGGAAGTGATCATTGTATCAGCATCTGCTGGGAGT
GAGGACTTGCATTGTGAAAGCTTTGCTGTCCTTGATGTGATCATGGAATCTTTTTCTCAC
TAGAGTCTATGTCACTCATTATACTCTGTCGAATGTCATTGAATGTCTTTACATGGGCTT
GTATGCCTATGAAAATTGTAATACAACTTTCAGCAACGGATCTCTTGGCTCTCGCATCGA
TGAAGAACGCAGCGAAATGCGATAAGTAATGTGAATTGCAGAATTCAGTGAATCATCGAA
TCTTTGAACGCATCTTGCGCTCCTTGGTATTCCGAGGAGCATGCCTGTTTGAGTGTCATT
AAATTCTCAACTCTCTTATACTTTTTTGTAAAAGAGAGCTTGGACTGTGGAGGCTTGCTG
GCCACTTTTTGGGGTCAGCTCCTCTGAAATGCATTAGCGGAACCGTTTGCGATCTGCCAC
AAGTGTGATAAGTTATCTACACTGGCGAGGGGATTGCTCTCTGTAATGTTCAGCTTCTAA
TTGTCTCTACTTTGTGAGACTACTTTTGAATGCTTGACCTCAAATCAGGTAGGACTACCC
GCTGAACTTAA
>A6_70
TTTCCGTAGGTGAACCTGCGGAAGGATCATTATTGAATTATGTTTCTAGATAGGTTGTAG
CTGGCTCTTTTAGAGCATGTGCACGCCTGTTTGGACTTCATTTTCATCCACCTGTGCACC
TATTGTAGTCTTTGGTTGGGTTAGGAGGAAGTGATCATTGTATCAGCATCTGCTGGGAGT
GAGGACTTGCATTGTGAAAGCTTTGCTGTCCTTGATGTGATCATGGAATCTTTTTCTCAC
TAGAGTCTATGTCACTCATTATACTCTGTCGAATGTCATTGAATGTCTTTACATGGGCTT
GTATGCCTATGAAAATTGTAATACAACTTTCAGCAACGGATCTCTTGGCTCTCGCATCGA
TGAAGAACGCAGCGAAATGCGATAAGTAATGTGAATTGCAGAATTCAGTGAATCATCGAA
TCTTTGAACGCATCTTGCGCTCCTTGGTATTCCGAGGAGCATGCCTGTTTGAGTGTCATT
AAATTCTCAACTCTCTTATACTTTTTTGTAAAAGAGAGCTTGGACTGTGGAGGCTTGCTG
GCCACTTTTTGGGGTCAGCTCCTCTGAAATGCATTAGCGGAACCGTTTGCGATCTGCCAC
AAGTGTGATAAGTTATCTACACTGGCGAGGGGATTGCTCTCTGTAATGTTCAGCTTCTAA
TTGTCTCTACTTTGTGAGACTACTTTTGAATGCTTGACCTCAAATCAGGTAGGACTACCC
GCTGAACTTAA
>A7_55
TTTCCGTAGGTGAACCTGCGGAAGGATCATTATTGAATTATGTTTCTAGATAGGTTGTAG
CTGGCTCTTTTAGAGCATGTGCACGCCTGTTTGGACTTCATTTTCATCCACCTGTGCACC
TATTGTAGTCTTTGGTTGGGTTAGGAGGAAGTGATCATTGTATCAGCATCTGCTGGGAGT
GAGGACTTGCATTGTGAAAGCTTTGCTGTCCTTGATGTGATCATGGAATCTTTTTCTCAC
TAGAGTCTATGTCACTCATTATACTCTGTCGAATGTCATTGAATGTCTTTACATGGGCTT
GTATGCCTATGAAAATTGTAATACAACTTTCAGCAACGGATCTCTTGGCTCTCGCATCGA
TGAAGAACGCAGCGAAATGCGATAAGTAATGTGAATTGCAGAATTCAGTGAATCATCGAA
TCTTTGAACGCATCTTGCGCTCCTTGGTATTCCGAGGAGCATGCCTGTTTGAGTGTCATT
AAATTCTCAACTCTCTTATACTTTTTTGTAAAAGAGAGCTTGGACTGTGGAGGCTTGCTG
GCCACTTTTTGGGGTCAGCTCCTCTGAAATGCATTAGCGGAACCGTTTGCGATCTGCCAC
AAGTGTGATAAGTTATCTACACTGGCGAGGGGATTGCTCTCTGTAATGTTCAGCTTCTAA
TTGTCTCTACTTTGTGAGACTACTTTTGAATGCTTGACCTCAAATCAGGTAGGACTACCC
GCTGAACTTAA

>A10_6
TTTCCGTAGGTGAACCTGCGGAAGGATCATTATTGAATTATGTTTCTAGATAGGTTGTAG
CTGGCTCTTTTAGAGCATGTGCACGCCTGTTTGGACTTCATTTTCATCCACCTGTGCACC
TATTGTAGTCTTTGGTTGGGTTAGGAGGAAGTGATCATTGTATCAGCATCTGCTGGGAGT
GAGGACTTGCATTGTGAAAGCTTTGCTGTCCTTGATGTGATCATGGAATCTTTTTCTCAC
TAGAGTCTATGTCACTCATTATACTCTGTCGAATGTCATTGAATGTCTTTACATGGGCTT
GTATGCCTATGAAAATTGTAATACAACTTTCAGCAACGGATCTCTTGGCTCTCGCATCGA
TGAAGAACGCAGCGAAATGCGATAAGTAATGTGAATTGCAGAATTCAGTGAATCATCGAA
TCTTTGAACGCATCTTGCGCTCCTTGGTATTCCGAGGAGCATGCCTGTTTGAGTGTCATT
AAATTCTCAACTCTCTTATACTTTTTTGTAAAAGAGAGCTTGGACTGTGGAGGCTTGCTG
GCCACTTTTTGGGGTCAGCTCCTCTGAAATGCATTAGCGGAACCGTTTGCGATCTGCCAC
AAGTGTGATAAGTTATCTACACTGGCGAGGGGATTGCTCTCTGTAATGTTCAGCTTCTAA
TTGTCTCTACTTTGTGAGACTACTTTTGAATGCTTGACCTCAAATCAGGTAGGACTACCC
GCTGAACTTAA
>A12_5
TTTCCGTAGGTGAACCTGCGGAAGGATCATTATTGAATTATGTTTCTAGATAGGTTGTAG
CTGGCTCTTTTAGAGCATGTGCACGCCTGTTTGGACTTCATTTTCATCCACCTGTGCACC
TATTGTAGTCTTTGGTTGGGTTAGGAGGAAGTGATCATTGTATCAGCATCTGCTGGGAGT
GAGGACTTGCATTGTGAAAGCTTTGCTGTCCTTGATGTGATCATGGAATCTTTTTCTCAC
TAGAGTCTATGTCACTCATTATACTCTGTCGAATGTCATTGAATGTCTTTACATGGGCTT
GTATGCCTATGAAAATTGTAATACAACTTTCAGCAACGGATCTCTTGGCTCTCGCATCGA
TGAAGAACGCAGCGAAATGCGATAAGTAATGTGAATTGCAGAATTCAGTGAATCATCGAA
TCTTTGAACGCATCTTGCGCTCCTTGGTATTCCGAGGAGCATGCCTGTTTGAGTGTCATT
AAATTCTCAACTCTCTTATACTTTTTTGTAAAAGAGAGCTTGGACTGTGGAGGCTTGCTG
GCCACTTTTTGGGGTCAGCTCCTCTGAAATGCATTAGCGGAACCGTTTGCGATCTGCCAC
AAGTGTGATAAGTTATCTACACTGGCGAGGGGATTGCTCTCTGTAATGTTCAGCTTCTAA
TTGTCTCTACTTTGTGAGACTACTTTTGAATGCTTGACCTCAAATCAGGTAGGACTACCC
GCTGAACTTAA
>A12_64
TTTCCGTAGGTGAACCTGCGGAAGGATCATTATTGAATTATGTTTCTAGATAGGTTGTAG
CTGGCTCTTTTAGAGCATGTGCACGCCTGTTTGGACTTCATTTTCATCCACCTGTGCACC
TATTGTAGTCTTTGGTTGGGTTAGGAGGAAGTGATCATTGTATCAGCATCTGCTGGGAGT
GAGGACTTGCATTGTGAAAGCTTTGCTGTCCTTGATGTGATCATGGAATCTTTTTCTCAC
TAGAGTCTATGTCACTCATTATACTCTGTCGAATGTCATTGAATGTCTTTACATGGGCTT
GTATGCCTATGAAAATTGTAATACAACTTTCAGCAACGGATCTCTTGGCTCTCGCATCGA
TGAAGAACGCAGCGAAATGCGATAAGTAATGTGAATTGCAGAATTCAGTGAATCATCGAA
TCTTTGAACGCATCTTGCGCTCCTTGGTATTCCGAGGAGCATGCCTGTTTGAGTGTCATT
AAATTCTCAACTCTCTTATACTTTTTTGTAAAAGAGAGCTTGGACTGTGGAGGCTTGCTG
GCCACTTTTTGGGGTCAGCTCCTCTGAAATGCATTAGCGGAACCGTTTGCGATCTGCCAC
AAGTGTGATAAGTTATCTACACTGGCGAGGGGATTGCTCTCTGTAATGTTCAGCTTCTAA
TTGTCTCTACTTTGTGAGACTACTTTTGAATGCTTGACCTCAAATCAGGTAGGACTACCC
GCTGAACTTAA
