# Supplementary material for: Patterns of PCR Amplification Artifacts of the Fungal Barcode Marker in a Hybrid Mushroom
Source: Front Microbiol. 2019 Nov 19;10:2686. doi: 10.3389/fmicb.2019.02686 (PMC6877668; doi:10.3389/fmicb.2019.02686)
Supplement: Supplementary file 3 [file Data_Sheet_3.PDF]

>A1
TTTCCGTAGGTGAACCTGCGGAAGGATCATTATTGAATTATGTTTCTAGATAGGTTGTAG
CTGGCTCTTTTAGAGCATGTGCACGCCTGTTTGGACTTCATTTTCATCCACCTGTGCACC
TATTGTAGTCTTTGGTTGGGTTAGGAGGAAGTGATCATTGTATCAGCATCTGCTGGGAGT
GAGGACTTGCATTGTGAAAGCTTTGCTGTCCTTGATGTGATCATGGAATCTTTTTCTCAC
TAGAGTCTATGTCACTCATTATACTCTGTCGAATGTCATTGAATGTCTTTACATGGGCTT
GTATGCCTATGAAAATTGTAATACAACTTTCAGCAACGGATCTCTTGGCTCTCGCATCGA
TGAAGAACGCAGCGAAATGCGATAAGTAATGTGAATTGCAGAATTCAGTGAATCATCGAA
TCTTTGAACGCATCTTGCGCTCCTTGGTATTCCGAGGAGCATGCCTGTTTGAGTGTCATT
AAATTCTCAACTCTCTTATACTTTTTTGTAAAAGAGAGCTTGGACTGTGGAGGCTTGCTG
GCCACTTTTTGGGGTCAGCTCCTCTGAAATGCATTAGCGGAACCGTTTGCGATCTGCCAC
AAGTGTGATAAGTTATCTACACTGGCGAGGGGATTGCTCTCTGTAATGTTCAGCTTCTAA
TTGTCTCTACTTTGTGAGACTACTTTTGAATGCTTGACCTCAAATCAGGTAGGACTACCC
GCTGAACTTAA
>A2
TTTCCGTAGGTGAACCTGCGGAAGGATCATTATTGAATTATGTTTCTAGATAGGTTGTAG
CTGGCTCTTTTAGAGCATGTGCACGCCTGTTTGGACTTCATTTTCATCCACCTGTGCACC
TATTGTAGTCTTTGGTTGGGTTAGGAGGAAGTGATCATTGTATCAGCATCTGCTGGGAGT
GAGGACTTGCATTGTGAAAGCTTTGCTGTCCTTGATGTGATCATGGAATCTTTTTCTCAC
TAGAGTCTATGTCACTCATTATACTCTGTCGAATGTCATTGAATGTCTTTACATGGGCTT
GTATGCCTATGAAAATTGTAATACAACTTTCAGCAACGGATCTCTTGGCTCTCGCATCGA
TGAAGAACGCAGCGAAATGCGATAAGTAATGTGAATTGCAGAATTCAGTGAATCATCGAA
TCTTTGAACGCATCTTGCGCTCCTTGGTATTCCGAGGAGCATGCCTGTTTGAGTGTCATT
AAATTCTCAACTCTCTTATACTTTTTTGTAAAAGAGAGCTTGGACTGTGGAGGCTTGCTG
GCCACTTTTTGGGGTCAGCTCCTCTGAAATGCATTAGCGGAACCGTTTGCGATCTGCCAC
AAGTGTGATAAGTTATCTACACTGGCGAGGGGATTGCTCTCTGTAATGTTCAGCTTCTAA
TTGTCTCTACTTTGTGAGACTACTTTTGAATGCTTGACCTCAAATCAGGTAGGACTACCC
GCTGAACTTAA
>A3
TTTCCGTAGGTGAACCTGCGGAAGGATCATTATTGAATTATGTTTCTAGATAGGTTGTAG
CTGGCTCTTTTAGAGCATGTGCACGCCTGTTTGGACTTCATTTTCATCCACCTGTGCACC
TATTGTAGTCTTTGGTTGGGTTAGGAGGAAGTGATCATTGTATCAGCATCTGCTGGGAGT
GAGGACTTGCATTGTGAAAGCTTTGCTGTCCTTGATGTGATCATGGAATCTTTTTCTCAC
TAGAGTCTATGTCACTCATTATACTCTGTCGAATGTCATTGAATGTCTTTACATGGGCTT
GTATGCCTATGAAAATTGTAATACAACTTTCAGCAACGGATCTCTTGGCTCTCGCATCGA
TGAAGAACGCAGCGAAATGCGATAAGTAATGTGAATTGCAGAATTCAGTGAATCATCGAA
TCTTTGAACGCATCTTGCGCTCCTTGGTATTCCGAGGAGCATGCCTGTTTGAGTGTCATT
AAATTCTCAACTCTCTTATACTTTTTTGTAAAAGAGAGCTTGGACTGTGGAGGCTTGCTG
GCCACTTTTTGGGGTCAGCTCCTCTGAAATGCATTAGCGGAACCGTTTGCGATCTGCCAC
AAGTGTGATAAGTTATCTACACTGGCGAGGGGATTGCTCTCTGTAATGTTCAGCTTCTAA
TTGTCTCTACTTTGTGAGACTACTTTTGAATGCTTGACCTCAAATCAGGTAGGACTACCC
GCTGAACTTAA
>A4
TTTCCGTAGGTGAACCTGCGGAAGGATCATTATTGAATTATGTTTCTAGATAGGTTGTAG
CTGGCTCTTTTAGAGCATGTGCACGCCTGTTTGGACTTCATTTTCATCCACCTGTGCACC
TATTGTAGTCTTTGGTTGGGTTAGGAGGAAGTGATCATTGTATCAGCATCTGCTGGGAGT
GAGGACTTGCATTGTGAAAGCTTTGCTGTCCTTGATGTGATCATGGAATCTTTTTCTCAC
TAGAGTCTATGTCACTCATTATACTCTGTCGAATGTCATTGAATGTCTTTACATGGGCTT
GTATGCCTATGAAAATTGTAATACAACTTTCAGCAACGGATCTCTTGGCTCTCGCATCGA
TGAAGAACGCAGCGAAATGCGATAAGTAATGTGAATTGCAGAATTCAGTGAATCATCGAA

TCTTTGAACGCATCTTGCGCTCCTTGGTATTCCGAGGAGCATGCCTGTTTGAGTGTCATT
AAATTCTCAACTCTCTTATACTTTTTTGTAAAAGAGAGCTTGGACTGTGGAGGCTTGCTG
GCCACTTTTTGGGGTCAGCTCCTCTGAAATGCATTAGCGGAACCGTTTGCGATCTGCCAC
AAGTGTGATAAGTTATCTACACTGGCGAGGGGATTGCTCTCTGTAATGTTCAGCTTCTAA
TTGTCTCTACTTTGTGAGACTACTTTTGAATGCTTGACCTCAAATCAGGTAGGACTACCC
GCTGAACTTAA
>A5
TTTCCGTAGGTGAACCTGCGGAAGGATCATTATTGAATTATGTTTCTAGATAGGTTGTAG
CTGGCTCTTTTAGAGCATGTGCACGCCTGTTTGGACTTCATTTTCATCCACCTGTGCACC
TATTGTAGTCTTTGGTTGGGTTAGGAGGAAGTGATCATTGTATCAGCATCTGCTGGGAGT
GAGGACTTGCATTGTGAAAGCTTTGCTGTCCTTGATGTGATCATGGAATCTTTTTCTCAC
TAGAGTCTATGTCACTCATTATACTCTGTCGAATGTCATTGAATGTCTTTACATGGGCTT
GTATGCCTATGAAAATTGTAATACAACTTTCAGCAACGGATCTCTTGGCTCTCGCATCGA
TGAAGAACGCAGCGAAATGCGATAAGTAATGTGAATTGCAGAATTCAGTGAATCATCGAA
TCTTTGAACGCATCTTGCGCTCCTTGGTATTCCGAGGAGCATGCCTGTTTGAGTGTCATT
AAATTCTCAACTCTCTTATACTTTTTTGTAAAAGAGAGCTTGGACTGTGGAGGCTTGCTG
GCCACTTTTTGGGGTCAGCTCCTCTGAAATGCATTAGCGGAACCGTTTGCGATCTGCCAC
AAGTGTGATAAGTTATCTACACTGGCGAGGGGATTGCTCTCTGTAATGTTCAGCTTCTAA
TTGTCTCTACTTTGTGAGACTACTTTTGAATGCTTGACCTCAAATCAGGTAGGACTACCC
GCTGAACTTAA
>A6
TTTCCGTAGGTGAACCTGCGGAAGGATCATTATTGAATTATGTTTCTAGATAGGTTGTAG
CTGGCTCTTTTAGAGCATGTGCACGCCTGTTTGGACTTCATTTTCATCCACCTGTGCACC
TATTGTAGTCTTTGGTTGGGTTAGGAGGAAGTGATCATTGTATCAGCATCTGCTGGGAGT
GAGGACTTGCATTGTGAAAGCTTTGCTGTCCTTGATGTGATCATGGAATCTTTTTCTCAC
TAGAGTCTATGTCACTCATTATACTCTGTCGAATGTCATTGAATGTCTTTACATGGGCTT
GTATGCCTATGAAAATTGTAATACAACTTTCAGCAACGGATCTCTTGGCTCTCGCATCGA
TGAAGAACGCAGCGAAATGCGATAAGTAATGTGAATTGCAGAATTCAGTGAATCATCGAA
TCTTTGAACGCATCTTGCGCTCCTTGGTATTCCGAGGAGCATGCCTGTTTGAGTGTCATT
AAATTCTCAACTCTCTTATACTTTTTTGTAAAAGAGAGCTTGGACTGTGGAGGCTTGCTG
GCCACTTTTTGGGGTCAGCTCCTCTGAAATGCATTAGCGGAACCGTTTGCGATCTGCCAC
AAGTGTGATAAGTTATCTACACTGGCGAGGGGATTGCTCTCTGTAATGTTCAGCTTCTAA
TTGTCTCTACTTTGTGAGACTACTTTTGAATGCTTGACCTCAAATCAGGTAGGACTACCC
GCTGAACTTAA
>A7
TTTCCGTAGGTGAACCTGCGGAAGGATCATTATTGAATTATGTTTCTAGATAGGTTGTAG
CTGGCTCTTTTAGAGCATGTGCACGCCTGTTTGGACTTCATTTTCATCCACCTGTGCACC
TATTGTAGTCTTTGGTTGGGTTAGGAGGAAGTGATCATTGTATCAGCATCTGCTGGGAGT
GAGGACTTGCATTGTGAAAGCTTTGCTGTCCTTGATGTGATCATGGAATCTTTTTCTCAC
TAGAGTCTATGTCACTCATTATACTCTGTCGAATGTCATTGAATGTCTTTACATGGGCTT
GTATGCCTATGAAAATTGTAATACAACTTTCAGCAACGGATCTCTTGGCTCTCGCATCGA
TGAAGAACGCAGCGAAATGCGATAAGTAATGTGAATTGCAGAATTCAGTGAATCATCGAA
TCTTTGAACGCATCTTGCGCTCCTTGGTATTCCGAGGAGCATGCCTGTTTGAGTGTCATT
AAATTCTCAACTCTCTTATACTTTTTTGTAAAAGAGAGCTTGGACTGTGGAGGCTTGCTG
GCCACTTTTTGGGGTCAGCTCCTCTGAAATGCATTAGCGGAACCGTTTGCGATCTGCCAC
AAGTGTGATAAGTTATCTACACTGGCGAGGGGATTGCTCTCTGTAATGTTCAGCTTCTAA
TTGTCTCTACTTTGTGAGACTACTTTTGAATGCTTGACCTCAAATCAGGTAGGACTACCC
GCTGAACTTAA
>A8
TTTCCGTAGGTGAACCTGCGGAAGGATCATTATTGAATTATGTTTCTAGATAGGTTGTAG

CTGGCTCTTTTAGAGCATGTGCACGCCTGTTTGGACTTCATTTTCATCCACCTGTGCACC
TATTGTAGTCTTTGGTTGGGTTAGGAGGAAGTGATCATTGTATCAGCATCTGCTGGGAGT
GAGGACTTGCATTGTGAAAGCTTTGCTGTCCTTGATGTGATCATGGAATCTTTTTCTCAC
TAGAGTCTATGTCACTCATTATACTCTGTCGAATGTCATTGAATGTCTTTACATGGGCTT
GTATGCCTATGAAAATTGTAATACAACTTTCAGCAACGGATCTCTTGGCTCTCGCATCGA
TGAAGAACGCAGCGAAATGCGATAAGTAATGTGAATTGCAGAATTCAGTGAATCATCGAA
TCTTTGAACGCATCTTGCGCTCCTTGGTATTCCGAGGAGCATGCCTGTTTGAGTGTCATT
AAATTCTCAACTCTCTTATACTTTTTTGTAAAAGAGAGCTTGGACTGTGGAGGCTTGCTG
GCCACTTTTTGGGGTCAGCTCCTCTGAAATGCATTAGCGGAACCGTTTGCGATCTGCCAC
AAGTGTGATAAGTTATCTACACTGGCGAGGGGATTGCTCTCTGTAATGTTCAGCTTCTAA
TTGTCTCTACTTTGTGAGACTACTTTTGAATGCTTGACCTCAAATCAGGTAGGACTACCC
GCTGAACTTAA
>A9
TTTCCGTAGGTGAACCTGCGGAAGGATCATTATTGAATTATGTTTCTAGATAGGTTGTAG
CTGGCTCTTTTAGAGCATGTGCACGCCTGTTTGGACTTCATTTTCATCCACCTGTGCACC
TATTGTAGTCTTTGGTTGGGTTAGGAGGAAGTGATCATTGTATCAGCATCTGCTGGGAGT
GAGGACTTGCATTGTGAAAGCTTTGCTGTCCTTGATGTGATCATGGAATCTTTTTCTCAC
TAGAGTCTATGTCACTCATTATACTCTGTCGAATGTCATTGAATGTCTTTACATGGGCTT
GTATGCCTATGAAAATTGTAATACAACTTTCAGCAACGGATCTCTTGGCTCTCGCATCGA
TGAAGAACGCAGCGAAATGCGATAAGTAATGTGAATTGCAGAATTCAGTGAATCATCGAA
TCTTTGAACGCATCTTGCGCTCCTTGGTATTCCGAGGAGCATGCCTGTTTGAGTGTCATT
AAATTCTCAACTCTCTTATACTTTTTTGTAAAAGAGAGCTTGGACTGTGGAGGCTTGCTG
GCCACTTTTTGGGGTCAGCTCCTCTGAAATGCATTAGCGGAACCGTTTGCGATCTGCCAC
AAGTGTGATAAGTTATCTACACTGGCGAGGGGATTGCTCTCTGTAATGTTCAGCTTCTAA
TTGTCTCTACTTTGTGAGACTACTTTTGAATGCTTGACCTCAAATCAGGTAGGACTACCC
GCTGAACTTAA
>A10
TTTCCGTAGGTGAACCTGCGGAAGGATCATTATTGAATTATGTTTCTAGATAGGTTGTAG
CTGGCTCTTTTAGAGCATGTGCACGCCTGTTTGGACTTCATTTTCATCCACCTGTGCACC
TATTGTAGTCTTTGGTTGGGTTAGGAGGAAGTGATCATTGTATCAGCATCTGCTGGGAGT
GAGGACTTGCATTGTGAAAGCTTTGCTGTCCTTGATGTGATCATGGAATCTTTTTCTCAC
TAGAGTCTATGTCACTCATTATACTCTGTCGAATGTCATTGAATGTCTTTACATGGGCTT
GTATGCCTATGAAAATTGTAATACAACTTTCAGCAACGGATCTCTTGGCTCTCGCATCGA
TGAAGAACGCAGCGAAATGCGATAAGTAATGTGAATTGCAGAATTCAGTGAATCATCGAA
TCTTTGAACGCATCTTGCGCTCCTTGGTATTCCGAGGAGCATGCCTGTTTGAGTGTCATT
AAATTCTCAACTCTCTTATACTTTTTTGTAAAAGAGAGCTTGGACTGTGGAGGCTTGCTG
GCCACTTTTTGGGGTCAGCTCCTCTGAAATGCATTAGCGGAACCGTTTGCGATCTGCCAC
AAGTGTGATAAGTTATCTACACTGGCGAGGGGATTGCTCTCTGTAATGTTCAGCTTCTAA
TTGTCTCTACTTTGTGAGACTACTTTTGAATGCTTGACCTCAAATCAGGTAGGACTACCC
GCTGAACTTAA
>A11
TTTCCGTAGGTGAACCTGCGGAAGGATCATTATTGAATTATGTTTCTAGATAGGTTGTAG
CTGGCTCTTTTAGAGCATGTGCACGCCTGTTTGGACTTCATTTTCATCCACCTGTGCACC
TATTGTAGTCTTTGGTTGGGTTAGGAGGAAGTGATCATTGTATCAGCATCTGCTGGGAGT
GAGGACTTGCATTGTGAAAGCTTTGCTGTCCTTGATGTGATCATGGAATCTTTTTCTCAC
TAGAGTCTATGTCACTCATTATACTCTGTCGAATGTCATTGAATGTCTTTACATGGGCTT
GTATGCCTATGAAAATTGTAATACAACTTTCAGCAACGGATCTCTTGGCTCTCGCATCGA
TGAAGAACGCAGCGAAATGCGATAAGTAATGTGAATTGCAGAATTCAGTGAATCATCGAA
TCTTTGAACGCATCTTGCGCTCCTTGGTATTCCGAGGAGCATGCCTGTTTGAGTGTCATT
AAATTCTCAACTCTCTTATACTTTTTTGTAAAAGAGAGCTTGGACTGTGGAGGCTTGCTG

GCCACTTTTTGGGGTCAGCTCCTCTGAAATGCATTAGCGGAACCGTTTGCGATCTGCCAC
AAGTGTGATAAGTTATCTACACTGGCGAGGGGATTGCTCTCTGTAATGTTCAGCTTCTAA
TTGTCTCTACTTTGTGAGACTACTTTTGAATGCTTGACCTCAAATCAGGTAGGACTACCC
GCTGAACTTAA
>A12
TTTCCGTAGGTGAACCTGCGGAAGGATCATTATTGAATTATGTTTCTAGATAGGTTGTAG
CTGGCTCTTTTAGAGCATGTGCACGCCTGTTTGGACTTCATTTTCATCCACCTGTGCACC
TATTGTAGTCTTTGGTTGGGTTAGGAGGAAGTGATCATTGTATCAGCATCTGCTGGGAGT
GAGGACTTGCATTGTGAAAGCTTTGCTGTCCTTGATGTGATCATGGAATCTTTTTCTCAC
TAGAGTCTATGTCACTCATTATACTCTGTCGAATGTCATTGAATGTCTTTACATGGGCTT
GTATGCCTATGAAAATTGTAATACAACTTTCAGCAACGGATCTCTTGGCTCTCGCATCGA
TGAAGAACGCAGCGAAATGCGATAAGTAATGTGAATTGCAGAATTCAGTGAATCATCGAA
TCTTTGAACGCATCTTGCGCTCCTTGGTATTCCGAGGAGCATGCCTGTTTGAGTGTCATT
AAATTCTCAACTCTCTTATACTTTTTTGTAAAAGAGAGCTTGGACTGTGGAGGCTTGCTG
GCCACTTTTTGGGGTCAGCTCCTCTGAAATGCATTAGCGGAACCGTTTGCGATCTGCCAC
AAGTGTGATAAGTTATCTACACTGGCGAGGGGATTGCTCTCTGTAATGTTCAGCTTCTAA
TTGTCTCTACTTTGTGAGACTACTTTTGAATGCTTGACCTCAAATCAGGTAGGACTACCC
GCTGAACTTAA
>A13
TTTCCGTAGGTGAACCTGCGGAAGGATCATTATTGAATTATGTTTCTAGATAGGTTGTAG
CTGGCTCTTTTAGAGCATGTGCACGCCTGTTTGGACTTCATTTTCATCCACCTGTGCACC
TATTGTAGTCTTTGGTTGGGTTAGGAGGAAGTGATCATTGTATCAGCATCTGCTGGGAGT
GAGGACTTGCATTGTGAAAGCTTTGCTGTCCTTGATGTGATCATGGAATCTTTTTCTCAC
TAGAGTCTATGTCACTCATTATACTCTGTCGAATGTCATTGAATGTCTTTACATGGGCTT
GTATGCCTATGAAAATTGTAATACAACTTTCAGCAACGGATCTCTTGGCTCTCGCATCGA
TGAAGAACGCAGCGAAATGCGATAAGTAATGTGAATTGCAGAATTCAGTGAATCATCGAA
TCTTTGAACGCATCTTGCGCTCCTTGGTATTCCGAGGAGCATGCCTGTTTGAGTGTCATT
AAATTCTCAACTCTCTTATACTTTTTTGTAAAAGAGAGCTTGGACTGTGGAGGCTTGCTG
GCCACTTTTTGGGGTCAGCTCCTCTGAAATGCATTAGCGGAACCGTTTGCGATCTGCCAC
AAGTGTGATAAGTTATCTACACTGGCGAGGGGATTGCTCTCTGTAATGTTCAGCTTCTAA
TTGTCTCTACTTTGTGAGACTACTTTTGAATGCTTGACCTCAAATCAGGTAGGACTACCC
GCTGAACTTAA
>A14
TTTCCGTAGGTGAACCTGCGGAAGGATCATTATTGAATTATGTTTCTAGATAGGTTGTAG
CTGGCTCTTTTAGAGCATGTGCACGCCTGTTTGGACTTCATTTTCATCCACCTGTGCACC
TATTGTAGTCTTTGGTTGGGTTAGGAGGAAGTGATCATTGTATCAGCATCTGCTGGGAGT
GAGGACTTGCATTGTGAAAGCTTTGCTGTCCTTGATGTGATCATGGAATCTTTTTCTCAC
TAGAGTCTATGTCACTCATTATACTCTGTCGAATGTCATTGAATGTCTTTACATGGGCTT
GTATGCCTATGAAAATTGTAATACAACTTTCAGCAACGGATCTCTTGGCTCTCGCATCGA
TGAAGAACGCAGCGAAATGCGATAAGTAATGTGAATTGCAGAATTCAGTGAATCATCGAA
TCTTTGAACGCATCTTGCGCTCCTTGGTATTCCGAGGAGCATGCCTGTTTGAGTGTCATT
AAATTCTCAACTCTCTTATACTTTTTTGTAAAAGAGAGCTTGGACTGTGGAGGCTTGCTG
GCCACTTTTTGGGGTCAGCTCCTCTGAAATGCATTAGCGGAACCGTTTGCGATCTGCCAC
AAGTGTGATAAGTTATCTACACTGGCGAGGGGATTGCTCTCTGTAATGTTCAGCTTCTAA
TTGTCTCTACTTTGTGAGACTACTTTTGAATGCTTGACCTCAAATCAGGTAGGACTACCC
GCTGAACTTAA
>A15
TTTCCGTAGGTGAACCTGCGGAAGGATCATTATTGAATTATGTTTCTAGATAGGTTGTAG
CTGGCTCTTTTAGAGCATGTGCACGCCTGTTTGGACTTCATTTTCATCCACCTGTGCACC
TATTGTAGTCTTTGGTTGGGTTAGGAGGAAGTGATCATTGTATCAGCATCTGCTGGGAGT

GAGGACTTGCATTGTGAAAGCTTTGCTGTCCTTGATGTGATCATGGAATCTTTTTCTCAC
TAGAGTCTATGTCACTCATTATACTCTGTCGAATGTCATTGAATGTCTTTACATGGGCTT
GTATGCCTATGAAAATTGTAATACAACTTTCAGCAACGGATCTCTTGGCTCTCGCATCGA
TGAAGAACGCAGCGAAATGCGATAAGTAATGTGAATTGCAGAATTCAGTGAATCATCGAA
TCTTTGAACGCATCTTGCGCTCCTTGGTATTCCGAGGAGCATGCCTGTTTGAGTGTCATT
AAATTCTCAACTCTCTTATACTTTTTTGTAAAAGAGAGCTTGGACTGTGGAGGCTTGCTG
GCCACTTTTTGGGGTCAGCTCCTCTGAAATGCATTAGCGGAACCGTTTGCGATCTGCCAC
AAGTGTGATAAGTTATCTACACTGGCGAGGGGATTGCTCTCTGTAATGTTCAGCTTCTAA
TTGTCTCTACTTTGTGAGACTACTTTTGAATGCTTGACCTCAAATCAGGTAGGACTACCC
GCTGAACTTAA
>A16
TTTCCGTAGGTGAACCTGCGGAAGGATCATTATTGAATTATGTTTCTAGATAGGTTGTAG
CTGGCTCTTTTAGAGCATGTGCACGCCTGTTTGGACTTCATTTTCATCCACCTGTGCACC
TATTGTAGTCTTTGGTTGGGTTAGGAGGAAGTGATCATTGTATCAGCATCTGCTGGGAGT
GAGGACTTGCATTGTGAAAGCTTTGCTGTCCTTGATGTGATCATGGAATCTTTTTCTCAC
TAGAGTCTATGTCACTCATTATACTCTGTCGAATGTCATTGAATGTCTTTACATGGGCTT
GTATGCCTATGAAAATTGTAATACAACTTTCAGCAACGGATCTCTTGGCTCTCGCATCGA
TGAAGAACGCAGCGAAATGCGATAAGTAATGTGAATTGCAGAATTCAGTGAATCATCGAA
TCTTTGAACGCATCTTGCGCTCCTTGGTATTCCGAGGAGCATGCCTGTTTGAGTGTCATT
AAATTCTCAACTCTCTTATACTTTTTTGTAAAAGAGAGCTTGGACTGTGGAGGCTTGCTG
GCCACTTTTTGGGGTCAGCTCCTCTGAAATGCATTAGCGGAACCGTTTGCGATCTGCCAC
AAGTGTGATAAGTTATCTACACTGGCGAGGGGATTGCTCTCTGTAATGTTCAGCTTCTAA
TTGTCTCTACTTTGTGAGACTACTTTTGAATGCTTGACCTCAAATCAGGTAGGACTACCC
GCTGAACTTAA
>A17
TTTCCGTAGGTGAACCTGCGGAAGGATCATTATTGAATTATGTTTCTAGATAGGTTGTAG
CTGGCTCTTTTAGAGCATGTGCACGCCTGTTTGGACTTCATTTTCATCCACCTGTGCACC
TATTGTAGTCTTTGGTTGGGTTAGGAGGAAGTGATCATTGTATCAGCATCTGCTGGGAGT
GAGGACTTGCATTGTGAAAGCTTTGCTGTCCTTGATGTGATCATGGAATCTTTTTCTCAC
TAGAGTCTATGTCACTCATTATACTCTGTCGAATGTCATTGAATGTCTTTACATGGGCTT
GTATGCCTATGAAAATTGTAATACAACTTTCAGCAACGGATCTCTTGGCTCTCGCATCGA
TGAAGAACGCAGCGAAATGCGATAAGTAATGTGAATTGCAGAATTCAGTGAATCATCGAA
TCTTTGAACGCATCTTGCGCTCCTTGGTATTCCGAGGAGCATGCCTGTTTGAGTGTCATT
AAATTCTCAACTCTCTTATACTTTTTTGTAAAAGAGAGCTTGGACTGTGGAGGCTTGCTG
GCCACTTTTTGGGGTCAGCTCCTCTGAAATGCATTAGCGGAACCGTTTGCGATCTGCCAC
AAGTGTGATAAGTTATCTACACTGGCGAGGGGATTGCTCTCTGTAATGTTCAGCTTCTAA
TTGTCTCTACTTTGTGAGACTACTTTTGAATGCTTGACCTCAAATCAGGTAGGACTACCC
GCTGAACTTAA
>A18
TTTCCGTAGGTGAACCTGCGGAAGGATCATTATTGAATTATGTTTCTAGATAGGTTGTAG
CTGGCTCTTTTAGAGCATGTGCACGCCTGTTTGGACTTCATTTTCATCCACCTGTGCACC
TATTGTAGTCTTTGGTTGGGTTAGGAGGAAGTGATCATTGTATCAGCATCTGCTGGGAGT
GAGGACTTGCATTGTGAAAGCTTTGCTGTCCTTGATGTGATCATGGAATCTTTTTCTCAC
TAGAGTCTATGTCACTCATTATACTCTGTCGAATGTCATTGAATGTCTTTACATGGGCTT
GTATGCCTATGAAAATTGTAATACAACTTTCAGCAACGGATCTCTTGGCTCTCGCATCGA
TGAAGAACGCAGCGAAATGCGATAAGTAATGTGAATTGCAGAATTCAGTGAATCATCGAA
TCTTTGAACGCATCTTGCGCTCCTTGGTATTCCGAGGAGCATGCCTGTTTGAGTGTCATT
AAATTCTCAACTCTCTTATACTTTTTTGTAAAAGAGAGCTTGGACTGTGGAGGCTTGCTG
GCCACTTTTTGGGGTCAGCTCCTCTGAAATGCATTAGCGGAACCGTTTGCGATCTGCCAC
AAGTGTGATAAGTTATCTACACTGGCGAGGGGATTGCTCTCTGTAATGTTCAGCTTCTAA

TTGTCTCTACTTTGTGAGACTACTTTTGAATGCTTGACCTCAAATCAGGTAGGACTACCC
GCTGAACTTAA
>A19
TTTCCGTAGGTGAACCTGCGGAAGGATCATTATTGAATTATGTTTCTAGATAGGTTGTAG
CTGGCTCTTTTAGAGCATGTGCACGCCTGTTTGGACTTCATTTTCATCCACCTGTGCACC
TATTGTAGTCTTTGGTTGGGTTAGGAGGAAGTGATCATTGTATCAGCATCTGCTGGGAGT
GAGGACTTGCATTGTGAAAGCTTTGCTGTCCTTGATGTGATCATGGAATCTTTTTCTCAC
TAGAGTCTATGTCACTCATTATACTCTGTCGAATGTCATTGAATGTCTTTACATGGGCTT
GTATGCCTATGAAAATTGTAATACAACTTTCAGCAACGGATCTCTTGGCTCTCGCATCGA
TGAAGAACGCAGCGAAATGCGATAAGTAATGTGAATTGCAGAATTCAGTGAATCATCGAA
TCTTTGAACGCATCTTGCGCTCCTTGGTATTCCGAGGAGCATGCCTGTTTGAGTGTCATT
AAATTCTCAACTCTCTTATACTTTTTTGTAAAAGAGAGCTTGGACTGTGGAGGCTTGCTG
GCCACTTTTTGGGGTCAGCTCCTCTGAAATGCATTAGCGGAACCGTTTGCGATCTGCCAC
AAGTGTGATAAGTTATCTACACTGGCGAGGGGATTGCTCTCTGTAATGTTCAGCTTCTAA
TTGTCTCTACTTTGTGAGACTACTTTTGAATGCTTGACCTCAAATCAGGTAGGACTACCC
GCTGAACTTAA
>A20
TTTCCGTAGGTGAACCTGCGGAAGGATCATTATTGAATTATGTTTCTAGATAGGTTGTAG
CTGGCTCTTTTAGAGCATGTGCACGCCTGTTTGGACTTCATTTTCATCCACCTGTGCACC
TATTGTAGTCTTTGGTTGGGTTAGGAGGAAGTGATCATTGTATCAGCATCTGCTGGGAGT
GAGGACTTGCATTGTGAAAGCTTTGCTGTCCTTGATGTGATCATGGAATCTTTTTCTCAC
TAGAGTCTATGTCACTCATTATACTCTGTCGAATGTCATTGAATGTCTTTACATGGGCTT
GTATGCCTATGAAAATTGTAATACAACTTTCAGCAACGGATCTCTTGGCTCTCGCATCGA
TGAAGAACGCAGCGAAATGCGATAAGTAATGTGAATTGCAGAATTCAGTGAATCATCGAA
TCTTTGAACGCATCTTGCGCTCCTTGGTATTCCGAGGAGCATGCCTGTTTGAGTGTCATT
AAATTCTCAACTCTCTTATACTTTTTTGTAAAAGAGAGCTTGGACTGTGGAGGCTTGCTG
GCCACTTTTTGGGGTCAGCTCCTCTGAAATGCATTAGCGGAACCGTTTGCGATCTGCCAC
AAGTGTGATAAGTTATCTACACTGGCGAGGGGATTGCTCTCTGTAATGTTCAGCTTCTAA
TTGTCTCTACTTTGTGAGACTACTTTTGAATGCTTGACCTCAAATCAGGTAGGACTACCC
GCTGAACTTAA
>A21
TTTCCGTAGGTGAACCTGCGGAAGGATCATTATTGAATTATGTTTCTAGATAGGTTGTAG
CTGGCTCTTTTAGAGCATGTGCACGCCTGTTTGGACTTCATTTTCATCCACCTGTGCACC
TATTGTAGTCTTTGGTTGGGTTAGGAGGAAGTGATCATTGTATCAGCATCTGCTGGGAGT
GAGGACTTGCATTGTGAAAGCTTTGCTGTCCTTGATGTGATCATGGAATCTTTTTCTCAC
TAGAGTCTATGTCACTCATTATACTCTGTCGAATGTCATTGAATGTCTTTACATGGGCTT
GTATGCCTATGAAAATTGTAATACAACTTTCAGCAACGGATCTCTTGGCTCTCGCATCGA
TGAAGAACGCAGCGAAATGCGATAAGTAATGTGAATTGCAGAATTCAGTGAATCATCGAA
TCTTTGAACGCATCTTGCGCTCCTTGGTATTCCGAGGAGCATGCCTGTTTGAGTGTCATT
AAATTCTCAACTCTCTTATACTTTTTTGTAAAAGAGAGCTTGGACTGTGGAGGCTTGCTG
GCCACTTTTTGGGGTCAGCTCCTCTGAAATGCATTAGCGGAACCGTTTGCGATCTGCCAC
AAGTGTGATAAGTTATCTACACTGGCGAGGGGATTGCTCTCTGTAATGTTCAGCTTCTAA
TTGTCTCTACTTTGTGAGACTACTTTTGAATGCTTGACCTCAAATCAGGTAGGACTACCC
GCTGAACTTAA
>A22
TTTCCGTAGGTGAACCTGCGGAAGGATCATTATTGAATTATGTTTCTAGATAGGTTGTAG
CTGGCTCTTTTAGAGCATGTGCACGCCTGTTTGGACTTCATTTTCATCCACCTGTGCACC
TATTGTAGTCTTTGGTTGGGTTAGGAGGAAGTGATCATTGTATCAGCATCTGCTGGGAGT
GAGGACTTGCATTGTGAAAGCTTTGCTGTCCTTGATGTGATCATGGAATCTTTTTCTCAC
TAGAGTCTATGTCACTCATTATACTCTGTCGAATGTCATTGAATGTCTTTACATGGGCTT

GTATGCCTATGAAAATTGTAATACAACTTTCAGCAACGGATCTCTTGGCTCTCGCATCGA
TGAAGAACGCAGCGAAATGCGATAAGTAATGTGAATTGCAGAATTCAGTGAATCATCGAA
TCTTTGAACGCATCTTGCGCTCCTTGGTATTCCGAGGAGCATGCCTGTTTGAGTGTCATT
AAATTCTCAACTCTCTTATACTTTTTTGTAAAAGAGAGCTTGGACTGTGGAGGCTTGCTG
GCCACTTTTTGGGGTCAGCTCCTCTGAAATGCATTAGCGGAACCGTTTGCGATCTGCCAC
AAGTGTGATAAGTTATCTACACTGGCGAGGGGATTGCTCTCTGTAATGTTCAGCTTCTAA
TTGTCTCTACTTTGTGAGACTACTTTTGAATGCTTGACCTCAAATCAGGTAGGACTACCC
GCTGAACTTAA
>A23
TTTCCGTAGGTGAACCTGCGGAAGGATCATTATTGAATTATGTTTCTAGATAGGTTGTAG
CTGGCTCTTTTAGAGCATGTGCACGCCTGTTTGGACTTCATTTTCATCCACCTGTGCACC
TATTGTAGTCTTTGGTTGGGTTAGGAGGAAGTGATCATTGTATCAGCATCTGCTGGGAGT
GAGGACTTGCATTGTGAAAGCTTTGCTGTCCTTGATGTGATCATGGAATCTTTTTCTCAC
TAGAGTCTATGTCACTCATTATACTCTGTCGAATGTCATTGAATGTCTTTACATGGGCTT
GTATGCCTATGAAAATTGTAATACAACTTTCAGCAACGGATCTCTTGGCTCTCGCATCGA
TGAAGAACGCAGCGAAATGCGATAAGTAATGTGAATTGCAGAATTCAGTGAATCATCGAA
TCTTTGAACGCATCTTGCGCTCCTTGGTATTCCGAGGAGCATGCCTGTTTGAGTGTCATT
AAATTCTCAACTCTCTTATACTTTTTTGTAAAAGAGAGCTTGGACTGTGGAGGCTTGCTG
GCCACTTTTTGGGGTCAGCTCCTCTGAAATGCATTAGCGGAACCGTTTGCGATCTGCCAC
AAGTGTGATAAGTTATCTACACTGGCGAGGGGATTGCTCTCTGTAATGTTCAGCTTCTAA
TTGTCTCTACTTTGTGAGACTACTTTTGAATGCTTGACCTCAAATCAGGTAGGACTACCC
GCTGAACTTAA
>A24
TTTCCGTAGGTGAACCTGCGGAAGGATCATTATTGAATTATGTTTCTAGATAGGTTGTAG
CTGGCTCTTTTAGAGCATGTGCACGCCTGTTTGGACTTCATTTTCATCCACCTGTGCACC
TATTGTAGTCTTTGGTTGGGTTAGGAGGAAGTGATCATTGTATCAGCATCTGCTGGGAGT
GAGGACTTGCATTGTGAAAGCTTTGCTGTCCTTGATGTGATCATGGAATCTTTTTCTCAC
TAGAGTCTATGTCACTCATTATACTCTGTCGAATGTCATTGAATGTCTTTACATGGGCTT
GTATGCCTATGAAAATTGTAATACAACTTTCAGCAACGGATCTCTTGGCTCTCGCATCGA
TGAAGAACGCAGCGAAATGCGATAAGTAATGTGAATTGCAGAATTCAGTGAATCATCGAA
TCTTTGAACGCATCTTGCGCTCCTTGGTATTCCGAGGAGCATGCCTGTTTGAGTGTCATT
AAATTCTCAACTCTCTTATACTTTTTTGTAAAAGAGAGCTTGGACTGTGGAGGCTTGCTG
GCCACTTTTTGGGGTCAGCTCCTCTGAAATGCATTAGCGGAACCGTTTGCGATCTGCCAC
AAGTGTGATAAGTTATCTACACTGGCGAGGGGATTGCTCTCTGTAATGTTCAGCTTCTAA
TTGTCTCTACTTTGTGAGACTACTTTTGAATGCTTGACCTCAAATCAGGTAGGACTACCC
GCTGAACTTAA
>A25
TTTCCGTAGGTGAACCTGCGGAAGGATCATTATTGAATTATGTTTCTAGATAGGTTGTAG
CTGGCTCTTTTAGAGCATGTGCACGCCTGTTTGGACTTCATTTTCATCCACCTGTGCACC
TATTGTAGTCTTTGGTTGGGTTAGGAGGAAGTGATCATTGTATCAGCATCTGCTGGGAGT
GAGGACTTGCATTGTGAAAGCTTTGCTGTCCTTGATGTGATCATGGAATCTTTTTCTCAC
TAGAGTCTATGTCACTCATTATACTCTGTCGAATGTCATTGAATGTCTTTACATGGGCTT
GTATGCCTATGAAAATTGTAATACAACTTTCAGCAACGGATCTCTTGGCTCTCGCATCGA
TGAAGAACGCAGCGAAATGCGATAAGTAATGTGAATTGCAGAATTCAGTGAATCATCGAA
TCTTTGAACGCATCTTGCGCTCCTTGGTATTCCGAGGAGCATGCCTGTTTGAGTGTCATT
AAATTCTCAACTCTCTTATACTTTTTTGTAAAAGAGAGCTTGGACTGTGGAGGCTTGCTG
GCCACTTTTTGGGGTCAGCTCCTCTGAAATGCATTAGCGGAACCGTTTGCGATCTGCCAC
AAGTGTGATAAGTTATCTACACTGGCGAGGGGATTGCTCTCTGTAATGTTCAGCTTCTAA
TTGTCTCTACTTTGTGAGACTACTTTTGAATGCTTGACCTCAAATCAGGTAGGACTACCC
GCTGAACTTAA

>A26
TTTCCGTAGGTGAACCTGCGGAAGGATCATTATTGAATTATGTTTCTAGATAGGTTGTAG
CTGGCTCTTTTAGAGCATGTGCACGCCTGTTTGGACTTCATTTTCATCCACCTGTGCACC
TATTGTAGTCTTTGGTTGGGTTAGGAGGAAGTGATCATTGTATCAGCATCTGCTGGGAGT
GAGGACTTGCATTGTGAAAGCTTTGCTGTCCTTGATGTGATCATGGAATCTTTTTCTCAC
TAGAGTCTATGTCACTCATTATACTCTGTCGAATGTCATTGAATGTCTTTACATGGGCTT
GTATGCCTATGAAAATTGTAATACAACTTTCAGCAACGGATCTCTTGGCTCTCGCATCGA
TGAAGAACGCAGCGAAATGCGATAAGTAATGTGAATTGCAGAATTCAGTGAATCATCGAA
TCTTTGAACGCATCTTGCGCTCCTTGGTATTCCGAGGAGCATGCCTGTTTGAGTGTCATT
AAATTCTCAACTCTCTTATACTTTTTTGTAAAAGAGAGCTTGGACTGTGGAGGCTTGCTG
GCCACTTTTTGGGGTCAGCTCCTCTGAAATGCATTAGCGGAACCGTTTGCGATCTGCCAC
AAGTGTGATAAGTTATCTACACTGGCGAGGGGATTGCTCTCTGTAATGTTCAGCTTCTAA
TTGTCTCTACTTTGTGAGACTACTTTTGAATGCTTGACCTCAAATCAGGTAGGACTACCC
GCTGAACTTAA
>A27
TTTCCGTAGGTGAACCTGCGGAAGGATCATTATTGAATTATGTTTCTAGATAGGTTGTAG
CTGGCTCTTTTAGAGCATGTGCACGCCTGTTTGGACTTCATTTTCATCCACCTGTGCACC
TATTGTAGTCTTTGGTTGGGTTAGGAGGAAGTGATCATTGTATCAGCATCTGCTGGGAGT
GAGGACTTGCATTGTGAAAGCTTTGCTGTCCTTGATGTGATCATGGAATCTTTTTCTCAC
TAGAGTCTATGTCACTCATTATACTCTGTCGAATGTCATTGAATGTCTTTACATGGGCTT
GTATGCCTATGAAAATTGTAATACAACTTTCAGCAACGGATCTCTTGGCTCTCGCATCGA
TGAAGAACGCAGCGAAATGCGATAAGTAATGTGAATTGCAGAATTCAGTGAATCATCGAA
TCTTTGAACGCATCTTGCGCTCCTTGGTATTCCGAGGAGCATGCCTGTTTGAGTGTCATT
AAATTCTCAACTCTCTTATACTTTTTTGTAAAAGAGAGCTTGGACTGTGGAGGCTTGCTG
GCCACTTTTTGGGGTCAGCTCCTCTGAAATGCATTAGCGGAACCGTTTGCGATCTGCCAC
AAGTGTGATAAGTTATCTACACTGGCGAGGGGATTGCTCTCTGTAATGTTCAGCTTCTAA
TTGTCTCTACTTTGTGAGACTACTTTTGAATGCTTGACCTCAAATCAGGTAGGACTACCC
GCTGAACTTAA
>A28
TTTCCGTAGGTGAACCTGCGGAAGGATCATTATTGAATTATGTTTCTAGATAGGTTGTAG
CTGGCTCTTTTAGAGCATGTGCACGCCTGTTTGGACTTCATTTTCATCCACCTGTGCACC
TATTGTAGTCTTTGGTTGGGTTAGGAGGAAGTGATCATTGTATCAGCATCTGCTGGGAGT
GAGGACTTGCATTGTGAAAGCTTTGCTGTCCTTGATGTGATCATGGAATCTTTTTCTCAC
TAGAGTCTATGTCACTCATTATACTCTGTCGAATGTCATTGAATGTCTTTACATGGGCTT
GTATGCCTATGAAAATTGTAATACAACTTTCAGCAACGGATCTCTTGGCTCTCGCATCGA
TGAAGAACGCAGCGAAATGCGATAAGTAATGTGAATTGCAGAATTCAGTGAATCATCGAA
TCTTTGAACGCATCTTGCGCTCCTTGGTATTCCGAGGAGCATGCCTGTTTGAGTGTCATT
AAATTCTCAACTCTCTTATACTTTTTTGTAAAAGAGAGCTTGGACTGTGGAGGCTTGCTG
GCCACTTTTTGGGGTCAGCTCCTCTGAAATGCATTAGCGGAACCGTTTGCGATCTGCCAC
AAGTGTGATAAGTTATCTACACTGGCGAGGGGATTGCTCTCTGTAATGTTCAGCTTCTAA
TTGTCTCTACTTTGTGAGACTACTTTTGAATGCTTGACCTCAAATCAGGTAGGACTACCC
GCTGAACTTAA
>A29
TTTCCGTAGGTGAACCTGCGGAAGGATCATTATTGAATTATGTTTCTAGATAGGTTGTAG
CTGGCTCTTTTAGAGCATGTGCACGCCTGTTTGGACTTCATTTTCATCCACCTGTGCACC
TATTGTAGTCTTTGGTTGGGTTAGGAGGAAGTGATCATTGTATCAGCATCTGCTGGGAGT
GAGGACTTGCATTGTGAAAGCTTTGCTGTCCTTGATGTGATCATGGAATCTTTTTCTCAC
TAGAGTCTATGTCACTCATTATACTCTGTCGAATGTCATTGAATGTCTTTACATGGGCTT
GTATGCCTATGAAAATTGTAATACAACTTTCAGCAACGGATCTCTTGGCTCTCGCATCGA
TGAAGAACGCAGCGAAATGCGATAAGTAATGTGAATTGCAGAATTCAGTGAATCATCGAA

TCTTTGAACGCATCTTGCGCTCCTTGGTATTCCGAGGAGCATGCCTGTTTGAGTGTCATT
AAATTCTCAACTCTCTTATACTTTTTTGTAAAAGAGAGCTTGGACTGTGGAGGCTTGCTG
GCCACTTTTTGGGGTCAGCTCCTCTGAAATGCATTAGCGGAACCGTTTGCGATCTGCCAC
AAGTGTGATAAGTTATCTACACTGGCGAGGGGATTGCTCTCTGTAATGTTCAGCTTCTAA
TTGTCTCTACTTTGTGAGACTACTTTTGAATGCTTGACCTCAAATCAGGTAGGACTACCC
GCTGAACTTAA
>A30
TTTCCGTAGGTGAACCTGCGGAAGGATCATTATTGAATTATGTTTCTAGATAGGTTGTAG
CTGGCTCTTTTAGAGCATGTGCACGCCTGTTTGGACTTCATTTTCATCCACCTGTGCACC
TATTGTAGTCTTTGGTTGGGTTAGGAGGAAGTGATCATTGTATCAGCATCTGCTGGGAGT
GAGGACTTGCATTGTGAAAGCTTTGCTGTCCTTGATGTGATCATGGAATCTTTTTCTCAC
TAGAGTCTATGTCACTCATTATACTCTGTCGAATGTCATTGAATGTCTTTACATGGGCTT
GTATGCCTATGAAAATTGTAATACAACTTTCAGCAACGGATCTCTTGGCTCTCGCATCGA
TGAAGAACGCAGCGAAATGCGATAAGTAATGTGAATTGCAGAATTCAGTGAATCATCGAA
TCTTTGAACGCATCTTGCGCTCCTTGGTATTCCGAGGAGCATGCCTGTTTGAGTGTCATT
AAATTCTCAACTCTCTTATACTTTTTTGTAAAAGAGAGCTTGGACTGTGGAGGCTTGCTG
GCCACTTTTTGGGGTCAGCTCCTCTGAAATGCATTAGCGGAACCGTTTGCGATCTGCCAC
AAGTGTGATAAGTTATCTACACTGGCGAGGGGATTGCTCTCTGTAATGTTCAGCTTCTAA
TTGTCTCTACTTTGTGAGACTACTTTTGAATGCTTGACCTCAAATCAGGTAGGACTACCC
GCTGAACTTAA
>A31
TTTCCGTAGGTGAACCTGCGGAAGGATCATTATTGAATTATGTTTCTAGATAGGTTGTAG
CTGGCTCTTTTAGAGCATGTGCACGCCTGTTTGGACTTCATTTTCATCCACCTGTGCACC
TATTGTAGTCTTTGGTTGGGTTAGGAGGAAGTGATCATTGTATCAGCATCTGCTGGGAGT
GAGGACTTGCATTGTGAAAGCTTTGCTGTCCTTGATGTGATCATGGAATCTTTTTCTCAC
TAGAGTCTATGTCACTCATTATACTCTGTCGAATGTCATTGAATGTCTTTACATGGGCTT
GTATGCCTATGAAAATTGTAATACAACTTTCAGCAACGGATCTCTTGGCTCTCGCATCGA
TGAAGAACGCAGCGAAATGCGATAAGTAATGTGAATTGCAGAATTCAGTGAATCATCGAA
TCTTTGAACGCATCTTGCGCTCCTTGGTATTCCGAGGAGCATGCCTGTTTGAGTGTCATT
AAATTCTCAACTCTCTTATACTTTTTTGTAAAAGAGAGCTTGGACTGTGGAGGCTTGCTG
GCCACTTTTTGGGGTCAGCTCCTCTGAAATGCATTAGCGGAACCGTTTGCGATCTGCCAC
AAGTGTGATAAGTTATCTACACTGGCGAGGGGATTGCTCTCTGTAATGTTCAGCTTCTAA
TTGTCTCTACTTTGTGAGACTACTTTTGAATGCTTGACCTCAAATCAGGTAGGACTACCC
GCTGAACTTAA
>A32
TTTCCGTAGGTGAACCTGCGGAAGGATCATTATTGAATTATGTTTCTAGATAGGTTGTAG
CTGGCTCTTTTAGAGCATGTGCACGCCTGTTTGGACTTCATTTTCATCCACCTGTGCACC
TATTGTAGTCTTTGGTTGGGTTAGGAGGAAGTGATCATTGTATCAGCATCTGCTGGGAGT
GAGGACTTGCATTGTGAAAGCTTTGCTGTCCTTGATGTGATCATGGAATCTTTTTCTCAC
TAGAGTCTATGTCACTCATTATACTCTGTCGAATGTCATTGAATGTCTTTACATGGGCTT
GTATGCCTATGAAAATTGTAATACAACTTTCAGCAACGGATCTCTTGGCTCTCGCATCGA
TGAAGAACGCAGCGAAATGCGATAAGTAATGTGAATTGCAGAATTCAGTGAATCATCGAA
TCTTTGAACGCATCTTGCGCTCCTTGGTATTCCGAGGAGCATGCCTGTTTGAGTGTCATT
AAATTCTCAACTCTCTTATACTTTTTTGTAAAAGAGAGCTTGGACTGTGGAGGCTTGCTG
GCCACTTTTTGGGGTCAGCTCCTCTGAAATGCATTAGCGGAACCGTTTGCGATCTGCCAC
AAGTGTGATAAGTTATCTACACTGGCGAGGGGATTGCTCTCTGTAATGTTCAGCTTCTAA
TTGTCTCTACTTTGTGAGACTACTTTTGAATGCTTGACCTCAAATCAGGTAGGACTACCC
GCTGAACTTAA
>A33
TTTCCGTAGGTGAACCTGCGGAAGGATCATTATTGAATTATGTTTCTAGATAGGTTGTAG

CTGGCTCTTTTAGAGCATGTGCACGCCTGTTTGGACTTCATTTTCATCCACCTGTGCACC
TATTGTAGTCTTTGGTTGGGTTAGGAGGAAGTGATCATTGTATCAGCATCTGCTGGGAGT
GAGGACTTGCATTGTGAAAGCTTTGCTGTCCTTGATGTGATCATGGAATCTTTTTCTCAC
TAGAGTCTATGTCACTCATTATACTCTGTCGAATGTCATTGAATGTCTTTACATGGGCTT
GTATGCCTATGAAAATTGTAATACAACTTTCAGCAACGGATCTCTTGGCTCTCGCATCGA
TGAAGAACGCAGCGAAATGCGATAAGTAATGTGAATTGCAGAATTCAGTGAATCATCGAA
TCTTTGAACGCATCTTGCGCTCCTTGGTATTCCGAGGAGCATGCCTGTTTGAGTGTCATT
AAATTCTCAACTCTCTTATACTTTTTTGTAAAAGAGAGCTTGGACTGTGGAGGCTTGCTG
GCCACTTTTTGGGGTCAGCTCCTCTGAAATGCATTAGCGGAACCGTTTGCGATCTGCCAC
AAGTGTGATAAGTTATCTACACTGGCGAGGGGATTGCTCTCTGTAATGTTCAGCTTCTAA
TTGTCTCTACTTTGTGAGACTACTTTTGAATGCTTGACCTCAAATCAGGTAGGACTACCC
GCTGAACTTAA
>A34
TTTCCGTAGGTGAACCTGCGGAAGGATCATTATTGAATTATGTTTCTAGATAGGTTGTAG
CTGGCTCTTTTAGAGCATGTGCACGCCTGTTTGGACTTCATTTTCATCCACCTGTGCACC
TATTGTAGTCTTTGGTTGGGTTAGGAGGAAGTGATCATTGTATCAGCATCTGCTGGGAGT
GAGGACTTGCATTGTGAAAGCTTTGCTGTCCTTGATGTGATCATGGAATCTTTTTCTCAC
TAGAGTCTATGTCACTCATTATACTCTGTCGAATGTCATTGAATGTCTTTACATGGGCTT
GTATGCCTATGAAAATTGTAATACAACTTTCAGCAACGGATCTCTTGGCTCTCGCATCGA
TGAAGAACGCAGCGAAATGCGATAAGTAATGTGAATTGCAGAATTCAGTGAATCATCGAA
TCTTTGAACGCATCTTGCGCTCCTTGGTATTCCGAGGAGCATGCCTGTTTGAGTGTCATT
AAATTCTCAACTCTCTTATACTTTTTTGTAAAAGAGAGCTTGGACTGTGGAGGCTTGCTG
GCCACTTTTTGGGGTCAGCTCCTCTGAAATGCATTAGCGGAACCGTTTGCGATCTGCCAC
AAGTGTGATAAGTTATCTACACTGGCGAGGGGATTGCTCTCTGTAATGTTCAGCTTCTAA
TTGTCTCTACTTTGTGAGACTACTTTTGAATGCTTGACCTCAAATCAGGTAGGACTACCC
GCTGAACTTAA
>A35
TTTCCGTAGGTGAACCTGCGGAAGGATCATTATTGAATTATGTTTCTAGATAGGTTGTAG
CTGGCTCTTTTAGAGCATGTGCACGCCTGTTTGGACTTCATTTTCATCCACCTGTGCACC
TATTGTAGTCTTTGGTTGGGTTAGGAGGAAGTGATCATTGTATCAGCATCTGCTGGGAGT
GAGGACTTGCATTGTGAAAGCTTTGCTGTCCTTGATGTGATCATGGAATCTTTTTCTCAC
TAGAGTCTATGTCACTCATTATACTCTGTCGAATGTCATTGAATGTCTTTACATGGGCTT
GTATGCCTATGAAAATTGTAATACAACTTTCAGCAACGGATCTCTTGGCTCTCGCATCGA
TGAAGAACGCAGCGAAATGCGATAAGTAATGTGAATTGCAGAATTCAGTGAATCATCGAA
TCTTTGAACGCATCTTGCGCTCCTTGGTATTCCGAGGAGCATGCCTGTTTGAGTGTCATT
AAATTCTCAACTCTCTTATACTTTTTTGTAAAAGAGAGCTTGGACTGTGGAGGCTTGCTG
GCCACTTTTTGGGGTCAGCTCCTCTGAAATGCATTAGCGGAACCGTTTGCGATCTGCCAC
AAGTGTGATAAGTTATCTACACTGGCGAGGGGATTGCTCTCTGTAATGTTCAGCTTCTAA
TTGTCTCTACTTTGTGAGACTACTTTTGAATGCTTGACCTCAAATCAGGTAGGACTACCC
GCTGAACTTAA
>A36
TTTCCGTAGGTGAACCTGCGGAAGGATCATTATTGAATTATGTTTCTAGATAGGTTGTAG
CTGGCTCTTTTAGAGCATGTGCACGCCTGTTTGGACTTCATTTTCATCCACCTGTGCACC
TATTGTAGTCTTTGGTTGGGTTAGGAGGAAGTGATCATTGTATCAGCATCTGCTGGGAGT
GAGGACTTGCATTGTGAAAGCTTTGCTGTCCTTGATGTGATCATGGAATCTTTTTCTCAC
TAGAGTCTATGTCACTCATTATACTCTGTCGAATGTCATTGAATGTCTTTACATGGGCTT
GTATGCCTATGAAAATTGTAATACAACTTTCAGCAACGGATCTCTTGGCTCTCGCATCGA
TGAAGAACGCAGCGAAATGCGATAAGTAATGTGAATTGCAGAATTCAGTGAATCATCGAA
TCTTTGAACGCATCTTGCGCTCCTTGGTATTCCGAGGAGCATGCCTGTTTGAGTGTCATT
AAATTCTCAACTCTCTTATACTTTTTTGTAAAAGAGAGCTTGGACTGTGGAGGCTTGCTG

GCCACTTTTTGGGGTCAGCTCCTCTGAAATGCATTAGCGGAACCGTTTGCGATCTGCCAC
AAGTGTGATAAGTTATCTACACTGGCGAGGGGATTGCTCTCTGTAATGTTCAGCTTCTAA
TTGTCTCTACTTTGTGAGACTACTTTTGAATGCTTGACCTCAAATCAGGTAGGACTACCC
GCTGAACTTAA
>A37
TTTCCGTAGGTGAACCTGCGGAAGGATCATTATTGAATTATGTTTCTAGATAGGTTGTAG
CTGGCTCTTTTAGAGCATGTGCACGCCTGTTTGGACTTCATTTTCATCCACCTGTGCACC
TATTGTAGTCTTTGGTTGGGTTAGGAGGAAGTGATCATTGTATCAGCATCTGCTGGGAGT
GAGGACTTGCATTGTGAAAGCTTTGCTGTCCTTGATGTGATCATGGAATCTTTTTCTCAC
TAGAGTCTATGTCACTCATTATACTCTGTCGAATGTCATTGAATGTCTTTACATGGGCTT
GTATGCCTATGAAAATTGTAATACAACTTTCAGCAACGGATCTCTTGGCTCTCGCATCGA
TGAAGAACGCAGCGAAATGCGATAAGTAATGTGAATTGCAGAATTCAGTGAATCATCGAA
TCTTTGAACGCATCTTGCGCTCCTTGGTATTCCGAGGAGCATGCCTGTTTGAGTGTCATT
AAATTCTCAACTCTCTTATACTTTTTGTAAAAGAGAGCTTGGACTGTGGAGGCTTGCTG
GCCACTTTTTGGGGTCAGCTCCTCTGAAATGCATTAGCGGAACCGTTTGCGATCTGCCAC
AAGTGTGATAAGTTATCTACACTGGCGAGGGGATTGCTCTCTGTAATGTTCAGCTTCTAA
TTGTCTCTACTTTGTGAGACTACTTTTGAATGCTTGACCTCAAATCAGGTAGGACTACCC
GCTGAACTTAA
>A38
TTTCCGTAGGTGAACCTGCGGAAGGATCATTATTGAATTATGTTTCTAGATAGGTTGTAG
CTGGCTCTTTTAGAGCATGTGCACGCCTGTTTGGACTTCATTTTCATCCACCTGTGCACC
TATTGTAGTCTTTGGTTGGGTTAGGAGGAAGTGATCATTGTATCAGCATCTGCTGGGAGT
GAGGACTTGCATTGTGAAAGCTTTGCTGTCCTTGATGTGATCATGGAATCTTTTTCTCAC
TAGAGTCTATGTCACTCATTATACTCTGTCGAATGTCATTGAATGTCTTTACATGGGCTT
GTATGCCTATGAAAATTGTAATACAACTTTCAGCAACGGATCTCTTGGCTCTCGCATCGA
TGAAGAACGCAGCGAAATGCGATAAGTAATGTGAATTGCAGAATTCAGTGAATCATCGAA
TCTTTGAACGCATCTTGCGCTCCTTGGTATTCCGAGGAGCATGCCTGTTTGAGTGTCATT
AAATTCTCAACTCTCTTATACTTTTTGTAAAAGAGAGCTTGGACTGTGGAGGCTTGCTG
GCCACTTTTTGGGGTCAGCTCCTCTGAAATGCATTAGCGGAACCGTTTGCGATCTGCCAC
AAGTGTGATAAGTTATCTACACTGGCGAGGGGATTGCTCTCTGTAATGTTCAGCTTCTAA
TTGTCTCTACTTTGTGAGACTACTTTTGAATGCTTGACCTCAAATCAGGTAGGACTACCC
GCTGAACTTAA
>A39
TTTCCGTAGGTGAACCTGCGGAAGGATCATTATTGAATTATGTTTCTAGATAGGTTGTAG
CTGGCTCTTTTAGAGCATGTGCACGCCTGTTTGGACTTCATTTTCATCCACCTGTGCACC
TATTGTAGTCTTTGGTTGGGTTAGGAGGAAGTGATCATTGTATCAGCATCTGCTGGGAGT
GAGGACTTGCATTGTGAAAGCTTTGCTGTCCTTGATGTGATCATGGAATCTTTTTCTCAC
TAGAGTCTATGTCACTCATTATACTCTGTCGAATGTCATTGAATGTCTTTACATGGGCTT
GTATGCCTATGAAAATTGTAATACAACTTTCAGCAACGGATCTCTTGGCTCTCGCATCGA
TGAAGAACGCAGCGAAATGCGATAAGTAATGTGAATTGCAGAATTCAGTGAATCATCGAA
TCTTTGAACGCATCTTGCGCTCCTTGGTATTCCGAGGAGCATGCCTGTTTGAGTGTCATT
AAATTCTCAACTCTCTTATACTTTTTGTAAAAGAGAGCTTGGACTGTGGAGGCTTGCTG
GCCACTTTTTGGGGTCAGCTCCTCTGAAATGCATTAGCGGAACCGTTTGCGATCTGCCAC
AAGTGTGATAAGTTATCTACACTGGCGAGGGGATTGCTCTCTGTAATGTTCAGCTTCTAA
TTGTCTCTACTTTGTGAGACTACTTTTGAATGCTTGACCTCAAATCAGGTAGGACTACCC
GCTGAACTTAA
>A40
TTTCCGTAGGTGAACCTGCGGAAGGATCATTATTGAATTATGTTTCTAGATAGGTTGTAG
CTGGCTCTTTTAGAGCATGTGCACGCCTGTTTGGACTTCATTTTCATCCACCTGTGCACC
TATTGTAGTCTTTGGTTGGGTTAGGAGGAAGTGATCATTGTATCAGCATCTGCTGGGAGT

GAGGACTTGCATTGTGAAAGCTTTGCTGTCCTTGATGTGATCATGGAATCTTTTTCTCAC
TAGAGTCTATGTCACTCATTATACTCTGTCGAATGTCATTGAATGTCTTTACATGGGCTT
GTATGCCTATGAAAATTGTAATACAACTTTCAGCAACGGATCTCTTGGCTCTCGCATCGA
TGAAGAACGCAGCGAAATGCGATAAGTAATGTGAATTGCAGAATTCAGTGAATCATCGAA
TCTTTGAACGCATCTTGCGCTCCTTGGTATTCCGAGGAGCATGCCTGTTTGAGTGTCATT
AAATTCTCAACTCTCTTATACTTTTTTGTAAAAGAGAGCTTGGACTGTGGAGGCTTGCTG
GCCACTTTTTGGGGTCAGCTCCTCTGAAATGCATTAGCGGAACCGTTTGCGATCTGCCAC
AAGTGTGATAAGTTATCTACACTGGCGAGGGGATTGCTCTCTGTAATGTTCAGCTTCTAA
TTGTCTCTACTTTGTGAGACTACTTTTGAATGCTTGACCTCAAATCAGGTAGGACTACCC
GCTGAACTTAA
>A41
TTTCCGTAGGTGAACCTGCGGAAGGATCATTATTGAATTATGTTTCTAGATAGGTTGTAG
CTGGCTCTTTTAGAGCATGTGCACGCCTGTTTGGACTTCATTTTCATCCACCTGTGCACC
TATTGTAGTCTTTGGTTGGGTTAGGAGGAAGTGATCATTGTATCAGCATCTGCTGGGAGT
GAGGACTTGCATTGTGAAAGCTTTGCTGTCCTTGATGTGATCATGGAATCTTTTTCTCAC
TAGAGTCTATGTCACTCATTATACTCTGTCGAATGTCATTGAATGTCTTTACATGGGCTT
GTATGCCTATGAAAATTGTAATACAACTTTCAGCAACGGATCTCTTGGCTCTCGCATCGA
TGAAGAACGCAGCGAAATGCGATAAGTAATGTGAATTGCAGAATTCAGTGAATCATCGAA
TCTTTGAACGCATCTTGCGCTCCTTGGTATTCCGAGGAGCATGCCTGTTTGAGTGTCATT
AAATTCTCAACTCTCTTATACTTTTTTGTAAAAGAGAGCTTGGACTGTGGAGGCTTGCTG
GCCACTTTTTGGGGTCAGCTCCTCTGAAATGCATTAGCGGAACCGTTTGCGATCTGCCAC
AAGTGTGATAAGTTATCTACACTGGCGAGGGGATTGCTCTCTGTAATGTTCAGCTTCTAA
TTGTCTCTACTTTGTGAGACTACTTTTGAATGCTTGACCTCAAATCAGGTAGGACTACCC
GCTGAACTTAA
>A42
TTTCCGTAGGTGAACCTGCGGAAGGATCATTATTGAATTATGTTTCTAGATAGGTTGTAG
CTGGCTCTTTTAGAGCATGTGCACGCCTGTTTGGACTTCATTTTCATCCACCTGTGCACC
TATTGTAGTCTTTGGTTGGGTTAGGAGGAAGTGATCATTGTATCAGCATCTGCTGGGAGT
GAGGACTTGCATTGTGAAAGCTTTGCTGTCCTTGATGTGATCATGGAATCTTTTTCTCAC
TAGAGTCTATGTCACTCATTATACTCTGTCGAATGTCATTGAATGTCTTTACATGGGCTT
GTATGCCTATGAAAATTGTAATACAACTTTCAGCAACGGATCTCTTGGCTCTCGCATCGA
TGAAGAACGCAGCGAAATGCGATAAGTAATGTGAATTGCAGAATTCAGTGAATCATCGAA
TCTTTGAACGCATCTTGCGCTCCTTGGTATTCCGAGGAGCATGCCTGTTTGAGTGTCATT
AAATTCTCAACTCTCTTATACTTTTTTGTAAAAGAGAGCTTGGACTGTGGAGGCTTGCTG
GCCACTTTTTGGGGTCAGCTCCTCTGAAATGCATTAGCGGAACCGTTTGCGATCTGCCAC
AAGTGTGATAAGTTATCTACACTGGCGAGGGGATTGCTCTCTGTAATGTTCAGCTTCTAA
TTGTCTCTACTTTGTGAGACTACTTTTGAATGCTTGACCTCAAATCAGGTAGGACTACCC
GCTGAACTTAA
>A43
TTTCCGTAGGTGAACCTGCGGAAGGATCATTATTGAATTATGTTTCTAGATAGGTTGTAG
CTGGCTCTTTTAGAGCATGTGCACGCCTGTTTGGACTTCATTTTCATCCACCTGTGCACC
TATTGTAGTCTTTGGTTGGGTTAGGAGGAAGTGATCATTGTATCAGCATCTGCTGGGAGT
GAGGACTTGCATTGTGAAAGCTTTGCTGTCCTTGATGTGATCATGGAATCTTTTTCTCAC
TAGAGTCTATGTCACTCATTATACTCTGTCGAATGTCATTGAATGTCTTTACATGGGCTT
GTATGCCTATGAAAATTGTAATACAACTTTCAGCAACGGATCTCTTGGCTCTCGCATCGA
TGAAGAACGCAGCGAAATGCGATAAGTAATGTGAATTGCAGAATTCAGTGAATCATCGAA
TCTTTGAACGCATCTTGCGCTCCTTGGTATTCCGAGGAGCATGCCTGTTTGAGTGTCATT
AAATTCTCAACTCTCTTATACTTTTTTGTAAAAGAGAGCTTGGACTGTGGAGGCTTGCTG
GCCACTTTTTGGGGTCAGCTCCTCTGAAATGCATTAGCGGAACCGTTTGCGATCTGCCAC
AAGTGTGATAAGTTATCTACACTGGCGAGGGGATTGCTCTCTGTAATGTTCAGCTTCTAA

TTGTCTCTACTTTGTGAGACTACTTTTGAATGCTTGACCTCAAATCAGGTAGGACTACCC
GCTGAACTTAA
>A44
TTTCCGTAGGTGAACCTGCGGAAGGATCATTATTGAATTATGTTTCTAGATAGGTTGTAG
CTGGCTCTTTTAGAGCATGTGCACGCCTGTTTGGACTTCATTTTCATCCACCTGTGCACC
TATTGTAGTCTTTGGTTGGGTTAGGAGGAAGTGATCATTGTATCAGCATCTGCTGGGAGT
GAGGACTTGCATTGTGAAAGCTTTGCTGTCCTTGATGTGATCATGGAATCTTTTTCTCAC
TAGAGTCTATGTCACTCATTATACTCTGTCGAATGTCATTGAATGTCTTTACATGGGCTT
GTATGCCTATGAAAATTGTAATACAACTTTCAGCAACGGATCTCTTGGCTCTCGCATCGA
TGAAGAACGCAGCGAAATGCGATAAGTAATGTGAATTGCAGAATTCAGTGAATCATCGAA
TCTTTGAACGCATCTTGCGCTCCTTGGTATTCCGAGGAGCATGCCTGTTTGAGTGTCATT
AAATTCTCAACTCTCTTATACTTTTTTGTAAAAGAGAGCTTGGACTGTGGAGGCTTGCTG
GCCACTTTTTGGGGTCAGCTCCTCTGAAATGCATTAGCGGAACCGTTTGCGATCTGCCAC
AAGTGTGATAAGTTATCTACACTGGCGAGGGGATTGCTCTCTGTAATGTTCAGCTTCTAA
TTGTCTCTACTTTGTGAGACTACTTTTGAATGCTTGACCTCAAATCAGGTAGGACTACCC
GCTGAACTTAA
>A45
TTTCCGTAGGTGAACCTGCGGAAGGATCATTATTGAATTATGTTTCTAGATAGGTTGTAG
CTGGCTCTTTTAGAGCATGTGCACGCCTGTTTGGACTTCATTTTCATCCACCTGTGCACC
TATTGTAGTCTTTGGTTGGGTTAGGAGGAAGTGATCATTGTATCAGCATCTGCTGGGAGT
GAGGACTTGCATTGTGAAAGCTTTGCTGTCCTTGATGTGATCATGGAATCTTTTTCTCAC
TAGAGTCTATGTCACTCATTATACTCTGTCGAATGTCATTGAATGTCTTTACATGGGCTT
GTATGCCTATGAAAATTGTAATACAACTTTCAGCAACGGATCTCTTGGCTCTCGCATCGA
TGAAGAACGCAGCGAAATGCGATAAGTAATGTGAATTGCAGAATTCAGTGAATCATCGAA
TCTTTGAACGCATCTTGCGCTCCTTGGTATTCCGAGGAGCATGCCTGTTTGAGTGTCATT
AAATTCTCAACTCTCTTATACTTTTTTGTAAAAGAGAGCTTGGACTGTGGAGGCTTGCTG
GCCACTTTTTGGGGTCAGCTCCTCTGAAATGCATTAGCGGAACCGTTTGCGATCTGCCAC
AAGTGTGATAAGTTATCTACACTGGCGAGGGGATTGCTCTCTGTAATGTTCAGCTTCTAA
TTGTCTCTACTTTGTGAGACTACTTTTGAATGCTTGACCTCAAATCAGGTAGGACTACCC
GCTGAACTTAA
>A46
TTTCCGTAGGTGAACCTGCGGAAGGATCATTATTGAATTATGTTTCTAGATAGGTTGTAG
CTGGCTCTTTTAGAGCATGTGCACGCCTGTTTGGACTTCATTTTCATCCACCTGTGCACC
TATTGTAGTCTTTGGTTGGGTTAGGAGGAAGTGATCATTGTATCAGCATCTGCTGGGAGT
GAGGACTTGCATTGTGAAAGCTTTGCTGTCCTTGATGTGATCATGGAATCTTTTTCTCAC
TAGAGTCTATGTCACTCATTATACTCTGTCGAATGTCATTGAATGTCTTTACATGGGCTT
GTATGCCTATGAAAATTGTAATACAACTTTCAGCAACGGATCTCTTGGCTCTCGCATCGA
TGAAGAACGCAGCGAAATGCGATAAGTAATGTGAATTGCAGAATTCAGTGAATCATCGAA
TCTTTGAACGCATCTTGCGCTCCTTGGTATTCCGAGGAGCATGCCTGTTTGAGTGTCATT
AAATTCTCAACTCTCTTATACTTTTTTGTAAAAGAGAGCTTGGACTGTGGAGGCTTGCTG
GCCACTTTTTGGGGTCAGCTCCTCTGAAATGCATTAGCGGAACCGTTTGCGATCTGCCAC
AAGTGTGATAAGTTATCTACACTGGCGAGGGGATTGCTCTCTGTAATGTTCAGCTTCTAA
TTGTCTCTACTTTGTGAGACTACTTTTGAATGCTTGACCTCAAATCAGGTAGGACTACCC
GCTGAACTTAA
>A47
TTTCCGTAGGTGAACCTGCGGAAGGATCATTATTGAATTATGTTTCTAGATAGGTTGTAG
CTGGCTCTTTTAGAGCATGTGCACGCCTGTTTGGACTTCATTTTCATCCACCTGTGCACC
TATTGTAGTCTTTGGTTGGGTTAGGAGGAAGTGATCATTGTATCAGCATCTGCTGGGAGT
GAGGACTTGCATTGTGAAAGCTTTGCTGTCCTTGATGTGATCATGGAATCTTTTTCTCAC
TAGAGTCTATGTCACTCATTATACTCTGTCGAATGTCATTGAATGTCTTTACATGGGCTT

GTATGCCTATGAAAATTGTAATACAACTTTCAGCAACGGATCTCTTGGCTCTCGCATCGA
TGAAGAACGCAGCGAAATGCGATAAGTAATGTGAATTGCAGAATTCAGTGAATCATCGAA
TCTTTGAACGCATCTTGCGCTCCTTGGTATTCCGAGGAGCATGCCTGTTTGAGTGTCATT
AAATTCTCAACTCTCTTATACTTTTTTGTAAAAGAGAGCTTGGACTGTGGAGGCTTGCTG
GCCACTTTTTGGGGTCAGCTCCTCTGAAATGCATTAGCGGAACCGTTTGCGATCTGCCAC
AAGTGTGATAAGTTATCTACACTGGCGAGGGGATTGCTCTCTGTAATGTTCAGCTTCTAA
TTGTCTCTACTTTGTGAGACTACTTTTGAATGCTTGACCTCAAATCAGGTAGGACTACCC
GCTGAACTTAA
>A48
TTTCCGTAGGTGAACCTGCGGAAGGATCATTATTGAATTATGTTTCTAGATAGGTTGTAG
CTGGCTCTTTTAGAGCATGTGCACGCCTGTTTGGACTTCATTTTCATCCACCTGTGCACC
TATTGTAGTCTTTGGTTGGGTTAGGAGGAAGTGATCATTGTATCAGCATCTGCTGGGAGT
GAGGACTTGCATTGTGAAAGCTTTGCTGTCCTTGATGTGATCATGGAATCTTTTTCTCAC
TAGAGTCTATGTCACTCATTATACTCTGTCGAATGTCATTGAATGTCTTTACATGGGCTT
GTATGCCTATGAAAATTGTAATACAACTTTCAGCAACGGATCTCTTGGCTCTCGCATCGA
TGAAGAACGCAGCGAAATGCGATAAGTAATGTGAATTGCAGAATTCAGTGAATCATCGAA
TCTTTGAACGCATCTTGCGCTCCTTGGTATTCCGAGGAGCATGCCTGTTTGAGTGTCATT
AAATTCTCAACTCTCTTATACTTTTTTGTAAAAGAGAGCTTGGACTGTGGAGGCTTGCTG
GCCACTTTTTGGGGTCAGCTCCTCTGAAATGCATTAGCGGAACCGTTTGCGATCTGCCAC
AAGTGTGATAAGTTATCTACACTGGCGAGGGGATTGCTCTCTGTAATGTTCAGCTTCTAA
TTGTCTCTACTTTGTGAGACTACTTTTGAATGCTTGACCTCAAATCAGGTAGGACTACCC
GCTGAACTTAA
>A49
TTTCCGTAGGTGAACCTGCGGAAGGATCATTATTGAATTATGTTTCTAGATAGGTTGTAG
CTGGCTCTTTTAGAGCATGTGCACGCCTGTTTGGACTTCATTTTCATCCACCTGTGCACC
TATTGTAGTCTTTGGTTGGGTTAGGAGGAAGTGATCATTGTATCAGCATCTGCTGGGAGT
GAGGACTTGCATTGTGAAAGCTTTGCTGTCCTTGATGTGATCATGGAATCTTTTTCTCAC
TAGAGTCTATGTCACTCATTATACTCTGTCGAATGTCATTGAATGTCTTTACATGGGCTT
GTATGCCTATGAAAATTGTAATACAACTTTCAGCAACGGATCTCTTGGCTCTCGCATCGA
TGAAGAACGCAGCGAAATGCGATAAGTAATGTGAATTGCAGAATTCAGTGAATCATCGAA
TCTTTGAACGCATCTTGCGCTCCTTGGTATTCCGAGGAGCATGCCTGTTTGAGTGTCATT
AAATTCTCAACTCTCTTATACTTTTTTGTAAAAGAGAGCTTGGACTGTGGAGGCTTGCTG
GCCACTTTTTGGGGTCAGCTCCTCTGAAATGCATTAGCGGAACCGTTTGCGATCTGCCAC
AAGTGTGATAAGTTATCTACACTGGCGAGGGGATTGCTCTCTGTAATGTTCAGCTTCTAA
TTGTCTCTACTTTGTGAGACTACTTTTGAATGCTTGACCTCAAATCAGGTAGGACTACCC
GCTGAACTTAA
>A50
TTTCCGTAGGTGAACCTGCGGAAGGATCATTATTGAATTATGTTTCTAGATAGGTTGTAG
CTGGCTCTTTTAGAGCATGTGCACGCCTGTTTGGACTTCATTTTCATCCACCTGTGCACC
TATTGTAGTCTTTGGTTGGGTTAGGAGGAAGTGATCATTGTATCAGCATCTGCTGGGAGT
GAGGACTTGCATTGTGAAAGCTTTGCTGTCCTTGATGTGATCATGGAATCTTTTTCTCAC
TAGAGTCTATGTCACTCATTATACTCTGTCGAATGTCATTGAATGTCTTTACATGGGCTT
GTATGCCTATGAAAATTGTAATACAACTTTCAGCAACGGATCTCTTGGCTCTCGCATCGA
TGAAGAACGCAGCGAAATGCGATAAGTAATGTGAATTGCAGAATTCAGTGAATCATCGAA
TCTTTGAACGCATCTTGCGCTCCTTGGTATTCCGAGGAGCATGCCTGTTTGAGTGTCATT
AAATTCTCAACTCTCTTATACTTTTTTGTAAAAGAGAGCTTGGACTGTGGAGGCTTGCTG
GCCACTTTTTGGGGTCAGCTCCTCTGAAATGCATTAGCGGAACCGTTTGCGATCTGCCAC
AAGTGTGATAAGTTATCTACACTGGCGAGGGGATTGCTCTCTGTAATGTTCAGCTTCTAA
TTGTCTCTACTTTGTGAGACTACTTTTGAATGCTTGACCTCAAATCAGGTAGGACTACCC
GCTGAACTTAA

>A51
TTTCCGTAGGTGAACCTGCGGAAGGATCATTATTGAATTATGTTTCTAGATAGGTTGTAG
CTGGCTCTTTTAGAGCATGTGCACGCCTGTTTGGACTTCATTTTCATCCACCTGTGCACC
TATTGTAGTCTTTGGTTGGGTTAGGAGGAAGTGATCATTGTATCAGCATCTGCTGGGAGT
GAGGACTTGCATTGTGAAAGCTTTGCTGTCCTTGATGTGATCATGGAATCTTTTTCTCAC
TAGAGTCTATGTCACTCATTATACTCTGTCGAATGTCATTGAATGTCTTTACATGGGCTT
GTATGCCTATGAAAATTGTAATACAACTTTCAGCAACGGATCTCTTGGCTCTCGCATCGA
TGAAGAACGCAGCGAAATGCGATAAGTAATGTGAATTGCAGAATTCAGTGAATCATCGAA
TCTTTGAACGCATCTTGCGCTCCTTGGTATTCCGAGGAGCATGCCTGTTTGAGTGTCATT
AAATTCTCAACTCTCTTATACTTTTTTGTAAAAGAGAGCTTGGACTGTGGAGGCTTGCTG
GCCACTTTTTGGGGTCAGCTCCTCTGAAATGCATTAGCGGAACCGTTTGCGATCTGCCAC
AAGTGTGATAAGTTATCTACACTGGCGAGGGGATTGCTCTCTGTAATGTTCAGCTTCTAA
TTGTCTCTACTTTGTGAGACTACTTTTGAATGCTTGACCTCAAATCAGGTAGGACTACCC
GCTGAACTTAA
>A52
TTTCCGTAGGTGAACCTGCGGAAGGATCATTATTGAATTATGTTTCTAGATAGGTTGTAG
CTGGCTCTTTTAGAGCATGTGCACGCCTGTTTGGACTTCATTTTCATCCACCTGTGCACC
TATTGTAGTCTTTGGTTGGGTTAGGAGGAAGTGATCATTGTATCAGCATCTGCTGGGAGT
GAGGACTTGCATTGTGAAAGCTTTGCTGTCCTTGATGTGATCATGGAATCTTTTTCTCAC
TAGAGTCTATGTCACTCATTATACTCTGTCGAATGTCATTGAATGTCTTTACATGGGCTT
GTATGCCTATGAAAATTGTAATACAACTTTCAGCAACGGATCTCTTGGCTCTCGCATCGA
TGAAGAACGCAGCGAAATGCGATAAGTAATGTGAATTGCAGAATTCAGTGAATCATCGAA
TCTTTGAACGCATCTTGCGCTCCTTGGTATTCCGAGGAGCATGCCTGTTTGAGTGTCATT
AAATTCTCAACTCTCTTATACTTTTTTGTAAAAGAGAGCTTGGACTGTGGAGGCTTGCTG
GCCACTTTTTGGGGTCAGCTCCTCTGAAATGCATTAGCGGAACCGTTTGCGATCTGCCAC
AAGTGTGATAAGTTATCTACACTGGCGAGGGGATTGCTCTCTGTAATGTTCAGCTTCTAA
TTGTCTCTACTTTGTGAGACTACTTTTGAATGCTTGACCTCAAATCAGGTAGGACTACCC
GCTGAACTTAA
>A53
TTTCCGTAGGTGAACCTGCGGAAGGATCATTATTGAATTATGTTTCTAGATAGGTTGTAG
CTGGCTCTTTTAGAGCATGTGCACGCCTGTTTGGACTTCATTTTCATCCACCTGTGCACC
TATTGTAGTCTTTGGTTGGGTTAGGAGGAAGTGATCATTGTATCAGCATCTGCTGGGAGT
GAGGACTTGCATTGTGAAAGCTTTGCTGTCCTTGATGTGATCATGGAATCTTTTTCTCAC
TAGAGTCTATGTCACTCATTATACTCTGTCGAATGTCATTGAATGTCTTTACATGGGCTT
GTATGCCTATGAAAATTGTAATACAACTTTCAGCAACGGATCTCTTGGCTCTCGCATCGA
TGAAGAACGCAGCGAAATGCGATAAGTAATGTGAATTGCAGAATTCAGTGAATCATCGAA
TCTTTGAACGCATCTTGCGCTCCTTGGTATTCCGAGGAGCATGCCTGTTTGAGTGTCATT
AAATTCTCAACTCTCTTATACTTTTTTGTAAAAGAGAGCTTGGACTGTGGAGGCTTGCTG
GCCACTTTTTGGGGTCAGCTCCTCTGAAATGCATTAGCGGAACCGTTTGCGATCTGCCAC
AAGTGTGATAAGTTATCTACACTGGCGAGGGGATTGCTCTCTGTAATGTTCAGCTTCTAA
TTGTCTCTACTTTGTGAGACTACTTTTGAATGCTTGACCTCAAATCAGGTAGGACTACCC
GCTGAACTTAA
>A54
TTTCCGTAGGTGAACCTGCGGAAGGATCATTATTGAATTATGTTTCTAGATAGGTTGTAG
CTGGCTCTTTTAGAGCATGTGCACGCCTGTTTGGACTTCATTTTCATCCACCTGTGCACC
TATTGTAGTCTTTGGTTGGGTTAGGAGGAAGTGATCATTGTATCAGCATCTGCTGGGAGT
GAGGACTTGCATTGTGAAAGCTTTGCTGTCCTTGATGTGATCATGGAATCTTTTTCTCAC
TAGAGTCTATGTCACTCATTATACTCTGTCGAATGTCATTGAATGTCTTTACATGGGCTT
GTATGCCTATGAAAATTGTAATACAACTTTCAGCAACGGATCTCTTGGCTCTCGCATCGA
TGAAGAACGCAGCGAAATGCGATAAGTAATGTGAATTGCAGAATTCAGTGAATCATCGAA

TCTTTGAACGCATCTTGCGCTCCTTGGTATTCCGAGGAGCATGCCTGTTTGAGTGTCATT
AAATTCTCAACTCTCTTATACTTTTTTGTAAAAGAGAGCTTGGACTGTGGAGGCTTGCTG
GCCACTTTTTGGGGTCAGCTCCTCTGAAATGCATTAGCGGAACCGTTTGCGATCTGCCAC
AAGTGTGATAAGTTATCTACACTGGCGAGGGGATTGCTCTCTGTAATGTTCAGCTTCTAA
TTGTCTCTACTTTGTGAGACTACTTTTGAATGCTTGACCTCAAATCAGGTAGGACTACCC
GCTGAACTTAA
>A55
TTTCCGTAGGTGAACCTGCGGAAGGATCATTATTGAATTATGTTTCTAGATAGGTTGTAG
CTGGCTCTTTTAGAGCATGTGCACGCCTGTTTGGACTTCATTTTCATCCACCTGTGCACC
TATTGTAGTCTTTGGTTGGGTTAGGAGGAAGTGATCATTGTATCAGCATCTGCTGGGAGT
GAGGACTTGCATTGTGAAAGCTTTGCTGTCCTTGATGTGATCATGGAATCTTTTTCTCAC
TAGAGTCTATGTCACTCATTATACTCTGTCGAATGTCATTGAATGTCTTTACATGGGCTT
GTATGCCTATGAAAATTGTAATACAACTTTCAGCAACGGATCTCTTGGCTCTCGCATCGA
TGAAGAACGCAGCGAAATGCGATAAGTAATGTGAATTGCAGAATTCAGTGAATCATCGAA
TCTTTGAACGCATCTTGCGCTCCTTGGTATTCCGAGGAGCATGCCTGTTTGAGTGTCATT
AAATTCTCAACTCTCTTATACTTTTTTGTAAAAGAGAGCTTGGACTGTGGAGGCTTGCTG
GCCACTTTTTGGGGTCAGCTCCTCTGAAATGCATTAGCGGAACCGTTTGCGATCTGCCAC
AAGTGTGATAAGTTATCTACACTGGCGAGGGGATTGCTCTCTGTAATGTTCAGCTTCTAA
TTGTCTCTACTTTGTGAGACTACTTTTGAATGCTTGACCTCAAATCAGGTAGGACTACCC
GCTGAACTTAA
>A56
TTTCCGTAGGTGAACCTGCGGAAGGATCATTATTGAATTATGTTTCTAGATAGGTTGTAG
CTGGCTCTTTTAGAGCATGTGCACGCCTGTTTGGACTTCATTTTCATCCACCTGTGCACC
TATTGTAGTCTTTGGTTGGGTTAGGAGGAAGTGATCATTGTATCAGCATCTGCTGGGAGT
GAGGACTTGCATTGTGAAAGCTTTGCTGTCCTTGATGTGATCATGGAATCTTTTTCTCAC
TAGAGTCTATGTCACTCATTATACTCTGTCGAATGTCATTGAATGTCTTTACATGGGCTT
GTATGCCTATGAAAATTGTAATACAACTTTCAGCAACGGATCTCTTGGCTCTCGCATCGA
TGAAGAACGCAGCGAAATGCGATAAGTAATGTGAATTGCAGAATTCAGTGAATCATCGAA
TCTTTGAACGCATCTTGCGCTCCTTGGTATTCCGAGGAGCATGCCTGTTTGAGTGTCATT
AAATTCTCAACTCTCTTATACTTTTTTGTAAAAGAGAGCTTGGACTGTGGAGGCTTGCTG
GCCACTTTTTGGGGTCAGCTCCTCTGAAATGCATTAGCGGAACCGTTTGCGATCTGCCAC
AAGTGTGATAAGTTATCTACACTGGCGAGGGGATTGCTCTCTGTAATGTTCAGCTTCTAA
TTGTCTCTACTTTGTGAGACTACTTTTGAATGCTTGACCTCAAATCAGGTAGGACTACCC
GCTGAACTTAA
>A57
TTTCCGTAGGTGAACCTGCGGAAGGATCATTATTGAATTATGTTTCTAGATAGGTTGTAG
CTGGCTCTTTTAGAGCATGTGCACGCCTGTTTGGACTTCATTTTCATCCACCTGTGCACC
TATTGTAGTCTTTGGTTGGGTTAGGAGGAAGTGATCATTGTATCAGCATCTGCTGGGAGT
GAGGACTTGCATTGTGAAAGCTTTGCTGTCCTTGATGTGATCATGGAATCTTTTTCTCAC
TAGAGTCTATGTCACTCATTATACTCTGTCGAATGTCATTGAATGTCTTTACATGGGCTT
GTATGCCTATGAAAATTGTAATACAACTTTCAGCAACGGATCTCTTGGCTCTCGCATCGA
TGAAGAACGCAGCGAAATGCGATAAGTAATGTGAATTGCAGAATTCAGTGAATCATCGAA
TCTTTGAACGCATCTTGCGCTCCTTGGTATTCCGAGGAGCATGCCTGTTTGAGTGTCATT
AAATTCTCAACTCTCTTATACTTTTTTGTAAAAGAGAGCTTGGACTGTGGAGGCTTGCTG
GCCACTTTTTGGGGTCAGCTCCTCTGAAATGCATTAGCGGAACCGTTTGCGATCTGCCAC
AAGTGTGATAAGTTATCTACACTGGCGAGGGGATTGCTCTCTGTAATGTTCAGCTTCTAA
TTGTCTCTACTTTGTGAGACTACTTTTGAATGCTTGACCTCAAATCAGGTAGGACTACCC
GCTGAACTTAA
>A58
TTTCCGTAGGTGAACCTGCGGAAGGATCATTATTGAATTATGTTTCTAGATAGGTTGTAG

CTGGCTCTTTTAGAGCATGTGCACGCCTGTTTGGACTTCATTTTCATCCACCTGTGCACC
TATTGTAGTCTTTGGTTGGGTTAGGAGGAAGTGATCATTGTATCAGCATCTGCTGGGAGT
GAGGACTTGCATTGTGAAAGCTTTGCTGTCCTTGATGTGATCATGGAATCTTTTTCTCAC
TAGAGTCTATGTCACTCATTATACTCTGTCGAATGTCATTGAATGTCTTTACATGGGCTT
GTATGCCTATGAAAATTGTAATACAACTTTCAGCAACGGATCTCTTGGCTCTCGCATCGA
TGAAGAACGCAGCGAAATGCGATAAGTAATGTGAATTGCAGAATTCAGTGAATCATCGAA
TCTTTGAACGCATCTTGCGCTCCTTGGTATTCCGAGGAGCATGCCTGTTTGAGTGTCATT
AAATTCTCAACTCTCTTATACTTTTTTGTAAAAGAGAGCTTGGACTGTGGAGGCTTGCTG
GCCACTTTTTGGGGTCAGCTCCTCTGAAATGCATTAGCGGAACCGTTTGCGATCTGCCAC
AAGTGTGATAAGTTATCTACACTGGCGAGGGGATTGCTCTCTGTAATGTTCAGCTTCTAA
TTGTCTCTACTTTGTGAGACTACTTTTGAATGCTTGACCTCAAATCAGGTAGGACTACCC
GCTGAACTTAA
>A59
TTTCCGTAGGTGAACCTGCGGAAGGATCATTATTGAATTATGTTTCTAGATAGGTTGTAG
CTGGCTCTTTTAGAGCATGTGCACGCCTGTTTGGACTTCATTTTCATCCACCTGTGCACC
TATTGTAGTCTTTGGTTGGGTTAGGAGGAAGTGATCATTGTATCAGCATCTGCTGGGAGT
GAGGACTTGCATTGTGAAAGCTTTGCTGTCCTTGATGTGATCATGGAATCTTTTTCTCAC
TAGAGTCTATGTCACTCATTATACTCTGTCGAATGTCATTGAATGTCTTTACATGGGCTT
GTATGCCTATGAAAATTGTAATACAACTTTCAGCAACGGATCTCTTGGCTCTCGCATCGA
TGAAGAACGCAGCGAAATGCGATAAGTAATGTGAATTGCAGAATTCAGTGAATCATCGAA
TCTTTGAACGCATCTTGCGCTCCTTGGTATTCCGAGGAGCATGCCTGTTTGAGTGTCATT
AAATTCTCAACTCTCTTATACTTTTTTGTAAAAGAGAGCTTGGACTGTGGAGGCTTGCTG
GCCACTTTTTGGGGTCAGCTCCTCTGAAATGCATTAGCGGAACCGTTTGCGATCTGCCAC
AAGTGTGATAAGTTATCTACACTGGCGAGGGGATTGCTCTCTGTAATGTTCAGCTTCTAA
TTGTCTCTACTTTGTGAGACTACTTTTGAATGCTTGACCTCAAATCAGGTAGGACTACCC
GCTGAACTTAA
>A60
TTTCCGTAGGTGAACCTGCGGAAGGATCATTATTGAATTATGTTTCTAGATAGGTTGTAG
CTGGCTCTTTTAGAGCATGTGCACGCCTGTTTGGACTTCATTTTCATCCACCTGTGCACC
TATTGTAGTCTTTGGTTGGGTTAGGAGGAAGTGATCATTGTATCAGCATCTGCTGGGAGT
GAGGACTTGCATTGTGAAAGCTTTGCTGTCCTTGATGTGATCATGGAATCTTTTTCTCAC
TAGAGTCTATGTCACTCATTATACTCTGTCGAATGTCATTGAATGTCTTTACATGGGCTT
GTATGCCTATGAAAATTGTAATACAACTTTCAGCAACGGATCTCTTGGCTCTCGCATCGA
TGAAGAACGCAGCGAAATGCGATAAGTAATGTGAATTGCAGAATTCAGTGAATCATCGAA
TCTTTGAACGCATCTTGCGCTCCTTGGTATTCCGAGGAGCATGCCTGTTTGAGTGTCATT
AAATTCTCAACTCTCTTATACTTTTTTGTAAAAGAGAGCTTGGACTGTGGAGGCTTGCTG
GCCACTTTTTGGGGTCAGCTCCTCTGAAATGCATTAGCGGAACCGTTTGCGATCTGCCAC
AAGTGTGATAAGTTATCTACACTGGCGAGGGGATTGCTCTCTGTAATGTTCAGCTTCTAA
TTGTCTCTACTTTGTGAGACTACTTTTGAATGCTTGACCTCAAATCAGGTAGGACTACCC
GCTGAACTTAA
>A61
TTTCCGTAGGTGAACCTGCGGAAGGATCATTATTGAATTATGTTTCTAGATAGGTTGTAG
CTGGCTCTTTTAGAGCATGTGCACGCCTGTTTGGACTTCATTTTCATCCACCTGTGCACC
TATTGTAGTCTTTGGTTGGGTTAGGAGGAAGTGATCATTGTATCAGCATCTGCTGGGAGT
GAGGACTTGCATTGTGAAAGCTTTGCTGTCCTTGATGTGATCATGGAATCTTTTTCTCAC
TAGAGTCTATGTCACTCATTATACTCTGTCGAATGTCATTGAATGTCTTTACATGGGCTT
GTATGCCTATGAAAATTGTAATACAACTTTCAGCAACGGATCTCTTGGCTCTCGCATCGA
TGAAGAACGCAGCGAAATGCGATAAGTAATGTGAATTGCAGAATTCAGTGAATCATCGAA
TCTTTGAACGCATCTTGCGCTCCTTGGTATTCCGAGGAGCATGCCTGTTTGAGTGTCATT
AAATTCTCAACTCTCTTATACTTTTTTGTAAAAGAGAGCTTGGACTGTGGAGGCTTGCTG

GCCACTTTTTGGGGTCAGCTCCTCTGAAATGCATTAGCGGAACCGTTTGCGATCTGCCAC
AAGTGTGATAAGTTATCTACACTGGCGAGGGGATTGCTCTCTGTAATGTTCAGCTTCTAA
TTGTCTCTACTTTGTGAGACTACTTTTGAATGCTTGACCTCAAATCAGGTAGGACTACCC
GCTGAACTTAA
>A62
TTTCCGTAGGTGAACCTGCGGAAGGATCATTATTGAATTATGTTTCTAGATAGGTTGTAG
CTGGCTCTTTTAGAGCATGTGCACGCCTGTTTGGACTTCATTTTCATCCACCTGTGCACC
TATTGTAGTCTTTGGTTGGGTTAGGAGGAAGTGATCATTGTATCAGCATCTGCTGGGAGT
GAGGACTTGCATTGTGAAAGCTTTGCTGTCCTTGATGTGATCATGGAATCTTTTTCTCAC
TAGAGTCTATGTCACTCATTATACTCTGTCGAATGTCATTGAATGTCTTTACATGGGCTT
GTATGCCTATGAAAATTGTAATACAACTTTCAGCAACGGATCTCTTGGCTCTCGCATCGA
TGAAGAACGCAGCGAAATGCGATAAGTAATGTGAATTGCAGAATTCAGTGAATCATCGAA
TCTTTGAACGCATCTTGCGCTCCTTGGTATTCCGAGGAGCATGCCTGTTTGAGTGTCATT
AAATTCTCAACTCTCTTATACTTTTTTGTAAAAGAGAGCTTGGACTGTGGAGGCTTGCTG
GCCACTTTTTGGGGTCAGCTCCTCTGAAATGCATTAGCGGAACCGTTTGCGATCTGCCAC
AAGTGTGATAAGTTATCTACACTGGCGAGGGGATTGCTCTCTGTAATGTTCAGCTTCTAA
TTGTCTCTACTTTGTGAGACTACTTTTGAATGCTTGACCTCAAATCAGGTAGGACTACCC
GCTGAACTTAA
>A63
TTTCCGTAGGTGAACCTGCGGAAGGATCATTATTGAATTATGTTTCTAGATAGGTTGTAG
CTGGCTCTTTTAGAGCATGTGCACGCCTGTTTGGACTTCATTTTCATCCACCTGTGCACC
TATTGTAGTCTTTGGTTGGGTTAGGAGGAAGTGATCATTGTATCAGCATCTGCTGGGAGT
GAGGACTTGCATTGTGAAAGCTTTGCTGTCCTTGATGTGATCATGGAATCTTTTTCTCAC
TAGAGTCTATGTCACTCATTATACTCTGTCGAATGTCATTGAATGTCTTTACATGGGCTT
GTATGCCTATGAAAATTGTAATACAACTTTCAGCAACGGATCTCTTGGCTCTCGCATCGA
TGAAGAACGCAGCGAAATGCGATAAGTAATGTGAATTGCAGAATTCAGTGAATCATCGAA
TCTTTGAACGCATCTTGCGCTCCTTGGTATTCCGAGGAGCATGCCTGTTTGAGTGTCATT
AAATTCTCAACTCTCTTATACTTTTTTGTAAAAGAGAGCTTGGACTGTGGAGGCTTGCTG
GCCACTTTTTGGGGTCAGCTCCTCTGAAATGCATTAGCGGAACCGTTTGCGATCTGCCAC
AAGTGTGATAAGTTATCTACACTGGCGAGGGGATTGCTCTCTGTAATGTTCAGCTTCTAA
TTGTCTCTACTTTGTGAGACTACTTTTGAATGCTTGACCTCAAATCAGGTAGGACTACCC
GCTGAACTTAA
>A64
TTTCCGTAGGTGAACCTGCGGAAGGATCATTATTGAATTATGTTTCTAGATAGGTTGTAG
CTGGCTCTTTTAGAGCATGTGCACGCCTGTTTGGACTTCATTTTCATCCACCTGTGCACC
TATTGTAGTCTTTGGTTGGGTTAGGAGGAAGTGATCATTGTATCAGCATCTGCTGGGAGT
GAGGACTTGCATTGTGAAAGCTTTGCTGTCCTTGATGTGATCATGGAATCTTTTTCTCAC
TAGAGTCTATGTCACTCATTATACTCTGTCGAATGTCATTGAATGTCTTTACATGGGCTT
GTATGCCTATGAAAATTGTAATACAACTTTCAGCAACGGATCTCTTGGCTCTCGCATCGA
TGAAGAACGCAGCGAAATGCGATAAGTAATGTGAATTGCAGAATTCAGTGAATCATCGAA
TCTTTGAACGCATCTTGCGCTCCTTGGTATTCCGAGGAGCATGCCTGTTTGAGTGTCATT
AAATTCTCAACTCTCTTATACTTTTTTGTAAAAGAGAGCTTGGACTGTGGAGGCTTGCTG
GCCACTTTTTGGGGTCAGCTCCTCTGAAATGCATTAGCGGAACCGTTTGCGATCTGCCAC
AAGTGTGATAAGTTATCTACACTGGCGAGGGGATTGCTCTCTGTAATGTTCAGCTTCTAA
TTGTCTCTACTTTGTGAGACTACTTTTGAATGCTTGACCTCAAATCAGGTAGGACTACCC
GCTGAACTTAA
>A65
TTTCCGTAGGTGAACCTGCGGAAGGATCATTATTGAATTATGTTTCTAGATAGGTTGTAG
CTGGCTCTTTTAGAGCATGTGCACGCCTGTTTGGACTTCATTTTCATCCACCTGTGCACC
TATTGTAGTCTTTGGTTGGGTTAGGAGGAAGTGATCATTGTATCAGCATCTGCTGGGAGT

GAGGACTTGCATTGTGAAAGCTTTGCTGTCCTTGATGTGATCATGGAATCTTTTTCTCAC
TAGAGTCTATGTCACTCATTATACTCTGTCGAATGTCATTGAATGTCTTTACATGGGCTT
GTATGCCTATGAAAATTGTAATACAACTTTCAGCAACGGATCTCTTGGCTCTCGCATCGA
TGAAGAACGCAGCGAAATGCGATAAGTAATGTGAATTGCAGAATTCAGTGAATCATCGAA
TCTTTGAACGCATCTTGCGCTCCTTGGTATTCCGAGGAGCATGCCTGTTTGAGTGTCATT
AAATTCTCAACTCTCTTATACTTTTTTGTAAAAGAGAGCTTGGACTGTGGAGGCTTGCTG
GCCACTTTTTGGGGTCAGCTCCTCTGAAATGCATTAGCGGAACCGTTTGCGATCTGCCAC
AAGTGTGATAAGTTATCTACACTGGCGAGGGGATTGCTCTCTGTAATGTTCAGCTTCTAA
TTGTCTCTACTTTGTGAGACTACTTTTGAATGCTTGACCTCAAATCAGGTAGGACTACCC
GCTGAACTTAA
>A66
TTTCCGTAGGTGAACCTGCGGAAGGATCATTATTGAATTATGTTTCTAGATAGGTTGTAG
CTGGCTCTTTTAGAGCATGTGCACGCCTGTTTGGACTTCATTTTCATCCACCTGTGCACC
TATTGTAGTCTTTGGTTGGGTTAGGAGGAAGTGATCATTGTATCAGCATCTGCTGGGAGT
GAGGACTTGCATTGTGAAAGCTTTGCTGTCCTTGATGTGATCATGGAATCTTTTTCTCAC
TAGAGTCTATGTCACTCATTATACTCTGTCGAATGTCATTGAATGTCTTTACATGGGCTT
GTATGCCTATGAAAATTGTAATACAACTTTCAGCAACGGATCTCTTGGCTCTCGCATCGA
TGAAGAACGCAGCGAAATGCGATAAGTAATGTGAATTGCAGAATTCAGTGAATCATCGAA
TCTTTGAACGCATCTTGCGCTCCTTGGTATTCCGAGGAGCATGCCTGTTTGAGTGTCATT
AAATTCTCAACTCTCTTATACTTTTTTGTAAAAGAGAGCTTGGACTGTGGAGGCTTGCTG
GCCACTTTTTGGGGTCAGCTCCTCTGAAATGCATTAGCGGAACCGTTTGCGATCTGCCAC
AAGTGTGATAAGTTATCTACACTGGCGAGGGGATTGCTCTCTGTAATGTTCAGCTTCTAA
TTGTCTCTACTTTGTGAGACTACTTTTGAATGCTTGACCTCAAATCAGGTAGGACTACCC
GCTGAACTTAA
>A67
TTTCCGTAGGTGAACCTGCGGAAGGATCATTATTGAATTATGTTTCTAGATAGGTTGTAG
CTGGCTCTTTTAGAGCATGTGCACGCCTGTTTGGACTTCATTTTCATCCACCTGTGCACC
TATTGTAGTCTTTGGTTGGGTTAGGAGGAAGTGATCATTGTATCAGCATCTGCTGGGAGT
GAGGACTTGCATTGTGAAAGCTTTGCTGTCCTTGATGTGATCATGGAATCTTTTTCTCAC
TAGAGTCTATGTCACTCATTATACTCTGTCGAATGTCATTGAATGTCTTTACATGGGCTT
GTATGCCTATGAAAATTGTAATACAACTTTCAGCAACGGATCTCTTGGCTCTCGCATCGA
TGAAGAACGCAGCGAAATGCGATAAGTAATGTGAATTGCAGAATTCAGTGAATCATCGAA
TCTTTGAACGCATCTTGCGCTCCTTGGTATTCCGAGGAGCATGCCTGTTTGAGTGTCATT
AAATTCTCAACTCTCTTATACTTTTTTGTAAAAGAGAGCTTGGACTGTGGAGGCTTGCTG
GCCACTTTTTGGGGTCAGCTCCTCTGAAATGCATTAGCGGAACCGTTTGCGATCTGCCAC
AAGTGTGATAAGTTATCTACACTGGCGAGGGGATTGCTCTCTGTAATGTTCAGCTTCTAA
TTGTCTCTACTTTGTGAGACTACTTTTGAATGCTTGACCTCAAATCAGGTAGGACTACCC
GCTGAACTTAA
>A68
TTTCCGTAGGTGAACCTGCGGAAGGATCATTATTGAATTATGTTTCTAGATAGGTTGTAG
CTGGCTCTTTTAGAGCATGTGCACGCCTGTTTGGACTTCATTTTCATCCACCTGTGCACC
TATTGTAGTCTTTGGTTGGGTTAGGAGGAAGTGATCATTGTATCAGCATCTGCTGGGAGT
GAGGACTTGCATTGTGAAAGCTTTGCTGTCCTTGATGTGATCATGGAATCTTTTTCTCAC
TAGAGTCTATGTCACTCATTATACTCTGTCGAATGTCATTGAATGTCTTTACATGGGCTT
GTATGCCTATGAAAATTGTAATACAACTTTCAGCAACGGATCTCTTGGCTCTCGCATCGA
TGAAGAACGCAGCGAAATGCGATAAGTAATGTGAATTGCAGAATTCAGTGAATCATCGAA
TCTTTGAACGCATCTTGCGCTCCTTGGTATTCCGAGGAGCATGCCTGTTTGAGTGTCATT
AAATTCTCAACTCTCTTATACTTTTTTGTAAAAGAGAGCTTGGACTGTGGAGGCTTGCTG
GCCACTTTTTGGGGTCAGCTCCTCTGAAATGCATTAGCGGAACCGTTTGCGATCTGCCAC
AAGTGTGATAAGTTATCTACACTGGCGAGGGGATTGCTCTCTGTAATGTTCAGCTTCTAA

TTGTCTCTACTTTGTGAGACTACTTTTGAATGCTTGACCTCAAATCAGGTAGGACTACCC
GCTGAACTTAA
>A69
TTTCCGTAGGTGAACCTGCGGAAGGATCATTATTGAATTATGTTTCTAGATAGGTTGTAG
CTGGCTCTTTTAGAGCATGTGCACGCCTGTTTGGACTTCATTTTCATCCACCTGTGCACC
TATTGTAGTCTTTGGTTGGGTTAGGAGGAAGTGATCATTGTATCAGCATCTGCTGGGAGT
GAGGACTTGCATTGTGAAAGCTTTGCTGTCCTTGATGTGATCATGGAATCTTTTTCTCAC
TAGAGTCTATGTCACTCATTATACTCTGTCGAATGTCATTGAATGTCTTTACATGGGCTT
GTATGCCTATGAAAATTGTAATACAACTTTCAGCAACGGATCTCTTGGCTCTCGCATCGA
TGAAGAACGCAGCGAAATGCGATAAGTAATGTGAATTGCAGAATTCAGTGAATCATCGAA
TCTTTGAACGCATCTTGCGCTCCTTGGTATTCCGAGGAGCATGCCTGTTTGAGTGTCATT
AAATTCTCAACTCTCTTATACTTTTTTGTAAAAGAGAGCTTGGACTGTGGAGGCTTGCTG
GCCACTTTTTGGGGTCAGCTCCTCTGAAATGCATTAGCGGAACCGTTTGCGATCTGCCAC
AAGTGTGATAAGTTATCTACACTGGCGAGGGGATTGCTCTCTGTAATGTTCAGCTTCTAA
TTGTCTCTACTTTGTGAGACTACTTTTGAATGCTTGACCTCAAATCAGGTAGGACTACCC
GCTGAACTTAA
>A70
TTTCCGTAGGTGAACCTGCGGAAGGATCATTATTGAATTATGTTTCTAGATAGGTTGTAG
CTGGCTCTTTTAGAGCATGTGCACGCCTGTTTGGACTTCATTTTCATCCACCTGTGCACC
TATTGTAGTCTTTGGTTGGGTTAGGAGGAAGTGATCATTGTATCAGCATCTGCTGGGAGT
GAGGACTTGCATTGTGAAAGCTTTGCTGTCCTTGATGTGATCATGGAATCTTTTTCTCAC
TAGAGTCTATGTCACTCATTATACTCTGTCGAATGTCATTGAATGTCTTTACATGGGCTT
GTATGCCTATGAAAATTGTAATACAACTTTCAGCAACGGATCTCTTGGCTCTCGCATCGA
TGAAGAACGCAGCGAAATGCGATAAGTAATGTGAATTGCAGAATTCAGTGAATCATCGAA
TCTTTGAACGCATCTTGCGCTCCTTGGTATTCCGAGGAGCATGCCTGTTTGAGTGTCATT
AAATTCTCAACTCTCTTATACTTTTTTGTAAAAGAGAGCTTGGACTGTGGAGGCTTGCTG
GCCACTTTTTGGGGTCAGCTCCTCTGAAATGCATTAGCGGAACCGTTTGCGATCTGCCAC
AAGTGTGATAAGTTATCTACACTGGCGAGGGGATTGCTCTCTGTAATGTTCAGCTTCTAA
TTGTCTCTACTTTGTGAGACTACTTTTGAATGCTTGACCTCAAATCAGGTAGGACTACCC
GCTGAACTTAA
>A71
TTTCCGTAGGTGAACCTGCGGAAGGATCATTATTGAATTATGTTTCTAGATAGGTTGTAG
CTGGCTCTTTTAGAGCATGTGCACGCCTGTTTGGACTTCATTTTCATCCACCTGTGCACC
TATTGTAGTCTTTGGTTGGGTTAGGAGGAAGTGATCATTGTATCAGCATCTGCTGGGAGT
GAGGACTTGCATTGTGAAAGCTTTGCTGTCCTTGATGTGATCATGGAATCTTTTTCTCAC
TAGAGTCTATGTCACTCATTATACTCTGTCGAATGTCATTGAATGTCTTTACATGGGCTT
GTATGCCTATGAAAATTGTAATACAACTTTCAGCAACGGATCTCTTGGCTCTCGCATCGA
TGAAGAACGCAGCGAAATGCGATAAGTAATGTGAATTGCAGAATTCAGTGAATCATCGAA
TCTTTGAACGCATCTTGCGCTCCTTGGTATTCCGAGGAGCATGCCTGTTTGAGTGTCATT
AAATTCTCAACTCTCTTATACTTTTTTGTAAAAGAGAGCTTGGACTGTGGAGGCTTGCTG
GCCACTTTTTGGGGTCAGCTCCTCTGAAATGCATTAGCGGAACCGTTTGCGATCTGCCAC
AAGTGTGATAAGTTATCTACACTGGCGAGGGGATTGCTCTCTGTAATGTTCAGCTTCTAA
TTGTCTCTACTTTGTGAGACTACTTTTGAATGCTTGACCTCAAATCAGGTAGGACTACCC
GCTGAACTTAA
>A72
TTTCCGTAGGTGAACCTGCGGAAGGATCATTATTGAATTATGTTTCTAGATAGGTTGTAG
CTGGCTCTTTTAGAGCATGTGCACGCCTGTTTGGACTTCATTTTCATCCACCTGTGCACC
TATTGTAGTCTTTGGTTGGGTTAGGAGGAAGTGATCATTGTATCAGCATCTGCTGGGAGT
GAGGACTTGCATTGTGAAAGCTTTGCTGTCCTTGATGTGATCATGGAATCTTTTTCTCAC
TAGAGTCTATGTCACTCATTATACTCTGTCGAATGTCATTGAATGTCTTTACATGGGCTT

GTATGCCTATGAAAATTGTAATACAACTTTCAGCAACGGATCTCTTGGCTCTCGCATCGA
TGAAGAACGCAGCGAAATGCGATAAGTAATGTGAATTGCAGAATTCAGTGAATCATCGAA
TCTTTGAACGCATCTTGCGCTCCTTGGTATTCCGAGGAGCATGCCTGTTTGAGTGTCATT
AAATTCTCAACTCTCTTATACTTTTTTGTAAAAGAGAGCTTGGACTGTGGAGGCTTGCTG
GCCACTTTTTGGGGTCAGCTCCTCTGAAATGCATTAGCGGAACCGTTTGCGATCTGCCAC
AAGTGTGATAAGTTATCTACACTGGCGAGGGGATTGCTCTCTGTAATGTTCAGCTTCTAA
TTGTCTCTACTTTGTGAGACTACTTTTGAATGCTTGACCTCAAATCAGGTAGGACTACCC
GCTGAACTTAA
>A73
TTTCCGTAGGTGAACCTGCGGAAGGATCATTATTGAATTATGTTTCTAGATAGGTTGTAG
CTGGCTCTTTTAGAGCATGTGCACGCCTGTTTGGACTTCATTTTCATCCACCTGTGCACC
TATTGTAGTCTTTGGTTGGGTTAGGAGGAAGTGATCATTGTATCAGCATCTGCTGGGAGT
GAGGACTTGCATTGTGAAAGCTTTGCTGTCCTTGATGTGATCATGGAATCTTTTTCTCAC
TAGAGTCTATGTCACTCATTATACTCTGTCGAATGTCATTGAATGTCTTTACATGGGCTT
GTATGCCTATGAAAATTGTAATACAACTTTCAGCAACGGATCTCTTGGCTCTCGCATCGA
TGAAGAACGCAGCGAAATGCGATAAGTAATGTGAATTGCAGAATTCAGTGAATCATCGAA
TCTTTGAACGCATCTTGCGCTCCTTGGTATTCCGAGGAGCATGCCTGTTTGAGTGTCATT
AAATTCTCAACTCTCTTATACTTTTTTGTAAAAGAGAGCTTGGACTGTGGAGGCTTGCTG
GCCACTTTTTGGGGTCAGCTCCTCTGAAATGCATTAGCGGAACCGTTTGCGATCTGCCAC
AAGTGTGATAAGTTATCTACACTGGCGAGGGGATTGCTCTCTGTAATGTTCAGCTTCTAA
TTGTCTCTACTTTGTGAGACTACTTTTGAATGCTTGACCTCAAATCAGGTAGGACTACCC
GCTGAACTTAA
>A74
TTTCCGTAGGTGAACCTGCGGAAGGATCATTATTGAATTATGTTTCTAGATAGGTTGTAG
CTGGCTCTTTTAGAGCATGTGCACGCCTGTTTGGACTTCATTTTCATCCACCTGTGCACC
TATTGTAGTCTTTGGTTGGGTTAGGAGGAAGTGATCATTGTATCAGCATCTGCTGGGAGT
GAGGACTTGCATTGTGAAAGCTTTGCTGTCCTTGATGTGATCATGGAATCTTTTTCTCAC
TAGAGTCTATGTCACTCATTATACTCTGTCGAATGTCATTGAATGTCTTTACATGGGCTT
GTATGCCTATGAAAATTGTAATACAACTTTCAGCAACGGATCTCTTGGCTCTCGCATCGA
TGAAGAACGCAGCGAAATGCGATAAGTAATGTGAATTGCAGAATTCAGTGAATCATCGAA
TCTTTGAACGCATCTTGCGCTCCTTGGTATTCCGAGGAGCATGCCTGTTTGAGTGTCATT
AAATTCTCAACTCTCTTATACTTTTTTGTAAAAGAGAGCTTGGACTGTGGAGGCTTGCTG
GCCACTTTTTGGGGTCAGCTCCTCTGAAATGCATTAGCGGAACCGTTTGCGATCTGCCAC
AAGTGTGATAAGTTATCTACACTGGCGAGGGGATTGCTCTCTGTAATGTTCAGCTTCTAA
TTGTCTCTACTTTGTGAGACTACTTTTGAATGCTTGACCTCAAATCAGGTAGGACTACCC
GCTGAACTTAA
>A75
TTTCCGTAGGTGAACCTGCGGAAGGATCATTATTGAATTATGTTTCTAGATAGGTTGTAG
CTGGCTCTTTTAGAGCATGTGCACGCCTGTTTGGACTTCATTTTCATCCACCTGTGCACC
TATTGTAGTCTTTGGTTGGGTTAGGAGGAAGTGATCATTGTATCAGCATCTGCTGGGAGT
GAGGACTTGCATTGTGAAAGCTTTGCTGTCCTTGATGTGATCATGGAATCTTTTTCTCAC
TAGAGTCTATGTCACTCATTATACTCTGTCGAATGTCATTGAATGTCTTTACATGGGCTT
GTATGCCTATGAAAATTGTAATACAACTTTCAGCAACGGATCTCTTGGCTCTCGCATCGA
TGAAGAACGCAGCGAAATGCGATAAGTAATGTGAATTGCAGAATTCAGTGAATCATCGAA
TCTTTGAACGCATCTTGCGCTCCTTGGTATTCCGAGGAGCATGCCTGTTTGAGTGTCATT
AAATTCTCAACTCTCTTATACTTTTTTGTAAAAGAGAGCTTGGACTGTGGAGGCTTGCTG
GCCACTTTTTGGGGTCAGCTCCTCTGAAATGCATTAGCGGAACCGTTTGCGATCTGCCAC
AAGTGTGATAAGTTATCTACACTGGCGAGGGGATTGCTCTCTGTAATGTTCAGCTTCTAA
TTGTCTCTACTTTGTGAGACTACTTTTGAATGCTTGACCTCAAATCAGGTAGGACTACCC
GCTGAACTTAA

>A76
TTTCCGTAGGTGAACCTGCGGAAGGATCATTATTGAATTATGTTTCTAGATAGGTTGTAG
CTGGCTCTTTTAGAGCATGTGCACGCCTGTTTGGACTTCATTTTCATCCACCTGTGCACC
TATTGTAGTCTTTGGTTGGGTTAGGAGGAAGTGATCATTGTATCAGCATCTGCTGGGAGT
GAGGACTTGCATTGTGAAAGCTTTGCTGTCCTTGATGTGATCATGGAATCTTTTTCTCAC
TAGAGTCTATGTCACTCATTATACTCTGTCGAATGTCATTGAATGTCTTTACATGGGCTT
GTATGCCTATGAAAATTGTAATACAACTTTCAGCAACGGATCTCTTGGCTCTCGCATCGA
TGAAGAACGCAGCGAAATGCGATAAGTAATGTGAATTGCAGAATTCAGTGAATCATCGAA
TCTTTGAACGCATCTTGCGCTCCTTGGTATTCCGAGGAGCATGCCTGTTTGAGTGTCATT
AAATTCTCAACTCTCTTATACTTTTTTGTAAAAGAGAGCTTGGACTGTGGAGGCTTGCTG
GCCACTTTTTGGGGTCAGCTCCTCTGAAATGCATTAGCGGAACCGTTTGCGATCTGCCAC
AAGTGTGATAAGTTATCTACACTGGCGAGGGGATTGCTCTCTGTAATGTTCAGCTTCTAA
TTGTCTCTACTTTGTGAGACTACTTTTGAATGCTTGACCTCAAATCAGGTAGGACTACCC
GCTGAACTTAA
>A77
TTTCCGTAGGTGAACCTGCGGAAGGATCATTATTGAATTATGTTTCTAGATAGGTTGTAG
CTGGCTCTTTTAGAGCATGTGCACGCCTGTTTGGACTTCATTTTCATCCACCTGTGCACC
TATTGTAGTCTTTGGTTGGGTTAGGAGGAAGTGATCATTGTATCAGCATCTGCTGGGAGT
GAGGACTTGCATTGTGAAAGCTTTGCTGTCCTTGATGTGATCATGGAATCTTTTTCTCAC
TAGAGTCTATGTCACTCATTATACTCTGTCGAATGTCATTGAATGTCTTTACATGGGCTT
GTATGCCTATGAAAATTGTAATACAACTTTCAGCAACGGATCTCTTGGCTCTCGCATCGA
TGAAGAACGCAGCGAAATGCGATAAGTAATGTGAATTGCAGAATTCAGTGAATCATCGAA
TCTTTGAACGCATCTTGCGCTCCTTGGTATTCCGAGGAGCATGCCTGTTTGAGTGTCATT
AAATTCTCAACTCTCTTATACTTTTTTGTAAAAGAGAGCTTGGACTGTGGAGGCTTGCTG
GCCACTTTTTGGGGTCAGCTCCTCTGAAATGCATTAGCGGAACCGTTTGCGATCTGCCAC
AAGTGTGATAAGTTATCTACACTGGCGAGGGGATTGCTCTCTGTAATGTTCAGCTTCTAA
TTGTCTCTACTTTGTGAGACTACTTTTGAATGCTTGACCTCAAATCAGGTAGGACTACCC
GCTGAACTTAA
>A78
TTTCCGTAGGTGAACCTGCGGAAGGATCATTATTGAATTATGTTTCTAGATAGGTTGTAG
CTGGCTCTTTTAGAGCATGTGCACGCCTGTTTGGACTTCATTTTCATCCACCTGTGCACC
TATTGTAGTCTTTGGTTGGGTTAGGAGGAAGTGATCATTGTATCAGCATCTGCTGGGAGT
GAGGACTTGCATTGTGAAAGCTTTGCTGTCCTTGATGTGATCATGGAATCTTTTTCTCAC
TAGAGTCTATGTCACTCATTATACTCTGTCGAATGTCATTGAATGTCTTTACATGGGCTT
GTATGCCTATGAAAATTGTAATACAACTTTCAGCAACGGATCTCTTGGCTCTCGCATCGA
TGAAGAACGCAGCGAAATGCGATAAGTAATGTGAATTGCAGAATTCAGTGAATCATCGAA
TCTTTGAACGCATCTTGCGCTCCTTGGTATTCCGAGGAGCATGCCTGTTTGAGTGTCATT
AAATTCTCAACTCTCTTATACTTTTTTGTAAAAGAGAGCTTGGACTGTGGAGGCTTGCTG
GCCACTTTTTGGGGTCAGCTCCTCTGAAATGCATTAGCGGAACCGTTTGCGATCTGCCAC
AAGTGTGATAAGTTATCTACACTGGCGAGGGGATTGCTCTCTGTAATGTTCAGCTTCTAA
TTGTCTCTACTTTGTGAGACTACTTTTGAATGCTTGACCTCAAATCAGGTAGGACTACCC
GCTGAACTTAA
>A79
TTTCCGTAGGTGAACCTGCGGAAGGATCATTATTGAATTATGTTTCTAGATAGGTTGTAG
CTGGCTCTTTTAGAGCATGTGCACGCCTGTTTGGACTTCATTTTCATCCACCTGTGCACC
TATTGTAGTCTTTGGTTGGGTTAGGAGGAAGTGATCATTGTATCAGCATCTGCTGGGAGT
GAGGACTTGCATTGTGAAAGCTTTGCTGTCCTTGATGTGATCATGGAATCTTTTTCTCAC
TAGAGTCTATGTCACTCATTATACTCTGTCGAATGTCATTGAATGTCTTTACATGGGCTT
GTATGCCTATGAAAATTGTAATACAACTTTCAGCAACGGATCTCTTGGCTCTCGCATCGA
TGAAGAACGCAGCGAAATGCGATAAGTAATGTGAATTGCAGAATTCAGTGAATCATCGAA

TCTTTGAACGCATCTTGCGCTCCTTGGTATTCCGAGGAGCATGCCTGTTTGAGTGTCATT
AAATTCTCAACTCTCTTATACTTTTTTGTAAAAGAGAGCTTGGACTGTGGAGGCTTGCTG
GCCACTTTTTGGGGTCAGCTCCTCTGAAATGCATTAGCGGAACCGTTTGCGATCTGCCAC
AAGTGTGATAAGTTATCTACACTGGCGAGGGGATTGCTCTCTGTAATGTTCAGCTTCTAA
TTGTCTCTACTTTGTGAGACTACTTTTGAATGCTTGACCTCAAATCAGGTAGGACTACCC
GCTGAACTTAA
>A80
TTTCCGTAGGTGAACCTGCGGAAGGATCATTATTGAATTATGTTTCTAGATAGGTTGTAG
CTGGCTCTTTTAGAGCATGTGCACGCCTGTTTGGACTTCATTTTCATCCACCTGTGCACC
TATTGTAGTCTTTGGTTGGGTTAGGAGGAAGTGATCATTGTATCAGCATCTGCTGGGAGT
GAGGACTTGCATTGTGAAAGCTTTGCTGTCCTTGATGTGATCATGGAATCTTTTTCTCAC
TAGAGTCTATGTCACTCATTATACTCTGTCGAATGTCATTGAATGTCTTTACATGGGCTT
GTATGCCTATGAAAATTGTAATACAACTTTCAGCAACGGATCTCTTGGCTCTCGCATCGA
TGAAGAACGCAGCGAAATGCGATAAGTAATGTGAATTGCAGAATTCAGTGAATCATCGAA
TCTTTGAACGCATCTTGCGCTCCTTGGTATTCCGAGGAGCATGCCTGTTTGAGTGTCATT
AAATTCTCAACTCTCTTATACTTTTTTGTAAAAGAGAGCTTGGACTGTGGAGGCTTGCTG
GCCACTTTTTGGGGTCAGCTCCTCTGAAATGCATTAGCGGAACCGTTTGCGATCTGCCAC
AAGTGTGATAAGTTATCTACACTGGCGAGGGGATTGCTCTCTGTAATGTTCAGCTTCTAA
TTGTCTCTACTTTGTGAGACTACTTTTGAATGCTTGACCTCAAATCAGGTAGGACTACCC
GCTGAACTTAA
>A81
TTTCCGTAGGTGAACCTGCGGAAGGATCATTATTGAATTATGTTTCTAGATAGGTTGTAG
CTGGCTCTTTTAGAGCATGTGCACGCCTGTTTGGACTTCATTTTCATCCACCTGTGCACC
TATTGTAGTCTTTGGTTGGGTTAGGAGGAAGTGATCATTGTATCAGCATCTGCTGGGAGT
GAGGACTTGCATTGTGAAAGCTTTGCTGTCCTTGATGTGATCATGGAATCTTTTTCTCAC
TAGAGTCTATGTCACTCATTATACTCTGTCGAATGTCATTGAATGTCTTTACATGGGCTT
GTATGCCTATGAAAATTGTAATACAACTTTCAGCAACGGATCTCTTGGCTCTCGCATCGA
TGAAGAACGCAGCGAAATGCGATAAGTAATGTGAATTGCAGAATTCAGTGAATCATCGAA
TCTTTGAACGCATCTTGCGCTCCTTGGTATTCCGAGGAGCATGCCTGTTTGAGTGTCATT
AAATTCTCAACTCTCTTATACTTTTTTGTAAAAGAGAGCTTGGACTGTGGAGGCTTGCTG
GCCACTTTTTGGGGTCAGCTCCTCTGAAATGCATTAGCGGAACCGTTTGCGATCTGCCAC
AAGTGTGATAAGTTATCTACACTGGCGAGGGGATTGCTCTCTGTAATGTTCAGCTTCTAA
TTGTCTCTACTTTGTGAGACTACTTTTGAATGCTTGACCTCAAATCAGGTAGGACTACCC
GCTGAACTTAA
>A82
TTTCCGTAGGTGAACCTGCGGAAGGATCATTATTGAATTATGTTTCTAGATAGGTTGTAG
CTGGCTCTTTTAGAGCATGTGCACGCCTGTTTGGACTTCATTTTCATCCACCTGTGCACC
TATTGTAGTCTTTGGTTGGGTTAGGAGGAAGTGATCATTGTATCAGCATCTGCTGGGAGT
GAGGACTTGCATTGTGAAAGCTTTGCTGTCCTTGATGTGATCATGGAATCTTTTTCTCAC
TAGAGTCTATGTCACTCATTATACTCTGTCGAATGTCATTGAATGTCTTTACATGGGCTT
GTATGCCTATGAAAATTGTAATACAACTTTCAGCAACGGATCTCTTGGCTCTCGCATCGA
TGAAGAACGCAGCGAAATGCGATAAGTAATGTGAATTGCAGAATTCAGTGAATCATCGAA
TCTTTGAACGCATCTTGCGCTCCTTGGTATTCCGAGGAGCATGCCTGTTTGAGTGTCATT
AAATTCTCAACTCTCTTATACTTTTTTGTAAAAGAGAGCTTGGACTGTGGAGGCTTGCTG
GCCACTTTTTGGGGTCAGCTCCTCTGAAATGCATTAGCGGAACCGTTTGCGATCTGCCAC
AAGTGTGATAAGTTATCTACACTGGCGAGGGGATTGCTCTCTGTAATGTTCAGCTTCTAA
TTGTCTCTACTTTGTGAGACTACTTTTGAATGCTTGACCTCAAATCAGGTAGGACTACCC
GCTGAACTTAA
>A83
TTTCCGTAGGTGAACCTGCGGAAGGATCATTATTGAATTATGTTTCTAGATAGGTTGTAG

CTGGCTCTTTTAGAGCATGTGCACGCCTGTTTGGACTTCATTTTCATCCACCTGTGCACC
TATTGTAGTCTTTGGTTGGGTTAGGAGGAAGTGATCATTGTATCAGCATCTGCTGGGAGT
GAGGACTTGCATTGTGAAAGCTTTGCTGTCCTTGATGTGATCATGGAATCTTTTTCTCAC
TAGAGTCTATGTCACTCATTATACTCTGTCGAATGTCATTGAATGTCTTTACATGGGCTT
GTATGCCTATGAAAATTGTAATACAACTTTCAGCAACGGATCTCTTGGCTCTCGCATCGA
TGAAGAACGCAGCGAAATGCGATAAGTAATGTGAATTGCAGAATTCAGTGAATCATCGAA
TCTTTGAACGCATCTTGCGCTCCTTGGTATTCCGAGGAGCATGCCTGTTTGAGTGTCATT
AAATTCTCAACTCTCTTATACTTTTTTGTAAAAGAGAGCTTGGACTGTGGAGGCTTGCTG
GCCACTTTTTGGGGTCAGCTCCTCTGAAATGCATTAGCGGAACCGTTTGCGATCTGCCAC
AAGTGTGATAAGTTATCTACACTGGCGAGGGGATTGCTCTCTGTAATGTTCAGCTTCTAA
TTGTCTCTACTTTGTGAGACTACTTTTGAATGCTTGACCTCAAATCAGGTAGGACTACCC
GCTGAACTTAA
>A84
TTTCCGTAGGTGAACCTGCGGAAGGATCATTATTGAATTATGTTTCTAGATAGGTTGTAG
CTGGCTCTTTTAGAGCATGTGCACGCCTGTTTGGACTTCATTTTCATCCACCTGTGCACC
TATTGTAGTCTTTGGTTGGGTTAGGAGGAAGTGATCATTGTATCAGCATCTGCTGGGAGT
GAGGACTTGCATTGTGAAAGCTTTGCTGTCCTTGATGTGATCATGGAATCTTTTTCTCAC
TAGAGTCTATGTCACTCATTATACTCTGTCGAATGTCATTGAATGTCTTTACATGGGCTT
GTATGCCTATGAAAATTGTAATACAACTTTCAGCAACGGATCTCTTGGCTCTCGCATCGA
TGAAGAACGCAGCGAAATGCGATAAGTAATGTGAATTGCAGAATTCAGTGAATCATCGAA
TCTTTGAACGCATCTTGCGCTCCTTGGTATTCCGAGGAGCATGCCTGTTTGAGTGTCATT
AAATTCTCAACTCTCTTATACTTTTTTGTAAAAGAGAGCTTGGACTGTGGAGGCTTGCTG
GCCACTTTTTGGGGTCAGCTCCTCTGAAATGCATTAGCGGAACCGTTTGCGATCTGCCAC
AAGTGTGATAAGTTATCTACACTGGCGAGGGGATTGCTCTCTGTAATGTTCAGCTTCTAA
TTGTCTCTACTTTGTGAGACTACTTTTGAATGCTTGACCTCAAATCAGGTAGGACTACCC
GCTGAACTTAA
>A85
TTTCCGTAGGTGAACCTGCGGAAGGATCATTATTGAATTATGTTTCTAGATAGGTTGTAG
CTGGCTCTTTTAGAGCATGTGCACGCCTGTTTGGACTTCATTTTCATCCACCTGTGCACC
TATTGTAGTCTTTGGTTGGGTTAGGAGGAAGTGATCATTGTATCAGCATCTGCTGGGAGT
GAGGACTTGCATTGTGAAAGCTTTGCTGTCCTTGATGTGATCATGGAATCTTTTTCTCAC
TAGAGTCTATGTCACTCATTATACTCTGTCGAATGTCATTGAATGTCTTTACATGGGCTT
GTATGCCTATGAAAATTGTAATACAACTTTCAGCAACGGATCTCTTGGCTCTCGCATCGA
TGAAGAACGCAGCGAAATGCGATAAGTAATGTGAATTGCAGAATTCAGTGAATCATCGAA
TCTTTGAACGCATCTTGCGCTCCTTGGTATTCCGAGGAGCATGCCTGTTTGAGTGTCATT
AAATTCTCAACTCTCTTATACTTTTTTGTAAAAGAGAGCTTGGACTGTGGAGGCTTGCTG
GCCACTTTTTGGGGTCAGCTCCTCTGAAATGCATTAGCGGAACCGTTTGCGATCTGCCAC
AAGTGTGATAAGTTATCTACACTGGCGAGGGGATTGCTCTCTGTAATGTTCAGCTTCTAA
TTGTCTCTACTTTGTGAGACTACTTTTGAATGCTTGACCTCAAATCAGGTAGGACTACCC
GCTGAACTTAA
>A86
TTTCCGTAGGTGAACCTGCGGAAGGATCATTATTGAATTATGTTTCTAGATAGGTTGTAG
CTGGCTCTTTTAGAGCATGTGCACGCCTGTTTGGACTTCATTTTCATCCACCTGTGCACC
TATTGTAGTCTTTGGTTGGGTTAGGAGGAAGTGATCATTGTATCAGCATCTGCTGGGAGT
GAGGACTTGCATTGTGAAAGCTTTGCTGTCCTTGATGTGATCATGGAATCTTTTTCTCAC
TAGAGTCTATGTCACTCATTATACTCTGTCGAATGTCATTGAATGTCTTTACATGGGCTT
GTATGCCTATGAAAATTGTAATACAACTTTCAGCAACGGATCTCTTGGCTCTCGCATCGA
TGAAGAACGCAGCGAAATGCGATAAGTAATGTGAATTGCAGAATTCAGTGAATCATCGAA
TCTTTGAACGCATCTTGCGCTCCTTGGTATTCCGAGGAGCATGCCTGTTTGAGTGTCATT
AAATTCTCAACTCTCTTATACTTTTTTGTAAAAGAGAGCTTGGACTGTGGAGGCTTGCTG

GCCACTTTTTGGGGTCAGCTCCTCTGAAATGCATTAGCGGAACCGTTTGCGATCTGCCAC
AAGTGTGATAAGTTATCTACACTGGCGAGGGGATTGCTCTCTGTAATGTTCAGCTTCTAA
TTGTCTCTACTTTGTGAGACTACTTTTGAATGCTTGACCTCAAATCAGGTAGGACTACCC
GCTGAACTTAA
>A87
TTTCCGTAGGTGAACCTGCGGAAGGATCATTATTGAATTATGTTTCTAGATAGGTTGTAG
CTGGCTCTTTTAGAGCATGTGCACGCCTGTTTGGACTTCATTTTCATCCACCTGTGCACC
TATTGTAGTCTTTGGTTGGGTTAGGAGGAAGTGATCATTGTATCAGCATCTGCTGGGAGT
GAGGACTTGCATTGTGAAAGCTTTGCTGTCCTTGATGTGATCATGGAATCTTTTTCTCAC
TAGAGTCTATGTCACTCATTATACTCTGTCGAATGTCATTGAATGTCTTTACATGGGCTT
GTATGCCTATGAAAATTGTAATACAACTTTCAGCAACGGATCTCTTGGCTCTCGCATCGA
TGAAGAACGCAGCGAAATGCGATAAGTAATGTGAATTGCAGAATTCAGTGAATCATCGAA
TCTTTGAACGCATCTTGCGCTCCTTGGTATTCCGAGGAGCATGCCTGTTTGAGTGTCATT
AAATTCTCAACTCTCTTATACTTTTTTGTAAAAGAGAGCTTGGACTGTGGAGGCTTGCTG
GCCACTTTTTGGGGTCAGCTCCTCTGAAATGCATTAGCGGAACCGTTTGCGATCTGCCAC
AAGTGTGATAAGTTATCTACACTGGCGAGGGGATTGCTCTCTGTAATGTTCAGCTTCTAA
TTGTCTCTACTTTGTGAGACTACTTTTGAATGCTTGACCTCAAATCAGGTAGGACTACCC
GCTGAACTTAA
>A88
TTTCCGTAGGTGAACCTGCGGAAGGATCATTATTGAATTATGTTTCTAGATAGGTTGTAG
CTGGCTCTTTTAGAGCATGTGCACGCCTGTTTGGACTTCATTTTCATCCACCTGTGCACC
TATTGTAGTCTTTGGTTGGGTTAGGAGGAAGTGATCATTGTATCAGCATCTGCTGGGAGT
GAGGACTTGCATTGTGAAAGCTTTGCTGTCCTTGATGTGATCATGGAATCTTTTTCTCAC
TAGAGTCTATGTCACTCATTATACTCTGTCGAATGTCATTGAATGTCTTTACATGGGCTT
GTATGCCTATGAAAATTGTAATACAACTTTCAGCAACGGATCTCTTGGCTCTCGCATCGA
TGAAGAACGCAGCGAAATGCGATAAGTAATGTGAATTGCAGAATTCAGTGAATCATCGAA
TCTTTGAACGCATCTTGCGCTCCTTGGTATTCCGAGGAGCATGCCTGTTTGAGTGTCATT
AAATTCTCAACTCTCTTATACTTTTTTGTAAAAGAGAGCTTGGACTGTGGAGGCTTGCTG
GCCACTTTTTGGGGTCAGCTCCTCTGAAATGCATTAGCGGAACCGTTTGCGATCTGCCAC
AAGTGTGATAAGTTATCTACACTGGCGAGGGGATTGCTCTCTGTAATGTTCAGCTTCTAA
TTGTCTCTACTTTGTGAGACTACTTTTGAATGCTTGACCTCAAATCAGGTAGGACTACCC
GCTGAACTTAA
>A89
TTTCCGTAGGTGAACCTGCGGAAGGATCATTATTGAATTATGTTTCTAGATAGGTTGTAG
CTGGCTCTTTTAGAGCATGTGCACGCCTGTTTGGACTTCATTTTCATCCACCTGTGCACC
TATTGTAGTCTTTGGTTGGGTTAGGAGGAAGTGATCATTGTATCAGCATCTGCTGGGAGT
GAGGACTTGCATTGTGAAAGCTTTGCTGTCCTTGATGTGATCATGGAATCTTTTTCTCAC
TAGAGTCTATGTCACTCATTATACTCTGTCGAATGTCATTGAATGTCTTTACATGGGCTT
GTATGCCTATGAAAATTGTAATACAACTTTCAGCAACGGATCTCTTGGCTCTCGCATCGA
TGAAGAACGCAGCGAAATGCGATAAGTAATGTGAATTGCAGAATTCAGTGAATCATCGAA
TCTTTGAACGCATCTTGCGCTCCTTGGTATTCCGAGGAGCATGCCTGTTTGAGTGTCATT
AAATTCTCAACTCTCTTATACTTTTTTGTAAAAGAGAGCTTGGACTGTGGAGGCTTGCTG
GCCACTTTTTGGGGTCAGCTCCTCTGAAATGCATTAGCGGAACCGTTTGCGATCTGCCAC
AAGTGTGATAAGTTATCTACACTGGCGAGGGGATTGCTCTCTGTAATGTTCAGCTTCTAA
TTGTCTCTACTTTGTGAGACTACTTTTGAATGCTTGACCTCAAATCAGGTAGGACTACCC
GCTGAACTTAA
>A90
TTTCCGTAGGTGAACCTGCGGAAGGATCATTATTGAATTATGTTTCTAGATAGGTTGTAG
CTGGCTCTTTTAGAGCATGTGCACGCCTGTTTGGACTTCATTTTCATCCACCTGTGCACC
TATTGTAGTCTTTGGTTGGGTTAGGAGGAAGTGATCATTGTATCAGCATCTGCTGGGAGT

GAGGACTTGCATTGTGAAAGCTTTGCTGTCCTTGATGTGATCATGGAATCTTTTTCTCAC
TAGAGTCTATGTCACTCATTATACTCTGTCGAATGTCATTGAATGTCTTTACATGGGCTT
GTATGCCTATGAAAATTGTAATACAACTTTCAGCAACGGATCTCTTGGCTCTCGCATCGA
TGAAGAACGCAGCGAAATGCGATAAGTAATGTGAATTGCAGAATTCAGTGAATCATCGAA
TCTTTGAACGCATCTTGCGCTCCTTGGTATTCCGAGGAGCATGCCTGTTTGAGTGTCATT
AAATTCTCAACTCTCTTATACTTTTTTGTAAAAGAGAGCTTGGACTGTGGAGGCTTGCTG
GCCACTTTTTGGGGTCAGCTCCTCTGAAATGCATTAGCGGAACCGTTTGCGATCTGCCAC
AAGTGTGATAAGTTATCTACACTGGCGAGGGGATTGCTCTCTGTAATGTTCAGCTTCTAA
TTGTCTCTACTTTGTGAGACTACTTTTGAATGCTTGACCTCAAATCAGGTAGGACTACCC
GCTGAACTTAA
>A91
TTTCCGTAGGTGAACCTGCGGAAGGATCATTATTGAATTATGTTTCTAGATAGGTTGTAG
CTGGCTCTTTTAGAGCATGTGCACGCCTGTTTGGACTTCATTTTCATCCACCTGTGCACC
TATTGTAGTCTTTGGTTGGGTTAGGAGGAAGTGATCATTGTATCAGCATCTGCTGGGAGT
GAGGACTTGCATTGTGAAAGCTTTGCTGTCCTTGATGTGATCATGGAATCTTTTTCTCAC
TAGAGTCTATGTCACTCATTATACTCTGTCGAATGTCATTGAATGTCTTTACATGGGCTT
GTATGCCTATGAAAATTGTAATACAACTTTCAGCAACGGATCTCTTGGCTCTCGCATCGA
TGAAGAACGCAGCGAAATGCGATAAGTAATGTGAATTGCAGAATTCAGTGAATCATCGAA
TCTTTGAACGCATCTTGCGCTCCTTGGTATTCCGAGGAGCATGCCTGTTTGAGTGTCATT
AAATTCTCAACTCTCTTATACTTTTTTGTAAAAGAGAGCTTGGACTGTGGAGGCTTGCTG
GCCACTTTTTGGGGTCAGCTCCTCTGAAATGCATTAGCGGAACCGTTTGCGATCTGCCAC
AAGTGTGATAAGTTATCTACACTGGCGAGGGGATTGCTCTCTGTAATGTTCAGCTTCTAA
TTGTCTCTACTTTGTGAGACTACTTTTGAATGCTTGACCTCAAATCAGGTAGGACTACCC
GCTGAACTTAA
>A92
TTTCCGTAGGTGAACCTGCGGAAGGATCATTATTGAATTATGTTTCTAGATAGGTTGTAG
CTGGCTCTTTTAGAGCATGTGCACGCCTGTTTGGACTTCATTTTCATCCACCTGTGCACC
TATTGTAGTCTTTGGTTGGGTTAGGAGGAAGTGATCATTGTATCAGCATCTGCTGGGAGT
GAGGACTTGCATTGTGAAAGCTTTGCTGTCCTTGATGTGATCATGGAATCTTTTTCTCAC
TAGAGTCTATGTCACTCATTATACTCTGTCGAATGTCATTGAATGTCTTTACATGGGCTT
GTATGCCTATGAAAATTGTAATACAACTTTCAGCAACGGATCTCTTGGCTCTCGCATCGA
TGAAGAACGCAGCGAAATGCGATAAGTAATGTGAATTGCAGAATTCAGTGAATCATCGAA
TCTTTGAACGCATCTTGCGCTCCTTGGTATTCCGAGGAGCATGCCTGTTTGAGTGTCATT
AAATTCTCAACTCTCTTATACTTTTTTGTAAAAGAGAGCTTGGACTGTGGAGGCTTGCTG
GCCACTTTTTGGGGTCAGCTCCTCTGAAATGCATTAGCGGAACCGTTTGCGATCTGCCAC
AAGTGTGATAAGTTATCTACACTGGCGAGGGGATTGCTCTCTGTAATGTTCAGCTTCTAA
TTGTCTCTACTTTGTGAGACTACTTTTGAATGCTTGACCTCAAATCAGGTAGGACTACCC
GCTGAACTTAA
>A93
TTTCCGTAGGTGAACCTGCGGAAGGATCATTATTGAATTATGTTTCTAGATAGGTTGTAG
CTGGCTCTTTTAGAGCATGTGCACGCCTGTTTGGACTTCATTTTCATCCACCTGTGCACC
TATTGTAGTCTTTGGTTGGGTTAGGAGGAAGTGATCATTGTATCAGCATCTGCTGGGAGT
GAGGACTTGCATTGTGAAAGCTTTGCTGTCCTTGATGTGATCATGGAATCTTTTTCTCAC
TAGAGTCTATGTCACTCATTATACTCTGTCGAATGTCATTGAATGTCTTTACATGGGCTT
GTATGCCTATGAAAATTGTAATACAACTTTCAGCAACGGATCTCTTGGCTCTCGCATCGA
TGAAGAACGCAGCGAAATGCGATAAGTAATGTGAATTGCAGAATTCAGTGAATCATCGAA
TCTTTGAACGCATCTTGCGCTCCTTGGTATTCCGAGGAGCATGCCTGTTTGAGTGTCATT
AAATTCTCAACTCTCTTATACTTTTTTGTAAAAGAGAGCTTGGACTGTGGAGGCTTGCTG
GCCACTTTTTGGGGTCAGCTCCTCTGAAATGCATTAGCGGAACCGTTTGCGATCTGCCAC
AAGTGTGATAAGTTATCTACACTGGCGAGGGGATTGCTCTCTGTAATGTTCAGCTTCTAA

TTGTCTCTACTTTGTGAGACTACTTTTGAATGCTTGACCTCAAATCAGGTAGGACTACCC
GCTGAACTTAA
>A94
TTTCCGTAGGTGAACCTGCGGAAGGATCATTATTGAATTATGTTTCTAGATAGGTTGTAG
CTGGCTCTTTTAGAGCATGTGCACGCCTGTTTGGACTTCATTTTCATCCACCTGTGCACC
TATTGTAGTCTTTGGTTGGGTTAGGAGGAAGTGATCATTGTATCAGCATCTGCTGGGAGT
GAGGACTTGCATTGTGAAAGCTTTGCTGTCCTTGATGTGATCATGGAATCTTTTTCTCAC
TAGAGTCTATGTCACTCATTATACTCTGTCGAATGTCATTGAATGTCTTTACATGGGCTT
GTATGCCTATGAAAATTGTAATACAACTTTCAGCAACGGATCTCTTGGCTCTCGCATCGA
TGAAGAACGCAGCGAAATGCGATAAGTAATGTGAATTGCAGAATTCAGTGAATCATCGAA
TCTTTGAACGCATCTTGCGCTCCTTGGTATTCCGAGGAGCATGCCTGTTTGAGTGTCATT
AAATTCTCAACTCTCTTATACTTTTTTGTAAAAGAGAGCTTGGACTGTGGAGGCTTGCTG
GCCACTTTTTGGGGTCAGCTCCTCTGAAATGCATTAGCGGAACCGTTTGCGATCTGCCAC
AAGTGTGATAAGTTATCTACACTGGCGAGGGGATTGCTCTCTGTAATGTTCAGCTTCTAA
TTGTCTCTACTTTGTGAGACTACTTTTGAATGCTTGACCTCAAATCAGGTAGGACTACCC
GCTGAACTTAA
>A95
TTTCCGTAGGTGAACCTGCGGAAGGATCATTATTGAATTATGTTTCTAGATAGGTTGTAG
CTGGCTCTTTTAGAGCATGTGCACGCCTGTTTGGACTTCATTTTCATCCACCTGTGCACC
TATTGTAGTCTTTGGTTGGGTTAGGAGGAAGTGATCATTGTATCAGCATCTGCTGGGAGT
GAGGACTTGCATTGTGAAAGCTTTGCTGTCCTTGATGTGATCATGGAATCTTTTTCTCAC
TAGAGTCTATGTCACTCATTATACTCTGTCGAATGTCATTGAATGTCTTTACATGGGCTT
GTATGCCTATGAAAATTGTAATACAACTTTCAGCAACGGATCTCTTGGCTCTCGCATCGA
TGAAGAACGCAGCGAAATGCGATAAGTAATGTGAATTGCAGAATTCAGTGAATCATCGAA
TCTTTGAACGCATCTTGCGCTCCTTGGTATTCCGAGGAGCATGCCTGTTTGAGTGTCATT
AAATTCTCAACTCTCTTATACTTTTTTGTAAAAGAGAGCTTGGACTGTGGAGGCTTGCTG
GCCACTTTTTGGGGTCAGCTCCTCTGAAATGCATTAGCGGAACCGTTTGCGATCTGCCAC
AAGTGTGATAAGTTATCTACACTGGCGAGGGGATTGCTCTCTGTAATGTTCAGCTTCTAA
TTGTCTCTACTTTGTGAGACTACTTTTGAATGCTTGACCTCAAATCAGGTAGGACTACCC
GCTGAACTTAA
>A96
TTTCCGTAGGTGAACCTGCGGAAGGATCATTATTGAATTATGTTTCTAGATAGGTTGTAG
CTGGCTCTTTTAGAGCATGTGCACGCCTGTTTGGACTTCATTTTCATCCACCTGTGCACC
TATTGTAGTCTTTGGTTGGGTTAGGAGGAAGTGATCATTGTATCAGCATCTGCTGGGAGT
GAGGACTTGCATTGTGAAAGCTTTGCTGTCCTTGATGTGATCATGGAATCTTTTTCTCAC
TAGAGTCTATGTCACTCATTATACTCTGTCGAATGTCATTGAATGTCTTTACATGGGCTT
GTATGCCTATGAAAATTGTAATACAACTTTCAGCAACGGATCTCTTGGCTCTCGCATCGA
TGAAGAACGCAGCGAAATGCGATAAGTAATGTGAATTGCAGAATTCAGTGAATCATCGAA
TCTTTGAACGCATCTTGCGCTCCTTGGTATTCCGAGGAGCATGCCTGTTTGAGTGTCATT
AAATTCTCAACTCTCTTATACTTTTTTGTAAAAGAGAGCTTGGACTGTGGAGGCTTGCTG
GCCACTTTTTGGGGTCAGCTCCTCTGAAATGCATTAGCGGAACCGTTTGCGATCTGCCAC
AAGTGTGATAAGTTATCTACACTGGCGAGGGGATTGCTCTCTGTAATGTTCAGCTTCTAA
TTGTCTCTACTTTGTGAGACTACTTTTGAATGCTTGACCTCAAATCAGGTAGGACTACCC
GCTGAACTTAA
>A97
TTTCCGTAGGTGAACCTGCGGAAGGATCATTATTGAATTATGTTTCTAGATAGGTTGTAG
CTGGCTCTTTTAGAGCATGTGCACGCCTGTTTGGACTTCATTTTCATCCACCTGTGCACC
TATTGTAGTCTTTGGTTGGGTTAGGAGGAAGTGATCATTGTATCAGCATCTGCTGGGAGT
GAGGACTTGCATTGTGAAAGCTTTGCTGTCCTTGATGTGATCATGGAATCTTTTTCTCAC
TAGAGTCTATGTCACTCATTATACTCTGTCGAATGTCATTGAATGTCTTTACATGGGCTT

GTATGCCTATGAAAATTGTAATACAACTTTCAGCAACGGATCTCTTGGCTCTCGCATCGA
TGAAGAACGCAGCGAAATGCGATAAGTAATGTGAATTGCAGAATTCAGTGAATCATCGAA
TCTTTGAACGCATCTTGCGCTCCTTGGTATTCCGAGGAGCATGCCTGTTTGAGTGTCATT
AAATTCTCAACTCTCTTATACTTTTTTGTAAAAGAGAGCTTGGACTGTGGAGGCTTGCTG
GCCACTTTTTGGGGTCAGCTCCTCTGAAATGCATTAGCGGAACCGTTTGCGATCTGCCAC
AAGTGTGATAAGTTATCTACACTGGCGAGGGGATTGCTCTCTGTAATGTTCAGCTTCTAA
TTGTCTCTACTTTGTGAGACTACTTTTGAATGCTTGACCTCAAATCAGGTAGGACTACCC
GCTGAACTTAA
>A98
TTTCCGTAGGTGAACCTGCGGAAGGATCATTATTGAATTATGTTTCTAGATAGGTTGTAG
CTGGCTCTTTTAGAGCATGTGCACGCCTGTTTGGACTTCATTTTCATCCACCTGTGCACC
TATTGTAGTCTTTGGTTGGGTTAGGAGGAAGTGATCATTGTATCAGCATCTGCTGGGAGT
GAGGACTTGCATTGTGAAAGCTTTGCTGTCCTTGATGTGATCATGGAATCTTTTTCTCAC
TAGAGTCTATGTCACTCATTATACTCTGTCGAATGTCATTGAATGTCTTTACATGGGCTT
GTATGCCTATGAAAATTGTAATACAACTTTCAGCAACGGATCTCTTGGCTCTCGCATCGA
TGAAGAACGCAGCGAAATGCGATAAGTAATGTGAATTGCAGAATTCAGTGAATCATCGAA
TCTTTGAACGCATCTTGCGCTCCTTGGTATTCCGAGGAGCATGCCTGTTTGAGTGTCATT
AAATTCTCAACTCTCTTATACTTTTTTGTAAAAGAGAGCTTGGACTGTGGAGGCTTGCTG
GCCACTTTTTGGGGTCAGCTCCTCTGAAATGCATTAGCGGAACCGTTTGCGATCTGCCAC
AAGTGTGATAAGTTATCTACACTGGCGAGGGGATTGCTCTCTGTAATGTTCAGCTTCTAA
TTGTCTCTACTTTGTGAGACTACTTTTGAATGCTTGACCTCAAATCAGGTAGGACTACCC
GCTGAACTTAA
>A99
TTTCCGTAGGTGAACCTGCGGAAGGATCATTATTGAATTATGTTTCTAGATAGGTTGTAG
CTGGCTCTTTTAGAGCATGTGCACGCCTGTTTGGACTTCATTTTCATCCACCTGTGCACC
TATTGTAGTCTTTGGTTGGGTTAGGAGGAAGTGATCATTGTATCAGCATCTGCTGGGAGT
GAGGACTTGCATTGTGAAAGCTTTGCTGTCCTTGATGTGATCATGGAATCTTTTTCTCAC
TAGAGTCTATGTCACTCATTATACTCTGTCGAATGTCATTGAATGTCTTTACATGGGCTT
GTATGCCTATGAAAATTGTAATACAACTTTCAGCAACGGATCTCTTGGCTCTCGCATCGA
TGAAGAACGCAGCGAAATGCGATAAGTAATGTGAATTGCAGAATTCAGTGAATCATCGAA
TCTTTGAACGCATCTTGCGCTCCTTGGTATTCCGAGGAGCATGCCTGTTTGAGTGTCATT
AAATTCTCAACTCTCTTATACTTTTTTGTAAAAGAGAGCTTGGACTGTGGAGGCTTGCTG
GCCACTTTTTGGGGTCAGCTCCTCTGAAATGCATTAGCGGAACCGTTTGCGATCTGCCAC
AAGTGTGATAAGTTATCTACACTGGCGAGGGGATTGCTCTCTGTAATGTTCAGCTTCTAA
TTGTCTCTACTTTGTGAGACTACTTTTGAATGCTTGACCTCAAATCAGGTAGGACTACCC
GCTGAACTTAA
>A100
TTTCCGTAGGTGAACCTGCGGAAGGATCATTATTGAATTATGTTTCTAGATAGGTTGTAG
CTGGCTCTTTTAGAGCATGTGCACGCCTGTTTGGACTTCATTTTCATCCACCTGTGCACC
TATTGTAGTCTTTGGTTGGGTTAGGAGGAAGTGATCATTGTATCAGCATCTGCTGGGAGT
GAGGACTTGCATTGTGAAAGCTTTGCTGTCCTTGATGTGATCATGGAATCTTTTTCTCAC
TAGAGTCTATGTCACTCATTATACTCTGTCGAATGTCATTGAATGTCTTTACATGGGCTT
GTATGCCTATGAAAATTGTAATACAACTTTCAGCAACGGATCTCTTGGCTCTCGCATCGA
TGAAGAACGCAGCGAAATGCGATAAGTAATGTGAATTGCAGAATTCAGTGAATCATCGAA
TCTTTGAACGCATCTTGCGCTCCTTGGTATTCCGAGGAGCATGCCTGTTTGAGTGTCATT
AAATTCTCAACTCTCTTATACTTTTTTGTAAAAGAGAGCTTGGACTGTGGAGGCTTGCTG
GCCACTTTTTGGGGTCAGCTCCTCTGAAATGCATTAGCGGAACCGTTTGCGATCTGCCAC
AAGTGTGATAAGTTATCTACACTGGCGAGGGGATTGCTCTCTGTAATGTTCAGCTTCTAA
TTGTCTCTACTTTGTGAGACTACTTTTGAATGCTTGACCTCAAATCAGGTAGGACTACCC
GCTGAACTTAA

>A101
TTTCCGTAGGTGAACCTGCGGAAGGATCATTATTGAATTATGTTTCTAGATAGGTTGTAG
CTGGCTCTTTTAGAGCATGTGCACGCCTGTTTGGACTTCATTTTCATCCACCTGTGCACC
TATTGTAGTCTTTGGTTGGGTTAGGAGGAAGTGATCATTGTATCAGCATCTGCTGGGAGT
GAGGACTTGCATTGTGAAAGCTTTGCTGTCCTTGATGTGATCATGGAATCTTTTTCTCAC
TAGAGTCTATGTCACTCATTATACTCTGTCGAATGTCATTGAATGTCTTTACATGGGCTT
GTATGCCTATGAAAATTGTAATACAACTTTCAGCAACGGATCTCTTGGCTCTCGCATCGA
TGAAGAACGCAGCGAAATGCGATAAGTAATGTGAATTGCAGAATTCAGTGAATCATCGAA
TCTTTGAACGCATCTTGCGCTCCTTGGTATTCCGAGGAGCATGCCTGTTTGAGTGTCATT
AAATTCTCAACTCTCTTATACTTTTTTGTAAAAGAGAGCTTGGACTGTGGAGGCTTGCTG
GCCACTTTTTGGGGTCAGCTCCTCTGAAATGCATTAGCGGAACCGTTTGCGATCTGCCAC
AAGTGTGATAAGTTATCTACACTGGCGAGGGGATTGCTCTCTGTAATGTTCAGCTTCTAA
TTGTCTCTACTTTGTGAGACTACTTTTGAATGCTTGACCTCAAATCAGGTAGGACTACCC
GCTGAACTTAA
>A102
TTTCCGTAGGTGAACCTGCGGAAGGATCATTATTGAATTATGTTTCTAGATAGGTTGTAG
CTGGCTCTTTTAGAGCATGTGCACGCCTGTTTGGACTTCATTTTCATCCACCTGTGCACC
TATTGTAGTCTTTGGTTGGGTTAGGAGGAAGTGATCATTGTATCAGCATCTGCTGGGAGT
GAGGACTTGCATTGTGAAAGCTTTGCTGTCCTTGATGTGATCATGGAATCTTTTTCTCAC
TAGAGTCTATGTCACTCATTATACTCTGTCGAATGTCATTGAATGTCTTTACATGGGCTT
GTATGCCTATGAAAATTGTAATACAACTTTCAGCAACGGATCTCTTGGCTCTCGCATCGA
TGAAGAACGCAGCGAAATGCGATAAGTAATGTGAATTGCAGAATTCAGTGAATCATCGAA
TCTTTGAACGCATCTTGCGCTCCTTGGTATTCCGAGGAGCATGCCTGTTTGAGTGTCATT
AAATTCTCAACTCTCTTATACTTTTTTGTAAAAGAGAGCTTGGACTGTGGAGGCTTGCTG
GCCACTTTTTGGGGTCAGCTCCTCTGAAATGCATTAGCGGAACCGTTTGCGATCTGCCAC
AAGTGTGATAAGTTATCTACACTGGCGAGGGGATTGCTCTCTGTAATGTTCAGCTTCTAA
TTGTCTCTACTTTGTGAGACTACTTTTGAATGCTTGACCTCAAATCAGGTAGGACTACCC
GCTGAACTTAA
>A103
TTTCCGTAGGTGAACCTGCGGAAGGATCATTATTGAATTATGTTTCTAGATAGGTTGTAG
CTGGCTCTTTTAGAGCATGTGCACGCCTGTTTGGACTTCATTTTCATCCACCTGTGCACC
TATTGTAGTCTTTGGTTGGGTTAGGAGGAAGTGATCATTGTATCAGCATCTGCTGGGAGT
GAGGACTTGCATTGTGAAAGCTTTGCTGTCCTTGATGTGATCATGGAATCTTTTTCTCAC
TAGAGTCTATGTCACTCATTATACTCTGTCGAATGTCATTGAATGTCTTTACATGGGCTT
GTATGCCTATGAAAATTGTAATACAACTTTCAGCAACGGATCTCTTGGCTCTCGCATCGA
TGAAGAACGCAGCGAAATGCGATAAGTAATGTGAATTGCAGAATTCAGTGAATCATCGAA
TCTTTGAACGCATCTTGCGCTCCTTGGTATTCCGAGGAGCATGCCTGTTTGAGTGTCATT
AAATTCTCAACTCTCTTATACTTTTTTGTAAAAGAGAGCTTGGACTGTGGAGGCTTGCTG
GCCACTTTTTGGGGTCAGCTCCTCTGAAATGCATTAGCGGAACCGTTTGCGATCTGCCAC
AAGTGTGATAAGTTATCTACACTGGCGAGGGGATTGCTCTCTGTAATGTTCAGCTTCTAA
TTGTCTCTACTTTGTGAGACTACTTTTGAATGCTTGACCTCAAATCAGGTAGGACTACCC
GCTGAACTTAA
>A104
TTTCCGTAGGTGAACCTGCGGAAGGATCATTATTGAATTATGTTTCTAGATAGGTTGTAG
CTGGCTCTTTTAGAGCATGTGCACGCCTGTTTGGACTTCATTTTCATCCACCTGTGCACC
TATTGTAGTCTTTGGTTGGGTTAGGAGGAAGTGATCATTGTATCAGCATCTGCTGGGAGT
GAGGACTTGCATTGTGAAAGCTTTGCTGTCCTTGATGTGATCATGGAATCTTTTTCTCAC
TAGAGTCTATGTCACTCATTATACTCTGTCGAATGTCATTGAATGTCTTTACATGGGCTT
GTATGCCTATGAAAATTGTAATACAACTTTCAGCAACGGATCTCTTGGCTCTCGCATCGA
TGAAGAACGCAGCGAAATGCGATAAGTAATGTGAATTGCAGAATTCAGTGAATCATCGAA

TCTTTGAACGCATCTTGCGCTCCTTGGTATTCCGAGGAGCATGCCTGTTTGAGTGTCATT
AAATTCTCAACTCTCTTATACTTTTTTGTAAAAGAGAGCTTGGACTGTGGAGGCTTGCTG
GCCACTTTTTGGGGTCAGCTCCTCTGAAATGCATTAGCGGAACCGTTTGCGATCTGCCAC
AAGTGTGATAAGTTATCTACACTGGCGAGGGGATTGCTCTCTGTAATGTTCAGCTTCTAA
TTGTCTCTACTTTGTGAGACTACTTTTGAATGCTTGACCTCAAATCAGGTAGGACTACCC
GCTGAACTTAA
>A105
TTTCCGTAGGTGAACCTGCGGAAGGATCATTATTGAATTATGTTTCTAGATAGGTTGTAG
CTGGCTCTTTTAGAGCATGTGCACGCCTGTTTGGACTTCATTTTCATCCACCTGTGCACC
TATTGTAGTCTTTGGTTGGGTTAGGAGGAAGTGATCATTGTATCAGCATCTGCTGGGAGT
GAGGACTTGCATTGTGAAAGCTTTGCTGTCCTTGATGTGATCATGGAATCTTTTTCTCAC
TAGAGTCTATGTCACTCATTATACTCTGTCGAATGTCATTGAATGTCTTTACATGGGCTT
GTATGCCTATGAAAATTGTAATACAACTTTCAGCAACGGATCTCTTGGCTCTCGCATCGA
TGAAGAACGCAGCGAAATGCGATAAGTAATGTGAATTGCAGAATTCAGTGAATCATCGAA
TCTTTGAACGCATCTTGCGCTCCTTGGTATTCCGAGGAGCATGCCTGTTTGAGTGTCATT
AAATTCTCAACTCTCTTATACTTTTTTGTAAAAGAGAGCTTGGACTGTGGAGGCTTGCTG
GCCACTTTTTGGGGTCAGCTCCTCTGAAATGCATTAGCGGAACCGTTTGCGATCTGCCAC
AAGTGTGATAAGTTATCTACACTGGCGAGGGGATTGCTCTCTGTAATGTTCAGCTTCTAA
TTGTCTCTACTTTGTGAGACTACTTTTGAATGCTTGACCTCAAATCAGGTAGGACTACCC
GCTGAACTTAA
>A106
TTTCCGTAGGTGAACCTGCGGAAGGATCATTATTGAATTATGTTTCTAGATAGGTTGTAG
CTGGCTCTTTTAGAGCATGTGCACGCCTGTTTGGACTTCATTTTCATCCACCTGTGCACC
TATTGTAGTCTTTGGTTGGGTTAGGAGGAAGTGATCATTGTATCAGCATCTGCTGGGAGT
GAGGACTTGCATTGTGAAAGCTTTGCTGTCCTTGATGTGATCATGGAATCTTTTTCTCAC
TAGAGTCTATGTCACTCATTATACTCTGTCGAATGTCATTGAATGTCTTTACATGGGCTT
GTATGCCTATGAAAATTGTAATACAACTTTCAGCAACGGATCTCTTGGCTCTCGCATCGA
TGAAGAACGCAGCGAAATGCGATAAGTAATGTGAATTGCAGAATTCAGTGAATCATCGAA
TCTTTGAACGCATCTTGCGCTCCTTGGTATTCCGAGGAGCATGCCTGTTTGAGTGTCATT
AAATTCTCAACTCTCTTATACTTTTTTGTAAAAGAGAGCTTGGACTGTGGAGGCTTGCTG
GCCACTTTTTGGGGTCAGCTCCTCTGAAATGCATTAGCGGAACCGTTTGCGATCTGCCAC
AAGTGTGATAAGTTATCTACACTGGCGAGGGGATTGCTCTCTGTAATGTTCAGCTTCTAA
TTGTCTCTACTTTGTGAGACTACTTTTGAATGCTTGACCTCAAATCAGGTAGGACTACCC
GCTGAACTTAA
>A107
TTTCCGTAGGTGAACCTGCGGAAGGATCATTATTGAATTATGTTTCTAGATAGGTTGTAG
CTGGCTCTTTTAGAGCATGTGCACGCCTGTTTGGACTTCATTTTCATCCACCTGTGCACC
TATTGTAGTCTTTGGTTGGGTTAGGAGGAAGTGATCATTGTATCAGCATCTGCTGGGAGT
GAGGACTTGCATTGTGAAAGCTTTGCTGTCCTTGATGTGATCATGGAATCTTTTTCTCAC
TAGAGTCTATGTCACTCATTATACTCTGTCGAATGTCATTGAATGTCTTTACATGGGCTT
GTATGCCTATGAAAATTGTAATACAACTTTCAGCAACGGATCTCTTGGCTCTCGCATCGA
TGAAGAACGCAGCGAAATGCGATAAGTAATGTGAATTGCAGAATTCAGTGAATCATCGAA
TCTTTGAACGCATCTTGCGCTCCTTGGTATTCCGAGGAGCATGCCTGTTTGAGTGTCATT
AAATTCTCAACTCTCTTATACTTTTTTGTAAAAGAGAGCTTGGACTGTGGAGGCTTGCTG
GCCACTTTTTGGGGTCAGCTCCTCTGAAATGCATTAGCGGAACCGTTTGCGATCTGCCAC
AAGTGTGATAAGTTATCTACACTGGCGAGGGGATTGCTCTCTGTAATGTTCAGCTTCTAA
TTGTCTCTACTTTGTGAGACTACTTTTGAATGCTTGACCTCAAATCAGGTAGGACTACCC
GCTGAACTTAA
>A108
TTTCCGTAGGTGAACCTGCGGAAGGATCATTATTGAATTATGTTTCTAGATAGGTTGTAG

CTGGCTCTTTTAGAGCATGTGCACGCCTGTTTGGACTTCATTTTCATCCACCTGTGCACC
TATTGTAGTCTTTGGTTGGGTTAGGAGGAAGTGATCATTGTATCAGCATCTGCTGGGAGT
GAGGACTTGCATTGTGAAAGCTTTGCTGTCCTTGATGTGATCATGGAATCTTTTTCTCAC
TAGAGTCTATGTCACTCATTATACTCTGTCGAATGTCATTGAATGTCTTTACATGGGCTT
GTATGCCTATGAAAATTGTAATACAACTTTCAGCAACGGATCTCTTGGCTCTCGCATCGA
TGAAGAACGCAGCGAAATGCGATAAGTAATGTGAATTGCAGAATTCAGTGAATCATCGAA
TCTTTGAACGCATCTTGCGCTCCTTGGTATTCCGAGGAGCATGCCTGTTTGAGTGTCATT
AAATTCTCAACTCTCTTATACTTTTTTGTAAAAGAGAGCTTGGACTGTGGAGGCTTGCTG
GCCACTTTTTGGGGTCAGCTCCTCTGAAATGCATTAGCGGAACCGTTTGCGATCTGCCAC
AAGTGTGATAAGTTATCTACACTGGCGAGGGGATTGCTCTCTGTAATGTTCAGCTTCTAA
TTGTCTCTACTTTGTGAGACTACTTTTGAATGCTTGACCTCAAATCAGGTAGGACTACCC
GCTGAACTTAA
>A109
TTTCCGTAGGTGAACCTGCGGAAGGATCATTATTGAATTATGTTTCTAGATAGGTTGTAG
CTGGCTCTTTTAGAGCATGTGCACGCCTGTTTGGACTTCATTTTCATCCACCTGTGCACC
TATTGTAGTCTTTGGTTGGGTTAGGAGGAAGTGATCATTGTATCAGCATCTGCTGGGAGT
GAGGACTTGCATTGTGAAAGCTTTGCTGTCCTTGATGTGATCATGGAATCTTTTTCTCAC
TAGAGTCTATGTCACTCATTATACTCTGTCGAATGTCATTGAATGTCTTTACATGGGCTT
GTATGCCTATGAAAATTGTAATACAACTTTCAGCAACGGATCTCTTGGCTCTCGCATCGA
TGAAGAACGCAGCGAAATGCGATAAGTAATGTGAATTGCAGAATTCAGTGAATCATCGAA
TCTTTGAACGCATCTTGCGCTCCTTGGTATTCCGAGGAGCATGCCTGTTTGAGTGTCATT
AAATTCTCAACTCTCTTATACTTTTTTGTAAAAGAGAGCTTGGACTGTGGAGGCTTGCTG
GCCACTTTTTGGGGTCAGCTCCTCTGAAATGCATTAGCGGAACCGTTTGCGATCTGCCAC
AAGTGTGATAAGTTATCTACACTGGCGAGGGGATTGCTCTCTGTAATGTTCAGCTTCTAA
TTGTCTCTACTTTGTGAGACTACTTTTGAATGCTTGACCTCAAATCAGGTAGGACTACCC
GCTGAACTTAA
>A110
TTTCCGTAGGTGAACCTGCGGAAGGATCATTATTGAATTATGTTTCTAGATAGGTTGTAG
CTGGCTCTTTTAGAGCATGTGCACGCCTGTTTGGACTTCATTTTCATCCACCTGTGCACC
TATTGTAGTCTTTGGTTGGGTTAGGAGGAAGTGATCATTGTATCAGCATCTGCTGGGAGT
GAGGACTTGCATTGTGAAAGCTTTGCTGTCCTTGATGTGATCATGGAATCTTTTTCTCAC
TAGAGTCTATGTCACTCATTATACTCTGTCGAATGTCATTGAATGTCTTTACATGGGCTT
GTATGCCTATGAAAATTGTAATACAACTTTCAGCAACGGATCTCTTGGCTCTCGCATCGA
TGAAGAACGCAGCGAAATGCGATAAGTAATGTGAATTGCAGAATTCAGTGAATCATCGAA
TCTTTGAACGCATCTTGCGCTCCTTGGTATTCCGAGGAGCATGCCTGTTTGAGTGTCATT
AAATTCTCAACTCTCTTATACTTTTTTGTAAAAGAGAGCTTGGACTGTGGAGGCTTGCTG
GCCACTTTTTGGGGTCAGCTCCTCTGAAATGCATTAGCGGAACCGTTTGCGATCTGCCAC
AAGTGTGATAAGTTATCTACACTGGCGAGGGGATTGCTCTCTGTAATGTTCAGCTTCTAA
TTGTCTCTACTTTGTGAGACTACTTTTGAATGCTTGACCTCAAATCAGGTAGGACTACCC
GCTGAACTTAA
