# Supplementary material for: Patterns of PCR Amplification Artifacts of the Fungal Barcode Marker in a Hybrid Mushroom
Source: Front Microbiol. 2019 Nov 19;10:2686. doi: 10.3389/fmicb.2019.02686 (PMC6877668; doi:10.3389/fmicb.2019.02686)
Supplement: Supplementary file 4 [file Data_Sheet_4.PDF]

>C1
TTTCCGTAGGTGAACCTGCGGAAGGATCATTATTGAATTATGTTTCTAGATAGGTTGTAG
CTGGCTCTTTAGAGCATGTGCACGCCTGTTTGGACTTCATTTTCATCCACCTGTGCACCT
ATTGTAGTCTTTGGTTGGGTTAGGAGGAAGTGGTCATTGTGTCAGCATCTGCTGGATGTG
AGGACTTGCATTGTGAAAGCTTTGCTGTCCTTGATGTGATCATGGAATCTCTTTCTCACT
AGAGTCTATGTCACTCATTATACTCTGTCGAATGTCATTGAATGTCTTTACATGGGCTTA
TATGCCTATGAAAATTGTAATACAACTTTCAGCAACGGATCTCTTGGCTCTCGCATCGAT
GAAGAACGCAGCGAAATGCGATAAGTAATGTGAATTGCAGAATTCAGTGAATCATCGAAT
CTTTGAACGCATCTTGCGCTCCTTGGTATTCCGAGGAGCATGCCTGTTTGAGTGTCATTA
AATTCTCAACTCTCTTCTACTTTTTGTAAAAGAGAGCTTGGACTGTGGAGGCTTGCTGGC
CACTTTTTGGGGTCAGCTCCTCTGAAATGCATTAGCGGAACCGTTTGCGATCTGCCACAA
GTGTGATAAGTTATCTACACTGGCGAGGGGATTGCTCTCTGTAATGTTCAGCTTCTAATT
GTCTCTACTTTGTGAGACTACTTTTGAATGCTTGACCTCAAATCAGGTAGGACTACCCGC
TGAACTTAA
>C2
TTTCCGTAGGTGAACCTGCGGAAGGATCATTATTGAATTATGTTTCTAGATAGGTTGTAG
CTGGCTCTTTAGAGCATGTGCACGCCTGTTTGGACTTCATTTTCATCCACCTGTGCACCT
ATTGTAGTCTTTGGTTGGGTTAGGAGGAAGTGGTCATTGTGTCAGCATCTGCTGGATGTG
AGGACTTGCATTGTGAAAGCTTTGCTGTCCTTGATGTGATCATGGAATCTCTTTCTCACT
AGAGTCTATGTCACTCATTATACTCTGTCGAATGTCATTGAATGTCTTTACATGGGCTTA
TATGCCTATGAAAATTGTAATACAACTTTCAGCAACGGATCTCTTGGCTCTCGCATCGAT
GAAGAACGCAGCGAAATGCGATAAGTAATGTGAATTGCAGAATTCAGTGAATCATCGAAT
CTTTGAACGCATCTTGCGCTCCTTGGTATTCCGAGGAGCATGCCTGTTTGAGTGTCATTA
AATTCTCAACTCTCTTCTACTTTTTGTAAAAGAGAGCTTGGACTGTGGAGGCTTGCTGGC
CACTTTTTGGGGTCAGCTCCTCTGAAATGCATTAGCGGAACCGTTTGCGATCTGCCACAA
GTGTGATAAGTTATCTACACTGGCGAGGGGATTGCTCTCTGTAATGTTCAGCTTCTAATT
GTCTCTACTTTGTGAGACTACTTTTGAATGCTTGACCTCAAATCAGGTAGGACTACCCGC
TGAACTTAA
>C3
TTTCCGTAGGTGAACCTGCGGAAGGATCATTATTGAATTATGTTTCTAGATAGGTTGTAG
CTGGCTCTTTAGAGCATGTGCACGCCTGTTTGGACTTCATTTTCATCCACCTGTGCACCT
ATTGTAGTCTTTGGTTGGGTTAGGAGGAAGTGGTCATTGTGTCAGCATCTGCTGGATGTG
AGGACTTGCATTGTGAAAGCTTTGCTGTCCTTGATGTGATCATGGAATCTCTTTCTCACT
AGAGTCTATGTCACTCATTATACTCTGTCGAATGTCATTGAATGTCTTTACATGGGCTTA
TATGCCTATGAAAATTGTAATACAACTTTCAGCAACGGATCTCTTGGCTCTCGCATCGAT
GAAGAACGCAGCGAAATGCGATAAGTAATGTGAATTGCAGAATTCAGTGAATCATCGAAT
CTTTGAACGCATCTTGCGCTCCTTGGTATTCCGAGGAGCATGCCTGTTTGAGTGTCATTA
AATTCTCAACTCTCTTCTACTTTTTGTAAAAGAGAGCTTGGACTGTGGAGGCTTGCTGGC
CACTTTTTGGGGTCAGCTCCTCTGAAATGCATTAGCGGAACCGTTTGCGATCTGCCACAA
GTGTGATAAGTTATCTACACTGGCGAGGGGATTGCTCTCTGTAATGTTCAGCTTCTAATT
GTCTCTACTTTGTGAGACTACTTTTGAATGCTTGACCTCAAATCAGGTAGGACTACCCGC
TGAACTTAA
>C4
TTTCCGTAGGTGAACCTGCGGAAGGATCATTATTGAATTATGTTTCTAGATAGGTTGTAG
CTGGCTCTTTAGAGCATGTGCACGCCTGTTTGGACTTCATTTTCATCCACCTGTGCACCT
ATTGTAGTCTTTGGTTGGGTTAGGAGGAAGTGGTCATTGTGTCAGCATCTGCTGGATGTG
AGGACTTGCATTGTGAAAGCTTTGCTGTCCTTGATGTGATCATGGAATCTCTTTCTCACT
AGAGTCTATGTCACTCATTATACTCTGTCGAATGTCATTGAATGTCTTTACATGGGCTTA
TATGCCTATGAAAATTGTAATACAACTTTCAGCAACGGATCTCTTGGCTCTCGCATCGAT
GAAGAACGCAGCGAAATGCGATAAGTAATGTGAATTGCAGAATTCAGTGAATCATCGAAT

CTTTGAACGCATCTTGCGCTCCTTGGTATTCCGAGGAGCATGCCTGTTTGAGTGTCATTA
AATTCTCAACTCTCTTCTACTTTTTGTAAAAGAGAGCTTGGACTGTGGAGGCTTGCTGGC
CACTTTTTGGGGTCAGCTCCTCTGAAATGCATTAGCGGAACCGTTTGCGATCTGCCACAA
GTGTGATAAGTTATCTACACTGGCGAGGGGATTGCTCTCTGTAATGTTCAGCTTCTAATT
GTCTCTACTTTGTGAGACTACTTTTGAATGCTTGACCTCAAATCAGGTAGGACTACCCGC
TGAACTTAA
>C5
TTTCCGTAGGTGAACCTGCGGAAGGATCATTATTGAATTATGTTTCTAGATAGGTTGTAG
CTGGCTCTTTAGAGCATGTGCACGCCTGTTTGGACTTCATTTTCATCCACCTGTGCACCT
ATTGTAGTCTTTGGTTGGGTTAGGAGGAAGTGGTCATTGTGTCAGCATCTGCTGGATGTG
AGGACTTGCATTGTGAAAGCTTTGCTGTCCTTGATGTGATCATGGAATCTCTTTCTCACT
AGAGTCTATGTCACTCATTATACTCTGTCGAATGTCATTGAATGTCTTTACATGGGCTTA
TATGCCTATGAAAATTGTAATACAACTTTCAGCAACGGATCTCTTGGCTCTCGCATCGAT
GAAGAACGCAGCGAAATGCGATAAGTAATGTGAATTGCAGAATTCAGTGAATCATCGAAT
CTTTGAACGCATCTTGCGCTCCTTGGTATTCCGAGGAGCATGCCTGTTTGAGTGTCATTA
AATTCTCAACTCTCTTCTACTTTTTGTAAAAGAGAGCTTGGACTGTGGAGGCTTGCTGGC
CACTTTTTGGGGTCAGCTCCTCTGAAATGCATTAGCGGAACCGTTTGCGATCTGCCACAA
GTGTGATAAGTTATCTACACTGGCGAGGGGATTGCTCTCTGTAATGTTCAGCTTCTAATT
GTCTCTACTTTGTGAGACTACTTTTGAATGCTTGACCTCAAATCAGGTAGGACTACCCGC
TGAACTTAA
>C6
TTTCCGTAGGTGAACCTGCGGAAGGATCATTATTGAATTATGTTTCTAGATAGGTTGTAG
CTGGCTCTTTAGAGCATGTGCACGCCTGTTTGGACTTCATTTTCATCCACCTGTGCACCT
ATTGTAGTCTTTGGTTGGGTTAGGAGGAAGTGGTCATTGTGTCAGCATCTGCTGGATGTG
AGGACTTGCATTGTGAAAGCTTTGCTGTCCTTGATGTGATCATGGAATCTCTTTCTCACT
AGAGTCTATGTCACTCATTATACTCTGTCGAATGTCATTGAATGTCTTTACATGGGCTTA
TATGCCTATGAAAATTGTAATACAACTTTCAGCAACGGATCTCTTGGCTCTCGCATCGAT
GAAGAACGCAGCGAAATGCGATAAGTAATGTGAATTGCAGAATTCAGTGAATCATCGAAT
CTTTGAACGCATCTTGCGCTCCTTGGTATTCCGAGGAGCATGCCTGTTTGAGTGTCATTA
AATTCTCAACTCTCTTCTACTTTTTGTAAAAGAGAGCTTGGACTGTGGAGGCTTGCTGGC
CACTTTTTGGGGTCAGCTCCTCTGAAATGCATTAGCGGAACCGTTTGCGATCTGCCACAA
GTGTGATAAGTTATCTACACTGGCGAGGGGATTGCTCTCTGTAATGTTCAGCTTCTAATT
GTCTCTACTTTGTGAGACTACTTTTGAATGCTTGACCTCAAATCAGGTAGGACTACCCGC
TGAACTTAA
>C7
TTTCCGTAGGTGAACCTGCGGAAGGATCATTATTGAATTATGTTTCTAGATAGGTTGTAG
CTGGCTCTTTAGAGCATGTGCACGCCTGTTTGGACTTCATTTTCATCCACCTGTGCACCT
ATTGTAGTCTTTGGTTGGGTTAGGAGGAAGTGGTCATTGTGTCAGCATCTGCTGGATGTG
AGGACTTGCATTGTGAAAGCTTTGCTGTCCTTGATGTGATCATGGAATCTCTTTCTCACT
AGAGTCTATGTCACTCATTATACTCTGTCGAATGTCATTGAATGTCTTTACATGGGCTTA
TATGCCTATGAAAATTGTAATACAACTTTCAGCAACGGATCTCTTGGCTCTCGCATCGAT
GAAGAACGCAGCGAAATGCGATAAGTAATGTGAATTGCAGAATTCAGTGAATCATCGAAT
CTTTGAACGCATCTTGCGCTCCTTGGTATTCCGAGGAGCATGCCTGTTTGAGTGTCATTA
AATTCTCAACTCTCTTCTACTTTTTGTAAAAGAGAGCTTGGACTGTGGAGGCTTGCTGGC
CACTTTTTGGGGTCAGCTCCTCTGAAATGCATTAGCGGAACCGTTTGCGATCTGCCACAA
GTGTGATAAGTTATCTACACTGGCGAGGGGATTGCTCTCTGTAATGTTCAGCTTCTAATT
GTCTCTACTTTGTGAGACTACTTTTGAATGCTTGACCTCAAATCAGGTAGGACTACCCGC
TGAACTTAA
>C8
TTTCCGTAGGTGAACCTGCGGAAGGATCATTATTGAATTATGTTTCTAGATAGGTTGTAG

CTGGCTCTTTAGAGCATGTGCACGCCTGTTTGGACTTCATTTTCATCCACCTGTGCACCT
ATTGTAGTCTTTGGTTGGGTTAGGAGGAAGTGGTCATTGTGTCAGCATCTGCTGGATGTG
AGGACTTGCATTGTGAAAGCTTTGCTGTCCTTGATGTGATCATGGAATCTCTTTCTCACT
AGAGTCTATGTCACTCATTATACTCTGTCGAATGTCATTGAATGTCTTTACATGGGCTTA
TATGCCTATGAAAATTGTAATACAACTTTCAGCAACGGATCTCTTGGCTCTCGCATCGAT
GAAGAACGCAGCGAAATGCGATAAGTAATGTGAATTGCAGAATTCAGTGAATCATCGAAT
CTTTGAACGCATCTTGCGCTCCTTGGTATTCCGAGGAGCATGCCTGTTTGAGTGTCATTA
AATTCTCAACTCTCTTCTACTTTTTGTAAAAGAGAGCTTGGACTGTGGAGGCTTGCTGGC
CACTTTTTGGGGTCAGCTCCTCTGAAATGCATTAGCGGAACCGTTTGCGATCTGCCACAA
GTGTGATAAGTTATCTACACTGGCGAGGGGATTGCTCTCTGTAATGTTCAGCTTCTAATT
GTCTCTACTTTGTGAGACTACTTTTGAATGCTTGACCTCAAATCAGGTAGGACTACCCGC
TGAACTTAA
>C9
TTTCCGTAGGTGAACCTGCGGAAGGATCATTATTGAATTATGTTTCTAGATAGGTTGTAG
CTGGCTCTTTAGAGCATGTGCACGCCTGTTTGGACTTCATTTTCATCCACCTGTGCACCT
ATTGTAGTCTTTGGTTGGGTTAGGAGGAAGTGGTCATTGTGTCAGCATCTGCTGGATGTG
AGGACTTGCATTGTGAAAGCTTTGCTGTCCTTGATGTGATCATGGAATCTCTTTCTCACT
AGAGTCTATGTCACTCATTATACTCTGTCGAATGTCATTGAATGTCTTTACATGGGCTTA
TATGCCTATGAAAATTGTAATACAACTTTCAGCAACGGATCTCTTGGCTCTCGCATCGAT
GAAGAACGCAGCGAAATGCGATAAGTAATGTGAATTGCAGAATTCAGTGAATCATCGAAT
CTTTGAACGCATCTTGCGCTCCTTGGTATTCCGAGGAGCATGCCTGTTTGAGTGTCATTA
AATTCTCAACTCTCTTCTACTTTTTGTAAAAGAGAGCTTGGACTGTGGAGGCTTGCTGGC
CACTTTTTGGGGTCAGCTCCTCTGAAATGCATTAGCGGAACCGTTTGCGATCTGCCACAA
GTGTGATAAGTTATCTACACTGGCGAGGGGATTGCTCTCTGTAATGTTCAGCTTCTAATT
GTCTCTACTTTGTGAGACTACTTTTGAATGCTTGACCTCAAATCAGGTAGGACTACCCGC
TGAACTTAA
>C10
TTTCCGTAGGTGAACCTGCGGAAGGATCATTATTGAATTATGTTTCTAGATAGGTTGTAG
CTGGCTCTTTAGAGCATGTGCACGCCTGTTTGGACTTCATTTTCATCCACCTGTGCACCT
ATTGTAGTCTTTGGTTGGGTTAGGAGGAAGTGGTCATTGTGTCAGCATCTGCTGGATGTG
AGGACTTGCATTGTGAAAGCTTTGCTGTCCTTGATGTGATCATGGAATCTCTTTCTCACT
AGAGTCTATGTCACTCATTATACTCTGTCGAATGTCATTGAATGTCTTTACATGGGCTTA
TATGCCTATGAAAATTGTAATACAACTTTCAGCAACGGATCTCTTGGCTCTCGCATCGAT
GAAGAACGCAGCGAAATGCGATAAGTAATGTGAATTGCAGAATTCAGTGAATCATCGAAT
CTTTGAACGCATCTTGCGCTCCTTGGTATTCCGAGGAGCATGCCTGTTTGAGTGTCATTA
AATTCTCAACTCTCTTCTACTTTTTGTAAAAGAGAGCTTGGACTGTGGAGGCTTGCTGGC
CACTTTTTGGGGTCAGCTCCTCTGAAATGCATTAGCGGAACCGTTTGCGATCTGCCACAA
GTGTGATAAGTTATCTACACTGGCGAGGGGATTGCTCTCTGTAATGTTCAGCTTCTAATT
GTCTCTACTTTGTGAGACTACTTTTGAATGCTTGACCTCAAATCAGGTAGGACTACCCGC
TGAACTTAA
>C11
TTTCCGTAGGTGAACCTGCGGAAGGATCATTATTGAATTATGTTTCTAGATAGGTTGTAG
CTGGCTCTTTAGAGCATGTGCACGCCTGTTTGGACTTCATTTTCATCCACCTGTGCACCT
ATTGTAGTCTTTGGTTGGGTTAGGAGGAAGTGGTCATTGTGTCAGCATCTGCTGGATGTG
AGGACTTGCATTGTGAAAGCTTTGCTGTCCTTGATGTGATCATGGAATCTCTTTCTCACT
AGAGTCTATGTCACTCATTATACTCTGTCGAATGTCATTGAATGTCTTTACATGGGCTTA
TATGCCTATGAAAATTGTAATACAACTTTCAGCAACGGATCTCTTGGCTCTCGCATCGAT
GAAGAACGCAGCGAAATGCGATAAGTAATGTGAATTGCAGAATTCAGTGAATCATCGAAT
CTTTGAACGCATCTTGCGCTCCTTGGTATTCCGAGGAGCATGCCTGTTTGAGTGTCATTA
AATTCTCAACTCTCTTCTACTTTTTGTAAAAGAGAGCTTGGACTGTGGAGGCTTGCTGGC

CACTTTTTGGGGTCAGCTCCTCTGAAATGCATTAGCGGAACCGTTTGCGATCTGCCACAA
GTGTGATAAGTTATCTACACTGGCGAGGGGATTGCTCTCTGTAATGTTCAGCTTCTAATT
GTCTCTACTTTGTGAGACTACTTTTGAATGCTTGACCTCAAATCAGGTAGGACTACCCGC
TGAACTTAA
>C12
TTTCCGTAGGTGAACCTGCGGAAGGATCATTATTGAATTATGTTTCTAGATAGGTTGTAG
CTGGCTCTTTAGAGCATGTGCACGCCTGTTTGGACTTCATTTTCATCCACCTGTGCACCT
ATTGTAGTCTTTGGTTGGGTTAGGAGGAAGTGGTCATTGTGTCAGCATCTGCTGGATGTG
AGGACTTGCATTGTGAAAGCTTTGCTGTCCTTGATGTGATCATGGAATCTCTTTCTCACT
AGAGTCTATGTCACTCATTATACTCTGTCGAATGTCATTGAATGTCTTTACATGGGCTTA
TATGCCTATGAAAATTGTAATACAACTTTCAGCAACGGATCTCTTGGCTCTCGCATCGAT
GAAGAACGCAGCGAAATGCGATAAGTAATGTGAATTGCAGAATTCAGTGAATCATCGAAT
CTTTGAACGCATCTTGCGCTCCTTGGTATTCCGAGGAGCATGCCTGTTTGAGTGTCATTA
AATTCTCAACTCTCTTCTACTTTTTGTAAAAGAGAGCTTGGACTGTGGAGGCTTGCTGGC
CACTTTTTGGGGTCAGCTCCTCTGAAATGCATTAGCGGAACCGTTTGCGATCTGCCACAA
GTGTGATAAGTTATCTACACTGGCGAGGGGATTGCTCTCTGTAATGTTCAGCTTCTAATT
GTCTCTACTTTGTGAGACTACTTTTGAATGCTTGACCTCAAATCAGGTAGGACTACCCGC
TGAACTTAA
>C13
TTTCCGTAGGTGAACCTGCGGAAGGATCATTATTGAATTATGTTTCTAGATAGGTTGTAG
CTGGCTCTTTAGAGCATGTGCACGCCTGTTTGGACTTCATTTTCATCCACCTGTGCACCT
ATTGTAGTCTTTGGTTGGGTTAGGAGGAAGTGGTCATTGTGTCAGCATCTGCTGGATGTG
AGGACTTGCATTGTGAAAGCTTTGCTGTCCTTGATGTGATCATGGAATCTCTTTCTCACT
AGAGTCTATGTCACTCATTATACTCTGTCGAATGTCATTGAATGTCTTTACATGGGCTTA
TATGCCTATGAAAATTGTAATACAACTTTCAGCAACGGATCTCTTGGCTCTCGCATCGAT
GAAGAACGCAGCGAAATGCGATAAGTAATGTGAATTGCAGAATTCAGTGAATCATCGAAT
CTTTGAACGCATCTTGCGCTCCTTGGTATTCCGAGGAGCATGCCTGTTTGAGTGTCATTA
AATTCTCAACTCTCTTCTACTTTTTGTAAAAGAGAGCTTGGACTGTGGAGGCTTGCTGGC
CACTTTTTGGGGTCAGCTCCTCTGAAATGCATTAGCGGAACCGTTTGCGATCTGCCACAA
GTGTGATAAGTTATCTACACTGGCGAGGGGATTGCTCTCTGTAATGTTCAGCTTCTAATT
GTCTCTACTTTGTGAGACTACTTTTGAATGCTTGACCTCAAATCAGGTAGGACTACCCGC
TGAACTTAA
>C14
TTTCCGTAGGTGAACCTGCGGAAGGATCATTATTGAATTATGTTTCTAGATAGGTTGTAG
CTGGCTCTTTAGAGCATGTGCACGCCTGTTTGGACTTCATTTTCATCCACCTGTGCACCT
ATTGTAGTCTTTGGTTGGGTTAGGAGGAAGTGGTCATTGTGTCAGCATCTGCTGGATGTG
AGGACTTGCATTGTGAAAGCTTTGCTGTCCTTGATGTGATCATGGAATCTCTTTCTCACT
AGAGTCTATGTCACTCATTATACTCTGTCGAATGTCATTGAATGTCTTTACATGGGCTTA
TATGCCTATGAAAATTGTAATACAACTTTCAGCAACGGATCTCTTGGCTCTCGCATCGAT
GAAGAACGCAGCGAAATGCGATAAGTAATGTGAATTGCAGAATTCAGTGAATCATCGAAT
CTTTGAACGCATCTTGCGCTCCTTGGTATTCCGAGGAGCATGCCTGTTTGAGTGTCATTA
AATTCTCAACTCTCTTCTACTTTTTGTAAAAGAGAGCTTGGACTGTGGAGGCTTGCTGGC
CACTTTTTGGGGTCAGCTCCTCTGAAATGCATTAGCGGAACCGTTTGCGATCTGCCACAA
GTGTGATAAGTTATCTACACTGGCGAGGGGATTGCTCTCTGTAATGTTCAGCTTCTAATT
GTCTCTACTTTGTGAGACTACTTTTGAATGCTTGACCTCAAATCAGGTAGGACTACCCGC
TGAACTTAA
>C15
TTTCCGTAGGTGAACCTGCGGAAGGATCATTATTGAATTATGTTTCTAGATAGGTTGTAG
CTGGCTCTTTAGAGCATGTGCACGCCTGTTTGGACTTCATTTTCATCCACCTGTGCACCT
ATTGTAGTCTTTGGTTGGGTTAGGAGGAAGTGGTCATTGTGTCAGCATCTGCTGGATGTG

AGGACTTGCATTGTGAAAGCTTTGCTGTCCTTGATGTGATCATGGAATCTCTTTCTCACT
AGAGTCTATGTCACTCATTATACTCTGTCGAATGTCATTGAATGTCTTTACATGGGCTTA
TATGCCTATGAAAATTGTAATACAACTTTCAGCAACGGATCTCTTGGCTCTCGCATCGAT
GAAGAACGCAGCGAAATGCGATAAGTAATGTGAATTGCAGAATTCAGTGAATCATCGAAT
CTTTGAACGCATCTTGCGCTCCTTGGTATTCCGAGGAGCATGCCTGTTTGAGTGTCATTA
AATTCTCAACTCTCTTCTACTTTTTGTAAAAGAGAGCTTGGACTGTGGAGGCTTGCTGGC
CACTTTTTGGGGTCAGCTCCTCTGAAATGCATTAGCGGAACCGTTTGCGATCTGCCACAA
GTGTGATAAGTTATCTACACTGGCGAGGGGATTGCTCTCTGTAATGTTCAGCTTCTAATT
GTCTCTACTTTGTGAGACTACTTTTGAATGCTTGACCTCAAATCAGGTAGGACTACCCGC
TGAACTTAA
>C16
TTTCCGTAGGTGAACCTGCGGAAGGATCATTATTGAATTATGTTTCTAGATAGGTTGTAG
CTGGCTCTTTAGAGCATGTGCACGCCTGTTTGGACTTCATTTTCATCCACCTGTGCACCT
ATTGTAGTCTTTGGTTGGGTTAGGAGGAAGTGGTCATTGTGTCAGCATCTGCTGGATGTG
AGGACTTGCATTGTGAAAGCTTTGCTGTCCTTGATGTGATCATGGAATCTCTTTCTCACT
AGAGTCTATGTCACTCATTATACTCTGTCGAATGTCATTGAATGTCTTTACATGGGCTTA
TATGCCTATGAAAATTGTAATACAACTTTCAGCAACGGATCTCTTGGCTCTCGCATCGAT
GAAGAACGCAGCGAAATGCGATAAGTAATGTGAATTGCAGAATTCAGTGAATCATCGAAT
CTTTGAACGCATCTTGCGCTCCTTGGTATTCCGAGGAGCATGCCTGTTTGAGTGTCATTA
AATTCTCAACTCTCTTCTACTTTTTGTAAAAGAGAGCTTGGACTGTGGAGGCTTGCTGGC
CACTTTTTGGGGTCAGCTCCTCTGAAATGCATTAGCGGAACCGTTTGCGATCTGCCACAA
GTGTGATAAGTTATCTACACTGGCGAGGGGATTGCTCTCTGTAATGTTCAGCTTCTAATT
GTCTCTACTTTGTGAGACTACTTTTGAATGCTTGACCTCAAATCAGGTAGGACTACCCGC
TGAACTTAA
>C17
TTTCCGTAGGTGAACCTGCGGAAGGATCATTATTGAATTATGTTTCTAGATAGGTTGTAG
CTGGCTCTTTAGAGCATGTGCACGCCTGTTTGGACTTCATTTTCATCCACCTGTGCACCT
ATTGTAGTCTTTGGTTGGGTTAGGAGGAAGTGGTCATTGTGTCAGCATCTGCTGGATGTG
AGGACTTGCATTGTGAAAGCTTTGCTGTCCTTGATGTGATCATGGAATCTCTTTCTCACT
AGAGTCTATGTCACTCATTATACTCTGTCGAATGTCATTGAATGTCTTTACATGGGCTTA
TATGCCTATGAAAATTGTAATACAACTTTCAGCAACGGATCTCTTGGCTCTCGCATCGAT
GAAGAACGCAGCGAAATGCGATAAGTAATGTGAATTGCAGAATTCAGTGAATCATCGAAT
CTTTGAACGCATCTTGCGCTCCTTGGTATTCCGAGGAGCATGCCTGTTTGAGTGTCATTA
AATTCTCAACTCTCTTCTACTTTTTGTAAAAGAGAGCTTGGACTGTGGAGGCTTGCTGGC
CACTTTTTGGGGTCAGCTCCTCTGAAATGCATTAGCGGAACCGTTTGCGATCTGCCACAA
GTGTGATAAGTTATCTACACTGGCGAGGGGATTGCTCTCTGTAATGTTCAGCTTCTAATT
GTCTCTACTTTGTGAGACTACTTTTGAATGCTTGACCTCAAATCAGGTAGGACTACCCGC
TGAACTTAA
>C18
TTTCCGTAGGTGAACCTGCGGAAGGATCATTATTGAATTATGTTTCTAGATAGGTTGTAG
CTGGCTCTTTAGAGCATGTGCACGCCTGTTTGGACTTCATTTTCATCCACCTGTGCACCT
ATTGTAGTCTTTGGTTGGGTTAGGAGGAAGTGGTCATTGTGTCAGCATCTGCTGGATGTG
AGGACTTGCATTGTGAAAGCTTTGCTGTCCTTGATGTGATCATGGAATCTCTTTCTCACT
AGAGTCTATGTCACTCATTATACTCTGTCGAATGTCATTGAATGTCTTTACATGGGCTTA
TATGCCTATGAAAATTGTAATACAACTTTCAGCAACGGATCTCTTGGCTCTCGCATCGAT
GAAGAACGCAGCGAAATGCGATAAGTAATGTGAATTGCAGAATTCAGTGAATCATCGAAT
CTTTGAACGCATCTTGCGCTCCTTGGTATTCCGAGGAGCATGCCTGTTTGAGTGTCATTA
AATTCTCAACTCTCTTCTACTTTTTGTAAAAGAGAGCTTGGACTGTGGAGGCTTGCTGGC
CACTTTTTGGGGTCAGCTCCTCTGAAATGCATTAGCGGAACCGTTTGCGATCTGCCACAA
GTGTGATAAGTTATCTACACTGGCGAGGGGATTGCTCTCTGTAATGTTCAGCTTCTAATT

GTCTCTACTTTGTGAGACTACTTTTGAATGCTTGACCTCAAATCAGGTAGGACTACCCGC
TGAACTTAA
>C19
TTTCCGTAGGTGAACCTGCGGAAGGATCATTATTGAATTATGTTTCTAGATAGGTTGTAG
CTGGCTCTTTAGAGCATGTGCACGCCTGTTTGGACTTCATTTTCATCCACCTGTGCACCT
ATTGTAGTCTTTGGTTGGGTTAGGAGGAAGTGGTCATTGTGTCAGCATCTGCTGGATGTG
AGGACTTGCATTGTGAAAGCTTTGCTGTCCTTGATGTGATCATGGAATCTCTTTCTCACT
AGAGTCTATGTCACTCATTATACTCTGTCGAATGTCATTGAATGTCTTTACATGGGCTTA
TATGCCTATGAAAATTGTAATACAACTTTCAGCAACGGATCTCTTGGCTCTCGCATCGAT
GAAGAACGCAGCGAAATGCGATAAGTAATGTGAATTGCAGAATTCAGTGAATCATCGAAT
CTTTGAACGCATCTTGCGCTCCTTGGTATTCCGAGGAGCATGCCTGTTTGAGTGTCATTA
AATTCTCAACTCTCTTCTACTTTTTGTAAAAGAGAGCTTGGACTGTGGAGGCTTGCTGGC
CACTTTTTGGGGTCAGCTCCTCTGAAATGCATTAGCGGAACCGTTTGCGATCTGCCACAA
GTGTGATAAGTTATCTACACTGGCGAGGGGATTGCTCTCTGTAATGTTCAGCTTCTAATT
GTCTCTACTTTGTGAGACTACTTTTGAATGCTTGACCTCAAATCAGGTAGGACTACCCGC
TGAACTTAA
>C20
TTTCCGTAGGTGAACCTGCGGAAGGATCATTATTGAATTATGTTTCTAGATAGGTTGTAG
CTGGCTCTTTAGAGCATGTGCACGCCTGTTTGGACTTCATTTTCATCCACCTGTGCACCT
ATTGTAGTCTTTGGTTGGGTTAGGAGGAAGTGGTCATTGTGTCAGCATCTGCTGGATGTG
AGGACTTGCATTGTGAAAGCTTTGCTGTCCTTGATGTGATCATGGAATCTCTTTCTCACT
AGAGTCTATGTCACTCATTATACTCTGTCGAATGTCATTGAATGTCTTTACATGGGCTTA
TATGCCTATGAAAATTGTAATACAACTTTCAGCAACGGATCTCTTGGCTCTCGCATCGAT
GAAGAACGCAGCGAAATGCGATAAGTAATGTGAATTGCAGAATTCAGTGAATCATCGAAT
CTTTGAACGCATCTTGCGCTCCTTGGTATTCCGAGGAGCATGCCTGTTTGAGTGTCATTA
AATTCTCAACTCTCTTCTACTTTTTGTAAAAGAGAGCTTGGACTGTGGAGGCTTGCTGGC
CACTTTTTGGGGTCAGCTCCTCTGAAATGCATTAGCGGAACCGTTTGCGATCTGCCACAA
GTGTGATAAGTTATCTACACTGGCGAGGGGATTGCTCTCTGTAATGTTCAGCTTCTAATT
GTCTCTACTTTGTGAGACTACTTTTGAATGCTTGACCTCAAATCAGGTAGGACTACCCGC
TGAACTTAA
>C21
TTTCCGTAGGTGAACCTGCGGAAGGATCATTATTGAATTATGTTTCTAGATAGGTTGTAG
CTGGCTCTTTAGAGCATGTGCACGCCTGTTTGGACTTCATTTTCATCCACCTGTGCACCT
ATTGTAGTCTTTGGTTGGGTTAGGAGGAAGTGGTCATTGTGTCAGCATCTGCTGGATGTG
AGGACTTGCATTGTGAAAGCTTTGCTGTCCTTGATGTGATCATGGAATCTCTTTCTCACT
AGAGTCTATGTCACTCATTATACTCTGTCGAATGTCATTGAATGTCTTTACATGGGCTTA
TATGCCTATGAAAATTGTAATACAACTTTCAGCAACGGATCTCTTGGCTCTCGCATCGAT
GAAGAACGCAGCGAAATGCGATAAGTAATGTGAATTGCAGAATTCAGTGAATCATCGAAT
CTTTGAACGCATCTTGCGCTCCTTGGTATTCCGAGGAGCATGCCTGTTTGAGTGTCATTA
AATTCTCAACTCTCTTCTACTTTTTGTAAAAGAGAGCTTGGACTGTGGAGGCTTGCTGGC
CACTTTTTGGGGTCAGCTCCTCTGAAATGCATTAGCGGAACCGTTTGCGATCTGCCACAA
GTGTGATAAGTTATCTACACTGGCGAGGGGATTGCTCTCTGTAATGTTCAGCTTCTAATT
GTCTCTACTTTGTGAGACTACTTTTGAATGCTTGACCTCAAATCAGGTAGGACTACCCGC
TGAACTTAA
>C22
TTTCCGTAGGTGAACCTGCGGAAGGATCATTATTGAATTATGTTTCTAGATAGGTTGTAG
CTGGCTCTTTAGAGCATGTGCACGCCTGTTTGGACTTCATTTTCATCCACCTGTGCACCT
ATTGTAGTCTTTGGTTGGGTTAGGAGGAAGTGGTCATTGTGTCAGCATCTGCTGGATGTG
AGGACTTGCATTGTGAAAGCTTTGCTGTCCTTGATGTGATCATGGAATCTCTTTCTCACT
AGAGTCTATGTCACTCATTATACTCTGTCGAATGTCATTGAATGTCTTTACATGGGCTTA

TATGCCTATGAAAATTGTAATACAACTTTCAGCAACGGATCTCTTGGCTCTCGCATCGAT
GAAGAACGCAGCGAAATGCGATAAGTAATGTGAATTGCAGAATTCAGTGAATCATCGAAT
CTTTGAACGCATCTTGCGCTCCTTGGTATTCCGAGGAGCATGCCTGTTTGAGTGTCATTA
AATTCTCAACTCTCTTCTACTTTTTGTAAAAGAGAGCTTGGACTGTGGAGGCTTGCTGGC
CACTTTTTGGGGTCAGCTCCTCTGAAATGCATTAGCGGAACCGTTTGCGATCTGCCACAA
GTGTGATAAGTTATCTACACTGGCGAGGGGATTGCTCTCTGTAATGTTCAGCTTCTAATT
GTCTCTACTTTGTGAGACTACTTTTGAATGCTTGACCTCAAATCAGGTAGGACTACCCGC
TGAACTTAA
>C23
TTTCCGTAGGTGAACCTGCGGAAGGATCATTATTGAATTATGTTTCTAGATAGGTTGTAG
CTGGCTCTTTAGAGCATGTGCACGCCTGTTTGGACTTCATTTTCATCCACCTGTGCACCT
ATTGTAGTCTTTGGTTGGGTTAGGAGGAAGTGGTCATTGTGTCAGCATCTGCTGGATGTG
AGGACTTGCATTGTGAAAGCTTTGCTGTCCTTGATGTGATCATGGAATCTCTTTCTCACT
AGAGTCTATGTCACTCATTATACTCTGTCGAATGTCATTGAATGTCTTTACATGGGCTTA
TATGCCTATGAAAATTGTAATACAACTTTCAGCAACGGATCTCTTGGCTCTCGCATCGAT
GAAGAACGCAGCGAAATGCGATAAGTAATGTGAATTGCAGAATTCAGTGAATCATCGAAT
CTTTGAACGCATCTTGCGCTCCTTGGTATTCCGAGGAGCATGCCTGTTTGAGTGTCATTA
AATTCTCAACTCTCTTCTACTTTTTGTAAAAGAGAGCTTGGACTGTGGAGGCTTGCTGGC
CACTTTTTGGGGTCAGCTCCTCTGAAATGCATTAGCGGAACCGTTTGCGATCTGCCACAA
GTGTGATAAGTTATCTACACTGGCGAGGGGATTGCTCTCTGTAATGTTCAGCTTCTAATT
GTCTCTACTTTGTGAGACTACTTTTGAATGCTTGACCTCAAATCAGGTAGGACTACCCGC
TGAACTTAA
>C24
TTTCCGTAGGTGAACCTGCGGAAGGATCATTATTGAATTATGTTTCTAGATAGGTTGTAG
CTGGCTCTTTAGAGCATGTGCACGCCTGTTTGGACTTCATTTTCATCCACCTGTGCACCT
ATTGTAGTCTTTGGTTGGGTTAGGAGGAAGTGGTCATTGTGTCAGCATCTGCTGGATGTG
AGGACTTGCATTGTGAAAGCTTTGCTGTCCTTGATGTGATCATGGAATCTCTTTCTCACT
AGAGTCTATGTCACTCATTATACTCTGTCGAATGTCATTGAATGTCTTTACATGGGCTTA
TATGCCTATGAAAATTGTAATACAACTTTCAGCAACGGATCTCTTGGCTCTCGCATCGAT
GAAGAACGCAGCGAAATGCGATAAGTAATGTGAATTGCAGAATTCAGTGAATCATCGAAT
CTTTGAACGCATCTTGCGCTCCTTGGTATTCCGAGGAGCATGCCTGTTTGAGTGTCATTA
AATTCTCAACTCTCTTCTACTTTTTGTAAAAGAGAGCTTGGACTGTGGAGGCTTGCTGGC
CACTTTTTGGGGTCAGCTCCTCTGAAATGCATTAGCGGAACCGTTTGCGATCTGCCACAA
GTGTGATAAGTTATCTACACTGGCGAGGGGATTGCTCTCTGTAATGTTCAGCTTCTAATT
GTCTCTACTTTGTGAGACTACTTTTGAATGCTTGACCTCAAATCAGGTAGGACTACCCGC
TGAACTTAA
>C25
TTTCCGTAGGTGAACCTGCGGAAGGATCATTATTGAATTATGTTTCTAGATAGGTTGTAG
CTGGCTCTTTAGAGCATGTGCACGCCTGTTTGGACTTCATTTTCATCCACCTGTGCACCT
ATTGTAGTCTTTGGTTGGGTTAGGAGGAAGTGGTCATTGTGTCAGCATCTGCTGGATGTG
AGGACTTGCATTGTGAAAGCTTTGCTGTCCTTGATGTGATCATGGAATCTCTTTCTCACT
AGAGTCTATGTCACTCATTATACTCTGTCGAATGTCATTGAATGTCTTTACATGGGCTTA
TATGCCTATGAAAATTGTAATACAACTTTCAGCAACGGATCTCTTGGCTCTCGCATCGAT
GAAGAACGCAGCGAAATGCGATAAGTAATGTGAATTGCAGAATTCAGTGAATCATCGAAT
CTTTGAACGCATCTTGCGCTCCTTGGTATTCCGAGGAGCATGCCTGTTTGAGTGTCATTA
AATTCTCAACTCTCTTCTACTTTTTGTAAAAGAGAGCTTGGACTGTGGAGGCTTGCTGGC
CACTTTTTGGGGTCAGCTCCTCTGAAATGCATTAGCGGAACCGTTTGCGATCTGCCACAA
GTGTGATAAGTTATCTACACTGGCGAGGGGATTGCTCTCTGTAATGTTCAGCTTCTAATT
GTCTCTACTTTGTGAGACTACTTTTGAATGCTTGACCTCAAATCAGGTAGGACTACCCGC
TGAACTTAA

>C26
TTTCCGTAGGTGAACCTGCGGAAGGATCATTATTGAATTATGTTTCTAGATAGGTTGTAG
CTGGCTCTTTAGAGCATGTGCACGCCTGTTTGGACTTCATTTTCATCCACCTGTGCACCT
ATTGTAGTCTTTGGTTGGGTTAGGAGGAAGTGGTCATTGTGTCAGCATCTGCTGGATGTG
AGGACTTGCATTGTGAAAGCTTTGCTGTCCTTGATGTGATCATGGAATCTCTTTCTCACT
AGAGTCTATGTCACTCATTATACTCTGTCGAATGTCATTGAATGTCTTTACATGGGCTTA
TATGCCTATGAAAATTGTAATACAACTTTCAGCAACGGATCTCTTGGCTCTCGCATCGAT
GAAGAACGCAGCGAAATGCGATAAGTAATGTGAATTGCAGAATTCAGTGAATCATCGAAT
CTTTGAACGCATCTTGCGCTCCTTGGTATTCCGAGGAGCATGCCTGTTTGAGTGTCATTA
AATTCTCAACTCTCTTCTACTTTTTGTAAAAGAGAGCTTGGACTGTGGAGGCTTGCTGGC
CACTTTTTGGGGTCAGCTCCTCTGAAATGCATTAGCGGAACCGTTTGCGATCTGCCACAA
GTGTGATAAGTTATCTACACTGGCGAGGGGATTGCTCTCTGTAATGTTCAGCTTCTAATT
GTCTCTACTTTGTGAGACTACTTTTGAATGCTTGACCTCAAATCAGGTAGGACTACCCGC
TGAACTTAA
>C27
TTTCCGTAGGTGAACCTGCGGAAGGATCATTATTGAATTATGTTTCTAGATAGGTTGTAG
CTGGCTCTTTAGAGCATGTGCACGCCTGTTTGGACTTCATTTTCATCCACCTGTGCACCT
ATTGTAGTCTTTGGTTGGGTTAGGAGGAAGTGGTCATTGTGTCAGCATCTGCTGGATGTG
AGGACTTGCATTGTGAAAGCTTTGCTGTCCTTGATGTGATCATGGAATCTCTTTCTCACT
AGAGTCTATGTCACTCATTATACTCTGTCGAATGTCATTGAATGTCTTTACATGGGCTTA
TATGCCTATGAAAATTGTAATACAACTTTCAGCAACGGATCTCTTGGCTCTCGCATCGAT
GAAGAACGCAGCGAAATGCGATAAGTAATGTGAATTGCAGAATTCAGTGAATCATCGAAT
CTTTGAACGCATCTTGCGCTCCTTGGTATTCCGAGGAGCATGCCTGTTTGAGTGTCATTA
AATTCTCAACTCTCTTCTACTTTTTGTAAAAGAGAGCTTGGACTGTGGAGGCTTGCTGGC
CACTTTTTGGGGTCAGCTCCTCTGAAATGCATTAGCGGAACCGTTTGCGATCTGCCACAA
GTGTGATAAGTTATCTACACTGGCGAGGGGATTGCTCTCTGTAATGTTCAGCTTCTAATT
GTCTCTACTTTGTGAGACTACTTTTGAATGCTTGACCTCAAATCAGGTAGGACTACCCGC
TGAACTTAA
>C28
TTTCCGTAGGTGAACCTGCGGAAGGATCATTATTGAATTATGTTTCTAGATAGGTTGTAG
CTGGCTCTTTAGAGCATGTGCACGCCTGTTTGGACTTCATTTTCATCCACCTGTGCACCT
ATTGTAGTCTTTGGTTGGGTTAGGAGGAAGTGGTCATTGTGTCAGCATCTGCTGGATGTG
AGGACTTGCATTGTGAAAGCTTTGCTGTCCTTGATGTGATCATGGAATCTCTTTCTCACT
AGAGTCTATGTCACTCATTATACTCTGTCGAATGTCATTGAATGTCTTTACATGGGCTTA
TATGCCTATGAAAATTGTAATACAACTTTCAGCAACGGATCTCTTGGCTCTCGCATCGAT
GAAGAACGCAGCGAAATGCGATAAGTAATGTGAATTGCAGAATTCAGTGAATCATCGAAT
CTTTGAACGCATCTTGCGCTCCTTGGTATTCCGAGGAGCATGCCTGTTTGAGTGTCATTA
AATTCTCAACTCTCTTCTACTTTTTGTAAAAGAGAGCTTGGACTGTGGAGGCTTGCTGGC
CACTTTTTGGGGTCAGCTCCTCTGAAATGCATTAGCGGAACCGTTTGCGATCTGCCACAA
GTGTGATAAGTTATCTACACTGGCGAGGGGATTGCTCTCTGTAATGTTCAGCTTCTAATT
GTCTCTACTTTGTGAGACTACTTTTGAATGCTTGACCTCAAATCAGGTAGGACTACCCGC
TGAACTTAA
>C29
TTTCCGTAGGTGAACCTGCGGAAGGATCATTATTGAATTATGTTTCTAGATAGGTTGTAG
CTGGCTCTTTAGAGCATGTGCACGCCTGTTTGGACTTCATTTTCATCCACCTGTGCACCT
ATTGTAGTCTTTGGTTGGGTTAGGAGGAAGTGGTCATTGTGTCAGCATCTGCTGGATGTG
AGGACTTGCATTGTGAAAGCTTTGCTGTCCTTGATGTGATCATGGAATCTCTTTCTCACT
AGAGTCTATGTCACTCATTATACTCTGTCGAATGTCATTGAATGTCTTTACATGGGCTTA
TATGCCTATGAAAATTGTAATACAACTTTCAGCAACGGATCTCTTGGCTCTCGCATCGAT
GAAGAACGCAGCGAAATGCGATAAGTAATGTGAATTGCAGAATTCAGTGAATCATCGAAT

CTTTGAACGCATCTTGCGCTCCTTGGTATTCCGAGGAGCATGCCTGTTTGAGTGTCATTA
AATTCTCAACTCTCTTCTACTTTTTGTAAAAGAGAGCTTGGACTGTGGAGGCTTGCTGGC
CACTTTTTGGGGTCAGCTCCTCTGAAATGCATTAGCGGAACCGTTTGCGATCTGCCACAA
GTGTGATAAGTTATCTACACTGGCGAGGGGATTGCTCTCTGTAATGTTCAGCTTCTAATT
GTCTCTACTTTGTGAGACTACTTTTGAATGCTTGACCTCAAATCAGGTAGGACTACCCGC
TGAACTTAA
>C30
TTTCCGTAGGTGAACCTGCGGAAGGATCATTATTGAATTATGTTTCTAGATAGGTTGTAG
CTGGCTCTTTAGAGCATGTGCACGCCTGTTTGGACTTCATTTTCATCCACCTGTGCACCT
ATTGTAGTCTTTGGTTGGGTTAGGAGGAAGTGGTCATTGTGTCAGCATCTGCTGGATGTG
AGGACTTGCATTGTGAAAGCTTTGCTGTCCTTGATGTGATCATGGAATCTCTTTCTCACT
AGAGTCTATGTCACTCATTATACTCTGTCGAATGTCATTGAATGTCTTTACATGGGCTTA
TATGCCTATGAAAATTGTAATACAACTTTCAGCAACGGATCTCTTGGCTCTCGCATCGAT
GAAGAACGCAGCGAAATGCGATAAGTAATGTGAATTGCAGAATTCAGTGAATCATCGAAT
CTTTGAACGCATCTTGCGCTCCTTGGTATTCCGAGGAGCATGCCTGTTTGAGTGTCATTA
AATTCTCAACTCTCTTCTACTTTTTGTAAAAGAGAGCTTGGACTGTGGAGGCTTGCTGGC
CACTTTTTGGGGTCAGCTCCTCTGAAATGCATTAGCGGAACCGTTTGCGATCTGCCACAA
GTGTGATAAGTTATCTACACTGGCGAGGGGATTGCTCTCTGTAATGTTCAGCTTCTAATT
GTCTCTACTTTGTGAGACTACTTTTGAATGCTTGACCTCAAATCAGGTAGGACTACCCGC
TGAACTTAA
>C31
TTTCCGTAGGTGAACCTGCGGAAGGATCATTATTGAATTATGTTTCTAGATAGGTTGTAG
CTGGCTCTTTAGAGCATGTGCACGCCTGTTTGGACTTCATTTTCATCCACCTGTGCACCT
ATTGTAGTCTTTGGTTGGGTTAGGAGGAAGTGGTCATTGTGTCAGCATCTGCTGGATGTG
AGGACTTGCATTGTGAAAGCTTTGCTGTCCTTGATGTGATCATGGAATCTCTTTCTCACT
AGAGTCTATGTCACTCATTATACTCTGTCGAATGTCATTGAATGTCTTTACATGGGCTTA
TATGCCTATGAAAATTGTAATACAACTTTCAGCAACGGATCTCTTGGCTCTCGCATCGAT
GAAGAACGCAGCGAAATGCGATAAGTAATGTGAATTGCAGAATTCAGTGAATCATCGAAT
CTTTGAACGCATCTTGCGCTCCTTGGTATTCCGAGGAGCATGCCTGTTTGAGTGTCATTA
AATTCTCAACTCTCTTCTACTTTTTGTAAAAGAGAGCTTGGACTGTGGAGGCTTGCTGGC
CACTTTTTGGGGTCAGCTCCTCTGAAATGCATTAGCGGAACCGTTTGCGATCTGCCACAA
GTGTGATAAGTTATCTACACTGGCGAGGGGATTGCTCTCTGTAATGTTCAGCTTCTAATT
GTCTCTACTTTGTGAGACTACTTTTGAATGCTTGACCTCAAATCAGGTAGGACTACCCGC
TGAACTTAA
>C32
TTTCCGTAGGTGAACCTGCGGAAGGATCATTATTGAATTATGTTTCTAGATAGGTTGTAG
CTGGCTCTTTAGAGCATGTGCACGCCTGTTTGGACTTCATTTTCATCCACCTGTGCACCT
ATTGTAGTCTTTGGTTGGGTTAGGAGGAAGTGGTCATTGTGTCAGCATCTGCTGGATGTG
AGGACTTGCATTGTGAAAGCTTTGCTGTCCTTGATGTGATCATGGAATCTCTTTCTCACT
AGAGTCTATGTCACTCATTATACTCTGTCGAATGTCATTGAATGTCTTTACATGGGCTTA
TATGCCTATGAAAATTGTAATACAACTTTCAGCAACGGATCTCTTGGCTCTCGCATCGAT
GAAGAACGCAGCGAAATGCGATAAGTAATGTGAATTGCAGAATTCAGTGAATCATCGAAT
CTTTGAACGCATCTTGCGCTCCTTGGTATTCCGAGGAGCATGCCTGTTTGAGTGTCATTA
AATTCTCAACTCTCTTCTACTTTTTGTAAAAGAGAGCTTGGACTGTGGAGGCTTGCTGGC
CACTTTTTGGGGTCAGCTCCTCTGAAATGCATTAGCGGAACCGTTTGCGATCTGCCACAA
GTGTGATAAGTTATCTACACTGGCGAGGGGATTGCTCTCTGTAATGTTCAGCTTCTAATT
GTCTCTACTTTGTGAGACTACTTTTGAATGCTTGACCTCAAATCAGGTAGGACTACCCGC
TGAACTTAA
>C33
TTTCCGTAGGTGAACCTGCGGAAGGATCATTATTGAATTATGTTTCTAGATAGGTTGTAG

CTGGCTCTTTAGAGCATGTGCACGCCTGTTTGGACTTCATTTTCATCCACCTGTGCACCT
ATTGTAGTCTTTGGTTGGGTTAGGAGGAAGTGGTCATTGTGTCAGCATCTGCTGGATGTG
AGGACTTGCATTGTGAAAGCTTTGCTGTCCTTGATGTGATCATGGAATCTCTTTCTCACT
AGAGTCTATGTCACTCATTATACTCTGTCGAATGTCATTGAATGTCTTTACATGGGCTTA
TATGCCTATGAAAATTGTAATACAACTTTCAGCAACGGATCTCTTGGCTCTCGCATCGAT
GAAGAACGCAGCGAAATGCGATAAGTAATGTGAATTGCAGAATTCAGTGAATCATCGAAT
CTTTGAACGCATCTTGCGCTCCTTGGTATTCCGAGGAGCATGCCTGTTTGAGTGTCATTA
AATTCTCAACTCTCTTCTACTTTTTGTAAAAGAGAGCTTGGACTGTGGAGGCTTGCTGGC
CACTTTTTGGGGTCAGCTCCTCTGAAATGCATTAGCGGAACCGTTTGCGATCTGCCACAA
GTGTGATAAGTTATCTACACTGGCGAGGGGATTGCTCTCTGTAATGTTCAGCTTCTAATT
GTCTCTACTTTGTGAGACTACTTTTGAATGCTTGACCTCAAATCAGGTAGGACTACCCGC
TGAACTTAA
>C34
TTTCCGTAGGTGAACCTGCGGAAGGATCATTATTGAATTATGTTTCTAGATAGGTTGTAG
CTGGCTCTTTAGAGCATGTGCACGCCTGTTTGGACTTCATTTTCATCCACCTGTGCACCT
ATTGTAGTCTTTGGTTGGGTTAGGAGGAAGTGGTCATTGTGTCAGCATCTGCTGGATGTG
AGGACTTGCATTGTGAAAGCTTTGCTGTCCTTGATGTGATCATGGAATCTCTTTCTCACT
AGAGTCTATGTCACTCATTATACTCTGTCGAATGTCATTGAATGTCTTTACATGGGCTTA
TATGCCTATGAAAATTGTAATACAACTTTCAGCAACGGATCTCTTGGCTCTCGCATCGAT
GAAGAACGCAGCGAAATGCGATAAGTAATGTGAATTGCAGAATTCAGTGAATCATCGAAT
CTTTGAACGCATCTTGCGCTCCTTGGTATTCCGAGGAGCATGCCTGTTTGAGTGTCATTA
AATTCTCAACTCTCTTCTACTTTTTGTAAAAGAGAGCTTGGACTGTGGAGGCTTGCTGGC
CACTTTTTGGGGTCAGCTCCTCTGAAATGCATTAGCGGAACCGTTTGCGATCTGCCACAA
GTGTGATAAGTTATCTACACTGGCGAGGGGATTGCTCTCTGTAATGTTCAGCTTCTAATT
GTCTCTACTTTGTGAGACTACTTTTGAATGCTTGACCTCAAATCAGGTAGGACTACCCGC
TGAACTTAA
>C35
TTTCCGTAGGTGAACCTGCGGAAGGATCATTATTGAATTATGTTTCTAGATAGGTTGTAG
CTGGCTCTTTAGAGCATGTGCACGCCTGTTTGGACTTCATTTTCATCCACCTGTGCACCT
ATTGTAGTCTTTGGTTGGGTTAGGAGGAAGTGGTCATTGTGTCAGCATCTGCTGGATGTG
AGGACTTGCATTGTGAAAGCTTTGCTGTCCTTGATGTGATCATGGAATCTCTTTCTCACT
AGAGTCTATGTCACTCATTATACTCTGTCGAATGTCATTGAATGTCTTTACATGGGCTTA
TATGCCTATGAAAATTGTAATACAACTTTCAGCAACGGATCTCTTGGCTCTCGCATCGAT
GAAGAACGCAGCGAAATGCGATAAGTAATGTGAATTGCAGAATTCAGTGAATCATCGAAT
CTTTGAACGCATCTTGCGCTCCTTGGTATTCCGAGGAGCATGCCTGTTTGAGTGTCATTA
AATTCTCAACTCTCTTCTACTTTTTGTAAAAGAGAGCTTGGACTGTGGAGGCTTGCTGGC
CACTTTTTGGGGTCAGCTCCTCTGAAATGCATTAGCGGAACCGTTTGCGATCTGCCACAA
GTGTGATAAGTTATCTACACTGGCGAGGGGATTGCTCTCTGTAATGTTCAGCTTCTAATT
GTCTCTACTTTGTGAGACTACTTTTGAATGCTTGACCTCAAATCAGGTAGGACTACCCGC
TGAACTTAA
>C36
TTTCCGTAGGTGAACCTGCGGAAGGATCATTATTGAATTATGTTTCTAGATAGGTTGTAG
CTGGCTCTTTAGAGCATGTGCACGCCTGTTTGGACTTCATTTTCATCCACCTGTGCACCT
ATTGTAGTCTTTGGTTGGGTTAGGAGGAAGTGGTCATTGTGTCAGCATCTGCTGGATGTG
AGGACTTGCATTGTGAAAGCTTTGCTGTCCTTGATGTGATCATGGAATCTCTTTCTCACT
AGAGTCTATGTCACTCATTATACTCTGTCGAATGTCATTGAATGTCTTTACATGGGCTTA
TATGCCTATGAAAATTGTAATACAACTTTCAGCAACGGATCTCTTGGCTCTCGCATCGAT
GAAGAACGCAGCGAAATGCGATAAGTAATGTGAATTGCAGAATTCAGTGAATCATCGAAT
CTTTGAACGCATCTTGCGCTCCTTGGTATTCCGAGGAGCATGCCTGTTTGAGTGTCATTA
AATTCTCAACTCTCTTCTACTTTTTGTAAAAGAGAGCTTGGACTGTGGAGGCTTGCTGGC

CACTTTTTGGGGTCAGCTCCTCTGAAATGCATTAGCGGAACCGTTTGCGATCTGCCACAA
GTGTGATAAGTTATCTACACTGGCGAGGGGATTGCTCTCTGTAATGTTCAGCTTCTAATT
GTCTCTACTTTGTGAGACTACTTTTGAATGCTTGACCTCAAATCAGGTAGGACTACCCGC
TGAACTTAA
>C37
TTTCCGTAGGTGAACCTGCGGAAGGATCATTATTGAATTATGTTTCTAGATAGGTTGTAG
CTGGCTCTTTAGAGCATGTGCACGCCTGTTTGGACTTCATTTTCATCCACCTGTGCACCT
ATTGTAGTCTTTGGTTGGGTTAGGAGGAAGTGGTCATTGTGTCAGCATCTGCTGGATGTG
AGGACTTGCATTGTGAAAGCTTTGCTGTCCTTGATGTGATCATGGAATCTCTTTCTCACT
AGAGTCTATGTCACTCATTATACTCTGTCGAATGTCATTGAATGTCTTTACATGGGCTTA
TATGCCTATGAAAATTGTAATACAACTTTCAGCAACGGATCTCTTGGCTCTCGCATCGAT
GAAGAACGCAGCGAAATGCGATAAGTAATGTGAATTGCAGAATTCAGTGAATCATCGAAT
CTTTGAACGCATCTTGCGCTCCTTGGTATTCCGAGGAGCATGCCTGTTTGAGTGTCATTA
AATTCTCAACTCTCTTCTACTTTTTGTAAAAGAGAGCTTGGACTGTGGAGGCTTGCTGGC
CACTTTTTGGGGTCAGCTCCTCTGAAATGCATTAGCGGAACCGTTTGCGATCTGCCACAA
GTGTGATAAGTTATCTACACTGGCGAGGGGATTGCTCTCTGTAATGTTCAGCTTCTAATT
GTCTCTACTTTGTGAGACTACTTTTGAATGCTTGACCTCAAATCAGGTAGGACTACCCGC
TGAACTTAA
>C38
TTTCCGTAGGTGAACCTGCGGAAGGATCATTATTGAATTATGTTTCTAGATAGGTTGTAG
CTGGCTCTTTAGAGCATGTGCACGCCTGTTTGGACTTCATTTTCATCCACCTGTGCACCT
ATTGTAGTCTTTGGTTGGGTTAGGAGGAAGTGGTCATTGTGTCAGCATCTGCTGGATGTG
AGGACTTGCATTGTGAAAGCTTTGCTGTCCTTGATGTGATCATGGAATCTCTTTCTCACT
AGAGTCTATGTCACTCATTATACTCTGTCGAATGTCATTGAATGTCTTTACATGGGCTTA
TATGCCTATGAAAATTGTAATACAACTTTCAGCAACGGATCTCTTGGCTCTCGCATCGAT
GAAGAACGCAGCGAAATGCGATAAGTAATGTGAATTGCAGAATTCAGTGAATCATCGAAT
CTTTGAACGCATCTTGCGCTCCTTGGTATTCCGAGGAGCATGCCTGTTTGAGTGTCATTA
AATTCTCAACTCTCTTCTACTTTTTGTAAAAGAGAGCTTGGACTGTGGAGGCTTGCTGGC
CACTTTTTGGGGTCAGCTCCTCTGAAATGCATTAGCGGAACCGTTTGCGATCTGCCACAA
GTGTGATAAGTTATCTACACTGGCGAGGGGATTGCTCTCTGTAATGTTCAGCTTCTAATT
GTCTCTACTTTGTGAGACTACTTTTGAATGCTTGACCTCAAATCAGGTAGGACTACCCGC
TGAACTTAA
>C39
TTTCCGTAGGTGAACCTGCGGAAGGATCATTATTGAATTATGTTTCTAGATAGGTTGTAG
CTGGCTCTTTAGAGCATGTGCACGCCTGTTTGGACTTCATTTTCATCCACCTGTGCACCT
ATTGTAGTCTTTGGTTGGGTTAGGAGGAAGTGGTCATTGTGTCAGCATCTGCTGGATGTG
AGGACTTGCATTGTGAAAGCTTTGCTGTCCTTGATGTGATCATGGAATCTCTTTCTCACT
AGAGTCTATGTCACTCATTATACTCTGTCGAATGTCATTGAATGTCTTTACATGGGCTTA
TATGCCTATGAAAATTGTAATACAACTTTCAGCAACGGATCTCTTGGCTCTCGCATCGAT
GAAGAACGCAGCGAAATGCGATAAGTAATGTGAATTGCAGAATTCAGTGAATCATCGAAT
CTTTGAACGCATCTTGCGCTCCTTGGTATTCCGAGGAGCATGCCTGTTTGAGTGTCATTA
AATTCTCAACTCTCTTCTACTTTTTGTAAAAGAGAGCTTGGACTGTGGAGGCTTGCTGGC
CACTTTTTGGGGTCAGCTCCTCTGAAATGCATTAGCGGAACCGTTTGCGATCTGCCACAA
GTGTGATAAGTTATCTACACTGGCGAGGGGATTGCTCTCTGTAATGTTCAGCTTCTAATT
GTCTCTACTTTGTGAGACTACTTTTGAATGCTTGACCTCAAATCAGGTAGGACTACCCGC
TGAACTTAA
>C40
TTTCCGTAGGTGAACCTGCGGAAGGATCATTATTGAATTATGTTTCTAGATAGGTTGTAG
CTGGCTCTTTAGAGCATGTGCACGCCTGTTTGGACTTCATTTTCATCCACCTGTGCACCT
ATTGTAGTCTTTGGTTGGGTTAGGAGGAAGTGGTCATTGTGTCAGCATCTGCTGGATGTG

AGGACTTGCATTGTGAAAGCTTTGCTGTCCTTGATGTGATCATGGAATCTCTTTCTCACT
AGAGTCTATGTCACTCATTATACTCTGTCGAATGTCATTGAATGTCTTTACATGGGCTTA
TATGCCTATGAAAATTGTAATACAACTTTCAGCAACGGATCTCTTGGCTCTCGCATCGAT
GAAGAACGCAGCGAAATGCGATAAGTAATGTGAATTGCAGAATTCAGTGAATCATCGAAT
CTTTGAACGCATCTTGCGCTCCTTGGTATTCCGAGGAGCATGCCTGTTTGAGTGTCATTA
AATTCTCAACTCTCTTCTACTTTTTGTAAAAGAGAGCTTGGACTGTGGAGGCTTGCTGGC
CACTTTTTGGGGTCAGCTCCTCTGAAATGCATTAGCGGAACCGTTTGCGATCTGCCACAA
GTGTGATAAGTTATCTACACTGGCGAGGGGATTGCTCTCTGTAATGTTCAGCTTCTAATT
GTCTCTACTTTGTGAGACTACTTTTGAATGCTTGACCTCAAATCAGGTAGGACTACCCGC
TGAACTTAA
>C41
TTTCCGTAGGTGAACCTGCGGAAGGATCATTATTGAATTATGTTTCTAGATAGGTTGTAG
CTGGCTCTTTAGAGCATGTGCACGCCTGTTTGGACTTCATTTTCATCCACCTGTGCACCT
ATTGTAGTCTTTGGTTGGGTTAGGAGGAAGTGGTCATTGTGTCAGCATCTGCTGGATGTG
AGGACTTGCATTGTGAAAGCTTTGCTGTCCTTGATGTGATCATGGAATCTCTTTCTCACT
AGAGTCTATGTCACTCATTATACTCTGTCGAATGTCATTGAATGTCTTTACATGGGCTTA
TATGCCTATGAAAATTGTAATACAACTTTCAGCAACGGATCTCTTGGCTCTCGCATCGAT
GAAGAACGCAGCGAAATGCGATAAGTAATGTGAATTGCAGAATTCAGTGAATCATCGAAT
CTTTGAACGCATCTTGCGCTCCTTGGTATTCCGAGGAGCATGCCTGTTTGAGTGTCATTA
AATTCTCAACTCTCTTCTACTTTTTGTAAAAGAGAGCTTGGACTGTGGAGGCTTGCTGGC
CACTTTTTGGGGTCAGCTCCTCTGAAATGCATTAGCGGAACCGTTTGCGATCTGCCACAA
GTGTGATAAGTTATCTACACTGGCGAGGGGATTGCTCTCTGTAATGTTCAGCTTCTAATT
GTCTCTACTTTGTGAGACTACTTTTGAATGCTTGACCTCAAATCAGGTAGGACTACCCGC
TGAACTTAA
>C42
TTTCCGTAGGTGAACCTGCGGAAGGATCATTATTGAATTATGTTTCTAGATAGGTTGTAG
CTGGCTCTTTAGAGCATGTGCACGCCTGTTTGGACTTCATTTTCATCCACCTGTGCACCT
ATTGTAGTCTTTGGTTGGGTTAGGAGGAAGTGGTCATTGTGTCAGCATCTGCTGGATGTG
AGGACTTGCATTGTGAAAGCTTTGCTGTCCTTGATGTGATCATGGAATCTCTTTCTCACT
AGAGTCTATGTCACTCATTATACTCTGTCGAATGTCATTGAATGTCTTTACATGGGCTTA
TATGCCTATGAAAATTGTAATACAACTTTCAGCAACGGATCTCTTGGCTCTCGCATCGAT
GAAGAACGCAGCGAAATGCGATAAGTAATGTGAATTGCAGAATTCAGTGAATCATCGAAT
CTTTGAACGCATCTTGCGCTCCTTGGTATTCCGAGGAGCATGCCTGTTTGAGTGTCATTA
AATTCTCAACTCTCTTCTACTTTTTGTAAAAGAGAGCTTGGACTGTGGAGGCTTGCTGGC
CACTTTTTGGGGTCAGCTCCTCTGAAATGCATTAGCGGAACCGTTTGCGATCTGCCACAA
GTGTGATAAGTTATCTACACTGGCGAGGGGATTGCTCTCTGTAATGTTCAGCTTCTAATT
GTCTCTACTTTGTGAGACTACTTTTGAATGCTTGACCTCAAATCAGGTAGGACTACCCGC
TGAACTTAA
>C43
TTTCCGTAGGTGAACCTGCGGAAGGATCATTATTGAATTATGTTTCTAGATAGGTTGTAG
CTGGCTCTTTAGAGCATGTGCACGCCTGTTTGGACTTCATTTTCATCCACCTGTGCACCT
ATTGTAGTCTTTGGTTGGGTTAGGAGGAAGTGGTCATTGTGTCAGCATCTGCTGGATGTG
AGGACTTGCATTGTGAAAGCTTTGCTGTCCTTGATGTGATCATGGAATCTCTTTCTCACT
AGAGTCTATGTCACTCATTATACTCTGTCGAATGTCATTGAATGTCTTTACATGGGCTTA
TATGCCTATGAAAATTGTAATACAACTTTCAGCAACGGATCTCTTGGCTCTCGCATCGAT
GAAGAACGCAGCGAAATGCGATAAGTAATGTGAATTGCAGAATTCAGTGAATCATCGAAT
CTTTGAACGCATCTTGCGCTCCTTGGTATTCCGAGGAGCATGCCTGTTTGAGTGTCATTA
AATTCTCAACTCTCTTCTACTTTTTGTAAAAGAGAGCTTGGACTGTGGAGGCTTGCTGGC
CACTTTTTGGGGTCAGCTCCTCTGAAATGCATTAGCGGAACCGTTTGCGATCTGCCACAA
GTGTGATAAGTTATCTACACTGGCGAGGGGATTGCTCTCTGTAATGTTCAGCTTCTAATT

GTCTCTACTTTGTGAGACTACTTTTGAATGCTTGACCTCAAATCAGGTAGGACTACCCGC
TGAACTTAA
>C44
TTTCCGTAGGTGAACCTGCGGAAGGATCATTATTGAATTATGTTTCTAGATAGGTTGTAG
CTGGCTCTTTAGAGCATGTGCACGCCTGTTTGGACTTCATTTTCATCCACCTGTGCACCT
ATTGTAGTCTTTGGTTGGGTTAGGAGGAAGTGGTCATTGTGTCAGCATCTGCTGGATGTG
AGGACTTGCATTGTGAAAGCTTTGCTGTCCTTGATGTGATCATGGAATCTCTTTCTCACT
AGAGTCTATGTCACTCATTATACTCTGTCGAATGTCATTGAATGTCTTTACATGGGCTTA
TATGCCTATGAAAATTGTAATACAACTTTCAGCAACGGATCTCTTGGCTCTCGCATCGAT
GAAGAACGCAGCGAAATGCGATAAGTAATGTGAATTGCAGAATTCAGTGAATCATCGAAT
CTTTGAACGCATCTTGCGCTCCTTGGTATTCCGAGGAGCATGCCTGTTTGAGTGTCATTA
AATTCTCAACTCTCTTCTACTTTTTGTAAAAGAGAGCTTGGACTGTGGAGGCTTGCTGGC
CACTTTTTGGGGTCAGCTCCTCTGAAATGCATTAGCGGAACCGTTTGCGATCTGCCACAA
GTGTGATAAGTTATCTACACTGGCGAGGGGATTGCTCTCTGTAATGTTCAGCTTCTAATT
GTCTCTACTTTGTGAGACTACTTTTGAATGCTTGACCTCAAATCAGGTAGGACTACCCGC
TGAACTTAA
>C45
TTTCCGTAGGTGAACCTGCGGAAGGATCATTATTGAATTATGTTTCTAGATAGGTTGTAG
CTGGCTCTTTAGAGCATGTGCACGCCTGTTTGGACTTCATTTTCATCCACCTGTGCACCT
ATTGTAGTCTTTGGTTGGGTTAGGAGGAAGTGGTCATTGTGTCAGCATCTGCTGGATGTG
AGGACTTGCATTGTGAAAGCTTTGCTGTCCTTGATGTGATCATGGAATCTCTTTCTCACT
AGAGTCTATGTCACTCATTATACTCTGTCGAATGTCATTGAATGTCTTTACATGGGCTTA
TATGCCTATGAAAATTGTAATACAACTTTCAGCAACGGATCTCTTGGCTCTCGCATCGAT
GAAGAACGCAGCGAAATGCGATAAGTAATGTGAATTGCAGAATTCAGTGAATCATCGAAT
CTTTGAACGCATCTTGCGCTCCTTGGTATTCCGAGGAGCATGCCTGTTTGAGTGTCATTA
AATTCTCAACTCTCTTCTACTTTTTGTAAAAGAGAGCTTGGACTGTGGAGGCTTGCTGGC
CACTTTTTGGGGTCAGCTCCTCTGAAATGCATTAGCGGAACCGTTTGCGATCTGCCACAA
GTGTGATAAGTTATCTACACTGGCGAGGGGATTGCTCTCTGTAATGTTCAGCTTCTAATT
GTCTCTACTTTGTGAGACTACTTTTGAATGCTTGACCTCAAATCAGGTAGGACTACCCGC
TGAACTTAA
>C46
TTTCCGTAGGTGAACCTGCGGAAGGATCATTATTGAATTATGTTTCTAGATAGGTTGTAG
CTGGCTCTTTAGAGCATGTGCACGCCTGTTTGGACTTCATTTTCATCCACCTGTGCACCT
ATTGTAGTCTTTGGTTGGGTTAGGAGGAAGTGGTCATTGTGTCAGCATCTGCTGGATGTG
AGGACTTGCATTGTGAAAGCTTTGCTGTCCTTGATGTGATCATGGAATCTCTTTCTCACT
AGAGTCTATGTCACTCATTATACTCTGTCGAATGTCATTGAATGTCTTTACATGGGCTTA
TATGCCTATGAAAATTGTAATACAACTTTCAGCAACGGATCTCTTGGCTCTCGCATCGAT
GAAGAACGCAGCGAAATGCGATAAGTAATGTGAATTGCAGAATTCAGTGAATCATCGAAT
CTTTGAACGCATCTTGCGCTCCTTGGTATTCCGAGGAGCATGCCTGTTTGAGTGTCATTA
AATTCTCAACTCTCTTCTACTTTTTGTAAAAGAGAGCTTGGACTGTGGAGGCTTGCTGGC
CACTTTTTGGGGTCAGCTCCTCTGAAATGCATTAGCGGAACCGTTTGCGATCTGCCACAA
GTGTGATAAGTTATCTACACTGGCGAGGGGATTGCTCTCTGTAATGTTCAGCTTCTAATT
GTCTCTACTTTGTGAGACTACTTTTGAATGCTTGACCTCAAATCAGGTAGGACTACCCGC
TGAACTTAA
>C47
TTTCCGTAGGTGAACCTGCGGAAGGATCATTATTGAATTATGTTTCTAGATAGGTTGTAG
CTGGCTCTTTAGAGCATGTGCACGCCTGTTTGGACTTCATTTTCATCCACCTGTGCACCT
ATTGTAGTCTTTGGTTGGGTTAGGAGGAAGTGGTCATTGTGTCAGCATCTGCTGGATGTG
AGGACTTGCATTGTGAAAGCTTTGCTGTCCTTGATGTGATCATGGAATCTCTTTCTCACT
AGAGTCTATGTCACTCATTATACTCTGTCGAATGTCATTGAATGTCTTTACATGGGCTTA

TATGCCTATGAAAATTGTAATACAACTTTCAGCAACGGATCTCTTGGCTCTCGCATCGAT
GAAGAACGCAGCGAAATGCGATAAGTAATGTGAATTGCAGAATTCAGTGAATCATCGAAT
CTTTGAACGCATCTTGCGCTCCTTGGTATTCCGAGGAGCATGCCTGTTTGAGTGTCATTA
AATTCTCAACTCTCTTCTACTTTTTGTAAAAGAGAGCTTGGACTGTGGAGGCTTGCTGGC
CACTTTTTGGGGTCAGCTCCTCTGAAATGCATTAGCGGAACCGTTTGCGATCTGCCACAA
GTGTGATAAGTTATCTACACTGGCGAGGGGATTGCTCTCTGTAATGTTCAGCTTCTAATT
GTCTCTACTTTGTGAGACTACTTTTGAATGCTTGACCTCAAATCAGGTAGGACTACCCGC
TGAACTTAA
>C48
TTTCCGTAGGTGAACCTGCGGAAGGATCATTATTGAATTATGTTTCTAGATAGGTTGTAG
CTGGCTCTTTAGAGCATGTGCACGCCTGTTTGGACTTCATTTTCATCCACCTGTGCACCT
ATTGTAGTCTTTGGTTGGGTTAGGAGGAAGTGGTCATTGTGTCAGCATCTGCTGGATGTG
AGGACTTGCATTGTGAAAGCTTTGCTGTCCTTGATGTGATCATGGAATCTCTTTCTCACT
AGAGTCTATGTCACTCATTATACTCTGTCGAATGTCATTGAATGTCTTTACATGGGCTTA
TATGCCTATGAAAATTGTAATACAACTTTCAGCAACGGATCTCTTGGCTCTCGCATCGAT
GAAGAACGCAGCGAAATGCGATAAGTAATGTGAATTGCAGAATTCAGTGAATCATCGAAT
CTTTGAACGCATCTTGCGCTCCTTGGTATTCCGAGGAGCATGCCTGTTTGAGTGTCATTA
AATTCTCAACTCTCTTCTACTTTTTGTAAAAGAGAGCTTGGACTGTGGAGGCTTGCTGGC
CACTTTTTGGGGTCAGCTCCTCTGAAATGCATTAGCGGAACCGTTTGCGATCTGCCACAA
GTGTGATAAGTTATCTACACTGGCGAGGGGATTGCTCTCTGTAATGTTCAGCTTCTAATT
GTCTCTACTTTGTGAGACTACTTTTGAATGCTTGACCTCAAATCAGGTAGGACTACCCGC
TGAACTTAA
>C49
TTTCCGTAGGTGAACCTGCGGAAGGATCATTATTGAATTATGTTTCTAGATAGGTTGTAG
CTGGCTCTTTAGAGCATGTGCACGCCTGTTTGGACTTCATTTTCATCCACCTGTGCACCT
ATTGTAGTCTTTGGTTGGGTTAGGAGGAAGTGGTCATTGTGTCAGCATCTGCTGGATGTG
AGGACTTGCATTGTGAAAGCTTTGCTGTCCTTGATGTGATCATGGAATCTCTTTCTCACT
AGAGTCTATGTCACTCATTATACTCTGTCGAATGTCATTGAATGTCTTTACATGGGCTTA
TATGCCTATGAAAATTGTAATACAACTTTCAGCAACGGATCTCTTGGCTCTCGCATCGAT
GAAGAACGCAGCGAAATGCGATAAGTAATGTGAATTGCAGAATTCAGTGAATCATCGAAT
CTTTGAACGCATCTTGCGCTCCTTGGTATTCCGAGGAGCATGCCTGTTTGAGTGTCATTA
AATTCTCAACTCTCTTCTACTTTTTGTAAAAGAGAGCTTGGACTGTGGAGGCTTGCTGGC
CACTTTTTGGGGTCAGCTCCTCTGAAATGCATTAGCGGAACCGTTTGCGATCTGCCACAA
GTGTGATAAGTTATCTACACTGGCGAGGGGATTGCTCTCTGTAATGTTCAGCTTCTAATT
GTCTCTACTTTGTGAGACTACTTTTGAATGCTTGACCTCAAATCAGGTAGGACTACCCGC
TGAACTTAA
>C50
TTTCCGTAGGTGAACCTGCGGAAGGATCATTATTGAATTATGTTTCTAGATAGGTTGTAG
CTGGCTCTTTAGAGCATGTGCACGCCTGTTTGGACTTCATTTTCATCCACCTGTGCACCT
ATTGTAGTCTTTGGTTGGGTTAGGAGGAAGTGGTCATTGTGTCAGCATCTGCTGGATGTG
AGGACTTGCATTGTGAAAGCTTTGCTGTCCTTGATGTGATCATGGAATCTCTTTCTCACT
AGAGTCTATGTCACTCATTATACTCTGTCGAATGTCATTGAATGTCTTTACATGGGCTTA
TATGCCTATGAAAATTGTAATACAACTTTCAGCAACGGATCTCTTGGCTCTCGCATCGAT
GAAGAACGCAGCGAAATGCGATAAGTAATGTGAATTGCAGAATTCAGTGAATCATCGAAT
CTTTGAACGCATCTTGCGCTCCTTGGTATTCCGAGGAGCATGCCTGTTTGAGTGTCATTA
AATTCTCAACTCTCTTCTACTTTTTGTAAAAGAGAGCTTGGACTGTGGAGGCTTGCTGGC
CACTTTTTGGGGTCAGCTCCTCTGAAATGCATTAGCGGAACCGTTTGCGATCTGCCACAA
GTGTGATAAGTTATCTACACTGGCGAGGGGATTGCTCTCTGTAATGTTCAGCTTCTAATT
GTCTCTACTTTGTGAGACTACTTTTGAATGCTTGACCTCAAATCAGGTAGGACTACCCGC
TGAACTTAA

>C51
TTTCCGTAGGTGAACCTGCGGAAGGATCATTATTGAATTATGTTTCTAGATAGGTTGTAG
CTGGCTCTTTAGAGCATGTGCACGCCTGTTTGGACTTCATTTTCATCCACCTGTGCACCT
ATTGTAGTCTTTGGTTGGGTTAGGAGGAAGTGGTCATTGTGTCAGCATCTGCTGGATGTG
AGGACTTGCATTGTGAAAGCTTTGCTGTCCTTGATGTGATCATGGAATCTCTTTCTCACT
AGAGTCTATGTCACTCATTATACTCTGTCGAATGTCATTGAATGTCTTTACATGGGCTTA
TATGCCTATGAAAATTGTAATACAACTTTCAGCAACGGATCTCTTGGCTCTCGCATCGAT
GAAGAACGCAGCGAAATGCGATAAGTAATGTGAATTGCAGAATTCAGTGAATCATCGAAT
CTTTGAACGCATCTTGCGCTCCTTGGTATTCCGAGGAGCATGCCTGTTTGAGTGTCATTA
AATTCTCAACTCTCTTCTACTTTTTGTAAAAGAGAGCTTGGACTGTGGAGGCTTGCTGGC
CACTTTTTGGGGTCAGCTCCTCTGAAATGCATTAGCGGAACCGTTTGCGATCTGCCACAA
GTGTGATAAGTTATCTACACTGGCGAGGGGATTGCTCTCTGTAATGTTCAGCTTCTAATT
GTCTCTACTTTGTGAGACTACTTTTGAATGCTTGACCTCAAATCAGGTAGGACTACCCGC
TGAACTTAA
>C52
TTTCCGTAGGTGAACCTGCGGAAGGATCATTATTGAATTATGTTTCTAGATAGGTTGTAG
CTGGCTCTTTAGAGCATGTGCACGCCTGTTTGGACTTCATTTTCATCCACCTGTGCACCT
ATTGTAGTCTTTGGTTGGGTTAGGAGGAAGTGGTCATTGTGTCAGCATCTGCTGGATGTG
AGGACTTGCATTGTGAAAGCTTTGCTGTCCTTGATGTGATCATGGAATCTCTTTCTCACT
AGAGTCTATGTCACTCATTATACTCTGTCGAATGTCATTGAATGTCTTTACATGGGCTTA
TATGCCTATGAAAATTGTAATACAACTTTCAGCAACGGATCTCTTGGCTCTCGCATCGAT
GAAGAACGCAGCGAAATGCGATAAGTAATGTGAATTGCAGAATTCAGTGAATCATCGAAT
CTTTGAACGCATCTTGCGCTCCTTGGTATTCCGAGGAGCATGCCTGTTTGAGTGTCATTA
AATTCTCAACTCTCTTCTACTTTTTGTAAAAGAGAGCTTGGACTGTGGAGGCTTGCTGGC
CACTTTTTGGGGTCAGCTCCTCTGAAATGCATTAGCGGAACCGTTTGCGATCTGCCACAA
GTGTGATAAGTTATCTACACTGGCGAGGGGATTGCTCTCTGTAATGTTCAGCTTCTAATT
GTCTCTACTTTGTGAGACTACTTTTGAATGCTTGACCTCAAATCAGGTAGGACTACCCGC
TGAACTTAA
>C53
TTTCCGTAGGTGAACCTGCGGAAGGATCATTATTGAATTATGTTTCTAGATAGGTTGTAG
CTGGCTCTTTAGAGCATGTGCACGCCTGTTTGGACTTCATTTTCATCCACCTGTGCACCT
ATTGTAGTCTTTGGTTGGGTTAGGAGGAAGTGGTCATTGTGTCAGCATCTGCTGGATGTG
AGGACTTGCATTGTGAAAGCTTTGCTGTCCTTGATGTGATCATGGAATCTCTTTCTCACT
AGAGTCTATGTCACTCATTATACTCTGTCGAATGTCATTGAATGTCTTTACATGGGCTTA
TATGCCTATGAAAATTGTAATACAACTTTCAGCAACGGATCTCTTGGCTCTCGCATCGAT
GAAGAACGCAGCGAAATGCGATAAGTAATGTGAATTGCAGAATTCAGTGAATCATCGAAT
CTTTGAACGCATCTTGCGCTCCTTGGTATTCCGAGGAGCATGCCTGTTTGAGTGTCATTA
AATTCTCAACTCTCTTCTACTTTTTGTAAAAGAGAGCTTGGACTGTGGAGGCTTGCTGGC
CACTTTTTGGGGTCAGCTCCTCTGAAATGCATTAGCGGAACCGTTTGCGATCTGCCACAA
GTGTGATAAGTTATCTACACTGGCGAGGGGATTGCTCTCTGTAATGTTCAGCTTCTAATT
GTCTCTACTTTGTGAGACTACTTTTGAATGCTTGACCTCAAATCAGGTAGGACTACCCGC
TGAACTTAA
>C54
TTTCCGTAGGTGAACCTGCGGAAGGATCATTATTGAATTATGTTTCTAGATAGGTTGTAG
CTGGCTCTTTAGAGCATGTGCACGCCTGTTTGGACTTCATTTTCATCCACCTGTGCACCT
ATTGTAGTCTTTGGTTGGGTTAGGAGGAAGTGGTCATTGTGTCAGCATCTGCTGGATGTG
AGGACTTGCATTGTGAAAGCTTTGCTGTCCTTGATGTGATCATGGAATCTCTTTCTCACT
AGAGTCTATGTCACTCATTATACTCTGTCGAATGTCATTGAATGTCTTTACATGGGCTTA
TATGCCTATGAAAATTGTAATACAACTTTCAGCAACGGATCTCTTGGCTCTCGCATCGAT
GAAGAACGCAGCGAAATGCGATAAGTAATGTGAATTGCAGAATTCAGTGAATCATCGAAT

CTTTGAACGCATCTTGCGCTCCTTGGTATTCCGAGGAGCATGCCTGTTTGAGTGTCATTA
AATTCTCAACTCTCTTCTACTTTTTGTAAAAGAGAGCTTGGACTGTGGAGGCTTGCTGGC
CACTTTTTGGGGTCAGCTCCTCTGAAATGCATTAGCGGAACCGTTTGCGATCTGCCACAA
GTGTGATAAGTTATCTACACTGGCGAGGGGATTGCTCTCTGTAATGTTCAGCTTCTAATT
GTCTCTACTTTGTGAGACTACTTTTGAATGCTTGACCTCAAATCAGGTAGGACTACCCGC
TGAACTTAA
>C55
TTTCCGTAGGTGAACCTGCGGAAGGATCATTATTGAATTATGTTTCTAGATAGGTTGTAG
CTGGCTCTTTAGAGCATGTGCACGCCTGTTTGGACTTCATTTTCATCCACCTGTGCACCT
ATTGTAGTCTTTGGTTGGGTTAGGAGGAAGTGGTCATTGTGTCAGCATCTGCTGGATGTG
AGGACTTGCATTGTGAAAGCTTTGCTGTCCTTGATGTGATCATGGAATCTCTTTCTCACT
AGAGTCTATGTCACTCATTATACTCTGTCGAATGTCATTGAATGTCTTTACATGGGCTTA
TATGCCTATGAAAATTGTAATACAACTTTCAGCAACGGATCTCTTGGCTCTCGCATCGAT
GAAGAACGCAGCGAAATGCGATAAGTAATGTGAATTGCAGAATTCAGTGAATCATCGAAT
CTTTGAACGCATCTTGCGCTCCTTGGTATTCCGAGGAGCATGCCTGTTTGAGTGTCATTA
AATTCTCAACTCTCTTCTACTTTTTGTAAAAGAGAGCTTGGACTGTGGAGGCTTGCTGGC
CACTTTTTGGGGTCAGCTCCTCTGAAATGCATTAGCGGAACCGTTTGCGATCTGCCACAA
GTGTGATAAGTTATCTACACTGGCGAGGGGATTGCTCTCTGTAATGTTCAGCTTCTAATT
GTCTCTACTTTGTGAGACTACTTTTGAATGCTTGACCTCAAATCAGGTAGGACTACCCGC
TGAACTTAA
>C56
TTTCCGTAGGTGAACCTGCGGAAGGATCATTATTGAATTATGTTTCTAGATAGGTTGTAG
CTGGCTCTTTAGAGCATGTGCACGCCTGTTTGGACTTCATTTTCATCCACCTGTGCACCT
ATTGTAGTCTTTGGTTGGGTTAGGAGGAAGTGGTCATTGTGTCAGCATCTGCTGGATGTG
AGGACTTGCATTGTGAAAGCTTTGCTGTCCTTGATGTGATCATGGAATCTCTTTCTCACT
AGAGTCTATGTCACTCATTATACTCTGTCGAATGTCATTGAATGTCTTTACATGGGCTTA
TATGCCTATGAAAATTGTAATACAACTTTCAGCAACGGATCTCTTGGCTCTCGCATCGAT
GAAGAACGCAGCGAAATGCGATAAGTAATGTGAATTGCAGAATTCAGTGAATCATCGAAT
CTTTGAACGCATCTTGCGCTCCTTGGTATTCCGAGGAGCATGCCTGTTTGAGTGTCATTA
AATTCTCAACTCTCTTCTACTTTTTGTAAAAGAGAGCTTGGACTGTGGAGGCTTGCTGGC
CACTTTTTGGGGTCAGCTCCTCTGAAATGCATTAGCGGAACCGTTTGCGATCTGCCACAA
GTGTGATAAGTTATCTACACTGGCGAGGGGATTGCTCTCTGTAATGTTCAGCTTCTAATT
GTCTCTACTTTGTGAGACTACTTTTGAATGCTTGACCTCAAATCAGGTAGGACTACCCGC
TGAACTTAA
>C57
TTTCCGTAGGTGAACCTGCGGAAGGATCATTATTGAATTATGTTTCTAGATAGGTTGTAG
CTGGCTCTTTAGAGCATGTGCACGCCTGTTTGGACTTCATTTTCATCCACCTGTGCACCT
ATTGTAGTCTTTGGTTGGGTTAGGAGGAAGTGGTCATTGTGTCAGCATCTGCTGGATGTG
AGGACTTGCATTGTGAAAGCTTTGCTGTCCTTGATGTGATCATGGAATCTCTTTCTCACT
AGAGTCTATGTCACTCATTATACTCTGTCGAATGTCATTGAATGTCTTTACATGGGCTTA
TATGCCTATGAAAATTGTAATACAACTTTCAGCAACGGATCTCTTGGCTCTCGCATCGAT
GAAGAACGCAGCGAAATGCGATAAGTAATGTGAATTGCAGAATTCAGTGAATCATCGAAT
CTTTGAACGCATCTTGCGCTCCTTGGTATTCCGAGGAGCATGCCTGTTTGAGTGTCATTA
AATTCTCAACTCTCTTCTACTTTTTGTAAAAGAGAGCTTGGACTGTGGAGGCTTGCTGGC
CACTTTTTGGGGTCAGCTCCTCTGAAATGCATTAGCGGAACCGTTTGCGATCTGCCACAA
GTGTGATAAGTTATCTACACTGGCGAGGGGATTGCTCTCTGTAATGTTCAGCTTCTAATT
GTCTCTACTTTGTGAGACTACTTTTGAATGCTTGACCTCAAATCAGGTAGGACTACCCGC
TGAACTTAA
>C58
TTTCCGTAGGTGAACCTGCGGAAGGATCATTATTGAATTATGTTTCTAGATAGGTTGTAG

CTGGCTCTTTAGAGCATGTGCACGCCTGTTTGGACTTCATTTTCATCCACCTGTGCACCT
ATTGTAGTCTTTGGTTGGGTTAGGAGGAAGTGGTCATTGTGTCAGCATCTGCTGGATGTG
AGGACTTGCATTGTGAAAGCTTTGCTGTCCTTGATGTGATCATGGAATCTCTTTCTCACT
AGAGTCTATGTCACTCATTATACTCTGTCGAATGTCATTGAATGTCTTTACATGGGCTTA
TATGCCTATGAAAATTGTAATACAACTTTCAGCAACGGATCTCTTGGCTCTCGCATCGAT
GAAGAACGCAGCGAAATGCGATAAGTAATGTGAATTGCAGAATTCAGTGAATCATCGAAT
CTTTGAACGCATCTTGCGCTCCTTGGTATTCCGAGGAGCATGCCTGTTTGAGTGTCATTA
AATTCTCAACTCTCTTCTACTTTTTGTAAAAGAGAGCTTGGACTGTGGAGGCTTGCTGGC
CACTTTTTGGGGTCAGCTCCTCTGAAATGCATTAGCGGAACCGTTTGCGATCTGCCACAA
GTGTGATAAGTTATCTACACTGGCGAGGGGATTGCTCTCTGTAATGTTCAGCTTCTAATT
GTCTCTACTTTGTGAGACTACTTTTGAATGCTTGACCTCAAATCAGGTAGGACTACCCGC
TGAACTTAA
>C59
TTTCCGTAGGTGAACCTGCGGAAGGATCATTATTGAATTATGTTTCTAGATAGGTTGTAG
CTGGCTCTTTAGAGCATGTGCACGCCTGTTTGGACTTCATTTTCATCCACCTGTGCACCT
ATTGTAGTCTTTGGTTGGGTTAGGAGGAAGTGGTCATTGTGTCAGCATCTGCTGGATGTG
AGGACTTGCATTGTGAAAGCTTTGCTGTCCTTGATGTGATCATGGAATCTCTTTCTCACT
AGAGTCTATGTCACTCATTATACTCTGTCGAATGTCATTGAATGTCTTTACATGGGCTTA
TATGCCTATGAAAATTGTAATACAACTTTCAGCAACGGATCTCTTGGCTCTCGCATCGAT
GAAGAACGCAGCGAAATGCGATAAGTAATGTGAATTGCAGAATTCAGTGAATCATCGAAT
CTTTGAACGCATCTTGCGCTCCTTGGTATTCCGAGGAGCATGCCTGTTTGAGTGTCATTA
AATTCTCAACTCTCTTCTACTTTTTGTAAAAGAGAGCTTGGACTGTGGAGGCTTGCTGGC
CACTTTTTGGGGTCAGCTCCTCTGAAATGCATTAGCGGAACCGTTTGCGATCTGCCACAA
GTGTGATAAGTTATCTACACTGGCGAGGGGATTGCTCTCTGTAATGTTCAGCTTCTAATT
GTCTCTACTTTGTGAGACTACTTTTGAATGCTTGACCTCAAATCAGGTAGGACTACCCGC
TGAACTTAA
>C60
TTTCCGTAGGTGAACCTGCGGAAGGATCATTATTGAATTATGTTTCTAGATAGGTTGTAG
CTGGCTCTTTAGAGCATGTGCACGCCTGTTTGGACTTCATTTTCATCCACCTGTGCACCT
ATTGTAGTCTTTGGTTGGGTTAGGAGGAAGTGGTCATTGTGTCAGCATCTGCTGGATGTG
AGGACTTGCATTGTGAAAGCTTTGCTGTCCTTGATGTGATCATGGAATCTCTTTCTCACT
AGAGTCTATGTCACTCATTATACTCTGTCGAATGTCATTGAATGTCTTTACATGGGCTTA
TATGCCTATGAAAATTGTAATACAACTTTCAGCAACGGATCTCTTGGCTCTCGCATCGAT
GAAGAACGCAGCGAAATGCGATAAGTAATGTGAATTGCAGAATTCAGTGAATCATCGAAT
CTTTGAACGCATCTTGCGCTCCTTGGTATTCCGAGGAGCATGCCTGTTTGAGTGTCATTA
AATTCTCAACTCTCTTCTACTTTTTGTAAAAGAGAGCTTGGACTGTGGAGGCTTGCTGGC
CACTTTTTGGGGTCAGCTCCTCTGAAATGCATTAGCGGAACCGTTTGCGATCTGCCACAA
GTGTGATAAGTTATCTACACTGGCGAGGGGATTGCTCTCTGTAATGTTCAGCTTCTAATT
GTCTCTACTTTGTGAGACTACTTTTGAATGCTTGACCTCAAATCAGGTAGGACTACCCGC
TGAACTTAA
>C61
TTTCCGTAGGTGAACCTGCGGAAGGATCATTATTGAATTATGTTTCTAGATAGGTTGTAG
CTGGCTCTTTAGAGCATGTGCACGCCTGTTTGGACTTCATTTTCATCCACCTGTGCACCT
ATTGTAGTCTTTGGTTGGGTTAGGAGGAAGTGGTCATTGTGTCAGCATCTGCTGGATGTG
AGGACTTGCATTGTGAAAGCTTTGCTGTCCTTGATGTGATCATGGAATCTCTTTCTCACT
AGAGTCTATGTCACTCATTATACTCTGTCGAATGTCATTGAATGTCTTTACATGGGCTTA
TATGCCTATGAAAATTGTAATACAACTTTCAGCAACGGATCTCTTGGCTCTCGCATCGAT
GAAGAACGCAGCGAAATGCGATAAGTAATGTGAATTGCAGAATTCAGTGAATCATCGAAT
CTTTGAACGCATCTTGCGCTCCTTGGTATTCCGAGGAGCATGCCTGTTTGAGTGTCATTA
AATTCTCAACTCTCTTCTACTTTTTGTAAAAGAGAGCTTGGACTGTGGAGGCTTGCTGGC

CACTTTTTGGGGTCAGCTCCTCTGAAATGCATTAGCGGAACCGTTTGCGATCTGCCACAA
GTGTGATAAGTTATCTACACTGGCGAGGGGATTGCTCTCTGTAATGTTCAGCTTCTAATT
GTCTCTACTTTGTGAGACTACTTTTGAATGCTTGACCTCAAATCAGGTAGGACTACCCGC
TGAACTTAA
>C62
TTTCCGTAGGTGAACCTGCGGAAGGATCATTATTGAATTATGTTTCTAGATAGGTTGTAG
CTGGCTCTTTAGAGCATGTGCACGCCTGTTTGGACTTCATTTTCATCCACCTGTGCACCT
ATTGTAGTCTTTGGTTGGGTTAGGAGGAAGTGGTCATTGTGTCAGCATCTGCTGGATGTG
AGGACTTGCATTGTGAAAGCTTTGCTGTCCTTGATGTGATCATGGAATCTCTTTCTCACT
AGAGTCTATGTCACTCATTATACTCTGTCGAATGTCATTGAATGTCTTTACATGGGCTTA
TATGCCTATGAAAATTGTAATACAACTTTCAGCAACGGATCTCTTGGCTCTCGCATCGAT
GAAGAACGCAGCGAAATGCGATAAGTAATGTGAATTGCAGAATTCAGTGAATCATCGAAT
CTTTGAACGCATCTTGCGCTCCTTGGTATTCCGAGGAGCATGCCTGTTTGAGTGTCATTA
AATTCTCAACTCTCTTCTACTTTTTGTAAAAGAGAGCTTGGACTGTGGAGGCTTGCTGGC
CACTTTTTGGGGTCAGCTCCTCTGAAATGCATTAGCGGAACCGTTTGCGATCTGCCACAA
GTGTGATAAGTTATCTACACTGGCGAGGGGATTGCTCTCTGTAATGTTCAGCTTCTAATT
GTCTCTACTTTGTGAGACTACTTTTGAATGCTTGACCTCAAATCAGGTAGGACTACCCGC
TGAACTTAA
>C63
TTTCCGTAGGTGAACCTGCGGAAGGATCATTATTGAATTATGTTTCTAGATAGGTTGTAG
CTGGCTCTTTAGAGCATGTGCACGCCTGTTTGGACTTCATTTTCATCCACCTGTGCACCT
ATTGTAGTCTTTGGTTGGGTTAGGAGGAAGTGGTCATTGTGTCAGCATCTGCTGGATGTG
AGGACTTGCATTGTGAAAGCTTTGCTGTCCTTGATGTGATCATGGAATCTCTTTCTCACT
AGAGTCTATGTCACTCATTATACTCTGTCGAATGTCATTGAATGTCTTTACATGGGCTTA
TATGCCTATGAAAATTGTAATACAACTTTCAGCAACGGATCTCTTGGCTCTCGCATCGAT
GAAGAACGCAGCGAAATGCGATAAGTAATGTGAATTGCAGAATTCAGTGAATCATCGAAT
CTTTGAACGCATCTTGCGCTCCTTGGTATTCCGAGGAGCATGCCTGTTTGAGTGTCATTA
AATTCTCAACTCTCTTCTACTTTTTGTAAAAGAGAGCTTGGACTGTGGAGGCTTGCTGGC
CACTTTTTGGGGTCAGCTCCTCTGAAATGCATTAGCGGAACCGTTTGCGATCTGCCACAA
GTGTGATAAGTTATCTACACTGGCGAGGGGATTGCTCTCTGTAATGTTCAGCTTCTAATT
GTCTCTACTTTGTGAGACTACTTTTGAATGCTTGACCTCAAATCAGGTAGGACTACCCGC
TGAACTTAA
>C64
TTTCCGTAGGTGAACCTGCGGAAGGATCATTATTGAATTATGTTTCTAGATAGGTTGTAG
CTGGCTCTTTAGAGCATGTGCACGCCTGTTTGGACTTCATTTTCATCCACCTGTGCACCT
ATTGTAGTCTTTGGTTGGGTTAGGAGGAAGTGGTCATTGTGTCAGCATCTGCTGGATGTG
AGGACTTGCATTGTGAAAGCTTTGCTGTCCTTGATGTGATCATGGAATCTCTTTCTCACT
AGAGTCTATGTCACTCATTATACTCTGTCGAATGTCATTGAATGTCTTTACATGGGCTTA
TATGCCTATGAAAATTGTAATACAACTTTCAGCAACGGATCTCTTGGCTCTCGCATCGAT
GAAGAACGCAGCGAAATGCGATAAGTAATGTGAATTGCAGAATTCAGTGAATCATCGAAT
CTTTGAACGCATCTTGCGCTCCTTGGTATTCCGAGGAGCATGCCTGTTTGAGTGTCATTA
AATTCTCAACTCTCTTCTACTTTTTGTAAAAGAGAGCTTGGACTGTGGAGGCTTGCTGGC
CACTTTTTGGGGTCAGCTCCTCTGAAATGCATTAGCGGAACCGTTTGCGATCTGCCACAA
GTGTGATAAGTTATCTACACTGGCGAGGGGATTGCTCTCTGTAATGTTCAGCTTCTAATT
GTCTCTACTTTGTGAGACTACTTTTGAATGCTTGACCTCAAATCAGGTAGGACTACCCGC
TGAACTTAA
>C65
TTTCCGTAGGTGAACCTGCGGAAGGATCATTATTGAATTATGTTTCTAGATAGGTTGTAG
CTGGCTCTTTAGAGCATGTGCACGCCTGTTTGGACTTCATTTTCATCCACCTGTGCACCT
ATTGTAGTCTTTGGTTGGGTTAGGAGGAAGTGGTCATTGTGTCAGCATCTGCTGGATGTG

AGGACTTGCATTGTGAAAGCTTTGCTGTCCTTGATGTGATCATGGAATCTCTTTCTCACT
AGAGTCTATGTCACTCATTATACTCTGTCGAATGTCATTGAATGTCTTTACATGGGCTTA
TATGCCTATGAAAATTGTAATACAACTTTCAGCAACGGATCTCTTGGCTCTCGCATCGAT
GAAGAACGCAGCGAAATGCGATAAGTAATGTGAATTGCAGAATTCAGTGAATCATCGAAT
CTTTGAACGCATCTTGCGCTCCTTGGTATTCCGAGGAGCATGCCTGTTTGAGTGTCATTA
AATTCTCAACTCTCTTCTACTTTTTGTAAAAGAGAGCTTGGACTGTGGAGGCTTGCTGGC
CACTTTTTGGGGTCAGCTCCTCTGAAATGCATTAGCGGAACCGTTTGCGATCTGCCACAA
GTGTGATAAGTTATCTACACTGGCGAGGGGATTGCTCTCTGTAATGTTCAGCTTCTAATT
GTCTCTACTTTGTGAGACTACTTTTGAATGCTTGACCTCAAATCAGGTAGGACTACCCGC
TGAACTTAA
>C66
TTTCCGTAGGTGAACCTGCGGAAGGATCATTATTGAATTATGTTTCTAGATAGGTTGTAG
CTGGCTCTTTAGAGCATGTGCACGCCTGTTTGGACTTCATTTTCATCCACCTGTGCACCT
ATTGTAGTCTTTGGTTGGGTTAGGAGGAAGTGGTCATTGTGTCAGCATCTGCTGGATGTG
AGGACTTGCATTGTGAAAGCTTTGCTGTCCTTGATGTGATCATGGAATCTCTTTCTCACT
AGAGTCTATGTCACTCATTATACTCTGTCGAATGTCATTGAATGTCTTTACATGGGCTTA
TATGCCTATGAAAATTGTAATACAACTTTCAGCAACGGATCTCTTGGCTCTCGCATCGAT
GAAGAACGCAGCGAAATGCGATAAGTAATGTGAATTGCAGAATTCAGTGAATCATCGAAT
CTTTGAACGCATCTTGCGCTCCTTGGTATTCCGAGGAGCATGCCTGTTTGAGTGTCATTA
AATTCTCAACTCTCTTCTACTTTTTGTAAAAGAGAGCTTGGACTGTGGAGGCTTGCTGGC
CACTTTTTGGGGTCAGCTCCTCTGAAATGCATTAGCGGAACCGTTTGCGATCTGCCACAA
GTGTGATAAGTTATCTACACTGGCGAGGGGATTGCTCTCTGTAATGTTCAGCTTCTAATT
GTCTCTACTTTGTGAGACTACTTTTGAATGCTTGACCTCAAATCAGGTAGGACTACCCGC
TGAACTTAA
>C67
TTTCCGTAGGTGAACCTGCGGAAGGATCATTATTGAATTATGTTTCTAGATAGGTTGTAG
CTGGCTCTTTAGAGCATGTGCACGCCTGTTTGGACTTCATTTTCATCCACCTGTGCACCT
ATTGTAGTCTTTGGTTGGGTTAGGAGGAAGTGGTCATTGTGTCAGCATCTGCTGGATGTG
AGGACTTGCATTGTGAAAGCTTTGCTGTCCTTGATGTGATCATGGAATCTCTTTCTCACT
AGAGTCTATGTCACTCATTATACTCTGTCGAATGTCATTGAATGTCTTTACATGGGCTTA
TATGCCTATGAAAATTGTAATACAACTTTCAGCAACGGATCTCTTGGCTCTCGCATCGAT
GAAGAACGCAGCGAAATGCGATAAGTAATGTGAATTGCAGAATTCAGTGAATCATCGAAT
CTTTGAACGCATCTTGCGCTCCTTGGTATTCCGAGGAGCATGCCTGTTTGAGTGTCATTA
AATTCTCAACTCTCTTCTACTTTTTGTAAAAGAGAGCTTGGACTGTGGAGGCTTGCTGGC
CACTTTTTGGGGTCAGCTCCTCTGAAATGCATTAGCGGAACCGTTTGCGATCTGCCACAA
GTGTGATAAGTTATCTACACTGGCGAGGGGATTGCTCTCTGTAATGTTCAGCTTCTAATT
GTCTCTACTTTGTGAGACTACTTTTGAATGCTTGACCTCAAATCAGGTAGGACTACCCGC
TGAACTTAA
>C68
TTTCCGTAGGTGAACCTGCGGAAGGATCATTATTGAATTATGTTTCTAGATAGGTTGTAG
CTGGCTCTTTAGAGCATGTGCACGCCTGTTTGGACTTCATTTTCATCCACCTGTGCACCT
ATTGTAGTCTTTGGTTGGGTTAGGAGGAAGTGGTCATTGTGTCAGCATCTGCTGGATGTG
AGGACTTGCATTGTGAAAGCTTTGCTGTCCTTGATGTGATCATGGAATCTCTTTCTCACT
AGAGTCTATGTCACTCATTATACTCTGTCGAATGTCATTGAATGTCTTTACATGGGCTTA
TATGCCTATGAAAATTGTAATACAACTTTCAGCAACGGATCTCTTGGCTCTCGCATCGAT
GAAGAACGCAGCGAAATGCGATAAGTAATGTGAATTGCAGAATTCAGTGAATCATCGAAT
CTTTGAACGCATCTTGCGCTCCTTGGTATTCCGAGGAGCATGCCTGTTTGAGTGTCATTA
AATTCTCAACTCTCTTCTACTTTTTGTAAAAGAGAGCTTGGACTGTGGAGGCTTGCTGGC
CACTTTTTGGGGTCAGCTCCTCTGAAATGCATTAGCGGAACCGTTTGCGATCTGCCACAA
GTGTGATAAGTTATCTACACTGGCGAGGGGATTGCTCTCTGTAATGTTCAGCTTCTAATT

GTCTCTACTTTGTGAGACTACTTTTGAATGCTTGACCTCAAATCAGGTAGGACTACCCGC
TGAACTTAA
>C69
TTTCCGTAGGTGAACCTGCGGAAGGATCATTATTGAATTATGTTTCTAGATAGGTTGTAG
CTGGCTCTTTAGAGCATGTGCACGCCTGTTTGGACTTCATTTTCATCCACCTGTGCACCT
ATTGTAGTCTTTGGTTGGGTTAGGAGGAAGTGGTCATTGTGTCAGCATCTGCTGGATGTG
AGGACTTGCATTGTGAAAGCTTTGCTGTCCTTGATGTGATCATGGAATCTCTTTCTCACT
AGAGTCTATGTCACTCATTATACTCTGTCGAATGTCATTGAATGTCTTTACATGGGCTTA
TATGCCTATGAAAATTGTAATACAACTTTCAGCAACGGATCTCTTGGCTCTCGCATCGAT
GAAGAACGCAGCGAAATGCGATAAGTAATGTGAATTGCAGAATTCAGTGAATCATCGAAT
CTTTGAACGCATCTTGCGCTCCTTGGTATTCCGAGGAGCATGCCTGTTTGAGTGTCATTA
AATTCTCAACTCTCTTCTACTTTTTGTAAAAGAGAGCTTGGACTGTGGAGGCTTGCTGGC
CACTTTTTGGGGTCAGCTCCTCTGAAATGCATTAGCGGAACCGTTTGCGATCTGCCACAA
GTGTGATAAGTTATCTACACTGGCGAGGGGATTGCTCTCTGTAATGTTCAGCTTCTAATT
GTCTCTACTTTGTGAGACTACTTTTGAATGCTTGACCTCAAATCAGGTAGGACTACCCGC
TGAACTTAA
>C70
TTTCCGTAGGTGAACCTGCGGAAGGATCATTATTGAATTATGTTTCTAGATAGGTTGTAG
CTGGCTCTTTAGAGCATGTGCACGCCTGTTTGGACTTCATTTTCATCCACCTGTGCACCT
ATTGTAGTCTTTGGTTGGGTTAGGAGGAAGTGGTCATTGTGTCAGCATCTGCTGGATGTG
AGGACTTGCATTGTGAAAGCTTTGCTGTCCTTGATGTGATCATGGAATCTCTTTCTCACT
AGAGTCTATGTCACTCATTATACTCTGTCGAATGTCATTGAATGTCTTTACATGGGCTTA
TATGCCTATGAAAATTGTAATACAACTTTCAGCAACGGATCTCTTGGCTCTCGCATCGAT
GAAGAACGCAGCGAAATGCGATAAGTAATGTGAATTGCAGAATTCAGTGAATCATCGAAT
CTTTGAACGCATCTTGCGCTCCTTGGTATTCCGAGGAGCATGCCTGTTTGAGTGTCATTA
AATTCTCAACTCTCTTCTACTTTTTGTAAAAGAGAGCTTGGACTGTGGAGGCTTGCTGGC
CACTTTTTGGGGTCAGCTCCTCTGAAATGCATTAGCGGAACCGTTTGCGATCTGCCACAA
GTGTGATAAGTTATCTACACTGGCGAGGGGATTGCTCTCTGTAATGTTCAGCTTCTAATT
GTCTCTACTTTGTGAGACTACTTTTGAATGCTTGACCTCAAATCAGGTAGGACTACCCGC
TGAACTTAA
>C71
TTTCCGTAGGTGAACCTGCGGAAGGATCATTATTGAATTATGTTTCTAGATAGGTTGTAG
CTGGCTCTTTAGAGCATGTGCACGCCTGTTTGGACTTCATTTTCATCCACCTGTGCACCT
ATTGTAGTCTTTGGTTGGGTTAGGAGGAAGTGGTCATTGTGTCAGCATCTGCTGGATGTG
AGGACTTGCATTGTGAAAGCTTTGCTGTCCTTGATGTGATCATGGAATCTCTTTCTCACT
AGAGTCTATGTCACTCATTATACTCTGTCGAATGTCATTGAATGTCTTTACATGGGCTTA
TATGCCTATGAAAATTGTAATACAACTTTCAGCAACGGATCTCTTGGCTCTCGCATCGAT
GAAGAACGCAGCGAAATGCGATAAGTAATGTGAATTGCAGAATTCAGTGAATCATCGAAT
CTTTGAACGCATCTTGCGCTCCTTGGTATTCCGAGGAGCATGCCTGTTTGAGTGTCATTA
AATTCTCAACTCTCTTCTACTTTTTGTAAAAGAGAGCTTGGACTGTGGAGGCTTGCTGGC
CACTTTTTGGGGTCAGCTCCTCTGAAATGCATTAGCGGAACCGTTTGCGATCTGCCACAA
GTGTGATAAGTTATCTACACTGGCGAGGGGATTGCTCTCTGTAATGTTCAGCTTCTAATT
GTCTCTACTTTGTGAGACTACTTTTGAATGCTTGACCTCAAATCAGGTAGGACTACCCGC
TGAACTTAA
>C72
TTTCCGTAGGTGAACCTGCGGAAGGATCATTATTGAATTATGTTTCTAGATAGGTTGTAG
CTGGCTCTTTAGAGCATGTGCACGCCTGTTTGGACTTCATTTTCATCCACCTGTGCACCT
ATTGTAGTCTTTGGTTGGGTTAGGAGGAAGTGGTCATTGTGTCAGCATCTGCTGGATGTG
AGGACTTGCATTGTGAAAGCTTTGCTGTCCTTGATGTGATCATGGAATCTCTTTCTCACT
AGAGTCTATGTCACTCATTATACTCTGTCGAATGTCATTGAATGTCTTTACATGGGCTTA

TATGCCTATGAAAATTGTAATACAACTTTCAGCAACGGATCTCTTGGCTCTCGCATCGAT
GAAGAACGCAGCGAAATGCGATAAGTAATGTGAATTGCAGAATTCAGTGAATCATCGAAT
CTTTGAACGCATCTTGCGCTCCTTGGTATTCCGAGGAGCATGCCTGTTTGAGTGTCATTA
AATTCTCAACTCTCTTCTACTTTTTGTAAAAGAGAGCTTGGACTGTGGAGGCTTGCTGGC
CACTTTTTGGGGTCAGCTCCTCTGAAATGCATTAGCGGAACCGTTTGCGATCTGCCACAA
GTGTGATAAGTTATCTACACTGGCGAGGGGATTGCTCTCTGTAATGTTCAGCTTCTAATT
GTCTCTACTTTGTGAGACTACTTTTGAATGCTTGACCTCAAATCAGGTAGGACTACCCGC
TGAACTTAA
>C73
TTTCCGTAGGTGAACCTGCGGAAGGATCATTATTGAATTATGTTTCTAGATAGGTTGTAG
CTGGCTCTTTAGAGCATGTGCACGCCTGTTTGGACTTCATTTTCATCCACCTGTGCACCT
ATTGTAGTCTTTGGTTGGGTTAGGAGGAAGTGGTCATTGTGTCAGCATCTGCTGGATGTG
AGGACTTGCATTGTGAAAGCTTTGCTGTCCTTGATGTGATCATGGAATCTCTTTCTCACT
AGAGTCTATGTCACTCATTATACTCTGTCGAATGTCATTGAATGTCTTTACATGGGCTTA
TATGCCTATGAAAATTGTAATACAACTTTCAGCAACGGATCTCTTGGCTCTCGCATCGAT
GAAGAACGCAGCGAAATGCGATAAGTAATGTGAATTGCAGAATTCAGTGAATCATCGAAT
CTTTGAACGCATCTTGCGCTCCTTGGTATTCCGAGGAGCATGCCTGTTTGAGTGTCATTA
AATTCTCAACTCTCTTCTACTTTTTGTAAAAGAGAGCTTGGACTGTGGAGGCTTGCTGGC
CACTTTTTGGGGTCAGCTCCTCTGAAATGCATTAGCGGAACCGTTTGCGATCTGCCACAA
GTGTGATAAGTTATCTACACTGGCGAGGGGATTGCTCTCTGTAATGTTCAGCTTCTAATT
GTCTCTACTTTGTGAGACTACTTTTGAATGCTTGACCTCAAATCAGGTAGGACTACCCGC
TGAACTTAA
>C74
TTTCCGTAGGTGAACCTGCGGAAGGATCATTATTGAATTATGTTTCTAGATAGGTTGTAG
CTGGCTCTTTAGAGCATGTGCACGCCTGTTTGGACTTCATTTTCATCCACCTGTGCACCT
ATTGTAGTCTTTGGTTGGGTTAGGAGGAAGTGGTCATTGTGTCAGCATCTGCTGGATGTG
AGGACTTGCATTGTGAAAGCTTTGCTGTCCTTGATGTGATCATGGAATCTCTTTCTCACT
AGAGTCTATGTCACTCATTATACTCTGTCGAATGTCATTGAATGTCTTTACATGGGCTTA
TATGCCTATGAAAATTGTAATACAACTTTCAGCAACGGATCTCTTGGCTCTCGCATCGAT
GAAGAACGCAGCGAAATGCGATAAGTAATGTGAATTGCAGAATTCAGTGAATCATCGAAT
CTTTGAACGCATCTTGCGCTCCTTGGTATTCCGAGGAGCATGCCTGTTTGAGTGTCATTA
AATTCTCAACTCTCTTCTACTTTTTGTAAAAGAGAGCTTGGACTGTGGAGGCTTGCTGGC
CACTTTTTGGGGTCAGCTCCTCTGAAATGCATTAGCGGAACCGTTTGCGATCTGCCACAA
GTGTGATAAGTTATCTACACTGGCGAGGGGATTGCTCTCTGTAATGTTCAGCTTCTAATT
GTCTCTACTTTGTGAGACTACTTTTGAATGCTTGACCTCAAATCAGGTAGGACTACCCGC
TGAACTTAA
>C75
TTTCCGTAGGTGAACCTGCGGAAGGATCATTATTGAATTATGTTTCTAGATAGGTTGTAG
CTGGCTCTTTAGAGCATGTGCACGCCTGTTTGGACTTCATTTTCATCCACCTGTGCACCT
ATTGTAGTCTTTGGTTGGGTTAGGAGGAAGTGGTCATTGTGTCAGCATCTGCTGGATGTG
AGGACTTGCATTGTGAAAGCTTTGCTGTCCTTGATGTGATCATGGAATCTCTTTCTCACT
AGAGTCTATGTCACTCATTATACTCTGTCGAATGTCATTGAATGTCTTTACATGGGCTTA
TATGCCTATGAAAATTGTAATACAACTTTCAGCAACGGATCTCTTGGCTCTCGCATCGAT
GAAGAACGCAGCGAAATGCGATAAGTAATGTGAATTGCAGAATTCAGTGAATCATCGAAT
CTTTGAACGCATCTTGCGCTCCTTGGTATTCCGAGGAGCATGCCTGTTTGAGTGTCATTA
AATTCTCAACTCTCTTCTACTTTTTGTAAAAGAGAGCTTGGACTGTGGAGGCTTGCTGGC
CACTTTTTGGGGTCAGCTCCTCTGAAATGCATTAGCGGAACCGTTTGCGATCTGCCACAA
GTGTGATAAGTTATCTACACTGGCGAGGGGATTGCTCTCTGTAATGTTCAGCTTCTAATT
GTCTCTACTTTGTGAGACTACTTTTGAATGCTTGACCTCAAATCAGGTAGGACTACCCGC
TGAACTTAA

>C76
TTTCCGTAGGTGAACCTGCGGAAGGATCATTATTGAATTATGTTTCTAGATAGGTTGTAG
CTGGCTCTTTAGAGCATGTGCACGCCTGTTTGGACTTCATTTTCATCCACCTGTGCACCT
ATTGTAGTCTTTGGTTGGGTTAGGAGGAAGTGGTCATTGTGTCAGCATCTGCTGGATGTG
AGGACTTGCATTGTGAAAGCTTTGCTGTCCTTGATGTGATCATGGAATCTCTTTCTCACT
AGAGTCTATGTCACTCATTATACTCTGTCGAATGTCATTGAATGTCTTTACATGGGCTTA
TATGCCTATGAAAATTGTAATACAACTTTCAGCAACGGATCTCTTGGCTCTCGCATCGAT
GAAGAACGCAGCGAAATGCGATAAGTAATGTGAATTGCAGAATTCAGTGAATCATCGAAT
CTTTGAACGCATCTTGCGCTCCTTGGTATTCCGAGGAGCATGCCTGTTTGAGTGTCATTA
AATTCTCAACTCTCTTCTACTTTTTGTAAAAGAGAGCTTGGACTGTGGAGGCTTGCTGGC
CACTTTTTGGGGTCAGCTCCTCTGAAATGCATTAGCGGAACCGTTTGCGATCTGCCACAA
GTGTGATAAGTTATCTACACTGGCGAGGGGATTGCTCTCTGTAATGTTCAGCTTCTAATT
GTCTCTACTTTGTGAGACTACTTTTGAATGCTTGACCTCAAATCAGGTAGGACTACCCGC
TGAACTTAA
>C77
TTTCCGTAGGTGAACCTGCGGAAGGATCATTATTGAATTATGTTTCTAGATAGGTTGTAG
CTGGCTCTTTAGAGCATGTGCACGCCTGTTTGGACTTCATTTTCATCCACCTGTGCACCT
ATTGTAGTCTTTGGTTGGGTTAGGAGGAAGTGGTCATTGTGTCAGCATCTGCTGGATGTG
AGGACTTGCATTGTGAAAGCTTTGCTGTCCTTGATGTGATCATGGAATCTCTTTCTCACT
AGAGTCTATGTCACTCATTATACTCTGTCGAATGTCATTGAATGTCTTTACATGGGCTTA
TATGCCTATGAAAATTGTAATACAACTTTCAGCAACGGATCTCTTGGCTCTCGCATCGAT
GAAGAACGCAGCGAAATGCGATAAGTAATGTGAATTGCAGAATTCAGTGAATCATCGAAT
CTTTGAACGCATCTTGCGCTCCTTGGTATTCCGAGGAGCATGCCTGTTTGAGTGTCATTA
AATTCTCAACTCTCTTCTACTTTTTGTAAAAGAGAGCTTGGACTGTGGAGGCTTGCTGGC
CACTTTTTGGGGTCAGCTCCTCTGAAATGCATTAGCGGAACCGTTTGCGATCTGCCACAA
GTGTGATAAGTTATCTACACTGGCGAGGGGATTGCTCTCTGTAATGTTCAGCTTCTAATT
GTCTCTACTTTGTGAGACTACTTTTGAATGCTTGACCTCAAATCAGGTAGGACTACCCGC
TGAACTTAA
>C78
TTTCCGTAGGTGAACCTGCGGAAGGATCATTATTGAATTATGTTTCTAGATAGGTTGTAG
CTGGCTCTTTAGAGCATGTGCACGCCTGTTTGGACTTCATTTTCATCCACCTGTGCACCT
ATTGTAGTCTTTGGTTGGGTTAGGAGGAAGTGGTCATTGTGTCAGCATCTGCTGGATGTG
AGGACTTGCATTGTGAAAGCTTTGCTGTCCTTGATGTGATCATGGAATCTCTTTCTCACT
AGAGTCTATGTCACTCATTATACTCTGTCGAATGTCATTGAATGTCTTTACATGGGCTTA
TATGCCTATGAAAATTGTAATACAACTTTCAGCAACGGATCTCTTGGCTCTCGCATCGAT
GAAGAACGCAGCGAAATGCGATAAGTAATGTGAATTGCAGAATTCAGTGAATCATCGAAT
CTTTGAACGCATCTTGCGCTCCTTGGTATTCCGAGGAGCATGCCTGTTTGAGTGTCATTA
AATTCTCAACTCTCTTCTACTTTTTGTAAAAGAGAGCTTGGACTGTGGAGGCTTGCTGGC
CACTTTTTGGGGTCAGCTCCTCTGAAATGCATTAGCGGAACCGTTTGCGATCTGCCACAA
GTGTGATAAGTTATCTACACTGGCGAGGGGATTGCTCTCTGTAATGTTCAGCTTCTAATT
GTCTCTACTTTGTGAGACTACTTTTGAATGCTTGACCTCAAATCAGGTAGGACTACCCGC
TGAACTTAA
>C79
TTTCCGTAGGTGAACCTGCGGAAGGATCATTATTGAATTATGTTTCTAGATAGGTTGTAG
CTGGCTCTTTAGAGCATGTGCACGCCTGTTTGGACTTCATTTTCATCCACCTGTGCACCT
ATTGTAGTCTTTGGTTGGGTTAGGAGGAAGTGGTCATTGTGTCAGCATCTGCTGGATGTG
AGGACTTGCATTGTGAAAGCTTTGCTGTCCTTGATGTGATCATGGAATCTCTTTCTCACT
AGAGTCTATGTCACTCATTATACTCTGTCGAATGTCATTGAATGTCTTTACATGGGCTTA
TATGCCTATGAAAATTGTAATACAACTTTCAGCAACGGATCTCTTGGCTCTCGCATCGAT
GAAGAACGCAGCGAAATGCGATAAGTAATGTGAATTGCAGAATTCAGTGAATCATCGAAT

CTTTGAACGCATCTTGCGCTCCTTGGTATTCCGAGGAGCATGCCTGTTTGAGTGTCATTA
AATTCTCAACTCTCTTCTACTTTTTGTAAAAGAGAGCTTGGACTGTGGAGGCTTGCTGGC
CACTTTTTGGGGTCAGCTCCTCTGAAATGCATTAGCGGAACCGTTTGCGATCTGCCACAA
GTGTGATAAGTTATCTACACTGGCGAGGGGATTGCTCTCTGTAATGTTCAGCTTCTAATT
GTCTCTACTTTGTGAGACTACTTTTGAATGCTTGACCTCAAATCAGGTAGGACTACCCGC
TGAACTTAA
>C80
TTTCCGTAGGTGAACCTGCGGAAGGATCATTATTGAATTATGTTTCTAGATAGGTTGTAG
CTGGCTCTTTAGAGCATGTGCACGCCTGTTTGGACTTCATTTTCATCCACCTGTGCACCT
ATTGTAGTCTTTGGTTGGGTTAGGAGGAAGTGGTCATTGTGTCAGCATCTGCTGGATGTG
AGGACTTGCATTGTGAAAGCTTTGCTGTCCTTGATGTGATCATGGAATCTCTTTCTCACT
AGAGTCTATGTCACTCATTATACTCTGTCGAATGTCATTGAATGTCTTTACATGGGCTTA
TATGCCTATGAAAATTGTAATACAACTTTCAGCAACGGATCTCTTGGCTCTCGCATCGAT
GAAGAACGCAGCGAAATGCGATAAGTAATGTGAATTGCAGAATTCAGTGAATCATCGAAT
CTTTGAACGCATCTTGCGCTCCTTGGTATTCCGAGGAGCATGCCTGTTTGAGTGTCATTA
AATTCTCAACTCTCTTCTACTTTTTGTAAAAGAGAGCTTGGACTGTGGAGGCTTGCTGGC
CACTTTTTGGGGTCAGCTCCTCTGAAATGCATTAGCGGAACCGTTTGCGATCTGCCACAA
GTGTGATAAGTTATCTACACTGGCGAGGGGATTGCTCTCTGTAATGTTCAGCTTCTAATT
GTCTCTACTTTGTGAGACTACTTTTGAATGCTTGACCTCAAATCAGGTAGGACTACCCGC
TGAACTTAA
>C81
TTTCCGTAGGTGAACCTGCGGAAGGATCATTATTGAATTATGTTTCTAGATAGGTTGTAG
CTGGCTCTTTAGAGCATGTGCACGCCTGTTTGGACTTCATTTTCATCCACCTGTGCACCT
ATTGTAGTCTTTGGTTGGGTTAGGAGGAAGTGGTCATTGTGTCAGCATCTGCTGGATGTG
AGGACTTGCATTGTGAAAGCTTTGCTGTCCTTGATGTGATCATGGAATCTCTTTCTCACT
AGAGTCTATGTCACTCATTATACTCTGTCGAATGTCATTGAATGTCTTTACATGGGCTTA
TATGCCTATGAAAATTGTAATACAACTTTCAGCAACGGATCTCTTGGCTCTCGCATCGAT
GAAGAACGCAGCGAAATGCGATAAGTAATGTGAATTGCAGAATTCAGTGAATCATCGAAT
CTTTGAACGCATCTTGCGCTCCTTGGTATTCCGAGGAGCATGCCTGTTTGAGTGTCATTA
AATTCTCAACTCTCTTCTACTTTTTGTAAAAGAGAGCTTGGACTGTGGAGGCTTGCTGGC
CACTTTTTGGGGTCAGCTCCTCTGAAATGCATTAGCGGAACCGTTTGCGATCTGCCACAA
GTGTGATAAGTTATCTACACTGGCGAGGGGATTGCTCTCTGTAATGTTCAGCTTCTAATT
GTCTCTACTTTGTGAGACTACTTTTGAATGCTTGACCTCAAATCAGGTAGGACTACCCGC
TGAACTTAA
>C82
TTTCCGTAGGTGAACCTGCGGAAGGATCATTATTGAATTATGTTTCTAGATAGGTTGTAG
CTGGCTCTTTAGAGCATGTGCACGCCTGTTTGGACTTCATTTTCATCCACCTGTGCACCT
ATTGTAGTCTTTGGTTGGGTTAGGAGGAAGTGGTCATTGTGTCAGCATCTGCTGGATGTG
AGGACTTGCATTGTGAAAGCTTTGCTGTCCTTGATGTGATCATGGAATCTCTTTCTCACT
AGAGTCTATGTCACTCATTATACTCTGTCGAATGTCATTGAATGTCTTTACATGGGCTTA
TATGCCTATGAAAATTGTAATACAACTTTCAGCAACGGATCTCTTGGCTCTCGCATCGAT
GAAGAACGCAGCGAAATGCGATAAGTAATGTGAATTGCAGAATTCAGTGAATCATCGAAT
CTTTGAACGCATCTTGCGCTCCTTGGTATTCCGAGGAGCATGCCTGTTTGAGTGTCATTA
AATTCTCAACTCTCTTCTACTTTTTGTAAAAGAGAGCTTGGACTGTGGAGGCTTGCTGGC
CACTTTTTGGGGTCAGCTCCTCTGAAATGCATTAGCGGAACCGTTTGCGATCTGCCACAA
GTGTGATAAGTTATCTACACTGGCGAGGGGATTGCTCTCTGTAATGTTCAGCTTCTAATT
GTCTCTACTTTGTGAGACTACTTTTGAATGCTTGACCTCAAATCAGGTAGGACTACCCGC
TGAACTTAA
>C83
TTTCCGTAGGTGAACCTGCGGAAGGATCATTATTGAATTATGTTTCTAGATAGGTTGTAG

CTGGCTCTTTAGAGCATGTGCACGCCTGTTTGGACTTCATTTTCATCCACCTGTGCACCT
ATTGTAGTCTTTGGTTGGGTTAGGAGGAAGTGGTCATTGTGTCAGCATCTGCTGGATGTG
AGGACTTGCATTGTGAAAGCTTTGCTGTCCTTGATGTGATCATGGAATCTCTTTCTCACT
AGAGTCTATGTCACTCATTATACTCTGTCGAATGTCATTGAATGTCTTTACATGGGCTTA
TATGCCTATGAAAATTGTAATACAACTTTCAGCAACGGATCTCTTGGCTCTCGCATCGAT
GAAGAACGCAGCGAAATGCGATAAGTAATGTGAATTGCAGAATTCAGTGAATCATCGAAT
CTTTGAACGCATCTTGCGCTCCTTGGTATTCCGAGGAGCATGCCTGTTTGAGTGTCATTA
AATTCTCAACTCTCTTCTACTTTTTGTAAAAGAGAGCTTGGACTGTGGAGGCTTGCTGGC
CACTTTTTGGGGTCAGCTCCTCTGAAATGCATTAGCGGAACCGTTTGCGATCTGCCACAA
GTGTGATAAGTTATCTACACTGGCGAGGGGATTGCTCTCTGTAATGTTCAGCTTCTAATT
GTCTCTACTTTGTGAGACTACTTTTGAATGCTTGACCTCAAATCAGGTAGGACTACCCGC
TGAACTTAA
>C84
TTTCCGTAGGTGAACCTGCGGAAGGATCATTATTGAATTATGTTTCTAGATAGGTTGTAG
CTGGCTCTTTAGAGCATGTGCACGCCTGTTTGGACTTCATTTTCATCCACCTGTGCACCT
ATTGTAGTCTTTGGTTGGGTTAGGAGGAAGTGGTCATTGTGTCAGCATCTGCTGGATGTG
AGGACTTGCATTGTGAAAGCTTTGCTGTCCTTGATGTGATCATGGAATCTCTTTCTCACT
AGAGTCTATGTCACTCATTATACTCTGTCGAATGTCATTGAATGTCTTTACATGGGCTTA
TATGCCTATGAAAATTGTAATACAACTTTCAGCAACGGATCTCTTGGCTCTCGCATCGAT
GAAGAACGCAGCGAAATGCGATAAGTAATGTGAATTGCAGAATTCAGTGAATCATCGAAT
CTTTGAACGCATCTTGCGCTCCTTGGTATTCCGAGGAGCATGCCTGTTTGAGTGTCATTA
AATTCTCAACTCTCTTCTACTTTTTGTAAAAGAGAGCTTGGACTGTGGAGGCTTGCTGGC
CACTTTTTGGGGTCAGCTCCTCTGAAATGCATTAGCGGAACCGTTTGCGATCTGCCACAA
GTGTGATAAGTTATCTACACTGGCGAGGGGATTGCTCTCTGTAATGTTCAGCTTCTAATT
GTCTCTACTTTGTGAGACTACTTTTGAATGCTTGACCTCAAATCAGGTAGGACTACCCGC
TGAACTTAA
>C85
TTTCCGTAGGTGAACCTGCGGAAGGATCATTATTGAATTATGTTTCTAGATAGGTTGTAG
CTGGCTCTTTAGAGCATGTGCACGCCTGTTTGGACTTCATTTTCATCCACCTGTGCACCT
ATTGTAGTCTTTGGTTGGGTTAGGAGGAAGTGGTCATTGTGTCAGCATCTGCTGGATGTG
AGGACTTGCATTGTGAAAGCTTTGCTGTCCTTGATGTGATCATGGAATCTCTTTCTCACT
AGAGTCTATGTCACTCATTATACTCTGTCGAATGTCATTGAATGTCTTTACATGGGCTTA
TATGCCTATGAAAATTGTAATACAACTTTCAGCAACGGATCTCTTGGCTCTCGCATCGAT
GAAGAACGCAGCGAAATGCGATAAGTAATGTGAATTGCAGAATTCAGTGAATCATCGAAT
CTTTGAACGCATCTTGCGCTCCTTGGTATTCCGAGGAGCATGCCTGTTTGAGTGTCATTA
AATTCTCAACTCTCTTCTACTTTTTGTAAAAGAGAGCTTGGACTGTGGAGGCTTGCTGGC
CACTTTTTGGGGTCAGCTCCTCTGAAATGCATTAGCGGAACCGTTTGCGATCTGCCACAA
GTGTGATAAGTTATCTACACTGGCGAGGGGATTGCTCTCTGTAATGTTCAGCTTCTAATT
GTCTCTACTTTGTGAGACTACTTTTGAATGCTTGACCTCAAATCAGGTAGGACTACCCGC
TGAACTTAA
>C86
TTTCCGTAGGTGAACCTGCGGAAGGATCATTATTGAATTATGTTTCTAGATAGGTTGTAG
CTGGCTCTTTAGAGCATGTGCACGCCTGTTTGGACTTCATTTTCATCCACCTGTGCACCT
ATTGTAGTCTTTGGTTGGGTTAGGAGGAAGTGGTCATTGTGTCAGCATCTGCTGGATGTG
AGGACTTGCATTGTGAAAGCTTTGCTGTCCTTGATGTGATCATGGAATCTCTTTCTCACT
AGAGTCTATGTCACTCATTATACTCTGTCGAATGTCATTGAATGTCTTTACATGGGCTTA
TATGCCTATGAAAATTGTAATACAACTTTCAGCAACGGATCTCTTGGCTCTCGCATCGAT
GAAGAACGCAGCGAAATGCGATAAGTAATGTGAATTGCAGAATTCAGTGAATCATCGAAT
CTTTGAACGCATCTTGCGCTCCTTGGTATTCCGAGGAGCATGCCTGTTTGAGTGTCATTA
AATTCTCAACTCTCTTCTACTTTTTGTAAAAGAGAGCTTGGACTGTGGAGGCTTGCTGGC

CACTTTTTGGGGTCAGCTCCTCTGAAATGCATTAGCGGAACCGTTTGCGATCTGCCACAA
GTGTGATAAGTTATCTACACTGGCGAGGGGATTGCTCTCTGTAATGTTCAGCTTCTAATT
GTCTCTACTTTGTGAGACTACTTTTGAATGCTTGACCTCAAATCAGGTAGGACTACCCGC
TGAACTTAA
>C87
TTTCCGTAGGTGAACCTGCGGAAGGATCATTATTGAATTATGTTTCTAGATAGGTTGTAG
CTGGCTCTTTAGAGCATGTGCACGCCTGTTTGGACTTCATTTTCATCCACCTGTGCACCT
ATTGTAGTCTTTGGTTGGGTTAGGAGGAAGTGGTCATTGTGTCAGCATCTGCTGGATGTG
AGGACTTGCATTGTGAAAGCTTTGCTGTCCTTGATGTGATCATGGAATCTCTTTCTCACT
AGAGTCTATGTCACTCATTATACTCTGTCGAATGTCATTGAATGTCTTTACATGGGCTTA
TATGCCTATGAAAATTGTAATACAACTTTCAGCAACGGATCTCTTGGCTCTCGCATCGAT
GAAGAACGCAGCGAAATGCGATAAGTAATGTGAATTGCAGAATTCAGTGAATCATCGAAT
CTTTGAACGCATCTTGCGCTCCTTGGTATTCCGAGGAGCATGCCTGTTTGAGTGTCATTA
AATTCTCAACTCTCTTCTACTTTTTGTAAAAGAGAGCTTGGACTGTGGAGGCTTGCTGGC
CACTTTTTGGGGTCAGCTCCTCTGAAATGCATTAGCGGAACCGTTTGCGATCTGCCACAA
GTGTGATAAGTTATCTACACTGGCGAGGGGATTGCTCTCTGTAATGTTCAGCTTCTAATT
GTCTCTACTTTGTGAGACTACTTTTGAATGCTTGACCTCAAATCAGGTAGGACTACCCGC
TGAACTTAA
>C88
TTTCCGTAGGTGAACCTGCGGAAGGATCATTATTGAATTATGTTTCTAGATAGGTTGTAG
CTGGCTCTTTAGAGCATGTGCACGCCTGTTTGGACTTCATTTTCATCCACCTGTGCACCT
ATTGTAGTCTTTGGTTGGGTTAGGAGGAAGTGGTCATTGTGTCAGCATCTGCTGGATGTG
AGGACTTGCATTGTGAAAGCTTTGCTGTCCTTGATGTGATCATGGAATCTCTTTCTCACT
AGAGTCTATGTCACTCATTATACTCTGTCGAATGTCATTGAATGTCTTTACATGGGCTTA
TATGCCTATGAAAATTGTAATACAACTTTCAGCAACGGATCTCTTGGCTCTCGCATCGAT
GAAGAACGCAGCGAAATGCGATAAGTAATGTGAATTGCAGAATTCAGTGAATCATCGAAT
CTTTGAACGCATCTTGCGCTCCTTGGTATTCCGAGGAGCATGCCTGTTTGAGTGTCATTA
AATTCTCAACTCTCTTCTACTTTTTGTAAAAGAGAGCTTGGACTGTGGAGGCTTGCTGGC
CACTTTTTGGGGTCAGCTCCTCTGAAATGCATTAGCGGAACCGTTTGCGATCTGCCACAA
GTGTGATAAGTTATCTACACTGGCGAGGGGATTGCTCTCTGTAATGTTCAGCTTCTAATT
GTCTCTACTTTGTGAGACTACTTTTGAATGCTTGACCTCAAATCAGGTAGGACTACCCGC
TGAACTTAA
>C89
TTTCCGTAGGTGAACCTGCGGAAGGATCATTATTGAATTATGTTTCTAGATAGGTTGTAG
CTGGCTCTTTAGAGCATGTGCACGCCTGTTTGGACTTCATTTTCATCCACCTGTGCACCT
ATTGTAGTCTTTGGTTGGGTTAGGAGGAAGTGGTCATTGTGTCAGCATCTGCTGGATGTG
AGGACTTGCATTGTGAAAGCTTTGCTGTCCTTGATGTGATCATGGAATCTCTTTCTCACT
AGAGTCTATGTCACTCATTATACTCTGTCGAATGTCATTGAATGTCTTTACATGGGCTTA
TATGCCTATGAAAATTGTAATACAACTTTCAGCAACGGATCTCTTGGCTCTCGCATCGAT
GAAGAACGCAGCGAAATGCGATAAGTAATGTGAATTGCAGAATTCAGTGAATCATCGAAT
CTTTGAACGCATCTTGCGCTCCTTGGTATTCCGAGGAGCATGCCTGTTTGAGTGTCATTA
AATTCTCAACTCTCTTCTACTTTTTGTAAAAGAGAGCTTGGACTGTGGAGGCTTGCTGGC
CACTTTTTGGGGTCAGCTCCTCTGAAATGCATTAGCGGAACCGTTTGCGATCTGCCACAA
GTGTGATAAGTTATCTACACTGGCGAGGGGATTGCTCTCTGTAATGTTCAGCTTCTAATT
GTCTCTACTTTGTGAGACTACTTTTGAATGCTTGACCTCAAATCAGGTAGGACTACCCGC
TGAACTTAA
>C90
TTTCCGTAGGTGAACCTGCGGAAGGATCATTATTGAATTATGTTTCTAGATAGGTTGTAG
CTGGCTCTTTAGAGCATGTGCACGCCTGTTTGGACTTCATTTTCATCCACCTGTGCACCT
ATTGTAGTCTTTGGTTGGGTTAGGAGGAAGTGGTCATTGTGTCAGCATCTGCTGGATGTG

AGGACTTGCATTGTGAAAGCTTTGCTGTCCTTGATGTGATCATGGAATCTCTTTCTCACT
AGAGTCTATGTCACTCATTATACTCTGTCGAATGTCATTGAATGTCTTTACATGGGCTTA
TATGCCTATGAAAATTGTAATACAACTTTCAGCAACGGATCTCTTGGCTCTCGCATCGAT
GAAGAACGCAGCGAAATGCGATAAGTAATGTGAATTGCAGAATTCAGTGAATCATCGAAT
CTTTGAACGCATCTTGCGCTCCTTGGTATTCCGAGGAGCATGCCTGTTTGAGTGTCATTA
AATTCTCAACTCTCTTCTACTTTTTGTAAAAGAGAGCTTGGACTGTGGAGGCTTGCTGGC
CACTTTTTGGGGTCAGCTCCTCTGAAATGCATTAGCGGAACCGTTTGCGATCTGCCACAA
GTGTGATAAGTTATCTACACTGGCGAGGGGATTGCTCTCTGTAATGTTCAGCTTCTAATT
GTCTCTACTTTGTGAGACTACTTTTGAATGCTTGACCTCAAATCAGGTAGGACTACCCGC
TGAACTTAA
>C91
TTTCCGTAGGTGAACCTGCGGAAGGATCATTATTGAATTATGTTTCTAGATAGGTTGTAG
CTGGCTCTTTAGAGCATGTGCACGCCTGTTTGGACTTCATTTTCATCCACCTGTGCACCT
ATTGTAGTCTTTGGTTGGGTTAGGAGGAAGTGGTCATTGTGTCAGCATCTGCTGGATGTG
AGGACTTGCATTGTGAAAGCTTTGCTGTCCTTGATGTGATCATGGAATCTCTTTCTCACT
AGAGTCTATGTCACTCATTATACTCTGTCGAATGTCATTGAATGTCTTTACATGGGCTTA
TATGCCTATGAAAATTGTAATACAACTTTCAGCAACGGATCTCTTGGCTCTCGCATCGAT
GAAGAACGCAGCGAAATGCGATAAGTAATGTGAATTGCAGAATTCAGTGAATCATCGAAT
CTTTGAACGCATCTTGCGCTCCTTGGTATTCCGAGGAGCATGCCTGTTTGAGTGTCATTA
AATTCTCAACTCTCTTCTACTTTTTGTAAAAGAGAGCTTGGACTGTGGAGGCTTGCTGGC
CACTTTTTGGGGTCAGCTCCTCTGAAATGCATTAGCGGAACCGTTTGCGATCTGCCACAA
GTGTGATAAGTTATCTACACTGGCGAGGGGATTGCTCTCTGTAATGTTCAGCTTCTAATT
GTCTCTACTTTGTGAGACTACTTTTGAATGCTTGACCTCAAATCAGGTAGGACTACCCGC
TGAACTTAA
>C92
TTTCCGTAGGTGAACCTGCGGAAGGATCATTATTGAATTATGTTTCTAGATAGGTTGTAG
CTGGCTCTTTAGAGCATGTGCACGCCTGTTTGGACTTCATTTTCATCCACCTGTGCACCT
ATTGTAGTCTTTGGTTGGGTTAGGAGGAAGTGGTCATTGTGTCAGCATCTGCTGGATGTG
AGGACTTGCATTGTGAAAGCTTTGCTGTCCTTGATGTGATCATGGAATCTCTTTCTCACT
AGAGTCTATGTCACTCATTATACTCTGTCGAATGTCATTGAATGTCTTTACATGGGCTTA
TATGCCTATGAAAATTGTAATACAACTTTCAGCAACGGATCTCTTGGCTCTCGCATCGAT
GAAGAACGCAGCGAAATGCGATAAGTAATGTGAATTGCAGAATTCAGTGAATCATCGAAT
CTTTGAACGCATCTTGCGCTCCTTGGTATTCCGAGGAGCATGCCTGTTTGAGTGTCATTA
AATTCTCAACTCTCTTCTACTTTTTGTAAAAGAGAGCTTGGACTGTGGAGGCTTGCTGGC
CACTTTTTGGGGTCAGCTCCTCTGAAATGCATTAGCGGAACCGTTTGCGATCTGCCACAA
GTGTGATAAGTTATCTACACTGGCGAGGGGATTGCTCTCTGTAATGTTCAGCTTCTAATT
GTCTCTACTTTGTGAGACTACTTTTGAATGCTTGACCTCAAATCAGGTAGGACTACCCGC
TGAACTTAA
>C93
TTTCCGTAGGTGAACCTGCGGAAGGATCATTATTGAATTATGTTTCTAGATAGGTTGTAG
CTGGCTCTTTAGAGCATGTGCACGCCTGTTTGGACTTCATTTTCATCCACCTGTGCACCT
ATTGTAGTCTTTGGTTGGGTTAGGAGGAAGTGGTCATTGTGTCAGCATCTGCTGGATGTG
AGGACTTGCATTGTGAAAGCTTTGCTGTCCTTGATGTGATCATGGAATCTCTTTCTCACT
AGAGTCTATGTCACTCATTATACTCTGTCGAATGTCATTGAATGTCTTTACATGGGCTTA
TATGCCTATGAAAATTGTAATACAACTTTCAGCAACGGATCTCTTGGCTCTCGCATCGAT
GAAGAACGCAGCGAAATGCGATAAGTAATGTGAATTGCAGAATTCAGTGAATCATCGAAT
CTTTGAACGCATCTTGCGCTCCTTGGTATTCCGAGGAGCATGCCTGTTTGAGTGTCATTA
AATTCTCAACTCTCTTCTACTTTTTGTAAAAGAGAGCTTGGACTGTGGAGGCTTGCTGGC
CACTTTTTGGGGTCAGCTCCTCTGAAATGCATTAGCGGAACCGTTTGCGATCTGCCACAA
GTGTGATAAGTTATCTACACTGGCGAGGGGATTGCTCTCTGTAATGTTCAGCTTCTAATT

GTCTCTACTTTGTGAGACTACTTTTGAATGCTTGACCTCAAATCAGGTAGGACTACCCGC
TGAACTTAA
>C94
TTTCCGTAGGTGAACCTGCGGAAGGATCATTATTGAATTATGTTTCTAGATAGGTTGTAG
CTGGCTCTTTAGAGCATGTGCACGCCTGTTTGGACTTCATTTTCATCCACCTGTGCACCT
ATTGTAGTCTTTGGTTGGGTTAGGAGGAAGTGGTCATTGTGTCAGCATCTGCTGGATGTG
AGGACTTGCATTGTGAAAGCTTTGCTGTCCTTGATGTGATCATGGAATCTCTTTCTCACT
AGAGTCTATGTCACTCATTATACTCTGTCGAATGTCATTGAATGTCTTTACATGGGCTTA
TATGCCTATGAAAATTGTAATACAACTTTCAGCAACGGATCTCTTGGCTCTCGCATCGAT
GAAGAACGCAGCGAAATGCGATAAGTAATGTGAATTGCAGAATTCAGTGAATCATCGAAT
CTTTGAACGCATCTTGCGCTCCTTGGTATTCCGAGGAGCATGCCTGTTTGAGTGTCATTA
AATTCTCAACTCTCTTCTACTTTTTGTAAAAGAGAGCTTGGACTGTGGAGGCTTGCTGGC
CACTTTTTGGGGTCAGCTCCTCTGAAATGCATTAGCGGAACCGTTTGCGATCTGCCACAA
GTGTGATAAGTTATCTACACTGGCGAGGGGATTGCTCTCTGTAATGTTCAGCTTCTAATT
GTCTCTACTTTGTGAGACTACTTTTGAATGCTTGACCTCAAATCAGGTAGGACTACCCGC
TGAACTTAA
>C95
TTTCCGTAGGTGAACCTGCGGAAGGATCATTATTGAATTATGTTTCTAGATAGGTTGTAG
CTGGCTCTTTAGAGCATGTGCACGCCTGTTTGGACTTCATTTTCATCCACCTGTGCACCT
ATTGTAGTCTTTGGTTGGGTTAGGAGGAAGTGGTCATTGTGTCAGCATCTGCTGGATGTG
AGGACTTGCATTGTGAAAGCTTTGCTGTCCTTGATGTGATCATGGAATCTCTTTCTCACT
AGAGTCTATGTCACTCATTATACTCTGTCGAATGTCATTGAATGTCTTTACATGGGCTTA
TATGCCTATGAAAATTGTAATACAACTTTCAGCAACGGATCTCTTGGCTCTCGCATCGAT
GAAGAACGCAGCGAAATGCGATAAGTAATGTGAATTGCAGAATTCAGTGAATCATCGAAT
CTTTGAACGCATCTTGCGCTCCTTGGTATTCCGAGGAGCATGCCTGTTTGAGTGTCATTA
AATTCTCAACTCTCTTCTACTTTTTGTAAAAGAGAGCTTGGACTGTGGAGGCTTGCTGGC
CACTTTTTGGGGTCAGCTCCTCTGAAATGCATTAGCGGAACCGTTTGCGATCTGCCACAA
GTGTGATAAGTTATCTACACTGGCGAGGGGATTGCTCTCTGTAATGTTCAGCTTCTAATT
GTCTCTACTTTGTGAGACTACTTTTGAATGCTTGACCTCAAATCAGGTAGGACTACCCGC
TGAACTTAA
>C96
TTTCCGTAGGTGAACCTGCGGAAGGATCATTATTGAATTATGTTTCTAGATAGGTTGTAG
CTGGCTCTTTAGAGCATGTGCACGCCTGTTTGGACTTCATTTTCATCCACCTGTGCACCT
ATTGTAGTCTTTGGTTGGGTTAGGAGGAAGTGGTCATTGTGTCAGCATCTGCTGGATGTG
AGGACTTGCATTGTGAAAGCTTTGCTGTCCTTGATGTGATCATGGAATCTCTTTCTCACT
AGAGTCTATGTCACTCATTATACTCTGTCGAATGTCATTGAATGTCTTTACATGGGCTTA
TATGCCTATGAAAATTGTAATACAACTTTCAGCAACGGATCTCTTGGCTCTCGCATCGAT
GAAGAACGCAGCGAAATGCGATAAGTAATGTGAATTGCAGAATTCAGTGAATCATCGAAT
CTTTGAACGCATCTTGCGCTCCTTGGTATTCCGAGGAGCATGCCTGTTTGAGTGTCATTA
AATTCTCAACTCTCTTCTACTTTTTGTAAAAGAGAGCTTGGACTGTGGAGGCTTGCTGGC
CACTTTTTGGGGTCAGCTCCTCTGAAATGCATTAGCGGAACCGTTTGCGATCTGCCACAA
GTGTGATAAGTTATCTACACTGGCGAGGGGATTGCTCTCTGTAATGTTCAGCTTCTAATT
GTCTCTACTTTGTGAGACTACTTTTGAATGCTTGACCTCAAATCAGGTAGGACTACCCGC
TGAACTTAA
>C97
TTTCCGTAGGTGAACCTGCGGAAGGATCATTATTGAATTATGTTTCTAGATAGGTTGTAG
CTGGCTCTTTAGAGCATGTGCACGCCTGTTTGGACTTCATTTTCATCCACCTGTGCACCT
ATTGTAGTCTTTGGTTGGGTTAGGAGGAAGTGGTCATTGTGTCAGCATCTGCTGGATGTG
AGGACTTGCATTGTGAAAGCTTTGCTGTCCTTGATGTGATCATGGAATCTCTTTCTCACT
AGAGTCTATGTCACTCATTATACTCTGTCGAATGTCATTGAATGTCTTTACATGGGCTTA

TATGCCTATGAAAATTGTAATACAACTTTCAGCAACGGATCTCTTGGCTCTCGCATCGAT
GAAGAACGCAGCGAAATGCGATAAGTAATGTGAATTGCAGAATTCAGTGAATCATCGAAT
CTTTGAACGCATCTTGCGCTCCTTGGTATTCCGAGGAGCATGCCTGTTTGAGTGTCATTA
AATTCTCAACTCTCTTCTACTTTTTGTAAAAGAGAGCTTGGACTGTGGAGGCTTGCTGGC
CACTTTTTGGGGTCAGCTCCTCTGAAATGCATTAGCGGAACCGTTTGCGATCTGCCACAA
GTGTGATAAGTTATCTACACTGGCGAGGGGATTGCTCTCTGTAATGTTCAGCTTCTAATT
GTCTCTACTTTGTGAGACTACTTTTGAATGCTTGACCTCAAATCAGGTAGGACTACCCGC
TGAACTTAA
>C98
TTTCCGTAGGTGAACCTGCGGAAGGATCATTATTGAATTATGTTTCTAGATAGGTTGTAG
CTGGCTCTTTAGAGCATGTGCACGCCTGTTTGGACTTCATTTTCATCCACCTGTGCACCT
ATTGTAGTCTTTGGTTGGGTTAGGAGGAAGTGGTCATTGTGTCAGCATCTGCTGGATGTG
AGGACTTGCATTGTGAAAGCTTTGCTGTCCTTGATGTGATCATGGAATCTCTTTCTCACT
AGAGTCTATGTCACTCATTATACTCTGTCGAATGTCATTGAATGTCTTTACATGGGCTTA
TATGCCTATGAAAATTGTAATACAACTTTCAGCAACGGATCTCTTGGCTCTCGCATCGAT
GAAGAACGCAGCGAAATGCGATAAGTAATGTGAATTGCAGAATTCAGTGAATCATCGAAT
CTTTGAACGCATCTTGCGCTCCTTGGTATTCCGAGGAGCATGCCTGTTTGAGTGTCATTA
AATTCTCAACTCTCTTCTACTTTTTGTAAAAGAGAGCTTGGACTGTGGAGGCTTGCTGGC
CACTTTTTGGGGTCAGCTCCTCTGAAATGCATTAGCGGAACCGTTTGCGATCTGCCACAA
GTGTGATAAGTTATCTACACTGGCGAGGGGATTGCTCTCTGTAATGTTCAGCTTCTAATT
GTCTCTACTTTGTGAGACTACTTTTGAATGCTTGACCTCAAATCAGGTAGGACTACCCGC
TGAACTTAA
>C99
TTTCCGTAGGTGAACCTGCGGAAGGATCATTATTGAATTATGTTTCTAGATAGGTTGTAG
CTGGCTCTTTAGAGCATGTGCACGCCTGTTTGGACTTCATTTTCATCCACCTGTGCACCT
ATTGTAGTCTTTGGTTGGGTTAGGAGGAAGTGGTCATTGTGTCAGCATCTGCTGGATGTG
AGGACTTGCATTGTGAAAGCTTTGCTGTCCTTGATGTGATCATGGAATCTCTTTCTCACT
AGAGTCTATGTCACTCATTATACTCTGTCGAATGTCATTGAATGTCTTTACATGGGCTTA
TATGCCTATGAAAATTGTAATACAACTTTCAGCAACGGATCTCTTGGCTCTCGCATCGAT
GAAGAACGCAGCGAAATGCGATAAGTAATGTGAATTGCAGAATTCAGTGAATCATCGAAT
CTTTGAACGCATCTTGCGCTCCTTGGTATTCCGAGGAGCATGCCTGTTTGAGTGTCATTA
AATTCTCAACTCTCTTCTACTTTTTGTAAAAGAGAGCTTGGACTGTGGAGGCTTGCTGGC
CACTTTTTGGGGTCAGCTCCTCTGAAATGCATTAGCGGAACCGTTTGCGATCTGCCACAA
GTGTGATAAGTTATCTACACTGGCGAGGGGATTGCTCTCTGTAATGTTCAGCTTCTAATT
GTCTCTACTTTGTGAGACTACTTTTGAATGCTTGACCTCAAATCAGGTAGGACTACCCGC
TGAACTTAA
>C100
TTTCCGTAGGTGAACCTGCGGAAGGATCATTATTGAATTATGTTTCTAGATAGGTTGTAG
CTGGCTCTTTAGAGCATGTGCACGCCTGTTTGGACTTCATTTTCATCCACCTGTGCACCT
ATTGTAGTCTTTGGTTGGGTTAGGAGGAAGTGGTCATTGTGTCAGCATCTGCTGGATGTG
AGGACTTGCATTGTGAAAGCTTTGCTGTCCTTGATGTGATCATGGAATCTCTTTCTCACT
AGAGTCTATGTCACTCATTATACTCTGTCGAATGTCATTGAATGTCTTTACATGGGCTTA
TATGCCTATGAAAATTGTAATACAACTTTCAGCAACGGATCTCTTGGCTCTCGCATCGAT
GAAGAACGCAGCGAAATGCGATAAGTAATGTGAATTGCAGAATTCAGTGAATCATCGAAT
CTTTGAACGCATCTTGCGCTCCTTGGTATTCCGAGGAGCATGCCTGTTTGAGTGTCATTA
AATTCTCAACTCTCTTCTACTTTTTGTAAAAGAGAGCTTGGACTGTGGAGGCTTGCTGGC
CACTTTTTGGGGTCAGCTCCTCTGAAATGCATTAGCGGAACCGTTTGCGATCTGCCACAA
GTGTGATAAGTTATCTACACTGGCGAGGGGATTGCTCTCTGTAATGTTCAGCTTCTAATT
GTCTCTACTTTGTGAGACTACTTTTGAATGCTTGACCTCAAATCAGGTAGGACTACCCGC
TGAACTTAA

>C101
TTTCCGTAGGTGAACCTGCGGAAGGATCATTATTGAATTATGTTTCTAGATAGGTTGTAG
CTGGCTCTTTAGAGCATGTGCACGCCTGTTTGGACTTCATTTTCATCCACCTGTGCACCT
ATTGTAGTCTTTGGTTGGGTTAGGAGGAAGTGGTCATTGTGTCAGCATCTGCTGGATGTG
AGGACTTGCATTGTGAAAGCTTTGCTGTCCTTGATGTGATCATGGAATCTCTTTCTCACT
AGAGTCTATGTCACTCATTATACTCTGTCGAATGTCATTGAATGTCTTTACATGGGCTTA
TATGCCTATGAAAATTGTAATACAACTTTCAGCAACGGATCTCTTGGCTCTCGCATCGAT
GAAGAACGCAGCGAAATGCGATAAGTAATGTGAATTGCAGAATTCAGTGAATCATCGAAT
CTTTGAACGCATCTTGCGCTCCTTGGTATTCCGAGGAGCATGCCTGTTTGAGTGTCATTA
AATTCTCAACTCTCTTCTACTTTTTGTAAAAGAGAGCTTGGACTGTGGAGGCTTGCTGGC
CACTTTTTGGGGTCAGCTCCTCTGAAATGCATTAGCGGAACCGTTTGCGATCTGCCACAA
GTGTGATAAGTTATCTACACTGGCGAGGGGATTGCTCTCTGTAATGTTCAGCTTCTAATT
GTCTCTACTTTGTGAGACTACTTTTGAATGCTTGACCTCAAATCAGGTAGGACTACCCGC
TGAACTTAA
>C102
TTTCCGTAGGTGAACCTGCGGAAGGATCATTATTGAATTATGTTTCTAGATAGGTTGTAG
CTGGCTCTTTAGAGCATGTGCACGCCTGTTTGGACTTCATTTTCATCCACCTGTGCACCT
ATTGTAGTCTTTGGTTGGGTTAGGAGGAAGTGGTCATTGTGTCAGCATCTGCTGGATGTG
AGGACTTGCATTGTGAAAGCTTTGCTGTCCTTGATGTGATCATGGAATCTCTTTCTCACT
AGAGTCTATGTCACTCATTATACTCTGTCGAATGTCATTGAATGTCTTTACATGGGCTTA
TATGCCTATGAAAATTGTAATACAACTTTCAGCAACGGATCTCTTGGCTCTCGCATCGAT
GAAGAACGCAGCGAAATGCGATAAGTAATGTGAATTGCAGAATTCAGTGAATCATCGAAT
CTTTGAACGCATCTTGCGCTCCTTGGTATTCCGAGGAGCATGCCTGTTTGAGTGTCATTA
AATTCTCAACTCTCTTCTACTTTTTGTAAAAGAGAGCTTGGACTGTGGAGGCTTGCTGGC
CACTTTTTGGGGTCAGCTCCTCTGAAATGCATTAGCGGAACCGTTTGCGATCTGCCACAA
GTGTGATAAGTTATCTACACTGGCGAGGGGATTGCTCTCTGTAATGTTCAGCTTCTAATT
GTCTCTACTTTGTGAGACTACTTTTGAATGCTTGACCTCAAATCAGGTAGGACTACCCGC
TGAACTTAA
>C103
TTTCCGTAGGTGAACCTGCGGAAGGATCATTATTGAATTATGTTTCTAGATAGGTTGTAG
CTGGCTCTTTAGAGCATGTGCACGCCTGTTTGGACTTCATTTTCATCCACCTGTGCACCT
ATTGTAGTCTTTGGTTGGGTTAGGAGGAAGTGGTCATTGTGTCAGCATCTGCTGGATGTG
AGGACTTGCATTGTGAAAGCTTTGCTGTCCTTGATGTGATCATGGAATCTCTTTCTCACT
AGAGTCTATGTCACTCATTATACTCTGTCGAATGTCATTGAATGTCTTTACATGGGCTTA
TATGCCTATGAAAATTGTAATACAACTTTCAGCAACGGATCTCTTGGCTCTCGCATCGAT
GAAGAACGCAGCGAAATGCGATAAGTAATGTGAATTGCAGAATTCAGTGAATCATCGAAT
CTTTGAACGCATCTTGCGCTCCTTGGTATTCCGAGGAGCATGCCTGTTTGAGTGTCATTA
AATTCTCAACTCTCTTCTACTTTTTGTAAAAGAGAGCTTGGACTGTGGAGGCTTGCTGGC
CACTTTTTGGGGTCAGCTCCTCTGAAATGCATTAGCGGAACCGTTTGCGATCTGCCACAA
GTGTGATAAGTTATCTACACTGGCGAGGGGATTGCTCTCTGTAATGTTCAGCTTCTAATT
GTCTCTACTTTGTGAGACTACTTTTGAATGCTTGACCTCAAATCAGGTAGGACTACCCGC
TGAACTTAA
>C104
TTTCCGTAGGTGAACCTGCGGAAGGATCATTATTGAATTATGTTTCTAGATAGGTTGTAG
CTGGCTCTTTAGAGCATGTGCACGCCTGTTTGGACTTCATTTTCATCCACCTGTGCACCT
ATTGTAGTCTTTGGTTGGGTTAGGAGGAAGTGGTCATTGTGTCAGCATCTGCTGGATGTG
AGGACTTGCATTGTGAAAGCTTTGCTGTCCTTGATGTGATCATGGAATCTCTTTCTCACT
AGAGTCTATGTCACTCATTATACTCTGTCGAATGTCATTGAATGTCTTTACATGGGCTTA
TATGCCTATGAAAATTGTAATACAACTTTCAGCAACGGATCTCTTGGCTCTCGCATCGAT
GAAGAACGCAGCGAAATGCGATAAGTAATGTGAATTGCAGAATTCAGTGAATCATCGAAT

CTTTGAACGCATCTTGCGCTCCTTGGTATTCCGAGGAGCATGCCTGTTTGAGTGTCATTA
AATTCTCAACTCTCTTCTACTTTTTGTAAAAGAGAGCTTGGACTGTGGAGGCTTGCTGGC
CACTTTTTGGGGTCAGCTCCTCTGAAATGCATTAGCGGAACCGTTTGCGATCTGCCACAA
GTGTGATAAGTTATCTACACTGGCGAGGGGATTGCTCTCTGTAATGTTCAGCTTCTAATT
GTCTCTACTTTGTGAGACTACTTTTGAATGCTTGACCTCAAATCAGGTAGGACTACCCGC
TGAACTTAA
>C105
TTTCCGTAGGTGAACCTGCGGAAGGATCATTATTGAATTATGTTTCTAGATAGGTTGTAG
CTGGCTCTTTAGAGCATGTGCACGCCTGTTTGGACTTCATTTTCATCCACCTGTGCACCT
ATTGTAGTCTTTGGTTGGGTTAGGAGGAAGTGGTCATTGTGTCAGCATCTGCTGGATGTG
AGGACTTGCATTGTGAAAGCTTTGCTGTCCTTGATGTGATCATGGAATCTCTTTCTCACT
AGAGTCTATGTCACTCATTATACTCTGTCGAATGTCATTGAATGTCTTTACATGGGCTTA
TATGCCTATGAAAATTGTAATACAACTTTCAGCAACGGATCTCTTGGCTCTCGCATCGAT
GAAGAACGCAGCGAAATGCGATAAGTAATGTGAATTGCAGAATTCAGTGAATCATCGAAT
CTTTGAACGCATCTTGCGCTCCTTGGTATTCCGAGGAGCATGCCTGTTTGAGTGTCATTA
AATTCTCAACTCTCTTCTACTTTTTGTAAAAGAGAGCTTGGACTGTGGAGGCTTGCTGGC
CACTTTTTGGGGTCAGCTCCTCTGAAATGCATTAGCGGAACCGTTTGCGATCTGCCACAA
GTGTGATAAGTTATCTACACTGGCGAGGGGATTGCTCTCTGTAATGTTCAGCTTCTAATT
GTCTCTACTTTGTGAGACTACTTTTGAATGCTTGACCTCAAATCAGGTAGGACTACCCGC
TGAACTTAA
>C106
TTTCCGTAGGTGAACCTGCGGAAGGATCATTATTGAATTATGTTTCTAGATAGGTTGTAG
CTGGCTCTTTAGAGCATGTGCACGCCTGTTTGGACTTCATTTTCATCCACCTGTGCACCT
ATTGTAGTCTTTGGTTGGGTTAGGAGGAAGTGGTCATTGTGTCAGCATCTGCTGGATGTG
AGGACTTGCATTGTGAAAGCTTTGCTGTCCTTGATGTGATCATGGAATCTCTTTCTCACT
AGAGTCTATGTCACTCATTATACTCTGTCGAATGTCATTGAATGTCTTTACATGGGCTTA
TATGCCTATGAAAATTGTAATACAACTTTCAGCAACGGATCTCTTGGCTCTCGCATCGAT
GAAGAACGCAGCGAAATGCGATAAGTAATGTGAATTGCAGAATTCAGTGAATCATCGAAT
CTTTGAACGCATCTTGCGCTCCTTGGTATTCCGAGGAGCATGCCTGTTTGAGTGTCATTA
AATTCTCAACTCTCTTCTACTTTTTGTAAAAGAGAGCTTGGACTGTGGAGGCTTGCTGGC
CACTTTTTGGGGTCAGCTCCTCTGAAATGCATTAGCGGAACCGTTTGCGATCTGCCACAA
GTGTGATAAGTTATCTACACTGGCGAGGGGATTGCTCTCTGTAATGTTCAGCTTCTAATT
GTCTCTACTTTGTGAGACTACTTTTGAATGCTTGACCTCAAATCAGGTAGGACTACCCGC
TGAACTTAA
>C107
TTTCCGTAGGTGAACCTGCGGAAGGATCATTATTGAATTATGTTTCTAGATAGGTTGTAG
CTGGCTCTTTAGAGCATGTGCACGCCTGTTTGGACTTCATTTTCATCCACCTGTGCACCT
ATTGTAGTCTTTGGTTGGGTTAGGAGGAAGTGGTCATTGTGTCAGCATCTGCTGGATGTG
AGGACTTGCATTGTGAAAGCTTTGCTGTCCTTGATGTGATCATGGAATCTCTTTCTCACT
AGAGTCTATGTCACTCATTATACTCTGTCGAATGTCATTGAATGTCTTTACATGGGCTTA
TATGCCTATGAAAATTGTAATACAACTTTCAGCAACGGATCTCTTGGCTCTCGCATCGAT
GAAGAACGCAGCGAAATGCGATAAGTAATGTGAATTGCAGAATTCAGTGAATCATCGAAT
CTTTGAACGCATCTTGCGCTCCTTGGTATTCCGAGGAGCATGCCTGTTTGAGTGTCATTA
AATTCTCAACTCTCTTCTACTTTTTGTAAAAGAGAGCTTGGACTGTGGAGGCTTGCTGGC
CACTTTTTGGGGTCAGCTCCTCTGAAATGCATTAGCGGAACCGTTTGCGATCTGCCACAA
GTGTGATAAGTTATCTACACTGGCGAGGGGATTGCTCTCTGTAATGTTCAGCTTCTAATT
GTCTCTACTTTGTGAGACTACTTTTGAATGCTTGACCTCAAATCAGGTAGGACTACCCGC
TGAACTTAA
>C108
TTTCCGTAGGTGAACCTGCGGAAGGATCATTATTGAATTATGTTTCTAGATAGGTTGTAG

CTGGCTCTTTAGAGCATGTGCACGCCTGTTTGGACTTCATTTTCATCCACCTGTGCACCT
ATTGTAGTCTTTGGTTGGGTTAGGAGGAAGTGGTCATTGTGTCAGCATCTGCTGGATGTG
AGGACTTGCATTGTGAAAGCTTTGCTGTCCTTGATGTGATCATGGAATCTCTTTCTCACT
AGAGTCTATGTCACTCATTATACTCTGTCGAATGTCATTGAATGTCTTTACATGGGCTTA
TATGCCTATGAAAATTGTAATACAACTTTCAGCAACGGATCTCTTGGCTCTCGCATCGAT
GAAGAACGCAGCGAAATGCGATAAGTAATGTGAATTGCAGAATTCAGTGAATCATCGAAT
CTTTGAACGCATCTTGCGCTCCTTGGTATTCCGAGGAGCATGCCTGTTTGAGTGTCATTA
AATTCTCAACTCTCTTCTACTTTTTGTAAAAGAGAGCTTGGACTGTGGAGGCTTGCTGGC
CACTTTTTGGGGTCAGCTCCTCTGAAATGCATTAGCGGAACCGTTTGCGATCTGCCACAA
GTGTGATAAGTTATCTACACTGGCGAGGGGATTGCTCTCTGTAATGTTCAGCTTCTAATT
GTCTCTACTTTGTGAGACTACTTTTGAATGCTTGACCTCAAATCAGGTAGGACTACCCGC
TGAACTTAA
>C109
TTTCCGTAGGTGAACCTGCGGAAGGATCATTATTGAATTATGTTTCTAGATAGGTTGTAG
CTGGCTCTTTAGAGCATGTGCACGCCTGTTTGGACTTCATTTTCATCCACCTGTGCACCT
ATTGTAGTCTTTGGTTGGGTTAGGAGGAAGTGGTCATTGTGTCAGCATCTGCTGGATGTG
AGGACTTGCATTGTGAAAGCTTTGCTGTCCTTGATGTGATCATGGAATCTCTTTCTCACT
AGAGTCTATGTCACTCATTATACTCTGTCGAATGTCATTGAATGTCTTTACATGGGCTTA
TATGCCTATGAAAATTGTAATACAACTTTCAGCAACGGATCTCTTGGCTCTCGCATCGAT
GAAGAACGCAGCGAAATGCGATAAGTAATGTGAATTGCAGAATTCAGTGAATCATCGAAT
CTTTGAACGCATCTTGCGCTCCTTGGTATTCCGAGGAGCATGCCTGTTTGAGTGTCATTA
AATTCTCAACTCTCTTCTACTTTTTGTAAAAGAGAGCTTGGACTGTGGAGGCTTGCTGGC
CACTTTTTGGGGTCAGCTCCTCTGAAATGCATTAGCGGAACCGTTTGCGATCTGCCACAA
GTGTGATAAGTTATCTACACTGGCGAGGGGATTGCTCTCTGTAATGTTCAGCTTCTAATT
GTCTCTACTTTGTGAGACTACTTTTGAATGCTTGACCTCAAATCAGGTAGGACTACCCGC
TGAACTTAA
>C110
TTTCCGTAGGTGAACCTGCGGAAGGATCATTATTGAATTATGTTTCTAGATAGGTTGTAG
CTGGCTCTTTAGAGCATGTGCACGCCTGTTTGGACTTCATTTTCATCCACCTGTGCACCT
ATTGTAGTCTTTGGTTGGGTTAGGAGGAAGTGGTCATTGTGTCAGCATCTGCTGGATGTG
AGGACTTGCATTGTGAAAGCTTTGCTGTCCTTGATGTGATCATGGAATCTCTTTCTCACT
AGAGTCTATGTCACTCATTATACTCTGTCGAATGTCATTGAATGTCTTTACATGGGCTTA
TATGCCTATGAAAATTGTAATACAACTTTCAGCAACGGATCTCTTGGCTCTCGCATCGAT
GAAGAACGCAGCGAAATGCGATAAGTAATGTGAATTGCAGAATTCAGTGAATCATCGAAT
CTTTGAACGCATCTTGCGCTCCTTGGTATTCCGAGGAGCATGCCTGTTTGAGTGTCATTA
AATTCTCAACTCTCTTCTACTTTTTGTAAAAGAGAGCTTGGACTGTGGAGGCTTGCTGGC
CACTTTTTGGGGTCAGCTCCTCTGAAATGCATTAGCGGAACCGTTTGCGATCTGCCACAA
GTGTGATAAGTTATCTACACTGGCGAGGGGATTGCTCTCTGTAATGTTCAGCTTCTAATT
GTCTCTACTTTGTGAGACTACTTTTGAATGCTTGACCTCAAATCAGGTAGGACTACCCGC
TGAACTTAA
