# Supplementary material for: Patterns of PCR Amplification Artifacts of the Fungal Barcode Marker in a Hybrid Mushroom
Source: Front Microbiol. 2019 Nov 19;10:2686. doi: 10.3389/fmicb.2019.02686 (PMC6877668; doi:10.3389/fmicb.2019.02686)
Supplement: Supplementary file 7 [file Data_Sheet_7.PDF]

>BC1-13
TTTCCGTAGGTGAACCTGCGGAAGGATCATTATTGAATTATGTTTCTAGATAGGTTGTAG
CTGGCTCTTTAGAGCATGTGCACGCCTGTTTGGACTTCATTTTCATCCACCTGTGCACCT
ATTGTAGTCTTTGGTTGGGTTAGGGGGAAGTGGTCATTGTGTCAGCATCTGCTGGATGTG
AGGACTTGCATTGTGAAAGCTTTGCTGTCCTTGATGTGATCATGGAATCTCTTTCTCACT
AGAGTCTATGTCACTCATTATACTCTGTCGAATGTCATTGAATGTCTTTACATGGGCTTG
TATGCCTATGAAAATTGTAATACAACTTTCAGCAACGGATCTCTTGGCTCTCGCATCGAT
GAAGGACGCAGCGAAATGCGATAAGTAATGTGAATTGCAGAATTCAGTGAATCATCGAAT
CTTTGAACGCATCTTGCGCTCCTTGGTATTCCGAGGAGCATGCCTGTTTGAGTGTCATTA
AATTCTCAACTCTCTTATACTTTTTTGTAAAAGAGAGCTTGGACTGTGGAGGCTTGCTGG
CCACTTTTTGGGGTCAGCTCCTCTGAAATGCATTAGCGGAACCGTTTGCAATCTGCCACA
AGTGTGATAAGTTATCTACACTGGCGAGGGGATTGCTCTCTGTAATGTTCAGCTTCTAAT
TGTCTCTACTTTGTGAGACAACTTTTGAATGCTTGACCTCAAATCAGGTAGGACTACCC-
GCTGAACTTAA
>BC1-2
TTTCCGTAGGTGAACCTGCGGAAGGATCATTATTGAATTATGTTTCTAGATAGGTTGTAG
CTGGCTCTTTAGAGCATGTGCACGCCTGTTTGGACTTCATTTTCATCCACCTGTGCACCT
ATTGTAGTCTTTGGTTGGGTTAGGGGGAAGTGGTCATTGTGTCAGCATCTGCTGGATGTG
AGGACTTGCATTGTGAAAGCTTTGCTGTCCTTGATGTGATCATGGAATCTCTTTCTCACT
AGAGTCTATGTCACTCATTATACTCTGTCGAATGTCATTGAATGTCTTTACATGGGCTTG
TATGCCTATGAAAATTGTAATACAACTTTCAGCAACGGATCTCTTGGCTCTCGCATCGAT
GAAGGACGCAGCGAAATGCGATAAGTAATGTGAATTGCAGAATTCAGTGAATCATCGAAT
CTTTGAACGCATCTTGCGCTCCTTGGTATTCCGAGGAGCATGCCTGTTTGAGTGTCATTA
AATTCTCAACTCTCTTATACTTTTTTGTAAAAGAGAGCTTGGACTGTGGAGGCTTGCTGG
CCACTTTTTGGGGTCAGCTCCTCTGAAATGCATTAGCGGAACCGTTTGCAATCTGCCACA
AGTGTGATAAGTTATCTACACTGGCGAGGGGATTGCTCTCTGTAATGTTCAGCTTCTAAT
TGTCTCTACTTTGTGAGACAACTTTTGAATGCTTGACCTCAAATCAGGTAGGACTACCC-
GCTGAACTTAA
>BC1-3
TTTCCGTAGGTGAACCTGCGGAAGGATCATTATTGAATTATGTTTCTAGATAGGTTGTAG
CTGGCTCTTTAGAGCATGTGCACGCCTGTTTGGACTTCATTTTCATCCACCTGTGCACCT
ATTGTAGTCTTTGGTTGGGTTAGGGGGAAGTGGTCATTGTGTCAGCATCTGCTGGATGTG
AGGACTTGCATTGTGAAAGCTTTGCTGTCCTTGATGTGATCATGGAATCTCTTTCTCACT
AGAGTCTATGTCACTCATTATACTCTGTCGAATGTCATTGAATGTCTTTACATGGGCTTG
TATGCCTATGAAAATTGTAATACAACTTTCAGCAACGGATCTCTTGGCTCTCGCATCGAT
GAAGGACGCAGCGAAATGCGATAAGTAATGTGAATTGCAGAATTCAGTGAATCATCGAAT
CTTTGAACGCATCTTGCGCTCCTTGGTATTCCGAGGAGCATGCCTGTTTGAGTGTCATTA
AATTCTCAACTCTCTTATACTTTTTTGTAAAAGAGAGCTTGGACTGTGGAGGCTTGCTGG
CCACTTTTTGGGGTCAGCTCCTCTGAAATGCATTAGCGGAACCGTTTGCAATCTGCCACA
AGTGTGATAAGTTATCTACACTGGCGAGGGGATTGCTCTCTGTAATGTTCAGCTTCTAAT
TGTCTCTACTTTGTGAGACAACTTTTGAATGCTTGACCTCAAATCAGGTAGGACTACCC-
GCTGAACTTAA
>BC1-4
TTTCCGTAGGTGAACCTGCGGAAGGATCATTATTGAATTATGTTTCTAGATAGGTTGTAG
CTGGCTCTTTAGAGCATGTGCACGCCTGTTTGGACTTCATTTTCATCCACCTGTGCACCT
ATTGTAGTCTTTGGTTGGGTTAGGGGGAAGTGGTCATTGTGTCAGCATCTGCTGGATGTG
AGGACTTGCATTGTGAAAGCTTTGCTGTCCTTGATGTGATCATGGAATCTCTTTCTCACT
AGAGTCTATGTCACTCATTATACTCTGTCGAATGTCATTGAATGTCTTTACATGGGCTTG
TATGCCTATGAAAATTGTAATACAACTTTCAGCAACGGATCTCTTGGCTCTCGCATCGAT
GAAGGACGCAGCGAAATGCGATAAGTAATGTGAATTGCAGAATTCAGTGAATCATCGAAT

CTTTGAACGCATCTTGCGCTCCTTGGTATTCCGAGGAGCATGCCTGTTTGAGTGTCATTA
AATTCTCAACTCTCTTATACTTTTTTGTAAAAGAGAGCTTGGACTGTGGAGGCTTGCTGG
CCACTTTTTGGGGTCAGCTCCTCTGAAATGCATTAGCGGAACCGTTTGCAATCTGCCACA
AGTGTGATAAGTTATCTACACTGGCGAGGGGATTGCTCTCTGTAATGTTCAGCTTCTAAT
TGTCTCTACTTTGTGAGACAACTTTTGAATGCTTGACCTCAAATCAGGTAGGACTACCC-
GCTGAACTTAA
>BC6-1
TTTCCGTAGGTGAACCTGCGGAAGGATCATTATTGAATTATGTTTCTAGATAGGTTGTAG
CTGGCTCTTTAGAGCATGTGCACGCCTGTTTGGACTTCATTTTCATCCACCTGTGCACCT
ATTGTAGTCTTTGGTTGGGTTAGGGGGAAGTGGTCATTGTGTCAGCATCTGCTGGATGTG
AGGACTTGCATTGTGAAAGCTTTGCTGTCCTTGATGTGATCATGGAATCTCTTTCTCACT
AGAGTCTATGTCACTCATTATACTCTGTCGAATGTCATTGAATGTCTTTACATGGGCTTG
TATGCCTATGAAAATTGTAATACAACTTTCAGCAACGGATCTCTTGGCTCTCGCATCGAT
GAAGGACGCAGCGAAATGCGATAAGTAATGTGAATTGCAGAATTCAGTGAATCATCGAAT
CTTTGAACGCATCTTGCGCTCCTTGGTATTCCGAGGAGCATGCCTGTTTGAGTGTCATTA
AATTCTCAACTCTCTTATACTTTTTTGTAAAAGAGAGCTTGGACTGTGGAGGCTTGCTGG
CCACTTTTTGGGGTCAGCTCCTCTGAAATGCATTAGCGGAACCGTTTGCAATCTGCCACA
AGTGTGATAAGTTATCTACACTGGCGAGGGGATTGCTCTCTGTAATGTTCAGCTTCTAAT
TGTCTCTACTTTGTGAGACAACTTTTGAATGCTTGACCTCAAATCAGGTAGGACTACCC-
GCTGAACTTAA
>BC10-53
TTTCCGTAGGTGAACCTGCGGAAGGATCATTATTGAATTATGTTTCTAGATAGGTTGTAG
CTGGCTCTTTAGAGCATGTGCACGCCTGTTTGGACTTCATTTTCATCCACCTGTGCACCT
ATTGTAGTCTTTGGTTGGGTTAGGGGGAAGTGGTCATTGTGTCAGCATCTGCTGGATGTG
AGGACTTGCATTGTGAAAGCTTTGCTGTCCTTGATGTGATCATGGAATCTCTTTCTCACT
AGAGTCTATGTCACTCATTATACTCTGTCGAATGTCATTGAATGTCTTTACATGGGCTTG
TATGCCTATGAAAATTGTAATACAACTTTCAGCAACGGATCTCTTGGCTCTCGCATCGAT
GAAGGACGCAGCGAAATGCGATAAGTAATGTGAATTGCAGAATTCAGTGAATCATCGAAT
CTTTGAACGCATCTTGCGCTCCTTGGTATTCCGAGGAGCATGCCTGTTTGAGTGTCATTA
AATTCTCAACTCTCTTATACTTTTTTGTAAAAGAGAGCTTGGACTGTGGAGGCTTGCTGG
CCACTTTTTGGGGTCAGCTCCTCTGAAATGCATTAGCGGAACCGTTTGCAATCTGCCACA
AGTGTGATAAGTTATCTACACTGGCGAGGGGATTGCTCTCTGTAATGTTCAGCTTCTAAT
TGTCTCTACTTTGTGAGACAACTTTTGAATGCTTGACCTCAAATCAGGTAGGACTACCC-
GCTGAACTTAA
>BC10-54
TTTCCGTAGGTGAACCTGCGGAAGGATCATTATTGAATTATGTTTCTAGATAGGTTGTAG
CTGGCTCTTTAGAGCATGTGCACGCCTGTTTGGACTTCATTTTCATCCACCTGTGCACCT
ATTGTAGTCTTTGGTTGGGTTAGGGGGAAGTGGTCATTGTGTCAGCATCTGCTGGATGTG
AGGACTTGCATTGTGAAAGCTTTGCTGTCCTTGATGTGATCATGGAATCTCTTTCTCACT
AGAGTCTATGTCACTCATTATACTCTGTCGAATGTCATTGAATGTCTTTACATGGGCTTG
TATGCCTATGAAAATTGTAATACAACTTTCAGCAACGGATCTCTTGGCTCTCGCATCGAT
GAAGGACGCAGCGAAATGCGATAAGTAATGTGAATTGCAGAATTCAGTGAATCATCGAAT
CTTTGAACGCATCTTGCGCTCCTTGGTATTCCGAGGAGCATGCCTGTTTGAGTGTCATTA
AATTCTCAACTCTCTTATACTTTTTTGTAAAAGAGAGCTTGGACTGTGGAGGCTTGCTGG
CCACTTTTTGGGGTCAGCTCCTCTGAAATGCATTAGCGGAACCGTTTGCAATCTGCCACA
AGTGTGATAAGTTATCTACACTGGCGAGGGGATTGCTCTCTGTAATGTTCAGCTTCTAAT
TGTCTCTACTTTGTGAGACAACTTTTGAATGCTTGACCTCAAATCAGGTAGGACTACCC-
GCTGAACTTAA
>BC1-53
TTTCCGTAGGTGAACCTGCGGAAGGATCATTATTGAATTATGTTTCTAGATAGGTTGTAG

CTGGCTCTTTAGAGCATGTGCACGCCTGTTTGGACTTCATTTTCATCCACCTGTGCACCT
ATTGTAGTCTTTGGTTGGGTTAGGGGGAAGTGGTCATTGTGTCAGCATCTGCTGGATGTG
AGGACTTGCATTGTGAAAGCTTTGCTGTCCTTGATGTGATCATGGAATCTCTTTCTCACT
AGAGTCTATGTCACTCATTATACTCTGTCGAATGTCATTGAATGTCTTTACATGGGCTTG
TATGCCTATGAAAATTGTAATACAACTTTCAGCAACGGATCTCTTGGCTCTCGCATCGAT
GAAGGACGCAGCGAAATGCGATAAGTAATGTGAATTGCAGAATTCAGTGAATCATCGAAT
CTTTGAACGCATCTTGCGCTCCTTGGTATTCCGAGGAGCATGCCTGTTTGAGTGTCATTA
AATTCTCAACTCTCTTATACTTTTTTGTAAAAGAGAGCTTGGACTGTGGAGGCTTGCTGG
CCACTTTTTGGGGTCAGCTCCTCTGAAATGCATTAGCGGAACCGTTTGCAATCTGCCACA
AGTGTGATAAGTTATCTACACTGGCGAGGGGATTGCTCTCTGTAATGTTCAGCTTCTAAT
TGTCTCTACTTTGTGAGACAACTTTTGAATGCTTGACCTCAAATCAGGTAGGACTACCC-
GCTGAACTTAA
>BC1-55
TTTCCGTAGGTGAACCTGCGGAAGGATCATTATTGAATTATGTTTCTAGATAGGTTGTAG
CTGGCTCTTTAGAGCATGTGCACGCCTGTTTGGACTTCATTTTCATCCACCTGTGCACCT
ATTGTAGTCTTTGGTTGGGTTAGGGGGAAGTGGTCATTGTGTCAGCATCTGCTGGATGTG
AGGACTTGCATTGTGAAAGCTTTGCTGTCCTTGATGTGATCATGGAATCTCTTTCTCACT
AGAGTCTATGTCACTCATTATACTCTGTCGAATGTCATTGAATGTCTTTACATGGGCTTG
TATGCCTATGAAAATTGTAATACAACTTTCAGCAACGGATCTCTTGGCTCTCGCATCGAT
GAAGGACGCAGCGAAATGCGATAAGTAATGTGAATTGCAGAATTCAGTGAATCATCGAAT
CTTTGAACGCATCTTGCGCTCCTTGGTATTCCGAGGAGCATGCCTGTTTGAGTGTCATTA
AATTCTCAACTCTCTTATACTTTTTTGTAAAAGAGAGCTTGGACTGTGGAGGCTTGCTGG
CCACTTTTTGGGGTCAGCTCCTCTGAAATGCATTAGCGGAACCGTTTGCAATCTGCCACA
AGTGTGATAAGTTATCTACACTGGCGAGGGGATTGCTCTCTGTAATGTTCAGCTTCTAAT
TGTCTCTACTTTGTGAGACAACTTTTGAATGCTTGACCTCAAATCAGGTAGGACTACCC-
GCTGAACTTAA
>BC1-56
TTTCCGTAGGTGAACCTGCGGAAGGATCATTATTGAATTATGTTTCTAGATAGGTTGTAG
CTGGCTCTTTAGAGCATGTGCACGCCTGTTTGGACTTCATTTTCATCCACCTGTGCACCT
ATTGTAGTCTTTGGTTGGGTTAGGGGGAAGTGGTCATTGTGTCAGCATCTGCTGGATGTG
AGGACTTGCATTGTGAAAGCTTTGCTGTCCTTGATGTGATCATGGAATCTCTTTCTCACT
AGAGTCTATGTCACTCATTATACTCTGTCGAATGTCATTGAATGTCTTTACATGGGCTTG
TATGCCTATGAAAATTGTAATACAACTTTCAGCAACGGATCTCTTGGCTCTCGCATCGAT
GAAGGACGCAGCGAAATGCGATAAGTAATGTGAATTGCAGAATTCAGTGAATCATCGAAT
CTTTGAACGCATCTTGCGCTCCTTGGTATTCCGAGGAGCATGCCTGTTTGAGTGTCATTA
AATTCTCAACTCTCTTATACTTTTTTGTAAAAGAGAGCTTGGACTGTGGAGGCTTGCTGG
CCACTTTTTGGGGTCAGCTCCTCTGAAATGCATTAGCGGAACCGTTTGCAATCTGCCACA
AGTGTGATAAGTTATCTACACTGGCGAGGGGATTGCTCTCTGTAATGTTCAGCTTCTAAT
TGTCTCTACTTTGTGAGACAACTTTTGAATGCTTGACCTCAAATCAGGTAGGACTACCC-
GCTGAACTTAA
>BC1-57
TTTCCGTAGGTGAACCTGCGGAAGGATCATTATTGAATTATGTTTCTAGATAGGTTGTAG
CTGGCTCTTTAGAGCATGTGCACGCCTGTTTGGACTTCATTTTCATCCACCTGTGCACCT
ATTGTAGTCTTTGGTTGGGTTAGGGGGAAGTGGTCATTGTGTCAGCATCTGCTGGATGTG
AGGACTTGCATTGTGAAAGCTTTGCTGTCCTTGATGTGATCATGGAATCTCTTTCTCACT
AGAGTCTATGTCACTCATTATACTCTGTCGAATGTCATTGAATGTCTTTACATGGGCTTG
TATGCCTATGAAAATTGTAATACAACTTTCAGCAACGGATCTCTTGGCTCTCGCATCGAT
GAAGGACGCAGCGAAATGCGATAAGTAATGTGAATTGCAGAATTCAGTGAATCATCGAAT
CTTTGAACGCATCTTGCGCTCCTTGGTATTCCGAGGAGCATGCCTGTTTGAGTGTCATTA
AATTCTCAACTCTCTTATACTTTTTTGTAAAAGAGAGCTTGGACTGTGGAGGCTTGCTGG

CCACTTTTTGGGGTCAGCTCCTCTGAAATGCATTAGCGGAACCGTTTGCAATCTGCCACA
AGTGTGATAAGTTATCTACACTGGCGAGGGGATTGCTCTCTGTAATGTTCAGCTTCTAAT
TGTCTCTACTTTGTGAGACAACTTTTGAATGCTTGACCTCAAATCAGGTAGGACTACCC-
GCTGAACTTAA
>BC2-60
TTTCCGTAGGTGAACCTGCGGAAGGATCATTATTGAATTATGTTTCTAGATAGGTTGTAG
CTGGCTCTTTAGAGCATGTGCACGCCTGTTTGGACTTCATTTTCATCCACCTGTGCACCT
ATTGTAGTCTTTGGTTGGGTTAGGGGGAAGTGGTCATTGTGTCAGCATCTGCTGGATGTG
AGGACTTGCATTGTGAAAGCTTTGCTGTCCTTGATGTGATCATGGAATCTCTTTCTCACT
AGAGTCTATGTCACTCATTATACTCTGTCGAATGTCATTGAATGTCTTTACATGGGCTTG
TATGCCTATGAAAATTGTAATACAACTTTCAGCAACGGATCTCTTGGCTCTCGCATCGAT
GAAGGACGCAGCGAAATGCGATAAGTAATGTGAATTGCAGAATTCAGTGAATCATCGAAT
CTTTGAACGCATCTTGCGCTCCTTGGTATTCCGAGGAGCATGCCTGTTTGAGTGTCATTA
AATTCTCAACTCTCTTATACTTTTTTGTAAAAGAGAGCTTGGACTGTGGAGGCTTGCTGG
CCACTTTTTGGGGTCAGCTCCTCTGAAATGCATTAGCGGAACCGTTTGCAATCTGCCACA
AGTGTGATAAGTTATCTACACTGGCGAGGGGATTGCTCTCTGTAATGTTCAGCTTCTAAT
TGTCTCTACTTTGTGAGACAACTTTTGAATGCTTGACCTCAAATCAGGTAGGACTACCC-
GCTGAACTTAA
>BC3-57
TTTCCGTAGGTGAACCTGCGGAAGGATCATTATTGAATTATGTTTCTAGATAGGTTGTAG
CTGGCTCTTTAGAGCATGTGCACGCCTGTTTGGACTTCATTTTCATCCACCTGTGCACCT
ATTGTAGTCTTTGGTTGGGTTAGGGGGAAGTGGTCATTGTGTCAGCATCTGCTGGATGTG
AGGACTTGCATTGTGAAAGCTTTGCTGTCCTTGATGTGATCATGGAATCTCTTTCTCACT
AGAGTCTATGTCACTCATTATACTCTGTCGAATGTCATTGAATGTCTTTACATGGGCTTG
TATGCCTATGAAAATTGTAATACAACTTTCAGCAACGGATCTCTTGGCTCTCGCATCGAT
GAAGGACGCAGCGAAATGCGATAAGTAATGTGAATTGCAGAATTCAGTGAATCATCGAAT
CTTTGAACGCATCTTGCGCTCCTTGGTATTCCGAGGAGCATGCCTGTTTGAGTGTCATTA
AATTCTCAACTCTCTTATACTTTTTTGTAAAAGAGAGCTTGGACTGTGGAGGCTTGCTGG
CCACTTTTTGGGGTCAGCTCCTCTGAAATGCATTAGCGGAACCGTTTGCAATCTGCCACA
AGTGTGATAAGTTATCTACACTGGCGAGGGGATTGCTCTCTGTAATGTTCAGCTTCTAAT
TGTCTCTACTTTGTGAGACAACTTTTGAATGCTTGACCTCAAATCAGGTAGGACTACCC-
GCTGAACTTAA
>BC7-56
TTTCCGTAGGTGAACCTGCGGAAGGATCATTATTGAATTATGTTTCTAGATAGGTTGTAG
CTGGCTCTTTAGAGCATGTGCACGCCTGTTTGGACTTCATTTTCATCCACCTGTGCACCT
ATTGTAGTCTTTGGTTGGGTTAGGGGGAAGTGGTCATTGTGTCAGCATCTGCTGGATGTG
AGGACTTGCATTGTGAAAGCTTTGCTGTCCTTGATGTGATCATGGAATCTCTTTCTCACT
AGAGTCTATGTCACTCATTATACTCTGTCGAATGTCATTGAATGTCTTTACATGGGCTTG
TATGCCTATGAAAATTGTAATACAACTTTCAGCAACGGATCTCTTGGCTCTCGCATCGAT
GAAGGACGCAGCGAAATGCGATAAGTAATGTGAATTGCAGAATTCAGTGAATCATCGAAT
CTTTGAACGCATCTTGCGCTCCTTGGTATTCCGAGGAGCATGCCTGTTTGAGTGTCATTA
AATTCTCAACTCTCTTATACTTTTTTGTAAAAGAGAGCTTGGACTGTGGAGGCTTGCTGG
CCACTTTTTGGGGTCAGCTCCTCTGAAATGCATTAGCGGAACCGTTTGCAATCTGCCACA
AGTGTGATAAGTTATCTACACTGGCGAGGGGATTGCTCTCTGTAATGTTCAGCTTCTAAT
TGTCTCTACTTTGTGAGACAACTTTTGAATGCTTGACCTCAAATCAGGTAGGACTACCC-
GCTGAACTTAA
>BC9-58
TTTCCGTAGGTGAACCTGCGGAAGGATCATTATTGAATTATGTTTCTAGATAGGTTGTAG
CTGGCTCTTTAGAGCATGTGCACGCCTGTTTGGACTTCATTTTCATCCACCTGTGCACCT
ATTGTAGTCTTTGGTTGGGTTAGGGGGAAGTGGTCATTGTGTCAGCATCTGCTGGATGTG

AGGACTTGCATTGTGAAAGCTTTGCTGTCCTTGATGTGATCATGGAATCTCTTTCTCACT
AGAGTCTATGTCACTCATTATACTCTGTCGAATGTCATTGAATGTCTTTACATGGGCTTG
TATGCCTATGAAAATTGTAATACAACTTTCAGCAACGGATCTCTTGGCTCTCGCATCGAT
GAAGGACGCAGCGAAATGCGATAAGTAATGTGAATTGCAGAATTCAGTGAATCATCGAAT
CTTTGAACGCATCTTGCGCTCCTTGGTATTCCGAGGAGCATGCCTGTTTGAGTGTCATTA
AATTCTCAACTCTCTTATACTTTTTTGTAAAAGAGAGCTTGGACTGTGGAGGCTTGCTGG
CCACTTTTTGGGGTCAGCTCCTCTGAAATGCATTAGCGGAACCGTTTGCAATCTGCCACA
AGTGTGATAAGTTATCTACACTGGCGAGGGGATTGCTCTCTGTAATGTTCAGCTTCTAAT
TGTCTCTACTTTGTGAGACAACTTTTGAATGCTTGACCTCAAATCAGGTAGGACTACCC-
GCTGAACTTAA
>BC10-55
TTTCCGTAGGTGAACCTGCGGAAGGATCATTATTGAATTATGTTTCTAGATAGGTTGTAG
CTGGCTCTTTAGAGCATGTGCACGCCTGTTTGGACTTCATTTTCATCCACCTGTGCACCT
ATTGTAGTCTTTGGTTGGGTTAGGGGGAAGTGGTCATTGTGTCAGCATCTGCTGGATGTG
AGGACTTGCATTGTGAAAGCTTTGCTGTCCTTGATGTGATCATGGAATCTCTTTCTCACT
AGAGTCTATGTCACTCATTATACTCTGTCGAATGTCATTGAATGTCTTTACATGGGCTTG
TATGCCTATGAAAATTGTAATACAACTTTCAGCAACGGATCTCTTGGCTCTCGCATCGAT
GAAGGACGCAGCGAAATGCGATAAGTAATGTGAATTGCAGAATTCAGTGAATCATCGAAT
CTTTGAACGCATCTTGCGCTCCTTGGTATTCCGAGGAGCATGCCTGTTTGAGTGTCATTA
AATTCTCAACTCTCTTATACTTTTTTGTAAAAGAGAGCTTGGACTGTGGAGGCTTGCTGG
CCACTTTTTGGGGTCAGCTCCTCTGAAATGCATTAGCGGAACCGTTTGCAATCTGCCACA
AGTGTGATAAGTTATCTACACTGGCGAGGGGATTGCTCTCTGTAATGTTCAGCTTCTAAT
TGTCTCTACTTTGTGAGACAACTTTTGAATGCTTGACCTCAAATCAGGTAGGACTACCC-
GCTGAACTTAA
>BC9-60
TTTCCGTAGGTGAACCTGCGGAAGGATCATTATTGAATTATGTTTCTAGATAGGTTGTAG
CTGGCTCTTTAGAGCATGTGCACGCCTGTTTGGACTTCATTTTCATCCACCTGTGCACCT
ATTGTAGTCTTTGGTTGGGTTAGGGGGAAGTGGTCATTGTGTCAGCATCTGCTGGATGTG
AGGACTTGCATTGTGAAAGCTTTGCTGTCCTTGATGTGATCATGGAATCTCTTTCTCACT
AGAGTCTATGTCACTCATTATACTCTGTCGAATGTCATTGAATGTCTTTACATGGGCTTG
TATGCCTATGAAAATTGTAATACAACTTTCAGCAACGGATCTCTTGGCTCTCGCATCGAT
GAAGGACGCAGCGAAATGCGATAAGTAATGTGAATTGCAGAATTCAGTGAATCATCGAAT
CTTTGAACGCATCTTGCGCTCCTTGGTATTCCGAGGAGCATGCCTGTTTGAGTGTCATTA
AATTCTCAACTCTCTTATACTTTTTTGTAAAAGAGAGCTTGGACTGTGGAGGCTTGCTGG
CCACTTTTTGGGGTCAGCTCCTCTGAAATGCATTAGCGGAACCGTTTGCAATCTGCCACA
AGTGTGATAAGTTATCTACACTGGCGAGGGGATTGCTCTCTGTAATGTTCAGCTTCTAAT
TGTCTCTACTTTGTGAGACAACTTTTGAATGCTTGACCTCAAATCAGGTAGGACTACCC-
GCTGAACTTAA
>BC11-60
TTTCCGTAGGTGAACCTGCGGAAGGATCATTATTGAATTATGTTTCTAGATAGGTTGTAG
CTGGCTCTTTAGAGCATGTGCACGCCTGTTTGGACTTCATTTTCATCCACCTGTGCACCT
ATTGTAGTCTTTGGTTGGGTTAGGGGGAAGTGGTCATTGTGTCAGCATCTGCTGGATGTG
AGGACTTGCATTGTGAAAGCTTTGCTGTCCTTGATGTGATCATGGAATCTCTTTCTCACT
AGAGTCTATGTCACTCATTATACTCTGTCGAATGTCATTGAATGTCTTTACATGGGCTTG
TATGCCTATGAAAATTGTAATACAACTTTCAGCAACGGATCTCTTGGCTCTCGCATCGAT
GAAGGACGCAGCGAAATGCGATAAGTAATGTGAATTGCAGAATTCAGTGAATCATCGAAT
CTTTGAACGCATCTTGCGCTCCTTGGTATTCCGAGGAGCATGCCTGTTTGAGTGTCATTA
AATTCTCAACTCTCTTATACTTTTTTGTAAAAGAGAGCTTGGACTGTGGAGGCTTGCTGG
CCACTTTTTGGGGTCAGCTCCTCTGAAATGCATTAGCGGAACCGTTTGCAATCTGCCACA
AGTGTGATAAGTTATCTACACTGGCGAGGGGATTGCTCTCTGTAATGTTCAGCTTCTAAT

TGTCTCTACTTTGTGAGACAACTTTTGAATGCTTGACCTCAAATCAGGTAGGACTACCC-
GCTGAACTTAA
>BC10-56
TTTCCGTAGGTGAACCTGCGGAAGGATCATTATTGAATTATGTTTCTAGATAGGTTGTAG
CTGGCTCTTTAGAGCATGTGCACGCCTGTTTGGACTTCATTTTCATCCACCTGTGCACCT
ATTGTAGTCTTTGGTTGGGTTAGGGGGAAGTGGTCATTGTGTCAGCATCTGCTGGATGTG
AGGACTTGCATTGTGAAAGCTTTGCTGTCCTTGATGTGATCATGGAATCTCTTTCTCACT
AGAGTCTATGTCACTCATTATACTCTGTCGAATGTCATTGAATGTCTTTACATGGGCTTG
TATGCCTATGAAAATTGTAATACAACTTTCAGCAACGGATCTCTTGGCTCTCGCATCGAT
GAAGGACGCAGCGAAATGCGATAAGTAATGTGAATTGCAGAATTCAGTGAATCATCGAAT
CTTTGAACGCATCTTGCGCTCCTTGGTATTCCGAGGAGCATGCCTGTTTGAGTGTCATTA
AATTCTCAACTCTCTTATACTTTTTTGTAAAAGAGAGCTTGGACTGTGGAGGCTTGCTGG
CCACTTTTTGGGGTCAGCTCCTCTGAAATGCATTAGCGGAACCGTTTGCAATCTGCCACA
AGTGTGATAAGTTATCTACACTGGCGAGGGGATTGCTCTCTGTAATGTTCAGCTTCTAAT
TGTCTCTACTTTGTGAGACAACTTTTGAATGCTTGACCTCAAATCAGGTAGGACTACCC-
GCTGAACTTAA
>BC8-60
TTTCCGTAGGTGAACCTGCGGAAGGATCATTATTGAATTATGTTTCTAGATAGGTTGTAG
CTGGCTCTTTAGAGCATGTGCACGCCTGTTTGGACTTCATTTTCATCCACCTGTGCACCT
ATTGTAGTCTTTGGTTGGGTTAGGGGGAAGTGGTCATTGTGTCAGCATCTGCTGGATGTG
AGGACTTGCATTGTGAAAGCTTTGCTGTCCTTGATGTGATCATGGAATCTCTTTCTCACT
AGAGTCTATGTCACTCATTATACTCTGTCGAATGTCATTGAATGTCTTTACATGGGCTTG
TATGCCTATGAAAATTGTAATACAACTTTCAGCAACGGATCTCTTGGCTCTCGCATCGAT
GAAGGACGCAGCGAAATGCGATAAGTAATGTGAATTGCAGAATTCAGTGAATCATCGAAT
CTTTGAACGCATCTTGCGCTCCTTGGTATTCCGAGGAGCATGCCTGTTTGAGTGTCATTA
AATTCTCAACTCTCTTATACTTTTTTGTAAAAGAGAGCTTGGACTGTGGAGGCTTGCTGG
CCACTTTTTGGGGTCAGCTCCTCTGAAATGCATTAGCGGAACCGTTTGCAATCTGCCACA
AGTGTGATAAGTTATCTACACTGGCGAGGGGATTGCTCTCTGTAATGTTCAGCTTCTAAT
TGTCTCTACTTTGTGAGACAACTTTTGAATGCTTGACCTCAAATCAGGTAGGACTACCC-
GCTGAACTTAA
>BC10_15
TTTCCGTAGGTGAACCTGCGGAAGGATCATTATTGAATTATGTTTCTAGATAGGTTGTAG
CTGGCTCTTTAGAGCATGTGCACGCCTGTTTGGACTTCATTTTCATCCACCTGTGCACCT
ATTGTAGTCTTTGGTTGGGTTAGGGGGAAGTGGTCATTGTGTCAGCATCTGCTGGATGTG
AGGACTTGCATTGTGAAAGCTTTGCTGTCCTTGATGTGATCATGGAATCTCTTTCTCACT
AGAGTCTATGTCACTCATTATACTCTGTCGAATGTCATTGAATGTCTTTACATGGGCTTG
TATGCCTATGAAAATTGTAATACAACTTTCAGCAACGGATCTCTTGGCTCTCGCATCGAT
GAAGGACGCAGCGAAATGCGATAAGTAATGTGAATTGCAGAATTCAGTGAATCATCGAAT
CTTTGAACGCATCTTGCGCTCCTTGGTATTCCGAGGAGCATGCCTGTTTGAGTGTCATTA
AATTCTCAACTCTCTTATACTTTTTTGTAAAAGAGAGCTTGGACTGTGGAGGCTTGCTGG
CCACTTTTTGGGGTCAGCTCCTCTGAAATGCATTAGCGGAACCGTTTGCAATCTGCCACA
AGTGTGATAAGTTATCTACACTGGCGAGGGGATTGCTCTCTGTAATGTTCAGCTTCTAAT
TGTCTCTACTTTGTGAGACAACTTTTGAATGCTTGACCTCAAATCAGGTAGGACTACCC-
GCTGAACTTAA
>BC3-34
TTTCCGTAGGTGAACCTGCGGAAGGATCATTATTGAATTATGTTTCTAGATAGGTTGTAG
CTGGCTCTTTAGAGCATGTGCACGCCTGTTTGGACTTCATTTTCATCCACCTGTGCACCT
ATTGTAGTCTTTGGTTGGGTTAGGGGGAAGTGGTCATTGTGTCAGCATCTGCTGGATGTG
AGGACTTGCATTGTGAAAGCTTTGCTGTCCTTGATGTGATCATGGAATCTCTTTCTCACT
AGAGTCTATGTCACTCATTATACTCTGTCGAATGTCATTGAATGTCTTTACATGGGCTTG

TATGCCTATGAAAATTGTAATACAACTTTCAGCAACGGATCTCTTGGCTCTCGCATCGAT
GAAGGACGCAGCGAAATGCGATAAGTAATGTGAATTGCAGAATTCAGTGAATCATCGAAT
CTTTGAACGCATCTTGCGCTCCTTGGTATTCCGAGGAGCATGCCTGTTTGAGTGTCATTA
AATTCTCAACTCTCTTATACTTTTTTGTAAAAGAGAGCTTGGACTGTGGAGGCTTGCTGG
CCACTTTTTGGGGTCAGCTCCTCTGAAATGCATTAGCGGAACCGTTTGCAATCTGCCACA
AGTGTGATAAGTTATCTACACTGGCGAGGGGATTGCTCTCTGTAATGTTCAGCTTCTAAT
TGTCTCTACTTTGTGAGACAACTTTTGAATGCTTGACCTCAAATCAGGTAGGACTACCC-
GCTGAACTTAA
>BC9-34
TTTCCGTAGGTGAACCTGCGGAAGGATCATTATTGAATTATGTTTCTAGATAGGTTGTAG
CTGGCTCTTTAGAGCATGTGCACGCCTGTTTGGACTTCATTTTCATCCACCTGTGCACCT
ATTGTAGTCTTTGGTTGGGTTAGGGGGAAGTGGTCATTGTGTCAGCATCTGCTGGATGTG
AGGACTTGCATTGTGAAAGCTTTGCTGTCCTTGATGTGATCATGGAATCTCTTTCTCACT
AGAGTCTATGTCACTCATTATACTCTGTCGAATGTCATTGAATGTCTTTACATGGGCTTG
TATGCCTATGAAAATTGTAATACAACTTTCAGCAACGGATCTCTTGGCTCTCGCATCGAT
GAAGGACGCAGCGAAATGCGATAAGTAATGTGAATTGCAGAATTCAGTGAATCATCGAAT
CTTTGAACGCATCTTGCGCTCCTTGGTATTCCGAGGAGCATGCCTGTTTGAGTGTCATTA
AATTCTCAACTCTCTTATACTTTTTTGTAAAAGAGAGCTTGGACTGTGGAGGCTTGCTGG
CCACTTTTTGGGGTCAGCTCCTCTGAAATGCATTAGCGGAACCGTTTGCAATCTGCCACA
AGTGTGATAAGTTATCTACACTGGCGAGGGGATTGCTCTCTGTAATGTTCAGCTTCTAAT
TGTCTCTACTTTGTGAGACAACTTTTGAATGCTTGACCTCAAATCAGGTAGGACTACCC-
GCTGAACTTAA
>BC12_26
TTTCCGTAGGTGAACCTGCGGAAGGATCATTATTGAATTATGTTTCTAGATAGGTTGTAG
CTGGCTCTTTAGAGCATGTGCACGCCTGTTTGGACTTCATTTTCATCCACCTGTGCACCT
ATTGTAGTCTTTGGTTGGGTTAGGGGGAAGTGGTCATTGTGTCAGCATCTGCTGGATGTG
AGGACTTGCATTGTGAAAGCTTTGCTGTCCTTGATGTGATCATGGAATCTCTTTCTCACT
AGAGTCTATGTCACTCATTATACTCTGTCGAATGTCATTGAATGTCTTTACATGGGCTTG
TATGCCTATGAAAATTGTAATACAACTTTCAGCAACGGATCTCTTGGCTCTCGCATCGAT
GAAGGACGCAGCGAAATGCGATAAGTAATGTGAATTGCAGAATTCAGTGAATCATCGAAT
CTTTGAACGCATCTTGCGCTCCTTGGTATTCCGAGGAGCATGCCTGTTTGAGTGTCATTA
AATTCTCAACTCTCTTATACTTTTTTGTAAAAGAGAGCTTGGACTGTGGAGGCTTGCTGG
CCACTTTTTGGGGTCAGCTCCTCTGAAATGCATTAGCGGAACCGTTTGCAATCTGCCACA
AGTGTGATAAGTTATCTACACTGGCGAGGGGATTGCTCTCTGTAATGTTCAGCTTCTAAT
TGTCTCTACTTTGTGAGACAACTTTTGAATGCTTGACCTCAAATCAGGTAGGACTACCC-
GCTGAACTTAA
>BC4-55
TTTCCGTAGGTGAACCTGCGGAAGGATCATTATTGAATTATGTTTCTAGATAGGTTGTAG
CTGGCTCTTTAGAGCATGTGCACGCCTGTTTGGACTTCATTTTCATCCACCTGTGCACCT
ATTGTAGTCTTTGGTTGGGTTAGGGGGAAGTGGTCATTGTGTCAGCATCTGCTGGATGTG
AGGACTTGCATTGTGAAAGCTTTGCTGTCCTTGATGTGATCATGGAATCTCTTTCTCACT
AGAGTCTATGTCACTCATTATACTCTGTCGAATGTCATTGAATGTCTTTACATGGGCTTG
TATGCCTATGAAAATTGTAATACAACTTTCAGCAACGGATCTCTTGGCTCTCGCATCGAT
GAAGGACGCAGCGAAATGCGATAAGTAATGTGAATTGCAGAATTCAGTGAATCATCGAAT
CTTTGAACGCATCTTGCGCTCCTTGGTATTCCGAGGAGCATGCCTGTTTGAGTGTCATTA
AATTCTCAACTCTCTTATACTTTTTTGTAAAAGAGAGCTTGGACTGTGGAGGCTTGCTGG
CCACTTTTTGGGGTCAGCTCCTCTGAAATGCATTAGCGGAACCGTTTGCAATCTGCCACA
AGTGTGATAAGTTATCTACACTGGCGAGGGGATTGCTCTCTGTAATGTTCAGCTTCTAAT
TGTCTCTACTTTGTGAGACAACTTTTGAATGCTTGACCTCAAATCAGGTAGGACTACCC-
GCTGAACTTAA

>BC5–80
TTTCCGTAGGTGAACCTGCGGAAGGATCATTATTGAATTATGTTTCTAGATAGGTTGTAG
CTGGCTCTTTAGAGCATGTGCACGCCTGTTTGGACTTCATTTTCATCCACCTGTGCACCT
ATTGTAGTCTTTGGTTGGGTTAGGGGGAAGTGGTCATTGTGTCAGCATCTGCTGGATGTG
AGGACTTGCATTGTGAAAGCTTTGCTGTCCTTGATGTGATCATGGAATCTCTTTCTCACT
AGAGTCTATGTCACTCATTATACTCTGTCGAATGTCATTGAATGTCTTTACATGGGCTTG
TATGCCTATGAAAATTGTAATACAACTTTCAGCAACGGATCTCTTGGCTCTCGCATCGAT
GAAGGACGCAGCGAAATGCGATAAGTAATGTGAATTGCAGAATTCAGTGAATCATCGAAT
CTTTGAACGCATCTTGCGCTCCTTGGTATTCCGAGGAGCATGCCTGTTTGAGTGTCATTA
AATTCTCAACTCTCTTATACTTTTTTGTAAAAGAGAGCTTGGACTGTGGAGGCTTGCTGG
CCACTTTTTGGGGTCAGCTCCTCTGAAATGCATTAGCGGAACCGTTTGCAATCTGCCACA
AGTGTGATAAGTTATCTACACTGGCGAGGGGATTGCTCTCTGTAATGTTCAGCTTCTAAT
TGTCTCTACTTTGTGAGACAACTTTTGAATGCTTGACCTCAAATCAGGTAGGACTACCC–
GCTGAACTTAA
>BC7–53
TTTCCGTAGGTGAACCTGCGGAAGGATCATTATTGAATTATGTTTCTAGATAGGTTGTAG
CTGGCTCTTTAGAGCATGTGCACGCCTGTTTGGACTTCATTTTCATCCACCTGTGCACCT
ATTGTAGTCTTTGGTTGGGTTAGGGGGAAGTGGTCATTGTGTCAGCATCTGCTGGATGTG
AGGACTTGCATTGTGAAAGCTTTGCTGTCCTTGATGTGATCATGGAATCTCTTTCTCACT
AGAGTCTATGTCACTCATTATACTCTGTCGAATGTCATTGAATGTCTTTACATGGGCTTG
TATGCCTATGAAAATTGTAATACAACTTTCAGCAACGGATCTCTTGGCTCTCGCATCGAT
GAAGGACGCAGCGAAATGCGATAAGTAATGTGAATTGCAGAATTCAGTGAATCATCGAAT
CTTTGAACGCATCTTGCGCTCCTTGGTATTCCGAGGAGCATGCCTGTTTGAGTGTCATTA
AATTCTCAACTCTCTTATACTTTTTTGTAAAAGAGAGCTTGGACTGTGGAGGCTTGCTGG
CCACTTTTTGGGGTCAGCTCCTCTGAAATGCATTAGCGGAACCGTTTGCAATCTGCCACA
AGTGTGATAAGTTATCTACACTGGCGAGGGGATTGCTCTCTGTAATGTTCAGCTTCTAAT
TGTCTCTACTTTGTGAGACAACTTTTGAATGCTTGACCTCAAATCAGGTAGGACTACCC–
GCTGAACTTAA
>BC5–128
TTTCCGTAGGTGAACCTGCGGAAGGATCATTATTGAATTATGTTTCTAGATAGGTTGTAG
CTGGCTCTTTAGAGCATGTGCACGCCTGTTTGGACTTCATTTTCATCCACCTGTGCACCT
ATTGTAGTCTTTGGTTGGGTTAGGGGGAAGTGGTCATTGTGTCAGCATCTGCTGGATGTG
AGGACTTGCATTGTGAAAGCTTTGCTGTCCTTGATGTGATCATGGAATCTCTTTCTCACT
AGAGTCTATGTCACTCATTATACTCTGTCGAATGTCATTGAATGTCTTTACATGGGCTTG
TATGCCTATGAAAATTGTAATACAACTTTCAGCAACGGATCTCTTGGCTCTCGCATCGAT
GAAGGACGCAGCGAAATGCGATAAGTAATGTGAATTGCAGAATTCAGTGAATCATCGAAT
CTTTGAACGCATCTTGCGCTCCTTGGTATTCCGAGGAGCATGCCTGTTTGAGTGTCATTA
AATTCTCAACTCTCTTATACTTTTTTGTAAAAGAGAGCTTGGACTGTGGAGGCTTGCTGG
CCACTTTTTGGGGTCAGCTCCTCTGAAATGCATTAGCGGAACCGTTTGCAATCTGCCACA
AGTGTGATAAGTTATCTACACTGGCGAGGGGATTGCTCTCTGTAATGTTCAGCTTCTAAT
TGTCTCTACTTTGTGAGACAACTTTTGAATGCTTGACCTCAAATCAGGTAGGACTACCC–
GCTGAACTTAA
>BC8–48
TTTCCGTAGGTGAACCTGCGGAAGGATCATTATTGAATTATGTTTCTAGATAGGTTGTAG
CTGGCTCTTTAGAGCATGTGCACGCCTGTTTGGACTTCATTTTCATCCACCTGTGCACCT
ATTGTAGTCTTTGGTTGGGTTAGGGGGAAGTGGTCATTGTGTCAGCATCTGCTGGATGTG
AGGACTTGCATTGTGAAAGCTTTGCTGTCCTTGATGTGATCATGGAATCTCTTTCTCACT
AGAGTCTATGTCACTCATTATACTCTGTCGAATGTCATTGAATGTCTTTACATGGGCTTG
TATGCCTATGAAAATTGTAATACAACTTTCAGCAACGGATCTCTTGGCTCTCGCATCGAT
GAAGGACGCAGCGAAATGCGATAAGTAATGTGAATTGCAGAATTCAGTGAATCATCGAAT

CTTTGAACGCATCTTGCGCTCCTTGGTATTCCGAGGAGCATGCCTGTTTGAGTGTCATTA
AATTCTCAACTCTCTTATACTTTTTTGTAAAAGAGAGCTTGGACTGTGGAGGCTTGCTGG
CCACTTTTTGGGGTCAGCTCCTCTGAAATGCATTAGCGGAACCGTTTGCAATCTGCCACA
AGTGTGATAAGTTATCTACACTGGCGAGGGGATTGCTCTCTGTAATGTTCAGCTTCTAAT
TGTCTCTACTTTGTGAGACAACTTTTGAATGCTTGACCTCAAATCAGGTAGGACTACCC-
GCTGAACTTAA
>BC9-41
TTTCCGTAGGTGAACCTGCGGAAGGATCATTATTGAATTATGTTTCTAGATAGGTTGTAG
CTGGCTCTTTAGAGCATGTGCACGCCTGTTTGGACTTCATTTTCATCCACCTGTGCACCT
ATTGTAGTCTTTGGTTGGGTTAGGGGGAAGTGGTCATTGTGTCAGCATCTGCTGGATGTG
AGGACTTGCATTGTGAAAGCTTTGCTGTCCTTGATGTGATCATGGAATCTCTTTCTCACT
AGAGTCTATGTCACTCATTATACTCTGTCGAATGTCATTGAATGTCTTTACATGGGCTTG
TATGCCTATGAAAATTGTAATACAACTTTCAGCAACGGATCTCTTGGCTCTCGCATCGAT
GAAGGACGCAGCGAAATGCGATAAGTAATGTGAATTGCAGAATTCAGTGAATCATCGAAT
CTTTGAACGCATCTTGCGCTCCTTGGTATTCCGAGGAGCATGCCTGTTTGAGTGTCATTA
AATTCTCAACTCTCTTATACTTTTTTGTAAAAGAGAGCTTGGACTGTGGAGGCTTGCTGG
CCACTTTTTGGGGTCAGCTCCTCTGAAATGCATTAGCGGAACCGTTTGCAATCTGCCACA
AGTGTGATAAGTTATCTACACTGGCGAGGGGATTGCTCTCTGTAATGTTCAGCTTCTAAT
TGTCTCTACTTTGTGAGACAACTTTTGAATGCTTGACCTCAAATCAGGTAGGACTACCC-
GCTGAACTTAA
>BC9-19
TTTCCGTAGGTGAACCTGCGGAAGGATCATTATTGAATTATGTTTCTAGATAGGTTGTAG
CTGGCTCTTTAGAGCATGTGCACGCCTGTTTGGACTTCATTTTCATCCACCTGTGCACCT
ATTGTAGTCTTTGGTTGGGTTAGGGGGAAGTGGTCATTGTGTCAGCATCTGCTGGATGTG
AGGACTTGCATTGTGAAAGCTTTGCTGTCCTTGATGTGATCATGGAATCTCTTTCTCACT
AGAGTCTATGTCACTCATTATACTCTGTCGAATGTCATTGAATGTCTTTACATGGGCTTG
TATGCCTATGAAAATTGTAATACAACTTTCAGCAACGGATCTCTTGGCTCTCGCATCGAT
GAAGGACGCAGCGAAATGCGATAAGTAATGTGAATTGCAGAATTCAGTGAATCATCGAAT
CTTTGAACGCATCTTGCGCTCCTTGGTATTCCGAGGAGCATGCCTGTTTGAGTGTCATTA
AATTCTCAACTCTCTTATACTTTTTTGTAAAAGAGAGCTTGGACTGTGGAGGCTTGCTGG
CCACTTTTTGGGGTCAGCTCCTCTGAAATGCATTAGCGGAACCGTTTGCAATCTGCCACA
AGTGTGATAAGTTATCTACACTGGCGAGGGGATTGCTCTCTGTAATGTTCAGCTTCTAAT
TGTCTCTACTTTGTGAGACAACTTTTGAATGCTTGACCTCAAATCAGGTAGGACTACCC-
GCTGAACTTAA
>BC8-4
TTTCCGTAGGTGAACCTGCGGAAGGATCATTATTGAATTATGTTTCTAGATAGGTTGTAG
CTGGCTCTTTAGAGCATGTGCACGCCTGTTTGGACTTCATTTTCATCCACCTGTGCACCT
ATTGTAGTCTTTGGTTGGGTTAGGGGGAAGTGGTCATTGTGTCAGCATCTGCTGGATGTG
AGGACTTGCATTGTGAAAGCTTTGCTGTCCTTGATGTGATCATGGAATCTCTTTCTCACT
AGAGTCTATGTCACTCATTATACTCTGTCGAATGTCATTGAATGTCTTTACATGGGCTTG
TATGCCTATGAAAATTGTAATACAACTTTCAGCAACGGATCTCTTGGCTCTCGCATCGAT
GAAGGACGCAGCGAAATGCGATAAGTAATGTGAATTGCAGAATTCAGTGAATCATCGAAT
CTTTGAACGCATCTTGCGCTCCTTGGTATTCCGAGGAGCATGCCTGTTTGAGTGTCATTA
AATTCTCAACTCTCTTATACTTTTTTGTAAAAGAGAGCTTGGACTGTGGAGGCTTGCTGG
CCACTTTTTGGGGTCAGCTCCTCTGAAATGCATTAGCGGAACCGTTTGCAATCTGCCACA
AGTGTGATAAGTTATCTACACTGGCGAGGGGATTGCTCTCTGTAATGTTCAGCTTCTAAT
TGTCTCTACTTTGTGAGACAACTTTTGAATGCTTGACCTCAAATCAGGTAGGACTACCC-
GCTGAACTTAA
>BC10_51
TTTCCGTAGGTGAACCTGCGGAAGGATCATTATTGAATTATGTTTCTAGATAGGTTGTAG

CTGGCTCTTTAGAGCATGTGCACGCCTGTTTGGACTTCATTTTCATCCACCTGTGCACCT
ATTGTAGTCTTTGGTTGGGTTAGGGGGAAGTGGTCATTGTGTCAGCATCTGCTGGATGTG
AGGACTTGCATTGTGAAAGCTTTGCTGTCCTTGATGTGATCATGGAATCTCTTTCTCACT
AGAGTCTATGTCACTCATTATACTCTGTCGAATGTCATTGAATGTCTTTACATGGGCTTG
TATGCCTATGAAAATTGTAATACAACTTTCAGCAACGGATCTCTTGGCTCTCGCATCGAT
GAAGGACGCAGCGAAATGCGATAAGTAATGTGAATTGCAGAATTCAGTGAATCATCGAAT
CTTTGAACGCATCTTGCGCTCCTTGGTATTCCGAGGAGCATGCCTGTTTGAGTGTCATTA
AATTCTCAACTCTCTTATACTTTTTTGTAAAAGAGAGCTTGGACTGTGGAGGCTTGCTGG
CCACTTTTTGGGGTCAGCTCCTCTGAAATGCATTAGCGGAACCGTTTGCAATCTGCCACA
AGTGTGATAAGTTATCTACACTGGCGAGGGGATTGCTCTCTGTAATGTTCAGCTTCTAAT
TGTCTCTACTTTGTGAGACAACTTTTGAATGCTTGACCTCAAATCAGGTAGGACTACCC-
GCTGAACTTAA
>BC8-23
TTTCCGTAGGTGAACCTGCGGAAGGATCATTATTGAATTATGTTTCTAGATAGGTTGTAG
CTGGCTCTTTAGAGCATGTGCACGCCTGTTTGGACTTCATTTTCATCCACCTGTGCACCT
ATTGTAGTCTTTGGTTGGGTTAGGGGGAAGTGGTCATTGTGTCAGCATCTGCTGGATGTG
AGGACTTGCATTGTGAAAGCTTTGCTGTCCTTGATGTGATCATGGAATCTCTTTCTCACT
AGAGTCTATGTCACTCATTATACTCTGTCGAATGTCATTGAATGTCTTTACATGGGCTTG
TATGCCTATGAAAATTGTAATACAACTTTCAGCAACGGATCTCTTGGCTCTCGCATCGAT
GAAGGACGCAGCGAAATGCGATAAGTAATGTGAATTGCAGAATTCAGTGAATCATCGAAT
CTTTGAACGCATCTTGCGCTCCTTGGTATTCCGAGGAGCATGCCTGTTTGAGTGTCATTA
AATTCTCAACTCTCTTATACTTTTTTGTAAAAGAGAGCTTGGACTGTGGAGGCTTGCTGG
CCACTTTTTGGGGTCAGCTCCTCTGAAATGCATTAGCGGAACCGTTTGCAATCTGCCACA
AGTGTGATAAGTTATCTACACTGGCGAGGGGATTGCTCTCTGTAATGTTCAGCTTCTAAT
TGTCTCTACTTTGTGAGACAACTTTTGAATGCTTGACCTCAAATCAGGTAGGACTACCC-
GCTGAACTTAA
>BC7-11
TTTCCGTAGGTGAACCTGCGGAAGGATCATTATTGAATTATGTTTCTAGATAGGTTGTAG
CTGGCTCTTTAGAGCATGTGCACGCCTGTTTGGACTTCATTTTCATCCACCTGTGCACCT
ATTGTAGTCTTTGGTTGGGTTAGGGGGAAGTGGTCATTGTGTCAGCATCTGCTGGATGTG
AGGACTTGCATTGTGAAAGCTTTGCTGTCCTTGATGTGATCATGGAATCTCTTTCTCACT
AGAGTCTATGTCACTCATTATACTCTGTCGAATGTCATTGAATGTCTTTACATGGGCTTG
TATGCCTATGAAAATTGTAATACAACTTTCAGCAACGGATCTCTTGGCTCTCGCATCGAT
GAAGGACGCAGCGAAATGCGATAAGTAATGTGAATTGCAGAATTCAGTGAATCATCGAAT
CTTTGAACGCATCTTGCGCTCCTTGGTATTCCGAGGAGCATGCCTGTTTGAGTGTCATTA
AATTCTCAACTCTCTTATACTTTTTTGTAAAAGAGAGCTTGGACTGTGGAGGCTTGCTGG
CCACTTTTTGGGGTCAGCTCCTCTGAAATGCATTAGCGGAACCGTTTGCAATCTGCCACA
AGTGTGATAAGTTATCTACACTGGCGAGGGGATTGCTCTCTGTAATGTTCAGCTTCTAAT
TGTCTCTACTTTGTGAGACAACTTTTGAATGCTTGACCTCAAATCAGGTAGGACTACCC-
GCTGAACTTAA
>BC8-2
TTTCCGTAGGTGAACCTGCGGAAGGATCATTATTGAATTATGTTTCTAGATAGGTTGTAG
CTGGCTCTTTAGAGCATGTGCACGCCTGTTTGGACTTCATTTTCATCCACCTGTGCACCT
ATTGTAGTCTTTGGTTGGGTTAGGGGGAAGTGGTCATTGTGTCAGCATCTGCTGGATGTG
AGGACTTGCATTGTGAAAGCTTTGCTGTCCTTGATGTGATCATGGAATCTCTTTCTCACT
AGAGTCTATGTCACTCATTATACTCTGTCGAATGTCATTGAATGTCTTTACATGGGCTTG
TATGCCTATGAAAATTGTAATACAACTTTCAGCAACGGATCTCTTGGCTCTCGCATCGAT
GAAGGACGCAGCGAAATGCGATAAGTAATGTGAATTGCAGAATTCAGTGAATCATCGAAT
CTTTGAACGCATCTTGCGCTCCTTGGTATTCCGAGGAGCATGCCTGTTTGAGTGTCATTA
AATTCTCAACTCTCTTATACTTTTTTGTAAAAGAGAGCTTGGACTGTGGAGGCTTGCTGG

CCACTTTTTGGGGTCAGCTCCTCTGAAATGCATTAGCGGAACCGTTTGCAATCTGCCACA
AGTGTGATAAGTTATCTACACTGGCGAGGGGATTGCTCTCTGTAATGTTCAGCTTCTAAT
TGTCTCTACTTTGTGAGACAACTTTTGAATGCTTGACCTCAAATCAGGTAGGACTACCC-
GCTGAACTTAA
>BC11_17
TTTCCGTAGGTGAACCTGCGGAAGGATCATTATTGAATTATGTTTCTAGATAGGTTGTAG
CTGGCTCTTTAGAGCATGTGCACGCCTGTTTGGACTTCATTTTCATCCACCTGTGCACCT
ATTGTAGTCTTTGGTTGGGTTAGGGGGAAGTGGTCATTGTGTCAGCATCTGCTGGATGTG
AGGACTTGCATTGTGAAAGCTTTGCTGTCCTTGATGTGATCATGGAATCTCTTTCTCACT
AGAGTCTATGTCACTCATTATACTCTGTCGAATGTCATTGAATGTCTTTACATGGGCTTG
TATGCCTATGAAAATTGTAATACAACTTTCAGCAACGGATCTCTTGGCTCTCGCATCGAT
GAAGGACGCAGCGAAATGCGATAAGTAATGTGAATTGCAGAATTCAGTGAATCATCGAAT
CTTTGAACGCATCTTGCGCTCCTTGGTATTCCGAGGAGCATGCCTGTTTGAGTGTCATTA
AATTCTCAACTCTCTTATACTTTTTTGTAAAAGAGAGCTTGGACTGTGGAGGCTTGCTGG
CCACTTTTTGGGGTCAGCTCCTCTGAAATGCATTAGCGGAACCGTTTGCAATCTGCCACA
AGTGTGATAAGTTATCTACACTGGCGAGGGGATTGCTCTCTGTAATGTTCAGCTTCTAAT
TGTCTCTACTTTGTGAGACAACTTTTGAATGCTTGACCTCAAATCAGGTAGGACTACCC-
GCTGAACTTAA
>BC11_33
TTTCCGTAGGTGAACCTGCGGAAGGATCATTATTGAATTATGTTTCTAGATAGGTTGTAG
CTGGCTCTTTAGAGCATGTGCACGCCTGTTTGGACTTCATTTTCATCCACCTGTGCACCT
ATTGTAGTCTTTGGTTGGGTTAGGGGGAAGTGGTCATTGTGTCAGCATCTGCTGGATGTG
AGGACTTGCATTGTGAAAGCTTTGCTGTCCTTGATGTGATCATGGAATCTCTTTCTCACT
AGAGTCTATGTCACTCATTATACTCTGTCGAATGTCATTGAATGTCTTTACATGGGCTTG
TATGCCTATGAAAATTGTAATACAACTTTCAGCAACGGATCTCTTGGCTCTCGCATCGAT
GAAGGACGCAGCGAAATGCGATAAGTAATGTGAATTGCAGAATTCAGTGAATCATCGAAT
CTTTGAACGCATCTTGCGCTCCTTGGTATTCCGAGGAGCATGCCTGTTTGAGTGTCATTA
AATTCTCAACTCTCTTATACTTTTTTGTAAAAGAGAGCTTGGACTGTGGAGGCTTGCTGG
CCACTTTTTGGGGTCAGCTCCTCTGAAATGCATTAGCGGAACCGTTTGCAATCTGCCACA
AGTGTGATAAGTTATCTACACTGGCGAGGGGATTGCTCTCTGTAATGTTCAGCTTCTAAT
TGTCTCTACTTTGTGAGACAACTTTTGAATGCTTGACCTCAAATCAGGTAGGACTACCC-
GCTGAACTTAA
>BC5-48
TTTCCGTAGGTGAACCTGCGGAAGGATCATTATTGAATTATGTTTCTAGATAGGTTGTAG
CTGGCTCTTTAGAGCATGTGCACGCCTGTTTGGACTTCATTTTCATCCACCTGTGCACCT
ATTGTAGTCTTTGGTTGGGTTAGGGGGAAGTGGTCATTGTGTCAGCATCTGCTGGATGTG
AGGACTTGCATTGTGAAAGCTTTGCTGTCCTTGATGTGATCATGGAATCTCTTTCTCACT
AGAGTCTATGTCACTCATTATACTCTGTCGAATGTCATTGAATGTCTTTACATGGGCTTG
TATGCCTATGAAAATTGTAATACAACTTTCAGCAACGGATCTCTTGGCTCTCGCATCGAT
GAAGGACGCAGCGAAATGCGATAAGTAATGTGAATTGCAGAATTCAGTGAATCATCGAAT
CTTTGAACGCATCTTGCGCTCCTTGGTATTCCGAGGAGCATGCCTGTTTGAGTGTCATTA
AATTCTCAACTCTCTTATACTTTTTTGTAAAAGAGAGCTTGGACTGTGGAGGCTTGCTGG
CCACTTTTTGGGGTCAGCTCCTCTGAAATGCATTAGCGGAACCGTTTGCAATCTGCCACA
AGTGTGATAAGTTATCTACACTGGCGAGGGGATTGCTCTCTGTAATGTTCAGCTTCTAAT
TGTCTCTACTTTGTGAGACAACTTTTGAATGCTTGACCTCAAATCAGGTAGGACTACCC-
GCTGAACTTAA
>BC8-30
TTTCCGTAGGTGAACCTGCGGAAGGATCATTATTGAATTATGTTTCTAGATAGGTTGTAG
CTGGCTCTTTAGAGCATGTGCACGCCTGTTTGGACTTCATTTTCATCCACCTGTGCACCT
ATTGTAGTCTTTGGTTGGGTTAGGGGGAAGTGGTCATTGTGTCAGCATCTGCTGGATGTG

AGGACTTGCATTGTGAAAGCTTTGCTGTCCTTGATGTGATCATGGAATCTCTTTCTCACT
AGAGTCTATGTCACTCATTATACTCTGTCGAATGTCATTGAATGTCTTTACATGGGCTTG
TATGCCTATGAAAATTGTAATACAACTTTCAGCAACGGATCTCTTGGCTCTCGCATCGAT
GAAGGACGCAGCGAAATGCGATAAGTAATGTGAATTGCAGAATTCAGTGAATCATCGAAT
CTTTGAACGCATCTTGCGCTCCTTGGTATTCCGAGGAGCATGCCTGTTTGAGTGTCATTA
AATTCTCAACTCTCTTATACTTTTTTGTAAAAGAGAGCTTGGACTGTGGAGGCTTGCTGG
CCACTTTTTGGGGTCAGCTCCTCTGAAATGCATTAGCGGAACCGTTTGCAATCTGCCACA
AGTGTGATAAGTTATCTACACTGGCGAGGGGATTGCTCTCTGTAATGTTCAGCTTCTAAT
TGTCTCTACTTTGTGAGACAACTTTTGAATGCTTGACCTCAAATCAGGTAGGACTACCC-
GCTGAACTTAA
>BC9-40
TTTCCGTAGGTGAACCTGCGGAAGGATCATTATTGAATTATGTTTCTAGATAGGTTGTAG
CTGGCTCTTTAGAGCATGTGCACGCCTGTTTGGACTTCATTTTCATCCACCTGTGCACCT
ATTGTAGTCTTTGGTTGGGTTAGGGGGAAGTGGTCATTGTGTCAGCATCTGCTGGATGTG
AGGACTTGCATTGTGAAAGCTTTGCTGTCCTTGATGTGATCATGGAATCTCTTTCTCACT
AGAGTCTATGTCACTCATTATACTCTGTCGAATGTCATTGAATGTCTTTACATGGGCTTG
TATGCCTATGAAAATTGTAATACAACTTTCAGCAACGGATCTCTTGGCTCTCGCATCGAT
GAAGGACGCAGCGAAATGCGATAAGTAATGTGAATTGCAGAATTCAGTGAATCATCGAAT
CTTTGAACGCATCTTGCGCTCCTTGGTATTCCGAGGAGCATGCCTGTTTGAGTGTCATTA
AATTCTCAACTCTCTTATACTTTTTTGTAAAAGAGAGCTTGGACTGTGGAGGCTTGCTGG
CCACTTTTTGGGGTCAGCTCCTCTGAAATGCATTAGCGGAACCGTTTGCAATCTGCCACA
AGTGTGATAAGTTATCTACACTGGCGAGGGGATTGCTCTCTGTAATGTTCAGCTTCTAAT
TGTCTCTACTTTGTGAGACAACTTTTGAATGCTTGACCTCAAATCAGGTAGGACTACCC-
GCTGAACTTAA
>BC3-13
TTTCCGTAGGTGAACCTGCGGAAGGATCATTATTGAATTATGTTTCTAGATAGGTTGTAG
CTGGCTCTTTAGAGCATGTGCACGCCTGTTTGGACTTCATTTTCATCCACCTGTGCACCT
ATTGTAGTCTTTGGTTGGGTTAGGGGGAAGTGGTCATTGTGTCAGCATCTGCTGGATGTG
AGGACTTGCATTGTGAAAGCTTTGCTGTCCTTGATGTGATCATGGAATCTCTTTCTCACT
AGAGTCTATGTCACTCATTATACTCTGTCGAATGTCATTGAATGTCTTTACATGGGCTTG
TATGCCTATGAAAATTGTAATACAACTTTCAGCAACGGATCTCTTGGCTCTCGCATCGAT
GAAGGACGCAGCGAAATGCGATAAGTAATGTGAATTGCAGAATTCAGTGAATCATCGAAT
CTTTGAACGCATCTTGCGCTCCTTGGTATTCCGAGGAGCATGCCTGTTTGAGTGTCATTA
AATTCTCAACTCTCTTATACTTTTTTGTAAAAGAGAGCTTGGACTGTGGAGGCTTGCTGG
CCACTTTTTGGGGTCAGCTCCTCTGAAATGCATTAGCGGAACCGTTTGCAATCTGCCACA
AGTGTGATAAGTTATCTACACTGGCGAGGGGATTGCTCTCTGTAATGTTCAGCTTCTAAT
TGTCTCTACTTTGTGAGACAACTTTTGAATGCTTGACCTCAAATCAGGTAGGACTACCC-
GCTGAACTTAA
>BC3-38
TTTCCGTAGGTGAACCTGCGGAAGGATCATTATTGAATTATGTTTCTAGATAGGTTGTAG
CTGGCTCTTTAGAGCATGTGCACGCCTGTTTGGACTTCATTTTCATCCACCTGTGCACCT
ATTGTAGTCTTTGGTTGGGTTAGGGGGAAGTGGTCATTGTGTCAGCATCTGCTGGATGTG
AGGACTTGCATTGTGAAAGCTTTGCTGTCCTTGATGTGATCATGGAATCTCTTTCTCACT
AGAGTCTATGTCACTCATTATACTCTGTCGAATGTCATTGAATGTCTTTACATGGGCTTG
TATGCCTATGAAAATTGTAATACAACTTTCAGCAACGGATCTCTTGGCTCTCGCATCGAT
GAAGGACGCAGCGAAATGCGATAAGTAATGTGAATTGCAGAATTCAGTGAATCATCGAAT
CTTTGAACGCATCTTGCGCTCCTTGGTATTCCGAGGAGCATGCCTGTTTGAGTGTCATTA
AATTCTCAACTCTCTTATACTTTTTTGTAAAAGAGAGCTTGGACTGTGGAGGCTTGCTGG
CCACTTTTTGGGGTCAGCTCCTCTGAAATGCATTAGCGGAACCGTTTGCAATCTGCCACA
AGTGTGATAAGTTATCTACACTGGCGAGGGGATTGCTCTCTGTAATGTTCAGCTTCTAAT

TGTCTCTACTTTGTGAGACAACTTTTGAATGCTTGACCTCAAATCAGGTAGGACTACCC-
GCTGAACTTAA
>BC3-40
TTTCCGTAGGTGAACCTGCGGAAGGATCATTATTGAATTATGTTTCTAGATAGGTTGTAG
CTGGCTCTTTAGAGCATGTGCACGCCTGTTTGGACTTCATTTTCATCCACCTGTGCACCT
ATTGTAGTCTTTGGTTGGGTTAGGGGGAAGTGGTCATTGTGTCAGCATCTGCTGGATGTG
AGGACTTGCATTGTGAAAGCTTTGCTGTCCTTGATGTGATCATGGAATCTCTTTCTCACT
AGAGTCTATGTCACTCATTATACTCTGTCGAATGTCATTGAATGTCTTTACATGGGCTTG
TATGCCTATGAAAATTGTAATACAACTTTCAGCAACGGATCTCTTGGCTCTCGCATCGAT
GAAGGACGCAGCGAAATGCGATAAGTAATGTGAATTGCAGAATTCAGTGAATCATCGAAT
CTTTGAACGCATCTTGCGCTCCTTGGTATTCCGAGGAGCATGCCTGTTTGAGTGTCATTA
AATTCTCAACTCTCTTATACTTTTTTGTAAAAGAGAGCTTGGACTGTGGAGGCTTGCTGG
CCACTTTTTGGGGTCAGCTCCTCTGAAATGCATTAGCGGAACCGTTTGCAATCTGCCACA
AGTGTGATAAGTTATCTACACTGGCGAGGGGATTGCTCTCTGTAATGTTCAGCTTCTAAT
TGTCTCTACTTTGTGAGACAACTTTTGAATGCTTGACCTCAAATCAGGTAGGACTACCC-
GCTGAACTTAA
>BC5-60
TTTCCGTAGGTGAACCTGCGGAAGGATCATTATTGAATTATGTTTCTAGATAGGTTGTAG
CTGGCTCTTTAGAGCATGTGCACGCCTGTTTGGACTTCATTTTCATCCACCTGTGCACCT
ATTGTAGTCTTTGGTTGGGTTAGGGGGAAGTGGTCATTGTGTCAGCATCTGCTGGATGTG
AGGACTTGCATTGTGAAAGCTTTGCTGTCCTTGATGTGATCATGGAATCTCTTTCTCACT
AGAGTCTATGTCACTCATTATACTCTGTCGAATGTCATTGAATGTCTTTACATGGGCTTG
TATGCCTATGAAAATTGTAATACAACTTTCAGCAACGGATCTCTTGGCTCTCGCATCGAT
GAAGGACGCAGCGAAATGCGATAAGTAATGTGAATTGCAGAATTCAGTGAATCATCGAAT
CTTTGAACGCATCTTGCGCTCCTTGGTATTCCGAGGAGCATGCCTGTTTGAGTGTCATTA
AATTCTCAACTCTCTTATACTTTTTTGTAAAAGAGAGCTTGGACTGTGGAGGCTTGCTGG
CCACTTTTTGGGGTCAGCTCCTCTGAAATGCATTAGCGGAACCGTTTGCAATCTGCCACA
AGTGTGATAAGTTATCTACACTGGCGAGGGGATTGCTCTCTGTAATGTTCAGCTTCTAAT
TGTCTCTACTTTGTGAGACAACTTTTGAATGCTTGACCTCAAATCAGGTAGGACTACCC-
GCTGAACTTAA
>BC6-17
TTTCCGTAGGTGAACCTGCGGAAGGATCATTATTGAATTATGTTTCTAGATAGGTTGTAG
CTGGCTCTTTAGAGCATGTGCACGCCTGTTTGGACTTCATTTTCATCCACCTGTGCACCT
ATTGTAGTCTTTGGTTGGGTTAGGGGGAAGTGGTCATTGTGTCAGCATCTGCTGGATGTG
AGGACTTGCATTGTGAAAGCTTTGCTGTCCTTGATGTGATCATGGAATCTCTTTCTCACT
AGAGTCTATGTCACTCATTATACTCTGTCGAATGTCATTGAATGTCTTTACATGGGCTTG
TATGCCTATGAAAATTGTAATACAACTTTCAGCAACGGATCTCTTGGCTCTCGCATCGAT
GAAGGACGCAGCGAAATGCGATAAGTAATGTGAATTGCAGAATTCAGTGAATCATCGAAT
CTTTGAACGCATCTTGCGCTCCTTGGTATTCCGAGGAGCATGCCTGTTTGAGTGTCATTA
AATTCTCAACTCTCTTATACTTTTTTGTAAAAGAGAGCTTGGACTGTGGAGGCTTGCTGG
CCACTTTTTGGGGTCAGCTCCTCTGAAATGCATTAGCGGAACCGTTTGCAATCTGCCACA
AGTGTGATAAGTTATCTACACTGGCGAGGGGATTGCTCTCTGTAATGTTCAGCTTCTAAT
TGTCTCTACTTTGTGAGACAACTTTTGAATGCTTGACCTCAAATCAGGTAGGACTACCC-
GCTGAACTTAA
>BC9-13
TTTCCGTAGGTGAACCTGCGGAAGGATCATTATTGAATTATGTTTCTAGATAGGTTGTAG
CTGGCTCTTTAGAGCATGTGCACGCCTGTTTGGACTTCATTTTCATCCACCTGTGCACCT
ATTGTAGTCTTTGGTTGGGTTAGGGGGAAGTGGTCATTGTGTCAGCATCTGCTGGATGTG
AGGACTTGCATTGTGAAAGCTTTGCTGTCCTTGATGTGATCATGGAATCTCTTTCTCACT
AGAGTCTATGTCACTCATTATACTCTGTCGAATGTCATTGAATGTCTTTACATGGGCTTG

TATGCCTATGAAAATTGTAATACAACTTTCAGCAACGGATCTCTTGGCTCTCGCATCGAT
GAAGGACGCAGCGAAATGCGATAAGTAATGTGAATTGCAGAATTCAGTGAATCATCGAAT
CTTTGAACGCATCTTGCGCTCCTTGGTATTCCGAGGAGCATGCCTGTTTGAGTGTCATTA
AATTCTCAACTCTCTTATACTTTTTTGTAAAAGAGAGCTTGGACTGTGGAGGCTTGCTGG
CCACTTTTTGGGGTCAGCTCCTCTGAAATGCATTAGCGGAACCGTTTGCAATCTGCCACA
AGTGTGATAAGTTATCTACACTGGCGAGGGGATTGCTCTCTGTAATGTTCAGCTTCTAAT
TGTCTCTACTTTGTGAGACAACTTTTGAATGCTTGACCTCAAATCAGGTAGGACTACCC-
GCTGAACTTAA
>BC12_12
TTTCCGTAGGTGAACCTGCGGAAGGATCATTATTGAATTATGTTTCTAGATAGGTTGTAG
CTGGCTCTTTAGAGCATGTGCACGCCTGTTTGGACTTCATTTTCATCCACCTGTGCACCT
ATTGTAGTCTTTGGTTGGGTTAGGGGGAAGTGGTCATTGTGTCAGCATCTGCTGGATGTG
AGGACTTGCATTGTGAAAGCTTTGCTGTCCTTGATGTGATCATGGAATCTCTTTCTCACT
AGAGTCTATGTCACTCATTATACTCTGTCGAATGTCATTGAATGTCTTTACATGGGCTTG
TATGCCTATGAAAATTGTAATACAACTTTCAGCAACGGATCTCTTGGCTCTCGCATCGAT
GAAGGACGCAGCGAAATGCGATAAGTAATGTGAATTGCAGAATTCAGTGAATCATCGAAT
CTTTGAACGCATCTTGCGCTCCTTGGTATTCCGAGGAGCATGCCTGTTTGAGTGTCATTA
AATTCTCAACTCTCTTATACTTTTTTGTAAAAGAGAGCTTGGACTGTGGAGGCTTGCTGG
CCACTTTTTGGGGTCAGCTCCTCTGAAATGCATTAGCGGAACCGTTTGCAATCTGCCACA
AGTGTGATAAGTTATCTACACTGGCGAGGGGATTGCTCTCTGTAATGTTCAGCTTCTAAT
TGTCTCTACTTTGTGAGACAACTTTTGAATGCTTGACCTCAAATCAGGTAGGACTACCC-
GCTGAACTTAA
>BC6-22
TTTCCGTAGGTGAACCTGCGGAAGGATCATTATTGAATTATGTTTCTAGATAGGTTGTAG
CTGGCTCTTTAGAGCATGTGCACGCCTGTTTGGACTTCATTTTCATCCACCTGTGCACCT
ATTGTAGTCTTTGGTTGGGTTAGGGGGAAGTGGTCATTGTGTCAGCATCTGCTGGATGTG
AGGACTTGCATTGTGAAAGCTTTGCTGTCCTTGATGTGATCATGGAATCTCTTTCTCACT
AGAGTCTATGTCACTCATTATACTCTGTCGAATGTCATTGAATGTCTTTACATGGGCTTG
TATGCCTATGAAAATTGTAATACAACTTTCAGCAACGGATCTCTTGGCTCTCGCATCGAT
GAAGGACGCAGCGAAATGCGATAAGTAATGTGAATTGCAGAATTCAGTGAATCATCGAAT
CTTTGAACGCATCTTGCGCTCCTTGGTATTCCGAGGAGCATGCCTGTTTGAGTGTCATTA
AATTCTCAACTCTCTTATACTTTTTTGTAAAAGAGAGCTTGGACTGTGGAGGCTTGCTGG
CCACTTTTTGGGGTCAGCTCCTCTGAAATGCATTAGCGGAACCGTTTGCAATCTGCCACA
AGTGTGATAAGTTATCTACACTGGCGAGGGGATTGCTCTCTGTAATGTTCAGCTTCTAAT
TGTCTCTACTTTGTGAGACAACTTTTGAATGCTTGACCTCAAATCAGGTAGGACTACCC-
GCTGAACTTAA
>BC4-33
TTTCCGTAGGTGAACCTGCGGAAGGATCATTATTGAATTATGTTTCTAGATAGGTTGTAG
CTGGCTCTTTAGAGCATGTGCACGCCTGTTTGGACTTCATTTTCATCCACCTGTGCACCT
ATTGTAGTCTTTGGTTGGGTTAGGGGGAAGTGGTCATTGTGTCAGCATCTGCTGGATGTG
AGGACTTGCATTGTGAAAGCTTTGCTGTCCTTGATGTGATCATGGAATCTCTTTCTCACT
AGAGTCTATGTCACTCATTATACTCTGTCGAATGTCATTGAATGTCTTTACATGGGCTTG
TATGCCTATGAAAATTGTAATACAACTTTCAGCAACGGATCTCTTGGCTCTCGCATCGAT
GAAGGACGCAGCGAAATGCGATAAGTAATGTGAATTGCAGAATTCAGTGAATCATCGAAT
CTTTGAACGCATCTTGCGCTCCTTGGTATTCCGAGGAGCATGCCTGTTTGAGTGTCATTA
AATTCTCAACTCTCTTATACTTTTTTGTAAAAGAGAGCTTGGACTGTGGAGGCTTGCTGG
CCACTTTTTGGGGTCAGCTCCTCTGAAATGCATTAGCGGAACCGTTTGCAATCTGCCACA
AGTGTGATAAGTTATCTACACTGGCGAGGGGATTGCTCTCTGTAATGTTCAGCTTCTAAT
TGTCTCTACTTTGTGAGACAACTTTTGAATGCTTGACCTCAAATCAGGTAGGACTACCC-
GCTGAACTTAA

>BC7–1
TTTCCGTAGGTGAACCTGCGGAAGGATCATTATTGAATTATGTTTCTAGATAGGTTGTAG
CTGGCTCTTTAGAGCATGTGCACGCCTGTTTGGACTTCATTTTCATCCACCTGTGCACCT
ATTGTAGTCTTTGGTTGGGTTAGGGGGAAGTGGTCATTGTGTCAGCATCTGCTGGATGTG
AGGACTTGCATTGTGAAAGCTTTGCTGTCCTTGATGTGATCATGGAATCTCTTTCTCACT
AGAGTCTATGTCACTCATTATACTCTGTCGAATGTCATTGAATGTCTTTACATGGGCTTG
TATGCCTATGAAAATTGTAATACAACTTTCAGCAACGGATCTCTTGGCTCTCGCATCGAT
GAAGGACGCAGCGAAATGCGATAAGTAATGTGAATTGCAGAATTCAGTGAATCATCGAAT
CTTTGAACGCATCTTGCGCTCCTTGGTATTCCGAGGAGCATGCCTGTTTGAGTGTCATTA
AATTCTCAACTCTCTTATACTTTTTTGTAAAAGAGAGCTTGGACTGTGGAGGCTTGCTGG
CCACTTTTTGGGGTCAGCTCCTCTGAAATGCATTAGCGGAACCGTTTGCAATCTGCCACA
AGTGTGATAAGTTATCTACACTGGCGAGGGGATTGCTCTCTGTAATGTTCAGCTTCTAAT
TGTCTCTACTTTGTGAGACAACTTTTGAATGCTTGACCTCAAATCAGGTAGGACTACCC–
GCTGAACTTAA
>BC7–23
TTTCCGTAGGTGAACCTGCGGAAGGATCATTATTGAATTATGTTTCTAGATAGGTTGTAG
CTGGCTCTTTAGAGCATGTGCACGCCTGTTTGGACTTCATTTTCATCCACCTGTGCACCT
ATTGTAGTCTTTGGTTGGGTTAGGGGGAAGTGGTCATTGTGTCAGCATCTGCTGGATGTG
AGGACTTGCATTGTGAAAGCTTTGCTGTCCTTGATGTGATCATGGAATCTCTTTCTCACT
AGAGTCTATGTCACTCATTATACTCTGTCGAATGTCATTGAATGTCTTTACATGGGCTTG
TATGCCTATGAAAATTGTAATACAACTTTCAGCAACGGATCTCTTGGCTCTCGCATCGAT
GAAGGACGCAGCGAAATGCGATAAGTAATGTGAATTGCAGAATTCAGTGAATCATCGAAT
CTTTGAACGCATCTTGCGCTCCTTGGTATTCCGAGGAGCATGCCTGTTTGAGTGTCATTA
AATTCTCAACTCTCTTATACTTTTTTGTAAAAGAGAGCTTGGACTGTGGAGGCTTGCTGG
CCACTTTTTGGGGTCAGCTCCTCTGAAATGCATTAGCGGAACCGTTTGCAATCTGCCACA
AGTGTGATAAGTTATCTACACTGGCGAGGGGATTGCTCTCTGTAATGTTCAGCTTCTAAT
TGTCTCTACTTTGTGAGACAACTTTTGAATGCTTGACCTCAAATCAGGTAGGACTACCC–
GCTGAACTTAA
>BC8–17
TTTCCGTAGGTGAACCTGCGGAAGGATCATTATTGAATTATGTTTCTAGATAGGTTGTAG
CTGGCTCTTTAGAGCATGTGCACGCCTGTTTGGACTTCATTTTCATCCACCTGTGCACCT
ATTGTAGTCTTTGGTTGGGTTAGGGGGAAGTGGTCATTGTGTCAGCATCTGCTGGATGTG
AGGACTTGCATTGTGAAAGCTTTGCTGTCCTTGATGTGATCATGGAATCTCTTTCTCACT
AGAGTCTATGTCACTCATTATACTCTGTCGAATGTCATTGAATGTCTTTACATGGGCTTG
TATGCCTATGAAAATTGTAATACAACTTTCAGCAACGGATCTCTTGGCTCTCGCATCGAT
GAAGGACGCAGCGAAATGCGATAAGTAATGTGAATTGCAGAATTCAGTGAATCATCGAAT
CTTTGAACGCATCTTGCGCTCCTTGGTATTCCGAGGAGCATGCCTGTTTGAGTGTCATTA
AATTCTCAACTCTCTTATACTTTTTTGTAAAAGAGAGCTTGGACTGTGGAGGCTTGCTGG
CCACTTTTTGGGGTCAGCTCCTCTGAAATGCATTAGCGGAACCGTTTGCAATCTGCCACA
AGTGTGATAAGTTATCTACACTGGCGAGGGGATTGCTCTCTGTAATGTTCAGCTTCTAAT
TGTCTCTACTTTGTGAGACAACTTTTGAATGCTTGACCTCAAATCAGGTAGGACTACCC–
GCTGAACTTAA
>BC10_35
TTTCCGTAGGTGAACCTGCGGAAGGATCATTATTGAATTATGTTTCTAGATAGGTTGTAG
CTGGCTCTTTAGAGCATGTGCACGCCTGTTTGGACTTCATTTTCATCCACCTGTGCACCT
ATTGTAGTCTTTGGTTGGGTTAGGGGGAAGTGGTCATTGTGTCAGCATCTGCTGGATGTG
AGGACTTGCATTGTGAAAGCTTTGCTGTCCTTGATGTGATCATGGAATCTCTTTCTCACT
AGAGTCTATGTCACTCATTATACTCTGTCGAATGTCATTGAATGTCTTTACATGGGCTTG
TATGCCTATGAAAATTGTAATACAACTTTCAGCAACGGATCTCTTGGCTCTCGCATCGAT
GAAGGACGCAGCGAAATGCGATAAGTAATGTGAATTGCAGAATTCAGTGAATCATCGAAT

CTTTGAACGCATCTTGCGCTCCTTGGTATTCCGAGGAGCATGCCTGTTTGAGTGTCATTA
AATTCTCAACTCTCTTATACTTTTTTGTAAAAGAGAGCTTGGACTGTGGAGGCTTGCTGG
CCACTTTTTGGGGTCAGCTCCTCTGAAATGCATTAGCGGAACCGTTTGCAATCTGCCACA
AGTGTGATAAGTTATCTACACTGGCGAGGGGATTGCTCTCTGTAATGTTCAGCTTCTAAT
TGTCTCTACTTTGTGAGACAACTTTTGAATGCTTGACCTCAAATCAGGTAGGACTACCC-
GCTGAACTTAA
>BC10_47
TTTCCGTAGGTGAACCTGCGGAAGGATCATTATTGAATTATGTTTCTAGATAGGTTGTAG
CTGGCTCTTTAGAGCATGTGCACGCCTGTTTGGACTTCATTTTCATCCACCTGTGCACCT
ATTGTAGTCTTTGGTTGGGTTAGGGGGAAGTGGTCATTGTGTCAGCATCTGCTGGATGTG
AGGACTTGCATTGTGAAAGCTTTGCTGTCCTTGATGTGATCATGGAATCTCTTTCTCACT
AGAGTCTATGTCACTCATTATACTCTGTCGAATGTCATTGAATGTCTTTACATGGGCTTG
TATGCCTATGAAAATTGTAATACAACTTTCAGCAACGGATCTCTTGGCTCTCGCATCGAT
GAAGGACGCAGCGAAATGCGATAAGTAATGTGAATTGCAGAATTCAGTGAATCATCGAAT
CTTTGAACGCATCTTGCGCTCCTTGGTATTCCGAGGAGCATGCCTGTTTGAGTGTCATTA
AATTCTCAACTCTCTTATACTTTTTTGTAAAAGAGAGCTTGGACTGTGGAGGCTTGCTGG
CCACTTTTTGGGGTCAGCTCCTCTGAAATGCATTAGCGGAACCGTTTGCAATCTGCCACA
AGTGTGATAAGTTATCTACACTGGCGAGGGGATTGCTCTCTGTAATGTTCAGCTTCTAAT
TGTCTCTACTTTGTGAGACAACTTTTGAATGCTTGACCTCAAATCAGGTAGGACTACCC-
GCTGAACTTAA
>BC3-32
TTTCCGTAGGTGAACCTGCGGAAGGATCATTATTGAATTATGTTTCTAGATAGGTTGTAG
CTGGCTCTTTAGAGCATGTGCACGCCTGTTTGGACTTCATTTTCATCCACCTGTGCACCT
ATTGTAGTCTTTGGTTGGGTTAGGGGGAAGTGGTCATTGTGTCAGCATCTGCTGGATGTG
AGGACTTGCATTGTGAAAGCTTTGCTGTCCTTGATGTGATCATGGAATCTCTTTCTCACT
AGAGTCTATGTCACTCATTATACTCTGTCGAATGTCATTGAATGTCTTTACATGGGCTTG
TATGCCTATGAAAATTGTAATACAACTTTCAGCAACGGATCTCTTGGCTCTCGCATCGAT
GAAGGACGCAGCGAAATGCGATAAGTAATGTGAATTGCAGAATTCAGTGAATCATCGAAT
CTTTGAACGCATCTTGCGCTCCTTGGTATTCCGAGGAGCATGCCTGTTTGAGTGTCATTA
AATTCTCAACTCTCTTATACTTTTTTGTAAAAGAGAGCTTGGACTGTGGAGGCTTGCTGG
CCACTTTTTGGGGTCAGCTCCTCTGAAATGCATTAGCGGAACCGTTTGCAATCTGCCACA
AGTGTGATAAGTTATCTACACTGGCGAGGGGATTGCTCTCTGTAATGTTCAGCTTCTAAT
TGTCTCTACTTTGTGAGACAACTTTTGAATGCTTGACCTCAAATCAGGTAGGACTACCC-
GCTGAACTTAA
>BC4-58
TTTCCGTAGGTGAACCTGCGGAAGGATCATTATTGAATTATGTTTCTAGATAGGTTGTAG
CTGGCTCTTTAGAGCATGTGCACGCCTGTTTGGACTTCATTTTCATCCACCTGTGCACCT
ATTGTAGTCTTTGGTTGGGTTAGGGGGAAGTGGTCATTGTGTCAGCATCTGCTGGATGTG
AGGACTTGCATTGTGAAAGCTTTGCTGTCCTTGATGTGATCATGGAATCTCTTTCTCACT
AGAGTCTATGTCACTCATTATACTCTGTCGAATGTCATTGAATGTCTTTACATGGGCTTG
TATGCCTATGAAAATTGTAATACAACTTTCAGCAACGGATCTCTTGGCTCTCGCATCGAT
GAAGGACGCAGCGAAATGCGATAAGTAATGTGAATTGCAGAATTCAGTGAATCATCGAAT
CTTTGAACGCATCTTGCGCTCCTTGGTATTCCGAGGAGCATGCCTGTTTGAGTGTCATTA
AATTCTCAACTCTCTTATACTTTTTTGTAAAAGAGAGCTTGGACTGTGGAGGCTTGCTGG
CCACTTTTTGGGGTCAGCTCCTCTGAAATGCATTAGCGGAACCGTTTGCAATCTGCCACA
AGTGTGATAAGTTATCTACACTGGCGAGGGGATTGCTCTCTGTAATGTTCAGCTTCTAAT
TGTCTCTACTTTGTGAGACAACTTTTGAATGCTTGACCTCAAATCAGGTAGGACTACCC-
GCTGAACTTAA
>BC4-67
TTTCCGTAGGTGAACCTGCGGAAGGATCATTATTGAATTATGTTTCTAGATAGGTTGTAG

CTGGCTCTTTAGAGCATGTGCACGCCTGTTTGGACTTCATTTTCATCCACCTGTGCACCT
ATTGTAGTCTTTGGTTGGGTTAGGGGGAAGTGGTCATTGTGTCAGCATCTGCTGGATGTG
AGGACTTGCATTGTGAAAGCTTTGCTGTCCTTGATGTGATCATGGAATCTCTTTCTCACT
AGAGTCTATGTCACTCATTATACTCTGTCGAATGTCATTGAATGTCTTTACATGGGCTTG
TATGCCTATGAAAATTGTAATACAACTTTCAGCAACGGATCTCTTGGCTCTCGCATCGAT
GAAGGACGCAGCGAAATGCGATAAGTAATGTGAATTGCAGAATTCAGTGAATCATCGAAT
CTTTGAACGCATCTTGCGCTCCTTGGTATTCCGAGGAGCATGCCTGTTTGAGTGTCATTA
AATTCTCAACTCTCTTATACTTTTTTGTAAAAGAGAGCTTGGACTGTGGAGGCTTGCTGG
CCACTTTTTGGGGTCAGCTCCTCTGAAATGCATTAGCGGAACCGTTTGCAATCTGCCACA
AGTGTGATAAGTTATCTACACTGGCGAGGGGATTGCTCTCTGTAATGTTCAGCTTCTAAT
TGTCTCTACTTTGTGAGACAACTTTTGAATGCTTGACCTCAAATCAGGTAGGACTACCC-
GCTGAACTTAA
>BC4-68
TTTCCGTAGGTGAACCTGCGGAAGGATCATTATTGAATTATGTTTCTAGATAGGTTGTAG
CTGGCTCTTTAGAGCATGTGCACGCCTGTTTGGACTTCATTTTCATCCACCTGTGCACCT
ATTGTAGTCTTTGGTTGGGTTAGGGGGAAGTGGTCATTGTGTCAGCATCTGCTGGATGTG
AGGACTTGCATTGTGAAAGCTTTGCTGTCCTTGATGTGATCATGGAATCTCTTTCTCACT
AGAGTCTATGTCACTCATTATACTCTGTCGAATGTCATTGAATGTCTTTACATGGGCTTG
TATGCCTATGAAAATTGTAATACAACTTTCAGCAACGGATCTCTTGGCTCTCGCATCGAT
GAAGGACGCAGCGAAATGCGATAAGTAATGTGAATTGCAGAATTCAGTGAATCATCGAAT
CTTTGAACGCATCTTGCGCTCCTTGGTATTCCGAGGAGCATGCCTGTTTGAGTGTCATTA
AATTCTCAACTCTCTTATACTTTTTTGTAAAAGAGAGCTTGGACTGTGGAGGCTTGCTGG
CCACTTTTTGGGGTCAGCTCCTCTGAAATGCATTAGCGGAACCGTTTGCAATCTGCCACA
AGTGTGATAAGTTATCTACACTGGCGAGGGGATTGCTCTCTGTAATGTTCAGCTTCTAAT
TGTCTCTACTTTGTGAGACAACTTTTGAATGCTTGACCTCAAATCAGGTAGGACTACCC-
GCTGAACTTAA
>BC1-1
TTTCCGTAGGTGAACCTGCGGAAGGATCATTATTGAATTATGTTTCTAGATAGGTTGTAG
CTGGCTCTTTAGAGCATGTGCACGCCTGTTTGGACTTCATTTTCATCCACCTGTGCACCT
ATTGTAGTCTTTGGTTGGGTTAGGGGGAAGTGGTCATTGTGTCAGCATCTGCTGGATGTG
AGGACTTGCATTGTGAAAGCTTTGCTGTCCTTGATGTGATCATGGAATCTCTTTCTCACT
AGAGTCTATGTCACTCATTATACTCTGTCGAATGTCATTGAATGTCTTTACATGGGCTTG
TATGCCTATGAAAATTGTAATACAACTTTCAGCAACGGATCTCTTGGCTCTCGCATCGAT
GAAGGACGCAGCGAAATGCGATAAGTAATGTGAATTGCAGAATTCAGTGAATCATCGAAT
CTTTGAACGCATCTTGCGCTCCTTGGTATTCCGAGGAGCATGCCTGTTTGAGTGTCATTA
AATTCTCAACTCTCTTATACTTTTTTGTAAAAGAGAGCTTGGACTGTGGAGGCTTGCTGG
CCACTTTTTGGGGTCAGCTCCTCTGAAATGCATTAGCGGAACCGTTTGCAATCTGCCACA
AGTGTGATAAGTTATCTACACTGGCGAGGGGATTGCTCTCTGTAATGTTCAGCTTCTAAT
TGTCTCTACTTTGTGAGACAACTTTTGAATGCTTGACCTCAAATCAGGTAGGACTACCC-
GCTGAACTTAA
>BC2-18
TTTCCGTAGGTGAACCTGCGGAAGGATCATTATTGAATTATGTTTCTAGATAGGTTGTAG
CTGGCTCTTTAGAGCATGTGCACGCCTGTTTGGACTTCATTTTCATCCACCTGTGCACCT
ATTGTAGTCTTTGGTTGGGTTAGGGGGAAGTGGTCATTGTGTCAGCATCTGCTGGATGTG
AGGACTTGCATTGTGAAAGCTTTGCTGTCCTTGATGTGATCATGGAATCTCTTTCTCACT
AGAGTCTATGTCACTCATTATACTCTGTCGAATGTCATTGAATGTCTTTACATGGGCTTG
TATGCCTATGAAAATTGTAATACAACTTTCAGCAACGGATCTCTTGGCTCTCGCATCGAT
GAAGGACGCAGCGAAATGCGATAAGTAATGTGAATTGCAGAATTCAGTGAATCATCGAAT
CTTTGAACGCATCTTGCGCTCCTTGGTATTCCGAGGAGCATGCCTGTTTGAGTGTCATTA
AATTCTCAACTCTCTTATACTTTTTTGTAAAAGAGAGCTTGGACTGTGGAGGCTTGCTGG

CCACTTTTTGGGGTCAGCTCCTCTGAAATGCATTAGCGGAACCGTTTGCAATCTGCCACA
AGTGTGATAAGTTATCTACACTGGCGAGGGGATTGCTCTCTGTAATGTTCAGCTTCTAAT
TGTCTCTACTTTGTGAGACAACTTTTGAATGCTTGACCTCAAATCAGGTAGGACTACCC-
GCTGAACTTAA
>BC2-42
TTTCCGTAGGTGAACCTGCGGAAGGATCATTATTGAATTATGTTTCTAGATAGGTTGTAG
CTGGCTCTTTAGAGCATGTGCACGCCTGTTTGGACTTCATTTTCATCCACCTGTGCACCT
ATTGTAGTCTTTGGTTGGGTTAGGGGGAAGTGGTCATTGTGTCAGCATCTGCTGGATGTG
AGGACTTGCATTGTGAAAGCTTTGCTGTCCTTGATGTGATCATGGAATCTCTTTCTCACT
AGAGTCTATGTCACTCATTATACTCTGTCGAATGTCATTGAATGTCTTTACATGGGCTTG
TATGCCTATGAAAATTGTAATACAACTTTCAGCAACGGATCTCTTGGCTCTCGCATCGAT
GAAGGACGCAGCGAAATGCGATAAGTAATGTGAATTGCAGAATTCAGTGAATCATCGAAT
CTTTGAACGCATCTTGCGCTCCTTGGTATTCCGAGGAGCATGCCTGTTTGAGTGTCATTA
AATTCTCAACTCTCTTATACTTTTTTGTAAAAGAGAGCTTGGACTGTGGAGGCTTGCTGG
CCACTTTTTGGGGTCAGCTCCTCTGAAATGCATTAGCGGAACCGTTTGCAATCTGCCACA
AGTGTGATAAGTTATCTACACTGGCGAGGGGATTGCTCTCTGTAATGTTCAGCTTCTAAT
TGTCTCTACTTTGTGAGACAACTTTTGAATGCTTGACCTCAAATCAGGTAGGACTACCC-
GCTGAACTTAA
>BC3-19
TTTCCGTAGGTGAACCTGCGGAAGGATCATTATTGAATTATGTTTCTAGATAGGTTGTAG
CTGGCTCTTTAGAGCATGTGCACGCCTGTTTGGACTTCATTTTCATCCACCTGTGCACCT
ATTGTAGTCTTTGGTTGGGTTAGGGGGAAGTGGTCATTGTGTCAGCATCTGCTGGATGTG
AGGACTTGCATTGTGAAAGCTTTGCTGTCCTTGATGTGATCATGGAATCTCTTTCTCACT
AGAGTCTATGTCACTCATTATACTCTGTCGAATGTCATTGAATGTCTTTACATGGGCTTG
TATGCCTATGAAAATTGTAATACAACTTTCAGCAACGGATCTCTTGGCTCTCGCATCGAT
GAAGGACGCAGCGAAATGCGATAAGTAATGTGAATTGCAGAATTCAGTGAATCATCGAAT
CTTTGAACGCATCTTGCGCTCCTTGGTATTCCGAGGAGCATGCCTGTTTGAGTGTCATTA
AATTCTCAACTCTCTTATACTTTTTTGTAAAAGAGAGCTTGGACTGTGGAGGCTTGCTGG
CCACTTTTTGGGGTCAGCTCCTCTGAAATGCATTAGCGGAACCGTTTGCAATCTGCCACA
AGTGTGATAAGTTATCTACACTGGCGAGGGGATTGCTCTCTGTAATGTTCAGCTTCTAAT
TGTCTCTACTTTGTGAGACAACTTTTGAATGCTTGACCTCAAATCAGGTAGGACTACCC-
GCTGAACTTAA
>BC4-20
TTTCCGTAGGTGAACCTGCGGAAGGATCATTATTGAATTATGTTTCTAGATAGGTTGTAG
CTGGCTCTTTAGAGCATGTGCACGCCTGTTTGGACTTCATTTTCATCCACCTGTGCACCT
ATTGTAGTCTTTGGTTGGGTTAGGGGGAAGTGGTCATTGTGTCAGCATCTGCTGGATGTG
AGGACTTGCATTGTGAAAGCTTTGCTGTCCTTGATGTGATCATGGAATCTCTTTCTCACT
AGAGTCTATGTCACTCATTATACTCTGTCGAATGTCATTGAATGTCTTTACATGGGCTTG
TATGCCTATGAAAATTGTAATACAACTTTCAGCAACGGATCTCTTGGCTCTCGCATCGAT
GAAGGACGCAGCGAAATGCGATAAGTAATGTGAATTGCAGAATTCAGTGAATCATCGAAT
CTTTGAACGCATCTTGCGCTCCTTGGTATTCCGAGGAGCATGCCTGTTTGAGTGTCATTA
AATTCTCAACTCTCTTATACTTTTTTGTAAAAGAGAGCTTGGACTGTGGAGGCTTGCTGG
CCACTTTTTGGGGTCAGCTCCTCTGAAATGCATTAGCGGAACCGTTTGCAATCTGCCACA
AGTGTGATAAGTTATCTACACTGGCGAGGGGATTGCTCTCTGTAATGTTCAGCTTCTAAT
TGTCTCTACTTTGTGAGACAACTTTTGAATGCTTGACCTCAAATCAGGTAGGACTACCC-
GCTGAACTTAA
>BC5-31
TTTCCGTAGGTGAACCTGCGGAAGGATCATTATTGAATTATGTTTCTAGATAGGTTGTAG
CTGGCTCTTTAGAGCATGTGCACGCCTGTTTGGACTTCATTTTCATCCACCTGTGCACCT
ATTGTAGTCTTTGGTTGGGTTAGGGGGAAGTGGTCATTGTGTCAGCATCTGCTGGATGTG

AGGACTTGCATTGTGAAAGCTTTGCTGTCCTTGATGTGATCATGGAATCTCTTTCTCACT
AGAGTCTATGTCACTCATTATACTCTGTCGAATGTCATTGAATGTCTTTACATGGGCTTG
TATGCCTATGAAAATTGTAATACAACTTTCAGCAACGGATCTCTTGGCTCTCGCATCGAT
GAAGGACGCAGCGAAATGCGATAAGTAATGTGAATTGCAGAATTCAGTGAATCATCGAAT
CTTTGAACGCATCTTGCGCTCCTTGGTATTCCGAGGAGCATGCCTGTTTGAGTGTCATTA
AATTCTCAACTCTCTTATACTTTTTTGTAAAAGAGAGCTTGGACTGTGGAGGCTTGCTGG
CCACTTTTTGGGGTCAGCTCCTCTGAAATGCATTAGCGGAACCGTTTGCAATCTGCCACA
AGTGTGATAAGTTATCTACACTGGCGAGGGGATTGCTCTCTGTAATGTTCAGCTTCTAAT
TGTCTCTACTTTGTGAGACAACTTTTGAATGCTTGACCTCAAATCAGGTAGGACTACCC-
GCTGAACTTAA
>BC5-105
TTTCCGTAGGTGAACCTGCGGAAGGATCATTATTGAATTATGTTTCTAGATAGGTTGTAG
CTGGCTCTTTAGAGCATGTGCACGCCTGTTTGGACTTCATTTTCATCCACCTGTGCACCT
ATTGTAGTCTTTGGTTGGGTTAGGGGGAAGTGGTCATTGTGTCAGCATCTGCTGGATGTG
AGGACTTGCATTGTGAAAGCTTTGCTGTCCTTGATGTGATCATGGAATCTCTTTCTCACT
AGAGTCTATGTCACTCATTATACTCTGTCGAATGTCATTGAATGTCTTTACATGGGCTTG
TATGCCTATGAAAATTGTAATACAACTTTCAGCAACGGATCTCTTGGCTCTCGCATCGAT
GAAGGACGCAGCGAAATGCGATAAGTAATGTGAATTGCAGAATTCAGTGAATCATCGAAT
CTTTGAACGCATCTTGCGCTCCTTGGTATTCCGAGGAGCATGCCTGTTTGAGTGTCATTA
AATTCTCAACTCTCTTATACTTTTTTGTAAAAGAGAGCTTGGACTGTGGAGGCTTGCTGG
CCACTTTTTGGGGTCAGCTCCTCTGAAATGCATTAGCGGAACCGTTTGCAATCTGCCACA
AGTGTGATAAGTTATCTACACTGGCGAGGGGATTGCTCTCTGTAATGTTCAGCTTCTAAT
TGTCTCTACTTTGTGAGACAACTTTTGAATGCTTGACCTCAAATCAGGTAGGACTACCC-
GCTGAACTTAA
>BC6-15
TTTCCGTAGGTGAACCTGCGGAAGGATCATTATTGAATTATGTTTCTAGATAGGTTGTAG
CTGGCTCTTTAGAGCATGTGCACGCCTGTTTGGACTTCATTTTCATCCACCTGTGCACCT
ATTGTAGTCTTTGGTTGGGTTAGGGGGAAGTGGTCATTGTGTCAGCATCTGCTGGATGTG
AGGACTTGCATTGTGAAAGCTTTGCTGTCCTTGATGTGATCATGGAATCTCTTTCTCACT
AGAGTCTATGTCACTCATTATACTCTGTCGAATGTCATTGAATGTCTTTACATGGGCTTG
TATGCCTATGAAAATTGTAATACAACTTTCAGCAACGGATCTCTTGGCTCTCGCATCGAT
GAAGGACGCAGCGAAATGCGATAAGTAATGTGAATTGCAGAATTCAGTGAATCATCGAAT
CTTTGAACGCATCTTGCGCTCCTTGGTATTCCGAGGAGCATGCCTGTTTGAGTGTCATTA
AATTCTCAACTCTCTTATACTTTTTTGTAAAAGAGAGCTTGGACTGTGGAGGCTTGCTGG
CCACTTTTTGGGGTCAGCTCCTCTGAAATGCATTAGCGGAACCGTTTGCAATCTGCCACA
AGTGTGATAAGTTATCTACACTGGCGAGGGGATTGCTCTCTGTAATGTTCAGCTTCTAAT
TGTCTCTACTTTGTGAGACAACTTTTGAATGCTTGACCTCAAATCAGGTAGGACTACCC-
GCTGAACTTAA
>BC11_23
TTTCCGTAGGTGAACCTGCGGAAGGATCATTATTGAATTATGTTTCTAGATAGGTTGTAG
CTGGCTCTTTAGAGCATGTGCACGCCTGTTTGGACTTCATTTTCATCCACCTGTGCACCT
ATTGTAGTCTTTGGTTGGGTTAGGGGGAAGTGGTCATTGTGTCAGCATCTGCTGGATGTG
AGGACTTGCATTGTGAAAGCTTTGCTGTCCTTGATGTGATCATGGAATCTCTTTCTCACT
AGAGTCTATGTCACTCATTATACTCTGTCGAATGTCATTGAATGTCTTTACATGGGCTTG
TATGCCTATGAAAATTGTAATACAACTTTCAGCAACGGATCTCTTGGCTCTCGCATCGAT
GAAGGACGCAGCGAAATGCGATAAGTAATGTGAATTGCAGAATTCAGTGAATCATCGAAT
CTTTGAACGCATCTTGCGCTCCTTGGTATTCCGAGGAGCATGCCTGTTTGAGTGTCATTA
AATTCTCAACTCTCTTATACTTTTTTGTAAAAGAGAGCTTGGACTGTGGAGGCTTGCTGG
CCACTTTTTGGGGTCAGCTCCTCTGAAATGCATTAGCGGAACCGTTTGCAATCTGCCACA
AGTGTGATAAGTTATCTACACTGGCGAGGGGATTGCTCTCTGTAATGTTCAGCTTCTAAT

TGTCTCTACTTTGTGAGACAACTTTTGAATGCTTGACCTCAAATCAGGTAGGACTACCC-
GCTGAACTTAA
>BC1-24
TTTCCGTAGGTGAACCTGCGGAAGGATCATTATTGAATTATGTTTCTAGATAGGTTGTAG
CTGGCTCTTTAGAGCATGTGCACGCCTGTTTGGACTTCATTTTCATCCACCTGTGCACCT
ATTGTAGTCTTTGGTTGGGTTAGGGGGAAGTGGTCATTGTGTCAGCATCTGCTGGATGTG
AGGACTTGCATTGTGAAAGCTTTGCTGTCCTTGATGTGATCATGGAATCTCTTTCTCACT
AGAGTCTATGTCACTCATTATACTCTGTCGAATGTCATTGAATGTCTTTACATGGGCTTG
TATGCCTATGAAAATTGTAATACAACTTTCAGCAACGGATCTCTTGGCTCTCGCATCGAT
GAAGGACGCAGCGAAATGCGATAAGTAATGTGAATTGCAGAATTCAGTGAATCATCGAAT
CTTTGAACGCATCTTGCGCTCCTTGGTATTCCGAGGAGCATGCCTGTTTGAGTGTCATTA
AATTCTCAACTCTCTTATACTTTTTTGTAAAAGAGAGCTTGGACTGTGGAGGCTTGCTGG
CCACTTTTTGGGGTCAGCTCCTCTGAAATGCATTAGCGGAACCGTTTGCAATCTGCCACA
AGTGTGATAAGTTATCTACACTGGCGAGGGGATTGCTCTCTGTAATGTTCAGCTTCTAAT
TGTCTCTACTTTGTGAGACAACTTTTGAATGCTTGACCTCAAATCAGGTAGGACTACCC-
GCTGAACTTAA
>BC9-22
TTTCCGTAGGTGAACCTGCGGAAGGATCATTATTGAATTATGTTTCTAGATAGGTTGTAG
CTGGCTCTTTAGAGCATGTGCACGCCTGTTTGGACTTCATTTTCATCCACCTGTGCACCT
ATTGTAGTCTTTGGTTGGGTTAGGGGGAAGTGGTCATTGTGTCAGCATCTGCTGGATGTG
AGGACTTGCATTGTGAAAGCTTTGCTGTCCTTGATGTGATCATGGAATCTCTTTCTCACT
AGAGTCTATGTCACTCATTATACTCTGTCGAATGTCATTGAATGTCTTTACATGGGCTTG
TATGCCTATGAAAATTGTAATACAACTTTCAGCAACGGATCTCTTGGCTCTCGCATCGAT
GAAGGACGCAGCGAAATGCGATAAGTAATGTGAATTGCAGAATTCAGTGAATCATCGAAT
CTTTGAACGCATCTTGCGCTCCTTGGTATTCCGAGGAGCATGCCTGTTTGAGTGTCATTA
AATTCTCAACTCTCTTATACTTTTTTGTAAAAGAGAGCTTGGACTGTGGAGGCTTGCTGG
CCACTTTTTGGGGTCAGCTCCTCTGAAATGCATTAGCGGAACCGTTTGCAATCTGCCACA
AGTGTGATAAGTTATCTACACTGGCGAGGGGATTGCTCTCTGTAATGTTCAGCTTCTAAT
TGTCTCTACTTTGTGAGACAACTTTTGAATGCTTGACCTCAAATCAGGTAGGACTACCC-
GCTGAACTTAA
>BC9-56
TTTCCGTAGGTGAACCTGCGGAAGGATCATTATTGAATTATGTTTCTAGATAGGTTGTAG
CTGGCTCTTTAGAGCATGTGCACGCCTGTTTGGACTTCATTTTCATCCACCTGTGCACCT
ATTGTAGTCTTTGGTTGGGTTAGGGGGAAGTGGTCATTGTGTCAGCATCTGCTGGATGTG
AGGACTTGCATTGTGAAAGCTTTGCTGTCCTTGATGTGATCATGGAATCTCTTTCTCACT
AGAGTCTATGTCACTCATTATACTCTGTCGAATGTCATTGAATGTCTTTACATGGGCTTG
TATGCCTATGAAAATTGTAATACAACTTTCAGCAACGGATCTCTTGGCTCTCGCATCGAT
GAAGGACGCAGCGAAATGCGATAAGTAATGTGAATTGCAGAATTCAGTGAATCATCGAAT
CTTTGAACGCATCTTGCGCTCCTTGGTATTCCGAGGAGCATGCCTGTTTGAGTGTCATTA
AATTCTCAACTCTCTTATACTTTTTTGTAAAAGAGAGCTTGGACTGTGGAGGCTTGCTGG
CCACTTTTTGGGGTCAGCTCCTCTGAAATGCATTAGCGGAACCGTTTGCAATCTGCCACA
AGTGTGATAAGTTATCTACACTGGCGAGGGGATTGCTCTCTGTAATGTTCAGCTTCTAAT
TGTCTCTACTTTGTGAGACAACTTTTGAATGCTTGACCTCAAATCAGGTAGGACTACCC-
GCTGAACTTAA
>BC3-24
TTTCCGTAGGTGAACCTGCGGAAGGATCATTATTGAATTATGTTTCTAGATAGGTTGTAG
CTGGCTCTTTAGAGCATGTGCACGCCTGTTTGGACTTCATTTTCATCCACCTGTGCACCT
ATTGTAGTCTTTGGTTGGGTTAGGGGGAAGTGGTCATTGTGTCAGCATCTGCTGGATGTG
AGGACTTGCATTGTGAAAGCTTTGCTGTCCTTGATGTGATCATGGAATCTCTTTCTCACT
AGAGTCTATGTCACTCATTATACTCTGTCGAATGTCATTGAATGTCTTTACATGGGCTTG

TATGCCTATGAAAATTGTAATACAACTTTCAGCAACGGATCTCTTGGCTCTCGCATCGAT
GAAGGACGCAGCGAAATGCGATAAGTAATGTGAATTGCAGAATTCAGTGAATCATCGAAT
CTTTGAACGCATCTTGCGCTCCTTGGTATTCCGAGGAGCATGCCTGTTTGAGTGTCATTA
AATTCTCAACTCTCTTATACTTTTTTGTAAAAGAGAGCTTGGACTGTGGAGGCTTGCTGG
CCACTTTTTGGGGTCAGCTCCTCTGAAATGCATTAGCGGAACCGTTTGCAATCTGCCACA
AGTGTGATAAGTTATCTACACTGGCGAGGGGATTGCTCTCTGTAATGTTCAGCTTCTAAT
TGTCTCTACTTTGTGAGACAACTTTTGAATGCTTGACCTCAAATCAGGTAGGACTACCC-
GCTGAACTTAA
>BC5-45
TTTCCGTAGGTGAACCTGCGGAAGGATCATTATTGAATTATGTTTCTAGATAGGTTGTAG
CTGGCTCTTTAGAGCATGTGCACGCCTGTTTGGACTTCATTTTCATCCACCTGTGCACCT
ATTGTAGTCTTTGGTTGGGTTAGGGGGAAGTGGTCATTGTGTCAGCATCTGCTGGATGTG
AGGACTTGCATTGTGAAAGCTTTGCTGTCCTTGATGTGATCATGGAATCTCTTTCTCACT
AGAGTCTATGTCACTCATTATACTCTGTCGAATGTCATTGAATGTCTTTACATGGGCTTG
TATGCCTATGAAAATTGTAATACAACTTTCAGCAACGGATCTCTTGGCTCTCGCATCGAT
GAAGGACGCAGCGAAATGCGATAAGTAATGTGAATTGCAGAATTCAGTGAATCATCGAAT
CTTTGAACGCATCTTGCGCTCCTTGGTATTCCGAGGAGCATGCCTGTTTGAGTGTCATTA
AATTCTCAACTCTCTTATACTTTTTTGTAAAAGAGAGCTTGGACTGTGGAGGCTTGCTGG
CCACTTTTTGGGGTCAGCTCCTCTGAAATGCATTAGCGGAACCGTTTGCAATCTGCCACA
AGTGTGATAAGTTATCTACACTGGCGAGGGGATTGCTCTCTGTAATGTTCAGCTTCTAAT
TGTCTCTACTTTGTGAGACAACTTTTGAATGCTTGACCTCAAATCAGGTAGGACTACCC-
GCTGAACTTAA
>BC9-29
TTTCCGTAGGTGAACCTGCGGAAGGATCATTATTGAATTATGTTTCTAGATAGGTTGTAG
CTGGCTCTTTAGAGCATGTGCACGCCTGTTTGGACTTCATTTTCATCCACCTGTGCACCT
ATTGTAGTCTTTGGTTGGGTTAGGGGGAAGTGGTCATTGTGTCAGCATCTGCTGGATGTG
AGGACTTGCATTGTGAAAGCTTTGCTGTCCTTGATGTGATCATGGAATCTCTTTCTCACT
AGAGTCTATGTCACTCATTATACTCTGTCGAATGTCATTGAATGTCTTTACATGGGCTTG
TATGCCTATGAAAATTGTAATACAACTTTCAGCAACGGATCTCTTGGCTCTCGCATCGAT
GAAGGACGCAGCGAAATGCGATAAGTAATGTGAATTGCAGAATTCAGTGAATCATCGAAT
CTTTGAACGCATCTTGCGCTCCTTGGTATTCCGAGGAGCATGCCTGTTTGAGTGTCATTA
AATTCTCAACTCTCTTATACTTTTTTGTAAAAGAGAGCTTGGACTGTGGAGGCTTGCTGG
CCACTTTTTGGGGTCAGCTCCTCTGAAATGCATTAGCGGAACCGTTTGCAATCTGCCACA
AGTGTGATAAGTTATCTACACTGGCGAGGGGATTGCTCTCTGTAATGTTCAGCTTCTAAT
TGTCTCTACTTTGTGAGACAACTTTTGAATGCTTGACCTCAAATCAGGTAGGACTACCC-
GCTGAACTTAA
>BC6-33
TTTCCGTAGGTGAACCTGCGGAAGGATCATTATTGAATTATGTTTCTAGATAGGTTGTAG
CTGGCTCTTTAGAGCATGTGCACGCCTGTTTGGACTTCATTTTCATCCACCTGTGCACCT
ATTGTAGTCTTTGGTTGGGTTAGGGGGAAGTGGTCATTGTGTCAGCATCTGCTGGATGTG
AGGACTTGCATTGTGAAAGCTTTGCTGTCCTTGATGTGATCATGGAATCTCTTTCTCACT
AGAGTCTATGTCACTCATTATACTCTGTCGAATGTCATTGAATGTCTTTACATGGGCTTG
TATGCCTATGAAAATTGTAATACAACTTTCAGCAACGGATCTCTTGGCTCTCGCATCGAT
GAAGGACGCAGCGAAATGCGATAAGTAATGTGAATTGCAGAATTCAGTGAATCATCGAAT
CTTTGAACGCATCTTGCGCTCCTTGGTATTCCGAGGAGCATGCCTGTTTGAGTGTCATTA
AATTCTCAACTCTCTTATACTTTTTTGTAAAAGAGAGCTTGGACTGTGGAGGCTTGCTGG
CCACTTTTTGGGGTCAGCTCCTCTGAAATGCATTAGCGGAACCGTTTGCAATCTGCCACA
AGTGTGATAAGTTATCTACACTGGCGAGGGGATTGCTCTCTGTAATGTTCAGCTTCTAAT
TGTCTCTACTTTGTGAGACAACTTTTGAATGCTTGACCTCAAATCAGGTAGGACTACCC-
GCTGAACTTAA

>BC10_24
TTTCCGTAGGTGAACCTGCGGAAGGATCATTATTGAATTATGTTTCTAGATAGGTTGTAG
CTGGCTCTTTAGAGCATGTGCACGCCTGTTTGGACTTCATTTTCATCCACCTGTGCACCT
ATTGTAGTCTTTGGTTGGGTTAGGGGGAAGTGGTCATTGTGTCAGCATCTGCTGGATGTG
AGGACTTGCATTGTGAAAGCTTTGCTGTCCTTGATGTGATCATGGAATCTCTTTCTCACT
AGAGTCTATGTCACTCATTATACTCTGTCGAATGTCATTGAATGTCTTTACATGGGCTTG
TATGCCTATGAAAATTGTAATACAACTTTCAGCAACGGATCTCTTGGCTCTCGCATCGAT
GAAGGACGCAGCGAAATGCGATAAGTAATGTGAATTGCAGAATTCAGTGAATCATCGAAT
CTTTGAACGCATCTTGCGCTCCTTGGTATTCCGAGGAGCATGCCTGTTTGAGTGTCATTA
AATTCTCAACTCTCTTATACTTTTTTGTAAAAGAGAGCTTGGACTGTGGAGGCTTGCTGG
CCACTTTTTGGGGTCAGCTCCTCTGAAATGCATTAGCGGAACCGTTTGCAATCTGCCACA
AGTGTGATAAGTTATCTACACTGGCGAGGGGATTGCTCTCTGTAATGTTCAGCTTCTAAT
TGTCTCTACTTTGTGAGACAACTTTTGAATGCTTGACCTCAAATCAGGTAGGACTACCC-
GCTGAACTTAA
>BC11_45
TTTCCGTAGGTGAACCTGCGGAAGGATCATTATTGAATTATGTTTCTAGATAGGTTGTAG
CTGGCTCTTTAGAGCATGTGCACGCCTGTTTGGACTTCATTTTCATCCACCTGTGCACCT
ATTGTAGTCTTTGGTTGGGTTAGGGGGAAGTGGTCATTGTGTCAGCATCTGCTGGATGTG
AGGACTTGCATTGTGAAAGCTTTGCTGTCCTTGATGTGATCATGGAATCTCTTTCTCACT
AGAGTCTATGTCACTCATTATACTCTGTCGAATGTCATTGAATGTCTTTACATGGGCTTG
TATGCCTATGAAAATTGTAATACAACTTTCAGCAACGGATCTCTTGGCTCTCGCATCGAT
GAAGGACGCAGCGAAATGCGATAAGTAATGTGAATTGCAGAATTCAGTGAATCATCGAAT
CTTTGAACGCATCTTGCGCTCCTTGGTATTCCGAGGAGCATGCCTGTTTGAGTGTCATTA
AATTCTCAACTCTCTTATACTTTTTTGTAAAAGAGAGCTTGGACTGTGGAGGCTTGCTGG
CCACTTTTTGGGGTCAGCTCCTCTGAAATGCATTAGCGGAACCGTTTGCAATCTGCCACA
AGTGTGATAAGTTATCTACACTGGCGAGGGGATTGCTCTCTGTAATGTTCAGCTTCTAAT
TGTCTCTACTTTGTGAGACAACTTTTGAATGCTTGACCTCAAATCAGGTAGGACTACCC-
GCTGAACTTAA
>BC4-36
TTTCCGTAGGTGAACCTGCGGAAGGATCATTATTGAATTATGTTTCTAGATAGGTTGTAG
CTGGCTCTTTAGAGCATGTGCACGCCTGTTTGGACTTCATTTTCATCCACCTGTGCACCT
ATTGTAGTCTTTGGTTGGGTTAGGGGGAAGTGGTCATTGTGTCAGCATCTGCTGGATGTG
AGGACTTGCATTGTGAAAGCTTTGCTGTCCTTGATGTGATCATGGAATCTCTTTCTCACT
AGAGTCTATGTCACTCATTATACTCTGTCGAATGTCATTGAATGTCTTTACATGGGCTTG
TATGCCTATGAAAATTGTAATACAACTTTCAGCAACGGATCTCTTGGCTCTCGCATCGAT
GAAGGACGCAGCGAAATGCGATAAGTAATGTGAATTGCAGAATTCAGTGAATCATCGAAT
CTTTGAACGCATCTTGCGCTCCTTGGTATTCCGAGGAGCATGCCTGTTTGAGTGTCATTA
AATTCTCAACTCTCTTATACTTTTTTGTAAAAGAGAGCTTGGACTGTGGAGGCTTGCTGG
CCACTTTTTGGGGTCAGCTCCTCTGAAATGCATTAGCGGAACCGTTTGCAATCTGCCACA
AGTGTGATAAGTTATCTACACTGGCGAGGGGATTGCTCTCTGTAATGTTCAGCTTCTAAT
TGTCTCTACTTTGTGAGACAACTTTTGAATGCTTGACCTCAAATCAGGTAGGACTACCC-
GCTGAACTTAA
>BC10_31
TTTCCGTAGGTGAACCTGCGGAAGGATCATTATTGAATTATGTTTCTAGATAGGTTGTAG
CTGGCTCTTTAGAGCATGTGCACGCCTGTTTGGACTTCATTTTCATCCACCTGTGCACCT
ATTGTAGTCTTTGGTTGGGTTAGGGGGAAGTGGTCATTGTGTCAGCATCTGCTGGATGTG
AGGACTTGCATTGTGAAAGCTTTGCTGTCCTTGATGTGATCATGGAATCTCTTTCTCACT
AGAGTCTATGTCACTCATTATACTCTGTCGAATGTCATTGAATGTCTTTACATGGGCTTG
TATGCCTATGAAAATTGTAATACAACTTTCAGCAACGGATCTCTTGGCTCTCGCATCGAT
GAAGGACGCAGCGAAATGCGATAAGTAATGTGAATTGCAGAATTCAGTGAATCATCGAAT

CTTTGAACGCATCTTGCGCTCCTTGGTATTCCGAGGAGCATGCCTGTTTGAGTGTCATTA
AATTCTCAACTCTCTTATACTTTTTTGTAAAAGAGAGCTTGGACTGTGGAGGCTTGCTGG
CCACTTTTTGGGGTCAGCTCCTCTGAAATGCATTAGCGGAACCGTTTGCAATCTGCCACA
AGTGTGATAAGTTATCTACACTGGCGAGGGGATTGCTCTCTGTAATGTTCAGCTTCTAAT
TGTCTCTACTTTGTGAGACAACTTTTGAATGCTTGACCTCAAATCAGGTAGGACTACCC-
GCTGAACTTAA
>BC3-3
TTTCCGTAGGTGAACCTGCGGAAGGATCATTATTGAATTATGTTTCTAGATAGGTTGTAG
CTGGCTCTTTAGAGCATGTGCACGCCTGTTTGGACTTCATTTTCATCCACCTGTGCACCT
ATTGTAGTCTTTGGTTGGGTTAGGGGGAAGTGGTCATTGTGTCAGCATCTGCTGGATGTG
AGGACTTGCATTGTGAAAGCTTTGCTGTCCTTGATGTGATCATGGAATCTCTTTCTCACT
AGAGTCTATGTCACTCATTATACTCTGTCGAATGTCATTGAATGTCTTTACATGGGCTTG
TATGCCTATGAAAATTGTAATACAACTTTCAGCAACGGATCTCTTGGCTCTCGCATCGAT
GAAGGACGCAGCGAAATGCGATAAGTAATGTGAATTGCAGAATTCAGTGAATCATCGAAT
CTTTGAACGCATCTTGCGCTCCTTGGTATTCCGAGGAGCATGCCTGTTTGAGTGTCATTA
AATTCTCAACTCTCTTATACTTTTTTGTAAAAGAGAGCTTGGACTGTGGAGGCTTGCTGG
CCACTTTTTGGGGTCAGCTCCTCTGAAATGCATTAGCGGAACCGTTTGCAATCTGCCACA
AGTGTGATAAGTTATCTACACTGGCGAGGGGATTGCTCTCTGTAATGTTCAGCTTCTAAT
TGTCTCTACTTTGTGAGACAACTTTTGAATGCTTGACCTCAAATCAGGTAGGACTACCC-
GCTGAACTTAA
>BC5-96
TTTCCGTAGGTGAACCTGCGGAAGGATCATTATTGAATTATGTTTCTAGATAGGTTGTAG
CTGGCTCTTTAGAGCATGTGCACGCCTGTTTGGACTTCATTTTCATCCACCTGTGCACCT
ATTGTAGTCTTTGGTTGGGTTAGGGGGAAGTGGTCATTGTGTCAGCATCTGCTGGATGTG
AGGACTTGCATTGTGAAAGCTTTGCTGTCCTTGATGTGATCATGGAATCTCTTTCTCACT
AGAGTCTATGTCACTCATTATACTCTGTCGAATGTCATTGAATGTCTTTACATGGGCTTG
TATGCCTATGAAAATTGTAATACAACTTTCAGCAACGGATCTCTTGGCTCTCGCATCGAT
GAAGGACGCAGCGAAATGCGATAAGTAATGTGAATTGCAGAATTCAGTGAATCATCGAAT
CTTTGAACGCATCTTGCGCTCCTTGGTATTCCGAGGAGCATGCCTGTTTGAGTGTCATTA
AATTCTCAACTCTCTTATACTTTTTTGTAAAAGAGAGCTTGGACTGTGGAGGCTTGCTGG
CCACTTTTTGGGGTCAGCTCCTCTGAAATGCATTAGCGGAACCGTTTGCAATCTGCCACA
AGTGTGATAAGTTATCTACACTGGCGAGGGGATTGCTCTCTGTAATGTTCAGCTTCTAAT
TGTCTCTACTTTGTGAGACAACTTTTGAATGCTTGACCTCAAATCAGGTAGGACTACCC-
GCTGAACTTAA
>BC6-23
TTTCCGTAGGTGAACCTGCGGAAGGATCATTATTGAATTATGTTTCTAGATAGGTTGTAG
CTGGCTCTTTAGAGCATGTGCACGCCTGTTTGGACTTCATTTTCATCCACCTGTGCACCT
ATTGTAGTCTTTGGTTGGGTTAGGGGGAAGTGGTCATTGTGTCAGCATCTGCTGGATGTG
AGGACTTGCATTGTGAAAGCTTTGCTGTCCTTGATGTGATCATGGAATCTCTTTCTCACT
AGAGTCTATGTCACTCATTATACTCTGTCGAATGTCATTGAATGTCTTTACATGGGCTTG
TATGCCTATGAAAATTGTAATACAACTTTCAGCAACGGATCTCTTGGCTCTCGCATCGAT
GAAGGACGCAGCGAAATGCGATAAGTAATGTGAATTGCAGAATTCAGTGAATCATCGAAT
CTTTGAACGCATCTTGCGCTCCTTGGTATTCCGAGGAGCATGCCTGTTTGAGTGTCATTA
AATTCTCAACTCTCTTATACTTTTTTGTAAAAGAGAGCTTGGACTGTGGAGGCTTGCTGG
CCACTTTTTGGGGTCAGCTCCTCTGAAATGCATTAGCGGAACCGTTTGCAATCTGCCACA
AGTGTGATAAGTTATCTACACTGGCGAGGGGATTGCTCTCTGTAATGTTCAGCTTCTAAT
TGTCTCTACTTTGTGAGACAACTTTTGAATGCTTGACCTCAAATCAGGTAGGACTACCC-
GCTGAACTTAA
>BC12_3
TTTCCGTAGGTGAACCTGCGGAAGGATCATTATTGAATTATGTTTCTAGATAGGTTGTAG

CTGGCTCTTTAGAGCATGTGCACGCCTGTTTGGACTTCATTTTCATCCACCTGTGCACCT
ATTGTAGTCTTTGGTTGGGTTAGGGGGAAGTGGTCATTGTGTCAGCATCTGCTGGATGTG
AGGACTTGCATTGTGAAAGCTTTGCTGTCCTTGATGTGATCATGGAATCTCTTTCTCACT
AGAGTCTATGTCACTCATTATACTCTGTCGAATGTCATTGAATGTCTTTACATGGGCTTG
TATGCCTATGAAAATTGTAATACAACTTTCAGCAACGGATCTCTTGGCTCTCGCATCGAT
GAAGGACGCAGCGAAATGCGATAAGTAATGTGAATTGCAGAATTCAGTGAATCATCGAAT
CTTTGAACGCATCTTGCGCTCCTTGGTATTCCGAGGAGCATGCCTGTTTGAGTGTCATTA
AATTCTCAACTCTCTTATACTTTTTTGTAAAAGAGAGCTTGGACTGTGGAGGCTTGCTGG
CCACTTTTTGGGGTCAGCTCCTCTGAAATGCATTAGCGGAACCGTTTGCAATCTGCCACA
AGTGTGATAAGTTATCTACACTGGCGAGGGGATTGCTCTCTGTAATGTTCAGCTTCTAAT
TGTCTCTACTTTGTGAGACAACTTTTGAATGCTTGACCTCAAATCAGGTAGGACTACCC-
GCTGAACTTAA
>BC6-19
TTTCCGTAGGTGAACCTGCGGAAGGATCATTATTGAATTATGTTTCTAGATAGGTTGTAG
CTGGCTCTTTAGAGCATGTGCACGCCTGTTTGGACTTCATTTTCATCCACCTGTGCACCT
ATTGTAGTCTTTGGTTGGGTTAGGGGGAAGTGGTCATTGTGTCAGCATCTGCTGGATGTG
AGGACTTGCATTGTGAAAGCTTTGCTGTCCTTGATGTGATCATGGAATCTCTTTCTCACT
AGAGTCTATGTCACTCATTATACTCTGTCGAATGTCATTGAATGTCTTTACATGGGCTTG
TATGCCTATGAAAATTGTAATACAACTTTCAGCAACGGATCTCTTGGCTCTCGCATCGAT
GAAGGACGCAGCGAAATGCGATAAGTAATGTGAATTGCAGAATTCAGTGAATCATCGAAT
CTTTGAACGCATCTTGCGCTCCTTGGTATTCCGAGGAGCATGCCTGTTTGAGTGTCATTA
AATTCTCAACTCTCTTATACTTTTTTGTAAAAGAGAGCTTGGACTGTGGAGGCTTGCTGG
CCACTTTTTGGGGTCAGCTCCTCTGAAATGCATTAGCGGAACCGTTTGCAATCTGCCACA
AGTGTGATAAGTTATCTACACTGGCGAGGGGATTGCTCTCTGTAATGTTCAGCTTCTAAT
TGTCTCTACTTTGTGAGACAACTTTTGAATGCTTGACCTCAAATCAGGTAGGACTACCC-
GCTGAACTTAA
>BC12_1
TTTCCGTAGGTGAACCTGCGGAAGGATCATTATTGAATTATGTTTCTAGATAGGTTGTAG
CTGGCTCTTTAGAGCATGTGCACGCCTGTTTGGACTTCATTTTCATCCACCTGTGCACCT
ATTGTAGTCTTTGGTTGGGTTAGGGGGAAGTGGTCATTGTGTCAGCATCTGCTGGATGTG
AGGACTTGCATTGTGAAAGCTTTGCTGTCCTTGATGTGATCATGGAATCTCTTTCTCACT
AGAGTCTATGTCACTCATTATACTCTGTCGAATGTCATTGAATGTCTTTACATGGGCTTG
TATGCCTATGAAAATTGTAATACAACTTTCAGCAACGGATCTCTTGGCTCTCGCATCGAT
GAAGGACGCAGCGAAATGCGATAAGTAATGTGAATTGCAGAATTCAGTGAATCATCGAAT
CTTTGAACGCATCTTGCGCTCCTTGGTATTCCGAGGAGCATGCCTGTTTGAGTGTCATTA
AATTCTCAACTCTCTTATACTTTTTTGTAAAAGAGAGCTTGGACTGTGGAGGCTTGCTGG
CCACTTTTTGGGGTCAGCTCCTCTGAAATGCATTAGCGGAACCGTTTGCAATCTGCCACA
AGTGTGATAAGTTATCTACACTGGCGAGGGGATTGCTCTCTGTAATGTTCAGCTTCTAAT
TGTCTCTACTTTGTGAGACAACTTTTGAATGCTTGACCTCAAATCAGGTAGGACTACCC-
GCTGAACTTAA
>BC7-50
TTTCCGTAGGTGAACCTGCGGAAGGATCATTATTGAATTATGTTTCTAGATAGGTTGTAG
CTGGCTCTTTAGAGCATGTGCACGCCTGTTTGGACTTCATTTTCATCCACCTGTGCACCT
ATTGTAGTCTTTGGTTGGGTTAGGGGGAAGTGGTCATTGTGTCAGCATCTGCTGGATGTG
AGGACTTGCATTGTGAAAGCTTTGCTGTCCTTGATGTGATCATGGAATCTCTTTCTCACT
AGAGTCTATGTCACTCATTATACTCTGTCGAATGTCATTGAATGTCTTTACATGGGCTTG
TATGCCTATGAAAATTGTAATACAACTTTCAGCAACGGATCTCTTGGCTCTCGCATCGAT
GAAGGACGCAGCGAAATGCGATAAGTAATGTGAATTGCAGAATTCAGTGAATCATCGAAT
CTTTGAACGCATCTTGCGCTCCTTGGTATTCCGAGGAGCATGCCTGTTTGAGTGTCATTA
AATTCTCAACTCTCTTATACTTTTTTGTAAAAGAGAGCTTGGACTGTGGAGGCTTGCTGG

CCACTTTTTGGGGTCAGCTCCTCTGAAATGCATTAGCGGAACCGTTTGCAATCTGCCACA
AGTGTGATAAGTTATCTACACTGGCGAGGGGATTGCTCTCTGTAATGTTCAGCTTCTAAT
TGTCTCTACTTTGTGAGACAACTTTTGAATGCTTGACCTCAAATCAGGTAGGACTACCC-
GCTGAACTTAA
>BC4-70
TTTCCGTAGGTGAACCTGCGGAAGGATCATTATTGAATTATGTTTCTAGATAGGTTGTAG
CTGGCTCTTTAGAGCATGTGCACGCCTGTTTGGACTTCATTTTCATCCACCTGTGCACCT
ATTGTAGTCTTTGGTTGGGTTAGGGGGAAGTGGTCATTGTGTCAGCATCTGCTGGATGTG
AGGACTTGCATTGTGAAAGCTTTGCTGTCCTTGATGTGATCATGGAATCTCTTTCTCACT
AGAGTCTATGTCACTCATTATACTCTGTCGAATGTCATTGAATGTCTTTACATGGGCTTG
TATGCCTATGAAAATTGTAATACAACTTTCAGCAACGGATCTCTTGGCTCTCGCATCGAT
GAAGGACGCAGCGAAATGCGATAAGTAATGTGAATTGCAGAATTCAGTGAATCATCGAAT
CTTTGAACGCATCTTGCGCTCCTTGGTATTCCGAGGAGCATGCCTGTTTGAGTGTCATTA
AATTCTCAACTCTCTTATACTTTTTTGTAAAAGAGAGCTTGGACTGTGGAGGCTTGCTGG
CCACTTTTTGGGGTCAGCTCCTCTGAAATGCATTAGCGGAACCGTTTGCAATCTGCCACA
AGTGTGATAAGTTATCTACACTGGCGAGGGGATTGCTCTCTGTAATGTTCAGCTTCTAAT
TGTCTCTACTTTGTGAGACAACTTTTGAATGCTTGACCTCAAATCAGGTAGGACTACCC-
GCTGAACTTAA
>BC5-27
TTTCCGTAGGTGAACCTGCGGAAGGATCATTATTGAATTATGTTTCTAGATAGGTTGTAG
CTGGCTCTTTAGAGCATGTGCACGCCTGTTTGGACTTCATTTTCATCCACCTGTGCACCT
ATTGTAGTCTTTGGTTGGGTTAGGGGGAAGTGGTCATTGTGTCAGCATCTGCTGGATGTG
AGGACTTGCATTGTGAAAGCTTTGCTGTCCTTGATGTGATCATGGAATCTCTTTCTCACT
AGAGTCTATGTCACTCATTATACTCTGTCGAATGTCATTGAATGTCTTTACATGGGCTTG
TATGCCTATGAAAATTGTAATACAACTTTCAGCAACGGATCTCTTGGCTCTCGCATCGAT
GAAGGACGCAGCGAAATGCGATAAGTAATGTGAATTGCAGAATTCAGTGAATCATCGAAT
CTTTGAACGCATCTTGCGCTCCTTGGTATTCCGAGGAGCATGCCTGTTTGAGTGTCATTA
AATTCTCAACTCTCTTATACTTTTTTGTAAAAGAGAGCTTGGACTGTGGAGGCTTGCTGG
CCACTTTTTGGGGTCAGCTCCTCTGAAATGCATTAGCGGAACCGTTTGCAATCTGCCACA
AGTGTGATAAGTTATCTACACTGGCGAGGGGATTGCTCTCTGTAATGTTCAGCTTCTAAT
TGTCTCTACTTTGTGAGACAACTTTTGAATGCTTGACCTCAAATCAGGTAGGACTACCC-
GCTGAACTTAA
>BC5-35
TTTCCGTAGGTGAACCTGCGGAAGGATCATTATTGAATTATGTTTCTAGATAGGTTGTAG
CTGGCTCTTTAGAGCATGTGCACGCCTGTTTGGACTTCATTTTCATCCACCTGTGCACCT
ATTGTAGTCTTTGGTTGGGTTAGGGGGAAGTGGTCATTGTGTCAGCATCTGCTGGATGTG
AGGACTTGCATTGTGAAAGCTTTGCTGTCCTTGATGTGATCATGGAATCTCTTTCTCACT
AGAGTCTATGTCACTCATTATACTCTGTCGAATGTCATTGAATGTCTTTACATGGGCTTG
TATGCCTATGAAAATTGTAATACAACTTTCAGCAACGGATCTCTTGGCTCTCGCATCGAT
GAAGGACGCAGCGAAATGCGATAAGTAATGTGAATTGCAGAATTCAGTGAATCATCGAAT
CTTTGAACGCATCTTGCGCTCCTTGGTATTCCGAGGAGCATGCCTGTTTGAGTGTCATTA
AATTCTCAACTCTCTTATACTTTTTTGTAAAAGAGAGCTTGGACTGTGGAGGCTTGCTGG
CCACTTTTTGGGGTCAGCTCCTCTGAAATGCATTAGCGGAACCGTTTGCAATCTGCCACA
AGTGTGATAAGTTATCTACACTGGCGAGGGGATTGCTCTCTGTAATGTTCAGCTTCTAAT
TGTCTCTACTTTGTGAGACAACTTTTGAATGCTTGACCTCAAATCAGGTAGGACTACCC-
GCTGAACTTAA
>BC6-42
TTTCCGTAGGTGAACCTGCGGAAGGATCATTATTGAATTATGTTTCTAGATAGGTTGTAG
CTGGCTCTTTAGAGCATGTGCACGCCTGTTTGGACTTCATTTTCATCCACCTGTGCACCT
ATTGTAGTCTTTGGTTGGGTTAGGGGGAAGTGGTCATTGTGTCAGCATCTGCTGGATGTG

AGGACTTGCATTGTGAAAGCTTTGCTGTCCTTGATGTGATCATGGAATCTCTTTCTCACT
AGAGTCTATGTCACTCATTATACTCTGTCGAATGTCATTGAATGTCTTTACATGGGCTTG
TATGCCTATGAAAATTGTAATACAACTTTCAGCAACGGATCTCTTGGCTCTCGCATCGAT
GAAGGACGCAGCGAAATGCGATAAGTAATGTGAATTGCAGAATTCAGTGAATCATCGAAT
CTTTGAACGCATCTTGCGCTCCTTGGTATTCCGAGGAGCATGCCTGTTTGAGTGTCATTA
AATTCTCAACTCTCTTATACTTTTTTGTAAAAGAGAGCTTGGACTGTGGAGGCTTGCTGG
CCACTTTTTGGGGTCAGCTCCTCTGAAATGCATTAGCGGAACCGTTTGCAATCTGCCACA
AGTGTGATAAGTTATCTACACTGGCGAGGGGATTGCTCTCTGTAATGTTCAGCTTCTAAT
TGTCTCTACTTTGTGAGACAACTTTTGAATGCTTGACCTCAAATCAGGTAGGACTACCC-
GCTGAACTTAA
>BC8-12
TTTCCGTAGGTGAACCTGCGGAAGGATCATTATTGAATTATGTTTCTAGATAGGTTGTAG
CTGGCTCTTTAGAGCATGTGCACGCCTGTTTGGACTTCATTTTCATCCACCTGTGCACCT
ATTGTAGTCTTTGGTTGGGTTAGGGGGAAGTGGTCATTGTGTCAGCATCTGCTGGATGTG
AGGACTTGCATTGTGAAAGCTTTGCTGTCCTTGATGTGATCATGGAATCTCTTTCTCACT
AGAGTCTATGTCACTCATTATACTCTGTCGAATGTCATTGAATGTCTTTACATGGGCTTG
TATGCCTATGAAAATTGTAATACAACTTTCAGCAACGGATCTCTTGGCTCTCGCATCGAT
GAAGGACGCAGCGAAATGCGATAAGTAATGTGAATTGCAGAATTCAGTGAATCATCGAAT
CTTTGAACGCATCTTGCGCTCCTTGGTATTCCGAGGAGCATGCCTGTTTGAGTGTCATTA
AATTCTCAACTCTCTTATACTTTTTTGTAAAAGAGAGCTTGGACTGTGGAGGCTTGCTGG
CCACTTTTTGGGGTCAGCTCCTCTGAAATGCATTAGCGGAACCGTTTGCAATCTGCCACA
AGTGTGATAAGTTATCTACACTGGCGAGGGGATTGCTCTCTGTAATGTTCAGCTTCTAAT
TGTCTCTACTTTGTGAGACAACTTTTGAATGCTTGACCTCAAATCAGGTAGGACTACCC-
GCTGAACTTAA
>BC8-28
TTTCCGTAGGTGAACCTGCGGAAGGATCATTATTGAATTATGTTTCTAGATAGGTTGTAG
CTGGCTCTTTAGAGCATGTGCACGCCTGTTTGGACTTCATTTTCATCCACCTGTGCACCT
ATTGTAGTCTTTGGTTGGGTTAGGGGGAAGTGGTCATTGTGTCAGCATCTGCTGGATGTG
AGGACTTGCATTGTGAAAGCTTTGCTGTCCTTGATGTGATCATGGAATCTCTTTCTCACT
AGAGTCTATGTCACTCATTATACTCTGTCGAATGTCATTGAATGTCTTTACATGGGCTTG
TATGCCTATGAAAATTGTAATACAACTTTCAGCAACGGATCTCTTGGCTCTCGCATCGAT
GAAGGACGCAGCGAAATGCGATAAGTAATGTGAATTGCAGAATTCAGTGAATCATCGAAT
CTTTGAACGCATCTTGCGCTCCTTGGTATTCCGAGGAGCATGCCTGTTTGAGTGTCATTA
AATTCTCAACTCTCTTATACTTTTTTGTAAAAGAGAGCTTGGACTGTGGAGGCTTGCTGG
CCACTTTTTGGGGTCAGCTCCTCTGAAATGCATTAGCGGAACCGTTTGCAATCTGCCACA
AGTGTGATAAGTTATCTACACTGGCGAGGGGATTGCTCTCTGTAATGTTCAGCTTCTAAT
TGTCTCTACTTTGTGAGACAACTTTTGAATGCTTGACCTCAAATCAGGTAGGACTACCC-
GCTGAACTTAA
>BC12_41
TTTCCGTAGGTGAACCTGCGGAAGGATCATTATTGAATTATGTTTCTAGATAGGTTGTAG
CTGGCTCTTTAGAGCATGTGCACGCCTGTTTGGACTTCATTTTCATCCACCTGTGCACCT
ATTGTAGTCTTTGGTTGGGTTAGGGGGAAGTGGTCATTGTGTCAGCATCTGCTGGATGTG
AGGACTTGCATTGTGAAAGCTTTGCTGTCCTTGATGTGATCATGGAATCTCTTTCTCACT
AGAGTCTATGTCACTCATTATACTCTGTCGAATGTCATTGAATGTCTTTACATGGGCTTG
TATGCCTATGAAAATTGTAATACAACTTTCAGCAACGGATCTCTTGGCTCTCGCATCGAT
GAAGGACGCAGCGAAATGCGATAAGTAATGTGAATTGCAGAATTCAGTGAATCATCGAAT
CTTTGAACGCATCTTGCGCTCCTTGGTATTCCGAGGAGCATGCCTGTTTGAGTGTCATTA
AATTCTCAACTCTCTTATACTTTTTTGTAAAAGAGAGCTTGGACTGTGGAGGCTTGCTGG
CCACTTTTTGGGGTCAGCTCCTCTGAAATGCATTAGCGGAACCGTTTGCAATCTGCCACA
AGTGTGATAAGTTATCTACACTGGCGAGGGGATTGCTCTCTGTAATGTTCAGCTTCTAAT

TGTCTCTACTTTGTGAGACAACTTTTGAATGCTTGACCTCAAATCAGGTAGGACTACCC-
GCTGAACTTAA
>BC9-53
TTTCCGTAGGTGAACCTGCGGAAGGATCATTATTGAATTATGTTTCTAGATAGGTTGTAG
CTGGCTCTTTAGAGCATGTGCACGCCTGTTTGGACTTCATTTTCATCCACCTGTGCACCT
ATTGTAGTCTTTGGTTGGGTTAGGGGGAAGTGGTCATTGTGTCAGCATCTGCTGGATGTG
AGGACTTGCATTGTGAAAGCTTTGCTGTCCTTGATGTGATCATGGAATCTCTTTCTCACT
AGAGTCTATGTCACTCATTATACTCTGTCGAATGTCATTGAATGTCTTTACATGGGCTTG
TATGCCTATGAAAATTGTAATACAACTTTCAGCAACGGATCTCTTGGCTCTCGCATCGAT
GAAGGACGCAGCGAAATGCGATAAGTAATGTGAATTGCAGAATTCAGTGAATCATCGAAT
CTTTGAACGCATCTTGCGCTCCTTGGTATTCCGAGGAGCATGCCTGTTTGAGTGTCATTA
AATTCTCAACTCTCTTATACTTTTTTGTAAAAGAGAGCTTGGACTGTGGAGGCTTGCTGG
CCACTTTTTGGGGTCAGCTCCTCTGAAATGCATTAGCGGAACCGTTTGCAATCTGCCACA
AGTGTGATAAGTTATCTACACTGGCGAGGGGATTGCTCTCTGTAATGTTCAGCTTCTAAT
TGTCTCTACTTTGTGAGACAACTTTTGAATGCTTGACCTCAAATCAGGTAGGACTACCC-
GCTGAACTTAA
>BC6-8
TTTCCGTAGGTGAACCTGCGGAAGGATCATTATTGAATTATGTTTCTAGATAGGTTGTAG
CTGGCTCTTTAGAGCATGTGCACGCCTGTTTGGACTTCATTTTCATCCACCTGTGCACCT
ATTGTAGTCTTTGGTTGGGTTAGGGGGAAGTGGTCATTGTGTCAGCATCTGCTGGATGTG
AGGACTTGCATTGTGAAAGCTTTGCTGTCCTTGATGTGATCATGGAATCTCTTTCTCACT
AGAGTCTATGTCACTCATTATACTCTGTCGAATGTCATTGAATGTCTTTACATGGGCTTG
TATGCCTATGAAAATTGTAATACAACTTTCAGCAACGGATCTCTTGGCTCTCGCATCGAT
GAAGGACGCAGCGAAATGCGATAAGTAATGTGAATTGCAGAATTCAGTGAATCATCGAAT
CTTTGAACGCATCTTGCGCTCCTTGGTATTCCGAGGAGCATGCCTGTTTGAGTGTCATTA
AATTCTCAACTCTCTTATACTTTTTTGTAAAAGAGAGCTTGGACTGTGGAGGCTTGCTGG
CCACTTTTTGGGGTCAGCTCCTCTGAAATGCATTAGCGGAACCGTTTGCAATCTGCCACA
AGTGTGATAAGTTATCTACACTGGCGAGGGGATTGCTCTCTGTAATGTTCAGCTTCTAAT
TGTCTCTACTTTGTGAGACAACTTTTGAATGCTTGACCTCAAATCAGGTAGGACTACCC-
GCTGAACTTAA
>BC12_34
TTTCCGTAGGTGAACCTGCGGAAGGATCATTATTGAATTATGTTTCTAGATAGGTTGTAG
CTGGCTCTTTAGAGCATGTGCACGCCTGTTTGGACTTCATTTTCATCCACCTGTGCACCT
ATTGTAGTCTTTGGTTGGGTTAGGGGGAAGTGGTCATTGTGTCAGCATCTGCTGGATGTG
AGGACTTGCATTGTGAAAGCTTTGCTGTCCTTGATGTGATCATGGAATCTCTTTCTCACT
AGAGTCTATGTCACTCATTATACTCTGTCGAATGTCATTGAATGTCTTTACATGGGCTTG
TATGCCTATGAAAATTGTAATACAACTTTCAGCAACGGATCTCTTGGCTCTCGCATCGAT
GAAGGACGCAGCGAAATGCGATAAGTAATGTGAATTGCAGAATTCAGTGAATCATCGAAT
CTTTGAACGCATCTTGCGCTCCTTGGTATTCCGAGGAGCATGCCTGTTTGAGTGTCATTA
AATTCTCAACTCTCTTATACTTTTTTGTAAAAGAGAGCTTGGACTGTGGAGGCTTGCTGG
CCACTTTTTGGGGTCAGCTCCTCTGAAATGCATTAGCGGAACCGTTTGCAATCTGCCACA
AGTGTGATAAGTTATCTACACTGGCGAGGGGATTGCTCTCTGTAATGTTCAGCTTCTAAT
TGTCTCTACTTTGTGAGACAACTTTTGAATGCTTGACCTCAAATCAGGTAGGACTACCC-
GCTGAACTTAA
>BC7-8
TTTCCGTAGGTGAACCTGCGGAAGGATCATTATTGAATTATGTTTCTAGATAGGTTGTAG
CTGGCTCTTTAGAGCATGTGCACGCCTGTTTGGACTTCATTTTCATCCACCTGTGCACCT
ATTGTAGTCTTTGGTTGGGTTAGGGGGAAGTGGTCATTGTGTCAGCATCTGCTGGATGTG
AGGACTTGCATTGTGAAAGCTTTGCTGTCCTTGATGTGATCATGGAATCTCTTTCTCACT
AGAGTCTATGTCACTCATTATACTCTGTCGAATGTCATTGAATGTCTTTACATGGGCTTG

TATGCCTATGAAAATTGTAATACAACTTTCAGCAACGGATCTCTTGGCTCTCGCATCGAT
GAAGGACGCAGCGAAATGCGATAAGTAATGTGAATTGCAGAATTCAGTGAATCATCGAAT
CTTTGAACGCATCTTGCGCTCCTTGGTATTCCGAGGAGCATGCCTGTTTGAGTGTCATTA
AATTCTCAACTCTCTTATACTTTTTTGTAAAAGAGAGCTTGGACTGTGGAGGCTTGCTGG
CCACTTTTTGGGGTCAGCTCCTCTGAAATGCATTAGCGGAACCGTTTGCAATCTGCCACA
AGTGTGATAAGTTATCTACACTGGCGAGGGGATTGCTCTCTGTAATGTTCAGCTTCTAAT
TGTCTCTACTTTGTGAGACAACTTTTGAATGCTTGACCTCAAATCAGGTAGGACTACCC-
GCTGAACTTAA
>BC7-17
TTTCCGTAGGTGAACCTGCGGAAGGATCATTATTGAATTATGTTTCTAGATAGGTTGTAG
CTGGCTCTTTAGAGCATGTGCACGCCTGTTTGGACTTCATTTTCATCCACCTGTGCACCT
ATTGTAGTCTTTGGTTGGGTTAGGGGGAAGTGGTCATTGTGTCAGCATCTGCTGGATGTG
AGGACTTGCATTGTGAAAGCTTTGCTGTCCTTGATGTGATCATGGAATCTCTTTCTCACT
AGAGTCTATGTCACTCATTATACTCTGTCGAATGTCATTGAATGTCTTTACATGGGCTTG
TATGCCTATGAAAATTGTAATACAACTTTCAGCAACGGATCTCTTGGCTCTCGCATCGAT
GAAGGACGCAGCGAAATGCGATAAGTAATGTGAATTGCAGAATTCAGTGAATCATCGAAT
CTTTGAACGCATCTTGCGCTCCTTGGTATTCCGAGGAGCATGCCTGTTTGAGTGTCATTA
AATTCTCAACTCTCTTATACTTTTTTGTAAAAGAGAGCTTGGACTGTGGAGGCTTGCTGG
CCACTTTTTGGGGTCAGCTCCTCTGAAATGCATTAGCGGAACCGTTTGCAATCTGCCACA
AGTGTGATAAGTTATCTACACTGGCGAGGGGATTGCTCTCTGTAATGTTCAGCTTCTAAT
TGTCTCTACTTTGTGAGACAACTTTTGAATGCTTGACCTCAAATCAGGTAGGACTACCC-
GCTGAACTTAA
>BC2-28
TTTCCGTAGGTGAACCTGCGGAAGGATCATTATTGAATTATGTTTCTAGATAGGTTGTAG
CTGGCTCTTTAGAGCATGTGCACGCCTGTTTGGACTTCATTTTCATCCACCTGTGCACCT
ATTGTAGTCTTTGGTTGGGTTAGGGGGAAGTGGTCATTGTGTCAGCATCTGCTGGATGTG
AGGACTTGCATTGTGAAAGCTTTGCTGTCCTTGATGTGATCATGGAATCTCTTTCTCACT
AGAGTCTATGTCACTCATTATACTCTGTCGAATGTCATTGAATGTCTTTACATGGGCTTG
TATGCCTATGAAAATTGTAATACAACTTTCAGCAACGGATCTCTTGGCTCTCGCATCGAT
GAAGGACGCAGCGAAATGCGATAAGTAATGTGAATTGCAGAATTCAGTGAATCATCGAAT
CTTTGAACGCATCTTGCGCTCCTTGGTATTCCGAGGAGCATGCCTGTTTGAGTGTCATTA
AATTCTCAACTCTCTTATACTTTTTTGTAAAAGAGAGCTTGGACTGTGGAGGCTTGCTGG
CCACTTTTTGGGGTCAGCTCCTCTGAAATGCATTAGCGGAACCGTTTGCAATCTGCCACA
AGTGTGATAAGTTATCTACACTGGCGAGGGGATTGCTCTCTGTAATGTTCAGCTTCTAAT
TGTCTCTACTTTGTGAGACAACTTTTGAATGCTTGACCTCAAATCAGGTAGGACTACCC-
GCTGAACTTAA
>BC2-30
TTTCCGTAGGTGAACCTGCGGAAGGATCATTATTGAATTATGTTTCTAGATAGGTTGTAG
CTGGCTCTTTAGAGCATGTGCACGCCTGTTTGGACTTCATTTTCATCCACCTGTGCACCT
ATTGTAGTCTTTGGTTGGGTTAGGGGGAAGTGGTCATTGTGTCAGCATCTGCTGGATGTG
AGGACTTGCATTGTGAAAGCTTTGCTGTCCTTGATGTGATCATGGAATCTCTTTCTCACT
AGAGTCTATGTCACTCATTATACTCTGTCGAATGTCATTGAATGTCTTTACATGGGCTTG
TATGCCTATGAAAATTGTAATACAACTTTCAGCAACGGATCTCTTGGCTCTCGCATCGAT
GAAGGACGCAGCGAAATGCGATAAGTAATGTGAATTGCAGAATTCAGTGAATCATCGAAT
CTTTGAACGCATCTTGCGCTCCTTGGTATTCCGAGGAGCATGCCTGTTTGAGTGTCATTA
AATTCTCAACTCTCTTATACTTTTTTGTAAAAGAGAGCTTGGACTGTGGAGGCTTGCTGG
CCACTTTTTGGGGTCAGCTCCTCTGAAATGCATTAGCGGAACCGTTTGCAATCTGCCACA
AGTGTGATAAGTTATCTACACTGGCGAGGGGATTGCTCTCTGTAATGTTCAGCTTCTAAT
TGTCTCTACTTTGTGAGACAACTTTTGAATGCTTGACCTCAAATCAGGTAGGACTACCC-
GCTGAACTTAA

>BC2–33
TTTCCGTAGGTGAACCTGCGGAAGGATCATTATTGAATTATGTTTCTAGATAGGTTGTAG
CTGGCTCTTTAGAGCATGTGCACGCCTGTTTGGACTTCATTTTCATCCACCTGTGCACCT
ATTGTAGTCTTTGGTTGGGTTAGGGGGAAGTGGTCATTGTGTCAGCATCTGCTGGATGTG
AGGACTTGCATTGTGAAAGCTTTGCTGTCCTTGATGTGATCATGGAATCTCTTTCTCACT
AGAGTCTATGTCACTCATTATACTCTGTCGAATGTCATTGAATGTCTTTACATGGGCTTG
TATGCCTATGAAAATTGTAATACAACTTTCAGCAACGGATCTCTTGGCTCTCGCATCGAT
GAAGGACGCAGCGAAATGCGATAAGTAATGTGAATTGCAGAATTCAGTGAATCATCGAAT
CTTTGAACGCATCTTGCGCTCCTTGGTATTCCGAGGAGCATGCCTGTTTGAGTGTCATTA
AATTCTCAACTCTCTTATACTTTTTTGTAAAAGAGAGCTTGGACTGTGGAGGCTTGCTGG
CCACTTTTTGGGGTCAGCTCCTCTGAAATGCATTAGCGGAACCGTTTGCAATCTGCCACA
AGTGTGATAAGTTATCTACACTGGCGAGGGGATTGCTCTCTGTAATGTTCAGCTTCTAAT
TGTCTCTACTTTGTGAGACAACTTTTGAATGCTTGACCTCAAATCAGGTAGGACTACCC–
GCTGAACTTAA
>BC3–51
TTTCCGTAGGTGAACCTGCGGAAGGATCATTATTGAATTATGTTTCTAGATAGGTTGTAG
CTGGCTCTTTAGAGCATGTGCACGCCTGTTTGGACTTCATTTTCATCCACCTGTGCACCT
ATTGTAGTCTTTGGTTGGGTTAGGGGGAAGTGGTCATTGTGTCAGCATCTGCTGGATGTG
AGGACTTGCATTGTGAAAGCTTTGCTGTCCTTGATGTGATCATGGAATCTCTTTCTCACT
AGAGTCTATGTCACTCATTATACTCTGTCGAATGTCATTGAATGTCTTTACATGGGCTTG
TATGCCTATGAAAATTGTAATACAACTTTCAGCAACGGATCTCTTGGCTCTCGCATCGAT
GAAGGACGCAGCGAAATGCGATAAGTAATGTGAATTGCAGAATTCAGTGAATCATCGAAT
CTTTGAACGCATCTTGCGCTCCTTGGTATTCCGAGGAGCATGCCTGTTTGAGTGTCATTA
AATTCTCAACTCTCTTATACTTTTTTGTAAAAGAGAGCTTGGACTGTGGAGGCTTGCTGG
CCACTTTTTGGGGTCAGCTCCTCTGAAATGCATTAGCGGAACCGTTTGCAATCTGCCACA
AGTGTGATAAGTTATCTACACTGGCGAGGGGATTGCTCTCTGTAATGTTCAGCTTCTAAT
TGTCTCTACTTTGTGAGACAACTTTTGAATGCTTGACCTCAAATCAGGTAGGACTACCC–
GCTGAACTTAA
>BC4–25
TTTCCGTAGGTGAACCTGCGGAAGGATCATTATTGAATTATGTTTCTAGATAGGTTGTAG
CTGGCTCTTTAGAGCATGTGCACGCCTGTTTGGACTTCATTTTCATCCACCTGTGCACCT
ATTGTAGTCTTTGGTTGGGTTAGGGGGAAGTGGTCATTGTGTCAGCATCTGCTGGATGTG
AGGACTTGCATTGTGAAAGCTTTGCTGTCCTTGATGTGATCATGGAATCTCTTTCTCACT
AGAGTCTATGTCACTCATTATACTCTGTCGAATGTCATTGAATGTCTTTACATGGGCTTG
TATGCCTATGAAAATTGTAATACAACTTTCAGCAACGGATCTCTTGGCTCTCGCATCGAT
GAAGGACGCAGCGAAATGCGATAAGTAATGTGAATTGCAGAATTCAGTGAATCATCGAAT
CTTTGAACGCATCTTGCGCTCCTTGGTATTCCGAGGAGCATGCCTGTTTGAGTGTCATTA
AATTCTCAACTCTCTTATACTTTTTTGTAAAAGAGAGCTTGGACTGTGGAGGCTTGCTGG
CCACTTTTTGGGGTCAGCTCCTCTGAAATGCATTAGCGGAACCGTTTGCAATCTGCCACA
AGTGTGATAAGTTATCTACACTGGCGAGGGGATTGCTCTCTGTAATGTTCAGCTTCTAAT
TGTCTCTACTTTGTGAGACAACTTTTGAATGCTTGACCTCAAATCAGGTAGGACTACCC–
GCTGAACTTAA
>BC4–50
TTTCCGTAGGTGAACCTGCGGAAGGATCATTATTGAATTATGTTTCTAGATAGGTTGTAG
CTGGCTCTTTAGAGCATGTGCACGCCTGTTTGGACTTCATTTTCATCCACCTGTGCACCT
ATTGTAGTCTTTGGTTGGGTTAGGGGGAAGTGGTCATTGTGTCAGCATCTGCTGGATGTG
AGGACTTGCATTGTGAAAGCTTTGCTGTCCTTGATGTGATCATGGAATCTCTTTCTCACT
AGAGTCTATGTCACTCATTATACTCTGTCGAATGTCATTGAATGTCTTTACATGGGCTTG
TATGCCTATGAAAATTGTAATACAACTTTCAGCAACGGATCTCTTGGCTCTCGCATCGAT
GAAGGACGCAGCGAAATGCGATAAGTAATGTGAATTGCAGAATTCAGTGAATCATCGAAT

CTTTGAACGCATCTTGCGCTCCTTGGTATTCCGAGGAGCATGCCTGTTTGAGTGTCATTA
AATTCTCAACTCTCTTATACTTTTTTGTAAAAGAGAGCTTGGACTGTGGAGGCTTGCTGG
CCACTTTTTGGGGTCAGCTCCTCTGAAATGCATTAGCGGAACCGTTTGCAATCTGCCACA
AGTGTGATAAGTTATCTACACTGGCGAGGGGATTGCTCTCTGTAATGTTCAGCTTCTAAT
TGTCTCTACTTTGTGAGACAACTTTTGAATGCTTGACCTCAAATCAGGTAGGACTACCC-
GCTGAACTTAA
>BC5-9
TTTCCGTAGGTGAACCTGCGGAAGGATCATTATTGAATTATGTTTCTAGATAGGTTGTAG
CTGGCTCTTTAGAGCATGTGCACGCCTGTTTGGACTTCATTTTCATCCACCTGTGCACCT
ATTGTAGTCTTTGGTTGGGTTAGGGGGAAGTGGTCATTGTGTCAGCATCTGCTGGATGTG
AGGACTTGCATTGTGAAAGCTTTGCTGTCCTTGATGTGATCATGGAATCTCTTTCTCACT
AGAGTCTATGTCACTCATTATACTCTGTCGAATGTCATTGAATGTCTTTACATGGGCTTG
TATGCCTATGAAAATTGTAATACAACTTTCAGCAACGGATCTCTTGGCTCTCGCATCGAT
GAAGGACGCAGCGAAATGCGATAAGTAATGTGAATTGCAGAATTCAGTGAATCATCGAAT
CTTTGAACGCATCTTGCGCTCCTTGGTATTCCGAGGAGCATGCCTGTTTGAGTGTCATTA
AATTCTCAACTCTCTTATACTTTTTTGTAAAAGAGAGCTTGGACTGTGGAGGCTTGCTGG
CCACTTTTTGGGGTCAGCTCCTCTGAAATGCATTAGCGGAACCGTTTGCAATCTGCCACA
AGTGTGATAAGTTATCTACACTGGCGAGGGGATTGCTCTCTGTAATGTTCAGCTTCTAAT
TGTCTCTACTTTGTGAGACAACTTTTGAATGCTTGACCTCAAATCAGGTAGGACTACCC-
GCTGAACTTAA
>BC5-25
TTTCCGTAGGTGAACCTGCGGAAGGATCATTATTGAATTATGTTTCTAGATAGGTTGTAG
CTGGCTCTTTAGAGCATGTGCACGCCTGTTTGGACTTCATTTTCATCCACCTGTGCACCT
ATTGTAGTCTTTGGTTGGGTTAGGGGGAAGTGGTCATTGTGTCAGCATCTGCTGGATGTG
AGGACTTGCATTGTGAAAGCTTTGCTGTCCTTGATGTGATCATGGAATCTCTTTCTCACT
AGAGTCTATGTCACTCATTATACTCTGTCGAATGTCATTGAATGTCTTTACATGGGCTTG
TATGCCTATGAAAATTGTAATACAACTTTCAGCAACGGATCTCTTGGCTCTCGCATCGAT
GAAGGACGCAGCGAAATGCGATAAGTAATGTGAATTGCAGAATTCAGTGAATCATCGAAT
CTTTGAACGCATCTTGCGCTCCTTGGTATTCCGAGGAGCATGCCTGTTTGAGTGTCATTA
AATTCTCAACTCTCTTATACTTTTTTGTAAAAGAGAGCTTGGACTGTGGAGGCTTGCTGG
CCACTTTTTGGGGTCAGCTCCTCTGAAATGCATTAGCGGAACCGTTTGCAATCTGCCACA
AGTGTGATAAGTTATCTACACTGGCGAGGGGATTGCTCTCTGTAATGTTCAGCTTCTAAT
TGTCTCTACTTTGTGAGACAACTTTTGAATGCTTGACCTCAAATCAGGTAGGACTACCC-
GCTGAACTTAA
>BC5-41
TTTCCGTAGGTGAACCTGCGGAAGGATCATTATTGAATTATGTTTCTAGATAGGTTGTAG
CTGGCTCTTTAGAGCATGTGCACGCCTGTTTGGACTTCATTTTCATCCACCTGTGCACCT
ATTGTAGTCTTTGGTTGGGTTAGGGGGAAGTGGTCATTGTGTCAGCATCTGCTGGATGTG
AGGACTTGCATTGTGAAAGCTTTGCTGTCCTTGATGTGATCATGGAATCTCTTTCTCACT
AGAGTCTATGTCACTCATTATACTCTGTCGAATGTCATTGAATGTCTTTACATGGGCTTG
TATGCCTATGAAAATTGTAATACAACTTTCAGCAACGGATCTCTTGGCTCTCGCATCGAT
GAAGGACGCAGCGAAATGCGATAAGTAATGTGAATTGCAGAATTCAGTGAATCATCGAAT
CTTTGAACGCATCTTGCGCTCCTTGGTATTCCGAGGAGCATGCCTGTTTGAGTGTCATTA
AATTCTCAACTCTCTTATACTTTTTTGTAAAAGAGAGCTTGGACTGTGGAGGCTTGCTGG
CCACTTTTTGGGGTCAGCTCCTCTGAAATGCATTAGCGGAACCGTTTGCAATCTGCCACA
AGTGTGATAAGTTATCTACACTGGCGAGGGGATTGCTCTCTGTAATGTTCAGCTTCTAAT
TGTCTCTACTTTGTGAGACAACTTTTGAATGCTTGACCTCAAATCAGGTAGGACTACCC-
GCTGAACTTAA
>BC5-56
TTTCCGTAGGTGAACCTGCGGAAGGATCATTATTGAATTATGTTTCTAGATAGGTTGTAG

CTGGCTCTTTAGAGCATGTGCACGCCTGTTTGGACTTCATTTTCATCCACCTGTGCACCT
ATTGTAGTCTTTGGTTGGGTTAGGGGGAAGTGGTCATTGTGTCAGCATCTGCTGGATGTG
AGGACTTGCATTGTGAAAGCTTTGCTGTCCTTGATGTGATCATGGAATCTCTTTCTCACT
AGAGTCTATGTCACTCATTATACTCTGTCGAATGTCATTGAATGTCTTTACATGGGCTTG
TATGCCTATGAAAATTGTAATACAACTTTCAGCAACGGATCTCTTGGCTCTCGCATCGAT
GAAGGACGCAGCGAAATGCGATAAGTAATGTGAATTGCAGAATTCAGTGAATCATCGAAT
CTTTGAACGCATCTTGCGCTCCTTGGTATTCCGAGGAGCATGCCTGTTTGAGTGTCATTA
AATTCTCAACTCTCTTATACTTTTTTGTAAAAGAGAGCTTGGACTGTGGAGGCTTGCTGG
CCACTTTTTGGGGTCAGCTCCTCTGAAATGCATTAGCGGAACCGTTTGCAATCTGCCACA
AGTGTGATAAGTTATCTACACTGGCGAGGGGATTGCTCTCTGTAATGTTCAGCTTCTAAT
TGTCTCTACTTTGTGAGACAACTTTTGAATGCTTGACCTCAAATCAGGTAGGACTACCC-
GCTGAACTTAA
>BC5-77
TTTCCGTAGGTGAACCTGCGGAAGGATCATTATTGAATTATGTTTCTAGATAGGTTGTAG
CTGGCTCTTTAGAGCATGTGCACGCCTGTTTGGACTTCATTTTCATCCACCTGTGCACCT
ATTGTAGTCTTTGGTTGGGTTAGGGGGAAGTGGTCATTGTGTCAGCATCTGCTGGATGTG
AGGACTTGCATTGTGAAAGCTTTGCTGTCCTTGATGTGATCATGGAATCTCTTTCTCACT
AGAGTCTATGTCACTCATTATACTCTGTCGAATGTCATTGAATGTCTTTACATGGGCTTG
TATGCCTATGAAAATTGTAATACAACTTTCAGCAACGGATCTCTTGGCTCTCGCATCGAT
GAAGGACGCAGCGAAATGCGATAAGTAATGTGAATTGCAGAATTCAGTGAATCATCGAAT
CTTTGAACGCATCTTGCGCTCCTTGGTATTCCGAGGAGCATGCCTGTTTGAGTGTCATTA
AATTCTCAACTCTCTTATACTTTTTTGTAAAAGAGAGCTTGGACTGTGGAGGCTTGCTGG
CCACTTTTTGGGGTCAGCTCCTCTGAAATGCATTAGCGGAACCGTTTGCAATCTGCCACA
AGTGTGATAAGTTATCTACACTGGCGAGGGGATTGCTCTCTGTAATGTTCAGCTTCTAAT
TGTCTCTACTTTGTGAGACAACTTTTGAATGCTTGACCTCAAATCAGGTAGGACTACCC-
GCTGAACTTAA
>BC5-108
TTTCCGTAGGTGAACCTGCGGAAGGATCATTATTGAATTATGTTTCTAGATAGGTTGTAG
CTGGCTCTTTAGAGCATGTGCACGCCTGTTTGGACTTCATTTTCATCCACCTGTGCACCT
ATTGTAGTCTTTGGTTGGGTTAGGGGGAAGTGGTCATTGTGTCAGCATCTGCTGGATGTG
AGGACTTGCATTGTGAAAGCTTTGCTGTCCTTGATGTGATCATGGAATCTCTTTCTCACT
AGAGTCTATGTCACTCATTATACTCTGTCGAATGTCATTGAATGTCTTTACATGGGCTTG
TATGCCTATGAAAATTGTAATACAACTTTCAGCAACGGATCTCTTGGCTCTCGCATCGAT
GAAGGACGCAGCGAAATGCGATAAGTAATGTGAATTGCAGAATTCAGTGAATCATCGAAT
CTTTGAACGCATCTTGCGCTCCTTGGTATTCCGAGGAGCATGCCTGTTTGAGTGTCATTA
AATTCTCAACTCTCTTATACTTTTTTGTAAAAGAGAGCTTGGACTGTGGAGGCTTGCTGG
CCACTTTTTGGGGTCAGCTCCTCTGAAATGCATTAGCGGAACCGTTTGCAATCTGCCACA
AGTGTGATAAGTTATCTACACTGGCGAGGGGATTGCTCTCTGTAATGTTCAGCTTCTAAT
TGTCTCTACTTTGTGAGACAACTTTTGAATGCTTGACCTCAAATCAGGTAGGACTACCC-
GCTGAACTTAA
>BC5-125
TTTCCGTAGGTGAACCTGCGGAAGGATCATTATTGAATTATGTTTCTAGATAGGTTGTAG
CTGGCTCTTTAGAGCATGTGCACGCCTGTTTGGACTTCATTTTCATCCACCTGTGCACCT
ATTGTAGTCTTTGGTTGGGTTAGGGGGAAGTGGTCATTGTGTCAGCATCTGCTGGATGTG
AGGACTTGCATTGTGAAAGCTTTGCTGTCCTTGATGTGATCATGGAATCTCTTTCTCACT
AGAGTCTATGTCACTCATTATACTCTGTCGAATGTCATTGAATGTCTTTACATGGGCTTG
TATGCCTATGAAAATTGTAATACAACTTTCAGCAACGGATCTCTTGGCTCTCGCATCGAT
GAAGGACGCAGCGAAATGCGATAAGTAATGTGAATTGCAGAATTCAGTGAATCATCGAAT
CTTTGAACGCATCTTGCGCTCCTTGGTATTCCGAGGAGCATGCCTGTTTGAGTGTCATTA
AATTCTCAACTCTCTTATACTTTTTTGTAAAAGAGAGCTTGGACTGTGGAGGCTTGCTGG

CCACTTTTTGGGGTCAGCTCCTCTGAAATGCATTAGCGGAACCGTTTGCAATCTGCCACA
AGTGTGATAAGTTATCTACACTGGCGAGGGGATTGCTCTCTGTAATGTTCAGCTTCTAAT
TGTCTCTACTTTGTGAGACAACTTTTGAATGCTTGACCTCAAATCAGGTAGGACTACCC-
GCTGAACTTAA
>BC6-18
TTTCCGTAGGTGAACCTGCGGAAGGATCATTATTGAATTATGTTTCTAGATAGGTTGTAG
CTGGCTCTTTAGAGCATGTGCACGCCTGTTTGGACTTCATTTTCATCCACCTGTGCACCT
ATTGTAGTCTTTGGTTGGGTTAGGGGGAAGTGGTCATTGTGTCAGCATCTGCTGGATGTG
AGGACTTGCATTGTGAAAGCTTTGCTGTCCTTGATGTGATCATGGAATCTCTTTCTCACT
AGAGTCTATGTCACTCATTATACTCTGTCGAATGTCATTGAATGTCTTTACATGGGCTTG
TATGCCTATGAAAATTGTAATACAACTTTCAGCAACGGATCTCTTGGCTCTCGCATCGAT
GAAGGACGCAGCGAAATGCGATAAGTAATGTGAATTGCAGAATTCAGTGAATCATCGAAT
CTTTGAACGCATCTTGCGCTCCTTGGTATTCCGAGGAGCATGCCTGTTTGAGTGTCATTA
AATTCTCAACTCTCTTATACTTTTTTGTAAAAGAGAGCTTGGACTGTGGAGGCTTGCTGG
CCACTTTTTGGGGTCAGCTCCTCTGAAATGCATTAGCGGAACCGTTTGCAATCTGCCACA
AGTGTGATAAGTTATCTACACTGGCGAGGGGATTGCTCTCTGTAATGTTCAGCTTCTAAT
TGTCTCTACTTTGTGAGACAACTTTTGAATGCTTGACCTCAAATCAGGTAGGACTACCC-
GCTGAACTTAA
>BC6-45
TTTCCGTAGGTGAACCTGCGGAAGGATCATTATTGAATTATGTTTCTAGATAGGTTGTAG
CTGGCTCTTTAGAGCATGTGCACGCCTGTTTGGACTTCATTTTCATCCACCTGTGCACCT
ATTGTAGTCTTTGGTTGGGTTAGGGGGAAGTGGTCATTGTGTCAGCATCTGCTGGATGTG
AGGACTTGCATTGTGAAAGCTTTGCTGTCCTTGATGTGATCATGGAATCTCTTTCTCACT
AGAGTCTATGTCACTCATTATACTCTGTCGAATGTCATTGAATGTCTTTACATGGGCTTG
TATGCCTATGAAAATTGTAATACAACTTTCAGCAACGGATCTCTTGGCTCTCGCATCGAT
GAAGGACGCAGCGAAATGCGATAAGTAATGTGAATTGCAGAATTCAGTGAATCATCGAAT
CTTTGAACGCATCTTGCGCTCCTTGGTATTCCGAGGAGCATGCCTGTTTGAGTGTCATTA
AATTCTCAACTCTCTTATACTTTTTTGTAAAAGAGAGCTTGGACTGTGGAGGCTTGCTGG
CCACTTTTTGGGGTCAGCTCCTCTGAAATGCATTAGCGGAACCGTTTGCAATCTGCCACA
AGTGTGATAAGTTATCTACACTGGCGAGGGGATTGCTCTCTGTAATGTTCAGCTTCTAAT
TGTCTCTACTTTGTGAGACAACTTTTGAATGCTTGACCTCAAATCAGGTAGGACTACCC-
GCTGAACTTAA
>BC6-49
TTTCCGTAGGTGAACCTGCGGAAGGATCATTATTGAATTATGTTTCTAGATAGGTTGTAG
CTGGCTCTTTAGAGCATGTGCACGCCTGTTTGGACTTCATTTTCATCCACCTGTGCACCT
ATTGTAGTCTTTGGTTGGGTTAGGGGGAAGTGGTCATTGTGTCAGCATCTGCTGGATGTG
AGGACTTGCATTGTGAAAGCTTTGCTGTCCTTGATGTGATCATGGAATCTCTTTCTCACT
AGAGTCTATGTCACTCATTATACTCTGTCGAATGTCATTGAATGTCTTTACATGGGCTTG
TATGCCTATGAAAATTGTAATACAACTTTCAGCAACGGATCTCTTGGCTCTCGCATCGAT
GAAGGACGCAGCGAAATGCGATAAGTAATGTGAATTGCAGAATTCAGTGAATCATCGAAT
CTTTGAACGCATCTTGCGCTCCTTGGTATTCCGAGGAGCATGCCTGTTTGAGTGTCATTA
AATTCTCAACTCTCTTATACTTTTTTGTAAAAGAGAGCTTGGACTGTGGAGGCTTGCTGG
CCACTTTTTGGGGTCAGCTCCTCTGAAATGCATTAGCGGAACCGTTTGCAATCTGCCACA
AGTGTGATAAGTTATCTACACTGGCGAGGGGATTGCTCTCTGTAATGTTCAGCTTCTAAT
TGTCTCTACTTTGTGAGACAACTTTTGAATGCTTGACCTCAAATCAGGTAGGACTACCC-
GCTGAACTTAA
>BC7-29
TTTCCGTAGGTGAACCTGCGGAAGGATCATTATTGAATTATGTTTCTAGATAGGTTGTAG
CTGGCTCTTTAGAGCATGTGCACGCCTGTTTGGACTTCATTTTCATCCACCTGTGCACCT
ATTGTAGTCTTTGGTTGGGTTAGGGGGAAGTGGTCATTGTGTCAGCATCTGCTGGATGTG

AGGACTTGCATTGTGAAAGCTTTGCTGTCCTTGATGTGATCATGGAATCTCTTTCTCACT
AGAGTCTATGTCACTCATTATACTCTGTCGAATGTCATTGAATGTCTTTACATGGGCTTG
TATGCCTATGAAAATTGTAATACAACTTTCAGCAACGGATCTCTTGGCTCTCGCATCGAT
GAAGGACGCAGCGAAATGCGATAAGTAATGTGAATTGCAGAATTCAGTGAATCATCGAAT
CTTTGAACGCATCTTGCGCTCCTTGGTATTCCGAGGAGCATGCCTGTTTGAGTGTCATTA
AATTCTCAACTCTCTTATACTTTTTTGTAAAAGAGAGCTTGGACTGTGGAGGCTTGCTGG
CCACTTTTTGGGGTCAGCTCCTCTGAAATGCATTAGCGGAACCGTTTGCAATCTGCCACA
AGTGTGATAAGTTATCTACACTGGCGAGGGGATTGCTCTCTGTAATGTTCAGCTTCTAAT
TGTCTCTACTTTGTGAGACAACTTTTGAATGCTTGACCTCAAATCAGGTAGGACTACCC-
GCTGAACTTAA
>BC7-38
TTTCCGTAGGTGAACCTGCGGAAGGATCATTATTGAATTATGTTTCTAGATAGGTTGTAG
CTGGCTCTTTAGAGCATGTGCACGCCTGTTTGGACTTCATTTTCATCCACCTGTGCACCT
ATTGTAGTCTTTGGTTGGGTTAGGGGGAAGTGGTCATTGTGTCAGCATCTGCTGGATGTG
AGGACTTGCATTGTGAAAGCTTTGCTGTCCTTGATGTGATCATGGAATCTCTTTCTCACT
AGAGTCTATGTCACTCATTATACTCTGTCGAATGTCATTGAATGTCTTTACATGGGCTTG
TATGCCTATGAAAATTGTAATACAACTTTCAGCAACGGATCTCTTGGCTCTCGCATCGAT
GAAGGACGCAGCGAAATGCGATAAGTAATGTGAATTGCAGAATTCAGTGAATCATCGAAT
CTTTGAACGCATCTTGCGCTCCTTGGTATTCCGAGGAGCATGCCTGTTTGAGTGTCATTA
AATTCTCAACTCTCTTATACTTTTTTGTAAAAGAGAGCTTGGACTGTGGAGGCTTGCTGG
CCACTTTTTGGGGTCAGCTCCTCTGAAATGCATTAGCGGAACCGTTTGCAATCTGCCACA
AGTGTGATAAGTTATCTACACTGGCGAGGGGATTGCTCTCTGTAATGTTCAGCTTCTAAT
TGTCTCTACTTTGTGAGACAACTTTTGAATGCTTGACCTCAAATCAGGTAGGACTACCC-
GCTGAACTTAA
>BC8-25
TTTCCGTAGGTGAACCTGCGGAAGGATCATTATTGAATTATGTTTCTAGATAGGTTGTAG
CTGGCTCTTTAGAGCATGTGCACGCCTGTTTGGACTTCATTTTCATCCACCTGTGCACCT
ATTGTAGTCTTTGGTTGGGTTAGGGGGAAGTGGTCATTGTGTCAGCATCTGCTGGATGTG
AGGACTTGCATTGTGAAAGCTTTGCTGTCCTTGATGTGATCATGGAATCTCTTTCTCACT
AGAGTCTATGTCACTCATTATACTCTGTCGAATGTCATTGAATGTCTTTACATGGGCTTG
TATGCCTATGAAAATTGTAATACAACTTTCAGCAACGGATCTCTTGGCTCTCGCATCGAT
GAAGGACGCAGCGAAATGCGATAAGTAATGTGAATTGCAGAATTCAGTGAATCATCGAAT
CTTTGAACGCATCTTGCGCTCCTTGGTATTCCGAGGAGCATGCCTGTTTGAGTGTCATTA
AATTCTCAACTCTCTTATACTTTTTTGTAAAAGAGAGCTTGGACTGTGGAGGCTTGCTGG
CCACTTTTTGGGGTCAGCTCCTCTGAAATGCATTAGCGGAACCGTTTGCAATCTGCCACA
AGTGTGATAAGTTATCTACACTGGCGAGGGGATTGCTCTCTGTAATGTTCAGCTTCTAAT
TGTCTCTACTTTGTGAGACAACTTTTGAATGCTTGACCTCAAATCAGGTAGGACTACCC-
GCTGAACTTAA
>BC8-56
TTTCCGTAGGTGAACCTGCGGAAGGATCATTATTGAATTATGTTTCTAGATAGGTTGTAG
CTGGCTCTTTAGAGCATGTGCACGCCTGTTTGGACTTCATTTTCATCCACCTGTGCACCT
ATTGTAGTCTTTGGTTGGGTTAGGGGGAAGTGGTCATTGTGTCAGCATCTGCTGGATGTG
AGGACTTGCATTGTGAAAGCTTTGCTGTCCTTGATGTGATCATGGAATCTCTTTCTCACT
AGAGTCTATGTCACTCATTATACTCTGTCGAATGTCATTGAATGTCTTTACATGGGCTTG
TATGCCTATGAAAATTGTAATACAACTTTCAGCAACGGATCTCTTGGCTCTCGCATCGAT
GAAGGACGCAGCGAAATGCGATAAGTAATGTGAATTGCAGAATTCAGTGAATCATCGAAT
CTTTGAACGCATCTTGCGCTCCTTGGTATTCCGAGGAGCATGCCTGTTTGAGTGTCATTA
AATTCTCAACTCTCTTATACTTTTTTGTAAAAGAGAGCTTGGACTGTGGAGGCTTGCTGG
CCACTTTTTGGGGTCAGCTCCTCTGAAATGCATTAGCGGAACCGTTTGCAATCTGCCACA
AGTGTGATAAGTTATCTACACTGGCGAGGGGATTGCTCTCTGTAATGTTCAGCTTCTAAT

TGTCTCTACTTTGTGAGACAACTTTTGAATGCTTGACCTCAAATCAGGTAGGACTACCC-
GCTGAACTTAA
>BC9-14
TTTCCGTAGGTGAACCTGCGGAAGGATCATTATTGAATTATGTTTCTAGATAGGTTGTAG
CTGGCTCTTTAGAGCATGTGCACGCCTGTTTGGACTTCATTTTCATCCACCTGTGCACCT
ATTGTAGTCTTTGGTTGGGTTAGGGGGAAGTGGTCATTGTGTCAGCATCTGCTGGATGTG
AGGACTTGCATTGTGAAAGCTTTGCTGTCCTTGATGTGATCATGGAATCTCTTTCTCACT
AGAGTCTATGTCACTCATTATACTCTGTCGAATGTCATTGAATGTCTTTACATGGGCTTG
TATGCCTATGAAAATTGTAATACAACTTTCAGCAACGGATCTCTTGGCTCTCGCATCGAT
GAAGGACGCAGCGAAATGCGATAAGTAATGTGAATTGCAGAATTCAGTGAATCATCGAAT
CTTTGAACGCATCTTGCGCTCCTTGGTATTCCGAGGAGCATGCCTGTTTGAGTGTCATTA
AATTCTCAACTCTCTTATACTTTTTTGTAAAAGAGAGCTTGGACTGTGGAGGCTTGCTGG
CCACTTTTTGGGGTCAGCTCCTCTGAAATGCATTAGCGGAACCGTTTGCAATCTGCCACA
AGTGTGATAAGTTATCTACACTGGCGAGGGGATTGCTCTCTGTAATGTTCAGCTTCTAAT
TGTCTCTACTTTGTGAGACAACTTTTGAATGCTTGACCTCAAATCAGGTAGGACTACCC-
GCTGAACTTAA
>BC9-36
TTTCCGTAGGTGAACCTGCGGAAGGATCATTATTGAATTATGTTTCTAGATAGGTTGTAG
CTGGCTCTTTAGAGCATGTGCACGCCTGTTTGGACTTCATTTTCATCCACCTGTGCACCT
ATTGTAGTCTTTGGTTGGGTTAGGGGGAAGTGGTCATTGTGTCAGCATCTGCTGGATGTG
AGGACTTGCATTGTGAAAGCTTTGCTGTCCTTGATGTGATCATGGAATCTCTTTCTCACT
AGAGTCTATGTCACTCATTATACTCTGTCGAATGTCATTGAATGTCTTTACATGGGCTTG
TATGCCTATGAAAATTGTAATACAACTTTCAGCAACGGATCTCTTGGCTCTCGCATCGAT
GAAGGACGCAGCGAAATGCGATAAGTAATGTGAATTGCAGAATTCAGTGAATCATCGAAT
CTTTGAACGCATCTTGCGCTCCTTGGTATTCCGAGGAGCATGCCTGTTTGAGTGTCATTA
AATTCTCAACTCTCTTATACTTTTTTGTAAAAGAGAGCTTGGACTGTGGAGGCTTGCTGG
CCACTTTTTGGGGTCAGCTCCTCTGAAATGCATTAGCGGAACCGTTTGCAATCTGCCACA
AGTGTGATAAGTTATCTACACTGGCGAGGGGATTGCTCTCTGTAATGTTCAGCTTCTAAT
TGTCTCTACTTTGTGAGACAACTTTTGAATGCTTGACCTCAAATCAGGTAGGACTACCC-
GCTGAACTTAA
>BC9-45
TTTCCGTAGGTGAACCTGCGGAAGGATCATTATTGAATTATGTTTCTAGATAGGTTGTAG
CTGGCTCTTTAGAGCATGTGCACGCCTGTTTGGACTTCATTTTCATCCACCTGTGCACCT
ATTGTAGTCTTTGGTTGGGTTAGGGGGAAGTGGTCATTGTGTCAGCATCTGCTGGATGTG
AGGACTTGCATTGTGAAAGCTTTGCTGTCCTTGATGTGATCATGGAATCTCTTTCTCACT
AGAGTCTATGTCACTCATTATACTCTGTCGAATGTCATTGAATGTCTTTACATGGGCTTG
TATGCCTATGAAAATTGTAATACAACTTTCAGCAACGGATCTCTTGGCTCTCGCATCGAT
GAAGGACGCAGCGAAATGCGATAAGTAATGTGAATTGCAGAATTCAGTGAATCATCGAAT
CTTTGAACGCATCTTGCGCTCCTTGGTATTCCGAGGAGCATGCCTGTTTGAGTGTCATTA
AATTCTCAACTCTCTTATACTTTTTTGTAAAAGAGAGCTTGGACTGTGGAGGCTTGCTGG
CCACTTTTTGGGGTCAGCTCCTCTGAAATGCATTAGCGGAACCGTTTGCAATCTGCCACA
AGTGTGATAAGTTATCTACACTGGCGAGGGGATTGCTCTCTGTAATGTTCAGCTTCTAAT
TGTCTCTACTTTGTGAGACAACTTTTGAATGCTTGACCTCAAATCAGGTAGGACTACCC-
GCTGAACTTAA
>BC10_1
TTTCCGTAGGTGAACCTGCGGAAGGATCATTATTGAATTATGTTTCTAGATAGGTTGTAG
CTGGCTCTTTAGAGCATGTGCACGCCTGTTTGGACTTCATTTTCATCCACCTGTGCACCT
ATTGTAGTCTTTGGTTGGGTTAGGGGGAAGTGGTCATTGTGTCAGCATCTGCTGGATGTG
AGGACTTGCATTGTGAAAGCTTTGCTGTCCTTGATGTGATCATGGAATCTCTTTCTCACT
AGAGTCTATGTCACTCATTATACTCTGTCGAATGTCATTGAATGTCTTTACATGGGCTTG

TATGCCTATGAAAATTGTAATACAACTTTCAGCAACGGATCTCTTGGCTCTCGCATCGAT
GAAGGACGCAGCGAAATGCGATAAGTAATGTGAATTGCAGAATTCAGTGAATCATCGAAT
CTTTGAACGCATCTTGCGCTCCTTGGTATTCCGAGGAGCATGCCTGTTTGAGTGTCATTA
AATTCTCAACTCTCTTATACTTTTTTGTAAAAGAGAGCTTGGACTGTGGAGGCTTGCTGG
CCACTTTTTGGGGTCAGCTCCTCTGAAATGCATTAGCGGAACCGTTTGCAATCTGCCACA
AGTGTGATAAGTTATCTACACTGGCGAGGGGATTGCTCTCTGTAATGTTCAGCTTCTAAT
TGTCTCTACTTTGTGAGACAACTTTTGAATGCTTGACCTCAAATCAGGTAGGACTACCC-
GCTGAACTTAA
>BC10_16
TTTCCGTAGGTGAACCTGCGGAAGGATCATTATTGAATTATGTTTCTAGATAGGTTGTAG
CTGGCTCTTTAGAGCATGTGCACGCCTGTTTGGACTTCATTTTCATCCACCTGTGCACCT
ATTGTAGTCTTTGGTTGGGTTAGGGGGAAGTGGTCATTGTGTCAGCATCTGCTGGATGTG
AGGACTTGCATTGTGAAAGCTTTGCTGTCCTTGATGTGATCATGGAATCTCTTTCTCACT
AGAGTCTATGTCACTCATTATACTCTGTCGAATGTCATTGAATGTCTTTACATGGGCTTG
TATGCCTATGAAAATTGTAATACAACTTTCAGCAACGGATCTCTTGGCTCTCGCATCGAT
GAAGGACGCAGCGAAATGCGATAAGTAATGTGAATTGCAGAATTCAGTGAATCATCGAAT
CTTTGAACGCATCTTGCGCTCCTTGGTATTCCGAGGAGCATGCCTGTTTGAGTGTCATTA
AATTCTCAACTCTCTTATACTTTTTTGTAAAAGAGAGCTTGGACTGTGGAGGCTTGCTGG
CCACTTTTTGGGGTCAGCTCCTCTGAAATGCATTAGCGGAACCGTTTGCAATCTGCCACA
AGTGTGATAAGTTATCTACACTGGCGAGGGGATTGCTCTCTGTAATGTTCAGCTTCTAAT
TGTCTCTACTTTGTGAGACAACTTTTGAATGCTTGACCTCAAATCAGGTAGGACTACCC-
GCTGAACTTAA
>BC10_30
TTTCCGTAGGTGAACCTGCGGAAGGATCATTATTGAATTATGTTTCTAGATAGGTTGTAG
CTGGCTCTTTAGAGCATGTGCACGCCTGTTTGGACTTCATTTTCATCCACCTGTGCACCT
ATTGTAGTCTTTGGTTGGGTTAGGGGGAAGTGGTCATTGTGTCAGCATCTGCTGGATGTG
AGGACTTGCATTGTGAAAGCTTTGCTGTCCTTGATGTGATCATGGAATCTCTTTCTCACT
AGAGTCTATGTCACTCATTATACTCTGTCGAATGTCATTGAATGTCTTTACATGGGCTTG
TATGCCTATGAAAATTGTAATACAACTTTCAGCAACGGATCTCTTGGCTCTCGCATCGAT
GAAGGACGCAGCGAAATGCGATAAGTAATGTGAATTGCAGAATTCAGTGAATCATCGAAT
CTTTGAACGCATCTTGCGCTCCTTGGTATTCCGAGGAGCATGCCTGTTTGAGTGTCATTA
AATTCTCAACTCTCTTATACTTTTTTGTAAAAGAGAGCTTGGACTGTGGAGGCTTGCTGG
CCACTTTTTGGGGTCAGCTCCTCTGAAATGCATTAGCGGAACCGTTTGCAATCTGCCACA
AGTGTGATAAGTTATCTACACTGGCGAGGGGATTGCTCTCTGTAATGTTCAGCTTCTAAT
TGTCTCTACTTTGTGAGACAACTTTTGAATGCTTGACCTCAAATCAGGTAGGACTACCC-
GCTGAACTTAA
>BC11_18
TTTCCGTAGGTGAACCTGCGGAAGGATCATTATTGAATTATGTTTCTAGATAGGTTGTAG
CTGGCTCTTTAGAGCATGTGCACGCCTGTTTGGACTTCATTTTCATCCACCTGTGCACCT
ATTGTAGTCTTTGGTTGGGTTAGGGGGAAGTGGTCATTGTGTCAGCATCTGCTGGATGTG
AGGACTTGCATTGTGAAAGCTTTGCTGTCCTTGATGTGATCATGGAATCTCTTTCTCACT
AGAGTCTATGTCACTCATTATACTCTGTCGAATGTCATTGAATGTCTTTACATGGGCTTG
TATGCCTATGAAAATTGTAATACAACTTTCAGCAACGGATCTCTTGGCTCTCGCATCGAT
GAAGGACGCAGCGAAATGCGATAAGTAATGTGAATTGCAGAATTCAGTGAATCATCGAAT
CTTTGAACGCATCTTGCGCTCCTTGGTATTCCGAGGAGCATGCCTGTTTGAGTGTCATTA
AATTCTCAACTCTCTTATACTTTTTTGTAAAAGAGAGCTTGGACTGTGGAGGCTTGCTGG
CCACTTTTTGGGGTCAGCTCCTCTGAAATGCATTAGCGGAACCGTTTGCAATCTGCCACA
AGTGTGATAAGTTATCTACACTGGCGAGGGGATTGCTCTCTGTAATGTTCAGCTTCTAAT
TGTCTCTACTTTGTGAGACAACTTTTGAATGCTTGACCTCAAATCAGGTAGGACTACCC-
GCTGAACTTAA

>BC11_53
TTTCCGTAGGTGAACCTGCGGAAGGATCATTATTGAATTATGTTTCTAGATAGGTTGTAG
CTGGCTCTTTAGAGCATGTGCACGCCTGTTTGGACTTCATTTTCATCCACCTGTGCACCT
ATTGTAGTCTTTGGTTGGGTTAGGGGGAAGTGGTCATTGTGTCAGCATCTGCTGGATGTG
AGGACTTGCATTGTGAAAGCTTTGCTGTCCTTGATGTGATCATGGAATCTCTTTCTCACT
AGAGTCTATGTCACTCATTATACTCTGTCGAATGTCATTGAATGTCTTTACATGGGCTTG
TATGCCTATGAAAATTGTAATACAACTTTCAGCAACGGATCTCTTGGCTCTCGCATCGAT
GAAGGACGCAGCGAAATGCGATAAGTAATGTGAATTGCAGAATTCAGTGAATCATCGAAT
CTTTGAACGCATCTTGCGCTCCTTGGTATTCCGAGGAGCATGCCTGTTTGAGTGTCATTA
AATTCTCAACTCTCTTATACTTTTTTGTAAAAGAGAGCTTGGACTGTGGAGGCTTGCTGG
CCACTTTTTGGGGTCAGCTCCTCTGAAATGCATTAGCGGAACCGTTTGCAATCTGCCACA
AGTGTGATAAGTTATCTACACTGGCGAGGGGATTGCTCTCTGTAATGTTCAGCTTCTAAT
TGTCTCTACTTTGTGAGACAACTTTTGAATGCTTGACCTCAAATCAGGTAGGACTACCC-
GCTGAACTTAA
>BC12_33
TTTCCGTAGGTGAACCTGCGGAAGGATCATTATTGAATTATGTTTCTAGATAGGTTGTAG
CTGGCTCTTTAGAGCATGTGCACGCCTGTTTGGACTTCATTTTCATCCACCTGTGCACCT
ATTGTAGTCTTTGGTTGGGTTAGGGGGAAGTGGTCATTGTGTCAGCATCTGCTGGATGTG
AGGACTTGCATTGTGAAAGCTTTGCTGTCCTTGATGTGATCATGGAATCTCTTTCTCACT
AGAGTCTATGTCACTCATTATACTCTGTCGAATGTCATTGAATGTCTTTACATGGGCTTG
TATGCCTATGAAAATTGTAATACAACTTTCAGCAACGGATCTCTTGGCTCTCGCATCGAT
GAAGGACGCAGCGAAATGCGATAAGTAATGTGAATTGCAGAATTCAGTGAATCATCGAAT
CTTTGAACGCATCTTGCGCTCCTTGGTATTCCGAGGAGCATGCCTGTTTGAGTGTCATTA
AATTCTCAACTCTCTTATACTTTTTTGTAAAAGAGAGCTTGGACTGTGGAGGCTTGCTGG
CCACTTTTTGGGGTCAGCTCCTCTGAAATGCATTAGCGGAACCGTTTGCAATCTGCCACA
AGTGTGATAAGTTATCTACACTGGCGAGGGGATTGCTCTCTGTAATGTTCAGCTTCTAAT
TGTCTCTACTTTGTGAGACAACTTTTGAATGCTTGACCTCAAATCAGGTAGGACTACCC-
GCTGAACTTAA
>BC12_40
TTTCCGTAGGTGAACCTGCGGAAGGATCATTATTGAATTATGTTTCTAGATAGGTTGTAG
CTGGCTCTTTAGAGCATGTGCACGCCTGTTTGGACTTCATTTTCATCCACCTGTGCACCT
ATTGTAGTCTTTGGTTGGGTTAGGGGGAAGTGGTCATTGTGTCAGCATCTGCTGGATGTG
AGGACTTGCATTGTGAAAGCTTTGCTGTCCTTGATGTGATCATGGAATCTCTTTCTCACT
AGAGTCTATGTCACTCATTATACTCTGTCGAATGTCATTGAATGTCTTTACATGGGCTTG
TATGCCTATGAAAATTGTAATACAACTTTCAGCAACGGATCTCTTGGCTCTCGCATCGAT
GAAGGACGCAGCGAAATGCGATAAGTAATGTGAATTGCAGAATTCAGTGAATCATCGAAT
CTTTGAACGCATCTTGCGCTCCTTGGTATTCCGAGGAGCATGCCTGTTTGAGTGTCATTA
AATTCTCAACTCTCTTATACTTTTTTGTAAAAGAGAGCTTGGACTGTGGAGGCTTGCTGG
CCACTTTTTGGGGTCAGCTCCTCTGAAATGCATTAGCGGAACCGTTTGCAATCTGCCACA
AGTGTGATAAGTTATCTACACTGGCGAGGGGATTGCTCTCTGTAATGTTCAGCTTCTAAT
TGTCTCTACTTTGTGAGACAACTTTTGAATGCTTGACCTCAAATCAGGTAGGACTACCC-
GCTGAACTTAA
>BC2-57
TTTCCGTAGGTGAACCTGCGGAAGGATCATTATTGAATTATGTTTCTAGATAGGTTGTAG
CTGGCTCTTTAGAGCATGTGCACGCCTGTTTGGACTTCATTTTCATCCACCTGTGCACCT
ATTGTAGTCTTTGGTTGGGTTAGGGGGAAGTGGTCATTGTGTCAGCATCTGCTGGATGTG
AGGACTTGCATTGTGAAAGCTTTGCTGTCCTTGATGTGATCATGGAATCTCTTTCTCACT
AGAGTCTATGTCACTCATTATACTCTGTCGAATGTCATTGAATGTCTTTACATGGGCTTG
TATGCCTATGAAAATTGTAATACAACTTTCAGCAACGGATCTCTTGGCTCTCGCATCGAT
GAAGGACGCAGCGAAATGCGATAAGTAATGTGAATTGCAGAATTCAGTGAATCATCGAAT

CTTTGAACGCATCTTGCGCTCCTTGGTATTCCGAGGAGCATGCCTGTTTGAGTGTCATTA
AATTCTCAACTCTCTTATACTTTTTTGTAAAAGAGAGCTTGGACTGTGGAGGCTTGCTGG
CCACTTTTTGGGGTCAGCTCCTCTGAAATGCATTAGCGGAACCGTTTGCAATCTGCCACA
AGTGTGATAAGTTATCTACACTGGCGAGGGGATTGCTCTCTGTAATGTTCAGCTTCTAAT
TGTCTCTACTTTGTGAGACAACTTTTGAATGCTTGACCTCAAATCAGGTAGGACTACCC-
GCTGAACTTAA
>BC11_13
TTTCCGTAGGTGAACCTGCGGAAGGATCATTATTGAATTATGTTTCTAGATAGGTTGTAG
CTGGCTCTTTAGAGCATGTGCACGCCTGTTTGGACTTCATTTTCATCCACCTGTGCACCT
ATTGTAGTCTTTGGTTGGGTTAGGGGGAAGTGGTCATTGTGTCAGCATCTGCTGGATGTG
AGGACTTGCATTGTGAAAGCTTTGCTGTCCTTGATGTGATCATGGAATCTCTTTCTCACT
AGAGTCTATGTCACTCATTATACTCTGTCGAATGTCATTGAATGTCTTTACATGGGCTTG
TATGCCTATGAAAATTGTAATACAACTTTCAGCAACGGATCTCTTGGCTCTCGCATCGAT
GAAGGACGCAGCGAAATGCGATAAGTAATGTGAATTGCAGAATTCAGTGAATCATCGAAT
CTTTGAACGCATCTTGCGCTCCTTGGTATTCCGAGGAGCATGCCTGTTTGAGTGTCATTA
AATTCTCAACTCTCTTATACTTTTTTGTAAAAGAGAGCTTGGACTGTGGAGGCTTGCTGG
CCACTTTTTGGGGTCAGCTCCTCTGAAATGCATTAGCGGAACCGTTTGCAATCTGCCACA
AGTGTGATAAGTTATCTACACTGGCGAGGGGATTGCTCTCTGTAATGTTCAGCTTCTAAT
TGTCTCTACTTTGTGAGACAACTTTTGAATGCTTGACCTCAAATCAGGTAGGACTACCC-
GCTGAACTTAA
>BC12_49
TTTCCGTAGGTGAACCTGCGGAAGGATCATTATTGAATTATGTTTCTAGATAGGTTGTAG
CTGGCTCTTTAGAGCATGTGCACGCCTGTTTGGACTTCATTTTCATCCACCTGTGCACCT
ATTGTAGTCTTTGGTTGGGTTAGGGGGAAGTGGTCATTGTGTCAGCATCTGCTGGATGTG
AGGACTTGCATTGTGAAAGCTTTGCTGTCCTTGATGTGATCATGGAATCTCTTTCTCACT
AGAGTCTATGTCACTCATTATACTCTGTCGAATGTCATTGAATGTCTTTACATGGGCTTG
TATGCCTATGAAAATTGTAATACAACTTTCAGCAACGGATCTCTTGGCTCTCGCATCGAT
GAAGGACGCAGCGAAATGCGATAAGTAATGTGAATTGCAGAATTCAGTGAATCATCGAAT
CTTTGAACGCATCTTGCGCTCCTTGGTATTCCGAGGAGCATGCCTGTTTGAGTGTCATTA
AATTCTCAACTCTCTTATACTTTTTTGTAAAAGAGAGCTTGGACTGTGGAGGCTTGCTGG
CCACTTTTTGGGGTCAGCTCCTCTGAAATGCATTAGCGGAACCGTTTGCAATCTGCCACA
AGTGTGATAAGTTATCTACACTGGCGAGGGGATTGCTCTCTGTAATGTTCAGCTTCTAAT
TGTCTCTACTTTGTGAGACAACTTTTGAATGCTTGACCTCAAATCAGGTAGGACTACCC-
GCTGAACTTAA
>BC1-29
TTTCCGTAGGTGAACCTGCGGAAGGATCATTATTGAATTATGTTTCTAGATAGGTTGTAG
CTGGCTCTTTAGAGCATGTGCACGCCTGTTTGGACTTCATTTTCATCCACCTGTGCACCT
ATTGTAGTCTTTGGTTGGGTTAGGGGGAAGTGGTCATTGTGTCAGCATCTGCTGGATGTG
AGGACTTGCATTGTGAAAGCTTTGCTGTCCTTGATGTGATCATGGAATCTCTTTCTCACT
AGAGTCTATGTCACTCATTATACTCTGTCGAATGTCATTGAATGTCTTTACATGGGCTTG
TATGCCTATGAAAATTGTAATACAACTTTCAGCAACGGATCTCTTGGCTCTCGCATCGAT
GAAGGACGCAGCGAAATGCGATAAGTAATGTGAATTGCAGAATTCAGTGAATCATCGAAT
CTTTGAACGCATCTTGCGCTCCTTGGTATTCCGAGGAGCATGCCTGTTTGAGTGTCATTA
AATTCTCAACTCTCTTATACTTTTTTGTAAAAGAGAGCTTGGACTGTGGAGGCTTGCTGG
CCACTTTTTGGGGTCAGCTCCTCTGAAATGCATTAGCGGAACCGTTTGCAATCTGCCACA
AGTGTGATAAGTTATCTACACTGGCGAGGGGATTGCTCTCTGTAATGTTCAGCTTCTAAT
TGTCTCTACTTTGTGAGACAACTTTTGAATGCTTGACCTCAAATCAGGTAGGACTACCC-
GCTGAACTTAA
>BC1-39
TTTCCGTAGGTGAACCTGCGGAAGGATCATTATTGAATTATGTTTCTAGATAGGTTGTAG

CTGGCTCTTTAGAGCATGTGCACGCCTGTTTGGACTTCATTTTCATCCACCTGTGCACCT
ATTGTAGTCTTTGGTTGGGTTAGGGGGAAGTGGTCATTGTGTCAGCATCTGCTGGATGTG
AGGACTTGCATTGTGAAAGCTTTGCTGTCCTTGATGTGATCATGGAATCTCTTTCTCACT
AGAGTCTATGTCACTCATTATACTCTGTCGAATGTCATTGAATGTCTTTACATGGGCTTG
TATGCCTATGAAAATTGTAATACAACTTTCAGCAACGGATCTCTTGGCTCTCGCATCGAT
GAAGGACGCAGCGAAATGCGATAAGTAATGTGAATTGCAGAATTCAGTGAATCATCGAAT
CTTTGAACGCATCTTGCGCTCCTTGGTATTCCGAGGAGCATGCCTGTTTGAGTGTCATTA
AATTCTCAACTCTCTTATACTTTTTTGTAAAAGAGAGCTTGGACTGTGGAGGCTTGCTGG
CCACTTTTTGGGGTCAGCTCCTCTGAAATGCATTAGCGGAACCGTTTGCAATCTGCCACA
AGTGTGATAAGTTATCTACACTGGCGAGGGGATTGCTCTCTGTAATGTTCAGCTTCTAAT
TGTCTCTACTTTGTGAGACAACTTTTGAATGCTTGACCTCAAATCAGGTAGGACTACCC-
GCTGAACTTAA
>BC2-12
TTTCCGTAGGTGAACCTGCGGAAGGATCATTATTGAATTATGTTTCTAGATAGGTTGTAG
CTGGCTCTTTAGAGCATGTGCACGCCTGTTTGGACTTCATTTTCATCCACCTGTGCACCT
ATTGTAGTCTTTGGTTGGGTTAGGGGGAAGTGGTCATTGTGTCAGCATCTGCTGGATGTG
AGGACTTGCATTGTGAAAGCTTTGCTGTCCTTGATGTGATCATGGAATCTCTTTCTCACT
AGAGTCTATGTCACTCATTATACTCTGTCGAATGTCATTGAATGTCTTTACATGGGCTTG
TATGCCTATGAAAATTGTAATACAACTTTCAGCAACGGATCTCTTGGCTCTCGCATCGAT
GAAGGACGCAGCGAAATGCGATAAGTAATGTGAATTGCAGAATTCAGTGAATCATCGAAT
CTTTGAACGCATCTTGCGCTCCTTGGTATTCCGAGGAGCATGCCTGTTTGAGTGTCATTA
AATTCTCAACTCTCTTATACTTTTTTGTAAAAGAGAGCTTGGACTGTGGAGGCTTGCTGG
CCACTTTTTGGGGTCAGCTCCTCTGAAATGCATTAGCGGAACCGTTTGCAATCTGCCACA
AGTGTGATAAGTTATCTACACTGGCGAGGGGATTGCTCTCTGTAATGTTCAGCTTCTAAT
TGTCTCTACTTTGTGAGACAACTTTTGAATGCTTGACCTCAAATCAGGTAGGACTACCC-
GCTGAACTTAA
>BC2-23
TTTCCGTAGGTGAACCTGCGGAAGGATCATTATTGAATTATGTTTCTAGATAGGTTGTAG
CTGGCTCTTTAGAGCATGTGCACGCCTGTTTGGACTTCATTTTCATCCACCTGTGCACCT
ATTGTAGTCTTTGGTTGGGTTAGGGGGAAGTGGTCATTGTGTCAGCATCTGCTGGATGTG
AGGACTTGCATTGTGAAAGCTTTGCTGTCCTTGATGTGATCATGGAATCTCTTTCTCACT
AGAGTCTATGTCACTCATTATACTCTGTCGAATGTCATTGAATGTCTTTACATGGGCTTG
TATGCCTATGAAAATTGTAATACAACTTTCAGCAACGGATCTCTTGGCTCTCGCATCGAT
GAAGGACGCAGCGAAATGCGATAAGTAATGTGAATTGCAGAATTCAGTGAATCATCGAAT
CTTTGAACGCATCTTGCGCTCCTTGGTATTCCGAGGAGCATGCCTGTTTGAGTGTCATTA
AATTCTCAACTCTCTTATACTTTTTTGTAAAAGAGAGCTTGGACTGTGGAGGCTTGCTGG
CCACTTTTTGGGGTCAGCTCCTCTGAAATGCATTAGCGGAACCGTTTGCAATCTGCCACA
AGTGTGATAAGTTATCTACACTGGCGAGGGGATTGCTCTCTGTAATGTTCAGCTTCTAAT
TGTCTCTACTTTGTGAGACAACTTTTGAATGCTTGACCTCAAATCAGGTAGGACTACCC-
GCTGAACTTAA
>BC2-29
TTTCCGTAGGTGAACCTGCGGAAGGATCATTATTGAATTATGTTTCTAGATAGGTTGTAG
CTGGCTCTTTAGAGCATGTGCACGCCTGTTTGGACTTCATTTTCATCCACCTGTGCACCT
ATTGTAGTCTTTGGTTGGGTTAGGGGGAAGTGGTCATTGTGTCAGCATCTGCTGGATGTG
AGGACTTGCATTGTGAAAGCTTTGCTGTCCTTGATGTGATCATGGAATCTCTTTCTCACT
AGAGTCTATGTCACTCATTATACTCTGTCGAATGTCATTGAATGTCTTTACATGGGCTTG
TATGCCTATGAAAATTGTAATACAACTTTCAGCAACGGATCTCTTGGCTCTCGCATCGAT
GAAGGACGCAGCGAAATGCGATAAGTAATGTGAATTGCAGAATTCAGTGAATCATCGAAT
CTTTGAACGCATCTTGCGCTCCTTGGTATTCCGAGGAGCATGCCTGTTTGAGTGTCATTA
AATTCTCAACTCTCTTATACTTTTTTGTAAAAGAGAGCTTGGACTGTGGAGGCTTGCTGG

CCACTTTTTGGGGTCAGCTCCTCTGAAATGCATTAGCGGAACCGTTTGCAATCTGCCACA
AGTGTGATAAGTTATCTACACTGGCGAGGGGATTGCTCTCTGTAATGTTCAGCTTCTAAT
TGTCTCTACTTTGTGAGACAACTTTTGAATGCTTGACCTCAAATCAGGTAGGACTACCC-
GCTGAACTTAA
>BC3-1
TTTCCGTAGGTGAACCTGCGGAAGGATCATTATTGAATTATGTTTCTAGATAGGTTGTAG
CTGGCTCTTTAGAGCATGTGCACGCCTGTTTGGACTTCATTTTCATCCACCTGTGCACCT
ATTGTAGTCTTTGGTTGGGTTAGGGGGAAGTGGTCATTGTGTCAGCATCTGCTGGATGTG
AGGACTTGCATTGTGAAAGCTTTGCTGTCCTTGATGTGATCATGGAATCTCTTTCTCACT
AGAGTCTATGTCACTCATTATACTCTGTCGAATGTCATTGAATGTCTTTACATGGGCTTG
TATGCCTATGAAAATTGTAATACAACTTTCAGCAACGGATCTCTTGGCTCTCGCATCGAT
GAAGGACGCAGCGAAATGCGATAAGTAATGTGAATTGCAGAATTCAGTGAATCATCGAAT
CTTTGAACGCATCTTGCGCTCCTTGGTATTCCGAGGAGCATGCCTGTTTGAGTGTCATTA
AATTCTCAACTCTCTTATACTTTTTTGTAAAAGAGAGCTTGGACTGTGGAGGCTTGCTGG
CCACTTTTTGGGGTCAGCTCCTCTGAAATGCATTAGCGGAACCGTTTGCAATCTGCCACA
AGTGTGATAAGTTATCTACACTGGCGAGGGGATTGCTCTCTGTAATGTTCAGCTTCTAAT
TGTCTCTACTTTGTGAGACAACTTTTGAATGCTTGACCTCAAATCAGGTAGGACTACCC-
GCTGAACTTAA
>BC3-7
TTTCCGTAGGTGAACCTGCGGAAGGATCATTATTGAATTATGTTTCTAGATAGGTTGTAG
CTGGCTCTTTAGAGCATGTGCACGCCTGTTTGGACTTCATTTTCATCCACCTGTGCACCT
ATTGTAGTCTTTGGTTGGGTTAGGGGGAAGTGGTCATTGTGTCAGCATCTGCTGGATGTG
AGGACTTGCATTGTGAAAGCTTTGCTGTCCTTGATGTGATCATGGAATCTCTTTCTCACT
AGAGTCTATGTCACTCATTATACTCTGTCGAATGTCATTGAATGTCTTTACATGGGCTTG
TATGCCTATGAAAATTGTAATACAACTTTCAGCAACGGATCTCTTGGCTCTCGCATCGAT
GAAGGACGCAGCGAAATGCGATAAGTAATGTGAATTGCAGAATTCAGTGAATCATCGAAT
CTTTGAACGCATCTTGCGCTCCTTGGTATTCCGAGGAGCATGCCTGTTTGAGTGTCATTA
AATTCTCAACTCTCTTATACTTTTTTGTAAAAGAGAGCTTGGACTGTGGAGGCTTGCTGG
CCACTTTTTGGGGTCAGCTCCTCTGAAATGCATTAGCGGAACCGTTTGCAATCTGCCACA
AGTGTGATAAGTTATCTACACTGGCGAGGGGATTGCTCTCTGTAATGTTCAGCTTCTAAT
TGTCTCTACTTTGTGAGACAACTTTTGAATGCTTGACCTCAAATCAGGTAGGACTACCC-
GCTGAACTTAA
>BC3-33
TTTCCGTAGGTGAACCTGCGGAAGGATCATTATTGAATTATGTTTCTAGATAGGTTGTAG
CTGGCTCTTTAGAGCATGTGCACGCCTGTTTGGACTTCATTTTCATCCACCTGTGCACCT
ATTGTAGTCTTTGGTTGGGTTAGGGGGAAGTGGTCATTGTGTCAGCATCTGCTGGATGTG
AGGACTTGCATTGTGAAAGCTTTGCTGTCCTTGATGTGATCATGGAATCTCTTTCTCACT
AGAGTCTATGTCACTCATTATACTCTGTCGAATGTCATTGAATGTCTTTACATGGGCTTG
TATGCCTATGAAAATTGTAATACAACTTTCAGCAACGGATCTCTTGGCTCTCGCATCGAT
GAAGGACGCAGCGAAATGCGATAAGTAATGTGAATTGCAGAATTCAGTGAATCATCGAAT
CTTTGAACGCATCTTGCGCTCCTTGGTATTCCGAGGAGCATGCCTGTTTGAGTGTCATTA
AATTCTCAACTCTCTTATACTTTTTTGTAAAAGAGAGCTTGGACTGTGGAGGCTTGCTGG
CCACTTTTTGGGGTCAGCTCCTCTGAAATGCATTAGCGGAACCGTTTGCAATCTGCCACA
AGTGTGATAAGTTATCTACACTGGCGAGGGGATTGCTCTCTGTAATGTTCAGCTTCTAAT
TGTCTCTACTTTGTGAGACAACTTTTGAATGCTTGACCTCAAATCAGGTAGGACTACCC-
GCTGAACTTAA
>BC3-39
TTTCCGTAGGTGAACCTGCGGAAGGATCATTATTGAATTATGTTTCTAGATAGGTTGTAG
CTGGCTCTTTAGAGCATGTGCACGCCTGTTTGGACTTCATTTTCATCCACCTGTGCACCT
ATTGTAGTCTTTGGTTGGGTTAGGGGGAAGTGGTCATTGTGTCAGCATCTGCTGGATGTG

AGGACTTGCATTGTGAAAGCTTTGCTGTCCTTGATGTGATCATGGAATCTCTTTCTCACT
AGAGTCTATGTCACTCATTATACTCTGTCGAATGTCATTGAATGTCTTTACATGGGCTTG
TATGCCTATGAAAATTGTAATACAACTTTCAGCAACGGATCTCTTGGCTCTCGCATCGAT
GAAGGACGCAGCGAAATGCGATAAGTAATGTGAATTGCAGAATTCAGTGAATCATCGAAT
CTTTGAACGCATCTTGCGCTCCTTGGTATTCCGAGGAGCATGCCTGTTTGAGTGTCATTA
AATTCTCAACTCTCTTATACTTTTTTGTAAAAGAGAGCTTGGACTGTGGAGGCTTGCTGG
CCACTTTTTGGGGTCAGCTCCTCTGAAATGCATTAGCGGAACCGTTTGCAATCTGCCACA
AGTGTGATAAGTTATCTACACTGGCGAGGGGATTGCTCTCTGTAATGTTCAGCTTCTAAT
TGTCTCTACTTTGTGAGACAACTTTTGAATGCTTGACCTCAAATCAGGTAGGACTACCC-
GCTGAACTTAA
>BC3-44
TTTCCGTAGGTGAACCTGCGGAAGGATCATTATTGAATTATGTTTCTAGATAGGTTGTAG
CTGGCTCTTTAGAGCATGTGCACGCCTGTTTGGACTTCATTTTCATCCACCTGTGCACCT
ATTGTAGTCTTTGGTTGGGTTAGGGGGAAGTGGTCATTGTGTCAGCATCTGCTGGATGTG
AGGACTTGCATTGTGAAAGCTTTGCTGTCCTTGATGTGATCATGGAATCTCTTTCTCACT
AGAGTCTATGTCACTCATTATACTCTGTCGAATGTCATTGAATGTCTTTACATGGGCTTG
TATGCCTATGAAAATTGTAATACAACTTTCAGCAACGGATCTCTTGGCTCTCGCATCGAT
GAAGGACGCAGCGAAATGCGATAAGTAATGTGAATTGCAGAATTCAGTGAATCATCGAAT
CTTTGAACGCATCTTGCGCTCCTTGGTATTCCGAGGAGCATGCCTGTTTGAGTGTCATTA
AATTCTCAACTCTCTTATACTTTTTTGTAAAAGAGAGCTTGGACTGTGGAGGCTTGCTGG
CCACTTTTTGGGGTCAGCTCCTCTGAAATGCATTAGCGGAACCGTTTGCAATCTGCCACA
AGTGTGATAAGTTATCTACACTGGCGAGGGGATTGCTCTCTGTAATGTTCAGCTTCTAAT
TGTCTCTACTTTGTGAGACAACTTTTGAATGCTTGACCTCAAATCAGGTAGGACTACCC-
GCTGAACTTAA
>BC3-45
TTTCCGTAGGTGAACCTGCGGAAGGATCATTATTGAATTATGTTTCTAGATAGGTTGTAG
CTGGCTCTTTAGAGCATGTGCACGCCTGTTTGGACTTCATTTTCATCCACCTGTGCACCT
ATTGTAGTCTTTGGTTGGGTTAGGGGGAAGTGGTCATTGTGTCAGCATCTGCTGGATGTG
AGGACTTGCATTGTGAAAGCTTTGCTGTCCTTGATGTGATCATGGAATCTCTTTCTCACT
AGAGTCTATGTCACTCATTATACTCTGTCGAATGTCATTGAATGTCTTTACATGGGCTTG
TATGCCTATGAAAATTGTAATACAACTTTCAGCAACGGATCTCTTGGCTCTCGCATCGAT
GAAGGACGCAGCGAAATGCGATAAGTAATGTGAATTGCAGAATTCAGTGAATCATCGAAT
CTTTGAACGCATCTTGCGCTCCTTGGTATTCCGAGGAGCATGCCTGTTTGAGTGTCATTA
AATTCTCAACTCTCTTATACTTTTTTGTAAAAGAGAGCTTGGACTGTGGAGGCTTGCTGG
CCACTTTTTGGGGTCAGCTCCTCTGAAATGCATTAGCGGAACCGTTTGCAATCTGCCACA
AGTGTGATAAGTTATCTACACTGGCGAGGGGATTGCTCTCTGTAATGTTCAGCTTCTAAT
TGTCTCTACTTTGTGAGACAACTTTTGAATGCTTGACCTCAAATCAGGTAGGACTACCC-
GCTGAACTTAA
>BC3-49
TTTCCGTAGGTGAACCTGCGGAAGGATCATTATTGAATTATGTTTCTAGATAGGTTGTAG
CTGGCTCTTTAGAGCATGTGCACGCCTGTTTGGACTTCATTTTCATCCACCTGTGCACCT
ATTGTAGTCTTTGGTTGGGTTAGGGGGAAGTGGTCATTGTGTCAGCATCTGCTGGATGTG
AGGACTTGCATTGTGAAAGCTTTGCTGTCCTTGATGTGATCATGGAATCTCTTTCTCACT
AGAGTCTATGTCACTCATTATACTCTGTCGAATGTCATTGAATGTCTTTACATGGGCTTG
TATGCCTATGAAAATTGTAATACAACTTTCAGCAACGGATCTCTTGGCTCTCGCATCGAT
GAAGGACGCAGCGAAATGCGATAAGTAATGTGAATTGCAGAATTCAGTGAATCATCGAAT
CTTTGAACGCATCTTGCGCTCCTTGGTATTCCGAGGAGCATGCCTGTTTGAGTGTCATTA
AATTCTCAACTCTCTTATACTTTTTTGTAAAAGAGAGCTTGGACTGTGGAGGCTTGCTGG
CCACTTTTTGGGGTCAGCTCCTCTGAAATGCATTAGCGGAACCGTTTGCAATCTGCCACA
AGTGTGATAAGTTATCTACACTGGCGAGGGGATTGCTCTCTGTAATGTTCAGCTTCTAAT

TGTCTCTACTTTGTGAGACAACTTTTGAATGCTTGACCTCAAATCAGGTAGGACTACCC−
GCTGAACTTAA
>BC4−6
TTTCCGTAGGTGAACCTGCGGAAGGATCATTATTGAATTATGTTTCTAGATAGGTTGTAG
CTGGCTCTTTAGAGCATGTGCACGCCTGTTTGGACTTCATTTTCATCCACCTGTGCACCT
ATTGTAGTCTTTGGTTGGGTTAGGGGGAAGTGGTCATTGTGTCAGCATCTGCTGGATGTG
AGGACTTGCATTGTGAAAGCTTTGCTGTCCTTGATGTGATCATGGAATCTCTTTCTCACT
AGAGTCTATGTCACTCATTATACTCTGTCGAATGTCATTGAATGTCTTTACATGGGCTTG
TATGCCTATGAAAATTGTAATACAACTTTCAGCAACGGATCTCTTGGCTCTCGCATCGAT
GAAGGACGCAGCGAAATGCGATAAGTAATGTGAATTGCAGAATTCAGTGAATCATCGAAT
CTTTGAACGCATCTTGCGCTCCTTGGTATTCCGAGGAGCATGCCTGTTTGAGTGTCATTA
AATTCTCAACTCTCTTATACTTTTTTGTAAAAGAGAGCTTGGACTGTGGAGGCTTGCTGG
CCACTTTTTGGGGTCAGCTCCTCTGAAATGCATTAGCGGAACCGTTTGCAATCTGCCACA
AGTGTGATAAGTTATCTACACTGGCGAGGGGATTGCTCTCTGTAATGTTCAGCTTCTAAT
TGTCTCTACTTTGTGAGACAACTTTTGAATGCTTGACCTCAAATCAGGTAGGACTACCC−
GCTGAACTTAA
>BC4−26
TTTCCGTAGGTGAACCTGCGGAAGGATCATTATTGAATTATGTTTCTAGATAGGTTGTAG
CTGGCTCTTTAGAGCATGTGCACGCCTGTTTGGACTTCATTTTCATCCACCTGTGCACCT
ATTGTAGTCTTTGGTTGGGTTAGGGGGAAGTGGTCATTGTGTCAGCATCTGCTGGATGTG
AGGACTTGCATTGTGAAAGCTTTGCTGTCCTTGATGTGATCATGGAATCTCTTTCTCACT
AGAGTCTATGTCACTCATTATACTCTGTCGAATGTCATTGAATGTCTTTACATGGGCTTG
TATGCCTATGAAAATTGTAATACAACTTTCAGCAACGGATCTCTTGGCTCTCGCATCGAT
GAAGGACGCAGCGAAATGCGATAAGTAATGTGAATTGCAGAATTCAGTGAATCATCGAAT
CTTTGAACGCATCTTGCGCTCCTTGGTATTCCGAGGAGCATGCCTGTTTGAGTGTCATTA
AATTCTCAACTCTCTTATACTTTTTTGTAAAAGAGAGCTTGGACTGTGGAGGCTTGCTGG
CCACTTTTTGGGGTCAGCTCCTCTGAAATGCATTAGCGGAACCGTTTGCAATCTGCCACA
AGTGTGATAAGTTATCTACACTGGCGAGGGGATTGCTCTCTGTAATGTTCAGCTTCTAAT
TGTCTCTACTTTGTGAGACAACTTTTGAATGCTTGACCTCAAATCAGGTAGGACTACCC−
GCTGAACTTAA
>BC4−30
TTTCCGTAGGTGAACCTGCGGAAGGATCATTATTGAATTATGTTTCTAGATAGGTTGTAG
CTGGCTCTTTAGAGCATGTGCACGCCTGTTTGGACTTCATTTTCATCCACCTGTGCACCT
ATTGTAGTCTTTGGTTGGGTTAGGGGGAAGTGGTCATTGTGTCAGCATCTGCTGGATGTG
AGGACTTGCATTGTGAAAGCTTTGCTGTCCTTGATGTGATCATGGAATCTCTTTCTCACT
AGAGTCTATGTCACTCATTATACTCTGTCGAATGTCATTGAATGTCTTTACATGGGCTTG
TATGCCTATGAAAATTGTAATACAACTTTCAGCAACGGATCTCTTGGCTCTCGCATCGAT
GAAGGACGCAGCGAAATGCGATAAGTAATGTGAATTGCAGAATTCAGTGAATCATCGAAT
CTTTGAACGCATCTTGCGCTCCTTGGTATTCCGAGGAGCATGCCTGTTTGAGTGTCATTA
AATTCTCAACTCTCTTATACTTTTTTGTAAAAGAGAGCTTGGACTGTGGAGGCTTGCTGG
CCACTTTTTGGGGTCAGCTCCTCTGAAATGCATTAGCGGAACCGTTTGCAATCTGCCACA
AGTGTGATAAGTTATCTACACTGGCGAGGGGATTGCTCTCTGTAATGTTCAGCTTCTAAT
TGTCTCTACTTTGTGAGACAACTTTTGAATGCTTGACCTCAAATCAGGTAGGACTACCC−
GCTGAACTTAA
>BC4−69
TTTCCGTAGGTGAACCTGCGGAAGGATCATTATTGAATTATGTTTCTAGATAGGTTGTAG
CTGGCTCTTTAGAGCATGTGCACGCCTGTTTGGACTTCATTTTCATCCACCTGTGCACCT
ATTGTAGTCTTTGGTTGGGTTAGGGGGAAGTGGTCATTGTGTCAGCATCTGCTGGATGTG
AGGACTTGCATTGTGAAAGCTTTGCTGTCCTTGATGTGATCATGGAATCTCTTTCTCACT
AGAGTCTATGTCACTCATTATACTCTGTCGAATGTCATTGAATGTCTTTACATGGGCTTG

TATGCCTATGAAAATTGTAATACAACTTTCAGCAACGGATCTCTTGGCTCTCGCATCGAT
GAAGGACGCAGCGAAATGCGATAAGTAATGTGAATTGCAGAATTCAGTGAATCATCGAAT
CTTTGAACGCATCTTGCGCTCCTTGGTATTCCGAGGAGCATGCCTGTTTGAGTGTCATTA
AATTCTCAACTCTCTTATACTTTTTTGTAAAAGAGAGCTTGGACTGTGGAGGCTTGCTGG
CCACTTTTTGGGGTCAGCTCCTCTGAAATGCATTAGCGGAACCGTTTGCAATCTGCCACA
AGTGTGATAAGTTATCTACACTGGCGAGGGGATTGCTCTCTGTAATGTTCAGCTTCTAAT
TGTCTCTACTTTGTGAGACAACTTTTGAATGCTTGACCTCAAATCAGGTAGGACTACCC-
GCTGAACTTAA
>BC4-43
TTTCCGTAGGTGAACCTGCGGAAGGATCATTATTGAATTATGTTTCTAGATAGGTTGTAG
CTGGCTCTTTAGAGCATGTGCACGCCTGTTTGGACTTCATTTTCATCCACCTGTGCACCT
ATTGTAGTCTTTGGTTGGGTTAGGGGGAAGTGGTCATTGTGTCAGCATCTGCTGGATGTG
AGGACTTGCATTGTGAAAGCTTTGCTGTCCTTGATGTGATCATGGAATCTCTTTCTCACT
AGAGTCTATGTCACTCATTATACTCTGTCGAATGTCATTGAATGTCTTTACATGGGCTTG
TATGCCTATGAAAATTGTAATACAACTTTCAGCAACGGATCTCTTGGCTCTCGCATCGAT
GAAGGACGCAGCGAAATGCGATAAGTAATGTGAATTGCAGAATTCAGTGAATCATCGAAT
CTTTGAACGCATCTTGCGCTCCTTGGTATTCCGAGGAGCATGCCTGTTTGAGTGTCATTA
AATTCTCAACTCTCTTATACTTTTTTGTAAAAGAGAGCTTGGACTGTGGAGGCTTGCTGG
CCACTTTTTGGGGTCAGCTCCTCTGAAATGCATTAGCGGAACCGTTTGCAATCTGCCACA
AGTGTGATAAGTTATCTACACTGGCGAGGGGATTGCTCTCTGTAATGTTCAGCTTCTAAT
TGTCTCTACTTTGTGAGACAACTTTTGAATGCTTGACCTCAAATCAGGTAGGACTACCC-
GCTGAACTTAA
>BC4-49
TTTCCGTAGGTGAACCTGCGGAAGGATCATTATTGAATTATGTTTCTAGATAGGTTGTAG
CTGGCTCTTTAGAGCATGTGCACGCCTGTTTGGACTTCATTTTCATCCACCTGTGCACCT
ATTGTAGTCTTTGGTTGGGTTAGGGGGAAGTGGTCATTGTGTCAGCATCTGCTGGATGTG
AGGACTTGCATTGTGAAAGCTTTGCTGTCCTTGATGTGATCATGGAATCTCTTTCTCACT
AGAGTCTATGTCACTCATTATACTCTGTCGAATGTCATTGAATGTCTTTACATGGGCTTG
TATGCCTATGAAAATTGTAATACAACTTTCAGCAACGGATCTCTTGGCTCTCGCATCGAT
GAAGGACGCAGCGAAATGCGATAAGTAATGTGAATTGCAGAATTCAGTGAATCATCGAAT
CTTTGAACGCATCTTGCGCTCCTTGGTATTCCGAGGAGCATGCCTGTTTGAGTGTCATTA
AATTCTCAACTCTCTTATACTTTTTTGTAAAAGAGAGCTTGGACTGTGGAGGCTTGCTGG
CCACTTTTTGGGGTCAGCTCCTCTGAAATGCATTAGCGGAACCGTTTGCAATCTGCCACA
AGTGTGATAAGTTATCTACACTGGCGAGGGGATTGCTCTCTGTAATGTTCAGCTTCTAAT
TGTCTCTACTTTGTGAGACAACTTTTGAATGCTTGACCTCAAATCAGGTAGGACTACCC-
GCTGAACTTAA
>BC4-64
TTTCCGTAGGTGAACCTGCGGAAGGATCATTATTGAATTATGTTTCTAGATAGGTTGTAG
CTGGCTCTTTAGAGCATGTGCACGCCTGTTTGGACTTCATTTTCATCCACCTGTGCACCT
ATTGTAGTCTTTGGTTGGGTTAGGGGGAAGTGGTCATTGTGTCAGCATCTGCTGGATGTG
AGGACTTGCATTGTGAAAGCTTTGCTGTCCTTGATGTGATCATGGAATCTCTTTCTCACT
AGAGTCTATGTCACTCATTATACTCTGTCGAATGTCATTGAATGTCTTTACATGGGCTTG
TATGCCTATGAAAATTGTAATACAACTTTCAGCAACGGATCTCTTGGCTCTCGCATCGAT
GAAGGACGCAGCGAAATGCGATAAGTAATGTGAATTGCAGAATTCAGTGAATCATCGAAT
CTTTGAACGCATCTTGCGCTCCTTGGTATTCCGAGGAGCATGCCTGTTTGAGTGTCATTA
AATTCTCAACTCTCTTATACTTTTTTGTAAAAGAGAGCTTGGACTGTGGAGGCTTGCTGG
CCACTTTTTGGGGTCAGCTCCTCTGAAATGCATTAGCGGAACCGTTTGCAATCTGCCACA
AGTGTGATAAGTTATCTACACTGGCGAGGGGATTGCTCTCTGTAATGTTCAGCTTCTAAT
TGTCTCTACTTTGTGAGACAACTTTTGAATGCTTGACCTCAAATCAGGTAGGACTACCC-
GCTGAACTTAA

>BC5-30
TTTCCGTAGGTGAACCTGCGGAAGGATCATTATTGAATTATGTTTCTAGATAGGTTGTAG
CTGGCTCTTTAGAGCATGTGCACGCCTGTTTGGACTTCATTTTCATCCACCTGTGCACCT
ATTGTAGTCTTTGGTTGGGTTAGGGGGAAGTGGTCATTGTGTCAGCATCTGCTGGATGTG
AGGACTTGCATTGTGAAAGCTTTGCTGTCCTTGATGTGATCATGGAATCTCTTTCTCACT
AGAGTCTATGTCACTCATTATACTCTGTCGAATGTCATTGAATGTCTTTACATGGGCTTG
TATGCCTATGAAAATTGTAATACAACTTTCAGCAACGGATCTCTTGGCTCTCGCATCGAT
GAAGGACGCAGCGAAATGCGATAAGTAATGTGAATTGCAGAATTCAGTGAATCATCGAAT
CTTTGAACGCATCTTGCGCTCCTTGGTATTCCGAGGAGCATGCCTGTTTGAGTGTCATTA
AATTCTCAACTCTCTTATACTTTTTTGTAAAAGAGAGCTTGGACTGTGGAGGCTTGCTGG
CCACTTTTTGGGGTCAGCTCCTCTGAAATGCATTAGCGGAACCGTTTGCAATCTGCCACA
AGTGTGATAAGTTATCTACACTGGCGAGGGGATTGCTCTCTGTAATGTTCAGCTTCTAAT
TGTCTCTACTTTGTGAGACAACTTTTGAATGCTTGACCTCAAATCAGGTAGGACTACCC-
GCTGAACTTAA
>BC5-37
TTTCCGTAGGTGAACCTGCGGAAGGATCATTATTGAATTATGTTTCTAGATAGGTTGTAG
CTGGCTCTTTAGAGCATGTGCACGCCTGTTTGGACTTCATTTTCATCCACCTGTGCACCT
ATTGTAGTCTTTGGTTGGGTTAGGGGGAAGTGGTCATTGTGTCAGCATCTGCTGGATGTG
AGGACTTGCATTGTGAAAGCTTTGCTGTCCTTGATGTGATCATGGAATCTCTTTCTCACT
AGAGTCTATGTCACTCATTATACTCTGTCGAATGTCATTGAATGTCTTTACATGGGCTTG
TATGCCTATGAAAATTGTAATACAACTTTCAGCAACGGATCTCTTGGCTCTCGCATCGAT
GAAGGACGCAGCGAAATGCGATAAGTAATGTGAATTGCAGAATTCAGTGAATCATCGAAT
CTTTGAACGCATCTTGCGCTCCTTGGTATTCCGAGGAGCATGCCTGTTTGAGTGTCATTA
AATTCTCAACTCTCTTATACTTTTTTGTAAAAGAGAGCTTGGACTGTGGAGGCTTGCTGG
CCACTTTTTGGGGTCAGCTCCTCTGAAATGCATTAGCGGAACCGTTTGCAATCTGCCACA
AGTGTGATAAGTTATCTACACTGGCGAGGGGATTGCTCTCTGTAATGTTCAGCTTCTAAT
TGTCTCTACTTTGTGAGACAACTTTTGAATGCTTGACCTCAAATCAGGTAGGACTACCC-
GCTGAACTTAA
>BC5-44
TTTCCGTAGGTGAACCTGCGGAAGGATCATTATTGAATTATGTTTCTAGATAGGTTGTAG
CTGGCTCTTTAGAGCATGTGCACGCCTGTTTGGACTTCATTTTCATCCACCTGTGCACCT
ATTGTAGTCTTTGGTTGGGTTAGGGGGAAGTGGTCATTGTGTCAGCATCTGCTGGATGTG
AGGACTTGCATTGTGAAAGCTTTGCTGTCCTTGATGTGATCATGGAATCTCTTTCTCACT
AGAGTCTATGTCACTCATTATACTCTGTCGAATGTCATTGAATGTCTTTACATGGGCTTG
TATGCCTATGAAAATTGTAATACAACTTTCAGCAACGGATCTCTTGGCTCTCGCATCGAT
GAAGGACGCAGCGAAATGCGATAAGTAATGTGAATTGCAGAATTCAGTGAATCATCGAAT
CTTTGAACGCATCTTGCGCTCCTTGGTATTCCGAGGAGCATGCCTGTTTGAGTGTCATTA
AATTCTCAACTCTCTTATACTTTTTTGTAAAAGAGAGCTTGGACTGTGGAGGCTTGCTGG
CCACTTTTTGGGGTCAGCTCCTCTGAAATGCATTAGCGGAACCGTTTGCAATCTGCCACA
AGTGTGATAAGTTATCTACACTGGCGAGGGGATTGCTCTCTGTAATGTTCAGCTTCTAAT
TGTCTCTACTTTGTGAGACAACTTTTGAATGCTTGACCTCAAATCAGGTAGGACTACCC-
GCTGAACTTAA
>BC5-49
TTTCCGTAGGTGAACCTGCGGAAGGATCATTATTGAATTATGTTTCTAGATAGGTTGTAG
CTGGCTCTTTAGAGCATGTGCACGCCTGTTTGGACTTCATTTTCATCCACCTGTGCACCT
ATTGTAGTCTTTGGTTGGGTTAGGGGGAAGTGGTCATTGTGTCAGCATCTGCTGGATGTG
AGGACTTGCATTGTGAAAGCTTTGCTGTCCTTGATGTGATCATGGAATCTCTTTCTCACT
AGAGTCTATGTCACTCATTATACTCTGTCGAATGTCATTGAATGTCTTTACATGGGCTTG
TATGCCTATGAAAATTGTAATACAACTTTCAGCAACGGATCTCTTGGCTCTCGCATCGAT
GAAGGACGCAGCGAAATGCGATAAGTAATGTGAATTGCAGAATTCAGTGAATCATCGAAT

CTTTGAACGCATCTTGCGCTCCTTGGTATTCCGAGGAGCATGCCTGTTTGAGTGTCATTA
AATTCTCAACTCTCTTATACTTTTTTGTAAAAGAGAGCTTGGACTGTGGAGGCTTGCTGG
CCACTTTTTGGGGTCAGCTCCTCTGAAATGCATTAGCGGAACCGTTTGCAATCTGCCACA
AGTGTGATAAGTTATCTACACTGGCGAGGGGATTGCTCTCTGTAATGTTCAGCTTCTAAT
TGTCTCTACTTTGTGAGACAACTTTTGAATGCTTGACCTCAAATCAGGTAGGACTACCC-
GCTGAACTTAA
>BC5-54
TTTCCGTAGGTGAACCTGCGGAAGGATCATTATTGAATTATGTTTCTAGATAGGTTGTAG
CTGGCTCTTTAGAGCATGTGCACGCCTGTTTGGACTTCATTTTCATCCACCTGTGCACCT
ATTGTAGTCTTTGGTTGGGTTAGGGGGAAGTGGTCATTGTGTCAGCATCTGCTGGATGTG
AGGACTTGCATTGTGAAAGCTTTGCTGTCCTTGATGTGATCATGGAATCTCTTTCTCACT
AGAGTCTATGTCACTCATTATACTCTGTCGAATGTCATTGAATGTCTTTACATGGGCTTG
TATGCCTATGAAAATTGTAATACAACTTTCAGCAACGGATCTCTTGGCTCTCGCATCGAT
GAAGGACGCAGCGAAATGCGATAAGTAATGTGAATTGCAGAATTCAGTGAATCATCGAAT
CTTTGAACGCATCTTGCGCTCCTTGGTATTCCGAGGAGCATGCCTGTTTGAGTGTCATTA
AATTCTCAACTCTCTTATACTTTTTTGTAAAAGAGAGCTTGGACTGTGGAGGCTTGCTGG
CCACTTTTTGGGGTCAGCTCCTCTGAAATGCATTAGCGGAACCGTTTGCAATCTGCCACA
AGTGTGATAAGTTATCTACACTGGCGAGGGGATTGCTCTCTGTAATGTTCAGCTTCTAAT
TGTCTCTACTTTGTGAGACAACTTTTGAATGCTTGACCTCAAATCAGGTAGGACTACCC-
GCTGAACTTAA
>BC5-62
TTTCCGTAGGTGAACCTGCGGAAGGATCATTATTGAATTATGTTTCTAGATAGGTTGTAG
CTGGCTCTTTAGAGCATGTGCACGCCTGTTTGGACTTCATTTTCATCCACCTGTGCACCT
ATTGTAGTCTTTGGTTGGGTTAGGGGGAAGTGGTCATTGTGTCAGCATCTGCTGGATGTG
AGGACTTGCATTGTGAAAGCTTTGCTGTCCTTGATGTGATCATGGAATCTCTTTCTCACT
AGAGTCTATGTCACTCATTATACTCTGTCGAATGTCATTGAATGTCTTTACATGGGCTTG
TATGCCTATGAAAATTGTAATACAACTTTCAGCAACGGATCTCTTGGCTCTCGCATCGAT
GAAGGACGCAGCGAAATGCGATAAGTAATGTGAATTGCAGAATTCAGTGAATCATCGAAT
CTTTGAACGCATCTTGCGCTCCTTGGTATTCCGAGGAGCATGCCTGTTTGAGTGTCATTA
AATTCTCAACTCTCTTATACTTTTTTGTAAAAGAGAGCTTGGACTGTGGAGGCTTGCTGG
CCACTTTTTGGGGTCAGCTCCTCTGAAATGCATTAGCGGAACCGTTTGCAATCTGCCACA
AGTGTGATAAGTTATCTACACTGGCGAGGGGATTGCTCTCTGTAATGTTCAGCTTCTAAT
TGTCTCTACTTTGTGAGACAACTTTTGAATGCTTGACCTCAAATCAGGTAGGACTACCC-
GCTGAACTTAA
>BC5-71
TTTCCGTAGGTGAACCTGCGGAAGGATCATTATTGAATTATGTTTCTAGATAGGTTGTAG
CTGGCTCTTTAGAGCATGTGCACGCCTGTTTGGACTTCATTTTCATCCACCTGTGCACCT
ATTGTAGTCTTTGGTTGGGTTAGGGGGAAGTGGTCATTGTGTCAGCATCTGCTGGATGTG
AGGACTTGCATTGTGAAAGCTTTGCTGTCCTTGATGTGATCATGGAATCTCTTTCTCACT
AGAGTCTATGTCACTCATTATACTCTGTCGAATGTCATTGAATGTCTTTACATGGGCTTG
TATGCCTATGAAAATTGTAATACAACTTTCAGCAACGGATCTCTTGGCTCTCGCATCGAT
GAAGGACGCAGCGAAATGCGATAAGTAATGTGAATTGCAGAATTCAGTGAATCATCGAAT
CTTTGAACGCATCTTGCGCTCCTTGGTATTCCGAGGAGCATGCCTGTTTGAGTGTCATTA
AATTCTCAACTCTCTTATACTTTTTTGTAAAAGAGAGCTTGGACTGTGGAGGCTTGCTGG
CCACTTTTTGGGGTCAGCTCCTCTGAAATGCATTAGCGGAACCGTTTGCAATCTGCCACA
AGTGTGATAAGTTATCTACACTGGCGAGGGGATTGCTCTCTGTAATGTTCAGCTTCTAAT
TGTCTCTACTTTGTGAGACAACTTTTGAATGCTTGACCTCAAATCAGGTAGGACTACCC-
GCTGAACTTAA
>BC5-73
TTTCCGTAGGTGAACCTGCGGAAGGATCATTATTGAATTATGTTTCTAGATAGGTTGTAG

CTGGCTCTTTAGAGCATGTGCACGCCTGTTTGGACTTCATTTTCATCCACCTGTGCACCT
ATTGTAGTCTTTGGTTGGGTTAGGGGGAAGTGGTCATTGTGTCAGCATCTGCTGGATGTG
AGGACTTGCATTGTGAAAGCTTTGCTGTCCTTGATGTGATCATGGAATCTCTTTCTCACT
AGAGTCTATGTCACTCATTATACTCTGTCGAATGTCATTGAATGTCTTTACATGGGCTTG
TATGCCTATGAAAATTGTAATACAACTTTCAGCAACGGATCTCTTGGCTCTCGCATCGAT
GAAGGACGCAGCGAAATGCGATAAGTAATGTGAATTGCAGAATTCAGTGAATCATCGAAT
CTTTGAACGCATCTTGCGCTCCTTGGTATTCCGAGGAGCATGCCTGTTTGAGTGTCATTA
AATTCTCAACTCTCTTATACTTTTTTGTAAAAGAGAGCTTGGACTGTGGAGGCTTGCTGG
CCACTTTTTGGGGTCAGCTCCTCTGAAATGCATTAGCGGAACCGTTTGCAATCTGCCACA
AGTGTGATAAGTTATCTACACTGGCGAGGGGATTGCTCTCTGTAATGTTCAGCTTCTAAT
TGTCTCTACTTTGTGAGACAACTTTTGAATGCTTGACCTCAAATCAGGTAGGACTACCC-
GCTGAACTTAA
>BC5-88
TTTCCGTAGGTGAACCTGCGGAAGGATCATTATTGAATTATGTTTCTAGATAGGTTGTAG
CTGGCTCTTTAGAGCATGTGCACGCCTGTTTGGACTTCATTTTCATCCACCTGTGCACCT
ATTGTAGTCTTTGGTTGGGTTAGGGGGAAGTGGTCATTGTGTCAGCATCTGCTGGATGTG
AGGACTTGCATTGTGAAAGCTTTGCTGTCCTTGATGTGATCATGGAATCTCTTTCTCACT
AGAGTCTATGTCACTCATTATACTCTGTCGAATGTCATTGAATGTCTTTACATGGGCTTG
TATGCCTATGAAAATTGTAATACAACTTTCAGCAACGGATCTCTTGGCTCTCGCATCGAT
GAAGGACGCAGCGAAATGCGATAAGTAATGTGAATTGCAGAATTCAGTGAATCATCGAAT
CTTTGAACGCATCTTGCGCTCCTTGGTATTCCGAGGAGCATGCCTGTTTGAGTGTCATTA
AATTCTCAACTCTCTTATACTTTTTTGTAAAAGAGAGCTTGGACTGTGGAGGCTTGCTGG
CCACTTTTTGGGGTCAGCTCCTCTGAAATGCATTAGCGGAACCGTTTGCAATCTGCCACA
AGTGTGATAAGTTATCTACACTGGCGAGGGGATTGCTCTCTGTAATGTTCAGCTTCTAAT
TGTCTCTACTTTGTGAGACAACTTTTGAATGCTTGACCTCAAATCAGGTAGGACTACCC-
GCTGAACTTAA
>BC5-124
TTTCCGTAGGTGAACCTGCGGAAGGATCATTATTGAATTATGTTTCTAGATAGGTTGTAG
CTGGCTCTTTAGAGCATGTGCACGCCTGTTTGGACTTCATTTTCATCCACCTGTGCACCT
ATTGTAGTCTTTGGTTGGGTTAGGGGGAAGTGGTCATTGTGTCAGCATCTGCTGGATGTG
AGGACTTGCATTGTGAAAGCTTTGCTGTCCTTGATGTGATCATGGAATCTCTTTCTCACT
AGAGTCTATGTCACTCATTATACTCTGTCGAATGTCATTGAATGTCTTTACATGGGCTTG
TATGCCTATGAAAATTGTAATACAACTTTCAGCAACGGATCTCTTGGCTCTCGCATCGAT
GAAGGACGCAGCGAAATGCGATAAGTAATGTGAATTGCAGAATTCAGTGAATCATCGAAT
CTTTGAACGCATCTTGCGCTCCTTGGTATTCCGAGGAGCATGCCTGTTTGAGTGTCATTA
AATTCTCAACTCTCTTATACTTTTTTGTAAAAGAGAGCTTGGACTGTGGAGGCTTGCTGG
CCACTTTTTGGGGTCAGCTCCTCTGAAATGCATTAGCGGAACCGTTTGCAATCTGCCACA
AGTGTGATAAGTTATCTACACTGGCGAGGGGATTGCTCTCTGTAATGTTCAGCTTCTAAT
TGTCTCTACTTTGTGAGACAACTTTTGAATGCTTGACCTCAAATCAGGTAGGACTACCC-
GCTGAACTTAA
>BC6-6
TTTCCGTAGGTGAACCTGCGGAAGGATCATTATTGAATTATGTTTCTAGATAGGTTGTAG
CTGGCTCTTTAGAGCATGTGCACGCCTGTTTGGACTTCATTTTCATCCACCTGTGCACCT
ATTGTAGTCTTTGGTTGGGTTAGGGGGAAGTGGTCATTGTGTCAGCATCTGCTGGATGTG
AGGACTTGCATTGTGAAAGCTTTGCTGTCCTTGATGTGATCATGGAATCTCTTTCTCACT
AGAGTCTATGTCACTCATTATACTCTGTCGAATGTCATTGAATGTCTTTACATGGGCTTG
TATGCCTATGAAAATTGTAATACAACTTTCAGCAACGGATCTCTTGGCTCTCGCATCGAT
GAAGGACGCAGCGAAATGCGATAAGTAATGTGAATTGCAGAATTCAGTGAATCATCGAAT
CTTTGAACGCATCTTGCGCTCCTTGGTATTCCGAGGAGCATGCCTGTTTGAGTGTCATTA
AATTCTCAACTCTCTTATACTTTTTTGTAAAAGAGAGCTTGGACTGTGGAGGCTTGCTGG

CCACTTTTTGGGGTCAGCTCCTCTGAAATGCATTAGCGGAACCGTTTGCAATCTGCCACA
AGTGTGATAAGTTATCTACACTGGCGAGGGGATTGCTCTCTGTAATGTTCAGCTTCTAAT
TGTCTCTACTTTGTGAGACAACTTTTGAATGCTTGACCTCAAATCAGGTAGGACTACCC-
GCTGAACTTAA
>BC6-14
TTTCCGTAGGTGAACCTGCGGAAGGATCATTATTGAATTATGTTTCTAGATAGGTTGTAG
CTGGCTCTTTAGAGCATGTGCACGCCTGTTTGGACTTCATTTTCATCCACCTGTGCACCT
ATTGTAGTCTTTGGTTGGGTTAGGGGGAAGTGGTCATTGTGTCAGCATCTGCTGGATGTG
AGGACTTGCATTGTGAAAGCTTTGCTGTCCTTGATGTGATCATGGAATCTCTTTCTCACT
AGAGTCTATGTCACTCATTATACTCTGTCGAATGTCATTGAATGTCTTTACATGGGCTTG
TATGCCTATGAAAATTGTAATACAACTTTCAGCAACGGATCTCTTGGCTCTCGCATCGAT
GAAGGACGCAGCGAAATGCGATAAGTAATGTGAATTGCAGAATTCAGTGAATCATCGAAT
CTTTGAACGCATCTTGCGCTCCTTGGTATTCCGAGGAGCATGCCTGTTTGAGTGTCATTA
AATTCTCAACTCTCTTATACTTTTTTGTAAAAGAGAGCTTGGACTGTGGAGGCTTGCTGG
CCACTTTTTGGGGTCAGCTCCTCTGAAATGCATTAGCGGAACCGTTTGCAATCTGCCACA
AGTGTGATAAGTTATCTACACTGGCGAGGGGATTGCTCTCTGTAATGTTCAGCTTCTAAT
TGTCTCTACTTTGTGAGACAACTTTTGAATGCTTGACCTCAAATCAGGTAGGACTACCC-
GCTGAACTTAA
>BC6-34
TTTCCGTAGGTGAACCTGCGGAAGGATCATTATTGAATTATGTTTCTAGATAGGTTGTAG
CTGGCTCTTTAGAGCATGTGCACGCCTGTTTGGACTTCATTTTCATCCACCTGTGCACCT
ATTGTAGTCTTTGGTTGGGTTAGGGGGAAGTGGTCATTGTGTCAGCATCTGCTGGATGTG
AGGACTTGCATTGTGAAAGCTTTGCTGTCCTTGATGTGATCATGGAATCTCTTTCTCACT
AGAGTCTATGTCACTCATTATACTCTGTCGAATGTCATTGAATGTCTTTACATGGGCTTG
TATGCCTATGAAAATTGTAATACAACTTTCAGCAACGGATCTCTTGGCTCTCGCATCGAT
GAAGGACGCAGCGAAATGCGATAAGTAATGTGAATTGCAGAATTCAGTGAATCATCGAAT
CTTTGAACGCATCTTGCGCTCCTTGGTATTCCGAGGAGCATGCCTGTTTGAGTGTCATTA
AATTCTCAACTCTCTTATACTTTTTTGTAAAAGAGAGCTTGGACTGTGGAGGCTTGCTGG
CCACTTTTTGGGGTCAGCTCCTCTGAAATGCATTAGCGGAACCGTTTGCAATCTGCCACA
AGTGTGATAAGTTATCTACACTGGCGAGGGGATTGCTCTCTGTAATGTTCAGCTTCTAAT
TGTCTCTACTTTGTGAGACAACTTTTGAATGCTTGACCTCAAATCAGGTAGGACTACCC-
GCTGAACTTAA
>BC6-36
TTTCCGTAGGTGAACCTGCGGAAGGATCATTATTGAATTATGTTTCTAGATAGGTTGTAG
CTGGCTCTTTAGAGCATGTGCACGCCTGTTTGGACTTCATTTTCATCCACCTGTGCACCT
ATTGTAGTCTTTGGTTGGGTTAGGGGGAAGTGGTCATTGTGTCAGCATCTGCTGGATGTG
AGGACTTGCATTGTGAAAGCTTTGCTGTCCTTGATGTGATCATGGAATCTCTTTCTCACT
AGAGTCTATGTCACTCATTATACTCTGTCGAATGTCATTGAATGTCTTTACATGGGCTTG
TATGCCTATGAAAATTGTAATACAACTTTCAGCAACGGATCTCTTGGCTCTCGCATCGAT
GAAGGACGCAGCGAAATGCGATAAGTAATGTGAATTGCAGAATTCAGTGAATCATCGAAT
CTTTGAACGCATCTTGCGCTCCTTGGTATTCCGAGGAGCATGCCTGTTTGAGTGTCATTA
AATTCTCAACTCTCTTATACTTTTTTGTAAAAGAGAGCTTGGACTGTGGAGGCTTGCTGG
CCACTTTTTGGGGTCAGCTCCTCTGAAATGCATTAGCGGAACCGTTTGCAATCTGCCACA
AGTGTGATAAGTTATCTACACTGGCGAGGGGATTGCTCTCTGTAATGTTCAGCTTCTAAT
TGTCTCTACTTTGTGAGACAACTTTTGAATGCTTGACCTCAAATCAGGTAGGACTACCC-
GCTGAACTTAA
>BC6-41
TTTCCGTAGGTGAACCTGCGGAAGGATCATTATTGAATTATGTTTCTAGATAGGTTGTAG
CTGGCTCTTTAGAGCATGTGCACGCCTGTTTGGACTTCATTTTCATCCACCTGTGCACCT
ATTGTAGTCTTTGGTTGGGTTAGGGGGAAGTGGTCATTGTGTCAGCATCTGCTGGATGTG

AGGACTTGCATTGTGAAAGCTTTGCTGTCCTTGATGTGATCATGGAATCTCTTTCTCACT
AGAGTCTATGTCACTCATTATACTCTGTCGAATGTCATTGAATGTCTTTACATGGGCTTG
TATGCCTATGAAAATTGTAATACAACTTTCAGCAACGGATCTCTTGGCTCTCGCATCGAT
GAAGGACGCAGCGAAATGCGATAAGTAATGTGAATTGCAGAATTCAGTGAATCATCGAAT
CTTTGAACGCATCTTGCGCTCCTTGGTATTCCGAGGAGCATGCCTGTTTGAGTGTCATTA
AATTCTCAACTCTCTTATACTTTTTTGTAAAAGAGAGCTTGGACTGTGGAGGCTTGCTGG
CCACTTTTTGGGGTCAGCTCCTCTGAAATGCATTAGCGGAACCGTTTGCAATCTGCCACA
AGTGTGATAAGTTATCTACACTGGCGAGGGGATTGCTCTCTGTAATGTTCAGCTTCTAAT
TGTCTCTACTTTGTGAGACAACTTTTGAATGCTTGACCTCAAATCAGGTAGGACTACCC-
GCTGAACTTAA
>BC6-59
TTTCCGTAGGTGAACCTGCGGAAGGATCATTATTGAATTATGTTTCTAGATAGGTTGTAG
CTGGCTCTTTAGAGCATGTGCACGCCTGTTTGGACTTCATTTTCATCCACCTGTGCACCT
ATTGTAGTCTTTGGTTGGGTTAGGGGGAAGTGGTCATTGTGTCAGCATCTGCTGGATGTG
AGGACTTGCATTGTGAAAGCTTTGCTGTCCTTGATGTGATCATGGAATCTCTTTCTCACT
AGAGTCTATGTCACTCATTATACTCTGTCGAATGTCATTGAATGTCTTTACATGGGCTTG
TATGCCTATGAAAATTGTAATACAACTTTCAGCAACGGATCTCTTGGCTCTCGCATCGAT
GAAGGACGCAGCGAAATGCGATAAGTAATGTGAATTGCAGAATTCAGTGAATCATCGAAT
CTTTGAACGCATCTTGCGCTCCTTGGTATTCCGAGGAGCATGCCTGTTTGAGTGTCATTA
AATTCTCAACTCTCTTATACTTTTTTGTAAAAGAGAGCTTGGACTGTGGAGGCTTGCTGG
CCACTTTTTGGGGTCAGCTCCTCTGAAATGCATTAGCGGAACCGTTTGCAATCTGCCACA
AGTGTGATAAGTTATCTACACTGGCGAGGGGATTGCTCTCTGTAATGTTCAGCTTCTAAT
TGTCTCTACTTTGTGAGACAACTTTTGAATGCTTGACCTCAAATCAGGTAGGACTACCC-
GCTGAACTTAA
>BC6-60
TTTCCGTAGGTGAACCTGCGGAAGGATCATTATTGAATTATGTTTCTAGATAGGTTGTAG
CTGGCTCTTTAGAGCATGTGCACGCCTGTTTGGACTTCATTTTCATCCACCTGTGCACCT
ATTGTAGTCTTTGGTTGGGTTAGGGGGAAGTGGTCATTGTGTCAGCATCTGCTGGATGTG
AGGACTTGCATTGTGAAAGCTTTGCTGTCCTTGATGTGATCATGGAATCTCTTTCTCACT
AGAGTCTATGTCACTCATTATACTCTGTCGAATGTCATTGAATGTCTTTACATGGGCTTG
TATGCCTATGAAAATTGTAATACAACTTTCAGCAACGGATCTCTTGGCTCTCGCATCGAT
GAAGGACGCAGCGAAATGCGATAAGTAATGTGAATTGCAGAATTCAGTGAATCATCGAAT
CTTTGAACGCATCTTGCGCTCCTTGGTATTCCGAGGAGCATGCCTGTTTGAGTGTCATTA
AATTCTCAACTCTCTTATACTTTTTTGTAAAAGAGAGCTTGGACTGTGGAGGCTTGCTGG
CCACTTTTTGGGGTCAGCTCCTCTGAAATGCATTAGCGGAACCGTTTGCAATCTGCCACA
AGTGTGATAAGTTATCTACACTGGCGAGGGGATTGCTCTCTGTAATGTTCAGCTTCTAAT
TGTCTCTACTTTGTGAGACAACTTTTGAATGCTTGACCTCAAATCAGGTAGGACTACCC-
GCTGAACTTAA
>BC7-3
TTTCCGTAGGTGAACCTGCGGAAGGATCATTATTGAATTATGTTTCTAGATAGGTTGTAG
CTGGCTCTTTAGAGCATGTGCACGCCTGTTTGGACTTCATTTTCATCCACCTGTGCACCT
ATTGTAGTCTTTGGTTGGGTTAGGGGGAAGTGGTCATTGTGTCAGCATCTGCTGGATGTG
AGGACTTGCATTGTGAAAGCTTTGCTGTCCTTGATGTGATCATGGAATCTCTTTCTCACT
AGAGTCTATGTCACTCATTATACTCTGTCGAATGTCATTGAATGTCTTTACATGGGCTTG
TATGCCTATGAAAATTGTAATACAACTTTCAGCAACGGATCTCTTGGCTCTCGCATCGAT
GAAGGACGCAGCGAAATGCGATAAGTAATGTGAATTGCAGAATTCAGTGAATCATCGAAT
CTTTGAACGCATCTTGCGCTCCTTGGTATTCCGAGGAGCATGCCTGTTTGAGTGTCATTA
AATTCTCAACTCTCTTATACTTTTTTGTAAAAGAGAGCTTGGACTGTGGAGGCTTGCTGG
CCACTTTTTGGGGTCAGCTCCTCTGAAATGCATTAGCGGAACCGTTTGCAATCTGCCACA
AGTGTGATAAGTTATCTACACTGGCGAGGGGATTGCTCTCTGTAATGTTCAGCTTCTAAT

TGTCTCTACTTTGTGAGACAACTTTTGAATGCTTGACCTCAAATCAGGTAGGACTACCC-
GCTGAACTTAA
>BC7-12
TTTCCGTAGGTGAACCTGCGGAAGGATCATTATTGAATTATGTTTCTAGATAGGTTGTAG
CTGGCTCTTTAGAGCATGTGCACGCCTGTTTGGACTTCATTTTCATCCACCTGTGCACCT
ATTGTAGTCTTTGGTTGGGTTAGGGGGAAGTGGTCATTGTGTCAGCATCTGCTGGATGTG
AGGACTTGCATTGTGAAAGCTTTGCTGTCCTTGATGTGATCATGGAATCTCTTTCTCACT
AGAGTCTATGTCACTCATTATACTCTGTCGAATGTCATTGAATGTCTTTACATGGGCTTG
TATGCCTATGAAAATTGTAATACAACTTTCAGCAACGGATCTCTTGGCTCTCGCATCGAT
GAAGGACGCAGCGAAATGCGATAAGTAATGTGAATTGCAGAATTCAGTGAATCATCGAAT
CTTTGAACGCATCTTGCGCTCCTTGGTATTCCGAGGAGCATGCCTGTTTGAGTGTCATTA
AATTCTCAACTCTCTTATACTTTTTTGTAAAAGAGAGCTTGGACTGTGGAGGCTTGCTGG
CCACTTTTTGGGGTCAGCTCCTCTGAAATGCATTAGCGGAACCGTTTGCAATCTGCCACA
AGTGTGATAAGTTATCTACACTGGCGAGGGGATTGCTCTCTGTAATGTTCAGCTTCTAAT
TGTCTCTACTTTGTGAGACAACTTTTGAATGCTTGACCTCAAATCAGGTAGGACTACCC-
GCTGAACTTAA
>BC7-18
TTTCCGTAGGTGAACCTGCGGAAGGATCATTATTGAATTATGTTTCTAGATAGGTTGTAG
CTGGCTCTTTAGAGCATGTGCACGCCTGTTTGGACTTCATTTTCATCCACCTGTGCACCT
ATTGTAGTCTTTGGTTGGGTTAGGGGGAAGTGGTCATTGTGTCAGCATCTGCTGGATGTG
AGGACTTGCATTGTGAAAGCTTTGCTGTCCTTGATGTGATCATGGAATCTCTTTCTCACT
AGAGTCTATGTCACTCATTATACTCTGTCGAATGTCATTGAATGTCTTTACATGGGCTTG
TATGCCTATGAAAATTGTAATACAACTTTCAGCAACGGATCTCTTGGCTCTCGCATCGAT
GAAGGACGCAGCGAAATGCGATAAGTAATGTGAATTGCAGAATTCAGTGAATCATCGAAT
CTTTGAACGCATCTTGCGCTCCTTGGTATTCCGAGGAGCATGCCTGTTTGAGTGTCATTA
AATTCTCAACTCTCTTATACTTTTTTGTAAAAGAGAGCTTGGACTGTGGAGGCTTGCTGG
CCACTTTTTGGGGTCAGCTCCTCTGAAATGCATTAGCGGAACCGTTTGCAATCTGCCACA
AGTGTGATAAGTTATCTACACTGGCGAGGGGATTGCTCTCTGTAATGTTCAGCTTCTAAT
TGTCTCTACTTTGTGAGACAACTTTTGAATGCTTGACCTCAAATCAGGTAGGACTACCC-
GCTGAACTTAA
>BC7-21
TTTCCGTAGGTGAACCTGCGGAAGGATCATTATTGAATTATGTTTCTAGATAGGTTGTAG
CTGGCTCTTTAGAGCATGTGCACGCCTGTTTGGACTTCATTTTCATCCACCTGTGCACCT
ATTGTAGTCTTTGGTTGGGTTAGGGGGAAGTGGTCATTGTGTCAGCATCTGCTGGATGTG
AGGACTTGCATTGTGAAAGCTTTGCTGTCCTTGATGTGATCATGGAATCTCTTTCTCACT
AGAGTCTATGTCACTCATTATACTCTGTCGAATGTCATTGAATGTCTTTACATGGGCTTG
TATGCCTATGAAAATTGTAATACAACTTTCAGCAACGGATCTCTTGGCTCTCGCATCGAT
GAAGGACGCAGCGAAATGCGATAAGTAATGTGAATTGCAGAATTCAGTGAATCATCGAAT
CTTTGAACGCATCTTGCGCTCCTTGGTATTCCGAGGAGCATGCCTGTTTGAGTGTCATTA
AATTCTCAACTCTCTTATACTTTTTTGTAAAAGAGAGCTTGGACTGTGGAGGCTTGCTGG
CCACTTTTTGGGGTCAGCTCCTCTGAAATGCATTAGCGGAACCGTTTGCAATCTGCCACA
AGTGTGATAAGTTATCTACACTGGCGAGGGGATTGCTCTCTGTAATGTTCAGCTTCTAAT
TGTCTCTACTTTGTGAGACAACTTTTGAATGCTTGACCTCAAATCAGGTAGGACTACCC-
GCTGAACTTAA
>BC7-30
TTTCCGTAGGTGAACCTGCGGAAGGATCATTATTGAATTATGTTTCTAGATAGGTTGTAG
CTGGCTCTTTAGAGCATGTGCACGCCTGTTTGGACTTCATTTTCATCCACCTGTGCACCT
ATTGTAGTCTTTGGTTGGGTTAGGGGGAAGTGGTCATTGTGTCAGCATCTGCTGGATGTG
AGGACTTGCATTGTGAAAGCTTTGCTGTCCTTGATGTGATCATGGAATCTCTTTCTCACT
AGAGTCTATGTCACTCATTATACTCTGTCGAATGTCATTGAATGTCTTTACATGGGCTTG

TATGCCTATGAAAATTGTAATACAACTTTCAGCAACGGATCTCTTGGCTCTCGCATCGAT
GAAGGACGCAGCGAAATGCGATAAGTAATGTGAATTGCAGAATTCAGTGAATCATCGAAT
CTTTGAACGCATCTTGCGCTCCTTGGTATTCCGAGGAGCATGCCTGTTTGAGTGTCATTA
AATTCTCAACTCTCTTATACTTTTTTGTAAAAGAGAGCTTGGACTGTGGAGGCTTGCTGG
CCACTTTTTGGGGTCAGCTCCTCTGAAATGCATTAGCGGAACCGTTTGCAATCTGCCACA
AGTGTGATAAGTTATCTACACTGGCGAGGGGATTGCTCTCTGTAATGTTCAGCTTCTAAT
TGTCTCTACTTTGTGAGACAACTTTTGAATGCTTGACCTCAAATCAGGTAGGACTACCC-
GCTGAACTTAA
>BC7-39
TTTCCGTAGGTGAACCTGCGGAAGGATCATTATTGAATTATGTTTCTAGATAGGTTGTAG
CTGGCTCTTTAGAGCATGTGCACGCCTGTTTGGACTTCATTTTCATCCACCTGTGCACCT
ATTGTAGTCTTTGGTTGGGTTAGGGGGAAGTGGTCATTGTGTCAGCATCTGCTGGATGTG
AGGACTTGCATTGTGAAAGCTTTGCTGTCCTTGATGTGATCATGGAATCTCTTTCTCACT
AGAGTCTATGTCACTCATTATACTCTGTCGAATGTCATTGAATGTCTTTACATGGGCTTG
TATGCCTATGAAAATTGTAATACAACTTTCAGCAACGGATCTCTTGGCTCTCGCATCGAT
GAAGGACGCAGCGAAATGCGATAAGTAATGTGAATTGCAGAATTCAGTGAATCATCGAAT
CTTTGAACGCATCTTGCGCTCCTTGGTATTCCGAGGAGCATGCCTGTTTGAGTGTCATTA
AATTCTCAACTCTCTTATACTTTTTTGTAAAAGAGAGCTTGGACTGTGGAGGCTTGCTGG
CCACTTTTTGGGGTCAGCTCCTCTGAAATGCATTAGCGGAACCGTTTGCAATCTGCCACA
AGTGTGATAAGTTATCTACACTGGCGAGGGGATTGCTCTCTGTAATGTTCAGCTTCTAAT
TGTCTCTACTTTGTGAGACAACTTTTGAATGCTTGACCTCAAATCAGGTAGGACTACCC-
GCTGAACTTAA
>BC7-49
TTTCCGTAGGTGAACCTGCGGAAGGATCATTATTGAATTATGTTTCTAGATAGGTTGTAG
CTGGCTCTTTAGAGCATGTGCACGCCTGTTTGGACTTCATTTTCATCCACCTGTGCACCT
ATTGTAGTCTTTGGTTGGGTTAGGGGGAAGTGGTCATTGTGTCAGCATCTGCTGGATGTG
AGGACTTGCATTGTGAAAGCTTTGCTGTCCTTGATGTGATCATGGAATCTCTTTCTCACT
AGAGTCTATGTCACTCATTATACTCTGTCGAATGTCATTGAATGTCTTTACATGGGCTTG
TATGCCTATGAAAATTGTAATACAACTTTCAGCAACGGATCTCTTGGCTCTCGCATCGAT
GAAGGACGCAGCGAAATGCGATAAGTAATGTGAATTGCAGAATTCAGTGAATCATCGAAT
CTTTGAACGCATCTTGCGCTCCTTGGTATTCCGAGGAGCATGCCTGTTTGAGTGTCATTA
AATTCTCAACTCTCTTATACTTTTTTGTAAAAGAGAGCTTGGACTGTGGAGGCTTGCTGG
CCACTTTTTGGGGTCAGCTCCTCTGAAATGCATTAGCGGAACCGTTTGCAATCTGCCACA
AGTGTGATAAGTTATCTACACTGGCGAGGGGATTGCTCTCTGTAATGTTCAGCTTCTAAT
TGTCTCTACTTTGTGAGACAACTTTTGAATGCTTGACCTCAAATCAGGTAGGACTACCC-
GCTGAACTTAA
>BC7-54
TTTCCGTAGGTGAACCTGCGGAAGGATCATTATTGAATTATGTTTCTAGATAGGTTGTAG
CTGGCTCTTTAGAGCATGTGCACGCCTGTTTGGACTTCATTTTCATCCACCTGTGCACCT
ATTGTAGTCTTTGGTTGGGTTAGGGGGAAGTGGTCATTGTGTCAGCATCTGCTGGATGTG
AGGACTTGCATTGTGAAAGCTTTGCTGTCCTTGATGTGATCATGGAATCTCTTTCTCACT
AGAGTCTATGTCACTCATTATACTCTGTCGAATGTCATTGAATGTCTTTACATGGGCTTG
TATGCCTATGAAAATTGTAATACAACTTTCAGCAACGGATCTCTTGGCTCTCGCATCGAT
GAAGGACGCAGCGAAATGCGATAAGTAATGTGAATTGCAGAATTCAGTGAATCATCGAAT
CTTTGAACGCATCTTGCGCTCCTTGGTATTCCGAGGAGCATGCCTGTTTGAGTGTCATTA
AATTCTCAACTCTCTTATACTTTTTTGTAAAAGAGAGCTTGGACTGTGGAGGCTTGCTGG
CCACTTTTTGGGGTCAGCTCCTCTGAAATGCATTAGCGGAACCGTTTGCAATCTGCCACA
AGTGTGATAAGTTATCTACACTGGCGAGGGGATTGCTCTCTGTAATGTTCAGCTTCTAAT
TGTCTCTACTTTGTGAGACAACTTTTGAATGCTTGACCTCAAATCAGGTAGGACTACCC-
GCTGAACTTAA

>BC7–55
TTTCCGTAGGTGAACCTGCGGAAGGATCATTATTGAATTATGTTTCTAGATAGGTTGTAG
CTGGCTCTTTAGAGCATGTGCACGCCTGTTTGGACTTCATTTTCATCCACCTGTGCACCT
ATTGTAGTCTTTGGTTGGGTTAGGGGGAAGTGGTCATTGTGTCAGCATCTGCTGGATGTG
AGGACTTGCATTGTGAAAGCTTTGCTGTCCTTGATGTGATCATGGAATCTCTTTCTCACT
AGAGTCTATGTCACTCATTATACTCTGTCGAATGTCATTGAATGTCTTTACATGGGCTTG
TATGCCTATGAAAATTGTAATACAACTTTCAGCAACGGATCTCTTGGCTCTCGCATCGAT
GAAGGACGCAGCGAAATGCGATAAGTAATGTGAATTGCAGAATTCAGTGAATCATCGAAT
CTTTGAACGCATCTTGCGCTCCTTGGTATTCCGAGGAGCATGCCTGTTTGAGTGTCATTA
AATTCTCAACTCTCTTATACTTTTTTGTAAAAGAGAGCTTGGACTGTGGAGGCTTGCTGG
CCACTTTTTGGGGTCAGCTCCTCTGAAATGCATTAGCGGAACCGTTTGCAATCTGCCACA
AGTGTGATAAGTTATCTACACTGGCGAGGGGATTGCTCTCTGTAATGTTCAGCTTCTAAT
TGTCTCTACTTTGTGAGACAACTTTTGAATGCTTGACCTCAAATCAGGTAGGACTACCC–
GCTGAACTTAA
>BC8–7
TTTCCGTAGGTGAACCTGCGGAAGGATCATTATTGAATTATGTTTCTAGATAGGTTGTAG
CTGGCTCTTTAGAGCATGTGCACGCCTGTTTGGACTTCATTTTCATCCACCTGTGCACCT
ATTGTAGTCTTTGGTTGGGTTAGGGGGAAGTGGTCATTGTGTCAGCATCTGCTGGATGTG
AGGACTTGCATTGTGAAAGCTTTGCTGTCCTTGATGTGATCATGGAATCTCTTTCTCACT
AGAGTCTATGTCACTCATTATACTCTGTCGAATGTCATTGAATGTCTTTACATGGGCTTG
TATGCCTATGAAAATTGTAATACAACTTTCAGCAACGGATCTCTTGGCTCTCGCATCGAT
GAAGGACGCAGCGAAATGCGATAAGTAATGTGAATTGCAGAATTCAGTGAATCATCGAAT
CTTTGAACGCATCTTGCGCTCCTTGGTATTCCGAGGAGCATGCCTGTTTGAGTGTCATTA
AATTCTCAACTCTCTTATACTTTTTTGTAAAAGAGAGCTTGGACTGTGGAGGCTTGCTGG
CCACTTTTTGGGGTCAGCTCCTCTGAAATGCATTAGCGGAACCGTTTGCAATCTGCCACA
AGTGTGATAAGTTATCTACACTGGCGAGGGGATTGCTCTCTGTAATGTTCAGCTTCTAAT
TGTCTCTACTTTGTGAGACAACTTTTGAATGCTTGACCTCAAATCAGGTAGGACTACCC–
GCTGAACTTAA
>BC8–9
TTTCCGTAGGTGAACCTGCGGAAGGATCATTATTGAATTATGTTTCTAGATAGGTTGTAG
CTGGCTCTTTAGAGCATGTGCACGCCTGTTTGGACTTCATTTTCATCCACCTGTGCACCT
ATTGTAGTCTTTGGTTGGGTTAGGGGGAAGTGGTCATTGTGTCAGCATCTGCTGGATGTG
AGGACTTGCATTGTGAAAGCTTTGCTGTCCTTGATGTGATCATGGAATCTCTTTCTCACT
AGAGTCTATGTCACTCATTATACTCTGTCGAATGTCATTGAATGTCTTTACATGGGCTTG
TATGCCTATGAAAATTGTAATACAACTTTCAGCAACGGATCTCTTGGCTCTCGCATCGAT
GAAGGACGCAGCGAAATGCGATAAGTAATGTGAATTGCAGAATTCAGTGAATCATCGAAT
CTTTGAACGCATCTTGCGCTCCTTGGTATTCCGAGGAGCATGCCTGTTTGAGTGTCATTA
AATTCTCAACTCTCTTATACTTTTTTGTAAAAGAGAGCTTGGACTGTGGAGGCTTGCTGG
CCACTTTTTGGGGTCAGCTCCTCTGAAATGCATTAGCGGAACCGTTTGCAATCTGCCACA
AGTGTGATAAGTTATCTACACTGGCGAGGGGATTGCTCTCTGTAATGTTCAGCTTCTAAT
TGTCTCTACTTTGTGAGACAACTTTTGAATGCTTGACCTCAAATCAGGTAGGACTACCC–
GCTGAACTTAA
>BC8–15
TTTCCGTAGGTGAACCTGCGGAAGGATCATTATTGAATTATGTTTCTAGATAGGTTGTAG
CTGGCTCTTTAGAGCATGTGCACGCCTGTTTGGACTTCATTTTCATCCACCTGTGCACCT
ATTGTAGTCTTTGGTTGGGTTAGGGGGAAGTGGTCATTGTGTCAGCATCTGCTGGATGTG
AGGACTTGCATTGTGAAAGCTTTGCTGTCCTTGATGTGATCATGGAATCTCTTTCTCACT
AGAGTCTATGTCACTCATTATACTCTGTCGAATGTCATTGAATGTCTTTACATGGGCTTG
TATGCCTATGAAAATTGTAATACAACTTTCAGCAACGGATCTCTTGGCTCTCGCATCGAT
GAAGGACGCAGCGAAATGCGATAAGTAATGTGAATTGCAGAATTCAGTGAATCATCGAAT

CTTTGAACGCATCTTGCGCTCCTTGGTATTCCGAGGAGCATGCCTGTTTGAGTGTCATTA
AATTCTCAACTCTCTTATACTTTTTTGTAAAAGAGAGCTTGGACTGTGGAGGCTTGCTGG
CCACTTTTTGGGGTCAGCTCCTCTGAAATGCATTAGCGGAACCGTTTGCAATCTGCCACA
AGTGTGATAAGTTATCTACACTGGCGAGGGGATTGCTCTCTGTAATGTTCAGCTTCTAAT
TGTCTCTACTTTGTGAGACAACTTTTGAATGCTTGACCTCAAATCAGGTAGGACTACCC-
GCTGAACTTAA
>BC8-16
TTTCCGTAGGTGAACCTGCGGAAGGATCATTATTGAATTATGTTTCTAGATAGGTTGTAG
CTGGCTCTTTAGAGCATGTGCACGCCTGTTTGGACTTCATTTTCATCCACCTGTGCACCT
ATTGTAGTCTTTGGTTGGGTTAGGGGGAAGTGGTCATTGTGTCAGCATCTGCTGGATGTG
AGGACTTGCATTGTGAAAGCTTTGCTGTCCTTGATGTGATCATGGAATCTCTTTCTCACT
AGAGTCTATGTCACTCATTATACTCTGTCGAATGTCATTGAATGTCTTTACATGGGCTTG
TATGCCTATGAAAATTGTAATACAACTTTCAGCAACGGATCTCTTGGCTCTCGCATCGAT
GAAGGACGCAGCGAAATGCGATAAGTAATGTGAATTGCAGAATTCAGTGAATCATCGAAT
CTTTGAACGCATCTTGCGCTCCTTGGTATTCCGAGGAGCATGCCTGTTTGAGTGTCATTA
AATTCTCAACTCTCTTATACTTTTTTGTAAAAGAGAGCTTGGACTGTGGAGGCTTGCTGG
CCACTTTTTGGGGTCAGCTCCTCTGAAATGCATTAGCGGAACCGTTTGCAATCTGCCACA
AGTGTGATAAGTTATCTACACTGGCGAGGGGATTGCTCTCTGTAATGTTCAGCTTCTAAT
TGTCTCTACTTTGTGAGACAACTTTTGAATGCTTGACCTCAAATCAGGTAGGACTACCC-
GCTGAACTTAA
>BC8-18
TTTCCGTAGGTGAACCTGCGGAAGGATCATTATTGAATTATGTTTCTAGATAGGTTGTAG
CTGGCTCTTTAGAGCATGTGCACGCCTGTTTGGACTTCATTTTCATCCACCTGTGCACCT
ATTGTAGTCTTTGGTTGGGTTAGGGGGAAGTGGTCATTGTGTCAGCATCTGCTGGATGTG
AGGACTTGCATTGTGAAAGCTTTGCTGTCCTTGATGTGATCATGGAATCTCTTTCTCACT
AGAGTCTATGTCACTCATTATACTCTGTCGAATGTCATTGAATGTCTTTACATGGGCTTG
TATGCCTATGAAAATTGTAATACAACTTTCAGCAACGGATCTCTTGGCTCTCGCATCGAT
GAAGGACGCAGCGAAATGCGATAAGTAATGTGAATTGCAGAATTCAGTGAATCATCGAAT
CTTTGAACGCATCTTGCGCTCCTTGGTATTCCGAGGAGCATGCCTGTTTGAGTGTCATTA
AATTCTCAACTCTCTTATACTTTTTTGTAAAAGAGAGCTTGGACTGTGGAGGCTTGCTGG
CCACTTTTTGGGGTCAGCTCCTCTGAAATGCATTAGCGGAACCGTTTGCAATCTGCCACA
AGTGTGATAAGTTATCTACACTGGCGAGGGGATTGCTCTCTGTAATGTTCAGCTTCTAAT
TGTCTCTACTTTGTGAGACAACTTTTGAATGCTTGACCTCAAATCAGGTAGGACTACCC-
GCTGAACTTAA
>BC8-27
TTTCCGTAGGTGAACCTGCGGAAGGATCATTATTGAATTATGTTTCTAGATAGGTTGTAG
CTGGCTCTTTAGAGCATGTGCACGCCTGTTTGGACTTCATTTTCATCCACCTGTGCACCT
ATTGTAGTCTTTGGTTGGGTTAGGGGGAAGTGGTCATTGTGTCAGCATCTGCTGGATGTG
AGGACTTGCATTGTGAAAGCTTTGCTGTCCTTGATGTGATCATGGAATCTCTTTCTCACT
AGAGTCTATGTCACTCATTATACTCTGTCGAATGTCATTGAATGTCTTTACATGGGCTTG
TATGCCTATGAAAATTGTAATACAACTTTCAGCAACGGATCTCTTGGCTCTCGCATCGAT
GAAGGACGCAGCGAAATGCGATAAGTAATGTGAATTGCAGAATTCAGTGAATCATCGAAT
CTTTGAACGCATCTTGCGCTCCTTGGTATTCCGAGGAGCATGCCTGTTTGAGTGTCATTA
AATTCTCAACTCTCTTATACTTTTTTGTAAAAGAGAGCTTGGACTGTGGAGGCTTGCTGG
CCACTTTTTGGGGTCAGCTCCTCTGAAATGCATTAGCGGAACCGTTTGCAATCTGCCACA
AGTGTGATAAGTTATCTACACTGGCGAGGGGATTGCTCTCTGTAATGTTCAGCTTCTAAT
TGTCTCTACTTTGTGAGACAACTTTTGAATGCTTGACCTCAAATCAGGTAGGACTACCC-
GCTGAACTTAA
>BC8-49
TTTCCGTAGGTGAACCTGCGGAAGGATCATTATTGAATTATGTTTCTAGATAGGTTGTAG

CTGGCTCTTTAGAGCATGTGCACGCCTGTTTGGACTTCATTTTCATCCACCTGTGCACCT
ATTGTAGTCTTTGGTTGGGTTAGGGGGAAGTGGTCATTGTGTCAGCATCTGCTGGATGTG
AGGACTTGCATTGTGAAAGCTTTGCTGTCCTTGATGTGATCATGGAATCTCTTTCTCACT
AGAGTCTATGTCACTCATTATACTCTGTCGAATGTCATTGAATGTCTTTACATGGGCTTG
TATGCCTATGAAAATTGTAATACAACTTTCAGCAACGGATCTCTTGGCTCTCGCATCGAT
GAAGGACGCAGCGAAATGCGATAAGTAATGTGAATTGCAGAATTCAGTGAATCATCGAAT
CTTTGAACGCATCTTGCGCTCCTTGGTATTCCGAGGAGCATGCCTGTTTGAGTGTCATTA
AATTCTCAACTCTCTTATACTTTTTTGTAAAAGAGAGCTTGGACTGTGGAGGCTTGCTGG
CCACTTTTTGGGGTCAGCTCCTCTGAAATGCATTAGCGGAACCGTTTGCAATCTGCCACA
AGTGTGATAAGTTATCTACACTGGCGAGGGGATTGCTCTCTGTAATGTTCAGCTTCTAAT
TGTCTCTACTTTGTGAGACAACTTTTGAATGCTTGACCTCAAATCAGGTAGGACTACCC-
GCTGAACTTAA
>BC9-5
TTTCCGTAGGTGAACCTGCGGAAGGATCATTATTGAATTATGTTTCTAGATAGGTTGTAG
CTGGCTCTTTAGAGCATGTGCACGCCTGTTTGGACTTCATTTTCATCCACCTGTGCACCT
ATTGTAGTCTTTGGTTGGGTTAGGGGGAAGTGGTCATTGTGTCAGCATCTGCTGGATGTG
AGGACTTGCATTGTGAAAGCTTTGCTGTCCTTGATGTGATCATGGAATCTCTTTCTCACT
AGAGTCTATGTCACTCATTATACTCTGTCGAATGTCATTGAATGTCTTTACATGGGCTTG
TATGCCTATGAAAATTGTAATACAACTTTCAGCAACGGATCTCTTGGCTCTCGCATCGAT
GAAGGACGCAGCGAAATGCGATAAGTAATGTGAATTGCAGAATTCAGTGAATCATCGAAT
CTTTGAACGCATCTTGCGCTCCTTGGTATTCCGAGGAGCATGCCTGTTTGAGTGTCATTA
AATTCTCAACTCTCTTATACTTTTTTGTAAAAGAGAGCTTGGACTGTGGAGGCTTGCTGG
CCACTTTTTGGGGTCAGCTCCTCTGAAATGCATTAGCGGAACCGTTTGCAATCTGCCACA
AGTGTGATAAGTTATCTACACTGGCGAGGGGATTGCTCTCTGTAATGTTCAGCTTCTAAT
TGTCTCTACTTTGTGAGACAACTTTTGAATGCTTGACCTCAAATCAGGTAGGACTACCC-
GCTGAACTTAA
>BC9-12
TTTCCGTAGGTGAACCTGCGGAAGGATCATTATTGAATTATGTTTCTAGATAGGTTGTAG
CTGGCTCTTTAGAGCATGTGCACGCCTGTTTGGACTTCATTTTCATCCACCTGTGCACCT
ATTGTAGTCTTTGGTTGGGTTAGGGGGAAGTGGTCATTGTGTCAGCATCTGCTGGATGTG
AGGACTTGCATTGTGAAAGCTTTGCTGTCCTTGATGTGATCATGGAATCTCTTTCTCACT
AGAGTCTATGTCACTCATTATACTCTGTCGAATGTCATTGAATGTCTTTACATGGGCTTG
TATGCCTATGAAAATTGTAATACAACTTTCAGCAACGGATCTCTTGGCTCTCGCATCGAT
GAAGGACGCAGCGAAATGCGATAAGTAATGTGAATTGCAGAATTCAGTGAATCATCGAAT
CTTTGAACGCATCTTGCGCTCCTTGGTATTCCGAGGAGCATGCCTGTTTGAGTGTCATTA
AATTCTCAACTCTCTTATACTTTTTTGTAAAAGAGAGCTTGGACTGTGGAGGCTTGCTGG
CCACTTTTTGGGGTCAGCTCCTCTGAAATGCATTAGCGGAACCGTTTGCAATCTGCCACA
AGTGTGATAAGTTATCTACACTGGCGAGGGGATTGCTCTCTGTAATGTTCAGCTTCTAAT
TGTCTCTACTTTGTGAGACAACTTTTGAATGCTTGACCTCAAATCAGGTAGGACTACCC-
GCTGAACTTAA
>BC9-30
TTTCCGTAGGTGAACCTGCGGAAGGATCATTATTGAATTATGTTTCTAGATAGGTTGTAG
CTGGCTCTTTAGAGCATGTGCACGCCTGTTTGGACTTCATTTTCATCCACCTGTGCACCT
ATTGTAGTCTTTGGTTGGGTTAGGGGGAAGTGGTCATTGTGTCAGCATCTGCTGGATGTG
AGGACTTGCATTGTGAAAGCTTTGCTGTCCTTGATGTGATCATGGAATCTCTTTCTCACT
AGAGTCTATGTCACTCATTATACTCTGTCGAATGTCATTGAATGTCTTTACATGGGCTTG
TATGCCTATGAAAATTGTAATACAACTTTCAGCAACGGATCTCTTGGCTCTCGCATCGAT
GAAGGACGCAGCGAAATGCGATAAGTAATGTGAATTGCAGAATTCAGTGAATCATCGAAT
CTTTGAACGCATCTTGCGCTCCTTGGTATTCCGAGGAGCATGCCTGTTTGAGTGTCATTA
AATTCTCAACTCTCTTATACTTTTTTGTAAAAGAGAGCTTGGACTGTGGAGGCTTGCTGG

CCACTTTTTGGGGTCAGCTCCTCTGAAATGCATTAGCGGAACCGTTTGCAATCTGCCACA
AGTGTGATAAGTTATCTACACTGGCGAGGGGATTGCTCTCTGTAATGTTCAGCTTCTAAT
TGTCTCTACTTTGTGAGACAACTTTTGAATGCTTGACCTCAAATCAGGTAGGACTACCC-
GCTGAACTTAA
>BC10_2
TTTCCGTAGGTGAACCTGCGGAAGGATCATTATTGAATTATGTTTCTAGATAGGTTGTAG
CTGGCTCTTTAGAGCATGTGCACGCCTGTTTGGACTTCATTTTCATCCACCTGTGCACCT
ATTGTAGTCTTTGGTTGGGTTAGGGGGAAGTGGTCATTGTGTCAGCATCTGCTGGATGTG
AGGACTTGCATTGTGAAAGCTTTGCTGTCCTTGATGTGATCATGGAATCTCTTTCTCACT
AGAGTCTATGTCACTCATTATACTCTGTCGAATGTCATTGAATGTCTTTACATGGGCTTG
TATGCCTATGAAAATTGTAATACAACTTTCAGCAACGGATCTCTTGGCTCTCGCATCGAT
GAAGGACGCAGCGAAATGCGATAAGTAATGTGAATTGCAGAATTCAGTGAATCATCGAAT
CTTTGAACGCATCTTGCGCTCCTTGGTATTCCGAGGAGCATGCCTGTTTGAGTGTCATTA
AATTCTCAACTCTCTTATACTTTTTTGTAAAAGAGAGCTTGGACTGTGGAGGCTTGCTGG
CCACTTTTTGGGGTCAGCTCCTCTGAAATGCATTAGCGGAACCGTTTGCAATCTGCCACA
AGTGTGATAAGTTATCTACACTGGCGAGGGGATTGCTCTCTGTAATGTTCAGCTTCTAAT
TGTCTCTACTTTGTGAGACAACTTTTGAATGCTTGACCTCAAATCAGGTAGGACTACCC-
GCTGAACTTAA
>BC10_11
TTTCCGTAGGTGAACCTGCGGAAGGATCATTATTGAATTATGTTTCTAGATAGGTTGTAG
CTGGCTCTTTAGAGCATGTGCACGCCTGTTTGGACTTCATTTTCATCCACCTGTGCACCT
ATTGTAGTCTTTGGTTGGGTTAGGGGGAAGTGGTCATTGTGTCAGCATCTGCTGGATGTG
AGGACTTGCATTGTGAAAGCTTTGCTGTCCTTGATGTGATCATGGAATCTCTTTCTCACT
AGAGTCTATGTCACTCATTATACTCTGTCGAATGTCATTGAATGTCTTTACATGGGCTTG
TATGCCTATGAAAATTGTAATACAACTTTCAGCAACGGATCTCTTGGCTCTCGCATCGAT
GAAGGACGCAGCGAAATGCGATAAGTAATGTGAATTGCAGAATTCAGTGAATCATCGAAT
CTTTGAACGCATCTTGCGCTCCTTGGTATTCCGAGGAGCATGCCTGTTTGAGTGTCATTA
AATTCTCAACTCTCTTATACTTTTTTGTAAAAGAGAGCTTGGACTGTGGAGGCTTGCTGG
CCACTTTTTGGGGTCAGCTCCTCTGAAATGCATTAGCGGAACCGTTTGCAATCTGCCACA
AGTGTGATAAGTTATCTACACTGGCGAGGGGATTGCTCTCTGTAATGTTCAGCTTCTAAT
TGTCTCTACTTTGTGAGACAACTTTTGAATGCTTGACCTCAAATCAGGTAGGACTACCC-
GCTGAACTTAA
>BC10_13
TTTCCGTAGGTGAACCTGCGGAAGGATCATTATTGAATTATGTTTCTAGATAGGTTGTAG
CTGGCTCTTTAGAGCATGTGCACGCCTGTTTGGACTTCATTTTCATCCACCTGTGCACCT
ATTGTAGTCTTTGGTTGGGTTAGGGGGAAGTGGTCATTGTGTCAGCATCTGCTGGATGTG
AGGACTTGCATTGTGAAAGCTTTGCTGTCCTTGATGTGATCATGGAATCTCTTTCTCACT
AGAGTCTATGTCACTCATTATACTCTGTCGAATGTCATTGAATGTCTTTACATGGGCTTG
TATGCCTATGAAAATTGTAATACAACTTTCAGCAACGGATCTCTTGGCTCTCGCATCGAT
GAAGGACGCAGCGAAATGCGATAAGTAATGTGAATTGCAGAATTCAGTGAATCATCGAAT
CTTTGAACGCATCTTGCGCTCCTTGGTATTCCGAGGAGCATGCCTGTTTGAGTGTCATTA
AATTCTCAACTCTCTTATACTTTTTTGTAAAAGAGAGCTTGGACTGTGGAGGCTTGCTGG
CCACTTTTTGGGGTCAGCTCCTCTGAAATGCATTAGCGGAACCGTTTGCAATCTGCCACA
AGTGTGATAAGTTATCTACACTGGCGAGGGGATTGCTCTCTGTAATGTTCAGCTTCTAAT
TGTCTCTACTTTGTGAGACAACTTTTGAATGCTTGACCTCAAATCAGGTAGGACTACCC-
GCTGAACTTAA
>BC10_19
TTTCCGTAGGTGAACCTGCGGAAGGATCATTATTGAATTATGTTTCTAGATAGGTTGTAG
CTGGCTCTTTAGAGCATGTGCACGCCTGTTTGGACTTCATTTTCATCCACCTGTGCACCT
ATTGTAGTCTTTGGTTGGGTTAGGGGGAAGTGGTCATTGTGTCAGCATCTGCTGGATGTG

AGGACTTGCATTGTGAAAGCTTTGCTGTCCTTGATGTGATCATGGAATCTCTTTCTCACT
AGAGTCTATGTCACTCATTATACTCTGTCGAATGTCATTGAATGTCTTTACATGGGCTTG
TATGCCTATGAAAATTGTAATACAACTTTCAGCAACGGATCTCTTGGCTCTCGCATCGAT
GAAGGACGCAGCGAAATGCGATAAGTAATGTGAATTGCAGAATTCAGTGAATCATCGAAT
CTTTGAACGCATCTTGCGCTCCTTGGTATTCCGAGGAGCATGCCTGTTTGAGTGTCATTA
AATTCTCAACTCTCTTATACTTTTTTGTAAAAGAGAGCTTGGACTGTGGAGGCTTGCTGG
CCACTTTTTGGGGTCAGCTCCTCTGAAATGCATTAGCGGAACCGTTTGCAATCTGCCACA
AGTGTGATAAGTTATCTACACTGGCGAGGGGATTGCTCTCTGTAATGTTCAGCTTCTAAT
TGTCTCTACTTTGTGAGACAACTTTTGAATGCTTGACCTCAAATCAGGTAGGACTACCC-
GCTGAACTTAA
>BC10_20
TTTCCGTAGGTGAACCTGCGGAAGGATCATTATTGAATTATGTTTCTAGATAGGTTGTAG
CTGGCTCTTTAGAGCATGTGCACGCCTGTTTGGACTTCATTTTCATCCACCTGTGCACCT
ATTGTAGTCTTTGGTTGGGTTAGGGGGAAGTGGTCATTGTGTCAGCATCTGCTGGATGTG
AGGACTTGCATTGTGAAAGCTTTGCTGTCCTTGATGTGATCATGGAATCTCTTTCTCACT
AGAGTCTATGTCACTCATTATACTCTGTCGAATGTCATTGAATGTCTTTACATGGGCTTG
TATGCCTATGAAAATTGTAATACAACTTTCAGCAACGGATCTCTTGGCTCTCGCATCGAT
GAAGGACGCAGCGAAATGCGATAAGTAATGTGAATTGCAGAATTCAGTGAATCATCGAAT
CTTTGAACGCATCTTGCGCTCCTTGGTATTCCGAGGAGCATGCCTGTTTGAGTGTCATTA
AATTCTCAACTCTCTTATACTTTTTTGTAAAAGAGAGCTTGGACTGTGGAGGCTTGCTGG
CCACTTTTTGGGGTCAGCTCCTCTGAAATGCATTAGCGGAACCGTTTGCAATCTGCCACA
AGTGTGATAAGTTATCTACACTGGCGAGGGGATTGCTCTCTGTAATGTTCAGCTTCTAAT
TGTCTCTACTTTGTGAGACAACTTTTGAATGCTTGACCTCAAATCAGGTAGGACTACCC-
GCTGAACTTAA
>BC10_25
TTTCCGTAGGTGAACCTGCGGAAGGATCATTATTGAATTATGTTTCTAGATAGGTTGTAG
CTGGCTCTTTAGAGCATGTGCACGCCTGTTTGGACTTCATTTTCATCCACCTGTGCACCT
ATTGTAGTCTTTGGTTGGGTTAGGGGGAAGTGGTCATTGTGTCAGCATCTGCTGGATGTG
AGGACTTGCATTGTGAAAGCTTTGCTGTCCTTGATGTGATCATGGAATCTCTTTCTCACT
AGAGTCTATGTCACTCATTATACTCTGTCGAATGTCATTGAATGTCTTTACATGGGCTTG
TATGCCTATGAAAATTGTAATACAACTTTCAGCAACGGATCTCTTGGCTCTCGCATCGAT
GAAGGACGCAGCGAAATGCGATAAGTAATGTGAATTGCAGAATTCAGTGAATCATCGAAT
CTTTGAACGCATCTTGCGCTCCTTGGTATTCCGAGGAGCATGCCTGTTTGAGTGTCATTA
AATTCTCAACTCTCTTATACTTTTTTGTAAAAGAGAGCTTGGACTGTGGAGGCTTGCTGG
CCACTTTTTGGGGTCAGCTCCTCTGAAATGCATTAGCGGAACCGTTTGCAATCTGCCACA
AGTGTGATAAGTTATCTACACTGGCGAGGGGATTGCTCTCTGTAATGTTCAGCTTCTAAT
TGTCTCTACTTTGTGAGACAACTTTTGAATGCTTGACCTCAAATCAGGTAGGACTACCC-
GCTGAACTTAA
>BC10_42
TTTCCGTAGGTGAACCTGCGGAAGGATCATTATTGAATTATGTTTCTAGATAGGTTGTAG
CTGGCTCTTTAGAGCATGTGCACGCCTGTTTGGACTTCATTTTCATCCACCTGTGCACCT
ATTGTAGTCTTTGGTTGGGTTAGGGGGAAGTGGTCATTGTGTCAGCATCTGCTGGATGTG
AGGACTTGCATTGTGAAAGCTTTGCTGTCCTTGATGTGATCATGGAATCTCTTTCTCACT
AGAGTCTATGTCACTCATTATACTCTGTCGAATGTCATTGAATGTCTTTACATGGGCTTG
TATGCCTATGAAAATTGTAATACAACTTTCAGCAACGGATCTCTTGGCTCTCGCATCGAT
GAAGGACGCAGCGAAATGCGATAAGTAATGTGAATTGCAGAATTCAGTGAATCATCGAAT
CTTTGAACGCATCTTGCGCTCCTTGGTATTCCGAGGAGCATGCCTGTTTGAGTGTCATTA
AATTCTCAACTCTCTTATACTTTTTTGTAAAAGAGAGCTTGGACTGTGGAGGCTTGCTGG
CCACTTTTTGGGGTCAGCTCCTCTGAAATGCATTAGCGGAACCGTTTGCAATCTGCCACA
AGTGTGATAAGTTATCTACACTGGCGAGGGGATTGCTCTCTGTAATGTTCAGCTTCTAAT

TGTCTCTACTTTGTGAGACAACTTTTGAATGCTTGACCTCAAATCAGGTAGGACTACCC-
GCTGAACTTAA
>BC10_49
TTTCCGTAGGTGAACCTGCGGAAGGATCATTATTGAATTATGTTTCTAGATAGGTTGTAG
CTGGCTCTTTAGAGCATGTGCACGCCTGTTTGGACTTCATTTTCATCCACCTGTGCACCT
ATTGTAGTCTTTGGTTGGGTTAGGGGGAAGTGGTCATTGTGTCAGCATCTGCTGGATGTG
AGGACTTGCATTGTGAAAGCTTTGCTGTCCTTGATGTGATCATGGAATCTCTTTCTCACT
AGAGTCTATGTCACTCATTATACTCTGTCGAATGTCATTGAATGTCTTTACATGGGCTTG
TATGCCTATGAAAATTGTAATACAACTTTCAGCAACGGATCTCTTGGCTCTCGCATCGAT
GAAGGACGCAGCGAAATGCGATAAGTAATGTGAATTGCAGAATTCAGTGAATCATCGAAT
CTTTGAACGCATCTTGCGCTCCTTGGTATTCCGAGGAGCATGCCTGTTTGAGTGTCATTA
AATTCTCAACTCTCTTATACTTTTTTGTAAAAGAGAGCTTGGACTGTGGAGGCTTGCTGG
CCACTTTTTGGGGTCAGCTCCTCTGAAATGCATTAGCGGAACCGTTTGCAATCTGCCACA
AGTGTGATAAGTTATCTACACTGGCGAGGGGATTGCTCTCTGTAATGTTCAGCTTCTAAT
TGTCTCTACTTTGTGAGACAACTTTTGAATGCTTGACCTCAAATCAGGTAGGACTACCC-
GCTGAACTTAA
>BC11_6
TTTCCGTAGGTGAACCTGCGGAAGGATCATTATTGAATTATGTTTCTAGATAGGTTGTAG
CTGGCTCTTTAGAGCATGTGCACGCCTGTTTGGACTTCATTTTCATCCACCTGTGCACCT
ATTGTAGTCTTTGGTTGGGTTAGGGGGAAGTGGTCATTGTGTCAGCATCTGCTGGATGTG
AGGACTTGCATTGTGAAAGCTTTGCTGTCCTTGATGTGATCATGGAATCTCTTTCTCACT
AGAGTCTATGTCACTCATTATACTCTGTCGAATGTCATTGAATGTCTTTACATGGGCTTG
TATGCCTATGAAAATTGTAATACAACTTTCAGCAACGGATCTCTTGGCTCTCGCATCGAT
GAAGGACGCAGCGAAATGCGATAAGTAATGTGAATTGCAGAATTCAGTGAATCATCGAAT
CTTTGAACGCATCTTGCGCTCCTTGGTATTCCGAGGAGCATGCCTGTTTGAGTGTCATTA
AATTCTCAACTCTCTTATACTTTTTTGTAAAAGAGAGCTTGGACTGTGGAGGCTTGCTGG
CCACTTTTTGGGGTCAGCTCCTCTGAAATGCATTAGCGGAACCGTTTGCAATCTGCCACA
AGTGTGATAAGTTATCTACACTGGCGAGGGGATTGCTCTCTGTAATGTTCAGCTTCTAAT
TGTCTCTACTTTGTGAGACAACTTTTGAATGCTTGACCTCAAATCAGGTAGGACTACCC-
GCTGAACTTAA
>BC11_14
TTTCCGTAGGTGAACCTGCGGAAGGATCATTATTGAATTATGTTTCTAGATAGGTTGTAG
CTGGCTCTTTAGAGCATGTGCACGCCTGTTTGGACTTCATTTTCATCCACCTGTGCACCT
ATTGTAGTCTTTGGTTGGGTTAGGGGGAAGTGGTCATTGTGTCAGCATCTGCTGGATGTG
AGGACTTGCATTGTGAAAGCTTTGCTGTCCTTGATGTGATCATGGAATCTCTTTCTCACT
AGAGTCTATGTCACTCATTATACTCTGTCGAATGTCATTGAATGTCTTTACATGGGCTTG
TATGCCTATGAAAATTGTAATACAACTTTCAGCAACGGATCTCTTGGCTCTCGCATCGAT
GAAGGACGCAGCGAAATGCGATAAGTAATGTGAATTGCAGAATTCAGTGAATCATCGAAT
CTTTGAACGCATCTTGCGCTCCTTGGTATTCCGAGGAGCATGCCTGTTTGAGTGTCATTA
AATTCTCAACTCTCTTATACTTTTTTGTAAAAGAGAGCTTGGACTGTGGAGGCTTGCTGG
CCACTTTTTGGGGTCAGCTCCTCTGAAATGCATTAGCGGAACCGTTTGCAATCTGCCACA
AGTGTGATAAGTTATCTACACTGGCGAGGGGATTGCTCTCTGTAATGTTCAGCTTCTAAT
TGTCTCTACTTTGTGAGACAACTTTTGAATGCTTGACCTCAAATCAGGTAGGACTACCC-
GCTGAACTTAA
>BC11_52
TTTCCGTAGGTGAACCTGCGGAAGGATCATTATTGAATTATGTTTCTAGATAGGTTGTAG
CTGGCTCTTTAGAGCATGTGCACGCCTGTTTGGACTTCATTTTCATCCACCTGTGCACCT
ATTGTAGTCTTTGGTTGGGTTAGGGGGAAGTGGTCATTGTGTCAGCATCTGCTGGATGTG
AGGACTTGCATTGTGAAAGCTTTGCTGTCCTTGATGTGATCATGGAATCTCTTTCTCACT
AGAGTCTATGTCACTCATTATACTCTGTCGAATGTCATTGAATGTCTTTACATGGGCTTG

TATGCCTATGAAAATTGTAATACAACTTTCAGCAACGGATCTCTTGGCTCTCGCATCGAT
GAAGGACGCAGCGAAATGCGATAAGTAATGTGAATTGCAGAATTCAGTGAATCATCGAAT
CTTTGAACGCATCTTGCGCTCCTTGGTATTCCGAGGAGCATGCCTGTTTGAGTGTCATTA
AATTCTCAACTCTCTTATACTTTTTTGTAAAAGAGAGCTTGGACTGTGGAGGCTTGCTGG
CCACTTTTTGGGGTCAGCTCCTCTGAAATGCATTAGCGGAACCGTTTGCAATCTGCCACA
AGTGTGATAAGTTATCTACACTGGCGAGGGGATTGCTCTCTGTAATGTTCAGCTTCTAAT
TGTCTCTACTTTGTGAGACAACTTTTGAATGCTTGACCTCAAATCAGGTAGGACTACCC-
GCTGAACTTAA
>BC12_4
TTTCCGTAGGTGAACCTGCGGAAGGATCATTATTGAATTATGTTTCTAGATAGGTTGTAG
CTGGCTCTTTAGAGCATGTGCACGCCTGTTTGGACTTCATTTTCATCCACCTGTGCACCT
ATTGTAGTCTTTGGTTGGGTTAGGGGGAAGTGGTCATTGTGTCAGCATCTGCTGGATGTG
AGGACTTGCATTGTGAAAGCTTTGCTGTCCTTGATGTGATCATGGAATCTCTTTCTCACT
AGAGTCTATGTCACTCATTATACTCTGTCGAATGTCATTGAATGTCTTTACATGGGCTTG
TATGCCTATGAAAATTGTAATACAACTTTCAGCAACGGATCTCTTGGCTCTCGCATCGAT
GAAGGACGCAGCGAAATGCGATAAGTAATGTGAATTGCAGAATTCAGTGAATCATCGAAT
CTTTGAACGCATCTTGCGCTCCTTGGTATTCCGAGGAGCATGCCTGTTTGAGTGTCATTA
AATTCTCAACTCTCTTATACTTTTTTGTAAAAGAGAGCTTGGACTGTGGAGGCTTGCTGG
CCACTTTTTGGGGTCAGCTCCTCTGAAATGCATTAGCGGAACCGTTTGCAATCTGCCACA
AGTGTGATAAGTTATCTACACTGGCGAGGGGATTGCTCTCTGTAATGTTCAGCTTCTAAT
TGTCTCTACTTTGTGAGACAACTTTTGAATGCTTGACCTCAAATCAGGTAGGACTACCC-
GCTGAACTTAA
>BC12_11
TTTCCGTAGGTGAACCTGCGGAAGGATCATTATTGAATTATGTTTCTAGATAGGTTGTAG
CTGGCTCTTTAGAGCATGTGCACGCCTGTTTGGACTTCATTTTCATCCACCTGTGCACCT
ATTGTAGTCTTTGGTTGGGTTAGGGGGAAGTGGTCATTGTGTCAGCATCTGCTGGATGTG
AGGACTTGCATTGTGAAAGCTTTGCTGTCCTTGATGTGATCATGGAATCTCTTTCTCACT
AGAGTCTATGTCACTCATTATACTCTGTCGAATGTCATTGAATGTCTTTACATGGGCTTG
TATGCCTATGAAAATTGTAATACAACTTTCAGCAACGGATCTCTTGGCTCTCGCATCGAT
GAAGGACGCAGCGAAATGCGATAAGTAATGTGAATTGCAGAATTCAGTGAATCATCGAAT
CTTTGAACGCATCTTGCGCTCCTTGGTATTCCGAGGAGCATGCCTGTTTGAGTGTCATTA
AATTCTCAACTCTCTTATACTTTTTTGTAAAAGAGAGCTTGGACTGTGGAGGCTTGCTGG
CCACTTTTTGGGGTCAGCTCCTCTGAAATGCATTAGCGGAACCGTTTGCAATCTGCCACA
AGTGTGATAAGTTATCTACACTGGCGAGGGGATTGCTCTCTGTAATGTTCAGCTTCTAAT
TGTCTCTACTTTGTGAGACAACTTTTGAATGCTTGACCTCAAATCAGGTAGGACTACCC-
GCTGAACTTAA
>BC12_14
TTTCCGTAGGTGAACCTGCGGAAGGATCATTATTGAATTATGTTTCTAGATAGGTTGTAG
CTGGCTCTTTAGAGCATGTGCACGCCTGTTTGGACTTCATTTTCATCCACCTGTGCACCT
ATTGTAGTCTTTGGTTGGGTTAGGGGGAAGTGGTCATTGTGTCAGCATCTGCTGGATGTG
AGGACTTGCATTGTGAAAGCTTTGCTGTCCTTGATGTGATCATGGAATCTCTTTCTCACT
AGAGTCTATGTCACTCATTATACTCTGTCGAATGTCATTGAATGTCTTTACATGGGCTTG
TATGCCTATGAAAATTGTAATACAACTTTCAGCAACGGATCTCTTGGCTCTCGCATCGAT
GAAGGACGCAGCGAAATGCGATAAGTAATGTGAATTGCAGAATTCAGTGAATCATCGAAT
CTTTGAACGCATCTTGCGCTCCTTGGTATTCCGAGGAGCATGCCTGTTTGAGTGTCATTA
AATTCTCAACTCTCTTATACTTTTTTGTAAAAGAGAGCTTGGACTGTGGAGGCTTGCTGG
CCACTTTTTGGGGTCAGCTCCTCTGAAATGCATTAGCGGAACCGTTTGCAATCTGCCACA
AGTGTGATAAGTTATCTACACTGGCGAGGGGATTGCTCTCTGTAATGTTCAGCTTCTAAT
TGTCTCTACTTTGTGAGACAACTTTTGAATGCTTGACCTCAAATCAGGTAGGACTACCC-
GCTGAACTTAA

>BC12_16
TTTCCGTAGGTGAACCTGCGGAAGGATCATTATTGAATTATGTTTCTAGATAGGTTGTAG
CTGGCTCTTTAGAGCATGTGCACGCCTGTTTGGACTTCATTTTCATCCACCTGTGCACCT
ATTGTAGTCTTTGGTTGGGTTAGGGGGAAGTGGTCATTGTGTCAGCATCTGCTGGATGTG
AGGACTTGCATTGTGAAAGCTTTGCTGTCCTTGATGTGATCATGGAATCTCTTTCTCACT
AGAGTCTATGTCACTCATTATACTCTGTCGAATGTCATTGAATGTCTTTACATGGGCTTG
TATGCCTATGAAAATTGTAATACAACTTTCAGCAACGGATCTCTTGGCTCTCGCATCGAT
GAAGGACGCAGCGAAATGCGATAAGTAATGTGAATTGCAGAATTCAGTGAATCATCGAAT
CTTTGAACGCATCTTGCGCTCCTTGGTATTCCGAGGAGCATGCCTGTTTGAGTGTCATTA
AATTCTCAACTCTCTTATACTTTTTTGTAAAAGAGAGCTTGGACTGTGGAGGCTTGCTGG
CCACTTTTTGGGGTCAGCTCCTCTGAAATGCATTAGCGGAACCGTTTGCAATCTGCCACA
AGTGTGATAAGTTATCTACACTGGCGAGGGGATTGCTCTCTGTAATGTTCAGCTTCTAAT
TGTCTCTACTTTGTGAGACAACTTTTGAATGCTTGACCTCAAATCAGGTAGGACTACCC-
GCTGAACTTAA
>BC12_21
TTTCCGTAGGTGAACCTGCGGAAGGATCATTATTGAATTATGTTTCTAGATAGGTTGTAG
CTGGCTCTTTAGAGCATGTGCACGCCTGTTTGGACTTCATTTTCATCCACCTGTGCACCT
ATTGTAGTCTTTGGTTGGGTTAGGGGGAAGTGGTCATTGTGTCAGCATCTGCTGGATGTG
AGGACTTGCATTGTGAAAGCTTTGCTGTCCTTGATGTGATCATGGAATCTCTTTCTCACT
AGAGTCTATGTCACTCATTATACTCTGTCGAATGTCATTGAATGTCTTTACATGGGCTTG
TATGCCTATGAAAATTGTAATACAACTTTCAGCAACGGATCTCTTGGCTCTCGCATCGAT
GAAGGACGCAGCGAAATGCGATAAGTAATGTGAATTGCAGAATTCAGTGAATCATCGAAT
CTTTGAACGCATCTTGCGCTCCTTGGTATTCCGAGGAGCATGCCTGTTTGAGTGTCATTA
AATTCTCAACTCTCTTATACTTTTTTGTAAAAGAGAGCTTGGACTGTGGAGGCTTGCTGG
CCACTTTTTGGGGTCAGCTCCTCTGAAATGCATTAGCGGAACCGTTTGCAATCTGCCACA
AGTGTGATAAGTTATCTACACTGGCGAGGGGATTGCTCTCTGTAATGTTCAGCTTCTAAT
TGTCTCTACTTTGTGAGACAACTTTTGAATGCTTGACCTCAAATCAGGTAGGACTACCC-
GCTGAACTTAA
>BC12_30
TTTCCGTAGGTGAACCTGCGGAAGGATCATTATTGAATTATGTTTCTAGATAGGTTGTAG
CTGGCTCTTTAGAGCATGTGCACGCCTGTTTGGACTTCATTTTCATCCACCTGTGCACCT
ATTGTAGTCTTTGGTTGGGTTAGGGGGAAGTGGTCATTGTGTCAGCATCTGCTGGATGTG
AGGACTTGCATTGTGAAAGCTTTGCTGTCCTTGATGTGATCATGGAATCTCTTTCTCACT
AGAGTCTATGTCACTCATTATACTCTGTCGAATGTCATTGAATGTCTTTACATGGGCTTG
TATGCCTATGAAAATTGTAATACAACTTTCAGCAACGGATCTCTTGGCTCTCGCATCGAT
GAAGGACGCAGCGAAATGCGATAAGTAATGTGAATTGCAGAATTCAGTGAATCATCGAAT
CTTTGAACGCATCTTGCGCTCCTTGGTATTCCGAGGAGCATGCCTGTTTGAGTGTCATTA
AATTCTCAACTCTCTTATACTTTTTTGTAAAAGAGAGCTTGGACTGTGGAGGCTTGCTGG
CCACTTTTTGGGGTCAGCTCCTCTGAAATGCATTAGCGGAACCGTTTGCAATCTGCCACA
AGTGTGATAAGTTATCTACACTGGCGAGGGGATTGCTCTCTGTAATGTTCAGCTTCTAAT
TGTCTCTACTTTGTGAGACAACTTTTGAATGCTTGACCTCAAATCAGGTAGGACTACCC-
GCTGAACTTAA
>BC5-82
TTTCCGTAGGTGAACCTGCGGAAGGATCATTATTGAATTATGTTTCTAGATAGGTTGTAG
CTGGCTCTTTAGAGCATGTGCACGCCTGTTTGGACTTCATTTTCATCCACCTGTGCACCT
ATTGTAGTCTTTGGTTGGGTTAGGGGGAAGTGGTCATTGTGTCAGCATCTGCTGGATGTG
AGGACTTGCATTGTGAAAGCTTTGCTGTCCTTGATGTGATCATGGAATCTCTTTCTCACT
AGAGTCTATGTCACTCATTATACTCTGTCGAATGTCATTGAATGTCTTTACATGGGCTTG
TATGCCTATGAAAATTGTAATACAACTTTCAGCAACGGATCTCTTGGCTCTCGCATCGAT
GAAGGACGCAGCGAAATGCGATAAGTAATGTGAATTGCAGAATTCAGTGAATCATCGAAT

CTTTGAACGCATCTTGCGCTCCTTGGTATTCCGAGGAGCATGCCTGTTTGAGTGTCATTA
AATTCTCAACTCTCTTATACTTTTTTGTAAAAGAGAGCTTGGACTGTGGAGGCTTGCTGG
CCACTTTTTGGGGTCAGCTCCTCTGAAATGCATTAGCGGAACCGTTTGCAATCTGCCACA
AGTGTGATAAGTTATCTACACTGGCGAGGGGATTGCTCTCTGTAATGTTCAGCTTCTAAT
TGTCTCTACTTTGTGAGACAACTTTTGAATGCTTGACCTCAAATCAGGTAGGACTACCC–
GCTGAACTTAA
>BC12_29
TTTCCGTAGGTGAACCTGCGGAAGGATCATTATTGAATTATGTTTCTAGATAGGTTGTAG
CTGGCTCTTTAGAGCATGTGCACGCCTGTTTGGACTTCATTTTCATCCACCTGTGCACCT
ATTGTAGTCTTTGGTTGGGTTAGGAGGAAGTGGTCATTGTGTCAGCATCTGCTGGATGTG
AGGACTTGCATTGTGAAAGCTTTGCTGTCCTTGATGTGATCATGGAATCTCTTTCTCACT
AGAGTCTATGTCACTCATTATACTCTGTCGAATGTCATTGAATGTCTTTACATGGGCTTG
TATGCCTATGAAAATTGTAATACAACTTTCAGCAACGGATCTCTTGGCTCTCGCATCGAT
GAAGGACGCAGCGAAATGCGATAAGTAATGTGAATTGCAGAATTCAGTGAATCATCGAAT
CTTTGAACGCATCTTGCGCTCCTTGGTATTCCGAGGAGCATGCCTGTTTGAGTGTCATTA
AATTCTCAACTCTCTTATACTTTTTTGTAAAAGAGAGCTTGGACTGTGGAGGCTTGCTGG
CCACTTTTTGGGGTCAGCTCCTCTGAAATGCATTAGCGGAACCGTTTGCAATCTGCCACA
AGTGTGATAAGTTATCTACACTGGCGAGGGGATTGCTCTCTGTAATGTTCAGCTTCTAAT
TGTCTCTACTTTGTGAGACAACTTTTGAATGCTTGACCTCAAATCAGGTAGGACTACCC–
GCTGAACTTAA
>BC1–23
TTTCCGTAGGTGAACCTGCGGAAGGATCATTATTGAATTATGTTTCTAGATAGGTTGTAG
CTGGCTCTTTAGAGCATGTGCACGCCTGTTTGGACTTCATTTTCATCCACCTGTGCACCT
ATTGTAGTCTTTGGTTGGGTTAGGAGGAAGTGGTCATTGTGTCAGCATCTGCTGGATGTG
AGGACTTGCATTGTGAAAGCTTTGCTGTCCTTGATGTGATCATGGAATCTCTTTCTCACT
AGAGTCTATGTCACTCATTATACTCTGTCGAATGTCATTGAATGTCTTTACATGGGCTTG
TATGCCTATGAAAATTGTAATACAACTTTCAGCAACGGATCTCTTGGCTCTCGCATCGAT
GAAGGACGCAGCGAAATGCGATAAGTAATGTGAATTGCAGAATTCAGTGAATCATCGAAT
CTTTGAACGCATCTTGCGCTCCTTGGTATTCCGAGGAGCATGCCTGTTTGAGTGTCATTA
AATTCTCAACTCTCTTATACTTTTTTGTAAAAGAGAGCTTGGACTGTGGAGGCTTGCTGG
CCACTTTTTGGGGTCAGCTCCTCTGAAATGCATTAGCGGAACCGTTTGCAATCTGCCACA
AGTGTGATAAGTTATCTACACTGGCGAGGGGATTGCTCTCTGTAATGTTCAGCTTCTAAT
TGTCTCTACTTTGTGAGACAACTTTTGAATGCTTGACCTCAAATCAGGTAGGACTACCC–
GCTGAACTTAA
>BC3–18
TTTCCGTAGGTGAACCTGCGGAAGGATCATTATTGAATTATGTTTCTAGATAGGTTGTAG
CTGGCTCTTTAGAGCATGTGCACGCCTGTTTGGACTTCATTTTCATCCACCTGTGCACCT
ATTGTAGTCTTTGGTTGGGTTAGGAGGAAGTGGTCATTGTGTCAGCATCTGCTGGATGTG
AGGACTTGCATTGTGAAAGCTTTGCTGTCCTTGATGTGATCATGGAATCTCTTTCTCACT
AGAGTCTATGTCACTCATTATACTCTGTCGAATGTCATTGAATGTCTTTACATGGGCTTG
TATGCCTATGAAAATTGTAATACAACTTTCAGCAACGGATCTCTTGGCTCTCGCATCGAT
GAAGGACGCAGCGAAATGCGATAAGTAATGTGAATTGCAGAATTCAGTGAATCATCGAAT
CTTTGAACGCATCTTGCGCTCCTTGGTATTCCGAGGAGCATGCCTGTTTGAGTGTCATTA
AATTCTCAACTCTCTTATACTTTTTTGTAAAAGAGAGCTTGGACTGTGGAGGCTTGCTGG
CCACTTTTTGGGGTCAGCTCCTCTGAAATGCATTAGCGGAACCGTTTGCAATCTGCCACA
AGTGTGATAAGTTATCTACACTGGCGAGGGGATTGCTCTCTGTAATGTTCAGCTTCTAAT
TGTCTCTACTTTGTGAGACAACTTTTGAATGCTTGACCTCAAATCAGGTAGGACTACCC–
GCTGAACTTAA
>BC4–45
TTTCCGTAGGTGAACCTGCGGAAGGATCATTATTGAATTATGTTTCTAGATAGGTTGTAG

CTGGCTCTTTAGAGCATGTGCACGCCTGTTTGGACTTCATTTTCATCCACCTGTGCACCT
ATTGTAGTCTTTGGTTGGGTTAGGAGGAAGTGGTCATTGTGTCAGCATCTGCTGGATGTG
AGGACTTGCATTGTGAAAGCTTTGCTGTCCTTGATGTGATCATGGAATCTCTTTCTCACT
AGAGTCTATGTCACTCATTATACTCTGTCGAATGTCATTGAATGTCTTTACATGGGCTTG
TATGCCTATGAAAATTGTAATACAACTTTCAGCAACGGATCTCTTGGCTCTCGCATCGAT
GAAGGACGCAGCGAAATGCGATAAGTAATGTGAATTGCAGAATTCAGTGAATCATCGAAT
CTTTGAACGCATCTTGCGCTCCTTGGTATTCCGAGGAGCATGCCTGTTTGAGTGTCATTA
AATTCTCAACTCTCTTATACTTTTTTGTAAAAGAGAGCTTGGACTGTGGAGGCTTGCTGG
CCACTTTTTGGGGTCAGCTCCTCTGAAATGCATTAGCGGAACCGTTTGCAATCTGCCACA
AGTGTGATAAGTTATCTACACTGGCGAGGGGATTGCTCTCTGTAATGTTCAGCTTCTAAT
TGTCTCTACTTTGTGAGACAACTTTTGAATGCTTGACCTCAAATCAGGTAGGACTACCC-
GCTGAACTTAA
>BC5-26
TTTCCGTAGGTGAACCTGCGGAAGGATCATTATTGAATTATGTTTCTAGATAGGTTGTAG
CTGGCTCTTTAGAGCATGTGCACGCCTGTTTGGACTTCATTTTCATCCACCTGTGCACCT
ATTGTAGTCTTTGGTTGGGTTAGGAGGAAGTGGTCATTGTGTCAGCATCTGCTGGATGTG
AGGACTTGCATTGTGAAAGCTTTGCTGTCCTTGATGTGATCATGGAATCTCTTTCTCACT
AGAGTCTATGTCACTCATTATACTCTGTCGAATGTCATTGAATGTCTTTACATGGGCTTG
TATGCCTATGAAAATTGTAATACAACTTTCAGCAACGGATCTCTTGGCTCTCGCATCGAT
GAAGGACGCAGCGAAATGCGATAAGTAATGTGAATTGCAGAATTCAGTGAATCATCGAAT
CTTTGAACGCATCTTGCGCTCCTTGGTATTCCGAGGAGCATGCCTGTTTGAGTGTCATTA
AATTCTCAACTCTCTTATACTTTTTTGTAAAAGAGAGCTTGGACTGTGGAGGCTTGCTGG
CCACTTTTTGGGGTCAGCTCCTCTGAAATGCATTAGCGGAACCGTTTGCAATCTGCCACA
AGTGTGATAAGTTATCTACACTGGCGAGGGGATTGCTCTCTGTAATGTTCAGCTTCTAAT
TGTCTCTACTTTGTGAGACAACTTTTGAATGCTTGACCTCAAATCAGGTAGGACTACCC-
GCTGAACTTAA
>BC8-22
TTTCCGTAGGTGAACCTGCGGAAGGATCATTATTGAATTATGTTTCTAGATAGGTTGTAG
CTGGCTCTTTAGAGCATGTGCACGCCTGTTTGGACTTCATTTTCATCCACCTGTGCACCT
ATTGTAGTCTTTGGTTGGGTTAGGAGGAAGTGGTCATTGTGTCAGCATCTGCTGGATGTG
AGGACTTGCATTGTGAAAGCTTTGCTGTCCTTGATGTGATCATGGAATCTCTTTCTCACT
AGAGTCTATGTCACTCATTATACTCTGTCGAATGTCATTGAATGTCTTTACATGGGCTTG
TATGCCTATGAAAATTGTAATACAACTTTCAGCAACGGATCTCTTGGCTCTCGCATCGAT
GAAGGACGCAGCGAAATGCGATAAGTAATGTGAATTGCAGAATTCAGTGAATCATCGAAT
CTTTGAACGCATCTTGCGCTCCTTGGTATTCCGAGGAGCATGCCTGTTTGAGTGTCATTA
AATTCTCAACTCTCTTATACTTTTTTGTAAAAGAGAGCTTGGACTGTGGAGGCTTGCTGG
CCACTTTTTGGGGTCAGCTCCTCTGAAATGCATTAGCGGAACCGTTTGCAATCTGCCACA
AGTGTGATAAGTTATCTACACTGGCGAGGGGATTGCTCTCTGTAATGTTCAGCTTCTAAT
TGTCTCTACTTTGTGAGACAACTTTTGAATGCTTGACCTCAAATCAGGTAGGACTACCC-
GCTGAACTTAA
>BC9-7
TTTCCGTAGGTGAACCTGCGGAAGGATCATTATTGAATTATGTTTCTAGATAGGTTGTAG
CTGGCTCTTTAGAGCATGTGCACGCCTGTTTGGACTTCATTTTCATCCACCTGTGCACCT
ATTGTAGTCTTTGGTTGGGTTAGGAGGAAGTGGTCATTGTGTCAGCATCTGCTGGATGTG
AGGACTTGCATTGTGAAAGCTTTGCTGTCCTTGATGTGATCATGGAATCTCTTTCTCACT
AGAGTCTATGTCACTCATTATACTCTGTCGAATGTCATTGAATGTCTTTACATGGGCTTG
TATGCCTATGAAAATTGTAATACAACTTTCAGCAACGGATCTCTTGGCTCTCGCATCGAT
GAAGGACGCAGCGAAATGCGATAAGTAATGTGAATTGCAGAATTCAGTGAATCATCGAAT
CTTTGAACGCATCTTGCGCTCCTTGGTATTCCGAGGAGCATGCCTGTTTGAGTGTCATTA
AATTCTCAACTCTCTTATACTTTTTTGTAAAAGAGAGCTTGGACTGTGGAGGCTTGCTGG

CCACTTTTTGGGGTCAGCTCCTCTGAAATGCATTAGCGGAACCGTTTGCAATCTGCCACA
AGTGTGATAAGTTATCTACACTGGCGAGGGGATTGCTCTCTGTAATGTTCAGCTTCTAAT
TGTCTCTACTTTGTGAGACAACTTTTGAATGCTTGACCTCAAATCAGGTAGGACTACCC-
GCTGAACTTAA
>BC10_39
TTTCCGTAGGTGAACCTGCGGAAGGATCATTATTGAATTATGTTTCTAGATAGGTTGTAG
CTGGCTCTTTAGAGCATGTGCACGCCTGTTTGGACTTCATTTTCATCCACCTGTGCACCT
ATTGTAGTCTTTGGTTGGGTTAGGAGGAAGTGGTCATTGTGTCAGCATCTGCTGGATGTG
AGGACTTGCATTGTGAAAGCTTTGCTGTCCTTGATGTGATCATGGAATCTCTTTCTCACT
AGAGTCTATGTCACTCATTATACTCTGTCGAATGTCATTGAATGTCTTTACATGGGCTTG
TATGCCTATGAAAATTGTAATACAACTTTCAGCAACGGATCTCTTGGCTCTCGCATCGAT
GAAGGACGCAGCGAAATGCGATAAGTAATGTGAATTGCAGAATTCAGTGAATCATCGAAT
CTTTGAACGCATCTTGCGCTCCTTGGTATTCCGAGGAGCATGCCTGTTTGAGTGTCATTA
AATTCTCAACTCTCTTATACTTTTTTGTAAAAGAGAGCTTGGACTGTGGAGGCTTGCTGG
CCACTTTTTGGGGTCAGCTCCTCTGAAATGCATTAGCGGAACCGTTTGCAATCTGCCACA
AGTGTGATAAGTTATCTACACTGGCGAGGGGATTGCTCTCTGTAATGTTCAGCTTCTAAT
TGTCTCTACTTTGTGAGACAACTTTTGAATGCTTGACCTCAAATCAGGTAGGACTACCC-
GCTGAACTTAA
>BC11_1
TTTCCGTAGGTGAACCTGCGGAAGGATCATTATTGAATTATGTTTCTAGATAGGTTGTAG
CTGGCTCTTTAGAGCATGTGCACGCCTGTTTGGACTTCATTTTCATCCACCTGTGCACCT
ATTGTAGTCTTTGGTTGGGTTAGGAGGAAGTGGTCATTGTGTCAGCATCTGCTGGATGTG
AGGACTTGCATTGTGAAAGCTTTGCTGTCCTTGATGTGATCATGGAATCTCTTTCTCACT
AGAGTCTATGTCACTCATTATACTCTGTCGAATGTCATTGAATGTCTTTACATGGGCTTG
TATGCCTATGAAAATTGTAATACAACTTTCAGCAACGGATCTCTTGGCTCTCGCATCGAT
GAAGGACGCAGCGAAATGCGATAAGTAATGTGAATTGCAGAATTCAGTGAATCATCGAAT
CTTTGAACGCATCTTGCGCTCCTTGGTATTCCGAGGAGCATGCCTGTTTGAGTGTCATTA
AATTCTCAACTCTCTTATACTTTTTTGTAAAAGAGAGCTTGGACTGTGGAGGCTTGCTGG
CCACTTTTTGGGGTCAGCTCCTCTGAAATGCATTAGCGGAACCGTTTGCAATCTGCCACA
AGTGTGATAAGTTATCTACACTGGCGAGGGGATTGCTCTCTGTAATGTTCAGCTTCTAAT
TGTCTCTACTTTGTGAGACAACTTTTGAATGCTTGACCTCAAATCAGGTAGGACTACCC-
GCTGAACTTAA
>BC12_31
TTTCCGTAGGTGAACCTGCGGAAGGATCATTATTGAATTATGTTTCTAGATAGGTTGTAG
CTGGCTCTTTAGAGCATGTGCACGCCTGTTTGGACTTCATTTTCATCCACCTGTGCACCT
ATTGTAGTCTTTGGTTGGGTTAGGAGGAAGTGGTCATTGTGTCAGCATCTGCTGGATGTG
AGGACTTGCATTGTGAAAGCTTTGCTGTCCTTGATGTGATCATGGAATCTCTTTCTCACT
AGAGTCTATGTCACTCATTATACTCTGTCGAATGTCATTGAATGTCTTTACATGGGCTTG
TATGCCTATGAAAATTGTAATACAACTTTCAGCAACGGATCTCTTGGCTCTCGCATCGAT
GAAGGACGCAGCGAAATGCGATAAGTAATGTGAATTGCAGAATTCAGTGAATCATCGAAT
CTTTGAACGCATCTTGCGCTCCTTGGTATTCCGAGGAGCATGCCTGTTTGAGTGTCATTA
AATTCTCAACTCTCTTATACTTTTTTGTAAAAGAGAGCTTGGACTGTGGAGGCTTGCTGG
CCACTTTTTGGGGTCAGCTCCTCTGAAATGCATTAGCGGAACCGTTTGCAATCTGCCACA
AGTGTGATAAGTTATCTACACTGGCGAGGGGATTGCTCTCTGTAATGTTCAGCTTCTAAT
TGTCTCTACTTTGTGAGACAACTTTTGAATGCTTGACCTCAAATCAGGTAGGACTACCC-
GCTGAACTTAA
>BC6-3
TTTCCGTAGGTGAACCTGCGGAAGGATCATTATTGAATTATGTTTCTAGATAGGTTGTAG
CTGGCTCTTTAGAGCATGTGCACGCCTGTTTGGACTTCATTTTCATCCACCTGTGCACCT
ATTGTAGTCTTTGGTTGGGTTAGGGGGAAGTGGTCATTGTGTCAGCATCTGCTGGATGTG

AGGACTTGCATTGTGAAAGCTTTGCTGTCCTTGATGTGATCATGGAATCTCTTTCTCACT
AGAGTCTATGTCACTCATTATACTCTGTCGAATGTCATTGAATGTCTTTACATGGGCTTG
TATGCCTATGAAAATTGTAATACAACTTTCAGCAACGGATCTCTTGGCTCTCGCATCGAT
GAAGGACGCAGCGAAATGCGATAAGTAATGTGAATTGCAGAATTCAGTGAATCATCGAAT
CTTTGAACGCATCTTGCGCTCCTTGGTATTCCGAGGAGCATGCCTGTTTGAGTGTCATTA
AATTCTCAACTCTCTTATACTTTTTTGTAAAAGAGAGCTTGGACTGTGGAGGCTTGCTGG
CCACTTTTTGGGGTCAGCTCCTCTGAAATGCATTAGCGGAACCGTTTGCGATCTGCCACA
AGTGTGATAAGTTATCTACACTGGCGAGGGGATTGCTCTCTGTAATGTTCAGCTTCTAAT
TGTCTCTACTTTGTGAGACAACTTTTGAATGCTTGACCTCAAATCAGGTAGGACTACCC-
GCTGAACTTAA
>BC11_47
TTTCCGTAGGTGAACCTGCGGAAGGATCATTATTGAATTATGTTTCTAGATAGGTTGTAG
CTGGCTCTTTAGAGCATGTGCACGCCTGTTTGGACTTCATTTTCATCCACCTGTGCACCT
ATTGTAGTCTTTGGTTGGGTTAGGGGGAAGTGGTCATTGTGTCAGCATCTGCTGGATGTG
AGGACTTGCATTGTGAAAGCTTTGCTGTCCTTGATGTGATCATGGAATCTCTTTCTCACT
AGAGTCTATGTCACTCATTATACTCTGTCGAATGTCATTGAATGTCTTTACATGGGCTTG
TATGCCTATGAAAATTGTAATACAACTTTCAGCAACGGATCTCTTGGCTCTCGCATCGAT
GAAGAACGCAGCGAAATGCGATAAGTAATGTGAATTGCAGAATTCAGTGAATCATCGAAT
CTTTGAACGCATCTTGCGCTCCTTGGTATTCCGAGGAGCATGCCTGTTTGAGTGTCATTA
AATTCTCAACTCTCTTATACTTTTTTGTAAAAGAGAGCTTGGACTGTGGAGGCTTGCTGG
CCACTTTTTGGGGTCAGCTCCTCTGAAATGCATTAGCGGAACCGTTTGCAATCTGCCACA
AGTGTGATAAGTTATCTACACTGGCGAGGGGATTGCTCTCTGTAATGTTCAGCTTCTAAT
TGTCTCTACTTTGTGAGACAACTTTTGAATGCTTGACCTCAAATCAGGTAGGACTACCC-
GCTGAACTTAA
>BC7-20
TTTCCGTAGGTGAACCTGCGGAAGGATCATTATTGAATTATGTTTCTAGATAGGTTGTAG
CTGGCTCTTTAGAGCATGTGCACGCCTGTTTGGACTTCATTTTCATCCACCTGTGCACCT
ATTGTAGTCTTTGGTTGGGTTAGGGGGAAGTGGTCATTGTGTCGGCATCTGCTGGATGTG
AGGACTTGCATTGTGAAAGCTTTGCTGTCCTTGATGTGATCATGGAATCTCTTTCTCACT
AGAGTCTATGTCACTCATTATACTCTGTCGAATGTCATTGAATGTCTTTACATGGGCTTG
TATGCCTATGAAAATTGTAATACAACTTTCAGCAACGGATCTCTTGGCTCTCGCATCGAT
GAAGGACGCAGCGAAATGCGATAAGTAATGTGAATTGCAGAATTCAGTGAATCATCGAAT
CTTTGAACGCATCTTGCGCTCCTTGGTATTCCGAGGAGCATGCCTGTTTGAGTGTCATTA
AATTCTCAACTCTCTTATACTTTTTTGTAAAAGAGAGCTTGGACTGTGGAGGCTTGCTGG
CCACTTTTTGGGGTCAGCTCCTCTGAAATGCATTAGCGGAACCGTTTGCAATCTGCCACA
AGTGTGATAAGTTATCTACACTGGCGAGGGGATTGCTCTCTGTAATGTTCAGCTTCTAAT
TGTCTCTACTTTGTGAGACAACTTTTGAATGCTTGACCTCAAATCAGGTAGGACTACCC-
GCTGAACTTAA
>BC11_5
TTTCCGTAGGTGAACCTGCGGAAGGATCATTATTGAATTATGTTTCTAGATAGGTTGTAG
CTGGCTCTTTAGAGCATGTGCACGCCTGTTTGGACTTCATTTTCATCCACCTGTGCACCT
ATTGTAGTCTTTGGTTGGGTTAGGGGGAAGTGGTCATTGTGTCAGCATCTGCTGGATGTG
AGGACTTGCATTGTGAAAGCTTTGCTGTCCTTGATGTGATCATGGAATCTCTTTCTCACT
AGAGTCTATGTCACTCATTATACTCTGTCGAATGTCATTGAATGTCTTTACATGGGCTTG
TATGCCTATGAAAATTGTAATACAACTTTCAGCAACGGATCTCTTGGCTCTCGCATCGAT
GAAGGACGCAGCGAAATGCGATAAGTAATGTGAATTGCAGAATTCAGTGAATCATCGAAT
CTTTGAACGCATCTTGCGCTCCTTGGTATTCCGAGGAGCATGCCTGTTTGAGTGTCATTA
AATTCTCAACTCTCTTATACTTTTTTGTAAAAGAGAGCTTGGACTGTGGAGGCTTGCTGG
CCACTTTTTGGGGTCAGCTCCTCTGAAATGCATTAGCGGAACCGTTTGCAATCTACCACA
AGTGTGATAAGTTATCTACACTGGCGAGGGGATTGCTCTCTGTAATGTTCAGCTTCTAAT

TGTCTCTACTTTGTGAGACAACTTTTGAATGCTTGACCTCAAATCAGGTAGGACTACCC-
GCTGAACTTAA
>BC3-43
TTTCCGTAGGTGAACCTGCGGAAGGATCATTATTGAATTATGTTTCTAGATAGGTTGTAG
CTGGCTCTTTAGAGCATGTGCACGCCTGTTTGGACTTCATTTTCATCCACCTGTGCACCT
ATTGTAGTCTTTGGTTGGGTTAGGGGGAAGTGGTCATTGTGTCAGCATCTGCTGGATGTG
AGGACTTGCATTGTGAAAGCTTTGCTGTCCTTGATGTGATCATGGAATCTCTTTCTCACT
AGAGTCTATGTCACTCATTATACTCTGTCGAATGTCATTGAATGTCTTTACATGGGCTTG
TATGCCTATGAAAATTGTAATACAACTTTCAGCAACGGATCTCTTGGCTCTCGCATCGAT
GAAGGACGCAGCGAAATGCGATAAGTAATGTGAATTGCAGAATTCAGTGAATCATCGAAT
CTTTGAACGCATCTTGCGCTCCTTGGTATTCCGAGGAGCATGCCTGTTTGAGTGTCATTA
AATTCTCAACTCTCTTATACTTTTTTGTAAAAGAGAGCTTGGACTGTGGAGGCTTGTTGG
CCACTTTTTGGGGTCAGCTCCTCTGAAATGCATTAGCGGAACCGTTTGCAATCTGCCACA
AGTGTGATAAGTTATCTACACTGGCGAGGGGATTGCTCTCTGTAATGTTCAGCTTCTAAT
TGTCTCTACTTTGTGAGACAACTTTTGAATGCTTGACCTCAAATCAGGTAGGACTACCC-
GCTGAACTTAA
>BC6-37
TTTCCGTAGGTGAACCTGCGGAAGGATCATTATTGAATTATGTTTCTAGATAGGTTGTAG
CTGGCTCTTTAGAGCATGTGCACGCCTGTTTGGACTTCATTTTCATCCACCTGTGCACCT
ATTGTAGTCTTTGGTTGGGTTAGGGGGAAGTGGTCATTGTGTCAGCATCTGCTGGATGTG
AGGACTTGCATTGTGAAAGCTTTGCTGTCCTTGATGTGATCATGGAATCTCTTTCTCACT
AGAGTCTATGTCACTCATTATACTCTGTCGAATGTCATTGAATGTCTTTACATGGGCTTG
TATGCCTATGAAAATTGTAATACAACTTTCAGCAACGGATCTCTTGGCTCTCGCATCGAT
GAAGGACGCAGCGAAATGCGATAAGTAATGTGAATTGCAGAATTCAGTGAATCATCGAAT
CTTTGAACGCATCTTGCGCTCCTTGGTATTCCGAGGAGCATGTCTGTTTGAGTGTCATTA
AATTCTCAACTCTCTTATACTTTTTTGTAAAAGAGAGCTTGGACTGTGGAGGCTTGCTGG
CCACTTTTTGGGGTCAGCTCCTCTGAAATGCATTAGCGGAACCGTTTGCAATCTGCCACA
AGTGTGATAAGTTATCTACACTGGCGAGGGGATTGCTCTCTGTAATGTTCAGCTTCTAAT
TGTCTCTACTTTGTGAGACAACTTTTGAATGCTTGACCTCAAATCAGGTAGGACTACCC-
GCTGAACTTAA
>BC2-1
TTTCCGTAGGTGAACCTGCGGAAGGATCATTATTGAATTATGTTTCTAGATAGGTTGTAG
CTGGCTCTTTAGAGCATGTGCACGCCTGTTTGGACTTCATTTTCATCCACCTGTGCACCT
ATTGTAGTCTTTGGTTGGGTTAGGAGGAAGTGGTCATTGTGTCAGCATCTGCTGGATGTG
AGGACTTGCATTGTGAAAGCTTTGCTGTCCTTGATGTGATCATGGAATCTCTTTCTCACT
AGAGTCTATGTCACTCATTATACTCTGTCGAATGTCATTGAATGTCTTTACATGGGCTTA
TATGCCTATGAAAATTGTAATACAACTTTCAGCAACGGATCTCTTGGCTCTCGCATCGAT
GAAGAACGCAGCGAAATGCGATAAGTAATGTGAATTGCAGAATTCAGTGAATCATCGAAT
CTTTGAACGCATCTTGCGCTCCTTGGTATTCCGAGGAGCATGCCTGTTTGAGTGTCATTA
AATTCTCAACTCTCTTATACTTTTTTGTAAAAGAGAGCTTGGACTGTGGAGGCTTGCTGG
CCACTTTTTGGGGTCAGCTCCTCTGAAATGCATTAGCGGAACCGTTTGCAATCTGCCACA
AGTGTGATAAGTTATCTACACTGGCGAGGGGATTGCTCTCTGTAATGTTCAGCTTCTAAT
TGTCTCTACTTTGTGAGACAACTTTTGAATGCTTGACCTCAAATCAGGTAGGACTACCC-
GCTGAACTTAA
>BC3-46
TTTCCGTAGGTGAACCTGCGGAAGGATCATTATTGAATTATGTTTCTAGATAGGTTGTAG
CTGGCTCTTTAGAGCATGTGCACGCCTGTTTGGACTTCATTTTCATCCACCTGTGCACCT
ATTGTAGTCTTTGGTTGGGTTAGGAGGAAGTGGTCATTGTGTCAGCATCTGCTGGATGTG
AGGACTTGCATTGTGAAAGCTTTGCTGTCCTTGATGTGATCATGGAATCTCTTTCTCACT
AGAGTCTATGTCACTCATTATACTCTGTCGAATGTCATTGAATGTCTTTACATGGGCTTA

TATGCCTATGAAAATTGTAATACAACTTTCAGCAACGGATCTCTTGGCTCTCGCATCGAT
GAAGAACGCAGCGAAATGCGATAAGTAATGTGAATTGCAGAATTCAGTGAATCATCGAAT
CTTTGAACGCATCTTGCGCTCCTTGGTATTCCGAGGAGCATGCCTGTTTGAGTGTCATTA
AATTCTCAACTCTCTTATACTTTTTTGTAAAAGAGAGCTTGGACTGTGGAGGCTTGCTGG
CCACTTTTTGGGGTCAGCTCCTCTGAAATGCATTAGCGGAACCGTTTGCAATCTGCCACA
AGTGTGATAAGTTATCTACACTGGCGAGGGGATTGCTCTCTGTAATGTTCAGCTTCTAAT
TGTCTCTACTTTGTGAGACAACTTTTGAATGCTTGACCTCAAATCAGGTAGGACTACCC-
GCTGAACTTAA
>BC6-58
TTTCCGTAGGTGAACCTGCGGAAGGATCATTATTGAATTATGTTTCTAGATAGGTTGTAG
CTGGCTCTTTAGAGCATGTGCACGCCTGTTTGGACTTCATTTTCATCCACCTGTGCACCT
ATTGTAGTCTTTGGTTGGGTTAGGAGGAAGTGGTCATTGTGTCAGCATCTGCTGGATGTG
AGGACTTGCATTGTGAAAGCTTTGCTGTCCTTGATGTGATCATGGAATCTCTTTCTCACT
AGAGTCTATGTCACTCATTATACTCTGTCGAATGTCATTGAATGTCTTTACATGGGCTTA
TATGCCTATGAAAATTGTAATACAACTTTCAGCAACGGATCTCTTGGCTCTCGCATCGAT
GAAGAACGCAGCGAAATGCGATAAGTAATGTGAATTGCAGAATTCAGTGAATCATCGAAT
CTTTGAACGCATCTTGCGCTCCTTGGTATTCCGAGGAGCATGCCTGTTTGAGTGTCATTA
AATTCTCAACTCTCTTATACTTTTTTGTAAAAGAGAGCTTGGACTGTGGAGGCTTGCTGG
CCACTTTTTGGGGTCAGCTCCTCTGAAATGCATTAGCGGAACCGTTTGCAATCTGCCACA
AGTGTGATAAGTTATCTACACTGGCGAGGGGATTGCTCTCTGTAATGTTCAGCTTCTAAT
TGTCTCTACTTTGTGAGACAACTTTTGAATGCTTGACCTCAAATCAGGTAGGACTACCC-
GCTGAACTTAA
>BC8-59
TTTCCGTAGGTGAACCTGCGGAAGGATCATTATTGAATTATGTTTCTAGATAGGTTGTAG
CTGGCTCTTTAGAGCATGTGCACGCCTGTTTGGACTTCATTTTCATCCACCTGTGCACCT
ATTGTAGTCTTTGGTTGGGTTAGGAGGAAGTGGTCATTGTGTCAGCATCTGCTGGATGTG
AGGACTTGCATTGTGAAAGCTTTGCTGTCCTTGATGTGATCATGGAATCTCTTTCTCACT
AGAGTCTATGTCACTCATTATACTCTGTCGAATGTCATTGAATGTCTTTACATGGGCTTA
TATGCCTATGAAAATTGTAATACAACTTTCAGCAACGGATCTCTTGGCTCTCGCATCGAT
GAAGAACGCAGCGAAATGCGATAAGTAATGTGAATTGCAGAATTCAGTGAATCATCGAAT
CTTTGAACGCATCTTGCGCTCCTTGGTATTCCGAGGAGCATGCCTGTTTGAGTGTCATTA
AATTCTCAACTCTCTTATACTTTTTTGTAAAAGAGAGCTTGGACTGTGGAGGCTTGCTGG
CCACTTTTTGGGGTCAGCTCCTCTGAAATGCATTAGCGGAACCGTTTGCAATCTGCCACA
AGTGTGATAAGTTATCTACACTGGCGAGGGGATTGCTCTCTGTAATGTTCAGCTTCTAAT
TGTCTCTACTTTGTGAGACAACTTTTGAATGCTTGACCTCAAATCAGGTAGGACTACCC-
GCTGAACTTAA
>BC9-4
TTTCCGTAGGTGAACCTGCGGAAGGATCATTATTGAATTATGTTTCTAGATAGGTTGTAG
CTGGCTCTTTAGAGCATGTGCACGCCTGTTTGGACTTCATTTTCATCCACCTGTGCACCT
ATTGTAGTCTTTGGTTGGGTTAGGAGGAAGTGGTCATTGTGTCAGCATCTGCTGGATGTG
AGGACTTGCATTGTGAAAGCTTTGCTGTCCTTGATGTGATCATGGAATCTCTTTCTCACT
AGAGTCTATGTCACTCATTATACTCTGTCGAATGTCATTGAATGTCTTTACATGGGCTTA
TATGCCTATGAAAATTGTAATACAACTTTCAGCAACGGATCTCTTGGCTCTCGCATCGAT
GAAGAACGCAGCGAAATGCGATAAGTAATGTGAATTGCAGAATTCAGTGAATCATCGAAT
CTTTGAACGCATCTTGCGCTCCTTGGTATTCCGAGGAGCATGCCTGTTTGAGTGTCATTA
AATTCTCAACTCTCTTATACTTTTTTGTAAAAGAGAGCTTGGACTGTGGAGGCTTGCTGG
CCACTTTTTGGGGTCAGCTCCTCTGAAATGCATTAGCGGAACCGTTTGCAATCTGCCACA
AGTGTGATAAGTTATCTACACTGGCGAGGGGATTGCTCTCTGTAATGTTCAGCTTCTAAT
TGTCTCTACTTTGTGAGACAACTTTTGAATGCTTGACCTCAAATCAGGTAGGACTACCC-
GCTGAACTTAA

>BC9–18
TTTCCGTAGGTGAACCTGCGGAAGGATCATTATTGAATTATGTTTCTAGATAGGTTGTAG
CTGGCTCTTTAGAGCATGTGCACGCCTGTTTGGACTTCATTTTCATCCACCTGTGCACCT
ATTGTAGTCTTTGGTTGGGTTAGGAGGAAGTGGTCATTGTGTCAGCATCTGCTGGATGTG
AGGACTTGCATTGTGAAAGCTTTGCTGTCCTTGATGTGATCATGGAATCTCTTTCTCACT
AGAGTCTATGTCACTCATTATACTCTGTCGAATGTCATTGAATGTCTTTACATGGGCTTA
TATGCCTATGAAAATTGTAATACAACTTTCAGCAACGGATCTCTTGGCTCTCGCATCGAT
GAAGAACGCAGCGAAATGCGATAAGTAATGTGAATTGCAGAATTCAGTGAATCATCGAAT
CTTTGAACGCATCTTGCGCTCCTTGGTATTCCGAGGAGCATGCCTGTTTGAGTGTCATTA
AATTCTCAACTCTCTTATACTTTTTTGTAAAAGAGAGCTTGGACTGTGGAGGCTTGCTGG
CCACTTTTTGGGGTCAGCTCCTCTGAAATGCATTAGCGGAACCGTTTGCAATCTGCCACA
AGTGTGATAAGTTATCTACACTGGCGAGGGGATTGCTCTCTGTAATGTTCAGCTTCTAAT
TGTCTCTACTTTGTGAGACAACTTTTGAATGCTTGACCTCAAATCAGGTAGGACTACCC–
GCTGAACTTAA
>BC10_6
TTTCCGTAGGTGAACCTGCGGAAGGATCATTATTGAATTATGTTTCTAGATAGGTTGTAG
CTGGCTCTTTAGAGCATGTGCACGCCTGTTTGGACTTCATTTTCATCCACCTGTGCACCT
ATTGTAGTCTTTGGTTGGGTTAGGAGGAAGTGGTCATTGTGTCAGCATCTGCTGGATGTG
AGGACTTGCATTGTGAAAGCTTTGCTGTCCTTGATGTGATCATGGAATCTCTTTCTCACT
AGAGTCTATGTCACTCATTATACTCTGTCGAATGTCATTGAATGTCTTTACATGGGCTTA
TATGCCTATGAAAATTGTAATACAACTTTCAGCAACGGATCTCTTGGCTCTCGCATCGAT
GAAGAACGCAGCGAAATGCGATAAGTAATGTGAATTGCAGAATTCAGTGAATCATCGAAT
CTTTGAACGCATCTTGCGCTCCTTGGTATTCCGAGGAGCATGCCTGTTTGAGTGTCATTA
AATTCTCAACTCTCTTATACTTTTTTGTAAAAGAGAGCTTGGACTGTGGAGGCTTGCTGG
CCACTTTTTGGGGTCAGCTCCTCTGAAATGCATTAGCGGAACCGTTTGCAATCTGCCACA
AGTGTGATAAGTTATCTACACTGGCGAGGGGATTGCTCTCTGTAATGTTCAGCTTCTAAT
TGTCTCTACTTTGTGAGACAACTTTTGAATGCTTGACCTCAAATCAGGTAGGACTACCC–
GCTGAACTTAA
>BC11_25
TTTCCGTAGGTGAACCTGCGGAAGGATCATTATTGAATTATGTTTCTAGATAGGTTGTAG
CTGGCTCTTTAGAGCATGTGCACGCCTGTTTGGACTTCATTTTCATCCACCTGTGCACCT
ATTGTAGTCTTTGGTTGGGTTAGGAGGAAGTGGTCATTGTGTCAGCATCTGCTGGATGTG
AGGACTTGCATTGTGAAAGCTTTGCTGTCCTTGATGTGATCATGGAATCTCTTTCTCACT
AGAGTCTATGTCACTCATTATACTCTGTCGAATGTCATTGAATGTCTTTACATGGGCTTA
TATGCCTATGAAAATTGTAATACAACTTTCAGCAACGGATCTCTTGGCTCTCGCATCGAT
GAAGAACGCAGCGAAATGCGATAAGTAATGTGAATTGCAGAATTCAGTGAATCATCGAAT
CTTTGAACGCATCTTGCGCTCCTTGGTATTCCGAGGAGCATGCCTGTTTGAGTGTCATTA
AATTCTCAACTCTCTTATACTTTTTTGTAAAAGAGAGCTTGGACTGTGGAGGCTTGCTGG
CCACTTTTTGGGGTCAGCTCCTCTGAAATGCATTAGCGGAACCGTTTGCAATCTGCCACA
AGTGTGATAAGTTATCTACACTGGCGAGGGGATTGCTCTCTGTAATGTTCAGCTTCTAAT
TGTCTCTACTTTGTGAGACAACTTTTGAATGCTTGACCTCAAATCAGGTAGGACTACCC–
GCTGAACTTAA
>BC11_28
TTTCCGTAGGTGAACCTGCGGAAGGATCATTATTGAATTATGTTTCTAGATAGGTTGTAG
CTGGCTCTTTAGAGCATGTGCACGCCTGTTTGGACTTCATTTTCATCCACCTGTGCACCT
ATTGTAGTCTTTGGTTGGGTTAGGAGGAAGTGGTCATTGTGTCAGCATCTGCTGGATGTG
AGGACTTGCATTGTGAAAGCTTTGCTGTCCTTGATGTGATCATGGAATCTCTTTCTCACT
AGAGTCTATGTCACTCATTATACTCTGTCGAATGTCATTGAATGTCTTTACATGGGCTTA
TATGCCTATGAAAATTGTAATACAACTTTCAGCAACGGATCTCTTGGCTCTCGCATCGAT
GAAGAACGCAGCGAAATGCGATAAGTAATGTGAATTGCAGAATTCAGTGAATCATCGAAT

CTTTGAACGCATCTTGCGCTCCTTGGTATTCCGAGGAGCATGCCTGTTTGAGTGTCATTA
AATTCTCAACTCTCTTATACTTTTTTGTAAAAGAGAGCTTGGACTGTGGAGGCTTGCTGG
CCACTTTTTGGGGTCAGCTCCTCTGAAATGCATTAGCGGAACCGTTTGCAATCTGCCACA
AGTGTGATAAGTTATCTACACTGGCGAGGGGATTGCTCTCTGTAATGTTCAGCTTCTAAT
TGTCTCTACTTTGTGAGACAACTTTTGAATGCTTGACCTCAAATCAGGTAGGACTACCC-
GCTGAACTTAA
>BC11_58
TTTCCGTAGGTGAACCTGCGGAAGGATCATTATTGAATTATGTTTCTAGATAGGTTGTAG
CTGGCTCTTTAGAGCATGTGCACGCCTGTTTGGACTTCATTTTCATCCACCTGTGCACCT
ATTGTAGTCTTTGGTTGGGTTAGGAGGAAGTGGTCATTGTGTCAGCATCTGCTGGATGTG
AGGACTTGCATTGTGAAAGCTTTGCTGTCCTTGATGTGATCATGGAATCTCTTTCTCACT
AGAGTCTATGTCACTCATTATACTCTGTCGAATGTCATTGAATGTCTTTACATGGGCTTA
TATGCCTATGAAAATTGTAATACAACTTTCAGCAACGGATCTCTTGGCTCTCGCATCGAT
GAAGAACGCAGCGAAATGCGATAAGTAATGTGAATTGCAGAATTCAGTGAATCATCGAAT
CTTTGAACGCATCTTGCGCTCCTTGGTATTCCGAGGAGCATGCCTGTTTGAGTGTCATTA
AATTCTCAACTCTCTTATACTTTTTTGTAAAAGAGAGCTTGGACTGTGGAGGCTTGCTGG
CCACTTTTTGGGGTCAGCTCCTCTGAAATGCATTAGCGGAACCGTTTGCAATCTGCCACA
AGTGTGATAAGTTATCTACACTGGCGAGGGGATTGCTCTCTGTAATGTTCAGCTTCTAAT
TGTCTCTACTTTGTGAGACAACTTTTGAATGCTTGACCTCAAATCAGGTAGGACTACCC-
GCTGAACTTAA
>BC8-34
TTTCCGTAGGTGAACCTGCGGAAGGATCATTATTGAATTATGTTTCTAGATAGGTTGTAG
CTGGCTCTTTAGAGCATGTGCACGCCTGTTTGGACTTCATTTTCATCCACCTGTGCACCT
ATTGTAGTCTTTGGTTGGGTTAGGAGGAAGTGGTCATTGTGTCAGCATCTGCTGGATGTG
AGGACTTGCATTGTGAAAGCTTTGCTGTCCTTGATGTGATCATGGAATCTCTTTCTCACT
AGAGTCTATGTCACTCATTATACTCTGTCGAATGTCATTGAATGTCTTTACATGGGCTTA
TATGCCTATGAAAATTGTAATACAACTTTCAGCAACGGATCTCTTGGCTCTCGCATCGAT
GAAGAACGCAGCGAAATGCGATAAGTAATGTGAATTGCAGAATTCAGTGAATCATCGAAT
CTTTGAACGCATCTTGCGCTCCTTGGTATTCCGAGGAGCATGCCTGTTTGAGTGTCATTA
AATTCTCAACTCTCTTATACTTTTTTGTAAAAGAGAGCTTGGACTGTGGAGGCTTGCTGG
CCACTTTTTGGGGTCAGCTCCTCTGAAATGCATTAGCGGAACCGTTTGCAATCTGCCACA
AGTGTGATAAGTTATCTACACTGGCGAGGGGATTGCTCTCTGTAATGTTCAGCTTCTAAT
TGTCTCTACTTTGTGAGACAACTTTTGAATGCTTGACCTCAAATCAGGTAGGACTACCC-
GCTGAACTTAA
>BC4-9
TTTCCGTAGGTGAACCTGCGGAAGGATCATTATTGAATTATGTTTCTAGATAGGTTGTAG
CTGGCTCTTTAGAGCATGTGCACGCCTGTTTGGACTTCATTTTCATCCACCTGTGCACCT
ATTGTAGTCTTTGGTTGGGTTAGGAGGAAGTGGTCATTGTGTCAGCATCTGCTGGATGTG
AGGACTTGCATTGTGAAAGCTTTGCTGTCCTTGATGTGATCATGGAATCTCTTTCTCACT
AGAGTCTATGTCACTCATTATACTCTGTCGAATGTCATTGAATGTCTTTACATGGGCTTA
TATGCCTATGAAAATTGTAATACAACTTTCAGCAACGGATCTCTTGGCTCTCGCATCGAT
GAAGGACGCAGCGAAATGCGATAAGTAATGTGAATTGCAGAATTCAGTGAATCATCGAAT
CTTTGAACGCATCTTGCGCTCCTTGGTATTCCGAGGAGCATGCCTGTTTGAGTGTCATTA
AATTCTCAACTCTCTTATACTTTTTTGTAAAAGAGAGCTTGGACTGTGGAGGCTTGCTGG
CCACTTTTTGGGGTCAGCTCCTCTGAAATGCATTAGCGGAACCGTTTGCAATCTGCCACA
AGTGTGATAAGTTATCTACACTGGCGAGGGGATTGCTCTCTGTAATGTTCAGCTTCTAAT
TGTCTCTACTTTGTGAGACAACTTTTGAATGCTTGACCTCAAATCAGGTAGGACTACCC-
GCTGAACTTAA
>BC5-21
TTTCCGTAGGTGAACCTGCGGAAGGATCATTATTGAATTATGTTTCTAGATAGGTTGTAG

CTGGCTCTTTAGAGCATGTGCACGCCTGTTTGGACTTCATTTTCATCCACCTGTGCACCT
ATTGTAGTCTTTGGTTGGGTTAGGAGGAAGTGGTCATTGTGTCAGCATCTGCTGGATGTG
AGGACTTGCATTGTGAAAGCTTTGCTGTCCTTGATGTGATCATGGAATCTCTTTCTCACT
AGAGTCTATGTCACTCATTATACTCTGTCGAATGTCATTGAATGTCTTTACATGGGCTTA
TATGCCTATGAAAATTGTAATACAACTTTCAGCAACGGATCTCTTGGCTCTCGCATCGAT
GAAGGACGCAGCGAAATGCGATAAGTAATGTGAATTGCAGAATTCAGTGAATCATCGAAT
CTTTGAACGCATCTTGCGCTCCTTGGTATTCCGAGGAGCATGCCTGTTTGAGTGTCATTA
AATTCTCAACTCTCTTATACTTTTTTGTAAAAGAGAGCTTGGACTGTGGAGGCTTGCTGG
CCACTTTTTGGGGTCAGCTCCTCTGAAATGCATTAGCGGAACCGTTTGCAATCTGCCACA
AGTGTGATAAGTTATCTACACTGGCGAGGGGATTGCTCTCTGTAATGTTCAGCTTCTAAT
TGTCTCTACTTTGTGAGACAACTTTTGAATGCTTGACCTCAAATCAGGTAGGACTACCC-
GCTGAACTTAA
>BC5-24
TTTCCGTAGGTGAACCTGCGGAAGGATCATTATTGAATTATGTTTCTAGATAGGTTGTAG
CTGGCTCTTTAGAGCATGTGCACGCCTGTTTGGACTTCATTTTCATCCACCTGTGCACCT
ATTGTAGTCTTTGGTTGGGTTAGGAGGAAGTGGTCATTGTGTCAGCATCTGCTGGATGTG
AGGACTTGCATTGTGAAAGCTTTGCTGTCCTTGATGTGATCATGGAATCTCTTTCTCACT
AGAGTCTATGTCACTCATTATACTCTGTCGAATGTCATTGAATGTCTTTACATGGGCTTA
TATGCCTATGAAAATTGTAATACAACTTTCAGCAACGGATCTCTTGGCTCTCGCATCGAT
GAAGGACGCAGCGAAATGCGATAAGTAATGTGAATTGCAGAATTCAGTGAATCATCGAAT
CTTTGAACGCATCTTGCGCTCCTTGGTATTCCGAGGAGCATGCCTGTTTGAGTGTCATTA
AATTCTCAACTCTCTTATACTTTTTTGTAAAAGAGAGCTTGGACTGTGGAGGCTTGCTGG
CCACTTTTTGGGGTCAGCTCCTCTGAAATGCATTAGCGGAACCGTTTGCAATCTGCCACA
AGTGTGATAAGTTATCTACACTGGCGAGGGGATTGCTCTCTGTAATGTTCAGCTTCTAAT
TGTCTCTACTTTGTGAGACAACTTTTGAATGCTTGACCTCAAATCAGGTAGGACTACCC-
GCTGAACTTAA
>BC11_15
TTTCCGTAGGTGAACCTGCGGAAGGATCATTATTGAATTATGTTTCTAGATAGGTTGTAG
CTGGCTCTTTAGAGCATGTGCACGCCTGTTTGGACTTCATTTTCATCCACCTGTGCACCT
ATTGTAGTCTTTGGTTGGGTTAGGAGGAAGTGGTCATTGTGTCAGCATCTGCTGGATGTG
AGGACTTGCATTGTGAAAGCTTTGCTGTCCTTGATGTGATCATGGAATCTCTTTCTCACT
AGAGTCTATGTCACTCATTATACTCTGTCGAATGTCATTGAATGTCTTTACATGGGCTTA
TATGCCTATGAAAATTGTAATACAACTTTCAGCAACGGATCTCTTGGCTCTCGCATCGAT
GAAGGACGCAGCGAAATGCGATAAGTAATGTGAATTGCAGAATTCAGTGAATCATCGAAT
CTTTGAACGCATCTTGCGCTCCTTGGTATTCCGAGGAGCATGCCTGTTTGAGTGTCATTA
AATTCTCAACTCTCTTATACTTTTTTGTAAAAGAGAGCTTGGACTGTGGAGGCTTGCTGG
CCACTTTTTGGGGTCAGCTCCTCTGAAATGCATTAGCGGAACCGTTTGCAATCTGCCACA
AGTGTGATAAGTTATCTACACTGGCGAGGGGATTGCTCTCTGTAATGTTCAGCTTCTAAT
TGTCTCTACTTTGTGAGACAACTTTTGAATGCTTGACCTCAAATCAGGTAGGACTACCC-
GCTGAACTTAA
>BC10_29
TTTCCGTAGGTGAACCTGCGGAAGGATCATTATTGAATTATGTTTCTAGATAGGTTGTAG
CTGGCTCTTTAGAGCATGTGCACGCCTGTTTGGACTTCATTTTCATCCACCTGTGCACCT
ATTGTAGTCTTTGGTTGGGTTAGGAGGAAGTGGTCATTGTGTCAGCATCTGCTGGATGTG
AGGACTTGCATTGTGAAAGCTTTGCTGTCCTTGATGTGATCATGGAATCTCTTTCTCACT
AGAGTCTATGTCACTCATTATACTCTGTCGAATGTCATTGAATGTCTTTACATGGGCTTA
TATGCCTATGAAAATTGTAATACAACTTTCAGCAACGGATCTCTTGGCTCTCGCATCGAT
GAAGGACGCAGCGAAATGCGATAAGTAATGTGAATTGCAGAATTCAGTGAATCATCGAAT
CTTTGAACGCATCTTGCGCTCCTTGGTATTCCGAGGAGCATGCCTGTTTGAGTGTCATTA
AATTCTCAACTCTCTTATACTTTTTTGTAAAAGAGAGCTTGGACTGTGGAGGCTTGCTGG

CCACTTTTTGGGGTCAGCTCCTCTGAAATGCATTAGCGGAACCGTTTGCAATCTGCCACA
AGTGTGATAAGTTATCTACACTGGCGAGGGGATTGCTCTCTGTAATGTTCAGCTTCTAAT
TGTCTCTACTTTGTGAGACAACTTTTGAATGCTTGACCTCAAATCAGGTAGGACTACCC-
GCTGAACTTAA
>BC8-33
TTTCCGTAGGTGAACCTGCGGAAGGATCATTATTGAATTATGTTTCTAGATAGGTTGTAG
CTGGCTCTTTAGAGCATGTGCACGCCTGTTTGGACTTCATTTTCATCCACCTGTGCACCT
ATTGTAGTCTTTGGTTGGGTTAGGGGGAAGTGGTCATTGTGTCAGCATCTGCTGGATGTG
AGGACTTGCATTGTGAAAGCTTTGCTGTCCTTGATGTGATCATGGAATCTCTTTCTCACT
AGAGTCTATGTCACTCATTATACTCTGTCGAATGTCATTGAATGTCTTTACATGGGCTTA
TATGCCTATGAAAATTGTAATACAACTTTCAGCAACGGATCTCTTGGCTCTCGCATCGAT
GAAGGACGCAGCGAAATGCGATAAGTAATGTGAATTGCAGAATTCAGTGAATCATCGAAT
CTTTGAACGCATCTTGCGCTCCTTGGTATTCCGAGGAGCATGCCTGTTTGAGTGTCATTA
AATTCTCAACTCTCTTATACTTTTTTGTAAAAGAGAGCTTGGACTGTGGAGGCTTGCTGG
CCACTTTTTGGGGTCAGCTCCTCTGAAATGCATTAGCGGAACCGTTTGCAATCTGCCACA
AGTGTGATAAGTTATCTACACTGGCGAGGGGATTGCTCTCTGTAATGTTCAGCTTCTAAT
TGTCTCTACTTTGTGAGACAACTTTTGAATGCTTGACCTCAAATCAGGTAGGACTACCC-
GCTGAACTTAA
>BC11_16
TTTCCGTAGGTGAACCTGCGGAAGGATCATTATTGAATTATGTTTCTAGATAGGTTGTAG
CTGGCTCTTTAGAGCATGTGCACGCCTGTTTGGACTTCATTTTCATCCACCTGTGCACCT
ATTGTAGTCTTTGGTTGGGTTAGGGGGAAGTGGTCATTGTGTCAGCATCTGCTGGATGTG
AGGACTTGCATTGTGAAAGCTTTGCTGTCCTTGATGTGATCATGGAATCTCTTTCTCACT
AGAGTCTATGTCACTCATTATACTCTGTCGAATGTCATTGAATGTCTTTACATGGGCTTA
TATGCCTATGAAAATTGTAATACAACTTTCAGCAACGGATCTCTTGGCTCTCGCATCGAT
GAAGGACGCAGCGAAATGCGATAAGTAATGTGAATTGCAGAATTCAGTGAATCATCGAAT
CTTTGAACGCATCTTGCGCTCCTTGGTATTCCGAGGAGCATGCCTGTTTGAGTGTCATTA
AATTCTCAACTCTCTTATACTTTTTTGTAAAAGAGAGCTTGGACTGTGGAGGCTTGCTGG
CCACTTTTTGGGGTCAGCTCCTCTGAAATGCATTAGCGGAACCGTTTGCAATCTGCCACA
AGTGTGATAAGTTATCTACACTGGCGAGGGGATTGCTCTCTGTAATGTTCAGCTTCTAAT
TGTCTCTACTTTGTGAGACAACTTTTGAATGCTTGACCTCAAATCAGGTAGGACTACCC-
GCTGAACTTAA
>BC8-55
TTTCCGTAGGTGAACCTGCGGAAGGATCATTATTGAATTATGTTTCTAGATAGGTTGTAG
CTGGCTCTTTAGAGCATGTGCACGCCTGTTTGGACTTCATTTTCATCCACCTGTGCACCT
ATTGTAGTCTTTGGTTGGGTTAGGGGGAAGTGGTCATTGTGTCAGCATCTGCTGGATGTG
AGGACTTGCATTGTGAAAGCTTTGCTGTCCTTGATGTGATCATGGAATCTCTTTCTCACT
AGAGTCTATGTCACTCATTATACTCTGTCGAATGTCATTGAATGTCTTTACATGGGCTTA
TATGCCTATGAAAATTGTAATACAACTTTCAGCAACGGATCTCTTGGCTCTCGCATCGAT
GAAGAACGCAGCGAAATGCGATAAGTAATGTGAATTGCAGAATTCAGTGAATCATCGAAT
CTTTGAACGCATCTTGCGCTCCTTGGTATTCCGAGGAGCATGCCTGTTTGAGTGTCATTA
AATTCTCAACTCTCTTATACTTTTTTGTAAAAGAGAGCTTGGACTGTGGAGGCTTGCTGG
CCACTTTTTGGGGTCAGCTCCTCTGAAATGCATTAGCGGAACCGTTTGCAATCTGCCACA
AGTGTGATAAGTTATCTACACTGGCGAGGGGATTGCTCTCTGTAATGTTCAGCTTCTAAT
TGTCTCTACTTTGTGAGACAACTTTTGAATGCTTGACCTCAAATCAGGTAGGACTACCC-
GCTGAACTTAA
>BC9-27
TTTCCGTAGGTGAACCTGCGGAAGGATCATTATTGAATTATGTTTCTAGATAGGTTGTAG
CTGGCTCTTTAGAGCATGTGCACGCCTGTTTGGACTTCATTTTCATCCACCTGTGCACCT
ATTGTAGTCTTTGGTTGGGTTAGGAGGAAGTGGTCATTGTGTCAGCATCTGCTGGATGTG

AGGACTTGCATTGTGAAAGCTTTGCTGTCCTTGATGTGATCATGGAATCTCTTTCTCACT
AGAGTCTATGTCACTCATTATACTCTGTCGAATGTCATTGAATGTCTTTACATGGGCTTA
TATGCCTATGAAAATTGTAATACAACTTTCAGCAACGGATCTCTTGGCTCTCGCATCAAT
GAAGGACGCAGCGAAATGCGATAAGTAATGTGAATTGCAGAATTCAGTGAATCATCGAAT
CTTTGAACGCATCTTGCGCTCCTTGGTATTCCGAGGAGCATGCCTGTTTGAGTGTCATTA
AATTCTCAACTCTCTTATACTTTTTTGTAAAAGAGAGCTTGGACTGTGGAGGCTTGCTGG
CCACTTTTTGGGGTCAGCTCCTCTGAAATGCATTAGCGGAACCGTTTGCAATCTGCCACA
AGTGTGATAAGTTATCTACACTGGCGAGGGGATTGCTCTCTGTAATGTTCAGCTTCTAAT
TGTCTCTACTTTGTGAGACAACTTTTGAATGCTTGACCTCAAATCAGGTAGGACTACCC-
GCTGAACTTAA
>BC8-6
TTTCCGTAGGTGAACCTGCGGAAGGATCATTATTGAATTATGTTTCTAGATAGGTTGTAG
CTGGCTCTTTAGAGCATGTGCACGCCTGTTTGGACTTCATTTTCATCCACCTGTGCACCT
ATTGTAGTCTTTGGTTGGGTTAGGAGGAAGTGGTCATTGTGTCAGCATCTGCTGGATGTG
AGGACTTGCATTGTGAAAGCTTTGCTGTCCTTGATGTGATCATGGAATCTCTTTCTCACT
AGAGTCTATGTCACTCATTATACTCTGTCGAATGTCATTGAATGTCTTTACATGGGCTTA
TATGCCTATGAAAATTGTAATACAACTTTCAGCAACGGATCTCTTGGCTCTCGCATCGAT
GAAGAACGCAGCGAAATGCGATAAGTAATGTGAATTGCAGAATTCAGTGAATCATCGAAT
CTTTGAACGCATCTTGCGCTCCTTGGTATTCCGAGGAGCATGCCTGTTTGAGTGTCATTA
AATTCTCAACTCTCTTATACTTTTTTGTAAAAGAGAGCTTGGACTGTGGAGGCTTGCTGG
CCACTTTTTGGGGTCAGCTCCTCTGAAATGCATTAGCGGAACCGTTTGCGATCTGCCACA
AGTGTGATAAGTTATCTACACTGGCGAGGGGATTGCTCTCTGTAATGTTCAGCTTCTAAT
TGTCTCTACTTTGTGAGACAACTTTTGAATGCTTGACCTCAAATCAGGTAGGACTACCC-
GCTGAACTTAA
>BC8-26
TTTCCGTAGGTGAACCTGCGGAAGGATCATTATTGAATTATGTTTCTAGATAGGTTGTAG
CTGGCTCTTTAGAGCATGTGCACGCCTGTTTGGACTTCATTTTCATCCACCTGTGCACCT
ATTGTAGTCTTTGGTTGGGTTAGGAGGAAGTGGTCATTGTGTCAGCATCTGCTGGATGTG
AGGACTTGCATTGTGAAAGCTTTGCTGTCCTTGATGTGATCATGGAATCTCTTTCTCACT
AGAGTCTATGTCACTCATTATACTCTGTCGAATGTCATTGAATGTCTTTACATGGGCTTA
TATGCCTATGAAAATTGTAATACAACTTTCAGCAACGGATCTCTTGGCTCTCGCATCGAT
GAAGGACGCAGCGAAATGCGATAAGTAATGTGAATTGCAGAATTCAGTGAATCATCGAAT
CTTTGAACGCATCTTGCGCTCCTTGGTATTCCGAGGAGCATGCCTGTTTGAGTGTCATTA
AATTCTCAACTCTCTTATACTTTTTTGTAAAAGAGAGCTTGGACTGTGGAGGCTTGCTGG
CCACTTTTTGGGGTCAGCTCCTCTGAAATGCATTAGCGGAACCGTTTGCGATCTGCCACA
AGTGTGATAAGTTATCTACACTGGCGAGGGGATTGCTCTCTGTAATGTTCAGCTTCTAAT
TGTCTCTACTTTGTGAGACAACTTTTGAATGCTTGACCTCAAATCAGGTAGGACTACCC-
GCTGAACTTAA
>BC7-13
TTTCCGTAGGTGAACCTGCGGAAGGATCATTATTGAATTATGTTTCTAGATAGGTTGTAG
CTGGCTCTTTAGAGCATGTGCACGCCTGTTTGGACTTCATTTTCATCCACCTGTGCACCT
ATTGTAGTCTTTGGTTGGGTTAGGAGGAAGTGGTCATTGTGTCAGCATCTGCTGGATGTG
AGGACTTGCATTGTGAAAGCTTTGCTGTCCTTGATGTGATCATGGAATCTCTTTCTCACT
AGAGTCTATGTCACTCATTATACTCTGTCGAATGTCATTGAATGTCTTTACATGGGCTTG
TATGCCTATGAAAATTGTAATACAACTTTCAGCAACGGATCTCTTGGCTCTCGCATCGAT
GAAGAACGCAGCGAAATGCGATAAGTAATGTGAATTGCAGAATTCAGTGAATCATCGAAT
CTTTGAACGCATCTTGCGCTCCTTGGTATTCCGAGGAGCATGCCTGTTTGAGTGTCATTA
AATTCTCAACTCTCTTATACTTTTTTGTAAAAGAGAGCTTGGACTGTGGAGGCTTGCTGG
CCACTTTTTGGGGTCAGCTCCTCTGAAATGCATTAGCGGAACCGTTTGCGATCTGCCACA
AGTGTGATAAGTTATCTACACTGGCGAGGGGATTGCTCTCTGTAATGTTCAGCTTCTAAT

TGTCTCTACTTTGTGAGACAACTTTTGAATGCTTGACCTCAAATCAGGTAGGACTACCC–
GCTGAACTTAA
>BC1–20
TTTCCGTAGGTGAACCTGCGGAAGGATCATTATTGAATTATGTTTCTAGATAGGTTGTAG
CTGGCTCTTTAGAGCATGTGCACGCCTGTTTGGACTTCATTTTCATCCACCTGTGCACCT
ATTGTAGTCTTTGGTTGGGTTAGGGGGAAGTGGTCATTGTGTCAGCATCTGCTGGATGTG
AGGACTTGCATTGTGAAAGCTTTGCTGTCCTTGATGTGATCATGGAATCTCTTTCTCACT
AGAGTCTATGTCACTCATTATACTCTGTCGAATGTCATTGAATGTCTTTACATGGGCTTG
TATGCCTATGAAAATTGTAATACAACTTTCAGCAACGGATCTCTTGGCTCTCGCATCGAT
GAAGGACGCAGCGAAATGCGATAAGTAATGTGAATTGCAGAATTCAGTGAATCATCGAAT
CTTTGAACGCATCTTGCGCTCCTTGGTATTCCGAGGAGCATGCCTGTTTGAGTGTCATTA
AATTCTCAACTCTCTTATACTTTTTTGTAAAAGAGAGCTTGGACTGTGGAGGCTTGCTGG
CCACTTTTTGGGGTCAGCTCCTCTGAAATGCATTAGCGGAACCGTTTGCAATCTGCCACA
AGTGTGATAAGTTATCTACACTGGCGAGGGGATTGCTCTCTGTAATGTTCAGCTTCTAAT
TGTCTCTACTTTGTGAGACTACTTTTGAATGCTTGACCTCAAATCAGGTAGGACTACCC–
GCTGAACTTAA
>BC3–22
TTTCCGTAGGTGAACCTGCGGAAGGATCATTATTGAATTATGTTTCTAGATAGGTTGTAG
CTGGCTCTTTAGAGCATGTGCACGCCTGTTTGGACTTCATTTTCATCCACCTGTGCACCT
ATTGTAGTCTTTGGTTGGGTTAGGGGGAAGTGGTCATTGTGTCAGCATCTGCTGGATGTG
AGGACTTGCATTGTGAAAGCTTTGCTGTCCTTGATGTGATCATGGAATCTCTTTCTCACT
AGAGTCTATGTCACTCATTATACTCTGTCGAATGTCATTGAATGTCTTTACATGGGCTTG
TATGCCTATGAAAATTGTAATACAACTTTCAGCAACGGATCTCTTGGCTCTCGCATCGAT
GAAGGACGCAGCGAAATGCGATAAGTAATGTGAATTGCAGAATTCAGTGAATCATCGAAT
CTTTGAACGCATCTTGCGCTCCTTGGTATTCCGAGGAGCATGCCTGTTTGAGTGTCATTA
AATTCTCAACTCTCTTATACTTTTTTGTAAAAGAGAGCTTGGACTGTGGAGGCTTGCTGG
CCACTTTTTGGGGTCAGCTCCTCTGAAATGCATTAGCGGAACCGTTTGCAATCTGCCACA
AGTGTGATAAGTTATCTACACTGGCGAGGGGATTGCTCTCTGTAATGTTCAGCTTCTAAT
TGTCTCTACTTTGTGAGACTACTTTTGAATGCTTGACCTCAAATCAGGTAGGACTACCC–
GCTGAACTTAA
>BC4–16
TTTCCGTAGGTGAACCTGCGGAAGGATCATTATTGAATTATGTTTCTAGATAGGTTGTAG
CTGGCTCTTTAGAGCATGTGCACGCCTGTTTGGACTTCATTTTCATCCACCTGTGCACCT
ATTGTAGTCTTTGGTTGGGTTAGGGGGAAGTGGTCATTGTGTCAGCATCTGCTGGATGTG
AGGACTTGCATTGTGAAAGCTTTGCTGTCCTTGATGTGATCATGGAATCTCTTTCTCACT
AGAGTCTATGTCACTCATTATACTCTGTCGAATGTCATTGAATGTCTTTACATGGGCTTG
TATGCCTATGAAAATTGTAATACAACTTTCAGCAACGGATCTCTTGGCTCTCGCATCGAT
GAAGGACGCAGCGAAATGCGATAAGTAATGTGAATTGCAGAATTCAGTGAATCATCGAAT
CTTTGAACGCATCTTGCGCTCCTTGGTATTCCGAGGAGCATGCCTGTTTGAGTGTCATTA
AATTCTCAACTCTCTTATACTTTTTTGTAAAAGAGAGCTTGGACTGTGGAGGCTTGCTGG
CCACTTTTTGGGGTCAGCTCCTCTGAAATGCATTAGCGGAACCGTTTGCAATCTGCCACA
AGTGTGATAAGTTATCTACACTGGCGAGGGGATTGCTCTCTGTAATGTTCAGCTTCTAAT
TGTCTCTACTTTGTGAGACTACTTTTGAATGCTTGACCTCAAATCAGGTAGGACTACCC–
GCTGAACTTAA
>BC5–58
TTTCCGTAGGTGAACCTGCGGAAGGATCATTATTGAATTATGTTTCTAGATAGGTTGTAG
CTGGCTCTTTAGAGCATGTGCACGCCTGTTTGGACTTCATTTTCATCCACCTGTGCACCT
ATTGTAGTCTTTGGTTGGGTTAGGGGGAAGTGGTCATTGTGTCAGCATCTGCTGGATGTG
AGGACTTGCATTGTGAAAGCTTTGCTGTCCTTGATGTGATCATGGAATCTCTTTCTCACT
AGAGTCTATGTCACTCATTATACTCTGTCGAATGTCATTGAATGTCTTTACATGGGCTTG

TATGCCTATGAAAATTGTAATACAACTTTCAGCAACGGATCTCTTGGCTCTCGCATCGAT
GAAGGACGCAGCGAAATGCGATAAGTAATGTGAATTGCAGAATTCAGTGAATCATCGAAT
CTTTGAACGCATCTTGCGCTCCTTGGTATTCCGAGGAGCATGCCTGTTTGAGTGTCATTA
AATTCTCAACTCTCTTATACTTTTTTGTAAAAGAGAGCTTGGACTGTGGAGGCTTGCTGG
CCACTTTTTGGGGTCAGCTCCTCTGAAATGCATTAGCGGAACCGTTTGCAATCTGCCACA
AGTGTGATAAGTTATCTACACTGGCGAGGGGATTGCTCTCTGTAATGTTCAGCTTCTAAT
TGTCTCTACTTTGTGAGACTACTTTTGAATGCTTGACCTCAAATCAGGTAGGACTACCC-
GCTGAACTTAA
>BC5-102
TTTCCGTAGGTGAACCTGCGGAAGGATCATTATTGAATTATGTTTCTAGATAGGTTGTAG
CTGGCTCTTTAGAGCATGTGCACGCCTGTTTGGACTTCATTTTCATCCACCTGTGCACCT
ATTGTAGTCTTTGGTTGGGTTAGGGGGAAGTGGTCATTGTGTCAGCATCTGCTGGATGTG
AGGACTTGCATTGTGAAAGCTTTGCTGTCCTTGATGTGATCATGGAATCTCTTTCTCACT
AGAGTCTATGTCACTCATTATACTCTGTCGAATGTCATTGAATGTCTTTACATGGGCTTG
TATGCCTATGAAAATTGTAATACAACTTTCAGCAACGGATCTCTTGGCTCTCGCATCGAT
GAAGGACGCAGCGAAATGCGATAAGTAATGTGAATTGCAGAATTCAGTGAATCATCGAAT
CTTTGAACGCATCTTGCGCTCCTTGGTATTCCGAGGAGCATGCCTGTTTGAGTGTCATTA
AATTCTCAACTCTCTTATACTTTTTTGTAAAAGAGAGCTTGGACTGTGGAGGCTTGCTGG
CCACTTTTTGGGGTCAGCTCCTCTGAAATGCATTAGCGGAACCGTTTGCAATCTGCCACA
AGTGTGATAAGTTATCTACACTGGCGAGGGGATTGCTCTCTGTAATGTTCAGCTTCTAAT
TGTCTCTACTTTGTGAGACTACTTTTGAATGCTTGACCTCAAATCAGGTAGGACTACCC-
GCTGAACTTAA
>BC6-11
TTTCCGTAGGTGAACCTGCGGAAGGATCATTATTGAATTATGTTTCTAGATAGGTTGTAG
CTGGCTCTTTAGAGCATGTGCACGCCTGTTTGGACTTCATTTTCATCCACCTGTGCACCT
ATTGTAGTCTTTGGTTGGGTTAGGGGGAAGTGGTCATTGTGTCAGCATCTGCTGGATGTG
AGGACTTGCATTGTGAAAGCTTTGCTGTCCTTGATGTGATCATGGAATCTCTTTCTCACT
AGAGTCTATGTCACTCATTATACTCTGTCGAATGTCATTGAATGTCTTTACATGGGCTTG
TATGCCTATGAAAATTGTAATACAACTTTCAGCAACGGATCTCTTGGCTCTCGCATCGAT
GAAGGACGCAGCGAAATGCGATAAGTAATGTGAATTGCAGAATTCAGTGAATCATCGAAT
CTTTGAACGCATCTTGCGCTCCTTGGTATTCCGAGGAGCATGCCTGTTTGAGTGTCATTA
AATTCTCAACTCTCTTATACTTTTTTGTAAAAGAGAGCTTGGACTGTGGAGGCTTGCTGG
CCACTTTTTGGGGTCAGCTCCTCTGAAATGCATTAGCGGAACCGTTTGCAATCTGCCACA
AGTGTGATAAGTTATCTACACTGGCGAGGGGATTGCTCTCTGTAATGTTCAGCTTCTAAT
TGTCTCTACTTTGTGAGACTACTTTTGAATGCTTGACCTCAAATCAGGTAGGACTACCC-
GCTGAACTTAA
>BC7-14
TTTCCGTAGGTGAACCTGCGGAAGGATCATTATTGAATTATGTTTCTAGATAGGTTGTAG
CTGGCTCTTTAGAGCATGTGCACGCCTGTTTGGACTTCATTTTCATCCACCTGTGCACCT
ATTGTAGTCTTTGGTTGGGTTAGGGGGAAGTGGTCATTGTGTCAGCATCTGCTGGATGTG
AGGACTTGCATTGTGAAAGCTTTGCTGTCCTTGATGTGATCATGGAATCTCTTTCTCACT
AGAGTCTATGTCACTCATTATACTCTGTCGAATGTCATTGAATGTCTTTACATGGGCTTG
TATGCCTATGAAAATTGTAATACAACTTTCAGCAACGGATCTCTTGGCTCTCGCATCGAT
GAAGGACGCAGCGAAATGCGATAAGTAATGTGAATTGCAGAATTCAGTGAATCATCGAAT
CTTTGAACGCATCTTGCGCTCCTTGGTATTCCGAGGAGCATGCCTGTTTGAGTGTCATTA
AATTCTCAACTCTCTTATACTTTTTTGTAAAAGAGAGCTTGGACTGTGGAGGCTTGCTGG
CCACTTTTTGGGGTCAGCTCCTCTGAAATGCATTAGCGGAACCGTTTGCAATCTGCCACA
AGTGTGATAAGTTATCTACACTGGCGAGGGGATTGCTCTCTGTAATGTTCAGCTTCTAAT
TGTCTCTACTTTGTGAGACTACTTTTGAATGCTTGACCTCAAATCAGGTAGGACTACCC-
GCTGAACTTAA

>BC11_43
TTTCCGTAGGTGAACCTGCGGAAGGATCATTATTGAATTATGTTTCTAGATAGGTTGTAG
CTGGCTCTTTAGAGCATGTGCACGCCTGTTTGGACTTCATTTTCATCCACCTGTGCACCT
ATTGTAGTCTTTGGTTGGGTTAGGGGGAAGTGGTCATTGTGTCAGCATCTGCTGGATGTG
AGGACTTGCATTGTGAAAGCTTTGCTGTCCTTGATGTGATCATGGAATCTCTTTCTCACT
AGAGTCTATGTCACTCATTATACTCTGTCGAATGTCATTGAATGTCTTTACATGGGCTTG
TATGCCTATGAAAATTGTAATACAACTTTCAGCAACGGATCTCTTGGCTCTCGCATCGAT
GAAGGACGCAGCGAAATGCGATAAGTAATGTGAATTGCAGAATTCAGTGAATCATCGAAT
CTTTGAACGCATCTTGCGCTCCTTGGTATTCCGAGGAGCATGCCTGTTTGAGTGTCATTA
AATTCTCAACTCTCTTATACTTTTTTGTAAAAGAGAGCTTGGACTGTGGAGGCTTGCTGG
CCACTTTTTGGGGTCAGCTCCTCTGAAATGCATTAGCGGAACCGTTTGCAATCTGCCACA
AGTGTGATAAGTTATCTACACTGGCGAGGGGATTGCTCTCTGTAATGTTCAGCTTCTAAT
TGTCTCTACTTTGTGAGACTACTTTTGAATGCTTGACCTCAAATCAGGTAGGACTACCC-
GCTGAACTTAA
>BC4-41
TTTCCGTAGGTGAACCTGCGGAAGGATCATTATTGAATTATGTTTCTAGATAGGTTGTAG
CTGGCTCTTTAGAGCATGTGCACGCCTGTTTGGACTTCATTTTCATCCACCTGTGCACCT
ATTGTAGTCTTTGGTTGGGTTAGGGGGAAGTGGTCATTGTGTCAGCATCTGCTGGATGTG
AGGACTTGCATTGTGAAAGCTTTGCTGTCCTTGATGTGATCATGGAATCTCTTTCTCACT
AGAGTCTATGTCACTCATTATACTCTGTCGAATGTCATTGAATGTCTTTACATGGGCTTG
TATGCCTATGAAAATTGTAATACAACTTTCAGCAACGGATCTCTTGGCTCTCGCATCGAT
GAAGGACGCAGCGAAATGCGATAAGTAATGTGAATTGCAGAATTCAGTGAATCATCGAAT
CTTTGAACGCATCTTGCGCTCCTTGGTATTCCGAGGAGCATGCCTGTTTGAGTGTCATTA
AATTCTCAACTCTCTTATACTTTTTTGTAAAAGAGAGCTTGGACTGTGGAGGCTTGCTGG
CCACTTTTTGGGGTCAGCTCCTCTGAAATGCATTAGCGGAACCGTTTGCGATCTGCCACA
AGTGTGATAAGTTATCTACACTGGCGAGGGGATTGCTCTCTGTAATGTTCAGCTTCTAAT
TGTCTCTACTTTGTGAGACTACTTTTGAATGCTTGACCTCAAATCAGGTAGGACTACCC-
GCTGAACTTAA
>BC4-60
TTTCCGTAGGTGAACCTGCGGAAGGATCATTATTGAATTATGTTTCTAGATAGGTTGTAG
CTGGCTCTTTAGAGCATGTGCACGCCTGTTTGGACTTCATTTTCATCCACCTGTGCACCT
ATTGTAGTCTTTGGTTGGGTTAGGGGGAAGTGGTCATTGTGTCAGCATCTGCTGGATGTG
AGGACTTGCATTGTGAAAGCTTTGCTGTCCTTGATGTGATCATGGAATCTCTTTCTCACT
AGAGTCTATGTCACTCATTATACTCTGTCGAATGTCATTGAATGTCTTTACATGGGCTTG
TATGCCTATGAAAATTGTAATACAACTTTCAGCAACGGATCTCTTGGCTCTCGCATCGAT
GAAGGACGCAGCGAAATGCGATAAGTAATGTGAATTGCAGAATTCAGTGAATCATCGAAT
CTTTGAACGCATCTTGCGCTCCTTGGTATTCCGAGGAGCATGCCTGTTTGAGTGTCATTA
AATTCTCAACTCTCTTATACTTTTTTGTAAAAGAGAGCTTGGACTGTGGAGGCTTGCTGG
CCACTTTTTGGGGTCAGCTCCTCTGAAATGCATTAGCGGAACCGTTTGCGATCTGCCACA
AGTGTGATAAGTTATCTACACTGGCGAGGGGATTGCTCTCTGTAATGTTCAGCTTCTAAT
TGTCTCTACTTTGTGAGACTACTTTTGAATGCTTGACCTCAAATCAGGTAGGACTACCC-
GCTGAACTTAA
>BC5-107
TTTCCGTAGGTGAACCTGCGGAAGGATCATTATTGAATTATGTTTCTAGATAGGTTGTAG
CTGGCTCTTTAGAGCATGTGCACGCCTGTTTGGACTTCATTTTCATCCACCTGTGCACCT
ATTGTAGTCTTTGGTTGGGTTAGGGGGAAGTGGTCATTGTGTCAGCATCTGCTGGATGTG
AGGACTTGCATTGTGAAAGCTTTGCTGTCCTTGATGTGATCATGGAATCTCTTTCTCACT
AGAGTCTATGTCACTCATTATACTCTGTCGAATGTCATTGAATGTCTTTACATGGGCTTG
TATGCCTATGAAAATTGTAATACAACTTTCAGCAACGGATCTCTTGGCTCTCGCATCGAT
GAAGGACGCAGCGAAATGCGATAAGTAATGTGAATTGCAGAATTCAGTGAATCATCGAAT

CTTTGAACGCATCTTGCGCTCCTTGGTATTCCGAGGAGCATGCCTGTTTGAGTGTCATTA
AATTCTCAACTCTCTTATACTTTTTTGTAAAAGAGAGCTTGGACTGTGGAGGCTTGCTGG
CCACTTTTTGGGGTCAGCTCCTCTGAAATGCATTAGCGGAACCGTTTGCGATCTGCCACA
AGTGTGATAAGTTATCTACACTGGCGAGGGGATTGCTCTCTGTAATGTTCAGCTTCTAAT
TGTCTCTACTTTGTGAGACTACTTTTGAATGCTTGACCTCAAATCAGGTAGGACTACCC-
GCTGAACTTAA
>BC7-22
TTTCCGTAGGTGAACCTGCGGAAGGATCATTATTGAATTATGTTTCTAGATAGGTTGTAG
CTGGCTCTTTAGAGCATGTGCACGCCTGTTTGGACTTCATTTTCATCCACCTGTGCACCT
ATTGTAGTCTTTGGTTGGGTTAGGGGGAAGTGGTCATTGTGTCAGCATCTGCTGGATGTG
AGGACTTGCATTGTGAAAGCTTTGCTGTCCTTGATGTGATCATGGAATCTCTTTCTCACT
AGAGTCTATGTCACTCATTATACTCTGTCGAATGTCATTGAATGTCTTTACATGGGCTTG
TATGCCTATGAAAATTGTAATACAACTTTCAGCAACGGATCTCTTGGCTCTCGCATCGAT
GAAGGACGCAGCGAAATGCGATAAGTAATGTGAATTGCAGAATTCAGTGAATCATCGAAT
CTTTGAACGCATCTTGCGCTCCTTGGTATTCCGAGGAGCATGCCTGTTTGAGTGTCATTA
AATTCTCAACTCTCTTATACTTTTTTGTAAAAGAGAGCTTGGACTGTGGAGGCTTGCTGG
CCACTTTTTGGGGTCAGCTCCTCTGAAATGCATTAGCGGAACCGTTTGCGATCTGCCACA
AGTGTGATAAGTTATCTACACTGGCGAGGGGATTGCTCTCTGTAATGTTCAGCTTCTAAT
TGTCTCTACTTTGTGAGACTACTTTTGAATGCTTGACCTCAAATCAGGTAGGACTACCC-
GCTGAACTTAA
>BC8-39
TTTCCGTAGGTGAACCTGCGGAAGGATCATTATTGAATTATGTTTCTAGATAGGTTGTAG
CTGGCTCTTTAGAGCATGTGCACGCCTGTTTGGACTTCATTTTCATCCACCTGTGCACCT
ATTGTAGTCTTTGGTTGGGTTAGGGGGAAGTGGTCATTGTGTCAGCATCTGCTGGATGTG
AGGACTTGCATTGTGAAAGCTTTGCTGTCCTTGATGTGATCATGGAATCTCTTTCTCACT
AGAGTCTATGTCACTCATTATACTCTGTCGAATGTCATTGAATGTCTTTACATGGGCTTG
TATGCCTATGAAAATTGTAATACAACTTTCAGCAACGGATCTCTTGGCTCTCGCATCGAT
GAAGGACGCAGCGAAATGCGATAAGTAATGTGAATTGCAGAATTCAGTGAATCATCGAAT
CTTTGAACGCATCTTGCGCTCCTTGGTATTCCGAGGAGCATGCCTGTTTGAGTGTCATTA
AATTCTCAACTCTCTTATACTTTTTTGTAAAAGAGAGCTTGGACTGTGGAGGCTTGCTGG
CCACTTTTTGGGGTCAGCTCCTCTGAAATGCATTAGCGGAACCGTTTGCGATCTGCCACA
AGTGTGATAAGTTATCTACACTGGCGAGGGGATTGCTCTCTGTAATGTTCAGCTTCTAAT
TGTCTCTACTTTGTGAGACTACTTTTGAATGCTTGACCTCAAATCAGGTAGGACTACCC-
GCTGAACTTAA
>BC10_32
TTTCCGTAGGTGAACCTGCGGAAGGATCATTATTGAATTATGTTTCTAGATAGGTTGTAG
CTGGCTCTTTAGAGCATGTGCACGCCTGTTTGGACTTCATTTTCATCCACCTGTGCACCT
ATTGTAGTCTTTGGTTGGGTTAGGGGGAAGTGGTCATTGTGTCAGCATCTGCTGGATGTG
AGGACTTGCATTGTGAAAGCTTTGCTGTCCTTGATGTGATCATGGAATCTCTTTCTCACT
AGAGTCTATGTCACTCATTATACTCTGTCGAATGTCATTGAATGTCTTTACATGGGCTTG
TATGCCTATGAAAATTGTAATACAACTTTCAGCAACGGATCTCTTGGCTCTCGCATCGAT
GAAGGACGCAGCGAAATGCGATAAGTAATGTGAATTGCAGAATTCAGTGAATCATCGAAT
CTTTGAACGCATCTTGCGCTCCTTGGTATTCCGAGGAGCATGCCTGTTTGAGTGTCATTA
AATTCTCAACTCTCTTATACTTTTTTGTAAAAGAGAGCTTGGACTGTGGAGGCTTGCTGG
CCACTTTTTGGGGTCAGCTCCTCTGAAATGCATTAGCGGAACCGTTTGCGATCTGCCACA
AGTGTGATAAGTTATCTACACTGGCGAGGGGATTGCTCTCTGTAATGTTCAGCTTCTAAT
TGTCTCTACTTTGTGAGACTACTTTTGAATGCTTGACCTCAAATCAGGTAGGACTACCC-
GCTGAACTTAA
>BC11_24
TTTCCGTAGGTGAACCTGCGGAAGGATCATTATTGAATTATGTTTCTAGATAGGTTGTAG

CTGGCTCTTTAGAGCATGTGCACGCCTGTTTGGACTTCATTTTCATCCACCTGTGCACCT
ATTGTAGTCTTTGGTTGGGTTAGGGGGAAGTGGTCATTGTGTCAGCATCTGCTGGATGTG
AGGACTTGCATTGTGAAAGCTTTGCTGTCCTTGATGTGATCATGGAATCTCTTTCTCACT
AGAGTCTATGTCACTCATTATACTCTGTCGAATGTCATTGAATGTCTTTACATGGGCTTG
TATGCCTATGAAAATTGTAATACAACTTTCAGCAACGGATCTCTTGGCTCTCGCATCGAT
GAAGGACGCAGCGAAATGCGATAAGTAATGTGAATTGCAGAATTCAGTGAATCATCGAAT
CTTTGAACGCATCTTGCGCTCCTTGGTATTCCGAGGAGCATGCCTGTTTGAGTGTCATTA
AATTCTCAACTCTCTTATACTTTTTTGTAAAAGAGAGCTTGGACTGTGGAGGCTTGCTGG
CCACTTTTTGGGGTCAGCTCCTCTGAAATGCATTAGCGGAACCGTTTGCGATCTGCCACA
AGTGTGATAAGTTATCTACACTGGCGAGGGGATTGCTCTCTGTAATGTTCAGCTTCTAAT
TGTCTCTACTTTGTGAGACTACTTTTGAATGCTTGACCTCAAATCAGGTAGGACTACCC-
GCTGAACTTAA
>BC8-40
TTTCCGTAGGTGAACCTGCGGAAGGATCATTATTGAATTATGTTTCTAGATAGGTTGTAG
CTGGCTCTTTAGAGCATGTGCACGCCTGTTTGGACTTCATTTTCATCCACCTGTGCACCT
ATTGTAGTCTTTGGTTGGGTTAGGAGGAAGTGGTCATTGTGTCAGCATCTGCTGGATGTG
AGGACTTGCATTGTGAAAGCTTTGCTGTCCTTGATGTGATCATGGAATCTCTTTCTCACT
AGAGTCTATGTCACTCATTATACTCTGTCGAATGTCATTGAATGTCTTTACATGGGCTTG
TATGCCTATGAAAATTGTAATACAACTTTCAGCAACGGATCTCTTGGCTCTCGCATCGAT
GAAGGACGCAGCGAAATGCGATAAGTAATGTGAATTGCAGAATTCAGTGAATCATCGAAT
CTTTGAACGCATCTTGCGCTCCTTGGTATTCCGAGGAGCATGCCTGTTTGAGTGTCATTA
AATTCTCAACTCTCTTATACTTTTTTGTAAAAGAGAGCTTGGACTGTGGAGGCTTGCTGG
CCACTTTTTGGGGTCAGCTCCTCTGAAATGCATTAGCGGAACCGTTTGCAATCTGCCACA
AGTGTGATAAGTTATCTACACTGGCGAGGGGATTGCTCTCTGTAATGTTCAGCTTCTAAT
TGTCTCTACTTTGTGAGACTACTTTTGAATGCTTGACCTCAAATCAGGTAGGACTACCC-
GCTGAACTTAA
>BC9-21
TTTCCGTAGGTGAACCTGCGGAAGGATCATTATTGAATTATGTTTCTAGATAGGTTGTAG
CTGGCTCTTTAGAGCATGTGCACGCCTGTTTGGACTTCATTTTCATCCACCTGTGCACCT
ATTGTAGTCTTTGGTTGGGTTAGGAGGAAGTGGTCATTGTGTCAGCATCTGCTGGATGTG
AGGACTTGCATTGTGAAAGCTTTGCTGTCCTTGATGTGATCATGGAATCTCTTTCTCACT
AGAGTCTATGTCACTCATTATACTCTGTCGAATGTCATTGAATGTCTTTACATGGGCTTG
TATGCCTATGAAAATTGTAATACAACTTTCAGCAACGGATCTCTTGGCTCTCGCATCGAT
GAAGGACGCAGCGAAATGCGATAAGTAATGTGAATTGCAGAATTCAGTGAATCATCGAAT
CTTTGAACGCATCTTGCGCTCCTTGGTATTCCGAGGAGCATGCCTGTTTGAGTGTCATTA
AATTCTCAACTCTCTTATACTTTTTTGTAAAAGAGAGCTTGGACTGTGGAGGCTTGCTGG
CCACTTTTTGGGGTCAGCTCCTCTGAAATGCATTAGCGGAACCGTTTGCAATCTGCCACA
AGTGTGATAAGTTATCTACACTGGCGAGGGGATTGCTCTCTGTAATGTTCAGCTTCTAAT
TGTCTCTACTTTGTGAGACTACTTTTGAATGCTTGACCTCAAATCAGGTAGGACTACCC-
GCTGAACTTAA
>BC10_46
TTTCCGTAGGTGAACCTGCGGAAGGATCATTATTGAATTATGTTTCTAGATAGGTTGTAG
CTGGCTCTTTAGAGCATGTGCACGCCTGTTTGGACTTCATTTTCATCCACCTGTGCACCT
ATTGTAGTCTTTGGTTGGGTTAGGAGGAAGTGGTCATTGTGTCAGCATCTGCTGGATGTG
AGGACTTGCATTGTGAAAGCTTTGCTGTCCTTGATGTGATCATGGAATCTCTTTCTCACT
AGAGTCTATGTCACTCATTATACTCTGTCGAATGTCATTGAATGTCTTTACATGGGCTTG
TATGCCTATGAAAATTGTAATACAACTTTCAGCAACGGATCTCTTGGCTCTCGCATCGAT
GAAGGACGCAGCGAAATGCGATAAGTAATGTGAATTGCAGAATTCAGTGAATCATCGAAT
CTTTGAACGCATCTTGCGCTCCTTGGTATTCCGAGGAGCATGCCTGTTTGAGTGTCATTA
AATTCTCAACTCTCTTATACTTTTTTGTAAAAGAGAGCTTGGACTGTGGAGGCTTGCTGG

CCACTTTTTGGGGTCAGCTCCTCTGAAATGCATTAGCGGAACCGTTTGCAATCTGCCACA
AGTGTGATAAGTTATCTACACTGGCGAGGGGATTGCTCTCTGTAATGTTCAGCTTCTAAT
TGTCTCTACTTTGTGAGACTACTTTTGAATGCTTGACCTCAAATCAGGTAGGACTACCC-
GCTGAACTTAA
>BC9-8
TTTCCGTAGGTGAACCTGCGGAAGGATCATTATTGAATTATGTTTCTAGATAGGTTGTAG
CTGGCTCTTTAGAGCATGTGCACGCCTGTTTGGACTTCATTTTCATCCACCTGTGCACCT
ATTGTAGTCTTTGGTTGGGTTAGGAGGAAGTGGTCATTGTGTCAGCATCTGCTGGATGTG
AGGACTTGCATTGTGAAAGCTTTGCTGTCCTTGATGTGATCATGGAATCTCTTTCTCACT
AGAGTCTATGTCACTCATTATACTCTGTCGAATGTCATTGAATGTCTTTACATGGGCTTG
TATGCCTATGAAAATTGTAATACAACTTTCAGCAACGGATCTCTTGGCTCTCGCATCGAT
GAAGGACGCAGCGAAATGCGATAAGTAATGTGAATTGCAGAATTCAGTGAATCATCGAAT
CTTTGAACGCATCTTGCGCTCCTTGGTATTCCGAGGAGCATGCCTGTTTGAGTGTCATTA
AATTCTCAACTCTCTTATACTTTTTTGTAAAAGAGAGCTTGGACTGTGGAGGCTTGCTGG
CCACTTTTTGGGGTCAGCTCCTCTGAAATGCATTAGCGGAACCGTTTGCGATCTGCCACA
AGTGTGATAAGTTATCTACACTGGCGAGGGGATTGCTCTCTGTAATGTTCAGCTTCTAAT
TGTCTCTACTTTGTGAGACTACTTTTGAATGCTTGACCTCAAATCAGGTAGGACTACCC-
GCTGAACTTAA
>BC11_55
TTTCCGTAGGTGAACCTGCGGAAGGATCATTATTGAATTATGTTTCTAGATAGGTTGTAG
CTGGCTCTTTAGAGCATGTGCACGCCTGTTTGGACTTCATTTTCATCCACCTGTGCACCT
ATTGTAGTCTTTGGTTGGGTTAGGAGGAAGTGGTCATTGTGTCAGCATCTGCTGGATGTG
AGGACTTGCATTGTGAAAGCTTTGCTGTCCTTGATGTGATCATGGAATCTCTTTCTCACT
AGAGTCTATGTCACTCATTATACTCTGTCGAATGTCATTGAATGTCTTTACATGGGCTTG
TATGCCTATGAAAATTGTAATACAACTTTCAGCAACGGATCTCTTGGCTCTCGCATCGAT
GAAGGACGCAGCGAAATGCGATAAGTAATGTGAATTGCAGAATTCAGTGAATCATCGAAT
CTTTGAACGCATCTTGCGCTCCTTGGTATTCCGAGGAGCATGCCTGTTTGAGTGTCATTA
AATTCTCAACTCTCTTATACTTTTTTGTAAAAGAGAGCTTGGACTGTGGAGGCTTGCTGG
CCACTTTTTGGGGTCAGCTCCTCTGAAATGCATTAGCGGAACCGTTTGCGATCTGCCACA
AGTGTGATAAGTTATCTACACTGGCGAGGGGATTGCTCTCTGTAATGTTCAGCTTCTAAT
TGTCTCTACTTTGTGAGACTACTTTTGAATGCTTGACCTCAAATCAGGTAGGACTACCC-
GCTGAACTTAA
>BC4-23
TTTCCGTAGGTGAACCTGCGGAAGGATCATTATTGAATTATGTTTCTAGATAGGTTGTAG
CTGGCTCTTTAGAGCATGTGCACGCCTGTTTGGACTTCATTTTCATCCACCTGTGCACCT
ATTGTAGTCTTTGGTTGGGTTAGGGGGAAGTGGTCATTGTGTCAGCATCTGCTGGATGTG
AGGACTTGCATTGTGAAAGCTTTGCTGTCCTTGATGTGATCATGGAATCTCTTTCTCACT
AGAGTCTATGTCACTCATTATACTCTGTCGAATGTCATTGAATGTCTTTACATGGGCTTG
TATGCCTATGAAAATTGTAATACAACTTTCAGCAACGGATCTCTTGGCTCTCGCATCGAT
GAAGAACGCAGCGAAATGCGATAAGTAATGTGAATTGCAGAATTCAGTGAATCATCGAAT
CTTTGAACGCATCTTGCGCTCCTTGGTATTCCGAGGAGCATGCCTGTTTGAGTGTCATTA
AATTCTCAACTCTCTTATACTTTTTTGTAAAAGAGAGCTTGGACTGTGGAGGCTTGCTGG
CCACTTTTTGGGGTCAGCTCCTCTGAAATGCATTAGCGGAACCGTTTGCAATCTGCCACA
AGTGTGATAAGTTATCTACACTGGCGAGGGGATTGCTCTCTGTAATGTTCAGCTTCTAAT
TGTCTCTACTTTGTGAGACTACTTTTGAATGCTTGACCTCAAATCAGGTAGGACTACCC-
GCTGAACTTAA
>BC12_7
TTTCCGTAGGTGAACCTGCGGAAGGATCATTATTGAATTATGTTTCTAGATAGGTTGTAG
CTGGCTCTTTAGAGCATGTGCACGCCTGTTTGGACTTCATTTTCATCCACCTGTGCACCT
ATTGTAGTCTTTGGTTGGGTTAGGGGGAAGTGGTCATTGTGTCAGCATCTGCTGGATGTG

AGGACTTGCATTGTGAAAGCTTTGCTGTCCTTGATGTGATCATGGAATCTCTTTCTCACT
AGAGTCTATGTCACTCATTATACTCTGTCGAATGTCATTGAATGTCTTTACATGGGCTTG
TATGCCTATGAAAATTGTAATACAACTTTCAGCAACGGATCTCTTGGCTCTCGCATCGAT
GAAGAACGCAGCGAAATGCGATAAGTAATGTGAATTGCAGAATTCAGTGAATCATCGAAT
CTTTGAACGCATCTTGCGCTCCTTGGTATTCCGAGGAGCATGCCTGTTTGAGTGTCATTA
AATTCTCAACTCTCTTATACTTTTTTGTAAAAGAGAGCTTGGACTGTGGAGGCTTGCTGG
CCACTTTTTGGGGTCAGCTCCTCTGAAATGCATTAGCGGAACCGTTTGCGATCTGCCACA
AGTGTGATAAGTTATCTACACTGGCGAGGGGATTGCTCTCTGTAATGTTCAGCTTCTAAT
TGTCTCTACTTTGTGAGACTACTTTTGAATGCTTGACCTCAAATCAGGTAGGACTACCC-
GCTGAACTTAA
>BC6-30
TTTCCGTAGGTGAACCTGCGGAAGGATCATTATTGAATTATGTTTCTAGATAGGTTGTAG
CTGGCTCTTTAGAGCATGTGCACGCCTGTTTGGACTTCATTTTCATCCACCTGTGCACCT
ATTGTAGTCTTTGGTTGGGTTAGGGGGAAGTGGTCATTGTGTCAGCATCTGCTGGATGTG
AGGACTTGCATTGTGAAAGCTTTGCTGTCCTTGATGTGATCATGGAATCTCTTTCTCACT
AGAGTCTATGTCACTCATTATACTCTGTCGAATGTCATTGAATGTCTTTACATGGGCTTA
TATGCCTATGAAAATTGTAATACAACTTTCAGCAACGGATCTCTTGGCTCTCGCATCGAT
GAAGAACGCAGCGAAATGCGATAAGTAATGTGAATTGCAGAATTCAGTGAATCATCGAAT
CTTTGAACGCATCTTGCGCTCCTTGGTATTCCGAGGAGCATGCCTGTTTGAGTGTCATTA
AATTCTCAACTCTCTTATACTTTTTTGTAAAAGAGAGCTTGGACTGTGGAGGCTTGCTGG
CCACTTTTTGGGGTCAGCTCCTCTGAAATGCATTAGCGGAACCGTTTGCAATCTGCCACA
AGTGTGATAAGTTATCTACACTGGCGAGGGGATTGCTCTCTGTAATGTTCAGCTTCTAAT
TGTCTCTACTTTGTGAGACTACTTTTGAATGCTTGACCTCAAATCAGGTAGGACTACCC-
GCTGAACTTAA
>BC7-27
TTTCCGTAGGTGAACCTGCGGAAGGATCATTATTGAATTATGTTTCTAGATAGGTTGTAG
CTGGCTCTTTAGAGCATGTGCACGCCTGTTTGGACTTCATTTTCATCCACCTGTGCACCT
ATTGTAGTCTTTGGTTGGGTTAGGAGGAAGTGGTCATTGTGTCAGCATCTGCTGGATGTG
AGGACTTGCATTGTGAAAGCTTTGCTGTCCTTGATGTGATCATGGAATCTCTTTCTCACT
AGAGTCTATGTCACTCATTATACTCTGTCGAATGTCATTGAATGTCTTTACATGGGCTTG
TATGCCTATGAAAATTGTAATACAACTTTCAGCAACGGATCTCTTGGCTCTCGCATCGAT
GAAGAACGCAGCGAAATGCGATAAGTAATGTGAATTGCAGAATTCAGTGAATCATCGAAT
CTTTGAACGCATCTTGCGCTCCTTGGTATTCCGAGGAGCATGCCTGTTTGAGTGTCATTA
AATTCTCAACTCTCTTATACTTTTTTGTAAAAGAGAGCTTGGACTGTGGAGGCTTGCTGG
CCACTTTTTGGGGTCAGCTCCTCTGAAATGCATTAGCGGAACCGTTTGCAATCTGCCACA
AGTGTGATAAGTTATCTACACTGGCGAGGGGATTGCTCTCTGTAATGTTCAGCTTCTAAT
TGTCTCTACTTTGTGAGACTACTTTTGAATGCTTGACCTCAAATCAGGTAGGACTACCC-
GCTGAACTTAA
>BC1-27
TTTCCGTAGGTGAACCTGCGGAAGGATCATTATTGAATTATGTTTCTAGATAGGTTGTAG
CTGGCTCTTTAGAGCATGTGCACGCCTGTTTGGACTTCATTTTCATCCACCTGTGCACCT
ATTGTAGTCTTTGGTTGGGTTAGGAGGAAGTGGTCATTGTGTCAGCATCTGCTGGATGTG
AGGACTTGCATTGTGAAAGCTTTGCTGTCCTTGATGTGATCATGGAATCTCTTTCTCACT
AGAGTCTATGTCACTCATTATACTCTGTCGAATGTCATTGAATGTCTTTACATGGGCTTA
TATGCCTATGAAAATTGTAATACAACTTTCAGCAACGGATCTCTTGGCTCTCGCATCGAT
GAAGGACGCAGCGAAATGCGATAAGTAATGTGAATTGCAGAATTCAGTGAATCATCGAAT
CTTTGAACGCATCTTGCGCTCCTTGGTATTCCGAGGAGCATGCCTGTTTGAGTGTCATTA
AATTCTCAACTCTCTTATACTTTTTTGTAAAAGAGAGCTTGGACTGTGGAGGCTTGCTGG
CCACTTTTTGGGGTCAGCTCCTCTGAAATGCATTAGCGGAACCGTTTGCGATCTGCCACA
AGTGTGATAAGTTATCTACACTGGCGAGGGGATTGCTCTCTGTAATGTTCAGCTTCTAAT

TGTCTCTACTTTGTGAGACTACTTTTGAATGCTTGACCTCAAATCAGGTAGGACTACCC-
GCTGAACTTAA
>BC8-32
TTTCCGTAGGTGAACCTGCGGAAGGATCATTATTGAATTATGTTTCTAGATAGGTTGTAG
CTGGCTCTTTAGAGCATGTGCACGCCTGTTTGGACTTCATTTTCATCCACCTGTGCACCT
ATTGTAGTCTTTGGTTGGGTTAGGAGGAAGTGGTCATTGTGTCAGCATCTGCTGGATGTG
AGGACTTGCATTGTGAAAGCTTTGCTGTCCTTGATGTGATCATGGAATCTCTTTCTCACT
AGAGTCTATGTCACTCATTATACTCTGTCGAATGTCATTGAATGTCTTTACATGGGCTTA
TATGCCTATGAAAATTGTAATACAACTTTCAGCAACGGATCTCTTGGCTCTCGCATCGAT
GAAGGACGCAGCGAAATGCGATAAGTAATGTGAATTGCAGAATTCAGTGAATCATCGAAT
CTTTGAACGCATCTTGCGCTCCTTGGTATTCCGAGGAGCATGCCTGTTTGAGTGTCATTA
AATTCTCAACTCTCTTATACTTTTTTGTAAAAGAGAGCTTGGACTGTGGAGGCTTGCTGG
CCACTTTTTGGGGTCAGCTCCTCTGAAATGCATTAGCGGAACCGTTTGCGATCTGCCACA
AGTGTGATAAGTTATCTACACTGGCGAGGGGATTGCTCTCTGTAATGTTCAGCTTCTAAT
TGTCTCTACTTTGTGAGACTACTTTTGAATGCTTGACCTCAAATCAGGTAGGACTACCC-
GCTGAACTTAA
>BC8-14
TTTCCGTAGGTGAACCTGCGGAAGGATCATTATTGAATTATGTTTCTAGATAGGTTGTAG
CTGGCTCTTTAGAGCATGTGCACGCCTGTTTGGACTTCATTTTCATCCACCTGTGCACCT
ATTGTAGTCTTTGGTTGGGTTAGGAGGAAGTGGTCATTGTGTCAGCATCTGCTGGATGTG
AGGACTTGCATTGTGAAAGCTTTGCTGTCCTTGATGTGATCATGGAATCTCTTTCTCACT
AGAGTCTATGTCACTCATTATACTCTGTCGAATGTCATTGAATGTCTTTACATGGGCTTA
TATGCCTATGAAAATTGTAATACAACTTTCAGCAACGGATCTCTTGGCTCTCGCATCGAT
GAAGGACGCAGCGAAATGCGATAAGTAATGTGAATTGCAGAATTCAGTGAATCATCGAAT
CTTTGAACGCATCTTGCGCTCCTTGGTATTCCGAGGAGCATGCCTGTTTGAGTGTCATTA
AATTCTCAACTCTCTTATACTTTTTTGTAAAAGAGAGCTTGGACTGTGGAGGCTTGCTGG
CCACTTTTTGGGGTCAGCTCCTCTGAAATGCATTAGCGGAACCGTTTGCGATCTGCCACA
AGTGTGATAAGTTATCTACACTGGCGAGGGGATTGCTCTCTGTAATGTTCAGCTTCTAAT
TGTCTCTACTTTGTGAGACTACTTTTGAATGCTTGACCTCAAATCAGGTAGGACTACCC-
GCTGAACTTAA
>BC11_7
TTTCCGTAGGTGAACCTGCGGAAGGATCATTATTGAATTATGTTTCTAGATAGGTTGTAG
CTGGCTCTTTAGAGCATGTGCACGCCTGTTTGGACTTCATTTTCATCCACCTGTGCACCT
ATTGTAGTCTTTGGTTGGGTTAGGAGGAAGTGGTCATTGTGTCAGCATCTGCTGGATGTG
AGGACTTGCATTGTGAAAGCTTTGCTGTCCTTGATGTGATCATGGAATCTCTTTCTCACT
AGAGTCTATGTCACTCATTATACTCTGTCGAATGTCATTGAATGTCTTTACATGGGCTTA
TATGCCTATGAAAATTGTAATACAACTTTCAGCAACGGATCTCTTGGCTCTCGCATCGAT
GAAGGACGCAGCGAAATGCGATAAGTAATGTGAATTGCAGAATTCAGTGAATCATCGAAT
CTTTGAACGCATCTTGCGCTCCTTGGTATTCCGAGGAGCATGCCTGTTTGAGTGTCATTA
AATTCTCAACTCTCTTATACTTTTTTGTAAAAGAGAGCTTGGACTGTGGAGGCTTGCTGG
CCACTTTTTGGGGTCAGCTCCTCTGAAATGCATTAGCGGAACCGTTTGCGATCTGCCACA
AGTGTGATAAGTTATCTACACTGGCGAGGGGATTGCTCTCTGTAATGTTCAGCTTCTAAT
TGTCTCTACTTTGTGAGACTACTTTTGAATGCTTGACCTCAAATCAGGTAGGACTACCC-
GCTGAACTTAA
>BC4-35
TTTCCGTAGGTGAACCTGCGGAAGGATCATTATTGAATTATGTTTCTAGATAGGTTGTAG
CTGGCTCTTTAGAGCATGTGCACGCCTGTTTGGACTTCATTTTCATCCACCTGTGCACCT
ATTGTAGTCTTTGGTTGGGTTAGGAGGAAGTGGTCATTGTGTCAGCATCTGCTGGATGTG
AGGACTTGCATTGTGAAAGCTTTGCTGTCCTTGATGTGATCATGGAATCTCTTTCTCACT
AGAGTCTATGTCACTCATTATACTCTGTCGAATGTCATTGAATGTCTTTACATGGGCTTA

TATGCCTATGAAAATTGTAATACAACTTTCAGCAACGGATCTCTTGGCTCTCGCATCGAT
GAAGAACGCAGCGAAATGCGATAAGTAATGTGAATTGCAGAATTCAGTGAATCATCGAAT
CTTTGAACGCATCTTGCGCTCCTTGGTATTCCGAGGAGCATGCCTGTTTGAGTGTCATTA
AATTCTCAACTCTCTTATACTTTTTTGTAAAAGAGAGCTTGGACTGTGGAGGCTTGCTGG
CCACTTTTTGGGGTCAGCTCCTCTGAAATGCATTAGCGGAACCGTTTGCGATCTGCCACA
AGTGTGATAAGTTATCTACACTGGCGAGGGGATTGCTCTCTGTAATGTTCAGCTTCTAAT
TGTCTCTACTTTGTGAGACTACTTTTGAATGCTTGACCTCAAATCAGGTAGGACTACCC-
GCTGAACTTAA
>BC6-56
TTTCCGTAGGTGAACCTGCGGAAGGATCATTATTGAATTATGTTTCTAGATAGGTTGTAG
CTGGCTCTTTAGAGCATGTGCACGCCTGTTTGGACTTCATTTTCATCCACCTGTGCACCT
ATTGTAGTCTTTGGTTGGGTTAGGAGGAAGTGGTCATTGTGTCAGCATCTGCTGGATGTG
AGGACTTGCATTGTGAAAGCTTTGCTGTCCTTGATGTGATCATGGAATCTCTTTCTCACT
AGAGTCTATGTCACTCATTATACTCTGTCGAATGTCATTGAATGTCTTTACATGGGCTTA
TATGCCTATGAAAATTGTAATACAACTTTCAGCAACGGATCTCTTGGCTCTCGCATCGAT
GAAGAACGCAGCGAAATGCGATAAGTAATGTGAATTGCAGAATTCAGTGAATCATCGAAT
CTTTGAACGCATCTTGCGCTCCTTGGTATTCCGAGGAGCATGCCTGTTTGAGTGTCATTA
AATTCTCAACTCTCTTATACTTTTTTGTAAAAGAGAGCTTGGACTGTGGAGGCTTGCTGG
CCACTTTTTGGGGTCAGCTCCTCTGAAATGCATTAGCGGAACCGTTTGCGATCTGCCACA
AGTGTGATAAGTTATCTACACTGGCGAGGGGATTGCTCTCTGTAATGTTCAGCTTCTAAT
TGTCTCTACTTTGTGAGACTACTTTTGAATGCTTGACCTCAAATCAGGTAGGACTACCC-
GCTGAACTTAA
>BC7-16
TTTCCGTAGGTGAACCTGCGGAAGGATCATTATTGAATTATGTTTCTAGATAGGTTGTAG
CTGGCTCTTTAGAGCATGTGCACGCCTGTTTGGACTTCATTTTCATCCACCTGTGCACCT
ATTGTAGTCTTTGGTTGGGTTAGGAGGAAGTGGTCATTGTGTCAGCATCTGCTGGATGTG
AGGACTTGCATTGTGAAAGCTTTGCTGTCCTTGATGTGATCATGGAATCTCTTTCTCACT
AGAGTCTATGTCACTCATTATACTCTGTCGAATGTCATTGAATGTCTTTACATGGGCTTA
TATGCCTATGAAAATTGTAATACAACTTTCAGCAACGGATCTCTTGGCTCTCGCATCGAT
GAAGAACGCAGCGAAATGCGATAAGTAATGTGAATTGCAGAATTCAGTGAATCATCGAAT
CTTTGAACGCATCTTGCGCTCCTTGGTATTCCGAGGAGCATGCCTGTTTGAGTGTCATTA
AATTCTCAACTCTCTTATACTTTTTTGTAAAAGAGAGCTTGGACTGTGGAGGCTTGCTGG
CCACTTTTTGGGGTCAGCTCCTCTGAAATGCATTAGCGGAACCGTTTGCGATCTGCCACA
AGTGTGATAAGTTATCTACACTGGCGAGGGGATTGCTCTCTGTAATGTTCAGCTTCTAAT
TGTCTCTACTTTGTGAGACTACTTTTGAATGCTTGACCTCAAATCAGGTAGGACTACCC-
GCTGAACTTAA
>BC10_5
TTTCCGTAGGTGAACCTGCGGAAGGATCATTATTGAATTATGTTTCTAGATAGGTTGTAG
CTGGCTCTTTAGAGCATGTGCACGCCTGTTTGGACTTCATTTTCATCCACCTGTGCACCT
ATTGTAGTCTTTGGTTGGGTTAGGAGGAAGTGGTCATTGTGTCAGCATCTGCTGGATGTG
AGGACTTGCATTGTGAAAGCTTTGCTGTCCTTGATGTGATCATGGAATCTCTTTCTCACT
AGAGTCTATGTCACTCATTATACTCTGTCGAATGTCATTGAATGTCTTTACATGGGCTTA
TATGCCTATGAAAATTGTAATACAACTTTCAGCAACGGATCTCTTGGCTCTCGCATCGAT
GAAGAACGCAGCGAAATGCGATAAGTAATGTGAATTGCAGAATTCAGTGAATCATCGAAT
CTTTGAACGCATCTTGCGCTCCTTGGTATTCCGAGGAGCATGCCTGTTTGAGTGTCATTA
AATTCTCAACTCTCTTATACTTTTTTGTAAAAGAGAGCTTGGACTGTGGAGGCTTGCTGG
CCACTTTTTGGGGTCAGCTCCTCTGAAATGCATTAGCGGAACCGTTTGCGATCTGCCACA
AGTGTGATAAGTTATCTACACTGGCGAGGGGATTGCTCTCTGTAATGTTCAGCTTCTAAT
TGTCTCTACTTTGTGAGACTACTTTTGAATGCTTGACCTCAAATCAGGTAGGACTACCC-
GCTGAACTTAA

>BC6–31
TTTCCGTAGGTGAACCTGCGGAAGGATCATTATTGAATTATGTTTCTAGATAGGTTGTAG
CTGGCTCTTTAGAGCATGTGCACGCCTGTTTGGACTTCATTTTCATCCACCTGTGCACCT
ATTGTAGTCTTTGGTTGGGTTAGGAGGAAGTGGTCATTGTGTCAGCATCTGCTGGATGTG
AGGACTTGCATTGTGAAAGCTTTGCTGTCCTTGATGTGATCATGGAATCTCTTTCTCACT
AGAGTCTATGTCACTCATTATACTCTGTCGAATGTCATTGAATGTCTTTACATGGGCTTA
TATGCCTATGAAAATTGTAATACAACTTTCAGCAACGGATCTCTTGGCTCTCGCATCGAT
GAAGGACGCAGCGAAATGCGATAAGTAATGTGAATTGCAGAATTCAGTGAATCATCGAAT
CTTTGAACGCATCTTGCGCTCCTTGGTATTCCGAGGAGCATGCCTGTTTGAGTGTCATTA
AATTCTCAACTCTCTTATACTTTTTTGTAAAAGAGAGCTTGGACTGTGGAGGCTTGCTGG
CCACTTTTTGGGGTCAGCTCCTCTGAAATGCATTAGCGGAACCGTTTGCAATCTGCCACA
AGTGTGATAAGTTATCTACACTGGCGAGGGGATTGCTCTCTGTAATGTTCAGCTTCTAAT
TGTCTCTACTTTGTGAGACTACTTTTGAATGCTTGACCTCAAATCAGGTAGGACTACCC–
GCTGAACTTAA
>BC11_19
TTTCCGTAGGTGAACCTGCGGAAGGATCATTATTGAATTATGTTTCTAGATAGGTTGTAG
CTGGCTCTTTAGAGCATGTGCACGCCTGTTTGGACTTCATTTTCATCCACCTGTGCACCT
ATTGTAGTCTTTGGTTGGGTTAGGAGGAAGTGGTCATTGTGTCAGCATCTGCTGGATGTG
AGGACTTGCATTGTGAAAGCTTTGCTGTCCTTGATGTGATCATGGAATCTCTTTCTCACT
AGAGTCTATGTCACTCATTATACTCTGTCGAATGTCATTGAATGTCTTTACATGGGCTTA
TATGCCTATGAAAATTGTAATACAACTTTCAGCAACGGATCTCTTGGCTCTCGCATCGAT
GAAGGACGCAGCGAAATGCGATAAGTAATGTGAATTGCAGAATTCAGTGAATCATCGAAT
CTTTGAACGCATCTTGCGCTCCTTGGTATTCCGAGGAGCATGCCTGTTTGAGTGTCATTA
AATTCTCAACTCTCTTATACTTTTTTGTAAAAGAGAGCTTGGACTGTGGAGGCTTGCTGG
CCACTTTTTGGGGTCAGCTCCTCTGAAATGCATTAGCGGAACCGTTTGCAATCTGCCACA
AGTGTGATAAGTTATCTACACTGGCGAGGGGATTGCTCTCTGTAATGTTCAGCTTCTAAT
TGTCTCTACTTTGTGAGACTACTTTTGAATGCTTGACCTCAAATCAGGTAGGACTACCC–
GCTGAACTTAA
>BC3–16
TTTCCGTAGGTGAACCTGCGGAAGGATCATTATTGAATTATGTTTCTAGATAGGTTGTAG
CTGGCTCTTTAGAGCATGTGCACGCCTGTTTGGACTTCATTTTCATCCACCTGTGCACCT
ATTGTAGTCTTTGGTTGGGTTAGGAGGAAGTGGTCATTGTGTCAGCATCTGCTGGATGTG
AGGACTTGCATTGTGAAAGCTTTGCTGTCCTTGATGTGATCATGGAATCTCTTTCTCACT
AGAGTCTATGTCACTCATTATACTCTGTCGAATGTCATTGAATGTCTTTACATGGGCTTA
TATGCCTATGAAAATTGTAATACAACTTTCAGCAACGGATCTCTTGGCTCTCGCATCGAT
GAAGAACGCAGCGAAATGCGATAAGTAATGTGAATTGCAGAATTCAGTGAATCATCGAAT
CTTTGAACGCATCTTGCGCTCCTTGGTATTCCGAGGAGCATGCCTGTTTGAGTGTCATTA
AATTCTCAACTCTCTTATACTTTTTTGTAAAAGAGAGCTTGGACTGTGGAGGCTTGCTGG
CCACTTTTTGGGGTCAGCTCCTCTGAAATGCATTAGCGGAACCGTTTGCAATCTGCCACA
AGTGTGATAAGTTATCTACACTGGCGAGGGGATTGCTCTCTGTAATGTTCAGCTTCTAAT
TGTCTCTACTTTGTGAGACTACTTTTGAATGCTTGACCTCAAATCAGGTAGGACTACCC–
GCTGAACTTAA
>BC6–4
TTTCCGTAGGTGAACCTGCGGAAGGATCATTATTGAATTATGTTTCTAGATAGGTTGTAG
CTGGCTCTTTAGAGCATGTGCACGCCTGTTTGGACTTCATTTTCATCCACCTGTGCACCT
ATTGTAGTCTTTGGTTGGGTTAGGAGGAAGTGGTCATTGTGTCAGCATCTGCTGGATGTG
AGGACTTGCATTGTGAAAGCTTTGCTGTCCTTGATGTGATCATGGAATCTCTTTCTCACT
AGAGTCTATGTCACTCATTATACTCTGTCGAATGTCATTGAATGTCTTTACATGGGCTTA
TATGCCTATGAAAATTGTAATACAACTTTCAGCAACGGATCTCTTGGCTCTCGCATCGAT
GAAGAACGCAGCGAAATGCGATAAGTAATGTGAATTGCAGAATTCAGTGAATCATCGAAT

CTTTGAACGCATCTTGCGCTCCTTGGTATTCCGAGGAGCATGCCTGTTTGAGTGTCATTA
AATTCTCAACTCTCTTATACTTTTTTGTAAAAGAGAGCTTGGACTGTGGAGGCTTGCTGG
CCACTTTTTGGGGTCAGCTCCTCTGAAATGCATTAGCGGAACCGTTTGCAATCTGCCACA
AGTGTGATAAGTTATCTACACTGGCGAGGGGATTGCTCTCTGTAATGTTCAGCTTCTAAT
TGTCTCTACTTTGTGAGACTACTTTTGAATGCTTGACCTCAAATCAGGTAGGACTACCC-
GCTGAACTTAA
>BC9-48
TTTCCGTAGGTGAACCTGCGGAAGGATCATTATTGAATTATGTTTCTAGATAGGTTGTAG
CTGGCTCTTTAGAGCATGTGCACGCCTGTTTGGACTTCATTTTCATCCACCTGTGCACCT
ATTGTAGTCTTTGGTTGGGTTAGGAGGAAGTGGTCATTGTGTCAGCATCTGCTGGATGTG
AGGACTTGCATTGTGAAAGCTTTGCTGTCCTTGATGTGATCATGGAATCTCTTTCTCACT
AGAGTCTATGTCACTCATTATACTCTGTCGAATGTCATTGAATGTCTTTACATGGGCTTA
TATGCCTATGAAAATTGTAATACAACTTTCAGCAACGGATCTCTTGGCTCTCGCATCGAT
GAAGAACGCAGCGAAATGCGATAAGTAATGTGAATTGCAGAATTCAGTGAATCATCGAAT
CTTTGAACGCATCTTGCGCTCCTTGGTATTCCGAGGAGCATGCCTGTTTGAGTGTCATTA
AATTCTCAACTCTCTTATACTTTTTTGTAAAAGAGAGCTTGGACTGTGGAGGCTTGCTGG
CCACTTTTTGGGGTCAGCTCCTCTGAAATGCATTAGCGGAACCGTTTGCAATCTGCCACA
AGTGTGATAAGTTATCTACACTGGCGAGGGGATTGCTCTCTGTAATGTTCAGCTTCTAAT
TGTCTCTACTTTGTGAGACTACTTTTGAATGCTTGACCTCAAATCAGGTAGGACTACCC-
GCTGAACTTAA
>BC11_36
TTTCCGTAGGTGAACCTGCGGAAGGATCATTATTGAATTATGTTTCTAGATAGGTTGTAG
CTGGCTCTTTAGAGCATGTGCACGCCTGTTTGGACTTCATTTTCATCCACCTGTGCACCT
ATTGTAGTCTTTGGTTGGGTTAGGAGGAAGTGGTCATTGTGTCAGCATCTGCTGGATGTG
AGGACTTGCATTGTGAAAGCTTTGCTGTCCTTGATGTGATCATGGAATCTCTTTCTCACT
AGAGTCTATGTCACTCATTATACTCTGTCGAATGTCATTGAATGTCTTTACATGGGCTTA
TATGCCTATGAAAATTGTAATACAACTTTCAGCAACGGATCTCTTGGCTCTCGCATCGAT
GAAGAACGCAGCGAAATGCGATAAGTAATGTGAATTGCAGAATTCAGTGAATCATCGAAT
CTTTGAACGCATCTTGCGCTCCTTGGTATTCCGAGGAGCATGCCTGTTTGAGTGTCATTA
AATTCTCAACTCTCTTATACTTTTTTGTAAAAGAGAGCTTGGACTGTGGAGGCTTGCTGG
CCACTTTTTGGGGTCAGCTCCTCTGAAATGCATTAGCGGAACCGTTTGCAATCTGCCACA
AGTGTGATAAGTTATCTACACTGGCGAGGGGATTGCTCTCTGTAATGTTCAGCTTCTAAT
TGTCTCTACTTTGTGAGACTACTTTTGAATGCTTGACCTCAAATCAGGTAGGACTACCC-
GCTGAACTTAA
>BC8-58
TTTCCGTAGGTGAACCTGCGGAAGGATCATTATTGAATTATGTTTCTAGATAGGTTGTAG
CTGGCTCTTTAGAGCATGTGCACGCCTGTTTGGACTTCATTTTCATCCACCTGTGCACCT
ATTGTAGTCTTTGGTTGGGTTAGGGGGAAGTGGTCATTGTGTCAGCATCTGCTGGATGTG
AGGACTTGCATTGTGAAAGCTTTGCTGTCCTTGATGTGATCATGGAATCTCTTTCTCACT
AGAGTCTATGTCACTCATTATACTCTGTCGAATGTCATTGAATGTCTTTACATGGGCTTG
TATGCCTATGAAAATTGTAATACAACTTTCAGCAACGGATCTCTTGGCTCTCGCATCGAT
GAAGGACGCAGCGAAATGCGATAAGTAATGTGAATTGCAGAATTCAGTGAATCATCGAAT
CTTTGAACGCATCTTGCGCTCCTTGGTATTCCGAGGAGCATGCCTGTTTGAGTGTCATTA
AATTCTCAACTCTCTTATACTTTTTTGTAAAAGAGAGCTTGGACTGTGGAGGCTTGCTGG
CCACTTTTTGGGGTCAGCTCCTCTGAAATGCATTAGCGGAACCGTTTGCGATCTGCCACA
TGTGTGATAAGTTATCTACACTGGCGAGGGGATTGCTCTCTGTAATGTTCAGCTTCTAAT
TGTCTCTACTTTGTGAGACTACTTTTGAATGCTTGACCTCAAATCAGGTAGGACTACCC-
GCTGAACTTAA
>BC5-18
TTTCCGTAGGTGAACCTGCGGAAGGATCATTATTGAATTATGTTTCTAGATAGGTTGTAG

CTGGCTCTTTAGAGCATGTGCACGCCTGTTTGGACTTCATTTTCATCCACCTGTGCACCT
ATTGTAGTCTTTGGTTGGGTTAGGGGGAAGTGGTCATTGTGTCAGCATCTGCTGGATGTG
AGGACTTGCATTGTGAAAGCTTTGCTGTCCTTGATGTGATCATGGAATCTCTTTCTCACT
AGAGTCTATGTCACTCATTATACTCTGTCGAATGTCATTGAATGTCTTTACATGGGCTTG
TATGCCTATGAAAATTGTAATACAACTTTCAGCAACGGATCTCTTGGCTCTCGCATCGAT
GAAGGACGCAGCGAAATGCGATAAGTAATGTGAATTGCAGAATTCAGTGAATCATCGAAT
CTTTGAACGCATCTTGCGCTCCTTGGTATTCCGAGGAGCATGCCTGTTTGAGTGTCATTA
AATTCTCAACGCTCTTATACTTTTTTGTAAAAGAGAGCTTGGACTGTGGAGGCTTGCTGG
CCACTTTTTGGGGTCAGCTCCTCTGAAATGCATTAGCGGAACCGTTTGCAATCTGCCACA
AGTGTGATAAGTTATCTACACTGGCGAGGGGATTGCTCTCTGTAATGTTCAGCTTCTAAT
TGTCTCTACTTTGTGAGACAACTTTTGAATGCTTGACCTCAAATCAGGTAGGACTACCC-
GCTGAACTTAA
>BC4-1
TTTCCGTAGGTGAACCTGCGGAAGGATCATTATTGAATTATGTTTCTAGATAGGTTGTAG
CTGGCTCTTTAGAGCATGTGCACGCCTGTTTGGACTTCATTTTCATCCACCTGTGCACCT
ATTGTAGTCTTTGGTTGGGTTAGGGGGAAGTGGTCATTGTGTCAGCATCTGCTGGATGTG
AGGACTTGCATTGTGAAAGCTTTGCTGTCCTTGATGTGATCATGGAATCTCTTTCTCACT
AGAGTCTATGTCACTCATTATACTCTGTCGAATGTCATTGAATGTCTTTACATGGGCTTG
TATGCCTATGAAAATTGTAATACAACTTTCAGCAACGGATCTCTTGGCTCTCGCATCGAT
GAAGGACGCAGCGAAATGCGATAAGTAATGTGAATTGCAGAATTCAGTGAATCATCGAAT
CTTTGAACGCATCTTGCGCTCCTTGGTATTCCGAGGAGCATGCCTGTTTGAGTGTCATTA
AATTCTCAACTCTCTTATACTTTTTTGTAAAAGAGAGCTTGGACTGTGGAGGCTTGCTGG
CCACTTTTTGGGGTCAGCTCCTCTGAAATGCATTAGCGGAACCGTTTGCAATCTGCCACC
AGTGTGATAAGTTATCTACACTGGCGAGGGGATTGCTCTCTGTAATGTTCAGCTTCTAAT
TGTCTCTACTTTGTGAGACAACTTTTGAATGCTTGACCTCAAATCAGGTAGGACTACCC-
GCTGAACTTAA
>BC8-42
TTTCCGTAGGTGAACCTGCGGAAGGATCATTATTGAATTATGTTTCTAGATAGGTTGTAG
CTGGCTCTTTAGAGCATGTGCACGCCTGTTTGGACTTCATTTTCATCCACCTGTGCACCT
ATTGTAGTCTTTGGTTGGGTTAGGGGGAAGTGGTCATTGTGTCAGCATCTGCTGGATGTG
AGGACTTGCATTGTGAAAGCTTTGCTGTCCTTGATGTGATCATGGAATCTCTTTCTCACT
AGAGTCTATGTCACTCATTATACTCTGTCGAATGTCATTGAATGTCTTTACATGGGCTTG
TATGCCTATGAAAATTGTAATACAACTTTCAGCAACGGATCTCTTGGCTCTCGCATCGAT
GAAGGACGCAGCGAAATGCGATAAGTAATGTGAATTGCAGAATTCAGTGAATCATCGAAT
CTTTGAACGCATCTTGCGCTCCTTGGTATTCCGAGGAGCATGCCTGTTTGAGTGTCATTA
AATTCTCAACTCTCTTATACTTTTTTGTAAAAGAGAGCTTGGACTGTGGAGGCTTGCTGG
CCACTTTTTGGGGTCAGCTCCTCTGAAATGCATTAGCGGAACCGTTTGCAATCTGCCACA
AGTGTGATAAGTTATCTACACTGGCGAGGGGATTGCTCTCTGTAATGTTCAGCTTCTAAT
TGTCTCTACTTTGTGAGACAAATTTTGAATGCTTGACCTCAAATCAGGTAGGACTACCC-
GCTGAACTTAA
>BC12_6
TTTCCGTAGGTGAACCTGCGGAAGGATCATTATTGAATTATGTTTCTAGATAGGTTGTAG
CTGGCTCTTTAGAGCATGTGCACGCCTGTTTGGACTTCATTTTCATCCACCTGTGCACCT
ATTGTAGTCTTTGGTTGGGTTAGGAGGAAGTGGTCATTGTGTCAGCATCTGCTGGATGTG
AGGACTTGCATTGTGAAAGCTTTGCTGTCCTTGATGTGATCATGGAATCTCTTTCTCACT
AGAGTCTATGTCACTCATTATACTCTGTCGAATGTCATTGAATGTCTTTACATGGGCTTA
TATGCCTATGAAAATTGTAATACAACTTTCAGCAACGGATCTCTTGGCTCTCGCATCGAT
GAAGAACGCAGCGAAATGCGATAAGTAATGTGAATTGCAGAATTCAGTGAATCATCGAAT
CTTTGAACGCATCTTGCGCTCCTTGGTATTCCGAGGAGCATGCCTGTTTGAGTGTCATTA
AATTCTCAACTCTCTTCTACTTTTTTGTAAAAGAGAGCTTGGACTGTGGAGGCTTGCTGG

CCACTTTTTGGGGTCAGCTCCTCTGAAATGCATTAGCGGAACCGTTTGCAATCTGCCACA
AGTGTGATAAGTTATCTACACTGGCGAGGGGATTGCTCTCTGTAATGTTCAGCTTCTAAT
TGTCTCTACTTTGTGAGACAACTTTTGAATGCTTGACCTCAAATCAGGTAGGACTACCC-
GCTGAACTTAA
>BC8-37
TTTCCGTAGGTGAACCTGCGGAAGGATCATTATTGAATTATGTTTCTAGATAGGTTGTAG
CTGGCTCTTTAGAGCATGTGCACGCCTGTTTGGACTTCATTTTCATCCACCTGTGAACCT
ATTGTAGTCTTTGGTTGGGTTAGGAGGAAGTGGTCATTGTGTCAGCATCTGCTGGATGTG
AGGACTTGCATTGTGAAAGCTTTGCTGTCCTTGATGTGATCATGGAATCTCTTTCTCACT
AGAGTCTATGTCACTCATTATACTCTGTCGAATGTCATTGAATGTCTTTACATGGGCATA
TATGCCTATGAAAATTGTAATACAACTTTCAGCAACGGATCTCTTGGCTCTCGCATCGAT
GAAGAACGCAGCGAAATGCGATAAGTAATGTGAATTGCAGAATTCAGTGAATCATCGAAT
CTTTGAACGCATCTTGCGCTCCTTGGTATTCCGAGGAGCATGCCTGTTTGAGTGTCATTA
AATTCTCAACTCTCTTATACTTTTTTGTAAAAGAGAGCTTGGACTGTGGAGGCTTGCTGG
CCACTTTTTGGGGTCAGCTCCTCTGAAATGCATTAGCGGAACCGTTTGCGATCTGCCACA
AGTGTGATAAGTTATCTACACTGGCGAGGGGATTGCTCTCTGTAATGTTCAGCTTCTAAT
TGTCTCTACTTTGTGAGACAACTTTTGAATGCTTGACCTCAAATCAGGTAGGACTACCC-
GCTGAACTTAA
>BC5-90
TTTCCGTAGGTGAACCTGCGGAAGGATCATTATTGAATTATGTTTCTAGATAGGTTGTAG
CTGGCTCTTTAGAGCATGTGCACGCCTGTTTGGACTTCATTTTCATCCACCTGTGCACCT
ATTGTAGTCTTTGGTTGGGTTAGGGGGAAGTGGTCATTGTGTCAGCATCTGCTGGATGTG
AGGACTTGCATTGTGAAAGCTTTGCTGTCCTTGATGTGATCATGGAATCTCTTTCTCACT
AGAGTCTATGTCACTCATTATACTCTGTCGAATGTCATTGAATGTCTTTACATGGGCTTG
TATGCCTATGAAAATTGTAATACAACTTTCAGCAACGGATCTCTTGGCTCTCGCATCGAT
GAAGGACGCAGCGAAATGCGATAAGTAATGTGAATTGCAGAATTCAGTGAATCATCGAAT
CTTTGAACGCATCTTGCGCTCCTTGGTATTCCGAGGAGCATGCCTGTTTGAGTGTCATTA
AATTCTCAACTCTCTTATACTTTTTTGTAAAAGAGAGCTTGGACTGTGGAGGCTTGCTGG
CCAC-TTTTGGGGTCAGCTCCTCTGAAATGCATTAGCGGAACCGTTTGCAATCTGCCACA
AGTGTGATAAGTTATCTACACTGGCGAGGGGATTGCTCTCTGTAATGTTCAGCTTCTAAT
TGTCTCTACTTTGTGAGACAACTTTTGAATGCTTGACCTCAAATCAGGTAGGACTACCC-
GCTGAACTTAA
>BC1-5
TTTCCGTAGGTGAACCTGCGGAAGGATCATTATTGAATTATGTTTCTAGATAGGTTGTAG
CTGGCTCTTTAGAGCATGTGCACGCCTGTTTGGACTTCATTTTCATCCACCTGTGCACCT
ATTGTAGTCTTTGGTTGGGTTAGGAGGAAGTGGTCATTGTGTCAGCATCTGCTGGATGTG
AGGACTTGCATTGTGAAAGCTTTGCTGTCCTTGATGTGATCATGGAATCTCTTTCTCACT
AGAGTCTATGTCACTCATTATACTCTGTCGAATGTCATTGAATGTCTTTACATGGGCTTA
TATGCCTATGAAAATTGTAATACAACTTTCAGCAACGGATCTCTTGGCTCTCGCATCGAT
GAAGAACGCAGCGAAATGCGATAAGTAATGTGAATTGCAGAATTCAGTGAATCATCGAAT
CTTTGAACGCATCTTGCGCTCCTTGGTATTCCGAGGAGCATGCCTGTTTGAGTGTCATTA
AATTCTCAACTCTCTTCTAC-TTTTTGTAAAAGAGAGCTTGGACTGTGGAGGCTTGCTGG
CCACTTTTTGGGGTCAGCTCCTCTGAAATGCATTAGCGGAACCGTTTGCGATCTGCCACA
AGTGTGATAAGTTATCTACACTGGCGAGGGGATTGCTCTCTGTAATGTTCAGCTTCTAAT
TGTCTCTACTTTGTGAGACTACTTTTGAATGCTTGACCTCAAATCAGGTAGGACTACCC-
GCTGAACTTAA
>BC1-7
TTTCCGTAGGTGAACCTGCGGAAGGATCATTATTGAATTATGTTTCTAGATAGGTTGTAG
CTGGCTCTTTAGAGCATGTGCACGCCTGTTTGGACTTCATTTTCATCCACCTGTGCACCT
ATTGTAGTCTTTGGTTGGGTTAGGAGGAAGTGGTCATTGTGTCAGCATCTGCTGGATGTG

AGGACTTGCATTGTGAAAGCTTTGCTGTCCTTGATGTGATCATGGAATCTCTTTCTCACT
AGAGTCTATGTCACTCATTATACTCTGTCGAATGTCATTGAATGTCTTTACATGGGCTTA
TATGCCTATGAAAATTGTAATACAACTTTCAGCAACGGATCTCTTGGCTCTCGCATCGAT
GAAGAACGCAGCGAAATGCGATAAGTAATGTGAATTGCAGAATTCAGTGAATCATCGAAT
CTTTGAACGCATCTTGCGCTCCTTGGTATTCCGAGGAGCATGCCTGTTTGAGTGTCATTA
AATTCTCAACTCTCTTCTAC-TTTTTGTAAAAGAGAGCTTGGACTGTGGAGGCTTGCTGG
CCACTTTTTGGGGTCAGCTCCTCTGAAATGCATTAGCGGAACCGTTTGCGATCTGCCACA
AGTGTGATAAGTTATCTACACTGGCGAGGGGATTGCTCTCTGTAATGTTCAGCTTCTAAT
TGTCTCTACTTTGTGAGACTACTTTTGAATGCTTGACCTCAAATCAGGTAGGACTACCC-
GCTGAACTTAA
>BC1-12
TTTCCGTAGGTGAACCTGCGGAAGGATCATTATTGAATTATGTTTCTAGATAGGTTGTAG
CTGGCTCTTTAGAGCATGTGCACGCCTGTTTGGACTTCATTTTCATCCACCTGTGCACCT
ATTGTAGTCTTTGGTTGGGTTAGGAGGAAGTGGTCATTGTGTCAGCATCTGCTGGATGTG
AGGACTTGCATTGTGAAAGCTTTGCTGTCCTTGATGTGATCATGGAATCTCTTTCTCACT
AGAGTCTATGTCACTCATTATACTCTGTCGAATGTCATTGAATGTCTTTACATGGGCTTA
TATGCCTATGAAAATTGTAATACAACTTTCAGCAACGGATCTCTTGGCTCTCGCATCGAT
GAAGAACGCAGCGAAATGCGATAAGTAATGTGAATTGCAGAATTCAGTGAATCATCGAAT
CTTTGAACGCATCTTGCGCTCCTTGGTATTCCGAGGAGCATGCCTGTTTGAGTGTCATTA
AATTCTCAACTCTCTTCTAC-TTTTTGTAAAAGAGAGCTTGGACTGTGGAGGCTTGCTGG
CCACTTTTTGGGGTCAGCTCCTCTGAAATGCATTAGCGGAACCGTTTGCGATCTGCCACA
AGTGTGATAAGTTATCTACACTGGCGAGGGGATTGCTCTCTGTAATGTTCAGCTTCTAAT
TGTCTCTACTTTGTGAGACTACTTTTGAATGCTTGACCTCAAATCAGGTAGGACTACCC-
GCTGAACTTAA
>BC1-21
TTTCCGTAGGTGAACCTGCGGAAGGATCATTATTGAATTATGTTTCTAGATAGGTTGTAG
CTGGCTCTTTAGAGCATGTGCACGCCTGTTTGGACTTCATTTTCATCCACCTGTGCACCT
ATTGTAGTCTTTGGTTGGGTTAGGAGGAAGTGGTCATTGTGTCAGCATCTGCTGGATGTG
AGGACTTGCATTGTGAAAGCTTTGCTGTCCTTGATGTGATCATGGAATCTCTTTCTCACT
AGAGTCTATGTCACTCATTATACTCTGTCGAATGTCATTGAATGTCTTTACATGGGCTTA
TATGCCTATGAAAATTGTAATACAACTTTCAGCAACGGATCTCTTGGCTCTCGCATCGAT
GAAGAACGCAGCGAAATGCGATAAGTAATGTGAATTGCAGAATTCAGTGAATCATCGAAT
CTTTGAACGCATCTTGCGCTCCTTGGTATTCCGAGGAGCATGCCTGTTTGAGTGTCATTA
AATTCTCAACTCTCTTCTAC-TTTTTGTAAAAGAGAGCTTGGACTGTGGAGGCTTGCTGG
CCACTTTTTGGGGTCAGCTCCTCTGAAATGCATTAGCGGAACCGTTTGCGATCTGCCACA
AGTGTGATAAGTTATCTACACTGGCGAGGGGATTGCTCTCTGTAATGTTCAGCTTCTAAT
TGTCTCTACTTTGTGAGACTACTTTTGAATGCTTGACCTCAAATCAGGTAGGACTACCC-
GCTGAACTTAA
>BC1-22
TTTCCGTAGGTGAACCTGCGGAAGGATCATTATTGAATTATGTTTCTAGATAGGTTGTAG
CTGGCTCTTTAGAGCATGTGCACGCCTGTTTGGACTTCATTTTCATCCACCTGTGCACCT
ATTGTAGTCTTTGGTTGGGTTAGGAGGAAGTGGTCATTGTGTCAGCATCTGCTGGATGTG
AGGACTTGCATTGTGAAAGCTTTGCTGTCCTTGATGTGATCATGGAATCTCTTTCTCACT
AGAGTCTATGTCACTCATTATACTCTGTCGAATGTCATTGAATGTCTTTACATGGGCTTA
TATGCCTATGAAAATTGTAATACAACTTTCAGCAACGGATCTCTTGGCTCTCGCATCGAT
GAAGAACGCAGCGAAATGCGATAAGTAATGTGAATTGCAGAATTCAGTGAATCATCGAAT
CTTTGAACGCATCTTGCGCTCCTTGGTATTCCGAGGAGCATGCCTGTTTGAGTGTCATTA
AATTCTCAACTCTCTTCTAC-TTTTTGTAAAAGAGAGCTTGGACTGTGGAGGCTTGCTGG
CCACTTTTTGGGGTCAGCTCCTCTGAAATGCATTAGCGGAACCGTTTGCGATCTGCCACA
AGTGTGATAAGTTATCTACACTGGCGAGGGGATTGCTCTCTGTAATGTTCAGCTTCTAAT

TGTCTCTACTTTGTGAGACTACTTTTGAATGCTTGACCTCAAATCAGGTAGGACTACCC-
GCTGAACTTAA
>BC1-28
TTTCCGTAGGTGAACCTGCGGAAGGATCATTATTGAATTATGTTTCTAGATAGGTTGTAG
CTGGCTCTTTAGAGCATGTGCACGCCTGTTTGGACTTCATTTTCATCCACCTGTGCACCT
ATTGTAGTCTTTGGTTGGGTTAGGAGGAAGTGGTCATTGTGTCAGCATCTGCTGGATGTG
AGGACTTGCATTGTGAAAGCTTTGCTGTCCTTGATGTGATCATGGAATCTCTTTCTCACT
AGAGTCTATGTCACTCATTATACTCTGTCGAATGTCATTGAATGTCTTTACATGGGCTTA
TATGCCTATGAAAATTGTAATACAACTTTCAGCAACGGATCTCTTGGCTCTCGCATCGAT
GAAGAACGCAGCGAAATGCGATAAGTAATGTGAATTGCAGAATTCAGTGAATCATCGAAT
CTTTGAACGCATCTTGCGCTCCTTGGTATTCCGAGGAGCATGCCTGTTTGAGTGTCATTA
AATTCTCAACTCTCTTCTAC-TTTTTGTAAAAGAGAGCTTGGACTGTGGAGGCTTGCTGG
CCACTTTTTGGGGTCAGCTCCTCTGAAATGCATTAGCGGAACCGTTTGCGATCTGCCACA
AGTGTGATAAGTTATCTACACTGGCGAGGGGATTGCTCTCTGTAATGTTCAGCTTCTAAT
TGTCTCTACTTTGTGAGACTACTTTTGAATGCTTGACCTCAAATCAGGTAGGACTACCC-
GCTGAACTTAA
>BC1-30
TTTCCGTAGGTGAACCTGCGGAAGGATCATTATTGAATTATGTTTCTAGATAGGTTGTAG
CTGGCTCTTTAGAGCATGTGCACGCCTGTTTGGACTTCATTTTCATCCACCTGTGCACCT
ATTGTAGTCTTTGGTTGGGTTAGGAGGAAGTGGTCATTGTGTCAGCATCTGCTGGATGTG
AGGACTTGCATTGTGAAAGCTTTGCTGTCCTTGATGTGATCATGGAATCTCTTTCTCACT
AGAGTCTATGTCACTCATTATACTCTGTCGAATGTCATTGAATGTCTTTACATGGGCTTA
TATGCCTATGAAAATTGTAATACAACTTTCAGCAACGGATCTCTTGGCTCTCGCATCGAT
GAAGAACGCAGCGAAATGCGATAAGTAATGTGAATTGCAGAATTCAGTGAATCATCGAAT
CTTTGAACGCATCTTGCGCTCCTTGGTATTCCGAGGAGCATGCCTGTTTGAGTGTCATTA
AATTCTCAACTCTCTTCTAC-TTTTTGTAAAAGAGAGCTTGGACTGTGGAGGCTTGCTGG
CCACTTTTTGGGGTCAGCTCCTCTGAAATGCATTAGCGGAACCGTTTGCGATCTGCCACA
AGTGTGATAAGTTATCTACACTGGCGAGGGGATTGCTCTCTGTAATGTTCAGCTTCTAAT
TGTCTCTACTTTGTGAGACTACTTTTGAATGCTTGACCTCAAATCAGGTAGGACTACCC-
GCTGAACTTAA
>BC1-36
TTTCCGTAGGTGAACCTGCGGAAGGATCATTATTGAATTATGTTTCTAGATAGGTTGTAG
CTGGCTCTTTAGAGCATGTGCACGCCTGTTTGGACTTCATTTTCATCCACCTGTGCACCT
ATTGTAGTCTTTGGTTGGGTTAGGAGGAAGTGGTCATTGTGTCAGCATCTGCTGGATGTG
AGGACTTGCATTGTGAAAGCTTTGCTGTCCTTGATGTGATCATGGAATCTCTTTCTCACT
AGAGTCTATGTCACTCATTATACTCTGTCGAATGTCATTGAATGTCTTTACATGGGCTTA
TATGCCTATGAAAATTGTAATACAACTTTCAGCAACGGATCTCTTGGCTCTCGCATCGAT
GAAGAACGCAGCGAAATGCGATAAGTAATGTGAATTGCAGAATTCAGTGAATCATCGAAT
CTTTGAACGCATCTTGCGCTCCTTGGTATTCCGAGGAGCATGCCTGTTTGAGTGTCATTA
AATTCTCAACTCTCTTCTAC-TTTTTGTAAAAGAGAGCTTGGACTGTGGAGGCTTGCTGG
CCACTTTTTGGGGTCAGCTCCTCTGAAATGCATTAGCGGAACCGTTTGCGATCTGCCACA
AGTGTGATAAGTTATCTACACTGGCGAGGGGATTGCTCTCTGTAATGTTCAGCTTCTAAT
TGTCTCTACTTTGTGAGACTACTTTTGAATGCTTGACCTCAAATCAGGTAGGACTACCC-
GCTGAACTTAA
>BC2-2
TTTCCGTAGGTGAACCTGCGGAAGGATCATTATTGAATTATGTTTCTAGATAGGTTGTAG
CTGGCTCTTTAGAGCATGTGCACGCCTGTTTGGACTTCATTTTCATCCACCTGTGCACCT
ATTGTAGTCTTTGGTTGGGTTAGGAGGAAGTGGTCATTGTGTCAGCATCTGCTGGATGTG
AGGACTTGCATTGTGAAAGCTTTGCTGTCCTTGATGTGATCATGGAATCTCTTTCTCACT
AGAGTCTATGTCACTCATTATACTCTGTCGAATGTCATTGAATGTCTTTACATGGGCTTA

TATGCCTATGAAAATTGTAATACAACTTTCAGCAACGGATCTCTTGGCTCTCGCATCGAT
GAAGAACGCAGCGAAATGCGATAAGTAATGTGAATTGCAGAATTCAGTGAATCATCGAAT
CTTTGAACGCATCTTGCGCTCCTTGGTATTCCGAGGAGCATGCCTGTTTGAGTGTCATTA
AATTCTCAACTCTCTTCTAC-TTTTTGTAAAAGAGAGCTTGGACTGTGGAGGCTTGCTGG
CCACTTTTTGGGGTCAGCTCCTCTGAAATGCATTAGCGGAACCGTTTGCGATCTGCCACA
AGTGTGATAAGTTATCTACACTGGCGAGGGGATTGCTCTCTGTAATGTTCAGCTTCTAAT
TGTCTCTACTTTGTGAGACTACTTTTGAATGCTTGACCTCAAATCAGGTAGGACTACCC-
GCTGAACTTAA
>BC2-3
TTTCCGTAGGTGAACCTGCGGAAGGATCATTATTGAATTATGTTTCTAGATAGGTTGTAG
CTGGCTCTTTAGAGCATGTGCACGCCTGTTTGGACTTCATTTTCATCCACCTGTGCACCT
ATTGTAGTCTTTGGTTGGGTTAGGAGGAAGTGGTCATTGTGTCAGCATCTGCTGGATGTG
AGGACTTGCATTGTGAAAGCTTTGCTGTCCTTGATGTGATCATGGAATCTCTTTCTCACT
AGAGTCTATGTCACTCATTATACTCTGTCGAATGTCATTGAATGTCTTTACATGGGCTTA
TATGCCTATGAAAATTGTAATACAACTTTCAGCAACGGATCTCTTGGCTCTCGCATCGAT
GAAGAACGCAGCGAAATGCGATAAGTAATGTGAATTGCAGAATTCAGTGAATCATCGAAT
CTTTGAACGCATCTTGCGCTCCTTGGTATTCCGAGGAGCATGCCTGTTTGAGTGTCATTA
AATTCTCAACTCTCTTCTAC-TTTTTGTAAAAGAGAGCTTGGACTGTGGAGGCTTGCTGG
CCACTTTTTGGGGTCAGCTCCTCTGAAATGCATTAGCGGAACCGTTTGCGATCTGCCACA
AGTGTGATAAGTTATCTACACTGGCGAGGGGATTGCTCTCTGTAATGTTCAGCTTCTAAT
TGTCTCTACTTTGTGAGACTACTTTTGAATGCTTGACCTCAAATCAGGTAGGACTACCC-
GCTGAACTTAA
>BC2-5
TTTCCGTAGGTGAACCTGCGGAAGGATCATTATTGAATTATGTTTCTAGATAGGTTGTAG
CTGGCTCTTTAGAGCATGTGCACGCCTGTTTGGACTTCATTTTCATCCACCTGTGCACCT
ATTGTAGTCTTTGGTTGGGTTAGGAGGAAGTGGTCATTGTGTCAGCATCTGCTGGATGTG
AGGACTTGCATTGTGAAAGCTTTGCTGTCCTTGATGTGATCATGGAATCTCTTTCTCACT
AGAGTCTATGTCACTCATTATACTCTGTCGAATGTCATTGAATGTCTTTACATGGGCTTA
TATGCCTATGAAAATTGTAATACAACTTTCAGCAACGGATCTCTTGGCTCTCGCATCGAT
GAAGAACGCAGCGAAATGCGATAAGTAATGTGAATTGCAGAATTCAGTGAATCATCGAAT
CTTTGAACGCATCTTGCGCTCCTTGGTATTCCGAGGAGCATGCCTGTTTGAGTGTCATTA
AATTCTCAACTCTCTTCTAC-TTTTTGTAAAAGAGAGCTTGGACTGTGGAGGCTTGCTGG
CCACTTTTTGGGGTCAGCTCCTCTGAAATGCATTAGCGGAACCGTTTGCGATCTGCCACA
AGTGTGATAAGTTATCTACACTGGCGAGGGGATTGCTCTCTGTAATGTTCAGCTTCTAAT
TGTCTCTACTTTGTGAGACTACTTTTGAATGCTTGACCTCAAATCAGGTAGGACTACCC-
GCTGAACTTAA
>BC2-6
TTTCCGTAGGTGAACCTGCGGAAGGATCATTATTGAATTATGTTTCTAGATAGGTTGTAG
CTGGCTCTTTAGAGCATGTGCACGCCTGTTTGGACTTCATTTTCATCCACCTGTGCACCT
ATTGTAGTCTTTGGTTGGGTTAGGAGGAAGTGGTCATTGTGTCAGCATCTGCTGGATGTG
AGGACTTGCATTGTGAAAGCTTTGCTGTCCTTGATGTGATCATGGAATCTCTTTCTCACT
AGAGTCTATGTCACTCATTATACTCTGTCGAATGTCATTGAATGTCTTTACATGGGCTTA
TATGCCTATGAAAATTGTAATACAACTTTCAGCAACGGATCTCTTGGCTCTCGCATCGAT
GAAGAACGCAGCGAAATGCGATAAGTAATGTGAATTGCAGAATTCAGTGAATCATCGAAT
CTTTGAACGCATCTTGCGCTCCTTGGTATTCCGAGGAGCATGCCTGTTTGAGTGTCATTA
AATTCTCAACTCTCTTCTAC-TTTTTGTAAAAGAGAGCTTGGACTGTGGAGGCTTGCTGG
CCACTTTTTGGGGTCAGCTCCTCTGAAATGCATTAGCGGAACCGTTTGCGATCTGCCACA
AGTGTGATAAGTTATCTACACTGGCGAGGGGATTGCTCTCTGTAATGTTCAGCTTCTAAT
TGTCTCTACTTTGTGAGACTACTTTTGAATGCTTGACCTCAAATCAGGTAGGACTACCC-
GCTGAACTTAA

>BC2-7
TTTCCGTAGGTGAACCTGCGGAAGGATCATTATTGAATTATGTTTCTAGATAGGTTGTAG
CTGGCTCTTTAGAGCATGTGCACGCCTGTTTGGACTTCATTTTCATCCACCTGTGCACCT
ATTGTAGTCTTTGGTTGGGTTAGGAGGAAGTGGTCATTGTGTCAGCATCTGCTGGATGTG
AGGACTTGCATTGTGAAAGCTTTGCTGTCCTTGATGTGATCATGGAATCTCTTTCTCACT
AGAGTCTATGTCACTCATTATACTCTGTCGAATGTCATTGAATGTCTTTACATGGGCTTA
TATGCCTATGAAAATTGTAATACAACTTTCAGCAACGGATCTCTTGGCTCTCGCATCGAT
GAAGAACGCAGCGAAATGCGATAAGTAATGTGAATTGCAGAATTCAGTGAATCATCGAAT
CTTTGAACGCATCTTGCGCTCCTTGGTATTCCGAGGAGCATGCCTGTTTGAGTGTCATTA
AATTCTCAACTCTCTTCTAC-TTTTTGTAAAAGAGAGCTTGGACTGTGGAGGCTTGCTGG
CCACTTTTTGGGGTCAGCTCCTCTGAAATGCATTAGCGGAACCGTTTGCGATCTGCCACA
AGTGTGATAAGTTATCTACACTGGCGAGGGGATTGCTCTCTGTAATGTTCAGCTTCTAAT
TGTCTCTACTTTGTGAGACTACTTTTGAATGCTTGACCTCAAATCAGGTAGGACTACCC-
GCTGAACTTAA
>BC2-8
TTTCCGTAGGTGAACCTGCGGAAGGATCATTATTGAATTATGTTTCTAGATAGGTTGTAG
CTGGCTCTTTAGAGCATGTGCACGCCTGTTTGGACTTCATTTTCATCCACCTGTGCACCT
ATTGTAGTCTTTGGTTGGGTTAGGAGGAAGTGGTCATTGTGTCAGCATCTGCTGGATGTG
AGGACTTGCATTGTGAAAGCTTTGCTGTCCTTGATGTGATCATGGAATCTCTTTCTCACT
AGAGTCTATGTCACTCATTATACTCTGTCGAATGTCATTGAATGTCTTTACATGGGCTTA
TATGCCTATGAAAATTGTAATACAACTTTCAGCAACGGATCTCTTGGCTCTCGCATCGAT
GAAGAACGCAGCGAAATGCGATAAGTAATGTGAATTGCAGAATTCAGTGAATCATCGAAT
CTTTGAACGCATCTTGCGCTCCTTGGTATTCCGAGGAGCATGCCTGTTTGAGTGTCATTA
AATTCTCAACTCTCTTCTAC-TTTTTGTAAAAGAGAGCTTGGACTGTGGAGGCTTGCTGG
CCACTTTTTGGGGTCAGCTCCTCTGAAATGCATTAGCGGAACCGTTTGCGATCTGCCACA
AGTGTGATAAGTTATCTACACTGGCGAGGGGATTGCTCTCTGTAATGTTCAGCTTCTAAT
TGTCTCTACTTTGTGAGACTACTTTTGAATGCTTGACCTCAAATCAGGTAGGACTACCC-
GCTGAACTTAA
>BC2-9
TTTCCGTAGGTGAACCTGCGGAAGGATCATTATTGAATTATGTTTCTAGATAGGTTGTAG
CTGGCTCTTTAGAGCATGTGCACGCCTGTTTGGACTTCATTTTCATCCACCTGTGCACCT
ATTGTAGTCTTTGGTTGGGTTAGGAGGAAGTGGTCATTGTGTCAGCATCTGCTGGATGTG
AGGACTTGCATTGTGAAAGCTTTGCTGTCCTTGATGTGATCATGGAATCTCTTTCTCACT
AGAGTCTATGTCACTCATTATACTCTGTCGAATGTCATTGAATGTCTTTACATGGGCTTA
TATGCCTATGAAAATTGTAATACAACTTTCAGCAACGGATCTCTTGGCTCTCGCATCGAT
GAAGAACGCAGCGAAATGCGATAAGTAATGTGAATTGCAGAATTCAGTGAATCATCGAAT
CTTTGAACGCATCTTGCGCTCCTTGGTATTCCGAGGAGCATGCCTGTTTGAGTGTCATTA
AATTCTCAACTCTCTTCTAC-TTTTTGTAAAAGAGAGCTTGGACTGTGGAGGCTTGCTGG
CCACTTTTTGGGGTCAGCTCCTCTGAAATGCATTAGCGGAACCGTTTGCGATCTGCCACA
AGTGTGATAAGTTATCTACACTGGCGAGGGGATTGCTCTCTGTAATGTTCAGCTTCTAAT
TGTCTCTACTTTGTGAGACTACTTTTGAATGCTTGACCTCAAATCAGGTAGGACTACCC-
GCTGAACTTAA
>BC2-10
TTTCCGTAGGTGAACCTGCGGAAGGATCATTATTGAATTATGTTTCTAGATAGGTTGTAG
CTGGCTCTTTAGAGCATGTGCACGCCTGTTTGGACTTCATTTTCATCCACCTGTGCACCT
ATTGTAGTCTTTGGTTGGGTTAGGAGGAAGTGGTCATTGTGTCAGCATCTGCTGGATGTG
AGGACTTGCATTGTGAAAGCTTTGCTGTCCTTGATGTGATCATGGAATCTCTTTCTCACT
AGAGTCTATGTCACTCATTATACTCTGTCGAATGTCATTGAATGTCTTTACATGGGCTTA
TATGCCTATGAAAATTGTAATACAACTTTCAGCAACGGATCTCTTGGCTCTCGCATCGAT
GAAGAACGCAGCGAAATGCGATAAGTAATGTGAATTGCAGAATTCAGTGAATCATCGAAT

CTTTGAACGCATCTTGCGCTCCTTGGTATTCCGAGGAGCATGCCTGTTTGAGTGTCATTA
AATTCTCAACTCTCTTCTAC-TTTTTGTAAAAGAGAGCTTGGACTGTGGAGGCTTGCTGG
CCACTTTTTGGGGTCAGCTCCTCTGAAATGCATTAGCGGAACCGTTTGCGATCTGCCACA
AGTGTGATAAGTTATCTACACTGGCGAGGGGATTGCTCTCTGTAATGTTCAGCTTCTAAT
TGTCTCTACTTTGTGAGACTACTTTTGAATGCTTGACCTCAAATCAGGTAGGACTACCC-
GCTGAACTTAA
>BC2-11
TTTCCGTAGGTGAACCTGCGGAAGGATCATTATTGAATTATGTTTCTAGATAGGTTGTAG
CTGGCTCTTTAGAGCATGTGCACGCCTGTTTGGACTTCATTTTCATCCACCTGTGCACCT
ATTGTAGTCTTTGGTTGGGTTAGGAGGAAGTGGTCATTGTGTCAGCATCTGCTGGATGTG
AGGACTTGCATTGTGAAAGCTTTGCTGTCCTTGATGTGATCATGGAATCTCTTTCTCACT
AGAGTCTATGTCACTCATTATACTCTGTCGAATGTCATTGAATGTCTTTACATGGGCTTA
TATGCCTATGAAAATTGTAATACAACTTTCAGCAACGGATCTCTTGGCTCTCGCATCGAT
GAAGAACGCAGCGAAATGCGATAAGTAATGTGAATTGCAGAATTCAGTGAATCATCGAAT
CTTTGAACGCATCTTGCGCTCCTTGGTATTCCGAGGAGCATGCCTGTTTGAGTGTCATTA
AATTCTCAACTCTCTTCTAC-TTTTTGTAAAAGAGAGCTTGGACTGTGGAGGCTTGCTGG
CCACTTTTTGGGGTCAGCTCCTCTGAAATGCATTAGCGGAACCGTTTGCGATCTGCCACA
AGTGTGATAAGTTATCTACACTGGCGAGGGGATTGCTCTCTGTAATGTTCAGCTTCTAAT
TGTCTCTACTTTGTGAGACTACTTTTGAATGCTTGACCTCAAATCAGGTAGGACTACCC-
GCTGAACTTAA
>BC2-13
TTTCCGTAGGTGAACCTGCGGAAGGATCATTATTGAATTATGTTTCTAGATAGGTTGTAG
CTGGCTCTTTAGAGCATGTGCACGCCTGTTTGGACTTCATTTTCATCCACCTGTGCACCT
ATTGTAGTCTTTGGTTGGGTTAGGAGGAAGTGGTCATTGTGTCAGCATCTGCTGGATGTG
AGGACTTGCATTGTGAAAGCTTTGCTGTCCTTGATGTGATCATGGAATCTCTTTCTCACT
AGAGTCTATGTCACTCATTATACTCTGTCGAATGTCATTGAATGTCTTTACATGGGCTTA
TATGCCTATGAAAATTGTAATACAACTTTCAGCAACGGATCTCTTGGCTCTCGCATCGAT
GAAGAACGCAGCGAAATGCGATAAGTAATGTGAATTGCAGAATTCAGTGAATCATCGAAT
CTTTGAACGCATCTTGCGCTCCTTGGTATTCCGAGGAGCATGCCTGTTTGAGTGTCATTA
AATTCTCAACTCTCTTCTAC-TTTTTGTAAAAGAGAGCTTGGACTGTGGAGGCTTGCTGG
CCACTTTTTGGGGTCAGCTCCTCTGAAATGCATTAGCGGAACCGTTTGCGATCTGCCACA
AGTGTGATAAGTTATCTACACTGGCGAGGGGATTGCTCTCTGTAATGTTCAGCTTCTAAT
TGTCTCTACTTTGTGAGACTACTTTTGAATGCTTGACCTCAAATCAGGTAGGACTACCC-
GCTGAACTTAA
>BC2-17
TTTCCGTAGGTGAACCTGCGGAAGGATCATTATTGAATTATGTTTCTAGATAGGTTGTAG
CTGGCTCTTTAGAGCATGTGCACGCCTGTTTGGACTTCATTTTCATCCACCTGTGCACCT
ATTGTAGTCTTTGGTTGGGTTAGGAGGAAGTGGTCATTGTGTCAGCATCTGCTGGATGTG
AGGACTTGCATTGTGAAAGCTTTGCTGTCCTTGATGTGATCATGGAATCTCTTTCTCACT
AGAGTCTATGTCACTCATTATACTCTGTCGAATGTCATTGAATGTCTTTACATGGGCTTA
TATGCCTATGAAAATTGTAATACAACTTTCAGCAACGGATCTCTTGGCTCTCGCATCGAT
GAAGAACGCAGCGAAATGCGATAAGTAATGTGAATTGCAGAATTCAGTGAATCATCGAAT
CTTTGAACGCATCTTGCGCTCCTTGGTATTCCGAGGAGCATGCCTGTTTGAGTGTCATTA
AATTCTCAACTCTCTTCTAC-TTTTTGTAAAAGAGAGCTTGGACTGTGGAGGCTTGCTGG
CCACTTTTTGGGGTCAGCTCCTCTGAAATGCATTAGCGGAACCGTTTGCGATCTGCCACA
AGTGTGATAAGTTATCTACACTGGCGAGGGGATTGCTCTCTGTAATGTTCAGCTTCTAAT
TGTCTCTACTTTGTGAGACTACTTTTGAATGCTTGACCTCAAATCAGGTAGGACTACCC-
GCTGAACTTAA
>BC2-19
TTTCCGTAGGTGAACCTGCGGAAGGATCATTATTGAATTATGTTTCTAGATAGGTTGTAG

CTGGCTCTTTAGAGCATGTGCACGCCTGTTTGGACTTCATTTTCATCCACCTGTGCACCT
ATTGTAGTCTTTGGTTGGGTTAGGAGGAAGTGGTCATTGTGTCAGCATCTGCTGGATGTG
AGGACTTGCATTGTGAAAGCTTTGCTGTCCTTGATGTGATCATGGAATCTCTTTCTCACT
AGAGTCTATGTCACTCATTATACTCTGTCGAATGTCATTGAATGTCTTTACATGGGCTTA
TATGCCTATGAAAATTGTAATACAACTTTCAGCAACGGATCTCTTGGCTCTCGCATCGAT
GAAGAACGCAGCGAAATGCGATAAGTAATGTGAATTGCAGAATTCAGTGAATCATCGAAT
CTTTGAACGCATCTTGCGCTCCTTGGTATTCCGAGGAGCATGCCTGTTTGAGTGTCATTA
AATTCTCAACTCTCTTCTAC-TTTTTGTAAAAGAGAGCTTGGACTGTGGAGGCTTGCTGG
CCACTTTTTGGGGTCAGCTCCTCTGAAATGCATTAGCGGAACCGTTTGCGATCTGCCACA
AGTGTGATAAGTTATCTACACTGGCGAGGGGATTGCTCTCTGTAATGTTCAGCTTCTAAT
TGTCTCTACTTTGTGAGACTACTTTTGAATGCTTGACCTCAAATCAGGTAGGACTACCC-
GCTGAACTTAA
>BC2-20
TTTCCGTAGGTGAACCTGCGGAAGGATCATTATTGAATTATGTTTCTAGATAGGTTGTAG
CTGGCTCTTTAGAGCATGTGCACGCCTGTTTGGACTTCATTTTCATCCACCTGTGCACCT
ATTGTAGTCTTTGGTTGGGTTAGGAGGAAGTGGTCATTGTGTCAGCATCTGCTGGATGTG
AGGACTTGCATTGTGAAAGCTTTGCTGTCCTTGATGTGATCATGGAATCTCTTTCTCACT
AGAGTCTATGTCACTCATTATACTCTGTCGAATGTCATTGAATGTCTTTACATGGGCTTA
TATGCCTATGAAAATTGTAATACAACTTTCAGCAACGGATCTCTTGGCTCTCGCATCGAT
GAAGAACGCAGCGAAATGCGATAAGTAATGTGAATTGCAGAATTCAGTGAATCATCGAAT
CTTTGAACGCATCTTGCGCTCCTTGGTATTCCGAGGAGCATGCCTGTTTGAGTGTCATTA
AATTCTCAACTCTCTTCTAC-TTTTTGTAAAAGAGAGCTTGGACTGTGGAGGCTTGCTGG
CCACTTTTTGGGGTCAGCTCCTCTGAAATGCATTAGCGGAACCGTTTGCGATCTGCCACA
AGTGTGATAAGTTATCTACACTGGCGAGGGGATTGCTCTCTGTAATGTTCAGCTTCTAAT
TGTCTCTACTTTGTGAGACTACTTTTGAATGCTTGACCTCAAATCAGGTAGGACTACCC-
GCTGAACTTAA
>BC2-21
TTTCCGTAGGTGAACCTGCGGAAGGATCATTATTGAATTATGTTTCTAGATAGGTTGTAG
CTGGCTCTTTAGAGCATGTGCACGCCTGTTTGGACTTCATTTTCATCCACCTGTGCACCT
ATTGTAGTCTTTGGTTGGGTTAGGAGGAAGTGGTCATTGTGTCAGCATCTGCTGGATGTG
AGGACTTGCATTGTGAAAGCTTTGCTGTCCTTGATGTGATCATGGAATCTCTTTCTCACT
AGAGTCTATGTCACTCATTATACTCTGTCGAATGTCATTGAATGTCTTTACATGGGCTTA
TATGCCTATGAAAATTGTAATACAACTTTCAGCAACGGATCTCTTGGCTCTCGCATCGAT
GAAGAACGCAGCGAAATGCGATAAGTAATGTGAATTGCAGAATTCAGTGAATCATCGAAT
CTTTGAACGCATCTTGCGCTCCTTGGTATTCCGAGGAGCATGCCTGTTTGAGTGTCATTA
AATTCTCAACTCTCTTCTAC-TTTTTGTAAAAGAGAGCTTGGACTGTGGAGGCTTGCTGG
CCACTTTTTGGGGTCAGCTCCTCTGAAATGCATTAGCGGAACCGTTTGCGATCTGCCACA
AGTGTGATAAGTTATCTACACTGGCGAGGGGATTGCTCTCTGTAATGTTCAGCTTCTAAT
TGTCTCTACTTTGTGAGACTACTTTTGAATGCTTGACCTCAAATCAGGTAGGACTACCC-
GCTGAACTTAA
>BC2-22
TTTCCGTAGGTGAACCTGCGGAAGGATCATTATTGAATTATGTTTCTAGATAGGTTGTAG
CTGGCTCTTTAGAGCATGTGCACGCCTGTTTGGACTTCATTTTCATCCACCTGTGCACCT
ATTGTAGTCTTTGGTTGGGTTAGGAGGAAGTGGTCATTGTGTCAGCATCTGCTGGATGTG
AGGACTTGCATTGTGAAAGCTTTGCTGTCCTTGATGTGATCATGGAATCTCTTTCTCACT
AGAGTCTATGTCACTCATTATACTCTGTCGAATGTCATTGAATGTCTTTACATGGGCTTA
TATGCCTATGAAAATTGTAATACAACTTTCAGCAACGGATCTCTTGGCTCTCGCATCGAT
GAAGAACGCAGCGAAATGCGATAAGTAATGTGAATTGCAGAATTCAGTGAATCATCGAAT
CTTTGAACGCATCTTGCGCTCCTTGGTATTCCGAGGAGCATGCCTGTTTGAGTGTCATTA
AATTCTCAACTCTCTTCTAC-TTTTTGTAAAAGAGAGCTTGGACTGTGGAGGCTTGCTGG

CCACTTTTTGGGGTCAGCTCCTCTGAAATGCATTAGCGGAACCGTTTGCGATCTGCCACA
AGTGTGATAAGTTATCTACACTGGCGAGGGGATTGCTCTCTGTAATGTTCAGCTTCTAAT
TGTCTCTACTTTGTGAGACTACTTTTGAATGCTTGACCTCAAATCAGGTAGGACTACCC-
GCTGAACTTAA
>BC2-25
TTTCCGTAGGTGAACCTGCGGAAGGATCATTATTGAATTATGTTTCTAGATAGGTTGTAG
CTGGCTCTTTAGAGCATGTGCACGCCTGTTTGGACTTCATTTTCATCCACCTGTGCACCT
ATTGTAGTCTTTGGTTGGGTTAGGAGGAAGTGGTCATTGTGTCAGCATCTGCTGGATGTG
AGGACTTGCATTGTGAAAGCTTTGCTGTCCTTGATGTGATCATGGAATCTCTTTCTCACT
AGAGTCTATGTCACTCATTATACTCTGTCGAATGTCATTGAATGTCTTTACATGGGCTTA
TATGCCTATGAAAATTGTAATACAACTTTCAGCAACGGATCTCTTGGCTCTCGCATCGAT
GAAGAACGCAGCGAAATGCGATAAGTAATGTGAATTGCAGAATTCAGTGAATCATCGAAT
CTTTGAACGCATCTTGCGCTCCTTGGTATTCCGAGGAGCATGCCTGTTTGAGTGTCATTA
AATTCTCAACTCTCTTCTAC-TTTTTGTAAAAGAGAGCTTGGACTGTGGAGGCTTGCTGG
CCACTTTTTGGGGTCAGCTCCTCTGAAATGCATTAGCGGAACCGTTTGCGATCTGCCACA
AGTGTGATAAGTTATCTACACTGGCGAGGGGATTGCTCTCTGTAATGTTCAGCTTCTAAT
TGTCTCTACTTTGTGAGACTACTTTTGAATGCTTGACCTCAAATCAGGTAGGACTACCC-
GCTGAACTTAA
>BC2-27
TTTCCGTAGGTGAACCTGCGGAAGGATCATTATTGAATTATGTTTCTAGATAGGTTGTAG
CTGGCTCTTTAGAGCATGTGCACGCCTGTTTGGACTTCATTTTCATCCACCTGTGCACCT
ATTGTAGTCTTTGGTTGGGTTAGGAGGAAGTGGTCATTGTGTCAGCATCTGCTGGATGTG
AGGACTTGCATTGTGAAAGCTTTGCTGTCCTTGATGTGATCATGGAATCTCTTTCTCACT
AGAGTCTATGTCACTCATTATACTCTGTCGAATGTCATTGAATGTCTTTACATGGGCTTA
TATGCCTATGAAAATTGTAATACAACTTTCAGCAACGGATCTCTTGGCTCTCGCATCGAT
GAAGAACGCAGCGAAATGCGATAAGTAATGTGAATTGCAGAATTCAGTGAATCATCGAAT
CTTTGAACGCATCTTGCGCTCCTTGGTATTCCGAGGAGCATGCCTGTTTGAGTGTCATTA
AATTCTCAACTCTCTTCTAC-TTTTTGTAAAAGAGAGCTTGGACTGTGGAGGCTTGCTGG
CCACTTTTTGGGGTCAGCTCCTCTGAAATGCATTAGCGGAACCGTTTGCGATCTGCCACA
AGTGTGATAAGTTATCTACACTGGCGAGGGGATTGCTCTCTGTAATGTTCAGCTTCTAAT
TGTCTCTACTTTGTGAGACTACTTTTGAATGCTTGACCTCAAATCAGGTAGGACTACCC-
GCTGAACTTAA
>BC2-31
TTTCCGTAGGTGAACCTGCGGAAGGATCATTATTGAATTATGTTTCTAGATAGGTTGTAG
CTGGCTCTTTAGAGCATGTGCACGCCTGTTTGGACTTCATTTTCATCCACCTGTGCACCT
ATTGTAGTCTTTGGTTGGGTTAGGAGGAAGTGGTCATTGTGTCAGCATCTGCTGGATGTG
AGGACTTGCATTGTGAAAGCTTTGCTGTCCTTGATGTGATCATGGAATCTCTTTCTCACT
AGAGTCTATGTCACTCATTATACTCTGTCGAATGTCATTGAATGTCTTTACATGGGCTTA
TATGCCTATGAAAATTGTAATACAACTTTCAGCAACGGATCTCTTGGCTCTCGCATCGAT
GAAGAACGCAGCGAAATGCGATAAGTAATGTGAATTGCAGAATTCAGTGAATCATCGAAT
CTTTGAACGCATCTTGCGCTCCTTGGTATTCCGAGGAGCATGCCTGTTTGAGTGTCATTA
AATTCTCAACTCTCTTCTAC-TTTTTGTAAAAGAGAGCTTGGACTGTGGAGGCTTGCTGG
CCACTTTTTGGGGTCAGCTCCTCTGAAATGCATTAGCGGAACCGTTTGCGATCTGCCACA
AGTGTGATAAGTTATCTACACTGGCGAGGGGATTGCTCTCTGTAATGTTCAGCTTCTAAT
TGTCTCTACTTTGTGAGACTACTTTTGAATGCTTGACCTCAAATCAGGTAGGACTACCC-
GCTGAACTTAA
>BC2-32
TTTCCGTAGGTGAACCTGCGGAAGGATCATTATTGAATTATGTTTCTAGATAGGTTGTAG
CTGGCTCTTTAGAGCATGTGCACGCCTGTTTGGACTTCATTTTCATCCACCTGTGCACCT
ATTGTAGTCTTTGGTTGGGTTAGGAGGAAGTGGTCATTGTGTCAGCATCTGCTGGATGTG

AGGACTTGCATTGTGAAAGCTTTGCTGTCCTTGATGTGATCATGGAATCTCTTTCTCACT
AGAGTCTATGTCACTCATTATACTCTGTCGAATGTCATTGAATGTCTTTACATGGGCTTA
TATGCCTATGAAAATTGTAATACAACTTTCAGCAACGGATCTCTTGGCTCTCGCATCGAT
GAAGAACGCAGCGAAATGCGATAAGTAATGTGAATTGCAGAATTCAGTGAATCATCGAAT
CTTTGAACGCATCTTGCGCTCCTTGGTATTCCGAGGAGCATGCCTGTTTGAGTGTCATTA
AATTCTCAACTCTCTTCTAC-TTTTTGTAAAAGAGAGCTTGGACTGTGGAGGCTTGCTGG
CCACTTTTTGGGGTCAGCTCCTCTGAAATGCATTAGCGGAACCGTTTGCGATCTGCCACA
AGTGTGATAAGTTATCTACACTGGCGAGGGGATTGCTCTCTGTAATGTTCAGCTTCTAAT
TGTCTCTACTTTGTGAGACTACTTTTGAATGCTTGACCTCAAATCAGGTAGGACTACCC-
GCTGAACTTAA
>BC2-36
TTTCCGTAGGTGAACCTGCGGAAGGATCATTATTGAATTATGTTTCTAGATAGGTTGTAG
CTGGCTCTTTAGAGCATGTGCACGCCTGTTTGGACTTCATTTTCATCCACCTGTGCACCT
ATTGTAGTCTTTGGTTGGGTTAGGAGGAAGTGGTCATTGTGTCAGCATCTGCTGGATGTG
AGGACTTGCATTGTGAAAGCTTTGCTGTCCTTGATGTGATCATGGAATCTCTTTCTCACT
AGAGTCTATGTCACTCATTATACTCTGTCGAATGTCATTGAATGTCTTTACATGGGCTTA
TATGCCTATGAAAATTGTAATACAACTTTCAGCAACGGATCTCTTGGCTCTCGCATCGAT
GAAGAACGCAGCGAAATGCGATAAGTAATGTGAATTGCAGAATTCAGTGAATCATCGAAT
CTTTGAACGCATCTTGCGCTCCTTGGTATTCCGAGGAGCATGCCTGTTTGAGTGTCATTA
AATTCTCAACTCTCTTCTAC-TTTTTGTAAAAGAGAGCTTGGACTGTGGAGGCTTGCTGG
CCACTTTTTGGGGTCAGCTCCTCTGAAATGCATTAGCGGAACCGTTTGCGATCTGCCACA
AGTGTGATAAGTTATCTACACTGGCGAGGGGATTGCTCTCTGTAATGTTCAGCTTCTAAT
TGTCTCTACTTTGTGAGACTACTTTTGAATGCTTGACCTCAAATCAGGTAGGACTACCC-
GCTGAACTTAA
>BC2-37
TTTCCGTAGGTGAACCTGCGGAAGGATCATTATTGAATTATGTTTCTAGATAGGTTGTAG
CTGGCTCTTTAGAGCATGTGCACGCCTGTTTGGACTTCATTTTCATCCACCTGTGCACCT
ATTGTAGTCTTTGGTTGGGTTAGGAGGAAGTGGTCATTGTGTCAGCATCTGCTGGATGTG
AGGACTTGCATTGTGAAAGCTTTGCTGTCCTTGATGTGATCATGGAATCTCTTTCTCACT
AGAGTCTATGTCACTCATTATACTCTGTCGAATGTCATTGAATGTCTTTACATGGGCTTA
TATGCCTATGAAAATTGTAATACAACTTTCAGCAACGGATCTCTTGGCTCTCGCATCGAT
GAAGAACGCAGCGAAATGCGATAAGTAATGTGAATTGCAGAATTCAGTGAATCATCGAAT
CTTTGAACGCATCTTGCGCTCCTTGGTATTCCGAGGAGCATGCCTGTTTGAGTGTCATTA
AATTCTCAACTCTCTTCTAC-TTTTTGTAAAAGAGAGCTTGGACTGTGGAGGCTTGCTGG
CCACTTTTTGGGGTCAGCTCCTCTGAAATGCATTAGCGGAACCGTTTGCGATCTGCCACA
AGTGTGATAAGTTATCTACACTGGCGAGGGGATTGCTCTCTGTAATGTTCAGCTTCTAAT
TGTCTCTACTTTGTGAGACTACTTTTGAATGCTTGACCTCAAATCAGGTAGGACTACCC-
GCTGAACTTAA
>BC2-39
TTTCCGTAGGTGAACCTGCGGAAGGATCATTATTGAATTATGTTTCTAGATAGGTTGTAG
CTGGCTCTTTAGAGCATGTGCACGCCTGTTTGGACTTCATTTTCATCCACCTGTGCACCT
ATTGTAGTCTTTGGTTGGGTTAGGAGGAAGTGGTCATTGTGTCAGCATCTGCTGGATGTG
AGGACTTGCATTGTGAAAGCTTTGCTGTCCTTGATGTGATCATGGAATCTCTTTCTCACT
AGAGTCTATGTCACTCATTATACTCTGTCGAATGTCATTGAATGTCTTTACATGGGCTTA
TATGCCTATGAAAATTGTAATACAACTTTCAGCAACGGATCTCTTGGCTCTCGCATCGAT
GAAGAACGCAGCGAAATGCGATAAGTAATGTGAATTGCAGAATTCAGTGAATCATCGAAT
CTTTGAACGCATCTTGCGCTCCTTGGTATTCCGAGGAGCATGCCTGTTTGAGTGTCATTA
AATTCTCAACTCTCTTCTAC-TTTTTGTAAAAGAGAGCTTGGACTGTGGAGGCTTGCTGG
CCACTTTTTGGGGTCAGCTCCTCTGAAATGCATTAGCGGAACCGTTTGCGATCTGCCACA
AGTGTGATAAGTTATCTACACTGGCGAGGGGATTGCTCTCTGTAATGTTCAGCTTCTAAT

TGTCTCTACTTTGTGAGACTACTTTTGAATGCTTGACCTCAAATCAGGTAGGACTACCC-
GCTGAACTTAA
>BC2-40
TTTCCGTAGGTGAACCTGCGGAAGGATCATTATTGAATTATGTTTCTAGATAGGTTGTAG
CTGGCTCTTTAGAGCATGTGCACGCCTGTTTGGACTTCATTTTCATCCACCTGTGCACCT
ATTGTAGTCTTTGGTTGGGTTAGGAGGAAGTGGTCATTGTGTCAGCATCTGCTGGATGTG
AGGACTTGCATTGTGAAAGCTTTGCTGTCCTTGATGTGATCATGGAATCTCTTTCTCACT
AGAGTCTATGTCACTCATTATACTCTGTCGAATGTCATTGAATGTCTTTACATGGGCTTA
TATGCCTATGAAAATTGTAATACAACTTTCAGCAACGGATCTCTTGGCTCTCGCATCGAT
GAAGAACGCAGCGAAATGCGATAAGTAATGTGAATTGCAGAATTCAGTGAATCATCGAAT
CTTTGAACGCATCTTGCGCTCCTTGGTATTCCGAGGAGCATGCCTGTTTGAGTGTCATTA
AATTCTCAACTCTCTTCTAC-TTTTTGTAAAAGAGAGCTTGGACTGTGGAGGCTTGCTGG
CCACTTTTTGGGGTCAGCTCCTCTGAAATGCATTAGCGGAACCGTTTGCGATCTGCCACA
AGTGTGATAAGTTATCTACACTGGCGAGGGGATTGCTCTCTGTAATGTTCAGCTTCTAAT
TGTCTCTACTTTGTGAGACTACTTTTGAATGCTTGACCTCAAATCAGGTAGGACTACCC-
GCTGAACTTAA
>BC2-41
TTTCCGTAGGTGAACCTGCGGAAGGATCATTATTGAATTATGTTTCTAGATAGGTTGTAG
CTGGCTCTTTAGAGCATGTGCACGCCTGTTTGGACTTCATTTTCATCCACCTGTGCACCT
ATTGTAGTCTTTGGTTGGGTTAGGAGGAAGTGGTCATTGTGTCAGCATCTGCTGGATGTG
AGGACTTGCATTGTGAAAGCTTTGCTGTCCTTGATGTGATCATGGAATCTCTTTCTCACT
AGAGTCTATGTCACTCATTATACTCTGTCGAATGTCATTGAATGTCTTTACATGGGCTTA
TATGCCTATGAAAATTGTAATACAACTTTCAGCAACGGATCTCTTGGCTCTCGCATCGAT
GAAGAACGCAGCGAAATGCGATAAGTAATGTGAATTGCAGAATTCAGTGAATCATCGAAT
CTTTGAACGCATCTTGCGCTCCTTGGTATTCCGAGGAGCATGCCTGTTTGAGTGTCATTA
AATTCTCAACTCTCTTCTAC-TTTTTGTAAAAGAGAGCTTGGACTGTGGAGGCTTGCTGG
CCACTTTTTGGGGTCAGCTCCTCTGAAATGCATTAGCGGAACCGTTTGCGATCTGCCACA
AGTGTGATAAGTTATCTACACTGGCGAGGGGATTGCTCTCTGTAATGTTCAGCTTCTAAT
TGTCTCTACTTTGTGAGACTACTTTTGAATGCTTGACCTCAAATCAGGTAGGACTACCC-
GCTGAACTTAA
>BC2-46
TTTCCGTAGGTGAACCTGCGGAAGGATCATTATTGAATTATGTTTCTAGATAGGTTGTAG
CTGGCTCTTTAGAGCATGTGCACGCCTGTTTGGACTTCATTTTCATCCACCTGTGCACCT
ATTGTAGTCTTTGGTTGGGTTAGGAGGAAGTGGTCATTGTGTCAGCATCTGCTGGATGTG
AGGACTTGCATTGTGAAAGCTTTGCTGTCCTTGATGTGATCATGGAATCTCTTTCTCACT
AGAGTCTATGTCACTCATTATACTCTGTCGAATGTCATTGAATGTCTTTACATGGGCTTA
TATGCCTATGAAAATTGTAATACAACTTTCAGCAACGGATCTCTTGGCTCTCGCATCGAT
GAAGAACGCAGCGAAATGCGATAAGTAATGTGAATTGCAGAATTCAGTGAATCATCGAAT
CTTTGAACGCATCTTGCGCTCCTTGGTATTCCGAGGAGCATGCCTGTTTGAGTGTCATTA
AATTCTCAACTCTCTTCTAC-TTTTTGTAAAAGAGAGCTTGGACTGTGGAGGCTTGCTGG
CCACTTTTTGGGGTCAGCTCCTCTGAAATGCATTAGCGGAACCGTTTGCGATCTGCCACA
AGTGTGATAAGTTATCTACACTGGCGAGGGGATTGCTCTCTGTAATGTTCAGCTTCTAAT
TGTCTCTACTTTGTGAGACTACTTTTGAATGCTTGACCTCAAATCAGGTAGGACTACCC-
GCTGAACTTAA
>BC2-48
TTTCCGTAGGTGAACCTGCGGAAGGATCATTATTGAATTATGTTTCTAGATAGGTTGTAG
CTGGCTCTTTAGAGCATGTGCACGCCTGTTTGGACTTCATTTTCATCCACCTGTGCACCT
ATTGTAGTCTTTGGTTGGGTTAGGAGGAAGTGGTCATTGTGTCAGCATCTGCTGGATGTG
AGGACTTGCATTGTGAAAGCTTTGCTGTCCTTGATGTGATCATGGAATCTCTTTCTCACT
AGAGTCTATGTCACTCATTATACTCTGTCGAATGTCATTGAATGTCTTTACATGGGCTTA

TATGCCTATGAAAATTGTAATACAACTTTCAGCAACGGATCTCTTGGCTCTCGCATCGAT
GAAGAACGCAGCGAAATGCGATAAGTAATGTGAATTGCAGAATTCAGTGAATCATCGAAT
CTTTGAACGCATCTTGCGCTCCTTGGTATTCCGAGGAGCATGCCTGTTTGAGTGTCATTA
AATTCTCAACTCTCTTCTAC-TTTTTGTAAAAGAGAGCTTGGACTGTGGAGGCTTGCTGG
CCACTTTTTGGGGTCAGCTCCTCTGAAATGCATTAGCGGAACCGTTTGCGATCTGCCACA
AGTGTGATAAGTTATCTACACTGGCGAGGGGATTGCTCTCTGTAATGTTCAGCTTCTAAT
TGTCTCTACTTTGTGAGACTACTTTTGAATGCTTGACCTCAAATCAGGTAGGACTACCC-
GCTGAACTTAA
>BC2-58
TTTCCGTAGGTGAACCTGCGGAAGGATCATTATTGAATTATGTTTCTAGATAGGTTGTAG
CTGGCTCTTTAGAGCATGTGCACGCCTGTTTGGACTTCATTTTCATCCACCTGTGCACCT
ATTGTAGTCTTTGGTTGGGTTAGGAGGAAGTGGTCATTGTGTCAGCATCTGCTGGATGTG
AGGACTTGCATTGTGAAAGCTTTGCTGTCCTTGATGTGATCATGGAATCTCTTTCTCACT
AGAGTCTATGTCACTCATTATACTCTGTCGAATGTCATTGAATGTCTTTACATGGGCTTA
TATGCCTATGAAAATTGTAATACAACTTTCAGCAACGGATCTCTTGGCTCTCGCATCGAT
GAAGAACGCAGCGAAATGCGATAAGTAATGTGAATTGCAGAATTCAGTGAATCATCGAAT
CTTTGAACGCATCTTGCGCTCCTTGGTATTCCGAGGAGCATGCCTGTTTGAGTGTCATTA
AATTCTCAACTCTCTTCTAC-TTTTTGTAAAAGAGAGCTTGGACTGTGGAGGCTTGCTGG
CCACTTTTTGGGGTCAGCTCCTCTGAAATGCATTAGCGGAACCGTTTGCGATCTGCCACA
AGTGTGATAAGTTATCTACACTGGCGAGGGGATTGCTCTCTGTAATGTTCAGCTTCTAAT
TGTCTCTACTTTGTGAGACTACTTTTGAATGCTTGACCTCAAATCAGGTAGGACTACCC-
GCTGAACTTAA
>BC2-51
TTTCCGTAGGTGAACCTGCGGAAGGATCATTATTGAATTATGTTTCTAGATAGGTTGTAG
CTGGCTCTTTAGAGCATGTGCACGCCTGTTTGGACTTCATTTTCATCCACCTGTGCACCT
ATTGTAGTCTTTGGTTGGGTTAGGAGGAAGTGGTCATTGTGTCAGCATCTGCTGGATGTG
AGGACTTGCATTGTGAAAGCTTTGCTGTCCTTGATGTGATCATGGAATCTCTTTCTCACT
AGAGTCTATGTCACTCATTATACTCTGTCGAATGTCATTGAATGTCTTTACATGGGCTTA
TATGCCTATGAAAATTGTAATACAACTTTCAGCAACGGATCTCTTGGCTCTCGCATCGAT
GAAGAACGCAGCGAAATGCGATAAGTAATGTGAATTGCAGAATTCAGTGAATCATCGAAT
CTTTGAACGCATCTTGCGCTCCTTGGTATTCCGAGGAGCATGCCTGTTTGAGTGTCATTA
AATTCTCAACTCTCTTCTAC-TTTTTGTAAAAGAGAGCTTGGACTGTGGAGGCTTGCTGG
CCACTTTTTGGGGTCAGCTCCTCTGAAATGCATTAGCGGAACCGTTTGCGATCTGCCACA
AGTGTGATAAGTTATCTACACTGGCGAGGGGATTGCTCTCTGTAATGTTCAGCTTCTAAT
TGTCTCTACTTTGTGAGACTACTTTTGAATGCTTGACCTCAAATCAGGTAGGACTACCC-
GCTGAACTTAA
>BC2-54
TTTCCGTAGGTGAACCTGCGGAAGGATCATTATTGAATTATGTTTCTAGATAGGTTGTAG
CTGGCTCTTTAGAGCATGTGCACGCCTGTTTGGACTTCATTTTCATCCACCTGTGCACCT
ATTGTAGTCTTTGGTTGGGTTAGGAGGAAGTGGTCATTGTGTCAGCATCTGCTGGATGTG
AGGACTTGCATTGTGAAAGCTTTGCTGTCCTTGATGTGATCATGGAATCTCTTTCTCACT
AGAGTCTATGTCACTCATTATACTCTGTCGAATGTCATTGAATGTCTTTACATGGGCTTA
TATGCCTATGAAAATTGTAATACAACTTTCAGCAACGGATCTCTTGGCTCTCGCATCGAT
GAAGAACGCAGCGAAATGCGATAAGTAATGTGAATTGCAGAATTCAGTGAATCATCGAAT
CTTTGAACGCATCTTGCGCTCCTTGGTATTCCGAGGAGCATGCCTGTTTGAGTGTCATTA
AATTCTCAACTCTCTTCTAC-TTTTTGTAAAAGAGAGCTTGGACTGTGGAGGCTTGCTGG
CCACTTTTTGGGGTCAGCTCCTCTGAAATGCATTAGCGGAACCGTTTGCGATCTGCCACA
AGTGTGATAAGTTATCTACACTGGCGAGGGGATTGCTCTCTGTAATGTTCAGCTTCTAAT
TGTCTCTACTTTGTGAGACTACTTTTGAATGCTTGACCTCAAATCAGGTAGGACTACCC-
GCTGAACTTAA

>BC2-56
TTTCCGTAGGTGAACCTGCGGAAGGATCATTATTGAATTATGTTTCTAGATAGGTTGTAG
CTGGCTCTTTAGAGCATGTGCACGCCTGTTTGGACTTCATTTTCATCCACCTGTGCACCT
ATTGTAGTCTTTGGTTGGGTTAGGAGGAAGTGGTCATTGTGTCAGCATCTGCTGGATGTG
AGGACTTGCATTGTGAAAGCTTTGCTGTCCTTGATGTGATCATGGAATCTCTTTCTCACT
AGAGTCTATGTCACTCATTATACTCTGTCGAATGTCATTGAATGTCTTTACATGGGCTTA
TATGCCTATGAAAATTGTAATACAACTTTCAGCAACGGATCTCTTGGCTCTCGCATCGAT
GAAGAACGCAGCGAAATGCGATAAGTAATGTGAATTGCAGAATTCAGTGAATCATCGAAT
CTTTGAACGCATCTTGCGCTCCTTGGTATTCCGAGGAGCATGCCTGTTTGAGTGTCATTA
AATTCTCAACTCTCTTCTAC-TTTTTGTAAAAGAGAGCTTGGACTGTGGAGGCTTGCTGG
CCACTTTTTGGGGTCAGCTCCTCTGAAATGCATTAGCGGAACCGTTTGCGATCTGCCACA
AGTGTGATAAGTTATCTACACTGGCGAGGGGATTGCTCTCTGTAATGTTCAGCTTCTAAT
TGTCTCTACTTTGTGAGACTACTTTTGAATGCTTGACCTCAAATCAGGTAGGACTACCC-
GCTGAACTTAA
>BC3-4
TTTCCGTAGGTGAACCTGCGGAAGGATCATTATTGAATTATGTTTCTAGATAGGTTGTAG
CTGGCTCTTTAGAGCATGTGCACGCCTGTTTGGACTTCATTTTCATCCACCTGTGCACCT
ATTGTAGTCTTTGGTTGGGTTAGGAGGAAGTGGTCATTGTGTCAGCATCTGCTGGATGTG
AGGACTTGCATTGTGAAAGCTTTGCTGTCCTTGATGTGATCATGGAATCTCTTTCTCACT
AGAGTCTATGTCACTCATTATACTCTGTCGAATGTCATTGAATGTCTTTACATGGGCTTA
TATGCCTATGAAAATTGTAATACAACTTTCAGCAACGGATCTCTTGGCTCTCGCATCGAT
GAAGAACGCAGCGAAATGCGATAAGTAATGTGAATTGCAGAATTCAGTGAATCATCGAAT
CTTTGAACGCATCTTGCGCTCCTTGGTATTCCGAGGAGCATGCCTGTTTGAGTGTCATTA
AATTCTCAACTCTCTTCTAC-TTTTTGTAAAAGAGAGCTTGGACTGTGGAGGCTTGCTGG
CCACTTTTTGGGGTCAGCTCCTCTGAAATGCATTAGCGGAACCGTTTGCGATCTGCCACA
AGTGTGATAAGTTATCTACACTGGCGAGGGGATTGCTCTCTGTAATGTTCAGCTTCTAAT
TGTCTCTACTTTGTGAGACTACTTTTGAATGCTTGACCTCAAATCAGGTAGGACTACCC-
GCTGAACTTAA
>BC3-8
TTTCCGTAGGTGAACCTGCGGAAGGATCATTATTGAATTATGTTTCTAGATAGGTTGTAG
CTGGCTCTTTAGAGCATGTGCACGCCTGTTTGGACTTCATTTTCATCCACCTGTGCACCT
ATTGTAGTCTTTGGTTGGGTTAGGAGGAAGTGGTCATTGTGTCAGCATCTGCTGGATGTG
AGGACTTGCATTGTGAAAGCTTTGCTGTCCTTGATGTGATCATGGAATCTCTTTCTCACT
AGAGTCTATGTCACTCATTATACTCTGTCGAATGTCATTGAATGTCTTTACATGGGCTTA
TATGCCTATGAAAATTGTAATACAACTTTCAGCAACGGATCTCTTGGCTCTCGCATCGAT
GAAGAACGCAGCGAAATGCGATAAGTAATGTGAATTGCAGAATTCAGTGAATCATCGAAT
CTTTGAACGCATCTTGCGCTCCTTGGTATTCCGAGGAGCATGCCTGTTTGAGTGTCATTA
AATTCTCAACTCTCTTCTAC-TTTTTGTAAAAGAGAGCTTGGACTGTGGAGGCTTGCTGG
CCACTTTTTGGGGTCAGCTCCTCTGAAATGCATTAGCGGAACCGTTTGCGATCTGCCACA
AGTGTGATAAGTTATCTACACTGGCGAGGGGATTGCTCTCTGTAATGTTCAGCTTCTAAT
TGTCTCTACTTTGTGAGACTACTTTTGAATGCTTGACCTCAAATCAGGTAGGACTACCC-
GCTGAACTTAA
>BC3-10
TTTCCGTAGGTGAACCTGCGGAAGGATCATTATTGAATTATGTTTCTAGATAGGTTGTAG
CTGGCTCTTTAGAGCATGTGCACGCCTGTTTGGACTTCATTTTCATCCACCTGTGCACCT
ATTGTAGTCTTTGGTTGGGTTAGGAGGAAGTGGTCATTGTGTCAGCATCTGCTGGATGTG
AGGACTTGCATTGTGAAAGCTTTGCTGTCCTTGATGTGATCATGGAATCTCTTTCTCACT
AGAGTCTATGTCACTCATTATACTCTGTCGAATGTCATTGAATGTCTTTACATGGGCTTA
TATGCCTATGAAAATTGTAATACAACTTTCAGCAACGGATCTCTTGGCTCTCGCATCGAT
GAAGAACGCAGCGAAATGCGATAAGTAATGTGAATTGCAGAATTCAGTGAATCATCGAAT

CTTTGAACGCATCTTGCGCTCCTTGGTATTCCGAGGAGCATGCCTGTTTGAGTGTCATTA
AATTCTCAACTCTCTTCTAC-TTTTTGTAAAAGAGAGCTTGGACTGTGGAGGCTTGCTGG
CCACTTTTTGGGGTCAGCTCCTCTGAAATGCATTAGCGGAACCGTTTGCGATCTGCCACA
AGTGTGATAAGTTATCTACACTGGCGAGGGGATTGCTCTCTGTAATGTTCAGCTTCTAAT
TGTCTCTACTTTGTGAGACTACTTTTGAATGCTTGACCTCAAATCAGGTAGGACTACCC-
GCTGAACTTAA
>BC3-11
TTTCCGTAGGTGAACCTGCGGAAGGATCATTATTGAATTATGTTTCTAGATAGGTTGTAG
CTGGCTCTTTAGAGCATGTGCACGCCTGTTTGGACTTCATTTTCATCCACCTGTGCACCT
ATTGTAGTCTTTGGTTGGGTTAGGAGGAAGTGGTCATTGTGTCAGCATCTGCTGGATGTG
AGGACTTGCATTGTGAAAGCTTTGCTGTCCTTGATGTGATCATGGAATCTCTTTCTCACT
AGAGTCTATGTCACTCATTATACTCTGTCGAATGTCATTGAATGTCTTTACATGGGCTTA
TATGCCTATGAAAATTGTAATACAACTTTCAGCAACGGATCTCTTGGCTCTCGCATCGAT
GAAGAACGCAGCGAAATGCGATAAGTAATGTGAATTGCAGAATTCAGTGAATCATCGAAT
CTTTGAACGCATCTTGCGCTCCTTGGTATTCCGAGGAGCATGCCTGTTTGAGTGTCATTA
AATTCTCAACTCTCTTCTAC-TTTTTGTAAAAGAGAGCTTGGACTGTGGAGGCTTGCTGG
CCACTTTTTGGGGTCAGCTCCTCTGAAATGCATTAGCGGAACCGTTTGCGATCTGCCACA
AGTGTGATAAGTTATCTACACTGGCGAGGGGATTGCTCTCTGTAATGTTCAGCTTCTAAT
TGTCTCTACTTTGTGAGACTACTTTTGAATGCTTGACCTCAAATCAGGTAGGACTACCC-
GCTGAACTTAA
>BC3-12
TTTCCGTAGGTGAACCTGCGGAAGGATCATTATTGAATTATGTTTCTAGATAGGTTGTAG
CTGGCTCTTTAGAGCATGTGCACGCCTGTTTGGACTTCATTTTCATCCACCTGTGCACCT
ATTGTAGTCTTTGGTTGGGTTAGGAGGAAGTGGTCATTGTGTCAGCATCTGCTGGATGTG
AGGACTTGCATTGTGAAAGCTTTGCTGTCCTTGATGTGATCATGGAATCTCTTTCTCACT
AGAGTCTATGTCACTCATTATACTCTGTCGAATGTCATTGAATGTCTTTACATGGGCTTA
TATGCCTATGAAAATTGTAATACAACTTTCAGCAACGGATCTCTTGGCTCTCGCATCGAT
GAAGAACGCAGCGAAATGCGATAAGTAATGTGAATTGCAGAATTCAGTGAATCATCGAAT
CTTTGAACGCATCTTGCGCTCCTTGGTATTCCGAGGAGCATGCCTGTTTGAGTGTCATTA
AATTCTCAACTCTCTTCTAC-TTTTTGTAAAAGAGAGCTTGGACTGTGGAGGCTTGCTGG
CCACTTTTTGGGGTCAGCTCCTCTGAAATGCATTAGCGGAACCGTTTGCGATCTGCCACA
AGTGTGATAAGTTATCTACACTGGCGAGGGGATTGCTCTCTGTAATGTTCAGCTTCTAAT
TGTCTCTACTTTGTGAGACTACTTTTGAATGCTTGACCTCAAATCAGGTAGGACTACCC-
GCTGAACTTAA
>BC3-15
TTTCCGTAGGTGAACCTGCGGAAGGATCATTATTGAATTATGTTTCTAGATAGGTTGTAG
CTGGCTCTTTAGAGCATGTGCACGCCTGTTTGGACTTCATTTTCATCCACCTGTGCACCT
ATTGTAGTCTTTGGTTGGGTTAGGAGGAAGTGGTCATTGTGTCAGCATCTGCTGGATGTG
AGGACTTGCATTGTGAAAGCTTTGCTGTCCTTGATGTGATCATGGAATCTCTTTCTCACT
AGAGTCTATGTCACTCATTATACTCTGTCGAATGTCATTGAATGTCTTTACATGGGCTTA
TATGCCTATGAAAATTGTAATACAACTTTCAGCAACGGATCTCTTGGCTCTCGCATCGAT
GAAGAACGCAGCGAAATGCGATAAGTAATGTGAATTGCAGAATTCAGTGAATCATCGAAT
CTTTGAACGCATCTTGCGCTCCTTGGTATTCCGAGGAGCATGCCTGTTTGAGTGTCATTA
AATTCTCAACTCTCTTCTAC-TTTTTGTAAAAGAGAGCTTGGACTGTGGAGGCTTGCTGG
CCACTTTTTGGGGTCAGCTCCTCTGAAATGCATTAGCGGAACCGTTTGCGATCTGCCACA
AGTGTGATAAGTTATCTACACTGGCGAGGGGATTGCTCTCTGTAATGTTCAGCTTCTAAT
TGTCTCTACTTTGTGAGACTACTTTTGAATGCTTGACCTCAAATCAGGTAGGACTACCC-
GCTGAACTTAA
>BC3-17
TTTCCGTAGGTGAACCTGCGGAAGGATCATTATTGAATTATGTTTCTAGATAGGTTGTAG

CTGGCTCTTTAGAGCATGTGCACGCCTGTTTGGACTTCATTTTCATCCACCTGTGCACCT
ATTGTAGTCTTTGGTTGGGTTAGGAGGAAGTGGTCATTGTGTCAGCATCTGCTGGATGTG
AGGACTTGCATTGTGAAAGCTTTGCTGTCCTTGATGTGATCATGGAATCTCTTTCTCACT
AGAGTCTATGTCACTCATTATACTCTGTCGAATGTCATTGAATGTCTTTACATGGGCTTA
TATGCCTATGAAAATTGTAATACAACTTTCAGCAACGGATCTCTTGGCTCTCGCATCGAT
GAAGAACGCAGCGAAATGCGATAAGTAATGTGAATTGCAGAATTCAGTGAATCATCGAAT
CTTTGAACGCATCTTGCGCTCCTTGGTATTCCGAGGAGCATGCCTGTTTGAGTGTCATTA
AATTCTCAACTCTCTTCTAC-TTTTTGTAAAAGAGAGCTTGGACTGTGGAGGCTTGCTGG
CCACTTTTTGGGGTCAGCTCCTCTGAAATGCATTAGCGGAACCGTTTGCGATCTGCCACA
AGTGTGATAAGTTATCTACACTGGCGAGGGGATTGCTCTCTGTAATGTTCAGCTTCTAAT
TGTCTCTACTTTGTGAGACTACTTTTGAATGCTTGACCTCAAATCAGGTAGGACTACCC-
GCTGAACTTAA
>BC3-20
TTTCCGTAGGTGAACCTGCGGAAGGATCATTATTGAATTATGTTTCTAGATAGGTTGTAG
CTGGCTCTTTAGAGCATGTGCACGCCTGTTTGGACTTCATTTTCATCCACCTGTGCACCT
ATTGTAGTCTTTGGTTGGGTTAGGAGGAAGTGGTCATTGTGTCAGCATCTGCTGGATGTG
AGGACTTGCATTGTGAAAGCTTTGCTGTCCTTGATGTGATCATGGAATCTCTTTCTCACT
AGAGTCTATGTCACTCATTATACTCTGTCGAATGTCATTGAATGTCTTTACATGGGCTTA
TATGCCTATGAAAATTGTAATACAACTTTCAGCAACGGATCTCTTGGCTCTCGCATCGAT
GAAGAACGCAGCGAAATGCGATAAGTAATGTGAATTGCAGAATTCAGTGAATCATCGAAT
CTTTGAACGCATCTTGCGCTCCTTGGTATTCCGAGGAGCATGCCTGTTTGAGTGTCATTA
AATTCTCAACTCTCTTCTAC-TTTTTGTAAAAGAGAGCTTGGACTGTGGAGGCTTGCTGG
CCACTTTTTGGGGTCAGCTCCTCTGAAATGCATTAGCGGAACCGTTTGCGATCTGCCACA
AGTGTGATAAGTTATCTACACTGGCGAGGGGATTGCTCTCTGTAATGTTCAGCTTCTAAT
TGTCTCTACTTTGTGAGACTACTTTTGAATGCTTGACCTCAAATCAGGTAGGACTACCC-
GCTGAACTTAA
>BC3-23
TTTCCGTAGGTGAACCTGCGGAAGGATCATTATTGAATTATGTTTCTAGATAGGTTGTAG
CTGGCTCTTTAGAGCATGTGCACGCCTGTTTGGACTTCATTTTCATCCACCTGTGCACCT
ATTGTAGTCTTTGGTTGGGTTAGGAGGAAGTGGTCATTGTGTCAGCATCTGCTGGATGTG
AGGACTTGCATTGTGAAAGCTTTGCTGTCCTTGATGTGATCATGGAATCTCTTTCTCACT
AGAGTCTATGTCACTCATTATACTCTGTCGAATGTCATTGAATGTCTTTACATGGGCTTA
TATGCCTATGAAAATTGTAATACAACTTTCAGCAACGGATCTCTTGGCTCTCGCATCGAT
GAAGAACGCAGCGAAATGCGATAAGTAATGTGAATTGCAGAATTCAGTGAATCATCGAAT
CTTTGAACGCATCTTGCGCTCCTTGGTATTCCGAGGAGCATGCCTGTTTGAGTGTCATTA
AATTCTCAACTCTCTTCTAC-TTTTTGTAAAAGAGAGCTTGGACTGTGGAGGCTTGCTGG
CCACTTTTTGGGGTCAGCTCCTCTGAAATGCATTAGCGGAACCGTTTGCGATCTGCCACA
AGTGTGATAAGTTATCTACACTGGCGAGGGGATTGCTCTCTGTAATGTTCAGCTTCTAAT
TGTCTCTACTTTGTGAGACTACTTTTGAATGCTTGACCTCAAATCAGGTAGGACTACCC-
GCTGAACTTAA
>BC3-25
TTTCCGTAGGTGAACCTGCGGAAGGATCATTATTGAATTATGTTTCTAGATAGGTTGTAG
CTGGCTCTTTAGAGCATGTGCACGCCTGTTTGGACTTCATTTTCATCCACCTGTGCACCT
ATTGTAGTCTTTGGTTGGGTTAGGAGGAAGTGGTCATTGTGTCAGCATCTGCTGGATGTG
AGGACTTGCATTGTGAAAGCTTTGCTGTCCTTGATGTGATCATGGAATCTCTTTCTCACT
AGAGTCTATGTCACTCATTATACTCTGTCGAATGTCATTGAATGTCTTTACATGGGCTTA
TATGCCTATGAAAATTGTAATACAACTTTCAGCAACGGATCTCTTGGCTCTCGCATCGAT
GAAGAACGCAGCGAAATGCGATAAGTAATGTGAATTGCAGAATTCAGTGAATCATCGAAT
CTTTGAACGCATCTTGCGCTCCTTGGTATTCCGAGGAGCATGCCTGTTTGAGTGTCATTA
AATTCTCAACTCTCTTCTAC-TTTTTGTAAAAGAGAGCTTGGACTGTGGAGGCTTGCTGG

CCACTTTTTGGGGTCAGCTCCTCTGAAATGCATTAGCGGAACCGTTTGCGATCTGCCACA
AGTGTGATAAGTTATCTACACTGGCGAGGGGATTGCTCTCTGTAATGTTCAGCTTCTAAT
TGTCTCTACTTTGTGAGACTACTTTTGAATGCTTGACCTCAAATCAGGTAGGACTACCC-
GCTGAACTTAA
>BC3-28
TTTCCGTAGGTGAACCTGCGGAAGGATCATTATTGAATTATGTTTCTAGATAGGTTGTAG
CTGGCTCTTTAGAGCATGTGCACGCCTGTTTGGACTTCATTTTCATCCACCTGTGCACCT
ATTGTAGTCTTTGGTTGGGTTAGGAGGAAGTGGTCATTGTGTCAGCATCTGCTGGATGTG
AGGACTTGCATTGTGAAAGCTTTGCTGTCCTTGATGTGATCATGGAATCTCTTTCTCACT
AGAGTCTATGTCACTCATTATACTCTGTCGAATGTCATTGAATGTCTTTACATGGGCTTA
TATGCCTATGAAAATTGTAATACAACTTTCAGCAACGGATCTCTTGGCTCTCGCATCGAT
GAAGAACGCAGCGAAATGCGATAAGTAATGTGAATTGCAGAATTCAGTGAATCATCGAAT
CTTTGAACGCATCTTGCGCTCCTTGGTATTCCGAGGAGCATGCCTGTTTGAGTGTCATTA
AATTCTCAACTCTCTTCTAC-TTTTTGTAAAAGAGAGCTTGGACTGTGGAGGCTTGCTGG
CCACTTTTTGGGGTCAGCTCCTCTGAAATGCATTAGCGGAACCGTTTGCGATCTGCCACA
AGTGTGATAAGTTATCTACACTGGCGAGGGGATTGCTCTCTGTAATGTTCAGCTTCTAAT
TGTCTCTACTTTGTGAGACTACTTTTGAATGCTTGACCTCAAATCAGGTAGGACTACCC-
GCTGAACTTAA
>BC3-30
TTTCCGTAGGTGAACCTGCGGAAGGATCATTATTGAATTATGTTTCTAGATAGGTTGTAG
CTGGCTCTTTAGAGCATGTGCACGCCTGTTTGGACTTCATTTTCATCCACCTGTGCACCT
ATTGTAGTCTTTGGTTGGGTTAGGAGGAAGTGGTCATTGTGTCAGCATCTGCTGGATGTG
AGGACTTGCATTGTGAAAGCTTTGCTGTCCTTGATGTGATCATGGAATCTCTTTCTCACT
AGAGTCTATGTCACTCATTATACTCTGTCGAATGTCATTGAATGTCTTTACATGGGCTTA
TATGCCTATGAAAATTGTAATACAACTTTCAGCAACGGATCTCTTGGCTCTCGCATCGAT
GAAGAACGCAGCGAAATGCGATAAGTAATGTGAATTGCAGAATTCAGTGAATCATCGAAT
CTTTGAACGCATCTTGCGCTCCTTGGTATTCCGAGGAGCATGCCTGTTTGAGTGTCATTA
AATTCTCAACTCTCTTCTAC-TTTTTGTAAAAGAGAGCTTGGACTGTGGAGGCTTGCTGG
CCACTTTTTGGGGTCAGCTCCTCTGAAATGCATTAGCGGAACCGTTTGCGATCTGCCACA
AGTGTGATAAGTTATCTACACTGGCGAGGGGATTGCTCTCTGTAATGTTCAGCTTCTAAT
TGTCTCTACTTTGTGAGACTACTTTTGAATGCTTGACCTCAAATCAGGTAGGACTACCC-
GCTGAACTTAA
>BC3-31
TTTCCGTAGGTGAACCTGCGGAAGGATCATTATTGAATTATGTTTCTAGATAGGTTGTAG
CTGGCTCTTTAGAGCATGTGCACGCCTGTTTGGACTTCATTTTCATCCACCTGTGCACCT
ATTGTAGTCTTTGGTTGGGTTAGGAGGAAGTGGTCATTGTGTCAGCATCTGCTGGATGTG
AGGACTTGCATTGTGAAAGCTTTGCTGTCCTTGATGTGATCATGGAATCTCTTTCTCACT
AGAGTCTATGTCACTCATTATACTCTGTCGAATGTCATTGAATGTCTTTACATGGGCTTA
TATGCCTATGAAAATTGTAATACAACTTTCAGCAACGGATCTCTTGGCTCTCGCATCGAT
GAAGAACGCAGCGAAATGCGATAAGTAATGTGAATTGCAGAATTCAGTGAATCATCGAAT
CTTTGAACGCATCTTGCGCTCCTTGGTATTCCGAGGAGCATGCCTGTTTGAGTGTCATTA
AATTCTCAACTCTCTTCTAC-TTTTTGTAAAAGAGAGCTTGGACTGTGGAGGCTTGCTGG
CCACTTTTTGGGGTCAGCTCCTCTGAAATGCATTAGCGGAACCGTTTGCGATCTGCCACA
AGTGTGATAAGTTATCTACACTGGCGAGGGGATTGCTCTCTGTAATGTTCAGCTTCTAAT
TGTCTCTACTTTGTGAGACTACTTTTGAATGCTTGACCTCAAATCAGGTAGGACTACCC-
GCTGAACTTAA
>BC3-36
TTTCCGTAGGTGAACCTGCGGAAGGATCATTATTGAATTATGTTTCTAGATAGGTTGTAG
CTGGCTCTTTAGAGCATGTGCACGCCTGTTTGGACTTCATTTTCATCCACCTGTGCACCT
ATTGTAGTCTTTGGTTGGGTTAGGAGGAAGTGGTCATTGTGTCAGCATCTGCTGGATGTG

AGGACTTGCATTGTGAAAGCTTTGCTGTCCTTGATGTGATCATGGAATCTCTTTCTCACT
AGAGTCTATGTCACTCATTATACTCTGTCGAATGTCATTGAATGTCTTTACATGGGCTTA
TATGCCTATGAAAATTGTAATACAACTTTCAGCAACGGATCTCTTGGCTCTCGCATCGAT
GAAGAACGCAGCGAAATGCGATAAGTAATGTGAATTGCAGAATTCAGTGAATCATCGAAT
CTTTGAACGCATCTTGCGCTCCTTGGTATTCCGAGGAGCATGCCTGTTTGAGTGTCATTA
AATTCTCAACTCTCTTCTAC-TTTTTGTAAAAGAGAGCTTGGACTGTGGAGGCTTGCTGG
CCACTTTTTGGGGTCAGCTCCTCTGAAATGCATTAGCGGAACCGTTTGCGATCTGCCACA
AGTGTGATAAGTTATCTACACTGGCGAGGGGATTGCTCTCTGTAATGTTCAGCTTCTAAT
TGTCTCTACTTTGTGAGACTACTTTTGAATGCTTGACCTCAAATCAGGTAGGACTACCC-
GCTGAACTTAA
>BC3-37
TTTCCGTAGGTGAACCTGCGGAAGGATCATTATTGAATTATGTTTCTAGATAGGTTGTAG
CTGGCTCTTTAGAGCATGTGCACGCCTGTTTGGACTTCATTTTCATCCACCTGTGCACCT
ATTGTAGTCTTTGGTTGGGTTAGGAGGAAGTGGTCATTGTGTCAGCATCTGCTGGATGTG
AGGACTTGCATTGTGAAAGCTTTGCTGTCCTTGATGTGATCATGGAATCTCTTTCTCACT
AGAGTCTATGTCACTCATTATACTCTGTCGAATGTCATTGAATGTCTTTACATGGGCTTA
TATGCCTATGAAAATTGTAATACAACTTTCAGCAACGGATCTCTTGGCTCTCGCATCGAT
GAAGAACGCAGCGAAATGCGATAAGTAATGTGAATTGCAGAATTCAGTGAATCATCGAAT
CTTTGAACGCATCTTGCGCTCCTTGGTATTCCGAGGAGCATGCCTGTTTGAGTGTCATTA
AATTCTCAACTCTCTTCTAC-TTTTTGTAAAAGAGAGCTTGGACTGTGGAGGCTTGCTGG
CCACTTTTTGGGGTCAGCTCCTCTGAAATGCATTAGCGGAACCGTTTGCGATCTGCCACA
AGTGTGATAAGTTATCTACACTGGCGAGGGGATTGCTCTCTGTAATGTTCAGCTTCTAAT
TGTCTCTACTTTGTGAGACTACTTTTGAATGCTTGACCTCAAATCAGGTAGGACTACCC-
GCTGAACTTAA
>BC3-42
TTTCCGTAGGTGAACCTGCGGAAGGATCATTATTGAATTATGTTTCTAGATAGGTTGTAG
CTGGCTCTTTAGAGCATGTGCACGCCTGTTTGGACTTCATTTTCATCCACCTGTGCACCT
ATTGTAGTCTTTGGTTGGGTTAGGAGGAAGTGGTCATTGTGTCAGCATCTGCTGGATGTG
AGGACTTGCATTGTGAAAGCTTTGCTGTCCTTGATGTGATCATGGAATCTCTTTCTCACT
AGAGTCTATGTCACTCATTATACTCTGTCGAATGTCATTGAATGTCTTTACATGGGCTTA
TATGCCTATGAAAATTGTAATACAACTTTCAGCAACGGATCTCTTGGCTCTCGCATCGAT
GAAGAACGCAGCGAAATGCGATAAGTAATGTGAATTGCAGAATTCAGTGAATCATCGAAT
CTTTGAACGCATCTTGCGCTCCTTGGTATTCCGAGGAGCATGCCTGTTTGAGTGTCATTA
AATTCTCAACTCTCTTCTAC-TTTTTGTAAAAGAGAGCTTGGACTGTGGAGGCTTGCTGG
CCACTTTTTGGGGTCAGCTCCTCTGAAATGCATTAGCGGAACCGTTTGCGATCTGCCACA
AGTGTGATAAGTTATCTACACTGGCGAGGGGATTGCTCTCTGTAATGTTCAGCTTCTAAT
TGTCTCTACTTTGTGAGACTACTTTTGAATGCTTGACCTCAAATCAGGTAGGACTACCC-
GCTGAACTTAA
>BC3-47
TTTCCGTAGGTGAACCTGCGGAAGGATCATTATTGAATTATGTTTCTAGATAGGTTGTAG
CTGGCTCTTTAGAGCATGTGCACGCCTGTTTGGACTTCATTTTCATCCACCTGTGCACCT
ATTGTAGTCTTTGGTTGGGTTAGGAGGAAGTGGTCATTGTGTCAGCATCTGCTGGATGTG
AGGACTTGCATTGTGAAAGCTTTGCTGTCCTTGATGTGATCATGGAATCTCTTTCTCACT
AGAGTCTATGTCACTCATTATACTCTGTCGAATGTCATTGAATGTCTTTACATGGGCTTA
TATGCCTATGAAAATTGTAATACAACTTTCAGCAACGGATCTCTTGGCTCTCGCATCGAT
GAAGAACGCAGCGAAATGCGATAAGTAATGTGAATTGCAGAATTCAGTGAATCATCGAAT
CTTTGAACGCATCTTGCGCTCCTTGGTATTCCGAGGAGCATGCCTGTTTGAGTGTCATTA
AATTCTCAACTCTCTTCTAC-TTTTTGTAAAAGAGAGCTTGGACTGTGGAGGCTTGCTGG
CCACTTTTTGGGGTCAGCTCCTCTGAAATGCATTAGCGGAACCGTTTGCGATCTGCCACA
AGTGTGATAAGTTATCTACACTGGCGAGGGGATTGCTCTCTGTAATGTTCAGCTTCTAAT

TGTCTCTACTTTGTGAGACTACTTTTGAATGCTTGACCTCAAATCAGGTAGGACTACCC-
GCTGAACTTAA
>BC3-48
TTTCCGTAGGTGAACCTGCGGAAGGATCATTATTGAATTATGTTTCTAGATAGGTTGTAG
CTGGCTCTTTAGAGCATGTGCACGCCTGTTTGGACTTCATTTTCATCCACCTGTGCACCT
ATTGTAGTCTTTGGTTGGGTTAGGAGGAAGTGGTCATTGTGTCAGCATCTGCTGGATGTG
AGGACTTGCATTGTGAAAGCTTTGCTGTCCTTGATGTGATCATGGAATCTCTTTCTCACT
AGAGTCTATGTCACTCATTATACTCTGTCGAATGTCATTGAATGTCTTTACATGGGCTTA
TATGCCTATGAAAATTGTAATACAACTTTCAGCAACGGATCTCTTGGCTCTCGCATCGAT
GAAGAACGCAGCGAAATGCGATAAGTAATGTGAATTGCAGAATTCAGTGAATCATCGAAT
CTTTGAACGCATCTTGCGCTCCTTGGTATTCCGAGGAGCATGCCTGTTTGAGTGTCATTA
AATTCTCAACTCTCTTCTAC-TTTTTGTAAAAGAGAGCTTGGACTGTGGAGGCTTGCTGG
CCACTTTTTGGGGTCAGCTCCTCTGAAATGCATTAGCGGAACCGTTTGCGATCTGCCACA
AGTGTGATAAGTTATCTACACTGGCGAGGGGATTGCTCTCTGTAATGTTCAGCTTCTAAT
TGTCTCTACTTTGTGAGACTACTTTTGAATGCTTGACCTCAAATCAGGTAGGACTACCC-
GCTGAACTTAA
>BC3-52
TTTCCGTAGGTGAACCTGCGGAAGGATCATTATTGAATTATGTTTCTAGATAGGTTGTAG
CTGGCTCTTTAGAGCATGTGCACGCCTGTTTGGACTTCATTTTCATCCACCTGTGCACCT
ATTGTAGTCTTTGGTTGGGTTAGGAGGAAGTGGTCATTGTGTCAGCATCTGCTGGATGTG
AGGACTTGCATTGTGAAAGCTTTGCTGTCCTTGATGTGATCATGGAATCTCTTTCTCACT
AGAGTCTATGTCACTCATTATACTCTGTCGAATGTCATTGAATGTCTTTACATGGGCTTA
TATGCCTATGAAAATTGTAATACAACTTTCAGCAACGGATCTCTTGGCTCTCGCATCGAT
GAAGAACGCAGCGAAATGCGATAAGTAATGTGAATTGCAGAATTCAGTGAATCATCGAAT
CTTTGAACGCATCTTGCGCTCCTTGGTATTCCGAGGAGCATGCCTGTTTGAGTGTCATTA
AATTCTCAACTCTCTTCTAC-TTTTTGTAAAAGAGAGCTTGGACTGTGGAGGCTTGCTGG
CCACTTTTTGGGGTCAGCTCCTCTGAAATGCATTAGCGGAACCGTTTGCGATCTGCCACA
AGTGTGATAAGTTATCTACACTGGCGAGGGGATTGCTCTCTGTAATGTTCAGCTTCTAAT
TGTCTCTACTTTGTGAGACTACTTTTGAATGCTTGACCTCAAATCAGGTAGGACTACCC-
GCTGAACTTAA
>BC3-53
TTTCCGTAGGTGAACCTGCGGAAGGATCATTATTGAATTATGTTTCTAGATAGGTTGTAG
CTGGCTCTTTAGAGCATGTGCACGCCTGTTTGGACTTCATTTTCATCCACCTGTGCACCT
ATTGTAGTCTTTGGTTGGGTTAGGAGGAAGTGGTCATTGTGTCAGCATCTGCTGGATGTG
AGGACTTGCATTGTGAAAGCTTTGCTGTCCTTGATGTGATCATGGAATCTCTTTCTCACT
AGAGTCTATGTCACTCATTATACTCTGTCGAATGTCATTGAATGTCTTTACATGGGCTTA
TATGCCTATGAAAATTGTAATACAACTTTCAGCAACGGATCTCTTGGCTCTCGCATCGAT
GAAGAACGCAGCGAAATGCGATAAGTAATGTGAATTGCAGAATTCAGTGAATCATCGAAT
CTTTGAACGCATCTTGCGCTCCTTGGTATTCCGAGGAGCATGCCTGTTTGAGTGTCATTA
AATTCTCAACTCTCTTCTAC-TTTTTGTAAAAGAGAGCTTGGACTGTGGAGGCTTGCTGG
CCACTTTTTGGGGTCAGCTCCTCTGAAATGCATTAGCGGAACCGTTTGCGATCTGCCACA
AGTGTGATAAGTTATCTACACTGGCGAGGGGATTGCTCTCTGTAATGTTCAGCTTCTAAT
TGTCTCTACTTTGTGAGACTACTTTTGAATGCTTGACCTCAAATCAGGTAGGACTACCC-
GCTGAACTTAA
>BC3-56
TTTCCGTAGGTGAACCTGCGGAAGGATCATTATTGAATTATGTTTCTAGATAGGTTGTAG
CTGGCTCTTTAGAGCATGTGCACGCCTGTTTGGACTTCATTTTCATCCACCTGTGCACCT
ATTGTAGTCTTTGGTTGGGTTAGGAGGAAGTGGTCATTGTGTCAGCATCTGCTGGATGTG
AGGACTTGCATTGTGAAAGCTTTGCTGTCCTTGATGTGATCATGGAATCTCTTTCTCACT
AGAGTCTATGTCACTCATTATACTCTGTCGAATGTCATTGAATGTCTTTACATGGGCTTA

TATGCCTATGAAAATTGTAATACAACTTTCAGCAACGGATCTCTTGGCTCTCGCATCGAT
GAAGAACGCAGCGAAATGCGATAAGTAATGTGAATTGCAGAATTCAGTGAATCATCGAAT
CTTTGAACGCATCTTGCGCTCCTTGGTATTCCGAGGAGCATGCCTGTTTGAGTGTCATTA
AATTCTCAACTCTCTTCTAC-TTTTTGTAAAAGAGAGCTTGGACTGTGGAGGCTTGCTGG
CCACTTTTTGGGGTCAGCTCCTCTGAAATGCATTAGCGGAACCGTTTGCGATCTGCCACA
AGTGTGATAAGTTATCTACACTGGCGAGGGGATTGCTCTCTGTAATGTTCAGCTTCTAAT
TGTCTCTACTTTGTGAGACTACTTTTGAATGCTTGACCTCAAATCAGGTAGGACTACCC-
GCTGAACTTAA
>BC4-7
TTTCCGTAGGTGAACCTGCGGAAGGATCATTATTGAATTATGTTTCTAGATAGGTTGTAG
CTGGCTCTTTAGAGCATGTGCACGCCTGTTTGGACTTCATTTTCATCCACCTGTGCACCT
ATTGTAGTCTTTGGTTGGGTTAGGAGGAAGTGGTCATTGTGTCAGCATCTGCTGGATGTG
AGGACTTGCATTGTGAAAGCTTTGCTGTCCTTGATGTGATCATGGAATCTCTTTCTCACT
AGAGTCTATGTCACTCATTATACTCTGTCGAATGTCATTGAATGTCTTTACATGGGCTTA
TATGCCTATGAAAATTGTAATACAACTTTCAGCAACGGATCTCTTGGCTCTCGCATCGAT
GAAGAACGCAGCGAAATGCGATAAGTAATGTGAATTGCAGAATTCAGTGAATCATCGAAT
CTTTGAACGCATCTTGCGCTCCTTGGTATTCCGAGGAGCATGCCTGTTTGAGTGTCATTA
AATTCTCAACTCTCTTCTAC-TTTTTGTAAAAGAGAGCTTGGACTGTGGAGGCTTGCTGG
CCACTTTTTGGGGTCAGCTCCTCTGAAATGCATTAGCGGAACCGTTTGCGATCTGCCACA
AGTGTGATAAGTTATCTACACTGGCGAGGGGATTGCTCTCTGTAATGTTCAGCTTCTAAT
TGTCTCTACTTTGTGAGACTACTTTTGAATGCTTGACCTCAAATCAGGTAGGACTACCC-
GCTGAACTTAA
>BC4-11
TTTCCGTAGGTGAACCTGCGGAAGGATCATTATTGAATTATGTTTCTAGATAGGTTGTAG
CTGGCTCTTTAGAGCATGTGCACGCCTGTTTGGACTTCATTTTCATCCACCTGTGCACCT
ATTGTAGTCTTTGGTTGGGTTAGGAGGAAGTGGTCATTGTGTCAGCATCTGCTGGATGTG
AGGACTTGCATTGTGAAAGCTTTGCTGTCCTTGATGTGATCATGGAATCTCTTTCTCACT
AGAGTCTATGTCACTCATTATACTCTGTCGAATGTCATTGAATGTCTTTACATGGGCTTA
TATGCCTATGAAAATTGTAATACAACTTTCAGCAACGGATCTCTTGGCTCTCGCATCGAT
GAAGAACGCAGCGAAATGCGATAAGTAATGTGAATTGCAGAATTCAGTGAATCATCGAAT
CTTTGAACGCATCTTGCGCTCCTTGGTATTCCGAGGAGCATGCCTGTTTGAGTGTCATTA
AATTCTCAACTCTCTTCTAC-TTTTTGTAAAAGAGAGCTTGGACTGTGGAGGCTTGCTGG
CCACTTTTTGGGGTCAGCTCCTCTGAAATGCATTAGCGGAACCGTTTGCGATCTGCCACA
AGTGTGATAAGTTATCTACACTGGCGAGGGGATTGCTCTCTGTAATGTTCAGCTTCTAAT
TGTCTCTACTTTGTGAGACTACTTTTGAATGCTTGACCTCAAATCAGGTAGGACTACCC-
GCTGAACTTAA
>BC4-12
TTTCCGTAGGTGAACCTGCGGAAGGATCATTATTGAATTATGTTTCTAGATAGGTTGTAG
CTGGCTCTTTAGAGCATGTGCACGCCTGTTTGGACTTCATTTTCATCCACCTGTGCACCT
ATTGTAGTCTTTGGTTGGGTTAGGAGGAAGTGGTCATTGTGTCAGCATCTGCTGGATGTG
AGGACTTGCATTGTGAAAGCTTTGCTGTCCTTGATGTGATCATGGAATCTCTTTCTCACT
AGAGTCTATGTCACTCATTATACTCTGTCGAATGTCATTGAATGTCTTTACATGGGCTTA
TATGCCTATGAAAATTGTAATACAACTTTCAGCAACGGATCTCTTGGCTCTCGCATCGAT
GAAGAACGCAGCGAAATGCGATAAGTAATGTGAATTGCAGAATTCAGTGAATCATCGAAT
CTTTGAACGCATCTTGCGCTCCTTGGTATTCCGAGGAGCATGCCTGTTTGAGTGTCATTA
AATTCTCAACTCTCTTCTAC-TTTTTGTAAAAGAGAGCTTGGACTGTGGAGGCTTGCTGG
CCACTTTTTGGGGTCAGCTCCTCTGAAATGCATTAGCGGAACCGTTTGCGATCTGCCACA
AGTGTGATAAGTTATCTACACTGGCGAGGGGATTGCTCTCTGTAATGTTCAGCTTCTAAT
TGTCTCTACTTTGTGAGACTACTTTTGAATGCTTGACCTCAAATCAGGTAGGACTACCC-
GCTGAACTTAA

>BC4-15
TTTCCGTAGGTGAACCTGCGGAAGGATCATTATTGAATTATGTTTCTAGATAGGTTGTAG
CTGGCTCTTTAGAGCATGTGCACGCCTGTTTGGACTTCATTTTCATCCACCTGTGCACCT
ATTGTAGTCTTTGGTTGGGTTAGGAGGAAGTGGTCATTGTGTCAGCATCTGCTGGATGTG
AGGACTTGCATTGTGAAAGCTTTGCTGTCCTTGATGTGATCATGGAATCTCTTTCTCACT
AGAGTCTATGTCACTCATTATACTCTGTCGAATGTCATTGAATGTCTTTACATGGGCTTA
TATGCCTATGAAAATTGTAATACAACTTTCAGCAACGGATCTCTTGGCTCTCGCATCGAT
GAAGAACGCAGCGAAATGCGATAAGTAATGTGAATTGCAGAATTCAGTGAATCATCGAAT
CTTTGAACGCATCTTGCGCTCCTTGGTATTCCGAGGAGCATGCCTGTTTGAGTGTCATTA
AATTCTCAACTCTCTTCTAC-TTTTTGTAAAAGAGAGCTTGGACTGTGGAGGCTTGCTGG
CCACTTTTTGGGGTCAGCTCCTCTGAAATGCATTAGCGGAACCGTTTGCGATCTGCCACA
AGTGTGATAAGTTATCTACACTGGCGAGGGGATTGCTCTCTGTAATGTTCAGCTTCTAAT
TGTCTCTACTTTGTGAGACTACTTTTGAATGCTTGACCTCAAATCAGGTAGGACTACCC-
GCTGAACTTAA
>BC4-18
TTTCCGTAGGTGAACCTGCGGAAGGATCATTATTGAATTATGTTTCTAGATAGGTTGTAG
CTGGCTCTTTAGAGCATGTGCACGCCTGTTTGGACTTCATTTTCATCCACCTGTGCACCT
ATTGTAGTCTTTGGTTGGGTTAGGAGGAAGTGGTCATTGTGTCAGCATCTGCTGGATGTG
AGGACTTGCATTGTGAAAGCTTTGCTGTCCTTGATGTGATCATGGAATCTCTTTCTCACT
AGAGTCTATGTCACTCATTATACTCTGTCGAATGTCATTGAATGTCTTTACATGGGCTTA
TATGCCTATGAAAATTGTAATACAACTTTCAGCAACGGATCTCTTGGCTCTCGCATCGAT
GAAGAACGCAGCGAAATGCGATAAGTAATGTGAATTGCAGAATTCAGTGAATCATCGAAT
CTTTGAACGCATCTTGCGCTCCTTGGTATTCCGAGGAGCATGCCTGTTTGAGTGTCATTA
AATTCTCAACTCTCTTCTAC-TTTTTGTAAAAGAGAGCTTGGACTGTGGAGGCTTGCTGG
CCACTTTTTGGGGTCAGCTCCTCTGAAATGCATTAGCGGAACCGTTTGCGATCTGCCACA
AGTGTGATAAGTTATCTACACTGGCGAGGGGATTGCTCTCTGTAATGTTCAGCTTCTAAT
TGTCTCTACTTTGTGAGACTACTTTTGAATGCTTGACCTCAAATCAGGTAGGACTACCC-
GCTGAACTTAA
>BC4-19
TTTCCGTAGGTGAACCTGCGGAAGGATCATTATTGAATTATGTTTCTAGATAGGTTGTAG
CTGGCTCTTTAGAGCATGTGCACGCCTGTTTGGACTTCATTTTCATCCACCTGTGCACCT
ATTGTAGTCTTTGGTTGGGTTAGGAGGAAGTGGTCATTGTGTCAGCATCTGCTGGATGTG
AGGACTTGCATTGTGAAAGCTTTGCTGTCCTTGATGTGATCATGGAATCTCTTTCTCACT
AGAGTCTATGTCACTCATTATACTCTGTCGAATGTCATTGAATGTCTTTACATGGGCTTA
TATGCCTATGAAAATTGTAATACAACTTTCAGCAACGGATCTCTTGGCTCTCGCATCGAT
GAAGAACGCAGCGAAATGCGATAAGTAATGTGAATTGCAGAATTCAGTGAATCATCGAAT
CTTTGAACGCATCTTGCGCTCCTTGGTATTCCGAGGAGCATGCCTGTTTGAGTGTCATTA
AATTCTCAACTCTCTTCTAC-TTTTTGTAAAAGAGAGCTTGGACTGTGGAGGCTTGCTGG
CCACTTTTTGGGGTCAGCTCCTCTGAAATGCATTAGCGGAACCGTTTGCGATCTGCCACA
AGTGTGATAAGTTATCTACACTGGCGAGGGGATTGCTCTCTGTAATGTTCAGCTTCTAAT
TGTCTCTACTTTGTGAGACTACTTTTGAATGCTTGACCTCAAATCAGGTAGGACTACCC-
GCTGAACTTAA
>BC4-22
TTTCCGTAGGTGAACCTGCGGAAGGATCATTATTGAATTATGTTTCTAGATAGGTTGTAG
CTGGCTCTTTAGAGCATGTGCACGCCTGTTTGGACTTCATTTTCATCCACCTGTGCACCT
ATTGTAGTCTTTGGTTGGGTTAGGAGGAAGTGGTCATTGTGTCAGCATCTGCTGGATGTG
AGGACTTGCATTGTGAAAGCTTTGCTGTCCTTGATGTGATCATGGAATCTCTTTCTCACT
AGAGTCTATGTCACTCATTATACTCTGTCGAATGTCATTGAATGTCTTTACATGGGCTTA
TATGCCTATGAAAATTGTAATACAACTTTCAGCAACGGATCTCTTGGCTCTCGCATCGAT
GAAGAACGCAGCGAAATGCGATAAGTAATGTGAATTGCAGAATTCAGTGAATCATCGAAT

CTTTGAACGCATCTTGCGCTCCTTGGTATTCCGAGGAGCATGCCTGTTTGAGTGTCATTA
AATTCTCAACTCTCTTCTAC-TTTTTGTAAAAGAGAGCTTGGACTGTGGAGGCTTGCTGG
CCACTTTTTGGGGTCAGCTCCTCTGAAATGCATTAGCGGAACCGTTTGCGATCTGCCACA
AGTGTGATAAGTTATCTACACTGGCGAGGGGATTGCTCTCTGTAATGTTCAGCTTCTAAT
TGTCTCTACTTTGTGAGACTACTTTTGAATGCTTGACCTCAAATCAGGTAGGACTACCC-
GCTGAACTTAA
>BC4-27
TTTCCGTAGGTGAACCTGCGGAAGGATCATTATTGAATTATGTTTCTAGATAGGTTGTAG
CTGGCTCTTTAGAGCATGTGCACGCCTGTTTGGACTTCATTTTCATCCACCTGTGCACCT
ATTGTAGTCTTTGGTTGGGTTAGGAGGAAGTGGTCATTGTGTCAGCATCTGCTGGATGTG
AGGACTTGCATTGTGAAAGCTTTGCTGTCCTTGATGTGATCATGGAATCTCTTTCTCACT
AGAGTCTATGTCACTCATTATACTCTGTCGAATGTCATTGAATGTCTTTACATGGGCTTA
TATGCCTATGAAAATTGTAATACAACTTTCAGCAACGGATCTCTTGGCTCTCGCATCGAT
GAAGAACGCAGCGAAATGCGATAAGTAATGTGAATTGCAGAATTCAGTGAATCATCGAAT
CTTTGAACGCATCTTGCGCTCCTTGGTATTCCGAGGAGCATGCCTGTTTGAGTGTCATTA
AATTCTCAACTCTCTTCTAC-TTTTTGTAAAAGAGAGCTTGGACTGTGGAGGCTTGCTGG
CCACTTTTTGGGGTCAGCTCCTCTGAAATGCATTAGCGGAACCGTTTGCGATCTGCCACA
AGTGTGATAAGTTATCTACACTGGCGAGGGGATTGCTCTCTGTAATGTTCAGCTTCTAAT
TGTCTCTACTTTGTGAGACTACTTTTGAATGCTTGACCTCAAATCAGGTAGGACTACCC-
GCTGAACTTAA
>BC4-28
TTTCCGTAGGTGAACCTGCGGAAGGATCATTATTGAATTATGTTTCTAGATAGGTTGTAG
CTGGCTCTTTAGAGCATGTGCACGCCTGTTTGGACTTCATTTTCATCCACCTGTGCACCT
ATTGTAGTCTTTGGTTGGGTTAGGAGGAAGTGGTCATTGTGTCAGCATCTGCTGGATGTG
AGGACTTGCATTGTGAAAGCTTTGCTGTCCTTGATGTGATCATGGAATCTCTTTCTCACT
AGAGTCTATGTCACTCATTATACTCTGTCGAATGTCATTGAATGTCTTTACATGGGCTTA
TATGCCTATGAAAATTGTAATACAACTTTCAGCAACGGATCTCTTGGCTCTCGCATCGAT
GAAGAACGCAGCGAAATGCGATAAGTAATGTGAATTGCAGAATTCAGTGAATCATCGAAT
CTTTGAACGCATCTTGCGCTCCTTGGTATTCCGAGGAGCATGCCTGTTTGAGTGTCATTA
AATTCTCAACTCTCTTCTAC-TTTTTGTAAAAGAGAGCTTGGACTGTGGAGGCTTGCTGG
CCACTTTTTGGGGTCAGCTCCTCTGAAATGCATTAGCGGAACCGTTTGCGATCTGCCACA
AGTGTGATAAGTTATCTACACTGGCGAGGGGATTGCTCTCTGTAATGTTCAGCTTCTAAT
TGTCTCTACTTTGTGAGACTACTTTTGAATGCTTGACCTCAAATCAGGTAGGACTACCC-
GCTGAACTTAA
>BC4-31
TTTCCGTAGGTGAACCTGCGGAAGGATCATTATTGAATTATGTTTCTAGATAGGTTGTAG
CTGGCTCTTTAGAGCATGTGCACGCCTGTTTGGACTTCATTTTCATCCACCTGTGCACCT
ATTGTAGTCTTTGGTTGGGTTAGGAGGAAGTGGTCATTGTGTCAGCATCTGCTGGATGTG
AGGACTTGCATTGTGAAAGCTTTGCTGTCCTTGATGTGATCATGGAATCTCTTTCTCACT
AGAGTCTATGTCACTCATTATACTCTGTCGAATGTCATTGAATGTCTTTACATGGGCTTA
TATGCCTATGAAAATTGTAATACAACTTTCAGCAACGGATCTCTTGGCTCTCGCATCGAT
GAAGAACGCAGCGAAATGCGATAAGTAATGTGAATTGCAGAATTCAGTGAATCATCGAAT
CTTTGAACGCATCTTGCGCTCCTTGGTATTCCGAGGAGCATGCCTGTTTGAGTGTCATTA
AATTCTCAACTCTCTTCTAC-TTTTTGTAAAAGAGAGCTTGGACTGTGGAGGCTTGCTGG
CCACTTTTTGGGGTCAGCTCCTCTGAAATGCATTAGCGGAACCGTTTGCGATCTGCCACA
AGTGTGATAAGTTATCTACACTGGCGAGGGGATTGCTCTCTGTAATGTTCAGCTTCTAAT
TGTCTCTACTTTGTGAGACTACTTTTGAATGCTTGACCTCAAATCAGGTAGGACTACCC-
GCTGAACTTAA
>BC4-34
TTTCCGTAGGTGAACCTGCGGAAGGATCATTATTGAATTATGTTTCTAGATAGGTTGTAG

CTGGCTCTTTAGAGCATGTGCACGCCTGTTTGGACTTCATTTTCATCCACCTGTGCACCT
ATTGTAGTCTTTGGTTGGGTTAGGAGGAAGTGGTCATTGTGTCAGCATCTGCTGGATGTG
AGGACTTGCATTGTGAAAGCTTTGCTGTCCTTGATGTGATCATGGAATCTCTTTCTCACT
AGAGTCTATGTCACTCATTATACTCTGTCGAATGTCATTGAATGTCTTTACATGGGCTTA
TATGCCTATGAAAATTGTAATACAACTTTCAGCAACGGATCTCTTGGCTCTCGCATCGAT
GAAGAACGCAGCGAAATGCGATAAGTAATGTGAATTGCAGAATTCAGTGAATCATCGAAT
CTTTGAACGCATCTTGCGCTCCTTGGTATTCCGAGGAGCATGCCTGTTTGAGTGTCATTA
AATTCTCAACTCTCTTCTAC-TTTTTGTAAAAGAGAGCTTGGACTGTGGAGGCTTGCTGG
CCACTTTTTGGGGTCAGCTCCTCTGAAATGCATTAGCGGAACCGTTTGCGATCTGCCACA
AGTGTGATAAGTTATCTACACTGGCGAGGGGATTGCTCTCTGTAATGTTCAGCTTCTAAT
TGTCTCTACTTTGTGAGACTACTTTTGAATGCTTGACCTCAAATCAGGTAGGACTACCC-
GCTGAACTTAA
>BC4-46
TTTCCGTAGGTGAACCTGCGGAAGGATCATTATTGAATTATGTTTCTAGATAGGTTGTAG
CTGGCTCTTTAGAGCATGTGCACGCCTGTTTGGACTTCATTTTCATCCACCTGTGCACCT
ATTGTAGTCTTTGGTTGGGTTAGGAGGAAGTGGTCATTGTGTCAGCATCTGCTGGATGTG
AGGACTTGCATTGTGAAAGCTTTGCTGTCCTTGATGTGATCATGGAATCTCTTTCTCACT
AGAGTCTATGTCACTCATTATACTCTGTCGAATGTCATTGAATGTCTTTACATGGGCTTA
TATGCCTATGAAAATTGTAATACAACTTTCAGCAACGGATCTCTTGGCTCTCGCATCGAT
GAAGAACGCAGCGAAATGCGATAAGTAATGTGAATTGCAGAATTCAGTGAATCATCGAAT
CTTTGAACGCATCTTGCGCTCCTTGGTATTCCGAGGAGCATGCCTGTTTGAGTGTCATTA
AATTCTCAACTCTCTTCTAC-TTTTTGTAAAAGAGAGCTTGGACTGTGGAGGCTTGCTGG
CCACTTTTTGGGGTCAGCTCCTCTGAAATGCATTAGCGGAACCGTTTGCGATCTGCCACA
AGTGTGATAAGTTATCTACACTGGCGAGGGGATTGCTCTCTGTAATGTTCAGCTTCTAAT
TGTCTCTACTTTGTGAGACTACTTTTGAATGCTTGACCTCAAATCAGGTAGGACTACCC-
GCTGAACTTAA
>BC4-47
TTTCCGTAGGTGAACCTGCGGAAGGATCATTATTGAATTATGTTTCTAGATAGGTTGTAG
CTGGCTCTTTAGAGCATGTGCACGCCTGTTTGGACTTCATTTTCATCCACCTGTGCACCT
ATTGTAGTCTTTGGTTGGGTTAGGAGGAAGTGGTCATTGTGTCAGCATCTGCTGGATGTG
AGGACTTGCATTGTGAAAGCTTTGCTGTCCTTGATGTGATCATGGAATCTCTTTCTCACT
AGAGTCTATGTCACTCATTATACTCTGTCGAATGTCATTGAATGTCTTTACATGGGCTTA
TATGCCTATGAAAATTGTAATACAACTTTCAGCAACGGATCTCTTGGCTCTCGCATCGAT
GAAGAACGCAGCGAAATGCGATAAGTAATGTGAATTGCAGAATTCAGTGAATCATCGAAT
CTTTGAACGCATCTTGCGCTCCTTGGTATTCCGAGGAGCATGCCTGTTTGAGTGTCATTA
AATTCTCAACTCTCTTCTAC-TTTTTGTAAAAGAGAGCTTGGACTGTGGAGGCTTGCTGG
CCACTTTTTGGGGTCAGCTCCTCTGAAATGCATTAGCGGAACCGTTTGCGATCTGCCACA
AGTGTGATAAGTTATCTACACTGGCGAGGGGATTGCTCTCTGTAATGTTCAGCTTCTAAT
TGTCTCTACTTTGTGAGACTACTTTTGAATGCTTGACCTCAAATCAGGTAGGACTACCC-
GCTGAACTTAA
>BC4-48
TTTCCGTAGGTGAACCTGCGGAAGGATCATTATTGAATTATGTTTCTAGATAGGTTGTAG
CTGGCTCTTTAGAGCATGTGCACGCCTGTTTGGACTTCATTTTCATCCACCTGTGCACCT
ATTGTAGTCTTTGGTTGGGTTAGGAGGAAGTGGTCATTGTGTCAGCATCTGCTGGATGTG
AGGACTTGCATTGTGAAAGCTTTGCTGTCCTTGATGTGATCATGGAATCTCTTTCTCACT
AGAGTCTATGTCACTCATTATACTCTGTCGAATGTCATTGAATGTCTTTACATGGGCTTA
TATGCCTATGAAAATTGTAATACAACTTTCAGCAACGGATCTCTTGGCTCTCGCATCGAT
GAAGAACGCAGCGAAATGCGATAAGTAATGTGAATTGCAGAATTCAGTGAATCATCGAAT
CTTTGAACGCATCTTGCGCTCCTTGGTATTCCGAGGAGCATGCCTGTTTGAGTGTCATTA
AATTCTCAACTCTCTTCTAC-TTTTTGTAAAAGAGAGCTTGGACTGTGGAGGCTTGCTGG

CCACTTTTTGGGGTCAGCTCCTCTGAAATGCATTAGCGGAACCGTTTGCGATCTGCCACA
AGTGTGATAAGTTATCTACACTGGCGAGGGGATTGCTCTCTGTAATGTTCAGCTTCTAAT
TGTCTCTACTTTGTGAGACTACTTTTGAATGCTTGACCTCAAATCAGGTAGGACTACCC-
GCTGAACTTAA
>BC4-51
TTTCCGTAGGTGAACCTGCGGAAGGATCATTATTGAATTATGTTTCTAGATAGGTTGTAG
CTGGCTCTTTAGAGCATGTGCACGCCTGTTTGGACTTCATTTTCATCCACCTGTGCACCT
ATTGTAGTCTTTGGTTGGGTTAGGAGGAAGTGGTCATTGTGTCAGCATCTGCTGGATGTG
AGGACTTGCATTGTGAAAGCTTTGCTGTCCTTGATGTGATCATGGAATCTCTTTCTCACT
AGAGTCTATGTCACTCATTATACTCTGTCGAATGTCATTGAATGTCTTTACATGGGCTTA
TATGCCTATGAAAATTGTAATACAACTTTCAGCAACGGATCTCTTGGCTCTCGCATCGAT
GAAGAACGCAGCGAAATGCGATAAGTAATGTGAATTGCAGAATTCAGTGAATCATCGAAT
CTTTGAACGCATCTTGCGCTCCTTGGTATTCCGAGGAGCATGCCTGTTTGAGTGTCATTA
AATTCTCAACTCTCTTCTAC-TTTTTGTAAAAGAGAGCTTGGACTGTGGAGGCTTGCTGG
CCACTTTTTGGGGTCAGCTCCTCTGAAATGCATTAGCGGAACCGTTTGCGATCTGCCACA
AGTGTGATAAGTTATCTACACTGGCGAGGGGATTGCTCTCTGTAATGTTCAGCTTCTAAT
TGTCTCTACTTTGTGAGACTACTTTTGAATGCTTGACCTCAAATCAGGTAGGACTACCC-
GCTGAACTTAA
>BC4-52
TTTCCGTAGGTGAACCTGCGGAAGGATCATTATTGAATTATGTTTCTAGATAGGTTGTAG
CTGGCTCTTTAGAGCATGTGCACGCCTGTTTGGACTTCATTTTCATCCACCTGTGCACCT
ATTGTAGTCTTTGGTTGGGTTAGGAGGAAGTGGTCATTGTGTCAGCATCTGCTGGATGTG
AGGACTTGCATTGTGAAAGCTTTGCTGTCCTTGATGTGATCATGGAATCTCTTTCTCACT
AGAGTCTATGTCACTCATTATACTCTGTCGAATGTCATTGAATGTCTTTACATGGGCTTA
TATGCCTATGAAAATTGTAATACAACTTTCAGCAACGGATCTCTTGGCTCTCGCATCGAT
GAAGAACGCAGCGAAATGCGATAAGTAATGTGAATTGCAGAATTCAGTGAATCATCGAAT
CTTTGAACGCATCTTGCGCTCCTTGGTATTCCGAGGAGCATGCCTGTTTGAGTGTCATTA
AATTCTCAACTCTCTTCTAC-TTTTTGTAAAAGAGAGCTTGGACTGTGGAGGCTTGCTGG
CCACTTTTTGGGGTCAGCTCCTCTGAAATGCATTAGCGGAACCGTTTGCGATCTGCCACA
AGTGTGATAAGTTATCTACACTGGCGAGGGGATTGCTCTCTGTAATGTTCAGCTTCTAAT
TGTCTCTACTTTGTGAGACTACTTTTGAATGCTTGACCTCAAATCAGGTAGGACTACCC-
GCTGAACTTAA
>BC4-56
TTTCCGTAGGTGAACCTGCGGAAGGATCATTATTGAATTATGTTTCTAGATAGGTTGTAG
CTGGCTCTTTAGAGCATGTGCACGCCTGTTTGGACTTCATTTTCATCCACCTGTGCACCT
ATTGTAGTCTTTGGTTGGGTTAGGAGGAAGTGGTCATTGTGTCAGCATCTGCTGGATGTG
AGGACTTGCATTGTGAAAGCTTTGCTGTCCTTGATGTGATCATGGAATCTCTTTCTCACT
AGAGTCTATGTCACTCATTATACTCTGTCGAATGTCATTGAATGTCTTTACATGGGCTTA
TATGCCTATGAAAATTGTAATACAACTTTCAGCAACGGATCTCTTGGCTCTCGCATCGAT
GAAGAACGCAGCGAAATGCGATAAGTAATGTGAATTGCAGAATTCAGTGAATCATCGAAT
CTTTGAACGCATCTTGCGCTCCTTGGTATTCCGAGGAGCATGCCTGTTTGAGTGTCATTA
AATTCTCAACTCTCTTCTAC-TTTTTGTAAAAGAGAGCTTGGACTGTGGAGGCTTGCTGG
CCACTTTTTGGGGTCAGCTCCTCTGAAATGCATTAGCGGAACCGTTTGCGATCTGCCACA
AGTGTGATAAGTTATCTACACTGGCGAGGGGATTGCTCTCTGTAATGTTCAGCTTCTAAT
TGTCTCTACTTTGTGAGACTACTTTTGAATGCTTGACCTCAAATCAGGTAGGACTACCC-
GCTGAACTTAA
>BC4-59
TTTCCGTAGGTGAACCTGCGGAAGGATCATTATTGAATTATGTTTCTAGATAGGTTGTAG
CTGGCTCTTTAGAGCATGTGCACGCCTGTTTGGACTTCATTTTCATCCACCTGTGCACCT
ATTGTAGTCTTTGGTTGGGTTAGGAGGAAGTGGTCATTGTGTCAGCATCTGCTGGATGTG

AGGACTTGCATTGTGAAAGCTTTGCTGTCCTTGATGTGATCATGGAATCTCTTTCTCACT
AGAGTCTATGTCACTCATTATACTCTGTCGAATGTCATTGAATGTCTTTACATGGGCTTA
TATGCCTATGAAAATTGTAATACAACTTTCAGCAACGGATCTCTTGGCTCTCGCATCGAT
GAAGAACGCAGCGAAATGCGATAAGTAATGTGAATTGCAGAATTCAGTGAATCATCGAAT
CTTTGAACGCATCTTGCGCTCCTTGGTATTCCGAGGAGCATGCCTGTTTGAGTGTCATTA
AATTCTCAACTCTCTTCTAC-TTTTTGTAAAAGAGAGCTTGGACTGTGGAGGCTTGCTGG
CCACTTTTTGGGGTCAGCTCCTCTGAAATGCATTAGCGGAACCGTTTGCGATCTGCCACA
AGTGTGATAAGTTATCTACACTGGCGAGGGGATTGCTCTCTGTAATGTTCAGCTTCTAAT
TGTCTCTACTTTGTGAGACTACTTTTGAATGCTTGACCTCAAATCAGGTAGGACTACCC-
GCTGAACTTAA
>BC4-65
TTTCCGTAGGTGAACCTGCGGAAGGATCATTATTGAATTATGTTTCTAGATAGGTTGTAG
CTGGCTCTTTAGAGCATGTGCACGCCTGTTTGGACTTCATTTTCATCCACCTGTGCACCT
ATTGTAGTCTTTGGTTGGGTTAGGAGGAAGTGGTCATTGTGTCAGCATCTGCTGGATGTG
AGGACTTGCATTGTGAAAGCTTTGCTGTCCTTGATGTGATCATGGAATCTCTTTCTCACT
AGAGTCTATGTCACTCATTATACTCTGTCGAATGTCATTGAATGTCTTTACATGGGCTTA
TATGCCTATGAAAATTGTAATACAACTTTCAGCAACGGATCTCTTGGCTCTCGCATCGAT
GAAGAACGCAGCGAAATGCGATAAGTAATGTGAATTGCAGAATTCAGTGAATCATCGAAT
CTTTGAACGCATCTTGCGCTCCTTGGTATTCCGAGGAGCATGCCTGTTTGAGTGTCATTA
AATTCTCAACTCTCTTCTAC-TTTTTGTAAAAGAGAGCTTGGACTGTGGAGGCTTGCTGG
CCACTTTTTGGGGTCAGCTCCTCTGAAATGCATTAGCGGAACCGTTTGCGATCTGCCACA
AGTGTGATAAGTTATCTACACTGGCGAGGGGATTGCTCTCTGTAATGTTCAGCTTCTAAT
TGTCTCTACTTTGTGAGACTACTTTTGAATGCTTGACCTCAAATCAGGTAGGACTACCC-
GCTGAACTTAA
>BC5-2
TTTCCGTAGGTGAACCTGCGGAAGGATCATTATTGAATTATGTTTCTAGATAGGTTGTAG
CTGGCTCTTTAGAGCATGTGCACGCCTGTTTGGACTTCATTTTCATCCACCTGTGCACCT
ATTGTAGTCTTTGGTTGGGTTAGGAGGAAGTGGTCATTGTGTCAGCATCTGCTGGATGTG
AGGACTTGCATTGTGAAAGCTTTGCTGTCCTTGATGTGATCATGGAATCTCTTTCTCACT
AGAGTCTATGTCACTCATTATACTCTGTCGAATGTCATTGAATGTCTTTACATGGGCTTA
TATGCCTATGAAAATTGTAATACAACTTTCAGCAACGGATCTCTTGGCTCTCGCATCGAT
GAAGAACGCAGCGAAATGCGATAAGTAATGTGAATTGCAGAATTCAGTGAATCATCGAAT
CTTTGAACGCATCTTGCGCTCCTTGGTATTCCGAGGAGCATGCCTGTTTGAGTGTCATTA
AATTCTCAACTCTCTTCTAC-TTTTTGTAAAAGAGAGCTTGGACTGTGGAGGCTTGCTGG
CCACTTTTTGGGGTCAGCTCCTCTGAAATGCATTAGCGGAACCGTTTGCGATCTGCCACA
AGTGTGATAAGTTATCTACACTGGCGAGGGGATTGCTCTCTGTAATGTTCAGCTTCTAAT
TGTCTCTACTTTGTGAGACTACTTTTGAATGCTTGACCTCAAATCAGGTAGGACTACCC-
GCTGAACTTAA
>BC5-4
TTTCCGTAGGTGAACCTGCGGAAGGATCATTATTGAATTATGTTTCTAGATAGGTTGTAG
CTGGCTCTTTAGAGCATGTGCACGCCTGTTTGGACTTCATTTTCATCCACCTGTGCACCT
ATTGTAGTCTTTGGTTGGGTTAGGAGGAAGTGGTCATTGTGTCAGCATCTGCTGGATGTG
AGGACTTGCATTGTGAAAGCTTTGCTGTCCTTGATGTGATCATGGAATCTCTTTCTCACT
AGAGTCTATGTCACTCATTATACTCTGTCGAATGTCATTGAATGTCTTTACATGGGCTTA
TATGCCTATGAAAATTGTAATACAACTTTCAGCAACGGATCTCTTGGCTCTCGCATCGAT
GAAGAACGCAGCGAAATGCGATAAGTAATGTGAATTGCAGAATTCAGTGAATCATCGAAT
CTTTGAACGCATCTTGCGCTCCTTGGTATTCCGAGGAGCATGCCTGTTTGAGTGTCATTA
AATTCTCAACTCTCTTCTAC-TTTTTGTAAAAGAGAGCTTGGACTGTGGAGGCTTGCTGG
CCACTTTTTGGGGTCAGCTCCTCTGAAATGCATTAGCGGAACCGTTTGCGATCTGCCACA
AGTGTGATAAGTTATCTACACTGGCGAGGGGATTGCTCTCTGTAATGTTCAGCTTCTAAT

TGTCTCTACTTTGTGAGACTACTTTTGAATGCTTGACCTCAAATCAGGTAGGACTACCC-
GCTGAACTTAA
>BC5-5
TTTCCGTAGGTGAACCTGCGGAAGGATCATTATTGAATTATGTTTCTAGATAGGTTGTAG
CTGGCTCTTTAGAGCATGTGCACGCCTGTTTGGACTTCATTTTCATCCACCTGTGCACCT
ATTGTAGTCTTTGGTTGGGTTAGGAGGAAGTGGTCATTGTGTCAGCATCTGCTGGATGTG
AGGACTTGCATTGTGAAAGCTTTGCTGTCCTTGATGTGATCATGGAATCTCTTTCTCACT
AGAGTCTATGTCACTCATTATACTCTGTCGAATGTCATTGAATGTCTTTACATGGGCTTA
TATGCCTATGAAAATTGTAATACAACTTTCAGCAACGGATCTCTTGGCTCTCGCATCGAT
GAAGAACGCAGCGAAATGCGATAAGTAATGTGAATTGCAGAATTCAGTGAATCATCGAAT
CTTTGAACGCATCTTGCGCTCCTTGGTATTCCGAGGAGCATGCCTGTTTGAGTGTCATTA
AATTCTCAACTCTCTTCTAC-TTTTTGTAAAAGAGAGCTTGGACTGTGGAGGCTTGCTGG
CCACTTTTTGGGGTCAGCTCCTCTGAAATGCATTAGCGGAACCGTTTGCGATCTGCCACA
AGTGTGATAAGTTATCTACACTGGCGAGGGGATTGCTCTCTGTAATGTTCAGCTTCTAAT
TGTCTCTACTTTGTGAGACTACTTTTGAATGCTTGACCTCAAATCAGGTAGGACTACCC-
GCTGAACTTAA
>BC5-6
TTTCCGTAGGTGAACCTGCGGAAGGATCATTATTGAATTATGTTTCTAGATAGGTTGTAG
CTGGCTCTTTAGAGCATGTGCACGCCTGTTTGGACTTCATTTTCATCCACCTGTGCACCT
ATTGTAGTCTTTGGTTGGGTTAGGAGGAAGTGGTCATTGTGTCAGCATCTGCTGGATGTG
AGGACTTGCATTGTGAAAGCTTTGCTGTCCTTGATGTGATCATGGAATCTCTTTCTCACT
AGAGTCTATGTCACTCATTATACTCTGTCGAATGTCATTGAATGTCTTTACATGGGCTTA
TATGCCTATGAAAATTGTAATACAACTTTCAGCAACGGATCTCTTGGCTCTCGCATCGAT
GAAGAACGCAGCGAAATGCGATAAGTAATGTGAATTGCAGAATTCAGTGAATCATCGAAT
CTTTGAACGCATCTTGCGCTCCTTGGTATTCCGAGGAGCATGCCTGTTTGAGTGTCATTA
AATTCTCAACTCTCTTCTAC-TTTTTGTAAAAGAGAGCTTGGACTGTGGAGGCTTGCTGG
CCACTTTTTGGGGTCAGCTCCTCTGAAATGCATTAGCGGAACCGTTTGCGATCTGCCACA
AGTGTGATAAGTTATCTACACTGGCGAGGGGATTGCTCTCTGTAATGTTCAGCTTCTAAT
TGTCTCTACTTTGTGAGACTACTTTTGAATGCTTGACCTCAAATCAGGTAGGACTACCC-
GCTGAACTTAA
>BC5-7
TTTCCGTAGGTGAACCTGCGGAAGGATCATTATTGAATTATGTTTCTAGATAGGTTGTAG
CTGGCTCTTTAGAGCATGTGCACGCCTGTTTGGACTTCATTTTCATCCACCTGTGCACCT
ATTGTAGTCTTTGGTTGGGTTAGGAGGAAGTGGTCATTGTGTCAGCATCTGCTGGATGTG
AGGACTTGCATTGTGAAAGCTTTGCTGTCCTTGATGTGATCATGGAATCTCTTTCTCACT
AGAGTCTATGTCACTCATTATACTCTGTCGAATGTCATTGAATGTCTTTACATGGGCTTA
TATGCCTATGAAAATTGTAATACAACTTTCAGCAACGGATCTCTTGGCTCTCGCATCGAT
GAAGAACGCAGCGAAATGCGATAAGTAATGTGAATTGCAGAATTCAGTGAATCATCGAAT
CTTTGAACGCATCTTGCGCTCCTTGGTATTCCGAGGAGCATGCCTGTTTGAGTGTCATTA
AATTCTCAACTCTCTTCTAC-TTTTTGTAAAAGAGAGCTTGGACTGTGGAGGCTTGCTGG
CCACTTTTTGGGGTCAGCTCCTCTGAAATGCATTAGCGGAACCGTTTGCGATCTGCCACA
AGTGTGATAAGTTATCTACACTGGCGAGGGGATTGCTCTCTGTAATGTTCAGCTTCTAAT
TGTCTCTACTTTGTGAGACTACTTTTGAATGCTTGACCTCAAATCAGGTAGGACTACCC-
GCTGAACTTAA
>BC5-8
TTTCCGTAGGTGAACCTGCGGAAGGATCATTATTGAATTATGTTTCTAGATAGGTTGTAG
CTGGCTCTTTAGAGCATGTGCACGCCTGTTTGGACTTCATTTTCATCCACCTGTGCACCT
ATTGTAGTCTTTGGTTGGGTTAGGAGGAAGTGGTCATTGTGTCAGCATCTGCTGGATGTG
AGGACTTGCATTGTGAAAGCTTTGCTGTCCTTGATGTGATCATGGAATCTCTTTCTCACT
AGAGTCTATGTCACTCATTATACTCTGTCGAATGTCATTGAATGTCTTTACATGGGCTTA

TATGCCTATGAAAATTGTAATACAACTTTCAGCAACGGATCTCTTGGCTCTCGCATCGAT
GAAGAACGCAGCGAAATGCGATAAGTAATGTGAATTGCAGAATTCAGTGAATCATCGAAT
CTTTGAACGCATCTTGCGCTCCTTGGTATTCCGAGGAGCATGCCTGTTTGAGTGTCATTA
AATTCTCAACTCTCTTCTAC-TTTTTGTAAAAGAGAGCTTGGACTGTGGAGGCTTGCTGG
CCACTTTTTGGGGTCAGCTCCTCTGAAATGCATTAGCGGAACCGTTTGCGATCTGCCACA
AGTGTGATAAGTTATCTACACTGGCGAGGGGATTGCTCTCTGTAATGTTCAGCTTCTAAT
TGTCTCTACTTTGTGAGACTACTTTTGAATGCTTGACCTCAAATCAGGTAGGACTACCC-
GCTGAACTTAA
>BC5-10
TTTCCGTAGGTGAACCTGCGGAAGGATCATTATTGAATTATGTTTCTAGATAGGTTGTAG
CTGGCTCTTTAGAGCATGTGCACGCCTGTTTGGACTTCATTTTCATCCACCTGTGCACCT
ATTGTAGTCTTTGGTTGGGTTAGGAGGAAGTGGTCATTGTGTCAGCATCTGCTGGATGTG
AGGACTTGCATTGTGAAAGCTTTGCTGTCCTTGATGTGATCATGGAATCTCTTTCTCACT
AGAGTCTATGTCACTCATTATACTCTGTCGAATGTCATTGAATGTCTTTACATGGGCTTA
TATGCCTATGAAAATTGTAATACAACTTTCAGCAACGGATCTCTTGGCTCTCGCATCGAT
GAAGAACGCAGCGAAATGCGATAAGTAATGTGAATTGCAGAATTCAGTGAATCATCGAAT
CTTTGAACGCATCTTGCGCTCCTTGGTATTCCGAGGAGCATGCCTGTTTGAGTGTCATTA
AATTCTCAACTCTCTTCTAC-TTTTTGTAAAAGAGAGCTTGGACTGTGGAGGCTTGCTGG
CCACTTTTTGGGGTCAGCTCCTCTGAAATGCATTAGCGGAACCGTTTGCGATCTGCCACA
AGTGTGATAAGTTATCTACACTGGCGAGGGGATTGCTCTCTGTAATGTTCAGCTTCTAAT
TGTCTCTACTTTGTGAGACTACTTTTGAATGCTTGACCTCAAATCAGGTAGGACTACCC-
GCTGAACTTAA
>BC5-11
TTTCCGTAGGTGAACCTGCGGAAGGATCATTATTGAATTATGTTTCTAGATAGGTTGTAG
CTGGCTCTTTAGAGCATGTGCACGCCTGTTTGGACTTCATTTTCATCCACCTGTGCACCT
ATTGTAGTCTTTGGTTGGGTTAGGAGGAAGTGGTCATTGTGTCAGCATCTGCTGGATGTG
AGGACTTGCATTGTGAAAGCTTTGCTGTCCTTGATGTGATCATGGAATCTCTTTCTCACT
AGAGTCTATGTCACTCATTATACTCTGTCGAATGTCATTGAATGTCTTTACATGGGCTTA
TATGCCTATGAAAATTGTAATACAACTTTCAGCAACGGATCTCTTGGCTCTCGCATCGAT
GAAGAACGCAGCGAAATGCGATAAGTAATGTGAATTGCAGAATTCAGTGAATCATCGAAT
CTTTGAACGCATCTTGCGCTCCTTGGTATTCCGAGGAGCATGCCTGTTTGAGTGTCATTA
AATTCTCAACTCTCTTCTAC-TTTTTGTAAAAGAGAGCTTGGACTGTGGAGGCTTGCTGG
CCACTTTTTGGGGTCAGCTCCTCTGAAATGCATTAGCGGAACCGTTTGCGATCTGCCACA
AGTGTGATAAGTTATCTACACTGGCGAGGGGATTGCTCTCTGTAATGTTCAGCTTCTAAT
TGTCTCTACTTTGTGAGACTACTTTTGAATGCTTGACCTCAAATCAGGTAGGACTACCC-
GCTGAACTTAA
>BC5-12
TTTCCGTAGGTGAACCTGCGGAAGGATCATTATTGAATTATGTTTCTAGATAGGTTGTAG
CTGGCTCTTTAGAGCATGTGCACGCCTGTTTGGACTTCATTTTCATCCACCTGTGCACCT
ATTGTAGTCTTTGGTTGGGTTAGGAGGAAGTGGTCATTGTGTCAGCATCTGCTGGATGTG
AGGACTTGCATTGTGAAAGCTTTGCTGTCCTTGATGTGATCATGGAATCTCTTTCTCACT
AGAGTCTATGTCACTCATTATACTCTGTCGAATGTCATTGAATGTCTTTACATGGGCTTA
TATGCCTATGAAAATTGTAATACAACTTTCAGCAACGGATCTCTTGGCTCTCGCATCGAT
GAAGAACGCAGCGAAATGCGATAAGTAATGTGAATTGCAGAATTCAGTGAATCATCGAAT
CTTTGAACGCATCTTGCGCTCCTTGGTATTCCGAGGAGCATGCCTGTTTGAGTGTCATTA
AATTCTCAACTCTCTTCTAC-TTTTTGTAAAAGAGAGCTTGGACTGTGGAGGCTTGCTGG
CCACTTTTTGGGGTCAGCTCCTCTGAAATGCATTAGCGGAACCGTTTGCGATCTGCCACA
AGTGTGATAAGTTATCTACACTGGCGAGGGGATTGCTCTCTGTAATGTTCAGCTTCTAAT
TGTCTCTACTTTGTGAGACTACTTTTGAATGCTTGACCTCAAATCAGGTAGGACTACCC-
GCTGAACTTAA

>BC5-13
TTTCCGTAGGTGAACCTGCGGAAGGATCATTATTGAATTATGTTTCTAGATAGGTTGTAG
CTGGCTCTTTAGAGCATGTGCACGCCTGTTTGGACTTCATTTTCATCCACCTGTGCACCT
ATTGTAGTCTTTGGTTGGGTTAGGAGGAAGTGGTCATTGTGTCAGCATCTGCTGGATGTG
AGGACTTGCATTGTGAAAGCTTTGCTGTCCTTGATGTGATCATGGAATCTCTTTCTCACT
AGAGTCTATGTCACTCATTATACTCTGTCGAATGTCATTGAATGTCTTTACATGGGCTTA
TATGCCTATGAAAATTGTAATACAACTTTCAGCAACGGATCTCTTGGCTCTCGCATCGAT
GAAGAACGCAGCGAAATGCGATAAGTAATGTGAATTGCAGAATTCAGTGAATCATCGAAT
CTTTGAACGCATCTTGCGCTCCTTGGTATTCCGAGGAGCATGCCTGTTTGAGTGTCATTA
AATTCTCAACTCTCTTCTAC-TTTTTGTAAAAGAGAGCTTGGACTGTGGAGGCTTGCTGG
CCACTTTTTGGGGTCAGCTCCTCTGAAATGCATTAGCGGAACCGTTTGCGATCTGCCACA
AGTGTGATAAGTTATCTACACTGGCGAGGGGATTGCTCTCTGTAATGTTCAGCTTCTAAT
TGTCTCTACTTTGTGAGACTACTTTTGAATGCTTGACCTCAAATCAGGTAGGACTACCC-
GCTGAACTTAA
>BC5-17
TTTCCGTAGGTGAACCTGCGGAAGGATCATTATTGAATTATGTTTCTAGATAGGTTGTAG
CTGGCTCTTTAGAGCATGTGCACGCCTGTTTGGACTTCATTTTCATCCACCTGTGCACCT
ATTGTAGTCTTTGGTTGGGTTAGGAGGAAGTGGTCATTGTGTCAGCATCTGCTGGATGTG
AGGACTTGCATTGTGAAAGCTTTGCTGTCCTTGATGTGATCATGGAATCTCTTTCTCACT
AGAGTCTATGTCACTCATTATACTCTGTCGAATGTCATTGAATGTCTTTACATGGGCTTA
TATGCCTATGAAAATTGTAATACAACTTTCAGCAACGGATCTCTTGGCTCTCGCATCGAT
GAAGAACGCAGCGAAATGCGATAAGTAATGTGAATTGCAGAATTCAGTGAATCATCGAAT
CTTTGAACGCATCTTGCGCTCCTTGGTATTCCGAGGAGCATGCCTGTTTGAGTGTCATTA
AATTCTCAACTCTCTTCTAC-TTTTTGTAAAAGAGAGCTTGGACTGTGGAGGCTTGCTGG
CCACTTTTTGGGGTCAGCTCCTCTGAAATGCATTAGCGGAACCGTTTGCGATCTGCCACA
AGTGTGATAAGTTATCTACACTGGCGAGGGGATTGCTCTCTGTAATGTTCAGCTTCTAAT
TGTCTCTACTTTGTGAGACTACTTTTGAATGCTTGACCTCAAATCAGGTAGGACTACCC-
GCTGAACTTAA
>BC5-19
TTTCCGTAGGTGAACCTGCGGAAGGATCATTATTGAATTATGTTTCTAGATAGGTTGTAG
CTGGCTCTTTAGAGCATGTGCACGCCTGTTTGGACTTCATTTTCATCCACCTGTGCACCT
ATTGTAGTCTTTGGTTGGGTTAGGAGGAAGTGGTCATTGTGTCAGCATCTGCTGGATGTG
AGGACTTGCATTGTGAAAGCTTTGCTGTCCTTGATGTGATCATGGAATCTCTTTCTCACT
AGAGTCTATGTCACTCATTATACTCTGTCGAATGTCATTGAATGTCTTTACATGGGCTTA
TATGCCTATGAAAATTGTAATACAACTTTCAGCAACGGATCTCTTGGCTCTCGCATCGAT
GAAGAACGCAGCGAAATGCGATAAGTAATGTGAATTGCAGAATTCAGTGAATCATCGAAT
CTTTGAACGCATCTTGCGCTCCTTGGTATTCCGAGGAGCATGCCTGTTTGAGTGTCATTA
AATTCTCAACTCTCTTCTAC-TTTTTGTAAAAGAGAGCTTGGACTGTGGAGGCTTGCTGG
CCACTTTTTGGGGTCAGCTCCTCTGAAATGCATTAGCGGAACCGTTTGCGATCTGCCACA
AGTGTGATAAGTTATCTACACTGGCGAGGGGATTGCTCTCTGTAATGTTCAGCTTCTAAT
TGTCTCTACTTTGTGAGACTACTTTTGAATGCTTGACCTCAAATCAGGTAGGACTACCC-
GCTGAACTTAA
>BC5-20
TTTCCGTAGGTGAACCTGCGGAAGGATCATTATTGAATTATGTTTCTAGATAGGTTGTAG
CTGGCTCTTTAGAGCATGTGCACGCCTGTTTGGACTTCATTTTCATCCACCTGTGCACCT
ATTGTAGTCTTTGGTTGGGTTAGGAGGAAGTGGTCATTGTGTCAGCATCTGCTGGATGTG
AGGACTTGCATTGTGAAAGCTTTGCTGTCCTTGATGTGATCATGGAATCTCTTTCTCACT
AGAGTCTATGTCACTCATTATACTCTGTCGAATGTCATTGAATGTCTTTACATGGGCTTA
TATGCCTATGAAAATTGTAATACAACTTTCAGCAACGGATCTCTTGGCTCTCGCATCGAT
GAAGAACGCAGCGAAATGCGATAAGTAATGTGAATTGCAGAATTCAGTGAATCATCGAAT

CTTTGAACGCATCTTGCGCTCCTTGGTATTCCGAGGAGCATGCCTGTTTGAGTGTCATTA
AATTCTCAACTCTCTTCTAC-TTTTTGTAAAAGAGAGCTTGGACTGTGGAGGCTTGCTGG
CCACTTTTTGGGGTCAGCTCCTCTGAAATGCATTAGCGGAACCGTTTGCGATCTGCCACA
AGTGTGATAAGTTATCTACACTGGCGAGGGGATTGCTCTCTGTAATGTTCAGCTTCTAAT
TGTCTCTACTTTGTGAGACTACTTTTGAATGCTTGACCTCAAATCAGGTAGGACTACCC-
GCTGAACTTAA
>BC5-22
TTTCCGTAGGTGAACCTGCGGAAGGATCATTATTGAATTATGTTTCTAGATAGGTTGTAG
CTGGCTCTTTAGAGCATGTGCACGCCTGTTTGGACTTCATTTTCATCCACCTGTGCACCT
ATTGTAGTCTTTGGTTGGGTTAGGAGGAAGTGGTCATTGTGTCAGCATCTGCTGGATGTG
AGGACTTGCATTGTGAAAGCTTTGCTGTCCTTGATGTGATCATGGAATCTCTTTCTCACT
AGAGTCTATGTCACTCATTATACTCTGTCGAATGTCATTGAATGTCTTTACATGGGCTTA
TATGCCTATGAAAATTGTAATACAACTTTCAGCAACGGATCTCTTGGCTCTCGCATCGAT
GAAGAACGCAGCGAAATGCGATAAGTAATGTGAATTGCAGAATTCAGTGAATCATCGAAT
CTTTGAACGCATCTTGCGCTCCTTGGTATTCCGAGGAGCATGCCTGTTTGAGTGTCATTA
AATTCTCAACTCTCTTCTAC-TTTTTGTAAAAGAGAGCTTGGACTGTGGAGGCTTGCTGG
CCACTTTTTGGGGTCAGCTCCTCTGAAATGCATTAGCGGAACCGTTTGCGATCTGCCACA
AGTGTGATAAGTTATCTACACTGGCGAGGGGATTGCTCTCTGTAATGTTCAGCTTCTAAT
TGTCTCTACTTTGTGAGACTACTTTTGAATGCTTGACCTCAAATCAGGTAGGACTACCC-
GCTGAACTTAA
>BC5-23
TTTCCGTAGGTGAACCTGCGGAAGGATCATTATTGAATTATGTTTCTAGATAGGTTGTAG
CTGGCTCTTTAGAGCATGTGCACGCCTGTTTGGACTTCATTTTCATCCACCTGTGCACCT
ATTGTAGTCTTTGGTTGGGTTAGGAGGAAGTGGTCATTGTGTCAGCATCTGCTGGATGTG
AGGACTTGCATTGTGAAAGCTTTGCTGTCCTTGATGTGATCATGGAATCTCTTTCTCACT
AGAGTCTATGTCACTCATTATACTCTGTCGAATGTCATTGAATGTCTTTACATGGGCTTA
TATGCCTATGAAAATTGTAATACAACTTTCAGCAACGGATCTCTTGGCTCTCGCATCGAT
GAAGAACGCAGCGAAATGCGATAAGTAATGTGAATTGCAGAATTCAGTGAATCATCGAAT
CTTTGAACGCATCTTGCGCTCCTTGGTATTCCGAGGAGCATGCCTGTTTGAGTGTCATTA
AATTCTCAACTCTCTTCTAC-TTTTTGTAAAAGAGAGCTTGGACTGTGGAGGCTTGCTGG
CCACTTTTTGGGGTCAGCTCCTCTGAAATGCATTAGCGGAACCGTTTGCGATCTGCCACA
AGTGTGATAAGTTATCTACACTGGCGAGGGGATTGCTCTCTGTAATGTTCAGCTTCTAAT
TGTCTCTACTTTGTGAGACTACTTTTGAATGCTTGACCTCAAATCAGGTAGGACTACCC-
GCTGAACTTAA
>BC5-29
TTTCCGTAGGTGAACCTGCGGAAGGATCATTATTGAATTATGTTTCTAGATAGGTTGTAG
CTGGCTCTTTAGAGCATGTGCACGCCTGTTTGGACTTCATTTTCATCCACCTGTGCACCT
ATTGTAGTCTTTGGTTGGGTTAGGAGGAAGTGGTCATTGTGTCAGCATCTGCTGGATGTG
AGGACTTGCATTGTGAAAGCTTTGCTGTCCTTGATGTGATCATGGAATCTCTTTCTCACT
AGAGTCTATGTCACTCATTATACTCTGTCGAATGTCATTGAATGTCTTTACATGGGCTTA
TATGCCTATGAAAATTGTAATACAACTTTCAGCAACGGATCTCTTGGCTCTCGCATCGAT
GAAGAACGCAGCGAAATGCGATAAGTAATGTGAATTGCAGAATTCAGTGAATCATCGAAT
CTTTGAACGCATCTTGCGCTCCTTGGTATTCCGAGGAGCATGCCTGTTTGAGTGTCATTA
AATTCTCAACTCTCTTCTAC-TTTTTGTAAAAGAGAGCTTGGACTGTGGAGGCTTGCTGG
CCACTTTTTGGGGTCAGCTCCTCTGAAATGCATTAGCGGAACCGTTTGCGATCTGCCACA
AGTGTGATAAGTTATCTACACTGGCGAGGGGATTGCTCTCTGTAATGTTCAGCTTCTAAT
TGTCTCTACTTTGTGAGACTACTTTTGAATGCTTGACCTCAAATCAGGTAGGACTACCC-
GCTGAACTTAA
>BC5-33
TTTCCGTAGGTGAACCTGCGGAAGGATCATTATTGAATTATGTTTCTAGATAGGTTGTAG

CTGGCTCTTTAGAGCATGTGCACGCCTGTTTGGACTTCATTTTCATCCACCTGTGCACCT
ATTGTAGTCTTTGGTTGGGTTAGGAGGAAGTGGTCATTGTGTCAGCATCTGCTGGATGTG
AGGACTTGCATTGTGAAAGCTTTGCTGTCCTTGATGTGATCATGGAATCTCTTTCTCACT
AGAGTCTATGTCACTCATTATACTCTGTCGAATGTCATTGAATGTCTTTACATGGGCTTA
TATGCCTATGAAAATTGTAATACAACTTTCAGCAACGGATCTCTTGGCTCTCGCATCGAT
GAAGAACGCAGCGAAATGCGATAAGTAATGTGAATTGCAGAATTCAGTGAATCATCGAAT
CTTTGAACGCATCTTGCGCTCCTTGGTATTCCGAGGAGCATGCCTGTTTGAGTGTCATTA
AATTCTCAACTCTCTTCTAC-TTTTTGTAAAAGAGAGCTTGGACTGTGGAGGCTTGCTGG
CCACTTTTTGGGGTCAGCTCCTCTGAAATGCATTAGCGGAACCGTTTGCGATCTGCCACA
AGTGTGATAAGTTATCTACACTGGCGAGGGGATTGCTCTCTGTAATGTTCAGCTTCTAAT
TGTCTCTACTTTGTGAGACTACTTTTGAATGCTTGACCTCAAATCAGGTAGGACTACCC-
GCTGAACTTAA
>BC5-34
TTTCCGTAGGTGAACCTGCGGAAGGATCATTATTGAATTATGTTTCTAGATAGGTTGTAG
CTGGCTCTTTAGAGCATGTGCACGCCTGTTTGGACTTCATTTTCATCCACCTGTGCACCT
ATTGTAGTCTTTGGTTGGGTTAGGAGGAAGTGGTCATTGTGTCAGCATCTGCTGGATGTG
AGGACTTGCATTGTGAAAGCTTTGCTGTCCTTGATGTGATCATGGAATCTCTTTCTCACT
AGAGTCTATGTCACTCATTATACTCTGTCGAATGTCATTGAATGTCTTTACATGGGCTTA
TATGCCTATGAAAATTGTAATACAACTTTCAGCAACGGATCTCTTGGCTCTCGCATCGAT
GAAGAACGCAGCGAAATGCGATAAGTAATGTGAATTGCAGAATTCAGTGAATCATCGAAT
CTTTGAACGCATCTTGCGCTCCTTGGTATTCCGAGGAGCATGCCTGTTTGAGTGTCATTA
AATTCTCAACTCTCTTCTAC-TTTTTGTAAAAGAGAGCTTGGACTGTGGAGGCTTGCTGG
CCACTTTTTGGGGTCAGCTCCTCTGAAATGCATTAGCGGAACCGTTTGCGATCTGCCACA
AGTGTGATAAGTTATCTACACTGGCGAGGGGATTGCTCTCTGTAATGTTCAGCTTCTAAT
TGTCTCTACTTTGTGAGACTACTTTTGAATGCTTGACCTCAAATCAGGTAGGACTACCC-
GCTGAACTTAA
>BC5-39
TTTCCGTAGGTGAACCTGCGGAAGGATCATTATTGAATTATGTTTCTAGATAGGTTGTAG
CTGGCTCTTTAGAGCATGTGCACGCCTGTTTGGACTTCATTTTCATCCACCTGTGCACCT
ATTGTAGTCTTTGGTTGGGTTAGGAGGAAGTGGTCATTGTGTCAGCATCTGCTGGATGTG
AGGACTTGCATTGTGAAAGCTTTGCTGTCCTTGATGTGATCATGGAATCTCTTTCTCACT
AGAGTCTATGTCACTCATTATACTCTGTCGAATGTCATTGAATGTCTTTACATGGGCTTA
TATGCCTATGAAAATTGTAATACAACTTTCAGCAACGGATCTCTTGGCTCTCGCATCGAT
GAAGAACGCAGCGAAATGCGATAAGTAATGTGAATTGCAGAATTCAGTGAATCATCGAAT
CTTTGAACGCATCTTGCGCTCCTTGGTATTCCGAGGAGCATGCCTGTTTGAGTGTCATTA
AATTCTCAACTCTCTTCTAC-TTTTTGTAAAAGAGAGCTTGGACTGTGGAGGCTTGCTGG
CCACTTTTTGGGGTCAGCTCCTCTGAAATGCATTAGCGGAACCGTTTGCGATCTGCCACA
AGTGTGATAAGTTATCTACACTGGCGAGGGGATTGCTCTCTGTAATGTTCAGCTTCTAAT
TGTCTCTACTTTGTGAGACTACTTTTGAATGCTTGACCTCAAATCAGGTAGGACTACCC-
GCTGAACTTAA
>BC5-40
TTTCCGTAGGTGAACCTGCGGAAGGATCATTATTGAATTATGTTTCTAGATAGGTTGTAG
CTGGCTCTTTAGAGCATGTGCACGCCTGTTTGGACTTCATTTTCATCCACCTGTGCACCT
ATTGTAGTCTTTGGTTGGGTTAGGAGGAAGTGGTCATTGTGTCAGCATCTGCTGGATGTG
AGGACTTGCATTGTGAAAGCTTTGCTGTCCTTGATGTGATCATGGAATCTCTTTCTCACT
AGAGTCTATGTCACTCATTATACTCTGTCGAATGTCATTGAATGTCTTTACATGGGCTTA
TATGCCTATGAAAATTGTAATACAACTTTCAGCAACGGATCTCTTGGCTCTCGCATCGAT
GAAGAACGCAGCGAAATGCGATAAGTAATGTGAATTGCAGAATTCAGTGAATCATCGAAT
CTTTGAACGCATCTTGCGCTCCTTGGTATTCCGAGGAGCATGCCTGTTTGAGTGTCATTA
AATTCTCAACTCTCTTCTAC-TTTTTGTAAAAGAGAGCTTGGACTGTGGAGGCTTGCTGG

CCACTTTTTGGGGTCAGCTCCTCTGAAATGCATTAGCGGAACCGTTTGCGATCTGCCACA
AGTGTGATAAGTTATCTACACTGGCGAGGGGATTGCTCTCTGTAATGTTCAGCTTCTAAT
TGTCTCTACTTTGTGAGACTACTTTTGAATGCTTGACCTCAAATCAGGTAGGACTACCC-
GCTGAACTTAA
>BC5-42
TTTCCGTAGGTGAACCTGCGGAAGGATCATTATTGAATTATGTTTCTAGATAGGTTGTAG
CTGGCTCTTTAGAGCATGTGCACGCCTGTTTGGACTTCATTTTCATCCACCTGTGCACCT
ATTGTAGTCTTTGGTTGGGTTAGGAGGAAGTGGTCATTGTGTCAGCATCTGCTGGATGTG
AGGACTTGCATTGTGAAAGCTTTGCTGTCCTTGATGTGATCATGGAATCTCTTTCTCACT
AGAGTCTATGTCACTCATTATACTCTGTCGAATGTCATTGAATGTCTTTACATGGGCTTA
TATGCCTATGAAAATTGTAATACAACTTTCAGCAACGGATCTCTTGGCTCTCGCATCGAT
GAAGAACGCAGCGAAATGCGATAAGTAATGTGAATTGCAGAATTCAGTGAATCATCGAAT
CTTTGAACGCATCTTGCGCTCCTTGGTATTCCGAGGAGCATGCCTGTTTGAGTGTCATTA
AATTCTCAACTCTCTTCTAC-TTTTTGTAAAAGAGAGCTTGGACTGTGGAGGCTTGCTGG
CCACTTTTTGGGGTCAGCTCCTCTGAAATGCATTAGCGGAACCGTTTGCGATCTGCCACA
AGTGTGATAAGTTATCTACACTGGCGAGGGGATTGCTCTCTGTAATGTTCAGCTTCTAAT
TGTCTCTACTTTGTGAGACTACTTTTGAATGCTTGACCTCAAATCAGGTAGGACTACCC-
GCTGAACTTAA
>BC5-43
TTTCCGTAGGTGAACCTGCGGAAGGATCATTATTGAATTATGTTTCTAGATAGGTTGTAG
CTGGCTCTTTAGAGCATGTGCACGCCTGTTTGGACTTCATTTTCATCCACCTGTGCACCT
ATTGTAGTCTTTGGTTGGGTTAGGAGGAAGTGGTCATTGTGTCAGCATCTGCTGGATGTG
AGGACTTGCATTGTGAAAGCTTTGCTGTCCTTGATGTGATCATGGAATCTCTTTCTCACT
AGAGTCTATGTCACTCATTATACTCTGTCGAATGTCATTGAATGTCTTTACATGGGCTTA
TATGCCTATGAAAATTGTAATACAACTTTCAGCAACGGATCTCTTGGCTCTCGCATCGAT
GAAGAACGCAGCGAAATGCGATAAGTAATGTGAATTGCAGAATTCAGTGAATCATCGAAT
CTTTGAACGCATCTTGCGCTCCTTGGTATTCCGAGGAGCATGCCTGTTTGAGTGTCATTA
AATTCTCAACTCTCTTCTAC-TTTTTGTAAAAGAGAGCTTGGACTGTGGAGGCTTGCTGG
CCACTTTTTGGGGTCAGCTCCTCTGAAATGCATTAGCGGAACCGTTTGCGATCTGCCACA
AGTGTGATAAGTTATCTACACTGGCGAGGGGATTGCTCTCTGTAATGTTCAGCTTCTAAT
TGTCTCTACTTTGTGAGACTACTTTTGAATGCTTGACCTCAAATCAGGTAGGACTACCC-
GCTGAACTTAA
>BC5-46
TTTCCGTAGGTGAACCTGCGGAAGGATCATTATTGAATTATGTTTCTAGATAGGTTGTAG
CTGGCTCTTTAGAGCATGTGCACGCCTGTTTGGACTTCATTTTCATCCACCTGTGCACCT
ATTGTAGTCTTTGGTTGGGTTAGGAGGAAGTGGTCATTGTGTCAGCATCTGCTGGATGTG
AGGACTTGCATTGTGAAAGCTTTGCTGTCCTTGATGTGATCATGGAATCTCTTTCTCACT
AGAGTCTATGTCACTCATTATACTCTGTCGAATGTCATTGAATGTCTTTACATGGGCTTA
TATGCCTATGAAAATTGTAATACAACTTTCAGCAACGGATCTCTTGGCTCTCGCATCGAT
GAAGAACGCAGCGAAATGCGATAAGTAATGTGAATTGCAGAATTCAGTGAATCATCGAAT
CTTTGAACGCATCTTGCGCTCCTTGGTATTCCGAGGAGCATGCCTGTTTGAGTGTCATTA
AATTCTCAACTCTCTTCTAC-TTTTTGTAAAAGAGAGCTTGGACTGTGGAGGCTTGCTGG
CCACTTTTTGGGGTCAGCTCCTCTGAAATGCATTAGCGGAACCGTTTGCGATCTGCCACA
AGTGTGATAAGTTATCTACACTGGCGAGGGGATTGCTCTCTGTAATGTTCAGCTTCTAAT
TGTCTCTACTTTGTGAGACTACTTTTGAATGCTTGACCTCAAATCAGGTAGGACTACCC-
GCTGAACTTAA
>BC5-47
TTTCCGTAGGTGAACCTGCGGAAGGATCATTATTGAATTATGTTTCTAGATAGGTTGTAG
CTGGCTCTTTAGAGCATGTGCACGCCTGTTTGGACTTCATTTTCATCCACCTGTGCACCT
ATTGTAGTCTTTGGTTGGGTTAGGAGGAAGTGGTCATTGTGTCAGCATCTGCTGGATGTG

AGGACTTGCATTGTGAAAGCTTTGCTGTCCTTGATGTGATCATGGAATCTCTTTCTCACT
AGAGTCTATGTCACTCATTATACTCTGTCGAATGTCATTGAATGTCTTTACATGGGCTTA
TATGCCTATGAAAATTGTAATACAACTTTCAGCAACGGATCTCTTGGCTCTCGCATCGAT
GAAGAACGCAGCGAAATGCGATAAGTAATGTGAATTGCAGAATTCAGTGAATCATCGAAT
CTTTGAACGCATCTTGCGCTCCTTGGTATTCCGAGGAGCATGCCTGTTTGAGTGTCATTA
AATTCTCAACTCTCTTCTAC-TTTTTGTAAAAGAGAGCTTGGACTGTGGAGGCTTGCTGG
CCACTTTTTGGGGTCAGCTCCTCTGAAATGCATTAGCGGAACCGTTTGCGATCTGCCACA
AGTGTGATAAGTTATCTACACTGGCGAGGGGATTGCTCTCTGTAATGTTCAGCTTCTAAT
TGTCTCTACTTTGTGAGACTACTTTTGAATGCTTGACCTCAAATCAGGTAGGACTACCC-
GCTGAACTTAA
>BC5-50
TTTCCGTAGGTGAACCTGCGGAAGGATCATTATTGAATTATGTTTCTAGATAGGTTGTAG
CTGGCTCTTTAGAGCATGTGCACGCCTGTTTGGACTTCATTTTCATCCACCTGTGCACCT
ATTGTAGTCTTTGGTTGGGTTAGGAGGAAGTGGTCATTGTGTCAGCATCTGCTGGATGTG
AGGACTTGCATTGTGAAAGCTTTGCTGTCCTTGATGTGATCATGGAATCTCTTTCTCACT
AGAGTCTATGTCACTCATTATACTCTGTCGAATGTCATTGAATGTCTTTACATGGGCTTA
TATGCCTATGAAAATTGTAATACAACTTTCAGCAACGGATCTCTTGGCTCTCGCATCGAT
GAAGAACGCAGCGAAATGCGATAAGTAATGTGAATTGCAGAATTCAGTGAATCATCGAAT
CTTTGAACGCATCTTGCGCTCCTTGGTATTCCGAGGAGCATGCCTGTTTGAGTGTCATTA
AATTCTCAACTCTCTTCTAC-TTTTTGTAAAAGAGAGCTTGGACTGTGGAGGCTTGCTGG
CCACTTTTTGGGGTCAGCTCCTCTGAAATGCATTAGCGGAACCGTTTGCGATCTGCCACA
AGTGTGATAAGTTATCTACACTGGCGAGGGGATTGCTCTCTGTAATGTTCAGCTTCTAAT
TGTCTCTACTTTGTGAGACTACTTTTGAATGCTTGACCTCAAATCAGGTAGGACTACCC-
GCTGAACTTAA
>BC5-51
TTTCCGTAGGTGAACCTGCGGAAGGATCATTATTGAATTATGTTTCTAGATAGGTTGTAG
CTGGCTCTTTAGAGCATGTGCACGCCTGTTTGGACTTCATTTTCATCCACCTGTGCACCT
ATTGTAGTCTTTGGTTGGGTTAGGAGGAAGTGGTCATTGTGTCAGCATCTGCTGGATGTG
AGGACTTGCATTGTGAAAGCTTTGCTGTCCTTGATGTGATCATGGAATCTCTTTCTCACT
AGAGTCTATGTCACTCATTATACTCTGTCGAATGTCATTGAATGTCTTTACATGGGCTTA
TATGCCTATGAAAATTGTAATACAACTTTCAGCAACGGATCTCTTGGCTCTCGCATCGAT
GAAGAACGCAGCGAAATGCGATAAGTAATGTGAATTGCAGAATTCAGTGAATCATCGAAT
CTTTGAACGCATCTTGCGCTCCTTGGTATTCCGAGGAGCATGCCTGTTTGAGTGTCATTA
AATTCTCAACTCTCTTCTAC-TTTTTGTAAAAGAGAGCTTGGACTGTGGAGGCTTGCTGG
CCACTTTTTGGGGTCAGCTCCTCTGAAATGCATTAGCGGAACCGTTTGCGATCTGCCACA
AGTGTGATAAGTTATCTACACTGGCGAGGGGATTGCTCTCTGTAATGTTCAGCTTCTAAT
TGTCTCTACTTTGTGAGACTACTTTTGAATGCTTGACCTCAAATCAGGTAGGACTACCC-
GCTGAACTTAA
>BC5-52
TTTCCGTAGGTGAACCTGCGGAAGGATCATTATTGAATTATGTTTCTAGATAGGTTGTAG
CTGGCTCTTTAGAGCATGTGCACGCCTGTTTGGACTTCATTTTCATCCACCTGTGCACCT
ATTGTAGTCTTTGGTTGGGTTAGGAGGAAGTGGTCATTGTGTCAGCATCTGCTGGATGTG
AGGACTTGCATTGTGAAAGCTTTGCTGTCCTTGATGTGATCATGGAATCTCTTTCTCACT
AGAGTCTATGTCACTCATTATACTCTGTCGAATGTCATTGAATGTCTTTACATGGGCTTA
TATGCCTATGAAAATTGTAATACAACTTTCAGCAACGGATCTCTTGGCTCTCGCATCGAT
GAAGAACGCAGCGAAATGCGATAAGTAATGTGAATTGCAGAATTCAGTGAATCATCGAAT
CTTTGAACGCATCTTGCGCTCCTTGGTATTCCGAGGAGCATGCCTGTTTGAGTGTCATTA
AATTCTCAACTCTCTTCTAC-TTTTTGTAAAAGAGAGCTTGGACTGTGGAGGCTTGCTGG
CCACTTTTTGGGGTCAGCTCCTCTGAAATGCATTAGCGGAACCGTTTGCGATCTGCCACA
AGTGTGATAAGTTATCTACACTGGCGAGGGGATTGCTCTCTGTAATGTTCAGCTTCTAAT

TGTCTCTACTTTGTGAGACTACTTTTGAATGCTTGACCTCAAATCAGGTAGGACTACCC-
GCTGAACTTAA
>BC5-55
TTTCCGTAGGTGAACCTGCGGAAGGATCATTATTGAATTATGTTTCTAGATAGGTTGTAG
CTGGCTCTTTAGAGCATGTGCACGCCTGTTTGGACTTCATTTTCATCCACCTGTGCACCT
ATTGTAGTCTTTGGTTGGGTTAGGAGGAAGTGGTCATTGTGTCAGCATCTGCTGGATGTG
AGGACTTGCATTGTGAAAGCTTTGCTGTCCTTGATGTGATCATGGAATCTCTTTCTCACT
AGAGTCTATGTCACTCATTATACTCTGTCGAATGTCATTGAATGTCTTTACATGGGCTTA
TATGCCTATGAAAATTGTAATACAACTTTCAGCAACGGATCTCTTGGCTCTCGCATCGAT
GAAGAACGCAGCGAAATGCGATAAGTAATGTGAATTGCAGAATTCAGTGAATCATCGAAT
CTTTGAACGCATCTTGCGCTCCTTGGTATTCCGAGGAGCATGCCTGTTTGAGTGTCATTA
AATTCTCAACTCTCTTCTAC-TTTTTGTAAAAGAGAGCTTGGACTGTGGAGGCTTGCTGG
CCACTTTTTGGGGTCAGCTCCTCTGAAATGCATTAGCGGAACCGTTTGCGATCTGCCACA
AGTGTGATAAGTTATCTACACTGGCGAGGGGATTGCTCTCTGTAATGTTCAGCTTCTAAT
TGTCTCTACTTTGTGAGACTACTTTTGAATGCTTGACCTCAAATCAGGTAGGACTACCC-
GCTGAACTTAA
>BC5-59
TTTCCGTAGGTGAACCTGCGGAAGGATCATTATTGAATTATGTTTCTAGATAGGTTGTAG
CTGGCTCTTTAGAGCATGTGCACGCCTGTTTGGACTTCATTTTCATCCACCTGTGCACCT
ATTGTAGTCTTTGGTTGGGTTAGGAGGAAGTGGTCATTGTGTCAGCATCTGCTGGATGTG
AGGACTTGCATTGTGAAAGCTTTGCTGTCCTTGATGTGATCATGGAATCTCTTTCTCACT
AGAGTCTATGTCACTCATTATACTCTGTCGAATGTCATTGAATGTCTTTACATGGGCTTA
TATGCCTATGAAAATTGTAATACAACTTTCAGCAACGGATCTCTTGGCTCTCGCATCGAT
GAAGAACGCAGCGAAATGCGATAAGTAATGTGAATTGCAGAATTCAGTGAATCATCGAAT
CTTTGAACGCATCTTGCGCTCCTTGGTATTCCGAGGAGCATGCCTGTTTGAGTGTCATTA
AATTCTCAACTCTCTTCTAC-TTTTTGTAAAAGAGAGCTTGGACTGTGGAGGCTTGCTGG
CCACTTTTTGGGGTCAGCTCCTCTGAAATGCATTAGCGGAACCGTTTGCGATCTGCCACA
AGTGTGATAAGTTATCTACACTGGCGAGGGGATTGCTCTCTGTAATGTTCAGCTTCTAAT
TGTCTCTACTTTGTGAGACTACTTTTGAATGCTTGACCTCAAATCAGGTAGGACTACCC-
GCTGAACTTAA
>BC5-61
TTTCCGTAGGTGAACCTGCGGAAGGATCATTATTGAATTATGTTTCTAGATAGGTTGTAG
CTGGCTCTTTAGAGCATGTGCACGCCTGTTTGGACTTCATTTTCATCCACCTGTGCACCT
ATTGTAGTCTTTGGTTGGGTTAGGAGGAAGTGGTCATTGTGTCAGCATCTGCTGGATGTG
AGGACTTGCATTGTGAAAGCTTTGCTGTCCTTGATGTGATCATGGAATCTCTTTCTCACT
AGAGTCTATGTCACTCATTATACTCTGTCGAATGTCATTGAATGTCTTTACATGGGCTTA
TATGCCTATGAAAATTGTAATACAACTTTCAGCAACGGATCTCTTGGCTCTCGCATCGAT
GAAGAACGCAGCGAAATGCGATAAGTAATGTGAATTGCAGAATTCAGTGAATCATCGAAT
CTTTGAACGCATCTTGCGCTCCTTGGTATTCCGAGGAGCATGCCTGTTTGAGTGTCATTA
AATTCTCAACTCTCTTCTAC-TTTTTGTAAAAGAGAGCTTGGACTGTGGAGGCTTGCTGG
CCACTTTTTGGGGTCAGCTCCTCTGAAATGCATTAGCGGAACCGTTTGCGATCTGCCACA
AGTGTGATAAGTTATCTACACTGGCGAGGGGATTGCTCTCTGTAATGTTCAGCTTCTAAT
TGTCTCTACTTTGTGAGACTACTTTTGAATGCTTGACCTCAAATCAGGTAGGACTACCC-
GCTGAACTTAA
>BC5-63
TTTCCGTAGGTGAACCTGCGGAAGGATCATTATTGAATTATGTTTCTAGATAGGTTGTAG
CTGGCTCTTTAGAGCATGTGCACGCCTGTTTGGACTTCATTTTCATCCACCTGTGCACCT
ATTGTAGTCTTTGGTTGGGTTAGGAGGAAGTGGTCATTGTGTCAGCATCTGCTGGATGTG
AGGACTTGCATTGTGAAAGCTTTGCTGTCCTTGATGTGATCATGGAATCTCTTTCTCACT
AGAGTCTATGTCACTCATTATACTCTGTCGAATGTCATTGAATGTCTTTACATGGGCTTA

TATGCCTATGAAAATTGTAATACAACTTTCAGCAACGGATCTCTTGGCTCTCGCATCGAT
GAAGAACGCAGCGAAATGCGATAAGTAATGTGAATTGCAGAATTCAGTGAATCATCGAAT
CTTTGAACGCATCTTGCGCTCCTTGGTATTCCGAGGAGCATGCCTGTTTGAGTGTCATTA
AATTCTCAACTCTCTTCTAC-TTTTTGTAAAAGAGAGCTTGGACTGTGGAGGCTTGCTGG
CCACTTTTTGGGGTCAGCTCCTCTGAAATGCATTAGCGGAACCGTTTGCGATCTGCCACA
AGTGTGATAAGTTATCTACACTGGCGAGGGGATTGCTCTCTGTAATGTTCAGCTTCTAAT
TGTCTCTACTTTGTGAGACTACTTTTGAATGCTTGACCTCAAATCAGGTAGGACTACCC-
GCTGAACTTAA
>BC5-72
TTTCCGTAGGTGAACCTGCGGAAGGATCATTATTGAATTATGTTTCTAGATAGGTTGTAG
CTGGCTCTTTAGAGCATGTGCACGCCTGTTTGGACTTCATTTTCATCCACCTGTGCACCT
ATTGTAGTCTTTGGTTGGGTTAGGAGGAAGTGGTCATTGTGTCAGCATCTGCTGGATGTG
AGGACTTGCATTGTGAAAGCTTTGCTGTCCTTGATGTGATCATGGAATCTCTTTCTCACT
AGAGTCTATGTCACTCATTATACTCTGTCGAATGTCATTGAATGTCTTTACATGGGCTTA
TATGCCTATGAAAATTGTAATACAACTTTCAGCAACGGATCTCTTGGCTCTCGCATCGAT
GAAGAACGCAGCGAAATGCGATAAGTAATGTGAATTGCAGAATTCAGTGAATCATCGAAT
CTTTGAACGCATCTTGCGCTCCTTGGTATTCCGAGGAGCATGCCTGTTTGAGTGTCATTA
AATTCTCAACTCTCTTCTAC-TTTTTGTAAAAGAGAGCTTGGACTGTGGAGGCTTGCTGG
CCACTTTTTGGGGTCAGCTCCTCTGAAATGCATTAGCGGAACCGTTTGCGATCTGCCACA
AGTGTGATAAGTTATCTACACTGGCGAGGGGATTGCTCTCTGTAATGTTCAGCTTCTAAT
TGTCTCTACTTTGTGAGACTACTTTTGAATGCTTGACCTCAAATCAGGTAGGACTACCC-
GCTGAACTTAA
>BC5-78
TTTCCGTAGGTGAACCTGCGGAAGGATCATTATTGAATTATGTTTCTAGATAGGTTGTAG
CTGGCTCTTTAGAGCATGTGCACGCCTGTTTGGACTTCATTTTCATCCACCTGTGCACCT
ATTGTAGTCTTTGGTTGGGTTAGGAGGAAGTGGTCATTGTGTCAGCATCTGCTGGATGTG
AGGACTTGCATTGTGAAAGCTTTGCTGTCCTTGATGTGATCATGGAATCTCTTTCTCACT
AGAGTCTATGTCACTCATTATACTCTGTCGAATGTCATTGAATGTCTTTACATGGGCTTA
TATGCCTATGAAAATTGTAATACAACTTTCAGCAACGGATCTCTTGGCTCTCGCATCGAT
GAAGAACGCAGCGAAATGCGATAAGTAATGTGAATTGCAGAATTCAGTGAATCATCGAAT
CTTTGAACGCATCTTGCGCTCCTTGGTATTCCGAGGAGCATGCCTGTTTGAGTGTCATTA
AATTCTCAACTCTCTTCTAC-TTTTTGTAAAAGAGAGCTTGGACTGTGGAGGCTTGCTGG
CCACTTTTTGGGGTCAGCTCCTCTGAAATGCATTAGCGGAACCGTTTGCGATCTGCCACA
AGTGTGATAAGTTATCTACACTGGCGAGGGGATTGCTCTCTGTAATGTTCAGCTTCTAAT
TGTCTCTACTTTGTGAGACTACTTTTGAATGCTTGACCTCAAATCAGGTAGGACTACCC-
GCTGAACTTAA
>BC5-79
TTTCCGTAGGTGAACCTGCGGAAGGATCATTATTGAATTATGTTTCTAGATAGGTTGTAG
CTGGCTCTTTAGAGCATGTGCACGCCTGTTTGGACTTCATTTTCATCCACCTGTGCACCT
ATTGTAGTCTTTGGTTGGGTTAGGAGGAAGTGGTCATTGTGTCAGCATCTGCTGGATGTG
AGGACTTGCATTGTGAAAGCTTTGCTGTCCTTGATGTGATCATGGAATCTCTTTCTCACT
AGAGTCTATGTCACTCATTATACTCTGTCGAATGTCATTGAATGTCTTTACATGGGCTTA
TATGCCTATGAAAATTGTAATACAACTTTCAGCAACGGATCTCTTGGCTCTCGCATCGAT
GAAGAACGCAGCGAAATGCGATAAGTAATGTGAATTGCAGAATTCAGTGAATCATCGAAT
CTTTGAACGCATCTTGCGCTCCTTGGTATTCCGAGGAGCATGCCTGTTTGAGTGTCATTA
AATTCTCAACTCTCTTCTAC-TTTTTGTAAAAGAGAGCTTGGACTGTGGAGGCTTGCTGG
CCACTTTTTGGGGTCAGCTCCTCTGAAATGCATTAGCGGAACCGTTTGCGATCTGCCACA
AGTGTGATAAGTTATCTACACTGGCGAGGGGATTGCTCTCTGTAATGTTCAGCTTCTAAT
TGTCTCTACTTTGTGAGACTACTTTTGAATGCTTGACCTCAAATCAGGTAGGACTACCC-
GCTGAACTTAA

>BC5-81
TTTCCGTAGGTGAACCTGCGGAAGGATCATTATTGAATTATGTTTCTAGATAGGTTGTAG
CTGGCTCTTTAGAGCATGTGCACGCCTGTTTGGACTTCATTTTCATCCACCTGTGCACCT
ATTGTAGTCTTTGGTTGGGTTAGGAGGAAGTGGTCATTGTGTCAGCATCTGCTGGATGTG
AGGACTTGCATTGTGAAAGCTTTGCTGTCCTTGATGTGATCATGGAATCTCTTTCTCACT
AGAGTCTATGTCACTCATTATACTCTGTCGAATGTCATTGAATGTCTTTACATGGGCTTA
TATGCCTATGAAAATTGTAATACAACTTTCAGCAACGGATCTCTTGGCTCTCGCATCGAT
GAAGAACGCAGCGAAATGCGATAAGTAATGTGAATTGCAGAATTCAGTGAATCATCGAAT
CTTTGAACGCATCTTGCGCTCCTTGGTATTCCGAGGAGCATGCCTGTTTGAGTGTCATTA
AATTCTCAACTCTCTTCTAC-TTTTTGTAAAAGAGAGCTTGGACTGTGGAGGCTTGCTGG
CCACTTTTTGGGGTCAGCTCCTCTGAAATGCATTAGCGGAACCGTTTGCGATCTGCCACA
AGTGTGATAAGTTATCTACACTGGCGAGGGGATTGCTCTCTGTAATGTTCAGCTTCTAAT
TGTCTCTACTTTGTGAGACTACTTTTGAATGCTTGACCTCAAATCAGGTAGGACTACCC-
GCTGAACTTAA
>BC5-92
TTTCCGTAGGTGAACCTGCGGAAGGATCATTATTGAATTATGTTTCTAGATAGGTTGTAG
CTGGCTCTTTAGAGCATGTGCACGCCTGTTTGGACTTCATTTTCATCCACCTGTGCACCT
ATTGTAGTCTTTGGTTGGGTTAGGAGGAAGTGGTCATTGTGTCAGCATCTGCTGGATGTG
AGGACTTGCATTGTGAAAGCTTTGCTGTCCTTGATGTGATCATGGAATCTCTTTCTCACT
AGAGTCTATGTCACTCATTATACTCTGTCGAATGTCATTGAATGTCTTTACATGGGCTTA
TATGCCTATGAAAATTGTAATACAACTTTCAGCAACGGATCTCTTGGCTCTCGCATCGAT
GAAGAACGCAGCGAAATGCGATAAGTAATGTGAATTGCAGAATTCAGTGAATCATCGAAT
CTTTGAACGCATCTTGCGCTCCTTGGTATTCCGAGGAGCATGCCTGTTTGAGTGTCATTA
AATTCTCAACTCTCTTCTAC-TTTTTGTAAAAGAGAGCTTGGACTGTGGAGGCTTGCTGG
CCACTTTTTGGGGTCAGCTCCTCTGAAATGCATTAGCGGAACCGTTTGCGATCTGCCACA
AGTGTGATAAGTTATCTACACTGGCGAGGGGATTGCTCTCTGTAATGTTCAGCTTCTAAT
TGTCTCTACTTTGTGAGACTACTTTTGAATGCTTGACCTCAAATCAGGTAGGACTACCC-
GCTGAACTTAA
>BC5-93
TTTCCGTAGGTGAACCTGCGGAAGGATCATTATTGAATTATGTTTCTAGATAGGTTGTAG
CTGGCTCTTTAGAGCATGTGCACGCCTGTTTGGACTTCATTTTCATCCACCTGTGCACCT
ATTGTAGTCTTTGGTTGGGTTAGGAGGAAGTGGTCATTGTGTCAGCATCTGCTGGATGTG
AGGACTTGCATTGTGAAAGCTTTGCTGTCCTTGATGTGATCATGGAATCTCTTTCTCACT
AGAGTCTATGTCACTCATTATACTCTGTCGAATGTCATTGAATGTCTTTACATGGGCTTA
TATGCCTATGAAAATTGTAATACAACTTTCAGCAACGGATCTCTTGGCTCTCGCATCGAT
GAAGAACGCAGCGAAATGCGATAAGTAATGTGAATTGCAGAATTCAGTGAATCATCGAAT
CTTTGAACGCATCTTGCGCTCCTTGGTATTCCGAGGAGCATGCCTGTTTGAGTGTCATTA
AATTCTCAACTCTCTTCTAC-TTTTTGTAAAAGAGAGCTTGGACTGTGGAGGCTTGCTGG
CCACTTTTTGGGGTCAGCTCCTCTGAAATGCATTAGCGGAACCGTTTGCGATCTGCCACA
AGTGTGATAAGTTATCTACACTGGCGAGGGGATTGCTCTCTGTAATGTTCAGCTTCTAAT
TGTCTCTACTTTGTGAGACTACTTTTGAATGCTTGACCTCAAATCAGGTAGGACTACCC-
GCTGAACTTAA
>BC5-103
TTTCCGTAGGTGAACCTGCGGAAGGATCATTATTGAATTATGTTTCTAGATAGGTTGTAG
CTGGCTCTTTAGAGCATGTGCACGCCTGTTTGGACTTCATTTTCATCCACCTGTGCACCT
ATTGTAGTCTTTGGTTGGGTTAGGAGGAAGTGGTCATTGTGTCAGCATCTGCTGGATGTG
AGGACTTGCATTGTGAAAGCTTTGCTGTCCTTGATGTGATCATGGAATCTCTTTCTCACT
AGAGTCTATGTCACTCATTATACTCTGTCGAATGTCATTGAATGTCTTTACATGGGCTTA
TATGCCTATGAAAATTGTAATACAACTTTCAGCAACGGATCTCTTGGCTCTCGCATCGAT
GAAGAACGCAGCGAAATGCGATAAGTAATGTGAATTGCAGAATTCAGTGAATCATCGAAT

CTTTGAACGCATCTTGCGCTCCTTGGTATTCCGAGGAGCATGCCTGTTTGAGTGTCATTA
AATTCTCAACTCTCTTCTAC-TTTTTGTAAAAGAGAGCTTGGACTGTGGAGGCTTGCTGG
CCACTTTTTGGGGTCAGCTCCTCTGAAATGCATTAGCGGAACCGTTTGCGATCTGCCACA
AGTGTGATAAGTTATCTACACTGGCGAGGGGATTGCTCTCTGTAATGTTCAGCTTCTAAT
TGTCTCTACTTTGTGAGACTACTTTTGAATGCTTGACCTCAAATCAGGTAGGACTACCC-
GCTGAACTTAA
>BC5-110
TTTCCGTAGGTGAACCTGCGGAAGGATCATTATTGAATTATGTTTCTAGATAGGTTGTAG
CTGGCTCTTTAGAGCATGTGCACGCCTGTTTGGACTTCATTTTCATCCACCTGTGCACCT
ATTGTAGTCTTTGGTTGGGTTAGGAGGAAGTGGTCATTGTGTCAGCATCTGCTGGATGTG
AGGACTTGCATTGTGAAAGCTTTGCTGTCCTTGATGTGATCATGGAATCTCTTTCTCACT
AGAGTCTATGTCACTCATTATACTCTGTCGAATGTCATTGAATGTCTTTACATGGGCTTA
TATGCCTATGAAAATTGTAATACAACTTTCAGCAACGGATCTCTTGGCTCTCGCATCGAT
GAAGAACGCAGCGAAATGCGATAAGTAATGTGAATTGCAGAATTCAGTGAATCATCGAAT
CTTTGAACGCATCTTGCGCTCCTTGGTATTCCGAGGAGCATGCCTGTTTGAGTGTCATTA
AATTCTCAACTCTCTTCTAC-TTTTTGTAAAAGAGAGCTTGGACTGTGGAGGCTTGCTGG
CCACTTTTTGGGGTCAGCTCCTCTGAAATGCATTAGCGGAACCGTTTGCGATCTGCCACA
AGTGTGATAAGTTATCTACACTGGCGAGGGGATTGCTCTCTGTAATGTTCAGCTTCTAAT
TGTCTCTACTTTGTGAGACTACTTTTGAATGCTTGACCTCAAATCAGGTAGGACTACCC-
GCTGAACTTAA
>BC5-112
TTTCCGTAGGTGAACCTGCGGAAGGATCATTATTGAATTATGTTTCTAGATAGGTTGTAG
CTGGCTCTTTAGAGCATGTGCACGCCTGTTTGGACTTCATTTTCATCCACCTGTGCACCT
ATTGTAGTCTTTGGTTGGGTTAGGAGGAAGTGGTCATTGTGTCAGCATCTGCTGGATGTG
AGGACTTGCATTGTGAAAGCTTTGCTGTCCTTGATGTGATCATGGAATCTCTTTCTCACT
AGAGTCTATGTCACTCATTATACTCTGTCGAATGTCATTGAATGTCTTTACATGGGCTTA
TATGCCTATGAAAATTGTAATACAACTTTCAGCAACGGATCTCTTGGCTCTCGCATCGAT
GAAGAACGCAGCGAAATGCGATAAGTAATGTGAATTGCAGAATTCAGTGAATCATCGAAT
CTTTGAACGCATCTTGCGCTCCTTGGTATTCCGAGGAGCATGCCTGTTTGAGTGTCATTA
AATTCTCAACTCTCTTCTAC-TTTTTGTAAAAGAGAGCTTGGACTGTGGAGGCTTGCTGG
CCACTTTTTGGGGTCAGCTCCTCTGAAATGCATTAGCGGAACCGTTTGCGATCTGCCACA
AGTGTGATAAGTTATCTACACTGGCGAGGGGATTGCTCTCTGTAATGTTCAGCTTCTAAT
TGTCTCTACTTTGTGAGACTACTTTTGAATGCTTGACCTCAAATCAGGTAGGACTACCC-
GCTGAACTTAA
>BC5-113
TTTCCGTAGGTGAACCTGCGGAAGGATCATTATTGAATTATGTTTCTAGATAGGTTGTAG
CTGGCTCTTTAGAGCATGTGCACGCCTGTTTGGACTTCATTTTCATCCACCTGTGCACCT
ATTGTAGTCTTTGGTTGGGTTAGGAGGAAGTGGTCATTGTGTCAGCATCTGCTGGATGTG
AGGACTTGCATTGTGAAAGCTTTGCTGTCCTTGATGTGATCATGGAATCTCTTTCTCACT
AGAGTCTATGTCACTCATTATACTCTGTCGAATGTCATTGAATGTCTTTACATGGGCTTA
TATGCCTATGAAAATTGTAATACAACTTTCAGCAACGGATCTCTTGGCTCTCGCATCGAT
GAAGAACGCAGCGAAATGCGATAAGTAATGTGAATTGCAGAATTCAGTGAATCATCGAAT
CTTTGAACGCATCTTGCGCTCCTTGGTATTCCGAGGAGCATGCCTGTTTGAGTGTCATTA
AATTCTCAACTCTCTTCTAC-TTTTTGTAAAAGAGAGCTTGGACTGTGGAGGCTTGCTGG
CCACTTTTTGGGGTCAGCTCCTCTGAAATGCATTAGCGGAACCGTTTGCGATCTGCCACA
AGTGTGATAAGTTATCTACACTGGCGAGGGGATTGCTCTCTGTAATGTTCAGCTTCTAAT
TGTCTCTACTTTGTGAGACTACTTTTGAATGCTTGACCTCAAATCAGGTAGGACTACCC-
GCTGAACTTAA
>BC5-130
TTTCCGTAGGTGAACCTGCGGAAGGATCATTATTGAATTATGTTTCTAGATAGGTTGTAG

CTGGCTCTTTAGAGCATGTGCACGCCTGTTTGGACTTCATTTTCATCCACCTGTGCACCT
ATTGTAGTCTTTGGTTGGGTTAGGAGGAAGTGGTCATTGTGTCAGCATCTGCTGGATGTG
AGGACTTGCATTGTGAAAGCTTTGCTGTCCTTGATGTGATCATGGAATCTCTTTCTCACT
AGAGTCTATGTCACTCATTATACTCTGTCGAATGTCATTGAATGTCTTTACATGGGCTTA
TATGCCTATGAAAATTGTAATACAACTTTCAGCAACGGATCTCTTGGCTCTCGCATCGAT
GAAGAACGCAGCGAAATGCGATAAGTAATGTGAATTGCAGAATTCAGTGAATCATCGAAT
CTTTGAACGCATCTTGCGCTCCTTGGTATTCCGAGGAGCATGCCTGTTTGAGTGTCATTA
AATTCTCAACTCTCTTCTAC-TTTTTGTAAAAGAGAGCTTGGACTGTGGAGGCTTGCTGG
CCACTTTTTGGGGTCAGCTCCTCTGAAATGCATTAGCGGAACCGTTTGCGATCTGCCACA
AGTGTGATAAGTTATCTACACTGGCGAGGGGATTGCTCTCTGTAATGTTCAGCTTCTAAT
TGTCTCTACTTTGTGAGACTACTTTTGAATGCTTGACCTCAAATCAGGTAGGACTACCC-
GCTGAACTTAA
>BC6-2
TTTCCGTAGGTGAACCTGCGGAAGGATCATTATTGAATTATGTTTCTAGATAGGTTGTAG
CTGGCTCTTTAGAGCATGTGCACGCCTGTTTGGACTTCATTTTCATCCACCTGTGCACCT
ATTGTAGTCTTTGGTTGGGTTAGGAGGAAGTGGTCATTGTGTCAGCATCTGCTGGATGTG
AGGACTTGCATTGTGAAAGCTTTGCTGTCCTTGATGTGATCATGGAATCTCTTTCTCACT
AGAGTCTATGTCACTCATTATACTCTGTCGAATGTCATTGAATGTCTTTACATGGGCTTA
TATGCCTATGAAAATTGTAATACAACTTTCAGCAACGGATCTCTTGGCTCTCGCATCGAT
GAAGAACGCAGCGAAATGCGATAAGTAATGTGAATTGCAGAATTCAGTGAATCATCGAAT
CTTTGAACGCATCTTGCGCTCCTTGGTATTCCGAGGAGCATGCCTGTTTGAGTGTCATTA
AATTCTCAACTCTCTTCTAC-TTTTTGTAAAAGAGAGCTTGGACTGTGGAGGCTTGCTGG
CCACTTTTTGGGGTCAGCTCCTCTGAAATGCATTAGCGGAACCGTTTGCGATCTGCCACA
AGTGTGATAAGTTATCTACACTGGCGAGGGGATTGCTCTCTGTAATGTTCAGCTTCTAAT
TGTCTCTACTTTGTGAGACTACTTTTGAATGCTTGACCTCAAATCAGGTAGGACTACCC-
GCTGAACTTAA
>BC6-10
TTTCCGTAGGTGAACCTGCGGAAGGATCATTATTGAATTATGTTTCTAGATAGGTTGTAG
CTGGCTCTTTAGAGCATGTGCACGCCTGTTTGGACTTCATTTTCATCCACCTGTGCACCT
ATTGTAGTCTTTGGTTGGGTTAGGAGGAAGTGGTCATTGTGTCAGCATCTGCTGGATGTG
AGGACTTGCATTGTGAAAGCTTTGCTGTCCTTGATGTGATCATGGAATCTCTTTCTCACT
AGAGTCTATGTCACTCATTATACTCTGTCGAATGTCATTGAATGTCTTTACATGGGCTTA
TATGCCTATGAAAATTGTAATACAACTTTCAGCAACGGATCTCTTGGCTCTCGCATCGAT
GAAGAACGCAGCGAAATGCGATAAGTAATGTGAATTGCAGAATTCAGTGAATCATCGAAT
CTTTGAACGCATCTTGCGCTCCTTGGTATTCCGAGGAGCATGCCTGTTTGAGTGTCATTA
AATTCTCAACTCTCTTCTAC-TTTTTGTAAAAGAGAGCTTGGACTGTGGAGGCTTGCTGG
CCACTTTTTGGGGTCAGCTCCTCTGAAATGCATTAGCGGAACCGTTTGCGATCTGCCACA
AGTGTGATAAGTTATCTACACTGGCGAGGGGATTGCTCTCTGTAATGTTCAGCTTCTAAT
TGTCTCTACTTTGTGAGACTACTTTTGAATGCTTGACCTCAAATCAGGTAGGACTACCC-
GCTGAACTTAA
>BC6-12
TTTCCGTAGGTGAACCTGCGGAAGGATCATTATTGAATTATGTTTCTAGATAGGTTGTAG
CTGGCTCTTTAGAGCATGTGCACGCCTGTTTGGACTTCATTTTCATCCACCTGTGCACCT
ATTGTAGTCTTTGGTTGGGTTAGGAGGAAGTGGTCATTGTGTCAGCATCTGCTGGATGTG
AGGACTTGCATTGTGAAAGCTTTGCTGTCCTTGATGTGATCATGGAATCTCTTTCTCACT
AGAGTCTATGTCACTCATTATACTCTGTCGAATGTCATTGAATGTCTTTACATGGGCTTA
TATGCCTATGAAAATTGTAATACAACTTTCAGCAACGGATCTCTTGGCTCTCGCATCGAT
GAAGAACGCAGCGAAATGCGATAAGTAATGTGAATTGCAGAATTCAGTGAATCATCGAAT
CTTTGAACGCATCTTGCGCTCCTTGGTATTCCGAGGAGCATGCCTGTTTGAGTGTCATTA
AATTCTCAACTCTCTTCTAC-TTTTTGTAAAAGAGAGCTTGGACTGTGGAGGCTTGCTGG

CCACTTTTTGGGGTCAGCTCCTCTGAAATGCATTAGCGGAACCGTTTGCGATCTGCCACA
AGTGTGATAAGTTATCTACACTGGCGAGGGGATTGCTCTCTGTAATGTTCAGCTTCTAAT
TGTCTCTACTTTGTGAGACTACTTTTGAATGCTTGACCTCAAATCAGGTAGGACTACCC-
GCTGAACTTAA
>BC6-16
TTTCCGTAGGTGAACCTGCGGAAGGATCATTATTGAATTATGTTTCTAGATAGGTTGTAG
CTGGCTCTTTAGAGCATGTGCACGCCTGTTTGGACTTCATTTTCATCCACCTGTGCACCT
ATTGTAGTCTTTGGTTGGGTTAGGAGGAAGTGGTCATTGTGTCAGCATCTGCTGGATGTG
AGGACTTGCATTGTGAAAGCTTTGCTGTCCTTGATGTGATCATGGAATCTCTTTCTCACT
AGAGTCTATGTCACTCATTATACTCTGTCGAATGTCATTGAATGTCTTTACATGGGCTTA
TATGCCTATGAAAATTGTAATACAACTTTCAGCAACGGATCTCTTGGCTCTCGCATCGAT
GAAGAACGCAGCGAAATGCGATAAGTAATGTGAATTGCAGAATTCAGTGAATCATCGAAT
CTTTGAACGCATCTTGCGCTCCTTGGTATTCCGAGGAGCATGCCTGTTTGAGTGTCATTA
AATTCTCAACTCTCTTCTAC-TTTTTGTAAAAGAGAGCTTGGACTGTGGAGGCTTGCTGG
CCACTTTTTGGGGTCAGCTCCTCTGAAATGCATTAGCGGAACCGTTTGCGATCTGCCACA
AGTGTGATAAGTTATCTACACTGGCGAGGGGATTGCTCTCTGTAATGTTCAGCTTCTAAT
TGTCTCTACTTTGTGAGACTACTTTTGAATGCTTGACCTCAAATCAGGTAGGACTACCC-
GCTGAACTTAA
>BC6-27
TTTCCGTAGGTGAACCTGCGGAAGGATCATTATTGAATTATGTTTCTAGATAGGTTGTAG
CTGGCTCTTTAGAGCATGTGCACGCCTGTTTGGACTTCATTTTCATCCACCTGTGCACCT
ATTGTAGTCTTTGGTTGGGTTAGGAGGAAGTGGTCATTGTGTCAGCATCTGCTGGATGTG
AGGACTTGCATTGTGAAAGCTTTGCTGTCCTTGATGTGATCATGGAATCTCTTTCTCACT
AGAGTCTATGTCACTCATTATACTCTGTCGAATGTCATTGAATGTCTTTACATGGGCTTA
TATGCCTATGAAAATTGTAATACAACTTTCAGCAACGGATCTCTTGGCTCTCGCATCGAT
GAAGAACGCAGCGAAATGCGATAAGTAATGTGAATTGCAGAATTCAGTGAATCATCGAAT
CTTTGAACGCATCTTGCGCTCCTTGGTATTCCGAGGAGCATGCCTGTTTGAGTGTCATTA
AATTCTCAACTCTCTTCTAC-TTTTTGTAAAAGAGAGCTTGGACTGTGGAGGCTTGCTGG
CCACTTTTTGGGGTCAGCTCCTCTGAAATGCATTAGCGGAACCGTTTGCGATCTGCCACA
AGTGTGATAAGTTATCTACACTGGCGAGGGGATTGCTCTCTGTAATGTTCAGCTTCTAAT
TGTCTCTACTTTGTGAGACTACTTTTGAATGCTTGACCTCAAATCAGGTAGGACTACCC-
GCTGAACTTAA
>BC6-32
TTTCCGTAGGTGAACCTGCGGAAGGATCATTATTGAATTATGTTTCTAGATAGGTTGTAG
CTGGCTCTTTAGAGCATGTGCACGCCTGTTTGGACTTCATTTTCATCCACCTGTGCACCT
ATTGTAGTCTTTGGTTGGGTTAGGAGGAAGTGGTCATTGTGTCAGCATCTGCTGGATGTG
AGGACTTGCATTGTGAAAGCTTTGCTGTCCTTGATGTGATCATGGAATCTCTTTCTCACT
AGAGTCTATGTCACTCATTATACTCTGTCGAATGTCATTGAATGTCTTTACATGGGCTTA
TATGCCTATGAAAATTGTAATACAACTTTCAGCAACGGATCTCTTGGCTCTCGCATCGAT
GAAGAACGCAGCGAAATGCGATAAGTAATGTGAATTGCAGAATTCAGTGAATCATCGAAT
CTTTGAACGCATCTTGCGCTCCTTGGTATTCCGAGGAGCATGCCTGTTTGAGTGTCATTA
AATTCTCAACTCTCTTCTAC-TTTTTGTAAAAGAGAGCTTGGACTGTGGAGGCTTGCTGG
CCACTTTTTGGGGTCAGCTCCTCTGAAATGCATTAGCGGAACCGTTTGCGATCTGCCACA
AGTGTGATAAGTTATCTACACTGGCGAGGGGATTGCTCTCTGTAATGTTCAGCTTCTAAT
TGTCTCTACTTTGTGAGACTACTTTTGAATGCTTGACCTCAAATCAGGTAGGACTACCC-
GCTGAACTTAA
>BC6-38
TTTCCGTAGGTGAACCTGCGGAAGGATCATTATTGAATTATGTTTCTAGATAGGTTGTAG
CTGGCTCTTTAGAGCATGTGCACGCCTGTTTGGACTTCATTTTCATCCACCTGTGCACCT
ATTGTAGTCTTTGGTTGGGTTAGGAGGAAGTGGTCATTGTGTCAGCATCTGCTGGATGTG

AGGACTTGCATTGTGAAAGCTTTGCTGTCCTTGATGTGATCATGGAATCTCTTTCTCACT
AGAGTCTATGTCACTCATTATACTCTGTCGAATGTCATTGAATGTCTTTACATGGGCTTA
TATGCCTATGAAAATTGTAATACAACTTTCAGCAACGGATCTCTTGGCTCTCGCATCGAT
GAAGAACGCAGCGAAATGCGATAAGTAATGTGAATTGCAGAATTCAGTGAATCATCGAAT
CTTTGAACGCATCTTGCGCTCCTTGGTATTCCGAGGAGCATGCCTGTTTGAGTGTCATTA
AATTCTCAACTCTCTTCTAC-TTTTTGTAAAAGAGAGCTTGGACTGTGGAGGCTTGCTGG
CCACTTTTTGGGGTCAGCTCCTCTGAAATGCATTAGCGGAACCGTTTGCGATCTGCCACA
AGTGTGATAAGTTATCTACACTGGCGAGGGGATTGCTCTCTGTAATGTTCAGCTTCTAAT
TGTCTCTACTTTGTGAGACTACTTTTGAATGCTTGACCTCAAATCAGGTAGGACTACCC-
GCTGAACTTAA
>BC6-40
TTTCCGTAGGTGAACCTGCGGAAGGATCATTATTGAATTATGTTTCTAGATAGGTTGTAG
CTGGCTCTTTAGAGCATGTGCACGCCTGTTTGGACTTCATTTTCATCCACCTGTGCACCT
ATTGTAGTCTTTGGTTGGGTTAGGAGGAAGTGGTCATTGTGTCAGCATCTGCTGGATGTG
AGGACTTGCATTGTGAAAGCTTTGCTGTCCTTGATGTGATCATGGAATCTCTTTCTCACT
AGAGTCTATGTCACTCATTATACTCTGTCGAATGTCATTGAATGTCTTTACATGGGCTTA
TATGCCTATGAAAATTGTAATACAACTTTCAGCAACGGATCTCTTGGCTCTCGCATCGAT
GAAGAACGCAGCGAAATGCGATAAGTAATGTGAATTGCAGAATTCAGTGAATCATCGAAT
CTTTGAACGCATCTTGCGCTCCTTGGTATTCCGAGGAGCATGCCTGTTTGAGTGTCATTA
AATTCTCAACTCTCTTCTAC-TTTTTGTAAAAGAGAGCTTGGACTGTGGAGGCTTGCTGG
CCACTTTTTGGGGTCAGCTCCTCTGAAATGCATTAGCGGAACCGTTTGCGATCTGCCACA
AGTGTGATAAGTTATCTACACTGGCGAGGGGATTGCTCTCTGTAATGTTCAGCTTCTAAT
TGTCTCTACTTTGTGAGACTACTTTTGAATGCTTGACCTCAAATCAGGTAGGACTACCC-
GCTGAACTTAA
>BC6-43
TTTCCGTAGGTGAACCTGCGGAAGGATCATTATTGAATTATGTTTCTAGATAGGTTGTAG
CTGGCTCTTTAGAGCATGTGCACGCCTGTTTGGACTTCATTTTCATCCACCTGTGCACCT
ATTGTAGTCTTTGGTTGGGTTAGGAGGAAGTGGTCATTGTGTCAGCATCTGCTGGATGTG
AGGACTTGCATTGTGAAAGCTTTGCTGTCCTTGATGTGATCATGGAATCTCTTTCTCACT
AGAGTCTATGTCACTCATTATACTCTGTCGAATGTCATTGAATGTCTTTACATGGGCTTA
TATGCCTATGAAAATTGTAATACAACTTTCAGCAACGGATCTCTTGGCTCTCGCATCGAT
GAAGAACGCAGCGAAATGCGATAAGTAATGTGAATTGCAGAATTCAGTGAATCATCGAAT
CTTTGAACGCATCTTGCGCTCCTTGGTATTCCGAGGAGCATGCCTGTTTGAGTGTCATTA
AATTCTCAACTCTCTTCTAC-TTTTTGTAAAAGAGAGCTTGGACTGTGGAGGCTTGCTGG
CCACTTTTTGGGGTCAGCTCCTCTGAAATGCATTAGCGGAACCGTTTGCGATCTGCCACA
AGTGTGATAAGTTATCTACACTGGCGAGGGGATTGCTCTCTGTAATGTTCAGCTTCTAAT
TGTCTCTACTTTGTGAGACTACTTTTGAATGCTTGACCTCAAATCAGGTAGGACTACCC-
GCTGAACTTAA
>BC6-46
TTTCCGTAGGTGAACCTGCGGAAGGATCATTATTGAATTATGTTTCTAGATAGGTTGTAG
CTGGCTCTTTAGAGCATGTGCACGCCTGTTTGGACTTCATTTTCATCCACCTGTGCACCT
ATTGTAGTCTTTGGTTGGGTTAGGAGGAAGTGGTCATTGTGTCAGCATCTGCTGGATGTG
AGGACTTGCATTGTGAAAGCTTTGCTGTCCTTGATGTGATCATGGAATCTCTTTCTCACT
AGAGTCTATGTCACTCATTATACTCTGTCGAATGTCATTGAATGTCTTTACATGGGCTTA
TATGCCTATGAAAATTGTAATACAACTTTCAGCAACGGATCTCTTGGCTCTCGCATCGAT
GAAGAACGCAGCGAAATGCGATAAGTAATGTGAATTGCAGAATTCAGTGAATCATCGAAT
CTTTGAACGCATCTTGCGCTCCTTGGTATTCCGAGGAGCATGCCTGTTTGAGTGTCATTA
AATTCTCAACTCTCTTCTAC-TTTTTGTAAAAGAGAGCTTGGACTGTGGAGGCTTGCTGG
CCACTTTTTGGGGTCAGCTCCTCTGAAATGCATTAGCGGAACCGTTTGCGATCTGCCACA
AGTGTGATAAGTTATCTACACTGGCGAGGGGATTGCTCTCTGTAATGTTCAGCTTCTAAT

TGTCTCTACTTTGTGAGACTACTTTTGAATGCTTGACCTCAAATCAGGTAGGACTACCC-
GCTGAACTTAA
>BC6-47
TTTCCGTAGGTGAACCTGCGGAAGGATCATTATTGAATTATGTTTCTAGATAGGTTGTAG
CTGGCTCTTTAGAGCATGTGCACGCCTGTTTGGACTTCATTTTCATCCACCTGTGCACCT
ATTGTAGTCTTTGGTTGGGTTAGGAGGAAGTGGTCATTGTGTCAGCATCTGCTGGATGTG
AGGACTTGCATTGTGAAAGCTTTGCTGTCCTTGATGTGATCATGGAATCTCTTTCTCACT
AGAGTCTATGTCACTCATTATACTCTGTCGAATGTCATTGAATGTCTTTACATGGGCTTA
TATGCCTATGAAAATTGTAATACAACTTTCAGCAACGGATCTCTTGGCTCTCGCATCGAT
GAAGAACGCAGCGAAATGCGATAAGTAATGTGAATTGCAGAATTCAGTGAATCATCGAAT
CTTTGAACGCATCTTGCGCTCCTTGGTATTCCGAGGAGCATGCCTGTTTGAGTGTCATTA
AATTCTCAACTCTCTTCTAC-TTTTTGTAAAAGAGAGCTTGGACTGTGGAGGCTTGCTGG
CCACTTTTTGGGGTCAGCTCCTCTGAAATGCATTAGCGGAACCGTTTGCGATCTGCCACA
AGTGTGATAAGTTATCTACACTGGCGAGGGGATTGCTCTCTGTAATGTTCAGCTTCTAAT
TGTCTCTACTTTGTGAGACTACTTTTGAATGCTTGACCTCAAATCAGGTAGGACTACCC-
GCTGAACTTAA
>BC6-50
TTTCCGTAGGTGAACCTGCGGAAGGATCATTATTGAATTATGTTTCTAGATAGGTTGTAG
CTGGCTCTTTAGAGCATGTGCACGCCTGTTTGGACTTCATTTTCATCCACCTGTGCACCT
ATTGTAGTCTTTGGTTGGGTTAGGAGGAAGTGGTCATTGTGTCAGCATCTGCTGGATGTG
AGGACTTGCATTGTGAAAGCTTTGCTGTCCTTGATGTGATCATGGAATCTCTTTCTCACT
AGAGTCTATGTCACTCATTATACTCTGTCGAATGTCATTGAATGTCTTTACATGGGCTTA
TATGCCTATGAAAATTGTAATACAACTTTCAGCAACGGATCTCTTGGCTCTCGCATCGAT
GAAGAACGCAGCGAAATGCGATAAGTAATGTGAATTGCAGAATTCAGTGAATCATCGAAT
CTTTGAACGCATCTTGCGCTCCTTGGTATTCCGAGGAGCATGCCTGTTTGAGTGTCATTA
AATTCTCAACTCTCTTCTAC-TTTTTGTAAAAGAGAGCTTGGACTGTGGAGGCTTGCTGG
CCACTTTTTGGGGTCAGCTCCTCTGAAATGCATTAGCGGAACCGTTTGCGATCTGCCACA
AGTGTGATAAGTTATCTACACTGGCGAGGGGATTGCTCTCTGTAATGTTCAGCTTCTAAT
TGTCTCTACTTTGTGAGACTACTTTTGAATGCTTGACCTCAAATCAGGTAGGACTACCC-
GCTGAACTTAA
>BC6-51
TTTCCGTAGGTGAACCTGCGGAAGGATCATTATTGAATTATGTTTCTAGATAGGTTGTAG
CTGGCTCTTTAGAGCATGTGCACGCCTGTTTGGACTTCATTTTCATCCACCTGTGCACCT
ATTGTAGTCTTTGGTTGGGTTAGGAGGAAGTGGTCATTGTGTCAGCATCTGCTGGATGTG
AGGACTTGCATTGTGAAAGCTTTGCTGTCCTTGATGTGATCATGGAATCTCTTTCTCACT
AGAGTCTATGTCACTCATTATACTCTGTCGAATGTCATTGAATGTCTTTACATGGGCTTA
TATGCCTATGAAAATTGTAATACAACTTTCAGCAACGGATCTCTTGGCTCTCGCATCGAT
GAAGAACGCAGCGAAATGCGATAAGTAATGTGAATTGCAGAATTCAGTGAATCATCGAAT
CTTTGAACGCATCTTGCGCTCCTTGGTATTCCGAGGAGCATGCCTGTTTGAGTGTCATTA
AATTCTCAACTCTCTTCTAC-TTTTTGTAAAAGAGAGCTTGGACTGTGGAGGCTTGCTGG
CCACTTTTTGGGGTCAGCTCCTCTGAAATGCATTAGCGGAACCGTTTGCGATCTGCCACA
AGTGTGATAAGTTATCTACACTGGCGAGGGGATTGCTCTCTGTAATGTTCAGCTTCTAAT
TGTCTCTACTTTGTGAGACTACTTTTGAATGCTTGACCTCAAATCAGGTAGGACTACCC-
GCTGAACTTAA
>BC6-57
TTTCCGTAGGTGAACCTGCGGAAGGATCATTATTGAATTATGTTTCTAGATAGGTTGTAG
CTGGCTCTTTAGAGCATGTGCACGCCTGTTTGGACTTCATTTTCATCCACCTGTGCACCT
ATTGTAGTCTTTGGTTGGGTTAGGAGGAAGTGGTCATTGTGTCAGCATCTGCTGGATGTG
AGGACTTGCATTGTGAAAGCTTTGCTGTCCTTGATGTGATCATGGAATCTCTTTCTCACT
AGAGTCTATGTCACTCATTATACTCTGTCGAATGTCATTGAATGTCTTTACATGGGCTTA

TATGCCTATGAAAATTGTAATACAACTTTCAGCAACGGATCTCTTGGCTCTCGCATCGAT
GAAGAACGCAGCGAAATGCGATAAGTAATGTGAATTGCAGAATTCAGTGAATCATCGAAT
CTTTGAACGCATCTTGCGCTCCTTGGTATTCCGAGGAGCATGCCTGTTTGAGTGTCATTA
AATTCTCAACTCTCTTCTAC-TTTTTGTAAAAGAGAGCTTGGACTGTGGAGGCTTGCTGG
CCACTTTTTGGGGTCAGCTCCTCTGAAATGCATTAGCGGAACCGTTTGCGATCTGCCACA
AGTGTGATAAGTTATCTACACTGGCGAGGGGATTGCTCTCTGTAATGTTCAGCTTCTAAT
TGTCTCTACTTTGTGAGACTACTTTTGAATGCTTGACCTCAAATCAGGTAGGACTACCC-
GCTGAACTTAA
>BC7-6
TTTCCGTAGGTGAACCTGCGGAAGGATCATTATTGAATTATGTTTCTAGATAGGTTGTAG
CTGGCTCTTTAGAGCATGTGCACGCCTGTTTGGACTTCATTTTCATCCACCTGTGCACCT
ATTGTAGTCTTTGGTTGGGTTAGGAGGAAGTGGTCATTGTGTCAGCATCTGCTGGATGTG
AGGACTTGCATTGTGAAAGCTTTGCTGTCCTTGATGTGATCATGGAATCTCTTTCTCACT
AGAGTCTATGTCACTCATTATACTCTGTCGAATGTCATTGAATGTCTTTACATGGGCTTA
TATGCCTATGAAAATTGTAATACAACTTTCAGCAACGGATCTCTTGGCTCTCGCATCGAT
GAAGAACGCAGCGAAATGCGATAAGTAATGTGAATTGCAGAATTCAGTGAATCATCGAAT
CTTTGAACGCATCTTGCGCTCCTTGGTATTCCGAGGAGCATGCCTGTTTGAGTGTCATTA
AATTCTCAACTCTCTTCTAC-TTTTTGTAAAAGAGAGCTTGGACTGTGGAGGCTTGCTGG
CCACTTTTTGGGGTCAGCTCCTCTGAAATGCATTAGCGGAACCGTTTGCGATCTGCCACA
AGTGTGATAAGTTATCTACACTGGCGAGGGGATTGCTCTCTGTAATGTTCAGCTTCTAAT
TGTCTCTACTTTGTGAGACTACTTTTGAATGCTTGACCTCAAATCAGGTAGGACTACCC-
GCTGAACTTAA
>BC7-9
TTTCCGTAGGTGAACCTGCGGAAGGATCATTATTGAATTATGTTTCTAGATAGGTTGTAG
CTGGCTCTTTAGAGCATGTGCACGCCTGTTTGGACTTCATTTTCATCCACCTGTGCACCT
ATTGTAGTCTTTGGTTGGGTTAGGAGGAAGTGGTCATTGTGTCAGCATCTGCTGGATGTG
AGGACTTGCATTGTGAAAGCTTTGCTGTCCTTGATGTGATCATGGAATCTCTTTCTCACT
AGAGTCTATGTCACTCATTATACTCTGTCGAATGTCATTGAATGTCTTTACATGGGCTTA
TATGCCTATGAAAATTGTAATACAACTTTCAGCAACGGATCTCTTGGCTCTCGCATCGAT
GAAGAACGCAGCGAAATGCGATAAGTAATGTGAATTGCAGAATTCAGTGAATCATCGAAT
CTTTGAACGCATCTTGCGCTCCTTGGTATTCCGAGGAGCATGCCTGTTTGAGTGTCATTA
AATTCTCAACTCTCTTCTAC-TTTTTGTAAAAGAGAGCTTGGACTGTGGAGGCTTGCTGG
CCACTTTTTGGGGTCAGCTCCTCTGAAATGCATTAGCGGAACCGTTTGCGATCTGCCACA
AGTGTGATAAGTTATCTACACTGGCGAGGGGATTGCTCTCTGTAATGTTCAGCTTCTAAT
TGTCTCTACTTTGTGAGACTACTTTTGAATGCTTGACCTCAAATCAGGTAGGACTACCC-
GCTGAACTTAA
>BC7-15
TTTCCGTAGGTGAACCTGCGGAAGGATCATTATTGAATTATGTTTCTAGATAGGTTGTAG
CTGGCTCTTTAGAGCATGTGCACGCCTGTTTGGACTTCATTTTCATCCACCTGTGCACCT
ATTGTAGTCTTTGGTTGGGTTAGGAGGAAGTGGTCATTGTGTCAGCATCTGCTGGATGTG
AGGACTTGCATTGTGAAAGCTTTGCTGTCCTTGATGTGATCATGGAATCTCTTTCTCACT
AGAGTCTATGTCACTCATTATACTCTGTCGAATGTCATTGAATGTCTTTACATGGGCTTA
TATGCCTATGAAAATTGTAATACAACTTTCAGCAACGGATCTCTTGGCTCTCGCATCGAT
GAAGAACGCAGCGAAATGCGATAAGTAATGTGAATTGCAGAATTCAGTGAATCATCGAAT
CTTTGAACGCATCTTGCGCTCCTTGGTATTCCGAGGAGCATGCCTGTTTGAGTGTCATTA
AATTCTCAACTCTCTTCTAC-TTTTTGTAAAAGAGAGCTTGGACTGTGGAGGCTTGCTGG
CCACTTTTTGGGGTCAGCTCCTCTGAAATGCATTAGCGGAACCGTTTGCGATCTGCCACA
AGTGTGATAAGTTATCTACACTGGCGAGGGGATTGCTCTCTGTAATGTTCAGCTTCTAAT
TGTCTCTACTTTGTGAGACTACTTTTGAATGCTTGACCTCAAATCAGGTAGGACTACCC-
GCTGAACTTAA

>BC7–19
TTTCCGTAGGTGAACCTGCGGAAGGATCATTATTGAATTATGTTTCTAGATAGGTTGTAG
CTGGCTCTTTAGAGCATGTGCACGCCTGTTTGGACTTCATTTTCATCCACCTGTGCACCT
ATTGTAGTCTTTGGTTGGGTTAGGAGGAAGTGGTCATTGTGTCAGCATCTGCTGGATGTG
AGGACTTGCATTGTGAAAGCTTTGCTGTCCTTGATGTGATCATGGAATCTCTTTCTCACT
AGAGTCTATGTCACTCATTATACTCTGTCGAATGTCATTGAATGTCTTTACATGGGCTTA
TATGCCTATGAAAATTGTAATACAACTTTCAGCAACGGATCTCTTGGCTCTCGCATCGAT
GAAGAACGCAGCGAAATGCGATAAGTAATGTGAATTGCAGAATTCAGTGAATCATCGAAT
CTTTGAACGCATCTTGCGCTCCTTGGTATTCCGAGGAGCATGCCTGTTTGAGTGTCATTA
AATTCTCAACTCTCTTCTAC–TTTTTGTAAAAGAGAGCTTGGACTGTGGAGGCTTGCTGG
CCACTTTTTGGGGTCAGCTCCTCTGAAATGCATTAGCGGAACCGTTTGCGATCTGCCACA
AGTGTGATAAGTTATCTACACTGGCGAGGGGATTGCTCTCTGTAATGTTCAGCTTCTAAT
TGTCTCTACTTTGTGAGACTACTTTTGAATGCTTGACCTCAAATCAGGTAGGACTACCC–
GCTGAACTTAA
>BC7–24
TTTCCGTAGGTGAACCTGCGGAAGGATCATTATTGAATTATGTTTCTAGATAGGTTGTAG
CTGGCTCTTTAGAGCATGTGCACGCCTGTTTGGACTTCATTTTCATCCACCTGTGCACCT
ATTGTAGTCTTTGGTTGGGTTAGGAGGAAGTGGTCATTGTGTCAGCATCTGCTGGATGTG
AGGACTTGCATTGTGAAAGCTTTGCTGTCCTTGATGTGATCATGGAATCTCTTTCTCACT
AGAGTCTATGTCACTCATTATACTCTGTCGAATGTCATTGAATGTCTTTACATGGGCTTA
TATGCCTATGAAAATTGTAATACAACTTTCAGCAACGGATCTCTTGGCTCTCGCATCGAT
GAAGAACGCAGCGAAATGCGATAAGTAATGTGAATTGCAGAATTCAGTGAATCATCGAAT
CTTTGAACGCATCTTGCGCTCCTTGGTATTCCGAGGAGCATGCCTGTTTGAGTGTCATTA
AATTCTCAACTCTCTTCTAC–TTTTTGTAAAAGAGAGCTTGGACTGTGGAGGCTTGCTGG
CCACTTTTTGGGGTCAGCTCCTCTGAAATGCATTAGCGGAACCGTTTGCGATCTGCCACA
AGTGTGATAAGTTATCTACACTGGCGAGGGGATTGCTCTCTGTAATGTTCAGCTTCTAAT
TGTCTCTACTTTGTGAGACTACTTTTGAATGCTTGACCTCAAATCAGGTAGGACTACCC–
GCTGAACTTAA
>BC7–26
TTTCCGTAGGTGAACCTGCGGAAGGATCATTATTGAATTATGTTTCTAGATAGGTTGTAG
CTGGCTCTTTAGAGCATGTGCACGCCTGTTTGGACTTCATTTTCATCCACCTGTGCACCT
ATTGTAGTCTTTGGTTGGGTTAGGAGGAAGTGGTCATTGTGTCAGCATCTGCTGGATGTG
AGGACTTGCATTGTGAAAGCTTTGCTGTCCTTGATGTGATCATGGAATCTCTTTCTCACT
AGAGTCTATGTCACTCATTATACTCTGTCGAATGTCATTGAATGTCTTTACATGGGCTTA
TATGCCTATGAAAATTGTAATACAACTTTCAGCAACGGATCTCTTGGCTCTCGCATCGAT
GAAGAACGCAGCGAAATGCGATAAGTAATGTGAATTGCAGAATTCAGTGAATCATCGAAT
CTTTGAACGCATCTTGCGCTCCTTGGTATTCCGAGGAGCATGCCTGTTTGAGTGTCATTA
AATTCTCAACTCTCTTCTAC–TTTTTGTAAAAGAGAGCTTGGACTGTGGAGGCTTGCTGG
CCACTTTTTGGGGTCAGCTCCTCTGAAATGCATTAGCGGAACCGTTTGCGATCTGCCACA
AGTGTGATAAGTTATCTACACTGGCGAGGGGATTGCTCTCTGTAATGTTCAGCTTCTAAT
TGTCTCTACTTTGTGAGACTACTTTTGAATGCTTGACCTCAAATCAGGTAGGACTACCC–
GCTGAACTTAA
>BC7–31
TTTCCGTAGGTGAACCTGCGGAAGGATCATTATTGAATTATGTTTCTAGATAGGTTGTAG
CTGGCTCTTTAGAGCATGTGCACGCCTGTTTGGACTTCATTTTCATCCACCTGTGCACCT
ATTGTAGTCTTTGGTTGGGTTAGGAGGAAGTGGTCATTGTGTCAGCATCTGCTGGATGTG
AGGACTTGCATTGTGAAAGCTTTGCTGTCCTTGATGTGATCATGGAATCTCTTTCTCACT
AGAGTCTATGTCACTCATTATACTCTGTCGAATGTCATTGAATGTCTTTACATGGGCTTA
TATGCCTATGAAAATTGTAATACAACTTTCAGCAACGGATCTCTTGGCTCTCGCATCGAT
GAAGAACGCAGCGAAATGCGATAAGTAATGTGAATTGCAGAATTCAGTGAATCATCGAAT

CTTTGAACGCATCTTGCGCTCCTTGGTATTCCGAGGAGCATGCCTGTTTGAGTGTCATTA
AATTCTCAACTCTCTTCTAC-TTTTTGTAAAAGAGAGCTTGGACTGTGGAGGCTTGCTGG
CCACTTTTTGGGGTCAGCTCCTCTGAAATGCATTAGCGGAACCGTTTGCGATCTGCCACA
AGTGTGATAAGTTATCTACACTGGCGAGGGGATTGCTCTCTGTAATGTTCAGCTTCTAAT
TGTCTCTACTTTGTGAGACTACTTTTGAATGCTTGACCTCAAATCAGGTAGGACTACCC-
GCTGAACTTAA
>BC7-32
TTTCCGTAGGTGAACCTGCGGAAGGATCATTATTGAATTATGTTTCTAGATAGGTTGTAG
CTGGCTCTTTAGAGCATGTGCACGCCTGTTTGGACTTCATTTTCATCCACCTGTGCACCT
ATTGTAGTCTTTGGTTGGGTTAGGAGGAAGTGGTCATTGTGTCAGCATCTGCTGGATGTG
AGGACTTGCATTGTGAAAGCTTTGCTGTCCTTGATGTGATCATGGAATCTCTTTCTCACT
AGAGTCTATGTCACTCATTATACTCTGTCGAATGTCATTGAATGTCTTTACATGGGCTTA
TATGCCTATGAAAATTGTAATACAACTTTCAGCAACGGATCTCTTGGCTCTCGCATCGAT
GAAGAACGCAGCGAAATGCGATAAGTAATGTGAATTGCAGAATTCAGTGAATCATCGAAT
CTTTGAACGCATCTTGCGCTCCTTGGTATTCCGAGGAGCATGCCTGTTTGAGTGTCATTA
AATTCTCAACTCTCTTCTAC-TTTTTGTAAAAGAGAGCTTGGACTGTGGAGGCTTGCTGG
CCACTTTTTGGGGTCAGCTCCTCTGAAATGCATTAGCGGAACCGTTTGCGATCTGCCACA
AGTGTGATAAGTTATCTACACTGGCGAGGGGATTGCTCTCTGTAATGTTCAGCTTCTAAT
TGTCTCTACTTTGTGAGACTACTTTTGAATGCTTGACCTCAAATCAGGTAGGACTACCC-
GCTGAACTTAA
>BC7-37
TTTCCGTAGGTGAACCTGCGGAAGGATCATTATTGAATTATGTTTCTAGATAGGTTGTAG
CTGGCTCTTTAGAGCATGTGCACGCCTGTTTGGACTTCATTTTCATCCACCTGTGCACCT
ATTGTAGTCTTTGGTTGGGTTAGGAGGAAGTGGTCATTGTGTCAGCATCTGCTGGATGTG
AGGACTTGCATTGTGAAAGCTTTGCTGTCCTTGATGTGATCATGGAATCTCTTTCTCACT
AGAGTCTATGTCACTCATTATACTCTGTCGAATGTCATTGAATGTCTTTACATGGGCTTA
TATGCCTATGAAAATTGTAATACAACTTTCAGCAACGGATCTCTTGGCTCTCGCATCGAT
GAAGAACGCAGCGAAATGCGATAAGTAATGTGAATTGCAGAATTCAGTGAATCATCGAAT
CTTTGAACGCATCTTGCGCTCCTTGGTATTCCGAGGAGCATGCCTGTTTGAGTGTCATTA
AATTCTCAACTCTCTTCTAC-TTTTTGTAAAAGAGAGCTTGGACTGTGGAGGCTTGCTGG
CCACTTTTTGGGGTCAGCTCCTCTGAAATGCATTAGCGGAACCGTTTGCGATCTGCCACA
AGTGTGATAAGTTATCTACACTGGCGAGGGGATTGCTCTCTGTAATGTTCAGCTTCTAAT
TGTCTCTACTTTGTGAGACTACTTTTGAATGCTTGACCTCAAATCAGGTAGGACTACCC-
GCTGAACTTAA
>BC7-41
TTTCCGTAGGTGAACCTGCGGAAGGATCATTATTGAATTATGTTTCTAGATAGGTTGTAG
CTGGCTCTTTAGAGCATGTGCACGCCTGTTTGGACTTCATTTTCATCCACCTGTGCACCT
ATTGTAGTCTTTGGTTGGGTTAGGAGGAAGTGGTCATTGTGTCAGCATCTGCTGGATGTG
AGGACTTGCATTGTGAAAGCTTTGCTGTCCTTGATGTGATCATGGAATCTCTTTCTCACT
AGAGTCTATGTCACTCATTATACTCTGTCGAATGTCATTGAATGTCTTTACATGGGCTTA
TATGCCTATGAAAATTGTAATACAACTTTCAGCAACGGATCTCTTGGCTCTCGCATCGAT
GAAGAACGCAGCGAAATGCGATAAGTAATGTGAATTGCAGAATTCAGTGAATCATCGAAT
CTTTGAACGCATCTTGCGCTCCTTGGTATTCCGAGGAGCATGCCTGTTTGAGTGTCATTA
AATTCTCAACTCTCTTCTAC-TTTTTGTAAAAGAGAGCTTGGACTGTGGAGGCTTGCTGG
CCACTTTTTGGGGTCAGCTCCTCTGAAATGCATTAGCGGAACCGTTTGCGATCTGCCACA
AGTGTGATAAGTTATCTACACTGGCGAGGGGATTGCTCTCTGTAATGTTCAGCTTCTAAT
TGTCTCTACTTTGTGAGACTACTTTTGAATGCTTGACCTCAAATCAGGTAGGACTACCC-
GCTGAACTTAA
>BC7-45
TTTCCGTAGGTGAACCTGCGGAAGGATCATTATTGAATTATGTTTCTAGATAGGTTGTAG

CTGGCTCTTTAGAGCATGTGCACGCCTGTTTGGACTTCATTTTCATCCACCTGTGCACCT
ATTGTAGTCTTTGGTTGGGTTAGGAGGAAGTGGTCATTGTGTCAGCATCTGCTGGATGTG
AGGACTTGCATTGTGAAAGCTTTGCTGTCCTTGATGTGATCATGGAATCTCTTTCTCACT
AGAGTCTATGTCACTCATTATACTCTGTCGAATGTCATTGAATGTCTTTACATGGGCTTA
TATGCCTATGAAAATTGTAATACAACTTTCAGCAACGGATCTCTTGGCTCTCGCATCGAT
GAAGAACGCAGCGAAATGCGATAAGTAATGTGAATTGCAGAATTCAGTGAATCATCGAAT
CTTTGAACGCATCTTGCGCTCCTTGGTATTCCGAGGAGCATGCCTGTTTGAGTGTCATTA
AATTCTCAACTCTCTTCTAC-TTTTTGTAAAAGAGAGCTTGGACTGTGGAGGCTTGCTGG
CCACTTTTTGGGGTCAGCTCCTCTGAAATGCATTAGCGGAACCGTTTGCGATCTGCCACA
AGTGTGATAAGTTATCTACACTGGCGAGGGGATTGCTCTCTGTAATGTTCAGCTTCTAAT
TGTCTCTACTTTGTGAGACTACTTTTGAATGCTTGACCTCAAATCAGGTAGGACTACCC-
GCTGAACTTAA
>BC7-47
TTTCCGTAGGTGAACCTGCGGAAGGATCATTATTGAATTATGTTTCTAGATAGGTTGTAG
CTGGCTCTTTAGAGCATGTGCACGCCTGTTTGGACTTCATTTTCATCCACCTGTGCACCT
ATTGTAGTCTTTGGTTGGGTTAGGAGGAAGTGGTCATTGTGTCAGCATCTGCTGGATGTG
AGGACTTGCATTGTGAAAGCTTTGCTGTCCTTGATGTGATCATGGAATCTCTTTCTCACT
AGAGTCTATGTCACTCATTATACTCTGTCGAATGTCATTGAATGTCTTTACATGGGCTTA
TATGCCTATGAAAATTGTAATACAACTTTCAGCAACGGATCTCTTGGCTCTCGCATCGAT
GAAGAACGCAGCGAAATGCGATAAGTAATGTGAATTGCAGAATTCAGTGAATCATCGAAT
CTTTGAACGCATCTTGCGCTCCTTGGTATTCCGAGGAGCATGCCTGTTTGAGTGTCATTA
AATTCTCAACTCTCTTCTAC-TTTTTGTAAAAGAGAGCTTGGACTGTGGAGGCTTGCTGG
CCACTTTTTGGGGTCAGCTCCTCTGAAATGCATTAGCGGAACCGTTTGCGATCTGCCACA
AGTGTGATAAGTTATCTACACTGGCGAGGGGATTGCTCTCTGTAATGTTCAGCTTCTAAT
TGTCTCTACTTTGTGAGACTACTTTTGAATGCTTGACCTCAAATCAGGTAGGACTACCC-
GCTGAACTTAA
>BC7-48
TTTCCGTAGGTGAACCTGCGGAAGGATCATTATTGAATTATGTTTCTAGATAGGTTGTAG
CTGGCTCTTTAGAGCATGTGCACGCCTGTTTGGACTTCATTTTCATCCACCTGTGCACCT
ATTGTAGTCTTTGGTTGGGTTAGGAGGAAGTGGTCATTGTGTCAGCATCTGCTGGATGTG
AGGACTTGCATTGTGAAAGCTTTGCTGTCCTTGATGTGATCATGGAATCTCTTTCTCACT
AGAGTCTATGTCACTCATTATACTCTGTCGAATGTCATTGAATGTCTTTACATGGGCTTA
TATGCCTATGAAAATTGTAATACAACTTTCAGCAACGGATCTCTTGGCTCTCGCATCGAT
GAAGAACGCAGCGAAATGCGATAAGTAATGTGAATTGCAGAATTCAGTGAATCATCGAAT
CTTTGAACGCATCTTGCGCTCCTTGGTATTCCGAGGAGCATGCCTGTTTGAGTGTCATTA
AATTCTCAACTCTCTTCTAC-TTTTTGTAAAAGAGAGCTTGGACTGTGGAGGCTTGCTGG
CCACTTTTTGGGGTCAGCTCCTCTGAAATGCATTAGCGGAACCGTTTGCGATCTGCCACA
AGTGTGATAAGTTATCTACACTGGCGAGGGGATTGCTCTCTGTAATGTTCAGCTTCTAAT
TGTCTCTACTTTGTGAGACTACTTTTGAATGCTTGACCTCAAATCAGGTAGGACTACCC-
GCTGAACTTAA
>BC7-52
TTTCCGTAGGTGAACCTGCGGAAGGATCATTATTGAATTATGTTTCTAGATAGGTTGTAG
CTGGCTCTTTAGAGCATGTGCACGCCTGTTTGGACTTCATTTTCATCCACCTGTGCACCT
ATTGTAGTCTTTGGTTGGGTTAGGAGGAAGTGGTCATTGTGTCAGCATCTGCTGGATGTG
AGGACTTGCATTGTGAAAGCTTTGCTGTCCTTGATGTGATCATGGAATCTCTTTCTCACT
AGAGTCTATGTCACTCATTATACTCTGTCGAATGTCATTGAATGTCTTTACATGGGCTTA
TATGCCTATGAAAATTGTAATACAACTTTCAGCAACGGATCTCTTGGCTCTCGCATCGAT
GAAGAACGCAGCGAAATGCGATAAGTAATGTGAATTGCAGAATTCAGTGAATCATCGAAT
CTTTGAACGCATCTTGCGCTCCTTGGTATTCCGAGGAGCATGCCTGTTTGAGTGTCATTA
AATTCTCAACTCTCTTCTAC-TTTTTGTAAAAGAGAGCTTGGACTGTGGAGGCTTGCTGG

CCACTTTTTGGGGTCAGCTCCTCTGAAATGCATTAGCGGAACCGTTTGCGATCTGCCACA
AGTGTGATAAGTTATCTACACTGGCGAGGGGATTGCTCTCTGTAATGTTCAGCTTCTAAT
TGTCTCTACTTTGTGAGACTACTTTTGAATGCTTGACCTCAAATCAGGTAGGACTACCC-
GCTGAACTTAA
>BC8-1
TTTCCGTAGGTGAACCTGCGGAAGGATCATTATTGAATTATGTTTCTAGATAGGTTGTAG
CTGGCTCTTTAGAGCATGTGCACGCCTGTTTGGACTTCATTTTCATCCACCTGTGCACCT
ATTGTAGTCTTTGGTTGGGTTAGGAGGAAGTGGTCATTGTGTCAGCATCTGCTGGATGTG
AGGACTTGCATTGTGAAAGCTTTGCTGTCCTTGATGTGATCATGGAATCTCTTTCTCACT
AGAGTCTATGTCACTCATTATACTCTGTCGAATGTCATTGAATGTCTTTACATGGGCTTA
TATGCCTATGAAAATTGTAATACAACTTTCAGCAACGGATCTCTTGGCTCTCGCATCGAT
GAAGAACGCAGCGAAATGCGATAAGTAATGTGAATTGCAGAATTCAGTGAATCATCGAAT
CTTTGAACGCATCTTGCGCTCCTTGGTATTCCGAGGAGCATGCCTGTTTGAGTGTCATTA
AATTCTCAACTCTCTTCTAC-TTTTTGTAAAAGAGAGCTTGGACTGTGGAGGCTTGCTGG
CCACTTTTTGGGGTCAGCTCCTCTGAAATGCATTAGCGGAACCGTTTGCGATCTGCCACA
AGTGTGATAAGTTATCTACACTGGCGAGGGGATTGCTCTCTGTAATGTTCAGCTTCTAAT
TGTCTCTACTTTGTGAGACTACTTTTGAATGCTTGACCTCAAATCAGGTAGGACTACCC-
GCTGAACTTAA
>BC8-3
TTTCCGTAGGTGAACCTGCGGAAGGATCATTATTGAATTATGTTTCTAGATAGGTTGTAG
CTGGCTCTTTAGAGCATGTGCACGCCTGTTTGGACTTCATTTTCATCCACCTGTGCACCT
ATTGTAGTCTTTGGTTGGGTTAGGAGGAAGTGGTCATTGTGTCAGCATCTGCTGGATGTG
AGGACTTGCATTGTGAAAGCTTTGCTGTCCTTGATGTGATCATGGAATCTCTTTCTCACT
AGAGTCTATGTCACTCATTATACTCTGTCGAATGTCATTGAATGTCTTTACATGGGCTTA
TATGCCTATGAAAATTGTAATACAACTTTCAGCAACGGATCTCTTGGCTCTCGCATCGAT
GAAGAACGCAGCGAAATGCGATAAGTAATGTGAATTGCAGAATTCAGTGAATCATCGAAT
CTTTGAACGCATCTTGCGCTCCTTGGTATTCCGAGGAGCATGCCTGTTTGAGTGTCATTA
AATTCTCAACTCTCTTCTAC-TTTTTGTAAAAGAGAGCTTGGACTGTGGAGGCTTGCTGG
CCACTTTTTGGGGTCAGCTCCTCTGAAATGCATTAGCGGAACCGTTTGCGATCTGCCACA
AGTGTGATAAGTTATCTACACTGGCGAGGGGATTGCTCTCTGTAATGTTCAGCTTCTAAT
TGTCTCTACTTTGTGAGACTACTTTTGAATGCTTGACCTCAAATCAGGTAGGACTACCC-
GCTGAACTTAA
>BC8-19
TTTCCGTAGGTGAACCTGCGGAAGGATCATTATTGAATTATGTTTCTAGATAGGTTGTAG
CTGGCTCTTTAGAGCATGTGCACGCCTGTTTGGACTTCATTTTCATCCACCTGTGCACCT
ATTGTAGTCTTTGGTTGGGTTAGGAGGAAGTGGTCATTGTGTCAGCATCTGCTGGATGTG
AGGACTTGCATTGTGAAAGCTTTGCTGTCCTTGATGTGATCATGGAATCTCTTTCTCACT
AGAGTCTATGTCACTCATTATACTCTGTCGAATGTCATTGAATGTCTTTACATGGGCTTA
TATGCCTATGAAAATTGTAATACAACTTTCAGCAACGGATCTCTTGGCTCTCGCATCGAT
GAAGAACGCAGCGAAATGCGATAAGTAATGTGAATTGCAGAATTCAGTGAATCATCGAAT
CTTTGAACGCATCTTGCGCTCCTTGGTATTCCGAGGAGCATGCCTGTTTGAGTGTCATTA
AATTCTCAACTCTCTTCTAC-TTTTTGTAAAAGAGAGCTTGGACTGTGGAGGCTTGCTGG
CCACTTTTTGGGGTCAGCTCCTCTGAAATGCATTAGCGGAACCGTTTGCGATCTGCCACA
AGTGTGATAAGTTATCTACACTGGCGAGGGGATTGCTCTCTGTAATGTTCAGCTTCTAAT
TGTCTCTACTTTGTGAGACTACTTTTGAATGCTTGACCTCAAATCAGGTAGGACTACCC-
GCTGAACTTAA
>BC8-31
TTTCCGTAGGTGAACCTGCGGAAGGATCATTATTGAATTATGTTTCTAGATAGGTTGTAG
CTGGCTCTTTAGAGCATGTGCACGCCTGTTTGGACTTCATTTTCATCCACCTGTGCACCT
ATTGTAGTCTTTGGTTGGGTTAGGAGGAAGTGGTCATTGTGTCAGCATCTGCTGGATGTG

AGGACTTGCATTGTGAAAGCTTTGCTGTCCTTGATGTGATCATGGAATCTCTTTCTCACT
AGAGTCTATGTCACTCATTATACTCTGTCGAATGTCATTGAATGTCTTTACATGGGCTTA
TATGCCTATGAAAATTGTAATACAACTTTCAGCAACGGATCTCTTGGCTCTCGCATCGAT
GAAGAACGCAGCGAAATGCGATAAGTAATGTGAATTGCAGAATTCAGTGAATCATCGAAT
CTTTGAACGCATCTTGCGCTCCTTGGTATTCCGAGGAGCATGCCTGTTTGAGTGTCATTA
AATTCTCAACTCTCTTCTAC-TTTTTGTAAAAGAGAGCTTGGACTGTGGAGGCTTGCTGG
CCACTTTTTGGGGTCAGCTCCTCTGAAATGCATTAGCGGAACCGTTTGCGATCTGCCACA
AGTGTGATAAGTTATCTACACTGGCGAGGGGATTGCTCTCTGTAATGTTCAGCTTCTAAT
TGTCTCTACTTTGTGAGACTACTTTTGAATGCTTGACCTCAAATCAGGTAGGACTACCC-
GCTGAACTTAA
>BC8-35
TTTCCGTAGGTGAACCTGCGGAAGGATCATTATTGAATTATGTTTCTAGATAGGTTGTAG
CTGGCTCTTTAGAGCATGTGCACGCCTGTTTGGACTTCATTTTCATCCACCTGTGCACCT
ATTGTAGTCTTTGGTTGGGTTAGGAGGAAGTGGTCATTGTGTCAGCATCTGCTGGATGTG
AGGACTTGCATTGTGAAAGCTTTGCTGTCCTTGATGTGATCATGGAATCTCTTTCTCACT
AGAGTCTATGTCACTCATTATACTCTGTCGAATGTCATTGAATGTCTTTACATGGGCTTA
TATGCCTATGAAAATTGTAATACAACTTTCAGCAACGGATCTCTTGGCTCTCGCATCGAT
GAAGAACGCAGCGAAATGCGATAAGTAATGTGAATTGCAGAATTCAGTGAATCATCGAAT
CTTTGAACGCATCTTGCGCTCCTTGGTATTCCGAGGAGCATGCCTGTTTGAGTGTCATTA
AATTCTCAACTCTCTTCTAC-TTTTTGTAAAAGAGAGCTTGGACTGTGGAGGCTTGCTGG
CCACTTTTTGGGGTCAGCTCCTCTGAAATGCATTAGCGGAACCGTTTGCGATCTGCCACA
AGTGTGATAAGTTATCTACACTGGCGAGGGGATTGCTCTCTGTAATGTTCAGCTTCTAAT
TGTCTCTACTTTGTGAGACTACTTTTGAATGCTTGACCTCAAATCAGGTAGGACTACCC-
GCTGAACTTAA
>BC8-36
TTTCCGTAGGTGAACCTGCGGAAGGATCATTATTGAATTATGTTTCTAGATAGGTTGTAG
CTGGCTCTTTAGAGCATGTGCACGCCTGTTTGGACTTCATTTTCATCCACCTGTGCACCT
ATTGTAGTCTTTGGTTGGGTTAGGAGGAAGTGGTCATTGTGTCAGCATCTGCTGGATGTG
AGGACTTGCATTGTGAAAGCTTTGCTGTCCTTGATGTGATCATGGAATCTCTTTCTCACT
AGAGTCTATGTCACTCATTATACTCTGTCGAATGTCATTGAATGTCTTTACATGGGCTTA
TATGCCTATGAAAATTGTAATACAACTTTCAGCAACGGATCTCTTGGCTCTCGCATCGAT
GAAGAACGCAGCGAAATGCGATAAGTAATGTGAATTGCAGAATTCAGTGAATCATCGAAT
CTTTGAACGCATCTTGCGCTCCTTGGTATTCCGAGGAGCATGCCTGTTTGAGTGTCATTA
AATTCTCAACTCTCTTCTAC-TTTTTGTAAAAGAGAGCTTGGACTGTGGAGGCTTGCTGG
CCACTTTTTGGGGTCAGCTCCTCTGAAATGCATTAGCGGAACCGTTTGCGATCTGCCACA
AGTGTGATAAGTTATCTACACTGGCGAGGGGATTGCTCTCTGTAATGTTCAGCTTCTAAT
TGTCTCTACTTTGTGAGACTACTTTTGAATGCTTGACCTCAAATCAGGTAGGACTACCC-
GCTGAACTTAA
>BC8-38
TTTCCGTAGGTGAACCTGCGGAAGGATCATTATTGAATTATGTTTCTAGATAGGTTGTAG
CTGGCTCTTTAGAGCATGTGCACGCCTGTTTGGACTTCATTTTCATCCACCTGTGCACCT
ATTGTAGTCTTTGGTTGGGTTAGGAGGAAGTGGTCATTGTGTCAGCATCTGCTGGATGTG
AGGACTTGCATTGTGAAAGCTTTGCTGTCCTTGATGTGATCATGGAATCTCTTTCTCACT
AGAGTCTATGTCACTCATTATACTCTGTCGAATGTCATTGAATGTCTTTACATGGGCTTA
TATGCCTATGAAAATTGTAATACAACTTTCAGCAACGGATCTCTTGGCTCTCGCATCGAT
GAAGAACGCAGCGAAATGCGATAAGTAATGTGAATTGCAGAATTCAGTGAATCATCGAAT
CTTTGAACGCATCTTGCGCTCCTTGGTATTCCGAGGAGCATGCCTGTTTGAGTGTCATTA
AATTCTCAACTCTCTTCTAC-TTTTTGTAAAAGAGAGCTTGGACTGTGGAGGCTTGCTGG
CCACTTTTTGGGGTCAGCTCCTCTGAAATGCATTAGCGGAACCGTTTGCGATCTGCCACA
AGTGTGATAAGTTATCTACACTGGCGAGGGGATTGCTCTCTGTAATGTTCAGCTTCTAAT

TGTCTCTACTTTGTGAGACTACTTTTGAATGCTTGACCTCAAATCAGGTAGGACTACCC-
GCTGAACTTAA
>BC8-41
TTTCCGTAGGTGAACCTGCGGAAGGATCATTATTGAATTATGTTTCTAGATAGGTTGTAG
CTGGCTCTTTAGAGCATGTGCACGCCTGTTTGGACTTCATTTTCATCCACCTGTGCACCT
ATTGTAGTCTTTGGTTGGGTTAGGAGGAAGTGGTCATTGTGTCAGCATCTGCTGGATGTG
AGGACTTGCATTGTGAAAGCTTTGCTGTCCTTGATGTGATCATGGAATCTCTTTCTCACT
AGAGTCTATGTCACTCATTATACTCTGTCGAATGTCATTGAATGTCTTTACATGGGCTTA
TATGCCTATGAAAATTGTAATACAACTTTCAGCAACGGATCTCTTGGCTCTCGCATCGAT
GAAGAACGCAGCGAAATGCGATAAGTAATGTGAATTGCAGAATTCAGTGAATCATCGAAT
CTTTGAACGCATCTTGCGCTCCTTGGTATTCCGAGGAGCATGCCTGTTTGAGTGTCATTA
AATTCTCAACTCTCTTCTAC-TTTTTGTAAAAGAGAGCTTGGACTGTGGAGGCTTGCTGG
CCACTTTTTGGGGTCAGCTCCTCTGAAATGCATTAGCGGAACCGTTTGCGATCTGCCACA
AGTGTGATAAGTTATCTACACTGGCGAGGGGATTGCTCTCTGTAATGTTCAGCTTCTAAT
TGTCTCTACTTTGTGAGACTACTTTTGAATGCTTGACCTCAAATCAGGTAGGACTACCC-
GCTGAACTTAA
>BC8-44
TTTCCGTAGGTGAACCTGCGGAAGGATCATTATTGAATTATGTTTCTAGATAGGTTGTAG
CTGGCTCTTTAGAGCATGTGCACGCCTGTTTGGACTTCATTTTCATCCACCTGTGCACCT
ATTGTAGTCTTTGGTTGGGTTAGGAGGAAGTGGTCATTGTGTCAGCATCTGCTGGATGTG
AGGACTTGCATTGTGAAAGCTTTGCTGTCCTTGATGTGATCATGGAATCTCTTTCTCACT
AGAGTCTATGTCACTCATTATACTCTGTCGAATGTCATTGAATGTCTTTACATGGGCTTA
TATGCCTATGAAAATTGTAATACAACTTTCAGCAACGGATCTCTTGGCTCTCGCATCGAT
GAAGAACGCAGCGAAATGCGATAAGTAATGTGAATTGCAGAATTCAGTGAATCATCGAAT
CTTTGAACGCATCTTGCGCTCCTTGGTATTCCGAGGAGCATGCCTGTTTGAGTGTCATTA
AATTCTCAACTCTCTTCTAC-TTTTTGTAAAAGAGAGCTTGGACTGTGGAGGCTTGCTGG
CCACTTTTTGGGGTCAGCTCCTCTGAAATGCATTAGCGGAACCGTTTGCGATCTGCCACA
AGTGTGATAAGTTATCTACACTGGCGAGGGGATTGCTCTCTGTAATGTTCAGCTTCTAAT
TGTCTCTACTTTGTGAGACTACTTTTGAATGCTTGACCTCAAATCAGGTAGGACTACCC-
GCTGAACTTAA
>BC8-52
TTTCCGTAGGTGAACCTGCGGAAGGATCATTATTGAATTATGTTTCTAGATAGGTTGTAG
CTGGCTCTTTAGAGCATGTGCACGCCTGTTTGGACTTCATTTTCATCCACCTGTGCACCT
ATTGTAGTCTTTGGTTGGGTTAGGAGGAAGTGGTCATTGTGTCAGCATCTGCTGGATGTG
AGGACTTGCATTGTGAAAGCTTTGCTGTCCTTGATGTGATCATGGAATCTCTTTCTCACT
AGAGTCTATGTCACTCATTATACTCTGTCGAATGTCATTGAATGTCTTTACATGGGCTTA
TATGCCTATGAAAATTGTAATACAACTTTCAGCAACGGATCTCTTGGCTCTCGCATCGAT
GAAGAACGCAGCGAAATGCGATAAGTAATGTGAATTGCAGAATTCAGTGAATCATCGAAT
CTTTGAACGCATCTTGCGCTCCTTGGTATTCCGAGGAGCATGCCTGTTTGAGTGTCATTA
AATTCTCAACTCTCTTCTAC-TTTTTGTAAAAGAGAGCTTGGACTGTGGAGGCTTGCTGG
CCACTTTTTGGGGTCAGCTCCTCTGAAATGCATTAGCGGAACCGTTTGCGATCTGCCACA
AGTGTGATAAGTTATCTACACTGGCGAGGGGATTGCTCTCTGTAATGTTCAGCTTCTAAT
TGTCTCTACTTTGTGAGACTACTTTTGAATGCTTGACCTCAAATCAGGTAGGACTACCC-
GCTGAACTTAA
>BC8-54
TTTCCGTAGGTGAACCTGCGGAAGGATCATTATTGAATTATGTTTCTAGATAGGTTGTAG
CTGGCTCTTTAGAGCATGTGCACGCCTGTTTGGACTTCATTTTCATCCACCTGTGCACCT
ATTGTAGTCTTTGGTTGGGTTAGGAGGAAGTGGTCATTGTGTCAGCATCTGCTGGATGTG
AGGACTTGCATTGTGAAAGCTTTGCTGTCCTTGATGTGATCATGGAATCTCTTTCTCACT
AGAGTCTATGTCACTCATTATACTCTGTCGAATGTCATTGAATGTCTTTACATGGGCTTA

TATGCCTATGAAAATTGTAATACAACTTTCAGCAACGGATCTCTTGGCTCTCGCATCGAT
GAAGAACGCAGCGAAATGCGATAAGTAATGTGAATTGCAGAATTCAGTGAATCATCGAAT
CTTTGAACGCATCTTGCGCTCCTTGGTATTCCGAGGAGCATGCCTGTTTGAGTGTCATTA
AATTCTCAACTCTCTTCTAC-TTTTTGTAAAAGAGAGCTTGGACTGTGGAGGCTTGCTGG
CCACTTTTTGGGGTCAGCTCCTCTGAAATGCATTAGCGGAACCGTTTGCGATCTGCCACA
AGTGTGATAAGTTATCTACACTGGCGAGGGGATTGCTCTCTGTAATGTTCAGCTTCTAAT
TGTCTCTACTTTGTGAGACTACTTTTGAATGCTTGACCTCAAATCAGGTAGGACTACCC-
GCTGAACTTAA
>BC8-57
TTTCCGTAGGTGAACCTGCGGAAGGATCATTATTGAATTATGTTTCTAGATAGGTTGTAG
CTGGCTCTTTAGAGCATGTGCACGCCTGTTTGGACTTCATTTTCATCCACCTGTGCACCT
ATTGTAGTCTTTGGTTGGGTTAGGAGGAAGTGGTCATTGTGTCAGCATCTGCTGGATGTG
AGGACTTGCATTGTGAAAGCTTTGCTGTCCTTGATGTGATCATGGAATCTCTTTCTCACT
AGAGTCTATGTCACTCATTATACTCTGTCGAATGTCATTGAATGTCTTTACATGGGCTTA
TATGCCTATGAAAATTGTAATACAACTTTCAGCAACGGATCTCTTGGCTCTCGCATCGAT
GAAGAACGCAGCGAAATGCGATAAGTAATGTGAATTGCAGAATTCAGTGAATCATCGAAT
CTTTGAACGCATCTTGCGCTCCTTGGTATTCCGAGGAGCATGCCTGTTTGAGTGTCATTA
AATTCTCAACTCTCTTCTAC-TTTTTGTAAAAGAGAGCTTGGACTGTGGAGGCTTGCTGG
CCACTTTTTGGGGTCAGCTCCTCTGAAATGCATTAGCGGAACCGTTTGCGATCTGCCACA
AGTGTGATAAGTTATCTACACTGGCGAGGGGATTGCTCTCTGTAATGTTCAGCTTCTAAT
TGTCTCTACTTTGTGAGACTACTTTTGAATGCTTGACCTCAAATCAGGTAGGACTACCC-
GCTGAACTTAA
>BC9-1
TTTCCGTAGGTGAACCTGCGGAAGGATCATTATTGAATTATGTTTCTAGATAGGTTGTAG
CTGGCTCTTTAGAGCATGTGCACGCCTGTTTGGACTTCATTTTCATCCACCTGTGCACCT
ATTGTAGTCTTTGGTTGGGTTAGGAGGAAGTGGTCATTGTGTCAGCATCTGCTGGATGTG
AGGACTTGCATTGTGAAAGCTTTGCTGTCCTTGATGTGATCATGGAATCTCTTTCTCACT
AGAGTCTATGTCACTCATTATACTCTGTCGAATGTCATTGAATGTCTTTACATGGGCTTA
TATGCCTATGAAAATTGTAATACAACTTTCAGCAACGGATCTCTTGGCTCTCGCATCGAT
GAAGAACGCAGCGAAATGCGATAAGTAATGTGAATTGCAGAATTCAGTGAATCATCGAAT
CTTTGAACGCATCTTGCGCTCCTTGGTATTCCGAGGAGCATGCCTGTTTGAGTGTCATTA
AATTCTCAACTCTCTTCTAC-TTTTTGTAAAAGAGAGCTTGGACTGTGGAGGCTTGCTGG
CCACTTTTTGGGGTCAGCTCCTCTGAAATGCATTAGCGGAACCGTTTGCGATCTGCCACA
AGTGTGATAAGTTATCTACACTGGCGAGGGGATTGCTCTCTGTAATGTTCAGCTTCTAAT
TGTCTCTACTTTGTGAGACTACTTTTGAATGCTTGACCTCAAATCAGGTAGGACTACCC-
GCTGAACTTAA
>BC9-9
TTTCCGTAGGTGAACCTGCGGAAGGATCATTATTGAATTATGTTTCTAGATAGGTTGTAG
CTGGCTCTTTAGAGCATGTGCACGCCTGTTTGGACTTCATTTTCATCCACCTGTGCACCT
ATTGTAGTCTTTGGTTGGGTTAGGAGGAAGTGGTCATTGTGTCAGCATCTGCTGGATGTG
AGGACTTGCATTGTGAAAGCTTTGCTGTCCTTGATGTGATCATGGAATCTCTTTCTCACT
AGAGTCTATGTCACTCATTATACTCTGTCGAATGTCATTGAATGTCTTTACATGGGCTTA
TATGCCTATGAAAATTGTAATACAACTTTCAGCAACGGATCTCTTGGCTCTCGCATCGAT
GAAGAACGCAGCGAAATGCGATAAGTAATGTGAATTGCAGAATTCAGTGAATCATCGAAT
CTTTGAACGCATCTTGCGCTCCTTGGTATTCCGAGGAGCATGCCTGTTTGAGTGTCATTA
AATTCTCAACTCTCTTCTAC-TTTTTGTAAAAGAGAGCTTGGACTGTGGAGGCTTGCTGG
CCACTTTTTGGGGTCAGCTCCTCTGAAATGCATTAGCGGAACCGTTTGCGATCTGCCACA
AGTGTGATAAGTTATCTACACTGGCGAGGGGATTGCTCTCTGTAATGTTCAGCTTCTAAT
TGTCTCTACTTTGTGAGACTACTTTTGAATGCTTGACCTCAAATCAGGTAGGACTACCC-
GCTGAACTTAA

>BC9−11
TTTCCGTAGGTGAACCTGCGGAAGGATCATTATTGAATTATGTTTCTAGATAGGTTGTAG
CTGGCTCTTTAGAGCATGTGCACGCCTGTTTGGACTTCATTTTCATCCACCTGTGCACCT
ATTGTAGTCTTTGGTTGGGTTAGGAGGAAGTGGTCATTGTGTCAGCATCTGCTGGATGTG
AGGACTTGCATTGTGAAAGCTTTGCTGTCCTTGATGTGATCATGGAATCTCTTTCTCACT
AGAGTCTATGTCACTCATTATACTCTGTCGAATGTCATTGAATGTCTTTACATGGGCTTA
TATGCCTATGAAAATTGTAATACAACTTTCAGCAACGGATCTCTTGGCTCTCGCATCGAT
GAAGAACGCAGCGAAATGCGATAAGTAATGTGAATTGCAGAATTCAGTGAATCATCGAAT
CTTTGAACGCATCTTGCGCTCCTTGGTATTCCGAGGAGCATGCCTGTTTGAGTGTCATTA
AATTCTCAACTCTCTTCTAC−TTTTTGTAAAAGAGAGCTTGGACTGTGGAGGCTTGCTGG
CCACTTTTTGGGGTCAGCTCCTCTGAAATGCATTAGCGGAACCGTTTGCGATCTGCCACA
AGTGTGATAAGTTATCTACACTGGCGAGGGGATTGCTCTCTGTAATGTTCAGCTTCTAAT
TGTCTCTACTTTGTGAGACTACTTTTGAATGCTTGACCTCAAATCAGGTAGGACTACCC−
GCTGAACTTAA
>BC9−23
TTTCCGTAGGTGAACCTGCGGAAGGATCATTATTGAATTATGTTTCTAGATAGGTTGTAG
CTGGCTCTTTAGAGCATGTGCACGCCTGTTTGGACTTCATTTTCATCCACCTGTGCACCT
ATTGTAGTCTTTGGTTGGGTTAGGAGGAAGTGGTCATTGTGTCAGCATCTGCTGGATGTG
AGGACTTGCATTGTGAAAGCTTTGCTGTCCTTGATGTGATCATGGAATCTCTTTCTCACT
AGAGTCTATGTCACTCATTATACTCTGTCGAATGTCATTGAATGTCTTTACATGGGCTTA
TATGCCTATGAAAATTGTAATACAACTTTCAGCAACGGATCTCTTGGCTCTCGCATCGAT
GAAGAACGCAGCGAAATGCGATAAGTAATGTGAATTGCAGAATTCAGTGAATCATCGAAT
CTTTGAACGCATCTTGCGCTCCTTGGTATTCCGAGGAGCATGCCTGTTTGAGTGTCATTA
AATTCTCAACTCTCTTCTAC−TTTTTGTAAAAGAGAGCTTGGACTGTGGAGGCTTGCTGG
CCACTTTTTGGGGTCAGCTCCTCTGAAATGCATTAGCGGAACCGTTTGCGATCTGCCACA
AGTGTGATAAGTTATCTACACTGGCGAGGGGATTGCTCTCTGTAATGTTCAGCTTCTAAT
TGTCTCTACTTTGTGAGACTACTTTTGAATGCTTGACCTCAAATCAGGTAGGACTACCC−
GCTGAACTTAA
>BC9−24
TTTCCGTAGGTGAACCTGCGGAAGGATCATTATTGAATTATGTTTCTAGATAGGTTGTAG
CTGGCTCTTTAGAGCATGTGCACGCCTGTTTGGACTTCATTTTCATCCACCTGTGCACCT
ATTGTAGTCTTTGGTTGGGTTAGGAGGAAGTGGTCATTGTGTCAGCATCTGCTGGATGTG
AGGACTTGCATTGTGAAAGCTTTGCTGTCCTTGATGTGATCATGGAATCTCTTTCTCACT
AGAGTCTATGTCACTCATTATACTCTGTCGAATGTCATTGAATGTCTTTACATGGGCTTA
TATGCCTATGAAAATTGTAATACAACTTTCAGCAACGGATCTCTTGGCTCTCGCATCGAT
GAAGAACGCAGCGAAATGCGATAAGTAATGTGAATTGCAGAATTCAGTGAATCATCGAAT
CTTTGAACGCATCTTGCGCTCCTTGGTATTCCGAGGAGCATGCCTGTTTGAGTGTCATTA
AATTCTCAACTCTCTTCTAC−TTTTTGTAAAAGAGAGCTTGGACTGTGGAGGCTTGCTGG
CCACTTTTTGGGGTCAGCTCCTCTGAAATGCATTAGCGGAACCGTTTGCGATCTGCCACA
AGTGTGATAAGTTATCTACACTGGCGAGGGGATTGCTCTCTGTAATGTTCAGCTTCTAAT
TGTCTCTACTTTGTGAGACTACTTTTGAATGCTTGACCTCAAATCAGGTAGGACTACCC−
GCTGAACTTAA
>BC9−26
TTTCCGTAGGTGAACCTGCGGAAGGATCATTATTGAATTATGTTTCTAGATAGGTTGTAG
CTGGCTCTTTAGAGCATGTGCACGCCTGTTTGGACTTCATTTTCATCCACCTGTGCACCT
ATTGTAGTCTTTGGTTGGGTTAGGAGGAAGTGGTCATTGTGTCAGCATCTGCTGGATGTG
AGGACTTGCATTGTGAAAGCTTTGCTGTCCTTGATGTGATCATGGAATCTCTTTCTCACT
AGAGTCTATGTCACTCATTATACTCTGTCGAATGTCATTGAATGTCTTTACATGGGCTTA
TATGCCTATGAAAATTGTAATACAACTTTCAGCAACGGATCTCTTGGCTCTCGCATCGAT
GAAGAACGCAGCGAAATGCGATAAGTAATGTGAATTGCAGAATTCAGTGAATCATCGAAT

CTTTGAACGCATCTTGCGCTCCTTGGTATTCCGAGGAGCATGCCTGTTTGAGTGTCATTA
AATTCTCAACTCTCTTCTAC-TTTTTGTAAAAGAGAGCTTGGACTGTGGAGGCTTGCTGG
CCACTTTTTGGGGTCAGCTCCTCTGAAATGCATTAGCGGAACCGTTTGCGATCTGCCACA
AGTGTGATAAGTTATCTACACTGGCGAGGGGATTGCTCTCTGTAATGTTCAGCTTCTAAT
TGTCTCTACTTTGTGAGACTACTTTTGAATGCTTGACCTCAAATCAGGTAGGACTACCC-
GCTGAACTTAA
>BC9-35
TTTCCGTAGGTGAACCTGCGGAAGGATCATTATTGAATTATGTTTCTAGATAGGTTGTAG
CTGGCTCTTTAGAGCATGTGCACGCCTGTTTGGACTTCATTTTCATCCACCTGTGCACCT
ATTGTAGTCTTTGGTTGGGTTAGGAGGAAGTGGTCATTGTGTCAGCATCTGCTGGATGTG
AGGACTTGCATTGTGAAAGCTTTGCTGTCCTTGATGTGATCATGGAATCTCTTTCTCACT
AGAGTCTATGTCACTCATTATACTCTGTCGAATGTCATTGAATGTCTTTACATGGGCTTA
TATGCCTATGAAAATTGTAATACAACTTTCAGCAACGGATCTCTTGGCTCTCGCATCGAT
GAAGAACGCAGCGAAATGCGATAAGTAATGTGAATTGCAGAATTCAGTGAATCATCGAAT
CTTTGAACGCATCTTGCGCTCCTTGGTATTCCGAGGAGCATGCCTGTTTGAGTGTCATTA
AATTCTCAACTCTCTTCTAC-TTTTTGTAAAAGAGAGCTTGGACTGTGGAGGCTTGCTGG
CCACTTTTTGGGGTCAGCTCCTCTGAAATGCATTAGCGGAACCGTTTGCGATCTGCCACA
AGTGTGATAAGTTATCTACACTGGCGAGGGGATTGCTCTCTGTAATGTTCAGCTTCTAAT
TGTCTCTACTTTGTGAGACTACTTTTGAATGCTTGACCTCAAATCAGGTAGGACTACCC-
GCTGAACTTAA
>BC9-37
TTTCCGTAGGTGAACCTGCGGAAGGATCATTATTGAATTATGTTTCTAGATAGGTTGTAG
CTGGCTCTTTAGAGCATGTGCACGCCTGTTTGGACTTCATTTTCATCCACCTGTGCACCT
ATTGTAGTCTTTGGTTGGGTTAGGAGGAAGTGGTCATTGTGTCAGCATCTGCTGGATGTG
AGGACTTGCATTGTGAAAGCTTTGCTGTCCTTGATGTGATCATGGAATCTCTTTCTCACT
AGAGTCTATGTCACTCATTATACTCTGTCGAATGTCATTGAATGTCTTTACATGGGCTTA
TATGCCTATGAAAATTGTAATACAACTTTCAGCAACGGATCTCTTGGCTCTCGCATCGAT
GAAGAACGCAGCGAAATGCGATAAGTAATGTGAATTGCAGAATTCAGTGAATCATCGAAT
CTTTGAACGCATCTTGCGCTCCTTGGTATTCCGAGGAGCATGCCTGTTTGAGTGTCATTA
AATTCTCAACTCTCTTCTAC-TTTTTGTAAAAGAGAGCTTGGACTGTGGAGGCTTGCTGG
CCACTTTTTGGGGTCAGCTCCTCTGAAATGCATTAGCGGAACCGTTTGCGATCTGCCACA
AGTGTGATAAGTTATCTACACTGGCGAGGGGATTGCTCTCTGTAATGTTCAGCTTCTAAT
TGTCTCTACTTTGTGAGACTACTTTTGAATGCTTGACCTCAAATCAGGTAGGACTACCC-
GCTGAACTTAA
>BC9-43
TTTCCGTAGGTGAACCTGCGGAAGGATCATTATTGAATTATGTTTCTAGATAGGTTGTAG
CTGGCTCTTTAGAGCATGTGCACGCCTGTTTGGACTTCATTTTCATCCACCTGTGCACCT
ATTGTAGTCTTTGGTTGGGTTAGGAGGAAGTGGTCATTGTGTCAGCATCTGCTGGATGTG
AGGACTTGCATTGTGAAAGCTTTGCTGTCCTTGATGTGATCATGGAATCTCTTTCTCACT
AGAGTCTATGTCACTCATTATACTCTGTCGAATGTCATTGAATGTCTTTACATGGGCTTA
TATGCCTATGAAAATTGTAATACAACTTTCAGCAACGGATCTCTTGGCTCTCGCATCGAT
GAAGAACGCAGCGAAATGCGATAAGTAATGTGAATTGCAGAATTCAGTGAATCATCGAAT
CTTTGAACGCATCTTGCGCTCCTTGGTATTCCGAGGAGCATGCCTGTTTGAGTGTCATTA
AATTCTCAACTCTCTTCTAC-TTTTTGTAAAAGAGAGCTTGGACTGTGGAGGCTTGCTGG
CCACTTTTTGGGGTCAGCTCCTCTGAAATGCATTAGCGGAACCGTTTGCGATCTGCCACA
AGTGTGATAAGTTATCTACACTGGCGAGGGGATTGCTCTCTGTAATGTTCAGCTTCTAAT
TGTCTCTACTTTGTGAGACTACTTTTGAATGCTTGACCTCAAATCAGGTAGGACTACCC-
GCTGAACTTAA
>BC9-44
TTTCCGTAGGTGAACCTGCGGAAGGATCATTATTGAATTATGTTTCTAGATAGGTTGTAG

CTGGCTCTTTAGAGCATGTGCACGCCTGTTTGGACTTCATTTTCATCCACCTGTGCACCT
ATTGTAGTCTTTGGTTGGGTTAGGAGGAAGTGGTCATTGTGTCAGCATCTGCTGGATGTG
AGGACTTGCATTGTGAAAGCTTTGCTGTCCTTGATGTGATCATGGAATCTCTTTCTCACT
AGAGTCTATGTCACTCATTATACTCTGTCGAATGTCATTGAATGTCTTTACATGGGCTTA
TATGCCTATGAAAATTGTAATACAACTTTCAGCAACGGATCTCTTGGCTCTCGCATCGAT
GAAGAACGCAGCGAAATGCGATAAGTAATGTGAATTGCAGAATTCAGTGAATCATCGAAT
CTTTGAACGCATCTTGCGCTCCTTGGTATTCCGAGGAGCATGCCTGTTTGAGTGTCATTA
AATTCTCAACTCTCTTCTAC-TTTTTGTAAAAGAGAGCTTGGACTGTGGAGGCTTGCTGG
CCACTTTTTGGGGTCAGCTCCTCTGAAATGCATTAGCGGAACCGTTTGCGATCTGCCACA
AGTGTGATAAGTTATCTACACTGGCGAGGGGATTGCTCTCTGTAATGTTCAGCTTCTAAT
TGTCTCTACTTTGTGAGACTACTTTTGAATGCTTGACCTCAAATCAGGTAGGACTACCC-
GCTGAACTTAA
>BC9-49
TTTCCGTAGGTGAACCTGCGGAAGGATCATTATTGAATTATGTTTCTAGATAGGTTGTAG
CTGGCTCTTTAGAGCATGTGCACGCCTGTTTGGACTTCATTTTCATCCACCTGTGCACCT
ATTGTAGTCTTTGGTTGGGTTAGGAGGAAGTGGTCATTGTGTCAGCATCTGCTGGATGTG
AGGACTTGCATTGTGAAAGCTTTGCTGTCCTTGATGTGATCATGGAATCTCTTTCTCACT
AGAGTCTATGTCACTCATTATACTCTGTCGAATGTCATTGAATGTCTTTACATGGGCTTA
TATGCCTATGAAAATTGTAATACAACTTTCAGCAACGGATCTCTTGGCTCTCGCATCGAT
GAAGAACGCAGCGAAATGCGATAAGTAATGTGAATTGCAGAATTCAGTGAATCATCGAAT
CTTTGAACGCATCTTGCGCTCCTTGGTATTCCGAGGAGCATGCCTGTTTGAGTGTCATTA
AATTCTCAACTCTCTTCTAC-TTTTTGTAAAAGAGAGCTTGGACTGTGGAGGCTTGCTGG
CCACTTTTTGGGGTCAGCTCCTCTGAAATGCATTAGCGGAACCGTTTGCGATCTGCCACA
AGTGTGATAAGTTATCTACACTGGCGAGGGGATTGCTCTCTGTAATGTTCAGCTTCTAAT
TGTCTCTACTTTGTGAGACTACTTTTGAATGCTTGACCTCAAATCAGGTAGGACTACCC-
GCTGAACTTAA
>BC9-50
TTTCCGTAGGTGAACCTGCGGAAGGATCATTATTGAATTATGTTTCTAGATAGGTTGTAG
CTGGCTCTTTAGAGCATGTGCACGCCTGTTTGGACTTCATTTTCATCCACCTGTGCACCT
ATTGTAGTCTTTGGTTGGGTTAGGAGGAAGTGGTCATTGTGTCAGCATCTGCTGGATGTG
AGGACTTGCATTGTGAAAGCTTTGCTGTCCTTGATGTGATCATGGAATCTCTTTCTCACT
AGAGTCTATGTCACTCATTATACTCTGTCGAATGTCATTGAATGTCTTTACATGGGCTTA
TATGCCTATGAAAATTGTAATACAACTTTCAGCAACGGATCTCTTGGCTCTCGCATCGAT
GAAGAACGCAGCGAAATGCGATAAGTAATGTGAATTGCAGAATTCAGTGAATCATCGAAT
CTTTGAACGCATCTTGCGCTCCTTGGTATTCCGAGGAGCATGCCTGTTTGAGTGTCATTA
AATTCTCAACTCTCTTCTAC-TTTTTGTAAAAGAGAGCTTGGACTGTGGAGGCTTGCTGG
CCACTTTTTGGGGTCAGCTCCTCTGAAATGCATTAGCGGAACCGTTTGCGATCTGCCACA
AGTGTGATAAGTTATCTACACTGGCGAGGGGATTGCTCTCTGTAATGTTCAGCTTCTAAT
TGTCTCTACTTTGTGAGACTACTTTTGAATGCTTGACCTCAAATCAGGTAGGACTACCC-
GCTGAACTTAA
>BC9-55
TTTCCGTAGGTGAACCTGCGGAAGGATCATTATTGAATTATGTTTCTAGATAGGTTGTAG
CTGGCTCTTTAGAGCATGTGCACGCCTGTTTGGACTTCATTTTCATCCACCTGTGCACCT
ATTGTAGTCTTTGGTTGGGTTAGGAGGAAGTGGTCATTGTGTCAGCATCTGCTGGATGTG
AGGACTTGCATTGTGAAAGCTTTGCTGTCCTTGATGTGATCATGGAATCTCTTTCTCACT
AGAGTCTATGTCACTCATTATACTCTGTCGAATGTCATTGAATGTCTTTACATGGGCTTA
TATGCCTATGAAAATTGTAATACAACTTTCAGCAACGGATCTCTTGGCTCTCGCATCGAT
GAAGAACGCAGCGAAATGCGATAAGTAATGTGAATTGCAGAATTCAGTGAATCATCGAAT
CTTTGAACGCATCTTGCGCTCCTTGGTATTCCGAGGAGCATGCCTGTTTGAGTGTCATTA
AATTCTCAACTCTCTTCTAC-TTTTTGTAAAAGAGAGCTTGGACTGTGGAGGCTTGCTGG

CCACTTTTTGGGGTCAGCTCCTCTGAAATGCATTAGCGGAACCGTTTGCGATCTGCCACA
AGTGTGATAAGTTATCTACACTGGCGAGGGGATTGCTCTCTGTAATGTTCAGCTTCTAAT
TGTCTCTACTTTGTGAGACTACTTTTGAATGCTTGACCTCAAATCAGGTAGGACTACCC-
GCTGAACTTAA
>BC10_3
TTTCCGTAGGTGAACCTGCGGAAGGATCATTATTGAATTATGTTTCTAGATAGGTTGTAG
CTGGCTCTTTAGAGCATGTGCACGCCTGTTTGGACTTCATTTTCATCCACCTGTGCACCT
ATTGTAGTCTTTGGTTGGGTTAGGAGGAAGTGGTCATTGTGTCAGCATCTGCTGGATGTG
AGGACTTGCATTGTGAAAGCTTTGCTGTCCTTGATGTGATCATGGAATCTCTTTCTCACT
AGAGTCTATGTCACTCATTATACTCTGTCGAATGTCATTGAATGTCTTTACATGGGCTTA
TATGCCTATGAAAATTGTAATACAACTTTCAGCAACGGATCTCTTGGCTCTCGCATCGAT
GAAGAACGCAGCGAAATGCGATAAGTAATGTGAATTGCAGAATTCAGTGAATCATCGAAT
CTTTGAACGCATCTTGCGCTCCTTGGTATTCCGAGGAGCATGCCTGTTTGAGTGTCATTA
AATTCTCAACTCTCTTCTAC-TTTTTGTAAAAGAGAGCTTGGACTGTGGAGGCTTGCTGG
CCACTTTTTGGGGTCAGCTCCTCTGAAATGCATTAGCGGAACCGTTTGCGATCTGCCACA
AGTGTGATAAGTTATCTACACTGGCGAGGGGATTGCTCTCTGTAATGTTCAGCTTCTAAT
TGTCTCTACTTTGTGAGACTACTTTTGAATGCTTGACCTCAAATCAGGTAGGACTACCC-
GCTGAACTTAA
>BC10_8
TTTCCGTAGGTGAACCTGCGGAAGGATCATTATTGAATTATGTTTCTAGATAGGTTGTAG
CTGGCTCTTTAGAGCATGTGCACGCCTGTTTGGACTTCATTTTCATCCACCTGTGCACCT
ATTGTAGTCTTTGGTTGGGTTAGGAGGAAGTGGTCATTGTGTCAGCATCTGCTGGATGTG
AGGACTTGCATTGTGAAAGCTTTGCTGTCCTTGATGTGATCATGGAATCTCTTTCTCACT
AGAGTCTATGTCACTCATTATACTCTGTCGAATGTCATTGAATGTCTTTACATGGGCTTA
TATGCCTATGAAAATTGTAATACAACTTTCAGCAACGGATCTCTTGGCTCTCGCATCGAT
GAAGAACGCAGCGAAATGCGATAAGTAATGTGAATTGCAGAATTCAGTGAATCATCGAAT
CTTTGAACGCATCTTGCGCTCCTTGGTATTCCGAGGAGCATGCCTGTTTGAGTGTCATTA
AATTCTCAACTCTCTTCTAC-TTTTTGTAAAAGAGAGCTTGGACTGTGGAGGCTTGCTGG
CCACTTTTTGGGGTCAGCTCCTCTGAAATGCATTAGCGGAACCGTTTGCGATCTGCCACA
AGTGTGATAAGTTATCTACACTGGCGAGGGGATTGCTCTCTGTAATGTTCAGCTTCTAAT
TGTCTCTACTTTGTGAGACTACTTTTGAATGCTTGACCTCAAATCAGGTAGGACTACCC-
GCTGAACTTAA
>BC10_9
TTTCCGTAGGTGAACCTGCGGAAGGATCATTATTGAATTATGTTTCTAGATAGGTTGTAG
CTGGCTCTTTAGAGCATGTGCACGCCTGTTTGGACTTCATTTTCATCCACCTGTGCACCT
ATTGTAGTCTTTGGTTGGGTTAGGAGGAAGTGGTCATTGTGTCAGCATCTGCTGGATGTG
AGGACTTGCATTGTGAAAGCTTTGCTGTCCTTGATGTGATCATGGAATCTCTTTCTCACT
AGAGTCTATGTCACTCATTATACTCTGTCGAATGTCATTGAATGTCTTTACATGGGCTTA
TATGCCTATGAAAATTGTAATACAACTTTCAGCAACGGATCTCTTGGCTCTCGCATCGAT
GAAGAACGCAGCGAAATGCGATAAGTAATGTGAATTGCAGAATTCAGTGAATCATCGAAT
CTTTGAACGCATCTTGCGCTCCTTGGTATTCCGAGGAGCATGCCTGTTTGAGTGTCATTA
AATTCTCAACTCTCTTCTAC-TTTTTGTAAAAGAGAGCTTGGACTGTGGAGGCTTGCTGG
CCACTTTTTGGGGTCAGCTCCTCTGAAATGCATTAGCGGAACCGTTTGCGATCTGCCACA
AGTGTGATAAGTTATCTACACTGGCGAGGGGATTGCTCTCTGTAATGTTCAGCTTCTAAT
TGTCTCTACTTTGTGAGACTACTTTTGAATGCTTGACCTCAAATCAGGTAGGACTACCC-
GCTGAACTTAA
>BC10_14
TTTCCGTAGGTGAACCTGCGGAAGGATCATTATTGAATTATGTTTCTAGATAGGTTGTAG
CTGGCTCTTTAGAGCATGTGCACGCCTGTTTGGACTTCATTTTCATCCACCTGTGCACCT
ATTGTAGTCTTTGGTTGGGTTAGGAGGAAGTGGTCATTGTGTCAGCATCTGCTGGATGTG

AGGACTTGCATTGTGAAAGCTTTGCTGTCCTTGATGTGATCATGGAATCTCTTTCTCACT
AGAGTCTATGTCACTCATTATACTCTGTCGAATGTCATTGAATGTCTTTACATGGGCTTA
TATGCCTATGAAAATTGTAATACAACTTTCAGCAACGGATCTCTTGGCTCTCGCATCGAT
GAAGAACGCAGCGAAATGCGATAAGTAATGTGAATTGCAGAATTCAGTGAATCATCGAAT
CTTTGAACGCATCTTGCGCTCCTTGGTATTCCGAGGAGCATGCCTGTTTGAGTGTCATTA
AATTCTCAACTCTCTTCTAC-TTTTTGTAAAAGAGAGCTTGGACTGTGGAGGCTTGCTGG
CCACTTTTTGGGGTCAGCTCCTCTGAAATGCATTAGCGGAACCGTTTGCGATCTGCCACA
AGTGTGATAAGTTATCTACACTGGCGAGGGGATTGCTCTCTGTAATGTTCAGCTTCTAAT
TGTCTCTACTTTGTGAGACTACTTTTGAATGCTTGACCTCAAATCAGGTAGGACTACCC-
GCTGAACTTAA
>BC10_17
TTTCCGTAGGTGAACCTGCGGAAGGATCATTATTGAATTATGTTTCTAGATAGGTTGTAG
CTGGCTCTTTAGAGCATGTGCACGCCTGTTTGGACTTCATTTTCATCCACCTGTGCACCT
ATTGTAGTCTTTGGTTGGGTTAGGAGGAAGTGGTCATTGTGTCAGCATCTGCTGGATGTG
AGGACTTGCATTGTGAAAGCTTTGCTGTCCTTGATGTGATCATGGAATCTCTTTCTCACT
AGAGTCTATGTCACTCATTATACTCTGTCGAATGTCATTGAATGTCTTTACATGGGCTTA
TATGCCTATGAAAATTGTAATACAACTTTCAGCAACGGATCTCTTGGCTCTCGCATCGAT
GAAGAACGCAGCGAAATGCGATAAGTAATGTGAATTGCAGAATTCAGTGAATCATCGAAT
CTTTGAACGCATCTTGCGCTCCTTGGTATTCCGAGGAGCATGCCTGTTTGAGTGTCATTA
AATTCTCAACTCTCTTCTAC-TTTTTGTAAAAGAGAGCTTGGACTGTGGAGGCTTGCTGG
CCACTTTTTGGGGTCAGCTCCTCTGAAATGCATTAGCGGAACCGTTTGCGATCTGCCACA
AGTGTGATAAGTTATCTACACTGGCGAGGGGATTGCTCTCTGTAATGTTCAGCTTCTAAT
TGTCTCTACTTTGTGAGACTACTTTTGAATGCTTGACCTCAAATCAGGTAGGACTACCC-
GCTGAACTTAA
>BC10_21
TTTCCGTAGGTGAACCTGCGGAAGGATCATTATTGAATTATGTTTCTAGATAGGTTGTAG
CTGGCTCTTTAGAGCATGTGCACGCCTGTTTGGACTTCATTTTCATCCACCTGTGCACCT
ATTGTAGTCTTTGGTTGGGTTAGGAGGAAGTGGTCATTGTGTCAGCATCTGCTGGATGTG
AGGACTTGCATTGTGAAAGCTTTGCTGTCCTTGATGTGATCATGGAATCTCTTTCTCACT
AGAGTCTATGTCACTCATTATACTCTGTCGAATGTCATTGAATGTCTTTACATGGGCTTA
TATGCCTATGAAAATTGTAATACAACTTTCAGCAACGGATCTCTTGGCTCTCGCATCGAT
GAAGAACGCAGCGAAATGCGATAAGTAATGTGAATTGCAGAATTCAGTGAATCATCGAAT
CTTTGAACGCATCTTGCGCTCCTTGGTATTCCGAGGAGCATGCCTGTTTGAGTGTCATTA
AATTCTCAACTCTCTTCTAC-TTTTTGTAAAAGAGAGCTTGGACTGTGGAGGCTTGCTGG
CCACTTTTTGGGGTCAGCTCCTCTGAAATGCATTAGCGGAACCGTTTGCGATCTGCCACA
AGTGTGATAAGTTATCTACACTGGCGAGGGGATTGCTCTCTGTAATGTTCAGCTTCTAAT
TGTCTCTACTTTGTGAGACTACTTTTGAATGCTTGACCTCAAATCAGGTAGGACTACCC-
GCTGAACTTAA
>BC10_22
TTTCCGTAGGTGAACCTGCGGAAGGATCATTATTGAATTATGTTTCTAGATAGGTTGTAG
CTGGCTCTTTAGAGCATGTGCACGCCTGTTTGGACTTCATTTTCATCCACCTGTGCACCT
ATTGTAGTCTTTGGTTGGGTTAGGAGGAAGTGGTCATTGTGTCAGCATCTGCTGGATGTG
AGGACTTGCATTGTGAAAGCTTTGCTGTCCTTGATGTGATCATGGAATCTCTTTCTCACT
AGAGTCTATGTCACTCATTATACTCTGTCGAATGTCATTGAATGTCTTTACATGGGCTTA
TATGCCTATGAAAATTGTAATACAACTTTCAGCAACGGATCTCTTGGCTCTCGCATCGAT
GAAGAACGCAGCGAAATGCGATAAGTAATGTGAATTGCAGAATTCAGTGAATCATCGAAT
CTTTGAACGCATCTTGCGCTCCTTGGTATTCCGAGGAGCATGCCTGTTTGAGTGTCATTA
AATTCTCAACTCTCTTCTAC-TTTTTGTAAAAGAGAGCTTGGACTGTGGAGGCTTGCTGG
CCACTTTTTGGGGTCAGCTCCTCTGAAATGCATTAGCGGAACCGTTTGCGATCTGCCACA
AGTGTGATAAGTTATCTACACTGGCGAGGGGATTGCTCTCTGTAATGTTCAGCTTCTAAT

TGTCTCTACTTTGTGAGACTACTTTTGAATGCTTGACCTCAAATCAGGTAGGACTACCC-
GCTGAACTTAA
>BC10_26
TTTCCGTAGGTGAACCTGCGGAAGGATCATTATTGAATTATGTTTCTAGATAGGTTGTAG
CTGGCTCTTTAGAGCATGTGCACGCCTGTTTGGACTTCATTTTCATCCACCTGTGCACCT
ATTGTAGTCTTTGGTTGGGTTAGGAGGAAGTGGTCATTGTGTCAGCATCTGCTGGATGTG
AGGACTTGCATTGTGAAAGCTTTGCTGTCCTTGATGTGATCATGGAATCTCTTTCTCACT
AGAGTCTATGTCACTCATTATACTCTGTCGAATGTCATTGAATGTCTTTACATGGGCTTA
TATGCCTATGAAAATTGTAATACAACTTTCAGCAACGGATCTCTTGGCTCTCGCATCGAT
GAAGAACGCAGCGAAATGCGATAAGTAATGTGAATTGCAGAATTCAGTGAATCATCGAAT
CTTTGAACGCATCTTGCGCTCCTTGGTATTCCGAGGAGCATGCCTGTTTGAGTGTCATTA
AATTCTCAACTCTCTTCTAC-TTTTTGTAAAAGAGAGCTTGGACTGTGGAGGCTTGCTGG
CCACTTTTTGGGGTCAGCTCCTCTGAAATGCATTAGCGGAACCGTTTGCGATCTGCCACA
AGTGTGATAAGTTATCTACACTGGCGAGGGGATTGCTCTCTGTAATGTTCAGCTTCTAAT
TGTCTCTACTTTGTGAGACTACTTTTGAATGCTTGACCTCAAATCAGGTAGGACTACCC-
GCTGAACTTAA
>BC10_27
TTTCCGTAGGTGAACCTGCGGAAGGATCATTATTGAATTATGTTTCTAGATAGGTTGTAG
CTGGCTCTTTAGAGCATGTGCACGCCTGTTTGGACTTCATTTTCATCCACCTGTGCACCT
ATTGTAGTCTTTGGTTGGGTTAGGAGGAAGTGGTCATTGTGTCAGCATCTGCTGGATGTG
AGGACTTGCATTGTGAAAGCTTTGCTGTCCTTGATGTGATCATGGAATCTCTTTCTCACT
AGAGTCTATGTCACTCATTATACTCTGTCGAATGTCATTGAATGTCTTTACATGGGCTTA
TATGCCTATGAAAATTGTAATACAACTTTCAGCAACGGATCTCTTGGCTCTCGCATCGAT
GAAGAACGCAGCGAAATGCGATAAGTAATGTGAATTGCAGAATTCAGTGAATCATCGAAT
CTTTGAACGCATCTTGCGCTCCTTGGTATTCCGAGGAGCATGCCTGTTTGAGTGTCATTA
AATTCTCAACTCTCTTCTAC-TTTTTGTAAAAGAGAGCTTGGACTGTGGAGGCTTGCTGG
CCACTTTTTGGGGTCAGCTCCTCTGAAATGCATTAGCGGAACCGTTTGCGATCTGCCACA
AGTGTGATAAGTTATCTACACTGGCGAGGGGATTGCTCTCTGTAATGTTCAGCTTCTAAT
TGTCTCTACTTTGTGAGACTACTTTTGAATGCTTGACCTCAAATCAGGTAGGACTACCC-
GCTGAACTTAA
>BC10_28
TTTCCGTAGGTGAACCTGCGGAAGGATCATTATTGAATTATGTTTCTAGATAGGTTGTAG
CTGGCTCTTTAGAGCATGTGCACGCCTGTTTGGACTTCATTTTCATCCACCTGTGCACCT
ATTGTAGTCTTTGGTTGGGTTAGGAGGAAGTGGTCATTGTGTCAGCATCTGCTGGATGTG
AGGACTTGCATTGTGAAAGCTTTGCTGTCCTTGATGTGATCATGGAATCTCTTTCTCACT
AGAGTCTATGTCACTCATTATACTCTGTCGAATGTCATTGAATGTCTTTACATGGGCTTA
TATGCCTATGAAAATTGTAATACAACTTTCAGCAACGGATCTCTTGGCTCTCGCATCGAT
GAAGAACGCAGCGAAATGCGATAAGTAATGTGAATTGCAGAATTCAGTGAATCATCGAAT
CTTTGAACGCATCTTGCGCTCCTTGGTATTCCGAGGAGCATGCCTGTTTGAGTGTCATTA
AATTCTCAACTCTCTTCTAC-TTTTTGTAAAAGAGAGCTTGGACTGTGGAGGCTTGCTGG
CCACTTTTTGGGGTCAGCTCCTCTGAAATGCATTAGCGGAACCGTTTGCGATCTGCCACA
AGTGTGATAAGTTATCTACACTGGCGAGGGGATTGCTCTCTGTAATGTTCAGCTTCTAAT
TGTCTCTACTTTGTGAGACTACTTTTGAATGCTTGACCTCAAATCAGGTAGGACTACCC-
GCTGAACTTAA
>BC10_34
TTTCCGTAGGTGAACCTGCGGAAGGATCATTATTGAATTATGTTTCTAGATAGGTTGTAG
CTGGCTCTTTAGAGCATGTGCACGCCTGTTTGGACTTCATTTTCATCCACCTGTGCACCT
ATTGTAGTCTTTGGTTGGGTTAGGAGGAAGTGGTCATTGTGTCAGCATCTGCTGGATGTG
AGGACTTGCATTGTGAAAGCTTTGCTGTCCTTGATGTGATCATGGAATCTCTTTCTCACT
AGAGTCTATGTCACTCATTATACTCTGTCGAATGTCATTGAATGTCTTTACATGGGCTTA

TATGCCTATGAAAATTGTAATACAACTTTCAGCAACGGATCTCTTGGCTCTCGCATCGAT
GAAGAACGCAGCGAAATGCGATAAGTAATGTGAATTGCAGAATTCAGTGAATCATCGAAT
CTTTGAACGCATCTTGCGCTCCTTGGTATTCCGAGGAGCATGCCTGTTTGAGTGTCATTA
AATTCTCAACTCTCTTCTAC-TTTTTGTAAAAGAGAGCTTGGACTGTGGAGGCTTGCTGG
CCACTTTTTGGGGTCAGCTCCTCTGAAATGCATTAGCGGAACCGTTTGCGATCTGCCACA
AGTGTGATAAGTTATCTACACTGGCGAGGGGATTGCTCTCTGTAATGTTCAGCTTCTAAT
TGTCTCTACTTTGTGAGACTACTTTTGAATGCTTGACCTCAAATCAGGTAGGACTACCC-
GCTGAACTTAA
>BC10_36
TTTCCGTAGGTGAACCTGCGGAAGGATCATTATTGAATTATGTTTCTAGATAGGTTGTAG
CTGGCTCTTTAGAGCATGTGCACGCCTGTTTGGACTTCATTTTCATCCACCTGTGCACCT
ATTGTAGTCTTTGGTTGGGTTAGGAGGAAGTGGTCATTGTGTCAGCATCTGCTGGATGTG
AGGACTTGCATTGTGAAAGCTTTGCTGTCCTTGATGTGATCATGGAATCTCTTTCTCACT
AGAGTCTATGTCACTCATTATACTCTGTCGAATGTCATTGAATGTCTTTACATGGGCTTA
TATGCCTATGAAAATTGTAATACAACTTTCAGCAACGGATCTCTTGGCTCTCGCATCGAT
GAAGAACGCAGCGAAATGCGATAAGTAATGTGAATTGCAGAATTCAGTGAATCATCGAAT
CTTTGAACGCATCTTGCGCTCCTTGGTATTCCGAGGAGCATGCCTGTTTGAGTGTCATTA
AATTCTCAACTCTCTTCTAC-TTTTTGTAAAAGAGAGCTTGGACTGTGGAGGCTTGCTGG
CCACTTTTTGGGGTCAGCTCCTCTGAAATGCATTAGCGGAACCGTTTGCGATCTGCCACA
AGTGTGATAAGTTATCTACACTGGCGAGGGGATTGCTCTCTGTAATGTTCAGCTTCTAAT
TGTCTCTACTTTGTGAGACTACTTTTGAATGCTTGACCTCAAATCAGGTAGGACTACCC-
GCTGAACTTAA
>BC10_37
TTTCCGTAGGTGAACCTGCGGAAGGATCATTATTGAATTATGTTTCTAGATAGGTTGTAG
CTGGCTCTTTAGAGCATGTGCACGCCTGTTTGGACTTCATTTTCATCCACCTGTGCACCT
ATTGTAGTCTTTGGTTGGGTTAGGAGGAAGTGGTCATTGTGTCAGCATCTGCTGGATGTG
AGGACTTGCATTGTGAAAGCTTTGCTGTCCTTGATGTGATCATGGAATCTCTTTCTCACT
AGAGTCTATGTCACTCATTATACTCTGTCGAATGTCATTGAATGTCTTTACATGGGCTTA
TATGCCTATGAAAATTGTAATACAACTTTCAGCAACGGATCTCTTGGCTCTCGCATCGAT
GAAGAACGCAGCGAAATGCGATAAGTAATGTGAATTGCAGAATTCAGTGAATCATCGAAT
CTTTGAACGCATCTTGCGCTCCTTGGTATTCCGAGGAGCATGCCTGTTTGAGTGTCATTA
AATTCTCAACTCTCTTCTAC-TTTTTGTAAAAGAGAGCTTGGACTGTGGAGGCTTGCTGG
CCACTTTTTGGGGTCAGCTCCTCTGAAATGCATTAGCGGAACCGTTTGCGATCTGCCACA
AGTGTGATAAGTTATCTACACTGGCGAGGGGATTGCTCTCTGTAATGTTCAGCTTCTAAT
TGTCTCTACTTTGTGAGACTACTTTTGAATGCTTGACCTCAAATCAGGTAGGACTACCC-
GCTGAACTTAA
>BC10_38
TTTCCGTAGGTGAACCTGCGGAAGGATCATTATTGAATTATGTTTCTAGATAGGTTGTAG
CTGGCTCTTTAGAGCATGTGCACGCCTGTTTGGACTTCATTTTCATCCACCTGTGCACCT
ATTGTAGTCTTTGGTTGGGTTAGGAGGAAGTGGTCATTGTGTCAGCATCTGCTGGATGTG
AGGACTTGCATTGTGAAAGCTTTGCTGTCCTTGATGTGATCATGGAATCTCTTTCTCACT
AGAGTCTATGTCACTCATTATACTCTGTCGAATGTCATTGAATGTCTTTACATGGGCTTA
TATGCCTATGAAAATTGTAATACAACTTTCAGCAACGGATCTCTTGGCTCTCGCATCGAT
GAAGAACGCAGCGAAATGCGATAAGTAATGTGAATTGCAGAATTCAGTGAATCATCGAAT
CTTTGAACGCATCTTGCGCTCCTTGGTATTCCGAGGAGCATGCCTGTTTGAGTGTCATTA
AATTCTCAACTCTCTTCTAC-TTTTTGTAAAAGAGAGCTTGGACTGTGGAGGCTTGCTGG
CCACTTTTTGGGGTCAGCTCCTCTGAAATGCATTAGCGGAACCGTTTGCGATCTGCCACA
AGTGTGATAAGTTATCTACACTGGCGAGGGGATTGCTCTCTGTAATGTTCAGCTTCTAAT
TGTCTCTACTTTGTGAGACTACTTTTGAATGCTTGACCTCAAATCAGGTAGGACTACCC-
GCTGAACTTAA

>BC10_41
TTTCCGTAGGTGAACCTGCGGAAGGATCATTATTGAATTATGTTTCTAGATAGGTTGTAG
CTGGCTCTTTAGAGCATGTGCACGCCTGTTTGGACTTCATTTTCATCCACCTGTGCACCT
ATTGTAGTCTTTGGTTGGGTTAGGAGGAAGTGGTCATTGTGTCAGCATCTGCTGGATGTG
AGGACTTGCATTGTGAAAGCTTTGCTGTCCTTGATGTGATCATGGAATCTCTTTCTCACT
AGAGTCTATGTCACTCATTATACTCTGTCGAATGTCATTGAATGTCTTTACATGGGCTTA
TATGCCTATGAAAATTGTAATACAACTTTCAGCAACGGATCTCTTGGCTCTCGCATCGAT
GAAGAACGCAGCGAAATGCGATAAGTAATGTGAATTGCAGAATTCAGTGAATCATCGAAT
CTTTGAACGCATCTTGCGCTCCTTGGTATTCCGAGGAGCATGCCTGTTTGAGTGTCATTA
AATTCTCAACTCTCTTCTAC-TTTTTGTAAAAGAGAGCTTGGACTGTGGAGGCTTGCTGG
CCACTTTTTGGGGTCAGCTCCTCTGAAATGCATTAGCGGAACCGTTTGCGATCTGCCACA
AGTGTGATAAGTTATCTACACTGGCGAGGGGATTGCTCTCTGTAATGTTCAGCTTCTAAT
TGTCTCTACTTTGTGAGACTACTTTTGAATGCTTGACCTCAAATCAGGTAGGACTACCC-
GCTGAACTTAA
>BC10_45
TTTCCGTAGGTGAACCTGCGGAAGGATCATTATTGAATTATGTTTCTAGATAGGTTGTAG
CTGGCTCTTTAGAGCATGTGCACGCCTGTTTGGACTTCATTTTCATCCACCTGTGCACCT
ATTGTAGTCTTTGGTTGGGTTAGGAGGAAGTGGTCATTGTGTCAGCATCTGCTGGATGTG
AGGACTTGCATTGTGAAAGCTTTGCTGTCCTTGATGTGATCATGGAATCTCTTTCTCACT
AGAGTCTATGTCACTCATTATACTCTGTCGAATGTCATTGAATGTCTTTACATGGGCTTA
TATGCCTATGAAAATTGTAATACAACTTTCAGCAACGGATCTCTTGGCTCTCGCATCGAT
GAAGAACGCAGCGAAATGCGATAAGTAATGTGAATTGCAGAATTCAGTGAATCATCGAAT
CTTTGAACGCATCTTGCGCTCCTTGGTATTCCGAGGAGCATGCCTGTTTGAGTGTCATTA
AATTCTCAACTCTCTTCTAC-TTTTTGTAAAAGAGAGCTTGGACTGTGGAGGCTTGCTGG
CCACTTTTTGGGGTCAGCTCCTCTGAAATGCATTAGCGGAACCGTTTGCGATCTGCCACA
AGTGTGATAAGTTATCTACACTGGCGAGGGGATTGCTCTCTGTAATGTTCAGCTTCTAAT
TGTCTCTACTTTGTGAGACTACTTTTGAATGCTTGACCTCAAATCAGGTAGGACTACCC-
GCTGAACTTAA
>BC10_50
TTTCCGTAGGTGAACCTGCGGAAGGATCATTATTGAATTATGTTTCTAGATAGGTTGTAG
CTGGCTCTTTAGAGCATGTGCACGCCTGTTTGGACTTCATTTTCATCCACCTGTGCACCT
ATTGTAGTCTTTGGTTGGGTTAGGAGGAAGTGGTCATTGTGTCAGCATCTGCTGGATGTG
AGGACTTGCATTGTGAAAGCTTTGCTGTCCTTGATGTGATCATGGAATCTCTTTCTCACT
AGAGTCTATGTCACTCATTATACTCTGTCGAATGTCATTGAATGTCTTTACATGGGCTTA
TATGCCTATGAAAATTGTAATACAACTTTCAGCAACGGATCTCTTGGCTCTCGCATCGAT
GAAGAACGCAGCGAAATGCGATAAGTAATGTGAATTGCAGAATTCAGTGAATCATCGAAT
CTTTGAACGCATCTTGCGCTCCTTGGTATTCCGAGGAGCATGCCTGTTTGAGTGTCATTA
AATTCTCAACTCTCTTCTAC-TTTTTGTAAAAGAGAGCTTGGACTGTGGAGGCTTGCTGG
CCACTTTTTGGGGTCAGCTCCTCTGAAATGCATTAGCGGAACCGTTTGCGATCTGCCACA
AGTGTGATAAGTTATCTACACTGGCGAGGGGATTGCTCTCTGTAATGTTCAGCTTCTAAT
TGTCTCTACTTTGTGAGACTACTTTTGAATGCTTGACCTCAAATCAGGTAGGACTACCC-
GCTGAACTTAA
>BC10_52
TTTCCGTAGGTGAACCTGCGGAAGGATCATTATTGAATTATGTTTCTAGATAGGTTGTAG
CTGGCTCTTTAGAGCATGTGCACGCCTGTTTGGACTTCATTTTCATCCACCTGTGCACCT
ATTGTAGTCTTTGGTTGGGTTAGGAGGAAGTGGTCATTGTGTCAGCATCTGCTGGATGTG
AGGACTTGCATTGTGAAAGCTTTGCTGTCCTTGATGTGATCATGGAATCTCTTTCTCACT
AGAGTCTATGTCACTCATTATACTCTGTCGAATGTCATTGAATGTCTTTACATGGGCTTA
TATGCCTATGAAAATTGTAATACAACTTTCAGCAACGGATCTCTTGGCTCTCGCATCGAT
GAAGAACGCAGCGAAATGCGATAAGTAATGTGAATTGCAGAATTCAGTGAATCATCGAAT

CTTTGAACGCATCTTGCGCTCCTTGGTATTCCGAGGAGCATGCCTGTTTGAGTGTCATTA
AATTCTCAACTCTCTTCTAC-TTTTTGTAAAAGAGAGCTTGGACTGTGGAGGCTTGCTGG
CCACTTTTTGGGGTCAGCTCCTCTGAAATGCATTAGCGGAACCGTTTGCGATCTGCCACA
AGTGTGATAAGTTATCTACACTGGCGAGGGGATTGCTCTCTGTAATGTTCAGCTTCTAAT
TGTCTCTACTTTGTGAGACTACTTTTGAATGCTTGACCTCAAATCAGGTAGGACTACCC-
GCTGAACTTAA
>BC11_2
TTTCCGTAGGTGAACCTGCGGAAGGATCATTATTGAATTATGTTTCTAGATAGGTTGTAG
CTGGCTCTTTAGAGCATGTGCACGCCTGTTTGGACTTCATTTTCATCCACCTGTGCACCT
ATTGTAGTCTTTGGTTGGGTTAGGAGGAAGTGGTCATTGTGTCAGCATCTGCTGGATGTG
AGGACTTGCATTGTGAAAGCTTTGCTGTCCTTGATGTGATCATGGAATCTCTTTCTCACT
AGAGTCTATGTCACTCATTATACTCTGTCGAATGTCATTGAATGTCTTTACATGGGCTTA
TATGCCTATGAAAATTGTAATACAACTTTCAGCAACGGATCTCTTGGCTCTCGCATCGAT
GAAGAACGCAGCGAAATGCGATAAGTAATGTGAATTGCAGAATTCAGTGAATCATCGAAT
CTTTGAACGCATCTTGCGCTCCTTGGTATTCCGAGGAGCATGCCTGTTTGAGTGTCATTA
AATTCTCAACTCTCTTCTAC-TTTTTGTAAAAGAGAGCTTGGACTGTGGAGGCTTGCTGG
CCACTTTTTGGGGTCAGCTCCTCTGAAATGCATTAGCGGAACCGTTTGCGATCTGCCACA
AGTGTGATAAGTTATCTACACTGGCGAGGGGATTGCTCTCTGTAATGTTCAGCTTCTAAT
TGTCTCTACTTTGTGAGACTACTTTTGAATGCTTGACCTCAAATCAGGTAGGACTACCC-
GCTGAACTTAA
>BC11_4
TTTCCGTAGGTGAACCTGCGGAAGGATCATTATTGAATTATGTTTCTAGATAGGTTGTAG
CTGGCTCTTTAGAGCATGTGCACGCCTGTTTGGACTTCATTTTCATCCACCTGTGCACCT
ATTGTAGTCTTTGGTTGGGTTAGGAGGAAGTGGTCATTGTGTCAGCATCTGCTGGATGTG
AGGACTTGCATTGTGAAAGCTTTGCTGTCCTTGATGTGATCATGGAATCTCTTTCTCACT
AGAGTCTATGTCACTCATTATACTCTGTCGAATGTCATTGAATGTCTTTACATGGGCTTA
TATGCCTATGAAAATTGTAATACAACTTTCAGCAACGGATCTCTTGGCTCTCGCATCGAT
GAAGAACGCAGCGAAATGCGATAAGTAATGTGAATTGCAGAATTCAGTGAATCATCGAAT
CTTTGAACGCATCTTGCGCTCCTTGGTATTCCGAGGAGCATGCCTGTTTGAGTGTCATTA
AATTCTCAACTCTCTTCTAC-TTTTTGTAAAAGAGAGCTTGGACTGTGGAGGCTTGCTGG
CCACTTTTTGGGGTCAGCTCCTCTGAAATGCATTAGCGGAACCGTTTGCGATCTGCCACA
AGTGTGATAAGTTATCTACACTGGCGAGGGGATTGCTCTCTGTAATGTTCAGCTTCTAAT
TGTCTCTACTTTGTGAGACTACTTTTGAATGCTTGACCTCAAATCAGGTAGGACTACCC-
GCTGAACTTAA
>BC11_8
TTTCCGTAGGTGAACCTGCGGAAGGATCATTATTGAATTATGTTTCTAGATAGGTTGTAG
CTGGCTCTTTAGAGCATGTGCACGCCTGTTTGGACTTCATTTTCATCCACCTGTGCACCT
ATTGTAGTCTTTGGTTGGGTTAGGAGGAAGTGGTCATTGTGTCAGCATCTGCTGGATGTG
AGGACTTGCATTGTGAAAGCTTTGCTGTCCTTGATGTGATCATGGAATCTCTTTCTCACT
AGAGTCTATGTCACTCATTATACTCTGTCGAATGTCATTGAATGTCTTTACATGGGCTTA
TATGCCTATGAAAATTGTAATACAACTTTCAGCAACGGATCTCTTGGCTCTCGCATCGAT
GAAGAACGCAGCGAAATGCGATAAGTAATGTGAATTGCAGAATTCAGTGAATCATCGAAT
CTTTGAACGCATCTTGCGCTCCTTGGTATTCCGAGGAGCATGCCTGTTTGAGTGTCATTA
AATTCTCAACTCTCTTCTAC-TTTTTGTAAAAGAGAGCTTGGACTGTGGAGGCTTGCTGG
CCACTTTTTGGGGTCAGCTCCTCTGAAATGCATTAGCGGAACCGTTTGCGATCTGCCACA
AGTGTGATAAGTTATCTACACTGGCGAGGGGATTGCTCTCTGTAATGTTCAGCTTCTAAT
TGTCTCTACTTTGTGAGACTACTTTTGAATGCTTGACCTCAAATCAGGTAGGACTACCC-
GCTGAACTTAA
>BC11_9
TTTCCGTAGGTGAACCTGCGGAAGGATCATTATTGAATTATGTTTCTAGATAGGTTGTAG

CTGGCTCTTTAGAGCATGTGCACGCCTGTTTGGACTTCATTTTCATCCACCTGTGCACCT
ATTGTAGTCTTTGGTTGGGTTAGGAGGAAGTGGTCATTGTGTCAGCATCTGCTGGATGTG
AGGACTTGCATTGTGAAAGCTTTGCTGTCCTTGATGTGATCATGGAATCTCTTTCTCACT
AGAGTCTATGTCACTCATTATACTCTGTCGAATGTCATTGAATGTCTTTACATGGGCTTA
TATGCCTATGAAAATTGTAATACAACTTTCAGCAACGGATCTCTTGGCTCTCGCATCGAT
GAAGAACGCAGCGAAATGCGATAAGTAATGTGAATTGCAGAATTCAGTGAATCATCGAAT
CTTTGAACGCATCTTGCGCTCCTTGGTATTCCGAGGAGCATGCCTGTTTGAGTGTCATTA
AATTCTCAACTCTCTTCTAC-TTTTTGTAAAAGAGAGCTTGGACTGTGGAGGCTTGCTGG
CCACTTTTTGGGGTCAGCTCCTCTGAAATGCATTAGCGGAACCGTTTGCGATCTGCCACA
AGTGTGATAAGTTATCTACACTGGCGAGGGGATTGCTCTCTGTAATGTTCAGCTTCTAAT
TGTCTCTACTTTGTGAGACTACTTTTGAATGCTTGACCTCAAATCAGGTAGGACTACCC-
GCTGAACTTAA
>BC11_10
TTTCCGTAGGTGAACCTGCGGAAGGATCATTATTGAATTATGTTTCTAGATAGGTTGTAG
CTGGCTCTTTAGAGCATGTGCACGCCTGTTTGGACTTCATTTTCATCCACCTGTGCACCT
ATTGTAGTCTTTGGTTGGGTTAGGAGGAAGTGGTCATTGTGTCAGCATCTGCTGGATGTG
AGGACTTGCATTGTGAAAGCTTTGCTGTCCTTGATGTGATCATGGAATCTCTTTCTCACT
AGAGTCTATGTCACTCATTATACTCTGTCGAATGTCATTGAATGTCTTTACATGGGCTTA
TATGCCTATGAAAATTGTAATACAACTTTCAGCAACGGATCTCTTGGCTCTCGCATCGAT
GAAGAACGCAGCGAAATGCGATAAGTAATGTGAATTGCAGAATTCAGTGAATCATCGAAT
CTTTGAACGCATCTTGCGCTCCTTGGTATTCCGAGGAGCATGCCTGTTTGAGTGTCATTA
AATTCTCAACTCTCTTCTAC-TTTTTGTAAAAGAGAGCTTGGACTGTGGAGGCTTGCTGG
CCACTTTTTGGGGTCAGCTCCTCTGAAATGCATTAGCGGAACCGTTTGCGATCTGCCACA
AGTGTGATAAGTTATCTACACTGGCGAGGGGATTGCTCTCTGTAATGTTCAGCTTCTAAT
TGTCTCTACTTTGTGAGACTACTTTTGAATGCTTGACCTCAAATCAGGTAGGACTACCC-
GCTGAACTTAA
>BC11_20
TTTCCGTAGGTGAACCTGCGGAAGGATCATTATTGAATTATGTTTCTAGATAGGTTGTAG
CTGGCTCTTTAGAGCATGTGCACGCCTGTTTGGACTTCATTTTCATCCACCTGTGCACCT
ATTGTAGTCTTTGGTTGGGTTAGGAGGAAGTGGTCATTGTGTCAGCATCTGCTGGATGTG
AGGACTTGCATTGTGAAAGCTTTGCTGTCCTTGATGTGATCATGGAATCTCTTTCTCACT
AGAGTCTATGTCACTCATTATACTCTGTCGAATGTCATTGAATGTCTTTACATGGGCTTA
TATGCCTATGAAAATTGTAATACAACTTTCAGCAACGGATCTCTTGGCTCTCGCATCGAT
GAAGAACGCAGCGAAATGCGATAAGTAATGTGAATTGCAGAATTCAGTGAATCATCGAAT
CTTTGAACGCATCTTGCGCTCCTTGGTATTCCGAGGAGCATGCCTGTTTGAGTGTCATTA
AATTCTCAACTCTCTTCTAC-TTTTTGTAAAAGAGAGCTTGGACTGTGGAGGCTTGCTGG
CCACTTTTTGGGGTCAGCTCCTCTGAAATGCATTAGCGGAACCGTTTGCGATCTGCCACA
AGTGTGATAAGTTATCTACACTGGCGAGGGGATTGCTCTCTGTAATGTTCAGCTTCTAAT
TGTCTCTACTTTGTGAGACTACTTTTGAATGCTTGACCTCAAATCAGGTAGGACTACCC-
GCTGAACTTAA
>BC11_22
TTTCCGTAGGTGAACCTGCGGAAGGATCATTATTGAATTATGTTTCTAGATAGGTTGTAG
CTGGCTCTTTAGAGCATGTGCACGCCTGTTTGGACTTCATTTTCATCCACCTGTGCACCT
ATTGTAGTCTTTGGTTGGGTTAGGAGGAAGTGGTCATTGTGTCAGCATCTGCTGGATGTG
AGGACTTGCATTGTGAAAGCTTTGCTGTCCTTGATGTGATCATGGAATCTCTTTCTCACT
AGAGTCTATGTCACTCATTATACTCTGTCGAATGTCATTGAATGTCTTTACATGGGCTTA
TATGCCTATGAAAATTGTAATACAACTTTCAGCAACGGATCTCTTGGCTCTCGCATCGAT
GAAGAACGCAGCGAAATGCGATAAGTAATGTGAATTGCAGAATTCAGTGAATCATCGAAT
CTTTGAACGCATCTTGCGCTCCTTGGTATTCCGAGGAGCATGCCTGTTTGAGTGTCATTA
AATTCTCAACTCTCTTCTAC-TTTTTGTAAAAGAGAGCTTGGACTGTGGAGGCTTGCTGG

CCACTTTTTGGGGTCAGCTCCTCTGAAATGCATTAGCGGAACCGTTTGCGATCTGCCACA
AGTGTGATAAGTTATCTACACTGGCGAGGGGATTGCTCTCTGTAATGTTCAGCTTCTAAT
TGTCTCTACTTTGTGAGACTACTTTTGAATGCTTGACCTCAAATCAGGTAGGACTACCC-
GCTGAACTTAA
>BC11_27
TTTCCGTAGGTGAACCTGCGGAAGGATCATTATTGAATTATGTTTCTAGATAGGTTGTAG
CTGGCTCTTTAGAGCATGTGCACGCCTGTTTGGACTTCATTTTCATCCACCTGTGCACCT
ATTGTAGTCTTTGGTTGGGTTAGGAGGAAGTGGTCATTGTGTCAGCATCTGCTGGATGTG
AGGACTTGCATTGTGAAAGCTTTGCTGTCCTTGATGTGATCATGGAATCTCTTTCTCACT
AGAGTCTATGTCACTCATTATACTCTGTCGAATGTCATTGAATGTCTTTACATGGGCTTA
TATGCCTATGAAAATTGTAATACAACTTTCAGCAACGGATCTCTTGGCTCTCGCATCGAT
GAAGAACGCAGCGAAATGCGATAAGTAATGTGAATTGCAGAATTCAGTGAATCATCGAAT
CTTTGAACGCATCTTGCGCTCCTTGGTATTCCGAGGAGCATGCCTGTTTGAGTGTCATTA
AATTCTCAACTCTCTTCTAC-TTTTTGTAAAAGAGAGCTTGGACTGTGGAGGCTTGCTGG
CCACTTTTTGGGGTCAGCTCCTCTGAAATGCATTAGCGGAACCGTTTGCGATCTGCCACA
AGTGTGATAAGTTATCTACACTGGCGAGGGGATTGCTCTCTGTAATGTTCAGCTTCTAAT
TGTCTCTACTTTGTGAGACTACTTTTGAATGCTTGACCTCAAATCAGGTAGGACTACCC-
GCTGAACTTAA
>BC11_32
TTTCCGTAGGTGAACCTGCGGAAGGATCATTATTGAATTATGTTTCTAGATAGGTTGTAG
CTGGCTCTTTAGAGCATGTGCACGCCTGTTTGGACTTCATTTTCATCCACCTGTGCACCT
ATTGTAGTCTTTGGTTGGGTTAGGAGGAAGTGGTCATTGTGTCAGCATCTGCTGGATGTG
AGGACTTGCATTGTGAAAGCTTTGCTGTCCTTGATGTGATCATGGAATCTCTTTCTCACT
AGAGTCTATGTCACTCATTATACTCTGTCGAATGTCATTGAATGTCTTTACATGGGCTTA
TATGCCTATGAAAATTGTAATACAACTTTCAGCAACGGATCTCTTGGCTCTCGCATCGAT
GAAGAACGCAGCGAAATGCGATAAGTAATGTGAATTGCAGAATTCAGTGAATCATCGAAT
CTTTGAACGCATCTTGCGCTCCTTGGTATTCCGAGGAGCATGCCTGTTTGAGTGTCATTA
AATTCTCAACTCTCTTCTAC-TTTTTGTAAAAGAGAGCTTGGACTGTGGAGGCTTGCTGG
CCACTTTTTGGGGTCAGCTCCTCTGAAATGCATTAGCGGAACCGTTTGCGATCTGCCACA
AGTGTGATAAGTTATCTACACTGGCGAGGGGATTGCTCTCTGTAATGTTCAGCTTCTAAT
TGTCTCTACTTTGTGAGACTACTTTTGAATGCTTGACCTCAAATCAGGTAGGACTACCC-
GCTGAACTTAA
>BC11_35
TTTCCGTAGGTGAACCTGCGGAAGGATCATTATTGAATTATGTTTCTAGATAGGTTGTAG
CTGGCTCTTTAGAGCATGTGCACGCCTGTTTGGACTTCATTTTCATCCACCTGTGCACCT
ATTGTAGTCTTTGGTTGGGTTAGGAGGAAGTGGTCATTGTGTCAGCATCTGCTGGATGTG
AGGACTTGCATTGTGAAAGCTTTGCTGTCCTTGATGTGATCATGGAATCTCTTTCTCACT
AGAGTCTATGTCACTCATTATACTCTGTCGAATGTCATTGAATGTCTTTACATGGGCTTA
TATGCCTATGAAAATTGTAATACAACTTTCAGCAACGGATCTCTTGGCTCTCGCATCGAT
GAAGAACGCAGCGAAATGCGATAAGTAATGTGAATTGCAGAATTCAGTGAATCATCGAAT
CTTTGAACGCATCTTGCGCTCCTTGGTATTCCGAGGAGCATGCCTGTTTGAGTGTCATTA
AATTCTCAACTCTCTTCTAC-TTTTTGTAAAAGAGAGCTTGGACTGTGGAGGCTTGCTGG
CCACTTTTTGGGGTCAGCTCCTCTGAAATGCATTAGCGGAACCGTTTGCGATCTGCCACA
AGTGTGATAAGTTATCTACACTGGCGAGGGGATTGCTCTCTGTAATGTTCAGCTTCTAAT
TGTCTCTACTTTGTGAGACTACTTTTGAATGCTTGACCTCAAATCAGGTAGGACTACCC-
GCTGAACTTAA
>BC11_42
TTTCCGTAGGTGAACCTGCGGAAGGATCATTATTGAATTATGTTTCTAGATAGGTTGTAG
CTGGCTCTTTAGAGCATGTGCACGCCTGTTTGGACTTCATTTTCATCCACCTGTGCACCT
ATTGTAGTCTTTGGTTGGGTTAGGAGGAAGTGGTCATTGTGTCAGCATCTGCTGGATGTG

AGGACTTGCATTGTGAAAGCTTTGCTGTCCTTGATGTGATCATGGAATCTCTTTCTCACT
AGAGTCTATGTCACTCATTATACTCTGTCGAATGTCATTGAATGTCTTTACATGGGCTTA
TATGCCTATGAAAATTGTAATACAACTTTCAGCAACGGATCTCTTGGCTCTCGCATCGAT
GAAGAACGCAGCGAAATGCGATAAGTAATGTGAATTGCAGAATTCAGTGAATCATCGAAT
CTTTGAACGCATCTTGCGCTCCTTGGTATTCCGAGGAGCATGCCTGTTTGAGTGTCATTA
AATTCTCAACTCTCTTCTAC-TTTTTGTAAAAGAGAGCTTGGACTGTGGAGGCTTGCTGG
CCACTTTTTGGGGTCAGCTCCTCTGAAATGCATTAGCGGAACCGTTTGCGATCTGCCACA
AGTGTGATAAGTTATCTACACTGGCGAGGGGATTGCTCTCTGTAATGTTCAGCTTCTAAT
TGTCTCTACTTTGTGAGACTACTTTTGAATGCTTGACCTCAAATCAGGTAGGACTACCC-
GCTGAACTTAA
>BC11_56
TTTCCGTAGGTGAACCTGCGGAAGGATCATTATTGAATTATGTTTCTAGATAGGTTGTAG
CTGGCTCTTTAGAGCATGTGCACGCCTGTTTGGACTTCATTTTCATCCACCTGTGCACCT
ATTGTAGTCTTTGGTTGGGTTAGGAGGAAGTGGTCATTGTGTCAGCATCTGCTGGATGTG
AGGACTTGCATTGTGAAAGCTTTGCTGTCCTTGATGTGATCATGGAATCTCTTTCTCACT
AGAGTCTATGTCACTCATTATACTCTGTCGAATGTCATTGAATGTCTTTACATGGGCTTA
TATGCCTATGAAAATTGTAATACAACTTTCAGCAACGGATCTCTTGGCTCTCGCATCGAT
GAAGAACGCAGCGAAATGCGATAAGTAATGTGAATTGCAGAATTCAGTGAATCATCGAAT
CTTTGAACGCATCTTGCGCTCCTTGGTATTCCGAGGAGCATGCCTGTTTGAGTGTCATTA
AATTCTCAACTCTCTTCTAC-TTTTTGTAAAAGAGAGCTTGGACTGTGGAGGCTTGCTGG
CCACTTTTTGGGGTCAGCTCCTCTGAAATGCATTAGCGGAACCGTTTGCGATCTGCCACA
AGTGTGATAAGTTATCTACACTGGCGAGGGGATTGCTCTCTGTAATGTTCAGCTTCTAAT
TGTCTCTACTTTGTGAGACTACTTTTGAATGCTTGACCTCAAATCAGGTAGGACTACCC-
GCTGAACTTAA
>BC12_8
TTTCCGTAGGTGAACCTGCGGAAGGATCATTATTGAATTATGTTTCTAGATAGGTTGTAG
CTGGCTCTTTAGAGCATGTGCACGCCTGTTTGGACTTCATTTTCATCCACCTGTGCACCT
ATTGTAGTCTTTGGTTGGGTTAGGAGGAAGTGGTCATTGTGTCAGCATCTGCTGGATGTG
AGGACTTGCATTGTGAAAGCTTTGCTGTCCTTGATGTGATCATGGAATCTCTTTCTCACT
AGAGTCTATGTCACTCATTATACTCTGTCGAATGTCATTGAATGTCTTTACATGGGCTTA
TATGCCTATGAAAATTGTAATACAACTTTCAGCAACGGATCTCTTGGCTCTCGCATCGAT
GAAGAACGCAGCGAAATGCGATAAGTAATGTGAATTGCAGAATTCAGTGAATCATCGAAT
CTTTGAACGCATCTTGCGCTCCTTGGTATTCCGAGGAGCATGCCTGTTTGAGTGTCATTA
AATTCTCAACTCTCTTCTAC-TTTTTGTAAAAGAGAGCTTGGACTGTGGAGGCTTGCTGG
CCACTTTTTGGGGTCAGCTCCTCTGAAATGCATTAGCGGAACCGTTTGCGATCTGCCACA
AGTGTGATAAGTTATCTACACTGGCGAGGGGATTGCTCTCTGTAATGTTCAGCTTCTAAT
TGTCTCTACTTTGTGAGACTACTTTTGAATGCTTGACCTCAAATCAGGTAGGACTACCC-
GCTGAACTTAA
>BC12_10
TTTCCGTAGGTGAACCTGCGGAAGGATCATTATTGAATTATGTTTCTAGATAGGTTGTAG
CTGGCTCTTTAGAGCATGTGCACGCCTGTTTGGACTTCATTTTCATCCACCTGTGCACCT
ATTGTAGTCTTTGGTTGGGTTAGGAGGAAGTGGTCATTGTGTCAGCATCTGCTGGATGTG
AGGACTTGCATTGTGAAAGCTTTGCTGTCCTTGATGTGATCATGGAATCTCTTTCTCACT
AGAGTCTATGTCACTCATTATACTCTGTCGAATGTCATTGAATGTCTTTACATGGGCTTA
TATGCCTATGAAAATTGTAATACAACTTTCAGCAACGGATCTCTTGGCTCTCGCATCGAT
GAAGAACGCAGCGAAATGCGATAAGTAATGTGAATTGCAGAATTCAGTGAATCATCGAAT
CTTTGAACGCATCTTGCGCTCCTTGGTATTCCGAGGAGCATGCCTGTTTGAGTGTCATTA
AATTCTCAACTCTCTTCTAC-TTTTTGTAAAAGAGAGCTTGGACTGTGGAGGCTTGCTGG
CCACTTTTTGGGGTCAGCTCCTCTGAAATGCATTAGCGGAACCGTTTGCGATCTGCCACA
AGTGTGATAAGTTATCTACACTGGCGAGGGGATTGCTCTCTGTAATGTTCAGCTTCTAAT

TGTCTCTACTTTGTGAGACTACTTTTGAATGCTTGACCTCAAATCAGGTAGGACTACCC-
GCTGAACTTAA
>BC12_20
TTTCCGTAGGTGAACCTGCGGAAGGATCATTATTGAATTATGTTTCTAGATAGGTTGTAG
CTGGCTCTTTAGAGCATGTGCACGCCTGTTTGGACTTCATTTTCATCCACCTGTGCACCT
ATTGTAGTCTTTGGTTGGGTTAGGAGGAAGTGGTCATTGTGTCAGCATCTGCTGGATGTG
AGGACTTGCATTGTGAAAGCTTTGCTGTCCTTGATGTGATCATGGAATCTCTTTCTCACT
AGAGTCTATGTCACTCATTATACTCTGTCGAATGTCATTGAATGTCTTTACATGGGCTTA
TATGCCTATGAAAATTGTAATACAACTTTCAGCAACGGATCTCTTGGCTCTCGCATCGAT
GAAGAACGCAGCGAAATGCGATAAGTAATGTGAATTGCAGAATTCAGTGAATCATCGAAT
CTTTGAACGCATCTTGCGCTCCTTGGTATTCCGAGGAGCATGCCTGTTTGAGTGTCATTA
AATTCTCAACTCTCTTCTAC-TTTTTGTAAAAGAGAGCTTGGACTGTGGAGGCTTGCTGG
CCACTTTTTGGGGTCAGCTCCTCTGAAATGCATTAGCGGAACCGTTTGCGATCTGCCACA
AGTGTGATAAGTTATCTACACTGGCGAGGGGATTGCTCTCTGTAATGTTCAGCTTCTAAT
TGTCTCTACTTTGTGAGACTACTTTTGAATGCTTGACCTCAAATCAGGTAGGACTACCC-
GCTGAACTTAA
>BC12_22
TTTCCGTAGGTGAACCTGCGGAAGGATCATTATTGAATTATGTTTCTAGATAGGTTGTAG
CTGGCTCTTTAGAGCATGTGCACGCCTGTTTGGACTTCATTTTCATCCACCTGTGCACCT
ATTGTAGTCTTTGGTTGGGTTAGGAGGAAGTGGTCATTGTGTCAGCATCTGCTGGATGTG
AGGACTTGCATTGTGAAAGCTTTGCTGTCCTTGATGTGATCATGGAATCTCTTTCTCACT
AGAGTCTATGTCACTCATTATACTCTGTCGAATGTCATTGAATGTCTTTACATGGGCTTA
TATGCCTATGAAAATTGTAATACAACTTTCAGCAACGGATCTCTTGGCTCTCGCATCGAT
GAAGAACGCAGCGAAATGCGATAAGTAATGTGAATTGCAGAATTCAGTGAATCATCGAAT
CTTTGAACGCATCTTGCGCTCCTTGGTATTCCGAGGAGCATGCCTGTTTGAGTGTCATTA
AATTCTCAACTCTCTTCTAC-TTTTTGTAAAAGAGAGCTTGGACTGTGGAGGCTTGCTGG
CCACTTTTTGGGGTCAGCTCCTCTGAAATGCATTAGCGGAACCGTTTGCGATCTGCCACA
AGTGTGATAAGTTATCTACACTGGCGAGGGGATTGCTCTCTGTAATGTTCAGCTTCTAAT
TGTCTCTACTTTGTGAGACTACTTTTGAATGCTTGACCTCAAATCAGGTAGGACTACCC-
GCTGAACTTAA
>BC12_24
TTTCCGTAGGTGAACCTGCGGAAGGATCATTATTGAATTATGTTTCTAGATAGGTTGTAG
CTGGCTCTTTAGAGCATGTGCACGCCTGTTTGGACTTCATTTTCATCCACCTGTGCACCT
ATTGTAGTCTTTGGTTGGGTTAGGAGGAAGTGGTCATTGTGTCAGCATCTGCTGGATGTG
AGGACTTGCATTGTGAAAGCTTTGCTGTCCTTGATGTGATCATGGAATCTCTTTCTCACT
AGAGTCTATGTCACTCATTATACTCTGTCGAATGTCATTGAATGTCTTTACATGGGCTTA
TATGCCTATGAAAATTGTAATACAACTTTCAGCAACGGATCTCTTGGCTCTCGCATCGAT
GAAGAACGCAGCGAAATGCGATAAGTAATGTGAATTGCAGAATTCAGTGAATCATCGAAT
CTTTGAACGCATCTTGCGCTCCTTGGTATTCCGAGGAGCATGCCTGTTTGAGTGTCATTA
AATTCTCAACTCTCTTCTAC-TTTTTGTAAAAGAGAGCTTGGACTGTGGAGGCTTGCTGG
CCACTTTTTGGGGTCAGCTCCTCTGAAATGCATTAGCGGAACCGTTTGCGATCTGCCACA
AGTGTGATAAGTTATCTACACTGGCGAGGGGATTGCTCTCTGTAATGTTCAGCTTCTAAT
TGTCTCTACTTTGTGAGACTACTTTTGAATGCTTGACCTCAAATCAGGTAGGACTACCC-
GCTGAACTTAA
>BC12_36
TTTCCGTAGGTGAACCTGCGGAAGGATCATTATTGAATTATGTTTCTAGATAGGTTGTAG
CTGGCTCTTTAGAGCATGTGCACGCCTGTTTGGACTTCATTTTCATCCACCTGTGCACCT
ATTGTAGTCTTTGGTTGGGTTAGGAGGAAGTGGTCATTGTGTCAGCATCTGCTGGATGTG
AGGACTTGCATTGTGAAAGCTTTGCTGTCCTTGATGTGATCATGGAATCTCTTTCTCACT
AGAGTCTATGTCACTCATTATACTCTGTCGAATGTCATTGAATGTCTTTACATGGGCTTA

TATGCCTATGAAAATTGTAATACAACTTTCAGCAACGGATCTCTTGGCTCTCGCATCGAT
GAAGAACGCAGCGAAATGCGATAAGTAATGTGAATTGCAGAATTCAGTGAATCATCGAAT
CTTTGAACGCATCTTGCGCTCCTTGGTATTCCGAGGAGCATGCCTGTTTGAGTGTCATTA
AATTCTCAACTCTCTTCTAC-TTTTTGTAAAAGAGAGCTTGGACTGTGGAGGCTTGCTGG
CCACTTTTTGGGGTCAGCTCCTCTGAAATGCATTAGCGGAACCGTTTGCGATCTGCCACA
AGTGTGATAAGTTATCTACACTGGCGAGGGGATTGCTCTCTGTAATGTTCAGCTTCTAAT
TGTCTCTACTTTGTGAGACTACTTTTGAATGCTTGACCTCAAATCAGGTAGGACTACCC-
GCTGAACTTAA
>BC12_37
TTTCCGTAGGTGAACCTGCGGAAGGATCATTATTGAATTATGTTTCTAGATAGGTTGTAG
CTGGCTCTTTAGAGCATGTGCACGCCTGTTTGGACTTCATTTTCATCCACCTGTGCACCT
ATTGTAGTCTTTGGTTGGGTTAGGAGGAAGTGGTCATTGTGTCAGCATCTGCTGGATGTG
AGGACTTGCATTGTGAAAGCTTTGCTGTCCTTGATGTGATCATGGAATCTCTTTCTCACT
AGAGTCTATGTCACTCATTATACTCTGTCGAATGTCATTGAATGTCTTTACATGGGCTTA
TATGCCTATGAAAATTGTAATACAACTTTCAGCAACGGATCTCTTGGCTCTCGCATCGAT
GAAGAACGCAGCGAAATGCGATAAGTAATGTGAATTGCAGAATTCAGTGAATCATCGAAT
CTTTGAACGCATCTTGCGCTCCTTGGTATTCCGAGGAGCATGCCTGTTTGAGTGTCATTA
AATTCTCAACTCTCTTCTAC-TTTTTGTAAAAGAGAGCTTGGACTGTGGAGGCTTGCTGG
CCACTTTTTGGGGTCAGCTCCTCTGAAATGCATTAGCGGAACCGTTTGCGATCTGCCACA
AGTGTGATAAGTTATCTACACTGGCGAGGGGATTGCTCTCTGTAATGTTCAGCTTCTAAT
TGTCTCTACTTTGTGAGACTACTTTTGAATGCTTGACCTCAAATCAGGTAGGACTACCC-
GCTGAACTTAA
>BC12_38
TTTCCGTAGGTGAACCTGCGGAAGGATCATTATTGAATTATGTTTCTAGATAGGTTGTAG
CTGGCTCTTTAGAGCATGTGCACGCCTGTTTGGACTTCATTTTCATCCACCTGTGCACCT
ATTGTAGTCTTTGGTTGGGTTAGGAGGAAGTGGTCATTGTGTCAGCATCTGCTGGATGTG
AGGACTTGCATTGTGAAAGCTTTGCTGTCCTTGATGTGATCATGGAATCTCTTTCTCACT
AGAGTCTATGTCACTCATTATACTCTGTCGAATGTCATTGAATGTCTTTACATGGGCTTA
TATGCCTATGAAAATTGTAATACAACTTTCAGCAACGGATCTCTTGGCTCTCGCATCGAT
GAAGAACGCAGCGAAATGCGATAAGTAATGTGAATTGCAGAATTCAGTGAATCATCGAAT
CTTTGAACGCATCTTGCGCTCCTTGGTATTCCGAGGAGCATGCCTGTTTGAGTGTCATTA
AATTCTCAACTCTCTTCTAC-TTTTTGTAAAAGAGAGCTTGGACTGTGGAGGCTTGCTGG
CCACTTTTTGGGGTCAGCTCCTCTGAAATGCATTAGCGGAACCGTTTGCGATCTGCCACA
AGTGTGATAAGTTATCTACACTGGCGAGGGGATTGCTCTCTGTAATGTTCAGCTTCTAAT
TGTCTCTACTTTGTGAGACTACTTTTGAATGCTTGACCTCAAATCAGGTAGGACTACCC-
GCTGAACTTAA
>BC12_39
TTTCCGTAGGTGAACCTGCGGAAGGATCATTATTGAATTATGTTTCTAGATAGGTTGTAG
CTGGCTCTTTAGAGCATGTGCACGCCTGTTTGGACTTCATTTTCATCCACCTGTGCACCT
ATTGTAGTCTTTGGTTGGGTTAGGAGGAAGTGGTCATTGTGTCAGCATCTGCTGGATGTG
AGGACTTGCATTGTGAAAGCTTTGCTGTCCTTGATGTGATCATGGAATCTCTTTCTCACT
AGAGTCTATGTCACTCATTATACTCTGTCGAATGTCATTGAATGTCTTTACATGGGCTTA
TATGCCTATGAAAATTGTAATACAACTTTCAGCAACGGATCTCTTGGCTCTCGCATCGAT
GAAGAACGCAGCGAAATGCGATAAGTAATGTGAATTGCAGAATTCAGTGAATCATCGAAT
CTTTGAACGCATCTTGCGCTCCTTGGTATTCCGAGGAGCATGCCTGTTTGAGTGTCATTA
AATTCTCAACTCTCTTCTAC-TTTTTGTAAAAGAGAGCTTGGACTGTGGAGGCTTGCTGG
CCACTTTTTGGGGTCAGCTCCTCTGAAATGCATTAGCGGAACCGTTTGCGATCTGCCACA
AGTGTGATAAGTTATCTACACTGGCGAGGGGATTGCTCTCTGTAATGTTCAGCTTCTAAT
TGTCTCTACTTTGTGAGACTACTTTTGAATGCTTGACCTCAAATCAGGTAGGACTACCC-
GCTGAACTTAA

>BC12_43
TTTCCGTAGGTGAACCTGCGGAAGGATCATTATTGAATTATGTTTCTAGATAGGTTGTAG
CTGGCTCTTTAGAGCATGTGCACGCCTGTTTGGACTTCATTTTCATCCACCTGTGCACCT
ATTGTAGTCTTTGGTTGGGTTAGGAGGAAGTGGTCATTGTGTCAGCATCTGCTGGATGTG
AGGACTTGCATTGTGAAAGCTTTGCTGTCCTTGATGTGATCATGGAATCTCTTTCTCACT
AGAGTCTATGTCACTCATTATACTCTGTCGAATGTCATTGAATGTCTTTACATGGGCTTA
TATGCCTATGAAAATTGTAATACAACTTTCAGCAACGGATCTCTTGGCTCTCGCATCGAT
GAAGAACGCAGCGAAATGCGATAAGTAATGTGAATTGCAGAATTCAGTGAATCATCGAAT
CTTTGAACGCATCTTGCGCTCCTTGGTATTCCGAGGAGCATGCCTGTTTGAGTGTCATTA
AATTCTCAACTCTCTTCTAC-TTTTTGTAAAAGAGAGCTTGGACTGTGGAGGCTTGCTGG
CCACTTTTTGGGGTCAGCTCCTCTGAAATGCATTAGCGGAACCGTTTGCGATCTGCCACA
AGTGTGATAAGTTATCTACACTGGCGAGGGGATTGCTCTCTGTAATGTTCAGCTTCTAAT
TGTCTCTACTTTGTGAGACTACTTTTGAATGCTTGACCTCAAATCAGGTAGGACTACCC-
GCTGAACTTAA
>BC12_44
TTTCCGTAGGTGAACCTGCGGAAGGATCATTATTGAATTATGTTTCTAGATAGGTTGTAG
CTGGCTCTTTAGAGCATGTGCACGCCTGTTTGGACTTCATTTTCATCCACCTGTGCACCT
ATTGTAGTCTTTGGTTGGGTTAGGAGGAAGTGGTCATTGTGTCAGCATCTGCTGGATGTG
AGGACTTGCATTGTGAAAGCTTTGCTGTCCTTGATGTGATCATGGAATCTCTTTCTCACT
AGAGTCTATGTCACTCATTATACTCTGTCGAATGTCATTGAATGTCTTTACATGGGCTTA
TATGCCTATGAAAATTGTAATACAACTTTCAGCAACGGATCTCTTGGCTCTCGCATCGAT
GAAGAACGCAGCGAAATGCGATAAGTAATGTGAATTGCAGAATTCAGTGAATCATCGAAT
CTTTGAACGCATCTTGCGCTCCTTGGTATTCCGAGGAGCATGCCTGTTTGAGTGTCATTA
AATTCTCAACTCTCTTCTAC-TTTTTGTAAAAGAGAGCTTGGACTGTGGAGGCTTGCTGG
CCACTTTTTGGGGTCAGCTCCTCTGAAATGCATTAGCGGAACCGTTTGCGATCTGCCACA
AGTGTGATAAGTTATCTACACTGGCGAGGGGATTGCTCTCTGTAATGTTCAGCTTCTAAT
TGTCTCTACTTTGTGAGACTACTTTTGAATGCTTGACCTCAAATCAGGTAGGACTACCC-
GCTGAACTTAA
>BC12_45
TTTCCGTAGGTGAACCTGCGGAAGGATCATTATTGAATTATGTTTCTAGATAGGTTGTAG
CTGGCTCTTTAGAGCATGTGCACGCCTGTTTGGACTTCATTTTCATCCACCTGTGCACCT
ATTGTAGTCTTTGGTTGGGTTAGGAGGAAGTGGTCATTGTGTCAGCATCTGCTGGATGTG
AGGACTTGCATTGTGAAAGCTTTGCTGTCCTTGATGTGATCATGGAATCTCTTTCTCACT
AGAGTCTATGTCACTCATTATACTCTGTCGAATGTCATTGAATGTCTTTACATGGGCTTA
TATGCCTATGAAAATTGTAATACAACTTTCAGCAACGGATCTCTTGGCTCTCGCATCGAT
GAAGAACGCAGCGAAATGCGATAAGTAATGTGAATTGCAGAATTCAGTGAATCATCGAAT
CTTTGAACGCATCTTGCGCTCCTTGGTATTCCGAGGAGCATGCCTGTTTGAGTGTCATTA
AATTCTCAACTCTCTTCTAC-TTTTTGTAAAAGAGAGCTTGGACTGTGGAGGCTTGCTGG
CCACTTTTTGGGGTCAGCTCCTCTGAAATGCATTAGCGGAACCGTTTGCGATCTGCCACA
AGTGTGATAAGTTATCTACACTGGCGAGGGGATTGCTCTCTGTAATGTTCAGCTTCTAAT
TGTCTCTACTTTGTGAGACTACTTTTGAATGCTTGACCTCAAATCAGGTAGGACTACCC-
GCTGAACTTAA
>BC12_46
TTTCCGTAGGTGAACCTGCGGAAGGATCATTATTGAATTATGTTTCTAGATAGGTTGTAG
CTGGCTCTTTAGAGCATGTGCACGCCTGTTTGGACTTCATTTTCATCCACCTGTGCACCT
ATTGTAGTCTTTGGTTGGGTTAGGAGGAAGTGGTCATTGTGTCAGCATCTGCTGGATGTG
AGGACTTGCATTGTGAAAGCTTTGCTGTCCTTGATGTGATCATGGAATCTCTTTCTCACT
AGAGTCTATGTCACTCATTATACTCTGTCGAATGTCATTGAATGTCTTTACATGGGCTTA
TATGCCTATGAAAATTGTAATACAACTTTCAGCAACGGATCTCTTGGCTCTCGCATCGAT
GAAGAACGCAGCGAAATGCGATAAGTAATGTGAATTGCAGAATTCAGTGAATCATCGAAT

CTTTGAACGCATCTTGCGCTCCTTGGTATTCCGAGGAGCATGCCTGTTTGAGTGTCATTA
AATTCTCAACTCTCTTCTAC-TTTTTGTAAAAGAGAGCTTGGACTGTGGAGGCTTGCTGG
CCACTTTTTGGGGTCAGCTCCTCTGAAATGCATTAGCGGAACCGTTTGCGATCTGCCACA
AGTGTGATAAGTTATCTACACTGGCGAGGGGATTGCTCTCTGTAATGTTCAGCTTCTAAT
TGTCTCTACTTTGTGAGACTACTTTTGAATGCTTGACCTCAAATCAGGTAGGACTACCC-
GCTGAACTTAA
>BC12_47
TTTCCGTAGGTGAACCTGCGGAAGGATCATTATTGAATTATGTTTCTAGATAGGTTGTAG
CTGGCTCTTTAGAGCATGTGCACGCCTGTTTGGACTTCATTTTCATCCACCTGTGCACCT
ATTGTAGTCTTTGGTTGGGTTAGGAGGAAGTGGTCATTGTGTCAGCATCTGCTGGATGTG
AGGACTTGCATTGTGAAAGCTTTGCTGTCCTTGATGTGATCATGGAATCTCTTTCTCACT
AGAGTCTATGTCACTCATTATACTCTGTCGAATGTCATTGAATGTCTTTACATGGGCTTA
TATGCCTATGAAAATTGTAATACAACTTTCAGCAACGGATCTCTTGGCTCTCGCATCGAT
GAAGAACGCAGCGAAATGCGATAAGTAATGTGAATTGCAGAATTCAGTGAATCATCGAAT
CTTTGAACGCATCTTGCGCTCCTTGGTATTCCGAGGAGCATGCCTGTTTGAGTGTCATTA
AATTCTCAACTCTCTTCTAC-TTTTTGTAAAAGAGAGCTTGGACTGTGGAGGCTTGCTGG
CCACTTTTTGGGGTCAGCTCCTCTGAAATGCATTAGCGGAACCGTTTGCGATCTGCCACA
AGTGTGATAAGTTATCTACACTGGCGAGGGGATTGCTCTCTGTAATGTTCAGCTTCTAAT
TGTCTCTACTTTGTGAGACTACTTTTGAATGCTTGACCTCAAATCAGGTAGGACTACCC-
GCTGAACTTAA
>BC12_50
TTTCCGTAGGTGAACCTGCGGAAGGATCATTATTGAATTATGTTTCTAGATAGGTTGTAG
CTGGCTCTTTAGAGCATGTGCACGCCTGTTTGGACTTCATTTTCATCCACCTGTGCACCT
ATTGTAGTCTTTGGTTGGGTTAGGAGGAAGTGGTCATTGTGTCAGCATCTGCTGGATGTG
AGGACTTGCATTGTGAAAGCTTTGCTGTCCTTGATGTGATCATGGAATCTCTTTCTCACT
AGAGTCTATGTCACTCATTATACTCTGTCGAATGTCATTGAATGTCTTTACATGGGCTTA
TATGCCTATGAAAATTGTAATACAACTTTCAGCAACGGATCTCTTGGCTCTCGCATCGAT
GAAGAACGCAGCGAAATGCGATAAGTAATGTGAATTGCAGAATTCAGTGAATCATCGAAT
CTTTGAACGCATCTTGCGCTCCTTGGTATTCCGAGGAGCATGCCTGTTTGAGTGTCATTA
AATTCTCAACTCTCTTCTAC-TTTTTGTAAAAGAGAGCTTGGACTGTGGAGGCTTGCTGG
CCACTTTTTGGGGTCAGCTCCTCTGAAATGCATTAGCGGAACCGTTTGCGATCTGCCACA
AGTGTGATAAGTTATCTACACTGGCGAGGGGATTGCTCTCTGTAATGTTCAGCTTCTAAT
TGTCTCTACTTTGTGAGACTACTTTTGAATGCTTGACCTCAAATCAGGTAGGACTACCC-
GCTGAACTTAA
>BC12_51
TTTCCGTAGGTGAACCTGCGGAAGGATCATTATTGAATTATGTTTCTAGATAGGTTGTAG
CTGGCTCTTTAGAGCATGTGCACGCCTGTTTGGACTTCATTTTCATCCACCTGTGCACCT
ATTGTAGTCTTTGGTTGGGTTAGGAGGAAGTGGTCATTGTGTCAGCATCTGCTGGATGTG
AGGACTTGCATTGTGAAAGCTTTGCTGTCCTTGATGTGATCATGGAATCTCTTTCTCACT
AGAGTCTATGTCACTCATTATACTCTGTCGAATGTCATTGAATGTCTTTACATGGGCTTA
TATGCCTATGAAAATTGTAATACAACTTTCAGCAACGGATCTCTTGGCTCTCGCATCGAT
GAAGAACGCAGCGAAATGCGATAAGTAATGTGAATTGCAGAATTCAGTGAATCATCGAAT
CTTTGAACGCATCTTGCGCTCCTTGGTATTCCGAGGAGCATGCCTGTTTGAGTGTCATTA
AATTCTCAACTCTCTTCTAC-TTTTTGTAAAAGAGAGCTTGGACTGTGGAGGCTTGCTGG
CCACTTTTTGGGGTCAGCTCCTCTGAAATGCATTAGCGGAACCGTTTGCGATCTGCCACA
AGTGTGATAAGTTATCTACACTGGCGAGGGGATTGCTCTCTGTAATGTTCAGCTTCTAAT
TGTCTCTACTTTGTGAGACTACTTTTGAATGCTTGACCTCAAATCAGGTAGGACTACCC-
GCTGAACTTAA
>BC12_52
TTTCCGTAGGTGAACCTGCGGAAGGATCATTATTGAATTATGTTTCTAGATAGGTTGTAG

CTGGCTCTTTAGAGCATGTGCACGCCTGTTTGGACTTCATTTTCATCCACCTGTGCACCT
ATTGTAGTCTTTGGTTGGGTTAGGAGGAAGTGGTCATTGTGTCAGCATCTGCTGGATGTG
AGGACTTGCATTGTGAAAGCTTTGCTGTCCTTGATGTGATCATGGAATCTCTTTCTCACT
AGAGTCTATGTCACTCATTATACTCTGTCGAATGTCATTGAATGTCTTTACATGGGCTTA
TATGCCTATGAAAATTGTAATACAACTTTCAGCAACGGATCTCTTGGCTCTCGCATCGAT
GAAGAACGCAGCGAAATGCGATAAGTAATGTGAATTGCAGAATTCAGTGAATCATCGAAT
CTTTGAACGCATCTTGCGCTCCTTGGTATTCCGAGGAGCATGCCTGTTTGAGTGTCATTA
AATTCTCAACTCTCTTCTAC-TTTTTGTAAAAGAGAGCTTGGACTGTGGAGGCTTGCTGG
CCACTTTTTGGGGTCAGCTCCTCTGAAATGCATTAGCGGAACCGTTTGCGATCTGCCACA
AGTGTGATAAGTTATCTACACTGGCGAGGGGATTGCTCTCTGTAATGTTCAGCTTCTAAT
TGTCTCTACTTTGTGAGACTACTTTTGAATGCTTGACCTCAAATCAGGTAGGACTACCC-
GCTGAACTTAA
>BC12_53
TTTCCGTAGGTGAACCTGCGGAAGGATCATTATTGAATTATGTTTCTAGATAGGTTGTAG
CTGGCTCTTTAGAGCATGTGCACGCCTGTTTGGACTTCATTTTCATCCACCTGTGCACCT
ATTGTAGTCTTTGGTTGGGTTAGGAGGAAGTGGTCATTGTGTCAGCATCTGCTGGATGTG
AGGACTTGCATTGTGAAAGCTTTGCTGTCCTTGATGTGATCATGGAATCTCTTTCTCACT
AGAGTCTATGTCACTCATTATACTCTGTCGAATGTCATTGAATGTCTTTACATGGGCTTA
TATGCCTATGAAAATTGTAATACAACTTTCAGCAACGGATCTCTTGGCTCTCGCATCGAT
GAAGAACGCAGCGAAATGCGATAAGTAATGTGAATTGCAGAATTCAGTGAATCATCGAAT
CTTTGAACGCATCTTGCGCTCCTTGGTATTCCGAGGAGCATGCCTGTTTGAGTGTCATTA
AATTCTCAACTCTCTTCTAC-TTTTTGTAAAAGAGAGCTTGGACTGTGGAGGCTTGCTGG
CCACTTTTTGGGGTCAGCTCCTCTGAAATGCATTAGCGGAACCGTTTGCGATCTGCCACA
AGTGTGATAAGTTATCTACACTGGCGAGGGGATTGCTCTCTGTAATGTTCAGCTTCTAAT
TGTCTCTACTTTGTGAGACTACTTTTGAATGCTTGACCTCAAATCAGGTAGGACTACCC-
GCTGAACTTAA
>BC3-50
TTTCCGTAGGTGAACCTGCGGAAGGATCATTATTGAATTATGTTTCTAGATAGGTTGTAG
CTGGCTCTTTAGAGCATGTGCACGCCTGTTTGGACTTCATTTTCATCCACCTGTGCACCT
ATTGTAGTCTTTGGTTGGGTTAGGGGGAAGTGGTCATTGTGTCAGCATCTGCTGGATGTG
AGGACTTGCATTGTGAAAGCTTTGCTGTCCTTGATGTGATCATGGAATCTCTTTCTCACT
AGAGTCTATGTCACTCATTATACTCTGTCGAATGTCATTGAATGTCTTTACATGGGCTTA
TATGCCTATGAAAATTGTAATACAACTTTCAGCAACGGATCTCTTGGCTCTCGCATCGAT
GAAGAACGCAGCGAAATGCGATAAGTAATGTGAATTGCAGAATTCAGTGAATCATCGAAT
CTTTGAACGCATCTTGCGCTCCTTGGTATTCCGAGGAGCATGCCTGTTTGAGTGTCATTA
AATTCTCAACTCTCTTCTAC-TTTTTGTAAAAGAGAGCTTGGACTGTGGAGGCTTGCTGG
CCACTTTTTGGGGTCAGCTCCTCTGAAATGCATTAGCGGAACCGTTTGCGATCTGCCACA
AGTGTGATAAGTTATCTACACTGGCGAGGGGATTGCTCTCTGTAATGTTCAGCTTCTAAT
TGTCTCTACTTTGTGAGACTACTTTTGAATGCTTGACCTCAAATCAGGTAGGACTACCC-
GCTGAACTTAA
>BC2-52
TTTCCGTAGGTGAACCTGCGGAAGGATCATTATTGAATTATGTTTCTAGATAGGTTGTAG
CTGGCTCTTTAGAGCATGTGCACGCCTGTTTGGACTTCATTTTCATCCACCTGTGCACCT
ATTGTAGTCTTTGGTTGGGTTAGGGGGAAGTGGTCATTGTGTCAGCATCTGCTGGATGTG
AGGACTTGCATTGTGAAAGCTTTGCTGTCCTTGATGTGATCATGGAATCTCTTTCTCACT
AGAGTCTATGTCACTCATTATACTCTGTCGAATGTCATTGAATGTCTTTACATGGGCTTA
TATGCCTATGAAAATTGTAATACAACTTTCAGCAACGGATCTCTTGGCTCTCGCATCGAT
GAAGAACGCAGCGAAATGCGATAAGTAATGTGAATTGCAGAATTCAGTGAATCATCGAAT
CTTTGAACGCATCTTGCGCTCCTTGGTATTCCGAGGAGCATGCCTGTTTGAGTGTCATTA
AATTCTCAACTCTCTTCTAC-TTTTTGTAAAAGAGAGCTTGGACTGTGGAGGCTTGCTGG

CCACTTTTTGGGGTCAGCTCCTCTGAAATGCATTAGCGGAACCGTTTGCGATCTGCCACA
AGTGTGATAAGTTATCTACACTGGCGAGGGGATTGCTCTCTGTAATGTTCAGCTTCTAAT
TGTCTCTACTTTGTGAGACTACTTTTGAATGCTTGACCTCAAATCAGGTAGGACTACCC-
GCTGAACTTAA
>BC4-61
TTTCCGTAGGTGAACCTGCGGAAGGATCATTATTGAATTATGTTTCTAGATAGGTTGTAG
CTGGCTCTTTAGAGCATGTGCACGCCTGTTTGGACTTCATTTTCATCCACCTGTGCACCT
ATTGTAGTCTTTGGTTGGGTTAGGGGGAAGTGGTCATTGTGTCAGCATCTGCTGGATGTG
AGGACTTGCATTGTGAAAGCTTTGCTGTCCTTGATGTGATCATGGAATCTCTTTCTCACT
AGAGTCTATGTCACTCATTATACTCTGTCGAATGTCATTGAATGTCTTTACATGGGCTTA
TATGCCTATGAAAATTGTAATACAACTTTCAGCAACGGATCTCTTGGCTCTCGCATCGAT
GAAGAACGCAGCGAAATGCGATAAGTAATGTGAATTGCAGAATTCAGTGAATCATCGAAT
CTTTGAACGCATCTTGCGCTCCTTGGTATTCCGAGGAGCATGCCTGTTTGAGTGTCATTA
AATTCTCAACTCTCTTCTAC-TTTTTGTAAAAGAGAGCTTGGACTGTGGAGGCTTGCTGG
CCACTTTTTGGGGTCAGCTCCTCTGAAATGCATTAGCGGAACCGTTTGCGATCTGCCACA
AGTGTGATAAGTTATCTACACTGGCGAGGGGATTGCTCTCTGTAATGTTCAGCTTCTAAT
TGTCTCTACTTTGTGAGACTACTTTTGAATGCTTGACCTCAAATCAGGTAGGACTACCC-
GCTGAACTTAA
>BAC5-3
TTTCCGTAGGTGAACCTGCGGAAGGATCATTATTGAATTATGTTTCTAGATAGGTTGTAG
CTGGCTCTTTAGAGCATGTGCACGCCTGTTTGGACTTCATTTTCATCCACCTGTGCACCT
ATTGTAGTCTTTGGTTGGGTTAGGGGGAAGTGGTCATTGTGTCAGCATCTGCTGGATGTG
AGGACTTGCATTGTGAAAGCTTTGCTGTCCTTGATGTGATCATGGAATCTCTTTCTCACT
AGAGTCTATGTCACTCATTATACTCTGTCGAATGTCATTGAATGTCTTTACATGGGCTTA
TATGCCTATGAAAATTGTAATACAACTTTCAGCAACGGATCTCTTGGCTCTCGCATCGAT
GAAGAACGCAGCGAAATGCGATAAGTAATGTGAATTGCAGAATTCAGTGAATCATCGAAT
CTTTGAACGCATCTTGCGCTCCTTGGTATTCCGAGGAGCATGCCTGTTTGAGTGTCATTA
AATTCTCAACTCTCTTCTAC-TTTTTGTAAAAGAGAGCTTGGACTGTGGAGGCTTGCTGG
CCACTTTTTGGGGTCAGCTCCTCTGAAATGCATTAGCGGAACCGTTTGCGATCTGCCACA
AGTGTGATAAGTTATCTACACTGGCGAGGGGATTGCTCTCTGTAATGTTCAGCTTCTAAT
TGTCTCTACTTTGTGAGACTACTTTTGAATGCTTGACCTCAAATCAGGTAGGACTACCC-
GCTGAACTTAA
>BC6-48
TTTCCGTAGGTGAACCTGCGGAAGGATCATTATTGAATTATGTTTCTAGATAGGTTGTAG
CTGGCTCTTTAGAGCATGTGCACGCCTGTTTGGACTTCATTTTCATCCACCTGTGCACCT
ATTGTAGTCTTTGGTTGGGTTAGGGGGAAGTGGTCATTGTGTCAGCATCTGCTGGATGTG
AGGACTTGCATTGTGAAAGCTTTGCTGTCCTTGATGTGATCATGGAATCTCTTTCTCACT
AGAGTCTATGTCACTCATTATACTCTGTCGAATGTCATTGAATGTCTTTACATGGGCTTA
TATGCCTATGAAAATTGTAATACAACTTTCAGCAACGGATCTCTTGGCTCTCGCATCGAT
GAAGAACGCAGCGAAATGCGATAAGTAATGTGAATTGCAGAATTCAGTGAATCATCGAAT
CTTTGAACGCATCTTGCGCTCCTTGGTATTCCGAGGAGCATGCCTGTTTGAGTGTCATTA
AATTCTCAACTCTCTTCTAC-TTTTTGTAAAAGAGAGCTTGGACTGTGGAGGCTTGCTGG
CCACTTTTTGGGGTCAGCTCCTCTGAAATGCATTAGCGGAACCGTTTGCGATCTGCCACA
AGTGTGATAAGTTATCTACACTGGCGAGGGGATTGCTCTCTGTAATGTTCAGCTTCTAAT
TGTCTCTACTTTGTGAGACTACTTTTGAATGCTTGACCTCAAATCAGGTAGGACTACCC-
GCTGAACTTAA
>BC7-2
TTTCCGTAGGTGAACCTGCGGAAGGATCATTATTGAATTATGTTTCTAGATAGGTTGTAG
CTGGCTCTTTAGAGCATGTGCACGCCTGTTTGGACTTCATTTTCATCCACCTGTGCACCT
ATTGTAGTCTTTGGTTGGGTTAGGGGGAAGTGGTCATTGTGTCAGCATCTGCTGGATGTG

AGGACTTGCATTGTGAAAGCTTTGCTGTCCTTGATGTGATCATGGAATCTCTTTCTCACT
AGAGTCTATGTCACTCATTATACTCTGTCGAATGTCATTGAATGTCTTTACATGGGCTTA
TATGCCTATGAAAATTGTAATACAACTTTCAGCAACGGATCTCTTGGCTCTCGCATCGAT
GAAGAACGCAGCGAAATGCGATAAGTAATGTGAATTGCAGAATTCAGTGAATCATCGAAT
CTTTGAACGCATCTTGCGCTCCTTGGTATTCCGAGGAGCATGCCTGTTTGAGTGTCATTA
AATTCTCAACTCTCTTCTAC-TTTTTGTAAAAGAGAGCTTGGACTGTGGAGGCTTGCTGG
CCACTTTTTGGGGTCAGCTCCTCTGAAATGCATTAGCGGAACCGTTTGCGATCTGCCACA
AGTGTGATAAGTTATCTACACTGGCGAGGGGATTGCTCTCTGTAATGTTCAGCTTCTAAT
TGTCTCTACTTTGTGAGACTACTTTTGAATGCTTGACCTCAAATCAGGTAGGACTACCC-
GCTGAACTTAA
>BC7-44
TTTCCGTAGGTGAACCTGCGGAAGGATCATTATTGAATTATGTTTCTAGATAGGTTGTAG
CTGGCTCTTTAGAGCATGTGCACGCCTGTTTGGACTTCATTTTCATCCACCTGTGCACCT
ATTGTAGTCTTTGGTTGGGTTAGGGGGAAGTGGTCATTGTGTCAGCATCTGCTGGATGTG
AGGACTTGCATTGTGAAAGCTTTGCTGTCCTTGATGTGATCATGGAATCTCTTTCTCACT
AGAGTCTATGTCACTCATTATACTCTGTCGAATGTCATTGAATGTCTTTACATGGGCTTA
TATGCCTATGAAAATTGTAATACAACTTTCAGCAACGGATCTCTTGGCTCTCGCATCGAT
GAAGAACGCAGCGAAATGCGATAAGTAATGTGAATTGCAGAATTCAGTGAATCATCGAAT
CTTTGAACGCATCTTGCGCTCCTTGGTATTCCGAGGAGCATGCCTGTTTGAGTGTCATTA
AATTCTCAACTCTCTTCTAC-TTTTTGTAAAAGAGAGCTTGGACTGTGGAGGCTTGCTGG
CCACTTTTTGGGGTCAGCTCCTCTGAAATGCATTAGCGGAACCGTTTGCGATCTGCCACA
AGTGTGATAAGTTATCTACACTGGCGAGGGGATTGCTCTCTGTAATGTTCAGCTTCTAAT
TGTCTCTACTTTGTGAGACTACTTTTGAATGCTTGACCTCAAATCAGGTAGGACTACCC-
GCTGAACTTAA
>BC8-13
TTTCCGTAGGTGAACCTGCGGAAGGATCATTATTGAATTATGTTTCTAGATAGGTTGTAG
CTGGCTCTTTAGAGCATGTGCACGCCTGTTTGGACTTCATTTTCATCCACCTGTGCACCT
ATTGTAGTCTTTGGTTGGGTTAGGGGGAAGTGGTCATTGTGTCAGCATCTGCTGGATGTG
AGGACTTGCATTGTGAAAGCTTTGCTGTCCTTGATGTGATCATGGAATCTCTTTCTCACT
AGAGTCTATGTCACTCATTATACTCTGTCGAATGTCATTGAATGTCTTTACATGGGCTTA
TATGCCTATGAAAATTGTAATACAACTTTCAGCAACGGATCTCTTGGCTCTCGCATCGAT
GAAGAACGCAGCGAAATGCGATAAGTAATGTGAATTGCAGAATTCAGTGAATCATCGAAT
CTTTGAACGCATCTTGCGCTCCTTGGTATTCCGAGGAGCATGCCTGTTTGAGTGTCATTA
AATTCTCAACTCTCTTCTAC-TTTTTGTAAAAGAGAGCTTGGACTGTGGAGGCTTGCTGG
CCACTTTTTGGGGTCAGCTCCTCTGAAATGCATTAGCGGAACCGTTTGCGATCTGCCACA
AGTGTGATAAGTTATCTACACTGGCGAGGGGATTGCTCTCTGTAATGTTCAGCTTCTAAT
TGTCTCTACTTTGTGAGACTACTTTTGAATGCTTGACCTCAAATCAGGTAGGACTACCC-
GCTGAACTTAA
>BC9-57
TTTCCGTAGGTGAACCTGCGGAAGGATCATTATTGAATTATGTTTCTAGATAGGTTGTAG
CTGGCTCTTTAGAGCATGTGCACGCCTGTTTGGACTTCATTTTCATCCACCTGTGCACCT
ATTGTAGTCTTTGGTTGGGTTAGGGGGAAGTGGTCATTGTGTCAGCATCTGCTGGATGTG
AGGACTTGCATTGTGAAAGCTTTGCTGTCCTTGATGTGATCATGGAATCTCTTTCTCACT
AGAGTCTATGTCACTCATTATACTCTGTCGAATGTCATTGAATGTCTTTACATGGGCTTA
TATGCCTATGAAAATTGTAATACAACTTTCAGCAACGGATCTCTTGGCTCTCGCATCGAT
GAAGAACGCAGCGAAATGCGATAAGTAATGTGAATTGCAGAATTCAGTGAATCATCGAAT
CTTTGAACGCATCTTGCGCTCCTTGGTATTCCGAGGAGCATGCCTGTTTGAGTGTCATTA
AATTCTCAACTCTCTTCTAC-TTTTTGTAAAAGAGAGCTTGGACTGTGGAGGCTTGCTGG
CCACTTTTTGGGGTCAGCTCCTCTGAAATGCATTAGCGGAACCGTTTGCGATCTGCCACA
AGTGTGATAAGTTATCTACACTGGCGAGGGGATTGCTCTCTGTAATGTTCAGCTTCTAAT

TGTCTCTACTTTGTGAGACTACTTTTGAATGCTTGACCTCAAATCAGGTAGGACTACCC-
GCTGAACTTAA
>BC10_33
TTTCCGTAGGTGAACCTGCGGAAGGATCATTATTGAATTATGTTTCTAGATAGGTTGTAG
CTGGCTCTTTAGAGCATGTGCACGCCTGTTTGGACTTCATTTTCATCCACCTGTGCACCT
ATTGTAGTCTTTGGTTGGGTTAGGGGGAAGTGGTCATTGTGTCAGCATCTGCTGGATGTG
AGGACTTGCATTGTGAAAGCTTTGCTGTCCTTGATGTGATCATGGAATCTCTTTCTCACT
AGAGTCTATGTCACTCATTATACTCTGTCGAATGTCATTGAATGTCTTTACATGGGCTTA
TATGCCTATGAAAATTGTAATACAACTTTCAGCAACGGATCTCTTGGCTCTCGCATCGAT
GAAGAACGCAGCGAAATGCGATAAGTAATGTGAATTGCAGAATTCAGTGAATCATCGAAT
CTTTGAACGCATCTTGCGCTCCTTGGTATTCCGAGGAGCATGCCTGTTTGAGTGTCATTA
AATTCTCAACTCTCTTCTAC-TTTTTGTAAAAGAGAGCTTGGACTGTGGAGGCTTGCTGG
CCACTTTTTGGGGTCAGCTCCTCTGAAATGCATTAGCGGAACCGTTTGCGATCTGCCACA
AGTGTGATAAGTTATCTACACTGGCGAGGGGATTGCTCTCTGTAATGTTCAGCTTCTAAT
TGTCTCTACTTTGTGAGACTACTTTTGAATGCTTGACCTCAAATCAGGTAGGACTACCC-
GCTGAACTTAA
>BC10_44
TTTCCGTAGGTGAACCTGCGGAAGGATCATTATTGAATTATGTTTCTAGATAGGTTGTAG
CTGGCTCTTTAGAGCATGTGCACGCCTGTTTGGACTTCATTTTCATCCACCTGTGCACCT
ATTGTAGTCTTTGGTTGGGTTAGGGGGAAGTGGTCATTGTGTCAGCATCTGCTGGATGTG
AGGACTTGCATTGTGAAAGCTTTGCTGTCCTTGATGTGATCATGGAATCTCTTTCTCACT
AGAGTCTATGTCACTCATTATACTCTGTCGAATGTCATTGAATGTCTTTACATGGGCTTA
TATGCCTATGAAAATTGTAATACAACTTTCAGCAACGGATCTCTTGGCTCTCGCATCGAT
GAAGAACGCAGCGAAATGCGATAAGTAATGTGAATTGCAGAATTCAGTGAATCATCGAAT
CTTTGAACGCATCTTGCGCTCCTTGGTATTCCGAGGAGCATGCCTGTTTGAGTGTCATTA
AATTCTCAACTCTCTTCTAC-TTTTTGTAAAAGAGAGCTTGGACTGTGGAGGCTTGCTGG
CCACTTTTTGGGGTCAGCTCCTCTGAAATGCATTAGCGGAACCGTTTGCGATCTGCCACA
AGTGTGATAAGTTATCTACACTGGCGAGGGGATTGCTCTCTGTAATGTTCAGCTTCTAAT
TGTCTCTACTTTGTGAGACTACTTTTGAATGCTTGACCTCAAATCAGGTAGGACTACCC-
GCTGAACTTAA
>BC12_2
TTTCCGTAGGTGAACCTGCGGAAGGATCATTATTGAATTATGTTTCTAGATAGGTTGTAG
CTGGCTCTTTAGAGCATGTGCACGCCTGTTTGGACTTCATTTTCATCCACCTGTGCACCT
ATTGTAGTCTTTGGTTGGGTTAGGGGGAAGTGGTCATTGTGTCAGCATCTGCTGGATGTG
AGGACTTGCATTGTGAAAGCTTTGCTGTCCTTGATGTGATCATGGAATCTCTTTCTCACT
AGAGTCTATGTCACTCATTATACTCTGTCGAATGTCATTGAATGTCTTTACATGGGCTTA
TATGCCTATGAAAATTGTAATACAACTTTCAGCAACGGATCTCTTGGCTCTCGCATCGAT
GAAGAACGCAGCGAAATGCGATAAGTAATGTGAATTGCAGAATTCAGTGAATCATCGAAT
CTTTGAACGCATCTTGCGCTCCTTGGTATTCCGAGGAGCATGCCTGTTTGAGTGTCATTA
AATTCTCAACTCTCTTCTAC-TTTTTGTAAAAGAGAGCTTGGACTGTGGAGGCTTGCTGG
CCACTTTTTGGGGTCAGCTCCTCTGAAATGCATTAGCGGAACCGTTTGCGATCTGCCACA
AGTGTGATAAGTTATCTACACTGGCGAGGGGATTGCTCTCTGTAATGTTCAGCTTCTAAT
TGTCTCTACTTTGTGAGACTACTTTTGAATGCTTGACCTCAAATCAGGTAGGACTACCC-
GCTGAACTTAA
>BC1-9
TTTCCGTAGGTGAACCTGCGGAAGGATCATTATTGAATTATGTTTCTAGATAGGTTGTAG
CTGGCTCTTTAGAGCATGTGCACGCCTGTTTGGACTTCATTTTCATCCACCTGTGCACCT
ATTGTAGTCTTTGGTTGGGTTAGGAGGAAGTGGTCATTGTGTCAGCATCTGCTGGATGTG
AGGACTTGCATTGTGAAAGCTTTGCTGTCCTTGATGTGATCATGGAATCTCTTTCTCACT
AGAGTCTATGTCACTCATTATACTCTGTCGAATGTCATTGAATGTCTTTACATGGGCTTG

TATGCCTATGAAAATTGTAATACAACTTTCAGCAACGGATCTCTTGGCTCTCGCATCGAT
GAAGAACGCAGCGAAATGCGATAAGTAATGTGAATTGCAGAATTCAGTGAATCATCGAAT
CTTTGAACGCATCTTGCGCTCCTTGGTATTCCGAGGAGCATGCCTGTTTGAGTGTCATTA
AATTCTCAACTCTCTTCTAC-TTTTTGTAAAAGAGAGCTTGGACTGTGGAGGCTTGCTGG
CCACTTTTTGGGGTCAGCTCCTCTGAAATGCATTAGCGGAACCGTTTGCGATCTGCCACA
AGTGTGATAAGTTATCTACACTGGCGAGGGGATTGCTCTCTGTAATGTTCAGCTTCTAAT
TGTCTCTACTTTGTGAGACTACTTTTGAATGCTTGACCTCAAATCAGGTAGGACTACCC-
GCTGAACTTAA
>BC5-98
TTTCCGTAGGTGAACCTGCGGAAGGATCATTATTGAATTATGTTTCTAGATAGGTTGTAG
CTGGCTCTTTAGAGCATGTGCACGCCTGTTTGGACTTCATTTTCATCCACCTGTGCACCT
ATTGTAGTCTTTGGTTGGGTTAGGAGGAAGTGGTCATTGTGTCAGCATCTGCTGGATGTG
AGGACTTGCATTGTGAAAGCTTTGCTGTCCTTGATGTGATCATGGAATCTCTTTCTCACT
AGAGTCTATGTCACTCATTATACTCTGTCGAATGTCATTGAATGTCTTTACATGGGCTTG
TATGCCTATGAAAATTGTAATACAACTTTCAGCAACGGATCTCTTGGCTCTCGCATCGAT
GAAGAACGCAGCGAAATGCGATAAGTAATGTGAATTGCAGAATTCAGTGAATCATCGAAT
CTTTGAACGCATCTTGCGCTCCTTGGTATTCCGAGGAGCATGCCTGTTTGAGTGTCATTA
AATTCTCAACTCTCTTCTAC-TTTTTGTAAAAGAGAGCTTGGACTGTGGAGGCTTGCTGG
CCACTTTTTGGGGTCAGCTCCTCTGAAATGCATTAGCGGAACCGTTTGCGATCTGCCACA
AGTGTGATAAGTTATCTACACTGGCGAGGGGATTGCTCTCTGTAATGTTCAGCTTCTAAT
TGTCTCTACTTTGTGAGACTACTTTTGAATGCTTGACCTCAAATCAGGTAGGACTACCC-
GCTGAACTTAA
>BC6-55
TTTCCGTAGGTGAACCTGCGGAAGGATCATTATTGAATTATGTTTCTAGATAGGTTGTAG
CTGGCTCTTTAGAGCATGTGCACGCCTGTTTGGACTTCATTTTCATCCACCTGTGCACCT
ATTGTAGTCTTTGGTTGGGTTAGGAGGAAGTGGTCATTGTGTCAGCATCTGCTGGATGTG
AGGACTTGCATTGTGAAAGCTTTGCTGTCCTTGATGTGATCATGGAATCTCTTTCTCACT
AGAGTCTATGTCACTCATTATACTCTGTCGAATGTCATTGAATGTCTTTACATGGGCTTG
TATGCCTATGAAAATTGTAATACAACTTTCAGCAACGGATCTCTTGGCTCTCGCATCGAT
GAAGAACGCAGCGAAATGCGATAAGTAATGTGAATTGCAGAATTCAGTGAATCATCGAAT
CTTTGAACGCATCTTGCGCTCCTTGGTATTCCGAGGAGCATGCCTGTTTGAGTGTCATTA
AATTCTCAACTCTCTTCTAC-TTTTTGTAAAAGAGAGCTTGGACTGTGGAGGCTTGCTGG
CCACTTTTTGGGGTCAGCTCCTCTGAAATGCATTAGCGGAACCGTTTGCGATCTGCCACA
AGTGTGATAAGTTATCTACACTGGCGAGGGGATTGCTCTCTGTAATGTTCAGCTTCTAAT
TGTCTCTACTTTGTGAGACTACTTTTGAATGCTTGACCTCAAATCAGGTAGGACTACCC-
GCTGAACTTAA
>BC12_27
TTTCCGTAGGTGAACCTGCGGAAGGATCATTATTGAATTATGTTTCTAGATAGGTTGTAG
CTGGCTCTTTAGAGCATGTGCACGCCTGTTTGGACTTCATTTTCATCCACCTGTGCACCT
ATTGTAGTCTTTGGTTGGGTTAGGAGGAAGTGGTCATTGTGTCAGCATCTGCTGGATGTG
AGGACTTGCATTGTGAAAGCTTTGCTGTCCTTGATGTGATCATGGAATCTCTTTCTCACT
AGAGTCTATGTCACTCATTATACTCTGTCGAATGTCATTGAATGTCTTTACATGGGCTTG
TATGCCTATGAAAATTGTAATACAACTTTCAGCAACGGATCTCTTGGCTCTCGCATCGAT
GAAGAACGCAGCGAAATGCGATAAGTAATGTGAATTGCAGAATTCAGTGAATCATCGAAT
CTTTGAACGCATCTTGCGCTCCTTGGTATTCCGAGGAGCATGCCTGTTTGAGTGTCATTA
AATTCTCAACTCTCTTCTAC-TTTTTGTAAAAGAGAGCTTGGACTGTGGAGGCTTGCTGG
CCACTTTTTGGGGTCAGCTCCTCTGAAATGCATTAGCGGAACCGTTTGCGATCTGCCACA
AGTGTGATAAGTTATCTACACTGGCGAGGGGATTGCTCTCTGTAATGTTCAGCTTCTAAT
TGTCTCTACTTTGTGAGACTACTTTTGAATGCTTGACCTCAAATCAGGTAGGACTACCC-
GCTGAACTTAA

>BC7-5
TTTCCGTAGGTGAACCTGCGGAAGGATCATTATTGAATTATGTTTCTAGATAGGTTGTAG
CTGGCTCTTTAGAGCATGTGCACGCCTGTTTGGACTTCATTTTCATCCACCTGTGCACCT
ATTGTAGTCTTTGGTTGGGTTAGGAGGAAGTGGTCATTGTGTCAGCATCTGCTGGATGTG
AGGACTTGCATTGTGAAAGCTTTGCTGTCCTTGATGTGATCATGGAATCTCTTTCTCACT
AGAGTCTATGTCACTCATTATACTCTGTCGAATGTCATTGAATGTCTTTACATGGGCTTG
TATGCCTATGAAAATTGTAATACAACTTTCAGCAACGGATCTCTTGGCTCTCGCATCGAT
GAAGAACGCAGCGAAATGCGATAAGTAATGTGAATTGCAGAATTCAGTGAATCATCGAAT
CTTTGAACGCATCTTGCGCTCCTTGGTATTCCGAGGAGCATGCCTGTTTGAGTGTCATTA
AATTCTCAACTCTCTTCTAC-TTTTTGTAAAAGAGAGCTTGGACTGTGGAGGCTTGCTGG
CCACTTTTTGGGGTCAGCTCCTCTGAAATGCATTAGCGGAACCGTTTGCGATCTGCCACA
AGTGTGATAAGTTATCTACACTGGCGAGGGGATTGCTCTCTGTAATGTTCAGCTTCTAAT
TGTCTCTACTTTGTGAGACTACTTTTGAATGCTTGACCTCAAATCAGGTAGGACTACCC-
GCTGAACTTAA
>BC1-6
TTTCCGTAGGTGAACCTGCGGAAGGATCATTATTGAATTATGTTTCTAGATAGGTTGTAG
CTGGCTCTTTAGAGCATGTGCACGCCTGTTTGGACTTCATTTTCATCCACCTGTGCACCT
ATTGTAGTCTTTGGTTGGGTTAGGGGGAAGTGGTCATTGTGTCAGCATCTGCTGGATGTG
AGGACTTGCATTGTGAAAGCTTTGCTGTCCTTGATGTGATCATGGAATCTCTTTCTCACT
AGAGTCTATGTCACTCATTATACTCTGTCGAATGTCATTGAATGTCTTTACATGGGCTTG
TATGCCTATGAAAATTGTAATACAACTTTCAGCAACGGATCTCTTGGCTCTCGCATCGAT
GAAGGACGCAGCGAAATGCGATAAGTAATGTGAATTGCAGAATTCAGTGAATCATCGAAT
CTTTGAACGCATCTTGCGCTCCTTGGTATTCCGAGGAGCATGCCTGTTTGAGTGTCATTA
AATTCTCAACTCTCTTCTAC-TTTTTGTAAAAGAGAGCTTGGACTGTGGAGGCTTGCTGG
CCACTTTTTGGGGTCAGCTCCTCTGAAATGCATTAGCGGAACCGTTTGCGATCTGCCACA
AGTGTGATAAGTTATCTACACTGGCGAGGGGATTGCTCTCTGTAATGTTCAGCTTCTAAT
TGTCTCTACTTTGTGAGACTACTTTTGAATGCTTGACCTCAAATCAGGTAGGACTACCC-
GCTGAACTTAA
>BC3-27
TTTCCGTAGGTGAACCTGCGGAAGGATCATTATTGAATTATGTTTCTAGATAGGTTGTAG
CTGGCTCTTTAGAGCATGTGCACGCCTGTTTGGACTTCATTTTCATCCACCTGTGCACCT
ATTGTAGTCTTTGGTTGGGTTAGGGGGAAGTGGTCATTGTGTCAGCATCTGCTGGATGTG
AGGACTTGCATTGTGAAAGCTTTGCTGTCCTTGATGTGATCATGGAATCTCTTTCTCACT
AGAGTCTATGTCACTCATTATACTCTGTCGAATGTCATTGAATGTCTTTACATGGGCTTG
TATGCCTATGAAAATTGTAATACAACTTTCAGCAACGGATCTCTTGGCTCTCGCATCGAT
GAAGGACGCAGCGAAATGCGATAAGTAATGTGAATTGCAGAATTCAGTGAATCATCGAAT
CTTTGAACGCATCTTGCGCTCCTTGGTATTCCGAGGAGCATGCCTGTTTGAGTGTCATTA
AATTCTCAACTCTCTTCTAC-TTTTTGTAAAAGAGAGCTTGGACTGTGGAGGCTTGCTGG
CCACTTTTTGGGGTCAGCTCCTCTGAAATGCATTAGCGGAACCGTTTGCGATCTGCCACA
AGTGTGATAAGTTATCTACACTGGCGAGGGGATTGCTCTCTGTAATGTTCAGCTTCTAAT
TGTCTCTACTTTGTGAGACTACTTTTGAATGCTTGACCTCAAATCAGGTAGGACTACCC-
GCTGAACTTAA
>BC3-35
TTTCCGTAGGTGAACCTGCGGAAGGATCATTATTGAATTATGTTTCTAGATAGGTTGTAG
CTGGCTCTTTAGAGCATGTGCACGCCTGTTTGGACTTCATTTTCATCCACCTGTGCACCT
ATTGTAGTCTTTGGTTGGGTTAGGGGGAAGTGGTCATTGTGTCAGCATCTGCTGGATGTG
AGGACTTGCATTGTGAAAGCTTTGCTGTCCTTGATGTGATCATGGAATCTCTTTCTCACT
AGAGTCTATGTCACTCATTATACTCTGTCGAATGTCATTGAATGTCTTTACATGGGCTTG
TATGCCTATGAAAATTGTAATACAACTTTCAGCAACGGATCTCTTGGCTCTCGCATCGAT
GAAGGACGCAGCGAAATGCGATAAGTAATGTGAATTGCAGAATTCAGTGAATCATCGAAT

CTTTGAACGCATCTTGCGCTCCTTGGTATTCCGAGGAGCATGCCTGTTTGAGTGTCATTA
AATTCTCAACTCTCTTCTAC-TTTTTGTAAAAGAGAGCTTGGACTGTGGAGGCTTGCTGG
CCACTTTTTGGGGTCAGCTCCTCTGAAATGCATTAGCGGAACCGTTTGCGATCTGCCACA
AGTGTGATAAGTTATCTACACTGGCGAGGGGATTGCTCTCTGTAATGTTCAGCTTCTAAT
TGTCTCTACTTTGTGAGACTACTTTTGAATGCTTGACCTCAAATCAGGTAGGACTACCC-
GCTGAACTTAA
>BC4-62
TTTCCGTAGGTGAACCTGCGGAAGGATCATTATTGAATTATGTTTCTAGATAGGTTGTAG
CTGGCTCTTTAGAGCATGTGCACGCCTGTTTGGACTTCATTTTCATCCACCTGTGCACCT
ATTGTAGTCTTTGGTTGGGTTAGGGGGAAGTGGTCATTGTGTCAGCATCTGCTGGATGTG
AGGACTTGCATTGTGAAAGCTTTGCTGTCCTTGATGTGATCATGGAATCTCTTTCTCACT
AGAGTCTATGTCACTCATTATACTCTGTCGAATGTCATTGAATGTCTTTACATGGGCTTG
TATGCCTATGAAAATTGTAATACAACTTTCAGCAACGGATCTCTTGGCTCTCGCATCGAT
GAAGGACGCAGCGAAATGCGATAAGTAATGTGAATTGCAGAATTCAGTGAATCATCGAAT
CTTTGAACGCATCTTGCGCTCCTTGGTATTCCGAGGAGCATGCCTGTTTGAGTGTCATTA
AATTCTCAACTCTCTTCTAC-TTTTTGTAAAAGAGAGCTTGGACTGTGGAGGCTTGCTGG
CCACTTTTTGGGGTCAGCTCCTCTGAAATGCATTAGCGGAACCGTTTGCGATCTGCCACA
AGTGTGATAAGTTATCTACACTGGCGAGGGGATTGCTCTCTGTAATGTTCAGCTTCTAAT
TGTCTCTACTTTGTGAGACTACTTTTGAATGCTTGACCTCAAATCAGGTAGGACTACCC-
GCTGAACTTAA
>BC5-36
TTTCCGTAGGTGAACCTGCGGAAGGATCATTATTGAATTATGTTTCTAGATAGGTTGTAG
CTGGCTCTTTAGAGCATGTGCACGCCTGTTTGGACTTCATTTTCATCCACCTGTGCACCT
ATTGTAGTCTTTGGTTGGGTTAGGGGGAAGTGGTCATTGTGTCAGCATCTGCTGGATGTG
AGGACTTGCATTGTGAAAGCTTTGCTGTCCTTGATGTGATCATGGAATCTCTTTCTCACT
AGAGTCTATGTCACTCATTATACTCTGTCGAATGTCATTGAATGTCTTTACATGGGCTTG
TATGCCTATGAAAATTGTAATACAACTTTCAGCAACGGATCTCTTGGCTCTCGCATCGAT
GAAGGACGCAGCGAAATGCGATAAGTAATGTGAATTGCAGAATTCAGTGAATCATCGAAT
CTTTGAACGCATCTTGCGCTCCTTGGTATTCCGAGGAGCATGCCTGTTTGAGTGTCATTA
AATTCTCAACTCTCTTCTAC-TTTTTGTAAAAGAGAGCTTGGACTGTGGAGGCTTGCTGG
CCACTTTTTGGGGTCAGCTCCTCTGAAATGCATTAGCGGAACCGTTTGCGATCTGCCACA
AGTGTGATAAGTTATCTACACTGGCGAGGGGATTGCTCTCTGTAATGTTCAGCTTCTAAT
TGTCTCTACTTTGTGAGACTACTTTTGAATGCTTGACCTCAAATCAGGTAGGACTACCC-
GCTGAACTTAA
>BC6-44
TTTCCGTAGGTGAACCTGCGGAAGGATCATTATTGAATTATGTTTCTAGATAGGTTGTAG
CTGGCTCTTTAGAGCATGTGCACGCCTGTTTGGACTTCATTTTCATCCACCTGTGCACCT
ATTGTAGTCTTTGGTTGGGTTAGGGGGAAGTGGTCATTGTGTCAGCATCTGCTGGATGTG
AGGACTTGCATTGTGAAAGCTTTGCTGTCCTTGATGTGATCATGGAATCTCTTTCTCACT
AGAGTCTATGTCACTCATTATACTCTGTCGAATGTCATTGAATGTCTTTACATGGGCTTG
TATGCCTATGAAAATTGTAATACAACTTTCAGCAACGGATCTCTTGGCTCTCGCATCGAT
GAAGGACGCAGCGAAATGCGATAAGTAATGTGAATTGCAGAATTCAGTGAATCATCGAAT
CTTTGAACGCATCTTGCGCTCCTTGGTATTCCGAGGAGCATGCCTGTTTGAGTGTCATTA
AATTCTCAACTCTCTTCTAC-TTTTTGTAAAAGAGAGCTTGGACTGTGGAGGCTTGCTGG
CCACTTTTTGGGGTCAGCTCCTCTGAAATGCATTAGCGGAACCGTTTGCGATCTGCCACA
AGTGTGATAAGTTATCTACACTGGCGAGGGGATTGCTCTCTGTAATGTTCAGCTTCTAAT
TGTCTCTACTTTGTGAGACTACTTTTGAATGCTTGACCTCAAATCAGGTAGGACTACCC-
GCTGAACTTAA
>BC7-33
TTTCCGTAGGTGAACCTGCGGAAGGATCATTATTGAATTATGTTTCTAGATAGGTTGTAG

CTGGCTCTTTAGAGCATGTGCACGCCTGTTTGGACTTCATTTTCATCCACCTGTGCACCT
ATTGTAGTCTTTGGTTGGGTTAGGGGGAAGTGGTCATTGTGTCAGCATCTGCTGGATGTG
AGGACTTGCATTGTGAAAGCTTTGCTGTCCTTGATGTGATCATGGAATCTCTTTCTCACT
AGAGTCTATGTCACTCATTATACTCTGTCGAATGTCATTGAATGTCTTTACATGGGCTTG
TATGCCTATGAAAATTGTAATACAACTTTCAGCAACGGATCTCTTGGCTCTCGCATCGAT
GAAGGACGCAGCGAAATGCGATAAGTAATGTGAATTGCAGAATTCAGTGAATCATCGAAT
CTTTGAACGCATCTTGCGCTCCTTGGTATTCCGAGGAGCATGCCTGTTTGAGTGTCATTA
AATTCTCAACTCTCTTCTAC-TTTTTGTAAAAGAGAGCTTGGACTGTGGAGGCTTGCTGG
CCACTTTTTGGGGTCAGCTCCTCTGAAATGCATTAGCGGAACCGTTTGCGATCTGCCACA
AGTGTGATAAGTTATCTACACTGGCGAGGGGATTGCTCTCTGTAATGTTCAGCTTCTAAT
TGTCTCTACTTTGTGAGACTACTTTTGAATGCTTGACCTCAAATCAGGTAGGACTACCC-
GCTGAACTTAA
>BC8-11
TTTCCGTAGGTGAACCTGCGGAAGGATCATTATTGAATTATGTTTCTAGATAGGTTGTAG
CTGGCTCTTTAGAGCATGTGCACGCCTGTTTGGACTTCATTTTCATCCACCTGTGCACCT
ATTGTAGTCTTTGGTTGGGTTAGGGGGAAGTGGTCATTGTGTCAGCATCTGCTGGATGTG
AGGACTTGCATTGTGAAAGCTTTGCTGTCCTTGATGTGATCATGGAATCTCTTTCTCACT
AGAGTCTATGTCACTCATTATACTCTGTCGAATGTCATTGAATGTCTTTACATGGGCTTG
TATGCCTATGAAAATTGTAATACAACTTTCAGCAACGGATCTCTTGGCTCTCGCATCGAT
GAAGGACGCAGCGAAATGCGATAAGTAATGTGAATTGCAGAATTCAGTGAATCATCGAAT
CTTTGAACGCATCTTGCGCTCCTTGGTATTCCGAGGAGCATGCCTGTTTGAGTGTCATTA
AATTCTCAACTCTCTTCTAC-TTTTTGTAAAAGAGAGCTTGGACTGTGGAGGCTTGCTGG
CCACTTTTTGGGGTCAGCTCCTCTGAAATGCATTAGCGGAACCGTTTGCGATCTGCCACA
AGTGTGATAAGTTATCTACACTGGCGAGGGGATTGCTCTCTGTAATGTTCAGCTTCTAAT
TGTCTCTACTTTGTGAGACTACTTTTGAATGCTTGACCTCAAATCAGGTAGGACTACCC-
GCTGAACTTAA
>BC9-33
TTTCCGTAGGTGAACCTGCGGAAGGATCATTATTGAATTATGTTTCTAGATAGGTTGTAG
CTGGCTCTTTAGAGCATGTGCACGCCTGTTTGGACTTCATTTTCATCCACCTGTGCACCT
ATTGTAGTCTTTGGTTGGGTTAGGGGGAAGTGGTCATTGTGTCAGCATCTGCTGGATGTG
AGGACTTGCATTGTGAAAGCTTTGCTGTCCTTGATGTGATCATGGAATCTCTTTCTCACT
AGAGTCTATGTCACTCATTATACTCTGTCGAATGTCATTGAATGTCTTTACATGGGCTTG
TATGCCTATGAAAATTGTAATACAACTTTCAGCAACGGATCTCTTGGCTCTCGCATCGAT
GAAGGACGCAGCGAAATGCGATAAGTAATGTGAATTGCAGAATTCAGTGAATCATCGAAT
CTTTGAACGCATCTTGCGCTCCTTGGTATTCCGAGGAGCATGCCTGTTTGAGTGTCATTA
AATTCTCAACTCTCTTCTAC-TTTTTGTAAAAGAGAGCTTGGACTGTGGAGGCTTGCTGG
CCACTTTTTGGGGTCAGCTCCTCTGAAATGCATTAGCGGAACCGTTTGCGATCTGCCACA
AGTGTGATAAGTTATCTACACTGGCGAGGGGATTGCTCTCTGTAATGTTCAGCTTCTAAT
TGTCTCTACTTTGTGAGACTACTTTTGAATGCTTGACCTCAAATCAGGTAGGACTACCC-
GCTGAACTTAA
>BC9-52
TTTCCGTAGGTGAACCTGCGGAAGGATCATTATTGAATTATGTTTCTAGATAGGTTGTAG
CTGGCTCTTTAGAGCATGTGCACGCCTGTTTGGACTTCATTTTCATCCACCTGTGCACCT
ATTGTAGTCTTTGGTTGGGTTAGGGGGAAGTGGTCATTGTGTCAGCATCTGCTGGATGTG
AGGACTTGCATTGTGAAAGCTTTGCTGTCCTTGATGTGATCATGGAATCTCTTTCTCACT
AGAGTCTATGTCACTCATTATACTCTGTCGAATGTCATTGAATGTCTTTACATGGGCTTG
TATGCCTATGAAAATTGTAATACAACTTTCAGCAACGGATCTCTTGGCTCTCGCATCGAT
GAAGGACGCAGCGAAATGCGATAAGTAATGTGAATTGCAGAATTCAGTGAATCATCGAAT
CTTTGAACGCATCTTGCGCTCCTTGGTATTCCGAGGAGCATGCCTGTTTGAGTGTCATTA
AATTCTCAACTCTCTTCTAC-TTTTTGTAAAAGAGAGCTTGGACTGTGGAGGCTTGCTGG

CCACTTTTTGGGGTCAGCTCCTCTGAAATGCATTAGCGGAACCGTTTGCGATCTGCCACA
AGTGTGATAAGTTATCTACACTGGCGAGGGGATTGCTCTCTGTAATGTTCAGCTTCTAAT
TGTCTCTACTTTGTGAGACTACTTTTGAATGCTTGACCTCAAATCAGGTAGGACTACCC-
GCTGAACTTAA
>BC10_23
TTTCCGTAGGTGAACCTGCGGAAGGATCATTATTGAATTATGTTTCTAGATAGGTTGTAG
CTGGCTCTTTAGAGCATGTGCACGCCTGTTTGGACTTCATTTTCATCCACCTGTGCACCT
ATTGTAGTCTTTGGTTGGGTTAGGGGGAAGTGGTCATTGTGTCAGCATCTGCTGGATGTG
AGGACTTGCATTGTGAAAGCTTTGCTGTCCTTGATGTGATCATGGAATCTCTTTCTCACT
AGAGTCTATGTCACTCATTATACTCTGTCGAATGTCATTGAATGTCTTTACATGGGCTTG
TATGCCTATGAAAATTGTAATACAACTTTCAGCAACGGATCTCTTGGCTCTCGCATCGAT
GAAGGACGCAGCGAAATGCGATAAGTAATGTGAATTGCAGAATTCAGTGAATCATCGAAT
CTTTGAACGCATCTTGCGCTCCTTGGTATTCCGAGGAGCATGCCTGTTTGAGTGTCATTA
AATTCTCAACTCTCTTCTAC-TTTTTGTAAAAGAGAGCTTGGACTGTGGAGGCTTGCTGG
CCACTTTTTGGGGTCAGCTCCTCTGAAATGCATTAGCGGAACCGTTTGCGATCTGCCACA
AGTGTGATAAGTTATCTACACTGGCGAGGGGATTGCTCTCTGTAATGTTCAGCTTCTAAT
TGTCTCTACTTTGTGAGACTACTTTTGAATGCTTGACCTCAAATCAGGTAGGACTACCC-
GCTGAACTTAA
>BC11_57
TTTCCGTAGGTGAACCTGCGGAAGGATCATTATTGAATTATGTTTCTAGATAGGTTGTAG
CTGGCTCTTTAGAGCATGTGCACGCCTGTTTGGACTTCATTTTCATCCACCTGTGCACCT
ATTGTAGTCTTTGGTTGGGTTAGGGGGAAGTGGTCATTGTGTCAGCATCTGCTGGATGTG
AGGACTTGCATTGTGAAAGCTTTGCTGTCCTTGATGTGATCATGGAATCTCTTTCTCACT
AGAGTCTATGTCACTCATTATACTCTGTCGAATGTCATTGAATGTCTTTACATGGGCTTG
TATGCCTATGAAAATTGTAATACAACTTTCAGCAACGGATCTCTTGGCTCTCGCATCGAT
GAAGGACGCAGCGAAATGCGATAAGTAATGTGAATTGCAGAATTCAGTGAATCATCGAAT
CTTTGAACGCATCTTGCGCTCCTTGGTATTCCGAGGAGCATGCCTGTTTGAGTGTCATTA
AATTCTCAACTCTCTTCTAC-TTTTTGTAAAAGAGAGCTTGGACTGTGGAGGCTTGCTGG
CCACTTTTTGGGGTCAGCTCCTCTGAAATGCATTAGCGGAACCGTTTGCGATCTGCCACA
AGTGTGATAAGTTATCTACACTGGCGAGGGGATTGCTCTCTGTAATGTTCAGCTTCTAAT
TGTCTCTACTTTGTGAGACTACTTTTGAATGCTTGACCTCAAATCAGGTAGGACTACCC-
GCTGAACTTAA
>BC12_17
TTTCCGTAGGTGAACCTGCGGAAGGATCATTATTGAATTATGTTTCTAGATAGGTTGTAG
CTGGCTCTTTAGAGCATGTGCACGCCTGTTTGGACTTCATTTTCATCCACCTGTGCACCT
ATTGTAGTCTTTGGTTGGGTTAGGGGGAAGTGGTCATTGTGTCAGCATCTGCTGGATGTG
AGGACTTGCATTGTGAAAGCTTTGCTGTCCTTGATGTGATCATGGAATCTCTTTCTCACT
AGAGTCTATGTCACTCATTATACTCTGTCGAATGTCATTGAATGTCTTTACATGGGCTTG
TATGCCTATGAAAATTGTAATACAACTTTCAGCAACGGATCTCTTGGCTCTCGCATCGAT
GAAGGACGCAGCGAAATGCGATAAGTAATGTGAATTGCAGAATTCAGTGAATCATCGAAT
CTTTGAACGCATCTTGCGCTCCTTGGTATTCCGAGGAGCATGCCTGTTTGAGTGTCATTA
AATTCTCAACTCTCTTCTAC-TTTTTGTAAAAGAGAGCTTGGACTGTGGAGGCTTGCTGG
CCACTTTTTGGGGTCAGCTCCTCTGAAATGCATTAGCGGAACCGTTTGCGATCTGCCACA
AGTGTGATAAGTTATCTACACTGGCGAGGGGATTGCTCTCTGTAATGTTCAGCTTCTAAT
TGTCTCTACTTTGTGAGACTACTTTTGAATGCTTGACCTCAAATCAGGTAGGACTACCC-
GCTGAACTTAA
>BC2-34
TTTCCGTAGGTGAACCTGCGGAAGGATCATTATTGAATTATGTTTCTAGATAGGTTGTAG
CTGGCTCTTTAGAGCATGTGCACGCCTGTTTGGACTTCATTTTCATCCACCTGTGCACCT
ATTGTAGTCTTTGGTTGGGTTAGGGGGAAGTGGTCATTGTGTCAGCATCTGCTGGATGTG

AGGACTTGCATTGTGAAAGCTTTGCTGTCCTTGATGTGATCATGGAATCTCTTTCTCACT
AGAGTCTATGTCACTCATTATACTCTGTCGAATGTCATTGAATGTCTTTACATGGGCTTG
TATGCCTATGAAAATTGTAATACAACTTTCAGCAACGGATCTCTTGGCTCTCGCATCGAT
GAAGGACGCAGCGAAATGCGATAAGTAATGTGAATTGCAGAATTCAGTGAATCATCGAAT
CTTTGAACGCATCTTGCGCTCCTTGGTATTCCGAGGAGCATGCCTGTTTGAGTGTCATTA
AATTCTCAACTCTCTTCTAC-TTTTTGTAAAAGAGAGCTTGGACTGTGGAGGCTTGCTGG
CCACTTTTTGGGGTCAGCTCCTCTGAAATGCATTAGCGGAACCGTTTGCGATCTGCCACA
AGTGTGATAAGTTATCTACACTGGCGAGGGGATTGCTCTCTGTAATGTTCAGCTTCTAAT
TGTCTCTACTTTGTGAGACTACTTTTGAATGCTTGACCTCAAATCAGGTAGGACTACCC-
GCTGAACTTAA
>BC12_18
TTTCCGTAGGTGAACCTGCGGAAGGATCATTATTGAATTATGTTTCTAGATAGGTTGTAG
CTGGCTCTTTAGAGCATGTGCACGCCTGTTTGGACTTCATTTTCATCCACCTGTGCACCT
ATTGTAGTCTTTGGTTGGGTTAGGGGGAAGTGGTCATTGTGTCAGCATCTGCTGGATGTG
AGGACTTGCATTGTGAAAGCTTTGCTGTCCTTGATGTGATCATGGAATCTCTTTCTCACT
AGAGTCTATGTCACTCATTATACTCTGTCGAATGTCATTGAATGTCTTTACATGGGCTTG
TATGCCTATGAAAATTGTAATACAACTTTCAGCAACGGATCTCTTGGCTCTCGCATCGAT
GAAGGACGCAGCGAAATGCGATAAGTAATGTGAATTGCAGAATTCAGTGAATCATCGAAT
CTTTGAACGCATCTTGCGCTCCTTGGTATTCCGAGGAGCATGCCTGTTTGAGTGTCATTA
AATTCTCAACTCTCTTCTAC-TTTTTGTAAAAGAGAGCTTGGACTGTGGAGGCTTGCTGG
CCACTTTTTGGGGTCAGCTCCTCTGAAATGCATTAGCGGAACCGTTTGCGATCTGCCACA
AGTGTGATAAGTTATCTACACTGGCGAGGGGATTGCTCTCTGTAATGTTCAGCTTCTAAT
TGTCTCTACTTTGTGAGACTACTTTTGAATGCTTGACCTCAAATCAGGTAGGACTACCC-
GCTGAACTTAA
>BC6-39
TTTCCGTAGGTGAACCTGCGGAAGGATCATTATTGAATTATGTTTCTAGATAGGTTGTAG
CTGGCTCTTTAGAGCATGTGCACGCCTGTTTGGACTTCATTTTCATCCACCTGTGCACCT
ATTGTAGTCTTTGGTTGGGTTAGGGGGAAGTGGTCATTGTGTCAGCATCTGCTGGATGTG
AGGACTTGCATTGTGAAAGCTTTGCTGTCCTTGATGTGATCATGGAATCTCTTTCTCACT
AGAGTCTATGTCACTCATTATACTCTGTCGAATGTCATTGAATGTCTTTACATGGGCTTG
TATGCCTATGAAAATTGTAATACAACTTTCAGCAACGGATCTCTTGGCTCTCGCATCGAT
GAAGGACGCAGCGAAATGCGATAAGTAATGTGAATTGCAGAATTCAGTGAATCATCGAAT
CTTTGAACGCATCTTGCGCTCCTTGGTATTCCGAGGAGCATGCCTGTTTGAGTGTCATTA
AATTCTCAACTCTCTTCTAC-TTTTTGTAAAAGAGAGCTTGGACTGTGGAGGCTTGCTGG
CCACTTTTTGGGGTCAGCTCCTCTGAAATGCATTAGCGGAACCGTTTGCGATCTGCCACA
AGTGTGATAAGTTATCTACACTGGCGAGGGGATTGCTCTCTGTAATGTTCAGCTTCTAAT
TGTCTCTACTTTGTGAGACTACTTTTGAATGCTTGACCTCAAATCAGGTAGGACTACCC-
GCTGAACTTAA
>BC3-29
TTTCCGTAGGTGAACCTGCGGAAGGATCATTATTGAATTATGTTTCTAGATAGGTTGTAG
CTGGCTCTTTAGAGCATGTGCACGCCTGTTTGGACTTCATTTTCATCCACCTGTGCACCT
ATTGTAGTCTTTGGTTGGGTTAGGGGGAAGTGGTCATTGTGTCAGCATCTGCTGGATGTG
AGGACTTGCATTGTGAAAGCTTTGCTGTCCTTGATGTGATCATGGAATCTCTTTCTCACT
AGAGTCTATGTCACTCATTATACTCTGTCGAATGTCATTGAATGTCTTTACATGGGCTTG
TATGCCTATGAAAATTGTAATACAACTTTCAGCAACGGATCTCTTGGCTCTCGCATCGAT
GAAGAACGCAGCGAAATGCGATAAGTAATGTGAATTGCAGAATTCAGTGAATCATCGAAT
CTTTGAACGCATCTTGCGCTCCTTGGTATTCCGAGGAGCATGCCTGTTTGAGTGTCATTA
AATTCTCAACTCTCTTCTAC-TTTTTGTAAAAGAGAGCTTGGACTGTGGAGGCTTGCTGG
CCACTTTTTGGGGTCAGCTCCTCTGAAATGCATTAGCGGAACCGTTTGCGATCTGCCACA
AGTGTGATAAGTTATCTACACTGGCGAGGGGATTGCTCTCTGTAATGTTCAGCTTCTAAT

TGTCTCTACTTTGTGAGACTACTTTTGAATGCTTGACCTCAAATCAGGTAGGACTACCC-
GCTGAACTTAA
>BC4-10
TTTCCGTAGGTGAACCTGCGGAAGGATCATTATTGAATTATGTTTCTAGATAGGTTGTAG
CTGGCTCTTTAGAGCATGTGCACGCCTGTTTGGACTTCATTTTCATCCACCTGTGCACCT
ATTGTAGTCTTTGGTTGGGTTAGGGGGAAGTGGTCATTGTGTCAGCATCTGCTGGATGTG
AGGACTTGCATTGTGAAAGCTTTGCTGTCCTTGATGTGATCATGGAATCTCTTTCTCACT
AGAGTCTATGTCACTCATTATACTCTGTCGAATGTCATTGAATGTCTTTACATGGGCTTG
TATGCCTATGAAAATTGTAATACAACTTTCAGCAACGGATCTCTTGGCTCTCGCATCGAT
GAAGAACGCAGCGAAATGCGATAAGTAATGTGAATTGCAGAATTCAGTGAATCATCGAAT
CTTTGAACGCATCTTGCGCTCCTTGGTATTCCGAGGAGCATGCCTGTTTGAGTGTCATTA
AATTCTCAACTCTCTTCTAC-TTTTTGTAAAAGAGAGCTTGGACTGTGGAGGCTTGCTGG
CCACTTTTTGGGGTCAGCTCCTCTGAAATGCATTAGCGGAACCGTTTGCGATCTGCCACA
AGTGTGATAAGTTATCTACACTGGCGAGGGGATTGCTCTCTGTAATGTTCAGCTTCTAAT
TGTCTCTACTTTGTGAGACTACTTTTGAATGCTTGACCTCAAATCAGGTAGGACTACCC-
GCTGAACTTAA
>BC4-63
TTTCCGTAGGTGAACCTGCGGAAGGATCATTATTGAATTATGTTTCTAGATAGGTTGTAG
CTGGCTCTTTAGAGCATGTGCACGCCTGTTTGGACTTCATTTTCATCCACCTGTGCACCT
ATTGTAGTCTTTGGTTGGGTTAGGGGGAAGTGGTCATTGTGTCAGCATCTGCTGGATGTG
AGGACTTGCATTGTGAAAGCTTTGCTGTCCTTGATGTGATCATGGAATCTCTTTCTCACT
AGAGTCTATGTCACTCATTATACTCTGTCGAATGTCATTGAATGTCTTTACATGGGCTTG
TATGCCTATGAAAATTGTAATACAACTTTCAGCAACGGATCTCTTGGCTCTCGCATCGAT
GAAGAACGCAGCGAAATGCGATAAGTAATGTGAATTGCAGAATTCAGTGAATCATCGAAT
CTTTGAACGCATCTTGCGCTCCTTGGTATTCCGAGGAGCATGCCTGTTTGAGTGTCATTA
AATTCTCAACTCTCTTCTAC-TTTTTGTAAAAGAGAGCTTGGACTGTGGAGGCTTGCTGG
CCACTTTTTGGGGTCAGCTCCTCTGAAATGCATTAGCGGAACCGTTTGCGATCTGCCACA
AGTGTGATAAGTTATCTACACTGGCGAGGGGATTGCTCTCTGTAATGTTCAGCTTCTAAT
TGTCTCTACTTTGTGAGACTACTTTTGAATGCTTGACCTCAAATCAGGTAGGACTACCC-
GCTGAACTTAA
>BC5-38
TTTCCGTAGGTGAACCTGCGGAAGGATCATTATTGAATTATGTTTCTAGATAGGTTGTAG
CTGGCTCTTTAGAGCATGTGCACGCCTGTTTGGACTTCATTTTCATCCACCTGTGCACCT
ATTGTAGTCTTTGGTTGGGTTAGGGGGAAGTGGTCATTGTGTCAGCATCTGCTGGATGTG
AGGACTTGCATTGTGAAAGCTTTGCTGTCCTTGATGTGATCATGGAATCTCTTTCTCACT
AGAGTCTATGTCACTCATTATACTCTGTCGAATGTCATTGAATGTCTTTACATGGGCTTG
TATGCCTATGAAAATTGTAATACAACTTTCAGCAACGGATCTCTTGGCTCTCGCATCGAT
GAAGAACGCAGCGAAATGCGATAAGTAATGTGAATTGCAGAATTCAGTGAATCATCGAAT
CTTTGAACGCATCTTGCGCTCCTTGGTATTCCGAGGAGCATGCCTGTTTGAGTGTCATTA
AATTCTCAACTCTCTTCTAC-TTTTTGTAAAAGAGAGCTTGGACTGTGGAGGCTTGCTGG
CCACTTTTTGGGGTCAGCTCCTCTGAAATGCATTAGCGGAACCGTTTGCGATCTGCCACA
AGTGTGATAAGTTATCTACACTGGCGAGGGGATTGCTCTCTGTAATGTTCAGCTTCTAAT
TGTCTCTACTTTGTGAGACTACTTTTGAATGCTTGACCTCAAATCAGGTAGGACTACCC-
GCTGAACTTAA
>BC7-40
TTTCCGTAGGTGAACCTGCGGAAGGATCATTATTGAATTATGTTTCTAGATAGGTTGTAG
CTGGCTCTTTAGAGCATGTGCACGCCTGTTTGGACTTCATTTTCATCCACCTGTGCACCT
ATTGTAGTCTTTGGTTGGGTTAGGGGGAAGTGGTCATTGTGTCAGCATCTGCTGGATGTG
AGGACTTGCATTGTGAAAGCTTTGCTGTCCTTGATGTGATCATGGAATCTCTTTCTCACT
AGAGTCTATGTCACTCATTATACTCTGTCGAATGTCATTGAATGTCTTTACATGGGCTTG

TATGCCTATGAAAATTGTAATACAACTTTCAGCAACGGATCTCTTGGCTCTCGCATCGAT
GAAGAACGCAGCGAAATGCGATAAGTAATGTGAATTGCAGAATTCAGTGAATCATCGAAT
CTTTGAACGCATCTTGCGCTCCTTGGTATTCCGAGGAGCATGCCTGTTTGAGTGTCATTA
AATTCTCAACTCTCTTCTAC-TTTTTGTAAAAGAGAGCTTGGACTGTGGAGGCTTGCTGG
CCACTTTTTGGGGTCAGCTCCTCTGAAATGCATTAGCGGAACCGTTTGCGATCTGCCACA
AGTGTGATAAGTTATCTACACTGGCGAGGGGATTGCTCTCTGTAATGTTCAGCTTCTAAT
TGTCTCTACTTTGTGAGACTACTTTTGAATGCTTGACCTCAAATCAGGTAGGACTACCC-
GCTGAACTTAA
>BC10_48
TTTCCGTAGGTGAACCTGCGGAAGGATCATTATTGAATTATGTTTCTAGATAGGTTGTAG
CTGGCTCTTTAGAGCATGTGCACGCCTGTTTGGACTTCATTTTCATCCACCTGTGCACCT
ATTGTAGTCTTTGGTTGGGTTAGGGGGAAGTGGTCATTGTGTCAGCATCTGCTGGATGTG
AGGACTTGCATTGTGAAAGCTTTGCTGTCCTTGATGTGATCATGGAATCTCTTTCTCACT
AGAGTCTATGTCACTCATTATACTCTGTCGAATGTCATTGAATGTCTTTACATGGGCTTG
TATGCCTATGAAAATTGTAATACAACTTTCAGCAACGGATCTCTTGGCTCTCGCATCGAT
GAAGAACGCAGCGAAATGCGATAAGTAATGTGAATTGCAGAATTCAGTGAATCATCGAAT
CTTTGAACGCATCTTGCGCTCCTTGGTATTCCGAGGAGCATGCCTGTTTGAGTGTCATTA
AATTCTCAACTCTCTTCTAC-TTTTTGTAAAAGAGAGCTTGGACTGTGGAGGCTTGCTGG
CCACTTTTTGGGGTCAGCTCCTCTGAAATGCATTAGCGGAACCGTTTGCGATCTGCCACA
AGTGTGATAAGTTATCTACACTGGCGAGGGGATTGCTCTCTGTAATGTTCAGCTTCTAAT
TGTCTCTACTTTGTGAGACTACTTTTGAATGCTTGACCTCAAATCAGGTAGGACTACCC-
GCTGAACTTAA
>BC11_31
TTTCCGTAGGTGAACCTGCGGAAGGATCATTATTGAATTATGTTTCTAGATAGGTTGTAG
CTGGCTCTTTAGAGCATGTGCACGCCTGTTTGGACTTCATTTTCATCCACCTGTGCACCT
ATTGTAGTCTTTGGTTGGGTTAGGGGGAAGTGGTCATTGTGTCAGCATCTGCTGGATGTG
AGGACTTGCATTGTGAAAGCTTTGCTGTCCTTGATGTGATCATGGAATCTCTTTCTCACT
AGAGTCTATGTCACTCATTATACTCTGTCGAATGTCATTGAATGTCTTTACATGGGCTTG
TATGCCTATGAAAATTGTAATACAACTTTCAGCAACGGATCTCTTGGCTCTCGCATCGAT
GAAGAACGCAGCGAAATGCGATAAGTAATGTGAATTGCAGAATTCAGTGAATCATCGAAT
CTTTGAACGCATCTTGCGCTCCTTGGTATTCCGAGGAGCATGCCTGTTTGAGTGTCATTA
AATTCTCAACTCTCTTCTAC-TTTTTGTAAAAGAGAGCTTGGACTGTGGAGGCTTGCTGG
CCACTTTTTGGGGTCAGCTCCTCTGAAATGCATTAGCGGAACCGTTTGCGATCTGCCACA
AGTGTGATAAGTTATCTACACTGGCGAGGGGATTGCTCTCTGTAATGTTCAGCTTCTAAT
TGTCTCTACTTTGTGAGACTACTTTTGAATGCTTGACCTCAAATCAGGTAGGACTACCC-
GCTGAACTTAA
>BC10_4
TTTCCGTAGGTGAACCTGCGGAAGGATCATTATTGAATTATGTTTCTAGATAGGTTGTAG
CTGGCTCTTTAGAGCATGTGCACGCCTGTTTGGACTTCATTTTCATCCACCTGTGCACCT
ATTGTAGTCTTTGGTTGGGTTAGGGGGAAGTGGTCATTGTGTCAGCATCTGCTGGATGTG
AGGACTTGCATTGTGAAAGCTTTGCTGTCCTTGATGTGATCATGGAATCTCTTTCTCACT
AGAGTCTATGTCACTCATTATACTCTGTCGAATGTCATTGAATGTCTTTACATGGGCTTG
TATGCCTATGAAAATTGTAATACAACTTTCAGCAACGGATCTCTTGGCTCTCGCATCGAT
GAAGAACGCAGCGAAATGCGATAAGTAATGTGAATTGCAGAATTCAGTGAATCATCGAAT
CTTTGAACGCATCTTGCGCTCCTTGGTATTCCGAGGAGCATGCCTGTTTGAGTGTCATTA
AATTCTCAACTCTCTTCTAC-TTTTTGTAAAAGAGAGCTTGGACTGTGGAGGCTTGCTGG
CCACTTTTTGGGGTCAGCTCCTCTGAAATGCATTAGCGGAACCGTTTGCGATCTGCCACA
AGTGTGATAAGTTATCTACACTGGCGAGGGGATTGCTCTCTGTAATGTTCAGCTTCTAAT
TGTCTCTACTTTGTGAGACTACTTTTGAATGCTTGACCTCAAATCAGGTAGGACTACCC-
GCTGAACTTAA

>BC9-3
TTTCCGTAGGTGAACCTGCGGAAGGATCATTATTGAATTATGTTTCTAGATAGGTTGTAG
CTGGCTCTTTAGAGCATGTGCACGCCTGTTTGGACTTCATTTTCATCCACCTGTGCACCT
ATTGTAGTCTTTGGTTGGGTTAGGGGGAAGTGGTCATTGTGTCAGCATCTGCTGGATGTG
AGGACTTGCATTGTGAAAGCTTTGCTGTCCTTGATGTGATCATGGAATCTCTTTCTCACT
AGAGTCTATGTCACTCATTATACTCTGTCGAATGTCATTGAATGTCTTTACATGGGCTTA
TATGCCTATGAAAATTGTAATACAACTTTCAGCAACGGATCTCTTGGCTCTCGCATCGAT
GAAGGACGCAGCGAAATGCGATAAGTAATGTGAATTGCAGAATTCAGTGAATCATCGAAT
CTTTGAACGCATCTTGCGCTCCTTGGTATTCCGAGGAGCATGCCTGTTTGAGTGTCATTA
AATTCTCAACTCTCTTCTAC-TTTTTGTAAAAGAGAGCTTGGACTGTGGAGGCTTGCTGG
CCACTTTTTGGGGTCAGCTCCTCTGAAATGCATTAGCGGAACCGTTTGCGATCTGCCACA
AGTGTGATAAGTTATCTACACTGGCGAGGGGATTGCTCTCTGTAATGTTCAGCTTCTAAT
TGTCTCTACTTTGTGAGACTACTTTTGAATGCTTGACCTCAAATCAGGTAGGACTACCC-
GCTGAACTTAA
>BC4-66
TTTCCGTAGGTGAACCTGCGGAAGGATCATTATTGAATTATGTTTCTAGATAGGTTGTAG
CTGGCTCTTTAGAGCATGTGCACGCCTGTTTGGACTTCATTTTCATCCACCTGTGCACCT
ATTGTAGTCTTTGGTTGGGTTAGGGGGAAGTGGTCATTGTGTCAGCATCTGCTGGATGTG
AGGACTTGCATTGTGAAAGCTTTGCTGTCCTTGATGTGATCATGGAATCTCTTTCTCACT
AGAGTCTATGTCACTCATTATACTCTGTCGAATGTCATTGAATGTCTTTACATGGGCTTA
TATGCCTATGAAAATTGTAATACAACTTTCAGCAACGGATCTCTTGGCTCTCGCATCGAT
GAAGGACGCAGCGAAATGCGATAAGTAATGTGAATTGCAGAATTCAGTGAATCATCGAAT
CTTTGAACGCATCTTGCGCTCCTTGGTATTCCGAGGAGCATGCCTGTTTGAGTGTCATTA
AATTCTCAACTCTCTTCTAC-TTTTTGTAAAAGAGAGCTTGGACTGTGGAGGCTTGCTGG
CCACTTTTTGGGGTCAGCTCCTCTGAAATGCATTAGCGGAACCGTTTGCGATCTGCCACA
AGTGTGATAAGTTATCTACACTGGCGAGGGGATTGCTCTCTGTAATGTTCAGCTTCTAAT
TGTCTCTACTTTGTGAGACTACTTTTGAATGCTTGACCTCAAATCAGGTAGGACTACCC-
GCTGAACTTAA
>BC2-26
TTTCCGTAGGTGAACCTGCGGAAGGATCATTATTGAATTATGTTTCTAGATAGGTTGTAG
CTGGCTCTTTAGAGCATGTGCACGCCTGTTTGGACTTCATTTTCATCCACCTGTGCACCT
ATTGTAGTCTTTGGTTGGGTTAGGAGGAAGTGGTCATTGTGTCAGCATCTGCTGGATGTG
AGGACTTGCATTGTGAAAGCTTTGCTGTCCTTGATGTGATCATGGAATCTCTTTCTCACT
AGAGTCTATGTCACTCATTATACTCTGTCGAATGTCATTGAATGTCTTTACATGGGCTTA
TATGCCTATGAAAATTGTAATACAACTTTCAGCAACGGATCTCTTGGCTCTCGCATCGAT
GAAGGACGCAGCGAAATGCGATAAGTAATGTGAATTGCAGAATTCAGTGAATCATCGAAT
CTTTGAACGCATCTTGCGCTCCTTGGTATTCCGAGGAGCATGCCTGTTTGAGTGTCATTA
AATTCTCAACTCTCTTCTAC-TTTTTGTAAAAGAGAGCTTGGACTGTGGAGGCTTGCTGG
CCACTTTTTGGGGTCAGCTCCTCTGAAATGCATTAGCGGAACCGTTTGCGATCTGCCACA
AGTGTGATAAGTTATCTACACTGGCGAGGGGATTGCTCTCTGTAATGTTCAGCTTCTAAT
TGTCTCTACTTTGTGAGACTACTTTTGAATGCTTGACCTCAAATCAGGTAGGACTACCC-
GCTGAACTTAA
>BC3-2
TTTCCGTAGGTGAACCTGCGGAAGGATCATTATTGAATTATGTTTCTAGATAGGTTGTAG
CTGGCTCTTTAGAGCATGTGCACGCCTGTTTGGACTTCATTTTCATCCACCTGTGCACCT
ATTGTAGTCTTTGGTTGGGTTAGGAGGAAGTGGTCATTGTGTCAGCATCTGCTGGATGTG
AGGACTTGCATTGTGAAAGCTTTGCTGTCCTTGATGTGATCATGGAATCTCTTTCTCACT
AGAGTCTATGTCACTCATTATACTCTGTCGAATGTCATTGAATGTCTTTACATGGGCTTA
TATGCCTATGAAAATTGTAATACAACTTTCAGCAACGGATCTCTTGGCTCTCGCATCGAT
GAAGGACGCAGCGAAATGCGATAAGTAATGTGAATTGCAGAATTCAGTGAATCATCGAAT

CTTTGAACGCATCTTGCGCTCCTTGGTATTCCGAGGAGCATGCCTGTTTGAGTGTCATTA
AATTCTCAACTCTCTTCTAC-TTTTTGTAAAAGAGAGCTTGGACTGTGGAGGCTTGCTGG
CCACTTTTTGGGGTCAGCTCCTCTGAAATGCATTAGCGGAACCGTTTGCGATCTGCCACA
AGTGTGATAAGTTATCTACACTGGCGAGGGGATTGCTCTCTGTAATGTTCAGCTTCTAAT
TGTCTCTACTTTGTGAGACTACTTTTGAATGCTTGACCTCAAATCAGGTAGGACTACCC-
GCTGAACTTAA
>BC8-5
TTTCCGTAGGTGAACCTGCGGAAGGATCATTATTGAATTATGTTTCTAGATAGGTTGTAG
CTGGCTCTTTAGAGCATGTGCACGCCTGTTTGGACTTCATTTTCATCCACCTGTGCACCT
ATTGTAGTCTTTGGTTGGGTTAGGAGGAAGTGGTCATTGTGTCAGCATCTGCTGGATGTG
AGGACTTGCATTGTGAAAGCTTTGCTGTCCTTGATGTGATCATGGAATCTCTTTCTCACT
AGAGTCTATGTCACTCATTATACTCTGTCGAATGTCATTGAATGTCTTTACATGGGCTTA
TATGCCTATGAAAATTGTAATACAACTTTCAGCAACGGATCTCTTGGCTCTCGCATCGAT
GAAGGACGCAGCGAAATGCGATAAGTAATGTGAATTGCAGAATTCAGTGAATCATCGAAT
CTTTGAACGCATCTTGCGCTCCTTGGTATTCCGAGGAGCATGCCTGTTTGAGTGTCATTA
AATTCTCAACTCTCTTCTAC-TTTTTGTAAAAGAGAGCTTGGACTGTGGAGGCTTGCTGG
CCACTTTTTGGGGTCAGCTCCTCTGAAATGCATTAGCGGAACCGTTTGCGATCTGCCACA
AGTGTGATAAGTTATCTACACTGGCGAGGGGATTGCTCTCTGTAATGTTCAGCTTCTAAT
TGTCTCTACTTTGTGAGACTACTTTTGAATGCTTGACCTCAAATCAGGTAGGACTACCC-
GCTGAACTTAA
>BC10_18
TTTCCGTAGGTGAACCTGCGGAAGGATCATTATTGAATTATGTTTCTAGATAGGTTGTAG
CTGGCTCTTTAGAGCATGTGCACGCCTGTTTGGACTTCATTTTCATCCACCTGTGCACCT
ATTGTAGTCTTTGGTTGGGTTAGGAGGAAGTGGTCATTGTGTCAGCATCTGCTGGATGTG
AGGACTTGCATTGTGAAAGCTTTGCTGTCCTTGATGTGATCATGGAATCTCTTTCTCACT
AGAGTCTATGTCACTCATTATACTCTGTCGAATGTCATTGAATGTCTTTACATGGGCTTA
TATGCCTATGAAAATTGTAATACAACTTTCAGCAACGGATCTCTTGGCTCTCGCATCGAT
GAAGGACGCAGCGAAATGCGATAAGTAATGTGAATTGCAGAATTCAGTGAATCATCGAAT
CTTTGAACGCATCTTGCGCTCCTTGGTATTCCGAGGAGCATGCCTGTTTGAGTGTCATTA
AATTCTCAACTCTCTTCTAC-TTTTTGTAAAAGAGAGCTTGGACTGTGGAGGCTTGCTGG
CCACTTTTTGGGGTCAGCTCCTCTGAAATGCATTAGCGGAACCGTTTGCGATCTGCCACA
AGTGTGATAAGTTATCTACACTGGCGAGGGGATTGCTCTCTGTAATGTTCAGCTTCTAAT
TGTCTCTACTTTGTGAGACTACTTTTGAATGCTTGACCTCAAATCAGGTAGGACTACCC-
GCTGAACTTAA
>BC11_11
TTTCCGTAGGTGAACCTGCGGAAGGATCATTATTGAATTATGTTTCTAGATAGGTTGTAG
CTGGCTCTTTAGAGCATGTGCACGCCTGTTTGGACTTCATTTTCATCCACCTGTGCACCT
ATTGTAGTCTTTGGTTGGGTTAGGAGGAAGTGGTCATTGTGTCAGCATCTGCTGGATGTG
AGGACTTGCATTGTGAAAGCTTTGCTGTCCTTGATGTGATCATGGAATCTCTTTCTCACT
AGAGTCTATGTCACTCATTATACTCTGTCGAATGTCATTGAATGTCTTTACATGGGCTTA
TATGCCTATGAAAATTGTAATACAACTTTCAGCAACGGATCTCTTGGCTCTCGCATCGAT
GAAGGACGCAGCGAAATGCGATAAGTAATGTGAATTGCAGAATTCAGTGAATCATCGAAT
CTTTGAACGCATCTTGCGCTCCTTGGTATTCCGAGGAGCATGCCTGTTTGAGTGTCATTA
AATTCTCAACTCTCTTCTAC-TTTTTGTAAAAGAGAGCTTGGACTGTGGAGGCTTGCTGG
CCACTTTTTGGGGTCAGCTCCTCTGAAATGCATTAGCGGAACCGTTTGCGATCTGCCACA
AGTGTGATAAGTTATCTACACTGGCGAGGGGATTGCTCTCTGTAATGTTCAGCTTCTAAT
TGTCTCTACTTTGTGAGACTACTTTTGAATGCTTGACCTCAAATCAGGTAGGACTACCC-
GCTGAACTTAA
>BC7-43
TTTCCGTAGGTGAACCTGCGGAAGGATCATTATTGAATTATGTTTCTAGATAGGTTGTAG

CTGGCTCTTTAGAGCATGTGCACGCCTGTTTGGACTTCATTTTCATCCACCTGTGCACCT
ATTGTAGTCTTTGGTTGGGTTAGGAGGAAGTGGTCATTGTGTCAGCATCTGCTGGATGTG
AGGACTTGCATTGTGAAAGCTTTGCTGTCCTTGATGTGATCATGGAATCTCTTTCTCACT
AGAGTCTATGTCACTCATTATACTCTGTCGAATGTCATTGAATGTCTTTACATGGGCTTG
TATGCCTATGAAAATTGTAATACAACTTTCAGCAACGGATCTCTTGGCTCTCGCATCGAT
GAAGGACGCAGCGAAATGCGATAAGTAATGTGAATTGCAGAATTCAGTGAATCATCGAAT
CTTTGAACGCATCTTGCGCTCCTTGGTATTCCGAGGAGCATGCCTGTTTGAGTGTCATTA
AATTCTCAACTCTCTTCTAC-TTTTTGTAAAAGAGAGCTTGGACTGTGGAGGCTTGCTGG
CCACTTTTTGGGGTCAGCTCCTCTGAAATGCATTAGCGGAACCGTTTGCGATCTGCCACA
AGTGTGATAAGTTATCTACACTGGCGAGGGGATTGCTCTCTGTAATGTTCAGCTTCTAAT
TGTCTCTACTTTGTGAGACTACTTTTGAATGCTTGACCTCAAATCAGGTAGGACTACCC-
GCTGAACTTAA
>BC11_30
TTTCCGTAGGTGAACCTGCGGAAGGATCATTATTGAATTATGTTTCTAGATAGGTTGTAG
CTGGCTCTTTAGAGCATGTGCACGCCTGTTTGGACTTCATTTTCATCCACCTGTGCACCT
ATTGTAGTCTTTGGTTGGGTTAGGAGGAAGTGGTCATTGTGTCAGCATCTGCTGGATGTG
AGGACTTGCATTGTGAAAGCTTTGCTGTCCTTGATGTGATCATGGAATCTCTTTCTCACT
AGAGTCTATGTCACTCATTATACTCTGTCGAATGTCATTGAATGTCTTTACATGGGCTTG
TATGCCTATGAAAATTGTAATACAACTTTCAGCAACGGATCTCTTGGCTCTCGCATCGAT
GAAGGACGCAGCGAAATGCGATAAGTAATGTGAATTGCAGAATTCAGTGAATCATCGAAT
CTTTGAACGCATCTTGCGCTCCTTGGTATTCCGAGGAGCATGCCTGTTTGAGTGTCATTA
AATTCTCAACTCTCTTCTAC-TTTTTGTAAAAGAGAGCTTGGACTGTGGAGGCTTGCTGG
CCACTTTTTGGGGTCAGCTCCTCTGAAATGCATTAGCGGAACCGTTTGCGATCTGCCACA
AGTGTGATAAGTTATCTACACTGGCGAGGGGATTGCTCTCTGTAATGTTCAGCTTCTAAT
TGTCTCTACTTTGTGAGACTACTTTTGAATGCTTGACCTCAAATCAGGTAGGACTACCC-
GCTGAACTTAA
>BC1-11
TTTCCGTAGGTGAACCTGCGGAAGGATCATTATTGAATTATGTTTCTAGATAGGTTGTAG
CTGGCTCTTTAGAGCATGTGCACGCCTGTTTGGACTTCATTTTCATCCACCTGTGCACCT
ATTGTAGTCTTTGGTTGGGTTAGGAGGAAGTGGTCATTGTGTCAGCATCTGCTGGATGTG
AGGACTTGCATTGTGAAAGCTTTGCTGTCCTTGATGTGATCATGGAATCTCTTTCTCACT
AGAGTCTATGTCACTCATTATACTCTGTCGAATGTCATTGAATGTCTTTACATGGGCTTA
TATGCCTATGAAAATTGTAATACAACTTTCAGCAACGGATCTCTTGGCTCTCGCATCGAT
GAAGGACGCAGCGAAATGCGATAAGTAATGTGAATTGCAGAATTCAGTGAATCATCGAAT
CTTTGAACGCATCTTGCGCTCCTTGGTATTCCGAGGAGCATGCCTGTTTGAGTGTCATTA
AATTCTCAACTCTCTTCTAC-TTTTTGTAAAAGAGAGCTTGGACTGTGGAGGCTTGCTGG
CCACTTTTTGGGGTCAGCTCCTCTGAAATGCATTAGCGGAACCGTTTGCAATCTGCCACA
AGTGTGATAAGTTATCTACACTGGCGAGGGGATTGCTCTCTGTAATGTTCAGCTTCTAAT
TGTCTCTACTTTGTGAGACTACTTTTGAATGCTTGACCTCAAATCAGGTAGGACTACCC-
GCTGAACTTAA
>BC4-42
TTTCCGTAGGTGAACCTGCGGAAGGATCATTATTGAATTATGTTTCTAGATAGGTTGTAG
CTGGCTCTTTAGAGCATGTGCACGCCTGTTTGGACTTCATTTTCATCCACCTGTGCACCT
ATTGTAGTCTTTGGTTGGGTTAGGAGGAAGTGGTCATTGTGTCAGCATCTGCTGGATGTG
AGGACTTGCATTGTGAAAGCTTTGCTGTCCTTGATGTGATCATGGAATCTCTTTCTCACT
AGAGTCTATGTCACTCATTATACTCTGTCGAATGTCATTGAATGTCTTTACATGGGCTTA
TATGCCTATGAAAATTGTAATACAACTTTCAGCAACGGATCTCTTGGCTCTCGCATCGAT
GAAGAACGCAGCGAAATGCGATAAGTAATGTGAATTGCAGAATTCAGTGAATCATCGAAT
CTTTGAACGCATCTTGCGCTCCTTGGTATTCCGAGGAGCATGCCTGTTTGAGTGTCATTA
AATTCTCAACTCTCTTCTAC-TTTTTGTAAAAGAGAGCTTGGACTGTGGAGGCTTGCTGG

```
CCACTTTTTGGGGTCAGCTCCTCTGAAATGCATTAGCGGAACCGTTTGCAATCTGCCACA
AGTGTGATAAGTTATCTACACTGGCGAGGGGATTGCTCTCTGTAATGTTCAGCTTCTAAT
TGTCTCTACTTTGTGAGACTACTTTTGAATGCTTGACCTCAAATCAGGTAGGACTACCC-
GCTGAACTTAA
>BC12_25
TTTCCGTAGGTGAACCTGCGGAAGGATCATTATTGAATTATGTTTCTAGATAGGTTGTAG
CTGGCTCTTTAGAGCATGTGCACGCCTGTTTGGACTTCATTTTCATCCACCTGTGCACCT
ATTGTAGTCTTTGGTTGGGTTAGGAGGAAGTGGTCATTGTGTCAGCATCTGCTGGATGTG
AGGACTTGCATTGTGAAAGCTTTGCTGTCCTTGATGTGATCATGGAATCTCTTTCTCACT
AGAGTCTATGTCACTCATTATACTCTGTCGAATGTCATTGAATGTCTTTACATGGGCTTA
TATGCCTATGAAAATTGTAATACAACTTTCAGCAACGGATCTCTTGGCTCTCGCATCGAT
GAAGAACGCAGCGAAATGCGATAAGTAATGTGAATTGCAGAATTCAGTGAATCATCGAAT
CTTTGAACGCATCTTGCGCTCCTTGGTATTCCGAGGAGCATGCCTGTTTGAGTGTCATTA
AATTCTCAACTCTCTTCTAC-TTTTTGTAAAAGAGAGCTTGGACTGTGGAGGCTTGCTGG
CCACTTTTTGGGGTCAGCTCCTCTGAAATGCATTAGCGGAACCGTTTGCAATCTGCCACA
AGTGTGATAAGTTATCTACACTGGCGAGGGGATTGCTCTCTGTAATGTTCAGCTTCTAAT
TGTCTCTACTTTGTGAGACTACTTTTGAATGCTTGACCTCAAATCAGGTAGGACTACCC-
GCTGAACTTAA
>BC9-17
TTTCCGTAGGTGAACCTGCGGAAGGATCATTATTGAATTATGTTTCTAGATAGGTTGTAG
CTGGCTCTTTAGAGCATGTGCACGCCTGTTTGGACTTCATTTTCATCCACCTGTGCACCT
ATTGTAGTCTTTGGTTGGGTTAGGGGGAAGTGGTCATTGTGTCAGCATCTGCTGGATGTG
AGGACTTGCATTGTGAAAGCTTTGCTGTCCTTGATGTGATCATGGAATCTCTTTCTCACT
AGAGTCTATGTCACTCATTATACTCTGTCGAATGTCATTGAATGTCTTTACATGGGCTTA
TATGCCTATGAAAATTGTAATACAACTTTCAGCAACGGATCTCTTGGCTCTCGCATCGAT
GAAGAACGCAGCGAAATGCGATAAGTAATGTGAATTGCAGAATTCAGTGAATCATCGAAT
CTTTGAACGCATCTTGCGCTCCTTGGTATTCCGAGGAGCATGCCTGTTTGAGTGTCATTA
AATTCTCAACTCTCTTCTAC-TTTTTGTAAAAGAGAGCTTGGACTGTGGAGGCTTGCTGG
CCACTTTTTGGGGTCAGCTCCTCTGAAATGCATTAGCGGAACCGTTTGCAATCTGCCACA
AGTGTGATAAGTTATCTACACTGGCGAGGGGATTGCTCTCTGTAATGTTCAGCTTCTAAT
TGTCTCTACTTTGTGAGACTACTTTTGAATGCTTGACCTCAAATCAGGTAGGACTACCC-
GCTGAACTTAA
>BC7-7
TTTCCGTAGGTGAACCTGCGGAAGGATCATTATTGAATTATGTTTCTAGATAGGTTGTAG
CTGGCTCTTTAGAGCATGTGCACGCCTGTTTGGACTTCATTTTCATCCACCTGTGCACCT
ATTGTAGTCTTTGGTTGGGTTAGGGGGAAGTGGTCATTGTGTCAGCATCTGCTGGATGTG
AGGACTTGCATTGTGAAAGCTTTGCTGTCCTTGATGTGATCATGGAATCTCTTTCTCACT
AGAGTCTATGTCACTCATTATACTCTGTCGAATGTCATTGAATGTCTTTACATGGGCTTG
TATGCCTATGAAAATTGTAATACAACTTTCAGCAACGGATCTCTTGGCTCTCGCATCGAT
GAAGGACGCAGCGAAATGCGATAAGTAATGTGAATTGCAGAATTCAGTGAATCATCGAAT
CTTTGAACGCATCTTGCGCTCCTTGGTATTCCGAGGAGCATGCCTGTTTGAGTGTCATTA
AATTCTCAACTCTCTTCTAC-TTTTTGTAAAAGAGAGCTTGGACTGTGGAGGCTTGCTGG
CCACTTTTTGGGGTCAGCTCCTCTGAAATGCATTAGCGGAACCGTTTGCAATCTGCCACA
AGTGTGATAAGTTATCTACACTGGCGAGGGGATTGCTCTCTGTAATGTTCAGCTTCTAAT
TGTCTCTACTTTGTGAGACTACTTTTGAATGCTTGACCTCAAATCAGGTAGGACTACCC-
GCTGAACTTAA
>BC10_43
TTTCCGTAGGTGAACCTGCGGAAGGATCATTATTGAATTATGTTTCTAGATAGGTTGTAG
CTGGCTCTTTAGAGCATGTGCACGCCTGTTTGGACTTCATTTTCATCCACCTGTGCACCT
ATTGTAGTCTTTGGTTGGGTTAGGGGGAAGTGGTCATTGTGTCAGCATCTGCTGGATGTG
```

AGGACTTGCATTGTGAAAGCTTTGCTGTCCTTGATGTGATCATGGAATCTCTTTCTCACT
AGAGTCTATGTCACTCATTATACTCTGTCGAATGTCATTGAATGTCTTTACATGGGCTTG
TATGCCTATGAAAATTGTAATACAACTTTCAGCAACGGATCTCTTGGCTCTCGCATCGAT
GAAGGACGCAGCGAAATGCGATAAGTAATGTGAATTGCAGAATTCAGTGAATCATCGAAT
CTTTGAACGCATCTTGCGCTCCTTGGTATTCCGAGGAGCATGCCTGTTTGAGTGTCATTA
AATTCTCAACTCTCTTCTAC-TTTTTGTAAAAGAGAGCTTGGACTGTGGAGGCTTGCTGG
CCACTTTTTGGGGTCAGCTCCTCTGAAATGCATTAGCGGAACCGTTTGCAATCTGCCACA
AGTGTGATAAGTTATCTACACTGGCGAGGGGATTGCTCTCTGTAATGTTCAGCTTCTAAT
TGTCTCTACTTTGTGAGACTACTTTTGAATGCTTGACCTCAAATCAGGTAGGACTACCC-
GCTGAACTTAA
>BC11_39
TTTCCGTAGGTGAACCTGCGGAAGGATCATTATTGAATTATGTTTCTAGATAGGTTGTAG
CTGGCTCTTTAGAGCATGTGCACGCCTGTTTGGACTTCATTTTCATCCACCTGTGCACCT
ATTGTAGTCTTTGGTTGGGTTAGGGGGAAGTGGTCATTGTGTCAGCATCTGCTGGATGTG
AGGACTTGCATTGTGAAAGCTTTGCTGTCCTTGATGTGATCATGGAATCTCTTTCTCACT
AGAGTCTATGTCACTCATTATACTCTGTCGAATGTCATTGAATGTCTTTACATGGGCTTG
TATGCCTATGAAAATTGTAATACAACTTTCAGCAACGGATCTCTTGGCTCTCGCATCGAT
GAAGAACGCAGCGAAATGCGATAAGTAATGTGAATTGCAGAATTCAGTGAATCATCGAAT
CTTTGAACGCATCTTGCGCTCCTTGGTATTCCGAGGAGCATGCCTGTTTGAGTGTCATTA
AATTCTCAACTCTCTTCTAC-TTTTTGTAAAAGAGAGCTTGGACTGTGGAGGCTTGCTGG
CCACTTTTTGGGGTCAGCTCCTCTGAAATGCATTAGCGGAACCGTTTGCAATCTGCCACA
AGTGTGATAAGTTATCTACACTGGCGAGGGGATTGCTCTCTGTAATGTTCAGCTTCTAAT
TGTCTCTACTTTGTGAGACTACTTTTGAATGCTTGACCTCAAATCAGGTAGGACTACCC-
GCTGAACTTAA
>BC10_7
TTTCCGTAGGTGAACCTGCGGAAGGATCATTATTGAATTATGTTTCTAGATAGGTTGTAG
CTGGCTCTTTAGAGCATGTGCACGCCTGTTTGGACTTCATTTTCATCCACCTGTGCACCT
ATTGTAGTCTTTGGTTGGGTTAGGAGGAAGTGGTCATTGTGTCAGCATCTGCTGGATGTG
AGGACTTGCATTGTGAAAGCTTTGCTGTCCTTGATGTGATCATGGAATCTCTTTCTCACT
AGAGTCTATGTCACTCATTATACTCTGTCGAATGTCATTGAATGTCTTTACATGGGCTTA
TATGCCTATGAAAATTGTAATACAACTTTCAGCAACGGATCTCTTGGCTCTCGCATCGAT
GAAGAACGCAGCGAAATGCGATAAGTAATGTGAATTGCAGAATTCAGTGAATCATCGAAT
CTTTGAACGCATCTTGCGCTCCTTGGTATTCCGAGGAGCATGCCTGTTTGAGTGTCATTA
AATTCTCAACTCTCTTCTAC-TTTTTGTAAAAGAGAGCTTGGACTGTGGAGGCTTGCTGA
CCACTTTTTGGGGTCAGCTCCTCTGAAATGCATTAGCGGAACCGTTTGCGATCTGCCACA
AGTGTGATAAGTTATCTACACTGGCGAGGGGATTGCTCTCTGTAATGTTCAGCTTCTAAT
TGTCTCTACTTTGTGAGACTACTTTTGAATGCTTGACCTCAAATCAGGTAGGACTACCC-
GCTGAACTTAA
>BC7-35
TTTCCGTAGGTGAACCTGCGGAAGGATCATTATTGAATTATGTTTCTAGATAGGTTGTAG
CTGGCTCTTTAGAGCATGTGCACGCCTGTTTGGACTTCATTTTCATCCACCTGTGCACCT
ATTGTAGTCTTTGGTTGGGTTAGGAGGAAGTGGTCATTGTGTCAGCATCTGCTGGATGTG
AGGACTTGCATTGTGAAAGCTTTGCTGTCCTTGATGTGATCATGGAATCTCTTTCTCACT
AGAGTCTATGTCACTCATTATACTCTGTCGAATGTCATTGAATGTCTTTACATGGGCTTA
TATGCCTATGAAAATTGTAATACAACTTTCAGCAACGGATCTCTTGGCTCTCGCATCGAT
GAAGAACGCAGCGAAATGCGATAAGTAATGTGAATTGCAGAATTCAGTGAATCATCGAAT
CTTTGAACGCATCTTGCGCTCCTTGGTATTCCGAGGAGCATGCCTGTTTGAGTGTCATTA
AATTCTCAACTCTCTTCTAC-TTTTTGTAAAAGAGAGCTTGGACTGTGGAGGCTTGCTGG
CCACTTTTTGGGATCAGCTCCTCTGAAATGCATTAGCGGAACCGTTTGCGATCTGCCACA
AGTGTGATAAGTTATCTACACTGGCGAGGGGATTGCTCTCTGTAATGTTCAGCTTCTAAT

TGTCTCTACTTTGTGAGACTACTTTTGAATGCTTGACCTCAAATCAGGTAGGACTACCC-
GCTGAACTTAA
>BC12_42
TTTCCGTAGGTGAACCTGCGGAAGGATCATTATTGAATTATGTTTCTAGATAGGTTGTAG
CTGGCTCTTTAGAGCATGTGCACGCCTGTTTGGACTTCATTTTCATCCACCTGTGCACCT
ATTGTAGTCTTTGGTTGGGTTAGGAGGAAGTGGTCATTGTGTCAGCATCTGCTGGATGTG
AGGACTTGCATTGTGAAAGCTTTGCTGTCCTTGATGTGATCATGGAATCTCTTTCTCACT
AGAGTCTATGTCACTCATTATACTCTGTCGAATGTCATTGAATGTCTTTACATGGGCTTA
TATGCCTATGAAAATTGTAATACAACTTTCAGCAACGGATCTCTTGGCTCTCGCATCGAT
GAAGAACGTAGCGAAATGCGATAAGTAATGTGAATTGCAGAATTCAGTGAATCATCGAAT
CTTTGAACGCATCTTGCGCTCCTTGGTATTCCGAGGAGCATGCCTGTTTGAGTGTCATTA
AATTCTCAACTCTCTTCTAC-TTTTTGTAAAAGAGAGCTTGGACTGTGGAGGCTTGCTGG
CCACTTTTTGGGGTCAGCTCCTCTGAAATGCATTAGCGGAACCGTTTGCGATCTGCCACA
AGTGTGATAAGTTATCTACACTGGCGAGGGGATTGCTCTCTGTAATGTTCAGCTTCTAAT
TGTCTCTACTTTGTGAGACTACTTTTGAATGCTTGACCTCAAATCAGGTAGGACTACCC-
GCTGAACTTAA
>BC5-53
TTTCCGTAGGTGAACCTGCGGAAGGATCATTATTGAATTATGTTTCTAGATAGGTTGTAG
CTGGCTCTTTAGAGCATGTGCACGCCTGTTTGGACTTCATTTTCATCCACCTGTGCACCT
ATTGTAGTCTTTGGTTGGGTTAGGGGGAAGTGGTCATTGTGTCAGCATCTGTTGGATGTG
AGGACTTGCATTGTGAAAGCTTTGCTGTCCTTGATGTGATCATGGAATCTCTTTCTCACT
AGAGTCTATGTCACTCATTATACTCTGTCGAATGTCATTGAATGTCTTTACATGGGCTTG
TATGCCTATGAAAATTGTAATACAACTTTCAGCAACGGATCTCTTGGCTCTCGCATCGAT
GAAGGACGCAGCGAAATGCGATAAGTAATGTGAATTGCAGAATTCAGTGAATCATCGAAT
CTTTGAACGCATCTTGCGCTCCTTGGTATTCCGAGGAGCATGCCTGTTTGAGTGTCATTA
AATTCTCAACTCTCTTCTAC-TTTTTGTAAAAGAGAGCTTGGACTGTGGAGGCTTGCTGG
CCACTTTTTGGGGTCAGCTCCTCTGAAATGCATTAGCGGAACCGTTTGCGATCTGCCACA
AGTGTGATAAGTTATCTACACTGGCGAGGGGATTGCTCTCTGTAATGTTCAGCTTCTAAT
TGTCTCTACTTTGTGAGACTACTTTTGAATGCTTGACCTCAAATCAGGTAGGACTACCC-
GCTGAACTTAA
>BC6-25
TTTCCGTAGGTGAACCTGCGGAAGGATCATTATTGAATTATGTTTCTAGATAGGTTGTAG
CTGGCTCTTTAGAGCATGTGCACGCCTGTTTGGACTTCATTTTCATCCACCTGTGCACCT
ATTGTAGTCTTTGGTTGGGTTAGGGGGAAGTGGTCATTGTGTCAGCATCTGCTGGATGTG
AGGACTTGCATTGTGAAAGCTTTGCTGTCCTTGATGTGATCATGGAATCTCTTTCTCACT
AGAGTCTATGTCACTCATTATACTCTGTCGAATGTCATTGAATGTCTTTACATGGGCTTG
TATGCCTATGAAAATTGTAATACAACTTTCAGCAACGGATCTCTTGGCTCTCGCATCGAT
GAAGGACGCAGCGAAATGCGATAAGTAATGTGAATTGCAGAATTCAGTGAATCATCGAAT
CTTTGAACGCATCTTGCGCTCCTTGGTATTCCGAGGAGCATGCCTGTTTGAGTGTCATTA
AATTCTCAACTCTCTTATAC-TTTTTGTAAAAGAGAGCTTGGACTGTGGAGGCTTGCTGG
CCACTTTTTGGGGTCAGCTCCTCTGAAATGCATTAGCGGAACCGTTTGCGATCTGCCACA
AGTGTGATAAGTTATCTACACTGGCGAGGGGATTGCTCTCTGTAATGTTCAGCTTCTAAT
TGTCTCTACTTTGTGAGACTACTTTTGAATGCTTGACCTCAAATCAGGTAGGACTACCC-
GCTGAACTTAA
>BC9-10
TTTCCGTAGGTGAACCTGCGGAAGGATCATTATTGAATTATGTTTCTAGATAGGTTGTAG
CTGGCTCTTTAGAGCATGTGCACGCCTGTTTGGACTTCATTTTCATCCACCTGTGCACCT
ATTGTAGTCTTTGGTTGGGTTAGGGGGAAGTGGTCATTGTGTCAGCATCTGCTGGATGTG
AGGACTTGCATTGTGAAAGCTTTGCTGTCCTTGATGTGATCATGGAATCTCTTTCTCACT
AGAGTCTATGTCACTCATTATACTCTGTCGAATGTCATTGAATGTCTTTACATGGGCTTG

TATGCCTATGAAAATTGTAATACAACTTTCAGCAACGGATCTCTTGGCTCTCGCATCGAT
GAAGGACGCAGCGAAATGCGATAAGTAATGTGAATTGCAGAATTCAGTGAATCATCGAAT
CTTTGAACGCATCTTGCGCTCCTTGGTATTCCGAGGAGCATGCCTGTTTGAGTGTCATTA
AATTCTCAACTCTCTTATAC-TTTTTGTAAAAGAGAGCTTGGACTGTGGAGGCTTGCTGG
CCACTTTTTGGGGTCAGCTCCTCTGAAATGCATTAGCGGAACCGTTTGCGATCTGCCACA
AGTGTGATAAGTTATCTACACTGGCGAGGGGATTGCTCTCTGTAATGTTCAGCTTCTAAT
TGTCTCTACTTTGTGAGACTACTTTTGAATGCTTGACCTCAAATCAGGTAGGACTACCC-
GCTGAACTTAA
>BC2-43
TTTCCGTAGGTGAACCTGCGGAAGGATCATTATTGAATTATGTTTCTAGATAGGTTGTAG
CTGGCTCTTTAGAGCATGTGCACGCCTGTTTGGACTTCATTTTCATCCACCTGTGCACCT
ATTGTAGTCTTTGGTTGGGTTAGGAGGAAGTGGTCATTGTGTCAGCATCTGCTGGATGTG
AGGACTTGCATTGTGAAAGCTTTGCTGTCCTTGATGTGATCATGGAATCTCTTTCTCACT
AGAGTCTATGTCACTCATTATACTCTGTCGAATGTCATTGAATGTCTTTACATGGGCTTA
TATGCCTATGAAAATTGTAATACAACTTTCAGCAACGGATCTCTTGGCTCTCGCATCGAT
GAAGAACGCAGCGAAATGCGATAAGTAATGTGAATTGCAGAATTCAGTGAATCATCGAAT
CTTTGAACGCATCTTGCGCTCCTTGGTATTCCGAGGAGCATGCCTGTTTGAGTGTCATTA
AATTCTCAACTCTCTTATAC-TTTTTGTAAAAGAGAGCTTGGACTGTGGAGGCTTGCTGG
CCACTTTTTGGGGTCAGCTCCTCTGAAATGCATTAGCGGAACCGTTTGCGATCTGCCACA
AGTGTGATAAGTTATCTACACTGGCGAGGGGATTGCTCTCTGTAATGTTCAGCTTCTAAT
TGTCTCTACTTTGTGAGACTACTTTTGAATGCTTGACCTCAAATCAGGTAGGACTACCC-
GCTGAACTTAA
>BC11_51
TTTCCGTAGGTGAACCTGCGGAAGGATCATTATTGAATTATGTTTCTAGATAGGTTGTAG
CTGGCTCTTTAGAGCATGTGCACGCCTGTTTGGACTTCATTTTCATCCACCTGTGCACCT
ATTGTAGTCTTTGGTTGGGTTAGGAGGAAGTGGTCATTGTGTCAGCATCTGCTGGATGTG
AGGACTTGCATTGTGAAAGCTTTGCTGTCCTTGATGTGATCATGGAATCTCTTTCTCACT
AGAGTCTATGTCACTCATTATACTCTGTCGAATGTCATTGAATGTCTTTACATGGGCTTA
TATGCCTATGAAAATTGTAATACAACTTTCAGCAACGGATCTCTTGGCTCTCGCATCGAT
GAAGAACGCAGCGAAATGCGATAAGTAATGTGAATTGCAGAATTCAGTGAATCATCGAAT
CTTTGAACGCATCTTGCGCTCCTTGGTATTCCGAGGAGCATGCCTGTTTGAGTGTCATTA
AATTCTCAACTCTCTTATAC-TTTTTGTAAAAGAGAGCTTGGACTGTGGAGGCTTGCTGG
CCACTTTTTGGGGTCAGCTCCTCTGAAATGCATTAGCGGAACCGTTTGCGATCTGCCACA
AGTGTGATAAGTTATCTACACTGGCGAGGGGATTGCTCTCTGTAATGTTCAGCTTCTAAT
TGTCTCTACTTTGTGAGACTACTTTTGAATGCTTGACCTCAAATCAGGTAGGACTACCC-
GCTGAACTTAA
>BC5-28
TTTCCGTAGGTGAACCTGCGGAAGGATCATTATTGAATTATGTTTCTAGATAGGTTGTAG
CTGGCTCTTTAGAGCATGTGCACGCCTGTTTGGACTTCATTTTCATCCACCTGTGCACCT
ATTGTAGTCTTTGGTTGGGTTAGGAGGAAGTGGTCATTGTGTCAGCATCTGCTGGATGTG
AGGACTTGCATTGTGAAAGCTTTGCTGTCCTTGATGTGATCATGGAATCTCTTTCTCACT
AGAGTCTATGTCACTCATTATACTCTGTCGAATGTCATTGAATGTCTTTACATGGGCTTA
TATGCCTATGAAAATTGTAATACAACTTTCAGCAACGGATCTCTTGGCTCTCGCATCGAT
GAAGGACGCAGCGAAATGCGATAAGTAATGTGAATTGCAGAATTCAGTGAATCATCGAAT
CTTTGAACGCATCTTGCGCTCCTTGGTATTCCGAGGAGCATGCCTGTTTGAGTGTCATTA
AATTCTCAACTCTCTTATAC-TTTTTGTAAAAGAGAGCTTGGACTGTGGAGGCTTGCTGG
CCACTTTTTGGGGTCAGCTCCTCTGAAATGCATTAGCGGAACCGTTTGCGATCTGCCACA
AGTGTGATAAGTTATCTACACTGGCGAGGGGATTGCTCTCTGTAATGTTCAGCTTCTAAT
TGTCTCTACTTTGTGAGACTACTTTTGAATGCTTGACCTCAAATCAGGTAGGACTACCC-
GCTGAACTTAA

>BC5–1
TTTCCGTAGGTGAACCTGCGGAAGGATCATTATTGAATTATGTTTCTAGATAGGTTGTAG
CTGGCTCTTTAGAGCATGTGCACGCCTGTTTGGACTTCATTTTCATCCACCTGTGCACCT
ATTGTAGTCTTTGGTTGGGTTAGGGGGAAGTGGTCATTGTGTCAGCATCTGCTGGATGTG
AGGACTTGCATTGTGAAAGCTTTGCTGTCCTTGATGTGATCATGGAATCTCTTTCTCACT
AGAGTCTATGTCACTCATTATACTCTGTCGAATGTCATTGAATGTCTTTACATGGGCTTG
TATGCCTATGAAAATTGTAATACAACTTTCAGCAACGGATCTCTTGGCTCTCGCATCGAT
GAAGGACGCAGCGAAATGCGATAAGTAATGTGAATTGCAGAATTCAGTGAATCATCGAAT
CTTTGAACGCATCTTGCGCTCCTTGGTATTCCGAGGAGCATGCCTGTTTGAGTGTCATTA
AATTCTCAACTCTCTTATAC–TTTTTGTAAAAGAGAGCTTGGACTGTGGAGGCTTGCTGG
CCACTTTTTGGGGTCAGCTCCTCTGAAATGCATTAGCGGAACCGTTTGCAATCTGCCACA
AGTGTGATAAGTTATCTACACTGGCGAGGGGATTGCTCTCTGTAATGTTCAGCTTCTAAT
TGTCTCTACTTTGTGAGACAACTTTTGAATGCTTGACCTCAAATCAGGTAGGACTACCC–
GCTGAACTTAA
>BC11_21
TTTCCGTAGGTGAACCTGCGGAAGGATCATTATTGAATTATGTTTCTAGATAGGTTGTAG
CTGGCTCTTTAGAGCATGTGCACGCCTGTTTGGACTTCATTTTCATCCACCTGTGCACCT
ATTGTAGTCTTTGGTTGGGTTAGGAGGAAGTGGTCATTGTGTCAGCATCTGCTGGATGTG
AGGACTTGCATTGTGAAAGCTTTGCTGTCCTTGATGTGATCATGGAATCTCTTTCTCACT
AGAGTCTATGTCACTCATTATACTCTGTCGAATGTCATTGAATGTCTTTACATGGGCTTG
TATGCCTATGAAAATTGTAATACAACTTTCAGCAACGGATCTCTTGGCTCTCGCATCGAT
GAAGGACGCAGCGAAATGCGATAAGTAATGTGAATTGCAGAATTCAGTGAATCATCGAAT
CTTTGAACGCATCTTGCGCTCCTTGGTATTCCGAGGAGCATGCCTGTTTGAGTGTCATTA
AATTCTCAACTCTCTTATAC–TTTTTGTAAAAGAGAGCTTGGACTGTGGAGGCTTGCTGG
CCACTTTTTGGGGTCAGCTCCTCTGAAATGCATTAGCGGAACCGTTTGCGATCTGCCACA
AGTGTGATAAGTTATCTACACTGGCGAGGGGATTGCTCTCTGTAATGTTCAGCTTCTAAT
TGTCTCTACTTTGTGAGACAACTTTTGAATGCTTGACCTCAAATCAGGTAGGACTACCC–
GCTGAACTTAA
>BC1–19
TTTCCGTAGGTGAACCTGCGGAAGGATCATTATTGAATTATGTTTCTAGATAGGTTGTAG
CTGGCTCTTTAGAGCATGTGCACGCCTGTTTGGACTTCATTTTCATCCACCTGTGCACCT
ATTGTAGTCTTTGGTTGGGTTAGGAGGAAGTGGTCATTGTGTCAGCATCTGCTGGATGTG
AGGACTTGCATTGTGAAAGCTTTGCTGTCCTTGATGTGATCATGGAATCTCTTTCTCACT
AGAGTCTATGTCACTCATTATACTCTGTCGAATGTCATTGAATGTCTTTACATGGGCTTA
TATGCCTATGAAAATTGTAATACAACTTTCAGCAACGGATCTCTTGGCTCTCGCATCGAT
GAAGGACGCAGCGAAATGCGATAAGTAATGTGAATTGCAGAATTCAGTGAATCATCGAAT
CTTTGAACGCATCTTGCGCTCCTTGGTATTCCGAGGAGCATGCCTGTTTGAGTGTCATTA
AATTCTCAACTCTCTTATAC–TTTTTGTAAAAGAGAGCTTGGACTGTGGAGGCTTGCTGG
CCACTTTTTGGGGTCAGCTCCTCTGAAATGCATTAGCGGAACCGTTTGCAATCTGCCACA
AGTGTGATAAGTTATCTACACTGGCGAGGGGATTGCTCTCTGTAATGTTCAGCTTCTAAT
TGTCTCTACTTTGTGAGACAACTTTTGAATGCTTGACCTCAAATCAGGTAgGACTACCC–
GCTGAACTTAA
>BC4–3
TTTCCGTAGGTGAACCTGCGGAAGGATCATTATTGAATTATGTTTCTAGATAGGTTGTAG
CTGGCTCTTTAGAGCATGTGCACGCCTGTTTGGACTTCATTTTCATCCACCTGTGCACCT
ATTGTAGTCTTTGGTTGGGTTAGGAGGAAGTGGTCATTGTGTCAGCATCTGCTGGATGTG
AGGACTTGCATTGTGAAAGCTTTGCTGTCCTTGATGTGATCATGGAATCTCTTTCTCACT
AGAGTCTATGTCACTCATTATACTCTGTCGAATGTCATTGAATGTCTTTACATGGGCTTA
TATGCCTATGAAAATTGTAATACAACTTTCAGCAACGGATCTCTTGGCTCTCGCATCGAT
GAAGAACGCAGCGAAATGCGATAAGTAATGTGAATTGCAGAATTCAGTGAATCATCGAAT

CTTTGAACGCATCTTGCGCTCCTTGGTATTCCGAGGAGCATGCCTGTTTGAGTGTCATTA
AATTCTCAACTCTCTTATAC-TTTTTGTAAAAGAGAGCTTGGACTGTGGAGGCTTGCTGG
CCACTTTTTGGGGTCAGCTCCTCTGAAATGCATTAGCGGAACCGTTTGCGATCTGCCACA
AGTGTGATAAGTTATCTACACTGGCGAGGGGATTGCTCTCTGTAATGTTCAGCTTCTAAT
TGTCTCTACTTTGTGAGACAACTTTTGAATGCTTGACCTCAAATCAGGTAGGACTACCC-
GCTGAACTTAA
>BC2-4
TTTCCGTAGGTGAACCTGCGGAAGGATCATTATTGAATTATGTTTCTAGATAGGTTGTAG
CTGGCTCTTTAGAGCATGTGCACGCCTGTTTGGACTTCATTTTCATCCACCTGTGCACCT
ATTGTAGTCTTTGGTTGGGTTAGGAGGAAGTGGTCATTGTGTCAGCATCTGCTGGATGTG
AGGACTTGCATTGTGAAAGCTTTGCTGTCCTTGATGTGATCATGGAATCTCTTTCTCACT
AGAGTCTATGTCACTCATTATACTCTGTCGAATGTCATTGAATGTCTTTACATGGGCTTA
TATGCCTATGAAAATTGTAATACAACTTTCAGCAACGGATCTCTTGGCTCTCGCATCGAT
GAAGAACGCAGCGAAATGCGATAAGTAATGTGAATTGCAGAATTCAGTGAATCATCGAAT
CTTTGAACGCATCTTGCGCTCCTTGGTATTCCGAGGAGCATGCCTGTTTGAGTGTCATTA
AATTCTCAACTCTCTTCTAC-TTTTTGTAAAAGAGAGCTTGGACTGTGGAGGCTTGCTGG
CCACTTTTTGGGGTCAGCTCCTCTGAAATGCATTAGCGGAACCGTTTGCGATCTGCCACA
AGTGTGATAAGTTATCTACACTGGCGAGGGGATTGCTCTCTGTAATGTTCAGCTTCTAAT
TGTCTCTACTTTGTGAGACAACTTTTGAATGCTTGACCTCAAATCAGGTAGGACTACCC-
GCTGAACTTAA
>BC2-24
TTTCCGTAGGTGAACCTGCGGAAGGATCATTATTGAATTATGTTTCTAGATAGGTTGTAG
CTGGCTCTTTAGAGCATGTGCACGCCTGTTTGGACTTCATTTTCATCCACCTGTGCACCT
ATTGTAGTCTTTGGTTGGGTTAGGAGGAAGTGGTCATTGTGTCAGCATCTGCTGGATGTG
AGGACTTGCATTGTGAAAGCTTTGCTGTCCTTGATGTGATCATGGAATCTCTTTCTCACT
AGAGTCTATGTCACTCATTATACTCTGTCGAATGTCATTGAATGTCTTTACATGGGCTTA
TATGCCTATGAAAATTGTAATACAACTTTCAGCAACGGATCTCTTGGCTCTCGCATCGAT
GAAGAACGCAGCGAAATGCGATAAGTAATGTGAATTGCAGAATTCAGTGAATCATCGAAT
CTTTGAACGCATCTTGCGCTCCTTGGTATTCCGAGGAGCATGCCTGTTTGAGTGTCATTA
AATTCTCAACTCTCTTCTAC-TTTTTGTAAAAGAGAGCTTGGACTGTGGAGGCTTGCTGG
CCACTTTTTGGGGTCAGCTCCTCTGAAATGCATTAGCGGAACCGTTTGCGATCTGCCACA
AGTGTGATAAGTTATCTACACTGGCGAGGGGATTGCTCTCTGTAATGTTCAGCTTCTAAT
TGTCTCTACTTTGTGAGACAACTTTTGAATGCTTGACCTCAAATCAGGTAGGACTACCC-
GCTGAACTTAA
>BC2-45
TTTCCGTAGGTGAACCTGCGGAAGGATCATTATTGAATTATGTTTCTAGATAGGTTGTAG
CTGGCTCTTTAGAGCATGTGCACGCCTGTTTGGACTTCATTTTCATCCACCTGTGCACCT
ATTGTAGTCTTTGGTTGGGTTAGGAGGAAGTGGTCATTGTGTCAGCATCTGCTGGATGTG
AGGACTTGCATTGTGAAAGCTTTGCTGTCCTTGATGTGATCATGGAATCTCTTTCTCACT
AGAGTCTATGTCACTCATTATACTCTGTCGAATGTCATTGAATGTCTTTACATGGGCTTA
TATGCCTATGAAAATTGTAATACAACTTTCAGCAACGGATCTCTTGGCTCTCGCATCGAT
GAAGAACGCAGCGAAATGCGATAAGTAATGTGAATTGCAGAATTCAGTGAATCATCGAAT
CTTTGAACGCATCTTGCGCTCCTTGGTATTCCGAGGAGCATGCCTGTTTGAGTGTCATTA
AATTCTCAACTCTCTTCTAC-TTTTTGTAAAAGAGAGCTTGGACTGTGGAGGCTTGCTGG
CCACTTTTTGGGGTCAGCTCCTCTGAAATGCATTAGCGGAACCGTTTGCGATCTGCCACA
AGTGTGATAAGTTATCTACACTGGCGAGGGGATTGCTCTCTGTAATGTTCAGCTTCTAAT
TGTCTCTACTTTGTGAGACAACTTTTGAATGCTTGACCTCAAATCAGGTAGGACTACCC-
GCTGAACTTAA
>BC2-50
TTTCCGTAGGTGAACCTGCGGAAGGATCATTATTGAATTATGTTTCTAGATAGGTTGTAG

CTGGCTCTTTAGAGCATGTGCACGCCTGTTTGGACTTCATTTTCATCCACCTGTGCACCT
ATTGTAGTCTTTGGTTGGGTTAGGAGGAAGTGGTCATTGTGTCAGCATCTGCTGGATGTG
AGGACTTGCATTGTGAAAGCTTTGCTGTCCTTGATGTGATCATGGAATCTCTTTCTCACT
AGAGTCTATGTCACTCATTATACTCTGTCGAATGTCATTGAATGTCTTTACATGGGCTTA
TATGCCTATGAAAATTGTAATACAACTTTCAGCAACGGATCTCTTGGCTCTCGCATCGAT
GAAGAACGCAGCGAAATGCGATAAGTAATGTGAATTGCAGAATTCAGTGAATCATCGAAT
CTTTGAACGCATCTTGCGCTCCTTGGTATTCCGAGGAGCATGCCTGTTTGAGTGTCATTA
AATTCTCAACTCTCTTCTAC-TTTTTGTAAAAGAGAGCTTGGACTGTGGAGGCTTGCTGG
CCACTTTTTGGGGTCAGCTCCTCTGAAATGCATTAGCGGAACCGTTTGCGATCTGCCACA
AGTGTGATAAGTTATCTACACTGGCGAGGGGATTGCTCTCTGTAATGTTCAGCTTCTAAT
TGTCTCTACTTTGTGAGACAACTTTTGAATGCTTGACCTCAAATCAGGTAGGACTACCC-
GCTGAACTTAA
>BC3-21
TTTCCGTAGGTGAACCTGCGGAAGGATCATTATTGAATTATGTTTCTAGATAGGTTGTAG
CTGGCTCTTTAGAGCATGTGCACGCCTGTTTGGACTTCATTTTCATCCACCTGTGCACCT
ATTGTAGTCTTTGGTTGGGTTAGGAGGAAGTGGTCATTGTGTCAGCATCTGCTGGATGTG
AGGACTTGCATTGTGAAAGCTTTGCTGTCCTTGATGTGATCATGGAATCTCTTTCTCACT
AGAGTCTATGTCACTCATTATACTCTGTCGAATGTCATTGAATGTCTTTACATGGGCTTA
TATGCCTATGAAAATTGTAATACAACTTTCAGCAACGGATCTCTTGGCTCTCGCATCGAT
GAAGAACGCAGCGAAATGCGATAAGTAATGTGAATTGCAGAATTCAGTGAATCATCGAAT
CTTTGAACGCATCTTGCGCTCCTTGGTATTCCGAGGAGCATGCCTGTTTGAGTGTCATTA
AATTCTCAACTCTCTTCTAC-TTTTTGTAAAAGAGAGCTTGGACTGTGGAGGCTTGCTGG
CCACTTTTTGGGGTCAGCTCCTCTGAAATGCATTAGCGGAACCGTTTGCGATCTGCCACA
AGTGTGATAAGTTATCTACACTGGCGAGGGGATTGCTCTCTGTAATGTTCAGCTTCTAAT
TGTCTCTACTTTGTGAGACAACTTTTGAATGCTTGACCTCAAATCAGGTAGGACTACCC-
GCTGAACTTAA
>BC4-13
TTTCCGTAGGTGAACCTGCGGAAGGATCATTATTGAATTATGTTTCTAGATAGGTTGTAG
CTGGCTCTTTAGAGCATGTGCACGCCTGTTTGGACTTCATTTTCATCCACCTGTGCACCT
ATTGTAGTCTTTGGTTGGGTTAGGAGGAAGTGGTCATTGTGTCAGCATCTGCTGGATGTG
AGGACTTGCATTGTGAAAGCTTTGCTGTCCTTGATGTGATCATGGAATCTCTTTCTCACT
AGAGTCTATGTCACTCATTATACTCTGTCGAATGTCATTGAATGTCTTTACATGGGCTTA
TATGCCTATGAAAATTGTAATACAACTTTCAGCAACGGATCTCTTGGCTCTCGCATCGAT
GAAGAACGCAGCGAAATGCGATAAGTAATGTGAATTGCAGAATTCAGTGAATCATCGAAT
CTTTGAACGCATCTTGCGCTCCTTGGTATTCCGAGGAGCATGCCTGTTTGAGTGTCATTA
AATTCTCAACTCTCTTCTAC-TTTTTGTAAAAGAGAGCTTGGACTGTGGAGGCTTGCTGG
CCACTTTTTGGGGTCAGCTCCTCTGAAATGCATTAGCGGAACCGTTTGCGATCTGCCACA
AGTGTGATAAGTTATCTACACTGGCGAGGGGATTGCTCTCTGTAATGTTCAGCTTCTAAT
TGTCTCTACTTTGTGAGACAACTTTTGAATGCTTGACCTCAAATCAGGTAGGACTACCC-
GCTGAACTTAA
>BC4-24
TTTCCGTAGGTGAACCTGCGGAAGGATCATTATTGAATTATGTTTCTAGATAGGTTGTAG
CTGGCTCTTTAGAGCATGTGCACGCCTGTTTGGACTTCATTTTCATCCACCTGTGCACCT
ATTGTAGTCTTTGGTTGGGTTAGGAGGAAGTGGTCATTGTGTCAGCATCTGCTGGATGTG
AGGACTTGCATTGTGAAAGCTTTGCTGTCCTTGATGTGATCATGGAATCTCTTTCTCACT
AGAGTCTATGTCACTCATTATACTCTGTCGAATGTCATTGAATGTCTTTACATGGGCTTA
TATGCCTATGAAAATTGTAATACAACTTTCAGCAACGGATCTCTTGGCTCTCGCATCGAT
GAAGAACGCAGCGAAATGCGATAAGTAATGTGAATTGCAGAATTCAGTGAATCATCGAAT
CTTTGAACGCATCTTGCGCTCCTTGGTATTCCGAGGAGCATGCCTGTTTGAGTGTCATTA
AATTCTCAACTCTCTTCTAC-TTTTTGTAAAAGAGAGCTTGGACTGTGGAGGCTTGCTGG

CCACTTTTTGGGGTCAGCTCCTCTGAAATGCATTAGCGGAACCGTTTGCGATCTGCCACA
AGTGTGATAAGTTATCTACACTGGCGAGGGGATTGCTCTCTGTAATGTTCAGCTTCTAAT
TGTCTCTACTTTGTGAGACAACTTTTGAATGCTTGACCTCAAATCAGGTAGGACTACCC-
GCTGAACTTAA
>BC5-57
TTTCCGTAGGTGAACCTGCGGAAGGATCATTATTGAATTATGTTTCTAGATAGGTTGTAG
CTGGCTCTTTAGAGCATGTGCACGCCTGTTTGGACTTCATTTTCATCCACCTGTGCACCT
ATTGTAGTCTTTGGTTGGGTTAGGAGGAAGTGGTCATTGTGTCAGCATCTGCTGGATGTG
AGGACTTGCATTGTGAAAGCTTTGCTGTCCTTGATGTGATCATGGAATCTCTTTCTCACT
AGAGTCTATGTCACTCATTATACTCTGTCGAATGTCATTGAATGTCTTTACATGGGCTTA
TATGCCTATGAAAATTGTAATACAACTTTCAGCAACGGATCTCTTGGCTCTCGCATCGAT
GAAGAACGCAGCGAAATGCGATAAGTAATGTGAATTGCAGAATTCAGTGAATCATCGAAT
CTTTGAACGCATCTTGCGCTCCTTGGTATTCCGAGGAGCATGCCTGTTTGAGTGTCATTA
AATTCTCAACTCTCTTCTAC-TTTTTGTAAAAGAGAGCTTGGACTGTGGAGGCTTGCTGG
CCACTTTTTGGGGTCAGCTCCTCTGAAATGCATTAGCGGAACCGTTTGCGATCTGCCACA
AGTGTGATAAGTTATCTACACTGGCGAGGGGATTGCTCTCTGTAATGTTCAGCTTCTAAT
TGTCTCTACTTTGTGAGACAACTTTTGAATGCTTGACCTCAAATCAGGTAGGACTACCC-
GCTGAACTTAA
>BC5-97
TTTCCGTAGGTGAACCTGCGGAAGGATCATTATTGAATTATGTTTCTAGATAGGTTGTAG
CTGGCTCTTTAGAGCATGTGCACGCCTGTTTGGACTTCATTTTCATCCACCTGTGCACCT
ATTGTAGTCTTTGGTTGGGTTAGGAGGAAGTGGTCATTGTGTCAGCATCTGCTGGATGTG
AGGACTTGCATTGTGAAAGCTTTGCTGTCCTTGATGTGATCATGGAATCTCTTTCTCACT
AGAGTCTATGTCACTCATTATACTCTGTCGAATGTCATTGAATGTCTTTACATGGGCTTA
TATGCCTATGAAAATTGTAATACAACTTTCAGCAACGGATCTCTTGGCTCTCGCATCGAT
GAAGAACGCAGCGAAATGCGATAAGTAATGTGAATTGCAGAATTCAGTGAATCATCGAAT
CTTTGAACGCATCTTGCGCTCCTTGGTATTCCGAGGAGCATGCCTGTTTGAGTGTCATTA
AATTCTCAACTCTCTTCTAC-TTTTTGTAAAAGAGAGCTTGGACTGTGGAGGCTTGCTGG
CCACTTTTTGGGGTCAGCTCCTCTGAAATGCATTAGCGGAACCGTTTGCGATCTGCCACA
AGTGTGATAAGTTATCTACACTGGCGAGGGGATTGCTCTCTGTAATGTTCAGCTTCTAAT
TGTCTCTACTTTGTGAGACAACTTTTGAATGCTTGACCTCAAATCAGGTAGGACTACCC-
GCTGAACTTAA
>BC5-106
TTTCCGTAGGTGAACCTGCGGAAGGATCATTATTGAATTATGTTTCTAGATAGGTTGTAG
CTGGCTCTTTAGAGCATGTGCACGCCTGTTTGGACTTCATTTTCATCCACCTGTGCACCT
ATTGTAGTCTTTGGTTGGGTTAGGAGGAAGTGGTCATTGTGTCAGCATCTGCTGGATGTG
AGGACTTGCATTGTGAAAGCTTTGCTGTCCTTGATGTGATCATGGAATCTCTTTCTCACT
AGAGTCTATGTCACTCATTATACTCTGTCGAATGTCATTGAATGTCTTTACATGGGCTTA
TATGCCTATGAAAATTGTAATACAACTTTCAGCAACGGATCTCTTGGCTCTCGCATCGAT
GAAGAACGCAGCGAAATGCGATAAGTAATGTGAATTGCAGAATTCAGTGAATCATCGAAT
CTTTGAACGCATCTTGCGCTCCTTGGTATTCCGAGGAGCATGCCTGTTTGAGTGTCATTA
AATTCTCAACTCTCTTCTAC-TTTTTGTAAAAGAGAGCTTGGACTGTGGAGGCTTGCTGG
CCACTTTTTGGGGTCAGCTCCTCTGAAATGCATTAGCGGAACCGTTTGCGATCTGCCACA
AGTGTGATAAGTTATCTACACTGGCGAGGGGATTGCTCTCTGTAATGTTCAGCTTCTAAT
TGTCTCTACTTTGTGAGACAACTTTTGAATGCTTGACCTCAAATCAGGTAGGACTACCC-
GCTGAACTTAA
>BC6-13
TTTCCGTAGGTGAACCTGCGGAAGGATCATTATTGAATTATGTTTCTAGATAGGTTGTAG
CTGGCTCTTTAGAGCATGTGCACGCCTGTTTGGACTTCATTTTCATCCACCTGTGCACCT
ATTGTAGTCTTTGGTTGGGTTAGGAGGAAGTGGTCATTGTGTCAGCATCTGCTGGATGTG

AGGACTTGCATTGTGAAAGCTTTGCTGTCCTTGATGTGATCATGGAATCTCTTTCTCACT
AGAGTCTATGTCACTCATTATACTCTGTCGAATGTCATTGAATGTCTTTACATGGGCTTA
TATGCCTATGAAAATTGTAATACAACTTTCAGCAACGGATCTCTTGGCTCTCGCATCGAT
GAAGAACGCAGCGAAATGCGATAAGTAATGTGAATTGCAGAATTCAGTGAATCATCGAAT
CTTTGAACGCATCTTGCGCTCCTTGGTATTCCGAGGAGCATGCCTGTTTGAGTGTCATTA
AATTCTCAACTCTCTTCTAC-TTTTTGTAAAAGAGAGCTTGGACTGTGGAGGCTTGCTGG
CCACTTTTTGGGGTCAGCTCCTCTGAAATGCATTAGCGGAACCGTTTGCGATCTGCCACA
AGTGTGATAAGTTATCTACACTGGCGAGGGGATTGCTCTCTGTAATGTTCAGCTTCTAAT
TGTCTCTACTTTGTGAGACAACTTTTGAATGCTTGACCTCAAATCAGGTAGGACTACCC-
GCTGAACTTAA
>BC8-45
TTTCCGTAGGTGAACCTGCGGAAGGATCATTATTGAATTATGTTTCTAGATAGGTTGTAG
CTGGCTCTTTAGAGCATGTGCACGCCTGTTTGGACTTCATTTTCATCCACCTGTGCACCT
ATTGTAGTCTTTGGTTGGGTTAGGAGGAAGTGGTCATTGTGTCAGCATCTGCTGGATGTG
AGGACTTGCATTGTGAAAGCTTTGCTGTCCTTGATGTGATCATGGAATCTCTTTCTCACT
AGAGTCTATGTCACTCATTATACTCTGTCGAATGTCATTGAATGTCTTTACATGGGCTTA
TATGCCTATGAAAATTGTAATACAACTTTCAGCAACGGATCTCTTGGCTCTCGCATCGAT
GAAGAACGCAGCGAAATGCGATAAGTAATGTGAATTGCAGAATTCAGTGAATCATCGAAT
CTTTGAACGCATCTTGCGCTCCTTGGTATTCCGAGGAGCATGCCTGTTTGAGTGTCATTA
AATTCTCAACTCTCTTCTAC-TTTTTGTAAAAGAGAGCTTGGACTGTGGAGGCTTGCTGG
CCACTTTTTGGGGTCAGCTCCTCTGAAATGCATTAGCGGAACCGTTTGCGATCTGCCACA
AGTGTGATAAGTTATCTACACTGGCGAGGGGATTGCTCTCTGTAATGTTCAGCTTCTAAT
TGTCTCTACTTTGTGAGACAACTTTTGAATGCTTGACCTCAAATCAGGTAGGACTACCC-
GCTGAACTTAA
>BC9-31
TTTCCGTAGGTGAACCTGCGGAAGGATCATTATTGAATTATGTTTCTAGATAGGTTGTAG
CTGGCTCTTTAGAGCATGTGCACGCCTGTTTGGACTTCATTTTCATCCACCTGTGCACCT
ATTGTAGTCTTTGGTTGGGTTAGGAGGAAGTGGTCATTGTGTCAGCATCTGCTGGATGTG
AGGACTTGCATTGTGAAAGCTTTGCTGTCCTTGATGTGATCATGGAATCTCTTTCTCACT
AGAGTCTATGTCACTCATTATACTCTGTCGAATGTCATTGAATGTCTTTACATGGGCTTA
TATGCCTATGAAAATTGTAATACAACTTTCAGCAACGGATCTCTTGGCTCTCGCATCGAT
GAAGAACGCAGCGAAATGCGATAAGTAATGTGAATTGCAGAATTCAGTGAATCATCGAAT
CTTTGAACGCATCTTGCGCTCCTTGGTATTCCGAGGAGCATGCCTGTTTGAGTGTCATTA
AATTCTCAACTCTCTTCTAC-TTTTTGTAAAAGAGAGCTTGGACTGTGGAGGCTTGCTGG
CCACTTTTTGGGGTCAGCTCCTCTGAAATGCATTAGCGGAACCGTTTGCGATCTGCCACA
AGTGTGATAAGTTATCTACACTGGCGAGGGGATTGCTCTCTGTAATGTTCAGCTTCTAAT
TGTCTCTACTTTGTGAGACAACTTTTGAATGCTTGACCTCAAATCAGGTAGGACTACCC-
GCTGAACTTAA
>BC11_49
TTTCCGTAGGTGAACCTGCGGAAGGATCATTATTGAATTATGTTTCTAGATAGGTTGTAG
CTGGCTCTTTAGAGCATGTGCACGCCTGTTTGGACTTCATTTTCATCCACCTGTGCACCT
ATTGTAGTCTTTGGTTGGGTTAGGAGGAAGTGGTCATTGTGTCAGCATCTGCTGGATGTG
AGGACTTGCATTGTGAAAGCTTTGCTGTCCTTGATGTGATCATGGAATCTCTTTCTCACT
AGAGTCTATGTCACTCATTATACTCTGTCGAATGTCATTGAATGTCTTTACATGGGCTTA
TATGCCTATGAAAATTGTAATACAACTTTCAGCAACGGATCTCTTGGCTCTCGCATCGAT
GAAGAACGCAGCGAAATGCGATAAGTAATGTGAATTGCAGAATTCAGTGAATCATCGAAT
CTTTGAACGCATCTTGCGCTCCTTGGTATTCCGAGGAGCATGCCTGTTTGAGTGTCATTA
AATTCTCAACTCTCTTCTAC-TTTTTGTAAAAGAGAGCTTGGACTGTGGAGGCTTGCTGG
CCACTTTTTGGGGTCAGCTCCTCTGAAATGCATTAGCGGAACCGTTTGCGATCTGCCACA
AGTGTGATAAGTTATCTACACTGGCGAGGGGATTGCTCTCTGTAATGTTCAGCTTCTAAT

TGTCTCTACTTTGTGAGACAACTTTTGAATGCTTGACCTCAAATCAGGTAGGACTACCC-
GCTGAACTTAA
>BC12_32
TTTCCGTAGGTGAACCTGCGGAAGGATCATTATTGAATTATGTTTCTAGATAGGTTGTAG
CTGGCTCTTTAGAGCATGTGCACGCCTGTTTGGACTTCATTTTCATCCACCTGTGCACCT
ATTGTAGTCTTTGGTTGGGTTAGGAGGAAGTGGTCATTGTGTCAGCATCTGCTGGATGTG
AGGACTTGCATTGTGAAAGCTTTGCTGTCCTTGATGTGATCATGGAATCTCTTTCTCACT
AGAGTCTATGTCACTCATTATACTCTGTCGAATGTCATTGAATGTCTTTACATGGGCTTA
TATGCCTATGAAAATTGTAATACAACTTTCAGCAACGGATCTCTTGGCTCTCGCATCGAT
GAAGAACGCAGCGAAATGCGATAAGTAATGTGAATTGCAGAATTCAGTGAATCATCGAAT
CTTTGAACGCATCTTGCGCTCCTTGGTATTCCGAGGAGCATGCCTGTTTGAGTGTCATTA
AATTCTCAACTCTCTTCTAC-TTTTTGTAAAAGAGAGCTTGGACTGTGGAGGCTTGCTGG
CCACTTTTTGGGGTCAGCTCCTCTGAAATGCATTAGCGGAACCGTTTGCGATCTGCCACA
AGTGTGATAAGTTATCTACACTGGCGAGGGGATTGCTCTCTGTAATGTTCAGCTTCTAAT
TGTCTCTACTTTGTGAGACAACTTTTGAATGCTTGACCTCAAATCAGGTAGGACTACCC-
GCTGAACTTAA
>BC10_12
TTTCCGTAGGTGAACCTGCGGAAGGATCATTATTGAATTATGTTTCTAGATAGGTTGTAG
CTGGCTCTTTAGAGCATGTGCACGCCTGTTTGGACTTCATTTTCATCCACCTGTGCACCT
ATTGTAGTCTTTGGTTGGGTTAGGAGGAAGTGGTCATTGTGTCAGCATCTGCTGGATGTG
AGGACTTGCATTGTGAAAGCTTTGCTGTCCTTGATGTGATCATGGAATCTCTTTCTCACT
AGAGTCTATGTCACTCATTATACTCTGTCGAATGTCATTGAATGTCTTTACATGGGCTTA
TATGCCTATGAAAATTGTAATACAACTTTCAGCAACGGATCTCTTGGCTCTCGCATCGAT
GAAGAACGCAGCGAAATGCGATAAGTAATGTGAATTGCAGAATTCAGTGAATCATCGAAT
CTTTGAACGCATCTTGCGCTCCTTGGTATTCCGAGGAGCATGCCTGTTTGAGTGTCATTA
AATTCTCAACTCTCTTCTAC-TTTTTGTAAAAGAGAGCTTGGACTGTGGAGGCTTGCTGG
CCACTTTTTGGGGTCAGCTCCTCTGAAATGCATTAGCGGAACCGTTTGCGATCTGCCACA
AGTGTGATAAGTTATCTACACTGGCGAGGGGATTGCTCTCTGTAATGTTCAGCTTCTAAT
TGTCTCTACTTTGTGAGACAACTTTTGAATGCTTGACCTCAAATCAGGTAGGACTACCC-
GCTGAACTTAA
>BC1-17
TTTCCGTAGGTGAACCTGCGGAAGGATCATTATTGAATTATGTTTCTAGATAGGTTGTAG
CTGGCTCTTTAGAGCATGTGCACGCCTGTTTGGACTTCATTTTCATCCACCTGTGCACCT
ATTGTAGTCTTTGGTTGGGTTAGGAGGAAGTGGTCATTGTGTCAGCATCTGCTGGATGTG
AGGACTTGCATTGTGAAAGCTTTGCTGTCCTTGATGTGATCATGGAATCTCTTTCTCACT
AGAGTCTATGTCACTCATTATACTCTGTCGAATGTCATTGAATGTCTTTACATGGGCTTA
TATGCCTATGAAAATTGTAATACAACTTTCAGCAACGGATCTCTTGGCTCTCGCATCGAT
GAAGAACGCAGCGAAATGCGATAAGTAATGTGAATTGCAGAATTCAGTGAATCATCGAAT
CTTTGAACGCATCTTGCGCTCCTTGGTATTCCGAGGAGCATGCCTGTTTGAGTGTCATTA
AATTCTCAACTCTCTTCTAC-TTTTTGTAAAAGAGAGCTTGGACTGTGGAGGCTTGCTGG
CCACTTTTTGGGGTCAGCTCCTCTGAAATGCATTAGCGGAACCGTTTGCAATCTGCCACA
AGTGTGATAAGTTATCTACACTGGCGAGGGGATTGCTCTCTGTAATGTTCAGCTTCTAAT
TGTCTCTACTTTGTGAGACAACTTTTGAATGCTTGACCTCAAATCAGGTAGGACTACCC-
GCTGAACTTAA
>BC1-26
TTTCCGTAGGTGAACCTGCGGAAGGATCATTATTGAATTATGTTTCTAGATAGGTTGTAG
CTGGCTCTTTAGAGCATGTGCACGCCTGTTTGGACTTCATTTTCATCCACCTGTGCACCT
ATTGTAGTCTTTGGTTGGGTTAGGAGGAAGTGGTCATTGTGTCAGCATCTGCTGGATGTG
AGGACTTGCATTGTGAAAGCTTTGCTGTCCTTGATGTGATCATGGAATCTCTTTCTCACT
AGAGTCTATGTCACTCATTATACTCTGTCGAATGTCATTGAATGTCTTTACATGGGCTTA

TATGCCTATGAAAATTGTAATACAACTTTCAGCAACGGATCTCTTGGCTCTCGCATCGAT
GAAGAACGCAGCGAAATGCGATAAGTAATGTGAATTGCAGAATTCAGTGAATCATCGAAT
CTTTGAACGCATCTTGCGCTCCTTGGTATTCCGAGGAGCATGCCTGTTTGAGTGTCATTA
AATTCTCAACTCTCTTCTAC-TTTTTGTAAAAGAGAGCTTGGACTGTGGAGGCTTGCTGG
CCACTTTTTGGGGTCAGCTCCTCTGAAATGCATTAGCGGAACCGTTTGCAATCTGCCACA
AGTGTGATAAGTTATCTACACTGGCGAGGGGATTGCTCTCTGTAATGTTCAGCTTCTAAT
TGTCTCTACTTTGTGAGACAACTTTTGAATGCTTGACCTCAAATCAGGTAGGACTACCC-
GCTGAACTTAA
>BC6-21
TTTCCGTAGGTGAACCTGCGGAAGGATCATTATTGAATTATGTTTCTAGATAGGTTGTAG
CTGGCTCTTTAGAGCATGTGCACGCCTGTTTGGACTTCATTTTCATCCACCTGTGCACCT
ATTGTAGTCTTTGGTTGGGTTAGGAGGAAGTGGTCATTGTGTCAGCATCTGCTGGATGTG
AGGACTTGCATTGTGAAAGCTTTGCTGTCCTTGATGTGATCATGGAATCTCTTTCTCACT
AGAGTCTATGTCACTCATTATACTCTGTCGAATGTCATTGAATGTCTTTACATGGGCTTA
TATGCCTATGAAAATTGTAATACAACTTTCAGCAACGGATCTCTTGGCTCTCGCATCGAT
GAAGAACGCAGCGAAATGCGATAAGTAATGTGAATTGCAGAATTCAGTGAATCATCGAAT
CTTTGAACGCATCTTGCGCTCCTTGGTATTCCGAGGAGCATGCCTGTTTGAGTGTCATTA
AATTCTCAACTCTCTTCTAC-TTTTTGTAAAAGAGAGCTTGGACTGTGGAGGCTTGCTGG
CCACTTTTTGGGGTCAGCTCCTCTGAAATGCATTAGCGGAACCGTTTGCAATCTGCCACA
AGTGTGATAAGTTATCTACACTGGCGAGGGGATTGCTCTCTGTAATGTTCAGCTTCTAAT
TGTCTCTACTTTGTGAGACAACTTTTGAATGCTTGACCTCAAATCAGGTAGGACTACCC-
GCTGAACTTAA
>BC7-28
TTTCCGTAGGTGAACCTGCGGAAGGATCATTATTGAATTATGTTTCTAGATAGGTTGTAG
CTGGCTCTTTAGAGCATGTGCACGCCTGTTTGGACTTCATTTTCATCCACCTGTGCACCT
ATTGTAGTCTTTGGTTGGGTTAGGAGGAAGTGGTCATTGTGTCAGCATCTGCTGGATGTG
AGGACTTGCATTGTGAAAGCTTTGCTGTCCTTGATGTGATCATGGAATCTCTTTCTCACT
AGAGTCTATGTCACTCATTATACTCTGTCGAATGTCATTGAATGTCTTTACATGGGCTTA
TATGCCTATGAAAATTGTAATACAACTTTCAGCAACGGATCTCTTGGCTCTCGCATCGAT
GAAGAACGCAGCGAAATGCGATAAGTAATGTGAATTGCAGAATTCAGTGAATCATCGAAT
CTTTGAACGCATCTTGCGCTCCTTGGTATTCCGAGGAGCATGCCTGTTTGAGTGTCATTA
AATTCTCAACTCTCTTCTAC-TTTTTGTAAAAGAGAGCTTGGACTGTGGAGGCTTGCTGG
CCACTTTTTGGGGTCAGCTCCTCTGAAATGCATTAGCGGAACCGTTTGCAATCTGCCACA
AGTGTGATAAGTTATCTACACTGGCGAGGGGATTGCTCTCTGTAATGTTCAGCTTCTAAT
TGTCTCTACTTTGTGAGACAACTTTTGAATGCTTGACCTCAAATCAGGTAGGACTACCC-
GCTGAACTTAA
>BC9-38
TTTCCGTAGGTGAACCTGCGGAAGGATCATTATTGAATTATGTTTCTAGATAGGTTGTAG
CTGGCTCTTTAGAGCATGTGCACGCCTGTTTGGACTTCATTTTCATCCACCTGTGCACCT
ATTGTAGTCTTTGGTTGGGTTAGGAGGAAGTGGTCATTGTGTCAGCATCTGCTGGATGTG
AGGACTTGCATTGTGAAAGCTTTGCTGTCCTTGATGTGATCATGGAATCTCTTTCTCACT
AGAGTCTATGTCACTCATTATACTCTGTCGAATGTCATTGAATGTCTTTACATGGGCTTA
TATGCCTATGAAAATTGTAATACAACTTTCAGCAACGGATCTCTTGGCTCTCGCATCGAT
GAAGAACGCAGCGAAATGCGATAAGTAATGTGAATTGCAGAATTCAGTGAATCATCGAAT
CTTTGAACGCATCTTGCGCTCCTTGGTATTCCGAGGAGCATGCCTGTTTGAGTGTCATTA
AATTCTCAACTCTCTTCTAC-TTTTTGTAAAAGAGAGCTTGGACTGTGGAGGCTTGCTGG
CCACTTTTTGGGGTCAGCTCCTCTGAAATGCATTAGCGGAACCGTTTGCAATCTGCCACA
AGTGTGATAAGTTATCTACACTGGCGAGGGGATTGCTCTCTGTAATGTTCAGCTTCTAAT
TGTCTCTACTTTGTGAGACAACTTTTGAATGCTTGACCTCAAATCAGGTAGGACTACCC-
GCTGAACTTAA

>BC9-47
TTTCCGTAGGTGAACCTGCGGAAGGATCATTATTGAATTATGTTTCTAGATAGGTTGTAG
CTGGCTCTTTAGAGCATGTGCACGCCTGTTTGGACTTCATTTTCATCCACCTGTGCACCT
ATTGTAGTCTTTGGTTGGGTTAGGAGGAAGTGGTCATTGTGTCAGCATCTGCTGGATGTG
AGGACTTGCATTGTGAAAGCTTTGCTGTCCTTGATGTGATCATGGAATCTCTTTCTCACT
AGAGTCTATGTCACTCATTATACTCTGTCGAATGTCATTGAATGTCTTTACATGGGCTTA
TATGCCTATGAAAATTGTAATACAACTTTCAGCAACGGATCTCTTGGCTCTCGCATCGAT
GAAGAACGCAGCGAAATGCGATAAGTAATGTGAATTGCAGAATTCAGTGAATCATCGAAT
CTTTGAACGCATCTTGCGCTCCTTGGTATTCCGAGGAGCATGCCTGTTTGAGTGTCATTA
AATTCTCAACTCTCTTCTAC-TTTTTGTAAAAGAGAGCTTGGACTGTGGAGGCTTGCTGG
CCACTTTTTGGGGTCAGCTCCTCTGAAATGCATTAGCGGAACCGTTTGCAATCTGCCACA
AGTGTGATAAGTTATCTACACTGGCGAGGGGATTGCTCTCTGTAATGTTCAGCTTCTAAT
TGTCTCTACTTTGTGAGACAACTTTTGAATGCTTGACCTCAAATCAGGTAGGACTACCC-
GCTGAACTTAA
>BC11_34
TTTCCGTAGGTGAACCTGCGGAAGGATCATTATTGAATTATGTTTCTAGATAGGTTGTAG
CTGGCTCTTTAGAGCATGTGCACGCCTGTTTGGACTTCATTTTCATCCACCTGTGCACCT
ATTGTAGTCTTTGGTTGGGTTAGGAGGAAGTGGTCATTGTGTCAGCATCTGCTGGATGTG
AGGACTTGCATTGTGAAAGCTTTGCTGTCCTTGATGTGATCATGGAATCTCTTTCTCACT
AGAGTCTATGTCACTCATTATACTCTGTCGAATGTCATTGAATGTCTTTACATGGGCTTA
TATGCCTATGAAAATTGTAATACAACTTTCAGCAACGGATCTCTTGGCTCTCGCATCGAT
GAAGAACGCAGCGAAATGCGATAAGTAATGTGAATTGCAGAATTCAGTGAATCATCGAAT
CTTTGAACGCATCTTGCGCTCCTTGGTATTCCGAGGAGCATGCCTGTTTGAGTGTCATTA
AATTCTCAACTCTCTTCTAC-TTTTTGTAAAAGAGAGCTTGGACTGTGGAGGCTTGCTGG
CCACTTTTTGGGGTCAGCTCCTCTGAAATGCATTAGCGGAACCGTTTGCAATCTGCCACA
AGTGTGATAAGTTATCTACACTGGCGAGGGGATTGCTCTCTGTAATGTTCAGCTTCTAAT
TGTCTCTACTTTGTGAGACAACTTTTGAATGCTTGACCTCAAATCAGGTAGGACTACCC-
GCTGAACTTAA
>BC12_23
TTTCCGTAGGTGAACCTGCGGAAGGATCATTATTGAATTATGTTTCTAGATAGGTTGTAG
CTGGCTCTTTAGAGCATGTGCACGCCTGTTTGGACTTCATTTTCATCCACCTGTGCACCT
ATTGTAGTCTTTGGTTGGGTTAGGAGGAAGTGGTCATTGTGTCAGCATCTGCTGGATGTG
AGGACTTGCATTGTGAAAGCTTTGCTGTCCTTGATGTGATCATGGAATCTCTTTCTCACT
AGAGTCTATGTCACTCATTATACTCTGTCGAATGTCATTGAATGTCTTTACATGGGCTTA
TATGCCTATGAAAATTGTAATACAACTTTCAGCAACGGATCTCTTGGCTCTCGCATCGAT
GAAGAACGCAGCGAAATGCGATAAGTAATGTGAATTGCAGAATTCAGTGAATCATCGAAT
CTTTGAACGCATCTTGCGCTCCTTGGTATTCCGAGGAGCATGCCTGTTTGAGTGTCATTA
AATTCTCAACTCTCTTCTAC-TTTTTGTAAAAGAGAGCTTGGACTGTGGAGGCTTGCTGG
CCACTTTTTGGGGTCAGCTCCTCTGAAATGCATTAGCGGAACCGTTTGCAATCTGCCACA
AGTGTGATAAGTTATCTACACTGGCGAGGGGATTGCTCTCTGTAATGTTCAGCTTCTAAT
TGTCTCTACTTTGTGAGACAACTTTTGAATGCTTGACCTCAAATCAgGTAGGACTACCC-
GCTGAACTTAA
>BC3-6
TTTCCGTAGGTGAACCTGCGGAAGGATCATTATTGAATTATGTTTCTAGATAGGTTGTAG
CTGGCTCTTTAGAGCATGTGCACGCCTGTTTGGACTTCATTTTCATCCACCTGTGCACCT
ATTGTAGTCTTTGGTTGGGTTAGGAGGAAGTGGTCATTGTGTCAGCATCTGCTGGATGTG
AGGACTTGCATTGTGAAAGCTTTGCTGTCCTTGATGTGATCATGGAATCTCTTTCTCACT
AGAGTCTATGTCACTCATTATACTCTGTCGAATGTCATTGAATGTCTTTACATGGGCTTA
TATGCCTATGAAAATTGTAATACAACTTTCAGCAACGGATCTCTTGGCTCTCGCATCGAT
GAAGAACGCAGCGAAATGCGATAAGTAATGTGAATTGCAGAATTCAGTGAATCATCGAAT

CTTTGAACGCATCTTGCGCTCCTTGGTATTCCGAGGAGCATGCCTGTTTGAGTGTCATTA
AATTCTCAACTCTCTTCTAC–TTTTTGTAAAAGAGAGCTTGGACTGTGGAGGCTTGCTGG
CCACTTTTTGGGGTCAGCTCCTCTGAAATGCATTAGCGGAACCGTTTGCAATCTGCCACA
AGTGTGATAAGTTATCTACACTGGCGAGGGGATTGCTCTCTGTAATGTTCAGCTTCTAAT
TGTCTCTACTTTGTGAGACAACTTTTGAATGCTTGACCTCAAATCAGGTAGGACTACCC–
GCTGAACTTAA
>BC3–41
TTTCCGTAGGTGAACCTGCGGAAGGATCATTATTGAATTATGTTTCTAGATAGGTTGTAG
CTGGCTCTTTAGAGCATGTGCACGCCTGTTTGGACTTCATTTTCATCCACCTGTGCACCT
ATTGTAGTCTTTGGTTGGGTTAGGGGGAAGTGGTCATTGTGTCAGCATCTGCTGGATGTG
AGGACTTGCATTGTGAAAGCTTTGCTGTCCTTGATGTGATCATGGAATCTCTTTCTCACT
AGAGTCTATGTCACTCATTATACTCTGTCGAATGTCATTGAATGTCTTTACATGGGCTTA
TATGCCTATGAAAATTGTAATACAACTTTCAGCAACGGATCTCTTGGCTCTCGCATCGAT
GAAGAACGCAGCGAAATGCGATAAGTAATGTGAATTGCAGAATTCAGTGAATCATCGAAT
CTTTGAACGCATCTTGCGCTCCTTGGTATTCCGAGGAGCATGCCTGTTTGAGTGTCATTA
AATTCTCAACTCTCTTCTAC–TTTTTGTAAAAGAGAGCTTGGACTGTGGAGGCTTGCTGG
CCACTTTTTGGGGTCAGCTCCTCTGAAATGCATTAGCGGAACCGTTTGCAATCTGCCACA
AGTGTGATAAGTTATCTACACTGGCGAGGGGATTGCTCTCTGTAATGTTCAGCTTCTAAT
TGTCTCTACTTTGTGAGACAACTTTTGAATGCTTGACCTCAAATCAGGTAGGACTACCC–
GCTGAACTTAA
>BC6–20
TTTCCGTAGGTGAACCTGCGGAAGGATCATTATTGAATTATGTTTCTAGATAGGTTGTAG
CTGGCTCTTTAGAGCATGTGCACGCCTGTTTGGACTTCATTTTCATCCACCTGTGCACCT
ATTGTAGTCTTTGGTTGGGTTAGGGGGAAGTGGTCATTGTGTCAGCATCTGCTGGATGTG
AGGACTTGCATTGTGAAAGCTTTGCTGTCCTTGATGTGATCATGGAATCTCTTTCTCACT
AGAGTCTATGTCACTCATTATACTCTGTCGAATGTCATTGAATGTCTTTACATGGGCTTA
TATGCCTATGAAAATTGTAATACAACTTTCAGCAACGGATCTCTTGGCTCTCGCATCGAT
GAAGAACGCAGCGAAATGCGATAAGTAATGTGAATTGCAGAATTCAGTGAATCATCGAAT
CTTTGAACGCATCTTGCGCTCCTTGGTATTCCGAGGAGCATGCCTGTTTGAGTGTCATTA
AATTCTCAACTCTCTTCTAC–TTTTTGTAAAAGAGAGCTTGGACTGTGGAGGCTTGCTGG
CCACTTTTTGGGGTCAGCTCCTCTGAAATGCATTAGCGGAACCGTTTGCAATCTGCCACA
AGTGTGATAAGTTATCTACACTGGCGAGGGGATTGCTCTCTGTAATGTTCAGCTTCTAAT
TGTCTCTACTTTGTGAGACAACTTTTGAATGCTTGACCTCAAATCAGGTAGGACTACCC–
GCTGAACTTAA
>BC7–34
TTTCCGTAGGTGAACCTGCGGAAGGATCATTATTGAATTATGTTTCTAGATAGGTTGTAG
CTGGCTCTTTAGAGCATGTGCACGCCTGTTTGGACTTCATTTTCATCCACCTGTGCACCT
ATTGTAGTCTTTGGTTGGGTTAGGGGGAAGTGGTCATTGTGTCAGCATCTGCTGGATGTG
AGGACTTGCATTGTGAAAGCTTTGCTGTCCTTGATGTGATCATGGAATCTCTTTCTCACT
AGAGTCTATGTCACTCATTATACTCTGTCGAATGTCATTGAATGTCTTTACATGGGCTTA
TATGCCTATGAAAATTGTAATACAACTTTCAGCAACGGATCTCTTGGCTCTCGCATCGAT
GAAGAACGCAGCGAAATGCGATAAGTAATGTGAATTGCAGAATTCAGTGAATCATCGAAT
CTTTGAACGCATCTTGCGCTCCTTGGTATTCCGAGGAGCATGCCTGTTTGAGTGTCATTA
AATTCTCAACTCTCTTCTAC–TTTTTGTAAAAGAGAGCTTGGACTGTGGAGGCTTGCTGG
CCACTTTTTGGGGTCAGCTCCTCTGAAATGCATTAGCGGAACCGTTTGCGATCTGCCACA
AGTGTGATAAGTTATCTACACTGGCGAGGGGATTGCTCTCTGTAATGTTCAGCTTCTAAT
TGTCTCTACTTTGTGAGACAACTTTTGAATGCTTGACCTCAAATCAGGTAGGACTACCC–
GCTGAACTTAA
>BC8–8
TTTCCGTAGGTGAACCTGCGGAAGGATCATTATTGAATTATGTTTCTAGATAGGTTGTAG

CTGGCTCTTTAGAGCATGTGCACGCCTGTTTGGACTTCATTTTCATCCACCTGTGCACCT
ATTGTAGTCTTTGGTTGGGTTAGGGGGAAGTGGTCATTGTGTCAGCATCTGCTGGATGTG
AGGACTTGCATTGTGAAAGCTTTGCTGTCCTTGATGTGATCATGGAATCTCTTTCTCACT
AGAGTCTATGTCACTCATTATACTCTGTCGAATGTCATTGAATGTCTTTACATGGGCTTA
TATGCCTATGAAAATTGTAATACAACTTTCAGCAACGGATCTCTTGGCTCTCGCATCGAT
GAAGAACGCAGCGAAATGCGATAAGTAATGTGAATTGCAGAATTCAGTGAATCATCGAAT
CTTTGAACGCATCTTGCGCTCCTTGGTATTCCGAGGAGCATGCCTGTTTGAGTGTCATTA
AATTCTCAACTCTCTTCTAC-TTTTTGTAAAAGAGAGCTTGGACTGTGGAGGCTTGCTGG
CCACTTTTTGGGGTCAGCTCCTCTGAAATGCATTAGCGGAACCGTTTGCGATCTGCCACA
AGTGTGATAAGTTATCTACACTGGCGAGGGGATTGCTCTCTGTAATGTTCAGCTTCTAAT
TGTCTCTACTTTGTGAGACAACTTTTGAATGCTTGACCTCAAATCAGGTAGGACTACCC-
GCTGAACTTAA
>BC9-54
TTTCCGTAGGTGAACCTGCGGAAGGATCATTATTGAATTATGTTTCTAGATAGGTTGTAG
CTGGCTCTTTAGAGCATGTGCACGCCTGTTTGGACTTCATTTTCATCCACCTGTGCACCT
ATTGTAGTCTTTGGTTGGGTTAGGGGGAAGTGGTCATTGTGTCAGCATCTGCTGGATGTG
AGGACTTGCATTGTGAAAGCTTTGCTGTCCTTGATGTGATCATGGAATCTCTTTCTCACT
AGAGTCTATGTCACTCATTATACTCTGTCGAATGTCATTGAATGTCTTTACATGGGCTTA
TATGCCTATGAAAATTGTAATACAACTTTCAGCAACGGATCTCTTGGCTCTCGCATCGAT
GAAGAACGCAGCGAAATGCGATAAGTAATGTGAATTGCAGAATTCAGTGAATCATCGAAT
CTTTGAACGCATCTTGCGCTCCTTGGTATTCCGAGGAGCATGCCTGTTTGAGTGTCATTA
AATTCTCAACTCTCTTCTAC-TTTTTGTAAAAGAGAGCTTGGACTGTGGAGGCTTGCTGG
CCACTTTTTGGGGTCAGCTCCTCTGAAATGCATTAGCGGAACCGTTTGCGATCTGCCACA
AGTGTGATAAGTTATCTACACTGGCGAGGGGATTGCTCTCTGTAATGTTCAGCTTCTAAT
TGTCTCTACTTTGTGAGACAACTTTTGAATGCTTGACCTCAAATCAGGTAGGACTACCC-
GCTGAACTTAA
>BC9-20
TTTCCGTAGGTGAACCTGCGGAAGGATCATTATTGAATTATGTTTCTAGATAGGTTGTAG
CTGGCTCTTTAGAGCATGTGCACGCCTGTTTGGACTTCATTTTCATCCACCTGTGCACCT
ATTGTAGTCTTTGGTTGGGTTAGGAGGAAGTGGTCATTGTGTCAGCATCTGCTGGATGTG
AGGACTTGCATTGTGAAAGCTTTGCTGTCCTTGATGTGATCATGGAATCTCTTTCTCACT
AGAGTCTATGTCACTCATTATACTCTGTCGAATGTCATTGAATGTCTTTACATGGGCTTA
TATGCCTATGAAAATTGTAATACAACTTTCAGCAACGGATCTCTTGGCTCTCGCATCGAT
GAAGAACGCAGCGAAATGCGATAAGTAATGTGAATTGCAGAATTCAGTGAATCATCGAAT
CTTTGAACGCATCTTGCGCTCCTTGGTATTCCGAGGAGCATGCCTGTTTGAGTGTCATTA
AATTCTCAACTCTCTTCTAC-TTTTTGTAAAAGAGAGCTTGGACTGTGGGGGCTTGCTGG
CCACTTTTTGGGGTCAGCTCCTCTGAAATGCATTAGCGGAACCGTTTGCGATCTGCCACA
AGTGTGATAAGTTATCTACACTGGCGAGGGGATTGCTCTCTGTAATGTTCAGCTTCTAAT
TGTCTCTACTTTGTGAGACAACTTTTGAATGCTTGACCTCAAATCAGGTAGGACTACCC-
GCTGAACTTAA
>BC11_29
TTTCCGTAGGTGAACCTGCGGAAGGATCATTATTGAATTATGTTTCTAGATAGGTTGTAG
CTGACTCTTTAGAGCATGTGCACGCCTGTTTGGACTTCATTTTCATCCACCTGTGCACCT
ATTGTAGTCTTTGGTTGGGTTAGGAGGAAGTGGTCATTGTGTCAGCATCTGCTGGATGTG
AGGACTTGCATTGTGAAAGCTTTGCTGTCCTTGATGTGATCATGGAATCTCTTTCTCACT
AGAGTCTATGTCACTCATTATACTCTGTCGAATGTCATTGAATGTCTTTACATGGGCTTA
TATGCCTATGAAAATTGTAATACAACTTTCAGCAACGGATCTCTTGGCTCTCGCATCGAT
GAAGAACGCAGCGAAATGCGATAAGTAATGTGAATTGCAGAATTCAGTGAATCATCGAAT
CTTTGAACGCATCTTGCGCTCCTTGGTATTCCGAGGAGCATGCCTGTTTGAGTGTCATTA
AATTCTCAACTCTCTTCTAC-TTTTTGTAAAAGAGAGCTTGGACTGTGGAGGCTTGCTGG

CCACTTTTTGGGGTCAGCTCCTCTGAAATGCATTAGCGGAACCGTTTGCGATCTGCCACA
AGTGTGATAAGTTATCTACACTGGCGAGGGGATTGCTCTCTGTAATGTTCAGCTTCTAAT
TGTCTCTACTTTGTGAGACAACTTTTGAATGCTTGACCTCAAATCAGGTAGGACTACCC-
GCTGAACTTAA
>BC4-53
TTTCCGTAGGTGAACCTGCGGAAGGATCATTATTGAATTATGTTTCTAGATAGGTTGTAG
CTGGCTCTTTAGAGCATGTGCACGCCTGTTTGGACTTCATTTTCATCCACCTGTGCACCT
ATTGTAGTCTTTGGTTGGGTTAGGGGGAAGTGGTCATTGTGTCAGCATCTGCTGGATGTG
AGGACTTGCATTGTGAAAGCTTTGCTGTCCTTGATGTGATCATGGAATCTCTTTCTCACT
AGAGTCTATGTCACTCATTATACTCTGTCGAATGTCATTGAATGTCTTTACATGGGCTTG
TATGCCTATGAAAATTGTAATACAACTTTCAGCAACGGATCTCTTGGCTCTCGCATCGAT
GAAGAACGCAGCGAAATGCGATAAGTAATGTGAATTGCAGAATTCAGTGAATCATCGAAT
CTTTGAACGCATCTTGCGCTCCTTGGTATTCCGAGGAGCATGCCTGTTTGAGTGTCATTA
AATTCTCAACTCTCTTCTAC-TTTTTGTAAAAGAGAGCTTGGACTGTGGAGGCTTGCTGG
CCACTTTTTGGGGTCAGCTCCTCTGAAATGCATTAGCGGAACCGTTTGCGATCTGCCACA
AGTGTGATAAGTTATCTACACTGGCGAGGGGATTGCTCTCTGTAATGTTCAGCTTCTAAT
TGTCTCTACTTTGTGAGACAACTTTTGAATGCTTGACCTCAAATCAGGTAGGACTACCC-
GCTGAACTTAA
>BC8-20
TTTCCGTAGGTGAACCTGCGGAAGGATCATTATTGAATTATGTTTCTAGATAGGTTGTAG
CTGGCTCTTTAGAGCATGTGCACGCCTGTTTGGACTTCATTTTCATCCACCTGTGCACCT
ATTGTAGTCTTTGGTTGGGTTAGGGGGAAGTGGTCATTGTGTCAGCATCTGCTGGATGTG
AGGACTTGCATTGTGAAAGCTTTGCTGTCCTTGATGTGATCATGGAATCTCTTTCTCACT
AGAGTCTATGTCACTCATTATACTCTGTCGAATGTCATTGAATGTCTTTACATGGGCTTG
TATGCCTATGAAAATTGTAATACAACTTTCAGCAACGGATCTCTTGGCTCTCGCATCGAT
GAAGGACGCAGCGAAATGCGATAAGTAATGTGAATTGCAGAATTCAGTGAATCATCGAAT
CTTTGAACGCATCTTGCGCTCCTTGGTATTCCGAGGAGCATGCCTGTTTGAGTGTCATTA
AATTCTCAACTCTCTTCTAC-TTTTTGTAAAAGAGAGCTTGGACTGTGGAGGCTTGCTGG
CCACTTTTTGGGGTCAGCTCCTCTGAAATGCATTAGCGGAACCGTTTGCGATCTGCCACA
AGTGTGATAAGTTATCTACACTGGCGAGGGGATTGCTCTCTGTAATGTTCAGCTTCTAAT
TGTCTCTACTTTGTGAGACAACTTTTGAATGCTTGACCTCAAATCAGGTAGGACTACCC-
GCTGAACTTAA
>BC9-42
TTTCCGTAGGTGAACCTGCGGAAGGATCATTATTGAATTATGTTTCTAGATAGGTTGTAG
CTGGCTCTTTAGAGCATGTGCACGCCTGTTTGGACTTCATTTTCATCCACCTGTGCACCT
ATTGTAGTCTTTGGTTGGGTTAGGAGGAAGTGGTCATTGTGTCAGCATCTGCTGGATGTG
AGGACTTGCATTGTGAAAGCTTTGCTGTCCTTGATGTGATCATGGAATCTCTTTCTCACT
AGAGTCTATGTCACTCATTATACTCTGTCGAATGTCATTGAATGTCTTTACATGGGCTTG
TATGCCTATGAAAATTGTAATACAACTTTCAGCAACGGATCTCTTGGCTCTCGCATCGAT
GAAGGACGCAGCGAAATGCGATAAGTAATGTGAATTGCAGAATTCAGTGAATCATCGAAT
CTTTGAACGCATCTTGCGCTCCTTGGTATTCCGAGGAGCATGCCTGTTTGAGTGTCATTA
AATTCTCAACTCTCTTCTAC-TTTTTGTAAAAGAGAGCTTGGACTGTGGAGGCTTGCTGG
CCACTTTTTGGGGTCAGCTCCTCTGAAATGCATTAGCGGAACCGTTTGCGATCTGCCACA
AGTGTGATAAGTTATCTACACTGGCGAGGGGATTGCTCTCTGTAATGTTCAGCTTCTAAT
TGTCTCTACTTTGTGAGACAACTTTTGAATGCTTGACCTCAAATCAGGTAGGACTACCC-
GCTGAACTTAA
>BC11_46
TTTCCGTAGGTGAACCTGCGGAAGGATCATTATTGAATTATGTTTCTAGATAGGTTGTAG
CTGGCTCTTTAGAGCATGTGCACGCCTGTTTGGACTTCATTTTCATCCACCTGTGCACCT
ATTGTAGTCTTTGGTTGGGTTAGGAGGAAGTGGTCATTGTGTCAGCATCTGCTGGATGTG

AGGACTTGCATTGTGAAAGCTTTGCTGTCCTTGATGTGATCATGGAATCTCTTTCTCACT
AGAGTCTATGTCACTCATTATACTCTGTCGAATGTCATTGAATGTCTTTACATGGGCTTG
TATGCCTATGAAAATTGTAATACAACTTTCAGCAACGGATCTCTTGGCTCTCGCATCGAT
GAAGGACGCAGCGAAATGCGATAAGTAATGTGAATTGCAGAATTCAGTGAATCATCGAAT
CTTTGAACGCATCTTGCGCTCCTTGGTATTCCGAGGAGCATGCCTGTTTGAGTGTCATTA
AATTCTCAACTCTCTTCTAC–TTTTTGTAAAAGAGAGCTTGGACTGTGGAGGCTTGCTGG
CCACTTTTTGGGGTCAGCTCCTCTGAAATGCATTAGCGGAACCGTTTGCGATCTGCCACA
AGTGTGATAAGTTATCTACACTGGCGAGGGGATTGCTCTCTGTAATGTTCAGCTTCTAAT
TGTCTCTACTTTGTGAGACAACTTTTGAATGCTTGACCTCAAATCAGGTAGGACTACCC–
GCTGAACTTAA
>BC12_19
TTTCCGTAGGTGAACCTGCGGAAGGATCATTATTGAATTATGTTTCTAGATAGGTTGTAG
CTGGCTCTTTAGAGCATGTGCACGCCTGTTTGGACTTCATTTTCATCCACCTGTGCACCT
ATTGTAGTCTTTGGTTGGGTTAGGAGGAAGTGGTCATTGTGTCAGCATCTGCTGGATGTG
AGGACTTGCATTGTGAAAGCTTTGCTGTCCTTGATGTGATCATGGAATCTCTTTCTCACT
AGAGTCTATGTCACTCATTATACTCTGTCGAATGTCATTGAATGTCTTTACATGGGCTTG
TATGCCTATGAAAATTGTAATACAACTTTCAGCAACGGATCTCTTGGCTCTCGCATCGAT
GAAGGACGCAGCGAAATGCGATAAGTAATGTGAATTGCAGAATTCAGTGAATCATCGAAT
CTTTGAACGCATCTTGCGCTCCTTGGTATTCCGAGGAGCATGCCTGTTTGAGTGTCATTA
AATTCTCAACTCTCTTCTAC–TTTTTGTAAAAGAGAGCTTGGACTGTGGAGGCTTGCTGG
CCACTTTTTGGGGTCAGCTCCTCTGAAATGCATTAGCGGAACCGTTTGCGATCTGCCACA
AGTGTGATAAGTTATCTACACTGGCGAGGGGATTGCTCTCTGTAATGTTCAGCTTCTAAT
TGTCTCTACTTTGTGAGACAACTTTTGAATGCTTGACCTCAAATCAGGTAGGACTACCC–
GCTGAACTTAA
>BC2–47
TTTCCGTAGGTGAACCTGCGGAAGGATCATTATTGAATTATGTTTCTAGATAGGTTGTAG
CTGGCTCTTTAGAGCATGTGCACGCCTGTTTGGACTTCATTTTCATCCACCTGTGCACCT
ATTGTAGTCTTTGGTTGGGTTAGGAGGAAGTGGTCATTGTGTCAGCATCTGCTGGATGTG
AGGACTTGCATTGTGAAAGCTTTGCTGTCCTTGATGTGATCATGGAATCTCTTTCTCACT
AGAGTCTATGTCACTCATTATACTCTGTCGAATGTCATTGAATGTCTTTACATGGGCTTG
TATGCCTATGAAAATTGTAATACAACTTTCAGCAACGGATCTCTTGGCTCTCGCATCGAT
GAAGGACGCAGCGAAATGCGATAAGTAATGTGAATTGCAGAATTCAGTGAATCATCGAAT
CTTTGAACGCATCTTGCGCTCCTTGGTATTCCGAGGAGCATGCCTGTTTGAGTGTCATTA
AATTCTCAACTCTCTTCTAC–TTTTTGTAAAAGAGAGCTTGGACTGTGGAGGCTTGCTGG
CCACTTTTTGGGGTCAGCTCCTCTGAAATGCATTAGCGGAACCGTTTGCGATCTGCCACA
AGTGTGATAAGTTATCTACACTGGCGAGGGGATTGCTCTCTGTAATGTTCAGCTTCTAAT
TGTCTCTACTTTGTGAGACAACTTTTGAATGCTTGACCTCAAATCAGGTAGGACTACCC–
GCTGAACTTAA
>BC4–14
TTTCCGTAGGTGAACCTGCGGAAGGATCATTATTGAATTATGTTTCTAGATAGGTTGTAG
CTGGCTCTTTAGAGCATGTGCACGCCTGTTTGGACTTCATTTTCATCCACCTGTGCACCT
ATTGTAGTCTTTGGTTGGGTTAGGAGGAAGTGGTCATTGTGTCAGCATCTGCTGGATGTG
AGGACTTGCATTGTGAAAGCTTTGCTGTCCTTGATGTGATCATGGAATCTCTTTCTCACT
AGAGTCTATGTCACTCATTATACTCTGTCGAATGTCATTGAATGTCTTTACATGGGCTTG
TATGCCTATGAAAATTGTAATACAACTTTCAGCAACGGATCTCTTGGCTCTCGCATCGAT
GAAGAACGCAGCGAAATGCGATAAGTAATGTGAATTGCAGAATTCAGTGAATCATCGAAT
CTTTGAACGCATCTTGCGCTCCTTGGTATTCCGAGGAGCATGCCTGTTTGAGTGTCATTA
AATTCTCAACTCTCTTCTAC–TTTTTGTAAAAGAGAGCTTGGACTGTGGAGGCTTGCTGG
CCACTTTTTGGGGTCAGCTCCTCTGAAATGCATTAGCGGAACCGTTTGCGATCTGCCACA
AGTGTGATAAGTTATCTACACTGGCGAGGGGATTGCTCTCTGTAATGTTCAGCTTCTAAT

TGTCTCTACTTTGTGAGACAACTTTTGAATGCTTGACCTCAAATCAGGTAGGACTACCC-
GCTGAACTTAA
>BC7-4
TTTCCGTAGGTGAACCTGCGGAAGGATCATTATTGAATTATGTTTCTAGATAGGTTGTAG
CTGGCTCTTTAGAGCATGTGCACGCCTGTTTGGACTTCATTTTCATCCACCTGTGCACCT
ATTGTAGTCTTTGGTTGGGTTAGGGGGAAGTGGTCATTGTGTCAGCATCTGCTGGATGTG
AGGACTTGCATTGTGAAAGCTTTGCTGTCCTTGATGTGATCATGGAATCTCTTTCTCACT
AGAGTCTATGTCACTCATTATACTCTGTCGAATGTCATTGAATGTCTTTACATGGGCTTG
TATGCCTATGAAAATTGTAATACAACTTTCAGCAACGGATCTCTTGGCTCTCGCATCGAT
GAAGGACGCAGCGAAATGCGATAAGTAATGTGAATTGCAGAATTCAGTGAATCATCGAAT
CTTTGAACGCATCTTGCGCTCCTTGGTATTCCGAGGAGCATGCCTGTTTGAGTGTCATTA
AATTCTCAACTCTCTTCTAC-TTTTTGTAAAAGAGAGCTTGGACTGTGGAGGCTTGCTGG
CCACTTTTTGGGGTCAGCTCCTCTGAAATGCATTAGCGGAACCGTTTGCAATCTGCCACA
AGTGTGATAAGTTATCTACACTGGCGAGGGGATTGCTCTCTGTAATGTTCAGCTTCTAAT
TGTCTCTACTTTGTGAGACAACTTTTGAATGCTTGACCTCAAATCAGGTAGGACTACCC-
GCTGAACTTAA
>BC11_50
TTTCCGTAGGTGAACCTGCGGAAGGATCATTATTGAATTATGTTTCTAGATAGGTTGTAG
CTGGCTCTTTAGAGCATGTGCACGCCTGTTTGGACTTCATTTTCATCCACCTGTGCACCT
ATTGTAGTCTTTGGTTGGGTTAGGGGGAAGTGGTCATTGTGTCAGCATCTGCTGGATGTG
AGGACTTGCATTGTGAAAGCTTTGCTGTCCTTGATGTGATCATGGAATCTCTTTCTCACT
AGAGTCTATGTCACTCATTATACTCTGTCGAATGTCATTGAATGTCTTTACATGGGCTTG
TATGCCTATGAAAATTGTAATACAACTTTCAGCAACGGATCTCTTGGCTCTCGCATCGAT
GAAGGACGCAGCGAAATGCGATAAGTAATGTGAATTGCAGAATTCAGTGAATCATCGAAT
CTTTGAACGCATCTTGCGCTCCTTGGTATTCCGAGGAGCATGCCTGTTTGAGTGTCATTA
AATTCTCAACTCTCTTCTAC-TTTTTGTAAAAGAGAGCTTGGACTGTGGAGGCTTGCTGG
CCACTTTTTGGGGTCAGCTCCTCTGAAATGCATTAGCGGAACCGTTTGCAATCTGCCACA
AGTGTGATAAGTTATCTACACTGGCGAGGGGATTGCTCTCTGTAATGTTCAGCTTCTAAT
TGTCTCTACTTTGTGAGACAACTTTTGAATGCTTGACCTCAAATCAGGTAGGACTACCC-
GCTGAACTTAA
>BC6-7
TTTCCGTAGGTGAACCTGCGGAAGGATCATTATTGAATTATGTTTCTAGATAGGTTGTAG
CTGGCTCTTTAGAGCATGTGCACGCCTGTTTGGACTTCATTTTCATCCACCTGTGCACCT
ATTGTAGTCTTTGGTTGGGTTAGGAGGAAGTGGTCATTGTGTCAGCATCTGCTGGATGTG
AGGACTTGCATTGTGAAAGCTTTGCTGTCCTTGATGTGATCATGGAATCTCTTTCTCACT
AGAGTCTATGTCACTCATTATACTCTGTCGAATGTCATTGAATGTCTTTACATGGGCTTG
TATGCCTATGAAAATTGTAATACAACTTTCAGCAACGGATCTCTTGGCTCTCGCATCGAT
GAAGGACGCAGCGAAATGCGATAAGTAATGTGAATTGCAGAATTCAGTGAATCATCGAAT
CTTTGAACGCATCTTGCGCTCCTTGGTATTCCGAGGAGCATGCCTGTTTGAGTGTCATTA
AATTCTCAACTCTCTTCTAC-TTTTTGTAAAAGAGAGCTTGGACTGTGGAGGCTTGCTGG
CCACTTTTTGGGGTCAGCTCCTCTGAAATGCATTAGCGGAACCGTTTGCAATCTGCCACA
AGTGTGATAAGTTATCTACACTGGCGAGGGGATTGCTCTCTGTAATGTTCAGCTTCTAAT
TGTCTCTACTTTGTGAGACAACTTTTGAATGCTTGACCTCAAATCAGGTAGGACTACCC-
GCTGAACTTAA
>BC11_12
TTTCCGTAGGTGAACCTGCGGAAGGATCATTATTGAATTATGTTTCTAGATAGGTTGTAG
CTGGCTCTTTAGAGCATGTGCACGCCTGTTTGGACTTCATTTTCATCCACCTGTGCACCT
ATTGTAGTCTTTGGTTGGGTTAGGAGGAAGTGGTCATTGTGTCAGCATCTGCTGGATGTG
AGGACTTGCATTGTGAAAGCTTTGCTGTCCTTGATGTGATCATGGAATCTCTTTCTCACT
AGAGTCTATGTCACTCATTATACTCTGTCGAATGTCATTGAATGTCTTTACATGGGCTTG

TATGCCTATGAAAATTGTAATACAACTTTCAGCAACGGATCTCTTGGCTCTCGCATCGAT
GAAGGACGCAGCGAAATGCGATAAGTAATGTGAATTGCAGAATTCAGTGAATCATCGAAT
CTTTGAACGCATCTTGCGCTCCTTGGTATTCCGAGGAGCATGCCTGTTTGAGTGTCATTA
AATTCTCAACTCTCTTCTAC-TTTTTGTAAAAGAGAGCTTGGACTGTGGAGGCTTGCTGG
CCACTTTTTGGGGTCAGCTCCTCTGAAATGCATTAGCGGAACCGTTTGCAATCTGCCACA
AGTGTGATAAGTTATCTACACTGGCGAGGGGATTGCTCTCTGTAATGTTCAGCTTCTAAT
TGTCTCTACTTTGTGAGACAACTTTTGAATGCTTGACCTCAAATCAGGTAGGACTACCC-
GCTGAACTTAA
>BC11_54
TTTCCGTAGGTGAACCTGCGGAAGGATCATTATTGAATTATGTTTCTAGATAGGTTGTAG
CTGGCTCTTTAGAGCATGTGCACGCCTGTTTGGACTTCATTTTCATCCACCTGTGCACCT
ATTGTAGTCTTTGGTTGGGTTAGGAGGAAGTGGTCATTGTGTCAGCATCTGCTGGATGTG
AGGACTTGCATTGTGAAAGCTTTGCTGTCCTTGATGTGATCATGGAATCTCTTTCTCACT
AGAGTCTATGTCACTCATTATACTCTGTCGAATGTCATTGAATGTCTTTACATGGGCTTG
TATGCCTATGAAAATTGTAATACAACTTTCAGCAACGGATCTCTTGGCTCTCGCATCGAT
GAAGGACGCAGCGAAATGCGATAAGTAATGTGAATTGCAGAATTCAGTGAATCATCGAAT
CTTTGAACGCATCTTGCGCTCCTTGGTATTCCGAGGAGCATGCCTGTTTGAGTGTCATTA
AATTCTCAACTCTCTTCTAC-TTTTTGTAAAAGAGAGCTTGGACTGTGGAGGCTTGCTGG
CCACTTTTTGGGGTCAGCTCCTCTGAAATGCATTAGCGGAACCGTTTGCAATCTGCCACA
AGTGTGATAAGTTATCTACACTGGCGAGGGGATTGCTCTCTGTAATGTTCAGCTTCTAAT
TGTCTCTACTTTGTGAGACAACTTTTGAATGCTTGACCTCAAATCAGGTAGGACTACCC-
GCTGAACTTAA
>BC12_9
TTTCCGTAGGTGAACCTGCGGAAGGATCATTATTGAATTATGTTTCTAGATAGGTTGTAG
CTGGCTCTTTAGAGCATGTGCACGCCTGTTTGGACTTCATTTTCATCCACCTGTGCACCT
ATTGTAGTCTTTGGTTGGGTTAGGGGGAAGTGGTCATTGTGTCAGCATCTGCTGGATGTG
AGGACTTGCATTGTGAAAGCTTTGCTGTCCTTGATGTGATCATGGAATCTCTTTCTCACT
AGAGTCCATGTCACTCATTATACTCTGTCGAATGTCATTGAATGTCTTTACATGGGCTTG
TATGCCTATGAAAATTGTAATACAACTTTCAGCAACGGATCTCTTGGCTCTCGCATCGAT
GAAGGACGCAGCGAAATGCGATAAGTAATGTGAATTGCAGAATTCAGTGAATCATCGAAT
CTTTGAACGCATCTTGCGCTCCTTGGTATTCCGAGGAGCATGCCTGTTTGAGTGTCATTA
AATTCTCAACTCTCTTCTAC-TTTTTGTAAAAGAGAGCTTGGACTGTGGAGGCTTGCTGG
CCACTTTTTGGGGTCAGCTCCTCTGAAATGCATTAGCGGAACCGTTTGCGATCTGCCACA
AGTGTGATAAGTTATCTACACTGGCGAGGGGATTGCTCTCTGTAATGTTCAGCTTCTAAT
TGTCTCTACTTTGTGAGACAACTTTTGAATGCTTGACCTCAAATCAGGTAGGACTACCC-
GCTGAACTTAA
>BC12_5
TTTCCGTAGGTGAACCTGCGGAAGGATCATTATTGAATTATGTTTCTAGATAGGTTGTAG
CTGGCTCTTTAGAGCATGTGCACGCCTGTTTGGACTTCATTTTCATCCACCTGTGCACCT
ATTGTAGTCTTTGGTTGGGTTAGGAGGAAGTGGTCATTGTGTCAGCATCTGCTGGATGTG
AGGACTTGCATTGTGAAAGCTTTGCTGTCCTTGATGTGATCATGGAATCTCTTTCTCACT
AGAGTCTATGTCACTCATTATACTCTGTCGAATGTCATTGAATGTCTTTACATGGGCTTA
TATGCCTATGAAAATTGTAATACAACTTTCAGCAACGGATCTCTTGACTCTCGCATCGAT
GAAGAACGCAGCGAAATGCGATAAGTAATGTGAATTGTAGAATTCAGTGAATCATCGAAT
CTTTGAACGCATCTTGCGCTCCTTGGTATTCCGAGGAGCATGCCTGTTTGAGTGTCATTA
AATTCTCAACTCTCTTCTAC-TTTTTGTAAAAGAGAGCTTGGACTGTGGAGGCTTGCTGG
CCACTTTTTGGGGTCAGCTCCTCTGAAATGCATTAGCGGAACCGTTTGCGATCTGCCACA
AGTGTGATAAGTTATCTACACTGGCGAGGGGATTGCTCTCTGTAATGTTCAGCTTCTAAT
TGTCTCTACTTTGTGAGACTACTTTTGAATGCTTGACCTCAAATCAGGTAGGACTACCC-
GCTGAACTTAA

>BC9–39
TTTCCGTAGGTGAACCTGCGGAAGGATCATTATTGAATTATGTTTCTAGATAGGTTGTAG
CTGGCTCTTTAGAGCATGTGCACGCCTGTTTGGACTTCATTTTCATCCACCTGTGCACCT
ATTGTAGTCTTTGGTTGGGTTAGGAGGAAGTGGTCATTGTGTCAGCATCTGCTGGATGTG
AGGACTTGCATTGTGAAAGCTTTGTTGTCCTTGATGTGATCATGGAATCTCTTTCTCACT
AGAGTCTATGTCACTCATTATACTCTGTCGAATGTCATTGAATGTCTTTACATGGGCTTA
TATGCCTATGAAAATTGTAATACAACTTTCAGCAACGGATCTCTTGGCTCTCGCATCGAT
GAAGGACGCAGCGAAATGCGATAAGTAATGTGAATTGCAGAATTCAGTGAATCATCGAAT
CTTTGAACGCATCTTGCGCTCCTTGGTATTCCGAGGAGCATGCCTGTTTGAGTGCCATTA
AATTCTCAACTCTCTTCTAC–TTTTTGTAAAAGAGAGCTTGGACTGTGGAGGCTTGCTGG
CCACTTTTTGGGGTCAGCTCCTCTGAAATGCATTAGCGGAACCGTTTGCGATCTGCCACA
AGTGTGATAAGTTATCTACACTGGCGAGGGGATTGCTCTCTGTAATGTTCAGCTTCTAAT
TGTCTCTACTTTGTGAGACTACTTTTGAATGCTTGACCTCAAATCAGGTAGGACTACCC–
GCTGAACTTAA
>BC9–46
TTTCCGTAGGTGAACCTGCGGAAGGATCATTATTGAATTATGTTTCTAGATAGGTTGTAG
CTGGCTCTTTAGAGCATGTGCACGCCTGTTTGGACTTCATTTTCATCCACCTGTGCACCT
ATTGTAGTCTTTGGTTGGGTTAGGAGGAAGTGGTCATTGTGTCAGCATCTGCTGGATGTG
AGGACTTGCATTGTGAAAGCTTTGCTGTCCTTGATGTGATCATGGAATCTCTTTCTCACT
AGAGTCTATGTCACTCATTATACTCTGTCGAATGTCATTGAATGTCTTTACATGGGCTTA
TATGCCTATGAAAATTGTAATACAACTTTCAGCAACGGATCTCTTGGCTCTCGCATCGAT
GAAGAACGCAGCGAAATGCGATAAGTAATGTGAATTGCAGAATTCAGTGAATCATCGAAT
CTTTGAACGCATCTTGCGCTCCTTGGTATTCCGAGGAGCATGCCTGTTTGAGTGTCATTA
AATTCTCAACTCTCTTCTAC–TTTTTGTAAAAGAGAGCTTGGACTGTGGAGGCTTGCTGG
CCAC–TTTTGGGGTCAGCTCCTCTGAAATGCATTAGCGGAACCGTTTGCGATCTGCCACA
AGTGTGATAAGTTATCTACACTGGCGAGGGGATTGCTCTCTGTAATGTTCAGCTTCTAAT
TGTCTCTACTTTGTGAGACAACTTTTGAATGCTTGACCTCAAATCAGGTAGGACTACCC–
GCTGAACTTAA
>BC7–10
TTTCCGTAGGTGAACCTGCGGAAGGATCATTATTGAATTATGTTTCTAGATAGGTTGTAG
CTGGCTCTTTAGAGCATGTGCACGCCTGTTTGGACTTCATTTTCATCCACCTGTGCACCT
ATTGTAGTCTTTGGTTGGGTTAGGAGGAAGTGGTCATTGTGTCAGCATCTGCTGGATGTG
AGGACTTGCATTGTGAAAGCTTTGCTGTCCTTGATGTGATCATGGAATCTCTTTCTCACT
AGAGTCTATGTCACTCATTATACTCTGTCGAATGTCATTGAATGTCTTTACATGGGCTTA
TATGCCTATGAAAATTGTAATACAACTTTCAGCAACGGATCTCTTGGCTCTCGCATCGAT
GAAGGACGCAGCGAAATGCGATAAGTAATGTGAATTGCAGAATTCAGTGAATCATCGAAT
CTTTGAACGCATCTTGCGCTCCTTGGTATTCCGAGGAGCATGCCTGTTTGAGTGTCATTA
AATTCTCAACTCTCTTCTAC–TTTTTGTAAAAGAGAGCTTGGACTGTGGAGGCTTGCTGG
CCAC–TTTTGGGGTCAGCTCCTCTGAAATGCATTAGCGGAACCGTTTGCGATCTGCCACA
AGTGTGATAAGTTATCTACACTGGCGAGGGGATTGCTCTCTGTAATGTTCAGCTTCTAAT
TGTCTCTACTTTGTGAGACTACTTTTGAATGCTTGACCTCAAATCAGGTAGGACTACCC–
GCTGAACTTAA
>BC12_13
TTTCCGTAGGTGAACCTGCGGAAGGATCATTATTGAATTATGTTTCTAGATAGGTTGTAG
CTGGCTCTTTAGAGCATGTGCACGCCTGTTTGGACTTCATTTTCATCCACCTGTGCACCT
ATTGTAGTCTTTGGTTGGGTTAGGAGGAAGTGGTCATTGTGTCAGCATCTGCTGGATGTG
AGGACTTGCATTGTGAAAGCTTTGCTGTCCTTGATGTGATCATGGAATCTCTTTCTCACT
AGAGTCTATGTCACTCATTATACTCTGTCGAATGTCATTGAATGTCTTTACATGGGCTTG
TATGCCTATGAAAATTGTAATACAACTTTCAGCAACGGATCTCTTGGCTCTCGCATCGAT
GAAGAACGCAGCGAAATGCGATAAGTAATGTGAATTGCAGAATTCAGTGAATCATCGAAT

```
CTTTGAACGCATCTTGCGCTCCTTGGTATTCCGAGGAGCATGCCTGTTTGAGTGTCATTA
AATTCTCAACTCTCTTCTAC-TTTTTGTAAAAGAGAGCTTGGACTGTGGAGGCTTGCTGG
CCACTTTTTGGGGTCAGCTCCTCTGAAATGCATTAGCGGAACCGTTTGCAATCTGCCACA
AGTGTGATAAGTTATCTACACTGGCGAGGGGATTGCTCTCTGTAATGTTCAGCTTCTAAT
TGTCTCTACTTTGTGAGACAACTTTTGAATGCTTGACCTCAAATCA-GTAGGACTACCC-
GCTGAACTTAA
>BC5-95
TTTCCGTAGGTGAACCTGCGGAAGGATCATTATTGAATTATGTTTCTAGATAGGTTGTAG
CTGGCTCTTTAGAGCATGTGCACGCCTGTTTGGACTTCATTTTCATCCACCTGTGCACCT
ATTGTAGTCTTTGGTTGGGTTAGGAGGAAGTGGTCATTGTGTCAGCATCTGCTGGATGTG
AGGACTTGCATTGTGAAAGCTTTGCTGTCCTTGATGTGATCATGGAATCTCTTTCTCACT
AGAGTCTATGTCACTCATTATACTCTGTCGAATGTCATTGAATGTCTTTACATGGGCTTA
TATGCCTATGAAAATTGTAATACAACTTTCAGCAACGGATCTCTTGGCTCTCGCATCGAT
GAAGAACGCAGCGAAATGCGATAAGTAATGTGAATTGCAGAATTCAGTGAATCATCGAAT
CTTTGAACGCATCTTGCGCTCCTTGGTATTCCGAGGAGCATGTCTGTTTGAGTGTCATTA
AATTCTCAACTCTCTTCTAC-TTTTTGTAAAAGAGAGCTTGGACTGTGGAGGCTTGCTGG
CCACTTTTTGGGGTCAGCTCCTCTGAAATGCATTAGCGGAACCGTTTGCGATCTGCCACA
AGTGTGATAAGTTATCTACACTGGCGAGGGGATTGCTCTCTGTAATGTTCAGCTTCTAAT
TGTCTCTACTTTGTGAGACTACTTTTGAATGCTTGACCTCAAATCAGGTAGGACTACCCG
GCTGAACTTAA
```
