# Supplementary material for: Patterns of PCR Amplification Artifacts of the Fungal Barcode Marker in a Hybrid Mushroom
Source: Front Microbiol. 2019 Nov 19;10:2686. doi: 10.3389/fmicb.2019.02686 (PMC6877668; doi:10.3389/fmicb.2019.02686)
Supplement: Supplementary file 8 [file Data_Sheet_8.PDF]

>B1_1
TTTCCGTAGGTGAACCTGCGGAAGGATCATTATTGAATTATGTTTCTAGATAGGTTGTAG
CTGGCTCTTTAGAGCATGTGCACGCCTGTTTGGACTTCATTTTCATCCACCTGTGCACCT
ATTGTAGTCTTTGGTTGGGTTAGGGGGAAGTGGTCATTGTGTCAGCATCTGCTGGATGTG
AGGACTTGCATTGTGAAAGCTTTGCTGTCCTTGATGTGATCATGGAATCTCTTTCTCACT
AGAGTCTATGTCACTCATTATACTCTGTCGAATGTCATTGAATGTCTTTACATGGGCTTG
TATGCCTATGAAAATTGTAATACAACTTTCAGCAACGGATCTCTTGGCTCTCGCATCGAT
GAAGGACGCAGCGAAATGCGATAAGTAATGTGAATTGCAGAATTCAGTGAATCATCGAAT
CTTTGAACGCATCTTGCGCTCCTTGGTATTCCGAGGAGCATGCCTGTTTGAGTGTCATTA
AATTCTCAACTCTCTTATACTTTTTTGTAAAAGAGAGCTTGGACTGTGGAGGCTTGCTGG
CCACTTTTTGGGGTCAGCTCCTCTGAAATGCATTAGCGGAACCGTTTGCAATCTGCCACA
AGTGTGATAAGTTATCTACACTGGCGAGGGGATTGCTCTCTGTAATGTTCAGCTTCTAAT
TGTCTCTACTTTGTGAGACAACTTTTGAATGCTTGACCTCAAATCAGGTAGGACTACCCG
CTGAACTTAA
>B6_33
TTTCCGTAGGTGAACCTGCGGAAGGATCATTATTGAATTATGTTTCTAGATAGGTTGTAG
CTGGCTCTTTAGAGCATGTGCACGCCTGTTTGGACTTCATTTTCATCCACCTGTGCACCT
ATTGTAGTCTTTGGTTGGGTTAGGGGGAAGTGGTCATTGTGTCAGCATCTGCTGGATGTG
AGGACTTGCATTGTGAAAGCTTTGCTGTCCTTGATGTGATCATGGAATCTCTTTCTCACT
AGAGTCTATGTCACTCATTATACTCTGTCGAATGTCATTGAATGTCTTTACATGGGCTTG
TATGCCTATGAAAATTGTAATACAACTTTCAGCAACGGATCTCTTGGCTCTCGCATCGAT
GAAGGACGCAGCGAAATGCGATAAGTAATGTGAATTGCAGAATTCAGTGAATCATCGAAT
CTTTGAACGCATCTTGCGCTCCTTGGTATTCCGAGGAGCATGCCTGTTTGAGTGTCATTA
AATTCTCAACTCTCTTATACTTTTTTGTAAAAGAGAGCTTGGACTGTGGAGGCTTGCTGG
CCACTTTTTGGGGTCAGCTCCTCTGAAATGCATTAGCGGAACCGTTTGCAATCTGCCACA
AGTGTGATAAGTTATCTACACTGGCGAGGGGATTGCTCTCTGTAATGTTCAGCTTCTAAT
TGTCTCTACTTTGTGAGACAACTTTTGAATGCTTGACCTCAAATCAGGTAGGACTACCCG
CTGAACTTAA
>B7_3
TTTCCGTAGGTGAACCTGCGGAAGGATCATTATTGAATTATGTTTCTAGATAGGTTGTAG
CTGGCTCTTTAGAGCATGTGCACGCCTGTTTGGACTTCATTTTCATCCACCTGTGCACCT
ATTGTAGTCTTTGGTTGGGTTAGGGGGAAGTGGTCATTGTGTCAGCATCTGCTGGATGTG
AGGACTTGCATTGTGAAAGCTTTGCTGTCCTTGATGTGATCATGGAATCTCTTTCTCACT
AGAGTCTATGTCACTCATTATACTCTGTCGAATGTCATTGAATGTCTTTACATGGGCTTG
TATGCCTATGAAAATTGTAATACAACTTTCAGCAACGGATCTCTTGGCTCTCGCATCGAT
GAAGGACGCAGCGAAATGCGATAAGTAATGTGAATTGCAGAATTCAGTGAATCATCGAAT
CTTTGAACGCATCTTGCGCTCCTTGGTATTCCGAGGAGCATGCCTGTTTGAGTGTCATTA
AATTCTCAACTCTCTTATACTTTTTTGTAAAAGAGAGCTTGGACTGTGGAGGCTTGCTGG
CCACTTTTTGGGGTCAGCTCCTCTGAAATGCATTAGCGGAACCGTTTGCAATCTGCCACA
AGTGTGATAAGTTATCTACACTGGCGAGGGGATTGCTCTCTGTAATGTTCAGCTTCTAAT
TGTCTCTACTTTGTGAGACAACTTTTGAATGCTTGACCTCAAATCAGGTAGGACTACCCG
CTGAACTTAA
>B7_36
TTTCCGTAGGTGAACCTGCGGAAGGATCATTATTGAATTATGTTTCTAGATAGGTTGTAG
CTGGCTCTTTAGAGCATGTGCACGCCTGTTTGGACTTCATTTTCATCCACCTGTGCACCT
ATTGTAGTCTTTGGTTGGGTTAGGGGGAAGTGGTCATTGTGTCAGCATCTGCTGGATGTG
AGGACTTGCATTGTGAAAGCTTTGCTGTCCTTGATGTGATCATGGAATCTCTTTCTCACT
AGAGTCTATGTCACTCATTATACTCTGTCGAATGTCATTGAATGTCTTTACATGGGCTTG
TATGCCTATGAAAATTGTAATACAACTTTCAGCAACGGATCTCTTGGCTCTCGCATCGAT
GAAGGACGCAGCGAAATGCGATAAGTAATGTGAATTGCAGAATTCAGTGAATCATCGAAT

CTTTGAACGCATCTTGCGCTCCTTGGTATTCCGAGGAGCATGCCTGTTTGAGTGTCATTA
AATTCTCAACTCTCTTATACTTTTTTGTAAAAGAGAGCTTGGACTGTGGAGGCTTGCTGG
CCACTTTTTGGGGTCAGCTCCTCTGAAATGCATTAGCGGAACCGTTTGCAATCTGCCACA
AGTGTGATAAGTTATCTACACTGGCGAGGGGATTGCTCTCTGTAATGTTCAGCTTCTAAT
TGTCTCTACTTTGTGAGACAACTTTTGAATGCTTGACCTCAAATCAGGTAGGACTACCCG
CTGAACTTAA
>B7_42
TTTCCGTAGGTGAACCTGCGGAAGGATCATTATTGAATTATGTTTCTAGATAGGTTGTAG
CTGGCTCTTTAGAGCATGTGCACGCCTGTTTGGACTTCATTTTCATCCACCTGTGCACCT
ATTGTAGTCTTTGGTTGGGTTAGGGGGAAGTGGTCATTGTGTCAGCATCTGCTGGATGTG
AGGACTTGCATTGTGAAAGCTTTGCTGTCCTTGATGTGATCATGGAATCTCTTTCTCACT
AGAGTCTATGTCACTCATTATACTCTGTCGAATGTCATTGAATGTCTTTACATGGGCTTG
TATGCCTATGAAAATTGTAATACAACTTTCAGCAACGGATCTCTTGGCTCTCGCATCGAT
GAAGGACGCAGCGAAATGCGATAAGTAATGTGAATTGCAGAATTCAGTGAATCATCGAAT
CTTTGAACGCATCTTGCGCTCCTTGGTATTCCGAGGAGCATGCCTGTTTGAGTGTCATTA
AATTCTCAACTCTCTTATACTTTTTTGTAAAAGAGAGCTTGGACTGTGGAGGCTTGCTGG
CCACTTTTTGGGGTCAGCTCCTCTGAAATGCATTAGCGGAACCGTTTGCAATCTGCCACA
AGTGTGATAAGTTATCTACACTGGCGAGGGGATTGCTCTCTGTAATGTTCAGCTTCTAAT
TGTCTCTACTTTGTGAGACAACTTTTGAATGCTTGACCTCAAATCAGGTAGGACTACCCG
CTGAACTTAA
>B7_61
TTTCCGTAGGTGAACCTGCGGAAGGATCATTATTGAATTATGTTTCTAGATAGGTTGTAG
CTGGCTCTTTAGAGCATGTGCACGCCTGTTTGGACTTCATTTTCATCCACCTGTGCACCT
ATTGTAGTCTTTGGTTGGGTTAGGGGGAAGTGGTCATTGTGTCAGCATCTGCTGGATGTG
AGGACTTGCATTGTGAAAGCTTTGCTGTCCTTGATGTGATCATGGAATCTCTTTCTCACT
AGAGTCTATGTCACTCATTATACTCTGTCGAATGTCATTGAATGTCTTTACATGGGCTTG
TATGCCTATGAAAATTGTAATACAACTTTCAGCAACGGATCTCTTGGCTCTCGCATCGAT
GAAGGACGCAGCGAAATGCGATAAGTAATGTGAATTGCAGAATTCAGTGAATCATCGAAT
CTTTGAACGCATCTTGCGCTCCTTGGTATTCCGAGGAGCATGCCTGTTTGAGTGTCATTA
AATTCTCAACTCTCTTATACTTTTTTGTAAAAGAGAGCTTGGACTGTGGAGGCTTGCTGG
CCACTTTTTGGGGTCAGCTCCTCTGAAATGCATTAGCGGAACCGTTTGCAATCTGCCACA
AGTGTGATAAGTTATCTACACTGGCGAGGGGATTGCTCTCTGTAATGTTCAGCTTCTAAT
TGTCTCTACTTTGTGAGACAACTTTTGAATGCTTGACCTCAAATCAGGTAGGACTACCCG
CTGAACTTAA
>B7_73
TTTCCGTAGGTGAACCTGCGGAAGGATCATTATTGAATTATGTTTCTAGATAGGTTGTAG
CTGGCTCTTTAGAGCATGTGCACGCCTGTTTGGACTTCATTTTCATCCACCTGTGCACCT
ATTGTAGTCTTTGGTTGGGTTAGGGGGAAGTGGTCATTGTGTCAGCATCTGCTGGATGTG
AGGACTTGCATTGTGAAAGCTTTGCTGTCCTTGATGTGATCATGGAATCTCTTTCTCACT
AGAGTCTATGTCACTCATTATACTCTGTCGAATGTCATTGAATGTCTTTACATGGGCTTG
TATGCCTATGAAAATTGTAATACAACTTTCAGCAACGGATCTCTTGGCTCTCGCATCGAT
GAAGGACGCAGCGAAATGCGATAAGTAATGTGAATTGCAGAATTCAGTGAATCATCGAAT
CTTTGAACGCATCTTGCGCTCCTTGGTATTCCGAGGAGCATGCCTGTTTGAGTGTCATTA
AATTCTCAACTCTCTTATACTTTTTTGTAAAAGAGAGCTTGGACTGTGGAGGCTTGCTGG
CCACTTTTTGGGGTCAGCTCCTCTGAAATGCATTAGCGGAACCGTTTGCAATCTGCCACA
AGTGTGATAAGTTATCTACACTGGCGAGGGGATTGCTCTCTGTAATGTTCAGCTTCTAAT
TGTCTCTACTTTGTGAGACAACTTTTGAATGCTTGACCTCAAATCAGGTAGGACTACCCG
CTGAACTTAA
>B7_75
TTTCCGTAGGTGAACCTGCGGAAGGATCATTATTGAATTATGTTTCTAGATAGGTTGTAG

CTGGCTCTTTAGAGCATGTGCACGCCTGTTTGGACTTCATTTTCATCCACCTGTGCACCT
ATTGTAGTCTTTGGTTGGGTTAGGGGGAAGTGGTCATTGTGTCAGCATCTGCTGGATGTG
AGGACTTGCATTGTGAAAGCTTTGCTGTCCTTGATGTGATCATGGAATCTCTTTCTCACT
AGAGTCTATGTCACTCATTATACTCTGTCGAATGTCATTGAATGTCTTTACATGGGCTTG
TATGCCTATGAAAATTGTAATACAACTTTCAGCAACGGATCTCTTGGCTCTCGCATCGAT
GAAGGACGCAGCGAAATGCGATAAGTAATGTGAATTGCAGAATTCAGTGAATCATCGAAT
CTTTGAACGCATCTTGCGCTCCTTGGTATTCCGAGGAGCATGCCTGTTTGAGTGTCATTA
AATTCTCAACTCTCTTATACTTTTTTGTAAAAGAGAGCTTGGACTGTGGAGGCTTGCTGG
CCACTTTTTGGGGTCAGCTCCTCTGAAATGCATTAGCGGAACCGTTTGCAATCTGCCACA
AGTGTGATAAGTTATCTACACTGGCGAGGGGATTGCTCTCTGTAATGTTCAGCTTCTAAT
TGTCTCTACTTTGTGAGACAACTTTTGAATGCTTGACCTCAAATCAGGTAGGACTACCCG
CTGAACTTAA
>B9_22
TTTCCGTAGGTGAACCTGCGGAAGGATCATTATTGAATTATGTTTCTAGATAGGTTGTAG
CTGGCTCTTTAGAGCATGTGCACGCCTGTTTGGACTTCATTTTCATCCACCTGTGCACCT
ATTGTAGTCTTTGGTTGGGTTAGGGGGAAGTGGTCATTGTGTCAGCATCTGCTGGATGTG
AGGACTTGCATTGTGAAAGCTTTGCTGTCCTTGATGTGATCATGGAATCTCTTTCTCACT
AGAGTCTATGTCACTCATTATACTCTGTCGAATGTCATTGAATGTCTTTACATGGGCTTG
TATGCCTATGAAAATTGTAATACAACTTTCAGCAACGGATCTCTTGGCTCTCGCATCGAT
GAAGGACGCAGCGAAATGCGATAAGTAATGTGAATTGCAGAATTCAGTGAATCATCGAAT
CTTTGAACGCATCTTGCGCTCCTTGGTATTCCGAGGAGCATGCCTGTTTGAGTGTCATTA
AATTCTCAACTCTCTTATACTTTTTTGTAAAAGAGAGCTTGGACTGTGGAGGCTTGCTGG
CCACTTTTTGGGGTCAGCTCCTCTGAAATGCATTAGCGGAACCGTTTGCAATCTGCCACA
AGTGTGATAAGTTATCTACACTGGCGAGGGGATTGCTCTCTGTAATGTTCAGCTTCTAAT
TGTCTCTACTTTGTGAGACAACTTTTGAATGCTTGACCTCAAATCAGGTAGGACTACCCG
CTGAACTTAA
>B11_59
TTTCCGTAGGTGAACCTGCGGAAGGATCATTATTGAATTATGTTTCTAGATAGGTTGTAG
CTGGCTCTTTAGAGCATGTGCACGCCTGTTTGGACTTCATTTTCATCCACCTGTGCACCT
ATTGTAGTCTTTGGTTGGGTTAGGGGGAAGTGGTCATTGTGTCAGCATCTGCTGGATGTG
AGGACTTGCATTGTGAAAGCTTTGCTGTCCTTGATGTGATCATGGAATCTCTTTCTCACT
AGAGTCTATGTCACTCATTATACTCTGTCGAATGTCATTGAATGTCTTTACATGGGCTTG
TATGCCTATGAAAATTGTAATACAACTTTCAGCAACGGATCTCTTGGCTCTCGCATCGAT
GAAGGACGCAGCGAAATGCGATAAGTAATGTGAATTGCAGAATTCAGTGAATCATCGAAT
CTTTGAACGCATCTTGCGCTCCTTGGTATTCCGAGGAGCATGCCTGTTTGAGTGTCATTA
AATTCTCAACTCTCTTATACTTTTTTGTAAAAGAGAGCTTGGACTGTGGAGGCTTGCTGG
CCACTTTTTGGGGTCAGCTCCTCTGAAATGCATTAGCGGAACCGTTTGCAATCTGCCACA
AGTGTGATAAGTTATCTACACTGGCGAGGGGATTGCTCTCTGTAATGTTCAGCTTCTAAT
TGTCTCTACTTTGTGAGACAACTTTTGAATGCTTGACCTCAAATCAGGTAGGACTACCCG
CTGAACTTAA
>B7_44
TTTCCGTAGGTGAACCTGCGGAAGGATCATTATTGAATTATGTTTCTAGATAGGTTGTAG
CTGGCTCTTTAGAGCATGTGCACGCCTGTTTGGACTTCATTTTCATCCACCTGTGCACCT
ATTGTAGTCTTTGGTTGGGTTAGGGGGAAGTGGTCATTGTGTCAGCATCTGCTGGATGTG
AGGACTTGCATTGTGAAAGCTTTGCTGTCCTTGATGTGATCATGGAATCTCTTTCTCACT
AGAGTCTATGTCACTCATTATACTCTGTCGAATGTCATTGAATGTCTTTACATGGGCTTG
TATGCCTATGAAAATTGTAATACAACTTTCAGCAACGGATCTCTTGGCTCTCGCATCGAT
GAAGGACGCAGCGAAATGCGATAAGTAATGTGAATTGCAGAATTCAGTGAATCATCGAAT
CTTTGAACGCATCTTGCGCTCCTTGGTATTCCGAGGAGCATGCCTGTTTGAGTGTCATTA
AATTCTCAACTCTCTTATACTTTTTTGTAAAAGAGAGCTTGGACTGTGGAGGCTTGCTGG

CCACTTTTTGGGGTCAGCTCCTCTGAAATGCATTAGCGGAACCGTTTGCAATCTGCCACA
AGTGTGATAAGTTATCTACACTGGCGAGGGGATTGCTCTCTGTAATGTTCAGCTTCTAAT
TGTCTCTACTTTGTGAGACAACTTTTGAATGCTTGACCTCAAATCAGGTAGGACTACCCG
CTGAACTTAA
>B9_44
TTTCCGTAGGTGAACCTGCGGAAGGATCATTATTGAATTATGTTTCTAGATAGGTTGTAG
CTGGCTCTTTAGAGCATGTGCACGCCTGTTTGGACTTCATTTTCATCCACCTGTGCACCT
ATTGTAGTCTTTGGTTGGGTTAGGGGGAAGTGGTCATTGTGTCAGCATCTGCTGGATGTG
AGGACTTGCATTGTGAAAGCTTTGCTGTCCTTGATGTGATCATGGAATCTCTTTCTCACT
AGAGTCTATGTCACTCATTATACTCTGTCGAATGTCATTGAATGTCTTTACATGGGCTTG
TATGCCTATGAAAATTGTAATACAACTTTCAGCAACGGATCTCTTGGCTCTCGCATCGAT
GAAGGACGCAGCGAAATGCGATAAGTAATGTGAATTGCAGAATTCAGTGAATCATCGAAT
CTTTGAACGCATCTTGCGCTCCTTGGTATTCCGAGGAGCATGCCTGTTTGAGTGTCATTA
AATTCTCAACTCTCTTATACTTTTTTGTAAAAGAGAGCTTGGACTGTGGAGGCTTGCTGG
CCACTTTTTGGGGTCAGCTCCTCTGAAATGCATTAGCGGAACCGTTTGCAATCTGCCACA
AGTGTGATAAGTTATCTACACTGGCGAGGGGATTGCTCTCTGTAATGTTCAGCTTCTAAT
TGTCTCTACTTTGTGAGACAACTTTTGAATGCTTGACCTCAAATCAGGTAGGACTACCCG
CTGAACTTAA
>B2_11
TTTCCGTAGGTGAACCTGCGGAAGGATCATTATTGAATTATGTTTCTAGATAGGTTGTAG
CTGGCTCTTTAGAGCATGTGCACGCCTGTTTGGACTTCATTTTCATCCACCTGTGCACCT
ATTGTAGTCTTTGGTTGGGTTAGGGGGAAGTGGTCATTGTGTCAGCATCTGCTGGATGTG
AGGACTTGCATTGTGAAAGCTTTGCTGTCCTTGATGTGATCATGGAATCTCTTTCTCACT
AGAGTCTATGTCACTCATTATACTCTGTCGAATGTCATTGAATGTCTTTACATGGGCTTG
TATGCCTATGAAAATTGTAATACAACTTTCAGCAACGGATCTCTTGGCTCTCGCATCGAT
GAAGGACGCAGCGAAATGCGATAAGTAATGTGAATTGCAGAATTCAGTGAATCATCGAAT
CTTTGAACGCATCTTGCGCTCCTTGGTATTCCGAGGAGCATGCCTGTTTGAGTGTCATTA
AATTCTCAACTCTCTTATACTTTTTTGTAAAAGAGAGCTTGGACTGTGGAGGCTTGCTGG
CCACTTTTTGGGGTCAGCTCCTCTGAAATGCATTAGCGGAACCGTTTGCAATCTGCCACA
AGTGTGATAAGTTATCTACACTGGCGAGGGGATTGCTCTCTGTAATGTTCAGCTTCTAAT
TGTCTCTACTTTGTGAGACAACTTTTGAATGCTTGACCTCAAATCAGGTAGGACTACCCG
CTGAACTTAA
>B2_25
TTTCCGTAGGTGAACCTGCGGAAGGATCATTATTGAATTATGTTTCTAGATAGGTTGTAG
CTGGCTCTTTAGAGCATGTGCACGCCTGTTTGGACTTCATTTTCATCCACCTGTGCACCT
ATTGTAGTCTTTGGTTGGGTTAGGGGGAAGTGGTCATTGTGTCAGCATCTGCTGGATGTG
AGGACTTGCATTGTGAAAGCTTTGCTGTCCTTGATGTGATCATGGAATCTCTTTCTCACT
AGAGTCTATGTCACTCATTATACTCTGTCGAATGTCATTGAATGTCTTTACATGGGCTTG
TATGCCTATGAAAATTGTAATACAACTTTCAGCAACGGATCTCTTGGCTCTCGCATCGAT
GAAGGACGCAGCGAAATGCGATAAGTAATGTGAATTGCAGAATTCAGTGAATCATCGAAT
CTTTGAACGCATCTTGCGCTCCTTGGTATTCCGAGGAGCATGCCTGTTTGAGTGTCATTA
AATTCTCAACTCTCTTATACTTTTTTGTAAAAGAGAGCTTGGACTGTGGAGGCTTGCTGG
CCACTTTTTGGGGTCAGCTCCTCTGAAATGCATTAGCGGAACCGTTTGCAATCTGCCACA
AGTGTGATAAGTTATCTACACTGGCGAGGGGATTGCTCTCTGTAATGTTCAGCTTCTAAT
TGTCTCTACTTTGTGAGACAACTTTTGAATGCTTGACCTCAAATCAGGTAGGACTACCCG
CTGAACTTAA
>B2_32
TTTCCGTAGGTGAACCTGCGGAAGGATCATTATTGAATTATGTTTCTAGATAGGTTGTAG
CTGGCTCTTTAGAGCATGTGCACGCCTGTTTGGACTTCATTTTCATCCACCTGTGCACCT
ATTGTAGTCTTTGGTTGGGTTAGGGGGAAGTGGTCATTGTGTCAGCATCTGCTGGATGTG

AGGACTTGCATTGTGAAAGCTTTGCTGTCCTTGATGTGATCATGGAATCTCTTTCTCACT
AGAGTCTATGTCACTCATTATACTCTGTCGAATGTCATTGAATGTCTTTACATGGGCTTG
TATGCCTATGAAAATTGTAATACAACTTTCAGCAACGGATCTCTTGGCTCTCGCATCGAT
GAAGGACGCAGCGAAATGCGATAAGTAATGTGAATTGCAGAATTCAGTGAATCATCGAAT
CTTTGAACGCATCTTGCGCTCCTTGGTATTCCGAGGAGCATGCCTGTTTGAGTGTCATTA
AATTCTCAACTCTCTTATACTTTTTTGTAAAAGAGAGCTTGGACTGTGGAGGCTTGCTGG
CCACTTTTTGGGGTCAGCTCCTCTGAAATGCATTAGCGGAACCGTTTGCAATCTGCCACA
AGTGTGATAAGTTATCTACACTGGCGAGGGGATTGCTCTCTGTAATGTTCAGCTTCTAAT
TGTCTCTACTTTGTGAGACAACTTTTGAATGCTTGACCTCAAATCAGGTAGGACTACCCG
CTGAACTTAA
>B2−70
TTTCCGTAGGTGAACCTGCGGAAGGATCATTATTGAATTATGTTTCTAGATAGGTTGTAG
CTGGCTCTTTAGAGCATGTGCACGCCTGTTTGGACTTCATTTTCATCCACCTGTGCACCT
ATTGTAGTCTTTGGTTGGGTTAGGGGGAAGTGGTCATTGTGTCAGCATCTGCTGGATGTG
AGGACTTGCATTGTGAAAGCTTTGCTGTCCTTGATGTGATCATGGAATCTCTTTCTCACT
AGAGTCTATGTCACTCATTATACTCTGTCGAATGTCATTGAATGTCTTTACATGGGCTTG
TATGCCTATGAAAATTGTAATACAACTTTCAGCAACGGATCTCTTGGCTCTCGCATCGAT
GAAGGACGCAGCGAAATGCGATAAGTAATGTGAATTGCAGAATTCAGTGAATCATCGAAT
CTTTGAACGCATCTTGCGCTCCTTGGTATTCCGAGGAGCATGCCTGTTTGAGTGTCATTA
AATTCTCAACTCTCTTATACTTTTTTGTAAAAGAGAGCTTGGACTGTGGAGGCTTGCTGG
CCACTTTTTGGGGTCAGCTCCTCTGAAATGCATTAGCGGAACCGTTTGCAATCTGCCACA
AGTGTGATAAGTTATCTACACTGGCGAGGGGATTGCTCTCTGTAATGTTCAGCTTCTAAT
TGTCTCTACTTTGTGAGACAACTTTTGAATGCTTGACCTCAAATCAGGTAGGACTACCCG
CTGAACTTAA
>B5_17
TTTCCGTAGGTGAACCTGCGGAAGGATCATTATTGAATTATGTTTCTAGATAGGTTGTAG
CTGGCTCTTTAGAGCATGTGCACGCCTGTTTGGACTTCATTTTCATCCACCTGTGCACCT
ATTGTAGTCTTTGGTTGGGTTAGGGGGAAGTGGTCATTGTGTCAGCATCTGCTGGATGTG
AGGACTTGCATTGTGAAAGCTTTGCTGTCCTTGATGTGATCATGGAATCTCTTTCTCACT
AGAGTCTATGTCACTCATTATACTCTGTCGAATGTCATTGAATGTCTTTACATGGGCTTG
TATGCCTATGAAAATTGTAATACAACTTTCAGCAACGGATCTCTTGGCTCTCGCATCGAT
GAAGGACGCAGCGAAATGCGATAAGTAATGTGAATTGCAGAATTCAGTGAATCATCGAAT
CTTTGAACGCATCTTGCGCTCCTTGGTATTCCGAGGAGCATGCCTGTTTGAGTGTCATTA
AATTCTCAACTCTCTTATACTTTTTTGTAAAAGAGAGCTTGGACTGTGGAGGCTTGCTGG
CCACTTTTTGGGGTCAGCTCCTCTGAAATGCATTAGCGGAACCGTTTGCAATCTGCCACA
AGTGTGATAAGTTATCTACACTGGCGAGGGGATTGCTCTCTGTAATGTTCAGCTTCTAAT
TGTCTCTACTTTGTGAGACAACTTTTGAATGCTTGACCTCAAATCAGGTAGGACTACCCG
CTGAACTTAA
>B6_39
TTTCCGTAGGTGAACCTGCGGAAGGATCATTATTGAATTATGTTTCTAGATAGGTTGTAG
CTGGCTCTTTAGAGCATGTGCACGCCTGTTTGGACTTCATTTTCATCCACCTGTGCACCT
ATTGTAGTCTTTGGTTGGGTTAGGGGGAAGTGGTCATTGTGTCAGCATCTGCTGGATGTG
AGGACTTGCATTGTGAAAGCTTTGCTGTCCTTGATGTGATCATGGAATCTCTTTCTCACT
AGAGTCTATGTCACTCATTATACTCTGTCGAATGTCATTGAATGTCTTTACATGGGCTTG
TATGCCTATGAAAATTGTAATACAACTTTCAGCAACGGATCTCTTGGCTCTCGCATCGAT
GAAGGACGCAGCGAAATGCGATAAGTAATGTGAATTGCAGAATTCAGTGAATCATCGAAT
CTTTGAACGCATCTTGCGCTCCTTGGTATTCCGAGGAGCATGCCTGTTTGAGTGTCATTA
AATTCTCAACTCTCTTATACTTTTTTGTAAAAGAGAGCTTGGACTGTGGAGGCTTGCTGG
CCACTTTTTGGGGTCAGCTCCTCTGAAATGCATTAGCGGAACCGTTTGCAATCTGCCACA
AGTGTGATAAGTTATCTACACTGGCGAGGGGATTGCTCTCTGTAATGTTCAGCTTCTAAT

TGTCTCTACTTTGTGAGACAACTTTTGAATGCTTGACCTCAAATCAGGTAGGACTACCCG
CTGAACTTAA
>B6_53
TTTCCGTAGGTGAACCTGCGGAAGGATCATTATTGAATTATGTTTCTAGATAGGTTGTAG
CTGGCTCTTTAGAGCATGTGCACGCCTGTTTGGACTTCATTTTCATCCACCTGTGCACCT
ATTGTAGTCTTTGGTTGGGTTAGGGGGAAGTGGTCATTGTGTCAGCATCTGCTGGATGTG
AGGACTTGCATTGTGAAAGCTTTGCTGTCCTTGATGTGATCATGGAATCTCTTTCTCACT
AGAGTCTATGTCACTCATTATACTCTGTCGAATGTCATTGAATGTCTTTACATGGGCTTG
TATGCCTATGAAAATTGTAATACAACTTTCAGCAACGGATCTCTTGGCTCTCGCATCGAT
GAAGGACGCAGCGAAATGCGATAAGTAATGTGAATTGCAGAATTCAGTGAATCATCGAAT
CTTTGAACGCATCTTGCGCTCCTTGGTATTCCGAGGAGCATGCCTGTTTGAGTGTCATTA
AATTCTCAACTCTCTTATACTTTTTTGTAAAAGAGAGCTTGGACTGTGGAGGCTTGCTGG
CCACTTTTTGGGGTCAGCTCCTCTGAAATGCATTAGCGGAACCGTTTGCAATCTGCCACA
AGTGTGATAAGTTATCTACACTGGCGAGGGGATTGCTCTCTGTAATGTTCAGCTTCTAAT
TGTCTCTACTTTGTGAGACAACTTTTGAATGCTTGACCTCAAATCAGGTAGGACTACCCG
CTGAACTTAA
>B2-55
TTTCCGTAGGTGAACCTGCGGAAGGATCATTATTGAATTATGTTTCTAGATAGGTTGTAG
CTGGCTCTTTAGAGCATGTGCACGCCTGTTTGGACTTCATTTTCATCCACCTGTGCACCT
ATTGTAGTCTTTGGTTGGGTTAGGGGGAAGTGGTCATTGTGTCAGCATCTGCTGGATGTG
AGGACTTGCATTGTGAAAGCTTTGCTGTCCTTGATGTGATCATGGAATCTCTTTCTCACT
AGAGTCTATGTCACTCATTATACTCTGTCGAATGTCATTGAATGTCTTTACATGGGCTTG
TATGCCTATGAAAATTGTAATACAACTTTCAGCAACGGATCTCTTGGCTCTCGCATCGAT
GAAGGACGCAGCGAAATGCGATAAGTAATGTGAATTGCAGAATTCAGTGAATCATCGAAT
CTTTGAACGCATCTTGCGCTCCTTGGTATTCCGAGGAGCATGCCTGTTTGAGTGTCATTA
AATTCTCAACTCTCTTATACTTTTTTGTAAAAGAGAGCTTGGACTGTGGAGGCTTGCTGG
CCACTTTTTGGGGTCAGCTCCTCTGAAATGCATTAGCGGAACCGTTTGCAATCTGCCACA
AGTGTGATAAGTTATCTACACTGGCGAGGGGATTGCTCTCTGTAATGTTCAGCTTCTAAT
TGTCTCTACTTTGTGAGACAACTTTTGAATGCTTGACCTCAAATCAGGTAGGACTACCCG
CTGAACTTAA
>B6_58
TTTCCGTAGGTGAACCTGCGGAAGGATCATTATTGAATTATGTTTCTAGATAGGTTGTAG
CTGGCTCTTTAGAGCATGTGCACGCCTGTTTGGACTTCATTTTCATCCACCTGTGCACCT
ATTGTAGTCTTTGGTTGGGTTAGGGGGAAGTGGTCATTGTGTCAGCATCTGCTGGATGTG
AGGACTTGCATTGTGAAAGCTTTGCTGTCCTTGATGTGATCATGGAATCTCTTTCTCACT
AGAGTCTATGTCACTCATTATACTCTGTCGAATGTCATTGAATGTCTTTACATGGGCTTG
TATGCCTATGAAAATTGTAATACAACTTTCAGCAACGGATCTCTTGGCTCTCGCATCGAT
GAAGGACGCAGCGAAATGCGATAAGTAATGTGAATTGCAGAATTCAGTGAATCATCGAAT
CTTTGAACGCATCTTGCGCTCCTTGGTATTCCGAGGAGCATGCCTGTTTGAGTGTCATTA
AATTCTCAACTCTCTTATACTTTTTTGTAAAAGAGAGCTTGGACTGTGGAGGCTTGCTGG
CCACTTTTTGGGGTCAGCTCCTCTGAAATGCATTAGCGGAACCGTTTGCAATCTGCCACA
AGTGTGATAAGTTATCTACACTGGCGAGGGGATTGCTCTCTGTAATGTTCAGCTTCTAAT
TGTCTCTACTTTGTGAGACAACTTTTGAATGCTTGACCTCAAATCAGGTAGGACTACCCG
CTGAACTTAA
>B7_45
TTTCCGTAGGTGAACCTGCGGAAGGATCATTATTGAATTATGTTTCTAGATAGGTTGTAG
CTGGCTCTTTAGAGCATGTGCACGCCTGTTTGGACTTCATTTTCATCCACCTGTGCACCT
ATTGTAGTCTTTGGTTGGGTTAGGGGGAAGTGGTCATTGTGTCAGCATCTGCTGGATGTG
AGGACTTGCATTGTGAAAGCTTTGCTGTCCTTGATGTGATCATGGAATCTCTTTCTCACT
AGAGTCTATGTCACTCATTATACTCTGTCGAATGTCATTGAATGTCTTTACATGGGCTTG

TATGCCTATGAAAATTGTAATACAACTTTCAGCAACGGATCTCTTGGCTCTCGCATCGAT
GAAGGACGCAGCGAAATGCGATAAGTAATGTGAATTGCAGAATTCAGTGAATCATCGAAT
CTTTGAACGCATCTTGCGCTCCTTGGTATTCCGAGGAGCATGCCTGTTTGAGTGTCATTA
AATTCTCAACTCTCTTATACTTTTTTGTAAAAGAGAGCTTGGACTGTGGAGGCTTGCTGG
CCACTTTTTGGGGTCAGCTCCTCTGAAATGCATTAGCGGAACCGTTTGCAATCTGCCACA
AGTGTGATAAGTTATCTACACTGGCGAGGGGATTGCTCTCTGTAATGTTCAGCTTCTAAT
TGTCTCTACTTTGTGAGACAACTTTTGAATGCTTGACCTCAAATCAGGTAGGACTACCCG
CTGAACTTAA
>B1_9
TTTCCGTAGGTGAACCTGCGGAAGGATCATTATTGAATTATGTTTCTAGATAGGTTGTAG
CTGGCTCTTTAGAGCATGTGCACGCCTGTTTGGACTTCATTTTCATCCACCTGTGCACCT
ATTGTAGTCTTTGGTTGGGTTAGGGGGAAGTGGTCATTGTGTCAGCATCTGCTGGATGTG
AGGACTTGCATTGTGAAAGCTTTGCTGTCCTTGATGTGATCATGGAATCTCTTTCTCACT
AGAGTCTATGTCACTCATTATACTCTGTCGAATGTCATTGAATGTCTTTACATGGGCTTG
TATGCCTATGAAAATTGTAATACAACTTTCAGCAACGGATCTCTTGGCTCTCGCATCGAT
GAAGGACGCAGCGAAATGCGATAAGTAATGTGAATTGCAGAATTCAGTGAATCATCGAAT
CTTTGAACGCATCTTGCGCTCCTTGGTATTCCGAGGAGCATGCCTGTTTGAGTGTCATTA
AATTCTCAACTCTCTTATACTTTTTTGTAAAAGAGAGCTTGGACTGTGGAGGCTTGCTGG
CCACTTTTTGGGGTCAGCTCCTCTGAAATGCATTAGCGGAACCGTTTGCAATCTGCCACA
AGTGTGATAAGTTATCTACACTGGCGAGGGGATTGCTCTCTGTAATGTTCAGCTTCTAAT
TGTCTCTACTTTGTGAGACAACTTTTGAATGCTTGACCTCAAATCAGGTAGGACTACCCG
CTGAACTTAA
>B7_47
TTTCCGTAGGTGAACCTGCGGAAGGATCATTATTGAATTATGTTTCTAGATAGGTTGTAG
CTGGCTCTTTAGAGCATGTGCACGCCTGTTTGGACTTCATTTTCATCCACCTGTGCACCT
ATTGTAGTCTTTGGTTGGGTTAGGGGGAAGTGGTCATTGTGTCAGCATCTGCTGGATGTG
AGGACTTGCATTGTGAAAGCTTTGCTGTCCTTGATGTGATCATGGAATCTCTTTCTCACT
AGAGTCTATGTCACTCATTATACTCTGTCGAATGTCATTGAATGTCTTTACATGGGCTTG
TATGCCTATGAAAATTGTAATACAACTTTCAGCAACGGATCTCTTGGCTCTCGCATCGAT
GAAGGACGCAGCGAAATGCGATAAGTAATGTGAATTGCAGAATTCAGTGAATCATCGAAT
CTTTGAACGCATCTTGCGCTCCTTGGTATTCCGAGGAGCATGCCTGTTTGAGTGTCATTA
AATTCTCAACTCTCTTATACTTTTTTGTAAAAGAGAGCTTGGACTGTGGAGGCTTGCTGG
CCACTTTTTGGGGTCAGCTCCTCTGAAATGCATTAGCGGAACCGTTTGCAATCTGCCACA
AGTGTGATAAGTTATCTACACTGGCGAGGGGATTGCTCTCTGTAATGTTCAGCTTCTAAT
TGTCTCTACTTTGTGAGACAACTTTTGAATGCTTGACCTCAAATCAGGTAGGACTACCCG
CTGAACTTAA
>B1_2
TTTCCGTAGGTGAACCTGCGGAAGGATCATTATTGAATTATGTTTCTAGATAGGTTGTAG
CTGGCTCTTTAGAGCATGTGCACGCCTGTTTGGACTTCATTTTCATCCACCTGTGCACCT
ATTGTAGTCTTTGGTTGGGTTAGGGGGAAGTGGTCATTGTGTCAGCATCTGCTGGATGTG
AGGACTTGCATTGTGAAAGCTTTGCTGTCCTTGATGTGATCATGGAATCTCTTTCTCACT
AGAGTCTATGTCACTCATTATACTCTGTCGAATGTCATTGAATGTCTTTACATGGGCTTG
TATGCCTATGAAAATTGTAATACAACTTTCAGCAACGGATCTCTTGGCTCTCGCATCGAT
GAAGGACGCAGCGAAATGCGATAAGTAATGTGAATTGCAGAATTCAGTGAATCATCGAAT
CTTTGAACGCATCTTGCGCTCCTTGGTATTCCGAGGAGCATGCCTGTTTGAGTGTCATTA
AATTCTCAACTCTCTTATACTTTTTTGTAAAAGAGAGCTTGGACTGTGGAGGCTTGCTGG
CCACTTTTTGGGGTCAGCTCCTCTGAAATGCATTAGCGGAACCGTTTGCAATCTGCCACA
AGTGTGATAAGTTATCTACACTGGCGAGGGGATTGCTCTCTGTAATGTTCAGCTTCTAAT
TGTCTCTACTTTGTGAGACAACTTTTGAATGCTTGACCTCAAATCAGGTAGGACTACCCG
CTGAACTTAA

>B1_3
TTTCCGTAGGTGAACCTGCGGAAGGATCATTATTGAATTATGTTTCTAGATAGGTTGTAG
CTGGCTCTTTAGAGCATGTGCACGCCTGTTTGGACTTCATTTTCATCCACCTGTGCACCT
ATTGTAGTCTTTGGTTGGGTTAGGGGGAAGTGGTCATTGTGTCAGCATCTGCTGGATGTG
AGGACTTGCATTGTGAAAGCTTTGCTGTCCTTGATGTGATCATGGAATCTCTTTCTCACT
AGAGTCTATGTCACTCATTATACTCTGTCGAATGTCATTGAATGTCTTTACATGGGCTTG
TATGCCTATGAAAATTGTAATACAACTTTCAGCAACGGATCTCTTGGCTCTCGCATCGAT
GAAGGACGCAGCGAAATGCGATAAGTAATGTGAATTGCAGAATTCAGTGAATCATCGAAT
CTTTGAACGCATCTTGCGCTCCTTGGTATTCCGAGGAGCATGCCTGTTTGAGTGTCATTA
AATTCTCAACTCTCTTATACTTTTTTGTAAAAGAGAGCTTGGACTGTGGAGGCTTGCTGG
CCACTTTTTGGGGTCAGCTCCTCTGAAATGCATTAGCGGAACCGTTTGCAATCTGCCACA
AGTGTGATAAGTTATCTACACTGGCGAGGGGATTGCTCTCTGTAATGTTCAGCTTCTAAT
TGTCTCTACTTTGTGAGACAACTTTTGAATGCTTGACCTCAAATCAGGTAGGACTACCCG
CTGAACTTAA
>B1_4
TTTCCGTAGGTGAACCTGCGGAAGGATCATTATTGAATTATGTTTCTAGATAGGTTGTAG
CTGGCTCTTTAGAGCATGTGCACGCCTGTTTGGACTTCATTTTCATCCACCTGTGCACCT
ATTGTAGTCTTTGGTTGGGTTAGGGGGAAGTGGTCATTGTGTCAGCATCTGCTGGATGTG
AGGACTTGCATTGTGAAAGCTTTGCTGTCCTTGATGTGATCATGGAATCTCTTTCTCACT
AGAGTCTATGTCACTCATTATACTCTGTCGAATGTCATTGAATGTCTTTACATGGGCTTG
TATGCCTATGAAAATTGTAATACAACTTTCAGCAACGGATCTCTTGGCTCTCGCATCGAT
GAAGGACGCAGCGAAATGCGATAAGTAATGTGAATTGCAGAATTCAGTGAATCATCGAAT
CTTTGAACGCATCTTGCGCTCCTTGGTATTCCGAGGAGCATGCCTGTTTGAGTGTCATTA
AATTCTCAACTCTCTTATACTTTTTTGTAAAAGAGAGCTTGGACTGTGGAGGCTTGCTGG
CCACTTTTTGGGGTCAGCTCCTCTGAAATGCATTAGCGGAACCGTTTGCAATCTGCCACA
AGTGTGATAAGTTATCTACACTGGCGAGGGGATTGCTCTCTGTAATGTTCAGCTTCTAAT
TGTCTCTACTTTGTGAGACAACTTTTGAATGCTTGACCTCAAATCAGGTAGGACTACCCG
CTGAACTTAA
>B1_5
TTTCCGTAGGTGAACCTGCGGAAGGATCATTATTGAATTATGTTTCTAGATAGGTTGTAG
CTGGCTCTTTAGAGCATGTGCACGCCTGTTTGGACTTCATTTTCATCCACCTGTGCACCT
ATTGTAGTCTTTGGTTGGGTTAGGGGGAAGTGGTCATTGTGTCAGCATCTGCTGGATGTG
AGGACTTGCATTGTGAAAGCTTTGCTGTCCTTGATGTGATCATGGAATCTCTTTCTCACT
AGAGTCTATGTCACTCATTATACTCTGTCGAATGTCATTGAATGTCTTTACATGGGCTTG
TATGCCTATGAAAATTGTAATACAACTTTCAGCAACGGATCTCTTGGCTCTCGCATCGAT
GAAGGACGCAGCGAAATGCGATAAGTAATGTGAATTGCAGAATTCAGTGAATCATCGAAT
CTTTGAACGCATCTTGCGCTCCTTGGTATTCCGAGGAGCATGCCTGTTTGAGTGTCATTA
AATTCTCAACTCTCTTATACTTTTTTGTAAAAGAGAGCTTGGACTGTGGAGGCTTGCTGG
CCACTTTTTGGGGTCAGCTCCTCTGAAATGCATTAGCGGAACCGTTTGCAATCTGCCACA
AGTGTGATAAGTTATCTACACTGGCGAGGGGATTGCTCTCTGTAATGTTCAGCTTCTAAT
TGTCTCTACTTTGTGAGACAACTTTTGAATGCTTGACCTCAAATCAGGTAGGACTACCCG
CTGAACTTAA
>B1_6
TTTCCGTAGGTGAACCTGCGGAAGGATCATTATTGAATTATGTTTCTAGATAGGTTGTAG
CTGGCTCTTTAGAGCATGTGCACGCCTGTTTGGACTTCATTTTCATCCACCTGTGCACCT
ATTGTAGTCTTTGGTTGGGTTAGGGGGAAGTGGTCATTGTGTCAGCATCTGCTGGATGTG
AGGACTTGCATTGTGAAAGCTTTGCTGTCCTTGATGTGATCATGGAATCTCTTTCTCACT
AGAGTCTATGTCACTCATTATACTCTGTCGAATGTCATTGAATGTCTTTACATGGGCTTG
TATGCCTATGAAAATTGTAATACAACTTTCAGCAACGGATCTCTTGGCTCTCGCATCGAT
GAAGGACGCAGCGAAATGCGATAAGTAATGTGAATTGCAGAATTCAGTGAATCATCGAAT

CTTTGAACGCATCTTGCGCTCCTTGGTATTCCGAGGAGCATGCCTGTTTGAGTGTCATTA
AATTCTCAACTCTCTTATACTTTTTTGTAAAAGAGAGCTTGGACTGTGGAGGCTTGCTGG
CCACTTTTTGGGGTCAGCTCCTCTGAAATGCATTAGCGGAACCGTTTGCAATCTGCCACA
AGTGTGATAAGTTATCTACACTGGCGAGGGGATTGCTCTCTGTAATGTTCAGCTTCTAAT
TGTCTCTACTTTGTGAGACAACTTTTGAATGCTTGACCTCAAATCAGGTAGGACTACCCG
CTGAACTTAA
>B1_7
TTTCCGTAGGTGAACCTGCGGAAGGATCATTATTGAATTATGTTTCTAGATAGGTTGTAG
CTGGCTCTTTAGAGCATGTGCACGCCTGTTTGGACTTCATTTTCATCCACCTGTGCACCT
ATTGTAGTCTTTGGTTGGGTTAGGGGGAAGTGGTCATTGTGTCAGCATCTGCTGGATGTG
AGGACTTGCATTGTGAAAGCTTTGCTGTCCTTGATGTGATCATGGAATCTCTTTCTCACT
AGAGTCTATGTCACTCATTATACTCTGTCGAATGTCATTGAATGTCTTTACATGGGCTTG
TATGCCTATGAAAATTGTAATACAACTTTCAGCAACGGATCTCTTGGCTCTCGCATCGAT
GAAGGACGCAGCGAAATGCGATAAGTAATGTGAATTGCAGAATTCAGTGAATCATCGAAT
CTTTGAACGCATCTTGCGCTCCTTGGTATTCCGAGGAGCATGCCTGTTTGAGTGTCATTA
AATTCTCAACTCTCTTATACTTTTTTGTAAAAGAGAGCTTGGACTGTGGAGGCTTGCTGG
CCACTTTTTGGGGTCAGCTCCTCTGAAATGCATTAGCGGAACCGTTTGCAATCTGCCACA
AGTGTGATAAGTTATCTACACTGGCGAGGGGATTGCTCTCTGTAATGTTCAGCTTCTAAT
TGTCTCTACTTTGTGAGACAACTTTTGAATGCTTGACCTCAAATCAGGTAGGACTACCCG
CTGAACTTAA
>B1_8
TTTCCGTAGGTGAACCTGCGGAAGGATCATTATTGAATTATGTTTCTAGATAGGTTGTAG
CTGGCTCTTTAGAGCATGTGCACGCCTGTTTGGACTTCATTTTCATCCACCTGTGCACCT
ATTGTAGTCTTTGGTTGGGTTAGGGGGAAGTGGTCATTGTGTCAGCATCTGCTGGATGTG
AGGACTTGCATTGTGAAAGCTTTGCTGTCCTTGATGTGATCATGGAATCTCTTTCTCACT
AGAGTCTATGTCACTCATTATACTCTGTCGAATGTCATTGAATGTCTTTACATGGGCTTG
TATGCCTATGAAAATTGTAATACAACTTTCAGCAACGGATCTCTTGGCTCTCGCATCGAT
GAAGGACGCAGCGAAATGCGATAAGTAATGTGAATTGCAGAATTCAGTGAATCATCGAAT
CTTTGAACGCATCTTGCGCTCCTTGGTATTCCGAGGAGCATGCCTGTTTGAGTGTCATTA
AATTCTCAACTCTCTTATACTTTTTTGTAAAAGAGAGCTTGGACTGTGGAGGCTTGCTGG
CCACTTTTTGGGGTCAGCTCCTCTGAAATGCATTAGCGGAACCGTTTGCAATCTGCCACA
AGTGTGATAAGTTATCTACACTGGCGAGGGGATTGCTCTCTGTAATGTTCAGCTTCTAAT
TGTCTCTACTTTGTGAGACAACTTTTGAATGCTTGACCTCAAATCAGGTAGGACTACCCG
CTGAACTTAA
>B1_10
TTTCCGTAGGTGAACCTGCGGAAGGATCATTATTGAATTATGTTTCTAGATAGGTTGTAG
CTGGCTCTTTAGAGCATGTGCACGCCTGTTTGGACTTCATTTTCATCCACCTGTGCACCT
ATTGTAGTCTTTGGTTGGGTTAGGGGGAAGTGGTCATTGTGTCAGCATCTGCTGGATGTG
AGGACTTGCATTGTGAAAGCTTTGCTGTCCTTGATGTGATCATGGAATCTCTTTCTCACT
AGAGTCTATGTCACTCATTATACTCTGTCGAATGTCATTGAATGTCTTTACATGGGCTTG
TATGCCTATGAAAATTGTAATACAACTTTCAGCAACGGATCTCTTGGCTCTCGCATCGAT
GAAGGACGCAGCGAAATGCGATAAGTAATGTGAATTGCAGAATTCAGTGAATCATCGAAT
CTTTGAACGCATCTTGCGCTCCTTGGTATTCCGAGGAGCATGCCTGTTTGAGTGTCATTA
AATTCTCAACTCTCTTATACTTTTTTGTAAAAGAGAGCTTGGACTGTGGAGGCTTGCTGG
CCACTTTTTGGGGTCAGCTCCTCTGAAATGCATTAGCGGAACCGTTTGCAATCTGCCACA
AGTGTGATAAGTTATCTACACTGGCGAGGGGATTGCTCTCTGTAATGTTCAGCTTCTAAT
TGTCTCTACTTTGTGAGACAACTTTTGAATGCTTGACCTCAAATCAGGTAGGACTACCCG
CTGAACTTAA
>B1_12
TTTCCGTAGGTGAACCTGCGGAAGGATCATTATTGAATTATGTTTCTAGATAGGTTGTAG

CTGGCTCTTTAGAGCATGTGCACGCCTGTTTGGACTTCATTTTCATCCACCTGTGCACCT
ATTGTAGTCTTTGGTTGGGTTAGGGGGAAGTGGTCATTGTGTCAGCATCTGCTGGATGTG
AGGACTTGCATTGTGAAAGCTTTGCTGTCCTTGATGTGATCATGGAATCTCTTTCTCACT
AGAGTCTATGTCACTCATTATACTCTGTCGAATGTCATTGAATGTCTTTACATGGGCTTG
TATGCCTATGAAAATTGTAATACAACTTTCAGCAACGGATCTCTTGGCTCTCGCATCGAT
GAAGGACGCAGCGAAATGCGATAAGTAATGTGAATTGCAGAATTCAGTGAATCATCGAAT
CTTTGAACGCATCTTGCGCTCCTTGGTATTCCGAGGAGCATGCCTGTTTGAGTGTCATTA
AATTCTCAACTCTCTTATACTTTTTTGTAAAAGAGAGCTTGGACTGTGGAGGCTTGCTGG
CCACTTTTTGGGGTCAGCTCCTCTGAAATGCATTAGCGGAACCGTTTGCAATCTGCCACA
AGTGTGATAAGTTATCTACACTGGCGAGGGGATTGCTCTCTGTAATGTTCAGCTTCTAAT
TGTCTCTACTTTGTGAGACAACTTTTGAATGCTTGACCTCAAATCAGGTAGGACTACCCG
CTGAACTTAA
>B1_14
TTTCCGTAGGTGAACCTGCGGAAGGATCATTATTGAATTATGTTTCTAGATAGGTTGTAG
CTGGCTCTTTAGAGCATGTGCACGCCTGTTTGGACTTCATTTTCATCCACCTGTGCACCT
ATTGTAGTCTTTGGTTGGGTTAGGGGGAAGTGGTCATTGTGTCAGCATCTGCTGGATGTG
AGGACTTGCATTGTGAAAGCTTTGCTGTCCTTGATGTGATCATGGAATCTCTTTCTCACT
AGAGTCTATGTCACTCATTATACTCTGTCGAATGTCATTGAATGTCTTTACATGGGCTTG
TATGCCTATGAAAATTGTAATACAACTTTCAGCAACGGATCTCTTGGCTCTCGCATCGAT
GAAGGACGCAGCGAAATGCGATAAGTAATGTGAATTGCAGAATTCAGTGAATCATCGAAT
CTTTGAACGCATCTTGCGCTCCTTGGTATTCCGAGGAGCATGCCTGTTTGAGTGTCATTA
AATTCTCAACTCTCTTATACTTTTTTGTAAAAGAGAGCTTGGACTGTGGAGGCTTGCTGG
CCACTTTTTGGGGTCAGCTCCTCTGAAATGCATTAGCGGAACCGTTTGCAATCTGCCACA
AGTGTGATAAGTTATCTACACTGGCGAGGGGATTGCTCTCTGTAATGTTCAGCTTCTAAT
TGTCTCTACTTTGTGAGACAACTTTTGAATGCTTGACCTCAAATCAGGTAGGACTACCCG
CTGAACTTAA
>B1_15
TTTCCGTAGGTGAACCTGCGGAAGGATCATTATTGAATTATGTTTCTAGATAGGTTGTAG
CTGGCTCTTTAGAGCATGTGCACGCCTGTTTGGACTTCATTTTCATCCACCTGTGCACCT
ATTGTAGTCTTTGGTTGGGTTAGGGGGAAGTGGTCATTGTGTCAGCATCTGCTGGATGTG
AGGACTTGCATTGTGAAAGCTTTGCTGTCCTTGATGTGATCATGGAATCTCTTTCTCACT
AGAGTCTATGTCACTCATTATACTCTGTCGAATGTCATTGAATGTCTTTACATGGGCTTG
TATGCCTATGAAAATTGTAATACAACTTTCAGCAACGGATCTCTTGGCTCTCGCATCGAT
GAAGGACGCAGCGAAATGCGATAAGTAATGTGAATTGCAGAATTCAGTGAATCATCGAAT
CTTTGAACGCATCTTGCGCTCCTTGGTATTCCGAGGAGCATGCCTGTTTGAGTGTCATTA
AATTCTCAACTCTCTTATACTTTTTTGTAAAAGAGAGCTTGGACTGTGGAGGCTTGCTGG
CCACTTTTTGGGGTCAGCTCCTCTGAAATGCATTAGCGGAACCGTTTGCAATCTGCCACA
AGTGTGATAAGTTATCTACACTGGCGAGGGGATTGCTCTCTGTAATGTTCAGCTTCTAAT
TGTCTCTACTTTGTGAGACAACTTTTGAATGCTTGACCTCAAATCAGGTAGGACTACCCG
CTGAACTTAA
>B1_16
TTTCCGTAGGTGAACCTGCGGAAGGATCATTATTGAATTATGTTTCTAGATAGGTTGTAG
CTGGCTCTTTAGAGCATGTGCACGCCTGTTTGGACTTCATTTTCATCCACCTGTGCACCT
ATTGTAGTCTTTGGTTGGGTTAGGGGGAAGTGGTCATTGTGTCAGCATCTGCTGGATGTG
AGGACTTGCATTGTGAAAGCTTTGCTGTCCTTGATGTGATCATGGAATCTCTTTCTCACT
AGAGTCTATGTCACTCATTATACTCTGTCGAATGTCATTGAATGTCTTTACATGGGCTTG
TATGCCTATGAAAATTGTAATACAACTTTCAGCAACGGATCTCTTGGCTCTCGCATCGAT
GAAGGACGCAGCGAAATGCGATAAGTAATGTGAATTGCAGAATTCAGTGAATCATCGAAT
CTTTGAACGCATCTTGCGCTCCTTGGTATTCCGAGGAGCATGCCTGTTTGAGTGTCATTA
AATTCTCAACTCTCTTATACTTTTTTGTAAAAGAGAGCTTGGACTGTGGAGGCTTGCTGG

CCACTTTTTGGGGTCAGCTCCTCTGAAATGCATTAGCGGAACCGTTTGCAATCTGCCACA
AGTGTGATAAGTTATCTACACTGGCGAGGGGATTGCTCTCTGTAATGTTCAGCTTCTAAT
TGTCTCTACTTTGTGAGACAACTTTTGAATGCTTGACCTCAAATCAGGTAGGACTACCCG
CTGAACTTAA
>B1_17
TTTCCGTAGGTGAACCTGCGGAAGGATCATTATTGAATTATGTTTCTAGATAGGTTGTAG
CTGGCTCTTTAGAGCATGTGCACGCCTGTTTGGACTTCATTTTCATCCACCTGTGCACCT
ATTGTAGTCTTTGGTTGGGTTAGGGGGAAGTGGTCATTGTGTCAGCATCTGCTGGATGTG
AGGACTTGCATTGTGAAAGCTTTGCTGTCCTTGATGTGATCATGGAATCTCTTTCTCACT
AGAGTCTATGTCACTCATTATACTCTGTCGAATGTCATTGAATGTCTTTACATGGGCTTG
TATGCCTATGAAAATTGTAATACAACTTTCAGCAACGGATCTCTTGGCTCTCGCATCGAT
GAAGGACGCAGCGAAATGCGATAAGTAATGTGAATTGCAGAATTCAGTGAATCATCGAAT
CTTTGAACGCATCTTGCGCTCCTTGGTATTCCGAGGAGCATGCCTGTTTGAGTGTCATTA
AATTCTCAACTCTCTTATACTTTTTTGTAAAAGAGAGCTTGGACTGTGGAGGCTTGCTGG
CCACTTTTTGGGGTCAGCTCCTCTGAAATGCATTAGCGGAACCGTTTGCAATCTGCCACA
AGTGTGATAAGTTATCTACACTGGCGAGGGGATTGCTCTCTGTAATGTTCAGCTTCTAAT
TGTCTCTACTTTGTGAGACAACTTTTGAATGCTTGACCTCAAATCAGGTAGGACTACCCG
CTGAACTTAA
>B1_18
TTTCCGTAGGTGAACCTGCGGAAGGATCATTATTGAATTATGTTTCTAGATAGGTTGTAG
CTGGCTCTTTAGAGCATGTGCACGCCTGTTTGGACTTCATTTTCATCCACCTGTGCACCT
ATTGTAGTCTTTGGTTGGGTTAGGGGGAAGTGGTCATTGTGTCAGCATCTGCTGGATGTG
AGGACTTGCATTGTGAAAGCTTTGCTGTCCTTGATGTGATCATGGAATCTCTTTCTCACT
AGAGTCTATGTCACTCATTATACTCTGTCGAATGTCATTGAATGTCTTTACATGGGCTTG
TATGCCTATGAAAATTGTAATACAACTTTCAGCAACGGATCTCTTGGCTCTCGCATCGAT
GAAGGACGCAGCGAAATGCGATAAGTAATGTGAATTGCAGAATTCAGTGAATCATCGAAT
CTTTGAACGCATCTTGCGCTCCTTGGTATTCCGAGGAGCATGCCTGTTTGAGTGTCATTA
AATTCTCAACTCTCTTATACTTTTTTGTAAAAGAGAGCTTGGACTGTGGAGGCTTGCTGG
CCACTTTTTGGGGTCAGCTCCTCTGAAATGCATTAGCGGAACCGTTTGCAATCTGCCACA
AGTGTGATAAGTTATCTACACTGGCGAGGGGATTGCTCTCTGTAATGTTCAGCTTCTAAT
TGTCTCTACTTTGTGAGACAACTTTTGAATGCTTGACCTCAAATCAGGTAGGACTACCCG
CTGAACTTAA
>B1_20
TTTCCGTAGGTGAACCTGCGGAAGGATCATTATTGAATTATGTTTCTAGATAGGTTGTAG
CTGGCTCTTTAGAGCATGTGCACGCCTGTTTGGACTTCATTTTCATCCACCTGTGCACCT
ATTGTAGTCTTTGGTTGGGTTAGGGGGAAGTGGTCATTGTGTCAGCATCTGCTGGATGTG
AGGACTTGCATTGTGAAAGCTTTGCTGTCCTTGATGTGATCATGGAATCTCTTTCTCACT
AGAGTCTATGTCACTCATTATACTCTGTCGAATGTCATTGAATGTCTTTACATGGGCTTG
TATGCCTATGAAAATTGTAATACAACTTTCAGCAACGGATCTCTTGGCTCTCGCATCGAT
GAAGGACGCAGCGAAATGCGATAAGTAATGTGAATTGCAGAATTCAGTGAATCATCGAAT
CTTTGAACGCATCTTGCGCTCCTTGGTATTCCGAGGAGCATGCCTGTTTGAGTGTCATTA
AATTCTCAACTCTCTTATACTTTTTTGTAAAAGAGAGCTTGGACTGTGGAGGCTTGCTGG
CCACTTTTTGGGGTCAGCTCCTCTGAAATGCATTAGCGGAACCGTTTGCAATCTGCCACA
AGTGTGATAAGTTATCTACACTGGCGAGGGGATTGCTCTCTGTAATGTTCAGCTTCTAAT
TGTCTCTACTTTGTGAGACAACTTTTGAATGCTTGACCTCAAATCAGGTAGGACTACCCG
CTGAACTTAA
>B1_21
TTTCCGTAGGTGAACCTGCGGAAGGATCATTATTGAATTATGTTTCTAGATAGGTTGTAG
CTGGCTCTTTAGAGCATGTGCACGCCTGTTTGGACTTCATTTTCATCCACCTGTGCACCT
ATTGTAGTCTTTGGTTGGGTTAGGGGGAAGTGGTCATTGTGTCAGCATCTGCTGGATGTG

AGGACTTGCATTGTGAAAGCTTTGCTGTCCTTGATGTGATCATGGAATCTCTTTCTCACT
AGAGTCTATGTCACTCATTATACTCTGTCGAATGTCATTGAATGTCTTTACATGGGCTTG
TATGCCTATGAAAATTGTAATACAACTTTCAGCAACGGATCTCTTGGCTCTCGCATCGAT
GAAGGACGCAGCGAAATGCGATAAGTAATGTGAATTGCAGAATTCAGTGAATCATCGAAT
CTTTGAACGCATCTTGCGCTCCTTGGTATTCCGAGGAGCATGCCTGTTTGAGTGTCATTA
AATTCTCAACTCTCTTATACTTTTTTGTAAAAGAGAGCTTGGACTGTGGAGGCTTGCTGG
CCACTTTTTGGGGTCAGCTCCTCTGAAATGCATTAGCGGAACCGTTTGCAATCTGCCACA
AGTGTGATAAGTTATCTACACTGGCGAGGGGATTGCTCTCTGTAATGTTCAGCTTCTAAT
TGTCTCTACTTTGTGAGACAACTTTTGAATGCTTGACCTCAAATCAGGTAGGACTACCCG
CTGAACTTAA
>B1_22
TTTCCGTAGGTGAACCTGCGGAAGGATCATTATTGAATTATGTTTCTAGATAGGTTGTAG
CTGGCTCTTTAGAGCATGTGCACGCCTGTTTGGACTTCATTTTCATCCACCTGTGCACCT
ATTGTAGTCTTTGGTTGGGTTAGGGGGAAGTGGTCATTGTGTCAGCATCTGCTGGATGTG
AGGACTTGCATTGTGAAAGCTTTGCTGTCCTTGATGTGATCATGGAATCTCTTTCTCACT
AGAGTCTATGTCACTCATTATACTCTGTCGAATGTCATTGAATGTCTTTACATGGGCTTG
TATGCCTATGAAAATTGTAATACAACTTTCAGCAACGGATCTCTTGGCTCTCGCATCGAT
GAAGGACGCAGCGAAATGCGATAAGTAATGTGAATTGCAGAATTCAGTGAATCATCGAAT
CTTTGAACGCATCTTGCGCTCCTTGGTATTCCGAGGAGCATGCCTGTTTGAGTGTCATTA
AATTCTCAACTCTCTTATACTTTTTTGTAAAAGAGAGCTTGGACTGTGGAGGCTTGCTGG
CCACTTTTTGGGGTCAGCTCCTCTGAAATGCATTAGCGGAACCGTTTGCAATCTGCCACA
AGTGTGATAAGTTATCTACACTGGCGAGGGGATTGCTCTCTGTAATGTTCAGCTTCTAAT
TGTCTCTACTTTGTGAGACAACTTTTGAATGCTTGACCTCAAATCAGGTAGGACTACCCG
CTGAACTTAA
>B1_23
TTTCCGTAGGTGAACCTGCGGAAGGATCATTATTGAATTATGTTTCTAGATAGGTTGTAG
CTGGCTCTTTAGAGCATGTGCACGCCTGTTTGGACTTCATTTTCATCCACCTGTGCACCT
ATTGTAGTCTTTGGTTGGGTTAGGGGGAAGTGGTCATTGTGTCAGCATCTGCTGGATGTG
AGGACTTGCATTGTGAAAGCTTTGCTGTCCTTGATGTGATCATGGAATCTCTTTCTCACT
AGAGTCTATGTCACTCATTATACTCTGTCGAATGTCATTGAATGTCTTTACATGGGCTTG
TATGCCTATGAAAATTGTAATACAACTTTCAGCAACGGATCTCTTGGCTCTCGCATCGAT
GAAGGACGCAGCGAAATGCGATAAGTAATGTGAATTGCAGAATTCAGTGAATCATCGAAT
CTTTGAACGCATCTTGCGCTCCTTGGTATTCCGAGGAGCATGCCTGTTTGAGTGTCATTA
AATTCTCAACTCTCTTATACTTTTTTGTAAAAGAGAGCTTGGACTGTGGAGGCTTGCTGG
CCACTTTTTGGGGTCAGCTCCTCTGAAATGCATTAGCGGAACCGTTTGCAATCTGCCACA
AGTGTGATAAGTTATCTACACTGGCGAGGGGATTGCTCTCTGTAATGTTCAGCTTCTAAT
TGTCTCTACTTTGTGAGACAACTTTTGAATGCTTGACCTCAAATCAGGTAGGACTACCCG
CTGAACTTAA
>B1_24
TTTCCGTAGGTGAACCTGCGGAAGGATCATTATTGAATTATGTTTCTAGATAGGTTGTAG
CTGGCTCTTTAGAGCATGTGCACGCCTGTTTGGACTTCATTTTCATCCACCTGTGCACCT
ATTGTAGTCTTTGGTTGGGTTAGGGGGAAGTGGTCATTGTGTCAGCATCTGCTGGATGTG
AGGACTTGCATTGTGAAAGCTTTGCTGTCCTTGATGTGATCATGGAATCTCTTTCTCACT
AGAGTCTATGTCACTCATTATACTCTGTCGAATGTCATTGAATGTCTTTACATGGGCTTG
TATGCCTATGAAAATTGTAATACAACTTTCAGCAACGGATCTCTTGGCTCTCGCATCGAT
GAAGGACGCAGCGAAATGCGATAAGTAATGTGAATTGCAGAATTCAGTGAATCATCGAAT
CTTTGAACGCATCTTGCGCTCCTTGGTATTCCGAGGAGCATGCCTGTTTGAGTGTCATTA
AATTCTCAACTCTCTTATACTTTTTTGTAAAAGAGAGCTTGGACTGTGGAGGCTTGCTGG
CCACTTTTTGGGGTCAGCTCCTCTGAAATGCATTAGCGGAACCGTTTGCAATCTGCCACA
AGTGTGATAAGTTATCTACACTGGCGAGGGGATTGCTCTCTGTAATGTTCAGCTTCTAAT

TGTCTCTACTTTGTGAGACAACTTTTGAATGCTTGACCTCAAATCAGGTAGGACTACCCG
CTGAACTTAA
>B1_25
TTTCCGTAGGTGAACCTGCGGAAGGATCATTATTGAATTATGTTTCTAGATAGGTTGTAG
CTGGCTCTTTAGAGCATGTGCACGCCTGTTTGGACTTCATTTTCATCCACCTGTGCACCT
ATTGTAGTCTTTGGTTGGGTTAGGGGGAAGTGGTCATTGTGTCAGCATCTGCTGGATGTG
AGGACTTGCATTGTGAAAGCTTTGCTGTCCTTGATGTGATCATGGAATCTCTTTCTCACT
AGAGTCTATGTCACTCATTATACTCTGTCGAATGTCATTGAATGTCTTTACATGGGCTTG
TATGCCTATGAAAATTGTAATACAACTTTCAGCAACGGATCTCTTGGCTCTCGCATCGAT
GAAGGACGCAGCGAAATGCGATAAGTAATGTGAATTGCAGAATTCAGTGAATCATCGAAT
CTTTGAACGCATCTTGCGCTCCTTGGTATTCCGAGGAGCATGCCTGTTTGAGTGTCATTA
AATTCTCAACTCTCTTATACTTTTTTGTAAAAGAGAGCTTGGACTGTGGAGGCTTGCTGG
CCACTTTTTGGGGTCAGCTCCTCTGAAATGCATTAGCGGAACCGTTTGCAATCTGCCACA
AGTGTGATAAGTTATCTACACTGGCGAGGGGATTGCTCTCTGTAATGTTCAGCTTCTAAT
TGTCTCTACTTTGTGAGACAACTTTTGAATGCTTGACCTCAAATCAGGTAGGACTACCCG
CTGAACTTAA
>B1_26
TTTCCGTAGGTGAACCTGCGGAAGGATCATTATTGAATTATGTTTCTAGATAGGTTGTAG
CTGGCTCTTTAGAGCATGTGCACGCCTGTTTGGACTTCATTTTCATCCACCTGTGCACCT
ATTGTAGTCTTTGGTTGGGTTAGGGGGAAGTGGTCATTGTGTCAGCATCTGCTGGATGTG
AGGACTTGCATTGTGAAAGCTTTGCTGTCCTTGATGTGATCATGGAATCTCTTTCTCACT
AGAGTCTATGTCACTCATTATACTCTGTCGAATGTCATTGAATGTCTTTACATGGGCTTG
TATGCCTATGAAAATTGTAATACAACTTTCAGCAACGGATCTCTTGGCTCTCGCATCGAT
GAAGGACGCAGCGAAATGCGATAAGTAATGTGAATTGCAGAATTCAGTGAATCATCGAAT
CTTTGAACGCATCTTGCGCTCCTTGGTATTCCGAGGAGCATGCCTGTTTGAGTGTCATTA
AATTCTCAACTCTCTTATACTTTTTTGTAAAAGAGAGCTTGGACTGTGGAGGCTTGCTGG
CCACTTTTTGGGGTCAGCTCCTCTGAAATGCATTAGCGGAACCGTTTGCAATCTGCCACA
AGTGTGATAAGTTATCTACACTGGCGAGGGGATTGCTCTCTGTAATGTTCAGCTTCTAAT
TGTCTCTACTTTGTGAGACAACTTTTGAATGCTTGACCTCAAATCAGGTAGGACTACCCG
CTGAACTTAA
>B1_27
TTTCCGTAGGTGAACCTGCGGAAGGATCATTATTGAATTATGTTTCTAGATAGGTTGTAG
CTGGCTCTTTAGAGCATGTGCACGCCTGTTTGGACTTCATTTTCATCCACCTGTGCACCT
ATTGTAGTCTTTGGTTGGGTTAGGGGGAAGTGGTCATTGTGTCAGCATCTGCTGGATGTG
AGGACTTGCATTGTGAAAGCTTTGCTGTCCTTGATGTGATCATGGAATCTCTTTCTCACT
AGAGTCTATGTCACTCATTATACTCTGTCGAATGTCATTGAATGTCTTTACATGGGCTTG
TATGCCTATGAAAATTGTAATACAACTTTCAGCAACGGATCTCTTGGCTCTCGCATCGAT
GAAGGACGCAGCGAAATGCGATAAGTAATGTGAATTGCAGAATTCAGTGAATCATCGAAT
CTTTGAACGCATCTTGCGCTCCTTGGTATTCCGAGGAGCATGCCTGTTTGAGTGTCATTA
AATTCTCAACTCTCTTATACTTTTTTGTAAAAGAGAGCTTGGACTGTGGAGGCTTGCTGG
CCACTTTTTGGGGTCAGCTCCTCTGAAATGCATTAGCGGAACCGTTTGCAATCTGCCACA
AGTGTGATAAGTTATCTACACTGGCGAGGGGATTGCTCTCTGTAATGTTCAGCTTCTAAT
TGTCTCTACTTTGTGAGACAACTTTTGAATGCTTGACCTCAAATCAGGTAGGACTACCCG
CTGAACTTAA
>B1_28
TTTCCGTAGGTGAACCTGCGGAAGGATCATTATTGAATTATGTTTCTAGATAGGTTGTAG
CTGGCTCTTTAGAGCATGTGCACGCCTGTTTGGACTTCATTTTCATCCACCTGTGCACCT
ATTGTAGTCTTTGGTTGGGTTAGGGGGAAGTGGTCATTGTGTCAGCATCTGCTGGATGTG
AGGACTTGCATTGTGAAAGCTTTGCTGTCCTTGATGTGATCATGGAATCTCTTTCTCACT
AGAGTCTATGTCACTCATTATACTCTGTCGAATGTCATTGAATGTCTTTACATGGGCTTG

TATGCCTATGAAAATTGTAATACAACTTTCAGCAACGGATCTCTTGGCTCTCGCATCGAT
GAAGGACGCAGCGAAATGCGATAAGTAATGTGAATTGCAGAATTCAGTGAATCATCGAAT
CTTTGAACGCATCTTGCGCTCCTTGGTATTCCGAGGAGCATGCCTGTTTGAGTGTCATTA
AATTCTCAACTCTCTTATACTTTTTTGTAAAAGAGAGCTTGGACTGTGGAGGCTTGCTGG
CCACTTTTTGGGGTCAGCTCCTCTGAAATGCATTAGCGGAACCGTTTGCAATCTGCCACA
AGTGTGATAAGTTATCTACACTGGCGAGGGGATTGCTCTCTGTAATGTTCAGCTTCTAAT
TGTCTCTACTTTGTGAGACAACTTTTGAATGCTTGACCTCAAATCAGGTAGGACTACCCG
CTGAACTTAA
>B1_29
TTTCCGTAGGTGAACCTGCGGAAGGATCATTATTGAATTATGTTTCTAGATAGGTTGTAG
CTGGCTCTTTAGAGCATGTGCACGCCTGTTTGGACTTCATTTTCATCCACCTGTGCACCT
ATTGTAGTCTTTGGTTGGGTTAGGGGGAAGTGGTCATTGTGTCAGCATCTGCTGGATGTG
AGGACTTGCATTGTGAAAGCTTTGCTGTCCTTGATGTGATCATGGAATCTCTTTCTCACT
AGAGTCTATGTCACTCATTATACTCTGTCGAATGTCATTGAATGTCTTTACATGGGCTTG
TATGCCTATGAAAATTGTAATACAACTTTCAGCAACGGATCTCTTGGCTCTCGCATCGAT
GAAGGACGCAGCGAAATGCGATAAGTAATGTGAATTGCAGAATTCAGTGAATCATCGAAT
CTTTGAACGCATCTTGCGCTCCTTGGTATTCCGAGGAGCATGCCTGTTTGAGTGTCATTA
AATTCTCAACTCTCTTATACTTTTTTGTAAAAGAGAGCTTGGACTGTGGAGGCTTGCTGG
CCACTTTTTGGGGTCAGCTCCTCTGAAATGCATTAGCGGAACCGTTTGCAATCTGCCACA
AGTGTGATAAGTTATCTACACTGGCGAGGGGATTGCTCTCTGTAATGTTCAGCTTCTAAT
TGTCTCTACTTTGTGAGACAACTTTTGAATGCTTGACCTCAAATCAGGTAGGACTACCCG
CTGAACTTAA
>B1_30
TTTCCGTAGGTGAACCTGCGGAAGGATCATTATTGAATTATGTTTCTAGATAGGTTGTAG
CTGGCTCTTTAGAGCATGTGCACGCCTGTTTGGACTTCATTTTCATCCACCTGTGCACCT
ATTGTAGTCTTTGGTTGGGTTAGGGGGAAGTGGTCATTGTGTCAGCATCTGCTGGATGTG
AGGACTTGCATTGTGAAAGCTTTGCTGTCCTTGATGTGATCATGGAATCTCTTTCTCACT
AGAGTCTATGTCACTCATTATACTCTGTCGAATGTCATTGAATGTCTTTACATGGGCTTG
TATGCCTATGAAAATTGTAATACAACTTTCAGCAACGGATCTCTTGGCTCTCGCATCGAT
GAAGGACGCAGCGAAATGCGATAAGTAATGTGAATTGCAGAATTCAGTGAATCATCGAAT
CTTTGAACGCATCTTGCGCTCCTTGGTATTCCGAGGAGCATGCCTGTTTGAGTGTCATTA
AATTCTCAACTCTCTTATACTTTTTTGTAAAAGAGAGCTTGGACTGTGGAGGCTTGCTGG
CCACTTTTTGGGGTCAGCTCCTCTGAAATGCATTAGCGGAACCGTTTGCAATCTGCCACA
AGTGTGATAAGTTATCTACACTGGCGAGGGGATTGCTCTCTGTAATGTTCAGCTTCTAAT
TGTCTCTACTTTGTGAGACAACTTTTGAATGCTTGACCTCAAATCAGGTAGGACTACCCG
CTGAACTTAA
>B1_31
TTTCCGTAGGTGAACCTGCGGAAGGATCATTATTGAATTATGTTTCTAGATAGGTTGTAG
CTGGCTCTTTAGAGCATGTGCACGCCTGTTTGGACTTCATTTTCATCCACCTGTGCACCT
ATTGTAGTCTTTGGTTGGGTTAGGGGGAAGTGGTCATTGTGTCAGCATCTGCTGGATGTG
AGGACTTGCATTGTGAAAGCTTTGCTGTCCTTGATGTGATCATGGAATCTCTTTCTCACT
AGAGTCTATGTCACTCATTATACTCTGTCGAATGTCATTGAATGTCTTTACATGGGCTTG
TATGCCTATGAAAATTGTAATACAACTTTCAGCAACGGATCTCTTGGCTCTCGCATCGAT
GAAGGACGCAGCGAAATGCGATAAGTAATGTGAATTGCAGAATTCAGTGAATCATCGAAT
CTTTGAACGCATCTTGCGCTCCTTGGTATTCCGAGGAGCATGCCTGTTTGAGTGTCATTA
AATTCTCAACTCTCTTATACTTTTTTGTAAAAGAGAGCTTGGACTGTGGAGGCTTGCTGG
CCACTTTTTGGGGTCAGCTCCTCTGAAATGCATTAGCGGAACCGTTTGCAATCTGCCACA
AGTGTGATAAGTTATCTACACTGGCGAGGGGATTGCTCTCTGTAATGTTCAGCTTCTAAT
TGTCTCTACTTTGTGAGACAACTTTTGAATGCTTGACCTCAAATCAGGTAGGACTACCCG
CTGAACTTAA

>B1_33
TTTCCGTAGGTGAACCTGCGGAAGGATCATTATTGAATTATGTTTCTAGATAGGTTGTAG
CTGGCTCTTTAGAGCATGTGCACGCCTGTTTGGACTTCATTTTCATCCACCTGTGCACCT
ATTGTAGTCTTTGGTTGGGTTAGGGGGAAGTGGTCATTGTGTCAGCATCTGCTGGATGTG
AGGACTTGCATTGTGAAAGCTTTGCTGTCCTTGATGTGATCATGGAATCTCTTTCTCACT
AGAGTCTATGTCACTCATTATACTCTGTCGAATGTCATTGAATGTCTTTACATGGGCTTG
TATGCCTATGAAAATTGTAATACAACTTTCAGCAACGGATCTCTTGGCTCTCGCATCGAT
GAAGGACGCAGCGAAATGCGATAAGTAATGTGAATTGCAGAATTCAGTGAATCATCGAAT
CTTTGAACGCATCTTGCGCTCCTTGGTATTCCGAGGAGCATGCCTGTTTGAGTGTCATTA
AATTCTCAACTCTCTTATACTTTTTTGTAAAAGAGAGCTTGGACTGTGGAGGCTTGCTGG
CCACTTTTTGGGGTCAGCTCCTCTGAAATGCATTAGCGGAACCGTTTGCAATCTGCCACA
AGTGTGATAAGTTATCTACACTGGCGAGGGGATTGCTCTCTGTAATGTTCAGCTTCTAAT
TGTCTCTACTTTGTGAGACAACTTTTGAATGCTTGACCTCAAATCAGGTAGGACTACCCG
CTGAACTTAA
>B1_34
TTTCCGTAGGTGAACCTGCGGAAGGATCATTATTGAATTATGTTTCTAGATAGGTTGTAG
CTGGCTCTTTAGAGCATGTGCACGCCTGTTTGGACTTCATTTTCATCCACCTGTGCACCT
ATTGTAGTCTTTGGTTGGGTTAGGGGGAAGTGGTCATTGTGTCAGCATCTGCTGGATGTG
AGGACTTGCATTGTGAAAGCTTTGCTGTCCTTGATGTGATCATGGAATCTCTTTCTCACT
AGAGTCTATGTCACTCATTATACTCTGTCGAATGTCATTGAATGTCTTTACATGGGCTTG
TATGCCTATGAAAATTGTAATACAACTTTCAGCAACGGATCTCTTGGCTCTCGCATCGAT
GAAGGACGCAGCGAAATGCGATAAGTAATGTGAATTGCAGAATTCAGTGAATCATCGAAT
CTTTGAACGCATCTTGCGCTCCTTGGTATTCCGAGGAGCATGCCTGTTTGAGTGTCATTA
AATTCTCAACTCTCTTATACTTTTTTGTAAAAGAGAGCTTGGACTGTGGAGGCTTGCTGG
CCACTTTTTGGGGTCAGCTCCTCTGAAATGCATTAGCGGAACCGTTTGCAATCTGCCACA
AGTGTGATAAGTTATCTACACTGGCGAGGGGATTGCTCTCTGTAATGTTCAGCTTCTAAT
TGTCTCTACTTTGTGAGACAACTTTTGAATGCTTGACCTCAAATCAGGTAGGACTACCCG
CTGAACTTAA
>B1_35
TTTCCGTAGGTGAACCTGCGGAAGGATCATTATTGAATTATGTTTCTAGATAGGTTGTAG
CTGGCTCTTTAGAGCATGTGCACGCCTGTTTGGACTTCATTTTCATCCACCTGTGCACCT
ATTGTAGTCTTTGGTTGGGTTAGGGGGAAGTGGTCATTGTGTCAGCATCTGCTGGATGTG
AGGACTTGCATTGTGAAAGCTTTGCTGTCCTTGATGTGATCATGGAATCTCTTTCTCACT
AGAGTCTATGTCACTCATTATACTCTGTCGAATGTCATTGAATGTCTTTACATGGGCTTG
TATGCCTATGAAAATTGTAATACAACTTTCAGCAACGGATCTCTTGGCTCTCGCATCGAT
GAAGGACGCAGCGAAATGCGATAAGTAATGTGAATTGCAGAATTCAGTGAATCATCGAAT
CTTTGAACGCATCTTGCGCTCCTTGGTATTCCGAGGAGCATGCCTGTTTGAGTGTCATTA
AATTCTCAACTCTCTTATACTTTTTTGTAAAAGAGAGCTTGGACTGTGGAGGCTTGCTGG
CCACTTTTTGGGGTCAGCTCCTCTGAAATGCATTAGCGGAACCGTTTGCAATCTGCCACA
AGTGTGATAAGTTATCTACACTGGCGAGGGGATTGCTCTCTGTAATGTTCAGCTTCTAAT
TGTCTCTACTTTGTGAGACAACTTTTGAATGCTTGACCTCAAATCAGGTAGGACTACCCG
CTGAACTTAA
>B1_36
TTTCCGTAGGTGAACCTGCGGAAGGATCATTATTGAATTATGTTTCTAGATAGGTTGTAG
CTGGCTCTTTAGAGCATGTGCACGCCTGTTTGGACTTCATTTTCATCCACCTGTGCACCT
ATTGTAGTCTTTGGTTGGGTTAGGGGGAAGTGGTCATTGTGTCAGCATCTGCTGGATGTG
AGGACTTGCATTGTGAAAGCTTTGCTGTCCTTGATGTGATCATGGAATCTCTTTCTCACT
AGAGTCTATGTCACTCATTATACTCTGTCGAATGTCATTGAATGTCTTTACATGGGCTTG
TATGCCTATGAAAATTGTAATACAACTTTCAGCAACGGATCTCTTGGCTCTCGCATCGAT
GAAGGACGCAGCGAAATGCGATAAGTAATGTGAATTGCAGAATTCAGTGAATCATCGAAT

CTTTGAACGCATCTTGCGCTCCTTGGTATTCCGAGGAGCATGCCTGTTTGAGTGTCATTA
AATTCTCAACTCTCTTATACTTTTTTGTAAAAGAGAGCTTGGACTGTGGAGGCTTGCTGG
CCACTTTTTGGGGTCAGCTCCTCTGAAATGCATTAGCGGAACCGTTTGCAATCTGCCACA
AGTGTGATAAGTTATCTACACTGGCGAGGGGATTGCTCTCTGTAATGTTCAGCTTCTAAT
TGTCTCTACTTTGTGAGACAACTTTTGAATGCTTGACCTCAAATCAGGTAGGACTACCCG
CTGAACTTAA
>B1_37
TTTCCGTAGGTGAACCTGCGGAAGGATCATTATTGAATTATGTTTCTAGATAGGTTGTAG
CTGGCTCTTTAGAGCATGTGCACGCCTGTTTGGACTTCATTTTCATCCACCTGTGCACCT
ATTGTAGTCTTTGGTTGGGTTAGGGGGAAGTGGTCATTGTGTCAGCATCTGCTGGATGTG
AGGACTTGCATTGTGAAAGCTTTGCTGTCCTTGATGTGATCATGGAATCTCTTTCTCACT
AGAGTCTATGTCACTCATTATACTCTGTCGAATGTCATTGAATGTCTTTACATGGGCTTG
TATGCCTATGAAAATTGTAATACAACTTTCAGCAACGGATCTCTTGGCTCTCGCATCGAT
GAAGGACGCAGCGAAATGCGATAAGTAATGTGAATTGCAGAATTCAGTGAATCATCGAAT
CTTTGAACGCATCTTGCGCTCCTTGGTATTCCGAGGAGCATGCCTGTTTGAGTGTCATTA
AATTCTCAACTCTCTTATACTTTTTTGTAAAAGAGAGCTTGGACTGTGGAGGCTTGCTGG
CCACTTTTTGGGGTCAGCTCCTCTGAAATGCATTAGCGGAACCGTTTGCAATCTGCCACA
AGTGTGATAAGTTATCTACACTGGCGAGGGGATTGCTCTCTGTAATGTTCAGCTTCTAAT
TGTCTCTACTTTGTGAGACAACTTTTGAATGCTTGACCTCAAATCAGGTAGGACTACCCG
CTGAACTTAA
>B1_38
TTTCCGTAGGTGAACCTGCGGAAGGATCATTATTGAATTATGTTTCTAGATAGGTTGTAG
CTGGCTCTTTAGAGCATGTGCACGCCTGTTTGGACTTCATTTTCATCCACCTGTGCACCT
ATTGTAGTCTTTGGTTGGGTTAGGGGGAAGTGGTCATTGTGTCAGCATCTGCTGGATGTG
AGGACTTGCATTGTGAAAGCTTTGCTGTCCTTGATGTGATCATGGAATCTCTTTCTCACT
AGAGTCTATGTCACTCATTATACTCTGTCGAATGTCATTGAATGTCTTTACATGGGCTTG
TATGCCTATGAAAATTGTAATACAACTTTCAGCAACGGATCTCTTGGCTCTCGCATCGAT
GAAGGACGCAGCGAAATGCGATAAGTAATGTGAATTGCAGAATTCAGTGAATCATCGAAT
CTTTGAACGCATCTTGCGCTCCTTGGTATTCCGAGGAGCATGCCTGTTTGAGTGTCATTA
AATTCTCAACTCTCTTATACTTTTTTGTAAAAGAGAGCTTGGACTGTGGAGGCTTGCTGG
CCACTTTTTGGGGTCAGCTCCTCTGAAATGCATTAGCGGAACCGTTTGCAATCTGCCACA
AGTGTGATAAGTTATCTACACTGGCGAGGGGATTGCTCTCTGTAATGTTCAGCTTCTAAT
TGTCTCTACTTTGTGAGACAACTTTTGAATGCTTGACCTCAAATCAGGTAGGACTACCCG
CTGAACTTAA
>B1_41
TTTCCGTAGGTGAACCTGCGGAAGGATCATTATTGAATTATGTTTCTAGATAGGTTGTAG
CTGGCTCTTTAGAGCATGTGCACGCCTGTTTGGACTTCATTTTCATCCACCTGTGCACCT
ATTGTAGTCTTTGGTTGGGTTAGGGGGAAGTGGTCATTGTGTCAGCATCTGCTGGATGTG
AGGACTTGCATTGTGAAAGCTTTGCTGTCCTTGATGTGATCATGGAATCTCTTTCTCACT
AGAGTCTATGTCACTCATTATACTCTGTCGAATGTCATTGAATGTCTTTACATGGGCTTG
TATGCCTATGAAAATTGTAATACAACTTTCAGCAACGGATCTCTTGGCTCTCGCATCGAT
GAAGGACGCAGCGAAATGCGATAAGTAATGTGAATTGCAGAATTCAGTGAATCATCGAAT
CTTTGAACGCATCTTGCGCTCCTTGGTATTCCGAGGAGCATGCCTGTTTGAGTGTCATTA
AATTCTCAACTCTCTTATACTTTTTTGTAAAAGAGAGCTTGGACTGTGGAGGCTTGCTGG
CCACTTTTTGGGGTCAGCTCCTCTGAAATGCATTAGCGGAACCGTTTGCAATCTGCCACA
AGTGTGATAAGTTATCTACACTGGCGAGGGGATTGCTCTCTGTAATGTTCAGCTTCTAAT
TGTCTCTACTTTGTGAGACAACTTTTGAATGCTTGACCTCAAATCAGGTAGGACTACCCG
CTGAACTTAA
>B1_43
TTTCCGTAGGTGAACCTGCGGAAGGATCATTATTGAATTATGTTTCTAGATAGGTTGTAG

CTGGCTCTTTAGAGCATGTGCACGCCTGTTTGGACTTCATTTTCATCCACCTGTGCACCT
ATTGTAGTCTTTGGTTGGGTTAGGGGGAAGTGGTCATTGTGTCAGCATCTGCTGGATGTG
AGGACTTGCATTGTGAAAGCTTTGCTGTCCTTGATGTGATCATGGAATCTCTTTCTCACT
AGAGTCTATGTCACTCATTATACTCTGTCGAATGTCATTGAATGTCTTTACATGGGCTTG
TATGCCTATGAAAATTGTAATACAACTTTCAGCAACGGATCTCTTGGCTCTCGCATCGAT
GAAGGACGCAGCGAAATGCGATAAGTAATGTGAATTGCAGAATTCAGTGAATCATCGAAT
CTTTGAACGCATCTTGCGCTCCTTGGTATTCCGAGGAGCATGCCTGTTTGAGTGTCATTA
AATTCTCAACTCTCTTATACTTTTTTGTAAAAGAGAGCTTGGACTGTGGAGGCTTGCTGG
CCACTTTTTGGGGTCAGCTCCTCTGAAATGCATTAGCGGAACCGTTTGCAATCTGCCACA
AGTGTGATAAGTTATCTACACTGGCGAGGGGATTGCTCTCTGTAATGTTCAGCTTCTAAT
TGTCTCTACTTTGTGAGACAACTTTTGAATGCTTGACCTCAAATCAGGTAGGACTACCCG
CTGAACTTAA
>B1_44
TTTCCGTAGGTGAACCTGCGGAAGGATCATTATTGAATTATGTTTCTAGATAGGTTGTAG
CTGGCTCTTTAGAGCATGTGCACGCCTGTTTGGACTTCATTTTCATCCACCTGTGCACCT
ATTGTAGTCTTTGGTTGGGTTAGGGGGAAGTGGTCATTGTGTCAGCATCTGCTGGATGTG
AGGACTTGCATTGTGAAAGCTTTGCTGTCCTTGATGTGATCATGGAATCTCTTTCTCACT
AGAGTCTATGTCACTCATTATACTCTGTCGAATGTCATTGAATGTCTTTACATGGGCTTG
TATGCCTATGAAAATTGTAATACAACTTTCAGCAACGGATCTCTTGGCTCTCGCATCGAT
GAAGGACGCAGCGAAATGCGATAAGTAATGTGAATTGCAGAATTCAGTGAATCATCGAAT
CTTTGAACGCATCTTGCGCTCCTTGGTATTCCGAGGAGCATGCCTGTTTGAGTGTCATTA
AATTCTCAACTCTCTTATACTTTTTTGTAAAAGAGAGCTTGGACTGTGGAGGCTTGCTGG
CCACTTTTTGGGGTCAGCTCCTCTGAAATGCATTAGCGGAACCGTTTGCAATCTGCCACA
AGTGTGATAAGTTATCTACACTGGCGAGGGGATTGCTCTCTGTAATGTTCAGCTTCTAAT
TGTCTCTACTTTGTGAGACAACTTTTGAATGCTTGACCTCAAATCAGGTAGGACTACCCG
CTGAACTTAA
>B1_45
TTTCCGTAGGTGAACCTGCGGAAGGATCATTATTGAATTATGTTTCTAGATAGGTTGTAG
CTGGCTCTTTAGAGCATGTGCACGCCTGTTTGGACTTCATTTTCATCCACCTGTGCACCT
ATTGTAGTCTTTGGTTGGGTTAGGGGGAAGTGGTCATTGTGTCAGCATCTGCTGGATGTG
AGGACTTGCATTGTGAAAGCTTTGCTGTCCTTGATGTGATCATGGAATCTCTTTCTCACT
AGAGTCTATGTCACTCATTATACTCTGTCGAATGTCATTGAATGTCTTTACATGGGCTTG
TATGCCTATGAAAATTGTAATACAACTTTCAGCAACGGATCTCTTGGCTCTCGCATCGAT
GAAGGACGCAGCGAAATGCGATAAGTAATGTGAATTGCAGAATTCAGTGAATCATCGAAT
CTTTGAACGCATCTTGCGCTCCTTGGTATTCCGAGGAGCATGCCTGTTTGAGTGTCATTA
AATTCTCAACTCTCTTATACTTTTTTGTAAAAGAGAGCTTGGACTGTGGAGGCTTGCTGG
CCACTTTTTGGGGTCAGCTCCTCTGAAATGCATTAGCGGAACCGTTTGCAATCTGCCACA
AGTGTGATAAGTTATCTACACTGGCGAGGGGATTGCTCTCTGTAATGTTCAGCTTCTAAT
TGTCTCTACTTTGTGAGACAACTTTTGAATGCTTGACCTCAAATCAGGTAGGACTACCCG
CTGAACTTAA
>B1_46
TTTCCGTAGGTGAACCTGCGGAAGGATCATTATTGAATTATGTTTCTAGATAGGTTGTAG
CTGGCTCTTTAGAGCATGTGCACGCCTGTTTGGACTTCATTTTCATCCACCTGTGCACCT
ATTGTAGTCTTTGGTTGGGTTAGGGGGAAGTGGTCATTGTGTCAGCATCTGCTGGATGTG
AGGACTTGCATTGTGAAAGCTTTGCTGTCCTTGATGTGATCATGGAATCTCTTTCTCACT
AGAGTCTATGTCACTCATTATACTCTGTCGAATGTCATTGAATGTCTTTACATGGGCTTG
TATGCCTATGAAAATTGTAATACAACTTTCAGCAACGGATCTCTTGGCTCTCGCATCGAT
GAAGGACGCAGCGAAATGCGATAAGTAATGTGAATTGCAGAATTCAGTGAATCATCGAAT
CTTTGAACGCATCTTGCGCTCCTTGGTATTCCGAGGAGCATGCCTGTTTGAGTGTCATTA
AATTCTCAACTCTCTTATACTTTTTTGTAAAAGAGAGCTTGGACTGTGGAGGCTTGCTGG

CCACTTTTTGGGGTCAGCTCCTCTGAAATGCATTAGCGGAACCGTTTGCAATCTGCCACA
AGTGTGATAAGTTATCTACACTGGCGAGGGGATTGCTCTCTGTAATGTTCAGCTTCTAAT
TGTCTCTACTTTGTGAGACAACTTTTGAATGCTTGACCTCAAATCAGGTAGGACTACCCG
CTGAACTTAA
>B1_47
TTTCCGTAGGTGAACCTGCGGAAGGATCATTATTGAATTATGTTTCTAGATAGGTTGTAG
CTGGCTCTTTAGAGCATGTGCACGCCTGTTTGGACTTCATTTTCATCCACCTGTGCACCT
ATTGTAGTCTTTGGTTGGGTTAGGGGGAAGTGGTCATTGTGTCAGCATCTGCTGGATGTG
AGGACTTGCATTGTGAAAGCTTTGCTGTCCTTGATGTGATCATGGAATCTCTTTCTCACT
AGAGTCTATGTCACTCATTATACTCTGTCGAATGTCATTGAATGTCTTTACATGGGCTTG
TATGCCTATGAAAATTGTAATACAACTTTCAGCAACGGATCTCTTGGCTCTCGCATCGAT
GAAGGACGCAGCGAAATGCGATAAGTAATGTGAATTGCAGAATTCAGTGAATCATCGAAT
CTTTGAACGCATCTTGCGCTCCTTGGTATTCCGAGGAGCATGCCTGTTTGAGTGTCATTA
AATTCTCAACTCTCTTATACTTTTTTGTAAAAGAGAGCTTGGACTGTGGAGGCTTGCTGG
CCACTTTTTGGGGTCAGCTCCTCTGAAATGCATTAGCGGAACCGTTTGCAATCTGCCACA
AGTGTGATAAGTTATCTACACTGGCGAGGGGATTGCTCTCTGTAATGTTCAGCTTCTAAT
TGTCTCTACTTTGTGAGACAACTTTTGAATGCTTGACCTCAAATCAGGTAGGACTACCCG
CTGAACTTAA
>B1_48
TTTCCGTAGGTGAACCTGCGGAAGGATCATTATTGAATTATGTTTCTAGATAGGTTGTAG
CTGGCTCTTTAGAGCATGTGCACGCCTGTTTGGACTTCATTTTCATCCACCTGTGCACCT
ATTGTAGTCTTTGGTTGGGTTAGGGGGAAGTGGTCATTGTGTCAGCATCTGCTGGATGTG
AGGACTTGCATTGTGAAAGCTTTGCTGTCCTTGATGTGATCATGGAATCTCTTTCTCACT
AGAGTCTATGTCACTCATTATACTCTGTCGAATGTCATTGAATGTCTTTACATGGGCTTG
TATGCCTATGAAAATTGTAATACAACTTTCAGCAACGGATCTCTTGGCTCTCGCATCGAT
GAAGGACGCAGCGAAATGCGATAAGTAATGTGAATTGCAGAATTCAGTGAATCATCGAAT
CTTTGAACGCATCTTGCGCTCCTTGGTATTCCGAGGAGCATGCCTGTTTGAGTGTCATTA
AATTCTCAACTCTCTTATACTTTTTTGTAAAAGAGAGCTTGGACTGTGGAGGCTTGCTGG
CCACTTTTTGGGGTCAGCTCCTCTGAAATGCATTAGCGGAACCGTTTGCAATCTGCCACA
AGTGTGATAAGTTATCTACACTGGCGAGGGGATTGCTCTCTGTAATGTTCAGCTTCTAAT
TGTCTCTACTTTGTGAGACAACTTTTGAATGCTTGACCTCAAATCAGGTAGGACTACCCG
CTGAACTTAA
>B1_49
TTTCCGTAGGTGAACCTGCGGAAGGATCATTATTGAATTATGTTTCTAGATAGGTTGTAG
CTGGCTCTTTAGAGCATGTGCACGCCTGTTTGGACTTCATTTTCATCCACCTGTGCACCT
ATTGTAGTCTTTGGTTGGGTTAGGGGGAAGTGGTCATTGTGTCAGCATCTGCTGGATGTG
AGGACTTGCATTGTGAAAGCTTTGCTGTCCTTGATGTGATCATGGAATCTCTTTCTCACT
AGAGTCTATGTCACTCATTATACTCTGTCGAATGTCATTGAATGTCTTTACATGGGCTTG
TATGCCTATGAAAATTGTAATACAACTTTCAGCAACGGATCTCTTGGCTCTCGCATCGAT
GAAGGACGCAGCGAAATGCGATAAGTAATGTGAATTGCAGAATTCAGTGAATCATCGAAT
CTTTGAACGCATCTTGCGCTCCTTGGTATTCCGAGGAGCATGCCTGTTTGAGTGTCATTA
AATTCTCAACTCTCTTATACTTTTTTGTAAAAGAGAGCTTGGACTGTGGAGGCTTGCTGG
CCACTTTTTGGGGTCAGCTCCTCTGAAATGCATTAGCGGAACCGTTTGCAATCTGCCACA
AGTGTGATAAGTTATCTACACTGGCGAGGGGATTGCTCTCTGTAATGTTCAGCTTCTAAT
TGTCTCTACTTTGTGAGACAACTTTTGAATGCTTGACCTCAAATCAGGTAGGACTACCCG
CTGAACTTAA
>B1–54
TTTCCGTAGGTGAACCTGCGGAAGGATCATTATTGAATTATGTTTCTAGATAGGTTGTAG
CTGGCTCTTTAGAGCATGTGCACGCCTGTTTGGACTTCATTTTCATCCACCTGTGCACCT
ATTGTAGTCTTTGGTTGGGTTAGGGGGAAGTGGTCATTGTGTCAGCATCTGCTGGATGTG

AGGACTTGCATTGTGAAAGCTTTGCTGTCCTTGATGTGATCATGGAATCTCTTTCTCACT
AGAGTCTATGTCACTCATTATACTCTGTCGAATGTCATTGAATGTCTTTACATGGGCTTG
TATGCCTATGAAAATTGTAATACAACTTTCAGCAACGGATCTCTTGGCTCTCGCATCGAT
GAAGGACGCAGCGAAATGCGATAAGTAATGTGAATTGCAGAATTCAGTGAATCATCGAAT
CTTTGAACGCATCTTGCGCTCCTTGGTATTCCGAGGAGCATGCCTGTTTGAGTGTCATTA
AATTCTCAACTCTCTTATACTTTTTTGTAAAAGAGAGCTTGGACTGTGGAGGCTTGCTGG
CCACTTTTTGGGGTCAGCTCCTCTGAAATGCATTAGCGGAACCGTTTGCAATCTGCCACA
AGTGTGATAAGTTATCTACACTGGCGAGGGGATTGCTCTCTGTAATGTTCAGCTTCTAAT
TGTCTCTACTTTGTGAGACAACTTTTGAATGCTTGACCTCAAATCAGGTAGGACTACCCG
CTGAACTTAA
>B1-56
TTTCCGTAGGTGAACCTGCGGAAGGATCATTATTGAATTATGTTTCTAGATAGGTTGTAG
CTGGCTCTTTAGAGCATGTGCACGCCTGTTTGGACTTCATTTTCATCCACCTGTGCACCT
ATTGTAGTCTTTGGTTGGGTTAGGGGGAAGTGGTCATTGTGTCAGCATCTGCTGGATGTG
AGGACTTGCATTGTGAAAGCTTTGCTGTCCTTGATGTGATCATGGAATCTCTTTCTCACT
AGAGTCTATGTCACTCATTATACTCTGTCGAATGTCATTGAATGTCTTTACATGGGCTTG
TATGCCTATGAAAATTGTAATACAACTTTCAGCAACGGATCTCTTGGCTCTCGCATCGAT
GAAGGACGCAGCGAAATGCGATAAGTAATGTGAATTGCAGAATTCAGTGAATCATCGAAT
CTTTGAACGCATCTTGCGCTCCTTGGTATTCCGAGGAGCATGCCTGTTTGAGTGTCATTA
AATTCTCAACTCTCTTATACTTTTTTGTAAAAGAGAGCTTGGACTGTGGAGGCTTGCTGG
CCACTTTTTGGGGTCAGCTCCTCTGAAATGCATTAGCGGAACCGTTTGCAATCTGCCACA
AGTGTGATAAGTTATCTACACTGGCGAGGGGATTGCTCTCTGTAATGTTCAGCTTCTAAT
TGTCTCTACTTTGTGAGACAACTTTTGAATGCTTGACCTCAAATCAGGTAGGACTACCCG
CTGAACTTAA
>B1-57
TTTCCGTAGGTGAACCTGCGGAAGGATCATTATTGAATTATGTTTCTAGATAGGTTGTAG
CTGGCTCTTTAGAGCATGTGCACGCCTGTTTGGACTTCATTTTCATCCACCTGTGCACCT
ATTGTAGTCTTTGGTTGGGTTAGGGGGAAGTGGTCATTGTGTCAGCATCTGCTGGATGTG
AGGACTTGCATTGTGAAAGCTTTGCTGTCCTTGATGTGATCATGGAATCTCTTTCTCACT
AGAGTCTATGTCACTCATTATACTCTGTCGAATGTCATTGAATGTCTTTACATGGGCTTG
TATGCCTATGAAAATTGTAATACAACTTTCAGCAACGGATCTCTTGGCTCTCGCATCGAT
GAAGGACGCAGCGAAATGCGATAAGTAATGTGAATTGCAGAATTCAGTGAATCATCGAAT
CTTTGAACGCATCTTGCGCTCCTTGGTATTCCGAGGAGCATGCCTGTTTGAGTGTCATTA
AATTCTCAACTCTCTTATACTTTTTTGTAAAAGAGAGCTTGGACTGTGGAGGCTTGCTGG
CCACTTTTTGGGGTCAGCTCCTCTGAAATGCATTAGCGGAACCGTTTGCAATCTGCCACA
AGTGTGATAAGTTATCTACACTGGCGAGGGGATTGCTCTCTGTAATGTTCAGCTTCTAAT
TGTCTCTACTTTGTGAGACAACTTTTGAATGCTTGACCTCAAATCAGGTAGGACTACCCG
CTGAACTTAA
>B1-58
TTTCCGTAGGTGAACCTGCGGAAGGATCATTATTGAATTATGTTTCTAGATAGGTTGTAG
CTGGCTCTTTAGAGCATGTGCACGCCTGTTTGGACTTCATTTTCATCCACCTGTGCACCT
ATTGTAGTCTTTGGTTGGGTTAGGGGGAAGTGGTCATTGTGTCAGCATCTGCTGGATGTG
AGGACTTGCATTGTGAAAGCTTTGCTGTCCTTGATGTGATCATGGAATCTCTTTCTCACT
AGAGTCTATGTCACTCATTATACTCTGTCGAATGTCATTGAATGTCTTTACATGGGCTTG
TATGCCTATGAAAATTGTAATACAACTTTCAGCAACGGATCTCTTGGCTCTCGCATCGAT
GAAGGACGCAGCGAAATGCGATAAGTAATGTGAATTGCAGAATTCAGTGAATCATCGAAT
CTTTGAACGCATCTTGCGCTCCTTGGTATTCCGAGGAGCATGCCTGTTTGAGTGTCATTA
AATTCTCAACTCTCTTATACTTTTTTGTAAAAGAGAGCTTGGACTGTGGAGGCTTGCTGG
CCACTTTTTGGGGTCAGCTCCTCTGAAATGCATTAGCGGAACCGTTTGCAATCTGCCACA
AGTGTGATAAGTTATCTACACTGGCGAGGGGATTGCTCTCTGTAATGTTCAGCTTCTAAT

TGTCTCTACTTTGTGAGACAACTTTTGAATGCTTGACCTCAAATCAGGTAGGACTACCCG
CTGAACTTAA
>B1-59
TTTCCGTAGGTGAACCTGCGGAAGGATCATTATTGAATTATGTTTCTAGATAGGTTGTAG
CTGGCTCTTTAGAGCATGTGCACGCCTGTTTGGACTTCATTTTCATCCACCTGTGCACCT
ATTGTAGTCTTTGGTTGGGTTAGGGGGAAGTGGTCATTGTGTCAGCATCTGCTGGATGTG
AGGACTTGCATTGTGAAAGCTTTGCTGTCCTTGATGTGATCATGGAATCTCTTTCTCACT
AGAGTCTATGTCACTCATTATACTCTGTCGAATGTCATTGAATGTCTTTACATGGGCTTG
TATGCCTATGAAAATTGTAATACAACTTTCAGCAACGGATCTCTTGGCTCTCGCATCGAT
GAAGGACGCAGCGAAATGCGATAAGTAATGTGAATTGCAGAATTCAGTGAATCATCGAAT
CTTTGAACGCATCTTGCGCTCCTTGGTATTCCGAGGAGCATGCCTGTTTGAGTGTCATTA
AATTCTCAACTCTCTTATACTTTTTTGTAAAAGAGAGCTTGGACTGTGGAGGCTTGCTGG
CCACTTTTTGGGGTCAGCTCCTCTGAAATGCATTAGCGGAACCGTTTGCAATCTGCCACA
AGTGTGATAAGTTATCTACACTGGCGAGGGGATTGCTCTCTGTAATGTTCAGCTTCTAAT
TGTCTCTACTTTGTGAGACAACTTTTGAATGCTTGACCTCAAATCAGGTAGGACTACCCG
CTGAACTTAA
>B1-60
TTTCCGTAGGTGAACCTGCGGAAGGATCATTATTGAATTATGTTTCTAGATAGGTTGTAG
CTGGCTCTTTAGAGCATGTGCACGCCTGTTTGGACTTCATTTTCATCCACCTGTGCACCT
ATTGTAGTCTTTGGTTGGGTTAGGGGGAAGTGGTCATTGTGTCAGCATCTGCTGGATGTG
AGGACTTGCATTGTGAAAGCTTTGCTGTCCTTGATGTGATCATGGAATCTCTTTCTCACT
AGAGTCTATGTCACTCATTATACTCTGTCGAATGTCATTGAATGTCTTTACATGGGCTTG
TATGCCTATGAAAATTGTAATACAACTTTCAGCAACGGATCTCTTGGCTCTCGCATCGAT
GAAGGACGCAGCGAAATGCGATAAGTAATGTGAATTGCAGAATTCAGTGAATCATCGAAT
CTTTGAACGCATCTTGCGCTCCTTGGTATTCCGAGGAGCATGCCTGTTTGAGTGTCATTA
AATTCTCAACTCTCTTATACTTTTTTGTAAAAGAGAGCTTGGACTGTGGAGGCTTGCTGG
CCACTTTTTGGGGTCAGCTCCTCTGAAATGCATTAGCGGAACCGTTTGCAATCTGCCACA
AGTGTGATAAGTTATCTACACTGGCGAGGGGATTGCTCTCTGTAATGTTCAGCTTCTAAT
TGTCTCTACTTTGTGAGACAACTTTTGAATGCTTGACCTCAAATCAGGTAGGACTACCCG
CTGAACTTAA
>B1-61
TTTCCGTAGGTGAACCTGCGGAAGGATCATTATTGAATTATGTTTCTAGATAGGTTGTAG
CTGGCTCTTTAGAGCATGTGCACGCCTGTTTGGACTTCATTTTCATCCACCTGTGCACCT
ATTGTAGTCTTTGGTTGGGTTAGGGGGAAGTGGTCATTGTGTCAGCATCTGCTGGATGTG
AGGACTTGCATTGTGAAAGCTTTGCTGTCCTTGATGTGATCATGGAATCTCTTTCTCACT
AGAGTCTATGTCACTCATTATACTCTGTCGAATGTCATTGAATGTCTTTACATGGGCTTG
TATGCCTATGAAAATTGTAATACAACTTTCAGCAACGGATCTCTTGGCTCTCGCATCGAT
GAAGGACGCAGCGAAATGCGATAAGTAATGTGAATTGCAGAATTCAGTGAATCATCGAAT
CTTTGAACGCATCTTGCGCTCCTTGGTATTCCGAGGAGCATGCCTGTTTGAGTGTCATTA
AATTCTCAACTCTCTTATACTTTTTTGTAAAAGAGAGCTTGGACTGTGGAGGCTTGCTGG
CCACTTTTTGGGGTCAGCTCCTCTGAAATGCATTAGCGGAACCGTTTGCAATCTGCCACA
AGTGTGATAAGTTATCTACACTGGCGAGGGGATTGCTCTCTGTAATGTTCAGCTTCTAAT
TGTCTCTACTTTGTGAGACAACTTTTGAATGCTTGACCTCAAATCAGGTAGGACTACCCG
CTGAACTTAA
>B1-62
TTTCCGTAGGTGAACCTGCGGAAGGATCATTATTGAATTATGTTTCTAGATAGGTTGTAG
CTGGCTCTTTAGAGCATGTGCACGCCTGTTTGGACTTCATTTTCATCCACCTGTGCACCT
ATTGTAGTCTTTGGTTGGGTTAGGGGGAAGTGGTCATTGTGTCAGCATCTGCTGGATGTG
AGGACTTGCATTGTGAAAGCTTTGCTGTCCTTGATGTGATCATGGAATCTCTTTCTCACT
AGAGTCTATGTCACTCATTATACTCTGTCGAATGTCATTGAATGTCTTTACATGGGCTTG

TATGCCTATGAAAATTGTAATACAACTTTCAGCAACGGATCTCTTGGCTCTCGCATCGAT
GAAGGACGCAGCGAAATGCGATAAGTAATGTGAATTGCAGAATTCAGTGAATCATCGAAT
CTTTGAACGCATCTTGCGCTCCTTGGTATTCCGAGGAGCATGCCTGTTTGAGTGTCATTA
AATTCTCAACTCTCTTATACTTTTTTGTAAAAGAGAGCTTGGACTGTGGAGGCTTGCTGG
CCACTTTTTGGGGTCAGCTCCTCTGAAATGCATTAGCGGAACCGTTTGCAATCTGCCACA
AGTGTGATAAGTTATCTACACTGGCGAGGGGATTGCTCTCTGTAATGTTCAGCTTCTAAT
TGTCTCTACTTTGTGAGACAACTTTTGAATGCTTGACCTCAAATCAGGTAGGACTACCCG
CTGAACTTAA
>B1-63
TTTCCGTAGGTGAACCTGCGGAAGGATCATTATTGAATTATGTTTCTAGATAGGTTGTAG
CTGGCTCTTTAGAGCATGTGCACGCCTGTTTGGACTTCATTTTCATCCACCTGTGCACCT
ATTGTAGTCTTTGGTTGGGTTAGGGGGAAGTGGTCATTGTGTCAGCATCTGCTGGATGTG
AGGACTTGCATTGTGAAAGCTTTGCTGTCCTTGATGTGATCATGGAATCTCTTTCTCACT
AGAGTCTATGTCACTCATTATACTCTGTCGAATGTCATTGAATGTCTTTACATGGGCTTG
TATGCCTATGAAAATTGTAATACAACTTTCAGCAACGGATCTCTTGGCTCTCGCATCGAT
GAAGGACGCAGCGAAATGCGATAAGTAATGTGAATTGCAGAATTCAGTGAATCATCGAAT
CTTTGAACGCATCTTGCGCTCCTTGGTATTCCGAGGAGCATGCCTGTTTGAGTGTCATTA
AATTCTCAACTCTCTTATACTTTTTTGTAAAAGAGAGCTTGGACTGTGGAGGCTTGCTGG
CCACTTTTTGGGGTCAGCTCCTCTGAAATGCATTAGCGGAACCGTTTGCAATCTGCCACA
AGTGTGATAAGTTATCTACACTGGCGAGGGGATTGCTCTCTGTAATGTTCAGCTTCTAAT
TGTCTCTACTTTGTGAGACAACTTTTGAATGCTTGACCTCAAATCAGGTAGGACTACCCG
CTGAACTTAA
>B1-64
TTTCCGTAGGTGAACCTGCGGAAGGATCATTATTGAATTATGTTTCTAGATAGGTTGTAG
CTGGCTCTTTAGAGCATGTGCACGCCTGTTTGGACTTCATTTTCATCCACCTGTGCACCT
ATTGTAGTCTTTGGTTGGGTTAGGGGGAAGTGGTCATTGTGTCAGCATCTGCTGGATGTG
AGGACTTGCATTGTGAAAGCTTTGCTGTCCTTGATGTGATCATGGAATCTCTTTCTCACT
AGAGTCTATGTCACTCATTATACTCTGTCGAATGTCATTGAATGTCTTTACATGGGCTTG
TATGCCTATGAAAATTGTAATACAACTTTCAGCAACGGATCTCTTGGCTCTCGCATCGAT
GAAGGACGCAGCGAAATGCGATAAGTAATGTGAATTGCAGAATTCAGTGAATCATCGAAT
CTTTGAACGCATCTTGCGCTCCTTGGTATTCCGAGGAGCATGCCTGTTTGAGTGTCATTA
AATTCTCAACTCTCTTATACTTTTTTGTAAAAGAGAGCTTGGACTGTGGAGGCTTGCTGG
CCACTTTTTGGGGTCAGCTCCTCTGAAATGCATTAGCGGAACCGTTTGCAATCTGCCACA
AGTGTGATAAGTTATCTACACTGGCGAGGGGATTGCTCTCTGTAATGTTCAGCTTCTAAT
TGTCTCTACTTTGTGAGACAACTTTTGAATGCTTGACCTCAAATCAGGTAGGACTACCCG
CTGAACTTAA
>B1-67
TTTCCGTAGGTGAACCTGCGGAAGGATCATTATTGAATTATGTTTCTAGATAGGTTGTAG
CTGGCTCTTTAGAGCATGTGCACGCCTGTTTGGACTTCATTTTCATCCACCTGTGCACCT
ATTGTAGTCTTTGGTTGGGTTAGGGGGAAGTGGTCATTGTGTCAGCATCTGCTGGATGTG
AGGACTTGCATTGTGAAAGCTTTGCTGTCCTTGATGTGATCATGGAATCTCTTTCTCACT
AGAGTCTATGTCACTCATTATACTCTGTCGAATGTCATTGAATGTCTTTACATGGGCTTG
TATGCCTATGAAAATTGTAATACAACTTTCAGCAACGGATCTCTTGGCTCTCGCATCGAT
GAAGGACGCAGCGAAATGCGATAAGTAATGTGAATTGCAGAATTCAGTGAATCATCGAAT
CTTTGAACGCATCTTGCGCTCCTTGGTATTCCGAGGAGCATGCCTGTTTGAGTGTCATTA
AATTCTCAACTCTCTTATACTTTTTTGTAAAAGAGAGCTTGGACTGTGGAGGCTTGCTGG
CCACTTTTTGGGGTCAGCTCCTCTGAAATGCATTAGCGGAACCGTTTGCAATCTGCCACA
AGTGTGATAAGTTATCTACACTGGCGAGGGGATTGCTCTCTGTAATGTTCAGCTTCTAAT
TGTCTCTACTTTGTGAGACAACTTTTGAATGCTTGACCTCAAATCAGGTAGGACTACCCG
CTGAACTTAA

>B1-68
TTTCCGTAGGTGAACCTGCGGAAGGATCATTATTGAATTATGTTTCTAGATAGGTTGTAG
CTGGCTCTTTAGAGCATGTGCACGCCTGTTTGGACTTCATTTTCATCCACCTGTGCACCT
ATTGTAGTCTTTGGTTGGGTTAGGGGGAAGTGGTCATTGTGTCAGCATCTGCTGGATGTG
AGGACTTGCATTGTGAAAGCTTTGCTGTCCTTGATGTGATCATGGAATCTCTTTCTCACT
AGAGTCTATGTCACTCATTATACTCTGTCGAATGTCATTGAATGTCTTTACATGGGCTTG
TATGCCTATGAAAATTGTAATACAACTTTCAGCAACGGATCTCTTGGCTCTCGCATCGAT
GAAGGACGCAGCGAAATGCGATAAGTAATGTGAATTGCAGAATTCAGTGAATCATCGAAT
CTTTGAACGCATCTTGCGCTCCTTGGTATTCCGAGGAGCATGCCTGTTTGAGTGTCATTA
AATTCTCAACTCTCTTATACTTTTTTGTAAAAGAGAGCTTGGACTGTGGAGGCTTGCTGG
CCACTTTTTGGGGTCAGCTCCTCTGAAATGCATTAGCGGAACCGTTTGCAATCTGCCACA
AGTGTGATAAGTTATCTACACTGGCGAGGGGATTGCTCTCTGTAATGTTCAGCTTCTAAT
TGTCTCTACTTTGTGAGACAACTTTTGAATGCTTGACCTCAAATCAGGTAGGACTACCCG
CTGAACTTAA
>B1-70
TTTCCGTAGGTGAACCTGCGGAAGGATCATTATTGAATTATGTTTCTAGATAGGTTGTAG
CTGGCTCTTTAGAGCATGTGCACGCCTGTTTGGACTTCATTTTCATCCACCTGTGCACCT
ATTGTAGTCTTTGGTTGGGTTAGGGGGAAGTGGTCATTGTGTCAGCATCTGCTGGATGTG
AGGACTTGCATTGTGAAAGCTTTGCTGTCCTTGATGTGATCATGGAATCTCTTTCTCACT
AGAGTCTATGTCACTCATTATACTCTGTCGAATGTCATTGAATGTCTTTACATGGGCTTG
TATGCCTATGAAAATTGTAATACAACTTTCAGCAACGGATCTCTTGGCTCTCGCATCGAT
GAAGGACGCAGCGAAATGCGATAAGTAATGTGAATTGCAGAATTCAGTGAATCATCGAAT
CTTTGAACGCATCTTGCGCTCCTTGGTATTCCGAGGAGCATGCCTGTTTGAGTGTCATTA
AATTCTCAACTCTCTTATACTTTTTTGTAAAAGAGAGCTTGGACTGTGGAGGCTTGCTGG
CCACTTTTTGGGGTCAGCTCCTCTGAAATGCATTAGCGGAACCGTTTGCAATCTGCCACA
AGTGTGATAAGTTATCTACACTGGCGAGGGGATTGCTCTCTGTAATGTTCAGCTTCTAAT
TGTCTCTACTTTGTGAGACAACTTTTGAATGCTTGACCTCAAATCAGGTAGGACTACCCG
CTGAACTTAA
>B2_1
TTTCCGTAGGTGAACCTGCGGAAGGATCATTATTGAATTATGTTTCTAGATAGGTTGTAG
CTGGCTCTTTAGAGCATGTGCACGCCTGTTTGGACTTCATTTTCATCCACCTGTGCACCT
ATTGTAGTCTTTGGTTGGGTTAGGGGGAAGTGGTCATTGTGTCAGCATCTGCTGGATGTG
AGGACTTGCATTGTGAAAGCTTTGCTGTCCTTGATGTGATCATGGAATCTCTTTCTCACT
AGAGTCTATGTCACTCATTATACTCTGTCGAATGTCATTGAATGTCTTTACATGGGCTTG
TATGCCTATGAAAATTGTAATACAACTTTCAGCAACGGATCTCTTGGCTCTCGCATCGAT
GAAGGACGCAGCGAAATGCGATAAGTAATGTGAATTGCAGAATTCAGTGAATCATCGAAT
CTTTGAACGCATCTTGCGCTCCTTGGTATTCCGAGGAGCATGCCTGTTTGAGTGTCATTA
AATTCTCAACTCTCTTATACTTTTTTGTAAAAGAGAGCTTGGACTGTGGAGGCTTGCTGG
CCACTTTTTGGGGTCAGCTCCTCTGAAATGCATTAGCGGAACCGTTTGCAATCTGCCACA
AGTGTGATAAGTTATCTACACTGGCGAGGGGATTGCTCTCTGTAATGTTCAGCTTCTAAT
TGTCTCTACTTTGTGAGACAACTTTTGAATGCTTGACCTCAAATCAGGTAGGACTACCCG
CTGAACTTAA
>B2_2
TTTCCGTAGGTGAACCTGCGGAAGGATCATTATTGAATTATGTTTCTAGATAGGTTGTAG
CTGGCTCTTTAGAGCATGTGCACGCCTGTTTGGACTTCATTTTCATCCACCTGTGCACCT
ATTGTAGTCTTTGGTTGGGTTAGGGGGAAGTGGTCATTGTGTCAGCATCTGCTGGATGTG
AGGACTTGCATTGTGAAAGCTTTGCTGTCCTTGATGTGATCATGGAATCTCTTTCTCACT
AGAGTCTATGTCACTCATTATACTCTGTCGAATGTCATTGAATGTCTTTACATGGGCTTG
TATGCCTATGAAAATTGTAATACAACTTTCAGCAACGGATCTCTTGGCTCTCGCATCGAT
GAAGGACGCAGCGAAATGCGATAAGTAATGTGAATTGCAGAATTCAGTGAATCATCGAAT

CTTTGAACGCATCTTGCGCTCCTTGGTATTCCGAGGAGCATGCCTGTTTGAGTGTCATTA
AATTCTCAACTCTCTTATACTTTTTTGTAAAAGAGAGCTTGGACTGTGGAGGCTTGCTGG
CCACTTTTTGGGGTCAGCTCCTCTGAAATGCATTAGCGGAACCGTTTGCAATCTGCCACA
AGTGTGATAAGTTATCTACACTGGCGAGGGGATTGCTCTCTGTAATGTTCAGCTTCTAAT
TGTCTCTACTTTGTGAGACAACTTTTGAATGCTTGACCTCAAATCAGGTAGGACTACCCG
CTGAACTTAA
>B2_3
TTTCCGTAGGTGAACCTGCGGAAGGATCATTATTGAATTATGTTTCTAGATAGGTTGTAG
CTGGCTCTTTAGAGCATGTGCACGCCTGTTTGGACTTCATTTTCATCCACCTGTGCACCT
ATTGTAGTCTTTGGTTGGGTTAGGGGGAAGTGGTCATTGTGTCAGCATCTGCTGGATGTG
AGGACTTGCATTGTGAAAGCTTTGCTGTCCTTGATGTGATCATGGAATCTCTTTCTCACT
AGAGTCTATGTCACTCATTATACTCTGTCGAATGTCATTGAATGTCTTTACATGGGCTTG
TATGCCTATGAAAATTGTAATACAACTTTCAGCAACGGATCTCTTGGCTCTCGCATCGAT
GAAGGACGCAGCGAAATGCGATAAGTAATGTGAATTGCAGAATTCAGTGAATCATCGAAT
CTTTGAACGCATCTTGCGCTCCTTGGTATTCCGAGGAGCATGCCTGTTTGAGTGTCATTA
AATTCTCAACTCTCTTATACTTTTTTGTAAAAGAGAGCTTGGACTGTGGAGGCTTGCTGG
CCACTTTTTGGGGTCAGCTCCTCTGAAATGCATTAGCGGAACCGTTTGCAATCTGCCACA
AGTGTGATAAGTTATCTACACTGGCGAGGGGATTGCTCTCTGTAATGTTCAGCTTCTAAT
TGTCTCTACTTTGTGAGACAACTTTTGAATGCTTGACCTCAAATCAGGTAGGACTACCCG
CTGAACTTAA
>B2_4
TTTCCGTAGGTGAACCTGCGGAAGGATCATTATTGAATTATGTTTCTAGATAGGTTGTAG
CTGGCTCTTTAGAGCATGTGCACGCCTGTTTGGACTTCATTTTCATCCACCTGTGCACCT
ATTGTAGTCTTTGGTTGGGTTAGGGGGAAGTGGTCATTGTGTCAGCATCTGCTGGATGTG
AGGACTTGCATTGTGAAAGCTTTGCTGTCCTTGATGTGATCATGGAATCTCTTTCTCACT
AGAGTCTATGTCACTCATTATACTCTGTCGAATGTCATTGAATGTCTTTACATGGGCTTG
TATGCCTATGAAAATTGTAATACAACTTTCAGCAACGGATCTCTTGGCTCTCGCATCGAT
GAAGGACGCAGCGAAATGCGATAAGTAATGTGAATTGCAGAATTCAGTGAATCATCGAAT
CTTTGAACGCATCTTGCGCTCCTTGGTATTCCGAGGAGCATGCCTGTTTGAGTGTCATTA
AATTCTCAACTCTCTTATACTTTTTTGTAAAAGAGAGCTTGGACTGTGGAGGCTTGCTGG
CCACTTTTTGGGGTCAGCTCCTCTGAAATGCATTAGCGGAACCGTTTGCAATCTGCCACA
AGTGTGATAAGTTATCTACACTGGCGAGGGGATTGCTCTCTGTAATGTTCAGCTTCTAAT
TGTCTCTACTTTGTGAGACAACTTTTGAATGCTTGACCTCAAATCAGGTAGGACTACCCG
CTGAACTTAA
>B2_5
TTTCCGTAGGTGAACCTGCGGAAGGATCATTATTGAATTATGTTTCTAGATAGGTTGTAG
CTGGCTCTTTAGAGCATGTGCACGCCTGTTTGGACTTCATTTTCATCCACCTGTGCACCT
ATTGTAGTCTTTGGTTGGGTTAGGGGGAAGTGGTCATTGTGTCAGCATCTGCTGGATGTG
AGGACTTGCATTGTGAAAGCTTTGCTGTCCTTGATGTGATCATGGAATCTCTTTCTCACT
AGAGTCTATGTCACTCATTATACTCTGTCGAATGTCATTGAATGTCTTTACATGGGCTTG
TATGCCTATGAAAATTGTAATACAACTTTCAGCAACGGATCTCTTGGCTCTCGCATCGAT
GAAGGACGCAGCGAAATGCGATAAGTAATGTGAATTGCAGAATTCAGTGAATCATCGAAT
CTTTGAACGCATCTTGCGCTCCTTGGTATTCCGAGGAGCATGCCTGTTTGAGTGTCATTA
AATTCTCAACTCTCTTATACTTTTTTGTAAAAGAGAGCTTGGACTGTGGAGGCTTGCTGG
CCACTTTTTGGGGTCAGCTCCTCTGAAATGCATTAGCGGAACCGTTTGCAATCTGCCACA
AGTGTGATAAGTTATCTACACTGGCGAGGGGATTGCTCTCTGTAATGTTCAGCTTCTAAT
TGTCTCTACTTTGTGAGACAACTTTTGAATGCTTGACCTCAAATCAGGTAGGACTACCCG
CTGAACTTAA
>B2_6
TTTCCGTAGGTGAACCTGCGGAAGGATCATTATTGAATTATGTTTCTAGATAGGTTGTAG

CTGGCTCTTTAGAGCATGTGCACGCCTGTTTGGACTTCATTTTCATCCACCTGTGCACCT
ATTGTAGTCTTTGGTTGGGTTAGGGGGAAGTGGTCATTGTGTCAGCATCTGCTGGATGTG
AGGACTTGCATTGTGAAAGCTTTGCTGTCCTTGATGTGATCATGGAATCTCTTTCTCACT
AGAGTCTATGTCACTCATTATACTCTGTCGAATGTCATTGAATGTCTTTACATGGGCTTG
TATGCCTATGAAAATTGTAATACAACTTTCAGCAACGGATCTCTTGGCTCTCGCATCGAT
GAAGGACGCAGCGAAATGCGATAAGTAATGTGAATTGCAGAATTCAGTGAATCATCGAAT
CTTTGAACGCATCTTGCGCTCCTTGGTATTCCGAGGAGCATGCCTGTTTGAGTGTCATTA
AATTCTCAACTCTCTTATACTTTTTTGTAAAAGAGAGCTTGGACTGTGGAGGCTTGCTGG
CCACTTTTTGGGGTCAGCTCCTCTGAAATGCATTAGCGGAACCGTTTGCAATCTGCCACA
AGTGTGATAAGTTATCTACACTGGCGAGGGGATTGCTCTCTGTAATGTTCAGCTTCTAAT
TGTCTCTACTTTGTGAGACAACTTTTGAATGCTTGACCTCAAATCAGGTAGGACTACCCG
CTGAACTTAA
>B2_7
TTTCCGTAGGTGAACCTGCGGAAGGATCATTATTGAATTATGTTTCTAGATAGGTTGTAG
CTGGCTCTTTAGAGCATGTGCACGCCTGTTTGGACTTCATTTTCATCCACCTGTGCACCT
ATTGTAGTCTTTGGTTGGGTTAGGGGGAAGTGGTCATTGTGTCAGCATCTGCTGGATGTG
AGGACTTGCATTGTGAAAGCTTTGCTGTCCTTGATGTGATCATGGAATCTCTTTCTCACT
AGAGTCTATGTCACTCATTATACTCTGTCGAATGTCATTGAATGTCTTTACATGGGCTTG
TATGCCTATGAAAATTGTAATACAACTTTCAGCAACGGATCTCTTGGCTCTCGCATCGAT
GAAGGACGCAGCGAAATGCGATAAGTAATGTGAATTGCAGAATTCAGTGAATCATCGAAT
CTTTGAACGCATCTTGCGCTCCTTGGTATTCCGAGGAGCATGCCTGTTTGAGTGTCATTA
AATTCTCAACTCTCTTATACTTTTTTGTAAAAGAGAGCTTGGACTGTGGAGGCTTGCTGG
CCACTTTTTGGGGTCAGCTCCTCTGAAATGCATTAGCGGAACCGTTTGCAATCTGCCACA
AGTGTGATAAGTTATCTACACTGGCGAGGGGATTGCTCTCTGTAATGTTCAGCTTCTAAT
TGTCTCTACTTTGTGAGACAACTTTTGAATGCTTGACCTCAAATCAGGTAGGACTACCCG
CTGAACTTAA
>B2_8
TTTCCGTAGGTGAACCTGCGGAAGGATCATTATTGAATTATGTTTCTAGATAGGTTGTAG
CTGGCTCTTTAGAGCATGTGCACGCCTGTTTGGACTTCATTTTCATCCACCTGTGCACCT
ATTGTAGTCTTTGGTTGGGTTAGGGGGAAGTGGTCATTGTGTCAGCATCTGCTGGATGTG
AGGACTTGCATTGTGAAAGCTTTGCTGTCCTTGATGTGATCATGGAATCTCTTTCTCACT
AGAGTCTATGTCACTCATTATACTCTGTCGAATGTCATTGAATGTCTTTACATGGGCTTG
TATGCCTATGAAAATTGTAATACAACTTTCAGCAACGGATCTCTTGGCTCTCGCATCGAT
GAAGGACGCAGCGAAATGCGATAAGTAATGTGAATTGCAGAATTCAGTGAATCATCGAAT
CTTTGAACGCATCTTGCGCTCCTTGGTATTCCGAGGAGCATGCCTGTTTGAGTGTCATTA
AATTCTCAACTCTCTTATACTTTTTTGTAAAAGAGAGCTTGGACTGTGGAGGCTTGCTGG
CCACTTTTTGGGGTCAGCTCCTCTGAAATGCATTAGCGGAACCGTTTGCAATCTGCCACA
AGTGTGATAAGTTATCTACACTGGCGAGGGGATTGCTCTCTGTAATGTTCAGCTTCTAAT
TGTCTCTACTTTGTGAGACAACTTTTGAATGCTTGACCTCAAATCAGGTAGGACTACCCG
CTGAACTTAA
>B2_9
TTTCCGTAGGTGAACCTGCGGAAGGATCATTATTGAATTATGTTTCTAGATAGGTTGTAG
CTGGCTCTTTAGAGCATGTGCACGCCTGTTTGGACTTCATTTTCATCCACCTGTGCACCT
ATTGTAGTCTTTGGTTGGGTTAGGGGGAAGTGGTCATTGTGTCAGCATCTGCTGGATGTG
AGGACTTGCATTGTGAAAGCTTTGCTGTCCTTGATGTGATCATGGAATCTCTTTCTCACT
AGAGTCTATGTCACTCATTATACTCTGTCGAATGTCATTGAATGTCTTTACATGGGCTTG
TATGCCTATGAAAATTGTAATACAACTTTCAGCAACGGATCTCTTGGCTCTCGCATCGAT
GAAGGACGCAGCGAAATGCGATAAGTAATGTGAATTGCAGAATTCAGTGAATCATCGAAT
CTTTGAACGCATCTTGCGCTCCTTGGTATTCCGAGGAGCATGCCTGTTTGAGTGTCATTA
AATTCTCAACTCTCTTATACTTTTTTGTAAAAGAGAGCTTGGACTGTGGAGGCTTGCTGG

CCACTTTTTGGGGTCAGCTCCTCTGAAATGCATTAGCGGAACCGTTTGCAATCTGCCACA
AGTGTGATAAGTTATCTACACTGGCGAGGGGATTGCTCTCTGTAATGTTCAGCTTCTAAT
TGTCTCTACTTTGTGAGACAACTTTTGAATGCTTGACCTCAAATCAGGTAGGACTACCCG
CTGAACTTAA
>B2_10
TTTCCGTAGGTGAACCTGCGGAAGGATCATTATTGAATTATGTTTCTAGATAGGTTGTAG
CTGGCTCTTTAGAGCATGTGCACGCCTGTTTGGACTTCATTTTCATCCACCTGTGCACCT
ATTGTAGTCTTTGGTTGGGTTAGGGGGAAGTGGTCATTGTGTCAGCATCTGCTGGATGTG
AGGACTTGCATTGTGAAAGCTTTGCTGTCCTTGATGTGATCATGGAATCTCTTTCTCACT
AGAGTCTATGTCACTCATTATACTCTGTCGAATGTCATTGAATGTCTTTACATGGGCTTG
TATGCCTATGAAAATTGTAATACAACTTTCAGCAACGGATCTCTTGGCTCTCGCATCGAT
GAAGGACGCAGCGAAATGCGATAAGTAATGTGAATTGCAGAATTCAGTGAATCATCGAAT
CTTTGAACGCATCTTGCGCTCCTTGGTATTCCGAGGAGCATGCCTGTTTGAGTGTCATTA
AATTCTCAACTCTCTTATACTTTTTTGTAAAAGAGAGCTTGGACTGTGGAGGCTTGCTGG
CCACTTTTTGGGGTCAGCTCCTCTGAAATGCATTAGCGGAACCGTTTGCAATCTGCCACA
AGTGTGATAAGTTATCTACACTGGCGAGGGGATTGCTCTCTGTAATGTTCAGCTTCTAAT
TGTCTCTACTTTGTGAGACAACTTTTGAATGCTTGACCTCAAATCAGGTAGGACTACCCG
CTGAACTTAA
>B2_12
TTTCCGTAGGTGAACCTGCGGAAGGATCATTATTGAATTATGTTTCTAGATAGGTTGTAG
CTGGCTCTTTAGAGCATGTGCACGCCTGTTTGGACTTCATTTTCATCCACCTGTGCACCT
ATTGTAGTCTTTGGTTGGGTTAGGGGGAAGTGGTCATTGTGTCAGCATCTGCTGGATGTG
AGGACTTGCATTGTGAAAGCTTTGCTGTCCTTGATGTGATCATGGAATCTCTTTCTCACT
AGAGTCTATGTCACTCATTATACTCTGTCGAATGTCATTGAATGTCTTTACATGGGCTTG
TATGCCTATGAAAATTGTAATACAACTTTCAGCAACGGATCTCTTGGCTCTCGCATCGAT
GAAGGACGCAGCGAAATGCGATAAGTAATGTGAATTGCAGAATTCAGTGAATCATCGAAT
CTTTGAACGCATCTTGCGCTCCTTGGTATTCCGAGGAGCATGCCTGTTTGAGTGTCATTA
AATTCTCAACTCTCTTATACTTTTTTGTAAAAGAGAGCTTGGACTGTGGAGGCTTGCTGG
CCACTTTTTGGGGTCAGCTCCTCTGAAATGCATTAGCGGAACCGTTTGCAATCTGCCACA
AGTGTGATAAGTTATCTACACTGGCGAGGGGATTGCTCTCTGTAATGTTCAGCTTCTAAT
TGTCTCTACTTTGTGAGACAACTTTTGAATGCTTGACCTCAAATCAGGTAGGACTACCCG
CTGAACTTAA
>B2_13
TTTCCGTAGGTGAACCTGCGGAAGGATCATTATTGAATTATGTTTCTAGATAGGTTGTAG
CTGGCTCTTTAGAGCATGTGCACGCCTGTTTGGACTTCATTTTCATCCACCTGTGCACCT
ATTGTAGTCTTTGGTTGGGTTAGGGGGAAGTGGTCATTGTGTCAGCATCTGCTGGATGTG
AGGACTTGCATTGTGAAAGCTTTGCTGTCCTTGATGTGATCATGGAATCTCTTTCTCACT
AGAGTCTATGTCACTCATTATACTCTGTCGAATGTCATTGAATGTCTTTACATGGGCTTG
TATGCCTATGAAAATTGTAATACAACTTTCAGCAACGGATCTCTTGGCTCTCGCATCGAT
GAAGGACGCAGCGAAATGCGATAAGTAATGTGAATTGCAGAATTCAGTGAATCATCGAAT
CTTTGAACGCATCTTGCGCTCCTTGGTATTCCGAGGAGCATGCCTGTTTGAGTGTCATTA
AATTCTCAACTCTCTTATACTTTTTTGTAAAAGAGAGCTTGGACTGTGGAGGCTTGCTGG
CCACTTTTTGGGGTCAGCTCCTCTGAAATGCATTAGCGGAACCGTTTGCAATCTGCCACA
AGTGTGATAAGTTATCTACACTGGCGAGGGGATTGCTCTCTGTAATGTTCAGCTTCTAAT
TGTCTCTACTTTGTGAGACAACTTTTGAATGCTTGACCTCAAATCAGGTAGGACTACCCG
CTGAACTTAA
>B2_14
TTTCCGTAGGTGAACCTGCGGAAGGATCATTATTGAATTATGTTTCTAGATAGGTTGTAG
CTGGCTCTTTAGAGCATGTGCACGCCTGTTTGGACTTCATTTTCATCCACCTGTGCACCT
ATTGTAGTCTTTGGTTGGGTTAGGGGGAAGTGGTCATTGTGTCAGCATCTGCTGGATGTG

AGGACTTGCATTGTGAAAGCTTTGCTGTCCTTGATGTGATCATGGAATCTCTTTCTCACT
AGAGTCTATGTCACTCATTATACTCTGTCGAATGTCATTGAATGTCTTTACATGGGCTTG
TATGCCTATGAAAATTGTAATACAACTTTCAGCAACGGATCTCTTGGCTCTCGCATCGAT
GAAGGACGCAGCGAAATGCGATAAGTAATGTGAATTGCAGAATTCAGTGAATCATCGAAT
CTTTGAACGCATCTTGCGCTCCTTGGTATTCCGAGGAGCATGCCTGTTTGAGTGTCATTA
AATTCTCAACTCTCTTATACTTTTTTGTAAAAGAGAGCTTGGACTGTGGAGGCTTGCTGG
CCACTTTTTGGGGTCAGCTCCTCTGAAATGCATTAGCGGAACCGTTTGCAATCTGCCACA
AGTGTGATAAGTTATCTACACTGGCGAGGGGATTGCTCTCTGTAATGTTCAGCTTCTAAT
TGTCTCTACTTTGTGAGACAACTTTTGAATGCTTGACCTCAAATCAGGTAGGACTACCCG
CTGAACTTAA
>B2_15
TTTCCGTAGGTGAACCTGCGGAAGGATCATTATTGAATTATGTTTCTAGATAGGTTGTAG
CTGGCTCTTTAGAGCATGTGCACGCCTGTTTGGACTTCATTTTCATCCACCTGTGCACCT
ATTGTAGTCTTTGGTTGGGTTAGGGGGAAGTGGTCATTGTGTCAGCATCTGCTGGATGTG
AGGACTTGCATTGTGAAAGCTTTGCTGTCCTTGATGTGATCATGGAATCTCTTTCTCACT
AGAGTCTATGTCACTCATTATACTCTGTCGAATGTCATTGAATGTCTTTACATGGGCTTG
TATGCCTATGAAAATTGTAATACAACTTTCAGCAACGGATCTCTTGGCTCTCGCATCGAT
GAAGGACGCAGCGAAATGCGATAAGTAATGTGAATTGCAGAATTCAGTGAATCATCGAAT
CTTTGAACGCATCTTGCGCTCCTTGGTATTCCGAGGAGCATGCCTGTTTGAGTGTCATTA
AATTCTCAACTCTCTTATACTTTTTTGTAAAAGAGAGCTTGGACTGTGGAGGCTTGCTGG
CCACTTTTTGGGGTCAGCTCCTCTGAAATGCATTAGCGGAACCGTTTGCAATCTGCCACA
AGTGTGATAAGTTATCTACACTGGCGAGGGGATTGCTCTCTGTAATGTTCAGCTTCTAAT
TGTCTCTACTTTGTGAGACAACTTTTGAATGCTTGACCTCAAATCAGGTAGGACTACCCG
CTGAACTTAA
>B2_16
TTTCCGTAGGTGAACCTGCGGAAGGATCATTATTGAATTATGTTTCTAGATAGGTTGTAG
CTGGCTCTTTAGAGCATGTGCACGCCTGTTTGGACTTCATTTTCATCCACCTGTGCACCT
ATTGTAGTCTTTGGTTGGGTTAGGGGGAAGTGGTCATTGTGTCAGCATCTGCTGGATGTG
AGGACTTGCATTGTGAAAGCTTTGCTGTCCTTGATGTGATCATGGAATCTCTTTCTCACT
AGAGTCTATGTCACTCATTATACTCTGTCGAATGTCATTGAATGTCTTTACATGGGCTTG
TATGCCTATGAAAATTGTAATACAACTTTCAGCAACGGATCTCTTGGCTCTCGCATCGAT
GAAGGACGCAGCGAAATGCGATAAGTAATGTGAATTGCAGAATTCAGTGAATCATCGAAT
CTTTGAACGCATCTTGCGCTCCTTGGTATTCCGAGGAGCATGCCTGTTTGAGTGTCATTA
AATTCTCAACTCTCTTATACTTTTTTGTAAAAGAGAGCTTGGACTGTGGAGGCTTGCTGG
CCACTTTTTGGGGTCAGCTCCTCTGAAATGCATTAGCGGAACCGTTTGCAATCTGCCACA
AGTGTGATAAGTTATCTACACTGGCGAGGGGATTGCTCTCTGTAATGTTCAGCTTCTAAT
TGTCTCTACTTTGTGAGACAACTTTTGAATGCTTGACCTCAAATCAGGTAGGACTACCCG
CTGAACTTAA
>B2_17
TTTCCGTAGGTGAACCTGCGGAAGGATCATTATTGAATTATGTTTCTAGATAGGTTGTAG
CTGGCTCTTTAGAGCATGTGCACGCCTGTTTGGACTTCATTTTCATCCACCTGTGCACCT
ATTGTAGTCTTTGGTTGGGTTAGGGGGAAGTGGTCATTGTGTCAGCATCTGCTGGATGTG
AGGACTTGCATTGTGAAAGCTTTGCTGTCCTTGATGTGATCATGGAATCTCTTTCTCACT
AGAGTCTATGTCACTCATTATACTCTGTCGAATGTCATTGAATGTCTTTACATGGGCTTG
TATGCCTATGAAAATTGTAATACAACTTTCAGCAACGGATCTCTTGGCTCTCGCATCGAT
GAAGGACGCAGCGAAATGCGATAAGTAATGTGAATTGCAGAATTCAGTGAATCATCGAAT
CTTTGAACGCATCTTGCGCTCCTTGGTATTCCGAGGAGCATGCCTGTTTGAGTGTCATTA
AATTCTCAACTCTCTTATACTTTTTTGTAAAAGAGAGCTTGGACTGTGGAGGCTTGCTGG
CCACTTTTTGGGGTCAGCTCCTCTGAAATGCATTAGCGGAACCGTTTGCAATCTGCCACA
AGTGTGATAAGTTATCTACACTGGCGAGGGGATTGCTCTCTGTAATGTTCAGCTTCTAAT

TGTCTCTACTTTGTGAGACAACTTTTGAATGCTTGACCTCAAATCAGGTAGGACTACCCG
CTGAACTTAA
>B2_18
TTTCCGTAGGTGAACCTGCGGAAGGATCATTATTGAATTATGTTTCTAGATAGGTTGTAG
CTGGCTCTTTAGAGCATGTGCACGCCTGTTTGGACTTCATTTTCATCCACCTGTGCACCT
ATTGTAGTCTTTGGTTGGGTTAGGGGGAAGTGGTCATTGTGTCAGCATCTGCTGGATGTG
AGGACTTGCATTGTGAAAGCTTTGCTGTCCTTGATGTGATCATGGAATCTCTTTCTCACT
AGAGTCTATGTCACTCATTATACTCTGTCGAATGTCATTGAATGTCTTTACATGGGCTTG
TATGCCTATGAAAATTGTAATACAACTTTCAGCAACGGATCTCTTGGCTCTCGCATCGAT
GAAGGACGCAGCGAAATGCGATAAGTAATGTGAATTGCAGAATTCAGTGAATCATCGAAT
CTTTGAACGCATCTTGCGCTCCTTGGTATTCCGAGGAGCATGCCTGTTTGAGTGTCATTA
AATTCTCAACTCTCTTATACTTTTTTGTAAAAGAGAGCTTGGACTGTGGAGGCTTGCTGG
CCACTTTTTGGGGTCAGCTCCTCTGAAATGCATTAGCGGAACCGTTTGCAATCTGCCACA
AGTGTGATAAGTTATCTACACTGGCGAGGGGATTGCTCTCTGTAATGTTCAGCTTCTAAT
TGTCTCTACTTTGTGAGACAACTTTTGAATGCTTGACCTCAAATCAGGTAGGACTACCCG
CTGAACTTAA
>B2_19
TTTCCGTAGGTGAACCTGCGGAAGGATCATTATTGAATTATGTTTCTAGATAGGTTGTAG
CTGGCTCTTTAGAGCATGTGCACGCCTGTTTGGACTTCATTTTCATCCACCTGTGCACCT
ATTGTAGTCTTTGGTTGGGTTAGGGGGAAGTGGTCATTGTGTCAGCATCTGCTGGATGTG
AGGACTTGCATTGTGAAAGCTTTGCTGTCCTTGATGTGATCATGGAATCTCTTTCTCACT
AGAGTCTATGTCACTCATTATACTCTGTCGAATGTCATTGAATGTCTTTACATGGGCTTG
TATGCCTATGAAAATTGTAATACAACTTTCAGCAACGGATCTCTTGGCTCTCGCATCGAT
GAAGGACGCAGCGAAATGCGATAAGTAATGTGAATTGCAGAATTCAGTGAATCATCGAAT
CTTTGAACGCATCTTGCGCTCCTTGGTATTCCGAGGAGCATGCCTGTTTGAGTGTCATTA
AATTCTCAACTCTCTTATACTTTTTTGTAAAAGAGAGCTTGGACTGTGGAGGCTTGCTGG
CCACTTTTTGGGGTCAGCTCCTCTGAAATGCATTAGCGGAACCGTTTGCAATCTGCCACA
AGTGTGATAAGTTATCTACACTGGCGAGGGGATTGCTCTCTGTAATGTTCAGCTTCTAAT
TGTCTCTACTTTGTGAGACAACTTTTGAATGCTTGACCTCAAATCAGGTAGGACTACCCG
CTGAACTTAA
>B2_20
TTTCCGTAGGTGAACCTGCGGAAGGATCATTATTGAATTATGTTTCTAGATAGGTTGTAG
CTGGCTCTTTAGAGCATGTGCACGCCTGTTTGGACTTCATTTTCATCCACCTGTGCACCT
ATTGTAGTCTTTGGTTGGGTTAGGGGGAAGTGGTCATTGTGTCAGCATCTGCTGGATGTG
AGGACTTGCATTGTGAAAGCTTTGCTGTCCTTGATGTGATCATGGAATCTCTTTCTCACT
AGAGTCTATGTCACTCATTATACTCTGTCGAATGTCATTGAATGTCTTTACATGGGCTTG
TATGCCTATGAAAATTGTAATACAACTTTCAGCAACGGATCTCTTGGCTCTCGCATCGAT
GAAGGACGCAGCGAAATGCGATAAGTAATGTGAATTGCAGAATTCAGTGAATCATCGAAT
CTTTGAACGCATCTTGCGCTCCTTGGTATTCCGAGGAGCATGCCTGTTTGAGTGTCATTA
AATTCTCAACTCTCTTATACTTTTTTGTAAAAGAGAGCTTGGACTGTGGAGGCTTGCTGG
CCACTTTTTGGGGTCAGCTCCTCTGAAATGCATTAGCGGAACCGTTTGCAATCTGCCACA
AGTGTGATAAGTTATCTACACTGGCGAGGGGATTGCTCTCTGTAATGTTCAGCTTCTAAT
TGTCTCTACTTTGTGAGACAACTTTTGAATGCTTGACCTCAAATCAGGTAGGACTACCCG
CTGAACTTAA
>B2_21
TTTCCGTAGGTGAACCTGCGGAAGGATCATTATTGAATTATGTTTCTAGATAGGTTGTAG
CTGGCTCTTTAGAGCATGTGCACGCCTGTTTGGACTTCATTTTCATCCACCTGTGCACCT
ATTGTAGTCTTTGGTTGGGTTAGGGGGAAGTGGTCATTGTGTCAGCATCTGCTGGATGTG
AGGACTTGCATTGTGAAAGCTTTGCTGTCCTTGATGTGATCATGGAATCTCTTTCTCACT
AGAGTCTATGTCACTCATTATACTCTGTCGAATGTCATTGAATGTCTTTACATGGGCTTG

TATGCCTATGAAAATTGTAATACAACTTTCAGCAACGGATCTCTTGGCTCTCGCATCGAT
GAAGGACGCAGCGAAATGCGATAAGTAATGTGAATTGCAGAATTCAGTGAATCATCGAAT
CTTTGAACGCATCTTGCGCTCCTTGGTATTCCGAGGAGCATGCCTGTTTGAGTGTCATTA
AATTCTCAACTCTCTTATACTTTTTTGTAAAAGAGAGCTTGGACTGTGGAGGCTTGCTGG
CCACTTTTTGGGGTCAGCTCCTCTGAAATGCATTAGCGGAACCGTTTGCAATCTGCCACA
AGTGTGATAAGTTATCTACACTGGCGAGGGGATTGCTCTCTGTAATGTTCAGCTTCTAAT
TGTCTCTACTTTGTGAGACAACTTTTGAATGCTTGACCTCAAATCAGGTAGGACTACCCG
CTGAACTTAA
>B2_22
TTTCCGTAGGTGAACCTGCGGAAGGATCATTATTGAATTATGTTTCTAGATAGGTTGTAG
CTGGCTCTTTAGAGCATGTGCACGCCTGTTTGGACTTCATTTTCATCCACCTGTGCACCT
ATTGTAGTCTTTGGTTGGGTTAGGGGGAAGTGGTCATTGTGTCAGCATCTGCTGGATGTG
AGGACTTGCATTGTGAAAGCTTTGCTGTCCTTGATGTGATCATGGAATCTCTTTCTCACT
AGAGTCTATGTCACTCATTATACTCTGTCGAATGTCATTGAATGTCTTTACATGGGCTTG
TATGCCTATGAAAATTGTAATACAACTTTCAGCAACGGATCTCTTGGCTCTCGCATCGAT
GAAGGACGCAGCGAAATGCGATAAGTAATGTGAATTGCAGAATTCAGTGAATCATCGAAT
CTTTGAACGCATCTTGCGCTCCTTGGTATTCCGAGGAGCATGCCTGTTTGAGTGTCATTA
AATTCTCAACTCTCTTATACTTTTTTGTAAAAGAGAGCTTGGACTGTGGAGGCTTGCTGG
CCACTTTTTGGGGTCAGCTCCTCTGAAATGCATTAGCGGAACCGTTTGCAATCTGCCACA
AGTGTGATAAGTTATCTACACTGGCGAGGGGATTGCTCTCTGTAATGTTCAGCTTCTAAT
TGTCTCTACTTTGTGAGACAACTTTTGAATGCTTGACCTCAAATCAGGTAGGACTACCCG
CTGAACTTAA
>B2_23
TTTCCGTAGGTGAACCTGCGGAAGGATCATTATTGAATTATGTTTCTAGATAGGTTGTAG
CTGGCTCTTTAGAGCATGTGCACGCCTGTTTGGACTTCATTTTCATCCACCTGTGCACCT
ATTGTAGTCTTTGGTTGGGTTAGGGGGAAGTGGTCATTGTGTCAGCATCTGCTGGATGTG
AGGACTTGCATTGTGAAAGCTTTGCTGTCCTTGATGTGATCATGGAATCTCTTTCTCACT
AGAGTCTATGTCACTCATTATACTCTGTCGAATGTCATTGAATGTCTTTACATGGGCTTG
TATGCCTATGAAAATTGTAATACAACTTTCAGCAACGGATCTCTTGGCTCTCGCATCGAT
GAAGGACGCAGCGAAATGCGATAAGTAATGTGAATTGCAGAATTCAGTGAATCATCGAAT
CTTTGAACGCATCTTGCGCTCCTTGGTATTCCGAGGAGCATGCCTGTTTGAGTGTCATTA
AATTCTCAACTCTCTTATACTTTTTTGTAAAAGAGAGCTTGGACTGTGGAGGCTTGCTGG
CCACTTTTTGGGGTCAGCTCCTCTGAAATGCATTAGCGGAACCGTTTGCAATCTGCCACA
AGTGTGATAAGTTATCTACACTGGCGAGGGGATTGCTCTCTGTAATGTTCAGCTTCTAAT
TGTCTCTACTTTGTGAGACAACTTTTGAATGCTTGACCTCAAATCAGGTAGGACTACCCG
CTGAACTTAA
>B2_24
TTTCCGTAGGTGAACCTGCGGAAGGATCATTATTGAATTATGTTTCTAGATAGGTTGTAG
CTGGCTCTTTAGAGCATGTGCACGCCTGTTTGGACTTCATTTTCATCCACCTGTGCACCT
ATTGTAGTCTTTGGTTGGGTTAGGGGGAAGTGGTCATTGTGTCAGCATCTGCTGGATGTG
AGGACTTGCATTGTGAAAGCTTTGCTGTCCTTGATGTGATCATGGAATCTCTTTCTCACT
AGAGTCTATGTCACTCATTATACTCTGTCGAATGTCATTGAATGTCTTTACATGGGCTTG
TATGCCTATGAAAATTGTAATACAACTTTCAGCAACGGATCTCTTGGCTCTCGCATCGAT
GAAGGACGCAGCGAAATGCGATAAGTAATGTGAATTGCAGAATTCAGTGAATCATCGAAT
CTTTGAACGCATCTTGCGCTCCTTGGTATTCCGAGGAGCATGCCTGTTTGAGTGTCATTA
AATTCTCAACTCTCTTATACTTTTTTGTAAAAGAGAGCTTGGACTGTGGAGGCTTGCTGG
CCACTTTTTGGGGTCAGCTCCTCTGAAATGCATTAGCGGAACCGTTTGCAATCTGCCACA
AGTGTGATAAGTTATCTACACTGGCGAGGGGATTGCTCTCTGTAATGTTCAGCTTCTAAT
TGTCTCTACTTTGTGAGACAACTTTTGAATGCTTGACCTCAAATCAGGTAGGACTACCCG
CTGAACTTAA

>B2_26
TTTCCGTAGGTGAACCTGCGGAAGGATCATTATTGAATTATGTTTCTAGATAGGTTGTAG
CTGGCTCTTTAGAGCATGTGCACGCCTGTTTGGACTTCATTTTCATCCACCTGTGCACCT
ATTGTAGTCTTTGGTTGGGTTAGGGGGAAGTGGTCATTGTGTCAGCATCTGCTGGATGTG
AGGACTTGCATTGTGAAAGCTTTGCTGTCCTTGATGTGATCATGGAATCTCTTTCTCACT
AGAGTCTATGTCACTCATTATACTCTGTCGAATGTCATTGAATGTCTTTACATGGGCTTG
TATGCCTATGAAAATTGTAATACAACTTTCAGCAACGGATCTCTTGGCTCTCGCATCGAT
GAAGGACGCAGCGAAATGCGATAAGTAATGTGAATTGCAGAATTCAGTGAATCATCGAAT
CTTTGAACGCATCTTGCGCTCCTTGGTATTCCGAGGAGCATGCCTGTTTGAGTGTCATTA
AATTCTCAACTCTCTTATACTTTTTTGTAAAAGAGAGCTTGGACTGTGGAGGCTTGCTGG
CCACTTTTTGGGGTCAGCTCCTCTGAAATGCATTAGCGGAACCGTTTGCAATCTGCCACA
AGTGTGATAAGTTATCTACACTGGCGAGGGGATTGCTCTCTGTAATGTTCAGCTTCTAAT
TGTCTCTACTTTGTGAGACAACTTTTGAATGCTTGACCTCAAATCAGGTAGGACTACCCG
CTGAACTTAA
>B2_28
TTTCCGTAGGTGAACCTGCGGAAGGATCATTATTGAATTATGTTTCTAGATAGGTTGTAG
CTGGCTCTTTAGAGCATGTGCACGCCTGTTTGGACTTCATTTTCATCCACCTGTGCACCT
ATTGTAGTCTTTGGTTGGGTTAGGGGGAAGTGGTCATTGTGTCAGCATCTGCTGGATGTG
AGGACTTGCATTGTGAAAGCTTTGCTGTCCTTGATGTGATCATGGAATCTCTTTCTCACT
AGAGTCTATGTCACTCATTATACTCTGTCGAATGTCATTGAATGTCTTTACATGGGCTTG
TATGCCTATGAAAATTGTAATACAACTTTCAGCAACGGATCTCTTGGCTCTCGCATCGAT
GAAGGACGCAGCGAAATGCGATAAGTAATGTGAATTGCAGAATTCAGTGAATCATCGAAT
CTTTGAACGCATCTTGCGCTCCTTGGTATTCCGAGGAGCATGCCTGTTTGAGTGTCATTA
AATTCTCAACTCTCTTATACTTTTTTGTAAAAGAGAGCTTGGACTGTGGAGGCTTGCTGG
CCACTTTTTGGGGTCAGCTCCTCTGAAATGCATTAGCGGAACCGTTTGCAATCTGCCACA
AGTGTGATAAGTTATCTACACTGGCGAGGGGATTGCTCTCTGTAATGTTCAGCTTCTAAT
TGTCTCTACTTTGTGAGACAACTTTTGAATGCTTGACCTCAAATCAGGTAGGACTACCCG
CTGAACTTAA
>B2_29
TTTCCGTAGGTGAACCTGCGGAAGGATCATTATTGAATTATGTTTCTAGATAGGTTGTAG
CTGGCTCTTTAGAGCATGTGCACGCCTGTTTGGACTTCATTTTCATCCACCTGTGCACCT
ATTGTAGTCTTTGGTTGGGTTAGGGGGAAGTGGTCATTGTGTCAGCATCTGCTGGATGTG
AGGACTTGCATTGTGAAAGCTTTGCTGTCCTTGATGTGATCATGGAATCTCTTTCTCACT
AGAGTCTATGTCACTCATTATACTCTGTCGAATGTCATTGAATGTCTTTACATGGGCTTG
TATGCCTATGAAAATTGTAATACAACTTTCAGCAACGGATCTCTTGGCTCTCGCATCGAT
GAAGGACGCAGCGAAATGCGATAAGTAATGTGAATTGCAGAATTCAGTGAATCATCGAAT
CTTTGAACGCATCTTGCGCTCCTTGGTATTCCGAGGAGCATGCCTGTTTGAGTGTCATTA
AATTCTCAACTCTCTTATACTTTTTTGTAAAAGAGAGCTTGGACTGTGGAGGCTTGCTGG
CCACTTTTTGGGGTCAGCTCCTCTGAAATGCATTAGCGGAACCGTTTGCAATCTGCCACA
AGTGTGATAAGTTATCTACACTGGCGAGGGGATTGCTCTCTGTAATGTTCAGCTTCTAAT
TGTCTCTACTTTGTGAGACAACTTTTGAATGCTTGACCTCAAATCAGGTAGGACTACCCG
CTGAACTTAA
>B2_30
TTTCCGTAGGTGAACCTGCGGAAGGATCATTATTGAATTATGTTTCTAGATAGGTTGTAG
CTGGCTCTTTAGAGCATGTGCACGCCTGTTTGGACTTCATTTTCATCCACCTGTGCACCT
ATTGTAGTCTTTGGTTGGGTTAGGGGGAAGTGGTCATTGTGTCAGCATCTGCTGGATGTG
AGGACTTGCATTGTGAAAGCTTTGCTGTCCTTGATGTGATCATGGAATCTCTTTCTCACT
AGAGTCTATGTCACTCATTATACTCTGTCGAATGTCATTGAATGTCTTTACATGGGCTTG
TATGCCTATGAAAATTGTAATACAACTTTCAGCAACGGATCTCTTGGCTCTCGCATCGAT
GAAGGACGCAGCGAAATGCGATAAGTAATGTGAATTGCAGAATTCAGTGAATCATCGAAT

CTTTGAACGCATCTTGCGCTCCTTGGTATTCCGAGGAGCATGCCTGTTTGAGTGTCATTA
AATTCTCAACTCTCTTATACTTTTTTGTAAAAGAGAGCTTGGACTGTGGAGGCTTGCTGG
CCACTTTTTGGGGTCAGCTCCTCTGAAATGCATTAGCGGAACCGTTTGCAATCTGCCACA
AGTGTGATAAGTTATCTACACTGGCGAGGGGATTGCTCTCTGTAATGTTCAGCTTCTAAT
TGTCTCTACTTTGTGAGACAACTTTTGAATGCTTGACCTCAAATCAGGTAGGACTACCCG
CTGAACTTAA
>B2_31
TTTCCGTAGGTGAACCTGCGGAAGGATCATTATTGAATTATGTTTCTAGATAGGTTGTAG
CTGGCTCTTTAGAGCATGTGCACGCCTGTTTGGACTTCATTTTCATCCACCTGTGCACCT
ATTGTAGTCTTTGGTTGGGTTAGGGGGAAGTGGTCATTGTGTCAGCATCTGCTGGATGTG
AGGACTTGCATTGTGAAAGCTTTGCTGTCCTTGATGTGATCATGGAATCTCTTTCTCACT
AGAGTCTATGTCACTCATTATACTCTGTCGAATGTCATTGAATGTCTTTACATGGGCTTG
TATGCCTATGAAAATTGTAATACAACTTTCAGCAACGGATCTCTTGGCTCTCGCATCGAT
GAAGGACGCAGCGAAATGCGATAAGTAATGTGAATTGCAGAATTCAGTGAATCATCGAAT
CTTTGAACGCATCTTGCGCTCCTTGGTATTCCGAGGAGCATGCCTGTTTGAGTGTCATTA
AATTCTCAACTCTCTTATACTTTTTTGTAAAAGAGAGCTTGGACTGTGGAGGCTTGCTGG
CCACTTTTTGGGGTCAGCTCCTCTGAAATGCATTAGCGGAACCGTTTGCAATCTGCCACA
AGTGTGATAAGTTATCTACACTGGCGAGGGGATTGCTCTCTGTAATGTTCAGCTTCTAAT
TGTCTCTACTTTGTGAGACAACTTTTGAATGCTTGACCTCAAATCAGGTAGGACTACCCG
CTGAACTTAA
>B2_33
TTTCCGTAGGTGAACCTGCGGAAGGATCATTATTGAATTATGTTTCTAGATAGGTTGTAG
CTGGCTCTTTAGAGCATGTGCACGCCTGTTTGGACTTCATTTTCATCCACCTGTGCACCT
ATTGTAGTCTTTGGTTGGGTTAGGGGGAAGTGGTCATTGTGTCAGCATCTGCTGGATGTG
AGGACTTGCATTGTGAAAGCTTTGCTGTCCTTGATGTGATCATGGAATCTCTTTCTCACT
AGAGTCTATGTCACTCATTATACTCTGTCGAATGTCATTGAATGTCTTTACATGGGCTTG
TATGCCTATGAAAATTGTAATACAACTTTCAGCAACGGATCTCTTGGCTCTCGCATCGAT
GAAGGACGCAGCGAAATGCGATAAGTAATGTGAATTGCAGAATTCAGTGAATCATCGAAT
CTTTGAACGCATCTTGCGCTCCTTGGTATTCCGAGGAGCATGCCTGTTTGAGTGTCATTA
AATTCTCAACTCTCTTATACTTTTTTGTAAAAGAGAGCTTGGACTGTGGAGGCTTGCTGG
CCACTTTTTGGGGTCAGCTCCTCTGAAATGCATTAGCGGAACCGTTTGCAATCTGCCACA
AGTGTGATAAGTTATCTACACTGGCGAGGGGATTGCTCTCTGTAATGTTCAGCTTCTAAT
TGTCTCTACTTTGTGAGACAACTTTTGAATGCTTGACCTCAAATCAGGTAGGACTACCCG
CTGAACTTAA
>B2_34
TTTCCGTAGGTGAACCTGCGGAAGGATCATTATTGAATTATGTTTCTAGATAGGTTGTAG
CTGGCTCTTTAGAGCATGTGCACGCCTGTTTGGACTTCATTTTCATCCACCTGTGCACCT
ATTGTAGTCTTTGGTTGGGTTAGGGGGAAGTGGTCATTGTGTCAGCATCTGCTGGATGTG
AGGACTTGCATTGTGAAAGCTTTGCTGTCCTTGATGTGATCATGGAATCTCTTTCTCACT
AGAGTCTATGTCACTCATTATACTCTGTCGAATGTCATTGAATGTCTTTACATGGGCTTG
TATGCCTATGAAAATTGTAATACAACTTTCAGCAACGGATCTCTTGGCTCTCGCATCGAT
GAAGGACGCAGCGAAATGCGATAAGTAATGTGAATTGCAGAATTCAGTGAATCATCGAAT
CTTTGAACGCATCTTGCGCTCCTTGGTATTCCGAGGAGCATGCCTGTTTGAGTGTCATTA
AATTCTCAACTCTCTTATACTTTTTTGTAAAAGAGAGCTTGGACTGTGGAGGCTTGCTGG
CCACTTTTTGGGGTCAGCTCCTCTGAAATGCATTAGCGGAACCGTTTGCAATCTGCCACA
AGTGTGATAAGTTATCTACACTGGCGAGGGGATTGCTCTCTGTAATGTTCAGCTTCTAAT
TGTCTCTACTTTGTGAGACAACTTTTGAATGCTTGACCTCAAATCAGGTAGGACTACCCG
CTGAACTTAA
>B2_35
TTTCCGTAGGTGAACCTGCGGAAGGATCATTATTGAATTATGTTTCTAGATAGGTTGTAG

CTGGCTCTTTAGAGCATGTGCACGCCTGTTTGGACTTCATTTTCATCCACCTGTGCACCT
ATTGTAGTCTTTGGTTGGGTTAGGGGGAAGTGGTCATTGTGTCAGCATCTGCTGGATGTG
AGGACTTGCATTGTGAAAGCTTTGCTGTCCTTGATGTGATCATGGAATCTCTTTCTCACT
AGAGTCTATGTCACTCATTATACTCTGTCGAATGTCATTGAATGTCTTTACATGGGCTTG
TATGCCTATGAAAATTGTAATACAACTTTCAGCAACGGATCTCTTGGCTCTCGCATCGAT
GAAGGACGCAGCGAAATGCGATAAGTAATGTGAATTGCAGAATTCAGTGAATCATCGAAT
CTTTGAACGCATCTTGCGCTCCTTGGTATTCCGAGGAGCATGCCTGTTTGAGTGTCATTA
AATTCTCAACTCTCTTATACTTTTTTGTAAAAGAGAGCTTGGACTGTGGAGGCTTGCTGG
CCACTTTTTGGGGTCAGCTCCTCTGAAATGCATTAGCGGAACCGTTTGCAATCTGCCACA
AGTGTGATAAGTTATCTACACTGGCGAGGGGATTGCTCTCTGTAATGTTCAGCTTCTAAT
TGTCTCTACTTTGTGAGACAACTTTTGAATGCTTGACCTCAAATCAGGTAGGACTACCCG
CTGAACTTAA
>B2_37
TTTCCGTAGGTGAACCTGCGGAAGGATCATTATTGAATTATGTTTCTAGATAGGTTGTAG
CTGGCTCTTTAGAGCATGTGCACGCCTGTTTGGACTTCATTTTCATCCACCTGTGCACCT
ATTGTAGTCTTTGGTTGGGTTAGGGGGAAGTGGTCATTGTGTCAGCATCTGCTGGATGTG
AGGACTTGCATTGTGAAAGCTTTGCTGTCCTTGATGTGATCATGGAATCTCTTTCTCACT
AGAGTCTATGTCACTCATTATACTCTGTCGAATGTCATTGAATGTCTTTACATGGGCTTG
TATGCCTATGAAAATTGTAATACAACTTTCAGCAACGGATCTCTTGGCTCTCGCATCGAT
GAAGGACGCAGCGAAATGCGATAAGTAATGTGAATTGCAGAATTCAGTGAATCATCGAAT
CTTTGAACGCATCTTGCGCTCCTTGGTATTCCGAGGAGCATGCCTGTTTGAGTGTCATTA
AATTCTCAACTCTCTTATACTTTTTTGTAAAAGAGAGCTTGGACTGTGGAGGCTTGCTGG
CCACTTTTTGGGGTCAGCTCCTCTGAAATGCATTAGCGGAACCGTTTGCAATCTGCCACA
AGTGTGATAAGTTATCTACACTGGCGAGGGGATTGCTCTCTGTAATGTTCAGCTTCTAAT
TGTCTCTACTTTGTGAGACAACTTTTGAATGCTTGACCTCAAATCAGGTAGGACTACCCG
CTGAACTTAA
>B2_38
TTTCCGTAGGTGAACCTGCGGAAGGATCATTATTGAATTATGTTTCTAGATAGGTTGTAG
CTGGCTCTTTAGAGCATGTGCACGCCTGTTTGGACTTCATTTTCATCCACCTGTGCACCT
ATTGTAGTCTTTGGTTGGGTTAGGGGGAAGTGGTCATTGTGTCAGCATCTGCTGGATGTG
AGGACTTGCATTGTGAAAGCTTTGCTGTCCTTGATGTGATCATGGAATCTCTTTCTCACT
AGAGTCTATGTCACTCATTATACTCTGTCGAATGTCATTGAATGTCTTTACATGGGCTTG
TATGCCTATGAAAATTGTAATACAACTTTCAGCAACGGATCTCTTGGCTCTCGCATCGAT
GAAGGACGCAGCGAAATGCGATAAGTAATGTGAATTGCAGAATTCAGTGAATCATCGAAT
CTTTGAACGCATCTTGCGCTCCTTGGTATTCCGAGGAGCATGCCTGTTTGAGTGTCATTA
AATTCTCAACTCTCTTATACTTTTTTGTAAAAGAGAGCTTGGACTGTGGAGGCTTGCTGG
CCACTTTTTGGGGTCAGCTCCTCTGAAATGCATTAGCGGAACCGTTTGCAATCTGCCACA
AGTGTGATAAGTTATCTACACTGGCGAGGGGATTGCTCTCTGTAATGTTCAGCTTCTAAT
TGTCTCTACTTTGTGAGACAACTTTTGAATGCTTGACCTCAAATCAGGTAGGACTACCCG
CTGAACTTAA
>B2_39
TTTCCGTAGGTGAACCTGCGGAAGGATCATTATTGAATTATGTTTCTAGATAGGTTGTAG
CTGGCTCTTTAGAGCATGTGCACGCCTGTTTGGACTTCATTTTCATCCACCTGTGCACCT
ATTGTAGTCTTTGGTTGGGTTAGGGGGAAGTGGTCATTGTGTCAGCATCTGCTGGATGTG
AGGACTTGCATTGTGAAAGCTTTGCTGTCCTTGATGTGATCATGGAATCTCTTTCTCACT
AGAGTCTATGTCACTCATTATACTCTGTCGAATGTCATTGAATGTCTTTACATGGGCTTG
TATGCCTATGAAAATTGTAATACAACTTTCAGCAACGGATCTCTTGGCTCTCGCATCGAT
GAAGGACGCAGCGAAATGCGATAAGTAATGTGAATTGCAGAATTCAGTGAATCATCGAAT
CTTTGAACGCATCTTGCGCTCCTTGGTATTCCGAGGAGCATGCCTGTTTGAGTGTCATTA
AATTCTCAACTCTCTTATACTTTTTTGTAAAAGAGAGCTTGGACTGTGGAGGCTTGCTGG

CCACTTTTTGGGGTCAGCTCCTCTGAAATGCATTAGCGGAACCGTTTGCAATCTGCCACA
AGTGTGATAAGTTATCTACACTGGCGAGGGGATTGCTCTCTGTAATGTTCAGCTTCTAAT
TGTCTCTACTTTGTGAGACAACTTTTGAATGCTTGACCTCAAATCAGGTAGGACTACCCG
CTGAACTTAA
>B2_40
TTTCCGTAGGTGAACCTGCGGAAGGATCATTATTGAATTATGTTTCTAGATAGGTTGTAG
CTGGCTCTTTAGAGCATGTGCACGCCTGTTTGGACTTCATTTTCATCCACCTGTGCACCT
ATTGTAGTCTTTGGTTGGGTTAGGGGGAAGTGGTCATTGTGTCAGCATCTGCTGGATGTG
AGGACTTGCATTGTGAAAGCTTTGCTGTCCTTGATGTGATCATGGAATCTCTTTCTCACT
AGAGTCTATGTCACTCATTATACTCTGTCGAATGTCATTGAATGTCTTTACATGGGCTTG
TATGCCTATGAAAATTGTAATACAACTTTCAGCAACGGATCTCTTGGCTCTCGCATCGAT
GAAGGACGCAGCGAAATGCGATAAGTAATGTGAATTGCAGAATTCAGTGAATCATCGAAT
CTTTGAACGCATCTTGCGCTCCTTGGTATTCCGAGGAGCATGCCTGTTTGAGTGTCATTA
AATTCTCAACTCTCTTATACTTTTTTGTAAAAGAGAGCTTGGACTGTGGAGGCTTGCTGG
CCACTTTTTGGGGTCAGCTCCTCTGAAATGCATTAGCGGAACCGTTTGCAATCTGCCACA
AGTGTGATAAGTTATCTACACTGGCGAGGGGATTGCTCTCTGTAATGTTCAGCTTCTAAT
TGTCTCTACTTTGTGAGACAACTTTTGAATGCTTGACCTCAAATCAGGTAGGACTACCCG
CTGAACTTAA
>B2_41
TTTCCGTAGGTGAACCTGCGGAAGGATCATTATTGAATTATGTTTCTAGATAGGTTGTAG
CTGGCTCTTTAGAGCATGTGCACGCCTGTTTGGACTTCATTTTCATCCACCTGTGCACCT
ATTGTAGTCTTTGGTTGGGTTAGGGGGAAGTGGTCATTGTGTCAGCATCTGCTGGATGTG
AGGACTTGCATTGTGAAAGCTTTGCTGTCCTTGATGTGATCATGGAATCTCTTTCTCACT
AGAGTCTATGTCACTCATTATACTCTGTCGAATGTCATTGAATGTCTTTACATGGGCTTG
TATGCCTATGAAAATTGTAATACAACTTTCAGCAACGGATCTCTTGGCTCTCGCATCGAT
GAAGGACGCAGCGAAATGCGATAAGTAATGTGAATTGCAGAATTCAGTGAATCATCGAAT
CTTTGAACGCATCTTGCGCTCCTTGGTATTCCGAGGAGCATGCCTGTTTGAGTGTCATTA
AATTCTCAACTCTCTTATACTTTTTTGTAAAAGAGAGCTTGGACTGTGGAGGCTTGCTGG
CCACTTTTTGGGGTCAGCTCCTCTGAAATGCATTAGCGGAACCGTTTGCAATCTGCCACA
AGTGTGATAAGTTATCTACACTGGCGAGGGGATTGCTCTCTGTAATGTTCAGCTTCTAAT
TGTCTCTACTTTGTGAGACAACTTTTGAATGCTTGACCTCAAATCAGGTAGGACTACCCG
CTGAACTTAA
>B2_42
TTTCCGTAGGTGAACCTGCGGAAGGATCATTATTGAATTATGTTTCTAGATAGGTTGTAG
CTGGCTCTTTAGAGCATGTGCACGCCTGTTTGGACTTCATTTTCATCCACCTGTGCACCT
ATTGTAGTCTTTGGTTGGGTTAGGGGGAAGTGGTCATTGTGTCAGCATCTGCTGGATGTG
AGGACTTGCATTGTGAAAGCTTTGCTGTCCTTGATGTGATCATGGAATCTCTTTCTCACT
AGAGTCTATGTCACTCATTATACTCTGTCGAATGTCATTGAATGTCTTTACATGGGCTTG
TATGCCTATGAAAATTGTAATACAACTTTCAGCAACGGATCTCTTGGCTCTCGCATCGAT
GAAGGACGCAGCGAAATGCGATAAGTAATGTGAATTGCAGAATTCAGTGAATCATCGAAT
CTTTGAACGCATCTTGCGCTCCTTGGTATTCCGAGGAGCATGCCTGTTTGAGTGTCATTA
AATTCTCAACTCTCTTATACTTTTTTGTAAAAGAGAGCTTGGACTGTGGAGGCTTGCTGG
CCACTTTTTGGGGTCAGCTCCTCTGAAATGCATTAGCGGAACCGTTTGCAATCTGCCACA
AGTGTGATAAGTTATCTACACTGGCGAGGGGATTGCTCTCTGTAATGTTCAGCTTCTAAT
TGTCTCTACTTTGTGAGACAACTTTTGAATGCTTGACCTCAAATCAGGTAGGACTACCCG
CTGAACTTAA
>B2_43
TTTCCGTAGGTGAACCTGCGGAAGGATCATTATTGAATTATGTTTCTAGATAGGTTGTAG
CTGGCTCTTTAGAGCATGTGCACGCCTGTTTGGACTTCATTTTCATCCACCTGTGCACCT
ATTGTAGTCTTTGGTTGGGTTAGGGGGAAGTGGTCATTGTGTCAGCATCTGCTGGATGTG

AGGACTTGCATTGTGAAAGCTTTGCTGTCCTTGATGTGATCATGGAATCTCTTTCTCACT
AGAGTCTATGTCACTCATTATACTCTGTCGAATGTCATTGAATGTCTTTACATGGGCTTG
TATGCCTATGAAAATTGTAATACAACTTTCAGCAACGGATCTCTTGGCTCTCGCATCGAT
GAAGGACGCAGCGAAATGCGATAAGTAATGTGAATTGCAGAATTCAGTGAATCATCGAAT
CTTTGAACGCATCTTGCGCTCCTTGGTATTCCGAGGAGCATGCCTGTTTGAGTGTCATTA
AATTCTCAACTCTCTTATACTTTTTTGTAAAAGAGAGCTTGGACTGTGGAGGCTTGCTGG
CCACTTTTTGGGGTCAGCTCCTCTGAAATGCATTAGCGGAACCGTTTGCAATCTGCCACA
AGTGTGATAAGTTATCTACACTGGCGAGGGGATTGCTCTCTGTAATGTTCAGCTTCTAAT
TGTCTCTACTTTGTGAGACAACTTTTGAATGCTTGACCTCAAATCAGGTAGGACTACCCG
CTGAACTTAA
>B2_44
TTTCCGTAGGTGAACCTGCGGAAGGATCATTATTGAATTATGTTTCTAGATAGGTTGTAG
CTGGCTCTTTAGAGCATGTGCACGCCTGTTTGGACTTCATTTTCATCCACCTGTGCACCT
ATTGTAGTCTTTGGTTGGGTTAGGGGGAAGTGGTCATTGTGTCAGCATCTGCTGGATGTG
AGGACTTGCATTGTGAAAGCTTTGCTGTCCTTGATGTGATCATGGAATCTCTTTCTCACT
AGAGTCTATGTCACTCATTATACTCTGTCGAATGTCATTGAATGTCTTTACATGGGCTTG
TATGCCTATGAAAATTGTAATACAACTTTCAGCAACGGATCTCTTGGCTCTCGCATCGAT
GAAGGACGCAGCGAAATGCGATAAGTAATGTGAATTGCAGAATTCAGTGAATCATCGAAT
CTTTGAACGCATCTTGCGCTCCTTGGTATTCCGAGGAGCATGCCTGTTTGAGTGTCATTA
AATTCTCAACTCTCTTATACTTTTTTGTAAAAGAGAGCTTGGACTGTGGAGGCTTGCTGG
CCACTTTTTGGGGTCAGCTCCTCTGAAATGCATTAGCGGAACCGTTTGCAATCTGCCACA
AGTGTGATAAGTTATCTACACTGGCGAGGGGATTGCTCTCTGTAATGTTCAGCTTCTAAT
TGTCTCTACTTTGTGAGACAACTTTTGAATGCTTGACCTCAAATCAGGTAGGACTACCCG
CTGAACTTAA
>B2_45
TTTCCGTAGGTGAACCTGCGGAAGGATCATTATTGAATTATGTTTCTAGATAGGTTGTAG
CTGGCTCTTTAGAGCATGTGCACGCCTGTTTGGACTTCATTTTCATCCACCTGTGCACCT
ATTGTAGTCTTTGGTTGGGTTAGGGGGAAGTGGTCATTGTGTCAGCATCTGCTGGATGTG
AGGACTTGCATTGTGAAAGCTTTGCTGTCCTTGATGTGATCATGGAATCTCTTTCTCACT
AGAGTCTATGTCACTCATTATACTCTGTCGAATGTCATTGAATGTCTTTACATGGGCTTG
TATGCCTATGAAAATTGTAATACAACTTTCAGCAACGGATCTCTTGGCTCTCGCATCGAT
GAAGGACGCAGCGAAATGCGATAAGTAATGTGAATTGCAGAATTCAGTGAATCATCGAAT
CTTTGAACGCATCTTGCGCTCCTTGGTATTCCGAGGAGCATGCCTGTTTGAGTGTCATTA
AATTCTCAACTCTCTTATACTTTTTTGTAAAAGAGAGCTTGGACTGTGGAGGCTTGCTGG
CCACTTTTTGGGGTCAGCTCCTCTGAAATGCATTAGCGGAACCGTTTGCAATCTGCCACA
AGTGTGATAAGTTATCTACACTGGCGAGGGGATTGCTCTCTGTAATGTTCAGCTTCTAAT
TGTCTCTACTTTGTGAGACAACTTTTGAATGCTTGACCTCAAATCAGGTAGGACTACCCG
CTGAACTTAA
>B2_46
TTTCCGTAGGTGAACCTGCGGAAGGATCATTATTGAATTATGTTTCTAGATAGGTTGTAG
CTGGCTCTTTAGAGCATGTGCACGCCTGTTTGGACTTCATTTTCATCCACCTGTGCACCT
ATTGTAGTCTTTGGTTGGGTTAGGGGGAAGTGGTCATTGTGTCAGCATCTGCTGGATGTG
AGGACTTGCATTGTGAAAGCTTTGCTGTCCTTGATGTGATCATGGAATCTCTTTCTCACT
AGAGTCTATGTCACTCATTATACTCTGTCGAATGTCATTGAATGTCTTTACATGGGCTTG
TATGCCTATGAAAATTGTAATACAACTTTCAGCAACGGATCTCTTGGCTCTCGCATCGAT
GAAGGACGCAGCGAAATGCGATAAGTAATGTGAATTGCAGAATTCAGTGAATCATCGAAT
CTTTGAACGCATCTTGCGCTCCTTGGTATTCCGAGGAGCATGCCTGTTTGAGTGTCATTA
AATTCTCAACTCTCTTATACTTTTTTGTAAAAGAGAGCTTGGACTGTGGAGGCTTGCTGG
CCACTTTTTGGGGTCAGCTCCTCTGAAATGCATTAGCGGAACCGTTTGCAATCTGCCACA
AGTGTGATAAGTTATCTACACTGGCGAGGGGATTGCTCTCTGTAATGTTCAGCTTCTAAT

TGTCTCTACTTTGTGAGACAACTTTTGAATGCTTGACCTCAAATCAGGTAGGACTACCCG
CTGAACTTAA
>B2_47
TTTCCGTAGGTGAACCTGCGGAAGGATCATTATTGAATTATGTTTCTAGATAGGTTGTAG
CTGGCTCTTTAGAGCATGTGCACGCCTGTTTGGACTTCATTTTCATCCACCTGTGCACCT
ATTGTAGTCTTTGGTTGGGTTAGGGGGAAGTGGTCATTGTGTCAGCATCTGCTGGATGTG
AGGACTTGCATTGTGAAAGCTTTGCTGTCCTTGATGTGATCATGGAATCTCTTTCTCACT
AGAGTCTATGTCACTCATTATACTCTGTCGAATGTCATTGAATGTCTTTACATGGGCTTG
TATGCCTATGAAAATTGTAATACAACTTTCAGCAACGGATCTCTTGGCTCTCGCATCGAT
GAAGGACGCAGCGAAATGCGATAAGTAATGTGAATTGCAGAATTCAGTGAATCATCGAAT
CTTTGAACGCATCTTGCGCTCCTTGGTATTCCGAGGAGCATGCCTGTTTGAGTGTCATTA
AATTCTCAACTCTCTTATACTTTTTTGTAAAAGAGAGCTTGGACTGTGGAGGCTTGCTGG
CCACTTTTTGGGGTCAGCTCCTCTGAAATGCATTAGCGGAACCGTTTGCAATCTGCCACA
AGTGTGATAAGTTATCTACACTGGCGAGGGGATTGCTCTCTGTAATGTTCAGCTTCTAAT
TGTCTCTACTTTGTGAGACAACTTTTGAATGCTTGACCTCAAATCAGGTAGGACTACCCG
CTGAACTTAA
>B2_48
TTTCCGTAGGTGAACCTGCGGAAGGATCATTATTGAATTATGTTTCTAGATAGGTTGTAG
CTGGCTCTTTAGAGCATGTGCACGCCTGTTTGGACTTCATTTTCATCCACCTGTGCACCT
ATTGTAGTCTTTGGTTGGGTTAGGGGGAAGTGGTCATTGTGTCAGCATCTGCTGGATGTG
AGGACTTGCATTGTGAAAGCTTTGCTGTCCTTGATGTGATCATGGAATCTCTTTCTCACT
AGAGTCTATGTCACTCATTATACTCTGTCGAATGTCATTGAATGTCTTTACATGGGCTTG
TATGCCTATGAAAATTGTAATACAACTTTCAGCAACGGATCTCTTGGCTCTCGCATCGAT
GAAGGACGCAGCGAAATGCGATAAGTAATGTGAATTGCAGAATTCAGTGAATCATCGAAT
CTTTGAACGCATCTTGCGCTCCTTGGTATTCCGAGGAGCATGCCTGTTTGAGTGTCATTA
AATTCTCAACTCTCTTATACTTTTTTGTAAAAGAGAGCTTGGACTGTGGAGGCTTGCTGG
CCACTTTTTGGGGTCAGCTCCTCTGAAATGCATTAGCGGAACCGTTTGCAATCTGCCACA
AGTGTGATAAGTTATCTACACTGGCGAGGGGATTGCTCTCTGTAATGTTCAGCTTCTAAT
TGTCTCTACTTTGTGAGACAACTTTTGAATGCTTGACCTCAAATCAGGTAGGACTACCCG
CTGAACTTAA
>B2_49
TTTCCGTAGGTGAACCTGCGGAAGGATCATTATTGAATTATGTTTCTAGATAGGTTGTAG
CTGGCTCTTTAGAGCATGTGCACGCCTGTTTGGACTTCATTTTCATCCACCTGTGCACCT
ATTGTAGTCTTTGGTTGGGTTAGGGGGAAGTGGTCATTGTGTCAGCATCTGCTGGATGTG
AGGACTTGCATTGTGAAAGCTTTGCTGTCCTTGATGTGATCATGGAATCTCTTTCTCACT
AGAGTCTATGTCACTCATTATACTCTGTCGAATGTCATTGAATGTCTTTACATGGGCTTG
TATGCCTATGAAAATTGTAATACAACTTTCAGCAACGGATCTCTTGGCTCTCGCATCGAT
GAAGGACGCAGCGAAATGCGATAAGTAATGTGAATTGCAGAATTCAGTGAATCATCGAAT
CTTTGAACGCATCTTGCGCTCCTTGGTATTCCGAGGAGCATGCCTGTTTGAGTGTCATTA
AATTCTCAACTCTCTTATACTTTTTTGTAAAAGAGAGCTTGGACTGTGGAGGCTTGCTGG
CCACTTTTTGGGGTCAGCTCCTCTGAAATGCATTAGCGGAACCGTTTGCAATCTGCCACA
AGTGTGATAAGTTATCTACACTGGCGAGGGGATTGCTCTCTGTAATGTTCAGCTTCTAAT
TGTCTCTACTTTGTGAGACAACTTTTGAATGCTTGACCTCAAATCAGGTAGGACTACCCG
CTGAACTTAA
>B2_50
TTTCCGTAGGTGAACCTGCGGAAGGATCATTATTGAATTATGTTTCTAGATAGGTTGTAG
CTGGCTCTTTAGAGCATGTGCACGCCTGTTTGGACTTCATTTTCATCCACCTGTGCACCT
ATTGTAGTCTTTGGTTGGGTTAGGGGGAAGTGGTCATTGTGTCAGCATCTGCTGGATGTG
AGGACTTGCATTGTGAAAGCTTTGCTGTCCTTGATGTGATCATGGAATCTCTTTCTCACT
AGAGTCTATGTCACTCATTATACTCTGTCGAATGTCATTGAATGTCTTTACATGGGCTTG

TATGCCTATGAAAATTGTAATACAACTTTCAGCAACGGATCTCTTGGCTCTCGCATCGAT
GAAGGACGCAGCGAAATGCGATAAGTAATGTGAATTGCAGAATTCAGTGAATCATCGAAT
CTTTGAACGCATCTTGCGCTCCTTGGTATTCCGAGGAGCATGCCTGTTTGAGTGTCATTA
AATTCTCAACTCTCTTATACTTTTTTGTAAAAGAGAGCTTGGACTGTGGAGGCTTGCTGG
CCACTTTTTGGGGTCAGCTCCTCTGAAATGCATTAGCGGAACCGTTTGCAATCTGCCACA
AGTGTGATAAGTTATCTACACTGGCGAGGGGATTGCTCTCTGTAATGTTCAGCTTCTAAT
TGTCTCTACTTTGTGAGACAACTTTTGAATGCTTGACCTCAAATCAGGTAGGACTACCCG
CTGAACTTAA
>B2-54
TTTCCGTAGGTGAACCTGCGGAAGGATCATTATTGAATTATGTTTCTAGATAGGTTGTAG
CTGGCTCTTTAGAGCATGTGCACGCCTGTTTGGACTTCATTTTCATCCACCTGTGCACCT
ATTGTAGTCTTTGGTTGGGTTAGGGGGAAGTGGTCATTGTGTCAGCATCTGCTGGATGTG
AGGACTTGCATTGTGAAAGCTTTGCTGTCCTTGATGTGATCATGGAATCTCTTTCTCACT
AGAGTCTATGTCACTCATTATACTCTGTCGAATGTCATTGAATGTCTTTACATGGGCTTG
TATGCCTATGAAAATTGTAATACAACTTTCAGCAACGGATCTCTTGGCTCTCGCATCGAT
GAAGGACGCAGCGAAATGCGATAAGTAATGTGAATTGCAGAATTCAGTGAATCATCGAAT
CTTTGAACGCATCTTGCGCTCCTTGGTATTCCGAGGAGCATGCCTGTTTGAGTGTCATTA
AATTCTCAACTCTCTTATACTTTTTTGTAAAAGAGAGCTTGGACTGTGGAGGCTTGCTGG
CCACTTTTTGGGGTCAGCTCCTCTGAAATGCATTAGCGGAACCGTTTGCAATCTGCCACA
AGTGTGATAAGTTATCTACACTGGCGAGGGGATTGCTCTCTGTAATGTTCAGCTTCTAAT
TGTCTCTACTTTGTGAGACAACTTTTGAATGCTTGACCTCAAATCAGGTAGGACTACCCG
CTGAACTTAA
>B2-56
TTTCCGTAGGTGAACCTGCGGAAGGATCATTATTGAATTATGTTTCTAGATAGGTTGTAG
CTGGCTCTTTAGAGCATGTGCACGCCTGTTTGGACTTCATTTTCATCCACCTGTGCACCT
ATTGTAGTCTTTGGTTGGGTTAGGGGGAAGTGGTCATTGTGTCAGCATCTGCTGGATGTG
AGGACTTGCATTGTGAAAGCTTTGCTGTCCTTGATGTGATCATGGAATCTCTTTCTCACT
AGAGTCTATGTCACTCATTATACTCTGTCGAATGTCATTGAATGTCTTTACATGGGCTTG
TATGCCTATGAAAATTGTAATACAACTTTCAGCAACGGATCTCTTGGCTCTCGCATCGAT
GAAGGACGCAGCGAAATGCGATAAGTAATGTGAATTGCAGAATTCAGTGAATCATCGAAT
CTTTGAACGCATCTTGCGCTCCTTGGTATTCCGAGGAGCATGCCTGTTTGAGTGTCATTA
AATTCTCAACTCTCTTATACTTTTTTGTAAAAGAGAGCTTGGACTGTGGAGGCTTGCTGG
CCACTTTTTGGGGTCAGCTCCTCTGAAATGCATTAGCGGAACCGTTTGCAATCTGCCACA
AGTGTGATAAGTTATCTACACTGGCGAGGGGATTGCTCTCTGTAATGTTCAGCTTCTAAT
TGTCTCTACTTTGTGAGACAACTTTTGAATGCTTGACCTCAAATCAGGTAGGACTACCCG
CTGAACTTAA
>B2-59
TTTCCGTAGGTGAACCTGCGGAAGGATCATTATTGAATTATGTTTCTAGATAGGTTGTAG
CTGGCTCTTTAGAGCATGTGCACGCCTGTTTGGACTTCATTTTCATCCACCTGTGCACCT
ATTGTAGTCTTTGGTTGGGTTAGGGGGAAGTGGTCATTGTGTCAGCATCTGCTGGATGTG
AGGACTTGCATTGTGAAAGCTTTGCTGTCCTTGATGTGATCATGGAATCTCTTTCTCACT
AGAGTCTATGTCACTCATTATACTCTGTCGAATGTCATTGAATGTCTTTACATGGGCTTG
TATGCCTATGAAAATTGTAATACAACTTTCAGCAACGGATCTCTTGGCTCTCGCATCGAT
GAAGGACGCAGCGAAATGCGATAAGTAATGTGAATTGCAGAATTCAGTGAATCATCGAAT
CTTTGAACGCATCTTGCGCTCCTTGGTATTCCGAGGAGCATGCCTGTTTGAGTGTCATTA
AATTCTCAACTCTCTTATACTTTTTTGTAAAAGAGAGCTTGGACTGTGGAGGCTTGCTGG
CCACTTTTTGGGGTCAGCTCCTCTGAAATGCATTAGCGGAACCGTTTGCAATCTGCCACA
AGTGTGATAAGTTATCTACACTGGCGAGGGGATTGCTCTCTGTAATGTTCAGCTTCTAAT
TGTCTCTACTTTGTGAGACAACTTTTGAATGCTTGACCTCAAATCAGGTAGGACTACCCG
CTGAACTTAA

>B2-60
TTTCCGTAGGTGAACCTGCGGAAGGATCATTATTGAATTATGTTTCTAGATAGGTTGTAG
CTGGCTCTTTAGAGCATGTGCACGCCTGTTTGGACTTCATTTTCATCCACCTGTGCACCT
ATTGTAGTCTTTGGTTGGGTTAGGGGGAAGTGGTCATTGTGTCAGCATCTGCTGGATGTG
AGGACTTGCATTGTGAAAGCTTTGCTGTCCTTGATGTGATCATGGAATCTCTTTCTCACT
AGAGTCTATGTCACTCATTATACTCTGTCGAATGTCATTGAATGTCTTTACATGGGCTTG
TATGCCTATGAAAATTGTAATACAACTTTCAGCAACGGATCTCTTGGCTCTCGCATCGAT
GAAGGACGCAGCGAAATGCGATAAGTAATGTGAATTGCAGAATTCAGTGAATCATCGAAT
CTTTGAACGCATCTTGCGCTCCTTGGTATTCCGAGGAGCATGCCTGTTTGAGTGTCATTA
AATTCTCAACTCTCTTATACTTTTTTGTAAAAGAGAGCTTGGACTGTGGAGGCTTGCTGG
CCACTTTTTGGGGTCAGCTCCTCTGAAATGCATTAGCGGAACCGTTTGCAATCTGCCACA
AGTGTGATAAGTTATCTACACTGGCGAGGGGATTGCTCTCTGTAATGTTCAGCTTCTAAT
TGTCTCTACTTTGTGAGACAACTTTTGAATGCTTGACCTCAAATCAGGTAGGACTACCCG
CTGAACTTAA
>B2-61
TTTCCGTAGGTGAACCTGCGGAAGGATCATTATTGAATTATGTTTCTAGATAGGTTGTAG
CTGGCTCTTTAGAGCATGTGCACGCCTGTTTGGACTTCATTTTCATCCACCTGTGCACCT
ATTGTAGTCTTTGGTTGGGTTAGGGGGAAGTGGTCATTGTGTCAGCATCTGCTGGATGTG
AGGACTTGCATTGTGAAAGCTTTGCTGTCCTTGATGTGATCATGGAATCTCTTTCTCACT
AGAGTCTATGTCACTCATTATACTCTGTCGAATGTCATTGAATGTCTTTACATGGGCTTG
TATGCCTATGAAAATTGTAATACAACTTTCAGCAACGGATCTCTTGGCTCTCGCATCGAT
GAAGGACGCAGCGAAATGCGATAAGTAATGTGAATTGCAGAATTCAGTGAATCATCGAAT
CTTTGAACGCATCTTGCGCTCCTTGGTATTCCGAGGAGCATGCCTGTTTGAGTGTCATTA
AATTCTCAACTCTCTTATACTTTTTTGTAAAAGAGAGCTTGGACTGTGGAGGCTTGCTGG
CCACTTTTTGGGGTCAGCTCCTCTGAAATGCATTAGCGGAACCGTTTGCAATCTGCCACA
AGTGTGATAAGTTATCTACACTGGCGAGGGGATTGCTCTCTGTAATGTTCAGCTTCTAAT
TGTCTCTACTTTGTGAGACAACTTTTGAATGCTTGACCTCAAATCAGGTAGGACTACCCG
CTGAACTTAA
>B2-62
TTTCCGTAGGTGAACCTGCGGAAGGATCATTATTGAATTATGTTTCTAGATAGGTTGTAG
CTGGCTCTTTAGAGCATGTGCACGCCTGTTTGGACTTCATTTTCATCCACCTGTGCACCT
ATTGTAGTCTTTGGTTGGGTTAGGGGGAAGTGGTCATTGTGTCAGCATCTGCTGGATGTG
AGGACTTGCATTGTGAAAGCTTTGCTGTCCTTGATGTGATCATGGAATCTCTTTCTCACT
AGAGTCTATGTCACTCATTATACTCTGTCGAATGTCATTGAATGTCTTTACATGGGCTTG
TATGCCTATGAAAATTGTAATACAACTTTCAGCAACGGATCTCTTGGCTCTCGCATCGAT
GAAGGACGCAGCGAAATGCGATAAGTAATGTGAATTGCAGAATTCAGTGAATCATCGAAT
CTTTGAACGCATCTTGCGCTCCTTGGTATTCCGAGGAGCATGCCTGTTTGAGTGTCATTA
AATTCTCAACTCTCTTATACTTTTTTGTAAAAGAGAGCTTGGACTGTGGAGGCTTGCTGG
CCACTTTTTGGGGTCAGCTCCTCTGAAATGCATTAGCGGAACCGTTTGCAATCTGCCACA
AGTGTGATAAGTTATCTACACTGGCGAGGGGATTGCTCTCTGTAATGTTCAGCTTCTAAT
TGTCTCTACTTTGTGAGACAACTTTTGAATGCTTGACCTCAAATCAGGTAGGACTACCCG
CTGAACTTAA
>B2-63
TTTCCGTAGGTGAACCTGCGGAAGGATCATTATTGAATTATGTTTCTAGATAGGTTGTAG
CTGGCTCTTTAGAGCATGTGCACGCCTGTTTGGACTTCATTTTCATCCACCTGTGCACCT
ATTGTAGTCTTTGGTTGGGTTAGGGGGAAGTGGTCATTGTGTCAGCATCTGCTGGATGTG
AGGACTTGCATTGTGAAAGCTTTGCTGTCCTTGATGTGATCATGGAATCTCTTTCTCACT
AGAGTCTATGTCACTCATTATACTCTGTCGAATGTCATTGAATGTCTTTACATGGGCTTG
TATGCCTATGAAAATTGTAATACAACTTTCAGCAACGGATCTCTTGGCTCTCGCATCGAT
GAAGGACGCAGCGAAATGCGATAAGTAATGTGAATTGCAGAATTCAGTGAATCATCGAAT

CTTTGAACGCATCTTGCGCTCCTTGGTATTCCGAGGAGCATGCCTGTTTGAGTGTCATTA
AATTCTCAACTCTCTTATACTTTTTTGTAAAAGAGAGCTTGGACTGTGGAGGCTTGCTGG
CCACTTTTTGGGGTCAGCTCCTCTGAAATGCATTAGCGGAACCGTTTGCAATCTGCCACA
AGTGTGATAAGTTATCTACACTGGCGAGGGGATTGCTCTCTGTAATGTTCAGCTTCTAAT
TGTCTCTACTTTGTGAGACAACTTTTGAATGCTTGACCTCAAATCAGGTAGGACTACCCG
CTGAACTTAA
>B2-64
TTTCCGTAGGTGAACCTGCGGAAGGATCATTATTGAATTATGTTTCTAGATAGGTTGTAG
CTGGCTCTTTAGAGCATGTGCACGCCTGTTTGGACTTCATTTTCATCCACCTGTGCACCT
ATTGTAGTCTTTGGTTGGGTTAGGGGGAAGTGGTCATTGTGTCAGCATCTGCTGGATGTG
AGGACTTGCATTGTGAAAGCTTTGCTGTCCTTGATGTGATCATGGAATCTCTTTCTCACT
AGAGTCTATGTCACTCATTATACTCTGTCGAATGTCATTGAATGTCTTTACATGGGCTTG
TATGCCTATGAAAATTGTAATACAACTTTCAGCAACGGATCTCTTGGCTCTCGCATCGAT
GAAGGACGCAGCGAAATGCGATAAGTAATGTGAATTGCAGAATTCAGTGAATCATCGAAT
CTTTGAACGCATCTTGCGCTCCTTGGTATTCCGAGGAGCATGCCTGTTTGAGTGTCATTA
AATTCTCAACTCTCTTATACTTTTTTGTAAAAGAGAGCTTGGACTGTGGAGGCTTGCTGG
CCACTTTTTGGGGTCAGCTCCTCTGAAATGCATTAGCGGAACCGTTTGCAATCTGCCACA
AGTGTGATAAGTTATCTACACTGGCGAGGGGATTGCTCTCTGTAATGTTCAGCTTCTAAT
TGTCTCTACTTTGTGAGACAACTTTTGAATGCTTGACCTCAAATCAGGTAGGACTACCCG
CTGAACTTAA
>B2-65
TTTCCGTAGGTGAACCTGCGGAAGGATCATTATTGAATTATGTTTCTAGATAGGTTGTAG
CTGGCTCTTTAGAGCATGTGCACGCCTGTTTGGACTTCATTTTCATCCACCTGTGCACCT
ATTGTAGTCTTTGGTTGGGTTAGGGGGAAGTGGTCATTGTGTCAGCATCTGCTGGATGTG
AGGACTTGCATTGTGAAAGCTTTGCTGTCCTTGATGTGATCATGGAATCTCTTTCTCACT
AGAGTCTATGTCACTCATTATACTCTGTCGAATGTCATTGAATGTCTTTACATGGGCTTG
TATGCCTATGAAAATTGTAATACAACTTTCAGCAACGGATCTCTTGGCTCTCGCATCGAT
GAAGGACGCAGCGAAATGCGATAAGTAATGTGAATTGCAGAATTCAGTGAATCATCGAAT
CTTTGAACGCATCTTGCGCTCCTTGGTATTCCGAGGAGCATGCCTGTTTGAGTGTCATTA
AATTCTCAACTCTCTTATACTTTTTTGTAAAAGAGAGCTTGGACTGTGGAGGCTTGCTGG
CCACTTTTTGGGGTCAGCTCCTCTGAAATGCATTAGCGGAACCGTTTGCAATCTGCCACA
AGTGTGATAAGTTATCTACACTGGCGAGGGGATTGCTCTCTGTAATGTTCAGCTTCTAAT
TGTCTCTACTTTGTGAGACAACTTTTGAATGCTTGACCTCAAATCAGGTAGGACTACCCG
CTGAACTTAA
>B2-66
TTTCCGTAGGTGAACCTGCGGAAGGATCATTATTGAATTATGTTTCTAGATAGGTTGTAG
CTGGCTCTTTAGAGCATGTGCACGCCTGTTTGGACTTCATTTTCATCCACCTGTGCACCT
ATTGTAGTCTTTGGTTGGGTTAGGGGGAAGTGGTCATTGTGTCAGCATCTGCTGGATGTG
AGGACTTGCATTGTGAAAGCTTTGCTGTCCTTGATGTGATCATGGAATCTCTTTCTCACT
AGAGTCTATGTCACTCATTATACTCTGTCGAATGTCATTGAATGTCTTTACATGGGCTTG
TATGCCTATGAAAATTGTAATACAACTTTCAGCAACGGATCTCTTGGCTCTCGCATCGAT
GAAGGACGCAGCGAAATGCGATAAGTAATGTGAATTGCAGAATTCAGTGAATCATCGAAT
CTTTGAACGCATCTTGCGCTCCTTGGTATTCCGAGGAGCATGCCTGTTTGAGTGTCATTA
AATTCTCAACTCTCTTATACTTTTTTGTAAAAGAGAGCTTGGACTGTGGAGGCTTGCTGG
CCACTTTTTGGGGTCAGCTCCTCTGAAATGCATTAGCGGAACCGTTTGCAATCTGCCACA
AGTGTGATAAGTTATCTACACTGGCGAGGGGATTGCTCTCTGTAATGTTCAGCTTCTAAT
TGTCTCTACTTTGTGAGACAACTTTTGAATGCTTGACCTCAAATCAGGTAGGACTACCCG
CTGAACTTAA
>B2-67
TTTCCGTAGGTGAACCTGCGGAAGGATCATTATTGAATTATGTTTCTAGATAGGTTGTAG

CTGGCTCTTTAGAGCATGTGCACGCCTGTTTGGACTTCATTTTCATCCACCTGTGCACCT
ATTGTAGTCTTTGGTTGGGTTAGGGGGAAGTGGTCATTGTGTCAGCATCTGCTGGATGTG
AGGACTTGCATTGTGAAAGCTTTGCTGTCCTTGATGTGATCATGGAATCTCTTTCTCACT
AGAGTCTATGTCACTCATTATACTCTGTCGAATGTCATTGAATGTCTTTACATGGGCTTG
TATGCCTATGAAAATTGTAATACAACTTTCAGCAACGGATCTCTTGGCTCTCGCATCGAT
GAAGGACGCAGCGAAATGCGATAAGTAATGTGAATTGCAGAATTCAGTGAATCATCGAAT
CTTTGAACGCATCTTGCGCTCCTTGGTATTCCGAGGAGCATGCCTGTTTGAGTGTCATTA
AATTCTCAACTCTCTTATACTTTTTTGTAAAAGAGAGCTTGGACTGTGGAGGCTTGCTGG
CCACTTTTTGGGGTCAGCTCCTCTGAAATGCATTAGCGGAACCGTTTGCAATCTGCCACA
AGTGTGATAAGTTATCTACACTGGCGAGGGGATTGCTCTCTGTAATGTTCAGCTTCTAAT
TGTCTCTACTTTGTGAGACAACTTTTGAATGCTTGACCTCAAATCAGGTAGGACTACCCG
CTGAACTTAA
>B2-68
TTTCCGTAGGTGAACCTGCGGAAGGATCATTATTGAATTATGTTTCTAGATAGGTTGTAG
CTGGCTCTTTAGAGCATGTGCACGCCTGTTTGGACTTCATTTTCATCCACCTGTGCACCT
ATTGTAGTCTTTGGTTGGGTTAGGGGGAAGTGGTCATTGTGTCAGCATCTGCTGGATGTG
AGGACTTGCATTGTGAAAGCTTTGCTGTCCTTGATGTGATCATGGAATCTCTTTCTCACT
AGAGTCTATGTCACTCATTATACTCTGTCGAATGTCATTGAATGTCTTTACATGGGCTTG
TATGCCTATGAAAATTGTAATACAACTTTCAGCAACGGATCTCTTGGCTCTCGCATCGAT
GAAGGACGCAGCGAAATGCGATAAGTAATGTGAATTGCAGAATTCAGTGAATCATCGAAT
CTTTGAACGCATCTTGCGCTCCTTGGTATTCCGAGGAGCATGCCTGTTTGAGTGTCATTA
AATTCTCAACTCTCTTATACTTTTTTGTAAAAGAGAGCTTGGACTGTGGAGGCTTGCTGG
CCACTTTTTGGGGTCAGCTCCTCTGAAATGCATTAGCGGAACCGTTTGCAATCTGCCACA
AGTGTGATAAGTTATCTACACTGGCGAGGGGATTGCTCTCTGTAATGTTCAGCTTCTAAT
TGTCTCTACTTTGTGAGACAACTTTTGAATGCTTGACCTCAAATCAGGTAGGACTACCCG
CTGAACTTAA
>B2-69
TTTCCGTAGGTGAACCTGCGGAAGGATCATTATTGAATTATGTTTCTAGATAGGTTGTAG
CTGGCTCTTTAGAGCATGTGCACGCCTGTTTGGACTTCATTTTCATCCACCTGTGCACCT
ATTGTAGTCTTTGGTTGGGTTAGGGGGAAGTGGTCATTGTGTCAGCATCTGCTGGATGTG
AGGACTTGCATTGTGAAAGCTTTGCTGTCCTTGATGTGATCATGGAATCTCTTTCTCACT
AGAGTCTATGTCACTCATTATACTCTGTCGAATGTCATTGAATGTCTTTACATGGGCTTG
TATGCCTATGAAAATTGTAATACAACTTTCAGCAACGGATCTCTTGGCTCTCGCATCGAT
GAAGGACGCAGCGAAATGCGATAAGTAATGTGAATTGCAGAATTCAGTGAATCATCGAAT
CTTTGAACGCATCTTGCGCTCCTTGGTATTCCGAGGAGCATGCCTGTTTGAGTGTCATTA
AATTCTCAACTCTCTTATACTTTTTTGTAAAAGAGAGCTTGGACTGTGGAGGCTTGCTGG
CCACTTTTTGGGGTCAGCTCCTCTGAAATGCATTAGCGGAACCGTTTGCAATCTGCCACA
AGTGTGATAAGTTATCTACACTGGCGAGGGGATTGCTCTCTGTAATGTTCAGCTTCTAAT
TGTCTCTACTTTGTGAGACAACTTTTGAATGCTTGACCTCAAATCAGGTAGGACTACCCG
CTGAACTTAA
>B2-71
TTTCCGTAGGTGAACCTGCGGAAGGATCATTATTGAATTATGTTTCTAGATAGGTTGTAG
CTGGCTCTTTAGAGCATGTGCACGCCTGTTTGGACTTCATTTTCATCCACCTGTGCACCT
ATTGTAGTCTTTGGTTGGGTTAGGGGGAAGTGGTCATTGTGTCAGCATCTGCTGGATGTG
AGGACTTGCATTGTGAAAGCTTTGCTGTCCTTGATGTGATCATGGAATCTCTTTCTCACT
AGAGTCTATGTCACTCATTATACTCTGTCGAATGTCATTGAATGTCTTTACATGGGCTTG
TATGCCTATGAAAATTGTAATACAACTTTCAGCAACGGATCTCTTGGCTCTCGCATCGAT
GAAGGACGCAGCGAAATGCGATAAGTAATGTGAATTGCAGAATTCAGTGAATCATCGAAT
CTTTGAACGCATCTTGCGCTCCTTGGTATTCCGAGGAGCATGCCTGTTTGAGTGTCATTA
AATTCTCAACTCTCTTATACTTTTTTGTAAAAGAGAGCTTGGACTGTGGAGGCTTGCTGG

CCACTTTTTGGGGTCAGCTCCTCTGAAATGCATTAGCGGAACCGTTTGCAATCTGCCACA
AGTGTGATAAGTTATCTACACTGGCGAGGGGATTGCTCTCTGTAATGTTCAGCTTCTAAT
TGTCTCTACTTTGTGAGACAACTTTTGAATGCTTGACCTCAAATCAGGTAGGACTACCCG
CTGAACTTAA
>B2-72
TTTCCGTAGGTGAACCTGCGGAAGGATCATTATTGAATTATGTTTCTAGATAGGTTGTAG
CTGGCTCTTTAGAGCATGTGCACGCCTGTTTGGACTTCATTTTCATCCACCTGTGCACCT
ATTGTAGTCTTTGGTTGGGTTAGGGGGAAGTGGTCATTGTGTCAGCATCTGCTGGATGTG
AGGACTTGCATTGTGAAAGCTTTGCTGTCCTTGATGTGATCATGGAATCTCTTTCTCACT
AGAGTCTATGTCACTCATTATACTCTGTCGAATGTCATTGAATGTCTTTACATGGGCTTG
TATGCCTATGAAAATTGTAATACAACTTTCAGCAACGGATCTCTTGGCTCTCGCATCGAT
GAAGGACGCAGCGAAATGCGATAAGTAATGTGAATTGCAGAATTCAGTGAATCATCGAAT
CTTTGAACGCATCTTGCGCTCCTTGGTATTCCGAGGAGCATGCCTGTTTGAGTGTCATTA
AATTCTCAACTCTCTTATACTTTTTTGTAAAAGAGAGCTTGGACTGTGGAGGCTTGCTGG
CCACTTTTTGGGGTCAGCTCCTCTGAAATGCATTAGCGGAACCGTTTGCAATCTGCCACA
AGTGTGATAAGTTATCTACACTGGCGAGGGGATTGCTCTCTGTAATGTTCAGCTTCTAAT
TGTCTCTACTTTGTGAGACAACTTTTGAATGCTTGACCTCAAATCAGGTAGGACTACCCG
CTGAACTTAA
>B3_1
TTTCCGTAGGTGAACCTGCGGAAGGATCATTATTGAATTATGTTTCTAGATAGGTTGTAG
CTGGCTCTTTAGAGCATGTGCACGCCTGTTTGGACTTCATTTTCATCCACCTGTGCACCT
ATTGTAGTCTTTGGTTGGGTTAGGGGGAAGTGGTCATTGTGTCAGCATCTGCTGGATGTG
AGGACTTGCATTGTGAAAGCTTTGCTGTCCTTGATGTGATCATGGAATCTCTTTCTCACT
AGAGTCTATGTCACTCATTATACTCTGTCGAATGTCATTGAATGTCTTTACATGGGCTTG
TATGCCTATGAAAATTGTAATACAACTTTCAGCAACGGATCTCTTGGCTCTCGCATCGAT
GAAGGACGCAGCGAAATGCGATAAGTAATGTGAATTGCAGAATTCAGTGAATCATCGAAT
CTTTGAACGCATCTTGCGCTCCTTGGTATTCCGAGGAGCATGCCTGTTTGAGTGTCATTA
AATTCTCAACTCTCTTATACTTTTTTGTAAAAGAGAGCTTGGACTGTGGAGGCTTGCTGG
CCACTTTTTGGGGTCAGCTCCTCTGAAATGCATTAGCGGAACCGTTTGCAATCTGCCACA
AGTGTGATAAGTTATCTACACTGGCGAGGGGATTGCTCTCTGTAATGTTCAGCTTCTAAT
TGTCTCTACTTTGTGAGACAACTTTTGAATGCTTGACCTCAAATCAGGTAGGACTACCCG
CTGAACTTAA
>B3_2
TTTCCGTAGGTGAACCTGCGGAAGGATCATTATTGAATTATGTTTCTAGATAGGTTGTAG
CTGGCTCTTTAGAGCATGTGCACGCCTGTTTGGACTTCATTTTCATCCACCTGTGCACCT
ATTGTAGTCTTTGGTTGGGTTAGGGGGAAGTGGTCATTGTGTCAGCATCTGCTGGATGTG
AGGACTTGCATTGTGAAAGCTTTGCTGTCCTTGATGTGATCATGGAATCTCTTTCTCACT
AGAGTCTATGTCACTCATTATACTCTGTCGAATGTCATTGAATGTCTTTACATGGGCTTG
TATGCCTATGAAAATTGTAATACAACTTTCAGCAACGGATCTCTTGGCTCTCGCATCGAT
GAAGGACGCAGCGAAATGCGATAAGTAATGTGAATTGCAGAATTCAGTGAATCATCGAAT
CTTTGAACGCATCTTGCGCTCCTTGGTATTCCGAGGAGCATGCCTGTTTGAGTGTCATTA
AATTCTCAACTCTCTTATACTTTTTTGTAAAAGAGAGCTTGGACTGTGGAGGCTTGCTGG
CCACTTTTTGGGGTCAGCTCCTCTGAAATGCATTAGCGGAACCGTTTGCAATCTGCCACA
AGTGTGATAAGTTATCTACACTGGCGAGGGGATTGCTCTCTGTAATGTTCAGCTTCTAAT
TGTCTCTACTTTGTGAGACAACTTTTGAATGCTTGACCTCAAATCAGGTAGGACTACCCG
CTGAACTTAA
>B3_3
TTTCCGTAGGTGAACCTGCGGAAGGATCATTATTGAATTATGTTTCTAGATAGGTTGTAG
CTGGCTCTTTAGAGCATGTGCACGCCTGTTTGGACTTCATTTTCATCCACCTGTGCACCT
ATTGTAGTCTTTGGTTGGGTTAGGGGGAAGTGGTCATTGTGTCAGCATCTGCTGGATGTG

AGGACTTGCATTGTGAAAGCTTTGCTGTCCTTGATGTGATCATGGAATCTCTTTCTCACT
AGAGTCTATGTCACTCATTATACTCTGTCGAATGTCATTGAATGTCTTTACATGGGCTTG
TATGCCTATGAAAATTGTAATACAACTTTCAGCAACGGATCTCTTGGCTCTCGCATCGAT
GAAGGACGCAGCGAAATGCGATAAGTAATGTGAATTGCAGAATTCAGTGAATCATCGAAT
CTTTGAACGCATCTTGCGCTCCTTGGTATTCCGAGGAGCATGCCTGTTTGAGTGTCATTA
AATTCTCAACTCTCTTATACTTTTTTGTAAAAGAGAGCTTGGACTGTGGAGGCTTGCTGG
CCACTTTTTGGGGTCAGCTCCTCTGAAATGCATTAGCGGAACCGTTTGCAATCTGCCACA
AGTGTGATAAGTTATCTACACTGGCGAGGGGATTGCTCTCTGTAATGTTCAGCTTCTAAT
TGTCTCTACTTTGTGAGACAACTTTTGAATGCTTGACCTCAAATCAGGTAGGACTACCCG
CTGAACTTAA
>B3_4
TTTCCGTAGGTGAACCTGCGGAAGGATCATTATTGAATTATGTTTCTAGATAGGTTGTAG
CTGGCTCTTTAGAGCATGTGCACGCCTGTTTGGACTTCATTTTCATCCACCTGTGCACCT
ATTGTAGTCTTTGGTTGGGTTAGGGGGAAGTGGTCATTGTGTCAGCATCTGCTGGATGTG
AGGACTTGCATTGTGAAAGCTTTGCTGTCCTTGATGTGATCATGGAATCTCTTTCTCACT
AGAGTCTATGTCACTCATTATACTCTGTCGAATGTCATTGAATGTCTTTACATGGGCTTG
TATGCCTATGAAAATTGTAATACAACTTTCAGCAACGGATCTCTTGGCTCTCGCATCGAT
GAAGGACGCAGCGAAATGCGATAAGTAATGTGAATTGCAGAATTCAGTGAATCATCGAAT
CTTTGAACGCATCTTGCGCTCCTTGGTATTCCGAGGAGCATGCCTGTTTGAGTGTCATTA
AATTCTCAACTCTCTTATACTTTTTTGTAAAAGAGAGCTTGGACTGTGGAGGCTTGCTGG
CCACTTTTTGGGGTCAGCTCCTCTGAAATGCATTAGCGGAACCGTTTGCAATCTGCCACA
AGTGTGATAAGTTATCTACACTGGCGAGGGGATTGCTCTCTGTAATGTTCAGCTTCTAAT
TGTCTCTACTTTGTGAGACAACTTTTGAATGCTTGACCTCAAATCAGGTAGGACTACCCG
CTGAACTTAA
>B3_5
TTTCCGTAGGTGAACCTGCGGAAGGATCATTATTGAATTATGTTTCTAGATAGGTTGTAG
CTGGCTCTTTAGAGCATGTGCACGCCTGTTTGGACTTCATTTTCATCCACCTGTGCACCT
ATTGTAGTCTTTGGTTGGGTTAGGGGGAAGTGGTCATTGTGTCAGCATCTGCTGGATGTG
AGGACTTGCATTGTGAAAGCTTTGCTGTCCTTGATGTGATCATGGAATCTCTTTCTCACT
AGAGTCTATGTCACTCATTATACTCTGTCGAATGTCATTGAATGTCTTTACATGGGCTTG
TATGCCTATGAAAATTGTAATACAACTTTCAGCAACGGATCTCTTGGCTCTCGCATCGAT
GAAGGACGCAGCGAAATGCGATAAGTAATGTGAATTGCAGAATTCAGTGAATCATCGAAT
CTTTGAACGCATCTTGCGCTCCTTGGTATTCCGAGGAGCATGCCTGTTTGAGTGTCATTA
AATTCTCAACTCTCTTATACTTTTTTGTAAAAGAGAGCTTGGACTGTGGAGGCTTGCTGG
CCACTTTTTGGGGTCAGCTCCTCTGAAATGCATTAGCGGAACCGTTTGCAATCTGCCACA
AGTGTGATAAGTTATCTACACTGGCGAGGGGATTGCTCTCTGTAATGTTCAGCTTCTAAT
TGTCTCTACTTTGTGAGACAACTTTTGAATGCTTGACCTCAAATCAGGTAGGACTACCCG
CTGAACTTAA
>B3_6
TTTCCGTAGGTGAACCTGCGGAAGGATCATTATTGAATTATGTTTCTAGATAGGTTGTAG
CTGGCTCTTTAGAGCATGTGCACGCCTGTTTGGACTTCATTTTCATCCACCTGTGCACCT
ATTGTAGTCTTTGGTTGGGTTAGGGGGAAGTGGTCATTGTGTCAGCATCTGCTGGATGTG
AGGACTTGCATTGTGAAAGCTTTGCTGTCCTTGATGTGATCATGGAATCTCTTTCTCACT
AGAGTCTATGTCACTCATTATACTCTGTCGAATGTCATTGAATGTCTTTACATGGGCTTG
TATGCCTATGAAAATTGTAATACAACTTTCAGCAACGGATCTCTTGGCTCTCGCATCGAT
GAAGGACGCAGCGAAATGCGATAAGTAATGTGAATTGCAGAATTCAGTGAATCATCGAAT
CTTTGAACGCATCTTGCGCTCCTTGGTATTCCGAGGAGCATGCCTGTTTGAGTGTCATTA
AATTCTCAACTCTCTTATACTTTTTTGTAAAAGAGAGCTTGGACTGTGGAGGCTTGCTGG
CCACTTTTTGGGGTCAGCTCCTCTGAAATGCATTAGCGGAACCGTTTGCAATCTGCCACA
AGTGTGATAAGTTATCTACACTGGCGAGGGGATTGCTCTCTGTAATGTTCAGCTTCTAAT

TGTCTCTACTTTGTGAGACAACTTTTGAATGCTTGACCTCAAATCAGGTAGGACTACCCG
CTGAACTTAA
>B3_7
TTTCCGTAGGTGAACCTGCGGAAGGATCATTATTGAATTATGTTTCTAGATAGGTTGTAG
CTGGCTCTTTAGAGCATGTGCACGCCTGTTTGGACTTCATTTTCATCCACCTGTGCACCT
ATTGTAGTCTTTGGTTGGGTTAGGGGGAAGTGGTCATTGTGTCAGCATCTGCTGGATGTG
AGGACTTGCATTGTGAAAGCTTTGCTGTCCTTGATGTGATCATGGAATCTCTTTCTCACT
AGAGTCTATGTCACTCATTATACTCTGTCGAATGTCATTGAATGTCTTTACATGGGCTTG
TATGCCTATGAAAATTGTAATACAACTTTCAGCAACGGATCTCTTGGCTCTCGCATCGAT
GAAGGACGCAGCGAAATGCGATAAGTAATGTGAATTGCAGAATTCAGTGAATCATCGAAT
CTTTGAACGCATCTTGCGCTCCTTGGTATTCCGAGGAGCATGCCTGTTTGAGTGTCATTA
AATTCTCAACTCTCTTATACTTTTTTGTAAAAGAGAGCTTGGACTGTGGAGGCTTGCTGG
CCACTTTTTGGGGTCAGCTCCTCTGAAATGCATTAGCGGAACCGTTTGCAATCTGCCACA
AGTGTGATAAGTTATCTACACTGGCGAGGGGATTGCTCTCTGTAATGTTCAGCTTCTAAT
TGTCTCTACTTTGTGAGACAACTTTTGAATGCTTGACCTCAAATCAGGTAGGACTACCCG
CTGAACTTAA
>B3_8
TTTCCGTAGGTGAACCTGCGGAAGGATCATTATTGAATTATGTTTCTAGATAGGTTGTAG
CTGGCTCTTTAGAGCATGTGCACGCCTGTTTGGACTTCATTTTCATCCACCTGTGCACCT
ATTGTAGTCTTTGGTTGGGTTAGGGGGAAGTGGTCATTGTGTCAGCATCTGCTGGATGTG
AGGACTTGCATTGTGAAAGCTTTGCTGTCCTTGATGTGATCATGGAATCTCTTTCTCACT
AGAGTCTATGTCACTCATTATACTCTGTCGAATGTCATTGAATGTCTTTACATGGGCTTG
TATGCCTATGAAAATTGTAATACAACTTTCAGCAACGGATCTCTTGGCTCTCGCATCGAT
GAAGGACGCAGCGAAATGCGATAAGTAATGTGAATTGCAGAATTCAGTGAATCATCGAAT
CTTTGAACGCATCTTGCGCTCCTTGGTATTCCGAGGAGCATGCCTGTTTGAGTGTCATTA
AATTCTCAACTCTCTTATACTTTTTTGTAAAAGAGAGCTTGGACTGTGGAGGCTTGCTGG
CCACTTTTTGGGGTCAGCTCCTCTGAAATGCATTAGCGGAACCGTTTGCAATCTGCCACA
AGTGTGATAAGTTATCTACACTGGCGAGGGGATTGCTCTCTGTAATGTTCAGCTTCTAAT
TGTCTCTACTTTGTGAGACAACTTTTGAATGCTTGACCTCAAATCAGGTAGGACTACCCG
CTGAACTTAA
>B3_11
TTTCCGTAGGTGAACCTGCGGAAGGATCATTATTGAATTATGTTTCTAGATAGGTTGTAG
CTGGCTCTTTAGAGCATGTGCACGCCTGTTTGGACTTCATTTTCATCCACCTGTGCACCT
ATTGTAGTCTTTGGTTGGGTTAGGGGGAAGTGGTCATTGTGTCAGCATCTGCTGGATGTG
AGGACTTGCATTGTGAAAGCTTTGCTGTCCTTGATGTGATCATGGAATCTCTTTCTCACT
AGAGTCTATGTCACTCATTATACTCTGTCGAATGTCATTGAATGTCTTTACATGGGCTTG
TATGCCTATGAAAATTGTAATACAACTTTCAGCAACGGATCTCTTGGCTCTCGCATCGAT
GAAGGACGCAGCGAAATGCGATAAGTAATGTGAATTGCAGAATTCAGTGAATCATCGAAT
CTTTGAACGCATCTTGCGCTCCTTGGTATTCCGAGGAGCATGCCTGTTTGAGTGTCATTA
AATTCTCAACTCTCTTATACTTTTTTGTAAAAGAGAGCTTGGACTGTGGAGGCTTGCTGG
CCACTTTTTGGGGTCAGCTCCTCTGAAATGCATTAGCGGAACCGTTTGCAATCTGCCACA
AGTGTGATAAGTTATCTACACTGGCGAGGGGATTGCTCTCTGTAATGTTCAGCTTCTAAT
TGTCTCTACTTTGTGAGACAACTTTTGAATGCTTGACCTCAAATCAGGTAGGACTACCCG
CTGAACTTAA
>B3_12
TTTCCGTAGGTGAACCTGCGGAAGGATCATTATTGAATTATGTTTCTAGATAGGTTGTAG
CTGGCTCTTTAGAGCATGTGCACGCCTGTTTGGACTTCATTTTCATCCACCTGTGCACCT
ATTGTAGTCTTTGGTTGGGTTAGGGGGAAGTGGTCATTGTGTCAGCATCTGCTGGATGTG
AGGACTTGCATTGTGAAAGCTTTGCTGTCCTTGATGTGATCATGGAATCTCTTTCTCACT
AGAGTCTATGTCACTCATTATACTCTGTCGAATGTCATTGAATGTCTTTACATGGGCTTG

TATGCCTATGAAAATTGTAATACAACTTTCAGCAACGGATCTCTTGGCTCTCGCATCGAT
GAAGGACGCAGCGAAATGCGATAAGTAATGTGAATTGCAGAATTCAGTGAATCATCGAAT
CTTTGAACGCATCTTGCGCTCCTTGGTATTCCGAGGAGCATGCCTGTTTGAGTGTCATTA
AATTCTCAACTCTCTTATACTTTTTTGTAAAAGAGAGCTTGGACTGTGGAGGCTTGCTGG
CCACTTTTTGGGGTCAGCTCCTCTGAAATGCATTAGCGGAACCGTTTGCAATCTGCCACA
AGTGTGATAAGTTATCTACACTGGCGAGGGGATTGCTCTCTGTAATGTTCAGCTTCTAAT
TGTCTCTACTTTGTGAGACAACTTTTGAATGCTTGACCTCAAATCAGGTAGGACTACCCG
CTGAACTTAA
>B3_13
TTTCCGTAGGTGAACCTGCGGAAGGATCATTATTGAATTATGTTTCTAGATAGGTTGTAG
CTGGCTCTTTAGAGCATGTGCACGCCTGTTTGGACTTCATTTTCATCCACCTGTGCACCT
ATTGTAGTCTTTGGTTGGGTTAGGGGGAAGTGGTCATTGTGTCAGCATCTGCTGGATGTG
AGGACTTGCATTGTGAAAGCTTTGCTGTCCTTGATGTGATCATGGAATCTCTTTCTCACT
AGAGTCTATGTCACTCATTATACTCTGTCGAATGTCATTGAATGTCTTTACATGGGCTTG
TATGCCTATGAAAATTGTAATACAACTTTCAGCAACGGATCTCTTGGCTCTCGCATCGAT
GAAGGACGCAGCGAAATGCGATAAGTAATGTGAATTGCAGAATTCAGTGAATCATCGAAT
CTTTGAACGCATCTTGCGCTCCTTGGTATTCCGAGGAGCATGCCTGTTTGAGTGTCATTA
AATTCTCAACTCTCTTATACTTTTTTGTAAAAGAGAGCTTGGACTGTGGAGGCTTGCTGG
CCACTTTTTGGGGTCAGCTCCTCTGAAATGCATTAGCGGAACCGTTTGCAATCTGCCACA
AGTGTGATAAGTTATCTACACTGGCGAGGGGATTGCTCTCTGTAATGTTCAGCTTCTAAT
TGTCTCTACTTTGTGAGACAACTTTTGAATGCTTGACCTCAAATCAGGTAGGACTACCCG
CTGAACTTAA
>B3_14
TTTCCGTAGGTGAACCTGCGGAAGGATCATTATTGAATTATGTTTCTAGATAGGTTGTAG
CTGGCTCTTTAGAGCATGTGCACGCCTGTTTGGACTTCATTTTCATCCACCTGTGCACCT
ATTGTAGTCTTTGGTTGGGTTAGGGGGAAGTGGTCATTGTGTCAGCATCTGCTGGATGTG
AGGACTTGCATTGTGAAAGCTTTGCTGTCCTTGATGTGATCATGGAATCTCTTTCTCACT
AGAGTCTATGTCACTCATTATACTCTGTCGAATGTCATTGAATGTCTTTACATGGGCTTG
TATGCCTATGAAAATTGTAATACAACTTTCAGCAACGGATCTCTTGGCTCTCGCATCGAT
GAAGGACGCAGCGAAATGCGATAAGTAATGTGAATTGCAGAATTCAGTGAATCATCGAAT
CTTTGAACGCATCTTGCGCTCCTTGGTATTCCGAGGAGCATGCCTGTTTGAGTGTCATTA
AATTCTCAACTCTCTTATACTTTTTTGTAAAAGAGAGCTTGGACTGTGGAGGCTTGCTGG
CCACTTTTTGGGGTCAGCTCCTCTGAAATGCATTAGCGGAACCGTTTGCAATCTGCCACA
AGTGTGATAAGTTATCTACACTGGCGAGGGGATTGCTCTCTGTAATGTTCAGCTTCTAAT
TGTCTCTACTTTGTGAGACAACTTTTGAATGCTTGACCTCAAATCAGGTAGGACTACCCG
CTGAACTTAA
>B3_15
TTTCCGTAGGTGAACCTGCGGAAGGATCATTATTGAATTATGTTTCTAGATAGGTTGTAG
CTGGCTCTTTAGAGCATGTGCACGCCTGTTTGGACTTCATTTTCATCCACCTGTGCACCT
ATTGTAGTCTTTGGTTGGGTTAGGGGGAAGTGGTCATTGTGTCAGCATCTGCTGGATGTG
AGGACTTGCATTGTGAAAGCTTTGCTGTCCTTGATGTGATCATGGAATCTCTTTCTCACT
AGAGTCTATGTCACTCATTATACTCTGTCGAATGTCATTGAATGTCTTTACATGGGCTTG
TATGCCTATGAAAATTGTAATACAACTTTCAGCAACGGATCTCTTGGCTCTCGCATCGAT
GAAGGACGCAGCGAAATGCGATAAGTAATGTGAATTGCAGAATTCAGTGAATCATCGAAT
CTTTGAACGCATCTTGCGCTCCTTGGTATTCCGAGGAGCATGCCTGTTTGAGTGTCATTA
AATTCTCAACTCTCTTATACTTTTTTGTAAAAGAGAGCTTGGACTGTGGAGGCTTGCTGG
CCACTTTTTGGGGTCAGCTCCTCTGAAATGCATTAGCGGAACCGTTTGCAATCTGCCACA
AGTGTGATAAGTTATCTACACTGGCGAGGGGATTGCTCTCTGTAATGTTCAGCTTCTAAT
TGTCTCTACTTTGTGAGACAACTTTTGAATGCTTGACCTCAAATCAGGTAGGACTACCCG
CTGAACTTAA

>B3_16
TTTCCGTAGGTGAACCTGCGGAAGGATCATTATTGAATTATGTTTCTAGATAGGTTGTAG
CTGGCTCTTTAGAGCATGTGCACGCCTGTTTGGACTTCATTTTCATCCACCTGTGCACCT
ATTGTAGTCTTTGGTTGGGTTAGGGGGAAGTGGTCATTGTGTCAGCATCTGCTGGATGTG
AGGACTTGCATTGTGAAAGCTTTGCTGTCCTTGATGTGATCATGGAATCTCTTTCTCACT
AGAGTCTATGTCACTCATTATACTCTGTCGAATGTCATTGAATGTCTTTACATGGGCTTG
TATGCCTATGAAAATTGTAATACAACTTTCAGCAACGGATCTCTTGGCTCTCGCATCGAT
GAAGGACGCAGCGAAATGCGATAAGTAATGTGAATTGCAGAATTCAGTGAATCATCGAAT
CTTTGAACGCATCTTGCGCTCCTTGGTATTCCGAGGAGCATGCCTGTTTGAGTGTCATTA
AATTCTCAACTCTCTTATACTTTTTTGTAAAAGAGAGCTTGGACTGTGGAGGCTTGCTGG
CCACTTTTTGGGGTCAGCTCCTCTGAAATGCATTAGCGGAACCGTTTGCAATCTGCCACA
AGTGTGATAAGTTATCTACACTGGCGAGGGGATTGCTCTCTGTAATGTTCAGCTTCTAAT
TGTCTCTACTTTGTGAGACAACTTTTGAATGCTTGACCTCAAATCAGGTAGGACTACCCG
CTGAACTTAA
>B3_17
TTTCCGTAGGTGAACCTGCGGAAGGATCATTATTGAATTATGTTTCTAGATAGGTTGTAG
CTGGCTCTTTAGAGCATGTGCACGCCTGTTTGGACTTCATTTTCATCCACCTGTGCACCT
ATTGTAGTCTTTGGTTGGGTTAGGGGGAAGTGGTCATTGTGTCAGCATCTGCTGGATGTG
AGGACTTGCATTGTGAAAGCTTTGCTGTCCTTGATGTGATCATGGAATCTCTTTCTCACT
AGAGTCTATGTCACTCATTATACTCTGTCGAATGTCATTGAATGTCTTTACATGGGCTTG
TATGCCTATGAAAATTGTAATACAACTTTCAGCAACGGATCTCTTGGCTCTCGCATCGAT
GAAGGACGCAGCGAAATGCGATAAGTAATGTGAATTGCAGAATTCAGTGAATCATCGAAT
CTTTGAACGCATCTTGCGCTCCTTGGTATTCCGAGGAGCATGCCTGTTTGAGTGTCATTA
AATTCTCAACTCTCTTATACTTTTTTGTAAAAGAGAGCTTGGACTGTGGAGGCTTGCTGG
CCACTTTTTGGGGTCAGCTCCTCTGAAATGCATTAGCGGAACCGTTTGCAATCTGCCACA
AGTGTGATAAGTTATCTACACTGGCGAGGGGATTGCTCTCTGTAATGTTCAGCTTCTAAT
TGTCTCTACTTTGTGAGACAACTTTTGAATGCTTGACCTCAAATCAGGTAGGACTACCCG
CTGAACTTAA
>B3_18
TTTCCGTAGGTGAACCTGCGGAAGGATCATTATTGAATTATGTTTCTAGATAGGTTGTAG
CTGGCTCTTTAGAGCATGTGCACGCCTGTTTGGACTTCATTTTCATCCACCTGTGCACCT
ATTGTAGTCTTTGGTTGGGTTAGGGGGAAGTGGTCATTGTGTCAGCATCTGCTGGATGTG
AGGACTTGCATTGTGAAAGCTTTGCTGTCCTTGATGTGATCATGGAATCTCTTTCTCACT
AGAGTCTATGTCACTCATTATACTCTGTCGAATGTCATTGAATGTCTTTACATGGGCTTG
TATGCCTATGAAAATTGTAATACAACTTTCAGCAACGGATCTCTTGGCTCTCGCATCGAT
GAAGGACGCAGCGAAATGCGATAAGTAATGTGAATTGCAGAATTCAGTGAATCATCGAAT
CTTTGAACGCATCTTGCGCTCCTTGGTATTCCGAGGAGCATGCCTGTTTGAGTGTCATTA
AATTCTCAACTCTCTTATACTTTTTTGTAAAAGAGAGCTTGGACTGTGGAGGCTTGCTGG
CCACTTTTTGGGGTCAGCTCCTCTGAAATGCATTAGCGGAACCGTTTGCAATCTGCCACA
AGTGTGATAAGTTATCTACACTGGCGAGGGGATTGCTCTCTGTAATGTTCAGCTTCTAAT
TGTCTCTACTTTGTGAGACAACTTTTGAATGCTTGACCTCAAATCAGGTAGGACTACCCG
CTGAACTTAA
>B3_19
TTTCCGTAGGTGAACCTGCGGAAGGATCATTATTGAATTATGTTTCTAGATAGGTTGTAG
CTGGCTCTTTAGAGCATGTGCACGCCTGTTTGGACTTCATTTTCATCCACCTGTGCACCT
ATTGTAGTCTTTGGTTGGGTTAGGGGGAAGTGGTCATTGTGTCAGCATCTGCTGGATGTG
AGGACTTGCATTGTGAAAGCTTTGCTGTCCTTGATGTGATCATGGAATCTCTTTCTCACT
AGAGTCTATGTCACTCATTATACTCTGTCGAATGTCATTGAATGTCTTTACATGGGCTTG
TATGCCTATGAAAATTGTAATACAACTTTCAGCAACGGATCTCTTGGCTCTCGCATCGAT
GAAGGACGCAGCGAAATGCGATAAGTAATGTGAATTGCAGAATTCAGTGAATCATCGAAT

CTTTGAACGCATCTTGCGCTCCTTGGTATTCCGAGGAGCATGCCTGTTTGAGTGTCATTA
AATTCTCAACTCTCTTATACTTTTTTGTAAAAGAGAGCTTGGACTGTGGAGGCTTGCTGG
CCACTTTTTGGGGTCAGCTCCTCTGAAATGCATTAGCGGAACCGTTTGCAATCTGCCACA
AGTGTGATAAGTTATCTACACTGGCGAGGGGATTGCTCTCTGTAATGTTCAGCTTCTAAT
TGTCTCTACTTTGTGAGACAACTTTTGAATGCTTGACCTCAAATCAGGTAGGACTACCCG
CTGAACTTAA
>B3_21
TTTCCGTAGGTGAACCTGCGGAAGGATCATTATTGAATTATGTTTCTAGATAGGTTGTAG
CTGGCTCTTTAGAGCATGTGCACGCCTGTTTGGACTTCATTTTCATCCACCTGTGCACCT
ATTGTAGTCTTTGGTTGGGTTAGGGGGAAGTGGTCATTGTGTCAGCATCTGCTGGATGTG
AGGACTTGCATTGTGAAAGCTTTGCTGTCCTTGATGTGATCATGGAATCTCTTTCTCACT
AGAGTCTATGTCACTCATTATACTCTGTCGAATGTCATTGAATGTCTTTACATGGGCTTG
TATGCCTATGAAAATTGTAATACAACTTTCAGCAACGGATCTCTTGGCTCTCGCATCGAT
GAAGGACGCAGCGAAATGCGATAAGTAATGTGAATTGCAGAATTCAGTGAATCATCGAAT
CTTTGAACGCATCTTGCGCTCCTTGGTATTCCGAGGAGCATGCCTGTTTGAGTGTCATTA
AATTCTCAACTCTCTTATACTTTTTTGTAAAAGAGAGCTTGGACTGTGGAGGCTTGCTGG
CCACTTTTTGGGGTCAGCTCCTCTGAAATGCATTAGCGGAACCGTTTGCAATCTGCCACA
AGTGTGATAAGTTATCTACACTGGCGAGGGGATTGCTCTCTGTAATGTTCAGCTTCTAAT
TGTCTCTACTTTGTGAGACAACTTTTGAATGCTTGACCTCAAATCAGGTAGGACTACCCG
CTGAACTTAA
>B3_23
TTTCCGTAGGTGAACCTGCGGAAGGATCATTATTGAATTATGTTTCTAGATAGGTTGTAG
CTGGCTCTTTAGAGCATGTGCACGCCTGTTTGGACTTCATTTTCATCCACCTGTGCACCT
ATTGTAGTCTTTGGTTGGGTTAGGGGGAAGTGGTCATTGTGTCAGCATCTGCTGGATGTG
AGGACTTGCATTGTGAAAGCTTTGCTGTCCTTGATGTGATCATGGAATCTCTTTCTCACT
AGAGTCTATGTCACTCATTATACTCTGTCGAATGTCATTGAATGTCTTTACATGGGCTTG
TATGCCTATGAAAATTGTAATACAACTTTCAGCAACGGATCTCTTGGCTCTCGCATCGAT
GAAGGACGCAGCGAAATGCGATAAGTAATGTGAATTGCAGAATTCAGTGAATCATCGAAT
CTTTGAACGCATCTTGCGCTCCTTGGTATTCCGAGGAGCATGCCTGTTTGAGTGTCATTA
AATTCTCAACTCTCTTATACTTTTTTGTAAAAGAGAGCTTGGACTGTGGAGGCTTGCTGG
CCACTTTTTGGGGTCAGCTCCTCTGAAATGCATTAGCGGAACCGTTTGCAATCTGCCACA
AGTGTGATAAGTTATCTACACTGGCGAGGGGATTGCTCTCTGTAATGTTCAGCTTCTAAT
TGTCTCTACTTTGTGAGACAACTTTTGAATGCTTGACCTCAAATCAGGTAGGACTACCCG
CTGAACTTAA
>B3_24
TTTCCGTAGGTGAACCTGCGGAAGGATCATTATTGAATTATGTTTCTAGATAGGTTGTAG
CTGGCTCTTTAGAGCATGTGCACGCCTGTTTGGACTTCATTTTCATCCACCTGTGCACCT
ATTGTAGTCTTTGGTTGGGTTAGGGGGAAGTGGTCATTGTGTCAGCATCTGCTGGATGTG
AGGACTTGCATTGTGAAAGCTTTGCTGTCCTTGATGTGATCATGGAATCTCTTTCTCACT
AGAGTCTATGTCACTCATTATACTCTGTCGAATGTCATTGAATGTCTTTACATGGGCTTG
TATGCCTATGAAAATTGTAATACAACTTTCAGCAACGGATCTCTTGGCTCTCGCATCGAT
GAAGGACGCAGCGAAATGCGATAAGTAATGTGAATTGCAGAATTCAGTGAATCATCGAAT
CTTTGAACGCATCTTGCGCTCCTTGGTATTCCGAGGAGCATGCCTGTTTGAGTGTCATTA
AATTCTCAACTCTCTTATACTTTTTTGTAAAAGAGAGCTTGGACTGTGGAGGCTTGCTGG
CCACTTTTTGGGGTCAGCTCCTCTGAAATGCATTAGCGGAACCGTTTGCAATCTGCCACA
AGTGTGATAAGTTATCTACACTGGCGAGGGGATTGCTCTCTGTAATGTTCAGCTTCTAAT
TGTCTCTACTTTGTGAGACAACTTTTGAATGCTTGACCTCAAATCAGGTAGGACTACCCG
CTGAACTTAA
>B3_25
TTTCCGTAGGTGAACCTGCGGAAGGATCATTATTGAATTATGTTTCTAGATAGGTTGTAG

CTGGCTCTTTAGAGCATGTGCACGCCTGTTTGGACTTCATTTTCATCCACCTGTGCACCT
ATTGTAGTCTTTGGTTGGGTTAGGGGGAAGTGGTCATTGTGTCAGCATCTGCTGGATGTG
AGGACTTGCATTGTGAAAGCTTTGCTGTCCTTGATGTGATCATGGAATCTCTTTCTCACT
AGAGTCTATGTCACTCATTATACTCTGTCGAATGTCATTGAATGTCTTTACATGGGCTTG
TATGCCTATGAAAATTGTAATACAACTTTCAGCAACGGATCTCTTGGCTCTCGCATCGAT
GAAGGACGCAGCGAAATGCGATAAGTAATGTGAATTGCAGAATTCAGTGAATCATCGAAT
CTTTGAACGCATCTTGCGCTCCTTGGTATTCCGAGGAGCATGCCTGTTTGAGTGTCATTA
AATTCTCAACTCTCTTATACTTTTTTGTAAAAGAGAGCTTGGACTGTGGAGGCTTGCTGG
CCACTTTTTGGGGTCAGCTCCTCTGAAATGCATTAGCGGAACCGTTTGCAATCTGCCACA
AGTGTGATAAGTTATCTACACTGGCGAGGGGATTGCTCTCTGTAATGTTCAGCTTCTAAT
TGTCTCTACTTTGTGAGACAACTTTTGAATGCTTGACCTCAAATCAGGTAGGACTACCCG
CTGAACTTAA
>B3_27
TTTCCGTAGGTGAACCTGCGGAAGGATCATTATTGAATTATGTTTCTAGATAGGTTGTAG
CTGGCTCTTTAGAGCATGTGCACGCCTGTTTGGACTTCATTTTCATCCACCTGTGCACCT
ATTGTAGTCTTTGGTTGGGTTAGGGGGAAGTGGTCATTGTGTCAGCATCTGCTGGATGTG
AGGACTTGCATTGTGAAAGCTTTGCTGTCCTTGATGTGATCATGGAATCTCTTTCTCACT
AGAGTCTATGTCACTCATTATACTCTGTCGAATGTCATTGAATGTCTTTACATGGGCTTG
TATGCCTATGAAAATTGTAATACAACTTTCAGCAACGGATCTCTTGGCTCTCGCATCGAT
GAAGGACGCAGCGAAATGCGATAAGTAATGTGAATTGCAGAATTCAGTGAATCATCGAAT
CTTTGAACGCATCTTGCGCTCCTTGGTATTCCGAGGAGCATGCCTGTTTGAGTGTCATTA
AATTCTCAACTCTCTTATACTTTTTTGTAAAAGAGAGCTTGGACTGTGGAGGCTTGCTGG
CCACTTTTTGGGGTCAGCTCCTCTGAAATGCATTAGCGGAACCGTTTGCAATCTGCCACA
AGTGTGATAAGTTATCTACACTGGCGAGGGGATTGCTCTCTGTAATGTTCAGCTTCTAAT
TGTCTCTACTTTGTGAGACAACTTTTGAATGCTTGACCTCAAATCAGGTAGGACTACCCG
CTGAACTTAA
>B3_28
TTTCCGTAGGTGAACCTGCGGAAGGATCATTATTGAATTATGTTTCTAGATAGGTTGTAG
CTGGCTCTTTAGAGCATGTGCACGCCTGTTTGGACTTCATTTTCATCCACCTGTGCACCT
ATTGTAGTCTTTGGTTGGGTTAGGGGGAAGTGGTCATTGTGTCAGCATCTGCTGGATGTG
AGGACTTGCATTGTGAAAGCTTTGCTGTCCTTGATGTGATCATGGAATCTCTTTCTCACT
AGAGTCTATGTCACTCATTATACTCTGTCGAATGTCATTGAATGTCTTTACATGGGCTTG
TATGCCTATGAAAATTGTAATACAACTTTCAGCAACGGATCTCTTGGCTCTCGCATCGAT
GAAGGACGCAGCGAAATGCGATAAGTAATGTGAATTGCAGAATTCAGTGAATCATCGAAT
CTTTGAACGCATCTTGCGCTCCTTGGTATTCCGAGGAGCATGCCTGTTTGAGTGTCATTA
AATTCTCAACTCTCTTATACTTTTTTGTAAAAGAGAGCTTGGACTGTGGAGGCTTGCTGG
CCACTTTTTGGGGTCAGCTCCTCTGAAATGCATTAGCGGAACCGTTTGCAATCTGCCACA
AGTGTGATAAGTTATCTACACTGGCGAGGGGATTGCTCTCTGTAATGTTCAGCTTCTAAT
TGTCTCTACTTTGTGAGACAACTTTTGAATGCTTGACCTCAAATCAGGTAGGACTACCCG
CTGAACTTAA
>B3_29
TTTCCGTAGGTGAACCTGCGGAAGGATCATTATTGAATTATGTTTCTAGATAGGTTGTAG
CTGGCTCTTTAGAGCATGTGCACGCCTGTTTGGACTTCATTTTCATCCACCTGTGCACCT
ATTGTAGTCTTTGGTTGGGTTAGGGGGAAGTGGTCATTGTGTCAGCATCTGCTGGATGTG
AGGACTTGCATTGTGAAAGCTTTGCTGTCCTTGATGTGATCATGGAATCTCTTTCTCACT
AGAGTCTATGTCACTCATTATACTCTGTCGAATGTCATTGAATGTCTTTACATGGGCTTG
TATGCCTATGAAAATTGTAATACAACTTTCAGCAACGGATCTCTTGGCTCTCGCATCGAT
GAAGGACGCAGCGAAATGCGATAAGTAATGTGAATTGCAGAATTCAGTGAATCATCGAAT
CTTTGAACGCATCTTGCGCTCCTTGGTATTCCGAGGAGCATGCCTGTTTGAGTGTCATTA
AATTCTCAACTCTCTTATACTTTTTTGTAAAAGAGAGCTTGGACTGTGGAGGCTTGCTGG

CCACTTTTTGGGGTCAGCTCCTCTGAAATGCATTAGCGGAACCGTTTGCAATCTGCCACA
AGTGTGATAAGTTATCTACACTGGCGAGGGGATTGCTCTCTGTAATGTTCAGCTTCTAAT
TGTCTCTACTTTGTGAGACAACTTTTGAATGCTTGACCTCAAATCAGGTAGGACTACCCG
CTGAACTTAA
>B3_30
TTTCCGTAGGTGAACCTGCGGAAGGATCATTATTGAATTATGTTTCTAGATAGGTTGTAG
CTGGCTCTTTAGAGCATGTGCACGCCTGTTTGGACTTCATTTTCATCCACCTGTGCACCT
ATTGTAGTCTTTGGTTGGGTTAGGGGGAAGTGGTCATTGTGTCAGCATCTGCTGGATGTG
AGGACTTGCATTGTGAAAGCTTTGCTGTCCTTGATGTGATCATGGAATCTCTTTCTCACT
AGAGTCTATGTCACTCATTATACTCTGTCGAATGTCATTGAATGTCTTTACATGGGCTTG
TATGCCTATGAAAATTGTAATACAACTTTCAGCAACGGATCTCTTGGCTCTCGCATCGAT
GAAGGACGCAGCGAAATGCGATAAGTAATGTGAATTGCAGAATTCAGTGAATCATCGAAT
CTTTGAACGCATCTTGCGCTCCTTGGTATTCCGAGGAGCATGCCTGTTTGAGTGTCATTA
AATTCTCAACTCTCTTATACTTTTTTGTAAAAGAGAGCTTGGACTGTGGAGGCTTGCTGG
CCACTTTTTGGGGTCAGCTCCTCTGAAATGCATTAGCGGAACCGTTTGCAATCTGCCACA
AGTGTGATAAGTTATCTACACTGGCGAGGGGATTGCTCTCTGTAATGTTCAGCTTCTAAT
TGTCTCTACTTTGTGAGACAACTTTTGAATGCTTGACCTCAAATCAGGTAGGACTACCCG
CTGAACTTAA
>B3_31
TTTCCGTAGGTGAACCTGCGGAAGGATCATTATTGAATTATGTTTCTAGATAGGTTGTAG
CTGGCTCTTTAGAGCATGTGCACGCCTGTTTGGACTTCATTTTCATCCACCTGTGCACCT
ATTGTAGTCTTTGGTTGGGTTAGGGGGAAGTGGTCATTGTGTCAGCATCTGCTGGATGTG
AGGACTTGCATTGTGAAAGCTTTGCTGTCCTTGATGTGATCATGGAATCTCTTTCTCACT
AGAGTCTATGTCACTCATTATACTCTGTCGAATGTCATTGAATGTCTTTACATGGGCTTG
TATGCCTATGAAAATTGTAATACAACTTTCAGCAACGGATCTCTTGGCTCTCGCATCGAT
GAAGGACGCAGCGAAATGCGATAAGTAATGTGAATTGCAGAATTCAGTGAATCATCGAAT
CTTTGAACGCATCTTGCGCTCCTTGGTATTCCGAGGAGCATGCCTGTTTGAGTGTCATTA
AATTCTCAACTCTCTTATACTTTTTTGTAAAAGAGAGCTTGGACTGTGGAGGCTTGCTGG
CCACTTTTTGGGGTCAGCTCCTCTGAAATGCATTAGCGGAACCGTTTGCAATCTGCCACA
AGTGTGATAAGTTATCTACACTGGCGAGGGGATTGCTCTCTGTAATGTTCAGCTTCTAAT
TGTCTCTACTTTGTGAGACAACTTTTGAATGCTTGACCTCAAATCAGGTAGGACTACCCG
CTGAACTTAA
>B3_32
TTTCCGTAGGTGAACCTGCGGAAGGATCATTATTGAATTATGTTTCTAGATAGGTTGTAG
CTGGCTCTTTAGAGCATGTGCACGCCTGTTTGGACTTCATTTTCATCCACCTGTGCACCT
ATTGTAGTCTTTGGTTGGGTTAGGGGGAAGTGGTCATTGTGTCAGCATCTGCTGGATGTG
AGGACTTGCATTGTGAAAGCTTTGCTGTCCTTGATGTGATCATGGAATCTCTTTCTCACT
AGAGTCTATGTCACTCATTATACTCTGTCGAATGTCATTGAATGTCTTTACATGGGCTTG
TATGCCTATGAAAATTGTAATACAACTTTCAGCAACGGATCTCTTGGCTCTCGCATCGAT
GAAGGACGCAGCGAAATGCGATAAGTAATGTGAATTGCAGAATTCAGTGAATCATCGAAT
CTTTGAACGCATCTTGCGCTCCTTGGTATTCCGAGGAGCATGCCTGTTTGAGTGTCATTA
AATTCTCAACTCTCTTATACTTTTTTGTAAAAGAGAGCTTGGACTGTGGAGGCTTGCTGG
CCACTTTTTGGGGTCAGCTCCTCTGAAATGCATTAGCGGAACCGTTTGCAATCTGCCACA
AGTGTGATAAGTTATCTACACTGGCGAGGGGATTGCTCTCTGTAATGTTCAGCTTCTAAT
TGTCTCTACTTTGTGAGACAACTTTTGAATGCTTGACCTCAAATCAGGTAGGACTACCCG
CTGAACTTAA
>B3_33
TTTCCGTAGGTGAACCTGCGGAAGGATCATTATTGAATTATGTTTCTAGATAGGTTGTAG
CTGGCTCTTTAGAGCATGTGCACGCCTGTTTGGACTTCATTTTCATCCACCTGTGCACCT
ATTGTAGTCTTTGGTTGGGTTAGGGGGAAGTGGTCATTGTGTCAGCATCTGCTGGATGTG

AGGACTTGCATTGTGAAAGCTTTGCTGTCCTTGATGTGATCATGGAATCTCTTTCTCACT
AGAGTCTATGTCACTCATTATACTCTGTCGAATGTCATTGAATGTCTTTACATGGGCTTG
TATGCCTATGAAAATTGTAATACAACTTTCAGCAACGGATCTCTTGGCTCTCGCATCGAT
GAAGGACGCAGCGAAATGCGATAAGTAATGTGAATTGCAGAATTCAGTGAATCATCGAAT
CTTTGAACGCATCTTGCGCTCCTTGGTATTCCGAGGAGCATGCCTGTTTGAGTGTCATTA
AATTCTCAACTCTCTTATACTTTTTTGTAAAAGAGAGCTTGGACTGTGGAGGCTTGCTGG
CCACTTTTTGGGGTCAGCTCCTCTGAAATGCATTAGCGGAACCGTTTGCAATCTGCCACA
AGTGTGATAAGTTATCTACACTGGCGAGGGGATTGCTCTCTGTAATGTTCAGCTTCTAAT
TGTCTCTACTTTGTGAGACAACTTTTGAATGCTTGACCTCAAATCAGGTAGGACTACCCG
CTGAACTTAA
>B3_34
TTTCCGTAGGTGAACCTGCGGAAGGATCATTATTGAATTATGTTTCTAGATAGGTTGTAG
CTGGCTCTTTAGAGCATGTGCACGCCTGTTTGGACTTCATTTTCATCCACCTGTGCACCT
ATTGTAGTCTTTGGTTGGGTTAGGGGGAAGTGGTCATTGTGTCAGCATCTGCTGGATGTG
AGGACTTGCATTGTGAAAGCTTTGCTGTCCTTGATGTGATCATGGAATCTCTTTCTCACT
AGAGTCTATGTCACTCATTATACTCTGTCGAATGTCATTGAATGTCTTTACATGGGCTTG
TATGCCTATGAAAATTGTAATACAACTTTCAGCAACGGATCTCTTGGCTCTCGCATCGAT
GAAGGACGCAGCGAAATGCGATAAGTAATGTGAATTGCAGAATTCAGTGAATCATCGAAT
CTTTGAACGCATCTTGCGCTCCTTGGTATTCCGAGGAGCATGCCTGTTTGAGTGTCATTA
AATTCTCAACTCTCTTATACTTTTTTGTAAAAGAGAGCTTGGACTGTGGAGGCTTGCTGG
CCACTTTTTGGGGTCAGCTCCTCTGAAATGCATTAGCGGAACCGTTTGCAATCTGCCACA
AGTGTGATAAGTTATCTACACTGGCGAGGGGATTGCTCTCTGTAATGTTCAGCTTCTAAT
TGTCTCTACTTTGTGAGACAACTTTTGAATGCTTGACCTCAAATCAGGTAGGACTACCCG
CTGAACTTAA
>B3_35
TTTCCGTAGGTGAACCTGCGGAAGGATCATTATTGAATTATGTTTCTAGATAGGTTGTAG
CTGGCTCTTTAGAGCATGTGCACGCCTGTTTGGACTTCATTTTCATCCACCTGTGCACCT
ATTGTAGTCTTTGGTTGGGTTAGGGGGAAGTGGTCATTGTGTCAGCATCTGCTGGATGTG
AGGACTTGCATTGTGAAAGCTTTGCTGTCCTTGATGTGATCATGGAATCTCTTTCTCACT
AGAGTCTATGTCACTCATTATACTCTGTCGAATGTCATTGAATGTCTTTACATGGGCTTG
TATGCCTATGAAAATTGTAATACAACTTTCAGCAACGGATCTCTTGGCTCTCGCATCGAT
GAAGGACGCAGCGAAATGCGATAAGTAATGTGAATTGCAGAATTCAGTGAATCATCGAAT
CTTTGAACGCATCTTGCGCTCCTTGGTATTCCGAGGAGCATGCCTGTTTGAGTGTCATTA
AATTCTCAACTCTCTTATACTTTTTTGTAAAAGAGAGCTTGGACTGTGGAGGCTTGCTGG
CCACTTTTTGGGGTCAGCTCCTCTGAAATGCATTAGCGGAACCGTTTGCAATCTGCCACA
AGTGTGATAAGTTATCTACACTGGCGAGGGGATTGCTCTCTGTAATGTTCAGCTTCTAAT
TGTCTCTACTTTGTGAGACAACTTTTGAATGCTTGACCTCAAATCAGGTAGGACTACCCG
CTGAACTTAA
>B3_36
TTTCCGTAGGTGAACCTGCGGAAGGATCATTATTGAATTATGTTTCTAGATAGGTTGTAG
CTGGCTCTTTAGAGCATGTGCACGCCTGTTTGGACTTCATTTTCATCCACCTGTGCACCT
ATTGTAGTCTTTGGTTGGGTTAGGGGGAAGTGGTCATTGTGTCAGCATCTGCTGGATGTG
AGGACTTGCATTGTGAAAGCTTTGCTGTCCTTGATGTGATCATGGAATCTCTTTCTCACT
AGAGTCTATGTCACTCATTATACTCTGTCGAATGTCATTGAATGTCTTTACATGGGCTTG
TATGCCTATGAAAATTGTAATACAACTTTCAGCAACGGATCTCTTGGCTCTCGCATCGAT
GAAGGACGCAGCGAAATGCGATAAGTAATGTGAATTGCAGAATTCAGTGAATCATCGAAT
CTTTGAACGCATCTTGCGCTCCTTGGTATTCCGAGGAGCATGCCTGTTTGAGTGTCATTA
AATTCTCAACTCTCTTATACTTTTTTGTAAAAGAGAGCTTGGACTGTGGAGGCTTGCTGG
CCACTTTTTGGGGTCAGCTCCTCTGAAATGCATTAGCGGAACCGTTTGCAATCTGCCACA
AGTGTGATAAGTTATCTACACTGGCGAGGGGATTGCTCTCTGTAATGTTCAGCTTCTAAT

TGTCTCTACTTTGTGAGACAACTTTTGAATGCTTGACCTCAAATCAGGTAGGACTACCCG
CTGAACTTAA
>B3_37
TTTCCGTAGGTGAACCTGCGGAAGGATCATTATTGAATTATGTTTCTAGATAGGTTGTAG
CTGGCTCTTTAGAGCATGTGCACGCCTGTTTGGACTTCATTTTCATCCACCTGTGCACCT
ATTGTAGTCTTTGGTTGGGTTAGGGGGAAGTGGTCATTGTGTCAGCATCTGCTGGATGTG
AGGACTTGCATTGTGAAAGCTTTGCTGTCCTTGATGTGATCATGGAATCTCTTTCTCACT
AGAGTCTATGTCACTCATTATACTCTGTCGAATGTCATTGAATGTCTTTACATGGGCTTG
TATGCCTATGAAAATTGTAATACAACTTTCAGCAACGGATCTCTTGGCTCTCGCATCGAT
GAAGGACGCAGCGAAATGCGATAAGTAATGTGAATTGCAGAATTCAGTGAATCATCGAAT
CTTTGAACGCATCTTGCGCTCCTTGGTATTCCGAGGAGCATGCCTGTTTGAGTGTCATTA
AATTCTCAACTCTCTTATACTTTTTTGTAAAAGAGAGCTTGGACTGTGGAGGCTTGCTGG
CCACTTTTTGGGGTCAGCTCCTCTGAAATGCATTAGCGGAACCGTTTGCAATCTGCCACA
AGTGTGATAAGTTATCTACACTGGCGAGGGGATTGCTCTCTGTAATGTTCAGCTTCTAAT
TGTCTCTACTTTGTGAGACAACTTTTGAATGCTTGACCTCAAATCAGGTAGGACTACCCG
CTGAACTTAA
>B3_38
TTTCCGTAGGTGAACCTGCGGAAGGATCATTATTGAATTATGTTTCTAGATAGGTTGTAG
CTGGCTCTTTAGAGCATGTGCACGCCTGTTTGGACTTCATTTTCATCCACCTGTGCACCT
ATTGTAGTCTTTGGTTGGGTTAGGGGGAAGTGGTCATTGTGTCAGCATCTGCTGGATGTG
AGGACTTGCATTGTGAAAGCTTTGCTGTCCTTGATGTGATCATGGAATCTCTTTCTCACT
AGAGTCTATGTCACTCATTATACTCTGTCGAATGTCATTGAATGTCTTTACATGGGCTTG
TATGCCTATGAAAATTGTAATACAACTTTCAGCAACGGATCTCTTGGCTCTCGCATCGAT
GAAGGACGCAGCGAAATGCGATAAGTAATGTGAATTGCAGAATTCAGTGAATCATCGAAT
CTTTGAACGCATCTTGCGCTCCTTGGTATTCCGAGGAGCATGCCTGTTTGAGTGTCATTA
AATTCTCAACTCTCTTATACTTTTTTGTAAAAGAGAGCTTGGACTGTGGAGGCTTGCTGG
CCACTTTTTGGGGTCAGCTCCTCTGAAATGCATTAGCGGAACCGTTTGCAATCTGCCACA
AGTGTGATAAGTTATCTACACTGGCGAGGGGATTGCTCTCTGTAATGTTCAGCTTCTAAT
TGTCTCTACTTTGTGAGACAACTTTTGAATGCTTGACCTCAAATCAGGTAGGACTACCCG
CTGAACTTAA
>B3_39
TTTCCGTAGGTGAACCTGCGGAAGGATCATTATTGAATTATGTTTCTAGATAGGTTGTAG
CTGGCTCTTTAGAGCATGTGCACGCCTGTTTGGACTTCATTTTCATCCACCTGTGCACCT
ATTGTAGTCTTTGGTTGGGTTAGGGGGAAGTGGTCATTGTGTCAGCATCTGCTGGATGTG
AGGACTTGCATTGTGAAAGCTTTGCTGTCCTTGATGTGATCATGGAATCTCTTTCTCACT
AGAGTCTATGTCACTCATTATACTCTGTCGAATGTCATTGAATGTCTTTACATGGGCTTG
TATGCCTATGAAAATTGTAATACAACTTTCAGCAACGGATCTCTTGGCTCTCGCATCGAT
GAAGGACGCAGCGAAATGCGATAAGTAATGTGAATTGCAGAATTCAGTGAATCATCGAAT
CTTTGAACGCATCTTGCGCTCCTTGGTATTCCGAGGAGCATGCCTGTTTGAGTGTCATTA
AATTCTCAACTCTCTTATACTTTTTTGTAAAAGAGAGCTTGGACTGTGGAGGCTTGCTGG
CCACTTTTTGGGGTCAGCTCCTCTGAAATGCATTAGCGGAACCGTTTGCAATCTGCCACA
AGTGTGATAAGTTATCTACACTGGCGAGGGGATTGCTCTCTGTAATGTTCAGCTTCTAAT
TGTCTCTACTTTGTGAGACAACTTTTGAATGCTTGACCTCAAATCAGGTAGGACTACCCG
CTGAACTTAA
>B3_40
TTTCCGTAGGTGAACCTGCGGAAGGATCATTATTGAATTATGTTTCTAGATAGGTTGTAG
CTGGCTCTTTAGAGCATGTGCACGCCTGTTTGGACTTCATTTTCATCCACCTGTGCACCT
ATTGTAGTCTTTGGTTGGGTTAGGGGGAAGTGGTCATTGTGTCAGCATCTGCTGGATGTG
AGGACTTGCATTGTGAAAGCTTTGCTGTCCTTGATGTGATCATGGAATCTCTTTCTCACT
AGAGTCTATGTCACTCATTATACTCTGTCGAATGTCATTGAATGTCTTTACATGGGCTTG

TATGCCTATGAAAATTGTAATACAACTTTCAGCAACGGATCTCTTGGCTCTCGCATCGAT
GAAGGACGCAGCGAAATGCGATAAGTAATGTGAATTGCAGAATTCAGTGAATCATCGAAT
CTTTGAACGCATCTTGCGCTCCTTGGTATTCCGAGGAGCATGCCTGTTTGAGTGTCATTA
AATTCTCAACTCTCTTATACTTTTTTGTAAAAGAGAGCTTGGACTGTGGAGGCTTGCTGG
CCACTTTTTGGGGTCAGCTCCTCTGAAATGCATTAGCGGAACCGTTTGCAATCTGCCACA
AGTGTGATAAGTTATCTACACTGGCGAGGGGATTGCTCTCTGTAATGTTCAGCTTCTAAT
TGTCTCTACTTTGTGAGACAACTTTTGAATGCTTGACCTCAAATCAGGTAGGACTACCCG
CTGAACTTAA
>B3_41
TTTCCGTAGGTGAACCTGCGGAAGGATCATTATTGAATTATGTTTCTAGATAGGTTGTAG
CTGGCTCTTTAGAGCATGTGCACGCCTGTTTGGACTTCATTTTCATCCACCTGTGCACCT
ATTGTAGTCTTTGGTTGGGTTAGGGGGAAGTGGTCATTGTGTCAGCATCTGCTGGATGTG
AGGACTTGCATTGTGAAAGCTTTGCTGTCCTTGATGTGATCATGGAATCTCTTTCTCACT
AGAGTCTATGTCACTCATTATACTCTGTCGAATGTCATTGAATGTCTTTACATGGGCTTG
TATGCCTATGAAAATTGTAATACAACTTTCAGCAACGGATCTCTTGGCTCTCGCATCGAT
GAAGGACGCAGCGAAATGCGATAAGTAATGTGAATTGCAGAATTCAGTGAATCATCGAAT
CTTTGAACGCATCTTGCGCTCCTTGGTATTCCGAGGAGCATGCCTGTTTGAGTGTCATTA
AATTCTCAACTCTCTTATACTTTTTTGTAAAAGAGAGCTTGGACTGTGGAGGCTTGCTGG
CCACTTTTTGGGGTCAGCTCCTCTGAAATGCATTAGCGGAACCGTTTGCAATCTGCCACA
AGTGTGATAAGTTATCTACACTGGCGAGGGGATTGCTCTCTGTAATGTTCAGCTTCTAAT
TGTCTCTACTTTGTGAGACAACTTTTGAATGCTTGACCTCAAATCAGGTAGGACTACCCG
CTGAACTTAA
>B3_42
TTTCCGTAGGTGAACCTGCGGAAGGATCATTATTGAATTATGTTTCTAGATAGGTTGTAG
CTGGCTCTTTAGAGCATGTGCACGCCTGTTTGGACTTCATTTTCATCCACCTGTGCACCT
ATTGTAGTCTTTGGTTGGGTTAGGGGGAAGTGGTCATTGTGTCAGCATCTGCTGGATGTG
AGGACTTGCATTGTGAAAGCTTTGCTGTCCTTGATGTGATCATGGAATCTCTTTCTCACT
AGAGTCTATGTCACTCATTATACTCTGTCGAATGTCATTGAATGTCTTTACATGGGCTTG
TATGCCTATGAAAATTGTAATACAACTTTCAGCAACGGATCTCTTGGCTCTCGCATCGAT
GAAGGACGCAGCGAAATGCGATAAGTAATGTGAATTGCAGAATTCAGTGAATCATCGAAT
CTTTGAACGCATCTTGCGCTCCTTGGTATTCCGAGGAGCATGCCTGTTTGAGTGTCATTA
AATTCTCAACTCTCTTATACTTTTTTGTAAAAGAGAGCTTGGACTGTGGAGGCTTGCTGG
CCACTTTTTGGGGTCAGCTCCTCTGAAATGCATTAGCGGAACCGTTTGCAATCTGCCACA
AGTGTGATAAGTTATCTACACTGGCGAGGGGATTGCTCTCTGTAATGTTCAGCTTCTAAT
TGTCTCTACTTTGTGAGACAACTTTTGAATGCTTGACCTCAAATCAGGTAGGACTACCCG
CTGAACTTAA
>B3_43
TTTCCGTAGGTGAACCTGCGGAAGGATCATTATTGAATTATGTTTCTAGATAGGTTGTAG
CTGGCTCTTTAGAGCATGTGCACGCCTGTTTGGACTTCATTTTCATCCACCTGTGCACCT
ATTGTAGTCTTTGGTTGGGTTAGGGGGAAGTGGTCATTGTGTCAGCATCTGCTGGATGTG
AGGACTTGCATTGTGAAAGCTTTGCTGTCCTTGATGTGATCATGGAATCTCTTTCTCACT
AGAGTCTATGTCACTCATTATACTCTGTCGAATGTCATTGAATGTCTTTACATGGGCTTG
TATGCCTATGAAAATTGTAATACAACTTTCAGCAACGGATCTCTTGGCTCTCGCATCGAT
GAAGGACGCAGCGAAATGCGATAAGTAATGTGAATTGCAGAATTCAGTGAATCATCGAAT
CTTTGAACGCATCTTGCGCTCCTTGGTATTCCGAGGAGCATGCCTGTTTGAGTGTCATTA
AATTCTCAACTCTCTTATACTTTTTTGTAAAAGAGAGCTTGGACTGTGGAGGCTTGCTGG
CCACTTTTTGGGGTCAGCTCCTCTGAAATGCATTAGCGGAACCGTTTGCAATCTGCCACA
AGTGTGATAAGTTATCTACACTGGCGAGGGGATTGCTCTCTGTAATGTTCAGCTTCTAAT
TGTCTCTACTTTGTGAGACAACTTTTGAATGCTTGACCTCAAATCAGGTAGGACTACCCG
CTGAACTTAA

>B3_44
TTTCCGTAGGTGAACCTGCGGAAGGATCATTATTGAATTATGTTTCTAGATAGGTTGTAG
CTGGCTCTTTAGAGCATGTGCACGCCTGTTTGGACTTCATTTTCATCCACCTGTGCACCT
ATTGTAGTCTTTGGTTGGGTTAGGGGGAAGTGGTCATTGTGTCAGCATCTGCTGGATGTG
AGGACTTGCATTGTGAAAGCTTTGCTGTCCTTGATGTGATCATGGAATCTCTTTCTCACT
AGAGTCTATGTCACTCATTATACTCTGTCGAATGTCATTGAATGTCTTTACATGGGCTTG
TATGCCTATGAAAATTGTAATACAACTTTCAGCAACGGATCTCTTGGCTCTCGCATCGAT
GAAGGACGCAGCGAAATGCGATAAGTAATGTGAATTGCAGAATTCAGTGAATCATCGAAT
CTTTGAACGCATCTTGCGCTCCTTGGTATTCCGAGGAGCATGCCTGTTTGAGTGTCATTA
AATTCTCAACTCTCTTATACTTTTTTGTAAAAGAGAGCTTGGACTGTGGAGGCTTGCTGG
CCACTTTTTGGGGTCAGCTCCTCTGAAATGCATTAGCGGAACCGTTTGCAATCTGCCACA
AGTGTGATAAGTTATCTACACTGGCGAGGGGATTGCTCTCTGTAATGTTCAGCTTCTAAT
TGTCTCTACTTTGTGAGACAACTTTTGAATGCTTGACCTCAAATCAGGTAGGACTACCCG
CTGAACTTAA
>B3_45
TTTCCGTAGGTGAACCTGCGGAAGGATCATTATTGAATTATGTTTCTAGATAGGTTGTAG
CTGGCTCTTTAGAGCATGTGCACGCCTGTTTGGACTTCATTTTCATCCACCTGTGCACCT
ATTGTAGTCTTTGGTTGGGTTAGGGGGAAGTGGTCATTGTGTCAGCATCTGCTGGATGTG
AGGACTTGCATTGTGAAAGCTTTGCTGTCCTTGATGTGATCATGGAATCTCTTTCTCACT
AGAGTCTATGTCACTCATTATACTCTGTCGAATGTCATTGAATGTCTTTACATGGGCTTG
TATGCCTATGAAAATTGTAATACAACTTTCAGCAACGGATCTCTTGGCTCTCGCATCGAT
GAAGGACGCAGCGAAATGCGATAAGTAATGTGAATTGCAGAATTCAGTGAATCATCGAAT
CTTTGAACGCATCTTGCGCTCCTTGGTATTCCGAGGAGCATGCCTGTTTGAGTGTCATTA
AATTCTCAACTCTCTTATACTTTTTTGTAAAAGAGAGCTTGGACTGTGGAGGCTTGCTGG
CCACTTTTTGGGGTCAGCTCCTCTGAAATGCATTAGCGGAACCGTTTGCAATCTGCCACA
AGTGTGATAAGTTATCTACACTGGCGAGGGGATTGCTCTCTGTAATGTTCAGCTTCTAAT
TGTCTCTACTTTGTGAGACAACTTTTGAATGCTTGACCTCAAATCAGGTAGGACTACCCG
CTGAACTTAA
>B3_46
TTTCCGTAGGTGAACCTGCGGAAGGATCATTATTGAATTATGTTTCTAGATAGGTTGTAG
CTGGCTCTTTAGAGCATGTGCACGCCTGTTTGGACTTCATTTTCATCCACCTGTGCACCT
ATTGTAGTCTTTGGTTGGGTTAGGGGGAAGTGGTCATTGTGTCAGCATCTGCTGGATGTG
AGGACTTGCATTGTGAAAGCTTTGCTGTCCTTGATGTGATCATGGAATCTCTTTCTCACT
AGAGTCTATGTCACTCATTATACTCTGTCGAATGTCATTGAATGTCTTTACATGGGCTTG
TATGCCTATGAAAATTGTAATACAACTTTCAGCAACGGATCTCTTGGCTCTCGCATCGAT
GAAGGACGCAGCGAAATGCGATAAGTAATGTGAATTGCAGAATTCAGTGAATCATCGAAT
CTTTGAACGCATCTTGCGCTCCTTGGTATTCCGAGGAGCATGCCTGTTTGAGTGTCATTA
AATTCTCAACTCTCTTATACTTTTTTGTAAAAGAGAGCTTGGACTGTGGAGGCTTGCTGG
CCACTTTTTGGGGTCAGCTCCTCTGAAATGCATTAGCGGAACCGTTTGCAATCTGCCACA
AGTGTGATAAGTTATCTACACTGGCGAGGGGATTGCTCTCTGTAATGTTCAGCTTCTAAT
TGTCTCTACTTTGTGAGACAACTTTTGAATGCTTGACCTCAAATCAGGTAGGACTACCCG
CTGAACTTAA
>B3_47
TTTCCGTAGGTGAACCTGCGGAAGGATCATTATTGAATTATGTTTCTAGATAGGTTGTAG
CTGGCTCTTTAGAGCATGTGCACGCCTGTTTGGACTTCATTTTCATCCACCTGTGCACCT
ATTGTAGTCTTTGGTTGGGTTAGGGGGAAGTGGTCATTGTGTCAGCATCTGCTGGATGTG
AGGACTTGCATTGTGAAAGCTTTGCTGTCCTTGATGTGATCATGGAATCTCTTTCTCACT
AGAGTCTATGTCACTCATTATACTCTGTCGAATGTCATTGAATGTCTTTACATGGGCTTG
TATGCCTATGAAAATTGTAATACAACTTTCAGCAACGGATCTCTTGGCTCTCGCATCGAT
GAAGGACGCAGCGAAATGCGATAAGTAATGTGAATTGCAGAATTCAGTGAATCATCGAAT

CTTTGAACGCATCTTGCGCTCCTTGGTATTCCGAGGAGCATGCCTGTTTGAGTGTCATTA
AATTCTCAACTCTCTTATACTTTTTTGTAAAAGAGAGCTTGGACTGTGGAGGCTTGCTGG
CCACTTTTTGGGGTCAGCTCCTCTGAAATGCATTAGCGGAACCGTTTGCAATCTGCCACA
AGTGTGATAAGTTATCTACACTGGCGAGGGGATTGCTCTCTGTAATGTTCAGCTTCTAAT
TGTCTCTACTTTGTGAGACAACTTTTGAATGCTTGACCTCAAATCAGGTAGGACTACCCG
CTGAACTTAA
>B3_48
TTTCCGTAGGTGAACCTGCGGAAGGATCATTATTGAATTATGTTTCTAGATAGGTTGTAG
CTGGCTCTTTAGAGCATGTGCACGCCTGTTTGGACTTCATTTTCATCCACCTGTGCACCT
ATTGTAGTCTTTGGTTGGGTTAGGGGGAAGTGGTCATTGTGTCAGCATCTGCTGGATGTG
AGGACTTGCATTGTGAAAGCTTTGCTGTCCTTGATGTGATCATGGAATCTCTTTCTCACT
AGAGTCTATGTCACTCATTATACTCTGTCGAATGTCATTGAATGTCTTTACATGGGCTTG
TATGCCTATGAAAATTGTAATACAACTTTCAGCAACGGATCTCTTGGCTCTCGCATCGAT
GAAGGACGCAGCGAAATGCGATAAGTAATGTGAATTGCAGAATTCAGTGAATCATCGAAT
CTTTGAACGCATCTTGCGCTCCTTGGTATTCCGAGGAGCATGCCTGTTTGAGTGTCATTA
AATTCTCAACTCTCTTATACTTTTTTGTAAAAGAGAGCTTGGACTGTGGAGGCTTGCTGG
CCACTTTTTGGGGTCAGCTCCTCTGAAATGCATTAGCGGAACCGTTTGCAATCTGCCACA
AGTGTGATAAGTTATCTACACTGGCGAGGGGATTGCTCTCTGTAATGTTCAGCTTCTAAT
TGTCTCTACTTTGTGAGACAACTTTTGAATGCTTGACCTCAAATCAGGTAGGACTACCCG
CTGAACTTAA
>B3_50
TTTCCGTAGGTGAACCTGCGGAAGGATCATTATTGAATTATGTTTCTAGATAGGTTGTAG
CTGGCTCTTTAGAGCATGTGCACGCCTGTTTGGACTTCATTTTCATCCACCTGTGCACCT
ATTGTAGTCTTTGGTTGGGTTAGGGGGAAGTGGTCATTGTGTCAGCATCTGCTGGATGTG
AGGACTTGCATTGTGAAAGCTTTGCTGTCCTTGATGTGATCATGGAATCTCTTTCTCACT
AGAGTCTATGTCACTCATTATACTCTGTCGAATGTCATTGAATGTCTTTACATGGGCTTG
TATGCCTATGAAAATTGTAATACAACTTTCAGCAACGGATCTCTTGGCTCTCGCATCGAT
GAAGGACGCAGCGAAATGCGATAAGTAATGTGAATTGCAGAATTCAGTGAATCATCGAAT
CTTTGAACGCATCTTGCGCTCCTTGGTATTCCGAGGAGCATGCCTGTTTGAGTGTCATTA
AATTCTCAACTCTCTTATACTTTTTTGTAAAAGAGAGCTTGGACTGTGGAGGCTTGCTGG
CCACTTTTTGGGGTCAGCTCCTCTGAAATGCATTAGCGGAACCGTTTGCAATCTGCCACA
AGTGTGATAAGTTATCTACACTGGCGAGGGGATTGCTCTCTGTAATGTTCAGCTTCTAAT
TGTCTCTACTTTGTGAGACAACTTTTGAATGCTTGACCTCAAATCAGGTAGGACTACCCG
CTGAACTTAA
>B3-54
TTTCCGTAGGTGAACCTGCGGAAGGATCATTATTGAATTATGTTTCTAGATAGGTTGTAG
CTGGCTCTTTAGAGCATGTGCACGCCTGTTTGGACTTCATTTTCATCCACCTGTGCACCT
ATTGTAGTCTTTGGTTGGGTTAGGGGGAAGTGGTCATTGTGTCAGCATCTGCTGGATGTG
AGGACTTGCATTGTGAAAGCTTTGCTGTCCTTGATGTGATCATGGAATCTCTTTCTCACT
AGAGTCTATGTCACTCATTATACTCTGTCGAATGTCATTGAATGTCTTTACATGGGCTTG
TATGCCTATGAAAATTGTAATACAACTTTCAGCAACGGATCTCTTGGCTCTCGCATCGAT
GAAGGACGCAGCGAAATGCGATAAGTAATGTGAATTGCAGAATTCAGTGAATCATCGAAT
CTTTGAACGCATCTTGCGCTCCTTGGTATTCCGAGGAGCATGCCTGTTTGAGTGTCATTA
AATTCTCAACTCTCTTATACTTTTTTGTAAAAGAGAGCTTGGACTGTGGAGGCTTGCTGG
CCACTTTTTGGGGTCAGCTCCTCTGAAATGCATTAGCGGAACCGTTTGCAATCTGCCACA
AGTGTGATAAGTTATCTACACTGGCGAGGGGATTGCTCTCTGTAATGTTCAGCTTCTAAT
TGTCTCTACTTTGTGAGACAACTTTTGAATGCTTGACCTCAAATCAGGTAGGACTACCCG
CTGAACTTAA
>B3-55
TTTCCGTAGGTGAACCTGCGGAAGGATCATTATTGAATTATGTTTCTAGATAGGTTGTAG

CTGGCTCTTTAGAGCATGTGCACGCCTGTTTGGACTTCATTTTCATCCACCTGTGCACCT
ATTGTAGTCTTTGGTTGGGTTAGGGGGAAGTGGTCATTGTGTCAGCATCTGCTGGATGTG
AGGACTTGCATTGTGAAAGCTTTGCTGTCCTTGATGTGATCATGGAATCTCTTTCTCACT
AGAGTCTATGTCACTCATTATACTCTGTCGAATGTCATTGAATGTCTTTACATGGGCTTG
TATGCCTATGAAAATTGTAATACAACTTTCAGCAACGGATCTCTTGGCTCTCGCATCGAT
GAAGGACGCAGCGAAATGCGATAAGTAATGTGAATTGCAGAATTCAGTGAATCATCGAAT
CTTTGAACGCATCTTGCGCTCCTTGGTATTCCGAGGAGCATGCCTGTTTGAGTGTCATTA
AATTCTCAACTCTCTTATACTTTTTTGTAAAAGAGAGCTTGGACTGTGGAGGCTTGCTGG
CCACTTTTTGGGGTCAGCTCCTCTGAAATGCATTAGCGGAACCGTTTGCAATCTGCCACA
AGTGTGATAAGTTATCTACACTGGCGAGGGGATTGCTCTCTGTAATGTTCAGCTTCTAAT
TGTCTCTACTTTGTGAGACAACTTTTGAATGCTTGACCTCAAATCAGGTAGGACTACCCG
CTGAACTTAA
>B3-56
TTTCCGTAGGTGAACCTGCGGAAGGATCATTATTGAATTATGTTTCTAGATAGGTTGTAG
CTGGCTCTTTAGAGCATGTGCACGCCTGTTTGGACTTCATTTTCATCCACCTGTGCACCT
ATTGTAGTCTTTGGTTGGGTTAGGGGGAAGTGGTCATTGTGTCAGCATCTGCTGGATGTG
AGGACTTGCATTGTGAAAGCTTTGCTGTCCTTGATGTGATCATGGAATCTCTTTCTCACT
AGAGTCTATGTCACTCATTATACTCTGTCGAATGTCATTGAATGTCTTTACATGGGCTTG
TATGCCTATGAAAATTGTAATACAACTTTCAGCAACGGATCTCTTGGCTCTCGCATCGAT
GAAGGACGCAGCGAAATGCGATAAGTAATGTGAATTGCAGAATTCAGTGAATCATCGAAT
CTTTGAACGCATCTTGCGCTCCTTGGTATTCCGAGGAGCATGCCTGTTTGAGTGTCATTA
AATTCTCAACTCTCTTATACTTTTTTGTAAAAGAGAGCTTGGACTGTGGAGGCTTGCTGG
CCACTTTTTGGGGTCAGCTCCTCTGAAATGCATTAGCGGAACCGTTTGCAATCTGCCACA
AGTGTGATAAGTTATCTACACTGGCGAGGGGATTGCTCTCTGTAATGTTCAGCTTCTAAT
TGTCTCTACTTTGTGAGACAACTTTTGAATGCTTGACCTCAAATCAGGTAGGACTACCCG
CTGAACTTAA
>B3-57
TTTCCGTAGGTGAACCTGCGGAAGGATCATTATTGAATTATGTTTCTAGATAGGTTGTAG
CTGGCTCTTTAGAGCATGTGCACGCCTGTTTGGACTTCATTTTCATCCACCTGTGCACCT
ATTGTAGTCTTTGGTTGGGTTAGGGGGAAGTGGTCATTGTGTCAGCATCTGCTGGATGTG
AGGACTTGCATTGTGAAAGCTTTGCTGTCCTTGATGTGATCATGGAATCTCTTTCTCACT
AGAGTCTATGTCACTCATTATACTCTGTCGAATGTCATTGAATGTCTTTACATGGGCTTG
TATGCCTATGAAAATTGTAATACAACTTTCAGCAACGGATCTCTTGGCTCTCGCATCGAT
GAAGGACGCAGCGAAATGCGATAAGTAATGTGAATTGCAGAATTCAGTGAATCATCGAAT
CTTTGAACGCATCTTGCGCTCCTTGGTATTCCGAGGAGCATGCCTGTTTGAGTGTCATTA
AATTCTCAACTCTCTTATACTTTTTTGTAAAAGAGAGCTTGGACTGTGGAGGCTTGCTGG
CCACTTTTTGGGGTCAGCTCCTCTGAAATGCATTAGCGGAACCGTTTGCAATCTGCCACA
AGTGTGATAAGTTATCTACACTGGCGAGGGGATTGCTCTCTGTAATGTTCAGCTTCTAAT
TGTCTCTACTTTGTGAGACAACTTTTGAATGCTTGACCTCAAATCAGGTAGGACTACCCG
CTGAACTTAA
>B3-59
TTTCCGTAGGTGAACCTGCGGAAGGATCATTATTGAATTATGTTTCTAGATAGGTTGTAG
CTGGCTCTTTAGAGCATGTGCACGCCTGTTTGGACTTCATTTTCATCCACCTGTGCACCT
ATTGTAGTCTTTGGTTGGGTTAGGGGGAAGTGGTCATTGTGTCAGCATCTGCTGGATGTG
AGGACTTGCATTGTGAAAGCTTTGCTGTCCTTGATGTGATCATGGAATCTCTTTCTCACT
AGAGTCTATGTCACTCATTATACTCTGTCGAATGTCATTGAATGTCTTTACATGGGCTTG
TATGCCTATGAAAATTGTAATACAACTTTCAGCAACGGATCTCTTGGCTCTCGCATCGAT
GAAGGACGCAGCGAAATGCGATAAGTAATGTGAATTGCAGAATTCAGTGAATCATCGAAT
CTTTGAACGCATCTTGCGCTCCTTGGTATTCCGAGGAGCATGCCTGTTTGAGTGTCATTA
AATTCTCAACTCTCTTATACTTTTTTGTAAAAGAGAGCTTGGACTGTGGAGGCTTGCTGG

CCACTTTTTGGGGTCAGCTCCTCTGAAATGCATTAGCGGAACCGTTTGCAATCTGCCACA
AGTGTGATAAGTTATCTACACTGGCGAGGGGATTGCTCTCTGTAATGTTCAGCTTCTAAT
TGTCTCTACTTTGTGAGACAACTTTTGAATGCTTGACCTCAAATCAGGTAGGACTACCCG
CTGAACTTAA
>B3-60
TTTCCGTAGGTGAACCTGCGGAAGGATCATTATTGAATTATGTTTCTAGATAGGTTGTAG
CTGGCTCTTTAGAGCATGTGCACGCCTGTTTGGACTTCATTTTCATCCACCTGTGCACCT
ATTGTAGTCTTTGGTTGGGTTAGGGGGAAGTGGTCATTGTGTCAGCATCTGCTGGATGTG
AGGACTTGCATTGTGAAAGCTTTGCTGTCCTTGATGTGATCATGGAATCTCTTTCTCACT
AGAGTCTATGTCACTCATTATACTCTGTCGAATGTCATTGAATGTCTTTACATGGGCTTG
TATGCCTATGAAAATTGTAATACAACTTTCAGCAACGGATCTCTTGGCTCTCGCATCGAT
GAAGGACGCAGCGAAATGCGATAAGTAATGTGAATTGCAGAATTCAGTGAATCATCGAAT
CTTTGAACGCATCTTGCGCTCCTTGGTATTCCGAGGAGCATGCCTGTTTGAGTGTCATTA
AATTCTCAACTCTCTTATACTTTTTTGTAAAAGAGAGCTTGGACTGTGGAGGCTTGCTGG
CCACTTTTTGGGGTCAGCTCCTCTGAAATGCATTAGCGGAACCGTTTGCAATCTGCCACA
AGTGTGATAAGTTATCTACACTGGCGAGGGGATTGCTCTCTGTAATGTTCAGCTTCTAAT
TGTCTCTACTTTGTGAGACAACTTTTGAATGCTTGACCTCAAATCAGGTAGGACTACCCG
CTGAACTTAA
>B3-61
TTTCCGTAGGTGAACCTGCGGAAGGATCATTATTGAATTATGTTTCTAGATAGGTTGTAG
CTGGCTCTTTAGAGCATGTGCACGCCTGTTTGGACTTCATTTTCATCCACCTGTGCACCT
ATTGTAGTCTTTGGTTGGGTTAGGGGGAAGTGGTCATTGTGTCAGCATCTGCTGGATGTG
AGGACTTGCATTGTGAAAGCTTTGCTGTCCTTGATGTGATCATGGAATCTCTTTCTCACT
AGAGTCTATGTCACTCATTATACTCTGTCGAATGTCATTGAATGTCTTTACATGGGCTTG
TATGCCTATGAAAATTGTAATACAACTTTCAGCAACGGATCTCTTGGCTCTCGCATCGAT
GAAGGACGCAGCGAAATGCGATAAGTAATGTGAATTGCAGAATTCAGTGAATCATCGAAT
CTTTGAACGCATCTTGCGCTCCTTGGTATTCCGAGGAGCATGCCTGTTTGAGTGTCATTA
AATTCTCAACTCTCTTATACTTTTTTGTAAAAGAGAGCTTGGACTGTGGAGGCTTGCTGG
CCACTTTTTGGGGTCAGCTCCTCTGAAATGCATTAGCGGAACCGTTTGCAATCTGCCACA
AGTGTGATAAGTTATCTACACTGGCGAGGGGATTGCTCTCTGTAATGTTCAGCTTCTAAT
TGTCTCTACTTTGTGAGACAACTTTTGAATGCTTGACCTCAAATCAGGTAGGACTACCCG
CTGAACTTAA
>B3-63
TTTCCGTAGGTGAACCTGCGGAAGGATCATTATTGAATTATGTTTCTAGATAGGTTGTAG
CTGGCTCTTTAGAGCATGTGCACGCCTGTTTGGACTTCATTTTCATCCACCTGTGCACCT
ATTGTAGTCTTTGGTTGGGTTAGGGGGAAGTGGTCATTGTGTCAGCATCTGCTGGATGTG
AGGACTTGCATTGTGAAAGCTTTGCTGTCCTTGATGTGATCATGGAATCTCTTTCTCACT
AGAGTCTATGTCACTCATTATACTCTGTCGAATGTCATTGAATGTCTTTACATGGGCTTG
TATGCCTATGAAAATTGTAATACAACTTTCAGCAACGGATCTCTTGGCTCTCGCATCGAT
GAAGGACGCAGCGAAATGCGATAAGTAATGTGAATTGCAGAATTCAGTGAATCATCGAAT
CTTTGAACGCATCTTGCGCTCCTTGGTATTCCGAGGAGCATGCCTGTTTGAGTGTCATTA
AATTCTCAACTCTCTTATACTTTTTTGTAAAAGAGAGCTTGGACTGTGGAGGCTTGCTGG
CCACTTTTTGGGGTCAGCTCCTCTGAAATGCATTAGCGGAACCGTTTGCAATCTGCCACA
AGTGTGATAAGTTATCTACACTGGCGAGGGGATTGCTCTCTGTAATGTTCAGCTTCTAAT
TGTCTCTACTTTGTGAGACAACTTTTGAATGCTTGACCTCAAATCAGGTAGGACTACCCG
CTGAACTTAA
>B3-64
TTTCCGTAGGTGAACCTGCGGAAGGATCATTATTGAATTATGTTTCTAGATAGGTTGTAG
CTGGCTCTTTAGAGCATGTGCACGCCTGTTTGGACTTCATTTTCATCCACCTGTGCACCT
ATTGTAGTCTTTGGTTGGGTTAGGGGGAAGTGGTCATTGTGTCAGCATCTGCTGGATGTG

AGGACTTGCATTGTGAAAGCTTTGCTGTCCTTGATGTGATCATGGAATCTCTTTCTCACT
AGAGTCTATGTCACTCATTATACTCTGTCGAATGTCATTGAATGTCTTTACATGGGCTTG
TATGCCTATGAAAATTGTAATACAACTTTCAGCAACGGATCTCTTGGCTCTCGCATCGAT
GAAGGACGCAGCGAAATGCGATAAGTAATGTGAATTGCAGAATTCAGTGAATCATCGAAT
CTTTGAACGCATCTTGCGCTCCTTGGTATTCCGAGGAGCATGCCTGTTTGAGTGTCATTA
AATTCTCAACTCTCTTATACTTTTTTGTAAAAGAGAGCTTGGACTGTGGAGGCTTGCTGG
CCACTTTTTGGGGTCAGCTCCTCTGAAATGCATTAGCGGAACCGTTTGCAATCTGCCACA
AGTGTGATAAGTTATCTACACTGGCGAGGGGATTGCTCTCTGTAATGTTCAGCTTCTAAT
TGTCTCTACTTTGTGAGACAACTTTTGAATGCTTGACCTCAAATCAGGTAGGACTACCCG
CTGAACTTAA
>B3-66
TTTCCGTAGGTGAACCTGCGGAAGGATCATTATTGAATTATGTTTCTAGATAGGTTGTAG
CTGGCTCTTTAGAGCATGTGCACGCCTGTTTGGACTTCATTTTCATCCACCTGTGCACCT
ATTGTAGTCTTTGGTTGGGTTAGGGGGAAGTGGTCATTGTGTCAGCATCTGCTGGATGTG
AGGACTTGCATTGTGAAAGCTTTGCTGTCCTTGATGTGATCATGGAATCTCTTTCTCACT
AGAGTCTATGTCACTCATTATACTCTGTCGAATGTCATTGAATGTCTTTACATGGGCTTG
TATGCCTATGAAAATTGTAATACAACTTTCAGCAACGGATCTCTTGGCTCTCGCATCGAT
GAAGGACGCAGCGAAATGCGATAAGTAATGTGAATTGCAGAATTCAGTGAATCATCGAAT
CTTTGAACGCATCTTGCGCTCCTTGGTATTCCGAGGAGCATGCCTGTTTGAGTGTCATTA
AATTCTCAACTCTCTTATACTTTTTTGTAAAAGAGAGCTTGGACTGTGGAGGCTTGCTGG
CCACTTTTTGGGGTCAGCTCCTCTGAAATGCATTAGCGGAACCGTTTGCAATCTGCCACA
AGTGTGATAAGTTATCTACACTGGCGAGGGGATTGCTCTCTGTAATGTTCAGCTTCTAAT
TGTCTCTACTTTGTGAGACAACTTTTGAATGCTTGACCTCAAATCAGGTAGGACTACCCG
CTGAACTTAA
>B3-67
TTTCCGTAGGTGAACCTGCGGAAGGATCATTATTGAATTATGTTTCTAGATAGGTTGTAG
CTGGCTCTTTAGAGCATGTGCACGCCTGTTTGGACTTCATTTTCATCCACCTGTGCACCT
ATTGTAGTCTTTGGTTGGGTTAGGGGGAAGTGGTCATTGTGTCAGCATCTGCTGGATGTG
AGGACTTGCATTGTGAAAGCTTTGCTGTCCTTGATGTGATCATGGAATCTCTTTCTCACT
AGAGTCTATGTCACTCATTATACTCTGTCGAATGTCATTGAATGTCTTTACATGGGCTTG
TATGCCTATGAAAATTGTAATACAACTTTCAGCAACGGATCTCTTGGCTCTCGCATCGAT
GAAGGACGCAGCGAAATGCGATAAGTAATGTGAATTGCAGAATTCAGTGAATCATCGAAT
CTTTGAACGCATCTTGCGCTCCTTGGTATTCCGAGGAGCATGCCTGTTTGAGTGTCATTA
AATTCTCAACTCTCTTATACTTTTTTGTAAAAGAGAGCTTGGACTGTGGAGGCTTGCTGG
CCACTTTTTGGGGTCAGCTCCTCTGAAATGCATTAGCGGAACCGTTTGCAATCTGCCACA
AGTGTGATAAGTTATCTACACTGGCGAGGGGATTGCTCTCTGTAATGTTCAGCTTCTAAT
TGTCTCTACTTTGTGAGACAACTTTTGAATGCTTGACCTCAAATCAGGTAGGACTACCCG
CTGAACTTAA
>B3-68
TTTCCGTAGGTGAACCTGCGGAAGGATCATTATTGAATTATGTTTCTAGATAGGTTGTAG
CTGGCTCTTTAGAGCATGTGCACGCCTGTTTGGACTTCATTTTCATCCACCTGTGCACCT
ATTGTAGTCTTTGGTTGGGTTAGGGGGAAGTGGTCATTGTGTCAGCATCTGCTGGATGTG
AGGACTTGCATTGTGAAAGCTTTGCTGTCCTTGATGTGATCATGGAATCTCTTTCTCACT
AGAGTCTATGTCACTCATTATACTCTGTCGAATGTCATTGAATGTCTTTACATGGGCTTG
TATGCCTATGAAAATTGTAATACAACTTTCAGCAACGGATCTCTTGGCTCTCGCATCGAT
GAAGGACGCAGCGAAATGCGATAAGTAATGTGAATTGCAGAATTCAGTGAATCATCGAAT
CTTTGAACGCATCTTGCGCTCCTTGGTATTCCGAGGAGCATGCCTGTTTGAGTGTCATTA
AATTCTCAACTCTCTTATACTTTTTTGTAAAAGAGAGCTTGGACTGTGGAGGCTTGCTGG
CCACTTTTTGGGGTCAGCTCCTCTGAAATGCATTAGCGGAACCGTTTGCAATCTGCCACA
AGTGTGATAAGTTATCTACACTGGCGAGGGGATTGCTCTCTGTAATGTTCAGCTTCTAAT

TGTCTCTACTTTGTGAGACAACTTTTGAATGCTTGACCTCAAATCAGGTAGGACTACCCG
CTGAACTTAA
>B3-69
TTTCCGTAGGTGAACCTGCGGAAGGATCATTATTGAATTATGTTTCTAGATAGGTTGTAG
CTGGCTCTTTAGAGCATGTGCACGCCTGTTTGGACTTCATTTTCATCCACCTGTGCACCT
ATTGTAGTCTTTGGTTGGGTTAGGGGGAAGTGGTCATTGTGTCAGCATCTGCTGGATGTG
AGGACTTGCATTGTGAAAGCTTTGCTGTCCTTGATGTGATCATGGAATCTCTTTCTCACT
AGAGTCTATGTCACTCATTATACTCTGTCGAATGTCATTGAATGTCTTTACATGGGCTTG
TATGCCTATGAAAATTGTAATACAACTTTCAGCAACGGATCTCTTGGCTCTCGCATCGAT
GAAGGACGCAGCGAAATGCGATAAGTAATGTGAATTGCAGAATTCAGTGAATCATCGAAT
CTTTGAACGCATCTTGCGCTCCTTGGTATTCCGAGGAGCATGCCTGTTTGAGTGTCATTA
AATTCTCAACTCTCTTATACTTTTTTGTAAAAGAGAGCTTGGACTGTGGAGGCTTGCTGG
CCACTTTTTGGGGTCAGCTCCTCTGAAATGCATTAGCGGAACCGTTTGCAATCTGCCACA
AGTGTGATAAGTTATCTACACTGGCGAGGGGATTGCTCTCTGTAATGTTCAGCTTCTAAT
TGTCTCTACTTTGTGAGACAACTTTTGAATGCTTGACCTCAAATCAGGTAGGACTACCCG
CTGAACTTAA
>B3-70
TTTCCGTAGGTGAACCTGCGGAAGGATCATTATTGAATTATGTTTCTAGATAGGTTGTAG
CTGGCTCTTTAGAGCATGTGCACGCCTGTTTGGACTTCATTTTCATCCACCTGTGCACCT
ATTGTAGTCTTTGGTTGGGTTAGGGGGAAGTGGTCATTGTGTCAGCATCTGCTGGATGTG
AGGACTTGCATTGTGAAAGCTTTGCTGTCCTTGATGTGATCATGGAATCTCTTTCTCACT
AGAGTCTATGTCACTCATTATACTCTGTCGAATGTCATTGAATGTCTTTACATGGGCTTG
TATGCCTATGAAAATTGTAATACAACTTTCAGCAACGGATCTCTTGGCTCTCGCATCGAT
GAAGGACGCAGCGAAATGCGATAAGTAATGTGAATTGCAGAATTCAGTGAATCATCGAAT
CTTTGAACGCATCTTGCGCTCCTTGGTATTCCGAGGAGCATGCCTGTTTGAGTGTCATTA
AATTCTCAACTCTCTTATACTTTTTTGTAAAAGAGAGCTTGGACTGTGGAGGCTTGCTGG
CCACTTTTTGGGGTCAGCTCCTCTGAAATGCATTAGCGGAACCGTTTGCAATCTGCCACA
AGTGTGATAAGTTATCTACACTGGCGAGGGGATTGCTCTCTGTAATGTTCAGCTTCTAAT
TGTCTCTACTTTGTGAGACAACTTTTGAATGCTTGACCTCAAATCAGGTAGGACTACCCG
CTGAACTTAA
>B3-71
TTTCCGTAGGTGAACCTGCGGAAGGATCATTATTGAATTATGTTTCTAGATAGGTTGTAG
CTGGCTCTTTAGAGCATGTGCACGCCTGTTTGGACTTCATTTTCATCCACCTGTGCACCT
ATTGTAGTCTTTGGTTGGGTTAGGGGGAAGTGGTCATTGTGTCAGCATCTGCTGGATGTG
AGGACTTGCATTGTGAAAGCTTTGCTGTCCTTGATGTGATCATGGAATCTCTTTCTCACT
AGAGTCTATGTCACTCATTATACTCTGTCGAATGTCATTGAATGTCTTTACATGGGCTTG
TATGCCTATGAAAATTGTAATACAACTTTCAGCAACGGATCTCTTGGCTCTCGCATCGAT
GAAGGACGCAGCGAAATGCGATAAGTAATGTGAATTGCAGAATTCAGTGAATCATCGAAT
CTTTGAACGCATCTTGCGCTCCTTGGTATTCCGAGGAGCATGCCTGTTTGAGTGTCATTA
AATTCTCAACTCTCTTATACTTTTTTGTAAAAGAGAGCTTGGACTGTGGAGGCTTGCTGG
CCACTTTTTGGGGTCAGCTCCTCTGAAATGCATTAGCGGAACCGTTTGCAATCTGCCACA
AGTGTGATAAGTTATCTACACTGGCGAGGGGATTGCTCTCTGTAATGTTCAGCTTCTAAT
TGTCTCTACTTTGTGAGACAACTTTTGAATGCTTGACCTCAAATCAGGTAGGACTACCCG
CTGAACTTAA
>B3-72
TTTCCGTAGGTGAACCTGCGGAAGGATCATTATTGAATTATGTTTCTAGATAGGTTGTAG
CTGGCTCTTTAGAGCATGTGCACGCCTGTTTGGACTTCATTTTCATCCACCTGTGCACCT
ATTGTAGTCTTTGGTTGGGTTAGGGGGAAGTGGTCATTGTGTCAGCATCTGCTGGATGTG
AGGACTTGCATTGTGAAAGCTTTGCTGTCCTTGATGTGATCATGGAATCTCTTTCTCACT
AGAGTCTATGTCACTCATTATACTCTGTCGAATGTCATTGAATGTCTTTACATGGGCTTG

TATGCCTATGAAAATTGTAATACAACTTTCAGCAACGGATCTCTTGGCTCTCGCATCGAT
GAAGGACGCAGCGAAATGCGATAAGTAATGTGAATTGCAGAATTCAGTGAATCATCGAAT
CTTTGAACGCATCTTGCGCTCCTTGGTATTCCGAGGAGCATGCCTGTTTGAGTGTCATTA
AATTCTCAACTCTCTTATACTTTTTTGTAAAAGAGAGCTTGGACTGTGGAGGCTTGCTGG
CCACTTTTTGGGGTCAGCTCCTCTGAAATGCATTAGCGGAACCGTTTGCAATCTGCCACA
AGTGTGATAAGTTATCTACACTGGCGAGGGGATTGCTCTCTGTAATGTTCAGCTTCTAAT
TGTCTCTACTTTGTGAGACAACTTTTGAATGCTTGACCTCAAATCAGGTAGGACTACCCG
CTGAACTTAA
>B4_1
TTTCCGTAGGTGAACCTGCGGAAGGATCATTATTGAATTATGTTTCTAGATAGGTTGTAG
CTGGCTCTTTAGAGCATGTGCACGCCTGTTTGGACTTCATTTTCATCCACCTGTGCACCT
ATTGTAGTCTTTGGTTGGGTTAGGGGGAAGTGGTCATTGTGTCAGCATCTGCTGGATGTG
AGGACTTGCATTGTGAAAGCTTTGCTGTCCTTGATGTGATCATGGAATCTCTTTCTCACT
AGAGTCTATGTCACTCATTATACTCTGTCGAATGTCATTGAATGTCTTTACATGGGCTTG
TATGCCTATGAAAATTGTAATACAACTTTCAGCAACGGATCTCTTGGCTCTCGCATCGAT
GAAGGACGCAGCGAAATGCGATAAGTAATGTGAATTGCAGAATTCAGTGAATCATCGAAT
CTTTGAACGCATCTTGCGCTCCTTGGTATTCCGAGGAGCATGCCTGTTTGAGTGTCATTA
AATTCTCAACTCTCTTATACTTTTTTGTAAAAGAGAGCTTGGACTGTGGAGGCTTGCTGG
CCACTTTTTGGGGTCAGCTCCTCTGAAATGCATTAGCGGAACCGTTTGCAATCTGCCACA
AGTGTGATAAGTTATCTACACTGGCGAGGGGATTGCTCTCTGTAATGTTCAGCTTCTAAT
TGTCTCTACTTTGTGAGACAACTTTTGAATGCTTGACCTCAAATCAGGTAGGACTACCCG
CTGAACTTAA
>B4_2
TTTCCGTAGGTGAACCTGCGGAAGGATCATTATTGAATTATGTTTCTAGATAGGTTGTAG
CTGGCTCTTTAGAGCATGTGCACGCCTGTTTGGACTTCATTTTCATCCACCTGTGCACCT
ATTGTAGTCTTTGGTTGGGTTAGGGGGAAGTGGTCATTGTGTCAGCATCTGCTGGATGTG
AGGACTTGCATTGTGAAAGCTTTGCTGTCCTTGATGTGATCATGGAATCTCTTTCTCACT
AGAGTCTATGTCACTCATTATACTCTGTCGAATGTCATTGAATGTCTTTACATGGGCTTG
TATGCCTATGAAAATTGTAATACAACTTTCAGCAACGGATCTCTTGGCTCTCGCATCGAT
GAAGGACGCAGCGAAATGCGATAAGTAATGTGAATTGCAGAATTCAGTGAATCATCGAAT
CTTTGAACGCATCTTGCGCTCCTTGGTATTCCGAGGAGCATGCCTGTTTGAGTGTCATTA
AATTCTCAACTCTCTTATACTTTTTTGTAAAAGAGAGCTTGGACTGTGGAGGCTTGCTGG
CCACTTTTTGGGGTCAGCTCCTCTGAAATGCATTAGCGGAACCGTTTGCAATCTGCCACA
AGTGTGATAAGTTATCTACACTGGCGAGGGGATTGCTCTCTGTAATGTTCAGCTTCTAAT
TGTCTCTACTTTGTGAGACAACTTTTGAATGCTTGACCTCAAATCAGGTAGGACTACCCG
CTGAACTTAA
>B4_3
TTTCCGTAGGTGAACCTGCGGAAGGATCATTATTGAATTATGTTTCTAGATAGGTTGTAG
CTGGCTCTTTAGAGCATGTGCACGCCTGTTTGGACTTCATTTTCATCCACCTGTGCACCT
ATTGTAGTCTTTGGTTGGGTTAGGGGGAAGTGGTCATTGTGTCAGCATCTGCTGGATGTG
AGGACTTGCATTGTGAAAGCTTTGCTGTCCTTGATGTGATCATGGAATCTCTTTCTCACT
AGAGTCTATGTCACTCATTATACTCTGTCGAATGTCATTGAATGTCTTTACATGGGCTTG
TATGCCTATGAAAATTGTAATACAACTTTCAGCAACGGATCTCTTGGCTCTCGCATCGAT
GAAGGACGCAGCGAAATGCGATAAGTAATGTGAATTGCAGAATTCAGTGAATCATCGAAT
CTTTGAACGCATCTTGCGCTCCTTGGTATTCCGAGGAGCATGCCTGTTTGAGTGTCATTA
AATTCTCAACTCTCTTATACTTTTTTGTAAAAGAGAGCTTGGACTGTGGAGGCTTGCTGG
CCACTTTTTGGGGTCAGCTCCTCTGAAATGCATTAGCGGAACCGTTTGCAATCTGCCACA
AGTGTGATAAGTTATCTACACTGGCGAGGGGATTGCTCTCTGTAATGTTCAGCTTCTAAT
TGTCTCTACTTTGTGAGACAACTTTTGAATGCTTGACCTCAAATCAGGTAGGACTACCCG
CTGAACTTAA

>B4_5
TTTCCGTAGGTGAACCTGCGGAAGGATCATTATTGAATTATGTTTCTAGATAGGTTGTAG
CTGGCTCTTTAGAGCATGTGCACGCCTGTTTGGACTTCATTTTCATCCACCTGTGCACCT
ATTGTAGTCTTTGGTTGGGTTAGGGGGAAGTGGTCATTGTGTCAGCATCTGCTGGATGTG
AGGACTTGCATTGTGAAAGCTTTGCTGTCCTTGATGTGATCATGGAATCTCTTTCTCACT
AGAGTCTATGTCACTCATTATACTCTGTCGAATGTCATTGAATGTCTTTACATGGGCTTG
TATGCCTATGAAAATTGTAATACAACTTTCAGCAACGGATCTCTTGGCTCTCGCATCGAT
GAAGGACGCAGCGAAATGCGATAAGTAATGTGAATTGCAGAATTCAGTGAATCATCGAAT
CTTTGAACGCATCTTGCGCTCCTTGGTATTCCGAGGAGCATGCCTGTTTGAGTGTCATTA
AATTCTCAACTCTCTTATACTTTTTTGTAAAAGAGAGCTTGGACTGTGGAGGCTTGCTGG
CCACTTTTTGGGGTCAGCTCCTCTGAAATGCATTAGCGGAACCGTTTGCAATCTGCCACA
AGTGTGATAAGTTATCTACACTGGCGAGGGGATTGCTCTCTGTAATGTTCAGCTTCTAAT
TGTCTCTACTTTGTGAGACAACTTTTGAATGCTTGACCTCAAATCAGGTAGGACTACCCG
CTGAACTTAA
>B4_7
TTTCCGTAGGTGAACCTGCGGAAGGATCATTATTGAATTATGTTTCTAGATAGGTTGTAG
CTGGCTCTTTAGAGCATGTGCACGCCTGTTTGGACTTCATTTTCATCCACCTGTGCACCT
ATTGTAGTCTTTGGTTGGGTTAGGGGGAAGTGGTCATTGTGTCAGCATCTGCTGGATGTG
AGGACTTGCATTGTGAAAGCTTTGCTGTCCTTGATGTGATCATGGAATCTCTTTCTCACT
AGAGTCTATGTCACTCATTATACTCTGTCGAATGTCATTGAATGTCTTTACATGGGCTTG
TATGCCTATGAAAATTGTAATACAACTTTCAGCAACGGATCTCTTGGCTCTCGCATCGAT
GAAGGACGCAGCGAAATGCGATAAGTAATGTGAATTGCAGAATTCAGTGAATCATCGAAT
CTTTGAACGCATCTTGCGCTCCTTGGTATTCCGAGGAGCATGCCTGTTTGAGTGTCATTA
AATTCTCAACTCTCTTATACTTTTTTGTAAAAGAGAGCTTGGACTGTGGAGGCTTGCTGG
CCACTTTTTGGGGTCAGCTCCTCTGAAATGCATTAGCGGAACCGTTTGCAATCTGCCACA
AGTGTGATAAGTTATCTACACTGGCGAGGGGATTGCTCTCTGTAATGTTCAGCTTCTAAT
TGTCTCTACTTTGTGAGACAACTTTTGAATGCTTGACCTCAAATCAGGTAGGACTACCCG
CTGAACTTAA
>B4_8
TTTCCGTAGGTGAACCTGCGGAAGGATCATTATTGAATTATGTTTCTAGATAGGTTGTAG
CTGGCTCTTTAGAGCATGTGCACGCCTGTTTGGACTTCATTTTCATCCACCTGTGCACCT
ATTGTAGTCTTTGGTTGGGTTAGGGGGAAGTGGTCATTGTGTCAGCATCTGCTGGATGTG
AGGACTTGCATTGTGAAAGCTTTGCTGTCCTTGATGTGATCATGGAATCTCTTTCTCACT
AGAGTCTATGTCACTCATTATACTCTGTCGAATGTCATTGAATGTCTTTACATGGGCTTG
TATGCCTATGAAAATTGTAATACAACTTTCAGCAACGGATCTCTTGGCTCTCGCATCGAT
GAAGGACGCAGCGAAATGCGATAAGTAATGTGAATTGCAGAATTCAGTGAATCATCGAAT
CTTTGAACGCATCTTGCGCTCCTTGGTATTCCGAGGAGCATGCCTGTTTGAGTGTCATTA
AATTCTCAACTCTCTTATACTTTTTTGTAAAAGAGAGCTTGGACTGTGGAGGCTTGCTGG
CCACTTTTTGGGGTCAGCTCCTCTGAAATGCATTAGCGGAACCGTTTGCAATCTGCCACA
AGTGTGATAAGTTATCTACACTGGCGAGGGGATTGCTCTCTGTAATGTTCAGCTTCTAAT
TGTCTCTACTTTGTGAGACAACTTTTGAATGCTTGACCTCAAATCAGGTAGGACTACCCG
CTGAACTTAA
>B4-9
TTTCCGTAGGTGAACCTGCGGAAGGATCATTATTGAATTATGTTTCTAGATAGGTTGTAG
CTGGCTCTTTAGAGCATGTGCACGCCTGTTTGGACTTCATTTTCATCCACCTGTGCACCT
ATTGTAGTCTTTGGTTGGGTTAGGGGGAAGTGGTCATTGTGTCAGCATCTGCTGGATGTG
AGGACTTGCATTGTGAAAGCTTTGCTGTCCTTGATGTGATCATGGAATCTCTTTCTCACT
AGAGTCTATGTCACTCATTATACTCTGTCGAATGTCATTGAATGTCTTTACATGGGCTTG
TATGCCTATGAAAATTGTAATACAACTTTCAGCAACGGATCTCTTGGCTCTCGCATCGAT
GAAGGACGCAGCGAAATGCGATAAGTAATGTGAATTGCAGAATTCAGTGAATCATCGAAT

CTTTGAACGCATCTTGCGCTCCTTGGTATTCCGAGGAGCATGCCTGTTTGAGTGTCATTA
AATTCTCAACTCTCTTATACTTTTTTGTAAAAGAGAGCTTGGACTGTGGAGGCTTGCTGG
CCACTTTTTGGGGTCAGCTCCTCTGAAATGCATTAGCGGAACCGTTTGCAATCTGCCACA
AGTGTGATAAGTTATCTACACTGGCGAGGGGATTGCTCTCTGTAATGTTCAGCTTCTAAT
TGTCTCTACTTTGTGAGACAACTTTTGAATGCTTGACCTCAAATCAGGTAGGACTACCCG
CTGAACTTAA
>B4-10
TTTCCGTAGGTGAACCTGCGGAAGGATCATTATTGAATTATGTTTCTAGATAGGTTGTAG
CTGGCTCTTTAGAGCATGTGCACGCCTGTTTGGACTTCATTTTCATCCACCTGTGCACCT
ATTGTAGTCTTTGGTTGGGTTAGGGGGAAGTGGTCATTGTGTCAGCATCTGCTGGATGTG
AGGACTTGCATTGTGAAAGCTTTGCTGTCCTTGATGTGATCATGGAATCTCTTTCTCACT
AGAGTCTATGTCACTCATTATACTCTGTCGAATGTCATTGAATGTCTTTACATGGGCTTG
TATGCCTATGAAAATTGTAATACAACTTTCAGCAACGGATCTCTTGGCTCTCGCATCGAT
GAAGGACGCAGCGAAATGCGATAAGTAATGTGAATTGCAGAATTCAGTGAATCATCGAAT
CTTTGAACGCATCTTGCGCTCCTTGGTATTCCGAGGAGCATGCCTGTTTGAGTGTCATTA
AATTCTCAACTCTCTTATACTTTTTTGTAAAAGAGAGCTTGGACTGTGGAGGCTTGCTGG
CCACTTTTTGGGGTCAGCTCCTCTGAAATGCATTAGCGGAACCGTTTGCAATCTGCCACA
AGTGTGATAAGTTATCTACACTGGCGAGGGGATTGCTCTCTGTAATGTTCAGCTTCTAAT
TGTCTCTACTTTGTGAGACAACTTTTGAATGCTTGACCTCAAATCAGGTAGGACTACCCG
CTGAACTTAA
>B4-11
TTTCCGTAGGTGAACCTGCGGAAGGATCATTATTGAATTATGTTTCTAGATAGGTTGTAG
CTGGCTCTTTAGAGCATGTGCACGCCTGTTTGGACTTCATTTTCATCCACCTGTGCACCT
ATTGTAGTCTTTGGTTGGGTTAGGGGGAAGTGGTCATTGTGTCAGCATCTGCTGGATGTG
AGGACTTGCATTGTGAAAGCTTTGCTGTCCTTGATGTGATCATGGAATCTCTTTCTCACT
AGAGTCTATGTCACTCATTATACTCTGTCGAATGTCATTGAATGTCTTTACATGGGCTTG
TATGCCTATGAAAATTGTAATACAACTTTCAGCAACGGATCTCTTGGCTCTCGCATCGAT
GAAGGACGCAGCGAAATGCGATAAGTAATGTGAATTGCAGAATTCAGTGAATCATCGAAT
CTTTGAACGCATCTTGCGCTCCTTGGTATTCCGAGGAGCATGCCTGTTTGAGTGTCATTA
AATTCTCAACTCTCTTATACTTTTTTGTAAAAGAGAGCTTGGACTGTGGAGGCTTGCTGG
CCACTTTTTGGGGTCAGCTCCTCTGAAATGCATTAGCGGAACCGTTTGCAATCTGCCACA
AGTGTGATAAGTTATCTACACTGGCGAGGGGATTGCTCTCTGTAATGTTCAGCTTCTAAT
TGTCTCTACTTTGTGAGACAACTTTTGAATGCTTGACCTCAAATCAGGTAGGACTACCCG
CTGAACTTAA
>B4-12
TTTCCGTAGGTGAACCTGCGGAAGGATCATTATTGAATTATGTTTCTAGATAGGTTGTAG
CTGGCTCTTTAGAGCATGTGCACGCCTGTTTGGACTTCATTTTCATCCACCTGTGCACCT
ATTGTAGTCTTTGGTTGGGTTAGGGGGAAGTGGTCATTGTGTCAGCATCTGCTGGATGTG
AGGACTTGCATTGTGAAAGCTTTGCTGTCCTTGATGTGATCATGGAATCTCTTTCTCACT
AGAGTCTATGTCACTCATTATACTCTGTCGAATGTCATTGAATGTCTTTACATGGGCTTG
TATGCCTATGAAAATTGTAATACAACTTTCAGCAACGGATCTCTTGGCTCTCGCATCGAT
GAAGGACGCAGCGAAATGCGATAAGTAATGTGAATTGCAGAATTCAGTGAATCATCGAAT
CTTTGAACGCATCTTGCGCTCCTTGGTATTCCGAGGAGCATGCCTGTTTGAGTGTCATTA
AATTCTCAACTCTCTTATACTTTTTTGTAAAAGAGAGCTTGGACTGTGGAGGCTTGCTGG
CCACTTTTTGGGGTCAGCTCCTCTGAAATGCATTAGCGGAACCGTTTGCAATCTGCCACA
AGTGTGATAAGTTATCTACACTGGCGAGGGGATTGCTCTCTGTAATGTTCAGCTTCTAAT
TGTCTCTACTTTGTGAGACAACTTTTGAATGCTTGACCTCAAATCAGGTAGGACTACCCG
CTGAACTTAA
>B4-13
TTTCCGTAGGTGAACCTGCGGAAGGATCATTATTGAATTATGTTTCTAGATAGGTTGTAG

CTGGCTCTTTAGAGCATGTGCACGCCTGTTTGGACTTCATTTTCATCCACCTGTGCACCT
ATTGTAGTCTTTGGTTGGGTTAGGGGGAAGTGGTCATTGTGTCAGCATCTGCTGGATGTG
AGGACTTGCATTGTGAAAGCTTTGCTGTCCTTGATGTGATCATGGAATCTCTTTCTCACT
AGAGTCTATGTCACTCATTATACTCTGTCGAATGTCATTGAATGTCTTTACATGGGCTTG
TATGCCTATGAAAATTGTAATACAACTTTCAGCAACGGATCTCTTGGCTCTCGCATCGAT
GAAGGACGCAGCGAAATGCGATAAGTAATGTGAATTGCAGAATTCAGTGAATCATCGAAT
CTTTGAACGCATCTTGCGCTCCTTGGTATTCCGAGGAGCATGCCTGTTTGAGTGTCATTA
AATTCTCAACTCTCTTATACTTTTTTGTAAAAGAGAGCTTGGACTGTGGAGGCTTGCTGG
CCACTTTTTGGGGTCAGCTCCTCTGAAATGCATTAGCGGAACCGTTTGCAATCTGCCACA
AGTGTGATAAGTTATCTACACTGGCGAGGGGATTGCTCTCTGTAATGTTCAGCTTCTAAT
TGTCTCTACTTTGTGAGACAACTTTTGAATGCTTGACCTCAAATCAGGTAGGACTACCCG
CTGAACTTAA
>B4-14
TTTCCGTAGGTGAACCTGCGGAAGGATCATTATTGAATTATGTTTCTAGATAGGTTGTAG
CTGGCTCTTTAGAGCATGTGCACGCCTGTTTGGACTTCATTTTCATCCACCTGTGCACCT
ATTGTAGTCTTTGGTTGGGTTAGGGGGAAGTGGTCATTGTGTCAGCATCTGCTGGATGTG
AGGACTTGCATTGTGAAAGCTTTGCTGTCCTTGATGTGATCATGGAATCTCTTTCTCACT
AGAGTCTATGTCACTCATTATACTCTGTCGAATGTCATTGAATGTCTTTACATGGGCTTG
TATGCCTATGAAAATTGTAATACAACTTTCAGCAACGGATCTCTTGGCTCTCGCATCGAT
GAAGGACGCAGCGAAATGCGATAAGTAATGTGAATTGCAGAATTCAGTGAATCATCGAAT
CTTTGAACGCATCTTGCGCTCCTTGGTATTCCGAGGAGCATGCCTGTTTGAGTGTCATTA
AATTCTCAACTCTCTTATACTTTTTTGTAAAAGAGAGCTTGGACTGTGGAGGCTTGCTGG
CCACTTTTTGGGGTCAGCTCCTCTGAAATGCATTAGCGGAACCGTTTGCAATCTGCCACA
AGTGTGATAAGTTATCTACACTGGCGAGGGGATTGCTCTCTGTAATGTTCAGCTTCTAAT
TGTCTCTACTTTGTGAGACAACTTTTGAATGCTTGACCTCAAATCAGGTAGGACTACCCG
CTGAACTTAA
>B4_15
TTTCCGTAGGTGAACCTGCGGAAGGATCATTATTGAATTATGTTTCTAGATAGGTTGTAG
CTGGCTCTTTAGAGCATGTGCACGCCTGTTTGGACTTCATTTTCATCCACCTGTGCACCT
ATTGTAGTCTTTGGTTGGGTTAGGGGGAAGTGGTCATTGTGTCAGCATCTGCTGGATGTG
AGGACTTGCATTGTGAAAGCTTTGCTGTCCTTGATGTGATCATGGAATCTCTTTCTCACT
AGAGTCTATGTCACTCATTATACTCTGTCGAATGTCATTGAATGTCTTTACATGGGCTTG
TATGCCTATGAAAATTGTAATACAACTTTCAGCAACGGATCTCTTGGCTCTCGCATCGAT
GAAGGACGCAGCGAAATGCGATAAGTAATGTGAATTGCAGAATTCAGTGAATCATCGAAT
CTTTGAACGCATCTTGCGCTCCTTGGTATTCCGAGGAGCATGCCTGTTTGAGTGTCATTA
AATTCTCAACTCTCTTATACTTTTTTGTAAAAGAGAGCTTGGACTGTGGAGGCTTGCTGG
CCACTTTTTGGGGTCAGCTCCTCTGAAATGCATTAGCGGAACCGTTTGCAATCTGCCACA
AGTGTGATAAGTTATCTACACTGGCGAGGGGATTGCTCTCTGTAATGTTCAGCTTCTAAT
TGTCTCTACTTTGTGAGACAACTTTTGAATGCTTGACCTCAAATCAGGTAGGACTACCCG
CTGAACTTAA
>B4_16
TTTCCGTAGGTGAACCTGCGGAAGGATCATTATTGAATTATGTTTCTAGATAGGTTGTAG
CTGGCTCTTTAGAGCATGTGCACGCCTGTTTGGACTTCATTTTCATCCACCTGTGCACCT
ATTGTAGTCTTTGGTTGGGTTAGGGGGAAGTGGTCATTGTGTCAGCATCTGCTGGATGTG
AGGACTTGCATTGTGAAAGCTTTGCTGTCCTTGATGTGATCATGGAATCTCTTTCTCACT
AGAGTCTATGTCACTCATTATACTCTGTCGAATGTCATTGAATGTCTTTACATGGGCTTG
TATGCCTATGAAAATTGTAATACAACTTTCAGCAACGGATCTCTTGGCTCTCGCATCGAT
GAAGGACGCAGCGAAATGCGATAAGTAATGTGAATTGCAGAATTCAGTGAATCATCGAAT
CTTTGAACGCATCTTGCGCTCCTTGGTATTCCGAGGAGCATGCCTGTTTGAGTGTCATTA
AATTCTCAACTCTCTTATACTTTTTTGTAAAAGAGAGCTTGGACTGTGGAGGCTTGCTGG

CCACTTTTTGGGGTCAGCTCCTCTGAAATGCATTAGCGGAACCGTTTGCAATCTGCCACA
AGTGTGATAAGTTATCTACACTGGCGAGGGGATTGCTCTCTGTAATGTTCAGCTTCTAAT
TGTCTCTACTTTGTGAGACAACTTTTGAATGCTTGACCTCAAATCAGGTAGGACTACCCG
CTGAACTTAA
>B4_17
TTTCCGTAGGTGAACCTGCGGAAGGATCATTATTGAATTATGTTTCTAGATAGGTTGTAG
CTGGCTCTTTAGAGCATGTGCACGCCTGTTTGGACTTCATTTTCATCCACCTGTGCACCT
ATTGTAGTCTTTGGTTGGGTTAGGGGGAAGTGGTCATTGTGTCAGCATCTGCTGGATGTG
AGGACTTGCATTGTGAAAGCTTTGCTGTCCTTGATGTGATCATGGAATCTCTTTCTCACT
AGAGTCTATGTCACTCATTATACTCTGTCGAATGTCATTGAATGTCTTTACATGGGCTTG
TATGCCTATGAAAATTGTAATACAACTTTCAGCAACGGATCTCTTGGCTCTCGCATCGAT
GAAGGACGCAGCGAAATGCGATAAGTAATGTGAATTGCAGAATTCAGTGAATCATCGAAT
CTTTGAACGCATCTTGCGCTCCTTGGTATTCCGAGGAGCATGCCTGTTTGAGTGTCATTA
AATTCTCAACTCTCTTATACTTTTTTGTAAAAGAGAGCTTGGACTGTGGAGGCTTGCTGG
CCACTTTTTGGGGTCAGCTCCTCTGAAATGCATTAGCGGAACCGTTTGCAATCTGCCACA
AGTGTGATAAGTTATCTACACTGGCGAGGGGATTGCTCTCTGTAATGTTCAGCTTCTAAT
TGTCTCTACTTTGTGAGACAACTTTTGAATGCTTGACCTCAAATCAGGTAGGACTACCCG
CTGAACTTAA
>B4_18
TTTCCGTAGGTGAACCTGCGGAAGGATCATTATTGAATTATGTTTCTAGATAGGTTGTAG
CTGGCTCTTTAGAGCATGTGCACGCCTGTTTGGACTTCATTTTCATCCACCTGTGCACCT
ATTGTAGTCTTTGGTTGGGTTAGGGGGAAGTGGTCATTGTGTCAGCATCTGCTGGATGTG
AGGACTTGCATTGTGAAAGCTTTGCTGTCCTTGATGTGATCATGGAATCTCTTTCTCACT
AGAGTCTATGTCACTCATTATACTCTGTCGAATGTCATTGAATGTCTTTACATGGGCTTG
TATGCCTATGAAAATTGTAATACAACTTTCAGCAACGGATCTCTTGGCTCTCGCATCGAT
GAAGGACGCAGCGAAATGCGATAAGTAATGTGAATTGCAGAATTCAGTGAATCATCGAAT
CTTTGAACGCATCTTGCGCTCCTTGGTATTCCGAGGAGCATGCCTGTTTGAGTGTCATTA
AATTCTCAACTCTCTTATACTTTTTTGTAAAAGAGAGCTTGGACTGTGGAGGCTTGCTGG
CCACTTTTTGGGGTCAGCTCCTCTGAAATGCATTAGCGGAACCGTTTGCAATCTGCCACA
AGTGTGATAAGTTATCTACACTGGCGAGGGGATTGCTCTCTGTAATGTTCAGCTTCTAAT
TGTCTCTACTTTGTGAGACAACTTTTGAATGCTTGACCTCAAATCAGGTAGGACTACCCG
CTGAACTTAA
>B4_19
TTTCCGTAGGTGAACCTGCGGAAGGATCATTATTGAATTATGTTTCTAGATAGGTTGTAG
CTGGCTCTTTAGAGCATGTGCACGCCTGTTTGGACTTCATTTTCATCCACCTGTGCACCT
ATTGTAGTCTTTGGTTGGGTTAGGGGGAAGTGGTCATTGTGTCAGCATCTGCTGGATGTG
AGGACTTGCATTGTGAAAGCTTTGCTGTCCTTGATGTGATCATGGAATCTCTTTCTCACT
AGAGTCTATGTCACTCATTATACTCTGTCGAATGTCATTGAATGTCTTTACATGGGCTTG
TATGCCTATGAAAATTGTAATACAACTTTCAGCAACGGATCTCTTGGCTCTCGCATCGAT
GAAGGACGCAGCGAAATGCGATAAGTAATGTGAATTGCAGAATTCAGTGAATCATCGAAT
CTTTGAACGCATCTTGCGCTCCTTGGTATTCCGAGGAGCATGCCTGTTTGAGTGTCATTA
AATTCTCAACTCTCTTATACTTTTTTGTAAAAGAGAGCTTGGACTGTGGAGGCTTGCTGG
CCACTTTTTGGGGTCAGCTCCTCTGAAATGCATTAGCGGAACCGTTTGCAATCTGCCACA
AGTGTGATAAGTTATCTACACTGGCGAGGGGATTGCTCTCTGTAATGTTCAGCTTCTAAT
TGTCTCTACTTTGTGAGACAACTTTTGAATGCTTGACCTCAAATCAGGTAGGACTACCCG
CTGAACTTAA
>B4_20
TTTCCGTAGGTGAACCTGCGGAAGGATCATTATTGAATTATGTTTCTAGATAGGTTGTAG
CTGGCTCTTTAGAGCATGTGCACGCCTGTTTGGACTTCATTTTCATCCACCTGTGCACCT
ATTGTAGTCTTTGGTTGGGTTAGGGGGAAGTGGTCATTGTGTCAGCATCTGCTGGATGTG

AGGACTTGCATTGTGAAAGCTTTGCTGTCCTTGATGTGATCATGGAATCTCTTTCTCACT
AGAGTCTATGTCACTCATTATACTCTGTCGAATGTCATTGAATGTCTTTACATGGGCTTG
TATGCCTATGAAAATTGTAATACAACTTTCAGCAACGGATCTCTTGGCTCTCGCATCGAT
GAAGGACGCAGCGAAATGCGATAAGTAATGTGAATTGCAGAATTCAGTGAATCATCGAAT
CTTTGAACGCATCTTGCGCTCCTTGGTATTCCGAGGAGCATGCCTGTTTGAGTGTCATTA
AATTCTCAACTCTCTTATACTTTTTTGTAAAAGAGAGCTTGGACTGTGGAGGCTTGCTGG
CCACTTTTTGGGGTCAGCTCCTCTGAAATGCATTAGCGGAACCGTTTGCAATCTGCCACA
AGTGTGATAAGTTATCTACACTGGCGAGGGGATTGCTCTCTGTAATGTTCAGCTTCTAAT
TGTCTCTACTTTGTGAGACAACTTTTGAATGCTTGACCTCAAATCAGGTAGGACTACCCG
CTGAACTTAA
>B4_21
TTTCCGTAGGTGAACCTGCGGAAGGATCATTATTGAATTATGTTTCTAGATAGGTTGTAG
CTGGCTCTTTAGAGCATGTGCACGCCTGTTTGGACTTCATTTTCATCCACCTGTGCACCT
ATTGTAGTCTTTGGTTGGGTTAGGGGGAAGTGGTCATTGTGTCAGCATCTGCTGGATGTG
AGGACTTGCATTGTGAAAGCTTTGCTGTCCTTGATGTGATCATGGAATCTCTTTCTCACT
AGAGTCTATGTCACTCATTATACTCTGTCGAATGTCATTGAATGTCTTTACATGGGCTTG
TATGCCTATGAAAATTGTAATACAACTTTCAGCAACGGATCTCTTGGCTCTCGCATCGAT
GAAGGACGCAGCGAAATGCGATAAGTAATGTGAATTGCAGAATTCAGTGAATCATCGAAT
CTTTGAACGCATCTTGCGCTCCTTGGTATTCCGAGGAGCATGCCTGTTTGAGTGTCATTA
AATTCTCAACTCTCTTATACTTTTTTGTAAAAGAGAGCTTGGACTGTGGAGGCTTGCTGG
CCACTTTTTGGGGTCAGCTCCTCTGAAATGCATTAGCGGAACCGTTTGCAATCTGCCACA
AGTGTGATAAGTTATCTACACTGGCGAGGGGATTGCTCTCTGTAATGTTCAGCTTCTAAT
TGTCTCTACTTTGTGAGACAACTTTTGAATGCTTGACCTCAAATCAGGTAGGACTACCCG
CTGAACTTAA
>B4_22
TTTCCGTAGGTGAACCTGCGGAAGGATCATTATTGAATTATGTTTCTAGATAGGTTGTAG
CTGGCTCTTTAGAGCATGTGCACGCCTGTTTGGACTTCATTTTCATCCACCTGTGCACCT
ATTGTAGTCTTTGGTTGGGTTAGGGGGAAGTGGTCATTGTGTCAGCATCTGCTGGATGTG
AGGACTTGCATTGTGAAAGCTTTGCTGTCCTTGATGTGATCATGGAATCTCTTTCTCACT
AGAGTCTATGTCACTCATTATACTCTGTCGAATGTCATTGAATGTCTTTACATGGGCTTG
TATGCCTATGAAAATTGTAATACAACTTTCAGCAACGGATCTCTTGGCTCTCGCATCGAT
GAAGGACGCAGCGAAATGCGATAAGTAATGTGAATTGCAGAATTCAGTGAATCATCGAAT
CTTTGAACGCATCTTGCGCTCCTTGGTATTCCGAGGAGCATGCCTGTTTGAGTGTCATTA
AATTCTCAACTCTCTTATACTTTTTTGTAAAAGAGAGCTTGGACTGTGGAGGCTTGCTGG
CCACTTTTTGGGGTCAGCTCCTCTGAAATGCATTAGCGGAACCGTTTGCAATCTGCCACA
AGTGTGATAAGTTATCTACACTGGCGAGGGGATTGCTCTCTGTAATGTTCAGCTTCTAAT
TGTCTCTACTTTGTGAGACAACTTTTGAATGCTTGACCTCAAATCAGGTAGGACTACCCG
CTGAACTTAA
>B4_23
TTTCCGTAGGTGAACCTGCGGAAGGATCATTATTGAATTATGTTTCTAGATAGGTTGTAG
CTGGCTCTTTAGAGCATGTGCACGCCTGTTTGGACTTCATTTTCATCCACCTGTGCACCT
ATTGTAGTCTTTGGTTGGGTTAGGGGGAAGTGGTCATTGTGTCAGCATCTGCTGGATGTG
AGGACTTGCATTGTGAAAGCTTTGCTGTCCTTGATGTGATCATGGAATCTCTTTCTCACT
AGAGTCTATGTCACTCATTATACTCTGTCGAATGTCATTGAATGTCTTTACATGGGCTTG
TATGCCTATGAAAATTGTAATACAACTTTCAGCAACGGATCTCTTGGCTCTCGCATCGAT
GAAGGACGCAGCGAAATGCGATAAGTAATGTGAATTGCAGAATTCAGTGAATCATCGAAT
CTTTGAACGCATCTTGCGCTCCTTGGTATTCCGAGGAGCATGCCTGTTTGAGTGTCATTA
AATTCTCAACTCTCTTATACTTTTTTGTAAAAGAGAGCTTGGACTGTGGAGGCTTGCTGG
CCACTTTTTGGGGTCAGCTCCTCTGAAATGCATTAGCGGAACCGTTTGCAATCTGCCACA
AGTGTGATAAGTTATCTACACTGGCGAGGGGATTGCTCTCTGTAATGTTCAGCTTCTAAT

TGTCTCTACTTTGTGAGACAACTTTTGAATGCTTGACCTCAAATCAGGTAGGACTACCCG
CTGAACTTAA
>B4_24
TTTCCGTAGGTGAACCTGCGGAAGGATCATTATTGAATTATGTTTCTAGATAGGTTGTAG
CTGGCTCTTTAGAGCATGTGCACGCCTGTTTGGACTTCATTTTCATCCACCTGTGCACCT
ATTGTAGTCTTTGGTTGGGTTAGGGGGAAGTGGTCATTGTGTCAGCATCTGCTGGATGTG
AGGACTTGCATTGTGAAAGCTTTGCTGTCCTTGATGTGATCATGGAATCTCTTTCTCACT
AGAGTCTATGTCACTCATTATACTCTGTCGAATGTCATTGAATGTCTTTACATGGGCTTG
TATGCCTATGAAAATTGTAATACAACTTTCAGCAACGGATCTCTTGGCTCTCGCATCGAT
GAAGGACGCAGCGAAATGCGATAAGTAATGTGAATTGCAGAATTCAGTGAATCATCGAAT
CTTTGAACGCATCTTGCGCTCCTTGGTATTCCGAGGAGCATGCCTGTTTGAGTGTCATTA
AATTCTCAACTCTCTTATACTTTTTTGTAAAAGAGAGCTTGGACTGTGGAGGCTTGCTGG
CCACTTTTTGGGGTCAGCTCCTCTGAAATGCATTAGCGGAACCGTTTGCAATCTGCCACA
AGTGTGATAAGTTATCTACACTGGCGAGGGGATTGCTCTCTGTAATGTTCAGCTTCTAAT
TGTCTCTACTTTGTGAGACAACTTTTGAATGCTTGACCTCAAATCAGGTAGGACTACCCG
CTGAACTTAA
>B4_25
TTTCCGTAGGTGAACCTGCGGAAGGATCATTATTGAATTATGTTTCTAGATAGGTTGTAG
CTGGCTCTTTAGAGCATGTGCACGCCTGTTTGGACTTCATTTTCATCCACCTGTGCACCT
ATTGTAGTCTTTGGTTGGGTTAGGGGGAAGTGGTCATTGTGTCAGCATCTGCTGGATGTG
AGGACTTGCATTGTGAAAGCTTTGCTGTCCTTGATGTGATCATGGAATCTCTTTCTCACT
AGAGTCTATGTCACTCATTATACTCTGTCGAATGTCATTGAATGTCTTTACATGGGCTTG
TATGCCTATGAAAATTGTAATACAACTTTCAGCAACGGATCTCTTGGCTCTCGCATCGAT
GAAGGACGCAGCGAAATGCGATAAGTAATGTGAATTGCAGAATTCAGTGAATCATCGAAT
CTTTGAACGCATCTTGCGCTCCTTGGTATTCCGAGGAGCATGCCTGTTTGAGTGTCATTA
AATTCTCAACTCTCTTATACTTTTTTGTAAAAGAGAGCTTGGACTGTGGAGGCTTGCTGG
CCACTTTTTGGGGTCAGCTCCTCTGAAATGCATTAGCGGAACCGTTTGCAATCTGCCACA
AGTGTGATAAGTTATCTACACTGGCGAGGGGATTGCTCTCTGTAATGTTCAGCTTCTAAT
TGTCTCTACTTTGTGAGACAACTTTTGAATGCTTGACCTCAAATCAGGTAGGACTACCCG
CTGAACTTAA
>B4_26
TTTCCGTAGGTGAACCTGCGGAAGGATCATTATTGAATTATGTTTCTAGATAGGTTGTAG
CTGGCTCTTTAGAGCATGTGCACGCCTGTTTGGACTTCATTTTCATCCACCTGTGCACCT
ATTGTAGTCTTTGGTTGGGTTAGGGGGAAGTGGTCATTGTGTCAGCATCTGCTGGATGTG
AGGACTTGCATTGTGAAAGCTTTGCTGTCCTTGATGTGATCATGGAATCTCTTTCTCACT
AGAGTCTATGTCACTCATTATACTCTGTCGAATGTCATTGAATGTCTTTACATGGGCTTG
TATGCCTATGAAAATTGTAATACAACTTTCAGCAACGGATCTCTTGGCTCTCGCATCGAT
GAAGGACGCAGCGAAATGCGATAAGTAATGTGAATTGCAGAATTCAGTGAATCATCGAAT
CTTTGAACGCATCTTGCGCTCCTTGGTATTCCGAGGAGCATGCCTGTTTGAGTGTCATTA
AATTCTCAACTCTCTTATACTTTTTTGTAAAAGAGAGCTTGGACTGTGGAGGCTTGCTGG
CCACTTTTTGGGGTCAGCTCCTCTGAAATGCATTAGCGGAACCGTTTGCAATCTGCCACA
AGTGTGATAAGTTATCTACACTGGCGAGGGGATTGCTCTCTGTAATGTTCAGCTTCTAAT
TGTCTCTACTTTGTGAGACAACTTTTGAATGCTTGACCTCAAATCAGGTAGGACTACCCG
CTGAACTTAA
>B4_27
TTTCCGTAGGTGAACCTGCGGAAGGATCATTATTGAATTATGTTTCTAGATAGGTTGTAG
CTGGCTCTTTAGAGCATGTGCACGCCTGTTTGGACTTCATTTTCATCCACCTGTGCACCT
ATTGTAGTCTTTGGTTGGGTTAGGGGGAAGTGGTCATTGTGTCAGCATCTGCTGGATGTG
AGGACTTGCATTGTGAAAGCTTTGCTGTCCTTGATGTGATCATGGAATCTCTTTCTCACT
AGAGTCTATGTCACTCATTATACTCTGTCGAATGTCATTGAATGTCTTTACATGGGCTTG

TATGCCTATGAAAATTGTAATACAACTTTCAGCAACGGATCTCTTGGCTCTCGCATCGAT
GAAGGACGCAGCGAAATGCGATAAGTAATGTGAATTGCAGAATTCAGTGAATCATCGAAT
CTTTGAACGCATCTTGCGCTCCTTGGTATTCCGAGGAGCATGCCTGTTTGAGTGTCATTA
AATTCTCAACTCTCTTATACTTTTTTGTAAAAGAGAGCTTGGACTGTGGAGGCTTGCTGG
CCACTTTTTGGGGTCAGCTCCTCTGAAATGCATTAGCGGAACCGTTTGCAATCTGCCACA
AGTGTGATAAGTTATCTACACTGGCGAGGGGATTGCTCTCTGTAATGTTCAGCTTCTAAT
TGTCTCTACTTTGTGAGACAACTTTTGAATGCTTGACCTCAAATCAGGTAGGACTACCCG
CTGAACTTAA
>B4_28
TTTCCGTAGGTGAACCTGCGGAAGGATCATTATTGAATTATGTTTCTAGATAGGTTGTAG
CTGGCTCTTTAGAGCATGTGCACGCCTGTTTGGACTTCATTTTCATCCACCTGTGCACCT
ATTGTAGTCTTTGGTTGGGTTAGGGGGAAGTGGTCATTGTGTCAGCATCTGCTGGATGTG
AGGACTTGCATTGTGAAAGCTTTGCTGTCCTTGATGTGATCATGGAATCTCTTTCTCACT
AGAGTCTATGTCACTCATTATACTCTGTCGAATGTCATTGAATGTCTTTACATGGGCTTG
TATGCCTATGAAAATTGTAATACAACTTTCAGCAACGGATCTCTTGGCTCTCGCATCGAT
GAAGGACGCAGCGAAATGCGATAAGTAATGTGAATTGCAGAATTCAGTGAATCATCGAAT
CTTTGAACGCATCTTGCGCTCCTTGGTATTCCGAGGAGCATGCCTGTTTGAGTGTCATTA
AATTCTCAACTCTCTTATACTTTTTTGTAAAAGAGAGCTTGGACTGTGGAGGCTTGCTGG
CCACTTTTTGGGGTCAGCTCCTCTGAAATGCATTAGCGGAACCGTTTGCAATCTGCCACA
AGTGTGATAAGTTATCTACACTGGCGAGGGGATTGCTCTCTGTAATGTTCAGCTTCTAAT
TGTCTCTACTTTGTGAGACAACTTTTGAATGCTTGACCTCAAATCAGGTAGGACTACCCG
CTGAACTTAA
>B4_29
TTTCCGTAGGTGAACCTGCGGAAGGATCATTATTGAATTATGTTTCTAGATAGGTTGTAG
CTGGCTCTTTAGAGCATGTGCACGCCTGTTTGGACTTCATTTTCATCCACCTGTGCACCT
ATTGTAGTCTTTGGTTGGGTTAGGGGGAAGTGGTCATTGTGTCAGCATCTGCTGGATGTG
AGGACTTGCATTGTGAAAGCTTTGCTGTCCTTGATGTGATCATGGAATCTCTTTCTCACT
AGAGTCTATGTCACTCATTATACTCTGTCGAATGTCATTGAATGTCTTTACATGGGCTTG
TATGCCTATGAAAATTGTAATACAACTTTCAGCAACGGATCTCTTGGCTCTCGCATCGAT
GAAGGACGCAGCGAAATGCGATAAGTAATGTGAATTGCAGAATTCAGTGAATCATCGAAT
CTTTGAACGCATCTTGCGCTCCTTGGTATTCCGAGGAGCATGCCTGTTTGAGTGTCATTA
AATTCTCAACTCTCTTATACTTTTTTGTAAAAGAGAGCTTGGACTGTGGAGGCTTGCTGG
CCACTTTTTGGGGTCAGCTCCTCTGAAATGCATTAGCGGAACCGTTTGCAATCTGCCACA
AGTGTGATAAGTTATCTACACTGGCGAGGGGATTGCTCTCTGTAATGTTCAGCTTCTAAT
TGTCTCTACTTTGTGAGACAACTTTTGAATGCTTGACCTCAAATCAGGTAGGACTACCCG
CTGAACTTAA
>B4_30
TTTCCGTAGGTGAACCTGCGGAAGGATCATTATTGAATTATGTTTCTAGATAGGTTGTAG
CTGGCTCTTTAGAGCATGTGCACGCCTGTTTGGACTTCATTTTCATCCACCTGTGCACCT
ATTGTAGTCTTTGGTTGGGTTAGGGGGAAGTGGTCATTGTGTCAGCATCTGCTGGATGTG
AGGACTTGCATTGTGAAAGCTTTGCTGTCCTTGATGTGATCATGGAATCTCTTTCTCACT
AGAGTCTATGTCACTCATTATACTCTGTCGAATGTCATTGAATGTCTTTACATGGGCTTG
TATGCCTATGAAAATTGTAATACAACTTTCAGCAACGGATCTCTTGGCTCTCGCATCGAT
GAAGGACGCAGCGAAATGCGATAAGTAATGTGAATTGCAGAATTCAGTGAATCATCGAAT
CTTTGAACGCATCTTGCGCTCCTTGGTATTCCGAGGAGCATGCCTGTTTGAGTGTCATTA
AATTCTCAACTCTCTTATACTTTTTTGTAAAAGAGAGCTTGGACTGTGGAGGCTTGCTGG
CCACTTTTTGGGGTCAGCTCCTCTGAAATGCATTAGCGGAACCGTTTGCAATCTGCCACA
AGTGTGATAAGTTATCTACACTGGCGAGGGGATTGCTCTCTGTAATGTTCAGCTTCTAAT
TGTCTCTACTTTGTGAGACAACTTTTGAATGCTTGACCTCAAATCAGGTAGGACTACCCG
CTGAACTTAA

>B4_31
TTTCCGTAGGTGAACCTGCGGAAGGATCATTATTGAATTATGTTTCTAGATAGGTTGTAG
CTGGCTCTTTAGAGCATGTGCACGCCTGTTTGGACTTCATTTTCATCCACCTGTGCACCT
ATTGTAGTCTTTGGTTGGGTTAGGGGGAAGTGGTCATTGTGTCAGCATCTGCTGGATGTG
AGGACTTGCATTGTGAAAGCTTTGCTGTCCTTGATGTGATCATGGAATCTCTTTCTCACT
AGAGTCTATGTCACTCATTATACTCTGTCGAATGTCATTGAATGTCTTTACATGGGCTTG
TATGCCTATGAAAATTGTAATACAACTTTCAGCAACGGATCTCTTGGCTCTCGCATCGAT
GAAGGACGCAGCGAAATGCGATAAGTAATGTGAATTGCAGAATTCAGTGAATCATCGAAT
CTTTGAACGCATCTTGCGCTCCTTGGTATTCCGAGGAGCATGCCTGTTTGAGTGTCATTA
AATTCTCAACTCTCTTATACTTTTTTGTAAAAGAGAGCTTGGACTGTGGAGGCTTGCTGG
CCACTTTTTGGGGTCAGCTCCTCTGAAATGCATTAGCGGAACCGTTTGCAATCTGCCACA
AGTGTGATAAGTTATCTACACTGGCGAGGGGATTGCTCTCTGTAATGTTCAGCTTCTAAT
TGTCTCTACTTTGTGAGACAACTTTTGAATGCTTGACCTCAAATCAGGTAGGACTACCCG
CTGAACTTAA
>B4_32
TTTCCGTAGGTGAACCTGCGGAAGGATCATTATTGAATTATGTTTCTAGATAGGTTGTAG
CTGGCTCTTTAGAGCATGTGCACGCCTGTTTGGACTTCATTTTCATCCACCTGTGCACCT
ATTGTAGTCTTTGGTTGGGTTAGGGGGAAGTGGTCATTGTGTCAGCATCTGCTGGATGTG
AGGACTTGCATTGTGAAAGCTTTGCTGTCCTTGATGTGATCATGGAATCTCTTTCTCACT
AGAGTCTATGTCACTCATTATACTCTGTCGAATGTCATTGAATGTCTTTACATGGGCTTG
TATGCCTATGAAAATTGTAATACAACTTTCAGCAACGGATCTCTTGGCTCTCGCATCGAT
GAAGGACGCAGCGAAATGCGATAAGTAATGTGAATTGCAGAATTCAGTGAATCATCGAAT
CTTTGAACGCATCTTGCGCTCCTTGGTATTCCGAGGAGCATGCCTGTTTGAGTGTCATTA
AATTCTCAACTCTCTTATACTTTTTTGTAAAAGAGAGCTTGGACTGTGGAGGCTTGCTGG
CCACTTTTTGGGGTCAGCTCCTCTGAAATGCATTAGCGGAACCGTTTGCAATCTGCCACA
AGTGTGATAAGTTATCTACACTGGCGAGGGGATTGCTCTCTGTAATGTTCAGCTTCTAAT
TGTCTCTACTTTGTGAGACAACTTTTGAATGCTTGACCTCAAATCAGGTAGGACTACCCG
CTGAACTTAA
>B4_33
TTTCCGTAGGTGAACCTGCGGAAGGATCATTATTGAATTATGTTTCTAGATAGGTTGTAG
CTGGCTCTTTAGAGCATGTGCACGCCTGTTTGGACTTCATTTTCATCCACCTGTGCACCT
ATTGTAGTCTTTGGTTGGGTTAGGGGGAAGTGGTCATTGTGTCAGCATCTGCTGGATGTG
AGGACTTGCATTGTGAAAGCTTTGCTGTCCTTGATGTGATCATGGAATCTCTTTCTCACT
AGAGTCTATGTCACTCATTATACTCTGTCGAATGTCATTGAATGTCTTTACATGGGCTTG
TATGCCTATGAAAATTGTAATACAACTTTCAGCAACGGATCTCTTGGCTCTCGCATCGAT
GAAGGACGCAGCGAAATGCGATAAGTAATGTGAATTGCAGAATTCAGTGAATCATCGAAT
CTTTGAACGCATCTTGCGCTCCTTGGTATTCCGAGGAGCATGCCTGTTTGAGTGTCATTA
AATTCTCAACTCTCTTATACTTTTTTGTAAAAGAGAGCTTGGACTGTGGAGGCTTGCTGG
CCACTTTTTGGGGTCAGCTCCTCTGAAATGCATTAGCGGAACCGTTTGCAATCTGCCACA
AGTGTGATAAGTTATCTACACTGGCGAGGGGATTGCTCTCTGTAATGTTCAGCTTCTAAT
TGTCTCTACTTTGTGAGACAACTTTTGAATGCTTGACCTCAAATCAGGTAGGACTACCCG
CTGAACTTAA
>B4_34
TTTCCGTAGGTGAACCTGCGGAAGGATCATTATTGAATTATGTTTCTAGATAGGTTGTAG
CTGGCTCTTTAGAGCATGTGCACGCCTGTTTGGACTTCATTTTCATCCACCTGTGCACCT
ATTGTAGTCTTTGGTTGGGTTAGGGGGAAGTGGTCATTGTGTCAGCATCTGCTGGATGTG
AGGACTTGCATTGTGAAAGCTTTGCTGTCCTTGATGTGATCATGGAATCTCTTTCTCACT
AGAGTCTATGTCACTCATTATACTCTGTCGAATGTCATTGAATGTCTTTACATGGGCTTG
TATGCCTATGAAAATTGTAATACAACTTTCAGCAACGGATCTCTTGGCTCTCGCATCGAT
GAAGGACGCAGCGAAATGCGATAAGTAATGTGAATTGCAGAATTCAGTGAATCATCGAAT

CTTTGAACGCATCTTGCGCTCCTTGGTATTCCGAGGAGCATGCCTGTTTGAGTGTCATTA
AATTCTCAACTCTCTTATACTTTTTTGTAAAAGAGAGCTTGGACTGTGGAGGCTTGCTGG
CCACTTTTTGGGGTCAGCTCCTCTGAAATGCATTAGCGGAACCGTTTGCAATCTGCCACA
AGTGTGATAAGTTATCTACACTGGCGAGGGGATTGCTCTCTGTAATGTTCAGCTTCTAAT
TGTCTCTACTTTGTGAGACAACTTTTGAATGCTTGACCTCAAATCAGGTAGGACTACCCG
CTGAACTTAA
>B4_35
TTTCCGTAGGTGAACCTGCGGAAGGATCATTATTGAATTATGTTTCTAGATAGGTTGTAG
CTGGCTCTTTAGAGCATGTGCACGCCTGTTTGGACTTCATTTTCATCCACCTGTGCACCT
ATTGTAGTCTTTGGTTGGGTTAGGGGGAAGTGGTCATTGTGTCAGCATCTGCTGGATGTG
AGGACTTGCATTGTGAAAGCTTTGCTGTCCTTGATGTGATCATGGAATCTCTTTCTCACT
AGAGTCTATGTCACTCATTATACTCTGTCGAATGTCATTGAATGTCTTTACATGGGCTTG
TATGCCTATGAAAATTGTAATACAACTTTCAGCAACGGATCTCTTGGCTCTCGCATCGAT
GAAGGACGCAGCGAAATGCGATAAGTAATGTGAATTGCAGAATTCAGTGAATCATCGAAT
CTTTGAACGCATCTTGCGCTCCTTGGTATTCCGAGGAGCATGCCTGTTTGAGTGTCATTA
AATTCTCAACTCTCTTATACTTTTTTGTAAAAGAGAGCTTGGACTGTGGAGGCTTGCTGG
CCACTTTTTGGGGTCAGCTCCTCTGAAATGCATTAGCGGAACCGTTTGCAATCTGCCACA
AGTGTGATAAGTTATCTACACTGGCGAGGGGATTGCTCTCTGTAATGTTCAGCTTCTAAT
TGTCTCTACTTTGTGAGACAACTTTTGAATGCTTGACCTCAAATCAGGTAGGACTACCCG
CTGAACTTAA
>B4_36
TTTCCGTAGGTGAACCTGCGGAAGGATCATTATTGAATTATGTTTCTAGATAGGTTGTAG
CTGGCTCTTTAGAGCATGTGCACGCCTGTTTGGACTTCATTTTCATCCACCTGTGCACCT
ATTGTAGTCTTTGGTTGGGTTAGGGGGAAGTGGTCATTGTGTCAGCATCTGCTGGATGTG
AGGACTTGCATTGTGAAAGCTTTGCTGTCCTTGATGTGATCATGGAATCTCTTTCTCACT
AGAGTCTATGTCACTCATTATACTCTGTCGAATGTCATTGAATGTCTTTACATGGGCTTG
TATGCCTATGAAAATTGTAATACAACTTTCAGCAACGGATCTCTTGGCTCTCGCATCGAT
GAAGGACGCAGCGAAATGCGATAAGTAATGTGAATTGCAGAATTCAGTGAATCATCGAAT
CTTTGAACGCATCTTGCGCTCCTTGGTATTCCGAGGAGCATGCCTGTTTGAGTGTCATTA
AATTCTCAACTCTCTTATACTTTTTTGTAAAAGAGAGCTTGGACTGTGGAGGCTTGCTGG
CCACTTTTTGGGGTCAGCTCCTCTGAAATGCATTAGCGGAACCGTTTGCAATCTGCCACA
AGTGTGATAAGTTATCTACACTGGCGAGGGGATTGCTCTCTGTAATGTTCAGCTTCTAAT
TGTCTCTACTTTGTGAGACAACTTTTGAATGCTTGACCTCAAATCAGGTAGGACTACCCG
CTGAACTTAA
>B4_37
TTTCCGTAGGTGAACCTGCGGAAGGATCATTATTGAATTATGTTTCTAGATAGGTTGTAG
CTGGCTCTTTAGAGCATGTGCACGCCTGTTTGGACTTCATTTTCATCCACCTGTGCACCT
ATTGTAGTCTTTGGTTGGGTTAGGGGGAAGTGGTCATTGTGTCAGCATCTGCTGGATGTG
AGGACTTGCATTGTGAAAGCTTTGCTGTCCTTGATGTGATCATGGAATCTCTTTCTCACT
AGAGTCTATGTCACTCATTATACTCTGTCGAATGTCATTGAATGTCTTTACATGGGCTTG
TATGCCTATGAAAATTGTAATACAACTTTCAGCAACGGATCTCTTGGCTCTCGCATCGAT
GAAGGACGCAGCGAAATGCGATAAGTAATGTGAATTGCAGAATTCAGTGAATCATCGAAT
CTTTGAACGCATCTTGCGCTCCTTGGTATTCCGAGGAGCATGCCTGTTTGAGTGTCATTA
AATTCTCAACTCTCTTATACTTTTTTGTAAAAGAGAGCTTGGACTGTGGAGGCTTGCTGG
CCACTTTTTGGGGTCAGCTCCTCTGAAATGCATTAGCGGAACCGTTTGCAATCTGCCACA
AGTGTGATAAGTTATCTACACTGGCGAGGGGATTGCTCTCTGTAATGTTCAGCTTCTAAT
TGTCTCTACTTTGTGAGACAACTTTTGAATGCTTGACCTCAAATCAGGTAGGACTACCCG
CTGAACTTAA
>B4_38
TTTCCGTAGGTGAACCTGCGGAAGGATCATTATTGAATTATGTTTCTAGATAGGTTGTAG

CTGGCTCTTTAGAGCATGTGCACGCCTGTTTGGACTTCATTTTCATCCACCTGTGCACCT
ATTGTAGTCTTTGGTTGGGTTAGGGGGAAGTGGTCATTGTGTCAGCATCTGCTGGATGTG
AGGACTTGCATTGTGAAAGCTTTGCTGTCCTTGATGTGATCATGGAATCTCTTTCTCACT
AGAGTCTATGTCACTCATTATACTCTGTCGAATGTCATTGAATGTCTTTACATGGGCTTG
TATGCCTATGAAAATTGTAATACAACTTTCAGCAACGGATCTCTTGGCTCTCGCATCGAT
GAAGGACGCAGCGAAATGCGATAAGTAATGTGAATTGCAGAATTCAGTGAATCATCGAAT
CTTTGAACGCATCTTGCGCTCCTTGGTATTCCGAGGAGCATGCCTGTTTGAGTGTCATTA
AATTCTCAACTCTCTTATACTTTTTTGTAAAAGAGAGCTTGGACTGTGGAGGCTTGCTGG
CCACTTTTTGGGGTCAGCTCCTCTGAAATGCATTAGCGGAACCGTTTGCAATCTGCCACA
AGTGTGATAAGTTATCTACACTGGCGAGGGGATTGCTCTCTGTAATGTTCAGCTTCTAAT
TGTCTCTACTTTGTGAGACAACTTTTGAATGCTTGACCTCAAATCAGGTAGGACTACCCG
CTGAACTTAA
>B4_39
TTTCCGTAGGTGAACCTGCGGAAGGATCATTATTGAATTATGTTTCTAGATAGGTTGTAG
CTGGCTCTTTAGAGCATGTGCACGCCTGTTTGGACTTCATTTTCATCCACCTGTGCACCT
ATTGTAGTCTTTGGTTGGGTTAGGGGGAAGTGGTCATTGTGTCAGCATCTGCTGGATGTG
AGGACTTGCATTGTGAAAGCTTTGCTGTCCTTGATGTGATCATGGAATCTCTTTCTCACT
AGAGTCTATGTCACTCATTATACTCTGTCGAATGTCATTGAATGTCTTTACATGGGCTTG
TATGCCTATGAAAATTGTAATACAACTTTCAGCAACGGATCTCTTGGCTCTCGCATCGAT
GAAGGACGCAGCGAAATGCGATAAGTAATGTGAATTGCAGAATTCAGTGAATCATCGAAT
CTTTGAACGCATCTTGCGCTCCTTGGTATTCCGAGGAGCATGCCTGTTTGAGTGTCATTA
AATTCTCAACTCTCTTATACTTTTTTGTAAAAGAGAGCTTGGACTGTGGAGGCTTGCTGG
CCACTTTTTGGGGTCAGCTCCTCTGAAATGCATTAGCGGAACCGTTTGCAATCTGCCACA
AGTGTGATAAGTTATCTACACTGGCGAGGGGATTGCTCTCTGTAATGTTCAGCTTCTAAT
TGTCTCTACTTTGTGAGACAACTTTTGAATGCTTGACCTCAAATCAGGTAGGACTACCCG
CTGAACTTAA
>B4_40
TTTCCGTAGGTGAACCTGCGGAAGGATCATTATTGAATTATGTTTCTAGATAGGTTGTAG
CTGGCTCTTTAGAGCATGTGCACGCCTGTTTGGACTTCATTTTCATCCACCTGTGCACCT
ATTGTAGTCTTTGGTTGGGTTAGGGGGAAGTGGTCATTGTGTCAGCATCTGCTGGATGTG
AGGACTTGCATTGTGAAAGCTTTGCTGTCCTTGATGTGATCATGGAATCTCTTTCTCACT
AGAGTCTATGTCACTCATTATACTCTGTCGAATGTCATTGAATGTCTTTACATGGGCTTG
TATGCCTATGAAAATTGTAATACAACTTTCAGCAACGGATCTCTTGGCTCTCGCATCGAT
GAAGGACGCAGCGAAATGCGATAAGTAATGTGAATTGCAGAATTCAGTGAATCATCGAAT
CTTTGAACGCATCTTGCGCTCCTTGGTATTCCGAGGAGCATGCCTGTTTGAGTGTCATTA
AATTCTCAACTCTCTTATACTTTTTTGTAAAAGAGAGCTTGGACTGTGGAGGCTTGCTGG
CCACTTTTTGGGGTCAGCTCCTCTGAAATGCATTAGCGGAACCGTTTGCAATCTGCCACA
AGTGTGATAAGTTATCTACACTGGCGAGGGGATTGCTCTCTGTAATGTTCAGCTTCTAAT
TGTCTCTACTTTGTGAGACAACTTTTGAATGCTTGACCTCAAATCAGGTAGGACTACCCG
CTGAACTTAA
>B4_41
TTTCCGTAGGTGAACCTGCGGAAGGATCATTATTGAATTATGTTTCTAGATAGGTTGTAG
CTGGCTCTTTAGAGCATGTGCACGCCTGTTTGGACTTCATTTTCATCCACCTGTGCACCT
ATTGTAGTCTTTGGTTGGGTTAGGGGGAAGTGGTCATTGTGTCAGCATCTGCTGGATGTG
AGGACTTGCATTGTGAAAGCTTTGCTGTCCTTGATGTGATCATGGAATCTCTTTCTCACT
AGAGTCTATGTCACTCATTATACTCTGTCGAATGTCATTGAATGTCTTTACATGGGCTTG
TATGCCTATGAAAATTGTAATACAACTTTCAGCAACGGATCTCTTGGCTCTCGCATCGAT
GAAGGACGCAGCGAAATGCGATAAGTAATGTGAATTGCAGAATTCAGTGAATCATCGAAT
CTTTGAACGCATCTTGCGCTCCTTGGTATTCCGAGGAGCATGCCTGTTTGAGTGTCATTA
AATTCTCAACTCTCTTATACTTTTTTGTAAAAGAGAGCTTGGACTGTGGAGGCTTGCTGG

CCACTTTTTGGGGTCAGCTCCTCTGAAATGCATTAGCGGAACCGTTTGCAATCTGCCACA
AGTGTGATAAGTTATCTACACTGGCGAGGGGATTGCTCTCTGTAATGTTCAGCTTCTAAT
TGTCTCTACTTTGTGAGACAACTTTTGAATGCTTGACCTCAAATCAGGTAGGACTACCCG
CTGAACTTAA
>B4_42
TTTCCGTAGGTGAACCTGCGGAAGGATCATTATTGAATTATGTTTCTAGATAGGTTGTAG
CTGGCTCTTTAGAGCATGTGCACGCCTGTTTGGACTTCATTTTCATCCACCTGTGCACCT
ATTGTAGTCTTTGGTTGGGTTAGGGGGAAGTGGTCATTGTGTCAGCATCTGCTGGATGTG
AGGACTTGCATTGTGAAAGCTTTGCTGTCCTTGATGTGATCATGGAATCTCTTTCTCACT
AGAGTCTATGTCACTCATTATACTCTGTCGAATGTCATTGAATGTCTTTACATGGGCTTG
TATGCCTATGAAAATTGTAATACAACTTTCAGCAACGGATCTCTTGGCTCTCGCATCGAT
GAAGGACGCAGCGAAATGCGATAAGTAATGTGAATTGCAGAATTCAGTGAATCATCGAAT
CTTTGAACGCATCTTGCGCTCCTTGGTATTCCGAGGAGCATGCCTGTTTGAGTGTCATTA
AATTCTCAACTCTCTTATACTTTTTTGTAAAAGAGAGCTTGGACTGTGGAGGCTTGCTGG
CCACTTTTTGGGGTCAGCTCCTCTGAAATGCATTAGCGGAACCGTTTGCAATCTGCCACA
AGTGTGATAAGTTATCTACACTGGCGAGGGGATTGCTCTCTGTAATGTTCAGCTTCTAAT
TGTCTCTACTTTGTGAGACAACTTTTGAATGCTTGACCTCAAATCAGGTAGGACTACCCG
CTGAACTTAA
>B4_43
TTTCCGTAGGTGAACCTGCGGAAGGATCATTATTGAATTATGTTTCTAGATAGGTTGTAG
CTGGCTCTTTAGAGCATGTGCACGCCTGTTTGGACTTCATTTTCATCCACCTGTGCACCT
ATTGTAGTCTTTGGTTGGGTTAGGGGGAAGTGGTCATTGTGTCAGCATCTGCTGGATGTG
AGGACTTGCATTGTGAAAGCTTTGCTGTCCTTGATGTGATCATGGAATCTCTTTCTCACT
AGAGTCTATGTCACTCATTATACTCTGTCGAATGTCATTGAATGTCTTTACATGGGCTTG
TATGCCTATGAAAATTGTAATACAACTTTCAGCAACGGATCTCTTGGCTCTCGCATCGAT
GAAGGACGCAGCGAAATGCGATAAGTAATGTGAATTGCAGAATTCAGTGAATCATCGAAT
CTTTGAACGCATCTTGCGCTCCTTGGTATTCCGAGGAGCATGCCTGTTTGAGTGTCATTA
AATTCTCAACTCTCTTATACTTTTTTGTAAAAGAGAGCTTGGACTGTGGAGGCTTGCTGG
CCACTTTTTGGGGTCAGCTCCTCTGAAATGCATTAGCGGAACCGTTTGCAATCTGCCACA
AGTGTGATAAGTTATCTACACTGGCGAGGGGATTGCTCTCTGTAATGTTCAGCTTCTAAT
TGTCTCTACTTTGTGAGACAACTTTTGAATGCTTGACCTCAAATCAGGTAGGACTACCCG
CTGAACTTAA
>B4_44
TTTCCGTAGGTGAACCTGCGGAAGGATCATTATTGAATTATGTTTCTAGATAGGTTGTAG
CTGGCTCTTTAGAGCATGTGCACGCCTGTTTGGACTTCATTTTCATCCACCTGTGCACCT
ATTGTAGTCTTTGGTTGGGTTAGGGGGAAGTGGTCATTGTGTCAGCATCTGCTGGATGTG
AGGACTTGCATTGTGAAAGCTTTGCTGTCCTTGATGTGATCATGGAATCTCTTTCTCACT
AGAGTCTATGTCACTCATTATACTCTGTCGAATGTCATTGAATGTCTTTACATGGGCTTG
TATGCCTATGAAAATTGTAATACAACTTTCAGCAACGGATCTCTTGGCTCTCGCATCGAT
GAAGGACGCAGCGAAATGCGATAAGTAATGTGAATTGCAGAATTCAGTGAATCATCGAAT
CTTTGAACGCATCTTGCGCTCCTTGGTATTCCGAGGAGCATGCCTGTTTGAGTGTCATTA
AATTCTCAACTCTCTTATACTTTTTTGTAAAAGAGAGCTTGGACTGTGGAGGCTTGCTGG
CCACTTTTTGGGGTCAGCTCCTCTGAAATGCATTAGCGGAACCGTTTGCAATCTGCCACA
AGTGTGATAAGTTATCTACACTGGCGAGGGGATTGCTCTCTGTAATGTTCAGCTTCTAAT
TGTCTCTACTTTGTGAGACAACTTTTGAATGCTTGACCTCAAATCAGGTAGGACTACCCG
CTGAACTTAA
>B4_45
TTTCCGTAGGTGAACCTGCGGAAGGATCATTATTGAATTATGTTTCTAGATAGGTTGTAG
CTGGCTCTTTAGAGCATGTGCACGCCTGTTTGGACTTCATTTTCATCCACCTGTGCACCT
ATTGTAGTCTTTGGTTGGGTTAGGGGGAAGTGGTCATTGTGTCAGCATCTGCTGGATGTG

AGGACTTGCATTGTGAAAGCTTTGCTGTCCTTGATGTGATCATGGAATCTCTTTCTCACT
AGAGTCTATGTCACTCATTATACTCTGTCGAATGTCATTGAATGTCTTTACATGGGCTTG
TATGCCTATGAAAATTGTAATACAACTTTCAGCAACGGATCTCTTGGCTCTCGCATCGAT
GAAGGACGCAGCGAAATGCGATAAGTAATGTGAATTGCAGAATTCAGTGAATCATCGAAT
CTTTGAACGCATCTTGCGCTCCTTGGTATTCCGAGGAGCATGCCTGTTTGAGTGTCATTA
AATTCTCAACTCTCTTATACTTTTTTGTAAAAGAGAGCTTGGACTGTGGAGGCTTGCTGG
CCACTTTTTGGGGTCAGCTCCTCTGAAATGCATTAGCGGAACCGTTTGCAATCTGCCACA
AGTGTGATAAGTTATCTACACTGGCGAGGGGATTGCTCTCTGTAATGTTCAGCTTCTAAT
TGTCTCTACTTTGTGAGACAACTTTTGAATGCTTGACCTCAAATCAGGTAGGACTACCCG
CTGAACTTAA
>B4_46
TTTCCGTAGGTGAACCTGCGGAAGGATCATTATTGAATTATGTTTCTAGATAGGTTGTAG
CTGGCTCTTTAGAGCATGTGCACGCCTGTTTGGACTTCATTTTCATCCACCTGTGCACCT
ATTGTAGTCTTTGGTTGGGTTAGGGGGAAGTGGTCATTGTGTCAGCATCTGCTGGATGTG
AGGACTTGCATTGTGAAAGCTTTGCTGTCCTTGATGTGATCATGGAATCTCTTTCTCACT
AGAGTCTATGTCACTCATTATACTCTGTCGAATGTCATTGAATGTCTTTACATGGGCTTG
TATGCCTATGAAAATTGTAATACAACTTTCAGCAACGGATCTCTTGGCTCTCGCATCGAT
GAAGGACGCAGCGAAATGCGATAAGTAATGTGAATTGCAGAATTCAGTGAATCATCGAAT
CTTTGAACGCATCTTGCGCTCCTTGGTATTCCGAGGAGCATGCCTGTTTGAGTGTCATTA
AATTCTCAACTCTCTTATACTTTTTTGTAAAAGAGAGCTTGGACTGTGGAGGCTTGCTGG
CCACTTTTTGGGGTCAGCTCCTCTGAAATGCATTAGCGGAACCGTTTGCAATCTGCCACA
AGTGTGATAAGTTATCTACACTGGCGAGGGGATTGCTCTCTGTAATGTTCAGCTTCTAAT
TGTCTCTACTTTGTGAGACAACTTTTGAATGCTTGACCTCAAATCAGGTAGGACTACCCG
CTGAACTTAA
>B4_47
TTTCCGTAGGTGAACCTGCGGAAGGATCATTATTGAATTATGTTTCTAGATAGGTTGTAG
CTGGCTCTTTAGAGCATGTGCACGCCTGTTTGGACTTCATTTTCATCCACCTGTGCACCT
ATTGTAGTCTTTGGTTGGGTTAGGGGGAAGTGGTCATTGTGTCAGCATCTGCTGGATGTG
AGGACTTGCATTGTGAAAGCTTTGCTGTCCTTGATGTGATCATGGAATCTCTTTCTCACT
AGAGTCTATGTCACTCATTATACTCTGTCGAATGTCATTGAATGTCTTTACATGGGCTTG
TATGCCTATGAAAATTGTAATACAACTTTCAGCAACGGATCTCTTGGCTCTCGCATCGAT
GAAGGACGCAGCGAAATGCGATAAGTAATGTGAATTGCAGAATTCAGTGAATCATCGAAT
CTTTGAACGCATCTTGCGCTCCTTGGTATTCCGAGGAGCATGCCTGTTTGAGTGTCATTA
AATTCTCAACTCTCTTATACTTTTTTGTAAAAGAGAGCTTGGACTGTGGAGGCTTGCTGG
CCACTTTTTGGGGTCAGCTCCTCTGAAATGCATTAGCGGAACCGTTTGCAATCTGCCACA
AGTGTGATAAGTTATCTACACTGGCGAGGGGATTGCTCTCTGTAATGTTCAGCTTCTAAT
TGTCTCTACTTTGTGAGACAACTTTTGAATGCTTGACCTCAAATCAGGTAGGACTACCCG
CTGAACTTAA
>B4_48
TTTCCGTAGGTGAACCTGCGGAAGGATCATTATTGAATTATGTTTCTAGATAGGTTGTAG
CTGGCTCTTTAGAGCATGTGCACGCCTGTTTGGACTTCATTTTCATCCACCTGTGCACCT
ATTGTAGTCTTTGGTTGGGTTAGGGGGAAGTGGTCATTGTGTCAGCATCTGCTGGATGTG
AGGACTTGCATTGTGAAAGCTTTGCTGTCCTTGATGTGATCATGGAATCTCTTTCTCACT
AGAGTCTATGTCACTCATTATACTCTGTCGAATGTCATTGAATGTCTTTACATGGGCTTG
TATGCCTATGAAAATTGTAATACAACTTTCAGCAACGGATCTCTTGGCTCTCGCATCGAT
GAAGGACGCAGCGAAATGCGATAAGTAATGTGAATTGCAGAATTCAGTGAATCATCGAAT
CTTTGAACGCATCTTGCGCTCCTTGGTATTCCGAGGAGCATGCCTGTTTGAGTGTCATTA
AATTCTCAACTCTCTTATACTTTTTTGTAAAAGAGAGCTTGGACTGTGGAGGCTTGCTGG
CCACTTTTTGGGGTCAGCTCCTCTGAAATGCATTAGCGGAACCGTTTGCAATCTGCCACA
AGTGTGATAAGTTATCTACACTGGCGAGGGGATTGCTCTCTGTAATGTTCAGCTTCTAAT

TGTCTCTACTTTGTGAGACAACTTTTGAATGCTTGACCTCAAATCAGGTAGGACTACCCG
CTGAACTTAA
>B4_49
TTTCCGTAGGTGAACCTGCGGAAGGATCATTATTGAATTATGTTTCTAGATAGGTTGTAG
CTGGCTCTTTAGAGCATGTGCACGCCTGTTTGGACTTCATTTTCATCCACCTGTGCACCT
ATTGTAGTCTTTGGTTGGGTTAGGGGGAAGTGGTCATTGTGTCAGCATCTGCTGGATGTG
AGGACTTGCATTGTGAAAGCTTTGCTGTCCTTGATGTGATCATGGAATCTCTTTCTCACT
AGAGTCTATGTCACTCATTATACTCTGTCGAATGTCATTGAATGTCTTTACATGGGCTTG
TATGCCTATGAAAATTGTAATACAACTTTCAGCAACGGATCTCTTGGCTCTCGCATCGAT
GAAGGACGCAGCGAAATGCGATAAGTAATGTGAATTGCAGAATTCAGTGAATCATCGAAT
CTTTGAACGCATCTTGCGCTCCTTGGTATTCCGAGGAGCATGCCTGTTTGAGTGTCATTA
AATTCTCAACTCTCTTATACTTTTTTGTAAAAGAGAGCTTGGACTGTGGAGGCTTGCTGG
CCACTTTTTGGGGTCAGCTCCTCTGAAATGCATTAGCGGAACCGTTTGCAATCTGCCACA
AGTGTGATAAGTTATCTACACTGGCGAGGGGATTGCTCTCTGTAATGTTCAGCTTCTAAT
TGTCTCTACTTTGTGAGACAACTTTTGAATGCTTGACCTCAAATCAGGTAGGACTACCCG
CTGAACTTAA
>B4_50
TTTCCGTAGGTGAACCTGCGGAAGGATCATTATTGAATTATGTTTCTAGATAGGTTGTAG
CTGGCTCTTTAGAGCATGTGCACGCCTGTTTGGACTTCATTTTCATCCACCTGTGCACCT
ATTGTAGTCTTTGGTTGGGTTAGGGGGAAGTGGTCATTGTGTCAGCATCTGCTGGATGTG
AGGACTTGCATTGTGAAAGCTTTGCTGTCCTTGATGTGATCATGGAATCTCTTTCTCACT
AGAGTCTATGTCACTCATTATACTCTGTCGAATGTCATTGAATGTCTTTACATGGGCTTG
TATGCCTATGAAAATTGTAATACAACTTTCAGCAACGGATCTCTTGGCTCTCGCATCGAT
GAAGGACGCAGCGAAATGCGATAAGTAATGTGAATTGCAGAATTCAGTGAATCATCGAAT
CTTTGAACGCATCTTGCGCTCCTTGGTATTCCGAGGAGCATGCCTGTTTGAGTGTCATTA
AATTCTCAACTCTCTTATACTTTTTTGTAAAAGAGAGCTTGGACTGTGGAGGCTTGCTGG
CCACTTTTTGGGGTCAGCTCCTCTGAAATGCATTAGCGGAACCGTTTGCAATCTGCCACA
AGTGTGATAAGTTATCTACACTGGCGAGGGGATTGCTCTCTGTAATGTTCAGCTTCTAAT
TGTCTCTACTTTGTGAGACAACTTTTGAATGCTTGACCTCAAATCAGGTAGGACTACCCG
CTGAACTTAA
>B4−53
TTTCCGTAGGTGAACCTGCGGAAGGATCATTATTGAATTATGTTTCTAGATAGGTTGTAG
CTGGCTCTTTAGAGCATGTGCACGCCTGTTTGGACTTCATTTTCATCCACCTGTGCACCT
ATTGTAGTCTTTGGTTGGGTTAGGGGGAAGTGGTCATTGTGTCAGCATCTGCTGGATGTG
AGGACTTGCATTGTGAAAGCTTTGCTGTCCTTGATGTGATCATGGAATCTCTTTCTCACT
AGAGTCTATGTCACTCATTATACTCTGTCGAATGTCATTGAATGTCTTTACATGGGCTTG
TATGCCTATGAAAATTGTAATACAACTTTCAGCAACGGATCTCTTGGCTCTCGCATCGAT
GAAGGACGCAGCGAAATGCGATAAGTAATGTGAATTGCAGAATTCAGTGAATCATCGAAT
CTTTGAACGCATCTTGCGCTCCTTGGTATTCCGAGGAGCATGCCTGTTTGAGTGTCATTA
AATTCTCAACTCTCTTATACTTTTTTGTAAAAGAGAGCTTGGACTGTGGAGGCTTGCTGG
CCACTTTTTGGGGTCAGCTCCTCTGAAATGCATTAGCGGAACCGTTTGCAATCTGCCACA
AGTGTGATAAGTTATCTACACTGGCGAGGGGATTGCTCTCTGTAATGTTCAGCTTCTAAT
TGTCTCTACTTTGTGAGACAACTTTTGAATGCTTGACCTCAAATCAGGTAGGACTACCCG
CTGAACTTAA
>B4−54
TTTCCGTAGGTGAACCTGCGGAAGGATCATTATTGAATTATGTTTCTAGATAGGTTGTAG
CTGGCTCTTTAGAGCATGTGCACGCCTGTTTGGACTTCATTTTCATCCACCTGTGCACCT
ATTGTAGTCTTTGGTTGGGTTAGGGGGAAGTGGTCATTGTGTCAGCATCTGCTGGATGTG
AGGACTTGCATTGTGAAAGCTTTGCTGTCCTTGATGTGATCATGGAATCTCTTTCTCACT
AGAGTCTATGTCACTCATTATACTCTGTCGAATGTCATTGAATGTCTTTACATGGGCTTG

TATGCCTATGAAAATTGTAATACAACTTTCAGCAACGGATCTCTTGGCTCTCGCATCGAT
GAAGGACGCAGCGAAATGCGATAAGTAATGTGAATTGCAGAATTCAGTGAATCATCGAAT
CTTTGAACGCATCTTGCGCTCCTTGGTATTCCGAGGAGCATGCCTGTTTGAGTGTCATTA
AATTCTCAACTCTCTTATACTTTTTTGTAAAAGAGAGCTTGGACTGTGGAGGCTTGCTGG
CCACTTTTTGGGGTCAGCTCCTCTGAAATGCATTAGCGGAACCGTTTGCAATCTGCCACA
AGTGTGATAAGTTATCTACACTGGCGAGGGGATTGCTCTCTGTAATGTTCAGCTTCTAAT
TGTCTCTACTTTGTGAGACAACTTTTGAATGCTTGACCTCAAATCAGGTAGGACTACCCG
CTGAACTTAA
>B4-55
TTTCCGTAGGTGAACCTGCGGAAGGATCATTATTGAATTATGTTTCTAGATAGGTTGTAG
CTGGCTCTTTAGAGCATGTGCACGCCTGTTTGGACTTCATTTTCATCCACCTGTGCACCT
ATTGTAGTCTTTGGTTGGGTTAGGGGGAAGTGGTCATTGTGTCAGCATCTGCTGGATGTG
AGGACTTGCATTGTGAAAGCTTTGCTGTCCTTGATGTGATCATGGAATCTCTTTCTCACT
AGAGTCTATGTCACTCATTATACTCTGTCGAATGTCATTGAATGTCTTTACATGGGCTTG
TATGCCTATGAAAATTGTAATACAACTTTCAGCAACGGATCTCTTGGCTCTCGCATCGAT
GAAGGACGCAGCGAAATGCGATAAGTAATGTGAATTGCAGAATTCAGTGAATCATCGAAT
CTTTGAACGCATCTTGCGCTCCTTGGTATTCCGAGGAGCATGCCTGTTTGAGTGTCATTA
AATTCTCAACTCTCTTATACTTTTTTGTAAAAGAGAGCTTGGACTGTGGAGGCTTGCTGG
CCACTTTTTGGGGTCAGCTCCTCTGAAATGCATTAGCGGAACCGTTTGCAATCTGCCACA
AGTGTGATAAGTTATCTACACTGGCGAGGGGATTGCTCTCTGTAATGTTCAGCTTCTAAT
TGTCTCTACTTTGTGAGACAACTTTTGAATGCTTGACCTCAAATCAGGTAGGACTACCCG
CTGAACTTAA
>B4-56
TTTCCGTAGGTGAACCTGCGGAAGGATCATTATTGAATTATGTTTCTAGATAGGTTGTAG
CTGGCTCTTTAGAGCATGTGCACGCCTGTTTGGACTTCATTTTCATCCACCTGTGCACCT
ATTGTAGTCTTTGGTTGGGTTAGGGGGAAGTGGTCATTGTGTCAGCATCTGCTGGATGTG
AGGACTTGCATTGTGAAAGCTTTGCTGTCCTTGATGTGATCATGGAATCTCTTTCTCACT
AGAGTCTATGTCACTCATTATACTCTGTCGAATGTCATTGAATGTCTTTACATGGGCTTG
TATGCCTATGAAAATTGTAATACAACTTTCAGCAACGGATCTCTTGGCTCTCGCATCGAT
GAAGGACGCAGCGAAATGCGATAAGTAATGTGAATTGCAGAATTCAGTGAATCATCGAAT
CTTTGAACGCATCTTGCGCTCCTTGGTATTCCGAGGAGCATGCCTGTTTGAGTGTCATTA
AATTCTCAACTCTCTTATACTTTTTTGTAAAAGAGAGCTTGGACTGTGGAGGCTTGCTGG
CCACTTTTTGGGGTCAGCTCCTCTGAAATGCATTAGCGGAACCGTTTGCAATCTGCCACA
AGTGTGATAAGTTATCTACACTGGCGAGGGGATTGCTCTCTGTAATGTTCAGCTTCTAAT
TGTCTCTACTTTGTGAGACAACTTTTGAATGCTTGACCTCAAATCAGGTAGGACTACCCG
CTGAACTTAA
>B4-57
TTTCCGTAGGTGAACCTGCGGAAGGATCATTATTGAATTATGTTTCTAGATAGGTTGTAG
CTGGCTCTTTAGAGCATGTGCACGCCTGTTTGGACTTCATTTTCATCCACCTGTGCACCT
ATTGTAGTCTTTGGTTGGGTTAGGGGGAAGTGGTCATTGTGTCAGCATCTGCTGGATGTG
AGGACTTGCATTGTGAAAGCTTTGCTGTCCTTGATGTGATCATGGAATCTCTTTCTCACT
AGAGTCTATGTCACTCATTATACTCTGTCGAATGTCATTGAATGTCTTTACATGGGCTTG
TATGCCTATGAAAATTGTAATACAACTTTCAGCAACGGATCTCTTGGCTCTCGCATCGAT
GAAGGACGCAGCGAAATGCGATAAGTAATGTGAATTGCAGAATTCAGTGAATCATCGAAT
CTTTGAACGCATCTTGCGCTCCTTGGTATTCCGAGGAGCATGCCTGTTTGAGTGTCATTA
AATTCTCAACTCTCTTATACTTTTTTGTAAAAGAGAGCTTGGACTGTGGAGGCTTGCTGG
CCACTTTTTGGGGTCAGCTCCTCTGAAATGCATTAGCGGAACCGTTTGCAATCTGCCACA
AGTGTGATAAGTTATCTACACTGGCGAGGGGATTGCTCTCTGTAATGTTCAGCTTCTAAT
TGTCTCTACTTTGTGAGACAACTTTTGAATGCTTGACCTCAAATCAGGTAGGACTACCCG
CTGAACTTAA

>B4-58
TTTCCGTAGGTGAACCTGCGGAAGGATCATTATTGAATTATGTTTCTAGATAGGTTGTAG
CTGGCTCTTTAGAGCATGTGCACGCCTGTTTGGACTTCATTTTCATCCACCTGTGCACCT
ATTGTAGTCTTTGGTTGGGTTAGGGGGAAGTGGTCATTGTGTCAGCATCTGCTGGATGTG
AGGACTTGCATTGTGAAAGCTTTGCTGTCCTTGATGTGATCATGGAATCTCTTTCTCACT
AGAGTCTATGTCACTCATTATACTCTGTCGAATGTCATTGAATGTCTTTACATGGGCTTG
TATGCCTATGAAAATTGTAATACAACTTTCAGCAACGGATCTCTTGGCTCTCGCATCGAT
GAAGGACGCAGCGAAATGCGATAAGTAATGTGAATTGCAGAATTCAGTGAATCATCGAAT
CTTTGAACGCATCTTGCGCTCCTTGGTATTCCGAGGAGCATGCCTGTTTGAGTGTCATTA
AATTCTCAACTCTCTTATACTTTTTTGTAAAAGAGAGCTTGGACTGTGGAGGCTTGCTGG
CCACTTTTTGGGGTCAGCTCCTCTGAAATGCATTAGCGGAACCGTTTGCAATCTGCCACA
AGTGTGATAAGTTATCTACACTGGCGAGGGGATTGCTCTCTGTAATGTTCAGCTTCTAAT
TGTCTCTACTTTGTGAGACAACTTTTGAATGCTTGACCTCAAATCAGGTAGGACTACCCG
CTGAACTTAA
>B4-60
TTTCCGTAGGTGAACCTGCGGAAGGATCATTATTGAATTATGTTTCTAGATAGGTTGTAG
CTGGCTCTTTAGAGCATGTGCACGCCTGTTTGGACTTCATTTTCATCCACCTGTGCACCT
ATTGTAGTCTTTGGTTGGGTTAGGGGGAAGTGGTCATTGTGTCAGCATCTGCTGGATGTG
AGGACTTGCATTGTGAAAGCTTTGCTGTCCTTGATGTGATCATGGAATCTCTTTCTCACT
AGAGTCTATGTCACTCATTATACTCTGTCGAATGTCATTGAATGTCTTTACATGGGCTTG
TATGCCTATGAAAATTGTAATACAACTTTCAGCAACGGATCTCTTGGCTCTCGCATCGAT
GAAGGACGCAGCGAAATGCGATAAGTAATGTGAATTGCAGAATTCAGTGAATCATCGAAT
CTTTGAACGCATCTTGCGCTCCTTGGTATTCCGAGGAGCATGCCTGTTTGAGTGTCATTA
AATTCTCAACTCTCTTATACTTTTTTGTAAAAGAGAGCTTGGACTGTGGAGGCTTGCTGG
CCACTTTTTGGGGTCAGCTCCTCTGAAATGCATTAGCGGAACCGTTTGCAATCTGCCACA
AGTGTGATAAGTTATCTACACTGGCGAGGGGATTGCTCTCTGTAATGTTCAGCTTCTAAT
TGTCTCTACTTTGTGAGACAACTTTTGAATGCTTGACCTCAAATCAGGTAGGACTACCCG
CTGAACTTAA
>B4-61
TTTCCGTAGGTGAACCTGCGGAAGGATCATTATTGAATTATGTTTCTAGATAGGTTGTAG
CTGGCTCTTTAGAGCATGTGCACGCCTGTTTGGACTTCATTTTCATCCACCTGTGCACCT
ATTGTAGTCTTTGGTTGGGTTAGGGGGAAGTGGTCATTGTGTCAGCATCTGCTGGATGTG
AGGACTTGCATTGTGAAAGCTTTGCTGTCCTTGATGTGATCATGGAATCTCTTTCTCACT
AGAGTCTATGTCACTCATTATACTCTGTCGAATGTCATTGAATGTCTTTACATGGGCTTG
TATGCCTATGAAAATTGTAATACAACTTTCAGCAACGGATCTCTTGGCTCTCGCATCGAT
GAAGGACGCAGCGAAATGCGATAAGTAATGTGAATTGCAGAATTCAGTGAATCATCGAAT
CTTTGAACGCATCTTGCGCTCCTTGGTATTCCGAGGAGCATGCCTGTTTGAGTGTCATTA
AATTCTCAACTCTCTTATACTTTTTTGTAAAAGAGAGCTTGGACTGTGGAGGCTTGCTGG
CCACTTTTTGGGGTCAGCTCCTCTGAAATGCATTAGCGGAACCGTTTGCAATCTGCCACA
AGTGTGATAAGTTATCTACACTGGCGAGGGGATTGCTCTCTGTAATGTTCAGCTTCTAAT
TGTCTCTACTTTGTGAGACAACTTTTGAATGCTTGACCTCAAATCAGGTAGGACTACCCG
CTGAACTTAA
>B4-62
TTTCCGTAGGTGAACCTGCGGAAGGATCATTATTGAATTATGTTTCTAGATAGGTTGTAG
CTGGCTCTTTAGAGCATGTGCACGCCTGTTTGGACTTCATTTTCATCCACCTGTGCACCT
ATTGTAGTCTTTGGTTGGGTTAGGGGGAAGTGGTCATTGTGTCAGCATCTGCTGGATGTG
AGGACTTGCATTGTGAAAGCTTTGCTGTCCTTGATGTGATCATGGAATCTCTTTCTCACT
AGAGTCTATGTCACTCATTATACTCTGTCGAATGTCATTGAATGTCTTTACATGGGCTTG
TATGCCTATGAAAATTGTAATACAACTTTCAGCAACGGATCTCTTGGCTCTCGCATCGAT
GAAGGACGCAGCGAAATGCGATAAGTAATGTGAATTGCAGAATTCAGTGAATCATCGAAT

CTTTGAACGCATCTTGCGCTCCTTGGTATTCCGAGGAGCATGCCTGTTTGAGTGTCATTA
AATTCTCAACTCTCTTATACTTTTTTGTAAAAGAGAGCTTGGACTGTGGAGGCTTGCTGG
CCACTTTTTGGGGTCAGCTCCTCTGAAATGCATTAGCGGAACCGTTTGCAATCTGCCACA
AGTGTGATAAGTTATCTACACTGGCGAGGGGATTGCTCTCTGTAATGTTCAGCTTCTAAT
TGTCTCTACTTTGTGAGACAACTTTTGAATGCTTGACCTCAAATCAGGTAGGACTACCCG
CTGAACTTAA
>B4-63
TTTCCGTAGGTGAACCTGCGGAAGGATCATTATTGAATTATGTTTCTAGATAGGTTGTAG
CTGGCTCTTTAGAGCATGTGCACGCCTGTTTGGACTTCATTTTCATCCACCTGTGCACCT
ATTGTAGTCTTTGGTTGGGTTAGGGGGAAGTGGTCATTGTGTCAGCATCTGCTGGATGTG
AGGACTTGCATTGTGAAAGCTTTGCTGTCCTTGATGTGATCATGGAATCTCTTTCTCACT
AGAGTCTATGTCACTCATTATACTCTGTCGAATGTCATTGAATGTCTTTACATGGGCTTG
TATGCCTATGAAAATTGTAATACAACTTTCAGCAACGGATCTCTTGGCTCTCGCATCGAT
GAAGGACGCAGCGAAATGCGATAAGTAATGTGAATTGCAGAATTCAGTGAATCATCGAAT
CTTTGAACGCATCTTGCGCTCCTTGGTATTCCGAGGAGCATGCCTGTTTGAGTGTCATTA
AATTCTCAACTCTCTTATACTTTTTTGTAAAAGAGAGCTTGGACTGTGGAGGCTTGCTGG
CCACTTTTTGGGGTCAGCTCCTCTGAAATGCATTAGCGGAACCGTTTGCAATCTGCCACA
AGTGTGATAAGTTATCTACACTGGCGAGGGGATTGCTCTCTGTAATGTTCAGCTTCTAAT
TGTCTCTACTTTGTGAGACAACTTTTGAATGCTTGACCTCAAATCAGGTAGGACTACCCG
CTGAACTTAA
>B4-64
TTTCCGTAGGTGAACCTGCGGAAGGATCATTATTGAATTATGTTTCTAGATAGGTTGTAG
CTGGCTCTTTAGAGCATGTGCACGCCTGTTTGGACTTCATTTTCATCCACCTGTGCACCT
ATTGTAGTCTTTGGTTGGGTTAGGGGGAAGTGGTCATTGTGTCAGCATCTGCTGGATGTG
AGGACTTGCATTGTGAAAGCTTTGCTGTCCTTGATGTGATCATGGAATCTCTTTCTCACT
AGAGTCTATGTCACTCATTATACTCTGTCGAATGTCATTGAATGTCTTTACATGGGCTTG
TATGCCTATGAAAATTGTAATACAACTTTCAGCAACGGATCTCTTGGCTCTCGCATCGAT
GAAGGACGCAGCGAAATGCGATAAGTAATGTGAATTGCAGAATTCAGTGAATCATCGAAT
CTTTGAACGCATCTTGCGCTCCTTGGTATTCCGAGGAGCATGCCTGTTTGAGTGTCATTA
AATTCTCAACTCTCTTATACTTTTTTGTAAAAGAGAGCTTGGACTGTGGAGGCTTGCTGG
CCACTTTTTGGGGTCAGCTCCTCTGAAATGCATTAGCGGAACCGTTTGCAATCTGCCACA
AGTGTGATAAGTTATCTACACTGGCGAGGGGATTGCTCTCTGTAATGTTCAGCTTCTAAT
TGTCTCTACTTTGTGAGACAACTTTTGAATGCTTGACCTCAAATCAGGTAGGACTACCCG
CTGAACTTAA
>B4-65
TTTCCGTAGGTGAACCTGCGGAAGGATCATTATTGAATTATGTTTCTAGATAGGTTGTAG
CTGGCTCTTTAGAGCATGTGCACGCCTGTTTGGACTTCATTTTCATCCACCTGTGCACCT
ATTGTAGTCTTTGGTTGGGTTAGGGGGAAGTGGTCATTGTGTCAGCATCTGCTGGATGTG
AGGACTTGCATTGTGAAAGCTTTGCTGTCCTTGATGTGATCATGGAATCTCTTTCTCACT
AGAGTCTATGTCACTCATTATACTCTGTCGAATGTCATTGAATGTCTTTACATGGGCTTG
TATGCCTATGAAAATTGTAATACAACTTTCAGCAACGGATCTCTTGGCTCTCGCATCGAT
GAAGGACGCAGCGAAATGCGATAAGTAATGTGAATTGCAGAATTCAGTGAATCATCGAAT
CTTTGAACGCATCTTGCGCTCCTTGGTATTCCGAGGAGCATGCCTGTTTGAGTGTCATTA
AATTCTCAACTCTCTTATACTTTTTTGTAAAAGAGAGCTTGGACTGTGGAGGCTTGCTGG
CCACTTTTTGGGGTCAGCTCCTCTGAAATGCATTAGCGGAACCGTTTGCAATCTGCCACA
AGTGTGATAAGTTATCTACACTGGCGAGGGGATTGCTCTCTGTAATGTTCAGCTTCTAAT
TGTCTCTACTTTGTGAGACAACTTTTGAATGCTTGACCTCAAATCAGGTAGGACTACCCG
CTGAACTTAA
>B4-66
TTTCCGTAGGTGAACCTGCGGAAGGATCATTATTGAATTATGTTTCTAGATAGGTTGTAG

CTGGCTCTTTAGAGCATGTGCACGCCTGTTTGGACTTCATTTTCATCCACCTGTGCACCT
ATTGTAGTCTTTGGTTGGGTTAGGGGGAAGTGGTCATTGTGTCAGCATCTGCTGGATGTG
AGGACTTGCATTGTGAAAGCTTTGCTGTCCTTGATGTGATCATGGAATCTCTTTCTCACT
AGAGTCTATGTCACTCATTATACTCTGTCGAATGTCATTGAATGTCTTTACATGGGCTTG
TATGCCTATGAAAATTGTAATACAACTTTCAGCAACGGATCTCTTGGCTCTCGCATCGAT
GAAGGACGCAGCGAAATGCGATAAGTAATGTGAATTGCAGAATTCAGTGAATCATCGAAT
CTTTGAACGCATCTTGCGCTCCTTGGTATTCCGAGGAGCATGCCTGTTTGAGTGTCATTA
AATTCTCAACTCTCTTATACTTTTTTGTAAAAGAGAGCTTGGACTGTGGAGGCTTGCTGG
CCACTTTTTGGGGTCAGCTCCTCTGAAATGCATTAGCGGAACCGTTTGCAATCTGCCACA
AGTGTGATAAGTTATCTACACTGGCGAGGGGATTGCTCTCTGTAATGTTCAGCTTCTAAT
TGTCTCTACTTTGTGAGACAACTTTTGAATGCTTGACCTCAAATCAGGTAGGACTACCCG
CTGAACTTAA
>B4-67
TTTCCGTAGGTGAACCTGCGGAAGGATCATTATTGAATTATGTTTCTAGATAGGTTGTAG
CTGGCTCTTTAGAGCATGTGCACGCCTGTTTGGACTTCATTTTCATCCACCTGTGCACCT
ATTGTAGTCTTTGGTTGGGTTAGGGGGAAGTGGTCATTGTGTCAGCATCTGCTGGATGTG
AGGACTTGCATTGTGAAAGCTTTGCTGTCCTTGATGTGATCATGGAATCTCTTTCTCACT
AGAGTCTATGTCACTCATTATACTCTGTCGAATGTCATTGAATGTCTTTACATGGGCTTG
TATGCCTATGAAAATTGTAATACAACTTTCAGCAACGGATCTCTTGGCTCTCGCATCGAT
GAAGGACGCAGCGAAATGCGATAAGTAATGTGAATTGCAGAATTCAGTGAATCATCGAAT
CTTTGAACGCATCTTGCGCTCCTTGGTATTCCGAGGAGCATGCCTGTTTGAGTGTCATTA
AATTCTCAACTCTCTTATACTTTTTTGTAAAAGAGAGCTTGGACTGTGGAGGCTTGCTGG
CCACTTTTTGGGGTCAGCTCCTCTGAAATGCATTAGCGGAACCGTTTGCAATCTGCCACA
AGTGTGATAAGTTATCTACACTGGCGAGGGGATTGCTCTCTGTAATGTTCAGCTTCTAAT
TGTCTCTACTTTGTGAGACAACTTTTGAATGCTTGACCTCAAATCAGGTAGGACTACCCG
CTGAACTTAA
>B4-68
TTTCCGTAGGTGAACCTGCGGAAGGATCATTATTGAATTATGTTTCTAGATAGGTTGTAG
CTGGCTCTTTAGAGCATGTGCACGCCTGTTTGGACTTCATTTTCATCCACCTGTGCACCT
ATTGTAGTCTTTGGTTGGGTTAGGGGGAAGTGGTCATTGTGTCAGCATCTGCTGGATGTG
AGGACTTGCATTGTGAAAGCTTTGCTGTCCTTGATGTGATCATGGAATCTCTTTCTCACT
AGAGTCTATGTCACTCATTATACTCTGTCGAATGTCATTGAATGTCTTTACATGGGCTTG
TATGCCTATGAAAATTGTAATACAACTTTCAGCAACGGATCTCTTGGCTCTCGCATCGAT
GAAGGACGCAGCGAAATGCGATAAGTAATGTGAATTGCAGAATTCAGTGAATCATCGAAT
CTTTGAACGCATCTTGCGCTCCTTGGTATTCCGAGGAGCATGCCTGTTTGAGTGTCATTA
AATTCTCAACTCTCTTATACTTTTTTGTAAAAGAGAGCTTGGACTGTGGAGGCTTGCTGG
CCACTTTTTGGGGTCAGCTCCTCTGAAATGCATTAGCGGAACCGTTTGCAATCTGCCACA
AGTGTGATAAGTTATCTACACTGGCGAGGGGATTGCTCTCTGTAATGTTCAGCTTCTAAT
TGTCTCTACTTTGTGAGACAACTTTTGAATGCTTGACCTCAAATCAGGTAGGACTACCCG
CTGAACTTAA
>B4-69
TTTCCGTAGGTGAACCTGCGGAAGGATCATTATTGAATTATGTTTCTAGATAGGTTGTAG
CTGGCTCTTTAGAGCATGTGCACGCCTGTTTGGACTTCATTTTCATCCACCTGTGCACCT
ATTGTAGTCTTTGGTTGGGTTAGGGGGAAGTGGTCATTGTGTCAGCATCTGCTGGATGTG
AGGACTTGCATTGTGAAAGCTTTGCTGTCCTTGATGTGATCATGGAATCTCTTTCTCACT
AGAGTCTATGTCACTCATTATACTCTGTCGAATGTCATTGAATGTCTTTACATGGGCTTG
TATGCCTATGAAAATTGTAATACAACTTTCAGCAACGGATCTCTTGGCTCTCGCATCGAT
GAAGGACGCAGCGAAATGCGATAAGTAATGTGAATTGCAGAATTCAGTGAATCATCGAAT
CTTTGAACGCATCTTGCGCTCCTTGGTATTCCGAGGAGCATGCCTGTTTGAGTGTCATTA
AATTCTCAACTCTCTTATACTTTTTTGTAAAAGAGAGCTTGGACTGTGGAGGCTTGCTGG

CCACTTTTTGGGGTCAGCTCCTCTGAAATGCATTAGCGGAACCGTTTGCAATCTGCCACA
AGTGTGATAAGTTATCTACACTGGCGAGGGGATTGCTCTCTGTAATGTTCAGCTTCTAAT
TGTCTCTACTTTGTGAGACAACTTTTGAATGCTTGACCTCAAATCAGGTAGGACTACCCG
CTGAACTTAA
>B4-70
TTTCCGTAGGTGAACCTGCGGAAGGATCATTATTGAATTATGTTTCTAGATAGGTTGTAG
CTGGCTCTTTAGAGCATGTGCACGCCTGTTTGGACTTCATTTTCATCCACCTGTGCACCT
ATTGTAGTCTTTGGTTGGGTTAGGGGGAAGTGGTCATTGTGTCAGCATCTGCTGGATGTG
AGGACTTGCATTGTGAAAGCTTTGCTGTCCTTGATGTGATCATGGAATCTCTTTCTCACT
AGAGTCTATGTCACTCATTATACTCTGTCGAATGTCATTGAATGTCTTTACATGGGCTTG
TATGCCTATGAAAATTGTAATACAACTTTCAGCAACGGATCTCTTGGCTCTCGCATCGAT
GAAGGACGCAGCGAAATGCGATAAGTAATGTGAATTGCAGAATTCAGTGAATCATCGAAT
CTTTGAACGCATCTTGCGCTCCTTGGTATTCCGAGGAGCATGCCTGTTTGAGTGTCATTA
AATTCTCAACTCTCTTATACTTTTTTGTAAAAGAGAGCTTGGACTGTGGAGGCTTGCTGG
CCACTTTTTGGGGTCAGCTCCTCTGAAATGCATTAGCGGAACCGTTTGCAATCTGCCACA
AGTGTGATAAGTTATCTACACTGGCGAGGGGATTGCTCTCTGTAATGTTCAGCTTCTAAT
TGTCTCTACTTTGTGAGACAACTTTTGAATGCTTGACCTCAAATCAGGTAGGACTACCCG
CTGAACTTAA
>B4-71
TTTCCGTAGGTGAACCTGCGGAAGGATCATTATTGAATTATGTTTCTAGATAGGTTGTAG
CTGGCTCTTTAGAGCATGTGCACGCCTGTTTGGACTTCATTTTCATCCACCTGTGCACCT
ATTGTAGTCTTTGGTTGGGTTAGGGGGAAGTGGTCATTGTGTCAGCATCTGCTGGATGTG
AGGACTTGCATTGTGAAAGCTTTGCTGTCCTTGATGTGATCATGGAATCTCTTTCTCACT
AGAGTCTATGTCACTCATTATACTCTGTCGAATGTCATTGAATGTCTTTACATGGGCTTG
TATGCCTATGAAAATTGTAATACAACTTTCAGCAACGGATCTCTTGGCTCTCGCATCGAT
GAAGGACGCAGCGAAATGCGATAAGTAATGTGAATTGCAGAATTCAGTGAATCATCGAAT
CTTTGAACGCATCTTGCGCTCCTTGGTATTCCGAGGAGCATGCCTGTTTGAGTGTCATTA
AATTCTCAACTCTCTTATACTTTTTTGTAAAAGAGAGCTTGGACTGTGGAGGCTTGCTGG
CCACTTTTTGGGGTCAGCTCCTCTGAAATGCATTAGCGGAACCGTTTGCAATCTGCCACA
AGTGTGATAAGTTATCTACACTGGCGAGGGGATTGCTCTCTGTAATGTTCAGCTTCTAAT
TGTCTCTACTTTGTGAGACAACTTTTGAATGCTTGACCTCAAATCAGGTAGGACTACCCG
CTGAACTTAA
>B4-72
TTTCCGTAGGTGAACCTGCGGAAGGATCATTATTGAATTATGTTTCTAGATAGGTTGTAG
CTGGCTCTTTAGAGCATGTGCACGCCTGTTTGGACTTCATTTTCATCCACCTGTGCACCT
ATTGTAGTCTTTGGTTGGGTTAGGGGGAAGTGGTCATTGTGTCAGCATCTGCTGGATGTG
AGGACTTGCATTGTGAAAGCTTTGCTGTCCTTGATGTGATCATGGAATCTCTTTCTCACT
AGAGTCTATGTCACTCATTATACTCTGTCGAATGTCATTGAATGTCTTTACATGGGCTTG
TATGCCTATGAAAATTGTAATACAACTTTCAGCAACGGATCTCTTGGCTCTCGCATCGAT
GAAGGACGCAGCGAAATGCGATAAGTAATGTGAATTGCAGAATTCAGTGAATCATCGAAT
CTTTGAACGCATCTTGCGCTCCTTGGTATTCCGAGGAGCATGCCTGTTTGAGTGTCATTA
AATTCTCAACTCTCTTATACTTTTTTGTAAAAGAGAGCTTGGACTGTGGAGGCTTGCTGG
CCACTTTTTGGGGTCAGCTCCTCTGAAATGCATTAGCGGAACCGTTTGCAATCTGCCACA
AGTGTGATAAGTTATCTACACTGGCGAGGGGATTGCTCTCTGTAATGTTCAGCTTCTAAT
TGTCTCTACTTTGTGAGACAACTTTTGAATGCTTGACCTCAAATCAGGTAGGACTACCCG
CTGAACTTAA
>B5_1
TTTCCGTAGGTGAACCTGCGGAAGGATCATTATTGAATTATGTTTCTAGATAGGTTGTAG
CTGGCTCTTTAGAGCATGTGCACGCCTGTTTGGACTTCATTTTCATCCACCTGTGCACCT
ATTGTAGTCTTTGGTTGGGTTAGGGGGAAGTGGTCATTGTGTCAGCATCTGCTGGATGTG

AGGACTTGCATTGTGAAAGCTTTGCTGTCCTTGATGTGATCATGGAATCTCTTTCTCACT
AGAGTCTATGTCACTCATTATACTCTGTCGAATGTCATTGAATGTCTTTACATGGGCTTG
TATGCCTATGAAAATTGTAATACAACTTTCAGCAACGGATCTCTTGGCTCTCGCATCGAT
GAAGGACGCAGCGAAATGCGATAAGTAATGTGAATTGCAGAATTCAGTGAATCATCGAAT
CTTTGAACGCATCTTGCGCTCCTTGGTATTCCGAGGAGCATGCCTGTTTGAGTGTCATTA
AATTCTCAACTCTCTTATACTTTTTTGTAAAAGAGAGCTTGGACTGTGGAGGCTTGCTGG
CCACTTTTTGGGGTCAGCTCCTCTGAAATGCATTAGCGGAACCGTTTGCAATCTGCCACA
AGTGTGATAAGTTATCTACACTGGCGAGGGGATTGCTCTCTGTAATGTTCAGCTTCTAAT
TGTCTCTACTTTGTGAGACAACTTTTGAATGCTTGACCTCAAATCAGGTAGGACTACCCG
CTGAACTTAA
>B5_2
TTTCCGTAGGTGAACCTGCGGAAGGATCATTATTGAATTATGTTTCTAGATAGGTTGTAG
CTGGCTCTTTAGAGCATGTGCACGCCTGTTTGGACTTCATTTTCATCCACCTGTGCACCT
ATTGTAGTCTTTGGTTGGGTTAGGGGGAAGTGGTCATTGTGTCAGCATCTGCTGGATGTG
AGGACTTGCATTGTGAAAGCTTTGCTGTCCTTGATGTGATCATGGAATCTCTTTCTCACT
AGAGTCTATGTCACTCATTATACTCTGTCGAATGTCATTGAATGTCTTTACATGGGCTTG
TATGCCTATGAAAATTGTAATACAACTTTCAGCAACGGATCTCTTGGCTCTCGCATCGAT
GAAGGACGCAGCGAAATGCGATAAGTAATGTGAATTGCAGAATTCAGTGAATCATCGAAT
CTTTGAACGCATCTTGCGCTCCTTGGTATTCCGAGGAGCATGCCTGTTTGAGTGTCATTA
AATTCTCAACTCTCTTATACTTTTTTGTAAAAGAGAGCTTGGACTGTGGAGGCTTGCTGG
CCACTTTTTGGGGTCAGCTCCTCTGAAATGCATTAGCGGAACCGTTTGCAATCTGCCACA
AGTGTGATAAGTTATCTACACTGGCGAGGGGATTGCTCTCTGTAATGTTCAGCTTCTAAT
TGTCTCTACTTTGTGAGACAACTTTTGAATGCTTGACCTCAAATCAGGTAGGACTACCCG
CTGAACTTAA
>B5_3
TTTCCGTAGGTGAACCTGCGGAAGGATCATTATTGAATTATGTTTCTAGATAGGTTGTAG
CTGGCTCTTTAGAGCATGTGCACGCCTGTTTGGACTTCATTTTCATCCACCTGTGCACCT
ATTGTAGTCTTTGGTTGGGTTAGGGGGAAGTGGTCATTGTGTCAGCATCTGCTGGATGTG
AGGACTTGCATTGTGAAAGCTTTGCTGTCCTTGATGTGATCATGGAATCTCTTTCTCACT
AGAGTCTATGTCACTCATTATACTCTGTCGAATGTCATTGAATGTCTTTACATGGGCTTG
TATGCCTATGAAAATTGTAATACAACTTTCAGCAACGGATCTCTTGGCTCTCGCATCGAT
GAAGGACGCAGCGAAATGCGATAAGTAATGTGAATTGCAGAATTCAGTGAATCATCGAAT
CTTTGAACGCATCTTGCGCTCCTTGGTATTCCGAGGAGCATGCCTGTTTGAGTGTCATTA
AATTCTCAACTCTCTTATACTTTTTTGTAAAAGAGAGCTTGGACTGTGGAGGCTTGCTGG
CCACTTTTTGGGGTCAGCTCCTCTGAAATGCATTAGCGGAACCGTTTGCAATCTGCCACA
AGTGTGATAAGTTATCTACACTGGCGAGGGGATTGCTCTCTGTAATGTTCAGCTTCTAAT
TGTCTCTACTTTGTGAGACAACTTTTGAATGCTTGACCTCAAATCAGGTAGGACTACCCG
CTGAACTTAA
>B5_5
TTTCCGTAGGTGAACCTGCGGAAGGATCATTATTGAATTATGTTTCTAGATAGGTTGTAG
CTGGCTCTTTAGAGCATGTGCACGCCTGTTTGGACTTCATTTTCATCCACCTGTGCACCT
ATTGTAGTCTTTGGTTGGGTTAGGGGGAAGTGGTCATTGTGTCAGCATCTGCTGGATGTG
AGGACTTGCATTGTGAAAGCTTTGCTGTCCTTGATGTGATCATGGAATCTCTTTCTCACT
AGAGTCTATGTCACTCATTATACTCTGTCGAATGTCATTGAATGTCTTTACATGGGCTTG
TATGCCTATGAAAATTGTAATACAACTTTCAGCAACGGATCTCTTGGCTCTCGCATCGAT
GAAGGACGCAGCGAAATGCGATAAGTAATGTGAATTGCAGAATTCAGTGAATCATCGAAT
CTTTGAACGCATCTTGCGCTCCTTGGTATTCCGAGGAGCATGCCTGTTTGAGTGTCATTA
AATTCTCAACTCTCTTATACTTTTTTGTAAAAGAGAGCTTGGACTGTGGAGGCTTGCTGG
CCACTTTTTGGGGTCAGCTCCTCTGAAATGCATTAGCGGAACCGTTTGCAATCTGCCACA
AGTGTGATAAGTTATCTACACTGGCGAGGGGATTGCTCTCTGTAATGTTCAGCTTCTAAT

TGTCTCTACTTTGTGAGACAACTTTTGAATGCTTGACCTCAAATCAGGTAGGACTACCCG
CTGAACTTAA
>B5_6
TTTCCGTAGGTGAACCTGCGGAAGGATCATTATTGAATTATGTTTCTAGATAGGTTGTAG
CTGGCTCTTTAGAGCATGTGCACGCCTGTTTGGACTTCATTTTCATCCACCTGTGCACCT
ATTGTAGTCTTTGGTTGGGTTAGGGGGAAGTGGTCATTGTGTCAGCATCTGCTGGATGTG
AGGACTTGCATTGTGAAAGCTTTGCTGTCCTTGATGTGATCATGGAATCTCTTTCTCACT
AGAGTCTATGTCACTCATTATACTCTGTCGAATGTCATTGAATGTCTTTACATGGGCTTG
TATGCCTATGAAAATTGTAATACAACTTTCAGCAACGGATCTCTTGGCTCTCGCATCGAT
GAAGGACGCAGCGAAATGCGATAAGTAATGTGAATTGCAGAATTCAGTGAATCATCGAAT
CTTTGAACGCATCTTGCGCTCCTTGGTATTCCGAGGAGCATGCCTGTTTGAGTGTCATTA
AATTCTCAACTCTCTTATACTTTTTTGTAAAAGAGAGCTTGGACTGTGGAGGCTTGCTGG
CCACTTTTTGGGGTCAGCTCCTCTGAAATGCATTAGCGGAACCGTTTGCAATCTGCCACA
AGTGTGATAAGTTATCTACACTGGCGAGGGGATTGCTCTCTGTAATGTTCAGCTTCTAAT
TGTCTCTACTTTGTGAGACAACTTTTGAATGCTTGACCTCAAATCAGGTAGGACTACCCG
CTGAACTTAA
>B5_7
TTTCCGTAGGTGAACCTGCGGAAGGATCATTATTGAATTATGTTTCTAGATAGGTTGTAG
CTGGCTCTTTAGAGCATGTGCACGCCTGTTTGGACTTCATTTTCATCCACCTGTGCACCT
ATTGTAGTCTTTGGTTGGGTTAGGGGGAAGTGGTCATTGTGTCAGCATCTGCTGGATGTG
AGGACTTGCATTGTGAAAGCTTTGCTGTCCTTGATGTGATCATGGAATCTCTTTCTCACT
AGAGTCTATGTCACTCATTATACTCTGTCGAATGTCATTGAATGTCTTTACATGGGCTTG
TATGCCTATGAAAATTGTAATACAACTTTCAGCAACGGATCTCTTGGCTCTCGCATCGAT
GAAGGACGCAGCGAAATGCGATAAGTAATGTGAATTGCAGAATTCAGTGAATCATCGAAT
CTTTGAACGCATCTTGCGCTCCTTGGTATTCCGAGGAGCATGCCTGTTTGAGTGTCATTA
AATTCTCAACTCTCTTATACTTTTTTGTAAAAGAGAGCTTGGACTGTGGAGGCTTGCTGG
CCACTTTTTGGGGTCAGCTCCTCTGAAATGCATTAGCGGAACCGTTTGCAATCTGCCACA
AGTGTGATAAGTTATCTACACTGGCGAGGGGATTGCTCTCTGTAATGTTCAGCTTCTAAT
TGTCTCTACTTTGTGAGACAACTTTTGAATGCTTGACCTCAAATCAGGTAGGACTACCCG
CTGAACTTAA
>B5_8
TTTCCGTAGGTGAACCTGCGGAAGGATCATTATTGAATTATGTTTCTAGATAGGTTGTAG
CTGGCTCTTTAGAGCATGTGCACGCCTGTTTGGACTTCATTTTCATCCACCTGTGCACCT
ATTGTAGTCTTTGGTTGGGTTAGGGGGAAGTGGTCATTGTGTCAGCATCTGCTGGATGTG
AGGACTTGCATTGTGAAAGCTTTGCTGTCCTTGATGTGATCATGGAATCTCTTTCTCACT
AGAGTCTATGTCACTCATTATACTCTGTCGAATGTCATTGAATGTCTTTACATGGGCTTG
TATGCCTATGAAAATTGTAATACAACTTTCAGCAACGGATCTCTTGGCTCTCGCATCGAT
GAAGGACGCAGCGAAATGCGATAAGTAATGTGAATTGCAGAATTCAGTGAATCATCGAAT
CTTTGAACGCATCTTGCGCTCCTTGGTATTCCGAGGAGCATGCCTGTTTGAGTGTCATTA
AATTCTCAACTCTCTTATACTTTTTTGTAAAAGAGAGCTTGGACTGTGGAGGCTTGCTGG
CCACTTTTTGGGGTCAGCTCCTCTGAAATGCATTAGCGGAACCGTTTGCAATCTGCCACA
AGTGTGATAAGTTATCTACACTGGCGAGGGGATTGCTCTCTGTAATGTTCAGCTTCTAAT
TGTCTCTACTTTGTGAGACAACTTTTGAATGCTTGACCTCAAATCAGGTAGGACTACCCG
CTGAACTTAA
>B5_9
TTTCCGTAGGTGAACCTGCGGAAGGATCATTATTGAATTATGTTTCTAGATAGGTTGTAG
CTGGCTCTTTAGAGCATGTGCACGCCTGTTTGGACTTCATTTTCATCCACCTGTGCACCT
ATTGTAGTCTTTGGTTGGGTTAGGGGGAAGTGGTCATTGTGTCAGCATCTGCTGGATGTG
AGGACTTGCATTGTGAAAGCTTTGCTGTCCTTGATGTGATCATGGAATCTCTTTCTCACT
AGAGTCTATGTCACTCATTATACTCTGTCGAATGTCATTGAATGTCTTTACATGGGCTTG

TATGCCTATGAAAATTGTAATACAACTTTCAGCAACGGATCTCTTGGCTCTCGCATCGAT
GAAGGACGCAGCGAAATGCGATAAGTAATGTGAATTGCAGAATTCAGTGAATCATCGAAT
CTTTGAACGCATCTTGCGCTCCTTGGTATTCCGAGGAGCATGCCTGTTTGAGTGTCATTA
AATTCTCAACTCTCTTATACTTTTTTGTAAAAGAGAGCTTGGACTGTGGAGGCTTGCTGG
CCACTTTTTGGGGTCAGCTCCTCTGAAATGCATTAGCGGAACCGTTTGCAATCTGCCACA
AGTGTGATAAGTTATCTACACTGGCGAGGGGATTGCTCTCTGTAATGTTCAGCTTCTAAT
TGTCTCTACTTTGTGAGACAACTTTTGAATGCTTGACCTCAAATCAGGTAGGACTACCCG
CTGAACTTAA
>B5_10
TTTCCGTAGGTGAACCTGCGGAAGGATCATTATTGAATTATGTTTCTAGATAGGTTGTAG
CTGGCTCTTTAGAGCATGTGCACGCCTGTTTGGACTTCATTTTCATCCACCTGTGCACCT
ATTGTAGTCTTTGGTTGGGTTAGGGGGAAGTGGTCATTGTGTCAGCATCTGCTGGATGTG
AGGACTTGCATTGTGAAAGCTTTGCTGTCCTTGATGTGATCATGGAATCTCTTTCTCACT
AGAGTCTATGTCACTCATTATACTCTGTCGAATGTCATTGAATGTCTTTACATGGGCTTG
TATGCCTATGAAAATTGTAATACAACTTTCAGCAACGGATCTCTTGGCTCTCGCATCGAT
GAAGGACGCAGCGAAATGCGATAAGTAATGTGAATTGCAGAATTCAGTGAATCATCGAAT
CTTTGAACGCATCTTGCGCTCCTTGGTATTCCGAGGAGCATGCCTGTTTGAGTGTCATTA
AATTCTCAACTCTCTTATACTTTTTTGTAAAAGAGAGCTTGGACTGTGGAGGCTTGCTGG
CCACTTTTTGGGGTCAGCTCCTCTGAAATGCATTAGCGGAACCGTTTGCAATCTGCCACA
AGTGTGATAAGTTATCTACACTGGCGAGGGGATTGCTCTCTGTAATGTTCAGCTTCTAAT
TGTCTCTACTTTGTGAGACAACTTTTGAATGCTTGACCTCAAATCAGGTAGGACTACCCG
CTGAACTTAA
>B5_11
TTTCCGTAGGTGAACCTGCGGAAGGATCATTATTGAATTATGTTTCTAGATAGGTTGTAG
CTGGCTCTTTAGAGCATGTGCACGCCTGTTTGGACTTCATTTTCATCCACCTGTGCACCT
ATTGTAGTCTTTGGTTGGGTTAGGGGGAAGTGGTCATTGTGTCAGCATCTGCTGGATGTG
AGGACTTGCATTGTGAAAGCTTTGCTGTCCTTGATGTGATCATGGAATCTCTTTCTCACT
AGAGTCTATGTCACTCATTATACTCTGTCGAATGTCATTGAATGTCTTTACATGGGCTTG
TATGCCTATGAAAATTGTAATACAACTTTCAGCAACGGATCTCTTGGCTCTCGCATCGAT
GAAGGACGCAGCGAAATGCGATAAGTAATGTGAATTGCAGAATTCAGTGAATCATCGAAT
CTTTGAACGCATCTTGCGCTCCTTGGTATTCCGAGGAGCATGCCTGTTTGAGTGTCATTA
AATTCTCAACTCTCTTATACTTTTTTGTAAAAGAGAGCTTGGACTGTGGAGGCTTGCTGG
CCACTTTTTGGGGTCAGCTCCTCTGAAATGCATTAGCGGAACCGTTTGCAATCTGCCACA
AGTGTGATAAGTTATCTACACTGGCGAGGGGATTGCTCTCTGTAATGTTCAGCTTCTAAT
TGTCTCTACTTTGTGAGACAACTTTTGAATGCTTGACCTCAAATCAGGTAGGACTACCCG
CTGAACTTAA
>B5_12
TTTCCGTAGGTGAACCTGCGGAAGGATCATTATTGAATTATGTTTCTAGATAGGTTGTAG
CTGGCTCTTTAGAGCATGTGCACGCCTGTTTGGACTTCATTTTCATCCACCTGTGCACCT
ATTGTAGTCTTTGGTTGGGTTAGGGGGAAGTGGTCATTGTGTCAGCATCTGCTGGATGTG
AGGACTTGCATTGTGAAAGCTTTGCTGTCCTTGATGTGATCATGGAATCTCTTTCTCACT
AGAGTCTATGTCACTCATTATACTCTGTCGAATGTCATTGAATGTCTTTACATGGGCTTG
TATGCCTATGAAAATTGTAATACAACTTTCAGCAACGGATCTCTTGGCTCTCGCATCGAT
GAAGGACGCAGCGAAATGCGATAAGTAATGTGAATTGCAGAATTCAGTGAATCATCGAAT
CTTTGAACGCATCTTGCGCTCCTTGGTATTCCGAGGAGCATGCCTGTTTGAGTGTCATTA
AATTCTCAACTCTCTTATACTTTTTTGTAAAAGAGAGCTTGGACTGTGGAGGCTTGCTGG
CCACTTTTTGGGGTCAGCTCCTCTGAAATGCATTAGCGGAACCGTTTGCAATCTGCCACA
AGTGTGATAAGTTATCTACACTGGCGAGGGGATTGCTCTCTGTAATGTTCAGCTTCTAAT
TGTCTCTACTTTGTGAGACAACTTTTGAATGCTTGACCTCAAATCAGGTAGGACTACCCG
CTGAACTTAA

>B5_13
TTTCCGTAGGTGAACCTGCGGAAGGATCATTATTGAATTATGTTTCTAGATAGGTTGTAG
CTGGCTCTTTAGAGCATGTGCACGCCTGTTTGGACTTCATTTTCATCCACCTGTGCACCT
ATTGTAGTCTTTGGTTGGGTTAGGGGGAAGTGGTCATTGTGTCAGCATCTGCTGGATGTG
AGGACTTGCATTGTGAAAGCTTTGCTGTCCTTGATGTGATCATGGAATCTCTTTCTCACT
AGAGTCTATGTCACTCATTATACTCTGTCGAATGTCATTGAATGTCTTTACATGGGCTTG
TATGCCTATGAAAATTGTAATACAACTTTCAGCAACGGATCTCTTGGCTCTCGCATCGAT
GAAGGACGCAGCGAAATGCGATAAGTAATGTGAATTGCAGAATTCAGTGAATCATCGAAT
CTTTGAACGCATCTTGCGCTCCTTGGTATTCCGAGGAGCATGCCTGTTTGAGTGTCATTA
AATTCTCAACTCTCTTATACTTTTTTGTAAAAGAGAGCTTGGACTGTGGAGGCTTGCTGG
CCACTTTTTGGGGTCAGCTCCTCTGAAATGCATTAGCGGAACCGTTTGCAATCTGCCACA
AGTGTGATAAGTTATCTACACTGGCGAGGGGATTGCTCTCTGTAATGTTCAGCTTCTAAT
TGTCTCTACTTTGTGAGACAACTTTTGAATGCTTGACCTCAAATCAGGTAGGACTACCCG
CTGAACTTAA
>B5_14
TTTCCGTAGGTGAACCTGCGGAAGGATCATTATTGAATTATGTTTCTAGATAGGTTGTAG
CTGGCTCTTTAGAGCATGTGCACGCCTGTTTGGACTTCATTTTCATCCACCTGTGCACCT
ATTGTAGTCTTTGGTTGGGTTAGGGGGAAGTGGTCATTGTGTCAGCATCTGCTGGATGTG
AGGACTTGCATTGTGAAAGCTTTGCTGTCCTTGATGTGATCATGGAATCTCTTTCTCACT
AGAGTCTATGTCACTCATTATACTCTGTCGAATGTCATTGAATGTCTTTACATGGGCTTG
TATGCCTATGAAAATTGTAATACAACTTTCAGCAACGGATCTCTTGGCTCTCGCATCGAT
GAAGGACGCAGCGAAATGCGATAAGTAATGTGAATTGCAGAATTCAGTGAATCATCGAAT
CTTTGAACGCATCTTGCGCTCCTTGGTATTCCGAGGAGCATGCCTGTTTGAGTGTCATTA
AATTCTCAACTCTCTTATACTTTTTTGTAAAAGAGAGCTTGGACTGTGGAGGCTTGCTGG
CCACTTTTTGGGGTCAGCTCCTCTGAAATGCATTAGCGGAACCGTTTGCAATCTGCCACA
AGTGTGATAAGTTATCTACACTGGCGAGGGGATTGCTCTCTGTAATGTTCAGCTTCTAAT
TGTCTCTACTTTGTGAGACAACTTTTGAATGCTTGACCTCAAATCAGGTAGGACTACCCG
CTGAACTTAA
>B5_15
TTTCCGTAGGTGAACCTGCGGAAGGATCATTATTGAATTATGTTTCTAGATAGGTTGTAG
CTGGCTCTTTAGAGCATGTGCACGCCTGTTTGGACTTCATTTTCATCCACCTGTGCACCT
ATTGTAGTCTTTGGTTGGGTTAGGGGGAAGTGGTCATTGTGTCAGCATCTGCTGGATGTG
AGGACTTGCATTGTGAAAGCTTTGCTGTCCTTGATGTGATCATGGAATCTCTTTCTCACT
AGAGTCTATGTCACTCATTATACTCTGTCGAATGTCATTGAATGTCTTTACATGGGCTTG
TATGCCTATGAAAATTGTAATACAACTTTCAGCAACGGATCTCTTGGCTCTCGCATCGAT
GAAGGACGCAGCGAAATGCGATAAGTAATGTGAATTGCAGAATTCAGTGAATCATCGAAT
CTTTGAACGCATCTTGCGCTCCTTGGTATTCCGAGGAGCATGCCTGTTTGAGTGTCATTA
AATTCTCAACTCTCTTATACTTTTTTGTAAAAGAGAGCTTGGACTGTGGAGGCTTGCTGG
CCACTTTTTGGGGTCAGCTCCTCTGAAATGCATTAGCGGAACCGTTTGCAATCTGCCACA
AGTGTGATAAGTTATCTACACTGGCGAGGGGATTGCTCTCTGTAATGTTCAGCTTCTAAT
TGTCTCTACTTTGTGAGACAACTTTTGAATGCTTGACCTCAAATCAGGTAGGACTACCCG
CTGAACTTAA
>B5_16
TTTCCGTAGGTGAACCTGCGGAAGGATCATTATTGAATTATGTTTCTAGATAGGTTGTAG
CTGGCTCTTTAGAGCATGTGCACGCCTGTTTGGACTTCATTTTCATCCACCTGTGCACCT
ATTGTAGTCTTTGGTTGGGTTAGGGGGAAGTGGTCATTGTGTCAGCATCTGCTGGATGTG
AGGACTTGCATTGTGAAAGCTTTGCTGTCCTTGATGTGATCATGGAATCTCTTTCTCACT
AGAGTCTATGTCACTCATTATACTCTGTCGAATGTCATTGAATGTCTTTACATGGGCTTG
TATGCCTATGAAAATTGTAATACAACTTTCAGCAACGGATCTCTTGGCTCTCGCATCGAT
GAAGGACGCAGCGAAATGCGATAAGTAATGTGAATTGCAGAATTCAGTGAATCATCGAAT

CTTTGAACGCATCTTGCGCTCCTTGGTATTCCGAGGAGCATGCCTGTTTGAGTGTCATTA
AATTCTCAACTCTCTTATACTTTTTTGTAAAAGAGAGCTTGGACTGTGGAGGCTTGCTGG
CCACTTTTTGGGGTCAGCTCCTCTGAAATGCATTAGCGGAACCGTTTGCAATCTGCCACA
AGTGTGATAAGTTATCTACACTGGCGAGGGGATTGCTCTCTGTAATGTTCAGCTTCTAAT
TGTCTCTACTTTGTGAGACAACTTTTGAATGCTTGACCTCAAATCAGGTAGGACTACCCG
CTGAACTTAA
>B5_18
TTTCCGTAGGTGAACCTGCGGAAGGATCATTATTGAATTATGTTTCTAGATAGGTTGTAG
CTGGCTCTTTAGAGCATGTGCACGCCTGTTTGGACTTCATTTTCATCCACCTGTGCACCT
ATTGTAGTCTTTGGTTGGGTTAGGGGGAAGTGGTCATTGTGTCAGCATCTGCTGGATGTG
AGGACTTGCATTGTGAAAGCTTTGCTGTCCTTGATGTGATCATGGAATCTCTTTCTCACT
AGAGTCTATGTCACTCATTATACTCTGTCGAATGTCATTGAATGTCTTTACATGGGCTTG
TATGCCTATGAAAATTGTAATACAACTTTCAGCAACGGATCTCTTGGCTCTCGCATCGAT
GAAGGACGCAGCGAAATGCGATAAGTAATGTGAATTGCAGAATTCAGTGAATCATCGAAT
CTTTGAACGCATCTTGCGCTCCTTGGTATTCCGAGGAGCATGCCTGTTTGAGTGTCATTA
AATTCTCAACTCTCTTATACTTTTTTGTAAAAGAGAGCTTGGACTGTGGAGGCTTGCTGG
CCACTTTTTGGGGTCAGCTCCTCTGAAATGCATTAGCGGAACCGTTTGCAATCTGCCACA
AGTGTGATAAGTTATCTACACTGGCGAGGGGATTGCTCTCTGTAATGTTCAGCTTCTAAT
TGTCTCTACTTTGTGAGACAACTTTTGAATGCTTGACCTCAAATCAGGTAGGACTACCCG
CTGAACTTAA
>B5_19
TTTCCGTAGGTGAACCTGCGGAAGGATCATTATTGAATTATGTTTCTAGATAGGTTGTAG
CTGGCTCTTTAGAGCATGTGCACGCCTGTTTGGACTTCATTTTCATCCACCTGTGCACCT
ATTGTAGTCTTTGGTTGGGTTAGGGGGAAGTGGTCATTGTGTCAGCATCTGCTGGATGTG
AGGACTTGCATTGTGAAAGCTTTGCTGTCCTTGATGTGATCATGGAATCTCTTTCTCACT
AGAGTCTATGTCACTCATTATACTCTGTCGAATGTCATTGAATGTCTTTACATGGGCTTG
TATGCCTATGAAAATTGTAATACAACTTTCAGCAACGGATCTCTTGGCTCTCGCATCGAT
GAAGGACGCAGCGAAATGCGATAAGTAATGTGAATTGCAGAATTCAGTGAATCATCGAAT
CTTTGAACGCATCTTGCGCTCCTTGGTATTCCGAGGAGCATGCCTGTTTGAGTGTCATTA
AATTCTCAACTCTCTTATACTTTTTTGTAAAAGAGAGCTTGGACTGTGGAGGCTTGCTGG
CCACTTTTTGGGGTCAGCTCCTCTGAAATGCATTAGCGGAACCGTTTGCAATCTGCCACA
AGTGTGATAAGTTATCTACACTGGCGAGGGGATTGCTCTCTGTAATGTTCAGCTTCTAAT
TGTCTCTACTTTGTGAGACAACTTTTGAATGCTTGACCTCAAATCAGGTAGGACTACCCG
CTGAACTTAA
>B5_20
TTTCCGTAGGTGAACCTGCGGAAGGATCATTATTGAATTATGTTTCTAGATAGGTTGTAG
CTGGCTCTTTAGAGCATGTGCACGCCTGTTTGGACTTCATTTTCATCCACCTGTGCACCT
ATTGTAGTCTTTGGTTGGGTTAGGGGGAAGTGGTCATTGTGTCAGCATCTGCTGGATGTG
AGGACTTGCATTGTGAAAGCTTTGCTGTCCTTGATGTGATCATGGAATCTCTTTCTCACT
AGAGTCTATGTCACTCATTATACTCTGTCGAATGTCATTGAATGTCTTTACATGGGCTTG
TATGCCTATGAAAATTGTAATACAACTTTCAGCAACGGATCTCTTGGCTCTCGCATCGAT
GAAGGACGCAGCGAAATGCGATAAGTAATGTGAATTGCAGAATTCAGTGAATCATCGAAT
CTTTGAACGCATCTTGCGCTCCTTGGTATTCCGAGGAGCATGCCTGTTTGAGTGTCATTA
AATTCTCAACTCTCTTATACTTTTTTGTAAAAGAGAGCTTGGACTGTGGAGGCTTGCTGG
CCACTTTTTGGGGTCAGCTCCTCTGAAATGCATTAGCGGAACCGTTTGCAATCTGCCACA
AGTGTGATAAGTTATCTACACTGGCGAGGGGATTGCTCTCTGTAATGTTCAGCTTCTAAT
TGTCTCTACTTTGTGAGACAACTTTTGAATGCTTGACCTCAAATCAGGTAGGACTACCCG
CTGAACTTAA
>B5_21
TTTCCGTAGGTGAACCTGCGGAAGGATCATTATTGAATTATGTTTCTAGATAGGTTGTAG

CTGGCTCTTTAGAGCATGTGCACGCCTGTTTGGACTTCATTTTCATCCACCTGTGCACCT
ATTGTAGTCTTTGGTTGGGTTAGGGGGAAGTGGTCATTGTGTCAGCATCTGCTGGATGTG
AGGACTTGCATTGTGAAAGCTTTGCTGTCCTTGATGTGATCATGGAATCTCTTTCTCACT
AGAGTCTATGTCACTCATTATACTCTGTCGAATGTCATTGAATGTCTTTACATGGGCTTG
TATGCCTATGAAAATTGTAATACAACTTTCAGCAACGGATCTCTTGGCTCTCGCATCGAT
GAAGGACGCAGCGAAATGCGATAAGTAATGTGAATTGCAGAATTCAGTGAATCATCGAAT
CTTTGAACGCATCTTGCGCTCCTTGGTATTCCGAGGAGCATGCCTGTTTGAGTGTCATTA
AATTCTCAACTCTCTTATACTTTTTTGTAAAAGAGAGCTTGGACTGTGGAGGCTTGCTGG
CCACTTTTTGGGGTCAGCTCCTCTGAAATGCATTAGCGGAACCGTTTGCAATCTGCCACA
AGTGTGATAAGTTATCTACACTGGCGAGGGGATTGCTCTCTGTAATGTTCAGCTTCTAAT
TGTCTCTACTTTGTGAGACAACTTTTGAATGCTTGACCTCAAATCAGGTAGGACTACCCG
CTGAACTTAA
>B5_22
TTTCCGTAGGTGAACCTGCGGAAGGATCATTATTGAATTATGTTTCTAGATAGGTTGTAG
CTGGCTCTTTAGAGCATGTGCACGCCTGTTTGGACTTCATTTTCATCCACCTGTGCACCT
ATTGTAGTCTTTGGTTGGGTTAGGGGGAAGTGGTCATTGTGTCAGCATCTGCTGGATGTG
AGGACTTGCATTGTGAAAGCTTTGCTGTCCTTGATGTGATCATGGAATCTCTTTCTCACT
AGAGTCTATGTCACTCATTATACTCTGTCGAATGTCATTGAATGTCTTTACATGGGCTTG
TATGCCTATGAAAATTGTAATACAACTTTCAGCAACGGATCTCTTGGCTCTCGCATCGAT
GAAGGACGCAGCGAAATGCGATAAGTAATGTGAATTGCAGAATTCAGTGAATCATCGAAT
CTTTGAACGCATCTTGCGCTCCTTGGTATTCCGAGGAGCATGCCTGTTTGAGTGTCATTA
AATTCTCAACTCTCTTATACTTTTTTGTAAAAGAGAGCTTGGACTGTGGAGGCTTGCTGG
CCACTTTTTGGGGTCAGCTCCTCTGAAATGCATTAGCGGAACCGTTTGCAATCTGCCACA
AGTGTGATAAGTTATCTACACTGGCGAGGGGATTGCTCTCTGTAATGTTCAGCTTCTAAT
TGTCTCTACTTTGTGAGACAACTTTTGAATGCTTGACCTCAAATCAGGTAGGACTACCCG
CTGAACTTAA
>B5_23
TTTCCGTAGGTGAACCTGCGGAAGGATCATTATTGAATTATGTTTCTAGATAGGTTGTAG
CTGGCTCTTTAGAGCATGTGCACGCCTGTTTGGACTTCATTTTCATCCACCTGTGCACCT
ATTGTAGTCTTTGGTTGGGTTAGGGGGAAGTGGTCATTGTGTCAGCATCTGCTGGATGTG
AGGACTTGCATTGTGAAAGCTTTGCTGTCCTTGATGTGATCATGGAATCTCTTTCTCACT
AGAGTCTATGTCACTCATTATACTCTGTCGAATGTCATTGAATGTCTTTACATGGGCTTG
TATGCCTATGAAAATTGTAATACAACTTTCAGCAACGGATCTCTTGGCTCTCGCATCGAT
GAAGGACGCAGCGAAATGCGATAAGTAATGTGAATTGCAGAATTCAGTGAATCATCGAAT
CTTTGAACGCATCTTGCGCTCCTTGGTATTCCGAGGAGCATGCCTGTTTGAGTGTCATTA
AATTCTCAACTCTCTTATACTTTTTTGTAAAAGAGAGCTTGGACTGTGGAGGCTTGCTGG
CCACTTTTTGGGGTCAGCTCCTCTGAAATGCATTAGCGGAACCGTTTGCAATCTGCCACA
AGTGTGATAAGTTATCTACACTGGCGAGGGGATTGCTCTCTGTAATGTTCAGCTTCTAAT
TGTCTCTACTTTGTGAGACAACTTTTGAATGCTTGACCTCAAATCAGGTAGGACTACCCG
CTGAACTTAA
>B5_24
TTTCCGTAGGTGAACCTGCGGAAGGATCATTATTGAATTATGTTTCTAGATAGGTTGTAG
CTGGCTCTTTAGAGCATGTGCACGCCTGTTTGGACTTCATTTTCATCCACCTGTGCACCT
ATTGTAGTCTTTGGTTGGGTTAGGGGGAAGTGGTCATTGTGTCAGCATCTGCTGGATGTG
AGGACTTGCATTGTGAAAGCTTTGCTGTCCTTGATGTGATCATGGAATCTCTTTCTCACT
AGAGTCTATGTCACTCATTATACTCTGTCGAATGTCATTGAATGTCTTTACATGGGCTTG
TATGCCTATGAAAATTGTAATACAACTTTCAGCAACGGATCTCTTGGCTCTCGCATCGAT
GAAGGACGCAGCGAAATGCGATAAGTAATGTGAATTGCAGAATTCAGTGAATCATCGAAT
CTTTGAACGCATCTTGCGCTCCTTGGTATTCCGAGGAGCATGCCTGTTTGAGTGTCATTA
AATTCTCAACTCTCTTATACTTTTTTGTAAAAGAGAGCTTGGACTGTGGAGGCTTGCTGG

CCACTTTTTGGGGTCAGCTCCTCTGAAATGCATTAGCGGAACCGTTTGCAATCTGCCACA
AGTGTGATAAGTTATCTACACTGGCGAGGGGATTGCTCTCTGTAATGTTCAGCTTCTAAT
TGTCTCTACTTTGTGAGACAACTTTTGAATGCTTGACCTCAAATCAGGTAGGACTACCCG
CTGAACTTAA
>B5_25
TTTCCGTAGGTGAACCTGCGGAAGGATCATTATTGAATTATGTTTCTAGATAGGTTGTAG
CTGGCTCTTTAGAGCATGTGCACGCCTGTTTGGACTTCATTTTCATCCACCTGTGCACCT
ATTGTAGTCTTTGGTTGGGTTAGGGGGAAGTGGTCATTGTGTCAGCATCTGCTGGATGTG
AGGACTTGCATTGTGAAAGCTTTGCTGTCCTTGATGTGATCATGGAATCTCTTTCTCACT
AGAGTCTATGTCACTCATTATACTCTGTCGAATGTCATTGAATGTCTTTACATGGGCTTG
TATGCCTATGAAAATTGTAATACAACTTTCAGCAACGGATCTCTTGGCTCTCGCATCGAT
GAAGGACGCAGCGAAATGCGATAAGTAATGTGAATTGCAGAATTCAGTGAATCATCGAAT
CTTTGAACGCATCTTGCGCTCCTTGGTATTCCGAGGAGCATGCCTGTTTGAGTGTCATTA
AATTCTCAACTCTCTTATACTTTTTTGTAAAAGAGAGCTTGGACTGTGGAGGCTTGCTGG
CCACTTTTTGGGGTCAGCTCCTCTGAAATGCATTAGCGGAACCGTTTGCAATCTGCCACA
AGTGTGATAAGTTATCTACACTGGCGAGGGGATTGCTCTCTGTAATGTTCAGCTTCTAAT
TGTCTCTACTTTGTGAGACAACTTTTGAATGCTTGACCTCAAATCAGGTAGGACTACCCG
CTGAACTTAA
>B5_26
TTTCCGTAGGTGAACCTGCGGAAGGATCATTATTGAATTATGTTTCTAGATAGGTTGTAG
CTGGCTCTTTAGAGCATGTGCACGCCTGTTTGGACTTCATTTTCATCCACCTGTGCACCT
ATTGTAGTCTTTGGTTGGGTTAGGGGGAAGTGGTCATTGTGTCAGCATCTGCTGGATGTG
AGGACTTGCATTGTGAAAGCTTTGCTGTCCTTGATGTGATCATGGAATCTCTTTCTCACT
AGAGTCTATGTCACTCATTATACTCTGTCGAATGTCATTGAATGTCTTTACATGGGCTTG
TATGCCTATGAAAATTGTAATACAACTTTCAGCAACGGATCTCTTGGCTCTCGCATCGAT
GAAGGACGCAGCGAAATGCGATAAGTAATGTGAATTGCAGAATTCAGTGAATCATCGAAT
CTTTGAACGCATCTTGCGCTCCTTGGTATTCCGAGGAGCATGCCTGTTTGAGTGTCATTA
AATTCTCAACTCTCTTATACTTTTTTGTAAAAGAGAGCTTGGACTGTGGAGGCTTGCTGG
CCACTTTTTGGGGTCAGCTCCTCTGAAATGCATTAGCGGAACCGTTTGCAATCTGCCACA
AGTGTGATAAGTTATCTACACTGGCGAGGGGATTGCTCTCTGTAATGTTCAGCTTCTAAT
TGTCTCTACTTTGTGAGACAACTTTTGAATGCTTGACCTCAAATCAGGTAGGACTACCCG
CTGAACTTAA
>B5_27
TTTCCGTAGGTGAACCTGCGGAAGGATCATTATTGAATTATGTTTCTAGATAGGTTGTAG
CTGGCTCTTTAGAGCATGTGCACGCCTGTTTGGACTTCATTTTCATCCACCTGTGCACCT
ATTGTAGTCTTTGGTTGGGTTAGGGGGAAGTGGTCATTGTGTCAGCATCTGCTGGATGTG
AGGACTTGCATTGTGAAAGCTTTGCTGTCCTTGATGTGATCATGGAATCTCTTTCTCACT
AGAGTCTATGTCACTCATTATACTCTGTCGAATGTCATTGAATGTCTTTACATGGGCTTG
TATGCCTATGAAAATTGTAATACAACTTTCAGCAACGGATCTCTTGGCTCTCGCATCGAT
GAAGGACGCAGCGAAATGCGATAAGTAATGTGAATTGCAGAATTCAGTGAATCATCGAAT
CTTTGAACGCATCTTGCGCTCCTTGGTATTCCGAGGAGCATGCCTGTTTGAGTGTCATTA
AATTCTCAACTCTCTTATACTTTTTTGTAAAAGAGAGCTTGGACTGTGGAGGCTTGCTGG
CCACTTTTTGGGGTCAGCTCCTCTGAAATGCATTAGCGGAACCGTTTGCAATCTGCCACA
AGTGTGATAAGTTATCTACACTGGCGAGGGGATTGCTCTCTGTAATGTTCAGCTTCTAAT
TGTCTCTACTTTGTGAGACAACTTTTGAATGCTTGACCTCAAATCAGGTAGGACTACCCG
CTGAACTTAA
>B5_28
TTTCCGTAGGTGAACCTGCGGAAGGATCATTATTGAATTATGTTTCTAGATAGGTTGTAG
CTGGCTCTTTAGAGCATGTGCACGCCTGTTTGGACTTCATTTTCATCCACCTGTGCACCT
ATTGTAGTCTTTGGTTGGGTTAGGGGGAAGTGGTCATTGTGTCAGCATCTGCTGGATGTG

AGGACTTGCATTGTGAAAGCTTTGCTGTCCTTGATGTGATCATGGAATCTCTTTCTCACT
AGAGTCTATGTCACTCATTATACTCTGTCGAATGTCATTGAATGTCTTTACATGGGCTTG
TATGCCTATGAAAATTGTAATACAACTTTCAGCAACGGATCTCTTGGCTCTCGCATCGAT
GAAGGACGCAGCGAAATGCGATAAGTAATGTGAATTGCAGAATTCAGTGAATCATCGAAT
CTTTGAACGCATCTTGCGCTCCTTGGTATTCCGAGGAGCATGCCTGTTTGAGTGTCATTA
AATTCTCAACTCTCTTATACTTTTTTGTAAAAGAGAGCTTGGACTGTGGAGGCTTGCTGG
CCACTTTTTGGGGTCAGCTCCTCTGAAATGCATTAGCGGAACCGTTTGCAATCTGCCACA
AGTGTGATAAGTTATCTACACTGGCGAGGGGATTGCTCTCTGTAATGTTCAGCTTCTAAT
TGTCTCTACTTTGTGAGACAACTTTTGAATGCTTGACCTCAAATCAGGTAGGACTACCCG
CTGAACTTAA
>B5_29
TTTCCGTAGGTGAACCTGCGGAAGGATCATTATTGAATTATGTTTCTAGATAGGTTGTAG
CTGGCTCTTTAGAGCATGTGCACGCCTGTTTGGACTTCATTTTCATCCACCTGTGCACCT
ATTGTAGTCTTTGGTTGGGTTAGGGGGAAGTGGTCATTGTGTCAGCATCTGCTGGATGTG
AGGACTTGCATTGTGAAAGCTTTGCTGTCCTTGATGTGATCATGGAATCTCTTTCTCACT
AGAGTCTATGTCACTCATTATACTCTGTCGAATGTCATTGAATGTCTTTACATGGGCTTG
TATGCCTATGAAAATTGTAATACAACTTTCAGCAACGGATCTCTTGGCTCTCGCATCGAT
GAAGGACGCAGCGAAATGCGATAAGTAATGTGAATTGCAGAATTCAGTGAATCATCGAAT
CTTTGAACGCATCTTGCGCTCCTTGGTATTCCGAGGAGCATGCCTGTTTGAGTGTCATTA
AATTCTCAACTCTCTTATACTTTTTTGTAAAAGAGAGCTTGGACTGTGGAGGCTTGCTGG
CCACTTTTTGGGGTCAGCTCCTCTGAAATGCATTAGCGGAACCGTTTGCAATCTGCCACA
AGTGTGATAAGTTATCTACACTGGCGAGGGGATTGCTCTCTGTAATGTTCAGCTTCTAAT
TGTCTCTACTTTGTGAGACAACTTTTGAATGCTTGACCTCAAATCAGGTAGGACTACCCG
CTGAACTTAA
>B5_30
TTTCCGTAGGTGAACCTGCGGAAGGATCATTATTGAATTATGTTTCTAGATAGGTTGTAG
CTGGCTCTTTAGAGCATGTGCACGCCTGTTTGGACTTCATTTTCATCCACCTGTGCACCT
ATTGTAGTCTTTGGTTGGGTTAGGGGGAAGTGGTCATTGTGTCAGCATCTGCTGGATGTG
AGGACTTGCATTGTGAAAGCTTTGCTGTCCTTGATGTGATCATGGAATCTCTTTCTCACT
AGAGTCTATGTCACTCATTATACTCTGTCGAATGTCATTGAATGTCTTTACATGGGCTTG
TATGCCTATGAAAATTGTAATACAACTTTCAGCAACGGATCTCTTGGCTCTCGCATCGAT
GAAGGACGCAGCGAAATGCGATAAGTAATGTGAATTGCAGAATTCAGTGAATCATCGAAT
CTTTGAACGCATCTTGCGCTCCTTGGTATTCCGAGGAGCATGCCTGTTTGAGTGTCATTA
AATTCTCAACTCTCTTATACTTTTTTGTAAAAGAGAGCTTGGACTGTGGAGGCTTGCTGG
CCACTTTTTGGGGTCAGCTCCTCTGAAATGCATTAGCGGAACCGTTTGCAATCTGCCACA
AGTGTGATAAGTTATCTACACTGGCGAGGGGATTGCTCTCTGTAATGTTCAGCTTCTAAT
TGTCTCTACTTTGTGAGACAACTTTTGAATGCTTGACCTCAAATCAGGTAGGACTACCCG
CTGAACTTAA
>B5_32
TTTCCGTAGGTGAACCTGCGGAAGGATCATTATTGAATTATGTTTCTAGATAGGTTGTAG
CTGGCTCTTTAGAGCATGTGCACGCCTGTTTGGACTTCATTTTCATCCACCTGTGCACCT
ATTGTAGTCTTTGGTTGGGTTAGGGGGAAGTGGTCATTGTGTCAGCATCTGCTGGATGTG
AGGACTTGCATTGTGAAAGCTTTGCTGTCCTTGATGTGATCATGGAATCTCTTTCTCACT
AGAGTCTATGTCACTCATTATACTCTGTCGAATGTCATTGAATGTCTTTACATGGGCTTG
TATGCCTATGAAAATTGTAATACAACTTTCAGCAACGGATCTCTTGGCTCTCGCATCGAT
GAAGGACGCAGCGAAATGCGATAAGTAATGTGAATTGCAGAATTCAGTGAATCATCGAAT
CTTTGAACGCATCTTGCGCTCCTTGGTATTCCGAGGAGCATGCCTGTTTGAGTGTCATTA
AATTCTCAACTCTCTTATACTTTTTTGTAAAAGAGAGCTTGGACTGTGGAGGCTTGCTGG
CCACTTTTTGGGGTCAGCTCCTCTGAAATGCATTAGCGGAACCGTTTGCAATCTGCCACA
AGTGTGATAAGTTATCTACACTGGCGAGGGGATTGCTCTCTGTAATGTTCAGCTTCTAAT

TGTCTCTACTTTGTGAGACAACTTTTGAATGCTTGACCTCAAATCAGGTAGGACTACCCG
CTGAACTTAA
>B5_33
TTTCCGTAGGTGAACCTGCGGAAGGATCATTATTGAATTATGTTTCTAGATAGGTTGTAG
CTGGCTCTTTAGAGCATGTGCACGCCTGTTTGGACTTCATTTTCATCCACCTGTGCACCT
ATTGTAGTCTTTGGTTGGGTTAGGGGGAAGTGGTCATTGTGTCAGCATCTGCTGGATGTG
AGGACTTGCATTGTGAAAGCTTTGCTGTCCTTGATGTGATCATGGAATCTCTTTCTCACT
AGAGTCTATGTCACTCATTATACTCTGTCGAATGTCATTGAATGTCTTTACATGGGCTTG
TATGCCTATGAAAATTGTAATACAACTTTCAGCAACGGATCTCTTGGCTCTCGCATCGAT
GAAGGACGCAGCGAAATGCGATAAGTAATGTGAATTGCAGAATTCAGTGAATCATCGAAT
CTTTGAACGCATCTTGCGCTCCTTGGTATTCCGAGGAGCATGCCTGTTTGAGTGTCATTA
AATTCTCAACTCTCTTATACTTTTTTGTAAAAGAGAGCTTGGACTGTGGAGGCTTGCTGG
CCACTTTTTGGGGTCAGCTCCTCTGAAATGCATTAGCGGAACCGTTTGCAATCTGCCACA
AGTGTGATAAGTTATCTACACTGGCGAGGGGATTGCTCTCTGTAATGTTCAGCTTCTAAT
TGTCTCTACTTTGTGAGACAACTTTTGAATGCTTGACCTCAAATCAGGTAGGACTACCCG
CTGAACTTAA
>B5_34
TTTCCGTAGGTGAACCTGCGGAAGGATCATTATTGAATTATGTTTCTAGATAGGTTGTAG
CTGGCTCTTTAGAGCATGTGCACGCCTGTTTGGACTTCATTTTCATCCACCTGTGCACCT
ATTGTAGTCTTTGGTTGGGTTAGGGGGAAGTGGTCATTGTGTCAGCATCTGCTGGATGTG
AGGACTTGCATTGTGAAAGCTTTGCTGTCCTTGATGTGATCATGGAATCTCTTTCTCACT
AGAGTCTATGTCACTCATTATACTCTGTCGAATGTCATTGAATGTCTTTACATGGGCTTG
TATGCCTATGAAAATTGTAATACAACTTTCAGCAACGGATCTCTTGGCTCTCGCATCGAT
GAAGGACGCAGCGAAATGCGATAAGTAATGTGAATTGCAGAATTCAGTGAATCATCGAAT
CTTTGAACGCATCTTGCGCTCCTTGGTATTCCGAGGAGCATGCCTGTTTGAGTGTCATTA
AATTCTCAACTCTCTTATACTTTTTTGTAAAAGAGAGCTTGGACTGTGGAGGCTTGCTGG
CCACTTTTTGGGGTCAGCTCCTCTGAAATGCATTAGCGGAACCGTTTGCAATCTGCCACA
AGTGTGATAAGTTATCTACACTGGCGAGGGGATTGCTCTCTGTAATGTTCAGCTTCTAAT
TGTCTCTACTTTGTGAGACAACTTTTGAATGCTTGACCTCAAATCAGGTAGGACTACCCG
CTGAACTTAA
>B5_36
TTTCCGTAGGTGAACCTGCGGAAGGATCATTATTGAATTATGTTTCTAGATAGGTTGTAG
CTGGCTCTTTAGAGCATGTGCACGCCTGTTTGGACTTCATTTTCATCCACCTGTGCACCT
ATTGTAGTCTTTGGTTGGGTTAGGGGGAAGTGGTCATTGTGTCAGCATCTGCTGGATGTG
AGGACTTGCATTGTGAAAGCTTTGCTGTCCTTGATGTGATCATGGAATCTCTTTCTCACT
AGAGTCTATGTCACTCATTATACTCTGTCGAATGTCATTGAATGTCTTTACATGGGCTTG
TATGCCTATGAAAATTGTAATACAACTTTCAGCAACGGATCTCTTGGCTCTCGCATCGAT
GAAGGACGCAGCGAAATGCGATAAGTAATGTGAATTGCAGAATTCAGTGAATCATCGAAT
CTTTGAACGCATCTTGCGCTCCTTGGTATTCCGAGGAGCATGCCTGTTTGAGTGTCATTA
AATTCTCAACTCTCTTATACTTTTTTGTAAAAGAGAGCTTGGACTGTGGAGGCTTGCTGG
CCACTTTTTGGGGTCAGCTCCTCTGAAATGCATTAGCGGAACCGTTTGCAATCTGCCACA
AGTGTGATAAGTTATCTACACTGGCGAGGGGATTGCTCTCTGTAATGTTCAGCTTCTAAT
TGTCTCTACTTTGTGAGACAACTTTTGAATGCTTGACCTCAAATCAGGTAGGACTACCCG
CTGAACTTAA
>B5_37
TTTCCGTAGGTGAACCTGCGGAAGGATCATTATTGAATTATGTTTCTAGATAGGTTGTAG
CTGGCTCTTTAGAGCATGTGCACGCCTGTTTGGACTTCATTTTCATCCACCTGTGCACCT
ATTGTAGTCTTTGGTTGGGTTAGGGGGAAGTGGTCATTGTGTCAGCATCTGCTGGATGTG
AGGACTTGCATTGTGAAAGCTTTGCTGTCCTTGATGTGATCATGGAATCTCTTTCTCACT
AGAGTCTATGTCACTCATTATACTCTGTCGAATGTCATTGAATGTCTTTACATGGGCTTG

TATGCCTATGAAAATTGTAATACAACTTTCAGCAACGGATCTCTTGGCTCTCGCATCGAT
GAAGGACGCAGCGAAATGCGATAAGTAATGTGAATTGCAGAATTCAGTGAATCATCGAAT
CTTTGAACGCATCTTGCGCTCCTTGGTATTCCGAGGAGCATGCCTGTTTGAGTGTCATTA
AATTCTCAACTCTCTTATACTTTTTTGTAAAAGAGAGCTTGGACTGTGGAGGCTTGCTGG
CCACTTTTTGGGGTCAGCTCCTCTGAAATGCATTAGCGGAACCGTTTGCAATCTGCCACA
AGTGTGATAAGTTATCTACACTGGCGAGGGGATTGCTCTCTGTAATGTTCAGCTTCTAAT
TGTCTCTACTTTGTGAGACAACTTTTGAATGCTTGACCTCAAATCAGGTAGGACTACCCG
CTGAACTTAA
>B5_38
TTTCCGTAGGTGAACCTGCGGAAGGATCATTATTGAATTATGTTTCTAGATAGGTTGTAG
CTGGCTCTTTAGAGCATGTGCACGCCTGTTTGGACTTCATTTTCATCCACCTGTGCACCT
ATTGTAGTCTTTGGTTGGGTTAGGGGGAAGTGGTCATTGTGTCAGCATCTGCTGGATGTG
AGGACTTGCATTGTGAAAGCTTTGCTGTCCTTGATGTGATCATGGAATCTCTTTCTCACT
AGAGTCTATGTCACTCATTATACTCTGTCGAATGTCATTGAATGTCTTTACATGGGCTTG
TATGCCTATGAAAATTGTAATACAACTTTCAGCAACGGATCTCTTGGCTCTCGCATCGAT
GAAGGACGCAGCGAAATGCGATAAGTAATGTGAATTGCAGAATTCAGTGAATCATCGAAT
CTTTGAACGCATCTTGCGCTCCTTGGTATTCCGAGGAGCATGCCTGTTTGAGTGTCATTA
AATTCTCAACTCTCTTATACTTTTTTGTAAAAGAGAGCTTGGACTGTGGAGGCTTGCTGG
CCACTTTTTGGGGTCAGCTCCTCTGAAATGCATTAGCGGAACCGTTTGCAATCTGCCACA
AGTGTGATAAGTTATCTACACTGGCGAGGGGATTGCTCTCTGTAATGTTCAGCTTCTAAT
TGTCTCTACTTTGTGAGACAACTTTTGAATGCTTGACCTCAAATCAGGTAGGACTACCCG
CTGAACTTAA
>B5_39
TTTCCGTAGGTGAACCTGCGGAAGGATCATTATTGAATTATGTTTCTAGATAGGTTGTAG
CTGGCTCTTTAGAGCATGTGCACGCCTGTTTGGACTTCATTTTCATCCACCTGTGCACCT
ATTGTAGTCTTTGGTTGGGTTAGGGGGAAGTGGTCATTGTGTCAGCATCTGCTGGATGTG
AGGACTTGCATTGTGAAAGCTTTGCTGTCCTTGATGTGATCATGGAATCTCTTTCTCACT
AGAGTCTATGTCACTCATTATACTCTGTCGAATGTCATTGAATGTCTTTACATGGGCTTG
TATGCCTATGAAAATTGTAATACAACTTTCAGCAACGGATCTCTTGGCTCTCGCATCGAT
GAAGGACGCAGCGAAATGCGATAAGTAATGTGAATTGCAGAATTCAGTGAATCATCGAAT
CTTTGAACGCATCTTGCGCTCCTTGGTATTCCGAGGAGCATGCCTGTTTGAGTGTCATTA
AATTCTCAACTCTCTTATACTTTTTTGTAAAAGAGAGCTTGGACTGTGGAGGCTTGCTGG
CCACTTTTTGGGGTCAGCTCCTCTGAAATGCATTAGCGGAACCGTTTGCAATCTGCCACA
AGTGTGATAAGTTATCTACACTGGCGAGGGGATTGCTCTCTGTAATGTTCAGCTTCTAAT
TGTCTCTACTTTGTGAGACAACTTTTGAATGCTTGACCTCAAATCAGGTAGGACTACCCG
CTGAACTTAA
>B5_41
TTTCCGTAGGTGAACCTGCGGAAGGATCATTATTGAATTATGTTTCTAGATAGGTTGTAG
CTGGCTCTTTAGAGCATGTGCACGCCTGTTTGGACTTCATTTTCATCCACCTGTGCACCT
ATTGTAGTCTTTGGTTGGGTTAGGGGGAAGTGGTCATTGTGTCAGCATCTGCTGGATGTG
AGGACTTGCATTGTGAAAGCTTTGCTGTCCTTGATGTGATCATGGAATCTCTTTCTCACT
AGAGTCTATGTCACTCATTATACTCTGTCGAATGTCATTGAATGTCTTTACATGGGCTTG
TATGCCTATGAAAATTGTAATACAACTTTCAGCAACGGATCTCTTGGCTCTCGCATCGAT
GAAGGACGCAGCGAAATGCGATAAGTAATGTGAATTGCAGAATTCAGTGAATCATCGAAT
CTTTGAACGCATCTTGCGCTCCTTGGTATTCCGAGGAGCATGCCTGTTTGAGTGTCATTA
AATTCTCAACTCTCTTATACTTTTTTGTAAAAGAGAGCTTGGACTGTGGAGGCTTGCTGG
CCACTTTTTGGGGTCAGCTCCTCTGAAATGCATTAGCGGAACCGTTTGCAATCTGCCACA
AGTGTGATAAGTTATCTACACTGGCGAGGGGATTGCTCTCTGTAATGTTCAGCTTCTAAT
TGTCTCTACTTTGTGAGACAACTTTTGAATGCTTGACCTCAAATCAGGTAGGACTACCCG
CTGAACTTAA

>B5_42
TTTCCGTAGGTGAACCTGCGGAAGGATCATTATTGAATTATGTTTCTAGATAGGTTGTAG
CTGGCTCTTTAGAGCATGTGCACGCCTGTTTGGACTTCATTTTCATCCACCTGTGCACCT
ATTGTAGTCTTTGGTTGGGTTAGGGGGAAGTGGTCATTGTGTCAGCATCTGCTGGATGTG
AGGACTTGCATTGTGAAAGCTTTGCTGTCCTTGATGTGATCATGGAATCTCTTTCTCACT
AGAGTCTATGTCACTCATTATACTCTGTCGAATGTCATTGAATGTCTTTACATGGGCTTG
TATGCCTATGAAAATTGTAATACAACTTTCAGCAACGGATCTCTTGGCTCTCGCATCGAT
GAAGGACGCAGCGAAATGCGATAAGTAATGTGAATTGCAGAATTCAGTGAATCATCGAAT
CTTTGAACGCATCTTGCGCTCCTTGGTATTCCGAGGAGCATGCCTGTTTGAGTGTCATTA
AATTCTCAACTCTCTTATACTTTTTTGTAAAAGAGAGCTTGGACTGTGGAGGCTTGCTGG
CCACTTTTTGGGGTCAGCTCCTCTGAAATGCATTAGCGGAACCGTTTGCAATCTGCCACA
AGTGTGATAAGTTATCTACACTGGCGAGGGGATTGCTCTCTGTAATGTTCAGCTTCTAAT
TGTCTCTACTTTGTGAGACAACTTTTGAATGCTTGACCTCAAATCAGGTAGGACTACCCG
CTGAACTTAA
>B5_43
TTTCCGTAGGTGAACCTGCGGAAGGATCATTATTGAATTATGTTTCTAGATAGGTTGTAG
CTGGCTCTTTAGAGCATGTGCACGCCTGTTTGGACTTCATTTTCATCCACCTGTGCACCT
ATTGTAGTCTTTGGTTGGGTTAGGGGGAAGTGGTCATTGTGTCAGCATCTGCTGGATGTG
AGGACTTGCATTGTGAAAGCTTTGCTGTCCTTGATGTGATCATGGAATCTCTTTCTCACT
AGAGTCTATGTCACTCATTATACTCTGTCGAATGTCATTGAATGTCTTTACATGGGCTTG
TATGCCTATGAAAATTGTAATACAACTTTCAGCAACGGATCTCTTGGCTCTCGCATCGAT
GAAGGACGCAGCGAAATGCGATAAGTAATGTGAATTGCAGAATTCAGTGAATCATCGAAT
CTTTGAACGCATCTTGCGCTCCTTGGTATTCCGAGGAGCATGCCTGTTTGAGTGTCATTA
AATTCTCAACTCTCTTATACTTTTTTGTAAAAGAGAGCTTGGACTGTGGAGGCTTGCTGG
CCACTTTTTGGGGTCAGCTCCTCTGAAATGCATTAGCGGAACCGTTTGCAATCTGCCACA
AGTGTGATAAGTTATCTACACTGGCGAGGGGATTGCTCTCTGTAATGTTCAGCTTCTAAT
TGTCTCTACTTTGTGAGACAACTTTTGAATGCTTGACCTCAAATCAGGTAGGACTACCCG
CTGAACTTAA
>B5_44
TTTCCGTAGGTGAACCTGCGGAAGGATCATTATTGAATTATGTTTCTAGATAGGTTGTAG
CTGGCTCTTTAGAGCATGTGCACGCCTGTTTGGACTTCATTTTCATCCACCTGTGCACCT
ATTGTAGTCTTTGGTTGGGTTAGGGGGAAGTGGTCATTGTGTCAGCATCTGCTGGATGTG
AGGACTTGCATTGTGAAAGCTTTGCTGTCCTTGATGTGATCATGGAATCTCTTTCTCACT
AGAGTCTATGTCACTCATTATACTCTGTCGAATGTCATTGAATGTCTTTACATGGGCTTG
TATGCCTATGAAAATTGTAATACAACTTTCAGCAACGGATCTCTTGGCTCTCGCATCGAT
GAAGGACGCAGCGAAATGCGATAAGTAATGTGAATTGCAGAATTCAGTGAATCATCGAAT
CTTTGAACGCATCTTGCGCTCCTTGGTATTCCGAGGAGCATGCCTGTTTGAGTGTCATTA
AATTCTCAACTCTCTTATACTTTTTTGTAAAAGAGAGCTTGGACTGTGGAGGCTTGCTGG
CCACTTTTTGGGGTCAGCTCCTCTGAAATGCATTAGCGGAACCGTTTGCAATCTGCCACA
AGTGTGATAAGTTATCTACACTGGCGAGGGGATTGCTCTCTGTAATGTTCAGCTTCTAAT
TGTCTCTACTTTGTGAGACAACTTTTGAATGCTTGACCTCAAATCAGGTAGGACTACCCG
CTGAACTTAA
>B5_45
TTTCCGTAGGTGAACCTGCGGAAGGATCATTATTGAATTATGTTTCTAGATAGGTTGTAG
CTGGCTCTTTAGAGCATGTGCACGCCTGTTTGGACTTCATTTTCATCCACCTGTGCACCT
ATTGTAGTCTTTGGTTGGGTTAGGGGGAAGTGGTCATTGTGTCAGCATCTGCTGGATGTG
AGGACTTGCATTGTGAAAGCTTTGCTGTCCTTGATGTGATCATGGAATCTCTTTCTCACT
AGAGTCTATGTCACTCATTATACTCTGTCGAATGTCATTGAATGTCTTTACATGGGCTTG
TATGCCTATGAAAATTGTAATACAACTTTCAGCAACGGATCTCTTGGCTCTCGCATCGAT
GAAGGACGCAGCGAAATGCGATAAGTAATGTGAATTGCAGAATTCAGTGAATCATCGAAT

CTTTGAACGCATCTTGCGCTCCTTGGTATTCCGAGGAGCATGCCTGTTTGAGTGTCATTA
AATTCTCAACTCTCTTATACTTTTTTGTAAAAGAGAGCTTGGACTGTGGAGGCTTGCTGG
CCACTTTTTGGGGTCAGCTCCTCTGAAATGCATTAGCGGAACCGTTTGCAATCTGCCACA
AGTGTGATAAGTTATCTACACTGGCGAGGGGATTGCTCTCTGTAATGTTCAGCTTCTAAT
TGTCTCTACTTTGTGAGACAACTTTTGAATGCTTGACCTCAAATCAGGTAGGACTACCCG
CTGAACTTAA
>B5_46
TTTCCGTAGGTGAACCTGCGGAAGGATCATTATTGAATTATGTTTCTAGATAGGTTGTAG
CTGGCTCTTTAGAGCATGTGCACGCCTGTTTGGACTTCATTTTCATCCACCTGTGCACCT
ATTGTAGTCTTTGGTTGGGTTAGGGGGAAGTGGTCATTGTGTCAGCATCTGCTGGATGTG
AGGACTTGCATTGTGAAAGCTTTGCTGTCCTTGATGTGATCATGGAATCTCTTTCTCACT
AGAGTCTATGTCACTCATTATACTCTGTCGAATGTCATTGAATGTCTTTACATGGGCTTG
TATGCCTATGAAAATTGTAATACAACTTTCAGCAACGGATCTCTTGGCTCTCGCATCGAT
GAAGGACGCAGCGAAATGCGATAAGTAATGTGAATTGCAGAATTCAGTGAATCATCGAAT
CTTTGAACGCATCTTGCGCTCCTTGGTATTCCGAGGAGCATGCCTGTTTGAGTGTCATTA
AATTCTCAACTCTCTTATACTTTTTTGTAAAAGAGAGCTTGGACTGTGGAGGCTTGCTGG
CCACTTTTTGGGGTCAGCTCCTCTGAAATGCATTAGCGGAACCGTTTGCAATCTGCCACA
AGTGTGATAAGTTATCTACACTGGCGAGGGGATTGCTCTCTGTAATGTTCAGCTTCTAAT
TGTCTCTACTTTGTGAGACAACTTTTGAATGCTTGACCTCAAATCAGGTAGGACTACCCG
CTGAACTTAA
>B5_47
TTTCCGTAGGTGAACCTGCGGAAGGATCATTATTGAATTATGTTTCTAGATAGGTTGTAG
CTGGCTCTTTAGAGCATGTGCACGCCTGTTTGGACTTCATTTTCATCCACCTGTGCACCT
ATTGTAGTCTTTGGTTGGGTTAGGGGGAAGTGGTCATTGTGTCAGCATCTGCTGGATGTG
AGGACTTGCATTGTGAAAGCTTTGCTGTCCTTGATGTGATCATGGAATCTCTTTCTCACT
AGAGTCTATGTCACTCATTATACTCTGTCGAATGTCATTGAATGTCTTTACATGGGCTTG
TATGCCTATGAAAATTGTAATACAACTTTCAGCAACGGATCTCTTGGCTCTCGCATCGAT
GAAGGACGCAGCGAAATGCGATAAGTAATGTGAATTGCAGAATTCAGTGAATCATCGAAT
CTTTGAACGCATCTTGCGCTCCTTGGTATTCCGAGGAGCATGCCTGTTTGAGTGTCATTA
AATTCTCAACTCTCTTATACTTTTTTGTAAAAGAGAGCTTGGACTGTGGAGGCTTGCTGG
CCACTTTTTGGGGTCAGCTCCTCTGAAATGCATTAGCGGAACCGTTTGCAATCTGCCACA
AGTGTGATAAGTTATCTACACTGGCGAGGGGATTGCTCTCTGTAATGTTCAGCTTCTAAT
TGTCTCTACTTTGTGAGACAACTTTTGAATGCTTGACCTCAAATCAGGTAGGACTACCCG
CTGAACTTAA
>B5_48
TTTCCGTAGGTGAACCTGCGGAAGGATCATTATTGAATTATGTTTCTAGATAGGTTGTAG
CTGGCTCTTTAGAGCATGTGCACGCCTGTTTGGACTTCATTTTCATCCACCTGTGCACCT
ATTGTAGTCTTTGGTTGGGTTAGGGGGAAGTGGTCATTGTGTCAGCATCTGCTGGATGTG
AGGACTTGCATTGTGAAAGCTTTGCTGTCCTTGATGTGATCATGGAATCTCTTTCTCACT
AGAGTCTATGTCACTCATTATACTCTGTCGAATGTCATTGAATGTCTTTACATGGGCTTG
TATGCCTATGAAAATTGTAATACAACTTTCAGCAACGGATCTCTTGGCTCTCGCATCGAT
GAAGGACGCAGCGAAATGCGATAAGTAATGTGAATTGCAGAATTCAGTGAATCATCGAAT
CTTTGAACGCATCTTGCGCTCCTTGGTATTCCGAGGAGCATGCCTGTTTGAGTGTCATTA
AATTCTCAACTCTCTTATACTTTTTTGTAAAAGAGAGCTTGGACTGTGGAGGCTTGCTGG
CCACTTTTTGGGGTCAGCTCCTCTGAAATGCATTAGCGGAACCGTTTGCAATCTGCCACA
AGTGTGATAAGTTATCTACACTGGCGAGGGGATTGCTCTCTGTAATGTTCAGCTTCTAAT
TGTCTCTACTTTGTGAGACAACTTTTGAATGCTTGACCTCAAATCAGGTAGGACTACCCG
CTGAACTTAA
>B5_49
TTTCCGTAGGTGAACCTGCGGAAGGATCATTATTGAATTATGTTTCTAGATAGGTTGTAG

CTGGCTCTTTAGAGCATGTGCACGCCTGTTTGGACTTCATTTTCATCCACCTGTGCACCT
ATTGTAGTCTTTGGTTGGGTTAGGGGGAAGTGGTCATTGTGTCAGCATCTGCTGGATGTG
AGGACTTGCATTGTGAAAGCTTTGCTGTCCTTGATGTGATCATGGAATCTCTTTCTCACT
AGAGTCTATGTCACTCATTATACTCTGTCGAATGTCATTGAATGTCTTTACATGGGCTTG
TATGCCTATGAAAATTGTAATACAACTTTCAGCAACGGATCTCTTGGCTCTCGCATCGAT
GAAGGACGCAGCGAAATGCGATAAGTAATGTGAATTGCAGAATTCAGTGAATCATCGAAT
CTTTGAACGCATCTTGCGCTCCTTGGTATTCCGAGGAGCATGCCTGTTTGAGTGTCATTA
AATTCTCAACTCTCTTATACTTTTTTGTAAAAGAGAGCTTGGACTGTGGAGGCTTGCTGG
CCACTTTTTGGGGTCAGCTCCTCTGAAATGCATTAGCGGAACCGTTTGCAATCTGCCACA
AGTGTGATAAGTTATCTACACTGGCGAGGGGATTGCTCTCTGTAATGTTCAGCTTCTAAT
TGTCTCTACTTTGTGAGACAACTTTTGAATGCTTGACCTCAAATCAGGTAGGACTACCCG
CTGAACTTAA
>B5_50
TTTCCGTAGGTGAACCTGCGGAAGGATCATTATTGAATTATGTTTCTAGATAGGTTGTAG
CTGGCTCTTTAGAGCATGTGCACGCCTGTTTGGACTTCATTTTCATCCACCTGTGCACCT
ATTGTAGTCTTTGGTTGGGTTAGGGGGAAGTGGTCATTGTGTCAGCATCTGCTGGATGTG
AGGACTTGCATTGTGAAAGCTTTGCTGTCCTTGATGTGATCATGGAATCTCTTTCTCACT
AGAGTCTATGTCACTCATTATACTCTGTCGAATGTCATTGAATGTCTTTACATGGGCTTG
TATGCCTATGAAAATTGTAATACAACTTTCAGCAACGGATCTCTTGGCTCTCGCATCGAT
GAAGGACGCAGCGAAATGCGATAAGTAATGTGAATTGCAGAATTCAGTGAATCATCGAAT
CTTTGAACGCATCTTGCGCTCCTTGGTATTCCGAGGAGCATGCCTGTTTGAGTGTCATTA
AATTCTCAACTCTCTTATACTTTTTTGTAAAAGAGAGCTTGGACTGTGGAGGCTTGCTGG
CCACTTTTTGGGGTCAGCTCCTCTGAAATGCATTAGCGGAACCGTTTGCAATCTGCCACA
AGTGTGATAAGTTATCTACACTGGCGAGGGGATTGCTCTCTGTAATGTTCAGCTTCTAAT
TGTCTCTACTTTGTGAGACAACTTTTGAATGCTTGACCTCAAATCAGGTAGGACTACCCG
CTGAACTTAA
>B5−52
TTTCCGTAGGTGAACCTGCGGAAGGATCATTATTGAATTATGTTTCTAGATAGGTTGTAG
CTGGCTCTTTAGAGCATGTGCACGCCTGTTTGGACTTCATTTTCATCCACCTGTGCACCT
ATTGTAGTCTTTGGTTGGGTTAGGGGGAAGTGGTCATTGTGTCAGCATCTGCTGGATGTG
AGGACTTGCATTGTGAAAGCTTTGCTGTCCTTGATGTGATCATGGAATCTCTTTCTCACT
AGAGTCTATGTCACTCATTATACTCTGTCGAATGTCATTGAATGTCTTTACATGGGCTTG
TATGCCTATGAAAATTGTAATACAACTTTCAGCAACGGATCTCTTGGCTCTCGCATCGAT
GAAGGACGCAGCGAAATGCGATAAGTAATGTGAATTGCAGAATTCAGTGAATCATCGAAT
CTTTGAACGCATCTTGCGCTCCTTGGTATTCCGAGGAGCATGCCTGTTTGAGTGTCATTA
AATTCTCAACTCTCTTATACTTTTTTGTAAAAGAGAGCTTGGACTGTGGAGGCTTGCTGG
CCACTTTTTGGGGTCAGCTCCTCTGAAATGCATTAGCGGAACCGTTTGCAATCTGCCACA
AGTGTGATAAGTTATCTACACTGGCGAGGGGATTGCTCTCTGTAATGTTCAGCTTCTAAT
TGTCTCTACTTTGTGAGACAACTTTTGAATGCTTGACCTCAAATCAGGTAGGACTACCCG
CTGAACTTAA
>B5−53
TTTCCGTAGGTGAACCTGCGGAAGGATCATTATTGAATTATGTTTCTAGATAGGTTGTAG
CTGGCTCTTTAGAGCATGTGCACGCCTGTTTGGACTTCATTTTCATCCACCTGTGCACCT
ATTGTAGTCTTTGGTTGGGTTAGGGGGAAGTGGTCATTGTGTCAGCATCTGCTGGATGTG
AGGACTTGCATTGTGAAAGCTTTGCTGTCCTTGATGTGATCATGGAATCTCTTTCTCACT
AGAGTCTATGTCACTCATTATACTCTGTCGAATGTCATTGAATGTCTTTACATGGGCTTG
TATGCCTATGAAAATTGTAATACAACTTTCAGCAACGGATCTCTTGGCTCTCGCATCGAT
GAAGGACGCAGCGAAATGCGATAAGTAATGTGAATTGCAGAATTCAGTGAATCATCGAAT
CTTTGAACGCATCTTGCGCTCCTTGGTATTCCGAGGAGCATGCCTGTTTGAGTGTCATTA
AATTCTCAACTCTCTTATACTTTTTTGTAAAAGAGAGCTTGGACTGTGGAGGCTTGCTGG

CCACTTTTTGGGGTCAGCTCCTCTGAAATGCATTAGCGGAACCGTTTGCAATCTGCCACA
AGTGTGATAAGTTATCTACACTGGCGAGGGGATTGCTCTCTGTAATGTTCAGCTTCTAAT
TGTCTCTACTTTGTGAGACAACTTTTGAATGCTTGACCTCAAATCAGGTAGGACTACCCG
CTGAACTTAA
>B5-54
TTTCCGTAGGTGAACCTGCGGAAGGATCATTATTGAATTATGTTTCTAGATAGGTTGTAG
CTGGCTCTTTAGAGCATGTGCACGCCTGTTTGGACTTCATTTTCATCCACCTGTGCACCT
ATTGTAGTCTTTGGTTGGGTTAGGGGGAAGTGGTCATTGTGTCAGCATCTGCTGGATGTG
AGGACTTGCATTGTGAAAGCTTTGCTGTCCTTGATGTGATCATGGAATCTCTTTCTCACT
AGAGTCTATGTCACTCATTATACTCTGTCGAATGTCATTGAATGTCTTTACATGGGCTTG
TATGCCTATGAAAATTGTAATACAACTTTCAGCAACGGATCTCTTGGCTCTCGCATCGAT
GAAGGACGCAGCGAAATGCGATAAGTAATGTGAATTGCAGAATTCAGTGAATCATCGAAT
CTTTGAACGCATCTTGCGCTCCTTGGTATTCCGAGGAGCATGCCTGTTTGAGTGTCATTA
AATTCTCAACTCTCTTATACTTTTTTGTAAAAGAGAGCTTGGACTGTGGAGGCTTGCTGG
CCACTTTTTGGGGTCAGCTCCTCTGAAATGCATTAGCGGAACCGTTTGCAATCTGCCACA
AGTGTGATAAGTTATCTACACTGGCGAGGGGATTGCTCTCTGTAATGTTCAGCTTCTAAT
TGTCTCTACTTTGTGAGACAACTTTTGAATGCTTGACCTCAAATCAGGTAGGACTACCCG
CTGAACTTAA
>B5-55
TTTCCGTAGGTGAACCTGCGGAAGGATCATTATTGAATTATGTTTCTAGATAGGTTGTAG
CTGGCTCTTTAGAGCATGTGCACGCCTGTTTGGACTTCATTTTCATCCACCTGTGCACCT
ATTGTAGTCTTTGGTTGGGTTAGGGGGAAGTGGTCATTGTGTCAGCATCTGCTGGATGTG
AGGACTTGCATTGTGAAAGCTTTGCTGTCCTTGATGTGATCATGGAATCTCTTTCTCACT
AGAGTCTATGTCACTCATTATACTCTGTCGAATGTCATTGAATGTCTTTACATGGGCTTG
TATGCCTATGAAAATTGTAATACAACTTTCAGCAACGGATCTCTTGGCTCTCGCATCGAT
GAAGGACGCAGCGAAATGCGATAAGTAATGTGAATTGCAGAATTCAGTGAATCATCGAAT
CTTTGAACGCATCTTGCGCTCCTTGGTATTCCGAGGAGCATGCCTGTTTGAGTGTCATTA
AATTCTCAACTCTCTTATACTTTTTTGTAAAAGAGAGCTTGGACTGTGGAGGCTTGCTGG
CCACTTTTTGGGGTCAGCTCCTCTGAAATGCATTAGCGGAACCGTTTGCAATCTGCCACA
AGTGTGATAAGTTATCTACACTGGCGAGGGGATTGCTCTCTGTAATGTTCAGCTTCTAAT
TGTCTCTACTTTGTGAGACAACTTTTGAATGCTTGACCTCAAATCAGGTAGGACTACCCG
CTGAACTTAA
>B5-56
TTTCCGTAGGTGAACCTGCGGAAGGATCATTATTGAATTATGTTTCTAGATAGGTTGTAG
CTGGCTCTTTAGAGCATGTGCACGCCTGTTTGGACTTCATTTTCATCCACCTGTGCACCT
ATTGTAGTCTTTGGTTGGGTTAGGGGGAAGTGGTCATTGTGTCAGCATCTGCTGGATGTG
AGGACTTGCATTGTGAAAGCTTTGCTGTCCTTGATGTGATCATGGAATCTCTTTCTCACT
AGAGTCTATGTCACTCATTATACTCTGTCGAATGTCATTGAATGTCTTTACATGGGCTTG
TATGCCTATGAAAATTGTAATACAACTTTCAGCAACGGATCTCTTGGCTCTCGCATCGAT
GAAGGACGCAGCGAAATGCGATAAGTAATGTGAATTGCAGAATTCAGTGAATCATCGAAT
CTTTGAACGCATCTTGCGCTCCTTGGTATTCCGAGGAGCATGCCTGTTTGAGTGTCATTA
AATTCTCAACTCTCTTATACTTTTTTGTAAAAGAGAGCTTGGACTGTGGAGGCTTGCTGG
CCACTTTTTGGGGTCAGCTCCTCTGAAATGCATTAGCGGAACCGTTTGCAATCTGCCACA
AGTGTGATAAGTTATCTACACTGGCGAGGGGATTGCTCTCTGTAATGTTCAGCTTCTAAT
TGTCTCTACTTTGTGAGACAACTTTTGAATGCTTGACCTCAAATCAGGTAGGACTACCCG
CTGAACTTAA
>B5-57
TTTCCGTAGGTGAACCTGCGGAAGGATCATTATTGAATTATGTTTCTAGATAGGTTGTAG
CTGGCTCTTTAGAGCATGTGCACGCCTGTTTGGACTTCATTTTCATCCACCTGTGCACCT
ATTGTAGTCTTTGGTTGGGTTAGGGGGAAGTGGTCATTGTGTCAGCATCTGCTGGATGTG

AGGACTTGCATTGTGAAAGCTTTGCTGTCCTTGATGTGATCATGGAATCTCTTTCTCACT
AGAGTCTATGTCACTCATTATACTCTGTCGAATGTCATTGAATGTCTTTACATGGGCTTG
TATGCCTATGAAAATTGTAATACAACTTTCAGCAACGGATCTCTTGGCTCTCGCATCGAT
GAAGGACGCAGCGAAATGCGATAAGTAATGTGAATTGCAGAATTCAGTGAATCATCGAAT
CTTTGAACGCATCTTGCGCTCCTTGGTATTCCGAGGAGCATGCCTGTTTGAGTGTCATTA
AATTCTCAACTCTCTTATACTTTTTTGTAAAAGAGAGCTTGGACTGTGGAGGCTTGCTGG
CCACTTTTTGGGGTCAGCTCCTCTGAAATGCATTAGCGGAACCGTTTGCAATCTGCCACA
AGTGTGATAAGTTATCTACACTGGCGAGGGGATTGCTCTCTGTAATGTTCAGCTTCTAAT
TGTCTCTACTTTGTGAGACAACTTTTGAATGCTTGACCTCAAATCAGGTAGGACTACCCG
CTGAACTTAA
>B5-58
TTTCCGTAGGTGAACCTGCGGAAGGATCATTATTGAATTATGTTTCTAGATAGGTTGTAG
CTGGCTCTTTAGAGCATGTGCACGCCTGTTTGGACTTCATTTTCATCCACCTGTGCACCT
ATTGTAGTCTTTGGTTGGGTTAGGGGGAAGTGGTCATTGTGTCAGCATCTGCTGGATGTG
AGGACTTGCATTGTGAAAGCTTTGCTGTCCTTGATGTGATCATGGAATCTCTTTCTCACT
AGAGTCTATGTCACTCATTATACTCTGTCGAATGTCATTGAATGTCTTTACATGGGCTTG
TATGCCTATGAAAATTGTAATACAACTTTCAGCAACGGATCTCTTGGCTCTCGCATCGAT
GAAGGACGCAGCGAAATGCGATAAGTAATGTGAATTGCAGAATTCAGTGAATCATCGAAT
CTTTGAACGCATCTTGCGCTCCTTGGTATTCCGAGGAGCATGCCTGTTTGAGTGTCATTA
AATTCTCAACTCTCTTATACTTTTTTGTAAAAGAGAGCTTGGACTGTGGAGGCTTGCTGG
CCACTTTTTGGGGTCAGCTCCTCTGAAATGCATTAGCGGAACCGTTTGCAATCTGCCACA
AGTGTGATAAGTTATCTACACTGGCGAGGGGATTGCTCTCTGTAATGTTCAGCTTCTAAT
TGTCTCTACTTTGTGAGACAACTTTTGAATGCTTGACCTCAAATCAGGTAGGACTACCCG
CTGAACTTAA
>B5-59
TTTCCGTAGGTGAACCTGCGGAAGGATCATTATTGAATTATGTTTCTAGATAGGTTGTAG
CTGGCTCTTTAGAGCATGTGCACGCCTGTTTGGACTTCATTTTCATCCACCTGTGCACCT
ATTGTAGTCTTTGGTTGGGTTAGGGGGAAGTGGTCATTGTGTCAGCATCTGCTGGATGTG
AGGACTTGCATTGTGAAAGCTTTGCTGTCCTTGATGTGATCATGGAATCTCTTTCTCACT
AGAGTCTATGTCACTCATTATACTCTGTCGAATGTCATTGAATGTCTTTACATGGGCTTG
TATGCCTATGAAAATTGTAATACAACTTTCAGCAACGGATCTCTTGGCTCTCGCATCGAT
GAAGGACGCAGCGAAATGCGATAAGTAATGTGAATTGCAGAATTCAGTGAATCATCGAAT
CTTTGAACGCATCTTGCGCTCCTTGGTATTCCGAGGAGCATGCCTGTTTGAGTGTCATTA
AATTCTCAACTCTCTTATACTTTTTTGTAAAAGAGAGCTTGGACTGTGGAGGCTTGCTGG
CCACTTTTTGGGGTCAGCTCCTCTGAAATGCATTAGCGGAACCGTTTGCAATCTGCCACA
AGTGTGATAAGTTATCTACACTGGCGAGGGGATTGCTCTCTGTAATGTTCAGCTTCTAAT
TGTCTCTACTTTGTGAGACAACTTTTGAATGCTTGACCTCAAATCAGGTAGGACTACCCG
CTGAACTTAA
>B5-60
TTTCCGTAGGTGAACCTGCGGAAGGATCATTATTGAATTATGTTTCTAGATAGGTTGTAG
CTGGCTCTTTAGAGCATGTGCACGCCTGTTTGGACTTCATTTTCATCCACCTGTGCACCT
ATTGTAGTCTTTGGTTGGGTTAGGGGGAAGTGGTCATTGTGTCAGCATCTGCTGGATGTG
AGGACTTGCATTGTGAAAGCTTTGCTGTCCTTGATGTGATCATGGAATCTCTTTCTCACT
AGAGTCTATGTCACTCATTATACTCTGTCGAATGTCATTGAATGTCTTTACATGGGCTTG
TATGCCTATGAAAATTGTAATACAACTTTCAGCAACGGATCTCTTGGCTCTCGCATCGAT
GAAGGACGCAGCGAAATGCGATAAGTAATGTGAATTGCAGAATTCAGTGAATCATCGAAT
CTTTGAACGCATCTTGCGCTCCTTGGTATTCCGAGGAGCATGCCTGTTTGAGTGTCATTA
AATTCTCAACTCTCTTATACTTTTTTGTAAAAGAGAGCTTGGACTGTGGAGGCTTGCTGG
CCACTTTTTGGGGTCAGCTCCTCTGAAATGCATTAGCGGAACCGTTTGCAATCTGCCACA
AGTGTGATAAGTTATCTACACTGGCGAGGGGATTGCTCTCTGTAATGTTCAGCTTCTAAT

TGTCTCTACTTTGTGAGACAACTTTTGAATGCTTGACCTCAAATCAGGTAGGACTACCCG
CTGAACTTAA
>B5-61
TTTCCGTAGGTGAACCTGCGGAAGGATCATTATTGAATTATGTTTCTAGATAGGTTGTAG
CTGGCTCTTTAGAGCATGTGCACGCCTGTTTGGACTTCATTTTCATCCACCTGTGCACCT
ATTGTAGTCTTTGGTTGGGTTAGGGGGAAGTGGTCATTGTGTCAGCATCTGCTGGATGTG
AGGACTTGCATTGTGAAAGCTTTGCTGTCCTTGATGTGATCATGGAATCTCTTTCTCACT
AGAGTCTATGTCACTCATTATACTCTGTCGAATGTCATTGAATGTCTTTACATGGGCTTG
TATGCCTATGAAAATTGTAATACAACTTTCAGCAACGGATCTCTTGGCTCTCGCATCGAT
GAAGGACGCAGCGAAATGCGATAAGTAATGTGAATTGCAGAATTCAGTGAATCATCGAAT
CTTTGAACGCATCTTGCGCTCCTTGGTATTCCGAGGAGCATGCCTGTTTGAGTGTCATTA
AATTCTCAACTCTCTTATACTTTTTTGTAAAAGAGAGCTTGGACTGTGGAGGCTTGCTGG
CCACTTTTTGGGGTCAGCTCCTCTGAAATGCATTAGCGGAACCGTTTGCAATCTGCCACA
AGTGTGATAAGTTATCTACACTGGCGAGGGGATTGCTCTCTGTAATGTTCAGCTTCTAAT
TGTCTCTACTTTGTGAGACAACTTTTGAATGCTTGACCTCAAATCAGGTAGGACTACCCG
CTGAACTTAA
>B5-62
TTTCCGTAGGTGAACCTGCGGAAGGATCATTATTGAATTATGTTTCTAGATAGGTTGTAG
CTGGCTCTTTAGAGCATGTGCACGCCTGTTTGGACTTCATTTTCATCCACCTGTGCACCT
ATTGTAGTCTTTGGTTGGGTTAGGGGGAAGTGGTCATTGTGTCAGCATCTGCTGGATGTG
AGGACTTGCATTGTGAAAGCTTTGCTGTCCTTGATGTGATCATGGAATCTCTTTCTCACT
AGAGTCTATGTCACTCATTATACTCTGTCGAATGTCATTGAATGTCTTTACATGGGCTTG
TATGCCTATGAAAATTGTAATACAACTTTCAGCAACGGATCTCTTGGCTCTCGCATCGAT
GAAGGACGCAGCGAAATGCGATAAGTAATGTGAATTGCAGAATTCAGTGAATCATCGAAT
CTTTGAACGCATCTTGCGCTCCTTGGTATTCCGAGGAGCATGCCTGTTTGAGTGTCATTA
AATTCTCAACTCTCTTATACTTTTTTGTAAAAGAGAGCTTGGACTGTGGAGGCTTGCTGG
CCACTTTTTGGGGTCAGCTCCTCTGAAATGCATTAGCGGAACCGTTTGCAATCTGCCACA
AGTGTGATAAGTTATCTACACTGGCGAGGGGATTGCTCTCTGTAATGTTCAGCTTCTAAT
TGTCTCTACTTTGTGAGACAACTTTTGAATGCTTGACCTCAAATCAGGTAGGACTACCCG
CTGAACTTAA
>B5-63
TTTCCGTAGGTGAACCTGCGGAAGGATCATTATTGAATTATGTTTCTAGATAGGTTGTAG
CTGGCTCTTTAGAGCATGTGCACGCCTGTTTGGACTTCATTTTCATCCACCTGTGCACCT
ATTGTAGTCTTTGGTTGGGTTAGGGGGAAGTGGTCATTGTGTCAGCATCTGCTGGATGTG
AGGACTTGCATTGTGAAAGCTTTGCTGTCCTTGATGTGATCATGGAATCTCTTTCTCACT
AGAGTCTATGTCACTCATTATACTCTGTCGAATGTCATTGAATGTCTTTACATGGGCTTG
TATGCCTATGAAAATTGTAATACAACTTTCAGCAACGGATCTCTTGGCTCTCGCATCGAT
GAAGGACGCAGCGAAATGCGATAAGTAATGTGAATTGCAGAATTCAGTGAATCATCGAAT
CTTTGAACGCATCTTGCGCTCCTTGGTATTCCGAGGAGCATGCCTGTTTGAGTGTCATTA
AATTCTCAACTCTCTTATACTTTTTTGTAAAAGAGAGCTTGGACTGTGGAGGCTTGCTGG
CCACTTTTTGGGGTCAGCTCCTCTGAAATGCATTAGCGGAACCGTTTGCAATCTGCCACA
AGTGTGATAAGTTATCTACACTGGCGAGGGGATTGCTCTCTGTAATGTTCAGCTTCTAAT
TGTCTCTACTTTGTGAGACAACTTTTGAATGCTTGACCTCAAATCAGGTAGGACTACCCG
CTGAACTTAA
>B5-64
TTTCCGTAGGTGAACCTGCGGAAGGATCATTATTGAATTATGTTTCTAGATAGGTTGTAG
CTGGCTCTTTAGAGCATGTGCACGCCTGTTTGGACTTCATTTTCATCCACCTGTGCACCT
ATTGTAGTCTTTGGTTGGGTTAGGGGGAAGTGGTCATTGTGTCAGCATCTGCTGGATGTG
AGGACTTGCATTGTGAAAGCTTTGCTGTCCTTGATGTGATCATGGAATCTCTTTCTCACT
AGAGTCTATGTCACTCATTATACTCTGTCGAATGTCATTGAATGTCTTTACATGGGCTTG

TATGCCTATGAAAATTGTAATACAACTTTCAGCAACGGATCTCTTGGCTCTCGCATCGAT
GAAGGACGCAGCGAAATGCGATAAGTAATGTGAATTGCAGAATTCAGTGAATCATCGAAT
CTTTGAACGCATCTTGCGCTCCTTGGTATTCCGAGGAGCATGCCTGTTTGAGTGTCATTA
AATTCTCAACTCTCTTATACTTTTTTGTAAAAGAGAGCTTGGACTGTGGAGGCTTGCTGG
CCACTTTTTGGGGTCAGCTCCTCTGAAATGCATTAGCGGAACCGTTTGCAATCTGCCACA
AGTGTGATAAGTTATCTACACTGGCGAGGGGATTGCTCTCTGTAATGTTCAGCTTCTAAT
TGTCTCTACTTTGTGAGACAACTTTTGAATGCTTGACCTCAAATCAGGTAGGACTACCCG
CTGAACTTAA
>B5-65
TTTCCGTAGGTGAACCTGCGGAAGGATCATTATTGAATTATGTTTCTAGATAGGTTGTAG
CTGGCTCTTTAGAGCATGTGCACGCCTGTTTGGACTTCATTTTCATCCACCTGTGCACCT
ATTGTAGTCTTTGGTTGGGTTAGGGGGAAGTGGTCATTGTGTCAGCATCTGCTGGATGTG
AGGACTTGCATTGTGAAAGCTTTGCTGTCCTTGATGTGATCATGGAATCTCTTTCTCACT
AGAGTCTATGTCACTCATTATACTCTGTCGAATGTCATTGAATGTCTTTACATGGGCTTG
TATGCCTATGAAAATTGTAATACAACTTTCAGCAACGGATCTCTTGGCTCTCGCATCGAT
GAAGGACGCAGCGAAATGCGATAAGTAATGTGAATTGCAGAATTCAGTGAATCATCGAAT
CTTTGAACGCATCTTGCGCTCCTTGGTATTCCGAGGAGCATGCCTGTTTGAGTGTCATTA
AATTCTCAACTCTCTTATACTTTTTTGTAAAAGAGAGCTTGGACTGTGGAGGCTTGCTGG
CCACTTTTTGGGGTCAGCTCCTCTGAAATGCATTAGCGGAACCGTTTGCAATCTGCCACA
AGTGTGATAAGTTATCTACACTGGCGAGGGGATTGCTCTCTGTAATGTTCAGCTTCTAAT
TGTCTCTACTTTGTGAGACAACTTTTGAATGCTTGACCTCAAATCAGGTAGGACTACCCG
CTGAACTTAA
>B5-66
TTTCCGTAGGTGAACCTGCGGAAGGATCATTATTGAATTATGTTTCTAGATAGGTTGTAG
CTGGCTCTTTAGAGCATGTGCACGCCTGTTTGGACTTCATTTTCATCCACCTGTGCACCT
ATTGTAGTCTTTGGTTGGGTTAGGGGGAAGTGGTCATTGTGTCAGCATCTGCTGGATGTG
AGGACTTGCATTGTGAAAGCTTTGCTGTCCTTGATGTGATCATGGAATCTCTTTCTCACT
AGAGTCTATGTCACTCATTATACTCTGTCGAATGTCATTGAATGTCTTTACATGGGCTTG
TATGCCTATGAAAATTGTAATACAACTTTCAGCAACGGATCTCTTGGCTCTCGCATCGAT
GAAGGACGCAGCGAAATGCGATAAGTAATGTGAATTGCAGAATTCAGTGAATCATCGAAT
CTTTGAACGCATCTTGCGCTCCTTGGTATTCCGAGGAGCATGCCTGTTTGAGTGTCATTA
AATTCTCAACTCTCTTATACTTTTTTGTAAAAGAGAGCTTGGACTGTGGAGGCTTGCTGG
CCACTTTTTGGGGTCAGCTCCTCTGAAATGCATTAGCGGAACCGTTTGCAATCTGCCACA
AGTGTGATAAGTTATCTACACTGGCGAGGGGATTGCTCTCTGTAATGTTCAGCTTCTAAT
TGTCTCTACTTTGTGAGACAACTTTTGAATGCTTGACCTCAAATCAGGTAGGACTACCCG
CTGAACTTAA
>B5-67
TTTCCGTAGGTGAACCTGCGGAAGGATCATTATTGAATTATGTTTCTAGATAGGTTGTAG
CTGGCTCTTTAGAGCATGTGCACGCCTGTTTGGACTTCATTTTCATCCACCTGTGCACCT
ATTGTAGTCTTTGGTTGGGTTAGGGGGAAGTGGTCATTGTGTCAGCATCTGCTGGATGTG
AGGACTTGCATTGTGAAAGCTTTGCTGTCCTTGATGTGATCATGGAATCTCTTTCTCACT
AGAGTCTATGTCACTCATTATACTCTGTCGAATGTCATTGAATGTCTTTACATGGGCTTG
TATGCCTATGAAAATTGTAATACAACTTTCAGCAACGGATCTCTTGGCTCTCGCATCGAT
GAAGGACGCAGCGAAATGCGATAAGTAATGTGAATTGCAGAATTCAGTGAATCATCGAAT
CTTTGAACGCATCTTGCGCTCCTTGGTATTCCGAGGAGCATGCCTGTTTGAGTGTCATTA
AATTCTCAACTCTCTTATACTTTTTTGTAAAAGAGAGCTTGGACTGTGGAGGCTTGCTGG
CCACTTTTTGGGGTCAGCTCCTCTGAAATGCATTAGCGGAACCGTTTGCAATCTGCCACA
AGTGTGATAAGTTATCTACACTGGCGAGGGGATTGCTCTCTGTAATGTTCAGCTTCTAAT
TGTCTCTACTTTGTGAGACAACTTTTGAATGCTTGACCTCAAATCAGGTAGGACTACCCG
CTGAACTTAA

>B5–68
TTTCCGTAGGTGAACCTGCGGAAGGATCATTATTGAATTATGTTTCTAGATAGGTTGTAG
CTGGCTCTTTAGAGCATGTGCACGCCTGTTTGGACTTCATTTTCATCCACCTGTGCACCT
ATTGTAGTCTTTGGTTGGGTTAGGGGGAAGTGGTCATTGTGTCAGCATCTGCTGGATGTG
AGGACTTGCATTGTGAAAGCTTTGCTGTCCTTGATGTGATCATGGAATCTCTTTCTCACT
AGAGTCTATGTCACTCATTATACTCTGTCGAATGTCATTGAATGTCTTTACATGGGCTTG
TATGCCTATGAAAATTGTAATACAACTTTCAGCAACGGATCTCTTGGCTCTCGCATCGAT
GAAGGACGCAGCGAAATGCGATAAGTAATGTGAATTGCAGAATTCAGTGAATCATCGAAT
CTTTGAACGCATCTTGCGCTCCTTGGTATTCCGAGGAGCATGCCTGTTTGAGTGTCATTA
AATTCTCAACTCTCTTATACTTTTTTGTAAAAGAGAGCTTGGACTGTGGAGGCTTGCTGG
CCACTTTTTGGGGTCAGCTCCTCTGAAATGCATTAGCGGAACCGTTTGCAATCTGCCACA
AGTGTGATAAGTTATCTACACTGGCGAGGGGATTGCTCTCTGTAATGTTCAGCTTCTAAT
TGTCTCTACTTTGTGAGACAACTTTTGAATGCTTGACCTCAAATCAGGTAGGACTACCCG
CTGAACTTAA
>B8_52
TTTCCGTAGGTGAACCTGCGGAAGGATCATTATTGAATTATGTTTCTAGATAGGTTGTAG
CTGGCTCTTTAGAGCATGTGCACGCCTGTTTGGACTTCATTTTCATCCACCTGTGCACCT
ATTGTAGTCTTTGGTTGGGTTAGGGGGAAGTGGTCATTGTGTCAGCATCTGCTGGATGTG
AGGACTTGCATTGTGAAAGCTTTGCTGTCCTTGATGTGATCATGGAATCTCTTTCTCACT
AGAGTCTATGTCACTCATTATACTCTGTCGAATGTCATTGAATGTCTTTACATGGGCTTG
TATGCCTATGAAAATTGTAATACAACTTTCAGCAACGGATCTCTTGGCTCTCGCATCGAT
GAAGGACGCAGCGAAATGCGATAAGTAATGTGAATTGCAGAATTCAGTGAATCATCGAAT
CTTTGAACGCATCTTGCGCTCCTTGGTATTCCGAGGAGCATGCCTGTTTGAGTGTCATTA
AATTCTCAACTCTCTTATACTTTTTTGTAAAAGAGAGCTTGGACTGTGGAGGCTTGCTGG
CCACTTTTTGGGGTCAGCTCCTCTGAAATGCATTAGCGGAACCGTTTGCAATCTGCCACA
AGTGTGATAAGTTATCTACACTGGCGAGGGGATTGCTCTCTGTAATGTTCAGCTTCTAAT
TGTCTCTACTTTGTGAGACAACTTTTGAATGCTTGACCTCAAATCAGGTAGGACTACCCG
CTGAACTTAA
>B8_51
TTTCCGTAGGTGAACCTGCGGAAGGATCATTATTGAATTATGTTTCTAGATAGGTTGTAG
CTGGCTCTTTAGAGCATGTGCACGCCTGTTTGGACTTCATTTTCATCCACCTGTGCACCT
ATTGTAGTCTTTGGTTGGGTTAGGGGGAAGTGGTCATTGTGTCAGCATCTGCTGGATGTG
AGGACTTGCATTGTGAAAGCTTTGCTGTCCTTGATGTGATCATGGAATCTCTTTCTCACT
AGAGTCTATGTCACTCATTATACTCTGTCGAATGTCATTGAATGTCTTTACATGGGCTTG
TATGCCTATGAAAATTGTAATACAACTTTCAGCAACGGATCTCTTGGCTCTCGCATCGAT
GAAGGACGCAGCGAAATGCGATAAGTAATGTGAATTGCAGAATTCAGTGAATCATCGAAT
CTTTGAACGCATCTTGCGCTCCTTGGTATTCCGAGGAGCATGCCTGTTTGAGTGTCATTA
AATTCTCAACTCTCTTATACTTTTTTGTAAAAGAGAGCTTGGACTGTGGAGGCTTGCTGG
CCACTTTTTGGGGTCAGCTCCTCTGAAATGCATTAGCGGAACCGTTTGCAATCTGCCACA
AGTGTGATAAGTTATCTACACTGGCGAGGGGATTGCTCTCTGTAATGTTCAGCTTCTAAT
TGTCTCTACTTTGTGAGACAACTTTTGAATGCTTGACCTCAAATCAGGTAGGACTACCCG
CTGAACTTAA
>B8_50
TTTCCGTAGGTGAACCTGCGGAAGGATCATTATTGAATTATGTTTCTAGATAGGTTGTAG
CTGGCTCTTTAGAGCATGTGCACGCCTGTTTGGACTTCATTTTCATCCACCTGTGCACCT
ATTGTAGTCTTTGGTTGGGTTAGGGGGAAGTGGTCATTGTGTCAGCATCTGCTGGATGTG
AGGACTTGCATTGTGAAAGCTTTGCTGTCCTTGATGTGATCATGGAATCTCTTTCTCACT
AGAGTCTATGTCACTCATTATACTCTGTCGAATGTCATTGAATGTCTTTACATGGGCTTG
TATGCCTATGAAAATTGTAATACAACTTTCAGCAACGGATCTCTTGGCTCTCGCATCGAT
GAAGGACGCAGCGAAATGCGATAAGTAATGTGAATTGCAGAATTCAGTGAATCATCGAAT

CTTTGAACGCATCTTGCGCTCCTTGGTATTCCGAGGAGCATGCCTGTTTGAGTGTCATTA
AATTCTCAACTCTCTTATACTTTTTTGTAAAAGAGAGCTTGGACTGTGGAGGCTTGCTGG
CCACTTTTTGGGGTCAGCTCCTCTGAAATGCATTAGCGGAACCGTTTGCAATCTGCCACA
AGTGTGATAAGTTATCTACACTGGCGAGGGGATTGCTCTCTGTAATGTTCAGCTTCTAAT
TGTCTCTACTTTGTGAGACAACTTTTGAATGCTTGACCTCAAATCAGGTAGGACTACCCG
CTGAACTTAA
>B6_2
TTTCCGTAGGTGAACCTGCGGAAGGATCATTATTGAATTATGTTTCTAGATAGGTTGTAG
CTGGCTCTTTAGAGCATGTGCACGCCTGTTTGGACTTCATTTTCATCCACCTGTGCACCT
ATTGTAGTCTTTGGTTGGGTTAGGGGGAAGTGGTCATTGTGTCAGCATCTGCTGGATGTG
AGGACTTGCATTGTGAAAGCTTTGCTGTCCTTGATGTGATCATGGAATCTCTTTCTCACT
AGAGTCTATGTCACTCATTATACTCTGTCGAATGTCATTGAATGTCTTTACATGGGCTTG
TATGCCTATGAAAATTGTAATACAACTTTCAGCAACGGATCTCTTGGCTCTCGCATCGAT
GAAGGACGCAGCGAAATGCGATAAGTAATGTGAATTGCAGAATTCAGTGAATCATCGAAT
CTTTGAACGCATCTTGCGCTCCTTGGTATTCCGAGGAGCATGCCTGTTTGAGTGTCATTA
AATTCTCAACTCTCTTATACTTTTTTGTAAAAGAGAGCTTGGACTGTGGAGGCTTGCTGG
CCACTTTTTGGGGTCAGCTCCTCTGAAATGCATTAGCGGAACCGTTTGCAATCTGCCACA
AGTGTGATAAGTTATCTACACTGGCGAGGGGATTGCTCTCTGTAATGTTCAGCTTCTAAT
TGTCTCTACTTTGTGAGACAACTTTTGAATGCTTGACCTCAAATCAGGTAGGACTACCCG
CTGAACTTAA
>B6_4
TTTCCGTAGGTGAACCTGCGGAAGGATCATTATTGAATTATGTTTCTAGATAGGTTGTAG
CTGGCTCTTTAGAGCATGTGCACGCCTGTTTGGACTTCATTTTCATCCACCTGTGCACCT
ATTGTAGTCTTTGGTTGGGTTAGGGGGAAGTGGTCATTGTGTCAGCATCTGCTGGATGTG
AGGACTTGCATTGTGAAAGCTTTGCTGTCCTTGATGTGATCATGGAATCTCTTTCTCACT
AGAGTCTATGTCACTCATTATACTCTGTCGAATGTCATTGAATGTCTTTACATGGGCTTG
TATGCCTATGAAAATTGTAATACAACTTTCAGCAACGGATCTCTTGGCTCTCGCATCGAT
GAAGGACGCAGCGAAATGCGATAAGTAATGTGAATTGCAGAATTCAGTGAATCATCGAAT
CTTTGAACGCATCTTGCGCTCCTTGGTATTCCGAGGAGCATGCCTGTTTGAGTGTCATTA
AATTCTCAACTCTCTTATACTTTTTTGTAAAAGAGAGCTTGGACTGTGGAGGCTTGCTGG
CCACTTTTTGGGGTCAGCTCCTCTGAAATGCATTAGCGGAACCGTTTGCAATCTGCCACA
AGTGTGATAAGTTATCTACACTGGCGAGGGGATTGCTCTCTGTAATGTTCAGCTTCTAAT
TGTCTCTACTTTGTGAGACAACTTTTGAATGCTTGACCTCAAATCAGGTAGGACTACCCG
CTGAACTTAA
>B6_8
TTTCCGTAGGTGAACCTGCGGAAGGATCATTATTGAATTATGTTTCTAGATAGGTTGTAG
CTGGCTCTTTAGAGCATGTGCACGCCTGTTTGGACTTCATTTTCATCCACCTGTGCACCT
ATTGTAGTCTTTGGTTGGGTTAGGGGGAAGTGGTCATTGTGTCAGCATCTGCTGGATGTG
AGGACTTGCATTGTGAAAGCTTTGCTGTCCTTGATGTGATCATGGAATCTCTTTCTCACT
AGAGTCTATGTCACTCATTATACTCTGTCGAATGTCATTGAATGTCTTTACATGGGCTTG
TATGCCTATGAAAATTGTAATACAACTTTCAGCAACGGATCTCTTGGCTCTCGCATCGAT
GAAGGACGCAGCGAAATGCGATAAGTAATGTGAATTGCAGAATTCAGTGAATCATCGAAT
CTTTGAACGCATCTTGCGCTCCTTGGTATTCCGAGGAGCATGCCTGTTTGAGTGTCATTA
AATTCTCAACTCTCTTATACTTTTTTGTAAAAGAGAGCTTGGACTGTGGAGGCTTGCTGG
CCACTTTTTGGGGTCAGCTCCTCTGAAATGCATTAGCGGAACCGTTTGCAATCTGCCACA
AGTGTGATAAGTTATCTACACTGGCGAGGGGATTGCTCTCTGTAATGTTCAGCTTCTAAT
TGTCTCTACTTTGTGAGACAACTTTTGAATGCTTGACCTCAAATCAGGTAGGACTACCCG
CTGAACTTAA
>B6_11
TTTCCGTAGGTGAACCTGCGGAAGGATCATTATTGAATTATGTTTCTAGATAGGTTGTAG

CTGGCTCTTTAGAGCATGTGCACGCCTGTTTGGACTTCATTTTCATCCACCTGTGCACCT
ATTGTAGTCTTTGGTTGGGTTAGGGGGAAGTGGTCATTGTGTCAGCATCTGCTGGATGTG
AGGACTTGCATTGTGAAAGCTTTGCTGTCCTTGATGTGATCATGGAATCTCTTTCTCACT
AGAGTCTATGTCACTCATTATACTCTGTCGAATGTCATTGAATGTCTTTACATGGGCTTG
TATGCCTATGAAAATTGTAATACAACTTTCAGCAACGGATCTCTTGGCTCTCGCATCGAT
GAAGGACGCAGCGAAATGCGATAAGTAATGTGAATTGCAGAATTCAGTGAATCATCGAAT
CTTTGAACGCATCTTGCGCTCCTTGGTATTCCGAGGAGCATGCCTGTTTGAGTGTCATTA
AATTCTCAACTCTCTTATACTTTTTTGTAAAAGAGAGCTTGGACTGTGGAGGCTTGCTGG
CCACTTTTTGGGGTCAGCTCCTCTGAAATGCATTAGCGGAACCGTTTGCAATCTGCCACA
AGTGTGATAAGTTATCTACACTGGCGAGGGGATTGCTCTCTGTAATGTTCAGCTTCTAAT
TGTCTCTACTTTGTGAGACAACTTTTGAATGCTTGACCTCAAATCAGGTAGGACTACCCG
CTGAACTTAA
>B6_12
TTTCCGTAGGTGAACCTGCGGAAGGATCATTATTGAATTATGTTTCTAGATAGGTTGTAG
CTGGCTCTTTAGAGCATGTGCACGCCTGTTTGGACTTCATTTTCATCCACCTGTGCACCT
ATTGTAGTCTTTGGTTGGGTTAGGGGGAAGTGGTCATTGTGTCAGCATCTGCTGGATGTG
AGGACTTGCATTGTGAAAGCTTTGCTGTCCTTGATGTGATCATGGAATCTCTTTCTCACT
AGAGTCTATGTCACTCATTATACTCTGTCGAATGTCATTGAATGTCTTTACATGGGCTTG
TATGCCTATGAAAATTGTAATACAACTTTCAGCAACGGATCTCTTGGCTCTCGCATCGAT
GAAGGACGCAGCGAAATGCGATAAGTAATGTGAATTGCAGAATTCAGTGAATCATCGAAT
CTTTGAACGCATCTTGCGCTCCTTGGTATTCCGAGGAGCATGCCTGTTTGAGTGTCATTA
AATTCTCAACTCTCTTATACTTTTTTGTAAAAGAGAGCTTGGACTGTGGAGGCTTGCTGG
CCACTTTTTGGGGTCAGCTCCTCTGAAATGCATTAGCGGAACCGTTTGCAATCTGCCACA
AGTGTGATAAGTTATCTACACTGGCGAGGGGATTGCTCTCTGTAATGTTCAGCTTCTAAT
TGTCTCTACTTTGTGAGACAACTTTTGAATGCTTGACCTCAAATCAGGTAGGACTACCCG
CTGAACTTAA
>B6_14
TTTCCGTAGGTGAACCTGCGGAAGGATCATTATTGAATTATGTTTCTAGATAGGTTGTAG
CTGGCTCTTTAGAGCATGTGCACGCCTGTTTGGACTTCATTTTCATCCACCTGTGCACCT
ATTGTAGTCTTTGGTTGGGTTAGGGGGAAGTGGTCATTGTGTCAGCATCTGCTGGATGTG
AGGACTTGCATTGTGAAAGCTTTGCTGTCCTTGATGTGATCATGGAATCTCTTTCTCACT
AGAGTCTATGTCACTCATTATACTCTGTCGAATGTCATTGAATGTCTTTACATGGGCTTG
TATGCCTATGAAAATTGTAATACAACTTTCAGCAACGGATCTCTTGGCTCTCGCATCGAT
GAAGGACGCAGCGAAATGCGATAAGTAATGTGAATTGCAGAATTCAGTGAATCATCGAAT
CTTTGAACGCATCTTGCGCTCCTTGGTATTCCGAGGAGCATGCCTGTTTGAGTGTCATTA
AATTCTCAACTCTCTTATACTTTTTTGTAAAAGAGAGCTTGGACTGTGGAGGCTTGCTGG
CCACTTTTTGGGGTCAGCTCCTCTGAAATGCATTAGCGGAACCGTTTGCAATCTGCCACA
AGTGTGATAAGTTATCTACACTGGCGAGGGGATTGCTCTCTGTAATGTTCAGCTTCTAAT
TGTCTCTACTTTGTGAGACAACTTTTGAATGCTTGACCTCAAATCAGGTAGGACTACCCG
CTGAACTTAA
>B6_17
TTTCCGTAGGTGAACCTGCGGAAGGATCATTATTGAATTATGTTTCTAGATAGGTTGTAG
CTGGCTCTTTAGAGCATGTGCACGCCTGTTTGGACTTCATTTTCATCCACCTGTGCACCT
ATTGTAGTCTTTGGTTGGGTTAGGGGGAAGTGGTCATTGTGTCAGCATCTGCTGGATGTG
AGGACTTGCATTGTGAAAGCTTTGCTGTCCTTGATGTGATCATGGAATCTCTTTCTCACT
AGAGTCTATGTCACTCATTATACTCTGTCGAATGTCATTGAATGTCTTTACATGGGCTTG
TATGCCTATGAAAATTGTAATACAACTTTCAGCAACGGATCTCTTGGCTCTCGCATCGAT
GAAGGACGCAGCGAAATGCGATAAGTAATGTGAATTGCAGAATTCAGTGAATCATCGAAT
CTTTGAACGCATCTTGCGCTCCTTGGTATTCCGAGGAGCATGCCTGTTTGAGTGTCATTA
AATTCTCAACTCTCTTATACTTTTTTGTAAAAGAGAGCTTGGACTGTGGAGGCTTGCTGG

CCACTTTTTGGGGTCAGCTCCTCTGAAATGCATTAGCGGAACCGTTTGCAATCTGCCACA
AGTGTGATAAGTTATCTACACTGGCGAGGGGATTGCTCTCTGTAATGTTCAGCTTCTAAT
TGTCTCTACTTTGTGAGACAACTTTTGAATGCTTGACCTCAAATCAGGTAGGACTACCCG
CTGAACTTAA
>B6_21
TTTCCGTAGGTGAACCTGCGGAAGGATCATTATTGAATTATGTTTCTAGATAGGTTGTAG
CTGGCTCTTTAGAGCATGTGCACGCCTGTTTGGACTTCATTTTCATCCACCTGTGCACCT
ATTGTAGTCTTTGGTTGGGTTAGGGGGAAGTGGTCATTGTGTCAGCATCTGCTGGATGTG
AGGACTTGCATTGTGAAAGCTTTGCTGTCCTTGATGTGATCATGGAATCTCTTTCTCACT
AGAGTCTATGTCACTCATTATACTCTGTCGAATGTCATTGAATGTCTTTACATGGGCTTG
TATGCCTATGAAAATTGTAATACAACTTTCAGCAACGGATCTCTTGGCTCTCGCATCGAT
GAAGGACGCAGCGAAATGCGATAAGTAATGTGAATTGCAGAATTCAGTGAATCATCGAAT
CTTTGAACGCATCTTGCGCTCCTTGGTATTCCGAGGAGCATGCCTGTTTGAGTGTCATTA
AATTCTCAACTCTCTTATACTTTTTTGTAAAAGAGAGCTTGGACTGTGGAGGCTTGCTGG
CCACTTTTTGGGGTCAGCTCCTCTGAAATGCATTAGCGGAACCGTTTGCAATCTGCCACA
AGTGTGATAAGTTATCTACACTGGCGAGGGGATTGCTCTCTGTAATGTTCAGCTTCTAAT
TGTCTCTACTTTGTGAGACAACTTTTGAATGCTTGACCTCAAATCAGGTAGGACTACCCG
CTGAACTTAA
>B6_22
TTTCCGTAGGTGAACCTGCGGAAGGATCATTATTGAATTATGTTTCTAGATAGGTTGTAG
CTGGCTCTTTAGAGCATGTGCACGCCTGTTTGGACTTCATTTTCATCCACCTGTGCACCT
ATTGTAGTCTTTGGTTGGGTTAGGGGGAAGTGGTCATTGTGTCAGCATCTGCTGGATGTG
AGGACTTGCATTGTGAAAGCTTTGCTGTCCTTGATGTGATCATGGAATCTCTTTCTCACT
AGAGTCTATGTCACTCATTATACTCTGTCGAATGTCATTGAATGTCTTTACATGGGCTTG
TATGCCTATGAAAATTGTAATACAACTTTCAGCAACGGATCTCTTGGCTCTCGCATCGAT
GAAGGACGCAGCGAAATGCGATAAGTAATGTGAATTGCAGAATTCAGTGAATCATCGAAT
CTTTGAACGCATCTTGCGCTCCTTGGTATTCCGAGGAGCATGCCTGTTTGAGTGTCATTA
AATTCTCAACTCTCTTATACTTTTTTGTAAAAGAGAGCTTGGACTGTGGAGGCTTGCTGG
CCACTTTTTGGGGTCAGCTCCTCTGAAATGCATTAGCGGAACCGTTTGCAATCTGCCACA
AGTGTGATAAGTTATCTACACTGGCGAGGGGATTGCTCTCTGTAATGTTCAGCTTCTAAT
TGTCTCTACTTTGTGAGACAACTTTTGAATGCTTGACCTCAAATCAGGTAGGACTACCCG
CTGAACTTAA
>B6_23
TTTCCGTAGGTGAACCTGCGGAAGGATCATTATTGAATTATGTTTCTAGATAGGTTGTAG
CTGGCTCTTTAGAGCATGTGCACGCCTGTTTGGACTTCATTTTCATCCACCTGTGCACCT
ATTGTAGTCTTTGGTTGGGTTAGGGGGAAGTGGTCATTGTGTCAGCATCTGCTGGATGTG
AGGACTTGCATTGTGAAAGCTTTGCTGTCCTTGATGTGATCATGGAATCTCTTTCTCACT
AGAGTCTATGTCACTCATTATACTCTGTCGAATGTCATTGAATGTCTTTACATGGGCTTG
TATGCCTATGAAAATTGTAATACAACTTTCAGCAACGGATCTCTTGGCTCTCGCATCGAT
GAAGGACGCAGCGAAATGCGATAAGTAATGTGAATTGCAGAATTCAGTGAATCATCGAAT
CTTTGAACGCATCTTGCGCTCCTTGGTATTCCGAGGAGCATGCCTGTTTGAGTGTCATTA
AATTCTCAACTCTCTTATACTTTTTTGTAAAAGAGAGCTTGGACTGTGGAGGCTTGCTGG
CCACTTTTTGGGGTCAGCTCCTCTGAAATGCATTAGCGGAACCGTTTGCAATCTGCCACA
AGTGTGATAAGTTATCTACACTGGCGAGGGGATTGCTCTCTGTAATGTTCAGCTTCTAAT
TGTCTCTACTTTGTGAGACAACTTTTGAATGCTTGACCTCAAATCAGGTAGGACTACCCG
CTGAACTTAA
>B6_24
TTTCCGTAGGTGAACCTGCGGAAGGATCATTATTGAATTATGTTTCTAGATAGGTTGTAG
CTGGCTCTTTAGAGCATGTGCACGCCTGTTTGGACTTCATTTTCATCCACCTGTGCACCT
ATTGTAGTCTTTGGTTGGGTTAGGGGGAAGTGGTCATTGTGTCAGCATCTGCTGGATGTG

AGGACTTGCATTGTGAAAGCTTTGCTGTCCTTGATGTGATCATGGAATCTCTTTCTCACT
AGAGTCTATGTCACTCATTATACTCTGTCGAATGTCATTGAATGTCTTTACATGGGCTTG
TATGCCTATGAAAATTGTAATACAACTTTCAGCAACGGATCTCTTGGCTCTCGCATCGAT
GAAGGACGCAGCGAAATGCGATAAGTAATGTGAATTGCAGAATTCAGTGAATCATCGAAT
CTTTGAACGCATCTTGCGCTCCTTGGTATTCCGAGGAGCATGCCTGTTTGAGTGTCATTA
AATTCTCAACTCTCTTATACTTTTTTGTAAAAGAGAGCTTGGACTGTGGAGGCTTGCTGG
CCACTTTTTGGGGTCAGCTCCTCTGAAATGCATTAGCGGAACCGTTTGCAATCTGCCACA
AGTGTGATAAGTTATCTACACTGGCGAGGGGATTGCTCTCTGTAATGTTCAGCTTCTAAT
TGTCTCTACTTTGTGAGACAACTTTTGAATGCTTGACCTCAAATCAGGTAGGACTACCCG
CTGAACTTAA
>B6_25
TTTCCGTAGGTGAACCTGCGGAAGGATCATTATTGAATTATGTTTCTAGATAGGTTGTAG
CTGGCTCTTTAGAGCATGTGCACGCCTGTTTGGACTTCATTTTCATCCACCTGTGCACCT
ATTGTAGTCTTTGGTTGGGTTAGGGGGAAGTGGTCATTGTGTCAGCATCTGCTGGATGTG
AGGACTTGCATTGTGAAAGCTTTGCTGTCCTTGATGTGATCATGGAATCTCTTTCTCACT
AGAGTCTATGTCACTCATTATACTCTGTCGAATGTCATTGAATGTCTTTACATGGGCTTG
TATGCCTATGAAAATTGTAATACAACTTTCAGCAACGGATCTCTTGGCTCTCGCATCGAT
GAAGGACGCAGCGAAATGCGATAAGTAATGTGAATTGCAGAATTCAGTGAATCATCGAAT
CTTTGAACGCATCTTGCGCTCCTTGGTATTCCGAGGAGCATGCCTGTTTGAGTGTCATTA
AATTCTCAACTCTCTTATACTTTTTTGTAAAAGAGAGCTTGGACTGTGGAGGCTTGCTGG
CCACTTTTTGGGGTCAGCTCCTCTGAAATGCATTAGCGGAACCGTTTGCAATCTGCCACA
AGTGTGATAAGTTATCTACACTGGCGAGGGGATTGCTCTCTGTAATGTTCAGCTTCTAAT
TGTCTCTACTTTGTGAGACAACTTTTGAATGCTTGACCTCAAATCAGGTAGGACTACCCG
CTGAACTTAA
>B6_27
TTTCCGTAGGTGAACCTGCGGAAGGATCATTATTGAATTATGTTTCTAGATAGGTTGTAG
CTGGCTCTTTAGAGCATGTGCACGCCTGTTTGGACTTCATTTTCATCCACCTGTGCACCT
ATTGTAGTCTTTGGTTGGGTTAGGGGGAAGTGGTCATTGTGTCAGCATCTGCTGGATGTG
AGGACTTGCATTGTGAAAGCTTTGCTGTCCTTGATGTGATCATGGAATCTCTTTCTCACT
AGAGTCTATGTCACTCATTATACTCTGTCGAATGTCATTGAATGTCTTTACATGGGCTTG
TATGCCTATGAAAATTGTAATACAACTTTCAGCAACGGATCTCTTGGCTCTCGCATCGAT
GAAGGACGCAGCGAAATGCGATAAGTAATGTGAATTGCAGAATTCAGTGAATCATCGAAT
CTTTGAACGCATCTTGCGCTCCTTGGTATTCCGAGGAGCATGCCTGTTTGAGTGTCATTA
AATTCTCAACTCTCTTATACTTTTTTGTAAAAGAGAGCTTGGACTGTGGAGGCTTGCTGG
CCACTTTTTGGGGTCAGCTCCTCTGAAATGCATTAGCGGAACCGTTTGCAATCTGCCACA
AGTGTGATAAGTTATCTACACTGGCGAGGGGATTGCTCTCTGTAATGTTCAGCTTCTAAT
TGTCTCTACTTTGTGAGACAACTTTTGAATGCTTGACCTCAAATCAGGTAGGACTACCCG
CTGAACTTAA
>B6_28
TTTCCGTAGGTGAACCTGCGGAAGGATCATTATTGAATTATGTTTCTAGATAGGTTGTAG
CTGGCTCTTTAGAGCATGTGCACGCCTGTTTGGACTTCATTTTCATCCACCTGTGCACCT
ATTGTAGTCTTTGGTTGGGTTAGGGGGAAGTGGTCATTGTGTCAGCATCTGCTGGATGTG
AGGACTTGCATTGTGAAAGCTTTGCTGTCCTTGATGTGATCATGGAATCTCTTTCTCACT
AGAGTCTATGTCACTCATTATACTCTGTCGAATGTCATTGAATGTCTTTACATGGGCTTG
TATGCCTATGAAAATTGTAATACAACTTTCAGCAACGGATCTCTTGGCTCTCGCATCGAT
GAAGGACGCAGCGAAATGCGATAAGTAATGTGAATTGCAGAATTCAGTGAATCATCGAAT
CTTTGAACGCATCTTGCGCTCCTTGGTATTCCGAGGAGCATGCCTGTTTGAGTGTCATTA
AATTCTCAACTCTCTTATACTTTTTTGTAAAAGAGAGCTTGGACTGTGGAGGCTTGCTGG
CCACTTTTTGGGGTCAGCTCCTCTGAAATGCATTAGCGGAACCGTTTGCAATCTGCCACA
AGTGTGATAAGTTATCTACACTGGCGAGGGGATTGCTCTCTGTAATGTTCAGCTTCTAAT

TGTCTCTACTTTGTGAGACAACTTTTGAATGCTTGACCTCAAATCAGGTAGGACTACCCG
CTGAACTTAA
>B6_31
TTTCCGTAGGTGAACCTGCGGAAGGATCATTATTGAATTATGTTTCTAGATAGGTTGTAG
CTGGCTCTTTAGAGCATGTGCACGCCTGTTTGGACTTCATTTTCATCCACCTGTGCACCT
ATTGTAGTCTTTGGTTGGGTTAGGGGGAAGTGGTCATTGTGTCAGCATCTGCTGGATGTG
AGGACTTGCATTGTGAAAGCTTTGCTGTCCTTGATGTGATCATGGAATCTCTTTCTCACT
AGAGTCTATGTCACTCATTATACTCTGTCGAATGTCATTGAATGTCTTTACATGGGCTTG
TATGCCTATGAAAATTGTAATACAACTTTCAGCAACGGATCTCTTGGCTCTCGCATCGAT
GAAGGACGCAGCGAAATGCGATAAGTAATGTGAATTGCAGAATTCAGTGAATCATCGAAT
CTTTGAACGCATCTTGCGCTCCTTGGTATTCCGAGGAGCATGCCTGTTTGAGTGTCATTA
AATTCTCAACTCTCTTATACTTTTTTGTAAAAGAGAGCTTGGACTGTGGAGGCTTGCTGG
CCACTTTTTGGGGTCAGCTCCTCTGAAATGCATTAGCGGAACCGTTTGCAATCTGCCACA
AGTGTGATAAGTTATCTACACTGGCGAGGGGATTGCTCTCTGTAATGTTCAGCTTCTAAT
TGTCTCTACTTTGTGAGACAACTTTTGAATGCTTGACCTCAAATCAGGTAGGACTACCCG
CTGAACTTAA
>B6_32
TTTCCGTAGGTGAACCTGCGGAAGGATCATTATTGAATTATGTTTCTAGATAGGTTGTAG
CTGGCTCTTTAGAGCATGTGCACGCCTGTTTGGACTTCATTTTCATCCACCTGTGCACCT
ATTGTAGTCTTTGGTTGGGTTAGGGGGAAGTGGTCATTGTGTCAGCATCTGCTGGATGTG
AGGACTTGCATTGTGAAAGCTTTGCTGTCCTTGATGTGATCATGGAATCTCTTTCTCACT
AGAGTCTATGTCACTCATTATACTCTGTCGAATGTCATTGAATGTCTTTACATGGGCTTG
TATGCCTATGAAAATTGTAATACAACTTTCAGCAACGGATCTCTTGGCTCTCGCATCGAT
GAAGGACGCAGCGAAATGCGATAAGTAATGTGAATTGCAGAATTCAGTGAATCATCGAAT
CTTTGAACGCATCTTGCGCTCCTTGGTATTCCGAGGAGCATGCCTGTTTGAGTGTCATTA
AATTCTCAACTCTCTTATACTTTTTTGTAAAAGAGAGCTTGGACTGTGGAGGCTTGCTGG
CCACTTTTTGGGGTCAGCTCCTCTGAAATGCATTAGCGGAACCGTTTGCAATCTGCCACA
AGTGTGATAAGTTATCTACACTGGCGAGGGGATTGCTCTCTGTAATGTTCAGCTTCTAAT
TGTCTCTACTTTGTGAGACAACTTTTGAATGCTTGACCTCAAATCAGGTAGGACTACCCG
CTGAACTTAA
>B6_34
TTTCCGTAGGTGAACCTGCGGAAGGATCATTATTGAATTATGTTTCTAGATAGGTTGTAG
CTGGCTCTTTAGAGCATGTGCACGCCTGTTTGGACTTCATTTTCATCCACCTGTGCACCT
ATTGTAGTCTTTGGTTGGGTTAGGGGGAAGTGGTCATTGTGTCAGCATCTGCTGGATGTG
AGGACTTGCATTGTGAAAGCTTTGCTGTCCTTGATGTGATCATGGAATCTCTTTCTCACT
AGAGTCTATGTCACTCATTATACTCTGTCGAATGTCATTGAATGTCTTTACATGGGCTTG
TATGCCTATGAAAATTGTAATACAACTTTCAGCAACGGATCTCTTGGCTCTCGCATCGAT
GAAGGACGCAGCGAAATGCGATAAGTAATGTGAATTGCAGAATTCAGTGAATCATCGAAT
CTTTGAACGCATCTTGCGCTCCTTGGTATTCCGAGGAGCATGCCTGTTTGAGTGTCATTA
AATTCTCAACTCTCTTATACTTTTTTGTAAAAGAGAGCTTGGACTGTGGAGGCTTGCTGG
CCACTTTTTGGGGTCAGCTCCTCTGAAATGCATTAGCGGAACCGTTTGCAATCTGCCACA
AGTGTGATAAGTTATCTACACTGGCGAGGGGATTGCTCTCTGTAATGTTCAGCTTCTAAT
TGTCTCTACTTTGTGAGACAACTTTTGAATGCTTGACCTCAAATCAGGTAGGACTACCCG
CTGAACTTAA
>B6_35
TTTCCGTAGGTGAACCTGCGGAAGGATCATTATTGAATTATGTTTCTAGATAGGTTGTAG
CTGGCTCTTTAGAGCATGTGCACGCCTGTTTGGACTTCATTTTCATCCACCTGTGCACCT
ATTGTAGTCTTTGGTTGGGTTAGGGGGAAGTGGTCATTGTGTCAGCATCTGCTGGATGTG
AGGACTTGCATTGTGAAAGCTTTGCTGTCCTTGATGTGATCATGGAATCTCTTTCTCACT
AGAGTCTATGTCACTCATTATACTCTGTCGAATGTCATTGAATGTCTTTACATGGGCTTG

TATGCCTATGAAAATTGTAATACAACTTTCAGCAACGGATCTCTTGGCTCTCGCATCGAT
GAAGGACGCAGCGAAATGCGATAAGTAATGTGAATTGCAGAATTCAGTGAATCATCGAAT
CTTTGAACGCATCTTGCGCTCCTTGGTATTCCGAGGAGCATGCCTGTTTGAGTGTCATTA
AATTCTCAACTCTCTTATACTTTTTTGTAAAAGAGAGCTTGGACTGTGGAGGCTTGCTGG
CCACTTTTTGGGGTCAGCTCCTCTGAAATGCATTAGCGGAACCGTTTGCAATCTGCCACA
AGTGTGATAAGTTATCTACACTGGCGAGGGGATTGCTCTCTGTAATGTTCAGCTTCTAAT
TGTCTCTACTTTGTGAGACAACTTTTGAATGCTTGACCTCAAATCAGGTAGGACTACCCG
CTGAACTTAA
>B6_36
TTTCCGTAGGTGAACCTGCGGAAGGATCATTATTGAATTATGTTTCTAGATAGGTTGTAG
CTGGCTCTTTAGAGCATGTGCACGCCTGTTTGGACTTCATTTTCATCCACCTGTGCACCT
ATTGTAGTCTTTGGTTGGGTTAGGGGGAAGTGGTCATTGTGTCAGCATCTGCTGGATGTG
AGGACTTGCATTGTGAAAGCTTTGCTGTCCTTGATGTGATCATGGAATCTCTTTCTCACT
AGAGTCTATGTCACTCATTATACTCTGTCGAATGTCATTGAATGTCTTTACATGGGCTTG
TATGCCTATGAAAATTGTAATACAACTTTCAGCAACGGATCTCTTGGCTCTCGCATCGAT
GAAGGACGCAGCGAAATGCGATAAGTAATGTGAATTGCAGAATTCAGTGAATCATCGAAT
CTTTGAACGCATCTTGCGCTCCTTGGTATTCCGAGGAGCATGCCTGTTTGAGTGTCATTA
AATTCTCAACTCTCTTATACTTTTTTGTAAAAGAGAGCTTGGACTGTGGAGGCTTGCTGG
CCACTTTTTGGGGTCAGCTCCTCTGAAATGCATTAGCGGAACCGTTTGCAATCTGCCACA
AGTGTGATAAGTTATCTACACTGGCGAGGGGATTGCTCTCTGTAATGTTCAGCTTCTAAT
TGTCTCTACTTTGTGAGACAACTTTTGAATGCTTGACCTCAAATCAGGTAGGACTACCCG
CTGAACTTAA
>B6_38
TTTCCGTAGGTGAACCTGCGGAAGGATCATTATTGAATTATGTTTCTAGATAGGTTGTAG
CTGGCTCTTTAGAGCATGTGCACGCCTGTTTGGACTTCATTTTCATCCACCTGTGCACCT
ATTGTAGTCTTTGGTTGGGTTAGGGGGAAGTGGTCATTGTGTCAGCATCTGCTGGATGTG
AGGACTTGCATTGTGAAAGCTTTGCTGTCCTTGATGTGATCATGGAATCTCTTTCTCACT
AGAGTCTATGTCACTCATTATACTCTGTCGAATGTCATTGAATGTCTTTACATGGGCTTG
TATGCCTATGAAAATTGTAATACAACTTTCAGCAACGGATCTCTTGGCTCTCGCATCGAT
GAAGGACGCAGCGAAATGCGATAAGTAATGTGAATTGCAGAATTCAGTGAATCATCGAAT
CTTTGAACGCATCTTGCGCTCCTTGGTATTCCGAGGAGCATGCCTGTTTGAGTGTCATTA
AATTCTCAACTCTCTTATACTTTTTTGTAAAAGAGAGCTTGGACTGTGGAGGCTTGCTGG
CCACTTTTTGGGGTCAGCTCCTCTGAAATGCATTAGCGGAACCGTTTGCAATCTGCCACA
AGTGTGATAAGTTATCTACACTGGCGAGGGGATTGCTCTCTGTAATGTTCAGCTTCTAAT
TGTCTCTACTTTGTGAGACAACTTTTGAATGCTTGACCTCAAATCAGGTAGGACTACCCG
CTGAACTTAA
>B6_40
TTTCCGTAGGTGAACCTGCGGAAGGATCATTATTGAATTATGTTTCTAGATAGGTTGTAG
CTGGCTCTTTAGAGCATGTGCACGCCTGTTTGGACTTCATTTTCATCCACCTGTGCACCT
ATTGTAGTCTTTGGTTGGGTTAGGGGGAAGTGGTCATTGTGTCAGCATCTGCTGGATGTG
AGGACTTGCATTGTGAAAGCTTTGCTGTCCTTGATGTGATCATGGAATCTCTTTCTCACT
AGAGTCTATGTCACTCATTATACTCTGTCGAATGTCATTGAATGTCTTTACATGGGCTTG
TATGCCTATGAAAATTGTAATACAACTTTCAGCAACGGATCTCTTGGCTCTCGCATCGAT
GAAGGACGCAGCGAAATGCGATAAGTAATGTGAATTGCAGAATTCAGTGAATCATCGAAT
CTTTGAACGCATCTTGCGCTCCTTGGTATTCCGAGGAGCATGCCTGTTTGAGTGTCATTA
AATTCTCAACTCTCTTATACTTTTTTGTAAAAGAGAGCTTGGACTGTGGAGGCTTGCTGG
CCACTTTTTGGGGTCAGCTCCTCTGAAATGCATTAGCGGAACCGTTTGCAATCTGCCACA
AGTGTGATAAGTTATCTACACTGGCGAGGGGATTGCTCTCTGTAATGTTCAGCTTCTAAT
TGTCTCTACTTTGTGAGACAACTTTTGAATGCTTGACCTCAAATCAGGTAGGACTACCCG
CTGAACTTAA

>B6_43
TTTCCGTAGGTGAACCTGCGGAAGGATCATTATTGAATTATGTTTCTAGATAGGTTGTAG
CTGGCTCTTTAGAGCATGTGCACGCCTGTTTGGACTTCATTTTCATCCACCTGTGCACCT
ATTGTAGTCTTTGGTTGGGTTAGGGGGAAGTGGTCATTGTGTCAGCATCTGCTGGATGTG
AGGACTTGCATTGTGAAAGCTTTGCTGTCCTTGATGTGATCATGGAATCTCTTTCTCACT
AGAGTCTATGTCACTCATTATACTCTGTCGAATGTCATTGAATGTCTTTACATGGGCTTG
TATGCCTATGAAAATTGTAATACAACTTTCAGCAACGGATCTCTTGGCTCTCGCATCGAT
GAAGGACGCAGCGAAATGCGATAAGTAATGTGAATTGCAGAATTCAGTGAATCATCGAAT
CTTTGAACGCATCTTGCGCTCCTTGGTATTCCGAGGAGCATGCCTGTTTGAGTGTCATTA
AATTCTCAACTCTCTTATACTTTTTTGTAAAAGAGAGCTTGGACTGTGGAGGCTTGCTGG
CCACTTTTTGGGGTCAGCTCCTCTGAAATGCATTAGCGGAACCGTTTGCAATCTGCCACA
AGTGTGATAAGTTATCTACACTGGCGAGGGGATTGCTCTCTGTAATGTTCAGCTTCTAAT
TGTCTCTACTTTGTGAGACAACTTTTGAATGCTTGACCTCAAATCAGGTAGGACTACCCG
CTGAACTTAA
>B6_44
TTTCCGTAGGTGAACCTGCGGAAGGATCATTATTGAATTATGTTTCTAGATAGGTTGTAG
CTGGCTCTTTAGAGCATGTGCACGCCTGTTTGGACTTCATTTTCATCCACCTGTGCACCT
ATTGTAGTCTTTGGTTGGGTTAGGGGGAAGTGGTCATTGTGTCAGCATCTGCTGGATGTG
AGGACTTGCATTGTGAAAGCTTTGCTGTCCTTGATGTGATCATGGAATCTCTTTCTCACT
AGAGTCTATGTCACTCATTATACTCTGTCGAATGTCATTGAATGTCTTTACATGGGCTTG
TATGCCTATGAAAATTGTAATACAACTTTCAGCAACGGATCTCTTGGCTCTCGCATCGAT
GAAGGACGCAGCGAAATGCGATAAGTAATGTGAATTGCAGAATTCAGTGAATCATCGAAT
CTTTGAACGCATCTTGCGCTCCTTGGTATTCCGAGGAGCATGCCTGTTTGAGTGTCATTA
AATTCTCAACTCTCTTATACTTTTTTGTAAAAGAGAGCTTGGACTGTGGAGGCTTGCTGG
CCACTTTTTGGGGTCAGCTCCTCTGAAATGCATTAGCGGAACCGTTTGCAATCTGCCACA
AGTGTGATAAGTTATCTACACTGGCGAGGGGATTGCTCTCTGTAATGTTCAGCTTCTAAT
TGTCTCTACTTTGTGAGACAACTTTTGAATGCTTGACCTCAAATCAGGTAGGACTACCCG
CTGAACTTAA
>B6_45
TTTCCGTAGGTGAACCTGCGGAAGGATCATTATTGAATTATGTTTCTAGATAGGTTGTAG
CTGGCTCTTTAGAGCATGTGCACGCCTGTTTGGACTTCATTTTCATCCACCTGTGCACCT
ATTGTAGTCTTTGGTTGGGTTAGGGGGAAGTGGTCATTGTGTCAGCATCTGCTGGATGTG
AGGACTTGCATTGTGAAAGCTTTGCTGTCCTTGATGTGATCATGGAATCTCTTTCTCACT
AGAGTCTATGTCACTCATTATACTCTGTCGAATGTCATTGAATGTCTTTACATGGGCTTG
TATGCCTATGAAAATTGTAATACAACTTTCAGCAACGGATCTCTTGGCTCTCGCATCGAT
GAAGGACGCAGCGAAATGCGATAAGTAATGTGAATTGCAGAATTCAGTGAATCATCGAAT
CTTTGAACGCATCTTGCGCTCCTTGGTATTCCGAGGAGCATGCCTGTTTGAGTGTCATTA
AATTCTCAACTCTCTTATACTTTTTTGTAAAAGAGAGCTTGGACTGTGGAGGCTTGCTGG
CCACTTTTTGGGGTCAGCTCCTCTGAAATGCATTAGCGGAACCGTTTGCAATCTGCCACA
AGTGTGATAAGTTATCTACACTGGCGAGGGGATTGCTCTCTGTAATGTTCAGCTTCTAAT
TGTCTCTACTTTGTGAGACAACTTTTGAATGCTTGACCTCAAATCAGGTAGGACTACCCG
CTGAACTTAA
>B6_46
TTTCCGTAGGTGAACCTGCGGAAGGATCATTATTGAATTATGTTTCTAGATAGGTTGTAG
CTGGCTCTTTAGAGCATGTGCACGCCTGTTTGGACTTCATTTTCATCCACCTGTGCACCT
ATTGTAGTCTTTGGTTGGGTTAGGGGGAAGTGGTCATTGTGTCAGCATCTGCTGGATGTG
AGGACTTGCATTGTGAAAGCTTTGCTGTCCTTGATGTGATCATGGAATCTCTTTCTCACT
AGAGTCTATGTCACTCATTATACTCTGTCGAATGTCATTGAATGTCTTTACATGGGCTTG
TATGCCTATGAAAATTGTAATACAACTTTCAGCAACGGATCTCTTGGCTCTCGCATCGAT
GAAGGACGCAGCGAAATGCGATAAGTAATGTGAATTGCAGAATTCAGTGAATCATCGAAT

CTTTGAACGCATCTTGCGCTCCTTGGTATTCCGAGGAGCATGCCTGTTTGAGTGTCATTA
AATTCTCAACTCTCTTATACTTTTTTGTAAAAGAGAGCTTGGACTGTGGAGGCTTGCTGG
CCACTTTTTGGGGTCAGCTCCTCTGAAATGCATTAGCGGAACCGTTTGCAATCTGCCACA
AGTGTGATAAGTTATCTACACTGGCGAGGGGATTGCTCTCTGTAATGTTCAGCTTCTAAT
TGTCTCTACTTTGTGAGACAACTTTTGAATGCTTGACCTCAAATCAGGTAGGACTACCCG
CTGAACTTAA
>B6_47
TTTCCGTAGGTGAACCTGCGGAAGGATCATTATTGAATTATGTTTCTAGATAGGTTGTAG
CTGGCTCTTTAGAGCATGTGCACGCCTGTTTGGACTTCATTTTCATCCACCTGTGCACCT
ATTGTAGTCTTTGGTTGGGTTAGGGGGAAGTGGTCATTGTGTCAGCATCTGCTGGATGTG
AGGACTTGCATTGTGAAAGCTTTGCTGTCCTTGATGTGATCATGGAATCTCTTTCTCACT
AGAGTCTATGTCACTCATTATACTCTGTCGAATGTCATTGAATGTCTTTACATGGGCTTG
TATGCCTATGAAAATTGTAATACAACTTTCAGCAACGGATCTCTTGGCTCTCGCATCGAT
GAAGGACGCAGCGAAATGCGATAAGTAATGTGAATTGCAGAATTCAGTGAATCATCGAAT
CTTTGAACGCATCTTGCGCTCCTTGGTATTCCGAGGAGCATGCCTGTTTGAGTGTCATTA
AATTCTCAACTCTCTTATACTTTTTTGTAAAAGAGAGCTTGGACTGTGGAGGCTTGCTGG
CCACTTTTTGGGGTCAGCTCCTCTGAAATGCATTAGCGGAACCGTTTGCAATCTGCCACA
AGTGTGATAAGTTATCTACACTGGCGAGGGGATTGCTCTCTGTAATGTTCAGCTTCTAAT
TGTCTCTACTTTGTGAGACAACTTTTGAATGCTTGACCTCAAATCAGGTAGGACTACCCG
CTGAACTTAA
>B6_48
TTTCCGTAGGTGAACCTGCGGAAGGATCATTATTGAATTATGTTTCTAGATAGGTTGTAG
CTGGCTCTTTAGAGCATGTGCACGCCTGTTTGGACTTCATTTTCATCCACCTGTGCACCT
ATTGTAGTCTTTGGTTGGGTTAGGGGGAAGTGGTCATTGTGTCAGCATCTGCTGGATGTG
AGGACTTGCATTGTGAAAGCTTTGCTGTCCTTGATGTGATCATGGAATCTCTTTCTCACT
AGAGTCTATGTCACTCATTATACTCTGTCGAATGTCATTGAATGTCTTTACATGGGCTTG
TATGCCTATGAAAATTGTAATACAACTTTCAGCAACGGATCTCTTGGCTCTCGCATCGAT
GAAGGACGCAGCGAAATGCGATAAGTAATGTGAATTGCAGAATTCAGTGAATCATCGAAT
CTTTGAACGCATCTTGCGCTCCTTGGTATTCCGAGGAGCATGCCTGTTTGAGTGTCATTA
AATTCTCAACTCTCTTATACTTTTTTGTAAAAGAGAGCTTGGACTGTGGAGGCTTGCTGG
CCACTTTTTGGGGTCAGCTCCTCTGAAATGCATTAGCGGAACCGTTTGCAATCTGCCACA
AGTGTGATAAGTTATCTACACTGGCGAGGGGATTGCTCTCTGTAATGTTCAGCTTCTAAT
TGTCTCTACTTTGTGAGACAACTTTTGAATGCTTGACCTCAAATCAGGTAGGACTACCCG
CTGAACTTAA
>B6_49
TTTCCGTAGGTGAACCTGCGGAAGGATCATTATTGAATTATGTTTCTAGATAGGTTGTAG
CTGGCTCTTTAGAGCATGTGCACGCCTGTTTGGACTTCATTTTCATCCACCTGTGCACCT
ATTGTAGTCTTTGGTTGGGTTAGGGGGAAGTGGTCATTGTGTCAGCATCTGCTGGATGTG
AGGACTTGCATTGTGAAAGCTTTGCTGTCCTTGATGTGATCATGGAATCTCTTTCTCACT
AGAGTCTATGTCACTCATTATACTCTGTCGAATGTCATTGAATGTCTTTACATGGGCTTG
TATGCCTATGAAAATTGTAATACAACTTTCAGCAACGGATCTCTTGGCTCTCGCATCGAT
GAAGGACGCAGCGAAATGCGATAAGTAATGTGAATTGCAGAATTCAGTGAATCATCGAAT
CTTTGAACGCATCTTGCGCTCCTTGGTATTCCGAGGAGCATGCCTGTTTGAGTGTCATTA
AATTCTCAACTCTCTTATACTTTTTTGTAAAAGAGAGCTTGGACTGTGGAGGCTTGCTGG
CCACTTTTTGGGGTCAGCTCCTCTGAAATGCATTAGCGGAACCGTTTGCAATCTGCCACA
AGTGTGATAAGTTATCTACACTGGCGAGGGGATTGCTCTCTGTAATGTTCAGCTTCTAAT
TGTCTCTACTTTGTGAGACAACTTTTGAATGCTTGACCTCAAATCAGGTAGGACTACCCG
CTGAACTTAA
>B6_51
TTTCCGTAGGTGAACCTGCGGAAGGATCATTATTGAATTATGTTTCTAGATAGGTTGTAG

CTGGCTCTTTAGAGCATGTGCACGCCTGTTTGGACTTCATTTTCATCCACCTGTGCACCT
ATTGTAGTCTTTGGTTGGGTTAGGGGGAAGTGGTCATTGTGTCAGCATCTGCTGGATGTG
AGGACTTGCATTGTGAAAGCTTTGCTGTCCTTGATGTGATCATGGAATCTCTTTCTCACT
AGAGTCTATGTCACTCATTATACTCTGTCGAATGTCATTGAATGTCTTTACATGGGCTTG
TATGCCTATGAAAATTGTAATACAACTTTCAGCAACGGATCTCTTGGCTCTCGCATCGAT
GAAGGACGCAGCGAAATGCGATAAGTAATGTGAATTGCAGAATTCAGTGAATCATCGAAT
CTTTGAACGCATCTTGCGCTCCTTGGTATTCCGAGGAGCATGCCTGTTTGAGTGTCATTA
AATTCTCAACTCTCTTATACTTTTTTGTAAAAGAGAGCTTGGACTGTGGAGGCTTGCTGG
CCACTTTTTGGGGTCAGCTCCTCTGAAATGCATTAGCGGAACCGTTTGCAATCTGCCACA
AGTGTGATAAGTTATCTACACTGGCGAGGGGATTGCTCTCTGTAATGTTCAGCTTCTAAT
TGTCTCTACTTTGTGAGACAACTTTTGAATGCTTGACCTCAAATCAGGTAGGACTACCCG
CTGAACTTAA
>B6_54
TTTCCGTAGGTGAACCTGCGGAAGGATCATTATTGAATTATGTTTCTAGATAGGTTGTAG
CTGGCTCTTTAGAGCATGTGCACGCCTGTTTGGACTTCATTTTCATCCACCTGTGCACCT
ATTGTAGTCTTTGGTTGGGTTAGGGGGAAGTGGTCATTGTGTCAGCATCTGCTGGATGTG
AGGACTTGCATTGTGAAAGCTTTGCTGTCCTTGATGTGATCATGGAATCTCTTTCTCACT
AGAGTCTATGTCACTCATTATACTCTGTCGAATGTCATTGAATGTCTTTACATGGGCTTG
TATGCCTATGAAAATTGTAATACAACTTTCAGCAACGGATCTCTTGGCTCTCGCATCGAT
GAAGGACGCAGCGAAATGCGATAAGTAATGTGAATTGCAGAATTCAGTGAATCATCGAAT
CTTTGAACGCATCTTGCGCTCCTTGGTATTCCGAGGAGCATGCCTGTTTGAGTGTCATTA
AATTCTCAACTCTCTTATACTTTTTTGTAAAAGAGAGCTTGGACTGTGGAGGCTTGCTGG
CCACTTTTTGGGGTCAGCTCCTCTGAAATGCATTAGCGGAACCGTTTGCAATCTGCCACA
AGTGTGATAAGTTATCTACACTGGCGAGGGGATTGCTCTCTGTAATGTTCAGCTTCTAAT
TGTCTCTACTTTGTGAGACAACTTTTGAATGCTTGACCTCAAATCAGGTAGGACTACCCG
CTGAACTTAA
>B6_55
TTTCCGTAGGTGAACCTGCGGAAGGATCATTATTGAATTATGTTTCTAGATAGGTTGTAG
CTGGCTCTTTAGAGCATGTGCACGCCTGTTTGGACTTCATTTTCATCCACCTGTGCACCT
ATTGTAGTCTTTGGTTGGGTTAGGGGGAAGTGGTCATTGTGTCAGCATCTGCTGGATGTG
AGGACTTGCATTGTGAAAGCTTTGCTGTCCTTGATGTGATCATGGAATCTCTTTCTCACT
AGAGTCTATGTCACTCATTATACTCTGTCGAATGTCATTGAATGTCTTTACATGGGCTTG
TATGCCTATGAAAATTGTAATACAACTTTCAGCAACGGATCTCTTGGCTCTCGCATCGAT
GAAGGACGCAGCGAAATGCGATAAGTAATGTGAATTGCAGAATTCAGTGAATCATCGAAT
CTTTGAACGCATCTTGCGCTCCTTGGTATTCCGAGGAGCATGCCTGTTTGAGTGTCATTA
AATTCTCAACTCTCTTATACTTTTTTGTAAAAGAGAGCTTGGACTGTGGAGGCTTGCTGG
CCACTTTTTGGGGTCAGCTCCTCTGAAATGCATTAGCGGAACCGTTTGCAATCTGCCACA
AGTGTGATAAGTTATCTACACTGGCGAGGGGATTGCTCTCTGTAATGTTCAGCTTCTAAT
TGTCTCTACTTTGTGAGACAACTTTTGAATGCTTGACCTCAAATCAGGTAGGACTACCCG
CTGAACTTAA
>B6_56
TTTCCGTAGGTGAACCTGCGGAAGGATCATTATTGAATTATGTTTCTAGATAGGTTGTAG
CTGGCTCTTTAGAGCATGTGCACGCCTGTTTGGACTTCATTTTCATCCACCTGTGCACCT
ATTGTAGTCTTTGGTTGGGTTAGGGGGAAGTGGTCATTGTGTCAGCATCTGCTGGATGTG
AGGACTTGCATTGTGAAAGCTTTGCTGTCCTTGATGTGATCATGGAATCTCTTTCTCACT
AGAGTCTATGTCACTCATTATACTCTGTCGAATGTCATTGAATGTCTTTACATGGGCTTG
TATGCCTATGAAAATTGTAATACAACTTTCAGCAACGGATCTCTTGGCTCTCGCATCGAT
GAAGGACGCAGCGAAATGCGATAAGTAATGTGAATTGCAGAATTCAGTGAATCATCGAAT
CTTTGAACGCATCTTGCGCTCCTTGGTATTCCGAGGAGCATGCCTGTTTGAGTGTCATTA
AATTCTCAACTCTCTTATACTTTTTTGTAAAAGAGAGCTTGGACTGTGGAGGCTTGCTGG

CCACTTTTTGGGGTCAGCTCCTCTGAAATGCATTAGCGGAACCGTTTGCAATCTGCCACA
AGTGTGATAAGTTATCTACACTGGCGAGGGGATTGCTCTCTGTAATGTTCAGCTTCTAAT
TGTCTCTACTTTGTGAGACAACTTTTGAATGCTTGACCTCAAATCAGGTAGGACTACCCG
CTGAACTTAA
>B6_57
TTTCCGTAGGTGAACCTGCGGAAGGATCATTATTGAATTATGTTTCTAGATAGGTTGTAG
CTGGCTCTTTAGAGCATGTGCACGCCTGTTTGGACTTCATTTTCATCCACCTGTGCACCT
ATTGTAGTCTTTGGTTGGGTTAGGGGGAAGTGGTCATTGTGTCAGCATCTGCTGGATGTG
AGGACTTGCATTGTGAAAGCTTTGCTGTCCTTGATGTGATCATGGAATCTCTTTCTCACT
AGAGTCTATGTCACTCATTATACTCTGTCGAATGTCATTGAATGTCTTTACATGGGCTTG
TATGCCTATGAAAATTGTAATACAACTTTCAGCAACGGATCTCTTGGCTCTCGCATCGAT
GAAGGACGCAGCGAAATGCGATAAGTAATGTGAATTGCAGAATTCAGTGAATCATCGAAT
CTTTGAACGCATCTTGCGCTCCTTGGTATTCCGAGGAGCATGCCTGTTTGAGTGTCATTA
AATTCTCAACTCTCTTATACTTTTTTGTAAAAGAGAGCTTGGACTGTGGAGGCTTGCTGG
CCACTTTTTGGGGTCAGCTCCTCTGAAATGCATTAGCGGAACCGTTTGCAATCTGCCACA
AGTGTGATAAGTTATCTACACTGGCGAGGGGATTGCTCTCTGTAATGTTCAGCTTCTAAT
TGTCTCTACTTTGTGAGACAACTTTTGAATGCTTGACCTCAAATCAGGTAGGACTACCCG
CTGAACTTAA
>B6_59
TTTCCGTAGGTGAACCTGCGGAAGGATCATTATTGAATTATGTTTCTAGATAGGTTGTAG
CTGGCTCTTTAGAGCATGTGCACGCCTGTTTGGACTTCATTTTCATCCACCTGTGCACCT
ATTGTAGTCTTTGGTTGGGTTAGGGGGAAGTGGTCATTGTGTCAGCATCTGCTGGATGTG
AGGACTTGCATTGTGAAAGCTTTGCTGTCCTTGATGTGATCATGGAATCTCTTTCTCACT
AGAGTCTATGTCACTCATTATACTCTGTCGAATGTCATTGAATGTCTTTACATGGGCTTG
TATGCCTATGAAAATTGTAATACAACTTTCAGCAACGGATCTCTTGGCTCTCGCATCGAT
GAAGGACGCAGCGAAATGCGATAAGTAATGTGAATTGCAGAATTCAGTGAATCATCGAAT
CTTTGAACGCATCTTGCGCTCCTTGGTATTCCGAGGAGCATGCCTGTTTGAGTGTCATTA
AATTCTCAACTCTCTTATACTTTTTTGTAAAAGAGAGCTTGGACTGTGGAGGCTTGCTGG
CCACTTTTTGGGGTCAGCTCCTCTGAAATGCATTAGCGGAACCGTTTGCAATCTGCCACA
AGTGTGATAAGTTATCTACACTGGCGAGGGGATTGCTCTCTGTAATGTTCAGCTTCTAAT
TGTCTCTACTTTGTGAGACAACTTTTGAATGCTTGACCTCAAATCAGGTAGGACTACCCG
CTGAACTTAA
>B6_60
TTTCCGTAGGTGAACCTGCGGAAGGATCATTATTGAATTATGTTTCTAGATAGGTTGTAG
CTGGCTCTTTAGAGCATGTGCACGCCTGTTTGGACTTCATTTTCATCCACCTGTGCACCT
ATTGTAGTCTTTGGTTGGGTTAGGGGGAAGTGGTCATTGTGTCAGCATCTGCTGGATGTG
AGGACTTGCATTGTGAAAGCTTTGCTGTCCTTGATGTGATCATGGAATCTCTTTCTCACT
AGAGTCTATGTCACTCATTATACTCTGTCGAATGTCATTGAATGTCTTTACATGGGCTTG
TATGCCTATGAAAATTGTAATACAACTTTCAGCAACGGATCTCTTGGCTCTCGCATCGAT
GAAGGACGCAGCGAAATGCGATAAGTAATGTGAATTGCAGAATTCAGTGAATCATCGAAT
CTTTGAACGCATCTTGCGCTCCTTGGTATTCCGAGGAGCATGCCTGTTTGAGTGTCATTA
AATTCTCAACTCTCTTATACTTTTTTGTAAAAGAGAGCTTGGACTGTGGAGGCTTGCTGG
CCACTTTTTGGGGTCAGCTCCTCTGAAATGCATTAGCGGAACCGTTTGCAATCTGCCACA
AGTGTGATAAGTTATCTACACTGGCGAGGGGATTGCTCTCTGTAATGTTCAGCTTCTAAT
TGTCTCTACTTTGTGAGACAACTTTTGAATGCTTGACCTCAAATCAGGTAGGACTACCCG
CTGAACTTAA
>B6_61
TTTCCGTAGGTGAACCTGCGGAAGGATCATTATTGAATTATGTTTCTAGATAGGTTGTAG
CTGGCTCTTTAGAGCATGTGCACGCCTGTTTGGACTTCATTTTCATCCACCTGTGCACCT
ATTGTAGTCTTTGGTTGGGTTAGGGGGAAGTGGTCATTGTGTCAGCATCTGCTGGATGTG

AGGACTTGCATTGTGAAAGCTTTGCTGTCCTTGATGTGATCATGGAATCTCTTTCTCACT
AGAGTCTATGTCACTCATTATACTCTGTCGAATGTCATTGAATGTCTTTACATGGGCTTG
TATGCCTATGAAAATTGTAATACAACTTTCAGCAACGGATCTCTTGGCTCTCGCATCGAT
GAAGGACGCAGCGAAATGCGATAAGTAATGTGAATTGCAGAATTCAGTGAATCATCGAAT
CTTTGAACGCATCTTGCGCTCCTTGGTATTCCGAGGAGCATGCCTGTTTGAGTGTCATTA
AATTCTCAACTCTCTTATACTTTTTTGTAAAAGAGAGCTTGGACTGTGGAGGCTTGCTGG
CCACTTTTTGGGGTCAGCTCCTCTGAAATGCATTAGCGGAACCGTTTGCAATCTGCCACA
AGTGTGATAAGTTATCTACACTGGCGAGGGGATTGCTCTCTGTAATGTTCAGCTTCTAAT
TGTCTCTACTTTGTGAGACAACTTTTGAATGCTTGACCTCAAATCAGGTAGGACTACCCG
CTGAACTTAA
>B6_63
TTTCCGTAGGTGAACCTGCGGAAGGATCATTATTGAATTATGTTTCTAGATAGGTTGTAG
CTGGCTCTTTAGAGCATGTGCACGCCTGTTTGGACTTCATTTTCATCCACCTGTGCACCT
ATTGTAGTCTTTGGTTGGGTTAGGGGGAAGTGGTCATTGTGTCAGCATCTGCTGGATGTG
AGGACTTGCATTGTGAAAGCTTTGCTGTCCTTGATGTGATCATGGAATCTCTTTCTCACT
AGAGTCTATGTCACTCATTATACTCTGTCGAATGTCATTGAATGTCTTTACATGGGCTTG
TATGCCTATGAAAATTGTAATACAACTTTCAGCAACGGATCTCTTGGCTCTCGCATCGAT
GAAGGACGCAGCGAAATGCGATAAGTAATGTGAATTGCAGAATTCAGTGAATCATCGAAT
CTTTGAACGCATCTTGCGCTCCTTGGTATTCCGAGGAGCATGCCTGTTTGAGTGTCATTA
AATTCTCAACTCTCTTATACTTTTTTGTAAAAGAGAGCTTGGACTGTGGAGGCTTGCTGG
CCACTTTTTGGGGTCAGCTCCTCTGAAATGCATTAGCGGAACCGTTTGCAATCTGCCACA
AGTGTGATAAGTTATCTACACTGGCGAGGGGATTGCTCTCTGTAATGTTCAGCTTCTAAT
TGTCTCTACTTTGTGAGACAACTTTTGAATGCTTGACCTCAAATCAGGTAGGACTACCCG
CTGAACTTAA
>B6_64
TTTCCGTAGGTGAACCTGCGGAAGGATCATTATTGAATTATGTTTCTAGATAGGTTGTAG
CTGGCTCTTTAGAGCATGTGCACGCCTGTTTGGACTTCATTTTCATCCACCTGTGCACCT
ATTGTAGTCTTTGGTTGGGTTAGGGGGAAGTGGTCATTGTGTCAGCATCTGCTGGATGTG
AGGACTTGCATTGTGAAAGCTTTGCTGTCCTTGATGTGATCATGGAATCTCTTTCTCACT
AGAGTCTATGTCACTCATTATACTCTGTCGAATGTCATTGAATGTCTTTACATGGGCTTG
TATGCCTATGAAAATTGTAATACAACTTTCAGCAACGGATCTCTTGGCTCTCGCATCGAT
GAAGGACGCAGCGAAATGCGATAAGTAATGTGAATTGCAGAATTCAGTGAATCATCGAAT
CTTTGAACGCATCTTGCGCTCCTTGGTATTCCGAGGAGCATGCCTGTTTGAGTGTCATTA
AATTCTCAACTCTCTTATACTTTTTTGTAAAAGAGAGCTTGGACTGTGGAGGCTTGCTGG
CCACTTTTTGGGGTCAGCTCCTCTGAAATGCATTAGCGGAACCGTTTGCAATCTGCCACA
AGTGTGATAAGTTATCTACACTGGCGAGGGGATTGCTCTCTGTAATGTTCAGCTTCTAAT
TGTCTCTACTTTGTGAGACAACTTTTGAATGCTTGACCTCAAATCAGGTAGGACTACCCG
CTGAACTTAA
>B6_65
TTTCCGTAGGTGAACCTGCGGAAGGATCATTATTGAATTATGTTTCTAGATAGGTTGTAG
CTGGCTCTTTAGAGCATGTGCACGCCTGTTTGGACTTCATTTTCATCCACCTGTGCACCT
ATTGTAGTCTTTGGTTGGGTTAGGGGGAAGTGGTCATTGTGTCAGCATCTGCTGGATGTG
AGGACTTGCATTGTGAAAGCTTTGCTGTCCTTGATGTGATCATGGAATCTCTTTCTCACT
AGAGTCTATGTCACTCATTATACTCTGTCGAATGTCATTGAATGTCTTTACATGGGCTTG
TATGCCTATGAAAATTGTAATACAACTTTCAGCAACGGATCTCTTGGCTCTCGCATCGAT
GAAGGACGCAGCGAAATGCGATAAGTAATGTGAATTGCAGAATTCAGTGAATCATCGAAT
CTTTGAACGCATCTTGCGCTCCTTGGTATTCCGAGGAGCATGCCTGTTTGAGTGTCATTA
AATTCTCAACTCTCTTATACTTTTTTGTAAAAGAGAGCTTGGACTGTGGAGGCTTGCTGG
CCACTTTTTGGGGTCAGCTCCTCTGAAATGCATTAGCGGAACCGTTTGCAATCTGCCACA
AGTGTGATAAGTTATCTACACTGGCGAGGGGATTGCTCTCTGTAATGTTCAGCTTCTAAT

TGTCTCTACTTTGTGAGACAACTTTTGAATGCTTGACCTCAAATCAGGTAGGACTACCCG
CTGAACTTAA
>B7_1
TTTCCGTAGGTGAACCTGCGGAAGGATCATTATTGAATTATGTTTCTAGATAGGTTGTAG
CTGGCTCTTTAGAGCATGTGCACGCCTGTTTGGACTTCATTTTCATCCACCTGTGCACCT
ATTGTAGTCTTTGGTTGGGTTAGGGGGAAGTGGTCATTGTGTCAGCATCTGCTGGATGTG
AGGACTTGCATTGTGAAAGCTTTGCTGTCCTTGATGTGATCATGGAATCTCTTTCTCACT
AGAGTCTATGTCACTCATTATACTCTGTCGAATGTCATTGAATGTCTTTACATGGGCTTG
TATGCCTATGAAAATTGTAATACAACTTTCAGCAACGGATCTCTTGGCTCTCGCATCGAT
GAAGGACGCAGCGAAATGCGATAAGTAATGTGAATTGCAGAATTCAGTGAATCATCGAAT
CTTTGAACGCATCTTGCGCTCCTTGGTATTCCGAGGAGCATGCCTGTTTGAGTGTCATTA
AATTCTCAACTCTCTTATACTTTTTTGTAAAAGAGAGCTTGGACTGTGGAGGCTTGCTGG
CCACTTTTTGGGGTCAGCTCCTCTGAAATGCATTAGCGGAACCGTTTGCAATCTGCCACA
AGTGTGATAAGTTATCTACACTGGCGAGGGGATTGCTCTCTGTAATGTTCAGCTTCTAAT
TGTCTCTACTTTGTGAGACAACTTTTGAATGCTTGACCTCAAATCAGGTAGGACTACCCG
CTGAACTTAA
>B7_2
TTTCCGTAGGTGAACCTGCGGAAGGATCATTATTGAATTATGTTTCTAGATAGGTTGTAG
CTGGCTCTTTAGAGCATGTGCACGCCTGTTTGGACTTCATTTTCATCCACCTGTGCACCT
ATTGTAGTCTTTGGTTGGGTTAGGGGGAAGTGGTCATTGTGTCAGCATCTGCTGGATGTG
AGGACTTGCATTGTGAAAGCTTTGCTGTCCTTGATGTGATCATGGAATCTCTTTCTCACT
AGAGTCTATGTCACTCATTATACTCTGTCGAATGTCATTGAATGTCTTTACATGGGCTTG
TATGCCTATGAAAATTGTAATACAACTTTCAGCAACGGATCTCTTGGCTCTCGCATCGAT
GAAGGACGCAGCGAAATGCGATAAGTAATGTGAATTGCAGAATTCAGTGAATCATCGAAT
CTTTGAACGCATCTTGCGCTCCTTGGTATTCCGAGGAGCATGCCTGTTTGAGTGTCATTA
AATTCTCAACTCTCTTATACTTTTTTGTAAAAGAGAGCTTGGACTGTGGAGGCTTGCTGG
CCACTTTTTGGGGTCAGCTCCTCTGAAATGCATTAGCGGAACCGTTTGCAATCTGCCACA
AGTGTGATAAGTTATCTACACTGGCGAGGGGATTGCTCTCTGTAATGTTCAGCTTCTAAT
TGTCTCTACTTTGTGAGACAACTTTTGAATGCTTGACCTCAAATCAGGTAGGACTACCCG
CTGAACTTAA
>B7_4
TTTCCGTAGGTGAACCTGCGGAAGGATCATTATTGAATTATGTTTCTAGATAGGTTGTAG
CTGGCTCTTTAGAGCATGTGCACGCCTGTTTGGACTTCATTTTCATCCACCTGTGCACCT
ATTGTAGTCTTTGGTTGGGTTAGGGGGAAGTGGTCATTGTGTCAGCATCTGCTGGATGTG
AGGACTTGCATTGTGAAAGCTTTGCTGTCCTTGATGTGATCATGGAATCTCTTTCTCACT
AGAGTCTATGTCACTCATTATACTCTGTCGAATGTCATTGAATGTCTTTACATGGGCTTG
TATGCCTATGAAAATTGTAATACAACTTTCAGCAACGGATCTCTTGGCTCTCGCATCGAT
GAAGGACGCAGCGAAATGCGATAAGTAATGTGAATTGCAGAATTCAGTGAATCATCGAAT
CTTTGAACGCATCTTGCGCTCCTTGGTATTCCGAGGAGCATGCCTGTTTGAGTGTCATTA
AATTCTCAACTCTCTTATACTTTTTTGTAAAAGAGAGCTTGGACTGTGGAGGCTTGCTGG
CCACTTTTTGGGGTCAGCTCCTCTGAAATGCATTAGCGGAACCGTTTGCAATCTGCCACA
AGTGTGATAAGTTATCTACACTGGCGAGGGGATTGCTCTCTGTAATGTTCAGCTTCTAAT
TGTCTCTACTTTGTGAGACAACTTTTGAATGCTTGACCTCAAATCAGGTAGGACTACCCG
CTGAACTTAA
>B7_9
TTTCCGTAGGTGAACCTGCGGAAGGATCATTATTGAATTATGTTTCTAGATAGGTTGTAG
CTGGCTCTTTAGAGCATGTGCACGCCTGTTTGGACTTCATTTTCATCCACCTGTGCACCT
ATTGTAGTCTTTGGTTGGGTTAGGGGGAAGTGGTCATTGTGTCAGCATCTGCTGGATGTG
AGGACTTGCATTGTGAAAGCTTTGCTGTCCTTGATGTGATCATGGAATCTCTTTCTCACT
AGAGTCTATGTCACTCATTATACTCTGTCGAATGTCATTGAATGTCTTTACATGGGCTTG

TATGCCTATGAAAATTGTAATACAACTTTCAGCAACGGATCTCTTGGCTCTCGCATCGAT
GAAGGACGCAGCGAAATGCGATAAGTAATGTGAATTGCAGAATTCAGTGAATCATCGAAT
CTTTGAACGCATCTTGCGCTCCTTGGTATTCCGAGGAGCATGCCTGTTTGAGTGTCATTA
AATTCTCAACTCTCTTATACTTTTTTGTAAAAGAGAGCTTGGACTGTGGAGGCTTGCTGG
CCACTTTTTGGGGTCAGCTCCTCTGAAATGCATTAGCGGAACCGTTTGCAATCTGCCACA
AGTGTGATAAGTTATCTACACTGGCGAGGGGATTGCTCTCTGTAATGTTCAGCTTCTAAT
TGTCTCTACTTTGTGAGACAACTTTTGAATGCTTGACCTCAAATCAGGTAGGACTACCCG
CTGAACTTAA
>B7_10
TTTCCGTAGGTGAACCTGCGGAAGGATCATTATTGAATTATGTTTCTAGATAGGTTGTAG
CTGGCTCTTTAGAGCATGTGCACGCCTGTTTGGACTTCATTTTCATCCACCTGTGCACCT
ATTGTAGTCTTTGGTTGGGTTAGGGGGAAGTGGTCATTGTGTCAGCATCTGCTGGATGTG
AGGACTTGCATTGTGAAAGCTTTGCTGTCCTTGATGTGATCATGGAATCTCTTTCTCACT
AGAGTCTATGTCACTCATTATACTCTGTCGAATGTCATTGAATGTCTTTACATGGGCTTG
TATGCCTATGAAAATTGTAATACAACTTTCAGCAACGGATCTCTTGGCTCTCGCATCGAT
GAAGGACGCAGCGAAATGCGATAAGTAATGTGAATTGCAGAATTCAGTGAATCATCGAAT
CTTTGAACGCATCTTGCGCTCCTTGGTATTCCGAGGAGCATGCCTGTTTGAGTGTCATTA
AATTCTCAACTCTCTTATACTTTTTTGTAAAAGAGAGCTTGGACTGTGGAGGCTTGCTGG
CCACTTTTTGGGGTCAGCTCCTCTGAAATGCATTAGCGGAACCGTTTGCAATCTGCCACA
AGTGTGATAAGTTATCTACACTGGCGAGGGGATTGCTCTCTGTAATGTTCAGCTTCTAAT
TGTCTCTACTTTGTGAGACAACTTTTGAATGCTTGACCTCAAATCAGGTAGGACTACCCG
CTGAACTTAA
>B7_11
TTTCCGTAGGTGAACCTGCGGAAGGATCATTATTGAATTATGTTTCTAGATAGGTTGTAG
CTGGCTCTTTAGAGCATGTGCACGCCTGTTTGGACTTCATTTTCATCCACCTGTGCACCT
ATTGTAGTCTTTGGTTGGGTTAGGGGGAAGTGGTCATTGTGTCAGCATCTGCTGGATGTG
AGGACTTGCATTGTGAAAGCTTTGCTGTCCTTGATGTGATCATGGAATCTCTTTCTCACT
AGAGTCTATGTCACTCATTATACTCTGTCGAATGTCATTGAATGTCTTTACATGGGCTTG
TATGCCTATGAAAATTGTAATACAACTTTCAGCAACGGATCTCTTGGCTCTCGCATCGAT
GAAGGACGCAGCGAAATGCGATAAGTAATGTGAATTGCAGAATTCAGTGAATCATCGAAT
CTTTGAACGCATCTTGCGCTCCTTGGTATTCCGAGGAGCATGCCTGTTTGAGTGTCATTA
AATTCTCAACTCTCTTATACTTTTTTGTAAAAGAGAGCTTGGACTGTGGAGGCTTGCTGG
CCACTTTTTGGGGTCAGCTCCTCTGAAATGCATTAGCGGAACCGTTTGCAATCTGCCACA
AGTGTGATAAGTTATCTACACTGGCGAGGGGATTGCTCTCTGTAATGTTCAGCTTCTAAT
TGTCTCTACTTTGTGAGACAACTTTTGAATGCTTGACCTCAAATCAGGTAGGACTACCCG
CTGAACTTAA
>B7_12
TTTCCGTAGGTGAACCTGCGGAAGGATCATTATTGAATTATGTTTCTAGATAGGTTGTAG
CTGGCTCTTTAGAGCATGTGCACGCCTGTTTGGACTTCATTTTCATCCACCTGTGCACCT
ATTGTAGTCTTTGGTTGGGTTAGGGGGAAGTGGTCATTGTGTCAGCATCTGCTGGATGTG
AGGACTTGCATTGTGAAAGCTTTGCTGTCCTTGATGTGATCATGGAATCTCTTTCTCACT
AGAGTCTATGTCACTCATTATACTCTGTCGAATGTCATTGAATGTCTTTACATGGGCTTG
TATGCCTATGAAAATTGTAATACAACTTTCAGCAACGGATCTCTTGGCTCTCGCATCGAT
GAAGGACGCAGCGAAATGCGATAAGTAATGTGAATTGCAGAATTCAGTGAATCATCGAAT
CTTTGAACGCATCTTGCGCTCCTTGGTATTCCGAGGAGCATGCCTGTTTGAGTGTCATTA
AATTCTCAACTCTCTTATACTTTTTTGTAAAAGAGAGCTTGGACTGTGGAGGCTTGCTGG
CCACTTTTTGGGGTCAGCTCCTCTGAAATGCATTAGCGGAACCGTTTGCAATCTGCCACA
AGTGTGATAAGTTATCTACACTGGCGAGGGGATTGCTCTCTGTAATGTTCAGCTTCTAAT
TGTCTCTACTTTGTGAGACAACTTTTGAATGCTTGACCTCAAATCAGGTAGGACTACCCG
CTGAACTTAA

>B7_13
TTTCCGTAGGTGAACCTGCGGAAGGATCATTATTGAATTATGTTTCTAGATAGGTTGTAG
CTGGCTCTTTAGAGCATGTGCACGCCTGTTTGGACTTCATTTTCATCCACCTGTGCACCT
ATTGTAGTCTTTGGTTGGGTTAGGGGGAAGTGGTCATTGTGTCAGCATCTGCTGGATGTG
AGGACTTGCATTGTGAAAGCTTTGCTGTCCTTGATGTGATCATGGAATCTCTTTCTCACT
AGAGTCTATGTCACTCATTATACTCTGTCGAATGTCATTGAATGTCTTTACATGGGCTTG
TATGCCTATGAAAATTGTAATACAACTTTCAGCAACGGATCTCTTGGCTCTCGCATCGAT
GAAGGACGCAGCGAAATGCGATAAGTAATGTGAATTGCAGAATTCAGTGAATCATCGAAT
CTTTGAACGCATCTTGCGCTCCTTGGTATTCCGAGGAGCATGCCTGTTTGAGTGTCATTA
AATTCTCAACTCTCTTATACTTTTTTGTAAAAGAGAGCTTGGACTGTGGAGGCTTGCTGG
CCACTTTTTGGGGTCAGCTCCTCTGAAATGCATTAGCGGAACCGTTTGCAATCTGCCACA
AGTGTGATAAGTTATCTACACTGGCGAGGGGATTGCTCTCTGTAATGTTCAGCTTCTAAT
TGTCTCTACTTTGTGAGACAACTTTTGAATGCTTGACCTCAAATCAGGTAGGACTACCCG
CTGAACTTAA
>B7_15
TTTCCGTAGGTGAACCTGCGGAAGGATCATTATTGAATTATGTTTCTAGATAGGTTGTAG
CTGGCTCTTTAGAGCATGTGCACGCCTGTTTGGACTTCATTTTCATCCACCTGTGCACCT
ATTGTAGTCTTTGGTTGGGTTAGGGGGAAGTGGTCATTGTGTCAGCATCTGCTGGATGTG
AGGACTTGCATTGTGAAAGCTTTGCTGTCCTTGATGTGATCATGGAATCTCTTTCTCACT
AGAGTCTATGTCACTCATTATACTCTGTCGAATGTCATTGAATGTCTTTACATGGGCTTG
TATGCCTATGAAAATTGTAATACAACTTTCAGCAACGGATCTCTTGGCTCTCGCATCGAT
GAAGGACGCAGCGAAATGCGATAAGTAATGTGAATTGCAGAATTCAGTGAATCATCGAAT
CTTTGAACGCATCTTGCGCTCCTTGGTATTCCGAGGAGCATGCCTGTTTGAGTGTCATTA
AATTCTCAACTCTCTTATACTTTTTTGTAAAAGAGAGCTTGGACTGTGGAGGCTTGCTGG
CCACTTTTTGGGGTCAGCTCCTCTGAAATGCATTAGCGGAACCGTTTGCAATCTGCCACA
AGTGTGATAAGTTATCTACACTGGCGAGGGGATTGCTCTCTGTAATGTTCAGCTTCTAAT
TGTCTCTACTTTGTGAGACAACTTTTGAATGCTTGACCTCAAATCAGGTAGGACTACCCG
CTGAACTTAA
>B7_18
TTTCCGTAGGTGAACCTGCGGAAGGATCATTATTGAATTATGTTTCTAGATAGGTTGTAG
CTGGCTCTTTAGAGCATGTGCACGCCTGTTTGGACTTCATTTTCATCCACCTGTGCACCT
ATTGTAGTCTTTGGTTGGGTTAGGGGGAAGTGGTCATTGTGTCAGCATCTGCTGGATGTG
AGGACTTGCATTGTGAAAGCTTTGCTGTCCTTGATGTGATCATGGAATCTCTTTCTCACT
AGAGTCTATGTCACTCATTATACTCTGTCGAATGTCATTGAATGTCTTTACATGGGCTTG
TATGCCTATGAAAATTGTAATACAACTTTCAGCAACGGATCTCTTGGCTCTCGCATCGAT
GAAGGACGCAGCGAAATGCGATAAGTAATGTGAATTGCAGAATTCAGTGAATCATCGAAT
CTTTGAACGCATCTTGCGCTCCTTGGTATTCCGAGGAGCATGCCTGTTTGAGTGTCATTA
AATTCTCAACTCTCTTATACTTTTTTGTAAAAGAGAGCTTGGACTGTGGAGGCTTGCTGG
CCACTTTTTGGGGTCAGCTCCTCTGAAATGCATTAGCGGAACCGTTTGCAATCTGCCACA
AGTGTGATAAGTTATCTACACTGGCGAGGGGATTGCTCTCTGTAATGTTCAGCTTCTAAT
TGTCTCTACTTTGTGAGACAACTTTTGAATGCTTGACCTCAAATCAGGTAGGACTACCCG
CTGAACTTAA
>B7_25
TTTCCGTAGGTGAACCTGCGGAAGGATCATTATTGAATTATGTTTCTAGATAGGTTGTAG
CTGGCTCTTTAGAGCATGTGCACGCCTGTTTGGACTTCATTTTCATCCACCTGTGCACCT
ATTGTAGTCTTTGGTTGGGTTAGGGGGAAGTGGTCATTGTGTCAGCATCTGCTGGATGTG
AGGACTTGCATTGTGAAAGCTTTGCTGTCCTTGATGTGATCATGGAATCTCTTTCTCACT
AGAGTCTATGTCACTCATTATACTCTGTCGAATGTCATTGAATGTCTTTACATGGGCTTG
TATGCCTATGAAAATTGTAATACAACTTTCAGCAACGGATCTCTTGGCTCTCGCATCGAT
GAAGGACGCAGCGAAATGCGATAAGTAATGTGAATTGCAGAATTCAGTGAATCATCGAAT

CTTTGAACGCATCTTGCGCTCCTTGGTATTCCGAGGAGCATGCCTGTTTGAGTGTCATTA
AATTCTCAACTCTCTTATACTTTTTTGTAAAAGAGAGCTTGGACTGTGGAGGCTTGCTGG
CCACTTTTTGGGGTCAGCTCCTCTGAAATGCATTAGCGGAACCGTTTGCAATCTGCCACA
AGTGTGATAAGTTATCTACACTGGCGAGGGGATTGCTCTCTGTAATGTTCAGCTTCTAAT
TGTCTCTACTTTGTGAGACAACTTTTGAATGCTTGACCTCAAATCAGGTAGGACTACCCG
CTGAACTTAA
>B7_26
TTTCCGTAGGTGAACCTGCGGAAGGATCATTATTGAATTATGTTTCTAGATAGGTTGTAG
CTGGCTCTTTAGAGCATGTGCACGCCTGTTTGGACTTCATTTTCATCCACCTGTGCACCT
ATTGTAGTCTTTGGTTGGGTTAGGGGGAAGTGGTCATTGTGTCAGCATCTGCTGGATGTG
AGGACTTGCATTGTGAAAGCTTTGCTGTCCTTGATGTGATCATGGAATCTCTTTCTCACT
AGAGTCTATGTCACTCATTATACTCTGTCGAATGTCATTGAATGTCTTTACATGGGCTTG
TATGCCTATGAAAATTGTAATACAACTTTCAGCAACGGATCTCTTGGCTCTCGCATCGAT
GAAGGACGCAGCGAAATGCGATAAGTAATGTGAATTGCAGAATTCAGTGAATCATCGAAT
CTTTGAACGCATCTTGCGCTCCTTGGTATTCCGAGGAGCATGCCTGTTTGAGTGTCATTA
AATTCTCAACTCTCTTATACTTTTTTGTAAAAGAGAGCTTGGACTGTGGAGGCTTGCTGG
CCACTTTTTGGGGTCAGCTCCTCTGAAATGCATTAGCGGAACCGTTTGCAATCTGCCACA
AGTGTGATAAGTTATCTACACTGGCGAGGGGATTGCTCTCTGTAATGTTCAGCTTCTAAT
TGTCTCTACTTTGTGAGACAACTTTTGAATGCTTGACCTCAAATCAGGTAGGACTACCCG
CTGAACTTAA
>B7_27
TTTCCGTAGGTGAACCTGCGGAAGGATCATTATTGAATTATGTTTCTAGATAGGTTGTAG
CTGGCTCTTTAGAGCATGTGCACGCCTGTTTGGACTTCATTTTCATCCACCTGTGCACCT
ATTGTAGTCTTTGGTTGGGTTAGGGGGAAGTGGTCATTGTGTCAGCATCTGCTGGATGTG
AGGACTTGCATTGTGAAAGCTTTGCTGTCCTTGATGTGATCATGGAATCTCTTTCTCACT
AGAGTCTATGTCACTCATTATACTCTGTCGAATGTCATTGAATGTCTTTACATGGGCTTG
TATGCCTATGAAAATTGTAATACAACTTTCAGCAACGGATCTCTTGGCTCTCGCATCGAT
GAAGGACGCAGCGAAATGCGATAAGTAATGTGAATTGCAGAATTCAGTGAATCATCGAAT
CTTTGAACGCATCTTGCGCTCCTTGGTATTCCGAGGAGCATGCCTGTTTGAGTGTCATTA
AATTCTCAACTCTCTTATACTTTTTTGTAAAAGAGAGCTTGGACTGTGGAGGCTTGCTGG
CCACTTTTTGGGGTCAGCTCCTCTGAAATGCATTAGCGGAACCGTTTGCAATCTGCCACA
AGTGTGATAAGTTATCTACACTGGCGAGGGGATTGCTCTCTGTAATGTTCAGCTTCTAAT
TGTCTCTACTTTGTGAGACAACTTTTGAATGCTTGACCTCAAATCAGGTAGGACTACCCG
CTGAACTTAA
>B7_28
TTTCCGTAGGTGAACCTGCGGAAGGATCATTATTGAATTATGTTTCTAGATAGGTTGTAG
CTGGCTCTTTAGAGCATGTGCACGCCTGTTTGGACTTCATTTTCATCCACCTGTGCACCT
ATTGTAGTCTTTGGTTGGGTTAGGGGGAAGTGGTCATTGTGTCAGCATCTGCTGGATGTG
AGGACTTGCATTGTGAAAGCTTTGCTGTCCTTGATGTGATCATGGAATCTCTTTCTCACT
AGAGTCTATGTCACTCATTATACTCTGTCGAATGTCATTGAATGTCTTTACATGGGCTTG
TATGCCTATGAAAATTGTAATACAACTTTCAGCAACGGATCTCTTGGCTCTCGCATCGAT
GAAGGACGCAGCGAAATGCGATAAGTAATGTGAATTGCAGAATTCAGTGAATCATCGAAT
CTTTGAACGCATCTTGCGCTCCTTGGTATTCCGAGGAGCATGCCTGTTTGAGTGTCATTA
AATTCTCAACTCTCTTATACTTTTTTGTAAAAGAGAGCTTGGACTGTGGAGGCTTGCTGG
CCACTTTTTGGGGTCAGCTCCTCTGAAATGCATTAGCGGAACCGTTTGCAATCTGCCACA
AGTGTGATAAGTTATCTACACTGGCGAGGGGATTGCTCTCTGTAATGTTCAGCTTCTAAT
TGTCTCTACTTTGTGAGACAACTTTTGAATGCTTGACCTCAAATCAGGTAGGACTACCCG
CTGAACTTAA
>B7_29
TTTCCGTAGGTGAACCTGCGGAAGGATCATTATTGAATTATGTTTCTAGATAGGTTGTAG

CTGGCTCTTTAGAGCATGTGCACGCCTGTTTGGACTTCATTTTCATCCACCTGTGCACCT
ATTGTAGTCTTTGGTTGGGTTAGGGGGAAGTGGTCATTGTGTCAGCATCTGCTGGATGTG
AGGACTTGCATTGTGAAAGCTTTGCTGTCCTTGATGTGATCATGGAATCTCTTTCTCACT
AGAGTCTATGTCACTCATTATACTCTGTCGAATGTCATTGAATGTCTTTACATGGGCTTG
TATGCCTATGAAAATTGTAATACAACTTTCAGCAACGGATCTCTTGGCTCTCGCATCGAT
GAAGGACGCAGCGAAATGCGATAAGTAATGTGAATTGCAGAATTCAGTGAATCATCGAAT
CTTTGAACGCATCTTGCGCTCCTTGGTATTCCGAGGAGCATGCCTGTTTGAGTGTCATTA
AATTCTCAACTCTCTTATACTTTTTTGTAAAAGAGAGCTTGGACTGTGGAGGCTTGCTGG
CCACTTTTTGGGGTCAGCTCCTCTGAAATGCATTAGCGGAACCGTTTGCAATCTGCCACA
AGTGTGATAAGTTATCTACACTGGCGAGGGGATTGCTCTCTGTAATGTTCAGCTTCTAAT
TGTCTCTACTTTGTGAGACAACTTTTGAATGCTTGACCTCAAATCAGGTAGGACTACCCG
CTGAACTTAA
>B7_31
TTTCCGTAGGTGAACCTGCGGAAGGATCATTATTGAATTATGTTTCTAGATAGGTTGTAG
CTGGCTCTTTAGAGCATGTGCACGCCTGTTTGGACTTCATTTTCATCCACCTGTGCACCT
ATTGTAGTCTTTGGTTGGGTTAGGGGGAAGTGGTCATTGTGTCAGCATCTGCTGGATGTG
AGGACTTGCATTGTGAAAGCTTTGCTGTCCTTGATGTGATCATGGAATCTCTTTCTCACT
AGAGTCTATGTCACTCATTATACTCTGTCGAATGTCATTGAATGTCTTTACATGGGCTTG
TATGCCTATGAAAATTGTAATACAACTTTCAGCAACGGATCTCTTGGCTCTCGCATCGAT
GAAGGACGCAGCGAAATGCGATAAGTAATGTGAATTGCAGAATTCAGTGAATCATCGAAT
CTTTGAACGCATCTTGCGCTCCTTGGTATTCCGAGGAGCATGCCTGTTTGAGTGTCATTA
AATTCTCAACTCTCTTATACTTTTTTGTAAAAGAGAGCTTGGACTGTGGAGGCTTGCTGG
CCACTTTTTGGGGTCAGCTCCTCTGAAATGCATTAGCGGAACCGTTTGCAATCTGCCACA
AGTGTGATAAGTTATCTACACTGGCGAGGGGATTGCTCTCTGTAATGTTCAGCTTCTAAT
TGTCTCTACTTTGTGAGACAACTTTTGAATGCTTGACCTCAAATCAGGTAGGACTACCCG
CTGAACTTAA
>B7_33
TTTCCGTAGGTGAACCTGCGGAAGGATCATTATTGAATTATGTTTCTAGATAGGTTGTAG
CTGGCTCTTTAGAGCATGTGCACGCCTGTTTGGACTTCATTTTCATCCACCTGTGCACCT
ATTGTAGTCTTTGGTTGGGTTAGGGGGAAGTGGTCATTGTGTCAGCATCTGCTGGATGTG
AGGACTTGCATTGTGAAAGCTTTGCTGTCCTTGATGTGATCATGGAATCTCTTTCTCACT
AGAGTCTATGTCACTCATTATACTCTGTCGAATGTCATTGAATGTCTTTACATGGGCTTG
TATGCCTATGAAAATTGTAATACAACTTTCAGCAACGGATCTCTTGGCTCTCGCATCGAT
GAAGGACGCAGCGAAATGCGATAAGTAATGTGAATTGCAGAATTCAGTGAATCATCGAAT
CTTTGAACGCATCTTGCGCTCCTTGGTATTCCGAGGAGCATGCCTGTTTGAGTGTCATTA
AATTCTCAACTCTCTTATACTTTTTTGTAAAAGAGAGCTTGGACTGTGGAGGCTTGCTGG
CCACTTTTTGGGGTCAGCTCCTCTGAAATGCATTAGCGGAACCGTTTGCAATCTGCCACA
AGTGTGATAAGTTATCTACACTGGCGAGGGGATTGCTCTCTGTAATGTTCAGCTTCTAAT
TGTCTCTACTTTGTGAGACAACTTTTGAATGCTTGACCTCAAATCAGGTAGGACTACCCG
CTGAACTTAA
>B7_35
TTTCCGTAGGTGAACCTGCGGAAGGATCATTATTGAATTATGTTTCTAGATAGGTTGTAG
CTGGCTCTTTAGAGCATGTGCACGCCTGTTTGGACTTCATTTTCATCCACCTGTGCACCT
ATTGTAGTCTTTGGTTGGGTTAGGGGGAAGTGGTCATTGTGTCAGCATCTGCTGGATGTG
AGGACTTGCATTGTGAAAGCTTTGCTGTCCTTGATGTGATCATGGAATCTCTTTCTCACT
AGAGTCTATGTCACTCATTATACTCTGTCGAATGTCATTGAATGTCTTTACATGGGCTTG
TATGCCTATGAAAATTGTAATACAACTTTCAGCAACGGATCTCTTGGCTCTCGCATCGAT
GAAGGACGCAGCGAAATGCGATAAGTAATGTGAATTGCAGAATTCAGTGAATCATCGAAT
CTTTGAACGCATCTTGCGCTCCTTGGTATTCCGAGGAGCATGCCTGTTTGAGTGTCATTA
AATTCTCAACTCTCTTATACTTTTTTGTAAAAGAGAGCTTGGACTGTGGAGGCTTGCTGG

CCACTTTTTGGGGTCAGCTCCTCTGAAATGCATTAGCGGAACCGTTTGCAATCTGCCACA
AGTGTGATAAGTTATCTACACTGGCGAGGGGATTGCTCTCTGTAATGTTCAGCTTCTAAT
TGTCTCTACTTTGTGAGACAACTTTTGAATGCTTGACCTCAAATCAGGTAGGACTACCCG
CTGAACTTAA
>B7_38
TTTCCGTAGGTGAACCTGCGGAAGGATCATTATTGAATTATGTTTCTAGATAGGTTGTAG
CTGGCTCTTTAGAGCATGTGCACGCCTGTTTGGACTTCATTTTCATCCACCTGTGCACCT
ATTGTAGTCTTTGGTTGGGTTAGGGGGAAGTGGTCATTGTGTCAGCATCTGCTGGATGTG
AGGACTTGCATTGTGAAAGCTTTGCTGTCCTTGATGTGATCATGGAATCTCTTTCTCACT
AGAGTCTATGTCACTCATTATACTCTGTCGAATGTCATTGAATGTCTTTACATGGGCTTG
TATGCCTATGAAAATTGTAATACAACTTTCAGCAACGGATCTCTTGGCTCTCGCATCGAT
GAAGGACGCAGCGAAATGCGATAAGTAATGTGAATTGCAGAATTCAGTGAATCATCGAAT
CTTTGAACGCATCTTGCGCTCCTTGGTATTCCGAGGAGCATGCCTGTTTGAGTGTCATTA
AATTCTCAACTCTCTTATACTTTTTTGTAAAAGAGAGCTTGGACTGTGGAGGCTTGCTGG
CCACTTTTTGGGGTCAGCTCCTCTGAAATGCATTAGCGGAACCGTTTGCAATCTGCCACA
AGTGTGATAAGTTATCTACACTGGCGAGGGGATTGCTCTCTGTAATGTTCAGCTTCTAAT
TGTCTCTACTTTGTGAGACAACTTTTGAATGCTTGACCTCAAATCAGGTAGGACTACCCG
CTGAACTTAA
>B7_41
TTTCCGTAGGTGAACCTGCGGAAGGATCATTATTGAATTATGTTTCTAGATAGGTTGTAG
CTGGCTCTTTAGAGCATGTGCACGCCTGTTTGGACTTCATTTTCATCCACCTGTGCACCT
ATTGTAGTCTTTGGTTGGGTTAGGGGGAAGTGGTCATTGTGTCAGCATCTGCTGGATGTG
AGGACTTGCATTGTGAAAGCTTTGCTGTCCTTGATGTGATCATGGAATCTCTTTCTCACT
AGAGTCTATGTCACTCATTATACTCTGTCGAATGTCATTGAATGTCTTTACATGGGCTTG
TATGCCTATGAAAATTGTAATACAACTTTCAGCAACGGATCTCTTGGCTCTCGCATCGAT
GAAGGACGCAGCGAAATGCGATAAGTAATGTGAATTGCAGAATTCAGTGAATCATCGAAT
CTTTGAACGCATCTTGCGCTCCTTGGTATTCCGAGGAGCATGCCTGTTTGAGTGTCATTA
AATTCTCAACTCTCTTATACTTTTTTGTAAAAGAGAGCTTGGACTGTGGAGGCTTGCTGG
CCACTTTTTGGGGTCAGCTCCTCTGAAATGCATTAGCGGAACCGTTTGCAATCTGCCACA
AGTGTGATAAGTTATCTACACTGGCGAGGGGATTGCTCTCTGTAATGTTCAGCTTCTAAT
TGTCTCTACTTTGTGAGACAACTTTTGAATGCTTGACCTCAAATCAGGTAGGACTACCCG
CTGAACTTAA
>B7_46
TTTCCGTAGGTGAACCTGCGGAAGGATCATTATTGAATTATGTTTCTAGATAGGTTGTAG
CTGGCTCTTTAGAGCATGTGCACGCCTGTTTGGACTTCATTTTCATCCACCTGTGCACCT
ATTGTAGTCTTTGGTTGGGTTAGGGGGAAGTGGTCATTGTGTCAGCATCTGCTGGATGTG
AGGACTTGCATTGTGAAAGCTTTGCTGTCCTTGATGTGATCATGGAATCTCTTTCTCACT
AGAGTCTATGTCACTCATTATACTCTGTCGAATGTCATTGAATGTCTTTACATGGGCTTG
TATGCCTATGAAAATTGTAATACAACTTTCAGCAACGGATCTCTTGGCTCTCGCATCGAT
GAAGGACGCAGCGAAATGCGATAAGTAATGTGAATTGCAGAATTCAGTGAATCATCGAAT
CTTTGAACGCATCTTGCGCTCCTTGGTATTCCGAGGAGCATGCCTGTTTGAGTGTCATTA
AATTCTCAACTCTCTTATACTTTTTTGTAAAAGAGAGCTTGGACTGTGGAGGCTTGCTGG
CCACTTTTTGGGGTCAGCTCCTCTGAAATGCATTAGCGGAACCGTTTGCAATCTGCCACA
AGTGTGATAAGTTATCTACACTGGCGAGGGGATTGCTCTCTGTAATGTTCAGCTTCTAAT
TGTCTCTACTTTGTGAGACAACTTTTGAATGCTTGACCTCAAATCAGGTAGGACTACCCG
CTGAACTTAA
>B7_50
TTTCCGTAGGTGAACCTGCGGAAGGATCATTATTGAATTATGTTTCTAGATAGGTTGTAG
CTGGCTCTTTAGAGCATGTGCACGCCTGTTTGGACTTCATTTTCATCCACCTGTGCACCT
ATTGTAGTCTTTGGTTGGGTTAGGGGGAAGTGGTCATTGTGTCAGCATCTGCTGGATGTG

AGGACTTGCATTGTGAAAGCTTTGCTGTCCTTGATGTGATCATGGAATCTCTTTCTCACT
AGAGTCTATGTCACTCATTATACTCTGTCGAATGTCATTGAATGTCTTTACATGGGCTTG
TATGCCTATGAAAATTGTAATACAACTTTCAGCAACGGATCTCTTGGCTCTCGCATCGAT
GAAGGACGCAGCGAAATGCGATAAGTAATGTGAATTGCAGAATTCAGTGAATCATCGAAT
CTTTGAACGCATCTTGCGCTCCTTGGTATTCCGAGGAGCATGCCTGTTTGAGTGTCATTA
AATTCTCAACTCTCTTATACTTTTTTGTAAAAGAGAGCTTGGACTGTGGAGGCTTGCTGG
CCACTTTTTGGGGTCAGCTCCTCTGAAATGCATTAGCGGAACCGTTTGCAATCTGCCACA
AGTGTGATAAGTTATCTACACTGGCGAGGGGATTGCTCTCTGTAATGTTCAGCTTCTAAT
TGTCTCTACTTTGTGAGACAACTTTTGAATGCTTGACCTCAAATCAGGTAGGACTACCCG
CTGAACTTAA
>B7_51
TTTCCGTAGGTGAACCTGCGGAAGGATCATTATTGAATTATGTTTCTAGATAGGTTGTAG
CTGGCTCTTTAGAGCATGTGCACGCCTGTTTGGACTTCATTTTCATCCACCTGTGCACCT
ATTGTAGTCTTTGGTTGGGTTAGGGGGAAGTGGTCATTGTGTCAGCATCTGCTGGATGTG
AGGACTTGCATTGTGAAAGCTTTGCTGTCCTTGATGTGATCATGGAATCTCTTTCTCACT
AGAGTCTATGTCACTCATTATACTCTGTCGAATGTCATTGAATGTCTTTACATGGGCTTG
TATGCCTATGAAAATTGTAATACAACTTTCAGCAACGGATCTCTTGGCTCTCGCATCGAT
GAAGGACGCAGCGAAATGCGATAAGTAATGTGAATTGCAGAATTCAGTGAATCATCGAAT
CTTTGAACGCATCTTGCGCTCCTTGGTATTCCGAGGAGCATGCCTGTTTGAGTGTCATTA
AATTCTCAACTCTCTTATACTTTTTTGTAAAAGAGAGCTTGGACTGTGGAGGCTTGCTGG
CCACTTTTTGGGGTCAGCTCCTCTGAAATGCATTAGCGGAACCGTTTGCAATCTGCCACA
AGTGTGATAAGTTATCTACACTGGCGAGGGGATTGCTCTCTGTAATGTTCAGCTTCTAAT
TGTCTCTACTTTGTGAGACAACTTTTGAATGCTTGACCTCAAATCAGGTAGGACTACCCG
CTGAACTTAA
>B7_52
TTTCCGTAGGTGAACCTGCGGAAGGATCATTATTGAATTATGTTTCTAGATAGGTTGTAG
CTGGCTCTTTAGAGCATGTGCACGCCTGTTTGGACTTCATTTTCATCCACCTGTGCACCT
ATTGTAGTCTTTGGTTGGGTTAGGGGGAAGTGGTCATTGTGTCAGCATCTGCTGGATGTG
AGGACTTGCATTGTGAAAGCTTTGCTGTCCTTGATGTGATCATGGAATCTCTTTCTCACT
AGAGTCTATGTCACTCATTATACTCTGTCGAATGTCATTGAATGTCTTTACATGGGCTTG
TATGCCTATGAAAATTGTAATACAACTTTCAGCAACGGATCTCTTGGCTCTCGCATCGAT
GAAGGACGCAGCGAAATGCGATAAGTAATGTGAATTGCAGAATTCAGTGAATCATCGAAT
CTTTGAACGCATCTTGCGCTCCTTGGTATTCCGAGGAGCATGCCTGTTTGAGTGTCATTA
AATTCTCAACTCTCTTATACTTTTTTGTAAAAGAGAGCTTGGACTGTGGAGGCTTGCTGG
CCACTTTTTGGGGTCAGCTCCTCTGAAATGCATTAGCGGAACCGTTTGCAATCTGCCACA
AGTGTGATAAGTTATCTACACTGGCGAGGGGATTGCTCTCTGTAATGTTCAGCTTCTAAT
TGTCTCTACTTTGTGAGACAACTTTTGAATGCTTGACCTCAAATCAGGTAGGACTACCCG
CTGAACTTAA
>B7_53
TTTCCGTAGGTGAACCTGCGGAAGGATCATTATTGAATTATGTTTCTAGATAGGTTGTAG
CTGGCTCTTTAGAGCATGTGCACGCCTGTTTGGACTTCATTTTCATCCACCTGTGCACCT
ATTGTAGTCTTTGGTTGGGTTAGGGGGAAGTGGTCATTGTGTCAGCATCTGCTGGATGTG
AGGACTTGCATTGTGAAAGCTTTGCTGTCCTTGATGTGATCATGGAATCTCTTTCTCACT
AGAGTCTATGTCACTCATTATACTCTGTCGAATGTCATTGAATGTCTTTACATGGGCTTG
TATGCCTATGAAAATTGTAATACAACTTTCAGCAACGGATCTCTTGGCTCTCGCATCGAT
GAAGGACGCAGCGAAATGCGATAAGTAATGTGAATTGCAGAATTCAGTGAATCATCGAAT
CTTTGAACGCATCTTGCGCTCCTTGGTATTCCGAGGAGCATGCCTGTTTGAGTGTCATTA
AATTCTCAACTCTCTTATACTTTTTTGTAAAAGAGAGCTTGGACTGTGGAGGCTTGCTGG
CCACTTTTTGGGGTCAGCTCCTCTGAAATGCATTAGCGGAACCGTTTGCAATCTGCCACA
AGTGTGATAAGTTATCTACACTGGCGAGGGGATTGCTCTCTGTAATGTTCAGCTTCTAAT

TGTCTCTACTTTGTGAGACAACTTTTGAATGCTTGACCTCAAATCAGGTAGGACTACCCG
CTGAACTTAA
>B7_56
TTTCCGTAGGTGAACCTGCGGAAGGATCATTATTGAATTATGTTTCTAGATAGGTTGTAG
CTGGCTCTTTAGAGCATGTGCACGCCTGTTTGGACTTCATTTTCATCCACCTGTGCACCT
ATTGTAGTCTTTGGTTGGGTTAGGGGGAAGTGGTCATTGTGTCAGCATCTGCTGGATGTG
AGGACTTGCATTGTGAAAGCTTTGCTGTCCTTGATGTGATCATGGAATCTCTTTCTCACT
AGAGTCTATGTCACTCATTATACTCTGTCGAATGTCATTGAATGTCTTTACATGGGCTTG
TATGCCTATGAAAATTGTAATACAACTTTCAGCAACGGATCTCTTGGCTCTCGCATCGAT
GAAGGACGCAGCGAAATGCGATAAGTAATGTGAATTGCAGAATTCAGTGAATCATCGAAT
CTTTGAACGCATCTTGCGCTCCTTGGTATTCCGAGGAGCATGCCTGTTTGAGTGTCATTA
AATTCTCAACTCTCTTATACTTTTTTGTAAAAGAGAGCTTGGACTGTGGAGGCTTGCTGG
CCACTTTTTGGGGTCAGCTCCTCTGAAATGCATTAGCGGAACCGTTTGCAATCTGCCACA
AGTGTGATAAGTTATCTACACTGGCGAGGGGATTGCTCTCTGTAATGTTCAGCTTCTAAT
TGTCTCTACTTTGTGAGACAACTTTTGAATGCTTGACCTCAAATCAGGTAGGACTACCCG
CTGAACTTAA
>B7_57
TTTCCGTAGGTGAACCTGCGGAAGGATCATTATTGAATTATGTTTCTAGATAGGTTGTAG
CTGGCTCTTTAGAGCATGTGCACGCCTGTTTGGACTTCATTTTCATCCACCTGTGCACCT
ATTGTAGTCTTTGGTTGGGTTAGGGGGAAGTGGTCATTGTGTCAGCATCTGCTGGATGTG
AGGACTTGCATTGTGAAAGCTTTGCTGTCCTTGATGTGATCATGGAATCTCTTTCTCACT
AGAGTCTATGTCACTCATTATACTCTGTCGAATGTCATTGAATGTCTTTACATGGGCTTG
TATGCCTATGAAAATTGTAATACAACTTTCAGCAACGGATCTCTTGGCTCTCGCATCGAT
GAAGGACGCAGCGAAATGCGATAAGTAATGTGAATTGCAGAATTCAGTGAATCATCGAAT
CTTTGAACGCATCTTGCGCTCCTTGGTATTCCGAGGAGCATGCCTGTTTGAGTGTCATTA
AATTCTCAACTCTCTTATACTTTTTTGTAAAAGAGAGCTTGGACTGTGGAGGCTTGCTGG
CCACTTTTTGGGGTCAGCTCCTCTGAAATGCATTAGCGGAACCGTTTGCAATCTGCCACA
AGTGTGATAAGTTATCTACACTGGCGAGGGGATTGCTCTCTGTAATGTTCAGCTTCTAAT
TGTCTCTACTTTGTGAGACAACTTTTGAATGCTTGACCTCAAATCAGGTAGGACTACCCG
CTGAACTTAA
>B7_58
TTTCCGTAGGTGAACCTGCGGAAGGATCATTATTGAATTATGTTTCTAGATAGGTTGTAG
CTGGCTCTTTAGAGCATGTGCACGCCTGTTTGGACTTCATTTTCATCCACCTGTGCACCT
ATTGTAGTCTTTGGTTGGGTTAGGGGGAAGTGGTCATTGTGTCAGCATCTGCTGGATGTG
AGGACTTGCATTGTGAAAGCTTTGCTGTCCTTGATGTGATCATGGAATCTCTTTCTCACT
AGAGTCTATGTCACTCATTATACTCTGTCGAATGTCATTGAATGTCTTTACATGGGCTTG
TATGCCTATGAAAATTGTAATACAACTTTCAGCAACGGATCTCTTGGCTCTCGCATCGAT
GAAGGACGCAGCGAAATGCGATAAGTAATGTGAATTGCAGAATTCAGTGAATCATCGAAT
CTTTGAACGCATCTTGCGCTCCTTGGTATTCCGAGGAGCATGCCTGTTTGAGTGTCATTA
AATTCTCAACTCTCTTATACTTTTTTGTAAAAGAGAGCTTGGACTGTGGAGGCTTGCTGG
CCACTTTTTGGGGTCAGCTCCTCTGAAATGCATTAGCGGAACCGTTTGCAATCTGCCACA
AGTGTGATAAGTTATCTACACTGGCGAGGGGATTGCTCTCTGTAATGTTCAGCTTCTAAT
TGTCTCTACTTTGTGAGACAACTTTTGAATGCTTGACCTCAAATCAGGTAGGACTACCCG
CTGAACTTAA
>B7_60
TTTCCGTAGGTGAACCTGCGGAAGGATCATTATTGAATTATGTTTCTAGATAGGTTGTAG
CTGGCTCTTTAGAGCATGTGCACGCCTGTTTGGACTTCATTTTCATCCACCTGTGCACCT
ATTGTAGTCTTTGGTTGGGTTAGGGGGAAGTGGTCATTGTGTCAGCATCTGCTGGATGTG
AGGACTTGCATTGTGAAAGCTTTGCTGTCCTTGATGTGATCATGGAATCTCTTTCTCACT
AGAGTCTATGTCACTCATTATACTCTGTCGAATGTCATTGAATGTCTTTACATGGGCTTG

TATGCCTATGAAAATTGTAATACAACTTTCAGCAACGGATCTCTTGGCTCTCGCATCGAT
GAAGGACGCAGCGAAATGCGATAAGTAATGTGAATTGCAGAATTCAGTGAATCATCGAAT
CTTTGAACGCATCTTGCGCTCCTTGGTATTCCGAGGAGCATGCCTGTTTGAGTGTCATTA
AATTCTCAACTCTCTTATACTTTTTTGTAAAAGAGAGCTTGGACTGTGGAGGCTTGCTGG
CCACTTTTTGGGGTCAGCTCCTCTGAAATGCATTAGCGGAACCGTTTGCAATCTGCCACA
AGTGTGATAAGTTATCTACACTGGCGAGGGGATTGCTCTCTGTAATGTTCAGCTTCTAAT
TGTCTCTACTTTGTGAGACAACTTTTGAATGCTTGACCTCAAATCAGGTAGGACTACCCG
CTGAACTTAA
>B7_64
TTTCCGTAGGTGAACCTGCGGAAGGATCATTATTGAATTATGTTTCTAGATAGGTTGTAG
CTGGCTCTTTAGAGCATGTGCACGCCTGTTTGGACTTCATTTTCATCCACCTGTGCACCT
ATTGTAGTCTTTGGTTGGGTTAGGGGGAAGTGGTCATTGTGTCAGCATCTGCTGGATGTG
AGGACTTGCATTGTGAAAGCTTTGCTGTCCTTGATGTGATCATGGAATCTCTTTCTCACT
AGAGTCTATGTCACTCATTATACTCTGTCGAATGTCATTGAATGTCTTTACATGGGCTTG
TATGCCTATGAAAATTGTAATACAACTTTCAGCAACGGATCTCTTGGCTCTCGCATCGAT
GAAGGACGCAGCGAAATGCGATAAGTAATGTGAATTGCAGAATTCAGTGAATCATCGAAT
CTTTGAACGCATCTTGCGCTCCTTGGTATTCCGAGGAGCATGCCTGTTTGAGTGTCATTA
AATTCTCAACTCTCTTATACTTTTTTGTAAAAGAGAGCTTGGACTGTGGAGGCTTGCTGG
CCACTTTTTGGGGTCAGCTCCTCTGAAATGCATTAGCGGAACCGTTTGCAATCTGCCACA
AGTGTGATAAGTTATCTACACTGGCGAGGGGATTGCTCTCTGTAATGTTCAGCTTCTAAT
TGTCTCTACTTTGTGAGACAACTTTTGAATGCTTGACCTCAAATCAGGTAGGACTACCCG
CTGAACTTAA
>B7_65
TTTCCGTAGGTGAACCTGCGGAAGGATCATTATTGAATTATGTTTCTAGATAGGTTGTAG
CTGGCTCTTTAGAGCATGTGCACGCCTGTTTGGACTTCATTTTCATCCACCTGTGCACCT
ATTGTAGTCTTTGGTTGGGTTAGGGGGAAGTGGTCATTGTGTCAGCATCTGCTGGATGTG
AGGACTTGCATTGTGAAAGCTTTGCTGTCCTTGATGTGATCATGGAATCTCTTTCTCACT
AGAGTCTATGTCACTCATTATACTCTGTCGAATGTCATTGAATGTCTTTACATGGGCTTG
TATGCCTATGAAAATTGTAATACAACTTTCAGCAACGGATCTCTTGGCTCTCGCATCGAT
GAAGGACGCAGCGAAATGCGATAAGTAATGTGAATTGCAGAATTCAGTGAATCATCGAAT
CTTTGAACGCATCTTGCGCTCCTTGGTATTCCGAGGAGCATGCCTGTTTGAGTGTCATTA
AATTCTCAACTCTCTTATACTTTTTTGTAAAAGAGAGCTTGGACTGTGGAGGCTTGCTGG
CCACTTTTTGGGGTCAGCTCCTCTGAAATGCATTAGCGGAACCGTTTGCAATCTGCCACA
AGTGTGATAAGTTATCTACACTGGCGAGGGGATTGCTCTCTGTAATGTTCAGCTTCTAAT
TGTCTCTACTTTGTGAGACAACTTTTGAATGCTTGACCTCAAATCAGGTAGGACTACCCG
CTGAACTTAA
>B7_66
TTTCCGTAGGTGAACCTGCGGAAGGATCATTATTGAATTATGTTTCTAGATAGGTTGTAG
CTGGCTCTTTAGAGCATGTGCACGCCTGTTTGGACTTCATTTTCATCCACCTGTGCACCT
ATTGTAGTCTTTGGTTGGGTTAGGGGGAAGTGGTCATTGTGTCAGCATCTGCTGGATGTG
AGGACTTGCATTGTGAAAGCTTTGCTGTCCTTGATGTGATCATGGAATCTCTTTCTCACT
AGAGTCTATGTCACTCATTATACTCTGTCGAATGTCATTGAATGTCTTTACATGGGCTTG
TATGCCTATGAAAATTGTAATACAACTTTCAGCAACGGATCTCTTGGCTCTCGCATCGAT
GAAGGACGCAGCGAAATGCGATAAGTAATGTGAATTGCAGAATTCAGTGAATCATCGAAT
CTTTGAACGCATCTTGCGCTCCTTGGTATTCCGAGGAGCATGCCTGTTTGAGTGTCATTA
AATTCTCAACTCTCTTATACTTTTTTGTAAAAGAGAGCTTGGACTGTGGAGGCTTGCTGG
CCACTTTTTGGGGTCAGCTCCTCTGAAATGCATTAGCGGAACCGTTTGCAATCTGCCACA
AGTGTGATAAGTTATCTACACTGGCGAGGGGATTGCTCTCTGTAATGTTCAGCTTCTAAT
TGTCTCTACTTTGTGAGACAACTTTTGAATGCTTGACCTCAAATCAGGTAGGACTACCCG
CTGAACTTAA

>B8_49
TTTCCGTAGGTGAACCTGCGGAAGGATCATTATTGAATTATGTTTCTAGATAGGTTGTAG
CTGGCTCTTTAGAGCATGTGCACGCCTGTTTGGACTTCATTTTCATCCACCTGTGCACCT
ATTGTAGTCTTTGGTTGGGTTAGGGGGAAGTGGTCATTGTGTCAGCATCTGCTGGATGTG
AGGACTTGCATTGTGAAAGCTTTGCTGTCCTTGATGTGATCATGGAATCTCTTTCTCACT
AGAGTCTATGTCACTCATTATACTCTGTCGAATGTCATTGAATGTCTTTACATGGGCTTG
TATGCCTATGAAAATTGTAATACAACTTTCAGCAACGGATCTCTTGGCTCTCGCATCGAT
GAAGGACGCAGCGAAATGCGATAAGTAATGTGAATTGCAGAATTCAGTGAATCATCGAAT
CTTTGAACGCATCTTGCGCTCCTTGGTATTCCGAGGAGCATGCCTGTTTGAGTGTCATTA
AATTCTCAACTCTCTTATACTTTTTTGTAAAAGAGAGCTTGGACTGTGGAGGCTTGCTGG
CCACTTTTTGGGGTCAGCTCCTCTGAAATGCATTAGCGGAACCGTTTGCAATCTGCCACA
AGTGTGATAAGTTATCTACACTGGCGAGGGGATTGCTCTCTGTAATGTTCAGCTTCTAAT
TGTCTCTACTTTGTGAGACAACTTTTGAATGCTTGACCTCAAATCAGGTAGGACTACCCG
CTGAACTTAA
>B8_48
TTTCCGTAGGTGAACCTGCGGAAGGATCATTATTGAATTATGTTTCTAGATAGGTTGTAG
CTGGCTCTTTAGAGCATGTGCACGCCTGTTTGGACTTCATTTTCATCCACCTGTGCACCT
ATTGTAGTCTTTGGTTGGGTTAGGGGGAAGTGGTCATTGTGTCAGCATCTGCTGGATGTG
AGGACTTGCATTGTGAAAGCTTTGCTGTCCTTGATGTGATCATGGAATCTCTTTCTCACT
AGAGTCTATGTCACTCATTATACTCTGTCGAATGTCATTGAATGTCTTTACATGGGCTTG
TATGCCTATGAAAATTGTAATACAACTTTCAGCAACGGATCTCTTGGCTCTCGCATCGAT
GAAGGACGCAGCGAAATGCGATAAGTAATGTGAATTGCAGAATTCAGTGAATCATCGAAT
CTTTGAACGCATCTTGCGCTCCTTGGTATTCCGAGGAGCATGCCTGTTTGAGTGTCATTA
AATTCTCAACTCTCTTATACTTTTTTGTAAAAGAGAGCTTGGACTGTGGAGGCTTGCTGG
CCACTTTTTGGGGTCAGCTCCTCTGAAATGCATTAGCGGAACCGTTTGCAATCTGCCACA
AGTGTGATAAGTTATCTACACTGGCGAGGGGATTGCTCTCTGTAATGTTCAGCTTCTAAT
TGTCTCTACTTTGTGAGACAACTTTTGAATGCTTGACCTCAAATCAGGTAGGACTACCCG
CTGAACTTAA
>B8_47
TTTCCGTAGGTGAACCTGCGGAAGGATCATTATTGAATTATGTTTCTAGATAGGTTGTAG
CTGGCTCTTTAGAGCATGTGCACGCCTGTTTGGACTTCATTTTCATCCACCTGTGCACCT
ATTGTAGTCTTTGGTTGGGTTAGGGGGAAGTGGTCATTGTGTCAGCATCTGCTGGATGTG
AGGACTTGCATTGTGAAAGCTTTGCTGTCCTTGATGTGATCATGGAATCTCTTTCTCACT
AGAGTCTATGTCACTCATTATACTCTGTCGAATGTCATTGAATGTCTTTACATGGGCTTG
TATGCCTATGAAAATTGTAATACAACTTTCAGCAACGGATCTCTTGGCTCTCGCATCGAT
GAAGGACGCAGCGAAATGCGATAAGTAATGTGAATTGCAGAATTCAGTGAATCATCGAAT
CTTTGAACGCATCTTGCGCTCCTTGGTATTCCGAGGAGCATGCCTGTTTGAGTGTCATTA
AATTCTCAACTCTCTTATACTTTTTTGTAAAAGAGAGCTTGGACTGTGGAGGCTTGCTGG
CCACTTTTTGGGGTCAGCTCCTCTGAAATGCATTAGCGGAACCGTTTGCAATCTGCCACA
AGTGTGATAAGTTATCTACACTGGCGAGGGGATTGCTCTCTGTAATGTTCAGCTTCTAAT
TGTCTCTACTTTGTGAGACAACTTTTGAATGCTTGACCTCAAATCAGGTAGGACTACCCG
CTGAACTTAA
>B8_46
TTTCCGTAGGTGAACCTGCGGAAGGATCATTATTGAATTATGTTTCTAGATAGGTTGTAG
CTGGCTCTTTAGAGCATGTGCACGCCTGTTTGGACTTCATTTTCATCCACCTGTGCACCT
ATTGTAGTCTTTGGTTGGGTTAGGGGGAAGTGGTCATTGTGTCAGCATCTGCTGGATGTG
AGGACTTGCATTGTGAAAGCTTTGCTGTCCTTGATGTGATCATGGAATCTCTTTCTCACT
AGAGTCTATGTCACTCATTATACTCTGTCGAATGTCATTGAATGTCTTTACATGGGCTTG
TATGCCTATGAAAATTGTAATACAACTTTCAGCAACGGATCTCTTGGCTCTCGCATCGAT
GAAGGACGCAGCGAAATGCGATAAGTAATGTGAATTGCAGAATTCAGTGAATCATCGAAT

CTTTGAACGCATCTTGCGCTCCTTGGTATTCCGAGGAGCATGCCTGTTTGAGTGTCATTA
AATTCTCAACTCTCTTATACTTTTTTGTAAAAGAGAGCTTGGACTGTGGAGGCTTGCTGG
CCACTTTTTGGGGTCAGCTCCTCTGAAATGCATTAGCGGAACCGTTTGCAATCTGCCACA
AGTGTGATAAGTTATCTACACTGGCGAGGGGATTGCTCTCTGTAATGTTCAGCTTCTAAT
TGTCTCTACTTTGTGAGACAACTTTTGAATGCTTGACCTCAAATCAGGTAGGACTACCCG
CTGAACTTAA
>B8_45
TTTCCGTAGGTGAACCTGCGGAAGGATCATTATTGAATTATGTTTCTAGATAGGTTGTAG
CTGGCTCTTTAGAGCATGTGCACGCCTGTTTGGACTTCATTTTCATCCACCTGTGCACCT
ATTGTAGTCTTTGGTTGGGTTAGGGGGAAGTGGTCATTGTGTCAGCATCTGCTGGATGTG
AGGACTTGCATTGTGAAAGCTTTGCTGTCCTTGATGTGATCATGGAATCTCTTTCTCACT
AGAGTCTATGTCACTCATTATACTCTGTCGAATGTCATTGAATGTCTTTACATGGGCTTG
TATGCCTATGAAAATTGTAATACAACTTTCAGCAACGGATCTCTTGGCTCTCGCATCGAT
GAAGGACGCAGCGAAATGCGATAAGTAATGTGAATTGCAGAATTCAGTGAATCATCGAAT
CTTTGAACGCATCTTGCGCTCCTTGGTATTCCGAGGAGCATGCCTGTTTGAGTGTCATTA
AATTCTCAACTCTCTTATACTTTTTTGTAAAAGAGAGCTTGGACTGTGGAGGCTTGCTGG
CCACTTTTTGGGGTCAGCTCCTCTGAAATGCATTAGCGGAACCGTTTGCAATCTGCCACA
AGTGTGATAAGTTATCTACACTGGCGAGGGGATTGCTCTCTGTAATGTTCAGCTTCTAAT
TGTCTCTACTTTGTGAGACAACTTTTGAATGCTTGACCTCAAATCAGGTAGGACTACCCG
CTGAACTTAA
>B8_44
TTTCCGTAGGTGAACCTGCGGAAGGATCATTATTGAATTATGTTTCTAGATAGGTTGTAG
CTGGCTCTTTAGAGCATGTGCACGCCTGTTTGGACTTCATTTTCATCCACCTGTGCACCT
ATTGTAGTCTTTGGTTGGGTTAGGGGGAAGTGGTCATTGTGTCAGCATCTGCTGGATGTG
AGGACTTGCATTGTGAAAGCTTTGCTGTCCTTGATGTGATCATGGAATCTCTTTCTCACT
AGAGTCTATGTCACTCATTATACTCTGTCGAATGTCATTGAATGTCTTTACATGGGCTTG
TATGCCTATGAAAATTGTAATACAACTTTCAGCAACGGATCTCTTGGCTCTCGCATCGAT
GAAGGACGCAGCGAAATGCGATAAGTAATGTGAATTGCAGAATTCAGTGAATCATCGAAT
CTTTGAACGCATCTTGCGCTCCTTGGTATTCCGAGGAGCATGCCTGTTTGAGTGTCATTA
AATTCTCAACTCTCTTATACTTTTTTGTAAAAGAGAGCTTGGACTGTGGAGGCTTGCTGG
CCACTTTTTGGGGTCAGCTCCTCTGAAATGCATTAGCGGAACCGTTTGCAATCTGCCACA
AGTGTGATAAGTTATCTACACTGGCGAGGGGATTGCTCTCTGTAATGTTCAGCTTCTAAT
TGTCTCTACTTTGTGAGACAACTTTTGAATGCTTGACCTCAAATCAGGTAGGACTACCCG
CTGAACTTAA
>B9_1
TTTCCGTAGGTGAACCTGCGGAAGGATCATTATTGAATTATGTTTCTAGATAGGTTGTAG
CTGGCTCTTTAGAGCATGTGCACGCCTGTTTGGACTTCATTTTCATCCACCTGTGCACCT
ATTGTAGTCTTTGGTTGGGTTAGGGGGAAGTGGTCATTGTGTCAGCATCTGCTGGATGTG
AGGACTTGCATTGTGAAAGCTTTGCTGTCCTTGATGTGATCATGGAATCTCTTTCTCACT
AGAGTCTATGTCACTCATTATACTCTGTCGAATGTCATTGAATGTCTTTACATGGGCTTG
TATGCCTATGAAAATTGTAATACAACTTTCAGCAACGGATCTCTTGGCTCTCGCATCGAT
GAAGGACGCAGCGAAATGCGATAAGTAATGTGAATTGCAGAATTCAGTGAATCATCGAAT
CTTTGAACGCATCTTGCGCTCCTTGGTATTCCGAGGAGCATGCCTGTTTGAGTGTCATTA
AATTCTCAACTCTCTTATACTTTTTTGTAAAAGAGAGCTTGGACTGTGGAGGCTTGCTGG
CCACTTTTTGGGGTCAGCTCCTCTGAAATGCATTAGCGGAACCGTTTGCAATCTGCCACA
AGTGTGATAAGTTATCTACACTGGCGAGGGGATTGCTCTCTGTAATGTTCAGCTTCTAAT
TGTCTCTACTTTGTGAGACAACTTTTGAATGCTTGACCTCAAATCAGGTAGGACTACCCG
CTGAACTTAA
>B9_2
TTTCCGTAGGTGAACCTGCGGAAGGATCATTATTGAATTATGTTTCTAGATAGGTTGTAG

CTGGCTCTTTAGAGCATGTGCACGCCTGTTTGGACTTCATTTTCATCCACCTGTGCACCT
ATTGTAGTCTTTGGTTGGGTTAGGGGGAAGTGGTCATTGTGTCAGCATCTGCTGGATGTG
AGGACTTGCATTGTGAAAGCTTTGCTGTCCTTGATGTGATCATGGAATCTCTTTCTCACT
AGAGTCTATGTCACTCATTATACTCTGTCGAATGTCATTGAATGTCTTTACATGGGCTTG
TATGCCTATGAAAATTGTAATACAACTTTCAGCAACGGATCTCTTGGCTCTCGCATCGAT
GAAGGACGCAGCGAAATGCGATAAGTAATGTGAATTGCAGAATTCAGTGAATCATCGAAT
CTTTGAACGCATCTTGCGCTCCTTGGTATTCCGAGGAGCATGCCTGTTTGAGTGTCATTA
AATTCTCAACTCTCTTATACTTTTTTGTAAAAGAGAGCTTGGACTGTGGAGGCTTGCTGG
CCACTTTTTGGGGTCAGCTCCTCTGAAATGCATTAGCGGAACCGTTTGCAATCTGCCACA
AGTGTGATAAGTTATCTACACTGGCGAGGGGATTGCTCTCTGTAATGTTCAGCTTCTAAT
TGTCTCTACTTTGTGAGACAACTTTTGAATGCTTGACCTCAAATCAGGTAGGACTACCCG
CTGAACTTAA
>B9_3
TTTCCGTAGGTGAACCTGCGGAAGGATCATTATTGAATTATGTTTCTAGATAGGTTGTAG
CTGGCTCTTTAGAGCATGTGCACGCCTGTTTGGACTTCATTTTCATCCACCTGTGCACCT
ATTGTAGTCTTTGGTTGGGTTAGGGGGAAGTGGTCATTGTGTCAGCATCTGCTGGATGTG
AGGACTTGCATTGTGAAAGCTTTGCTGTCCTTGATGTGATCATGGAATCTCTTTCTCACT
AGAGTCTATGTCACTCATTATACTCTGTCGAATGTCATTGAATGTCTTTACATGGGCTTG
TATGCCTATGAAAATTGTAATACAACTTTCAGCAACGGATCTCTTGGCTCTCGCATCGAT
GAAGGACGCAGCGAAATGCGATAAGTAATGTGAATTGCAGAATTCAGTGAATCATCGAAT
CTTTGAACGCATCTTGCGCTCCTTGGTATTCCGAGGAGCATGCCTGTTTGAGTGTCATTA
AATTCTCAACTCTCTTATACTTTTTTGTAAAAGAGAGCTTGGACTGTGGAGGCTTGCTGG
CCACTTTTTGGGGTCAGCTCCTCTGAAATGCATTAGCGGAACCGTTTGCAATCTGCCACA
AGTGTGATAAGTTATCTACACTGGCGAGGGGATTGCTCTCTGTAATGTTCAGCTTCTAAT
TGTCTCTACTTTGTGAGACAACTTTTGAATGCTTGACCTCAAATCAGGTAGGACTACCCG
CTGAACTTAA
>B9_7
TTTCCGTAGGTGAACCTGCGGAAGGATCATTATTGAATTATGTTTCTAGATAGGTTGTAG
CTGGCTCTTTAGAGCATGTGCACGCCTGTTTGGACTTCATTTTCATCCACCTGTGCACCT
ATTGTAGTCTTTGGTTGGGTTAGGGGGAAGTGGTCATTGTGTCAGCATCTGCTGGATGTG
AGGACTTGCATTGTGAAAGCTTTGCTGTCCTTGATGTGATCATGGAATCTCTTTCTCACT
AGAGTCTATGTCACTCATTATACTCTGTCGAATGTCATTGAATGTCTTTACATGGGCTTG
TATGCCTATGAAAATTGTAATACAACTTTCAGCAACGGATCTCTTGGCTCTCGCATCGAT
GAAGGACGCAGCGAAATGCGATAAGTAATGTGAATTGCAGAATTCAGTGAATCATCGAAT
CTTTGAACGCATCTTGCGCTCCTTGGTATTCCGAGGAGCATGCCTGTTTGAGTGTCATTA
AATTCTCAACTCTCTTATACTTTTTTGTAAAAGAGAGCTTGGACTGTGGAGGCTTGCTGG
CCACTTTTTGGGGTCAGCTCCTCTGAAATGCATTAGCGGAACCGTTTGCAATCTGCCACA
AGTGTGATAAGTTATCTACACTGGCGAGGGGATTGCTCTCTGTAATGTTCAGCTTCTAAT
TGTCTCTACTTTGTGAGACAACTTTTGAATGCTTGACCTCAAATCAGGTAGGACTACCCG
CTGAACTTAA
>B9_8
TTTCCGTAGGTGAACCTGCGGAAGGATCATTATTGAATTATGTTTCTAGATAGGTTGTAG
CTGGCTCTTTAGAGCATGTGCACGCCTGTTTGGACTTCATTTTCATCCACCTGTGCACCT
ATTGTAGTCTTTGGTTGGGTTAGGGGGAAGTGGTCATTGTGTCAGCATCTGCTGGATGTG
AGGACTTGCATTGTGAAAGCTTTGCTGTCCTTGATGTGATCATGGAATCTCTTTCTCACT
AGAGTCTATGTCACTCATTATACTCTGTCGAATGTCATTGAATGTCTTTACATGGGCTTG
TATGCCTATGAAAATTGTAATACAACTTTCAGCAACGGATCTCTTGGCTCTCGCATCGAT
GAAGGACGCAGCGAAATGCGATAAGTAATGTGAATTGCAGAATTCAGTGAATCATCGAAT
CTTTGAACGCATCTTGCGCTCCTTGGTATTCCGAGGAGCATGCCTGTTTGAGTGTCATTA
AATTCTCAACTCTCTTATACTTTTTTGTAAAAGAGAGCTTGGACTGTGGAGGCTTGCTGG

CCACTTTTTGGGGTCAGCTCCTCTGAAATGCATTAGCGGAACCGTTTGCAATCTGCCACA
AGTGTGATAAGTTATCTACACTGGCGAGGGGATTGCTCTCTGTAATGTTCAGCTTCTAAT
TGTCTCTACTTTGTGAGACAACTTTTGAATGCTTGACCTCAAATCAGGTAGGACTACCCG
CTGAACTTAA
>B9_12
TTTCCGTAGGTGAACCTGCGGAAGGATCATTATTGAATTATGTTTCTAGATAGGTTGTAG
CTGGCTCTTTAGAGCATGTGCACGCCTGTTTGGACTTCATTTTCATCCACCTGTGCACCT
ATTGTAGTCTTTGGTTGGGTTAGGGGGAAGTGGTCATTGTGTCAGCATCTGCTGGATGTG
AGGACTTGCATTGTGAAAGCTTTGCTGTCCTTGATGTGATCATGGAATCTCTTTCTCACT
AGAGTCTATGTCACTCATTATACTCTGTCGAATGTCATTGAATGTCTTTACATGGGCTTG
TATGCCTATGAAAATTGTAATACAACTTTCAGCAACGGATCTCTTGGCTCTCGCATCGAT
GAAGGACGCAGCGAAATGCGATAAGTAATGTGAATTGCAGAATTCAGTGAATCATCGAAT
CTTTGAACGCATCTTGCGCTCCTTGGTATTCCGAGGAGCATGCCTGTTTGAGTGTCATTA
AATTCTCAACTCTCTTATACTTTTTTGTAAAAGAGAGCTTGGACTGTGGAGGCTTGCTGG
CCACTTTTTGGGGTCAGCTCCTCTGAAATGCATTAGCGGAACCGTTTGCAATCTGCCACA
AGTGTGATAAGTTATCTACACTGGCGAGGGGATTGCTCTCTGTAATGTTCAGCTTCTAAT
TGTCTCTACTTTGTGAGACAACTTTTGAATGCTTGACCTCAAATCAGGTAGGACTACCCG
CTGAACTTAA
>B9_13
TTTCCGTAGGTGAACCTGCGGAAGGATCATTATTGAATTATGTTTCTAGATAGGTTGTAG
CTGGCTCTTTAGAGCATGTGCACGCCTGTTTGGACTTCATTTTCATCCACCTGTGCACCT
ATTGTAGTCTTTGGTTGGGTTAGGGGGAAGTGGTCATTGTGTCAGCATCTGCTGGATGTG
AGGACTTGCATTGTGAAAGCTTTGCTGTCCTTGATGTGATCATGGAATCTCTTTCTCACT
AGAGTCTATGTCACTCATTATACTCTGTCGAATGTCATTGAATGTCTTTACATGGGCTTG
TATGCCTATGAAAATTGTAATACAACTTTCAGCAACGGATCTCTTGGCTCTCGCATCGAT
GAAGGACGCAGCGAAATGCGATAAGTAATGTGAATTGCAGAATTCAGTGAATCATCGAAT
CTTTGAACGCATCTTGCGCTCCTTGGTATTCCGAGGAGCATGCCTGTTTGAGTGTCATTA
AATTCTCAACTCTCTTATACTTTTTTGTAAAAGAGAGCTTGGACTGTGGAGGCTTGCTGG
CCACTTTTTGGGGTCAGCTCCTCTGAAATGCATTAGCGGAACCGTTTGCAATCTGCCACA
AGTGTGATAAGTTATCTACACTGGCGAGGGGATTGCTCTCTGTAATGTTCAGCTTCTAAT
TGTCTCTACTTTGTGAGACAACTTTTGAATGCTTGACCTCAAATCAGGTAGGACTACCCG
CTGAACTTAA
>B9_14
TTTCCGTAGGTGAACCTGCGGAAGGATCATTATTGAATTATGTTTCTAGATAGGTTGTAG
CTGGCTCTTTAGAGCATGTGCACGCCTGTTTGGACTTCATTTTCATCCACCTGTGCACCT
ATTGTAGTCTTTGGTTGGGTTAGGGGGAAGTGGTCATTGTGTCAGCATCTGCTGGATGTG
AGGACTTGCATTGTGAAAGCTTTGCTGTCCTTGATGTGATCATGGAATCTCTTTCTCACT
AGAGTCTATGTCACTCATTATACTCTGTCGAATGTCATTGAATGTCTTTACATGGGCTTG
TATGCCTATGAAAATTGTAATACAACTTTCAGCAACGGATCTCTTGGCTCTCGCATCGAT
GAAGGACGCAGCGAAATGCGATAAGTAATGTGAATTGCAGAATTCAGTGAATCATCGAAT
CTTTGAACGCATCTTGCGCTCCTTGGTATTCCGAGGAGCATGCCTGTTTGAGTGTCATTA
AATTCTCAACTCTCTTATACTTTTTTGTAAAAGAGAGCTTGGACTGTGGAGGCTTGCTGG
CCACTTTTTGGGGTCAGCTCCTCTGAAATGCATTAGCGGAACCGTTTGCAATCTGCCACA
AGTGTGATAAGTTATCTACACTGGCGAGGGGATTGCTCTCTGTAATGTTCAGCTTCTAAT
TGTCTCTACTTTGTGAGACAACTTTTGAATGCTTGACCTCAAATCAGGTAGGACTACCCG
CTGAACTTAA
>B9_15
TTTCCGTAGGTGAACCTGCGGAAGGATCATTATTGAATTATGTTTCTAGATAGGTTGTAG
CTGGCTCTTTAGAGCATGTGCACGCCTGTTTGGACTTCATTTTCATCCACCTGTGCACCT
ATTGTAGTCTTTGGTTGGGTTAGGGGGAAGTGGTCATTGTGTCAGCATCTGCTGGATGTG

AGGACTTGCATTGTGAAAGCTTTGCTGTCCTTGATGTGATCATGGAATCTCTTTCTCACT
AGAGTCTATGTCACTCATTATACTCTGTCGAATGTCATTGAATGTCTTTACATGGGCTTG
TATGCCTATGAAAATTGTAATACAACTTTCAGCAACGGATCTCTTGGCTCTCGCATCGAT
GAAGGACGCAGCGAAATGCGATAAGTAATGTGAATTGCAGAATTCAGTGAATCATCGAAT
CTTTGAACGCATCTTGCGCTCCTTGGTATTCCGAGGAGCATGCCTGTTTGAGTGTCATTA
AATTCTCAACTCTCTTATACTTTTTTGTAAAAGAGAGCTTGGACTGTGGAGGCTTGCTGG
CCACTTTTTGGGGTCAGCTCCTCTGAAATGCATTAGCGGAACCGTTTGCAATCTGCCACA
AGTGTGATAAGTTATCTACACTGGCGAGGGGATTGCTCTCTGTAATGTTCAGCTTCTAAT
TGTCTCTACTTTGTGAGACAACTTTTGAATGCTTGACCTCAAATCAGGTAGGACTACCCG
CTGAACTTAA
>B9_17
TTTCCGTAGGTGAACCTGCGGAAGGATCATTATTGAATTATGTTTCTAGATAGGTTGTAG
CTGGCTCTTTAGAGCATGTGCACGCCTGTTTGGACTTCATTTTCATCCACCTGTGCACCT
ATTGTAGTCTTTGGTTGGGTTAGGGGGAAGTGGTCATTGTGTCAGCATCTGCTGGATGTG
AGGACTTGCATTGTGAAAGCTTTGCTGTCCTTGATGTGATCATGGAATCTCTTTCTCACT
AGAGTCTATGTCACTCATTATACTCTGTCGAATGTCATTGAATGTCTTTACATGGGCTTG
TATGCCTATGAAAATTGTAATACAACTTTCAGCAACGGATCTCTTGGCTCTCGCATCGAT
GAAGGACGCAGCGAAATGCGATAAGTAATGTGAATTGCAGAATTCAGTGAATCATCGAAT
CTTTGAACGCATCTTGCGCTCCTTGGTATTCCGAGGAGCATGCCTGTTTGAGTGTCATTA
AATTCTCAACTCTCTTATACTTTTTTGTAAAAGAGAGCTTGGACTGTGGAGGCTTGCTGG
CCACTTTTTGGGGTCAGCTCCTCTGAAATGCATTAGCGGAACCGTTTGCAATCTGCCACA
AGTGTGATAAGTTATCTACACTGGCGAGGGGATTGCTCTCTGTAATGTTCAGCTTCTAAT
TGTCTCTACTTTGTGAGACAACTTTTGAATGCTTGACCTCAAATCAGGTAGGACTACCCG
CTGAACTTAA
>B9_18
TTTCCGTAGGTGAACCTGCGGAAGGATCATTATTGAATTATGTTTCTAGATAGGTTGTAG
CTGGCTCTTTAGAGCATGTGCACGCCTGTTTGGACTTCATTTTCATCCACCTGTGCACCT
ATTGTAGTCTTTGGTTGGGTTAGGGGGAAGTGGTCATTGTGTCAGCATCTGCTGGATGTG
AGGACTTGCATTGTGAAAGCTTTGCTGTCCTTGATGTGATCATGGAATCTCTTTCTCACT
AGAGTCTATGTCACTCATTATACTCTGTCGAATGTCATTGAATGTCTTTACATGGGCTTG
TATGCCTATGAAAATTGTAATACAACTTTCAGCAACGGATCTCTTGGCTCTCGCATCGAT
GAAGGACGCAGCGAAATGCGATAAGTAATGTGAATTGCAGAATTCAGTGAATCATCGAAT
CTTTGAACGCATCTTGCGCTCCTTGGTATTCCGAGGAGCATGCCTGTTTGAGTGTCATTA
AATTCTCAACTCTCTTATACTTTTTTGTAAAAGAGAGCTTGGACTGTGGAGGCTTGCTGG
CCACTTTTTGGGGTCAGCTCCTCTGAAATGCATTAGCGGAACCGTTTGCAATCTGCCACA
AGTGTGATAAGTTATCTACACTGGCGAGGGGATTGCTCTCTGTAATGTTCAGCTTCTAAT
TGTCTCTACTTTGTGAGACAACTTTTGAATGCTTGACCTCAAATCAGGTAGGACTACCCG
CTGAACTTAA
>B9_20
TTTCCGTAGGTGAACCTGCGGAAGGATCATTATTGAATTATGTTTCTAGATAGGTTGTAG
CTGGCTCTTTAGAGCATGTGCACGCCTGTTTGGACTTCATTTTCATCCACCTGTGCACCT
ATTGTAGTCTTTGGTTGGGTTAGGGGGAAGTGGTCATTGTGTCAGCATCTGCTGGATGTG
AGGACTTGCATTGTGAAAGCTTTGCTGTCCTTGATGTGATCATGGAATCTCTTTCTCACT
AGAGTCTATGTCACTCATTATACTCTGTCGAATGTCATTGAATGTCTTTACATGGGCTTG
TATGCCTATGAAAATTGTAATACAACTTTCAGCAACGGATCTCTTGGCTCTCGCATCGAT
GAAGGACGCAGCGAAATGCGATAAGTAATGTGAATTGCAGAATTCAGTGAATCATCGAAT
CTTTGAACGCATCTTGCGCTCCTTGGTATTCCGAGGAGCATGCCTGTTTGAGTGTCATTA
AATTCTCAACTCTCTTATACTTTTTTGTAAAAGAGAGCTTGGACTGTGGAGGCTTGCTGG
CCACTTTTTGGGGTCAGCTCCTCTGAAATGCATTAGCGGAACCGTTTGCAATCTGCCACA
AGTGTGATAAGTTATCTACACTGGCGAGGGGATTGCTCTCTGTAATGTTCAGCTTCTAAT

TGTCTCTACTTTGTGAGACAACTTTTGAATGCTTGACCTCAAATCAGGTAGGACTACCCG
CTGAACTTAA
>B9_21
TTTCCGTAGGTGAACCTGCGGAAGGATCATTATTGAATTATGTTTCTAGATAGGTTGTAG
CTGGCTCTTTAGAGCATGTGCACGCCTGTTTGGACTTCATTTTCATCCACCTGTGCACCT
ATTGTAGTCTTTGGTTGGGTTAGGGGGAAGTGGTCATTGTGTCAGCATCTGCTGGATGTG
AGGACTTGCATTGTGAAAGCTTTGCTGTCCTTGATGTGATCATGGAATCTCTTTCTCACT
AGAGTCTATGTCACTCATTATACTCTGTCGAATGTCATTGAATGTCTTTACATGGGCTTG
TATGCCTATGAAAATTGTAATACAACTTTCAGCAACGGATCTCTTGGCTCTCGCATCGAT
GAAGGACGCAGCGAAATGCGATAAGTAATGTGAATTGCAGAATTCAGTGAATCATCGAAT
CTTTGAACGCATCTTGCGCTCCTTGGTATTCCGAGGAGCATGCCTGTTTGAGTGTCATTA
AATTCTCAACTCTCTTATACTTTTTTGTAAAAGAGAGCTTGGACTGTGGAGGCTTGCTGG
CCACTTTTTGGGGTCAGCTCCTCTGAAATGCATTAGCGGAACCGTTTGCAATCTGCCACA
AGTGTGATAAGTTATCTACACTGGCGAGGGGATTGCTCTCTGTAATGTTCAGCTTCTAAT
TGTCTCTACTTTGTGAGACAACTTTTGAATGCTTGACCTCAAATCAGGTAGGACTACCCG
CTGAACTTAA
>B9_23
TTTCCGTAGGTGAACCTGCGGAAGGATCATTATTGAATTATGTTTCTAGATAGGTTGTAG
CTGGCTCTTTAGAGCATGTGCACGCCTGTTTGGACTTCATTTTCATCCACCTGTGCACCT
ATTGTAGTCTTTGGTTGGGTTAGGGGGAAGTGGTCATTGTGTCAGCATCTGCTGGATGTG
AGGACTTGCATTGTGAAAGCTTTGCTGTCCTTGATGTGATCATGGAATCTCTTTCTCACT
AGAGTCTATGTCACTCATTATACTCTGTCGAATGTCATTGAATGTCTTTACATGGGCTTG
TATGCCTATGAAAATTGTAATACAACTTTCAGCAACGGATCTCTTGGCTCTCGCATCGAT
GAAGGACGCAGCGAAATGCGATAAGTAATGTGAATTGCAGAATTCAGTGAATCATCGAAT
CTTTGAACGCATCTTGCGCTCCTTGGTATTCCGAGGAGCATGCCTGTTTGAGTGTCATTA
AATTCTCAACTCTCTTATACTTTTTTGTAAAAGAGAGCTTGGACTGTGGAGGCTTGCTGG
CCACTTTTTGGGGTCAGCTCCTCTGAAATGCATTAGCGGAACCGTTTGCAATCTGCCACA
AGTGTGATAAGTTATCTACACTGGCGAGGGGATTGCTCTCTGTAATGTTCAGCTTCTAAT
TGTCTCTACTTTGTGAGACAACTTTTGAATGCTTGACCTCAAATCAGGTAGGACTACCCG
CTGAACTTAA
>B9_24
TTTCCGTAGGTGAACCTGCGGAAGGATCATTATTGAATTATGTTTCTAGATAGGTTGTAG
CTGGCTCTTTAGAGCATGTGCACGCCTGTTTGGACTTCATTTTCATCCACCTGTGCACCT
ATTGTAGTCTTTGGTTGGGTTAGGGGGAAGTGGTCATTGTGTCAGCATCTGCTGGATGTG
AGGACTTGCATTGTGAAAGCTTTGCTGTCCTTGATGTGATCATGGAATCTCTTTCTCACT
AGAGTCTATGTCACTCATTATACTCTGTCGAATGTCATTGAATGTCTTTACATGGGCTTG
TATGCCTATGAAAATTGTAATACAACTTTCAGCAACGGATCTCTTGGCTCTCGCATCGAT
GAAGGACGCAGCGAAATGCGATAAGTAATGTGAATTGCAGAATTCAGTGAATCATCGAAT
CTTTGAACGCATCTTGCGCTCCTTGGTATTCCGAGGAGCATGCCTGTTTGAGTGTCATTA
AATTCTCAACTCTCTTATACTTTTTTGTAAAAGAGAGCTTGGACTGTGGAGGCTTGCTGG
CCACTTTTTGGGGTCAGCTCCTCTGAAATGCATTAGCGGAACCGTTTGCAATCTGCCACA
AGTGTGATAAGTTATCTACACTGGCGAGGGGATTGCTCTCTGTAATGTTCAGCTTCTAAT
TGTCTCTACTTTGTGAGACAACTTTTGAATGCTTGACCTCAAATCAGGTAGGACTACCCG
CTGAACTTAA
>B9_25
TTTCCGTAGGTGAACCTGCGGAAGGATCATTATTGAATTATGTTTCTAGATAGGTTGTAG
CTGGCTCTTTAGAGCATGTGCACGCCTGTTTGGACTTCATTTTCATCCACCTGTGCACCT
ATTGTAGTCTTTGGTTGGGTTAGGGGGAAGTGGTCATTGTGTCAGCATCTGCTGGATGTG
AGGACTTGCATTGTGAAAGCTTTGCTGTCCTTGATGTGATCATGGAATCTCTTTCTCACT
AGAGTCTATGTCACTCATTATACTCTGTCGAATGTCATTGAATGTCTTTACATGGGCTTG

TATGCCTATGAAAATTGTAATACAACTTTCAGCAACGGATCTCTTGGCTCTCGCATCGAT
GAAGGACGCAGCGAAATGCGATAAGTAATGTGAATTGCAGAATTCAGTGAATCATCGAAT
CTTTGAACGCATCTTGCGCTCCTTGGTATTCCGAGGAGCATGCCTGTTTGAGTGTCATTA
AATTCTCAACTCTCTTATACTTTTTTGTAAAAGAGAGCTTGGACTGTGGAGGCTTGCTGG
CCACTTTTTGGGGTCAGCTCCTCTGAAATGCATTAGCGGAACCGTTTGCAATCTGCCACA
AGTGTGATAAGTTATCTACACTGGCGAGGGGATTGCTCTCTGTAATGTTCAGCTTCTAAT
TGTCTCTACTTTGTGAGACAACTTTTGAATGCTTGACCTCAAATCAGGTAGGACTACCCG
CTGAACTTAA
>B9_27
TTTCCGTAGGTGAACCTGCGGAAGGATCATTATTGAATTATGTTTCTAGATAGGTTGTAG
CTGGCTCTTTAGAGCATGTGCACGCCTGTTTGGACTTCATTTTCATCCACCTGTGCACCT
ATTGTAGTCTTTGGTTGGGTTAGGGGGAAGTGGTCATTGTGTCAGCATCTGCTGGATGTG
AGGACTTGCATTGTGAAAGCTTTGCTGTCCTTGATGTGATCATGGAATCTCTTTCTCACT
AGAGTCTATGTCACTCATTATACTCTGTCGAATGTCATTGAATGTCTTTACATGGGCTTG
TATGCCTATGAAAATTGTAATACAACTTTCAGCAACGGATCTCTTGGCTCTCGCATCGAT
GAAGGACGCAGCGAAATGCGATAAGTAATGTGAATTGCAGAATTCAGTGAATCATCGAAT
CTTTGAACGCATCTTGCGCTCCTTGGTATTCCGAGGAGCATGCCTGTTTGAGTGTCATTA
AATTCTCAACTCTCTTATACTTTTTTGTAAAAGAGAGCTTGGACTGTGGAGGCTTGCTGG
CCACTTTTTGGGGTCAGCTCCTCTGAAATGCATTAGCGGAACCGTTTGCAATCTGCCACA
AGTGTGATAAGTTATCTACACTGGCGAGGGGATTGCTCTCTGTAATGTTCAGCTTCTAAT
TGTCTCTACTTTGTGAGACAACTTTTGAATGCTTGACCTCAAATCAGGTAGGACTACCCG
CTGAACTTAA
>B9_28
TTTCCGTAGGTGAACCTGCGGAAGGATCATTATTGAATTATGTTTCTAGATAGGTTGTAG
CTGGCTCTTTAGAGCATGTGCACGCCTGTTTGGACTTCATTTTCATCCACCTGTGCACCT
ATTGTAGTCTTTGGTTGGGTTAGGGGGAAGTGGTCATTGTGTCAGCATCTGCTGGATGTG
AGGACTTGCATTGTGAAAGCTTTGCTGTCCTTGATGTGATCATGGAATCTCTTTCTCACT
AGAGTCTATGTCACTCATTATACTCTGTCGAATGTCATTGAATGTCTTTACATGGGCTTG
TATGCCTATGAAAATTGTAATACAACTTTCAGCAACGGATCTCTTGGCTCTCGCATCGAT
GAAGGACGCAGCGAAATGCGATAAGTAATGTGAATTGCAGAATTCAGTGAATCATCGAAT
CTTTGAACGCATCTTGCGCTCCTTGGTATTCCGAGGAGCATGCCTGTTTGAGTGTCATTA
AATTCTCAACTCTCTTATACTTTTTTGTAAAAGAGAGCTTGGACTGTGGAGGCTTGCTGG
CCACTTTTTGGGGTCAGCTCCTCTGAAATGCATTAGCGGAACCGTTTGCAATCTGCCACA
AGTGTGATAAGTTATCTACACTGGCGAGGGGATTGCTCTCTGTAATGTTCAGCTTCTAAT
TGTCTCTACTTTGTGAGACAACTTTTGAATGCTTGACCTCAAATCAGGTAGGACTACCCG
CTGAACTTAA
>B9_29
TTTCCGTAGGTGAACCTGCGGAAGGATCATTATTGAATTATGTTTCTAGATAGGTTGTAG
CTGGCTCTTTAGAGCATGTGCACGCCTGTTTGGACTTCATTTTCATCCACCTGTGCACCT
ATTGTAGTCTTTGGTTGGGTTAGGGGGAAGTGGTCATTGTGTCAGCATCTGCTGGATGTG
AGGACTTGCATTGTGAAAGCTTTGCTGTCCTTGATGTGATCATGGAATCTCTTTCTCACT
AGAGTCTATGTCACTCATTATACTCTGTCGAATGTCATTGAATGTCTTTACATGGGCTTG
TATGCCTATGAAAATTGTAATACAACTTTCAGCAACGGATCTCTTGGCTCTCGCATCGAT
GAAGGACGCAGCGAAATGCGATAAGTAATGTGAATTGCAGAATTCAGTGAATCATCGAAT
CTTTGAACGCATCTTGCGCTCCTTGGTATTCCGAGGAGCATGCCTGTTTGAGTGTCATTA
AATTCTCAACTCTCTTATACTTTTTTGTAAAAGAGAGCTTGGACTGTGGAGGCTTGCTGG
CCACTTTTTGGGGTCAGCTCCTCTGAAATGCATTAGCGGAACCGTTTGCAATCTGCCACA
AGTGTGATAAGTTATCTACACTGGCGAGGGGATTGCTCTCTGTAATGTTCAGCTTCTAAT
TGTCTCTACTTTGTGAGACAACTTTTGAATGCTTGACCTCAAATCAGGTAGGACTACCCG
CTGAACTTAA

>B9_31
TTTCCGTAGGTGAACCTGCGGAAGGATCATTATTGAATTATGTTTCTAGATAGGTTGTAG
CTGGCTCTTTAGAGCATGTGCACGCCTGTTTGGACTTCATTTTCATCCACCTGTGCACCT
ATTGTAGTCTTTGGTTGGGTTAGGGGGAAGTGGTCATTGTGTCAGCATCTGCTGGATGTG
AGGACTTGCATTGTGAAAGCTTTGCTGTCCTTGATGTGATCATGGAATCTCTTTCTCACT
AGAGTCTATGTCACTCATTATACTCTGTCGAATGTCATTGAATGTCTTTACATGGGCTTG
TATGCCTATGAAAATTGTAATACAACTTTCAGCAACGGATCTCTTGGCTCTCGCATCGAT
GAAGGACGCAGCGAAATGCGATAAGTAATGTGAATTGCAGAATTCAGTGAATCATCGAAT
CTTTGAACGCATCTTGCGCTCCTTGGTATTCCGAGGAGCATGCCTGTTTGAGTGTCATTA
AATTCTCAACTCTCTTATACTTTTTTGTAAAAGAGAGCTTGGACTGTGGAGGCTTGCTGG
CCACTTTTTGGGGTCAGCTCCTCTGAAATGCATTAGCGGAACCGTTTGCAATCTGCCACA
AGTGTGATAAGTTATCTACACTGGCGAGGGGATTGCTCTCTGTAATGTTCAGCTTCTAAT
TGTCTCTACTTTGTGAGACAACTTTTGAATGCTTGACCTCAAATCAGGTAGGACTACCCG
CTGAACTTAA
>B9_32
TTTCCGTAGGTGAACCTGCGGAAGGATCATTATTGAATTATGTTTCTAGATAGGTTGTAG
CTGGCTCTTTAGAGCATGTGCACGCCTGTTTGGACTTCATTTTCATCCACCTGTGCACCT
ATTGTAGTCTTTGGTTGGGTTAGGGGGAAGTGGTCATTGTGTCAGCATCTGCTGGATGTG
AGGACTTGCATTGTGAAAGCTTTGCTGTCCTTGATGTGATCATGGAATCTCTTTCTCACT
AGAGTCTATGTCACTCATTATACTCTGTCGAATGTCATTGAATGTCTTTACATGGGCTTG
TATGCCTATGAAAATTGTAATACAACTTTCAGCAACGGATCTCTTGGCTCTCGCATCGAT
GAAGGACGCAGCGAAATGCGATAAGTAATGTGAATTGCAGAATTCAGTGAATCATCGAAT
CTTTGAACGCATCTTGCGCTCCTTGGTATTCCGAGGAGCATGCCTGTTTGAGTGTCATTA
AATTCTCAACTCTCTTATACTTTTTTGTAAAAGAGAGCTTGGACTGTGGAGGCTTGCTGG
CCACTTTTTGGGGTCAGCTCCTCTGAAATGCATTAGCGGAACCGTTTGCAATCTGCCACA
AGTGTGATAAGTTATCTACACTGGCGAGGGGATTGCTCTCTGTAATGTTCAGCTTCTAAT
TGTCTCTACTTTGTGAGACAACTTTTGAATGCTTGACCTCAAATCAGGTAGGACTACCCG
CTGAACTTAA
>B9_35
TTTCCGTAGGTGAACCTGCGGAAGGATCATTATTGAATTATGTTTCTAGATAGGTTGTAG
CTGGCTCTTTAGAGCATGTGCACGCCTGTTTGGACTTCATTTTCATCCACCTGTGCACCT
ATTGTAGTCTTTGGTTGGGTTAGGGGGAAGTGGTCATTGTGTCAGCATCTGCTGGATGTG
AGGACTTGCATTGTGAAAGCTTTGCTGTCCTTGATGTGATCATGGAATCTCTTTCTCACT
AGAGTCTATGTCACTCATTATACTCTGTCGAATGTCATTGAATGTCTTTACATGGGCTTG
TATGCCTATGAAAATTGTAATACAACTTTCAGCAACGGATCTCTTGGCTCTCGCATCGAT
GAAGGACGCAGCGAAATGCGATAAGTAATGTGAATTGCAGAATTCAGTGAATCATCGAAT
CTTTGAACGCATCTTGCGCTCCTTGGTATTCCGAGGAGCATGCCTGTTTGAGTGTCATTA
AATTCTCAACTCTCTTATACTTTTTTGTAAAAGAGAGCTTGGACTGTGGAGGCTTGCTGG
CCACTTTTTGGGGTCAGCTCCTCTGAAATGCATTAGCGGAACCGTTTGCAATCTGCCACA
AGTGTGATAAGTTATCTACACTGGCGAGGGGATTGCTCTCTGTAATGTTCAGCTTCTAAT
TGTCTCTACTTTGTGAGACAACTTTTGAATGCTTGACCTCAAATCAGGTAGGACTACCCG
CTGAACTTAA
>B9_37
TTTCCGTAGGTGAACCTGCGGAAGGATCATTATTGAATTATGTTTCTAGATAGGTTGTAG
CTGGCTCTTTAGAGCATGTGCACGCCTGTTTGGACTTCATTTTCATCCACCTGTGCACCT
ATTGTAGTCTTTGGTTGGGTTAGGGGGAAGTGGTCATTGTGTCAGCATCTGCTGGATGTG
AGGACTTGCATTGTGAAAGCTTTGCTGTCCTTGATGTGATCATGGAATCTCTTTCTCACT
AGAGTCTATGTCACTCATTATACTCTGTCGAATGTCATTGAATGTCTTTACATGGGCTTG
TATGCCTATGAAAATTGTAATACAACTTTCAGCAACGGATCTCTTGGCTCTCGCATCGAT
GAAGGACGCAGCGAAATGCGATAAGTAATGTGAATTGCAGAATTCAGTGAATCATCGAAT

CTTTGAACGCATCTTGCGCTCCTTGGTATTCCGAGGAGCATGCCTGTTTGAGTGTCATTA
AATTCTCAACTCTCTTATACTTTTTTGTAAAAGAGAGCTTGGACTGTGGAGGCTTGCTGG
CCACTTTTTGGGGTCAGCTCCTCTGAAATGCATTAGCGGAACCGTTTGCAATCTGCCACA
AGTGTGATAAGTTATCTACACTGGCGAGGGGATTGCTCTCTGTAATGTTCAGCTTCTAAT
TGTCTCTACTTTGTGAGACAACTTTTGAATGCTTGACCTCAAATCAGGTAGGACTACCCG
CTGAACTTAA
>B9_41
TTTCCGTAGGTGAACCTGCGGAAGGATCATTATTGAATTATGTTTCTAGATAGGTTGTAG
CTGGCTCTTTAGAGCATGTGCACGCCTGTTTGGACTTCATTTTCATCCACCTGTGCACCT
ATTGTAGTCTTTGGTTGGGTTAGGGGGAAGTGGTCATTGTGTCAGCATCTGCTGGATGTG
AGGACTTGCATTGTGAAAGCTTTGCTGTCCTTGATGTGATCATGGAATCTCTTTCTCACT
AGAGTCTATGTCACTCATTATACTCTGTCGAATGTCATTGAATGTCTTTACATGGGCTTG
TATGCCTATGAAAATTGTAATACAACTTTCAGCAACGGATCTCTTGGCTCTCGCATCGAT
GAAGGACGCAGCGAAATGCGATAAGTAATGTGAATTGCAGAATTCAGTGAATCATCGAAT
CTTTGAACGCATCTTGCGCTCCTTGGTATTCCGAGGAGCATGCCTGTTTGAGTGTCATTA
AATTCTCAACTCTCTTATACTTTTTTGTAAAAGAGAGCTTGGACTGTGGAGGCTTGCTGG
CCACTTTTTGGGGTCAGCTCCTCTGAAATGCATTAGCGGAACCGTTTGCAATCTGCCACA
AGTGTGATAAGTTATCTACACTGGCGAGGGGATTGCTCTCTGTAATGTTCAGCTTCTAAT
TGTCTCTACTTTGTGAGACAACTTTTGAATGCTTGACCTCAAATCAGGTAGGACTACCCG
CTGAACTTAA
>B9_42
TTTCCGTAGGTGAACCTGCGGAAGGATCATTATTGAATTATGTTTCTAGATAGGTTGTAG
CTGGCTCTTTAGAGCATGTGCACGCCTGTTTGGACTTCATTTTCATCCACCTGTGCACCT
ATTGTAGTCTTTGGTTGGGTTAGGGGGAAGTGGTCATTGTGTCAGCATCTGCTGGATGTG
AGGACTTGCATTGTGAAAGCTTTGCTGTCCTTGATGTGATCATGGAATCTCTTTCTCACT
AGAGTCTATGTCACTCATTATACTCTGTCGAATGTCATTGAATGTCTTTACATGGGCTTG
TATGCCTATGAAAATTGTAATACAACTTTCAGCAACGGATCTCTTGGCTCTCGCATCGAT
GAAGGACGCAGCGAAATGCGATAAGTAATGTGAATTGCAGAATTCAGTGAATCATCGAAT
CTTTGAACGCATCTTGCGCTCCTTGGTATTCCGAGGAGCATGCCTGTTTGAGTGTCATTA
AATTCTCAACTCTCTTATACTTTTTTGTAAAAGAGAGCTTGGACTGTGGAGGCTTGCTGG
CCACTTTTTGGGGTCAGCTCCTCTGAAATGCATTAGCGGAACCGTTTGCAATCTGCCACA
AGTGTGATAAGTTATCTACACTGGCGAGGGGATTGCTCTCTGTAATGTTCAGCTTCTAAT
TGTCTCTACTTTGTGAGACAACTTTTGAATGCTTGACCTCAAATCAGGTAGGACTACCCG
CTGAACTTAA
>B9_46
TTTCCGTAGGTGAACCTGCGGAAGGATCATTATTGAATTATGTTTCTAGATAGGTTGTAG
CTGGCTCTTTAGAGCATGTGCACGCCTGTTTGGACTTCATTTTCATCCACCTGTGCACCT
ATTGTAGTCTTTGGTTGGGTTAGGGGGAAGTGGTCATTGTGTCAGCATCTGCTGGATGTG
AGGACTTGCATTGTGAAAGCTTTGCTGTCCTTGATGTGATCATGGAATCTCTTTCTCACT
AGAGTCTATGTCACTCATTATACTCTGTCGAATGTCATTGAATGTCTTTACATGGGCTTG
TATGCCTATGAAAATTGTAATACAACTTTCAGCAACGGATCTCTTGGCTCTCGCATCGAT
GAAGGACGCAGCGAAATGCGATAAGTAATGTGAATTGCAGAATTCAGTGAATCATCGAAT
CTTTGAACGCATCTTGCGCTCCTTGGTATTCCGAGGAGCATGCCTGTTTGAGTGTCATTA
AATTCTCAACTCTCTTATACTTTTTTGTAAAAGAGAGCTTGGACTGTGGAGGCTTGCTGG
CCACTTTTTGGGGTCAGCTCCTCTGAAATGCATTAGCGGAACCGTTTGCAATCTGCCACA
AGTGTGATAAGTTATCTACACTGGCGAGGGGATTGCTCTCTGTAATGTTCAGCTTCTAAT
TGTCTCTACTTTGTGAGACAACTTTTGAATGCTTGACCTCAAATCAGGTAGGACTACCCG
CTGAACTTAA
>B9_47
TTTCCGTAGGTGAACCTGCGGAAGGATCATTATTGAATTATGTTTCTAGATAGGTTGTAG

CTGGCTCTTTAGAGCATGTGCACGCCTGTTTGGACTTCATTTTCATCCACCTGTGCACCT
ATTGTAGTCTTTGGTTGGGTTAGGGGGAAGTGGTCATTGTGTCAGCATCTGCTGGATGTG
AGGACTTGCATTGTGAAAGCTTTGCTGTCCTTGATGTGATCATGGAATCTCTTTCTCACT
AGAGTCTATGTCACTCATTATACTCTGTCGAATGTCATTGAATGTCTTTACATGGGCTTG
TATGCCTATGAAAATTGTAATACAACTTTCAGCAACGGATCTCTTGGCTCTCGCATCGAT
GAAGGACGCAGCGAAATGCGATAAGTAATGTGAATTGCAGAATTCAGTGAATCATCGAAT
CTTTGAACGCATCTTGCGCTCCTTGGTATTCCGAGGAGCATGCCTGTTTGAGTGTCATTA
AATTCTCAACTCTCTTATACTTTTTTGTAAAAGAGAGCTTGGACTGTGGAGGCTTGCTGG
CCACTTTTTGGGGTCAGCTCCTCTGAAATGCATTAGCGGAACCGTTTGCAATCTGCCACA
AGTGTGATAAGTTATCTACACTGGCGAGGGGATTGCTCTCTGTAATGTTCAGCTTCTAAT
TGTCTCTACTTTGTGAGACAACTTTTGAATGCTTGACCTCAAATCAGGTAGGACTACCCG
CTGAACTTAA
>B9_48
TTTCCGTAGGTGAACCTGCGGAAGGATCATTATTGAATTATGTTTCTAGATAGGTTGTAG
CTGGCTCTTTAGAGCATGTGCACGCCTGTTTGGACTTCATTTTCATCCACCTGTGCACCT
ATTGTAGTCTTTGGTTGGGTTAGGGGGAAGTGGTCATTGTGTCAGCATCTGCTGGATGTG
AGGACTTGCATTGTGAAAGCTTTGCTGTCCTTGATGTGATCATGGAATCTCTTTCTCACT
AGAGTCTATGTCACTCATTATACTCTGTCGAATGTCATTGAATGTCTTTACATGGGCTTG
TATGCCTATGAAAATTGTAATACAACTTTCAGCAACGGATCTCTTGGCTCTCGCATCGAT
GAAGGACGCAGCGAAATGCGATAAGTAATGTGAATTGCAGAATTCAGTGAATCATCGAAT
CTTTGAACGCATCTTGCGCTCCTTGGTATTCCGAGGAGCATGCCTGTTTGAGTGTCATTA
AATTCTCAACTCTCTTATACTTTTTTGTAAAAGAGAGCTTGGACTGTGGAGGCTTGCTGG
CCACTTTTTGGGGTCAGCTCCTCTGAAATGCATTAGCGGAACCGTTTGCAATCTGCCACA
AGTGTGATAAGTTATCTACACTGGCGAGGGGATTGCTCTCTGTAATGTTCAGCTTCTAAT
TGTCTCTACTTTGTGAGACAACTTTTGAATGCTTGACCTCAAATCAGGTAGGACTACCCG
CTGAACTTAA
>B9_50
TTTCCGTAGGTGAACCTGCGGAAGGATCATTATTGAATTATGTTTCTAGATAGGTTGTAG
CTGGCTCTTTAGAGCATGTGCACGCCTGTTTGGACTTCATTTTCATCCACCTGTGCACCT
ATTGTAGTCTTTGGTTGGGTTAGGGGGAAGTGGTCATTGTGTCAGCATCTGCTGGATGTG
AGGACTTGCATTGTGAAAGCTTTGCTGTCCTTGATGTGATCATGGAATCTCTTTCTCACT
AGAGTCTATGTCACTCATTATACTCTGTCGAATGTCATTGAATGTCTTTACATGGGCTTG
TATGCCTATGAAAATTGTAATACAACTTTCAGCAACGGATCTCTTGGCTCTCGCATCGAT
GAAGGACGCAGCGAAATGCGATAAGTAATGTGAATTGCAGAATTCAGTGAATCATCGAAT
CTTTGAACGCATCTTGCGCTCCTTGGTATTCCGAGGAGCATGCCTGTTTGAGTGTCATTA
AATTCTCAACTCTCTTATACTTTTTTGTAAAAGAGAGCTTGGACTGTGGAGGCTTGCTGG
CCACTTTTTGGGGTCAGCTCCTCTGAAATGCATTAGCGGAACCGTTTGCAATCTGCCACA
AGTGTGATAAGTTATCTACACTGGCGAGGGGATTGCTCTCTGTAATGTTCAGCTTCTAAT
TGTCTCTACTTTGTGAGACAACTTTTGAATGCTTGACCTCAAATCAGGTAGGACTACCCG
CTGAACTTAA
>B9_53
TTTCCGTAGGTGAACCTGCGGAAGGATCATTATTGAATTATGTTTCTAGATAGGTTGTAG
CTGGCTCTTTAGAGCATGTGCACGCCTGTTTGGACTTCATTTTCATCCACCTGTGCACCT
ATTGTAGTCTTTGGTTGGGTTAGGGGGAAGTGGTCATTGTGTCAGCATCTGCTGGATGTG
AGGACTTGCATTGTGAAAGCTTTGCTGTCCTTGATGTGATCATGGAATCTCTTTCTCACT
AGAGTCTATGTCACTCATTATACTCTGTCGAATGTCATTGAATGTCTTTACATGGGCTTG
TATGCCTATGAAAATTGTAATACAACTTTCAGCAACGGATCTCTTGGCTCTCGCATCGAT
GAAGGACGCAGCGAAATGCGATAAGTAATGTGAATTGCAGAATTCAGTGAATCATCGAAT
CTTTGAACGCATCTTGCGCTCCTTGGTATTCCGAGGAGCATGCCTGTTTGAGTGTCATTA
AATTCTCAACTCTCTTATACTTTTTTGTAAAAGAGAGCTTGGACTGTGGAGGCTTGCTGG

CCACTTTTTGGGGTCAGCTCCTCTGAAATGCATTAGCGGAACCGTTTGCAATCTGCCACA
AGTGTGATAAGTTATCTACACTGGCGAGGGGATTGCTCTCTGTAATGTTCAGCTTCTAAT
TGTCTCTACTTTGTGAGACAACTTTTGAATGCTTGACCTCAAATCAGGTAGGACTACCCG
CTGAACTTAA
>B9_54
TTTCCGTAGGTGAACCTGCGGAAGGATCATTATTGAATTATGTTTCTAGATAGGTTGTAG
CTGGCTCTTTAGAGCATGTGCACGCCTGTTTGGACTTCATTTTCATCCACCTGTGCACCT
ATTGTAGTCTTTGGTTGGGTTAGGGGGAAGTGGTCATTGTGTCAGCATCTGCTGGATGTG
AGGACTTGCATTGTGAAAGCTTTGCTGTCCTTGATGTGATCATGGAATCTCTTTCTCACT
AGAGTCTATGTCACTCATTATACTCTGTCGAATGTCATTGAATGTCTTTACATGGGCTTG
TATGCCTATGAAAATTGTAATACAACTTTCAGCAACGGATCTCTTGGCTCTCGCATCGAT
GAAGGACGCAGCGAAATGCGATAAGTAATGTGAATTGCAGAATTCAGTGAATCATCGAAT
CTTTGAACGCATCTTGCGCTCCTTGGTATTCCGAGGAGCATGCCTGTTTGAGTGTCATTA
AATTCTCAACTCTCTTATACTTTTTTGTAAAAGAGAGCTTGGACTGTGGAGGCTTGCTGG
CCACTTTTTGGGGTCAGCTCCTCTGAAATGCATTAGCGGAACCGTTTGCAATCTGCCACA
AGTGTGATAAGTTATCTACACTGGCGAGGGGATTGCTCTCTGTAATGTTCAGCTTCTAAT
TGTCTCTACTTTGTGAGACAACTTTTGAATGCTTGACCTCAAATCAGGTAGGACTACCCG
CTGAACTTAA
>B9_55
TTTCCGTAGGTGAACCTGCGGAAGGATCATTATTGAATTATGTTTCTAGATAGGTTGTAG
CTGGCTCTTTAGAGCATGTGCACGCCTGTTTGGACTTCATTTTCATCCACCTGTGCACCT
ATTGTAGTCTTTGGTTGGGTTAGGGGGAAGTGGTCATTGTGTCAGCATCTGCTGGATGTG
AGGACTTGCATTGTGAAAGCTTTGCTGTCCTTGATGTGATCATGGAATCTCTTTCTCACT
AGAGTCTATGTCACTCATTATACTCTGTCGAATGTCATTGAATGTCTTTACATGGGCTTG
TATGCCTATGAAAATTGTAATACAACTTTCAGCAACGGATCTCTTGGCTCTCGCATCGAT
GAAGGACGCAGCGAAATGCGATAAGTAATGTGAATTGCAGAATTCAGTGAATCATCGAAT
CTTTGAACGCATCTTGCGCTCCTTGGTATTCCGAGGAGCATGCCTGTTTGAGTGTCATTA
AATTCTCAACTCTCTTATACTTTTTTGTAAAAGAGAGCTTGGACTGTGGAGGCTTGCTGG
CCACTTTTTGGGGTCAGCTCCTCTGAAATGCATTAGCGGAACCGTTTGCAATCTGCCACA
AGTGTGATAAGTTATCTACACTGGCGAGGGGATTGCTCTCTGTAATGTTCAGCTTCTAAT
TGTCTCTACTTTGTGAGACAACTTTTGAATGCTTGACCTCAAATCAGGTAGGACTACCCG
CTGAACTTAA
>B9_57
TTTCCGTAGGTGAACCTGCGGAAGGATCATTATTGAATTATGTTTCTAGATAGGTTGTAG
CTGGCTCTTTAGAGCATGTGCACGCCTGTTTGGACTTCATTTTCATCCACCTGTGCACCT
ATTGTAGTCTTTGGTTGGGTTAGGGGGAAGTGGTCATTGTGTCAGCATCTGCTGGATGTG
AGGACTTGCATTGTGAAAGCTTTGCTGTCCTTGATGTGATCATGGAATCTCTTTCTCACT
AGAGTCTATGTCACTCATTATACTCTGTCGAATGTCATTGAATGTCTTTACATGGGCTTG
TATGCCTATGAAAATTGTAATACAACTTTCAGCAACGGATCTCTTGGCTCTCGCATCGAT
GAAGGACGCAGCGAAATGCGATAAGTAATGTGAATTGCAGAATTCAGTGAATCATCGAAT
CTTTGAACGCATCTTGCGCTCCTTGGTATTCCGAGGAGCATGCCTGTTTGAGTGTCATTA
AATTCTCAACTCTCTTATACTTTTTTGTAAAAGAGAGCTTGGACTGTGGAGGCTTGCTGG
CCACTTTTTGGGGTCAGCTCCTCTGAAATGCATTAGCGGAACCGTTTGCAATCTGCCACA
AGTGTGATAAGTTATCTACACTGGCGAGGGGATTGCTCTCTGTAATGTTCAGCTTCTAAT
TGTCTCTACTTTGTGAGACAACTTTTGAATGCTTGACCTCAAATCAGGTAGGACTACCCG
CTGAACTTAA
>B9_59
TTTCCGTAGGTGAACCTGCGGAAGGATCATTATTGAATTATGTTTCTAGATAGGTTGTAG
CTGGCTCTTTAGAGCATGTGCACGCCTGTTTGGACTTCATTTTCATCCACCTGTGCACCT
ATTGTAGTCTTTGGTTGGGTTAGGGGGAAGTGGTCATTGTGTCAGCATCTGCTGGATGTG

AGGACTTGCATTGTGAAAGCTTTGCTGTCCTTGATGTGATCATGGAATCTCTTTCTCACT
AGAGTCTATGTCACTCATTATACTCTGTCGAATGTCATTGAATGTCTTTACATGGGCTTG
TATGCCTATGAAAATTGTAATACAACTTTCAGCAACGGATCTCTTGGCTCTCGCATCGAT
GAAGGACGCAGCGAAATGCGATAAGTAATGTGAATTGCAGAATTCAGTGAATCATCGAAT
CTTTGAACGCATCTTGCGCTCCTTGGTATTCCGAGGAGCATGCCTGTTTGAGTGTCATTA
AATTCTCAACTCTCTTATACTTTTTTGTAAAAGAGAGCTTGGACTGTGGAGGCTTGCTGG
CCACTTTTTGGGGTCAGCTCCTCTGAAATGCATTAGCGGAACCGTTTGCAATCTGCCACA
AGTGTGATAAGTTATCTACACTGGCGAGGGGATTGCTCTCTGTAATGTTCAGCTTCTAAT
TGTCTCTACTTTGTGAGACAACTTTTGAATGCTTGACCTCAAATCAGGTAGGACTACCCG
CTGAACTTAA
>B9_61
TTTCCGTAGGTGAACCTGCGGAAGGATCATTATTGAATTATGTTTCTAGATAGGTTGTAG
CTGGCTCTTTAGAGCATGTGCACGCCTGTTTGGACTTCATTTTCATCCACCTGTGCACCT
ATTGTAGTCTTTGGTTGGGTTAGGGGGAAGTGGTCATTGTGTCAGCATCTGCTGGATGTG
AGGACTTGCATTGTGAAAGCTTTGCTGTCCTTGATGTGATCATGGAATCTCTTTCTCACT
AGAGTCTATGTCACTCATTATACTCTGTCGAATGTCATTGAATGTCTTTACATGGGCTTG
TATGCCTATGAAAATTGTAATACAACTTTCAGCAACGGATCTCTTGGCTCTCGCATCGAT
GAAGGACGCAGCGAAATGCGATAAGTAATGTGAATTGCAGAATTCAGTGAATCATCGAAT
CTTTGAACGCATCTTGCGCTCCTTGGTATTCCGAGGAGCATGCCTGTTTGAGTGTCATTA
AATTCTCAACTCTCTTATACTTTTTTGTAAAAGAGAGCTTGGACTGTGGAGGCTTGCTGG
CCACTTTTTGGGGTCAGCTCCTCTGAAATGCATTAGCGGAACCGTTTGCAATCTGCCACA
AGTGTGATAAGTTATCTACACTGGCGAGGGGATTGCTCTCTGTAATGTTCAGCTTCTAAT
TGTCTCTACTTTGTGAGACAACTTTTGAATGCTTGACCTCAAATCAGGTAGGACTACCCG
CTGAACTTAA
>B9_62
TTTCCGTAGGTGAACCTGCGGAAGGATCATTATTGAATTATGTTTCTAGATAGGTTGTAG
CTGGCTCTTTAGAGCATGTGCACGCCTGTTTGGACTTCATTTTCATCCACCTGTGCACCT
ATTGTAGTCTTTGGTTGGGTTAGGGGGAAGTGGTCATTGTGTCAGCATCTGCTGGATGTG
AGGACTTGCATTGTGAAAGCTTTGCTGTCCTTGATGTGATCATGGAATCTCTTTCTCACT
AGAGTCTATGTCACTCATTATACTCTGTCGAATGTCATTGAATGTCTTTACATGGGCTTG
TATGCCTATGAAAATTGTAATACAACTTTCAGCAACGGATCTCTTGGCTCTCGCATCGAT
GAAGGACGCAGCGAAATGCGATAAGTAATGTGAATTGCAGAATTCAGTGAATCATCGAAT
CTTTGAACGCATCTTGCGCTCCTTGGTATTCCGAGGAGCATGCCTGTTTGAGTGTCATTA
AATTCTCAACTCTCTTATACTTTTTTGTAAAAGAGAGCTTGGACTGTGGAGGCTTGCTGG
CCACTTTTTGGGGTCAGCTCCTCTGAAATGCATTAGCGGAACCGTTTGCAATCTGCCACA
AGTGTGATAAGTTATCTACACTGGCGAGGGGATTGCTCTCTGTAATGTTCAGCTTCTAAT
TGTCTCTACTTTGTGAGACAACTTTTGAATGCTTGACCTCAAATCAGGTAGGACTACCCG
CTGAACTTAA
>B9_63
TTTCCGTAGGTGAACCTGCGGAAGGATCATTATTGAATTATGTTTCTAGATAGGTTGTAG
CTGGCTCTTTAGAGCATGTGCACGCCTGTTTGGACTTCATTTTCATCCACCTGTGCACCT
ATTGTAGTCTTTGGTTGGGTTAGGGGGAAGTGGTCATTGTGTCAGCATCTGCTGGATGTG
AGGACTTGCATTGTGAAAGCTTTGCTGTCCTTGATGTGATCATGGAATCTCTTTCTCACT
AGAGTCTATGTCACTCATTATACTCTGTCGAATGTCATTGAATGTCTTTACATGGGCTTG
TATGCCTATGAAAATTGTAATACAACTTTCAGCAACGGATCTCTTGGCTCTCGCATCGAT
GAAGGACGCAGCGAAATGCGATAAGTAATGTGAATTGCAGAATTCAGTGAATCATCGAAT
CTTTGAACGCATCTTGCGCTCCTTGGTATTCCGAGGAGCATGCCTGTTTGAGTGTCATTA
AATTCTCAACTCTCTTATACTTTTTTGTAAAAGAGAGCTTGGACTGTGGAGGCTTGCTGG
CCACTTTTTGGGGTCAGCTCCTCTGAAATGCATTAGCGGAACCGTTTGCAATCTGCCACA
AGTGTGATAAGTTATCTACACTGGCGAGGGGATTGCTCTCTGTAATGTTCAGCTTCTAAT

TGTCTCTACTTTGTGAGACAACTTTTGAATGCTTGACCTCAAATCAGGTAGGACTACCCG
CTGAACTTAA
>B9_64
TTTCCGTAGGTGAACCTGCGGAAGGATCATTATTGAATTATGTTTCTAGATAGGTTGTAG
CTGGCTCTTTAGAGCATGTGCACGCCTGTTTGGACTTCATTTTCATCCACCTGTGCACCT
ATTGTAGTCTTTGGTTGGGTTAGGGGGAAGTGGTCATTGTGTCAGCATCTGCTGGATGTG
AGGACTTGCATTGTGAAAGCTTTGCTGTCCTTGATGTGATCATGGAATCTCTTTCTCACT
AGAGTCTATGTCACTCATTATACTCTGTCGAATGTCATTGAATGTCTTTACATGGGCTTG
TATGCCTATGAAAATTGTAATACAACTTTCAGCAACGGATCTCTTGGCTCTCGCATCGAT
GAAGGACGCAGCGAAATGCGATAAGTAATGTGAATTGCAGAATTCAGTGAATCATCGAAT
CTTTGAACGCATCTTGCGCTCCTTGGTATTCCGAGGAGCATGCCTGTTTGAGTGTCATTA
AATTCTCAACTCTCTTATACTTTTTTGTAAAAGAGAGCTTGGACTGTGGAGGCTTGCTGG
CCACTTTTTGGGGTCAGCTCCTCTGAAATGCATTAGCGGAACCGTTTGCAATCTGCCACA
AGTGTGATAAGTTATCTACACTGGCGAGGGGATTGCTCTCTGTAATGTTCAGCTTCTAAT
TGTCTCTACTTTGTGAGACAACTTTTGAATGCTTGACCTCAAATCAGGTAGGACTACCCG
CTGAACTTAA
>B9_66
TTTCCGTAGGTGAACCTGCGGAAGGATCATTATTGAATTATGTTTCTAGATAGGTTGTAG
CTGGCTCTTTAGAGCATGTGCACGCCTGTTTGGACTTCATTTTCATCCACCTGTGCACCT
ATTGTAGTCTTTGGTTGGGTTAGGGGGAAGTGGTCATTGTGTCAGCATCTGCTGGATGTG
AGGACTTGCATTGTGAAAGCTTTGCTGTCCTTGATGTGATCATGGAATCTCTTTCTCACT
AGAGTCTATGTCACTCATTATACTCTGTCGAATGTCATTGAATGTCTTTACATGGGCTTG
TATGCCTATGAAAATTGTAATACAACTTTCAGCAACGGATCTCTTGGCTCTCGCATCGAT
GAAGGACGCAGCGAAATGCGATAAGTAATGTGAATTGCAGAATTCAGTGAATCATCGAAT
CTTTGAACGCATCTTGCGCTCCTTGGTATTCCGAGGAGCATGCCTGTTTGAGTGTCATTA
AATTCTCAACTCTCTTATACTTTTTTGTAAAAGAGAGCTTGGACTGTGGAGGCTTGCTGG
CCACTTTTTGGGGTCAGCTCCTCTGAAATGCATTAGCGGAACCGTTTGCAATCTGCCACA
AGTGTGATAAGTTATCTACACTGGCGAGGGGATTGCTCTCTGTAATGTTCAGCTTCTAAT
TGTCTCTACTTTGTGAGACAACTTTTGAATGCTTGACCTCAAATCAGGTAGGACTACCCG
CTGAACTTAA
>B9_67
TTTCCGTAGGTGAACCTGCGGAAGGATCATTATTGAATTATGTTTCTAGATAGGTTGTAG
CTGGCTCTTTAGAGCATGTGCACGCCTGTTTGGACTTCATTTTCATCCACCTGTGCACCT
ATTGTAGTCTTTGGTTGGGTTAGGGGGAAGTGGTCATTGTGTCAGCATCTGCTGGATGTG
AGGACTTGCATTGTGAAAGCTTTGCTGTCCTTGATGTGATCATGGAATCTCTTTCTCACT
AGAGTCTATGTCACTCATTATACTCTGTCGAATGTCATTGAATGTCTTTACATGGGCTTG
TATGCCTATGAAAATTGTAATACAACTTTCAGCAACGGATCTCTTGGCTCTCGCATCGAT
GAAGGACGCAGCGAAATGCGATAAGTAATGTGAATTGCAGAATTCAGTGAATCATCGAAT
CTTTGAACGCATCTTGCGCTCCTTGGTATTCCGAGGAGCATGCCTGTTTGAGTGTCATTA
AATTCTCAACTCTCTTATACTTTTTTGTAAAAGAGAGCTTGGACTGTGGAGGCTTGCTGG
CCACTTTTTGGGGTCAGCTCCTCTGAAATGCATTAGCGGAACCGTTTGCAATCTGCCACA
AGTGTGATAAGTTATCTACACTGGCGAGGGGATTGCTCTCTGTAATGTTCAGCTTCTAAT
TGTCTCTACTTTGTGAGACAACTTTTGAATGCTTGACCTCAAATCAGGTAGGACTACCCG
CTGAACTTAA
>B9_68
TTTCCGTAGGTGAACCTGCGGAAGGATCATTATTGAATTATGTTTCTAGATAGGTTGTAG
CTGGCTCTTTAGAGCATGTGCACGCCTGTTTGGACTTCATTTTCATCCACCTGTGCACCT
ATTGTAGTCTTTGGTTGGGTTAGGGGGAAGTGGTCATTGTGTCAGCATCTGCTGGATGTG
AGGACTTGCATTGTGAAAGCTTTGCTGTCCTTGATGTGATCATGGAATCTCTTTCTCACT
AGAGTCTATGTCACTCATTATACTCTGTCGAATGTCATTGAATGTCTTTACATGGGCTTG

TATGCCTATGAAAATTGTAATACAACTTTCAGCAACGGATCTCTTGGCTCTCGCATCGAT
GAAGGACGCAGCGAAATGCGATAAGTAATGTGAATTGCAGAATTCAGTGAATCATCGAAT
CTTTGAACGCATCTTGCGCTCCTTGGTATTCCGAGGAGCATGCCTGTTTGAGTGTCATTA
AATTCTCAACTCTCTTATACTTTTTTGTAAAAGAGAGCTTGGACTGTGGAGGCTTGCTGG
CCACTTTTTGGGGTCAGCTCCTCTGAAATGCATTAGCGGAACCGTTTGCAATCTGCCACA
AGTGTGATAAGTTATCTACACTGGCGAGGGGATTGCTCTCTGTAATGTTCAGCTTCTAAT
TGTCTCTACTTTGTGAGACAACTTTTGAATGCTTGACCTCAAATCAGGTAGGACTACCCG
CTGAACTTAA
>B9_70
TTTCCGTAGGTGAACCTGCGGAAGGATCATTATTGAATTATGTTTCTAGATAGGTTGTAG
CTGGCTCTTTAGAGCATGTGCACGCCTGTTTGGACTTCATTTTCATCCACCTGTGCACCT
ATTGTAGTCTTTGGTTGGGTTAGGGGGAAGTGGTCATTGTGTCAGCATCTGCTGGATGTG
AGGACTTGCATTGTGAAAGCTTTGCTGTCCTTGATGTGATCATGGAATCTCTTTCTCACT
AGAGTCTATGTCACTCATTATACTCTGTCGAATGTCATTGAATGTCTTTACATGGGCTTG
TATGCCTATGAAAATTGTAATACAACTTTCAGCAACGGATCTCTTGGCTCTCGCATCGAT
GAAGGACGCAGCGAAATGCGATAAGTAATGTGAATTGCAGAATTCAGTGAATCATCGAAT
CTTTGAACGCATCTTGCGCTCCTTGGTATTCCGAGGAGCATGCCTGTTTGAGTGTCATTA
AATTCTCAACTCTCTTATACTTTTTTGTAAAAGAGAGCTTGGACTGTGGAGGCTTGCTGG
CCACTTTTTGGGGTCAGCTCCTCTGAAATGCATTAGCGGAACCGTTTGCAATCTGCCACA
AGTGTGATAAGTTATCTACACTGGCGAGGGGATTGCTCTCTGTAATGTTCAGCTTCTAAT
TGTCTCTACTTTGTGAGACAACTTTTGAATGCTTGACCTCAAATCAGGTAGGACTACCCG
CTGAACTTAA
>B9_71
TTTCCGTAGGTGAACCTGCGGAAGGATCATTATTGAATTATGTTTCTAGATAGGTTGTAG
CTGGCTCTTTAGAGCATGTGCACGCCTGTTTGGACTTCATTTTCATCCACCTGTGCACCT
ATTGTAGTCTTTGGTTGGGTTAGGGGGAAGTGGTCATTGTGTCAGCATCTGCTGGATGTG
AGGACTTGCATTGTGAAAGCTTTGCTGTCCTTGATGTGATCATGGAATCTCTTTCTCACT
AGAGTCTATGTCACTCATTATACTCTGTCGAATGTCATTGAATGTCTTTACATGGGCTTG
TATGCCTATGAAAATTGTAATACAACTTTCAGCAACGGATCTCTTGGCTCTCGCATCGAT
GAAGGACGCAGCGAAATGCGATAAGTAATGTGAATTGCAGAATTCAGTGAATCATCGAAT
CTTTGAACGCATCTTGCGCTCCTTGGTATTCCGAGGAGCATGCCTGTTTGAGTGTCATTA
AATTCTCAACTCTCTTATACTTTTTTGTAAAAGAGAGCTTGGACTGTGGAGGCTTGCTGG
CCACTTTTTGGGGTCAGCTCCTCTGAAATGCATTAGCGGAACCGTTTGCAATCTGCCACA
AGTGTGATAAGTTATCTACACTGGCGAGGGGATTGCTCTCTGTAATGTTCAGCTTCTAAT
TGTCTCTACTTTGTGAGACAACTTTTGAATGCTTGACCTCAAATCAGGTAGGACTACCCG
CTGAACTTAA
>B10_2
TTTCCGTAGGTGAACCTGCGGAAGGATCATTATTGAATTATGTTTCTAGATAGGTTGTAG
CTGGCTCTTTAGAGCATGTGCACGCCTGTTTGGACTTCATTTTCATCCACCTGTGCACCT
ATTGTAGTCTTTGGTTGGGTTAGGGGGAAGTGGTCATTGTGTCAGCATCTGCTGGATGTG
AGGACTTGCATTGTGAAAGCTTTGCTGTCCTTGATGTGATCATGGAATCTCTTTCTCACT
AGAGTCTATGTCACTCATTATACTCTGTCGAATGTCATTGAATGTCTTTACATGGGCTTG
TATGCCTATGAAAATTGTAATACAACTTTCAGCAACGGATCTCTTGGCTCTCGCATCGAT
GAAGGACGCAGCGAAATGCGATAAGTAATGTGAATTGCAGAATTCAGTGAATCATCGAAT
CTTTGAACGCATCTTGCGCTCCTTGGTATTCCGAGGAGCATGCCTGTTTGAGTGTCATTA
AATTCTCAACTCTCTTATACTTTTTTGTAAAAGAGAGCTTGGACTGTGGAGGCTTGCTGG
CCACTTTTTGGGGTCAGCTCCTCTGAAATGCATTAGCGGAACCGTTTGCAATCTGCCACA
AGTGTGATAAGTTATCTACACTGGCGAGGGGATTGCTCTCTGTAATGTTCAGCTTCTAAT
TGTCTCTACTTTGTGAGACAACTTTTGAATGCTTGACCTCAAATCAGGTAGGACTACCCG
CTGAACTTAA

>B10_3
TTTCCGTAGGTGAACCTGCGGAAGGATCATTATTGAATTATGTTTCTAGATAGGTTGTAG
CTGGCTCTTTAGAGCATGTGCACGCCTGTTTGGACTTCATTTTCATCCACCTGTGCACCT
ATTGTAGTCTTTGGTTGGGTTAGGGGGAAGTGGTCATTGTGTCAGCATCTGCTGGATGTG
AGGACTTGCATTGTGAAAGCTTTGCTGTCCTTGATGTGATCATGGAATCTCTTTCTCACT
AGAGTCTATGTCACTCATTATACTCTGTCGAATGTCATTGAATGTCTTTACATGGGCTTG
TATGCCTATGAAAATTGTAATACAACTTTCAGCAACGGATCTCTTGGCTCTCGCATCGAT
GAAGGACGCAGCGAAATGCGATAAGTAATGTGAATTGCAGAATTCAGTGAATCATCGAAT
CTTTGAACGCATCTTGCGCTCCTTGGTATTCCGAGGAGCATGCCTGTTTGAGTGTCATTA
AATTCTCAACTCTCTTATACTTTTTTGTAAAAGAGAGCTTGGACTGTGGAGGCTTGCTGG
CCACTTTTTGGGGTCAGCTCCTCTGAAATGCATTAGCGGAACCGTTTGCAATCTGCCACA
AGTGTGATAAGTTATCTACACTGGCGAGGGGATTGCTCTCTGTAATGTTCAGCTTCTAAT
TGTCTCTACTTTGTGAGACAACTTTTGAATGCTTGACCTCAAATCAGGTAGGACTACCCG
CTGAACTTAA
>B10_5
TTTCCGTAGGTGAACCTGCGGAAGGATCATTATTGAATTATGTTTCTAGATAGGTTGTAG
CTGGCTCTTTAGAGCATGTGCACGCCTGTTTGGACTTCATTTTCATCCACCTGTGCACCT
ATTGTAGTCTTTGGTTGGGTTAGGGGGAAGTGGTCATTGTGTCAGCATCTGCTGGATGTG
AGGACTTGCATTGTGAAAGCTTTGCTGTCCTTGATGTGATCATGGAATCTCTTTCTCACT
AGAGTCTATGTCACTCATTATACTCTGTCGAATGTCATTGAATGTCTTTACATGGGCTTG
TATGCCTATGAAAATTGTAATACAACTTTCAGCAACGGATCTCTTGGCTCTCGCATCGAT
GAAGGACGCAGCGAAATGCGATAAGTAATGTGAATTGCAGAATTCAGTGAATCATCGAAT
CTTTGAACGCATCTTGCGCTCCTTGGTATTCCGAGGAGCATGCCTGTTTGAGTGTCATTA
AATTCTCAACTCTCTTATACTTTTTTGTAAAAGAGAGCTTGGACTGTGGAGGCTTGCTGG
CCACTTTTTGGGGTCAGCTCCTCTGAAATGCATTAGCGGAACCGTTTGCAATCTGCCACA
AGTGTGATAAGTTATCTACACTGGCGAGGGGATTGCTCTCTGTAATGTTCAGCTTCTAAT
TGTCTCTACTTTGTGAGACAACTTTTGAATGCTTGACCTCAAATCAGGTAGGACTACCCG
CTGAACTTAA
>B10_6
TTTCCGTAGGTGAACCTGCGGAAGGATCATTATTGAATTATGTTTCTAGATAGGTTGTAG
CTGGCTCTTTAGAGCATGTGCACGCCTGTTTGGACTTCATTTTCATCCACCTGTGCACCT
ATTGTAGTCTTTGGTTGGGTTAGGGGGAAGTGGTCATTGTGTCAGCATCTGCTGGATGTG
AGGACTTGCATTGTGAAAGCTTTGCTGTCCTTGATGTGATCATGGAATCTCTTTCTCACT
AGAGTCTATGTCACTCATTATACTCTGTCGAATGTCATTGAATGTCTTTACATGGGCTTG
TATGCCTATGAAAATTGTAATACAACTTTCAGCAACGGATCTCTTGGCTCTCGCATCGAT
GAAGGACGCAGCGAAATGCGATAAGTAATGTGAATTGCAGAATTCAGTGAATCATCGAAT
CTTTGAACGCATCTTGCGCTCCTTGGTATTCCGAGGAGCATGCCTGTTTGAGTGTCATTA
AATTCTCAACTCTCTTATACTTTTTTGTAAAAGAGAGCTTGGACTGTGGAGGCTTGCTGG
CCACTTTTTGGGGTCAGCTCCTCTGAAATGCATTAGCGGAACCGTTTGCAATCTGCCACA
AGTGTGATAAGTTATCTACACTGGCGAGGGGATTGCTCTCTGTAATGTTCAGCTTCTAAT
TGTCTCTACTTTGTGAGACAACTTTTGAATGCTTGACCTCAAATCAGGTAGGACTACCCG
CTGAACTTAA
>B10_7
TTTCCGTAGGTGAACCTGCGGAAGGATCATTATTGAATTATGTTTCTAGATAGGTTGTAG
CTGGCTCTTTAGAGCATGTGCACGCCTGTTTGGACTTCATTTTCATCCACCTGTGCACCT
ATTGTAGTCTTTGGTTGGGTTAGGGGGAAGTGGTCATTGTGTCAGCATCTGCTGGATGTG
AGGACTTGCATTGTGAAAGCTTTGCTGTCCTTGATGTGATCATGGAATCTCTTTCTCACT
AGAGTCTATGTCACTCATTATACTCTGTCGAATGTCATTGAATGTCTTTACATGGGCTTG
TATGCCTATGAAAATTGTAATACAACTTTCAGCAACGGATCTCTTGGCTCTCGCATCGAT
GAAGGACGCAGCGAAATGCGATAAGTAATGTGAATTGCAGAATTCAGTGAATCATCGAAT

CTTTGAACGCATCTTGCGCTCCTTGGTATTCCGAGGAGCATGCCTGTTTGAGTGTCATTA
AATTCTCAACTCTCTTATACTTTTTTGTAAAAGAGAGCTTGGACTGTGGAGGCTTGCTGG
CCACTTTTTGGGGTCAGCTCCTCTGAAATGCATTAGCGGAACCGTTTGCAATCTGCCACA
AGTGTGATAAGTTATCTACACTGGCGAGGGGATTGCTCTCTGTAATGTTCAGCTTCTAAT
TGTCTCTACTTTGTGAGACAACTTTTGAATGCTTGACCTCAAATCAGGTAGGACTACCCG
CTGAACTTAA
>B10_9
TTTCCGTAGGTGAACCTGCGGAAGGATCATTATTGAATTATGTTTCTAGATAGGTTGTAG
CTGGCTCTTTAGAGCATGTGCACGCCTGTTTGGACTTCATTTTCATCCACCTGTGCACCT
ATTGTAGTCTTTGGTTGGGTTAGGGGGAAGTGGTCATTGTGTCAGCATCTGCTGGATGTG
AGGACTTGCATTGTGAAAGCTTTGCTGTCCTTGATGTGATCATGGAATCTCTTTCTCACT
AGAGTCTATGTCACTCATTATACTCTGTCGAATGTCATTGAATGTCTTTACATGGGCTTG
TATGCCTATGAAAATTGTAATACAACTTTCAGCAACGGATCTCTTGGCTCTCGCATCGAT
GAAGGACGCAGCGAAATGCGATAAGTAATGTGAATTGCAGAATTCAGTGAATCATCGAAT
CTTTGAACGCATCTTGCGCTCCTTGGTATTCCGAGGAGCATGCCTGTTTGAGTGTCATTA
AATTCTCAACTCTCTTATACTTTTTTGTAAAAGAGAGCTTGGACTGTGGAGGCTTGCTGG
CCACTTTTTGGGGTCAGCTCCTCTGAAATGCATTAGCGGAACCGTTTGCAATCTGCCACA
AGTGTGATAAGTTATCTACACTGGCGAGGGGATTGCTCTCTGTAATGTTCAGCTTCTAAT
TGTCTCTACTTTGTGAGACAACTTTTGAATGCTTGACCTCAAATCAGGTAGGACTACCCG
CTGAACTTAA
>B10_10
TTTCCGTAGGTGAACCTGCGGAAGGATCATTATTGAATTATGTTTCTAGATAGGTTGTAG
CTGGCTCTTTAGAGCATGTGCACGCCTGTTTGGACTTCATTTTCATCCACCTGTGCACCT
ATTGTAGTCTTTGGTTGGGTTAGGGGGAAGTGGTCATTGTGTCAGCATCTGCTGGATGTG
AGGACTTGCATTGTGAAAGCTTTGCTGTCCTTGATGTGATCATGGAATCTCTTTCTCACT
AGAGTCTATGTCACTCATTATACTCTGTCGAATGTCATTGAATGTCTTTACATGGGCTTG
TATGCCTATGAAAATTGTAATACAACTTTCAGCAACGGATCTCTTGGCTCTCGCATCGAT
GAAGGACGCAGCGAAATGCGATAAGTAATGTGAATTGCAGAATTCAGTGAATCATCGAAT
CTTTGAACGCATCTTGCGCTCCTTGGTATTCCGAGGAGCATGCCTGTTTGAGTGTCATTA
AATTCTCAACTCTCTTATACTTTTTTGTAAAAGAGAGCTTGGACTGTGGAGGCTTGCTGG
CCACTTTTTGGGGTCAGCTCCTCTGAAATGCATTAGCGGAACCGTTTGCAATCTGCCACA
AGTGTGATAAGTTATCTACACTGGCGAGGGGATTGCTCTCTGTAATGTTCAGCTTCTAAT
TGTCTCTACTTTGTGAGACAACTTTTGAATGCTTGACCTCAAATCAGGTAGGACTACCCG
CTGAACTTAA
>B10_11
TTTCCGTAGGTGAACCTGCGGAAGGATCATTATTGAATTATGTTTCTAGATAGGTTGTAG
CTGGCTCTTTAGAGCATGTGCACGCCTGTTTGGACTTCATTTTCATCCACCTGTGCACCT
ATTGTAGTCTTTGGTTGGGTTAGGGGGAAGTGGTCATTGTGTCAGCATCTGCTGGATGTG
AGGACTTGCATTGTGAAAGCTTTGCTGTCCTTGATGTGATCATGGAATCTCTTTCTCACT
AGAGTCTATGTCACTCATTATACTCTGTCGAATGTCATTGAATGTCTTTACATGGGCTTG
TATGCCTATGAAAATTGTAATACAACTTTCAGCAACGGATCTCTTGGCTCTCGCATCGAT
GAAGGACGCAGCGAAATGCGATAAGTAATGTGAATTGCAGAATTCAGTGAATCATCGAAT
CTTTGAACGCATCTTGCGCTCCTTGGTATTCCGAGGAGCATGCCTGTTTGAGTGTCATTA
AATTCTCAACTCTCTTATACTTTTTTGTAAAAGAGAGCTTGGACTGTGGAGGCTTGCTGG
CCACTTTTTGGGGTCAGCTCCTCTGAAATGCATTAGCGGAACCGTTTGCAATCTGCCACA
AGTGTGATAAGTTATCTACACTGGCGAGGGGATTGCTCTCTGTAATGTTCAGCTTCTAAT
TGTCTCTACTTTGTGAGACAACTTTTGAATGCTTGACCTCAAATCAGGTAGGACTACCCG
CTGAACTTAA
>B10_14
TTTCCGTAGGTGAACCTGCGGAAGGATCATTATTGAATTATGTTTCTAGATAGGTTGTAG

CTGGCTCTTTAGAGCATGTGCACGCCTGTTTGGACTTCATTTTCATCCACCTGTGCACCT
ATTGTAGTCTTTGGTTGGGTTAGGGGGAAGTGGTCATTGTGTCAGCATCTGCTGGATGTG
AGGACTTGCATTGTGAAAGCTTTGCTGTCCTTGATGTGATCATGGAATCTCTTTCTCACT
AGAGTCTATGTCACTCATTATACTCTGTCGAATGTCATTGAATGTCTTTACATGGGCTTG
TATGCCTATGAAAATTGTAATACAACTTTCAGCAACGGATCTCTTGGCTCTCGCATCGAT
GAAGGACGCAGCGAAATGCGATAAGTAATGTGAATTGCAGAATTCAGTGAATCATCGAAT
CTTTGAACGCATCTTGCGCTCCTTGGTATTCCGAGGAGCATGCCTGTTTGAGTGTCATTA
AATTCTCAACTCTCTTATACTTTTTTGTAAAAGAGAGCTTGGACTGTGGAGGCTTGCTGG
CCACTTTTTGGGGTCAGCTCCTCTGAAATGCATTAGCGGAACCGTTTGCAATCTGCCACA
AGTGTGATAAGTTATCTACACTGGCGAGGGGATTGCTCTCTGTAATGTTCAGCTTCTAAT
TGTCTCTACTTTGTGAGACAACTTTTGAATGCTTGACCTCAAATCAGGTAGGACTACCCG
CTGAACTTAA
>B10_15
TTTCCGTAGGTGAACCTGCGGAAGGATCATTATTGAATTATGTTTCTAGATAGGTTGTAG
CTGGCTCTTTAGAGCATGTGCACGCCTGTTTGGACTTCATTTTCATCCACCTGTGCACCT
ATTGTAGTCTTTGGTTGGGTTAGGGGGAAGTGGTCATTGTGTCAGCATCTGCTGGATGTG
AGGACTTGCATTGTGAAAGCTTTGCTGTCCTTGATGTGATCATGGAATCTCTTTCTCACT
AGAGTCTATGTCACTCATTATACTCTGTCGAATGTCATTGAATGTCTTTACATGGGCTTG
TATGCCTATGAAAATTGTAATACAACTTTCAGCAACGGATCTCTTGGCTCTCGCATCGAT
GAAGGACGCAGCGAAATGCGATAAGTAATGTGAATTGCAGAATTCAGTGAATCATCGAAT
CTTTGAACGCATCTTGCGCTCCTTGGTATTCCGAGGAGCATGCCTGTTTGAGTGTCATTA
AATTCTCAACTCTCTTATACTTTTTTGTAAAAGAGAGCTTGGACTGTGGAGGCTTGCTGG
CCACTTTTTGGGGTCAGCTCCTCTGAAATGCATTAGCGGAACCGTTTGCAATCTGCCACA
AGTGTGATAAGTTATCTACACTGGCGAGGGGATTGCTCTCTGTAATGTTCAGCTTCTAAT
TGTCTCTACTTTGTGAGACAACTTTTGAATGCTTGACCTCAAATCAGGTAGGACTACCCG
CTGAACTTAA
>B10_16
TTTCCGTAGGTGAACCTGCGGAAGGATCATTATTGAATTATGTTTCTAGATAGGTTGTAG
CTGGCTCTTTAGAGCATGTGCACGCCTGTTTGGACTTCATTTTCATCCACCTGTGCACCT
ATTGTAGTCTTTGGTTGGGTTAGGGGGAAGTGGTCATTGTGTCAGCATCTGCTGGATGTG
AGGACTTGCATTGTGAAAGCTTTGCTGTCCTTGATGTGATCATGGAATCTCTTTCTCACT
AGAGTCTATGTCACTCATTATACTCTGTCGAATGTCATTGAATGTCTTTACATGGGCTTG
TATGCCTATGAAAATTGTAATACAACTTTCAGCAACGGATCTCTTGGCTCTCGCATCGAT
GAAGGACGCAGCGAAATGCGATAAGTAATGTGAATTGCAGAATTCAGTGAATCATCGAAT
CTTTGAACGCATCTTGCGCTCCTTGGTATTCCGAGGAGCATGCCTGTTTGAGTGTCATTA
AATTCTCAACTCTCTTATACTTTTTTGTAAAAGAGAGCTTGGACTGTGGAGGCTTGCTGG
CCACTTTTTGGGGTCAGCTCCTCTGAAATGCATTAGCGGAACCGTTTGCAATCTGCCACA
AGTGTGATAAGTTATCTACACTGGCGAGGGGATTGCTCTCTGTAATGTTCAGCTTCTAAT
TGTCTCTACTTTGTGAGACAACTTTTGAATGCTTGACCTCAAATCAGGTAGGACTACCCG
CTGAACTTAA
>B10_17
TTTCCGTAGGTGAACCTGCGGAAGGATCATTATTGAATTATGTTTCTAGATAGGTTGTAG
CTGGCTCTTTAGAGCATGTGCACGCCTGTTTGGACTTCATTTTCATCCACCTGTGCACCT
ATTGTAGTCTTTGGTTGGGTTAGGGGGAAGTGGTCATTGTGTCAGCATCTGCTGGATGTG
AGGACTTGCATTGTGAAAGCTTTGCTGTCCTTGATGTGATCATGGAATCTCTTTCTCACT
AGAGTCTATGTCACTCATTATACTCTGTCGAATGTCATTGAATGTCTTTACATGGGCTTG
TATGCCTATGAAAATTGTAATACAACTTTCAGCAACGGATCTCTTGGCTCTCGCATCGAT
GAAGGACGCAGCGAAATGCGATAAGTAATGTGAATTGCAGAATTCAGTGAATCATCGAAT
CTTTGAACGCATCTTGCGCTCCTTGGTATTCCGAGGAGCATGCCTGTTTGAGTGTCATTA
AATTCTCAACTCTCTTATACTTTTTTGTAAAAGAGAGCTTGGACTGTGGAGGCTTGCTGG

CCACTTTTTGGGGTCAGCTCCTCTGAAATGCATTAGCGGAACCGTTTGCAATCTGCCACA
AGTGTGATAAGTTATCTACACTGGCGAGGGGATTGCTCTCTGTAATGTTCAGCTTCTAAT
TGTCTCTACTTTGTGAGACAACTTTTGAATGCTTGACCTCAAATCAGGTAGGACTACCCG
CTGAACTTAA
>B10_18
TTTCCGTAGGTGAACCTGCGGAAGGATCATTATTGAATTATGTTTCTAGATAGGTTGTAG
CTGGCTCTTTAGAGCATGTGCACGCCTGTTTGGACTTCATTTTCATCCACCTGTGCACCT
ATTGTAGTCTTTGGTTGGGTTAGGGGGAAGTGGTCATTGTGTCAGCATCTGCTGGATGTG
AGGACTTGCATTGTGAAAGCTTTGCTGTCCTTGATGTGATCATGGAATCTCTTTCTCACT
AGAGTCTATGTCACTCATTATACTCTGTCGAATGTCATTGAATGTCTTTACATGGGCTTG
TATGCCTATGAAAATTGTAATACAACTTTCAGCAACGGATCTCTTGGCTCTCGCATCGAT
GAAGGACGCAGCGAAATGCGATAAGTAATGTGAATTGCAGAATTCAGTGAATCATCGAAT
CTTTGAACGCATCTTGCGCTCCTTGGTATTCCGAGGAGCATGCCTGTTTGAGTGTCATTA
AATTCTCAACTCTCTTATACTTTTTTGTAAAAGAGAGCTTGGACTGTGGAGGCTTGCTGG
CCACTTTTTGGGGTCAGCTCCTCTGAAATGCATTAGCGGAACCGTTTGCAATCTGCCACA
AGTGTGATAAGTTATCTACACTGGCGAGGGGATTGCTCTCTGTAATGTTCAGCTTCTAAT
TGTCTCTACTTTGTGAGACAACTTTTGAATGCTTGACCTCAAATCAGGTAGGACTACCCG
CTGAACTTAA
>B10_19
TTTCCGTAGGTGAACCTGCGGAAGGATCATTATTGAATTATGTTTCTAGATAGGTTGTAG
CTGGCTCTTTAGAGCATGTGCACGCCTGTTTGGACTTCATTTTCATCCACCTGTGCACCT
ATTGTAGTCTTTGGTTGGGTTAGGGGGAAGTGGTCATTGTGTCAGCATCTGCTGGATGTG
AGGACTTGCATTGTGAAAGCTTTGCTGTCCTTGATGTGATCATGGAATCTCTTTCTCACT
AGAGTCTATGTCACTCATTATACTCTGTCGAATGTCATTGAATGTCTTTACATGGGCTTG
TATGCCTATGAAAATTGTAATACAACTTTCAGCAACGGATCTCTTGGCTCTCGCATCGAT
GAAGGACGCAGCGAAATGCGATAAGTAATGTGAATTGCAGAATTCAGTGAATCATCGAAT
CTTTGAACGCATCTTGCGCTCCTTGGTATTCCGAGGAGCATGCCTGTTTGAGTGTCATTA
AATTCTCAACTCTCTTATACTTTTTTGTAAAAGAGAGCTTGGACTGTGGAGGCTTGCTGG
CCACTTTTTGGGGTCAGCTCCTCTGAAATGCATTAGCGGAACCGTTTGCAATCTGCCACA
AGTGTGATAAGTTATCTACACTGGCGAGGGGATTGCTCTCTGTAATGTTCAGCTTCTAAT
TGTCTCTACTTTGTGAGACAACTTTTGAATGCTTGACCTCAAATCAGGTAGGACTACCCG
CTGAACTTAA
>B10_20
TTTCCGTAGGTGAACCTGCGGAAGGATCATTATTGAATTATGTTTCTAGATAGGTTGTAG
CTGGCTCTTTAGAGCATGTGCACGCCTGTTTGGACTTCATTTTCATCCACCTGTGCACCT
ATTGTAGTCTTTGGTTGGGTTAGGGGGAAGTGGTCATTGTGTCAGCATCTGCTGGATGTG
AGGACTTGCATTGTGAAAGCTTTGCTGTCCTTGATGTGATCATGGAATCTCTTTCTCACT
AGAGTCTATGTCACTCATTATACTCTGTCGAATGTCATTGAATGTCTTTACATGGGCTTG
TATGCCTATGAAAATTGTAATACAACTTTCAGCAACGGATCTCTTGGCTCTCGCATCGAT
GAAGGACGCAGCGAAATGCGATAAGTAATGTGAATTGCAGAATTCAGTGAATCATCGAAT
CTTTGAACGCATCTTGCGCTCCTTGGTATTCCGAGGAGCATGCCTGTTTGAGTGTCATTA
AATTCTCAACTCTCTTATACTTTTTTGTAAAAGAGAGCTTGGACTGTGGAGGCTTGCTGG
CCACTTTTTGGGGTCAGCTCCTCTGAAATGCATTAGCGGAACCGTTTGCAATCTGCCACA
AGTGTGATAAGTTATCTACACTGGCGAGGGGATTGCTCTCTGTAATGTTCAGCTTCTAAT
TGTCTCTACTTTGTGAGACAACTTTTGAATGCTTGACCTCAAATCAGGTAGGACTACCCG
CTGAACTTAA
>B10_21
TTTCCGTAGGTGAACCTGCGGAAGGATCATTATTGAATTATGTTTCTAGATAGGTTGTAG
CTGGCTCTTTAGAGCATGTGCACGCCTGTTTGGACTTCATTTTCATCCACCTGTGCACCT
ATTGTAGTCTTTGGTTGGGTTAGGGGGAAGTGGTCATTGTGTCAGCATCTGCTGGATGTG

AGGACTTGCATTGTGAAAGCTTTGCTGTCCTTGATGTGATCATGGAATCTCTTTCTCACT
AGAGTCTATGTCACTCATTATACTCTGTCGAATGTCATTGAATGTCTTTACATGGGCTTG
TATGCCTATGAAAATTGTAATACAACTTTCAGCAACGGATCTCTTGGCTCTCGCATCGAT
GAAGGACGCAGCGAAATGCGATAAGTAATGTGAATTGCAGAATTCAGTGAATCATCGAAT
CTTTGAACGCATCTTGCGCTCCTTGGTATTCCGAGGAGCATGCCTGTTTGAGTGTCATTA
AATTCTCAACTCTCTTATACTTTTTTGTAAAAGAGAGCTTGGACTGTGGAGGCTTGCTGG
CCACTTTTTGGGGTCAGCTCCTCTGAAATGCATTAGCGGAACCGTTTGCAATCTGCCACA
AGTGTGATAAGTTATCTACACTGGCGAGGGGATTGCTCTCTGTAATGTTCAGCTTCTAAT
TGTCTCTACTTTGTGAGACAACTTTTGAATGCTTGACCTCAAATCAGGTAGGACTACCCG
CTGAACTTAA
>B10_22
TTTCCGTAGGTGAACCTGCGGAAGGATCATTATTGAATTATGTTTCTAGATAGGTTGTAG
CTGGCTCTTTAGAGCATGTGCACGCCTGTTTGGACTTCATTTTCATCCACCTGTGCACCT
ATTGTAGTCTTTGGTTGGGTTAGGGGGAAGTGGTCATTGTGTCAGCATCTGCTGGATGTG
AGGACTTGCATTGTGAAAGCTTTGCTGTCCTTGATGTGATCATGGAATCTCTTTCTCACT
AGAGTCTATGTCACTCATTATACTCTGTCGAATGTCATTGAATGTCTTTACATGGGCTTG
TATGCCTATGAAAATTGTAATACAACTTTCAGCAACGGATCTCTTGGCTCTCGCATCGAT
GAAGGACGCAGCGAAATGCGATAAGTAATGTGAATTGCAGAATTCAGTGAATCATCGAAT
CTTTGAACGCATCTTGCGCTCCTTGGTATTCCGAGGAGCATGCCTGTTTGAGTGTCATTA
AATTCTCAACTCTCTTATACTTTTTTGTAAAAGAGAGCTTGGACTGTGGAGGCTTGCTGG
CCACTTTTTGGGGTCAGCTCCTCTGAAATGCATTAGCGGAACCGTTTGCAATCTGCCACA
AGTGTGATAAGTTATCTACACTGGCGAGGGGATTGCTCTCTGTAATGTTCAGCTTCTAAT
TGTCTCTACTTTGTGAGACAACTTTTGAATGCTTGACCTCAAATCAGGTAGGACTACCCG
CTGAACTTAA
>B10_23
TTTCCGTAGGTGAACCTGCGGAAGGATCATTATTGAATTATGTTTCTAGATAGGTTGTAG
CTGGCTCTTTAGAGCATGTGCACGCCTGTTTGGACTTCATTTTCATCCACCTGTGCACCT
ATTGTAGTCTTTGGTTGGGTTAGGGGGAAGTGGTCATTGTGTCAGCATCTGCTGGATGTG
AGGACTTGCATTGTGAAAGCTTTGCTGTCCTTGATGTGATCATGGAATCTCTTTCTCACT
AGAGTCTATGTCACTCATTATACTCTGTCGAATGTCATTGAATGTCTTTACATGGGCTTG
TATGCCTATGAAAATTGTAATACAACTTTCAGCAACGGATCTCTTGGCTCTCGCATCGAT
GAAGGACGCAGCGAAATGCGATAAGTAATGTGAATTGCAGAATTCAGTGAATCATCGAAT
CTTTGAACGCATCTTGCGCTCCTTGGTATTCCGAGGAGCATGCCTGTTTGAGTGTCATTA
AATTCTCAACTCTCTTATACTTTTTTGTAAAAGAGAGCTTGGACTGTGGAGGCTTGCTGG
CCACTTTTTGGGGTCAGCTCCTCTGAAATGCATTAGCGGAACCGTTTGCAATCTGCCACA
AGTGTGATAAGTTATCTACACTGGCGAGGGGATTGCTCTCTGTAATGTTCAGCTTCTAAT
TGTCTCTACTTTGTGAGACAACTTTTGAATGCTTGACCTCAAATCAGGTAGGACTACCCG
CTGAACTTAA
>B10_25
TTTCCGTAGGTGAACCTGCGGAAGGATCATTATTGAATTATGTTTCTAGATAGGTTGTAG
CTGGCTCTTTAGAGCATGTGCACGCCTGTTTGGACTTCATTTTCATCCACCTGTGCACCT
ATTGTAGTCTTTGGTTGGGTTAGGGGGAAGTGGTCATTGTGTCAGCATCTGCTGGATGTG
AGGACTTGCATTGTGAAAGCTTTGCTGTCCTTGATGTGATCATGGAATCTCTTTCTCACT
AGAGTCTATGTCACTCATTATACTCTGTCGAATGTCATTGAATGTCTTTACATGGGCTTG
TATGCCTATGAAAATTGTAATACAACTTTCAGCAACGGATCTCTTGGCTCTCGCATCGAT
GAAGGACGCAGCGAAATGCGATAAGTAATGTGAATTGCAGAATTCAGTGAATCATCGAAT
CTTTGAACGCATCTTGCGCTCCTTGGTATTCCGAGGAGCATGCCTGTTTGAGTGTCATTA
AATTCTCAACTCTCTTATACTTTTTTGTAAAAGAGAGCTTGGACTGTGGAGGCTTGCTGG
CCACTTTTTGGGGTCAGCTCCTCTGAAATGCATTAGCGGAACCGTTTGCAATCTGCCACA
AGTGTGATAAGTTATCTACACTGGCGAGGGGATTGCTCTCTGTAATGTTCAGCTTCTAAT

TGTCTCTACTTTGTGAGACAACTTTTGAATGCTTGACCTCAAATCAGGTAGGACTACCCG
CTGAACTTAA
>B10_26
TTTCCGTAGGTGAACCTGCGGAAGGATCATTATTGAATTATGTTTCTAGATAGGTTGTAG
CTGGCTCTTTAGAGCATGTGCACGCCTGTTTGGACTTCATTTTCATCCACCTGTGCACCT
ATTGTAGTCTTTGGTTGGGTTAGGGGGAAGTGGTCATTGTGTCAGCATCTGCTGGATGTG
AGGACTTGCATTGTGAAAGCTTTGCTGTCCTTGATGTGATCATGGAATCTCTTTCTCACT
AGAGTCTATGTCACTCATTATACTCTGTCGAATGTCATTGAATGTCTTTACATGGGCTTG
TATGCCTATGAAAATTGTAATACAACTTTCAGCAACGGATCTCTTGGCTCTCGCATCGAT
GAAGGACGCAGCGAAATGCGATAAGTAATGTGAATTGCAGAATTCAGTGAATCATCGAAT
CTTTGAACGCATCTTGCGCTCCTTGGTATTCCGAGGAGCATGCCTGTTTGAGTGTCATTA
AATTCTCAACTCTCTTATACTTTTTTGTAAAAGAGAGCTTGGACTGTGGAGGCTTGCTGG
CCACTTTTTGGGGTCAGCTCCTCTGAAATGCATTAGCGGAACCGTTTGCAATCTGCCACA
AGTGTGATAAGTTATCTACACTGGCGAGGGGATTGCTCTCTGTAATGTTCAGCTTCTAAT
TGTCTCTACTTTGTGAGACAACTTTTGAATGCTTGACCTCAAATCAGGTAGGACTACCCG
CTGAACTTAA
>B10_29
TTTCCGTAGGTGAACCTGCGGAAGGATCATTATTGAATTATGTTTCTAGATAGGTTGTAG
CTGGCTCTTTAGAGCATGTGCACGCCTGTTTGGACTTCATTTTCATCCACCTGTGCACCT
ATTGTAGTCTTTGGTTGGGTTAGGGGGAAGTGGTCATTGTGTCAGCATCTGCTGGATGTG
AGGACTTGCATTGTGAAAGCTTTGCTGTCCTTGATGTGATCATGGAATCTCTTTCTCACT
AGAGTCTATGTCACTCATTATACTCTGTCGAATGTCATTGAATGTCTTTACATGGGCTTG
TATGCCTATGAAAATTGTAATACAACTTTCAGCAACGGATCTCTTGGCTCTCGCATCGAT
GAAGGACGCAGCGAAATGCGATAAGTAATGTGAATTGCAGAATTCAGTGAATCATCGAAT
CTTTGAACGCATCTTGCGCTCCTTGGTATTCCGAGGAGCATGCCTGTTTGAGTGTCATTA
AATTCTCAACTCTCTTATACTTTTTTGTAAAAGAGAGCTTGGACTGTGGAGGCTTGCTGG
CCACTTTTTGGGGTCAGCTCCTCTGAAATGCATTAGCGGAACCGTTTGCAATCTGCCACA
AGTGTGATAAGTTATCTACACTGGCGAGGGGATTGCTCTCTGTAATGTTCAGCTTCTAAT
TGTCTCTACTTTGTGAGACAACTTTTGAATGCTTGACCTCAAATCAGGTAGGACTACCCG
CTGAACTTAA
>B10_30
TTTCCGTAGGTGAACCTGCGGAAGGATCATTATTGAATTATGTTTCTAGATAGGTTGTAG
CTGGCTCTTTAGAGCATGTGCACGCCTGTTTGGACTTCATTTTCATCCACCTGTGCACCT
ATTGTAGTCTTTGGTTGGGTTAGGGGGAAGTGGTCATTGTGTCAGCATCTGCTGGATGTG
AGGACTTGCATTGTGAAAGCTTTGCTGTCCTTGATGTGATCATGGAATCTCTTTCTCACT
AGAGTCTATGTCACTCATTATACTCTGTCGAATGTCATTGAATGTCTTTACATGGGCTTG
TATGCCTATGAAAATTGTAATACAACTTTCAGCAACGGATCTCTTGGCTCTCGCATCGAT
GAAGGACGCAGCGAAATGCGATAAGTAATGTGAATTGCAGAATTCAGTGAATCATCGAAT
CTTTGAACGCATCTTGCGCTCCTTGGTATTCCGAGGAGCATGCCTGTTTGAGTGTCATTA
AATTCTCAACTCTCTTATACTTTTTTGTAAAAGAGAGCTTGGACTGTGGAGGCTTGCTGG
CCACTTTTTGGGGTCAGCTCCTCTGAAATGCATTAGCGGAACCGTTTGCAATCTGCCACA
AGTGTGATAAGTTATCTACACTGGCGAGGGGATTGCTCTCTGTAATGTTCAGCTTCTAAT
TGTCTCTACTTTGTGAGACAACTTTTGAATGCTTGACCTCAAATCAGGTAGGACTACCCG
CTGAACTTAA
>B10_31
TTTCCGTAGGTGAACCTGCGGAAGGATCATTATTGAATTATGTTTCTAGATAGGTTGTAG
CTGGCTCTTTAGAGCATGTGCACGCCTGTTTGGACTTCATTTTCATCCACCTGTGCACCT
ATTGTAGTCTTTGGTTGGGTTAGGGGGAAGTGGTCATTGTGTCAGCATCTGCTGGATGTG
AGGACTTGCATTGTGAAAGCTTTGCTGTCCTTGATGTGATCATGGAATCTCTTTCTCACT
AGAGTCTATGTCACTCATTATACTCTGTCGAATGTCATTGAATGTCTTTACATGGGCTTG

TATGCCTATGAAAATTGTAATACAACTTTCAGCAACGGATCTCTTGGCTCTCGCATCGAT
GAAGGACGCAGCGAAATGCGATAAGTAATGTGAATTGCAGAATTCAGTGAATCATCGAAT
CTTTGAACGCATCTTGCGCTCCTTGGTATTCCGAGGAGCATGCCTGTTTGAGTGTCATTA
AATTCTCAACTCTCTTATACTTTTTTGTAAAAGAGAGCTTGGACTGTGGAGGCTTGCTGG
CCACTTTTTGGGGTCAGCTCCTCTGAAATGCATTAGCGGAACCGTTTGCAATCTGCCACA
AGTGTGATAAGTTATCTACACTGGCGAGGGGATTGCTCTCTGTAATGTTCAGCTTCTAAT
TGTCTCTACTTTGTGAGACAACTTTTGAATGCTTGACCTCAAATCAGGTAGGACTACCCG
CTGAACTTAA
>B10_32
TTTCCGTAGGTGAACCTGCGGAAGGATCATTATTGAATTATGTTTCTAGATAGGTTGTAG
CTGGCTCTTTAGAGCATGTGCACGCCTGTTTGGACTTCATTTTCATCCACCTGTGCACCT
ATTGTAGTCTTTGGTTGGGTTAGGGGGAAGTGGTCATTGTGTCAGCATCTGCTGGATGTG
AGGACTTGCATTGTGAAAGCTTTGCTGTCCTTGATGTGATCATGGAATCTCTTTCTCACT
AGAGTCTATGTCACTCATTATACTCTGTCGAATGTCATTGAATGTCTTTACATGGGCTTG
TATGCCTATGAAAATTGTAATACAACTTTCAGCAACGGATCTCTTGGCTCTCGCATCGAT
GAAGGACGCAGCGAAATGCGATAAGTAATGTGAATTGCAGAATTCAGTGAATCATCGAAT
CTTTGAACGCATCTTGCGCTCCTTGGTATTCCGAGGAGCATGCCTGTTTGAGTGTCATTA
AATTCTCAACTCTCTTATACTTTTTTGTAAAAGAGAGCTTGGACTGTGGAGGCTTGCTGG
CCACTTTTTGGGGTCAGCTCCTCTGAAATGCATTAGCGGAACCGTTTGCAATCTGCCACA
AGTGTGATAAGTTATCTACACTGGCGAGGGGATTGCTCTCTGTAATGTTCAGCTTCTAAT
TGTCTCTACTTTGTGAGACAACTTTTGAATGCTTGACCTCAAATCAGGTAGGACTACCCG
CTGAACTTAA
>B10_33
TTTCCGTAGGTGAACCTGCGGAAGGATCATTATTGAATTATGTTTCTAGATAGGTTGTAG
CTGGCTCTTTAGAGCATGTGCACGCCTGTTTGGACTTCATTTTCATCCACCTGTGCACCT
ATTGTAGTCTTTGGTTGGGTTAGGGGGAAGTGGTCATTGTGTCAGCATCTGCTGGATGTG
AGGACTTGCATTGTGAAAGCTTTGCTGTCCTTGATGTGATCATGGAATCTCTTTCTCACT
AGAGTCTATGTCACTCATTATACTCTGTCGAATGTCATTGAATGTCTTTACATGGGCTTG
TATGCCTATGAAAATTGTAATACAACTTTCAGCAACGGATCTCTTGGCTCTCGCATCGAT
GAAGGACGCAGCGAAATGCGATAAGTAATGTGAATTGCAGAATTCAGTGAATCATCGAAT
CTTTGAACGCATCTTGCGCTCCTTGGTATTCCGAGGAGCATGCCTGTTTGAGTGTCATTA
AATTCTCAACTCTCTTATACTTTTTTGTAAAAGAGAGCTTGGACTGTGGAGGCTTGCTGG
CCACTTTTTGGGGTCAGCTCCTCTGAAATGCATTAGCGGAACCGTTTGCAATCTGCCACA
AGTGTGATAAGTTATCTACACTGGCGAGGGGATTGCTCTCTGTAATGTTCAGCTTCTAAT
TGTCTCTACTTTGTGAGACAACTTTTGAATGCTTGACCTCAAATCAGGTAGGACTACCCG
CTGAACTTAA
>B10_35
TTTCCGTAGGTGAACCTGCGGAAGGATCATTATTGAATTATGTTTCTAGATAGGTTGTAG
CTGGCTCTTTAGAGCATGTGCACGCCTGTTTGGACTTCATTTTCATCCACCTGTGCACCT
ATTGTAGTCTTTGGTTGGGTTAGGGGGAAGTGGTCATTGTGTCAGCATCTGCTGGATGTG
AGGACTTGCATTGTGAAAGCTTTGCTGTCCTTGATGTGATCATGGAATCTCTTTCTCACT
AGAGTCTATGTCACTCATTATACTCTGTCGAATGTCATTGAATGTCTTTACATGGGCTTG
TATGCCTATGAAAATTGTAATACAACTTTCAGCAACGGATCTCTTGGCTCTCGCATCGAT
GAAGGACGCAGCGAAATGCGATAAGTAATGTGAATTGCAGAATTCAGTGAATCATCGAAT
CTTTGAACGCATCTTGCGCTCCTTGGTATTCCGAGGAGCATGCCTGTTTGAGTGTCATTA
AATTCTCAACTCTCTTATACTTTTTTGTAAAAGAGAGCTTGGACTGTGGAGGCTTGCTGG
CCACTTTTTGGGGTCAGCTCCTCTGAAATGCATTAGCGGAACCGTTTGCAATCTGCCACA
AGTGTGATAAGTTATCTACACTGGCGAGGGGATTGCTCTCTGTAATGTTCAGCTTCTAAT
TGTCTCTACTTTGTGAGACAACTTTTGAATGCTTGACCTCAAATCAGGTAGGACTACCCG
CTGAACTTAA

>B10_36
TTTCCGTAGGTGAACCTGCGGAAGGATCATTATTGAATTATGTTTCTAGATAGGTTGTAG
CTGGCTCTTTAGAGCATGTGCACGCCTGTTTGGACTTCATTTTCATCCACCTGTGCACCT
ATTGTAGTCTTTGGTTGGGTTAGGGGGAAGTGGTCATTGTGTCAGCATCTGCTGGATGTG
AGGACTTGCATTGTGAAAGCTTTGCTGTCCTTGATGTGATCATGGAATCTCTTTCTCACT
AGAGTCTATGTCACTCATTATACTCTGTCGAATGTCATTGAATGTCTTTACATGGGCTTG
TATGCCTATGAAAATTGTAATACAACTTTCAGCAACGGATCTCTTGGCTCTCGCATCGAT
GAAGGACGCAGCGAAATGCGATAAGTAATGTGAATTGCAGAATTCAGTGAATCATCGAAT
CTTTGAACGCATCTTGCGCTCCTTGGTATTCCGAGGAGCATGCCTGTTTGAGTGTCATTA
AATTCTCAACTCTCTTATACTTTTTTGTAAAAGAGAGCTTGGACTGTGGAGGCTTGCTGG
CCACTTTTTGGGGTCAGCTCCTCTGAAATGCATTAGCGGAACCGTTTGCAATCTGCCACA
AGTGTGATAAGTTATCTACACTGGCGAGGGGATTGCTCTCTGTAATGTTCAGCTTCTAAT
TGTCTCTACTTTGTGAGACAACTTTTGAATGCTTGACCTCAAATCAGGTAGGACTACCCG
CTGAACTTAA
>B10_37
TTTCCGTAGGTGAACCTGCGGAAGGATCATTATTGAATTATGTTTCTAGATAGGTTGTAG
CTGGCTCTTTAGAGCATGTGCACGCCTGTTTGGACTTCATTTTCATCCACCTGTGCACCT
ATTGTAGTCTTTGGTTGGGTTAGGGGGAAGTGGTCATTGTGTCAGCATCTGCTGGATGTG
AGGACTTGCATTGTGAAAGCTTTGCTGTCCTTGATGTGATCATGGAATCTCTTTCTCACT
AGAGTCTATGTCACTCATTATACTCTGTCGAATGTCATTGAATGTCTTTACATGGGCTTG
TATGCCTATGAAAATTGTAATACAACTTTCAGCAACGGATCTCTTGGCTCTCGCATCGAT
GAAGGACGCAGCGAAATGCGATAAGTAATGTGAATTGCAGAATTCAGTGAATCATCGAAT
CTTTGAACGCATCTTGCGCTCCTTGGTATTCCGAGGAGCATGCCTGTTTGAGTGTCATTA
AATTCTCAACTCTCTTATACTTTTTTGTAAAAGAGAGCTTGGACTGTGGAGGCTTGCTGG
CCACTTTTTGGGGTCAGCTCCTCTGAAATGCATTAGCGGAACCGTTTGCAATCTGCCACA
AGTGTGATAAGTTATCTACACTGGCGAGGGGATTGCTCTCTGTAATGTTCAGCTTCTAAT
TGTCTCTACTTTGTGAGACAACTTTTGAATGCTTGACCTCAAATCAGGTAGGACTACCCG
CTGAACTTAA
>B10_38
TTTCCGTAGGTGAACCTGCGGAAGGATCATTATTGAATTATGTTTCTAGATAGGTTGTAG
CTGGCTCTTTAGAGCATGTGCACGCCTGTTTGGACTTCATTTTCATCCACCTGTGCACCT
ATTGTAGTCTTTGGTTGGGTTAGGGGGAAGTGGTCATTGTGTCAGCATCTGCTGGATGTG
AGGACTTGCATTGTGAAAGCTTTGCTGTCCTTGATGTGATCATGGAATCTCTTTCTCACT
AGAGTCTATGTCACTCATTATACTCTGTCGAATGTCATTGAATGTCTTTACATGGGCTTG
TATGCCTATGAAAATTGTAATACAACTTTCAGCAACGGATCTCTTGGCTCTCGCATCGAT
GAAGGACGCAGCGAAATGCGATAAGTAATGTGAATTGCAGAATTCAGTGAATCATCGAAT
CTTTGAACGCATCTTGCGCTCCTTGGTATTCCGAGGAGCATGCCTGTTTGAGTGTCATTA
AATTCTCAACTCTCTTATACTTTTTTGTAAAAGAGAGCTTGGACTGTGGAGGCTTGCTGG
CCACTTTTTGGGGTCAGCTCCTCTGAAATGCATTAGCGGAACCGTTTGCAATCTGCCACA
AGTGTGATAAGTTATCTACACTGGCGAGGGGATTGCTCTCTGTAATGTTCAGCTTCTAAT
TGTCTCTACTTTGTGAGACAACTTTTGAATGCTTGACCTCAAATCAGGTAGGACTACCCG
CTGAACTTAA
>B10_39
TTTCCGTAGGTGAACCTGCGGAAGGATCATTATTGAATTATGTTTCTAGATAGGTTGTAG
CTGGCTCTTTAGAGCATGTGCACGCCTGTTTGGACTTCATTTTCATCCACCTGTGCACCT
ATTGTAGTCTTTGGTTGGGTTAGGGGGAAGTGGTCATTGTGTCAGCATCTGCTGGATGTG
AGGACTTGCATTGTGAAAGCTTTGCTGTCCTTGATGTGATCATGGAATCTCTTTCTCACT
AGAGTCTATGTCACTCATTATACTCTGTCGAATGTCATTGAATGTCTTTACATGGGCTTG
TATGCCTATGAAAATTGTAATACAACTTTCAGCAACGGATCTCTTGGCTCTCGCATCGAT
GAAGGACGCAGCGAAATGCGATAAGTAATGTGAATTGCAGAATTCAGTGAATCATCGAAT

CTTTGAACGCATCTTGCGCTCCTTGGTATTCCGAGGAGCATGCCTGTTTGAGTGTCATTA
AATTCTCAACTCTCTTATACTTTTTTGTAAAAGAGAGCTTGGACTGTGGAGGCTTGCTGG
CCACTTTTTGGGGTCAGCTCCTCTGAAATGCATTAGCGGAACCGTTTGCAATCTGCCACA
AGTGTGATAAGTTATCTACACTGGCGAGGGGATTGCTCTCTGTAATGTTCAGCTTCTAAT
TGTCTCTACTTTGTGAGACAACTTTTGAATGCTTGACCTCAAATCAGGTAGGACTACCCG
CTGAACTTAA
>B10_40
TTTCCGTAGGTGAACCTGCGGAAGGATCATTATTGAATTATGTTTCTAGATAGGTTGTAG
CTGGCTCTTTAGAGCATGTGCACGCCTGTTTGGACTTCATTTTCATCCACCTGTGCACCT
ATTGTAGTCTTTGGTTGGGTTAGGGGGAAGTGGTCATTGTGTCAGCATCTGCTGGATGTG
AGGACTTGCATTGTGAAAGCTTTGCTGTCCTTGATGTGATCATGGAATCTCTTTCTCACT
AGAGTCTATGTCACTCATTATACTCTGTCGAATGTCATTGAATGTCTTTACATGGGCTTG
TATGCCTATGAAAATTGTAATACAACTTTCAGCAACGGATCTCTTGGCTCTCGCATCGAT
GAAGGACGCAGCGAAATGCGATAAGTAATGTGAATTGCAGAATTCAGTGAATCATCGAAT
CTTTGAACGCATCTTGCGCTCCTTGGTATTCCGAGGAGCATGCCTGTTTGAGTGTCATTA
AATTCTCAACTCTCTTATACTTTTTTGTAAAAGAGAGCTTGGACTGTGGAGGCTTGCTGG
CCACTTTTTGGGGTCAGCTCCTCTGAAATGCATTAGCGGAACCGTTTGCAATCTGCCACA
AGTGTGATAAGTTATCTACACTGGCGAGGGGATTGCTCTCTGTAATGTTCAGCTTCTAAT
TGTCTCTACTTTGTGAGACAACTTTTGAATGCTTGACCTCAAATCAGGTAGGACTACCCG
CTGAACTTAA
>B10_41
TTTCCGTAGGTGAACCTGCGGAAGGATCATTATTGAATTATGTTTCTAGATAGGTTGTAG
CTGGCTCTTTAGAGCATGTGCACGCCTGTTTGGACTTCATTTTCATCCACCTGTGCACCT
ATTGTAGTCTTTGGTTGGGTTAGGGGGAAGTGGTCATTGTGTCAGCATCTGCTGGATGTG
AGGACTTGCATTGTGAAAGCTTTGCTGTCCTTGATGTGATCATGGAATCTCTTTCTCACT
AGAGTCTATGTCACTCATTATACTCTGTCGAATGTCATTGAATGTCTTTACATGGGCTTG
TATGCCTATGAAAATTGTAATACAACTTTCAGCAACGGATCTCTTGGCTCTCGCATCGAT
GAAGGACGCAGCGAAATGCGATAAGTAATGTGAATTGCAGAATTCAGTGAATCATCGAAT
CTTTGAACGCATCTTGCGCTCCTTGGTATTCCGAGGAGCATGCCTGTTTGAGTGTCATTA
AATTCTCAACTCTCTTATACTTTTTTGTAAAAGAGAGCTTGGACTGTGGAGGCTTGCTGG
CCACTTTTTGGGGTCAGCTCCTCTGAAATGCATTAGCGGAACCGTTTGCAATCTGCCACA
AGTGTGATAAGTTATCTACACTGGCGAGGGGATTGCTCTCTGTAATGTTCAGCTTCTAAT
TGTCTCTACTTTGTGAGACAACTTTTGAATGCTTGACCTCAAATCAGGTAGGACTACCCG
CTGAACTTAA
>B10_42
TTTCCGTAGGTGAACCTGCGGAAGGATCATTATTGAATTATGTTTCTAGATAGGTTGTAG
CTGGCTCTTTAGAGCATGTGCACGCCTGTTTGGACTTCATTTTCATCCACCTGTGCACCT
ATTGTAGTCTTTGGTTGGGTTAGGGGGAAGTGGTCATTGTGTCAGCATCTGCTGGATGTG
AGGACTTGCATTGTGAAAGCTTTGCTGTCCTTGATGTGATCATGGAATCTCTTTCTCACT
AGAGTCTATGTCACTCATTATACTCTGTCGAATGTCATTGAATGTCTTTACATGGGCTTG
TATGCCTATGAAAATTGTAATACAACTTTCAGCAACGGATCTCTTGGCTCTCGCATCGAT
GAAGGACGCAGCGAAATGCGATAAGTAATGTGAATTGCAGAATTCAGTGAATCATCGAAT
CTTTGAACGCATCTTGCGCTCCTTGGTATTCCGAGGAGCATGCCTGTTTGAGTGTCATTA
AATTCTCAACTCTCTTATACTTTTTTGTAAAAGAGAGCTTGGACTGTGGAGGCTTGCTGG
CCACTTTTTGGGGTCAGCTCCTCTGAAATGCATTAGCGGAACCGTTTGCAATCTGCCACA
AGTGTGATAAGTTATCTACACTGGCGAGGGGATTGCTCTCTGTAATGTTCAGCTTCTAAT
TGTCTCTACTTTGTGAGACAACTTTTGAATGCTTGACCTCAAATCAGGTAGGACTACCCG
CTGAACTTAA
>B10_43
TTTCCGTAGGTGAACCTGCGGAAGGATCATTATTGAATTATGTTTCTAGATAGGTTGTAG

CTGGCTCTTTAGAGCATGTGCACGCCTGTTTGGACTTCATTTTCATCCACCTGTGCACCT
ATTGTAGTCTTTGGTTGGGTTAGGGGGAAGTGGTCATTGTGTCAGCATCTGCTGGATGTG
AGGACTTGCATTGTGAAAGCTTTGCTGTCCTTGATGTGATCATGGAATCTCTTTCTCACT
AGAGTCTATGTCACTCATTATACTCTGTCGAATGTCATTGAATGTCTTTACATGGGCTTG
TATGCCTATGAAAATTGTAATACAACTTTCAGCAACGGATCTCTTGGCTCTCGCATCGAT
GAAGGACGCAGCGAAATGCGATAAGTAATGTGAATTGCAGAATTCAGTGAATCATCGAAT
CTTTGAACGCATCTTGCGCTCCTTGGTATTCCGAGGAGCATGCCTGTTTGAGTGTCATTA
AATTCTCAACTCTCTTATACTTTTTTGTAAAAGAGAGCTTGGACTGTGGAGGCTTGCTGG
CCACTTTTTGGGGTCAGCTCCTCTGAAATGCATTAGCGGAACCGTTTGCAATCTGCCACA
AGTGTGATAAGTTATCTACACTGGCGAGGGGATTGCTCTCTGTAATGTTCAGCTTCTAAT
TGTCTCTACTTTGTGAGACAACTTTTGAATGCTTGACCTCAAATCAGGTAGGACTACCCG
CTGAACTTAA
>B10_45
TTTCCGTAGGTGAACCTGCGGAAGGATCATTATTGAATTATGTTTCTAGATAGGTTGTAG
CTGGCTCTTTAGAGCATGTGCACGCCTGTTTGGACTTCATTTTCATCCACCTGTGCACCT
ATTGTAGTCTTTGGTTGGGTTAGGGGGAAGTGGTCATTGTGTCAGCATCTGCTGGATGTG
AGGACTTGCATTGTGAAAGCTTTGCTGTCCTTGATGTGATCATGGAATCTCTTTCTCACT
AGAGTCTATGTCACTCATTATACTCTGTCGAATGTCATTGAATGTCTTTACATGGGCTTG
TATGCCTATGAAAATTGTAATACAACTTTCAGCAACGGATCTCTTGGCTCTCGCATCGAT
GAAGGACGCAGCGAAATGCGATAAGTAATGTGAATTGCAGAATTCAGTGAATCATCGAAT
CTTTGAACGCATCTTGCGCTCCTTGGTATTCCGAGGAGCATGCCTGTTTGAGTGTCATTA
AATTCTCAACTCTCTTATACTTTTTTGTAAAAGAGAGCTTGGACTGTGGAGGCTTGCTGG
CCACTTTTTGGGGTCAGCTCCTCTGAAATGCATTAGCGGAACCGTTTGCAATCTGCCACA
AGTGTGATAAGTTATCTACACTGGCGAGGGGATTGCTCTCTGTAATGTTCAGCTTCTAAT
TGTCTCTACTTTGTGAGACAACTTTTGAATGCTTGACCTCAAATCAGGTAGGACTACCCG
CTGAACTTAA
>B10_46
TTTCCGTAGGTGAACCTGCGGAAGGATCATTATTGAATTATGTTTCTAGATAGGTTGTAG
CTGGCTCTTTAGAGCATGTGCACGCCTGTTTGGACTTCATTTTCATCCACCTGTGCACCT
ATTGTAGTCTTTGGTTGGGTTAGGGGGAAGTGGTCATTGTGTCAGCATCTGCTGGATGTG
AGGACTTGCATTGTGAAAGCTTTGCTGTCCTTGATGTGATCATGGAATCTCTTTCTCACT
AGAGTCTATGTCACTCATTATACTCTGTCGAATGTCATTGAATGTCTTTACATGGGCTTG
TATGCCTATGAAAATTGTAATACAACTTTCAGCAACGGATCTCTTGGCTCTCGCATCGAT
GAAGGACGCAGCGAAATGCGATAAGTAATGTGAATTGCAGAATTCAGTGAATCATCGAAT
CTTTGAACGCATCTTGCGCTCCTTGGTATTCCGAGGAGCATGCCTGTTTGAGTGTCATTA
AATTCTCAACTCTCTTATACTTTTTTGTAAAAGAGAGCTTGGACTGTGGAGGCTTGCTGG
CCACTTTTTGGGGTCAGCTCCTCTGAAATGCATTAGCGGAACCGTTTGCAATCTGCCACA
AGTGTGATAAGTTATCTACACTGGCGAGGGGATTGCTCTCTGTAATGTTCAGCTTCTAAT
TGTCTCTACTTTGTGAGACAACTTTTGAATGCTTGACCTCAAATCAGGTAGGACTACCCG
CTGAACTTAA
>B10_47
TTTCCGTAGGTGAACCTGCGGAAGGATCATTATTGAATTATGTTTCTAGATAGGTTGTAG
CTGGCTCTTTAGAGCATGTGCACGCCTGTTTGGACTTCATTTTCATCCACCTGTGCACCT
ATTGTAGTCTTTGGTTGGGTTAGGGGGAAGTGGTCATTGTGTCAGCATCTGCTGGATGTG
AGGACTTGCATTGTGAAAGCTTTGCTGTCCTTGATGTGATCATGGAATCTCTTTCTCACT
AGAGTCTATGTCACTCATTATACTCTGTCGAATGTCATTGAATGTCTTTACATGGGCTTG
TATGCCTATGAAAATTGTAATACAACTTTCAGCAACGGATCTCTTGGCTCTCGCATCGAT
GAAGGACGCAGCGAAATGCGATAAGTAATGTGAATTGCAGAATTCAGTGAATCATCGAAT
CTTTGAACGCATCTTGCGCTCCTTGGTATTCCGAGGAGCATGCCTGTTTGAGTGTCATTA
AATTCTCAACTCTCTTATACTTTTTTGTAAAAGAGAGCTTGGACTGTGGAGGCTTGCTGG

CCACTTTTTGGGGTCAGCTCCTCTGAAATGCATTAGCGGAACCGTTTGCAATCTGCCACA
AGTGTGATAAGTTATCTACACTGGCGAGGGGATTGCTCTCTGTAATGTTCAGCTTCTAAT
TGTCTCTACTTTGTGAGACAACTTTTGAATGCTTGACCTCAAATCAGGTAGGACTACCCG
CTGAACTTAA
>B10_48
TTTCCGTAGGTGAACCTGCGGAAGGATCATTATTGAATTATGTTTCTAGATAGGTTGTAG
CTGGCTCTTTAGAGCATGTGCACGCCTGTTTGGACTTCATTTTCATCCACCTGTGCACCT
ATTGTAGTCTTTGGTTGGGTTAGGGGGAAGTGGTCATTGTGTCAGCATCTGCTGGATGTG
AGGACTTGCATTGTGAAAGCTTTGCTGTCCTTGATGTGATCATGGAATCTCTTTCTCACT
AGAGTCTATGTCACTCATTATACTCTGTCGAATGTCATTGAATGTCTTTACATGGGCTTG
TATGCCTATGAAAATTGTAATACAACTTTCAGCAACGGATCTCTTGGCTCTCGCATCGAT
GAAGGACGCAGCGAAATGCGATAAGTAATGTGAATTGCAGAATTCAGTGAATCATCGAAT
CTTTGAACGCATCTTGCGCTCCTTGGTATTCCGAGGAGCATGCCTGTTTGAGTGTCATTA
AATTCTCAACTCTCTTATACTTTTTTGTAAAAGAGAGCTTGGACTGTGGAGGCTTGCTGG
CCACTTTTTGGGGTCAGCTCCTCTGAAATGCATTAGCGGAACCGTTTGCAATCTGCCACA
AGTGTGATAAGTTATCTACACTGGCGAGGGGATTGCTCTCTGTAATGTTCAGCTTCTAAT
TGTCTCTACTTTGTGAGACAACTTTTGAATGCTTGACCTCAAATCAGGTAGGACTACCCG
CTGAACTTAA
>B10_49
TTTCCGTAGGTGAACCTGCGGAAGGATCATTATTGAATTATGTTTCTAGATAGGTTGTAG
CTGGCTCTTTAGAGCATGTGCACGCCTGTTTGGACTTCATTTTCATCCACCTGTGCACCT
ATTGTAGTCTTTGGTTGGGTTAGGGGGAAGTGGTCATTGTGTCAGCATCTGCTGGATGTG
AGGACTTGCATTGTGAAAGCTTTGCTGTCCTTGATGTGATCATGGAATCTCTTTCTCACT
AGAGTCTATGTCACTCATTATACTCTGTCGAATGTCATTGAATGTCTTTACATGGGCTTG
TATGCCTATGAAAATTGTAATACAACTTTCAGCAACGGATCTCTTGGCTCTCGCATCGAT
GAAGGACGCAGCGAAATGCGATAAGTAATGTGAATTGCAGAATTCAGTGAATCATCGAAT
CTTTGAACGCATCTTGCGCTCCTTGGTATTCCGAGGAGCATGCCTGTTTGAGTGTCATTA
AATTCTCAACTCTCTTATACTTTTTTGTAAAAGAGAGCTTGGACTGTGGAGGCTTGCTGG
CCACTTTTTGGGGTCAGCTCCTCTGAAATGCATTAGCGGAACCGTTTGCAATCTGCCACA
AGTGTGATAAGTTATCTACACTGGCGAGGGGATTGCTCTCTGTAATGTTCAGCTTCTAAT
TGTCTCTACTTTGTGAGACAACTTTTGAATGCTTGACCTCAAATCAGGTAGGACTACCCG
CTGAACTTAA
>B10_50
TTTCCGTAGGTGAACCTGCGGAAGGATCATTATTGAATTATGTTTCTAGATAGGTTGTAG
CTGGCTCTTTAGAGCATGTGCACGCCTGTTTGGACTTCATTTTCATCCACCTGTGCACCT
ATTGTAGTCTTTGGTTGGGTTAGGGGGAAGTGGTCATTGTGTCAGCATCTGCTGGATGTG
AGGACTTGCATTGTGAAAGCTTTGCTGTCCTTGATGTGATCATGGAATCTCTTTCTCACT
AGAGTCTATGTCACTCATTATACTCTGTCGAATGTCATTGAATGTCTTTACATGGGCTTG
TATGCCTATGAAAATTGTAATACAACTTTCAGCAACGGATCTCTTGGCTCTCGCATCGAT
GAAGGACGCAGCGAAATGCGATAAGTAATGTGAATTGCAGAATTCAGTGAATCATCGAAT
CTTTGAACGCATCTTGCGCTCCTTGGTATTCCGAGGAGCATGCCTGTTTGAGTGTCATTA
AATTCTCAACTCTCTTATACTTTTTTGTAAAAGAGAGCTTGGACTGTGGAGGCTTGCTGG
CCACTTTTTGGGGTCAGCTCCTCTGAAATGCATTAGCGGAACCGTTTGCAATCTGCCACA
AGTGTGATAAGTTATCTACACTGGCGAGGGGATTGCTCTCTGTAATGTTCAGCTTCTAAT
TGTCTCTACTTTGTGAGACAACTTTTGAATGCTTGACCTCAAATCAGGTAGGACTACCCG
CTGAACTTAA
>B10_51
TTTCCGTAGGTGAACCTGCGGAAGGATCATTATTGAATTATGTTTCTAGATAGGTTGTAG
CTGGCTCTTTAGAGCATGTGCACGCCTGTTTGGACTTCATTTTCATCCACCTGTGCACCT
ATTGTAGTCTTTGGTTGGGTTAGGGGGAAGTGGTCATTGTGTCAGCATCTGCTGGATGTG

AGGACTTGCATTGTGAAAGCTTTGCTGTCCTTGATGTGATCATGGAATCTCTTTCTCACT
AGAGTCTATGTCACTCATTATACTCTGTCGAATGTCATTGAATGTCTTTACATGGGCTTG
TATGCCTATGAAAATTGTAATACAACTTTCAGCAACGGATCTCTTGGCTCTCGCATCGAT
GAAGGACGCAGCGAAATGCGATAAGTAATGTGAATTGCAGAATTCAGTGAATCATCGAAT
CTTTGAACGCATCTTGCGCTCCTTGGTATTCCGAGGAGCATGCCTGTTTGAGTGTCATTA
AATTCTCAACTCTCTTATACTTTTTTGTAAAAGAGAGCTTGGACTGTGGAGGCTTGCTGG
CCACTTTTTGGGGTCAGCTCCTCTGAAATGCATTAGCGGAACCGTTTGCAATCTGCCACA
AGTGTGATAAGTTATCTACACTGGCGAGGGGATTGCTCTCTGTAATGTTCAGCTTCTAAT
TGTCTCTACTTTGTGAGACAACTTTTGAATGCTTGACCTCAAATCAGGTAGGACTACCCG
CTGAACTTAA
>B10_52
TTTCCGTAGGTGAACCTGCGGAAGGATCATTATTGAATTATGTTTCTAGATAGGTTGTAG
CTGGCTCTTTAGAGCATGTGCACGCCTGTTTGGACTTCATTTTCATCCACCTGTGCACCT
ATTGTAGTCTTTGGTTGGGTTAGGGGGAAGTGGTCATTGTGTCAGCATCTGCTGGATGTG
AGGACTTGCATTGTGAAAGCTTTGCTGTCCTTGATGTGATCATGGAATCTCTTTCTCACT
AGAGTCTATGTCACTCATTATACTCTGTCGAATGTCATTGAATGTCTTTACATGGGCTTG
TATGCCTATGAAAATTGTAATACAACTTTCAGCAACGGATCTCTTGGCTCTCGCATCGAT
GAAGGACGCAGCGAAATGCGATAAGTAATGTGAATTGCAGAATTCAGTGAATCATCGAAT
CTTTGAACGCATCTTGCGCTCCTTGGTATTCCGAGGAGCATGCCTGTTTGAGTGTCATTA
AATTCTCAACTCTCTTATACTTTTTTGTAAAAGAGAGCTTGGACTGTGGAGGCTTGCTGG
CCACTTTTTGGGGTCAGCTCCTCTGAAATGCATTAGCGGAACCGTTTGCAATCTGCCACA
AGTGTGATAAGTTATCTACACTGGCGAGGGGATTGCTCTCTGTAATGTTCAGCTTCTAAT
TGTCTCTACTTTGTGAGACAACTTTTGAATGCTTGACCTCAAATCAGGTAGGACTACCCG
CTGAACTTAA
>B10_53
TTTCCGTAGGTGAACCTGCGGAAGGATCATTATTGAATTATGTTTCTAGATAGGTTGTAG
CTGGCTCTTTAGAGCATGTGCACGCCTGTTTGGACTTCATTTTCATCCACCTGTGCACCT
ATTGTAGTCTTTGGTTGGGTTAGGGGGAAGTGGTCATTGTGTCAGCATCTGCTGGATGTG
AGGACTTGCATTGTGAAAGCTTTGCTGTCCTTGATGTGATCATGGAATCTCTTTCTCACT
AGAGTCTATGTCACTCATTATACTCTGTCGAATGTCATTGAATGTCTTTACATGGGCTTG
TATGCCTATGAAAATTGTAATACAACTTTCAGCAACGGATCTCTTGGCTCTCGCATCGAT
GAAGGACGCAGCGAAATGCGATAAGTAATGTGAATTGCAGAATTCAGTGAATCATCGAAT
CTTTGAACGCATCTTGCGCTCCTTGGTATTCCGAGGAGCATGCCTGTTTGAGTGTCATTA
AATTCTCAACTCTCTTATACTTTTTTGTAAAAGAGAGCTTGGACTGTGGAGGCTTGCTGG
CCACTTTTTGGGGTCAGCTCCTCTGAAATGCATTAGCGGAACCGTTTGCAATCTGCCACA
AGTGTGATAAGTTATCTACACTGGCGAGGGGATTGCTCTCTGTAATGTTCAGCTTCTAAT
TGTCTCTACTTTGTGAGACAACTTTTGAATGCTTGACCTCAAATCAGGTAGGACTACCCG
CTGAACTTAA
>B10_54
TTTCCGTAGGTGAACCTGCGGAAGGATCATTATTGAATTATGTTTCTAGATAGGTTGTAG
CTGGCTCTTTAGAGCATGTGCACGCCTGTTTGGACTTCATTTTCATCCACCTGTGCACCT
ATTGTAGTCTTTGGTTGGGTTAGGGGGAAGTGGTCATTGTGTCAGCATCTGCTGGATGTG
AGGACTTGCATTGTGAAAGCTTTGCTGTCCTTGATGTGATCATGGAATCTCTTTCTCACT
AGAGTCTATGTCACTCATTATACTCTGTCGAATGTCATTGAATGTCTTTACATGGGCTTG
TATGCCTATGAAAATTGTAATACAACTTTCAGCAACGGATCTCTTGGCTCTCGCATCGAT
GAAGGACGCAGCGAAATGCGATAAGTAATGTGAATTGCAGAATTCAGTGAATCATCGAAT
CTTTGAACGCATCTTGCGCTCCTTGGTATTCCGAGGAGCATGCCTGTTTGAGTGTCATTA
AATTCTCAACTCTCTTATACTTTTTTGTAAAAGAGAGCTTGGACTGTGGAGGCTTGCTGG
CCACTTTTTGGGGTCAGCTCCTCTGAAATGCATTAGCGGAACCGTTTGCAATCTGCCACA
AGTGTGATAAGTTATCTACACTGGCGAGGGGATTGCTCTCTGTAATGTTCAGCTTCTAAT

TGTCTCTACTTTGTGAGACAACTTTTGAATGCTTGACCTCAAATCAGGTAGGACTACCCG
CTGAACTTAA
>B10_56
TTTCCGTAGGTGAACCTGCGGAAGGATCATTATTGAATTATGTTTCTAGATAGGTTGTAG
CTGGCTCTTTAGAGCATGTGCACGCCTGTTTGGACTTCATTTTCATCCACCTGTGCACCT
ATTGTAGTCTTTGGTTGGGTTAGGGGGAAGTGGTCATTGTGTCAGCATCTGCTGGATGTG
AGGACTTGCATTGTGAAAGCTTTGCTGTCCTTGATGTGATCATGGAATCTCTTTCTCACT
AGAGTCTATGTCACTCATTATACTCTGTCGAATGTCATTGAATGTCTTTACATGGGCTTG
TATGCCTATGAAAATTGTAATACAACTTTCAGCAACGGATCTCTTGGCTCTCGCATCGAT
GAAGGACGCAGCGAAATGCGATAAGTAATGTGAATTGCAGAATTCAGTGAATCATCGAAT
CTTTGAACGCATCTTGCGCTCCTTGGTATTCCGAGGAGCATGCCTGTTTGAGTGTCATTA
AATTCTCAACTCTCTTATACTTTTTTGTAAAAGAGAGCTTGGACTGTGGAGGCTTGCTGG
CCACTTTTTGGGGTCAGCTCCTCTGAAATGCATTAGCGGAACCGTTTGCAATCTGCCACA
AGTGTGATAAGTTATCTACACTGGCGAGGGGATTGCTCTCTGTAATGTTCAGCTTCTAAT
TGTCTCTACTTTGTGAGACAACTTTTGAATGCTTGACCTCAAATCAGGTAGGACTACCCG
CTGAACTTAA
>B10_57
TTTCCGTAGGTGAACCTGCGGAAGGATCATTATTGAATTATGTTTCTAGATAGGTTGTAG
CTGGCTCTTTAGAGCATGTGCACGCCTGTTTGGACTTCATTTTCATCCACCTGTGCACCT
ATTGTAGTCTTTGGTTGGGTTAGGGGGAAGTGGTCATTGTGTCAGCATCTGCTGGATGTG
AGGACTTGCATTGTGAAAGCTTTGCTGTCCTTGATGTGATCATGGAATCTCTTTCTCACT
AGAGTCTATGTCACTCATTATACTCTGTCGAATGTCATTGAATGTCTTTACATGGGCTTG
TATGCCTATGAAAATTGTAATACAACTTTCAGCAACGGATCTCTTGGCTCTCGCATCGAT
GAAGGACGCAGCGAAATGCGATAAGTAATGTGAATTGCAGAATTCAGTGAATCATCGAAT
CTTTGAACGCATCTTGCGCTCCTTGGTATTCCGAGGAGCATGCCTGTTTGAGTGTCATTA
AATTCTCAACTCTCTTATACTTTTTTGTAAAAGAGAGCTTGGACTGTGGAGGCTTGCTGG
CCACTTTTTGGGGTCAGCTCCTCTGAAATGCATTAGCGGAACCGTTTGCAATCTGCCACA
AGTGTGATAAGTTATCTACACTGGCGAGGGGATTGCTCTCTGTAATGTTCAGCTTCTAAT
TGTCTCTACTTTGTGAGACAACTTTTGAATGCTTGACCTCAAATCAGGTAGGACTACCCG
CTGAACTTAA
>B10_60
TTTCCGTAGGTGAACCTGCGGAAGGATCATTATTGAATTATGTTTCTAGATAGGTTGTAG
CTGGCTCTTTAGAGCATGTGCACGCCTGTTTGGACTTCATTTTCATCCACCTGTGCACCT
ATTGTAGTCTTTGGTTGGGTTAGGGGGAAGTGGTCATTGTGTCAGCATCTGCTGGATGTG
AGGACTTGCATTGTGAAAGCTTTGCTGTCCTTGATGTGATCATGGAATCTCTTTCTCACT
AGAGTCTATGTCACTCATTATACTCTGTCGAATGTCATTGAATGTCTTTACATGGGCTTG
TATGCCTATGAAAATTGTAATACAACTTTCAGCAACGGATCTCTTGGCTCTCGCATCGAT
GAAGGACGCAGCGAAATGCGATAAGTAATGTGAATTGCAGAATTCAGTGAATCATCGAAT
CTTTGAACGCATCTTGCGCTCCTTGGTATTCCGAGGAGCATGCCTGTTTGAGTGTCATTA
AATTCTCAACTCTCTTATACTTTTTTGTAAAAGAGAGCTTGGACTGTGGAGGCTTGCTGG
CCACTTTTTGGGGTCAGCTCCTCTGAAATGCATTAGCGGAACCGTTTGCAATCTGCCACA
AGTGTGATAAGTTATCTACACTGGCGAGGGGATTGCTCTCTGTAATGTTCAGCTTCTAAT
TGTCTCTACTTTGTGAGACAACTTTTGAATGCTTGACCTCAAATCAGGTAGGACTACCCG
CTGAACTTAA
>B11_2
TTTCCGTAGGTGAACCTGCGGAAGGATCATTATTGAATTATGTTTCTAGATAGGTTGTAG
CTGGCTCTTTAGAGCATGTGCACGCCTGTTTGGACTTCATTTTCATCCACCTGTGCACCT
ATTGTAGTCTTTGGTTGGGTTAGGGGGAAGTGGTCATTGTGTCAGCATCTGCTGGATGTG
AGGACTTGCATTGTGAAAGCTTTGCTGTCCTTGATGTGATCATGGAATCTCTTTCTCACT
AGAGTCTATGTCACTCATTATACTCTGTCGAATGTCATTGAATGTCTTTACATGGGCTTG

TATGCCTATGAAAATTGTAATACAACTTTCAGCAACGGATCTCTTGGCTCTCGCATCGAT
GAAGGACGCAGCGAAATGCGATAAGTAATGTGAATTGCAGAATTCAGTGAATCATCGAAT
CTTTGAACGCATCTTGCGCTCCTTGGTATTCCGAGGAGCATGCCTGTTTGAGTGTCATTA
AATTCTCAACTCTCTTATACTTTTTTGTAAAAGAGAGCTTGGACTGTGGAGGCTTGCTGG
CCACTTTTTGGGGTCAGCTCCTCTGAAATGCATTAGCGGAACCGTTTGCAATCTGCCACA
AGTGTGATAAGTTATCTACACTGGCGAGGGGATTGCTCTCTGTAATGTTCAGCTTCTAAT
TGTCTCTACTTTGTGAGACAACTTTTGAATGCTTGACCTCAAATCAGGTAGGACTACCCG
CTGAACTTAA
>B11_3
TTTCCGTAGGTGAACCTGCGGAAGGATCATTATTGAATTATGTTTCTAGATAGGTTGTAG
CTGGCTCTTTAGAGCATGTGCACGCCTGTTTGGACTTCATTTTCATCCACCTGTGCACCT
ATTGTAGTCTTTGGTTGGGTTAGGGGGAAGTGGTCATTGTGTCAGCATCTGCTGGATGTG
AGGACTTGCATTGTGAAAGCTTTGCTGTCCTTGATGTGATCATGGAATCTCTTTCTCACT
AGAGTCTATGTCACTCATTATACTCTGTCGAATGTCATTGAATGTCTTTACATGGGCTTG
TATGCCTATGAAAATTGTAATACAACTTTCAGCAACGGATCTCTTGGCTCTCGCATCGAT
GAAGGACGCAGCGAAATGCGATAAGTAATGTGAATTGCAGAATTCAGTGAATCATCGAAT
CTTTGAACGCATCTTGCGCTCCTTGGTATTCCGAGGAGCATGCCTGTTTGAGTGTCATTA
AATTCTCAACTCTCTTATACTTTTTTGTAAAAGAGAGCTTGGACTGTGGAGGCTTGCTGG
CCACTTTTTGGGGTCAGCTCCTCTGAAATGCATTAGCGGAACCGTTTGCAATCTGCCACA
AGTGTGATAAGTTATCTACACTGGCGAGGGGATTGCTCTCTGTAATGTTCAGCTTCTAAT
TGTCTCTACTTTGTGAGACAACTTTTGAATGCTTGACCTCAAATCAGGTAGGACTACCCG
CTGAACTTAA
>B11_4
TTTCCGTAGGTGAACCTGCGGAAGGATCATTATTGAATTATGTTTCTAGATAGGTTGTAG
CTGGCTCTTTAGAGCATGTGCACGCCTGTTTGGACTTCATTTTCATCCACCTGTGCACCT
ATTGTAGTCTTTGGTTGGGTTAGGGGGAAGTGGTCATTGTGTCAGCATCTGCTGGATGTG
AGGACTTGCATTGTGAAAGCTTTGCTGTCCTTGATGTGATCATGGAATCTCTTTCTCACT
AGAGTCTATGTCACTCATTATACTCTGTCGAATGTCATTGAATGTCTTTACATGGGCTTG
TATGCCTATGAAAATTGTAATACAACTTTCAGCAACGGATCTCTTGGCTCTCGCATCGAT
GAAGGACGCAGCGAAATGCGATAAGTAATGTGAATTGCAGAATTCAGTGAATCATCGAAT
CTTTGAACGCATCTTGCGCTCCTTGGTATTCCGAGGAGCATGCCTGTTTGAGTGTCATTA
AATTCTCAACTCTCTTATACTTTTTTGTAAAAGAGAGCTTGGACTGTGGAGGCTTGCTGG
CCACTTTTTGGGGTCAGCTCCTCTGAAATGCATTAGCGGAACCGTTTGCAATCTGCCACA
AGTGTGATAAGTTATCTACACTGGCGAGGGGATTGCTCTCTGTAATGTTCAGCTTCTAAT
TGTCTCTACTTTGTGAGACAACTTTTGAATGCTTGACCTCAAATCAGGTAGGACTACCCG
CTGAACTTAA
>B11_5
TTTCCGTAGGTGAACCTGCGGAAGGATCATTATTGAATTATGTTTCTAGATAGGTTGTAG
CTGGCTCTTTAGAGCATGTGCACGCCTGTTTGGACTTCATTTTCATCCACCTGTGCACCT
ATTGTAGTCTTTGGTTGGGTTAGGGGGAAGTGGTCATTGTGTCAGCATCTGCTGGATGTG
AGGACTTGCATTGTGAAAGCTTTGCTGTCCTTGATGTGATCATGGAATCTCTTTCTCACT
AGAGTCTATGTCACTCATTATACTCTGTCGAATGTCATTGAATGTCTTTACATGGGCTTG
TATGCCTATGAAAATTGTAATACAACTTTCAGCAACGGATCTCTTGGCTCTCGCATCGAT
GAAGGACGCAGCGAAATGCGATAAGTAATGTGAATTGCAGAATTCAGTGAATCATCGAAT
CTTTGAACGCATCTTGCGCTCCTTGGTATTCCGAGGAGCATGCCTGTTTGAGTGTCATTA
AATTCTCAACTCTCTTATACTTTTTTGTAAAAGAGAGCTTGGACTGTGGAGGCTTGCTGG
CCACTTTTTGGGGTCAGCTCCTCTGAAATGCATTAGCGGAACCGTTTGCAATCTGCCACA
AGTGTGATAAGTTATCTACACTGGCGAGGGGATTGCTCTCTGTAATGTTCAGCTTCTAAT
TGTCTCTACTTTGTGAGACAACTTTTGAATGCTTGACCTCAAATCAGGTAGGACTACCCG
CTGAACTTAA

>B11_8
TTTCCGTAGGTGAACCTGCGGAAGGATCATTATTGAATTATGTTTCTAGATAGGTTGTAG
CTGGCTCTTTAGAGCATGTGCACGCCTGTTTGGACTTCATTTTCATCCACCTGTGCACCT
ATTGTAGTCTTTGGTTGGGTTAGGGGGAAGTGGTCATTGTGTCAGCATCTGCTGGATGTG
AGGACTTGCATTGTGAAAGCTTTGCTGTCCTTGATGTGATCATGGAATCTCTTTCTCACT
AGAGTCTATGTCACTCATTATACTCTGTCGAATGTCATTGAATGTCTTTACATGGGCTTG
TATGCCTATGAAAATTGTAATACAACTTTCAGCAACGGATCTCTTGGCTCTCGCATCGAT
GAAGGACGCAGCGAAATGCGATAAGTAATGTGAATTGCAGAATTCAGTGAATCATCGAAT
CTTTGAACGCATCTTGCGCTCCTTGGTATTCCGAGGAGCATGCCTGTTTGAGTGTCATTA
AATTCTCAACTCTCTTATACTTTTTTGTAAAAGAGAGCTTGGACTGTGGAGGCTTGCTGG
CCACTTTTTGGGGTCAGCTCCTCTGAAATGCATTAGCGGAACCGTTTGCAATCTGCCACA
AGTGTGATAAGTTATCTACACTGGCGAGGGGATTGCTCTCTGTAATGTTCAGCTTCTAAT
TGTCTCTACTTTGTGAGACAACTTTTGAATGCTTGACCTCAAATCAGGTAGGACTACCCG
CTGAACTTAA
>B11_10
TTTCCGTAGGTGAACCTGCGGAAGGATCATTATTGAATTATGTTTCTAGATAGGTTGTAG
CTGGCTCTTTAGAGCATGTGCACGCCTGTTTGGACTTCATTTTCATCCACCTGTGCACCT
ATTGTAGTCTTTGGTTGGGTTAGGGGGAAGTGGTCATTGTGTCAGCATCTGCTGGATGTG
AGGACTTGCATTGTGAAAGCTTTGCTGTCCTTGATGTGATCATGGAATCTCTTTCTCACT
AGAGTCTATGTCACTCATTATACTCTGTCGAATGTCATTGAATGTCTTTACATGGGCTTG
TATGCCTATGAAAATTGTAATACAACTTTCAGCAACGGATCTCTTGGCTCTCGCATCGAT
GAAGGACGCAGCGAAATGCGATAAGTAATGTGAATTGCAGAATTCAGTGAATCATCGAAT
CTTTGAACGCATCTTGCGCTCCTTGGTATTCCGAGGAGCATGCCTGTTTGAGTGTCATTA
AATTCTCAACTCTCTTATACTTTTTTGTAAAAGAGAGCTTGGACTGTGGAGGCTTGCTGG
CCACTTTTTGGGGTCAGCTCCTCTGAAATGCATTAGCGGAACCGTTTGCAATCTGCCACA
AGTGTGATAAGTTATCTACACTGGCGAGGGGATTGCTCTCTGTAATGTTCAGCTTCTAAT
TGTCTCTACTTTGTGAGACAACTTTTGAATGCTTGACCTCAAATCAGGTAGGACTACCCG
CTGAACTTAA
>B11_11
TTTCCGTAGGTGAACCTGCGGAAGGATCATTATTGAATTATGTTTCTAGATAGGTTGTAG
CTGGCTCTTTAGAGCATGTGCACGCCTGTTTGGACTTCATTTTCATCCACCTGTGCACCT
ATTGTAGTCTTTGGTTGGGTTAGGGGGAAGTGGTCATTGTGTCAGCATCTGCTGGATGTG
AGGACTTGCATTGTGAAAGCTTTGCTGTCCTTGATGTGATCATGGAATCTCTTTCTCACT
AGAGTCTATGTCACTCATTATACTCTGTCGAATGTCATTGAATGTCTTTACATGGGCTTG
TATGCCTATGAAAATTGTAATACAACTTTCAGCAACGGATCTCTTGGCTCTCGCATCGAT
GAAGGACGCAGCGAAATGCGATAAGTAATGTGAATTGCAGAATTCAGTGAATCATCGAAT
CTTTGAACGCATCTTGCGCTCCTTGGTATTCCGAGGAGCATGCCTGTTTGAGTGTCATTA
AATTCTCAACTCTCTTATACTTTTTTGTAAAAGAGAGCTTGGACTGTGGAGGCTTGCTGG
CCACTTTTTGGGGTCAGCTCCTCTGAAATGCATTAGCGGAACCGTTTGCAATCTGCCACA
AGTGTGATAAGTTATCTACACTGGCGAGGGGATTGCTCTCTGTAATGTTCAGCTTCTAAT
TGTCTCTACTTTGTGAGACAACTTTTGAATGCTTGACCTCAAATCAGGTAGGACTACCCG
CTGAACTTAA
>B11_12
TTTCCGTAGGTGAACCTGCGGAAGGATCATTATTGAATTATGTTTCTAGATAGGTTGTAG
CTGGCTCTTTAGAGCATGTGCACGCCTGTTTGGACTTCATTTTCATCCACCTGTGCACCT
ATTGTAGTCTTTGGTTGGGTTAGGGGGAAGTGGTCATTGTGTCAGCATCTGCTGGATGTG
AGGACTTGCATTGTGAAAGCTTTGCTGTCCTTGATGTGATCATGGAATCTCTTTCTCACT
AGAGTCTATGTCACTCATTATACTCTGTCGAATGTCATTGAATGTCTTTACATGGGCTTG
TATGCCTATGAAAATTGTAATACAACTTTCAGCAACGGATCTCTTGGCTCTCGCATCGAT
GAAGGACGCAGCGAAATGCGATAAGTAATGTGAATTGCAGAATTCAGTGAATCATCGAAT

CTTTGAACGCATCTTGCGCTCCTTGGTATTCCGAGGAGCATGCCTGTTTGAGTGTCATTA
AATTCTCAACTCTCTTATACTTTTTTGTAAAAGAGAGCTTGGACTGTGGAGGCTTGCTGG
CCACTTTTTGGGGTCAGCTCCTCTGAAATGCATTAGCGGAACCGTTTGCAATCTGCCACA
AGTGTGATAAGTTATCTACACTGGCGAGGGGATTGCTCTCTGTAATGTTCAGCTTCTAAT
TGTCTCTACTTTGTGAGACAACTTTTGAATGCTTGACCTCAAATCAGGTAGGACTACCCG
CTGAACTTAA
>B11_13
TTTCCGTAGGTGAACCTGCGGAAGGATCATTATTGAATTATGTTTCTAGATAGGTTGTAG
CTGGCTCTTTAGAGCATGTGCACGCCTGTTTGGACTTCATTTTCATCCACCTGTGCACCT
ATTGTAGTCTTTGGTTGGGTTAGGGGGAAGTGGTCATTGTGTCAGCATCTGCTGGATGTG
AGGACTTGCATTGTGAAAGCTTTGCTGTCCTTGATGTGATCATGGAATCTCTTTCTCACT
AGAGTCTATGTCACTCATTATACTCTGTCGAATGTCATTGAATGTCTTTACATGGGCTTG
TATGCCTATGAAAATTGTAATACAACTTTCAGCAACGGATCTCTTGGCTCTCGCATCGAT
GAAGGACGCAGCGAAATGCGATAAGTAATGTGAATTGCAGAATTCAGTGAATCATCGAAT
CTTTGAACGCATCTTGCGCTCCTTGGTATTCCGAGGAGCATGCCTGTTTGAGTGTCATTA
AATTCTCAACTCTCTTATACTTTTTTGTAAAAGAGAGCTTGGACTGTGGAGGCTTGCTGG
CCACTTTTTGGGGTCAGCTCCTCTGAAATGCATTAGCGGAACCGTTTGCAATCTGCCACA
AGTGTGATAAGTTATCTACACTGGCGAGGGGATTGCTCTCTGTAATGTTCAGCTTCTAAT
TGTCTCTACTTTGTGAGACAACTTTTGAATGCTTGACCTCAAATCAGGTAGGACTACCCG
CTGAACTTAA
>B11_14
TTTCCGTAGGTGAACCTGCGGAAGGATCATTATTGAATTATGTTTCTAGATAGGTTGTAG
CTGGCTCTTTAGAGCATGTGCACGCCTGTTTGGACTTCATTTTCATCCACCTGTGCACCT
ATTGTAGTCTTTGGTTGGGTTAGGGGGAAGTGGTCATTGTGTCAGCATCTGCTGGATGTG
AGGACTTGCATTGTGAAAGCTTTGCTGTCCTTGATGTGATCATGGAATCTCTTTCTCACT
AGAGTCTATGTCACTCATTATACTCTGTCGAATGTCATTGAATGTCTTTACATGGGCTTG
TATGCCTATGAAAATTGTAATACAACTTTCAGCAACGGATCTCTTGGCTCTCGCATCGAT
GAAGGACGCAGCGAAATGCGATAAGTAATGTGAATTGCAGAATTCAGTGAATCATCGAAT
CTTTGAACGCATCTTGCGCTCCTTGGTATTCCGAGGAGCATGCCTGTTTGAGTGTCATTA
AATTCTCAACTCTCTTATACTTTTTTGTAAAAGAGAGCTTGGACTGTGGAGGCTTGCTGG
CCACTTTTTGGGGTCAGCTCCTCTGAAATGCATTAGCGGAACCGTTTGCAATCTGCCACA
AGTGTGATAAGTTATCTACACTGGCGAGGGGATTGCTCTCTGTAATGTTCAGCTTCTAAT
TGTCTCTACTTTGTGAGACAACTTTTGAATGCTTGACCTCAAATCAGGTAGGACTACCCG
CTGAACTTAA
>B11_15
TTTCCGTAGGTGAACCTGCGGAAGGATCATTATTGAATTATGTTTCTAGATAGGTTGTAG
CTGGCTCTTTAGAGCATGTGCACGCCTGTTTGGACTTCATTTTCATCCACCTGTGCACCT
ATTGTAGTCTTTGGTTGGGTTAGGGGGAAGTGGTCATTGTGTCAGCATCTGCTGGATGTG
AGGACTTGCATTGTGAAAGCTTTGCTGTCCTTGATGTGATCATGGAATCTCTTTCTCACT
AGAGTCTATGTCACTCATTATACTCTGTCGAATGTCATTGAATGTCTTTACATGGGCTTG
TATGCCTATGAAAATTGTAATACAACTTTCAGCAACGGATCTCTTGGCTCTCGCATCGAT
GAAGGACGCAGCGAAATGCGATAAGTAATGTGAATTGCAGAATTCAGTGAATCATCGAAT
CTTTGAACGCATCTTGCGCTCCTTGGTATTCCGAGGAGCATGCCTGTTTGAGTGTCATTA
AATTCTCAACTCTCTTATACTTTTTTGTAAAAGAGAGCTTGGACTGTGGAGGCTTGCTGG
CCACTTTTTGGGGTCAGCTCCTCTGAAATGCATTAGCGGAACCGTTTGCAATCTGCCACA
AGTGTGATAAGTTATCTACACTGGCGAGGGGATTGCTCTCTGTAATGTTCAGCTTCTAAT
TGTCTCTACTTTGTGAGACAACTTTTGAATGCTTGACCTCAAATCAGGTAGGACTACCCG
CTGAACTTAA
>B11_17
TTTCCGTAGGTGAACCTGCGGAAGGATCATTATTGAATTATGTTTCTAGATAGGTTGTAG

CTGGCTCTTTAGAGCATGTGCACGCCTGTTTGGACTTCATTTTCATCCACCTGTGCACCT
ATTGTAGTCTTTGGTTGGGTTAGGGGGAAGTGGTCATTGTGTCAGCATCTGCTGGATGTG
AGGACTTGCATTGTGAAAGCTTTGCTGTCCTTGATGTGATCATGGAATCTCTTTCTCACT
AGAGTCTATGTCACTCATTATACTCTGTCGAATGTCATTGAATGTCTTTACATGGGCTTG
TATGCCTATGAAAATTGTAATACAACTTTCAGCAACGGATCTCTTGGCTCTCGCATCGAT
GAAGGACGCAGCGAAATGCGATAAGTAATGTGAATTGCAGAATTCAGTGAATCATCGAAT
CTTTGAACGCATCTTGCGCTCCTTGGTATTCCGAGGAGCATGCCTGTTTGAGTGTCATTA
AATTCTCAACTCTCTTATACTTTTTTGTAAAAGAGAGCTTGGACTGTGGAGGCTTGCTGG
CCACTTTTTGGGGTCAGCTCCTCTGAAATGCATTAGCGGAACCGTTTGCAATCTGCCACA
AGTGTGATAAGTTATCTACACTGGCGAGGGGATTGCTCTCTGTAATGTTCAGCTTCTAAT
TGTCTCTACTTTGTGAGACAACTTTTGAATGCTTGACCTCAAATCAGGTAGGACTACCCG
CTGAACTTAA
>B11_19
TTTCCGTAGGTGAACCTGCGGAAGGATCATTATTGAATTATGTTTCTAGATAGGTTGTAG
CTGGCTCTTTAGAGCATGTGCACGCCTGTTTGGACTTCATTTTCATCCACCTGTGCACCT
ATTGTAGTCTTTGGTTGGGTTAGGGGGAAGTGGTCATTGTGTCAGCATCTGCTGGATGTG
AGGACTTGCATTGTGAAAGCTTTGCTGTCCTTGATGTGATCATGGAATCTCTTTCTCACT
AGAGTCTATGTCACTCATTATACTCTGTCGAATGTCATTGAATGTCTTTACATGGGCTTG
TATGCCTATGAAAATTGTAATACAACTTTCAGCAACGGATCTCTTGGCTCTCGCATCGAT
GAAGGACGCAGCGAAATGCGATAAGTAATGTGAATTGCAGAATTCAGTGAATCATCGAAT
CTTTGAACGCATCTTGCGCTCCTTGGTATTCCGAGGAGCATGCCTGTTTGAGTGTCATTA
AATTCTCAACTCTCTTATACTTTTTTGTAAAAGAGAGCTTGGACTGTGGAGGCTTGCTGG
CCACTTTTTGGGGTCAGCTCCTCTGAAATGCATTAGCGGAACCGTTTGCAATCTGCCACA
AGTGTGATAAGTTATCTACACTGGCGAGGGGATTGCTCTCTGTAATGTTCAGCTTCTAAT
TGTCTCTACTTTGTGAGACAACTTTTGAATGCTTGACCTCAAATCAGGTAGGACTACCCG
CTGAACTTAA
>B11_20
TTTCCGTAGGTGAACCTGCGGAAGGATCATTATTGAATTATGTTTCTAGATAGGTTGTAG
CTGGCTCTTTAGAGCATGTGCACGCCTGTTTGGACTTCATTTTCATCCACCTGTGCACCT
ATTGTAGTCTTTGGTTGGGTTAGGGGGAAGTGGTCATTGTGTCAGCATCTGCTGGATGTG
AGGACTTGCATTGTGAAAGCTTTGCTGTCCTTGATGTGATCATGGAATCTCTTTCTCACT
AGAGTCTATGTCACTCATTATACTCTGTCGAATGTCATTGAATGTCTTTACATGGGCTTG
TATGCCTATGAAAATTGTAATACAACTTTCAGCAACGGATCTCTTGGCTCTCGCATCGAT
GAAGGACGCAGCGAAATGCGATAAGTAATGTGAATTGCAGAATTCAGTGAATCATCGAAT
CTTTGAACGCATCTTGCGCTCCTTGGTATTCCGAGGAGCATGCCTGTTTGAGTGTCATTA
AATTCTCAACTCTCTTATACTTTTTTGTAAAAGAGAGCTTGGACTGTGGAGGCTTGCTGG
CCACTTTTTGGGGTCAGCTCCTCTGAAATGCATTAGCGGAACCGTTTGCAATCTGCCACA
AGTGTGATAAGTTATCTACACTGGCGAGGGGATTGCTCTCTGTAATGTTCAGCTTCTAAT
TGTCTCTACTTTGTGAGACAACTTTTGAATGCTTGACCTCAAATCAGGTAGGACTACCCG
CTGAACTTAA
>B11_21
TTTCCGTAGGTGAACCTGCGGAAGGATCATTATTGAATTATGTTTCTAGATAGGTTGTAG
CTGGCTCTTTAGAGCATGTGCACGCCTGTTTGGACTTCATTTTCATCCACCTGTGCACCT
ATTGTAGTCTTTGGTTGGGTTAGGGGGAAGTGGTCATTGTGTCAGCATCTGCTGGATGTG
AGGACTTGCATTGTGAAAGCTTTGCTGTCCTTGATGTGATCATGGAATCTCTTTCTCACT
AGAGTCTATGTCACTCATTATACTCTGTCGAATGTCATTGAATGTCTTTACATGGGCTTG
TATGCCTATGAAAATTGTAATACAACTTTCAGCAACGGATCTCTTGGCTCTCGCATCGAT
GAAGGACGCAGCGAAATGCGATAAGTAATGTGAATTGCAGAATTCAGTGAATCATCGAAT
CTTTGAACGCATCTTGCGCTCCTTGGTATTCCGAGGAGCATGCCTGTTTGAGTGTCATTA
AATTCTCAACTCTCTTATACTTTTTTGTAAAAGAGAGCTTGGACTGTGGAGGCTTGCTGG

CCACTTTTTGGGGTCAGCTCCTCTGAAATGCATTAGCGGAACCGTTTGCAATCTGCCACA
AGTGTGATAAGTTATCTACACTGGCGAGGGGATTGCTCTCTGTAATGTTCAGCTTCTAAT
TGTCTCTACTTTGTGAGACAACTTTTGAATGCTTGACCTCAAATCAGGTAGGACTACCCG
CTGAACTTAA
>B11_24
TTTCCGTAGGTGAACCTGCGGAAGGATCATTATTGAATTATGTTTCTAGATAGGTTGTAG
CTGGCTCTTTAGAGCATGTGCACGCCTGTTTGGACTTCATTTTCATCCACCTGTGCACCT
ATTGTAGTCTTTGGTTGGGTTAGGGGGAAGTGGTCATTGTGTCAGCATCTGCTGGATGTG
AGGACTTGCATTGTGAAAGCTTTGCTGTCCTTGATGTGATCATGGAATCTCTTTCTCACT
AGAGTCTATGTCACTCATTATACTCTGTCGAATGTCATTGAATGTCTTTACATGGGCTTG
TATGCCTATGAAAATTGTAATACAACTTTCAGCAACGGATCTCTTGGCTCTCGCATCGAT
GAAGGACGCAGCGAAATGCGATAAGTAATGTGAATTGCAGAATTCAGTGAATCATCGAAT
CTTTGAACGCATCTTGCGCTCCTTGGTATTCCGAGGAGCATGCCTGTTTGAGTGTCATTA
AATTCTCAACTCTCTTATACTTTTTTGTAAAAGAGAGCTTGGACTGTGGAGGCTTGCTGG
CCACTTTTTGGGGTCAGCTCCTCTGAAATGCATTAGCGGAACCGTTTGCAATCTGCCACA
AGTGTGATAAGTTATCTACACTGGCGAGGGGATTGCTCTCTGTAATGTTCAGCTTCTAAT
TGTCTCTACTTTGTGAGACAACTTTTGAATGCTTGACCTCAAATCAGGTAGGACTACCCG
CTGAACTTAA
>B11_27
TTTCCGTAGGTGAACCTGCGGAAGGATCATTATTGAATTATGTTTCTAGATAGGTTGTAG
CTGGCTCTTTAGAGCATGTGCACGCCTGTTTGGACTTCATTTTCATCCACCTGTGCACCT
ATTGTAGTCTTTGGTTGGGTTAGGGGGAAGTGGTCATTGTGTCAGCATCTGCTGGATGTG
AGGACTTGCATTGTGAAAGCTTTGCTGTCCTTGATGTGATCATGGAATCTCTTTCTCACT
AGAGTCTATGTCACTCATTATACTCTGTCGAATGTCATTGAATGTCTTTACATGGGCTTG
TATGCCTATGAAAATTGTAATACAACTTTCAGCAACGGATCTCTTGGCTCTCGCATCGAT
GAAGGACGCAGCGAAATGCGATAAGTAATGTGAATTGCAGAATTCAGTGAATCATCGAAT
CTTTGAACGCATCTTGCGCTCCTTGGTATTCCGAGGAGCATGCCTGTTTGAGTGTCATTA
AATTCTCAACTCTCTTATACTTTTTTGTAAAAGAGAGCTTGGACTGTGGAGGCTTGCTGG
CCACTTTTTGGGGTCAGCTCCTCTGAAATGCATTAGCGGAACCGTTTGCAATCTGCCACA
AGTGTGATAAGTTATCTACACTGGCGAGGGGATTGCTCTCTGTAATGTTCAGCTTCTAAT
TGTCTCTACTTTGTGAGACAACTTTTGAATGCTTGACCTCAAATCAGGTAGGACTACCCG
CTGAACTTAA
>B11_28
TTTCCGTAGGTGAACCTGCGGAAGGATCATTATTGAATTATGTTTCTAGATAGGTTGTAG
CTGGCTCTTTAGAGCATGTGCACGCCTGTTTGGACTTCATTTTCATCCACCTGTGCACCT
ATTGTAGTCTTTGGTTGGGTTAGGGGGAAGTGGTCATTGTGTCAGCATCTGCTGGATGTG
AGGACTTGCATTGTGAAAGCTTTGCTGTCCTTGATGTGATCATGGAATCTCTTTCTCACT
AGAGTCTATGTCACTCATTATACTCTGTCGAATGTCATTGAATGTCTTTACATGGGCTTG
TATGCCTATGAAAATTGTAATACAACTTTCAGCAACGGATCTCTTGGCTCTCGCATCGAT
GAAGGACGCAGCGAAATGCGATAAGTAATGTGAATTGCAGAATTCAGTGAATCATCGAAT
CTTTGAACGCATCTTGCGCTCCTTGGTATTCCGAGGAGCATGCCTGTTTGAGTGTCATTA
AATTCTCAACTCTCTTATACTTTTTTGTAAAAGAGAGCTTGGACTGTGGAGGCTTGCTGG
CCACTTTTTGGGGTCAGCTCCTCTGAAATGCATTAGCGGAACCGTTTGCAATCTGCCACA
AGTGTGATAAGTTATCTACACTGGCGAGGGGATTGCTCTCTGTAATGTTCAGCTTCTAAT
TGTCTCTACTTTGTGAGACAACTTTTGAATGCTTGACCTCAAATCAGGTAGGACTACCCG
CTGAACTTAA
>B11_31
TTTCCGTAGGTGAACCTGCGGAAGGATCATTATTGAATTATGTTTCTAGATAGGTTGTAG
CTGGCTCTTTAGAGCATGTGCACGCCTGTTTGGACTTCATTTTCATCCACCTGTGCACCT
ATTGTAGTCTTTGGTTGGGTTAGGGGGAAGTGGTCATTGTGTCAGCATCTGCTGGATGTG

AGGACTTGCATTGTGAAAGCTTTGCTGTCCTTGATGTGATCATGGAATCTCTTTCTCACT
AGAGTCTATGTCACTCATTATACTCTGTCGAATGTCATTGAATGTCTTTACATGGGCTTG
TATGCCTATGAAAATTGTAATACAACTTTCAGCAACGGATCTCTTGGCTCTCGCATCGAT
GAAGGACGCAGCGAAATGCGATAAGTAATGTGAATTGCAGAATTCAGTGAATCATCGAAT
CTTTGAACGCATCTTGCGCTCCTTGGTATTCCGAGGAGCATGCCTGTTTGAGTGTCATTA
AATTCTCAACTCTCTTATACTTTTTTGTAAAAGAGAGCTTGGACTGTGGAGGCTTGCTGG
CCACTTTTTGGGGTCAGCTCCTCTGAAATGCATTAGCGGAACCGTTTGCAATCTGCCACA
AGTGTGATAAGTTATCTACACTGGCGAGGGGATTGCTCTCTGTAATGTTCAGCTTCTAAT
TGTCTCTACTTTGTGAGACAACTTTTGAATGCTTGACCTCAAATCAGGTAGGACTACCCG
CTGAACTTAA
>B11_32
TTTCCGTAGGTGAACCTGCGGAAGGATCATTATTGAATTATGTTTCTAGATAGGTTGTAG
CTGGCTCTTTAGAGCATGTGCACGCCTGTTTGGACTTCATTTTCATCCACCTGTGCACCT
ATTGTAGTCTTTGGTTGGGTTAGGGGGAAGTGGTCATTGTGTCAGCATCTGCTGGATGTG
AGGACTTGCATTGTGAAAGCTTTGCTGTCCTTGATGTGATCATGGAATCTCTTTCTCACT
AGAGTCTATGTCACTCATTATACTCTGTCGAATGTCATTGAATGTCTTTACATGGGCTTG
TATGCCTATGAAAATTGTAATACAACTTTCAGCAACGGATCTCTTGGCTCTCGCATCGAT
GAAGGACGCAGCGAAATGCGATAAGTAATGTGAATTGCAGAATTCAGTGAATCATCGAAT
CTTTGAACGCATCTTGCGCTCCTTGGTATTCCGAGGAGCATGCCTGTTTGAGTGTCATTA
AATTCTCAACTCTCTTATACTTTTTTGTAAAAGAGAGCTTGGACTGTGGAGGCTTGCTGG
CCACTTTTTGGGGTCAGCTCCTCTGAAATGCATTAGCGGAACCGTTTGCAATCTGCCACA
AGTGTGATAAGTTATCTACACTGGCGAGGGGATTGCTCTCTGTAATGTTCAGCTTCTAAT
TGTCTCTACTTTGTGAGACAACTTTTGAATGCTTGACCTCAAATCAGGTAGGACTACCCG
CTGAACTTAA
>B11_37
TTTCCGTAGGTGAACCTGCGGAAGGATCATTATTGAATTATGTTTCTAGATAGGTTGTAG
CTGGCTCTTTAGAGCATGTGCACGCCTGTTTGGACTTCATTTTCATCCACCTGTGCACCT
ATTGTAGTCTTTGGTTGGGTTAGGGGGAAGTGGTCATTGTGTCAGCATCTGCTGGATGTG
AGGACTTGCATTGTGAAAGCTTTGCTGTCCTTGATGTGATCATGGAATCTCTTTCTCACT
AGAGTCTATGTCACTCATTATACTCTGTCGAATGTCATTGAATGTCTTTACATGGGCTTG
TATGCCTATGAAAATTGTAATACAACTTTCAGCAACGGATCTCTTGGCTCTCGCATCGAT
GAAGGACGCAGCGAAATGCGATAAGTAATGTGAATTGCAGAATTCAGTGAATCATCGAAT
CTTTGAACGCATCTTGCGCTCCTTGGTATTCCGAGGAGCATGCCTGTTTGAGTGTCATTA
AATTCTCAACTCTCTTATACTTTTTTGTAAAAGAGAGCTTGGACTGTGGAGGCTTGCTGG
CCACTTTTTGGGGTCAGCTCCTCTGAAATGCATTAGCGGAACCGTTTGCAATCTGCCACA
AGTGTGATAAGTTATCTACACTGGCGAGGGGATTGCTCTCTGTAATGTTCAGCTTCTAAT
TGTCTCTACTTTGTGAGACAACTTTTGAATGCTTGACCTCAAATCAGGTAGGACTACCCG
CTGAACTTAA
>B11_38
TTTCCGTAGGTGAACCTGCGGAAGGATCATTATTGAATTATGTTTCTAGATAGGTTGTAG
CTGGCTCTTTAGAGCATGTGCACGCCTGTTTGGACTTCATTTTCATCCACCTGTGCACCT
ATTGTAGTCTTTGGTTGGGTTAGGGGGAAGTGGTCATTGTGTCAGCATCTGCTGGATGTG
AGGACTTGCATTGTGAAAGCTTTGCTGTCCTTGATGTGATCATGGAATCTCTTTCTCACT
AGAGTCTATGTCACTCATTATACTCTGTCGAATGTCATTGAATGTCTTTACATGGGCTTG
TATGCCTATGAAAATTGTAATACAACTTTCAGCAACGGATCTCTTGGCTCTCGCATCGAT
GAAGGACGCAGCGAAATGCGATAAGTAATGTGAATTGCAGAATTCAGTGAATCATCGAAT
CTTTGAACGCATCTTGCGCTCCTTGGTATTCCGAGGAGCATGCCTGTTTGAGTGTCATTA
AATTCTCAACTCTCTTATACTTTTTTGTAAAAGAGAGCTTGGACTGTGGAGGCTTGCTGG
CCACTTTTTGGGGTCAGCTCCTCTGAAATGCATTAGCGGAACCGTTTGCAATCTGCCACA
AGTGTGATAAGTTATCTACACTGGCGAGGGGATTGCTCTCTGTAATGTTCAGCTTCTAAT

TGTCTCTACTTTGTGAGACAACTTTTGAATGCTTGACCTCAAATCAGGTAGGACTACCCG
CTGAACTTAA
>B11_39
TTTCCGTAGGTGAACCTGCGGAAGGATCATTATTGAATTATGTTTCTAGATAGGTTGTAG
CTGGCTCTTTAGAGCATGTGCACGCCTGTTTGGACTTCATTTTCATCCACCTGTGCACCT
ATTGTAGTCTTTGGTTGGGTTAGGGGGAAGTGGTCATTGTGTCAGCATCTGCTGGATGTG
AGGACTTGCATTGTGAAAGCTTTGCTGTCCTTGATGTGATCATGGAATCTCTTTCTCACT
AGAGTCTATGTCACTCATTATACTCTGTCGAATGTCATTGAATGTCTTTACATGGGCTTG
TATGCCTATGAAAATTGTAATACAACTTTCAGCAACGGATCTCTTGGCTCTCGCATCGAT
GAAGGACGCAGCGAAATGCGATAAGTAATGTGAATTGCAGAATTCAGTGAATCATCGAAT
CTTTGAACGCATCTTGCGCTCCTTGGTATTCCGAGGAGCATGCCTGTTTGAGTGTCATTA
AATTCTCAACTCTCTTATACTTTTTTGTAAAAGAGAGCTTGGACTGTGGAGGCTTGCTGG
CCACTTTTTGGGGTCAGCTCCTCTGAAATGCATTAGCGGAACCGTTTGCAATCTGCCACA
AGTGTGATAAGTTATCTACACTGGCGAGGGGATTGCTCTCTGTAATGTTCAGCTTCTAAT
TGTCTCTACTTTGTGAGACAACTTTTGAATGCTTGACCTCAAATCAGGTAGGACTACCCG
CTGAACTTAA
>B11_40
TTTCCGTAGGTGAACCTGCGGAAGGATCATTATTGAATTATGTTTCTAGATAGGTTGTAG
CTGGCTCTTTAGAGCATGTGCACGCCTGTTTGGACTTCATTTTCATCCACCTGTGCACCT
ATTGTAGTCTTTGGTTGGGTTAGGGGGAAGTGGTCATTGTGTCAGCATCTGCTGGATGTG
AGGACTTGCATTGTGAAAGCTTTGCTGTCCTTGATGTGATCATGGAATCTCTTTCTCACT
AGAGTCTATGTCACTCATTATACTCTGTCGAATGTCATTGAATGTCTTTACATGGGCTTG
TATGCCTATGAAAATTGTAATACAACTTTCAGCAACGGATCTCTTGGCTCTCGCATCGAT
GAAGGACGCAGCGAAATGCGATAAGTAATGTGAATTGCAGAATTCAGTGAATCATCGAAT
CTTTGAACGCATCTTGCGCTCCTTGGTATTCCGAGGAGCATGCCTGTTTGAGTGTCATTA
AATTCTCAACTCTCTTATACTTTTTTGTAAAAGAGAGCTTGGACTGTGGAGGCTTGCTGG
CCACTTTTTGGGGTCAGCTCCTCTGAAATGCATTAGCGGAACCGTTTGCAATCTGCCACA
AGTGTGATAAGTTATCTACACTGGCGAGGGGATTGCTCTCTGTAATGTTCAGCTTCTAAT
TGTCTCTACTTTGTGAGACAACTTTTGAATGCTTGACCTCAAATCAGGTAGGACTACCCG
CTGAACTTAA
>B11_42
TTTCCGTAGGTGAACCTGCGGAAGGATCATTATTGAATTATGTTTCTAGATAGGTTGTAG
CTGGCTCTTTAGAGCATGTGCACGCCTGTTTGGACTTCATTTTCATCCACCTGTGCACCT
ATTGTAGTCTTTGGTTGGGTTAGGGGGAAGTGGTCATTGTGTCAGCATCTGCTGGATGTG
AGGACTTGCATTGTGAAAGCTTTGCTGTCCTTGATGTGATCATGGAATCTCTTTCTCACT
AGAGTCTATGTCACTCATTATACTCTGTCGAATGTCATTGAATGTCTTTACATGGGCTTG
TATGCCTATGAAAATTGTAATACAACTTTCAGCAACGGATCTCTTGGCTCTCGCATCGAT
GAAGGACGCAGCGAAATGCGATAAGTAATGTGAATTGCAGAATTCAGTGAATCATCGAAT
CTTTGAACGCATCTTGCGCTCCTTGGTATTCCGAGGAGCATGCCTGTTTGAGTGTCATTA
AATTCTCAACTCTCTTATACTTTTTTGTAAAAGAGAGCTTGGACTGTGGAGGCTTGCTGG
CCACTTTTTGGGGTCAGCTCCTCTGAAATGCATTAGCGGAACCGTTTGCAATCTGCCACA
AGTGTGATAAGTTATCTACACTGGCGAGGGGATTGCTCTCTGTAATGTTCAGCTTCTAAT
TGTCTCTACTTTGTGAGACAACTTTTGAATGCTTGACCTCAAATCAGGTAGGACTACCCG
CTGAACTTAA
>B11_44
TTTCCGTAGGTGAACCTGCGGAAGGATCATTATTGAATTATGTTTCTAGATAGGTTGTAG
CTGGCTCTTTAGAGCATGTGCACGCCTGTTTGGACTTCATTTTCATCCACCTGTGCACCT
ATTGTAGTCTTTGGTTGGGTTAGGGGGAAGTGGTCATTGTGTCAGCATCTGCTGGATGTG
AGGACTTGCATTGTGAAAGCTTTGCTGTCCTTGATGTGATCATGGAATCTCTTTCTCACT
AGAGTCTATGTCACTCATTATACTCTGTCGAATGTCATTGAATGTCTTTACATGGGCTTG

TATGCCTATGAAAATTGTAATACAACTTTCAGCAACGGATCTCTTGGCTCTCGCATCGAT
GAAGGACGCAGCGAAATGCGATAAGTAATGTGAATTGCAGAATTCAGTGAATCATCGAAT
CTTTGAACGCATCTTGCGCTCCTTGGTATTCCGAGGAGCATGCCTGTTTGAGTGTCATTA
AATTCTCAACTCTCTTATACTTTTTTGTAAAAGAGAGCTTGGACTGTGGAGGCTTGCTGG
CCACTTTTTGGGGTCAGCTCCTCTGAAATGCATTAGCGGAACCGTTTGCAATCTGCCACA
AGTGTGATAAGTTATCTACACTGGCGAGGGGATTGCTCTCTGTAATGTTCAGCTTCTAAT
TGTCTCTACTTTGTGAGACAACTTTTGAATGCTTGACCTCAAATCAGGTAGGACTACCCG
CTGAACTTAA
>B11_45
TTTCCGTAGGTGAACCTGCGGAAGGATCATTATTGAATTATGTTTCTAGATAGGTTGTAG
CTGGCTCTTTAGAGCATGTGCACGCCTGTTTGGACTTCATTTTCATCCACCTGTGCACCT
ATTGTAGTCTTTGGTTGGGTTAGGGGGAAGTGGTCATTGTGTCAGCATCTGCTGGATGTG
AGGACTTGCATTGTGAAAGCTTTGCTGTCCTTGATGTGATCATGGAATCTCTTTCTCACT
AGAGTCTATGTCACTCATTATACTCTGTCGAATGTCATTGAATGTCTTTACATGGGCTTG
TATGCCTATGAAAATTGTAATACAACTTTCAGCAACGGATCTCTTGGCTCTCGCATCGAT
GAAGGACGCAGCGAAATGCGATAAGTAATGTGAATTGCAGAATTCAGTGAATCATCGAAT
CTTTGAACGCATCTTGCGCTCCTTGGTATTCCGAGGAGCATGCCTGTTTGAGTGTCATTA
AATTCTCAACTCTCTTATACTTTTTTGTAAAAGAGAGCTTGGACTGTGGAGGCTTGCTGG
CCACTTTTTGGGGTCAGCTCCTCTGAAATGCATTAGCGGAACCGTTTGCAATCTGCCACA
AGTGTGATAAGTTATCTACACTGGCGAGGGGATTGCTCTCTGTAATGTTCAGCTTCTAAT
TGTCTCTACTTTGTGAGACAACTTTTGAATGCTTGACCTCAAATCAGGTAGGACTACCCG
CTGAACTTAA
>B11_46
TTTCCGTAGGTGAACCTGCGGAAGGATCATTATTGAATTATGTTTCTAGATAGGTTGTAG
CTGGCTCTTTAGAGCATGTGCACGCCTGTTTGGACTTCATTTTCATCCACCTGTGCACCT
ATTGTAGTCTTTGGTTGGGTTAGGGGGAAGTGGTCATTGTGTCAGCATCTGCTGGATGTG
AGGACTTGCATTGTGAAAGCTTTGCTGTCCTTGATGTGATCATGGAATCTCTTTCTCACT
AGAGTCTATGTCACTCATTATACTCTGTCGAATGTCATTGAATGTCTTTACATGGGCTTG
TATGCCTATGAAAATTGTAATACAACTTTCAGCAACGGATCTCTTGGCTCTCGCATCGAT
GAAGGACGCAGCGAAATGCGATAAGTAATGTGAATTGCAGAATTCAGTGAATCATCGAAT
CTTTGAACGCATCTTGCGCTCCTTGGTATTCCGAGGAGCATGCCTGTTTGAGTGTCATTA
AATTCTCAACTCTCTTATACTTTTTTGTAAAAGAGAGCTTGGACTGTGGAGGCTTGCTGG
CCACTTTTTGGGGTCAGCTCCTCTGAAATGCATTAGCGGAACCGTTTGCAATCTGCCACA
AGTGTGATAAGTTATCTACACTGGCGAGGGGATTGCTCTCTGTAATGTTCAGCTTCTAAT
TGTCTCTACTTTGTGAGACAACTTTTGAATGCTTGACCTCAAATCAGGTAGGACTACCCG
CTGAACTTAA
>B11_48
TTTCCGTAGGTGAACCTGCGGAAGGATCATTATTGAATTATGTTTCTAGATAGGTTGTAG
CTGGCTCTTTAGAGCATGTGCACGCCTGTTTGGACTTCATTTTCATCCACCTGTGCACCT
ATTGTAGTCTTTGGTTGGGTTAGGGGGAAGTGGTCATTGTGTCAGCATCTGCTGGATGTG
AGGACTTGCATTGTGAAAGCTTTGCTGTCCTTGATGTGATCATGGAATCTCTTTCTCACT
AGAGTCTATGTCACTCATTATACTCTGTCGAATGTCATTGAATGTCTTTACATGGGCTTG
TATGCCTATGAAAATTGTAATACAACTTTCAGCAACGGATCTCTTGGCTCTCGCATCGAT
GAAGGACGCAGCGAAATGCGATAAGTAATGTGAATTGCAGAATTCAGTGAATCATCGAAT
CTTTGAACGCATCTTGCGCTCCTTGGTATTCCGAGGAGCATGCCTGTTTGAGTGTCATTA
AATTCTCAACTCTCTTATACTTTTTTGTAAAAGAGAGCTTGGACTGTGGAGGCTTGCTGG
CCACTTTTTGGGGTCAGCTCCTCTGAAATGCATTAGCGGAACCGTTTGCAATCTGCCACA
AGTGTGATAAGTTATCTACACTGGCGAGGGGATTGCTCTCTGTAATGTTCAGCTTCTAAT
TGTCTCTACTTTGTGAGACAACTTTTGAATGCTTGACCTCAAATCAGGTAGGACTACCCG
CTGAACTTAA

>B11_49
TTTCCGTAGGTGAACCTGCGGAAGGATCATTATTGAATTATGTTTCTAGATAGGTTGTAG
CTGGCTCTTTAGAGCATGTGCACGCCTGTTTGGACTTCATTTTCATCCACCTGTGCACCT
ATTGTAGTCTTTGGTTGGGTTAGGGGGAAGTGGTCATTGTGTCAGCATCTGCTGGATGTG
AGGACTTGCATTGTGAAAGCTTTGCTGTCCTTGATGTGATCATGGAATCTCTTTCTCACT
AGAGTCTATGTCACTCATTATACTCTGTCGAATGTCATTGAATGTCTTTACATGGGCTTG
TATGCCTATGAAAATTGTAATACAACTTTCAGCAACGGATCTCTTGGCTCTCGCATCGAT
GAAGGACGCAGCGAAATGCGATAAGTAATGTGAATTGCAGAATTCAGTGAATCATCGAAT
CTTTGAACGCATCTTGCGCTCCTTGGTATTCCGAGGAGCATGCCTGTTTGAGTGTCATTA
AATTCTCAACTCTCTTATACTTTTTTGTAAAAGAGAGCTTGGACTGTGGAGGCTTGCTGG
CCACTTTTTGGGGTCAGCTCCTCTGAAATGCATTAGCGGAACCGTTTGCAATCTGCCACA
AGTGTGATAAGTTATCTACACTGGCGAGGGGATTGCTCTCTGTAATGTTCAGCTTCTAAT
TGTCTCTACTTTGTGAGACAACTTTTGAATGCTTGACCTCAAATCAGGTAGGACTACCCG
CTGAACTTAA
>B11_50
TTTCCGTAGGTGAACCTGCGGAAGGATCATTATTGAATTATGTTTCTAGATAGGTTGTAG
CTGGCTCTTTAGAGCATGTGCACGCCTGTTTGGACTTCATTTTCATCCACCTGTGCACCT
ATTGTAGTCTTTGGTTGGGTTAGGGGGAAGTGGTCATTGTGTCAGCATCTGCTGGATGTG
AGGACTTGCATTGTGAAAGCTTTGCTGTCCTTGATGTGATCATGGAATCTCTTTCTCACT
AGAGTCTATGTCACTCATTATACTCTGTCGAATGTCATTGAATGTCTTTACATGGGCTTG
TATGCCTATGAAAATTGTAATACAACTTTCAGCAACGGATCTCTTGGCTCTCGCATCGAT
GAAGGACGCAGCGAAATGCGATAAGTAATGTGAATTGCAGAATTCAGTGAATCATCGAAT
CTTTGAACGCATCTTGCGCTCCTTGGTATTCCGAGGAGCATGCCTGTTTGAGTGTCATTA
AATTCTCAACTCTCTTATACTTTTTTGTAAAAGAGAGCTTGGACTGTGGAGGCTTGCTGG
CCACTTTTTGGGGTCAGCTCCTCTGAAATGCATTAGCGGAACCGTTTGCAATCTGCCACA
AGTGTGATAAGTTATCTACACTGGCGAGGGGATTGCTCTCTGTAATGTTCAGCTTCTAAT
TGTCTCTACTTTGTGAGACAACTTTTGAATGCTTGACCTCAAATCAGGTAGGACTACCCG
CTGAACTTAA
>B11_52
TTTCCGTAGGTGAACCTGCGGAAGGATCATTATTGAATTATGTTTCTAGATAGGTTGTAG
CTGGCTCTTTAGAGCATGTGCACGCCTGTTTGGACTTCATTTTCATCCACCTGTGCACCT
ATTGTAGTCTTTGGTTGGGTTAGGGGGAAGTGGTCATTGTGTCAGCATCTGCTGGATGTG
AGGACTTGCATTGTGAAAGCTTTGCTGTCCTTGATGTGATCATGGAATCTCTTTCTCACT
AGAGTCTATGTCACTCATTATACTCTGTCGAATGTCATTGAATGTCTTTACATGGGCTTG
TATGCCTATGAAAATTGTAATACAACTTTCAGCAACGGATCTCTTGGCTCTCGCATCGAT
GAAGGACGCAGCGAAATGCGATAAGTAATGTGAATTGCAGAATTCAGTGAATCATCGAAT
CTTTGAACGCATCTTGCGCTCCTTGGTATTCCGAGGAGCATGCCTGTTTGAGTGTCATTA
AATTCTCAACTCTCTTATACTTTTTTGTAAAAGAGAGCTTGGACTGTGGAGGCTTGCTGG
CCACTTTTTGGGGTCAGCTCCTCTGAAATGCATTAGCGGAACCGTTTGCAATCTGCCACA
AGTGTGATAAGTTATCTACACTGGCGAGGGGATTGCTCTCTGTAATGTTCAGCTTCTAAT
TGTCTCTACTTTGTGAGACAACTTTTGAATGCTTGACCTCAAATCAGGTAGGACTACCCG
CTGAACTTAA
>B11_53
TTTCCGTAGGTGAACCTGCGGAAGGATCATTATTGAATTATGTTTCTAGATAGGTTGTAG
CTGGCTCTTTAGAGCATGTGCACGCCTGTTTGGACTTCATTTTCATCCACCTGTGCACCT
ATTGTAGTCTTTGGTTGGGTTAGGGGGAAGTGGTCATTGTGTCAGCATCTGCTGGATGTG
AGGACTTGCATTGTGAAAGCTTTGCTGTCCTTGATGTGATCATGGAATCTCTTTCTCACT
AGAGTCTATGTCACTCATTATACTCTGTCGAATGTCATTGAATGTCTTTACATGGGCTTG
TATGCCTATGAAAATTGTAATACAACTTTCAGCAACGGATCTCTTGGCTCTCGCATCGAT
GAAGGACGCAGCGAAATGCGATAAGTAATGTGAATTGCAGAATTCAGTGAATCATCGAAT

CTTTGAACGCATCTTGCGCTCCTTGGTATTCCGAGGAGCATGCCTGTTTGAGTGTCATTA
AATTCTCAACTCTCTTATACTTTTTTGTAAAAGAGAGCTTGGACTGTGGAGGCTTGCTGG
CCACTTTTTGGGGTCAGCTCCTCTGAAATGCATTAGCGGAACCGTTTGCAATCTGCCACA
AGTGTGATAAGTTATCTACACTGGCGAGGGGATTGCTCTCTGTAATGTTCAGCTTCTAAT
TGTCTCTACTTTGTGAGACAACTTTTGAATGCTTGACCTCAAATCAGGTAGGACTACCCG
CTGAACTTAA
>B11_54
TTTCCGTAGGTGAACCTGCGGAAGGATCATTATTGAATTATGTTTCTAGATAGGTTGTAG
CTGGCTCTTTAGAGCATGTGCACGCCTGTTTGGACTTCATTTTCATCCACCTGTGCACCT
ATTGTAGTCTTTGGTTGGGTTAGGGGGAAGTGGTCATTGTGTCAGCATCTGCTGGATGTG
AGGACTTGCATTGTGAAAGCTTTGCTGTCCTTGATGTGATCATGGAATCTCTTTCTCACT
AGAGTCTATGTCACTCATTATACTCTGTCGAATGTCATTGAATGTCTTTACATGGGCTTG
TATGCCTATGAAAATTGTAATACAACTTTCAGCAACGGATCTCTTGGCTCTCGCATCGAT
GAAGGACGCAGCGAAATGCGATAAGTAATGTGAATTGCAGAATTCAGTGAATCATCGAAT
CTTTGAACGCATCTTGCGCTCCTTGGTATTCCGAGGAGCATGCCTGTTTGAGTGTCATTA
AATTCTCAACTCTCTTATACTTTTTTGTAAAAGAGAGCTTGGACTGTGGAGGCTTGCTGG
CCACTTTTTGGGGTCAGCTCCTCTGAAATGCATTAGCGGAACCGTTTGCAATCTGCCACA
AGTGTGATAAGTTATCTACACTGGCGAGGGGATTGCTCTCTGTAATGTTCAGCTTCTAAT
TGTCTCTACTTTGTGAGACAACTTTTGAATGCTTGACCTCAAATCAGGTAGGACTACCCG
CTGAACTTAA
>B11_56
TTTCCGTAGGTGAACCTGCGGAAGGATCATTATTGAATTATGTTTCTAGATAGGTTGTAG
CTGGCTCTTTAGAGCATGTGCACGCCTGTTTGGACTTCATTTTCATCCACCTGTGCACCT
ATTGTAGTCTTTGGTTGGGTTAGGGGGAAGTGGTCATTGTGTCAGCATCTGCTGGATGTG
AGGACTTGCATTGTGAAAGCTTTGCTGTCCTTGATGTGATCATGGAATCTCTTTCTCACT
AGAGTCTATGTCACTCATTATACTCTGTCGAATGTCATTGAATGTCTTTACATGGGCTTG
TATGCCTATGAAAATTGTAATACAACTTTCAGCAACGGATCTCTTGGCTCTCGCATCGAT
GAAGGACGCAGCGAAATGCGATAAGTAATGTGAATTGCAGAATTCAGTGAATCATCGAAT
CTTTGAACGCATCTTGCGCTCCTTGGTATTCCGAGGAGCATGCCTGTTTGAGTGTCATTA
AATTCTCAACTCTCTTATACTTTTTTGTAAAAGAGAGCTTGGACTGTGGAGGCTTGCTGG
CCACTTTTTGGGGTCAGCTCCTCTGAAATGCATTAGCGGAACCGTTTGCAATCTGCCACA
AGTGTGATAAGTTATCTACACTGGCGAGGGGATTGCTCTCTGTAATGTTCAGCTTCTAAT
TGTCTCTACTTTGTGAGACAACTTTTGAATGCTTGACCTCAAATCAGGTAGGACTACCCG
CTGAACTTAA
>B11_60
TTTCCGTAGGTGAACCTGCGGAAGGATCATTATTGAATTATGTTTCTAGATAGGTTGTAG
CTGGCTCTTTAGAGCATGTGCACGCCTGTTTGGACTTCATTTTCATCCACCTGTGCACCT
ATTGTAGTCTTTGGTTGGGTTAGGGGGAAGTGGTCATTGTGTCAGCATCTGCTGGATGTG
AGGACTTGCATTGTGAAAGCTTTGCTGTCCTTGATGTGATCATGGAATCTCTTTCTCACT
AGAGTCTATGTCACTCATTATACTCTGTCGAATGTCATTGAATGTCTTTACATGGGCTTG
TATGCCTATGAAAATTGTAATACAACTTTCAGCAACGGATCTCTTGGCTCTCGCATCGAT
GAAGGACGCAGCGAAATGCGATAAGTAATGTGAATTGCAGAATTCAGTGAATCATCGAAT
CTTTGAACGCATCTTGCGCTCCTTGGTATTCCGAGGAGCATGCCTGTTTGAGTGTCATTA
AATTCTCAACTCTCTTATACTTTTTTGTAAAAGAGAGCTTGGACTGTGGAGGCTTGCTGG
CCACTTTTTGGGGTCAGCTCCTCTGAAATGCATTAGCGGAACCGTTTGCAATCTGCCACA
AGTGTGATAAGTTATCTACACTGGCGAGGGGATTGCTCTCTGTAATGTTCAGCTTCTAAT
TGTCTCTACTTTGTGAGACAACTTTTGAATGCTTGACCTCAAATCAGGTAGGACTACCCG
CTGAACTTAA
>B11_61
TTTCCGTAGGTGAACCTGCGGAAGGATCATTATTGAATTATGTTTCTAGATAGGTTGTAG

CTGGCTCTTTAGAGCATGTGCACGCCTGTTTGGACTTCATTTTCATCCACCTGTGCACCT
ATTGTAGTCTTTGGTTGGGTTAGGGGGAAGTGGTCATTGTGTCAGCATCTGCTGGATGTG
AGGACTTGCATTGTGAAAGCTTTGCTGTCCTTGATGTGATCATGGAATCTCTTTCTCACT
AGAGTCTATGTCACTCATTATACTCTGTCGAATGTCATTGAATGTCTTTACATGGGCTTG
TATGCCTATGAAAATTGTAATACAACTTTCAGCAACGGATCTCTTGGCTCTCGCATCGAT
GAAGGACGCAGCGAAATGCGATAAGTAATGTGAATTGCAGAATTCAGTGAATCATCGAAT
CTTTGAACGCATCTTGCGCTCCTTGGTATTCCGAGGAGCATGCCTGTTTGAGTGTCATTA
AATTCTCAACTCTCTTATACTTTTTTGTAAAAGAGAGCTTGGACTGTGGAGGCTTGCTGG
CCACTTTTTGGGGTCAGCTCCTCTGAAATGCATTAGCGGAACCGTTTGCAATCTGCCACA
AGTGTGATAAGTTATCTACACTGGCGAGGGGATTGCTCTCTGTAATGTTCAGCTTCTAAT
TGTCTCTACTTTGTGAGACAACTTTTGAATGCTTGACCTCAAATCAGGTAGGACTACCCG
CTGAACTTAA
>B11_64
TTTCCGTAGGTGAACCTGCGGAAGGATCATTATTGAATTATGTTTCTAGATAGGTTGTAG
CTGGCTCTTTAGAGCATGTGCACGCCTGTTTGGACTTCATTTTCATCCACCTGTGCACCT
ATTGTAGTCTTTGGTTGGGTTAGGGGGAAGTGGTCATTGTGTCAGCATCTGCTGGATGTG
AGGACTTGCATTGTGAAAGCTTTGCTGTCCTTGATGTGATCATGGAATCTCTTTCTCACT
AGAGTCTATGTCACTCATTATACTCTGTCGAATGTCATTGAATGTCTTTACATGGGCTTG
TATGCCTATGAAAATTGTAATACAACTTTCAGCAACGGATCTCTTGGCTCTCGCATCGAT
GAAGGACGCAGCGAAATGCGATAAGTAATGTGAATTGCAGAATTCAGTGAATCATCGAAT
CTTTGAACGCATCTTGCGCTCCTTGGTATTCCGAGGAGCATGCCTGTTTGAGTGTCATTA
AATTCTCAACTCTCTTATACTTTTTTGTAAAAGAGAGCTTGGACTGTGGAGGCTTGCTGG
CCACTTTTTGGGGTCAGCTCCTCTGAAATGCATTAGCGGAACCGTTTGCAATCTGCCACA
AGTGTGATAAGTTATCTACACTGGCGAGGGGATTGCTCTCTGTAATGTTCAGCTTCTAAT
TGTCTCTACTTTGTGAGACAACTTTTGAATGCTTGACCTCAAATCAGGTAGGACTACCCG
CTGAACTTAA
>B11_65
TTTCCGTAGGTGAACCTGCGGAAGGATCATTATTGAATTATGTTTCTAGATAGGTTGTAG
CTGGCTCTTTAGAGCATGTGCACGCCTGTTTGGACTTCATTTTCATCCACCTGTGCACCT
ATTGTAGTCTTTGGTTGGGTTAGGGGGAAGTGGTCATTGTGTCAGCATCTGCTGGATGTG
AGGACTTGCATTGTGAAAGCTTTGCTGTCCTTGATGTGATCATGGAATCTCTTTCTCACT
AGAGTCTATGTCACTCATTATACTCTGTCGAATGTCATTGAATGTCTTTACATGGGCTTG
TATGCCTATGAAAATTGTAATACAACTTTCAGCAACGGATCTCTTGGCTCTCGCATCGAT
GAAGGACGCAGCGAAATGCGATAAGTAATGTGAATTGCAGAATTCAGTGAATCATCGAAT
CTTTGAACGCATCTTGCGCTCCTTGGTATTCCGAGGAGCATGCCTGTTTGAGTGTCATTA
AATTCTCAACTCTCTTATACTTTTTTGTAAAAGAGAGCTTGGACTGTGGAGGCTTGCTGG
CCACTTTTTGGGGTCAGCTCCTCTGAAATGCATTAGCGGAACCGTTTGCAATCTGCCACA
AGTGTGATAAGTTATCTACACTGGCGAGGGGATTGCTCTCTGTAATGTTCAGCTTCTAAT
TGTCTCTACTTTGTGAGACAACTTTTGAATGCTTGACCTCAAATCAGGTAGGACTACCCG
CTGAACTTAA
>B11_66
TTTCCGTAGGTGAACCTGCGGAAGGATCATTATTGAATTATGTTTCTAGATAGGTTGTAG
CTGGCTCTTTAGAGCATGTGCACGCCTGTTTGGACTTCATTTTCATCCACCTGTGCACCT
ATTGTAGTCTTTGGTTGGGTTAGGGGGAAGTGGTCATTGTGTCAGCATCTGCTGGATGTG
AGGACTTGCATTGTGAAAGCTTTGCTGTCCTTGATGTGATCATGGAATCTCTTTCTCACT
AGAGTCTATGTCACTCATTATACTCTGTCGAATGTCATTGAATGTCTTTACATGGGCTTG
TATGCCTATGAAAATTGTAATACAACTTTCAGCAACGGATCTCTTGGCTCTCGCATCGAT
GAAGGACGCAGCGAAATGCGATAAGTAATGTGAATTGCAGAATTCAGTGAATCATCGAAT
CTTTGAACGCATCTTGCGCTCCTTGGTATTCCGAGGAGCATGCCTGTTTGAGTGTCATTA
AATTCTCAACTCTCTTATACTTTTTTGTAAAAGAGAGCTTGGACTGTGGAGGCTTGCTGG

CCACTTTTTGGGGTCAGCTCCTCTGAAATGCATTAGCGGAACCGTTTGCAATCTGCCACA
AGTGTGATAAGTTATCTACACTGGCGAGGGGATTGCTCTCTGTAATGTTCAGCTTCTAAT
TGTCTCTACTTTGTGAGACAACTTTTGAATGCTTGACCTCAAATCAGGTAGGACTACCCG
CTGAACTTAA
>B11_68
TTTCCGTAGGTGAACCTGCGGAAGGATCATTATTGAATTATGTTTCTAGATAGGTTGTAG
CTGGCTCTTTAGAGCATGTGCACGCCTGTTTGGACTTCATTTTCATCCACCTGTGCACCT
ATTGTAGTCTTTGGTTGGGTTAGGGGGAAGTGGTCATTGTGTCAGCATCTGCTGGATGTG
AGGACTTGCATTGTGAAAGCTTTGCTGTCCTTGATGTGATCATGGAATCTCTTTCTCACT
AGAGTCTATGTCACTCATTATACTCTGTCGAATGTCATTGAATGTCTTTACATGGGCTTG
TATGCCTATGAAAATTGTAATACAACTTTCAGCAACGGATCTCTTGGCTCTCGCATCGAT
GAAGGACGCAGCGAAATGCGATAAGTAATGTGAATTGCAGAATTCAGTGAATCATCGAAT
CTTTGAACGCATCTTGCGCTCCTTGGTATTCCGAGGAGCATGCCTGTTTGAGTGTCATTA
AATTCTCAACTCTCTTATACTTTTTTGTAAAAGAGAGCTTGGACTGTGGAGGCTTGCTGG
CCACTTTTTGGGGTCAGCTCCTCTGAAATGCATTAGCGGAACCGTTTGCAATCTGCCACA
AGTGTGATAAGTTATCTACACTGGCGAGGGGATTGCTCTCTGTAATGTTCAGCTTCTAAT
TGTCTCTACTTTGTGAGACAACTTTTGAATGCTTGACCTCAAATCAGGTAGGACTACCCG
CTGAACTTAA
>B12_1
TTTCCGTAGGTGAACCTGCGGAAGGATCATTATTGAATTATGTTTCTAGATAGGTTGTAG
CTGGCTCTTTAGAGCATGTGCACGCCTGTTTGGACTTCATTTTCATCCACCTGTGCACCT
ATTGTAGTCTTTGGTTGGGTTAGGGGGAAGTGGTCATTGTGTCAGCATCTGCTGGATGTG
AGGACTTGCATTGTGAAAGCTTTGCTGTCCTTGATGTGATCATGGAATCTCTTTCTCACT
AGAGTCTATGTCACTCATTATACTCTGTCGAATGTCATTGAATGTCTTTACATGGGCTTG
TATGCCTATGAAAATTGTAATACAACTTTCAGCAACGGATCTCTTGGCTCTCGCATCGAT
GAAGGACGCAGCGAAATGCGATAAGTAATGTGAATTGCAGAATTCAGTGAATCATCGAAT
CTTTGAACGCATCTTGCGCTCCTTGGTATTCCGAGGAGCATGCCTGTTTGAGTGTCATTA
AATTCTCAACTCTCTTATACTTTTTTGTAAAAGAGAGCTTGGACTGTGGAGGCTTGCTGG
CCACTTTTTGGGGTCAGCTCCTCTGAAATGCATTAGCGGAACCGTTTGCAATCTGCCACA
AGTGTGATAAGTTATCTACACTGGCGAGGGGATTGCTCTCTGTAATGTTCAGCTTCTAAT
TGTCTCTACTTTGTGAGACAACTTTTGAATGCTTGACCTCAAATCAGGTAGGACTACCCG
CTGAACTTAA
>B12_2
TTTCCGTAGGTGAACCTGCGGAAGGATCATTATTGAATTATGTTTCTAGATAGGTTGTAG
CTGGCTCTTTAGAGCATGTGCACGCCTGTTTGGACTTCATTTTCATCCACCTGTGCACCT
ATTGTAGTCTTTGGTTGGGTTAGGGGGAAGTGGTCATTGTGTCAGCATCTGCTGGATGTG
AGGACTTGCATTGTGAAAGCTTTGCTGTCCTTGATGTGATCATGGAATCTCTTTCTCACT
AGAGTCTATGTCACTCATTATACTCTGTCGAATGTCATTGAATGTCTTTACATGGGCTTG
TATGCCTATGAAAATTGTAATACAACTTTCAGCAACGGATCTCTTGGCTCTCGCATCGAT
GAAGGACGCAGCGAAATGCGATAAGTAATGTGAATTGCAGAATTCAGTGAATCATCGAAT
CTTTGAACGCATCTTGCGCTCCTTGGTATTCCGAGGAGCATGCCTGTTTGAGTGTCATTA
AATTCTCAACTCTCTTATACTTTTTTGTAAAAGAGAGCTTGGACTGTGGAGGCTTGCTGG
CCACTTTTTGGGGTCAGCTCCTCTGAAATGCATTAGCGGAACCGTTTGCAATCTGCCACA
AGTGTGATAAGTTATCTACACTGGCGAGGGGATTGCTCTCTGTAATGTTCAGCTTCTAAT
TGTCTCTACTTTGTGAGACAACTTTTGAATGCTTGACCTCAAATCAGGTAGGACTACCCG
CTGAACTTAA
>B12_3
TTTCCGTAGGTGAACCTGCGGAAGGATCATTATTGAATTATGTTTCTAGATAGGTTGTAG
CTGGCTCTTTAGAGCATGTGCACGCCTGTTTGGACTTCATTTTCATCCACCTGTGCACCT
ATTGTAGTCTTTGGTTGGGTTAGGGGGAAGTGGTCATTGTGTCAGCATCTGCTGGATGTG

AGGACTTGCATTGTGAAAGCTTTGCTGTCCTTGATGTGATCATGGAATCTCTTTCTCACT
AGAGTCTATGTCACTCATTATACTCTGTCGAATGTCATTGAATGTCTTTACATGGGCTTG
TATGCCTATGAAAATTGTAATACAACTTTCAGCAACGGATCTCTTGGCTCTCGCATCGAT
GAAGGACGCAGCGAAATGCGATAAGTAATGTGAATTGCAGAATTCAGTGAATCATCGAAT
CTTTGAACGCATCTTGCGCTCCTTGGTATTCCGAGGAGCATGCCTGTTTGAGTGTCATTA
AATTCTCAACTCTCTTATACTTTTTTGTAAAAGAGAGCTTGGACTGTGGAGGCTTGCTGG
CCACTTTTTGGGGTCAGCTCCTCTGAAATGCATTAGCGGAACCGTTTGCAATCTGCCACA
AGTGTGATAAGTTATCTACACTGGCGAGGGGATTGCTCTCTGTAATGTTCAGCTTCTAAT
TGTCTCTACTTTGTGAGACAACTTTTGAATGCTTGACCTCAAATCAGGTAGGACTACCCG
CTGAACTTAA
>B12_5
TTTCCGTAGGTGAACCTGCGGAAGGATCATTATTGAATTATGTTTCTAGATAGGTTGTAG
CTGGCTCTTTAGAGCATGTGCACGCCTGTTTGGACTTCATTTTCATCCACCTGTGCACCT
ATTGTAGTCTTTGGTTGGGTTAGGGGGAAGTGGTCATTGTGTCAGCATCTGCTGGATGTG
AGGACTTGCATTGTGAAAGCTTTGCTGTCCTTGATGTGATCATGGAATCTCTTTCTCACT
AGAGTCTATGTCACTCATTATACTCTGTCGAATGTCATTGAATGTCTTTACATGGGCTTG
TATGCCTATGAAAATTGTAATACAACTTTCAGCAACGGATCTCTTGGCTCTCGCATCGAT
GAAGGACGCAGCGAAATGCGATAAGTAATGTGAATTGCAGAATTCAGTGAATCATCGAAT
CTTTGAACGCATCTTGCGCTCCTTGGTATTCCGAGGAGCATGCCTGTTTGAGTGTCATTA
AATTCTCAACTCTCTTATACTTTTTTGTAAAAGAGAGCTTGGACTGTGGAGGCTTGCTGG
CCACTTTTTGGGGTCAGCTCCTCTGAAATGCATTAGCGGAACCGTTTGCAATCTGCCACA
AGTGTGATAAGTTATCTACACTGGCGAGGGGATTGCTCTCTGTAATGTTCAGCTTCTAAT
TGTCTCTACTTTGTGAGACAACTTTTGAATGCTTGACCTCAAATCAGGTAGGACTACCCG
CTGAACTTAA
>B12_9
TTTCCGTAGGTGAACCTGCGGAAGGATCATTATTGAATTATGTTTCTAGATAGGTTGTAG
CTGGCTCTTTAGAGCATGTGCACGCCTGTTTGGACTTCATTTTCATCCACCTGTGCACCT
ATTGTAGTCTTTGGTTGGGTTAGGGGGAAGTGGTCATTGTGTCAGCATCTGCTGGATGTG
AGGACTTGCATTGTGAAAGCTTTGCTGTCCTTGATGTGATCATGGAATCTCTTTCTCACT
AGAGTCTATGTCACTCATTATACTCTGTCGAATGTCATTGAATGTCTTTACATGGGCTTG
TATGCCTATGAAAATTGTAATACAACTTTCAGCAACGGATCTCTTGGCTCTCGCATCGAT
GAAGGACGCAGCGAAATGCGATAAGTAATGTGAATTGCAGAATTCAGTGAATCATCGAAT
CTTTGAACGCATCTTGCGCTCCTTGGTATTCCGAGGAGCATGCCTGTTTGAGTGTCATTA
AATTCTCAACTCTCTTATACTTTTTTGTAAAAGAGAGCTTGGACTGTGGAGGCTTGCTGG
CCACTTTTTGGGGTCAGCTCCTCTGAAATGCATTAGCGGAACCGTTTGCAATCTGCCACA
AGTGTGATAAGTTATCTACACTGGCGAGGGGATTGCTCTCTGTAATGTTCAGCTTCTAAT
TGTCTCTACTTTGTGAGACAACTTTTGAATGCTTGACCTCAAATCAGGTAGGACTACCCG
CTGAACTTAA
>B12_10
TTTCCGTAGGTGAACCTGCGGAAGGATCATTATTGAATTATGTTTCTAGATAGGTTGTAG
CTGGCTCTTTAGAGCATGTGCACGCCTGTTTGGACTTCATTTTCATCCACCTGTGCACCT
ATTGTAGTCTTTGGTTGGGTTAGGGGGAAGTGGTCATTGTGTCAGCATCTGCTGGATGTG
AGGACTTGCATTGTGAAAGCTTTGCTGTCCTTGATGTGATCATGGAATCTCTTTCTCACT
AGAGTCTATGTCACTCATTATACTCTGTCGAATGTCATTGAATGTCTTTACATGGGCTTG
TATGCCTATGAAAATTGTAATACAACTTTCAGCAACGGATCTCTTGGCTCTCGCATCGAT
GAAGGACGCAGCGAAATGCGATAAGTAATGTGAATTGCAGAATTCAGTGAATCATCGAAT
CTTTGAACGCATCTTGCGCTCCTTGGTATTCCGAGGAGCATGCCTGTTTGAGTGTCATTA
AATTCTCAACTCTCTTATACTTTTTTGTAAAAGAGAGCTTGGACTGTGGAGGCTTGCTGG
CCACTTTTTGGGGTCAGCTCCTCTGAAATGCATTAGCGGAACCGTTTGCAATCTGCCACA
AGTGTGATAAGTTATCTACACTGGCGAGGGGATTGCTCTCTGTAATGTTCAGCTTCTAAT

TGTCTCTACTTTGTGAGACAACTTTTGAATGCTTGACCTCAAATCAGGTAGGACTACCCG
CTGAACTTAA
>B12_11
TTTCCGTAGGTGAACCTGCGGAAGGATCATTATTGAATTATGTTTCTAGATAGGTTGTAG
CTGGCTCTTTAGAGCATGTGCACGCCTGTTTGGACTTCATTTTCATCCACCTGTGCACCT
ATTGTAGTCTTTGGTTGGGTTAGGGGGAAGTGGTCATTGTGTCAGCATCTGCTGGATGTG
AGGACTTGCATTGTGAAAGCTTTGCTGTCCTTGATGTGATCATGGAATCTCTTTCTCACT
AGAGTCTATGTCACTCATTATACTCTGTCGAATGTCATTGAATGTCTTTACATGGGCTTG
TATGCCTATGAAAATTGTAATACAACTTTCAGCAACGGATCTCTTGGCTCTCGCATCGAT
GAAGGACGCAGCGAAATGCGATAAGTAATGTGAATTGCAGAATTCAGTGAATCATCGAAT
CTTTGAACGCATCTTGCGCTCCTTGGTATTCCGAGGAGCATGCCTGTTTGAGTGTCATTA
AATTCTCAACTCTCTTATACTTTTTTGTAAAAGAGAGCTTGGACTGTGGAGGCTTGCTGG
CCACTTTTTGGGGTCAGCTCCTCTGAAATGCATTAGCGGAACCGTTTGCAATCTGCCACA
AGTGTGATAAGTTATCTACACTGGCGAGGGGATTGCTCTCTGTAATGTTCAGCTTCTAAT
TGTCTCTACTTTGTGAGACAACTTTTGAATGCTTGACCTCAAATCAGGTAGGACTACCCG
CTGAACTTAA
>B12_13
TTTCCGTAGGTGAACCTGCGGAAGGATCATTATTGAATTATGTTTCTAGATAGGTTGTAG
CTGGCTCTTTAGAGCATGTGCACGCCTGTTTGGACTTCATTTTCATCCACCTGTGCACCT
ATTGTAGTCTTTGGTTGGGTTAGGGGGAAGTGGTCATTGTGTCAGCATCTGCTGGATGTG
AGGACTTGCATTGTGAAAGCTTTGCTGTCCTTGATGTGATCATGGAATCTCTTTCTCACT
AGAGTCTATGTCACTCATTATACTCTGTCGAATGTCATTGAATGTCTTTACATGGGCTTG
TATGCCTATGAAAATTGTAATACAACTTTCAGCAACGGATCTCTTGGCTCTCGCATCGAT
GAAGGACGCAGCGAAATGCGATAAGTAATGTGAATTGCAGAATTCAGTGAATCATCGAAT
CTTTGAACGCATCTTGCGCTCCTTGGTATTCCGAGGAGCATGCCTGTTTGAGTGTCATTA
AATTCTCAACTCTCTTATACTTTTTTGTAAAAGAGAGCTTGGACTGTGGAGGCTTGCTGG
CCACTTTTTGGGGTCAGCTCCTCTGAAATGCATTAGCGGAACCGTTTGCAATCTGCCACA
AGTGTGATAAGTTATCTACACTGGCGAGGGGATTGCTCTCTGTAATGTTCAGCTTCTAAT
TGTCTCTACTTTGTGAGACAACTTTTGAATGCTTGACCTCAAATCAGGTAGGACTACCCG
CTGAACTTAA
>B12_14
TTTCCGTAGGTGAACCTGCGGAAGGATCATTATTGAATTATGTTTCTAGATAGGTTGTAG
CTGGCTCTTTAGAGCATGTGCACGCCTGTTTGGACTTCATTTTCATCCACCTGTGCACCT
ATTGTAGTCTTTGGTTGGGTTAGGGGGAAGTGGTCATTGTGTCAGCATCTGCTGGATGTG
AGGACTTGCATTGTGAAAGCTTTGCTGTCCTTGATGTGATCATGGAATCTCTTTCTCACT
AGAGTCTATGTCACTCATTATACTCTGTCGAATGTCATTGAATGTCTTTACATGGGCTTG
TATGCCTATGAAAATTGTAATACAACTTTCAGCAACGGATCTCTTGGCTCTCGCATCGAT
GAAGGACGCAGCGAAATGCGATAAGTAATGTGAATTGCAGAATTCAGTGAATCATCGAAT
CTTTGAACGCATCTTGCGCTCCTTGGTATTCCGAGGAGCATGCCTGTTTGAGTGTCATTA
AATTCTCAACTCTCTTATACTTTTTTGTAAAAGAGAGCTTGGACTGTGGAGGCTTGCTGG
CCACTTTTTGGGGTCAGCTCCTCTGAAATGCATTAGCGGAACCGTTTGCAATCTGCCACA
AGTGTGATAAGTTATCTACACTGGCGAGGGGATTGCTCTCTGTAATGTTCAGCTTCTAAT
TGTCTCTACTTTGTGAGACAACTTTTGAATGCTTGACCTCAAATCAGGTAGGACTACCCG
CTGAACTTAA
>B12_15
TTTCCGTAGGTGAACCTGCGGAAGGATCATTATTGAATTATGTTTCTAGATAGGTTGTAG
CTGGCTCTTTAGAGCATGTGCACGCCTGTTTGGACTTCATTTTCATCCACCTGTGCACCT
ATTGTAGTCTTTGGTTGGGTTAGGGGGAAGTGGTCATTGTGTCAGCATCTGCTGGATGTG
AGGACTTGCATTGTGAAAGCTTTGCTGTCCTTGATGTGATCATGGAATCTCTTTCTCACT
AGAGTCTATGTCACTCATTATACTCTGTCGAATGTCATTGAATGTCTTTACATGGGCTTG

TATGCCTATGAAAATTGTAATACAACTTTCAGCAACGGATCTCTTGGCTCTCGCATCGAT
GAAGGACGCAGCGAAATGCGATAAGTAATGTGAATTGCAGAATTCAGTGAATCATCGAAT
CTTTGAACGCATCTTGCGCTCCTTGGTATTCCGAGGAGCATGCCTGTTTGAGTGTCATTA
AATTCTCAACTCTCTTATACTTTTTTGTAAAAGAGAGCTTGGACTGTGGAGGCTTGCTGG
CCACTTTTTGGGGTCAGCTCCTCTGAAATGCATTAGCGGAACCGTTTGCAATCTGCCACA
AGTGTGATAAGTTATCTACACTGGCGAGGGGATTGCTCTCTGTAATGTTCAGCTTCTAAT
TGTCTCTACTTTGTGAGACAACTTTTGAATGCTTGACCTCAAATCAGGTAGGACTACCCG
CTGAACTTAA
>B12_16
TTTCCGTAGGTGAACCTGCGGAAGGATCATTATTGAATTATGTTTCTAGATAGGTTGTAG
CTGGCTCTTTAGAGCATGTGCACGCCTGTTTGGACTTCATTTTCATCCACCTGTGCACCT
ATTGTAGTCTTTGGTTGGGTTAGGGGGAAGTGGTCATTGTGTCAGCATCTGCTGGATGTG
AGGACTTGCATTGTGAAAGCTTTGCTGTCCTTGATGTGATCATGGAATCTCTTTCTCACT
AGAGTCTATGTCACTCATTATACTCTGTCGAATGTCATTGAATGTCTTTACATGGGCTTG
TATGCCTATGAAAATTGTAATACAACTTTCAGCAACGGATCTCTTGGCTCTCGCATCGAT
GAAGGACGCAGCGAAATGCGATAAGTAATGTGAATTGCAGAATTCAGTGAATCATCGAAT
CTTTGAACGCATCTTGCGCTCCTTGGTATTCCGAGGAGCATGCCTGTTTGAGTGTCATTA
AATTCTCAACTCTCTTATACTTTTTTGTAAAAGAGAGCTTGGACTGTGGAGGCTTGCTGG
CCACTTTTTGGGGTCAGCTCCTCTGAAATGCATTAGCGGAACCGTTTGCAATCTGCCACA
AGTGTGATAAGTTATCTACACTGGCGAGGGGATTGCTCTCTGTAATGTTCAGCTTCTAAT
TGTCTCTACTTTGTGAGACAACTTTTGAATGCTTGACCTCAAATCAGGTAGGACTACCCG
CTGAACTTAA
>B12_17
TTTCCGTAGGTGAACCTGCGGAAGGATCATTATTGAATTATGTTTCTAGATAGGTTGTAG
CTGGCTCTTTAGAGCATGTGCACGCCTGTTTGGACTTCATTTTCATCCACCTGTGCACCT
ATTGTAGTCTTTGGTTGGGTTAGGGGGAAGTGGTCATTGTGTCAGCATCTGCTGGATGTG
AGGACTTGCATTGTGAAAGCTTTGCTGTCCTTGATGTGATCATGGAATCTCTTTCTCACT
AGAGTCTATGTCACTCATTATACTCTGTCGAATGTCATTGAATGTCTTTACATGGGCTTG
TATGCCTATGAAAATTGTAATACAACTTTCAGCAACGGATCTCTTGGCTCTCGCATCGAT
GAAGGACGCAGCGAAATGCGATAAGTAATGTGAATTGCAGAATTCAGTGAATCATCGAAT
CTTTGAACGCATCTTGCGCTCCTTGGTATTCCGAGGAGCATGCCTGTTTGAGTGTCATTA
AATTCTCAACTCTCTTATACTTTTTTGTAAAAGAGAGCTTGGACTGTGGAGGCTTGCTGG
CCACTTTTTGGGGTCAGCTCCTCTGAAATGCATTAGCGGAACCGTTTGCAATCTGCCACA
AGTGTGATAAGTTATCTACACTGGCGAGGGGATTGCTCTCTGTAATGTTCAGCTTCTAAT
TGTCTCTACTTTGTGAGACAACTTTTGAATGCTTGACCTCAAATCAGGTAGGACTACCCG
CTGAACTTAA
>B12_18
TTTCCGTAGGTGAACCTGCGGAAGGATCATTATTGAATTATGTTTCTAGATAGGTTGTAG
CTGGCTCTTTAGAGCATGTGCACGCCTGTTTGGACTTCATTTTCATCCACCTGTGCACCT
ATTGTAGTCTTTGGTTGGGTTAGGGGGAAGTGGTCATTGTGTCAGCATCTGCTGGATGTG
AGGACTTGCATTGTGAAAGCTTTGCTGTCCTTGATGTGATCATGGAATCTCTTTCTCACT
AGAGTCTATGTCACTCATTATACTCTGTCGAATGTCATTGAATGTCTTTACATGGGCTTG
TATGCCTATGAAAATTGTAATACAACTTTCAGCAACGGATCTCTTGGCTCTCGCATCGAT
GAAGGACGCAGCGAAATGCGATAAGTAATGTGAATTGCAGAATTCAGTGAATCATCGAAT
CTTTGAACGCATCTTGCGCTCCTTGGTATTCCGAGGAGCATGCCTGTTTGAGTGTCATTA
AATTCTCAACTCTCTTATACTTTTTTGTAAAAGAGAGCTTGGACTGTGGAGGCTTGCTGG
CCACTTTTTGGGGTCAGCTCCTCTGAAATGCATTAGCGGAACCGTTTGCAATCTGCCACA
AGTGTGATAAGTTATCTACACTGGCGAGGGGATTGCTCTCTGTAATGTTCAGCTTCTAAT
TGTCTCTACTTTGTGAGACAACTTTTGAATGCTTGACCTCAAATCAGGTAGGACTACCCG
CTGAACTTAA

>B12_19
TTTCCGTAGGTGAACCTGCGGAAGGATCATTATTGAATTATGTTTCTAGATAGGTTGTAG
CTGGCTCTTTAGAGCATGTGCACGCCTGTTTGGACTTCATTTTCATCCACCTGTGCACCT
ATTGTAGTCTTTGGTTGGGTTAGGGGGAAGTGGTCATTGTGTCAGCATCTGCTGGATGTG
AGGACTTGCATTGTGAAAGCTTTGCTGTCCTTGATGTGATCATGGAATCTCTTTCTCACT
AGAGTCTATGTCACTCATTATACTCTGTCGAATGTCATTGAATGTCTTTACATGGGCTTG
TATGCCTATGAAAATTGTAATACAACTTTCAGCAACGGATCTCTTGGCTCTCGCATCGAT
GAAGGACGCAGCGAAATGCGATAAGTAATGTGAATTGCAGAATTCAGTGAATCATCGAAT
CTTTGAACGCATCTTGCGCTCCTTGGTATTCCGAGGAGCATGCCTGTTTGAGTGTCATTA
AATTCTCAACTCTCTTATACTTTTTTGTAAAAGAGAGCTTGGACTGTGGAGGCTTGCTGG
CCACTTTTTGGGGTCAGCTCCTCTGAAATGCATTAGCGGAACCGTTTGCAATCTGCCACA
AGTGTGATAAGTTATCTACACTGGCGAGGGGATTGCTCTCTGTAATGTTCAGCTTCTAAT
TGTCTCTACTTTGTGAGACAACTTTTGAATGCTTGACCTCAAATCAGGTAGGACTACCCG
CTGAACTTAA
>B12_20
TTTCCGTAGGTGAACCTGCGGAAGGATCATTATTGAATTATGTTTCTAGATAGGTTGTAG
CTGGCTCTTTAGAGCATGTGCACGCCTGTTTGGACTTCATTTTCATCCACCTGTGCACCT
ATTGTAGTCTTTGGTTGGGTTAGGGGGAAGTGGTCATTGTGTCAGCATCTGCTGGATGTG
AGGACTTGCATTGTGAAAGCTTTGCTGTCCTTGATGTGATCATGGAATCTCTTTCTCACT
AGAGTCTATGTCACTCATTATACTCTGTCGAATGTCATTGAATGTCTTTACATGGGCTTG
TATGCCTATGAAAATTGTAATACAACTTTCAGCAACGGATCTCTTGGCTCTCGCATCGAT
GAAGGACGCAGCGAAATGCGATAAGTAATGTGAATTGCAGAATTCAGTGAATCATCGAAT
CTTTGAACGCATCTTGCGCTCCTTGGTATTCCGAGGAGCATGCCTGTTTGAGTGTCATTA
AATTCTCAACTCTCTTATACTTTTTTGTAAAAGAGAGCTTGGACTGTGGAGGCTTGCTGG
CCACTTTTTGGGGTCAGCTCCTCTGAAATGCATTAGCGGAACCGTTTGCAATCTGCCACA
AGTGTGATAAGTTATCTACACTGGCGAGGGGATTGCTCTCTGTAATGTTCAGCTTCTAAT
TGTCTCTACTTTGTGAGACAACTTTTGAATGCTTGACCTCAAATCAGGTAGGACTACCCG
CTGAACTTAA
>B12_21
TTTCCGTAGGTGAACCTGCGGAAGGATCATTATTGAATTATGTTTCTAGATAGGTTGTAG
CTGGCTCTTTAGAGCATGTGCACGCCTGTTTGGACTTCATTTTCATCCACCTGTGCACCT
ATTGTAGTCTTTGGTTGGGTTAGGGGGAAGTGGTCATTGTGTCAGCATCTGCTGGATGTG
AGGACTTGCATTGTGAAAGCTTTGCTGTCCTTGATGTGATCATGGAATCTCTTTCTCACT
AGAGTCTATGTCACTCATTATACTCTGTCGAATGTCATTGAATGTCTTTACATGGGCTTG
TATGCCTATGAAAATTGTAATACAACTTTCAGCAACGGATCTCTTGGCTCTCGCATCGAT
GAAGGACGCAGCGAAATGCGATAAGTAATGTGAATTGCAGAATTCAGTGAATCATCGAAT
CTTTGAACGCATCTTGCGCTCCTTGGTATTCCGAGGAGCATGCCTGTTTGAGTGTCATTA
AATTCTCAACTCTCTTATACTTTTTTGTAAAAGAGAGCTTGGACTGTGGAGGCTTGCTGG
CCACTTTTTGGGGTCAGCTCCTCTGAAATGCATTAGCGGAACCGTTTGCAATCTGCCACA
AGTGTGATAAGTTATCTACACTGGCGAGGGGATTGCTCTCTGTAATGTTCAGCTTCTAAT
TGTCTCTACTTTGTGAGACAACTTTTGAATGCTTGACCTCAAATCAGGTAGGACTACCCG
CTGAACTTAA
>B12_22
TTTCCGTAGGTGAACCTGCGGAAGGATCATTATTGAATTATGTTTCTAGATAGGTTGTAG
CTGGCTCTTTAGAGCATGTGCACGCCTGTTTGGACTTCATTTTCATCCACCTGTGCACCT
ATTGTAGTCTTTGGTTGGGTTAGGGGGAAGTGGTCATTGTGTCAGCATCTGCTGGATGTG
AGGACTTGCATTGTGAAAGCTTTGCTGTCCTTGATGTGATCATGGAATCTCTTTCTCACT
AGAGTCTATGTCACTCATTATACTCTGTCGAATGTCATTGAATGTCTTTACATGGGCTTG
TATGCCTATGAAAATTGTAATACAACTTTCAGCAACGGATCTCTTGGCTCTCGCATCGAT
GAAGGACGCAGCGAAATGCGATAAGTAATGTGAATTGCAGAATTCAGTGAATCATCGAAT

CTTTGAACGCATCTTGCGCTCCTTGGTATTCCGAGGAGCATGCCTGTTTGAGTGTCATTA
AATTCTCAACTCTCTTATACTTTTTTGTAAAAGAGAGCTTGGACTGTGGAGGCTTGCTGG
CCACTTTTTGGGGTCAGCTCCTCTGAAATGCATTAGCGGAACCGTTTGCAATCTGCCACA
AGTGTGATAAGTTATCTACACTGGCGAGGGGATTGCTCTCTGTAATGTTCAGCTTCTAAT
TGTCTCTACTTTGTGAGACAACTTTTGAATGCTTGACCTCAAATCAGGTAGGACTACCCG
CTGAACTTAA
>B12_23
TTTCCGTAGGTGAACCTGCGGAAGGATCATTATTGAATTATGTTTCTAGATAGGTTGTAG
CTGGCTCTTTAGAGCATGTGCACGCCTGTTTGGACTTCATTTTCATCCACCTGTGCACCT
ATTGTAGTCTTTGGTTGGGTTAGGGGGAAGTGGTCATTGTGTCAGCATCTGCTGGATGTG
AGGACTTGCATTGTGAAAGCTTTGCTGTCCTTGATGTGATCATGGAATCTCTTTCTCACT
AGAGTCTATGTCACTCATTATACTCTGTCGAATGTCATTGAATGTCTTTACATGGGCTTG
TATGCCTATGAAAATTGTAATACAACTTTCAGCAACGGATCTCTTGGCTCTCGCATCGAT
GAAGGACGCAGCGAAATGCGATAAGTAATGTGAATTGCAGAATTCAGTGAATCATCGAAT
CTTTGAACGCATCTTGCGCTCCTTGGTATTCCGAGGAGCATGCCTGTTTGAGTGTCATTA
AATTCTCAACTCTCTTATACTTTTTTGTAAAAGAGAGCTTGGACTGTGGAGGCTTGCTGG
CCACTTTTTGGGGTCAGCTCCTCTGAAATGCATTAGCGGAACCGTTTGCAATCTGCCACA
AGTGTGATAAGTTATCTACACTGGCGAGGGGATTGCTCTCTGTAATGTTCAGCTTCTAAT
TGTCTCTACTTTGTGAGACAACTTTTGAATGCTTGACCTCAAATCAGGTAGGACTACCCG
CTGAACTTAA
>B12_24
TTTCCGTAGGTGAACCTGCGGAAGGATCATTATTGAATTATGTTTCTAGATAGGTTGTAG
CTGGCTCTTTAGAGCATGTGCACGCCTGTTTGGACTTCATTTTCATCCACCTGTGCACCT
ATTGTAGTCTTTGGTTGGGTTAGGGGGAAGTGGTCATTGTGTCAGCATCTGCTGGATGTG
AGGACTTGCATTGTGAAAGCTTTGCTGTCCTTGATGTGATCATGGAATCTCTTTCTCACT
AGAGTCTATGTCACTCATTATACTCTGTCGAATGTCATTGAATGTCTTTACATGGGCTTG
TATGCCTATGAAAATTGTAATACAACTTTCAGCAACGGATCTCTTGGCTCTCGCATCGAT
GAAGGACGCAGCGAAATGCGATAAGTAATGTGAATTGCAGAATTCAGTGAATCATCGAAT
CTTTGAACGCATCTTGCGCTCCTTGGTATTCCGAGGAGCATGCCTGTTTGAGTGTCATTA
AATTCTCAACTCTCTTATACTTTTTTGTAAAAGAGAGCTTGGACTGTGGAGGCTTGCTGG
CCACTTTTTGGGGTCAGCTCCTCTGAAATGCATTAGCGGAACCGTTTGCAATCTGCCACA
AGTGTGATAAGTTATCTACACTGGCGAGGGGATTGCTCTCTGTAATGTTCAGCTTCTAAT
TGTCTCTACTTTGTGAGACAACTTTTGAATGCTTGACCTCAAATCAGGTAGGACTACCCG
CTGAACTTAA
>B12_25
TTTCCGTAGGTGAACCTGCGGAAGGATCATTATTGAATTATGTTTCTAGATAGGTTGTAG
CTGGCTCTTTAGAGCATGTGCACGCCTGTTTGGACTTCATTTTCATCCACCTGTGCACCT
ATTGTAGTCTTTGGTTGGGTTAGGGGGAAGTGGTCATTGTGTCAGCATCTGCTGGATGTG
AGGACTTGCATTGTGAAAGCTTTGCTGTCCTTGATGTGATCATGGAATCTCTTTCTCACT
AGAGTCTATGTCACTCATTATACTCTGTCGAATGTCATTGAATGTCTTTACATGGGCTTG
TATGCCTATGAAAATTGTAATACAACTTTCAGCAACGGATCTCTTGGCTCTCGCATCGAT
GAAGGACGCAGCGAAATGCGATAAGTAATGTGAATTGCAGAATTCAGTGAATCATCGAAT
CTTTGAACGCATCTTGCGCTCCTTGGTATTCCGAGGAGCATGCCTGTTTGAGTGTCATTA
AATTCTCAACTCTCTTATACTTTTTTGTAAAAGAGAGCTTGGACTGTGGAGGCTTGCTGG
CCACTTTTTGGGGTCAGCTCCTCTGAAATGCATTAGCGGAACCGTTTGCAATCTGCCACA
AGTGTGATAAGTTATCTACACTGGCGAGGGGATTGCTCTCTGTAATGTTCAGCTTCTAAT
TGTCTCTACTTTGTGAGACAACTTTTGAATGCTTGACCTCAAATCAGGTAGGACTACCCG
CTGAACTTAA
>B12_26
TTTCCGTAGGTGAACCTGCGGAAGGATCATTATTGAATTATGTTTCTAGATAGGTTGTAG

CTGGCTCTTTAGAGCATGTGCACGCCTGTTTGGACTTCATTTTCATCCACCTGTGCACCT
ATTGTAGTCTTTGGTTGGGTTAGGGGGAAGTGGTCATTGTGTCAGCATCTGCTGGATGTG
AGGACTTGCATTGTGAAAGCTTTGCTGTCCTTGATGTGATCATGGAATCTCTTTCTCACT
AGAGTCTATGTCACTCATTATACTCTGTCGAATGTCATTGAATGTCTTTACATGGGCTTG
TATGCCTATGAAAATTGTAATACAACTTTCAGCAACGGATCTCTTGGCTCTCGCATCGAT
GAAGGACGCAGCGAAATGCGATAAGTAATGTGAATTGCAGAATTCAGTGAATCATCGAAT
CTTTGAACGCATCTTGCGCTCCTTGGTATTCCGAGGAGCATGCCTGTTTGAGTGTCATTA
AATTCTCAACTCTCTTATACTTTTTTGTAAAAGAGAGCTTGGACTGTGGAGGCTTGCTGG
CCACTTTTTGGGGTCAGCTCCTCTGAAATGCATTAGCGGAACCGTTTGCAATCTGCCACA
AGTGTGATAAGTTATCTACACTGGCGAGGGGATTGCTCTCTGTAATGTTCAGCTTCTAAT
TGTCTCTACTTTGTGAGACAACTTTTGAATGCTTGACCTCAAATCAGGTAGGACTACCCG
CTGAACTTAA
>B12_28
TTTCCGTAGGTGAACCTGCGGAAGGATCATTATTGAATTATGTTTCTAGATAGGTTGTAG
CTGGCTCTTTAGAGCATGTGCACGCCTGTTTGGACTTCATTTTCATCCACCTGTGCACCT
ATTGTAGTCTTTGGTTGGGTTAGGGGGAAGTGGTCATTGTGTCAGCATCTGCTGGATGTG
AGGACTTGCATTGTGAAAGCTTTGCTGTCCTTGATGTGATCATGGAATCTCTTTCTCACT
AGAGTCTATGTCACTCATTATACTCTGTCGAATGTCATTGAATGTCTTTACATGGGCTTG
TATGCCTATGAAAATTGTAATACAACTTTCAGCAACGGATCTCTTGGCTCTCGCATCGAT
GAAGGACGCAGCGAAATGCGATAAGTAATGTGAATTGCAGAATTCAGTGAATCATCGAAT
CTTTGAACGCATCTTGCGCTCCTTGGTATTCCGAGGAGCATGCCTGTTTGAGTGTCATTA
AATTCTCAACTCTCTTATACTTTTTTGTAAAAGAGAGCTTGGACTGTGGAGGCTTGCTGG
CCACTTTTTGGGGTCAGCTCCTCTGAAATGCATTAGCGGAACCGTTTGCAATCTGCCACA
AGTGTGATAAGTTATCTACACTGGCGAGGGGATTGCTCTCTGTAATGTTCAGCTTCTAAT
TGTCTCTACTTTGTGAGACAACTTTTGAATGCTTGACCTCAAATCAGGTAGGACTACCCG
CTGAACTTAA
>B12_29
TTTCCGTAGGTGAACCTGCGGAAGGATCATTATTGAATTATGTTTCTAGATAGGTTGTAG
CTGGCTCTTTAGAGCATGTGCACGCCTGTTTGGACTTCATTTTCATCCACCTGTGCACCT
ATTGTAGTCTTTGGTTGGGTTAGGGGGAAGTGGTCATTGTGTCAGCATCTGCTGGATGTG
AGGACTTGCATTGTGAAAGCTTTGCTGTCCTTGATGTGATCATGGAATCTCTTTCTCACT
AGAGTCTATGTCACTCATTATACTCTGTCGAATGTCATTGAATGTCTTTACATGGGCTTG
TATGCCTATGAAAATTGTAATACAACTTTCAGCAACGGATCTCTTGGCTCTCGCATCGAT
GAAGGACGCAGCGAAATGCGATAAGTAATGTGAATTGCAGAATTCAGTGAATCATCGAAT
CTTTGAACGCATCTTGCGCTCCTTGGTATTCCGAGGAGCATGCCTGTTTGAGTGTCATTA
AATTCTCAACTCTCTTATACTTTTTTGTAAAAGAGAGCTTGGACTGTGGAGGCTTGCTGG
CCACTTTTTGGGGTCAGCTCCTCTGAAATGCATTAGCGGAACCGTTTGCAATCTGCCACA
AGTGTGATAAGTTATCTACACTGGCGAGGGGATTGCTCTCTGTAATGTTCAGCTTCTAAT
TGTCTCTACTTTGTGAGACAACTTTTGAATGCTTGACCTCAAATCAGGTAGGACTACCCG
CTGAACTTAA
>B12_30
TTTCCGTAGGTGAACCTGCGGAAGGATCATTATTGAATTATGTTTCTAGATAGGTTGTAG
CTGGCTCTTTAGAGCATGTGCACGCCTGTTTGGACTTCATTTTCATCCACCTGTGCACCT
ATTGTAGTCTTTGGTTGGGTTAGGGGGAAGTGGTCATTGTGTCAGCATCTGCTGGATGTG
AGGACTTGCATTGTGAAAGCTTTGCTGTCCTTGATGTGATCATGGAATCTCTTTCTCACT
AGAGTCTATGTCACTCATTATACTCTGTCGAATGTCATTGAATGTCTTTACATGGGCTTG
TATGCCTATGAAAATTGTAATACAACTTTCAGCAACGGATCTCTTGGCTCTCGCATCGAT
GAAGGACGCAGCGAAATGCGATAAGTAATGTGAATTGCAGAATTCAGTGAATCATCGAAT
CTTTGAACGCATCTTGCGCTCCTTGGTATTCCGAGGAGCATGCCTGTTTGAGTGTCATTA
AATTCTCAACTCTCTTATACTTTTTTGTAAAAGAGAGCTTGGACTGTGGAGGCTTGCTGG

CCACTTTTTGGGGTCAGCTCCTCTGAAATGCATTAGCGGAACCGTTTGCAATCTGCCACA
AGTGTGATAAGTTATCTACACTGGCGAGGGGATTGCTCTCTGTAATGTTCAGCTTCTAAT
TGTCTCTACTTTGTGAGACAACTTTTGAATGCTTGACCTCAAATCAGGTAGGACTACCCG
CTGAACTTAA
>B12_31
TTTCCGTAGGTGAACCTGCGGAAGGATCATTATTGAATTATGTTTCTAGATAGGTTGTAG
CTGGCTCTTTAGAGCATGTGCACGCCTGTTTGGACTTCATTTTCATCCACCTGTGCACCT
ATTGTAGTCTTTGGTTGGGTTAGGGGGAAGTGGTCATTGTGTCAGCATCTGCTGGATGTG
AGGACTTGCATTGTGAAAGCTTTGCTGTCCTTGATGTGATCATGGAATCTCTTTCTCACT
AGAGTCTATGTCACTCATTATACTCTGTCGAATGTCATTGAATGTCTTTACATGGGCTTG
TATGCCTATGAAAATTGTAATACAACTTTCAGCAACGGATCTCTTGGCTCTCGCATCGAT
GAAGGACGCAGCGAAATGCGATAAGTAATGTGAATTGCAGAATTCAGTGAATCATCGAAT
CTTTGAACGCATCTTGCGCTCCTTGGTATTCCGAGGAGCATGCCTGTTTGAGTGTCATTA
AATTCTCAACTCTCTTATACTTTTTTGTAAAAGAGAGCTTGGACTGTGGAGGCTTGCTGG
CCACTTTTTGGGGTCAGCTCCTCTGAAATGCATTAGCGGAACCGTTTGCAATCTGCCACA
AGTGTGATAAGTTATCTACACTGGCGAGGGGATTGCTCTCTGTAATGTTCAGCTTCTAAT
TGTCTCTACTTTGTGAGACAACTTTTGAATGCTTGACCTCAAATCAGGTAGGACTACCCG
CTGAACTTAA
>B12_34
TTTCCGTAGGTGAACCTGCGGAAGGATCATTATTGAATTATGTTTCTAGATAGGTTGTAG
CTGGCTCTTTAGAGCATGTGCACGCCTGTTTGGACTTCATTTTCATCCACCTGTGCACCT
ATTGTAGTCTTTGGTTGGGTTAGGGGGAAGTGGTCATTGTGTCAGCATCTGCTGGATGTG
AGGACTTGCATTGTGAAAGCTTTGCTGTCCTTGATGTGATCATGGAATCTCTTTCTCACT
AGAGTCTATGTCACTCATTATACTCTGTCGAATGTCATTGAATGTCTTTACATGGGCTTG
TATGCCTATGAAAATTGTAATACAACTTTCAGCAACGGATCTCTTGGCTCTCGCATCGAT
GAAGGACGCAGCGAAATGCGATAAGTAATGTGAATTGCAGAATTCAGTGAATCATCGAAT
CTTTGAACGCATCTTGCGCTCCTTGGTATTCCGAGGAGCATGCCTGTTTGAGTGTCATTA
AATTCTCAACTCTCTTATACTTTTTTGTAAAAGAGAGCTTGGACTGTGGAGGCTTGCTGG
CCACTTTTTGGGGTCAGCTCCTCTGAAATGCATTAGCGGAACCGTTTGCAATCTGCCACA
AGTGTGATAAGTTATCTACACTGGCGAGGGGATTGCTCTCTGTAATGTTCAGCTTCTAAT
TGTCTCTACTTTGTGAGACAACTTTTGAATGCTTGACCTCAAATCAGGTAGGACTACCCG
CTGAACTTAA
>B12_35
TTTCCGTAGGTGAACCTGCGGAAGGATCATTATTGAATTATGTTTCTAGATAGGTTGTAG
CTGGCTCTTTAGAGCATGTGCACGCCTGTTTGGACTTCATTTTCATCCACCTGTGCACCT
ATTGTAGTCTTTGGTTGGGTTAGGGGGAAGTGGTCATTGTGTCAGCATCTGCTGGATGTG
AGGACTTGCATTGTGAAAGCTTTGCTGTCCTTGATGTGATCATGGAATCTCTTTCTCACT
AGAGTCTATGTCACTCATTATACTCTGTCGAATGTCATTGAATGTCTTTACATGGGCTTG
TATGCCTATGAAAATTGTAATACAACTTTCAGCAACGGATCTCTTGGCTCTCGCATCGAT
GAAGGACGCAGCGAAATGCGATAAGTAATGTGAATTGCAGAATTCAGTGAATCATCGAAT
CTTTGAACGCATCTTGCGCTCCTTGGTATTCCGAGGAGCATGCCTGTTTGAGTGTCATTA
AATTCTCAACTCTCTTATACTTTTTTGTAAAAGAGAGCTTGGACTGTGGAGGCTTGCTGG
CCACTTTTTGGGGTCAGCTCCTCTGAAATGCATTAGCGGAACCGTTTGCAATCTGCCACA
AGTGTGATAAGTTATCTACACTGGCGAGGGGATTGCTCTCTGTAATGTTCAGCTTCTAAT
TGTCTCTACTTTGTGAGACAACTTTTGAATGCTTGACCTCAAATCAGGTAGGACTACCCG
CTGAACTTAA
>B12_36
TTTCCGTAGGTGAACCTGCGGAAGGATCATTATTGAATTATGTTTCTAGATAGGTTGTAG
CTGGCTCTTTAGAGCATGTGCACGCCTGTTTGGACTTCATTTTCATCCACCTGTGCACCT
ATTGTAGTCTTTGGTTGGGTTAGGGGGAAGTGGTCATTGTGTCAGCATCTGCTGGATGTG

AGGACTTGCATTGTGAAAGCTTTGCTGTCCTTGATGTGATCATGGAATCTCTTTCTCACT
AGAGTCTATGTCACTCATTATACTCTGTCGAATGTCATTGAATGTCTTTACATGGGCTTG
TATGCCTATGAAAATTGTAATACAACTTTCAGCAACGGATCTCTTGGCTCTCGCATCGAT
GAAGGACGCAGCGAAATGCGATAAGTAATGTGAATTGCAGAATTCAGTGAATCATCGAAT
CTTTGAACGCATCTTGCGCTCCTTGGTATTCCGAGGAGCATGCCTGTTTGAGTGTCATTA
AATTCTCAACTCTCTTATACTTTTTTGTAAAAGAGAGCTTGGACTGTGGAGGCTTGCTGG
CCACTTTTTGGGGTCAGCTCCTCTGAAATGCATTAGCGGAACCGTTTGCAATCTGCCACA
AGTGTGATAAGTTATCTACACTGGCGAGGGGATTGCTCTCTGTAATGTTCAGCTTCTAAT
TGTCTCTACTTTGTGAGACAACTTTTGAATGCTTGACCTCAAATCAGGTAGGACTACCCG
CTGAACTTAA
>B12_37
TTTCCGTAGGTGAACCTGCGGAAGGATCATTATTGAATTATGTTTCTAGATAGGTTGTAG
CTGGCTCTTTAGAGCATGTGCACGCCTGTTTGGACTTCATTTTCATCCACCTGTGCACCT
ATTGTAGTCTTTGGTTGGGTTAGGGGGAAGTGGTCATTGTGTCAGCATCTGCTGGATGTG
AGGACTTGCATTGTGAAAGCTTTGCTGTCCTTGATGTGATCATGGAATCTCTTTCTCACT
AGAGTCTATGTCACTCATTATACTCTGTCGAATGTCATTGAATGTCTTTACATGGGCTTG
TATGCCTATGAAAATTGTAATACAACTTTCAGCAACGGATCTCTTGGCTCTCGCATCGAT
GAAGGACGCAGCGAAATGCGATAAGTAATGTGAATTGCAGAATTCAGTGAATCATCGAAT
CTTTGAACGCATCTTGCGCTCCTTGGTATTCCGAGGAGCATGCCTGTTTGAGTGTCATTA
AATTCTCAACTCTCTTATACTTTTTTGTAAAAGAGAGCTTGGACTGTGGAGGCTTGCTGG
CCACTTTTTGGGGTCAGCTCCTCTGAAATGCATTAGCGGAACCGTTTGCAATCTGCCACA
AGTGTGATAAGTTATCTACACTGGCGAGGGGATTGCTCTCTGTAATGTTCAGCTTCTAAT
TGTCTCTACTTTGTGAGACAACTTTTGAATGCTTGACCTCAAATCAGGTAGGACTACCCG
CTGAACTTAA
>B12_38
TTTCCGTAGGTGAACCTGCGGAAGGATCATTATTGAATTATGTTTCTAGATAGGTTGTAG
CTGGCTCTTTAGAGCATGTGCACGCCTGTTTGGACTTCATTTTCATCCACCTGTGCACCT
ATTGTAGTCTTTGGTTGGGTTAGGGGGAAGTGGTCATTGTGTCAGCATCTGCTGGATGTG
AGGACTTGCATTGTGAAAGCTTTGCTGTCCTTGATGTGATCATGGAATCTCTTTCTCACT
AGAGTCTATGTCACTCATTATACTCTGTCGAATGTCATTGAATGTCTTTACATGGGCTTG
TATGCCTATGAAAATTGTAATACAACTTTCAGCAACGGATCTCTTGGCTCTCGCATCGAT
GAAGGACGCAGCGAAATGCGATAAGTAATGTGAATTGCAGAATTCAGTGAATCATCGAAT
CTTTGAACGCATCTTGCGCTCCTTGGTATTCCGAGGAGCATGCCTGTTTGAGTGTCATTA
AATTCTCAACTCTCTTATACTTTTTTGTAAAAGAGAGCTTGGACTGTGGAGGCTTGCTGG
CCACTTTTTGGGGTCAGCTCCTCTGAAATGCATTAGCGGAACCGTTTGCAATCTGCCACA
AGTGTGATAAGTTATCTACACTGGCGAGGGGATTGCTCTCTGTAATGTTCAGCTTCTAAT
TGTCTCTACTTTGTGAGACAACTTTTGAATGCTTGACCTCAAATCAGGTAGGACTACCCG
CTGAACTTAA
>B12_39
TTTCCGTAGGTGAACCTGCGGAAGGATCATTATTGAATTATGTTTCTAGATAGGTTGTAG
CTGGCTCTTTAGAGCATGTGCACGCCTGTTTGGACTTCATTTTCATCCACCTGTGCACCT
ATTGTAGTCTTTGGTTGGGTTAGGGGGAAGTGGTCATTGTGTCAGCATCTGCTGGATGTG
AGGACTTGCATTGTGAAAGCTTTGCTGTCCTTGATGTGATCATGGAATCTCTTTCTCACT
AGAGTCTATGTCACTCATTATACTCTGTCGAATGTCATTGAATGTCTTTACATGGGCTTG
TATGCCTATGAAAATTGTAATACAACTTTCAGCAACGGATCTCTTGGCTCTCGCATCGAT
GAAGGACGCAGCGAAATGCGATAAGTAATGTGAATTGCAGAATTCAGTGAATCATCGAAT
CTTTGAACGCATCTTGCGCTCCTTGGTATTCCGAGGAGCATGCCTGTTTGAGTGTCATTA
AATTCTCAACTCTCTTATACTTTTTTGTAAAAGAGAGCTTGGACTGTGGAGGCTTGCTGG
CCACTTTTTGGGGTCAGCTCCTCTGAAATGCATTAGCGGAACCGTTTGCAATCTGCCACA
AGTGTGATAAGTTATCTACACTGGCGAGGGGATTGCTCTCTGTAATGTTCAGCTTCTAAT

TGTCTCTACTTTGTGAGACAACTTTTGAATGCTTGACCTCAAATCAGGTAGGACTACCCG
CTGAACTTAA
>B12_40
TTTCCGTAGGTGAACCTGCGGAAGGATCATTATTGAATTATGTTTCTAGATAGGTTGTAG
CTGGCTCTTTAGAGCATGTGCACGCCTGTTTGGACTTCATTTTCATCCACCTGTGCACCT
ATTGTAGTCTTTGGTTGGGTTAGGGGGAAGTGGTCATTGTGTCAGCATCTGCTGGATGTG
AGGACTTGCATTGTGAAAGCTTTGCTGTCCTTGATGTGATCATGGAATCTCTTTCTCACT
AGAGTCTATGTCACTCATTATACTCTGTCGAATGTCATTGAATGTCTTTACATGGGCTTG
TATGCCTATGAAAATTGTAATACAACTTTCAGCAACGGATCTCTTGGCTCTCGCATCGAT
GAAGGACGCAGCGAAATGCGATAAGTAATGTGAATTGCAGAATTCAGTGAATCATCGAAT
CTTTGAACGCATCTTGCGCTCCTTGGTATTCCGAGGAGCATGCCTGTTTGAGTGTCATTA
AATTCTCAACTCTCTTATACTTTTTTGTAAAAGAGAGCTTGGACTGTGGAGGCTTGCTGG
CCACTTTTTGGGGTCAGCTCCTCTGAAATGCATTAGCGGAACCGTTTGCAATCTGCCACA
AGTGTGATAAGTTATCTACACTGGCGAGGGGATTGCTCTCTGTAATGTTCAGCTTCTAAT
TGTCTCTACTTTGTGAGACAACTTTTGAATGCTTGACCTCAAATCAGGTAGGACTACCCG
CTGAACTTAA
>B12_42
TTTCCGTAGGTGAACCTGCGGAAGGATCATTATTGAATTATGTTTCTAGATAGGTTGTAG
CTGGCTCTTTAGAGCATGTGCACGCCTGTTTGGACTTCATTTTCATCCACCTGTGCACCT
ATTGTAGTCTTTGGTTGGGTTAGGGGGAAGTGGTCATTGTGTCAGCATCTGCTGGATGTG
AGGACTTGCATTGTGAAAGCTTTGCTGTCCTTGATGTGATCATGGAATCTCTTTCTCACT
AGAGTCTATGTCACTCATTATACTCTGTCGAATGTCATTGAATGTCTTTACATGGGCTTG
TATGCCTATGAAAATTGTAATACAACTTTCAGCAACGGATCTCTTGGCTCTCGCATCGAT
GAAGGACGCAGCGAAATGCGATAAGTAATGTGAATTGCAGAATTCAGTGAATCATCGAAT
CTTTGAACGCATCTTGCGCTCCTTGGTATTCCGAGGAGCATGCCTGTTTGAGTGTCATTA
AATTCTCAACTCTCTTATACTTTTTTGTAAAAGAGAGCTTGGACTGTGGAGGCTTGCTGG
CCACTTTTTGGGGTCAGCTCCTCTGAAATGCATTAGCGGAACCGTTTGCAATCTGCCACA
AGTGTGATAAGTTATCTACACTGGCGAGGGGATTGCTCTCTGTAATGTTCAGCTTCTAAT
TGTCTCTACTTTGTGAGACAACTTTTGAATGCTTGACCTCAAATCAGGTAGGACTACCCG
CTGAACTTAA
>B12_43
TTTCCGTAGGTGAACCTGCGGAAGGATCATTATTGAATTATGTTTCTAGATAGGTTGTAG
CTGGCTCTTTAGAGCATGTGCACGCCTGTTTGGACTTCATTTTCATCCACCTGTGCACCT
ATTGTAGTCTTTGGTTGGGTTAGGGGGAAGTGGTCATTGTGTCAGCATCTGCTGGATGTG
AGGACTTGCATTGTGAAAGCTTTGCTGTCCTTGATGTGATCATGGAATCTCTTTCTCACT
AGAGTCTATGTCACTCATTATACTCTGTCGAATGTCATTGAATGTCTTTACATGGGCTTG
TATGCCTATGAAAATTGTAATACAACTTTCAGCAACGGATCTCTTGGCTCTCGCATCGAT
GAAGGACGCAGCGAAATGCGATAAGTAATGTGAATTGCAGAATTCAGTGAATCATCGAAT
CTTTGAACGCATCTTGCGCTCCTTGGTATTCCGAGGAGCATGCCTGTTTGAGTGTCATTA
AATTCTCAACTCTCTTATACTTTTTTGTAAAAGAGAGCTTGGACTGTGGAGGCTTGCTGG
CCACTTTTTGGGGTCAGCTCCTCTGAAATGCATTAGCGGAACCGTTTGCAATCTGCCACA
AGTGTGATAAGTTATCTACACTGGCGAGGGGATTGCTCTCTGTAATGTTCAGCTTCTAAT
TGTCTCTACTTTGTGAGACAACTTTTGAATGCTTGACCTCAAATCAGGTAGGACTACCCG
CTGAACTTAA
>B12_44
TTTCCGTAGGTGAACCTGCGGAAGGATCATTATTGAATTATGTTTCTAGATAGGTTGTAG
CTGGCTCTTTAGAGCATGTGCACGCCTGTTTGGACTTCATTTTCATCCACCTGTGCACCT
ATTGTAGTCTTTGGTTGGGTTAGGGGGAAGTGGTCATTGTGTCAGCATCTGCTGGATGTG
AGGACTTGCATTGTGAAAGCTTTGCTGTCCTTGATGTGATCATGGAATCTCTTTCTCACT
AGAGTCTATGTCACTCATTATACTCTGTCGAATGTCATTGAATGTCTTTACATGGGCTTG

TATGCCTATGAAAATTGTAATACAACTTTCAGCAACGGATCTCTTGGCTCTCGCATCGAT
GAAGGACGCAGCGAAATGCGATAAGTAATGTGAATTGCAGAATTCAGTGAATCATCGAAT
CTTTGAACGCATCTTGCGCTCCTTGGTATTCCGAGGAGCATGCCTGTTTGAGTGTCATTA
AATTCTCAACTCTCTTATACTTTTTTGTAAAAGAGAGCTTGGACTGTGGAGGCTTGCTGG
CCACTTTTTGGGGTCAGCTCCTCTGAAATGCATTAGCGGAACCGTTTGCAATCTGCCACA
AGTGTGATAAGTTATCTACACTGGCGAGGGGATTGCTCTCTGTAATGTTCAGCTTCTAAT
TGTCTCTACTTTGTGAGACAACTTTTGAATGCTTGACCTCAAATCAGGTAGGACTACCCG
CTGAACTTAA
>B12_45
TTTCCGTAGGTGAACCTGCGGAAGGATCATTATTGAATTATGTTTCTAGATAGGTTGTAG
CTGGCTCTTTAGAGCATGTGCACGCCTGTTTGGACTTCATTTTCATCCACCTGTGCACCT
ATTGTAGTCTTTGGTTGGGTTAGGGGGAAGTGGTCATTGTGTCAGCATCTGCTGGATGTG
AGGACTTGCATTGTGAAAGCTTTGCTGTCCTTGATGTGATCATGGAATCTCTTTCTCACT
AGAGTCTATGTCACTCATTATACTCTGTCGAATGTCATTGAATGTCTTTACATGGGCTTG
TATGCCTATGAAAATTGTAATACAACTTTCAGCAACGGATCTCTTGGCTCTCGCATCGAT
GAAGGACGCAGCGAAATGCGATAAGTAATGTGAATTGCAGAATTCAGTGAATCATCGAAT
CTTTGAACGCATCTTGCGCTCCTTGGTATTCCGAGGAGCATGCCTGTTTGAGTGTCATTA
AATTCTCAACTCTCTTATACTTTTTTGTAAAAGAGAGCTTGGACTGTGGAGGCTTGCTGG
CCACTTTTTGGGGTCAGCTCCTCTGAAATGCATTAGCGGAACCGTTTGCAATCTGCCACA
AGTGTGATAAGTTATCTACACTGGCGAGGGGATTGCTCTCTGTAATGTTCAGCTTCTAAT
TGTCTCTACTTTGTGAGACAACTTTTGAATGCTTGACCTCAAATCAGGTAGGACTACCCG
CTGAACTTAA
>B12_46
TTTCCGTAGGTGAACCTGCGGAAGGATCATTATTGAATTATGTTTCTAGATAGGTTGTAG
CTGGCTCTTTAGAGCATGTGCACGCCTGTTTGGACTTCATTTTCATCCACCTGTGCACCT
ATTGTAGTCTTTGGTTGGGTTAGGGGGAAGTGGTCATTGTGTCAGCATCTGCTGGATGTG
AGGACTTGCATTGTGAAAGCTTTGCTGTCCTTGATGTGATCATGGAATCTCTTTCTCACT
AGAGTCTATGTCACTCATTATACTCTGTCGAATGTCATTGAATGTCTTTACATGGGCTTG
TATGCCTATGAAAATTGTAATACAACTTTCAGCAACGGATCTCTTGGCTCTCGCATCGAT
GAAGGACGCAGCGAAATGCGATAAGTAATGTGAATTGCAGAATTCAGTGAATCATCGAAT
CTTTGAACGCATCTTGCGCTCCTTGGTATTCCGAGGAGCATGCCTGTTTGAGTGTCATTA
AATTCTCAACTCTCTTATACTTTTTTGTAAAAGAGAGCTTGGACTGTGGAGGCTTGCTGG
CCACTTTTTGGGGTCAGCTCCTCTGAAATGCATTAGCGGAACCGTTTGCAATCTGCCACA
AGTGTGATAAGTTATCTACACTGGCGAGGGGATTGCTCTCTGTAATGTTCAGCTTCTAAT
TGTCTCTACTTTGTGAGACAACTTTTGAATGCTTGACCTCAAATCAGGTAGGACTACCCG
CTGAACTTAA
>B12_47
TTTCCGTAGGTGAACCTGCGGAAGGATCATTATTGAATTATGTTTCTAGATAGGTTGTAG
CTGGCTCTTTAGAGCATGTGCACGCCTGTTTGGACTTCATTTTCATCCACCTGTGCACCT
ATTGTAGTCTTTGGTTGGGTTAGGGGGAAGTGGTCATTGTGTCAGCATCTGCTGGATGTG
AGGACTTGCATTGTGAAAGCTTTGCTGTCCTTGATGTGATCATGGAATCTCTTTCTCACT
AGAGTCTATGTCACTCATTATACTCTGTCGAATGTCATTGAATGTCTTTACATGGGCTTG
TATGCCTATGAAAATTGTAATACAACTTTCAGCAACGGATCTCTTGGCTCTCGCATCGAT
GAAGGACGCAGCGAAATGCGATAAGTAATGTGAATTGCAGAATTCAGTGAATCATCGAAT
CTTTGAACGCATCTTGCGCTCCTTGGTATTCCGAGGAGCATGCCTGTTTGAGTGTCATTA
AATTCTCAACTCTCTTATACTTTTTTGTAAAAGAGAGCTTGGACTGTGGAGGCTTGCTGG
CCACTTTTTGGGGTCAGCTCCTCTGAAATGCATTAGCGGAACCGTTTGCAATCTGCCACA
AGTGTGATAAGTTATCTACACTGGCGAGGGGATTGCTCTCTGTAATGTTCAGCTTCTAAT
TGTCTCTACTTTGTGAGACAACTTTTGAATGCTTGACCTCAAATCAGGTAGGACTACCCG
CTGAACTTAA

>B12_48
TTTCCGTAGGTGAACCTGCGGAAGGATCATTATTGAATTATGTTTCTAGATAGGTTGTAG
CTGGCTCTTTAGAGCATGTGCACGCCTGTTTGGACTTCATTTTCATCCACCTGTGCACCT
ATTGTAGTCTTTGGTTGGGTTAGGGGGAAGTGGTCATTGTGTCAGCATCTGCTGGATGTG
AGGACTTGCATTGTGAAAGCTTTGCTGTCCTTGATGTGATCATGGAATCTCTTTCTCACT
AGAGTCTATGTCACTCATTATACTCTGTCGAATGTCATTGAATGTCTTTACATGGGCTTG
TATGCCTATGAAAATTGTAATACAACTTTCAGCAACGGATCTCTTGGCTCTCGCATCGAT
GAAGGACGCAGCGAAATGCGATAAGTAATGTGAATTGCAGAATTCAGTGAATCATCGAAT
CTTTGAACGCATCTTGCGCTCCTTGGTATTCCGAGGAGCATGCCTGTTTGAGTGTCATTA
AATTCTCAACTCTCTTATACTTTTTTGTAAAAGAGAGCTTGGACTGTGGAGGCTTGCTGG
CCACTTTTTGGGGTCAGCTCCTCTGAAATGCATTAGCGGAACCGTTTGCAATCTGCCACA
AGTGTGATAAGTTATCTACACTGGCGAGGGGATTGCTCTCTGTAATGTTCAGCTTCTAAT
TGTCTCTACTTTGTGAGACAACTTTTGAATGCTTGACCTCAAATCAGGTAGGACTACCCG
CTGAACTTAA
>B12_49
TTTCCGTAGGTGAACCTGCGGAAGGATCATTATTGAATTATGTTTCTAGATAGGTTGTAG
CTGGCTCTTTAGAGCATGTGCACGCCTGTTTGGACTTCATTTTCATCCACCTGTGCACCT
ATTGTAGTCTTTGGTTGGGTTAGGGGGAAGTGGTCATTGTGTCAGCATCTGCTGGATGTG
AGGACTTGCATTGTGAAAGCTTTGCTGTCCTTGATGTGATCATGGAATCTCTTTCTCACT
AGAGTCTATGTCACTCATTATACTCTGTCGAATGTCATTGAATGTCTTTACATGGGCTTG
TATGCCTATGAAAATTGTAATACAACTTTCAGCAACGGATCTCTTGGCTCTCGCATCGAT
GAAGGACGCAGCGAAATGCGATAAGTAATGTGAATTGCAGAATTCAGTGAATCATCGAAT
CTTTGAACGCATCTTGCGCTCCTTGGTATTCCGAGGAGCATGCCTGTTTGAGTGTCATTA
AATTCTCAACTCTCTTATACTTTTTTGTAAAAGAGAGCTTGGACTGTGGAGGCTTGCTGG
CCACTTTTTGGGGTCAGCTCCTCTGAAATGCATTAGCGGAACCGTTTGCAATCTGCCACA
AGTGTGATAAGTTATCTACACTGGCGAGGGGATTGCTCTCTGTAATGTTCAGCTTCTAAT
TGTCTCTACTTTGTGAGACAACTTTTGAATGCTTGACCTCAAATCAGGTAGGACTACCCG
CTGAACTTAA
>B12_50
TTTCCGTAGGTGAACCTGCGGAAGGATCATTATTGAATTATGTTTCTAGATAGGTTGTAG
CTGGCTCTTTAGAGCATGTGCACGCCTGTTTGGACTTCATTTTCATCCACCTGTGCACCT
ATTGTAGTCTTTGGTTGGGTTAGGGGGAAGTGGTCATTGTGTCAGCATCTGCTGGATGTG
AGGACTTGCATTGTGAAAGCTTTGCTGTCCTTGATGTGATCATGGAATCTCTTTCTCACT
AGAGTCTATGTCACTCATTATACTCTGTCGAATGTCATTGAATGTCTTTACATGGGCTTG
TATGCCTATGAAAATTGTAATACAACTTTCAGCAACGGATCTCTTGGCTCTCGCATCGAT
GAAGGACGCAGCGAAATGCGATAAGTAATGTGAATTGCAGAATTCAGTGAATCATCGAAT
CTTTGAACGCATCTTGCGCTCCTTGGTATTCCGAGGAGCATGCCTGTTTGAGTGTCATTA
AATTCTCAACTCTCTTATACTTTTTTGTAAAAGAGAGCTTGGACTGTGGAGGCTTGCTGG
CCACTTTTTGGGGTCAGCTCCTCTGAAATGCATTAGCGGAACCGTTTGCAATCTGCCACA
AGTGTGATAAGTTATCTACACTGGCGAGGGGATTGCTCTCTGTAATGTTCAGCTTCTAAT
TGTCTCTACTTTGTGAGACAACTTTTGAATGCTTGACCTCAAATCAGGTAGGACTACCCG
CTGAACTTAA
>B8_1
TTTCCGTAGGTGAACCTGCGGAAGGATCATTATTGAATTATGTTTCTAGATAGGTTGTAG
CTGGCTCTTTAGAGCATGTGCACGCCTGTTTGGACTTCATTTTCATCCACCTGTGCACCT
ATTGTAGTCTTTGGTTGGGTTAGGGGGAAGTGGTCATTGTGTCAGCATCTGCTGGATGTG
AGGACTTGCATTGTGAAAGCTTTGCTGTCCTTGATGTGATCATGGAATCTCTTTCTCACT
AGAGTCTATGTCACTCATTATACTCTGTCGAATGTCATTGAATGTCTTTACATGGGCTTG
TATGCCTATGAAAATTGTAATACAACTTTCAGCAACGGATCTCTTGGCTCTCGCATCGAT
GAAGGACGCAGCGAAATGCGATAAGTAATGTGAATTGCAGAATTCAGTGAATCATCGAAT

CTTTGAACGCATCTTGCGCTCCTTGGTATTCCGAGGAGCATGCCTGTTTGAGTGTCATTA
AATTCTCAACTCTCTTATACTTTTTTGTAAAAGAGAGCTTGGACTGTGGAGGCTTGCTGG
CCACTTTTTGGGGTCAGCTCCTCTGAAATGCATTAGCGGAACCGTTTGCAATCTGCCACA
AGTGTGATAAGTTATCTACACTGGCGAGGGGATTGCTCTCTGTAATGTTCAGCTTCTAAT
TGTCTCTACTTTGTGAGACAACTTTTGAATGCTTGACCTCAAATCAGGTAGGACTACCCG
CTGAACTTAA
>B8_2
TTTCCGTAGGTGAACCTGCGGAAGGATCATTATTGAATTATGTTTCTAGATAGGTTGTAG
CTGGCTCTTTAGAGCATGTGCACGCCTGTTTGGACTTCATTTTCATCCACCTGTGCACCT
ATTGTAGTCTTTGGTTGGGTTAGGGGGAAGTGGTCATTGTGTCAGCATCTGCTGGATGTG
AGGACTTGCATTGTGAAAGCTTTGCTGTCCTTGATGTGATCATGGAATCTCTTTCTCACT
AGAGTCTATGTCACTCATTATACTCTGTCGAATGTCATTGAATGTCTTTACATGGGCTTG
TATGCCTATGAAAATTGTAATACAACTTTCAGCAACGGATCTCTTGGCTCTCGCATCGAT
GAAGGACGCAGCGAAATGCGATAAGTAATGTGAATTGCAGAATTCAGTGAATCATCGAAT
CTTTGAACGCATCTTGCGCTCCTTGGTATTCCGAGGAGCATGCCTGTTTGAGTGTCATTA
AATTCTCAACTCTCTTATACTTTTTTGTAAAAGAGAGCTTGGACTGTGGAGGCTTGCTGG
CCACTTTTTGGGGTCAGCTCCTCTGAAATGCATTAGCGGAACCGTTTGCAATCTGCCACA
AGTGTGATAAGTTATCTACACTGGCGAGGGGATTGCTCTCTGTAATGTTCAGCTTCTAAT
TGTCTCTACTTTGTGAGACAACTTTTGAATGCTTGACCTCAAATCAGGTAGGACTACCCG
CTGAACTTAA
>B8_3
TTTCCGTAGGTGAACCTGCGGAAGGATCATTATTGAATTATGTTTCTAGATAGGTTGTAG
CTGGCTCTTTAGAGCATGTGCACGCCTGTTTGGACTTCATTTTCATCCACCTGTGCACCT
ATTGTAGTCTTTGGTTGGGTTAGGGGGAAGTGGTCATTGTGTCAGCATCTGCTGGATGTG
AGGACTTGCATTGTGAAAGCTTTGCTGTCCTTGATGTGATCATGGAATCTCTTTCTCACT
AGAGTCTATGTCACTCATTATACTCTGTCGAATGTCATTGAATGTCTTTACATGGGCTTG
TATGCCTATGAAAATTGTAATACAACTTTCAGCAACGGATCTCTTGGCTCTCGCATCGAT
GAAGGACGCAGCGAAATGCGATAAGTAATGTGAATTGCAGAATTCAGTGAATCATCGAAT
CTTTGAACGCATCTTGCGCTCCTTGGTATTCCGAGGAGCATGCCTGTTTGAGTGTCATTA
AATTCTCAACTCTCTTATACTTTTTTGTAAAAGAGAGCTTGGACTGTGGAGGCTTGCTGG
CCACTTTTTGGGGTCAGCTCCTCTGAAATGCATTAGCGGAACCGTTTGCAATCTGCCACA
AGTGTGATAAGTTATCTACACTGGCGAGGGGATTGCTCTCTGTAATGTTCAGCTTCTAAT
TGTCTCTACTTTGTGAGACAACTTTTGAATGCTTGACCTCAAATCAGGTAGGACTACCCG
CTGAACTTAA
>B8_4
TTTCCGTAGGTGAACCTGCGGAAGGATCATTATTGAATTATGTTTCTAGATAGGTTGTAG
CTGGCTCTTTAGAGCATGTGCACGCCTGTTTGGACTTCATTTTCATCCACCTGTGCACCT
ATTGTAGTCTTTGGTTGGGTTAGGGGGAAGTGGTCATTGTGTCAGCATCTGCTGGATGTG
AGGACTTGCATTGTGAAAGCTTTGCTGTCCTTGATGTGATCATGGAATCTCTTTCTCACT
AGAGTCTATGTCACTCATTATACTCTGTCGAATGTCATTGAATGTCTTTACATGGGCTTG
TATGCCTATGAAAATTGTAATACAACTTTCAGCAACGGATCTCTTGGCTCTCGCATCGAT
GAAGGACGCAGCGAAATGCGATAAGTAATGTGAATTGCAGAATTCAGTGAATCATCGAAT
CTTTGAACGCATCTTGCGCTCCTTGGTATTCCGAGGAGCATGCCTGTTTGAGTGTCATTA
AATTCTCAACTCTCTTATACTTTTTTGTAAAAGAGAGCTTGGACTGTGGAGGCTTGCTGG
CCACTTTTTGGGGTCAGCTCCTCTGAAATGCATTAGCGGAACCGTTTGCAATCTGCCACA
AGTGTGATAAGTTATCTACACTGGCGAGGGGATTGCTCTCTGTAATGTTCAGCTTCTAAT
TGTCTCTACTTTGTGAGACAACTTTTGAATGCTTGACCTCAAATCAGGTAGGACTACCCG
CTGAACTTAA
>B8_5
TTTCCGTAGGTGAACCTGCGGAAGGATCATTATTGAATTATGTTTCTAGATAGGTTGTAG

CTGGCTCTTTAGAGCATGTGCACGCCTGTTTGGACTTCATTTTCATCCACCTGTGCACCT
ATTGTAGTCTTTGGTTGGGTTAGGGGGAAGTGGTCATTGTGTCAGCATCTGCTGGATGTG
AGGACTTGCATTGTGAAAGCTTTGCTGTCCTTGATGTGATCATGGAATCTCTTTCTCACT
AGAGTCTATGTCACTCATTATACTCTGTCGAATGTCATTGAATGTCTTTACATGGGCTTG
TATGCCTATGAAAATTGTAATACAACTTTCAGCAACGGATCTCTTGGCTCTCGCATCGAT
GAAGGACGCAGCGAAATGCGATAAGTAATGTGAATTGCAGAATTCAGTGAATCATCGAAT
CTTTGAACGCATCTTGCGCTCCTTGGTATTCCGAGGAGCATGCCTGTTTGAGTGTCATTA
AATTCTCAACTCTCTTATACTTTTTTGTAAAAGAGAGCTTGGACTGTGGAGGCTTGCTGG
CCACTTTTTGGGGTCAGCTCCTCTGAAATGCATTAGCGGAACCGTTTGCAATCTGCCACA
AGTGTGATAAGTTATCTACACTGGCGAGGGGATTGCTCTCTGTAATGTTCAGCTTCTAAT
TGTCTCTACTTTGTGAGACAACTTTTGAATGCTTGACCTCAAATCAGGTAGGACTACCCG
CTGAACTTAA
>B8_6
TTTCCGTAGGTGAACCTGCGGAAGGATCATTATTGAATTATGTTTCTAGATAGGTTGTAG
CTGGCTCTTTAGAGCATGTGCACGCCTGTTTGGACTTCATTTTCATCCACCTGTGCACCT
ATTGTAGTCTTTGGTTGGGTTAGGGGGAAGTGGTCATTGTGTCAGCATCTGCTGGATGTG
AGGACTTGCATTGTGAAAGCTTTGCTGTCCTTGATGTGATCATGGAATCTCTTTCTCACT
AGAGTCTATGTCACTCATTATACTCTGTCGAATGTCATTGAATGTCTTTACATGGGCTTG
TATGCCTATGAAAATTGTAATACAACTTTCAGCAACGGATCTCTTGGCTCTCGCATCGAT
GAAGGACGCAGCGAAATGCGATAAGTAATGTGAATTGCAGAATTCAGTGAATCATCGAAT
CTTTGAACGCATCTTGCGCTCCTTGGTATTCCGAGGAGCATGCCTGTTTGAGTGTCATTA
AATTCTCAACTCTCTTATACTTTTTTGTAAAAGAGAGCTTGGACTGTGGAGGCTTGCTGG
CCACTTTTTGGGGTCAGCTCCTCTGAAATGCATTAGCGGAACCGTTTGCAATCTGCCACA
AGTGTGATAAGTTATCTACACTGGCGAGGGGATTGCTCTCTGTAATGTTCAGCTTCTAAT
TGTCTCTACTTTGTGAGACAACTTTTGAATGCTTGACCTCAAATCAGGTAGGACTACCCG
CTGAACTTAA
>B8_8
TTTCCGTAGGTGAACCTGCGGAAGGATCATTATTGAATTATGTTTCTAGATAGGTTGTAG
CTGGCTCTTTAGAGCATGTGCACGCCTGTTTGGACTTCATTTTCATCCACCTGTGCACCT
ATTGTAGTCTTTGGTTGGGTTAGGGGGAAGTGGTCATTGTGTCAGCATCTGCTGGATGTG
AGGACTTGCATTGTGAAAGCTTTGCTGTCCTTGATGTGATCATGGAATCTCTTTCTCACT
AGAGTCTATGTCACTCATTATACTCTGTCGAATGTCATTGAATGTCTTTACATGGGCTTG
TATGCCTATGAAAATTGTAATACAACTTTCAGCAACGGATCTCTTGGCTCTCGCATCGAT
GAAGGACGCAGCGAAATGCGATAAGTAATGTGAATTGCAGAATTCAGTGAATCATCGAAT
CTTTGAACGCATCTTGCGCTCCTTGGTATTCCGAGGAGCATGCCTGTTTGAGTGTCATTA
AATTCTCAACTCTCTTATACTTTTTTGTAAAAGAGAGCTTGGACTGTGGAGGCTTGCTGG
CCACTTTTTGGGGTCAGCTCCTCTGAAATGCATTAGCGGAACCGTTTGCAATCTGCCACA
AGTGTGATAAGTTATCTACACTGGCGAGGGGATTGCTCTCTGTAATGTTCAGCTTCTAAT
TGTCTCTACTTTGTGAGACAACTTTTGAATGCTTGACCTCAAATCAGGTAGGACTACCCG
CTGAACTTAA
>B8_9
TTTCCGTAGGTGAACCTGCGGAAGGATCATTATTGAATTATGTTTCTAGATAGGTTGTAG
CTGGCTCTTTAGAGCATGTGCACGCCTGTTTGGACTTCATTTTCATCCACCTGTGCACCT
ATTGTAGTCTTTGGTTGGGTTAGGGGGAAGTGGTCATTGTGTCAGCATCTGCTGGATGTG
AGGACTTGCATTGTGAAAGCTTTGCTGTCCTTGATGTGATCATGGAATCTCTTTCTCACT
AGAGTCTATGTCACTCATTATACTCTGTCGAATGTCATTGAATGTCTTTACATGGGCTTG
TATGCCTATGAAAATTGTAATACAACTTTCAGCAACGGATCTCTTGGCTCTCGCATCGAT
GAAGGACGCAGCGAAATGCGATAAGTAATGTGAATTGCAGAATTCAGTGAATCATCGAAT
CTTTGAACGCATCTTGCGCTCCTTGGTATTCCGAGGAGCATGCCTGTTTGAGTGTCATTA
AATTCTCAACTCTCTTATACTTTTTTGTAAAAGAGAGCTTGGACTGTGGAGGCTTGCTGG

CCACTTTTTGGGGTCAGCTCCTCTGAAATGCATTAGCGGAACCGTTTGCAATCTGCCACA
AGTGTGATAAGTTATCTACACTGGCGAGGGGATTGCTCTCTGTAATGTTCAGCTTCTAAT
TGTCTCTACTTTGTGAGACAACTTTTGAATGCTTGACCTCAAATCAGGTAGGACTACCCG
CTGAACTTAA
>B8_10
TTTCCGTAGGTGAACCTGCGGAAGGATCATTATTGAATTATGTTTCTAGATAGGTTGTAG
CTGGCTCTTTAGAGCATGTGCACGCCTGTTTGGACTTCATTTTCATCCACCTGTGCACCT
ATTGTAGTCTTTGGTTGGGTTAGGGGGAAGTGGTCATTGTGTCAGCATCTGCTGGATGTG
AGGACTTGCATTGTGAAAGCTTTGCTGTCCTTGATGTGATCATGGAATCTCTTTCTCACT
AGAGTCTATGTCACTCATTATACTCTGTCGAATGTCATTGAATGTCTTTACATGGGCTTG
TATGCCTATGAAAATTGTAATACAACTTTCAGCAACGGATCTCTTGGCTCTCGCATCGAT
GAAGGACGCAGCGAAATGCGATAAGTAATGTGAATTGCAGAATTCAGTGAATCATCGAAT
CTTTGAACGCATCTTGCGCTCCTTGGTATTCCGAGGAGCATGCCTGTTTGAGTGTCATTA
AATTCTCAACTCTCTTATACTTTTTTGTAAAAGAGAGCTTGGACTGTGGAGGCTTGCTGG
CCACTTTTTGGGGTCAGCTCCTCTGAAATGCATTAGCGGAACCGTTTGCAATCTGCCACA
AGTGTGATAAGTTATCTACACTGGCGAGGGGATTGCTCTCTGTAATGTTCAGCTTCTAAT
TGTCTCTACTTTGTGAGACAACTTTTGAATGCTTGACCTCAAATCAGGTAGGACTACCCG
CTGAACTTAA
>B8_11
TTTCCGTAGGTGAACCTGCGGAAGGATCATTATTGAATTATGTTTCTAGATAGGTTGTAG
CTGGCTCTTTAGAGCATGTGCACGCCTGTTTGGACTTCATTTTCATCCACCTGTGCACCT
ATTGTAGTCTTTGGTTGGGTTAGGGGGAAGTGGTCATTGTGTCAGCATCTGCTGGATGTG
AGGACTTGCATTGTGAAAGCTTTGCTGTCCTTGATGTGATCATGGAATCTCTTTCTCACT
AGAGTCTATGTCACTCATTATACTCTGTCGAATGTCATTGAATGTCTTTACATGGGCTTG
TATGCCTATGAAAATTGTAATACAACTTTCAGCAACGGATCTCTTGGCTCTCGCATCGAT
GAAGGACGCAGCGAAATGCGATAAGTAATGTGAATTGCAGAATTCAGTGAATCATCGAAT
CTTTGAACGCATCTTGCGCTCCTTGGTATTCCGAGGAGCATGCCTGTTTGAGTGTCATTA
AATTCTCAACTCTCTTATACTTTTTTGTAAAAGAGAGCTTGGACTGTGGAGGCTTGCTGG
CCACTTTTTGGGGTCAGCTCCTCTGAAATGCATTAGCGGAACCGTTTGCAATCTGCCACA
AGTGTGATAAGTTATCTACACTGGCGAGGGGATTGCTCTCTGTAATGTTCAGCTTCTAAT
TGTCTCTACTTTGTGAGACAACTTTTGAATGCTTGACCTCAAATCAGGTAGGACTACCCG
CTGAACTTAA
>B8_12
TTTCCGTAGGTGAACCTGCGGAAGGATCATTATTGAATTATGTTTCTAGATAGGTTGTAG
CTGGCTCTTTAGAGCATGTGCACGCCTGTTTGGACTTCATTTTCATCCACCTGTGCACCT
ATTGTAGTCTTTGGTTGGGTTAGGGGGAAGTGGTCATTGTGTCAGCATCTGCTGGATGTG
AGGACTTGCATTGTGAAAGCTTTGCTGTCCTTGATGTGATCATGGAATCTCTTTCTCACT
AGAGTCTATGTCACTCATTATACTCTGTCGAATGTCATTGAATGTCTTTACATGGGCTTG
TATGCCTATGAAAATTGTAATACAACTTTCAGCAACGGATCTCTTGGCTCTCGCATCGAT
GAAGGACGCAGCGAAATGCGATAAGTAATGTGAATTGCAGAATTCAGTGAATCATCGAAT
CTTTGAACGCATCTTGCGCTCCTTGGTATTCCGAGGAGCATGCCTGTTTGAGTGTCATTA
AATTCTCAACTCTCTTATACTTTTTTGTAAAAGAGAGCTTGGACTGTGGAGGCTTGCTGG
CCACTTTTTGGGGTCAGCTCCTCTGAAATGCATTAGCGGAACCGTTTGCAATCTGCCACA
AGTGTGATAAGTTATCTACACTGGCGAGGGGATTGCTCTCTGTAATGTTCAGCTTCTAAT
TGTCTCTACTTTGTGAGACAACTTTTGAATGCTTGACCTCAAATCAGGTAGGACTACCCG
CTGAACTTAA
>B8_13
TTTCCGTAGGTGAACCTGCGGAAGGATCATTATTGAATTATGTTTCTAGATAGGTTGTAG
CTGGCTCTTTAGAGCATGTGCACGCCTGTTTGGACTTCATTTTCATCCACCTGTGCACCT
ATTGTAGTCTTTGGTTGGGTTAGGGGGAAGTGGTCATTGTGTCAGCATCTGCTGGATGTG

AGGACTTGCATTGTGAAAGCTTTGCTGTCCTTGATGTGATCATGGAATCTCTTTCTCACT
AGAGTCTATGTCACTCATTATACTCTGTCGAATGTCATTGAATGTCTTTACATGGGCTTG
TATGCCTATGAAAATTGTAATACAACTTTCAGCAACGGATCTCTTGGCTCTCGCATCGAT
GAAGGACGCAGCGAAATGCGATAAGTAATGTGAATTGCAGAATTCAGTGAATCATCGAAT
CTTTGAACGCATCTTGCGCTCCTTGGTATTCCGAGGAGCATGCCTGTTTGAGTGTCATTA
AATTCTCAACTCTCTTATACTTTTTTGTAAAAGAGAGCTTGGACTGTGGAGGCTTGCTGG
CCACTTTTTGGGGTCAGCTCCTCTGAAATGCATTAGCGGAACCGTTTGCAATCTGCCACA
AGTGTGATAAGTTATCTACACTGGCGAGGGGATTGCTCTCTGTAATGTTCAGCTTCTAAT
TGTCTCTACTTTGTGAGACAACTTTTGAATGCTTGACCTCAAATCAGGTAGGACTACCCG
CTGAACTTAA
>B8_14
TTTCCGTAGGTGAACCTGCGGAAGGATCATTATTGAATTATGTTTCTAGATAGGTTGTAG
CTGGCTCTTTAGAGCATGTGCACGCCTGTTTGGACTTCATTTTCATCCACCTGTGCACCT
ATTGTAGTCTTTGGTTGGGTTAGGGGGAAGTGGTCATTGTGTCAGCATCTGCTGGATGTG
AGGACTTGCATTGTGAAAGCTTTGCTGTCCTTGATGTGATCATGGAATCTCTTTCTCACT
AGAGTCTATGTCACTCATTATACTCTGTCGAATGTCATTGAATGTCTTTACATGGGCTTG
TATGCCTATGAAAATTGTAATACAACTTTCAGCAACGGATCTCTTGGCTCTCGCATCGAT
GAAGGACGCAGCGAAATGCGATAAGTAATGTGAATTGCAGAATTCAGTGAATCATCGAAT
CTTTGAACGCATCTTGCGCTCCTTGGTATTCCGAGGAGCATGCCTGTTTGAGTGTCATTA
AATTCTCAACTCTCTTATACTTTTTTGTAAAAGAGAGCTTGGACTGTGGAGGCTTGCTGG
CCACTTTTTGGGGTCAGCTCCTCTGAAATGCATTAGCGGAACCGTTTGCAATCTGCCACA
AGTGTGATAAGTTATCTACACTGGCGAGGGGATTGCTCTCTGTAATGTTCAGCTTCTAAT
TGTCTCTACTTTGTGAGACAACTTTTGAATGCTTGACCTCAAATCAGGTAGGACTACCCG
CTGAACTTAA
>B8_16
TTTCCGTAGGTGAACCTGCGGAAGGATCATTATTGAATTATGTTTCTAGATAGGTTGTAG
CTGGCTCTTTAGAGCATGTGCACGCCTGTTTGGACTTCATTTTCATCCACCTGTGCACCT
ATTGTAGTCTTTGGTTGGGTTAGGGGGAAGTGGTCATTGTGTCAGCATCTGCTGGATGTG
AGGACTTGCATTGTGAAAGCTTTGCTGTCCTTGATGTGATCATGGAATCTCTTTCTCACT
AGAGTCTATGTCACTCATTATACTCTGTCGAATGTCATTGAATGTCTTTACATGGGCTTG
TATGCCTATGAAAATTGTAATACAACTTTCAGCAACGGATCTCTTGGCTCTCGCATCGAT
GAAGGACGCAGCGAAATGCGATAAGTAATGTGAATTGCAGAATTCAGTGAATCATCGAAT
CTTTGAACGCATCTTGCGCTCCTTGGTATTCCGAGGAGCATGCCTGTTTGAGTGTCATTA
AATTCTCAACTCTCTTATACTTTTTTGTAAAAGAGAGCTTGGACTGTGGAGGCTTGCTGG
CCACTTTTTGGGGTCAGCTCCTCTGAAATGCATTAGCGGAACCGTTTGCAATCTGCCACA
AGTGTGATAAGTTATCTACACTGGCGAGGGGATTGCTCTCTGTAATGTTCAGCTTCTAAT
TGTCTCTACTTTGTGAGACAACTTTTGAATGCTTGACCTCAAATCAGGTAGGACTACCCG
CTGAACTTAA
>B8_17
TTTCCGTAGGTGAACCTGCGGAAGGATCATTATTGAATTATGTTTCTAGATAGGTTGTAG
CTGGCTCTTTAGAGCATGTGCACGCCTGTTTGGACTTCATTTTCATCCACCTGTGCACCT
ATTGTAGTCTTTGGTTGGGTTAGGGGGAAGTGGTCATTGTGTCAGCATCTGCTGGATGTG
AGGACTTGCATTGTGAAAGCTTTGCTGTCCTTGATGTGATCATGGAATCTCTTTCTCACT
AGAGTCTATGTCACTCATTATACTCTGTCGAATGTCATTGAATGTCTTTACATGGGCTTG
TATGCCTATGAAAATTGTAATACAACTTTCAGCAACGGATCTCTTGGCTCTCGCATCGAT
GAAGGACGCAGCGAAATGCGATAAGTAATGTGAATTGCAGAATTCAGTGAATCATCGAAT
CTTTGAACGCATCTTGCGCTCCTTGGTATTCCGAGGAGCATGCCTGTTTGAGTGTCATTA
AATTCTCAACTCTCTTATACTTTTTTGTAAAAGAGAGCTTGGACTGTGGAGGCTTGCTGG
CCACTTTTTGGGGTCAGCTCCTCTGAAATGCATTAGCGGAACCGTTTGCAATCTGCCACA
AGTGTGATAAGTTATCTACACTGGCGAGGGGATTGCTCTCTGTAATGTTCAGCTTCTAAT

TGTCTCTACTTTGTGAGACAACTTTTGAATGCTTGACCTCAAATCAGGTAGGACTACCCG
CTGAACTTAA
>B8_18
TTTCCGTAGGTGAACCTGCGGAAGGATCATTATTGAATTATGTTTCTAGATAGGTTGTAG
CTGGCTCTTTAGAGCATGTGCACGCCTGTTTGGACTTCATTTTCATCCACCTGTGCACCT
ATTGTAGTCTTTGGTTGGGTTAGGGGGAAGTGGTCATTGTGTCAGCATCTGCTGGATGTG
AGGACTTGCATTGTGAAAGCTTTGCTGTCCTTGATGTGATCATGGAATCTCTTTCTCACT
AGAGTCTATGTCACTCATTATACTCTGTCGAATGTCATTGAATGTCTTTACATGGGCTTG
TATGCCTATGAAAATTGTAATACAACTTTCAGCAACGGATCTCTTGGCTCTCGCATCGAT
GAAGGACGCAGCGAAATGCGATAAGTAATGTGAATTGCAGAATTCAGTGAATCATCGAAT
CTTTGAACGCATCTTGCGCTCCTTGGTATTCCGAGGAGCATGCCTGTTTGAGTGTCATTA
AATTCTCAACTCTCTTATACTTTTTTGTAAAAGAGAGCTTGGACTGTGGAGGCTTGCTGG
CCACTTTTTGGGGTCAGCTCCTCTGAAATGCATTAGCGGAACCGTTTGCAATCTGCCACA
AGTGTGATAAGTTATCTACACTGGCGAGGGGATTGCTCTCTGTAATGTTCAGCTTCTAAT
TGTCTCTACTTTGTGAGACAACTTTTGAATGCTTGACCTCAAATCAGGTAGGACTACCCG
CTGAACTTAA
>B8_19
TTTCCGTAGGTGAACCTGCGGAAGGATCATTATTGAATTATGTTTCTAGATAGGTTGTAG
CTGGCTCTTTAGAGCATGTGCACGCCTGTTTGGACTTCATTTTCATCCACCTGTGCACCT
ATTGTAGTCTTTGGTTGGGTTAGGGGGAAGTGGTCATTGTGTCAGCATCTGCTGGATGTG
AGGACTTGCATTGTGAAAGCTTTGCTGTCCTTGATGTGATCATGGAATCTCTTTCTCACT
AGAGTCTATGTCACTCATTATACTCTGTCGAATGTCATTGAATGTCTTTACATGGGCTTG
TATGCCTATGAAAATTGTAATACAACTTTCAGCAACGGATCTCTTGGCTCTCGCATCGAT
GAAGGACGCAGCGAAATGCGATAAGTAATGTGAATTGCAGAATTCAGTGAATCATCGAAT
CTTTGAACGCATCTTGCGCTCCTTGGTATTCCGAGGAGCATGCCTGTTTGAGTGTCATTA
AATTCTCAACTCTCTTATACTTTTTTGTAAAAGAGAGCTTGGACTGTGGAGGCTTGCTGG
CCACTTTTTGGGGTCAGCTCCTCTGAAATGCATTAGCGGAACCGTTTGCAATCTGCCACA
AGTGTGATAAGTTATCTACACTGGCGAGGGGATTGCTCTCTGTAATGTTCAGCTTCTAAT
TGTCTCTACTTTGTGAGACAACTTTTGAATGCTTGACCTCAAATCAGGTAGGACTACCCG
CTGAACTTAA
>B8_20
TTTCCGTAGGTGAACCTGCGGAAGGATCATTATTGAATTATGTTTCTAGATAGGTTGTAG
CTGGCTCTTTAGAGCATGTGCACGCCTGTTTGGACTTCATTTTCATCCACCTGTGCACCT
ATTGTAGTCTTTGGTTGGGTTAGGGGGAAGTGGTCATTGTGTCAGCATCTGCTGGATGTG
AGGACTTGCATTGTGAAAGCTTTGCTGTCCTTGATGTGATCATGGAATCTCTTTCTCACT
AGAGTCTATGTCACTCATTATACTCTGTCGAATGTCATTGAATGTCTTTACATGGGCTTG
TATGCCTATGAAAATTGTAATACAACTTTCAGCAACGGATCTCTTGGCTCTCGCATCGAT
GAAGGACGCAGCGAAATGCGATAAGTAATGTGAATTGCAGAATTCAGTGAATCATCGAAT
CTTTGAACGCATCTTGCGCTCCTTGGTATTCCGAGGAGCATGCCTGTTTGAGTGTCATTA
AATTCTCAACTCTCTTATACTTTTTTGTAAAAGAGAGCTTGGACTGTGGAGGCTTGCTGG
CCACTTTTTGGGGTCAGCTCCTCTGAAATGCATTAGCGGAACCGTTTGCAATCTGCCACA
AGTGTGATAAGTTATCTACACTGGCGAGGGGATTGCTCTCTGTAATGTTCAGCTTCTAAT
TGTCTCTACTTTGTGAGACAACTTTTGAATGCTTGACCTCAAATCAGGTAGGACTACCCG
CTGAACTTAA
>B8_21
TTTCCGTAGGTGAACCTGCGGAAGGATCATTATTGAATTATGTTTCTAGATAGGTTGTAG
CTGGCTCTTTAGAGCATGTGCACGCCTGTTTGGACTTCATTTTCATCCACCTGTGCACCT
ATTGTAGTCTTTGGTTGGGTTAGGGGGAAGTGGTCATTGTGTCAGCATCTGCTGGATGTG
AGGACTTGCATTGTGAAAGCTTTGCTGTCCTTGATGTGATCATGGAATCTCTTTCTCACT
AGAGTCTATGTCACTCATTATACTCTGTCGAATGTCATTGAATGTCTTTACATGGGCTTG

TATGCCTATGAAAATTGTAATACAACTTTCAGCAACGGATCTCTTGGCTCTCGCATCGAT
GAAGGACGCAGCGAAATGCGATAAGTAATGTGAATTGCAGAATTCAGTGAATCATCGAAT
CTTTGAACGCATCTTGCGCTCCTTGGTATTCCGAGGAGCATGCCTGTTTGAGTGTCATTA
AATTCTCAACTCTCTTATACTTTTTTGTAAAAGAGAGCTTGGACTGTGGAGGCTTGCTGG
CCACTTTTTGGGGTCAGCTCCTCTGAAATGCATTAGCGGAACCGTTTGCAATCTGCCACA
AGTGTGATAAGTTATCTACACTGGCGAGGGGATTGCTCTCTGTAATGTTCAGCTTCTAAT
TGTCTCTACTTTGTGAGACAACTTTTGAATGCTTGACCTCAAATCAGGTAGGACTACCCG
CTGAACTTAA
>B8_22
TTTCCGTAGGTGAACCTGCGGAAGGATCATTATTGAATTATGTTTCTAGATAGGTTGTAG
CTGGCTCTTTAGAGCATGTGCACGCCTGTTTGGACTTCATTTTCATCCACCTGTGCACCT
ATTGTAGTCTTTGGTTGGGTTAGGGGGAAGTGGTCATTGTGTCAGCATCTGCTGGATGTG
AGGACTTGCATTGTGAAAGCTTTGCTGTCCTTGATGTGATCATGGAATCTCTTTCTCACT
AGAGTCTATGTCACTCATTATACTCTGTCGAATGTCATTGAATGTCTTTACATGGGCTTG
TATGCCTATGAAAATTGTAATACAACTTTCAGCAACGGATCTCTTGGCTCTCGCATCGAT
GAAGGACGCAGCGAAATGCGATAAGTAATGTGAATTGCAGAATTCAGTGAATCATCGAAT
CTTTGAACGCATCTTGCGCTCCTTGGTATTCCGAGGAGCATGCCTGTTTGAGTGTCATTA
AATTCTCAACTCTCTTATACTTTTTTGTAAAAGAGAGCTTGGACTGTGGAGGCTTGCTGG
CCACTTTTTGGGGTCAGCTCCTCTGAAATGCATTAGCGGAACCGTTTGCAATCTGCCACA
AGTGTGATAAGTTATCTACACTGGCGAGGGGATTGCTCTCTGTAATGTTCAGCTTCTAAT
TGTCTCTACTTTGTGAGACAACTTTTGAATGCTTGACCTCAAATCAGGTAGGACTACCCG
CTGAACTTAA
>B8_23
TTTCCGTAGGTGAACCTGCGGAAGGATCATTATTGAATTATGTTTCTAGATAGGTTGTAG
CTGGCTCTTTAGAGCATGTGCACGCCTGTTTGGACTTCATTTTCATCCACCTGTGCACCT
ATTGTAGTCTTTGGTTGGGTTAGGGGGAAGTGGTCATTGTGTCAGCATCTGCTGGATGTG
AGGACTTGCATTGTGAAAGCTTTGCTGTCCTTGATGTGATCATGGAATCTCTTTCTCACT
AGAGTCTATGTCACTCATTATACTCTGTCGAATGTCATTGAATGTCTTTACATGGGCTTG
TATGCCTATGAAAATTGTAATACAACTTTCAGCAACGGATCTCTTGGCTCTCGCATCGAT
GAAGGACGCAGCGAAATGCGATAAGTAATGTGAATTGCAGAATTCAGTGAATCATCGAAT
CTTTGAACGCATCTTGCGCTCCTTGGTATTCCGAGGAGCATGCCTGTTTGAGTGTCATTA
AATTCTCAACTCTCTTATACTTTTTTGTAAAAGAGAGCTTGGACTGTGGAGGCTTGCTGG
CCACTTTTTGGGGTCAGCTCCTCTGAAATGCATTAGCGGAACCGTTTGCAATCTGCCACA
AGTGTGATAAGTTATCTACACTGGCGAGGGGATTGCTCTCTGTAATGTTCAGCTTCTAAT
TGTCTCTACTTTGTGAGACAACTTTTGAATGCTTGACCTCAAATCAGGTAGGACTACCCG
CTGAACTTAA
>B8_25
TTTCCGTAGGTGAACCTGCGGAAGGATCATTATTGAATTATGTTTCTAGATAGGTTGTAG
CTGGCTCTTTAGAGCATGTGCACGCCTGTTTGGACTTCATTTTCATCCACCTGTGCACCT
ATTGTAGTCTTTGGTTGGGTTAGGGGGAAGTGGTCATTGTGTCAGCATCTGCTGGATGTG
AGGACTTGCATTGTGAAAGCTTTGCTGTCCTTGATGTGATCATGGAATCTCTTTCTCACT
AGAGTCTATGTCACTCATTATACTCTGTCGAATGTCATTGAATGTCTTTACATGGGCTTG
TATGCCTATGAAAATTGTAATACAACTTTCAGCAACGGATCTCTTGGCTCTCGCATCGAT
GAAGGACGCAGCGAAATGCGATAAGTAATGTGAATTGCAGAATTCAGTGAATCATCGAAT
CTTTGAACGCATCTTGCGCTCCTTGGTATTCCGAGGAGCATGCCTGTTTGAGTGTCATTA
AATTCTCAACTCTCTTATACTTTTTTGTAAAAGAGAGCTTGGACTGTGGAGGCTTGCTGG
CCACTTTTTGGGGTCAGCTCCTCTGAAATGCATTAGCGGAACCGTTTGCAATCTGCCACA
AGTGTGATAAGTTATCTACACTGGCGAGGGGATTGCTCTCTGTAATGTTCAGCTTCTAAT
TGTCTCTACTTTGTGAGACAACTTTTGAATGCTTGACCTCAAATCAGGTAGGACTACCCG
CTGAACTTAA

>B8_26
TTTCCGTAGGTGAACCTGCGGAAGGATCATTATTGAATTATGTTTCTAGATAGGTTGTAG
CTGGCTCTTTAGAGCATGTGCACGCCTGTTTGGACTTCATTTTCATCCACCTGTGCACCT
ATTGTAGTCTTTGGTTGGGTTAGGGGGAAGTGGTCATTGTGTCAGCATCTGCTGGATGTG
AGGACTTGCATTGTGAAAGCTTTGCTGTCCTTGATGTGATCATGGAATCTCTTTCTCACT
AGAGTCTATGTCACTCATTATACTCTGTCGAATGTCATTGAATGTCTTTACATGGGCTTG
TATGCCTATGAAAATTGTAATACAACTTTCAGCAACGGATCTCTTGGCTCTCGCATCGAT
GAAGGACGCAGCGAAATGCGATAAGTAATGTGAATTGCAGAATTCAGTGAATCATCGAAT
CTTTGAACGCATCTTGCGCTCCTTGGTATTCCGAGGAGCATGCCTGTTTGAGTGTCATTA
AATTCTCAACTCTCTTATACTTTTTTGTAAAAGAGAGCTTGGACTGTGGAGGCTTGCTGG
CCACTTTTTGGGGTCAGCTCCTCTGAAATGCATTAGCGGAACCGTTTGCAATCTGCCACA
AGTGTGATAAGTTATCTACACTGGCGAGGGGATTGCTCTCTGTAATGTTCAGCTTCTAAT
TGTCTCTACTTTGTGAGACAACTTTTGAATGCTTGACCTCAAATCAGGTAGGACTACCCG
CTGAACTTAA
>B8_27
TTTCCGTAGGTGAACCTGCGGAAGGATCATTATTGAATTATGTTTCTAGATAGGTTGTAG
CTGGCTCTTTAGAGCATGTGCACGCCTGTTTGGACTTCATTTTCATCCACCTGTGCACCT
ATTGTAGTCTTTGGTTGGGTTAGGGGGAAGTGGTCATTGTGTCAGCATCTGCTGGATGTG
AGGACTTGCATTGTGAAAGCTTTGCTGTCCTTGATGTGATCATGGAATCTCTTTCTCACT
AGAGTCTATGTCACTCATTATACTCTGTCGAATGTCATTGAATGTCTTTACATGGGCTTG
TATGCCTATGAAAATTGTAATACAACTTTCAGCAACGGATCTCTTGGCTCTCGCATCGAT
GAAGGACGCAGCGAAATGCGATAAGTAATGTGAATTGCAGAATTCAGTGAATCATCGAAT
CTTTGAACGCATCTTGCGCTCCTTGGTATTCCGAGGAGCATGCCTGTTTGAGTGTCATTA
AATTCTCAACTCTCTTATACTTTTTTGTAAAAGAGAGCTTGGACTGTGGAGGCTTGCTGG
CCACTTTTTGGGGTCAGCTCCTCTGAAATGCATTAGCGGAACCGTTTGCAATCTGCCACA
AGTGTGATAAGTTATCTACACTGGCGAGGGGATTGCTCTCTGTAATGTTCAGCTTCTAAT
TGTCTCTACTTTGTGAGACAACTTTTGAATGCTTGACCTCAAATCAGGTAGGACTACCCG
CTGAACTTAA
>B8_28
TTTCCGTAGGTGAACCTGCGGAAGGATCATTATTGAATTATGTTTCTAGATAGGTTGTAG
CTGGCTCTTTAGAGCATGTGCACGCCTGTTTGGACTTCATTTTCATCCACCTGTGCACCT
ATTGTAGTCTTTGGTTGGGTTAGGGGGAAGTGGTCATTGTGTCAGCATCTGCTGGATGTG
AGGACTTGCATTGTGAAAGCTTTGCTGTCCTTGATGTGATCATGGAATCTCTTTCTCACT
AGAGTCTATGTCACTCATTATACTCTGTCGAATGTCATTGAATGTCTTTACATGGGCTTG
TATGCCTATGAAAATTGTAATACAACTTTCAGCAACGGATCTCTTGGCTCTCGCATCGAT
GAAGGACGCAGCGAAATGCGATAAGTAATGTGAATTGCAGAATTCAGTGAATCATCGAAT
CTTTGAACGCATCTTGCGCTCCTTGGTATTCCGAGGAGCATGCCTGTTTGAGTGTCATTA
AATTCTCAACTCTCTTATACTTTTTTGTAAAAGAGAGCTTGGACTGTGGAGGCTTGCTGG
CCACTTTTTGGGGTCAGCTCCTCTGAAATGCATTAGCGGAACCGTTTGCAATCTGCCACA
AGTGTGATAAGTTATCTACACTGGCGAGGGGATTGCTCTCTGTAATGTTCAGCTTCTAAT
TGTCTCTACTTTGTGAGACAACTTTTGAATGCTTGACCTCAAATCAGGTAGGACTACCCG
CTGAACTTAA
>B8_29
TTTCCGTAGGTGAACCTGCGGAAGGATCATTATTGAATTATGTTTCTAGATAGGTTGTAG
CTGGCTCTTTAGAGCATGTGCACGCCTGTTTGGACTTCATTTTCATCCACCTGTGCACCT
ATTGTAGTCTTTGGTTGGGTTAGGGGGAAGTGGTCATTGTGTCAGCATCTGCTGGATGTG
AGGACTTGCATTGTGAAAGCTTTGCTGTCCTTGATGTGATCATGGAATCTCTTTCTCACT
AGAGTCTATGTCACTCATTATACTCTGTCGAATGTCATTGAATGTCTTTACATGGGCTTG
TATGCCTATGAAAATTGTAATACAACTTTCAGCAACGGATCTCTTGGCTCTCGCATCGAT
GAAGGACGCAGCGAAATGCGATAAGTAATGTGAATTGCAGAATTCAGTGAATCATCGAAT

CTTTGAACGCATCTTGCGCTCCTTGGTATTCCGAGGAGCATGCCTGTTTGAGTGTCATTA
AATTCTCAACTCTCTTATACTTTTTTGTAAAAGAGAGCTTGGACTGTGGAGGCTTGCTGG
CCACTTTTTGGGGTCAGCTCCTCTGAAATGCATTAGCGGAACCGTTTGCAATCTGCCACA
AGTGTGATAAGTTATCTACACTGGCGAGGGGATTGCTCTCTGTAATGTTCAGCTTCTAAT
TGTCTCTACTTTGTGAGACAACTTTTGAATGCTTGACCTCAAATCAGGTAGGACTACCCG
CTGAACTTAA
>B8_30
TTTCCGTAGGTGAACCTGCGGAAGGATCATTATTGAATTATGTTTCTAGATAGGTTGTAG
CTGGCTCTTTAGAGCATGTGCACGCCTGTTTGGACTTCATTTTCATCCACCTGTGCACCT
ATTGTAGTCTTTGGTTGGGTTAGGGGGAAGTGGTCATTGTGTCAGCATCTGCTGGATGTG
AGGACTTGCATTGTGAAAGCTTTGCTGTCCTTGATGTGATCATGGAATCTCTTTCTCACT
AGAGTCTATGTCACTCATTATACTCTGTCGAATGTCATTGAATGTCTTTACATGGGCTTG
TATGCCTATGAAAATTGTAATACAACTTTCAGCAACGGATCTCTTGGCTCTCGCATCGAT
GAAGGACGCAGCGAAATGCGATAAGTAATGTGAATTGCAGAATTCAGTGAATCATCGAAT
CTTTGAACGCATCTTGCGCTCCTTGGTATTCCGAGGAGCATGCCTGTTTGAGTGTCATTA
AATTCTCAACTCTCTTATACTTTTTTGTAAAAGAGAGCTTGGACTGTGGAGGCTTGCTGG
CCACTTTTTGGGGTCAGCTCCTCTGAAATGCATTAGCGGAACCGTTTGCAATCTGCCACA
AGTGTGATAAGTTATCTACACTGGCGAGGGGATTGCTCTCTGTAATGTTCAGCTTCTAAT
TGTCTCTACTTTGTGAGACAACTTTTGAATGCTTGACCTCAAATCAGGTAGGACTACCCG
CTGAACTTAA
>B8_31
TTTCCGTAGGTGAACCTGCGGAAGGATCATTATTGAATTATGTTTCTAGATAGGTTGTAG
CTGGCTCTTTAGAGCATGTGCACGCCTGTTTGGACTTCATTTTCATCCACCTGTGCACCT
ATTGTAGTCTTTGGTTGGGTTAGGGGGAAGTGGTCATTGTGTCAGCATCTGCTGGATGTG
AGGACTTGCATTGTGAAAGCTTTGCTGTCCTTGATGTGATCATGGAATCTCTTTCTCACT
AGAGTCTATGTCACTCATTATACTCTGTCGAATGTCATTGAATGTCTTTACATGGGCTTG
TATGCCTATGAAAATTGTAATACAACTTTCAGCAACGGATCTCTTGGCTCTCGCATCGAT
GAAGGACGCAGCGAAATGCGATAAGTAATGTGAATTGCAGAATTCAGTGAATCATCGAAT
CTTTGAACGCATCTTGCGCTCCTTGGTATTCCGAGGAGCATGCCTGTTTGAGTGTCATTA
AATTCTCAACTCTCTTATACTTTTTTGTAAAAGAGAGCTTGGACTGTGGAGGCTTGCTGG
CCACTTTTTGGGGTCAGCTCCTCTGAAATGCATTAGCGGAACCGTTTGCAATCTGCCACA
AGTGTGATAAGTTATCTACACTGGCGAGGGGATTGCTCTCTGTAATGTTCAGCTTCTAAT
TGTCTCTACTTTGTGAGACAACTTTTGAATGCTTGACCTCAAATCAGGTAGGACTACCCG
CTGAACTTAA
>B8_32
TTTCCGTAGGTGAACCTGCGGAAGGATCATTATTGAATTATGTTTCTAGATAGGTTGTAG
CTGGCTCTTTAGAGCATGTGCACGCCTGTTTGGACTTCATTTTCATCCACCTGTGCACCT
ATTGTAGTCTTTGGTTGGGTTAGGGGGAAGTGGTCATTGTGTCAGCATCTGCTGGATGTG
AGGACTTGCATTGTGAAAGCTTTGCTGTCCTTGATGTGATCATGGAATCTCTTTCTCACT
AGAGTCTATGTCACTCATTATACTCTGTCGAATGTCATTGAATGTCTTTACATGGGCTTG
TATGCCTATGAAAATTGTAATACAACTTTCAGCAACGGATCTCTTGGCTCTCGCATCGAT
GAAGGACGCAGCGAAATGCGATAAGTAATGTGAATTGCAGAATTCAGTGAATCATCGAAT
CTTTGAACGCATCTTGCGCTCCTTGGTATTCCGAGGAGCATGCCTGTTTGAGTGTCATTA
AATTCTCAACTCTCTTATACTTTTTTGTAAAAGAGAGCTTGGACTGTGGAGGCTTGCTGG
CCACTTTTTGGGGTCAGCTCCTCTGAAATGCATTAGCGGAACCGTTTGCAATCTGCCACA
AGTGTGATAAGTTATCTACACTGGCGAGGGGATTGCTCTCTGTAATGTTCAGCTTCTAAT
TGTCTCTACTTTGTGAGACAACTTTTGAATGCTTGACCTCAAATCAGGTAGGACTACCCG
CTGAACTTAA
>B8_33
TTTCCGTAGGTGAACCTGCGGAAGGATCATTATTGAATTATGTTTCTAGATAGGTTGTAG

CTGGCTCTTTAGAGCATGTGCACGCCTGTTTGGACTTCATTTTCATCCACCTGTGCACCT
ATTGTAGTCTTTGGTTGGGTTAGGGGGAAGTGGTCATTGTGTCAGCATCTGCTGGATGTG
AGGACTTGCATTGTGAAAGCTTTGCTGTCCTTGATGTGATCATGGAATCTCTTTCTCACT
AGAGTCTATGTCACTCATTATACTCTGTCGAATGTCATTGAATGTCTTTACATGGGCTTG
TATGCCTATGAAAATTGTAATACAACTTTCAGCAACGGATCTCTTGGCTCTCGCATCGAT
GAAGGACGCAGCGAAATGCGATAAGTAATGTGAATTGCAGAATTCAGTGAATCATCGAAT
CTTTGAACGCATCTTGCGCTCCTTGGTATTCCGAGGAGCATGCCTGTTTGAGTGTCATTA
AATTCTCAACTCTCTTATACTTTTTTGTAAAAGAGAGCTTGGACTGTGGAGGCTTGCTGG
CCACTTTTTGGGGTCAGCTCCTCTGAAATGCATTAGCGGAACCGTTTGCAATCTGCCACA
AGTGTGATAAGTTATCTACACTGGCGAGGGGATTGCTCTCTGTAATGTTCAGCTTCTAAT
TGTCTCTACTTTGTGAGACAACTTTTGAATGCTTGACCTCAAATCAGGTAGGACTACCCG
CTGAACTTAA
>B8_34
TTTCCGTAGGTGAACCTGCGGAAGGATCATTATTGAATTATGTTTCTAGATAGGTTGTAG
CTGGCTCTTTAGAGCATGTGCACGCCTGTTTGGACTTCATTTTCATCCACCTGTGCACCT
ATTGTAGTCTTTGGTTGGGTTAGGGGGAAGTGGTCATTGTGTCAGCATCTGCTGGATGTG
AGGACTTGCATTGTGAAAGCTTTGCTGTCCTTGATGTGATCATGGAATCTCTTTCTCACT
AGAGTCTATGTCACTCATTATACTCTGTCGAATGTCATTGAATGTCTTTACATGGGCTTG
TATGCCTATGAAAATTGTAATACAACTTTCAGCAACGGATCTCTTGGCTCTCGCATCGAT
GAAGGACGCAGCGAAATGCGATAAGTAATGTGAATTGCAGAATTCAGTGAATCATCGAAT
CTTTGAACGCATCTTGCGCTCCTTGGTATTCCGAGGAGCATGCCTGTTTGAGTGTCATTA
AATTCTCAACTCTCTTATACTTTTTTGTAAAAGAGAGCTTGGACTGTGGAGGCTTGCTGG
CCACTTTTTGGGGTCAGCTCCTCTGAAATGCATTAGCGGAACCGTTTGCAATCTGCCACA
AGTGTGATAAGTTATCTACACTGGCGAGGGGATTGCTCTCTGTAATGTTCAGCTTCTAAT
TGTCTCTACTTTGTGAGACAACTTTTGAATGCTTGACCTCAAATCAGGTAGGACTACCCG
CTGAACTTAA
>B8_35
TTTCCGTAGGTGAACCTGCGGAAGGATCATTATTGAATTATGTTTCTAGATAGGTTGTAG
CTGGCTCTTTAGAGCATGTGCACGCCTGTTTGGACTTCATTTTCATCCACCTGTGCACCT
ATTGTAGTCTTTGGTTGGGTTAGGGGGAAGTGGTCATTGTGTCAGCATCTGCTGGATGTG
AGGACTTGCATTGTGAAAGCTTTGCTGTCCTTGATGTGATCATGGAATCTCTTTCTCACT
AGAGTCTATGTCACTCATTATACTCTGTCGAATGTCATTGAATGTCTTTACATGGGCTTG
TATGCCTATGAAAATTGTAATACAACTTTCAGCAACGGATCTCTTGGCTCTCGCATCGAT
GAAGGACGCAGCGAAATGCGATAAGTAATGTGAATTGCAGAATTCAGTGAATCATCGAAT
CTTTGAACGCATCTTGCGCTCCTTGGTATTCCGAGGAGCATGCCTGTTTGAGTGTCATTA
AATTCTCAACTCTCTTATACTTTTTTGTAAAAGAGAGCTTGGACTGTGGAGGCTTGCTGG
CCACTTTTTGGGGTCAGCTCCTCTGAAATGCATTAGCGGAACCGTTTGCAATCTGCCACA
AGTGTGATAAGTTATCTACACTGGCGAGGGGATTGCTCTCTGTAATGTTCAGCTTCTAAT
TGTCTCTACTTTGTGAGACAACTTTTGAATGCTTGACCTCAAATCAGGTAGGACTACCCG
CTGAACTTAA
>B8_36
TTTCCGTAGGTGAACCTGCGGAAGGATCATTATTGAATTATGTTTCTAGATAGGTTGTAG
CTGGCTCTTTAGAGCATGTGCACGCCTGTTTGGACTTCATTTTCATCCACCTGTGCACCT
ATTGTAGTCTTTGGTTGGGTTAGGGGGAAGTGGTCATTGTGTCAGCATCTGCTGGATGTG
AGGACTTGCATTGTGAAAGCTTTGCTGTCCTTGATGTGATCATGGAATCTCTTTCTCACT
AGAGTCTATGTCACTCATTATACTCTGTCGAATGTCATTGAATGTCTTTACATGGGCTTG
TATGCCTATGAAAATTGTAATACAACTTTCAGCAACGGATCTCTTGGCTCTCGCATCGAT
GAAGGACGCAGCGAAATGCGATAAGTAATGTGAATTGCAGAATTCAGTGAATCATCGAAT
CTTTGAACGCATCTTGCGCTCCTTGGTATTCCGAGGAGCATGCCTGTTTGAGTGTCATTA
AATTCTCAACTCTCTTATACTTTTTTGTAAAAGAGAGCTTGGACTGTGGAGGCTTGCTGG

CCACTTTTTGGGGTCAGCTCCTCTGAAATGCATTAGCGGAACCGTTTGCAATCTGCCACA
AGTGTGATAAGTTATCTACACTGGCGAGGGGATTGCTCTCTGTAATGTTCAGCTTCTAAT
TGTCTCTACTTTGTGAGACAACTTTTGAATGCTTGACCTCAAATCAGGTAGGACTACCCG
CTGAACTTAA
>B8_37
TTTCCGTAGGTGAACCTGCGGAAGGATCATTATTGAATTATGTTTCTAGATAGGTTGTAG
CTGGCTCTTTAGAGCATGTGCACGCCTGTTTGGACTTCATTTTCATCCACCTGTGCACCT
ATTGTAGTCTTTGGTTGGGTTAGGGGGAAGTGGTCATTGTGTCAGCATCTGCTGGATGTG
AGGACTTGCATTGTGAAAGCTTTGCTGTCCTTGATGTGATCATGGAATCTCTTTCTCACT
AGAGTCTATGTCACTCATTATACTCTGTCGAATGTCATTGAATGTCTTTACATGGGCTTG
TATGCCTATGAAAATTGTAATACAACTTTCAGCAACGGATCTCTTGGCTCTCGCATCGAT
GAAGGACGCAGCGAAATGCGATAAGTAATGTGAATTGCAGAATTCAGTGAATCATCGAAT
CTTTGAACGCATCTTGCGCTCCTTGGTATTCCGAGGAGCATGCCTGTTTGAGTGTCATTA
AATTCTCAACTCTCTTATACTTTTTTGTAAAAGAGAGCTTGGACTGTGGAGGCTTGCTGG
CCACTTTTTGGGGTCAGCTCCTCTGAAATGCATTAGCGGAACCGTTTGCAATCTGCCACA
AGTGTGATAAGTTATCTACACTGGCGAGGGGATTGCTCTCTGTAATGTTCAGCTTCTAAT
TGTCTCTACTTTGTGAGACAACTTTTGAATGCTTGACCTCAAATCAGGTAGGACTACCCG
CTGAACTTAA
>B8_38
TTTCCGTAGGTGAACCTGCGGAAGGATCATTATTGAATTATGTTTCTAGATAGGTTGTAG
CTGGCTCTTTAGAGCATGTGCACGCCTGTTTGGACTTCATTTTCATCCACCTGTGCACCT
ATTGTAGTCTTTGGTTGGGTTAGGGGGAAGTGGTCATTGTGTCAGCATCTGCTGGATGTG
AGGACTTGCATTGTGAAAGCTTTGCTGTCCTTGATGTGATCATGGAATCTCTTTCTCACT
AGAGTCTATGTCACTCATTATACTCTGTCGAATGTCATTGAATGTCTTTACATGGGCTTG
TATGCCTATGAAAATTGTAATACAACTTTCAGCAACGGATCTCTTGGCTCTCGCATCGAT
GAAGGACGCAGCGAAATGCGATAAGTAATGTGAATTGCAGAATTCAGTGAATCATCGAAT
CTTTGAACGCATCTTGCGCTCCTTGGTATTCCGAGGAGCATGCCTGTTTGAGTGTCATTA
AATTCTCAACTCTCTTATACTTTTTTGTAAAAGAGAGCTTGGACTGTGGAGGCTTGCTGG
CCACTTTTTGGGGTCAGCTCCTCTGAAATGCATTAGCGGAACCGTTTGCAATCTGCCACA
AGTGTGATAAGTTATCTACACTGGCGAGGGGATTGCTCTCTGTAATGTTCAGCTTCTAAT
TGTCTCTACTTTGTGAGACAACTTTTGAATGCTTGACCTCAAATCAGGTAGGACTACCCG
CTGAACTTAA
>B8_39
TTTCCGTAGGTGAACCTGCGGAAGGATCATTATTGAATTATGTTTCTAGATAGGTTGTAG
CTGGCTCTTTAGAGCATGTGCACGCCTGTTTGGACTTCATTTTCATCCACCTGTGCACCT
ATTGTAGTCTTTGGTTGGGTTAGGGGGAAGTGGTCATTGTGTCAGCATCTGCTGGATGTG
AGGACTTGCATTGTGAAAGCTTTGCTGTCCTTGATGTGATCATGGAATCTCTTTCTCACT
AGAGTCTATGTCACTCATTATACTCTGTCGAATGTCATTGAATGTCTTTACATGGGCTTG
TATGCCTATGAAAATTGTAATACAACTTTCAGCAACGGATCTCTTGGCTCTCGCATCGAT
GAAGGACGCAGCGAAATGCGATAAGTAATGTGAATTGCAGAATTCAGTGAATCATCGAAT
CTTTGAACGCATCTTGCGCTCCTTGGTATTCCGAGGAGCATGCCTGTTTGAGTGTCATTA
AATTCTCAACTCTCTTATACTTTTTTGTAAAAGAGAGCTTGGACTGTGGAGGCTTGCTGG
CCACTTTTTGGGGTCAGCTCCTCTGAAATGCATTAGCGGAACCGTTTGCAATCTGCCACA
AGTGTGATAAGTTATCTACACTGGCGAGGGGATTGCTCTCTGTAATGTTCAGCTTCTAAT
TGTCTCTACTTTGTGAGACAACTTTTGAATGCTTGACCTCAAATCAGGTAGGACTACCCG
CTGAACTTAA
>B8_40
TTTCCGTAGGTGAACCTGCGGAAGGATCATTATTGAATTATGTTTCTAGATAGGTTGTAG
CTGGCTCTTTAGAGCATGTGCACGCCTGTTTGGACTTCATTTTCATCCACCTGTGCACCT
ATTGTAGTCTTTGGTTGGGTTAGGGGGAAGTGGTCATTGTGTCAGCATCTGCTGGATGTG

AGGACTTGCATTGTGAAAGCTTTGCTGTCCTTGATGTGATCATGGAATCTCTTTCTCACT
AGAGTCTATGTCACTCATTATACTCTGTCGAATGTCATTGAATGTCTTTACATGGGCTTG
TATGCCTATGAAAATTGTAATACAACTTTCAGCAACGGATCTCTTGGCTCTCGCATCGAT
GAAGGACGCAGCGAAATGCGATAAGTAATGTGAATTGCAGAATTCAGTGAATCATCGAAT
CTTTGAACGCATCTTGCGCTCCTTGGTATTCCGAGGAGCATGCCTGTTTGAGTGTCATTA
AATTCTCAACTCTCTTATACTTTTTTGTAAAAGAGAGCTTGGACTGTGGAGGCTTGCTGG
CCACTTTTTGGGGTCAGCTCCTCTGAAATGCATTAGCGGAACCGTTTGCAATCTGCCACA
AGTGTGATAAGTTATCTACACTGGCGAGGGGATTGCTCTCTGTAATGTTCAGCTTCTAAT
TGTCTCTACTTTGTGAGACAACTTTTGAATGCTTGACCTCAAATCAGGTAGGACTACCCG
CTGAACTTAA
>B8_41
TTTCCGTAGGTGAACCTGCGGAAGGATCATTATTGAATTATGTTTCTAGATAGGTTGTAG
CTGGCTCTTTAGAGCATGTGCACGCCTGTTTGGACTTCATTTTCATCCACCTGTGCACCT
ATTGTAGTCTTTGGTTGGGTTAGGGGGAAGTGGTCATTGTGTCAGCATCTGCTGGATGTG
AGGACTTGCATTGTGAAAGCTTTGCTGTCCTTGATGTGATCATGGAATCTCTTTCTCACT
AGAGTCTATGTCACTCATTATACTCTGTCGAATGTCATTGAATGTCTTTACATGGGCTTG
TATGCCTATGAAAATTGTAATACAACTTTCAGCAACGGATCTCTTGGCTCTCGCATCGAT
GAAGGACGCAGCGAAATGCGATAAGTAATGTGAATTGCAGAATTCAGTGAATCATCGAAT
CTTTGAACGCATCTTGCGCTCCTTGGTATTCCGAGGAGCATGCCTGTTTGAGTGTCATTA
AATTCTCAACTCTCTTATACTTTTTTGTAAAAGAGAGCTTGGACTGTGGAGGCTTGCTGG
CCACTTTTTGGGGTCAGCTCCTCTGAAATGCATTAGCGGAACCGTTTGCAATCTGCCACA
AGTGTGATAAGTTATCTACACTGGCGAGGGGATTGCTCTCTGTAATGTTCAGCTTCTAAT
TGTCTCTACTTTGTGAGACAACTTTTGAATGCTTGACCTCAAATCAGGTAGGACTACCCG
CTGAACTTAA
>B8_42
TTTCCGTAGGTGAACCTGCGGAAGGATCATTATTGAATTATGTTTCTAGATAGGTTGTAG
CTGGCTCTTTAGAGCATGTGCACGCCTGTTTGGACTTCATTTTCATCCACCTGTGCACCT
ATTGTAGTCTTTGGTTGGGTTAGGGGGAAGTGGTCATTGTGTCAGCATCTGCTGGATGTG
AGGACTTGCATTGTGAAAGCTTTGCTGTCCTTGATGTGATCATGGAATCTCTTTCTCACT
AGAGTCTATGTCACTCATTATACTCTGTCGAATGTCATTGAATGTCTTTACATGGGCTTG
TATGCCTATGAAAATTGTAATACAACTTTCAGCAACGGATCTCTTGGCTCTCGCATCGAT
GAAGGACGCAGCGAAATGCGATAAGTAATGTGAATTGCAGAATTCAGTGAATCATCGAAT
CTTTGAACGCATCTTGCGCTCCTTGGTATTCCGAGGAGCATGCCTGTTTGAGTGTCATTA
AATTCTCAACTCTCTTATACTTTTTTGTAAAAGAGAGCTTGGACTGTGGAGGCTTGCTGG
CCACTTTTTGGGGTCAGCTCCTCTGAAATGCATTAGCGGAACCGTTTGCAATCTGCCACA
AGTGTGATAAGTTATCTACACTGGCGAGGGGATTGCTCTCTGTAATGTTCAGCTTCTAAT
TGTCTCTACTTTGTGAGACAACTTTTGAATGCTTGACCTCAAATCAGGTAGGACTACCCG
CTGAACTTAA
>B8_43
TTTCCGTAGGTGAACCTGCGGAAGGATCATTATTGAATTATGTTTCTAGATAGGTTGTAG
CTGGCTCTTTAGAGCATGTGCACGCCTGTTTGGACTTCATTTTCATCCACCTGTGCACCT
ATTGTAGTCTTTGGTTGGGTTAGGGGGAAGTGGTCATTGTGTCAGCATCTGCTGGATGTG
AGGACTTGCATTGTGAAAGCTTTGCTGTCCTTGATGTGATCATGGAATCTCTTTCTCACT
AGAGTCTATGTCACTCATTATACTCTGTCGAATGTCATTGAATGTCTTTACATGGGCTTG
TATGCCTATGAAAATTGTAATACAACTTTCAGCAACGGATCTCTTGGCTCTCGCATCGAT
GAAGGACGCAGCGAAATGCGATAAGTAATGTGAATTGCAGAATTCAGTGAATCATCGAAT
CTTTGAACGCATCTTGCGCTCCTTGGTATTCCGAGGAGCATGCCTGTTTGAGTGTCATTA
AATTCTCAACTCTCTTATACTTTTTTGTAAAAGAGAGCTTGGACTGTGGAGGCTTGCTGG
CCACTTTTTGGGGTCAGCTCCTCTGAAATGCATTAGCGGAACCGTTTGCAATCTGCCACA
AGTGTGATAAGTTATCTACACTGGCGAGGGGATTGCTCTCTGTAATGTTCAGCTTCTAAT

TGTCTCTACTTTGTGAGACAACTTTTGAATGCTTGACCTCAAATCAGGTAGGACTACCCG
CTGAACTTAA
>B6_15
TTTCCGTAGGTGAACCTGCGGAAGGATCATTATTGAATTATGTTTCTAGATAGGTTGTAG
CTGGCTCTTTAGAGCATGTGCACGCCTGTTTGGACTTCATTTTCATCCACCTGTGCACCT
ATTGTAGTCTTTGGTTGGGTTAGGGGGAAGTGGTCATTGTGTCAGCATCTGCTGGATGTG
AGGACTTGCATTGTGAAAGCTTTGCTGTCCTTGATGTGATCATGGAATCTCTTTCTCACT
AGAGTCTATGTCACTCATTATACTCTGTCGAATGTCATTGAATGTCTTTACATGGGCTTG
TATGCCTATGAAAATTGTAATACAACTTTCAGCAACGGATCTCTTGGCTCTCGCATCGAT
GAAGGACGCAGCGAAATGCGATAAGTAATGTGAATTGCAGAATTCAGTGAATCATCGAAT
CTTTGAACGCATCTTGCGCTCCTTGGTATTCCGAGGAGCATGCCTGTTTGAGTGTCATTA
AATTCTCAACTCTCTTATACTTTTTTGTAAAAGAGAGCTTGGACTGTGGAGGCTTGCTGG
CCACTTTTTGGGGTCAGCTCCTCTGAAATGCATTAGCGGAACCGTTTGCGATCTGCCACA
AGTGTGATAAGTTATCTACACTGGCGAGGGGATTGCTCTCTGTAATGTTCAGCTTCTAAT
TGTCTCTACTTTGTGAGACAACTTTTGAATGCTTGACCTCAAATCAGGTAGGACTACCCG
CTGAACTTAA
>B8_24
TTTCCGTAGGTGAACCTGCGGAAGGATCATTATTGAATTATGTTTCTAGATAGGTTGTAG
CTGGCTCTTTAGAGCATGTGCACGCCTGTTTGGACTTCATTTTCATCCACCTGTGCACCT
ATTGTAGTCTTTGGTTGGGTTAGGGGGAAGTGGTCATTGTGTCAGCATCTGCTGGATGTG
AGGACTTGCATTGTGAAAGCTTTGCTGTCCTTGATGTGATCATGGAATCTCTTTCTCACT
AGAGTCTATGTCACTCATTATACTCTGTCGAATGTCATTGAATGTCTTTACATGGGCTTG
TATGCCTATGAAAATTGTAATACAACTTTCAGCAACGGATCTCTTGGCTCTCGCATCGAT
GAAGGACGCAGCGAAATGCGATAAGTAATGCGAATTGCAGAATTCAGTGAATCATCGAAT
CTTTGAACGCATCTTGCGCTCCTTGGTATTCCGAGGAGCATGCCTGTTTGAGTGTCATTA
AATTCTCAACTCTCTTATACTTTTTTGTAAAAGAGAGCTTGGACTGTGGAGGCTTGCTGG
CCACTTTTTGGGGTCAGCTCCTCTGAAATGCATTAGCGGAACCGTTTGCAATCTGCCACA
AGTGTGATAAGTTATCTACACTGGCGAGGGGATTGCTCTCTGTAATGTTCAGCTTCTAAT
TGTCTCTACTTTGTGAGACAACTTTTGAATGCTTGACCTCAAATCAGGTAGGACTACCCG
CTGAACTTAA
>B12_6
TTTCCGTAGGTGAACCTGCGGAAGGATCATTATTGAATTATGTTTCTAGATAGGTTGTAG
CTGGCTCTTTAGAGCATGTGCACGCCTGTTTGGACTTCATTTTCATCCACCTGTGCACCT
ATTGTAGTCTTTGGTTGGGTTAGGGGGAAGTGGTCATTGTGTCAGCATCTGCTGGATGTT
AGGACTTGCATTGTGAAAGCTTTGCTGTCCTTGATGTGATCATGGAATCTCTTTCTCACT
AGAGTCTATGTCACTCATTATACTCTGTCGAATGTCATTGAATGTCTTTACATGGGCTTG
TATGCCTATGAAAATTGTAATACAACTTTCAGCAACGGATCTCTTGGCTCTCGCATCGAT
GAAGGACGCAGCGAAATGCGATAAGTAATGTGAATTGCAGAATTCAGTGAATCATCGAAT
CTTTGAACGCATCTTGCGCTCCTTGGTATTCCGAGGAGCATGCCTGTTTGAGTGTCATTA
AATTCTCAACTCTCTTATACTTTTTTGTAAAAGAGAGCTTGGACTGTGGAGGCTTGCTGG
CCACTTTTTGGGGTCAGCTCCTCTGAAATGCATTAGCGGAACCGTTTGCAATCTGCCACA
AGTGTGATAAGTTATCTACACTGGCGAGGGGATTGCTCTCTGTAATGTTCAGCTTCTAAT
TGTCTCTACTTTGTGAGACAACTTTTGAATGCTTGACCTCAAATCAGGTAGGACTACCCG
CTGAACTTAA
>B6_19
TTTCCGTAGGTGAACCTGCGGAAGGATCATTATTGAATTATGTTTCTAGATAGGTTGTAG
CTGGCTCTTTAGAGCATGTGCACGCATGTTTGGACTTCATTTTCATCCACCTGTGCACCT
ATTGTAGTCTTTGGTTGGGTTAGGGGGAAGTGGTCATTGTGTCAGCATCTGCTGGATGTG
AGGACTTGCATTGTGAAAGCTTTGCTGTCCTTGATGTGATCATGGAATCTCTTTCTCACT
AGAGTCTATGTCACTCATTATACTCTGTCGAATGTCATTGAATGTCTTTACATGGGCTTG

TATGCCTATGAAAATTGTAATACAACTTTCAGCAACGGATCTCTTGGCTCTCGCATCGAT
GAAGGACGCAGCGAAATGCGATAAGTAATGTGAATTGCAGAATTCAGTGAATCATCGAAT
CTTTGAACGCATCTTGCGCTCCTTGGTATTCCGAGGAGCATGCCTGTTTGAGTGTCATTA
AATTCTCAACTCTCTTATACTTTTTTGTAAAAGAGAGCTTGGACTGTGGAGGCTTGCTGG
CCACTTTTTGGGGTCAGCTCCTCTGAAATGCATTAGCGGAACCGTTTGCAATCTGCCACA
AGTGTGATAAGTTATCTACACTGGCGAGGGGATTGCTCTCTGTAATGTTCAGCTTCTAAT
TGTCTCTACTTTGTGAGACAACTTTTGAATGCTTGACCTCAAATCAGGTAGGACTACCCG
CTGAACTTAA
>B7_70
TTTCCGTAGGTGAACCTGCGGAAGGATCATTATTGAATTATGTTTCTAGATAGGTTGTAG
CTGGCTCTTTAGAGCATGTGCACGCCTGTTTGGACTTCATTTTCATCCACCTGTGCACCT
ATTGTAGTCTTTGGTTGGGTTAGGGGGAAGTGGTCATTGTGTCAGCATCTGCTGGATGTG
AGGACTTGCATTGTGAAAGCTGTGCTGTCCTTGATGTGATCATGGAATCTCTTTCTCACT
AGAGTCTATGTCACTCATTATACTCTGTCGAATGTCATTGAATGTCTTTACATGGGCTTG
TATGCCTATGAAAATTGTAATACAACTTTCAGCAACGGATCTCTTGGCTCTCGCATCGAT
GAAGGACGCAGCGAAATGCGATAAGTAATGTGAATTGCAGAATTCAGTGAATCATCGAAT
CTTTGAACGCATCTTGCGCTCCTTGGTATTCCGAGGAGCATGCCTGTTTGAGTGTCATTA
AATTCTCAACTCTCTTATACTTTTTTGTAAAAGAGAGCTTGGACTGTGGAGGCTTGCTGG
CCACTTTTTGGGGTCAGCTCCTCTGAAATGCATTAGCGGAACCGTTTGCAATCTGCCACA
AGTGTGATAAGTTATCTACACTGGCGAGGGGATTGCTCTCTGTAATGTTCAGCTTCTAAT
TGTCTCTACTTTGTGAGACAACTTTTGAATGCTTGACCTCAAATCAGGTAGGACTACCCG
CTGAACTTAA
>B9_60
TTTCCGTAGGTGAACCTGCGGAAGGATCATTATTGAATTATGTTTCTAGATAGGTTGTAG
CTGGCTCTTTAGAGCATGTGCACGCCTGTTTGGACTTCATTTTCATCCACCTGTGCACCT
ATTGTAGTCTTTGGTTGGGTTAGGGGGAAGTGGTCATTGTGTCAGCATCTGCTGGATGTG
AGGACTTGCTTTGTGAAAGCTTTGCTGTCCTTGATGTGATCATGGAATCTCTTTCTCACT
AGAGTCTATGTCACTCATTATACTCTGTCGAATGTCATTGAATGTCTTTACATGGGCTTG
TATGCCTATGAAAATTGTAATACAACTTTCAGCAACGGATCTCTTGGCTCTCGCATCGAT
GAAGGACGCAGCGAAATGCGATAAGTAATGTGAATTGCAGAATTCAGTGAATCATCGAAT
CTTTGAACGCATCTTGCGCTCCTTGGTATTCCGAGGAGCATGCCTGTTTGAGTGTCATTA
AATTCTCAACTCTCTTATACTTTTTTGTAAAAGAGAGCTTGGACTGTGGAGGCTTGCTGG
CCACTTTTTGGGGTCAGCTCCTCTGAAATGCATTAGCGGAACCGTTTGCAATCTGCCACA
AGTGTGATAAGTTATCTACACTGGCGAGGGGATTGCTCTCTGTAATGTTCAGCTTCTAAT
TGTCTCTACTTTGTGAGACAACTTTTGAATGCTTGACCTCAAATCAGGTAGGACTACCCG
CTGAACTTAA
>B1_11
TTTCCGTAGGTGAACCTGCGGAAGGATCATTATTGAATTATGTTTCTAGATAGGTTGTAG
CTGGCTCTTTAGAGCATGTGCACGCCTGTTTGGACTTCATTTTCATCCACCTGTGCACCT
ATTGTAGTCTTTGGTTGGGTTAGGGGGAAGTGGTCATTGTGTCAGCATCTGCTGGATGTG
AGGACTTGCATTGTGAAAGCTTTGCTGTCCTTGATGTGATCATGGAATCTCTTTCTCACT
AGAGTCTATGTCACTCATTATACTCTGTCGAATGTCATTGAATGTCTTTACATGGGCTTG
TATGCCTATGAAAATTGTAATACAACTTTCAGCAACGGATCTCTTGGCTCTCGCATCGAT
GAAGGACGCAGCGAAATGCGATAAGTAATGTGAATTGCAGAATTCAGTGAATCATCGAAT
CTTTGAACGCATCTTGCGCTCCTTGGTATTCCGAGGAGCATGCCTGTTTGAGTGTCATTA
AATTCTCAACTCTCTTATACTTTTTTGTAAAAGAGAGCTTGGACTGTGGAGGCTTGCTGG
CCACTTTTTGGGGTCAGCTCCTCTGAAATGCATTAGCGGAACCGTTTGCAATCTGCCACA
AGTGTGATAAGTTATCTACACTGGCGAGGGGATTGCTCTCTGTAATGTTCAGCTTCTAAT
TGTCTCTACTTTGTGAGACTACTTTTGAATGCTTGACCTCAAATCAGGTAGGACTACCCG
CTGAACTTAA

>B10_13
TTTCCGTAGGTGAACCTGCGGAAGGATCATTATTGAATTATGTTTCTAGATAGGTTGTAG
CTGGCTCTTTAGAGCATGTGCACGCCTGTTTGGACTACATTTTCATCCACCTGTGCACCT
ATTGTAGTCTTTGGTTGGGTTAGGGGGAAGTGGTCATTGTGTCAGCATCTGCTGGATGTG
AGGACTTGCATTGTGAAAGCTTTGCTGTCCTTGATGTGATCATGGAATCTCTTTCTCACT
AGAGTCTATGTCACTCATTATACTCTGTCGAATGTCATTGAATGTCTTTACATGGGCTTG
TATGCCTATGAAAATTGTAATACAACTTTCAGCAACGGATCTCTTGGCTCTCGCATCGAT
GAAGGACGCAGCGAAATGCGATAAGTAATGTGAATTGCAGAATTCAGTGAATCATCGAAT
CTTTGAACGCATCTTGCGCTCCTTGGTATTCCGAGGAGCATGCCTGTTTGAGTGTCATTA
AATTCTCAACTCTCTTATACTTTTTTGTAAAAGAGAGCTTGGACTGTGGAGGCTTGCTGG
CCACTTTTTGGGGTCAGCTCCTCTGAAATGCATTAGCGGAACCGTTTGCAATCTGCCACA
AGTGTGATAAGTTATCTACACTGGCGAGGGGATTGCTCTCTGTAATGTTCAGCTTCTAAT
TGTCTCTACTTTGTGAGACAACTTTTGAATGCTTGACCTCAAATCAGGTAGGACTACCCG
CTGAACTTAA
>B10_58
TTTCCGTAGGTGAACCTGCGGAAGGATCATTATTGAATTATGTTTCTAGATAGGTTGTAG
CTGGCTCTTTAGAGCATGTGCACGCCTGTTTGGACTTCATTTTCATCCACCTGTGCACCT
ATTGTAGTCTTTGGTTGGGTTAGGGGGAAGTGGTCATTGTGTCAGCATCTGCTGGATGTG
AGGACTTGCATTGTGAAAGCTTTGCTGTCCTTGATGTGATCATGGAATCTCTTTCTCACT
AGAGTCTATGTCACTCATTATACTCTGTCGAATGTCATTGAATGTCTTTACATGGGCTTG
TATGCCTATGAAAATTGTAATACAACTTTCAGCAACGGATCTCTTGGCTCTCGCATCGAT
GAAGGACGCAGCGAAATGCGATAAGTAATGTGAATTGCAGAATTCAGTGAATCATCGAAT
CTTTGAACGCATCTTGCGCTCCTTGGTATTCCGAGGAGCATGCCTGTTTGAGTGTCATTA
AATTCTCAACTCTCTTATACTTTTTTGTAAAAGAGAGCTTGGACTGTGGAGGCTTGCTGG
CCACTTTTTGGGGTCAGCTCCTCTGAAATGCATTAGCGGAACCGTTTGCAAACTGCCACA
AGTGTGATAAGTTATCTACACTGGCGAGGGGATTGCTCTCTGTAATGTTCAGCTTCTAAT
TGTCTCTACTTTGTGAGACAACTTTTGAATGCTTGACCTCAAATCAGGTAGGACTACCCG
CTGAACTTAA
>B8_7
TTTCCGTAGGTGAACCTGCGGAAGGATCATTATTGAATTATGTTTCTAGATAGGTTGTAG
CTGGCTCTTTAGAGCATGTGCACGCCTGTTTGGACTTCATTTTCATTCACCTGTGCACCT
ATTGTAGTCTTTGGTTGGGTTAGGGGGAAGTGGTCATTGTGTCAGCATCTGCTGGATGTG
AGGACTTGCATTGTGAAAGCTTTGCTGTCCTTGATGTGATCATGGAATCTCTTTCTCACT
AGAGTCTATGTCACTCATTATACTCTGTCGAATGTCATTGAATGTCTTTACATGGGCTTG
TATGCCTATGAAAATTGTAATACAACTTTCAGCAACGGATCTCTTGGCTCTCGCATCGAT
GAAGGACGCAGCGAAATGCGATAAGTAATGTGAATTGCAGAATTCAGTGAATCATCGAAT
CTTTGAACGCATCTTGCGCTCCTTGGTATTCCGAGGAGCATGCCTGTTTGAGTGTCATTA
AATTCTCAACTCTCTTATACTTTTTTGTAAAAGAGAGCTTGGACTGTGGAGGCTTGCTGG
CCACTTTTTGGGGTCAGCTCCTCTGAAATGCATTAGCGGAACCGTTTGCAATCTGCCACA
AGTGTGATAAGTTATCTACACTGGCGAGGGGATTGCTCTCTGTAATGTTCAGCTTCTAAT
TGTCTCTACTTTGTGAGACAACTTTTGAATGCTTGACCTCAAATCAGGTAGGACTACCCG
CTGAACTTAA
>B7_16
TTTCCGTAGGTGAACCTGCGGAAGGATCATTATTGAATTATGTTTCTAGATAGGTTGTAG
CTGGCTCTTTAGAGCATGTGCACGCCTGTTTGGACTTCATTTTCATCCACCTGTGCACCT
ATTGTAGTCTTTGGTTGGGTTAGGGGGAAGTGGTCATTGTATCAGCATCTGCTGGATGTG
AGGACTTGCATTGTGAAAGCTTTGCTGTCCTTGATGTGATCATGGAATCTCTTTCTCACT
AGAGTCTATGTCACTCATTATACTCTGTCGAATGTCATTGAATGTCTTTACATGGGCTTG
TATGCCTATGAAAATTGTAATACAACTTTCAGCAACGGATCTCTTGGCTCTCGCATCGAT
GAAGGACGCAGCGAAATGCGATAAGTAATGTGAATTGCAGAATTCAGTGAATCATCGAAT

CTTTGAACGCATCTTGCGCTCCTTGGTATTCCGAGGAGCATGCCTGTTTGAGTGTCATTA
AATTCTCAACTCTCTTATACTTTTTTGTAAAAGAGAGCTTGGACTGTGGAGGCTTGCTGG
CCACTTTTTGGGGTCAGCTCCTCTGAAATGCATTAGCGGAACCGTTTGCAATCTGCCACA
AGTGTGATAAGTTATCTACACTGGCGAGGGGATTGCTCTCTGTAATGTTCAGCTTCTAAT
TGTCTCTACTTTGTGAGACAACTTTTGAATGCTTGACCTCAAATCAGGTAGGACTACCCG
CTGAACTTAA
>B11_35
TTTCCGTAGGTGAACCTGCGGAAGGATCATTATTGAATTATGTTTCTAGATAGGTTGTAG
CTGGCTCTTTAGAGCATGTGCACGCCTGTTTGGACTTCATTTTCATCCACCTGTGCACCT
ATTGTAGTCTTTGGTTGGGTTAGGGGGAAGTGGTCATTGTGTCAGCATCTGCTGGATGTG
AGGACTTGCATTGTGAAAGCTTTGCTGTCCTTGATGTGATCATGGAATCTCTTTCTCACT
AGAGTCTATGTCACTCATTATACTCTGTCGAATGTCATTGAATGTCTTTACATGGACTTG
TATGCCTATGAAAATTGTAATACAACTTTCAGCAACGGATCTCTTGGCTCTCGCATCGAT
GAAGGACGCAGCGAAATGCGATAAGTAATGTGAATTGCAGAATTCAGTGAATCATCGAAT
CTTTGAACGCATCTTGCGCTCCTTGGTATTCCGAGGAGCATGCCTGTTTGAGTGTCATTA
AATTCTCAACTCTCTTATACTTTTTTGTAAAAGAGAGCTTGGACTGTGGAGGCTTGCTGG
CCACTTTTTGGGGTCAGCTCCTCTGAAATGCATTAGCGGAACCGTTTGCAATCTGCCACA
AGTGTGATAAGTTATCTACACTGGCGAGGGGATTGCTCTCTGTAATGTTCAGCTTCTAAT
TGTCTCTACTTTGTGAGACAACTTTTGAATGCTTGACCTCAAATCAGGTAGGACTACCCG
CTGAACTTAA
>B11_26
TTTCCGTAGGTGAACCTGCGGAAGGATCATTATTGAATTATGTTTCTAGATAGGTTGTAG
CTGGCTCTTTAGAGCATGTGCACGCCTGTTTGGACTTCATTTTCATCCACCTGTGCACCT
ATTGTAGTCTTTGGTTGGGTTAGGGGGAAGTGGTCATTGTGTCAGCATCTGCTGGATGTG
AGGACTTGCATTGTGAAAGCTTTGCTGTCCTTGATGTGATCATGGAATCTCTTTCTCACT
AGAGTCTATGTCACTCATTATACTCTGTCGAATGTCATTGAATGTCTTTACATGGGCTTG
TATGTCTATGAAAATTGTAATACAACTTTCAGCAACGGATCTCTTGGCTCTCGCATCGAT
GAAGGACGCAGCGAAATGCGATAAGTAATGTGAATTGCAGAATTCAGTGAATCATCGAAT
CTTTGAACGCATCTTGCGCTCCTTGGTATTCCGAGGAGCATGCCTGTTTGAGTGTCATTA
AATTCTCAACTCTCTTATACTTTTTTGTAAAAGAGAGCTTGGACTGTGGAGGCTTGCTGG
CCACTTTTTGGGGTCAGCTCCTCTGAAATGCATTAGCGGAACCGTTTGCAATCTGCCACA
AGTGTGATAAGTTATCTACACTGGCGAGGGGATTGCTCTCTGTAATGTTCAGCTTCTAAT
TGTCTCTACTTTGTGAGACAACTTTTGAATGCTTGACCTCAAATCAGGTAGGACTACCCG
CTGAACTTAA
>B11_1
TTTCCGTAGGTGAACCTGCGGAAGGATCATTATTGAATTATGTTTCTAGATAGGTTGTAG
CTGGCTCTTTAGAGCATGTGCACGCCTGTTTGGACTTCATTTTCATCCACCTGTGCACCT
ATTGTAGTCTTTGGTTGGGTTAGGGGGAAGTGGTCATTGTGTCAGCATCTGCTGGATGTG
AGGACTTGCATTGTGAAAGCTTTGCTGTCCTTGATGTGATCATGGAATCTCTTTCTCACT
AGAGTCTATGTCACTCATTATACTCTGTCGAATGTCATTGAATGTCTTTACATGGGCTTA
TATGCCTATGAAAATTGTAATACAACTTTCAGCAACGGATCTCTTGGCTCTCGCATCGAT
GAAGAACGCAGCGAAATGCGATAAGTAATGTGAATTGCAGAATTCAGTGAATCATCGAAT
CTTTGAACGCATCTTGCGCTCCTTGGTATTCCGAGGAGCATGCCTGTTTGAGTGTCATTA
AATTCTCAACTCTCTTCTACTTTTTTGTAAAAGAGAGCTTGGACTGTGGAGGCTTGCTGG
CCACTTTTTGGGGTCAGCTCCTCTGAAATGCATTAGCGGAACCGTTTGCGATCTGCCACA
AGTGTGATAAGTTATCTACACTGGCGAGGGGATTGCTCTCTGTAATGTTCAGCTTCTAAT
TGTCTCTACTTTGTGAGACAACTTTTGAATGCTTGACCTCAAATCAGGTAGGACTACCCG
CTGAACTTAA
>B2_36
TTTCCGTAGGTGAACCTGCGGAAGGATCATTATTGAATTATGTTTCTAGATAGGTTGTAG

CTGGCTCTTTAGAGCATGTGCACGCCTGTTTGGACTTCATTTTCATCCACCTGTGCACCT
ATTGTAGTCTTTGGTTGGGTTAGGGGGAAGTGGTCATTGTGTCAGCATCTGCTGGATGTG
AGGACTTGCATTGTGAAAGCTTTGCTGTCCTTGATGTGATCATGGAATCTCTTTCTCACT
AGAGTCTATGTCACTCATTATACTCTGTCGAATGTCATTGAATGTCTTTACATGGGCTTG
TATGCCTATGAAAATTGTAATACAACTTTCAGCAACGGATCTCTTGGCTCTCACATCGAT
GAAGGACGCAGCGAAATGCGATAAGTAATGTGAATTGCAGAATTCAGTGAATCATCGAAT
CTTTGAACGCATCTTGCGCTCCTTGGTATTCCGAGGAGCATGCCTGTTTGAGTGTCATTA
AATTCTCAACTCTCTTATACTTTTTTGTAAAAGAGAGCTTGGACTGTGGAGGCTTGCTGG
CCACTTTTTGGGGTCAGCTCCTCTGAAATGCATTAGCGGAACCGTTTGCAATCTGCCACA
AGTGTGATAAGTTATCTACACTGGCGAGGGGATTGCTCTCTGTAATGTTCAGCTTCTAAT
TGTCTCTACTTTGTGAGACAACTTTTGAATGCTTGACCTCAAATCAGGTAGGACTACCCG
CTGAACTTAA
>B4−59
TTTCCGTAGGTGAACCTGCGGAAGGATCATTATTGAATTATGTTTCTAGATAGGTTGTAG
CTGGCTCTTTAGAGCATGTGCACGCCTGTTTGGACTTCATTTTCATCCACCTGTGCACCT
ATTGTAGTCTTTGGTTGGGTTAGGGGGAAGTGGTCATTGTGTCAGCATCTGCTGGATGTG
AGGACTTGCATTGTGAAAGCTTTGCTGTCCTTGATGTGATCATGGAATCTCTTTCTCACT
AGAGTCTATGTCACTCATTATACTCTGTCGAATGTCATTGAATGTCTTTACATGGGCTTG
TATGCCTATGAAAATTGTAATACAACTTTCAGCAACGGATCTCTTGGCTCTCGCATCGAT
GAAGGACGCAGCGAAATGCGATAAGTAATGTGAATTGCAGAATTCAGTGAATCATCGAAT
CTTTGAACACATCTTGCGCTCCTTGGTATTCCGAGGAGCATGCCTGTTTGAGTGTCATTA
AATTCTCAACTCTCTTATACTTTTTTGTAAAAGAGAGCTTGGACTGTGGAGGCTTGCTGG
CCACTTTTTGGGGTCAGCTCCTCTGAAATGCATTAGCGGAACCGTTTGCAATCTGCCACA
AGTGTGATAAGTTATCTACACTGGCGAGGGGATTGCTCTCTGTAATGTTCAGCTTCTAAT
TGTCTCTACTTTGTGAGACAACTTTTGAATGCTTGACCTCAAATCAGGTAGGACTACCCG
CTGAACTTAA
>B5_4
TTTCCGTAGGTGAACCTGCGGAAGGATCATTATTGAATTATGTTTCTAGATAGGTTGTAG
CTGGCTCTTTAGAGCATGTGCACGCCTGTTTGGACTTCATTTTCATCCACCTGTGCACCT
ATTGTAGTCTTTGGTTGGGTTAGGGGGAAGTGGTCATTGTGTCAGCATCTGCTGGATGTG
AGGACTTGCATTGTGAAAGCTTTGCTGTCCTTGATGTGATCATGGAATCTCTTTCTCACT
AGAGTCTATGTCACTCATTATACTCTGTCGAATGTCATTGAATGTCTTTACATGGGCTTG
TATGCCTATGAAAATTGTAATACAACTTTCAGCAACGGATCTCTTGGCTCTCGCATCGAT
GAAGGACGCAGCGAAATGCGATAAGTAATGTGAATTGCAGAATTCAGTGAATCATCGAAT
CTTTGAATGCATCTTGCGCTCCTTGGTATTCCGAGGAGCATGCCTGTTTGAGTGTCATTA
AATTCTCAACTCTCTTATACTTTTTTGTAAAAGAGAGCTTGGACTGTGGAGGCTTGCTGG
CCACTTTTTGGGGTCAGCTCCTCTGAAATGCATTAGCGGAACCGTTTGCAATCTGCCACA
AGTGTGATAAGTTATCTACACTGGCGAGGGGATTGCTCTCTGTAATGTTCAGCTTCTAAT
TGTCTCTACTTTGTGAGACAACTTTTGAATGCTTGACCTCAAATCAGGTAGGACTACCCG
CTGAACTTAA
>B9_39
TTTCCGTAGGTGAACCTGCGGAAGGATCATTATTGAATTATGTTTCTAGATAGGTTGTAG
CTGGCTCTTTAGAGCATGTGCACGCCTGTTTGGACTTCATTTTCATCCACCTGTGCACCT
ATTGTAGTCTTTGGTTGGGTTAGGGGGAAGTGGTCATTGTGTCAGCATCTGCTGGATGTG
AGGACTTGCATTGTGAAAGCTTTGCTGTCCTTGATGTGATCATGGAATCTCTTTCTCACT
AGAGTCTATGTCACTCATTATACTCTGTCGAATGTCATTGAATGTCTTTACATGGGCTTG
TATGCCTATGAAAATTGTAATACAACTTTCAGCAACGGATCTCTTGGCTCTCGCATCGAT
GAAGGACGCAGCGAAATGCGATAAGTAATGTGAATTGCAGAATTCAGTGAATCATCGAAT
CTTTGAACGCATCTTGCGCTCCTTGGTATTCCGAGGAGCATGCCTGTTTGAGTGTCATTA
AATTCTCAACTCTCTTATACTTTTTTGTAAAAGAGAGCTTGGACTGTGGAGGCTTGCTGG

CCACTTTTTGGGATCAGCTCCTCTGAAATGCATTAGCGGAACCGTTTGCAATCTGCCACA
AGTGTGATAAGTTATCTACACTGGCGAGGGGATTGCTCTCTGTAATGTTCAGCTTCTAAT
TGTCTCTACTTTGTGAGACAACTTTTGAATGCTTGACCTCAAATCAGGTAGGACTACCCG
CTGAACTTAA
>B6_16
TTTCCGTAGGTGAACCTGCGGAAGGATCATTATTGAATTATGTTTCTAGATAGGTTGTAG
CTGGCTCTTTAGAGCATGTGCACGCCTGTTTGGACTTCATTTTCATCCACCTGTGCACCT
ATTGTAGTCTTTGGTTGGGTTAGGGGGAAGTGGTCATTGTGTCAGCATCTGCTGGATGTG
AGGACTTGCATTGTGAAAGCTTTGCTGTCCTTGATGTGATCATGGAATCTCTTTCTCACT
AGAGTCTATGTCACTCATTATACTCTGTCGAATGTCATTGAATGTCTTTACATGGGCTTG
TATGCCTATGAAAATTGTAATACAACTTTCAGCAACGGATCTCTTGGCTCTCGCATCGAT
GAAGGACGCAGCGAAATGCGATAAGTAATGTGAATTGCAGAATTCAGTGAATCATCGAAT
CTTTGAACGCATCTTGCGCTCCTTGGTATTCCGAGGAGCATGCCTGTTTGAGTGTCATTA
AATTCTCAACTCTCTTATACTTTTTTGTAAAAGAGAGCTTGGACTGTGGAGGCTTGCTGG
CCACTTTTTGGGGTCAGCTCCTCTGAAATGCATTAGCGGAACCGTTTGCAATCTACCACA
AGTGTGATAAGTTATCTACACTGGCGAGGGGATTGCTCTCTGTAATGTTCAGCTTCTAAT
TGTCTCTACTTTGTGAGACAACTTTTGAATGCTTGACCTCAAATCAGGTAGGACTACCCG
CTGAACTTAA
>B11_23
TTTCCGTAGGTGAACCTGCGGAAGGATCATTATTGAATTATGTTTCTAGATAGGTTGTAG
CTGGCTCTTTAGAGCATGTGCACGCCTGTTTGGACTTCATTTTCATCCACCTGTGCACCT
ATTGTAGTCTTTGGTTGGGTTAGGGGGAAGTGGTCATTGTGTCAGCATCTGCTGGATGTG
AGGACTTGCATTGTGAAAGCTTTGCTGTCCTTGATGTGATCATGGAATCTCTTTCTCACT
AGAGTCTATGTCACTCATTATACTCTGTCGAATGTCATTGAATGTCTTTACATGGGCTTG
TATGCCTATGAAAATTGTAATACAACTTTCAGCAACGGATCTCTTGGCTCTCGCATCGAT
GAAGGACGCAGCGAAATGCGATAAGTAATGTGAATTGCAGAATTCAGTGAATCATCGAAT
CTTTGAACGCATCTTGCGCTCCTTGGTATTCCGAGGAGCATGCCTGTTTGAGTGTCATTA
AATTCTCAACTCTCTTATACTTTTTTGTAAAAGAGAGCTTGGACTGTGGAGGCTTGCTGG
CCACTTTTTGGGGTCAGCTCCTCTGAAATGCATTAGCGGAACCGTTTGCAATCTACCACA
AGTGTGATAAGTTATCTACACTGGCGAGGGGATTGCTCTCTGTAATGTTCAGCTTCTAAT
TGTCTCTACTTTGTGAGACAACTTTTGAATGCTTGACCTCAAATCAGGTAGGACTACCCG
CTGAACTTAA
>B3–58
TTTCCGTAGGTGAACCTGCGGAAGGATCATTATTGAATTATGTTTCTAGATAGGTTGTAG
CTGGCTCTTTAGAGCATGTGCACGCCTGTTTGGACTTCATTTTCATCCACCTGTGCACCT
ATTGTAGTCTTTGGTTGGGTTAGGGGGAAGTGGTCATTGTGTCAGCATCTGCTGGATGTG
AGGACTTGCATTGTGAAAGCTTTGCTGTCCTTGATGTGATCATGGAATCTCTTTCTCACT
AGAGTCTATGTCACTCATTATACTCTGTCGAATGTCATTGAATGTCTTTACATGGGCTTG
TATGCCTATGAAAATTGTAATACAACTTTCAGCAACGGATCTCTTGGCTCTCGCATCGAT
GAAGGACGCAGCGAAATGCGATAAGTAATGTGAATTGCAGAATTCAGTGAATCATCGAAT
CTTTGAACGCATCTTGCGCTCCTTGGTATTCCGAGGAGCATGCCTGTTTGAGTGTCATTA
AATTCTCAACTCTCTTATACTTTTTTGTAAAAGAGAGCTTGGACTGTGGAGGCTTGCTGG
CCACTTTTTGGGGTCAGCTCCTCTGAAATGCATTAGCGGAACCGTTTGTAATCTGCCACA
AGTGTGATAAGTTATCTACACTGGCGAGGGGATTGCTCTCTGTAATGTTCAGCTTCTAAT
TGTCTCTACTTTGTGAGACAACTTTTGAATGCTTGACCTCAAATCAGGTAGGACTACCCG
CTGAACTTAA
>B1_40
TTTCCGTAGGTGAACCTGCGGAAGGATCATTATTGAATTATGTTTCTAGATAGGTTGTAG
CTGGCTCTTTAGAGCATGTGCACGCCTGTTTGGACTTCATTTTCATCCACCTGTGCACCT
ATTGTAGTCTTTGGTTGGGTTAGGGGGAAGTGGTCATTGTGTCAGCATCTGCTGGATGTG

AGGACTTGCATTGTGAAAGCTTTGCTGTCCTTGATGTGATCATGGAATCTCTTTCTCACT
AGAGTCTATGTCACTCATTATACTCTGTCGAATGTCATTGAATGTCTTTACATGGGCTTG
TATGCCTATGAAAATTGTAATACAACTTTCAGCAACGGATCTCTTGGCTCTCGCATCGAT
GAAGGACGCAGCGAAATGCGATAAGTAATGTGAATTGCAGAATTCAGTGAATCATCGAAT
CTTTGAACGCATCTTGCGCTCCTTGGTATTCCGAGGAGCATGCCTGTTTGAGTGTCATTA
AATTCTCAACTCTCTTATACTTTTTTGTAAAAGAGAGCTTGGACTGTGGAGGCTTGCTGG
CCACTTTTTGGGGTCAGCTCCTCTGAAATGCATTAGCGGAACCGTTTGTAATCTGCCACA
AGTGTGATAAGTTATCTACACTGGCGAGGGGATTGCTCTCTGTAATGTTCAGCTTCTAAT
TGTCTCTACTTTGTGAGACAACTTTTGAATGCTTGACCTCAAATCAGGTAGGACTACCCG
CTGAACTTAA
>B6_3
TTTCCGTAGGTGAACCTGCGGAAGGATCATTATTGAATTATGTTTCTAGATAGGTTGTAG
CTGGCTCTTTAGAGCATGTGCACGCCTGTTTGGACTTCATTTTCATCCACCTGTGCACCT
ATTGTAGTCTTTGGTTGGGTTAGGGGGAAGTGGTCATTGTGTCAGCATCTGCTGGATGTG
AGGACTTGCATTGTGAAAGCTTTGCTGTCCTTGATGTGATCATGGAATCTCTTTCTCACT
AGAGTCTATGTCACTCATTATACTCTGTCGAATGTCATTGAATGTCTTTACATGGGCTTG
TATGCCTATGAAAATTGTAATACAACTTTCAGCAACGGATCTCTTGGCTCTCGCATCGAT
GAAGGACGCAGCGAAATGCGATAAGTAATGTGAATTGCAGAATTCAGTGAATCATCGAAT
CTTTGAACGCATCTTGCGCTCCTTGGTATTCCGAGGAGCATGCCTGTTTGAGTGTCATTA
AATTCTCAACTCTCTTATACTTTTTTGTAAAAGAGAGCTTGGACTGTGGAGGCTTGCTGG
CCACTTTTTGGGGTCAGCTCCTCTGAAATGCATTAGTGGAACCGTTTGCAATCTGCCACA
AGTGTGATAAGTTATCTACACTGGCGAGGGGATTGCTCTCTGTAATGTTCAGCTTCTAAT
TGTCTCTACTTTGTGAGACAACTTTTGAATGCTTGACCTCAAATCAGGTAGGACTACCCG
CTGAACTTAA
