# Supplementary material for: Patterns of PCR Amplification Artifacts of the Fungal Barcode Marker in a Hybrid Mushroom
Source: Front Microbiol. 2019 Nov 19;10:2686. doi: 10.3389/fmicb.2019.02686 (PMC6877668; doi:10.3389/fmicb.2019.02686)
Supplement: Supplementary file 9 [file Data_Sheet_9.PDF]

```
>AC1-11
TTTCCGTAGGTGAACCTGCGGAAGGATCATTATTGAATTATGTTTCTAGATAGGTTGTAG
CTGGCTCTTTTAGAGCATGTGCACGCCTGTTTGGACTTCATTTTCATCCACCTGTGCACC
TATTGTAGTCTTTGGTTGGGTTAGGAGGAAGTGATCATTGTATCAGCATCTGCTGGGAGT
GAGGACTTGCATTGTGAAAGCTTTGCTGTCCTTGATGTGATCATGGAATCTTTTTC----
------------------------------------------------------------
------------------------------------------------------------
------------------------------------------------------------
------------------------------------------------------------
------------------------------------------------------------
------------------------------------------------------------
------------------------------------------------------------
------------------------------------------------------------
------------------------------------------------------------
------------------------------------------------------------
------------------------------------------------------------
------------------------------------------------------------
-------TCACTAGAGTCTATGTCACTCATTATACTCTGTCGAATGTCATTGAATGTCTT
TACATGGGCTTGTATGCCTATGAAAATTGTAATACAACTTTCAGCAACGGATCTCTTGGC
TCTCGCATCGATGAAGAACGCAGCGAAATGCGATAAGTAATGTGAATTGCAGAATTCAGT
GAATCATCGAATCTTTGAACGCATCTTGCGCTCCTTGGTATTCCGAGGAGCATGCCTGTT
TGAGTGTCATTAAATTCTCAACTCTCTTATACTTTTTTGTAAAAGAGAGCTTGGACTGTG
GAGGCTTGCTGGCCACTTTTTGGGGTCAGCTCCTCTGAAATGCATTAGCGGAACCGTTTG
CGATCTGCCACAAGTGTGATAAGTTATCTACACTGGCGAGGGGATTGCTCTCTGTAATGT
TCAGCTTCTAATTGTCTCTACTTTGTGAGACTACTTTTGAATGCTTGACCTCAAATCAGG
TAGGACTACCCGCTGAACTTAA
>AC4-76
TTTCCGTAGGTGAACCTGCGGAAGGATCATTATTGAATTATGTTTCTAGATAGGTTGTAG
CTGGCTCTTTTAGAGCATGTGCACGCCTGTTTGGACTTCATTTTCATCCACCTGTGCACC
TATTGTAGTCTTTGGTTGGGTTAGGAGGAAGTGATCATTGTATCAGCATCTGCTGGGAGT
GAGGACTTGCATTGTGAAAGCTTTGCTGTCCTTGATGTGATCATGGAATCTTTTTC----
------------------------------------------------------------
------------------------------------------------------------
------------------------------------------------------------
------------------------------------------------------------
------------------------------------------------------------
------------------------------------------------------------
------------------------------------------------------------
------------------------------------------------------------
------------------------------------------------------------
------------------------------------------------------------
------------------------------------------------------------
------------------------------------------------------------
-------TCACTAGAGTCTATGTCACTCATTATACTCTGTCGAATGTCATTGAATGTCTT
TACATGGGCTTGTATGCCTATGAAAATTGTAATACAACTTTCAGCAACGGATCTCTTGGC
TCTCGCATCGATGAAGAACGCAGCGAAATGCGATAAGTAATGTGAATTGCAGAATTCAGT
GAATCATCGAATCTTTGAACGCATCTTGCGCTCCTTGGTATTCCGAGGAGCATGCCTGTT
TGAGTGTCATTAAATTCTCAACTCTCTTATACTTTTTTGTAAAAGAGAGCTTGGACTGTG
GAGGCTTGCTGGCCACTTTTTGGGGTCAGCTCCTCTGAAATGCATTAGCGGAACCGTTTG
CGATCTGCCACAAGTGTGATAAGTTATCTACACTGGCGAGGGGATTGCTCTCTGTAATGT
```

TCAGCTTCTAATTGTCTCTACTTTGTGAGACTACTTTTGAATGCTTGACCTCAAATCAGG
TAGGACTACCCGCTGAACTTAA
>AC4-84
TTTCCGTAGGTGAACCTGCGGAAGGATCATTATTGAATTATGTTTCTAGATAGGTTGTAG
CTGGCTCTTTTAGAGCATGTGCACGCCTGTTTGGACTTCATTTTCATCCACCTGTGCACC
TATTGTAGTCTTTGGTTGGGTTAGGAGGAAGTGATCATTGTATCAGCATCTGCTGGGAGT
GAGGACTTGCATTGTGAAAGCTTTGCTGTCCTTGATGTGATCATGGAATCTTTTTC----

-------TCACTAGAGTCTATGTCACTCATTATACTCTGTCGAATGTCATTGAATGTCTT
TACATGGGCTTGTATGCCTATGAAAATTGTAATACAACTTTCAGCAACGGATCTCTTGGC
TCTCGCATCGATGAAGAACGCAGCGAAATGCGATAAGTAATGTGAATTGCAGAATTCAGT
GAATCATCGAATCTTTGAACGCATCTTGCGCTCCTTGGTATTCCGAGGAGCATGCCTGTT
TGAGTGTCATTAAATTCTCAACTCTCTTATACTTTTTTGTAAAAGAGAGCTTGGACTGTG
GAGGCTTGCTGGCCACTTTTTGGGGTCAGCTCCTCTGAAATGCATTAGCGGAACCGTTTG
CGATCTGCCACAAGTGTGATAAGTTATCTACACTGGCGAGGGGATTGCTCTCTGTAATGT
TCAGCTTCTAATTGTCTCTACTTTGTGAGACTACTTTTGAATGCTTGACCTCAAATCAGG
TAGGACTACCCGCTGAACTTAA
>AC6-47
TTTCCGTAGGTGAACCTGCGGAAGGATCATTATTGAATTATGTTTCTAGATAGGTTGTAG
CTGGCTCTTTTAGAGCATGTGCACGCCTGTTTGGACTTCATTTTCATCCACCTGTGCACC
TATTGTAGTCTTTGGTTGGGTTAGGAGGAAGTGATCATTGTATCAGCATCTGCTGGGAGT
GAGGACTTGCATTGTGAAAGCTTTGCTGTCCTTGATGTGATCATGGAATCTTTTTC----

-------TCACTAGAGTCTATGTCACTCATTATACTCTGTCGAATGTCATTGAATGTCTT
TACATGGGCTTGTATGCCTATGAAAATTGTAATACAACTTTCAGCAACGGATCTCTTGGC
TCTCGCATCGATGAAGAACGCAGCGAAATGCGATAAGTAATGTGAATTGCAGAATTCAGT
GAATCATCGAATCTTTGAACGCATCTTGCGCTCCTTGGTATTCCGAGGAGCATGCCTGTT
TGAGTGTCATTAAATTCTCAACTCTCTTATACTTTTTTGTAAAAGAGAGCTTGGACTGTG

GAGGCTTGCTGGCCACTTTTTGGGGTCAGCTCCTCTGAAATGCATTAGCGGAACCGTTTG
CGATCTGCCACAAGTGTGATAAGTTATCTACACTGGCGAGGGGATTGCTCTCTGTAATGT
TCAGCTTCTAATTGTCTCTACTTTGTGAGACTACTTTTGAATGCTTGACCTCAAATCAGG
TAGGACTACCCGCTGAACTTAA
>AC10-34
TTTCCGTAGGTGAACCTGCGGAAGGATCATTATTGAATTATGTTTCTAGATAGGTTGTAG
CTGGCTCTTTTAGAGCATGTGCACGCCTGTTTGGACTTCATTTTCATCCACCTGTGCACC
TATTGTAGTCTTTGGTTGGGTTAGGAGGAAGTGATCATTGTATCAGCATCTGCTGGGAGT
GAGGACTTGCATTGTGAAAGCTTTGCTGTCCTTGATGTGATCATGGAATCTTTTTC----

-------TCACTAGAGTCTATGTCACTCATTATACTCTGTCGAATGTCATTGAATGTCTT
TACATGGGCTTGTATGCCTATGAAAATTGTAATACAACTTTCAGCAACGGATCTCTTGGC
TCTCGCATCGATGAAGAACGCAGCGAAATGCGATAAGTAATGTGAATTGCAGAATTCAGT
GAATCATCGAATCTTTGAACGCATCTTGCGCTCCTTGGTATTCCGAGGAGCATGCCTGTT
TGAGTGTCATTAAATTCTCAACTCTCTTATACTTTTTTGTAAAAGAGAGCTTGGACTGTG
GAGGCTTGCTGGCCACTTTTTGGGGTCAGCTCCTCTGAAATGCATTAGCGGAACCGTTTG
CGATCTGCCACAAGTGTGATAAGTTATCTACACTGGCGAGGGGATTGCTCTCTGTAATGT
TCAGCTTCTAATTGTCTCTACTTTGTGAGACTACTTTTGAATGCTTGACCTCAAATCAGG
TAGGACTACCCGCTGAACTTAA
>AC3-3
TTTCCGTAGGTGAACCTGCGGAAGGATCATTATTGAATTATGTTTCTAGATAGGTTGTAG
CTGGCTCTTTTAGAGCATGTGCACGCCTGTTTGGACTTCATTTTCATCCACCTGTGCACC
TATTGTAGTCTTTGGTTGGGTTAGGAGGAAGTGATCATTGTATCAGCATCTGCTGGGAGT
GAGGACTTGCATTGTGAAAGCTTTGCTGTCCTTGATGTGATCATGGAATCTTTTTC----

-------TCACTAGAGTCTATGTCACTCATTATACTCTGTCGAATGTCATTGAATGTCTT
TACATGGGCTTGTATGCCTATGAAAATTGTAATACAACTTTCAGCAACGGATCTCTTGGC
TCTCGCATCGATGAAGAACGCAGCGAAATGCGATAAGTAATGTGAATTGCAGAATTCAGT

GAATCATCGAATCTTTGAACGCATCTTGCGCTCCTTGGTATTCCGAGGAGCATGCCTGTT
TGAGTGTCATTAAATTCTCAACTCTCTTATACTTTTTTGTAAAAGAGAGCTTGGACTGTG
GAGGCTTGCTGGCCACTTTTTGGGGTCAGCTCCTCTGAAATGCATTAGCGGAACCGTTTG
CGATCTGCCACAAGTGTGATAAGTTATCTACACTGGCGAGGGGATTGCTCTCTGTAATGT
TCAGCTTCTAATTGTCTCTACTTTGTGAGACTACTTTTGAATGCTTGACCTCAAATCAGG
TAGGACTACCCGCTGAACTTAA
>AC6-24
TTTCCGTAGGTGAACCTGCGGAAGGATCATTATTGAATTATGTTTCTAGATAGGTTGTAG
CTGGCTCTTTTAGAGCATGTGCACGCCTGTTTGGACTTCATTTTCATCCACCTGTGCACC
TATTGTAGTCTTTGGTTGGGTTAGGAGGAAGTGATCATTGTATCAGCATCTGCTGGGAGT
GAGGACTTGCATTGTGAAAGCTTTGCTGTCCTTGATGTGATCATGGAATCTTTTTC----

-------TCACTAGAGTCTATGTCACTCATTATACTCTGTCGAATGTCATTGAATGTCTT
TACATGGGCTTGTATGCCTATGAAAATTGTAATACAACTTTCAGCAACGGATCTCTTGGC
TCTCGCATCGATGAAGAACGCAGCGAAATGCGATAAGTAATGTGAATTGCAGAATTCAGT
GAATCATCGAATCTTTGAACGCATCTTGCGCTCCTTGGTATTCCGAGGAGCATGCCTGTT
TGAGTGTCATTAAATTCTCAACTCTCTTATACTTTTTTGTAAAAGAGAGCTTGGACTGTG
GAGGCTTGCTGGCCACTTTTTGGGGTCAGCTCCTCTGAAATGCATTAGCGGAACCGTTTG
CGATCTGCCACAAGTGTGATAAGTTATCTACACTGGCGAGGGGATTGCTCTCTGTAATGT
TCAGCTTCTAATTGTCTCTACTTTGTGAGACTACTTTTGAATGCTTGACCTCAAATCAGG
TAGGACTACCCGCTGAACTTAA
>AC1-56
TTTCCGTAGGTGAACCTGCGGAAGGATCATTATTGAATTATGTTTCTAGATAGGTTGTAG
CTGGCTCTTTTAGAGCATGTGCACGCCTGTTTGGACTTCATTTTCATCCACCTGTGCACC
TATTGTAGTCTTTGGTTGGGTTAGGAGGAAGTGATCATTGTATCAGCATCTGCTGGGAGT
GAGGACTTGCATTGTGAAAGCTTTGCTGTCCTTGATGTGATCATGGAATCTTTTTC----

-------TCACTAGAGTCTATGTCACTCATTATACTCTGTCGAATGTCATTGAATGTCTT

TACATGGGCTTGTATGCCTATGAAAATTGTAATACAACTTTCAGCAACGGATCTCTTGGC
TCTCGCATCGATGAAGAACGCAGCGAAATGCGATAAGTAATGTGAATTGCAGAATTCAGT
GAATCATCGAATCTTTGAACGCATCTTGCGCTCCTTGGTATTCCGAGGAGCATGCCTGTT
TGAGTGTCATTAAATTCTCAACTCTCTTATACTTTTTTGTAAAAGAGAGCTTGGACTGTG
GAGGCTTGCTGGCCACTTTTTGGGGTCAGCTCCTCTGAAATGCATTAGCGGAACCGTTTG
CGATCTGCCACAAGTGTGATAAGTTATCTACACTGGCGAGGGGATTGCTCTCTGTAATGT
TCAGCTTCTAATTGTCTCTACTTTGTGAGACTACTTTTGAATGCTTGACCTCAAATCAGG
TAGGACTACCCGCTGAACTTAA
>AC3-39
TTTCCGTAGGTGAACCTGCGGAAGGATCATTATTGAATTATGTTTCTAGATAGGTTGTAG
CTGGCTCTTTTAGAGCATGTGCACGCCTGTTTGGACTTCATTTTCATCCACCTGTGCACC
TATTGTAGTCTTTGGTTGGGTTAGGAGGAAGTGATCATTGTATCAGCATCTGCTGGGAGT
GAGGACTTGCATTGTGAAAGCTTTGCTGTCCTTGATGTGATCATGGAATCTTTTTC----

-------TCACTAGAGTCTATGTCACTCATTATACTCTGTCGAATGTCATTGAATGTCTT
TACATGGGCTTGTATGCCTATGAAAATTGTAATACAACTTTCAGCAACGGATCTCTTGGC
TCTCGCATCGATGAAGAACGCAGCGAAATGCGATAAGTAATGTGAATTGCAGAATTCAGT
GAATCATCGAATCTTTGAACGCATCTTGCGCTCCTTGGTATTCCGAGGAGCATGCCTGTT
TGAGTGTCATTAAATTCTCAACTCTCTTATACTTTTTTGTAAAAGAGAGCTTGGACTGTG
GAGGCTTGCTGGCCACTTTTTGGGGTCAGCTCCTCTGAAATGCATTAGCGGAACCGTTTG
CGATCTGCCACAAGTGTGATAAGTTATCTACACTGGCGAGGGGATTGCTCTCTGTAATGT
TCAGCTTCTAATTGTCTCTACTTTGTGAGACTACTTTTGAATGCTTGACCTCAAATCAGG
TAGGACTACCCGCTGAACTTAA
>AC3-46
TTTCCGTAGGTGAACCTGCGGAAGGATCATTATTGAATTATGTTTCTAGATAGGTTGTAG
CTGGCTCTTTTAGAGCATGTGCACGCCTGTTTGGACTTCATTTTCATCCACCTGTGCACC
TATTGTAGTCTTTGGTTGGGTTAGGAGGAAGTGATCATTGTATCAGCATCTGCTGGGAGT
GAGGACTTGCATTGTGAAAGCTTTGCTGTCCTTGATGTGATCATGGAATCTTTTTC----


```
------------------------------------------------------------
-------TCACTAGAGTCTATGTCACTCATTATACTCTGTCGAATGTCATTGAATGTCTT
TACATGGGCTTGTATGCCTATGAAAATTGTAATACAACTTTCAGCAACGGATCTCTTGGC
TCTCGCATCGATGAAGAACGCAGCGAAATGCGATAAGTAATGTGAATTGCAGAATTCAGT
GAATCATCGAATCTTTGAACGCATCTTGCGCTCCTTGGTATTCCGAGGAGCATGCCTGTT
TGAGTGTCATTAAATTCTCAACTCTCTTATACTTTTTTGTAAAAGAGAGCTTGGACTGTG
GAGGCTTGCTGGCCACTTTTTGGGGTCAGCTCCTCTGAAATGCATTAGCGGAACCGTTTG
CGATCTGCCACAAGTGTGATAAGTTATCTACACTGGCGAGGGGATTGCTCTCTGTAATGT
TCAGCTTCTAATTGTCTCTACTTTGTGAGACTACTTTTGAATGCTTGACCTCAAATCAGG
TAGGACTACCCGCTGAACTTAA
>AC4-23
TTTCCGTAGGTGAACCTGCGGAAGGATCATTATTGAATTATGTTTCTAGATAGGTTGTAG
CTGGCTCTTTTAGAGCATGTGCACGCCTGTTTGGACTTCATTTTCATCCACCTGTGCACC
TATTGTAGTCTTTGGTTGGGTTAGGAGGAAGTGATCATTGTATCAGCATCTGCTGGGAGT
GAGGACTTGCATTGTGAAAGCTTTGCTGTCCTTGATGTGATCATGGAATCTTTTTC----
------------------------------------------------------------
------------------------------------------------------------
------------------------------------------------------------
------------------------------------------------------------
------------------------------------------------------------
------------------------------------------------------------
------------------------------------------------------------
------------------------------------------------------------
------------------------------------------------------------
------------------------------------------------------------
------------------------------------------------------------
------------------------------------------------------------
-------TCACTAGAGTCTATGTCACTCATTATACTCTGTCGAATGTCATTGAATGTCTT
TACATGGGCTTGTATGCCTATGAAAATTGTAATACAACTTTCAGCAACGGATCTCTTGGC
TCTCGCATCGATGAAGAACGCAGCGAAATGCGATAAGTAATGTGAATTGCAGAATTCAGT
GAATCATCGAATCTTTGAACGCATCTTGCGCTCCTTGGTATTCCGAGGAGCATGCCTGTT
TGAGTGTCATTAAATTCTCAACTCTCTTATACTTTTTTGTAAAAGAGAGCTTGGACTGTG
GAGGCTTGCTGGCCACTTTTTGGGGTCAGCTCCTCTGAAATGCATTAGCGGAACCGTTTG
CGATCTGCCACAAGTGTGATAAGTTATCTACACTGGCGAGGGGATTGCTCTCTGTAATGT
TCAGCTTCTAATTGTCTCTACTTTGTGAGACTACTTTTGAATGCTTGACCTCAAATCAGG
TAGGACTACCCGCTGAACTTAA
>AC4-85
TTTCCGTAGGTGAACCTGCGGAAGGATCATTATTGAATTATGTTTCTAGATAGGTTGTAG
CTGGCTCTTTTAGAGCATGTGCACGCCTGTTTGGACTTCATTTTCATCCACCTGTGCACC
TATTGTAGTCTTTGGTTGGGTTAGGAGGAAGTGATCATTGTATCAGCATCTGCTGGGAGT
GAGGACTTGCATTGTGAAAGCTTTGCTGTCCTTGATGTGATCATGGAATCTTTTTC----
------------------------------------------------------------
------------------------------------------------------------
------------------------------------------------------------
------------------------------------------------------------
------------------------------------------------------------
------------------------------------------------------------
------------------------------------------------------------
------------------------------------------------------------
------------------------------------------------------------
```


-------TCACTAGAGTCTATGTCACTCATTATACTCTGTCGAATGTCATTGAATGTCTT
TACATGGGCTTGTATGCCTATGAAAATTGTAATACAACTTTCAGCAACGGATCTCTTGGC
TCTCGCATCGATGAAGAACGCAGCGAAATGCGATAAGTAATGTGAATTGCAGAATTCAGT
GAATCATCGAATCTTTGAACGCATCTTGCGCTCCTTGGTATTCCGAGGAGCATGCCTGTT
TGAGTGTCATTAAATTCTCAACTCTCTTATACTTTTTTGTAAAAGAGAGCTTGGACTGTG
GAGGCTTGCTGGCCACTTTTTGGGGTCAGCTCCTCTGAAATGCATTAGCGGAACCGTTTG
CGATCTGCCACAAGTGTGATAAGTTATCTACACTGGCGAGGGGATTGCTCTCTGTAATGT
TCAGCTTCTAATTGTCTCTACTTTGTGAGACTACTTTTGAATGCTTGACCTCAAATCAGG
TAGGACTACCCGCTGAACTTAA
>AC5-58
TTTCCGTAGGTGAACCTGCGGAAGGATCATTATTGAATTATGTTTCTAGATAGGTTGTAG
CTGGCTCTTTTAGAGCATGTGCACGCCTGTTTGGACTTCATTTTCATCCACCTGTGCACC
TATTGTAGTCTTTGGTTGGGTTAGGAGGAAGTGATCATTGTATCAGCATCTGCTGGGAGT
GAGGACTTGCATTGTGAAAGCTTTGCTGTCCTTGATGTGATCATGGAATCTTTTTC----

-------TCACTAGAGTCTATGTCACTCATTATACTCTGTCGAATGTCATTGAATGTCTT
TACATGGGCTTGTATGCCTATGAAAATTGTAATACAACTTTCAGCAACGGATCTCTTGGC
TCTCGCATCGATGAAGAACGCAGCGAAATGCGATAAGTAATGTGAATTGCAGAATTCAGT
GAATCATCGAATCTTTGAACGCATCTTGCGCTCCTTGGTATTCCGAGGAGCATGCCTGTT
TGAGTGTCATTAAATTCTCAACTCTCTTATACTTTTTTGTAAAAGAGAGCTTGGACTGTG
GAGGCTTGCTGGCCACTTTTTGGGGTCAGCTCCTCTGAAATGCATTAGCGGAACCGTTTG
CGATCTGCCACAAGTGTGATAAGTTATCTACACTGGCGAGGGGATTGCTCTCTGTAATGT
TCAGCTTCTAATTGTCTCTACTTTGTGAGACTACTTTTGAATGCTTGACCTCAAATCAGG
TAGGACTACCCGCTGAACTTAA
>AC6-39
TTTCCGTAGGTGAACCTGCGGAAGGATCATTATTGAATTATGTTTCTAGATAGGTTGTAG
CTGGCTCTTTTAGAGCATGTGCACGCCTGTTTGGACTTCATTTTCATCCACCTGTGCACC
TATTGTAGTCTTTGGTTGGGTTAGGAGGAAGTGATCATTGTATCAGCATCTGCTGGGAGT
GAGGACTTGCATTGTGAAAGCTTTGCTGTCCTTGATGTGATCATGGAATCTTTTTC----


```
------------------------------------------------------------
------------------------------------------------------------
------------------------------------------------------------
------------------------------------------------------------
------------------------------------------------------------
-------TCACTAGAGTCTATGTCACTCATTATACTCTGTCGAATGTCATTGAATGTCTT
TACATGGGCTTGTATGCCTATGAAAATTGTAATACAACTTTCAGCAACGGATCTCTTGGC
TCTCGCATCGATGAAGAACGCAGCGAAATGCGATAAGTAATGTGAATTGCAGAATTCAGT
GAATCATCGAATCTTTGAACGCATCTTGCGCTCCTTGGTATTCCGAGGAGCATGCCTGTT
TGAGTGTCATTAAATTCTCAACTCTCTTATACTTTTTTGTAAAAGAGAGCTTGGACTGTG
GAGGCTTGCTGGCCACTTTTTGGGGTCAGCTCCTCTGAAATGCATTAGCGGAACCGTTTG
CGATCTGCCACAAGTGTGATAAGTTATCTACACTGGCGAGGGGATTGCTCTCTGTAATGT
TCAGCTTCTAATTGTCTCTACTTTGTGAGACTACTTTTGAATGCTTGACCTCAAATCAGG
TAGGACTACCCGCTGAACTTAA
>AC11-20
TTTCCGTAGGTGAACCTGCGGAAGGATCATTATTGAATTATGTTTCTAGATAGGTTGTAG
CTGGCTCTTTTAGAGCATGTGCACGCCTGTTTGGACTTCATTTTCATCCACCTGTGCACC
TATTGTAGTCTTTGGTTGGGTTAGGAGGAAGTGATCATTGTATCAGCATCTGCTGGGAGT
GAGGACTTGCATTGTGAAAGCTTTGCTGTCCTTGATGTGATCATGGAATCTTTTTC----
------------------------------------------------------------
------------------------------------------------------------
------------------------------------------------------------
------------------------------------------------------------
------------------------------------------------------------
------------------------------------------------------------
------------------------------------------------------------
------------------------------------------------------------
------------------------------------------------------------
------------------------------------------------------------
------------------------------------------------------------
------------------------------------------------------------
-------TCACTAGAGTCTATGTCACTCATTATACTCTGTCGAATGTCATTGAATGTCTT
TACATGGGCTTGTATGCCTATGAAAATTGTAATACAACTTTCAGCAACGGATCTCTTGGC
TCTCGCATCGATGAAGAACGCAGCGAAATGCGATAAGTAATGTGAATTGCAGAATTCAGT
GAATCATCGAATCTTTGAACGCATCTTGCGCTCCTTGGTATTCCGAGGAGCATGCCTGTT
TGAGTGTCATTAAATTCTCAACTCTCTTATACTTTTTTGTAAAAGAGAGCTTGGACTGTG
GAGGCTTGCTGGCCACTTTTTGGGGTCAGCTCCTCTGAAATGCATTAGCGGAACCGTTTG
CGATCTGCCACAAGTGTGATAAGTTATCTACACTGGCGAGGGGATTGCTCTCTGTAATGT
TCAGCTTCTAATTGTCTCTACTTTGTGAGACTACTTTTGAATGCTTGACCTCAAATCAGG
TAGGACTACCCGCTGAACTTAA
>AC12-18
TTTCCGTAGGTGAACCTGCGGAAGGATCATTATTGAATTATGTTTCTAGATAGGTTGTAG
CTGGCTCTTTTAGAGCATGTGCACGCCTGTTTGGACTTCATTTTCATCCACCTGTGCACC
TATTGTAGTCTTTGGTTGGGTTAGGAGGAAGTGATCATTGTATCAGCATCTGCTGGGAGT
GAGGACTTGCATTGTGAAAGCTTTGCTGTCCTTGATGTGATCATGGAATCTTTTTC----
------------------------------------------------------------
------------------------------------------------------------
------------------------------------------------------------
------------------------------------------------------------
------------------------------------------------------------
```

```
------------------------------------------------------------
------------------------------------------------------------
------------------------------------------------------------
------------------------------------------------------------
------------------------------------------------------------
------------------------------------------------------------
------------------------------------------------------------
-------TCACTAGAGTCTATGTCACTCATTATACTCTGTCGAATGTCATTGAATGTCTT
TACATGGGCTTGTATGCCTATGAAAATTGTAATACAACTTTCAGCAACGGATCTCTTGGC
TCTCGCATCGATGAAGAACGCAGCGAAATGCGATAAGTAATGTGAATTGCAGAATTCAGT
GAATCATCGAATCTTTGAACGCATCTTGCGCTCCTTGGTATTCCGAGGAGCATGCCTGTT
TGAGTGTCATTAAATTCTCAACTCTCTTATACTTTTTTGTAAAAGAGAGCTTGGACTGTG
GAGGCTTGCTGGCCACTTTTTGGGGTCAGCTCCTCTGAAATGCATTAGCGGAACCGTTTG
CGATCTGCCACAAGTGTGATAAGTTATCTACACTGGCGAGGGGATTGCTCTCTGTAATGT
TCAGCTTCTAATTGTCTCTACTTTGTGAGACTACTTTTGAATGCTTGACCTCAAATCAGG
TAGGACTACCCGCTGAACTTAA
>AC12-45
TTTCCGTAGGTGAACCTGCGGAAGGATCATTATTGAATTATGTTTCTAGATAGGTTGTAG
CTGGCTCTTTTAGAGCATGTGCACGCCTGTTTGGACTTCATTTTCATCCACCTGTGCACC
TATTGTAGTCTTTGGTTGGGTTAGGAGGAAGTGATCATTGTATCAGCATCTGCTGGGAGT
GAGGACTTGCATTGTGAAAGCTTTGCTGTCCTTGATGTGATCATGGAATCTTTTTC----
------------------------------------------------------------
------------------------------------------------------------
------------------------------------------------------------
------------------------------------------------------------
------------------------------------------------------------
------------------------------------------------------------
------------------------------------------------------------
------------------------------------------------------------
------------------------------------------------------------
------------------------------------------------------------
------------------------------------------------------------
------------------------------------------------------------
-------TCACTAGAGTCTATGTCACTCATTATACTCTGTCGAATGTCATTGAATGTCTT
TACATGGGCTTGTATGCCTATGAAAATTGTAATACAACTTTCAGCAACGGATCTCTTGGC
TCTCGCATCGATGAAGAACGCAGCGAAATGCGATAAGTAATGTGAATTGCAGAATTCAGT
GAATCATCGAATCTTTGAACGCATCTTGCGCTCCTTGGTATTCCGAGGAGCATGCCTGTT
TGAGTGTCATTAAATTCTCAACTCTCTTATACTTTTTTGTAAAAGAGAGCTTGGACTGTG
GAGGCTTGCTGGCCACTTTTTGGGGTCAGCTCCTCTGAAATGCATTAGCGGAACCGTTTG
CGATCTGCCACAAGTGTGATAAGTTATCTACACTGGCGAGGGGATTGCTCTCTGTAATGT
TCAGCTTCTAATTGTCTCTACTTTGTGAGACTACTTTTGAATGCTTGACCTCAAATCAGG
TAGGACTACCCGCTGAACTTAA
>AC8-23
TTTCCGTAGGTGAACCTGCGGAAGGATCATTATTGAATTATGTTTCTAGATAGGTTGTAG
CTGGCTCTTTTAGAGCATGTGCACGCCTGTTTGGACTTCATTTTCATCCACCTGTGCACC
TATTGTAGTCTTTGGTTGGGTTAGGAGGAAGTGATCATTGTATCAGCATCTGCTGGGAGT
GAGGACTTGCATTGTGAAAGCTTTGCTGTCCTTGATGTGATCATGGAATCTTTTTC----
------------------------------------------------------------
------------------------------------------------------------
------------------------------------------------------------
```


-------TCACTAGAGTCTATGTCACTCATTATACTCTGTCGAATGTCATTGAATGTCTT
TACATGGGCTTGTATGCCTATGAAAATTGTAATACAACTTTCAGCAACGGATCTCTTGGC
TCTCGCATCGATGAAGAACGCAGCGAAATGCGATAAGTAATGTGAATTGCAGAATTCAGT
GAATCATCGAATCTTTGAACGCATCTTGCGCTCCTTGGTATTCCGAGGAGCATGCCTGTT
TGAGTGTCATTAAATTCTCAACTCTCTTATACTTTTTTGTAAAAGAGAGCTTGGACTGTG
GAGGCTTGCTGGCCACTTTTTGGGGTCAGCTCCTCTGAAATGCATTAGCGGAACCGTTTG
CGATCTGCCACAAGTGTGATAAGTTATCTACACTGGCGAGGGGATTGCTCTCTGTAATGT
TCAGCTTCTAATTGTCTCTACTTTGTGAGACTACTTTTGAATGCTTGACCTCAAATCAGG
TAGGACTACCCGCTGAACTTAA
>AC1-48
TTTCCGTAGGTGAACCTGCGGAAGGATCATTATTGAATTATGTTTCTAGATAGGTTGTAG
CTGGCTCTTTTAGAGCATGTGCACGCCTGTTTGGACTTCATTTTCATCCACCTGTGCACC
TATTGTAGTCTTTGGTTGGGTTAGGAGGAAGTGATCATTGTATCAGCATCTGCTGGGAGT
GAGGACTTGCATTGTGAAAGCTTTGCTGTCCTTGATGTGATCATGGAATCTTTTTC----

-------TCACTAGAGTCTATGTCACTCATTATACTCTGTCGAATGTCATTGAATGTCTT
TACATGGGCTTGTATGCCTATGAAAATTGTAATACAACTTTCAGCAACGGATCTCTTGGC
TCTCGCATCGATGAAGAACGCAGCGAAATGCGATAAGTAATGTGAATTGCAGAATTCAGT
GAATCATCGAATCTTTGAACGCATCTTGCGCTCCTTGGTATTCCGAGGAGCATGCCTGTT
TGAGTGTCATTAAATTCTCAACTCTCTTATACTTTTTTGTAAAAGAGAGCTTGGACTGTG
GAGGCTTGCTGGCCACTTTTTGGGGTCAGCTCCTCTGAAATGCATTAGCGGAACCGTTTG
CGATCTGCCACAAGTGTGATAAGTTATCTACACTGGCGAGGGGATTGCTCTCTGTAATGT
TCAGCTTCTAATTGTCTCTACTTTGTGAGACTACTTTTGAATGCTTGACCTCAAATCAGG
TAGGACTACCCGCTGAACTTAA
>AC7-4
TTTCCGTAGGTGAACCTGCGGAAGGATCATTATTGAATTATGTTTCTAGATAGGTTGTAG
CTGGCTCTTTTAGAGCATGTGCACGCCTGTTTGGACTTCATTTTCATCCACCTGTGCACC
TATTGTAGTCTTTGGTTGGGTTAGGAGGAAGTGATCATTGTATCAGCATCTGCTGGGAGT
GAGGACTTGCATTGTGAAAGCTTTGCTGTCCTTGATGTGATCATGGAATCTTTTTC----

-------TCACTAGAGTCTATGTCACTCATTATACTCTGTCGAATGTCATTGAATGTCTT
TACATGGGCTTGTATGCCTATGAAAATTGTAATACAACTTTCAGCAACGGATCTCTTGGC
TCTCGCATCGATGAAGAACGCAGCGAAATGCGATAAGTAATGTGAATTGCAGAATTCAGT
GAATCATCGAATCTTTGAACGCATCTTGCGCTCCTTGGTATTCCGAGGAGCATGCCTGTT
TGAGTGTCATTAAATTCTCAACTCTCTTATACTTTTTTGTAAAAGAGAGCTTGGACTGTG
GAGGCTTGCTGGCCACTTTTTGGGGTCAGCTCCTCTGAAATGCATTAGCGGAACCGTTTG
CGATCTGCCACAAGTGTGATAAGTTATCTACACTGGCGAGGGGATTGCTCTCTGTAATGT
TCAGCTTCTAATTGTCTCTACTTTGTGAGACTACTTTTGAATGCTTGACCTCAAATCAGG
TAGGACTACCCGCTGAACTTAA
>AC6-40
TTTCCGTAGGTGAACCTGCGGAAGGATCATTATTGAATTATGTTTCTAGATAGGTTGTAG
CTGGCTCTTTTAGAGCATGTGCACGCCTGTTTGGACTTCATTTTCATCCACCTGTGCACC
TATTGTAGTCTTTGGTTGGGTTAGGAGGAAGTGATCATTGTATCAGCATCTGCTGGGAGT
GAGGACTTGCATTGTGAAAGCTTTGCTGTCCTTGATGTGATCATGGAATCTTTTTC----

-------TCACTAGAGTCTATGTCACTCATTATACTCTGTCGAATGTCATTGAATGTCTT
TACATGGGCTTGTATGCCTATGAAAATTGTAATACAACTTTCAGCAACGGATCTCTTGGC
TCTCGCATCGATGAAGAACGCAGCGAAATGCGATAAGTAATGTGAATTGCAGAATTCAGT
GAATCATCGAATCTTTGAACGCATCTTGCGCTCCTTGGTATTCCGAGGAGCATGCCTGTT
TGAGTGTCATTAAATTCTCAACTCTCTTATACTTTTTTGTAAAAGAGAGCTTGGACTGTG
GAGGCTTGCTGGCCACTTTTTGGGGTCAGCTCCTCTGAAATGCATTAGCGGAACCGTTTG
CGATCTGCCACAAGTGTGATAAGTTATCTACACTGGCGAGGGGATTGCTCTCTGTAATGT
TCAGCTTCTAATTGTCTCTACTTTGTGAGACTACTTTTGAATGCTTGACCTCAAATCAGG
TAGGACTACCCGCTGAACTTAA
>AC9-16
TTTCCGTAGGTGAACCTGCGGAAGGATCATTATTGAATTATGTTTCTAGATAGGTTGTAG
CTGGCTCTTTTAGAGCATGTGCACGCCTGTTTGGACTTCATTTTCATCCACCTGTGCACC
TATTGTAGTCTTTGGTTGGGTTAGGAGGAAGTGATCATTGTATCAGCATCTGCTGGGAGT

GAGGACTTGCATTGTGAAAGCTTTGCTGTCCTTGATGTGATCATGGAATCTTTTTC----

-------TCACTAGAGTCTATGTCACTCATTATACTCTGTCGAATGTCATTGAATGTCTT
TACATGGGCTTGTATGCCTATGAAAATTGTAATACAACTTTCAGCAACGGATCTCTTGGC
TCTCGCATCGATGAAGAACGCAGCGAAATGCGATAAGTAATGTGAATTGCAGAATTCAGT
GAATCATCGAATCTTTGAACGCATCTTGCGCTCCTTGGTATTCCGAGGAGCATGCCTGTT
TGAGTGTCATTAAATTCTCAACTCTCTTATACTTTTTTGTAAAAGAGAGCTTGGACTGTG
GAGGCTTGCTGGCCACTTTTTGGGGTCAGCTCCTCTGAAATGCATTAGCGGAACCGTTTG
CGATCTGCCACAAGTGTGATAAGTTATCTACACTGGCGAGGGGATTGCTCTCTGTAATGT
TCAGCTTCTAATTGTCTCTACTTTGTGAGACTACTTTTGAATGCTTGACCTCAAATCAGG
TAGGACTACCCGCTGAACTTAA
>AC10-53
TTTCCGTAGGTGAACCTGCGGAAGGATCATTATTGAATTATGTTTCTAGATAGGTTGTAG
CTGGCTCTTTTAGAGCATGTGCACGCCTGTTTGGACTTCATTTTCATCCACCTGTGCACC
TATTGTAGTCTTTGGTTGGGTTAGGAGGAAGTGATCATTGTATCAGCATCTGCTGGGAGT
GAGGACTTGCATTGTGAAAGCTTTGCTGTCCTTGATGTGATCATGGAATCTTTTTC----

-------TCACTAGAGTCTATGTCACTCATTATACTCTGTCGAATGTCATTGAATGTCTT
TACATGGGCTTGTATGCCTATGAAAATTGTAATACAACTTTCAGCAACGGATCTCTTGGC
TCTCGCATCGATGAAGAACGCAGCGAAATGCGATAAGTAATGTGAATTGCAGAATTCAGT
GAATCATCGAATCTTTGAACGCATCTTGCGCTCCTTGGTATTCCGAGGAGCATGCCTGTT
TGAGTGTCATTAAATTCTCAACTCTCTTATACTTTTTTGTAAAAGAGAGCTTGGACTGTG
GAGGCTTGCTGGCCACTTTTTGGGGTCAGCTCCTCTGAAATGCATTAGCGGAACCGTTTG
CGATCTGCCACAAGTGTGATAAGTTATCTACACTGGCGAGGGGATTGCTCTCTGTAATGT
TCAGCTTCTAATTGTCTCTACTTTGTGAGACTACTTTTGAATGCTTGACCTCAAATCAGG
TAGGACTACCCGCTGAACTTAA
>AC4-86
TTTCCGTAGGTGAACCTGCGGAAGGATCATTATTGAATTATGTTTCTAGATAGGTTGTAG

CTGGCTCTTTTAGAGCATGTGCACGCCTGTTTGGACTTCATTTTCATCCACCTGTGCACC
TATTGTAGTCTTTGGTTGGGTTAGGAGGAAGTGATCATTGTATCAGCATCTGCTGGGAGT
GAGGACTTGCATTGTGAAAGCTTTGCTGTCCTTGATGTGATCATGGAATCTTTTTC----

-------TCACTAGAGTCTATGTCACTCATTATACTCTGTCGAATGTCATTGAATGTCTT
TACATGGGCTTGTATGCCTATGAAAATTGTAATACAACTTTCAGCAACGGATCTCTTGGC
TCTCGCATCGATGAAGAACGCAGCGAAATGCGATAAGTAATGTGAATTGCAGAATTCAGT
GAATCATCGAATCTTTGAACGCATCTTGCGCTCCTTGGTATTCCGAGGAGCATGCCTGTT
TGAGTGTCATTAAATTCTCAACTCTCTTATACTTTTTTGTAAAAGAGAGCTTGGACTGTG
GAGGCTTGCTGGCCACTTTTTGGGGTCAGCTCCTCTGAAATGCATTAGCGGAACCGTTTG
CGATCTGCCACAAGTGTGATAAGTTATCTACACTGGCGAGGGGATTGCTCTCTGTAATGT
TCAGCTTCTAATTGTCTCTACTTTGTGAGACTACTTTTGAATGCTTGACCTCAAATCAGG
TAGGACTACCCGCTGAACTTAA
>AC7-10
TTTCCGTAGGTGAACCTGCGGAAGGATCATTATTGAATTATGTTTCTAGATAGGTTGTAG
CTGGCTCTTTTAGAGCATGTGCACGCCTGTTTGGACTTCATTTTCATCCACCTGTGCACC
TATTGTAGTCTTTGGTTGGGTTAGGAGGAAGTGATCATTGTATCAGCATCTGCTGGGAGT
GAGGACTTGCATTGTGAAAGCTTTGCTGTCCTTGATGTGATCATGGAATCTTTTTC----

-------TCACTAGAGTCTATGTCACTCATTATACTCTGTCGAATGTCATTGAATGTCTT
TACATGGGCTTGTATGCCTATGAAAATTGTAATACAACTTTCAGCAACGGATCTCTTGGC
TCTCGCATCGATGAAGAACGCAGCGAAATGCGATAAGTAATGTGAATTGCAGAATTCAGT
GAATCATCGAATCTTTGAACGCATCTTGCGCTCCTTGGTATTCCGAGGAGCATGCCTGTT
TGAGTGTCATTAAATTCTCAACTCTCTTATACTTTTTTGTAAAAGAGAGCTTGGACTGTG
GAGGCTTGCTGGCCACTTTTTGGGGTCAGCTCCTCTGAAATGCATTAGCGGAACCGTTTG
CGATCTGCCACAAGTGTGATAAGTTATCTACACTGGCGAGGGGATTGCTCTCTGTAATGT
TCAGCTTCTAATTGTCTCTACTTTGTGAGACTACTTTTGAATGCTTGACCTCAAATCAGG
TAGGACTACCCGCTGAACTTAA

```
>AC10-10
TTTCCGTAGGTGAACCTGCGGAAGGATCATTATTGAATTATGTTTCTAGATAGGTTGTAG
CTGGCTCTTTTAGAGCATGTGCACGCCTGTTTGGACTTCATTTTCATCCACCTGTGCACC
TATTGTAGTCTTTGGTTGGGTTAGGAGGAAGTGATCATTGTATCAGCATCTGCTGGGAGT
GAGGACTTGCATTGTGAAAGCTTTGCTGTCCTTGATGTGATCATGGAATCTTTTTC----
------------------------------------------------------------
------------------------------------------------------------
------------------------------------------------------------
------------------------------------------------------------
------------------------------------------------------------
------------------------------------------------------------
------------------------------------------------------------
------------------------------------------------------------
------------------------------------------------------------
------------------------------------------------------------
------------------------------------------------------------
------------------------------------------------------------
-------TCACTAGAGTCTATGTCACTCATTATACTCTGTCGAATGTCATTGAATGTCTT
TACATGGGCTTGTATGCCTATGAAAATTGTAATACAACTTTCAGCAACGGATCTCTTGGC
TCTCGCATCGATGAAGAACGCAGCGAAATGCGATAAGTAATGTGAATTGCAGAATTCAGT
GAATCATCGAATCTTTGAACGCATCTTGCGCTCCTTGGTATTCCGAGGAGCATGCCTGTT
TGAGTGTCATTAAATTCTCAACTCTCTTATACTTTTTTGTAAAAGAGAGCTTGGACTGTG
GAGGCTTGCTGGCCACTTTTTGGGGTCAGCTCCTCTGAAATGCATTAGCGGAACCGTTTG
CGATCTGCCACAAGTGTGATAAGTTATCTACACTGGCGAGGGGATTGCTCTCTGTAATGT
TCAGCTTCTAATTGTCTCTACTTTGTGAGACTACTTTTGAATGCTTGACCTCAAATCAGG
TAGGACTACCCGCTGAACTTAA
>AC7-52
TTTCCGTAGGTGAACCTGCGGAAGGATCATTATTGAATTATGTTTCTAGATAGGTTGTAG
CTGGCTCTTTTAGAGCATGTGCACGCCTGTTTGGACTTCATTTTCATCCACCTGTGCACC
TATTGTAGTCTTTGGTTGGGTTAGGAGGAAGTGATCATTGTATCAGCATCTGCTGGGAGT
GAGGACTTGCATTGTGAAAGCTTTGCTGTCCTTGATGTGATCATGGAATCTTTTTC----
------------------------------------------------------------
------------------------------------------------------------
------------------------------------------------------------
------------------------------------------------------------
------------------------------------------------------------
------------------------------------------------------------
------------------------------------------------------------
------------------------------------------------------------
------------------------------------------------------------
------------------------------------------------------------
------------------------------------------------------------
------------------------------------------------------------
-------TCACTAGAGTCTATGTCACTCATTATACTCTGTCGAATGTCATTGAATGTCTT
TACATGGGCTTGTATGCCTATGAAAATTGTAATACAACTTTCAGCAACGGATCTCTTGGC
TCTCGCATCGATGAAGAACGCAGCGAAATGCGATAAGTAATGTGAATTGCAGAATTCAGT
GAATCATCGAATCTTTGAACGCATCTTGCGCTCCTTGGTATTCCGAGGAGCATGCCTGTT
TGAGTGTCATTAAATTCTCAACTCTCTTATACTTTTTTGTAAAAGAGAGCTTGGACTGTG
GAGGCTTGCTGGCCACTTTTTGGGGTCAGCTCCTCTGAAATGCATTAGCGGAACCGTTTG
CGATCTGCCACAAGTGTGATAAGTTATCTACACTGGCGAGGGGATTGCTCTCTGTAATGT
```

```
TCAGCTTCTAATTGTCTCTACTTTGTGAGACTACTTTTGAATGCTTGACCTCAAATCAGG
TAGGACTACCCGCTGAACTTAA
>AC9-43
TTTCCGTAGGTGAACCTGCGGAAGGATCATTATTGAATTATGTTTCTAGATAGGTTGTAG
CTGGCTCTTTTAGAGCATGTGCACGCCTGTTTGGACTTCATTTTCATCCACCTGTGCACC
TATTGTAGTCTTTGGTTGGGTTAGGAGGAAGTGATCATTGTATCAGCATCTGCTGGGAGT
GAGGACTTGCATTGTGAAAGCTTTGCTGTCCTTGATGTGATCATGGAATCTTTTTC----
------------------------------------------------------------
------------------------------------------------------------
------------------------------------------------------------
------------------------------------------------------------
------------------------------------------------------------
------------------------------------------------------------
------------------------------------------------------------
------------------------------------------------------------
------------------------------------------------------------
------------------------------------------------------------
------------------------------------------------------------
------------------------------------------------------------
-------TCACTAGAGTCTATGTCACTCATTATACTCTGTCGAATGTCATTGAATGTCTT
TACATGGGCTTGTATGCCTATGAAAATTGTAATACAACTTTCAGCAACGGATCTCTTGGC
TCTCGCATCGATGAAGAACGCAGCGAAATGCGATAAGTAATGTGAATTGCAGAATTCAGT
GAATCATCGAATCTTTGAACGCATCTTGCGCTCCTTGGTATTCCGAGGAGCATGCCTGTT
TGAGTGTCATTAAATTCTCAACTCTCTTATACTTTTTTGTAAAAGAGAGCTTGGACTGTG
GAGGCTTGCTGGCCACTTTTTGGGGTCAGCTCCTCTGAAATGCATTAGCGGAACCGTTTG
CGATCTGCCACAAGTGTGATAAGTTATCTACACTGGCGAGGGGATTGCTCTCTGTAATGT
TCAGCTTCTAATTGTCTCTACTTTGTGAGACTACTTTTGAATGCTTGACCTCAAATCAGG
TAGGACTACCCGCTGAACTTAA
>AC12-6
TTTCCGTAGGTGAACCTGCGGAAGGATCATTATTGAATTATGTTTCTAGATAGGTTGTAG
CTGGCTCTTTTAGAGCATGTGCACGCCTGTTTGGACTTCATTTTCATCCACCTGTGCACC
TATTGTAGTCTTTGGTTGGGTTAGGAGGAAGTGATCATTGTATCAGCATCTGCTGGGAGT
GAGGACTTGCATTGTGAAAGCTTTGCTGTCCTTGATGTGATCATGGAATCTTTTTC----
------------------------------------------------------------
------------------------------------------------------------
------------------------------------------------------------
------------------------------------------------------------
------------------------------------------------------------
------------------------------------------------------------
------------------------------------------------------------
------------------------------------------------------------
------------------------------------------------------------
------------------------------------------------------------
------------------------------------------------------------
------------------------------------------------------------
-------TCACTAGAGTCTATGTCACTCATTATACTCTGTCGAATGTCATTGAATGTCTT
TACATGGGCTTGTATGCCTATGAAAATTGTAATACAACTTTCAGCAACGGATCTCTTGGC
TCTCGCATCGATGAAGAACGCAGCGAAATGCGATAAGTAATGTGAATTGCAGAATTCAGT
GAATCATCGAATCTTTGAACGCATCTTGCGCTCCTTGGTATTCCGAGGAGCATGCCTGTT
TGAGTGTCATTAAATTCTCAACTCTCTTATACTTTTTTGTAAAAGAGAGCTTGGACTGTG
```

GAGGCTTGCTGGCCACTTTTTGGGGTCAGCTCCTCTGAAATGCATTAGCGGAACCGTTTG
CGATCTGCCACAAGTGTGATAAGTTATCTACACTGGCGAGGGGATTGCTCTCTGTAATGT
TCAGCTTCTAATTGTCTCTACTTTGTGAGACTACTTTTGAATGCTTGACCTCAAATCAGG
TAGGACTACCCGCTGAACTTAA
>AC11-34
TTTCCGTAGGTGAACCTGCGGAAGGATCATTATTGAATTATGTTTCTAGATAGGTTGTAG
CTGGCTCTTTTAGAGCATGTGCACGCCTGTTTGGACTTCATTTTCATCCACCTGTGCACC
TATTGTAGTCTTTGGTTGGGTTAGGAGGAAGTGATCATTGTATCAGCATCTGCTGGGAGT
GAGGACTTGCATTGTGAAAGCTTTGCTGTCCTTGATGTGATCATGGAATCTTTTTC----

-------TCACTAGAGTCTATGTCACTCATTATACTCTGTCGAATGTCATTGAATGTCTT
TACATGGGCTTGTATGCCTATGAAAATTGTAATACAACTTTCAGCAACGGATCTCTTGGC
TCTCGCATCGATGAAGAACGCAGCGAAATGCGATAAGTAATGTGAATTGCAGAATTCAGT
GAATCATCGAATCTTTGAACGCATCTTGCGCTCCTTGGTATTCCGAGGAGCATGCCTGTT
TGAGTGTCATTAAATTCTCAACTCTCTTATACTTTTTTGTAAAAGAGAGCTTGGACTGTG
GAGGCTTGCTGGCCACTTTTTGGGGTCAGCTCCTCTGAAATGCATTAGCGGAACCGTTTG
CGATCTGCCACAAGTGTGATAAGTTATCTACACTGGCGAGGGGATTGCTCTCTGTAATGT
TCAGCTTCTAATTGTCTCTACTTTGTGAGACTACTTTTGAATGCTTGACCTCAAATCAGG
TAGGACTACCCGCTGAACTTAA
>AC12-41
TTTCCGTAGGTGAACCTGCGGAAGGATCATTATTGAATTATGTTTCTAGATAGGTTGTAG
CTGGCTCTTTTAGAGCATGTGCACGCCTGTTTGGACTTCATTTTCATCCACCTGTGCACC
TATTGTAGTCTTTGGTTGGGTTAGGAGGAAGTGATCATTGTATCAGCATCTGCTGGGAGT
GAGGACTTGCATTGTGAAAGCTTTGCTGTCCTTGATGTGATCATGGAATCTTTTTC----

-------TCACTAGAGTCTATGTCACTCATTATACTCTGTCGAATGTCATTGAATGTCTT
TACATGGGCTTGTATGCCTATGAAAATTGTAATACAACTTTCAGCAACGGATCTCTTGGC
TCTCGCATCGATGAAGAACGCAGCGAAATGCGATAAGTAATGTGAATTGCAGAATTCAGT

GAATCATCGAATCTTTGAACGCATCTTGCGCTCCTTGGTATTCCGAGGAGCATGCCTGTT
TGAGTGTCATTAAATTCTCAACTCTCTTATACTTTTTTGTAAAAGAGAGCTTGGACTGTG
GAGGCTTGCTGGCCACTTTTTGGGGTCAGCTCCTCTGAAATGCATTAGCGGAACCGTTTG
CGATCTGCCACAAGTGTGATAAGTTATCTACACTGGCGAGGGGATTGCTCTCTGTAATGT
TCAGCTTCTAATTGTCTCTACTTTGTGAGACTACTTTTGAATGCTTGACCTCAAATCAGG
TAGGACTACCCGCTGAACTTAA
>AC8-16
TTTCCGTAGGTGAACCTGCGGAAGGATCATTATTGAATTATGTTTCTAGATAGGTTGTAG
CTGGCTCTTTTAGAGCATGTGCACGCCTGTTTGGACTTCATTTTCATCCACCTGTGCACC
TATTGTAGTCTTTGGTTGGGTTAGGAGGAAGTGATCATTGTATCAGCATCTGCTGGGAGT
GAGGACTTGCATTGTGAAAGCTTTGCTGTCCTTGATGTGATCATGGAATCTTTTTC----

-------TCACTAGAGTCTATGTCACTCATTATACTCTGTCGAATGTCATTGAATGTCTT
TACATGGGCTTGTATGCCTATGAAAATTGTAATACAACTTTCAGCAACGGATCTCTTGGC
TCTCGCATCGATGAAGAACGCAGCGAAATGCGATAAGTAATGTGAATTGCAGAATTCAGT
GAATCATCGAATCTTTGAACGCATCTTGCGCTCCTTGGTATTCCGAGGAGCATGCCTGTT
TGAGTGTCATTAAATTCTCAACTCTCTTATACTTTTTTGTAAAAGAGAGCTTGGACTGTG
GAGGCTTGCTGGCCACTTTTTGGGGTCAGCTCCTCTGAAATGCATTAGCGGAACCGTTTG
CGATCTGCCACAAGTGTGATAAGTTATCTACACTGGCGAGGGGATTGCTCTCTGTAATGT
TCAGCTTCTAATTGTCTCTACTTTGTGAGACTACTTTTGAATGCTTGACCTCAAATCAGG
TAGGACTACCCGCTGAACTTAA
>AC4-65
TTTCCGTAGGTGAACCTGCGGAAGGATCATTATTGAATTATGTTTCTAGATAGGTTGTAG
CTGGCTCTTTTAGAGCATGTGCACGCCTGTTTGGACTTCATTTTCATCCACCTGTGCACC
TATTGTAGTCTTTGGTTGGGTTAGGAGGAAGTGATCATTGTATCAGCATCTGCTGGGAGT
GAGGACTTGCATTGTGAAAGCTTTGCTGTCCTTGATGTGATCATGGAATCTTTTTC----

-------TCACTAGAGTCTATGTCACTCATTATACTCTGTCGAATGTCATTGAATGTCTT

TACATGGGCTTGTATGCCTATGAAAATTGTAATACAACTTTCAGCAACGGATCTCTTGGC
TCTCGCATCGATGAAGAACGCAGCGAAATGCGATAAGTAATGTGAATTGCAGAATTCAGT
GAATCATCGAATCTTTGAACGCATCTTGCGCTCCTTGGTATTCCGAGGAGCATGCCTGTT
TGAGTGTCATTAAATTCTCAACTCTCTTATACTTTTTTGTAAAAGAGAGCTTGGACTGTG
GAGGCTTGCTGGCCACTTTTTGGGGTCAGCTCCTCTGAAATGCATTAGCGGAACCGTTTG
CGATCTGCCACAAGTGTGATAAGTTATCTACACTGGCGAGGGGATTGCTCTCTGTAATGT
TCAGCTTCTAATTGTCTCTACTTTGTGAGACTACTTTTGAATGCTTGACCTCAAATCAGG
TAGGACTACCCGCTGAACTTAA
>AC11-18
TTTCCGTAGGTGAACCTGCGGAAGGATCATTATTGAATTATGTTTCTAGATAGGTTGTAG
CTGGCTCTTTTAGAGCATGTGCACGCCTGTTTGGACTTCATTTTCATCCACCTGTGCACC
TATTGTAGTCTTTGGTTGGGTTAGGAGGAAGTGATCATTGTATCAGCATCTGCTGGGAGT
GAGGACTTGCATTGTGAAAGCTTTGCTGTCCTTGATGTGATCATGGAATCTTTTTC----

-------TCACTAGAGTCTATGTCACTCATTATACTCTGTCGAATGTCATTGAATGTCTT
TACATGGGCTTGTATGCCTATGAAAATTGTAATACAACTTTCAGCAACGGATCTCTTGGC
TCTCGCATCGATGAAGAACGCAGCGAAATGCGATAAGTAATGTGAATTGCAGAATTCAGT
GAATCATCGAATCTTTGAACGCATCTTGCGCTCCTTGGTATTCCGAGGAGCATGCCTGTT
TGAGTGTCATTAAATTCTCAACTCTCTTATACTTTTTTGTAAAAGAGAGCTTGGACTGTG
GAGGCTTGCTGGCCACTTTTTGGGGTCAGCTCCTCTGAAATGCATTAGCGGAACCGTTTG
CGATCTGCCACAAGTGTGATAAGTTATCTACACTGGCGAGGGGATTGCTCTCTGTAATGT
TCAGCTTCTAATTGTCTCTACTTTGTGAGACTACTTTTGAATGCTTGACCTCAAATCAGG
TAGGACTACCCGCTGAACTTAA
>AC2-1
TTTCCGTAGGTGAACCTGCGGAAGGATCATTATTGAATTATGTTTCTAGATAGGTTGTAG
CTGGCTCTTTTAGAGCATGTGCACGCCTGTTTGGACTTCATTTTCATCCACCTGTGCACC
TATTGTAGTCTTTGGTTGGGTTAGGAGGAAGTGATCATTGTATCAGCATCTGCTGGGAGT
GAGGACTTGCATTGTGAAAGCTTTGCTGTCCTTGATGTGATCATGGAATCTTTTTC----


```
------------------------------------------------------------
-------TCACTAGAGTCTATGTCACTCATTATACTCTGTCGAATGTCATTGAATGTCTT
TACATGGGCTTGTATGCCTATGAAAATTGTAATACAACTTTCAGCAACGGATCTCTTGGC
TCTCGCATCGATGAAGAACGCAGCGAAATGCGATAAGTAATGTGAATTGCAGAATTCAGT
GAATCATCGAATCTTTGAACGCATCTTGCGCTCCTTGGTATTCCGAGGAGCATGCCTGTT
TGAGTGTCATTAAATTCTCAACTCTCTTATACTTTTTTGTAAAAGAGAGCTTGGACTGTG
GAGGCTTGCTGGCCACTTTTTGGGGTCAGCTCCTCTGAAATGCATTAGCGGAACCGTTTG
CGATCTGCCACAAGTGTGATAAGTTATCTACACTGGCGAGGGGATTGCTCTCTGTAATGT
TCAGCTTCTAATTGTCTCTACTTTGTGAGACTACTTTTGAATGCTTGACCTCAAATCAGG
TAGGACTACCCGCTGAACTTAA
>AC8-14
TTTCCGTAGGTGAACCTGCGGAAGGATCATTATTGAATTATGTTTCTAGATAGGTTGTAG
CTGGCTCTTTTAGAGCATGTGCACGCCTGTTTGGACTTCATTTTCATCCACCTGTGCACC
TATTGTAGTCTTTGGTTGGGTTAGGAGGAAGTGATCATTGTATCAGCATCTGCTGGGAGT
GAGGACTTGCATTGTGAAAGCTTTGCTGTCCTTGATGTGATCATGGAATCTTTTTC----
------------------------------------------------------------
------------------------------------------------------------
------------------------------------------------------------
------------------------------------------------------------
------------------------------------------------------------
------------------------------------------------------------
------------------------------------------------------------
------------------------------------------------------------
------------------------------------------------------------
------------------------------------------------------------
------------------------------------------------------------
------------------------------------------------------------
-------TCACTAGAGTCTATGTCACTCATTATACTCTGTCGAATGTCATTGAATGTCTT
TACATGGGCTTGTATGCCTATGAAAATTGTAATACAACTTTCAGCAACGGATCTCTTGGC
TCTCGCATCGATGAAGAACGCAGCGAAATGCGATAAGTAATGTGAATTGCAGAATTCAGT
GAATCATCGAATCTTTGAACGCATCTTGCGCTCCTTGGTATTCCGAGGAGCATGCCTGTT
TGAGTGTCATTAAATTCTCAACTCTCTTATACTTTTTTGTAAAAGAGAGCTTGGACTGTG
GAGGCTTGCTGGCCACTTTTTGGGGTCAGCTCCTCTGAAATGCATTAGCGGAACCGTTTG
CGATCTGCCACAAGTGTGATAAGTTATCTACACTGGCGAGGGGATTGCTCTCTGTAATGT
TCAGCTTCTAATTGTCTCTACTTTGTGAGACTACTTTTGAATGCTTGACCTCAAATCAGG
TAGGACTACCCGCTGAACTTAA
>AC10-31
TTTCCGTAGGTGAACCTGCGGAAGGATCATTATTGAATTATGTTTCTAGATAGGTTGTAG
CTGGCTCTTTTAGAGCATGTGCACGCCTGTTTGGACTTCATTTTCATCCACCTGTGCACC
TATTGTAGTCTTTGGTTGGGTTAGGAGGAAGTGATCATTGTATCAGCATCTGCTGGGAGT
GAGGACTTGCATTGTGAAAGCTTTGCTGTCCTTGATGTGATCATGGAATCTTTTTC----
------------------------------------------------------------
------------------------------------------------------------
------------------------------------------------------------
------------------------------------------------------------
------------------------------------------------------------
------------------------------------------------------------
------------------------------------------------------------
------------------------------------------------------------
------------------------------------------------------------
```


-------TCACTAGAGTCTATGTCACTCATTATACTCTGTCGAATGTCATTGAATGTCTT
TACATGGGCTTGTATGCCTATGAAAATTGTAATACAACTTTCAGCAACGGATCTCTTGGC
TCTCGCATCGATGAAGAACGCAGCGAAATGCGATAAGTAATGTGAATTGCAGAATTCAGT
GAATCATCGAATCTTTGAACGCATCTTGCGCTCCTTGGTATTCCGAGGAGCATGCCTGTT
TGAGTGTCATTAAATTCTCAACTCTCTTATACTTTTTTGTAAAAGAGAGCTTGGACTGTG
GAGGCTTGCTGGCCACTTTTTGGGGTCAGCTCCTCTGAAATGCATTAGCGGAACCGTTTG
CGATCTGCCACAAGTGTGATAAGTTATCTACACTGGCGAGGGGATTGCTCTCTGTAATGT
TCAGCTTCTAATTGTCTCTACTTTGTGAGACTACTTTTGAATGCTTGACCTCAAATCAGG
TAGGACTACCCGCTGAACTTAA
>AC11-11
TTTCCGTAGGTGAACCTGCGGAAGGATCATTATTGAATTATGTTTCTAGATAGGTTGTAG
CTGGCTCTTTTAGAGCATGTGCACGCCTGTTTGGACTTCATTTTCATCCACCTGTGCACC
TATTGTAGTCTTTGGTTGGGTTAGGAGGAAGTGATCATTGTATCAGCATCTGCTGGGAGT
GAGGACTTGCATTGTGAAAGCTTTGCTGTCCTTGATGTGATCATGGAATCTTTTTC----

-------TCACTAGAGTCTATGTCACTCATTATACTCTGTCGAATGTCATTGAATGTCTT
TACATGGGCTTGTATGCCTATGAAAATTGTAATACAACTTTCAGCAACGGATCTCTTGGC
TCTCGCATCGATGAAGAACGCAGCGAAATGCGATAAGTAATGTGAATTGCAGAATTCAGT
GAATCATCGAATCTTTGAACGCATCTTGCGCTCCTTGGTATTCCGAGGAGCATGCCTGTT
TGAGTGTCATTAAATTCTCAACTCTCTTATACTTTTTTGTAAAAGAGAGCTTGGACTGTG
GAGGCTTGCTGGCCACTTTTTGGGGTCAGCTCCTCTGAAATGCATTAGCGGAACCGTTTG
CGATCTGCCACAAGTGTGATAAGTTATCTACACTGGCGAGGGGATTGCTCTCTGTAATGT
TCAGCTTCTAATTGTCTCTACTTTGTGAGACTACTTTTGAATGCTTGACCTCAAATCAGG
TAGGACTACCCGCTGAACTTAA
>AC1-26
TTTCCGTAGGTGAACCTGCGGAAGGATCATTATTGAATTATGTTTCTAGATAGGTTGTAG
CTGGCTCTTTTAGAGCATGTGCACGCCTGTTTGGACTTCATTTTCATCCACCTGTGCACC
TATTGTAGTCTTTGGTTGGGTTAGGAGGAAGTGATCATTGTATCAGCATCTGCTGGGAGT
GAGGACTTGCATTGTGAAAGCTTTGCTGTCCTTGATGTGATCATGGAATCTTTTTC----

-------TCACTAGAGTCTATGTCACTCATTATACTCTGTCGAATGTCATTGAATGTCTT
TACATGGGCTTGTATGCCTATGAAAATTGTAATACAACTTTCAGCAACGGATCTCTTGGC
TCTCGCATCGATGAAGAACGCAGCGAAATGCGATAAGTAATGTGAATTGCAGAATTCAGT
GAATCATCGAATCTTTGAACGCATCTTGCGCTCCTTGGTATTCCGAGGAGCATGCCTGTT
TGAGTGTCATTAAATTCTCAACTCTCTTATACTTTTTTGTAAAAGAGAGCTTGGACTGTG
GAGGCTTGCTGGCCACTTTTTGGGGTCAGCTCCTCTGAAATGCATTAGCGGAACCGTTTG
CGATCTGCCACAAGTGTGATAAGTTATCTACACTGGCGAGGGGATTGCTCTCTGTAATGT
TCAGCTTCTAATTGTCTCTACTTTGTGAGACTACTTTTGAATGCTTGACCTCAAATCAGG
TAGGACTACCCGCTGAACTTAA
>AC1-32
TTTCCGTAGGTGAACCTGCGGAAGGATCATTATTGAATTATGTTTCTAGATAGGTTGTAG
CTGGCTCTTTTAGAGCATGTGCACGCCTGTTTGGACTTCATTTTCATCCACCTGTGCACC
TATTGTAGTCTTTGGTTGGGTTAGGAGGAAGTGATCATTGTATCAGCATCTGCTGGGAGT
GAGGACTTGCATTGTGAAAGCTTTGCTGTCCTTGATGTGATCATGGAATCTTTTTC----

-------TCACTAGAGTCTATGTCACTCATTATACTCTGTCGAATGTCATTGAATGTCTT
TACATGGGCTTGTATGCCTATGAAAATTGTAATACAACTTTCAGCAACGGATCTCTTGGC
TCTCGCATCGATGAAGAACGCAGCGAAATGCGATAAGTAATGTGAATTGCAGAATTCAGT
GAATCATCGAATCTTTGAACGCATCTTGCGCTCCTTGGTATTCCGAGGAGCATGCCTGTT
TGAGTGTCATTAAATTCTCAACTCTCTTATACTTTTTTGTAAAAGAGAGCTTGGACTGTG
GAGGCTTGCTGGCCACTTTTTGGGGTCAGCTCCTCTGAAATGCATTAGCGGAACCGTTTG
CGATCTGCCACAAGTGTGATAAGTTATCTACACTGGCGAGGGGATTGCTCTCTGTAATGT
TCAGCTTCTAATTGTCTCTACTTTGTGAGACTACTTTTGAATGCTTGACCTCAAATCAGG
TAGGACTACCCGCTGAACTTAA
>AC1-33
TTTCCGTAGGTGAACCTGCGGAAGGATCATTATTGAATTATGTTTCTAGATAGGTTGTAG
CTGGCTCTTTTAGAGCATGTGCACGCCTGTTTGGACTTCATTTTCATCCACCTGTGCACC
TATTGTAGTCTTTGGTTGGGTTAGGAGGAAGTGATCATTGTATCAGCATCTGCTGGGAGT
GAGGACTTGCATTGTGAAAGCTTTGCTGTCCTTGATGTGATCATGGAATCTTTTTC----

-------TCACTAGAGTCTATGTCACTCATTATACTCTGTCGAATGTCATTGAATGTCTT
TACATGGGCTTGTATGCCTATGAAAATTGTAATACAACTTTCAGCAACGGATCTCTTGGC
TCTCGCATCGATGAAGAACGCAGCGAAATGCGATAAGTAATGTGAATTGCAGAATTCAGT
GAATCATCGAATCTTTGAACGCATCTTGCGCTCCTTGGTATTCCGAGGAGCATGCCTGTT
TGAGTGTCATTAAATTCTCAACTCTCTTATACTTTTTTGTAAAAGAGAGCTTGGACTGTG
GAGGCTTGCTGGCCACTTTTTGGGGTCAGCTCCTCTGAAATGCATTAGCGGAACCGTTTG
CGATCTGCCACAAGTGTGATAAGTTATCTACACTGGCGAGGGGATTGCTCTCTGTAATGT
TCAGCTTCTAATTGTCTCTACTTTGTGAGACTACTTTTGAATGCTTGACCTCAAATCAGG
TAGGACTACCCGCTGAACTTAA
>AC1-37
TTTCCGTAGGTGAACCTGCGGAAGGATCATTATTGAATTATGTTTCTAGATAGGTTGTAG
CTGGCTCTTTTAGAGCATGTGCACGCCTGTTTGGACTTCATTTTCATCCACCTGTGCACC
TATTGTAGTCTTTGGTTGGGTTAGGAGGAAGTGATCATTGTATCAGCATCTGCTGGGAGT
GAGGACTTGCATTGTGAAAGCTTTGCTGTCCTTGATGTGATCATGGAATCTTTTTC----

-------TCACTAGAGTCTATGTCACTCATTATACTCTGTCGAATGTCATTGAATGTCTT
TACATGGGCTTGTATGCCTATGAAAATTGTAATACAACTTTCAGCAACGGATCTCTTGGC
TCTCGCATCGATGAAGAACGCAGCGAAATGCGATAAGTAATGTGAATTGCAGAATTCAGT
GAATCATCGAATCTTTGAACGCATCTTGCGCTCCTTGGTATTCCGAGGAGCATGCCTGTT
TGAGTGTCATTAAATTCTCAACTCTCTTATACTTTTTTGTAAAAGAGAGCTTGGACTGTG
GAGGCTTGCTGGCCACTTTTTGGGGTCAGCTCCTCTGAAATGCATTAGCGGAACCGTTTG
CGATCTGCCACAAGTGTGATAAGTTATCTACACTGGCGAGGGGATTGCTCTCTGTAATGT
TCAGCTTCTAATTGTCTCTACTTTGTGAGACTACTTTTGAATGCTTGACCTCAAATCAGG
TAGGACTACCCGCTGAACTTAA
>AC1-40
TTTCCGTAGGTGAACCTGCGGAAGGATCATTATTGAATTATGTTTCTAGATAGGTTGTAG
CTGGCTCTTTTAGAGCATGTGCACGCCTGTTTGGACTTCATTTTCATCCACCTGTGCACC
TATTGTAGTCTTTGGTTGGGTTAGGAGGAAGTGATCATTGTATCAGCATCTGCTGGGAGT
GAGGACTTGCATTGTGAAAGCTTTGCTGTCCTTGATGTGATCATGGAATCTTTTTC----


```
------------------------------------------------------------
------------------------------------------------------------
------------------------------------------------------------
------------------------------------------------------------
------------------------------------------------------------
------------------------------------------------------------
------------------------------------------------------------
------------------------------------------------------------
------------------------------------------------------------
-------TCACTAGAGTCTATGTCACTCATTATACTCTGTCGAATGTCATTGAATGTCTT
TACATGGGCTTGTATGCCTATGAAAATTGTAATACAACTTTCAGCAACGGATCTCTTGGC
TCTCGCATCGATGAAGAACGCAGCGAAATGCGATAAGTAATGTGAATTGCAGAATTCAGT
GAATCATCGAATCTTTGAACGCATCTTGCGCTCCTTGGTATTCCGAGGAGCATGCCTGTT
TGAGTGTCATTAAATTCTCAACTCTCTTATACTTTTTTGTAAAAGAGAGCTTGGACTGTG
GAGGCTTGCTGGCCACTTTTTGGGGTCAGCTCCTCTGAAATGCATTAGCGGAACCGTTTG
CGATCTGCCACAAGTGTGATAAGTTATCTACACTGGCGAGGGGATTGCTCTCTGTAATGT
TCAGCTTCTAATTGTCTCTACTTTGTGAGACTACTTTTGAATGCTTGACCTCAAATCAGG
TAGGACTACCCGCTGAACTTAA
>AC1-41
TTTCCGTAGGTGAACCTGCGGAAGGATCATTATTGAATTATGTTTCTAGATAGGTTGTAG
CTGGCTCTTTTAGAGCATGTGCACGCCTGTTTGGACTTCATTTTCATCCACCTGTGCACC
TATTGTAGTCTTTGGTTGGGTTAGGAGGAAGTGATCATTGTATCAGCATCTGCTGGGAGT
GAGGACTTGCATTGTGAAAGCTTTGCTGTCCTTGATGTGATCATGGAATCTTTTTC----
------------------------------------------------------------
------------------------------------------------------------
------------------------------------------------------------
------------------------------------------------------------
------------------------------------------------------------
------------------------------------------------------------
------------------------------------------------------------
------------------------------------------------------------
------------------------------------------------------------
------------------------------------------------------------
------------------------------------------------------------
------------------------------------------------------------
-------TCACTAGAGTCTATGTCACTCATTATACTCTGTCGAATGTCATTGAATGTCTT
TACATGGGCTTGTATGCCTATGAAAATTGTAATACAACTTTCAGCAACGGATCTCTTGGC
TCTCGCATCGATGAAGAACGCAGCGAAATGCGATAAGTAATGTGAATTGCAGAATTCAGT
GAATCATCGAATCTTTGAACGCATCTTGCGCTCCTTGGTATTCCGAGGAGCATGCCTGTT
TGAGTGTCATTAAATTCTCAACTCTCTTATACTTTTTTGTAAAAGAGAGCTTGGACTGTG
GAGGCTTGCTGGCCACTTTTTGGGGTCAGCTCCTCTGAAATGCATTAGCGGAACCGTTTG
CGATCTGCCACAAGTGTGATAAGTTATCTACACTGGCGAGGGGATTGCTCTCTGTAATGT
TCAGCTTCTAATTGTCTCTACTTTGTGAGACTACTTTTGAATGCTTGACCTCAAATCAGG
TAGGACTACCCGCTGAACTTAA
>AC1-51
TTTCCGTAGGTGAACCTGCGGAAGGATCATTATTGAATTATGTTTCTAGATAGGTTGTAG
CTGGCTCTTTTAGAGCATGTGCACGCCTGTTTGGACTTCATTTTCATCCACCTGTGCACC
TATTGTAGTCTTTGGTTGGGTTAGGAGGAAGTGATCATTGTATCAGCATCTGCTGGGAGT
GAGGACTTGCATTGTGAAAGCTTTGCTGTCCTTGATGTGATCATGGAATCTTTTTC----
------------------------------------------------------------
```


-------TCACTAGAGTCTATGTCACTCATTATACTCTGTCGAATGTCATTGAATGTCTT
TACATGGGCTTGTATGCCTATGAAAATTGTAATACAACTTTCAGCAACGGATCTCTTGGC
TCTCGCATCGATGAAGAACGCAGCGAAATGCGATAAGTAATGTGAATTGCAGAATTCAGT
GAATCATCGAATCTTTGAACGCATCTTGCGCTCCTTGGTATTCCGAGGAGCATGCCTGTT
TGAGTGTCATTAAATTCTCAACTCTCTTATACTTTTTTGTAAAAGAGAGCTTGGACTGTG
GAGGCTTGCTGGCCACTTTTTGGGGTCAGCTCCTCTGAAATGCATTAGCGGAACCGTTTG
CGATCTGCCACAAGTGTGATAAGTTATCTACACTGGCGAGGGGATTGCTCTCTGTAATGT
TCAGCTTCTAATTGTCTCTACTTTGTGAGACTACTTTTGAATGCTTGACCTCAAATCAGG
TAGGACTACCCGCTGAACTTAA
>AC1-52
TTTCCGTAGGTGAACCTGCGGAAGGATCATTATTGAATTATGTTTCTAGATAGGTTGTAG
CTGGCTCTTTTAGAGCATGTGCACGCCTGTTTGGACTTCATTTTCATCCACCTGTGCACC
TATTGTAGTCTTTGGTTGGGTTAGGAGGAAGTGATCATTGTATCAGCATCTGCTGGGAGT
GAGGACTTGCATTGTGAAAGCTTTGCTGTCCTTGATGTGATCATGGAATCTTTTTC----

-------TCACTAGAGTCTATGTCACTCATTATACTCTGTCGAATGTCATTGAATGTCTT
TACATGGGCTTGTATGCCTATGAAAATTGTAATACAACTTTCAGCAACGGATCTCTTGGC
TCTCGCATCGATGAAGAACGCAGCGAAATGCGATAAGTAATGTGAATTGCAGAATTCAGT
GAATCATCGAATCTTTGAACGCATCTTGCGCTCCTTGGTATTCCGAGGAGCATGCCTGTT
TGAGTGTCATTAAATTCTCAACTCTCTTATACTTTTTTGTAAAAGAGAGCTTGGACTGTG
GAGGCTTGCTGGCCACTTTTTGGGGTCAGCTCCTCTGAAATGCATTAGCGGAACCGTTTG
CGATCTGCCACAAGTGTGATAAGTTATCTACACTGGCGAGGGGATTGCTCTCTGTAATGT
TCAGCTTCTAATTGTCTCTACTTTGTGAGACTACTTTTGAATGCTTGACCTCAAATCAGG
TAGGACTACCCGCTGAACTTAA
>AC1-55
TTTCCGTAGGTGAACCTGCGGAAGGATCATTATTGAATTATGTTTCTAGATAGGTTGTAG
CTGGCTCTTTTAGAGCATGTGCACGCCTGTTTGGACTTCATTTTCATCCACCTGTGCACC
TATTGTAGTCTTTGGTTGGGTTAGGAGGAAGTGATCATTGTATCAGCATCTGCTGGGAGT

GAGGACTTGCATTGTGAAAGCTTTGCTGTCCTTGATGTGATCATGGAATCTTTTTC----

-------TCACTAGAGTCTATGTCACTCATTATACTCTGTCGAATGTCATTGAATGTCTT
TACATGGGCTTGTATGCCTATGAAAATTGTAATACAACTTTCAGCAACGGATCTCTTGGC
TCTCGCATCGATGAAGAACGCAGCGAAATGCGATAAGTAATGTGAATTGCAGAATTCAGT
GAATCATCGAATCTTTGAACGCATCTTGCGCTCCTTGGTATTCCGAGGAGCATGCCTGTT
TGAGTGTCATTAAATTCTCAACTCTCTTATACTTTTTTGTAAAAGAGAGCTTGGACTGTG
GAGGCTTGCTGGCCACTTTTTGGGGTCAGCTCCTCTGAAATGCATTAGCGGAACCGTTTG
CGATCTGCCACAAGTGTGATAAGTTATCTACACTGGCGAGGGGATTGCTCTCTGTAATGT
TCAGCTTCTAATTGTCTCTACTTTGTGAGACTACTTTTGAATGCTTGACCTCAAATCAGG
TAGGACTACCCGCTGAACTTAA
>AC1-61
TTTCCGTAGGTGAACCTGCGGAAGGATCATTATTGAATTATGTTTCTAGATAGGTTGTAG
CTGGCTCTTTTAGAGCATGTGCACGCCTGTTTGGACTTCATTTTCATCCACCTGTGCACC
TATTGTAGTCTTTGGTTGGGTTAGGAGGAAGTGATCATTGTATCAGCATCTGCTGGGAGT
GAGGACTTGCATTGTGAAAGCTTTGCTGTCCTTGATGTGATCATGGAATCTTTTTC----

-------TCACTAGAGTCTATGTCACTCATTATACTCTGTCGAATGTCATTGAATGTCTT
TACATGGGCTTGTATGCCTATGAAAATTGTAATACAACTTTCAGCAACGGATCTCTTGGC
TCTCGCATCGATGAAGAACGCAGCGAAATGCGATAAGTAATGTGAATTGCAGAATTCAGT
GAATCATCGAATCTTTGAACGCATCTTGCGCTCCTTGGTATTCCGAGGAGCATGCCTGTT
TGAGTGTCATTAAATTCTCAACTCTCTTATACTTTTTTGTAAAAGAGAGCTTGGACTGTG
GAGGCTTGCTGGCCACTTTTTGGGGTCAGCTCCTCTGAAATGCATTAGCGGAACCGTTTG
CGATCTGCCACAAGTGTGATAAGTTATCTACACTGGCGAGGGGATTGCTCTCTGTAATGT
TCAGCTTCTAATTGTCTCTACTTTGTGAGACTACTTTTGAATGCTTGACCTCAAATCAGG
TAGGACTACCCGCTGAACTTAA
>AC1-65
TTTCCGTAGGTGAACCTGCGGAAGGATCATTATTGAATTATGTTTCTAGATAGGTTGTAG

CTGGCTCTTTTAGAGCATGTGCACGCCTGTTTGGACTTCATTTTCATCCACCTGTGCACC
TATTGTAGTCTTTGGTTGGGTTAGGAGGAAGTGATCATTGTATCAGCATCTGCTGGGAGT
GAGGACTTGCATTGTGAAAGCTTTGCTGTCCTTGATGTGATCATGGAATCTTTTTC----

-------TCACTAGAGTCTATGTCACTCATTATACTCTGTCGAATGTCATTGAATGTCTT
TACATGGGCTTGTATGCCTATGAAAATTGTAATACAACTTTCAGCAACGGATCTCTTGGC
TCTCGCATCGATGAAGAACGCAGCGAAATGCGATAAGTAATGTGAATTGCAGAATTCAGT
GAATCATCGAATCTTTGAACGCATCTTGCGCTCCTTGGTATTCCGAGGAGCATGCCTGTT
TGAGTGTCATTAAATTCTCAACTCTCTTATACTTTTTTGTAAAAGAGAGCTTGGACTGTG
GAGGCTTGCTGGCCACTTTTTGGGGTCAGCTCCTCTGAAATGCATTAGCGGAACCGTTTG
CGATCTGCCACAAGTGTGATAAGTTATCTACACTGGCGAGGGGATTGCTCTCTGTAATGT
TCAGCTTCTAATTGTCTCTACTTTGTGAGACTACTTTTGAATGCTTGACCTCAAATCAGG
TAGGACTACCCGCTGAACTTAA
>AC2-3
TTTCCGTAGGTGAACCTGCGGAAGGATCATTATTGAATTATGTTTCTAGATAGGTTGTAG
CTGGCTCTTTTAGAGCATGTGCACGCCTGTTTGGACTTCATTTTCATCCACCTGTGCACC
TATTGTAGTCTTTGGTTGGGTTAGGAGGAAGTGATCATTGTATCAGCATCTGCTGGGAGT
GAGGACTTGCATTGTGAAAGCTTTGCTGTCCTTGATGTGATCATGGAATCTTTTTC----

-------TCACTAGAGTCTATGTCACTCATTATACTCTGTCGAATGTCATTGAATGTCTT
TACATGGGCTTGTATGCCTATGAAAATTGTAATACAACTTTCAGCAACGGATCTCTTGGC
TCTCGCATCGATGAAGAACGCAGCGAAATGCGATAAGTAATGTGAATTGCAGAATTCAGT
GAATCATCGAATCTTTGAACGCATCTTGCGCTCCTTGGTATTCCGAGGAGCATGCCTGTT
TGAGTGTCATTAAATTCTCAACTCTCTTATACTTTTTTGTAAAAGAGAGCTTGGACTGTG
GAGGCTTGCTGGCCACTTTTTGGGGTCAGCTCCTCTGAAATGCATTAGCGGAACCGTTTG
CGATCTGCCACAAGTGTGATAAGTTATCTACACTGGCGAGGGGATTGCTCTCTGTAATGT
TCAGCTTCTAATTGTCTCTACTTTGTGAGACTACTTTTGAATGCTTGACCTCAAATCAGG
TAGGACTACCCGCTGAACTTAA

>AC2-4
TTTCCGTAGGTGAACCTGCGGAAGGATCATTATTGAATTATGTTTCTAGATAGGTTGTAG
CTGGCTCTTTTAGAGCATGTGCACGCCTGTTTGGACTTCATTTTCATCCACCTGTGCACC
TATTGTAGTCTTTGGTTGGGTTAGGAGGAAGTGATCATTGTATCAGCATCTGCTGGGAGT
GAGGACTTGCATTGTGAAAGCTTTGCTGTCCTTGATGTGATCATGGAATCTTTTTC----

-------TCACTAGAGTCTATGTCACTCATTATACTCTGTCGAATGTCATTGAATGTCTT
TACATGGGCTTGTATGCCTATGAAAATTGTAATACAACTTTCAGCAACGGATCTCTTGGC
TCTCGCATCGATGAAGAACGCAGCGAAATGCGATAAGTAATGTGAATTGCAGAATTCAGT
GAATCATCGAATCTTTGAACGCATCTTGCGCTCCTTGGTATTCCGAGGAGCATGCCTGTT
TGAGTGTCATTAAATTCTCAACTCTCTTATACTTTTTTGTAAAAGAGAGCTTGGACTGTG
GAGGCTTGCTGGCCACTTTTTGGGGTCAGCTCCTCTGAAATGCATTAGCGGAACCGTTTG
CGATCTGCCACAAGTGTGATAAGTTATCTACACTGGCGAGGGGATTGCTCTCTGTAATGT
TCAGCTTCTAATTGTCTCTACTTTGTGAGACTACTTTTGAATGCTTGACCTCAAATCAGG
TAGGACTACCCGCTGAACTTAA
>AC2-8
TTTCCGTAGGTGAACCTGCGGAAGGATCATTATTGAATTATGTTTCTAGATAGGTTGTAG
CTGGCTCTTTTAGAGCATGTGCACGCCTGTTTGGACTTCATTTTCATCCACCTGTGCACC
TATTGTAGTCTTTGGTTGGGTTAGGAGGAAGTGATCATTGTATCAGCATCTGCTGGGAGT
GAGGACTTGCATTGTGAAAGCTTTGCTGTCCTTGATGTGATCATGGAATCTTTTTC----

-------TCACTAGAGTCTATGTCACTCATTATACTCTGTCGAATGTCATTGAATGTCTT
TACATGGGCTTGTATGCCTATGAAAATTGTAATACAACTTTCAGCAACGGATCTCTTGGC
TCTCGCATCGATGAAGAACGCAGCGAAATGCGATAAGTAATGTGAATTGCAGAATTCAGT
GAATCATCGAATCTTTGAACGCATCTTGCGCTCCTTGGTATTCCGAGGAGCATGCCTGTT
TGAGTGTCATTAAATTCTCAACTCTCTTATACTTTTTTGTAAAAGAGAGCTTGGACTGTG
GAGGCTTGCTGGCCACTTTTTGGGGTCAGCTCCTCTGAAATGCATTAGCGGAACCGTTTG
CGATCTGCCACAAGTGTGATAAGTTATCTACACTGGCGAGGGGATTGCTCTCTGTAATGT

TCAGCTTCTAATTGTCTCTACTTTGTGAGACTACTTTTGAATGCTTGACCTCAAATCAGG
TAGGACTACCCGCTGAACTTAA
>AC2-11
TTTCCGTAGGTGAACCTGCGGAAGGATCATTATTGAATTATGTTTCTAGATAGGTTGTAG
CTGGCTCTTTTAGAGCATGTGCACGCCTGTTTGGACTTCATTTTCATCCACCTGTGCACC
TATTGTAGTCTTTGGTTGGGTTAGGAGGAAGTGATCATTGTATCAGCATCTGCTGGGAGT
GAGGACTTGCATTGTGAAAGCTTTGCTGTCCTTGATGTGATCATGGAATCTTTTTC----

-------TCACTAGAGTCTATGTCACTCATTATACTCTGTCGAATGTCATTGAATGTCTT
TACATGGGCTTGTATGCCTATGAAAATTGTAATACAACTTTCAGCAACGGATCTCTTGGC
TCTCGCATCGATGAAGAACGCAGCGAAATGCGATAAGTAATGTGAATTGCAGAATTCAGT
GAATCATCGAATCTTTGAACGCATCTTGCGCTCCTTGGTATTCCGAGGAGCATGCCTGTT
TGAGTGTCATTAAATTCTCAACTCTCTTATACTTTTTTGTAAAAGAGAGCTTGGACTGTG
GAGGCTTGCTGGCCACTTTTTGGGGTCAGCTCCTCTGAAATGCATTAGCGGAACCGTTTG
CGATCTGCCACAAGTGTGATAAGTTATCTACACTGGCGAGGGGATTGCTCTCTGTAATGT
TCAGCTTCTAATTGTCTCTACTTTGTGAGACTACTTTTGAATGCTTGACCTCAAATCAGG
TAGGACTACCCGCTGAACTTAA
>AC2-12
TTTCCGTAGGTGAACCTGCGGAAGGATCATTATTGAATTATGTTTCTAGATAGGTTGTAG
CTGGCTCTTTTAGAGCATGTGCACGCCTGTTTGGACTTCATTTTCATCCACCTGTGCACC
TATTGTAGTCTTTGGTTGGGTTAGGAGGAAGTGATCATTGTATCAGCATCTGCTGGGAGT
GAGGACTTGCATTGTGAAAGCTTTGCTGTCCTTGATGTGATCATGGAATCTTTTTC----

-------TCACTAGAGTCTATGTCACTCATTATACTCTGTCGAATGTCATTGAATGTCTT
TACATGGGCTTGTATGCCTATGAAAATTGTAATACAACTTTCAGCAACGGATCTCTTGGC
TCTCGCATCGATGAAGAACGCAGCGAAATGCGATAAGTAATGTGAATTGCAGAATTCAGT
GAATCATCGAATCTTTGAACGCATCTTGCGCTCCTTGGTATTCCGAGGAGCATGCCTGTT
TGAGTGTCATTAAATTCTCAACTCTCTTATACTTTTTTGTAAAAGAGAGCTTGGACTGTG

```
GAGGCTTGCTGGCCACTTTTTGGGGTCAGCTCCTCTGAAATGCATTAGCGGAACCGTTTG
CGATCTGCCACAAGTGTGATAAGTTATCTACACTGGCGAGGGGATTGCTCTCTGTAATGT
TCAGCTTCTAATTGTCTCTACTTTGTGAGACTACTTTTGAATGCTTGACCTCAAATCAGG
TAGGACTACCCGCTGAACTTAA
>AC2-13
TTTCCGTAGGTGAACCTGCGGAAGGATCATTATTGAATTATGTTTCTAGATAGGTTGTAG
CTGGCTCTTTTAGAGCATGTGCACGCCTGTTTGGACTTCATTTTCATCCACCTGTGCACC
TATTGTAGTCTTTGGTTGGGTTAGGAGGAAGTGATCATTGTATCAGCATCTGCTGGGAGT
GAGGACTTGCATTGTGAAAGCTTTGCTGTCCTTGATGTGATCATGGAATCTTTTTC----
------------------------------------------------------------
------------------------------------------------------------
------------------------------------------------------------
------------------------------------------------------------
------------------------------------------------------------
------------------------------------------------------------
------------------------------------------------------------
------------------------------------------------------------
------------------------------------------------------------
------------------------------------------------------------
------------------------------------------------------------
------------------------------------------------------------
-------TCACTAGAGTCTATGTCACTCATTATACTCTGTCGAATGTCATTGAATGTCTT
TACATGGGCTTGTATGCCTATGAAAATTGTAATACAACTTTCAGCAACGGATCTCTTGGC
TCTCGCATCGATGAAGAACGCAGCGAAATGCGATAAGTAATGTGAATTGCAGAATTCAGT
GAATCATCGAATCTTTGAACGCATCTTGCGCTCCTTGGTATTCCGAGGAGCATGCCTGTT
TGAGTGTCATTAAATTCTCAACTCTCTTATACTTTTTTGTAAAAGAGAGCTTGGACTGTG
GAGGCTTGCTGGCCACTTTTTGGGGTCAGCTCCTCTGAAATGCATTAGCGGAACCGTTTG
CGATCTGCCACAAGTGTGATAAGTTATCTACACTGGCGAGGGGATTGCTCTCTGTAATGT
TCAGCTTCTAATTGTCTCTACTTTGTGAGACTACTTTTGAATGCTTGACCTCAAATCAGG
TAGGACTACCCGCTGAACTTAA
>AC2-21
TTTCCGTAGGTGAACCTGCGGAAGGATCATTATTGAATTATGTTTCTAGATAGGTTGTAG
CTGGCTCTTTTAGAGCATGTGCACGCCTGTTTGGACTTCATTTTCATCCACCTGTGCACC
TATTGTAGTCTTTGGTTGGGTTAGGAGGAAGTGATCATTGTATCAGCATCTGCTGGGAGT
GAGGACTTGCATTGTGAAAGCTTTGCTGTCCTTGATGTGATCATGGAATCTTTTTC----
------------------------------------------------------------
------------------------------------------------------------
------------------------------------------------------------
------------------------------------------------------------
------------------------------------------------------------
------------------------------------------------------------
------------------------------------------------------------
------------------------------------------------------------
------------------------------------------------------------
------------------------------------------------------------
------------------------------------------------------------
------------------------------------------------------------
-------TCACTAGAGTCTATGTCACTCATTATACTCTGTCGAATGTCATTGAATGTCTT
TACATGGGCTTGTATGCCTATGAAAATTGTAATACAACTTTCAGCAACGGATCTCTTGGC
TCTCGCATCGATGAAGAACGCAGCGAAATGCGATAAGTAATGTGAATTGCAGAATTCAGT
```

```
GAATCATCGAATCTTTGAACGCATCTTGCGCTCCTTGGTATTCCGAGGAGCATGCCTGTT
TGAGTGTCATTAAATTCTCAACTCTCTTATACTTTTTTGTAAAAGAGAGCTTGGACTGTG
GAGGCTTGCTGGCCACTTTTTGGGGTCAGCTCCTCTGAAATGCATTAGCGGAACCGTTTG
CGATCTGCCACAAGTGTGATAAGTTATCTACACTGGCGAGGGGATTGCTCTCTGTAATGT
TCAGCTTCTAATTGTCTCTACTTTGTGAGACTACTTTTGAATGCTTGACCTCAAATCAGG
TAGGACTACCCGCTGAACTTAA
>AC2-23
TTTCCGTAGGTGAACCTGCGGAAGGATCATTATTGAATTATGTTTCTAGATAGGTTGTAG
CTGGCTCTTTTAGAGCATGTGCACGCCTGTTTGGACTTCATTTTCATCCACCTGTGCACC
TATTGTAGTCTTTGGTTGGGTTAGGAGGAAGTGATCATTGTATCAGCATCTGCTGGGAGT
GAGGACTTGCATTGTGAAAGCTTTGCTGTCCTTGATGTGATCATGGAATCTTTTTC----
------------------------------------------------------------
------------------------------------------------------------
------------------------------------------------------------
------------------------------------------------------------
------------------------------------------------------------
------------------------------------------------------------
------------------------------------------------------------
------------------------------------------------------------
------------------------------------------------------------
------------------------------------------------------------
------------------------------------------------------------
------------------------------------------------------------
-------TCACTAGAGTCTATGTCACTCATTATACTCTGTCGAATGTCATTGAATGTCTT
TACATGGGCTTGTATGCCTATGAAAATTGTAATACAACTTTCAGCAACGGATCTCTTGGC
TCTCGCATCGATGAAGAACGCAGCGAAATGCGATAAGTAATGTGAATTGCAGAATTCAGT
GAATCATCGAATCTTTGAACGCATCTTGCGCTCCTTGGTATTCCGAGGAGCATGCCTGTT
TGAGTGTCATTAAATTCTCAACTCTCTTATACTTTTTTGTAAAAGAGAGCTTGGACTGTG
GAGGCTTGCTGGCCACTTTTTGGGGTCAGCTCCTCTGAAATGCATTAGCGGAACCGTTTG
CGATCTGCCACAAGTGTGATAAGTTATCTACACTGGCGAGGGGATTGCTCTCTGTAATGT
TCAGCTTCTAATTGTCTCTACTTTGTGAGACTACTTTTGAATGCTTGACCTCAAATCAGG
TAGGACTACCCGCTGAACTTAA
>AC2-25
TTTCCGTAGGTGAACCTGCGGAAGGATCATTATTGAATTATGTTTCTAGATAGGTTGTAG
CTGGCTCTTTTAGAGCATGTGCACGCCTGTTTGGACTTCATTTTCATCCACCTGTGCACC
TATTGTAGTCTTTGGTTGGGTTAGGAGGAAGTGATCATTGTATCAGCATCTGCTGGGAGT
GAGGACTTGCATTGTGAAAGCTTTGCTGTCCTTGATGTGATCATGGAATCTTTTTC----
------------------------------------------------------------
------------------------------------------------------------
------------------------------------------------------------
------------------------------------------------------------
------------------------------------------------------------
------------------------------------------------------------
------------------------------------------------------------
------------------------------------------------------------
------------------------------------------------------------
------------------------------------------------------------
------------------------------------------------------------
------------------------------------------------------------
-------TCACTAGAGTCTATGTCACTCATTATACTCTGTCGAATGTCATTGAATGTCTT
```

TACATGGGCTTGTATGCCTATGAAAATTGTAATACAACTTTCAGCAACGGATCTCTTGGC
TCTCGCATCGATGAAGAACGCAGCGAAATGCGATAAGTAATGTGAATTGCAGAATTCAGT
GAATCATCGAATCTTTGAACGCATCTTGCGCTCCTTGGTATTCCGAGGAGCATGCCTGTT
TGAGTGTCATTAAATTCTCAACTCTCTTATACTTTTTTGTAAAAGAGAGCTTGGACTGTG
GAGGCTTGCTGGCCACTTTTTGGGGTCAGCTCCTCTGAAATGCATTAGCGGAACCGTTTG
CGATCTGCCACAAGTGTGATAAGTTATCTACACTGGCGAGGGGATTGCTCTCTGTAATGT
TCAGCTTCTAATTGTCTCTACTTTGTGAGACTACTTTTGAATGCTTGACCTCAAATCAGG
TAGGACTACCCGCTGAACTTAA
>AC2-27
TTTCCGTAGGTGAACCTGCGGAAGGATCATTATTGAATTATGTTTCTAGATAGGTTGTAG
CTGGCTCTTTTAGAGCATGTGCACGCCTGTTTGGACTTCATTTTCATCCACCTGTGCACC
TATTGTAGTCTTTGGTTGGGTTAGGAGGAAGTGATCATTGTATCAGCATCTGCTGGGAGT
GAGGACTTGCATTGTGAAAGCTTTGCTGTCCTTGATGTGATCATGGAATCTTTTTC----

-------TCACTAGAGTCTATGTCACTCATTATACTCTGTCGAATGTCATTGAATGTCTT
TACATGGGCTTGTATGCCTATGAAAATTGTAATACAACTTTCAGCAACGGATCTCTTGGC
TCTCGCATCGATGAAGAACGCAGCGAAATGCGATAAGTAATGTGAATTGCAGAATTCAGT
GAATCATCGAATCTTTGAACGCATCTTGCGCTCCTTGGTATTCCGAGGAGCATGCCTGTT
TGAGTGTCATTAAATTCTCAACTCTCTTATACTTTTTTGTAAAAGAGAGCTTGGACTGTG
GAGGCTTGCTGGCCACTTTTTGGGGTCAGCTCCTCTGAAATGCATTAGCGGAACCGTTTG
CGATCTGCCACAAGTGTGATAAGTTATCTACACTGGCGAGGGGATTGCTCTCTGTAATGT
TCAGCTTCTAATTGTCTCTACTTTGTGAGACTACTTTTGAATGCTTGACCTCAAATCAGG
TAGGACTACCCGCTGAACTTAA
>AC2-28
TTTCCGTAGGTGAACCTGCGGAAGGATCATTATTGAATTATGTTTCTAGATAGGTTGTAG
CTGGCTCTTTTAGAGCATGTGCACGCCTGTTTGGACTTCATTTTCATCCACCTGTGCACC
TATTGTAGTCTTTGGTTGGGTTAGGAGGAAGTGATCATTGTATCAGCATCTGCTGGGAGT
GAGGACTTGCATTGTGAAAGCTTTGCTGTCCTTGATGTGATCATGGAATCTTTTTC----


```
------------------------------------------------------------
-------TCACTAGAGTCTATGTCACTCATTATACTCTGTCGAATGTCATTGAATGTCTT
TACATGGGCTTGTATGCCTATGAAAATTGTAATACAACTTTCAGCAACGGATCTCTTGGC
TCTCGCATCGATGAAGAACGCAGCGAAATGCGATAAGTAATGTGAATTGCAGAATTCAGT
GAATCATCGAATCTTTGAACGCATCTTGCGCTCCTTGGTATTCCGAGGAGCATGCCTGTT
TGAGTGTCATTAAATTCTCAACTCTCTTATACTTTTTTGTAAAAGAGAGCTTGGACTGTG
GAGGCTTGCTGGCCACTTTTTGGGGTCAGCTCCTCTGAAATGCATTAGCGGAACCGTTTG
CGATCTGCCACAAGTGTGATAAGTTATCTACACTGGCGAGGGGATTGCTCTCTGTAATGT
TCAGCTTCTAATTGTCTCTACTTTGTGAGACTACTTTTGAATGCTTGACCTCAAATCAGG
TAGGACTACCCGCTGAACTTAA
>AC2-38
TTTCCGTAGGTGAACCTGCGGAAGGATCATTATTGAATTATGTTTCTAGATAGGTTGTAG
CTGGCTCTTTTAGAGCATGTGCACGCCTGTTTGGACTTCATTTTCATCCACCTGTGCACC
TATTGTAGTCTTTGGTTGGGTTAGGAGGAAGTGATCATTGTATCAGCATCTGCTGGGAGT
GAGGACTTGCATTGTGAAAGCTTTGCTGTCCTTGATGTGATCATGGAATCTTTTTC----
------------------------------------------------------------
------------------------------------------------------------
------------------------------------------------------------
------------------------------------------------------------
------------------------------------------------------------
------------------------------------------------------------
------------------------------------------------------------
------------------------------------------------------------
------------------------------------------------------------
------------------------------------------------------------
------------------------------------------------------------
------------------------------------------------------------
-------TCACTAGAGTCTATGTCACTCATTATACTCTGTCGAATGTCATTGAATGTCTT
TACATGGGCTTGTATGCCTATGAAAATTGTAATACAACTTTCAGCAACGGATCTCTTGGC
TCTCGCATCGATGAAGAACGCAGCGAAATGCGATAAGTAATGTGAATTGCAGAATTCAGT
GAATCATCGAATCTTTGAACGCATCTTGCGCTCCTTGGTATTCCGAGGAGCATGCCTGTT
TGAGTGTCATTAAATTCTCAACTCTCTTATACTTTTTTGTAAAAGAGAGCTTGGACTGTG
GAGGCTTGCTGGCCACTTTTTGGGGTCAGCTCCTCTGAAATGCATTAGCGGAACCGTTTG
CGATCTGCCACAAGTGTGATAAGTTATCTACACTGGCGAGGGGATTGCTCTCTGTAATGT
TCAGCTTCTAATTGTCTCTACTTTGTGAGACTACTTTTGAATGCTTGACCTCAAATCAGG
TAGGACTACCCGCTGAACTTAA
>AC2-43
TTTCCGTAGGTGAACCTGCGGAAGGATCATTATTGAATTATGTTTCTAGATAGGTTGTAG
CTGGCTCTTTTAGAGCATGTGCACGCCTGTTTGGACTTCATTTTCATCCACCTGTGCACC
TATTGTAGTCTTTGGTTGGGTTAGGAGGAAGTGATCATTGTATCAGCATCTGCTGGGAGT
GAGGACTTGCATTGTGAAAGCTTTGCTGTCCTTGATGTGATCATGGAATCTTTTTC----
------------------------------------------------------------
------------------------------------------------------------
------------------------------------------------------------
------------------------------------------------------------
------------------------------------------------------------
------------------------------------------------------------
------------------------------------------------------------
------------------------------------------------------------
------------------------------------------------------------
```

```
------------------------------------------------------------
------------------------------------------------------------
------------------------------------------------------------
-------TCACTAGAGTCTATGTCACTCATTATACTCTGTCGAATGTCATTGAATGTCTT
TACATGGGCTTGTATGCCTATGAAAATTGTAATACAACTTTCAGCAACGGATCTCTTGGC
TCTCGCATCGATGAAGAACGCAGCGAAATGCGATAAGTAATGTGAATTGCAGAATTCAGT
GAATCATCGAATCTTTGAACGCATCTTGCGCTCCTTGGTATTCCGAGGAGCATGCCTGTT
TGAGTGTCATTAAATTCTCAACTCTCTTATACTTTTTTGTAAAAGAGAGCTTGGACTGTG
GAGGCTTGCTGGCCACTTTTTGGGGTCAGCTCCTCTGAAATGCATTAGCGGAACCGTTTG
CGATCTGCCACAAGTGTGATAAGTTATCTACACTGGCGAGGGGATTGCTCTCTGTAATGT
TCAGCTTCTAATTGTCTCTACTTTGTGAGACTACTTTTGAATGCTTGACCTCAAATCAGG
TAGGACTACCCGCTGAACTTAA
>AC2-46
TTTCCGTAGGTGAACCTGCGGAAGGATCATTATTGAATTATGTTTCTAGATAGGTTGTAG
CTGGCTCTTTTAGAGCATGTGCACGCCTGTTTGGACTTCATTTTCATCCACCTGTGCACC
TATTGTAGTCTTTGGTTGGGTTAGGAGGAAGTGATCATTGTATCAGCATCTGCTGGGAGT
GAGGACTTGCATTGTGAAAGCTTTGCTGTCCTTGATGTGATCATGGAATCTTTTTC----
------------------------------------------------------------
------------------------------------------------------------
------------------------------------------------------------
------------------------------------------------------------
------------------------------------------------------------
------------------------------------------------------------
------------------------------------------------------------
------------------------------------------------------------
------------------------------------------------------------
------------------------------------------------------------
------------------------------------------------------------
------------------------------------------------------------
-------TCACTAGAGTCTATGTCACTCATTATACTCTGTCGAATGTCATTGAATGTCTT
TACATGGGCTTGTATGCCTATGAAAATTGTAATACAACTTTCAGCAACGGATCTCTTGGC
TCTCGCATCGATGAAGAACGCAGCGAAATGCGATAAGTAATGTGAATTGCAGAATTCAGT
GAATCATCGAATCTTTGAACGCATCTTGCGCTCCTTGGTATTCCGAGGAGCATGCCTGTT
TGAGTGTCATTAAATTCTCAACTCTCTTATACTTTTTTGTAAAAGAGAGCTTGGACTGTG
GAGGCTTGCTGGCCACTTTTTGGGGTCAGCTCCTCTGAAATGCATTAGCGGAACCGTTTG
CGATCTGCCACAAGTGTGATAAGTTATCTACACTGGCGAGGGGATTGCTCTCTGTAATGT
TCAGCTTCTAATTGTCTCTACTTTGTGAGACTACTTTTGAATGCTTGACCTCAAATCAGG
TAGGACTACCCGCTGAACTTAA
>AC2-49
TTTCCGTAGGTGAACCTGCGGAAGGATCATTATTGAATTATGTTTCTAGATAGGTTGTAG
CTGGCTCTTTTAGAGCATGTGCACGCCTGTTTGGACTTCATTTTCATCCACCTGTGCACC
TATTGTAGTCTTTGGTTGGGTTAGGAGGAAGTGATCATTGTATCAGCATCTGCTGGGAGT
GAGGACTTGCATTGTGAAAGCTTTGCTGTCCTTGATGTGATCATGGAATCTTTTTC----
------------------------------------------------------------
------------------------------------------------------------
------------------------------------------------------------
------------------------------------------------------------
------------------------------------------------------------
------------------------------------------------------------
------------------------------------------------------------
```


-------TCACTAGAGTCTATGTCACTCATTATACTCTGTCGAATGTCATTGAATGTCTT
TACATGGGCTTGTATGCCTATGAAAATTGTAATACAACTTTCAGCAACGGATCTCTTGGC
TCTCGCATCGATGAAGAACGCAGCGAAATGCGATAAGTAATGTGAATTGCAGAATTCAGT
GAATCATCGAATCTTTGAACGCATCTTGCGCTCCTTGGTATTCCGAGGAGCATGCCTGTT
TGAGTGTCATTAAATTCTCAACTCTCTTATACTTTTTTGTAAAAGAGAGCTTGGACTGTG
GAGGCTTGCTGGCCACTTTTTGGGGTCAGCTCCTCTGAAATGCATTAGCGGAACCGTTTG
CGATCTGCCACAAGTGTGATAAGTTATCTACACTGGCGAGGGGATTGCTCTCTGTAATGT
TCAGCTTCTAATTGTCTCTACTTTGTGAGACTACTTTTGAATGCTTGACCTCAAATCAGG
TAGGACTACCCGCTGAACTTAA
>AC2-59
TTTCCGTAGGTGAACCTGCGGAAGGATCATTATTGAATTATGTTTCTAGATAGGTTGTAG
CTGGCTCTTTTAGAGCATGTGCACGCCTGTTTGGACTTCATTTTCATCCACCTGTGCACC
TATTGTAGTCTTTGGTTGGGTTAGGAGGAAGTGATCATTGTATCAGCATCTGCTGGGAGT
GAGGACTTGCATTGTGAAAGCTTTGCTGTCCTTGATGTGATCATGGAATCTTTTTC----

-------TCACTAGAGTCTATGTCACTCATTATACTCTGTCGAATGTCATTGAATGTCTT
TACATGGGCTTGTATGCCTATGAAAATTGTAATACAACTTTCAGCAACGGATCTCTTGGC
TCTCGCATCGATGAAGAACGCAGCGAAATGCGATAAGTAATGTGAATTGCAGAATTCAGT
GAATCATCGAATCTTTGAACGCATCTTGCGCTCCTTGGTATTCCGAGGAGCATGCCTGTT
TGAGTGTCATTAAATTCTCAACTCTCTTATACTTTTTTGTAAAAGAGAGCTTGGACTGTG
GAGGCTTGCTGGCCACTTTTTGGGGTCAGCTCCTCTGAAATGCATTAGCGGAACCGTTTG
CGATCTGCCACAAGTGTGATAAGTTATCTACACTGGCGAGGGGATTGCTCTCTGTAATGT
TCAGCTTCTAATTGTCTCTACTTTGTGAGACTACTTTTGAATGCTTGACCTCAAATCAGG
TAGGACTACCCGCTGAACTTAA
>AC2-61
TTTCCGTAGGTGAACCTGCGGAAGGATCATTATTGAATTATGTTTCTAGATAGGTTGTAG
CTGGCTCTTTTAGAGCATGTGCACGCCTGTTTGGACTTCATTTTCATCCACCTGTGCACC
TATTGTAGTCTTTGGTTGGGTTAGGAGGAAGTGATCATTGTATCAGCATCTGCTGGGAGT
GAGGACTTGCATTGTGAAAGCTTTGCTGTCCTTGATGTGATCATGGAATCTTTTTC----

-------TCACTAGAGTCTATGTCACTCATTATACTCTGTCGAATGTCATTGAATGTCTT
TACATGGGCTTGTATGCCTATGAAAATTGTAATACAACTTTCAGCAACGGATCTCTTGGC
TCTCGCATCGATGAAGAACGCAGCGAAATGCGATAAGTAATGTGAATTGCAGAATTCAGT
GAATCATCGAATCTTTGAACGCATCTTGCGCTCCTTGGTATTCCGAGGAGCATGCCTGTT
TGAGTGTCATTAAATTCTCAACTCTCTTATACTTTTTTGTAAAAGAGAGCTTGGACTGTG
GAGGCTTGCTGGCCACTTTTTGGGGTCAGCTCCTCTGAAATGCATTAGCGGAACCGTTTG
CGATCTGCCACAAGTGTGATAAGTTATCTACACTGGCGAGGGGATTGCTCTCTGTAATGT
TCAGCTTCTAATTGTCTCTACTTTGTGAGACTACTTTTGAATGCTTGACCTCAAATCAGG
TAGGACTACCCGCTGAACTTAA
>AC2-68
TTTCCGTAGGTGAACCTGCGGAAGGATCATTATTGAATTATGTTTCTAGATAGGTTGTAG
CTGGCTCTTTTAGAGCATGTGCACGCCTGTTTGGACTTCATTTTCATCCACCTGTGCACC
TATTGTAGTCTTTGGTTGGGTTAGGAGGAAGTGATCATTGTATCAGCATCTGCTGGGAGT
GAGGACTTGCATTGTGAAAGCTTTGCTGTCCTTGATGTGATCATGGAATCTTTTTC----

-------TCACTAGAGTCTATGTCACTCATTATACTCTGTCGAATGTCATTGAATGTCTT
TACATGGGCTTGTATGCCTATGAAAATTGTAATACAACTTTCAGCAACGGATCTCTTGGC
TCTCGCATCGATGAAGAACGCAGCGAAATGCGATAAGTAATGTGAATTGCAGAATTCAGT
GAATCATCGAATCTTTGAACGCATCTTGCGCTCCTTGGTATTCCGAGGAGCATGCCTGTT
TGAGTGTCATTAAATTCTCAACTCTCTTATACTTTTTTGTAAAAGAGAGCTTGGACTGTG
GAGGCTTGCTGGCCACTTTTTGGGGTCAGCTCCTCTGAAATGCATTAGCGGAACCGTTTG
CGATCTGCCACAAGTGTGATAAGTTATCTACACTGGCGAGGGGATTGCTCTCTGTAATGT
TCAGCTTCTAATTGTCTCTACTTTGTGAGACTACTTTTGAATGCTTGACCTCAAATCAGG
TAGGACTACCCGCTGAACTTAA
>AC2-75
TTTCCGTAGGTGAACCTGCGGAAGGATCATTATTGAATTATGTTTCTAGATAGGTTGTAG
CTGGCTCTTTTAGAGCATGTGCACGCCTGTTTGGACTTCATTTTCATCCACCTGTGCACC
TATTGTAGTCTTTGGTTGGGTTAGGAGGAAGTGATCATTGTATCAGCATCTGCTGGGAGT
GAGGACTTGCATTGTGAAAGCTTTGCTGTCCTTGATGTGATCATGGAATCTTTTTC----

-------TCACTAGAGTCTATGTCACTCATTATACTCTGTCGAATGTCATTGAATGTCTT
TACATGGGCTTGTATGCCTATGAAAATTGTAATACAACTTTCAGCAACGGATCTCTTGGC
TCTCGCATCGATGAAGAACGCAGCGAAATGCGATAAGTAATGTGAATTGCAGAATTCAGT
GAATCATCGAATCTTTGAACGCATCTTGCGCTCCTTGGTATTCCGAGGAGCATGCCTGTT
TGAGTGTCATTAAATTCTCAACTCTCTTATACTTTTTTGTAAAAGAGAGCTTGGACTGTG
GAGGCTTGCTGGCCACTTTTTGGGGTCAGCTCCTCTGAAATGCATTAGCGGAACCGTTTG
CGATCTGCCACAAGTGTGATAAGTTATCTACACTGGCGAGGGGATTGCTCTCTGTAATGT
TCAGCTTCTAATTGTCTCTACTTTGTGAGACTACTTTTGAATGCTTGACCTCAAATCAGG
TAGGACTACCCGCTGAACTTAA
>AC3-4
TTTCCGTAGGTGAACCTGCGGAAGGATCATTATTGAATTATGTTTCTAGATAGGTTGTAG
CTGGCTCTTTTAGAGCATGTGCACGCCTGTTTGGACTTCATTTTCATCCACCTGTGCACC
TATTGTAGTCTTTGGTTGGGTTAGGAGGAAGTGATCATTGTATCAGCATCTGCTGGGAGT
GAGGACTTGCATTGTGAAAGCTTTGCTGTCCTTGATGTGATCATGGAATCTTTTTC----

-------TCACTAGAGTCTATGTCACTCATTATACTCTGTCGAATGTCATTGAATGTCTT
TACATGGGCTTGTATGCCTATGAAAATTGTAATACAACTTTCAGCAACGGATCTCTTGGC
TCTCGCATCGATGAAGAACGCAGCGAAATGCGATAAGTAATGTGAATTGCAGAATTCAGT
GAATCATCGAATCTTTGAACGCATCTTGCGCTCCTTGGTATTCCGAGGAGCATGCCTGTT
TGAGTGTCATTAAATTCTCAACTCTCTTATACTTTTTTGTAAAAGAGAGCTTGGACTGTG
GAGGCTTGCTGGCCACTTTTTGGGGTCAGCTCCTCTGAAATGCATTAGCGGAACCGTTTG
CGATCTGCCACAAGTGTGATAAGTTATCTACACTGGCGAGGGGATTGCTCTCTGTAATGT
TCAGCTTCTAATTGTCTCTACTTTGTGAGACTACTTTTGAATGCTTGACCTCAAATCAGG
TAGGACTACCCGCTGAACTTAA
>AC3-6
TTTCCGTAGGTGAACCTGCGGAAGGATCATTATTGAATTATGTTTCTAGATAGGTTGTAG
CTGGCTCTTTTAGAGCATGTGCACGCCTGTTTGGACTTCATTTTCATCCACCTGTGCACC
TATTGTAGTCTTTGGTTGGGTTAGGAGGAAGTGATCATTGTATCAGCATCTGCTGGGAGT
GAGGACTTGCATTGTGAAAGCTTTGCTGTCCTTGATGTGATCATGGAATCTTTTTC----

-------TCACTAGAGTCTATGTCACTCATTATACTCTGTCGAATGTCATTGAATGTCTT
TACATGGGCTTGTATGCCTATGAAAATTGTAATACAACTTTCAGCAACGGATCTCTTGGC
TCTCGCATCGATGAAGAACGCAGCGAAATGCGATAAGTAATGTGAATTGCAGAATTCAGT
GAATCATCGAATCTTTGAACGCATCTTGCGCTCCTTGGTATTCCGAGGAGCATGCCTGTT
TGAGTGTCATTAAATTCTCAACTCTCTTATACTTTTTTGTAAAAGAGAGCTTGGACTGTG
GAGGCTTGCTGGCCACTTTTTGGGGTCAGCTCCTCTGAAATGCATTAGCGGAACCGTTTG
CGATCTGCCACAAGTGTGATAAGTTATCTACACTGGCGAGGGGATTGCTCTCTGTAATGT
TCAGCTTCTAATTGTCTCTACTTTGTGAGACTACTTTTGAATGCTTGACCTCAAATCAGG
TAGGACTACCCGCTGAACTTAA
>AC3-16
TTTCCGTAGGTGAACCTGCGGAAGGATCATTATTGAATTATGTTTCTAGATAGGTTGTAG
CTGGCTCTTTTAGAGCATGTGCACGCCTGTTTGGACTTCATTTTCATCCACCTGTGCACC
TATTGTAGTCTTTGGTTGGGTTAGGAGGAAGTGATCATTGTATCAGCATCTGCTGGGAGT
GAGGACTTGCATTGTGAAAGCTTTGCTGTCCTTGATGTGATCATGGAATCTTTTTC----

-------TCACTAGAGTCTATGTCACTCATTATACTCTGTCGAATGTCATTGAATGTCTT
TACATGGGCTTGTATGCCTATGAAAATTGTAATACAACTTTCAGCAACGGATCTCTTGGC
TCTCGCATCGATGAAGAACGCAGCGAAATGCGATAAGTAATGTGAATTGCAGAATTCAGT
GAATCATCGAATCTTTGAACGCATCTTGCGCTCCTTGGTATTCCGAGGAGCATGCCTGTT
TGAGTGTCATTAAATTCTCAACTCTCTTATACTTTTTTGTAAAAGAGAGCTTGGACTGTG
GAGGCTTGCTGGCCACTTTTTGGGGTCAGCTCCTCTGAAATGCATTAGCGGAACCGTTTG
CGATCTGCCACAAGTGTGATAAGTTATCTACACTGGCGAGGGGATTGCTCTCTGTAATGT
TCAGCTTCTAATTGTCTCTACTTTGTGAGACTACTTTTGAATGCTTGACCTCAAATCAGG
TAGGACTACCCGCTGAACTTAA
>AC3-18
TTTCCGTAGGTGAACCTGCGGAAGGATCATTATTGAATTATGTTTCTAGATAGGTTGTAG
CTGGCTCTTTTAGAGCATGTGCACGCCTGTTTGGACTTCATTTTCATCCACCTGTGCACC
TATTGTAGTCTTTGGTTGGGTTAGGAGGAAGTGATCATTGTATCAGCATCTGCTGGGAGT

GAGGACTTGCATTGTGAAAGCTTTGCTGTCCTTGATGTGATCATGGAATCTTTTTC----

-------TCACTAGAGTCTATGTCACTCATTATACTCTGTCGAATGTCATTGAATGTCTT
TACATGGGCTTGTATGCCTATGAAAATTGTAATACAACTTTCAGCAACGGATCTCTTGGC
TCTCGCATCGATGAAGAACGCAGCGAAATGCGATAAGTAATGTGAATTGCAGAATTCAGT
GAATCATCGAATCTTTGAACGCATCTTGCGCTCCTTGGTATTCCGAGGAGCATGCCTGTT
TGAGTGTCATTAAATTCTCAACTCTCTTATACTTTTTTGTAAAAGAGAGCTTGGACTGTG
GAGGCTTGCTGGCCACTTTTTGGGGTCAGCTCCTCTGAAATGCATTAGCGGAACCGTTTG
CGATCTGCCACAAGTGTGATAAGTTATCTACACTGGCGAGGGGATTGCTCTCTGTAATGT
TCAGCTTCTAATTGTCTCTACTTTGTGAGACTACTTTTGAATGCTTGACCTCAAATCAGG
TAGGACTACCCGCTGAACTTAA
>AC3-19
TTTCCGTAGGTGAACCTGCGGAAGGATCATTATTGAATTATGTTTCTAGATAGGTTGTAG
CTGGCTCTTTTAGAGCATGTGCACGCCTGTTTGGACTTCATTTTCATCCACCTGTGCACC
TATTGTAGTCTTTGGTTGGGTTAGGAGGAAGTGATCATTGTATCAGCATCTGCTGGGAGT
GAGGACTTGCATTGTGAAAGCTTTGCTGTCCTTGATGTGATCATGGAATCTTTTTC----

-------TCACTAGAGTCTATGTCACTCATTATACTCTGTCGAATGTCATTGAATGTCTT
TACATGGGCTTGTATGCCTATGAAAATTGTAATACAACTTTCAGCAACGGATCTCTTGGC
TCTCGCATCGATGAAGAACGCAGCGAAATGCGATAAGTAATGTGAATTGCAGAATTCAGT
GAATCATCGAATCTTTGAACGCATCTTGCGCTCCTTGGTATTCCGAGGAGCATGCCTGTT
TGAGTGTCATTAAATTCTCAACTCTCTTATACTTTTTTGTAAAAGAGAGCTTGGACTGTG
GAGGCTTGCTGGCCACTTTTTGGGGTCAGCTCCTCTGAAATGCATTAGCGGAACCGTTTG
CGATCTGCCACAAGTGTGATAAGTTATCTACACTGGCGAGGGGATTGCTCTCTGTAATGT
TCAGCTTCTAATTGTCTCTACTTTGTGAGACTACTTTTGAATGCTTGACCTCAAATCAGG
TAGGACTACCCGCTGAACTTAA
>AC3-27
TTTCCGTAGGTGAACCTGCGGAAGGATCATTATTGAATTATGTTTCTAGATAGGTTGTAG

```
CTGGCTCTTTTAGAGCATGTGCACGCCTGTTTGGACTTCATTTTCATCCACCTGTGCACC
TATTGTAGTCTTTGGTTGGGTTAGGAGGAAGTGATCATTGTATCAGCATCTGCTGGGAGT
GAGGACTTGCATTGTGAAAGCTTTGCTGTCCTTGATGTGATCATGGAATCTTTTTC----
------------------------------------------------------------
------------------------------------------------------------
------------------------------------------------------------
------------------------------------------------------------
------------------------------------------------------------
------------------------------------------------------------
------------------------------------------------------------
------------------------------------------------------------
------------------------------------------------------------
------------------------------------------------------------
------------------------------------------------------------
------------------------------------------------------------
-------TCACTAGAGTCTATGTCACTCATTATACTCTGTCGAATGTCATTGAATGTCTT
TACATGGGCTTGTATGCCTATGAAAATTGTAATACAACTTTCAGCAACGGATCTCTTGGC
TCTCGCATCGATGAAGAACGCAGCGAAATGCGATAAGTAATGTGAATTGCAGAATTCAGT
GAATCATCGAATCTTTGAACGCATCTTGCGCTCCTTGGTATTCCGAGGAGCATGCCTGTT
TGAGTGTCATTAAATTCTCAACTCTCTTATACTTTTTTGTAAAAGAGAGCTTGGACTGTG
GAGGCTTGCTGGCCACTTTTTGGGGTCAGCTCCTCTGAAATGCATTAGCGGAACCGTTTG
CGATCTGCCACAAGTGTGATAAGTTATCTACACTGGCGAGGGGATTGCTCTCTGTAATGT
TCAGCTTCTAATTGTCTCTACTTTGTGAGACTACTTTTGAATGCTTGACCTCAAATCAGG
TAGGACTACCCGCTGAACTTAA
>AC3-28
TTTCCGTAGGTGAACCTGCGGAAGGATCATTATTGAATTATGTTTCTAGATAGGTTGTAG
CTGGCTCTTTTAGAGCATGTGCACGCCTGTTTGGACTTCATTTTCATCCACCTGTGCACC
TATTGTAGTCTTTGGTTGGGTTAGGAGGAAGTGATCATTGTATCAGCATCTGCTGGGAGT
GAGGACTTGCATTGTGAAAGCTTTGCTGTCCTTGATGTGATCATGGAATCTTTTTC----
------------------------------------------------------------
------------------------------------------------------------
------------------------------------------------------------
------------------------------------------------------------
------------------------------------------------------------
------------------------------------------------------------
------------------------------------------------------------
------------------------------------------------------------
------------------------------------------------------------
------------------------------------------------------------
------------------------------------------------------------
------------------------------------------------------------
-------TCACTAGAGTCTATGTCACTCATTATACTCTGTCGAATGTCATTGAATGTCTT
TACATGGGCTTGTATGCCTATGAAAATTGTAATACAACTTTCAGCAACGGATCTCTTGGC
TCTCGCATCGATGAAGAACGCAGCGAAATGCGATAAGTAATGTGAATTGCAGAATTCAGT
GAATCATCGAATCTTTGAACGCATCTTGCGCTCCTTGGTATTCCGAGGAGCATGCCTGTT
TGAGTGTCATTAAATTCTCAACTCTCTTATACTTTTTTGTAAAAGAGAGCTTGGACTGTG
GAGGCTTGCTGGCCACTTTTTGGGGTCAGCTCCTCTGAAATGCATTAGCGGAACCGTTTG
CGATCTGCCACAAGTGTGATAAGTTATCTACACTGGCGAGGGGATTGCTCTCTGTAATGT
TCAGCTTCTAATTGTCTCTACTTTGTGAGACTACTTTTGAATGCTTGACCTCAAATCAGG
TAGGACTACCCGCTGAACTTAA
```

>AC3-29
TTTCCGTAGGTGAACCTGCGGAAGGATCATTATTGAATTATGTTTCTAGATAGGTTGTAG
CTGGCTCTTTTAGAGCATGTGCACGCCTGTTTGGACTTCATTTTCATCCACCTGTGCACC
TATTGTAGTCTTTGGTTGGGTTAGGAGGAAGTGATCATTGTATCAGCATCTGCTGGGAGT
GAGGACTTGCATTGTGAAAGCTTTGCTGTCCTTGATGTGATCATGGAATCTTTTTC----

-------TCACTAGAGTCTATGTCACTCATTATACTCTGTCGAATGTCATTGAATGTCTT
TACATGGGCTTGTATGCCTATGAAAATTGTAATACAACTTTCAGCAACGGATCTCTTGGC
TCTCGCATCGATGAAGAACGCAGCGAAATGCGATAAGTAATGTGAATTGCAGAATTCAGT
GAATCATCGAATCTTTGAACGCATCTTGCGCTCCTTGGTATTCCGAGGAGCATGCCTGTT
TGAGTGTCATTAAATTCTCAACTCTCTTATACTTTTTTGTAAAAGAGAGCTTGGACTGTG
GAGGCTTGCTGGCCACTTTTTGGGGTCAGCTCCTCTGAAATGCATTAGCGGAACCGTTTG
CGATCTGCCACAAGTGTGATAAGTTATCTACACTGGCGAGGGGATTGCTCTCTGTAATGT
TCAGCTTCTAATTGTCTCTACTTTGTGAGACTACTTTTGAATGCTTGACCTCAAATCAGG
TAGGACTACCCGCTGAACTTAA
>AC3-30
TTTCCGTAGGTGAACCTGCGGAAGGATCATTATTGAATTATGTTTCTAGATAGGTTGTAG
CTGGCTCTTTTAGAGCATGTGCACGCCTGTTTGGACTTCATTTTCATCCACCTGTGCACC
TATTGTAGTCTTTGGTTGGGTTAGGAGGAAGTGATCATTGTATCAGCATCTGCTGGGAGT
GAGGACTTGCATTGTGAAAGCTTTGCTGTCCTTGATGTGATCATGGAATCTTTTTC----

-------TCACTAGAGTCTATGTCACTCATTATACTCTGTCGAATGTCATTGAATGTCTT
TACATGGGCTTGTATGCCTATGAAAATTGTAATACAACTTTCAGCAACGGATCTCTTGGC
TCTCGCATCGATGAAGAACGCAGCGAAATGCGATAAGTAATGTGAATTGCAGAATTCAGT
GAATCATCGAATCTTTGAACGCATCTTGCGCTCCTTGGTATTCCGAGGAGCATGCCTGTT
TGAGTGTCATTAAATTCTCAACTCTCTTATACTTTTTTGTAAAAGAGAGCTTGGACTGTG
GAGGCTTGCTGGCCACTTTTTGGGGTCAGCTCCTCTGAAATGCATTAGCGGAACCGTTTG
CGATCTGCCACAAGTGTGATAAGTTATCTACACTGGCGAGGGGATTGCTCTCTGTAATGT

TCAGCTTCTAATTGTCTCTACTTTGTGAGACTACTTTTGAATGCTTGACCTCAAATCAGG
TAGGACTACCCGCTGAACTTAA
>AC3-36
TTTCCGTAGGTGAACCTGCGGAAGGATCATTATTGAATTATGTTTCTAGATAGGTTGTAG
CTGGCTCTTTTAGAGCATGTGCACGCCTGTTTGGACTTCATTTTCATCCACCTGTGCACC
TATTGTAGTCTTTGGTTGGGTTAGGAGGAAGTGATCATTGTATCAGCATCTGCTGGGAGT
GAGGACTTGCATTGTGAAAGCTTTGCTGTCCTTGATGTGATCATGGAATCTTTTTC----

-------TCACTAGAGTCTATGTCACTCATTATACTCTGTCGAATGTCATTGAATGTCTT
TACATGGGCTTGTATGCCTATGAAAATTGTAATACAACTTTCAGCAACGGATCTCTTGGC
TCTCGCATCGATGAAGAACGCAGCGAAATGCGATAAGTAATGTGAATTGCAGAATTCAGT
GAATCATCGAATCTTTGAACGCATCTTGCGCTCCTTGGTATTCCGAGGAGCATGCCTGTT
TGAGTGTCATTAAATTCTCAACTCTCTTATACTTTTTTGTAAAAGAGAGCTTGGACTGTG
GAGGCTTGCTGGCCACTTTTTGGGGTCAGCTCCTCTGAAATGCATTAGCGGAACCGTTTG
CGATCTGCCACAAGTGTGATAAGTTATCTACACTGGCGAGGGGATTGCTCTCTGTAATGT
TCAGCTTCTAATTGTCTCTACTTTGTGAGACTACTTTTGAATGCTTGACCTCAAATCAGG
TAGGACTACCCGCTGAACTTAA
>AC3-37
TTTCCGTAGGTGAACCTGCGGAAGGATCATTATTGAATTATGTTTCTAGATAGGTTGTAG
CTGGCTCTTTTAGAGCATGTGCACGCCTGTTTGGACTTCATTTTCATCCACCTGTGCACC
TATTGTAGTCTTTGGTTGGGTTAGGAGGAAGTGATCATTGTATCAGCATCTGCTGGGAGT
GAGGACTTGCATTGTGAAAGCTTTGCTGTCCTTGATGTGATCATGGAATCTTTTTC----

-------TCACTAGAGTCTATGTCACTCATTATACTCTGTCGAATGTCATTGAATGTCTT
TACATGGGCTTGTATGCCTATGAAAATTGTAATACAACTTTCAGCAACGGATCTCTTGGC
TCTCGCATCGATGAAGAACGCAGCGAAATGCGATAAGTAATGTGAATTGCAGAATTCAGT
GAATCATCGAATCTTTGAACGCATCTTGCGCTCCTTGGTATTCCGAGGAGCATGCCTGTT
TGAGTGTCATTAAATTCTCAACTCTCTTATACTTTTTTGTAAAAGAGAGCTTGGACTGTG

GAGGCTTGCTGGCCACTTTTTGGGGTCAGCTCCTCTGAAATGCATTAGCGGAACCGTTTG
CGATCTGCCACAAGTGTGATAAGTTATCTACACTGGCGAGGGGATTGCTCTCTGTAATGT
TCAGCTTCTAATTGTCTCTACTTTGTGAGACTACTTTTGAATGCTTGACCTCAAATCAGG
TAGGACTACCCGCTGAACTTAA
>AC3-41
TTTCCGTAGGTGAACCTGCGGAAGGATCATTATTGAATTATGTTTCTAGATAGGTTGTAG
CTGGCTCTTTTAGAGCATGTGCACGCCTGTTTGGACTTCATTTTCATCCACCTGTGCACC
TATTGTAGTCTTTGGTTGGGTTAGGAGGAAGTGATCATTGTATCAGCATCTGCTGGGAGT
GAGGACTTGCATTGTGAAAGCTTTGCTGTCCTTGATGTGATCATGGAATCTTTTTC----

-------TCACTAGAGTCTATGTCACTCATTATACTCTGTCGAATGTCATTGAATGTCTT
TACATGGGCTTGTATGCCTATGAAAATTGTAATACAACTTTCAGCAACGGATCTCTTGGC
TCTCGCATCGATGAAGAACGCAGCGAAATGCGATAAGTAATGTGAATTGCAGAATTCAGT
GAATCATCGAATCTTTGAACGCATCTTGCGCTCCTTGGTATTCCGAGGAGCATGCCTGTT
TGAGTGTCATTAAATTCTCAACTCTCTTATACTTTTTTGTAAAAGAGAGCTTGGACTGTG
GAGGCTTGCTGGCCACTTTTTGGGGTCAGCTCCTCTGAAATGCATTAGCGGAACCGTTTG
CGATCTGCCACAAGTGTGATAAGTTATCTACACTGGCGAGGGGATTGCTCTCTGTAATGT
TCAGCTTCTAATTGTCTCTACTTTGTGAGACTACTTTTGAATGCTTGACCTCAAATCAGG
TAGGACTACCCGCTGAACTTAA
>AC3-53
TTTCCGTAGGTGAACCTGCGGAAGGATCATTATTGAATTATGTTTCTAGATAGGTTGTAG
CTGGCTCTTTTAGAGCATGTGCACGCCTGTTTGGACTTCATTTTCATCCACCTGTGCACC
TATTGTAGTCTTTGGTTGGGTTAGGAGGAAGTGATCATTGTATCAGCATCTGCTGGGAGT
GAGGACTTGCATTGTGAAAGCTTTGCTGTCCTTGATGTGATCATGGAATCTTTTTC----

-------TCACTAGAGTCTATGTCACTCATTATACTCTGTCGAATGTCATTGAATGTCTT
TACATGGGCTTGTATGCCTATGAAAATTGTAATACAACTTTCAGCAACGGATCTCTTGGC
TCTCGCATCGATGAAGAACGCAGCGAAATGCGATAAGTAATGTGAATTGCAGAATTCAGT

GAATCATCGAATCTTTGAACGCATCTTGCGCTCCTTGGTATTCCGAGGAGCATGCCTGTT
TGAGTGTCATTAAATTCTCAACTCTCTTATACTTTTTTGTAAAAGAGAGCTTGGACTGTG
GAGGCTTGCTGGCCACTTTTTGGGGTCAGCTCCTCTGAAATGCATTAGCGGAACCGTTTG
CGATCTGCCACAAGTGTGATAAGTTATCTACACTGGCGAGGGGATTGCTCTCTGTAATGT
TCAGCTTCTAATTGTCTCTACTTTGTGAGACTACTTTTGAATGCTTGACCTCAAATCAGG
TAGGACTACCCGCTGAACTTAA
>AC3-56
TTTCCGTAGGTGAACCTGCGGAAGGATCATTATTGAATTATGTTTCTAGATAGGTTGTAG
CTGGCTCTTTTAGAGCATGTGCACGCCTGTTTGGACTTCATTTTCATCCACCTGTGCACC
TATTGTAGTCTTTGGTTGGGTTAGGAGGAAGTGATCATTGTATCAGCATCTGCTGGGAGT
GAGGACTTGCATTGTGAAAGCTTTGCTGTCCTTGATGTGATCATGGAATCTTTTTC----

-------TCACTAGAGTCTATGTCACTCATTATACTCTGTCGAATGTCATTGAATGTCTT
TACATGGGCTTGTATGCCTATGAAAATTGTAATACAACTTTCAGCAACGGATCTCTTGGC
TCTCGCATCGATGAAGAACGCAGCGAAATGCGATAAGTAATGTGAATTGCAGAATTCAGT
GAATCATCGAATCTTTGAACGCATCTTGCGCTCCTTGGTATTCCGAGGAGCATGCCTGTT
TGAGTGTCATTAAATTCTCAACTCTCTTATACTTTTTTGTAAAAGAGAGCTTGGACTGTG
GAGGCTTGCTGGCCACTTTTTGGGGTCAGCTCCTCTGAAATGCATTAGCGGAACCGTTTG
CGATCTGCCACAAGTGTGATAAGTTATCTACACTGGCGAGGGGATTGCTCTCTGTAATGT
TCAGCTTCTAATTGTCTCTACTTTGTGAGACTACTTTTGAATGCTTGACCTCAAATCAGG
TAGGACTACCCGCTGAACTTAA
>AC3-62
TTTCCGTAGGTGAACCTGCGGAAGGATCATTATTGAATTATGTTTCTAGATAGGTTGTAG
CTGGCTCTTTTAGAGCATGTGCACGCCTGTTTGGACTTCATTTTCATCCACCTGTGCACC
TATTGTAGTCTTTGGTTGGGTTAGGAGGAAGTGATCATTGTATCAGCATCTGCTGGGAGT
GAGGACTTGCATTGTGAAAGCTTTGCTGTCCTTGATGTGATCATGGAATCTTTTTC----

-------TCACTAGAGTCTATGTCACTCATTATACTCTGTCGAATGTCATTGAATGTCTT

TACATGGGCTTGTATGCCTATGAAAATTGTAATACAACTTTCAGCAACGGATCTCTTGGC
TCTCGCATCGATGAAGAACGCAGCGAAATGCGATAAGTAATGTGAATTGCAGAATTCAGT
GAATCATCGAATCTTTGAACGCATCTTGCGCTCCTTGGTATTCCGAGGAGCATGCCTGTT
TGAGTGTCATTAAATTCTCAACTCTCTTATACTTTTTTGTAAAAGAGAGCTTGGACTGTG
GAGGCTTGCTGGCCACTTTTTGGGGTCAGCTCCTCTGAAATGCATTAGCGGAACCGTTTG
CGATCTGCCACAAGTGTGATAAGTTATCTACACTGGCGAGGGGATTGCTCTCTGTAATGT
TCAGCTTCTAATTGTCTCTACTTTGTGAGACTACTTTTGAATGCTTGACCTCAAATCAGG
TAGGACTACCCGCTGAACTTAA
>AC3-64
TTTCCGTAGGTGAACCTGCGGAAGGATCATTATTGAATTATGTTTCTAGATAGGTTGTAG
CTGGCTCTTTTAGAGCATGTGCACGCCTGTTTGGACTTCATTTTCATCCACCTGTGCACC
TATTGTAGTCTTTGGTTGGGTTAGGAGGAAGTGATCATTGTATCAGCATCTGCTGGGAGT
GAGGACTTGCATTGTGAAAGCTTTGCTGTCCTTGATGTGATCATGGAATCTTTTTC----

-------TCACTAGAGTCTATGTCACTCATTATACTCTGTCGAATGTCATTGAATGTCTT
TACATGGGCTTGTATGCCTATGAAAATTGTAATACAACTTTCAGCAACGGATCTCTTGGC
TCTCGCATCGATGAAGAACGCAGCGAAATGCGATAAGTAATGTGAATTGCAGAATTCAGT
GAATCATCGAATCTTTGAACGCATCTTGCGCTCCTTGGTATTCCGAGGAGCATGCCTGTT
TGAGTGTCATTAAATTCTCAACTCTCTTATACTTTTTTGTAAAAGAGAGCTTGGACTGTG
GAGGCTTGCTGGCCACTTTTTGGGGTCAGCTCCTCTGAAATGCATTAGCGGAACCGTTTG
CGATCTGCCACAAGTGTGATAAGTTATCTACACTGGCGAGGGGATTGCTCTCTGTAATGT
TCAGCTTCTAATTGTCTCTACTTTGTGAGACTACTTTTGAATGCTTGACCTCAAATCAGG
TAGGACTACCCGCTGAACTTAA
>AC3-67
TTTCCGTAGGTGAACCTGCGGAAGGATCATTATTGAATTATGTTTCTAGATAGGTTGTAG
CTGGCTCTTTTAGAGCATGTGCACGCCTGTTTGGACTTCATTTTCATCCACCTGTGCACC
TATTGTAGTCTTTGGTTGGGTTAGGAGGAAGTGATCATTGTATCAGCATCTGCTGGGAGT
GAGGACTTGCATTGTGAAAGCTTTGCTGTCCTTGATGTGATCATGGAATCTTTTTC----

-------TCACTAGAGTCTATGTCACTCATTATACTCTGTCGAATGTCATTGAATGTCTT
TACATGGGCTTGTATGCCTATGAAAATTGTAATACAACTTTCAGCAACGGATCTCTTGGC
TCTCGCATCGATGAAGAACGCAGCGAAATGCGATAAGTAATGTGAATTGCAGAATTCAGT
GAATCATCGAATCTTTGAACGCATCTTGCGCTCCTTGGTATTCCGAGGAGCATGCCTGTT
TGAGTGTCATTAAATTCTCAACTCTCTTATACTTTTTTGTAAAAGAGAGCTTGGACTGTG
GAGGCTTGCTGGCCACTTTTTGGGGTCAGCTCCTCTGAAATGCATTAGCGGAACCGTTTG
CGATCTGCCACAAGTGTGATAAGTTATCTACACTGGCGAGGGGATTGCTCTCTGTAATGT
TCAGCTTCTAATTGTCTCTACTTTGTGAGACTACTTTTGAATGCTTGACCTCAAATCAGG
TAGGACTACCCGCTGAACTTAA
>AC4-6
TTTCCGTAGGTGAACCTGCGGAAGGATCATTATTGAATTATGTTTCTAGATAGGTTGTAG
CTGGCTCTTTTAGAGCATGTGCACGCCTGTTTGGACTTCATTTTCATCCACCTGTGCACC
TATTGTAGTCTTTGGTTGGGTTAGGAGGAAGTGATCATTGTATCAGCATCTGCTGGGAGT
GAGGACTTGCATTGTGAAAGCTTTGCTGTCCTTGATGTGATCATGGAATCTTTTTC----

-------TCACTAGAGTCTATGTCACTCATTATACTCTGTCGAATGTCATTGAATGTCTT
TACATGGGCTTGTATGCCTATGAAAATTGTAATACAACTTTCAGCAACGGATCTCTTGGC
TCTCGCATCGATGAAGAACGCAGCGAAATGCGATAAGTAATGTGAATTGCAGAATTCAGT
GAATCATCGAATCTTTGAACGCATCTTGCGCTCCTTGGTATTCCGAGGAGCATGCCTGTT
TGAGTGTCATTAAATTCTCAACTCTCTTATACTTTTTTGTAAAAGAGAGCTTGGACTGTG
GAGGCTTGCTGGCCACTTTTTGGGGTCAGCTCCTCTGAAATGCATTAGCGGAACCGTTTG
CGATCTGCCACAAGTGTGATAAGTTATCTACACTGGCGAGGGGATTGCTCTCTGTAATGT
TCAGCTTCTAATTGTCTCTACTTTGTGAGACTACTTTTGAATGCTTGACCTCAAATCAGG
TAGGACTACCCGCTGAACTTAA
>AC4-10
TTTCCGTAGGTGAACCTGCGGAAGGATCATTATTGAATTATGTTTCTAGATAGGTTGTAG
CTGGCTCTTTTAGAGCATGTGCACGCCTGTTTGGACTTCATTTTCATCCACCTGTGCACC
TATTGTAGTCTTTGGTTGGGTTAGGAGGAAGTGATCATTGTATCAGCATCTGCTGGGAGT
GAGGACTTGCATTGTGAAAGCTTTGCTGTCCTTGATGTGATCATGGAATCTTTTTC----

-------TCACTAGAGTCTATGTCACTCATTATACTCTGTCGAATGTCATTGAATGTCTT
TACATGGGCTTGTATGCCTATGAAAATTGTAATACAACTTTCAGCAACGGATCTCTTGGC
TCTCGCATCGATGAAGAACGCAGCGAAATGCGATAAGTAATGTGAATTGCAGAATTCAGT
GAATCATCGAATCTTTGAACGCATCTTGCGCTCCTTGGTATTCCGAGGAGCATGCCTGTT
TGAGTGTCATTAAATTCTCAACTCTCTTATACTTTTTTGTAAAAGAGAGCTTGGACTGTG
GAGGCTTGCTGGCCACTTTTTGGGGTCAGCTCCTCTGAAATGCATTAGCGGAACCGTTTG
CGATCTGCCACAAGTGTGATAAGTTATCTACACTGGCGAGGGGATTGCTCTCTGTAATGT
TCAGCTTCTAATTGTCTCTACTTTGTGAGACTACTTTTGAATGCTTGACCTCAAATCAGG
TAGGACTACCCGCTGAACTTAA
>AC4-25
TTTCCGTAGGTGAACCTGCGGAAGGATCATTATTGAATTATGTTTCTAGATAGGTTGTAG
CTGGCTCTTTTAGAGCATGTGCACGCCTGTTTGGACTTCATTTTCATCCACCTGTGCACC
TATTGTAGTCTTTGGTTGGGTTAGGAGGAAGTGATCATTGTATCAGCATCTGCTGGGAGT
GAGGACTTGCATTGTGAAAGCTTTGCTGTCCTTGATGTGATCATGGAATCTTTTTC----

-------TCACTAGAGTCTATGTCACTCATTATACTCTGTCGAATGTCATTGAATGTCTT
TACATGGGCTTGTATGCCTATGAAAATTGTAATACAACTTTCAGCAACGGATCTCTTGGC
TCTCGCATCGATGAAGAACGCAGCGAAATGCGATAAGTAATGTGAATTGCAGAATTCAGT
GAATCATCGAATCTTTGAACGCATCTTGCGCTCCTTGGTATTCCGAGGAGCATGCCTGTT
TGAGTGTCATTAAATTCTCAACTCTCTTATACTTTTTTGTAAAAGAGAGCTTGGACTGTG
GAGGCTTGCTGGCCACTTTTTGGGGTCAGCTCCTCTGAAATGCATTAGCGGAACCGTTTG
CGATCTGCCACAAGTGTGATAAGTTATCTACACTGGCGAGGGGATTGCTCTCTGTAATGT
TCAGCTTCTAATTGTCTCTACTTTGTGAGACTACTTTTGAATGCTTGACCTCAAATCAGG
TAGGACTACCCGCTGAACTTAA
>AC4-26
TTTCCGTAGGTGAACCTGCGGAAGGATCATTATTGAATTATGTTTCTAGATAGGTTGTAG
CTGGCTCTTTTAGAGCATGTGCACGCCTGTTTGGACTTCATTTTCATCCACCTGTGCACC
TATTGTAGTCTTTGGTTGGGTTAGGAGGAAGTGATCATTGTATCAGCATCTGCTGGGAGT
GAGGACTTGCATTGTGAAAGCTTTGCTGTCCTTGATGTGATCATGGAATCTTTTTC----


```
------------------------------------------------------------
------------------------------------------------------------
------------------------------------------------------------
------------------------------------------------------------
------------------------------------------------------------
-------TCACTAGAGTCTATGTCACTCATTATACTCTGTCGAATGTCATTGAATGTCTT
TACATGGGCTTGTATGCCTATGAAAATTGTAATACAACTTTCAGCAACGGATCTCTTGGC
TCTCGCATCGATGAAGAACGCAGCGAAATGCGATAAGTAATGTGAATTGCAGAATTCAGT
GAATCATCGAATCTTTGAACGCATCTTGCGCTCCTTGGTATTCCGAGGAGCATGCCTGTT
TGAGTGTCATTAAATTCTCAACTCTCTTATACTTTTTTGTAAAAGAGAGCTTGGACTGTG
GAGGCTTGCTGGCCACTTTTTGGGGTCAGCTCCTCTGAAATGCATTAGCGGAACCGTTTG
CGATCTGCCACAAGTGTGATAAGTTATCTACACTGGCGAGGGGATTGCTCTCTGTAATGT
TCAGCTTCTAATTGTCTCTACTTTGTGAGACTACTTTTGAATGCTTGACCTCAAATCAGG
TAGGACTACCCGCTGAACTTAA
>AC4-28
TTTCCGTAGGTGAACCTGCGGAAGGATCATTATTGAATTATGTTTCTAGATAGGTTGTAG
CTGGCTCTTTTAGAGCATGTGCACGCCTGTTTGGACTTCATTTTCATCCACCTGTGCACC
TATTGTAGTCTTTGGTTGGGTTAGGAGGAAGTGATCATTGTATCAGCATCTGCTGGGAGT
GAGGACTTGCATTGTGAAAGCTTTGCTGTCCTTGATGTGATCATGGAATCTTTTTC----
------------------------------------------------------------
------------------------------------------------------------
------------------------------------------------------------
------------------------------------------------------------
------------------------------------------------------------
------------------------------------------------------------
------------------------------------------------------------
------------------------------------------------------------
------------------------------------------------------------
------------------------------------------------------------
------------------------------------------------------------
------------------------------------------------------------
-------TCACTAGAGTCTATGTCACTCATTATACTCTGTCGAATGTCATTGAATGTCTT
TACATGGGCTTGTATGCCTATGAAAATTGTAATACAACTTTCAGCAACGGATCTCTTGGC
TCTCGCATCGATGAAGAACGCAGCGAAATGCGATAAGTAATGTGAATTGCAGAATTCAGT
GAATCATCGAATCTTTGAACGCATCTTGCGCTCCTTGGTATTCCGAGGAGCATGCCTGTT
TGAGTGTCATTAAATTCTCAACTCTCTTATACTTTTTTGTAAAAGAGAGCTTGGACTGTG
GAGGCTTGCTGGCCACTTTTTGGGGTCAGCTCCTCTGAAATGCATTAGCGGAACCGTTTG
CGATCTGCCACAAGTGTGATAAGTTATCTACACTGGCGAGGGGATTGCTCTCTGTAATGT
TCAGCTTCTAATTGTCTCTACTTTGTGAGACTACTTTTGAATGCTTGACCTCAAATCAGG
TAGGACTACCCGCTGAACTTAA
>AC4-29
TTTCCGTAGGTGAACCTGCGGAAGGATCATTATTGAATTATGTTTCTAGATAGGTTGTAG
CTGGCTCTTTTAGAGCATGTGCACGCCTGTTTGGACTTCATTTTCATCCACCTGTGCACC
TATTGTAGTCTTTGGTTGGGTTAGGAGGAAGTGATCATTGTATCAGCATCTGCTGGGAGT
GAGGACTTGCATTGTGAAAGCTTTGCTGTCCTTGATGTGATCATGGAATCTTTTTC----
------------------------------------------------------------
------------------------------------------------------------
------------------------------------------------------------
------------------------------------------------------------
------------------------------------------------------------
```


-------TCACTAGAGTCTATGTCACTCATTATACTCTGTCGAATGTCATTGAATGTCTT
TACATGGGCTTGTATGCCTATGAAAATTGTAATACAACTTTCAGCAACGGATCTCTTGGC
TCTCGCATCGATGAAGAACGCAGCGAAATGCGATAAGTAATGTGAATTGCAGAATTCAGT
GAATCATCGAATCTTTGAACGCATCTTGCGCTCCTTGGTATTCCGAGGAGCATGCCTGTT
TGAGTGTCATTAAATTCTCAACTCTCTTATACTTTTTTGTAAAAGAGAGCTTGGACTGTG
GAGGCTTGCTGGCCACTTTTTGGGGTCAGCTCCTCTGAAATGCATTAGCGGAACCGTTTG
CGATCTGCCACAAGTGTGATAAGTTATCTACACTGGCGAGGGGATTGCTCTCTGTAATGT
TCAGCTTCTAATTGTCTCTACTTTGTGAGACTACTTTTGAATGCTTGACCTCAAATCAGG
TAGGACTACCCGCTGAACTTAA
>AC4-31
TTTCCGTAGGTGAACCTGCGGAAGGATCATTATTGAATTATGTTTCTAGATAGGTTGTAG
CTGGCTCTTTTAGAGCATGTGCACGCCTGTTTGGACTTCATTTTCATCCACCTGTGCACC
TATTGTAGTCTTTGGTTGGGTTAGGAGGAAGTGATCATTGTATCAGCATCTGCTGGGAGT
GAGGACTTGCATTGTGAAAGCTTTGCTGTCCTTGATGTGATCATGGAATCTTTTTC----

-------TCACTAGAGTCTATGTCACTCATTATACTCTGTCGAATGTCATTGAATGTCTT
TACATGGGCTTGTATGCCTATGAAAATTGTAATACAACTTTCAGCAACGGATCTCTTGGC
TCTCGCATCGATGAAGAACGCAGCGAAATGCGATAAGTAATGTGAATTGCAGAATTCAGT
GAATCATCGAATCTTTGAACGCATCTTGCGCTCCTTGGTATTCCGAGGAGCATGCCTGTT
TGAGTGTCATTAAATTCTCAACTCTCTTATACTTTTTTGTAAAAGAGAGCTTGGACTGTG
GAGGCTTGCTGGCCACTTTTTGGGGTCAGCTCCTCTGAAATGCATTAGCGGAACCGTTTG
CGATCTGCCACAAGTGTGATAAGTTATCTACACTGGCGAGGGGATTGCTCTCTGTAATGT
TCAGCTTCTAATTGTCTCTACTTTGTGAGACTACTTTTGAATGCTTGACCTCAAATCAGG
TAGGACTACCCGCTGAACTTAA
>AC4-33
TTTCCGTAGGTGAACCTGCGGAAGGATCATTATTGAATTATGTTTCTAGATAGGTTGTAG
CTGGCTCTTTTAGAGCATGTGCACGCCTGTTTGGACTTCATTTTCATCCACCTGTGCACC
TATTGTAGTCTTTGGTTGGGTTAGGAGGAAGTGATCATTGTATCAGCATCTGCTGGGAGT
GAGGACTTGCATTGTGAAAGCTTTGCTGTCCTTGATGTGATCATGGAATCTTTTTC----

-------TCACTAGAGTCTATGTCACTCATTATACTCTGTCGAATGTCATTGAATGTCTT
TACATGGGCTTGTATGCCTATGAAAATTGTAATACAACTTTCAGCAACGGATCTCTTGGC
TCTCGCATCGATGAAGAACGCAGCGAAATGCGATAAGTAATGTGAATTGCAGAATTCAGT
GAATCATCGAATCTTTGAACGCATCTTGCGCTCCTTGGTATTCCGAGGAGCATGCCTGTT
TGAGTGTCATTAAATTCTCAACTCTCTTATACTTTTTTGTAAAAGAGAGCTTGGACTGTG
GAGGCTTGCTGGCCACTTTTTGGGGTCAGCTCCTCTGAAATGCATTAGCGGAACCGTTTG
CGATCTGCCACAAGTGTGATAAGTTATCTACACTGGCGAGGGGATTGCTCTCTGTAATGT
TCAGCTTCTAATTGTCTCTACTTTGTGAGACTACTTTTGAATGCTTGACCTCAAATCAGG
TAGGACTACCCGCTGAACTTAA
>AC4-38
TTTCCGTAGGTGAACCTGCGGAAGGATCATTATTGAATTATGTTTCTAGATAGGTTGTAG
CTGGCTCTTTTAGAGCATGTGCACGCCTGTTTGGACTTCATTTTCATCCACCTGTGCACC
TATTGTAGTCTTTGGTTGGGTTAGGAGGAAGTGATCATTGTATCAGCATCTGCTGGGAGT
GAGGACTTGCATTGTGAAAGCTTTGCTGTCCTTGATGTGATCATGGAATCTTTTTC----

-------TCACTAGAGTCTATGTCACTCATTATACTCTGTCGAATGTCATTGAATGTCTT
TACATGGGCTTGTATGCCTATGAAAATTGTAATACAACTTTCAGCAACGGATCTCTTGGC
TCTCGCATCGATGAAGAACGCAGCGAAATGCGATAAGTAATGTGAATTGCAGAATTCAGT
GAATCATCGAATCTTTGAACGCATCTTGCGCTCCTTGGTATTCCGAGGAGCATGCCTGTT
TGAGTGTCATTAAATTCTCAACTCTCTTATACTTTTTTGTAAAAGAGAGCTTGGACTGTG
GAGGCTTGCTGGCCACTTTTTGGGGTCAGCTCCTCTGAAATGCATTAGCGGAACCGTTTG
CGATCTGCCACAAGTGTGATAAGTTATCTACACTGGCGAGGGGATTGCTCTCTGTAATGT
TCAGCTTCTAATTGTCTCTACTTTGTGAGACTACTTTTGAATGCTTGACCTCAAATCAGG
TAGGACTACCCGCTGAACTTAA
>AC4-43
TTTCCGTAGGTGAACCTGCGGAAGGATCATTATTGAATTATGTTTCTAGATAGGTTGTAG
CTGGCTCTTTTAGAGCATGTGCACGCCTGTTTGGACTTCATTTTCATCCACCTGTGCACC
TATTGTAGTCTTTGGTTGGGTTAGGAGGAAGTGATCATTGTATCAGCATCTGCTGGGAGT
GAGGACTTGCATTGTGAAAGCTTTGCTGTCCTTGATGTGATCATGGAATCTTTTTC----

```
------------------------------------------------------------
------------------------------------------------------------
------------------------------------------------------------
------------------------------------------------------------
------------------------------------------------------------
------------------------------------------------------------
------------------------------------------------------------
------------------------------------------------------------
------------------------------------------------------------
------------------------------------------------------------
------------------------------------------------------------
-------TCACTAGAGTCTATGTCACTCATTATACTCTGTCGAATGTCATTGAATGTCTT
TACATGGGCTTGTATGCCTATGAAAATTGTAATACAACTTTCAGCAACGGATCTCTTGGC
TCTCGCATCGATGAAGAACGCAGCGAAATGCGATAAGTAATGTGAATTGCAGAATTCAGT
GAATCATCGAATCTTTGAACGCATCTTGCGCTCCTTGGTATTCCGAGGAGCATGCCTGTT
TGAGTGTCATTAAATTCTCAACTCTCTTATACTTTTTTGTAAAAGAGAGCTTGGACTGTG
GAGGCTTGCTGGCCACTTTTTGGGGTCAGCTCCTCTGAAATGCATTAGCGGAACCGTTTG
CGATCTGCCACAAGTGTGATAAGTTATCTACACTGGCGAGGGGATTGCTCTCTGTAATGT
TCAGCTTCTAATTGTCTCTACTTTGTGAGACTACTTTTGAATGCTTGACCTCAAATCAGG
TAGGACTACCCGCTGAACTTAA
>AC4-61
TTTCCGTAGGTGAACCTGCGGAAGGATCATTATTGAATTATGTTTCTAGATAGGTTGTAG
CTGGCTCTTTTAGAGCATGTGCACGCCTGTTTGGACTTCATTTTCATCCACCTGTGCACC
TATTGTAGTCTTTGGTTGGGTTAGGAGGAAGTGATCATTGTATCAGCATCTGCTGGGAGT
GAGGACTTGCATTGTGAAAGCTTTGCTGTCCTTGATGTGATCATGGAATCTTTTTC----
------------------------------------------------------------
------------------------------------------------------------
------------------------------------------------------------
------------------------------------------------------------
------------------------------------------------------------
------------------------------------------------------------
------------------------------------------------------------
------------------------------------------------------------
------------------------------------------------------------
------------------------------------------------------------
------------------------------------------------------------
------------------------------------------------------------
-------TCACTAGAGTCTATGTCACTCATTATACTCTGTCGAATGTCATTGAATGTCTT
TACATGGGCTTGTATGCCTATGAAAATTGTAATACAACTTTCAGCAACGGATCTCTTGGC
TCTCGCATCGATGAAGAACGCAGCGAAATGCGATAAGTAATGTGAATTGCAGAATTCAGT
GAATCATCGAATCTTTGAACGCATCTTGCGCTCCTTGGTATTCCGAGGAGCATGCCTGTT
TGAGTGTCATTAAATTCTCAACTCTCTTATACTTTTTTGTAAAAGAGAGCTTGGACTGTG
GAGGCTTGCTGGCCACTTTTTGGGGTCAGCTCCTCTGAAATGCATTAGCGGAACCGTTTG
CGATCTGCCACAAGTGTGATAAGTTATCTACACTGGCGAGGGGATTGCTCTCTGTAATGT
TCAGCTTCTAATTGTCTCTACTTTGTGAGACTACTTTTGAATGCTTGACCTCAAATCAGG
TAGGACTACCCGCTGAACTTAA
>AC4-64
TTTCCGTAGGTGAACCTGCGGAAGGATCATTATTGAATTATGTTTCTAGATAGGTTGTAG
CTGGCTCTTTTAGAGCATGTGCACGCCTGTTTGGACTTCATTTTCATCCACCTGTGCACC
TATTGTAGTCTTTGGTTGGGTTAGGAGGAAGTGATCATTGTATCAGCATCTGCTGGGAGT
```

GAGGACTTGCATTGTGAAAGCTTTGCTGTCCTTGATGTGATCATGGAATCTTTTTC----

-------TCACTAGAGTCTATGTCACTCATTATACTCTGTCGAATGTCATTGAATGTCTT
TACATGGGCTTGTATGCCTATGAAAATTGTAATACAACTTTCAGCAACGGATCTCTTGGC
TCTCGCATCGATGAAGAACGCAGCGAAATGCGATAAGTAATGTGAATTGCAGAATTCAGT
GAATCATCGAATCTTTGAACGCATCTTGCGCTCCTTGGTATTCCGAGGAGCATGCCTGTT
TGAGTGTCATTAAATTCTCAACTCTCTTATACTTTTTTGTAAAAGAGAGCTTGGACTGTG
GAGGCTTGCTGGCCACTTTTTGGGGTCAGCTCCTCTGAAATGCATTAGCGGAACCGTTTG
CGATCTGCCACAAGTGTGATAAGTTATCTACACTGGCGAGGGGATTGCTCTCTGTAATGT
TCAGCTTCTAATTGTCTCTACTTTGTGAGACTACTTTTGAATGCTTGACCTCAAATCAGG
TAGGACTACCCGCTGAACTTAA
>AC4-67
TTTCCGTAGGTGAACCTGCGGAAGGATCATTATTGAATTATGTTTCTAGATAGGTTGTAG
CTGGCTCTTTTAGAGCATGTGCACGCCTGTTTGGACTTCATTTTCATCCACCTGTGCACC
TATTGTAGTCTTTGGTTGGGTTAGGAGGAAGTGATCATTGTATCAGCATCTGCTGGGAGT
GAGGACTTGCATTGTGAAAGCTTTGCTGTCCTTGATGTGATCATGGAATCTTTTTC----

-------TCACTAGAGTCTATGTCACTCATTATACTCTGTCGAATGTCATTGAATGTCTT
TACATGGGCTTGTATGCCTATGAAAATTGTAATACAACTTTCAGCAACGGATCTCTTGGC
TCTCGCATCGATGAAGAACGCAGCGAAATGCGATAAGTAATGTGAATTGCAGAATTCAGT
GAATCATCGAATCTTTGAACGCATCTTGCGCTCCTTGGTATTCCGAGGAGCATGCCTGTT
TGAGTGTCATTAAATTCTCAACTCTCTTATACTTTTTTGTAAAAGAGAGCTTGGACTGTG
GAGGCTTGCTGGCCACTTTTTGGGGTCAGCTCCTCTGAAATGCATTAGCGGAACCGTTTG
CGATCTGCCACAAGTGTGATAAGTTATCTACACTGGCGAGGGGATTGCTCTCTGTAATGT
TCAGCTTCTAATTGTCTCTACTTTGTGAGACTACTTTTGAATGCTTGACCTCAAATCAGG
TAGGACTACCCGCTGAACTTAA
>AC4-69
TTTCCGTAGGTGAACCTGCGGAAGGATCATTATTGAATTATGTTTCTAGATAGGTTGTAG

CTGGCTCTTTTAGAGCATGTGCACGCCTGTTTGGACTTCATTTTCATCCACCTGTGCACC
TATTGTAGTCTTTGGTTGGGTTAGGAGGAAGTGATCATTGTATCAGCATCTGCTGGGAGT
GAGGACTTGCATTGTGAAAGCTTTGCTGTCCTTGATGTGATCATGGAATCTTTTTC----

-------TCACTAGAGTCTATGTCACTCATTATACTCTGTCGAATGTCATTGAATGTCTT
TACATGGGCTTGTATGCCTATGAAAATTGTAATACAACTTTCAGCAACGGATCTCTTGGC
TCTCGCATCGATGAAGAACGCAGCGAAATGCGATAAGTAATGTGAATTGCAGAATTCAGT
GAATCATCGAATCTTTGAACGCATCTTGCGCTCCTTGGTATTCCGAGGAGCATGCCTGTT
TGAGTGTCATTAAATTCTCAACTCTCTTATACTTTTTTGTAAAAGAGAGCTTGGACTGTG
GAGGCTTGCTGGCCACTTTTTGGGGTCAGCTCCTCTGAAATGCATTAGCGGAACCGTTTG
CGATCTGCCACAAGTGTGATAAGTTATCTACACTGGCGAGGGGATTGCTCTCTGTAATGT
TCAGCTTCTAATTGTCTCTACTTTGTGAGACTACTTTTGAATGCTTGACCTCAAATCAGG
TAGGACTACCCGCTGAACTTAA
>AC4-80
TTTCCGTAGGTGAACCTGCGGAAGGATCATTATTGAATTATGTTTCTAGATAGGTTGTAG
CTGGCTCTTTTAGAGCATGTGCACGCCTGTTTGGACTTCATTTTCATCCACCTGTGCACC
TATTGTAGTCTTTGGTTGGGTTAGGAGGAAGTGATCATTGTATCAGCATCTGCTGGGAGT
GAGGACTTGCATTGTGAAAGCTTTGCTGTCCTTGATGTGATCATGGAATCTTTTTC----

-------TCACTAGAGTCTATGTCACTCATTATACTCTGTCGAATGTCATTGAATGTCTT
TACATGGGCTTGTATGCCTATGAAAATTGTAATACAACTTTCAGCAACGGATCTCTTGGC
TCTCGCATCGATGAAGAACGCAGCGAAATGCGATAAGTAATGTGAATTGCAGAATTCAGT
GAATCATCGAATCTTTGAACGCATCTTGCGCTCCTTGGTATTCCGAGGAGCATGCCTGTT
TGAGTGTCATTAAATTCTCAACTCTCTTATACTTTTTTGTAAAAGAGAGCTTGGACTGTG
GAGGCTTGCTGGCCACTTTTTGGGGTCAGCTCCTCTGAAATGCATTAGCGGAACCGTTTG
CGATCTGCCACAAGTGTGATAAGTTATCTACACTGGCGAGGGGATTGCTCTCTGTAATGT
TCAGCTTCTAATTGTCTCTACTTTGTGAGACTACTTTTGAATGCTTGACCTCAAATCAGG
TAGGACTACCCGCTGAACTTAA

```
>AC4-82
TTTCCGTAGGTGAACCTGCGGAAGGATCATTATTGAATTATGTTTCTAGATAGGTTGTAG
CTGGCTCTTTTAGAGCATGTGCACGCCTGTTTGGACTTCATTTTCATCCACCTGTGCACC
TATTGTAGTCTTTGGTTGGGTTAGGAGGAAGTGATCATTGTATCAGCATCTGCTGGGAGT
GAGGACTTGCATTGTGAAAGCTTTGCTGTCCTTGATGTGATCATGGAATCTTTTTC----
------------------------------------------------------------
------------------------------------------------------------
------------------------------------------------------------
------------------------------------------------------------
------------------------------------------------------------
------------------------------------------------------------
------------------------------------------------------------
------------------------------------------------------------
------------------------------------------------------------
------------------------------------------------------------
------------------------------------------------------------
------------------------------------------------------------
-------TCACTAGAGTCTATGTCACTCATTATACTCTGTCGAATGTCATTGAATGTCTT
TACATGGGCTTGTATGCCTATGAAAATTGTAATACAACTTTCAGCAACGGATCTCTTGGC
TCTCGCATCGATGAAGAACGCAGCGAAATGCGATAAGTAATGTGAATTGCAGAATTCAGT
GAATCATCGAATCTTTGAACGCATCTTGCGCTCCTTGGTATTCCGAGGAGCATGCCTGTT
TGAGTGTCATTAAATTCTCAACTCTCTTATACTTTTTTGTAAAAGAGAGCTTGGACTGTG
GAGGCTTGCTGGCCACTTTTTGGGGTCAGCTCCTCTGAAATGCATTAGCGGAACCGTTTG
CGATCTGCCACAAGTGTGATAAGTTATCTACACTGGCGAGGGGATTGCTCTCTGTAATGT
TCAGCTTCTAATTGTCTCTACTTTGTGAGACTACTTTTGAATGCTTGACCTCAAATCAGG
TAGGACTACCCGCTGAACTTAA
>AC5-14
TTTCCGTAGGTGAACCTGCGGAAGGATCATTATTGAATTATGTTTCTAGATAGGTTGTAG
CTGGCTCTTTTAGAGCATGTGCACGCCTGTTTGGACTTCATTTTCATCCACCTGTGCACC
TATTGTAGTCTTTGGTTGGGTTAGGAGGAAGTGATCATTGTATCAGCATCTGCTGGGAGT
GAGGACTTGCATTGTGAAAGCTTTGCTGTCCTTGATGTGATCATGGAATCTTTTTC----
------------------------------------------------------------
------------------------------------------------------------
------------------------------------------------------------
------------------------------------------------------------
------------------------------------------------------------
------------------------------------------------------------
------------------------------------------------------------
------------------------------------------------------------
------------------------------------------------------------
------------------------------------------------------------
------------------------------------------------------------
------------------------------------------------------------
-------TCACTAGAGTCTATGTCACTCATTATACTCTGTCGAATGTCATTGAATGTCTT
TACATGGGCTTGTATGCCTATGAAAATTGTAATACAACTTTCAGCAACGGATCTCTTGGC
TCTCGCATCGATGAAGAACGCAGCGAAATGCGATAAGTAATGTGAATTGCAGAATTCAGT
GAATCATCGAATCTTTGAACGCATCTTGCGCTCCTTGGTATTCCGAGGAGCATGCCTGTT
TGAGTGTCATTAAATTCTCAACTCTCTTATACTTTTTTGTAAAAGAGAGCTTGGACTGTG
GAGGCTTGCTGGCCACTTTTTGGGGTCAGCTCCTCTGAAATGCATTAGCGGAACCGTTTG
CGATCTGCCACAAGTGTGATAAGTTATCTACACTGGCGAGGGGATTGCTCTCTGTAATGT
```

TCAGCTTCTAATTGTCTCTACTTTGTGAGACTACTTTTGAATGCTTGACCTCAAATCAGG
TAGGACTACCCGCTGAACTTAA
>AC5-17
TTTCCGTAGGTGAACCTGCGGAAGGATCATTATTGAATTATGTTTCTAGATAGGTTGTAG
CTGGCTCTTTTAGAGCATGTGCACGCCTGTTTGGACTTCATTTTCATCCACCTGTGCACC
TATTGTAGTCTTTGGTTGGGTTAGGAGGAAGTGATCATTGTATCAGCATCTGCTGGGAGT
GAGGACTTGCATTGTGAAAGCTTTGCTGTCCTTGATGTGATCATGGAATCTTTTTC----

-------TCACTAGAGTCTATGTCACTCATTATACTCTGTCGAATGTCATTGAATGTCTT
TACATGGGCTTGTATGCCTATGAAAATTGTAATACAACTTTCAGCAACGGATCTCTTGGC
TCTCGCATCGATGAAGAACGCAGCGAAATGCGATAAGTAATGTGAATTGCAGAATTCAGT
GAATCATCGAATCTTTGAACGCATCTTGCGCTCCTTGGTATTCCGAGGAGCATGCCTGTT
TGAGTGTCATTAAATTCTCAACTCTCTTATACTTTTTTGTAAAAGAGAGCTTGGACTGTG
GAGGCTTGCTGGCCACTTTTTGGGGTCAGCTCCTCTGAAATGCATTAGCGGAACCGTTTG
CGATCTGCCACAAGTGTGATAAGTTATCTACACTGGCGAGGGGATTGCTCTCTGTAATGT
TCAGCTTCTAATTGTCTCTACTTTGTGAGACTACTTTTGAATGCTTGACCTCAAATCAGG
TAGGACTACCCGCTGAACTTAA
>AC5-24
TTTCCGTAGGTGAACCTGCGGAAGGATCATTATTGAATTATGTTTCTAGATAGGTTGTAG
CTGGCTCTTTTAGAGCATGTGCACGCCTGTTTGGACTTCATTTTCATCCACCTGTGCACC
TATTGTAGTCTTTGGTTGGGTTAGGAGGAAGTGATCATTGTATCAGCATCTGCTGGGAGT
GAGGACTTGCATTGTGAAAGCTTTGCTGTCCTTGATGTGATCATGGAATCTTTTTC----

-------TCACTAGAGTCTATGTCACTCATTATACTCTGTCGAATGTCATTGAATGTCTT
TACATGGGCTTGTATGCCTATGAAAATTGTAATACAACTTTCAGCAACGGATCTCTTGGC
TCTCGCATCGATGAAGAACGCAGCGAAATGCGATAAGTAATGTGAATTGCAGAATTCAGT
GAATCATCGAATCTTTGAACGCATCTTGCGCTCCTTGGTATTCCGAGGAGCATGCCTGTT
TGAGTGTCATTAAATTCTCAACTCTCTTATACTTTTTTGTAAAAGAGAGCTTGGACTGTG

GAGGCTTGCTGGCCACTTTTTGGGGTCAGCTCCTCTGAAATGCATTAGCGGAACCGTTTG
CGATCTGCCACAAGTGTGATAAGTTATCTACACTGGCGAGGGGATTGCTCTCTGTAATGT
TCAGCTTCTAATTGTCTCTACTTTGTGAGACTACTTTTGAATGCTTGACCTCAAATCAGG
TAGGACTACCCGCTGAACTTAA
>AC5-26
TTTCCGTAGGTGAACCTGCGGAAGGATCATTATTGAATTATGTTTCTAGATAGGTTGTAG
CTGGCTCTTTTAGAGCATGTGCACGCCTGTTTGGACTTCATTTTCATCCACCTGTGCACC
TATTGTAGTCTTTGGTTGGGTTAGGAGGAAGTGATCATTGTATCAGCATCTGCTGGGAGT
GAGGACTTGCATTGTGAAAGCTTTGCTGTCCTTGATGTGATCATGGAATCTTTTTC----

-------TCACTAGAGTCTATGTCACTCATTATACTCTGTCGAATGTCATTGAATGTCTT
TACATGGGCTTGTATGCCTATGAAAATTGTAATACAACTTTCAGCAACGGATCTCTTGGC
TCTCGCATCGATGAAGAACGCAGCGAAATGCGATAAGTAATGTGAATTGCAGAATTCAGT
GAATCATCGAATCTTTGAACGCATCTTGCGCTCCTTGGTATTCCGAGGAGCATGCCTGTT
TGAGTGTCATTAAATTCTCAACTCTCTTATACTTTTTTGTAAAAGAGAGCTTGGACTGTG
GAGGCTTGCTGGCCACTTTTTGGGGTCAGCTCCTCTGAAATGCATTAGCGGAACCGTTTG
CGATCTGCCACAAGTGTGATAAGTTATCTACACTGGCGAGGGGATTGCTCTCTGTAATGT
TCAGCTTCTAATTGTCTCTACTTTGTGAGACTACTTTTGAATGCTTGACCTCAAATCAGG
TAGGACTACCCGCTGAACTTAA
>AC5-28
TTTCCGTAGGTGAACCTGCGGAAGGATCATTATTGAATTATGTTTCTAGATAGGTTGTAG
CTGGCTCTTTTAGAGCATGTGCACGCCTGTTTGGACTTCATTTTCATCCACCTGTGCACC
TATTGTAGTCTTTGGTTGGGTTAGGAGGAAGTGATCATTGTATCAGCATCTGCTGGGAGT
GAGGACTTGCATTGTGAAAGCTTTGCTGTCCTTGATGTGATCATGGAATCTTTTTC----

-------TCACTAGAGTCTATGTCACTCATTATACTCTGTCGAATGTCATTGAATGTCTT
TACATGGGCTTGTATGCCTATGAAAATTGTAATACAACTTTCAGCAACGGATCTCTTGGC
TCTCGCATCGATGAAGAACGCAGCGAAATGCGATAAGTAATGTGAATTGCAGAATTCAGT

GAATCATCGAATCTTTGAACGCATCTTGCGCTCCTTGGTATTCCGAGGAGCATGCCTGTT
TGAGTGTCATTAAATTCTCAACTCTCTTATACTTTTTTGTAAAAGAGAGCTTGGACTGTG
GAGGCTTGCTGGCCACTTTTTGGGGTCAGCTCCTCTGAAATGCATTAGCGGAACCGTTTG
CGATCTGCCACAAGTGTGATAAGTTATCTACACTGGCGAGGGGATTGCTCTCTGTAATGT
TCAGCTTCTAATTGTCTCTACTTTGTGAGACTACTTTTGAATGCTTGACCTCAAATCAGG
TAGGACTACCCGCTGAACTTAA
>AC5-29
TTTCCGTAGGTGAACCTGCGGAAGGATCATTATTGAATTATGTTTCTAGATAGGTTGTAG
CTGGCTCTTTTAGAGCATGTGCACGCCTGTTTGGACTTCATTTTCATCCACCTGTGCACC
TATTGTAGTCTTTGGTTGGGTTAGGAGGAAGTGATCATTGTATCAGCATCTGCTGGGAGT
GAGGACTTGCATTGTGAAAGCTTTGCTGTCCTTGATGTGATCATGGAATCTTTTTC----

-------TCACTAGAGTCTATGTCACTCATTATACTCTGTCGAATGTCATTGAATGTCTT
TACATGGGCTTGTATGCCTATGAAAATTGTAATACAACTTTCAGCAACGGATCTCTTGGC
TCTCGCATCGATGAAGAACGCAGCGAAATGCGATAAGTAATGTGAATTGCAGAATTCAGT
GAATCATCGAATCTTTGAACGCATCTTGCGCTCCTTGGTATTCCGAGGAGCATGCCTGTT
TGAGTGTCATTAAATTCTCAACTCTCTTATACTTTTTTGTAAAAGAGAGCTTGGACTGTG
GAGGCTTGCTGGCCACTTTTTGGGGTCAGCTCCTCTGAAATGCATTAGCGGAACCGTTTG
CGATCTGCCACAAGTGTGATAAGTTATCTACACTGGCGAGGGGATTGCTCTCTGTAATGT
TCAGCTTCTAATTGTCTCTACTTTGTGAGACTACTTTTGAATGCTTGACCTCAAATCAGG
TAGGACTACCCGCTGAACTTAA
>AC5-41
TTTCCGTAGGTGAACCTGCGGAAGGATCATTATTGAATTATGTTTCTAGATAGGTTGTAG
CTGGCTCTTTTAGAGCATGTGCACGCCTGTTTGGACTTCATTTTCATCCACCTGTGCACC
TATTGTAGTCTTTGGTTGGGTTAGGAGGAAGTGATCATTGTATCAGCATCTGCTGGGAGT
GAGGACTTGCATTGTGAAAGCTTTGCTGTCCTTGATGTGATCATGGAATCTTTTTC----

-------TCACTAGAGTCTATGTCACTCATTATACTCTGTCGAATGTCATTGAATGTCTT

TACATGGGCTTGTATGCCTATGAAAATTGTAATACAACTTTCAGCAACGGATCTCTTGGC
TCTCGCATCGATGAAGAACGCAGCGAAATGCGATAAGTAATGTGAATTGCAGAATTCAGT
GAATCATCGAATCTTTGAACGCATCTTGCGCTCCTTGGTATTCCGAGGAGCATGCCTGTT
TGAGTGTCATTAAATTCTCAACTCTCTTATACTTTTTTGTAAAAGAGAGCTTGGACTGTG
GAGGCTTGCTGGCCACTTTTTGGGGTCAGCTCCTCTGAAATGCATTAGCGGAACCGTTTG
CGATCTGCCACAAGTGTGATAAGTTATCTACACTGGCGAGGGGATTGCTCTCTGTAATGT
TCAGCTTCTAATTGTCTCTACTTTGTGAGACTACTTTTGAATGCTTGACCTCAAATCAGG
TAGGACTACCCGCTGAACTTAA
>AC5-52
TTTCCGTAGGTGAACCTGCGGAAGGATCATTATTGAATTATGTTTCTAGATAGGTTGTAG
CTGGCTCTTTTAGAGCATGTGCACGCCTGTTTGGACTTCATTTTCATCCACCTGTGCACC
TATTGTAGTCTTTGGTTGGGTTAGGAGGAAGTGATCATTGTATCAGCATCTGCTGGGAGT
GAGGACTTGCATTGTGAAAGCTTTGCTGTCCTTGATGTGATCATGGAATCTTTTTC----

-------TCACTAGAGTCTATGTCACTCATTATACTCTGTCGAATGTCATTGAATGTCTT
TACATGGGCTTGTATGCCTATGAAAATTGTAATACAACTTTCAGCAACGGATCTCTTGGC
TCTCGCATCGATGAAGAACGCAGCGAAATGCGATAAGTAATGTGAATTGCAGAATTCAGT
GAATCATCGAATCTTTGAACGCATCTTGCGCTCCTTGGTATTCCGAGGAGCATGCCTGTT
TGAGTGTCATTAAATTCTCAACTCTCTTATACTTTTTTGTAAAAGAGAGCTTGGACTGTG
GAGGCTTGCTGGCCACTTTTTGGGGTCAGCTCCTCTGAAATGCATTAGCGGAACCGTTTG
CGATCTGCCACAAGTGTGATAAGTTATCTACACTGGCGAGGGGATTGCTCTCTGTAATGT
TCAGCTTCTAATTGTCTCTACTTTGTGAGACTACTTTTGAATGCTTGACCTCAAATCAGG
TAGGACTACCCGCTGAACTTAA
>AC5-54
TTTCCGTAGGTGAACCTGCGGAAGGATCATTATTGAATTATGTTTCTAGATAGGTTGTAG
CTGGCTCTTTTAGAGCATGTGCACGCCTGTTTGGACTTCATTTTCATCCACCTGTGCACC
TATTGTAGTCTTTGGTTGGGTTAGGAGGAAGTGATCATTGTATCAGCATCTGCTGGGAGT
GAGGACTTGCATTGTGAAAGCTTTGCTGTCCTTGATGTGATCATGGAATCTTTTTC----

-------TCACTAGAGTCTATGTCACTCATTATACTCTGTCGAATGTCATTGAATGTCTT
TACATGGGCTTGTATGCCTATGAAAATTGTAATACAACTTTCAGCAACGGATCTCTTGGC
TCTCGCATCGATGAAGAACGCAGCGAAATGCGATAAGTAATGTGAATTGCAGAATTCAGT
GAATCATCGAATCTTTGAACGCATCTTGCGCTCCTTGGTATTCCGAGGAGCATGCCTGTT
TGAGTGTCATTAAATTCTCAACTCTCTTATACTTTTTTGTAAAAGAGAGCTTGGACTGTG
GAGGCTTGCTGGCCACTTTTTGGGGTCAGCTCCTCTGAAATGCATTAGCGGAACCGTTTG
CGATCTGCCACAAGTGTGATAAGTTATCTACACTGGCGAGGGGATTGCTCTCTGTAATGT
TCAGCTTCTAATTGTCTCTACTTTGTGAGACTACTTTTGAATGCTTGACCTCAAATCAGG
TAGGACTACCCGCTGAACTTAA
>AC5-57
TTTCCGTAGGTGAACCTGCGGAAGGATCATTATTGAATTATGTTTCTAGATAGGTTGTAG
CTGGCTCTTTTAGAGCATGTGCACGCCTGTTTGGACTTCATTTTCATCCACCTGTGCACC
TATTGTAGTCTTTGGTTGGGTTAGGAGGAAGTGATCATTGTATCAGCATCTGCTGGGAGT
GAGGACTTGCATTGTGAAAGCTTTGCTGTCCTTGATGTGATCATGGAATCTTTTTC----

-------TCACTAGAGTCTATGTCACTCATTATACTCTGTCGAATGTCATTGAATGTCTT
TACATGGGCTTGTATGCCTATGAAAATTGTAATACAACTTTCAGCAACGGATCTCTTGGC
TCTCGCATCGATGAAGAACGCAGCGAAATGCGATAAGTAATGTGAATTGCAGAATTCAGT
GAATCATCGAATCTTTGAACGCATCTTGCGCTCCTTGGTATTCCGAGGAGCATGCCTGTT
TGAGTGTCATTAAATTCTCAACTCTCTTATACTTTTTTGTAAAAGAGAGCTTGGACTGTG
GAGGCTTGCTGGCCACTTTTTGGGGTCAGCTCCTCTGAAATGCATTAGCGGAACCGTTTG
CGATCTGCCACAAGTGTGATAAGTTATCTACACTGGCGAGGGGATTGCTCTCTGTAATGT
TCAGCTTCTAATTGTCTCTACTTTGTGAGACTACTTTTGAATGCTTGACCTCAAATCAGG
TAGGACTACCCGCTGAACTTAA
>AC5-71
TTTCCGTAGGTGAACCTGCGGAAGGATCATTATTGAATTATGTTTCTAGATAGGTTGTAG
CTGGCTCTTTTAGAGCATGTGCACGCCTGTTTGGACTTCATTTTCATCCACCTGTGCACC
TATTGTAGTCTTTGGTTGGGTTAGGAGGAAGTGATCATTGTATCAGCATCTGCTGGGAGT
GAGGACTTGCATTGTGAAAGCTTTGCTGTCCTTGATGTGATCATGGAATCTTTTTC----

-------TCACTAGAGTCTATGTCACTCATTATACTCTGTCGAATGTCATTGAATGTCTT
TACATGGGCTTGTATGCCTATGAAAATTGTAATACAACTTTCAGCAACGGATCTCTTGGC
TCTCGCATCGATGAAGAACGCAGCGAAATGCGATAAGTAATGTGAATTGCAGAATTCAGT
GAATCATCGAATCTTTGAACGCATCTTGCGCTCCTTGGTATTCCGAGGAGCATGCCTGTT
TGAGTGTCATTAAATTCTCAACTCTCTTATACTTTTTTGTAAAAGAGAGCTTGGACTGTG
GAGGCTTGCTGGCCACTTTTTGGGGTCAGCTCCTCTGAAATGCATTAGCGGAACCGTTTG
CGATCTGCCACAAGTGTGATAAGTTATCTACACTGGCGAGGGGATTGCTCTCTGTAATGT
TCAGCTTCTAATTGTCTCTACTTTGTGAGACTACTTTTGAATGCTTGACCTCAAATCAGG
TAGGACTACCCGCTGAACTTAA
>AC5-74
TTTCCGTAGGTGAACCTGCGGAAGGATCATTATTGAATTATGTTTCTAGATAGGTTGTAG
CTGGCTCTTTTAGAGCATGTGCACGCCTGTTTGGACTTCATTTTCATCCACCTGTGCACC
TATTGTAGTCTTTGGTTGGGTTAGGAGGAAGTGATCATTGTATCAGCATCTGCTGGGAGT
GAGGACTTGCATTGTGAAAGCTTTGCTGTCCTTGATGTGATCATGGAATCTTTTTC----

-------TCACTAGAGTCTATGTCACTCATTATACTCTGTCGAATGTCATTGAATGTCTT
TACATGGGCTTGTATGCCTATGAAAATTGTAATACAACTTTCAGCAACGGATCTCTTGGC
TCTCGCATCGATGAAGAACGCAGCGAAATGCGATAAGTAATGTGAATTGCAGAATTCAGT
GAATCATCGAATCTTTGAACGCATCTTGCGCTCCTTGGTATTCCGAGGAGCATGCCTGTT
TGAGTGTCATTAAATTCTCAACTCTCTTATACTTTTTTGTAAAAGAGAGCTTGGACTGTG
GAGGCTTGCTGGCCACTTTTTGGGGTCAGCTCCTCTGAAATGCATTAGCGGAACCGTTTG
CGATCTGCCACAAGTGTGATAAGTTATCTACACTGGCGAGGGGATTGCTCTCTGTAATGT
TCAGCTTCTAATTGTCTCTACTTTGTGAGACTACTTTTGAATGCTTGACCTCAAATCAGG
TAGGACTACCCGCTGAACTTAA
>AC5-82
TTTCCGTAGGTGAACCTGCGGAAGGATCATTATTGAATTATGTTTCTAGATAGGTTGTAG
CTGGCTCTTTTAGAGCATGTGCACGCCTGTTTGGACTTCATTTTCATCCACCTGTGCACC
TATTGTAGTCTTTGGTTGGGTTAGGAGGAAGTGATCATTGTATCAGCATCTGCTGGGAGT
GAGGACTTGCATTGTGAAAGCTTTGCTGTCCTTGATGTGATCATGGAATCTTTTTC----

-------TCACTAGAGTCTATGTCACTCATTATACTCTGTCGAATGTCATTGAATGTCTT
TACATGGGCTTGTATGCCTATGAAAATTGTAATACAACTTTCAGCAACGGATCTCTTGGC
TCTCGCATCGATGAAGAACGCAGCGAAATGCGATAAGTAATGTGAATTGCAGAATTCAGT
GAATCATCGAATCTTTGAACGCATCTTGCGCTCCTTGGTATTCCGAGGAGCATGCCTGTT
TGAGTGTCATTAAATTCTCAACTCTCTTATACTTTTTTGTAAAAGAGAGCTTGGACTGTG
GAGGCTTGCTGGCCACTTTTTGGGGTCAGCTCCTCTGAAATGCATTAGCGGAACCGTTTG
CGATCTGCCACAAGTGTGATAAGTTATCTACACTGGCGAGGGGATTGCTCTCTGTAATGT
TCAGCTTCTAATTGTCTCTACTTTGTGAGACTACTTTTGAATGCTTGACCTCAAATCAGG
TAGGACTACCCGCTGAACTTAA
>AC5-85
TTTCCGTAGGTGAACCTGCGGAAGGATCATTATTGAATTATGTTTCTAGATAGGTTGTAG
CTGGCTCTTTTAGAGCATGTGCACGCCTGTTTGGACTTCATTTTCATCCACCTGTGCACC
TATTGTAGTCTTTGGTTGGGTTAGGAGGAAGTGATCATTGTATCAGCATCTGCTGGGAGT
GAGGACTTGCATTGTGAAAGCTTTGCTGTCCTTGATGTGATCATGGAATCTTTTTC----

-------TCACTAGAGTCTATGTCACTCATTATACTCTGTCGAATGTCATTGAATGTCTT
TACATGGGCTTGTATGCCTATGAAAATTGTAATACAACTTTCAGCAACGGATCTCTTGGC
TCTCGCATCGATGAAGAACGCAGCGAAATGCGATAAGTAATGTGAATTGCAGAATTCAGT
GAATCATCGAATCTTTGAACGCATCTTGCGCTCCTTGGTATTCCGAGGAGCATGCCTGTT
TGAGTGTCATTAAATTCTCAACTCTCTTATACTTTTTTGTAAAAGAGAGCTTGGACTGTG
GAGGCTTGCTGGCCACTTTTTGGGGTCAGCTCCTCTGAAATGCATTAGCGGAACCGTTTG
CGATCTGCCACAAGTGTGATAAGTTATCTACACTGGCGAGGGGATTGCTCTCTGTAATGT
TCAGCTTCTAATTGTCTCTACTTTGTGAGACTACTTTTGAATGCTTGACCTCAAATCAGG
TAGGACTACCCGCTGAACTTAA
>AC6-8
TTTCCGTAGGTGAACCTGCGGAAGGATCATTATTGAATTATGTTTCTAGATAGGTTGTAG
CTGGCTCTTTTAGAGCATGTGCACGCCTGTTTGGACTTCATTTTCATCCACCTGTGCACC
TATTGTAGTCTTTGGTTGGGTTAGGAGGAAGTGATCATTGTATCAGCATCTGCTGGGAGT
GAGGACTTGCATTGTGAAAGCTTTGCTGTCCTTGATGTGATCATGGAATCTTTTTC----

-------TCACTAGAGTCTATGTCACTCATTATACTCTGTCGAATGTCATTGAATGTCTT
TACATGGGCTTGTATGCCTATGAAAATTGTAATACAACTTTCAGCAACGGATCTCTTGGC
TCTCGCATCGATGAAGAACGCAGCGAAATGCGATAAGTAATGTGAATTGCAGAATTCAGT
GAATCATCGAATCTTTGAACGCATCTTGCGCTCCTTGGTATTCCGAGGAGCATGCCTGTT
TGAGTGTCATTAAATTCTCAACTCTCTTATACTTTTTTGTAAAAGAGAGCTTGGACTGTG
GAGGCTTGCTGGCCACTTTTTGGGGTCAGCTCCTCTGAAATGCATTAGCGGAACCGTTTG
CGATCTGCCACAAGTGTGATAAGTTATCTACACTGGCGAGGGGATTGCTCTCTGTAATGT
TCAGCTTCTAATTGTCTCTACTTTGTGAGACTACTTTTGAATGCTTGACCTCAAATCAGG
TAGGACTACCCGCTGAACTTAA
>AC6-10
TTTCCGTAGGTGAACCTGCGGAAGGATCATTATTGAATTATGTTTCTAGATAGGTTGTAG
CTGGCTCTTTTAGAGCATGTGCACGCCTGTTTGGACTTCATTTTCATCCACCTGTGCACC
TATTGTAGTCTTTGGTTGGGTTAGGAGGAAGTGATCATTGTATCAGCATCTGCTGGGAGT
GAGGACTTGCATTGTGAAAGCTTTGCTGTCCTTGATGTGATCATGGAATCTTTTTC----

-------TCACTAGAGTCTATGTCACTCATTATACTCTGTCGAATGTCATTGAATGTCTT
TACATGGGCTTGTATGCCTATGAAAATTGTAATACAACTTTCAGCAACGGATCTCTTGGC
TCTCGCATCGATGAAGAACGCAGCGAAATGCGATAAGTAATGTGAATTGCAGAATTCAGT
GAATCATCGAATCTTTGAACGCATCTTGCGCTCCTTGGTATTCCGAGGAGCATGCCTGTT
TGAGTGTCATTAAATTCTCAACTCTCTTATACTTTTTTGTAAAAGAGAGCTTGGACTGTG
GAGGCTTGCTGGCCACTTTTTGGGGTCAGCTCCTCTGAAATGCATTAGCGGAACCGTTTG
CGATCTGCCACAAGTGTGATAAGTTATCTACACTGGCGAGGGGATTGCTCTCTGTAATGT
TCAGCTTCTAATTGTCTCTACTTTGTGAGACTACTTTTGAATGCTTGACCTCAAATCAGG
TAGGACTACCCGCTGAACTTAA
>AC6-23
TTTCCGTAGGTGAACCTGCGGAAGGATCATTATTGAATTATGTTTCTAGATAGGTTGTAG
CTGGCTCTTTTAGAGCATGTGCACGCCTGTTTGGACTTCATTTTCATCCACCTGTGCACC
TATTGTAGTCTTTGGTTGGGTTAGGAGGAAGTGATCATTGTATCAGCATCTGCTGGGAGT
GAGGACTTGCATTGTGAAAGCTTTGCTGTCCTTGATGTGATCATGGAATCTTTTTC----

-------TCACTAGAGTCTATGTCACTCATTATACTCTGTCGAATGTCATTGAATGTCTT
TACATGGGCTTGTATGCCTATGAAAATTGTAATACAACTTTCAGCAACGGATCTCTTGGC
TCTCGCATCGATGAAGAACGCAGCGAAATGCGATAAGTAATGTGAATTGCAGAATTCAGT
GAATCATCGAATCTTTGAACGCATCTTGCGCTCCTTGGTATTCCGAGGAGCATGCCTGTT
TGAGTGTCATTAAATTCTCAACTCTCTTATACTTTTTTGTAAAAGAGAGCTTGGACTGTG
GAGGCTTGCTGGCCACTTTTTGGGGTCAGCTCCTCTGAAATGCATTAGCGGAACCGTTTG
CGATCTGCCACAAGTGTGATAAGTTATCTACACTGGCGAGGGGATTGCTCTCTGTAATGT
TCAGCTTCTAATTGTCTCTACTTTGTGAGACTACTTTTGAATGCTTGACCTCAAATCAGG
TAGGACTACCCGCTGAACTTAA
>AC6-27
TTTCCGTAGGTGAACCTGCGGAAGGATCATTATTGAATTATGTTTCTAGATAGGTTGTAG
CTGGCTCTTTTAGAGCATGTGCACGCCTGTTTGGACTTCATTTTCATCCACCTGTGCACC
TATTGTAGTCTTTGGTTGGGTTAGGAGGAAGTGATCATTGTATCAGCATCTGCTGGGAGT
GAGGACTTGCATTGTGAAAGCTTTGCTGTCCTTGATGTGATCATGGAATCTTTTTC----

-------TCACTAGAGTCTATGTCACTCATTATACTCTGTCGAATGTCATTGAATGTCTT
TACATGGGCTTGTATGCCTATGAAAATTGTAATACAACTTTCAGCAACGGATCTCTTGGC
TCTCGCATCGATGAAGAACGCAGCGAAATGCGATAAGTAATGTGAATTGCAGAATTCAGT
GAATCATCGAATCTTTGAACGCATCTTGCGCTCCTTGGTATTCCGAGGAGCATGCCTGTT
TGAGTGTCATTAAATTCTCAACTCTCTTATACTTTTTTGTAAAAGAGAGCTTGGACTGTG
GAGGCTTGCTGGCCACTTTTTGGGGTCAGCTCCTCTGAAATGCATTAGCGGAACCGTTTG
CGATCTGCCACAAGTGTGATAAGTTATCTACACTGGCGAGGGGATTGCTCTCTGTAATGT
TCAGCTTCTAATTGTCTCTACTTTGTGAGACTACTTTTGAATGCTTGACCTCAAATCAGG
TAGGACTACCCGCTGAACTTAA
>AC6-28
TTTCCGTAGGTGAACCTGCGGAAGGATCATTATTGAATTATGTTTCTAGATAGGTTGTAG
CTGGCTCTTTTAGAGCATGTGCACGCCTGTTTGGACTTCATTTTCATCCACCTGTGCACC
TATTGTAGTCTTTGGTTGGGTTAGGAGGAAGTGATCATTGTATCAGCATCTGCTGGGAGT
GAGGACTTGCATTGTGAAAGCTTTGCTGTCCTTGATGTGATCATGGAATCTTTTTC----

-------TCACTAGAGTCTATGTCACTCATTATACTCTGTCGAATGTCATTGAATGTCTT
TACATGGGCTTGTATGCCTATGAAAATTGTAATACAACTTTCAGCAACGGATCTCTTGGC
TCTCGCATCGATGAAGAACGCAGCGAAATGCGATAAGTAATGTGAATTGCAGAATTCAGT
GAATCATCGAATCTTTGAACGCATCTTGCGCTCCTTGGTATTCCGAGGAGCATGCCTGTT
TGAGTGTCATTAAATTCTCAACTCTCTTATACTTTTTTGTAAAAGAGAGCTTGGACTGTG
GAGGCTTGCTGGCCACTTTTTGGGGTCAGCTCCTCTGAAATGCATTAGCGGAACCGTTTG
CGATCTGCCACAAGTGTGATAAGTTATCTACACTGGCGAGGGGATTGCTCTCTGTAATGT
TCAGCTTCTAATTGTCTCTACTTTGTGAGACTACTTTTGAATGCTTGACCTCAAATCAGG
TAGGACTACCCGCTGAACTTAA
>AC6-29
TTTCCGTAGGTGAACCTGCGGAAGGATCATTATTGAATTATGTTTCTAGATAGGTTGTAG
CTGGCTCTTTTAGAGCATGTGCACGCCTGTTTGGACTTCATTTTCATCCACCTGTGCACC
TATTGTAGTCTTTGGTTGGGTTAGGAGGAAGTGATCATTGTATCAGCATCTGCTGGGAGT
GAGGACTTGCATTGTGAAAGCTTTGCTGTCCTTGATGTGATCATGGAATCTTTTTC----

-------TCACTAGAGTCTATGTCACTCATTATACTCTGTCGAATGTCATTGAATGTCTT
TACATGGGCTTGTATGCCTATGAAAATTGTAATACAACTTTCAGCAACGGATCTCTTGGC
TCTCGCATCGATGAAGAACGCAGCGAAATGCGATAAGTAATGTGAATTGCAGAATTCAGT
GAATCATCGAATCTTTGAACGCATCTTGCGCTCCTTGGTATTCCGAGGAGCATGCCTGTT
TGAGTGTCATTAAATTCTCAACTCTCTTATACTTTTTTGTAAAAGAGAGCTTGGACTGTG
GAGGCTTGCTGGCCACTTTTTGGGGTCAGCTCCTCTGAAATGCATTAGCGGAACCGTTTG
CGATCTGCCACAAGTGTGATAAGTTATCTACACTGGCGAGGGGATTGCTCTCTGTAATGT
TCAGCTTCTAATTGTCTCTACTTTGTGAGACTACTTTTGAATGCTTGACCTCAAATCAGG
TAGGACTACCCGCTGAACTTAA
>AC6-33
TTTCCGTAGGTGAACCTGCGGAAGGATCATTATTGAATTATGTTTCTAGATAGGTTGTAG
CTGGCTCTTTTAGAGCATGTGCACGCCTGTTTGGACTTCATTTTCATCCACCTGTGCACC
TATTGTAGTCTTTGGTTGGGTTAGGAGGAAGTGATCATTGTATCAGCATCTGCTGGGAGT

GAGGACTTGCATTGTGAAAGCTTTGCTGTCCTTGATGTGATCATGGAATCTTTTTC----

-------TCACTAGAGTCTATGTCACTCATTATACTCTGTCGAATGTCATTGAATGTCTT
TACATGGGCTTGTATGCCTATGAAAATTGTAATACAACTTTCAGCAACGGATCTCTTGGC
TCTCGCATCGATGAAGAACGCAGCGAAATGCGATAAGTAATGTGAATTGCAGAATTCAGT
GAATCATCGAATCTTTGAACGCATCTTGCGCTCCTTGGTATTCCGAGGAGCATGCCTGTT
TGAGTGTCATTAAATTCTCAACTCTCTTATACTTTTTTGTAAAAGAGAGCTTGGACTGTG
GAGGCTTGCTGGCCACTTTTTGGGGTCAGCTCCTCTGAAATGCATTAGCGGAACCGTTTG
CGATCTGCCACAAGTGTGATAAGTTATCTACACTGGCGAGGGGATTGCTCTCTGTAATGT
TCAGCTTCTAATTGTCTCTACTTTGTGAGACTACTTTTGAATGCTTGACCTCAAATCAGG
TAGGACTACCCGCTGAACTTAA
>AC6-43
TTTCCGTAGGTGAACCTGCGGAAGGATCATTATTGAATTATGTTTCTAGATAGGTTGTAG
CTGGCTCTTTTAGAGCATGTGCACGCCTGTTTGGACTTCATTTTCATCCACCTGTGCACC
TATTGTAGTCTTTGGTTGGGTTAGGAGGAAGTGATCATTGTATCAGCATCTGCTGGGAGT
GAGGACTTGCATTGTGAAAGCTTTGCTGTCCTTGATGTGATCATGGAATCTTTTTC----

-------TCACTAGAGTCTATGTCACTCATTATACTCTGTCGAATGTCATTGAATGTCTT
TACATGGGCTTGTATGCCTATGAAAATTGTAATACAACTTTCAGCAACGGATCTCTTGGC
TCTCGCATCGATGAAGAACGCAGCGAAATGCGATAAGTAATGTGAATTGCAGAATTCAGT
GAATCATCGAATCTTTGAACGCATCTTGCGCTCCTTGGTATTCCGAGGAGCATGCCTGTT
TGAGTGTCATTAAATTCTCAACTCTCTTATACTTTTTTGTAAAAGAGAGCTTGGACTGTG
GAGGCTTGCTGGCCACTTTTTGGGGTCAGCTCCTCTGAAATGCATTAGCGGAACCGTTTG
CGATCTGCCACAAGTGTGATAAGTTATCTACACTGGCGAGGGGATTGCTCTCTGTAATGT
TCAGCTTCTAATTGTCTCTACTTTGTGAGACTACTTTTGAATGCTTGACCTCAAATCAGG
TAGGACTACCCGCTGAACTTAA
>AC6-50
TTTCCGTAGGTGAACCTGCGGAAGGATCATTATTGAATTATGTTTCTAGATAGGTTGTAG

CTGGCTCTTTTAGAGCATGTGCACGCCTGTTTGGACTTCATTTTCATCCACCTGTGCACC
TATTGTAGTCTTTGGTTGGGTTAGGAGGAAGTGATCATTGTATCAGCATCTGCTGGGAGT
GAGGACTTGCATTGTGAAAGCTTTGCTGTCCTTGATGTGATCATGGAATCTTTTTC----

-------TCACTAGAGTCTATGTCACTCATTATACTCTGTCGAATGTCATTGAATGTCTT
TACATGGGCTTGTATGCCTATGAAAATTGTAATACAACTTTCAGCAACGGATCTCTTGGC
TCTCGCATCGATGAAGAACGCAGCGAAATGCGATAAGTAATGTGAATTGCAGAATTCAGT
GAATCATCGAATCTTTGAACGCATCTTGCGCTCCTTGGTATTCCGAGGAGCATGCCTGTT
TGAGTGTCATTAAATTCTCAACTCTCTTATACTTTTTTGTAAAAGAGAGCTTGGACTGTG
GAGGCTTGCTGGCCACTTTTTGGGGTCAGCTCCTCTGAAATGCATTAGCGGAACCGTTTG
CGATCTGCCACAAGTGTGATAAGTTATCTACACTGGCGAGGGGATTGCTCTCTGTAATGT
TCAGCTTCTAATTGTCTCTACTTTGTGAGACTACTTTTGAATGCTTGACCTCAAATCAGG
TAGGACTACCCGCTGAACTTAA
>AC6-52
TTTCCGTAGGTGAACCTGCGGAAGGATCATTATTGAATTATGTTTCTAGATAGGTTGTAG
CTGGCTCTTTTAGAGCATGTGCACGCCTGTTTGGACTTCATTTTCATCCACCTGTGCACC
TATTGTAGTCTTTGGTTGGGTTAGGAGGAAGTGATCATTGTATCAGCATCTGCTGGGAGT
GAGGACTTGCATTGTGAAAGCTTTGCTGTCCTTGATGTGATCATGGAATCTTTTTC----

-------TCACTAGAGTCTATGTCACTCATTATACTCTGTCGAATGTCATTGAATGTCTT
TACATGGGCTTGTATGCCTATGAAAATTGTAATACAACTTTCAGCAACGGATCTCTTGGC
TCTCGCATCGATGAAGAACGCAGCGAAATGCGATAAGTAATGTGAATTGCAGAATTCAGT
GAATCATCGAATCTTTGAACGCATCTTGCGCTCCTTGGTATTCCGAGGAGCATGCCTGTT
TGAGTGTCATTAAATTCTCAACTCTCTTATACTTTTTTGTAAAAGAGAGCTTGGACTGTG
GAGGCTTGCTGGCCACTTTTTGGGGTCAGCTCCTCTGAAATGCATTAGCGGAACCGTTTG
CGATCTGCCACAAGTGTGATAAGTTATCTACACTGGCGAGGGGATTGCTCTCTGTAATGT
TCAGCTTCTAATTGTCTCTACTTTGTGAGACTACTTTTGAATGCTTGACCTCAAATCAGG
TAGGACTACCCGCTGAACTTAA

>AC6-58
TTTCCGTAGGTGAACCTGCGGAAGGATCATTATTGAATTATGTTTCTAGATAGGTTGTAG
CTGGCTCTTTTAGAGCATGTGCACGCCTGTTTGGACTTCATTTTCATCCACCTGTGCACC
TATTGTAGTCTTTGGTTGGGTTAGGAGGAAGTGATCATTGTATCAGCATCTGCTGGGAGT
GAGGACTTGCATTGTGAAAGCTTTGCTGTCCTTGATGTGATCATGGAATCTTTTTC----

-------TCACTAGAGTCTATGTCACTCATTATACTCTGTCGAATGTCATTGAATGTCTT
TACATGGGCTTGTATGCCTATGAAAATTGTAATACAACTTTCAGCAACGGATCTCTTGGC
TCTCGCATCGATGAAGAACGCAGCGAAATGCGATAAGTAATGTGAATTGCAGAATTCAGT
GAATCATCGAATCTTTGAACGCATCTTGCGCTCCTTGGTATTCCGAGGAGCATGCCTGTT
TGAGTGTCATTAAATTCTCAACTCTCTTATACTTTTTTGTAAAAGAGAGCTTGGACTGTG
GAGGCTTGCTGGCCACTTTTTGGGGTCAGCTCCTCTGAAATGCATTAGCGGAACCGTTTG
CGATCTGCCACAAGTGTGATAAGTTATCTACACTGGCGAGGGGATTGCTCTCTGTAATGT
TCAGCTTCTAATTGTCTCTACTTTGTGAGACTACTTTTGAATGCTTGACCTCAAATCAGG
TAGGACTACCCGCTGAACTTAA
>AC6-60
TTTCCGTAGGTGAACCTGCGGAAGGATCATTATTGAATTATGTTTCTAGATAGGTTGTAG
CTGGCTCTTTTAGAGCATGTGCACGCCTGTTTGGACTTCATTTTCATCCACCTGTGCACC
TATTGTAGTCTTTGGTTGGGTTAGGAGGAAGTGATCATTGTATCAGCATCTGCTGGGAGT
GAGGACTTGCATTGTGAAAGCTTTGCTGTCCTTGATGTGATCATGGAATCTTTTTC----

-------TCACTAGAGTCTATGTCACTCATTATACTCTGTCGAATGTCATTGAATGTCTT
TACATGGGCTTGTATGCCTATGAAAATTGTAATACAACTTTCAGCAACGGATCTCTTGGC
TCTCGCATCGATGAAGAACGCAGCGAAATGCGATAAGTAATGTGAATTGCAGAATTCAGT
GAATCATCGAATCTTTGAACGCATCTTGCGCTCCTTGGTATTCCGAGGAGCATGCCTGTT
TGAGTGTCATTAAATTCTCAACTCTCTTATACTTTTTTGTAAAAGAGAGCTTGGACTGTG
GAGGCTTGCTGGCCACTTTTTGGGGTCAGCTCCTCTGAAATGCATTAGCGGAACCGTTTG
CGATCTGCCACAAGTGTGATAAGTTATCTACACTGGCGAGGGGATTGCTCTCTGTAATGT

TCAGCTTCTAATTGTCTCTACTTTGTGAGACTACTTTTGAATGCTTGACCTCAAATCAGG
TAGGACTACCCGCTGAACTTAA
>AC6-61
TTTCCGTAGGTGAACCTGCGGAAGGATCATTATTGAATTATGTTTCTAGATAGGTTGTAG
CTGGCTCTTTTAGAGCATGTGCACGCCTGTTTGGACTTCATTTTCATCCACCTGTGCACC
TATTGTAGTCTTTGGTTGGGTTAGGAGGAAGTGATCATTGTATCAGCATCTGCTGGGAGT
GAGGACTTGCATTGTGAAAGCTTTGCTGTCCTTGATGTGATCATGGAATCTTTTTC----

-------TCACTAGAGTCTATGTCACTCATTATACTCTGTCGAATGTCATTGAATGTCTT
TACATGGGCTTGTATGCCTATGAAAATTGTAATACAACTTTCAGCAACGGATCTCTTGGC
TCTCGCATCGATGAAGAACGCAGCGAAATGCGATAAGTAATGTGAATTGCAGAATTCAGT
GAATCATCGAATCTTTGAACGCATCTTGCGCTCCTTGGTATTCCGAGGAGCATGCCTGTT
TGAGTGTCATTAAATTCTCAACTCTCTTATACTTTTTTGTAAAAGAGAGCTTGGACTGTG
GAGGCTTGCTGGCCACTTTTTGGGGTCAGCTCCTCTGAAATGCATTAGCGGAACCGTTTG
CGATCTGCCACAAGTGTGATAAGTTATCTACACTGGCGAGGGGATTGCTCTCTGTAATGT
TCAGCTTCTAATTGTCTCTACTTTGTGAGACTACTTTTGAATGCTTGACCTCAAATCAGG
TAGGACTACCCGCTGAACTTAA
>AC7-1
TTTCCGTAGGTGAACCTGCGGAAGGATCATTATTGAATTATGTTTCTAGATAGGTTGTAG
CTGGCTCTTTTAGAGCATGTGCACGCCTGTTTGGACTTCATTTTCATCCACCTGTGCACC
TATTGTAGTCTTTGGTTGGGTTAGGAGGAAGTGATCATTGTATCAGCATCTGCTGGGAGT
GAGGACTTGCATTGTGAAAGCTTTGCTGTCCTTGATGTGATCATGGAATCTTTTTC----

-------TCACTAGAGTCTATGTCACTCATTATACTCTGTCGAATGTCATTGAATGTCTT
TACATGGGCTTGTATGCCTATGAAAATTGTAATACAACTTTCAGCAACGGATCTCTTGGC
TCTCGCATCGATGAAGAACGCAGCGAAATGCGATAAGTAATGTGAATTGCAGAATTCAGT
GAATCATCGAATCTTTGAACGCATCTTGCGCTCCTTGGTATTCCGAGGAGCATGCCTGTT
TGAGTGTCATTAAATTCTCAACTCTCTTATACTTTTTTGTAAAAGAGAGCTTGGACTGTG

```
GAGGCTTGCTGGCCACTTTTTGGGGTCAGCTCCTCTGAAATGCATTAGCGGAACCGTTTG
CGATCTGCCACAAGTGTGATAAGTTATCTACACTGGCGAGGGGATTGCTCTCTGTAATGT
TCAGCTTCTAATTGTCTCTACTTTGTGAGACTACTTTTGAATGCTTGACCTCAAATCAGG
TAGGACTACCCGCTGAACTTAA
>AC7-8
TTTCCGTAGGTGAACCTGCGGAAGGATCATTATTGAATTATGTTTCTAGATAGGTTGTAG
CTGGCTCTTTTAGAGCATGTGCACGCCTGTTTGGACTTCATTTTCATCCACCTGTGCACC
TATTGTAGTCTTTGGTTGGGTTAGGAGGAAGTGATCATTGTATCAGCATCTGCTGGGAGT
GAGGACTTGCATTGTGAAAGCTTTGCTGTCCTTGATGTGATCATGGAATCTTTTTC----
------------------------------------------------------------
------------------------------------------------------------
------------------------------------------------------------
------------------------------------------------------------
------------------------------------------------------------
------------------------------------------------------------
------------------------------------------------------------
------------------------------------------------------------
------------------------------------------------------------
------------------------------------------------------------
------------------------------------------------------------
------------------------------------------------------------
-------TCACTAGAGTCTATGTCACTCATTATACTCTGTCGAATGTCATTGAATGTCTT
TACATGGGCTTGTATGCCTATGAAAATTGTAATACAACTTTCAGCAACGGATCTCTTGGC
TCTCGCATCGATGAAGAACGCAGCGAAATGCGATAAGTAATGTGAATTGCAGAATTCAGT
GAATCATCGAATCTTTGAACGCATCTTGCGCTCCTTGGTATTCCGAGGAGCATGCCTGTT
TGAGTGTCATTAAATTCTCAACTCTCTTATACTTTTTTGTAAAAGAGAGCTTGGACTGTG
GAGGCTTGCTGGCCACTTTTTGGGGTCAGCTCCTCTGAAATGCATTAGCGGAACCGTTTG
CGATCTGCCACAAGTGTGATAAGTTATCTACACTGGCGAGGGGATTGCTCTCTGTAATGT
TCAGCTTCTAATTGTCTCTACTTTGTGAGACTACTTTTGAATGCTTGACCTCAAATCAGG
TAGGACTACCCGCTGAACTTAA
>AC7-11
TTTCCGTAGGTGAACCTGCGGAAGGATCATTATTGAATTATGTTTCTAGATAGGTTGTAG
CTGGCTCTTTTAGAGCATGTGCACGCCTGTTTGGACTTCATTTTCATCCACCTGTGCACC
TATTGTAGTCTTTGGTTGGGTTAGGAGGAAGTGATCATTGTATCAGCATCTGCTGGGAGT
GAGGACTTGCATTGTGAAAGCTTTGCTGTCCTTGATGTGATCATGGAATCTTTTTC----
------------------------------------------------------------
------------------------------------------------------------
------------------------------------------------------------
------------------------------------------------------------
------------------------------------------------------------
------------------------------------------------------------
------------------------------------------------------------
------------------------------------------------------------
------------------------------------------------------------
------------------------------------------------------------
------------------------------------------------------------
------------------------------------------------------------
-------TCACTAGAGTCTATGTCACTCATTATACTCTGTCGAATGTCATTGAATGTCTT
TACATGGGCTTGTATGCCTATGAAAATTGTAATACAACTTTCAGCAACGGATCTCTTGGC
TCTCGCATCGATGAAGAACGCAGCGAAATGCGATAAGTAATGTGAATTGCAGAATTCAGT
```

GAATCATCGAATCTTTGAACGCATCTTGCGCTCCTTGGTATTCCGAGGAGCATGCCTGTT
TGAGTGTCATTAAATTCTCAACTCTCTTATACTTTTTTGTAAAAGAGAGCTTGGACTGTG
GAGGCTTGCTGGCCACTTTTTGGGGTCAGCTCCTCTGAAATGCATTAGCGGAACCGTTTG
CGATCTGCCACAAGTGTGATAAGTTATCTACACTGGCGAGGGGATTGCTCTCTGTAATGT
TCAGCTTCTAATTGTCTCTACTTTGTGAGACTACTTTTGAATGCTTGACCTCAAATCAGG
TAGGACTACCCGCTGAACTTAA
>AC7-14
TTTCCGTAGGTGAACCTGCGGAAGGATCATTATTGAATTATGTTTCTAGATAGGTTGTAG
CTGGCTCTTTTAGAGCATGTGCACGCCTGTTTGGACTTCATTTTCATCCACCTGTGCACC
TATTGTAGTCTTTGGTTGGGTTAGGAGGAAGTGATCATTGTATCAGCATCTGCTGGGAGT
GAGGACTTGCATTGTGAAAGCTTTGCTGTCCTTGATGTGATCATGGAATCTTTTTC----

-------TCACTAGAGTCTATGTCACTCATTATACTCTGTCGAATGTCATTGAATGTCTT
TACATGGGCTTGTATGCCTATGAAAATTGTAATACAACTTTCAGCAACGGATCTCTTGGC
TCTCGCATCGATGAAGAACGCAGCGAAATGCGATAAGTAATGTGAATTGCAGAATTCAGT
GAATCATCGAATCTTTGAACGCATCTTGCGCTCCTTGGTATTCCGAGGAGCATGCCTGTT
TGAGTGTCATTAAATTCTCAACTCTCTTATACTTTTTTGTAAAAGAGAGCTTGGACTGTG
GAGGCTTGCTGGCCACTTTTTGGGGTCAGCTCCTCTGAAATGCATTAGCGGAACCGTTTG
CGATCTGCCACAAGTGTGATAAGTTATCTACACTGGCGAGGGGATTGCTCTCTGTAATGT
TCAGCTTCTAATTGTCTCTACTTTGTGAGACTACTTTTGAATGCTTGACCTCAAATCAGG
TAGGACTACCCGCTGAACTTAA
>AC7-15
TTTCCGTAGGTGAACCTGCGGAAGGATCATTATTGAATTATGTTTCTAGATAGGTTGTAG
CTGGCTCTTTTAGAGCATGTGCACGCCTGTTTGGACTTCATTTTCATCCACCTGTGCACC
TATTGTAGTCTTTGGTTGGGTTAGGAGGAAGTGATCATTGTATCAGCATCTGCTGGGAGT
GAGGACTTGCATTGTGAAAGCTTTGCTGTCCTTGATGTGATCATGGAATCTTTTTC----

-------TCACTAGAGTCTATGTCACTCATTATACTCTGTCGAATGTCATTGAATGTCTT

TACATGGGCTTGTATGCCTATGAAAATTGTAATACAACTTTCAGCAACGGATCTCTTGGC
TCTCGCATCGATGAAGAACGCAGCGAAATGCGATAAGTAATGTGAATTGCAGAATTCAGT
GAATCATCGAATCTTTGAACGCATCTTGCGCTCCTTGGTATTCCGAGGAGCATGCCTGTT
TGAGTGTCATTAAATTCTCAACTCTCTTATACTTTTTTGTAAAAGAGAGCTTGGACTGTG
GAGGCTTGCTGGCCACTTTTTGGGGTCAGCTCCTCTGAAATGCATTAGCGGAACCGTTTG
CGATCTGCCACAAGTGTGATAAGTTATCTACACTGGCGAGGGGATTGCTCTCTGTAATGT
TCAGCTTCTAATTGTCTCTACTTTGTGAGACTACTTTTGAATGCTTGACCTCAAATCAGG
TAGGACTACCCGCTGAACTTAA
>AC7-17
TTTCCGTAGGTGAACCTGCGGAAGGATCATTATTGAATTATGTTTCTAGATAGGTTGTAG
CTGGCTCTTTTAGAGCATGTGCACGCCTGTTTGGACTTCATTTTCATCCACCTGTGCACC
TATTGTAGTCTTTGGTTGGGTTAGGAGGAAGTGATCATTGTATCAGCATCTGCTGGGAGT
GAGGACTTGCATTGTGAAAGCTTTGCTGTCCTTGATGTGATCATGGAATCTTTTTC----

-------TCACTAGAGTCTATGTCACTCATTATACTCTGTCGAATGTCATTGAATGTCTT
TACATGGGCTTGTATGCCTATGAAAATTGTAATACAACTTTCAGCAACGGATCTCTTGGC
TCTCGCATCGATGAAGAACGCAGCGAAATGCGATAAGTAATGTGAATTGCAGAATTCAGT
GAATCATCGAATCTTTGAACGCATCTTGCGCTCCTTGGTATTCCGAGGAGCATGCCTGTT
TGAGTGTCATTAAATTCTCAACTCTCTTATACTTTTTTGTAAAAGAGAGCTTGGACTGTG
GAGGCTTGCTGGCCACTTTTTGGGGTCAGCTCCTCTGAAATGCATTAGCGGAACCGTTTG
CGATCTGCCACAAGTGTGATAAGTTATCTACACTGGCGAGGGGATTGCTCTCTGTAATGT
TCAGCTTCTAATTGTCTCTACTTTGTGAGACTACTTTTGAATGCTTGACCTCAAATCAGG
TAGGACTACCCGCTGAACTTAA
>AC7-22
TTTCCGTAGGTGAACCTGCGGAAGGATCATTATTGAATTATGTTTCTAGATAGGTTGTAG
CTGGCTCTTTTAGAGCATGTGCACGCCTGTTTGGACTTCATTTTCATCCACCTGTGCACC
TATTGTAGTCTTTGGTTGGGTTAGGAGGAAGTGATCATTGTATCAGCATCTGCTGGGAGT
GAGGACTTGCATTGTGAAAGCTTTGCTGTCCTTGATGTGATCATGGAATCTTTTTC----

-------TCACTAGAGTCTATGTCACTCATTATACTCTGTCGAATGTCATTGAATGTCTT
TACATGGGCTTGTATGCCTATGAAAATTGTAATACAACTTTCAGCAACGGATCTCTTGGC
TCTCGCATCGATGAAGAACGCAGCGAAATGCGATAAGTAATGTGAATTGCAGAATTCAGT
GAATCATCGAATCTTTGAACGCATCTTGCGCTCCTTGGTATTCCGAGGAGCATGCCTGTT
TGAGTGTCATTAAATTCTCAACTCTCTTATACTTTTTTGTAAAAGAGAGCTTGGACTGTG
GAGGCTTGCTGGCCACTTTTTGGGGTCAGCTCCTCTGAAATGCATTAGCGGAACCGTTTG
CGATCTGCCACAAGTGTGATAAGTTATCTACACTGGCGAGGGGATTGCTCTCTGTAATGT
TCAGCTTCTAATTGTCTCTACTTTGTGAGACTACTTTTGAATGCTTGACCTCAAATCAGG
TAGGACTACCCGCTGAACTTAA
>AC7-39
TTTCCGTAGGTGAACCTGCGGAAGGATCATTATTGAATTATGTTTCTAGATAGGTTGTAG
CTGGCTCTTTTAGAGCATGTGCACGCCTGTTTGGACTTCATTTTCATCCACCTGTGCACC
TATTGTAGTCTTTGGTTGGGTTAGGAGGAAGTGATCATTGTATCAGCATCTGCTGGGAGT
GAGGACTTGCATTGTGAAAGCTTTGCTGTCCTTGATGTGATCATGGAATCTTTTTC----

-------TCACTAGAGTCTATGTCACTCATTATACTCTGTCGAATGTCATTGAATGTCTT
TACATGGGCTTGTATGCCTATGAAAATTGTAATACAACTTTCAGCAACGGATCTCTTGGC
TCTCGCATCGATGAAGAACGCAGCGAAATGCGATAAGTAATGTGAATTGCAGAATTCAGT
GAATCATCGAATCTTTGAACGCATCTTGCGCTCCTTGGTATTCCGAGGAGCATGCCTGTT
TGAGTGTCATTAAATTCTCAACTCTCTTATACTTTTTTGTAAAAGAGAGCTTGGACTGTG
GAGGCTTGCTGGCCACTTTTTGGGGTCAGCTCCTCTGAAATGCATTAGCGGAACCGTTTG
CGATCTGCCACAAGTGTGATAAGTTATCTACACTGGCGAGGGGATTGCTCTCTGTAATGT
TCAGCTTCTAATTGTCTCTACTTTGTGAGACTACTTTTGAATGCTTGACCTCAAATCAGG
TAGGACTACCCGCTGAACTTAA
>AC7-43
TTTCCGTAGGTGAACCTGCGGAAGGATCATTATTGAATTATGTTTCTAGATAGGTTGTAG
CTGGCTCTTTTAGAGCATGTGCACGCCTGTTTGGACTTCATTTTCATCCACCTGTGCACC
TATTGTAGTCTTTGGTTGGGTTAGGAGGAAGTGATCATTGTATCAGCATCTGCTGGGAGT
GAGGACTTGCATTGTGAAAGCTTTGCTGTCCTTGATGTGATCATGGAATCTTTTTC----

-------TCACTAGAGTCTATGTCACTCATTATACTCTGTCGAATGTCATTGAATGTCTT
TACATGGGCTTGTATGCCTATGAAAATTGTAATACAACTTTCAGCAACGGATCTCTTGGC
TCTCGCATCGATGAAGAACGCAGCGAAATGCGATAAGTAATGTGAATTGCAGAATTCAGT
GAATCATCGAATCTTTGAACGCATCTTGCGCTCCTTGGTATTCCGAGGAGCATGCCTGTT
TGAGTGTCATTAAATTCTCAACTCTCTTATACTTTTTTGTAAAAGAGAGCTTGGACTGTG
GAGGCTTGCTGGCCACTTTTTGGGGTCAGCTCCTCTGAAATGCATTAGCGGAACCGTTTG
CGATCTGCCACAAGTGTGATAAGTTATCTACACTGGCGAGGGGATTGCTCTCTGTAATGT
TCAGCTTCTAATTGTCTCTACTTTGTGAGACTACTTTTGAATGCTTGACCTCAAATCAGG
TAGGACTACCCGCTGAACTTAA
>AC7-51
TTTCCGTAGGTGAACCTGCGGAAGGATCATTATTGAATTATGTTTCTAGATAGGTTGTAG
CTGGCTCTTTTAGAGCATGTGCACGCCTGTTTGGACTTCATTTTCATCCACCTGTGCACC
TATTGTAGTCTTTGGTTGGGTTAGGAGGAAGTGATCATTGTATCAGCATCTGCTGGGAGT
GAGGACTTGCATTGTGAAAGCTTTGCTGTCCTTGATGTGATCATGGAATCTTTTTC----

-------TCACTAGAGTCTATGTCACTCATTATACTCTGTCGAATGTCATTGAATGTCTT
TACATGGGCTTGTATGCCTATGAAAATTGTAATACAACTTTCAGCAACGGATCTCTTGGC
TCTCGCATCGATGAAGAACGCAGCGAAATGCGATAAGTAATGTGAATTGCAGAATTCAGT
GAATCATCGAATCTTTGAACGCATCTTGCGCTCCTTGGTATTCCGAGGAGCATGCCTGTT
TGAGTGTCATTAAATTCTCAACTCTCTTATACTTTTTTGTAAAAGAGAGCTTGGACTGTG
GAGGCTTGCTGGCCACTTTTTGGGGTCAGCTCCTCTGAAATGCATTAGCGGAACCGTTTG
CGATCTGCCACAAGTGTGATAAGTTATCTACACTGGCGAGGGGATTGCTCTCTGTAATGT
TCAGCTTCTAATTGTCTCTACTTTGTGAGACTACTTTTGAATGCTTGACCTCAAATCAGG
TAGGACTACCCGCTGAACTTAA
>AC7-54
TTTCCGTAGGTGAACCTGCGGAAGGATCATTATTGAATTATGTTTCTAGATAGGTTGTAG
CTGGCTCTTTTAGAGCATGTGCACGCCTGTTTGGACTTCATTTTCATCCACCTGTGCACC
TATTGTAGTCTTTGGTTGGGTTAGGAGGAAGTGATCATTGTATCAGCATCTGCTGGGAGT
GAGGACTTGCATTGTGAAAGCTTTGCTGTCCTTGATGTGATCATGGAATCTTTTTC----

-------TCACTAGAGTCTATGTCACTCATTATACTCTGTCGAATGTCATTGAATGTCTT
TACATGGGCTTGTATGCCTATGAAAATTGTAATACAACTTTCAGCAACGGATCTCTTGGC
TCTCGCATCGATGAAGAACGCAGCGAAATGCGATAAGTAATGTGAATTGCAGAATTCAGT
GAATCATCGAATCTTTGAACGCATCTTGCGCTCCTTGGTATTCCGAGGAGCATGCCTGTT
TGAGTGTCATTAAATTCTCAACTCTCTTATACTTTTTTGTAAAAGAGAGCTTGGACTGTG
GAGGCTTGCTGGCCACTTTTTGGGGTCAGCTCCTCTGAAATGCATTAGCGGAACCGTTTG
CGATCTGCCACAAGTGTGATAAGTTATCTACACTGGCGAGGGGATTGCTCTCTGTAATGT
TCAGCTTCTAATTGTCTCTACTTTGTGAGACTACTTTTGAATGCTTGACCTCAAATCAGG
TAGGACTACCCGCTGAACTTAA
>AC7-55
TTTCCGTAGGTGAACCTGCGGAAGGATCATTATTGAATTATGTTTCTAGATAGGTTGTAG
CTGGCTCTTTTAGAGCATGTGCACGCCTGTTTGGACTTCATTTTCATCCACCTGTGCACC
TATTGTAGTCTTTGGTTGGGTTAGGAGGAAGTGATCATTGTATCAGCATCTGCTGGGAGT
GAGGACTTGCATTGTGAAAGCTTTGCTGTCCTTGATGTGATCATGGAATCTTTTTC----

-------TCACTAGAGTCTATGTCACTCATTATACTCTGTCGAATGTCATTGAATGTCTT
TACATGGGCTTGTATGCCTATGAAAATTGTAATACAACTTTCAGCAACGGATCTCTTGGC
TCTCGCATCGATGAAGAACGCAGCGAAATGCGATAAGTAATGTGAATTGCAGAATTCAGT
GAATCATCGAATCTTTGAACGCATCTTGCGCTCCTTGGTATTCCGAGGAGCATGCCTGTT
TGAGTGTCATTAAATTCTCAACTCTCTTATACTTTTTTGTAAAAGAGAGCTTGGACTGTG
GAGGCTTGCTGGCCACTTTTTGGGGTCAGCTCCTCTGAAATGCATTAGCGGAACCGTTTG
CGATCTGCCACAAGTGTGATAAGTTATCTACACTGGCGAGGGGATTGCTCTCTGTAATGT
TCAGCTTCTAATTGTCTCTACTTTGTGAGACTACTTTTGAATGCTTGACCTCAAATCAGG
TAGGACTACCCGCTGAACTTAA
>AC7-60
TTTCCGTAGGTGAACCTGCGGAAGGATCATTATTGAATTATGTTTCTAGATAGGTTGTAG
CTGGCTCTTTTAGAGCATGTGCACGCCTGTTTGGACTTCATTTTCATCCACCTGTGCACC
TATTGTAGTCTTTGGTTGGGTTAGGAGGAAGTGATCATTGTATCAGCATCTGCTGGGAGT
GAGGACTTGCATTGTGAAAGCTTTGCTGTCCTTGATGTGATCATGGAATCTTTTTC----

-------TCACTAGAGTCTATGTCACTCATTATACTCTGTCGAATGTCATTGAATGTCTT
TACATGGGCTTGTATGCCTATGAAAATTGTAATACAACTTTCAGCAACGGATCTCTTGGC
TCTCGCATCGATGAAGAACGCAGCGAAATGCGATAAGTAATGTGAATTGCAGAATTCAGT
GAATCATCGAATCTTTGAACGCATCTTGCGCTCCTTGGTATTCCGAGGAGCATGCCTGTT
TGAGTGTCATTAAATTCTCAACTCTCTTATACTTTTTTGTAAAAGAGAGCTTGGACTGTG
GAGGCTTGCTGGCCACTTTTTGGGGTCAGCTCCTCTGAAATGCATTAGCGGAACCGTTTG
CGATCTGCCACAAGTGTGATAAGTTATCTACACTGGCGAGGGGATTGCTCTCTGTAATGT
TCAGCTTCTAATTGTCTCTACTTTGTGAGACTACTTTTGAATGCTTGACCTCAAATCAGG
TAGGACTACCCGCTGAACTTAA
>AC7-61
TTTCCGTAGGTGAACCTGCGGAAGGATCATTATTGAATTATGTTTCTAGATAGGTTGTAG
CTGGCTCTTTTAGAGCATGTGCACGCCTGTTTGGACTTCATTTTCATCCACCTGTGCACC
TATTGTAGTCTTTGGTTGGGTTAGGAGGAAGTGATCATTGTATCAGCATCTGCTGGGAGT
GAGGACTTGCATTGTGAAAGCTTTGCTGTCCTTGATGTGATCATGGAATCTTTTTC----

-------TCACTAGAGTCTATGTCACTCATTATACTCTGTCGAATGTCATTGAATGTCTT
TACATGGGCTTGTATGCCTATGAAAATTGTAATACAACTTTCAGCAACGGATCTCTTGGC
TCTCGCATCGATGAAGAACGCAGCGAAATGCGATAAGTAATGTGAATTGCAGAATTCAGT
GAATCATCGAATCTTTGAACGCATCTTGCGCTCCTTGGTATTCCGAGGAGCATGCCTGTT
TGAGTGTCATTAAATTCTCAACTCTCTTATACTTTTTTGTAAAAGAGAGCTTGGACTGTG
GAGGCTTGCTGGCCACTTTTTGGGGTCAGCTCCTCTGAAATGCATTAGCGGAACCGTTTG
CGATCTGCCACAAGTGTGATAAGTTATCTACACTGGCGAGGGGATTGCTCTCTGTAATGT
TCAGCTTCTAATTGTCTCTACTTTGTGAGACTACTTTTGAATGCTTGACCTCAAATCAGG
TAGGACTACCCGCTGAACTTAA
>AC7-62
TTTCCGTAGGTGAACCTGCGGAAGGATCATTATTGAATTATGTTTCTAGATAGGTTGTAG
CTGGCTCTTTTAGAGCATGTGCACGCCTGTTTGGACTTCATTTTCATCCACCTGTGCACC
TATTGTAGTCTTTGGTTGGGTTAGGAGGAAGTGATCATTGTATCAGCATCTGCTGGGAGT
GAGGACTTGCATTGTGAAAGCTTTGCTGTCCTTGATGTGATCATGGAATCTTTTTC----

-------TCACTAGAGTCTATGTCACTCATTATACTCTGTCGAATGTCATTGAATGTCTT
TACATGGGCTTGTATGCCTATGAAAATTGTAATACAACTTTCAGCAACGGATCTCTTGGC
TCTCGCATCGATGAAGAACGCAGCGAAATGCGATAAGTAATGTGAATTGCAGAATTCAGT
GAATCATCGAATCTTTGAACGCATCTTGCGCTCCTTGGTATTCCGAGGAGCATGCCTGTT
TGAGTGTCATTAAATTCTCAACTCTCTTATACTTTTTTGTAAAAGAGAGCTTGGACTGTG
GAGGCTTGCTGGCCACTTTTTGGGGTCAGCTCCTCTGAAATGCATTAGCGGAACCGTTTG
CGATCTGCCACAAGTGTGATAAGTTATCTACACTGGCGAGGGGATTGCTCTCTGTAATGT
TCAGCTTCTAATTGTCTCTACTTTGTGAGACTACTTTTGAATGCTTGACCTCAAATCAGG
TAGGACTACCCGCTGAACTTAA
>AC7-63
TTTCCGTAGGTGAACCTGCGGAAGGATCATTATTGAATTATGTTTCTAGATAGGTTGTAG
CTGGCTCTTTTAGAGCATGTGCACGCCTGTTTGGACTTCATTTTCATCCACCTGTGCACC
TATTGTAGTCTTTGGTTGGGTTAGGAGGAAGTGATCATTGTATCAGCATCTGCTGGGAGT
GAGGACTTGCATTGTGAAAGCTTTGCTGTCCTTGATGTGATCATGGAATCTTTTTC----

-------TCACTAGAGTCTATGTCACTCATTATACTCTGTCGAATGTCATTGAATGTCTT
TACATGGGCTTGTATGCCTATGAAAATTGTAATACAACTTTCAGCAACGGATCTCTTGGC
TCTCGCATCGATGAAGAACGCAGCGAAATGCGATAAGTAATGTGAATTGCAGAATTCAGT
GAATCATCGAATCTTTGAACGCATCTTGCGCTCCTTGGTATTCCGAGGAGCATGCCTGTT
TGAGTGTCATTAAATTCTCAACTCTCTTATACTTTTTTGTAAAAGAGAGCTTGGACTGTG
GAGGCTTGCTGGCCACTTTTTGGGGTCAGCTCCTCTGAAATGCATTAGCGGAACCGTTTG
CGATCTGCCACAAGTGTGATAAGTTATCTACACTGGCGAGGGGATTGCTCTCTGTAATGT
TCAGCTTCTAATTGTCTCTACTTTGTGAGACTACTTTTGAATGCTTGACCTCAAATCAGG
TAGGACTACCCGCTGAACTTAA
>AC8-2
TTTCCGTAGGTGAACCTGCGGAAGGATCATTATTGAATTATGTTTCTAGATAGGTTGTAG
CTGGCTCTTTTAGAGCATGTGCACGCCTGTTTGGACTTCATTTTCATCCACCTGTGCACC
TATTGTAGTCTTTGGTTGGGTTAGGAGGAAGTGATCATTGTATCAGCATCTGCTGGGAGT
GAGGACTTGCATTGTGAAAGCTTTGCTGTCCTTGATGTGATCATGGAATCTTTTTC----

-------TCACTAGAGTCTATGTCACTCATTATACTCTGTCGAATGTCATTGAATGTCTT
TACATGGGCTTGTATGCCTATGAAAATTGTAATACAACTTTCAGCAACGGATCTCTTGGC
TCTCGCATCGATGAAGAACGCAGCGAAATGCGATAAGTAATGTGAATTGCAGAATTCAGT
GAATCATCGAATCTTTGAACGCATCTTGCGCTCCTTGGTATTCCGAGGAGCATGCCTGTT
TGAGTGTCATTAAATTCTCAACTCTCTTATACTTTTTTGTAAAAGAGAGCTTGGACTGTG
GAGGCTTGCTGGCCACTTTTTGGGGTCAGCTCCTCTGAAATGCATTAGCGGAACCGTTTG
CGATCTGCCACAAGTGTGATAAGTTATCTACACTGGCGAGGGGATTGCTCTCTGTAATGT
TCAGCTTCTAATTGTCTCTACTTTGTGAGACTACTTTTGAATGCTTGACCTCAAATCAGG
TAGGACTACCCGCTGAACTTAA
>AC8-9
TTTCCGTAGGTGAACCTGCGGAAGGATCATTATTGAATTATGTTTCTAGATAGGTTGTAG
CTGGCTCTTTTAGAGCATGTGCACGCCTGTTTGGACTTCATTTTCATCCACCTGTGCACC
TATTGTAGTCTTTGGTTGGGTTAGGAGGAAGTGATCATTGTATCAGCATCTGCTGGGAGT
GAGGACTTGCATTGTGAAAGCTTTGCTGTCCTTGATGTGATCATGGAATCTTTTTC----

-------TCACTAGAGTCTATGTCACTCATTATACTCTGTCGAATGTCATTGAATGTCTT
TACATGGGCTTGTATGCCTATGAAAATTGTAATACAACTTTCAGCAACGGATCTCTTGGC
TCTCGCATCGATGAAGAACGCAGCGAAATGCGATAAGTAATGTGAATTGCAGAATTCAGT
GAATCATCGAATCTTTGAACGCATCTTGCGCTCCTTGGTATTCCGAGGAGCATGCCTGTT
TGAGTGTCATTAAATTCTCAACTCTCTTATACTTTTTTGTAAAAGAGAGCTTGGACTGTG
GAGGCTTGCTGGCCACTTTTTGGGGTCAGCTCCTCTGAAATGCATTAGCGGAACCGTTTG
CGATCTGCCACAAGTGTGATAAGTTATCTACACTGGCGAGGGGATTGCTCTCTGTAATGT
TCAGCTTCTAATTGTCTCTACTTTGTGAGACTACTTTTGAATGCTTGACCTCAAATCAGG
TAGGACTACCCGCTGAACTTAA
>AC8-17
TTTCCGTAGGTGAACCTGCGGAAGGATCATTATTGAATTATGTTTCTAGATAGGTTGTAG
CTGGCTCTTTTAGAGCATGTGCACGCCTGTTTGGACTTCATTTTCATCCACCTGTGCACC
TATTGTAGTCTTTGGTTGGGTTAGGAGGAAGTGATCATTGTATCAGCATCTGCTGGGAGT

GAGGACTTGCATTGTGAAAGCTTTGCTGTCCTTGATGTGATCATGGAATCTTTTTC----

-------TCACTAGAGTCTATGTCACTCATTATACTCTGTCGAATGTCATTGAATGTCTT
TACATGGGCTTGTATGCCTATGAAAATTGTAATACAACTTTCAGCAACGGATCTCTTGGC
TCTCGCATCGATGAAGAACGCAGCGAAATGCGATAAGTAATGTGAATTGCAGAATTCAGT
GAATCATCGAATCTTTGAACGCATCTTGCGCTCCTTGGTATTCCGAGGAGCATGCCTGTT
TGAGTGTCATTAAATTCTCAACTCTCTTATACTTTTTTGTAAAAGAGAGCTTGGACTGTG
GAGGCTTGCTGGCCACTTTTTGGGGTCAGCTCCTCTGAAATGCATTAGCGGAACCGTTTG
CGATCTGCCACAAGTGTGATAAGTTATCTACACTGGCGAGGGGATTGCTCTCTGTAATGT
TCAGCTTCTAATTGTCTCTACTTTGTGAGACTACTTTTGAATGCTTGACCTCAAATCAGG
TAGGACTACCCGCTGAACTTAA
>AC8-20
TTTCCGTAGGTGAACCTGCGGAAGGATCATTATTGAATTATGTTTCTAGATAGGTTGTAG
CTGGCTCTTTTAGAGCATGTGCACGCCTGTTTGGACTTCATTTTCATCCACCTGTGCACC
TATTGTAGTCTTTGGTTGGGTTAGGAGGAAGTGATCATTGTATCAGCATCTGCTGGGAGT
GAGGACTTGCATTGTGAAAGCTTTGCTGTCCTTGATGTGATCATGGAATCTTTTTC----

-------TCACTAGAGTCTATGTCACTCATTATACTCTGTCGAATGTCATTGAATGTCTT
TACATGGGCTTGTATGCCTATGAAAATTGTAATACAACTTTCAGCAACGGATCTCTTGGC
TCTCGCATCGATGAAGAACGCAGCGAAATGCGATAAGTAATGTGAATTGCAGAATTCAGT
GAATCATCGAATCTTTGAACGCATCTTGCGCTCCTTGGTATTCCGAGGAGCATGCCTGTT
TGAGTGTCATTAAATTCTCAACTCTCTTATACTTTTTTGTAAAAGAGAGCTTGGACTGTG
GAGGCTTGCTGGCCACTTTTTGGGGTCAGCTCCTCTGAAATGCATTAGCGGAACCGTTTG
CGATCTGCCACAAGTGTGATAAGTTATCTACACTGGCGAGGGGATTGCTCTCTGTAATGT
TCAGCTTCTAATTGTCTCTACTTTGTGAGACTACTTTTGAATGCTTGACCTCAAATCAGG
TAGGACTACCCGCTGAACTTAA
>AC8-25
TTTCCGTAGGTGAACCTGCGGAAGGATCATTATTGAATTATGTTTCTAGATAGGTTGTAG

CTGGCTCTTTTAGAGCATGTGCACGCCTGTTTGGACTTCATTTTCATCCACCTGTGCACC
TATTGTAGTCTTTGGTTGGGTTAGGAGGAAGTGATCATTGTATCAGCATCTGCTGGGAGT
GAGGACTTGCATTGTGAAAGCTTTGCTGTCCTTGATGTGATCATGGAATCTTTTTC----

-------TCACTAGAGTCTATGTCACTCATTATACTCTGTCGAATGTCATTGAATGTCTT
TACATGGGCTTGTATGCCTATGAAAATTGTAATACAACTTTCAGCAACGGATCTCTTGGC
TCTCGCATCGATGAAGAACGCAGCGAAATGCGATAAGTAATGTGAATTGCAGAATTCAGT
GAATCATCGAATCTTTGAACGCATCTTGCGCTCCTTGGTATTCCGAGGAGCATGCCTGTT
TGAGTGTCATTAAATTCTCAACTCTCTTATACTTTTTTGTAAAAGAGAGCTTGGACTGTG
GAGGCTTGCTGGCCACTTTTTGGGGTCAGCTCCTCTGAAATGCATTAGCGGAACCGTTTG
CGATCTGCCACAAGTGTGATAAGTTATCTACACTGGCGAGGGGATTGCTCTCTGTAATGT
TCAGCTTCTAATTGTCTCTACTTTGTGAGACTACTTTTGAATGCTTGACCTCAAATCAGG
TAGGACTACCCGCTGAACTTAA
>AC8-28
TTTCCGTAGGTGAACCTGCGGAAGGATCATTATTGAATTATGTTTCTAGATAGGTTGTAG
CTGGCTCTTTTAGAGCATGTGCACGCCTGTTTGGACTTCATTTTCATCCACCTGTGCACC
TATTGTAGTCTTTGGTTGGGTTAGGAGGAAGTGATCATTGTATCAGCATCTGCTGGGAGT
GAGGACTTGCATTGTGAAAGCTTTGCTGTCCTTGATGTGATCATGGAATCTTTTTC----

-------TCACTAGAGTCTATGTCACTCATTATACTCTGTCGAATGTCATTGAATGTCTT
TACATGGGCTTGTATGCCTATGAAAATTGTAATACAACTTTCAGCAACGGATCTCTTGGC
TCTCGCATCGATGAAGAACGCAGCGAAATGCGATAAGTAATGTGAATTGCAGAATTCAGT
GAATCATCGAATCTTTGAACGCATCTTGCGCTCCTTGGTATTCCGAGGAGCATGCCTGTT
TGAGTGTCATTAAATTCTCAACTCTCTTATACTTTTTTGTAAAAGAGAGCTTGGACTGTG
GAGGCTTGCTGGCCACTTTTTGGGGTCAGCTCCTCTGAAATGCATTAGCGGAACCGTTTG
CGATCTGCCACAAGTGTGATAAGTTATCTACACTGGCGAGGGGATTGCTCTCTGTAATGT
TCAGCTTCTAATTGTCTCTACTTTGTGAGACTACTTTTGAATGCTTGACCTCAAATCAGG
TAGGACTACCCGCTGAACTTAA

```
>AC8-46
TTTCCGTAGGTGAACCTGCGGAAGGATCATTATTGAATTATGTTTCTAGATAGGTTGTAG
CTGGCTCTTTTAGAGCATGTGCACGCCTGTTTGGACTTCATTTTCATCCACCTGTGCACC
TATTGTAGTCTTTGGTTGGGTTAGGAGGAAGTGATCATTGTATCAGCATCTGCTGGGAGT
GAGGACTTGCATTGTGAAAGCTTTGCTGTCCTTGATGTGATCATGGAATCTTTTTC----
------------------------------------------------------------
------------------------------------------------------------
------------------------------------------------------------
------------------------------------------------------------
------------------------------------------------------------
------------------------------------------------------------
------------------------------------------------------------
------------------------------------------------------------
------------------------------------------------------------
------------------------------------------------------------
------------------------------------------------------------
------------------------------------------------------------
-------TCACTAGAGTCTATGTCACTCATTATACTCTGTCGAATGTCATTGAATGTCTT
TACATGGGCTTGTATGCCTATGAAAATTGTAATACAACTTTCAGCAACGGATCTCTTGGC
TCTCGCATCGATGAAGAACGCAGCGAAATGCGATAAGTAATGTGAATTGCAGAATTCAGT
GAATCATCGAATCTTTGAACGCATCTTGCGCTCCTTGGTATTCCGAGGAGCATGCCTGTT
TGAGTGTCATTAAATTCTCAACTCTCTTATACTTTTTTGTAAAAGAGAGCTTGGACTGTG
GAGGCTTGCTGGCCACTTTTTGGGGTCAGCTCCTCTGAAATGCATTAGCGGAACCGTTTG
CGATCTGCCACAAGTGTGATAAGTTATCTACACTGGCGAGGGGATTGCTCTCTGTAATGT
TCAGCTTCTAATTGTCTCTACTTTGTGAGACTACTTTTGAATGCTTGACCTCAAATCAGG
TAGGACTACCCGCTGAACTTAA
>AC8-48
TTTCCGTAGGTGAACCTGCGGAAGGATCATTATTGAATTATGTTTCTAGATAGGTTGTAG
CTGGCTCTTTTAGAGCATGTGCACGCCTGTTTGGACTTCATTTTCATCCACCTGTGCACC
TATTGTAGTCTTTGGTTGGGTTAGGAGGAAGTGATCATTGTATCAGCATCTGCTGGGAGT
GAGGACTTGCATTGTGAAAGCTTTGCTGTCCTTGATGTGATCATGGAATCTTTTTC----
------------------------------------------------------------
------------------------------------------------------------
------------------------------------------------------------
------------------------------------------------------------
------------------------------------------------------------
------------------------------------------------------------
------------------------------------------------------------
------------------------------------------------------------
------------------------------------------------------------
------------------------------------------------------------
------------------------------------------------------------
------------------------------------------------------------
-------TCACTAGAGTCTATGTCACTCATTATACTCTGTCGAATGTCATTGAATGTCTT
TACATGGGCTTGTATGCCTATGAAAATTGTAATACAACTTTCAGCAACGGATCTCTTGGC
TCTCGCATCGATGAAGAACGCAGCGAAATGCGATAAGTAATGTGAATTGCAGAATTCAGT
GAATCATCGAATCTTTGAACGCATCTTGCGCTCCTTGGTATTCCGAGGAGCATGCCTGTT
TGAGTGTCATTAAATTCTCAACTCTCTTATACTTTTTTGTAAAAGAGAGCTTGGACTGTG
GAGGCTTGCTGGCCACTTTTTGGGGTCAGCTCCTCTGAAATGCATTAGCGGAACCGTTTG
CGATCTGCCACAAGTGTGATAAGTTATCTACACTGGCGAGGGGATTGCTCTCTGTAATGT
```

TCAGCTTCTAATTGTCTCTACTTTGTGAGACTACTTTTGAATGCTTGACCTCAAATCAGG
TAGGACTACCCGCTGAACTTAA
>AC8-49
TTTCCGTAGGTGAACCTGCGGAAGGATCATTATTGAATTATGTTTCTAGATAGGTTGTAG
CTGGCTCTTTTAGAGCATGTGCACGCCTGTTTGGACTTCATTTTCATCCACCTGTGCACC
TATTGTAGTCTTTGGTTGGGTTAGGAGGAAGTGATCATTGTATCAGCATCTGCTGGGAGT
GAGGACTTGCATTGTGAAAGCTTTGCTGTCCTTGATGTGATCATGGAATCTTTTTC----

-------TCACTAGAGTCTATGTCACTCATTATACTCTGTCGAATGTCATTGAATGTCTT
TACATGGGCTTGTATGCCTATGAAAATTGTAATACAACTTTCAGCAACGGATCTCTTGGC
TCTCGCATCGATGAAGAACGCAGCGAAATGCGATAAGTAATGTGAATTGCAGAATTCAGT
GAATCATCGAATCTTTGAACGCATCTTGCGCTCCTTGGTATTCCGAGGAGCATGCCTGTT
TGAGTGTCATTAAATTCTCAACTCTCTTATACTTTTTTGTAAAAGAGAGCTTGGACTGTG
GAGGCTTGCTGGCCACTTTTTGGGGTCAGCTCCTCTGAAATGCATTAGCGGAACCGTTTG
CGATCTGCCACAAGTGTGATAAGTTATCTACACTGGCGAGGGGATTGCTCTCTGTAATGT
TCAGCTTCTAATTGTCTCTACTTTGTGAGACTACTTTTGAATGCTTGACCTCAAATCAGG
TAGGACTACCCGCTGAACTTAA
>AC8-60
TTTCCGTAGGTGAACCTGCGGAAGGATCATTATTGAATTATGTTTCTAGATAGGTTGTAG
CTGGCTCTTTTAGAGCATGTGCACGCCTGTTTGGACTTCATTTTCATCCACCTGTGCACC
TATTGTAGTCTTTGGTTGGGTTAGGAGGAAGTGATCATTGTATCAGCATCTGCTGGGAGT
GAGGACTTGCATTGTGAAAGCTTTGCTGTCCTTGATGTGATCATGGAATCTTTTTC----

-------TCACTAGAGTCTATGTCACTCATTATACTCTGTCGAATGTCATTGAATGTCTT
TACATGGGCTTGTATGCCTATGAAAATTGTAATACAACTTTCAGCAACGGATCTCTTGGC
TCTCGCATCGATGAAGAACGCAGCGAAATGCGATAAGTAATGTGAATTGCAGAATTCAGT
GAATCATCGAATCTTTGAACGCATCTTGCGCTCCTTGGTATTCCGAGGAGCATGCCTGTT
TGAGTGTCATTAAATTCTCAACTCTCTTATACTTTTTTGTAAAAGAGAGCTTGGACTGTG

GAGGCTTGCTGGCCACTTTTTGGGGTCAGCTCCTCTGAAATGCATTAGCGGAACCGTTTG
CGATCTGCCACAAGTGTGATAAGTTATCTACACTGGCGAGGGGATTGCTCTCTGTAATGT
TCAGCTTCTAATTGTCTCTACTTTGTGAGACTACTTTTGAATGCTTGACCTCAAATCAGG
TAGGACTACCCGCTGAACTTAA
>AC9-4
TTTCCGTAGGTGAACCTGCGGAAGGATCATTATTGAATTATGTTTCTAGATAGGTTGTAG
CTGGCTCTTTTAGAGCATGTGCACGCCTGTTTGGACTTCATTTTCATCCACCTGTGCACC
TATTGTAGTCTTTGGTTGGGTTAGGAGGAAGTGATCATTGTATCAGCATCTGCTGGGAGT
GAGGACTTGCATTGTGAAAGCTTTGCTGTCCTTGATGTGATCATGGAATCTTTTTC----

-------TCACTAGAGTCTATGTCACTCATTATACTCTGTCGAATGTCATTGAATGTCTT
TACATGGGCTTGTATGCCTATGAAAATTGTAATACAACTTTCAGCAACGGATCTCTTGGC
TCTCGCATCGATGAAGAACGCAGCGAAATGCGATAAGTAATGTGAATTGCAGAATTCAGT
GAATCATCGAATCTTTGAACGCATCTTGCGCTCCTTGGTATTCCGAGGAGCATGCCTGTT
TGAGTGTCATTAAATTCTCAACTCTCTTATACTTTTTTGTAAAAGAGAGCTTGGACTGTG
GAGGCTTGCTGGCCACTTTTTGGGGTCAGCTCCTCTGAAATGCATTAGCGGAACCGTTTG
CGATCTGCCACAAGTGTGATAAGTTATCTACACTGGCGAGGGGATTGCTCTCTGTAATGT
TCAGCTTCTAATTGTCTCTACTTTGTGAGACTACTTTTGAATGCTTGACCTCAAATCAGG
TAGGACTACCCGCTGAACTTAA
>AC9-9
TTTCCGTAGGTGAACCTGCGGAAGGATCATTATTGAATTATGTTTCTAGATAGGTTGTAG
CTGGCTCTTTTAGAGCATGTGCACGCCTGTTTGGACTTCATTTTCATCCACCTGTGCACC
TATTGTAGTCTTTGGTTGGGTTAGGAGGAAGTGATCATTGTATCAGCATCTGCTGGGAGT
GAGGACTTGCATTGTGAAAGCTTTGCTGTCCTTGATGTGATCATGGAATCTTTTTC----

-------TCACTAGAGTCTATGTCACTCATTATACTCTGTCGAATGTCATTGAATGTCTT
TACATGGGCTTGTATGCCTATGAAAATTGTAATACAACTTTCAGCAACGGATCTCTTGGC
TCTCGCATCGATGAAGAACGCAGCGAAATGCGATAAGTAATGTGAATTGCAGAATTCAGT

```
GAATCATCGAATCTTTGAACGCATCTTGCGCTCCTTGGTATTCCGAGGAGCATGCCTGTT
TGAGTGTCATTAAATTCTCAACTCTCTTATACTTTTTTGTAAAAGAGAGCTTGGACTGTG
GAGGCTTGCTGGCCACTTTTTGGGGTCAGCTCCTCTGAAATGCATTAGCGGAACCGTTTG
CGATCTGCCACAAGTGTGATAAGTTATCTACACTGGCGAGGGGATTGCTCTCTGTAATGT
TCAGCTTCTAATTGTCTCTACTTTGTGAGACTACTTTTGAATGCTTGACCTCAAATCAGG
TAGGACTACCCGCTGAACTTAA
>AC9-15
TTTCCGTAGGTGAACCTGCGGAAGGATCATTATTGAATTATGTTTCTAGATAGGTTGTAG
CTGGCTCTTTTAGAGCATGTGCACGCCTGTTTGGACTTCATTTTCATCCACCTGTGCACC
TATTGTAGTCTTTGGTTGGGTTAGGAGGAAGTGATCATTGTATCAGCATCTGCTGGGAGT
GAGGACTTGCATTGTGAAAGCTTTGCTGTCCTTGATGTGATCATGGAATCTTTTTC----
------------------------------------------------------------
------------------------------------------------------------
------------------------------------------------------------
------------------------------------------------------------
------------------------------------------------------------
------------------------------------------------------------
------------------------------------------------------------
------------------------------------------------------------
------------------------------------------------------------
------------------------------------------------------------
------------------------------------------------------------
------------------------------------------------------------
-------TCACTAGAGTCTATGTCACTCATTATACTCTGTCGAATGTCATTGAATGTCTT
TACATGGGCTTGTATGCCTATGAAAATTGTAATACAACTTTCAGCAACGGATCTCTTGGC
TCTCGCATCGATGAAGAACGCAGCGAAATGCGATAAGTAATGTGAATTGCAGAATTCAGT
GAATCATCGAATCTTTGAACGCATCTTGCGCTCCTTGGTATTCCGAGGAGCATGCCTGTT
TGAGTGTCATTAAATTCTCAACTCTCTTATACTTTTTTGTAAAAGAGAGCTTGGACTGTG
GAGGCTTGCTGGCCACTTTTTGGGGTCAGCTCCTCTGAAATGCATTAGCGGAACCGTTTG
CGATCTGCCACAAGTGTGATAAGTTATCTACACTGGCGAGGGGATTGCTCTCTGTAATGT
TCAGCTTCTAATTGTCTCTACTTTGTGAGACTACTTTTGAATGCTTGACCTCAAATCAGG
TAGGACTACCCGCTGAACTTAA
>AC9-23
TTTCCGTAGGTGAACCTGCGGAAGGATCATTATTGAATTATGTTTCTAGATAGGTTGTAG
CTGGCTCTTTTAGAGCATGTGCACGCCTGTTTGGACTTCATTTTCATCCACCTGTGCACC
TATTGTAGTCTTTGGTTGGGTTAGGAGGAAGTGATCATTGTATCAGCATCTGCTGGGAGT
GAGGACTTGCATTGTGAAAGCTTTGCTGTCCTTGATGTGATCATGGAATCTTTTTC----
------------------------------------------------------------
------------------------------------------------------------
------------------------------------------------------------
------------------------------------------------------------
------------------------------------------------------------
------------------------------------------------------------
------------------------------------------------------------
------------------------------------------------------------
------------------------------------------------------------
------------------------------------------------------------
------------------------------------------------------------
------------------------------------------------------------
-------TCACTAGAGTCTATGTCACTCATTATACTCTGTCGAATGTCATTGAATGTCTT
```

TACATGGGCTTGTATGCCTATGAAAATTGTAATACAACTTTCAGCAACGGATCTCTTGGC
TCTCGCATCGATGAAGAACGCAGCGAAATGCGATAAGTAATGTGAATTGCAGAATTCAGT
GAATCATCGAATCTTTGAACGCATCTTGCGCTCCTTGGTATTCCGAGGAGCATGCCTGTT
TGAGTGTCATTAAATTCTCAACTCTCTTATACTTTTTTGTAAAAGAGAGCTTGGACTGTG
GAGGCTTGCTGGCCACTTTTTGGGGTCAGCTCCTCTGAAATGCATTAGCGGAACCGTTTG
CGATCTGCCACAAGTGTGATAAGTTATCTACACTGGCGAGGGGATTGCTCTCTGTAATGT
TCAGCTTCTAATTGTCTCTACTTTGTGAGACTACTTTTGAATGCTTGACCTCAAATCAGG
TAGGACTACCCGCTGAACTTAA
>AC9-24
TTTCCGTAGGTGAACCTGCGGAAGGATCATTATTGAATTATGTTTCTAGATAGGTTGTAG
CTGGCTCTTTTAGAGCATGTGCACGCCTGTTTGGACTTCATTTTCATCCACCTGTGCACC
TATTGTAGTCTTTGGTTGGGTTAGGAGGAAGTGATCATTGTATCAGCATCTGCTGGGAGT
GAGGACTTGCATTGTGAAAGCTTTGCTGTCCTTGATGTGATCATGGAATCTTTTTC----

-------TCACTAGAGTCTATGTCACTCATTATACTCTGTCGAATGTCATTGAATGTCTT
TACATGGGCTTGTATGCCTATGAAAATTGTAATACAACTTTCAGCAACGGATCTCTTGGC
TCTCGCATCGATGAAGAACGCAGCGAAATGCGATAAGTAATGTGAATTGCAGAATTCAGT
GAATCATCGAATCTTTGAACGCATCTTGCGCTCCTTGGTATTCCGAGGAGCATGCCTGTT
TGAGTGTCATTAAATTCTCAACTCTCTTATACTTTTTTGTAAAAGAGAGCTTGGACTGTG
GAGGCTTGCTGGCCACTTTTTGGGGTCAGCTCCTCTGAAATGCATTAGCGGAACCGTTTG
CGATCTGCCACAAGTGTGATAAGTTATCTACACTGGCGAGGGGATTGCTCTCTGTAATGT
TCAGCTTCTAATTGTCTCTACTTTGTGAGACTACTTTTGAATGCTTGACCTCAAATCAGG
TAGGACTACCCGCTGAACTTAA
>AC9-30
TTTCCGTAGGTGAACCTGCGGAAGGATCATTATTGAATTATGTTTCTAGATAGGTTGTAG
CTGGCTCTTTTAGAGCATGTGCACGCCTGTTTGGACTTCATTTTCATCCACCTGTGCACC
TATTGTAGTCTTTGGTTGGGTTAGGAGGAAGTGATCATTGTATCAGCATCTGCTGGGAGT
GAGGACTTGCATTGTGAAAGCTTTGCTGTCCTTGATGTGATCATGGAATCTTTTTC----

-------TCACTAGAGTCTATGTCACTCATTATACTCTGTCGAATGTCATTGAATGTCTT
TACATGGGCTTGTATGCCTATGAAAATTGTAATACAACTTTCAGCAACGGATCTCTTGGC
TCTCGCATCGATGAAGAACGCAGCGAAATGCGATAAGTAATGTGAATTGCAGAATTCAGT
GAATCATCGAATCTTTGAACGCATCTTGCGCTCCTTGGTATTCCGAGGAGCATGCCTGTT
TGAGTGTCATTAAATTCTCAACTCTCTTATACTTTTTTGTAAAAGAGAGCTTGGACTGTG
GAGGCTTGCTGGCCACTTTTTGGGGTCAGCTCCTCTGAAATGCATTAGCGGAACCGTTTG
CGATCTGCCACAAGTGTGATAAGTTATCTACACTGGCGAGGGGATTGCTCTCTGTAATGT
TCAGCTTCTAATTGTCTCTACTTTGTGAGACTACTTTTGAATGCTTGACCTCAAATCAGG
TAGGACTACCCGCTGAACTTAA
>AC9-35
TTTCCGTAGGTGAACCTGCGGAAGGATCATTATTGAATTATGTTTCTAGATAGGTTGTAG
CTGGCTCTTTTAGAGCATGTGCACGCCTGTTTGGACTTCATTTTCATCCACCTGTGCACC
TATTGTAGTCTTTGGTTGGGTTAGGAGGAAGTGATCATTGTATCAGCATCTGCTGGGAGT
GAGGACTTGCATTGTGAAAGCTTTGCTGTCCTTGATGTGATCATGGAATCTTTTTC----

-------TCACTAGAGTCTATGTCACTCATTATACTCTGTCGAATGTCATTGAATGTCTT
TACATGGGCTTGTATGCCTATGAAAATTGTAATACAACTTTCAGCAACGGATCTCTTGGC
TCTCGCATCGATGAAGAACGCAGCGAAATGCGATAAGTAATGTGAATTGCAGAATTCAGT
GAATCATCGAATCTTTGAACGCATCTTGCGCTCCTTGGTATTCCGAGGAGCATGCCTGTT
TGAGTGTCATTAAATTCTCAACTCTCTTATACTTTTTTGTAAAAGAGAGCTTGGACTGTG
GAGGCTTGCTGGCCACTTTTTGGGGTCAGCTCCTCTGAAATGCATTAGCGGAACCGTTTG
CGATCTGCCACAAGTGTGATAAGTTATCTACACTGGCGAGGGGATTGCTCTCTGTAATGT
TCAGCTTCTAATTGTCTCTACTTTGTGAGACTACTTTTGAATGCTTGACCTCAAATCAGG
TAGGACTACCCGCTGAACTTAA
>AC9-37
TTTCCGTAGGTGAACCTGCGGAAGGATCATTATTGAATTATGTTTCTAGATAGGTTGTAG
CTGGCTCTTTTAGAGCATGTGCACGCCTGTTTGGACTTCATTTTCATCCACCTGTGCACC
TATTGTAGTCTTTGGTTGGGTTAGGAGGAAGTGATCATTGTATCAGCATCTGCTGGGAGT
GAGGACTTGCATTGTGAAAGCTTTGCTGTCCTTGATGTGATCATGGAATCTTTTTC----

-------TCACTAGAGTCTATGTCACTCATTATACTCTGTCGAATGTCATTGAATGTCTT
TACATGGGCTTGTATGCCTATGAAAATTGTAATACAACTTTCAGCAACGGATCTCTTGGC
TCTCGCATCGATGAAGAACGCAGCGAAATGCGATAAGTAATGTGAATTGCAGAATTCAGT
GAATCATCGAATCTTTGAACGCATCTTGCGCTCCTTGGTATTCCGAGGAGCATGCCTGTT
TGAGTGTCATTAAATTCTCAACTCTCTTATACTTTTTTGTAAAAGAGAGCTTGGACTGTG
GAGGCTTGCTGGCCACTTTTTGGGGTCAGCTCCTCTGAAATGCATTAGCGGAACCGTTTG
CGATCTGCCACAAGTGTGATAAGTTATCTACACTGGCGAGGGGATTGCTCTCTGTAATGT
TCAGCTTCTAATTGTCTCTACTTTGTGAGACTACTTTTGAATGCTTGACCTCAAATCAGG
TAGGACTACCCGCTGAACTTAA
>AC9-40
TTTCCGTAGGTGAACCTGCGGAAGGATCATTATTGAATTATGTTTCTAGATAGGTTGTAG
CTGGCTCTTTTAGAGCATGTGCACGCCTGTTTGGACTTCATTTTCATCCACCTGTGCACC
TATTGTAGTCTTTGGTTGGGTTAGGAGGAAGTGATCATTGTATCAGCATCTGCTGGGAGT
GAGGACTTGCATTGTGAAAGCTTTGCTGTCCTTGATGTGATCATGGAATCTTTTTC----

-------TCACTAGAGTCTATGTCACTCATTATACTCTGTCGAATGTCATTGAATGTCTT
TACATGGGCTTGTATGCCTATGAAAATTGTAATACAACTTTCAGCAACGGATCTCTTGGC
TCTCGCATCGATGAAGAACGCAGCGAAATGCGATAAGTAATGTGAATTGCAGAATTCAGT
GAATCATCGAATCTTTGAACGCATCTTGCGCTCCTTGGTATTCCGAGGAGCATGCCTGTT
TGAGTGTCATTAAATTCTCAACTCTCTTATACTTTTTTGTAAAAGAGAGCTTGGACTGTG
GAGGCTTGCTGGCCACTTTTTGGGGTCAGCTCCTCTGAAATGCATTAGCGGAACCGTTTG
CGATCTGCCACAAGTGTGATAAGTTATCTACACTGGCGAGGGGATTGCTCTCTGTAATGT
TCAGCTTCTAATTGTCTCTACTTTGTGAGACTACTTTTGAATGCTTGACCTCAAATCAGG
TAGGACTACCCGCTGAACTTAA
>AC9-41
TTTCCGTAGGTGAACCTGCGGAAGGATCATTATTGAATTATGTTTCTAGATAGGTTGTAG
CTGGCTCTTTTAGAGCATGTGCACGCCTGTTTGGACTTCATTTTCATCCACCTGTGCACC
TATTGTAGTCTTTGGTTGGGTTAGGAGGAAGTGATCATTGTATCAGCATCTGCTGGGAGT
GAGGACTTGCATTGTGAAAGCTTTGCTGTCCTTGATGTGATCATGGAATCTTTTTC----

-------TCACTAGAGTCTATGTCACTCATTATACTCTGTCGAATGTCATTGAATGTCTT
TACATGGGCTTGTATGCCTATGAAAATTGTAATACAACTTTCAGCAACGGATCTCTTGGC
TCTCGCATCGATGAAGAACGCAGCGAAATGCGATAAGTAATGTGAATTGCAGAATTCAGT
GAATCATCGAATCTTTGAACGCATCTTGCGCTCCTTGGTATTCCGAGGAGCATGCCTGTT
TGAGTGTCATTAAATTCTCAACTCTCTTATACTTTTTTGTAAAAGAGAGCTTGGACTGTG
GAGGCTTGCTGGCCACTTTTTGGGGTCAGCTCCTCTGAAATGCATTAGCGGAACCGTTTG
CGATCTGCCACAAGTGTGATAAGTTATCTACACTGGCGAGGGGATTGCTCTCTGTAATGT
TCAGCTTCTAATTGTCTCTACTTTGTGAGACTACTTTTGAATGCTTGACCTCAAATCAGG
TAGGACTACCCGCTGAACTTAA
>AC9-42
TTTCCGTAGGTGAACCTGCGGAAGGATCATTATTGAATTATGTTTCTAGATAGGTTGTAG
CTGGCTCTTTTAGAGCATGTGCACGCCTGTTTGGACTTCATTTTCATCCACCTGTGCACC
TATTGTAGTCTTTGGTTGGGTTAGGAGGAAGTGATCATTGTATCAGCATCTGCTGGGAGT
GAGGACTTGCATTGTGAAAGCTTTGCTGTCCTTGATGTGATCATGGAATCTTTTTC----

-------TCACTAGAGTCTATGTCACTCATTATACTCTGTCGAATGTCATTGAATGTCTT
TACATGGGCTTGTATGCCTATGAAAATTGTAATACAACTTTCAGCAACGGATCTCTTGGC
TCTCGCATCGATGAAGAACGCAGCGAAATGCGATAAGTAATGTGAATTGCAGAATTCAGT
GAATCATCGAATCTTTGAACGCATCTTGCGCTCCTTGGTATTCCGAGGAGCATGCCTGTT
TGAGTGTCATTAAATTCTCAACTCTCTTATACTTTTTTGTAAAAGAGAGCTTGGACTGTG
GAGGCTTGCTGGCCACTTTTTGGGGTCAGCTCCTCTGAAATGCATTAGCGGAACCGTTTG
CGATCTGCCACAAGTGTGATAAGTTATCTACACTGGCGAGGGGATTGCTCTCTGTAATGT
TCAGCTTCTAATTGTCTCTACTTTGTGAGACTACTTTTGAATGCTTGACCTCAAATCAGG
TAGGACTACCCGCTGAACTTAA
>AC9-55
TTTCCGTAGGTGAACCTGCGGAAGGATCATTATTGAATTATGTTTCTAGATAGGTTGTAG
CTGGCTCTTTTAGAGCATGTGCACGCCTGTTTGGACTTCATTTTCATCCACCTGTGCACC
TATTGTAGTCTTTGGTTGGGTTAGGAGGAAGTGATCATTGTATCAGCATCTGCTGGGAGT
GAGGACTTGCATTGTGAAAGCTTTGCTGTCCTTGATGTGATCATGGAATCTTTTTC----

-------TCACTAGAGTCTATGTCACTCATTATACTCTGTCGAATGTCATTGAATGTCTT
TACATGGGCTTGTATGCCTATGAAAATTGTAATACAACTTTCAGCAACGGATCTCTTGGC
TCTCGCATCGATGAAGAACGCAGCGAAATGCGATAAGTAATGTGAATTGCAGAATTCAGT
GAATCATCGAATCTTTGAACGCATCTTGCGCTCCTTGGTATTCCGAGGAGCATGCCTGTT
TGAGTGTCATTAAATTCTCAACTCTCTTATACTTTTTTGTAAAAGAGAGCTTGGACTGTG
GAGGCTTGCTGGCCACTTTTTGGGGTCAGCTCCTCTGAAATGCATTAGCGGAACCGTTTG
CGATCTGCCACAAGTGTGATAAGTTATCTACACTGGCGAGGGGATTGCTCTCTGTAATGT
TCAGCTTCTAATTGTCTCTACTTTGTGAGACTACTTTTGAATGCTTGACCTCAAATCAGG
TAGGACTACCCGCTGAACTTAA
>AC9-59
TTTCCGTAGGTGAACCTGCGGAAGGATCATTATTGAATTATGTTTCTAGATAGGTTGTAG
CTGGCTCTTTTAGAGCATGTGCACGCCTGTTTGGACTTCATTTTCATCCACCTGTGCACC
TATTGTAGTCTTTGGTTGGGTTAGGAGGAAGTGATCATTGTATCAGCATCTGCTGGGAGT
GAGGACTTGCATTGTGAAAGCTTTGCTGTCCTTGATGTGATCATGGAATCTTTTTC----

-------TCACTAGAGTCTATGTCACTCATTATACTCTGTCGAATGTCATTGAATGTCTT
TACATGGGCTTGTATGCCTATGAAAATTGTAATACAACTTTCAGCAACGGATCTCTTGGC
TCTCGCATCGATGAAGAACGCAGCGAAATGCGATAAGTAATGTGAATTGCAGAATTCAGT
GAATCATCGAATCTTTGAACGCATCTTGCGCTCCTTGGTATTCCGAGGAGCATGCCTGTT
TGAGTGTCATTAAATTCTCAACTCTCTTATACTTTTTTGTAAAAGAGAGCTTGGACTGTG
GAGGCTTGCTGGCCACTTTTTGGGGTCAGCTCCTCTGAAATGCATTAGCGGAACCGTTTG
CGATCTGCCACAAGTGTGATAAGTTATCTACACTGGCGAGGGGATTGCTCTCTGTAATGT
TCAGCTTCTAATTGTCTCTACTTTGTGAGACTACTTTTGAATGCTTGACCTCAAATCAGG
TAGGACTACCCGCTGAACTTAA
>AC10-1
TTTCCGTAGGTGAACCTGCGGAAGGATCATTATTGAATTATGTTTCTAGATAGGTTGTAG
CTGGCTCTTTTAGAGCATGTGCACGCCTGTTTGGACTTCATTTTCATCCACCTGTGCACC
TATTGTAGTCTTTGGTTGGGTTAGGAGGAAGTGATCATTGTATCAGCATCTGCTGGGAGT
GAGGACTTGCATTGTGAAAGCTTTGCTGTCCTTGATGTGATCATGGAATCTTTTTC----

-------TCACTAGAGTCTATGTCACTCATTATACTCTGTCGAATGTCATTGAATGTCTT
TACATGGGCTTGTATGCCTATGAAAATTGTAATACAACTTTCAGCAACGGATCTCTTGGC
TCTCGCATCGATGAAGAACGCAGCGAAATGCGATAAGTAATGTGAATTGCAGAATTCAGT
GAATCATCGAATCTTTGAACGCATCTTGCGCTCCTTGGTATTCCGAGGAGCATGCCTGTT
TGAGTGTCATTAAATTCTCAACTCTCTTATACTTTTTTGTAAAAGAGAGCTTGGACTGTG
GAGGCTTGCTGGCCACTTTTTGGGGTCAGCTCCTCTGAAATGCATTAGCGGAACCGTTTG
CGATCTGCCACAAGTGTGATAAGTTATCTACACTGGCGAGGGGATTGCTCTCTGTAATGT
TCAGCTTCTAATTGTCTCTACTTTGTGAGACTACTTTTGAATGCTTGACCTCAAATCAGG
TAGGACTACCCGCTGAACTTAA
>AC10-13
TTTCCGTAGGTGAACCTGCGGAAGGATCATTATTGAATTATGTTTCTAGATAGGTTGTAG
CTGGCTCTTTTAGAGCATGTGCACGCCTGTTTGGACTTCATTTTCATCCACCTGTGCACC
TATTGTAGTCTTTGGTTGGGTTAGGAGGAAGTGATCATTGTATCAGCATCTGCTGGGAGT
GAGGACTTGCATTGTGAAAGCTTTGCTGTCCTTGATGTGATCATGGAATCTTTTTC----

-------TCACTAGAGTCTATGTCACTCATTATACTCTGTCGAATGTCATTGAATGTCTT
TACATGGGCTTGTATGCCTATGAAAATTGTAATACAACTTTCAGCAACGGATCTCTTGGC
TCTCGCATCGATGAAGAACGCAGCGAAATGCGATAAGTAATGTGAATTGCAGAATTCAGT
GAATCATCGAATCTTTGAACGCATCTTGCGCTCCTTGGTATTCCGAGGAGCATGCCTGTT
TGAGTGTCATTAAATTCTCAACTCTCTTATACTTTTTTGTAAAAGAGAGCTTGGACTGTG
GAGGCTTGCTGGCCACTTTTTGGGGTCAGCTCCTCTGAAATGCATTAGCGGAACCGTTTG
CGATCTGCCACAAGTGTGATAAGTTATCTACACTGGCGAGGGGATTGCTCTCTGTAATGT
TCAGCTTCTAATTGTCTCTACTTTGTGAGACTACTTTTGAATGCTTGACCTCAAATCAGG
TAGGACTACCCGCTGAACTTAA
>AC10-14
TTTCCGTAGGTGAACCTGCGGAAGGATCATTATTGAATTATGTTTCTAGATAGGTTGTAG
CTGGCTCTTTTAGAGCATGTGCACGCCTGTTTGGACTTCATTTTCATCCACCTGTGCACC
TATTGTAGTCTTTGGTTGGGTTAGGAGGAAGTGATCATTGTATCAGCATCTGCTGGGAGT
GAGGACTTGCATTGTGAAAGCTTTGCTGTCCTTGATGTGATCATGGAATCTTTTTC----

-------TCACTAGAGTCTATGTCACTCATTATACTCTGTCGAATGTCATTGAATGTCTT
TACATGGGCTTGTATGCCTATGAAAATTGTAATACAACTTTCAGCAACGGATCTCTTGGC
TCTCGCATCGATGAAGAACGCAGCGAAATGCGATAAGTAATGTGAATTGCAGAATTCAGT
GAATCATCGAATCTTTGAACGCATCTTGCGCTCCTTGGTATTCCGAGGAGCATGCCTGTT
TGAGTGTCATTAAATTCTCAACTCTCTTATACTTTTTTGTAAAAGAGAGCTTGGACTGTG
GAGGCTTGCTGGCCACTTTTTGGGGTCAGCTCCTCTGAAATGCATTAGCGGAACCGTTTG
CGATCTGCCACAAGTGTGATAAGTTATCTACACTGGCGAGGGGATTGCTCTCTGTAATGT
TCAGCTTCTAATTGTCTCTACTTTGTGAGACTACTTTTGAATGCTTGACCTCAAATCAGG
TAGGACTACCCGCTGAACTTAA
>AC10-15
TTTCCGTAGGTGAACCTGCGGAAGGATCATTATTGAATTATGTTTCTAGATAGGTTGTAG
CTGGCTCTTTTAGAGCATGTGCACGCCTGTTTGGACTTCATTTTCATCCACCTGTGCACC
TATTGTAGTCTTTGGTTGGGTTAGGAGGAAGTGATCATTGTATCAGCATCTGCTGGGAGT
GAGGACTTGCATTGTGAAAGCTTTGCTGTCCTTGATGTGATCATGGAATCTTTTTC----

-------TCACTAGAGTCTATGTCACTCATTATACTCTGTCGAATGTCATTGAATGTCTT
TACATGGGCTTGTATGCCTATGAAAATTGTAATACAACTTTCAGCAACGGATCTCTTGGC
TCTCGCATCGATGAAGAACGCAGCGAAATGCGATAAGTAATGTGAATTGCAGAATTCAGT
GAATCATCGAATCTTTGAACGCATCTTGCGCTCCTTGGTATTCCGAGGAGCATGCCTGTT
TGAGTGTCATTAAATTCTCAACTCTCTTATACTTTTTTGTAAAAGAGAGCTTGGACTGTG
GAGGCTTGCTGGCCACTTTTTGGGGTCAGCTCCTCTGAAATGCATTAGCGGAACCGTTTG
CGATCTGCCACAAGTGTGATAAGTTATCTACACTGGCGAGGGGATTGCTCTCTGTAATGT
TCAGCTTCTAATTGTCTCTACTTTGTGAGACTACTTTTGAATGCTTGACCTCAAATCAGG
TAGGACTACCCGCTGAACTTAA
>AC10-17
TTTCCGTAGGTGAACCTGCGGAAGGATCATTATTGAATTATGTTTCTAGATAGGTTGTAG
CTGGCTCTTTTAGAGCATGTGCACGCCTGTTTGGACTTCATTTTCATCCACCTGTGCACC
TATTGTAGTCTTTGGTTGGGTTAGGAGGAAGTGATCATTGTATCAGCATCTGCTGGGAGT

GAGGACTTGCATTGTGAAAGCTTTGCTGTCCTTGATGTGATCATGGAATCTTTTTC----

-------TCACTAGAGTCTATGTCACTCATTATACTCTGTCGAATGTCATTGAATGTCTT
TACATGGGCTTGTATGCCTATGAAAATTGTAATACAACTTTCAGCAACGGATCTCTTGGC
TCTCGCATCGATGAAGAACGCAGCGAAATGCGATAAGTAATGTGAATTGCAGAATTCAGT
GAATCATCGAATCTTTGAACGCATCTTGCGCTCCTTGGTATTCCGAGGAGCATGCCTGTT
TGAGTGTCATTAAATTCTCAACTCTCTTATACTTTTTTGTAAAAGAGAGCTTGGACTGTG
GAGGCTTGCTGGCCACTTTTTGGGGTCAGCTCCTCTGAAATGCATTAGCGGAACCGTTTG
CGATCTGCCACAAGTGTGATAAGTTATCTACACTGGCGAGGGGATTGCTCTCTGTAATGT
TCAGCTTCTAATTGTCTCTACTTTGTGAGACTACTTTTGAATGCTTGACCTCAAATCAGG
TAGGACTACCCGCTGAACTTAA
>AC10-18
TTTCCGTAGGTGAACCTGCGGAAGGATCATTATTGAATTATGTTTCTAGATAGGTTGTAG
CTGGCTCTTTTAGAGCATGTGCACGCCTGTTTGGACTTCATTTTCATCCACCTGTGCACC
TATTGTAGTCTTTGGTTGGGTTAGGAGGAAGTGATCATTGTATCAGCATCTGCTGGGAGT
GAGGACTTGCATTGTGAAAGCTTTGCTGTCCTTGATGTGATCATGGAATCTTTTTC----

-------TCACTAGAGTCTATGTCACTCATTATACTCTGTCGAATGTCATTGAATGTCTT
TACATGGGCTTGTATGCCTATGAAAATTGTAATACAACTTTCAGCAACGGATCTCTTGGC
TCTCGCATCGATGAAGAACGCAGCGAAATGCGATAAGTAATGTGAATTGCAGAATTCAGT
GAATCATCGAATCTTTGAACGCATCTTGCGCTCCTTGGTATTCCGAGGAGCATGCCTGTT
TGAGTGTCATTAAATTCTCAACTCTCTTATACTTTTTTGTAAAAGAGAGCTTGGACTGTG
GAGGCTTGCTGGCCACTTTTTGGGGTCAGCTCCTCTGAAATGCATTAGCGGAACCGTTTG
CGATCTGCCACAAGTGTGATAAGTTATCTACACTGGCGAGGGGATTGCTCTCTGTAATGT
TCAGCTTCTAATTGTCTCTACTTTGTGAGACTACTTTTGAATGCTTGACCTCAAATCAGG
TAGGACTACCCGCTGAACTTAA
>AC10-23
TTTCCGTAGGTGAACCTGCGGAAGGATCATTATTGAATTATGTTTCTAGATAGGTTGTAG

CTGGCTCTTTTAGAGCATGTGCACGCCTGTTTGGACTTCATTTTCATCCACCTGTGCACC
TATTGTAGTCTTTGGTTGGGTTAGGAGGAAGTGATCATTGTATCAGCATCTGCTGGGAGT
GAGGACTTGCATTGTGAAAGCTTTGCTGTCCTTGATGTGATCATGGAATCTTTTTC----

-------TCACTAGAGTCTATGTCACTCATTATACTCTGTCGAATGTCATTGAATGTCTT
TACATGGGCTTGTATGCCTATGAAAATTGTAATACAACTTTCAGCAACGGATCTCTTGGC
TCTCGCATCGATGAAGAACGCAGCGAAATGCGATAAGTAATGTGAATTGCAGAATTCAGT
GAATCATCGAATCTTTGAACGCATCTTGCGCTCCTTGGTATTCCGAGGAGCATGCCTGTT
TGAGTGTCATTAAATTCTCAACTCTCTTATACTTTTTTGTAAAAGAGAGCTTGGACTGTG
GAGGCTTGCTGGCCACTTTTTGGGGTCAGCTCCTCTGAAATGCATTAGCGGAACCGTTTG
CGATCTGCCACAAGTGTGATAAGTTATCTACACTGGCGAGGGGATTGCTCTCTGTAATGT
TCAGCTTCTAATTGTCTCTACTTTGTGAGACTACTTTTGAATGCTTGACCTCAAATCAGG
TAGGACTACCCGCTGAACTTAA
>AC10-24
TTTCCGTAGGTGAACCTGCGGAAGGATCATTATTGAATTATGTTTCTAGATAGGTTGTAG
CTGGCTCTTTTAGAGCATGTGCACGCCTGTTTGGACTTCATTTTCATCCACCTGTGCACC
TATTGTAGTCTTTGGTTGGGTTAGGAGGAAGTGATCATTGTATCAGCATCTGCTGGGAGT
GAGGACTTGCATTGTGAAAGCTTTGCTGTCCTTGATGTGATCATGGAATCTTTTTC----

-------TCACTAGAGTCTATGTCACTCATTATACTCTGTCGAATGTCATTGAATGTCTT
TACATGGGCTTGTATGCCTATGAAAATTGTAATACAACTTTCAGCAACGGATCTCTTGGC
TCTCGCATCGATGAAGAACGCAGCGAAATGCGATAAGTAATGTGAATTGCAGAATTCAGT
GAATCATCGAATCTTTGAACGCATCTTGCGCTCCTTGGTATTCCGAGGAGCATGCCTGTT
TGAGTGTCATTAAATTCTCAACTCTCTTATACTTTTTTGTAAAAGAGAGCTTGGACTGTG
GAGGCTTGCTGGCCACTTTTTGGGGTCAGCTCCTCTGAAATGCATTAGCGGAACCGTTTG
CGATCTGCCACAAGTGTGATAAGTTATCTACACTGGCGAGGGGATTGCTCTCTGTAATGT
TCAGCTTCTAATTGTCTCTACTTTGTGAGACTACTTTTGAATGCTTGACCTCAAATCAGG
TAGGACTACCCGCTGAACTTAA

>AC10-26
TTTCCGTAGGTGAACCTGCGGAAGGATCATTATTGAATTATGTTTCTAGATAGGTTGTAG
CTGGCTCTTTTAGAGCATGTGCACGCCTGTTTGGACTTCATTTTCATCCACCTGTGCACC
TATTGTAGTCTTTGGTTGGGTTAGGAGGAAGTGATCATTGTATCAGCATCTGCTGGGAGT
GAGGACTTGCATTGTGAAAGCTTTGCTGTCCTTGATGTGATCATGGAATCTTTTTC----

-------TCACTAGAGTCTATGTCACTCATTATACTCTGTCGAATGTCATTGAATGTCTT
TACATGGGCTTGTATGCCTATGAAAATTGTAATACAACTTTCAGCAACGGATCTCTTGGC
TCTCGCATCGATGAAGAACGCAGCGAAATGCGATAAGTAATGTGAATTGCAGAATTCAGT
GAATCATCGAATCTTTGAACGCATCTTGCGCTCCTTGGTATTCCGAGGAGCATGCCTGTT
TGAGTGTCATTAAATTCTCAACTCTCTTATACTTTTTTGTAAAAGAGAGCTTGGACTGTG
GAGGCTTGCTGGCCACTTTTTGGGGTCAGCTCCTCTGAAATGCATTAGCGGAACCGTTTG
CGATCTGCCACAAGTGTGATAAGTTATCTACACTGGCGAGGGGATTGCTCTCTGTAATGT
TCAGCTTCTAATTGTCTCTACTTTGTGAGACTACTTTTGAATGCTTGACCTCAAATCAGG
TAGGACTACCCGCTGAACTTAA
>AC10-27
TTTCCGTAGGTGAACCTGCGGAAGGATCATTATTGAATTATGTTTCTAGATAGGTTGTAG
CTGGCTCTTTTAGAGCATGTGCACGCCTGTTTGGACTTCATTTTCATCCACCTGTGCACC
TATTGTAGTCTTTGGTTGGGTTAGGAGGAAGTGATCATTGTATCAGCATCTGCTGGGAGT
GAGGACTTGCATTGTGAAAGCTTTGCTGTCCTTGATGTGATCATGGAATCTTTTTC----

-------TCACTAGAGTCTATGTCACTCATTATACTCTGTCGAATGTCATTGAATGTCTT
TACATGGGCTTGTATGCCTATGAAAATTGTAATACAACTTTCAGCAACGGATCTCTTGGC
TCTCGCATCGATGAAGAACGCAGCGAAATGCGATAAGTAATGTGAATTGCAGAATTCAGT
GAATCATCGAATCTTTGAACGCATCTTGCGCTCCTTGGTATTCCGAGGAGCATGCCTGTT
TGAGTGTCATTAAATTCTCAACTCTCTTATACTTTTTTGTAAAAGAGAGCTTGGACTGTG
GAGGCTTGCTGGCCACTTTTTGGGGTCAGCTCCTCTGAAATGCATTAGCGGAACCGTTTG
CGATCTGCCACAAGTGTGATAAGTTATCTACACTGGCGAGGGGATTGCTCTCTGTAATGT

TCAGCTTCTAATTGTCTCTACTTTGTGAGACTACTTTTGAATGCTTGACCTCAAATCAGG
TAGGACTACCCGCTGAACTTAA
>AC10-28
TTTCCGTAGGTGAACCTGCGGAAGGATCATTATTGAATTATGTTTCTAGATAGGTTGTAG
CTGGCTCTTTTAGAGCATGTGCACGCCTGTTTGGACTTCATTTTCATCCACCTGTGCACC
TATTGTAGTCTTTGGTTGGGTTAGGAGGAAGTGATCATTGTATCAGCATCTGCTGGGAGT
GAGGACTTGCATTGTGAAAGCTTTGCTGTCCTTGATGTGATCATGGAATCTTTTTC----

-------TCACTAGAGTCTATGTCACTCATTATACTCTGTCGAATGTCATTGAATGTCTT
TACATGGGCTTGTATGCCTATGAAAATTGTAATACAACTTTCAGCAACGGATCTCTTGGC
TCTCGCATCGATGAAGAACGCAGCGAAATGCGATAAGTAATGTGAATTGCAGAATTCAGT
GAATCATCGAATCTTTGAACGCATCTTGCGCTCCTTGGTATTCCGAGGAGCATGCCTGTT
TGAGTGTCATTAAATTCTCAACTCTCTTATACTTTTTTGTAAAAGAGAGCTTGGACTGTG
GAGGCTTGCTGGCCACTTTTTGGGGTCAGCTCCTCTGAAATGCATTAGCGGAACCGTTTG
CGATCTGCCACAAGTGTGATAAGTTATCTACACTGGCGAGGGGATTGCTCTCTGTAATGT
TCAGCTTCTAATTGTCTCTACTTTGTGAGACTACTTTTGAATGCTTGACCTCAAATCAGG
TAGGACTACCCGCTGAACTTAA
>AC10-39
TTTCCGTAGGTGAACCTGCGGAAGGATCATTATTGAATTATGTTTCTAGATAGGTTGTAG
CTGGCTCTTTTAGAGCATGTGCACGCCTGTTTGGACTTCATTTTCATCCACCTGTGCACC
TATTGTAGTCTTTGGTTGGGTTAGGAGGAAGTGATCATTGTATCAGCATCTGCTGGGAGT
GAGGACTTGCATTGTGAAAGCTTTGCTGTCCTTGATGTGATCATGGAATCTTTTTC----

-------TCACTAGAGTCTATGTCACTCATTATACTCTGTCGAATGTCATTGAATGTCTT
TACATGGGCTTGTATGCCTATGAAAATTGTAATACAACTTTCAGCAACGGATCTCTTGGC
TCTCGCATCGATGAAGAACGCAGCGAAATGCGATAAGTAATGTGAATTGCAGAATTCAGT
GAATCATCGAATCTTTGAACGCATCTTGCGCTCCTTGGTATTCCGAGGAGCATGCCTGTT
TGAGTGTCATTAAATTCTCAACTCTCTTATACTTTTTTGTAAAAGAGAGCTTGGACTGTG

GAGGCTTGCTGGCCACTTTTTGGGGTCAGCTCCTCTGAAATGCATTAGCGGAACCGTTTG
CGATCTGCCACAAGTGTGATAAGTTATCTACACTGGCGAGGGGATTGCTCTCTGTAATGT
TCAGCTTCTAATTGTCTCTACTTTGTGAGACTACTTTTGAATGCTTGACCTCAAATCAGG
TAGGACTACCCGCTGAACTTAA
>AC10-45
TTTCCGTAGGTGAACCTGCGGAAGGATCATTATTGAATTATGTTTCTAGATAGGTTGTAG
CTGGCTCTTTTAGAGCATGTGCACGCCTGTTTGGACTTCATTTTCATCCACCTGTGCACC
TATTGTAGTCTTTGGTTGGGTTAGGAGGAAGTGATCATTGTATCAGCATCTGCTGGGAGT
GAGGACTTGCATTGTGAAAGCTTTGCTGTCCTTGATGTGATCATGGAATCTTTTTC----

-------TCACTAGAGTCTATGTCACTCATTATACTCTGTCGAATGTCATTGAATGTCTT
TACATGGGCTTGTATGCCTATGAAAATTGTAATACAACTTTCAGCAACGGATCTCTTGGC
TCTCGCATCGATGAAGAACGCAGCGAAATGCGATAAGTAATGTGAATTGCAGAATTCAGT
GAATCATCGAATCTTTGAACGCATCTTGCGCTCCTTGGTATTCCGAGGAGCATGCCTGTT
TGAGTGTCATTAAATTCTCAACTCTCTTATACTTTTTTGTAAAAGAGAGCTTGGACTGTG
GAGGCTTGCTGGCCACTTTTTGGGGTCAGCTCCTCTGAAATGCATTAGCGGAACCGTTTG
CGATCTGCCACAAGTGTGATAAGTTATCTACACTGGCGAGGGGATTGCTCTCTGTAATGT
TCAGCTTCTAATTGTCTCTACTTTGTGAGACTACTTTTGAATGCTTGACCTCAAATCAGG
TAGGACTACCCGCTGAACTTAA
>AC10-58
TTTCCGTAGGTGAACCTGCGGAAGGATCATTATTGAATTATGTTTCTAGATAGGTTGTAG
CTGGCTCTTTTAGAGCATGTGCACGCCTGTTTGGACTTCATTTTCATCCACCTGTGCACC
TATTGTAGTCTTTGGTTGGGTTAGGAGGAAGTGATCATTGTATCAGCATCTGCTGGGAGT
GAGGACTTGCATTGTGAAAGCTTTGCTGTCCTTGATGTGATCATGGAATCTTTTTC----

-------TCACTAGAGTCTATGTCACTCATTATACTCTGTCGAATGTCATTGAATGTCTT
TACATGGGCTTGTATGCCTATGAAAATTGTAATACAACTTTCAGCAACGGATCTCTTGGC
TCTCGCATCGATGAAGAACGCAGCGAAATGCGATAAGTAATGTGAATTGCAGAATTCAGT

GAATCATCGAATCTTTGAACGCATCTTGCGCTCCTTGGTATTCCGAGGAGCATGCCTGTT
TGAGTGTCATTAAATTCTCAACTCTCTTATACTTTTTTGTAAAAGAGAGCTTGGACTGTG
GAGGCTTGCTGGCCACTTTTTGGGGTCAGCTCCTCTGAAATGCATTAGCGGAACCGTTTG
CGATCTGCCACAAGTGTGATAAGTTATCTACACTGGCGAGGGGATTGCTCTCTGTAATGT
TCAGCTTCTAATTGTCTCTACTTTGTGAGACTACTTTTGAATGCTTGACCTCAAATCAGG
TAGGACTACCCGCTGAACTTAA
>AC11-3
TTTCCGTAGGTGAACCTGCGGAAGGATCATTATTGAATTATGTTTCTAGATAGGTTGTAG
CTGGCTCTTTTAGAGCATGTGCACGCCTGTTTGGACTTCATTTTCATCCACCTGTGCACC
TATTGTAGTCTTTGGTTGGGTTAGGAGGAAGTGATCATTGTATCAGCATCTGCTGGGAGT
GAGGACTTGCATTGTGAAAGCTTTGCTGTCCTTGATGTGATCATGGAATCTTTTTC----

-------TCACTAGAGTCTATGTCACTCATTATACTCTGTCGAATGTCATTGAATGTCTT
TACATGGGCTTGTATGCCTATGAAAATTGTAATACAACTTTCAGCAACGGATCTCTTGGC
TCTCGCATCGATGAAGAACGCAGCGAAATGCGATAAGTAATGTGAATTGCAGAATTCAGT
GAATCATCGAATCTTTGAACGCATCTTGCGCTCCTTGGTATTCCGAGGAGCATGCCTGTT
TGAGTGTCATTAAATTCTCAACTCTCTTATACTTTTTTGTAAAAGAGAGCTTGGACTGTG
GAGGCTTGCTGGCCACTTTTTGGGGTCAGCTCCTCTGAAATGCATTAGCGGAACCGTTTG
CGATCTGCCACAAGTGTGATAAGTTATCTACACTGGCGAGGGGATTGCTCTCTGTAATGT
TCAGCTTCTAATTGTCTCTACTTTGTGAGACTACTTTTGAATGCTTGACCTCAAATCAGG
TAGGACTACCCGCTGAACTTAA
>AC11-4
TTTCCGTAGGTGAACCTGCGGAAGGATCATTATTGAATTATGTTTCTAGATAGGTTGTAG
CTGGCTCTTTTAGAGCATGTGCACGCCTGTTTGGACTTCATTTTCATCCACCTGTGCACC
TATTGTAGTCTTTGGTTGGGTTAGGAGGAAGTGATCATTGTATCAGCATCTGCTGGGAGT
GAGGACTTGCATTGTGAAAGCTTTGCTGTCCTTGATGTGATCATGGAATCTTTTTC----

-------TCACTAGAGTCTATGTCACTCATTATACTCTGTCGAATGTCATTGAATGTCTT

TACATGGGCTTGTATGCCTATGAAAATTGTAATACAACTTTCAGCAACGGATCTCTTGGC
TCTCGCATCGATGAAGAACGCAGCGAAATGCGATAAGTAATGTGAATTGCAGAATTCAGT
GAATCATCGAATCTTTGAACGCATCTTGCGCTCCTTGGTATTCCGAGGAGCATGCCTGTT
TGAGTGTCATTAAATTCTCAACTCTCTTATACTTTTTTGTAAAAGAGAGCTTGGACTGTG
GAGGCTTGCTGGCCACTTTTTGGGGTCAGCTCCTCTGAAATGCATTAGCGGAACCGTTTG
CGATCTGCCACAAGTGTGATAAGTTATCTACACTGGCGAGGGGATTGCTCTCTGTAATGT
TCAGCTTCTAATTGTCTCTACTTTGTGAGACTACTTTTGAATGCTTGACCTCAAATCAGG
TAGGACTACCCGCTGAACTTAA
>AC11-9
TTTCCGTAGGTGAACCTGCGGAAGGATCATTATTGAATTATGTTTCTAGATAGGTTGTAG
CTGGCTCTTTTAGAGCATGTGCACGCCTGTTTGGACTTCATTTTCATCCACCTGTGCACC
TATTGTAGTCTTTGGTTGGGTTAGGAGGAAGTGATCATTGTATCAGCATCTGCTGGGAGT
GAGGACTTGCATTGTGAAAGCTTTGCTGTCCTTGATGTGATCATGGAATCTTTTTC----

-------TCACTAGAGTCTATGTCACTCATTATACTCTGTCGAATGTCATTGAATGTCTT
TACATGGGCTTGTATGCCTATGAAAATTGTAATACAACTTTCAGCAACGGATCTCTTGGC
TCTCGCATCGATGAAGAACGCAGCGAAATGCGATAAGTAATGTGAATTGCAGAATTCAGT
GAATCATCGAATCTTTGAACGCATCTTGCGCTCCTTGGTATTCCGAGGAGCATGCCTGTT
TGAGTGTCATTAAATTCTCAACTCTCTTATACTTTTTTGTAAAAGAGAGCTTGGACTGTG
GAGGCTTGCTGGCCACTTTTTGGGGTCAGCTCCTCTGAAATGCATTAGCGGAACCGTTTG
CGATCTGCCACAAGTGTGATAAGTTATCTACACTGGCGAGGGGATTGCTCTCTGTAATGT
TCAGCTTCTAATTGTCTCTACTTTGTGAGACTACTTTTGAATGCTTGACCTCAAATCAGG
TAGGACTACCCGCTGAACTTAA
>AC11-21
TTTCCGTAGGTGAACCTGCGGAAGGATCATTATTGAATTATGTTTCTAGATAGGTTGTAG
CTGGCTCTTTTAGAGCATGTGCACGCCTGTTTGGACTTCATTTTCATCCACCTGTGCACC
TATTGTAGTCTTTGGTTGGGTTAGGAGGAAGTGATCATTGTATCAGCATCTGCTGGGAGT
GAGGACTTGCATTGTGAAAGCTTTGCTGTCCTTGATGTGATCATGGAATCTTTTTC----


```
------------------------------------------------------------
-------TCACTAGAGTCTATGTCACTCATTATACTCTGTCGAATGTCATTGAATGTCTT
TACATGGGCTTGTATGCCTATGAAAATTGTAATACAACTTTCAGCAACGGATCTCTTGGC
TCTCGCATCGATGAAGAACGCAGCGAAATGCGATAAGTAATGTGAATTGCAGAATTCAGT
GAATCATCGAATCTTTGAACGCATCTTGCGCTCCTTGGTATTCCGAGGAGCATGCCTGTT
TGAGTGTCATTAAATTCTCAACTCTCTTATACTTTTTTGTAAAAGAGAGCTTGGACTGTG
GAGGCTTGCTGGCCACTTTTTGGGGTCAGCTCCTCTGAAATGCATTAGCGGAACCGTTTG
CGATCTGCCACAAGTGTGATAAGTTATCTACACTGGCGAGGGGATTGCTCTCTGTAATGT
TCAGCTTCTAATTGTCTCTACTTTGTGAGACTACTTTTGAATGCTTGACCTCAAATCAGG
TAGGACTACCCGCTGAACTTAA
>AC11-22
TTTCCGTAGGTGAACCTGCGGAAGGATCATTATTGAATTATGTTTCTAGATAGGTTGTAG
CTGGCTCTTTTAGAGCATGTGCACGCCTGTTTGGACTTCATTTTCATCCACCTGTGCACC
TATTGTAGTCTTTGGTTGGGTTAGGAGGAAGTGATCATTGTATCAGCATCTGCTGGGAGT
GAGGACTTGCATTGTGAAAGCTTTGCTGTCCTTGATGTGATCATGGAATCTTTTTC----
------------------------------------------------------------
------------------------------------------------------------
------------------------------------------------------------
------------------------------------------------------------
------------------------------------------------------------
------------------------------------------------------------
------------------------------------------------------------
------------------------------------------------------------
------------------------------------------------------------
------------------------------------------------------------
------------------------------------------------------------
------------------------------------------------------------
-------TCACTAGAGTCTATGTCACTCATTATACTCTGTCGAATGTCATTGAATGTCTT
TACATGGGCTTGTATGCCTATGAAAATTGTAATACAACTTTCAGCAACGGATCTCTTGGC
TCTCGCATCGATGAAGAACGCAGCGAAATGCGATAAGTAATGTGAATTGCAGAATTCAGT
GAATCATCGAATCTTTGAACGCATCTTGCGCTCCTTGGTATTCCGAGGAGCATGCCTGTT
TGAGTGTCATTAAATTCTCAACTCTCTTATACTTTTTTGTAAAAGAGAGCTTGGACTGTG
GAGGCTTGCTGGCCACTTTTTGGGGTCAGCTCCTCTGAAATGCATTAGCGGAACCGTTTG
CGATCTGCCACAAGTGTGATAAGTTATCTACACTGGCGAGGGGATTGCTCTCTGTAATGT
TCAGCTTCTAATTGTCTCTACTTTGTGAGACTACTTTTGAATGCTTGACCTCAAATCAGG
TAGGACTACCCGCTGAACTTAA
>AC11-24
TTTCCGTAGGTGAACCTGCGGAAGGATCATTATTGAATTATGTTTCTAGATAGGTTGTAG
CTGGCTCTTTTAGAGCATGTGCACGCCTGTTTGGACTTCATTTTCATCCACCTGTGCACC
TATTGTAGTCTTTGGTTGGGTTAGGAGGAAGTGATCATTGTATCAGCATCTGCTGGGAGT
GAGGACTTGCATTGTGAAAGCTTTGCTGTCCTTGATGTGATCATGGAATCTTTTTC----
------------------------------------------------------------
------------------------------------------------------------
------------------------------------------------------------
------------------------------------------------------------
------------------------------------------------------------
------------------------------------------------------------
------------------------------------------------------------
------------------------------------------------------------
------------------------------------------------------------
```


-------TCACTAGAGTCTATGTCACTCATTATACTCTGTCGAATGTCATTGAATGTCTT
TACATGGGCTTGTATGCCTATGAAAATTGTAATACAACTTTCAGCAACGGATCTCTTGGC
TCTCGCATCGATGAAGAACGCAGCGAAATGCGATAAGTAATGTGAATTGCAGAATTCAGT
GAATCATCGAATCTTTGAACGCATCTTGCGCTCCTTGGTATTCCGAGGAGCATGCCTGTT
TGAGTGTCATTAAATTCTCAACTCTCTTATACTTTTTTGTAAAAGAGAGCTTGGACTGTG
GAGGCTTGCTGGCCACTTTTTGGGGTCAGCTCCTCTGAAATGCATTAGCGGAACCGTTTG
CGATCTGCCACAAGTGTGATAAGTTATCTACACTGGCGAGGGGATTGCTCTCTGTAATGT
TCAGCTTCTAATTGTCTCTACTTTGTGAGACTACTTTTGAATGCTTGACCTCAAATCAGG
TAGGACTACCCGCTGAACTTAA
>AC11-26
TTTCCGTAGGTGAACCTGCGGAAGGATCATTATTGAATTATGTTTCTAGATAGGTTGTAG
CTGGCTCTTTTAGAGCATGTGCACGCCTGTTTGGACTTCATTTTCATCCACCTGTGCACC
TATTGTAGTCTTTGGTTGGGTTAGGAGGAAGTGATCATTGTATCAGCATCTGCTGGGAGT
GAGGACTTGCATTGTGAAAGCTTTGCTGTCCTTGATGTGATCATGGAATCTTTTTC----

-------TCACTAGAGTCTATGTCACTCATTATACTCTGTCGAATGTCATTGAATGTCTT
TACATGGGCTTGTATGCCTATGAAAATTGTAATACAACTTTCAGCAACGGATCTCTTGGC
TCTCGCATCGATGAAGAACGCAGCGAAATGCGATAAGTAATGTGAATTGCAGAATTCAGT
GAATCATCGAATCTTTGAACGCATCTTGCGCTCCTTGGTATTCCGAGGAGCATGCCTGTT
TGAGTGTCATTAAATTCTCAACTCTCTTATACTTTTTTGTAAAAGAGAGCTTGGACTGTG
GAGGCTTGCTGGCCACTTTTTGGGGTCAGCTCCTCTGAAATGCATTAGCGGAACCGTTTG
CGATCTGCCACAAGTGTGATAAGTTATCTACACTGGCGAGGGGATTGCTCTCTGTAATGT
TCAGCTTCTAATTGTCTCTACTTTGTGAGACTACTTTTGAATGCTTGACCTCAAATCAGG
TAGGACTACCCGCTGAACTTAA
>AC11-30
TTTCCGTAGGTGAACCTGCGGAAGGATCATTATTGAATTATGTTTCTAGATAGGTTGTAG
CTGGCTCTTTTAGAGCATGTGCACGCCTGTTTGGACTTCATTTTCATCCACCTGTGCACC
TATTGTAGTCTTTGGTTGGGTTAGGAGGAAGTGATCATTGTATCAGCATCTGCTGGGAGT
GAGGACTTGCATTGTGAAAGCTTTGCTGTCCTTGATGTGATCATGGAATCTTTTTC----

-------TCACTAGAGTCTATGTCACTCATTATACTCTGTCGAATGTCATTGAATGTCTT
TACATGGGCTTGTATGCCTATGAAAATTGTAATACAACTTTCAGCAACGGATCTCTTGGC
TCTCGCATCGATGAAGAACGCAGCGAAATGCGATAAGTAATGTGAATTGCAGAATTCAGT
GAATCATCGAATCTTTGAACGCATCTTGCGCTCCTTGGTATTCCGAGGAGCATGCCTGTT
TGAGTGTCATTAAATTCTCAACTCTCTTATACTTTTTTGTAAAAGAGAGCTTGGACTGTG
GAGGCTTGCTGGCCACTTTTTGGGGTCAGCTCCTCTGAAATGCATTAGCGGAACCGTTTG
CGATCTGCCACAAGTGTGATAAGTTATCTACACTGGCGAGGGGATTGCTCTCTGTAATGT
TCAGCTTCTAATTGTCTCTACTTTGTGAGACTACTTTTGAATGCTTGACCTCAAATCAGG
TAGGACTACCCGCTGAACTTAA
>AC11-31
TTTCCGTAGGTGAACCTGCGGAAGGATCATTATTGAATTATGTTTCTAGATAGGTTGTAG
CTGGCTCTTTTAGAGCATGTGCACGCCTGTTTGGACTTCATTTTCATCCACCTGTGCACC
TATTGTAGTCTTTGGTTGGGTTAGGAGGAAGTGATCATTGTATCAGCATCTGCTGGGAGT
GAGGACTTGCATTGTGAAAGCTTTGCTGTCCTTGATGTGATCATGGAATCTTTTTC----

-------TCACTAGAGTCTATGTCACTCATTATACTCTGTCGAATGTCATTGAATGTCTT
TACATGGGCTTGTATGCCTATGAAAATTGTAATACAACTTTCAGCAACGGATCTCTTGGC
TCTCGCATCGATGAAGAACGCAGCGAAATGCGATAAGTAATGTGAATTGCAGAATTCAGT
GAATCATCGAATCTTTGAACGCATCTTGCGCTCCTTGGTATTCCGAGGAGCATGCCTGTT
TGAGTGTCATTAAATTCTCAACTCTCTTATACTTTTTTGTAAAAGAGAGCTTGGACTGTG
GAGGCTTGCTGGCCACTTTTTGGGGTCAGCTCCTCTGAAATGCATTAGCGGAACCGTTTG
CGATCTGCCACAAGTGTGATAAGTTATCTACACTGGCGAGGGGATTGCTCTCTGTAATGT
TCAGCTTCTAATTGTCTCTACTTTGTGAGACTACTTTTGAATGCTTGACCTCAAATCAGG
TAGGACTACCCGCTGAACTTAA
>AC11-32
TTTCCGTAGGTGAACCTGCGGAAGGATCATTATTGAATTATGTTTCTAGATAGGTTGTAG
CTGGCTCTTTTAGAGCATGTGCACGCCTGTTTGGACTTCATTTTCATCCACCTGTGCACC
TATTGTAGTCTTTGGTTGGGTTAGGAGGAAGTGATCATTGTATCAGCATCTGCTGGGAGT
GAGGACTTGCATTGTGAAAGCTTTGCTGTCCTTGATGTGATCATGGAATCTTTTTC----

-------TCACTAGAGTCTATGTCACTCATTATACTCTGTCGAATGTCATTGAATGTCTT
TACATGGGCTTGTATGCCTATGAAAATTGTAATACAACTTTCAGCAACGGATCTCTTGGC
TCTCGCATCGATGAAGAACGCAGCGAAATGCGATAAGTAATGTGAATTGCAGAATTCAGT
GAATCATCGAATCTTTGAACGCATCTTGCGCTCCTTGGTATTCCGAGGAGCATGCCTGTT
TGAGTGTCATTAAATTCTCAACTCTCTTATACTTTTTTGTAAAAGAGAGCTTGGACTGTG
GAGGCTTGCTGGCCACTTTTTGGGGTCAGCTCCTCTGAAATGCATTAGCGGAACCGTTTG
CGATCTGCCACAAGTGTGATAAGTTATCTACACTGGCGAGGGGATTGCTCTCTGTAATGT
TCAGCTTCTAATTGTCTCTACTTTGTGAGACTACTTTTGAATGCTTGACCTCAAATCAGG
TAGGACTACCCGCTGAACTTAA
>AC11-38
TTTCCGTAGGTGAACCTGCGGAAGGATCATTATTGAATTATGTTTCTAGATAGGTTGTAG
CTGGCTCTTTTAGAGCATGTGCACGCCTGTTTGGACTTCATTTTCATCCACCTGTGCACC
TATTGTAGTCTTTGGTTGGGTTAGGAGGAAGTGATCATTGTATCAGCATCTGCTGGGAGT
GAGGACTTGCATTGTGAAAGCTTTGCTGTCCTTGATGTGATCATGGAATCTTTTTC----

-------TCACTAGAGTCTATGTCACTCATTATACTCTGTCGAATGTCATTGAATGTCTT
TACATGGGCTTGTATGCCTATGAAAATTGTAATACAACTTTCAGCAACGGATCTCTTGGC
TCTCGCATCGATGAAGAACGCAGCGAAATGCGATAAGTAATGTGAATTGCAGAATTCAGT
GAATCATCGAATCTTTGAACGCATCTTGCGCTCCTTGGTATTCCGAGGAGCATGCCTGTT
TGAGTGTCATTAAATTCTCAACTCTCTTATACTTTTTTGTAAAAGAGAGCTTGGACTGTG
GAGGCTTGCTGGCCACTTTTTGGGGTCAGCTCCTCTGAAATGCATTAGCGGAACCGTTTG
CGATCTGCCACAAGTGTGATAAGTTATCTACACTGGCGAGGGGATTGCTCTCTGTAATGT
TCAGCTTCTAATTGTCTCTACTTTGTGAGACTACTTTTGAATGCTTGACCTCAAATCAGG
TAGGACTACCCGCTGAACTTAA
>AC11-41
TTTCCGTAGGTGAACCTGCGGAAGGATCATTATTGAATTATGTTTCTAGATAGGTTGTAG
CTGGCTCTTTTAGAGCATGTGCACGCCTGTTTGGACTTCATTTTCATCCACCTGTGCACC
TATTGTAGTCTTTGGTTGGGTTAGGAGGAAGTGATCATTGTATCAGCATCTGCTGGGAGT
GAGGACTTGCATTGTGAAAGCTTTGCTGTCCTTGATGTGATCATGGAATCTTTTTC----

-------TCACTAGAGTCTATGTCACTCATTATACTCTGTCGAATGTCATTGAATGTCTT
TACATGGGCTTGTATGCCTATGAAAATTGTAATACAACTTTCAGCAACGGATCTCTTGGC
TCTCGCATCGATGAAGAACGCAGCGAAATGCGATAAGTAATGTGAATTGCAGAATTCAGT
GAATCATCGAATCTTTGAACGCATCTTGCGCTCCTTGGTATTCCGAGGAGCATGCCTGTT
TGAGTGTCATTAAATTCTCAACTCTCTTATACTTTTTTGTAAAAGAGAGCTTGGACTGTG
GAGGCTTGCTGGCCACTTTTTGGGGTCAGCTCCTCTGAAATGCATTAGCGGAACCGTTTG
CGATCTGCCACAAGTGTGATAAGTTATCTACACTGGCGAGGGGATTGCTCTCTGTAATGT
TCAGCTTCTAATTGTCTCTACTTTGTGAGACTACTTTTGAATGCTTGACCTCAAATCAGG
TAGGACTACCCGCTGAACTTAA
>AC11-46
TTTCCGTAGGTGAACCTGCGGAAGGATCATTATTGAATTATGTTTCTAGATAGGTTGTAG
CTGGCTCTTTTAGAGCATGTGCACGCCTGTTTGGACTTCATTTTCATCCACCTGTGCACC
TATTGTAGTCTTTGGTTGGGTTAGGAGGAAGTGATCATTGTATCAGCATCTGCTGGGAGT
GAGGACTTGCATTGTGAAAGCTTTGCTGTCCTTGATGTGATCATGGAATCTTTTTC----

-------TCACTAGAGTCTATGTCACTCATTATACTCTGTCGAATGTCATTGAATGTCTT
TACATGGGCTTGTATGCCTATGAAAATTGTAATACAACTTTCAGCAACGGATCTCTTGGC
TCTCGCATCGATGAAGAACGCAGCGAAATGCGATAAGTAATGTGAATTGCAGAATTCAGT
GAATCATCGAATCTTTGAACGCATCTTGCGCTCCTTGGTATTCCGAGGAGCATGCCTGTT
TGAGTGTCATTAAATTCTCAACTCTCTTATACTTTTTTGTAAAAGAGAGCTTGGACTGTG
GAGGCTTGCTGGCCACTTTTTGGGGTCAGCTCCTCTGAAATGCATTAGCGGAACCGTTTG
CGATCTGCCACAAGTGTGATAAGTTATCTACACTGGCGAGGGGATTGCTCTCTGTAATGT
TCAGCTTCTAATTGTCTCTACTTTGTGAGACTACTTTTGAATGCTTGACCTCAAATCAGG
TAGGACTACCCGCTGAACTTAA
>AC11-48
TTTCCGTAGGTGAACCTGCGGAAGGATCATTATTGAATTATGTTTCTAGATAGGTTGTAG
CTGGCTCTTTTAGAGCATGTGCACGCCTGTTTGGACTTCATTTTCATCCACCTGTGCACC
TATTGTAGTCTTTGGTTGGGTTAGGAGGAAGTGATCATTGTATCAGCATCTGCTGGGAGT
GAGGACTTGCATTGTGAAAGCTTTGCTGTCCTTGATGTGATCATGGAATCTTTTTC----

-------TCACTAGAGTCTATGTCACTCATTATACTCTGTCGAATGTCATTGAATGTCTT
TACATGGGCTTGTATGCCTATGAAAATTGTAATACAACTTTCAGCAACGGATCTCTTGGC
TCTCGCATCGATGAAGAACGCAGCGAAATGCGATAAGTAATGTGAATTGCAGAATTCAGT
GAATCATCGAATCTTTGAACGCATCTTGCGCTCCTTGGTATTCCGAGGAGCATGCCTGTT
TGAGTGTCATTAAATTCTCAACTCTCTTATACTTTTTTGTAAAAGAGAGCTTGGACTGTG
GAGGCTTGCTGGCCACTTTTTGGGGTCAGCTCCTCTGAAATGCATTAGCGGAACCGTTTG
CGATCTGCCACAAGTGTGATAAGTTATCTACACTGGCGAGGGGATTGCTCTCTGTAATGT
TCAGCTTCTAATTGTCTCTACTTTGTGAGACTACTTTTGAATGCTTGACCTCAAATCAGG
TAGGACTACCCGCTGAACTTAA
>AC11-55
TTTCCGTAGGTGAACCTGCGGAAGGATCATTATTGAATTATGTTTCTAGATAGGTTGTAG
CTGGCTCTTTTAGAGCATGTGCACGCCTGTTTGGACTTCATTTTCATCCACCTGTGCACC
TATTGTAGTCTTTGGTTGGGTTAGGAGGAAGTGATCATTGTATCAGCATCTGCTGGGAGT
GAGGACTTGCATTGTGAAAGCTTTGCTGTCCTTGATGTGATCATGGAATCTTTTTC----

-------TCACTAGAGTCTATGTCACTCATTATACTCTGTCGAATGTCATTGAATGTCTT
TACATGGGCTTGTATGCCTATGAAAATTGTAATACAACTTTCAGCAACGGATCTCTTGGC
TCTCGCATCGATGAAGAACGCAGCGAAATGCGATAAGTAATGTGAATTGCAGAATTCAGT
GAATCATCGAATCTTTGAACGCATCTTGCGCTCCTTGGTATTCCGAGGAGCATGCCTGTT
TGAGTGTCATTAAATTCTCAACTCTCTTATACTTTTTTGTAAAAGAGAGCTTGGACTGTG
GAGGCTTGCTGGCCACTTTTTGGGGTCAGCTCCTCTGAAATGCATTAGCGGAACCGTTTG
CGATCTGCCACAAGTGTGATAAGTTATCTACACTGGCGAGGGGATTGCTCTCTGTAATGT
TCAGCTTCTAATTGTCTCTACTTTGTGAGACTACTTTTGAATGCTTGACCTCAAATCAGG
TAGGACTACCCGCTGAACTTAA
>AC12-1
TTTCCGTAGGTGAACCTGCGGAAGGATCATTATTGAATTATGTTTCTAGATAGGTTGTAG
CTGGCTCTTTTAGAGCATGTGCACGCCTGTTTGGACTTCATTTTCATCCACCTGTGCACC
TATTGTAGTCTTTGGTTGGGTTAGGAGGAAGTGATCATTGTATCAGCATCTGCTGGGAGT

GAGGACTTGCATTGTGAAAGCTTTGCTGTCCTTGATGTGATCATGGAATCTTTTTC----

-------TCACTAGAGTCTATGTCACTCATTATACTCTGTCGAATGTCATTGAATGTCTT
TACATGGGCTTGTATGCCTATGAAAATTGTAATACAACTTTCAGCAACGGATCTCTTGGC
TCTCGCATCGATGAAGAACGCAGCGAAATGCGATAAGTAATGTGAATTGCAGAATTCAGT
GAATCATCGAATCTTTGAACGCATCTTGCGCTCCTTGGTATTCCGAGGAGCATGCCTGTT
TGAGTGTCATTAAATTCTCAACTCTCTTATACTTTTTTGTAAAAGAGAGCTTGGACTGTG
GAGGCTTGCTGGCCACTTTTTGGGGTCAGCTCCTCTGAAATGCATTAGCGGAACCGTTTG
CGATCTGCCACAAGTGTGATAAGTTATCTACACTGGCGAGGGGATTGCTCTCTGTAATGT
TCAGCTTCTAATTGTCTCTACTTTGTGAGACTACTTTTGAATGCTTGACCTCAAATCAGG
TAGGACTACCCGCTGAACTTAA
>AC12-3
TTTCCGTAGGTGAACCTGCGGAAGGATCATTATTGAATTATGTTTCTAGATAGGTTGTAG
CTGGCTCTTTTAGAGCATGTGCACGCCTGTTTGGACTTCATTTTCATCCACCTGTGCACC
TATTGTAGTCTTTGGTTGGGTTAGGAGGAAGTGATCATTGTATCAGCATCTGCTGGGAGT
GAGGACTTGCATTGTGAAAGCTTTGCTGTCCTTGATGTGATCATGGAATCTTTTTC----

-------TCACTAGAGTCTATGTCACTCATTATACTCTGTCGAATGTCATTGAATGTCTT
TACATGGGCTTGTATGCCTATGAAAATTGTAATACAACTTTCAGCAACGGATCTCTTGGC
TCTCGCATCGATGAAGAACGCAGCGAAATGCGATAAGTAATGTGAATTGCAGAATTCAGT
GAATCATCGAATCTTTGAACGCATCTTGCGCTCCTTGGTATTCCGAGGAGCATGCCTGTT
TGAGTGTCATTAAATTCTCAACTCTCTTATACTTTTTTGTAAAAGAGAGCTTGGACTGTG
GAGGCTTGCTGGCCACTTTTTGGGGTCAGCTCCTCTGAAATGCATTAGCGGAACCGTTTG
CGATCTGCCACAAGTGTGATAAGTTATCTACACTGGCGAGGGGATTGCTCTCTGTAATGT
TCAGCTTCTAATTGTCTCTACTTTGTGAGACTACTTTTGAATGCTTGACCTCAAATCAGG
TAGGACTACCCGCTGAACTTAA
>AC12-4
TTTCCGTAGGTGAACCTGCGGAAGGATCATTATTGAATTATGTTTCTAGATAGGTTGTAG

CTGGCTCTTTTAGAGCATGTGCACGCCTGTTTGGACTTCATTTTCATCCACCTGTGCACC
TATTGTAGTCTTTGGTTGGGTTAGGAGGAAGTGATCATTGTATCAGCATCTGCTGGGAGT
GAGGACTTGCATTGTGAAAGCTTTGCTGTCCTTGATGTGATCATGGAATCTTTTTC----

-------TCACTAGAGTCTATGTCACTCATTATACTCTGTCGAATGTCATTGAATGTCTT
TACATGGGCTTGTATGCCTATGAAAATTGTAATACAACTTTCAGCAACGGATCTCTTGGC
TCTCGCATCGATGAAGAACGCAGCGAAATGCGATAAGTAATGTGAATTGCAGAATTCAGT
GAATCATCGAATCTTTGAACGCATCTTGCGCTCCTTGGTATTCCGAGGAGCATGCCTGTT
TGAGTGTCATTAAATTCTCAACTCTCTTATACTTTTTTGTAAAAGAGAGCTTGGACTGTG
GAGGCTTGCTGGCCACTTTTTGGGGTCAGCTCCTCTGAAATGCATTAGCGGAACCGTTTG
CGATCTGCCACAAGTGTGATAAGTTATCTACACTGGCGAGGGGATTGCTCTCTGTAATGT
TCAGCTTCTAATTGTCTCTACTTTGTGAGACTACTTTTGAATGCTTGACCTCAAATCAGG
TAGGACTACCCGCTGAACTTAA
>AC12-5
TTTCCGTAGGTGAACCTGCGGAAGGATCATTATTGAATTATGTTTCTAGATAGGTTGTAG
CTGGCTCTTTTAGAGCATGTGCACGCCTGTTTGGACTTCATTTTCATCCACCTGTGCACC
TATTGTAGTCTTTGGTTGGGTTAGGAGGAAGTGATCATTGTATCAGCATCTGCTGGGAGT
GAGGACTTGCATTGTGAAAGCTTTGCTGTCCTTGATGTGATCATGGAATCTTTTTC----

-------TCACTAGAGTCTATGTCACTCATTATACTCTGTCGAATGTCATTGAATGTCTT
TACATGGGCTTGTATGCCTATGAAAATTGTAATACAACTTTCAGCAACGGATCTCTTGGC
TCTCGCATCGATGAAGAACGCAGCGAAATGCGATAAGTAATGTGAATTGCAGAATTCAGT
GAATCATCGAATCTTTGAACGCATCTTGCGCTCCTTGGTATTCCGAGGAGCATGCCTGTT
TGAGTGTCATTAAATTCTCAACTCTCTTATACTTTTTTGTAAAAGAGAGCTTGGACTGTG
GAGGCTTGCTGGCCACTTTTTGGGGTCAGCTCCTCTGAAATGCATTAGCGGAACCGTTTG
CGATCTGCCACAAGTGTGATAAGTTATCTACACTGGCGAGGGGATTGCTCTCTGTAATGT
TCAGCTTCTAATTGTCTCTACTTTGTGAGACTACTTTTGAATGCTTGACCTCAAATCAGG
TAGGACTACCCGCTGAACTTAA

```
>AC12-8
TTTCCGTAGGTGAACCTGCGGAAGGATCATTATTGAATTATGTTTCTAGATAGGTTGTAG
CTGGCTCTTTTAGAGCATGTGCACGCCTGTTTGGACTTCATTTTCATCCACCTGTGCACC
TATTGTAGTCTTTGGTTGGGTTAGGAGGAAGTGATCATTGTATCAGCATCTGCTGGGAGT
GAGGACTTGCATTGTGAAAGCTTTGCTGTCCTTGATGTGATCATGGAATCTTTTTC----
------------------------------------------------------------
------------------------------------------------------------
------------------------------------------------------------
------------------------------------------------------------
------------------------------------------------------------
------------------------------------------------------------
------------------------------------------------------------
------------------------------------------------------------
------------------------------------------------------------
------------------------------------------------------------
------------------------------------------------------------
------------------------------------------------------------
-------TCACTAGAGTCTATGTCACTCATTATACTCTGTCGAATGTCATTGAATGTCTT
TACATGGGCTTGTATGCCTATGAAAATTGTAATACAACTTTCAGCAACGGATCTCTTGGC
TCTCGCATCGATGAAGAACGCAGCGAAATGCGATAAGTAATGTGAATTGCAGAATTCAGT
GAATCATCGAATCTTTGAACGCATCTTGCGCTCCTTGGTATTCCGAGGAGCATGCCTGTT
TGAGTGTCATTAAATTCTCAACTCTCTTATACTTTTTTGTAAAAGAGAGCTTGGACTGTG
GAGGCTTGCTGGCCACTTTTTGGGGTCAGCTCCTCTGAAATGCATTAGCGGAACCGTTTG
CGATCTGCCACAAGTGTGATAAGTTATCTACACTGGCGAGGGGATTGCTCTCTGTAATGT
TCAGCTTCTAATTGTCTCTACTTTGTGAGACTACTTTTGAATGCTTGACCTCAAATCAGG
TAGGACTACCCGCTGAACTTAA
>AC12-10
TTTCCGTAGGTGAACCTGCGGAAGGATCATTATTGAATTATGTTTCTAGATAGGTTGTAG
CTGGCTCTTTTAGAGCATGTGCACGCCTGTTTGGACTTCATTTTCATCCACCTGTGCACC
TATTGTAGTCTTTGGTTGGGTTAGGAGGAAGTGATCATTGTATCAGCATCTGCTGGGAGT
GAGGACTTGCATTGTGAAAGCTTTGCTGTCCTTGATGTGATCATGGAATCTTTTTC----
------------------------------------------------------------
------------------------------------------------------------
------------------------------------------------------------
------------------------------------------------------------
------------------------------------------------------------
------------------------------------------------------------
------------------------------------------------------------
------------------------------------------------------------
------------------------------------------------------------
------------------------------------------------------------
------------------------------------------------------------
------------------------------------------------------------
-------TCACTAGAGTCTATGTCACTCATTATACTCTGTCGAATGTCATTGAATGTCTT
TACATGGGCTTGTATGCCTATGAAAATTGTAATACAACTTTCAGCAACGGATCTCTTGGC
TCTCGCATCGATGAAGAACGCAGCGAAATGCGATAAGTAATGTGAATTGCAGAATTCAGT
GAATCATCGAATCTTTGAACGCATCTTGCGCTCCTTGGTATTCCGAGGAGCATGCCTGTT
TGAGTGTCATTAAATTCTCAACTCTCTTATACTTTTTTGTAAAAGAGAGCTTGGACTGTG
GAGGCTTGCTGGCCACTTTTTGGGGTCAGCTCCTCTGAAATGCATTAGCGGAACCGTTTG
CGATCTGCCACAAGTGTGATAAGTTATCTACACTGGCGAGGGGATTGCTCTCTGTAATGT
```

TCAGCTTCTAATTGTCTCTACTTTGTGAGACTACTTTTGAATGCTTGACCTCAAATCAGG
TAGGACTACCCGCTGAACTTAA
>AC12-11
TTTCCGTAGGTGAACCTGCGGAAGGATCATTATTGAATTATGTTTCTAGATAGGTTGTAG
CTGGCTCTTTTAGAGCATGTGCACGCCTGTTTGGACTTCATTTTCATCCACCTGTGCACC
TATTGTAGTCTTTGGTTGGGTTAGGAGGAAGTGATCATTGTATCAGCATCTGCTGGGAGT
GAGGACTTGCATTGTGAAAGCTTTGCTGTCCTTGATGTGATCATGGAATCTTTTTC----

-------TCACTAGAGTCTATGTCACTCATTATACTCTGTCGAATGTCATTGAATGTCTT
TACATGGGCTTGTATGCCTATGAAAATTGTAATACAACTTTCAGCAACGGATCTCTTGGC
TCTCGCATCGATGAAGAACGCAGCGAAATGCGATAAGTAATGTGAATTGCAGAATTCAGT
GAATCATCGAATCTTTGAACGCATCTTGCGCTCCTTGGTATTCCGAGGAGCATGCCTGTT
TGAGTGTCATTAAATTCTCAACTCTCTTATACTTTTTTGTAAAAGAGAGCTTGGACTGTG
GAGGCTTGCTGGCCACTTTTTGGGGTCAGCTCCTCTGAAATGCATTAGCGGAACCGTTTG
CGATCTGCCACAAGTGTGATAAGTTATCTACACTGGCGAGGGGATTGCTCTCTGTAATGT
TCAGCTTCTAATTGTCTCTACTTTGTGAGACTACTTTTGAATGCTTGACCTCAAATCAGG
TAGGACTACCCGCTGAACTTAA
>AC12-20
TTTCCGTAGGTGAACCTGCGGAAGGATCATTATTGAATTATGTTTCTAGATAGGTTGTAG
CTGGCTCTTTTAGAGCATGTGCACGCCTGTTTGGACTTCATTTTCATCCACCTGTGCACC
TATTGTAGTCTTTGGTTGGGTTAGGAGGAAGTGATCATTGTATCAGCATCTGCTGGGAGT
GAGGACTTGCATTGTGAAAGCTTTGCTGTCCTTGATGTGATCATGGAATCTTTTTC----

-------TCACTAGAGTCTATGTCACTCATTATACTCTGTCGAATGTCATTGAATGTCTT
TACATGGGCTTGTATGCCTATGAAAATTGTAATACAACTTTCAGCAACGGATCTCTTGGC
TCTCGCATCGATGAAGAACGCAGCGAAATGCGATAAGTAATGTGAATTGCAGAATTCAGT
GAATCATCGAATCTTTGAACGCATCTTGCGCTCCTTGGTATTCCGAGGAGCATGCCTGTT
TGAGTGTCATTAAATTCTCAACTCTCTTATACTTTTTTGTAAAAGAGAGCTTGGACTGTG

GAGGCTTGCTGGCCACTTTTTGGGGTCAGCTCCTCTGAAATGCATTAGCGGAACCGTTTG
CGATCTGCCACAAGTGTGATAAGTTATCTACACTGGCGAGGGGATTGCTCTCTGTAATGT
TCAGCTTCTAATTGTCTCTACTTTGTGAGACTACTTTTGAATGCTTGACCTCAAATCAGG
TAGGACTACCCGCTGAACTTAA
>AC12-22
TTTCCGTAGGTGAACCTGCGGAAGGATCATTATTGAATTATGTTTCTAGATAGGTTGTAG
CTGGCTCTTTTAGAGCATGTGCACGCCTGTTTGGACTTCATTTTCATCCACCTGTGCACC
TATTGTAGTCTTTGGTTGGGTTAGGAGGAAGTGATCATTGTATCAGCATCTGCTGGGAGT
GAGGACTTGCATTGTGAAAGCTTTGCTGTCCTTGATGTGATCATGGAATCTTTTTC----

-------TCACTAGAGTCTATGTCACTCATTATACTCTGTCGAATGTCATTGAATGTCTT
TACATGGGCTTGTATGCCTATGAAAATTGTAATACAACTTTCAGCAACGGATCTCTTGGC
TCTCGCATCGATGAAGAACGCAGCGAAATGCGATAAGTAATGTGAATTGCAGAATTCAGT
GAATCATCGAATCTTTGAACGCATCTTGCGCTCCTTGGTATTCCGAGGAGCATGCCTGTT
TGAGTGTCATTAAATTCTCAACTCTCTTATACTTTTTTGTAAAAGAGAGCTTGGACTGTG
GAGGCTTGCTGGCCACTTTTTGGGGTCAGCTCCTCTGAAATGCATTAGCGGAACCGTTTG
CGATCTGCCACAAGTGTGATAAGTTATCTACACTGGCGAGGGGATTGCTCTCTGTAATGT
TCAGCTTCTAATTGTCTCTACTTTGTGAGACTACTTTTGAATGCTTGACCTCAAATCAGG
TAGGACTACCCGCTGAACTTAA
>AC12-23
TTTCCGTAGGTGAACCTGCGGAAGGATCATTATTGAATTATGTTTCTAGATAGGTTGTAG
CTGGCTCTTTTAGAGCATGTGCACGCCTGTTTGGACTTCATTTTCATCCACCTGTGCACC
TATTGTAGTCTTTGGTTGGGTTAGGAGGAAGTGATCATTGTATCAGCATCTGCTGGGAGT
GAGGACTTGCATTGTGAAAGCTTTGCTGTCCTTGATGTGATCATGGAATCTTTTTC----

-------TCACTAGAGTCTATGTCACTCATTATACTCTGTCGAATGTCATTGAATGTCTT
TACATGGGCTTGTATGCCTATGAAAATTGTAATACAACTTTCAGCAACGGATCTCTTGGC
TCTCGCATCGATGAAGAACGCAGCGAAATGCGATAAGTAATGTGAATTGCAGAATTCAGT

GAATCATCGAATCTTTGAACGCATCTTGCGCTCCTTGGTATTCCGAGGAGCATGCCTGTT
TGAGTGTCATTAAATTCTCAACTCTCTTATACTTTTTTGTAAAAGAGAGCTTGGACTGTG
GAGGCTTGCTGGCCACTTTTTGGGGTCAGCTCCTCTGAAATGCATTAGCGGAACCGTTTG
CGATCTGCCACAAGTGTGATAAGTTATCTACACTGGCGAGGGGATTGCTCTCTGTAATGT
TCAGCTTCTAATTGTCTCTACTTTGTGAGACTACTTTTGAATGCTTGACCTCAAATCAGG
TAGGACTACCCGCTGAACTTAA
>AC12-24
TTTCCGTAGGTGAACCTGCGGAAGGATCATTATTGAATTATGTTTCTAGATAGGTTGTAG
CTGGCTCTTTTAGAGCATGTGCACGCCTGTTTGGACTTCATTTTCATCCACCTGTGCACC
TATTGTAGTCTTTGGTTGGGTTAGGAGGAAGTGATCATTGTATCAGCATCTGCTGGGAGT
GAGGACTTGCATTGTGAAAGCTTTGCTGTCCTTGATGTGATCATGGAATCTTTTTC----

-------TCACTAGAGTCTATGTCACTCATTATACTCTGTCGAATGTCATTGAATGTCTT
TACATGGGCTTGTATGCCTATGAAAATTGTAATACAACTTTCAGCAACGGATCTCTTGGC
TCTCGCATCGATGAAGAACGCAGCGAAATGCGATAAGTAATGTGAATTGCAGAATTCAGT
GAATCATCGAATCTTTGAACGCATCTTGCGCTCCTTGGTATTCCGAGGAGCATGCCTGTT
TGAGTGTCATTAAATTCTCAACTCTCTTATACTTTTTTGTAAAAGAGAGCTTGGACTGTG
GAGGCTTGCTGGCCACTTTTTGGGGTCAGCTCCTCTGAAATGCATTAGCGGAACCGTTTG
CGATCTGCCACAAGTGTGATAAGTTATCTACACTGGCGAGGGGATTGCTCTCTGTAATGT
TCAGCTTCTAATTGTCTCTACTTTGTGAGACTACTTTTGAATGCTTGACCTCAAATCAGG
TAGGACTACCCGCTGAACTTAA
>AC12-25
TTTCCGTAGGTGAACCTGCGGAAGGATCATTATTGAATTATGTTTCTAGATAGGTTGTAG
CTGGCTCTTTTAGAGCATGTGCACGCCTGTTTGGACTTCATTTTCATCCACCTGTGCACC
TATTGTAGTCTTTGGTTGGGTTAGGAGGAAGTGATCATTGTATCAGCATCTGCTGGGAGT
GAGGACTTGCATTGTGAAAGCTTTGCTGTCCTTGATGTGATCATGGAATCTTTTTC----

-------TCACTAGAGTCTATGTCACTCATTATACTCTGTCGAATGTCATTGAATGTCTT

TACATGGGCTTGTATGCCTATGAAAATTGTAATACAACTTTCAGCAACGGATCTCTTGGC
TCTCGCATCGATGAAGAACGCAGCGAAATGCGATAAGTAATGTGAATTGCAGAATTCAGT
GAATCATCGAATCTTTGAACGCATCTTGCGCTCCTTGGTATTCCGAGGAGCATGCCTGTT
TGAGTGTCATTAAATTCTCAACTCTCTTATACTTTTTTGTAAAAGAGAGCTTGGACTGTG
GAGGCTTGCTGGCCACTTTTTGGGGTCAGCTCCTCTGAAATGCATTAGCGGAACCGTTTG
CGATCTGCCACAAGTGTGATAAGTTATCTACACTGGCGAGGGGATTGCTCTCTGTAATGT
TCAGCTTCTAATTGTCTCTACTTTGTGAGACTACTTTTGAATGCTTGACCTCAAATCAGG
TAGGACTACCCGCTGAACTTAA
>AC12-27
TTTCCGTAGGTGAACCTGCGGAAGGATCATTATTGAATTATGTTTCTAGATAGGTTGTAG
CTGGCTCTTTTAGAGCATGTGCACGCCTGTTTGGACTTCATTTTCATCCACCTGTGCACC
TATTGTAGTCTTTGGTTGGGTTAGGAGGAAGTGATCATTGTATCAGCATCTGCTGGGAGT
GAGGACTTGCATTGTGAAAGCTTTGCTGTCCTTGATGTGATCATGGAATCTTTTTC----

-------TCACTAGAGTCTATGTCACTCATTATACTCTGTCGAATGTCATTGAATGTCTT
TACATGGGCTTGTATGCCTATGAAAATTGTAATACAACTTTCAGCAACGGATCTCTTGGC
TCTCGCATCGATGAAGAACGCAGCGAAATGCGATAAGTAATGTGAATTGCAGAATTCAGT
GAATCATCGAATCTTTGAACGCATCTTGCGCTCCTTGGTATTCCGAGGAGCATGCCTGTT
TGAGTGTCATTAAATTCTCAACTCTCTTATACTTTTTTGTAAAAGAGAGCTTGGACTGTG
GAGGCTTGCTGGCCACTTTTTGGGGTCAGCTCCTCTGAAATGCATTAGCGGAACCGTTTG
CGATCTGCCACAAGTGTGATAAGTTATCTACACTGGCGAGGGGATTGCTCTCTGTAATGT
TCAGCTTCTAATTGTCTCTACTTTGTGAGACTACTTTTGAATGCTTGACCTCAAATCAGG
TAGGACTACCCGCTGAACTTAA
>AC12-35
TTTCCGTAGGTGAACCTGCGGAAGGATCATTATTGAATTATGTTTCTAGATAGGTTGTAG
CTGGCTCTTTTAGAGCATGTGCACGCCTGTTTGGACTTCATTTTCATCCACCTGTGCACC
TATTGTAGTCTTTGGTTGGGTTAGGAGGAAGTGATCATTGTATCAGCATCTGCTGGGAGT
GAGGACTTGCATTGTGAAAGCTTTGCTGTCCTTGATGTGATCATGGAATCTTTTTC----

-------TCACTAGAGTCTATGTCACTCATTATACTCTGTCGAATGTCATTGAATGTCTT
TACATGGGCTTGTATGCCTATGAAAATTGTAATACAACTTTCAGCAACGGATCTCTTGGC
TCTCGCATCGATGAAGAACGCAGCGAAATGCGATAAGTAATGTGAATTGCAGAATTCAGT
GAATCATCGAATCTTTGAACGCATCTTGCGCTCCTTGGTATTCCGAGGAGCATGCCTGTT
TGAGTGTCATTAAATTCTCAACTCTCTTATACTTTTTTGTAAAAGAGAGCTTGGACTGTG
GAGGCTTGCTGGCCACTTTTTGGGGTCAGCTCCTCTGAAATGCATTAGCGGAACCGTTTG
CGATCTGCCACAAGTGTGATAAGTTATCTACACTGGCGAGGGGATTGCTCTCTGTAATGT
TCAGCTTCTAATTGTCTCTACTTTGTGAGACTACTTTTGAATGCTTGACCTCAAATCAGG
TAGGACTACCCGCTGAACTTAA
>AC12-53
TTTCCGTAGGTGAACCTGCGGAAGGATCATTATTGAATTATGTTTCTAGATAGGTTGTAG
CTGGCTCTTTTAGAGCATGTGCACGCCTGTTTGGACTTCATTTTCATCCACCTGTGCACC
TATTGTAGTCTTTGGTTGGGTTAGGAGGAAGTGATCATTGTATCAGCATCTGCTGGGAGT
GAGGACTTGCATTGTGAAAGCTTTGCTGTCCTTGATGTGATCATGGAATCTTTTTC----

-------TCACTAGAGTCTATGTCACTCATTATACTCTGTCGAATGTCATTGAATGTCTT
TACATGGGCTTGTATGCCTATGAAAATTGTAATACAACTTTCAGCAACGGATCTCTTGGC
TCTCGCATCGATGAAGAACGCAGCGAAATGCGATAAGTAATGTGAATTGCAGAATTCAGT
GAATCATCGAATCTTTGAACGCATCTTGCGCTCCTTGGTATTCCGAGGAGCATGCCTGTT
TGAGTGTCATTAAATTCTCAACTCTCTTATACTTTTTTGTAAAAGAGAGCTTGGACTGTG
GAGGCTTGCTGGCCACTTTTTGGGGTCAGCTCCTCTGAAATGCATTAGCGGAACCGTTTG
CGATCTGCCACAAGTGTGATAAGTTATCTACACTGGCGAGGGGATTGCTCTCTGTAATGT
TCAGCTTCTAATTGTCTCTACTTTGTGAGACTACTTTTGAATGCTTGACCTCAAATCAGG
TAGGACTACCCGCTGAACTTAA
>AC2-15
TTTCCGTAGGTGAACCTGCGGAAGGATCATTATTGAATTATGTTTCTAGATAGGTTGTAG
CTGGCTCTTTTAGAGCATGTGCACGCCTGTTTGGACTTCATTTTCATCCACCTGTGCACC
TATTGTAGTCTTTGGTTGGGTTAGGAGGAAGTGATCATTGTATCAGCATCTGCTGGGAGT
GAGGACTTGCATTGTGAAAGCTTTGCTGTCCTTGATGTGATCATGGAATCTTTTTC----


```
------------------------------------------------------------
------------------------------------------------------------
------------------------------------------------------------
-------TCACTAGAGTCTATGTCACTCATTATACTCTGTCGAATGTCATTGAATGTCTT
TACATGGGCTTGTATGCCTATGAAAATTGTAATACAACTTTCAGCAACGGATCTCTTGGC
TCTCGCATCGATGAAGAACGCAGCGAAATGCGATAAGTAATGTGAATTGCAGAATTCAGT
GAATCATCGAATCTTTGAACGCATCTTGCGCTCCTTGGTATTCCGAGGAGCATGCCTGTT
TGAGTGTCATTAAATTCTCAACTCTCTTATACTTTTTTGTAAAAGAGAGCTTGGACTGTG
GAGGCTTGCTGGCCACTTTTTGGGGTCAGCTCCTCTGAAATGCATTAGCGGAACCGTTTG
CGATCTGCCACAAGTGTGATAAGTTATCTACACTGGCGAGGGGATTGCTCTCTGTAATGT
TCAGCTTCTAATTGTCTCTACTTTGTGAGACTACTTTTGAATGCTTGACCTCAAATCAGG
TAGGACTACCCGCTGAACTTAA
>AC9-45
TTTCCGTAGGTGAACCTGCGGAAGGATCATTATTGAATTATGTTTCTAGATAGGTTGTAG
CTGGCTCTTTTAGAGCATGTGCACGCCTGTTTGGACTTCATTTTCATCCACCTGTGCACC
TATTGTAGTCTTTGGTTGGGTTAGGAGGAAGTGATCATTGTATCAGCATCTGCTGGGAGT
GAGGACTTGCATTGTGAAAGCTTTGCTGTCCTTGATGTGATCATGGAATCTTTTTC----
------------------------------------------------------------
------------------------------------------------------------
------------------------------------------------------------
------------------------------------------------------------
------------------------------------------------------------
------------------------------------------------------------
------------------------------------------------------------
------------------------------------------------------------
------------------------------------------------------------
------------------------------------------------------------
------------------------------------------------------------
------------------------------------------------------------
-------TCACTAGAGTCTATGTCACTCATTATACTCTGTCGAATGTCATTGAATGTCTT
TACATGGGCTTGTATGCCTATGAAAATTGTAATACAACTTTCAGCAACGGATCTCTTGGC
TCTCGCATCGATGAAGAACGCAGCGAAATGCGATAAGTAATGTGAATTGCAGAATTCAGT
GAATCATCGAATCTTTGAACGCATCTTGCGCTCCTTGGTATTCCGAGGAGCATGCCTGTT
TGAGTGTCATTAAATTCTCAACTCTCTTATACTTTTTTGTAAAAGAGAGCTTGGACTGTG
GAGGCTTGCTGGCCACTTTTTGGGGTCAGCTCCTCTGAAATGCATTAGCGGAACCGTTTG
CGATCTGCCACAAGTGTGATAAGTTATCTACACTGGCGAGGGGATTGCTCTCTGTAATGT
TCAGCTTCTAATTGTCTCTACTTTGTGAGACTACTTTTGAATGCTTGACCTCAAATCAGG
TAGGACTACCCGCTGAACTTAA
>AC11-13
TTTCCGTAGGTGAACCTGCGGAAGGATCATTATTGAATTATGTTTCTAGATAGGTTGTAG
CTGGCTCTTTTAGAGCATGTGCACGCCTGTTTGGACTTCATTTTCATCCACCTGTGCACC
TATTGTAGTCTTTGGTTGGGTTAGGAGGAAGTGATCATTGTATCAGCATCTGCTGGGAGT
GAGGACTTGCATTGTGAAAGCTTTGCTGTCCTTGATGTGATCATGGAATCTTTTTC----
------------------------------------------------------------
------------------------------------------------------------
------------------------------------------------------------
------------------------------------------------------------
------------------------------------------------------------
------------------------------------------------------------
------------------------------------------------------------
```

```
------------------------------------------------------------
------------------------------------------------------------
------------------------------------------------------------
------------------------------------------------------------
------------------------------------------------------------
-------TCACTAGAGTCTATGTCACTCATTATACTCTGTCGAATGTCATTGAATGTCTT
TACATGGGCTTGTATGCCTATGAAAATTGTAATACAACTTTCAGCAACGGATCTCTTGGC
TCTCGCATCGATGAAGAACGCAGCGAAATGCGATAAGTAATGTGAATTGCAGAATTCAGT
GAATCATCGAATCTTTGAACGCATCTTGCGCTCCTTGGTATTCCGAGGAGCATGCCTGTT
TGAGTGTCATTAAATTCTCAACTCTCTTATACTTTTTTGTAAAAGAGAGCTTGGACTGTG
GAGGCTTGCTGGCCACTTTTTGGGGTCAGCTCCTCTGAAATGCATTAGCGGAACCGTTTG
CGATCTGCCACAAGTGTGATAAGTTATCTACACTGGCGAGGGGATTGCTCTCTGTAATGT
TCAGCTTCTAATTGTCTCTACTTTGTGAGACTACTTTTGAATGCTTGACCTCAAATCAGG
TAGGACTACCCGCTGAACTTAA
>AC9-48
TTTCCGTAGGTGAACCTGCGGAAGGATCATTATTGAATTATGTTTCTAGATAGGTTGTAG
CTGGCTCTTTTAGAGCATGTGCACGCCTGTTTGGACTTCATTTTCATCCACCTGTGCACC
TATTGTAGTCTTTGGTTGGGTTAGGAGGAAGTGATCATTGTATCAGCATCTGCTGGGAGT
GAGGACTTGCATTGTGAAAGCTTTGCTGTCCTTGATGTGATCATGGAATCTTTTTC----
------------------------------------------------------------
------------------------------------------------------------
------------------------------------------------------------
------------------------------------------------------------
------------------------------------------------------------
------------------------------------------------------------
------------------------------------------------------------
------------------------------------------------------------
------------------------------------------------------------
------------------------------------------------------------
------------------------------------------------------------
------------------------------------------------------------
-------TCACTAGAGTCTATGTCACTCATTATACTCTGTCGAATGTCATTGAATGTCTT
TACATGGGCTTGTATGCCTATGAAAATTGTAATACAACTTTCAGCAACGGATCTCTTGGC
TCTCGCATCGATGAAGAACGCAGCGAAATGCGATAAGTAATGTGAATTGCAGAATTCAGT
GAATCATCGAATCTTTGAACGCATCTTGCGCTCCTTGGTATTCCGAGGAGCATGCCTGTT
TGAGTGTCATTAAATTCTCAACTCTCTTATACTTTTTTGTAAAAGAGAGCTTGGACTGTG
GAGGCTTGCTGGCCACTTTTTGGGGTCAGCTCCTCTGAAATGCATTAGCGGAACCGTTTG
CGATCTGCCACAAGTGTGATAAGTTATCTACACTGGCGAGGGGATTGCTCTCTGTAATGT
TCAGCTTCTAATTGTCTCTACTTTGTGAGACTACTTTTGAATGCTTGACCTCAAATCAGG
TAGGACTACCCGCTGAACTTAA
>AC3-52
TTTCCGTAGGTGAACCTGCGGAAGGATCATTATTGAATTATGTTTCTAGATAGGTTGTAG
CTGGCTCTTTTAGAGCATGTGCACGCCTGTTTGGACTTCATTTTCATCCACCTGTGCACC
TATTGTAGTCTTTGGTTGGGTTAGGAGGAAGTGATCATTGTATCAGCATCTGCTGGGAGT
GAGGACTTGCATTGTGAAAGCTTTGCTGTCCTTGATGTGATCATGGAATCTTTTTC----
------------------------------------------------------------
------------------------------------------------------------
------------------------------------------------------------
------------------------------------------------------------
------------------------------------------------------------
```


-------TCACTAGAGTCTATGTCACTCATTATACTCTGTCGAATGTCATTGAATGTCTT
TACATGGGCTTGTATGCCTATGAAAATTGTAATACAACTTTCAGCAACGGATCTCTTGGC
TCTCGCATCGATGAAGAACGCAGCGAAATGCGATAAGTAATGTGAATTGCAGAATTCAGT
GAATCATCGAATCTTTGAACGCATCTTGCGCTCCTTGGTATTCCGAGGAGCATGCCTGTT
TGAGTGTCATTAAATTCTCAACTCTCTTATACTTTTTTGTAAAAGAGAGCTTGGACTGTG
GAGGCTTGCTGGCCACTTTTTGGGGTCAGCTCCTCTGAAATGCATTAGCGGAACCGTTTG
CGATCTGCCACAAGTGTGATAAGTTATCTACACTGGCGAGGGGATTGCTCTCTGTAATGT
TCAGCTTCTAATTGTCTCTACTTTGTGAGACTACTTTTGAATGCTTGACCTCAAATCAGG
TAGGACTACCCGCTGAACTTAA
>AC3-44
TTTCCGTAGGTGAACCTGCGGAAGGATCATTATTGAATTATGTTTCTAGATAGGTTGTAG
CTGGCTCTTTTAGAGCATGTGCACGCCTGTTTGGACTTCATTTTCATCCACCTGTGCACC
TATTGTAGTCTTTGGTTGGGTTAGGAGGAAGTGATCATTGTATCAGCATCTGCTGGGAGT
GAGGACTTGCATTGTGAAAGCTTTGCTGTCCTTGATGTGATCATGGAATCTTTTTC----

-------TCACTAGAGTCTATGTCACTCATTATACTCTGTCGAATGTCATTGAATGTCTT
TACATGGGCTTATATGCCTATGAAAATTGTAATACAACTTTCAGCAACGGATCTCTTGGC
TCTCGCATCGATGAAGAACGCAGCGAAATGCGATAAGTAATGTGAATTGCAGAATTCAGT
GAATCATCGAATCTTTGAACGCATCTTGCGCTCCTTGGTATTCCGAGGAGCATGCCTGTT
TGAGTGTCATTAAATTCTCAACTCTCTTATACTTTTTTGTAAAAGAGAGCTTGGACTGTG
GAGGCTTGCTGGCCACTTTTTGGGGTCAGCTCCTCTGAAATGCATTAGCGGAACCGTTTG
CGATCTGCCACAAGTGTGATAAGTTATCTACACTGGCGAGGGGATTGCTCTCTGTAATGT
TCAGCTTCTAATTGTCTCTACTTTGTGAGACTACTTTTGAATGCTTGACCTCAAATCAGG
TAGGACTACCCGCTGAACTTAA
>AC5-43
TTTCCGTAGGTGAACCTGCGGAAGGATCATTATTGAATTATGTTTCTAGATAGGTTGTAG
CTGGCTCTTTTAGAGCATGTGCACGCCTGTTTGGACTTCATTTTCATCCACCTGTGCACC
TATTGTAGTCTTTGGTTGGGTTAGGAGGAAGTGATCATTGTATCAGCATCTGCTGGGAGT
GAGGACTTGCATTGTGAAAGCTTTGCTGTCCTTGATGTGATCATGGAATCTTTTTC----


```
------------------------------------------------------------
------------------------------------------------------------
------------------------------------------------------------
------------------------------------------------------------
------------------------------------------------------------
------------------------------------------------------------
------------------------------------------------------------
------------------------------------------------------------
------------------------------------------------------------
-------TCACTAGAGTCTATGTCACTCATTATACTCTGTCGAATGTCATTGAATGTCTT
TACATGGGCTTATATGCCTATGAAAATTGTAATACAACTTTCAGCAACGGATCTCTTGGC
TCTCGCATCGATGAAGAACGCAGCGAAATGCGATAAGTAATGTGAATTGCAGAATTCAGT
GAATCATCGAATCTTTGAACGCATCTTGCGCTCCTTGGTATTCCGAGGAGCATGCCTGTT
TGAGTGTCATTAAATTCTCAACTCTCTTATACTTTTTTGTAAAAGAGAGCTTGGACTGTG
GAGGCTTGCTGGCCACTTTTTGGGGTCAGCTCCTCTGAAATGCATTAGCGGAACCGTTTG
CGATCTGCCACAAGTGTGATAAGTTATCTACACTGGCGAGGGGATTGCTCTCTGTAATGT
TCAGCTTCTAATTGTCTCTACTTTGTGAGACTACTTTTGAATGCTTGACCTCAAATCAGG
TAGGACTACCCGCTGAACTTAA
>AC7-33
TTTCCGTAGGTGAACCTGCGGAAGGATCATTATTGAATTATGTTTCTAGATAGGTTGTAG
CTGGCTCTTTTAGAGCATGTGCACGCCTGTTTGGACTTCATTTTCATCCACCTGTGCACC
TATTGTAGTCTTTGGTTGGGTTAGGAGGAAGTGATCATTGTATCAGCATCTGCTGGGAGT
GAGGACTTGCATTGTGAAAGCTTTGCTGTCCTTGATGTGATCATGGAATCTTTTTC----
------------------------------------------------------------
------------------------------------------------------------
------------------------------------------------------------
------------------------------------------------------------
------------------------------------------------------------
------------------------------------------------------------
------------------------------------------------------------
------------------------------------------------------------
------------------------------------------------------------
------------------------------------------------------------
------------------------------------------------------------
------------------------------------------------------------
------------------------------------------------------------
-------TCACTAGAGTCTATGTCACTCATTATACTCTGTCGAATGTCATTGAATGTCTT
TACATGGGCTTATATGCCTATGAAAATTGTAATACAACTTTCAGCAACGGATCTCTTGGC
TCTCGCATCGATGAAGAACGCAGCGAAATGCGATAAGTAATGTGAATTGCAGAATTCAGT
GAATCATCGAATCTTTGAACGCATCTTGCGCTCCTTGGTATTCCGAGGAGCATGCCTGTT
TGAGTGTCATTAAATTCTCAACTCTCTTATACTTTTTTGTAAAAGAGAGCTTGGACTGTG
GAGGCTTGCTGGCCACTTTTTGGGGTCAGCTCCTCTGAAATGCATTAGCGGAACCGTTTG
CGATCTGCCACAAGTGTGATAAGTTATCTACACTGGCGAGGGGATTGCTCTCTGTAATGT
TCAGCTTCTAATTGTCTCTACTTTGTGAGACTACTTTTGAATGCTTGACCTCAAATCAGG
TAGGACTACCCGCTGAACTTAA
>AC8-8
TTTCCGTAGGTGAACCTGCGGAAGGATCATTATTGAATTATGTTTCTAGATAGGTTGTAG
CTGGCTCTTTTAGAGCATGTGCACGCCTGTTTGGACTTCATTTTCATCCACCTGTGCACC
TATTGTAGTCTTTGGTTGGGTTAGGAGGAAGTGATCATTGTATCAGCATCTGCTGGGAGT
GAGGACTTGCATTGTGAAAGCTTTGCTGTCCTTGATGTGATCATGGAATCTTTTTC----
------------------------------------------------------------
```


-------TCACTAGAGTCTATGTCACTCATTATACTCTGTCGAATGTCATTGAATGTCTT
TACATGGGCTTATATGCCTATGAAAATTGTAATACAACTTTCAGCAACGGATCTCTTGGC
TCTCGCATCGATGAAGAACGCAGCGAAATGCGATAAGTAATGTGAATTGCAGAATTCAGT
GAATCATCGAATCTTTGAACGCATCTTGCGCTCCTTGGTATTCCGAGGAGCATGCCTGTT
TGAGTGTCATTAAATTCTCAACTCTCTTATACTTTTTTGTAAAAGAGAGCTTGGACTGTG
GAGGCTTGCTGGCCACTTTTTGGGGTCAGCTCCTCTGAAATGCATTAGCGGAACCGTTTG
CGATCTGCCACAAGTGTGATAAGTTATCTACACTGGCGAGGGGATTGCTCTCTGTAATGT
TCAGCTTCTAATTGTCTCTACTTTGTGAGACTACTTTTGAATGCTTGACCTCAAATCAGG
TAGGACTACCCGCTGAACTTAA
>AC10-21
TTTCCGTAGGTGAACCTGCGGAAGGATCATTATTGAATTATGTTTCTAGATAGGTTGTAG
CTGGCTCTTTTAGAGCATGTGCACGCCTGTTTGGACTTCATTTTCATCCACCTGTGCACC
TATTGTAGTCTTTGGTTGGGTTAGGAGGAAGTGATCATTGTATCAGCATCTGCTGGGAGT
GAGGACTTGCATTGTGAAAGCTTTGCTGTCCTTGATGTGATCATGGAATCTTTTTC----

-------TCACTAGAGTCTATGTCACTCATTATACTCTGTCGAATGTCATTGAATGTCTT
TACATGGGCTTATATGCCTATGAAAATTGTAATACAACTTTCAGCAACGGATCTCTTGGC
TCTCGCATCGATGAAGAACGCAGCGAAATGCGATAAGTAATGTGAATTGCAGAATTCAGT
GAATCATCGAATCTTTGAACGCATCTTGCGCTCCTTGGTATTCCGAGGAGCATGCCTGTT
TGAGTGTCATTAAATTCTCAACTCTCTTATACTTTTTTGTAAAAGAGAGCTTGGACTGTG
GAGGCTTGCTGGCCACTTTTTGGGGTCAGCTCCTCTGAAATGCATTAGCGGAACCGTTTG
CGATCTGCCACAAGTGTGATAAGTTATCTACACTGGCGAGGGGATTGCTCTCTGTAATGT
TCAGCTTCTAATTGTCTCTACTTTGTGAGACTACTTTTGAATGCTTGACCTCAAATCAGG
TAGGACTACCCGCTGAACTTAA
>AC11-25
TTTCCGTAGGTGAACCTGCGGAAGGATCATTATTGAATTATGTTTCTAGATAGGTTGTAG
CTGGCTCTTTTAGAGCATGTGCACGCCTGTTTGGACTTCATTTTCATCCACCTGTGCACC
TATTGTAGTCTTTGGTTGGGTTAGGAGGAAGTGATCATTGTATCAGCATCTGCTGGGAGT

GAGGACTTGCATTGTGAAAGCTTTGCTGTCCTTGATGTGATCATGGAATCTTTTTC----

-------TCACTAGAGTCTATGTCACTCATTATACTCTGTCGAATGTCATTGAATGTCTT
TACATGGGCTTATATGCCTATGAAAATTGTAATACAACTTTCAGCAACGGATCTCTTGGC
TCTCGCATCGATGAAGAACGCAGCGAAATGCGATAAGTAATGTGAATTGCAGAATTCAGT
GAATCATCGAATCTTTGAACGCATCTTGCGCTCCTTGGTATTCCGAGGAGCATGCCTGTT
TGAGTGTCATTAAATTCTCAACTCTCTTATACTTTTTTGTAAAAGAGAGCTTGGACTGTG
GAGGCTTGCTGGCCACTTTTTGGGGTCAGCTCCTCTGAAATGCATTAGCGGAACCGTTTG
CGATCTGCCACAAGTGTGATAAGTTATCTACACTGGCGAGGGGATTGCTCTCTGTAATGT
TCAGCTTCTAATTGTCTCTACTTTGTGAGACTACTTTTGAATGCTTGACCTCAAATCAGG
TAGGACTACCCGCTGAACTTAA
>AC7-20
TTTCCGTAGGTGAACCTGCGGAAGGATCATTATTGAATTATGTTTCTAGATAGGTTGTAG
CTGGCTCTTTTAGAGCATGTGCACGCCTGTTTGGACTTCATTTTCATCCACCTGTGCACC
TATTGTAGTCTTTGGTTGGGTTAGGAGGAAGTGATCATTGTATCAGCATCTGCTGGGAGT
GAGGACTTGCATTGTGAAAGCTTTGCTGTCCTTGATGTGATCATGGAATCTCTTTC----

-------TCACTAGAGTCTATGTCACTCATTATACTCTGTCGAATGTCATTGAATGTCTT
TACATGGGCTTATATGCCTATGAAAATTGTAATACAACTTTCAGCAACGGATCTCTTGGC
TCTCGCATCGATGAAGAACGCAGCGAAATGCGATAAGTAATGTGAATTGCAGAATTCAGT
GAATCATCGAATCTTTGAACGCATCTTGCGCTCCTTGGTATTCCGAGGAGCATGCCTGTT
TGAGTGTCATTAAATTCTCAACTCTCTTATACTTTTTTGTAAAAGAGAGCTTGGACTGTG
GAGGCTTGCTGGCCACTTTTTGGGGTCAGCTCCTCTGAAATGCATTAGCGGAACCGTTTG
CGATCTGCCACAAGTGTGATAAGTTATCTACACTGGCGAGGGGATTGCTCTCTGTAATGT
TCAGCTTCTAATTGTCTCTACTTTGTGAGACTACTTTTGAATGCTTGACCTCAAATCAGG
TAGGACTACCCGCTGAACTTAA
>AC10-59
TTTCCGTAGGTGAACCTGCGGAAGGATCATTATTGAATTATGTTTCTAGATAGGTTGTAG

CTGGCTCTTTTAGAGCATGTGCACGCCTGTTTGGACTTCATTTTCATCCACCTGTGCACC
TATTGTAGTCTTTGGTTGGGTTAGGAGGAAGTGATCATTGTATCAGCATCTGCTGGGAGT
GAGGACTTGCATTGTGAAAGCTTTGCTGTCCTTGATGTGATCATGGAATCTCTTTC----

-------TCACTAGAGTCTATGTCACTCATTATACTCTGTCGAATGTCATTGAATGTCTT
TACATGGGCTTATATGCCTATGAAAATTGTAATACAACTTTCAGCAACGGATCTCTTGGC
TCTCGCATCGATGAAGAACGCAGCGAAATGCGATAAGTAATGTGAATTGCAGAATTCAGT
GAATCATCGAATCTTTGAACGCATCTTGCGCTCCTTGGTATTCCGAGGAGCATGCCTGTT
TGAGTGTCATTAAATTCTCAACTCTCTTATACTTTTTTGTAAAAGAGAGCTTGGACTGTG
GAGGCTTGCTGGCCACTTTTTGGGGTCAGCTCCTCTGAAATGCATTAGCGGAACCGTTTG
CGATCTGCCACAAGTGTGATAAGTTATCTACACTGGCGAGGGGATTGCTCTCTGTAATGT
TCAGCTTCTAATTGTCTCTACTTTGTGAGACTACTTTTGAATGCTTGACCTCAAATCAGG
TAGGACTACCCGCTGAACTTAA
>AC6-56
TTTCCGTAGGTGAACCTGCGGAAGGATCATTATTGAATTATGTTTCTAGATAGGTTGTAG
CTGGCTCTTTTAGAGCATGTGCACGCCTGTTTGGACTTCATTTTCATCCACCTGTGCACC
TATTGTAGTCTTTGGTTGGGTTAGGAGGAAGTGATCATTGTATCAGCATCTGCTGGGAGT
GAGGACTTGCATTGTGAAAGCTTTGCTGTCCTTGATGTGATCATGGAATCTCTTTC----

-------TCACTAGAGTCTATGTCACTCATTATACTCTGTCGAATGTCATTGAATGTCTT
TACATGGGCTTATATGCCTATGAAAATTGTAATACAACTTTCAGCAACGGATCTCTTGGC
TCTCGCATCGATGAAGAACGCAGCGAAATGCGATAAGTAATGTGAATTGCAGAATTCAGT
GAATCATCGAATCTTTGAACGCATCTTGCGCTCCTTGGTATTCCGAGGAGCATGCCTGTT
TGAGTGTCATTAAATTCTCAACTCTCTTATACTTTTTTGTAAAAGAGAGCTTGGACTGTG
GAGGCTTGCTGGCCACTTTTTGGGGTCAGCTCCTCTGAAATGCATTAGCGGAACCGTTTG
CGATCTGCCACAAGTGTGATAAGTTATCTACACTGGCGAGGGGATTGCTCTCTGTAATGT
TCAGCTTCTAATTGTCTCTACTTTGTGAGACTACTTTTGAATGCTTGACCTCAAATCAGG
TAGGACTACCCGCTGAACTTAA

>AC12-46
TTTCCGTAGGTGAACCTGCGGAAGGATCATTATTGAATTATGTTTCTAGATAGGTTGTAG
CTGGCTCTTTTAGAGCATGTGCACGCCTGTTTGGACTTCATTTTCATCCACCTGTGCACC
TATTGTAGTCTTTGGTTGGGTTAGGAGGAAGTGATCATTGTATCAGCATCTGCTGGGAGT
GAGGACTTGCATTGTGAAAGCTTTGCTGTCCTTGATGTGATCATGGAATCTCTTTC----

-------TCACTAGAGTCTATGTCACTCATTATACTCTGTCGAATGTCATTGAATGTCTT
TACATGGGCTTATATGCCTATGAAAATTGTAATACAACTTTCAGCAACGGATCTCTTGGC
TCTCGCATCGATGAAGAACGCAGCGAAATGCGATAAGTAATGTGAATTGCAGAATTCAGT
GAATCATCGAATCTTTGAACGCATCTTGCGCTCCTTGGTATTCCGAGGAGCATGCCTGTT
TGAGTGTCATTAAATTCTCAACTCTCTTATACTTTTTTGTAAAAGAGAGCTTGGACTGTG
GAGGCTTGCTGGCCACTTTTTGGGGTCAGCTCCTCTGAAATGCATTAGCGGAACCGTTTG
CGATCTGCCACAAGTGTGATAAGTTATCTACACTGGCGAGGGGATTGCTCTCTGTAATGT
TCAGCTTCTAATTGTCTCTACTTTGTGAGACTACTTTTGAATGCTTGACCTCAAATCAGG
TAGGACTACCCGCTGAACTTAA
>AC10-52
TTTCCGTAGGTGAACCTGCGGAAGGATCATTATTGAATTATGTTTCTAGATAGGTTGTAG
CTGGCTCTTTTAGAGCATGTGCACGCCTGTTTGGACTTCATTTTCATCCACCTGTGCACC
TATTGTAGTCTTTGGTTGGGTTAGGAGGAAGTGATCATTGTATCAGCATCTGCTGGGAGT
GAGGACTTGCATTGTGAAAGCTTTGCTGTCCTTGATGTGATCATGGAATCTTTTTC----

-------TCACTAGAGTCTATGTCACTCATTATACTCTGTCGAATGTCATTGAATGTCTT
TACATGGGCTTGTATGCCTATGAAAATTGTAATACAACTTTCAGCAACGGATCTCTTGGC
TCTCGCATCGATGAAGAACGCAGCGAAATGCGATAAGTAATGTGAATTGCAGAATTCAGT
GAATCACCGAATCTTTGAACGCATCTTGCGCTCCTTGGTATTCCGAGGAGCATGCCTGTT
TGAGTGTCATTAAATTCTCAACTCTCTTATACTTTTTTGTAAAAGAGAGCTTGGACTGTG
GAGGCTTGCTGGCCACTTTTTGGGGTCAGCTCCTCTGAAATGCATTAGCGGAACCGTTTG
CGATCTGCCACAAGTGTGATAAGTTATCTACACTGGCGAGGGGATTGCTCTCTGTAATGT

TCAGCTTCTAATTGTCTCTACTTTGTGAGACTACTTTTGAATGCTTGACCTCAAATCAGG
TAGGACTACCCGCTGAACTTAA
>AC1-27
TTTCCGTAGGTGAACCTGCGGAAGGATCATTATTGAATTATGTTTCTAGATAGGTTGTAG
CTGGCTCTTTTAGAGCATGTGCACGCCTGTTTGGACTTCATTTTCATCCACCTGTGCACC
TATTGTAGTCTTTGGTTGGGTTAGGAGGAAGTGATCATTGTATCAACATCTGCTGGGAGT
GAGGACTTGCATTGTGAAAGCTTTGCTGTCCTTGATGTGATCATGGAATCTTTTTC----

-------TCACTAGAGTCTATGTCACTCATTATACTCTGTCGAATGTCATTGAATGTCTT
TACATGGGCTTGTATGCCTATGAAAATTGTAATACAACTTTCAGCAACGGATCTCTTGGC
TCTCGCATCGATGAAGAACGCAGCGAAATGCGATAAGTAATGTGAATTGCAGAATTCAGT
GAATCATCGAATCTTTGAACGCATCTTGCGCTCCTTGGTATTCCGAGGAGCATGCCTGTT
TGAGTGTCATTAAATTCTCAACTCTCTTATACTTTTTTGTAAAAGAGAGCTTGGACTGTG
GAGGCTTGCTGGCCACTTTTTGGGGTCAGCTCCTCTGAAATGCATTAGCGGAACCGTTTG
CGATCTGCCACAAGTGTGATAAGTTATCTACACTGGCGAGGGGATTGCTCTCTGTAATGT
TCAGCTTCTAATTGTCTCTACTTTGTGAGACTACTTTTGAATGCTTGACCTCAAATCAGG
TAGGACTACCCGCTGAACTTAA
>AC8-5
TTTCCGTAGGTGAACCTGCGGAAGGATCATTATTGAATTATGTTTCTAGATAGGTTGTAG
CTGGCTCTTTTAGAGCATGTGCACGCCTGTTTGGACTTCATTTTCATCCACCTGTGCACC
TATTGTAGTCTTTGGTTGGGTTAGGAGGAAGTGATCATTGTATCAGCATCTGCTGGGAGT
GAGGACTTGCATTGTGAAAGCTTTGCTGTCCTTGATGTGATCATGGAATCTTTTTC----

-------TCACTAGAGTCTATGTCACTCATTATACTCTGTCGAATGTCATTGAATGTCTT
TACATGGGCTTGTATGCCTATGAAAATTGTAATACAACTTTCAGCAACGGATCTCTTGGC
TCTCACATCGATGAAGAACGCAGCGAAATGCGATAAGTAATGTGAATTGCAGAATTCAGT
GAATCATCGAATCTTTGAACGCATCTTGCGCTCCTTGGTATTCCGAGGAGCATGCCTGTT
TGAGTGTCATTAAATTCTCAACTCTCTTATACTTTTTTGTAAAAGAGAGCTTGGACTGTG

```
GAGGCTTGCTGGCCACTTTTTGGGGTCAGCTCCTCTGAAATGCATTAGCGGAACCGTTTG
CGATCTGCCACAAGTGTGATAAGTTATCTACACTGGCGAGGGGATTGCTCTCTGTAATGT
TCAGCTTCTAATTGTCTCTACTTTGTGAGACTACTTTTGAATGCTTGACCTCAAATCAGG
TAGGACTACCCGCTGAACTTAA
>AC11-6
TTTCCGTAGGTGAACCTGCGGAAGGATCATTATTGAATTATGTTTCTAGATAGGTTGTAG
CTGGCTCTTTTAGAGCATGTGCACGCCTGTTTGGACTTCATTTTCATCCACCTGTGCACC
TATTGTAGTCTTTGGTTGGGTTAGGAGGAAGTGATCATTGTATCAGCATCTGCTGGGAGT
GAGGACTTGCATTGTGAAAGCTTTGCTGTCCTTGATGTGATCATGGAATCTTTTTC----
------------------------------------------------------------
------------------------------------------------------------
------------------------------------------------------------
------------------------------------------------------------
------------------------------------------------------------
------------------------------------------------------------
------------------------------------------------------------
------------------------------------------------------------
------------------------------------------------------------
------------------------------------------------------------
------------------------------------------------------------
------------------------------------------------------------
-------TCACTAGAGTCTATGTCACTCATTATACTCTGTCGAATGTCATTGAATGTCTT
TACATGGGCTTGTATGCCTATGAAAATTGTAATACAACTTTCAGCAACGGATCTCTTGGC
TCTCGCATCGATGAAGAACGCAGCGAAATGCGATAAGTAATGTGAATTGCAGAATTCAGT
GAATCATCGAATCTTTGAACGCATCTTGCGCTCCTTGGTATTCCGAGGAGCATGCCTGTT
TGAGTGTCATTAAATTCTCAACTCTCTTATACTTTTTTGTAAAAGAGAGCTTGGACTGTG
GAGGCTTGCTGGCCACTTTTTGGGGTCAGCTCCTCTGAAATGCATTAGCGGAACCGTTTG
CGATCTACCACAAGTGTGATAAGTTATCTACACTGGCGAGGGGATTGCTCTCTGTAATGT
TCAGCTTCTAATTGTCTCTACTTTGTGAGACTACTTTTGAATGCTTGACCTCAAATCAGG
TAGGACTACCCGCTGAACTTAA
>AC8-1
TTTCCGTAGGTGAACCTGCGGAAGGATCATTATTGAATTATGTTTCTAGATAGGTTGTAG
CTGGCTCTTTTAGAGCATGTGCACGCCTGTTTGGACTTCATTTTCATCCACCTGTGCACC
TATTGTAGTCTTTGGTTGGGTTAGGAGGAAGTGATCATTGTATCAGCATCTGCTGGGAGT
GAGGACTTGCATTGTGAAAGCTTTGCTGTCCTTGATGTGATCATGGAATCTTTTTC----
------------------------------------------------------------
------------------------------------------------------------
------------------------------------------------------------
------------------------------------------------------------
------------------------------------------------------------
------------------------------------------------------------
------------------------------------------------------------
------------------------------------------------------------
------------------------------------------------------------
------------------------------------------------------------
------------------------------------------------------------
------------------------------------------------------------
-------TCACTAGAGTCTATGTCACTCATTATACTCTGTCGAATGTCATTGAATGTCTT
TACATGGGCTTGTATGCCTATGAAAATTGTAATACAACTTTCAGCAACGGATCTCTTGGC
TCTCGCATCGATGAAGAACGCAGCGAAATGTGATAAGTAATGTGAATTGCAGAATTCAGT
```

GAATCATCGAATCTTTGAACGCATCTTGCGCTCCTTGGTATTCCGAGGAGCATGCCTGTT
TGAGTGTCATTAAATTCTCAACTCTCTTATACTTTTTTGTAAAAGAGAGCTTGGACTGTG
GAGGCTTGCTGGCCACTTTTTGGGGTCAGCTCCTCTGAAATGCATTAGCGGAACCGTTTG
CGATCTGCCACAAGTGTGATAAGTTATCTACACTGGCGAGGGGATTGCTCTCTGTAATGT
TCAGCTTCTAATTGTCTCTACTTTGTGAGACTACTTTTGAATGCTTGACCTCAAATCAGG
TAGGACTACCCGCTGAACTTAA
>AC9-7
TTTCCGTAGGTGAACCTGCGGAAGGATCATTATTGAATTATGTTTCTAGATAGGTTGTAG
CTGGCTCTTTTAGAGCATGTGCACGCCTGTTTGGACTTCATTTTCATCCACCTGTGCACC
TATTGTAGTCTTTGGTTGGGTTAGGAGGAAGTGATCATTGTATCAGCATCTGCTGGGAGT
GAGGACTTGCATTGTGAAAGCTTTGCTGTCCTTGATGTGATCATGGAATCTTTTTC----

-------TCACTAGAGTCTATGTCACTCATTATACTCTGTCGAATGTCATTGAATGTCTT
TACATGGGCTTGTATGCCTATGAAAATTGTAATACAACTTTCAGCAACGGATCTCTTGGC
TCTCGCATCGATGAAGAACGCAGCGAAATGCGATAAGTAATGTGAATTGTAGAATTCAGT
GAATCATCGAATCTTTGAACGCATCTTGCGCTCCTTGGTATTCCGAGGAGCATGCCTGTT
TGAGTGTCATTAAATTCTCAACTCTCTTATACTTTTTTGTAAAAGAGAGCTTGGACTGTG
GAGGCTTGCTGGCCACTTTTTGGGGTCAGCTCCTCTGAAATGCATTAGCGGAACCGTTTG
CGATCTGCCACAAGTGTGATAAGTTATCTACACTGGCGAGGGGATTGCTCTCTGTAATGT
TCAGCTTCTAATTGTCTCTACTTTGTGAGACTACTTTTGAATGCTTGACCTCAAATCAGG
TAGGACTACCCGCTGAACTTAA
>AC9-47
TTTCCGTAGGTGAACCTGCGGAAGGATCATTATTGAATTATGTTTCTAGATAGGTTGTAG
CTGGCTCTTTTAGAGCATGTGCACGCCTGTTTGGACTTCATTTTCATCCACCTGTGCACC
TATTGTAGTCTTTGGTTGGGTTAGGAGGAAGTGATCATTGTATCAGCATCTGCTGGGAGT
GAGGACTTGCATTGTGAAAGCTTTGCTGTCCTTGATGTGATCATGGAATCTTTTTC----

-------TCACTAGAGTCTATGTCACTCATTATACTCTGTCGAATGTCATTGAATGTCTT

```
TACATGGGCTTGTATGTCTATGAAAATTGTAATACAACTTTCAGCAACGGATCTCTTGGC
TCTCGCATCGATGAAGAACGCAGCGAAATGCGATAAGTAATGTGAATTGCAGAATTCAGT
GAATCATCGAATCTTTGAACGCATCTTGCGCTCCTTGGTATTCCGAGGAGCATGCCTGTT
TGAGTGTCATTAAATTCTCAACTCTCTTATACTTTTTTGTAAAAGAGAGCTTGGACTGTG
GAGGCTTGCTGGCCACTTTTTGGGGTCAGCTCCTCTGAAATGCATTAGCGGAACCGTTTG
CGATCTGCCACAAGTGTGATAAGTTATCTACACTGGCGAGGGGATTGCTCTCTGTAATGT
TCAGCTTCTAATTGTCTCTACTTTGTGAGACTACTTTTGAATGCTTGACCTCAAATCAGG
TAGGACTACCCGCTGAACTTAA
>AC4-46
TTTCCGTAGGTGAACCTGCGGAAGGATCATTATTGAATTATGTTTCTAGATAGGTTGTAG
CTGGCTCTTTTAGAGCATGTGCACGCCTGTTTGGACTTCATTTTCATCCACCTGTGCACC
TATTGTAGTCTTTGGTTGGGTTAGGAGGAAGTGATCATTGTATCAGCATCTGCTGGGAGT
GAGGACTTGCATTGTGAAAGCTTTGCTGTCCTTGATGTGATCATGGAATCTTTTTC----
------------------------------------------------------------
------------------------------------------------------------
------------------------------------------------------------
------------------------------------------------------------
------------------------------------------------------------
------------------------------------------------------------
------------------------------------------------------------
------------------------------------------------------------
------------------------------------------------------------
------------------------------------------------------------
------------------------------------------------------------
------------------------------------------------------------
-------TCACTAGAGTCTATGTCACTCATTATACTCTGTCGAATGTCATTGAATGTCTT
TACATGGGCTTGTATGCCTATGAAAATTGTAATACAACTTTCAGCAACGGATCTCTTGGC
TCTCGCATCGATGAAGAACGCAGCGAAATGCGATAAGTAATGTGAATTGCAGAATTCAGT
GAATCATCGAATCTTTGAACGCATCTTGCGCTCCTTGGTATTCCGAGGAGCATGCCTGTT
TGAGTGTCATTAAATTCTCAACTCTCTTATACTTTTTTGTAAAAGAGAGCTTGGACTGTG
GAGGCTTGCTGACCACTTTTTGGGGTCAGCTCCTCTGAAATGCATTAGCGGAACCGTTTG
CGATCTGCCACAAGTGTGATAAGTTATCTACACTGGCGAGGGGATTGCTCTCTGTAATGT
TCAGCTTCTAATTGTCTCTACTTTGTGAGACTACTTTTGAATGCTTGACCTCAAATCAGG
TAGGACTACCCGCTGAACTTAA
>AC6-25
TTTCCGTAGGTGAACCTGCGGAAGGATCATTATTGAATTATGTTTCTAGATAGGTTGTAG
CTGGCTCTTTTAGAGCATGTGCACGCCTGTTTGGACTTCATTTTCATCCACCTGTGCACC
TATTGTAGTCTTTGGTTGGGTTAGGAGGAAGTGATCATTGTATCAGCATCTGCTGGGAGT
GAGGACTTGCATTGTGAAAGCTTTGCTGTCCTTGATGTGATCATGGAATCTTTTTC----
------------------------------------------------------------
------------------------------------------------------------
------------------------------------------------------------
------------------------------------------------------------
------------------------------------------------------------
------------------------------------------------------------
------------------------------------------------------------
------------------------------------------------------------
------------------------------------------------------------
------------------------------------------------------------
------------------------------------------------------------
```

```
------------------------------------------------------------
-------TCACTAGAGTCTATGTCACTCATTATACTCTGTCGAATGTCATTGAATGTCTT
TACATGGGCTTGTATGCCTATGAAAATTGTAATACAACTTTCAGCAACGGATCTCTTGGC
TCTCGCATCGATGAAGAACGCAGCGAAATGCGATAAGTAATGTGAATTGCAGAATTCAGT
GAATCATCGAATCTTTGAACGCATCTTGCGCTCCTTGGTATTCCGAGGAGCATGCCTGTT
TGAGTGTCATTAAATTCTCAACTCTCTTATACTTTTTTGTAAAAGAGAGCTTGGACTGTG
GAGGCTTGTTGACCACTTTTTGGGGTCAGCTCCTCTGAAATGCATTAGCGGAACCGTTTG
CGATCTGCCACAAGTGTGATAAGTTATCTACACTGGCGAGGGGATTGCTCTCTGTAATGT
TCAGCTTCTAATTGTCTCTACTTTGTGAGACTACTTTTGAATGCTTGACCTCAAATCAGG
TAGGACTACCCGCTGAACTTAA
>AC10-55
TTTCCGTAGGTGAACCTGCGGAAGGATCATTATTGAATTATGTTTCTAGATAGGTTGTAG
CTGGCTCTTTTAGAGCATGTGCACGCCTGTTTGGACTTCATTTTCATCCACCTGTGCACC
TATTGTAGTCTTTGGTTGGGTTAGGAGGAAGTGATCATTGTATCAGCATCTGCTGGGAGT
GAGGACTTGCATTGTGAAAGCTTTGCTGTCCTTGATGTGATCATGGAATCTTTTTC----
------------------------------------------------------------
------------------------------------------------------------
------------------------------------------------------------
------------------------------------------------------------
------------------------------------------------------------
------------------------------------------------------------
------------------------------------------------------------
------------------------------------------------------------
------------------------------------------------------------
------------------------------------------------------------
------------------------------------------------------------
------------------------------------------------------------
-------TCACTAGAGTCTATGTCACTCATTATACTCTGTCGAATGTCATTGAATGTCTT
TACATGGGCTTGTATGCCTATGAAAATTGTAATACAACTTTCAGCAACGGATCTCTTGGC
TCTCGCATCGATGAAGAACGCAGCGAAATGCGATAAGTAATGTGAATTGCAGAATTCAGT
GAATCATCGAATCTTTGAACGCATCTTGCGCTCCTTGGTATTCCGAGGAGCATGCCTGTT
TGAGTGTCATTAAATTCTCAACTCTCTTATACTTTTTTGTAAAAGAGAGCTTGGACTGTG
GAGGCTTGCTGGCCACTTTTTGGGGTCAGCTCCTCTGAAATGCATTCGCGGAACCGTTTG
CGATCTGCCACAAGTGTGATAAGTTATCTACACTGGCGAGGGGATTGCTCTCTGTAATGT
TCAGCTTCTAATTGTCTCTACTTTGTGAGACTACTTTTGAATGCTTGACCTCAAATCAGG
TAGGACTACCCGCTGAACTTAA
>AC1-38
TTTCCGTAGGTGAACCTGCGGAAGGATCATTATTGAATTATGTTTCTAGATAGGTTGTAG
CTGGCTCTTTTAGAGCATGTGCACGCCTGTTTGGACTTCATTTTCATCCACCTGTGCACC
TATTGTAGTCTTTGGTTGGGTTAGGAGGAAGTGATCATTGTGTCAGCATCTGCTGGATGT
GAGGACTTGCATTGTGAAAGCTTTGCTGTCCTTGATGTGATCATGGAATCTCTTTC----
------------------------------------------------------------
------------------------------------------------------------
------------------------------------------------------------
------------------------------------------------------------
------------------------------------------------------------
------------------------------------------------------------
------------------------------------------------------------
------------------------------------------------------------
------------------------------------------------------------
```


-------TCACTAGAGTCTATGTCACTCATTATACTCTGTCGAATGTCATTGAATGTCTT
TACATGGGCTTATATGCCTATGAAAATTGTAATACAACTTTCAGCAACGGATCTCTTGGC
TCTCGCATCGATGAAGAACGCAGCGAAATGCGATAAGTAATGTGAATTGCAGAATTCAGT
GAATCATCGAATCTTTGAACGCATCTTGCGCTCCTTGGTATTCCGAGGAGCATGCCTGTT
TGAGTGTCATTAAATTCTCAACTCTCTTATACTTTTTTGTAAAAGAGAGCTTGGACTGTG
GAGGCTTGCTGGCCACTTTTTGGGGTCAGCTCCTCTGAAATGCATTAGCGGAACCGTTTG
CGATCTGCCACAAGTGTGATAAGTTATCTACACTGGCGAGGGGATTGCTCTCTGTAATGT
TCAGCTTCTAATTGTCTCTACTTTGTGAGACTACTTTTGAATGCTTGACCTCAAATCAGG
TAGGACTACCCGCTGAACTTAA
>AC3-11
TTTCCGTAGGTGAACCTGCGGAAGGATCATTATTGAATTATGTTTCTAGATAGGTTGTAG
CTGGCTCTTTTAGAGCATGTGCACGCCTGTTTGGACTTCATTTTCATCCACCTGTGCACC
TATTGTAGTCTTTGGTTGGGTTAGGAGGAAGTGATCATTGTGTCAGCATCTGCTGGATGT
GAGGACTTGCATTGTGAAAGCTTTGCTGTCCTTGATGTGATCATGGAATCTCTTTC----

-------TCACTAGAGTCTATGTCACTCATTATACTCTGTCGAATGTCATTGAATGTCTT
TACATGGGCTTATATGCCTATGAAAATTGTAATACAACTTTCAGCAACGGATCTCTTGGC
TCTCGCATCGATGAAGAACGCAGCGAAATGCGATAAGTAATGTGAATTGCAGAATTCAGT
GAATCATCGAATCTTTGAACGCATCTTGCGCTCCTTGGTATTCCGAGGAGCATGCCTGTT
TGAGTGTCATTAAATTCTCAACTCTCTTATACTTTTTTGTAAAAGAGAGCTTGGACTGTG
GAGGCTTGCTGGCCACTTTTTGGGGTCAGCTCCTCTGAAATGCATTAGCGGAACCGTTTG
CGATCTGCCACAAGTGTGATAAGTTATCTACACTGGCGAGGGGATTGCTCTCTGTAATGT
TCAGCTTCTAATTGTCTCTACTTTGTGAGACTACTTTTGAATGCTTGACCTCAAATCAGG
TAGGACTACCCGCTGAACTTAA
>AC1-64
TTTCCGTAGGTGAACCTGCGGAAGGATCATTATTGAATTATGTTTCTAGATAGGTTGTAG
CTGGCTCTTTTAGAGCATGTGCACGCCTGTTTGGACTTCATTTTCATCCACCTGTGCACC
TATTGTAGTCTTTGGTTGGGTTAGGAGGAAGTGGTCATTGTGTCAGCATCTGCTGGATGT
GAGGACTTGCATTGTGAAAGCTTTGCTGTCCTTGATGTGATCATGGAATCTCTTTC----


```
------------------------------------------------------------
------------------------------------------------------------
------------------------------------------------------------
------------------------------------------------------------
------------------------------------------------------------
-------TCACTAGAGTCTATGTCACTCATTATACTCTGTCGAATGTCATTGAATGTCTT
TACATGGGCTTATATGCCTATGAAAATTGTAATACAACTTTCAGCAACGGATCTCTTGGC
TCTCGCATCGATGAAGAACGCAGCGAAATGCGATAAGTAATGTGAATTGCAGAATTCAGT
GAATCATCGAATCTTTGAACGCATCTTGCGCTCCTTGGTATTCCGAGGAGCATGCCTGTT
TGAGTGTCATTAAATTCTCAACTCTCTTATACTTTTTTGTAAAAGAGAGCTTGGACTGTG
GAGGCTTGCTGGCCACTTTTTGGGGTCAGCTCCTCTGAAATGCATTAGCGGAACCGTTTG
CGATCTGCCACAAGTGTGATAAGTTATCTACACTGGCGAGGGGATTGCTCTCTGTAATGT
TCAGCTTCTAATTGTCTCTACTTTGTGAGACTACTTTTGAATGCTTGACCTCAAATCAGG
TAGGACTACCCGCTGAACTTAA
>AC5-10
TTTCCGTAGGTGAACCTGCGGAAGGATCATTATTGAATTATGTTTCTAGATAGGTTGTAG
CTGGCTCTTTTAGAGCATGTGCACGCCTGTTTGGACTTCATTTTCATCCACCTGTGCACC
TATTGTAGTCTTTGGTTGGGTTAGGAGGAAGTGGTCATTGTGTCAGCATCTGCTGGATGT
GAGGACTTGCATTGTGAAAGCTTTGCTGTCCTTGATGTGATCATGGAATCTCTTTC----
------------------------------------------------------------
------------------------------------------------------------
------------------------------------------------------------
------------------------------------------------------------
------------------------------------------------------------
------------------------------------------------------------
------------------------------------------------------------
------------------------------------------------------------
------------------------------------------------------------
------------------------------------------------------------
------------------------------------------------------------
------------------------------------------------------------
-------TCACTAGAGTCTATGTCACTCATTATACTCTGTCGAATGTCATTGAATGTCTT
TACATGGGCTTATATGCCTATGAAAATTGTAATACAACTTTCAGCAACGGATCTCTTGGC
TCTCGCATCGATGAAGAACGCAGCGAAATGCGATAAGTAATGTGAATTGCAGAATTCAGT
GAATCATCGAATCTTTGAACGCATCTTGCGCTCCTTGGTATTCCGAGGAGCATGCCTGTT
TGAGTGTCATTAAATTCTCAACTCTCTTATACTTTTTTGTAAAAGAGAGCTTGGACTGTG
GAGGCTTGCTGGCCACTTTTTGGGGTCAGCTCCTCTGAAATGCATTAGCGGAACCGTTTG
CGATCTGCCACAAGTGTGATAAGTTATCTACACTGGCGAGGGGATTGCTCTCTGTAATGT
TCAGCTTCTAATTGTCTCTACTTTGTGAGACTACTTTTGAATGCTTGACCTCAAATCAGG
TAGGACTACCCGCTGAACTTAA
>AC9-13
TTTCCGTAGGTGAACCTGCGGAAGGATCATTATTGAATTATGTTTCTAGATAGGTTGTAG
CTGGCTCTTTTAGAGCATGTGCACGCCTGTTTGGACTTCATTTTCATCCACCTGTGCACC
TATTGTAGTCTTTGGTTGGGTTAGGAGGAAGTGGTCATTGTGTCAGCATCTGCTGGATGT
GAGGACTTGCATTGTGAAAGCTTTGCTGTCCTTGATGTGATCATGGAATCTCTTTC----
------------------------------------------------------------
------------------------------------------------------------
------------------------------------------------------------
------------------------------------------------------------
------------------------------------------------------------
```


-------TCACTAGAGTCTATGTCACTCATTATACTCTGTCGAATGTCATTGAATGTCTT
TACATGGGCTTATATGCCTATGAAAATTGTAATACAACTTTCAGCAACGGATCTCTTGGC
TCTCGCATCGATGAAGAACGCAGCGAAATGCGATAAGTAATGTGAATTGCAGAATTCAGT
GAATCATCGAATCTTTGAACGCATCTTGCGCTCCTTGGTATTCCGAGGAGCATGCCTGTT
TGAGTGTCATTAAATTCTCAACTCTCTTATACTTTTTTGTAAAAGAGAGCTTGGACTGTG
GAGGCTTGCTGGCCACTTTTTGGGGTCAGCTCCTCTGAAATGCATTAGCGGAACCGTTTG
CGATCTGCCACAAGTGTGATAAGTTATCTACACTGGCGAGGGGATTGCTCTCTGTAATGT
TCAGCTTCTAATTGTCTCTACTTTGTGAGACTACTTTTGAATGCTTGACCTCAAATCAGG
TAGGACTACCCGCTGAACTTAA
>AC12-42
TTTCCGTAGGTGAACCTGCGGAAGGATCATTATTGAATTATGTTTCTAGATAGGTTGTAG
CTGGCTCTTTTAGAGCATGTGCACGCCTGTTTGGACTTCATTTTCATCCACCTGTGCACC
TATTGTAGTCTTTGGTTGGGTTAGGAGGAAGTGGTCATTGTGTCAGCATCTGCTGGATGT
GAGGACTTGCATTGTGAAAGCTTTGCTGTCCTTGATGTGATCATGGAATCTCTTTC----

-------TCACTAGAGTCTATGTCACTCATTATACTCTGTCGAATGTCATTGAATGTCTT
TACATGGGCTTATATGCCTATGAAAATTGTAATACAACTTTCAGCAACGGATCTCTTGGC
TCTCGCATCGATGAAGAACGCAGCGAAATGCGATAAGTAATGTGAATTGCAGAATTCAGT
GAATCATCGAATCTTTGAACGCATCTTGCGCTCCTTGGTATTCCGAGGAGCATGCCTGTT
TGAGTGTCATTAAATTCTCAACTCTCTTATACTTTTTTGTAAAAGAGAGCTTGGACTGTG
GAGGCTTGCTGGCCACTTTTTGGGGTCAGCTCCTCTGAAATGCATTAGCGGAACCGTTTG
CGATCTGCCACAAGTGTGATAAGTTATCTACACTGGCGAGGGGATTGCTCTCTGTAATGT
TCAGCTTCTAATTGTCTCTACTTTGTGAGACTACTTTTGAATGCTTGACCTCAAATCAGG
TAGGACTACCCGCTGAACTTAA
>AC12-31
TTTCCGTAGGTGAACCTGCGGAAGGATCATTATTGAATTATGTTTCTAGATAGGTTGTAG
CTGGCTCTTTTAGAGCATGTGCACGCCTGTTTGGACTTCATTTTCATCCACCTGTGCACC
TATTGTAGTCTTTGGTTGGGTTAGGAGGAAGTGGTCATTGTGTCAGCATCTGCTGGATGT
GAGGACTTGCATTGTGAAAGCTTTGCTGTCCTTGATGTGATCATGGAATCTCTTTC----

-------TCACTAGAGTCTATGTCACTCATTATACTCTGTCGAATGTCATTGAATGTCTT
TACATGGGCTTATATGCCTATGAAAATTGTAATACAACTTTCAGCAACGGATCTCTTGGC
TCTCGCATCGATGAAGAACGCAGCGAAATGCGATAAGTAATGTGAATTGCAGAATTCAGT
GAATCATCGAATCTTTGAACGCATCTTGCGCTCCTTGGTATTCCGAGGAGCATGCCTGTT
TGAGTGTCATTAAATTCTCAACTCTCTTATACTTTTTTGTAAAAGAGAGCTTGGACTGTG
GAGGCTTGCTGGCCACTTTTTGGGGTCAGCTCCTCTGAAATGCATTAGCGGAACCGTTTG
CGATCTGCCACAAGTGTGATAAGTTATCTACACTGGCGAGGGGATTGCTCTCTGCAATGT
TCAGCTTCTAATTGTCTCTACTTTGTGAGACTACTTTTGAATGCTTGACCTCAAATCAGG
TAGGACTACCCGCTGAACTTAA
>AC10-12
TTTCCGTAGGTGAACCTGCGGAAGGATCATTATTGAATTATGTTTCTAGATAGGTTGTAG
CTGGCTCTTTTAGAGCATGTGCACGCCTGTTTGGACTTCATTTTCATCCACCTGTGCACC
TATTGTAGTCTTTGGTTGGGTTAGGAGGAAGTGGTCATTGTGTCAGCATCTGCTGGATGT
GAGGACTTGCATTGTGAAAGCTTTGCTGTCCTTGATGTGATCATGGAATCTTTTTC----

-------TCACTAGAGTCTATGTCACTCATTATACTCTGTCGAATGTCATTGAATGTCTT
TACATGGGCTTGTATGCCTATGAAAATTGTAATACAACTTTCAGCAACGGATCTCTTGGC
TCTCGCATCGATGAAGAACGCAGCGAAATGCGATAAGTAATGTGAATTGCAGAATTCAGT
GAATCATCGAATCTTTGAACGCATCTTGCGCTCCTTGGTATTCCGAGGAGCATGCCTGTT
TGAGTGTCATTAAATTCTCAACTCTCTTATACTTTTTTGTAAAAGAGAGCTTGGACTGTG
GAGGCTTGCTGGCCACTTTTTGGGGTCAGCTCCTCTGAAATGCATTAGCGGAACCGTTTG
CGATCTGCCACAAGTGTGATAAGTTATCTACACTGGCGAGGGGATTGCTCTCTGTAATGT
TCAGCTTCTAATTGTCTCTACTTTGTGAGACTACTTTTGAATGCTTGACCTCAAATCAGG
TAGGACTACCCGCTGAACTTAA
>AC11-28
TTTCCGTAGGTGAACCTGCGGAAGGATCATTATTGAATTATGTTTCTAGATAGGTTGTAG
CTGGCTCTTTTAGAGCATGTGCACGCCTGTTTGGACTTCATTTTCATCCACCTGTGCACC
TATTGTAGTCTTTGGTTGGGTTAGGAGGAAGTGGTCATTGTGTCAGCATCTGCTGGATGT
GAGGACTTGCATTGTGAAAGCTTTGCTGTCCTTGATGTGATCATGGAATCTTTTTC----

-------TCACTAGAGTCTATGTCACTCATTATACTCTGTCGAATGTCATTGAATGTCTT
TACATGGGCTTATATGCCTATGAAAATTGTAATACAACTTTCAGCAACGGATCTCTTGGC
TCTCGCATCGATGAAGAACGCAGCGAAATGCGATAAGTAATGTGAATTGCAGAATTCAGT
GAATCATCGAATCTTTGAACGCATCTTGCGCTCCTTGGTATTCCGAGGAGCATGCCTGTT
TGAGTGTCATTAAATTCTCAACTCTCTTATACTTTTTTGTAAAAGAGAGCTTGGACTGTG
GAGGCTTGCTGGCCACTTTTTGGGGTCAGCTCCTCTGAAATGCATTAGCGGAACCGTTTG
CGATCTGCCACAAGTGTGATAAGTTATCTACACTGGCGAGGGGATTGCTCTCTGTAATGT
TCAGCTTCTAATTGTCTCTACTTTGTGAGACTACTTTTGAATGCTTGACCTCAAATCAGG
TAGGACTACCCGCTGAACTTAA
>AC10-7
TTTCCGTAGGTGAACCTGCGGAAGGATCATTATTGAATTATGTTTCTAGATAGGTTGTAG
CTGGCTCTTTTAGAGCATGTGCACGCCTGTTTGGACTTCATTTTCATCCACCTGTGCACC
TATTGTAGTCTTTGGTTGGGTTAGGAGGAAGTGATCATTGTATCAGCATCTGCTGGATGT
GAGGACTTGCATTGTGAAAGCTTTGCTGTCCTTGATGTGATCATGGAATCTCTTTC----

-------TCACTAGAGTCTATGTCACTCATTATACTCTGTCGAATGTCATTGAATGTCTT
TACATGGGCTTATATGCCTATGAAAATTGTAATACAACTTTCAGCAACGGATCTCTTGGC
TCTCGCATCGATGAAGAACGCAGCGAAATGCGATAAGTAATGTGAATTGCAGAATTCAGT
GAATCATCGAATCTTTGAACGCATCTTGCGCTCCTTGGTATTCCGAGGAGCATGCCTGTT
TGAGTGTCATTAAATTCTCAACTCTCTTATACTTTTTTGTAAAAGAGAGCTTGGACTGTG
GAGGCTTGCTGGCCACTTTTTGGGGTCAGCTCCTCTGAAATGCATTAGCGGAACCGTTTG
CGATCTGCCACAAGTGTGATAAGTTATCTACACTGGCGAGGGGATTGCTCTCTGTAATGT
TCAGCTTCTAATTGTCTCTACTTTGTGAGACTACTTTTGAATGCTTGACCTCAAATCAGG
TAGGACTACCCGCTGAACTTAA
>AC6-13
TTTCCGTAGGTGAACCTGCGGAAGGATCATTATTGAATTATGTTTCTAGATAGGTTGTAG
CTGGCTCTTTTAGAGCATGTGCACGCCTGTTTGGACTTCATTTTCATCCACCTGTGCACC
TATTGTAGTCTTTGGTTGGGTTAGGAGGAAGTGATCATTGTATCAGCATCTGCTGGATGT

GAGGACTTGCATTGTGAAAGCTTTGCTGTCCTTGATGTGATCATGGAATCTCTTTC----

-------TCACTAGAGTCTATGTCACTCATTATACTCTGTCGAATGTCATTGAATGTCTT
TACATGGGCTTGTATGCCTATGAAAATTGTAATACAACTTTCAGCAACGGATCTCTTGGC
TCTCGCATCGATGAAGAACGCAGCGAAATGCGATAAGTAATGTGAATTGCAGAATTCAGT
GAATCATCGAATCTTTGAACGCATCTTGCGCTCCTTGGTATTCCGAGGAGCATGCCTGTT
TGAGTGTCATTAAATTCTCAACTCTCTTATACTTTTTTGTAAAAGAGAGCTTGGACTGTG
GAGGCTTGCTGGCCACTTTTTGGGGTCAGCTCCTCTGAAATGCATTAGCGGAACCGTTTG
CGATCTGCCACAAGTGTGATAAGTTATCTACACTGGCGAGGGGATTGCTCTCTGTAATGT
TCAGCTTCTAATTGTCTCTACTTTGTGAGACTACTTTTGAATGCTTGACCTCAAATCAGG
TAGGACTACCCGCTGAACTTAA
>AC8-40
TTTCCGTAGGTGAACCTGCGGAAGGATCATTATTGAATTATGTTTCTAGATAGGTTGTAG
CTGGCTCTTTTAGAGCATGTGCACGCCTGTTTGGACTTCATTTTCATCCACCTGTGCACC
TATTGTAGTCTTTGGTTGGGTTAGGAGGAAGTGATCATTGTATCAGCATCTGCTGGATGT
GAGGACTTGCATTGTGAAAGCTTTGCTGTCCTTGATGTGATCATGGAATCTCTTTC----

-------TCACTAGAGTCTATGTCACTCATTATACTCTGTCGAATGTCATTGAATGTCTT
TACATGGGCTTGTATGCCTATGAAAATTGTAATACAACTTTCAGCAACGGATCTCTTGGC
TCTCGCATCGATGAAGAACGCAGCGAAATGCGATAAGTAATGTGAATTGCAGAATTCAGT
GAATCATCGAATCTTTGAACGCATCTTGCGCTCCTTGGTATTCCGAGGAGCATGCCTGTT
TGAGTGTCATTAAATTCTCAACTCTCTTATACTTTTTTGTAAAAGAGAGCTTGGACTGTG
GAGGCTTGCTGGCCACTTTTTGGGGTCAGCTCCTCTGAAATGCATTAGCGGAACCGTTTG
CGATCTGCCACAAGTGTGATAAGTTATCTACACTGGCGAGGGGATTGCTCTCTGTAATGT
TCAGCTTCTAATTGTCTCTACTTTGTGAGACTACTTTTGAATGCTTGACCTCAAATCAGG
TAGGACTACCCGCTGAACTTAA
>AC12-12
TTTCCGTAGGTGAACCTGCGGAAGGATCATTATTGAATTATGTTTCTAGATAGGTTGTAG

CTGGCTCTTTTAGAGCATGTGCACGCCTGTTTGGACTTCATTTTCATCCACCTGTGCACC
TATTGTAGTCTTTGGTTGGGTTAGGAGGAAGTGATCATTGTATCAGCATCTGCTGGATGT
GAGGACTTGCATTGTGAAAGCTTTGCTGTCCTTGATGTGATCATGGAATCTCTTTC----

-------TCACTAGAGTCTATGTCACTCATTATACTCTGTCGAATGTCATTGAATGTCTT
TACATGGGCTTGTATGCCTATGAAAATTGTAATACAACTTTCAGCAACGGATCTCTTGGC
TCTCGCATCGATGAAGAACGCAGCGAAATGCGATAAGTAATGTGAATTGCAGAATTCAGT
GAATCATCGAATCTTTGAACGCATCTTGCGCTCCTTGGTATTCCGAGGAGCATGCCTGTT
TGAGTGTCATTAAATTCTCAACTCTCTTATACTTTTTTGTAAAAGAGAGCTTGGACTGTG
GAGGCTTGCTGGCCACTTTTTGGGGTCAGCTCCTCTGAAATGCATTAGCGGAACCGTTTG
CGATCTGCCACAAGTGTGATAAGTTATCTACACTGGCGAGGGGATTGCTCTCTGTAATGT
TCAGCTTCTAATTGTCTCTACTTTGTGAGACTACTTTTGAATGCTTGACCTCAAATCAGG
TAGGACTACCCGCTGAACTTAA
>AC6-49
TTTCCGTAGGTGAACCTGCGGAAGGATCATTATTGAATTATGTTTCTAGATAGGTTGTAG
CTGGCTCTTTTAGAGCATGTGCACGCCTGTTTGGACTTCATTTTCATCCACCTGTGCACC
TATTGTAGTCTTTGGTTGGGTTAGGAGGAAGTGATCATTGTATCAGCATCTGCTGGATGT
GAGGACTTGCATTGTGAAAGCTTTGCTGTCCTTGATGTGATCATGGAATCTTTTTC----

-------TCACTAGAGTCTATGTCACTCATTATACTCTGTCGAATGTCATTGAATGTCTT
TACATGGGCTTGTATGCCTATGAAAATTGTAATACAACTTTCAGCAACGGATCTCTTGGC
TCTCGCATCGATGAAGAACGCAGCGAAATGCGATAAGTAATGTGAATTGCAGAATTCAGT
GAATCATCGAATCTTTGAACGCATCTTGCGCTCCTTGGTATTCCGAGGAGCATGCCTGTT
TGAGTGTCATTAAATTCTCAACTCTCTTATACTTTTTTGTAAAAGAGAGCTTGGACTGTG
GAGGCTTGCTGGCCACTTTTTGGGGTCAGCTCCTCTGAAATGCATTAGCGGAACCGTTTG
CGATCTGCCACAAGTGTGATAAGTTATCTACACTGGCGAGGGGATTGCTCTCTGTAATGT
TCAGCTTCTAATTGTCTCTACTTTGTGAGACTACTTTTGAATGCTTGACCTCAAATCAGG
TAGGACTACCCGCTGAACTTAA

>AC4-47
TTTCCGTAGGTGAACCTGCGGAAGGATCATTATTGAATTATGTTTCTAGATAGGTTGTAG
CTGGCTCTTTTAGAGCATGTGCACGCCTGTTTGGACTTCATTTTCATCCACCTGTGCACC
TATTGTAGTCTTTGGTTGGGTTAGGAGGAAGTGATCATTGTATCAGCATCTGCTGGGTGT
GAGGACTTGCATTGTGAAAGCTTTGCTGTCCTTGATGTGATCATGGAATCTTTTTC----

-------TCACTAGAGTCTATGTCACTCATTATACTCTGTCGAATGTCATTGAATGTCTT
TACATGGGCTTATATGCCTATGAAAATTGTAATACAACTTTCAGCAACGGATCTCTTGGC
TCTCGCATCGATGAAGAACGCAGCGAAATGCGATAAGTAATGTGAATTGCAGAATTCAGT
GAATCATCGAATCTTTGAACGCATCTTGCGCTCCTTGGTATTCCGAGGAGCATGCCTGTT
TGAGTGTCATTAAATTCTCAACTCTCTTATACTTTTTTGTAAAAGAGAGCTTGGACTGTG
GAGGCTTGCTGGCCACTTTTTGGGGTCAGCTCCTCTGAAATGCATTAGCGGAACCGTTTG
CGATCTGCCACAAGTGTGATAAGTTATCTACACTGGCGAGGGGATTGCTCTCTGTAATGT
TCAGCTTCTAATTGTCTCTACTTTGTGAGACTACTTTTGAATGCTTGACCTCAAATCAGG
TAGGACTACCCGCTGAACTTAA
>AC11-29
TTTCCGTAGGTGAACCTGCGGAAGGATCATTATTGAATTATGTTTCTAGATAGGTTGTAG
CTGGCTCTTTTAGAGCATGTGCACGCCTGTTTGGACTTCATTTTCATCCACCTGTGCACC
TATTGTAGTCTTTGGTTGGGTTAGGAGGAAGTGATCATTGTATCAGCATCTGCTGGGAGT
GAGGACTTGCATTGTGAAAGCTTTGCTGTCCTTGATGTGATCATGGAATCTTTTTCTCAC
TAGAGTCTATGTCACTCATTATACTCTGTCGAATGTCATTGAATGTCTTTACATGGGCTT
GTATGCCTATGAAAATTGTAATACAACTTTCAGCAACGGATCTCTTGGCTCTCGCATCGA
TGAAGAACGCAGCGAAATGCGATAAGTAATGTGAATTGCAGAATTCAGTGAATCATCGAA
TCTTTGAACGCATCTTGCGCTCCTTGGTATTCCGAGGAGCATGCCTGTTTGAGTGTCATT
AAATTCTCAACTCTCTTATACTTTTTTGTAAAAGAGAGCTTGGACTGTGGAGGCTTGCTG
GCCACTTTTTGGGGTCAGCTCCTCTGAAATGCATTAGCGGAACCGTTTGCGATCTGCCAC
AAGTGTGATAAGTTATCTACACTGGCGAGGGGATTGCTCTCTGTAATGTTCAGCTTCTAA
TTGTCTCTACTTTGTGAGACTACTTTTGAATGCTTGACCTCAAATCAGGTAGGACTACCC
GCTGAACTTAATTTCCGTAGGTGAACCTGCGGAAGGATCATTATTGAATTATGTTTCTAG
ATAGGTTGTAGCTGGCTCTTTTAGAGCATGTGCACGCCTGTTTGGACTTCATTTTCATCC
ACCTGTGCACCTATTGTAGTCTTTGGTTGGGTTAGGAGGAAGTGATCATTGTATCAGCAT
CTGCTGGGAGTGAGGACTTGCATTGTGAAAGCTTTGCTGTCCTTGATGTGATCATGGAAT
CTTTTTCTCACTAGAGTCTATGTCACTCATTATACTCTGTCGAATGTCATTGAATGTCTT
TACATGGGCTTGTATGCCTATGAAAATTGTAATACAACTTTCAGCAACGGATCTCTTGGC
TCTCGCATCGATGAAGAACGCAGCGAAATGCGATAAGTAATGTGAATTGCAGAATTCAGT
GAATCATCGAATCTTTGAACGCATCTTGCGCTCCTTGGTATTCCGAGGAGCATGCCTGTT
TGAGTGTCATTAAATTCTCAACTCTCTTATACTTTTTTGTAAAAGAGAGCTTGGACTGTG
GAGGCTTGCTGGCCACTTTTTGGGGTCAGCTCCTCTGAAATGCATTAGCGGAACCGTTTG
CGATCTGCCACAAGTGTGATAAGTTATCTACACTGGCGAGGGGATTGCTCTCTGTAATGT

TCAGCTTCTAATTGTCTCTACTTTGTGAGACTACTTTTGAATGCTTGACCTCAAATCAGG
TAGGACTACCCGCTGAACTTAA
>AC1-24
TTTCCGTAGGTGAACCTGCGGAAGGATCATTATTGAATTATGTTTCTAGATAGGTTGTAG
CTGGCTC-TTTAGAGCATGTGCACGCCTGTTTGGACTTCATTTTCATCCACCTGTGCACC
TATTGTAGTCTTTGGTTGGGTTAGGAGGAAGTGGTCATTGTGTCAGCATCTGCTGGATGT
GAGGACTTGCATTGTGAAAGCTTTGCTGTCCTTGATGTGATCATGGAATCTCTTTC----

-------TCACTAGAGTCTATGTCACTCATTATACTCTGTCGAATGTCATTGAATGTCTT
TACATGGGCTTGTATGCCTATGAAAATTGTAATACAACTTTCAGCAACGGATCTCTTGGC
TCTCGCATCGATGAAGAACGCAGCGAAATGCGATAAGTAATGTGAATTGCAGAATTCAGT
GAATCATCGAATCTTTGAACGCATCTTGCGCTCCTTGGTATTCCGAGGAGCATGCCTGTT
TGAGTGTCATTAAATTCTCAACTCTCTTATACTTTTTTGTAAAAGAGAGCTTGGACTGTG
GAGGCTTGCTGGCCACTTTTTGGGGTCAGCTCCTCTGAAATGCATTAGCGGAACCGTTTG
CGATCTGCCACAAGTGTGATAAGTTATCTACACTGGCGAGGGGATTGCTCTCTGTAATGT
TCAGCTTCTAATTGTCTCTACTTTGTGAGACTACTTTTGAATGCTTGACCTCAAATCAGG
TAGGACTACCCGCTGAACTTAA
>AC3-25
TTTCCGTAGGTGAACCTGCGGAAGGATCATTATTGAATTATGTTTCTAGATAGGTTGTAG
CTGGCTC-TTTAGAGCATGTGCACGCCTGTTTGGACTTCATTTTCATCCACCTGTGCACC
TATTGTAGTCTTTGGTTGGGTTAGGAGGAAGTGGTCATTGTGTCAGCATCTGCTGGATGT
GAGGACTTGCATTGTGAAAGCTTTGCTGTCCTTGATGTGATCATGGAATCTCTTTC----

-------TCACTAGAGTCTATGTCACTCATTATACTCTGTCGAATGTCATTGAATGTCTT
TACATGGGCTTGTATGCCTATGAAAATTGTAATACAACTTTCAGCAACGGATCTCTTGGC
TCTCGCATCGATGAAGAACGCAGCGAAATGCGATAAGTAATGTGAATTGCAGAATTCAGT
GAATCATCGAATCTTTGAACGCATCTTGCGCTCCTTGGTATTCCGAGGAGCATGCCTGTT
TGAGTGTCATTAAATTCTCAACTCTCTTATACTTTTTTGTAAAAGAGAGCTTGGACTGTG

GAGGCTTGCTGGCCACTTTTTGGGGTCAGCTCCTCTGAAATGCATTAGCGGAACCGTTTG
CGATCTGCCACAAGTGTGATAAGTTATCTACACTGGCGAGGGGATTGCTCTCTGTAATGT
TCAGCTTCTAATTGTCTCTACTTTGTGAGACTACTTTTGAATGCTTGACCTCAAATCAGG
TAGGACTACCCGCTGAACTTAA
>AC4-56
TTTCCGTAGGTGAACCTGCGGAAGGATCATTATTGAATTATGTTTCTAGATAGGTTGTAG
CTGGCTC-TTTAGAGCATGTGCACGCCTGTTTGGACTTCATTTTCATCCACCTGTGCACC
TATTGTAGTCTTTGGTTGGGTTAGGAGGAAGTGGTCATTGTGTCAGCATCTGCTGGATGT
GAGGACTTGCATTGTGAAAGCTTTGCTGTCCTTGATGTGATCATGGAATCTCTTTC----

-------TCACTAGAGTCTATGTCACTCATTATACTCTGTCGAATGTCATTGAATGTCTT
TACATGGGCTTGTATGCCTATGAAAATTGTAATACAACTTTCAGCAACGGATCTCTTGGC
TCTCGCATCGATGAAGAACGCAGCGAAATGCGATAAGTAATGTGAATTGCAGAATTCAGT
GAATCATCGAATCTTTGAACGCATCTTGCGCTCCTTGGTATTCCGAGGAGCATGCCTGTT
TGAGTGTCATTAAATTCTCAACTCTCTTATACTTTTTTGTAAAAGAGAGCTTGGACTGTG
GAGGCTTGCTGGCCACTTTTTGGGGTCAGCTCCTCTGAAATGCATTAGCGGAACCGTTTG
CGATCTGCCACAAGTGTGATAAGTTATCTACACTGGCGAGGGGATTGCTCTCTGTAATGT
TCAGCTTCTAATTGTCTCTACTTTGTGAGACTACTTTTGAATGCTTGACCTCAAATCAGG
TAGGACTACCCGCTGAACTTAA
>AC4-71
TTTCCGTAGGTGAACCTGCGGAAGGATCATTATTGAATTATGTTTCTAGATAGGTTGTAG
CTGGCTC-TTTAGAGCATGTGCACGCCTGTTTGGACTTCATTTTCATCCACCTGTGCACC
TATTGTAGTCTTTGGTTGGGTTAGGAGGAAGTGGTCATTGTGTCAGCATCTGCTGGATGT
GAGGACTTGCATTGTGAAAGCTTTGCTGTCCTTGATGTGATCATGGAATCTCTTTC----

-------TCACTAGAGTCTATGTCACTCATTATACTCTGTCGAATGTCATTGAATGTCTT
TACATGGGCTTGTATGCCTATGAAAATTGTAATACAACTTTCAGCAACGGATCTCTTGGC
TCTCGCATCGATGAAGAACGCAGCGAAATGCGATAAGTAATGTGAATTGCAGAATTCAGT

```
GAATCATCGAATCTTTGAACGCATCTTGCGCTCCTTGGTATTCCGAGGAGCATGCCTGTT
TGAGTGTCATTAAATTCTCAACTCTCTTATACTTTTTTGTAAAAGAGAGCTTGGACTGTG
GAGGCTTGCTGGCCACTTTTTGGGGTCAGCTCCTCTGAAATGCATTAGCGGAACCGTTTG
CGATCTGCCACAAGTGTGATAAGTTATCTACACTGGCGAGGGGATTGCTCTCTGTAATGT
TCAGCTTCTAATTGTCTCTACTTTGTGAGACTACTTTTGAATGCTTGACCTCAAATCAGG
TAGGACTACCCGCTGAACTTAA
>AC5-48
TTTCCGTAGGTGAACCTGCGGAAGGATCATTATTGAATTATGTTTCTAGATAGGTTGTAG
CTGGCTC-TTTAGAGCATGTGCACGCCTGTTTGGACTTCATTTTCATCCACCTGTGCACC
TATTGTAGTCTTTGGTTGGGTTAGGAGGAAGTGGTCATTGTGTCAGCATCTGCTGGATGT
GAGGACTTGCATTGTGAAAGCTTTGCTGTCCTTGATGTGATCATGGAATCTCTTTC----
------------------------------------------------------------
------------------------------------------------------------
------------------------------------------------------------
------------------------------------------------------------
------------------------------------------------------------
------------------------------------------------------------
------------------------------------------------------------
------------------------------------------------------------
------------------------------------------------------------
------------------------------------------------------------
------------------------------------------------------------
------------------------------------------------------------
-------TCACTAGAGTCTATGTCACTCATTATACTCTGTCGAATGTCATTGAATGTCTT
TACATGGGCTTGTATGCCTATGAAAATTGTAATACAACTTTCAGCAACGGATCTCTTGGC
TCTCGCATCGATGAAGAACGCAGCGAAATGCGATAAGTAATGTGAATTGCAGAATTCAGT
GAATCATCGAATCTTTGAACGCATCTTGCGCTCCTTGGTATTCCGAGGAGCATGCCTGTT
TGAGTGTCATTAAATTCTCAACTCTCTTATACTTTTTTGTAAAAGAGAGCTTGGACTGTG
GAGGCTTGCTGGCCACTTTTTGGGGTCAGCTCCTCTGAAATGCATTAGCGGAACCGTTTG
CGATCTGCCACAAGTGTGATAAGTTATCTACACTGGCGAGGGGATTGCTCTCTGTAATGT
TCAGCTTCTAATTGTCTCTACTTTGTGAGACTACTTTTGAATGCTTGACCTCAAATCAGG
TAGGACTACCCGCTGAACTTAA
>AC6-45
TTTCCGTAGGTGAACCTGCGGAAGGATCATTATTGAATTATGTTTCTAGATAGGTTGTAG
CTGGCTC-TTTAGAGCATGTGCACGCCTGTTTGGACTTCATTTTCATCCACCTGTGCACC
TATTGTAGTCTTTGGTTGGGTTAGGAGGAAGTGGTCATTGTGTCAGCATCTGCTGGATGT
GAGGACTTGCATTGTGAAAGCTTTGCTGTCCTTGATGTGATCATGGAATCTCTTTC----
------------------------------------------------------------
------------------------------------------------------------
------------------------------------------------------------
------------------------------------------------------------
------------------------------------------------------------
------------------------------------------------------------
------------------------------------------------------------
------------------------------------------------------------
------------------------------------------------------------
------------------------------------------------------------
------------------------------------------------------------
------------------------------------------------------------
-------TCACTAGAGTCTATGTCACTCATTATACTCTGTCGAATGTCATTGAATGTCTT
```

```
TACATGGGCTTGTATGCCTATGAAAATTGTAATACAACTTTCAGCAACGGATCTCTTGGC
TCTCGCATCGATGAAGAACGCAGCGAAATGCGATAAGTAATGTGAATTGCAGAATTCAGT
GAATCATCGAATCTTTGAACGCATCTTGCGCTCCTTGGTATTCCGAGGAGCATGCCTGTT
TGAGTGTCATTAAATTCTCAACTCTCTTATACTTTTTTGTAAAAGAGAGCTTGGACTGTG
GAGGCTTGCTGGCCACTTTTTGGGGTCAGCTCCTCTGAAATGCATTAGCGGAACCGTTTG
CGATCTGCCACAAGTGTGATAAGTTATCTACACTGGCGAGGGGATTGCTCTCTGTAATGT
TCAGCTTCTAATTGTCTCTACTTTGTGAGACTACTTTTGAATGCTTGACCTCAAATCAGG
TAGGACTACCCGCTGAACTTAA
>AC10-48
TTTCCGTAGGTGAACCTGCGGAAGGATCATTATTGAATTATGTTTCTAGATAGGTTGTAG
CTGGCTC-TTTAGAGCATGTGCACGCCTGTTTGGACTTCATTTTCATCCACCTGTGCACC
TATTGTAGTCTTTGGTTGGGTTAGGAGGAAGTGGTCATTGTGTCAGCATCTGCTGGATGT
GAGGACTTGCATTGTGAAAGCTTTGCTGTCCTTGATGTGATCATGGAATCTCTTTC----
------------------------------------------------------------
------------------------------------------------------------
------------------------------------------------------------
------------------------------------------------------------
------------------------------------------------------------
------------------------------------------------------------
------------------------------------------------------------
------------------------------------------------------------
------------------------------------------------------------
------------------------------------------------------------
------------------------------------------------------------
------------------------------------------------------------
-------TCACTAGAGTCTATGTCACTCATTATACTCTGTCGAATGTCATTGAATGTCTT
TACATGGGCTTGTATGCCTATGAAAATTGTAATACAACTTTCAGCAACGGATCTCTTGGC
TCTCGCATCGATGAAGAACGCAGCGAAATGCGATAAGTAATGTGAATTGCAGAATTCAGT
GAATCATCGAATCTTTGAACGCATCTTGCGCTCCTTGGTATTCCGAGGAGCATGCCTGTT
TGAGTGTCATTAAATTCTCAACTCTCTTATACTTTTTTGTAAAAGAGAGCTTGGACTGTG
GAGGCTTGCTGGCCACTTTTTGGGGTCAGCTCCTCTGAAATGCATTAGCGGAACCGTTTG
CGATCTGCCACAAGTGTGATAAGTTATCTACACTGGCGAGGGGATTGCTCTCTGTAATGT
TCAGCTTCTAATTGTCTCTACTTTGTGAGACTACTTTTGAATGCTTGACCTCAAATCAGG
TAGGACTACCCGCTGAACTTAA
>AC5-4
TTTCCGTAGGTGAACCTGCGGAAGGATCATTATTGAATTATGTTTCTAGATAGGTTGTAG
CTGGCTC-TTTAGAGCATGTGCACGCCTGTTTGGACTTCATTTTCATCCACCTGTGCACC
TATTGTAGTCTTTGGTTGGGTTAGGAGGAAGTGGTCATTGTGTCAGCATCTGCTGGATGT
GAGGACTTGCATTGTGAAAGCTTTGCTGTCCTTGATGTGATCATGGAATCTCTTTC----
------------------------------------------------------------
------------------------------------------------------------
------------------------------------------------------------
------------------------------------------------------------
------------------------------------------------------------
------------------------------------------------------------
------------------------------------------------------------
------------------------------------------------------------
------------------------------------------------------------
------------------------------------------------------------
------------------------------------------------------------
```

```
------------------------------------------------------------
-------TCACTAGAGTCTATGTCACTCATTATACTCTGTCGAATGTCATTGAATGTCTT
TACATGGGCTTGTATGCCTATGAAAATTGTAATACAACTTTCAGCAACGGATCTCTTGGC
TCTCGCATCGATGAAGAACGCAGCGAAATGCGATAAGTAATGTGAATTGCAGAATTCAGT
GAATCATCGAATCTTTGAACGCATCTTGCGCTCCTTGGTATTCCGAGGAGCATGCCTGTT
TGAGTGTCATTAAATTCTCAACTCTCTTATACTTTTTTGTAAAAGAGAGCTTGGACTGTG
GAGGCTTGCTGGCCACTTTTTGGGGTCAGCTCCTCTGAAATGCATTAGCGGAACCGTTTG
CGATCTGCCACAAGTGTGATAAGTTATCTACACTGGCGAGGGGATTGCTCTCTGTAATGT
TCAGCTTCTAATTGTCTCTACTTTGTGAGACTACTTTTGAATGCTTGACCTCAAATCAGG
TAGGACTACCCGCTGAACTTAA
>AC3-72
TTTCCGTAGGTGAACCTGCGGAAGGATCATTATTGAATTATGTTTCTAGATAGGTTGTAG
CTGGCTC-TTTAGAGCATGTGCACGCCTGTTTGGACTTCATTTTCATCCACCTGTGCACC
TATTGTAGTCTTTGGTTGGGTTAGGAGGAAGTGGTCATTGTGTCAGCATCTGCTGGATGT
GAGGACTTGCATTGTGAAAGCTTTGCTGTCCTTGATGTGATCATGGAATCTTTTTC----
------------------------------------------------------------
------------------------------------------------------------
------------------------------------------------------------
------------------------------------------------------------
------------------------------------------------------------
------------------------------------------------------------
------------------------------------------------------------
------------------------------------------------------------
------------------------------------------------------------
------------------------------------------------------------
------------------------------------------------------------
------------------------------------------------------------
-------TCACTAGAGTCTATGTCACTCATTATACTCTGTCGAATGTCATTGAATGTCTT
TACATGGGCTTGTATGCCTATGAAAATTGTAATACAACTTTCAGCAACGGATCTCTTGGC
TCTCGCATCGATGAAGAACGCAGCGAAATGCGATAAGTAATGTGAATTGCAGAATTCAGT
GAATCATCGAATCTTTGAACGCATCTTGCGCTCCTTGGTATTCCGAGGAGCATGCCTGTT
TGAGTGTCATTAAATTCTCAACTCTCTTATACTTTTTTGTAAAAGAGAGCTTGGACTGTG
GAGGCTTGCTGGCCACTTTTTGGGGTCAGCTCCTCTGAAATGCATTAGCGGAACCGTTTG
CGATCTGCCACAAGTGTGATAAGTTATCTACACTGGCGAGGGGATTGCTCTCTGTAATGT
TCAGCTTCTAATTGTCTCTACTTTGTGAGACTACTTTTGAATGCTTGACCTCAAATCAGG
TAGGACTACCCGCTGAACTTAA
>AC6-14
TTTCCGTAGGTGAACCTGCGGAAGGATCATTATTGAATTATGTTTCTAGATAGGTTGTAG
CTGGCTC-TTTAGAGCATGTGCACGCCTGTTTGGACTTCATTTTCATCCACCTGTGCACC
TATTGTAGTCTTTGGTTGGGTTAGGAGGAAGTGGTCATTGTGTCAGCATCTGCTGGATGT
GAGGACTTGCATTGTGAAAGCTTTGCTGTCCTTGATGTGATCATGGAATCTTTTTC----
------------------------------------------------------------
------------------------------------------------------------
------------------------------------------------------------
------------------------------------------------------------
------------------------------------------------------------
------------------------------------------------------------
------------------------------------------------------------
------------------------------------------------------------
------------------------------------------------------------
```


-------TCACTAGAGTCTATGTCACTCATTATACTCTGTCGAATGTCATTGAATGTCTT
TACATGGGCTTGTATGCCTATGAAAATTGTAATACAACTTTCAGCAACGGATCTCTTGGC
TCTCGCATCGATGAAGAACGCAGCGAAATGCGATAAGTAATGTGAATTGCAGAATTCAGT
GAATCATCGAATCTTTGAACGCATCTTGCGCTCCTTGGTATTCCGAGGAGCATGCCTGTT
TGAGTGTCATTAAATTCTCAACTCTCTTATACTTTTTTGTAAAAGAGAGCTTGGACTGTG
GAGGCTTGCTGGCCACTTTTTGGGGTCAGCTCCTCTGAAATGCATTAGCGGAACCGTTTG
CGATCTGCCACAAGTGTGATAAGTTATCTACACTGGCGAGGGGATTGCTCTCTGTAATGT
TCAGCTTCTAATTGTCTCTACTTTGTGAGACTACTTTTGAATGCTTGACCTCAAATCAGG
TAGGACTACCCGCTGAACTTAA
>AC7-44
TTTCCGTAGGTGAACCTGCGGAAGGATCATTATTGAATTATGTTTCTAGATAGGTTGTAG
CTGGCTC-TTTAGAGCATGTGCACGCCTGTTTGGACTTCATTTTCATCCACCTGTGCACC
TATTGTAGTCTTTGGTTGGGTTAGGAGGAAGTGGTCATTGTGTCAGCATCTGCTGGATGT
GAGGACTTGCATTGTGAAAGCTTTGCTGTCCTTGATGTGATCATGGAATCTTTTTC----

-------TCACTAGAGTCTATGTCACTCATTATACTCTGTCGAATGTCATTGAATGTCTT
TACATGGGCTTGTATGCCTATGAAAATTGTAATACAACTTTCAGCAACGGATCTCTTGGC
TCTCGCATCGATGAAGAACGCAGCGAAATGCGATAAGTAATGTGAATTGCAGAATTCAGT
GAATCATCGAATCTTTGAACGCATCTTGCGCTCCTTGGTATTCCGAGGAGCATGCCTGTT
TGAGTGTCATTAAATTCTCAACTCTCTTATACTTTTTTGTAAAAGAGAGCTTGGACTGTG
GAGGCTTGCTGGCCACTTTTTGGGGTCAGCTCCTCTGAAATGCATTAGCGGAACCGTTTG
CGATCTGCCACAAGTGTGATAAGTTATCTACACTGGCGAGGGGATTGCTCTCTGTAATGT
TCAGCTTCTAATTGTCTCTACTTTGTGAGACTACTTTTGAATGCTTGACCTCAAATCAGG
TAGGACTACCCGCTGAACTTAA
>AC9-36
TTTCCGTAGGTGAACCTGCGGAAGGATCATTATTGAATTATGTTTCTAGATAGGTTGTAG
CTGGCTC-TTTAGAGCATGTGCACGCCTGTTTGGACTTCATTTTCATCCACCTGTGCACC
TATTGTAGTCTTTGGTTGGGTTAGGAGGAAGTGGTCATTGTGTCAGCATCTGCTGGATGT
GAGGACTTGCATTGTGAAAGCTTTGCTGTCCTTGATGTGATCATGGAATCTTTTTC----


```
------------------------------------------------------------
------------------------------------------------------------
------------------------------------------------------------
------------------------------------------------------------
------------------------------------------------------------
-------TCACTAGAGTCTATGTCACTCATTATACTCTGTCGAATGTCATTGAATGTCTT
TACATGGGCTTGTATGCCTATGAAAATTGTAATACAACTTTCAGCAACGGATCTCTTGGC
TCTCGCATCGATGAAGAACGCAGCGAAATGCGATAAGTAATGTGAATTGCAGAATTCAGT
GAATCATCGAATCTTTGAACGCATCTTGCGCTCCTTGGTATTCCGAGGAGCATGCCTGTT
TGAGTGTCATTAAATTCTCAACTCTCTTATACTTTTTTGTAAAAGAGAGCTTGGACTGTG
GAGGCTTGCTGGCCACTTTTTGGGGTCAGCTCCTCTGAAATGCATTAGCGGAACCGTTTG
CGATCTGCCACAAGTGTGATAAGTTATCTACACTGGCGAGGGGATTGCTCTCTGTAATGT
TCAGCTTCTAATTGTCTCTACTTTGTGAGACTACTTTTGAATGCTTGACCTCAAATCAGG
TAGGACTACCCGCTGAACTTAA
>AC11-53
TTTCCGTAGGTGAACCTGCGGAAGGATCATTATTGAATTATGTTTCTAGATAGGTTGTAG
CTGGCTC-TTTAGAGCATGTGCACGCCTGTTTGGACTTCATTTTCATCCACCTGTGCACC
TATTGTAGTCTTTGGTTGGGTTAGGAGGAAGTGGTCATTGTGTCAGCATCTGCTGGATGT
GAGGACTTGCATTGTGAAAGCTTTGCTGTCCTTGATGTGATCATGGAATCTTTTTC----
------------------------------------------------------------
------------------------------------------------------------
------------------------------------------------------------
------------------------------------------------------------
------------------------------------------------------------
------------------------------------------------------------
------------------------------------------------------------
------------------------------------------------------------
------------------------------------------------------------
------------------------------------------------------------
------------------------------------------------------------
------------------------------------------------------------
-------TCACTAGAGTCTATGTCACTCATTATACTCTGTCGAATGTCATTGAATGTCTT
TACATGGGCTTGTATGCCTATGAAAATTGTAATACAACTTTCAGCAACGGATCTCTTGGC
TCTCGCATCGATGAAGAACGCAGCGAAATGCGATAAGTAATGTGAATTGCAGAATTCAGT
GAATCATCGAATCTTTGAACGCATCTTGCGCTCCTTGGTATTCCGAGGAGCATGCCTGTT
TGAGTGTCATTAAATTCTCAACTCTCTTATACTTTTTTGTAAAAGAGAGCTTGGACTGTG
GAGGCTTGCTGGCCACTTTTTGGGGTCAGCTCCTCTGAAATGCATTAGCGGAACCGTTTG
CGATCTGCCACAAGTGTGATAAGTTATCTACACTGGCGAGGGGATTGCTCTCTGTAATGT
TCAGCTTCTAATTGTCTCTACTTTGTGAGACTACTTTTGAATGCTTGACCTCAAATCAGG
TAGGACTACCCGCTGAACTTAA
>AC12-50
TTTCCGTAGGTGAACCTGCGGAAGGATCATTATTGAATTATGTTTCTAGATAGGTTGTAG
CTGGCTC-TTTAGAGCATGTGCACGCCTGTTTGGACTTCATTTTCATCCACCTGTGCACC
TATTGTAGTCTTTGGTTGGGTTAGGAGGAAGTGGTCATTGTGTCAGCATCTGCTGGATGT
GAGGACTTGCATTGTGAAAGCTTTGCTGTCCTTGATGTGATCATGGAATCTTTTTC----
------------------------------------------------------------
------------------------------------------------------------
------------------------------------------------------------
------------------------------------------------------------
------------------------------------------------------------
```


-------TCACTAGAGTCTATGTCACTCATTATACTCTGTCGAATGTCATTGAATGTCTT
TACATGGGCTTGTATGCCTATGAAAATTGTAATACAACTTTCAGCAACGGATCTCTTGGC
TCTCGCATCGATGAAGAACGCAGCGAAATGCGATAAGTAATGTGAATTGCAGAATTCAGT
GAATCATCGAATCTTTGAACGCATCTTGCGCTCCTTGGTATTCCGAGGAGCATGCCTGTT
TGAGTGTCATTAAATTCTCAACTCTCTTATACTTTTTTGTAAAAGAGAGCTTGGACTGTG
GAGGCTTGCTGGCCACTTTTTGGGGTCAGCTCCTCTGAAATGCATTAGCGGAACCGTTTG
CGATCTGCCACAAGTGTGATAAGTTATCTACACTGGCGAGGGGATTGCTCTCTGTAATGT
TCAGCTTCTAATTGTCTCTACTTTGTGAGACTACTTTTGAATGCTTGACCTCAAATCAGG
TAGGACTACCCGCTGAACTTAA
>AC4-62
TTTCCGTAGGTGAACCTGCGGAAGGATCATTATTGAATTATGTTTCTAGATAGGTTGTAG
CTGGCTC-TTTAGAGCATGTGCACGCCTGTTTGGACTTCATTTTCATCCACCTGTGCACC
TATTGTAGTCTTTGGTTGGGTTAGGAGGAAGTGGTCATTGTGTCAGCATCTGCTGGATGT
GAGGACTTGCATTGTGAAAGCTTTGCTGTCCTTGATGTGATCATGGAATCTTTTTC----

-------TCACTAGAGTCTATGTCACTCATTATACTCTGTCGAATGTCATTGAATGTCTT
TACATGGGCTTGTATGCCTATGAAAATTGTAATACAACTTTCAGCAACGGATCTCTTGGC
TCTCGCATCGATGAAGAACGCAGCGAAATGCGATAAGTAATGTGAATTGCAGAATTCAGT
GAATCATCGAATCTTTGAACGCATCTTGCGCTCCTTGGTATTCCGAGGAGCATGCCTGTT
TGAGTGTCATTAAATTCTCAACTCTCTTATACTTTTTTGTAAAAGAGAGCTTGGACTGTG
GAGGCTTGCTGGCCACTTTTTGGGGTCAGCTCCTCTGAAATGCATTAGCGGAACCGTTTG
CGATCTGCCACAAGTGTGATAAGTTATCTACACTGGCGAGGGGATTGCTCTCTGTAATGT
TCAGCTTCTAATTGTCTCTACTTTGTGAGACTACTTTTGAATGCTTGACCTCAAATCAGG
TAGGACTACCCGCTGAACTTAA
>AC1-60
TTTCCGTAGGTGAACCTGCGGAAGGATCATTATTGAATTATGTTTCTAGATAGGTTGTAG
CTGGCTC-TTTAGAGCATGTGCACGCCTGTTTGGACTTCATTTTCATCCACCTGTGCACC
TATTGTAGTCTTTGGTTGGGTTAGGAGGAAGTGGTCATTGTGTCAGCATCTGCTGGATGT
GAGGACTTGCATTGTGAAAGCTTTGCTGTCCTTGATGTGATCATGGAATCTCTTTC----

-------TCACTAGAGTCTATGTCACTCATTATACTCTGTCGAATGTCATTGAATGTCTT
TACATGGGCTTATATGCCTATGAAAATTGTAATACAACTTTCAGCAACGGATCTCTTGGC
TCTCGCATCGATGAAGAACGCAGCGAAATGCGATAAGTAATGTGAATTGCAGAATTCAGT
GAATCATCGAATCTTTGAACGCATCTTGCGCTCCTTGGTATTCCGAGGAGCATGCCTGTT
TGAGTGTCATTAAATTCTCAACTCTCTTATACTTTTTTGTAAAAGAGAGCTTGGACTGTG
GAGGCTTGCTGGCCACTTTTTGGGGTCAGCTCCTCTGAAATGCATTAGCGGAACCGTTTG
CGATCTGCCACAAGTGTGATAAGTTATCTACACTGGCGAGGGGATTGCTCTCTGTAATGT
TCAGCTTCTAATTGTCTCTACTTTGTGAGACTACTTTTGAATGCTTGACCTCAAATCAGG
TAGGACTACCCGCTGAACTTAA
>AC3-17
TTTCCGTAGGTGAACCTGCGGAAGGATCATTATTGAATTATGTTTCTAGATAGGTTGTAG
CTGGCTC-TTTAGAGCATGTGCACGCCTGTTTGGACTTCATTTTCATCCACCTGTGCACC
TATTGTAGTCTTTGGTTGGGTTAGGAGGAAGTGGTCATTGTGTCAGCATCTGCTGGATGT
GAGGACTTGCATTGTGAAAGCTTTGCTGTCCTTGATGTGATCATGGAATCTCTTTC----

-------TCACTAGAGTCTATGTCACTCATTATACTCTGTCGAATGTCATTGAATGTCTT
TACATGGGCTTATATGCCTATGAAAATTGTAATACAACTTTCAGCAACGGATCTCTTGGC
TCTCGCATCGATGAAGAACGCAGCGAAATGCGATAAGTAATGTGAATTGCAGAATTCAGT
GAATCATCGAATCTTTGAACGCATCTTGCGCTCCTTGGTATTCCGAGGAGCATGCCTGTT
TGAGTGTCATTAAATTCTCAACTCTCTTATACTTTTTTGTAAAAGAGAGCTTGGACTGTG
GAGGCTTGCTGGCCACTTTTTGGGGTCAGCTCCTCTGAAATGCATTAGCGGAACCGTTTG
CGATCTGCCACAAGTGTGATAAGTTATCTACACTGGCGAGGGGATTGCTCTCTGTAATGT
TCAGCTTCTAATTGTCTCTACTTTGTGAGACTACTTTTGAATGCTTGACCTCAAATCAGG
TAGGACTACCCGCTGAACTTAA
>AC4-51
TTTCCGTAGGTGAACCTGCGGAAGGATCATTATTGAATTATGTTTCTAGATAGGTTGTAG
CTGGCTC-TTTAGAGCATGTGCACGCCTGTTTGGACTTCATTTTCATCCACCTGTGCACC
TATTGTAGTCTTTGGTTGGGTTAGGAGGAAGTGGTCATTGTGTCAGCATCTGCTGGATGT
GAGGACTTGCATTGTGAAAGCTTTGCTGTCCTTGATGTGATCATGGAATCTCTTTC----

-------TCACTAGAGTCTATGTCACTCATTATACTCTGTCGAATGTCATTGAATGTCTT
TACATGGGCTTATATGCCTATGAAAATTGTAATACAACTTTCAGCAACGGATCTCTTGGC
TCTCGCATCGATGAAGAACGCAGCGAAATGCGATAAGTAATGTGAATTGCAGAATTCAGT
GAATCATCGAATCTTTGAACGCATCTTGCGCTCCTTGGTATTCCGAGGAGCATGCCTGTT
TGAGTGTCATTAAATTCTCAACTCTCTTATACTTTTTTGTAAAAGAGAGCTTGGACTGTG
GAGGCTTGCTGGCCACTTTTTGGGGTCAGCTCCTCTGAAATGCATTAGCGGAACCGTTTG
CGATCTGCCACAAGTGTGATAAGTTATCTACACTGGCGAGGGGATTGCTCTCTGTAATGT
TCAGCTTCTAATTGTCTCTACTTTGTGAGACTACTTTTGAATGCTTGACCTCAAATCAGG
TAGGACTACCCGCTGAACTTAA
>AC4-83
TTTCCGTAGGTGAACCTGCGGAAGGATCATTATTGAATTATGTTTCTAGATAGGTTGTAG
CTGGCTC-TTTAGAGCATGTGCACGCCTGTTTGGACTTCATTTTCATCCACCTGTGCACC
TATTGTAGTCTTTGGTTGGGTTAGGAGGAAGTGGTCATTGTGTCAGCATCTGCTGGATGT
GAGGACTTGCATTGTGAAAGCTTTGCTGTCCTTGATGTGATCATGGAATCTCTTTC----

-------TCACTAGAGTCTATGTCACTCATTATACTCTGTCGAATGTCATTGAATGTCTT
TACATGGGCTTATATGCCTATGAAAATTGTAATACAACTTTCAGCAACGGATCTCTTGGC
TCTCGCATCGATGAAGAACGCAGCGAAATGCGATAAGTAATGTGAATTGCAGAATTCAGT
GAATCATCGAATCTTTGAACGCATCTTGCGCTCCTTGGTATTCCGAGGAGCATGCCTGTT
TGAGTGTCATTAAATTCTCAACTCTCTTATACTTTTTTGTAAAAGAGAGCTTGGACTGTG
GAGGCTTGCTGGCCACTTTTTGGGGTCAGCTCCTCTGAAATGCATTAGCGGAACCGTTTG
CGATCTGCCACAAGTGTGATAAGTTATCTACACTGGCGAGGGGATTGCTCTCTGTAATGT
TCAGCTTCTAATTGTCTCTACTTTGTGAGACTACTTTTGAATGCTTGACCTCAAATCAGG
TAGGACTACCCGCTGAACTTAA
>AC5-47
TTTCCGTAGGTGAACCTGCGGAAGGATCATTATTGAATTATGTTTCTAGATAGGTTGTAG
CTGGCTC-TTTAGAGCATGTGCACGCCTGTTTGGACTTCATTTTCATCCACCTGTGCACC
TATTGTAGTCTTTGGTTGGGTTAGGAGGAAGTGGTCATTGTGTCAGCATCTGCTGGATGT

GAGGACTTGCATTGTGAAAGCTTTGCTGTCCTTGATGTGATCATGGAATCTCTTTC----

-------TCACTAGAGTCTATGTCACTCATTATACTCTGTCGAATGTCATTGAATGTCTT
TACATGGGCTTATATGCCTATGAAAATTGTAATACAACTTTCAGCAACGGATCTCTTGGC
TCTCGCATCGATGAAGAACGCAGCGAAATGCGATAAGTAATGTGAATTGCAGAATTCAGT
GAATCATCGAATCTTTGAACGCATCTTGCGCTCCTTGGTATTCCGAGGAGCATGCCTGTT
TGAGTGTCATTAAATTCTCAACTCTCTTATACTTTTTTGTAAAAGAGAGCTTGGACTGTG
GAGGCTTGCTGGCCACTTTTTGGGGTCAGCTCCTCTGAAATGCATTAGCGGAACCGTTTG
CGATCTGCCACAAGTGTGATAAGTTATCTACACTGGCGAGGGGATTGCTCTCTGTAATGT
TCAGCTTCTAATTGTCTCTACTTTGTGAGACTACTTTTGAATGCTTGACCTCAAATCAGG
TAGGACTACCCGCTGAACTTAA
>AC5-75
TTTCCGTAGGTGAACCTGCGGAAGGATCATTATTGAATTATGTTTCTAGATAGGTTGTAG
CTGGCTC-TTTAGAGCATGTGCACGCCTGTTTGGACTTCATTTTCATCCACCTGTGCACC
TATTGTAGTCTTTGGTTGGGTTAGGAGGAAGTGGTCATTGTGTCAGCATCTGCTGGATGT
GAGGACTTGCATTGTGAAAGCTTTGCTGTCCTTGATGTGATCATGGAATCTCTTTC----

-------TCACTAGAGTCTATGTCACTCATTATACTCTGTCGAATGTCATTGAATGTCTT
TACATGGGCTTATATGCCTATGAAAATTGTAATACAACTTTCAGCAACGGATCTCTTGGC
TCTCGCATCGATGAAGAACGCAGCGAAATGCGATAAGTAATGTGAATTGCAGAATTCAGT
GAATCATCGAATCTTTGAACGCATCTTGCGCTCCTTGGTATTCCGAGGAGCATGCCTGTT
TGAGTGTCATTAAATTCTCAACTCTCTTATACTTTTTTGTAAAAGAGAGCTTGGACTGTG
GAGGCTTGCTGGCCACTTTTTGGGGTCAGCTCCTCTGAAATGCATTAGCGGAACCGTTTG
CGATCTGCCACAAGTGTGATAAGTTATCTACACTGGCGAGGGGATTGCTCTCTGTAATGT
TCAGCTTCTAATTGTCTCTACTTTGTGAGACTACTTTTGAATGCTTGACCTCAAATCAGG
TAGGACTACCCGCTGAACTTAA
>AC6-37
TTTCCGTAGGTGAACCTGCGGAAGGATCATTATTGAATTATGTTTCTAGATAGGTTGTAG

CTGGCTC-TTTAGAGCATGTGCACGCCTGTTTGGACTTCATTTTCATCCACCTGTGCACC
TATTGTAGTCTTTGGTTGGGTTAGGAGGAAGTGGTCATTGTGTCAGCATCTGCTGGATGT
GAGGACTTGCATTGTGAAAGCTTTGCTGTCCTTGATGTGATCATGGAATCTCTTTC----

-------TCACTAGAGTCTATGTCACTCATTATACTCTGTCGAATGTCATTGAATGTCTT
TACATGGGCTTATATGCCTATGAAAATTGTAATACAACTTTCAGCAACGGATCTCTTGGC
TCTCGCATCGATGAAGAACGCAGCGAAATGCGATAAGTAATGTGAATTGCAGAATTCAGT
GAATCATCGAATCTTTGAACGCATCTTGCGCTCCTTGGTATTCCGAGGAGCATGCCTGTT
TGAGTGTCATTAAATTCTCAACTCTCTTATACTTTTTTGTAAAAGAGAGCTTGGACTGTG
GAGGCTTGCTGGCCACTTTTTGGGGTCAGCTCCTCTGAAATGCATTAGCGGAACCGTTTG
CGATCTGCCACAAGTGTGATAAGTTATCTACACTGGCGAGGGGATTGCTCTCTGTAATGT
TCAGCTTCTAATTGTCTCTACTTTGTGAGACTACTTTTGAATGCTTGACCTCAAATCAGG
TAGGACTACCCGCTGAACTTAA
>AC8-55
TTTCCGTAGGTGAACCTGCGGAAGGATCATTATTGAATTATGTTTCTAGATAGGTTGTAG
CTGGCTC-TTTAGAGCATGTGCACGCCTGTTTGGACTTCATTTTCATCCACCTGTGCACC
TATTGTAGTCTTTGGTTGGGTTAGGAGGAAGTGGTCATTGTGTCAGCATCTGCTGGATGT
GAGGACTTGCATTGTGAAAGCTTTGCTGTCCTTGATGTGATCATGGAATCTCTTTC----

-------TCACTAGAGTCTATGTCACTCATTATACTCTGTCGAATGTCATTGAATGTCTT
TACATGGGCTTATATGCCTATGAAAATTGTAATACAACTTTCAGCAACGGATCTCTTGGC
TCTCGCATCGATGAAGAACGCAGCGAAATGCGATAAGTAATGTGAATTGCAGAATTCAGT
GAATCATCGAATCTTTGAACGCATCTTGCGCTCCTTGGTATTCCGAGGAGCATGCCTGTT
TGAGTGTCATTAAATTCTCAACTCTCTTATACTTTTTTGTAAAAGAGAGCTTGGACTGTG
GAGGCTTGCTGGCCACTTTTTGGGGTCAGCTCCTCTGAAATGCATTAGCGGAACCGTTTG
CGATCTGCCACAAGTGTGATAAGTTATCTACACTGGCGAGGGGATTGCTCTCTGTAATGT
TCAGCTTCTAATTGTCTCTACTTTGTGAGACTACTTTTGAATGCTTGACCTCAAATCAGG
TAGGACTACCCGCTGAACTTAA

>AC8-59
TTTCCGTAGGTGAACCTGCGGAAGGATCATTATTGAATTATGTTTCTAGATAGGTTGTAG
CTGGCTC-TTTAGAGCATGTGCACGCCTGTTTGGACTTCATTTTCATCCACCTGTGCACC
TATTGTAGTCTTTGGTTGGGTTAGGAGGAAGTGGTCATTGTGTCAGCATCTGCTGGATGT
GAGGACTTGCATTGTGAAAGCTTTGCTGTCCTTGATGTGATCATGGAATCTCTTTC----

-------TCACTAGAGTCTATGTCACTCATTATACTCTGTCGAATGTCATTGAATGTCTT
TACATGGGCTTATATGCCTATGAAAATTGTAATACAACTTTCAGCAACGGATCTCTTGGC
TCTCGCATCGATGAAGAACGCAGCGAAATGCGATAAGTAATGTGAATTGCAGAATTCAGT
GAATCATCGAATCTTTGAACGCATCTTGCGCTCCTTGGTATTCCGAGGAGCATGCCTGTT
TGAGTGTCATTAAATTCTCAACTCTCTTATACTTTTTTGTAAAAGAGAGCTTGGACTGTG
GAGGCTTGCTGGCCACTTTTTGGGGTCAGCTCCTCTGAAATGCATTAGCGGAACCGTTTG
CGATCTGCCACAAGTGTGATAAGTTATCTACACTGGCGAGGGGATTGCTCTCTGTAATGT
TCAGCTTCTAATTGTCTCTACTTTGTGAGACTACTTTTGAATGCTTGACCTCAAATCAGG
TAGGACTACCCGCTGAACTTAA
>AC9-3
TTTCCGTAGGTGAACCTGCGGAAGGATCATTATTGAATTATGTTTCTAGATAGGTTGTAG
CTGGCTC-TTTAGAGCATGTGCACGCCTGTTTGGACTTCATTTTCATCCACCTGTGCACC
TATTGTAGTCTTTGGTTGGGTTAGGAGGAAGTGGTCATTGTGTCAGCATCTGCTGGATGT
GAGGACTTGCATTGTGAAAGCTTTGCTGTCCTTGATGTGATCATGGAATCTCTTTC----

-------TCACTAGAGTCTATGTCACTCATTATACTCTGTCGAATGTCATTGAATGTCTT
TACATGGGCTTATATGCCTATGAAAATTGTAATACAACTTTCAGCAACGGATCTCTTGGC
TCTCGCATCGATGAAGAACGCAGCGAAATGCGATAAGTAATGTGAATTGCAGAATTCAGT
GAATCATCGAATCTTTGAACGCATCTTGCGCTCCTTGGTATTCCGAGGAGCATGCCTGTT
TGAGTGTCATTAAATTCTCAACTCTCTTATACTTTTTTGTAAAAGAGAGCTTGGACTGTG
GAGGCTTGCTGGCCACTTTTTGGGGTCAGCTCCTCTGAAATGCATTAGCGGAACCGTTTG
CGATCTGCCACAAGTGTGATAAGTTATCTACACTGGCGAGGGGATTGCTCTCTGTAATGT

TCAGCTTCTAATTGTCTCTACTTTGTGAGACTACTTTTGAATGCTTGACCTCAAATCAGG
TAGGACTACCCGCTGAACTTAA
>AC11-12
TTTCCGTAGGTGAACCTGCGGAAGGATCATTATTGAATTATGTTTCTAGATAGGTTGTAG
CTGGCTC-TTTAGAGCATGTGCACGCCTGTTTGGACTTCATTTTCATCCACCTGTGCACC
TATTGTAGTCTTTGGTTGGGTTAGGAGGAAGTGGTCATTGTGTCAGCATCTGCTGGATGT
GAGGACTTGCATTGTGAAAGCTTTGCTGTCCTTGATGTGATCATGGAATCTCTTTC----

-------TCACTAGAGTCTATGTCACTCATTATACTCTGTCGAATGTCATTGAATGTCTT
TACATGGGCTTATATGCCTATGAAAATTGTAATACAACTTTCAGCAACGGATCTCTTGGC
TCTCGCATCGATGAAGAACGCAGCGAAATGCGATAAGTAATGTGAATTGCAGAATTCAGT
GAATCATCGAATCTTTGAACGCATCTTGCGCTCCTTGGTATTCCGAGGAGCATGCCTGTT
TGAGTGTCATTAAATTCTCAACTCTCTTATACTTTTTTGTAAAAGAGAGCTTGGACTGTG
GAGGCTTGCTGGCCACTTTTTGGGGTCAGCTCCTCTGAAATGCATTAGCGGAACCGTTTG
CGATCTGCCACAAGTGTGATAAGTTATCTACACTGGCGAGGGGATTGCTCTCTGTAATGT
TCAGCTTCTAATTGTCTCTACTTTGTGAGACTACTTTTGAATGCTTGACCTCAAATCAGG
TAGGACTACCCGCTGAACTTAA
>AC11-43
TTTCCGTAGGTGAACCTGCGGAAGGATCATTATTGAATTATGTTTCTAGATAGGTTGTAG
CTGGCTC-TTTAGAGCATGTGCACGCCTGTTTGGACTTCATTTTCATCCACCTGTGCACC
TATTGTAGTCTTTGGTTGGGTTAGGAGGAAGTGGTCATTGTGTCAGCATCTGCTGGATGT
GAGGACTTGCATTGTGAAAGCTTTGCTGTCCTTGATGTGATCATGGAATCTCTTTC----

-------TCACTAGAGTCTATGTCACTCATTATACTCTGTCGAATGTCATTGAATGTCTT
TACATGGGCTTATATGCCTATGAAAATTGTAATACAACTTTCAGCAACGGATCTCTTGGC
TCTCGCATCGATGAAGAACGCAGCGAAATGCGATAAGTAATGTGAATTGCAGAATTCAGT
GAATCATCGAATCTTTGAACGCATCTTGCGCTCCTTGGTATTCCGAGGAGCATGCCTGTT
TGAGTGTCATTAAATTCTCAACTCTCTTATACTTTTTTGTAAAAGAGAGCTTGGACTGTG

GAGGCTTGCTGGCCACTTTTTGGGGTCAGCTCCTCTGAAATGCATTAGCGGAACCGTTTG
CGATCTGCCACAAGTGTGATAAGTTATCTACACTGGCGAGGGGATTGCTCTCTGTAATGT
TCAGCTTCTAATTGTCTCTACTTTGTGAGACTACTTTTGAATGCTTGACCTCAAATCAGG
TAGGACTACCCGCTGAACTTAA
>AC12-38
TTTCCGTAGGTGAACCTGCGGAAGGATCATTATTGAATTATGTTTCTAGATAGGTTGTAG
CTGGCTC-TTTAGAGCATGTGCACGCCTGTTTGGACTTCATTTTCATCCACCTGTGCACC
TATTGTAGTCTTTGGTTGGGTTAGGAGGAAGTGGTCATTGTGTCAGCATCTGCTGGATGT
GAGGACTTGCATTGTGAAAGCTTTGCTGTCCTTGATGTGATCATGGAATCTCTTTC----

-------TCACTAGAGTCTATGTCACTCATTATACTCTGTCGAATGTCATTGAATGTCTT
TACATGGGCTTATATGCCTATGAAAATTGTAATACAACTTTCAGCAACGGATCTCTTGGC
TCTCGCATCGATGAAGAACGCAGCGAAATGCGATAAGTAATGTGAATTGCAGAATTCAGT
GAATCATCGAATCTTTGAACGCATCTTGCGCTCCTTGGTATTCCGAGGAGCATGCCTGTT
TGAGTGTCATTAAATTCTCAACTCTCTTATACTTTTTTGTAAAAGAGAGCTTGGACTGTG
GAGGCTTGCTGGCCACTTTTTGGGGTCAGCTCCTCTGAAATGCATTAGCGGAACCGTTTG
CGATCTGCCACAAGTGTGATAAGTTATCTACACTGGCGAGGGGATTGCTCTCTGTAATGT
TCAGCTTCTAATTGTCTCTACTTTGTGAGACTACTTTTGAATGCTTGACCTCAAATCAGG
TAGGACTACCCGCTGAACTTAA
>AC11-47
TTTCCGTAGGTGAACCTGCGGAAGGATCATTATTGAATTATGTTTCTAGATAGGTTGTAG
CTGGCTC-TTTAGAGCATGTGCACGCCTGTTTGGACTTCATTTTCATCCACCTGTGCACC
TATTGTAGTCTTTGGTTGGGTTAGGAGGAAGTGGTCATTGTGTCAGCATCTGCTGGATGT
GAGGACTTGCATTGTGAAAGCTTTGCTGTCCTTGATGTGATCATGGAATCTCTTTC----

-------TCACTAGAGTCTATGTCACTCATTATACTCTGTCGAATGTCATTGAATGTCTT
TACATGGGCTTATATGCCTATGAAAATTGTAATACAACTTTCAGCAACGGATCTCTTGGC
TCTCGCATCGATGAAGAACGCAGCGAAATGCGATAAGTAATGTGAATTGCAGAATTCAGT

```
GAATCATCGAATCTTTGAACGCATCTTGCGCTCCTTGGTATTCCGAGGAGCATGCCTGTT
TGAGTGTCATTAAATTCTCAACTCTCTTATACTTTTTTGTAAAAGAGAGCTTGGACTGTG
GAGGCTTGCTGGCCACTTTTTGGGGTCAGCTCCTCTGAAATGCATTAGCGGAACCGTTTG
CGATCTGCCACAAGTGTGATAAGTTATCTACACTGGCGAGGGGATTGCTCTCTGTAATGT
TCAGCTTCTAATTGTCTCTACTTTGTGAGACTACTTTTGAATGCTTGACCTCAAATCAGG
TAGGACTACCCGCTGAACTTAA
>AC12-14
TTTCCGTAGGTGAACCTGCGGAAGGATCATTATTGAATTATGTTTCTAGATAGGTTGTAG
CTGGCTC-TTTAGAGCATGTGCACGCCTGTTTGGACTTCATTTTCATCCACCTGTGCACC
TATTGTAGTCTTTGGTTGGGTTAGGAGGAAGTGGTCATTGTGTCAGCATCTGCTGGATGT
GAGGACTTGCATTGTGAAAGCTTTGCTGTCCTTGATGTGATCATGGAATCTCTTTC----
------------------------------------------------------------
------------------------------------------------------------
------------------------------------------------------------
------------------------------------------------------------
------------------------------------------------------------
------------------------------------------------------------
------------------------------------------------------------
------------------------------------------------------------
------------------------------------------------------------
------------------------------------------------------------
------------------------------------------------------------
------------------------------------------------------------
-------TCACTAGAGTCTATGTCACTCATTATACTCTGTCGAATGTCATTGAATGTCTT
TACATGGGCTTATATGCCTATGAAAATTGTAATACAACTTTCAGCAACGGATCTCTTGGC
TCTCGCATCGATGAAGAACGCAGCGAAATGCGATAAGTAATGTGAATTGCAGAATTCAGT
GAATCATCGAATCTTTGAACGCATCTTGCGCTCCTTGGTATTCCGAGGAGCATGCCTGTT
TGAGTGTCATTAAATTCTCAACTCTCTTATACTTTTTTGTAAAAGAGAGCTTGGACTGTG
GAGGCTTGCTGGCCACTTTTTGGGGTCAGCTCCTCTGAAATGCATTAGCGGAACCGTTTG
CGATCTGCCACAAGTGTGATAAGTTATCTACACTGGCGAGGGGATTGCTCTCTGTAATGT
TCAGCTTCTAATTGTCTCTACTTTGTGAGACTACTTTTGAATGCTTGACCTCAAATCAGG
TAGGACTACCCGCTGAACTTAA
>AC6-15
TTTCCGTAGGTGAACCTGCGGAAGGATCATTATTGAATTATGTTTCTAGATAGGTTGTAG
CTGGCTC-TTTAGAGCATGTGCACGCCTGTTTGGACTTCATTTTCATCCACCTGTGCACC
TATTGTAGTCTTTGGTTGGGTTAGGAGGAAGTGGTCATTGTGTCAGCATCTGCTGGATGT
GAGGACTTGCATTGTGAAAGCTTTGCTGTCCTTGATGTGATCATGGAATCTTTTTC----
------------------------------------------------------------
------------------------------------------------------------
------------------------------------------------------------
------------------------------------------------------------
------------------------------------------------------------
------------------------------------------------------------
------------------------------------------------------------
------------------------------------------------------------
------------------------------------------------------------
------------------------------------------------------------
------------------------------------------------------------
------------------------------------------------------------
-------TCACTAGAGTCTATGTCACTCATTATACTCTGTCGAATGTCATTGAATGTCTT
```

TACATGGGCTTATATGCCTATGAAAATTGTAATACAACTTTCAGCAACGGATCTCTTGGC
TCTCGCATCGATGAAGAACGCAGCGAAATGCGATAAGTAATGTGAATTGCAGAATTCAGT
GAATCATCGAATCTTTGAACGCATCTTGCGCTCCTTGGTATTCCGAGGAGCATGCCTGTT
TGAGTGTCATTAAATTCTCAACTCTCTTATACTTTTTTGTAAAAGAGAGCTTGGACTGTG
GAGGCTTGCTGGCCACTTTTTGGGGTCAGCTCCTCTGAAATGCATTAGCGGAACCGTTTG
CGATCTGCCACAAGTGTGATAAGTTATCTACACTGGCGAGGGGATTGCTCTCTGTAATGT
TCAGCTTCTAATTGTCTCTACTTTGTGAGACTACTTTTGAATGCTTGACCTCAAATCAGG
TAGGACTACCCGCTGAACTTAA
>AC10-57
TTTCCGTAGGTGAACCTGCGGAAGGATCATTATTGAATTATGTTTCTAGATAGGTTGTAG
CTGGCTC-TTTAGAGCATGTGCACGCCTGTTTGGACTTCATTTTCATCCACCTGTGCACC
TATTGTAGTCTTTGGTTGGGTTAGGAGGAAGTGGTCATTGTGTCAGCATCTGCTGGATGT
GAGGACTTGCATTGTGAAAGCTTTGCTGTCCTTGATGTGATCATGGAATCTTTTTC----

-------TCACTAGAGTCTATGTCACTCATTATACTCTGTCGAATGTCATTGAATGTCTT
TACATGGGCTTATATGCCTATGAAAATTGTAATACAACTTTCAGCAACGGATCTCTTGGC
TCTCGCATCGATGAAGAACGCAGCGAAATGCGATAAGTAATGTGAATTGCAGAATTCAGT
GAATCATCGAATCTTTGAACGCATCTTGCGCTCCTTGGTATTCCGAGGAGCATGCCTGTT
TGAGTGTCATTAAATTCTCAACTCTCTTATACTTTTTTGTAAAAGAGAGCTTGGACTGTG
GAGGCTTGCTGGCCACTTTTTGGGGTCAGCTCCTCTGAAATGCATTAGCGGAACCGTTTG
CGATCTGCCACAAGTGTGATAAGTTATCTACACTGGCGAGGGGATTGCTCTCTGTAATGT
TCAGCTTCTAATTGTCTCTACTTTGTGAGACTACTTTTGAATGCTTGACCTCAAATCAGG
TAGGACTACCCGCTGAACTTAA
>AC3-51
TTTCCGTAGGTGAACCTGCGGAAGGATCATTATTGAATTATGTTTCTAGATAGGTTGTAG
CTGGCTC-TTTAGAGCATGTGCACGCCTGTTTGGACTTCATTTTCATCCACCTGTGCACC
TATTGTAGTCTTTGGTTGGGTTAGGAGGAAGTGGTCATTGTGTCAGCATCTGCTGGATGT
GAGGACTTGCATTGTGAAAGCTTTGCTGTCCTTGATGTGATCATGGAATCTCTTTC----


```
------------------------------------------------------------
-------TCACTAGAGTCTATGTCACTCATTATACTCTGTCGAATGTCATTGAATGTCTT
TACATGGGCTTGTATGCCTATGAAAATTGTAATACAACTTTCAGCAACGGATCTCTTGGC
TCTCGCATCGATGAAGGACGCAGCGAAATGCGATAAGTAATGTGAATTGCAGAATTCAGT
GAATCATCGAATCTTTGAACGCATCTTGCGCTCCTTGGTATTCCGAGGAGCATGCCTGTT
TGAGTGTCATTAAATTCTCAACTCTCTTATACTTTTTTGTAAAAGAGAGCTTGGACTGTG
GAGGCTTGCTGGCCACTTTTTGGGGTCAGCTCCTCTGAAATGCATTAGCGGAACCGTTTG
CGATCTGCCACAAGTGTGATAAGTTATCTACACTGGCGAGGGGATTGCTCTCTGTAATGT
TCAGCTTCTAATTGTCTCTACTTTGTGAGACTACTTTTGAATGCTTGACCTCAAATCAGG
TAGGACTACCCGCTGAACTTAA
>AC12-40
TTTCCGTAGGTGAACCTGCGGAAGGATCATTATTGAATTATGTTTCTAGATAGGTTGTAG
CTGGCTC-TTTAGAGCATGTGCACGCCTGTTTGGACTTCATTTTCATCCACCTGTGCACC
TATTGTAGTCTTTGGTTGGGTTAGGAGGAAGTGGTCATTGTGTCAGCATCTGCTGGATGT
GAGGACTTGCATTGTGAAAGCTTTGCTGTCCTTGATGTGATCATGGAATCTCTTTC----
------------------------------------------------------------
------------------------------------------------------------
------------------------------------------------------------
------------------------------------------------------------
------------------------------------------------------------
------------------------------------------------------------
------------------------------------------------------------
------------------------------------------------------------
------------------------------------------------------------
------------------------------------------------------------
------------------------------------------------------------
------------------------------------------------------------
-------TCACTAGAGTCTATGTCACTCATTATACTCTGTCGAATGTCATTGAATGTCTT
TACATGGGCTTGTATGCCTATGAAAATTGTAATACAACTTTCAGCAACGGATCTCTTGGC
TCTCGCATCGATGAAGAACGCAGCGAAATGCGATAAGTAATGTGAATTGCAGAATTCAGT
GAATCATCGAATCTTTGAACGCATCTTGCGCTCCTTGGTATTCCGAGGAGCATGCCTGTT
TGAGTGTCATTAAATTCTCAACTCTCTTATACTTTTTTGTAAAAGAGAGCTTGGACTGTG
GAGGCTTGCTGGTCACTTTTTGGGGTCAGCTCCTCTGAAATGCATTAGCGGAACCGTTTG
CGATCTGCCACAAGTGTGATAAGTTATCTACACTGGCGAGGGGATTGCTCTCTGTAATGT
TCAGCTTCTAATTGTCTCTACTTTGTGAGACTACTTTTGAATGCTTGACCTCAAATCAGG
TAGGACTACCCGCTGAACTTAA
>AC6-7
TTTCCGTAGGTGAACCTGCGGAAGGATCATTATTGAATTATGTTTCTAGATAGGTTGTAG
CTGGCTC-TTTAGAGCATGTGCACGCCTGTTTGGACTTCATTTTCATCCACCTGTGCACC
TATTGTAGTCTTTGGTTGGGTTAGGAGGAAGTGATCATTGTATCAGCATCTGCTGGATGT
GAGGACTTGCATTGTGAAAGCTTTGCTGTCCTTGATGTGATCATGGAATCTCTTTC----
------------------------------------------------------------
------------------------------------------------------------
------------------------------------------------------------
------------------------------------------------------------
------------------------------------------------------------
------------------------------------------------------------
------------------------------------------------------------
------------------------------------------------------------
------------------------------------------------------------
```

```
------------------------------------------------------------
------------------------------------------------------------
------------------------------------------------------------
-------TCACTAGAGTCTATGTCACTCATTATACTCTGTCGAATGTCATTGAATGTCTT
TACATGGGCTTGTATGCCTATGAAAATTGTAATACAACTTTCAGCAACGGATCTCTTGGC
TCTCGCATCGATGAAGAACGCAGCGAAATGCGATAAGTAATGTGAATTGCAGAATTCAGT
GAATCATCGAATCTTTGAACGCATCTTGCGCTCCTTGGTATTCCGAGGAGCATGCCTGTT
TGAGTGTCATTAAATTCTCAACTCTCTTATACTTTTTTGTAAAAGAGAGCTTGGACTGTG
GAGGCTTGCTGGCCACTTTTTGGGGTCAGCTCCTCTGAAATGCATTAGCGGAACCGTTTG
CGATCTGCCACAAGTGTGATAAGTTATCTACACTGGCGAGGGGATTGCTCTCTGTAATGT
TCAGCTTCTAATTGTCTCTACTTTGTGAGACTACTTTTGAATGCTTGACCTCAAATCAGG
TAGGACTACCCGCTGAACTTAA
>AC5-42
TTTCCGTAGGTGAACCTGCGGAAGGATCATTATTGAATTATGTTTCTAGATAGGTTGTAG
CTGGCTC-TTTAGAGCATGTGCACGCCTGTTTGGACTTCATTTTCATCCACCTGTGCACC
TATTGTAGTCTTTGGTTGGGTTAGGAGGAAGTGATCATTGTATCAGCATCTGCTGGATGT
GAGGACTTGCATTGTGAAAGCTTTGCTGTCCTTGATGTGATCATGGAATCTCTTTC----
------------------------------------------------------------
------------------------------------------------------------
------------------------------------------------------------
------------------------------------------------------------
------------------------------------------------------------
------------------------------------------------------------
------------------------------------------------------------
------------------------------------------------------------
------------------------------------------------------------
------------------------------------------------------------
------------------------------------------------------------
------------------------------------------------------------
-------TCACTAGAGTCTATGTCACTCATTATACTCTGTCGAATGTCATTGAATGTCTT
TACATGGGCTTATATGCCTATGAAAATTGTAATACAACTTTCAGCAACGGATCTCTTGGC
TCTCGCATCGATGAAGAACGCAGCGAAATGCGATAAGTAATGTGAATTGCAGAATTCAGT
GAATCATCGAATCTTTGAACGCATCTTGCGCTCCTTGGTATTCCGAGGAGCATGCCTGTT
TGAGTGTCATTAAATTCTCAACTCTCTTATACTTTTTTGTAAAAGAGAGCTTGGACTGTG
GAGGCTTGCTGGCCACTTTTTGGGGTCAGCTCCTCTGAAATGCATTAGCGGAACCGTTTG
CGATCTGCCACAAGTGTGATAAGTTATCTACACTGGCGAGGGGATTGCTCTCTGTAATGT
TCAGCTTCTAATTGTCTCTACTTTGTGAGACTACTTTTGAATGCTTGACCTCAAATCAGG
TAGGACTACCCGCTGAACTTAA
>AC8-47
TTTCCGTAGGTGAACCTGCGGAAGGATCATTATTGAATTATGTTTCTAGATAGGTTGTAG
CTGGCTC-TTTAGAGCATGTGCACGCCTGTTTGGACTTCATTTTCATCCACCTGTGCACC
TATTGTAGTCTTTGGTTGGGTTAGGAGGAAGTGATCATTGTGTCAGCATCTGCTGGATGT
GAGGACTTGCATTGTGAAAGCTTTGCTGTCCTTGATGTGATCATGGAATCTCTTTC----
------------------------------------------------------------
------------------------------------------------------------
------------------------------------------------------------
------------------------------------------------------------
------------------------------------------------------------
------------------------------------------------------------
------------------------------------------------------------
```


-------TCACTAGAGTCTATGTCACTCATTATACTCTGTCGAATGTCATTGAATGTCTT
TACATGGGCTTATATGCCTATGAAAATTGTAATACAACTTTCAGCAACGGATCTCTTGGC
TCTCGCATCGATGAAGAACGCAGCGAAATGCGATAAGTAATGTGAATTGCAGAATTCAGT
GAATCATCGAATCTTTGAACGCATCTTGCGCTCCTTGGTATTCCGAGGAGCATGCCTGTT
TGAGTGTCATTAAATTCTCAACTCTCTTATACTTTTTTGTAAAAGAGAGCTTGGACTGTG
GAGGCTTGCTGGCCACTTTTTGGGGTCAGCTCCTCTGAAATGCATTAGCGGAACCGTTTG
CGATCTGCCACAAGTGTGATAAGTTATCTACACTGGCGAGGGGATTGCTCTCTGTAATGT
TCAGCTTCTAATTGTCTCTACTTTGTGAGACTACTTTTGAATGCTTGACCTCAAATCAGG
TAGGACTACCCGCTGAACTTAA
>AC3-15
TTTCCGTAGGTGAACCTGCGGAAGGATCATTATTGAATTATGTTTCTAGATAGGTTGTAG
CTGGCTC-TTTAGAGCATGTGCACGCCTGTTTGGACTTCATTTTCATCCACCTGTGCACC
TATTGTAGTCTTTGGTTGGGTTAGGAGGAAGTGGTCATTGTGTCAGCATCTGCTGGATGT
GAGGACTTGCATTGTGAAAGCTTTGCTGTCCTTGATGTGATCATGGAATCTCTTTC----

-------TCACTAGAGTCTATGTCACTCATTATACTCTGTCGAATGTCATTGAATGTCTT
TACATGGGCTTGTATGCCTATGAAAATTGTAATACAACTTTCAGCAACGGATCTCTTGGC
TCTCGCATCGATGAAGAACGCAGCGAAATGCGATAAGTAATGTGAATTGCAGAATTCAGT
GAATCATCGAATCTTTGAACGCATCTTGCGCTCCTTGGTATTCCGAGGAGCATGCCTGTT
TGAGTGTCATTAAATTCTCAACTCTCTTCTACTTTTTTGTAAAAGAGAGCTTGGACTGTG
GAGGCTTGCTGGCCACTTTTTGGGGTCAGCTCCTCTGAAATGCATTAGCGGAACCGTTTG
CGATCTGCCACAAGTGTGATAAGTTATCTACACTGGCGAGGGGATTGCTCTCTGTAATGT
TCAGCTTCTAATTGTCTCTACTTTGTGAGACTACTTTTGAATGCTTGACCTCAAATCAGG
TAGGACTACCCGCTGAACTTAA
>AC4-54
TTTCCGTAGGTGAACCTGCGGAAGGATCATTATTGAATTATGTTTCTAGATAGGTTGTAG
CTGGCTC-TTTAGAGCATGTGCACGCCTGTTTGGACTTCATTTTCATCCACCTGTGCACC
TATTGTAGTCTTTGGTTGGGTTAGGAGGAAGTGGTCATTGTGTCAGCATCTGCTGGATGT
GAGGACTTGCATTGTGAAAGCTTTGCTATCCTTGATGTGATCATGGAATCTTTTTC----

-------TCACTAGAGTCTATGTCACTCATTATACTCTGTCGAATGTCATTGAATGTCTT
TACATGGGCTTGTATGCCTATGAAAATTGTAATACAACTTTCAGCAACGGATCTCTTGGC
TCTCGCATCGATGAAGAACGCAGCGAAATGCGATAAGTAATGTGAATTGCAGAATTCAGT
GAATCATCGAATCTTTGAACGCATCTTGCGCTCCTTGGTATTCCGAGGAGCATGCCTGTT
TGAGTGTCATTAAATTCTCAACTCTCTTATACTTTTTTGTAAAAGAGAGCTTGGACTGTG
GAGGCTTGCTGGCCACTTTTTGGGGTCAGCTCCTCTGAAATGCATTAGCGGAACCGTTTG
CGATTTGCCACAAGTGTGATAAGTTATCTACACTGGCGAGGGGATTGCTCTCTGTAATGT
TCAGCTTCTAATTGTCTCTACTTTGTGAGACTACTTTTGAATGCTTGACCTCAAATCAGG
TAGGACTACCCGCTGAACTTAA
>AC1-31
TTTCCGTAGGTGAACCTGCGGAAGGATCATTATTGAATTATGTTTCTAGATAGGTTGTAG
CTGGCTC-TTTAGAGCATGTGCACGCCTGTTTGGACTTCATTTTCATCCACCTGTGCACC
TATTGTAGTCTTTGGTTGGGTTAGGAGGAAGTGATCATTGTATCAGCATCTGCTGGGAGT
GAGGACTTGCATTGTGAAAGCTTTGCTGTCCTTGATGTGATCATGGAATCTCTTTC----

-------TCACTAGAGTCTATGTCACTCATTATACTCTGTCGAATGTCATTGAATGTCTT
TACATGGGCTTGTATGCCTATGAAAATTGTAATACAACTTTCAGCAACGGATCTCTTGGC
TCTCGCATCGATGAAGAACGCAGCGAAATGCGATAAGTAATGTGAATTGCAGAATTCAGT
GAATCATCGAATCTTTGAACGCATCTTGCGCTCCTTGGTATTCCGAGGAGCATGCCTGTT
TGAGTGTCATTAAATTCTCAACTCTCTTATACTTTTTTGTAAAAGAGAGCTTGGACTGTG
GAGGCTTGCTGGCCACTTTTTGGGGTCAGCTCCTCTGAAATGCATTAGCGGAACCGTTTG
CGATCTGCCACAAGTGTGATAAGTTATCTACACTGGCGAGGGGATTGCTCTCTGTAATGT
TCAGCTTCTAATTGTCTCTACTTTGTGAGACTACTTTTGAATGCTTGACCTCAAATCAGG
TAGGACTACCCGCTGAACTTAA
>AC2-54
TTTCCGTAGGTGAACCTGCGGAAGGATCATTATTGAATTATGTTTCTAGATAGGTTGTAG
CTGGCTC-TTTAGAGCATGTGCACGCCTGTTTGGACTTCATTTTCATCCACCTGTGCACC
TATTGTAGTCTTTGGTTGGGTTAGGAGGAAGTGATCATTGTATCAGCATCTGCTGGGAGT
GAGGACTTGCATTGTGAAAGCTTTGCTGTCCTTGATGTGATCATGGAATCTTTTTC----


```
------------------------------------------------------------
------------------------------------------------------------
------------------------------------------------------------
------------------------------------------------------------
------------------------------------------------------------
------------------------------------------------------------
------------------------------------------------------------
------------------------------------------------------------
------------------------------------------------------------
-------TCACTAGAGTCTATGTCACTCATTATACTCTGTCGAATGTCATTGAATGTCTT
TACATGGGCTTGTATGCCTATGAAAATTGTAATACAACTTTCAGCAACGGATCTCTTGGC
TCTCGCATCGATGAAGAACGCAGCGAAATGCGATAAGTAATGTGAATTGCAGAATTCAGT
GAATCATCGAATCTTTGAACGCATCTTGCGCTCCTTGGTATTCCGAGGAGCATGCCTGTT
TGAGTGTCATTAAATTCTCAACTCTCTTATACTTTTTTGTAAAAGAGAGCTTGGACTGTG
GAGGCTTGCTGGCCACTTTTTGGGGTCAGCTCCTCTGAAATGCATTAGCGGAACCGTTTG
CGATCTGCCACAAGTGTGATAAGTTATCTACACTGGCGAGGGGATTGCTCTCTGTAATGT
TCAGCTTCTAATTGTCTCTACTTTGTGAGACTACTTTTGAATGCTTGACCTCAAATCAGG
TAGGACTACCCGCTGAACTTAA
>AC3-26
TTTCCGTAGGTGAACCTGCGGAAGGATCATTATTGAATTATGTTTCTAGATAGGTTGTAG
CTGGCTC-TTTAGAGCATGTGCACGCCTGTTTGGACTTCATTTTCATCCACCTGTGCACC
TATTGTAGTCTTTGGTTGGGTTAGGAGGAAGTGATCATTGTATCAGCATCTGCTGGGAGT
GAGGACTTGCATTGTGAAAGCTTTGCTGTCCTTGATGTGATCATGGAATCTTTTTC----
------------------------------------------------------------
------------------------------------------------------------
------------------------------------------------------------
------------------------------------------------------------
------------------------------------------------------------
------------------------------------------------------------
------------------------------------------------------------
------------------------------------------------------------
------------------------------------------------------------
------------------------------------------------------------
------------------------------------------------------------
------------------------------------------------------------
-------TCACTAGAGTCTATGTCACTCATTATACTCTGTCGAATGTCATTGAATGTCTT
TACATGGGCTTGTATGCCTATGAAAATTGTAATACAACTTTCAGCAACGGATCTCTTGGC
TCTCGCATCGATGAAGAACGCAGCGAAATGCGATAAGTAATGTGAATTGCAGAATTCAGT
GAATCATCGAATCTTTGAACGCATCTTGCGCTCCTTGGTATTCCGAGGAGCATGCCTGTT
TGAGTGTCATTAAATTCTCAACTCTCTTATACTTTTTTGTAAAAGAGAGCTTGGACTGTG
GAGGCTTGCTGGCCACTTTTTGGGGTCAGCTCCTCTGAAATGCATTAGCGGAACCGTTTG
CGATCTGCCACAAGTGTGATAAGTTATCTACACTGGCGAGGGGATTGCTCTCTGTAATGT
TCAGCTTCTAATTGTCTCTACTTTGTGAGACTACTTTTGAATGCTTGACCTCAAATCAGG
TAGGACTACCCGCTGAACTTAA
>AC3-40
TTTCCGTAGGTGAACCTGCGGAAGGATCATTATTGAATTATGTTTCTAGATAGGTTGTAG
CTGGCTC-TTTAGAGCATGTGCACGCCTGTTTGGACTTCATTTTCATCCACCTGTGCACC
TATTGTAGTCTTTGGTTGGGTTAGGAGGAAGTGATCATTGTATCAGCATCTGCTGGGAGT
GAGGACTTGCATTGTGAAAGCTTTGCTGTCCTTGATGTGATCATGGAATCTTTTTC----
------------------------------------------------------------
```


-------TCACTAGAGTCTATGTCACTCATTATACTCTGTCGAATGTCATTGAATGTCTT
TACATGGGCTTGTATGCCTATGAAAATTGTAATACAACTTTCAGCAACGGATCTCTTGGC
TCTCGCATCGATGAAGAACGCAGCGAAATGCGATAAGTAATGTGAATTGCAGAATTCAGT
GAATCATCGAATCTTTGAACGCATCTTGCGCTCCTTGGTATTCCGAGGAGCATGCCTGTT
TGAGTGTCATTAAATTCTCAACTCTCTTATACTTTTTTGTAAAAGAGAGCTTGGACTGTG
GAGGCTTGCTGGCCACTTTTTGGGGTCAGCTCCTCTGAAATGCATTAGCGGAACCGTTTG
CGATCTGCCACAAGTGTGATAAGTTATCTACACTGGCGAGGGGATTGCTCTCTGTAATGT
TCAGCTTCTAATTGTCTCTACTTTGTGAGACTACTTTTGAATGCTTGACCTCAAATCAGG
TAGGACTACCCGCTGAACTTAA
>AC5-37
TTTCCGTAGGTGAACCTGCGGAAGGATCATTATTGAATTATGTTTCTAGATAGGTTGTAG
CTGGCTC-TTTAGAGCATGTGCACGCCTGTTTGGACTTCATTTTCATCCACCTGTGCACC
TATTGTAGTCTTTGGTTGGGTTAGGAGGAAGTGATCATTGTATCAGCATCTGCTGGGAGT
GAGGACTTGCATTGTGAAAGCTTTGCTGTCCTTGATGTGATCATGGAATCTTTTTC----

-------TCACTAGAGTCTATGTCACTCATTATACTCTGTCGAATGTCATTGAATGTCTT
TACATGGGCTTGTATGCCTATGAAAATTGTAATACAACTTTCAGCAACGGATCTCTTGGC
TCTCGCATCGATGAAGAACGCAGCGAAATGCGATAAGTAATGTGAATTGCAGAATTCAGT
GAATCATCGAATCTTTGAACGCATCTTGCGCTCCTTGGTATTCCGAGGAGCATGCCTGTT
TGAGTGTCATTAAATTCTCAACTCTCTTATACTTTTTTGTAAAAGAGAGCTTGGACTGTG
GAGGCTTGCTGGCCACTTTTTGGGGTCAGCTCCTCTGAAATGCATTAGCGGAACCGTTTG
CGATCTGCCACAAGTGTGATAAGTTATCTACACTGGCGAGGGGATTGCTCTCTGTAATGT
TCAGCTTCTAATTGTCTCTACTTTGTGAGACTACTTTTGAATGCTTGACCTCAAATCAGG
TAGGACTACCCGCTGAACTTAA
>AC5-44
TTTCCGTAGGTGAACCTGCGGAAGGATCATTATTGAATTATGTTTCTAGATAGGTTGTAG
CTGGCTC-TTTAGAGCATGTGCACGCCTGTTTGGACTTCATTTTCATCCACCTGTGCACC
TATTGTAGTCTTTGGTTGGGTTAGGAGGAAGTGATCATTGTATCAGCATCTGCTGGGAGT

```
GAGGACTTGCATTGTGAAAGCTTTGCTGTCCTTGATGTGATCATGGAATCTTTTTC----
------------------------------------------------------------
------------------------------------------------------------
------------------------------------------------------------
------------------------------------------------------------
------------------------------------------------------------
------------------------------------------------------------
------------------------------------------------------------
------------------------------------------------------------
------------------------------------------------------------
------------------------------------------------------------
------------------------------------------------------------
------------------------------------------------------------
-------TCACTAGAGTCTATGTCACTCATTATACTCTGTCGAATGTCATTGAATGTCTT
TACATGGGCTTGTATGCCTATGAAAATTGTAATACAACTTTCAGCAACGGATCTCTTGGC
TCTCGCATCGATGAAGAACGCAGCGAAATGCGATAAGTAATGTGAATTGCAGAATTCAGT
GAATCATCGAATCTTTGAACGCATCTTGCGCTCCTTGGTATTCCGAGGAGCATGCCTGTT
TGAGTGTCATTAAATTCTCAACTCTCTTATACTTTTTTGTAAAAGAGAGCTTGGACTGTG
GAGGCTTGCTGGCCACTTTTTGGGGTCAGCTCCTCTGAAATGCATTAGCGGAACCGTTTG
CGATCTGCCACAAGTGTGATAAGTTATCTACACTGGCGAGGGGATTGCTCTCTGTAATGT
TCAGCTTCTAATTGTCTCTACTTTGTGAGACTACTTTTGAATGCTTGACCTCAAATCAGG
TAGGACTACCCGCTGAACTTAA
>AC9-18
TTTCCGTAGGTGAACCTGCGGAAGGATCATTATTGAATTATGTTTCTAGATAGGTTGTAG
CTGGCTC-TTTAGAGCATGTGCACGCCTGTTTGGACTTCATTTTCATCCACCTGTGCACC
TATTGTAGTCTTTGGTTGGGTTAGGAGGAAGTGATCATTGTATCAGCATCTGCTGGGAGT
GAGGACTTGCATTGTGAAAGCTTTGCTGTCCTTGATGTGATCATGGAATCTTTTTC----
------------------------------------------------------------
------------------------------------------------------------
------------------------------------------------------------
------------------------------------------------------------
------------------------------------------------------------
------------------------------------------------------------
------------------------------------------------------------
------------------------------------------------------------
------------------------------------------------------------
------------------------------------------------------------
------------------------------------------------------------
------------------------------------------------------------
-------TCACTAGAGTCTATGTCACTCATTATACTCTGTCGAATGTCATTGAATGTCTT
TACATGGGCTTGTATGCCTATGAAAATTGTAATACAACTTTCAGCAACGGATCTCTTGGC
TCTCGCATCGATGAAGAACGCAGCGAAATGCGATAAGTAATGTGAATTGCAGAATTCAGT
GAATCATCGAATCTTTGAACGCATCTTGCGCTCCTTGGTATTCCGAGGAGCATGCCTGTT
TGAGTGTCATTAAATTCTCAACTCTCTTATACTTTTTTGTAAAAGAGAGCTTGGACTGTG
GAGGCTTGCTGGCCACTTTTTGGGGTCAGCTCCTCTGAAATGCATTAGCGGAACCGTTTG
CGATCTGCCACAAGTGTGATAAGTTATCTACACTGGCGAGGGGATTGCTCTCTGTAATGT
TCAGCTTCTAATTGTCTCTACTTTGTGAGACTACTTTTGAATGCTTGACCTCAAATCAGG
TAGGACTACCCGCTGAACTTAA
>AC12-16
TTTCCGTAGGTGAACCTGCGGAAGGATCATTATTGAATTATGTTTCTAGATAGGTTGTAG
```

CTGGCTC-TTTAGAGCATGTGCACGCCTGTTTGGACTTCATTTTCATCCACCTGTGCACC
TATTGTAGTCTTTGGTTGGGTTAGGAGGAAGTGATCATTGTATCAGCATCTGCTGGGAGT
GAGGACTTGCATTGTGAAAGCTTTGCTGTCCTTGATGTGATCATGGAATCTTTTTC----

-------TCACTAGAGTCTATGTCACTCATTATACTCTGTCGAATGTCATTGAATGTCTT
TACATGGGCTTGTATGCCTATGAAAATTGTAATACAACTTTCAGCAACGGATCTCTTGGC
TCTCGCATCGATGAAGAACGCAGCGAAATGCGATAAGTAATGTGAATTGCAGAATTCAGT
GAATCATCGAATCTTTGAACGCATCTTGCGCTCCTTGGTATTCCGAGGAGCATGCCTGTT
TGAGTGTCATTAAATTCTCAACTCTCTTATACTTTTTTGTAAAAGAGAGCTTGGACTGTG
GAGGCTTGCTGGCCACTTTTTGGGGTCAGCTCCTCTGAAATGCATTAGCGGAACCGTTTG
CGATCTGCCACAAGTGTGATAAGTTATCTACACTGGCGAGGGGATTGCTCTCTGTAATGT
TCAGCTTCTAATTGTCTCTACTTTGTGAGACTACTTTTGAATGCTTGACCTCAAATCAGG
TAGGACTACCCGCTGAACTTAA
>AC10-38
TTTCCGTAGGTGAACCTGCGGAAGGATCATTATTGAATTATGTTTCTAGATAGGTTGTAG
CTGGCTC-TTTAGAGCATGTGCACGCCTGTTTGGACTTCATTTTCATCCACCTGTGCACC
TATTGTAGTCTTTGGTTGGGTTAGGAGGAAGTGATCATTGTATCAGCATCTGCTGGGAGT
GAGGACTTGCATTGTGAAAGCTTTGCTGTCCTTGATGTGATCATGGAATCTTTTTC----

-------TCACTAGAGTCTATGTCACTCATTATACTCTGTCGAATGTCATTGAATGTCTT
TACATGGGCTTGTATGCCTATGAAAATTGTAATACAACTTTCAGCAACGGATCTCTTGGC
TCTCGCATCGATGAAGAACGCAGCGAAATGCGATAAGTAATGTGAATTGCAGAATTCAGT
GAATCATCGAATCTTTGAACGCATCTTGCGCTCCTTGGTATTCCGAGGAGCATGCCTGTT
TGAGTGTCATTAAATTCTCAACTCTCTTATACTTTTTTGTAAAAGAGAGCTTGGACTGTG
GAGGCTTGCTGGCCACTTTTTGGGGTCAGCTCCTCTGAAATGCATTAGCGGAACCGTTTG
CGATCTGCCACAAGTGTGATAAGTTATCTACACTGGCGAGGGGATTGCTCTCTGTAATGT
TCAGCTTCTAATTGTCTCTACTTTGTGAGACTACTTTTGAATGCTTGACCTCAAATCAGG
TAGGACTACCCGCTGAACTTAA

>AC8–56
TTTCCGTAGGTGAACCTGCGGAAGGATCATTATTGAATTATGTTTCTAGATAGGTTGTAG
CTGGCTC-TTTAGAGCATGTGCACGCCTGTTTGGACTTCATTTTCATCCACCTGTGCACC
TATTGTAGTCTTTGGTTGGGTTAGGAGGAAGTGATCATTGTATCAGCATCTGCTGGGAGT
GAGGACTTGCATTGTGAAAGCTTTGCTGTCCTTGATGTGATCATGGAATCTTTTTC----

-------TCACTAGAGTCTATGTCACTCATTATACTCTGTCGAATGTCATTGAATGTCTT
TACATGGGCTTATATGCCTATGAAAATTGTAATACAACTTTCAGCAACGGATCTCTTGGC
TCTCGCATCGATGAAGAACGCAGCGAAATGCGATAAGTAATGTGAATTGCAGAATTCAGT
GAATCATCGAATCTTTGAACGCATCTTGCGCTCCTTGGTATTCCGAGGAGCATGCCTGTT
TGAGTGTCATTAAATTCTCAACTCTCTTATACTTTTTTGTAAAAGAGAGCTTGGACTGTG
GAGGCTTGCTGGCCACTTTTTGGGGTCAGCTCCTCTGAAATGCATTAGCGGAACCGTTTG
CGATCTGCCACAAGTGTGATAAGTTATCTACACTGGCGAGGGGATTGCTCTCTGTAATGT
TCAGCTTCTAATTGTCTCTACTTTGTGAGACTACTTTTGAATGCTTGACCTCAAATCAGG
TAGGACTACCCGCTGAACTTAA
>AC12–13
TTTCCGTAGGTGAACCTGCGGAAGGATCATTATTGAATTATGTTTCTAGATAGGTTGTAG
CTGGCTC-TTTAGAGCATGTGCACGCCTGTTTGGACTTCATTTTCATCCACCTGTGCACC
TATTGTAGTCTTTGGTTGGGTTAGGAGGAAGTGATCATTGTATCAGCATCTGCTGGGAGT
GAGGACTTGCATTGTGAAAGCTTTGCTGTCCTTGATGTGATCATGGAATCTTTTTC----

-------TCACTAGAGTCTATGTCACTCATTATACTCTGTCGAATGTCATTGAATGTCTT
TACATGGGCTTATATGCCTATGAAAATTGTAATACAACTTTCAGCAACGGATCTCTTGGC
TCTCGCATCGATGAAGAACGCAGCGAAATGCGATAAGTAATGTGAATTGCAGAATTCAGT
GAATCATCGAATCTTTGAACGCATCTTGCGCTCCTTGGTATTCCGAGGAGCATGCCTGTT
TGAGTGTCATTAAATTCTCAACTCTCTTATACTTTTTTGTAAAAGAGAGCTTGGACTGTG
GAGGCTTGCTGGCCACTTTTTGGGGTCAGCTCCTCTGAAATGCATTAGCGGAACCGTTTG
CGATCTGCCACAAGTGTGATAAGTTATCTACACTGGCGAGGGGATTGCTCTCTGTAATGT

TCAGCTTCTAATTGTCTCTACTTTGTGAGACTACTTTTGAATGCTTGACCTCAAATCAGG
TAGGACTACCCGCTGAACTTAA
>AC12-37
TTTCCGTAGGTGAACCTGCGGAAGGATCATTATTGAATTATGTTTCTAGATAGGTTGTAG
CTGGCTC-TTTAGAGCATGTGCACGCCTGTTTGGACTTCATTTTCATCCACCTGTGCACC
TATTGTAGTCTTTGGTTGGGTTAGGAGGAAGTGATCATTGTATCAGCATCTGCTGGGAGT
GAGGACTTGCATTGTGAAAGCTTTGCTGTCCTTGATGTGATCATGGAATCTTTTTC----

-------TCACTAGAGTCTATGTCACTCATTATACTCTGTCGAATGTCATTGAATGTCTT
TACATGGGCTTATATGCCTATGAAAATTGTAATACAACTTTCAGCAACGGATCTCTTGGC
TCTCGCATCGATGAAGAACGCAGCGAAATGCGATAAGTAATGTGAATTGCAGAATTCAGT
GAATCATCGAATCTTTGAACGCATCTTGCGCTCCTTGGTATTCCGAGGAGCATGCCTGTT
TGAGTGTCATTAAATTCTCAACTCTCTTATACTTTTTTGTAAAAGAGAGCTTGGACTGTG
GAGGCTTGCTGGCCACTTTTTGGGGTCAGCTCCTCTGAAATGCATTAGCGGAACCGTTTG
CGATCTGCCACAAGTGTGATAAGTTATCTACACTGGCGAGGGGATTGCTCTCTGTAATGT
TCAGCTTCTAATTGTCTCTACTTTGTGAGACTACTTTTGAATGCTTGACCTCAAATCAGG
TAGGACTACCCGCTGAACTTAA
>AC6-51
TTTCCGTAGGTGAACCTGCGGAAGGATCATTATTGAATTATGTTTCTAGATAGGTTGTAG
CTGGCTC-TTTAGAGCATGTGCACGCCTGTTTGGACTTCATTTTCATCCACCTGTGCACC
TATTGTAGTCTTTGGTTGGGTTAGGAGGAAGTGGTCATTGTGTCAGCATCTGCTGGGAGT
GAGGACTTGCATTGTGAAAGCTTTGCTGTCCTTGATGTGATCATGGAATCTTTTTC----

-------TCACTAGAGTCTATGTCACTCATTATACTCTGTCGAATGTCATTGAATGTCTT
TACATGGGCTTGTATGCCTATGAAAATTGTAATACAACTTTCAGCAACGGATCTCTTGGC
TCTCGCATCGATGAAGAACGCAGCGAAATGCGATAAGTAATGTGAATTGCAGAATTCAGT
GAATCATCGAATCTTTGAACGCATCTTGCGCTCCTTGGTATTCCGAGGAGCATGCCTGTT
TGAGTGTCATTAAATTCTCAACTCTCTTATACTTTTTTGTAAAAGAGAGCTTGGACTGTG

GAGGCTTGCTGGCCACTTTTTGGGGTCAGCTCCTCTGAAATGCATTAGCGGAACCGTTTG
CGATCTGCCACAAGTGTGATAAGTTATCTACACTGGCGAGGGGATTGCTCTCTGTAATGT
TCAGCTTCTAATTGTCTCTACTTTGTGAGACTACTTTTGAATGCTTGACCTCAAATCAGG
TAGGACTACCCGCTGAACTTAA
>AC1-1
TTTCCGTAGGTGAACCTGCGGAAGGATCATTATTGAATTATGTTTCTAGATAGGTTGTAG
CTGGCTC-TTTAGAGCATGTGCACGCCTGTTTGGACTTCATTTTCATCCACCTGTGCACC
TATTGTAGTCTTTGGTTGGGTTAGGAGGAAGTGGTCATTGTGTCAGCATCTGCTGGATGT
GAGGACTTGCATTGTGAAAGCTTTGCTGTCCTTGATGTGATCATGGAATCTCTTTC----

-------TCACTAGAGTCTATGTCACTCATTATACTCTGTCGAATGTCATTGAATGTCTT
TACATGGGCTTATATGCCTATGAAAATTGTAATACAACTTTCAGCAACGGATCTCTTGGC
TCTCGCATCGATGAAGAACGCAGCGAAATGCGATAAGTAATGTGAATTGCAGAATTCAGT
GAATCATCGAATCTTTGAACGCATCTTGCGCTCCTTGGTATTCCGAGGAGCATGCCTGTT
TGAGTGTCATTAAATTCTCAACTCTCTTCTAC-TTTTTGTAAAAGAGAGCTTGGACTGTG
GAGGCTTGCTGGCCACTTTTTGGGGTCAGCTCCTCTGAAATGCATTAGCGGAACCGTTTG
CGATCTGCCACAAGTGTGATAAGTTATCTACACTGGCGAGGGGATTGCTCTCTGTAATGT
TCAGCTTCTAATTGTCTCTACTTTGTGAGACTACTTTTGAATGCTTGACCTCAAATCAGG
TAGGACTACCCGCTGAACTTAA
>AC9-57
TTTCCGTAGGTGAACCTGCGGAAGGATCATTATTGAATTATGTTTCTAGATAGGTTGTAG
CTGGCTC-TTTAGAGCATGTGCACGCCTGTTTGGACTTCATTTTCATCCACCTGTGCACC
TATTGTAGTCTTTGGTTGGGTTAGGAGGAAGTGGTCATTGTGTCAGCATCTGCTGGATGT
GAGGACTTGCATTGTGAAAGCTTTGCTGTCCTTGATGTGATCATGGAATCTCTTTC----

-------TCACTAGAGTCTATGTCACTCATTATACTCTGTCGAATGTCATTGAATGTCTT
TACATGGGCTTATATGCCTATGAAAATTGTAATACAACTTTCAGCAACGGATCTCTTGGC
TCTCGCATCGATGAAGAACGCAGCGAAATGCGATAAGTAATGTGAATTGCAGAATTCAGT

GAATCATCGAATCTTTGAACGCATCTTGCGCTCCTTGGTATTCCGAGGAGCATGCCTGTT
TGAGTGTCATTAAATTCTCAACTCTCTTCTAC-TTTTTGTAAAAGAGAGCTTGGACTGTG
GAGGCTTGCTGGCCACTTTTTGGGGTCAGCTCCTCTGAAATGCATTAGCGGAACCGTTTG
CGATCTGCCACAAGTGTGATAAGTTATCTACACTGGCGAGGGGATTGCTCTCTGTAATGT
TCAGCTTCTAATTGTCTCTACTTTGTGAGACTACTTTTGAATGCTTGACCTCAAATCAGG
TAGGACTACCCGCTGAACTTAA
>AC5-51
TTTCCGTAGGTGAACCTGCGGAAGGATCATTATTGAATTATGTTTCTAGATAGGTTGTAG
CTGGCTC-TTTAGAGCATGTGCACGCCTGTTTGGACTTCATTTTCATCCACCTGTGCACC
TATTGTAGTCTTTGGTTGGGTTAGGAGGAAGTGGTCATTGTGTCAGCATCTGCTGGATGT
GAGGACTTGCATTGTGAAAGCTTTGCTGTCCTTGATGTGATCATGGAATCTCTTTC----

-------TCACTAGAGTCTATGTCACTCATTATACTCTGTCGAATGTCATTGAATGTCTT
TACATGGGCTTATATGCCTATGAAAATTGTAATACAACTTTCAGCAACGGATCTCTTGGC
TCTCGCATCGATGAAGAACGCAGCGAAATGCGATAAGTAATGTGAATTGCAGAATTCAGT
GAATCATCGAATCTTTGAACGCATCTTGCGCTCCTTGGTATTCCGAGGAGCATGCCTGTT
TGAGTGTCATTAAATTCTCAACTCTCTTCTAC-TTTTTGTAAAAGAGAGCTTGGACTGTG
GAGGCTTGCTGGCCACTTTTTGGGGTCAGCTCCTCTGAAATGCATTAGCGGAACCGTTTG
CGATCTGCCACAAGTGTGATAAGTTATCTACACTGGCGAGGGGATTGCTCTCTGTAATGT
TCAGCTTCTAATTGTCTCTACTTTGTGAGACTACTTTTGAATGCTTGACCTCAAATCAGG
TAGGACTACCCGCTGAACTTAA
>AC11-45
TTTCCGTAGGTGAACCTGCGGAAGGATCATTATTGAATTATGTTTCTAGATAGGTTGTAG
CTGGCTC-TTTAGAGCATGTGCACGCCTGTTTGGACTTCATTTTCATCCACCTGTGCACC
TATTGTAGTCTTTGGTTGGGTTAGGAGGAAGTGGTCATTGTGTCAGCATCTGCTGGATGT
GAGGACTTGCATTGTGAAAGCTTTGCTGTCCTTGATGTGATCATGGAATCTCTTTC----

-------TCACTAGAGTCTATGTCACTCATTATACTCTGTCGAATGTCATTGAATGTCTT

TACATGGGCTTATATGCCTATGAAAATTGTAATACAACTTTCAGCAACGGATCTCTTGGC
TCTCGCATCGATGAAGAACGCAGCGAAATGCGATAAGTAATGTGAATTGCAGAATTCAGT
GAATCATCGAATCTTTGAACGCATCTTGCGCTCCTTGGTATTCCGAGGAGCATGCCTGTT
TGAGTGTCATTAAATTCTCAACTCTCTTCTAC-TTTTTGTAAAAGAGAGCTTGGACTGTG
GAGGCTTGCTGGCCACTTTTTGGGGTCAGCTCCTCTGAAATGCATTAGCGGAACCGTTTG
CGATCTGCCACAAGTGTGATAAGTTATCTACACTGGCGAGGGGATTGCTCTCTGTAATGT
TCAGCTTCTAATTGTCTCTACTTTGTGAGACTACTTTTGAATGCTTGACCTCAAATCAGG
TAGGACTACCCGCTGAACTTAA
>AC12-52
TTTCCGTAGGTGAACCTGCGGAAGGATCATTATTGAATTATGTTTCTAGATAGGTTGTAG
CTGGCTC-TTTAGAGCATGTGCACGCCTGTTTGGACTTCATTTTCATCCACCTGTGCACC
TATTGTAGTCTTTGGTTGGGTTAGGAGGAAGTGGTCATTGTGTCAGCATCTGCTGGATGT
GAGGACTTGCATTGTGAAAGCTTTGCTGTCCTTGATGTGATCATGGAATCTCTTTC----

-------TCACTAGAGTCTATGTCACTCATTATACTCTGTCGAATGTCATTGAATGTCTT
TACATGGGCTTATATGCCTATGAAAATTGTAATACAACTTTCAGCAACGGATCTCTTGGC
TCTCGCATCGATGAAGAACGCAGCGAAATGCGATAAGTAATGTGAATTGCAGAATTCAGT
GAATCATCGAATCTTTGAACGCATCTTGCGCTCCTTGGTATTCCGAGGAGCATGCCTGTT
TGAGTGTCATTAAATTCTCAACTCTCTTCTAC-TTTTTGTAAAAGAGAGCTTGGACTGTG
GAGGCTTGCTGGCCACTTTTTGGGGTCAGCTCCTCTGAAATGCATTAGCGGAACCGTTTG
CGATCTGCCACAAGTGTGATAAGTTATCTACACTGGCGAGGGGATTGCTCTCTGTAATGT
TCAGCTTCTAATTGTCTCTACTTTGTGAGACTACTTTTGAATGCTTGACCTCAAATCAGG
TAGGACTACCCGCTGAACTTAA
>AC6-1
TTTCCGTAGGTGAACCTGCGGAAGGATCATTATTGAATTATGTTTCTAGATAGGTTGTAG
CTGGCTC-TTTAGAGCATGTGCACGCCTGTTTGGACTTCATTTTCATCCACCTGTGCACC
TATTGTAGTCTTTGGTTGGGTTAGGAGGAAGTGGTCATTGTGTCAGCATCTGCTGGATGT
GAGGACTTGCATTGTGAAAGCTTTGCTGTCCTTGATGTGATCATGGAATCTCTTTC----

-------TCACTAGAGTCTATGTCACTCATTATACTCTGTCGAATGTCATTGAATGTCTT
TACATGGGCTTATATGCCTATGAAAATTGTAATACAACTTTCAGCAACGGATCTCTTGGC
TCTCGCATCGATGAAGAACGCAGCGAAATGCGATAAGTAATGTGAATTGCAGAATTCAGT
GAATCATCGAATCTTTGAACGCATCTTGCGCTCCTTGGTATTCCGAGGAGCATGCCTGTT
TGAGTGTCATTAAATTCTCAACTCTCTTCTAC-TTTTTGTAAAAGAGAGCTTGGACTGTG
GAGGCTTGCTGGCCACTTTTTGGGGTCAGCTCCTCTGAAATGCATTAGCGGAACCGTTTG
CGATCTGCCACAAGTGTGATAAGTTATCTACACTGGCGAGGGGATTGCTCTCTGTAATGT
TCAGCTTCTAATTGTCTCTACTTTGTGAGACTACTTTTGAATGCTTGACCTCAAATCAGG
TAGGACTACCCGCTGAACTTAA
>AC7-16
TTTCCGTAGGTGAACCTGCGGAAGGATCATTATTGAATTATGTTTCTAGATAGGTTGTAG
CTGGCTC-TTTAGAGCATGTGCACGCCTGTTTGGACTTCATTTTCATCCACCTGTGCACC
TATTGTAGTCTTTGGTTGGGTTAGGAGGAAGTGGTCATTGTGTCAGCATCTGCTGGATGT
GAGGACTTGCATTGTGAAAGCTTTGCTGTCCTTGATGTGATCATGGAATCTCTTTC----

-------TCACTAGAGTCTATGTCACTCATTATACTCTGTCGAATGTCATTGAATGTCTT
TACATGGGCTTATATGCCTATGAAAATTGTAATACAACTTTCAGCAACGGATCTCTTGGC
TCTCGCATCGATGAAGAACGCAGCGAAATGCGATAAGTAATGTGAATTGCAGAATTCAGT
GAATCATCGAATCTTTGAACGCATCTTGCGCTCCTTGGTATTCCGAGGAGCATGCCTGTT
TGAGTGTCATTAAATTCTCAACTCTCTTCTAC-TTTTTGTAAAAGAGAGCTTGGACTGTG
GAGGCTTGCTGGCCACTTTTTGGGGTCAGCTCCTCTGAAATGCATTAGCGGAACCGTTTG
CGATCTGCCACAAGTGTGATAAGTTATCTACACTGGCGAGGGGATTGCTCTCTGTAATGT
TCAGCTTCTAATTGTCTCTACTTTGTGAGACTACTTTTGAATGCTTGACCTCAAATCAGG
TAGGACTACCCGCTGAACTTAA
>AC8-15
TTTCCGTAGGTGAACCTGCGGAAGGATCATTATTGAATTATGTTTCTAGATAGGTTGTAG
CTGGCTC-TTTAGAGCATGTGCACGCCTGTTTGGACTTCATTTTCATCCACCTGTGCACC
TATTGTAGTCTTTGGTTGGGTTAGGAGGAAGTGGTCATTGTGTCAGCATCTGCTGGATGT
GAGGACTTGCATTGTGAAAGCTTTGCTGTCCTTGATGTGATCATGGAATCTCTTTC----


```
------------------------------------------------------------
------------------------------------------------------------
------------------------------------------------------------
-------TCACTAGAGTCTATGTCACTCATTATACTCTGTCGAATGTCATTGAATGTCTT
TACATGGGCTTATATGCCTATGAAAATTGTAATACAACTTTCAGCAACGGATCTCTTGGC
TCTCGCATCGATGAAGAACGCAGCGAAATGCGATAAGTAATGTGAATTGCAGAATTCAGT
GAATCATCGAATCTTTGAACGCATCTTGCGCTCCTTGGTATTCCGAGGAGCATGCCTGTT
TGAGTGTCATTAAATTCTCAACTCTCTTCTAC-TTTTTGTAAAAGAGAGCTTGGACTGTG
GAGGCTTGCTGGCCACTTTTTGGGGTCAGCTCCTCTGAAATGCATTAGCGGAACCGTTTG
CGATCTGCCACAAGTGTGATAAGTTATCTACACTGGCGAGGGGATTGCTCTCTGTAATGT
TCAGCTTCTAATTGTCTCTACTTTGTGAGACTACTTTTGAATGCTTGACCTCAAATCAGG
TAGGACTACCCGCTGAACTTAA
>AC10-32
TTTCCGTAGGTGAACCTGCGGAAGGATCATTATTGAATTATGTTTCTAGATAGGTTGTAG
CTGGCTC-TTTAGAGCATGTGCACGCCTGTTTGGACTTCATTTTCATCCACCTGTGCACC
TATTGTAGTCTTTGGTTGGGTTAGGAGGAAGTGGTCATTGTGTCAGCATCTGCTGGATGT
GAGGACTTGCATTGTGAAAGCTTTGCTGTCCTTGATGTGATCATGGAATCTCTTTC----
------------------------------------------------------------
------------------------------------------------------------
------------------------------------------------------------
------------------------------------------------------------
------------------------------------------------------------
------------------------------------------------------------
------------------------------------------------------------
------------------------------------------------------------
------------------------------------------------------------
------------------------------------------------------------
------------------------------------------------------------
------------------------------------------------------------
-------TCACTAGAGTCTATGTCACTCATTATACTCTGTCGAATGTCATTGAATGTCTT
TACATGGGCTTATATGCCTATGAAAATTGTAATACAACTTTCAGCAACGGATCTCTTGGC
TCTCGCATCGATGAAGAACGCAGCGAAATGCGATAAGTAATGTGAATTGCAGAATTCAGT
GAATCATCGAATCTTTGAACGCATCTTGCGCTCCTTGGTATTCCGAGGAGCATGCCTGTT
TGAGTGTCATTAAATTCTCAACTCTCTTCTAC-TTTTTGTAAAAGAGAGCTTGGACTGTG
GAGGCTTGCTGGCCACTTTTTGGGGTCAGCTCCTCTGAAATGCATTAGCGGAACCGTTTG
CGATCTGCCACAAGTGTGATAAGTTATCTACACTGGCGAGGGGATTGCTCTCTGTAATGT
TCAGCTTCTAATTGTCTCTACTTTGTGAGACTACTTTTGAATGCTTGACCTCAAATCAGG
TAGGACTACCCGCTGAACTTAA
>AC7-21
TTTCCGTAGGTGAACCTGCGGAAGGATCATTATTGAATTATGTTTCTAGATAGGTTGTAG
CTGGCTC-TTTAGAGCATGTGCACGCCTGTTTGGACTTCATTTTCATCCACCTGTGCACC
TATTGTAGTCTTTGGTTGGGTTAGGAGGAAGTGGTCATTGTGTCAGCATCTGCTGGATGT
GAGGACTTGCATTGTGAAAGCTTTGCTGTCCTTGATGTGATCATGGAATCTCTTTC----
------------------------------------------------------------
------------------------------------------------------------
------------------------------------------------------------
------------------------------------------------------------
------------------------------------------------------------
------------------------------------------------------------
------------------------------------------------------------
```


-------TCACTAGAGTCTATGTCACTCATTATACTCTGTCGAATGTCATTGAATGTCTT
TACATGGGCTTATATGCCTATGAAAATTGTAATACAACTTTCAGCAACGGATCTCTTGGC
TCTCGCATCGATGAAGAACGCAGCGAAATGCGATAAGTAATGTGAATTGCAGAATTCAGT
GAATCATCGAATCTTTGAACGCATCTTGCGCTCCTTGGTATTCCGAGGAGCATGCCTGTT
TGAGTGTCATTAAATTCTCAACTCTCTTCTAC-TTTTTGTAAAAGAGAGCTTGGACTGTG
GAGGCTTGCTGGCCACTTTTTGGGGTCAGCTCCTCTGAAATGCATTAGCGGAACCGTTTG
CGATCTGCCACAAGTGTGATAAGTTATCTACACTGGCGAGGGGATTGCTCTCTGTAATGT
TCAGCTTCTAATTGTCTCTACTTTGTGAGACTACTTTTGAATGCTTGACCTCAAATCAGG
TAGGACTACCCGCTGAACTTAA
>AC11-14
TTTCCGTAGGTGAACCTGCGGAAGGATCATTATTGAATTATGTTTCTAGATAGGTTGTAG
CTGGCTC-TTTAGAGCATGTGCACGCCTGTTTGGACTTCATTTTCATCCACCTGTGCACC
TATTGTAGTCTTTGGTTGGGTTAGGAGGAAGTGGTCATTGTGTCAGCATCTGCTGGATGT
GAGGACTTGCATTGTGAAAGCTTTGCTGTCCTTGATGTGATCATGGAATCTCTTTC----

-------TCACTAGAGTCTATGTCACTCATTATACTCTGTCGAATGTCATTGAATGTCTT
TACATGGGCTTATATGCCTATGAAAATTGTAATACAACTTTCAGCAACGGATCTCTTGGC
TCTCGCATCGATGAAGAACGCAGCGAAATGCGATAAGTAATGTGAATTGCAGAATTCAGT
GAATCATCGAATCTTTGAACGCATCTTGCGCTCCTTGGTATTCCGAGGAGCATGCCTGTT
TGAGTGTCATTAAATTCTCAACTCTCTTCTAC-TTTTTGTAAAAGAGAGCTTGGACTGTG
GAGGCTTGCTGGCCACTTTTTGGGGTCAGCTCCTCTGAAATGCATTAGCGGAACCGTTTG
CGATCTGCCACAAGTGTGATAAGTTATCTACACTGGCGAGGGGATTGCTCTCTGTAATGT
TCAGCTTCTAATTGTCTCTACTTTGTGAGACTACTTTTGAATGCTTGACCTCAAATCAGG
TAGGACTACCCGCTGAACTTAA
>AC12-34
TTTCCGTAGGTGAACCTGCGGAAGGATCATTATTGAATTATGTTTCTAGATAGGTTGTAG
CTGGCTC-TTTAGAGCATGTGCACGCCTGTTTGGACTTCATTTTCATCCACCTGTGCACC
TATTGTAGTCTTTGGTTGGGTTAGGAGGAAGTGGTCATTGTGTCAGCATCTGCTGGATGT
GAGGACTTGCATTGTGAAAGCTTTGCTGTCCTTGATGTGATCATGGAATCTCTTTC----

-------TCACTAGAGTCTATGTCACTCATTATACTCTGTCGAATGTCATTGAATGTCTT
TACATGGGCTTATATGCCTATGAAAATTGTAATACAACTTTCAGCAACGGATCTCTTGGC
TCTCGCATCGATGAAGAACGCAGCGAAATGCGATAAGTAATGTGAATTGCAGAATTCAGT
GAATCATCGAATCTTTGAACGCATCTTGCGCTCCTTGGTATTCCGAGGAGCATGCCTGTT
TGAGTGTCATTAAATTCTCAACTCTCTTCTAC-TTTTTGTAAAAGAGAGCTTGGACTGTG
GAGGCTTGCTGGCCACTTTTTGGGGTCAGCTCCTCTGAAATGCATTAGCGGAACCGTTTG
CGATCTGCCACAAGTGTGATAAGTTATCTACACTGGCGAGGGGATTGCTCTCTGTAATGT
TCAGCTTCTAATTGTCTCTACTTTGTGAGACTACTTTTGAATGCTTGACCTCAAATCAGG
TAGGACTACCCGCTGAACTTAA
>AC12-32
TTTCCGTAGGTGAACCTGCGGAAGGATCATTATTGAATTATGTTTCTAGATAGGTTGTAG
CTGGCTC-TTTAGAGCATGTGCACGCCTGTTTGGACTTCATTTTCATCCACCTGTGCACC
TATTGTAGTCTTTGGTTGGGTTAGGAGGAAGTGGTCATTGTGTCAGCATCTGCTGGATGT
GAGGACTTGCATTGTGAAAGCTTTGCTGTCCTTGATGTGATCATGGAATCTCTTTC----

-------TCACTAGAGTCTATGTCACTCATTATACTCTGTCGAATGTCATTGAATGTCTT
TACATGGGCTTATATGCCTATGAAAATTGTAATACAACTTTCAGCAACGGATCTCTTGGC
TCTCGCATCGATGAAGAACGCAGCGAAATGCGATAAGTAATGTGAATTGCAGAATTCAGT
GAATCATCGAATCTTTGAACGCATCTTGCGCTCCTTGGTATTCCGAGGAGCATGCCTGTT
TGAGTGTCATTAAATTCTCAACTCTCTTCTAC-TTTTTGTAAAAGAGAGCTTGGACTGTG
GAGGCTTGCTGGCCACTTTTTGGGGTCAGCTCCTCTGAAATGCATTAGCGGAACCGTTTG
CGATCTGCCACAAGTGTGATAAGTTATCTACACTGGCGAGGGGATTGCTCTCTGTAATGT
TCAGCTTCTAATTGTCTCTACTTTGTGAGACTACTTTTGAATGCTTGACCTCAAATCAGG
TAGGACTACCCGCTGAACTTAA
>AC7-41
TTTCCGTAGGTGAACCTGCGGAAGGATCATTATTGAATTATGTTTCTAGATAGGTTGTAG
CTGGCTC-TTTAGAGCATGTGCACGCCTGTTTGGACTTCATTTTCATCCACCTGTGCACC
TATTGTAGTCTTTGGTTGGGTTAGGAGGAAGTGGTCATTGTGTCAGCATCTGCTGGATGT
GAGGACTTGCATTGTGAAAGCTTTGCTGTCCTTGATGTGATCATGGAATCTCTTTC----

-------TCACTAGAGTCTATGTCACTCATTATACTCTGTCGAATGTCATTGAATGTCTT
TACATGGGCTTATATGCCTATGAAAATTGTAATACAACTTTCAGCAACGGATCTCTTGGC
TCTCGCATCGATGAAGAACGCAGCGAAATGCGATAAGTAATGTGAATTGCAGAATTCAGT
GAATCATCGAATCTTTGAACGCATCTTGCGCTCCTTGGTATTCCGAGGAGCATGCCTGTT
TGAGTGTCATTAAATTCTCAACTCTCTTCTAC-TTTTTGTAAAAGAGAGCTTGGACTGTG
GAGGCTTGCTGGCCACTTTTTGGGGTCAGCTCCTCTGAAATGCATTAGCGGAACCGTTTG
CGATCTGCCACAAGTGTGATAAGTTATCTACACTGGCGAGGGGATTGCTCTCTGTAATGT
TCAGCTTCTAATTGTCTCTACTTTGTGAGACTACTTTTGAATGCTTGACCTCAAATCAGG
TAGGACTACCCGCTGAACTTAA
>AC1-18
TTTCCGTAGGTGAACCTGCGGAAGGATCATTATTGAATTATGTTTCTAGATAGGTTGTAG
CTGGCTC-TTTAGAGCATGTGCACGCCTGTTTGGACTTCATTTTCATCCACCTGTGCACC
TATTGTAGTCTTTGGTTGGGTTAGGAGGAAGTGGTCATTGTGTCAGCATCTGCTGGATGT
GAGGACTTGCATTGTGAAAGCTTTGCTGTCCTTGATGTGATCATGGAATCTCTTTC----

-------TCACTAGAGTCTATGTCACTCATTATACTCTGTCGAATGTCATTGAATGTCTT
TACATGGGCTTATATGCCTATGAAAATTGTAATACAACTTTCAGCAACGGATCTCTTGGC
TCTCGCATCGATGAAGAACGCAGCGAAATGCGATAAGTAATGTGAATTGCAGAATTCAGT
GAATCATCGAATCTTTGAACGCATCTTGCGCTCCTTGGTATTCCGAGGAGCATGCCTGTT
TGAGTGTCATTAAATTCTCAACTCTCTTCTAC-TTTTTGTAAAAGAGAGCTTGGACTGTG
GAGGCTTGCTGGCCACTTTTTGGGGTCAGCTCCTCTGAAATGCATTAGCGGAACCGTTTG
CGATCTGCCACAAGTGTGATAAGTTATCTACACTGGCGAGGGGATTGCTCTCTGTAATGT
TCAGCTTCTAATTGTCTCTACTTTGTGAGACTACTTTTGAATGCTTGACCTCAAATCAGG
TAGGACTACCCGCTGAACTTAA
>AC2-33
TTTCCGTAGGTGAACCTGCGGAAGGATCATTATTGAATTATGTTTCTAGATAGGTTGTAG
CTGGCTC-TTTAGAGCATGTGCACGCCTGTTTGGACTTCATTTTCATCCACCTGTGCACC
TATTGTAGTCTTTGGTTGGGTTAGGAGGAAGTGGTCATTGTGTCAGCATCTGCTGGATGT
GAGGACTTGCATTGTGAAAGCTTTGCTGTCCTTGATGTGATCATGGAATCTCTTTC----

-------TCACTAGAGTCTATGTCACTCATTATACTCTGTCGAATGTCATTGAATGTCTT
TACATGGGCTTATATGCCTATGAAAATTGTAATACAACTTTCAGCAACGGATCTCTTGGC
TCTCGCATCGATGAAGAACGCAGCGAAATGCGATAAGTAATGTGAATTGCAGAATTCAGT
GAATCATCGAATCTTTGAACGCATCTTGCGCTCCTTGGTATTCCGAGGAGCATGCCTGTT
TGAGTGTCATTAAATTCTCAACTCTCTTCTAC-TTTTTGTAAAAGAGAGCTTGGACTGTG
GAGGCTTGCTGGCCACTTTTTGGGGTCAGCTCCTCTGAAATGCATTAGCGGAACCGTTTG
CGATCTGCCACAAGTGTGATAAGTTATCTACACTGGCGAGGGGATTGCTCTCTGTAATGT
TCAGCTTCTAATTGTCTCTACTTTGTGAGACTACTTTTGAATGCTTGACCTCAAATCAGG
TAGGACTACCCGCTGAACTTAA
>AC5-11
TTTCCGTAGGTGAACCTGCGGAAGGATCATTATTGAATTATGTTTCTAGATAGGTTGTAG
CTGGCTC-TTTAGAGCATGTGCACGCCTGTTTGGACTTCATTTTCATCCACCTGTGCACC
TATTGTAGTCTTTGGTTGGGTTAGGAGGAAGTGGTCATTGTGTCAGCATCTGCTGGATGT
GAGGACTTGCATTGTGAAAGCTTTGCTGTCCTTGATGTGATCATGGAATCTCTTTC----

-------TCACTAGAGTCTATGTCACTCATTATACTCTGTCGAATGTCATTGAATGTCTT
TACATGGGCTTATATGCCTATGAAAATTGTAATACAACTTTCAGCAACGGATCTCTTGGC
TCTCGCATCGATGAAGAACGCAGCGAAATGCGATAAGTAATGTGAATTGCAGAATTCAGT
GAATCATCGAATCTTTGAACGCATCTTGCGCTCCTTGGTATTCCGAGGAGCATGCCTGTT
TGAGTGTCATTAAATTCTCAACTCTCTTCTAC-TTTTTGTAAAAGAGAGCTTGGACTGTG
GAGGCTTGCTGGCCACTTTTTGGGGTCAGCTCCTCTGAAATGCATTAGCGGAACCGTTTG
CGATCTGCCACAAGTGTGATAAGTTATCTACACTGGCGAGGGGATTGCTCTCTGTAATGT
TCAGCTTCTAATTGTCTCTACTTTGTGAGACTACTTTTGAATGCTTGACCTCAAATCAGG
TAGGACTACCCGCTGAACTTAA
>AC7-27
TTTCCGTAGGTGAACCTGCGGAAGGATCATTATTGAATTATGTTTCTAGATAGGTTGTAG
CTGGCTC-TTTAGAGCATGTGCACGCCTGTTTGGACTTCATTTTCATCCACCTGTGCACC
TATTGTAGTCTTTGGTTGGGTTAGGAGGAAGTGGTCATTGTGTCAGCATCTGCTGGATGT

GAGGACTTGCATTGTGAAAGCTTTGCTGTCCTTGATGTGATCATGGAATCTCTTTC----

-------TCACTAGAGTCTATGTCACTCATTATACTCTGTCGAATGTCATTGAATGTCTT
TACATGGGCTTATATGCCTATGAAAATTGTAATACAACTTTCAGCAACGGATCTCTTGGC
TCTCGCATCGATGAAGAACGCAGCGAAATGCGATAAGTAATGTGAATTGCAGAATTCAGT
GAATCATCGAATCTTTGAACGCATCTTGCGCTCCTTGGTATTCCGAGGAGCATGCCTGTT
TGAGTGTCATTAAATTCTCAACTCTCTTCTAC-TTTTTGTAAAAGAGAGCTTGGACTGTG
GAGGCTTGCTGGCCACTTTTTGGGGTCAGCTCCTCTGAAATGCATTAGCGGAACCGTTTG
CGATCTGCCACAAGTGTGATAAGTTATCTACACTGGCGAGGGGATTGCTCTCTGTAATGT
TCAGCTTCTAATTGTCTCTACTTTGTGAGACTACTTTTGAATGCTTGACCTCAAATCAGG
TAGGACTACCCGCTGAACTTAA
>AC8-24
TTTCCGTAGGTGAACCTGCGGAAGGATCATTATTGAATTATGTTTCTAGATAGGTTGTAG
CTGGCTC-TTTAGAGCATGTGCACGCCTGTTTGGACTTCATTTTCATCCACCTGTGCACC
TATTGTAGTCTTTGGTTGGGTTAGGAGGAAGTGGTCATTGTGTCAGCATCTGCTGGATGT
GAGGACTTGCATTGTGAAAGCTTTGCTGTCCTTGATGTGATCATGGAATCTCTTTC----

-------TCACTAGAGTCTATGTCACTCATTATACTCTGTCGAATGTCATTGAATGTCTT
TACATGGGCTTATATGCCTATGAAAATTGTAATACAACTTTCAGCAACGGATCTCTTGGC
TCTCGCATCGATGAAGAACGCAGCGAAATGCGATAAGTAATGTGAATTGCAGAATTCAGT
GAATCATCGAATCTTTGAACGCATCTTGCGCTCCTTGGTATTCCGAGGAGCATGCCTGTT
TGAGTGTCATTAAATTCTCAACTCTCTTCTAC-TTTTTGTAAAAGAGAGCTTGGACTGTG
GAGGCTTGCTGGCCACTTTTTGGGGTCAGCTCCTCTGAAATGCATTAGCGGAACCGTTTG
CGATCTGCCACAAGTGTGATAAGTTATCTACACTGGCGAGGGGATTGCTCTCTGTAATGT
TCAGCTTCTAATTGTCTCTACTTTGTGAGACTACTTTTGAATGCTTGACCTCAAATCAGG
TAGGACTACCCGCTGAACTTAA
>AC8-31
TTTCCGTAGGTGAACCTGCGGAAGGATCATTATTGAATTATGTTTCTAGATAGGTTGTAG

CTGGCTC-TTTAGAGCATGTGCACGCCTGTTTGGACTTCATTTTCATCCACCTGTGCACC
TATTGTAGTCTTTGGTTGGGTTAGGAGGAAGTGGTCATTGTGTCAGCATCTGCTGGATGT
GAGGACTTGCATTGTGAAAGCTTTGCTGTCCTTGATGTGATCATGGAATCTCTTTC----

-------TCACTAGAGTCTATGTCACTCATTATACTCTGTCGAATGTCATTGAATGTCTT
TACATGGGCTTATATGCCTATGAAAATTGTAATACAACTTTCAGCAACGGATCTCTTGGC
TCTCGCATCGATGAAGAACGCAGCGAAATGCGATAAGTAATGTGAATTGCAGAATTCAGT
GAATCATCGAATCTTTGAACGCATCTTGCGCTCCTTGGTATTCCGAGGAGCATGCCTGTT
TGAGTGTCATTAAATTCTCAACTCTCTTCTAC-TTTTTGTAAAAGAGAGCTTGGACTGTG
GAGGCTTGCTGGCCACTTTTTGGGGTCAGCTCCTCTGAAATGCATTAGCGGAACCGTTTG
CGATCTGCCACAAGTGTGATAAGTTATCTACACTGGCGAGGGGATTGCTCTCTGTAATGT
TCAGCTTCTAATTGTCTCTACTTTGTGAGACTACTTTTGAATGCTTGACCTCAAATCAGG
TAGGACTACCCGCTGAACTTAA
>AC10-20
TTTCCGTAGGTGAACCTGCGGAAGGATCATTATTGAATTATGTTTCTAGATAGGTTGTAG
CTGGCTC-TTTAGAGCATGTGCACGCCTGTTTGGACTTCATTTTCATCCACCTGTGCACC
TATTGTAGTCTTTGGTTGGGTTAGGAGGAAGTGGTCATTGTGTCAGCATCTGCTGGATGT
GAGGACTTGCATTGTGAAAGCTTTGCTGTCCTTGATGTGATCATGGAATCTCTTTC----

-------TCACTAGAGTCTATGTCACTCATTATACTCTGTCGAATGTCATTGAATGTCTT
TACATGGGCTTATATGCCTATGAAAATTGTAATACAACTTTCAGCAACGGATCTCTTGGC
TCTCGCATCGATGAAGAACGCAGCGAAATGCGATAAGTAATGTGAATTGCAGAATTCAGT
GAATCATCGAATCTTTGAACGCATCTTGCGCTCCTTGGTATTCCGAGGAGCATGCCTGTT
TGAGTGTCATTAAATTCTCAACTCTCTTCTAC-TTTTTGTAAAAGAGAGCTTGGACTGTG
GAGGCTTGCTGGCCACTTTTTGGGGTCAGCTCCTCTGAAATGCATTAGCGGAACCGTTTG
CGATCTGCCACAAGTGTGATAAGTTATCTACACTGGCGAGGGGATTGCTCTCTGTAATGT
TCAGCTTCTAATTGTCTCTACTTTGTGAGACTACTTTTGAATGCTTGACCTCAAATCAGG
TAGGACTACCCGCTGAACTTAA

```
>AC10-51
TTTCCGTAGGTGAACCTGCGGAAGGATCATTATTGAATTATGTTTCTAGATAGGTTGTAG
CTGGCTC-TTTAGAGCATGTGCACGCCTGTTTGGACTTCATTTTCATCCACCTGTGCACC
TATTGTAGTCTTTGGTTGGGTTAGGAGGAAGTGGTCATTGTGTCAGCATCTGCTGGATGT
GAGGACTTGCATTGTGAAAGCTTTGCTGTCCTTGATGTGATCATGGAATCTCTTTC----
------------------------------------------------------------
------------------------------------------------------------
------------------------------------------------------------
------------------------------------------------------------
------------------------------------------------------------
------------------------------------------------------------
------------------------------------------------------------
------------------------------------------------------------
------------------------------------------------------------
------------------------------------------------------------
------------------------------------------------------------
------------------------------------------------------------
-------TCACTAGAGTCTATGTCACTCATTATACTCTGTCGAATGTCATTGAATGTCTT
TACATGGGCTTATATGCCTATGAAAATTGTAATACAACTTTCAGCAACGGATCTCTTGGC
TCTCGCATCGATGAAGAACGCAGCGAAATGCGATAAGTAATGTGAATTGCAGAATTCAGT
GAATCATCGAATCTTTGAACGCATCTTGCGCTCCTTGGTATTCCGAGGAGCATGCCTGTT
TGAGTGTCATTAAATTCTCAACTCTCTTCTAC-TTTTTGTAAAAGAGAGCTTGGACTGTG
GAGGCTTGCTGGCCACTTTTTGGGGTCAGCTCCTCTGAAATGCATTAGCGGAACCGTTTG
CGATCTGCCACAAGTGTGATAAGTTATCTACACTGGCGAGGGGATTGCTCTCTGTAATGT
TCAGCTTCTAATTGTCTCTACTTTGTGAGACTACTTTTGAATGCTTGACCTCAAATCAGG
TAGGACTACCCGCTGAACTTAA
>AC10-54
TTTCCGTAGGTGAACCTGCGGAAGGATCATTATTGAATTATGTTTCTAGATAGGTTGTAG
CTGGCTC-TTTAGAGCATGTGCACGCCTGTTTGGACTTCATTTTCATCCACCTGTGCACC
TATTGTAGTCTTTGGTTGGGTTAGGAGGAAGTGGTCATTGTGTCAGCATCTGCTGGATGT
GAGGACTTGCATTGTGAAAGCTTTGCTGTCCTTGATGTGATCATGGAATCTCTTTC----
------------------------------------------------------------
------------------------------------------------------------
------------------------------------------------------------
------------------------------------------------------------
------------------------------------------------------------
------------------------------------------------------------
------------------------------------------------------------
------------------------------------------------------------
------------------------------------------------------------
------------------------------------------------------------
------------------------------------------------------------
------------------------------------------------------------
-------TCACTAGAGTCTATGTCACTCATTATACTCTGTCGAATGTCATTGAATGTCTT
TACATGGGCTTATATGCCTATGAAAATTGTAATACAACTTTCAGCAACGGATCTCTTGGC
TCTCGCATCGATGAAGAACGCAGCGAAATGCGATAAGTAATGTGAATTGCAGAATTCAGT
GAATCATCGAATCTTTGAACGCATCTTGCGCTCCTTGGTATTCCGAGGAGCATGCCTGTT
TGAGTGTCATTAAATTCTCAACTCTCTTCTAC-TTTTTGTAAAAGAGAGCTTGGACTGTG
GAGGCTTGCTGGCCACTTTTTGGGGTCAGCTCCTCTGAAATGCATTAGCGGAACCGTTTG
CGATCTGCCACAAGTGTGATAAGTTATCTACACTGGCGAGGGGATTGCTCTCTGTAATGT
```

TCAGCTTCTAATTGTCTCTACTTTGTGAGACTACTTTTGAATGCTTGACCTCAAATCAGG
TAGGACTACCCGCTGAACTTAA
>AC11-2
TTTCCGTAGGTGAACCTGCGGAAGGATCATTATTGAATTATGTTTCTAGATAGGTTGTAG
CTGGCTC-TTTAGAGCATGTGCACGCCTGTTTGGACTTCATTTTCATCCACCTGTGCACC
TATTGTAGTCTTTGGTTGGGTTAGGAGGAAGTGGTCATTGTGTCAGCATCTGCTGGATGT
GAGGACTTGCATTGTGAAAGCTTTGCTGTCCTTGATGTGATCATGGAATCTCTTTC----

-------TCACTAGAGTCTATGTCACTCATTATACTCTGTCGAATGTCATTGAATGTCTT
TACATGGGCTTATATGCCTATGAAAATTGTAATACAACTTTCAGCAACGGATCTCTTGGC
TCTCGCATCGATGAAGAACGCAGCGAAATGCGATAAGTAATGTGAATTGCAGAATTCAGT
GAATCATCGAATCTTTGAACGCATCTTGCGCTCCTTGGTATTCCGAGGAGCATGCCTGTT
TGAGTGTCATTAAATTCTCAACTCTCTTCTAC-TTTTTGTAAAAGAGAGCTTGGACTGTG
GAGGCTTGCTGGCCACTTTTTGGGGTCAGCTCCTCTGAAATGCATTAGCGGAACCGTTTG
CGATCTGCCACAAGTGTGATAAGTTATCTACACTGGCGAGGGGATTGCTCTCTGTAATGT
TCAGCTTCTAATTGTCTCTACTTTGTGAGACTACTTTTGAATGCTTGACCTCAAATCAGG
TAGGACTACCCGCTGAACTTAA
>AC8-39
TTTCCGTAGGTGAACCTGCGGAAGGATCATTATTGAATTATGTTTCTAGATAGGTTGTAG
CTGGCTC-TTTAGAGCATGTGCACGCCTGTTTGGACTTCATTTTCATCCACCTGTGCACC
TATTGTAGTCTTTGGTTGGGTTAGGAGGAAGTGGTCATTGTGTCAGCATCTGCTGGATGT
GAGGACTTGCATTGTGAAAGCTTTGCTGTCCTTGATGTGATCATGGAATCTCTTTC----

-------TCACTAGAGTCTATGTCACTCATTATACTCTGTCGAATGTCATTGAATGTCTT
TACATGGGCTTATATGCCTATGAAAATTGTAATACAACTTTCAGCAACGGATCTCTTGGC
TCTCGCATCGATGAAGAACGCAGCGAAATGCGATAAGTAATGTGAATTGCAGAATTCAGT
GAATCATCGAATCTTTGAACGCATCTTGCGCTCCTTGGTATTCCGAGGAGCATGCCTGTT
TGAGTGTCATTAAATTCTCAACTCTCTTCTAC-TTTTTGTAAAAGAGAGCTTGGACTGTG

GAGGCTTGCTGGCCACTTTTTGGGGTCAGCTCCTCTGAAATGCATTAGCGGAACCGTTTG
CGATCTGCCACAAGTGTGATAAGTTATCTACACTGGCGAGGGGATTGCTCTCTGTAATGT
TCAGCTTCTAATTGTCTCTACTTTGTGAGACTACTTTTGAATGCTTGACCTCAAATCAGG
TAGGACTACCCGCTGAACTTAA
>AC9-21
TTTCCGTAGGTGAACCTGCGGAAGGATCATTATTGAATTATGTTTCTAGATAGGTTGTAG
CTGGCTC-TTTAGAGCATGTGCACGCCTGTTTGGACTTCATTTTCATCCACCTGTGCACC
TATTGTAGTCTTTGGTTGGGTTAGGAGGAAGTGGTCATTGTGTCAGCATCTGCTGGATGT
GAGGACTTGCATTGTGAAAGCTTTGCTGTCCTTGATGTGATCATGGAATCTCTTTC----

-------TCACTAGAGTCTATGTCACTCATTATACTCTGTCGAATGTCATTGAATGTCTT
TACATGGGCTTATATGCCTATGAAAATTGTAATACAACTTTCAGCAACGGATCTCTTGGC
TCTCGCATCGATGAAGAACGCAGCGAAATGCGATAAGTAATGTGAATTGCAGAATTCAGT
GAATCATCGAATCTTTGAACGCATCTTGCGCTCCTTGGTATTCCGAGGAGCATGCCTGTT
TGAGTGTCATTAAATTCTCAACTCTCTTCTAC-TTTTTGTAAAAGAGAGCTTGGACTGTG
GAGGCTTGCTGGCCACTTTTTGGGGTCAGCTCCTCTGAAATGCATTAGCGGAACCGTTTG
CGATCTGCCACAAGTGTGATAAGTTATCTACACTGGCGAGGGGATTGCTCTCTGTAATGT
TCAGCTTCTAATTGTCTCTACTTTGTGAGACTACTTTTGAATGCTTGACCTCAAATCAGG
TAGGACTACCCGCTGAACTTAA
>AC10-8
TTTCCGTAGGTGAACCTGCGGAAGGATCATTATTGAATTATGTTTCTAGATAGGTTGTAG
CTGGCTC-TTTAGAGCATGTGCACGCCTGTTTGGACTTCATTTTCATCCACCTGTGCACC
TATTGTAGTCTTTGGTTGGGTTAGGAGGAAGTGGTCATTGTGTCAGCATCTGCTGGATGT
GAGGACTTGCATTGTGAAAGCTTTGCTGTCCTTGATGTGATCATGGAATCTCTTTC----

-------TCACTAGAGTCTATGTCACTCATTATACTCTGTCGAATGTCATTGAATGTCTT
TACATGGGCTTATATGCCTATGAAAATTGTAATACAACTTTCAGCAACGGATCTCTTGGC
TCTCGCATCGATGAAGAACGCAGCGAAATGCGATAAGTAATGTGAATTGCAGAATTCAGT

GAATCATCGAATCTTTGAACGCATCTTGCGCTCCTTGGTATTCCGAGGAGCATGCCTGTT
TGAGTGTCATTAAATTCTCAACTCTCTTCTAC-TTTTTGTAAAAGAGAGCTTGGACTGTG
GAGGCTTGCTGGCCACTTTTTGGGGTCAGCTCCTCTGAAATGCATTAGCGGAACCGTTTG
CGATCTGCCACAAGTGTGATAAGTTATCTACACTGGCGAGGGGATTGCTCTCTGTAATGT
TCAGCTTCTAATTGTCTCTACTTTGTGAGACTACTTTTGAATGCTTGACCTCAAATCAGG
TAGGACTACCCGCTGAACTTAA
>AC1-2
TTTCCGTAGGTGAACCTGCGGAAGGATCATTATTGAATTATGTTTCTAGATAGGTTGTAG
CTGGCTC-TTTAGAGCATGTGCACGCCTGTTTGGACTTCATTTTCATCCACCTGTGCACC
TATTGTAGTCTTTGGTTGGGTTAGGAGGAAGTGGTCATTGTGTCAGCATCTGCTGGATGT
GAGGACTTGCATTGTGAAAGCTTTGCTGTCCTTGATGTGATCATGGAATCTCTTTC----

-------TCACTAGAGTCTATGTCACTCATTATACTCTGTCGAATGTCATTGAATGTCTT
TACATGGGCTTATATGCCTATGAAAATTGTAATACAACTTTCAGCAACGGATCTCTTGGC
TCTCGCATCGATGAAGAACGCAGCGAAATGCGATAAGTAATGTGAATTGCAGAATTCAGT
GAATCATCGAATCTTTGAACGCATCTTGCGCTCCTTGGTATTCCGAGGAGCATGCCTGTT
TGAGTGTCATTAAATTCTCAACTCTCTTCTAC-TTTTTGTAAAAGAGAGCTTGGACTGTG
GAGGCTTGCTGGCCACTTTTTGGGGTCAGCTCCTCTGAAATGCATTAGCGGAACCGTTTG
CGATCTGCCACAAGTGTGATAAGTTATCTACACTGGCGAGGGGATTGCTCTCTGTAATGT
TCAGCTTCTAATTGTCTCTACTTTGTGAGACTACTTTTGAATGCTTGACCTCAAATCAGG
TAGGACTACCCGCTGAACTTAA
>AC1-3
TTTCCGTAGGTGAACCTGCGGAAGGATCATTATTGAATTATGTTTCTAGATAGGTTGTAG
CTGGCTC-TTTAGAGCATGTGCACGCCTGTTTGGACTTCATTTTCATCCACCTGTGCACC
TATTGTAGTCTTTGGTTGGGTTAGGAGGAAGTGGTCATTGTGTCAGCATCTGCTGGATGT
GAGGACTTGCATTGTGAAAGCTTTGCTGTCCTTGATGTGATCATGGAATCTCTTTC----

-------TCACTAGAGTCTATGTCACTCATTATACTCTGTCGAATGTCATTGAATGTCTT

TACATGGGCTTATATGCCTATGAAAATTGTAATACAACTTTCAGCAACGGATCTCTTGGC
TCTCGCATCGATGAAGAACGCAGCGAAATGCGATAAGTAATGTGAATTGCAGAATTCAGT
GAATCATCGAATCTTTGAACGCATCTTGCGCTCCTTGGTATTCCGAGGAGCATGCCTGTT
TGAGTGTCATTAAATTCTCAACTCTCTTCTAC-TTTTTGTAAAAGAGAGCTTGGACTGTG
GAGGCTTGCTGGCCACTTTTTGGGGTCAGCTCCTCTGAAATGCATTAGCGGAACCGTTTG
CGATCTGCCACAAGTGTGATAAGTTATCTACACTGGCGAGGGGATTGCTCTCTGTAATGT
TCAGCTTCTAATTGTCTCTACTTTGTGAGACTACTTTTGAATGCTTGACCTCAAATCAGG
TAGGACTACCCGCTGAACTTAA
>AC1-5
TTTCCGTAGGTGAACCTGCGGAAGGATCATTATTGAATTATGTTTCTAGATAGGTTGTAG
CTGGCTC-TTTAGAGCATGTGCACGCCTGTTTGGACTTCATTTTCATCCACCTGTGCACC
TATTGTAGTCTTTGGTTGGGTTAGGAGGAAGTGGTCATTGTGTCAGCATCTGCTGGATGT
GAGGACTTGCATTGTGAAAGCTTTGCTGTCCTTGATGTGATCATGGAATCTCTTTC----

-------TCACTAGAGTCTATGTCACTCATTATACTCTGTCGAATGTCATTGAATGTCTT
TACATGGGCTTATATGCCTATGAAAATTGTAATACAACTTTCAGCAACGGATCTCTTGGC
TCTCGCATCGATGAAGAACGCAGCGAAATGCGATAAGTAATGTGAATTGCAGAATTCAGT
GAATCATCGAATCTTTGAACGCATCTTGCGCTCCTTGGTATTCCGAGGAGCATGCCTGTT
TGAGTGTCATTAAATTCTCAACTCTCTTCTAC-TTTTTGTAAAAGAGAGCTTGGACTGTG
GAGGCTTGCTGGCCACTTTTTGGGGTCAGCTCCTCTGAAATGCATTAGCGGAACCGTTTG
CGATCTGCCACAAGTGTGATAAGTTATCTACACTGGCGAGGGGATTGCTCTCTGTAATGT
TCAGCTTCTAATTGTCTCTACTTTGTGAGACTACTTTTGAATGCTTGACCTCAAATCAGG
TAGGACTACCCGCTGAACTTAA
>AC1-6
TTTCCGTAGGTGAACCTGCGGAAGGATCATTATTGAATTATGTTTCTAGATAGGTTGTAG
CTGGCTC-TTTAGAGCATGTGCACGCCTGTTTGGACTTCATTTTCATCCACCTGTGCACC
TATTGTAGTCTTTGGTTGGGTTAGGAGGAAGTGGTCATTGTGTCAGCATCTGCTGGATGT
GAGGACTTGCATTGTGAAAGCTTTGCTGTCCTTGATGTGATCATGGAATCTCTTTC----

-------TCACTAGAGTCTATGTCACTCATTATACTCTGTCGAATGTCATTGAATGTCTT
TACATGGGCTTATATGCCTATGAAAATTGTAATACAACTTTCAGCAACGGATCTCTTGGC
TCTCGCATCGATGAAGAACGCAGCGAAATGCGATAAGTAATGTGAATTGCAGAATTCAGT
GAATCATCGAATCTTTGAACGCATCTTGCGCTCCTTGGTATTCCGAGGAGCATGCCTGTT
TGAGTGTCATTAAATTCTCAACTCTCTTCTAC-TTTTTGTAAAAGAGAGCTTGGACTGTG
GAGGCTTGCTGGCCACTTTTTGGGGTCAGCTCCTCTGAAATGCATTAGCGGAACCGTTTG
CGATCTGCCACAAGTGTGATAAGTTATCTACACTGGCGAGGGGATTGCTCTCTGTAATGT
TCAGCTTCTAATTGTCTCTACTTTGTGAGACTACTTTTGAATGCTTGACCTCAAATCAGG
TAGGACTACCCGCTGAACTTAA
>AC1-12
TTTCCGTAGGTGAACCTGCGGAAGGATCATTATTGAATTATGTTTCTAGATAGGTTGTAG
CTGGCTC-TTTAGAGCATGTGCACGCCTGTTTGGACTTCATTTTCATCCACCTGTGCACC
TATTGTAGTCTTTGGTTGGGTTAGGAGGAAGTGGTCATTGTGTCAGCATCTGCTGGATGT
GAGGACTTGCATTGTGAAAGCTTTGCTGTCCTTGATGTGATCATGGAATCTCTTTC----

-------TCACTAGAGTCTATGTCACTCATTATACTCTGTCGAATGTCATTGAATGTCTT
TACATGGGCTTATATGCCTATGAAAATTGTAATACAACTTTCAGCAACGGATCTCTTGGC
TCTCGCATCGATGAAGAACGCAGCGAAATGCGATAAGTAATGTGAATTGCAGAATTCAGT
GAATCATCGAATCTTTGAACGCATCTTGCGCTCCTTGGTATTCCGAGGAGCATGCCTGTT
TGAGTGTCATTAAATTCTCAACTCTCTTCTAC-TTTTTGTAAAAGAGAGCTTGGACTGTG
GAGGCTTGCTGGCCACTTTTTGGGGTCAGCTCCTCTGAAATGCATTAGCGGAACCGTTTG
CGATCTGCCACAAGTGTGATAAGTTATCTACACTGGCGAGGGGATTGCTCTCTGTAATGT
TCAGCTTCTAATTGTCTCTACTTTGTGAGACTACTTTTGAATGCTTGACCTCAAATCAGG
TAGGACTACCCGCTGAACTTAA
>AC1-14
TTTCCGTAGGTGAACCTGCGGAAGGATCATTATTGAATTATGTTTCTAGATAGGTTGTAG
CTGGCTC-TTTAGAGCATGTGCACGCCTGTTTGGACTTCATTTTCATCCACCTGTGCACC
TATTGTAGTCTTTGGTTGGGTTAGGAGGAAGTGGTCATTGTGTCAGCATCTGCTGGATGT
GAGGACTTGCATTGTGAAAGCTTTGCTGTCCTTGATGTGATCATGGAATCTCTTTC----


```
------------------------------------------------------------
------------------------------------------------------------
------------------------------------------------------------
-------TCACTAGAGTCTATGTCACTCATTATACTCTGTCGAATGTCATTGAATGTCTT
TACATGGGCTTATATGCCTATGAAAATTGTAATACAACTTTCAGCAACGGATCTCTTGGC
TCTCGCATCGATGAAGAACGCAGCGAAATGCGATAAGTAATGTGAATTGCAGAATTCAGT
GAATCATCGAATCTTTGAACGCATCTTGCGCTCCTTGGTATTCCGAGGAGCATGCCTGTT
TGAGTGTCATTAAATTCTCAACTCTCTTCTAC-TTTTTGTAAAAGAGAGCTTGGACTGTG
GAGGCTTGCTGGCCACTTTTTGGGGTCAGCTCCTCTGAAATGCATTAGCGGAACCGTTTG
CGATCTGCCACAAGTGTGATAAGTTATCTACACTGGCGAGGGGATTGCTCTCTGTAATGT
TCAGCTTCTAATTGTCTCTACTTTGTGAGACTACTTTTGAATGCTTGACCTCAAATCAGG
TAGGACTACCCGCTGAACTTAA
>AC1-16
TTTCCGTAGGTGAACCTGCGGAAGGATCATTATTGAATTATGTTTCTAGATAGGTTGTAG
CTGGCTC-TTTAGAGCATGTGCACGCCTGTTTGGACTTCATTTTCATCCACCTGTGCACC
TATTGTAGTCTTTGGTTGGGTTAGGAGGAAGTGGTCATTGTGTCAGCATCTGCTGGATGT
GAGGACTTGCATTGTGAAAGCTTTGCTGTCCTTGATGTGATCATGGAATCTCTTTC----
------------------------------------------------------------
------------------------------------------------------------
------------------------------------------------------------
------------------------------------------------------------
------------------------------------------------------------
------------------------------------------------------------
------------------------------------------------------------
------------------------------------------------------------
------------------------------------------------------------
------------------------------------------------------------
------------------------------------------------------------
------------------------------------------------------------
-------TCACTAGAGTCTATGTCACTCATTATACTCTGTCGAATGTCATTGAATGTCTT
TACATGGGCTTATATGCCTATGAAAATTGTAATACAACTTTCAGCAACGGATCTCTTGGC
TCTCGCATCGATGAAGAACGCAGCGAAATGCGATAAGTAATGTGAATTGCAGAATTCAGT
GAATCATCGAATCTTTGAACGCATCTTGCGCTCCTTGGTATTCCGAGGAGCATGCCTGTT
TGAGTGTCATTAAATTCTCAACTCTCTTCTAC-TTTTTGTAAAAGAGAGCTTGGACTGTG
GAGGCTTGCTGGCCACTTTTTGGGGTCAGCTCCTCTGAAATGCATTAGCGGAACCGTTTG
CGATCTGCCACAAGTGTGATAAGTTATCTACACTGGCGAGGGGATTGCTCTCTGTAATGT
TCAGCTTCTAATTGTCTCTACTTTGTGAGACTACTTTTGAATGCTTGACCTCAAATCAGG
TAGGACTACCCGCTGAACTTAA
>AC1-21
TTTCCGTAGGTGAACCTGCGGAAGGATCATTATTGAATTATGTTTCTAGATAGGTTGTAG
CTGGCTC-TTTAGAGCATGTGCACGCCTGTTTGGACTTCATTTTCATCCACCTGTGCACC
TATTGTAGTCTTTGGTTGGGTTAGGAGGAAGTGGTCATTGTGTCAGCATCTGCTGGATGT
GAGGACTTGCATTGTGAAAGCTTTGCTGTCCTTGATGTGATCATGGAATCTCTTTC----
------------------------------------------------------------
------------------------------------------------------------
------------------------------------------------------------
------------------------------------------------------------
------------------------------------------------------------
------------------------------------------------------------
------------------------------------------------------------
```


-------TCACTAGAGTCTATGTCACTCATTATACTCTGTCGAATGTCATTGAATGTCTT
TACATGGGCTTATATGCCTATGAAAATTGTAATACAACTTTCAGCAACGGATCTCTTGGC
TCTCGCATCGATGAAGAACGCAGCGAAATGCGATAAGTAATGTGAATTGCAGAATTCAGT
GAATCATCGAATCTTTGAACGCATCTTGCGCTCCTTGGTATTCCGAGGAGCATGCCTGTT
TGAGTGTCATTAAATTCTCAACTCTCTTCTAC-TTTTTGTAAAAGAGAGCTTGGACTGTG
GAGGCTTGCTGGCCACTTTTTGGGGTCAGCTCCTCTGAAATGCATTAGCGGAACCGTTTG
CGATCTGCCACAAGTGTGATAAGTTATCTACACTGGCGAGGGGATTGCTCTCTGTAATGT
TCAGCTTCTAATTGTCTCTACTTTGTGAGACTACTTTTGAATGCTTGACCTCAAATCAGG
TAGGACTACCCGCTGAACTTAA
>AC1-22
TTTCCGTAGGTGAACCTGCGGAAGGATCATTATTGAATTATGTTTCTAGATAGGTTGTAG
CTGGCTC-TTTAGAGCATGTGCACGCCTGTTTGGACTTCATTTTCATCCACCTGTGCACC
TATTGTAGTCTTTGGTTGGGTTAGGAGGAAGTGGTCATTGTGTCAGCATCTGCTGGATGT
GAGGACTTGCATTGTGAAAGCTTTGCTGTCCTTGATGTGATCATGGAATCTCTTTC----

-------TCACTAGAGTCTATGTCACTCATTATACTCTGTCGAATGTCATTGAATGTCTT
TACATGGGCTTATATGCCTATGAAAATTGTAATACAACTTTCAGCAACGGATCTCTTGGC
TCTCGCATCGATGAAGAACGCAGCGAAATGCGATAAGTAATGTGAATTGCAGAATTCAGT
GAATCATCGAATCTTTGAACGCATCTTGCGCTCCTTGGTATTCCGAGGAGCATGCCTGTT
TGAGTGTCATTAAATTCTCAACTCTCTTCTAC-TTTTTGTAAAAGAGAGCTTGGACTGTG
GAGGCTTGCTGGCCACTTTTTGGGGTCAGCTCCTCTGAAATGCATTAGCGGAACCGTTTG
CGATCTGCCACAAGTGTGATAAGTTATCTACACTGGCGAGGGGATTGCTCTCTGTAATGT
TCAGCTTCTAATTGTCTCTACTTTGTGAGACTACTTTTGAATGCTTGACCTCAAATCAGG
TAGGACTACCCGCTGAACTTAA
>AC1-23
TTTCCGTAGGTGAACCTGCGGAAGGATCATTATTGAATTATGTTTCTAGATAGGTTGTAG
CTGGCTC-TTTAGAGCATGTGCACGCCTGTTTGGACTTCATTTTCATCCACCTGTGCACC
TATTGTAGTCTTTGGTTGGGTTAGGAGGAAGTGGTCATTGTGTCAGCATCTGCTGGATGT
GAGGACTTGCATTGTGAAAGCTTTGCTGTCCTTGATGTGATCATGGAATCTCTTTC----

-------TCACTAGAGTCTATGTCACTCATTATACTCTGTCGAATGTCATTGAATGTCTT
TACATGGGCTTATATGCCTATGAAAATTGTAATACAACTTTCAGCAACGGATCTCTTGGC
TCTCGCATCGATGAAGAACGCAGCGAAATGCGATAAGTAATGTGAATTGCAGAATTCAGT
GAATCATCGAATCTTTGAACGCATCTTGCGCTCCTTGGTATTCCGAGGAGCATGCCTGTT
TGAGTGTCATTAAATTCTCAACTCTCTTCTAC-TTTTTGTAAAAGAGAGCTTGGACTGTG
GAGGCTTGCTGGCCACTTTTTGGGGTCAGCTCCTCTGAAATGCATTAGCGGAACCGTTTG
CGATCTGCCACAAGTGTGATAAGTTATCTACACTGGCGAGGGGATTGCTCTCTGTAATGT
TCAGCTTCTAATTGTCTCTACTTTGTGAGACTACTTTTGAATGCTTGACCTCAAATCAGG
TAGGACTACCCGCTGAACTTAA
>AC1-25
TTTCCGTAGGTGAACCTGCGGAAGGATCATTATTGAATTATGTTTCTAGATAGGTTGTAG
CTGGCTC-TTTAGAGCATGTGCACGCCTGTTTGGACTTCATTTTCATCCACCTGTGCACC
TATTGTAGTCTTTGGTTGGGTTAGGAGGAAGTGGTCATTGTGTCAGCATCTGCTGGATGT
GAGGACTTGCATTGTGAAAGCTTTGCTGTCCTTGATGTGATCATGGAATCTCTTTC----

-------TCACTAGAGTCTATGTCACTCATTATACTCTGTCGAATGTCATTGAATGTCTT
TACATGGGCTTATATGCCTATGAAAATTGTAATACAACTTTCAGCAACGGATCTCTTGGC
TCTCGCATCGATGAAGAACGCAGCGAAATGCGATAAGTAATGTGAATTGCAGAATTCAGT
GAATCATCGAATCTTTGAACGCATCTTGCGCTCCTTGGTATTCCGAGGAGCATGCCTGTT
TGAGTGTCATTAAATTCTCAACTCTCTTCTAC-TTTTTGTAAAAGAGAGCTTGGACTGTG
GAGGCTTGCTGGCCACTTTTTGGGGTCAGCTCCTCTGAAATGCATTAGCGGAACCGTTTG
CGATCTGCCACAAGTGTGATAAGTTATCTACACTGGCGAGGGGATTGCTCTCTGTAATGT
TCAGCTTCTAATTGTCTCTACTTTGTGAGACTACTTTTGAATGCTTGACCTCAAATCAGG
TAGGACTACCCGCTGAACTTAA
>AC1-28
TTTCCGTAGGTGAACCTGCGGAAGGATCATTATTGAATTATGTTTCTAGATAGGTTGTAG
CTGGCTC-TTTAGAGCATGTGCACGCCTGTTTGGACTTCATTTTCATCCACCTGTGCACC
TATTGTAGTCTTTGGTTGGGTTAGGAGGAAGTGGTCATTGTGTCAGCATCTGCTGGATGT
GAGGACTTGCATTGTGAAAGCTTTGCTGTCCTTGATGTGATCATGGAATCTCTTTC----

-------TCACTAGAGTCTATGTCACTCATTATACTCTGTCGAATGTCATTGAATGTCTT
TACATGGGCTTATATGCCTATGAAAATTGTAATACAACTTTCAGCAACGGATCTCTTGGC
TCTCGCATCGATGAAGAACGCAGCGAAATGCGATAAGTAATGTGAATTGCAGAATTCAGT
GAATCATCGAATCTTTGAACGCATCTTGCGCTCCTTGGTATTCCGAGGAGCATGCCTGTT
TGAGTGTCATTAAATTCTCAACTCTCTTCTAC-TTTTTGTAAAAGAGAGCTTGGACTGTG
GAGGCTTGCTGGCCACTTTTTGGGGTCAGCTCCTCTGAAATGCATTAGCGGAACCGTTTG
CGATCTGCCACAAGTGTGATAAGTTATCTACACTGGCGAGGGGATTGCTCTCTGTAATGT
TCAGCTTCTAATTGTCTCTACTTTGTGAGACTACTTTTGAATGCTTGACCTCAAATCAGG
TAGGACTACCCGCTGAACTTAA
>AC1-39
TTTCCGTAGGTGAACCTGCGGAAGGATCATTATTGAATTATGTTTCTAGATAGGTTGTAG
CTGGCTC-TTTAGAGCATGTGCACGCCTGTTTGGACTTCATTTTCATCCACCTGTGCACC
TATTGTAGTCTTTGGTTGGGTTAGGAGGAAGTGGTCATTGTGTCAGCATCTGCTGGATGT
GAGGACTTGCATTGTGAAAGCTTTGCTGTCCTTGATGTGATCATGGAATCTCTTTC----

-------TCACTAGAGTCTATGTCACTCATTATACTCTGTCGAATGTCATTGAATGTCTT
TACATGGGCTTATATGCCTATGAAAATTGTAATACAACTTTCAGCAACGGATCTCTTGGC
TCTCGCATCGATGAAGAACGCAGCGAAATGCGATAAGTAATGTGAATTGCAGAATTCAGT
GAATCATCGAATCTTTGAACGCATCTTGCGCTCCTTGGTATTCCGAGGAGCATGCCTGTT
TGAGTGTCATTAAATTCTCAACTCTCTTCTAC-TTTTTGTAAAAGAGAGCTTGGACTGTG
GAGGCTTGCTGGCCACTTTTTGGGGTCAGCTCCTCTGAAATGCATTAGCGGAACCGTTTG
CGATCTGCCACAAGTGTGATAAGTTATCTACACTGGCGAGGGGATTGCTCTCTGTAATGT
TCAGCTTCTAATTGTCTCTACTTTGTGAGACTACTTTTGAATGCTTGACCTCAAATCAGG
TAGGACTACCCGCTGAACTTAA
>AC1-43
TTTCCGTAGGTGAACCTGCGGAAGGATCATTATTGAATTATGTTTCTAGATAGGTTGTAG
CTGGCTC-TTTAGAGCATGTGCACGCCTGTTTGGACTTCATTTTCATCCACCTGTGCACC
TATTGTAGTCTTTGGTTGGGTTAGGAGGAAGTGGTCATTGTGTCAGCATCTGCTGGATGT
GAGGACTTGCATTGTGAAAGCTTTGCTGTCCTTGATGTGATCATGGAATCTCTTTC----

-------TCACTAGAGTCTATGTCACTCATTATACTCTGTCGAATGTCATTGAATGTCTT
TACATGGGCTTATATGCCTATGAAAATTGTAATACAACTTTCAGCAACGGATCTCTTGGC
TCTCGCATCGATGAAGAACGCAGCGAAATGCGATAAGTAATGTGAATTGCAGAATTCAGT
GAATCATCGAATCTTTGAACGCATCTTGCGCTCCTTGGTATTCCGAGGAGCATGCCTGTT
TGAGTGTCATTAAATTCTCAACTCTCTTCTAC-TTTTTGTAAAAGAGAGCTTGGACTGTG
GAGGCTTGCTGGCCACTTTTTGGGGTCAGCTCCTCTGAAATGCATTAGCGGAACCGTTTG
CGATCTGCCACAAGTGTGATAAGTTATCTACACTGGCGAGGGGATTGCTCTCTGTAATGT
TCAGCTTCTAATTGTCTCTACTTTGTGAGACTACTTTTGAATGCTTGACCTCAAATCAGG
TAGGACTACCCGCTGAACTTAA
>AC1-44
TTTCCGTAGGTGAACCTGCGGAAGGATCATTATTGAATTATGTTTCTAGATAGGTTGTAG
CTGGCTC-TTTAGAGCATGTGCACGCCTGTTTGGACTTCATTTTCATCCACCTGTGCACC
TATTGTAGTCTTTGGTTGGGTTAGGAGGAAGTGGTCATTGTGTCAGCATCTGCTGGATGT
GAGGACTTGCATTGTGAAAGCTTTGCTGTCCTTGATGTGATCATGGAATCTCTTTC----

-------TCACTAGAGTCTATGTCACTCATTATACTCTGTCGAATGTCATTGAATGTCTT
TACATGGGCTTATATGCCTATGAAAATTGTAATACAACTTTCAGCAACGGATCTCTTGGC
TCTCGCATCGATGAAGAACGCAGCGAAATGCGATAAGTAATGTGAATTGCAGAATTCAGT
GAATCATCGAATCTTTGAACGCATCTTGCGCTCCTTGGTATTCCGAGGAGCATGCCTGTT
TGAGTGTCATTAAATTCTCAACTCTCTTCTAC-TTTTTGTAAAAGAGAGCTTGGACTGTG
GAGGCTTGCTGGCCACTTTTTGGGGTCAGCTCCTCTGAAATGCATTAGCGGAACCGTTTG
CGATCTGCCACAAGTGTGATAAGTTATCTACACTGGCGAGGGGATTGCTCTCTGTAATGT
TCAGCTTCTAATTGTCTCTACTTTGTGAGACTACTTTTGAATGCTTGACCTCAAATCAGG
TAGGACTACCCGCTGAACTTAA
>AC1-46
TTTCCGTAGGTGAACCTGCGGAAGGATCATTATTGAATTATGTTTCTAGATAGGTTGTAG
CTGGCTC-TTTAGAGCATGTGCACGCCTGTTTGGACTTCATTTTCATCCACCTGTGCACC
TATTGTAGTCTTTGGTTGGGTTAGGAGGAAGTGGTCATTGTGTCAGCATCTGCTGGATGT

GAGGACTTGCATTGTGAAAGCTTTGCTGTCCTTGATGTGATCATGGAATCTCTTTC----

-------TCACTAGAGTCTATGTCACTCATTATACTCTGTCGAATGTCATTGAATGTCTT
TACATGGGCTTATATGCCTATGAAAATTGTAATACAACTTTCAGCAACGGATCTCTTGGC
TCTCGCATCGATGAAGAACGCAGCGAAATGCGATAAGTAATGTGAATTGCAGAATTCAGT
GAATCATCGAATCTTTGAACGCATCTTGCGCTCCTTGGTATTCCGAGGAGCATGCCTGTT
TGAGTGTCATTAAATTCTCAACTCTCTTCTAC-TTTTTGTAAAAGAGAGCTTGGACTGTG
GAGGCTTGCTGGCCACTTTTTGGGGTCAGCTCCTCTGAAATGCATTAGCGGAACCGTTTG
CGATCTGCCACAAGTGTGATAAGTTATCTACACTGGCGAGGGGATTGCTCTCTGTAATGT
TCAGCTTCTAATTGTCTCTACTTTGTGAGACTACTTTTGAATGCTTGACCTCAAATCAGG
TAGGACTACCCGCTGAACTTAA
>AC1-50
TTTCCGTAGGTGAACCTGCGGAAGGATCATTATTGAATTATGTTTCTAGATAGGTTGTAG
CTGGCTC-TTTAGAGCATGTGCACGCCTGTTTGGACTTCATTTTCATCCACCTGTGCACC
TATTGTAGTCTTTGGTTGGGTTAGGAGGAAGTGGTCATTGTGTCAGCATCTGCTGGATGT
GAGGACTTGCATTGTGAAAGCTTTGCTGTCCTTGATGTGATCATGGAATCTCTTTC----

-------TCACTAGAGTCTATGTCACTCATTATACTCTGTCGAATGTCATTGAATGTCTT
TACATGGGCTTATATGCCTATGAAAATTGTAATACAACTTTCAGCAACGGATCTCTTGGC
TCTCGCATCGATGAAGAACGCAGCGAAATGCGATAAGTAATGTGAATTGCAGAATTCAGT
GAATCATCGAATCTTTGAACGCATCTTGCGCTCCTTGGTATTCCGAGGAGCATGCCTGTT
TGAGTGTCATTAAATTCTCAACTCTCTTCTAC-TTTTTGTAAAAGAGAGCTTGGACTGTG
GAGGCTTGCTGGCCACTTTTTGGGGTCAGCTCCTCTGAAATGCATTAGCGGAACCGTTTG
CGATCTGCCACAAGTGTGATAAGTTATCTACACTGGCGAGGGGATTGCTCTCTGTAATGT
TCAGCTTCTAATTGTCTCTACTTTGTGAGACTACTTTTGAATGCTTGACCTCAAATCAGG
TAGGACTACCCGCTGAACTTAA
>AC1-53
TTTCCGTAGGTGAACCTGCGGAAGGATCATTATTGAATTATGTTTCTAGATAGGTTGTAG

CTGGCTC-TTTAGAGCATGTGCACGCCTGTTTGGACTTCATTTTCATCCACCTGTGCACC
TATTGTAGTCTTTGGTTGGGTTAGGAGGAAGTGGTCATTGTGTCAGCATCTGCTGGATGT
GAGGACTTGCATTGTGAAAGCTTTGCTGTCCTTGATGTGATCATGGAATCTCTTTC----

-------TCACTAGAGTCTATGTCACTCATTATACTCTGTCGAATGTCATTGAATGTCTT
TACATGGGCTTATATGCCTATGAAAATTGTAATACAACTTTCAGCAACGGATCTCTTGGC
TCTCGCATCGATGAAGAACGCAGCGAAATGCGATAAGTAATGTGAATTGCAGAATTCAGT
GAATCATCGAATCTTTGAACGCATCTTGCGCTCCTTGGTATTCCGAGGAGCATGCCTGTT
TGAGTGTCATTAAATTCTCAACTCTCTTCTAC-TTTTTGTAAAAGAGAGCTTGGACTGTG
GAGGCTTGCTGGCCACTTTTTGGGGTCAGCTCCTCTGAAATGCATTAGCGGAACCGTTTG
CGATCTGCCACAAGTGTGATAAGTTATCTACACTGGCGAGGGGATTGCTCTCTGTAATGT
TCAGCTTCTAATTGTCTCTACTTTGTGAGACTACTTTTGAATGCTTGACCTCAAATCAGG
TAGGACTACCCGCTGAACTTAA
>AC1-58
TTTCCGTAGGTGAACCTGCGGAAGGATCATTATTGAATTATGTTTCTAGATAGGTTGTAG
CTGGCTC-TTTAGAGCATGTGCACGCCTGTTTGGACTTCATTTTCATCCACCTGTGCACC
TATTGTAGTCTTTGGTTGGGTTAGGAGGAAGTGGTCATTGTGTCAGCATCTGCTGGATGT
GAGGACTTGCATTGTGAAAGCTTTGCTGTCCTTGATGTGATCATGGAATCTCTTTC----

-------TCACTAGAGTCTATGTCACTCATTATACTCTGTCGAATGTCATTGAATGTCTT
TACATGGGCTTATATGCCTATGAAAATTGTAATACAACTTTCAGCAACGGATCTCTTGGC
TCTCGCATCGATGAAGAACGCAGCGAAATGCGATAAGTAATGTGAATTGCAGAATTCAGT
GAATCATCGAATCTTTGAACGCATCTTGCGCTCCTTGGTATTCCGAGGAGCATGCCTGTT
TGAGTGTCATTAAATTCTCAACTCTCTTCTAC-TTTTTGTAAAAGAGAGCTTGGACTGTG
GAGGCTTGCTGGCCACTTTTTGGGGTCAGCTCCTCTGAAATGCATTAGCGGAACCGTTTG
CGATCTGCCACAAGTGTGATAAGTTATCTACACTGGCGAGGGGATTGCTCTCTGTAATGT
TCAGCTTCTAATTGTCTCTACTTTGTGAGACTACTTTTGAATGCTTGACCTCAAATCAGG
TAGGACTACCCGCTGAACTTAA

>AC1-74
TTTCCGTAGGTGAACCTGCGGAAGGATCATTATTGAATTATGTTTCTAGATAGGTTGTAG
CTGGCTC-TTTAGAGCATGTGCACGCCTGTTTGGACTTCATTTTCATCCACCTGTGCACC
TATTGTAGTCTTTGGTTGGGTTAGGAGGAAGTGGTCATTGTGTCAGCATCTGCTGGATGT
GAGGACTTGCATTGTGAAAGCTTTGCTGTCCTTGATGTGATCATGGAATCTCTTTC----

-------TCACTAGAGTCTATGTCACTCATTATACTCTGTCGAATGTCATTGAATGTCTT
TACATGGGCTTATATGCCTATGAAAATTGTAATACAACTTTCAGCAACGGATCTCTTGGC
TCTCGCATCGATGAAGAACGCAGCGAAATGCGATAAGTAATGTGAATTGCAGAATTCAGT
GAATCATCGAATCTTTGAACGCATCTTGCGCTCCTTGGTATTCCGAGGAGCATGCCTGTT
TGAGTGTCATTAAATTCTCAACTCTCTTCTAC-TTTTTGTAAAAGAGAGCTTGGACTGTG
GAGGCTTGCTGGCCACTTTTTGGGGTCAGCTCCTCTGAAATGCATTAGCGGAACCGTTTG
CGATCTGCCACAAGTGTGATAAGTTATCTACACTGGCGAGGGGATTGCTCTCTGTAATGT
TCAGCTTCTAATTGTCTCTACTTTGTGAGACTACTTTTGAATGCTTGACCTCAAATCAGG
TAGGACTACCCGCTGAACTTAA
>AC1-75
TTTCCGTAGGTGAACCTGCGGAAGGATCATTATTGAATTATGTTTCTAGATAGGTTGTAG
CTGGCTC-TTTAGAGCATGTGCACGCCTGTTTGGACTTCATTTTCATCCACCTGTGCACC
TATTGTAGTCTTTGGTTGGGTTAGGAGGAAGTGGTCATTGTGTCAGCATCTGCTGGATGT
GAGGACTTGCATTGTGAAAGCTTTGCTGTCCTTGATGTGATCATGGAATCTCTTTC----

-------TCACTAGAGTCTATGTCACTCATTATACTCTGTCGAATGTCATTGAATGTCTT
TACATGGGCTTATATGCCTATGAAAATTGTAATACAACTTTCAGCAACGGATCTCTTGGC
TCTCGCATCGATGAAGAACGCAGCGAAATGCGATAAGTAATGTGAATTGCAGAATTCAGT
GAATCATCGAATCTTTGAACGCATCTTGCGCTCCTTGGTATTCCGAGGAGCATGCCTGTT
TGAGTGTCATTAAATTCTCAACTCTCTTCTAC-TTTTTGTAAAAGAGAGCTTGGACTGTG
GAGGCTTGCTGGCCACTTTTTGGGGTCAGCTCCTCTGAAATGCATTAGCGGAACCGTTTG
CGATCTGCCACAAGTGTGATAAGTTATCTACACTGGCGAGGGGATTGCTCTCTGTAATGT

TCAGCTTCTAATTGTCTCTACTTTGTGAGACTACTTTTGAATGCTTGACCTCAAATCAGG
TAGGACTACCCGCTGAACTTAA
>AC1-76
TTTCCGTAGGTGAACCTGCGGAAGGATCATTATTGAATTATGTTTCTAGATAGGTTGTAG
CTGGCTC-TTTAGAGCATGTGCACGCCTGTTTGGACTTCATTTTCATCCACCTGTGCACC
TATTGTAGTCTTTGGTTGGGTTAGGAGGAAGTGGTCATTGTGTCAGCATCTGCTGGATGT
GAGGACTTGCATTGTGAAAGCTTTGCTGTCCTTGATGTGATCATGGAATCTCTTTC----

-------TCACTAGAGTCTATGTCACTCATTATACTCTGTCGAATGTCATTGAATGTCTT
TACATGGGCTTATATGCCTATGAAAATTGTAATACAACTTTCAGCAACGGATCTCTTGGC
TCTCGCATCGATGAAGAACGCAGCGAAATGCGATAAGTAATGTGAATTGCAGAATTCAGT
GAATCATCGAATCTTTGAACGCATCTTGCGCTCCTTGGTATTCCGAGGAGCATGCCTGTT
TGAGTGTCATTAAATTCTCAACTCTCTTCTAC-TTTTTGTAAAAGAGAGCTTGGACTGTG
GAGGCTTGCTGGCCACTTTTTGGGGTCAGCTCCTCTGAAATGCATTAGCGGAACCGTTTG
CGATCTGCCACAAGTGTGATAAGTTATCTACACTGGCGAGGGGATTGCTCTCTGTAATGT
TCAGCTTCTAATTGTCTCTACTTTGTGAGACTACTTTTGAATGCTTGACCTCAAATCAGG
TAGGACTACCCGCTGAACTTAA
>AC2-7
TTTCCGTAGGTGAACCTGCGGAAGGATCATTATTGAATTATGTTTCTAGATAGGTTGTAG
CTGGCTC-TTTAGAGCATGTGCACGCCTGTTTGGACTTCATTTTCATCCACCTGTGCACC
TATTGTAGTCTTTGGTTGGGTTAGGAGGAAGTGGTCATTGTGTCAGCATCTGCTGGATGT
GAGGACTTGCATTGTGAAAGCTTTGCTGTCCTTGATGTGATCATGGAATCTCTTTC----

-------TCACTAGAGTCTATGTCACTCATTATACTCTGTCGAATGTCATTGAATGTCTT
TACATGGGCTTATATGCCTATGAAAATTGTAATACAACTTTCAGCAACGGATCTCTTGGC
TCTCGCATCGATGAAGAACGCAGCGAAATGCGATAAGTAATGTGAATTGCAGAATTCAGT
GAATCATCGAATCTTTGAACGCATCTTGCGCTCCTTGGTATTCCGAGGAGCATGCCTGTT
TGAGTGTCATTAAATTCTCAACTCTCTTCTAC-TTTTTGTAAAAGAGAGCTTGGACTGTG

GAGGCTTGCTGGCCACTTTTTGGGGTCAGCTCCTCTGAAATGCATTAGCGGAACCGTTTG
CGATCTGCCACAAGTGTGATAAGTTATCTACACTGGCGAGGGGATTGCTCTCTGTAATGT
TCAGCTTCTAATTGTCTCTACTTTGTGAGACTACTTTTGAATGCTTGACCTCAAATCAGG
TAGGACTACCCGCTGAACTTAA
>AC2-16
TTTCCGTAGGTGAACCTGCGGAAGGATCATTATTGAATTATGTTTCTAGATAGGTTGTAG
CTGGCTC-TTTAGAGCATGTGCACGCCTGTTTGGACTTCATTTTCATCCACCTGTGCACC
TATTGTAGTCTTTGGTTGGGTTAGGAGGAAGTGGTCATTGTGTCAGCATCTGCTGGATGT
GAGGACTTGCATTGTGAAAGCTTTGCTGTCCTTGATGTGATCATGGAATCTCTTTC----

-------TCACTAGAGTCTATGTCACTCATTATACTCTGTCGAATGTCATTGAATGTCTT
TACATGGGCTTATATGCCTATGAAAATTGTAATACAACTTTCAGCAACGGATCTCTTGGC
TCTCGCATCGATGAAGAACGCAGCGAAATGCGATAAGTAATGTGAATTGCAGAATTCAGT
GAATCATCGAATCTTTGAACGCATCTTGCGCTCCTTGGTATTCCGAGGAGCATGCCTGTT
TGAGTGTCATTAAATTCTCAACTCTCTTCTAC-TTTTTGTAAAAGAGAGCTTGGACTGTG
GAGGCTTGCTGGCCACTTTTTGGGGTCAGCTCCTCTGAAATGCATTAGCGGAACCGTTTG
CGATCTGCCACAAGTGTGATAAGTTATCTACACTGGCGAGGGGATTGCTCTCTGTAATGT
TCAGCTTCTAATTGTCTCTACTTTGTGAGACTACTTTTGAATGCTTGACCTCAAATCAGG
TAGGACTACCCGCTGAACTTAA
>AC2-17
TTTCCGTAGGTGAACCTGCGGAAGGATCATTATTGAATTATGTTTCTAGATAGGTTGTAG
CTGGCTC-TTTAGAGCATGTGCACGCCTGTTTGGACTTCATTTTCATCCACCTGTGCACC
TATTGTAGTCTTTGGTTGGGTTAGGAGGAAGTGGTCATTGTGTCAGCATCTGCTGGATGT
GAGGACTTGCATTGTGAAAGCTTTGCTGTCCTTGATGTGATCATGGAATCTCTTTC----

-------TCACTAGAGTCTATGTCACTCATTATACTCTGTCGAATGTCATTGAATGTCTT
TACATGGGCTTATATGCCTATGAAAATTGTAATACAACTTTCAGCAACGGATCTCTTGGC
TCTCGCATCGATGAAGAACGCAGCGAAATGCGATAAGTAATGTGAATTGCAGAATTCAGT

GAATCATCGAATCTTTGAACGCATCTTGCGCTCCTTGGTATTCCGAGGAGCATGCCTGTT
TGAGTGTCATTAAATTCTCAACTCTCTTCTAC-TTTTTGTAAAAGAGAGCTTGGACTGTG
GAGGCTTGCTGGCCACTTTTTGGGGTCAGCTCCTCTGAAATGCATTAGCGGAACCGTTTG
CGATCTGCCACAAGTGTGATAAGTTATCTACACTGGCGAGGGGATTGCTCTCTGTAATGT
TCAGCTTCTAATTGTCTCTACTTTGTGAGACTACTTTTGAATGCTTGACCTCAAATCAGG
TAGGACTACCCGCTGAACTTAA
>AC2-19
TTTCCGTAGGTGAACCTGCGGAAGGATCATTATTGAATTATGTTTCTAGATAGGTTGTAG
CTGGCTC-TTTAGAGCATGTGCACGCCTGTTTGGACTTCATTTTCATCCACCTGTGCACC
TATTGTAGTCTTTGGTTGGGTTAGGAGGAAGTGGTCATTGTGTCAGCATCTGCTGGATGT
GAGGACTTGCATTGTGAAAGCTTTGCTGTCCTTGATGTGATCATGGAATCTCTTTC----

-------TCACTAGAGTCTATGTCACTCATTATACTCTGTCGAATGTCATTGAATGTCTT
TACATGGGCTTATATGCCTATGAAAATTGTAATACAACTTTCAGCAACGGATCTCTTGGC
TCTCGCATCGATGAAGAACGCAGCGAAATGCGATAAGTAATGTGAATTGCAGAATTCAGT
GAATCATCGAATCTTTGAACGCATCTTGCGCTCCTTGGTATTCCGAGGAGCATGCCTGTT
TGAGTGTCATTAAATTCTCAACTCTCTTCTAC-TTTTTGTAAAAGAGAGCTTGGACTGTG
GAGGCTTGCTGGCCACTTTTTGGGGTCAGCTCCTCTGAAATGCATTAGCGGAACCGTTTG
CGATCTGCCACAAGTGTGATAAGTTATCTACACTGGCGAGGGGATTGCTCTCTGTAATGT
TCAGCTTCTAATTGTCTCTACTTTGTGAGACTACTTTTGAATGCTTGACCTCAAATCAGG
TAGGACTACCCGCTGAACTTAA
>AC2-29
TTTCCGTAGGTGAACCTGCGGAAGGATCATTATTGAATTATGTTTCTAGATAGGTTGTAG
CTGGCTC-TTTAGAGCATGTGCACGCCTGTTTGGACTTCATTTTCATCCACCTGTGCACC
TATTGTAGTCTTTGGTTGGGTTAGGAGGAAGTGGTCATTGTGTCAGCATCTGCTGGATGT
GAGGACTTGCATTGTGAAAGCTTTGCTGTCCTTGATGTGATCATGGAATCTCTTTC----

-------TCACTAGAGTCTATGTCACTCATTATACTCTGTCGAATGTCATTGAATGTCTT

```
TACATGGGCTTATATGCCTATGAAAATTGTAATACAACTTTCAGCAACGGATCTCTTGGC
TCTCGCATCGATGAAGAACGCAGCGAAATGCGATAAGTAATGTGAATTGCAGAATTCAGT
GAATCATCGAATCTTTGAACGCATCTTGCGCTCCTTGGTATTCCGAGGAGCATGCCTGTT
TGAGTGTCATTAAATTCTCAACTCTCTTCTAC-TTTTTGTAAAAGAGAGCTTGGACTGTG
GAGGCTTGCTGGCCACTTTTTGGGGTCAGCTCCTCTGAAATGCATTAGCGGAACCGTTTG
CGATCTGCCACAAGTGTGATAAGTTATCTACACTGGCGAGGGGATTGCTCTCTGTAATGT
TCAGCTTCTAATTGTCTCTACTTTGTGAGACTACTTTTGAATGCTTGACCTCAAATCAGG
TAGGACTACCCGCTGAACTTAA
>AC2-31
TTTCCGTAGGTGAACCTGCGGAAGGATCATTATTGAATTATGTTTCTAGATAGGTTGTAG
CTGGCTC-TTTAGAGCATGTGCACGCCTGTTTGGACTTCATTTTCATCCACCTGTGCACC
TATTGTAGTCTTTGGTTGGGTTAGGAGGAAGTGGTCATTGTGTCAGCATCTGCTGGATGT
GAGGACTTGCATTGTGAAAGCTTTGCTGTCCTTGATGTGATCATGGAATCTCTTTC----
------------------------------------------------------------
------------------------------------------------------------
------------------------------------------------------------
------------------------------------------------------------
------------------------------------------------------------
------------------------------------------------------------
------------------------------------------------------------
------------------------------------------------------------
------------------------------------------------------------
------------------------------------------------------------
------------------------------------------------------------
------------------------------------------------------------
-------TCACTAGAGTCTATGTCACTCATTATACTCTGTCGAATGTCATTGAATGTCTT
TACATGGGCTTATATGCCTATGAAAATTGTAATACAACTTTCAGCAACGGATCTCTTGGC
TCTCGCATCGATGAAGAACGCAGCGAAATGCGATAAGTAATGTGAATTGCAGAATTCAGT
GAATCATCGAATCTTTGAACGCATCTTGCGCTCCTTGGTATTCCGAGGAGCATGCCTGTT
TGAGTGTCATTAAATTCTCAACTCTCTTCTAC-TTTTTGTAAAAGAGAGCTTGGACTGTG
GAGGCTTGCTGGCCACTTTTTGGGGTCAGCTCCTCTGAAATGCATTAGCGGAACCGTTTG
CGATCTGCCACAAGTGTGATAAGTTATCTACACTGGCGAGGGGATTGCTCTCTGTAATGT
TCAGCTTCTAATTGTCTCTACTTTGTGAGACTACTTTTGAATGCTTGACCTCAAATCAGG
TAGGACTACCCGCTGAACTTAA
>AC2-32
TTTCCGTAGGTGAACCTGCGGAAGGATCATTATTGAATTATGTTTCTAGATAGGTTGTAG
CTGGCTC-TTTAGAGCATGTGCACGCCTGTTTGGACTTCATTTTCATCCACCTGTGCACC
TATTGTAGTCTTTGGTTGGGTTAGGAGGAAGTGGTCATTGTGTCAGCATCTGCTGGATGT
GAGGACTTGCATTGTGAAAGCTTTGCTGTCCTTGATGTGATCATGGAATCTCTTTC----
------------------------------------------------------------
------------------------------------------------------------
------------------------------------------------------------
------------------------------------------------------------
------------------------------------------------------------
------------------------------------------------------------
------------------------------------------------------------
------------------------------------------------------------
------------------------------------------------------------
------------------------------------------------------------
------------------------------------------------------------
```

-------TCACTAGAGTCTATGTCACTCATTATACTCTGTCGAATGTCATTGAATGTCTT
TACATGGGCTTATATGCCTATGAAAATTGTAATACAACTTTCAGCAACGGATCTCTTGGC
TCTCGCATCGATGAAGAACGCAGCGAAATGCGATAAGTAATGTGAATTGCAGAATTCAGT
GAATCATCGAATCTTTGAACGCATCTTGCGCTCCTTGGTATTCCGAGGAGCATGCCTGTT
TGAGTGTCATTAAATTCTCAACTCTCTTCTAC-TTTTTGTAAAAGAGAGCTTGGACTGTG
GAGGCTTGCTGGCCACTTTTTGGGGTCAGCTCCTCTGAAATGCATTAGCGGAACCGTTTG
CGATCTGCCACAAGTGTGATAAGTTATCTACACTGGCGAGGGGATTGCTCTCTGTAATGT
TCAGCTTCTAATTGTCTCTACTTTGTGAGACTACTTTTGAATGCTTGACCTCAAATCAGG
TAGGACTACCCGCTGAACTTAA
>AC2-34
TTTCCGTAGGTGAACCTGCGGAAGGATCATTATTGAATTATGTTTCTAGATAGGTTGTAG
CTGGCTC-TTTAGAGCATGTGCACGCCTGTTTGGACTTCATTTTCATCCACCTGTGCACC
TATTGTAGTCTTTGGTTGGGTTAGGAGGAAGTGGTCATTGTGTCAGCATCTGCTGGATGT
GAGGACTTGCATTGTGAAAGCTTTGCTGTCCTTGATGTGATCATGGAATCTCTTTC----

-------TCACTAGAGTCTATGTCACTCATTATACTCTGTCGAATGTCATTGAATGTCTT
TACATGGGCTTATATGCCTATGAAAATTGTAATACAACTTTCAGCAACGGATCTCTTGGC
TCTCGCATCGATGAAGAACGCAGCGAAATGCGATAAGTAATGTGAATTGCAGAATTCAGT
GAATCATCGAATCTTTGAACGCATCTTGCGCTCCTTGGTATTCCGAGGAGCATGCCTGTT
TGAGTGTCATTAAATTCTCAACTCTCTTCTAC-TTTTTGTAAAAGAGAGCTTGGACTGTG
GAGGCTTGCTGGCCACTTTTTGGGGTCAGCTCCTCTGAAATGCATTAGCGGAACCGTTTG
CGATCTGCCACAAGTGTGATAAGTTATCTACACTGGCGAGGGGATTGCTCTCTGTAATGT
TCAGCTTCTAATTGTCTCTACTTTGTGAGACTACTTTTGAATGCTTGACCTCAAATCAGG
TAGGACTACCCGCTGAACTTAA
>AC2-35
TTTCCGTAGGTGAACCTGCGGAAGGATCATTATTGAATTATGTTTCTAGATAGGTTGTAG
CTGGCTC-TTTAGAGCATGTGCACGCCTGTTTGGACTTCATTTTCATCCACCTGTGCACC
TATTGTAGTCTTTGGTTGGGTTAGGAGGAAGTGGTCATTGTGTCAGCATCTGCTGGATGT
GAGGACTTGCATTGTGAAAGCTTTGCTGTCCTTGATGTGATCATGGAATCTCTTTC----

-------TCACTAGAGTCTATGTCACTCATTATACTCTGTCGAATGTCATTGAATGTCTT
TACATGGGCTTATATGCCTATGAAAATTGTAATACAACTTTCAGCAACGGATCTCTTGGC
TCTCGCATCGATGAAGAACGCAGCGAAATGCGATAAGTAATGTGAATTGCAGAATTCAGT
GAATCATCGAATCTTTGAACGCATCTTGCGCTCCTTGGTATTCCGAGGAGCATGCCTGTT
TGAGTGTCATTAAATTCTCAACTCTCTTCTAC-TTTTTGTAAAAGAGAGCTTGGACTGTG
GAGGCTTGCTGGCCACTTTTTGGGGTCAGCTCCTCTGAAATGCATTAGCGGAACCGTTTG
CGATCTGCCACAAGTGTGATAAGTTATCTACACTGGCGAGGGGATTGCTCTCTGTAATGT
TCAGCTTCTAATTGTCTCTACTTTGTGAGACTACTTTTGAATGCTTGACCTCAAATCAGG
TAGGACTACCCGCTGAACTTAA
>AC2-37
TTTCCGTAGGTGAACCTGCGGAAGGATCATTATTGAATTATGTTTCTAGATAGGTTGTAG
CTGGCTC-TTTAGAGCATGTGCACGCCTGTTTGGACTTCATTTTCATCCACCTGTGCACC
TATTGTAGTCTTTGGTTGGGTTAGGAGGAAGTGGTCATTGTGTCAGCATCTGCTGGATGT
GAGGACTTGCATTGTGAAAGCTTTGCTGTCCTTGATGTGATCATGGAATCTCTTTC----

-------TCACTAGAGTCTATGTCACTCATTATACTCTGTCGAATGTCATTGAATGTCTT
TACATGGGCTTATATGCCTATGAAAATTGTAATACAACTTTCAGCAACGGATCTCTTGGC
TCTCGCATCGATGAAGAACGCAGCGAAATGCGATAAGTAATGTGAATTGCAGAATTCAGT
GAATCATCGAATCTTTGAACGCATCTTGCGCTCCTTGGTATTCCGAGGAGCATGCCTGTT
TGAGTGTCATTAAATTCTCAACTCTCTTCTAC-TTTTTGTAAAAGAGAGCTTGGACTGTG
GAGGCTTGCTGGCCACTTTTTGGGGTCAGCTCCTCTGAAATGCATTAGCGGAACCGTTTG
CGATCTGCCACAAGTGTGATAAGTTATCTACACTGGCGAGGGGATTGCTCTCTGTAATGT
TCAGCTTCTAATTGTCTCTACTTTGTGAGACTACTTTTGAATGCTTGACCTCAAATCAGG
TAGGACTACCCGCTGAACTTAA
>AC2-40
TTTCCGTAGGTGAACCTGCGGAAGGATCATTATTGAATTATGTTTCTAGATAGGTTGTAG
CTGGCTC-TTTAGAGCATGTGCACGCCTGTTTGGACTTCATTTTCATCCACCTGTGCACC
TATTGTAGTCTTTGGTTGGGTTAGGAGGAAGTGGTCATTGTGTCAGCATCTGCTGGATGT
GAGGACTTGCATTGTGAAAGCTTTGCTGTCCTTGATGTGATCATGGAATCTCTTTC----

-------TCACTAGAGTCTATGTCACTCATTATACTCTGTCGAATGTCATTGAATGTCTT
TACATGGGCTTATATGCCTATGAAAATTGTAATACAACTTTCAGCAACGGATCTCTTGGC
TCTCGCATCGATGAAGAACGCAGCGAAATGCGATAAGTAATGTGAATTGCAGAATTCAGT
GAATCATCGAATCTTTGAACGCATCTTGCGCTCCTTGGTATTCCGAGGAGCATGCCTGTT
TGAGTGTCATTAAATTCTCAACTCTCTTCTAC-TTTTTGTAAAAGAGAGCTTGGACTGTG
GAGGCTTGCTGGCCACTTTTTGGGGTCAGCTCCTCTGAAATGCATTAGCGGAACCGTTTG
CGATCTGCCACAAGTGTGATAAGTTATCTACACTGGCGAGGGGATTGCTCTCTGTAATGT
TCAGCTTCTAATTGTCTCTACTTTGTGAGACTACTTTTGAATGCTTGACCTCAAATCAGG
TAGGACTACCCGCTGAACTTAA
>AC2-41
TTTCCGTAGGTGAACCTGCGGAAGGATCATTATTGAATTATGTTTCTAGATAGGTTGTAG
CTGGCTC-TTTAGAGCATGTGCACGCCTGTTTGGACTTCATTTTCATCCACCTGTGCACC
TATTGTAGTCTTTGGTTGGGTTAGGAGGAAGTGGTCATTGTGTCAGCATCTGCTGGATGT
GAGGACTTGCATTGTGAAAGCTTTGCTGTCCTTGATGTGATCATGGAATCTCTTTC----

-------TCACTAGAGTCTATGTCACTCATTATACTCTGTCGAATGTCATTGAATGTCTT
TACATGGGCTTATATGCCTATGAAAATTGTAATACAACTTTCAGCAACGGATCTCTTGGC
TCTCGCATCGATGAAGAACGCAGCGAAATGCGATAAGTAATGTGAATTGCAGAATTCAGT
GAATCATCGAATCTTTGAACGCATCTTGCGCTCCTTGGTATTCCGAGGAGCATGCCTGTT
TGAGTGTCATTAAATTCTCAACTCTCTTCTAC-TTTTTGTAAAAGAGAGCTTGGACTGTG
GAGGCTTGCTGGCCACTTTTTGGGGTCAGCTCCTCTGAAATGCATTAGCGGAACCGTTTG
CGATCTGCCACAAGTGTGATAAGTTATCTACACTGGCGAGGGGATTGCTCTCTGTAATGT
TCAGCTTCTAATTGTCTCTACTTTGTGAGACTACTTTTGAATGCTTGACCTCAAATCAGG
TAGGACTACCCGCTGAACTTAA
>AC2-42
TTTCCGTAGGTGAACCTGCGGAAGGATCATTATTGAATTATGTTTCTAGATAGGTTGTAG
CTGGCTC-TTTAGAGCATGTGCACGCCTGTTTGGACTTCATTTTCATCCACCTGTGCACC
TATTGTAGTCTTTGGTTGGGTTAGGAGGAAGTGGTCATTGTGTCAGCATCTGCTGGATGT
GAGGACTTGCATTGTGAAAGCTTTGCTGTCCTTGATGTGATCATGGAATCTCTTTC----


```
------------------------------------------------------------
------------------------------------------------------------
------------------------------------------------------------
------------------------------------------------------------
------------------------------------------------------------
------------------------------------------------------------
------------------------------------------------------------
-------TCACTAGAGTCTATGTCACTCATTATACTCTGTCGAATGTCATTGAATGTCTT
TACATGGGCTTATATGCCTATGAAAATTGTAATACAACTTTCAGCAACGGATCTCTTGGC
TCTCGCATCGATGAAGAACGCAGCGAAATGCGATAAGTAATGTGAATTGCAGAATTCAGT
GAATCATCGAATCTTTGAACGCATCTTGCGCTCCTTGGTATTCCGAGGAGCATGCCTGTT
TGAGTGTCATTAAATTCTCAACTCTCTTCTAC-TTTTTGTAAAAGAGAGCTTGGACTGTG
GAGGCTTGCTGGCCACTTTTTGGGGTCAGCTCCTCTGAAATGCATTAGCGGAACCGTTTG
CGATCTGCCACAAGTGTGATAAGTTATCTACACTGGCGAGGGGATTGCTCTCTGTAATGT
TCAGCTTCTAATTGTCTCTACTTTGTGAGACTACTTTTGAATGCTTGACCTCAAATCAGG
TAGGACTACCCGCTGAACTTAA
>AC2-44
TTTCCGTAGGTGAACCTGCGGAAGGATCATTATTGAATTATGTTTCTAGATAGGTTGTAG
CTGGCTC-TTTAGAGCATGTGCACGCCTGTTTGGACTTCATTTTCATCCACCTGTGCACC
TATTGTAGTCTTTGGTTGGGTTAGGAGGAAGTGGTCATTGTGTCAGCATCTGCTGGATGT
GAGGACTTGCATTGTGAAAGCTTTGCTGTCCTTGATGTGATCATGGAATCTCTTTC----
------------------------------------------------------------
------------------------------------------------------------
------------------------------------------------------------
------------------------------------------------------------
------------------------------------------------------------
------------------------------------------------------------
------------------------------------------------------------
------------------------------------------------------------
------------------------------------------------------------
------------------------------------------------------------
------------------------------------------------------------
------------------------------------------------------------
-------TCACTAGAGTCTATGTCACTCATTATACTCTGTCGAATGTCATTGAATGTCTT
TACATGGGCTTATATGCCTATGAAAATTGTAATACAACTTTCAGCAACGGATCTCTTGGC
TCTCGCATCGATGAAGAACGCAGCGAAATGCGATAAGTAATGTGAATTGCAGAATTCAGT
GAATCATCGAATCTTTGAACGCATCTTGCGCTCCTTGGTATTCCGAGGAGCATGCCTGTT
TGAGTGTCATTAAATTCTCAACTCTCTTCTAC-TTTTTGTAAAAGAGAGCTTGGACTGTG
GAGGCTTGCTGGCCACTTTTTGGGGTCAGCTCCTCTGAAATGCATTAGCGGAACCGTTTG
CGATCTGCCACAAGTGTGATAAGTTATCTACACTGGCGAGGGGATTGCTCTCTGTAATGT
TCAGCTTCTAATTGTCTCTACTTTGTGAGACTACTTTTGAATGCTTGACCTCAAATCAGG
TAGGACTACCCGCTGAACTTAA
>AC2-45
TTTCCGTAGGTGAACCTGCGGAAGGATCATTATTGAATTATGTTTCTAGATAGGTTGTAG
CTGGCTC-TTTAGAGCATGTGCACGCCTGTTTGGACTTCATTTTCATCCACCTGTGCACC
TATTGTAGTCTTTGGTTGGGTTAGGAGGAAGTGGTCATTGTGTCAGCATCTGCTGGATGT
GAGGACTTGCATTGTGAAAGCTTTGCTGTCCTTGATGTGATCATGGAATCTCTTTC----
------------------------------------------------------------
------------------------------------------------------------
------------------------------------------------------------
```

```
------------------------------------------------------------
------------------------------------------------------------
------------------------------------------------------------
------------------------------------------------------------
------------------------------------------------------------
------------------------------------------------------------
------------------------------------------------------------
------------------------------------------------------------
------------------------------------------------------------
-------TCACTAGAGTCTATGTCACTCATTATACTCTGTCGAATGTCATTGAATGTCTT
TACATGGGCTTATATGCCTATGAAAATTGTAATACAACTTTCAGCAACGGATCTCTTGGC
TCTCGCATCGATGAAGAACGCAGCGAAATGCGATAAGTAATGTGAATTGCAGAATTCAGT
GAATCATCGAATCTTTGAACGCATCTTGCGCTCCTTGGTATTCCGAGGAGCATGCCTGTT
TGAGTGTCATTAAATTCTCAACTCTCTTCTAC-TTTTTGTAAAAGAGAGCTTGGACTGTG
GAGGCTTGCTGGCCACTTTTTGGGGTCAGCTCCTCTGAAATGCATTAGCGGAACCGTTTG
CGATCTGCCACAAGTGTGATAAGTTATCTACACTGGCGAGGGGATTGCTCTCTGTAATGT
TCAGCTTCTAATTGTCTCTACTTTGTGAGACTACTTTTGAATGCTTGACCTCAAATCAGG
TAGGACTACCCGCTGAACTTAA
>AC2-47
TTTCCGTAGGTGAACCTGCGGAAGGATCATTATTGAATTATGTTTCTAGATAGGTTGTAG
CTGGCTC-TTTAGAGCATGTGCACGCCTGTTTGGACTTCATTTTCATCCACCTGTGCACC
TATTGTAGTCTTTGGTTGGGTTAGGAGGAAGTGGTCATTGTGTCAGCATCTGCTGGATGT
GAGGACTTGCATTGTGAAAGCTTTGCTGTCCTTGATGTGATCATGGAATCTCTTTC----
------------------------------------------------------------
------------------------------------------------------------
------------------------------------------------------------
------------------------------------------------------------
------------------------------------------------------------
------------------------------------------------------------
------------------------------------------------------------
------------------------------------------------------------
------------------------------------------------------------
------------------------------------------------------------
------------------------------------------------------------
------------------------------------------------------------
------------------------------------------------------------
-------TCACTAGAGTCTATGTCACTCATTATACTCTGTCGAATGTCATTGAATGTCTT
TACATGGGCTTATATGCCTATGAAAATTGTAATACAACTTTCAGCAACGGATCTCTTGGC
TCTCGCATCGATGAAGAACGCAGCGAAATGCGATAAGTAATGTGAATTGCAGAATTCAGT
GAATCATCGAATCTTTGAACGCATCTTGCGCTCCTTGGTATTCCGAGGAGCATGCCTGTT
TGAGTGTCATTAAATTCTCAACTCTCTTCTAC-TTTTTGTAAAAGAGAGCTTGGACTGTG
GAGGCTTGCTGGCCACTTTTTGGGGTCAGCTCCTCTGAAATGCATTAGCGGAACCGTTTG
CGATCTGCCACAAGTGTGATAAGTTATCTACACTGGCGAGGGGATTGCTCTCTGTAATGT
TCAGCTTCTAATTGTCTCTACTTTGTGAGACTACTTTTGAATGCTTGACCTCAAATCAGG
TAGGACTACCCGCTGAACTTAA
>AC2-48
TTTCCGTAGGTGAACCTGCGGAAGGATCATTATTGAATTATGTTTCTAGATAGGTTGTAG
CTGGCTC-TTTAGAGCATGTGCACGCCTGTTTGGACTTCATTTTCATCCACCTGTGCACC
TATTGTAGTCTTTGGTTGGGTTAGGAGGAAGTGGTCATTGTGTCAGCATCTGCTGGATGT
GAGGACTTGCATTGTGAAAGCTTTGCTGTCCTTGATGTGATCATGGAATCTCTTTC----
------------------------------------------------------------
```


-------TCACTAGAGTCTATGTCACTCATTATACTCTGTCGAATGTCATTGAATGTCTT
TACATGGGCTTATATGCCTATGAAAATTGTAATACAACTTTCAGCAACGGATCTCTTGGC
TCTCGCATCGATGAAGAACGCAGCGAAATGCGATAAGTAATGTGAATTGCAGAATTCAGT
GAATCATCGAATCTTTGAACGCATCTTGCGCTCCTTGGTATTCCGAGGAGCATGCCTGTT
TGAGTGTCATTAAATTCTCAACTCTCTTCTAC-TTTTTGTAAAAGAGAGCTTGGACTGTG
GAGGCTTGCTGGCCACTTTTTGGGGTCAGCTCCTCTGAAATGCATTAGCGGAACCGTTTG
CGATCTGCCACAAGTGTGATAAGTTATCTACACTGGCGAGGGGATTGCTCTCTGTAATGT
TCAGCTTCTAATTGTCTCTACTTTGTGAGACTACTTTTGAATGCTTGACCTCAAATCAGG
TAGGACTACCCGCTGAACTTAA
>AC2-50
TTTCCGTAGGTGAACCTGCGGAAGGATCATTATTGAATTATGTTTCTAGATAGGTTGTAG
CTGGCTC-TTTAGAGCATGTGCACGCCTGTTTGGACTTCATTTTCATCCACCTGTGCACC
TATTGTAGTCTTTGGTTGGGTTAGGAGGAAGTGGTCATTGTGTCAGCATCTGCTGGATGT
GAGGACTTGCATTGTGAAAGCTTTGCTGTCCTTGATGTGATCATGGAATCTCTTTC----

-------TCACTAGAGTCTATGTCACTCATTATACTCTGTCGAATGTCATTGAATGTCTT
TACATGGGCTTATATGCCTATGAAAATTGTAATACAACTTTCAGCAACGGATCTCTTGGC
TCTCGCATCGATGAAGAACGCAGCGAAATGCGATAAGTAATGTGAATTGCAGAATTCAGT
GAATCATCGAATCTTTGAACGCATCTTGCGCTCCTTGGTATTCCGAGGAGCATGCCTGTT
TGAGTGTCATTAAATTCTCAACTCTCTTCTAC-TTTTTGTAAAAGAGAGCTTGGACTGTG
GAGGCTTGCTGGCCACTTTTTGGGGTCAGCTCCTCTGAAATGCATTAGCGGAACCGTTTG
CGATCTGCCACAAGTGTGATAAGTTATCTACACTGGCGAGGGGATTGCTCTCTGTAATGT
TCAGCTTCTAATTGTCTCTACTTTGTGAGACTACTTTTGAATGCTTGACCTCAAATCAGG
TAGGACTACCCGCTGAACTTAA
>AC2-51
TTTCCGTAGGTGAACCTGCGGAAGGATCATTATTGAATTATGTTTCTAGATAGGTTGTAG
CTGGCTC-TTTAGAGCATGTGCACGCCTGTTTGGACTTCATTTTCATCCACCTGTGCACC
TATTGTAGTCTTTGGTTGGGTTAGGAGGAAGTGGTCATTGTGTCAGCATCTGCTGGATGT

```
GAGGACTTGCATTGTGAAAGCTTTGCTGTCCTTGATGTGATCATGGAATCTCTTTC----
------------------------------------------------------------
------------------------------------------------------------
------------------------------------------------------------
------------------------------------------------------------
------------------------------------------------------------
------------------------------------------------------------
------------------------------------------------------------
------------------------------------------------------------
------------------------------------------------------------
------------------------------------------------------------
------------------------------------------------------------
------------------------------------------------------------
-------TCACTAGAGTCTATGTCACTCATTATACTCTGTCGAATGTCATTGAATGTCTT
TACATGGGCTTATATGCCTATGAAAATTGTAATACAACTTTCAGCAACGGATCTCTTGGC
TCTCGCATCGATGAAGAACGCAGCGAAATGCGATAAGTAATGTGAATTGCAGAATTCAGT
GAATCATCGAATCTTTGAACGCATCTTGCGCTCCTTGGTATTCCGAGGAGCATGCCTGTT
TGAGTGTCATTAAATTCTCAACTCTCTTCTAC-TTTTTGTAAAAGAGAGCTTGGACTGTG
GAGGCTTGCTGGCCACTTTTTGGGGTCAGCTCCTCTGAAATGCATTAGCGGAACCGTTTG
CGATCTGCCACAAGTGTGATAAGTTATCTACACTGGCGAGGGGATTGCTCTCTGTAATGT
TCAGCTTCTAATTGTCTCTACTTTGTGAGACTACTTTTGAATGCTTGACCTCAAATCAGG
TAGGACTACCCGCTGAACTTAA
>AC2-53
TTTCCGTAGGTGAACCTGCGGAAGGATCATTATTGAATTATGTTTCTAGATAGGTTGTAG
CTGGCTC-TTTAGAGCATGTGCACGCCTGTTTGGACTTCATTTTCATCCACCTGTGCACC
TATTGTAGTCTTTGGTTGGGTTAGGAGGAAGTGGTCATTGTGTCAGCATCTGCTGGATGT
GAGGACTTGCATTGTGAAAGCTTTGCTGTCCTTGATGTGATCATGGAATCTCTTTC----
------------------------------------------------------------
------------------------------------------------------------
------------------------------------------------------------
------------------------------------------------------------
------------------------------------------------------------
------------------------------------------------------------
------------------------------------------------------------
------------------------------------------------------------
------------------------------------------------------------
------------------------------------------------------------
------------------------------------------------------------
------------------------------------------------------------
-------TCACTAGAGTCTATGTCACTCATTATACTCTGTCGAATGTCATTGAATGTCTT
TACATGGGCTTATATGCCTATGAAAATTGTAATACAACTTTCAGCAACGGATCTCTTGGC
TCTCGCATCGATGAAGAACGCAGCGAAATGCGATAAGTAATGTGAATTGCAGAATTCAGT
GAATCATCGAATCTTTGAACGCATCTTGCGCTCCTTGGTATTCCGAGGAGCATGCCTGTT
TGAGTGTCATTAAATTCTCAACTCTCTTCTAC-TTTTTGTAAAAGAGAGCTTGGACTGTG
GAGGCTTGCTGGCCACTTTTTGGGGTCAGCTCCTCTGAAATGCATTAGCGGAACCGTTTG
CGATCTGCCACAAGTGTGATAAGTTATCTACACTGGCGAGGGGATTGCTCTCTGTAATGT
TCAGCTTCTAATTGTCTCTACTTTGTGAGACTACTTTTGAATGCTTGACCTCAAATCAGG
TAGGACTACCCGCTGAACTTAA
>AC2-57
TTTCCGTAGGTGAACCTGCGGAAGGATCATTATTGAATTATGTTTCTAGATAGGTTGTAG
```

CTGGCTC-TTTAGAGCATGTGCACGCCTGTTTGGACTTCATTTTCATCCACCTGTGCACC
TATTGTAGTCTTTGGTTGGGTTAGGAGGAAGTGGTCATTGTGTCAGCATCTGCTGGATGT
GAGGACTTGCATTGTGAAAGCTTTGCTGTCCTTGATGTGATCATGGAATCTCTTTC----

-------TCACTAGAGTCTATGTCACTCATTATACTCTGTCGAATGTCATTGAATGTCTT
TACATGGGCTTATATGCCTATGAAAATTGTAATACAACTTTCAGCAACGGATCTCTTGGC
TCTCGCATCGATGAAGAACGCAGCGAAATGCGATAAGTAATGTGAATTGCAGAATTCAGT
GAATCATCGAATCTTTGAACGCATCTTGCGCTCCTTGGTATTCCGAGGAGCATGCCTGTT
TGAGTGTCATTAAATTCTCAACTCTCTTCTAC-TTTTTGTAAAAGAGAGCTTGGACTGTG
GAGGCTTGCTGGCCACTTTTTGGGGTCAGCTCCTCTGAAATGCATTAGCGGAACCGTTTG
CGATCTGCCACAAGTGTGATAAGTTATCTACACTGGCGAGGGGATTGCTCTCTGTAATGT
TCAGCTTCTAATTGTCTCTACTTTGTGAGACTACTTTTGAATGCTTGACCTCAAATCAGG
TAGGACTACCCGCTGAACTTAA
>AC2-58
TTTCCGTAGGTGAACCTGCGGAAGGATCATTATTGAATTATGTTTCTAGATAGGTTGTAG
CTGGCTC-TTTAGAGCATGTGCACGCCTGTTTGGACTTCATTTTCATCCACCTGTGCACC
TATTGTAGTCTTTGGTTGGGTTAGGAGGAAGTGGTCATTGTGTCAGCATCTGCTGGATGT
GAGGACTTGCATTGTGAAAGCTTTGCTGTCCTTGATGTGATCATGGAATCTCTTTC----

-------TCACTAGAGTCTATGTCACTCATTATACTCTGTCGAATGTCATTGAATGTCTT
TACATGGGCTTATATGCCTATGAAAATTGTAATACAACTTTCAGCAACGGATCTCTTGGC
TCTCGCATCGATGAAGAACGCAGCGAAATGCGATAAGTAATGTGAATTGCAGAATTCAGT
GAATCATCGAATCTTTGAACGCATCTTGCGCTCCTTGGTATTCCGAGGAGCATGCCTGTT
TGAGTGTCATTAAATTCTCAACTCTCTTCTAC-TTTTTGTAAAAGAGAGCTTGGACTGTG
GAGGCTTGCTGGCCACTTTTTGGGGTCAGCTCCTCTGAAATGCATTAGCGGAACCGTTTG
CGATCTGCCACAAGTGTGATAAGTTATCTACACTGGCGAGGGGATTGCTCTCTGTAATGT
TCAGCTTCTAATTGTCTCTACTTTGTGAGACTACTTTTGAATGCTTGACCTCAAATCAGG
TAGGACTACCCGCTGAACTTAA

```
>AC2-62
TTTCCGTAGGTGAACCTGCGGAAGGATCATTATTGAATTATGTTTCTAGATAGGTTGTAG
CTGGCTC-TTTAGAGCATGTGCACGCCTGTTTGGACTTCATTTTCATCCACCTGTGCACC
TATTGTAGTCTTTGGTTGGGTTAGGAGGAAGTGGTCATTGTGTCAGCATCTGCTGGATGT
GAGGACTTGCATTGTGAAAGCTTTGCTGTCCTTGATGTGATCATGGAATCTCTTTC----
------------------------------------------------------------
------------------------------------------------------------
------------------------------------------------------------
------------------------------------------------------------
------------------------------------------------------------
------------------------------------------------------------
------------------------------------------------------------
------------------------------------------------------------
------------------------------------------------------------
------------------------------------------------------------
------------------------------------------------------------
------------------------------------------------------------
-------TCACTAGAGTCTATGTCACTCATTATACTCTGTCGAATGTCATTGAATGTCTT
TACATGGGCTTATATGCCTATGAAAATTGTAATACAACTTTCAGCAACGGATCTCTTGGC
TCTCGCATCGATGAAGAACGCAGCGAAATGCGATAAGTAATGTGAATTGCAGAATTCAGT
GAATCATCGAATCTTTGAACGCATCTTGCGCTCCTTGGTATTCCGAGGAGCATGCCTGTT
TGAGTGTCATTAAATTCTCAACTCTCTTCTAC-TTTTTGTAAAAGAGAGCTTGGACTGTG
GAGGCTTGCTGGCCACTTTTTGGGGTCAGCTCCTCTGAAATGCATTAGCGGAACCGTTTG
CGATCTGCCACAAGTGTGATAAGTTATCTACACTGGCGAGGGGATTGCTCTCTGTAATGT
TCAGCTTCTAATTGTCTCTACTTTGTGAGACTACTTTTGAATGCTTGACCTCAAATCAGG
TAGGACTACCCGCTGAACTTAA
>AC2-64
TTTCCGTAGGTGAACCTGCGGAAGGATCATTATTGAATTATGTTTCTAGATAGGTTGTAG
CTGGCTC-TTTAGAGCATGTGCACGCCTGTTTGGACTTCATTTTCATCCACCTGTGCACC
TATTGTAGTCTTTGGTTGGGTTAGGAGGAAGTGGTCATTGTGTCAGCATCTGCTGGATGT
GAGGACTTGCATTGTGAAAGCTTTGCTGTCCTTGATGTGATCATGGAATCTCTTTC----
------------------------------------------------------------
------------------------------------------------------------
------------------------------------------------------------
------------------------------------------------------------
------------------------------------------------------------
------------------------------------------------------------
------------------------------------------------------------
------------------------------------------------------------
------------------------------------------------------------
------------------------------------------------------------
------------------------------------------------------------
------------------------------------------------------------
-------TCACTAGAGTCTATGTCACTCATTATACTCTGTCGAATGTCATTGAATGTCTT
TACATGGGCTTATATGCCTATGAAAATTGTAATACAACTTTCAGCAACGGATCTCTTGGC
TCTCGCATCGATGAAGAACGCAGCGAAATGCGATAAGTAATGTGAATTGCAGAATTCAGT
GAATCATCGAATCTTTGAACGCATCTTGCGCTCCTTGGTATTCCGAGGAGCATGCCTGTT
TGAGTGTCATTAAATTCTCAACTCTCTTCTAC-TTTTTGTAAAAGAGAGCTTGGACTGTG
GAGGCTTGCTGGCCACTTTTTGGGGTCAGCTCCTCTGAAATGCATTAGCGGAACCGTTTG
CGATCTGCCACAAGTGTGATAAGTTATCTACACTGGCGAGGGGATTGCTCTCTGTAATGT
```

TCAGCTTCTAATTGTCTCTACTTTGTGAGACTACTTTTGAATGCTTGACCTCAAATCAGG
TAGGACTACCCGCTGAACTTAA
>AC2-66
TTTCCGTAGGTGAACCTGCGGAAGGATCATTATTGAATTATGTTTCTAGATAGGTTGTAG
CTGGCTC-TTTAGAGCATGTGCACGCCTGTTTGGACTTCATTTTCATCCACCTGTGCACC
TATTGTAGTCTTTGGTTGGGTTAGGAGGAAGTGGTCATTGTGTCAGCATCTGCTGGATGT
GAGGACTTGCATTGTGAAAGCTTTGCTGTCCTTGATGTGATCATGGAATCTCTTTC----

-------TCACTAGAGTCTATGTCACTCATTATACTCTGTCGAATGTCATTGAATGTCTT
TACATGGGCTTATATGCCTATGAAAATTGTAATACAACTTTCAGCAACGGATCTCTTGGC
TCTCGCATCGATGAAGAACGCAGCGAAATGCGATAAGTAATGTGAATTGCAGAATTCAGT
GAATCATCGAATCTTTGAACGCATCTTGCGCTCCTTGGTATTCCGAGGAGCATGCCTGTT
TGAGTGTCATTAAATTCTCAACTCTCTTCTAC-TTTTTGTAAAAGAGAGCTTGGACTGTG
GAGGCTTGCTGGCCACTTTTTGGGGTCAGCTCCTCTGAAATGCATTAGCGGAACCGTTTG
CGATCTGCCACAAGTGTGATAAGTTATCTACACTGGCGAGGGGATTGCTCTCTGTAATGT
TCAGCTTCTAATTGTCTCTACTTTGTGAGACTACTTTTGAATGCTTGACCTCAAATCAGG
TAGGACTACCCGCTGAACTTAA
>AC2-67
TTTCCGTAGGTGAACCTGCGGAAGGATCATTATTGAATTATGTTTCTAGATAGGTTGTAG
CTGGCTC-TTTAGAGCATGTGCACGCCTGTTTGGACTTCATTTTCATCCACCTGTGCACC
TATTGTAGTCTTTGGTTGGGTTAGGAGGAAGTGGTCATTGTGTCAGCATCTGCTGGATGT
GAGGACTTGCATTGTGAAAGCTTTGCTGTCCTTGATGTGATCATGGAATCTCTTTC----

-------TCACTAGAGTCTATGTCACTCATTATACTCTGTCGAATGTCATTGAATGTCTT
TACATGGGCTTATATGCCTATGAAAATTGTAATACAACTTTCAGCAACGGATCTCTTGGC
TCTCGCATCGATGAAGAACGCAGCGAAATGCGATAAGTAATGTGAATTGCAGAATTCAGT
GAATCATCGAATCTTTGAACGCATCTTGCGCTCCTTGGTATTCCGAGGAGCATGCCTGTT
TGAGTGTCATTAAATTCTCAACTCTCTTCTAC-TTTTTGTAAAAGAGAGCTTGGACTGTG

GAGGCTTGCTGGCCACTTTTTGGGGTCAGCTCCTCTGAAATGCATTAGCGGAACCGTTTG
CGATCTGCCACAAGTGTGATAAGTTATCTACACTGGCGAGGGGATTGCTCTCTGTAATGT
TCAGCTTCTAATTGTCTCTACTTTGTGAGACTACTTTTGAATGCTTGACCTCAAATCAGG
TAGGACTACCCGCTGAACTTAA
>AC2-73
TTTCCGTAGGTGAACCTGCGGAAGGATCATTATTGAATTATGTTTCTAGATAGGTTGTAG
CTGGCTC-TTTAGAGCATGTGCACGCCTGTTTGGACTTCATTTTCATCCACCTGTGCACC
TATTGTAGTCTTTGGTTGGGTTAGGAGGAAGTGGTCATTGTGTCAGCATCTGCTGGATGT
GAGGACTTGCATTGTGAAAGCTTTGCTGTCCTTGATGTGATCATGGAATCTCTTTC----

-------TCACTAGAGTCTATGTCACTCATTATACTCTGTCGAATGTCATTGAATGTCTT
TACATGGGCTTATATGCCTATGAAAATTGTAATACAACTTTCAGCAACGGATCTCTTGGC
TCTCGCATCGATGAAGAACGCAGCGAAATGCGATAAGTAATGTGAATTGCAGAATTCAGT
GAATCATCGAATCTTTGAACGCATCTTGCGCTCCTTGGTATTCCGAGGAGCATGCCTGTT
TGAGTGTCATTAAATTCTCAACTCTCTTCTAC-TTTTTGTAAAAGAGAGCTTGGACTGTG
GAGGCTTGCTGGCCACTTTTTGGGGTCAGCTCCTCTGAAATGCATTAGCGGAACCGTTTG
CGATCTGCCACAAGTGTGATAAGTTATCTACACTGGCGAGGGGATTGCTCTCTGTAATGT
TCAGCTTCTAATTGTCTCTACTTTGTGAGACTACTTTTGAATGCTTGACCTCAAATCAGG
TAGGACTACCCGCTGAACTTAA
>AC2-74
TTTCCGTAGGTGAACCTGCGGAAGGATCATTATTGAATTATGTTTCTAGATAGGTTGTAG
CTGGCTC-TTTAGAGCATGTGCACGCCTGTTTGGACTTCATTTTCATCCACCTGTGCACC
TATTGTAGTCTTTGGTTGGGTTAGGAGGAAGTGGTCATTGTGTCAGCATCTGCTGGATGT
GAGGACTTGCATTGTGAAAGCTTTGCTGTCCTTGATGTGATCATGGAATCTCTTTC----

-------TCACTAGAGTCTATGTCACTCATTATACTCTGTCGAATGTCATTGAATGTCTT
TACATGGGCTTATATGCCTATGAAAATTGTAATACAACTTTCAGCAACGGATCTCTTGGC
TCTCGCATCGATGAAGAACGCAGCGAAATGCGATAAGTAATGTGAATTGCAGAATTCAGT

```
GAATCATCGAATCTTTGAACGCATCTTGCGCTCCTTGGTATTCCGAGGAGCATGCCTGTT
TGAGTGTCATTAAATTCTCAACTCTCTTCTAC-TTTTTGTAAAAGAGAGCTTGGACTGTG
GAGGCTTGCTGGCCACTTTTTGGGGTCAGCTCCTCTGAAATGCATTAGCGGAACCGTTTG
CGATCTGCCACAAGTGTGATAAGTTATCTACACTGGCGAGGGGATTGCTCTCTGTAATGT
TCAGCTTCTAATTGTCTCTACTTTGTGAGACTACTTTTGAATGCTTGACCTCAAATCAGG
TAGGACTACCCGCTGAACTTAA
>AC2-76
TTTCCGTAGGTGAACCTGCGGAAGGATCATTATTGAATTATGTTTCTAGATAGGTTGTAG
CTGGCTC-TTTAGAGCATGTGCACGCCTGTTTGGACTTCATTTTCATCCACCTGTGCACC
TATTGTAGTCTTTGGTTGGGTTAGGAGGAAGTGGTCATTGTGTCAGCATCTGCTGGATGT
GAGGACTTGCATTGTGAAAGCTTTGCTGTCCTTGATGTGATCATGGAATCTCTTTC----
------------------------------------------------------------
------------------------------------------------------------
------------------------------------------------------------
------------------------------------------------------------
------------------------------------------------------------
------------------------------------------------------------
------------------------------------------------------------
------------------------------------------------------------
------------------------------------------------------------
------------------------------------------------------------
------------------------------------------------------------
------------------------------------------------------------
-------TCACTAGAGTCTATGTCACTCATTATACTCTGTCGAATGTCATTGAATGTCTT
TACATGGGCTTATATGCCTATGAAAATTGTAATACAACTTTCAGCAACGGATCTCTTGGC
TCTCGCATCGATGAAGAACGCAGCGAAATGCGATAAGTAATGTGAATTGCAGAATTCAGT
GAATCATCGAATCTTTGAACGCATCTTGCGCTCCTTGGTATTCCGAGGAGCATGCCTGTT
TGAGTGTCATTAAATTCTCAACTCTCTTCTAC-TTTTTGTAAAAGAGAGCTTGGACTGTG
GAGGCTTGCTGGCCACTTTTTGGGGTCAGCTCCTCTGAAATGCATTAGCGGAACCGTTTG
CGATCTGCCACAAGTGTGATAAGTTATCTACACTGGCGAGGGGATTGCTCTCTGTAATGT
TCAGCTTCTAATTGTCTCTACTTTGTGAGACTACTTTTGAATGCTTGACCTCAAATCAGG
TAGGACTACCCGCTGAACTTAA
>AC3-2
TTTCCGTAGGTGAACCTGCGGAAGGATCATTATTGAATTATGTTTCTAGATAGGTTGTAG
CTGGCTC-TTTAGAGCATGTGCACGCCTGTTTGGACTTCATTTTCATCCACCTGTGCACC
TATTGTAGTCTTTGGTTGGGTTAGGAGGAAGTGGTCATTGTGTCAGCATCTGCTGGATGT
GAGGACTTGCATTGTGAAAGCTTTGCTGTCCTTGATGTGATCATGGAATCTCTTTC----
------------------------------------------------------------
------------------------------------------------------------
------------------------------------------------------------
------------------------------------------------------------
------------------------------------------------------------
------------------------------------------------------------
------------------------------------------------------------
------------------------------------------------------------
------------------------------------------------------------
------------------------------------------------------------
------------------------------------------------------------
------------------------------------------------------------
-------TCACTAGAGTCTATGTCACTCATTATACTCTGTCGAATGTCATTGAATGTCTT
```

TACATGGGCTTATATGCCTATGAAAATTGTAATACAACTTTCAGCAACGGATCTCTTGGC
TCTCGCATCGATGAAGAACGCAGCGAAATGCGATAAGTAATGTGAATTGCAGAATTCAGT
GAATCATCGAATCTTTGAACGCATCTTGCGCTCCTTGGTATTCCGAGGAGCATGCCTGTT
TGAGTGTCATTAAATTCTCAACTCTCTTCTAC-TTTTTGTAAAAGAGAGCTTGGACTGTG
GAGGCTTGCTGGCCACTTTTTGGGGTCAGCTCCTCTGAAATGCATTAGCGGAACCGTTTG
CGATCTGCCACAAGTGTGATAAGTTATCTACACTGGCGAGGGGATTGCTCTCTGTAATGT
TCAGCTTCTAATTGTCTCTACTTTGTGAGACTACTTTTGAATGCTTGACCTCAAATCAGG
TAGGACTACCCGCTGAACTTAA
>AC3-7
TTTCCGTAGGTGAACCTGCGGAAGGATCATTATTGAATTATGTTTCTAGATAGGTTGTAG
CTGGCTC-TTTAGAGCATGTGCACGCCTGTTTGGACTTCATTTTCATCCACCTGTGCACC
TATTGTAGTCTTTGGTTGGGTTAGGAGGAAGTGGTCATTGTGTCAGCATCTGCTGGATGT
GAGGACTTGCATTGTGAAAGCTTTGCTGTCCTTGATGTGATCATGGAATCTCTTTC----

-------TCACTAGAGTCTATGTCACTCATTATACTCTGTCGAATGTCATTGAATGTCTT
TACATGGGCTTATATGCCTATGAAAATTGTAATACAACTTTCAGCAACGGATCTCTTGGC
TCTCGCATCGATGAAGAACGCAGCGAAATGCGATAAGTAATGTGAATTGCAGAATTCAGT
GAATCATCGAATCTTTGAACGCATCTTGCGCTCCTTGGTATTCCGAGGAGCATGCCTGTT
TGAGTGTCATTAAATTCTCAACTCTCTTCTAC-TTTTTGTAAAAGAGAGCTTGGACTGTG
GAGGCTTGCTGGCCACTTTTTGGGGTCAGCTCCTCTGAAATGCATTAGCGGAACCGTTTG
CGATCTGCCACAAGTGTGATAAGTTATCTACACTGGCGAGGGGATTGCTCTCTGTAATGT
TCAGCTTCTAATTGTCTCTACTTTGTGAGACTACTTTTGAATGCTTGACCTCAAATCAGG
TAGGACTACCCGCTGAACTTAA
>AC3-8
TTTCCGTAGGTGAACCTGCGGAAGGATCATTATTGAATTATGTTTCTAGATAGGTTGTAG
CTGGCTC-TTTAGAGCATGTGCACGCCTGTTTGGACTTCATTTTCATCCACCTGTGCACC
TATTGTAGTCTTTGGTTGGGTTAGGAGGAAGTGGTCATTGTGTCAGCATCTGCTGGATGT
GAGGACTTGCATTGTGAAAGCTTTGCTGTCCTTGATGTGATCATGGAATCTCTTTC----

-------TCACTAGAGTCTATGTCACTCATTATACTCTGTCGAATGTCATTGAATGTCTT
TACATGGGCTTATATGCCTATGAAAATTGTAATACAACTTTCAGCAACGGATCTCTTGGC
TCTCGCATCGATGAAGAACGCAGCGAAATGCGATAAGTAATGTGAATTGCAGAATTCAGT
GAATCATCGAATCTTTGAACGCATCTTGCGCTCCTTGGTATTCCGAGGAGCATGCCTGTT
TGAGTGTCATTAAATTCTCAACTCTCTTCTAC-TTTTTGTAAAAGAGAGCTTGGACTGTG
GAGGCTTGCTGGCCACTTTTTGGGGTCAGCTCCTCTGAAATGCATTAGCGGAACCGTTTG
CGATCTGCCACAAGTGTGATAAGTTATCTACACTGGCGAGGGGATTGCTCTCTGTAATGT
TCAGCTTCTAATTGTCTCTACTTTGTGAGACTACTTTTGAATGCTTGACCTCAAATCAGG
TAGGACTACCCGCTGAACTTAA
>AC3-9
TTTCCGTAGGTGAACCTGCGGAAGGATCATTATTGAATTATGTTTCTAGATAGGTTGTAG
CTGGCTC-TTTAGAGCATGTGCACGCCTGTTTGGACTTCATTTTCATCCACCTGTGCACC
TATTGTAGTCTTTGGTTGGGTTAGGAGGAAGTGGTCATTGTGTCAGCATCTGCTGGATGT
GAGGACTTGCATTGTGAAAGCTTTGCTGTCCTTGATGTGATCATGGAATCTCTTTC----

-------TCACTAGAGTCTATGTCACTCATTATACTCTGTCGAATGTCATTGAATGTCTT
TACATGGGCTTATATGCCTATGAAAATTGTAATACAACTTTCAGCAACGGATCTCTTGGC
TCTCGCATCGATGAAGAACGCAGCGAAATGCGATAAGTAATGTGAATTGCAGAATTCAGT
GAATCATCGAATCTTTGAACGCATCTTGCGCTCCTTGGTATTCCGAGGAGCATGCCTGTT
TGAGTGTCATTAAATTCTCAACTCTCTTCTAC-TTTTTGTAAAAGAGAGCTTGGACTGTG
GAGGCTTGCTGGCCACTTTTTGGGGTCAGCTCCTCTGAAATGCATTAGCGGAACCGTTTG
CGATCTGCCACAAGTGTGATAAGTTATCTACACTGGCGAGGGGATTGCTCTCTGTAATGT
TCAGCTTCTAATTGTCTCTACTTTGTGAGACTACTTTTGAATGCTTGACCTCAAATCAGG
TAGGACTACCCGCTGAACTTAA
>AC3-10
TTTCCGTAGGTGAACCTGCGGAAGGATCATTATTGAATTATGTTTCTAGATAGGTTGTAG
CTGGCTC-TTTAGAGCATGTGCACGCCTGTTTGGACTTCATTTTCATCCACCTGTGCACC
TATTGTAGTCTTTGGTTGGGTTAGGAGGAAGTGGTCATTGTGTCAGCATCTGCTGGATGT
GAGGACTTGCATTGTGAAAGCTTTGCTGTCCTTGATGTGATCATGGAATCTCTTTC----

-------TCACTAGAGTCTATGTCACTCATTATACTCTGTCGAATGTCATTGAATGTCTT
TACATGGGCTTATATGCCTATGAAAATTGTAATACAACTTTCAGCAACGGATCTCTTGGC
TCTCGCATCGATGAAGAACGCAGCGAAATGCGATAAGTAATGTGAATTGCAGAATTCAGT
GAATCATCGAATCTTTGAACGCATCTTGCGCTCCTTGGTATTCCGAGGAGCATGCCTGTT
TGAGTGTCATTAAATTCTCAACTCTCTTCTAC-TTTTTGTAAAAGAGAGCTTGGACTGTG
GAGGCTTGCTGGCCACTTTTTGGGGTCAGCTCCTCTGAAATGCATTAGCGGAACCGTTTG
CGATCTGCCACAAGTGTGATAAGTTATCTACACTGGCGAGGGGATTGCTCTCTGTAATGT
TCAGCTTCTAATTGTCTCTACTTTGTGAGACTACTTTTGAATGCTTGACCTCAAATCAGG
TAGGACTACCCGCTGAACTTAA
>AC3-23
TTTCCGTAGGTGAACCTGCGGAAGGATCATTATTGAATTATGTTTCTAGATAGGTTGTAG
CTGGCTC-TTTAGAGCATGTGCACGCCTGTTTGGACTTCATTTTCATCCACCTGTGCACC
TATTGTAGTCTTTGGTTGGGTTAGGAGGAAGTGGTCATTGTGTCAGCATCTGCTGGATGT
GAGGACTTGCATTGTGAAAGCTTTGCTGTCCTTGATGTGATCATGGAATCTCTTTC----

-------TCACTAGAGTCTATGTCACTCATTATACTCTGTCGAATGTCATTGAATGTCTT
TACATGGGCTTATATGCCTATGAAAATTGTAATACAACTTTCAGCAACGGATCTCTTGGC
TCTCGCATCGATGAAGAACGCAGCGAAATGCGATAAGTAATGTGAATTGCAGAATTCAGT
GAATCATCGAATCTTTGAACGCATCTTGCGCTCCTTGGTATTCCGAGGAGCATGCCTGTT
TGAGTGTCATTAAATTCTCAACTCTCTTCTAC-TTTTTGTAAAAGAGAGCTTGGACTGTG
GAGGCTTGCTGGCCACTTTTTGGGGTCAGCTCCTCTGAAATGCATTAGCGGAACCGTTTG
CGATCTGCCACAAGTGTGATAAGTTATCTACACTGGCGAGGGGATTGCTCTCTGTAATGT
TCAGCTTCTAATTGTCTCTACTTTGTGAGACTACTTTTGAATGCTTGACCTCAAATCAGG
TAGGACTACCCGCTGAACTTAA
>AC3-24
TTTCCGTAGGTGAACCTGCGGAAGGATCATTATTGAATTATGTTTCTAGATAGGTTGTAG
CTGGCTC-TTTAGAGCATGTGCACGCCTGTTTGGACTTCATTTTCATCCACCTGTGCACC
TATTGTAGTCTTTGGTTGGGTTAGGAGGAAGTGGTCATTGTGTCAGCATCTGCTGGATGT
GAGGACTTGCATTGTGAAAGCTTTGCTGTCCTTGATGTGATCATGGAATCTCTTTC----

-------TCACTAGAGTCTATGTCACTCATTATACTCTGTCGAATGTCATTGAATGTCTT
TACATGGGCTTATATGCCTATGAAAATTGTAATACAACTTTCAGCAACGGATCTCTTGGC
TCTCGCATCGATGAAGAACGCAGCGAAATGCGATAAGTAATGTGAATTGCAGAATTCAGT
GAATCATCGAATCTTTGAACGCATCTTGCGCTCCTTGGTATTCCGAGGAGCATGCCTGTT
TGAGTGTCATTAAATTCTCAACTCTCTTCTAC-TTTTTGTAAAAGAGAGCTTGGACTGTG
GAGGCTTGCTGGCCACTTTTTGGGGTCAGCTCCTCTGAAATGCATTAGCGGAACCGTTTG
CGATCTGCCACAAGTGTGATAAGTTATCTACACTGGCGAGGGGATTGCTCTCTGTAATGT
TCAGCTTCTAATTGTCTCTACTTTGTGAGACTACTTTTGAATGCTTGACCTCAAATCAGG
TAGGACTACCCGCTGAACTTAA
>AC3-33
TTTCCGTAGGTGAACCTGCGGAAGGATCATTATTGAATTATGTTTCTAGATAGGTTGTAG
CTGGCTC-TTTAGAGCATGTGCACGCCTGTTTGGACTTCATTTTCATCCACCTGTGCACC
TATTGTAGTCTTTGGTTGGGTTAGGAGGAAGTGGTCATTGTGTCAGCATCTGCTGGATGT
GAGGACTTGCATTGTGAAAGCTTTGCTGTCCTTGATGTGATCATGGAATCTCTTTC----

-------TCACTAGAGTCTATGTCACTCATTATACTCTGTCGAATGTCATTGAATGTCTT
TACATGGGCTTATATGCCTATGAAAATTGTAATACAACTTTCAGCAACGGATCTCTTGGC
TCTCGCATCGATGAAGAACGCAGCGAAATGCGATAAGTAATGTGAATTGCAGAATTCAGT
GAATCATCGAATCTTTGAACGCATCTTGCGCTCCTTGGTATTCCGAGGAGCATGCCTGTT
TGAGTGTCATTAAATTCTCAACTCTCTTCTAC-TTTTTGTAAAAGAGAGCTTGGACTGTG
GAGGCTTGCTGGCCACTTTTTGGGGTCAGCTCCTCTGAAATGCATTAGCGGAACCGTTTG
CGATCTGCCACAAGTGTGATAAGTTATCTACACTGGCGAGGGGATTGCTCTCTGTAATGT
TCAGCTTCTAATTGTCTCTACTTTGTGAGACTACTTTTGAATGCTTGACCTCAAATCAGG
TAGGACTACCCGCTGAACTTAA
>AC3-34
TTTCCGTAGGTGAACCTGCGGAAGGATCATTATTGAATTATGTTTCTAGATAGGTTGTAG
CTGGCTC-TTTAGAGCATGTGCACGCCTGTTTGGACTTCATTTTCATCCACCTGTGCACC
TATTGTAGTCTTTGGTTGGGTTAGGAGGAAGTGGTCATTGTGTCAGCATCTGCTGGATGT
GAGGACTTGCATTGTGAAAGCTTTGCTGTCCTTGATGTGATCATGGAATCTCTTTC----

-------TCACTAGAGTCTATGTCACTCATTATACTCTGTCGAATGTCATTGAATGTCTT
TACATGGGCTTATATGCCTATGAAAATTGTAATACAACTTTCAGCAACGGATCTCTTGGC
TCTCGCATCGATGAAGAACGCAGCGAAATGCGATAAGTAATGTGAATTGCAGAATTCAGT
GAATCATCGAATCTTTGAACGCATCTTGCGCTCCTTGGTATTCCGAGGAGCATGCCTGTT
TGAGTGTCATTAAATTCTCAACTCTCTTCTAC-TTTTTGTAAAAGAGAGCTTGGACTGTG
GAGGCTTGCTGGCCACTTTTTGGGGTCAGCTCCTCTGAAATGCATTAGCGGAACCGTTTG
CGATCTGCCACAAGTGTGATAAGTTATCTACACTGGCGAGGGGATTGCTCTCTGTAATGT
TCAGCTTCTAATTGTCTCTACTTTGTGAGACTACTTTTGAATGCTTGACCTCAAATCAGG
TAGGACTACCCGCTGAACTTAA
>AC3-38
TTTCCGTAGGTGAACCTGCGGAAGGATCATTATTGAATTATGTTTCTAGATAGGTTGTAG
CTGGCTC-TTTAGAGCATGTGCACGCCTGTTTGGACTTCATTTTCATCCACCTGTGCACC
TATTGTAGTCTTTGGTTGGGTTAGGAGGAAGTGGTCATTGTGTCAGCATCTGCTGGATGT
GAGGACTTGCATTGTGAAAGCTTTGCTGTCCTTGATGTGATCATGGAATCTCTTTC----

-------TCACTAGAGTCTATGTCACTCATTATACTCTGTCGAATGTCATTGAATGTCTT
TACATGGGCTTATATGCCTATGAAAATTGTAATACAACTTTCAGCAACGGATCTCTTGGC
TCTCGCATCGATGAAGAACGCAGCGAAATGCGATAAGTAATGTGAATTGCAGAATTCAGT
GAATCATCGAATCTTTGAACGCATCTTGCGCTCCTTGGTATTCCGAGGAGCATGCCTGTT
TGAGTGTCATTAAATTCTCAACTCTCTTCTAC-TTTTTGTAAAAGAGAGCTTGGACTGTG
GAGGCTTGCTGGCCACTTTTTGGGGTCAGCTCCTCTGAAATGCATTAGCGGAACCGTTTG
CGATCTGCCACAAGTGTGATAAGTTATCTACACTGGCGAGGGGATTGCTCTCTGTAATGT
TCAGCTTCTAATTGTCTCTACTTTGTGAGACTACTTTTGAATGCTTGACCTCAAATCAGG
TAGGACTACCCGCTGAACTTAA
>AC3-42
TTTCCGTAGGTGAACCTGCGGAAGGATCATTATTGAATTATGTTTCTAGATAGGTTGTAG
CTGGCTC-TTTAGAGCATGTGCACGCCTGTTTGGACTTCATTTTCATCCACCTGTGCACC
TATTGTAGTCTTTGGTTGGGTTAGGAGGAAGTGGTCATTGTGTCAGCATCTGCTGGATGT
GAGGACTTGCATTGTGAAAGCTTTGCTGTCCTTGATGTGATCATGGAATCTCTTTC----

-------TCACTAGAGTCTATGTCACTCATTATACTCTGTCGAATGTCATTGAATGTCTT
TACATGGGCTTATATGCCTATGAAAATTGTAATACAACTTTCAGCAACGGATCTCTTGGC
TCTCGCATCGATGAAGAACGCAGCGAAATGCGATAAGTAATGTGAATTGCAGAATTCAGT
GAATCATCGAATCTTTGAACGCATCTTGCGCTCCTTGGTATTCCGAGGAGCATGCCTGTT
TGAGTGTCATTAAATTCTCAACTCTCTTCTAC-TTTTTGTAAAAGAGAGCTTGGACTGTG
GAGGCTTGCTGGCCACTTTTTGGGGTCAGCTCCTCTGAAATGCATTAGCGGAACCGTTTG
CGATCTGCCACAAGTGTGATAAGTTATCTACACTGGCGAGGGGATTGCTCTCTGTAATGT
TCAGCTTCTAATTGTCTCTACTTTGTGAGACTACTTTTGAATGCTTGACCTCAAATCAGG
TAGGACTACCCGCTGAACTTAA
>AC3-43
TTTCCGTAGGTGAACCTGCGGAAGGATCATTATTGAATTATGTTTCTAGATAGGTTGTAG
CTGGCTC-TTTAGAGCATGTGCACGCCTGTTTGGACTTCATTTTCATCCACCTGTGCACC
TATTGTAGTCTTTGGTTGGGTTAGGAGGAAGTGGTCATTGTGTCAGCATCTGCTGGATGT
GAGGACTTGCATTGTGAAAGCTTTGCTGTCCTTGATGTGATCATGGAATCTCTTTC----

-------TCACTAGAGTCTATGTCACTCATTATACTCTGTCGAATGTCATTGAATGTCTT
TACATGGGCTTATATGCCTATGAAAATTGTAATACAACTTTCAGCAACGGATCTCTTGGC
TCTCGCATCGATGAAGAACGCAGCGAAATGCGATAAGTAATGTGAATTGCAGAATTCAGT
GAATCATCGAATCTTTGAACGCATCTTGCGCTCCTTGGTATTCCGAGGAGCATGCCTGTT
TGAGTGTCATTAAATTCTCAACTCTCTTCTAC-TTTTTGTAAAAGAGAGCTTGGACTGTG
GAGGCTTGCTGGCCACTTTTTGGGGTCAGCTCCTCTGAAATGCATTAGCGGAACCGTTTG
CGATCTGCCACAAGTGTGATAAGTTATCTACACTGGCGAGGGGATTGCTCTCTGTAATGT
TCAGCTTCTAATTGTCTCTACTTTGTGAGACTACTTTTGAATGCTTGACCTCAAATCAGG
TAGGACTACCCGCTGAACTTAA
>AC3-45
TTTCCGTAGGTGAACCTGCGGAAGGATCATTATTGAATTATGTTTCTAGATAGGTTGTAG
CTGGCTC-TTTAGAGCATGTGCACGCCTGTTTGGACTTCATTTTCATCCACCTGTGCACC
TATTGTAGTCTTTGGTTGGGTTAGGAGGAAGTGGTCATTGTGTCAGCATCTGCTGGATGT
GAGGACTTGCATTGTGAAAGCTTTGCTGTCCTTGATGTGATCATGGAATCTCTTTC----

```
------------------------------------------------------------
------------------------------------------------------------
------------------------------------------------------------
------------------------------------------------------------
------------------------------------------------------------
------------------------------------------------------------
------------------------------------------------------------
------------------------------------------------------------
------------------------------------------------------------
------------------------------------------------------------
------------------------------------------------------------
-------TCACTAGAGTCTATGTCACTCATTATACTCTGTCGAATGTCATTGAATGTCTT
TACATGGGCTTATATGCCTATGAAAATTGTAATACAACTTTCAGCAACGGATCTCTTGGC
TCTCGCATCGATGAAGAACGCAGCGAAATGCGATAAGTAATGTGAATTGCAGAATTCAGT
GAATCATCGAATCTTTGAACGCATCTTGCGCTCCTTGGTATTCCGAGGAGCATGCCTGTT
TGAGTGTCATTAAATTCTCAACTCTCTTCTAC-TTTTTGTAAAAGAGAGCTTGGACTGTG
GAGGCTTGCTGGCCACTTTTTGGGGTCAGCTCCTCTGAAATGCATTAGCGGAACCGTTTG
CGATCTGCCACAAGTGTGATAAGTTATCTACACTGGCGAGGGGATTGCTCTCTGTAATGT
TCAGCTTCTAATTGTCTCTACTTTGTGAGACTACTTTTGAATGCTTGACCTCAAATCAGG
TAGGACTACCCGCTGAACTTAA
>AC3-48
TTTCCGTAGGTGAACCTGCGGAAGGATCATTATTGAATTATGTTTCTAGATAGGTTGTAG
CTGGCTC-TTTAGAGCATGTGCACGCCTGTTTGGACTTCATTTTCATCCACCTGTGCACC
TATTGTAGTCTTTGGTTGGGTTAGGAGGAAGTGGTCATTGTGTCAGCATCTGCTGGATGT
GAGGACTTGCATTGTGAAAGCTTTGCTGTCCTTGATGTGATCATGGAATCTCTTTC----
------------------------------------------------------------
------------------------------------------------------------
------------------------------------------------------------
------------------------------------------------------------
------------------------------------------------------------
------------------------------------------------------------
------------------------------------------------------------
------------------------------------------------------------
------------------------------------------------------------
------------------------------------------------------------
------------------------------------------------------------
------------------------------------------------------------
-------TCACTAGAGTCTATGTCACTCATTATACTCTGTCGAATGTCATTGAATGTCTT
TACATGGGCTTATATGCCTATGAAAATTGTAATACAACTTTCAGCAACGGATCTCTTGGC
TCTCGCATCGATGAAGAACGCAGCGAAATGCGATAAGTAATGTGAATTGCAGAATTCAGT
GAATCATCGAATCTTTGAACGCATCTTGCGCTCCTTGGTATTCCGAGGAGCATGCCTGTT
TGAGTGTCATTAAATTCTCAACTCTCTTCTAC-TTTTTGTAAAAGAGAGCTTGGACTGTG
GAGGCTTGCTGGCCACTTTTTGGGGTCAGCTCCTCTGAAATGCATTAGCGGAACCGTTTG
CGATCTGCCACAAGTGTGATAAGTTATCTACACTGGCGAGGGGATTGCTCTCTGTAATGT
TCAGCTTCTAATTGTCTCTACTTTGTGAGACTACTTTTGAATGCTTGACCTCAAATCAGG
TAGGACTACCCGCTGAACTTAA
>AC3-49
TTTCCGTAGGTGAACCTGCGGAAGGATCATTATTGAATTATGTTTCTAGATAGGTTGTAG
CTGGCTC-TTTAGAGCATGTGCACGCCTGTTTGGACTTCATTTTCATCCACCTGTGCACC
TATTGTAGTCTTTGGTTGGGTTAGGAGGAAGTGGTCATTGTGTCAGCATCTGCTGGATGT
```

GAGGACTTGCATTGTGAAAGCTTTGCTGTCCTTGATGTGATCATGGAATCTCTTTC----

-------TCACTAGAGTCTATGTCACTCATTATACTCTGTCGAATGTCATTGAATGTCTT
TACATGGGCTTATATGCCTATGAAAATTGTAATACAACTTTCAGCAACGGATCTCTTGGC
TCTCGCATCGATGAAGAACGCAGCGAAATGCGATAAGTAATGTGAATTGCAGAATTCAGT
GAATCATCGAATCTTTGAACGCATCTTGCGCTCCTTGGTATTCCGAGGAGCATGCCTGTT
TGAGTGTCATTAAATTCTCAACTCTCTTCTAC-TTTTTGTAAAAGAGAGCTTGGACTGTG
GAGGCTTGCTGGCCACTTTTTGGGGTCAGCTCCTCTGAAATGCATTAGCGGAACCGTTTG
CGATCTGCCACAAGTGTGATAAGTTATCTACACTGGCGAGGGGATTGCTCTCTGTAATGT
TCAGCTTCTAATTGTCTCTACTTTGTGAGACTACTTTTGAATGCTTGACCTCAAATCAGG
TAGGACTACCCGCTGAACTTAA
>AC3-54
TTTCCGTAGGTGAACCTGCGGAAGGATCATTATTGAATTATGTTTCTAGATAGGTTGTAG
CTGGCTC-TTTAGAGCATGTGCACGCCTGTTTGGACTTCATTTTCATCCACCTGTGCACC
TATTGTAGTCTTTGGTTGGGTTAGGAGGAAGTGGTCATTGTGTCAGCATCTGCTGGATGT
GAGGACTTGCATTGTGAAAGCTTTGCTGTCCTTGATGTGATCATGGAATCTCTTTC----

-------TCACTAGAGTCTATGTCACTCATTATACTCTGTCGAATGTCATTGAATGTCTT
TACATGGGCTTATATGCCTATGAAAATTGTAATACAACTTTCAGCAACGGATCTCTTGGC
TCTCGCATCGATGAAGAACGCAGCGAAATGCGATAAGTAATGTGAATTGCAGAATTCAGT
GAATCATCGAATCTTTGAACGCATCTTGCGCTCCTTGGTATTCCGAGGAGCATGCCTGTT
TGAGTGTCATTAAATTCTCAACTCTCTTCTAC-TTTTTGTAAAAGAGAGCTTGGACTGTG
GAGGCTTGCTGGCCACTTTTTGGGGTCAGCTCCTCTGAAATGCATTAGCGGAACCGTTTG
CGATCTGCCACAAGTGTGATAAGTTATCTACACTGGCGAGGGGATTGCTCTCTGTAATGT
TCAGCTTCTAATTGTCTCTACTTTGTGAGACTACTTTTGAATGCTTGACCTCAAATCAGG
TAGGACTACCCGCTGAACTTAA
>AC3-58
TTTCCGTAGGTGAACCTGCGGAAGGATCATTATTGAATTATGTTTCTAGATAGGTTGTAG

```
CTGGCTC-TTTAGAGCATGTGCACGCCTGTTTGGACTTCATTTTCATCCACCTGTGCACC
TATTGTAGTCTTTGGTTGGGTTAGGAGGAAGTGGTCATTGTGTCAGCATCTGCTGGATGT
GAGGACTTGCATTGTGAAAGCTTTGCTGTCCTTGATGTGATCATGGAATCTCTTTC----
------------------------------------------------------------
------------------------------------------------------------
------------------------------------------------------------
------------------------------------------------------------
------------------------------------------------------------
------------------------------------------------------------
------------------------------------------------------------
------------------------------------------------------------
------------------------------------------------------------
------------------------------------------------------------
------------------------------------------------------------
------------------------------------------------------------
-------TCACTAGAGTCTATGTCACTCATTATACTCTGTCGAATGTCATTGAATGTCTT
TACATGGGCTTATATGCCTATGAAAATTGTAATACAACTTTCAGCAACGGATCTCTTGGC
TCTCGCATCGATGAAGAACGCAGCGAAATGCGATAAGTAATGTGAATTGCAGAATTCAGT
GAATCATCGAATCTTTGAACGCATCTTGCGCTCCTTGGTATTCCGAGGAGCATGCCTGTT
TGAGTGTCATTAAATTCTCAACTCTCTTCTAC-TTTTTGTAAAAGAGAGCTTGGACTGTG
GAGGCTTGCTGGCCACTTTTTGGGGTCAGCTCCTCTGAAATGCATTAGCGGAACCGTTTG
CGATCTGCCACAAGTGTGATAAGTTATCTACACTGGCGAGGGGATTGCTCTCTGTAATGT
TCAGCTTCTAATTGTCTCTACTTTGTGAGACTACTTTTGAATGCTTGACCTCAAATCAGG
TAGGACTACCCGCTGAACTTAA
>AC3-59
TTTCCGTAGGTGAACCTGCGGAAGGATCATTATTGAATTATGTTTCTAGATAGGTTGTAG
CTGGCTC-TTTAGAGCATGTGCACGCCTGTTTGGACTTCATTTTCATCCACCTGTGCACC
TATTGTAGTCTTTGGTTGGGTTAGGAGGAAGTGGTCATTGTGTCAGCATCTGCTGGATGT
GAGGACTTGCATTGTGAAAGCTTTGCTGTCCTTGATGTGATCATGGAATCTCTTTC----
------------------------------------------------------------
------------------------------------------------------------
------------------------------------------------------------
------------------------------------------------------------
------------------------------------------------------------
------------------------------------------------------------
------------------------------------------------------------
------------------------------------------------------------
------------------------------------------------------------
------------------------------------------------------------
------------------------------------------------------------
------------------------------------------------------------
-------TCACTAGAGTCTATGTCACTCATTATACTCTGTCGAATGTCATTGAATGTCTT
TACATGGGCTTATATGCCTATGAAAATTGTAATACAACTTTCAGCAACGGATCTCTTGGC
TCTCGCATCGATGAAGAACGCAGCGAAATGCGATAAGTAATGTGAATTGCAGAATTCAGT
GAATCATCGAATCTTTGAACGCATCTTGCGCTCCTTGGTATTCCGAGGAGCATGCCTGTT
TGAGTGTCATTAAATTCTCAACTCTCTTCTAC-TTTTTGTAAAAGAGAGCTTGGACTGTG
GAGGCTTGCTGGCCACTTTTTGGGGTCAGCTCCTCTGAAATGCATTAGCGGAACCGTTTG
CGATCTGCCACAAGTGTGATAAGTTATCTACACTGGCGAGGGGATTGCTCTCTGTAATGT
TCAGCTTCTAATTGTCTCTACTTTGTGAGACTACTTTTGAATGCTTGACCTCAAATCAGG
TAGGACTACCCGCTGAACTTAA
```

```
>AC3-60
TTTCCGTAGGTGAACCTGCGGAAGGATCATTATTGAATTATGTTTCTAGATAGGTTGTAG
CTGGCTC-TTTAGAGCATGTGCACGCCTGTTTGGACTTCATTTTCATCCACCTGTGCACC
TATTGTAGTCTTTGGTTGGGTTAGGAGGAAGTGGTCATTGTGTCAGCATCTGCTGGATGT
GAGGACTTGCATTGTGAAAGCTTTGCTGTCCTTGATGTGATCATGGAATCTCTTTC----
------------------------------------------------------------
------------------------------------------------------------
------------------------------------------------------------
------------------------------------------------------------
------------------------------------------------------------
------------------------------------------------------------
------------------------------------------------------------
------------------------------------------------------------
------------------------------------------------------------
------------------------------------------------------------
------------------------------------------------------------
------------------------------------------------------------
-------TCACTAGAGTCTATGTCACTCATTATACTCTGTCGAATGTCATTGAATGTCTT
TACATGGGCTTATATGCCTATGAAAATTGTAATACAACTTTCAGCAACGGATCTCTTGGC
TCTCGCATCGATGAAGAACGCAGCGAAATGCGATAAGTAATGTGAATTGCAGAATTCAGT
GAATCATCGAATCTTTGAACGCATCTTGCGCTCCTTGGTATTCCGAGGAGCATGCCTGTT
TGAGTGTCATTAAATTCTCAACTCTCTTCTAC-TTTTTGTAAAAGAGAGCTTGGACTGTG
GAGGCTTGCTGGCCACTTTTTGGGGTCAGCTCCTCTGAAATGCATTAGCGGAACCGTTTG
CGATCTGCCACAAGTGTGATAAGTTATCTACACTGGCGAGGGGATTGCTCTCTGTAATGT
TCAGCTTCTAATTGTCTCTACTTTGTGAGACTACTTTTGAATGCTTGACCTCAAATCAGG
TAGGACTACCCGCTGAACTTAA
>AC3-61
TTTCCGTAGGTGAACCTGCGGAAGGATCATTATTGAATTATGTTTCTAGATAGGTTGTAG
CTGGCTC-TTTAGAGCATGTGCACGCCTGTTTGGACTTCATTTTCATCCACCTGTGCACC
TATTGTAGTCTTTGGTTGGGTTAGGAGGAAGTGGTCATTGTGTCAGCATCTGCTGGATGT
GAGGACTTGCATTGTGAAAGCTTTGCTGTCCTTGATGTGATCATGGAATCTCTTTC----
------------------------------------------------------------
------------------------------------------------------------
------------------------------------------------------------
------------------------------------------------------------
------------------------------------------------------------
------------------------------------------------------------
------------------------------------------------------------
------------------------------------------------------------
------------------------------------------------------------
------------------------------------------------------------
------------------------------------------------------------
------------------------------------------------------------
-------TCACTAGAGTCTATGTCACTCATTATACTCTGTCGAATGTCATTGAATGTCTT
TACATGGGCTTATATGCCTATGAAAATTGTAATACAACTTTCAGCAACGGATCTCTTGGC
TCTCGCATCGATGAAGAACGCAGCGAAATGCGATAAGTAATGTGAATTGCAGAATTCAGT
GAATCATCGAATCTTTGAACGCATCTTGCGCTCCTTGGTATTCCGAGGAGCATGCCTGTT
TGAGTGTCATTAAATTCTCAACTCTCTTCTAC-TTTTTGTAAAAGAGAGCTTGGACTGTG
GAGGCTTGCTGGCCACTTTTTGGGGTCAGCTCCTCTGAAATGCATTAGCGGAACCGTTTG
CGATCTGCCACAAGTGTGATAAGTTATCTACACTGGCGAGGGGATTGCTCTCTGTAATGT
```

TCAGCTTCTAATTGTCTCTACTTTGTGAGACTACTTTTGAATGCTTGACCTCAAATCAGG
TAGGACTACCCGCTGAACTTAA
>AC3-65
TTTCCGTAGGTGAACCTGCGGAAGGATCATTATTGAATTATGTTTCTAGATAGGTTGTAG
CTGGCTC-TTTAGAGCATGTGCACGCCTGTTTGGACTTCATTTTCATCCACCTGTGCACC
TATTGTAGTCTTTGGTTGGGTTAGGAGGAAGTGGTCATTGTGTCAGCATCTGCTGGATGT
GAGGACTTGCATTGTGAAAGCTTTGCTGTCCTTGATGTGATCATGGAATCTCTTTC----

-------TCACTAGAGTCTATGTCACTCATTATACTCTGTCGAATGTCATTGAATGTCTT
TACATGGGCTTATATGCCTATGAAAATTGTAATACAACTTTCAGCAACGGATCTCTTGGC
TCTCGCATCGATGAAGAACGCAGCGAAATGCGATAAGTAATGTGAATTGCAGAATTCAGT
GAATCATCGAATCTTTGAACGCATCTTGCGCTCCTTGGTATTCCGAGGAGCATGCCTGTT
TGAGTGTCATTAAATTCTCAACTCTCTTCTAC-TTTTTGTAAAAGAGAGCTTGGACTGTG
GAGGCTTGCTGGCCACTTTTTGGGGTCAGCTCCTCTGAAATGCATTAGCGGAACCGTTTG
CGATCTGCCACAAGTGTGATAAGTTATCTACACTGGCGAGGGGATTGCTCTCTGTAATGT
TCAGCTTCTAATTGTCTCTACTTTGTGAGACTACTTTTGAATGCTTGACCTCAAATCAGG
TAGGACTACCCGCTGAACTTAA
>AC3-69
TTTCCGTAGGTGAACCTGCGGAAGGATCATTATTGAATTATGTTTCTAGATAGGTTGTAG
CTGGCTC-TTTAGAGCATGTGCACGCCTGTTTGGACTTCATTTTCATCCACCTGTGCACC
TATTGTAGTCTTTGGTTGGGTTAGGAGGAAGTGGTCATTGTGTCAGCATCTGCTGGATGT
GAGGACTTGCATTGTGAAAGCTTTGCTGTCCTTGATGTGATCATGGAATCTCTTTC----

-------TCACTAGAGTCTATGTCACTCATTATACTCTGTCGAATGTCATTGAATGTCTT
TACATGGGCTTATATGCCTATGAAAATTGTAATACAACTTTCAGCAACGGATCTCTTGGC
TCTCGCATCGATGAAGAACGCAGCGAAATGCGATAAGTAATGTGAATTGCAGAATTCAGT
GAATCATCGAATCTTTGAACGCATCTTGCGCTCCTTGGTATTCCGAGGAGCATGCCTGTT
TGAGTGTCATTAAATTCTCAACTCTCTTCTAC-TTTTTGTAAAAGAGAGCTTGGACTGTG

```
GAGGCTTGCTGGCCACTTTTTGGGGTCAGCTCCTCTGAAATGCATTAGCGGAACCGTTTG
CGATCTGCCACAAGTGTGATAAGTTATCTACACTGGCGAGGGGATTGCTCTCTGTAATGT
TCAGCTTCTAATTGTCTCTACTTTGTGAGACTACTTTTGAATGCTTGACCTCAAATCAGG
TAGGACTACCCGCTGAACTTAA
>AC3-71
TTTCCGTAGGTGAACCTGCGGAAGGATCATTATTGAATTATGTTTCTAGATAGGTTGTAG
CTGGCTC-TTTAGAGCATGTGCACGCCTGTTTGGACTTCATTTTCATCCACCTGTGCACC
TATTGTAGTCTTTGGTTGGGTTAGGAGGAAGTGGTCATTGTGTCAGCATCTGCTGGATGT
GAGGACTTGCATTGTGAAAGCTTTGCTGTCCTTGATGTGATCATGGAATCTCTTTC----
------------------------------------------------------------
------------------------------------------------------------
------------------------------------------------------------
------------------------------------------------------------
------------------------------------------------------------
------------------------------------------------------------
------------------------------------------------------------
------------------------------------------------------------
------------------------------------------------------------
------------------------------------------------------------
------------------------------------------------------------
------------------------------------------------------------
-------TCACTAGAGTCTATGTCACTCATTATACTCTGTCGAATGTCATTGAATGTCTT
TACATGGGCTTATATGCCTATGAAAATTGTAATACAACTTTCAGCAACGGATCTCTTGGC
TCTCGCATCGATGAAGAACGCAGCGAAATGCGATAAGTAATGTGAATTGCAGAATTCAGT
GAATCATCGAATCTTTGAACGCATCTTGCGCTCCTTGGTATTCCGAGGAGCATGCCTGTT
TGAGTGTCATTAAATTCTCAACTCTCTTCTAC-TTTTTGTAAAAGAGAGCTTGGACTGTG
GAGGCTTGCTGGCCACTTTTTGGGGTCAGCTCCTCTGAAATGCATTAGCGGAACCGTTTG
CGATCTGCCACAAGTGTGATAAGTTATCTACACTGGCGAGGGGATTGCTCTCTGTAATGT
TCAGCTTCTAATTGTCTCTACTTTGTGAGACTACTTTTGAATGCTTGACCTCAAATCAGG
TAGGACTACCCGCTGAACTTAA
>AC4-3
TTTCCGTAGGTGAACCTGCGGAAGGATCATTATTGAATTATGTTTCTAGATAGGTTGTAG
CTGGCTC-TTTAGAGCATGTGCACGCCTGTTTGGACTTCATTTTCATCCACCTGTGCACC
TATTGTAGTCTTTGGTTGGGTTAGGAGGAAGTGGTCATTGTGTCAGCATCTGCTGGATGT
GAGGACTTGCATTGTGAAAGCTTTGCTGTCCTTGATGTGATCATGGAATCTCTTTC----
------------------------------------------------------------
------------------------------------------------------------
------------------------------------------------------------
------------------------------------------------------------
------------------------------------------------------------
------------------------------------------------------------
------------------------------------------------------------
------------------------------------------------------------
------------------------------------------------------------
------------------------------------------------------------
------------------------------------------------------------
------------------------------------------------------------
-------TCACTAGAGTCTATGTCACTCATTATACTCTGTCGAATGTCATTGAATGTCTT
TACATGGGCTTATATGCCTATGAAAATTGTAATACAACTTTCAGCAACGGATCTCTTGGC
TCTCGCATCGATGAAGAACGCAGCGAAATGCGATAAGTAATGTGAATTGCAGAATTCAGT
```

GAATCATCGAATCTTTGAACGCATCTTGCGCTCCTTGGTATTCCGAGGAGCATGCCTGTT
TGAGTGTCATTAAATTCTCAACTCTCTTCTAC-TTTTTGTAAAAGAGAGCTTGGACTGTG
GAGGCTTGCTGGCCACTTTTTGGGGTCAGCTCCTCTGAAATGCATTAGCGGAACCGTTTG
CGATCTGCCACAAGTGTGATAAGTTATCTACACTGGCGAGGGGATTGCTCTCTGTAATGT
TCAGCTTCTAATTGTCTCTACTTTGTGAGACTACTTTTGAATGCTTGACCTCAAATCAGG
TAGGACTACCCGCTGAACTTAA
>AC4-7
TTTCCGTAGGTGAACCTGCGGAAGGATCATTATTGAATTATGTTTCTAGATAGGTTGTAG
CTGGCTC-TTTAGAGCATGTGCACGCCTGTTTGGACTTCATTTTCATCCACCTGTGCACC
TATTGTAGTCTTTGGTTGGGTTAGGAGGAAGTGGTCATTGTGTCAGCATCTGCTGGATGT
GAGGACTTGCATTGTGAAAGCTTTGCTGTCCTTGATGTGATCATGGAATCTCTTTC----

-------TCACTAGAGTCTATGTCACTCATTATACTCTGTCGAATGTCATTGAATGTCTT
TACATGGGCTTATATGCCTATGAAAATTGTAATACAACTTTCAGCAACGGATCTCTTGGC
TCTCGCATCGATGAAGAACGCAGCGAAATGCGATAAGTAATGTGAATTGCAGAATTCAGT
GAATCATCGAATCTTTGAACGCATCTTGCGCTCCTTGGTATTCCGAGGAGCATGCCTGTT
TGAGTGTCATTAAATTCTCAACTCTCTTCTAC-TTTTTGTAAAAGAGAGCTTGGACTGTG
GAGGCTTGCTGGCCACTTTTTGGGGTCAGCTCCTCTGAAATGCATTAGCGGAACCGTTTG
CGATCTGCCACAAGTGTGATAAGTTATCTACACTGGCGAGGGGATTGCTCTCTGTAATGT
TCAGCTTCTAATTGTCTCTACTTTGTGAGACTACTTTTGAATGCTTGACCTCAAATCAGG
TAGGACTACCCGCTGAACTTAA
>AC4-8
TTTCCGTAGGTGAACCTGCGGAAGGATCATTATTGAATTATGTTTCTAGATAGGTTGTAG
CTGGCTC-TTTAGAGCATGTGCACGCCTGTTTGGACTTCATTTTCATCCACCTGTGCACC
TATTGTAGTCTTTGGTTGGGTTAGGAGGAAGTGGTCATTGTGTCAGCATCTGCTGGATGT
GAGGACTTGCATTGTGAAAGCTTTGCTGTCCTTGATGTGATCATGGAATCTCTTTC----

-------TCACTAGAGTCTATGTCACTCATTATACTCTGTCGAATGTCATTGAATGTCTT

TACATGGGCTTATATGCCTATGAAAATTGTAATACAACTTTCAGCAACGGATCTCTTGGC
TCTCGCATCGATGAAGAACGCAGCGAAATGCGATAAGTAATGTGAATTGCAGAATTCAGT
GAATCATCGAATCTTTGAACGCATCTTGCGCTCCTTGGTATTCCGAGGAGCATGCCTGTT
TGAGTGTCATTAAATTCTCAACTCTCTTCTAC-TTTTTGTAAAAGAGAGCTTGGACTGTG
GAGGCTTGCTGGCCACTTTTTGGGGTCAGCTCCTCTGAAATGCATTAGCGGAACCGTTTG
CGATCTGCCACAAGTGTGATAAGTTATCTACACTGGCGAGGGGATTGCTCTCTGTAATGT
TCAGCTTCTAATTGTCTCTACTTTGTGAGACTACTTTTGAATGCTTGACCTCAAATCAGG
TAGGACTACCCGCTGAACTTAA
>AC4-24
TTTCCGTAGGTGAACCTGCGGAAGGATCATTATTGAATTATGTTTCTAGATAGGTTGTAG
CTGGCTC-TTTAGAGCATGTGCACGCCTGTTTGGACTTCATTTTCATCCACCTGTGCACC
TATTGTAGTCTTTGGTTGGGTTAGGAGGAAGTGGTCATTGTGTCAGCATCTGCTGGATGT
GAGGACTTGCATTGTGAAAGCTTTGCTGTCCTTGATGTGATCATGGAATCTCTTTC----

-------TCACTAGAGTCTATGTCACTCATTATACTCTGTCGAATGTCATTGAATGTCTT
TACATGGGCTTATATGCCTATGAAAATTGTAATACAACTTTCAGCAACGGATCTCTTGGC
TCTCGCATCGATGAAGAACGCAGCGAAATGCGATAAGTAATGTGAATTGCAGAATTCAGT
GAATCATCGAATCTTTGAACGCATCTTGCGCTCCTTGGTATTCCGAGGAGCATGCCTGTT
TGAGTGTCATTAAATTCTCAACTCTCTTCTAC-TTTTTGTAAAAGAGAGCTTGGACTGTG
GAGGCTTGCTGGCCACTTTTTGGGGTCAGCTCCTCTGAAATGCATTAGCGGAACCGTTTG
CGATCTGCCACAAGTGTGATAAGTTATCTACACTGGCGAGGGGATTGCTCTCTGTAATGT
TCAGCTTCTAATTGTCTCTACTTTGTGAGACTACTTTTGAATGCTTGACCTCAAATCAGG
TAGGACTACCCGCTGAACTTAA
>AC4-30
TTTCCGTAGGTGAACCTGCGGAAGGATCATTATTGAATTATGTTTCTAGATAGGTTGTAG
CTGGCTC-TTTAGAGCATGTGCACGCCTGTTTGGACTTCATTTTCATCCACCTGTGCACC
TATTGTAGTCTTTGGTTGGGTTAGGAGGAAGTGGTCATTGTGTCAGCATCTGCTGGATGT
GAGGACTTGCATTGTGAAAGCTTTGCTGTCCTTGATGTGATCATGGAATCTCTTTC----

-------TCACTAGAGTCTATGTCACTCATTATACTCTGTCGAATGTCATTGAATGTCTT
TACATGGGCTTATATGCCTATGAAAATTGTAATACAACTTTCAGCAACGGATCTCTTGGC
TCTCGCATCGATGAAGAACGCAGCGAAATGCGATAAGTAATGTGAATTGCAGAATTCAGT
GAATCATCGAATCTTTGAACGCATCTTGCGCTCCTTGGTATTCCGAGGAGCATGCCTGTT
TGAGTGTCATTAAATTCTCAACTCTCTTCTAC-TTTTTGTAAAAGAGAGCTTGGACTGTG
GAGGCTTGCTGGCCACTTTTTGGGGTCAGCTCCTCTGAAATGCATTAGCGGAACCGTTTG
CGATCTGCCACAAGTGTGATAAGTTATCTACACTGGCGAGGGGATTGCTCTCTGTAATGT
TCAGCTTCTAATTGTCTCTACTTTGTGAGACTACTTTTGAATGCTTGACCTCAAATCAGG
TAGGACTACCCGCTGAACTTAA
>AC4-35
TTTCCGTAGGTGAACCTGCGGAAGGATCATTATTGAATTATGTTTCTAGATAGGTTGTAG
CTGGCTC-TTTAGAGCATGTGCACGCCTGTTTGGACTTCATTTTCATCCACCTGTGCACC
TATTGTAGTCTTTGGTTGGGTTAGGAGGAAGTGGTCATTGTGTCAGCATCTGCTGGATGT
GAGGACTTGCATTGTGAAAGCTTTGCTGTCCTTGATGTGATCATGGAATCTCTTTC----

-------TCACTAGAGTCTATGTCACTCATTATACTCTGTCGAATGTCATTGAATGTCTT
TACATGGGCTTATATGCCTATGAAAATTGTAATACAACTTTCAGCAACGGATCTCTTGGC
TCTCGCATCGATGAAGAACGCAGCGAAATGCGATAAGTAATGTGAATTGCAGAATTCAGT
GAATCATCGAATCTTTGAACGCATCTTGCGCTCCTTGGTATTCCGAGGAGCATGCCTGTT
TGAGTGTCATTAAATTCTCAACTCTCTTCTAC-TTTTTGTAAAAGAGAGCTTGGACTGTG
GAGGCTTGCTGGCCACTTTTTGGGGTCAGCTCCTCTGAAATGCATTAGCGGAACCGTTTG
CGATCTGCCACAAGTGTGATAAGTTATCTACACTGGCGAGGGGATTGCTCTCTGTAATGT
TCAGCTTCTAATTGTCTCTACTTTGTGAGACTACTTTTGAATGCTTGACCTCAAATCAGG
TAGGACTACCCGCTGAACTTAA
>AC4-40
TTTCCGTAGGTGAACCTGCGGAAGGATCATTATTGAATTATGTTTCTAGATAGGTTGTAG
CTGGCTC-TTTAGAGCATGTGCACGCCTGTTTGGACTTCATTTTCATCCACCTGTGCACC
TATTGTAGTCTTTGGTTGGGTTAGGAGGAAGTGGTCATTGTGTCAGCATCTGCTGGATGT
GAGGACTTGCATTGTGAAAGCTTTGCTGTCCTTGATGTGATCATGGAATCTCTTTC----

-------TCACTAGAGTCTATGTCACTCATTATACTCTGTCGAATGTCATTGAATGTCTT
TACATGGGCTTATATGCCTATGAAAATTGTAATACAACTTTCAGCAACGGATCTCTTGGC
TCTCGCATCGATGAAGAACGCAGCGAAATGCGATAAGTAATGTGAATTGCAGAATTCAGT
GAATCATCGAATCTTTGAACGCATCTTGCGCTCCTTGGTATTCCGAGGAGCATGCCTGTT
TGAGTGTCATTAAATTCTCAACTCTCTTCTAC-TTTTTGTAAAAGAGAGCTTGGACTGTG
GAGGCTTGCTGGCCACTTTTTGGGGTCAGCTCCTCTGAAATGCATTAGCGGAACCGTTTG
CGATCTGCCACAAGTGTGATAAGTTATCTACACTGGCGAGGGGATTGCTCTCTGTAATGT
TCAGCTTCTAATTGTCTCTACTTTGTGAGACTACTTTTGAATGCTTGACCTCAAATCAGG
TAGGACTACCCGCTGAACTTAA
>AC4-41
TTTCCGTAGGTGAACCTGCGGAAGGATCATTATTGAATTATGTTTCTAGATAGGTTGTAG
CTGGCTC-TTTAGAGCATGTGCACGCCTGTTTGGACTTCATTTTCATCCACCTGTGCACC
TATTGTAGTCTTTGGTTGGGTTAGGAGGAAGTGGTCATTGTGTCAGCATCTGCTGGATGT
GAGGACTTGCATTGTGAAAGCTTTGCTGTCCTTGATGTGATCATGGAATCTCTTTC----

-------TCACTAGAGTCTATGTCACTCATTATACTCTGTCGAATGTCATTGAATGTCTT
TACATGGGCTTATATGCCTATGAAAATTGTAATACAACTTTCAGCAACGGATCTCTTGGC
TCTCGCATCGATGAAGAACGCAGCGAAATGCGATAAGTAATGTGAATTGCAGAATTCAGT
GAATCATCGAATCTTTGAACGCATCTTGCGCTCCTTGGTATTCCGAGGAGCATGCCTGTT
TGAGTGTCATTAAATTCTCAACTCTCTTCTAC-TTTTTGTAAAAGAGAGCTTGGACTGTG
GAGGCTTGCTGGCCACTTTTTGGGGTCAGCTCCTCTGAAATGCATTAGCGGAACCGTTTG
CGATCTGCCACAAGTGTGATAAGTTATCTACACTGGCGAGGGGATTGCTCTCTGTAATGT
TCAGCTTCTAATTGTCTCTACTTTGTGAGACTACTTTTGAATGCTTGACCTCAAATCAGG
TAGGACTACCCGCTGAACTTAA
>AC4-45
TTTCCGTAGGTGAACCTGCGGAAGGATCATTATTGAATTATGTTTCTAGATAGGTTGTAG
CTGGCTC-TTTAGAGCATGTGCACGCCTGTTTGGACTTCATTTTCATCCACCTGTGCACC
TATTGTAGTCTTTGGTTGGGTTAGGAGGAAGTGGTCATTGTGTCAGCATCTGCTGGATGT
GAGGACTTGCATTGTGAAAGCTTTGCTGTCCTTGATGTGATCATGGAATCTCTTTC----

-------TCACTAGAGTCTATGTCACTCATTATACTCTGTCGAATGTCATTGAATGTCTT
TACATGGGCTTATATGCCTATGAAAATTGTAATACAACTTTCAGCAACGGATCTCTTGGC
TCTCGCATCGATGAAGAACGCAGCGAAATGCGATAAGTAATGTGAATTGCAGAATTCAGT
GAATCATCGAATCTTTGAACGCATCTTGCGCTCCTTGGTATTCCGAGGAGCATGCCTGTT
TGAGTGTCATTAAATTCTCAACTCTCTTCTAC-TTTTTGTAAAAGAGAGCTTGGACTGTG
GAGGCTTGCTGGCCACTTTTTGGGGTCAGCTCCTCTGAAATGCATTAGCGGAACCGTTTG
CGATCTGCCACAAGTGTGATAAGTTATCTACACTGGCGAGGGGATTGCTCTCTGTAATGT
TCAGCTTCTAATTGTCTCTACTTTGTGAGACTACTTTTGAATGCTTGACCTCAAATCAGG
TAGGACTACCCGCTGAACTTAA
>AC4-49
TTTCCGTAGGTGAACCTGCGGAAGGATCATTATTGAATTATGTTTCTAGATAGGTTGTAG
CTGGCTC-TTTAGAGCATGTGCACGCCTGTTTGGACTTCATTTTCATCCACCTGTGCACC
TATTGTAGTCTTTGGTTGGGTTAGGAGGAAGTGGTCATTGTGTCAGCATCTGCTGGATGT
GAGGACTTGCATTGTGAAAGCTTTGCTGTCCTTGATGTGATCATGGAATCTCTTTC----

-------TCACTAGAGTCTATGTCACTCATTATACTCTGTCGAATGTCATTGAATGTCTT
TACATGGGCTTATATGCCTATGAAAATTGTAATACAACTTTCAGCAACGGATCTCTTGGC
TCTCGCATCGATGAAGAACGCAGCGAAATGCGATAAGTAATGTGAATTGCAGAATTCAGT
GAATCATCGAATCTTTGAACGCATCTTGCGCTCCTTGGTATTCCGAGGAGCATGCCTGTT
TGAGTGTCATTAAATTCTCAACTCTCTTCTAC-TTTTTGTAAAAGAGAGCTTGGACTGTG
GAGGCTTGCTGGCCACTTTTTGGGGTCAGCTCCTCTGAAATGCATTAGCGGAACCGTTTG
CGATCTGCCACAAGTGTGATAAGTTATCTACACTGGCGAGGGGATTGCTCTCTGTAATGT
TCAGCTTCTAATTGTCTCTACTTTGTGAGACTACTTTTGAATGCTTGACCTCAAATCAGG
TAGGACTACCCGCTGAACTTAA
>AC4-50
TTTCCGTAGGTGAACCTGCGGAAGGATCATTATTGAATTATGTTTCTAGATAGGTTGTAG
CTGGCTC-TTTAGAGCATGTGCACGCCTGTTTGGACTTCATTTTCATCCACCTGTGCACC
TATTGTAGTCTTTGGTTGGGTTAGGAGGAAGTGGTCATTGTGTCAGCATCTGCTGGATGT
GAGGACTTGCATTGTGAAAGCTTTGCTGTCCTTGATGTGATCATGGAATCTCTTTC----

-------TCACTAGAGTCTATGTCACTCATTATACTCTGTCGAATGTCATTGAATGTCTT
TACATGGGCTTATATGCCTATGAAAATTGTAATACAACTTTCAGCAACGGATCTCTTGGC
TCTCGCATCGATGAAGAACGCAGCGAAATGCGATAAGTAATGTGAATTGCAGAATTCAGT
GAATCATCGAATCTTTGAACGCATCTTGCGCTCCTTGGTATTCCGAGGAGCATGCCTGTT
TGAGTGTCATTAAATTCTCAACTCTCTTCTAC-TTTTTGTAAAAGAGAGCTTGGACTGTG
GAGGCTTGCTGGCCACTTTTTGGGGTCAGCTCCTCTGAAATGCATTAGCGGAACCGTTTG
CGATCTGCCACAAGTGTGATAAGTTATCTACACTGGCGAGGGGATTGCTCTCTGTAATGT
TCAGCTTCTAATTGTCTCTACTTTGTGAGACTACTTTTGAATGCTTGACCTCAAATCAGG
TAGGACTACCCGCTGAACTTAA
>AC4-55
TTTCCGTAGGTGAACCTGCGGAAGGATCATTATTGAATTATGTTTCTAGATAGGTTGTAG
CTGGCTC-TTTAGAGCATGTGCACGCCTGTTTGGACTTCATTTTCATCCACCTGTGCACC
TATTGTAGTCTTTGGTTGGGTTAGGAGGAAGTGGTCATTGTGTCAGCATCTGCTGGATGT
GAGGACTTGCATTGTGAAAGCTTTGCTGTCCTTGATGTGATCATGGAATCTCTTTC----

-------TCACTAGAGTCTATGTCACTCATTATACTCTGTCGAATGTCATTGAATGTCTT
TACATGGGCTTATATGCCTATGAAAATTGTAATACAACTTTCAGCAACGGATCTCTTGGC
TCTCGCATCGATGAAGAACGCAGCGAAATGCGATAAGTAATGTGAATTGCAGAATTCAGT
GAATCATCGAATCTTTGAACGCATCTTGCGCTCCTTGGTATTCCGAGGAGCATGCCTGTT
TGAGTGTCATTAAATTCTCAACTCTCTTCTAC-TTTTTGTAAAAGAGAGCTTGGACTGTG
GAGGCTTGCTGGCCACTTTTTGGGGTCAGCTCCTCTGAAATGCATTAGCGGAACCGTTTG
CGATCTGCCACAAGTGTGATAAGTTATCTACACTGGCGAGGGGATTGCTCTCTGTAATGT
TCAGCTTCTAATTGTCTCTACTTTGTGAGACTACTTTTGAATGCTTGACCTCAAATCAGG
TAGGACTACCCGCTGAACTTAA
>AC4-63
TTTCCGTAGGTGAACCTGCGGAAGGATCATTATTGAATTATGTTTCTAGATAGGTTGTAG
CTGGCTC-TTTAGAGCATGTGCACGCCTGTTTGGACTTCATTTTCATCCACCTGTGCACC
TATTGTAGTCTTTGGTTGGGTTAGGAGGAAGTGGTCATTGTGTCAGCATCTGCTGGATGT
GAGGACTTGCATTGTGAAAGCTTTGCTGTCCTTGATGTGATCATGGAATCTCTTTC----


```
------------------------------------------------------------
------------------------------------------------------------
------------------------------------------------------------
------------------------------------------------------------
------------------------------------------------------------
------------------------------------------------------------
------------------------------------------------------------
------------------------------------------------------------
------------------------------------------------------------
-------TCACTAGAGTCTATGTCACTCATTATACTCTGTCGAATGTCATTGAATGTCTT
TACATGGGCTTATATGCCTATGAAAATTGTAATACAACTTTCAGCAACGGATCTCTTGGC
TCTCGCATCGATGAAGAACGCAGCGAAATGCGATAAGTAATGTGAATTGCAGAATTCAGT
GAATCATCGAATCTTTGAACGCATCTTGCGCTCCTTGGTATTCCGAGGAGCATGCCTGTT
TGAGTGTCATTAAATTCTCAACTCTCTTCTAC-TTTTTGTAAAAGAGAGCTTGGACTGTG
GAGGCTTGCTGGCCACTTTTTGGGGTCAGCTCCTCTGAAATGCATTAGCGGAACCGTTTG
CGATCTGCCACAAGTGTGATAAGTTATCTACACTGGCGAGGGGATTGCTCTCTGTAATGT
TCAGCTTCTAATTGTCTCTACTTTGTGAGACTACTTTTGAATGCTTGACCTCAAATCAGG
TAGGACTACCCGCTGAACTTAA
>AC4-66
TTTCCGTAGGTGAACCTGCGGAAGGATCATTATTGAATTATGTTTCTAGATAGGTTGTAG
CTGGCTC-TTTAGAGCATGTGCACGCCTGTTTGGACTTCATTTTCATCCACCTGTGCACC
TATTGTAGTCTTTGGTTGGGTTAGGAGGAAGTGGTCATTGTGTCAGCATCTGCTGGATGT
GAGGACTTGCATTGTGAAAGCTTTGCTGTCCTTGATGTGATCATGGAATCTCTTTC----
------------------------------------------------------------
------------------------------------------------------------
------------------------------------------------------------
------------------------------------------------------------
------------------------------------------------------------
------------------------------------------------------------
------------------------------------------------------------
------------------------------------------------------------
------------------------------------------------------------
------------------------------------------------------------
------------------------------------------------------------
------------------------------------------------------------
-------TCACTAGAGTCTATGTCACTCATTATACTCTGTCGAATGTCATTGAATGTCTT
TACATGGGCTTATATGCCTATGAAAATTGTAATACAACTTTCAGCAACGGATCTCTTGGC
TCTCGCATCGATGAAGAACGCAGCGAAATGCGATAAGTAATGTGAATTGCAGAATTCAGT
GAATCATCGAATCTTTGAACGCATCTTGCGCTCCTTGGTATTCCGAGGAGCATGCCTGTT
TGAGTGTCATTAAATTCTCAACTCTCTTCTAC-TTTTTGTAAAAGAGAGCTTGGACTGTG
GAGGCTTGCTGGCCACTTTTTGGGGTCAGCTCCTCTGAAATGCATTAGCGGAACCGTTTG
CGATCTGCCACAAGTGTGATAAGTTATCTACACTGGCGAGGGGATTGCTCTCTGTAATGT
TCAGCTTCTAATTGTCTCTACTTTGTGAGACTACTTTTGAATGCTTGACCTCAAATCAGG
TAGGACTACCCGCTGAACTTAA
>AC4-68
TTTCCGTAGGTGAACCTGCGGAAGGATCATTATTGAATTATGTTTCTAGATAGGTTGTAG
CTGGCTC-TTTAGAGCATGTGCACGCCTGTTTGGACTTCATTTTCATCCACCTGTGCACC
TATTGTAGTCTTTGGTTGGGTTAGGAGGAAGTGGTCATTGTGTCAGCATCTGCTGGATGT
GAGGACTTGCATTGTGAAAGCTTTGCTGTCCTTGATGTGATCATGGAATCTCTTTC----
------------------------------------------------------------
```

```
------------------------------------------------------------
------------------------------------------------------------
------------------------------------------------------------
------------------------------------------------------------
------------------------------------------------------------
------------------------------------------------------------
------------------------------------------------------------
------------------------------------------------------------
------------------------------------------------------------
------------------------------------------------------------
------------------------------------------------------------
-------TCACTAGAGTCTATGTCACTCATTATACTCTGTCGAATGTCATTGAATGTCTT
TACATGGGCTTATATGCCTATGAAAATTGTAATACAACTTTCAGCAACGGATCTCTTGGC
TCTCGCATCGATGAAGAACGCAGCGAAATGCGATAAGTAATGTGAATTGCAGAATTCAGT
GAATCATCGAATCTTTGAACGCATCTTGCGCTCCTTGGTATTCCGAGGAGCATGCCTGTT
TGAGTGTCATTAAATTCTCAACTCTCTTCTAC-TTTTTGTAAAAGAGAGCTTGGACTGTG
GAGGCTTGCTGGCCACTTTTTGGGGTCAGCTCCTCTGAAATGCATTAGCGGAACCGTTTG
CGATCTGCCACAAGTGTGATAAGTTATCTACACTGGCGAGGGGATTGCTCTCTGTAATGT
TCAGCTTCTAATTGTCTCTACTTTGTGAGACTACTTTTGAATGCTTGACCTCAAATCAGG
TAGGACTACCCGCTGAACTTAA
>AC4-70
TTTCCGTAGGTGAACCTGCGGAAGGATCATTATTGAATTATGTTTCTAGATAGGTTGTAG
CTGGCTC-TTTAGAGCATGTGCACGCCTGTTTGGACTTCATTTTCATCCACCTGTGCACC
TATTGTAGTCTTTGGTTGGGTTAGGAGGAAGTGGTCATTGTGTCAGCATCTGCTGGATGT
GAGGACTTGCATTGTGAAAGCTTTGCTGTCCTTGATGTGATCATGGAATCTCTTTC----
------------------------------------------------------------
------------------------------------------------------------
------------------------------------------------------------
------------------------------------------------------------
------------------------------------------------------------
------------------------------------------------------------
------------------------------------------------------------
------------------------------------------------------------
------------------------------------------------------------
------------------------------------------------------------
------------------------------------------------------------
------------------------------------------------------------
-------TCACTAGAGTCTATGTCACTCATTATACTCTGTCGAATGTCATTGAATGTCTT
TACATGGGCTTATATGCCTATGAAAATTGTAATACAACTTTCAGCAACGGATCTCTTGGC
TCTCGCATCGATGAAGAACGCAGCGAAATGCGATAAGTAATGTGAATTGCAGAATTCAGT
GAATCATCGAATCTTTGAACGCATCTTGCGCTCCTTGGTATTCCGAGGAGCATGCCTGTT
TGAGTGTCATTAAATTCTCAACTCTCTTCTAC-TTTTTGTAAAAGAGAGCTTGGACTGTG
GAGGCTTGCTGGCCACTTTTTGGGGTCAGCTCCTCTGAAATGCATTAGCGGAACCGTTTG
CGATCTGCCACAAGTGTGATAAGTTATCTACACTGGCGAGGGGATTGCTCTCTGTAATGT
TCAGCTTCTAATTGTCTCTACTTTGTGAGACTACTTTTGAATGCTTGACCTCAAATCAGG
TAGGACTACCCGCTGAACTTAA
>AC4-74
TTTCCGTAGGTGAACCTGCGGAAGGATCATTATTGAATTATGTTTCTAGATAGGTTGTAG
CTGGCTC-TTTAGAGCATGTGCACGCCTGTTTGGACTTCATTTTCATCCACCTGTGCACC
TATTGTAGTCTTTGGTTGGGTTAGGAGGAAGTGGTCATTGTGTCAGCATCTGCTGGATGT
```

```
GAGGACTTGCATTGTGAAAGCTTTGCTGTCCTTGATGTGATCATGGAATCTCTTTC----
------------------------------------------------------------
------------------------------------------------------------
------------------------------------------------------------
------------------------------------------------------------
------------------------------------------------------------
------------------------------------------------------------
------------------------------------------------------------
------------------------------------------------------------
------------------------------------------------------------
------------------------------------------------------------
------------------------------------------------------------
------------------------------------------------------------
-------TCACTAGAGTCTATGTCACTCATTATACTCTGTCGAATGTCATTGAATGTCTT
TACATGGGCTTATATGCCTATGAAAATTGTAATACAACTTTCAGCAACGGATCTCTTGGC
TCTCGCATCGATGAAGAACGCAGCGAAATGCGATAAGTAATGTGAATTGCAGAATTCAGT
GAATCATCGAATCTTTGAACGCATCTTGCGCTCCTTGGTATTCCGAGGAGCATGCCTGTT
TGAGTGTCATTAAATTCTCAACTCTCTTCTAC-TTTTTGTAAAAGAGAGCTTGGACTGTG
GAGGCTTGCTGGCCACTTTTTGGGGTCAGCTCCTCTGAAATGCATTAGCGGAACCGTTTG
CGATCTGCCACAAGTGTGATAAGTTATCTACACTGGCGAGGGGATTGCTCTCTGTAATGT
TCAGCTTCTAATTGTCTCTACTTTGTGAGACTACTTTTGAATGCTTGACCTCAAATCAGG
TAGGACTACCCGCTGAACTTAA
>AC4-79
TTTCCGTAGGTGAACCTGCGGAAGGATCATTATTGAATTATGTTTCTAGATAGGTTGTAG
CTGGCTC-TTTAGAGCATGTGCACGCCTGTTTGGACTTCATTTTCATCCACCTGTGCACC
TATTGTAGTCTTTGGTTGGGTTAGGAGGAAGTGGTCATTGTGTCAGCATCTGCTGGATGT
GAGGACTTGCATTGTGAAAGCTTTGCTGTCCTTGATGTGATCATGGAATCTCTTTC----
------------------------------------------------------------
------------------------------------------------------------
------------------------------------------------------------
------------------------------------------------------------
------------------------------------------------------------
------------------------------------------------------------
------------------------------------------------------------
------------------------------------------------------------
------------------------------------------------------------
------------------------------------------------------------
------------------------------------------------------------
------------------------------------------------------------
-------TCACTAGAGTCTATGTCACTCATTATACTCTGTCGAATGTCATTGAATGTCTT
TACATGGGCTTATATGCCTATGAAAATTGTAATACAACTTTCAGCAACGGATCTCTTGGC
TCTCGCATCGATGAAGAACGCAGCGAAATGCGATAAGTAATGTGAATTGCAGAATTCAGT
GAATCATCGAATCTTTGAACGCATCTTGCGCTCCTTGGTATTCCGAGGAGCATGCCTGTT
TGAGTGTCATTAAATTCTCAACTCTCTTCTAC-TTTTTGTAAAAGAGAGCTTGGACTGTG
GAGGCTTGCTGGCCACTTTTTGGGGTCAGCTCCTCTGAAATGCATTAGCGGAACCGTTTG
CGATCTGCCACAAGTGTGATAAGTTATCTACACTGGCGAGGGGATTGCTCTCTGTAATGT
TCAGCTTCTAATTGTCTCTACTTTGTGAGACTACTTTTGAATGCTTGACCTCAAATCAGG
TAGGACTACCCGCTGAACTTAA
>AC5-5
TTTCCGTAGGTGAACCTGCGGAAGGATCATTATTGAATTATGTTTCTAGATAGGTTGTAG
```

CTGGCTC-TTTAGAGCATGTGCACGCCTGTTTGGACTTCATTTTCATCCACCTGTGCACC
TATTGTAGTCTTTGGTTGGGTTAGGAGGAAGTGGTCATTGTGTCAGCATCTGCTGGATGT
GAGGACTTGCATTGTGAAAGCTTTGCTGTCCTTGATGTGATCATGGAATCTCTTTC----

-------TCACTAGAGTCTATGTCACTCATTATACTCTGTCGAATGTCATTGAATGTCTT
TACATGGGCTTATATGCCTATGAAAATTGTAATACAACTTTCAGCAACGGATCTCTTGGC
TCTCGCATCGATGAAGAACGCAGCGAAATGCGATAAGTAATGTGAATTGCAGAATTCAGT
GAATCATCGAATCTTTGAACGCATCTTGCGCTCCTTGGTATTCCGAGGAGCATGCCTGTT
TGAGTGTCATTAAATTCTCAACTCTCTTCTAC-TTTTTGTAAAAGAGAGCTTGGACTGTG
GAGGCTTGCTGGCCACTTTTTGGGGTCAGCTCCTCTGAAATGCATTAGCGGAACCGTTTG
CGATCTGCCACAAGTGTGATAAGTTATCTACACTGGCGAGGGGATTGCTCTCTGTAATGT
TCAGCTTCTAATTGTCTCTACTTTGTGAGACTACTTTTGAATGCTTGACCTCAAATCAGG
TAGGACTACCCGCTGAACTTAA
>AC5-7
TTTCCGTAGGTGAACCTGCGGAAGGATCATTATTGAATTATGTTTCTAGATAGGTTGTAG
CTGGCTC-TTTAGAGCATGTGCACGCCTGTTTGGACTTCATTTTCATCCACCTGTGCACC
TATTGTAGTCTTTGGTTGGGTTAGGAGGAAGTGGTCATTGTGTCAGCATCTGCTGGATGT
GAGGACTTGCATTGTGAAAGCTTTGCTGTCCTTGATGTGATCATGGAATCTCTTTC----

-------TCACTAGAGTCTATGTCACTCATTATACTCTGTCGAATGTCATTGAATGTCTT
TACATGGGCTTATATGCCTATGAAAATTGTAATACAACTTTCAGCAACGGATCTCTTGGC
TCTCGCATCGATGAAGAACGCAGCGAAATGCGATAAGTAATGTGAATTGCAGAATTCAGT
GAATCATCGAATCTTTGAACGCATCTTGCGCTCCTTGGTATTCCGAGGAGCATGCCTGTT
TGAGTGTCATTAAATTCTCAACTCTCTTCTAC-TTTTTGTAAAAGAGAGCTTGGACTGTG
GAGGCTTGCTGGCCACTTTTTGGGGTCAGCTCCTCTGAAATGCATTAGCGGAACCGTTTG
CGATCTGCCACAAGTGTGATAAGTTATCTACACTGGCGAGGGGATTGCTCTCTGTAATGT
TCAGCTTCTAATTGTCTCTACTTTGTGAGACTACTTTTGAATGCTTGACCTCAAATCAGG
TAGGACTACCCGCTGAACTTAA

```
>AC5-8
TTTCCGTAGGTGAACCTGCGGAAGGATCATTATTGAATTATGTTTCTAGATAGGTTGTAG
CTGGCTC-TTTAGAGCATGTGCACGCCTGTTTGGACTTCATTTTCATCCACCTGTGCACC
TATTGTAGTCTTTGGTTGGGTTAGGAGGAAGTGGTCATTGTGTCAGCATCTGCTGGATGT
GAGGACTTGCATTGTGAAAGCTTTGCTGTCCTTGATGTGATCATGGAATCTCTTTC----
------------------------------------------------------------
------------------------------------------------------------
------------------------------------------------------------
------------------------------------------------------------
------------------------------------------------------------
------------------------------------------------------------
------------------------------------------------------------
------------------------------------------------------------
------------------------------------------------------------
------------------------------------------------------------
------------------------------------------------------------
------------------------------------------------------------
-------TCACTAGAGTCTATGTCACTCATTATACTCTGTCGAATGTCATTGAATGTCTT
TACATGGGCTTATATGCCTATGAAAATTGTAATACAACTTTCAGCAACGGATCTCTTGGC
TCTCGCATCGATGAAGAACGCAGCGAAATGCGATAAGTAATGTGAATTGCAGAATTCAGT
GAATCATCGAATCTTTGAACGCATCTTGCGCTCCTTGGTATTCCGAGGAGCATGCCTGTT
TGAGTGTCATTAAATTCTCAACTCTCTTCTAC-TTTTTGTAAAAGAGAGCTTGGACTGTG
GAGGCTTGCTGGCCACTTTTTGGGGTCAGCTCCTCTGAAATGCATTAGCGGAACCGTTTG
CGATCTGCCACAAGTGTGATAAGTTATCTACACTGGCGAGGGGATTGCTCTCTGTAATGT
TCAGCTTCTAATTGTCTCTACTTTGTGAGACTACTTTTGAATGCTTGACCTCAAATCAGG
TAGGACTACCCGCTGAACTTAA
>AC5-19
TTTCCGTAGGTGAACCTGCGGAAGGATCATTATTGAATTATGTTTCTAGATAGGTTGTAG
CTGGCTC-TTTAGAGCATGTGCACGCCTGTTTGGACTTCATTTTCATCCACCTGTGCACC
TATTGTAGTCTTTGGTTGGGTTAGGAGGAAGTGGTCATTGTGTCAGCATCTGCTGGATGT
GAGGACTTGCATTGTGAAAGCTTTGCTGTCCTTGATGTGATCATGGAATCTCTTTC----
------------------------------------------------------------
------------------------------------------------------------
------------------------------------------------------------
------------------------------------------------------------
------------------------------------------------------------
------------------------------------------------------------
------------------------------------------------------------
------------------------------------------------------------
------------------------------------------------------------
------------------------------------------------------------
------------------------------------------------------------
------------------------------------------------------------
-------TCACTAGAGTCTATGTCACTCATTATACTCTGTCGAATGTCATTGAATGTCTT
TACATGGGCTTATATGCCTATGAAAATTGTAATACAACTTTCAGCAACGGATCTCTTGGC
TCTCGCATCGATGAAGAACGCAGCGAAATGCGATAAGTAATGTGAATTGCAGAATTCAGT
GAATCATCGAATCTTTGAACGCATCTTGCGCTCCTTGGTATTCCGAGGAGCATGCCTGTT
TGAGTGTCATTAAATTCTCAACTCTCTTCTAC-TTTTTGTAAAAGAGAGCTTGGACTGTG
GAGGCTTGCTGGCCACTTTTTGGGGTCAGCTCCTCTGAAATGCATTAGCGGAACCGTTTG
CGATCTGCCACAAGTGTGATAAGTTATCTACACTGGCGAGGGGATTGCTCTCTGTAATGT
```

TCAGCTTCTAATTGTCTCTACTTTGTGAGACTACTTTTGAATGCTTGACCTCAAATCAGG
TAGGACTACCCGCTGAACTTAA
>AC5-30
TTTCCGTAGGTGAACCTGCGGAAGGATCATTATTGAATTATGTTTCTAGATAGGTTGTAG
CTGGCTC-TTTAGAGCATGTGCACGCCTGTTTGGACTTCATTTTCATCCACCTGTGCACC
TATTGTAGTCTTTGGTTGGGTTAGGAGGAAGTGGTCATTGTGTCAGCATCTGCTGGATGT
GAGGACTTGCATTGTGAAAGCTTTGCTGTCCTTGATGTGATCATGGAATCTCTTTC----

-------TCACTAGAGTCTATGTCACTCATTATACTCTGTCGAATGTCATTGAATGTCTT
TACATGGGCTTATATGCCTATGAAAATTGTAATACAACTTTCAGCAACGGATCTCTTGGC
TCTCGCATCGATGAAGAACGCAGCGAAATGCGATAAGTAATGTGAATTGCAGAATTCAGT
GAATCATCGAATCTTTGAACGCATCTTGCGCTCCTTGGTATTCCGAGGAGCATGCCTGTT
TGAGTGTCATTAAATTCTCAACTCTCTTCTAC-TTTTTGTAAAAGAGAGCTTGGACTGTG
GAGGCTTGCTGGCCACTTTTTGGGGTCAGCTCCTCTGAAATGCATTAGCGGAACCGTTTG
CGATCTGCCACAAGTGTGATAAGTTATCTACACTGGCGAGGGGATTGCTCTCTGTAATGT
TCAGCTTCTAATTGTCTCTACTTTGTGAGACTACTTTTGAATGCTTGACCTCAAATCAGG
TAGGACTACCCGCTGAACTTAA
>AC5-35
TTTCCGTAGGTGAACCTGCGGAAGGATCATTATTGAATTATGTTTCTAGATAGGTTGTAG
CTGGCTC-TTTAGAGCATGTGCACGCCTGTTTGGACTTCATTTTCATCCACCTGTGCACC
TATTGTAGTCTTTGGTTGGGTTAGGAGGAAGTGGTCATTGTGTCAGCATCTGCTGGATGT
GAGGACTTGCATTGTGAAAGCTTTGCTGTCCTTGATGTGATCATGGAATCTCTTTC----

-------TCACTAGAGTCTATGTCACTCATTATACTCTGTCGAATGTCATTGAATGTCTT
TACATGGGCTTATATGCCTATGAAAATTGTAATACAACTTTCAGCAACGGATCTCTTGGC
TCTCGCATCGATGAAGAACGCAGCGAAATGCGATAAGTAATGTGAATTGCAGAATTCAGT
GAATCATCGAATCTTTGAACGCATCTTGCGCTCCTTGGTATTCCGAGGAGCATGCCTGTT
TGAGTGTCATTAAATTCTCAACTCTCTTCTAC-TTTTTGTAAAAGAGAGCTTGGACTGTG

GAGGCTTGCTGGCCACTTTTTGGGGTCAGCTCCTCTGAAATGCATTAGCGGAACCGTTTG
CGATCTGCCACAAGTGTGATAAGTTATCTACACTGGCGAGGGGATTGCTCTCTGTAATGT
TCAGCTTCTAATTGTCTCTACTTTGTGAGACTACTTTTGAATGCTTGACCTCAAATCAGG
TAGGACTACCCGCTGAACTTAA
>AC5-36
TTTCCGTAGGTGAACCTGCGGAAGGATCATTATTGAATTATGTTTCTAGATAGGTTGTAG
CTGGCTC-TTTAGAGCATGTGCACGCCTGTTTGGACTTCATTTTCATCCACCTGTGCACC
TATTGTAGTCTTTGGTTGGGTTAGGAGGAAGTGGTCATTGTGTCAGCATCTGCTGGATGT
GAGGACTTGCATTGTGAAAGCTTTGCTGTCCTTGATGTGATCATGGAATCTCTTTC----

-------TCACTAGAGTCTATGTCACTCATTATACTCTGTCGAATGTCATTGAATGTCTT
TACATGGGCTTATATGCCTATGAAAATTGTAATACAACTTTCAGCAACGGATCTCTTGGC
TCTCGCATCGATGAAGAACGCAGCGAAATGCGATAAGTAATGTGAATTGCAGAATTCAGT
GAATCATCGAATCTTTGAACGCATCTTGCGCTCCTTGGTATTCCGAGGAGCATGCCTGTT
TGAGTGTCATTAAATTCTCAACTCTCTTCTAC-TTTTTGTAAAAGAGAGCTTGGACTGTG
GAGGCTTGCTGGCCACTTTTTGGGGTCAGCTCCTCTGAAATGCATTAGCGGAACCGTTTG
CGATCTGCCACAAGTGTGATAAGTTATCTACACTGGCGAGGGGATTGCTCTCTGTAATGT
TCAGCTTCTAATTGTCTCTACTTTGTGAGACTACTTTTGAATGCTTGACCTCAAATCAGG
TAGGACTACCCGCTGAACTTAA
>AC5-45
TTTCCGTAGGTGAACCTGCGGAAGGATCATTATTGAATTATGTTTCTAGATAGGTTGTAG
CTGGCTC-TTTAGAGCATGTGCACGCCTGTTTGGACTTCATTTTCATCCACCTGTGCACC
TATTGTAGTCTTTGGTTGGGTTAGGAGGAAGTGGTCATTGTGTCAGCATCTGCTGGATGT
GAGGACTTGCATTGTGAAAGCTTTGCTGTCCTTGATGTGATCATGGAATCTCTTTC----

-------TCACTAGAGTCTATGTCACTCATTATACTCTGTCGAATGTCATTGAATGTCTT
TACATGGGCTTATATGCCTATGAAAATTGTAATACAACTTTCAGCAACGGATCTCTTGGC
TCTCGCATCGATGAAGAACGCAGCGAAATGCGATAAGTAATGTGAATTGCAGAATTCAGT

GAATCATCGAATCTTTGAACGCATCTTGCGCTCCTTGGTATTCCGAGGAGCATGCCTGTT
TGAGTGTCATTAAATTCTCAACTCTCTTCTAC-TTTTTGTAAAAGAGAGCTTGGACTGTG
GAGGCTTGCTGGCCACTTTTTGGGGTCAGCTCCTCTGAAATGCATTAGCGGAACCGTTTG
CGATCTGCCACAAGTGTGATAAGTTATCTACACTGGCGAGGGGATTGCTCTCTGTAATGT
TCAGCTTCTAATTGTCTCTACTTTGTGAGACTACTTTTGAATGCTTGACCTCAAATCAGG
TAGGACTACCCGCTGAACTTAA
>AC5-50
TTTCCGTAGGTGAACCTGCGGAAGGATCATTATTGAATTATGTTTCTAGATAGGTTGTAG
CTGGCTC-TTTAGAGCATGTGCACGCCTGTTTGGACTTCATTTTCATCCACCTGTGCACC
TATTGTAGTCTTTGGTTGGGTTAGGAGGAAGTGGTCATTGTGTCAGCATCTGCTGGATGT
GAGGACTTGCATTGTGAAAGCTTTGCTGTCCTTGATGTGATCATGGAATCTCTTTC----

-------TCACTAGAGTCTATGTCACTCATTATACTCTGTCGAATGTCATTGAATGTCTT
TACATGGGCTTATATGCCTATGAAAATTGTAATACAACTTTCAGCAACGGATCTCTTGGC
TCTCGCATCGATGAAGAACGCAGCGAAATGCGATAAGTAATGTGAATTGCAGAATTCAGT
GAATCATCGAATCTTTGAACGCATCTTGCGCTCCTTGGTATTCCGAGGAGCATGCCTGTT
TGAGTGTCATTAAATTCTCAACTCTCTTCTAC-TTTTTGTAAAAGAGAGCTTGGACTGTG
GAGGCTTGCTGGCCACTTTTTGGGGTCAGCTCCTCTGAAATGCATTAGCGGAACCGTTTG
CGATCTGCCACAAGTGTGATAAGTTATCTACACTGGCGAGGGGATTGCTCTCTGTAATGT
TCAGCTTCTAATTGTCTCTACTTTGTGAGACTACTTTTGAATGCTTGACCTCAAATCAGG
TAGGACTACCCGCTGAACTTAA
>AC5-53
TTTCCGTAGGTGAACCTGCGGAAGGATCATTATTGAATTATGTTTCTAGATAGGTTGTAG
CTGGCTC-TTTAGAGCATGTGCACGCCTGTTTGGACTTCATTTTCATCCACCTGTGCACC
TATTGTAGTCTTTGGTTGGGTTAGGAGGAAGTGGTCATTGTGTCAGCATCTGCTGGATGT
GAGGACTTGCATTGTGAAAGCTTTGCTGTCCTTGATGTGATCATGGAATCTCTTTC----

-------TCACTAGAGTCTATGTCACTCATTATACTCTGTCGAATGTCATTGAATGTCTT

TACATGGGCTTATATGCCTATGAAAATTGTAATACAACTTTCAGCAACGGATCTCTTGGC
TCTCGCATCGATGAAGAACGCAGCGAAATGCGATAAGTAATGTGAATTGCAGAATTCAGT
GAATCATCGAATCTTTGAACGCATCTTGCGCTCCTTGGTATTCCGAGGAGCATGCCTGTT
TGAGTGTCATTAAATTCTCAACTCTCTTCTAC-TTTTTGTAAAAGAGAGCTTGGACTGTG
GAGGCTTGCTGGCCACTTTTTGGGGTCAGCTCCTCTGAAATGCATTAGCGGAACCGTTTG
CGATCTGCCACAAGTGTGATAAGTTATCTACACTGGCGAGGGGATTGCTCTCTGTAATGT
TCAGCTTCTAATTGTCTCTACTTTGTGAGACTACTTTTGAATGCTTGACCTCAAATCAGG
TAGGACTACCCGCTGAACTTAA
>AC5-56
TTTCCGTAGGTGAACCTGCGGAAGGATCATTATTGAATTATGTTTCTAGATAGGTTGTAG
CTGGCTC-TTTAGAGCATGTGCACGCCTGTTTGGACTTCATTTTCATCCACCTGTGCACC
TATTGTAGTCTTTGGTTGGGTTAGGAGGAAGTGGTCATTGTGTCAGCATCTGCTGGATGT
GAGGACTTGCATTGTGAAAGCTTTGCTGTCCTTGATGTGATCATGGAATCTCTTTC----

-------TCACTAGAGTCTATGTCACTCATTATACTCTGTCGAATGTCATTGAATGTCTT
TACATGGGCTTATATGCCTATGAAAATTGTAATACAACTTTCAGCAACGGATCTCTTGGC
TCTCGCATCGATGAAGAACGCAGCGAAATGCGATAAGTAATGTGAATTGCAGAATTCAGT
GAATCATCGAATCTTTGAACGCATCTTGCGCTCCTTGGTATTCCGAGGAGCATGCCTGTT
TGAGTGTCATTAAATTCTCAACTCTCTTCTAC-TTTTTGTAAAAGAGAGCTTGGACTGTG
GAGGCTTGCTGGCCACTTTTTGGGGTCAGCTCCTCTGAAATGCATTAGCGGAACCGTTTG
CGATCTGCCACAAGTGTGATAAGTTATCTACACTGGCGAGGGGATTGCTCTCTGTAATGT
TCAGCTTCTAATTGTCTCTACTTTGTGAGACTACTTTTGAATGCTTGACCTCAAATCAGG
TAGGACTACCCGCTGAACTTAA
>AC5-59
TTTCCGTAGGTGAACCTGCGGAAGGATCATTATTGAATTATGTTTCTAGATAGGTTGTAG
CTGGCTC-TTTAGAGCATGTGCACGCCTGTTTGGACTTCATTTTCATCCACCTGTGCACC
TATTGTAGTCTTTGGTTGGGTTAGGAGGAAGTGGTCATTGTGTCAGCATCTGCTGGATGT
GAGGACTTGCATTGTGAAAGCTTTGCTGTCCTTGATGTGATCATGGAATCTCTTTC----

-------TCACTAGAGTCTATGTCACTCATTATACTCTGTCGAATGTCATTGAATGTCTT
TACATGGGCTTATATGCCTATGAAAATTGTAATACAACTTTCAGCAACGGATCTCTTGGC
TCTCGCATCGATGAAGAACGCAGCGAAATGCGATAAGTAATGTGAATTGCAGAATTCAGT
GAATCATCGAATCTTTGAACGCATCTTGCGCTCCTTGGTATTCCGAGGAGCATGCCTGTT
TGAGTGTCATTAAATTCTCAACTCTCTTCTAC-TTTTTGTAAAAGAGAGCTTGGACTGTG
GAGGCTTGCTGGCCACTTTTTGGGGTCAGCTCCTCTGAAATGCATTAGCGGAACCGTTTG
CGATCTGCCACAAGTGTGATAAGTTATCTACACTGGCGAGGGGATTGCTCTCTGTAATGT
TCAGCTTCTAATTGTCTCTACTTTGTGAGACTACTTTTGAATGCTTGACCTCAAATCAGG
TAGGACTACCCGCTGAACTTAA
>AC5-60
TTTCCGTAGGTGAACCTGCGGAAGGATCATTATTGAATTATGTTTCTAGATAGGTTGTAG
CTGGCTC-TTTAGAGCATGTGCACGCCTGTTTGGACTTCATTTTCATCCACCTGTGCACC
TATTGTAGTCTTTGGTTGGGTTAGGAGGAAGTGGTCATTGTGTCAGCATCTGCTGGATGT
GAGGACTTGCATTGTGAAAGCTTTGCTGTCCTTGATGTGATCATGGAATCTCTTTC----

-------TCACTAGAGTCTATGTCACTCATTATACTCTGTCGAATGTCATTGAATGTCTT
TACATGGGCTTATATGCCTATGAAAATTGTAATACAACTTTCAGCAACGGATCTCTTGGC
TCTCGCATCGATGAAGAACGCAGCGAAATGCGATAAGTAATGTGAATTGCAGAATTCAGT
GAATCATCGAATCTTTGAACGCATCTTGCGCTCCTTGGTATTCCGAGGAGCATGCCTGTT
TGAGTGTCATTAAATTCTCAACTCTCTTCTAC-TTTTTGTAAAAGAGAGCTTGGACTGTG
GAGGCTTGCTGGCCACTTTTTGGGGTCAGCTCCTCTGAAATGCATTAGCGGAACCGTTTG
CGATCTGCCACAAGTGTGATAAGTTATCTACACTGGCGAGGGGATTGCTCTCTGTAATGT
TCAGCTTCTAATTGTCTCTACTTTGTGAGACTACTTTTGAATGCTTGACCTCAAATCAGG
TAGGACTACCCGCTGAACTTAA
>AC5-61
TTTCCGTAGGTGAACCTGCGGAAGGATCATTATTGAATTATGTTTCTAGATAGGTTGTAG
CTGGCTC-TTTAGAGCATGTGCACGCCTGTTTGGACTTCATTTTCATCCACCTGTGCACC
TATTGTAGTCTTTGGTTGGGTTAGGAGGAAGTGGTCATTGTGTCAGCATCTGCTGGATGT
GAGGACTTGCATTGTGAAAGCTTTGCTGTCCTTGATGTGATCATGGAATCTCTTTC----

-------TCACTAGAGTCTATGTCACTCATTATACTCTGTCGAATGTCATTGAATGTCTT
TACATGGGCTTATATGCCTATGAAAATTGTAATACAACTTTCAGCAACGGATCTCTTGGC
TCTCGCATCGATGAAGAACGCAGCGAAATGCGATAAGTAATGTGAATTGCAGAATTCAGT
GAATCATCGAATCTTTGAACGCATCTTGCGCTCCTTGGTATTCCGAGGAGCATGCCTGTT
TGAGTGTCATTAAATTCTCAACTCTCTTCTAC-TTTTTGTAAAAGAGAGCTTGGACTGTG
GAGGCTTGCTGGCCACTTTTTGGGGTCAGCTCCTCTGAAATGCATTAGCGGAACCGTTTG
CGATCTGCCACAAGTGTGATAAGTTATCTACACTGGCGAGGGGATTGCTCTCTGTAATGT
TCAGCTTCTAATTGTCTCTACTTTGTGAGACTACTTTTGAATGCTTGACCTCAAATCAGG
TAGGACTACCCGCTGAACTTAA
>AC5-72
TTTCCGTAGGTGAACCTGCGGAAGGATCATTATTGAATTATGTTTCTAGATAGGTTGTAG
CTGGCTC-TTTAGAGCATGTGCACGCCTGTTTGGACTTCATTTTCATCCACCTGTGCACC
TATTGTAGTCTTTGGTTGGGTTAGGAGGAAGTGGTCATTGTGTCAGCATCTGCTGGATGT
GAGGACTTGCATTGTGAAAGCTTTGCTGTCCTTGATGTGATCATGGAATCTCTTTC----

-------TCACTAGAGTCTATGTCACTCATTATACTCTGTCGAATGTCATTGAATGTCTT
TACATGGGCTTATATGCCTATGAAAATTGTAATACAACTTTCAGCAACGGATCTCTTGGC
TCTCGCATCGATGAAGAACGCAGCGAAATGCGATAAGTAATGTGAATTGCAGAATTCAGT
GAATCATCGAATCTTTGAACGCATCTTGCGCTCCTTGGTATTCCGAGGAGCATGCCTGTT
TGAGTGTCATTAAATTCTCAACTCTCTTCTAC-TTTTTGTAAAAGAGAGCTTGGACTGTG
GAGGCTTGCTGGCCACTTTTTGGGGTCAGCTCCTCTGAAATGCATTAGCGGAACCGTTTG
CGATCTGCCACAAGTGTGATAAGTTATCTACACTGGCGAGGGGATTGCTCTCTGTAATGT
TCAGCTTCTAATTGTCTCTACTTTGTGAGACTACTTTTGAATGCTTGACCTCAAATCAGG
TAGGACTACCCGCTGAACTTAA
>AC5-73
TTTCCGTAGGTGAACCTGCGGAAGGATCATTATTGAATTATGTTTCTAGATAGGTTGTAG
CTGGCTC-TTTAGAGCATGTGCACGCCTGTTTGGACTTCATTTTCATCCACCTGTGCACC
TATTGTAGTCTTTGGTTGGGTTAGGAGGAAGTGGTCATTGTGTCAGCATCTGCTGGATGT
GAGGACTTGCATTGTGAAAGCTTTGCTGTCCTTGATGTGATCATGGAATCTCTTTC----

-------TCACTAGAGTCTATGTCACTCATTATACTCTGTCGAATGTCATTGAATGTCTT
TACATGGGCTTATATGCCTATGAAAATTGTAATACAACTTTCAGCAACGGATCTCTTGGC
TCTCGCATCGATGAAGAACGCAGCGAAATGCGATAAGTAATGTGAATTGCAGAATTCAGT
GAATCATCGAATCTTTGAACGCATCTTGCGCTCCTTGGTATTCCGAGGAGCATGCCTGTT
TGAGTGTCATTAAATTCTCAACTCTCTTCTAC-TTTTTGTAAAAGAGAGCTTGGACTGTG
GAGGCTTGCTGGCCACTTTTTGGGGTCAGCTCCTCTGAAATGCATTAGCGGAACCGTTTG
CGATCTGCCACAAGTGTGATAAGTTATCTACACTGGCGAGGGGATTGCTCTCTGTAATGT
TCAGCTTCTAATTGTCTCTACTTTGTGAGACTACTTTTGAATGCTTGACCTCAAATCAGG
TAGGACTACCCGCTGAACTTAA
>AC5-78
TTTCCGTAGGTGAACCTGCGGAAGGATCATTATTGAATTATGTTTCTAGATAGGTTGTAG
CTGGCTC-TTTAGAGCATGTGCACGCCTGTTTGGACTTCATTTTCATCCACCTGTGCACC
TATTGTAGTCTTTGGTTGGGTTAGGAGGAAGTGGTCATTGTGTCAGCATCTGCTGGATGT
GAGGACTTGCATTGTGAAAGCTTTGCTGTCCTTGATGTGATCATGGAATCTCTTTC----

-------TCACTAGAGTCTATGTCACTCATTATACTCTGTCGAATGTCATTGAATGTCTT
TACATGGGCTTATATGCCTATGAAAATTGTAATACAACTTTCAGCAACGGATCTCTTGGC
TCTCGCATCGATGAAGAACGCAGCGAAATGCGATAAGTAATGTGAATTGCAGAATTCAGT
GAATCATCGAATCTTTGAACGCATCTTGCGCTCCTTGGTATTCCGAGGAGCATGCCTGTT
TGAGTGTCATTAAATTCTCAACTCTCTTCTAC-TTTTTGTAAAAGAGAGCTTGGACTGTG
GAGGCTTGCTGGCCACTTTTTGGGGTCAGCTCCTCTGAAATGCATTAGCGGAACCGTTTG
CGATCTGCCACAAGTGTGATAAGTTATCTACACTGGCGAGGGGATTGCTCTCTGTAATGT
TCAGCTTCTAATTGTCTCTACTTTGTGAGACTACTTTTGAATGCTTGACCTCAAATCAGG
TAGGACTACCCGCTGAACTTAA
>AC5-79
TTTCCGTAGGTGAACCTGCGGAAGGATCATTATTGAATTATGTTTCTAGATAGGTTGTAG
CTGGCTC-TTTAGAGCATGTGCACGCCTGTTTGGACTTCATTTTCATCCACCTGTGCACC
TATTGTAGTCTTTGGTTGGGTTAGGAGGAAGTGGTCATTGTGTCAGCATCTGCTGGATGT
GAGGACTTGCATTGTGAAAGCTTTGCTGTCCTTGATGTGATCATGGAATCTCTTTC----

-------TCACTAGAGTCTATGTCACTCATTATACTCTGTCGAATGTCATTGAATGTCTT
TACATGGGCTTATATGCCTATGAAAATTGTAATACAACTTTCAGCAACGGATCTCTTGGC
TCTCGCATCGATGAAGAACGCAGCGAAATGCGATAAGTAATGTGAATTGCAGAATTCAGT
GAATCATCGAATCTTTGAACGCATCTTGCGCTCCTTGGTATTCCGAGGAGCATGCCTGTT
TGAGTGTCATTAAATTCTCAACTCTCTTCTAC-TTTTTGTAAAAGAGAGCTTGGACTGTG
GAGGCTTGCTGGCCACTTTTTGGGGTCAGCTCCTCTGAAATGCATTAGCGGAACCGTTTG
CGATCTGCCACAAGTGTGATAAGTTATCTACACTGGCGAGGGGATTGCTCTCTGTAATGT
TCAGCTTCTAATTGTCTCTACTTTGTGAGACTACTTTTGAATGCTTGACCTCAAATCAGG
TAGGACTACCCGCTGAACTTAA
>AC5-81
TTTCCGTAGGTGAACCTGCGGAAGGATCATTATTGAATTATGTTTCTAGATAGGTTGTAG
CTGGCTC-TTTAGAGCATGTGCACGCCTGTTTGGACTTCATTTTCATCCACCTGTGCACC
TATTGTAGTCTTTGGTTGGGTTAGGAGGAAGTGGTCATTGTGTCAGCATCTGCTGGATGT
GAGGACTTGCATTGTGAAAGCTTTGCTGTCCTTGATGTGATCATGGAATCTCTTTC----

-------TCACTAGAGTCTATGTCACTCATTATACTCTGTCGAATGTCATTGAATGTCTT
TACATGGGCTTATATGCCTATGAAAATTGTAATACAACTTTCAGCAACGGATCTCTTGGC
TCTCGCATCGATGAAGAACGCAGCGAAATGCGATAAGTAATGTGAATTGCAGAATTCAGT
GAATCATCGAATCTTTGAACGCATCTTGCGCTCCTTGGTATTCCGAGGAGCATGCCTGTT
TGAGTGTCATTAAATTCTCAACTCTCTTCTAC-TTTTTGTAAAAGAGAGCTTGGACTGTG
GAGGCTTGCTGGCCACTTTTTGGGGTCAGCTCCTCTGAAATGCATTAGCGGAACCGTTTG
CGATCTGCCACAAGTGTGATAAGTTATCTACACTGGCGAGGGGATTGCTCTCTGTAATGT
TCAGCTTCTAATTGTCTCTACTTTGTGAGACTACTTTTGAATGCTTGACCTCAAATCAGG
TAGGACTACCCGCTGAACTTAA
>AC5-83
TTTCCGTAGGTGAACCTGCGGAAGGATCATTATTGAATTATGTTTCTAGATAGGTTGTAG
CTGGCTC-TTTAGAGCATGTGCACGCCTGTTTGGACTTCATTTTCATCCACCTGTGCACC
TATTGTAGTCTTTGGTTGGGTTAGGAGGAAGTGGTCATTGTGTCAGCATCTGCTGGATGT
GAGGACTTGCATTGTGAAAGCTTTGCTGTCCTTGATGTGATCATGGAATCTCTTTC----

-------TCACTAGAGTCTATGTCACTCATTATACTCTGTCGAATGTCATTGAATGTCTT
TACATGGGCTTATATGCCTATGAAAATTGTAATACAACTTTCAGCAACGGATCTCTTGGC
TCTCGCATCGATGAAGAACGCAGCGAAATGCGATAAGTAATGTGAATTGCAGAATTCAGT
GAATCATCGAATCTTTGAACGCATCTTGCGCTCCTTGGTATTCCGAGGAGCATGCCTGTT
TGAGTGTCATTAAATTCTCAACTCTCTTCTAC-TTTTTGTAAAAGAGAGCTTGGACTGTG
GAGGCTTGCTGGCCACTTTTTGGGGTCAGCTCCTCTGAAATGCATTAGCGGAACCGTTTG
CGATCTGCCACAAGTGTGATAAGTTATCTACACTGGCGAGGGGATTGCTCTCTGTAATGT
TCAGCTTCTAATTGTCTCTACTTTGTGAGACTACTTTTGAATGCTTGACCTCAAATCAGG
TAGGACTACCCGCTGAACTTAA
>AC5-86
TTTCCGTAGGTGAACCTGCGGAAGGATCATTATTGAATTATGTTTCTAGATAGGTTGTAG
CTGGCTC-TTTAGAGCATGTGCACGCCTGTTTGGACTTCATTTTCATCCACCTGTGCACC
TATTGTAGTCTTTGGTTGGGTTAGGAGGAAGTGGTCATTGTGTCAGCATCTGCTGGATGT
GAGGACTTGCATTGTGAAAGCTTTGCTGTCCTTGATGTGATCATGGAATCTCTTTC----

-------TCACTAGAGTCTATGTCACTCATTATACTCTGTCGAATGTCATTGAATGTCTT
TACATGGGCTTATATGCCTATGAAAATTGTAATACAACTTTCAGCAACGGATCTCTTGGC
TCTCGCATCGATGAAGAACGCAGCGAAATGCGATAAGTAATGTGAATTGCAGAATTCAGT
GAATCATCGAATCTTTGAACGCATCTTGCGCTCCTTGGTATTCCGAGGAGCATGCCTGTT
TGAGTGTCATTAAATTCTCAACTCTCTTCTAC-TTTTTGTAAAAGAGAGCTTGGACTGTG
GAGGCTTGCTGGCCACTTTTTGGGGTCAGCTCCTCTGAAATGCATTAGCGGAACCGTTTG
CGATCTGCCACAAGTGTGATAAGTTATCTACACTGGCGAGGGGATTGCTCTCTGTAATGT
TCAGCTTCTAATTGTCTCTACTTTGTGAGACTACTTTTGAATGCTTGACCTCAAATCAGG
TAGGACTACCCGCTGAACTTAA
>AC11
TTTCCGTAGGTGAACCTGCGGAAGGATCATTATTGAATTATGTTTCTAGATAGGTTGTAG
CTGGCTC-TTTAGAGCATGTGCACGCCTGTTTGGACTTCATTTTCATCCACCTGTGCACC
TATTGTAGTCTTTGGTTGGGTTAGGAGGAAGTGGTCATTGTGTCAGCATCTGCTGGATGT
GAGGACTTGCATTGTGAAAGCTTTGCTGTCCTTGATGTGATCATGGAATCTCTTTC----

-------TCACTAGAGTCTATGTCACTCATTATACTCTGTCGAATGTCATTGAATGTCTT
TACATGGGCTTATATGCCTATGAAAATTGTAATACAACTTTCAGCAACGGATCTCTTGGC
TCTCGCATCGATGAAGAACGCAGCGAAATGCGATAAGTAATGTGAATTGCAGAATTCAGT
GAATCATCGAATCTTTGAACGCATCTTGCGCTCCTTGGTATTCCGAGGAGCATGCCTGTT
TGAGTGTCATTAAATTCTCAACTCTCTTCTAC-TTTTTGTAAAAGAGAGCTTGGACTGTG
GAGGCTTGCTGGCCACTTTTTGGGGTCAGCTCCTCTGAAATGCATTAGCGGAACCGTTTG
CGATCTGCCACAAGTGTGATAAGTTATCTACACTGGCGAGGGGATTGCTCTCTGTAATGT
TCAGCTTCTAATTGTCTCTACTTTGTGAGACTACTTTTGAATGCTTGACCTCAAATCAGG
TAGGACTACCCGCTGAACTTAA
>AC27
TTTCCGTAGGTGAACCTGCGGAAGGATCATTATTGAATTATGTTTCTAGATAGGTTGTAG
CTGGCTC-TTTAGAGCATGTGCACGCCTGTTTGGACTTCATTTTCATCCACCTGTGCACC
TATTGTAGTCTTTGGTTGGGTTAGGAGGAAGTGGTCATTGTGTCAGCATCTGCTGGATGT
GAGGACTTGCATTGTGAAAGCTTTGCTGTCCTTGATGTGATCATGGAATCTCTTTC----

-------TCACTAGAGTCTATGTCACTCATTATACTCTGTCGAATGTCATTGAATGTCTT
TACATGGGCTTATATGCCTATGAAAATTGTAATACAACTTTCAGCAACGGATCTCTTGGC
TCTCGCATCGATGAAGAACGCAGCGAAATGCGATAAGTAATGTGAATTGCAGAATTCAGT
GAATCATCGAATCTTTGAACGCATCTTGCGCTCCTTGGTATTCCGAGGAGCATGCCTGTT
TGAGTGTCATTAAATTCTCAACTCTCTTCTAC-TTTTTGTAAAAGAGAGCTTGGACTGTG
GAGGCTTGCTGGCCACTTTTTGGGGTCAGCTCCTCTGAAATGCATTAGCGGAACCGTTTG
CGATCTGCCACAAGTGTGATAAGTTATCTACACTGGCGAGGGGATTGCTCTCTGTAATGT
TCAGCTTCTAATTGTCTCTACTTTGTGAGACTACTTTTGAATGCTTGACCTCAAATCAGG
TAGGACTACCCGCTGAACTTAA
>AC28
TTTCCGTAGGTGAACCTGCGGAAGGATCATTATTGAATTATGTTTCTAGATAGGTTGTAG
CTGGCTC-TTTAGAGCATGTGCACGCCTGTTTGGACTTCATTTTCATCCACCTGTGCACC
TATTGTAGTCTTTGGTTGGGTTAGGAGGAAGTGGTCATTGTGTCAGCATCTGCTGGATGT

```
GAGGACTTGCATTGTGAAAGCTTTGCTGTCCTTGATGTGATCATGGAATCTCTTTC----
------------------------------------------------------------
------------------------------------------------------------
------------------------------------------------------------
------------------------------------------------------------
------------------------------------------------------------
------------------------------------------------------------
------------------------------------------------------------
------------------------------------------------------------
------------------------------------------------------------
------------------------------------------------------------
------------------------------------------------------------
------------------------------------------------------------
-------TCACTAGAGTCTATGTCACTCATTATACTCTGTCGAATGTCATTGAATGTCTT
TACATGGGCTTATATGCCTATGAAAATTGTAATACAACTTTCAGCAACGGATCTCTTGGC
TCTCGCATCGATGAAGAACGCAGCGAAATGCGATAAGTAATGTGAATTGCAGAATTCAGT
GAATCATCGAATCTTTGAACGCATCTTGCGCTCCTTGGTATTCCGAGGAGCATGCCTGTT
TGAGTGTCATTAAATTCTCAACTCTCTTCTAC-TTTTTGTAAAAGAGAGCTTGGACTGTG
GAGGCTTGCTGGCCACTTTTTGGGGTCAGCTCCTCTGAAATGCATTAGCGGAACCGTTTG
CGATCTGCCACAAGTGTGATAAGTTATCTACACTGGCGAGGGGATTGCTCTCTGTAATGT
TCAGCTTCTAATTGTCTCTACTTTGTGAGACTACTTTTGAATGCTTGACCTCAAATCAGG
TAGGACTACCCGCTGAACTTAA
>AC6-3
TTTCCGTAGGTGAACCTGCGGAAGGATCATTATTGAATTATGTTTCTAGATAGGTTGTAG
CTGGCTC-TTTAGAGCATGTGCACGCCTGTTTGGACTTCATTTTCATCCACCTGTGCACC
TATTGTAGTCTTTGGTTGGGTTAGGAGGAAGTGGTCATTGTGTCAGCATCTGCTGGATGT
GAGGACTTGCATTGTGAAAGCTTTGCTGTCCTTGATGTGATCATGGAATCTCTTTC----
------------------------------------------------------------
------------------------------------------------------------
------------------------------------------------------------
------------------------------------------------------------
------------------------------------------------------------
------------------------------------------------------------
------------------------------------------------------------
------------------------------------------------------------
------------------------------------------------------------
------------------------------------------------------------
------------------------------------------------------------
------------------------------------------------------------
-------TCACTAGAGTCTATGTCACTCATTATACTCTGTCGAATGTCATTGAATGTCTT
TACATGGGCTTATATGCCTATGAAAATTGTAATACAACTTTCAGCAACGGATCTCTTGGC
TCTCGCATCGATGAAGAACGCAGCGAAATGCGATAAGTAATGTGAATTGCAGAATTCAGT
GAATCATCGAATCTTTGAACGCATCTTGCGCTCCTTGGTATTCCGAGGAGCATGCCTGTT
TGAGTGTCATTAAATTCTCAACTCTCTTCTAC-TTTTTGTAAAAGAGAGCTTGGACTGTG
GAGGCTTGCTGGCCACTTTTTGGGGTCAGCTCCTCTGAAATGCATTAGCGGAACCGTTTG
CGATCTGCCACAAGTGTGATAAGTTATCTACACTGGCGAGGGGATTGCTCTCTGTAATGT
TCAGCTTCTAATTGTCTCTACTTTGTGAGACTACTTTTGAATGCTTGACCTCAAATCAGG
TAGGACTACCCGCTGAACTTAA
>AC6-4
TTTCCGTAGGTGAACCTGCGGAAGGATCATTATTGAATTATGTTTCTAGATAGGTTGTAG
```

CTGGCTC-TTTAGAGCATGTGCACGCCTGTTTGGACTTCATTTTCATCCACCTGTGCACC
TATTGTAGTCTTTGGTTGGGTTAGGAGGAAGTGGTCATTGTGTCAGCATCTGCTGGATGT
GAGGACTTGCATTGTGAAAGCTTTGCTGTCCTTGATGTGATCATGGAATCTCTTTC----

-------TCACTAGAGTCTATGTCACTCATTATACTCTGTCGAATGTCATTGAATGTCTT
TACATGGGCTTATATGCCTATGAAAATTGTAATACAACTTTCAGCAACGGATCTCTTGGC
TCTCGCATCGATGAAGAACGCAGCGAAATGCGATAAGTAATGTGAATTGCAGAATTCAGT
GAATCATCGAATCTTTGAACGCATCTTGCGCTCCTTGGTATTCCGAGGAGCATGCCTGTT
TGAGTGTCATTAAATTCTCAACTCTCTTCTAC-TTTTTGTAAAAGAGAGCTTGGACTGTG
GAGGCTTGCTGGCCACTTTTTGGGGTCAGCTCCTCTGAAATGCATTAGCGGAACCGTTTG
CGATCTGCCACAAGTGTGATAAGTTATCTACACTGGCGAGGGGATTGCTCTCTGTAATGT
TCAGCTTCTAATTGTCTCTACTTTGTGAGACTACTTTTGAATGCTTGACCTCAAATCAGG
TAGGACTACCCGCTGAACTTAA
>AC6-9
TTTCCGTAGGTGAACCTGCGGAAGGATCATTATTGAATTATGTTTCTAGATAGGTTGTAG
CTGGCTC-TTTAGAGCATGTGCACGCCTGTTTGGACTTCATTTTCATCCACCTGTGCACC
TATTGTAGTCTTTGGTTGGGTTAGGAGGAAGTGGTCATTGTGTCAGCATCTGCTGGATGT
GAGGACTTGCATTGTGAAAGCTTTGCTGTCCTTGATGTGATCATGGAATCTCTTTC----

-------TCACTAGAGTCTATGTCACTCATTATACTCTGTCGAATGTCATTGAATGTCTT
TACATGGGCTTATATGCCTATGAAAATTGTAATACAACTTTCAGCAACGGATCTCTTGGC
TCTCGCATCGATGAAGAACGCAGCGAAATGCGATAAGTAATGTGAATTGCAGAATTCAGT
GAATCATCGAATCTTTGAACGCATCTTGCGCTCCTTGGTATTCCGAGGAGCATGCCTGTT
TGAGTGTCATTAAATTCTCAACTCTCTTCTAC-TTTTTGTAAAAGAGAGCTTGGACTGTG
GAGGCTTGCTGGCCACTTTTTGGGGTCAGCTCCTCTGAAATGCATTAGCGGAACCGTTTG
CGATCTGCCACAAGTGTGATAAGTTATCTACACTGGCGAGGGGATTGCTCTCTGTAATGT
TCAGCTTCTAATTGTCTCTACTTTGTGAGACTACTTTTGAATGCTTGACCTCAAATCAGG
TAGGACTACCCGCTGAACTTAA

```
>AC6-11
TTTCCGTAGGTGAACCTGCGGAAGGATCATTATTGAATTATGTTTCTAGATAGGTTGTAG
CTGGCTC-TTTAGAGCATGTGCACGCCTGTTTGGACTTCATTTTCATCCACCTGTGCACC
TATTGTAGTCTTTGGTTGGGTTAGGAGGAAGTGGTCATTGTGTCAGCATCTGCTGGATGT
GAGGACTTGCATTGTGAAAGCTTTGCTGTCCTTGATGTGATCATGGAATCTCTTTC----
------------------------------------------------------------
------------------------------------------------------------
------------------------------------------------------------
------------------------------------------------------------
------------------------------------------------------------
------------------------------------------------------------
------------------------------------------------------------
------------------------------------------------------------
------------------------------------------------------------
------------------------------------------------------------
------------------------------------------------------------
------------------------------------------------------------
-------TCACTAGAGTCTATGTCACTCATTATACTCTGTCGAATGTCATTGAATGTCTT
TACATGGGCTTATATGCCTATGAAAATTGTAATACAACTTTCAGCAACGGATCTCTTGGC
TCTCGCATCGATGAAGAACGCAGCGAAATGCGATAAGTAATGTGAATTGCAGAATTCAGT
GAATCATCGAATCTTTGAACGCATCTTGCGCTCCTTGGTATTCCGAGGAGCATGCCTGTT
TGAGTGTCATTAAATTCTCAACTCTCTTCTAC-TTTTTGTAAAAGAGAGCTTGGACTGTG
GAGGCTTGCTGGCCACTTTTTGGGGTCAGCTCCTCTGAAATGCATTAGCGGAACCGTTTG
CGATCTGCCACAAGTGTGATAAGTTATCTACACTGGCGAGGGGATTGCTCTCTGTAATGT
TCAGCTTCTAATTGTCTCTACTTTGTGAGACTACTTTTGAATGCTTGACCTCAAATCAGG
TAGGACTACCCGCTGAACTTAA
>AC6-12
TTTCCGTAGGTGAACCTGCGGAAGGATCATTATTGAATTATGTTTCTAGATAGGTTGTAG
CTGGCTC-TTTAGAGCATGTGCACGCCTGTTTGGACTTCATTTTCATCCACCTGTGCACC
TATTGTAGTCTTTGGTTGGGTTAGGAGGAAGTGGTCATTGTGTCAGCATCTGCTGGATGT
GAGGACTTGCATTGTGAAAGCTTTGCTGTCCTTGATGTGATCATGGAATCTCTTTC----
------------------------------------------------------------
------------------------------------------------------------
------------------------------------------------------------
------------------------------------------------------------
------------------------------------------------------------
------------------------------------------------------------
------------------------------------------------------------
------------------------------------------------------------
------------------------------------------------------------
------------------------------------------------------------
------------------------------------------------------------
------------------------------------------------------------
-------TCACTAGAGTCTATGTCACTCATTATACTCTGTCGAATGTCATTGAATGTCTT
TACATGGGCTTATATGCCTATGAAAATTGTAATACAACTTTCAGCAACGGATCTCTTGGC
TCTCGCATCGATGAAGAACGCAGCGAAATGCGATAAGTAATGTGAATTGCAGAATTCAGT
GAATCATCGAATCTTTGAACGCATCTTGCGCTCCTTGGTATTCCGAGGAGCATGCCTGTT
TGAGTGTCATTAAATTCTCAACTCTCTTCTAC-TTTTTGTAAAAGAGAGCTTGGACTGTG
GAGGCTTGCTGGCCACTTTTTGGGGTCAGCTCCTCTGAAATGCATTAGCGGAACCGTTTG
CGATCTGCCACAAGTGTGATAAGTTATCTACACTGGCGAGGGGATTGCTCTCTGTAATGT
```

TCAGCTTCTAATTGTCTCTACTTTGTGAGACTACTTTTGAATGCTTGACCTCAAATCAGG
TAGGACTACCCGCTGAACTTAA
>AC6-21
TTTCCGTAGGTGAACCTGCGGAAGGATCATTATTGAATTATGTTTCTAGATAGGTTGTAG
CTGGCTC-TTTAGAGCATGTGCACGCCTGTTTGGACTTCATTTTCATCCACCTGTGCACC
TATTGTAGTCTTTGGTTGGGTTAGGAGGAAGTGGTCATTGTGTCAGCATCTGCTGGATGT
GAGGACTTGCATTGTGAAAGCTTTGCTGTCCTTGATGTGATCATGGAATCTCTTTC----

-------TCACTAGAGTCTATGTCACTCATTATACTCTGTCGAATGTCATTGAATGTCTT
TACATGGGCTTATATGCCTATGAAAATTGTAATACAACTTTCAGCAACGGATCTCTTGGC
TCTCGCATCGATGAAGAACGCAGCGAAATGCGATAAGTAATGTGAATTGCAGAATTCAGT
GAATCATCGAATCTTTGAACGCATCTTGCGCTCCTTGGTATTCCGAGGAGCATGCCTGTT
TGAGTGTCATTAAATTCTCAACTCTCTTCTAC-TTTTTGTAAAAGAGAGCTTGGACTGTG
GAGGCTTGCTGGCCACTTTTTGGGGTCAGCTCCTCTGAAATGCATTAGCGGAACCGTTTG
CGATCTGCCACAAGTGTGATAAGTTATCTACACTGGCGAGGGGATTGCTCTCTGTAATGT
TCAGCTTCTAATTGTCTCTACTTTGTGAGACTACTTTTGAATGCTTGACCTCAAATCAGG
TAGGACTACCCGCTGAACTTAA
>AC6-38
TTTCCGTAGGTGAACCTGCGGAAGGATCATTATTGAATTATGTTTCTAGATAGGTTGTAG
CTGGCTC-TTTAGAGCATGTGCACGCCTGTTTGGACTTCATTTTCATCCACCTGTGCACC
TATTGTAGTCTTTGGTTGGGTTAGGAGGAAGTGGTCATTGTGTCAGCATCTGCTGGATGT
GAGGACTTGCATTGTGAAAGCTTTGCTGTCCTTGATGTGATCATGGAATCTCTTTC----

-------TCACTAGAGTCTATGTCACTCATTATACTCTGTCGAATGTCATTGAATGTCTT
TACATGGGCTTATATGCCTATGAAAATTGTAATACAACTTTCAGCAACGGATCTCTTGGC
TCTCGCATCGATGAAGAACGCAGCGAAATGCGATAAGTAATGTGAATTGCAGAATTCAGT
GAATCATCGAATCTTTGAACGCATCTTGCGCTCCTTGGTATTCCGAGGAGCATGCCTGTT
TGAGTGTCATTAAATTCTCAACTCTCTTCTAC-TTTTTGTAAAAGAGAGCTTGGACTGTG

GAGGCTTGCTGGCCACTTTTTGGGGTCAGCTCCTCTGAAATGCATTAGCGGAACCGTTTG
CGATCTGCCACAAGTGTGATAAGTTATCTACACTGGCGAGGGGATTGCTCTCTGTAATGT
TCAGCTTCTAATTGTCTCTACTTTGTGAGACTACTTTTGAATGCTTGACCTCAAATCAGG
TAGGACTACCCGCTGAACTTAA
>AC6-42
TTTCCGTAGGTGAACCTGCGGAAGGATCATTATTGAATTATGTTTCTAGATAGGTTGTAG
CTGGCTC-TTTAGAGCATGTGCACGCCTGTTTGGACTTCATTTTCATCCACCTGTGCACC
TATTGTAGTCTTTGGTTGGGTTAGGAGGAAGTGGTCATTGTGTCAGCATCTGCTGGATGT
GAGGACTTGCATTGTGAAAGCTTTGCTGTCCTTGATGTGATCATGGAATCTCTTTC----

-------TCACTAGAGTCTATGTCACTCATTATACTCTGTCGAATGTCATTGAATGTCTT
TACATGGGCTTATATGCCTATGAAAATTGTAATACAACTTTCAGCAACGGATCTCTTGGC
TCTCGCATCGATGAAGAACGCAGCGAAATGCGATAAGTAATGTGAATTGCAGAATTCAGT
GAATCATCGAATCTTTGAACGCATCTTGCGCTCCTTGGTATTCCGAGGAGCATGCCTGTT
TGAGTGTCATTAAATTCTCAACTCTCTTCTAC-TTTTTGTAAAAGAGAGCTTGGACTGTG
GAGGCTTGCTGGCCACTTTTTGGGGTCAGCTCCTCTGAAATGCATTAGCGGAACCGTTTG
CGATCTGCCACAAGTGTGATAAGTTATCTACACTGGCGAGGGGATTGCTCTCTGTAATGT
TCAGCTTCTAATTGTCTCTACTTTGTGAGACTACTTTTGAATGCTTGACCTCAAATCAGG
TAGGACTACCCGCTGAACTTAA
>AC6-46
TTTCCGTAGGTGAACCTGCGGAAGGATCATTATTGAATTATGTTTCTAGATAGGTTGTAG
CTGGCTC-TTTAGAGCATGTGCACGCCTGTTTGGACTTCATTTTCATCCACCTGTGCACC
TATTGTAGTCTTTGGTTGGGTTAGGAGGAAGTGGTCATTGTGTCAGCATCTGCTGGATGT
GAGGACTTGCATTGTGAAAGCTTTGCTGTCCTTGATGTGATCATGGAATCTCTTTC----

-------TCACTAGAGTCTATGTCACTCATTATACTCTGTCGAATGTCATTGAATGTCTT
TACATGGGCTTATATGCCTATGAAAATTGTAATACAACTTTCAGCAACGGATCTCTTGGC
TCTCGCATCGATGAAGAACGCAGCGAAATGCGATAAGTAATGTGAATTGCAGAATTCAGT

GAATCATCGAATCTTTGAACGCATCTTGCGCTCCTTGGTATTCCGAGGAGCATGCCTGTT
TGAGTGTCATTAAATTCTCAACTCTCTTCTAC-TTTTTGTAAAAGAGAGCTTGGACTGTG
GAGGCTTGCTGGCCACTTTTTGGGGTCAGCTCCTCTGAAATGCATTAGCGGAACCGTTTG
CGATCTGCCACAAGTGTGATAAGTTATCTACACTGGCGAGGGGATTGCTCTCTGTAATGT
TCAGCTTCTAATTGTCTCTACTTTGTGAGACTACTTTTGAATGCTTGACCTCAAATCAGG
TAGGACTACCCGCTGAACTTAA
>AC6-48
TTTCCGTAGGTGAACCTGCGGAAGGATCATTATTGAATTATGTTTCTAGATAGGTTGTAG
CTGGCTC-TTTAGAGCATGTGCACGCCTGTTTGGACTTCATTTTCATCCACCTGTGCACC
TATTGTAGTCTTTGGTTGGGTTAGGAGGAAGTGGTCATTGTGTCAGCATCTGCTGGATGT
GAGGACTTGCATTGTGAAAGCTTTGCTGTCCTTGATGTGATCATGGAATCTCTTTC----

-------TCACTAGAGTCTATGTCACTCATTATACTCTGTCGAATGTCATTGAATGTCTT
TACATGGGCTTATATGCCTATGAAAATTGTAATACAACTTTCAGCAACGGATCTCTTGGC
TCTCGCATCGATGAAGAACGCAGCGAAATGCGATAAGTAATGTGAATTGCAGAATTCAGT
GAATCATCGAATCTTTGAACGCATCTTGCGCTCCTTGGTATTCCGAGGAGCATGCCTGTT
TGAGTGTCATTAAATTCTCAACTCTCTTCTAC-TTTTTGTAAAAGAGAGCTTGGACTGTG
GAGGCTTGCTGGCCACTTTTTGGGGTCAGCTCCTCTGAAATGCATTAGCGGAACCGTTTG
CGATCTGCCACAAGTGTGATAAGTTATCTACACTGGCGAGGGGATTGCTCTCTGTAATGT
TCAGCTTCTAATTGTCTCTACTTTGTGAGACTACTTTTGAATGCTTGACCTCAAATCAGG
TAGGACTACCCGCTGAACTTAA
>AC6-53
TTTCCGTAGGTGAACCTGCGGAAGGATCATTATTGAATTATGTTTCTAGATAGGTTGTAG
CTGGCTC-TTTAGAGCATGTGCACGCCTGTTTGGACTTCATTTTCATCCACCTGTGCACC
TATTGTAGTCTTTGGTTGGGTTAGGAGGAAGTGGTCATTGTGTCAGCATCTGCTGGATGT
GAGGACTTGCATTGTGAAAGCTTTGCTGTCCTTGATGTGATCATGGAATCTCTTTC----

-------TCACTAGAGTCTATGTCACTCATTATACTCTGTCGAATGTCATTGAATGTCTT

TACATGGGCTTATATGCCTATGAAAATTGTAATACAACTTTCAGCAACGGATCTCTTGGC
TCTCGCATCGATGAAGAACGCAGCGAAATGCGATAAGTAATGTGAATTGCAGAATTCAGT
GAATCATCGAATCTTTGAACGCATCTTGCGCTCCTTGGTATTCCGAGGAGCATGCCTGTT
TGAGTGTCATTAAATTCTCAACTCTCTTCTAC-TTTTTGTAAAAGAGAGCTTGGACTGTG
GAGGCTTGCTGGCCACTTTTTGGGGTCAGCTCCTCTGAAATGCATTAGCGGAACCGTTTG
CGATCTGCCACAAGTGTGATAAGTTATCTACACTGGCGAGGGGATTGCTCTCTGTAATGT
TCAGCTTCTAATTGTCTCTACTTTGTGAGACTACTTTTGAATGCTTGACCTCAAATCAGG
TAGGACTACCCGCTGAACTTAA
>AC6-54
TTTCCGTAGGTGAACCTGCGGAAGGATCATTATTGAATTATGTTTCTAGATAGGTTGTAG
CTGGCTC-TTTAGAGCATGTGCACGCCTGTTTGGACTTCATTTTCATCCACCTGTGCACC
TATTGTAGTCTTTGGTTGGGTTAGGAGGAAGTGGTCATTGTGTCAGCATCTGCTGGATGT
GAGGACTTGCATTGTGAAAGCTTTGCTGTCCTTGATGTGATCATGGAATCTCTTTC----

-------TCACTAGAGTCTATGTCACTCATTATACTCTGTCGAATGTCATTGAATGTCTT
TACATGGGCTTATATGCCTATGAAAATTGTAATACAACTTTCAGCAACGGATCTCTTGGC
TCTCGCATCGATGAAGAACGCAGCGAAATGCGATAAGTAATGTGAATTGCAGAATTCAGT
GAATCATCGAATCTTTGAACGCATCTTGCGCTCCTTGGTATTCCGAGGAGCATGCCTGTT
TGAGTGTCATTAAATTCTCAACTCTCTTCTAC-TTTTTGTAAAAGAGAGCTTGGACTGTG
GAGGCTTGCTGGCCACTTTTTGGGGTCAGCTCCTCTGAAATGCATTAGCGGAACCGTTTG
CGATCTGCCACAAGTGTGATAAGTTATCTACACTGGCGAGGGGATTGCTCTCTGTAATGT
TCAGCTTCTAATTGTCTCTACTTTGTGAGACTACTTTTGAATGCTTGACCTCAAATCAGG
TAGGACTACCCGCTGAACTTAA
>AC6-55
TTTCCGTAGGTGAACCTGCGGAAGGATCATTATTGAATTATGTTTCTAGATAGGTTGTAG
CTGGCTC-TTTAGAGCATGTGCACGCCTGTTTGGACTTCATTTTCATCCACCTGTGCACC
TATTGTAGTCTTTGGTTGGGTTAGGAGGAAGTGGTCATTGTGTCAGCATCTGCTGGATGT
GAGGACTTGCATTGTGAAAGCTTTGCTGTCCTTGATGTGATCATGGAATCTCTTTC----


```
------------------------------------------------------------
-------TCACTAGAGTCTATGTCACTCATTATACTCTGTCGAATGTCATTGAATGTCTT
TACATGGGCTTATATGCCTATGAAAATTGTAATACAACTTTCAGCAACGGATCTCTTGGC
TCTCGCATCGATGAAGAACGCAGCGAAATGCGATAAGTAATGTGAATTGCAGAATTCAGT
GAATCATCGAATCTTTGAACGCATCTTGCGCTCCTTGGTATTCCGAGGAGCATGCCTGTT
TGAGTGTCATTAAATTCTCAACTCTCTTCTAC-TTTTTGTAAAAGAGAGCTTGGACTGTG
GAGGCTTGCTGGCCACTTTTTGGGGTCAGCTCCTCTGAAATGCATTAGCGGAACCGTTTG
CGATCTGCCACAAGTGTGATAAGTTATCTACACTGGCGAGGGGATTGCTCTCTGTAATGT
TCAGCTTCTAATTGTCTCTACTTTGTGAGACTACTTTTGAATGCTTGACCTCAAATCAGG
TAGGACTACCCGCTGAACTTAA
>AC6-59
TTTCCGTAGGTGAACCTGCGGAAGGATCATTATTGAATTATGTTTCTAGATAGGTTGTAG
CTGGCTC-TTTAGAGCATGTGCACGCCTGTTTGGACTTCATTTTCATCCACCTGTGCACC
TATTGTAGTCTTTGGTTGGGTTAGGAGGAAGTGGTCATTGTGTCAGCATCTGCTGGATGT
GAGGACTTGCATTGTGAAAGCTTTGCTGTCCTTGATGTGATCATGGAATCTCTTTC----
------------------------------------------------------------
------------------------------------------------------------
------------------------------------------------------------
------------------------------------------------------------
------------------------------------------------------------
------------------------------------------------------------
------------------------------------------------------------
------------------------------------------------------------
------------------------------------------------------------
------------------------------------------------------------
------------------------------------------------------------
------------------------------------------------------------
-------TCACTAGAGTCTATGTCACTCATTATACTCTGTCGAATGTCATTGAATGTCTT
TACATGGGCTTATATGCCTATGAAAATTGTAATACAACTTTCAGCAACGGATCTCTTGGC
TCTCGCATCGATGAAGAACGCAGCGAAATGCGATAAGTAATGTGAATTGCAGAATTCAGT
GAATCATCGAATCTTTGAACGCATCTTGCGCTCCTTGGTATTCCGAGGAGCATGCCTGTT
TGAGTGTCATTAAATTCTCAACTCTCTTCTAC-TTTTTGTAAAAGAGAGCTTGGACTGTG
GAGGCTTGCTGGCCACTTTTTGGGGTCAGCTCCTCTGAAATGCATTAGCGGAACCGTTTG
CGATCTGCCACAAGTGTGATAAGTTATCTACACTGGCGAGGGGATTGCTCTCTGTAATGT
TCAGCTTCTAATTGTCTCTACTTTGTGAGACTACTTTTGAATGCTTGACCTCAAATCAGG
TAGGACTACCCGCTGAACTTAA
>AC7-2
TTTCCGTAGGTGAACCTGCGGAAGGATCATTATTGAATTATGTTTCTAGATAGGTTGTAG
CTGGCTC-TTTAGAGCATGTGCACGCCTGTTTGGACTTCATTTTCATCCACCTGTGCACC
TATTGTAGTCTTTGGTTGGGTTAGGAGGAAGTGGTCATTGTGTCAGCATCTGCTGGATGT
GAGGACTTGCATTGTGAAAGCTTTGCTGTCCTTGATGTGATCATGGAATCTCTTTC----
------------------------------------------------------------
------------------------------------------------------------
------------------------------------------------------------
------------------------------------------------------------
------------------------------------------------------------
------------------------------------------------------------
------------------------------------------------------------
------------------------------------------------------------
------------------------------------------------------------
```


-------TCACTAGAGTCTATGTCACTCATTATACTCTGTCGAATGTCATTGAATGTCTT
TACATGGGCTTATATGCCTATGAAAATTGTAATACAACTTTCAGCAACGGATCTCTTGGC
TCTCGCATCGATGAAGAACGCAGCGAAATGCGATAAGTAATGTGAATTGCAGAATTCAGT
GAATCATCGAATCTTTGAACGCATCTTGCGCTCCTTGGTATTCCGAGGAGCATGCCTGTT
TGAGTGTCATTAAATTCTCAACTCTCTTCTAC-TTTTTGTAAAAGAGAGCTTGGACTGTG
GAGGCTTGCTGGCCACTTTTTGGGGTCAGCTCCTCTGAAATGCATTAGCGGAACCGTTTG
CGATCTGCCACAAGTGTGATAAGTTATCTACACTGGCGAGGGGATTGCTCTCTGTAATGT
TCAGCTTCTAATTGTCTCTACTTTGTGAGACTACTTTTGAATGCTTGACCTCAAATCAGG
TAGGACTACCCGCTGAACTTAA
>AC7-5
TTTCCGTAGGTGAACCTGCGGAAGGATCATTATTGAATTATGTTTCTAGATAGGTTGTAG
CTGGCTC-TTTAGAGCATGTGCACGCCTGTTTGGACTTCATTTTCATCCACCTGTGCACC
TATTGTAGTCTTTGGTTGGGTTAGGAGGAAGTGGTCATTGTGTCAGCATCTGCTGGATGT
GAGGACTTGCATTGTGAAAGCTTTGCTGTCCTTGATGTGATCATGGAATCTCTTTC----

-------TCACTAGAGTCTATGTCACTCATTATACTCTGTCGAATGTCATTGAATGTCTT
TACATGGGCTTATATGCCTATGAAAATTGTAATACAACTTTCAGCAACGGATCTCTTGGC
TCTCGCATCGATGAAGAACGCAGCGAAATGCGATAAGTAATGTGAATTGCAGAATTCAGT
GAATCATCGAATCTTTGAACGCATCTTGCGCTCCTTGGTATTCCGAGGAGCATGCCTGTT
TGAGTGTCATTAAATTCTCAACTCTCTTCTAC-TTTTTGTAAAAGAGAGCTTGGACTGTG
GAGGCTTGCTGGCCACTTTTTGGGGTCAGCTCCTCTGAAATGCATTAGCGGAACCGTTTG
CGATCTGCCACAAGTGTGATAAGTTATCTACACTGGCGAGGGGATTGCTCTCTGTAATGT
TCAGCTTCTAATTGTCTCTACTTTGTGAGACTACTTTTGAATGCTTGACCTCAAATCAGG
TAGGACTACCCGCTGAACTTAA
>AC7-7
TTTCCGTAGGTGAACCTGCGGAAGGATCATTATTGAATTATGTTTCTAGATAGGTTGTAG
CTGGCTC-TTTAGAGCATGTGCACGCCTGTTTGGACTTCATTTTCATCCACCTGTGCACC
TATTGTAGTCTTTGGTTGGGTTAGGAGGAAGTGGTCATTGTGTCAGCATCTGCTGGATGT
GAGGACTTGCATTGTGAAAGCTTTGCTGTCCTTGATGTGATCATGGAATCTCTTTC----


```
------------------------------------------------------------
------------------------------------------------------------
------------------------------------------------------------
------------------------------------------------------------
------------------------------------------------------------
-------TCACTAGAGTCTATGTCACTCATTATACTCTGTCGAATGTCATTGAATGTCTT
TACATGGGCTTATATGCCTATGAAAATTGTAATACAACTTTCAGCAACGGATCTCTTGGC
TCTCGCATCGATGAAGAACGCAGCGAAATGCGATAAGTAATGTGAATTGCAGAATTCAGT
GAATCATCGAATCTTTGAACGCATCTTGCGCTCCTTGGTATTCCGAGGAGCATGCCTGTT
TGAGTGTCATTAAATTCTCAACTCTCTTCTAC-TTTTTGTAAAAGAGAGCTTGGACTGTG
GAGGCTTGCTGGCCACTTTTTGGGGTCAGCTCCTCTGAAATGCATTAGCGGAACCGTTTG
CGATCTGCCACAAGTGTGATAAGTTATCTACACTGGCGAGGGGATTGCTCTCTGTAATGT
TCAGCTTCTAATTGTCTCTACTTTGTGAGACTACTTTTGAATGCTTGACCTCAAATCAGG
TAGGACTACCCGCTGAACTTAA
>AC7-9
TTTCCGTAGGTGAACCTGCGGAAGGATCATTATTGAATTATGTTTCTAGATAGGTTGTAG
CTGGCTC-TTTAGAGCATGTGCACGCCTGTTTGGACTTCATTTTCATCCACCTGTGCACC
TATTGTAGTCTTTGGTTGGGTTAGGAGGAAGTGGTCATTGTGTCAGCATCTGCTGGATGT
GAGGACTTGCATTGTGAAAGCTTTGCTGTCCTTGATGTGATCATGGAATCTCTTTC----
------------------------------------------------------------
------------------------------------------------------------
------------------------------------------------------------
------------------------------------------------------------
------------------------------------------------------------
------------------------------------------------------------
------------------------------------------------------------
------------------------------------------------------------
------------------------------------------------------------
------------------------------------------------------------
------------------------------------------------------------
------------------------------------------------------------
-------TCACTAGAGTCTATGTCACTCATTATACTCTGTCGAATGTCATTGAATGTCTT
TACATGGGCTTATATGCCTATGAAAATTGTAATACAACTTTCAGCAACGGATCTCTTGGC
TCTCGCATCGATGAAGAACGCAGCGAAATGCGATAAGTAATGTGAATTGCAGAATTCAGT
GAATCATCGAATCTTTGAACGCATCTTGCGCTCCTTGGTATTCCGAGGAGCATGCCTGTT
TGAGTGTCATTAAATTCTCAACTCTCTTCTAC-TTTTTGTAAAAGAGAGCTTGGACTGTG
GAGGCTTGCTGGCCACTTTTTGGGGTCAGCTCCTCTGAAATGCATTAGCGGAACCGTTTG
CGATCTGCCACAAGTGTGATAAGTTATCTACACTGGCGAGGGGATTGCTCTCTGTAATGT
TCAGCTTCTAATTGTCTCTACTTTGTGAGACTACTTTTGAATGCTTGACCTCAAATCAGG
TAGGACTACCCGCTGAACTTAA
>AC7-13
TTTCCGTAGGTGAACCTGCGGAAGGATCATTATTGAATTATGTTTCTAGATAGGTTGTAG
CTGGCTC-TTTAGAGCATGTGCACGCCTGTTTGGACTTCATTTTCATCCACCTGTGCACC
TATTGTAGTCTTTGGTTGGGTTAGGAGGAAGTGGTCATTGTGTCAGCATCTGCTGGATGT
GAGGACTTGCATTGTGAAAGCTTTGCTGTCCTTGATGTGATCATGGAATCTCTTTC----
------------------------------------------------------------
------------------------------------------------------------
------------------------------------------------------------
------------------------------------------------------------
------------------------------------------------------------
```


-------TCACTAGAGTCTATGTCACTCATTATACTCTGTCGAATGTCATTGAATGTCTT
TACATGGGCTTATATGCCTATGAAAATTGTAATACAACTTTCAGCAACGGATCTCTTGGC
TCTCGCATCGATGAAGAACGCAGCGAAATGCGATAAGTAATGTGAATTGCAGAATTCAGT
GAATCATCGAATCTTTGAACGCATCTTGCGCTCCTTGGTATTCCGAGGAGCATGCCTGTT
TGAGTGTCATTAAATTCTCAACTCTCTTCTAC-TTTTTGTAAAAGAGAGCTTGGACTGTG
GAGGCTTGCTGGCCACTTTTTGGGGTCAGCTCCTCTGAAATGCATTAGCGGAACCGTTTG
CGATCTGCCACAAGTGTGATAAGTTATCTACACTGGCGAGGGGATTGCTCTCTGTAATGT
TCAGCTTCTAATTGTCTCTACTTTGTGAGACTACTTTTGAATGCTTGACCTCAAATCAGG
TAGGACTACCCGCTGAACTTAA
>AC7-18
TTTCCGTAGGTGAACCTGCGGAAGGATCATTATTGAATTATGTTTCTAGATAGGTTGTAG
CTGGCTC-TTTAGAGCATGTGCACGCCTGTTTGGACTTCATTTTCATCCACCTGTGCACC
TATTGTAGTCTTTGGTTGGGTTAGGAGGAAGTGGTCATTGTGTCAGCATCTGCTGGATGT
GAGGACTTGCATTGTGAAAGCTTTGCTGTCCTTGATGTGATCATGGAATCTCTTTC----

-------TCACTAGAGTCTATGTCACTCATTATACTCTGTCGAATGTCATTGAATGTCTT
TACATGGGCTTATATGCCTATGAAAATTGTAATACAACTTTCAGCAACGGATCTCTTGGC
TCTCGCATCGATGAAGAACGCAGCGAAATGCGATAAGTAATGTGAATTGCAGAATTCAGT
GAATCATCGAATCTTTGAACGCATCTTGCGCTCCTTGGTATTCCGAGGAGCATGCCTGTT
TGAGTGTCATTAAATTCTCAACTCTCTTCTAC-TTTTTGTAAAAGAGAGCTTGGACTGTG
GAGGCTTGCTGGCCACTTTTTGGGGTCAGCTCCTCTGAAATGCATTAGCGGAACCGTTTG
CGATCTGCCACAAGTGTGATAAGTTATCTACACTGGCGAGGGGATTGCTCTCTGTAATGT
TCAGCTTCTAATTGTCTCTACTTTGTGAGACTACTTTTGAATGCTTGACCTCAAATCAGG
TAGGACTACCCGCTGAACTTAA
>AC7-19
TTTCCGTAGGTGAACCTGCGGAAGGATCATTATTGAATTATGTTTCTAGATAGGTTGTAG
CTGGCTC-TTTAGAGCATGTGCACGCCTGTTTGGACTTCATTTTCATCCACCTGTGCACC
TATTGTAGTCTTTGGTTGGGTTAGGAGGAAGTGGTCATTGTGTCAGCATCTGCTGGATGT
GAGGACTTGCATTGTGAAAGCTTTGCTGTCCTTGATGTGATCATGGAATCTCTTTC----

-------TCACTAGAGTCTATGTCACTCATTATACTCTGTCGAATGTCATTGAATGTCTT
TACATGGGCTTATATGCCTATGAAAATTGTAATACAACTTTCAGCAACGGATCTCTTGGC
TCTCGCATCGATGAAGAACGCAGCGAAATGCGATAAGTAATGTGAATTGCAGAATTCAGT
GAATCATCGAATCTTTGAACGCATCTTGCGCTCCTTGGTATTCCGAGGAGCATGCCTGTT
TGAGTGTCATTAAATTCTCAACTCTCTTCTAC-TTTTTGTAAAAGAGAGCTTGGACTGTG
GAGGCTTGCTGGCCACTTTTTGGGGTCAGCTCCTCTGAAATGCATTAGCGGAACCGTTTG
CGATCTGCCACAAGTGTGATAAGTTATCTACACTGGCGAGGGGATTGCTCTCTGTAATGT
TCAGCTTCTAATTGTCTCTACTTTGTGAGACTACTTTTGAATGCTTGACCTCAAATCAGG
TAGGACTACCCGCTGAACTTAA
>AC7-26
TTTCCGTAGGTGAACCTGCGGAAGGATCATTATTGAATTATGTTTCTAGATAGGTTGTAG
CTGGCTC-TTTAGAGCATGTGCACGCCTGTTTGGACTTCATTTTCATCCACCTGTGCACC
TATTGTAGTCTTTGGTTGGGTTAGGAGGAAGTGGTCATTGTGTCAGCATCTGCTGGATGT
GAGGACTTGCATTGTGAAAGCTTTGCTGTCCTTGATGTGATCATGGAATCTCTTTC----

-------TCACTAGAGTCTATGTCACTCATTATACTCTGTCGAATGTCATTGAATGTCTT
TACATGGGCTTATATGCCTATGAAAATTGTAATACAACTTTCAGCAACGGATCTCTTGGC
TCTCGCATCGATGAAGAACGCAGCGAAATGCGATAAGTAATGTGAATTGCAGAATTCAGT
GAATCATCGAATCTTTGAACGCATCTTGCGCTCCTTGGTATTCCGAGGAGCATGCCTGTT
TGAGTGTCATTAAATTCTCAACTCTCTTCTAC-TTTTTGTAAAAGAGAGCTTGGACTGTG
GAGGCTTGCTGGCCACTTTTTGGGGTCAGCTCCTCTGAAATGCATTAGCGGAACCGTTTG
CGATCTGCCACAAGTGTGATAAGTTATCTACACTGGCGAGGGGATTGCTCTCTGTAATGT
TCAGCTTCTAATTGTCTCTACTTTGTGAGACTACTTTTGAATGCTTGACCTCAAATCAGG
TAGGACTACCCGCTGAACTTAA
>AC7-28
TTTCCGTAGGTGAACCTGCGGAAGGATCATTATTGAATTATGTTTCTAGATAGGTTGTAG
CTGGCTC-TTTAGAGCATGTGCACGCCTGTTTGGACTTCATTTTCATCCACCTGTGCACC
TATTGTAGTCTTTGGTTGGGTTAGGAGGAAGTGGTCATTGTGTCAGCATCTGCTGGATGT
GAGGACTTGCATTGTGAAAGCTTTGCTGTCCTTGATGTGATCATGGAATCTCTTTC----

-------TCACTAGAGTCTATGTCACTCATTATACTCTGTCGAATGTCATTGAATGTCTT
TACATGGGCTTATATGCCTATGAAAATTGTAATACAACTTTCAGCAACGGATCTCTTGGC
TCTCGCATCGATGAAGAACGCAGCGAAATGCGATAAGTAATGTGAATTGCAGAATTCAGT
GAATCATCGAATCTTTGAACGCATCTTGCGCTCCTTGGTATTCCGAGGAGCATGCCTGTT
TGAGTGTCATTAAATTCTCAACTCTCTTCTAC-TTTTTGTAAAAGAGAGCTTGGACTGTG
GAGGCTTGCTGGCCACTTTTTGGGGTCAGCTCCTCTGAAATGCATTAGCGGAACCGTTTG
CGATCTGCCACAAGTGTGATAAGTTATCTACACTGGCGAGGGGATTGCTCTCTGTAATGT
TCAGCTTCTAATTGTCTCTACTTTGTGAGACTACTTTTGAATGCTTGACCTCAAATCAGG
TAGGACTACCCGCTGAACTTAA
>AC7-31
TTTCCGTAGGTGAACCTGCGGAAGGATCATTATTGAATTATGTTTCTAGATAGGTTGTAG
CTGGCTC-TTTAGAGCATGTGCACGCCTGTTTGGACTTCATTTTCATCCACCTGTGCACC
TATTGTAGTCTTTGGTTGGGTTAGGAGGAAGTGGTCATTGTGTCAGCATCTGCTGGATGT
GAGGACTTGCATTGTGAAAGCTTTGCTGTCCTTGATGTGATCATGGAATCTCTTTC----

-------TCACTAGAGTCTATGTCACTCATTATACTCTGTCGAATGTCATTGAATGTCTT
TACATGGGCTTATATGCCTATGAAAATTGTAATACAACTTTCAGCAACGGATCTCTTGGC
TCTCGCATCGATGAAGAACGCAGCGAAATGCGATAAGTAATGTGAATTGCAGAATTCAGT
GAATCATCGAATCTTTGAACGCATCTTGCGCTCCTTGGTATTCCGAGGAGCATGCCTGTT
TGAGTGTCATTAAATTCTCAACTCTCTTCTAC-TTTTTGTAAAAGAGAGCTTGGACTGTG
GAGGCTTGCTGGCCACTTTTTGGGGTCAGCTCCTCTGAAATGCATTAGCGGAACCGTTTG
CGATCTGCCACAAGTGTGATAAGTTATCTACACTGGCGAGGGGATTGCTCTCTGTAATGT
TCAGCTTCTAATTGTCTCTACTTTGTGAGACTACTTTTGAATGCTTGACCTCAAATCAGG
TAGGACTACCCGCTGAACTTAA
>AC7-38
TTTCCGTAGGTGAACCTGCGGAAGGATCATTATTGAATTATGTTTCTAGATAGGTTGTAG
CTGGCTC-TTTAGAGCATGTGCACGCCTGTTTGGACTTCATTTTCATCCACCTGTGCACC
TATTGTAGTCTTTGGTTGGGTTAGGAGGAAGTGGTCATTGTGTCAGCATCTGCTGGATGT

GAGGACTTGCATTGTGAAAGCTTTGCTGTCCTTGATGTGATCATGGAATCTCTTTC----

-------TCACTAGAGTCTATGTCACTCATTATACTCTGTCGAATGTCATTGAATGTCTT
TACATGGGCTTATATGCCTATGAAAATTGTAATACAACTTTCAGCAACGGATCTCTTGGC
TCTCGCATCGATGAAGAACGCAGCGAAATGCGATAAGTAATGTGAATTGCAGAATTCAGT
GAATCATCGAATCTTTGAACGCATCTTGCGCTCCTTGGTATTCCGAGGAGCATGCCTGTT
TGAGTGTCATTAAATTCTCAACTCTCTTCTAC-TTTTTGTAAAAGAGAGCTTGGACTGTG
GAGGCTTGCTGGCCACTTTTTGGGGTCAGCTCCTCTGAAATGCATTAGCGGAACCGTTTG
CGATCTGCCACAAGTGTGATAAGTTATCTACACTGGCGAGGGGATTGCTCTCTGTAATGT
TCAGCTTCTAATTGTCTCTACTTTGTGAGACTACTTTTGAATGCTTGACCTCAAATCAGG
TAGGACTACCCGCTGAACTTAA
>AC7-45
TTTCCGTAGGTGAACCTGCGGAAGGATCATTATTGAATTATGTTTCTAGATAGGTTGTAG
CTGGCTC-TTTAGAGCATGTGCACGCCTGTTTGGACTTCATTTTCATCCACCTGTGCACC
TATTGTAGTCTTTGGTTGGGTTAGGAGGAAGTGGTCATTGTGTCAGCATCTGCTGGATGT
GAGGACTTGCATTGTGAAAGCTTTGCTGTCCTTGATGTGATCATGGAATCTCTTTC----

-------TCACTAGAGTCTATGTCACTCATTATACTCTGTCGAATGTCATTGAATGTCTT
TACATGGGCTTATATGCCTATGAAAATTGTAATACAACTTTCAGCAACGGATCTCTTGGC
TCTCGCATCGATGAAGAACGCAGCGAAATGCGATAAGTAATGTGAATTGCAGAATTCAGT
GAATCATCGAATCTTTGAACGCATCTTGCGCTCCTTGGTATTCCGAGGAGCATGCCTGTT
TGAGTGTCATTAAATTCTCAACTCTCTTCTAC-TTTTTGTAAAAGAGAGCTTGGACTGTG
GAGGCTTGCTGGCCACTTTTTGGGGTCAGCTCCTCTGAAATGCATTAGCGGAACCGTTTG
CGATCTGCCACAAGTGTGATAAGTTATCTACACTGGCGAGGGGATTGCTCTCTGTAATGT
TCAGCTTCTAATTGTCTCTACTTTGTGAGACTACTTTTGAATGCTTGACCTCAAATCAGG
TAGGACTACCCGCTGAACTTAA
>AC7-58
TTTCCGTAGGTGAACCTGCGGAAGGATCATTATTGAATTATGTTTCTAGATAGGTTGTAG

CTGGCTC-TTTAGAGCATGTGCACGCCTGTTTGGACTTCATTTTCATCCACCTGTGCACC
TATTGTAGTCTTTGGTTGGGTTAGGAGGAAGTGGTCATTGTGTCAGCATCTGCTGGATGT
GAGGACTTGCATTGTGAAAGCTTTGCTGTCCTTGATGTGATCATGGAATCTCTTTC----

-------TCACTAGAGTCTATGTCACTCATTATACTCTGTCGAATGTCATTGAATGTCTT
TACATGGGCTTATATGCCTATGAAAATTGTAATACAACTTTCAGCAACGGATCTCTTGGC
TCTCGCATCGATGAAGAACGCAGCGAAATGCGATAAGTAATGTGAATTGCAGAATTCAGT
GAATCATCGAATCTTTGAACGCATCTTGCGCTCCTTGGTATTCCGAGGAGCATGCCTGTT
TGAGTGTCATTAAATTCTCAACTCTCTTCTAC-TTTTTGTAAAAGAGAGCTTGGACTGTG
GAGGCTTGCTGGCCACTTTTTGGGGTCAGCTCCTCTGAAATGCATTAGCGGAACCGTTTG
CGATCTGCCACAAGTGTGATAAGTTATCTACACTGGCGAGGGGATTGCTCTCTGTAATGT
TCAGCTTCTAATTGTCTCTACTTTGTGAGACTACTTTTGAATGCTTGACCTCAAATCAGG
TAGGACTACCCGCTGAACTTAA
>AC8-3
TTTCCGTAGGTGAACCTGCGGAAGGATCATTATTGAATTATGTTTCTAGATAGGTTGTAG
CTGGCTC-TTTAGAGCATGTGCACGCCTGTTTGGACTTCATTTTCATCCACCTGTGCACC
TATTGTAGTCTTTGGTTGGGTTAGGAGGAAGTGGTCATTGTGTCAGCATCTGCTGGATGT
GAGGACTTGCATTGTGAAAGCTTTGCTGTCCTTGATGTGATCATGGAATCTCTTTC----

-------TCACTAGAGTCTATGTCACTCATTATACTCTGTCGAATGTCATTGAATGTCTT
TACATGGGCTTATATGCCTATGAAAATTGTAATACAACTTTCAGCAACGGATCTCTTGGC
TCTCGCATCGATGAAGAACGCAGCGAAATGCGATAAGTAATGTGAATTGCAGAATTCAGT
GAATCATCGAATCTTTGAACGCATCTTGCGCTCCTTGGTATTCCGAGGAGCATGCCTGTT
TGAGTGTCATTAAATTCTCAACTCTCTTCTAC-TTTTTGTAAAAGAGAGCTTGGACTGTG
GAGGCTTGCTGGCCACTTTTTGGGGTCAGCTCCTCTGAAATGCATTAGCGGAACCGTTTG
CGATCTGCCACAAGTGTGATAAGTTATCTACACTGGCGAGGGGATTGCTCTCTGTAATGT
TCAGCTTCTAATTGTCTCTACTTTGTGAGACTACTTTTGAATGCTTGACCTCAAATCAGG
TAGGACTACCCGCTGAACTTAA

>AC8-4
TTTCCGTAGGTGAACCTGCGGAAGGATCATTATTGAATTATGTTTCTAGATAGGTTGTAG
CTGGCTC-TTTAGAGCATGTGCACGCCTGTTTGGACTTCATTTTCATCCACCTGTGCACC
TATTGTAGTCTTTGGTTGGGTTAGGAGGAAGTGGTCATTGTGTCAGCATCTGCTGGATGT
GAGGACTTGCATTGTGAAAGCTTTGCTGTCCTTGATGTGATCATGGAATCTCTTTC----

-------TCACTAGAGTCTATGTCACTCATTATACTCTGTCGAATGTCATTGAATGTCTT
TACATGGGCTTATATGCCTATGAAAATTGTAATACAACTTTCAGCAACGGATCTCTTGGC
TCTCGCATCGATGAAGAACGCAGCGAAATGCGATAAGTAATGTGAATTGCAGAATTCAGT
GAATCATCGAATCTTTGAACGCATCTTGCGCTCCTTGGTATTCCGAGGAGCATGCCTGTT
TGAGTGTCATTAAATTCTCAACTCTCTTCTAC-TTTTTGTAAAAGAGAGCTTGGACTGTG
GAGGCTTGCTGGCCACTTTTTGGGGTCAGCTCCTCTGAAATGCATTAGCGGAACCGTTTG
CGATCTGCCACAAGTGTGATAAGTTATCTACACTGGCGAGGGGATTGCTCTCTGTAATGT
TCAGCTTCTAATTGTCTCTACTTTGTGAGACTACTTTTGAATGCTTGACCTCAAATCAGG
TAGGACTACCCGCTGAACTTAA
>AC8-13
TTTCCGTAGGTGAACCTGCGGAAGGATCATTATTGAATTATGTTTCTAGATAGGTTGTAG
CTGGCTC-TTTAGAGCATGTGCACGCCTGTTTGGACTTCATTTTCATCCACCTGTGCACC
TATTGTAGTCTTTGGTTGGGTTAGGAGGAAGTGGTCATTGTGTCAGCATCTGCTGGATGT
GAGGACTTGCATTGTGAAAGCTTTGCTGTCCTTGATGTGATCATGGAATCTCTTTC----

-------TCACTAGAGTCTATGTCACTCATTATACTCTGTCGAATGTCATTGAATGTCTT
TACATGGGCTTATATGCCTATGAAAATTGTAATACAACTTTCAGCAACGGATCTCTTGGC
TCTCGCATCGATGAAGAACGCAGCGAAATGCGATAAGTAATGTGAATTGCAGAATTCAGT
GAATCATCGAATCTTTGAACGCATCTTGCGCTCCTTGGTATTCCGAGGAGCATGCCTGTT
TGAGTGTCATTAAATTCTCAACTCTCTTCTAC-TTTTTGTAAAAGAGAGCTTGGACTGTG
GAGGCTTGCTGGCCACTTTTTGGGGTCAGCTCCTCTGAAATGCATTAGCGGAACCGTTTG
CGATCTGCCACAAGTGTGATAAGTTATCTACACTGGCGAGGGGATTGCTCTCTGTAATGT

```
TCAGCTTCTAATTGTCTCTACTTTGTGAGACTACTTTTGAATGCTTGACCTCAAATCAGG
TAGGACTACCCGCTGAACTTAA
>AC8-18
TTTCCGTAGGTGAACCTGCGGAAGGATCATTATTGAATTATGTTTCTAGATAGGTTGTAG
CTGGCTC-TTTAGAGCATGTGCACGCCTGTTTGGACTTCATTTTCATCCACCTGTGCACC
TATTGTAGTCTTTGGTTGGGTTAGGAGGAAGTGGTCATTGTGTCAGCATCTGCTGGATGT
GAGGACTTGCATTGTGAAAGCTTTGCTGTCCTTGATGTGATCATGGAATCTCTTTC----
------------------------------------------------------------
------------------------------------------------------------
------------------------------------------------------------
------------------------------------------------------------
------------------------------------------------------------
------------------------------------------------------------
------------------------------------------------------------
------------------------------------------------------------
------------------------------------------------------------
------------------------------------------------------------
------------------------------------------------------------
------------------------------------------------------------
-------TCACTAGAGTCTATGTCACTCATTATACTCTGTCGAATGTCATTGAATGTCTT
TACATGGGCTTATATGCCTATGAAAATTGTAATACAACTTTCAGCAACGGATCTCTTGGC
TCTCGCATCGATGAAGAACGCAGCGAAATGCGATAAGTAATGTGAATTGCAGAATTCAGT
GAATCATCGAATCTTTGAACGCATCTTGCGCTCCTTGGTATTCCGAGGAGCATGCCTGTT
TGAGTGTCATTAAATTCTCAACTCTCTTCTAC-TTTTTGTAAAAGAGAGCTTGGACTGTG
GAGGCTTGCTGGCCACTTTTTGGGGTCAGCTCCTCTGAAATGCATTAGCGGAACCGTTTG
CGATCTGCCACAAGTGTGATAAGTTATCTACACTGGCGAGGGGATTGCTCTCTGTAATGT
TCAGCTTCTAATTGTCTCTACTTTGTGAGACTACTTTTGAATGCTTGACCTCAAATCAGG
TAGGACTACCCGCTGAACTTAA
>AC8-27
TTTCCGTAGGTGAACCTGCGGAAGGATCATTATTGAATTATGTTTCTAGATAGGTTGTAG
CTGGCTC-TTTAGAGCATGTGCACGCCTGTTTGGACTTCATTTTCATCCACCTGTGCACC
TATTGTAGTCTTTGGTTGGGTTAGGAGGAAGTGGTCATTGTGTCAGCATCTGCTGGATGT
GAGGACTTGCATTGTGAAAGCTTTGCTGTCCTTGATGTGATCATGGAATCTCTTTC----
------------------------------------------------------------
------------------------------------------------------------
------------------------------------------------------------
------------------------------------------------------------
------------------------------------------------------------
------------------------------------------------------------
------------------------------------------------------------
------------------------------------------------------------
------------------------------------------------------------
------------------------------------------------------------
------------------------------------------------------------
------------------------------------------------------------
-------TCACTAGAGTCTATGTCACTCATTATACTCTGTCGAATGTCATTGAATGTCTT
TACATGGGCTTATATGCCTATGAAAATTGTAATACAACTTTCAGCAACGGATCTCTTGGC
TCTCGCATCGATGAAGAACGCAGCGAAATGCGATAAGTAATGTGAATTGCAGAATTCAGT
GAATCATCGAATCTTTGAACGCATCTTGCGCTCCTTGGTATTCCGAGGAGCATGCCTGTT
TGAGTGTCATTAAATTCTCAACTCTCTTCTAC-TTTTTGTAAAAGAGAGCTTGGACTGTG
```

GAGGCTTGCTGGCCACTTTTTGGGGTCAGCTCCTCTGAAATGCATTAGCGGAACCGTTTG
CGATCTGCCACAAGTGTGATAAGTTATCTACACTGGCGAGGGGATTGCTCTCTGTAATGT
TCAGCTTCTAATTGTCTCTACTTTGTGAGACTACTTTTGAATGCTTGACCTCAAATCAGG
TAGGACTACCCGCTGAACTTAA
>AC8-30
TTTCCGTAGGTGAACCTGCGGAAGGATCATTATTGAATTATGTTTCTAGATAGGTTGTAG
CTGGCTC-TTTAGAGCATGTGCACGCCTGTTTGGACTTCATTTTCATCCACCTGTGCACC
TATTGTAGTCTTTGGTTGGGTTAGGAGGAAGTGGTCATTGTGTCAGCATCTGCTGGATGT
GAGGACTTGCATTGTGAAAGCTTTGCTGTCCTTGATGTGATCATGGAATCTCTTTC----

-------TCACTAGAGTCTATGTCACTCATTATACTCTGTCGAATGTCATTGAATGTCTT
TACATGGGCTTATATGCCTATGAAAATTGTAATACAACTTTCAGCAACGGATCTCTTGGC
TCTCGCATCGATGAAGAACGCAGCGAAATGCGATAAGTAATGTGAATTGCAGAATTCAGT
GAATCATCGAATCTTTGAACGCATCTTGCGCTCCTTGGTATTCCGAGGAGCATGCCTGTT
TGAGTGTCATTAAATTCTCAACTCTCTTCTAC-TTTTTGTAAAAGAGAGCTTGGACTGTG
GAGGCTTGCTGGCCACTTTTTGGGGTCAGCTCCTCTGAAATGCATTAGCGGAACCGTTTG
CGATCTGCCACAAGTGTGATAAGTTATCTACACTGGCGAGGGGATTGCTCTCTGTAATGT
TCAGCTTCTAATTGTCTCTACTTTGTGAGACTACTTTTGAATGCTTGACCTCAAATCAGG
TAGGACTACCCGCTGAACTTAA
>AC8-34
TTTCCGTAGGTGAACCTGCGGAAGGATCATTATTGAATTATGTTTCTAGATAGGTTGTAG
CTGGCTC-TTTAGAGCATGTGCACGCCTGTTTGGACTTCATTTTCATCCACCTGTGCACC
TATTGTAGTCTTTGGTTGGGTTAGGAGGAAGTGGTCATTGTGTCAGCATCTGCTGGATGT
GAGGACTTGCATTGTGAAAGCTTTGCTGTCCTTGATGTGATCATGGAATCTCTTTC----

-------TCACTAGAGTCTATGTCACTCATTATACTCTGTCGAATGTCATTGAATGTCTT
TACATGGGCTTATATGCCTATGAAAATTGTAATACAACTTTCAGCAACGGATCTCTTGGC
TCTCGCATCGATGAAGAACGCAGCGAAATGCGATAAGTAATGTGAATTGCAGAATTCAGT

GAATCATCGAATCTTTGAACGCATCTTGCGCTCCTTGGTATTCCGAGGAGCATGCCTGTT
TGAGTGTCATTAAATTCTCAACTCTCTTCTAC-TTTTTGTAAAAGAGAGCTTGGACTGTG
GAGGCTTGCTGGCCACTTTTTGGGGTCAGCTCCTCTGAAATGCATTAGCGGAACCGTTTG
CGATCTGCCACAAGTGTGATAAGTTATCTACACTGGCGAGGGGATTGCTCTCTGTAATGT
TCAGCTTCTAATTGTCTCTACTTTGTGAGACTACTTTTGAATGCTTGACCTCAAATCAGG
TAGGACTACCCGCTGAACTTAA
>AC8-36
TTTCCGTAGGTGAACCTGCGGAAGGATCATTATTGAATTATGTTTCTAGATAGGTTGTAG
CTGGCTC-TTTAGAGCATGTGCACGCCTGTTTGGACTTCATTTTCATCCACCTGTGCACC
TATTGTAGTCTTTGGTTGGGTTAGGAGGAAGTGGTCATTGTGTCAGCATCTGCTGGATGT
GAGGACTTGCATTGTGAAAGCTTTGCTGTCCTTGATGTGATCATGGAATCTCTTTC----

-------TCACTAGAGTCTATGTCACTCATTATACTCTGTCGAATGTCATTGAATGTCTT
TACATGGGCTTATATGCCTATGAAAATTGTAATACAACTTTCAGCAACGGATCTCTTGGC
TCTCGCATCGATGAAGAACGCAGCGAAATGCGATAAGTAATGTGAATTGCAGAATTCAGT
GAATCATCGAATCTTTGAACGCATCTTGCGCTCCTTGGTATTCCGAGGAGCATGCCTGTT
TGAGTGTCATTAAATTCTCAACTCTCTTCTAC-TTTTTGTAAAAGAGAGCTTGGACTGTG
GAGGCTTGCTGGCCACTTTTTGGGGTCAGCTCCTCTGAAATGCATTAGCGGAACCGTTTG
CGATCTGCCACAAGTGTGATAAGTTATCTACACTGGCGAGGGGATTGCTCTCTGTAATGT
TCAGCTTCTAATTGTCTCTACTTTGTGAGACTACTTTTGAATGCTTGACCTCAAATCAGG
TAGGACTACCCGCTGAACTTAA
>AC8-45
TTTCCGTAGGTGAACCTGCGGAAGGATCATTATTGAATTATGTTTCTAGATAGGTTGTAG
CTGGCTC-TTTAGAGCATGTGCACGCCTGTTTGGACTTCATTTTCATCCACCTGTGCACC
TATTGTAGTCTTTGGTTGGGTTAGGAGGAAGTGGTCATTGTGTCAGCATCTGCTGGATGT
GAGGACTTGCATTGTGAAAGCTTTGCTGTCCTTGATGTGATCATGGAATCTCTTTC----

-------TCACTAGAGTCTATGTCACTCATTATACTCTGTCGAATGTCATTGAATGTCTT

TACATGGGCTTATATGCCTATGAAAATTGTAATACAACTTTCAGCAACGGATCTCTTGGC
TCTCGCATCGATGAAGAACGCAGCGAAATGCGATAAGTAATGTGAATTGCAGAATTCAGT
GAATCATCGAATCTTTGAACGCATCTTGCGCTCCTTGGTATTCCGAGGAGCATGCCTGTT
TGAGTGTCATTAAATTCTCAACTCTCTTCTAC-TTTTTGTAAAAGAGAGCTTGGACTGTG
GAGGCTTGCTGGCCACTTTTTGGGGTCAGCTCCTCTGAAATGCATTAGCGGAACCGTTTG
CGATCTGCCACAAGTGTGATAAGTTATCTACACTGGCGAGGGGATTGCTCTCTGTAATGT
TCAGCTTCTAATTGTCTCTACTTTGTGAGACTACTTTTGAATGCTTGACCTCAAATCAGG
TAGGACTACCCGCTGAACTTAA
>AC9-1
TTTCCGTAGGTGAACCTGCGGAAGGATCATTATTGAATTATGTTTCTAGATAGGTTGTAG
CTGGCTC-TTTAGAGCATGTGCACGCCTGTTTGGACTTCATTTTCATCCACCTGTGCACC
TATTGTAGTCTTTGGTTGGGTTAGGAGGAAGTGGTCATTGTGTCAGCATCTGCTGGATGT
GAGGACTTGCATTGTGAAAGCTTTGCTGTCCTTGATGTGATCATGGAATCTCTTTC----

-------TCACTAGAGTCTATGTCACTCATTATACTCTGTCGAATGTCATTGAATGTCTT
TACATGGGCTTATATGCCTATGAAAATTGTAATACAACTTTCAGCAACGGATCTCTTGGC
TCTCGCATCGATGAAGAACGCAGCGAAATGCGATAAGTAATGTGAATTGCAGAATTCAGT
GAATCATCGAATCTTTGAACGCATCTTGCGCTCCTTGGTATTCCGAGGAGCATGCCTGTT
TGAGTGTCATTAAATTCTCAACTCTCTTCTAC-TTTTTGTAAAAGAGAGCTTGGACTGTG
GAGGCTTGCTGGCCACTTTTTGGGGTCAGCTCCTCTGAAATGCATTAGCGGAACCGTTTG
CGATCTGCCACAAGTGTGATAAGTTATCTACACTGGCGAGGGGATTGCTCTCTGTAATGT
TCAGCTTCTAATTGTCTCTACTTTGTGAGACTACTTTTGAATGCTTGACCTCAAATCAGG
TAGGACTACCCGCTGAACTTAA
>AC9-2
TTTCCGTAGGTGAACCTGCGGAAGGATCATTATTGAATTATGTTTCTAGATAGGTTGTAG
CTGGCTC-TTTAGAGCATGTGCACGCCTGTTTGGACTTCATTTTCATCCACCTGTGCACC
TATTGTAGTCTTTGGTTGGGTTAGGAGGAAGTGGTCATTGTGTCAGCATCTGCTGGATGT
GAGGACTTGCATTGTGAAAGCTTTGCTGTCCTTGATGTGATCATGGAATCTCTTTC----

-------TCACTAGAGTCTATGTCACTCATTATACTCTGTCGAATGTCATTGAATGTCTT
TACATGGGCTTATATGCCTATGAAAATTGTAATACAACTTTCAGCAACGGATCTCTTGGC
TCTCGCATCGATGAAGAACGCAGCGAAATGCGATAAGTAATGTGAATTGCAGAATTCAGT
GAATCATCGAATCTTTGAACGCATCTTGCGCTCCTTGGTATTCCGAGGAGCATGCCTGTT
TGAGTGTCATTAAATTCTCAACTCTCTTCTAC-TTTTTGTAAAAGAGAGCTTGGACTGTG
GAGGCTTGCTGGCCACTTTTTGGGGTCAGCTCCTCTGAAATGCATTAGCGGAACCGTTTG
CGATCTGCCACAAGTGTGATAAGTTATCTACACTGGCGAGGGGATTGCTCTCTGTAATGT
TCAGCTTCTAATTGTCTCTACTTTGTGAGACTACTTTTGAATGCTTGACCTCAAATCAGG
TAGGACTACCCGCTGAACTTAA
>AC9-10
TTTCCGTAGGTGAACCTGCGGAAGGATCATTATTGAATTATGTTTCTAGATAGGTTGTAG
CTGGCTC-TTTAGAGCATGTGCACGCCTGTTTGGACTTCATTTTCATCCACCTGTGCACC
TATTGTAGTCTTTGGTTGGGTTAGGAGGAAGTGGTCATTGTGTCAGCATCTGCTGGATGT
GAGGACTTGCATTGTGAAAGCTTTGCTGTCCTTGATGTGATCATGGAATCTCTTTC----

-------TCACTAGAGTCTATGTCACTCATTATACTCTGTCGAATGTCATTGAATGTCTT
TACATGGGCTTATATGCCTATGAAAATTGTAATACAACTTTCAGCAACGGATCTCTTGGC
TCTCGCATCGATGAAGAACGCAGCGAAATGCGATAAGTAATGTGAATTGCAGAATTCAGT
GAATCATCGAATCTTTGAACGCATCTTGCGCTCCTTGGTATTCCGAGGAGCATGCCTGTT
TGAGTGTCATTAAATTCTCAACTCTCTTCTAC-TTTTTGTAAAAGAGAGCTTGGACTGTG
GAGGCTTGCTGGCCACTTTTTGGGGTCAGCTCCTCTGAAATGCATTAGCGGAACCGTTTG
CGATCTGCCACAAGTGTGATAAGTTATCTACACTGGCGAGGGGATTGCTCTCTGTAATGT
TCAGCTTCTAATTGTCTCTACTTTGTGAGACTACTTTTGAATGCTTGACCTCAAATCAGG
TAGGACTACCCGCTGAACTTAA
>AC9-11
TTTCCGTAGGTGAACCTGCGGAAGGATCATTATTGAATTATGTTTCTAGATAGGTTGTAG
CTGGCTC-TTTAGAGCATGTGCACGCCTGTTTGGACTTCATTTTCATCCACCTGTGCACC
TATTGTAGTCTTTGGTTGGGTTAGGAGGAAGTGGTCATTGTGTCAGCATCTGCTGGATGT
GAGGACTTGCATTGTGAAAGCTTTGCTGTCCTTGATGTGATCATGGAATCTCTTTC----

-------TCACTAGAGTCTATGTCACTCATTATACTCTGTCGAATGTCATTGAATGTCTT
TACATGGGCTTATATGCCTATGAAAATTGTAATACAACTTTCAGCAACGGATCTCTTGGC
TCTCGCATCGATGAAGAACGCAGCGAAATGCGATAAGTAATGTGAATTGCAGAATTCAGT
GAATCATCGAATCTTTGAACGCATCTTGCGCTCCTTGGTATTCCGAGGAGCATGCCTGTT
TGAGTGTCATTAAATTCTCAACTCTCTTCTAC-TTTTTGTAAAAGAGAGCTTGGACTGTG
GAGGCTTGCTGGCCACTTTTTGGGGTCAGCTCCTCTGAAATGCATTAGCGGAACCGTTTG
CGATCTGCCACAAGTGTGATAAGTTATCTACACTGGCGAGGGGATTGCTCTCTGTAATGT
TCAGCTTCTAATTGTCTCTACTTTGTGAGACTACTTTTGAATGCTTGACCTCAAATCAGG
TAGGACTACCCGCTGAACTTAA
>AC9-12
TTTCCGTAGGTGAACCTGCGGAAGGATCATTATTGAATTATGTTTCTAGATAGGTTGTAG
CTGGCTC-TTTAGAGCATGTGCACGCCTGTTTGGACTTCATTTTCATCCACCTGTGCACC
TATTGTAGTCTTTGGTTGGGTTAGGAGGAAGTGGTCATTGTGTCAGCATCTGCTGGATGT
GAGGACTTGCATTGTGAAAGCTTTGCTGTCCTTGATGTGATCATGGAATCTCTTTC----

-------TCACTAGAGTCTATGTCACTCATTATACTCTGTCGAATGTCATTGAATGTCTT
TACATGGGCTTATATGCCTATGAAAATTGTAATACAACTTTCAGCAACGGATCTCTTGGC
TCTCGCATCGATGAAGAACGCAGCGAAATGCGATAAGTAATGTGAATTGCAGAATTCAGT
GAATCATCGAATCTTTGAACGCATCTTGCGCTCCTTGGTATTCCGAGGAGCATGCCTGTT
TGAGTGTCATTAAATTCTCAACTCTCTTCTAC-TTTTTGTAAAAGAGAGCTTGGACTGTG
GAGGCTTGCTGGCCACTTTTTGGGGTCAGCTCCTCTGAAATGCATTAGCGGAACCGTTTG
CGATCTGCCACAAGTGTGATAAGTTATCTACACTGGCGAGGGGATTGCTCTCTGTAATGT
TCAGCTTCTAATTGTCTCTACTTTGTGAGACTACTTTTGAATGCTTGACCTCAAATCAGG
TAGGACTACCCGCTGAACTTAA
>AC9-14
TTTCCGTAGGTGAACCTGCGGAAGGATCATTATTGAATTATGTTTCTAGATAGGTTGTAG
CTGGCTC-TTTAGAGCATGTGCACGCCTGTTTGGACTTCATTTTCATCCACCTGTGCACC
TATTGTAGTCTTTGGTTGGGTTAGGAGGAAGTGGTCATTGTGTCAGCATCTGCTGGATGT
GAGGACTTGCATTGTGAAAGCTTTGCTGTCCTTGATGTGATCATGGAATCTCTTTC----


```
------------------------------------------------------------
------------------------------------------------------------
------------------------------------------------------------
------------------------------------------------------------
------------------------------------------------------------
-------TCACTAGAGTCTATGTCACTCATTATACTCTGTCGAATGTCATTGAATGTCTT
TACATGGGCTTATATGCCTATGAAAATTGTAATACAACTTTCAGCAACGGATCTCTTGGC
TCTCGCATCGATGAAGAACGCAGCGAAATGCGATAAGTAATGTGAATTGCAGAATTCAGT
GAATCATCGAATCTTTGAACGCATCTTGCGCTCCTTGGTATTCCGAGGAGCATGCCTGTT
TGAGTGTCATTAAATTCTCAACTCTCTTCTAC-TTTTTGTAAAAGAGAGCTTGGACTGTG
GAGGCTTGCTGGCCACTTTTTGGGGTCAGCTCCTCTGAAATGCATTAGCGGAACCGTTTG
CGATCTGCCACAAGTGTGATAAGTTATCTACACTGGCGAGGGGATTGCTCTCTGTAATGT
TCAGCTTCTAATTGTCTCTACTTTGTGAGACTACTTTTGAATGCTTGACCTCAAATCAGG
TAGGACTACCCGCTGAACTTAA
>AC9-17
TTTCCGTAGGTGAACCTGCGGAAGGATCATTATTGAATTATGTTTCTAGATAGGTTGTAG
CTGGCTC-TTTAGAGCATGTGCACGCCTGTTTGGACTTCATTTTCATCCACCTGTGCACC
TATTGTAGTCTTTGGTTGGGTTAGGAGGAAGTGGTCATTGTGTCAGCATCTGCTGGATGT
GAGGACTTGCATTGTGAAAGCTTTGCTGTCCTTGATGTGATCATGGAATCTCTTTC----
------------------------------------------------------------
------------------------------------------------------------
------------------------------------------------------------
------------------------------------------------------------
------------------------------------------------------------
------------------------------------------------------------
------------------------------------------------------------
------------------------------------------------------------
------------------------------------------------------------
------------------------------------------------------------
------------------------------------------------------------
------------------------------------------------------------
-------TCACTAGAGTCTATGTCACTCATTATACTCTGTCGAATGTCATTGAATGTCTT
TACATGGGCTTATATGCCTATGAAAATTGTAATACAACTTTCAGCAACGGATCTCTTGGC
TCTCGCATCGATGAAGAACGCAGCGAAATGCGATAAGTAATGTGAATTGCAGAATTCAGT
GAATCATCGAATCTTTGAACGCATCTTGCGCTCCTTGGTATTCCGAGGAGCATGCCTGTT
TGAGTGTCATTAAATTCTCAACTCTCTTCTAC-TTTTTGTAAAAGAGAGCTTGGACTGTG
GAGGCTTGCTGGCCACTTTTTGGGGTCAGCTCCTCTGAAATGCATTAGCGGAACCGTTTG
CGATCTGCCACAAGTGTGATAAGTTATCTACACTGGCGAGGGGATTGCTCTCTGTAATGT
TCAGCTTCTAATTGTCTCTACTTTGTGAGACTACTTTTGAATGCTTGACCTCAAATCAGG
TAGGACTACCCGCTGAACTTAA
>AC9-20
TTTCCGTAGGTGAACCTGCGGAAGGATCATTATTGAATTATGTTTCTAGATAGGTTGTAG
CTGGCTC-TTTAGAGCATGTGCACGCCTGTTTGGACTTCATTTTCATCCACCTGTGCACC
TATTGTAGTCTTTGGTTGGGTTAGGAGGAAGTGGTCATTGTGTCAGCATCTGCTGGATGT
GAGGACTTGCATTGTGAAAGCTTTGCTGTCCTTGATGTGATCATGGAATCTCTTTC----
------------------------------------------------------------
------------------------------------------------------------
------------------------------------------------------------
------------------------------------------------------------
------------------------------------------------------------
```


-------TCACTAGAGTCTATGTCACTCATTATACTCTGTCGAATGTCATTGAATGTCTT
TACATGGGCTTATATGCCTATGAAAATTGTAATACAACTTTCAGCAACGGATCTCTTGGC
TCTCGCATCGATGAAGAACGCAGCGAAATGCGATAAGTAATGTGAATTGCAGAATTCAGT
GAATCATCGAATCTTTGAACGCATCTTGCGCTCCTTGGTATTCCGAGGAGCATGCCTGTT
TGAGTGTCATTAAATTCTCAACTCTCTTCTAC-TTTTTGTAAAAGAGAGCTTGGACTGTG
GAGGCTTGCTGGCCACTTTTTGGGGTCAGCTCCTCTGAAATGCATTAGCGGAACCGTTTG
CGATCTGCCACAAGTGTGATAAGTTATCTACACTGGCGAGGGGATTGCTCTCTGTAATGT
TCAGCTTCTAATTGTCTCTACTTTGTGAGACTACTTTTGAATGCTTGACCTCAAATCAGG
TAGGACTACCCGCTGAACTTAA
>AC9-22
TTTCCGTAGGTGAACCTGCGGAAGGATCATTATTGAATTATGTTTCTAGATAGGTTGTAG
CTGGCTC-TTTAGAGCATGTGCACGCCTGTTTGGACTTCATTTTCATCCACCTGTGCACC
TATTGTAGTCTTTGGTTGGGTTAGGAGGAAGTGGTCATTGTGTCAGCATCTGCTGGATGT
GAGGACTTGCATTGTGAAAGCTTTGCTGTCCTTGATGTGATCATGGAATCTCTTTC----

-------TCACTAGAGTCTATGTCACTCATTATACTCTGTCGAATGTCATTGAATGTCTT
TACATGGGCTTATATGCCTATGAAAATTGTAATACAACTTTCAGCAACGGATCTCTTGGC
TCTCGCATCGATGAAGAACGCAGCGAAATGCGATAAGTAATGTGAATTGCAGAATTCAGT
GAATCATCGAATCTTTGAACGCATCTTGCGCTCCTTGGTATTCCGAGGAGCATGCCTGTT
TGAGTGTCATTAAATTCTCAACTCTCTTCTAC-TTTTTGTAAAAGAGAGCTTGGACTGTG
GAGGCTTGCTGGCCACTTTTTGGGGTCAGCTCCTCTGAAATGCATTAGCGGAACCGTTTG
CGATCTGCCACAAGTGTGATAAGTTATCTACACTGGCGAGGGGATTGCTCTCTGTAATGT
TCAGCTTCTAATTGTCTCTACTTTGTGAGACTACTTTTGAATGCTTGACCTCAAATCAGG
TAGGACTACCCGCTGAACTTAA
>AC9-27
TTTCCGTAGGTGAACCTGCGGAAGGATCATTATTGAATTATGTTTCTAGATAGGTTGTAG
CTGGCTC-TTTAGAGCATGTGCACGCCTGTTTGGACTTCATTTTCATCCACCTGTGCACC
TATTGTAGTCTTTGGTTGGGTTAGGAGGAAGTGGTCATTGTGTCAGCATCTGCTGGATGT
GAGGACTTGCATTGTGAAAGCTTTGCTGTCCTTGATGTGATCATGGAATCTCTTTC----

-------TCACTAGAGTCTATGTCACTCATTATACTCTGTCGAATGTCATTGAATGTCTT
TACATGGGCTTATATGCCTATGAAAATTGTAATACAACTTTCAGCAACGGATCTCTTGGC
TCTCGCATCGATGAAGAACGCAGCGAAATGCGATAAGTAATGTGAATTGCAGAATTCAGT
GAATCATCGAATCTTTGAACGCATCTTGCGCTCCTTGGTATTCCGAGGAGCATGCCTGTT
TGAGTGTCATTAAATTCTCAACTCTCTTCTAC-TTTTTGTAAAAGAGAGCTTGGACTGTG
GAGGCTTGCTGGCCACTTTTTGGGGTCAGCTCCTCTGAAATGCATTAGCGGAACCGTTTG
CGATCTGCCACAAGTGTGATAAGTTATCTACACTGGCGAGGGGATTGCTCTCTGTAATGT
TCAGCTTCTAATTGTCTCTACTTTGTGAGACTACTTTTGAATGCTTGACCTCAAATCAGG
TAGGACTACCCGCTGAACTTAA
>AC9-34
TTTCCGTAGGTGAACCTGCGGAAGGATCATTATTGAATTATGTTTCTAGATAGGTTGTAG
CTGGCTC-TTTAGAGCATGTGCACGCCTGTTTGGACTTCATTTTCATCCACCTGTGCACC
TATTGTAGTCTTTGGTTGGGTTAGGAGGAAGTGGTCATTGTGTCAGCATCTGCTGGATGT
GAGGACTTGCATTGTGAAAGCTTTGCTGTCCTTGATGTGATCATGGAATCTCTTTC----

-------TCACTAGAGTCTATGTCACTCATTATACTCTGTCGAATGTCATTGAATGTCTT
TACATGGGCTTATATGCCTATGAAAATTGTAATACAACTTTCAGCAACGGATCTCTTGGC
TCTCGCATCGATGAAGAACGCAGCGAAATGCGATAAGTAATGTGAATTGCAGAATTCAGT
GAATCATCGAATCTTTGAACGCATCTTGCGCTCCTTGGTATTCCGAGGAGCATGCCTGTT
TGAGTGTCATTAAATTCTCAACTCTCTTCTAC-TTTTTGTAAAAGAGAGCTTGGACTGTG
GAGGCTTGCTGGCCACTTTTTGGGGTCAGCTCCTCTGAAATGCATTAGCGGAACCGTTTG
CGATCTGCCACAAGTGTGATAAGTTATCTACACTGGCGAGGGGATTGCTCTCTGTAATGT
TCAGCTTCTAATTGTCTCTACTTTGTGAGACTACTTTTGAATGCTTGACCTCAAATCAGG
TAGGACTACCCGCTGAACTTAA
>AC9-38
TTTCCGTAGGTGAACCTGCGGAAGGATCATTATTGAATTATGTTTCTAGATAGGTTGTAG
CTGGCTC-TTTAGAGCATGTGCACGCCTGTTTGGACTTCATTTTCATCCACCTGTGCACC
TATTGTAGTCTTTGGTTGGGTTAGGAGGAAGTGGTCATTGTGTCAGCATCTGCTGGATGT
GAGGACTTGCATTGTGAAAGCTTTGCTGTCCTTGATGTGATCATGGAATCTCTTTC----

-------TCACTAGAGTCTATGTCACTCATTATACTCTGTCGAATGTCATTGAATGTCTT
TACATGGGCTTATATGCCTATGAAAATTGTAATACAACTTTCAGCAACGGATCTCTTGGC
TCTCGCATCGATGAAGAACGCAGCGAAATGCGATAAGTAATGTGAATTGCAGAATTCAGT
GAATCATCGAATCTTTGAACGCATCTTGCGCTCCTTGGTATTCCGAGGAGCATGCCTGTT
TGAGTGTCATTAAATTCTCAACTCTCTTCTAC-TTTTTGTAAAAGAGAGCTTGGACTGTG
GAGGCTTGCTGGCCACTTTTTGGGGTCAGCTCCTCTGAAATGCATTAGCGGAACCGTTTG
CGATCTGCCACAAGTGTGATAAGTTATCTACACTGGCGAGGGGATTGCTCTCTGTAATGT
TCAGCTTCTAATTGTCTCTACTTTGTGAGACTACTTTTGAATGCTTGACCTCAAATCAGG
TAGGACTACCCGCTGAACTTAA
>AC9-39
TTTCCGTAGGTGAACCTGCGGAAGGATCATTATTGAATTATGTTTCTAGATAGGTTGTAG
CTGGCTC-TTTAGAGCATGTGCACGCCTGTTTGGACTTCATTTTCATCCACCTGTGCACC
TATTGTAGTCTTTGGTTGGGTTAGGAGGAAGTGGTCATTGTGTCAGCATCTGCTGGATGT
GAGGACTTGCATTGTGAAAGCTTTGCTGTCCTTGATGTGATCATGGAATCTCTTTC----

-------TCACTAGAGTCTATGTCACTCATTATACTCTGTCGAATGTCATTGAATGTCTT
TACATGGGCTTATATGCCTATGAAAATTGTAATACAACTTTCAGCAACGGATCTCTTGGC
TCTCGCATCGATGAAGAACGCAGCGAAATGCGATAAGTAATGTGAATTGCAGAATTCAGT
GAATCATCGAATCTTTGAACGCATCTTGCGCTCCTTGGTATTCCGAGGAGCATGCCTGTT
TGAGTGTCATTAAATTCTCAACTCTCTTCTAC-TTTTTGTAAAAGAGAGCTTGGACTGTG
GAGGCTTGCTGGCCACTTTTTGGGGTCAGCTCCTCTGAAATGCATTAGCGGAACCGTTTG
CGATCTGCCACAAGTGTGATAAGTTATCTACACTGGCGAGGGGATTGCTCTCTGTAATGT
TCAGCTTCTAATTGTCTCTACTTTGTGAGACTACTTTTGAATGCTTGACCTCAAATCAGG
TAGGACTACCCGCTGAACTTAA
>AC9-49
TTTCCGTAGGTGAACCTGCGGAAGGATCATTATTGAATTATGTTTCTAGATAGGTTGTAG
CTGGCTC-TTTAGAGCATGTGCACGCCTGTTTGGACTTCATTTTCATCCACCTGTGCACC
TATTGTAGTCTTTGGTTGGGTTAGGAGGAAGTGGTCATTGTGTCAGCATCTGCTGGATGT

GAGGACTTGCATTGTGAAAGCTTTGCTGTCCTTGATGTGATCATGGAATCTCTTTC----

-------TCACTAGAGTCTATGTCACTCATTATACTCTGTCGAATGTCATTGAATGTCTT
TACATGGGCTTATATGCCTATGAAAATTGTAATACAACTTTCAGCAACGGATCTCTTGGC
TCTCGCATCGATGAAGAACGCAGCGAAATGCGATAAGTAATGTGAATTGCAGAATTCAGT
GAATCATCGAATCTTTGAACGCATCTTGCGCTCCTTGGTATTCCGAGGAGCATGCCTGTT
TGAGTGTCATTAAATTCTCAACTCTCTTCTAC-TTTTTGTAAAAGAGAGCTTGGACTGTG
GAGGCTTGCTGGCCACTTTTTGGGGTCAGCTCCTCTGAAATGCATTAGCGGAACCGTTTG
CGATCTGCCACAAGTGTGATAAGTTATCTACACTGGCGAGGGGATTGCTCTCTGTAATGT
TCAGCTTCTAATTGTCTCTACTTTGTGAGACTACTTTTGAATGCTTGACCTCAAATCAGG
TAGGACTACCCGCTGAACTTAA
>AC9-50
TTTCCGTAGGTGAACCTGCGGAAGGATCATTATTGAATTATGTTTCTAGATAGGTTGTAG
CTGGCTC-TTTAGAGCATGTGCACGCCTGTTTGGACTTCATTTTCATCCACCTGTGCACC
TATTGTAGTCTTTGGTTGGGTTAGGAGGAAGTGGTCATTGTGTCAGCATCTGCTGGATGT
GAGGACTTGCATTGTGAAAGCTTTGCTGTCCTTGATGTGATCATGGAATCTCTTTC----

-------TCACTAGAGTCTATGTCACTCATTATACTCTGTCGAATGTCATTGAATGTCTT
TACATGGGCTTATATGCCTATGAAAATTGTAATACAACTTTCAGCAACGGATCTCTTGGC
TCTCGCATCGATGAAGAACGCAGCGAAATGCGATAAGTAATGTGAATTGCAGAATTCAGT
GAATCATCGAATCTTTGAACGCATCTTGCGCTCCTTGGTATTCCGAGGAGCATGCCTGTT
TGAGTGTCATTAAATTCTCAACTCTCTTCTAC-TTTTTGTAAAAGAGAGCTTGGACTGTG
GAGGCTTGCTGGCCACTTTTTGGGGTCAGCTCCTCTGAAATGCATTAGCGGAACCGTTTG
CGATCTGCCACAAGTGTGATAAGTTATCTACACTGGCGAGGGGATTGCTCTCTGTAATGT
TCAGCTTCTAATTGTCTCTACTTTGTGAGACTACTTTTGAATGCTTGACCTCAAATCAGG
TAGGACTACCCGCTGAACTTAA
>AC9-52
TTTCCGTAGGTGAACCTGCGGAAGGATCATTATTGAATTATGTTTCTAGATAGGTTGTAG

CTGGCTC-TTTAGAGCATGTGCACGCCTGTTTGGACTTCATTTTCATCCACCTGTGCACC
TATTGTAGTCTTTGGTTGGGTTAGGAGGAAGTGGTCATTGTGTCAGCATCTGCTGGATGT
GAGGACTTGCATTGTGAAAGCTTTGCTGTCCTTGATGTGATCATGGAATCTCTTTC----

-------TCACTAGAGTCTATGTCACTCATTATACTCTGTCGAATGTCATTGAATGTCTT
TACATGGGCTTATATGCCTATGAAAATTGTAATACAACTTTCAGCAACGGATCTCTTGGC
TCTCGCATCGATGAAGAACGCAGCGAAATGCGATAAGTAATGTGAATTGCAGAATTCAGT
GAATCATCGAATCTTTGAACGCATCTTGCGCTCCTTGGTATTCCGAGGAGCATGCCTGTT
TGAGTGTCATTAAATTCTCAACTCTCTTCTAC-TTTTTGTAAAAGAGAGCTTGGACTGTG
GAGGCTTGCTGGCCACTTTTTGGGGTCAGCTCCTCTGAAATGCATTAGCGGAACCGTTTG
CGATCTGCCACAAGTGTGATAAGTTATCTACACTGGCGAGGGGATTGCTCTCTGTAATGT
TCAGCTTCTAATTGTCTCTACTTTGTGAGACTACTTTTGAATGCTTGACCTCAAATCAGG
TAGGACTACCCGCTGAACTTAA
>AC10-6
TTTCCGTAGGTGAACCTGCGGAAGGATCATTATTGAATTATGTTTCTAGATAGGTTGTAG
CTGGCTC-TTTAGAGCATGTGCACGCCTGTTTGGACTTCATTTTCATCCACCTGTGCACC
TATTGTAGTCTTTGGTTGGGTTAGGAGGAAGTGGTCATTGTGTCAGCATCTGCTGGATGT
GAGGACTTGCATTGTGAAAGCTTTGCTGTCCTTGATGTGATCATGGAATCTCTTTC----

-------TCACTAGAGTCTATGTCACTCATTATACTCTGTCGAATGTCATTGAATGTCTT
TACATGGGCTTATATGCCTATGAAAATTGTAATACAACTTTCAGCAACGGATCTCTTGGC
TCTCGCATCGATGAAGAACGCAGCGAAATGCGATAAGTAATGTGAATTGCAGAATTCAGT
GAATCATCGAATCTTTGAACGCATCTTGCGCTCCTTGGTATTCCGAGGAGCATGCCTGTT
TGAGTGTCATTAAATTCTCAACTCTCTTCTAC-TTTTTGTAAAAGAGAGCTTGGACTGTG
GAGGCTTGCTGGCCACTTTTTGGGGTCAGCTCCTCTGAAATGCATTAGCGGAACCGTTTG
CGATCTGCCACAAGTGTGATAAGTTATCTACACTGGCGAGGGGATTGCTCTCTGTAATGT
TCAGCTTCTAATTGTCTCTACTTTGTGAGACTACTTTTGAATGCTTGACCTCAAATCAGG
TAGGACTACCCGCTGAACTTAA

>AC10-16
TTTCCGTAGGTGAACCTGCGGAAGGATCATTATTGAATTATGTTTCTAGATAGGTTGTAG
CTGGCTC-TTTAGAGCATGTGCACGCCTGTTTGGACTTCATTTTCATCCACCTGTGCACC
TATTGTAGTCTTTGGTTGGGTTAGGAGGAAGTGGTCATTGTGTCAGCATCTGCTGGATGT
GAGGACTTGCATTGTGAAAGCTTTGCTGTCCTTGATGTGATCATGGAATCTCTTTC----

-------TCACTAGAGTCTATGTCACTCATTATACTCTGTCGAATGTCATTGAATGTCTT
TACATGGGCTTATATGCCTATGAAAATTGTAATACAACTTTCAGCAACGGATCTCTTGGC
TCTCGCATCGATGAAGAACGCAGCGAAATGCGATAAGTAATGTGAATTGCAGAATTCAGT
GAATCATCGAATCTTTGAACGCATCTTGCGCTCCTTGGTATTCCGAGGAGCATGCCTGTT
TGAGTGTCATTAAATTCTCAACTCTCTTCTAC-TTTTTGTAAAAGAGAGCTTGGACTGTG
GAGGCTTGCTGGCCACTTTTTGGGGTCAGCTCCTCTGAAATGCATTAGCGGAACCGTTTG
CGATCTGCCACAAGTGTGATAAGTTATCTACACTGGCGAGGGGATTGCTCTCTGTAATGT
TCAGCTTCTAATTGTCTCTACTTTGTGAGACTACTTTTGAATGCTTGACCTCAAATCAGG
TAGGACTACCCGCTGAACTTAA
>AC10-22
TTTCCGTAGGTGAACCTGCGGAAGGATCATTATTGAATTATGTTTCTAGATAGGTTGTAG
CTGGCTC-TTTAGAGCATGTGCACGCCTGTTTGGACTTCATTTTCATCCACCTGTGCACC
TATTGTAGTCTTTGGTTGGGTTAGGAGGAAGTGGTCATTGTGTCAGCATCTGCTGGATGT
GAGGACTTGCATTGTGAAAGCTTTGCTGTCCTTGATGTGATCATGGAATCTCTTTC----

-------TCACTAGAGTCTATGTCACTCATTATACTCTGTCGAATGTCATTGAATGTCTT
TACATGGGCTTATATGCCTATGAAAATTGTAATACAACTTTCAGCAACGGATCTCTTGGC
TCTCGCATCGATGAAGAACGCAGCGAAATGCGATAAGTAATGTGAATTGCAGAATTCAGT
GAATCATCGAATCTTTGAACGCATCTTGCGCTCCTTGGTATTCCGAGGAGCATGCCTGTT
TGAGTGTCATTAAATTCTCAACTCTCTTCTAC-TTTTTGTAAAAGAGAGCTTGGACTGTG
GAGGCTTGCTGGCCACTTTTTGGGGTCAGCTCCTCTGAAATGCATTAGCGGAACCGTTTG
CGATCTGCCACAAGTGTGATAAGTTATCTACACTGGCGAGGGGATTGCTCTCTGTAATGT

TCAGCTTCTAATTGTCTCTACTTTGTGAGACTACTTTTGAATGCTTGACCTCAAATCAGG
TAGGACTACCCGCTGAACTTAA
>AC10-35
TTTCCGTAGGTGAACCTGCGGAAGGATCATTATTGAATTATGTTTCTAGATAGGTTGTAG
CTGGCTC-TTTAGAGCATGTGCACGCCTGTTTGGACTTCATTTTCATCCACCTGTGCACC
TATTGTAGTCTTTGGTTGGGTTAGGAGGAAGTGGTCATTGTGTCAGCATCTGCTGGATGT
GAGGACTTGCATTGTGAAAGCTTTGCTGTCCTTGATGTGATCATGGAATCTCTTTC----

-------TCACTAGAGTCTATGTCACTCATTATACTCTGTCGAATGTCATTGAATGTCTT
TACATGGGCTTATATGCCTATGAAAATTGTAATACAACTTTCAGCAACGGATCTCTTGGC
TCTCGCATCGATGAAGAACGCAGCGAAATGCGATAAGTAATGTGAATTGCAGAATTCAGT
GAATCATCGAATCTTTGAACGCATCTTGCGCTCCTTGGTATTCCGAGGAGCATGCCTGTT
TGAGTGTCATTAAATTCTCAACTCTCTTCTAC-TTTTTGTAAAAGAGAGCTTGGACTGTG
GAGGCTTGCTGGCCACTTTTTGGGGTCAGCTCCTCTGAAATGCATTAGCGGAACCGTTTG
CGATCTGCCACAAGTGTGATAAGTTATCTACACTGGCGAGGGGATTGCTCTCTGTAATGT
TCAGCTTCTAATTGTCTCTACTTTGTGAGACTACTTTTGAATGCTTGACCTCAAATCAGG
TAGGACTACCCGCTGAACTTAA
>AC10-37
TTTCCGTAGGTGAACCTGCGGAAGGATCATTATTGAATTATGTTTCTAGATAGGTTGTAG
CTGGCTC-TTTAGAGCATGTGCACGCCTGTTTGGACTTCATTTTCATCCACCTGTGCACC
TATTGTAGTCTTTGGTTGGGTTAGGAGGAAGTGGTCATTGTGTCAGCATCTGCTGGATGT
GAGGACTTGCATTGTGAAAGCTTTGCTGTCCTTGATGTGATCATGGAATCTCTTTC----

-------TCACTAGAGTCTATGTCACTCATTATACTCTGTCGAATGTCATTGAATGTCTT
TACATGGGCTTATATGCCTATGAAAATTGTAATACAACTTTCAGCAACGGATCTCTTGGC
TCTCGCATCGATGAAGAACGCAGCGAAATGCGATAAGTAATGTGAATTGCAGAATTCAGT
GAATCATCGAATCTTTGAACGCATCTTGCGCTCCTTGGTATTCCGAGGAGCATGCCTGTT
TGAGTGTCATTAAATTCTCAACTCTCTTCTAC-TTTTTGTAAAAGAGAGCTTGGACTGTG

GAGGCTTGCTGGCCACTTTTTGGGGTCAGCTCCTCTGAAATGCATTAGCGGAACCGTTTG
CGATCTGCCACAAGTGTGATAAGTTATCTACACTGGCGAGGGGATTGCTCTCTGTAATGT
TCAGCTTCTAATTGTCTCTACTTTGTGAGACTACTTTTGAATGCTTGACCTCAAATCAGG
TAGGACTACCCGCTGAACTTAA
>AC10-42
TTTCCGTAGGTGAACCTGCGGAAGGATCATTATTGAATTATGTTTCTAGATAGGTTGTAG
CTGGCTC-TTTAGAGCATGTGCACGCCTGTTTGGACTTCATTTTCATCCACCTGTGCACC
TATTGTAGTCTTTGGTTGGGTTAGGAGGAAGTGGTCATTGTGTCAGCATCTGCTGGATGT
GAGGACTTGCATTGTGAAAGCTTTGCTGTCCTTGATGTGATCATGGAATCTCTTTC----

-------TCACTAGAGTCTATGTCACTCATTATACTCTGTCGAATGTCATTGAATGTCTT
TACATGGGCTTATATGCCTATGAAAATTGTAATACAACTTTCAGCAACGGATCTCTTGGC
TCTCGCATCGATGAAGAACGCAGCGAAATGCGATAAGTAATGTGAATTGCAGAATTCAGT
GAATCATCGAATCTTTGAACGCATCTTGCGCTCCTTGGTATTCCGAGGAGCATGCCTGTT
TGAGTGTCATTAAATTCTCAACTCTCTTCTAC-TTTTTGTAAAAGAGAGCTTGGACTGTG
GAGGCTTGCTGGCCACTTTTTGGGGTCAGCTCCTCTGAAATGCATTAGCGGAACCGTTTG
CGATCTGCCACAAGTGTGATAAGTTATCTACACTGGCGAGGGGATTGCTCTCTGTAATGT
TCAGCTTCTAATTGTCTCTACTTTGTGAGACTACTTTTGAATGCTTGACCTCAAATCAGG
TAGGACTACCCGCTGAACTTAA
>AC10-47
TTTCCGTAGGTGAACCTGCGGAAGGATCATTATTGAATTATGTTTCTAGATAGGTTGTAG
CTGGCTC-TTTAGAGCATGTGCACGCCTGTTTGGACTTCATTTTCATCCACCTGTGCACC
TATTGTAGTCTTTGGTTGGGTTAGGAGGAAGTGGTCATTGTGTCAGCATCTGCTGGATGT
GAGGACTTGCATTGTGAAAGCTTTGCTGTCCTTGATGTGATCATGGAATCTCTTTC----

-------TCACTAGAGTCTATGTCACTCATTATACTCTGTCGAATGTCATTGAATGTCTT
TACATGGGCTTATATGCCTATGAAAATTGTAATACAACTTTCAGCAACGGATCTCTTGGC
TCTCGCATCGATGAAGAACGCAGCGAAATGCGATAAGTAATGTGAATTGCAGAATTCAGT

GAATCATCGAATCTTTGAACGCATCTTGCGCTCCTTGGTATTCCGAGGAGCATGCCTGTT
TGAGTGTCATTAAATTCTCAACTCTCTTCTAC-TTTTTGTAAAAGAGAGCTTGGACTGTG
GAGGCTTGCTGGCCACTTTTTGGGGTCAGCTCCTCTGAAATGCATTAGCGGAACCGTTTG
CGATCTGCCACAAGTGTGATAAGTTATCTACACTGGCGAGGGGATTGCTCTCTGTAATGT
TCAGCTTCTAATTGTCTCTACTTTGTGAGACTACTTTTGAATGCTTGACCTCAAATCAGG
TAGGACTACCCGCTGAACTTAA
>AC10-50
TTTCCGTAGGTGAACCTGCGGAAGGATCATTATTGAATTATGTTTCTAGATAGGTTGTAG
CTGGCTC-TTTAGAGCATGTGCACGCCTGTTTGGACTTCATTTTCATCCACCTGTGCACC
TATTGTAGTCTTTGGTTGGGTTAGGAGGAAGTGGTCATTGTGTCAGCATCTGCTGGATGT
GAGGACTTGCATTGTGAAAGCTTTGCTGTCCTTGATGTGATCATGGAATCTCTTTC----

-------TCACTAGAGTCTATGTCACTCATTATACTCTGTCGAATGTCATTGAATGTCTT
TACATGGGCTTATATGCCTATGAAAATTGTAATACAACTTTCAGCAACGGATCTCTTGGC
TCTCGCATCGATGAAGAACGCAGCGAAATGCGATAAGTAATGTGAATTGCAGAATTCAGT
GAATCATCGAATCTTTGAACGCATCTTGCGCTCCTTGGTATTCCGAGGAGCATGCCTGTT
TGAGTGTCATTAAATTCTCAACTCTCTTCTAC-TTTTTGTAAAAGAGAGCTTGGACTGTG
GAGGCTTGCTGGCCACTTTTTGGGGTCAGCTCCTCTGAAATGCATTAGCGGAACCGTTTG
CGATCTGCCACAAGTGTGATAAGTTATCTACACTGGCGAGGGGATTGCTCTCTGTAATGT
TCAGCTTCTAATTGTCTCTACTTTGTGAGACTACTTTTGAATGCTTGACCTCAAATCAGG
TAGGACTACCCGCTGAACTTAA
>AC11-1
TTTCCGTAGGTGAACCTGCGGAAGGATCATTATTGAATTATGTTTCTAGATAGGTTGTAG
CTGGCTC-TTTAGAGCATGTGCACGCCTGTTTGGACTTCATTTTCATCCACCTGTGCACC
TATTGTAGTCTTTGGTTGGGTTAGGAGGAAGTGGTCATTGTGTCAGCATCTGCTGGATGT
GAGGACTTGCATTGTGAAAGCTTTGCTGTCCTTGATGTGATCATGGAATCTCTTTC----

-------TCACTAGAGTCTATGTCACTCATTATACTCTGTCGAATGTCATTGAATGTCTT

```
TACATGGGCTTATATGCCTATGAAAATTGTAATACAACTTTCAGCAACGGATCTCTTGGC
TCTCGCATCGATGAAGAACGCAGCGAAATGCGATAAGTAATGTGAATTGCAGAATTCAGT
GAATCATCGAATCTTTGAACGCATCTTGCGCTCCTTGGTATTCCGAGGAGCATGCCTGTT
TGAGTGTCATTAAATTCTCAACTCTCTTCTAC-TTTTTGTAAAAGAGAGCTTGGACTGTG
GAGGCTTGCTGGCCACTTTTTGGGGTCAGCTCCTCTGAAATGCATTAGCGGAACCGTTTG
CGATCTGCCACAAGTGTGATAAGTTATCTACACTGGCGAGGGGATTGCTCTCTGTAATGT
TCAGCTTCTAATTGTCTCTACTTTGTGAGACTACTTTTGAATGCTTGACCTCAAATCAGG
TAGGACTACCCGCTGAACTTAA
>AC11-8
TTTCCGTAGGTGAACCTGCGGAAGGATCATTATTGAATTATGTTTCTAGATAGGTTGTAG
CTGGCTC-TTTAGAGCATGTGCACGCCTGTTTGGACTTCATTTTCATCCACCTGTGCACC
TATTGTAGTCTTTGGTTGGGTTAGGAGGAAGTGGTCATTGTGTCAGCATCTGCTGGATGT
GAGGACTTGCATTGTGAAAGCTTTGCTGTCCTTGATGTGATCATGGAATCTCTTTC----
------------------------------------------------------------
------------------------------------------------------------
------------------------------------------------------------
------------------------------------------------------------
------------------------------------------------------------
------------------------------------------------------------
------------------------------------------------------------
------------------------------------------------------------
------------------------------------------------------------
------------------------------------------------------------
------------------------------------------------------------
------------------------------------------------------------
-------TCACTAGAGTCTATGTCACTCATTATACTCTGTCGAATGTCATTGAATGTCTT
TACATGGGCTTATATGCCTATGAAAATTGTAATACAACTTTCAGCAACGGATCTCTTGGC
TCTCGCATCGATGAAGAACGCAGCGAAATGCGATAAGTAATGTGAATTGCAGAATTCAGT
GAATCATCGAATCTTTGAACGCATCTTGCGCTCCTTGGTATTCCGAGGAGCATGCCTGTT
TGAGTGTCATTAAATTCTCAACTCTCTTCTAC-TTTTTGTAAAAGAGAGCTTGGACTGTG
GAGGCTTGCTGGCCACTTTTTGGGGTCAGCTCCTCTGAAATGCATTAGCGGAACCGTTTG
CGATCTGCCACAAGTGTGATAAGTTATCTACACTGGCGAGGGGATTGCTCTCTGTAATGT
TCAGCTTCTAATTGTCTCTACTTTGTGAGACTACTTTTGAATGCTTGACCTCAAATCAGG
TAGGACTACCCGCTGAACTTAA
>AC11-10
TTTCCGTAGGTGAACCTGCGGAAGGATCATTATTGAATTATGTTTCTAGATAGGTTGTAG
CTGGCTC-TTTAGAGCATGTGCACGCCTGTTTGGACTTCATTTTCATCCACCTGTGCACC
TATTGTAGTCTTTGGTTGGGTTAGGAGGAAGTGGTCATTGTGTCAGCATCTGCTGGATGT
GAGGACTTGCATTGTGAAAGCTTTGCTGTCCTTGATGTGATCATGGAATCTCTTTC----
------------------------------------------------------------
------------------------------------------------------------
------------------------------------------------------------
------------------------------------------------------------
------------------------------------------------------------
------------------------------------------------------------
------------------------------------------------------------
------------------------------------------------------------
------------------------------------------------------------
------------------------------------------------------------
------------------------------------------------------------
```

-------TCACTAGAGTCTATGTCACTCATTATACTCTGTCGAATGTCATTGAATGTCTT
TACATGGGCTTATATGCCTATGAAAATTGTAATACAACTTTCAGCAACGGATCTCTTGGC
TCTCGCATCGATGAAGAACGCAGCGAAATGCGATAAGTAATGTGAATTGCAGAATTCAGT
GAATCATCGAATCTTTGAACGCATCTTGCGCTCCTTGGTATTCCGAGGAGCATGCCTGTT
TGAGTGTCATTAAATTCTCAACTCTCTTCTAC-TTTTTGTAAAAGAGAGCTTGGACTGTG
GAGGCTTGCTGGCCACTTTTTGGGGTCAGCTCCTCTGAAATGCATTAGCGGAACCGTTTG
CGATCTGCCACAAGTGTGATAAGTTATCTACACTGGCGAGGGGATTGCTCTCTGTAATGT
TCAGCTTCTAATTGTCTCTACTTTGTGAGACTACTTTTGAATGCTTGACCTCAAATCAGG
TAGGACTACCCGCTGAACTTAA
>AC11-16
TTTCCGTAGGTGAACCTGCGGAAGGATCATTATTGAATTATGTTTCTAGATAGGTTGTAG
CTGGCTC-TTTAGAGCATGTGCACGCCTGTTTGGACTTCATTTTCATCCACCTGTGCACC
TATTGTAGTCTTTGGTTGGGTTAGGAGGAAGTGGTCATTGTGTCAGCATCTGCTGGATGT
GAGGACTTGCATTGTGAAAGCTTTGCTGTCCTTGATGTGATCATGGAATCTCTTTC----

-------TCACTAGAGTCTATGTCACTCATTATACTCTGTCGAATGTCATTGAATGTCTT
TACATGGGCTTATATGCCTATGAAAATTGTAATACAACTTTCAGCAACGGATCTCTTGGC
TCTCGCATCGATGAAGAACGCAGCGAAATGCGATAAGTAATGTGAATTGCAGAATTCAGT
GAATCATCGAATCTTTGAACGCATCTTGCGCTCCTTGGTATTCCGAGGAGCATGCCTGTT
TGAGTGTCATTAAATTCTCAACTCTCTTCTAC-TTTTTGTAAAAGAGAGCTTGGACTGTG
GAGGCTTGCTGGCCACTTTTTGGGGTCAGCTCCTCTGAAATGCATTAGCGGAACCGTTTG
CGATCTGCCACAAGTGTGATAAGTTATCTACACTGGCGAGGGGATTGCTCTCTGTAATGT
TCAGCTTCTAATTGTCTCTACTTTGTGAGACTACTTTTGAATGCTTGACCTCAAATCAGG
TAGGACTACCCGCTGAACTTAA
>AC11-17
TTTCCGTAGGTGAACCTGCGGAAGGATCATTATTGAATTATGTTTCTAGATAGGTTGTAG
CTGGCTC-TTTAGAGCATGTGCACGCCTGTTTGGACTTCATTTTCATCCACCTGTGCACC
TATTGTAGTCTTTGGTTGGGTTAGGAGGAAGTGGTCATTGTGTCAGCATCTGCTGGATGT
GAGGACTTGCATTGTGAAAGCTTTGCTGTCCTTGATGTGATCATGGAATCTCTTTC----

-------TCACTAGAGTCTATGTCACTCATTATACTCTGTCGAATGTCATTGAATGTCTT
TACATGGGCTTATATGCCTATGAAAATTGTAATACAACTTTCAGCAACGGATCTCTTGGC
TCTCGCATCGATGAAGAACGCAGCGAAATGCGATAAGTAATGTGAATTGCAGAATTCAGT
GAATCATCGAATCTTTGAACGCATCTTGCGCTCCTTGGTATTCCGAGGAGCATGCCTGTT
TGAGTGTCATTAAATTCTCAACTCTCTTCTAC-TTTTTGTAAAAGAGAGCTTGGACTGTG
GAGGCTTGCTGGCCACTTTTTGGGGTCAGCTCCTCTGAAATGCATTAGCGGAACCGTTTG
CGATCTGCCACAAGTGTGATAAGTTATCTACACTGGCGAGGGGATTGCTCTCTGTAATGT
TCAGCTTCTAATTGTCTCTACTTTGTGAGACTACTTTTGAATGCTTGACCTCAAATCAGG
TAGGACTACCCGCTGAACTTAA
>AC11-33
TTTCCGTAGGTGAACCTGCGGAAGGATCATTATTGAATTATGTTTCTAGATAGGTTGTAG
CTGGCTC-TTTAGAGCATGTGCACGCCTGTTTGGACTTCATTTTCATCCACCTGTGCACC
TATTGTAGTCTTTGGTTGGGTTAGGAGGAAGTGGTCATTGTGTCAGCATCTGCTGGATGT
GAGGACTTGCATTGTGAAAGCTTTGCTGTCCTTGATGTGATCATGGAATCTCTTTC----

-------TCACTAGAGTCTATGTCACTCATTATACTCTGTCGAATGTCATTGAATGTCTT
TACATGGGCTTATATGCCTATGAAAATTGTAATACAACTTTCAGCAACGGATCTCTTGGC
TCTCGCATCGATGAAGAACGCAGCGAAATGCGATAAGTAATGTGAATTGCAGAATTCAGT
GAATCATCGAATCTTTGAACGCATCTTGCGCTCCTTGGTATTCCGAGGAGCATGCCTGTT
TGAGTGTCATTAAATTCTCAACTCTCTTCTAC-TTTTTGTAAAAGAGAGCTTGGACTGTG
GAGGCTTGCTGGCCACTTTTTGGGGTCAGCTCCTCTGAAATGCATTAGCGGAACCGTTTG
CGATCTGCCACAAGTGTGATAAGTTATCTACACTGGCGAGGGGATTGCTCTCTGTAATGT
TCAGCTTCTAATTGTCTCTACTTTGTGAGACTACTTTTGAATGCTTGACCTCAAATCAGG
TAGGACTACCCGCTGAACTTAA
>AC11-35
TTTCCGTAGGTGAACCTGCGGAAGGATCATTATTGAATTATGTTTCTAGATAGGTTGTAG
CTGGCTC-TTTAGAGCATGTGCACGCCTGTTTGGACTTCATTTTCATCCACCTGTGCACC
TATTGTAGTCTTTGGTTGGGTTAGGAGGAAGTGGTCATTGTGTCAGCATCTGCTGGATGT
GAGGACTTGCATTGTGAAAGCTTTGCTGTCCTTGATGTGATCATGGAATCTCTTTC----

-------TCACTAGAGTCTATGTCACTCATTATACTCTGTCGAATGTCATTGAATGTCTT
TACATGGGCTTATATGCCTATGAAAATTGTAATACAACTTTCAGCAACGGATCTCTTGGC
TCTCGCATCGATGAAGAACGCAGCGAAATGCGATAAGTAATGTGAATTGCAGAATTCAGT
GAATCATCGAATCTTTGAACGCATCTTGCGCTCCTTGGTATTCCGAGGAGCATGCCTGTT
TGAGTGTCATTAAATTCTCAACTCTCTTCTAC-TTTTTGTAAAAGAGAGCTTGGACTGTG
GAGGCTTGCTGGCCACTTTTTGGGGTCAGCTCCTCTGAAATGCATTAGCGGAACCGTTTG
CGATCTGCCACAAGTGTGATAAGTTATCTACACTGGCGAGGGGATTGCTCTCTGTAATGT
TCAGCTTCTAATTGTCTCTACTTTGTGAGACTACTTTTGAATGCTTGACCTCAAATCAGG
TAGGACTACCCGCTGAACTTAA
>AC11-44
TTTCCGTAGGTGAACCTGCGGAAGGATCATTATTGAATTATGTTTCTAGATAGGTTGTAG
CTGGCTC-TTTAGAGCATGTGCACGCCTGTTTGGACTTCATTTTCATCCACCTGTGCACC
TATTGTAGTCTTTGGTTGGGTTAGGAGGAAGTGGTCATTGTGTCAGCATCTGCTGGATGT
GAGGACTTGCATTGTGAAAGCTTTGCTGTCCTTGATGTGATCATGGAATCTCTTTC----

-------TCACTAGAGTCTATGTCACTCATTATACTCTGTCGAATGTCATTGAATGTCTT
TACATGGGCTTATATGCCTATGAAAATTGTAATACAACTTTCAGCAACGGATCTCTTGGC
TCTCGCATCGATGAAGAACGCAGCGAAATGCGATAAGTAATGTGAATTGCAGAATTCAGT
GAATCATCGAATCTTTGAACGCATCTTGCGCTCCTTGGTATTCCGAGGAGCATGCCTGTT
TGAGTGTCATTAAATTCTCAACTCTCTTCTAC-TTTTTGTAAAAGAGAGCTTGGACTGTG
GAGGCTTGCTGGCCACTTTTTGGGGTCAGCTCCTCTGAAATGCATTAGCGGAACCGTTTG
CGATCTGCCACAAGTGTGATAAGTTATCTACACTGGCGAGGGGATTGCTCTCTGTAATGT
TCAGCTTCTAATTGTCTCTACTTTGTGAGACTACTTTTGAATGCTTGACCTCAAATCAGG
TAGGACTACCCGCTGAACTTAA
>AC11-49
TTTCCGTAGGTGAACCTGCGGAAGGATCATTATTGAATTATGTTTCTAGATAGGTTGTAG
CTGGCTC-TTTAGAGCATGTGCACGCCTGTTTGGACTTCATTTTCATCCACCTGTGCACC
TATTGTAGTCTTTGGTTGGGTTAGGAGGAAGTGGTCATTGTGTCAGCATCTGCTGGATGT
GAGGACTTGCATTGTGAAAGCTTTGCTGTCCTTGATGTGATCATGGAATCTCTTTC----


```
------------------------------------------------------------
------------------------------------------------------------
------------------------------------------------------------
------------------------------------------------------------
------------------------------------------------------------
------------------------------------------------------------
------------------------------------------------------------
-------TCACTAGAGTCTATGTCACTCATTATACTCTGTCGAATGTCATTGAATGTCTT
TACATGGGCTTATATGCCTATGAAAATTGTAATACAACTTTCAGCAACGGATCTCTTGGC
TCTCGCATCGATGAAGAACGCAGCGAAATGCGATAAGTAATGTGAATTGCAGAATTCAGT
GAATCATCGAATCTTTGAACGCATCTTGCGCTCCTTGGTATTCCGAGGAGCATGCCTGTT
TGAGTGTCATTAAATTCTCAACTCTCTTCTAC-TTTTTGTAAAAGAGAGCTTGGACTGTG
GAGGCTTGCTGGCCACTTTTTGGGGTCAGCTCCTCTGAAATGCATTAGCGGAACCGTTTG
CGATCTGCCACAAGTGTGATAAGTTATCTACACTGGCGAGGGGATTGCTCTCTGTAATGT
TCAGCTTCTAATTGTCTCTACTTTGTGAGACTACTTTTGAATGCTTGACCTCAAATCAGG
TAGGACTACCCGCTGAACTTAA
>AC12-2
TTTCCGTAGGTGAACCTGCGGAAGGATCATTATTGAATTATGTTTCTAGATAGGTTGTAG
CTGGCTC-TTTAGAGCATGTGCACGCCTGTTTGGACTTCATTTTCATCCACCTGTGCACC
TATTGTAGTCTTTGGTTGGGTTAGGAGGAAGTGGTCATTGTGTCAGCATCTGCTGGATGT
GAGGACTTGCATTGTGAAAGCTTTGCTGTCCTTGATGTGATCATGGAATCTCTTTC----
------------------------------------------------------------
------------------------------------------------------------
------------------------------------------------------------
------------------------------------------------------------
------------------------------------------------------------
------------------------------------------------------------
------------------------------------------------------------
------------------------------------------------------------
------------------------------------------------------------
------------------------------------------------------------
------------------------------------------------------------
------------------------------------------------------------
-------TCACTAGAGTCTATGTCACTCATTATACTCTGTCGAATGTCATTGAATGTCTT
TACATGGGCTTATATGCCTATGAAAATTGTAATACAACTTTCAGCAACGGATCTCTTGGC
TCTCGCATCGATGAAGAACGCAGCGAAATGCGATAAGTAATGTGAATTGCAGAATTCAGT
GAATCATCGAATCTTTGAACGCATCTTGCGCTCCTTGGTATTCCGAGGAGCATGCCTGTT
TGAGTGTCATTAAATTCTCAACTCTCTTCTAC-TTTTTGTAAAAGAGAGCTTGGACTGTG
GAGGCTTGCTGGCCACTTTTTGGGGTCAGCTCCTCTGAAATGCATTAGCGGAACCGTTTG
CGATCTGCCACAAGTGTGATAAGTTATCTACACTGGCGAGGGGATTGCTCTCTGTAATGT
TCAGCTTCTAATTGTCTCTACTTTGTGAGACTACTTTTGAATGCTTGACCTCAAATCAGG
TAGGACTACCCGCTGAACTTAA
>AC12-9
TTTCCGTAGGTGAACCTGCGGAAGGATCATTATTGAATTATGTTTCTAGATAGGTTGTAG
CTGGCTC-TTTAGAGCATGTGCACGCCTGTTTGGACTTCATTTTCATCCACCTGTGCACC
TATTGTAGTCTTTGGTTGGGTTAGGAGGAAGTGGTCATTGTGTCAGCATCTGCTGGATGT
GAGGACTTGCATTGTGAAAGCTTTGCTGTCCTTGATGTGATCATGGAATCTCTTTC----
------------------------------------------------------------
------------------------------------------------------------
------------------------------------------------------------
```


-------TCACTAGAGTCTATGTCACTCATTATACTCTGTCGAATGTCATTGAATGTCTT
TACATGGGCTTATATGCCTATGAAAATTGTAATACAACTTTCAGCAACGGATCTCTTGGC
TCTCGCATCGATGAAGAACGCAGCGAAATGCGATAAGTAATGTGAATTGCAGAATTCAGT
GAATCATCGAATCTTTGAACGCATCTTGCGCTCCTTGGTATTCCGAGGAGCATGCCTGTT
TGAGTGTCATTAAATTCTCAACTCTCTTCTAC-TTTTTGTAAAAGAGAGCTTGGACTGTG
GAGGCTTGCTGGCCACTTTTTGGGGTCAGCTCCTCTGAAATGCATTAGCGGAACCGTTTG
CGATCTGCCACAAGTGTGATAAGTTATCTACACTGGCGAGGGGATTGCTCTCTGTAATGT
TCAGCTTCTAATTGTCTCTACTTTGTGAGACTACTTTTGAATGCTTGACCTCAAATCAGG
TAGGACTACCCGCTGAACTTAA
>AC12-15
TTTCCGTAGGTGAACCTGCGGAAGGATCATTATTGAATTATGTTTCTAGATAGGTTGTAG
CTGGCTC-TTTAGAGCATGTGCACGCCTGTTTGGACTTCATTTTCATCCACCTGTGCACC
TATTGTAGTCTTTGGTTGGGTTAGGAGGAAGTGGTCATTGTGTCAGCATCTGCTGGATGT
GAGGACTTGCATTGTGAAAGCTTTGCTGTCCTTGATGTGATCATGGAATCTCTTTC----

-------TCACTAGAGTCTATGTCACTCATTATACTCTGTCGAATGTCATTGAATGTCTT
TACATGGGCTTATATGCCTATGAAAATTGTAATACAACTTTCAGCAACGGATCTCTTGGC
TCTCGCATCGATGAAGAACGCAGCGAAATGCGATAAGTAATGTGAATTGCAGAATTCAGT
GAATCATCGAATCTTTGAACGCATCTTGCGCTCCTTGGTATTCCGAGGAGCATGCCTGTT
TGAGTGTCATTAAATTCTCAACTCTCTTCTAC-TTTTTGTAAAAGAGAGCTTGGACTGTG
GAGGCTTGCTGGCCACTTTTTGGGGTCAGCTCCTCTGAAATGCATTAGCGGAACCGTTTG
CGATCTGCCACAAGTGTGATAAGTTATCTACACTGGCGAGGGGATTGCTCTCTGTAATGT
TCAGCTTCTAATTGTCTCTACTTTGTGAGACTACTTTTGAATGCTTGACCTCAAATCAGG
TAGGACTACCCGCTGAACTTAA
>AC12-17
TTTCCGTAGGTGAACCTGCGGAAGGATCATTATTGAATTATGTTTCTAGATAGGTTGTAG
CTGGCTC-TTTAGAGCATGTGCACGCCTGTTTGGACTTCATTTTCATCCACCTGTGCACC
TATTGTAGTCTTTGGTTGGGTTAGGAGGAAGTGGTCATTGTGTCAGCATCTGCTGGATGT
GAGGACTTGCATTGTGAAAGCTTTGCTGTCCTTGATGTGATCATGGAATCTCTTTC----

```
------------------------------------------------------------
------------------------------------------------------------
------------------------------------------------------------
------------------------------------------------------------
------------------------------------------------------------
------------------------------------------------------------
------------------------------------------------------------
------------------------------------------------------------
------------------------------------------------------------
------------------------------------------------------------
------------------------------------------------------------
-------TCACTAGAGTCTATGTCACTCATTATACTCTGTCGAATGTCATTGAATGTCTT
TACATGGGCTTATATGCCTATGAAAATTGTAATACAACTTTCAGCAACGGATCTCTTGGC
TCTCGCATCGATGAAGAACGCAGCGAAATGCGATAAGTAATGTGAATTGCAGAATTCAGT
GAATCATCGAATCTTTGAACGCATCTTGCGCTCCTTGGTATTCCGAGGAGCATGCCTGTT
TGAGTGTCATTAAATTCTCAACTCTCTTCTAC-TTTTTGTAAAAGAGAGCTTGGACTGTG
GAGGCTTGCTGGCCACTTTTTGGGGTCAGCTCCTCTGAAATGCATTAGCGGAACCGTTTG
CGATCTGCCACAAGTGTGATAAGTTATCTACACTGGCGAGGGGATTGCTCTCTGTAATGT
TCAGCTTCTAATTGTCTCTACTTTGTGAGACTACTTTTGAATGCTTGACCTCAAATCAGG
TAGGACTACCCGCTGAACTTAA
>AC12-19
TTTCCGTAGGTGAACCTGCGGAAGGATCATTATTGAATTATGTTTCTAGATAGGTTGTAG
CTGGCTC-TTTAGAGCATGTGCACGCCTGTTTGGACTTCATTTTCATCCACCTGTGCACC
TATTGTAGTCTTTGGTTGGGTTAGGAGGAAGTGGTCATTGTGTCAGCATCTGCTGGATGT
GAGGACTTGCATTGTGAAAGCTTTGCTGTCCTTGATGTGATCATGGAATCTCTTTC----
------------------------------------------------------------
------------------------------------------------------------
------------------------------------------------------------
------------------------------------------------------------
------------------------------------------------------------
------------------------------------------------------------
------------------------------------------------------------
------------------------------------------------------------
------------------------------------------------------------
------------------------------------------------------------
------------------------------------------------------------
------------------------------------------------------------
-------TCACTAGAGTCTATGTCACTCATTATACTCTGTCGAATGTCATTGAATGTCTT
TACATGGGCTTATATGCCTATGAAAATTGTAATACAACTTTCAGCAACGGATCTCTTGGC
TCTCGCATCGATGAAGAACGCAGCGAAATGCGATAAGTAATGTGAATTGCAGAATTCAGT
GAATCATCGAATCTTTGAACGCATCTTGCGCTCCTTGGTATTCCGAGGAGCATGCCTGTT
TGAGTGTCATTAAATTCTCAACTCTCTTCTAC-TTTTTGTAAAAGAGAGCTTGGACTGTG
GAGGCTTGCTGGCCACTTTTTGGGGTCAGCTCCTCTGAAATGCATTAGCGGAACCGTTTG
CGATCTGCCACAAGTGTGATAAGTTATCTACACTGGCGAGGGGATTGCTCTCTGTAATGT
TCAGCTTCTAATTGTCTCTACTTTGTGAGACTACTTTTGAATGCTTGACCTCAAATCAGG
TAGGACTACCCGCTGAACTTAA
>AC12-21
TTTCCGTAGGTGAACCTGCGGAAGGATCATTATTGAATTATGTTTCTAGATAGGTTGTAG
CTGGCTC-TTTAGAGCATGTGCACGCCTGTTTGGACTTCATTTTCATCCACCTGTGCACC
TATTGTAGTCTTTGGTTGGGTTAGGAGGAAGTGGTCATTGTGTCAGCATCTGCTGGATGT
```

GAGGACTTGCATTGTGAAAGCTTTGCTGTCCTTGATGTGATCATGGAATCTCTTTC----

-------TCACTAGAGTCTATGTCACTCATTATACTCTGTCGAATGTCATTGAATGTCTT
TACATGGGCTTATATGCCTATGAAAATTGTAATACAACTTTCAGCAACGGATCTCTTGGC
TCTCGCATCGATGAAGAACGCAGCGAAATGCGATAAGTAATGTGAATTGCAGAATTCAGT
GAATCATCGAATCTTTGAACGCATCTTGCGCTCCTTGGTATTCCGAGGAGCATGCCTGTT
TGAGTGTCATTAAATTCTCAACTCTCTTCTAC-TTTTTGTAAAAGAGAGCTTGGACTGTG
GAGGCTTGCTGGCCACTTTTTGGGGTCAGCTCCTCTGAAATGCATTAGCGGAACCGTTTG
CGATCTGCCACAAGTGTGATAAGTTATCTACACTGGCGAGGGGATTGCTCTCTGTAATGT
TCAGCTTCTAATTGTCTCTACTTTGTGAGACTACTTTTGAATGCTTGACCTCAAATCAGG
TAGGACTACCCGCTGAACTTAA
>AC12-26
TTTCCGTAGGTGAACCTGCGGAAGGATCATTATTGAATTATGTTTCTAGATAGGTTGTAG
CTGGCTC-TTTAGAGCATGTGCACGCCTGTTTGGACTTCATTTTCATCCACCTGTGCACC
TATTGTAGTCTTTGGTTGGGTTAGGAGGAAGTGGTCATTGTGTCAGCATCTGCTGGATGT
GAGGACTTGCATTGTGAAAGCTTTGCTGTCCTTGATGTGATCATGGAATCTCTTTC----

-------TCACTAGAGTCTATGTCACTCATTATACTCTGTCGAATGTCATTGAATGTCTT
TACATGGGCTTATATGCCTATGAAAATTGTAATACAACTTTCAGCAACGGATCTCTTGGC
TCTCGCATCGATGAAGAACGCAGCGAAATGCGATAAGTAATGTGAATTGCAGAATTCAGT
GAATCATCGAATCTTTGAACGCATCTTGCGCTCCTTGGTATTCCGAGGAGCATGCCTGTT
TGAGTGTCATTAAATTCTCAACTCTCTTCTAC-TTTTTGTAAAAGAGAGCTTGGACTGTG
GAGGCTTGCTGGCCACTTTTTGGGGTCAGCTCCTCTGAAATGCATTAGCGGAACCGTTTG
CGATCTGCCACAAGTGTGATAAGTTATCTACACTGGCGAGGGGATTGCTCTCTGTAATGT
TCAGCTTCTAATTGTCTCTACTTTGTGAGACTACTTTTGAATGCTTGACCTCAAATCAGG
TAGGACTACCCGCTGAACTTAA
>AC12-29
TTTCCGTAGGTGAACCTGCGGAAGGATCATTATTGAATTATGTTTCTAGATAGGTTGTAG

CTGGCTC-TTTAGAGCATGTGCACGCCTGTTTGGACTTCATTTTCATCCACCTGTGCACC
TATTGTAGTCTTTGGTTGGGTTAGGAGGAAGTGGTCATTGTGTCAGCATCTGCTGGATGT
GAGGACTTGCATTGTGAAAGCTTTGCTGTCCTTGATGTGATCATGGAATCTCTTTC----

-------TCACTAGAGTCTATGTCACTCATTATACTCTGTCGAATGTCATTGAATGTCTT
TACATGGGCTTATATGCCTATGAAAATTGTAATACAACTTTCAGCAACGGATCTCTTGGC
TCTCGCATCGATGAAGAACGCAGCGAAATGCGATAAGTAATGTGAATTGCAGAATTCAGT
GAATCATCGAATCTTTGAACGCATCTTGCGCTCCTTGGTATTCCGAGGAGCATGCCTGTT
TGAGTGTCATTAAATTCTCAACTCTCTTCTAC-TTTTTGTAAAAGAGAGCTTGGACTGTG
GAGGCTTGCTGGCCACTTTTTGGGGTCAGCTCCTCTGAAATGCATTAGCGGAACCGTTTG
CGATCTGCCACAAGTGTGATAAGTTATCTACACTGGCGAGGGGATTGCTCTCTGTAATGT
TCAGCTTCTAATTGTCTCTACTTTGTGAGACTACTTTTGAATGCTTGACCTCAAATCAGG
TAGGACTACCCGCTGAACTTAA
>AC12-33
TTTCCGTAGGTGAACCTGCGGAAGGATCATTATTGAATTATGTTTCTAGATAGGTTGTAG
CTGGCTC-TTTAGAGCATGTGCACGCCTGTTTGGACTTCATTTTCATCCACCTGTGCACC
TATTGTAGTCTTTGGTTGGGTTAGGAGGAAGTGGTCATTGTGTCAGCATCTGCTGGATGT
GAGGACTTGCATTGTGAAAGCTTTGCTGTCCTTGATGTGATCATGGAATCTCTTTC----

-------TCACTAGAGTCTATGTCACTCATTATACTCTGTCGAATGTCATTGAATGTCTT
TACATGGGCTTATATGCCTATGAAAATTGTAATACAACTTTCAGCAACGGATCTCTTGGC
TCTCGCATCGATGAAGAACGCAGCGAAATGCGATAAGTAATGTGAATTGCAGAATTCAGT
GAATCATCGAATCTTTGAACGCATCTTGCGCTCCTTGGTATTCCGAGGAGCATGCCTGTT
TGAGTGTCATTAAATTCTCAACTCTCTTCTAC-TTTTTGTAAAAGAGAGCTTGGACTGTG
GAGGCTTGCTGGCCACTTTTTGGGGTCAGCTCCTCTGAAATGCATTAGCGGAACCGTTTG
CGATCTGCCACAAGTGTGATAAGTTATCTACACTGGCGAGGGGATTGCTCTCTGTAATGT
TCAGCTTCTAATTGTCTCTACTTTGTGAGACTACTTTTGAATGCTTGACCTCAAATCAGG
TAGGACTACCCGCTGAACTTAA

```
>AC12-39
TTTCCGTAGGTGAACCTGCGGAAGGATCATTATTGAATTATGTTTCTAGATAGGTTGTAG
CTGGCTC-TTTAGAGCATGTGCACGCCTGTTTGGACTTCATTTTCATCCACCTGTGCACC
TATTGTAGTCTTTGGTTGGGTTAGGAGGAAGTGGTCATTGTGTCAGCATCTGCTGGATGT
GAGGACTTGCATTGTGAAAGCTTTGCTGTCCTTGATGTGATCATGGAATCTCTTTC----
------------------------------------------------------------
------------------------------------------------------------
------------------------------------------------------------
------------------------------------------------------------
------------------------------------------------------------
------------------------------------------------------------
------------------------------------------------------------
------------------------------------------------------------
------------------------------------------------------------
------------------------------------------------------------
------------------------------------------------------------
------------------------------------------------------------
-------TCACTAGAGTCTATGTCACTCATTATACTCTGTCGAATGTCATTGAATGTCTT
TACATGGGCTTATATGCCTATGAAAATTGTAATACAACTTTCAGCAACGGATCTCTTGGC
TCTCGCATCGATGAAGAACGCAGCGAAATGCGATAAGTAATGTGAATTGCAGAATTCAGT
GAATCATCGAATCTTTGAACGCATCTTGCGCTCCTTGGTATTCCGAGGAGCATGCCTGTT
TGAGTGTCATTAAATTCTCAACTCTCTTCTAC-TTTTTGTAAAAGAGAGCTTGGACTGTG
GAGGCTTGCTGGCCACTTTTTGGGGTCAGCTCCTCTGAAATGCATTAGCGGAACCGTTTG
CGATCTGCCACAAGTGTGATAAGTTATCTACACTGGCGAGGGGATTGCTCTCTGTAATGT
TCAGCTTCTAATTGTCTCTACTTTGTGAGACTACTTTTGAATGCTTGACCTCAAATCAGG
TAGGACTACCCGCTGAACTTAA
>AC12-44
TTTCCGTAGGTGAACCTGCGGAAGGATCATTATTGAATTATGTTTCTAGATAGGTTGTAG
CTGGCTC-TTTAGAGCATGTGCACGCCTGTTTGGACTTCATTTTCATCCACCTGTGCACC
TATTGTAGTCTTTGGTTGGGTTAGGAGGAAGTGGTCATTGTGTCAGCATCTGCTGGATGT
GAGGACTTGCATTGTGAAAGCTTTGCTGTCCTTGATGTGATCATGGAATCTCTTTC----
------------------------------------------------------------
------------------------------------------------------------
------------------------------------------------------------
------------------------------------------------------------
------------------------------------------------------------
------------------------------------------------------------
------------------------------------------------------------
------------------------------------------------------------
------------------------------------------------------------
------------------------------------------------------------
------------------------------------------------------------
------------------------------------------------------------
-------TCACTAGAGTCTATGTCACTCATTATACTCTGTCGAATGTCATTGAATGTCTT
TACATGGGCTTATATGCCTATGAAAATTGTAATACAACTTTCAGCAACGGATCTCTTGGC
TCTCGCATCGATGAAGAACGCAGCGAAATGCGATAAGTAATGTGAATTGCAGAATTCAGT
GAATCATCGAATCTTTGAACGCATCTTGCGCTCCTTGGTATTCCGAGGAGCATGCCTGTT
TGAGTGTCATTAAATTCTCAACTCTCTTCTAC-TTTTTGTAAAAGAGAGCTTGGACTGTG
GAGGCTTGCTGGCCACTTTTTGGGGTCAGCTCCTCTGAAATGCATTAGCGGAACCGTTTG
CGATCTGCCACAAGTGTGATAAGTTATCTACACTGGCGAGGGGATTGCTCTCTGTAATGT
```

TCAGCTTCTAATTGTCTCTACTTTGTGAGACTACTTTTGAATGCTTGACCTCAAATCAGG
TAGGACTACCCGCTGAACTTAA
>AC10-29
TTTCCGTAGGTGAACCTGCGGAAGGATCATTATTGAATTATGTTTCTAGATAGGTTGTAG
CTGGCTC-TTTAGAGCATGTGCACGCCTGTTTGGACTTCATTTTCATCCACCTGTGCACC
TATTGTAGTCTTTGGTTGGGTTAGGAGGAAGTGGTCATTGTGTCAGCATCTGCTGGATGT
GAGGACTTGCATTGTGAAAGCTTTGCTGTCCTTGATGTGATCATGGAATCTCTTTC----

-------TCACTAGAGTCTATGTCACTCATTATACTCTGTCGAATGTCATTGAATGTCTT
TACATGGGCTTATATGCCTATGAAAATTGTAATACAACTTTCAGCAACGGATCTCTTGGC
TCTCGCATCGATGAAGAACGCAGCGAAATGCGATAAGTAATGTGAATTGCAGAATTCAGT
GAATCATCGAATCTTTGAACGCATCTTGCGCTCCTTGGTATTCCGAGGAGCATGCCTGTT
TGAGTGTCATTAAATTCTCAACTCTCTTCTAC-TTTTTGTAAAAGAGAGCTTGGACTGTG
GAGGCTTGCTGGCCACTTTTTGGGGTCAGCTCCTCTGAAATGCATTAGCGGAACCGTTTG
CGATCTGCCACAAGTGTGATAAGTTATCTACACTGGCGAGGGGATTGCTCTCTGTAATGT
TCAGCTTCTAATTGTCTCTACTTTGTGAGACTACTTTTGAATGCTTGACCTCAAATCAGG
TAGGACTACCCGCTGAACTTAA
>AC10-30
TTTCCGTAGGTGAACCTGCGGAAGGATCATTATTGAATTATGTTTCTAGATAGGTTGTAG
CTGGCTC-TTTAGAGCATGTGCACGCCTGTTTGGACTTCATTTTCATCCACCTGTGCACC
TATTGTAGTCTTTGGTTGGGTTAGGAGGAAGTGGTCATTGTGTCAGCATCTGCTGGATGT
GAGGACTTGCATTGTGAAAGCTTTGCTGTCCTTGATGTGATCATGGAATCTCTTTC----

-------TCACTAGAGTCTATGTCACTCATTATACTCTGTCGAATGTCATTGAATGTCTT
TACATGGGCTTATATGCCTATGAAAATTGTAATACAACTTTCAGCAACGGATCTCTTGGC
TCTCGCATCGATGAAGAACGCAGCGAAATGCGATAAGTAATGTGAATTGCAGAATTCAGT
GAATCATCGAATCTTTGAACGCATCTTGCGCTCCTTGGTATTCCGAGGAGCATGCCTGTT
TGAGTGTCATTAAATTCTCAACTCTCTTCTAC-TTTTTGTAAAAGAGAGCTTGGACTGTG

```
GAGGCTTGCTGGCCACTTTTTGGGGTCAGCTCCTCTGAAATGCATTAGCGGAACCGTTTG
CGATCTGCCACAAGTGTGATAAGTTATCTACACTGGCGAGGGGATTGCTCTCTGTAATGT
TCAGCTTCTAATTGTCTCTACTTTGTGAGACTACTTTTGAATGCTTGACCTCAAATCAGG
TAGGACTACCCGCTGAACTTAA
>AC10-19
TTTCCGTAGGTGAACCTGCGGAAGGATCATTATTGAATTATGTTTCTAGATAGGTTGTAG
CTGGCTC-TTTAGAGCATGTGCACGCCTGTTTGGACTTCATTTTCATCCACCTGTGCACC
TATTGTAGTCTTTGGTTGGGTTAGGAGGAAGTGGTCATTGTGTCAGCATCTGCTGGATGT
GAGGACTTGCATTGTGAAAGCTTTGCTGTCCTTGATGTGATCATGGAATCTCTTTC----
------------------------------------------------------------
------------------------------------------------------------
------------------------------------------------------------
------------------------------------------------------------
------------------------------------------------------------
------------------------------------------------------------
------------------------------------------------------------
------------------------------------------------------------
------------------------------------------------------------
------------------------------------------------------------
------------------------------------------------------------
------------------------------------------------------------
-------TCACTAGAGTCTATGTCACTCATTATACTCTGTCGAATGTCATTGAATGTCTT
TACATGGGCTTATATGCCTATGAAAATTGTAATACAACTTTCAGCAACGGATCTCTTGGC
TCTCGCATCGATGAAGAACGCAGCGAAATGCGATAAGTAATGTGAATTGCAGAATTCAGT
GAATCATCGAATCTTTGAACGCATCTTGCGCTCCTTGGTATTCCGAGGAGCATGCCTGTT
TGAGTGTCATTAAATTCTCAACTCTCTTCTAC-TTTTTGTAAAAGAGAGCTTGGACTGTG
GAGGCTTGCTGGCCACTTTTTGGGGTCAGCTCCTCTGAAATGCATTAGCGGAACCGTTTG
CGATCTGCCACAAGTGTGATAAGTTATCTACACTGGCGAGGGGATTGCTCTCTGTAATGT
TCAGCTTCTAATTGTCTCTACTTTGTGAGACTACTTTTGAATGCTTGACCTCAAATCAGG
TAGGACTACCCGCTGAACTTAA
>AC12-36
TTTCCGTAGGTGAACCTGCGGAAGGATCATTATTGAATTATGTTTCTAGATAGGTTGTAG
CTGGCTC-TTTAGAGCATGTGCACGCCTGTTTGGACTTCATTTTCATCCACCTGTGCACC
TATTGTAGTCTTTGGTTGGGTTAGGAGGAAGTGGTCATTGTGTCAGCATCTGCTGGATGT
GAGGACTTGCATTGTGAAAGCTTTGCTGTCCTTGATGTGATCATGGAATCTCTTTC----
------------------------------------------------------------
------------------------------------------------------------
------------------------------------------------------------
------------------------------------------------------------
------------------------------------------------------------
------------------------------------------------------------
------------------------------------------------------------
------------------------------------------------------------
------------------------------------------------------------
------------------------------------------------------------
------------------------------------------------------------
------------------------------------------------------------
-------TCACTAGAGTCTATGTCACTCATTATACTCTGTCGAATGTCATTGAATGTCTT
TACATGGGCTTATATGCCTATGAAAATTGTAATACAACTTTCAGCAACGGATCTCTTGGC
TCTCGCATCGATGAAGAACGCAGCGAAATGCGATAAGTAATGTGAATTGCAGAATTCAGT
```

```
GAATCATCGAATCTTTGAACGCATCTTGCGCTCCTTGGTATTCCGAGGAGCATGCCTGTT
TGAGTGTCATTAAATTCTCAACTCTCTTCTAC-TTTTTGTAAAAGAGAGCTTGGACTGTG
GAGGCTTGCTGGCCACTTTTTGGGGTCAGCTCCTCTGAAATGCATTAGCGGAACCGTTTG
CGATCTGCCACAAGTGTGATAAGTTATCTACACTGGCGAGGGGATTGCTCTCTGTAATGT
TCAGCTTCTAATTGTCTCTACTTTGTGAGACTACTTTTGAATGCTTGACCTCAAATCAGG
TAGGACTACCCGCTGAACTTAA
>AC3-47
TTTCCGTAGGTGAACCTGCGGAAGGATCATTATTGAATTATGTTTCTAGATAGGTTGTAG
CTGGCTC-TTTAGAGCATGTGCACGCCTGTTTGGACTTCATTTTCATCCACCTGTGCACC
TATTGTAGTCTTTGGTTGGGTTAGGAGGAAGTGGTCATTGTGTCAGCATCTGCTGGATGT
GAGGACTTGCATTGTGAAAGCTTTGCTGTCCTTGATGTGATCATGGAATCTCTTTC----
------------------------------------------------------------
------------------------------------------------------------
------------------------------------------------------------
------------------------------------------------------------
------------------------------------------------------------
------------------------------------------------------------
------------------------------------------------------------
------------------------------------------------------------
------------------------------------------------------------
------------------------------------------------------------
------------------------------------------------------------
------------------------------------------------------------
-------TCACTAGAGTCTATGTCACTCATTATACTCTGTCGAATGTCATTGAATGTCTT
TACATGGGCTTATATGCCTATGAAAATTGTAATACAACTTTCAGCAACGGATCTCTTGGC
TCTCGCATCGATGAAGAACGCAGCGAAATGCGATAAGTAATGTGAATTGCAGAATTCAGT
GAATCATCGAATCTTTGAACGCATCTTGCGCTCCTTGGTATTCCGAGGAGCATGCCTGTT
TGAGTGTCATTAAATTCTCAACTCTCTTCTAC-TTTTTGTAAAAGAGAGCTTGGACTGTG
GAGGCTTGCTGGCCACTTTTTGGGGTCAGCTCCTCTGAAATGCATTAGCGGAACCGTTTG
CGATCTGCCACAAGTGTGATAAGTTATCTACACTGGCGAGGGGATTGCTCTCTGTAATGT
TCAGCTTCTAATTGTCTCTACTTTGTGAGACTACTTTTGAATGCTTGACCTCAAATCAGG
TAGGACTACCCGCTGAACTTAA
>AC3-70
TTTCCGTAGGTGAACCTGCGGAAGGATCATTATTGAATTATGTTTCTAGATAGGTTGTAG
CTGGCTC-TTTAGAGCATGTGCACGCCTGTTTGGACTTCATTTTCATCCACCTGTGCACC
TATTGTAGTCTTTGGTTGGGTTAGGAGGAAGTGGTCATTGTGTCAGCATCTGCTGGATGT
GAGGACTTGCATTGTGAAAGCTTTGCTGTCCTTGATGTGATCATGGAATCTCTTTC----
------------------------------------------------------------
------------------------------------------------------------
------------------------------------------------------------
------------------------------------------------------------
------------------------------------------------------------
------------------------------------------------------------
------------------------------------------------------------
------------------------------------------------------------
------------------------------------------------------------
------------------------------------------------------------
------------------------------------------------------------
------------------------------------------------------------
-------TCACTAGAGTCTATGTCACTCATTATACTCTGTCGAATGTCATTGAATGTCTT
```

```
TACATGGGCTTATATGCCTATGAAAATTGTAATACAACTTTCAGCAACGGATCTCTTGGC
TCTCGCATCGATGAAGAACGCAGCGAAATGCGATAAGTAATGTGAATTGCAGAATTCAGT
GAATCATCGAATCTTTGAACGCATCTTGCGCTCCTTGGTATTCCGAGGAGCATGCCTGTT
TGAGTGTCATTAAATTCTCAACTCTCTTCTAC-TTTTTGTAAAAGAGAGCTTGGACTGTG
GAGGCTTGCTGGCCACTTTTTGGGGTCAGCTCCTCTGAAATGCATTAGCGGAACCGTTTG
CGATCTGCCACAAGTGTGATAAGTTATCTACACTGGCGAGGGGATTGCTCTCTGTAATGT
TCAGCTTCTAATTGTCTCTACTTTGTGAGACTACTTTTGAATGCTTGACCTCAAATCAGG
TAGGACTACCCGCTGAACTTAA
>AC2-55
TTTCCGTAGGTGAACCTGCGGAAGGATCATTATTGAATTATGTTTCTAGATAGGTTGTAG
CTGGCTC-TTTAGAGCATGTGCACGCCTGTTTGGACTTCATTTTCATCCACCTGTGCACC
TATTGTAGTCTTTGGTTGGGTTAGGAGGAAGTGGTCATTGTGTCAGCATCTGCTGGATGT
GAGGACTTGCATTGTGAAAGCTTTGCTGTCCTTGATGTGATCATGGAATCTCTTTC----
------------------------------------------------------------
------------------------------------------------------------
------------------------------------------------------------
------------------------------------------------------------
------------------------------------------------------------
------------------------------------------------------------
------------------------------------------------------------
------------------------------------------------------------
------------------------------------------------------------
------------------------------------------------------------
------------------------------------------------------------
------------------------------------------------------------
-------TCACTAGAGTCTATGTCACTCATTATACTCTGTCGAATGTCATTGAATGTCTT
TACATGGGCTTATATGCCTATGAAAATTGTAATACAACTTTCAGCAACGGATCTCTTGGC
TCTCGCATCGATGAAGAACGCAGCGAAATGCGATAAGTAATGTGAATTGCAGAATTCAGT
GAATCATCGAATCTTTGAACGCATCTTGCGCTCCTTGGTATTCCGAGGAGCATGCCTGTT
TGAGTGTCATTAAATTCTCAACTCTCTTCTAC-TTTTTGTAAAAGAGAGCTTGGACTGTG
GAGGCTTGCTGGCCACTTTTTGGGGTCAGCTCCTCTGAAATGCATTAGCGGAACCGTTTG
CGATCTGCCACAAGTGTGATAAGTTATCTACACTGGCGAGGGGATTGCTCTCTGTAATGT
TCAGCTTCTAATTGTCTCTACTTTGTGAGACTACTTTTGAATGCTTGACCTCAAATCAGG
TAGGACTACCCGCTGAACTTAA
>AC2-56
TTTCCGTAGGTGAACCTGCGGAAGGATCATTATTGAATTATGTTTCTAGATAGGTTGTAG
CTGGCTC-TTTAGAGCATGTGCACGCCTGTTTGGACTTCATTTTCATCCACCTGTGCACC
TATTGTAGTCTTTGGTTGGGTTAGGAGGAAGTGGTCATTGTGTCAGCATCTGCTGGATGT
GAGGACTTGCATTGTGAAAGCTTTGCTGTCCTTGATGTGATCATGGAATCTCTTTC----
------------------------------------------------------------
------------------------------------------------------------
------------------------------------------------------------
------------------------------------------------------------
------------------------------------------------------------
------------------------------------------------------------
------------------------------------------------------------
------------------------------------------------------------
------------------------------------------------------------
------------------------------------------------------------
------------------------------------------------------------
```

```
------------------------------------------------------------
-------TCACTAGAGTCTATGTCACTCATTATACTCTGTCGAATGTCATTGAATGTCTT
TACATGGGCTTATATGCCTATGAAAATTGTAATACAACTTTCAGCAACGGATCTCTTGGC
TCTCGCATCGATGAAGAACGCAGCGAAATGCGATAAGTAATGTGAATTGCAGAATTCAGT
GAATCATCGAATCTTTGAACGCATCTTGCGCTCCTTGGTATTCCGAGGAGCATGCCTGTT
TGAGTGTCATTAAATTCTCAACTCTCTTCTAC-TTTTTGTAAAAGAGAGCTTGGACTGTG
GAGGCTTGCTGGCCACTTTTTGGGGTCAGCTCCTCTGAAATGCATTAGCGGAACCGTTTG
CGATCTGCCACAAGTGTGATAAGTTATCTACACTGGCGAGGGGATTGCTCTCTGTAATGT
TCAGCTTCTAATTGTCTCTACTTTGTGAGACTACTTTTGAATGCTTGACCTCAAATCAGG
TAGGACTACCCGCTGAACTTAA
>AC3-31
TTTCCGTAGGTGAACCTGCGGAAGGATCATTATTGAATTATGTTTCTAGATAGGTTGTAG
CTGGCTC-TTTAGAGCATGTGCACGCCTGTTTGGACTTCATTTTCATCCACCTGTGCACC
TATTGTAGTCTTTGGTTGGGTTAGGAGGAAGTGGTCATTGTGTCAGCATCTGCTGGATGT
GAGGACTTGCATTGTGAAAGCTTTGCTGTCCTTGATGTGATCATGGAATCTTTTTC----
------------------------------------------------------------
------------------------------------------------------------
------------------------------------------------------------
------------------------------------------------------------
------------------------------------------------------------
------------------------------------------------------------
------------------------------------------------------------
------------------------------------------------------------
------------------------------------------------------------
------------------------------------------------------------
------------------------------------------------------------
------------------------------------------------------------
-------TCACTAGAGTCTATGTCACTCATTATACTCTGTCGAATGTCATTGAATGTCTT
TACATGGGCTTATATGCCTATGAAAATTGTAATACAACTTTCAGCAACGGATCTCTTGGC
TCTCGCATCGATGAAGAACGCAGCGAAATGCGATAAGTAATGTGAATTGCAGAATTCAGT
GAATCATCGAATCTTTGAACGCATCTTGCGCTCCTTGGTATTCCGAGGAGCATGCCTGTT
TGAGTGTCATTAAATTCTCAACTCTCTTCTAC-TTTTTGTAAAAGAGAGCTTGGACTGTG
GAGGCTTGCTGGCCACTTTTTGGGGTCAGCTCCTCTGAAATGCATTAGCGGAACCGTTTG
CGATCTGCCACAAGTGTGATAAGTTATCTACACTGGCGAGGGGATTGCTCTCTGTAATGT
TCAGCTTCTAATTGTCTCTACTTTGTGAGACTACTTTTGAATGCTTGACCTCAAATCAGG
TAGGACTACCCGCTGAACTTAA
>AC7-50
TTTCCGTAGGTGAACCTGCGGAAGGATCATTATTGAATTATGTTTCTAGATAGGTTGTAG
CTGGCTC-TTTAGAGCATGTGCACGCCTGTTTGGACTTCATTTTCATCCACCTGTGCACC
TATTGTAGTCTTTGGTTGGGTTAGGAGGAAGTGGTCATTGTGTCAGCATCTGCTGGATGT
GAGGACTTGCATTGTGAAAGCTTTGCTGTCCTTGATGTGATCATGGAATCTTTTTC----
------------------------------------------------------------
------------------------------------------------------------
------------------------------------------------------------
------------------------------------------------------------
------------------------------------------------------------
------------------------------------------------------------
------------------------------------------------------------
------------------------------------------------------------
------------------------------------------------------------
```


-------TCACTAGAGTCTATGTCACTCATTATACTCTGTCGAATGTCATTGAATGTCTT
TACATGGGCTTATATGCCTATGAAAATTGTAATACAACTTTCAGCAACGGATCTCTTGGC
TCTCGCATCGATGAAGAACGCAGCGAAATGCGATAAGTAATGTGAATTGCAGAATTCAGT
GAATCATCGAATCTTTGAACGCATCTTGCGCTCCTTGGTATTCCGAGGAGCATGCCTGTT
TGAGTGTCATTAAATTCTCAACTCTCTTCTAC-TTTTTGTAAAAGAGAGCTTGGACTGTG
GAGGCTTGCTGGCCACTTTTTGGGGTCAGCTCCTCTGAAATGCATTAGCGGAACCGTTTG
CGATCTGCCACAAGTGTGATAAGTTATCTACACTGGCGAGGGGATTGCTCTCTGTAATGT
TCAGCTTCTAATTGTCTCTACTTTGTGAGACTACTTTTGAATGCTTGACCTCAAATCAGG
TAGGACTACCCGCTGAACTTAA
>AC10-3
TTTCCGTAGGTGAACCTGCGGAAGGATCATTATTGAATTATGTTTCTAGATAGGTTGTAG
CTGGCTC-TTTAGAGCATGTGCACGCCTGTTTGGACTTCATTTTCATCCACCTGTGCACC
TATTGTAGTCTTTGGTTGGGTTAGGAGGAAGTGGTCATTGTGTCAGCATCTGCTGGATGT
GAGGACTTGCATTGTGAAAGCTTTGCTGTCCTTGATGTGATCATGGAATCTTTTTC----

-------TCACTAGAGTCTATGTCACTCATTATACTCTGTCGAATGTCATTGAATGTCTT
TACATGGGCTTATATGCCTATGAAAATTGTAATACAACTTTCAGCAACGGATCTCTTGGC
TCTCGCATCGATGAAGAACGCAGCGAAATGCGATAAGTAATGTGAATTGCAGAATTCAGT
GAATCATCGAATCTTTGAACGCATCTTGCGCTCCTTGGTATTCCGAGGAGCATGCCTGTT
TGAGTGTCATTAAATTCTCAACTCTCTTCTAC-TTTTTGTAAAAGAGAGCTTGGACTGTG
GAGGCTTGCTGGCCACTTTTTGGGGTCAGCTCCTCTGAAATGCATTAGCGGAACCGTTTG
CGATCTGCCACAAGTGTGATAAGTTATCTACACTGGCGAGGGGATTGCTCTCTGTAATGT
TCAGCTTCTAATTGTCTCTACTTTGTGAGACTACTTTTGAATGCTTGACCTCAAATCAGG
TAGGACTACCCGCTGAACTTAA
>AC4-53
TTTCCGTAGGTGAACCTGCGGAAGGATCATTATTGAATTATGTTTCTAGATAGGTTGTAG
CTGGCTC-TTTAGAGCATGTGCACGCCTGTTTGGACTTCATTTTCATCCACCTGTGCACC
TATTGTAGTCTTTGGTTGGGTTAGGAGGAAGTGGTCATTGTGTCAGCATCTGCTGGATGT
GAGGACTTGCATTGTGAAAGCTTTGCTGTCCTTGATGTGATCATGGAATCTTTTTC----

-------TCACTAGAGTCTATGTCACTCATTATACTCTGTCGAATGTCATTGAATGTCTT
TACATGGGCTTGTATGCCTATGAAAATTGTAATACAACTTTCAGCAACGGATCTCTTGGC
TCTCGCATCGATGAAGAACGCAGCGAAATGCGATAAGTAATGTGAATTGCAGAATTCAGT
GAATCATCGAATCTTTGAACGCATCTTGCGCTCCTTGGTATTCCGAGGAGCATGCCTGTT
TGAGTGTCATTAAATTCTCAACTCTCTTCTAC-TTTTTGTAAAAGAGAGCTTGGACTGTG
GAGGCTTGCTGGCCACTTTTTGGGGTCAGCTCCTCTGAAATGCATTAGCGGAACCGTTTG
CGATCTGCCACAAGTGTGATAAGTTATCTACACTGGCGAGGGGATTGCTCTCTGTAATGT
TCAGCTTCTAATTGTCTCTACTTTGTGAGACTACTTTTGAATGCTTGACCTCAAATCAGG
TAGGACTACCCGCTGAACTTAA
>AC7-32
TTTCCGTAGGTGAACCTGCGGAAGGATCATTATTGAATTATGTTTCTAGATAGGTTGTAG
CTGGCTC-TTTAGAGCATGTGCACGCCTGTTTGGACTTCATTTTCATCCACCTGTGCACC
TATTGTAGTCTTTGGTTGGGTTAGGAGGAAGTGGTCATTGTGTCAGCATCTGCTGGATGT
GAGGACTTGCATTGTGAAAGCTTTGCTGTCCTTGATGTGATCATGGAATCTTTTTC----

-------TCACTAGAGTCTATGTCACTCATTATACTCTGTCGAATGTCATTGAATGTCTT
TACATGGGCTTGTATGCCTATGAAAATTGTAATACAACTTTCAGCAACGGATCTCTTGGC
TCTCGCATCGATGAAGAACGCAGCGAAATGCGATAAGTAATGTGAATTGCAGAATTCAGT
GAATCATCGAATCTTTGAACGCATCTTGCGCTCCTTGGTATTCCGAGGAGCATGCCTGTT
TGAGTGTCATTAAATTCTCAACTCTCTTCTAC-TTTTTGTAAAAGAGAGCTTGGACTGTG
GAGGCTTGCTGGCCACTTTTTGGGGTCAGCTCCTCTGAAATGCATTAGCGGAACCGTTTG
CGATCTGCCACAAGTGTGATAAGTTATCTACACTGGCGAGGGGATTGCTCTCTGTAATGT
TCAGCTTCTAATTGTCTCTACTTTGTGAGACTACTTTTGAATGCTTGACCTCAAATCAGG
TAGGACTACCCGCTGAACTTAA
>AC6-2
TTTCCGTAGGTGAACCTGCGGAAGGATCATTATTGAATTATGTTTCTAGATAGGTTGTAG
CTGGCTC-TTTAGAGCATGTGCACGCCTGTTTGGACTTCATTTTCATCCACCTGTGCACC
TATTGTAGTCTTTGGTTGGGTTAGGAGGAAGTGGTCATTGTGTCAGCATCTGCTGGATGT
GAGGACTTGCATTGTGAAAGCTTTGCTGTCCTTGATGTGATCATGGAATCTCTTTC----

-------TCACTAGAGTCTATGTCACTCATTATACTCTGTCGAATGTCATTGAATGTCTT
TACATGGGCTTGTATGCCTATGAAAATTGTAATACAACTTTCAGCAACGGATCTCTTGGC
TCTCGCATCGATGAAGAACGCAGCGAAATGCGATAAGTAATGTGAATTGCAGAATTCAGT
GAATCATCGAATCTTTGAACGCATCTTGCGCTCCTTGGTATTCCGAGGAGCATGCCTGTT
TGAGTGTCATTAAATTCTCAACTCTCTTCTAC-TTTTTGTAAAAGAGAGCTTGGACTGTG
GAGGCTTGCTGGCCACTTTTTGGGGTCAGCTCCTCTGAAATGCATTAGCGGAACCGTTTG
CGATCTGCCACAAGTGTGATAAGTTATCTACACTGGCGAGGGGATTGCTCTCTGTAATGT
TCAGCTTCTAATTGTCTCTACTTTGTGAGACTACTTTTGAATGCTTGACCTCAAATCAGG
TAGGACTACCCGCTGAACTTAA
>AC7-12
TTTCCGTAGGTGAACCTGCGGAAGGATCATTATTGAATTATGTTTCTAGATAGGTTGTAG
CTGGCTC-TTTAGAGCATGTGCACGCCTGTTTGGACTTCATTTTCATCCACCTGTGCACC
TATTGTAGTCTTTGGTTGGGTTAGGAGGAAGTGGTCATTGTGTCAGCATCTGCTGGATGT
GAGGACTTGCATTGTGAAAGCTTTGCTGTCCTTGATGTGATCATGGAATCTCTTTC----

-------TCACTAGAGTCTATGTCACTCATTATACTCTGTCGAATGTCATTGAATGTCTT
TACATGGGCTTGTATGCCTATGAAAATTGTAATACAACTTTCAGCAACGGATCTCTTGGC
TCTCGCATCGATGAAGAACGCAGCGAAATGCGATAAGTAATGTGAATTGCAGAATTCAGT
GAATCATCGAATCTTTGAACGCATCTTGCGCTCCTTGGTATTCCGAGGAGCATGCCTGTT
TGAGTGTCATTAAATTCTCAACTCTCTTCTAC-TTTTTGTAAAAGAGAGCTTGGACTGTG
GAGGCTTGCTGGCCACTTTTTGGGGTCAGCTCCTCTGAAATGCATTAGCGGAACCGTTTG
CGATCTGCCACAAGTGTGATAAGTTATCTACACTGGCGAGGGGATTGCTCTCTGTAATGT
TCAGCTTCTAATTGTCTCTACTTTGTGAGACTACTTTTGAATGCTTGACCTCAAATCAGG
TAGGACTACCCGCTGAACTTAA
>AC8-53
TTTCCGTAGGTGAACCTGCGGAAGGATCATTATTGAATTATGTTTCTAGATAGGTTGTAG
CTGGCTC-TTTAGAGCATGTGCACGCCTGTTTGGACTTCATTTTCATCCACCTGTGCACC
TATTGTAGTCTTTGGTTGGGTTAGGAGGAAGTGGTCATTGTGTCAGCATCTGCTGGATGT
GAGGACTTGCATTGTGAAAGCTTTGCTGTCCTTGATGTGATCATGGAATCTCTTTC----

-------TCACTAGAGTCTATGTCACTCATTATACTCTGTCGAATGTCATTGAATGTCTT
TACATGGGCTTGTATGCCTATGAAAATTGTAATACAACTTTCAGCAACGGATCTCTTGGC
TCTCGCATCGATGAAGAACGCAGCGAAATGCGATAAGTAATGTGAATTGCAGAATTCAGT
GAATCATCGAATCTTTGAACGCATCTTGCGCTCCTTGGTATTCCGAGGAGCATGCCTGTT
TGAGTGTCATTAAATTCTCAACTCTCTTCTAC-TTTTTGTAAAAGAGAGCTTGGACTGTG
GAGGCTTGCTGGCCACTTTTTGGGGTCAGCTCCTCTGAAATGCATTAGCGGAACCGTTTG
CGATCTGCCACAAGTGTGATAAGTTATCTACACTGGCGAGGGGATTGCTCTCTGTAATGT
TCAGCTTCTAATTGTCTCTACTTTGTGAGACTACTTTTGAATGCTTGACCTCAAATCAGG
TAGGACTACCCGCTGAACTTAA
>AC11-40
TTTCCGTAGGTGAACCTGCGGAAGGATCATTATTGAATTATGTTTCTAGATAGGTTGTAG
CTGGCTC-TTTAGAGCATGTGCACGCCTGTTTGGACTTCATTTTCATCCACCTGTGCACC
TATTGTAGTCTTTGGTTGGGTTAGGAGGAAGTGGTCATTGTGTCAGCATCTGCTGGATGT
GAGGACTTGCATTGTGAAAGCTTTGCTGTCCTTGATGTGATCATGGAATCTCTTTC----

-------TCACTAGAGTCTATGTCACTCATTATACTCTGTCGAATGTCATTGAATGTCTT
TACATGGGCTTGTATGCCTATGAAAATTGTAATACAACTTTCAGCAACGGATCTCTTGGC
TCTCGCATCGATGAAGAACGCAGCGAAATGCGATAAGTAATGTGAATTGCAGAATTCAGT
GAATCATCGAATCTTTGAACGCATCTTGCGCTCCTTGGTATTCCGAGGAGCATGCCTGTT
TGAGTGTCATTAAATTCTCAACTCTCTTCTAC-TTTTTGTAAAAGAGAGCTTGGACTGTG
GAGGCTTGCTGGCCACTTTTTGGGGTCAGCTCCTCTGAAATGCATTAGCGGAACCGTTTG
CGATCTGCCACAAGTGTGATAAGTTATCTACACTGGCGAGGGGATTGCTCTCTGTAATGT
TCAGCTTCTAATTGTCTCTACTTTGTGAGACTACTTTTGAATGCTTGACCTCAAATCAGG
TAGGACTACCCGCTGAACTTAA
>AC12-51
TTTCCGTAGGTGAACCTGCGGAAGGATCATTATTGAATTATGTTTCTAGATAGGTTGTAG
CTGGCTC-TTTAGAGCATGTGCACGCCTGTTTGGACTTCATTTTCATCCACCTGTGCACC
TATTGTAGTCTTTGGTTGGGTTAGGAGGAAGTGGTCATTGTGTCAGCATCTGCTGGATGT
GAGGACTTGCATTGTGAAAGCTTTGCTGTCCTTGATGTGATCATGGAATCTCTTTC----

-------TCACTAGAGTCTATGTCACTCATTATACTCTGTCGAATGTCATTGAATGTCTT
TACATGGGCTTGTATGCCTATGAAAATTGTAATACAACTTTCAGCAACGGATCTCTTGGC
TCTCGCATCGATGAAGAACGCAGCGAAATGCGATAAGTAATGTGAATTGCAGAATTCAGT
GAATCATCGAATCTTTGAACGCATCTTGCGCTCCTTGGTATTCCGAGGAGCATGCCTGTT
TGAGTGTCATTAAATTCTCAACTCTCTTCTAC-TTTTTGTAAAAGAGAGCTTGGACTGTG
GAGGCTTGCTGGCCACTTTTTGGGGTCAGCTCCTCTGAAATGCATTAGCGGAACCGTTTG
CGATCTGCCACAAGTGTGATAAGTTATCTACACTGGCGAGGGGATTGCTCTCTGTAATGT
TCAGCTTCTAATTGTCTCTACTTTGTGAGACTACTTTTGAATGCTTGACCTCAAATCAGG
TAGGACTACCCGCTGAACTTAA
>AC11-19
TTTCCGTAGGTGAACCTGCGGAAGGATCATTATTGAATTATGTTTCTAGATAGGTTGTAG
CTGGCTC-TTTAGAGCATGTGCACGCCTGTTTGGACTTCATTTTCATCCACCTGTGCACC
TATTGTAGTCTTTGGTTGGGTTAGGAGGAAGTGGTCATTGTGTCAGCATCTGCTGGATGT
GAGGACTTGCATTGTGAAAGCTTTGCTGTCCTTGATGTGATCATGGAATCTCTTTC----

-------TCACTAGAGTCTATGTCACTCATTATACTCTGTCGAATGTCATTGAATGTCTT
TACATGGGCTTGTATGCCTATGAAAATTGTAATACAACTTTCAGCAACGGATCTCTTGGC
TCTCGCATCGATGAAGAACGCAGCGAAATGCGATAAGTAATGTGAATTGCAGAATTCAGT
GAATCATCGAATCTTTGAACGCATCTTGCGCTCCTTGGTATTCCGAGGAGCATGCCTGTT
TGAGTGTCATTAAATTCTCAACTCTCTTCTAC-TTTTTGTAAAAGAGAGCTTGGACTGTG
GAGGCTTGCTGGCCACTTTTTGGGGTCAGCTCCTCTGAAATGCATTAGCGGAACCGTTTG
CGATCTGCCACAAGTGTGATAAGTTATCTACACTGGCGAGGGGATTGCTCTCTGTAATGT
TCAGCTTCTAATTGTCTCTACTTTGTGAGACTACTTTTGAATGCTTGACCTCAAATCAGG
TAGGACTACCCGCTGAACTTAA
>AC2-39
TTTCCGTAGGTGAACCTGCGGAAGGATCATTATTGAATTATGTTTCTAGATAGGTTGTAG
CTGGCTC-TTTAGAGCATGTGCACGCCTGTTTGGACTTCATTTTCATCCACCTGTGCACC
TATTGTAGTCTTTGGTTGGGTTAGGAGGAAGTGGTCATTGTGTCAGCATCTGCTGGATGT

GAGGACTTGCATTGTGAAAGCTTTGCTGTCCTTGATGTGATCATGGAATCTCTTTC----

-------TCACTAGAGTCTATGTCACTCATTATACTCTGTCGAATGTCATTGAATGTCTT
TACATGGGCTTATATGCCTATGAAAATTGTAATACAACTTTCAGCAACGGATCTCTTGGC
TCTCGCATCGATGAAGAACGCAGCGAAATGCGATAAGTAATGTGAATTGCAGAATTCAGT
GAATCATCGAATCTTTGAACGCATCTTGCGCTCCTTGGTATTCCGAGGAGCATGCCTGTT
TGAGTGTCATTAAATTCTCAACTCTCTTCTAC-TTTTTGTAAAAGAGAGCTTGGACTGTG
GAGGCTTGCTGGTCACTTTTTGGGGTCAGCTCCTCTGAAATGCATTAGCGGAACCGTTTG
CGATCTGCCACAAGTGTGATAAGTTATCTACACTGGCGAGGGGATTGCTCTCTGTAATGT
TCAGCTTCTAATTGTCTCTACTTTGTGAGACTACTTTTGAATGCTTGACCTCAAATCAGG
TAGGACTACCCGCTGAACTTAA
>AC6-35
TTTCCGTAGGTGAACCTGCGGAAGGATCATTATTGAATTATGTTTCTAGATAGGTTGTAG
CTGGCTC-TTTAGAGCATGTGCACGCCTGTTTGGACTTCATTTTCATCCACCTGTGTACC
TATTGTAGTCTTTGGTTGGGTTAGGAGGAAGTGGTCATTGTGTCAGCATCTGCTGGATGT
GAGGACTTGCATTGTGAAAGCTTTGCTGTCCTTGATGTGATCATGGAATCTCTTTC----

-------TCACTAGAGTCTATGTCACTCATTATACTCTGTCGAATGTCATTGAATGTCTT
TACATGGGCTTATATGCCTATGAAAATTGTAATACAACTTTCAGCAACGGATCTCTTGGC
TCTCGCATCGATGAAGAACGCAGCGAAATGCGATAAGTAATGTGAATTGCAGAATTCAGT
GAATCATCGAATCTTTGAACGCATCTTGCGCTCCTTGGTATTCCGAGGAGCATGCCTGTT
TGAGTGTCATTAAATTCTCAACTCTCTTCTAC-TTTTTGTAAAAGAGAGCTTGGACTGTG
GAGGCTTGCTGGCCACTTTTTGGGGTCAGCTCCTCTGAAATGCATTAGCGGAACCGTTTG
CGATCTGCCACAAGTGTGATAAGTTATCTACACTGGCGAGGGGATTGCTCTCTGTAATGT
TCAGCTTCTAATTGTCTCTACTTTGTGAGACTACTTTTGAATGCTTGACCTCAAATCAGG
TAGGACTACCCGCTGAACTTAA
>AC8-54
TTTCCGTAGGTGAACCTGCGGAAGGATCATTATTGAATTATGTTTCTAGATAGGTTGTAG

CTGGCTC-TTTAGAGCATGTGCACGCCTGTTTGGACTTCATTTTCATCCACCTGTGCACC
TATTGTAGTCTTTGGTTGGGTTATGAGGAAGTGGTCATTGTGTCAGCATCTGCTGGATGT
GAGGACTTGCATTGTGAAAGCTTTGCTGTCCTTGATGTGATCATGGAATCTCTTTC----

-------TCACTAGAGTCTATGTCACTCATTATACTCTGTCGAATGTCATTGAATGTCTT
TACATGGGCTTATATGCCTATGAAAATTGTAATACAACTTTCAGCAACGGATCTCTTGGC
TCTCGCATCGATGAAGAACGCAGCGAAATGCGATAAGTAATGTGAATTGCAGAATTCAGT
GAATCATCGAATCTTTGAACGCATCTTGCGCTCCTTGGTATTCCGAGGAGCATGCCTGTT
TGAGTGTCATTAAATTCTCAACTCTCTTCTAC-TTTTTGTAAAAGAGAGCTTGGACTGTG
GAGGCTTGCTGGCCACTTTTTGGGGTCAGCTCCTCTGAAATGCATTAGCGGAACCGTTTG
CGATCTGCCACAAGTGTGATAAGTTATCTACACTGGCGAGGGGATTGCTCTCTGTAATGT
TCAGCTTCTAATTGTCTCTACTTTGTGAGACTACTTTTGAATGCTTGACCTCAAATCAGG
TAGGACTACCCGCTGAACTTAA
>AC9-28
TTTCCGTAGGTGAACCTGCGGAAGGATCATTATTGAATTATGTTTCTAGATAGGTTGTAG
CTGGCTC-TTTAGAGCATGTGCACGCCTGTTTGGACTTCATTTTCATCCACCTGTGCACC
TATTGTAGTCTTTGGTTGGGTTAGGAGGAAGTGGTCATTGTGTCAGCATCTGCTGGATGT
GAGGACTTGCATTGTGAAAGCTTTGCTGTCCTTGATGTGATCATGGAATCTCTTTC----

-------TCACTAGAGTCTATGTCACTCATTATACTCTGTCGAATGTCATTGAATGTCTT
TACATGGGCTTATATGCCTATGAAAATTGTAATACAACTTTCAGCAACGGATCTCTTGGC
TCTCGCATCGATGAAGAACGCAGCGAAATGCGATAAGTAATGTGAATTGCAGAATTCAGT
GAATCATCGAATCTTTGAACGCATCTTGCGCTCCTTGGTATTCCGAGGAGCATGCCTGTT
TGAGTGTCATTAAATTCTCAACTCTCTTCTAC-TTTTTGTAAAAGAGAGCTTGGACTGTG
GAGGCTTGATGGCCACTTTTTGGGGTCAGCTCCTCTGAAATGCATTAGCGGAACCGTTTG
CGATCTGCCACAAGTGTGATAAGTTATCTACACTGGCGAGGGGATTGCTCTCTGTAATGT
TCAGCTTCTAATTGTCTCTACTTTGTGAGACTACTTTTGAATGCTTGACCTCAAATCAGG
TAGGACTACCCGCTGAACTTAA

```
>AC5-87
TTTCCGTAGGTGAACCTGCGGAAGGATCATTATTGAATTATGTTTCTAGATAGGTTGTAG
CTGGCTC-TTTAGAGCATGTGCACGCCTGTTTGGACTTCATTTTCATCCACCAGTGCACC
TATTGTAGTCTTTGGTTGGGTTAGGAGGAAGTGGTCATTGTGTCAGCATCTGCTGGATGT
GAGGACTTGCATTGTGAAAGCTTTGCTGTCCTTGATGTGATCATGGAATCTCTTTC----
------------------------------------------------------------
------------------------------------------------------------
------------------------------------------------------------
------------------------------------------------------------
------------------------------------------------------------
------------------------------------------------------------
------------------------------------------------------------
------------------------------------------------------------
------------------------------------------------------------
------------------------------------------------------------
------------------------------------------------------------
------------------------------------------------------------
-------TCACTAGAGTCTATGTCACTCATTATACTCTGTCGAATGTCATTGAATGTCTT
TACATGGGCTTATATGCCTATGAAAATTGTAATACAACTTTCAGCAACGGATCTCTTGGC
TCTCGCATCGATGAAGAACGCAGCGAAATGCGATAAGTAATGTGAATTGCAGAATTCAGT
GAATCATCGAATCTTTGAACGCATCTTGCGCTCCTTGGTATTCCGAGGAGCATGCCTGTT
TGAGTGTCATTAAATTCTCAACTCTCTTCTAC-TTTTTGTAAAAGAGAGCTTGGACTGTG
GAGGCTTGCTGGCCACTTTTTGGGGTCAGCTCCTCTGAAATGCATTAGCGGAACCGTTTG
CGATCTGCCACAAGTGTGATAAGTTATCTACACTGGCGAGGGGATTGCTCTCTGTAATGT
TCAGCTTCTAATTGTCTCTACTTTGTGAGACTACTTTTGAATGCTTGACCTCAAATCAGG
TAGGACTACCCGCTGAACTTAA
>AC10-36
TTTCCGTAGGTGAACCTGCGGAAGGATCATTATTGAATTATGTTTCTAGATAGGTTGTAG
CTGGCTC-TTTAGAGCATGTGCACGCCTGTTTGGACTTCATTTTCATCCACCTGTGCACC
TATTGTAGTCTTTGGTTGGGTTAGGAGGAAGTGGTCATTGTGTCAGCATCTGCTGGATGT
GAGGACTTGCATTGTGAAAGCTTTGCTGTCCTTGATGTGATCATGGAATCTCTTTC----
------------------------------------------------------------
------------------------------------------------------------
------------------------------------------------------------
------------------------------------------------------------
------------------------------------------------------------
------------------------------------------------------------
------------------------------------------------------------
------------------------------------------------------------
------------------------------------------------------------
------------------------------------------------------------
------------------------------------------------------------
------------------------------------------------------------
-------TCACTAGAGTCTATGTCACTCATTATACTCTGTCGAATGTCATTGAATGTCTT
TACATGGGCTTGTATGCCTATGAAAATTGTAATACAACTTTCAGCAACGGATCTCTTGGC
TCTCGCATCGATGAAGAACGCAGCGAAATGCGATAAGTAATGTGAATTGCAGAATTCAGT
GAATCATCGAATCTTTGAACGCATCTTGCGCTCCTTGGTATTCCGAGGAGCATGCCTGTT
TGAGTGTCATTAAATTCTCAACTCTCTTATAC-TTTTTGTAAAAGAGAGCTTGGACTGTG
GAGGCTTGCTGGCCACTTTTTGGGGTCAGCTCCTCTGAAATGCATTAGCGGAACCGTTTG
CGATCTGCCACAAGTGTGATAAGTTATCTACACTGGCGAGGGGATTGCTCTCTGTAATGT
```

TCAGCTTCTAATTGTCTCTACTTTGTGAGACTACTTTTGAATGCTTGACCTCAAATCAGG
TAGGACTACCCGCTGAACTTAA
>AC3-63
TTTCCGTAGGTGAACCTGCGGAAGGATCATTATTGAATTATGTTTCTAGATAGGTTGTAG
CTGGCTC-TTTAGAGCATGTGCACGCCTGTTTGGACTTCATTTTCATCCACCTGTGCACC
TATTGTAGTCTTTGGTTGGGTTAGGAGGAAGTGATCATTGTATCAGCATCTGCTGGGAGT
GAGGACTTGCATTGTGAAAGCTTTGCTGTCCTTGATGTGATCATGGAATCTTTTTC----

-------TCACTAGAGTCTATGTCACTCATTATACTCTGTCGAATGTCATTGAATGTCTT
TACATGGGCTTGTATGCCTATGAAAATTGTAATACAACTTTCAGCAACGGATCTCTTGGC
TCTCGCATCGATGAAGAACGCAGCGAAATGCGATAAGTAATGTGAATTGCAGAATTCAGT
GAATCATCGAATCTTTGAACGCATCTTGCGCTCCTTGGTATTCCGAGGAGCATGCCTGTT
TGAGTGTCATTAAATTCTCAACTCTCTTCTAC-TTTTTGTAAAAGAGAGCTTGGACTGTG
GAGGCTTGCTGGCCACTTTTTGGGGTCAGCTCCTCTGAAATGCATTAGCGGAACCGTTTG
CGATCTGCCACAAGTGTGATAAGTTATCTACACTGGCGAGGGGATTGCTCTCTGTAATGT
TCAGCTTCTAATTGTCTCTACTTTGTGAGACTACTTTTGAATGCTTGACCTCAAATCAGG
TAGGACTACCCGCTGAACTTAA
>AC4-72
TTTCCGTAGGTGAACCTGCGGAAGGATCATTATTGAATTATGTTTCTAGATAGGTTGTAG
CTGGCTC-TTTAGAGCATGTGCACGCCTGTTTGGACTTCATTTTCATCCACCTGTGCACC
TATTGTAGTCTTTGGTTGGGTTAGGAGGAAGTGATCATTGTATCAGCATCTGCTGGGAGT
GAGGACTTGCATTGTGAAAGCTTTGCTGTCCTTGATGTGATCATGGAATCTTTTTC----

-------TCACTAGAGTCTATGTCACTCATTATACTCTGTCGAATGTCATTGAATGTCTT
TACATGGGCTTGTATGCCTATGAAAATTGTAATACAACTTTCAGCAACGGATCTCTTGGC
TCTCGCATCGATGAAGAACGCAGCGAAATGCGATAAGTAATGTGAATTGCAGAATTCAGT
GAATCATCGAATCTTTGAACGCATCTTGCGCTCCTTGGTATTCCGAGGAGCATGCCTGTT
TGAGTGTCATTAAATTCTCAACTCTCTTCTAC-TTTTTGTAAAAGAGAGCTTGGACTGTG

```
GAGGCTTGCTGGCCACTTTTTGGGGTCAGCTCCTCTGAAATGCATTAGCGGAACCGTTTG
CGATCTGCCACAAGTGTGATAAGTTATCTACACTGGCGAGGGGATTGCTCTCTGTAATGT
TCAGCTTCTAATTGTCTCTACTTTGTGAGACTACTTTTGAATGCTTGACCTCAAATCAGG
TAGGACTACCCGCTGAACTTAA
>AC6-17
TTTCCGTAGGTGAACCTGCGGAAGGATCATTATTGAATTATGTTTCTAGATAGGTTGTAG
CTGGCTC-TTTAGAGCATGTGCACGCCTGTTTGGACTTCATTTTCATCCACCTGTGCACC
TATTGTAGTCTTTGGTTGGGTTAGGAGGAAGTGATCATTGTATCAGCATCTGCTGGGAGT
GAGGACTTGCATTGTGAAAGCTTTGCTGTCCTTGATGTGATCATGGAATCTTTTTC----
------------------------------------------------------------
------------------------------------------------------------
------------------------------------------------------------
------------------------------------------------------------
------------------------------------------------------------
------------------------------------------------------------
------------------------------------------------------------
------------------------------------------------------------
------------------------------------------------------------
------------------------------------------------------------
------------------------------------------------------------
------------------------------------------------------------
-------TCACTAGAGTCTATGTCACTCATTATACTCTGTCGAATGTCATTGAATGTCTT
TACATGGGCTTGTATGCCTATGAAAATTGTAATACAACTTTCAGCAACGGATCTCTTGGC
TCTCGCATCGATGAAGAACGCAGCGAAATGCGATAAGTAATGTGAATTGCAGAATTCAGT
GAATCATCGAATCTTTGAACGCATCTTGCGCTCCTTGGTATTCCGAGGAGCATGCCTGTT
TGAGTGTCATTAAATTCTCAACTCTCTTCTAC-TTTTTGTAAAAGAGAGCTTGGACTGTG
GAGGCTTGCTGGCCACTTTTTGGGGTCAGCTCCTCTGAAATGCATTAGCGGAACCGTTTG
CGATCTGCCACAAGTGTGATAAGTTATCTACACTGGCGAGGGGATTGCTCTCTGTAATGT
TCAGCTTCTAATTGTCTCTACTTTGTGAGACTACTTTTGAATGCTTGACCTCAAATCAGG
TAGGACTACCCGCTGAACTTAA
>AC7-30
TTTCCGTAGGTGAACCTGCGGAAGGATCATTATTGAATTATGTTTCTAGATAGGTTGTAG
CTGGCTC-TTTAGAGCATGTGCACGCCTGTTTGGACTTCATTTTCATCCACCTGTGCACC
TATTGTAGTCTTTGGTTGGGTTAGGAGGAAGTGATCATTGTATCAGCATCTGCTGGGAGT
GAGGACTTGCATTGTGAAAGCTTTGCTGTCCTTGATGTGATCATGGAATCTTTTTC----
------------------------------------------------------------
------------------------------------------------------------
------------------------------------------------------------
------------------------------------------------------------
------------------------------------------------------------
------------------------------------------------------------
------------------------------------------------------------
------------------------------------------------------------
------------------------------------------------------------
------------------------------------------------------------
------------------------------------------------------------
------------------------------------------------------------
-------TCACTAGAGTCTATGTCACTCATTATACTCTGTCGAATGTCATTGAATGTCTT
TACATGGGCTTGTATGCCTATGAAAATTGTAATACAACTTTCAGCAACGGATCTCTTGGC
TCTCGCATCGATGAAGAACGCAGCGAAATGCGATAAGTAATGTGAATTGCAGAATTCAGT
```

GAATCATCGAATCTTTGAACGCATCTTGCGCTCCTTGGTATTCCGAGGAGCATGCCTGTT
TGAGTGTCATTAAATTCTCAACTCTCTTCTAC-TTTTTGTAAAAGAGAGCTTGGACTGTG
GAGGCTTGCTGGCCACTTTTTGGGGTCAGCTCCTCTGAAATGCATTAGCGGAACCGTTTG
CGATCTGCCACAAGTGTGATAAGTTATCTACACTGGCGAGGGGATTGCTCTCTGTAATGT
TCAGCTTCTAATTGTCTCTACTTTGTGAGACTACTTTTGAATGCTTGACCTCAAATCAGG
TAGGACTACCCGCTGAACTTAA
>AC9-51
TTTCCGTAGGTGAACCTGCGGAAGGATCATTATTGAATTATGTTTCTAGATAGGTTGTAG
CTGGCTC-TTTAGAGCATGTGCACGCCTGTTTGGACTTCATTTTCATCCACCTGTGCACC
TATTGTAGTCTTTGGTTGGGTTAGGAGGAAGTGATCATTGTATCAGCATCTGCTGGGAGT
GAGGACTTGCATTGTGAAAGCTTTGCTGTCCTTGATGTGATCATGGAATCTTTTTC----

-------TCACTAGAGTCTATGTCACTCATTATACTCTGTCGAATGTCATTGAATGTCTT
TACATGGGCTTGTATGCCTATGAAAATTGTAATACAACTTTCAGCAACGGATCTCTTGGC
TCTCGCATCGATGAAGAACGCAGCGAAATGCGATAAGTAATGTGAATTGCAGAATTCAGT
GAATCATCGAATCTTTGAACGCATCTTGCGCTCCTTGGTATTCCGAGGAGCATGCCTGTT
TGAGTGTCATTAAATTCTCAACTCTCTTCTAC-TTTTTGTAAAAGAGAGCTTGGACTGTG
GAGGCTTGCTGGCCACTTTTTGGGGTCAGCTCCTCTGAAATGCATTAGCGGAACCGTTTG
CGATCTGCCACAAGTGTGATAAGTTATCTACACTGGCGAGGGGATTGCTCTCTGTAATGT
TCAGCTTCTAATTGTCTCTACTTTGTGAGACTACTTTTGAATGCTTGACCTCAAATCAGG
TAGGACTACCCGCTGAACTTAA
>AC7-47
TTTCCGTAGGTGAACCTGCGGAAGGATCATTATTGAATTATGTTTCTAGATAGGTTGTAG
CTGGCTC-TTTAGAGCATGTGCACGCCTGTTTGGACTTCATTTTCATCCACCTGTGCACC
TATTGTAGTCTTTGGTTGGGTTAGGAGGAAGTGGTCATTGTGTCAGCATCTGCTGGGAGT
GAGGACTTGCATTGTGAAAGCTTTGCTGTCCTTGATGTGATCATGGAATCTTTTTC----

-------TCACTAGAGTCTATGTCACTCATTATACTCTGTCGAATGTCATTGAATGTCTT

TACATGGGCTTGTATGCCTATGAAAATTGTAATACAACTTTCAGCAACGGATCTCTTGGC
TCTCGCATCGATGAAGAACGCAGCGAAATGCGATAAGTAATGTGAATTGCAGAATTCAGT
GAATCATCGAATCTTTGAACGCATCTTGCGCTCCTTGGTATTCCGAGGAGCATGCCTGTT
TGAGTGTCATTAAATTCTCAACTCTCTTCTAC-TTTTTGTAAAAGAGAGCTTGGACTGTG
GAGGCTTGCTGGCCACTTTTTGGGGTCAGCTCCTCTGAAATGCATTAGCGGAACCGTTTG
CGATCTGCCACAAGTGTGATAAGTTATCTACACTGGCGAGGGGATTGCTCTCTGTAATGT
TCAGCTTCTAATTGTCTCTACTTTGTGAGACTACTTTTGAATGCTTGACCTCAAATCAGG
TAGGACTACCCGCTGAACTTAA
>AC9-44
TTTCCGTAGGTGAACCTGCGGAAGGATCATTATTGAATTATGTTTCTAGATAGGTTGTAG
CTGGCTC-TTTAGAGCATGTGCACGCCTGTTTGGACTTCATTTTCATCCACCTGTGCACC
TATTGTAGTCTTTGGTTGGGTTAGGAGGAAGTGGTCATTGTGTCAGCATCTGCTGGGAGT
GAGGACTTGCATTGTGAAAGCTTTGCTGTCCTTGATGTGATCATGGAATCTTTTTC----

-------TCACTAGAGTCTATGTCACTCATTATACTCTGTCGAATGTCATTGAATGTCTT
TACATGGGCTTGTATGCCTATGAAAATTGTAATACAACTTTCAGCAACGGATCTCTTGGC
TCTCGCATCGATGAAGAACGCAGCGAAATGCGATAAGTAATGTGAATTGCAGAATTCAGT
GAATCATCGAATCTTTGAACGCATCTTGCGCTCCTTGGTATTCCGAGGAGCATGCCTGTT
TGAGTGTCATTAAATTCTCAACTCTCTTCTAC-TTTTTGTAAAAGAGAGCTTGGACTGTG
GAGGCTTGCTGGCCACTTTTTGGGGTCAGCTCCTCTGAAATGCATTAGCGGAACCGTTTG
CGATCTGCCACAAGTGTGATAAGTTATCTACACTGGCGAGGGGATTGCTCTCTGTAATGT
TCAGCTTCTAATTGTCTCTACTTTGTGAGACTACTTTTGAATGCTTGACCTCAAATCAGG
TAGGACTACCCGCTGAACTTAA
>AC11-42
TTTCCGTAGGTGAACCTGCGGAAGGATCATTATTGAATTATGTTTCTAGATAGGTTGTAG
CTGGCTC-TTTAGAGCATGTGCACGCCTGTTTGGACTTCATTTTCATCCACCTGTGCACC
TATTGTAGTCTTTGGTTGGGTTAGGAGGAAGTGGTCATTGTGTCAGCATCTGCTGGGAGT
GAGGACTTGCATTGTGAAAGCTTTGCTGTCCTTGATGTGATCATGGAATCTCTTTC----

-------TCACTAGAGTCTATGTCACTCATTATACTCTGTCGAATGTCATTGAATGTCTT
TACATGGGCTTGTATGCCTATGAAAATTGTAATACAACTTTCAGCAACGGATCTCTTGGC
TCTCGCATCGATGAAGAACGCAGCGAAATGCGATAAGTAATGTGAATTGCAGAATTCAGT
GAATCATCGAATCTTTGAACGCATCTTGCGCTCCTTGGTATTCCGAGGAGCATGCCTGTT
TGAGTGTCATTAAATTCTCAACTCTCTTCTAC-TTTTTGTAAAAGAGAGCTTGGACTGTG
GAGGCTTGCTGGCCACTTTTTGGGGTCAGCTCCTCTGAAATGCATTAGCGGAACCGTTTG
CGATCTGCCACAAGTGTGATAAGTTATCTACACTGGCGAGGGGATTGCTCTCTGTAATGT
TCAGCTTCTAATTGTCTCTACTTTGTGAGACTACTTTTGAATGCTTGACCTCAAATCAGG
TAGGACTACCCGCTGAACTTAA
>AC8-50
TTTCCGTAGGTGAACCTGCGGAAGGATCATTATTGAATTATGTTTCTAGATAGGTTGTAG
CTGGCTC-TTTAGAGCATGTGCACGCCTGTTTGGACTTCATTTTCATCCACCTGTGCACC
TATTGTAGTCTTTGGTTGGGTTAGGAGGAAGTGGTCATTGTGTCAGCATCTGCTGGGAGT
GAGGACTTGCATTGTGAAAGCTTTGCTGTCCTTGATGTGATCATGGAATCTCTTTC----

-------TCACTAGAGTCTATGTCACTCATTATACTCTGTCGAATGTCATTGAATGTCTT
TACATGGGCTTATATGCCTATGAAAATTGTAATACAACTTTCAGCAACGGATCTCTTGGC
TCTCGCATCGATGAAGAACGCAGCGAAATGCGATAAGTAATGTGAATTGCAGAATTCAGT
GAATCATCGAATCTTTGAACGCATCTTGCGCTCCTTGGTATTCCGAGGAGCATGCCTGTT
TGAGTGTCATTAAATTCTCAACTCTCTTCTAC-TTTTTGTAAAAGAGAGCTTGGACTGTG
GAGGCTTGCTGGCCACTTTTTGGGGTCAGCTCCTCTGAAATGCATTAGCGGAACCGTTTG
CGATCTGCCACAAGTGTGATAAGTTATCTACACTGGCGAGGGGATTGCTCTCTGTAATGT
TCAGCTTCTAATTGTCTCTACTTTGTGAGACTACTTTTGAATGCTTGACCTCAAATCAGG
TAGGACTACCCGCTGAACTTAA
>AC1-15
TTTCCGTAGGTGAACCTGCGGAAGGATCATTATTGAATTATGTTTCTAGATAGGTTGTAG
CTGGCTCTTTTAGAGCATGTGCACGCCTGTTTGGACTTCATTTTCATCCACCTGTGCACC
TATTGTAGTCTTTGGTTGGGTTAGGAGGAAGTGATCATTGTATCAGCATCTGCTGGGAGT
GAGGACTTGCATTGTGAAAGCTTTGCTGTCCTTGATGTGATCATGGAATCTTTTTC----

-------TCACTAGAGTCTATGTCACTCATTATACTCTGTCGAATGTCATTGAATGTCTT
TACATGGGCTTGTATGCCTATGAAAATTGTAATACAACTTTCAGCAACGGATCTCTTGGC
TCTCGCATCGATGAAGAACGCAGCGAAATGCGATAAGTAATGTGAATTGCAGAATTCAGT
GAATCATCGAATCTTTGAACGCATCTTGCGCTCCTTGGTATTCCGAGGAGCATGCCTGTT
TGAGTGTCATTAAATTCTCAACTCTCTTCTAC-TTTTTGTAAAAGAGAGCTTGGACTGTG
GAGGCTTGCTGGCCACTTTTTGGGGTCAGCTCCTCTGAAATGCATTAGCGGAACCGTTTG
CGATCTGCCACAAGTGTGATAAGTTATCTACACTGGCGAGGGGATTGCTCTCTGTAATGT
TCAGCTTCTAATTGTCTCTACTTTGTGAGACTACTTTTGAATGCTTGACCTCAAATCAGG
TAGGACTACCCGCTGAACTTAA
>AC1-42
TTTCCGTAGGTGAACCTGCGGAAGGATCATTATTGAATTATGTTTCTAGATAGGTTGTAG
CTGGCTCTTTTAGAGCATGTGCACGCCTGTTTGGACTTCATTTTCATCCACCTGTGCACC
TATTGTAGTCTTTGGTTGGGTTAGGAGGAAGTGATCATTGTATCAGCATCTGCTGGGAGT
GAGGACTTGCATTGTGAAAGCTTTGCTGTCCTTGATGTGATCATGGAATCTTTTTC----

-------TCACTAGAGTCTATGTCACTCATTATACTCTGTCGAATGTCATTGAATGTCTT
TACATGGGCTTGTATGCCTATGAAAATTGTAATACAACTTTCAGCAACGGATCTCTTGGC
TCTCGCATCGATGAAGAACGCAGCGAAATGCGATAAGTAATGTGAATTGCAGAATTCAGT
GAATCATCGAATCTTTGAACGCATCTTGCGCTCCTTGGTATTCCGAGGAGCATGCCTGTT
TGAGTGTCATTAAATTCTCAACTCTCTTCTAC-TTTTTGTAAAAGAGAGCTTGGACTGTG
GAGGCTTGCTGGCCACTTTTTGGGGTCAGCTCCTCTGAAATGCATTAGCGGAACCGTTTG
CGATCTGCCACAAGTGTGATAAGTTATCTACACTGGCGAGGGGATTGCTCTCTGTAATGT
TCAGCTTCTAATTGTCTCTACTTTGTGAGACTACTTTTGAATGCTTGACCTCAAATCAGG
TAGGACTACCCGCTGAACTTAA
>AC1-54
TTTCCGTAGGTGAACCTGCGGAAGGATCATTATTGAATTATGTTTCTAGATAGGTTGTAG
CTGGCTCTTTTAGAGCATGTGCACGCCTGTTTGGACTTCATTTTCATCCACCTGTGCACC
TATTGTAGTCTTTGGTTGGGTTAGGAGGAAGTGATCATTGTATCAGCATCTGCTGGGAGT
GAGGACTTGCATTGTGAAAGCTTTGCTGTCCTTGATGTGATCATGGAATCTTTTTC----

-------TCACTAGAGTCTATGTCACTCATTATACTCTGTCGAATGTCATTGAATGTCTT
TACATGGGCTTGTATGCCTATGAAAATTGTAATACAACTTTCAGCAACGGATCTCTTGGC
TCTCGCATCGATGAAGAACGCAGCGAAATGCGATAAGTAATGTGAATTGCAGAATTCAGT
GAATCATCGAATCTTTGAACGCATCTTGCGCTCCTTGGTATTCCGAGGAGCATGCCTGTT
TGAGTGTCATTAAATTCTCAACTCTCTTCTAC-TTTTTGTAAAAGAGAGCTTGGACTGTG
GAGGCTTGCTGGCCACTTTTTGGGGTCAGCTCCTCTGAAATGCATTAGCGGAACCGTTTG
CGATCTGCCACAAGTGTGATAAGTTATCTACACTGGCGAGGGGATTGCTCTCTGTAATGT
TCAGCTTCTAATTGTCTCTACTTTGTGAGACTACTTTTGAATGCTTGACCTCAAATCAGG
TAGGACTACCCGCTGAACTTAA
>AC2-60
TTTCCGTAGGTGAACCTGCGGAAGGATCATTATTGAATTATGTTTCTAGATAGGTTGTAG
CTGGCTCTTTTAGAGCATGTGCACGCCTGTTTGGACTTCATTTTCATCCACCTGTGCACC
TATTGTAGTCTTTGGTTGGGTTAGGAGGAAGTGATCATTGTATCAGCATCTGCTGGGAGT
GAGGACTTGCATTGTGAAAGCTTTGCTGTCCTTGATGTGATCATGGAATCTTTTTC----

-------TCACTAGAGTCTATGTCACTCATTATACTCTGTCGAATGTCATTGAATGTCTT
TACATGGGCTTGTATGCCTATGAAAATTGTAATACAACTTTCAGCAACGGATCTCTTGGC
TCTCGCATCGATGAAGAACGCAGCGAAATGCGATAAGTAATGTGAATTGCAGAATTCAGT
GAATCATCGAATCTTTGAACGCATCTTGCGCTCCTTGGTATTCCGAGGAGCATGCCTGTT
TGAGTGTCATTAAATTCTCAACTCTCTTCTAC-TTTTTGTAAAAGAGAGCTTGGACTGTG
GAGGCTTGCTGGCCACTTTTTGGGGTCAGCTCCTCTGAAATGCATTAGCGGAACCGTTTG
CGATCTGCCACAAGTGTGATAAGTTATCTACACTGGCGAGGGGATTGCTCTCTGTAATGT
TCAGCTTCTAATTGTCTCTACTTTGTGAGACTACTTTTGAATGCTTGACCTCAAATCAGG
TAGGACTACCCGCTGAACTTAA
>AC3-13
TTTCCGTAGGTGAACCTGCGGAAGGATCATTATTGAATTATGTTTCTAGATAGGTTGTAG
CTGGCTCTTTTAGAGCATGTGCACGCCTGTTTGGACTTCATTTTCATCCACCTGTGCACC
TATTGTAGTCTTTGGTTGGGTTAGGAGGAAGTGATCATTGTATCAGCATCTGCTGGGAGT
GAGGACTTGCATTGTGAAAGCTTTGCTGTCCTTGATGTGATCATGGAATCTTTTTC----

-------TCACTAGAGTCTATGTCACTCATTATACTCTGTCGAATGTCATTGAATGTCTT
TACATGGGCTTGTATGCCTATGAAAATTGTAATACAACTTTCAGCAACGGATCTCTTGGC
TCTCGCATCGATGAAGAACGCAGCGAAATGCGATAAGTAATGTGAATTGCAGAATTCAGT
GAATCATCGAATCTTTGAACGCATCTTGCGCTCCTTGGTATTCCGAGGAGCATGCCTGTT
TGAGTGTCATTAAATTCTCAACTCTCTTCTAC-TTTTTGTAAAAGAGAGCTTGGACTGTG
GAGGCTTGCTGGCCACTTTTTGGGGTCAGCTCCTCTGAAATGCATTAGCGGAACCGTTTG
CGATCTGCCACAAGTGTGATAAGTTATCTACACTGGCGAGGGGATTGCTCTCTGTAATGT
TCAGCTTCTAATTGTCTCTACTTTGTGAGACTACTTTTGAATGCTTGACCTCAAATCAGG
TAGGACTACCCGCTGAACTTAA
>AC3-35
TTTCCGTAGGTGAACCTGCGGAAGGATCATTATTGAATTATGTTTCTAGATAGGTTGTAG
CTGGCTCTTTTAGAGCATGTGCACGCCTGTTTGGACTTCATTTTCATCCACCTGTGCACC
TATTGTAGTCTTTGGTTGGGTTAGGAGGAAGTGATCATTGTATCAGCATCTGCTGGGAGT
GAGGACTTGCATTGTGAAAGCTTTGCTGTCCTTGATGTGATCATGGAATCTTTTTC----

-------TCACTAGAGTCTATGTCACTCATTATACTCTGTCGAATGTCATTGAATGTCTT
TACATGGGCTTGTATGCCTATGAAAATTGTAATACAACTTTCAGCAACGGATCTCTTGGC
TCTCGCATCGATGAAGAACGCAGCGAAATGCGATAAGTAATGTGAATTGCAGAATTCAGT
GAATCATCGAATCTTTGAACGCATCTTGCGCTCCTTGGTATTCCGAGGAGCATGCCTGTT
TGAGTGTCATTAAATTCTCAACTCTCTTCTAC-TTTTTGTAAAAGAGAGCTTGGACTGTG
GAGGCTTGCTGGCCACTTTTTGGGGTCAGCTCCTCTGAAATGCATTAGCGGAACCGTTTG
CGATCTGCCACAAGTGTGATAAGTTATCTACACTGGCGAGGGGATTGCTCTCTGTAATGT
TCAGCTTCTAATTGTCTCTACTTTGTGAGACTACTTTTGAATGCTTGACCTCAAATCAGG
TAGGACTACCCGCTGAACTTAA
>AC4-52
TTTCCGTAGGTGAACCTGCGGAAGGATCATTATTGAATTATGTTTCTAGATAGGTTGTAG
CTGGCTCTTTTAGAGCATGTGCACGCCTGTTTGGACTTCATTTTCATCCACCTGTGCACC
TATTGTAGTCTTTGGTTGGGTTAGGAGGAAGTGATCATTGTATCAGCATCTGCTGGGAGT
GAGGACTTGCATTGTGAAAGCTTTGCTGTCCTTGATGTGATCATGGAATCTTTTTC----

-------TCACTAGAGTCTATGTCACTCATTATACTCTGTCGAATGTCATTGAATGTCTT
TACATGGGCTTGTATGCCTATGAAAATTGTAATACAACTTTCAGCAACGGATCTCTTGGC
TCTCGCATCGATGAAGAACGCAGCGAAATGCGATAAGTAATGTGAATTGCAGAATTCAGT
GAATCATCGAATCTTTGAACGCATCTTGCGCTCCTTGGTATTCCGAGGAGCATGCCTGTT
TGAGTGTCATTAAATTCTCAACTCTCTTCTAC-TTTTTGTAAAAGAGAGCTTGGACTGTG
GAGGCTTGCTGGCCACTTTTTGGGGTCAGCTCCTCTGAAATGCATTAGCGGAACCGTTTG
CGATCTGCCACAAGTGTGATAAGTTATCTACACTGGCGAGGGGATTGCTCTCTGTAATGT
TCAGCTTCTAATTGTCTCTACTTTGTGAGACTACTTTTGAATGCTTGACCTCAAATCAGG
TAGGACTACCCGCTGAACTTAA
>AC4-87
TTTCCGTAGGTGAACCTGCGGAAGGATCATTATTGAATTATGTTTCTAGATAGGTTGTAG
CTGGCTCTTTTAGAGCATGTGCACGCCTGTTTGGACTTCATTTTCATCCACCTGTGCACC
TATTGTAGTCTTTGGTTGGGTTAGGAGGAAGTGATCATTGTATCAGCATCTGCTGGGAGT
GAGGACTTGCATTGTGAAAGCTTTGCTGTCCTTGATGTGATCATGGAATCTTTTTC----

-------TCACTAGAGTCTATGTCACTCATTATACTCTGTCGAATGTCATTGAATGTCTT
TACATGGGCTTGTATGCCTATGAAAATTGTAATACAACTTTCAGCAACGGATCTCTTGGC
TCTCGCATCGATGAAGAACGCAGCGAAATGCGATAAGTAATGTGAATTGCAGAATTCAGT
GAATCATCGAATCTTTGAACGCATCTTGCGCTCCTTGGTATTCCGAGGAGCATGCCTGTT
TGAGTGTCATTAAATTCTCAACTCTCTTCTAC-TTTTTGTAAAAGAGAGCTTGGACTGTG
GAGGCTTGCTGGCCACTTTTTGGGGTCAGCTCCTCTGAAATGCATTAGCGGAACCGTTTG
CGATCTGCCACAAGTGTGATAAGTTATCTACACTGGCGAGGGGATTGCTCTCTGTAATGT
TCAGCTTCTAATTGTCTCTACTTTGTGAGACTACTTTTGAATGCTTGACCTCAAATCAGG
TAGGACTACCCGCTGAACTTAA
>AC5-1
TTTCCGTAGGTGAACCTGCGGAAGGATCATTATTGAATTATGTTTCTAGATAGGTTGTAG
CTGGCTCTTTTAGAGCATGTGCACGCCTGTTTGGACTTCATTTTCATCCACCTGTGCACC
TATTGTAGTCTTTGGTTGGGTTAGGAGGAAGTGATCATTGTATCAGCATCTGCTGGGAGT
GAGGACTTGCATTGTGAAAGCTTTGCTGTCCTTGATGTGATCATGGAATCTTTTTC----

-------TCACTAGAGTCTATGTCACTCATTATACTCTGTCGAATGTCATTGAATGTCTT
TACATGGGCTTGTATGCCTATGAAAATTGTAATACAACTTTCAGCAACGGATCTCTTGGC
TCTCGCATCGATGAAGAACGCAGCGAAATGCGATAAGTAATGTGAATTGCAGAATTCAGT
GAATCATCGAATCTTTGAACGCATCTTGCGCTCCTTGGTATTCCGAGGAGCATGCCTGTT
TGAGTGTCATTAAATTCTCAACTCTCTTCTAC-TTTTTGTAAAAGAGAGCTTGGACTGTG
GAGGCTTGCTGGCCACTTTTTGGGGTCAGCTCCTCTGAAATGCATTAGCGGAACCGTTTG
CGATCTGCCACAAGTGTGATAAGTTATCTACACTGGCGAGGGGATTGCTCTCTGTAATGT
TCAGCTTCTAATTGTCTCTACTTTGTGAGACTACTTTTGAATGCTTGACCTCAAATCAGG
TAGGACTACCCGCTGAACTTAA
>AC5-38
TTTCCGTAGGTGAACCTGCGGAAGGATCATTATTGAATTATGTTTCTAGATAGGTTGTAG
CTGGCTCTTTTAGAGCATGTGCACGCCTGTTTGGACTTCATTTTCATCCACCTGTGCACC
TATTGTAGTCTTTGGTTGGGTTAGGAGGAAGTGATCATTGTATCAGCATCTGCTGGGAGT
GAGGACTTGCATTGTGAAAGCTTTGCTGTCCTTGATGTGATCATGGAATCTTTTTC----

-------TCACTAGAGTCTATGTCACTCATTATACTCTGTCGAATGTCATTGAATGTCTT
TACATGGGCTTGTATGCCTATGAAAATTGTAATACAACTTTCAGCAACGGATCTCTTGGC
TCTCGCATCGATGAAGAACGCAGCGAAATGCGATAAGTAATGTGAATTGCAGAATTCAGT
GAATCATCGAATCTTTGAACGCATCTTGCGCTCCTTGGTATTCCGAGGAGCATGCCTGTT
TGAGTGTCATTAAATTCTCAACTCTCTTCTAC-TTTTTGTAAAAGAGAGCTTGGACTGTG
GAGGCTTGCTGGCCACTTTTTGGGGTCAGCTCCTCTGAAATGCATTAGCGGAACCGTTTG
CGATCTGCCACAAGTGTGATAAGTTATCTACACTGGCGAGGGGATTGCTCTCTGTAATGT
TCAGCTTCTAATTGTCTCTACTTTGTGAGACTACTTTTGAATGCTTGACCTCAAATCAGG
TAGGACTACCCGCTGAACTTAA
>AC5-40
TTTCCGTAGGTGAACCTGCGGAAGGATCATTATTGAATTATGTTTCTAGATAGGTTGTAG
CTGGCTCTTTTAGAGCATGTGCACGCCTGTTTGGACTTCATTTTCATCCACCTGTGCACC
TATTGTAGTCTTTGGTTGGGTTAGGAGGAAGTGATCATTGTATCAGCATCTGCTGGGAGT

GAGGACTTGCATTGTGAAAGCTTTGCTGTCCTTGATGTGATCATGGAATCTTTTTC----

-------TCACTAGAGTCTATGTCACTCATTATACTCTGTCGAATGTCATTGAATGTCTT
TACATGGGCTTGTATGCCTATGAAAATTGTAATACAACTTTCAGCAACGGATCTCTTGGC
TCTCGCATCGATGAAGAACGCAGCGAAATGCGATAAGTAATGTGAATTGCAGAATTCAGT
GAATCATCGAATCTTTGAACGCATCTTGCGCTCCTTGGTATTCCGAGGAGCATGCCTGTT
TGAGTGTCATTAAATTCTCAACTCTCTTCTAC-TTTTTGTAAAAGAGAGCTTGGACTGTG
GAGGCTTGCTGGCCACTTTTTGGGGTCAGCTCCTCTGAAATGCATTAGCGGAACCGTTTG
CGATCTGCCACAAGTGTGATAAGTTATCTACACTGGCGAGGGGATTGCTCTCTGTAATGT
TCAGCTTCTAATTGTCTCTACTTTGTGAGACTACTTTTGAATGCTTGACCTCAAATCAGG
TAGGACTACCCGCTGAACTTAA
>AC5-76
TTTCCGTAGGTGAACCTGCGGAAGGATCATTATTGAATTATGTTTCTAGATAGGTTGTAG
CTGGCTCTTTTAGAGCATGTGCACGCCTGTTTGGACTTCATTTTCATCCACCTGTGCACC
TATTGTAGTCTTTGGTTGGGTTAGGAGGAAGTGATCATTGTATCAGCATCTGCTGGGAGT
GAGGACTTGCATTGTGAAAGCTTTGCTGTCCTTGATGTGATCATGGAATCTTTTTC----

-------TCACTAGAGTCTATGTCACTCATTATACTCTGTCGAATGTCATTGAATGTCTT
TACATGGGCTTGTATGCCTATGAAAATTGTAATACAACTTTCAGCAACGGATCTCTTGGC
TCTCGCATCGATGAAGAACGCAGCGAAATGCGATAAGTAATGTGAATTGCAGAATTCAGT
GAATCATCGAATCTTTGAACGCATCTTGCGCTCCTTGGTATTCCGAGGAGCATGCCTGTT
TGAGTGTCATTAAATTCTCAACTCTCTTCTAC-TTTTTGTAAAAGAGAGCTTGGACTGTG
GAGGCTTGCTGGCCACTTTTTGGGGTCAGCTCCTCTGAAATGCATTAGCGGAACCGTTTG
CGATCTGCCACAAGTGTGATAAGTTATCTACACTGGCGAGGGGATTGCTCTCTGTAATGT
TCAGCTTCTAATTGTCTCTACTTTGTGAGACTACTTTTGAATGCTTGACCTCAAATCAGG
TAGGACTACCCGCTGAACTTAA
>AC5-80
TTTCCGTAGGTGAACCTGCGGAAGGATCATTATTGAATTATGTTTCTAGATAGGTTGTAG

CTGGCTCTTTTAGAGCATGTGCACGCCTGTTTGGACTTCATTTTCATCCACCTGTGCACC
TATTGTAGTCTTTGGTTGGGTTAGGAGGAAGTGATCATTGTATCAGCATCTGCTGGGAGT
GAGGACTTGCATTGTGAAAGCTTTGCTGTCCTTGATGTGATCATGGAATCTTTTTC----

-------TCACTAGAGTCTATGTCACTCATTATACTCTGTCGAATGTCATTGAATGTCTT
TACATGGGCTTGTATGCCTATGAAAATTGTAATACAACTTTCAGCAACGGATCTCTTGGC
TCTCGCATCGATGAAGAACGCAGCGAAATGCGATAAGTAATGTGAATTGCAGAATTCAGT
GAATCATCGAATCTTTGAACGCATCTTGCGCTCCTTGGTATTCCGAGGAGCATGCCTGTT
TGAGTGTCATTAAATTCTCAACTCTCTTCTAC-TTTTTGTAAAAGAGAGCTTGGACTGTG
GAGGCTTGCTGGCCACTTTTTGGGGTCAGCTCCTCTGAAATGCATTAGCGGAACCGTTTG
CGATCTGCCACAAGTGTGATAAGTTATCTACACTGGCGAGGGGATTGCTCTCTGTAATGT
TCAGCTTCTAATTGTCTCTACTTTGTGAGACTACTTTTGAATGCTTGACCTCAAATCAGG
TAGGACTACCCGCTGAACTTAA
>AC5-88
TTTCCGTAGGTGAACCTGCGGAAGGATCATTATTGAATTATGTTTCTAGATAGGTTGTAG
CTGGCTCTTTTAGAGCATGTGCACGCCTGTTTGGACTTCATTTTCATCCACCTGTGCACC
TATTGTAGTCTTTGGTTGGGTTAGGAGGAAGTGATCATTGTATCAGCATCTGCTGGGAGT
GAGGACTTGCATTGTGAAAGCTTTGCTGTCCTTGATGTGATCATGGAATCTTTTTC----

-------TCACTAGAGTCTATGTCACTCATTATACTCTGTCGAATGTCATTGAATGTCTT
TACATGGGCTTGTATGCCTATGAAAATTGTAATACAACTTTCAGCAACGGATCTCTTGGC
TCTCGCATCGATGAAGAACGCAGCGAAATGCGATAAGTAATGTGAATTGCAGAATTCAGT
GAATCATCGAATCTTTGAACGCATCTTGCGCTCCTTGGTATTCCGAGGAGCATGCCTGTT
TGAGTGTCATTAAATTCTCAACTCTCTTCTAC-TTTTTGTAAAAGAGAGCTTGGACTGTG
GAGGCTTGCTGGCCACTTTTTGGGGTCAGCTCCTCTGAAATGCATTAGCGGAACCGTTTG
CGATCTGCCACAAGTGTGATAAGTTATCTACACTGGCGAGGGGATTGCTCTCTGTAATGT
TCAGCTTCTAATTGTCTCTACTTTGTGAGACTACTTTTGAATGCTTGACCTCAAATCAGG
TAGGACTACCCGCTGAACTTAA

```
>AC6-16
TTTCCGTAGGTGAACCTGCGGAAGGATCATTATTGAATTATGTTTCTAGATAGGTTGTAG
CTGGCTCTTTTAGAGCATGTGCACGCCTGTTTGGACTTCATTTTCATCCACCTGTGCACC
TATTGTAGTCTTTGGTTGGGTTAGGAGGAAGTGATCATTGTATCAGCATCTGCTGGGAGT
GAGGACTTGCATTGTGAAAGCTTTGCTGTCCTTGATGTGATCATGGAATCTTTTTC----
------------------------------------------------------------
------------------------------------------------------------
------------------------------------------------------------
------------------------------------------------------------
------------------------------------------------------------
------------------------------------------------------------
------------------------------------------------------------
------------------------------------------------------------
------------------------------------------------------------
------------------------------------------------------------
------------------------------------------------------------
------------------------------------------------------------
-------TCACTAGAGTCTATGTCACTCATTATACTCTGTCGAATGTCATTGAATGTCTT
TACATGGGCTTGTATGCCTATGAAAATTGTAATACAACTTTCAGCAACGGATCTCTTGGC
TCTCGCATCGATGAAGAACGCAGCGAAATGCGATAAGTAATGTGAATTGCAGAATTCAGT
GAATCATCGAATCTTTGAACGCATCTTGCGCTCCTTGGTATTCCGAGGAGCATGCCTGTT
TGAGTGTCATTAAATTCTCAACTCTCTTCTAC-TTTTTGTAAAAGAGAGCTTGGACTGTG
GAGGCTTGCTGGCCACTTTTTGGGGTCAGCTCCTCTGAAATGCATTAGCGGAACCGTTTG
CGATCTGCCACAAGTGTGATAAGTTATCTACACTGGCGAGGGGATTGCTCTCTGTAATGT
TCAGCTTCTAATTGTCTCTACTTTGTGAGACTACTTTTGAATGCTTGACCTCAAATCAGG
TAGGACTACCCGCTGAACTTAA
>AC6-34
TTTCCGTAGGTGAACCTGCGGAAGGATCATTATTGAATTATGTTTCTAGATAGGTTGTAG
CTGGCTCTTTTAGAGCATGTGCACGCCTGTTTGGACTTCATTTTCATCCACCTGTGCACC
TATTGTAGTCTTTGGTTGGGTTAGGAGGAAGTGATCATTGTATCAGCATCTGCTGGGAGT
GAGGACTTGCATTGTGAAAGCTTTGCTGTCCTTGATGTGATCATGGAATCTTTTTC----
------------------------------------------------------------
------------------------------------------------------------
------------------------------------------------------------
------------------------------------------------------------
------------------------------------------------------------
------------------------------------------------------------
------------------------------------------------------------
------------------------------------------------------------
------------------------------------------------------------
------------------------------------------------------------
------------------------------------------------------------
------------------------------------------------------------
-------TCACTAGAGTCTATGTCACTCATTATACTCTGTCGAATGTCATTGAATGTCTT
TACATGGGCTTGTATGCCTATGAAAATTGTAATACAACTTTCAGCAACGGATCTCTTGGC
TCTCGCATCGATGAAGAACGCAGCGAAATGCGATAAGTAATGTGAATTGCAGAATTCAGT
GAATCATCGAATCTTTGAACGCATCTTGCGCTCCTTGGTATTCCGAGGAGCATGCCTGTT
TGAGTGTCATTAAATTCTCAACTCTCTTCTAC-TTTTTGTAAAAGAGAGCTTGGACTGTG
GAGGCTTGCTGGCCACTTTTTGGGGTCAGCTCCTCTGAAATGCATTAGCGGAACCGTTTG
CGATCTGCCACAAGTGTGATAAGTTATCTACACTGGCGAGGGGATTGCTCTCTGTAATGT
```

```
TCAGCTTCTAATTGTCTCTACTTTGTGAGACTACTTTTGAATGCTTGACCTCAAATCAGG
TAGGACTACCCGCTGAACTTAA
>AC7-34
TTTCCGTAGGTGAACCTGCGGAAGGATCATTATTGAATTATGTTTCTAGATAGGTTGTAG
CTGGCTCTTTTAGAGCATGTGCACGCCTGTTTGGACTTCATTTTCATCCACCTGTGCACC
TATTGTAGTCTTTGGTTGGGTTAGGAGGAAGTGATCATTGTATCAGCATCTGCTGGGAGT
GAGGACTTGCATTGTGAAAGCTTTGCTGTCCTTGATGTGATCATGGAATCTTTTTC----
------------------------------------------------------------
------------------------------------------------------------
------------------------------------------------------------
------------------------------------------------------------
------------------------------------------------------------
------------------------------------------------------------
------------------------------------------------------------
------------------------------------------------------------
------------------------------------------------------------
------------------------------------------------------------
------------------------------------------------------------
------------------------------------------------------------
-------TCACTAGAGTCTATGTCACTCATTATACTCTGTCGAATGTCATTGAATGTCTT
TACATGGGCTTGTATGCCTATGAAAATTGTAATACAACTTTCAGCAACGGATCTCTTGGC
TCTCGCATCGATGAAGAACGCAGCGAAATGCGATAAGTAATGTGAATTGCAGAATTCAGT
GAATCATCGAATCTTTGAACGCATCTTGCGCTCCTTGGTATTCCGAGGAGCATGCCTGTT
TGAGTGTCATTAAATTCTCAACTCTCTTCTAC-TTTTTGTAAAAGAGAGCTTGGACTGTG
GAGGCTTGCTGGCCACTTTTTGGGGTCAGCTCCTCTGAAATGCATTAGCGGAACCGTTTG
CGATCTGCCACAAGTGTGATAAGTTATCTACACTGGCGAGGGGATTGCTCTCTGTAATGT
TCAGCTTCTAATTGTCTCTACTTTGTGAGACTACTTTTGAATGCTTGACCTCAAATCAGG
TAGGACTACCCGCTGAACTTAA
>AC7-37
TTTCCGTAGGTGAACCTGCGGAAGGATCATTATTGAATTATGTTTCTAGATAGGTTGTAG
CTGGCTCTTTTAGAGCATGTGCACGCCTGTTTGGACTTCATTTTCATCCACCTGTGCACC
TATTGTAGTCTTTGGTTGGGTTAGGAGGAAGTGATCATTGTATCAGCATCTGCTGGGAGT
GAGGACTTGCATTGTGAAAGCTTTGCTGTCCTTGATGTGATCATGGAATCTTTTTC----
------------------------------------------------------------
------------------------------------------------------------
------------------------------------------------------------
------------------------------------------------------------
------------------------------------------------------------
------------------------------------------------------------
------------------------------------------------------------
------------------------------------------------------------
------------------------------------------------------------
------------------------------------------------------------
------------------------------------------------------------
------------------------------------------------------------
-------TCACTAGAGTCTATGTCACTCATTATACTCTGTCGAATGTCATTGAATGTCTT
TACATGGGCTTGTATGCCTATGAAAATTGTAATACAACTTTCAGCAACGGATCTCTTGGC
TCTCGCATCGATGAAGAACGCAGCGAAATGCGATAAGTAATGTGAATTGCAGAATTCAGT
GAATCATCGAATCTTTGAACGCATCTTGCGCTCCTTGGTATTCCGAGGAGCATGCCTGTT
TGAGTGTCATTAAATTCTCAACTCTCTTCTAC-TTTTTGTAAAAGAGAGCTTGGACTGTG
```

GAGGCTTGCTGGCCACTTTTTGGGGTCAGCTCCTCTGAAATGCATTAGCGGAACCGTTTG
CGATCTGCCACAAGTGTGATAAGTTATCTACACTGGCGAGGGGATTGCTCTCTGTAATGT
TCAGCTTCTAATTGTCTCTACTTTGTGAGACTACTTTTGAATGCTTGACCTCAAATCAGG
TAGGACTACCCGCTGAACTTAA
>AC8-6
TTTCCGTAGGTGAACCTGCGGAAGGATCATTATTGAATTATGTTTCTAGATAGGTTGTAG
CTGGCTCTTTTAGAGCATGTGCACGCCTGTTTGGACTTCATTTTCATCCACCTGTGCACC
TATTGTAGTCTTTGGTTGGGTTAGGAGGAAGTGATCATTGTATCAGCATCTGCTGGGAGT
GAGGACTTGCATTGTGAAAGCTTTGCTGTCCTTGATGTGATCATGGAATCTTTTTC----

-------TCACTAGAGTCTATGTCACTCATTATACTCTGTCGAATGTCATTGAATGTCTT
TACATGGGCTTGTATGCCTATGAAAATTGTAATACAACTTTCAGCAACGGATCTCTTGGC
TCTCGCATCGATGAAGAACGCAGCGAAATGCGATAAGTAATGTGAATTGCAGAATTCAGT
GAATCATCGAATCTTTGAACGCATCTTGCGCTCCTTGGTATTCCGAGGAGCATGCCTGTT
TGAGTGTCATTAAATTCTCAACTCTCTTCTAC-TTTTTGTAAAAGAGAGCTTGGACTGTG
GAGGCTTGCTGGCCACTTTTTGGGGTCAGCTCCTCTGAAATGCATTAGCGGAACCGTTTG
CGATCTGCCACAAGTGTGATAAGTTATCTACACTGGCGAGGGGATTGCTCTCTGTAATGT
TCAGCTTCTAATTGTCTCTACTTTGTGAGACTACTTTTGAATGCTTGACCTCAAATCAGG
TAGGACTACCCGCTGAACTTAA
>AC8-22
TTTCCGTAGGTGAACCTGCGGAAGGATCATTATTGAATTATGTTTCTAGATAGGTTGTAG
CTGGCTCTTTTAGAGCATGTGCACGCCTGTTTGGACTTCATTTTCATCCACCTGTGCACC
TATTGTAGTCTTTGGTTGGGTTAGGAGGAAGTGATCATTGTATCAGCATCTGCTGGGAGT
GAGGACTTGCATTGTGAAAGCTTTGCTGTCCTTGATGTGATCATGGAATCTTTTTC----

-------TCACTAGAGTCTATGTCACTCATTATACTCTGTCGAATGTCATTGAATGTCTT
TACATGGGCTTGTATGCCTATGAAAATTGTAATACAACTTTCAGCAACGGATCTCTTGGC
TCTCGCATCGATGAAGAACGCAGCGAAATGCGATAAGTAATGTGAATTGCAGAATTCAGT

GAATCATCGAATCTTTGAACGCATCTTGCGCTCCTTGGTATTCCGAGGAGCATGCCTGTT
TGAGTGTCATTAAATTCTCAACTCTCTTCTAC-TTTTTGTAAAAGAGAGCTTGGACTGTG
GAGGCTTGCTGGCCACTTTTTGGGGTCAGCTCCTCTGAAATGCATTAGCGGAACCGTTTG
CGATCTGCCACAAGTGTGATAAGTTATCTACACTGGCGAGGGGATTGCTCTCTGTAATGT
TCAGCTTCTAATTGTCTCTACTTTGTGAGACTACTTTTGAATGCTTGACCTCAAATCAGG
TAGGACTACCCGCTGAACTTAA
>AC8-29
TTTCCGTAGGTGAACCTGCGGAAGGATCATTATTGAATTATGTTTCTAGATAGGTTGTAG
CTGGCTCTTTTAGAGCATGTGCACGCCTGTTTGGACTTCATTTTCATCCACCTGTGCACC
TATTGTAGTCTTTGGTTGGGTTAGGAGGAAGTGATCATTGTATCAGCATCTGCTGGGAGT
GAGGACTTGCATTGTGAAAGCTTTGCTGTCCTTGATGTGATCATGGAATCTTTTTC----

-------TCACTAGAGTCTATGTCACTCATTATACTCTGTCGAATGTCATTGAATGTCTT
TACATGGGCTTGTATGCCTATGAAAATTGTAATACAACTTTCAGCAACGGATCTCTTGGC
TCTCGCATCGATGAAGAACGCAGCGAAATGCGATAAGTAATGTGAATTGCAGAATTCAGT
GAATCATCGAATCTTTGAACGCATCTTGCGCTCCTTGGTATTCCGAGGAGCATGCCTGTT
TGAGTGTCATTAAATTCTCAACTCTCTTCTAC-TTTTTGTAAAAGAGAGCTTGGACTGTG
GAGGCTTGCTGGCCACTTTTTGGGGTCAGCTCCTCTGAAATGCATTAGCGGAACCGTTTG
CGATCTGCCACAAGTGTGATAAGTTATCTACACTGGCGAGGGGATTGCTCTCTGTAATGT
TCAGCTTCTAATTGTCTCTACTTTGTGAGACTACTTTTGAATGCTTGACCTCAAATCAGG
TAGGACTACCCGCTGAACTTAA
>AC8-42
TTTCCGTAGGTGAACCTGCGGAAGGATCATTATTGAATTATGTTTCTAGATAGGTTGTAG
CTGGCTCTTTTAGAGCATGTGCACGCCTGTTTGGACTTCATTTTCATCCACCTGTGCACC
TATTGTAGTCTTTGGTTGGGTTAGGAGGAAGTGATCATTGTATCAGCATCTGCTGGGAGT
GAGGACTTGCATTGTGAAAGCTTTGCTGTCCTTGATGTGATCATGGAATCTTTTTC----

-------TCACTAGAGTCTATGTCACTCATTATACTCTGTCGAATGTCATTGAATGTCTT

TACATGGGCTTGTATGCCTATGAAAATTGTAATACAACTTTCAGCAACGGATCTCTTGGC
TCTCGCATCGATGAAGAACGCAGCGAAATGCGATAAGTAATGTGAATTGCAGAATTCAGT
GAATCATCGAATCTTTGAACGCATCTTGCGCTCCTTGGTATTCCGAGGAGCATGCCTGTT
TGAGTGTCATTAAATTCTCAACTCTCTTCTAC-TTTTTGTAAAAGAGAGCTTGGACTGTG
GAGGCTTGCTGGCCACTTTTTGGGGTCAGCTCCTCTGAAATGCATTAGCGGAACCGTTTG
CGATCTGCCACAAGTGTGATAAGTTATCTACACTGGCGAGGGGATTGCTCTCTGTAATGT
TCAGCTTCTAATTGTCTCTACTTTGTGAGACTACTTTTGAATGCTTGACCTCAAATCAGG
TAGGACTACCCGCTGAACTTAA
>AC8-58
TTTCCGTAGGTGAACCTGCGGAAGGATCATTATTGAATTATGTTTCTAGATAGGTTGTAG
CTGGCTCTTTTAGAGCATGTGCACGCCTGTTTGGACTTCATTTTCATCCACCTGTGCACC
TATTGTAGTCTTTGGTTGGGTTAGGAGGAAGTGATCATTGTATCAGCATCTGCTGGGAGT
GAGGACTTGCATTGTGAAAGCTTTGCTGTCCTTGATGTGATCATGGAATCTTTTTC----

-------TCACTAGAGTCTATGTCACTCATTATACTCTGTCGAATGTCATTGAATGTCTT
TACATGGGCTTGTATGCCTATGAAAATTGTAATACAACTTTCAGCAACGGATCTCTTGGC
TCTCGCATCGATGAAGAACGCAGCGAAATGCGATAAGTAATGTGAATTGCAGAATTCAGT
GAATCATCGAATCTTTGAACGCATCTTGCGCTCCTTGGTATTCCGAGGAGCATGCCTGTT
TGAGTGTCATTAAATTCTCAACTCTCTTCTAC-TTTTTGTAAAAGAGAGCTTGGACTGTG
GAGGCTTGCTGGCCACTTTTTGGGGTCAGCTCCTCTGAAATGCATTAGCGGAACCGTTTG
CGATCTGCCACAAGTGTGATAAGTTATCTACACTGGCGAGGGGATTGCTCTCTGTAATGT
TCAGCTTCTAATTGTCTCTACTTTGTGAGACTACTTTTGAATGCTTGACCTCAAATCAGG
TAGGACTACCCGCTGAACTTAA
>AC10-4
TTTCCGTAGGTGAACCTGCGGAAGGATCATTATTGAATTATGTTTCTAGATAGGTTGTAG
CTGGCTCTTTTAGAGCATGTGCACGCCTGTTTGGACTTCATTTTCATCCACCTGTGCACC
TATTGTAGTCTTTGGTTGGGTTAGGAGGAAGTGATCATTGTATCAGCATCTGCTGGGAGT
GAGGACTTGCATTGTGAAAGCTTTGCTGTCCTTGATGTGATCATGGAATCTTTTTC----

-------TCACTAGAGTCTATGTCACTCATTATACTCTGTCGAATGTCATTGAATGTCTT
TACATGGGCTTGTATGCCTATGAAAATTGTAATACAACTTTCAGCAACGGATCTCTTGGC
TCTCGCATCGATGAAGAACGCAGCGAAATGCGATAAGTAATGTGAATTGCAGAATTCAGT
GAATCATCGAATCTTTGAACGCATCTTGCGCTCCTTGGTATTCCGAGGAGCATGCCTGTT
TGAGTGTCATTAAATTCTCAACTCTCTTCTAC-TTTTTGTAAAAGAGAGCTTGGACTGTG
GAGGCTTGCTGGCCACTTTTTGGGGTCAGCTCCTCTGAAATGCATTAGCGGAACCGTTTG
CGATCTGCCACAAGTGTGATAAGTTATCTACACTGGCGAGGGGATTGCTCTCTGTAATGT
TCAGCTTCTAATTGTCTCTACTTTGTGAGACTACTTTTGAATGCTTGACCTCAAATCAGG
TAGGACTACCCGCTGAACTTAA
>AC10-40
TTTCCGTAGGTGAACCTGCGGAAGGATCATTATTGAATTATGTTTCTAGATAGGTTGTAG
CTGGCTCTTTTAGAGCATGTGCACGCCTGTTTGGACTTCATTTTCATCCACCTGTGCACC
TATTGTAGTCTTTGGTTGGGTTAGGAGGAAGTGATCATTGTATCAGCATCTGCTGGGAGT
GAGGACTTGCATTGTGAAAGCTTTGCTGTCCTTGATGTGATCATGGAATCTTTTTC----

-------TCACTAGAGTCTATGTCACTCATTATACTCTGTCGAATGTCATTGAATGTCTT
TACATGGGCTTGTATGCCTATGAAAATTGTAATACAACTTTCAGCAACGGATCTCTTGGC
TCTCGCATCGATGAAGAACGCAGCGAAATGCGATAAGTAATGTGAATTGCAGAATTCAGT
GAATCATCGAATCTTTGAACGCATCTTGCGCTCCTTGGTATTCCGAGGAGCATGCCTGTT
TGAGTGTCATTAAATTCTCAACTCTCTTCTAC-TTTTTGTAAAAGAGAGCTTGGACTGTG
GAGGCTTGCTGGCCACTTTTTGGGGTCAGCTCCTCTGAAATGCATTAGCGGAACCGTTTG
CGATCTGCCACAAGTGTGATAAGTTATCTACACTGGCGAGGGGATTGCTCTCTGTAATGT
TCAGCTTCTAATTGTCTCTACTTTGTGAGACTACTTTTGAATGCTTGACCTCAAATCAGG
TAGGACTACCCGCTGAACTTAA
>AC11-5
TTTCCGTAGGTGAACCTGCGGAAGGATCATTATTGAATTATGTTTCTAGATAGGTTGTAG
CTGGCTCTTTTAGAGCATGTGCACGCCTGTTTGGACTTCATTTTCATCCACCTGTGCACC
TATTGTAGTCTTTGGTTGGGTTAGGAGGAAGTGATCATTGTATCAGCATCTGCTGGGAGT
GAGGACTTGCATTGTGAAAGCTTTGCTGTCCTTGATGTGATCATGGAATCTTTTTC----

-------TCACTAGAGTCTATGTCACTCATTATACTCTGTCGAATGTCATTGAATGTCTT
TACATGGGCTTGTATGCCTATGAAAATTGTAATACAACTTTCAGCAACGGATCTCTTGGC
TCTCGCATCGATGAAGAACGCAGCGAAATGCGATAAGTAATGTGAATTGCAGAATTCAGT
GAATCATCGAATCTTTGAACGCATCTTGCGCTCCTTGGTATTCCGAGGAGCATGCCTGTT
TGAGTGTCATTAAATTCTCAACTCTCTTCTAC-TTTTTGTAAAAGAGAGCTTGGACTGTG
GAGGCTTGCTGGCCACTTTTTGGGGTCAGCTCCTCTGAAATGCATTAGCGGAACCGTTTG
CGATCTGCCACAAGTGTGATAAGTTATCTACACTGGCGAGGGGATTGCTCTCTGTAATGT
TCAGCTTCTAATTGTCTCTACTTTGTGAGACTACTTTTGAATGCTTGACCTCAAATCAGG
TAGGACTACCCGCTGAACTTAA
>AC11-37
TTTCCGTAGGTGAACCTGCGGAAGGATCATTATTGAATTATGTTTCTAGATAGGTTGTAG
CTGGCTCTTTTAGAGCATGTGCACGCCTGTTTGGACTTCATTTTCATCCACCTGTGCACC
TATTGTAGTCTTTGGTTGGGTTAGGAGGAAGTGATCATTGTATCAGCATCTGCTGGGAGT
GAGGACTTGCATTGTGAAAGCTTTGCTGTCCTTGATGTGATCATGGAATCTTTTTC----

-------TCACTAGAGTCTATGTCACTCATTATACTCTGTCGAATGTCATTGAATGTCTT
TACATGGGCTTGTATGCCTATGAAAATTGTAATACAACTTTCAGCAACGGATCTCTTGGC
TCTCGCATCGATGAAGAACGCAGCGAAATGCGATAAGTAATGTGAATTGCAGAATTCAGT
GAATCATCGAATCTTTGAACGCATCTTGCGCTCCTTGGTATTCCGAGGAGCATGCCTGTT
TGAGTGTCATTAAATTCTCAACTCTCTTCTAC-TTTTTGTAAAAGAGAGCTTGGACTGTG
GAGGCTTGCTGGCCACTTTTTGGGGTCAGCTCCTCTGAAATGCATTAGCGGAACCGTTTG
CGATCTGCCACAAGTGTGATAAGTTATCTACACTGGCGAGGGGATTGCTCTCTGTAATGT
TCAGCTTCTAATTGTCTCTACTTTGTGAGACTACTTTTGAATGCTTGACCTCAAATCAGG
TAGGACTACCCGCTGAACTTAA
>AC12-48
TTTCCGTAGGTGAACCTGCGGAAGGATCATTATTGAATTATGTTTCTAGATAGGTTGTAG
CTGGCTCTTTTAGAGCATGTGCACGCCTGTTTGGACTTCATTTTCATCCACCTGTGCACC
TATTGTAGTCTTTGGTTGGGTTAGGAGGAAGTGATCATTGTATCAGCATCTGCTGGGAGT
GAGGACTTGCATTGTGAAAGCTTTGCTGTCCTTGATGTGATCATGGAATCTTTTTC----

-------TCACTAGAGTCTATGTCACTCATTATACTCTGTCGAATGTCATTGAATGTCTT
TACATGGGCTTGTATGCCTATGAAAATTGTAATACAACTTTCAGCAACGGATCTCTTGGC
TCTCGCATCGATGAAGAACGCAGCGAAATGCGATAAGTAATGTGAATTGCAGAATTCAGT
GAATCATCGAATCTTTGAACGCATCTTGCGCTCCTTGGTATTCCGAGGAGCATGCCTGTT
TGAGTGTCATTAAATTCTCAACTCTCTTCTAC-TTTTTGTAAAAGAGAGCTTGGACTGTG
GAGGCTTGCTGGCCACTTTTTGGGGTCAGCTCCTCTGAAATGCATTAGCGGAACCGTTTG
CGATCTGCCACAAGTGTGATAAGTTATCTACACTGGCGAGGGGATTGCTCTCTGTAATGT
TCAGCTTCTAATTGTCTCTACTTTGTGAGACTACTTTTGAATGCTTGACCTCAAATCAGG
TAGGACTACCCGCTGAACTTAA
>AC12-54
TTTCCGTAGGTGAACCTGCGGAAGGATCATTATTGAATTATGTTTCTAGATAGGTTGTAG
CTGGCTCTTTTAGAGCATGTGCACGCCTGTTTGGACTTCATTTTCATCCACCTGTGCACC
TATTGTAGTCTTTGGTTGGGTTAGGAGGAAGTGATCATTGTATCAGCATCTGCTGGGAGT
GAGGACTTGCATTGTGAAAGCTTTGCTGTCCTTGATGTGATCATGGAATCTTTTTC----

-------TCACTAGAGTCTATGTCACTCATTATACTCTGTCGAATGTCATTGAATGTCTT
TACATGGGCTTGTATGCCTATGAAAATTGTAATACAACTTTCAGCAACGGATCTCTTGGC
TCTCGCATCGATGAAGAACGCAGCGAAATGCGATAAGTAATGTGAATTGCAGAATTCAGT
GAATCATCGAATCTTTGAACGCATCTTGCGCTCCTTGGTATTCCGAGGAGCATGCCTGTT
TGAGTGTCATTAAATTCTCAACTCTCTTCTAC-TTTTTGTAAAAGAGAGCTTGGACTGTG
GAGGCTTGCTGGCCACTTTTTGGGGTCAGCTCCTCTGAAATGCATTAGCGGAACCGTTTG
CGATCTGCCACAAGTGTGATAAGTTATCTACACTGGCGAGGGGATTGCTCTCTGTAATGT
TCAGCTTCTAATTGTCTCTACTTTGTGAGACTACTTTTGAATGCTTGACCTCAAATCAGG
TAGGACTACCCGCTGAACTTAA
>AC4-34
TTTCCGTAGGTGAACCTGCGGAAGGATCATTATTGAATTATGTTTCTAGATAGGTTGTAG
CTGGCTCTTTTAGAGCATGTGCACGCCTGTTTGGACTTCATTTTCATCCACCTGTGCACC
TATTGTAGTCTTTGGTTGGGTTAGGAGGAAGTGATCATTGTATCAGCATCTGCTGGGAGT
GAGGACTTGCATTGTGAAAGCTTTGCTGTCCTTGATGTGATCATGGAATCTTTTTC----

-------TCACTAGAGTCTATGTCACTCATTATACTCTGTCGAATGTCATTGAATGTCTT
TACATGGGCTTGTATGCCTATGAAAATTGTAATACAACTTTCAGCAACGGATCTCTTGGC
TCTCGCATCGATGAAGAACGCAGCGAAATGCGATAAGTAATGTGAATTGCAGAATTCAGT
GAATCATCGAATCTTTGAACGCATCTTGCGCTCCTTGGTATTCCGAGGAGCATGCCTGTT
TGAGTGTCATTAAATTCTCAACTCTCTTCTAC-TTTTTGTAAAAGAGAGCTTGGACTGTG
GAGGCTTGCTGGCCACTTTTTGGGGTCAGCTCCTCTGAAATGCATTAGCGGAACCGTTTG
CGATCTGCCACAAGTGTGATAAGTTATCTACACTGGCGAGGGGATTGCTCTCTGTAATGT
TCAGCTTCTAATTGTCTCTACTTTGTGAGACTACTTTTGAATGCTTGACCTCAAATCAGG
TAGGACTACCCGCTGAACTTAA
>AC7-46
TTTCCGTAGGTGAACCTGCGGAAGGATCATTATTGAATTATGTTTCTAGATAGGTTGTAG
CTGGCTCTTTTAGAGCATGTGCACGCCTGTTTGGACTTCATTTTCATCCACCTGTGCACC
TATTGTAGTCTTTGGTTGGGTTAGGAGGAAGTGATCATTGTATCAGCATCTGCTGGGAGT
GAGGACTTGCATTGTGAAAGCTTTGCTGTCCTTGATGTGATCATGGAATCTTTTTC----

-------TCACTAGAGTCTATGTCACTCATTATACTCTGTCGAATGTCATTGAATGTCTT
TACATGGGCTTGTATGCCTATGAAAATTGTAATACAACTTTCAGCAACGGATCTCTTGGC
TCTCGCATCGATGAAGAACGCAGCGAAATGCGATAAGTAATGTGAATTGCAGAATTCAGT
GAATCATCGAATCTTTGAACGCATCTTGCGCTCCTTGGTATTCCGAGGAGCATGCCTGTT
TGAGTGTCATTAAATTCTCAACTCTCTTCTAC-TTTTTGTAAAAGAGAGCTTGGACTGTG
GAGGCTTGCTGGCCACTTTTTGGGGTCAGCTCCTCTGAAATGCATTAGCGGAACCGTTTG
CGATCTGCCACAAGTGTGATAAGTTATCTACACTGGCGAGGGGATTGCTCTCTGTAATGT
TCAGCTTCTAATTGTCTCTACTTTGTGAGACTACTTTTGAATGCTTGACCTCAAATCAGG
TAGGACTACCCGCTGAACTTAA
>AC11-52
TTTCCGTAGGTGAACCTGCGGAAGGATCATTATTGAATTATGTTTCTAGATAGGTTGTAG
CTGGCTCTTTTAGAGCATGTGCACGCCTGTTTGGACTTCATTTTCATCCACCTGTGCACC
TATTGTAGTCTTTGGTTGGGTTAGGAGGAAGTGATCATTGTATCAGCATCTGCTGGGAGT
GAGGACTTGCATTGTGAAAGCTTTGCTGTCCTTGATGTGATCATGGAATCTTTTTC----

-------TCACTAGAGTCTATGTCACTCATTATACTCTGTCGAATGTCATTGAATGTCTT
TACATGGGCTTGTATGCCTATGAAAATTGTAATACAACTTTCAGCAACGGATCTCTTGGC
TCTCGCATCGATGAAGAACGCAGCGAAATGCGATAAGTAATGTGAATTGCAGAATTCAGT
GAATCATCGAATCTTTGAACGCATCTTGCGCTCCTTGGTATTCCGAGGAGCATGCCTGTT
TGAGTGTCATTAAATTCTCAACTCTCTTCTAC-TTTTTGTAAAAGAGAGCTTGGACTGTG
GAGGCTTGCTGGCCACTTTTTGGGGTCAGCTCCTCTGAAATGCATTAGCGGAACCGTTTG
CGATCTGCCACAAGTGTGATAAGTTATCTACACTGGCGAGGGGATTGCTCTCTGTAATGT
TCAGCTTCTAATTGTCTCTACTTTGTGAGACTACTTTTGAATGCTTGACCTCAAATCAGG
TAGGACTACCCGCTGAACTTAA
>AC11-23
TTTCCGTAGGTGAACCTGCGGAAGGATCATTATTGAATTATGTTTCTAGATAGGTTGTAG
CTGGCTCTTTTAGAGCATGTGCACGCCTGTTTGGACTTCATTTTCATCCACCTGTGCACC
TATTGTAGTCTTTGGTTGGGTTAGGAGGAAGTGATCATTGTATCAGCATCTGCTGGGAGT
GAGGACTTGCATTGTGAAAGCTTTGCTGTCCTTGATGTGATCATGGAATCTTTTTC----

-------TCACTAGAGTCTATGTCACTCATTATACTCTGTCGAATGTCATTGAATGTCTT
TACATGGGCTTGTATGCCTATGAAAATTGTAATACAACTTTCAGCAACGGATCTCTTGGC
TCTCGCATCGATGAAGAACGCAGCGAAATGCGATAAGTAATGTGAATTGCAGAATTCAGT
GAATCATCGAATCTTTGAACGCATCTTGCGCTCCTTGGTATTCCGAGGAGCATGCCTGTT
TGAGTGTCATTAAATTCTCAACTCTCTTCTAC-TTTTTGTAAAAGAGAGCTTGGACTGTG
GAGGCTTGCTGGCCACTTTTTGGGGTCAGCTCCTCTGAAATGCATTAGCGGAACCGTTTG
CGATCTGCCACAAGTGTGATAAGTTATCTACACTGGCGAGGGGATTGCTCTCTGTAATGT
TCAGCTTCTAATTGTCTCTACTTTGTGAGACTACTTTTGAATGCTTGACCTCAAATCAGG
TAGGACTACCCGCTGAACTTAA
>AC1-30
TTTCCGTAGGTGAACCTGCGGAAGGATCATTATTGAATTATGTTTCTAGATAGGTTGTAG
CTGGCTCTTTTAGAGCATGTGCACGCCTGTTTGGACTTCATTTTCATCCACCTGTGCACC
TATTGTAGTCTTTGGTTGGGTTAGGAGGAAGTGATCATTGTATCAGCATCTGCTGGGAGT
GAGGACTTGCATTGTGAAAGCTTTGCTGTCCTTGATGTGATCATGGAATCTTTTTC----

-------TCACTAGAGTCTATGTCACTCATTATACTCTGTCGAATGTCATTGAATGTCTT
TACATGGGCTTATATGCCTATGAAAATTGTAATACAACTTTCAGCAACGGATCTCTTGGC
TCTCGCATCGATGAAGAACGCAGCGAAATGCGATAAGTAATGTGAATTGCAGAATTCAGT
GAATCATCGAATCTTTGAACGCATCTTGCGCTCCTTGGTATTCCGAGGAGCATGCCTGTT
TGAGTGTCATTAAATTCTCAACTCTCTTCTAC-TTTTTGTAAAAGAGAGCTTGGACTGTG
GAGGCTTGCTGGCCACTTTTTGGGGTCAGCTCCTCTGAAATGCATTAGCGGAACCGTTTG
CGATCTGCCACAAGTGTGATAAGTTATCTACACTGGCGAGGGGATTGCTCTCTGTAATGT
TCAGCTTCTAATTGTCTCTACTTTGTGAGACTACTTTTGAATGCTTGACCTCAAATCAGG
TAGGACTACCCGCTGAACTTAA
>AC3-1
TTTCCGTAGGTGAACCTGCGGAAGGATCATTATTGAATTATGTTTCTAGATAGGTTGTAG
CTGGCTCTTTTAGAGCATGTGCACGCCTGTTTGGACTTCATTTTCATCCACCTGTGCACC
TATTGTAGTCTTTGGTTGGGTTAGGAGGAAGTGATCATTGTATCAGCATCTGCTGGGAGT
GAGGACTTGCATTGTGAAAGCTTTGCTGTCCTTGATGTGATCATGGAATCTTTTTC----

-------TCACTAGAGTCTATGTCACTCATTATACTCTGTCGAATGTCATTGAATGTCTT
TACATGGGCTTATATGCCTATGAAAATTGTAATACAACTTTCAGCAACGGATCTCTTGGC
TCTCGCATCGATGAAGAACGCAGCGAAATGCGATAAGTAATGTGAATTGCAGAATTCAGT
GAATCATCGAATCTTTGAACGCATCTTGCGCTCCTTGGTATTCCGAGGAGCATGCCTGTT
TGAGTGTCATTAAATTCTCAACTCTCTTCTAC-TTTTTGTAAAAGAGAGCTTGGACTGTG
GAGGCTTGCTGGCCACTTTTTGGGGTCAGCTCCTCTGAAATGCATTAGCGGAACCGTTTG
CGATCTGCCACAAGTGTGATAAGTTATCTACACTGGCGAGGGGATTGCTCTCTGTAATGT
TCAGCTTCTAATTGTCTCTACTTTGTGAGACTACTTTTGAATGCTTGACCTCAAATCAGG
TAGGACTACCCGCTGAACTTAA
>AC5-55
TTTCCGTAGGTGAACCTGCGGAAGGATCATTATTGAATTATGTTTCTAGATAGGTTGTAG
CTGGCTCTTTTAGAGCATGTGCACGCCTGTTTGGACTTCATTTTCATCCACCTGTGCACC
TATTGTAGTCTTTGGTTGGGTTAGGAGGAAGTGATCATTGTATCAGCATCTGCTGGGAGT

GAGGACTTGCATTGTGAAAGCTTTGCTGTCCTTGATGTGATCATGGAATCTTTTTC----

-------TCACTAGAGTCTATGTCACTCATTATACTCTGTCGAATGTCATTGAATGTCTT
TACATGGGCTTATATGCCTATGAAAATTGTAATACAACTTTCAGCAACGGATCTCTTGGC
TCTCGCATCGATGAAGAACGCAGCGAAATGCGATAAGTAATGTGAATTGCAGAATTCAGT
GAATCATCGAATCTTTGAACGCATCTTGCGCTCCTTGGTATTCCGAGGAGCATGCCTGTT
TGAGTGTCATTAAATTCTCAACTCTCTTCTAC-TTTTTGTAAAAGAGAGCTTGGACTGTG
GAGGCTTGCTGGCCACTTTTTGGGGTCAGCTCCTCTGAAATGCATTAGCGGAACCGTTTG
CGATCTGCCACAAGTGTGATAAGTTATCTACACTGGCGAGGGGATTGCTCTCTGTAATGT
TCAGCTTCTAATTGTCTCTACTTTGTGAGACTACTTTTGAATGCTTGACCTCAAATCAGG
TAGGACTACCCGCTGAACTTAA
>AC43
TTTCCGTAGGTGAACCTGCGGAAGGATCATTATTGAATTATGTTTCTAGATAGGTTGTAG
CTGGCTCTTTTAGAGCATGTGCACGCCTGTTTGGACTTCATTTTCATCCACCTGTGCACC
TATTGTAGTCTTTGGTTGGGTTAGGAGGAAGTGATCATTGTATCAGCATCTGCTGGGAGT
GAGGACTTGCATTGTGAAAGCTTTGCTGTCCTTGATGTGATCATGGAATCTTTTTC----

-------TCACTAGAGTCTATGTCACTCATTATACTCTGTCGAATGTCATTGAATGTCTT
TACATGGGCTTATATGCCTATGAAAATTGTAATACAACTTTCAGCAACGGATCTCTTGGC
TCTCGCATCGATGAAGAACGCAGCGAAATGCGATAAGTAATGTGAATTGCAGAATTCAGT
GAATCATCGAATCTTTGAACGCATCTTGCGCTCCTTGGTATTCCGAGGAGCATGCCTGTT
TGAGTGTCATTAAATTCTCAACTCTCTTCTAC-TTTTTGTAAAAGAGAGCTTGGACTGTG
GAGGCTTGCTGGCCACTTTTTGGGGTCAGCTCCTCTGAAATGCATTAGCGGAACCGTTTG
CGATCTGCCACAAGTGTGATAAGTTATCTACACTGGCGAGGGGATTGCTCTCTGTAATGT
TCAGCTTCTAATTGTCTCTACTTTGTGAGACTACTTTTGAATGCTTGACCTCAAATCAGG
TAGGACTACCCGCTGAACTTAA
>AC8-11
TTTCCGTAGGTGAACCTGCGGAAGGATCATTATTGAATTATGTTTCTAGATAGGTTGTAG

CTGGCTCTTTTAGAGCATGTGCACGCCTGTTTGGACTTCATTTTCATCCACCTGTGCACC
TATTGTAGTCTTTGGTTGGGTTAGGAGGAAGTGATCATTGTATCAGCATCTGCTGGGAGT
GAGGACTTGCATTGTGAAAGCTTTGCTGTCCTTGATGTGATCATGGAATCTTTTTC----

-------TCACTAGAGTCTATGTCACTCATTATACTCTGTCGAATGTCATTGAATGTCTT
TACATGGGCTTATATGCCTATGAAAATTGTAATACAACTTTCAGCAACGGATCTCTTGGC
TCTCGCATCGATGAAGAACGCAGCGAAATGCGATAAGTAATGTGAATTGCAGAATTCAGT
GAATCATCGAATCTTTGAACGCATCTTGCGCTCCTTGGTATTCCGAGGAGCATGCCTGTT
TGAGTGTCATTAAATTCTCAACTCTCTTCTAC-TTTTTGTAAAAGAGAGCTTGGACTGTG
GAGGCTTGCTGGCCACTTTTTGGGGTCAGCTCCTCTGAAATGCATTAGCGGAACCGTTTG
CGATCTGCCACAAGTGTGATAAGTTATCTACACTGGCGAGGGGATTGCTCTCTGTAATGT
TCAGCTTCTAATTGTCTCTACTTTGTGAGACTACTTTTGAATGCTTGACCTCAAATCAGG
TAGGACTACCCGCTGAACTTAA
>AC8-19
TTTCCGTAGGTGAACCTGCGGAAGGATCATTATTGAATTATGTTTCTAGATAGGTTGTAG
CTGGCTCTTTTAGAGCATGTGCACGCCTGTTTGGACTTCATTTTCATCCACCTGTGCACC
TATTGTAGTCTTTGGTTGGGTTAGGAGGAAGTGATCATTGTATCAGCATCTGCTGGGAGT
GAGGACTTGCATTGTGAAAGCTTTGCTGTCCTTGATGTGATCATGGAATCTTTTTC----

-------TCACTAGAGTCTATGTCACTCATTATACTCTGTCGAATGTCATTGAATGTCTT
TACATGGGCTTATATGCCTATGAAAATTGTAATACAACTTTCAGCAACGGATCTCTTGGC
TCTCGCATCGATGAAGAACGCAGCGAAATGCGATAAGTAATGTGAATTGCAGAATTCAGT
GAATCATCGAATCTTTGAACGCATCTTGCGCTCCTTGGTATTCCGAGGAGCATGCCTGTT
TGAGTGTCATTAAATTCTCAACTCTCTTCTAC-TTTTTGTAAAAGAGAGCTTGGACTGTG
GAGGCTTGCTGGCCACTTTTTGGGGTCAGCTCCTCTGAAATGCATTAGCGGAACCGTTTG
CGATCTGCCACAAGTGTGATAAGTTATCTACACTGGCGAGGGGATTGCTCTCTGTAATGT
TCAGCTTCTAATTGTCTCTACTTTGTGAGACTACTTTTGAATGCTTGACCTCAAATCAGG
TAGGACTACCCGCTGAACTTAA

>AC9-25
TTTCCGTAGGTGAACCTGCGGAAGGATCATTATTGAATTATGTTTCTAGATAGGTTGTAG
CTGGCTCTTTTAGAGCATGTGCACGCCTGTTTGGACTTCATTTTCATCCACCTGTGCACC
TATTGTAGTCTTTGGTTGGGTTAGGAGGAAGTGATCATTGTATCAGCATCTGCTGGGAGT
GAGGACTTGCATTGTGAAAGCTTTGCTGTCCTTGATGTGATCATGGAATCTTTTTC----

-------TCACTAGAGTCTATGTCACTCATTATACTCTGTCGAATGTCATTGAATGTCTT
TACATGGGCTTATATGCCTATGAAAATTGTAATACAACTTTCAGCAACGGATCTCTTGGC
TCTCGCATCGATGAAGAACGCAGCGAAATGCGATAAGTAATGTGAATTGCAGAATTCAGT
GAATCATCGAATCTTTGAACGCATCTTGCGCTCCTTGGTATTCCGAGGAGCATGCCTGTT
TGAGTGTCATTAAATTCTCAACTCTCTTCTAC-TTTTTGTAAAAGAGAGCTTGGACTGTG
GAGGCTTGCTGGCCACTTTTTGGGGTCAGCTCCTCTGAAATGCATTAGCGGAACCGTTTG
CGATCTGCCACAAGTGTGATAAGTTATCTACACTGGCGAGGGGATTGCTCTCTGTAATGT
TCAGCTTCTAATTGTCTCTACTTTGTGAGACTACTTTTGAATGCTTGACCTCAAATCAGG
TAGGACTACCCGCTGAACTTAA
>AC9-46
TTTCCGTAGGTGAACCTGCGGAAGGATCATTATTGAATTATGTTTCTAGATAGGTTGTAG
CTGGCTCTTTTAGAGCATGTGCACGCCTGTTTGGACTTCATTTTCATCCACCTGTGCACC
TATTGTAGTCTTTGGTTGGGTTAGGAGGAAGTGATCATTGTATCAGCATCTGCTGGGAGT
GAGGACTTGCATTGTGAAAGCTTTGCTGTCCTTGATGTGATCATGGAATCTTTTTC----

-------TCACTAGAGTCTATGTCACTCATTATACTCTGTCGAATGTCATTGAATGTCTT
TACATGGGCTTATATGCCTATGAAAATTGTAATACAACTTTCAGCAACGGATCTCTTGGC
TCTCGCATCGATGAAGAACGCAGCGAAATGCGATAAGTAATGTGAATTGCAGAATTCAGT
GAATCATCGAATCTTTGAACGCATCTTGCGCTCCTTGGTATTCCGAGGAGCATGCCTGTT
TGAGTGTCATTAAATTCTCAACTCTCTTCTAC-TTTTTGTAAAAGAGAGCTTGGACTGTG
GAGGCTTGCTGGCCACTTTTTGGGGTCAGCTCCTCTGAAATGCATTAGCGGAACCGTTTG
CGATCTGCCACAAGTGTGATAAGTTATCTACACTGGCGAGGGGATTGCTCTCTGTAATGT

TCAGCTTCTAATTGTCTCTACTTTGTGAGACTACTTTTGAATGCTTGACCTCAAATCAGG
TAGGACTACCCGCTGAACTTAA
>AC11-27
TTTCCGTAGGTGAACCTGCGGAAGGATCATTATTGAATTATGTTTCTAGATAGGTTGTAG
CTGGCTCTTTTAGAGCATGTGCACGCCTGTTTGGACTTCATTTTCATCCACCTGTGCACC
TATTGTAGTCTTTGGTTGGGTTAGGAGGAAGTGATCATTGTATCAGCATCTGCTGGGAGT
GAGGACTTGCATTGTGAAAGCTTTGCTGTCCTTGATGTGATCATGGAATCTTTTTC----

-------TCACTAGAGTCTATGTCACTCATTATACTCTGTCGAATGTCATTGAATGTCTT
TACATGGGCTTATATGCCTATGAAAATTGTAATACAACTTTCAGCAACGGATCTCTTGGC
TCTCGCATCGATGAAGAACGCAGCGAAATGCGATAAGTAATGTGAATTGCAGAATTCAGT
GAATCATCGAATCTTTGAACGCATCTTGCGCTCCTTGGTATTCCGAGGAGCATGCCTGTT
TGAGTGTCATTAAATTCTCAACTCTCTTCTAC-TTTTTGTAAAAGAGAGCTTGGACTGTG
GAGGCTTGCTGGCCACTTTTTGGGGTCAGCTCCTCTGAAATGCATTAGCGGAACCGTTTG
CGATCTGCCACAAGTGTGATAAGTTATCTACACTGGCGAGGGGATTGCTCTCTGTAATGT
TCAGCTTCTAATTGTCTCTACTTTGTGAGACTACTTTTGAATGCTTGACCTCAAATCAGG
TAGGACTACCCGCTGAACTTAA
>AC3-57
TTTCCGTAGGTGAACCTGCGGAAGGATCATTATTGAATTATGTTTCTAGATAGGTTGTAG
CTGGCTCTTTTAGAGCATGTGCACGCCTGTTTGGACTTCATTTTCATCCACCTGTGCACC
TATTGTAGTCTTTGGTTGGGTTAGGAGGAAGTGATCATTGTATCAGCATCTGCTGGGAGT
GAGGACTTGCATTGTGAAAGCTTTGCTGTCCTTGATGTGATCATGGAATCTCTTTC----

-------TCACTAGAGTCTATGTCACTCATTATACTCTGTCGAATGTCATTGAATGTCTT
TACATGGGCTTATATGCCTATGAAAATTGTAATACAACTTTCAGCAACGGATCTCTTGGC
TCTCGCATCGATGAAGAACGCAGCGAAATGCGATAAGTAATGTGAATTGCAGAATTCAGT
GAATCATCGAATCTTTGAACGCATCTTGCGCTCCTTGGTATTCCGAGGAGCATGCCTGTT
TGAGTGTCATTAAATTCTCAACTCTCTTCTAC-TTTTTGTAAAAGAGAGCTTGGACTGTG

GAGGCTTGCTGGCCACTTTTTGGGGTCAGCTCCTCTGAAATGCATTAGCGGAACCGTTTG
CGATCTGCCACAAGTGTGATAAGTTATCTACACTGGCGAGGGGATTGCTCTCTGTAATGT
TCAGCTTCTAATTGTCTCTACTTTGTGAGACTACTTTTGAATGCTTGACCTCAAATCAGG
TAGGACTACCCGCTGAACTTAA
>AC7-49
TTTCCGTAGGTGAACCTGCGGAAGGATCATTATTGAATTATGTTTCTAGATAGGTTGTAG
CTGGCTCTTTTAGAGCATGTGCACGCCTGTTTGGACTTCATTTTCATCCACCTGTGCACC
TATTGTAGTCTTTGGTTGGGTTAGGAGGAAGTGATCATTGTATCAGCATCTGCTGGGAGT
GAGGACTTGCATTGTGAAAGCTTTGCTGTCCTTGATGTGATCATGGAATCTCTTTC----

-------TCACTAGAGTCTATGTCACTCATTATACTCTGTCGAATGTCATTGAATGTCTT
TACATGGGCTTATATGCCTATGAAAATTGTAATACAACTTTCAGCAACGGATCTCTTGGC
TCTCGCATCGATGAAGAACGCAGCGAAATGCGATAAGTAATGTGAATTGCAGAATTCAGT
GAATCATCGAATCTTTGAACGCATCTTGCGCTCCTTGGTATTCCGAGGAGCATGCCTGTT
TGAGTGTCATTAAATTCTCAACTCTCTTCTAC-TTTTTGTAAAAGAGAGCTTGGACTGTG
GAGGCTTGCTGGCCACTTTTTGGGGTCAGCTCCTCTGAAATGCATTAGCGGAACCGTTTG
CGATCTGCCACAAGTGTGATAAGTTATCTACACTGGCGAGGGGATTGCTCTCTGTAATGT
TCAGCTTCTAATTGTCTCTACTTTGTGAGACTACTTTTGAATGCTTGACCTCAAATCAGG
TAGGACTACCCGCTGAACTTAA
>AC7-59
TTTCCGTAGGTGAACCTGCGGAAGGATCATTATTGAATTATGTTTCTAGATAGGTTGTAG
CTGGCTCTTTTAGAGCATGTGCACGCCTGTTTGGACTTCATTTTCATCCACCTGTGCACC
TATTGTAGTCTTTGGTTGGGTTAGGAGGAAGTGATCATTGTATCAGCATCTGCTGGGAGT
GAGGACTTGCATTGTGAAAGCTTTGCTGTCCTTGATGTGATCATGGAATCTCTTTC----

-------TCACTAGAGTCTATGTCACTCATTATACTCTGTCGAATGTCATTGAATGTCTT
TACATGGGCTTATATGCCTATGAAAATTGTAATACAACTTTCAGCAACGGATCTCTTGGC
TCTCGCATCGATGAAGAACGCAGCGAAATGCGATAAGTAATGTGAATTGCAGAATTCAGT

```
GAATCATCGAATCTTTGAACGCATCTTGCGCTCCTTGGTATTCCGAGGAGCATGCCTGTT
TGAGTGTCATTAAATTCTCAACTCTCTTCTAC-TTTTTGTAAAAGAGAGCTTGGACTGTG
GAGGCTTGCTGGCCACTTTTTGGGGTCAGCTCCTCTGAAATGCATTAGCGGAACCGTTTG
CGATCTGCCACAAGTGTGATAAGTTATCTACACTGGCGAGGGGATTGCTCTCTGTAATGT
TCAGCTTCTAATTGTCTCTACTTTGTGAGACTACTTTTGAATGCTTGACCTCAAATCAGG
TAGGACTACCCGCTGAACTTAA
>AC8-61
TTTCCGTAGGTGAACCTGCGGAAGGATCATTATTGAATTATGTTTCTAGATAGGTTGTAG
CTGGCTCTTTTAGAGCATGTGCACGCCTGTTTGGACTTCATTTTCATCCACCTGTGCACC
TATTGTAGTCTTTGGTTGGGTTAGGAGGAAGTGATCATTGTATCAGCATCTGCTGGGAGT
GAGGACTTGCATTGTGAAAGCTTTGCTGTCCTTGATGTGATCATGGAATCTCTTTC----
------------------------------------------------------------
------------------------------------------------------------
------------------------------------------------------------
------------------------------------------------------------
------------------------------------------------------------
------------------------------------------------------------
------------------------------------------------------------
------------------------------------------------------------
------------------------------------------------------------
------------------------------------------------------------
------------------------------------------------------------
------------------------------------------------------------
-------TCACTAGAGTCTATGTCACTCATTATACTCTGTCGAATGTCATTGAATGTCTT
TACATGGGCTTATATGCCTATGAAAATTGTAATACAACTTTCAGCAACGGATCTCTTGGC
TCTCGCATCGATGAAGAACGCAGCGAAATGCGATAAGTAATGTGAATTGCAGAATTCAGT
GAATCATCGAATCTTTGAACGCATCTTGCGCTCCTTGGTATTCCGAGGAGCATGCCTGTT
TGAGTGTCATTAAATTCTCAACTCTCTTCTAC-TTTTTGTAAAAGAGAGCTTGGACTGTG
GAGGCTTGCTGGCCACTTTTTGGGGTCAGCTCCTCTGAAATGCATTAGCGGAACCGTTTG
CGATCTGCCACAAGTGTGATAAGTTATCTACACTGGCGAGGGGATTGCTCTCTGTAATGT
TCAGCTTCTAATTGTCTCTACTTTGTGAGACTACTTTTGAATGCTTGACCTCAAATCAGG
TAGGACTACCCGCTGAACTTAA
>AC11-50
TTTCCGTAGGTGAACCTGCGGAAGGATCATTATTGAATTATGTTTCTAGATAGGTTGTAG
CTGGCTCTTTTAGAGCATGTGCACGCCTGTTTGGACTTCATTTTCATCCACCTGTGCACC
TATTGTAGTCTTTGGTTGGGTTAGGAGGAAGTGATCATTGTATCAGCATCTGCTGGGAGT
GAGGACTTGCATTGTGAAAGCTTTGCTGTCCTTGATGTGATCATGGAATCTCTTTC----
------------------------------------------------------------
------------------------------------------------------------
------------------------------------------------------------
------------------------------------------------------------
------------------------------------------------------------
------------------------------------------------------------
------------------------------------------------------------
------------------------------------------------------------
------------------------------------------------------------
------------------------------------------------------------
------------------------------------------------------------
------------------------------------------------------------
-------TCACTAGAGTCTATGTCACTCATTATACTCTGTCGAATGTCATTGAATGTCTT
```

```
TACATGGGCTTATATGCCTATGAAAATTGTAATACAACTTTCAGCAACGGATCTCTTGGC
TCTCGCATCGATGAAGAACGCAGCGAAATGCGATAAGTAATGTGAATTGCAGAATTCAGT
GAATCATCGAATCTTTGAACGCATCTTGCGCTCCTTGGTATTCCGAGGAGCATGCCTGTT
TGAGTGTCATTAAATTCTCAACTCTCTTCTAC-TTTTTGTAAAAGAGAGCTTGGACTGTG
GAGGCTTGCTGGCCACTTTTTGGGGTCAGCTCCTCTGAAATGCATTAGCGGAACCGTTTG
CGATCTGCCACAAGTGTGATAAGTTATCTACACTGGCGAGGGGATTGCTCTCTGTAATGT
TCAGCTTCTAATTGTCTCTACTTTGTGAGACTACTTTTGAATGCTTGACCTCAAATCAGG
TAGGACTACCCGCTGAACTTAA
>AC4-11
TTTCCGTAGGTGAACCTGCGGAAGGATCATTATTGAATTATGTTTCTAGATAGGTTGTAG
CTGGCTCTTTTAGAGCATGTGCACGCCTGTTTGGACTTCATTTTCATCCACCTGTGCACC
TATTGTAGTCTTTGGTTGGGTTAGGAGGAAGTGATCATTGTATCAGCATCTGCTGGGAGT
GAGGACTTGCATTGTGAAAGCTTTGCTGTCCTTGATGTGATCATGGAATCTCTTTC----
------------------------------------------------------------
------------------------------------------------------------
------------------------------------------------------------
------------------------------------------------------------
------------------------------------------------------------
------------------------------------------------------------
------------------------------------------------------------
------------------------------------------------------------
------------------------------------------------------------
------------------------------------------------------------
------------------------------------------------------------
------------------------------------------------------------
-------TCACTAGAGTCTATGTCACTCATTATACTCTGTCGAATGTCATTGAATGTCTT
TACATGGGCTTGTATGCCTATGAAAATTGTAATACAACTTTCAGCAACGGATCTCTTGGC
TCTCGCATCGATGAAGAACGCAGCGAAATGCGATAAGTAATGTGAATTGCAGAATTCAGT
GAATCATCGAATCTTTGAACGCATCTTGCGCTCCTTGGTATTCCGAGGAGCATGCCTGTT
TGAGTGTCATTAAATTCTCAACTCTCTTCTAC-TTTTTGTAAAAGAGAGCTTGGACTGTG
GAGGCTTGCTGGCCACTTTTTGGGGTCAGCTCCTCTGAAATGCATTAGCGGAACCGTTTG
CGATCTGCCACAAGTGTGATAAGTTATCTACACTGGCGAGGGGATTGCTCTCTGTAATGT
TCAGCTTCTAATTGTCTCTACTTTGTGAGACTACTTTTGAATGCTTGACCTCAAATCAGG
TAGGACTACCCGCTGAACTTAA
>AC4-22
TTTCCGTAGGTGAACCTGCGGAAGGATCATTATTGAATTATGTTTCTAGATAGGTTGTAG
CTGGCTCTTTTAGAGCATGTGCACGCCTGTTTGGACTTCATTTTCATCCACCTGTGCACC
TATTGTAGTCTTTGGTTGGGTTAGGAGGAAGTGATCATTGTATCAGCATCTGCTGGGAGT
GAGGACTTGCATTGTGAAAGCTTTGCTGTCCTTGATGTGATCATGGAATCTCTTTC----
------------------------------------------------------------
------------------------------------------------------------
------------------------------------------------------------
------------------------------------------------------------
------------------------------------------------------------
------------------------------------------------------------
------------------------------------------------------------
------------------------------------------------------------
------------------------------------------------------------
------------------------------------------------------------
------------------------------------------------------------
```

-------TCACTAGAGTCTATGTCACTCATTATACTCTGTCGAATGTCATTGAATGTCTT
TACATGGGCTTGTATGCCTATGAAAATTGTAATACAACTTTCAGCAACGGATCTCTTGGC
TCTCGCATCGATGAAGAACGCAGCGAAATGCGATAAGTAATGTGAATTGCAGAATTCAGT
GAATCATCGAATCTTTGAACGCATCTTGCGCTCCTTGGTATTCCGAGGAGCATGCCTGTT
TGAGTGTCATTAAATTCTCAACTCTCTTCTAC-TTTTTGTAAAAGAGAGCTTGGACTGTG
GAGGCTTGCTGGCCACTTTTTGGGGTCAGCTCCTCTGAAATGCATTAGCGGAACCGTTTG
CGATCTGCCACAAGTGTGATAAGTTATCTACACTGGCGAGGGGATTGCTCTCTGTAATGT
TCAGCTTCTAATTGTCTCTACTTTGTGAGACTACTTTTGAATGCTTGACCTCAAATCAGG
TAGGACTACCCGCTGAACTTAA
>AC5-34
TTTCCGTAGGTGAACCTGCGGAAGGATCATTATTGAATTATGTTTCTAGATAGGTTGTAG
CTGGCTCTTTTAGAGCATGTGCACGCCTGTTTGGACTTCATTTTCATCCACCTGTGCACC
TATTGTAGTCTTTGGTTGGGTTAGGAGGAAGTGATCATTGTATCAGCATCTGCTGGGAGT
GAGGACTTGCATTGTGAAAGCTTTGCTGTCCTTGATGTGATCATGGAATCTCTTTC----

-------TCACTAGAGTCTATGTCACTCATTATACTCTGTCGAATGTCATTGAATGTCTT
TACATGGGCTTGTATGCCTATGAAAATTGTAATACAACTTTCAGCAACGGATCTCTTGGC
TCTCGCATCGATGAAGAACGCAGCGAAATGCGATAAGTAATGTGAATTGCAGAATTCAGT
GAATCATCGAATCTTTGAACGCATCTTGCGCTCCTTGGTATTCCGAGGAGCATGCCTGTT
TGAGTGTCATTAAATTCTCAACTCTCTTCTAC-TTTTTGTAAAAGAGAGCTTGGACTGTG
GAGGCTTGCTGGCCACTTTTTGGGGTCAGCTCCTCTGAAATGCATTAGCGGAACCGTTTG
CGATCTGCCACAAGTGTGATAAGTTATCTACACTGGCGAGGGGATTGCTCTCTGTAATGT
TCAGCTTCTAATTGTCTCTACTTTGTGAGACTACTTTTGAATGCTTGACCTCAAATCAGG
TAGGACTACCCGCTGAACTTAA
>AC12-47
TTTCCGTAGGTGAACCTGCGGAAGGATCATTATTGAATTATGTTTCTAGATAGGTTGTAG
CTGGCTCTTTTAGAGCATGTGCACGCCTGTTTGGACTTCATTTTCATCCACCTGTGCACC
TATTGTAGTCTTTGGTTGGGTTAGGAGGAAGTGATCATTGTATCAGCATCTGCTGGGAGT
GAGGACTTGCATTGTGAAAGCTTTGCTGTCCTTGATGTGATCATGGAATCTTTTTC----

-------TCACTAGAGTCTATGTCACTCATTATACTCTGTCGAATGTCATTGAATGTCTT
TACATGGGCTTGTATGCCTATGAAAATTGTAATACAACTTTCAGCAACGGATCTCTTGGC
TCTCGCATCGATGAAGAACGCAGCGAAATGCGATAAGTAATGTGAATTGCAGAATTCAGT
GAATCATCGAATCTTTGAACGCATCTTGCGCTCCTTGGTATTCCGAGGAGCATGCCTGTT
TGAGTGTCATTAAATTCTCAACTCTCTTCTAC-TTTTTGTAAAAGAGAGCTTGGACTGTG
GAGGCTTGTTGGCCACTTTTTGGGGTCAGCTCCTCTGAAGTGCATTAGCGGAACCGTTTG
CGATCTGCCACAAGTGTGATAAGTTATCTACACTGGCGAGGGGATTGCTCTCTGTAATGT
TCAGCTTCTAATTGTCTCTACTTTGTGAGACTACTTTTGAATGCTTGACCTCAAATCAGG
TAGGACTACCCGCTGAACTTAA
>AC6-22
TTTCCGTAGGTGAACCTGCGGAAGGATCATTATTGAATTATGTTTCTAGATAGGTTGTAG
CTGGCTCTTTTAGAGCATGTGCACGCCTGTTTGGACTTCATTTTCATCCACCTGTGCACC
TATTGTAGTCTTTGGTTGGGTTAGGAGGAAGTGATCATTGTATCAGCATCTGCTGGGAGT
GAGGACTTGCATTGTGAAAGCTTTGCTGTCCTTGATGTGATCATGGAATCTTTTTC----

-------TCACTAGAGTCTATGTCACTCATTATACTCTGTCGAATGTCATTGAATGTCTT
TACATGGGCTTGTATGCCTATGAAAATTGTAATACAACTTTCAGCAACGGATCTCTTGGC
TCTCGCATCGATGAAGAACGCAGCGAAATGCGATAAGTAATGTGAATTGCAGAATTCAGT
GAATCATCGAATCTTTGAACGCATCTTGCGCTCCTTGGTATTCCGAGGAGCATGCCTGTT
TGAGTGTCATTAAATTCTCAACTCTCTTATAC-TTTTTGTAAAAGAGAGCTTGGACTGTG
GAGGCTTGCTGGCCACTTTTTGGGGTCAGCTCCTCTGAAATGCATTAGCGGAACCGTTTG
CGATCTGCCACAAGTGTGATAAGTTATCTACACTGGCGAGGGGATTGCTCTCTGTAATGT
TCAGCTTCTAATTGTCTCTACTTTGTGAGACTACTTTTGAATGCTTGACCTCAAATCAGG
TAGGACTACCCGCTGAACTTAA
>AC9-56
TTTCCGTAGGTGAACCTGCGGAAGGATCATTATTGAATTATGTTTCTAGATAGGTTGTAG
CTGGCTCTTTTAGAGCATGTGCACGCCTGTTTGGACTTCATTTTCATCCACCTGTGCACC
TATTGTAGTCTTTGGTTGGGTTAGGAGGAAGTGATCATTGTATCAGCATCTGCTGGGAGT
GAGGACTTGCATTGTGAAAGCTTTGCTGTCCTTGATGTGATCATGGAATCTTTTTC----

-------TCACTAGAGTCTATGTCACTCATTATACTCTGTCGAATGTCATTGAATGTCTT
TACATGGGCTTGTATGCCTATGAAAATTGTAATACAACTTTCAGCAACGGATCTCTTGGC
TCTCGCATCGATGAAGAACGCAGCGAAATGCGATAAGTAATGTGAATTGCAGAATTCAGT
GAATCATCGAATCTTTGAACGCATCTTGCGCTCCTTGGTATTCCGAGGAGCATGCCTGTT
TGAGTGTCATTAAATTCTCAACTCTCTTATAC-TTTTTGTAAAAGAGAGCTTGGACTGTG
GAGGCTTGCTGGCCACTTTTTGGGGTCAGCTCCTCTGAAATGCATTAGCGGAACCGTTTG
CGATCTGCCACAAGTGTGATAAGTTATCTACACTGGCGAGGGGATTGCTCTCTGTAATGT
TCAGCTTCTAATTGTCTCTACTTTGTGAGACTACTTTTGAATGCTTGACCTCAAATCAGG
TAGGACTACCCGCTGAACTTAA
>AC8-51
TTTCCGTAGGTGAACCTGCGGAAGGATCATTATTGAATTATGTTTCTAGATAGGTTGTAG
CTGGCTCTTTTAGAGCATGTGCACGCCTGTTTGGACTTCATTTTCATCCACCTGTGCACC
TATTGTAGTCTTTGGTTGGGTTAGGAGGAAGTGATCATTGTATCAGCATCTGCTGGGAGT
GAGGACTTGCATTGTGAAAGCTTTGCTGTCCTTGATGTGATCATGGAATCTTTTTC----

-------TCACTAGAGTCTATGTCACTCATTATACTCTGTCGAATGTCATTGAATGTCTT
TACATGGGCTTGTATGCCTATGAAAATTGTAATACAACTTTCAGCAACGGATCTCTTGGC
TCTCGCATCGATGAAGAACGCAGCGAAATGCGATAAGTAATGTGAATTGCAGAATTCAGT
GAATCATCGAATCTTTGAACGCATCTTGCGCTCCTTGGTATTCCGAGGAGCATGCCTGTT
TGAGTGTCATTAAATTCTCAACTCTCTTATAC-TTTTTGTAAAAGAGAGCTTGGACTGTG
GAGGCTTGCTGGCCACTTTTTGGGGTCAGCTCCTCTGAAATGCATTAGCGGAACCGTTTG
CGATCTGCCACAAGTGTGATAAGTTATCTACACTGGCGAGGGGATTGCTCTCTGTAATGT
TCAGCTTCTAATTGTCTCTACTTTGTGAGACTACTTTTGAATGCTTGACCTCAAATCAGG
TAGGACTACCCGCTGAACTTAA
>AC7-48
TTTCCGTAGGTGAACCTGCGGAAGGATCATTATTGAATTATGTTTCTAGATAGGTTGTAG
CTGGCTCTTTTAGAGCATGTGCACGCCTGTTTGGACTTCATTTTCATCCACCTGTGCACC
TATTGTAGTCTTTGGTTGGGTTAGGAGGAAGTGATCATTGTATCAGCATCTGCTGGGAGT
GAGGACTTGCATTGTGAAAGCTTTGCTGTCCTTGATGTGATCATGGAATCTTTTTC----

-------TCACTAGAGTCTATGTCACTCATTATACTCTGTCGAATGTCATTGAATGTCTT
TACATGGGCTTATATGCCTATGAAAATTGTAATACAACTTTCAGCAACGGATCTCTTGGC
TCTCGCATCGATGAAGAACGCAGCGAAATGCGATAAGTAATGTGAATTGCAGAATTCAGT
GAATCATCGAATCTTTGAACGCATCTTGCGCTCCTTGGTATTCCGAGGAGCATGCCTGTT
TGAGTGTCATTAAATTCTCAACTCTCTTATAC-TTTTTGTAAAAGAGAGCTTGGACTGTG
GAGGCTTGCTGGCCACTTTTTGGGGTCAGCTCCTCTGAAATGCATTAGCGGAACCGTTTG
CGATCTGCCACAAGTGTGATAAGTTATCTACACTGGCGAGGGGATTGCTCTCTGTAATGT
TCAGCTTCTAATTGTCTCTACTTTGTGAGACTACTTTTGAATGCTTGACCTCAAATCAGG
TAGGACTACCCGCTGAACTTAA
>AC1-35
TTTCCGTAGGTGAACCTGCGGAAGGATCATTATTGAATTATGTTTCTAGATAGGTTGTAG
CTGGCTCTTTTAGAGCATGTGCACGCCTGTTTGGACTTCATTTTCATCCACCTGTGCACC
TATTGTAGTCTTTGGTTGGGTTAGGAGGAAGTGGTCATTGTGTCAGCATCTGCTGGATGT
GAGGACTTGCATTGTGAAAGCTTTGCTGTCCTTGATGTGATCATGGAATCTCTTTC----

-------TCACTAGAGTCTATGTCACTCATTATACTCTGTCGAATGTCATTGAATGTCTT
TACATGGGCTTATATGCCTATGAAAATTGTAATACAACTTTCAGCAACGGATCTCTTGGC
TCTCGCATCGATGAAGAACGCAGCGAAATGCGATAAGTAATGTGAATTGCAGAATTCAGT
GAATCATCGAATCTTTGAACGCATCTTGCGCTCCTTGGTATTCCGAGGAGCATGCCTGTT
TGAGTGTCATTAAATTCTCAACTCTCTTCTAC-TTTTTGTAAAAGAGAGCTTGGACTGTG
GAGGCTTGCTGGCCACTTTTTGGGGTCAGCTCCTCTGAAATGCATTAGCGGAACCGTTTG
CGATCTGCCACAAGTGTGATAAGTTATCTACACTGGCGAGGGGATTGCTCTCTGTAATGT
TCAGCTTCTAATTGTCTCTACTTTGTGAGACTACTTTTGAATGCTTGACCTCAAATCAGG
TAGGACTACCCGCTGAACTTAA
>AC5-9
TTTCCGTAGGTGAACCTGCGGAAGGATCATTATTGAATTATGTTTCTAGATAGGTTGTAG
CTGGCTCTTTTAGAGCATGTGCACGCCTGTTTGGACTTCATTTTCATCCACCTGTGCACC
TATTGTAGTCTTTGGTTGGGTTAGGAGGAAGTGGTCATTGTGTCAGCATCTGCTGGATGT
GAGGACTTGCATTGTGAAAGCTTTGCTGTCCTTGATGTGATCATGGAATCTCTTTC----

-------TCACTAGAGTCTATGTCACTCATTATACTCTGTCGAATGTCATTGAATGTCTT
TACATGGGCTTATATGCCTATGAAAATTGTAATACAACTTTCAGCAACGGATCTCTTGGC
TCTCGCATCGATGAAGAACGCAGCGAAATGCGATAAGTAATGTGAATTGCAGAATTCAGT
GAATCATCGAATCTTTGAACGCATCTTGCGCTCCTTGGTATTCCGAGGAGCATGCCTGTT
TGAGTGTCATTAAATTCTCAACTCTCTTCTAC-TTTTTGTAAAAGAGAGCTTGGACTGTG
GAGGCTTGCTGGCCACTTTTTGGGGTCAGCTCCTCTGAAATGCATTAGCGGAACCGTTTG
CGATCTGCCACAAGTGTGATAAGTTATCTACACTGGCGAGGGGATTGCTCTCTGTAATGT
TCAGCTTCTAATTGTCTCTACTTTGTGAGACTACTTTTGAATGCTTGACCTCAAATCAGG
TAGGACTACCCGCTGAACTTAA
>AC6-6
TTTCCGTAGGTGAACCTGCGGAAGGATCATTATTGAATTATGTTTCTAGATAGGTTGTAG
CTGGCTCTTTTAGAGCATGTGCACGCCTGTTTGGACTTCATTTTCATCCACCTGTGCACC
TATTGTAGTCTTTGGTTGGGTTAGGAGGAAGTGGTCATTGTGTCAGCATCTGCTGGATGT
GAGGACTTGCATTGTGAAAGCTTTGCTGTCCTTGATGTGATCATGGAATCTCTTTC----

-------TCACTAGAGTCTATGTCACTCATTATACTCTGTCGAATGTCATTGAATGTCTT
TACATGGGCTTATATGCCTATGAAAATTGTAATACAACTTTCAGCAACGGATCTCTTGGC
TCTCGCATCGATGAAGAACGCAGCGAAATGCGATAAGTAATGTGAATTGCAGAATTCAGT
GAATCATCGAATCTTTGAACGCATCTTGCGCTCCTTGGTATTCCGAGGAGCATGCCTGTT
TGAGTGTCATTAAATTCTCAACTCTCTTCTAC-TTTTTGTAAAAGAGAGCTTGGACTGTG
GAGGCTTGCTGGCCACTTTTTGGGGTCAGCTCCTCTGAAATGCATTAGCGGAACCGTTTG
CGATCTGCCACAAGTGTGATAAGTTATCTACACTGGCGAGGGGATTGCTCTCTGTAATGT
TCAGCTTCTAATTGTCTCTACTTTGTGAGACTACTTTTGAATGCTTGACCTCAAATCAGG
TAGGACTACCCGCTGAACTTAA
>AC6-36
TTTCCGTAGGTGAACCTGCGGAAGGATCATTATTGAATTATGTTTCTAGATAGGTTGTAG
CTGGCTCTTTTAGAGCATGTGCACGCCTGTTTGGACTTCATTTTCATCCACCTGTGCACC
TATTGTAGTCTTTGGTTGGGTTAGGAGGAAGTGGTCATTGTGTCAGCATCTGCTGGATGT
GAGGACTTGCATTGTGAAAGCTTTGCTGTCCTTGATGTGATCATGGAATCTCTTTC----

-------TCACTAGAGTCTATGTCACTCATTATACTCTGTCGAATGTCATTGAATGTCTT
TACATGGGCTTATATGCCTATGAAAATTGTAATACAACTTTCAGCAACGGATCTCTTGGC
TCTCGCATCGATGAAGAACGCAGCGAAATGCGATAAGTAATGTGAATTGCAGAATTCAGT
GAATCATCGAATCTTTGAACGCATCTTGCGCTCCTTGGTATTCCGAGGAGCATGCCTGTT
TGAGTGTCATTAAATTCTCAACTCTCTTCTAC-TTTTTGTAAAAGAGAGCTTGGACTGTG
GAGGCTTGCTGGCCACTTTTTGGGGTCAGCTCCTCTGAAATGCATTAGCGGAACCGTTTG
CGATCTGCCACAAGTGTGATAAGTTATCTACACTGGCGAGGGGATTGCTCTCTGTAATGT
TCAGCTTCTAATTGTCTCTACTTTGTGAGACTACTTTTGAATGCTTGACCTCAAATCAGG
TAGGACTACCCGCTGAACTTAA
>AC8-7
TTTCCGTAGGTGAACCTGCGGAAGGATCATTATTGAATTATGTTTCTAGATAGGTTGTAG
CTGGCTCTTTTAGAGCATGTGCACGCCTGTTTGGACTTCATTTTCATCCACCTGTGCACC
TATTGTAGTCTTTGGTTGGGTTAGGAGGAAGTGGTCATTGTGTCAGCATCTGCTGGATGT
GAGGACTTGCATTGTGAAAGCTTTGCTGTCCTTGATGTGATCATGGAATCTCTTTC----

-------TCACTAGAGTCTATGTCACTCATTATACTCTGTCGAATGTCATTGAATGTCTT
TACATGGGCTTATATGCCTATGAAAATTGTAATACAACTTTCAGCAACGGATCTCTTGGC
TCTCGCATCGATGAAGAACGCAGCGAAATGCGATAAGTAATGTGAATTGCAGAATTCAGT
GAATCATCGAATCTTTGAACGCATCTTGCGCTCCTTGGTATTCCGAGGAGCATGCCTGTT
TGAGTGTCATTAAATTCTCAACTCTCTTCTAC-TTTTTGTAAAAGAGAGCTTGGACTGTG
GAGGCTTGCTGGCCACTTTTTGGGGTCAGCTCCTCTGAAATGCATTAGCGGAACCGTTTG
CGATCTGCCACAAGTGTGATAAGTTATCTACACTGGCGAGGGGATTGCTCTCTGTAATGT
TCAGCTTCTAATTGTCTCTACTTTGTGAGACTACTTTTGAATGCTTGACCTCAAATCAGG
TAGGACTACCCGCTGAACTTAA
>AC8-41
TTTCCGTAGGTGAACCTGCGGAAGGATCATTATTGAATTATGTTTCTAGATAGGTTGTAG
CTGGCTCTTTTAGAGCATGTGCACGCCTGTTTGGACTTCATTTTCATCCACCTGTGCACC
TATTGTAGTCTTTGGTTGGGTTAGGAGGAAGTGGTCATTGTGTCAGCATCTGCTGGATGT

GAGGACTTGCATTGTGAAAGCTTTGCTGTCCTTGATGTGATCATGGAATCTCTTTC----

-------TCACTAGAGTCTATGTCACTCATTATACTCTGTCGAATGTCATTGAATGTCTT
TACATGGGCTTATATGCCTATGAAAATTGTAATACAACTTTCAGCAACGGATCTCTTGGC
TCTCGCATCGATGAAGAACGCAGCGAAATGCGATAAGTAATGTGAATTGCAGAATTCAGT
GAATCATCGAATCTTTGAACGCATCTTGCGCTCCTTGGTATTCCGAGGAGCATGCCTGTT
TGAGTGTCATTAAATTCTCAACTCTCTTCTAC-TTTTTGTAAAAGAGAGCTTGGACTGTG
GAGGCTTGCTGGCCACTTTTTGGGGTCAGCTCCTCTGAAATGCATTAGCGGAACCGTTTG
CGATCTGCCACAAGTGTGATAAGTTATCTACACTGGCGAGGGGATTGCTCTCTGTAATGT
TCAGCTTCTAATTGTCTCTACTTTGTGAGACTACTTTTGAATGCTTGACCTCAAATCAGG
TAGGACTACCCGCTGAACTTAA
>AC8-52
TTTCCGTAGGTGAACCTGCGGAAGGATCATTATTGAATTATGTTTCTAGATAGGTTGTAG
CTGGCTCTTTTAGAGCATGTGCACGCCTGTTTGGACTTCATTTTCATCCACCTGTGCACC
TATTGTAGTCTTTGGTTGGGTTAGGAGGAAGTGGTCATTGTGTCAGCATCTGCTGGATGT
GAGGACTTGCATTGTGAAAGCTTTGCTGTCCTTGATGTGATCATGGAATCTCTTTC----

-------TCACTAGAGTCTATGTCACTCATTATACTCTGTCGAATGTCATTGAATGTCTT
TACATGGGCTTATATGCCTATGAAAATTGTAATACAACTTTCAGCAACGGATCTCTTGGC
TCTCGCATCGATGAAGAACGCAGCGAAATGCGATAAGTAATGTGAATTGCAGAATTCAGT
GAATCATCGAATCTTTGAACGCATCTTGCGCTCCTTGGTATTCCGAGGAGCATGCCTGTT
TGAGTGTCATTAAATTCTCAACTCTCTTCTAC-TTTTTGTAAAAGAGAGCTTGGACTGTG
GAGGCTTGCTGGCCACTTTTTGGGGTCAGCTCCTCTGAAATGCATTAGCGGAACCGTTTG
CGATCTGCCACAAGTGTGATAAGTTATCTACACTGGCGAGGGGATTGCTCTCTGTAATGT
TCAGCTTCTAATTGTCTCTACTTTGTGAGACTACTTTTGAATGCTTGACCTCAAATCAGG
TAGGACTACCCGCTGAACTTAA
>AC9-29
TTTCCGTAGGTGAACCTGCGGAAGGATCATTATTGAATTATGTTTCTAGATAGGTTGTAG

CTGGCTCTTTTAGAGCATGTGCACGCCTGTTTGGACTTCATTTTCATCCACCTGTGCACC
TATTGTAGTCTTTGGTTGGGTTAGGAGGAAGTGGTCATTGTGTCAGCATCTGCTGGATGT
GAGGACTTGCATTGTGAAAGCTTTGCTGTCCTTGATGTGATCATGGAATCTCTTTC----

-------TCACTAGAGTCTATGTCACTCATTATACTCTGTCGAATGTCATTGAATGTCTT
TACATGGGCTTATATGCCTATGAAAATTGTAATACAACTTTCAGCAACGGATCTCTTGGC
TCTCGCATCGATGAAGAACGCAGCGAAATGCGATAAGTAATGTGAATTGCAGAATTCAGT
GAATCATCGAATCTTTGAACGCATCTTGCGCTCCTTGGTATTCCGAGGAGCATGCCTGTT
TGAGTGTCATTAAATTCTCAACTCTCTTCTAC-TTTTTGTAAAAGAGAGCTTGGACTGTG
GAGGCTTGCTGGCCACTTTTTGGGGTCAGCTCCTCTGAAATGCATTAGCGGAACCGTTTG
CGATCTGCCACAAGTGTGATAAGTTATCTACACTGGCGAGGGGATTGCTCTCTGTAATGT
TCAGCTTCTAATTGTCTCTACTTTGTGAGACTACTTTTGAATGCTTGACCTCAAATCAGG
TAGGACTACCCGCTGAACTTAA
>AC10-2
TTTCCGTAGGTGAACCTGCGGAAGGATCATTATTGAATTATGTTTCTAGATAGGTTGTAG
CTGGCTCTTTTAGAGCATGTGCACGCCTGTTTGGACTTCATTTTCATCCACCTGTGCACC
TATTGTAGTCTTTGGTTGGGTTAGGAGGAAGTGGTCATTGTGTCAGCATCTGCTGGATGT
GAGGACTTGCATTGTGAAAGCTTTGCTGTCCTTGATGTGATCATGGAATCTCTTTC----

-------TCACTAGAGTCTATGTCACTCATTATACTCTGTCGAATGTCATTGAATGTCTT
TACATGGGCTTATATGCCTATGAAAATTGTAATACAACTTTCAGCAACGGATCTCTTGGC
TCTCGCATCGATGAAGAACGCAGCGAAATGCGATAAGTAATGTGAATTGCAGAATTCAGT
GAATCATCGAATCTTTGAACGCATCTTGCGCTCCTTGGTATTCCGAGGAGCATGCCTGTT
TGAGTGTCATTAAATTCTCAACTCTCTTCTAC-TTTTTGTAAAAGAGAGCTTGGACTGTG
GAGGCTTGCTGGCCACTTTTTGGGGTCAGCTCCTCTGAAATGCATTAGCGGAACCGTTTG
CGATCTGCCACAAGTGTGATAAGTTATCTACACTGGCGAGGGGATTGCTCTCTGTAATGT
TCAGCTTCTAATTGTCTCTACTTTGTGAGACTACTTTTGAATGCTTGACCTCAAATCAGG
TAGGACTACCCGCTGAACTTAA

```
>AC10-9
TTTCCGTAGGTGAACCTGCGGAAGGATCATTATTGAATTATGTTTCTAGATAGGTTGTAG
CTGGCTCTTTTAGAGCATGTGCACGCCTGTTTGGACTTCATTTTCATCCACCTGTGCACC
TATTGTAGTCTTTGGTTGGGTTAGGAGGAAGTGGTCATTGTGTCAGCATCTGCTGGATGT
GAGGACTTGCATTGTGAAAGCTTTGCTGTCCTTGATGTGATCATGGAATCTCTTTC----
------------------------------------------------------------
------------------------------------------------------------
------------------------------------------------------------
------------------------------------------------------------
------------------------------------------------------------
------------------------------------------------------------
------------------------------------------------------------
------------------------------------------------------------
------------------------------------------------------------
------------------------------------------------------------
------------------------------------------------------------
------------------------------------------------------------
-------TCACTAGAGTCTATGTCACTCATTATACTCTGTCGAATGTCATTGAATGTCTT
TACATGGGCTTATATGCCTATGAAAATTGTAATACAACTTTCAGCAACGGATCTCTTGGC
TCTCGCATCGATGAAGAACGCAGCGAAATGCGATAAGTAATGTGAATTGCAGAATTCAGT
GAATCATCGAATCTTTGAACGCATCTTGCGCTCCTTGGTATTCCGAGGAGCATGCCTGTT
TGAGTGTCATTAAATTCTCAACTCTCTTCTAC-TTTTTGTAAAAGAGAGCTTGGACTGTG
GAGGCTTGCTGGCCACTTTTTGGGGTCAGCTCCTCTGAAATGCATTAGCGGAACCGTTTG
CGATCTGCCACAAGTGTGATAAGTTATCTACACTGGCGAGGGGATTGCTCTCTGTAATGT
TCAGCTTCTAATTGTCTCTACTTTGTGAGACTACTTTTGAATGCTTGACCTCAAATCAGG
TAGGACTACCCGCTGAACTTAA
>AC12-7
TTTCCGTAGGTGAACCTGCGGAAGGATCATTATTGAATTATGTTTCTAGATAGGTTGTAG
CTGGCTCTTTTAGAGCATGTGCACGCCTGTTTGGACTTCATTTTCATCCACCTGTGCACC
TATTGTAGTCTTTGGTTGGGTTAGGAGGAAGTGGTCATTGTGTCAGCATCTGCTGGATGT
GAGGACTTGCATTGTGAAAGCTTTGCTGTCCTTGATGTGATCATGGAATCTCTTTC----
------------------------------------------------------------
------------------------------------------------------------
------------------------------------------------------------
------------------------------------------------------------
------------------------------------------------------------
------------------------------------------------------------
------------------------------------------------------------
------------------------------------------------------------
------------------------------------------------------------
------------------------------------------------------------
------------------------------------------------------------
------------------------------------------------------------
-------TCACTAGAGTCTATGTCACTCATTATACTCTGTCGAATGTCATTGAATGTCTT
TACATGGGCTTATATGCCTATGAAAATTGTAATACAACTTTCAGCAACGGATCTCTTGGC
TCTCGCATCGATGAAGAACGCAGCGAAATGCGATAAGTAATGTGAATTGCAGAATTCAGT
GAATCATCGAATCTTTGAACGCATCTTGCGCTCCTTGGTATTCCGAGGAGCATGCCTGTT
TGAGTGTCATTAAATTCTCAACTCTCTTCTAC-TTTTTGTAAAAGAGAGCTTGGACTGTG
GAGGCTTGCTGGCCACTTTTTGGGGTCAGCTCCTCTGAAATGCATTAGCGGAACCGTTTG
CGATCTGCCACAAGTGTGATAAGTTATCTACACTGGCGAGGGGATTGCTCTCTGTAATGT
```

TCAGCTTCTAATTGTCTCTACTTTGTGAGACTACTTTTGAATGCTTGACCTCAAATCAGG
TAGGACTACCCGCTGAACTTAA
>AC9-26
TTTCCGTAGGTGAACCTGCGGAAGGATCATTATTGAATTATGTTTCTAGATAGGTTGTAG
CTGGCTCTTTTAGAGCATGTGCACGCCTGTTTGGACTTCATTTTCATCCACCTGTGCACC
TATTGTAGTCTTTGGTTGGGTTAGGAGGAAGTGGTCATTGTGTCAGCATCTGCTGGATGT
GAGGACTTGCATTGTGAAAGCTTTGCTGTCCTTGATGTGATCATGGAATCTCTTTC----

-------TCACTAGAGTCTATGTCACTCATTATACTCTGTCGAATGTCATTGAATGTCTT
TACATGGGCTTATATGCCTATGAAAATTGTAATACAACTTTCAGCAACGGATCTCTTGGC
TCTCGCATCGATGAAGAACGCAGCGAAATGCGATAAGTAATGTGAATTGCAGAATTCAGT
GAATCATCGAATCTTTGAACGCATCTTGCGCTCCTTGGTATTCCGAGGAGCATGCCTGTT
TGAGTGTCATTAAATTCTCAACTCTCTTCTAC-TTTTTGTAAAAGAGAGCTTGGACTGTG
GAGGCTTGCTGGCCACTTTTTGGGGTCAGCTCCTCTGAAATGCATTAGCGGAACCGTTTG
CGATCTGCCACAAGTGTGATAAGTTATCTACACTGGCGAGGGGATTGCTCTCTGTAATGT
TCAGCTTCTAATTGTCTCTACTTTGTGAGACTACTTTTGAATGCTTGACCTCAAATCAGG
TAGGACTACCCGCTGAACTTAA
>AC8-26
TTTCCGTAGGTGAACCTGCGGAAGGATCATTATTGAATTATGTTTCTAGATAGGTTGTAG
CTGGCTCTTTTAGAGCATGTGCACGCCTGTTTGGACTTCATTTTCATCCACCTGTGCACC
TATTGTAGTCTTTGGTTGGGTTAGGAGGAAGTGATCATTGTGTCAGCATCTGCTGGATGT
GAGGACTTGCATTGTGAAAGCTTTGCTGTCCTTGATGTGATCATGGAATCTCTTTC----

-------TCACTAGAGTCTATGTCACTCATTATACTCTGTCGAATGTCATTGAATGTCTT
TACATGGGCTTATATGCCTATGAAAATTGTAATACAACTTTCAGCAACGGATCTCTTGGC
TCTCGCATCGATGAAGAACGCAGCGAAATGCGATAAGTAATGTGAATTGCAGAATTCAGT
GAATCATCGAATCTTTGAACGCATCTTGCGCTCCTTGGTATTCCGAGGAGCATGCCTGTT
TGAGTGTCATTAAATTCTCAACTCTCTTCTAC-TTTTTGTAAAAGAGAGCTTGGACTGTG

```
GAGGCTTGCTGGCCACTTTTTGGGGTCAGCTCCTCTGAAATGCATTAGCGGAACCGTTTG
CGATCTGCCACAAGTGTGATAAGTTATCTACACTGGCGAGGGGATTGCTCTCTGTAATGT
TCAGCTTCTAATTGTCTCTACTTTGTGAGACTACTTTTGAATGCTTGACCTCAAATCAGG
TAGGACTACCCGCTGAACTTAA
>AC10-11
TTTCCGTAGGTGAACCTGCGGAAGGATCATTATTGAATTATGTTTCTAGATAGGTTGTAG
CTGGCTCTTTTAGAGCATGTGCACGCCTGTTTGGACTTCATTTTCATCCACCTGTGCACC
TATTGTAGTCTTTGGTTGGGTTAGGAGGAAGTGATCATTGTATCAGCATCTGCTGGATGT
GAGGACTTGCATTGTGAAAGCTTTGCTGTCCTTGATGTGATCATGGAATCTCTTTC----
------------------------------------------------------------
------------------------------------------------------------
------------------------------------------------------------
------------------------------------------------------------
------------------------------------------------------------
------------------------------------------------------------
------------------------------------------------------------
------------------------------------------------------------
------------------------------------------------------------
------------------------------------------------------------
------------------------------------------------------------
------------------------------------------------------------
-------TCACTAGAGTCTATGTCACTCATTATACTCTGTCGAATGTCATTGAATGTCTT
TACATGGGCTTATATGCCTATGAAAATTGTAATACAACTTTCAGCAACGGATCTCTTGGC
TCTCGCATCGATGAAGAACGCAGCGAAATGCGATAAGTAATGTGAATTGCAGAATTCAGT
GAATCATCGAATCTTTGAACGCATCTTGCGCTCCTTGGTATTCCGAGGAGCATGCCTGTT
TGAGTGTCATTAAATTCTCAACTCTCTTCTAC-TTTTTGTAAAAGAGAGCTTGGACTGTG
GAGGCTTGCTGGCCACTTTTTGGGGTCAGCTCCTCTGAAATGCATTAGCGGAACCGTTTG
CGATCTGCCACAAGTGTGATAAGTTATCTACACTGGCGAGGGGATTGCTCTCTGTAATGT
TCAGCTTCTAATTGTCTCTACTTTGTGAGACTACTTTTGAATGCTTGACCTCAAATCAGG
TAGGACTACCCGCTGAACTTAA
>AC9-54
TTTCCGTAGGTGAACCTGCGGAAGGATCATTATTGAATTATGTTTCTAGATAGGTTGTAG
CTGGCTCTTTTAGAGCATGTGCACGCCTGTTTGGACTTCATTTTCATCCACCTGTGCACC
TATTGTAGTCTTTGGTTGGGTTAGGAGGAAGTGGTCATTGTGTCAGCATCTGCTGGATGT
GAGGACTTGCATTGTGAAAGCTTTGCTGTCCTTGATGTGATCATGGAATCTCTTTC----
------------------------------------------------------------
------------------------------------------------------------
------------------------------------------------------------
------------------------------------------------------------
------------------------------------------------------------
------------------------------------------------------------
------------------------------------------------------------
------------------------------------------------------------
------------------------------------------------------------
------------------------------------------------------------
------------------------------------------------------------
------------------------------------------------------------
-------TCACTAGAGTCTATGTCACTCATTATACTCTGTCGAATGTCATTGAATGTCTT
TACATGGGCTTGTATGCCTATGAAAATTGTAATACAACTTTCAGCAACGGATCTCTTGGC
TCTCGCATCGATGAAGAACGCAGCGAAATGCGATAAGTAATGTGAATTGCAGAATTCAGT
```

GAATCATCGAATCTTTGAACGCATCTTGCGCTCCTTGGTATTCCGAGGAGCATGCCTGTT
TGAGTGTCATTAAATTCTCAACTCTCTTCTAC-TTTTTGTAAAAGAGAGCTTGGACTGTG
GAGGCTTGCTGGCCACTTTTTGGGGTCAGCTCCTCTGAAATGCATTAGCGGAACCGTTTG
CGATCTGCCACAAGTGTGATAAGTTATCTACACTGGCGAGGGGATTGCTCTCTGTAATGT
TCAGCTTCTAATTGTCTCTACTTTGTGAGACTACTTTTGAATGCTTGACCTCAAATCAGG
TAGGACTACCCGCTGAACTTAA
>AC11-51
TTTCCGTAGGTGAACCTGCGGAAGGATCATTATTGAATTATGTTTCTAGATAGGTTGTAG
CTGGCTCTTTTAGAGCATGTGCACGCCTGTTTGGACTTCATTTTCATCCACCTGTGCACC
TATTGTAGTCTTTGGTTGGGTTAGGAGGAAGTGGTCATTGTGTCAGCATCTGCTGGATGT
GAGGACTTGCATTGTGAAAGCTTTGCTGTCCTTGATGTGATCATGGAATCTCTTTC----

-------TCACTAGAGTCTATGTCACTCATTATACTCTGTCGAATGTCATTGAATGTCTT
TACATGGGCTTGTATGCCTATGAAAATTGTAATACAACTTTCAGCAACGGATCTCTTGGC
TCTCGCATCGATGAAGAACGCAGCGAAATGCGATAAGTAATGTGAATTGCAGAATTCAGT
GAATCATCGAATCTTTGAACGCATCTTGCGCTCCTTGGTATTCCGAGGAGCATGCCTGTT
TGAGTGTCATTAAATTCTCAACTCTCTTCTAC-TTTTTGTAAAAGAGAGCTTGGACTGTG
GAGGCTTGCTGGCCACTTTTTGGGGTCAGCTCCTCTGAAATGCATTAGCGGAACCGTTTG
CGATCTGCCACAAGTGTGATAAGTTATCTACACTGGCGAGGGGATTGCTCTCTGTAATGT
TCAGCTTCTAATTGTCTCTACTTTGTGAGACTACTTTTGAATGCTTGACCTCAAATCAGG
TAGGACTACCCGCTGAACTTAA
>AC1-49
TTTCCGTAGGTGAACCTGCGGAAGGATCATTATTGAATTATGTTTCTAGATAGGTTGTAG
CTGGCTCtTTTAGAGCATGTGCACGCCTGTTTGGACTTCATTTTCATCCACCTGTGCACC
TATTGTAGTCTTTGGTTGGGTTAGGAGGAAGTGGTCATTGTGTCAGCATCTGCTGGATGT
GAGGACTTGCATTGTGAAAGCTTTGCTGTCCTTGATGTGATCATGGAATCTCTTTC----

-------TCACTAGAGTCTATGTCACTCATTATACTCTGTCGAATGTCATTGAATGTCTT

TACATGGGCTTGTATGCCTATGAAAATTGTAATACAACTTTCAGCAACGGATCTCTTGGC
TCTCGCATCGATGAAGAACGCAGCGAAATGCGATAAGTAATGTGAATTGCAGAATTCAGT
GAATCATCGAATCTTTGAACGCATCTTGCGCTCCTTGGTATTCCGAGGAGCATGCCTGTT
TGAGTGTCATTAAATTCTCAACTCTCTTCTAC-TTTTTGTAAAAGAGAGCTTGGACTGTG
GAGGCTTGCTGGCCACTTTTTGGGGTCAGCTCCTCTGAAATGCATTAGCGGAACCGTTTG
CGATCTGCCACAAGTGTGATAAGTTATCTACACTGGCGAGGGGATTGCTCTCTGTAATGT
TCAGCTTCTAATTGTCTCTACTTTGTGAGACTACTTTTGAATGCTTGACCTCAAATCAGG
TAGGACTACCCGCTGAACTTAA
>AC5-84
TTTCCGTAGGTGAACCTGCGGAAGGATCATTATTGAATTATGTTTCTAGATAGGTTGTAG
CTGGCTCTTTTAGAGCATGTGCACGCCTGTTTGGACTTCATTTTCATCCACCTGTGCACC
TATTGTAGTCTTTGGTTGGGTTAGGAGGAAGTGATCATTGTGTCAGCATCTGCTGGATGT
GAGGACTTGCATTGTGAAAGCTTTGCTGTCCTTGATGTGATCATGGAATCTTTTTC----

-------TCACTAGAGTCTATGTCACTCATTATACTCTGTCGAATGTCATTGAATGTCTT
TACATGGGCTTGTATGCCTATGAAAATTGTAATACAACTTTCAGCAACGGATCTCTTGGC
TCTCGCATCGATGAAGAACGCAGCGAAATGCGATAAGTAATGTGAATTGCAGAATTCAGT
GAATCATCGAATCTTTGAACGCATCTTGCGCTCCTTGGTATTCCGAGGAGCATGCCTGTT
TGAGTGTCATTAAATTCTCAACTCTCTTCTAC-TTTTTGTAAAAGAGAGCTTGGACTGTG
GAGGCTTGCTGGCCACTTTTTGGGGTCAGCTCCTCTGAAATGCATTAGCGGAACCGTTTG
CGATCTGCCACAAGTGTGATAAGTTATCTACACTGGCGAGGGGATTGCTCTCTGTAATGT
TCAGCTTCTAATTGTCTCTACTTTGTGAGACTACTTTTGAATGCTTGACCTCAAATCAGG
TAGGACTACCCGCTGAACTTAA
>AC8-10
TTTCCGTAGGTGAACCTGCGGAAGGATCATTATTGAATTATGTTTCTAGATAGGTTGTAG
CTGGCTCTTTTAGAGCATGTGCACGCCTGTTTGGACTTCATTTTCATCCACCTGTGCACC
TATTGTAGTCTTTGGTTGGGTTAGGAGGAAGTGATCATTGTATCAGCATCTGCTGGGAGT
GAGGACTTGCATTGTGAAAGCTTTGCTGTCCTTGATGTGATCATGGAATCTTTTTCTCAC
TAGAGTCTATGTCACTCATTATACTCTGTCGAATGTCATTGAATGTCTTTACATGGGCTT
GTATGCCTATGAAAATTGTAATACAACTTTCAGCAACGGATCTCTTGGCTCTCGCATCGA
TGAAGAACGCAGCGAAATGCGATAAGTAATGTGAATTGCAGAATTCAGTGAATCATCGAA
TCTTTGAACGCATCTTGCGCTCCTTGGTATTCCGAGGAGCATGCCTGTTTGAGTGTCATT
AAATTCTCAACTCTCTTATACTTTTTGTAAAAGAGAGCTTGGACTGTGGAGGCTTGCTG
GCCACTTTTTGGGGTCAGCTCCTCTGAAATGCATTAGCGGAACCGTTTGCGATCTGCCAC
AAGTGTGATAAGTTATCTACACTGGCGAGGGGATTGCTCTCTGTAATGTTCAGCTTCTAA
TTGTCTCTACTTTGTGAGACTACTTTTGAATGCTTGACCTCAAATCAGGTAGGACTACCC
GCTGAACTTAATTTCCGTAGGTGAACCTGCGGAAGGATCATTATTGAATTATGTTTCTAG
ATAGGTTGTAGCTGGCTCTTTTAGAGCATGTGCACGCCTGTTTGGACTTCATTTTCATCC
ACCTGTGCACCTATTGTAGTCTTTGGTTGGGTTAGGAGGAAGTGATCATTGTATCAGCAT

CTGCTGGGAGTGAGGACTTGCATTGTGAAAGCTTTGCTGTCCTTGATGTGATCATGGAAT
CTTTTTCTCACTAGAGTCTATGTCACTCATTATACTCTGTCGAATGTCATTGAATGTCTT
TACATGGGCTTGTATGCCTATGAAAATTGTAATACAACTTTCAGCAACGGATCTCTTGGC
TCTCGCATCGATGAAGAACGCAGCGAAATGCGATAAGTAATGTGAATTGCAGAATTCAGT
GAATCATCGAATCTTTGAACGCATCTTGCGCTCCTTGGTATTCCGAGGAGCATGCCTGTT
TGAGTGTCATTAAATTCTCAACTCTCTTATACTTTTTTGTAAAAGAGAGCTTGGACTGTG
GAGGCTTGCTGGCCACTTTTTGGGGTCAGCTCCTCTGAAATGCATTAGCGGAACCGTTTG
CGATCTGCCACAAGTGTGATAAGTTATCTACACTGGCGAGGGGATTGCTCTCTGTAATGT
TCAGCTTCTAATTGTCTCTACTTTGTGAGACTACTTTTGAATGCTTGACCTCAAATCAGG
TAGGACTACCCGCTGAACTTAA
