# Supplementary material for: Patterns of PCR Amplification Artifacts of the Fungal Barcode Marker in a Hybrid Mushroom
Source: Front Microbiol. 2019 Nov 19;10:2686. doi: 10.3389/fmicb.2019.02686 (PMC6877668; doi:10.3389/fmicb.2019.02686)
Supplement: Supplementary file 10 [file Data_Sheet_10.PDF]

>ABC1-15
TTTCCGTAGGTGAACCTGCGGAAGGATCATTATTGAATTATGTTTCTAGATAGGTTGTAG
CTGGCTCTTTTAGAGCATGTGCACGCCTGTTTGGACTTCATTTTCATCCACCTGTGCACC
TATTGTAGTCTTTGGTTGGGTTAGGAGGAAGTGATCATTGTATCAGCATCTGCTGGGAGT
GAGGACTTGCATTGTGAAAGCTTTGCTGTCCTTGATGTGATCATGGAATCTTTTTCTCAC
TAGAGTCTATGTCACTCATTATACTCTGTCGAATGTCATTGAATGTCTTTACATGGGCTT
GTATGCCTATGAAAATTGTAATACAACTTTCAGCAACGGATCTCTTGGCTCTCGCATCGA
TGAAGAACGCAGCGAAATGCGATAAGTAATGTGAATTGCAGAATTCAGTGAATCATCGAA
TCTTTGAACGCATCTTGCGCTCCTTGGTATTCCGAGGAGCATGCCTGTTTGAGTGTCATT
AAATTCTCAACTCTCTTATAC-TTTTTTGTAAAAGAGAGCTTGGACTGTGGAGGCTTGCT
GGCCACTTTTTGGGGTCAGCTCCTCTGAAATGCATTAGCGGAACCGTTTGCGATCTGCCA
CAAGTGTGATAAGTTATCTACACTGGCGAGGGGATTGCTCTCTGTAATGTTCAGCTTCTA
ATTGTCTCTACTTTGTGAGACTACTTTTGAATGCTTGACCTCAAATCAGGTAGGACTACC
CGCTGAACTTAA
>ABC2-3
TTTCCGTAGGTGAACCTGCGGAAGGATCATTATTGAATTATGTTTCTAGATAGGTTGTAG
CTGGCTCTTTTAGAGCATGTGCACGCCTGTTTGGACTTCATTTTCATCCACCTGTGCACC
TATTGTAGTCTTTGGTTGGGTTAGGAGGAAGTGATCATTGTATCAGCATCTGCTGGGAGT
GAGGACTTGCATTGTGAAAGCTTTGCTGTCCTTGATGTGATCATGGAATCTTTTTCTCAC
TAGAGTCTATGTCACTCATTATACTCTGTCGAATGTCATTGAATGTCTTTACATGGGCTT
GTATGCCTATGAAAATTGTAATACAACTTTCAGCAACGGATCTCTTGGCTCTCGCATCGA
TGAAGAACGCAGCGAAATGCGATAAGTAATGTGAATTGCAGAATTCAGTGAATCATCGAA
TCTTTGAACGCATCTTGCGCTCCTTGGTATTCCGAGGAGCATGCCTGTTTGAGTGTCATT
AAATTCTCAACTCTCTTATAC-TTTTTTGTAAAAGAGAGCTTGGACTGTGGAGGCTTGCT
GGCCACTTTTTGGGGTCAGCTCCTCTGAAATGCATTAGCGGAACCGTTTGCGATCTGCCA
CAAGTGTGATAAGTTATCTACACTGGCGAGGGGATTGCTCTCTGTAATGTTCAGCTTCTA
ATTGTCTCTACTTTGTGAGACTACTTTTGAATGCTTGACCTCAAATCAGGTAGGACTACC
CGCTGAACTTAA
>ABC2-8
TTTCCGTAGGTGAACCTGCGGAAGGATCATTATTGAATTATGTTTCTAGATAGGTTGTAG
CTGGCTCTTTTAGAGCATGTGCACGCCTGTTTGGACTTCATTTTCATCCACCTGTGCACC
TATTGTAGTCTTTGGTTGGGTTAGGAGGAAGTGATCATTGTATCAGCATCTGCTGGGAGT
GAGGACTTGCATTGTGAAAGCTTTGCTGTCCTTGATGTGATCATGGAATCTTTTTCTCAC
TAGAGTCTATGTCACTCATTATACTCTGTCGAATGTCATTGAATGTCTTTACATGGGCTT
GTATGCCTATGAAAATTGTAATACAACTTTCAGCAACGGATCTCTTGGCTCTCGCATCGA
TGAAGAACGCAGCGAAATGCGATAAGTAATGTGAATTGCAGAATTCAGTGAATCATCGAA
TCTTTGAACGCATCTTGCGCTCCTTGGTATTCCGAGGAGCATGCCTGTTTGAGTGTCATT
AAATTCTCAACTCTCTTATAC-TTTTTTGTAAAAGAGAGCTTGGACTGTGGAGGCTTGCT
GGCCACTTTTTGGGGTCAGCTCCTCTGAAATGCATTAGCGGAACCGTTTGCGATCTGCCA
CAAGTGTGATAAGTTATCTACACTGGCGAGGGGATTGCTCTCTGTAATGTTCAGCTTCTA
ATTGTCTCTACTTTGTGAGACTACTTTTGAATGCTTGACCTCAAATCAGGTAGGACTACC
CGCTGAACTTAA
>ABC2-25
TTTCCGTAGGTGAACCTGCGGAAGGATCATTATTGAATTATGTTTCTAGATAGGTTGTAG
CTGGCTCTTTTAGAGCATGTGCACGCCTGTTTGGACTTCATTTTCATCCACCTGTGCACC
TATTGTAGTCTTTGGTTGGGTTAGGAGGAAGTGATCATTGTATCAGCATCTGCTGGGAGT
GAGGACTTGCATTGTGAAAGCTTTGCTGTCCTTGATGTGATCATGGAATCTTTTTCTCAC
TAGAGTCTATGTCACTCATTATACTCTGTCGAATGTCATTGAATGTCTTTACATGGGCTT
GTATGCCTATGAAAATTGTAATACAACTTTCAGCAACGGATCTCTTGGCTCTCGCATCGA
TGAAGAACGCAGCGAAATGCGATAAGTAATGTGAATTGCAGAATTCAGTGAATCATCGAA

TCTTTGAACGCATCTTGCGCTCCTTGGTATTCCGAGGAGCATGCCTGTTTGAGTGTCATT
AAATTCTCAACTCTCTTATAC-TTTTTTGTAAAAGAGAGCTTGGACTGTGGAGGCTTGCT
GGCCACTTTTTGGGGTCAGCTCCTCTGAAATGCATTAGCGGAACCGTTTGCGATCTGCCA
CAAGTGTGATAAGTTATCTACACTGGCGAGGGGATTGCTCTCTGTAATGTTCAGCTTCTA
ATTGTCTCTACTTTGTGAGACTACTTTTGAATGCTTGACCTCAAATCAGGTAGGACTACC
CGCTGAACTTAA
>ABC2-76
TTTCCGTAGGTGAACCTGCGGAAGGATCATTATTGAATTATGTTTCTAGATAGGTTGTAG
CTGGCTCTTTTAGAGCATGTGCACGCCTGTTTGGACTTCATTTTCATCCACCTGTGCACC
TATTGTAGTCTTTGGTTGGGTTAGGAGGAAGTGATCATTGTATCAGCATCTGCTGGGAGT
GAGGACTTGCATTGTGAAAGCTTTGCTGTCCTTGATGTGATCATGGAATCTTTTTCTCAC
TAGAGTCTATGTCACTCATTATACTCTGTCGAATGTCATTGAATGTCTTTACATGGGCTT
GTATGCCTATGAAAATTGTAATACAACTTTCAGCAACGGATCTCTTGGCTCTCGCATCGA
TGAAGAACGCAGCGAAATGCGATAAGTAATGTGAATTGCAGAATTCAGTGAATCATCGAA
TCTTTGAACGCATCTTGCGCTCCTTGGTATTCCGAGGAGCATGCCTGTTTGAGTGTCATT
AAATTCTCAACTCTCTTATAC-TTTTTTGTAAAAGAGAGCTTGGACTGTGGAGGCTTGCT
GGCCACTTTTTGGGGTCAGCTCCTCTGAAATGCATTAGCGGAACCGTTTGCGATCTGCCA
CAAGTGTGATAAGTTATCTACACTGGCGAGGGGATTGCTCTCTGTAATGTTCAGCTTCTA
ATTGTCTCTACTTTGTGAGACTACTTTTGAATGCTTGACCTCAAATCAGGTAGGACTACC
CGCTGAACTTAA
>ABC5-37
TTTCCGTAGGTGAACCTGCGGAAGGATCATTATTGAATTATGTTTCTAGATAGGTTGTAG
CTGGCTCTTTTAGAGCATGTGCACGCCTGTTTGGACTTCATTTTCATCCACCTGTGCACC
TATTGTAGTCTTTGGTTGGGTTAGGAGGAAGTGATCATTGTATCAGCATCTGCTGGGAGT
GAGGACTTGCATTGTGAAAGCTTTGCTGTCCTTGATGTGATCATGGAATCTTTTTCTCAC
TAGAGTCTATGTCACTCATTATACTCTGTCGAATGTCATTGAATGTCTTTACATGGGCTT
GTATGCCTATGAAAATTGTAATACAACTTTCAGCAACGGATCTCTTGGCTCTCGCATCGA
TGAAGAACGCAGCGAAATGCGATAAGTAATGTGAATTGCAGAATTCAGTGAATCATCGAA
TCTTTGAACGCATCTTGCGCTCCTTGGTATTCCGAGGAGCATGCCTGTTTGAGTGTCATT
AAATTCTCAACTCTCTTATAC-TTTTTTGTAAAAGAGAGCTTGGACTGTGGAGGCTTGCT
GGCCACTTTTTGGGGTCAGCTCCTCTGAAATGCATTAGCGGAACCGTTTGCGATCTGCCA
CAAGTGTGATAAGTTATCTACACTGGCGAGGGGATTGCTCTCTGTAATGTTCAGCTTCTA
ATTGTCTCTACTTTGTGAGACTACTTTTGAATGCTTGACCTCAAATCAGGTAGGACTACC
CGCTGAACTTAA
>ABC7-44
TTTCCGTAGGTGAACCTGCGGAAGGATCATTATTGAATTATGTTTCTAGATAGGTTGTAG
CTGGCTCTTTTAGAGCATGTGCACGCCTGTTTGGACTTCATTTTCATCCACCTGTGCACC
TATTGTAGTCTTTGGTTGGGTTAGGAGGAAGTGATCATTGTATCAGCATCTGCTGGGAGT
GAGGACTTGCATTGTGAAAGCTTTGCTGTCCTTGATGTGATCATGGAATCTTTTTCTCAC
TAGAGTCTATGTCACTCATTATACTCTGTCGAATGTCATTGAATGTCTTTACATGGGCTT
GTATGCCTATGAAAATTGTAATACAACTTTCAGCAACGGATCTCTTGGCTCTCGCATCGA
TGAAGAACGCAGCGAAATGCGATAAGTAATGTGAATTGCAGAATTCAGTGAATCATCGAA
TCTTTGAACGCATCTTGCGCTCCTTGGTATTCCGAGGAGCATGCCTGTTTGAGTGTCATT
AAATTCTCAACTCTCTTATAC-TTTTTTGTAAAAGAGAGCTTGGACTGTGGAGGCTTGCT
GGCCACTTTTTGGGGTCAGCTCCTCTGAAATGCATTAGCGGAACCGTTTGCGATCTGCCA
CAAGTGTGATAAGTTATCTACACTGGCGAGGGGATTGCTCTCTGTAATGTTCAGCTTCTA
ATTGTCTCTACTTTGTGAGACTACTTTTGAATGCTTGACCTCAAATCAGGTAGGACTACC
CGCTGAACTTAA
>ABC1-16
TTTCCGTAGGTGAACCTGCGGAAGGATCATTATTGAATTATGTTTCTAGATAGGTTGTAG

CTGGCTCTTTTAGAGCATGTGCACGCCTGTTTGGACTTCATTTTCATCCACCTGTGCACC
TATTGTAGTCTTTGGTTGGGTTAGGAGGAAGTGATCATTGTATCAGCATCTGCTGGGAGT
GAGGACTTGCATTGTGAAAGCTTTGCTGTCCTTGATGTGATCATGGAATCTTTTTCTCAC
TAGAGTCTATGTCACTCATTATACTCTGTCGAATGTCATTGAATGTCTTTACATGGGCTT
GTATGCCTATGAAAATTGTAATACAACTTTCAGCAACGGATCTCTTGGCTCTCGCATCGA
TGAAGAACGCAGCGAAATGCGATAAGTAATGTGAATTGCAGAATTCAGTGAATCATCGAA
TCTTTGAACGCATCTTGCGCTCCTTGGTATTCCGAGGAGCATGCCTGTTTGAGTGTCATT
AAATTCTCAACTCTCTTATAC-TTTTTTGTAAAAGAGAGCTTGGACTGTGGAGGCTTGCT
GGCCACTTTTTGGGGTCAGCTCCTCTGAAATGCATTAGCGGAACCGTTTGCGATCTGCCA
CAAGTGTGATAAGTTATCTACACTGGCGAGGGGATTGCTCTCTGTAATGTTCAGCTTCTA
ATTGTCTCTACTTTGTGAGACTACTTTTGAATGCTTGACCTCAAATCAGGTAGGACTACC
CGCTGAACTTAA
>ABC1-22
TTTCCGTAGGTGAACCTGCGGAAGGATCATTATTGAATTATGTTTCTAGATAGGTTGTAG
CTGGCTCTTTTAGAGCATGTGCACGCCTGTTTGGACTTCATTTTCATCCACCTGTGCACC
TATTGTAGTCTTTGGTTGGGTTAGGAGGAAGTGATCATTGTATCAGCATCTGCTGGGAGT
GAGGACTTGCATTGTGAAAGCTTTGCTGTCCTTGATGTGATCATGGAATCTTTTTCTCAC
TAGAGTCTATGTCACTCATTATACTCTGTCGAATGTCATTGAATGTCTTTACATGGGCTT
GTATGCCTATGAAAATTGTAATACAACTTTCAGCAACGGATCTCTTGGCTCTCGCATCGA
TGAAGAACGCAGCGAAATGCGATAAGTAATGTGAATTGCAGAATTCAGTGAATCATCGAA
TCTTTGAACGCATCTTGCGCTCCTTGGTATTCCGAGGAGCATGCCTGTTTGAGTGTCATT
AAATTCTCAACTCTCTTATAC-TTTTTTGTAAAAGAGAGCTTGGACTGTGGAGGCTTGCT
GGCCACTTTTTGGGGTCAGCTCCTCTGAAATGCATTAGCGGAACCGTTTGCGATCTGCCA
CAAGTGTGATAAGTTATCTACACTGGCGAGGGGATTGCTCTCTGTAATGTTCAGCTTCTA
ATTGTCTCTACTTTGTGAGACTACTTTTGAATGCTTGACCTCAAATCAGGTAGGACTACC
CGCTGAACTTAA
>ABC1-24
TTTCCGTAGGTGAACCTGCGGAAGGATCATTATTGAATTATGTTTCTAGATAGGTTGTAG
CTGGCTCTTTTAGAGCATGTGCACGCCTGTTTGGACTTCATTTTCATCCACCTGTGCACC
TATTGTAGTCTTTGGTTGGGTTAGGAGGAAGTGATCATTGTATCAGCATCTGCTGGGAGT
GAGGACTTGCATTGTGAAAGCTTTGCTGTCCTTGATGTGATCATGGAATCTTTTTCTCAC
TAGAGTCTATGTCACTCATTATACTCTGTCGAATGTCATTGAATGTCTTTACATGGGCTT
GTATGCCTATGAAAATTGTAATACAACTTTCAGCAACGGATCTCTTGGCTCTCGCATCGA
TGAAGAACGCAGCGAAATGCGATAAGTAATGTGAATTGCAGAATTCAGTGAATCATCGAA
TCTTTGAACGCATCTTGCGCTCCTTGGTATTCCGAGGAGCATGCCTGTTTGAGTGTCATT
AAATTCTCAACTCTCTTATAC-TTTTTTGTAAAAGAGAGCTTGGACTGTGGAGGCTTGCT
GGCCACTTTTTGGGGTCAGCTCCTCTGAAATGCATTAGCGGAACCGTTTGCGATCTGCCA
CAAGTGTGATAAGTTATCTACACTGGCGAGGGGATTGCTCTCTGTAATGTTCAGCTTCTA
ATTGTCTCTACTTTGTGAGACTACTTTTGAATGCTTGACCTCAAATCAGGTAGGACTACC
CGCTGAACTTAA
>ABC1-25
TTTCCGTAGGTGAACCTGCGGAAGGATCATTATTGAATTATGTTTCTAGATAGGTTGTAG
CTGGCTCTTTTAGAGCATGTGCACGCCTGTTTGGACTTCATTTTCATCCACCTGTGCACC
TATTGTAGTCTTTGGTTGGGTTAGGAGGAAGTGATCATTGTATCAGCATCTGCTGGGAGT
GAGGACTTGCATTGTGAAAGCTTTGCTGTCCTTGATGTGATCATGGAATCTTTTTCTCAC
TAGAGTCTATGTCACTCATTATACTCTGTCGAATGTCATTGAATGTCTTTACATGGGCTT
GTATGCCTATGAAAATTGTAATACAACTTTCAGCAACGGATCTCTTGGCTCTCGCATCGA
TGAAGAACGCAGCGAAATGCGATAAGTAATGTGAATTGCAGAATTCAGTGAATCATCGAA
TCTTTGAACGCATCTTGCGCTCCTTGGTATTCCGAGGAGCATGCCTGTTTGAGTGTCATT
AAATTCTCAACTCTCTTATAC-TTTTTTGTAAAAGAGAGCTTGGACTGTGGAGGCTTGCT

GGCCACTTTTTGGGGTCAGCTCCTCTGAAATGCATTAGCGGAACCGTTTGCGATCTGCCA
CAAGTGTGATAAGTTATCTACACTGGCGAGGGGATTGCTCTCTGTAATGTTCAGCTTCTA
ATTGTCTCTACTTTGTGAGACTACTTTTGAATGCTTGACCTCAAATCAGGTAGGACTACC
CGCTGAACTTAA
>ABC1–27
TTTCCGTAGGTGAACCTGCGGAAGGATCATTATTGAATTATGTTTCTAGATAGGTTGTAG
CTGGCTCTTTTAGAGCATGTGCACGCCTGTTTGGACTTCATTTTCATCCACCTGTGCACC
TATTGTAGTCTTTGGTTGGGTTAGGAGGAAGTGATCATTGTATCAGCATCTGCTGGGAGT
GAGGACTTGCATTGTGAAAGCTTTGCTGTCCTTGATGTGATCATGGAATCTTTTTCTCAC
TAGAGTCTATGTCACTCATTATACTCTGTCGAATGTCATTGAATGTCTTTACATGGGCTT
GTATGCCTATGAAAATTGTAATACAACTTTCAGCAACGGATCTCTTGGCTCTCGCATCGA
TGAAGAACGCAGCGAAATGCGATAAGTAATGTGAATTGCAGAATTCAGTGAATCATCGAA
TCTTTGAACGCATCTTGCGCTCCTTGGTATTCCGAGGAGCATGCCTGTTTGAGTGTCATT
AAATTCTCAACTCTCTTATAC–TTTTTTGTAAAAGAGAGCTTGGACTGTGGAGGCTTGCT
GGCCACTTTTTGGGGTCAGCTCCTCTGAAATGCATTAGCGGAACCGTTTGCGATCTGCCA
CAAGTGTGATAAGTTATCTACACTGGCGAGGGGATTGCTCTCTGTAATGTTCAGCTTCTA
ATTGTCTCTACTTTGTGAGACTACTTTTGAATGCTTGACCTCAAATCAGGTAGGACTACC
CGCTGAACTTAA
>ABC1–30
TTTCCGTAGGTGAACCTGCGGAAGGATCATTATTGAATTATGTTTCTAGATAGGTTGTAG
CTGGCTCTTTTAGAGCATGTGCACGCCTGTTTGGACTTCATTTTCATCCACCTGTGCACC
TATTGTAGTCTTTGGTTGGGTTAGGAGGAAGTGATCATTGTATCAGCATCTGCTGGGAGT
GAGGACTTGCATTGTGAAAGCTTTGCTGTCCTTGATGTGATCATGGAATCTTTTTCTCAC
TAGAGTCTATGTCACTCATTATACTCTGTCGAATGTCATTGAATGTCTTTACATGGGCTT
GTATGCCTATGAAAATTGTAATACAACTTTCAGCAACGGATCTCTTGGCTCTCGCATCGA
TGAAGAACGCAGCGAAATGCGATAAGTAATGTGAATTGCAGAATTCAGTGAATCATCGAA
TCTTTGAACGCATCTTGCGCTCCTTGGTATTCCGAGGAGCATGCCTGTTTGAGTGTCATT
AAATTCTCAACTCTCTTATAC–TTTTTTGTAAAAGAGAGCTTGGACTGTGGAGGCTTGCT
GGCCACTTTTTGGGGTCAGCTCCTCTGAAATGCATTAGCGGAACCGTTTGCGATCTGCCA
CAAGTGTGATAAGTTATCTACACTGGCGAGGGGATTGCTCTCTGTAATGTTCAGCTTCTA
ATTGTCTCTACTTTGTGAGACTACTTTTGAATGCTTGACCTCAAATCAGGTAGGACTACC
CGCTGAACTTAA
>ABC1–33
TTTCCGTAGGTGAACCTGCGGAAGGATCATTATTGAATTATGTTTCTAGATAGGTTGTAG
CTGGCTCTTTTAGAGCATGTGCACGCCTGTTTGGACTTCATTTTCATCCACCTGTGCACC
TATTGTAGTCTTTGGTTGGGTTAGGAGGAAGTGATCATTGTATCAGCATCTGCTGGGAGT
GAGGACTTGCATTGTGAAAGCTTTGCTGTCCTTGATGTGATCATGGAATCTTTTTCTCAC
TAGAGTCTATGTCACTCATTATACTCTGTCGAATGTCATTGAATGTCTTTACATGGGCTT
GTATGCCTATGAAAATTGTAATACAACTTTCAGCAACGGATCTCTTGGCTCTCGCATCGA
TGAAGAACGCAGCGAAATGCGATAAGTAATGTGAATTGCAGAATTCAGTGAATCATCGAA
TCTTTGAACGCATCTTGCGCTCCTTGGTATTCCGAGGAGCATGCCTGTTTGAGTGTCATT
AAATTCTCAACTCTCTTATAC–TTTTTTGTAAAAGAGAGCTTGGACTGTGGAGGCTTGCT
GGCCACTTTTTGGGGTCAGCTCCTCTGAAATGCATTAGCGGAACCGTTTGCGATCTGCCA
CAAGTGTGATAAGTTATCTACACTGGCGAGGGGATTGCTCTCTGTAATGTTCAGCTTCTA
ATTGTCTCTACTTTGTGAGACTACTTTTGAATGCTTGACCTCAAATCAGGTAGGACTACC
CGCTGAACTTAA
>ABC1–36
TTTCCGTAGGTGAACCTGCGGAAGGATCATTATTGAATTATGTTTCTAGATAGGTTGTAG
CTGGCTCTTTTAGAGCATGTGCACGCCTGTTTGGACTTCATTTTCATCCACCTGTGCACC
TATTGTAGTCTTTGGTTGGGTTAGGAGGAAGTGATCATTGTATCAGCATCTGCTGGGAGT

GAGGACTTGCATTGTGAAAGCTTTGCTGTCCTTGATGTGATCATGGAATCTTTTTCTCAC
TAGAGTCTATGTCACTCATTATACTCTGTCGAATGTCATTGAATGTCTTTACATGGGCTT
GTATGCCTATGAAAATTGTAATACAACTTTCAGCAACGGATCTCTTGGCTCTCGCATCGA
TGAAGAACGCAGCGAAATGCGATAAGTAATGTGAATTGCAGAATTCAGTGAATCATCGAA
TCTTTGAACGCATCTTGCGCTCCTTGGTATTCCGAGGAGCATGCCTGTTTGAGTGTCATT
AAATTCTCAACTCTCTTATAC-TTTTTTGTAAAAGAGAGCTTGGACTGTGGAGGCTTGCT
GGCCACTTTTTGGGGTCAGCTCCTCTGAAATGCATTAGCGGAACCGTTTGCGATCTGCCA
CAAGTGTGATAAGTTATCTACACTGGCGAGGGGATTGCTCTCTGTAATGTTCAGCTTCTA
ATTGTCTCTACTTTGTGAGACTACTTTTGAATGCTTGACCTCAAATCAGGTAGGACTACC
CGCTGAACTTAA
>ABC1-43
TTTCCGTAGGTGAACCTGCGGAAGGATCATTATTGAATTATGTTTCTAGATAGGTTGTAG
CTGGCTCTTTTAGAGCATGTGCACGCCTGTTTGGACTTCATTTTCATCCACCTGTGCACC
TATTGTAGTCTTTGGTTGGGTTAGGAGGAAGTGATCATTGTATCAGCATCTGCTGGGAGT
GAGGACTTGCATTGTGAAAGCTTTGCTGTCCTTGATGTGATCATGGAATCTTTTTCTCAC
TAGAGTCTATGTCACTCATTATACTCTGTCGAATGTCATTGAATGTCTTTACATGGGCTT
GTATGCCTATGAAAATTGTAATACAACTTTCAGCAACGGATCTCTTGGCTCTCGCATCGA
TGAAGAACGCAGCGAAATGCGATAAGTAATGTGAATTGCAGAATTCAGTGAATCATCGAA
TCTTTGAACGCATCTTGCGCTCCTTGGTATTCCGAGGAGCATGCCTGTTTGAGTGTCATT
AAATTCTCAACTCTCTTATAC-TTTTTTGTAAAAGAGAGCTTGGACTGTGGAGGCTTGCT
GGCCACTTTTTGGGGTCAGCTCCTCTGAAATGCATTAGCGGAACCGTTTGCGATCTGCCA
CAAGTGTGATAAGTTATCTACACTGGCGAGGGGATTGCTCTCTGTAATGTTCAGCTTCTA
ATTGTCTCTACTTTGTGAGACTACTTTTGAATGCTTGACCTCAAATCAGGTAGGACTACC
CGCTGAACTTAA
>ABC1-46
TTTCCGTAGGTGAACCTGCGGAAGGATCATTATTGAATTATGTTTCTAGATAGGTTGTAG
CTGGCTCTTTTAGAGCATGTGCACGCCTGTTTGGACTTCATTTTCATCCACCTGTGCACC
TATTGTAGTCTTTGGTTGGGTTAGGAGGAAGTGATCATTGTATCAGCATCTGCTGGGAGT
GAGGACTTGCATTGTGAAAGCTTTGCTGTCCTTGATGTGATCATGGAATCTTTTTCTCAC
TAGAGTCTATGTCACTCATTATACTCTGTCGAATGTCATTGAATGTCTTTACATGGGCTT
GTATGCCTATGAAAATTGTAATACAACTTTCAGCAACGGATCTCTTGGCTCTCGCATCGA
TGAAGAACGCAGCGAAATGCGATAAGTAATGTGAATTGCAGAATTCAGTGAATCATCGAA
TCTTTGAACGCATCTTGCGCTCCTTGGTATTCCGAGGAGCATGCCTGTTTGAGTGTCATT
AAATTCTCAACTCTCTTATAC-TTTTTTGTAAAAGAGAGCTTGGACTGTGGAGGCTTGCT
GGCCACTTTTTGGGGTCAGCTCCTCTGAAATGCATTAGCGGAACCGTTTGCGATCTGCCA
CAAGTGTGATAAGTTATCTACACTGGCGAGGGGATTGCTCTCTGTAATGTTCAGCTTCTA
ATTGTCTCTACTTTGTGAGACTACTTTTGAATGCTTGACCTCAAATCAGGTAGGACTACC
CGCTGAACTTAA
>ABC1-48
TTTCCGTAGGTGAACCTGCGGAAGGATCATTATTGAATTATGTTTCTAGATAGGTTGTAG
CTGGCTCTTTTAGAGCATGTGCACGCCTGTTTGGACTTCATTTTCATCCACCTGTGCACC
TATTGTAGTCTTTGGTTGGGTTAGGAGGAAGTGATCATTGTATCAGCATCTGCTGGGAGT
GAGGACTTGCATTGTGAAAGCTTTGCTGTCCTTGATGTGATCATGGAATCTTTTTCTCAC
TAGAGTCTATGTCACTCATTATACTCTGTCGAATGTCATTGAATGTCTTTACATGGGCTT
GTATGCCTATGAAAATTGTAATACAACTTTCAGCAACGGATCTCTTGGCTCTCGCATCGA
TGAAGAACGCAGCGAAATGCGATAAGTAATGTGAATTGCAGAATTCAGTGAATCATCGAA
TCTTTGAACGCATCTTGCGCTCCTTGGTATTCCGAGGAGCATGCCTGTTTGAGTGTCATT
AAATTCTCAACTCTCTTATAC-TTTTTTGTAAAAGAGAGCTTGGACTGTGGAGGCTTGCT
GGCCACTTTTTGGGGTCAGCTCCTCTGAAATGCATTAGCGGAACCGTTTGCGATCTGCCA
CAAGTGTGATAAGTTATCTACACTGGCGAGGGGATTGCTCTCTGTAATGTTCAGCTTCTA

ATTGTCTCTACTTTGTGAGACTACTTTTGAATGCTTGACCTCAAATCAGGTAGGACTACC
CGCTGAACTTAA
>ABC1-50
TTTCCGTAGGTGAACCTGCGGAAGGATCATTATTGAATTATGTTTCTAGATAGGTTGTAG
CTGGCTCTTTTAGAGCATGTGCACGCCTGTTTGGACTTCATTTTCATCCACCTGTGCACC
TATTGTAGTCTTTGGTTGGGTTAGGAGGAAGTGATCATTGTATCAGCATCTGCTGGGAGT
GAGGACTTGCATTGTGAAAGCTTTGCTGTCCTTGATGTGATCATGGAATCTTTTTCTCAC
TAGAGTCTATGTCACTCATTATACTCTGTCGAATGTCATTGAATGTCTTTACATGGGCTT
GTATGCCTATGAAAATTGTAATACAACTTTCAGCAACGGATCTCTTGGCTCTCGCATCGA
TGAAGAACGCAGCGAAATGCGATAAGTAATGTGAATTGCAGAATTCAGTGAATCATCGAA
TCTTTGAACGCATCTTGCGCTCCTTGGTATTCCGAGGAGCATGCCTGTTTGAGTGTCATT
AAATTCTCAACTCTCTTATAC-TTTTTTGTAAAAGAGAGCTTGGACTGTGGAGGCTTGCT
GGCCACTTTTTGGGGTCAGCTCCTCTGAAATGCATTAGCGGAACCGTTTGCGATCTGCCA
CAAGTGTGATAAGTTATCTACACTGGCGAGGGGATTGCTCTCTGTAATGTTCAGCTTCTA
ATTGTCTCTACTTTGTGAGACTACTTTTGAATGCTTGACCTCAAATCAGGTAGGACTACC
CGCTGAACTTAA
>ABC1-62
TTTCCGTAGGTGAACCTGCGGAAGGATCATTATTGAATTATGTTTCTAGATAGGTTGTAG
CTGGCTCTTTTAGAGCATGTGCACGCCTGTTTGGACTTCATTTTCATCCACCTGTGCACC
TATTGTAGTCTTTGGTTGGGTTAGGAGGAAGTGATCATTGTATCAGCATCTGCTGGGAGT
GAGGACTTGCATTGTGAAAGCTTTGCTGTCCTTGATGTGATCATGGAATCTTTTTCTCAC
TAGAGTCTATGTCACTCATTATACTCTGTCGAATGTCATTGAATGTCTTTACATGGGCTT
GTATGCCTATGAAAATTGTAATACAACTTTCAGCAACGGATCTCTTGGCTCTCGCATCGA
TGAAGAACGCAGCGAAATGCGATAAGTAATGTGAATTGCAGAATTCAGTGAATCATCGAA
TCTTTGAACGCATCTTGCGCTCCTTGGTATTCCGAGGAGCATGCCTGTTTGAGTGTCATT
AAATTCTCAACTCTCTTATAC-TTTTTTGTAAAAGAGAGCTTGGACTGTGGAGGCTTGCT
GGCCACTTTTTGGGGTCAGCTCCTCTGAAATGCATTAGCGGAACCGTTTGCGATCTGCCA
CAAGTGTGATAAGTTATCTACACTGGCGAGGGGATTGCTCTCTGTAATGTTCAGCTTCTA
ATTGTCTCTACTTTGTGAGACTACTTTTGAATGCTTGACCTCAAATCAGGTAGGACTACC
CGCTGAACTTAA
>ABC1-64
TTTCCGTAGGTGAACCTGCGGAAGGATCATTATTGAATTATGTTTCTAGATAGGTTGTAG
CTGGCTCTTTTAGAGCATGTGCACGCCTGTTTGGACTTCATTTTCATCCACCTGTGCACC
TATTGTAGTCTTTGGTTGGGTTAGGAGGAAGTGATCATTGTATCAGCATCTGCTGGGAGT
GAGGACTTGCATTGTGAAAGCTTTGCTGTCCTTGATGTGATCATGGAATCTTTTTCTCAC
TAGAGTCTATGTCACTCATTATACTCTGTCGAATGTCATTGAATGTCTTTACATGGGCTT
GTATGCCTATGAAAATTGTAATACAACTTTCAGCAACGGATCTCTTGGCTCTCGCATCGA
TGAAGAACGCAGCGAAATGCGATAAGTAATGTGAATTGCAGAATTCAGTGAATCATCGAA
TCTTTGAACGCATCTTGCGCTCCTTGGTATTCCGAGGAGCATGCCTGTTTGAGTGTCATT
AAATTCTCAACTCTCTTATAC-TTTTTTGTAAAAGAGAGCTTGGACTGTGGAGGCTTGCT
GGCCACTTTTTGGGGTCAGCTCCTCTGAAATGCATTAGCGGAACCGTTTGCGATCTGCCA
CAAGTGTGATAAGTTATCTACACTGGCGAGGGGATTGCTCTCTGTAATGTTCAGCTTCTA
ATTGTCTCTACTTTGTGAGACTACTTTTGAATGCTTGACCTCAAATCAGGTAGGACTACC
CGCTGAACTTAA
>ABC1-66
TTTCCGTAGGTGAACCTGCGGAAGGATCATTATTGAATTATGTTTCTAGATAGGTTGTAG
CTGGCTCTTTTAGAGCATGTGCACGCCTGTTTGGACTTCATTTTCATCCACCTGTGCACC
TATTGTAGTCTTTGGTTGGGTTAGGAGGAAGTGATCATTGTATCAGCATCTGCTGGGAGT
GAGGACTTGCATTGTGAAAGCTTTGCTGTCCTTGATGTGATCATGGAATCTTTTTCTCAC
TAGAGTCTATGTCACTCATTATACTCTGTCGAATGTCATTGAATGTCTTTACATGGGCTT

GTATGCCTATGAAAATTGTAATACAACTTTCAGCAACGGATCTCTTGGCTCTCGCATCGA
TGAAGAACGCAGCGAAATGCGATAAGTAATGTGAATTGCAGAATTCAGTGAATCATCGAA
TCTTTGAACGCATCTTGCGCTCCTTGGTATTCCGAGGAGCATGCCTGTTTGAGTGTCATT
AAATTCTCAACTCTCTTATAC-TTTTTTGTAAAAGAGAGCTTGGACTGTGGAGGCTTGCT
GGCCACTTTTTGGGGTCAGCTCCTCTGAAATGCATTAGCGGAACCGTTTGCGATCTGCCA
CAAGTGTGATAAGTTATCTACACTGGCGAGGGGATTGCTCTCTGTAATGTTCAGCTTCTA
ATTGTCTCTACTTTGTGAGACTACTTTTGAATGCTTGACCTCAAATCAGGTAGGACTACC
CGCTGAACTTAA
>ABC1-73
TTTCCGTAGGTGAACCTGCGGAAGGATCATTATTGAATTATGTTTCTAGATAGGTTGTAG
CTGGCTCTTTTAGAGCATGTGCACGCCTGTTTGGACTTCATTTTCATCCACCTGTGCACC
TATTGTAGTCTTTGGTTGGGTTAGGAGGAAGTGATCATTGTATCAGCATCTGCTGGGAGT
GAGGACTTGCATTGTGAAAGCTTTGCTGTCCTTGATGTGATCATGGAATCTTTTTCTCAC
TAGAGTCTATGTCACTCATTATACTCTGTCGAATGTCATTGAATGTCTTTACATGGGCTT
GTATGCCTATGAAAATTGTAATACAACTTTCAGCAACGGATCTCTTGGCTCTCGCATCGA
TGAAGAACGCAGCGAAATGCGATAAGTAATGTGAATTGCAGAATTCAGTGAATCATCGAA
TCTTTGAACGCATCTTGCGCTCCTTGGTATTCCGAGGAGCATGCCTGTTTGAGTGTCATT
AAATTCTCAACTCTCTTATAC-TTTTTTGTAAAAGAGAGCTTGGACTGTGGAGGCTTGCT
GGCCACTTTTTGGGGTCAGCTCCTCTGAAATGCATTAGCGGAACCGTTTGCGATCTGCCA
CAAGTGTGATAAGTTATCTACACTGGCGAGGGGATTGCTCTCTGTAATGTTCAGCTTCTA
ATTGTCTCTACTTTGTGAGACTACTTTTGAATGCTTGACCTCAAATCAGGTAGGACTACC
CGCTGAACTTAA
>ABC1-75
TTTCCGTAGGTGAACCTGCGGAAGGATCATTATTGAATTATGTTTCTAGATAGGTTGTAG
CTGGCTCTTTTAGAGCATGTGCACGCCTGTTTGGACTTCATTTTCATCCACCTGTGCACC
TATTGTAGTCTTTGGTTGGGTTAGGAGGAAGTGATCATTGTATCAGCATCTGCTGGGAGT
GAGGACTTGCATTGTGAAAGCTTTGCTGTCCTTGATGTGATCATGGAATCTTTTTCTCAC
TAGAGTCTATGTCACTCATTATACTCTGTCGAATGTCATTGAATGTCTTTACATGGGCTT
GTATGCCTATGAAAATTGTAATACAACTTTCAGCAACGGATCTCTTGGCTCTCGCATCGA
TGAAGAACGCAGCGAAATGCGATAAGTAATGTGAATTGCAGAATTCAGTGAATCATCGAA
TCTTTGAACGCATCTTGCGCTCCTTGGTATTCCGAGGAGCATGCCTGTTTGAGTGTCATT
AAATTCTCAACTCTCTTATAC-TTTTTTGTAAAAGAGAGCTTGGACTGTGGAGGCTTGCT
GGCCACTTTTTGGGGTCAGCTCCTCTGAAATGCATTAGCGGAACCGTTTGCGATCTGCCA
CAAGTGTGATAAGTTATCTACACTGGCGAGGGGATTGCTCTCTGTAATGTTCAGCTTCTA
ATTGTCTCTACTTTGTGAGACTACTTTTGAATGCTTGACCTCAAATCAGGTAGGACTACC
CGCTGAACTTAA
>ABC1-76
TTTCCGTAGGTGAACCTGCGGAAGGATCATTATTGAATTATGTTTCTAGATAGGTTGTAG
CTGGCTCTTTTAGAGCATGTGCACGCCTGTTTGGACTTCATTTTCATCCACCTGTGCACC
TATTGTAGTCTTTGGTTGGGTTAGGAGGAAGTGATCATTGTATCAGCATCTGCTGGGAGT
GAGGACTTGCATTGTGAAAGCTTTGCTGTCCTTGATGTGATCATGGAATCTTTTTCTCAC
TAGAGTCTATGTCACTCATTATACTCTGTCGAATGTCATTGAATGTCTTTACATGGGCTT
GTATGCCTATGAAAATTGTAATACAACTTTCAGCAACGGATCTCTTGGCTCTCGCATCGA
TGAAGAACGCAGCGAAATGCGATAAGTAATGTGAATTGCAGAATTCAGTGAATCATCGAA
TCTTTGAACGCATCTTGCGCTCCTTGGTATTCCGAGGAGCATGCCTGTTTGAGTGTCATT
AAATTCTCAACTCTCTTATAC-TTTTTTGTAAAAGAGAGCTTGGACTGTGGAGGCTTGCT
GGCCACTTTTTGGGGTCAGCTCCTCTGAAATGCATTAGCGGAACCGTTTGCGATCTGCCA
CAAGTGTGATAAGTTATCTACACTGGCGAGGGGATTGCTCTCTGTAATGTTCAGCTTCTA
ATTGTCTCTACTTTGTGAGACTACTTTTGAATGCTTGACCTCAAATCAGGTAGGACTACC
CGCTGAACTTAA

>ABC1–78
TTTCCGTAGGTGAACCTGCGGAAGGATCATTATTGAATTATGTTTCTAGATAGGTTGTAG
CTGGCTCTTTTAGAGCATGTGCACGCCTGTTTGGACTTCATTTTCATCCACCTGTGCACC
TATTGTAGTCTTTGGTTGGGTTAGGAGGAAGTGATCATTGTATCAGCATCTGCTGGGAGT
GAGGACTTGCATTGTGAAAGCTTTGCTGTCCTTGATGTGATCATGGAATCTTTTTCTCAC
TAGAGTCTATGTCACTCATTATACTCTGTCGAATGTCATTGAATGTCTTTACATGGGCTT
GTATGCCTATGAAAATTGTAATACAACTTTCAGCAACGGATCTCTTGGCTCTCGCATCGA
TGAAGAACGCAGCGAAATGCGATAAGTAATGTGAATTGCAGAATTCAGTGAATCATCGAA
TCTTTGAACGCATCTTGCGCTCCTTGGTATTCCGAGGAGCATGCCTGTTTGAGTGTCATT
AAATTCTCAACTCTCTTATAC–TTTTTTGTAAAAGAGAGCTTGGACTGTGGAGGCTTGCT
GGCCACTTTTTGGGGTCAGCTCCTCTGAAATGCATTAGCGGAACCGTTTGCGATCTGCCA
CAAGTGTGATAAGTTATCTACACTGGCGAGGGGATTGCTCTCTGTAATGTTCAGCTTCTA
ATTGTCTCTACTTTGTGAGACTACTTTTGAATGCTTGACCTCAAATCAGGTAGGACTACC
CGCTGAACTTAA
>ABC1–79
TTTCCGTAGGTGAACCTGCGGAAGGATCATTATTGAATTATGTTTCTAGATAGGTTGTAG
CTGGCTCTTTTAGAGCATGTGCACGCCTGTTTGGACTTCATTTTCATCCACCTGTGCACC
TATTGTAGTCTTTGGTTGGGTTAGGAGGAAGTGATCATTGTATCAGCATCTGCTGGGAGT
GAGGACTTGCATTGTGAAAGCTTTGCTGTCCTTGATGTGATCATGGAATCTTTTTCTCAC
TAGAGTCTATGTCACTCATTATACTCTGTCGAATGTCATTGAATGTCTTTACATGGGCTT
GTATGCCTATGAAAATTGTAATACAACTTTCAGCAACGGATCTCTTGGCTCTCGCATCGA
TGAAGAACGCAGCGAAATGCGATAAGTAATGTGAATTGCAGAATTCAGTGAATCATCGAA
TCTTTGAACGCATCTTGCGCTCCTTGGTATTCCGAGGAGCATGCCTGTTTGAGTGTCATT
AAATTCTCAACTCTCTTATAC–TTTTTTGTAAAAGAGAGCTTGGACTGTGGAGGCTTGCT
GGCCACTTTTTGGGGTCAGCTCCTCTGAAATGCATTAGCGGAACCGTTTGCGATCTGCCA
CAAGTGTGATAAGTTATCTACACTGGCGAGGGGATTGCTCTCTGTAATGTTCAGCTTCTA
ATTGTCTCTACTTTGTGAGACTACTTTTGAATGCTTGACCTCAAATCAGGTAGGACTACC
CGCTGAACTTAA
>ABC2–5
TTTCCGTAGGTGAACCTGCGGAAGGATCATTATTGAATTATGTTTCTAGATAGGTTGTAG
CTGGCTCTTTTAGAGCATGTGCACGCCTGTTTGGACTTCATTTTCATCCACCTGTGCACC
TATTGTAGTCTTTGGTTGGGTTAGGAGGAAGTGATCATTGTATCAGCATCTGCTGGGAGT
GAGGACTTGCATTGTGAAAGCTTTGCTGTCCTTGATGTGATCATGGAATCTTTTTCTCAC
TAGAGTCTATGTCACTCATTATACTCTGTCGAATGTCATTGAATGTCTTTACATGGGCTT
GTATGCCTATGAAAATTGTAATACAACTTTCAGCAACGGATCTCTTGGCTCTCGCATCGA
TGAAGAACGCAGCGAAATGCGATAAGTAATGTGAATTGCAGAATTCAGTGAATCATCGAA
TCTTTGAACGCATCTTGCGCTCCTTGGTATTCCGAGGAGCATGCCTGTTTGAGTGTCATT
AAATTCTCAACTCTCTTATAC–TTTTTTGTAAAAGAGAGCTTGGACTGTGGAGGCTTGCT
GGCCACTTTTTGGGGTCAGCTCCTCTGAAATGCATTAGCGGAACCGTTTGCGATCTGCCA
CAAGTGTGATAAGTTATCTACACTGGCGAGGGGATTGCTCTCTGTAATGTTCAGCTTCTA
ATTGTCTCTACTTTGTGAGACTACTTTTGAATGCTTGACCTCAAATCAGGTAGGACTACC
CGCTGAACTTAA
>ABC2–6
TTTCCGTAGGTGAACCTGCGGAAGGATCATTATTGAATTATGTTTCTAGATAGGTTGTAG
CTGGCTCTTTTAGAGCATGTGCACGCCTGTTTGGACTTCATTTTCATCCACCTGTGCACC
TATTGTAGTCTTTGGTTGGGTTAGGAGGAAGTGATCATTGTATCAGCATCTGCTGGGAGT
GAGGACTTGCATTGTGAAAGCTTTGCTGTCCTTGATGTGATCATGGAATCTTTTTCTCAC
TAGAGTCTATGTCACTCATTATACTCTGTCGAATGTCATTGAATGTCTTTACATGGGCTT
GTATGCCTATGAAAATTGTAATACAACTTTCAGCAACGGATCTCTTGGCTCTCGCATCGA
TGAAGAACGCAGCGAAATGCGATAAGTAATGTGAATTGCAGAATTCAGTGAATCATCGAA

TCTTTGAACGCATCTTGCGCTCCTTGGTATTCCGAGGAGCATGCCTGTTTGAGTGTCATT
AAATTCTCAACTCTCTTATAC-TTTTTTGTAAAAGAGAGCTTGGACTGTGGAGGCTTGCT
GGCCACTTTTTGGGGTCAGCTCCTCTGAAATGCATTAGCGGAACCGTTTGCGATCTGCCA
CAAGTGTGATAAGTTATCTACACTGGCGAGGGGATTGCTCTCTGTAATGTTCAGCTTCTA
ATTGTCTCTACTTTGTGAGACTACTTTTGAATGCTTGACCTCAAATCAGGTAGGACTACC
CGCTGAACTTAA
>ABC2-11
TTTCCGTAGGTGAACCTGCGGAAGGATCATTATTGAATTATGTTTCTAGATAGGTTGTAG
CTGGCTCTTTTAGAGCATGTGCACGCCTGTTTGGACTTCATTTTCATCCACCTGTGCACC
TATTGTAGTCTTTGGTTGGGTTAGGAGGAAGTGATCATTGTATCAGCATCTGCTGGGAGT
GAGGACTTGCATTGTGAAAGCTTTGCTGTCCTTGATGTGATCATGGAATCTTTTTCTCAC
TAGAGTCTATGTCACTCATTATACTCTGTCGAATGTCATTGAATGTCTTTACATGGGCTT
GTATGCCTATGAAAATTGTAATACAACTTTCAGCAACGGATCTCTTGGCTCTCGCATCGA
TGAAGAACGCAGCGAAATGCGATAAGTAATGTGAATTGCAGAATTCAGTGAATCATCGAA
TCTTTGAACGCATCTTGCGCTCCTTGGTATTCCGAGGAGCATGCCTGTTTGAGTGTCATT
AAATTCTCAACTCTCTTATAC-TTTTTTGTAAAAGAGAGCTTGGACTGTGGAGGCTTGCT
GGCCACTTTTTGGGGTCAGCTCCTCTGAAATGCATTAGCGGAACCGTTTGCGATCTGCCA
CAAGTGTGATAAGTTATCTACACTGGCGAGGGGATTGCTCTCTGTAATGTTCAGCTTCTA
ATTGTCTCTACTTTGTGAGACTACTTTTGAATGCTTGACCTCAAATCAGGTAGGACTACC
CGCTGAACTTAA
>ABC2-13
TTTCCGTAGGTGAACCTGCGGAAGGATCATTATTGAATTATGTTTCTAGATAGGTTGTAG
CTGGCTCTTTTAGAGCATGTGCACGCCTGTTTGGACTTCATTTTCATCCACCTGTGCACC
TATTGTAGTCTTTGGTTGGGTTAGGAGGAAGTGATCATTGTATCAGCATCTGCTGGGAGT
GAGGACTTGCATTGTGAAAGCTTTGCTGTCCTTGATGTGATCATGGAATCTTTTTCTCAC
TAGAGTCTATGTCACTCATTATACTCTGTCGAATGTCATTGAATGTCTTTACATGGGCTT
GTATGCCTATGAAAATTGTAATACAACTTTCAGCAACGGATCTCTTGGCTCTCGCATCGA
TGAAGAACGCAGCGAAATGCGATAAGTAATGTGAATTGCAGAATTCAGTGAATCATCGAA
TCTTTGAACGCATCTTGCGCTCCTTGGTATTCCGAGGAGCATGCCTGTTTGAGTGTCATT
AAATTCTCAACTCTCTTATAC-TTTTTTGTAAAAGAGAGCTTGGACTGTGGAGGCTTGCT
GGCCACTTTTTGGGGTCAGCTCCTCTGAAATGCATTAGCGGAACCGTTTGCGATCTGCCA
CAAGTGTGATAAGTTATCTACACTGGCGAGGGGATTGCTCTCTGTAATGTTCAGCTTCTA
ATTGTCTCTACTTTGTGAGACTACTTTTGAATGCTTGACCTCAAATCAGGTAGGACTACC
CGCTGAACTTAA
>ABC2-17
TTTCCGTAGGTGAACCTGCGGAAGGATCATTATTGAATTATGTTTCTAGATAGGTTGTAG
CTGGCTCTTTTAGAGCATGTGCACGCCTGTTTGGACTTCATTTTCATCCACCTGTGCACC
TATTGTAGTCTTTGGTTGGGTTAGGAGGAAGTGATCATTGTATCAGCATCTGCTGGGAGT
GAGGACTTGCATTGTGAAAGCTTTGCTGTCCTTGATGTGATCATGGAATCTTTTTCTCAC
TAGAGTCTATGTCACTCATTATACTCTGTCGAATGTCATTGAATGTCTTTACATGGGCTT
GTATGCCTATGAAAATTGTAATACAACTTTCAGCAACGGATCTCTTGGCTCTCGCATCGA
TGAAGAACGCAGCGAAATGCGATAAGTAATGTGAATTGCAGAATTCAGTGAATCATCGAA
TCTTTGAACGCATCTTGCGCTCCTTGGTATTCCGAGGAGCATGCCTGTTTGAGTGTCATT
AAATTCTCAACTCTCTTATAC-TTTTTTGTAAAAGAGAGCTTGGACTGTGGAGGCTTGCT
GGCCACTTTTTGGGGTCAGCTCCTCTGAAATGCATTAGCGGAACCGTTTGCGATCTGCCA
CAAGTGTGATAAGTTATCTACACTGGCGAGGGGATTGCTCTCTGTAATGTTCAGCTTCTA
ATTGTCTCTACTTTGTGAGACTACTTTTGAATGCTTGACCTCAAATCAGGTAGGACTACC
CGCTGAACTTAA
>ABC2-19
TTTCCGTAGGTGAACCTGCGGAAGGATCATTATTGAATTATGTTTCTAGATAGGTTGTAG

CTGGCTCTTTTAGAGCATGTGCACGCCTGTTTGGACTTCATTTTCATCCACCTGTGCACC
TATTGTAGTCTTTGGTTGGGTTAGGAGGAAGTGATCATTGTATCAGCATCTGCTGGGAGT
GAGGACTTGCATTGTGAAAGCTTTGCTGTCCTTGATGTGATCATGGAATCTTTTTCTCAC
TAGAGTCTATGTCACTCATTATACTCTGTCGAATGTCATTGAATGTCTTTACATGGGCTT
GTATGCCTATGAAAATTGTAATACAACTTTCAGCAACGGATCTCTTGGCTCTCGCATCGA
TGAAGAACGCAGCGAAATGCGATAAGTAATGTGAATTGCAGAATTCAGTGAATCATCGAA
TCTTTGAACGCATCTTGCGCTCCTTGGTATTCCGAGGAGCATGCCTGTTTGAGTGTCATT
AAATTCTCAACTCTCTTATAC-TTTTTTGTAAAAGAGAGCTTGGACTGTGGAGGCTTGCT
GGCCACTTTTTGGGGTCAGCTCCTCTGAAATGCATTAGCGGAACCGTTTGCGATCTGCCA
CAAGTGTGATAAGTTATCTACACTGGCGAGGGGATTGCTCTCTGTAATGTTCAGCTTCTA
ATTGTCTCTACTTTGTGAGACTACTTTTGAATGCTTGACCTCAAATCAGGTAGGACTACC
CGCTGAACTTAA
>ABC2-21
TTTCCGTAGGTGAACCTGCGGAAGGATCATTATTGAATTATGTTTCTAGATAGGTTGTAG
CTGGCTCTTTTAGAGCATGTGCACGCCTGTTTGGACTTCATTTTCATCCACCTGTGCACC
TATTGTAGTCTTTGGTTGGGTTAGGAGGAAGTGATCATTGTATCAGCATCTGCTGGGAGT
GAGGACTTGCATTGTGAAAGCTTTGCTGTCCTTGATGTGATCATGGAATCTTTTTCTCAC
TAGAGTCTATGTCACTCATTATACTCTGTCGAATGTCATTGAATGTCTTTACATGGGCTT
GTATGCCTATGAAAATTGTAATACAACTTTCAGCAACGGATCTCTTGGCTCTCGCATCGA
TGAAGAACGCAGCGAAATGCGATAAGTAATGTGAATTGCAGAATTCAGTGAATCATCGAA
TCTTTGAACGCATCTTGCGCTCCTTGGTATTCCGAGGAGCATGCCTGTTTGAGTGTCATT
AAATTCTCAACTCTCTTATAC-TTTTTTGTAAAAGAGAGCTTGGACTGTGGAGGCTTGCT
GGCCACTTTTTGGGGTCAGCTCCTCTGAAATGCATTAGCGGAACCGTTTGCGATCTGCCA
CAAGTGTGATAAGTTATCTACACTGGCGAGGGGATTGCTCTCTGTAATGTTCAGCTTCTA
ATTGTCTCTACTTTGTGAGACTACTTTTGAATGCTTGACCTCAAATCAGGTAGGACTACC
CGCTGAACTTAA
>ABC2-26
TTTCCGTAGGTGAACCTGCGGAAGGATCATTATTGAATTATGTTTCTAGATAGGTTGTAG
CTGGCTCTTTTAGAGCATGTGCACGCCTGTTTGGACTTCATTTTCATCCACCTGTGCACC
TATTGTAGTCTTTGGTTGGGTTAGGAGGAAGTGATCATTGTATCAGCATCTGCTGGGAGT
GAGGACTTGCATTGTGAAAGCTTTGCTGTCCTTGATGTGATCATGGAATCTTTTTCTCAC
TAGAGTCTATGTCACTCATTATACTCTGTCGAATGTCATTGAATGTCTTTACATGGGCTT
GTATGCCTATGAAAATTGTAATACAACTTTCAGCAACGGATCTCTTGGCTCTCGCATCGA
TGAAGAACGCAGCGAAATGCGATAAGTAATGTGAATTGCAGAATTCAGTGAATCATCGAA
TCTTTGAACGCATCTTGCGCTCCTTGGTATTCCGAGGAGCATGCCTGTTTGAGTGTCATT
AAATTCTCAACTCTCTTATAC-TTTTTTGTAAAAGAGAGCTTGGACTGTGGAGGCTTGCT
GGCCACTTTTTGGGGTCAGCTCCTCTGAAATGCATTAGCGGAACCGTTTGCGATCTGCCA
CAAGTGTGATAAGTTATCTACACTGGCGAGGGGATTGCTCTCTGTAATGTTCAGCTTCTA
ATTGTCTCTACTTTGTGAGACTACTTTTGAATGCTTGACCTCAAATCAGGTAGGACTACC
CGCTGAACTTAA
>ABC2-31
TTTCCGTAGGTGAACCTGCGGAAGGATCATTATTGAATTATGTTTCTAGATAGGTTGTAG
CTGGCTCTTTTAGAGCATGTGCACGCCTGTTTGGACTTCATTTTCATCCACCTGTGCACC
TATTGTAGTCTTTGGTTGGGTTAGGAGGAAGTGATCATTGTATCAGCATCTGCTGGGAGT
GAGGACTTGCATTGTGAAAGCTTTGCTGTCCTTGATGTGATCATGGAATCTTTTTCTCAC
TAGAGTCTATGTCACTCATTATACTCTGTCGAATGTCATTGAATGTCTTTACATGGGCTT
GTATGCCTATGAAAATTGTAATACAACTTTCAGCAACGGATCTCTTGGCTCTCGCATCGA
TGAAGAACGCAGCGAAATGCGATAAGTAATGTGAATTGCAGAATTCAGTGAATCATCGAA
TCTTTGAACGCATCTTGCGCTCCTTGGTATTCCGAGGAGCATGCCTGTTTGAGTGTCATT
AAATTCTCAACTCTCTTATAC-TTTTTTGTAAAAGAGAGCTTGGACTGTGGAGGCTTGCT

GGCCACTTTTTGGGGTCAGCTCCTCTGAAATGCATTAGCGGAACCGTTTGCGATCTGCCA
CAAGTGTGATAAGTTATCTACACTGGCGAGGGGATTGCTCTCTGTAATGTTCAGCTTCTA
ATTGTCTCTACTTTGTGAGACTACTTTTGAATGCTTGACCTCAAATCAGGTAGGACTACC
CGCTGAACTTAA
>ABC2-35
TTTCCGTAGGTGAACCTGCGGAAGGATCATTATTGAATTATGTTTCTAGATAGGTTGTAG
CTGGCTCTTTTAGAGCATGTGCACGCCTGTTTGGACTTCATTTTCATCCACCTGTGCACC
TATTGTAGTCTTTGGTTGGGTTAGGAGGAAGTGATCATTGTATCAGCATCTGCTGGGAGT
GAGGACTTGCATTGTGAAAGCTTTGCTGTCCTTGATGTGATCATGGAATCTTTTTCTCAC
TAGAGTCTATGTCACTCATTATACTCTGTCGAATGTCATTGAATGTCTTTACATGGGCTT
GTATGCCTATGAAAATTGTAATACAACTTTCAGCAACGGATCTCTTGGCTCTCGCATCGA
TGAAGAACGCAGCGAAATGCGATAAGTAATGTGAATTGCAGAATTCAGTGAATCATCGAA
TCTTTGAACGCATCTTGCGCTCCTTGGTATTCCGAGGAGCATGCCTGTTTGAGTGTCATT
AAATTCTCAACTCTCTTATAC-TTTTTTGTAAAAGAGAGCTTGGACTGTGGAGGCTTGCT
GGCCACTTTTTGGGGTCAGCTCCTCTGAAATGCATTAGCGGAACCGTTTGCGATCTGCCA
CAAGTGTGATAAGTTATCTACACTGGCGAGGGGATTGCTCTCTGTAATGTTCAGCTTCTA
ATTGTCTCTACTTTGTGAGACTACTTTTGAATGCTTGACCTCAAATCAGGTAGGACTACC
CGCTGAACTTAA
>ABC2-37
TTTCCGTAGGTGAACCTGCGGAAGGATCATTATTGAATTATGTTTCTAGATAGGTTGTAG
CTGGCTCTTTTAGAGCATGTGCACGCCTGTTTGGACTTCATTTTCATCCACCTGTGCACC
TATTGTAGTCTTTGGTTGGGTTAGGAGGAAGTGATCATTGTATCAGCATCTGCTGGGAGT
GAGGACTTGCATTGTGAAAGCTTTGCTGTCCTTGATGTGATCATGGAATCTTTTTCTCAC
TAGAGTCTATGTCACTCATTATACTCTGTCGAATGTCATTGAATGTCTTTACATGGGCTT
GTATGCCTATGAAAATTGTAATACAACTTTCAGCAACGGATCTCTTGGCTCTCGCATCGA
TGAAGAACGCAGCGAAATGCGATAAGTAATGTGAATTGCAGAATTCAGTGAATCATCGAA
TCTTTGAACGCATCTTGCGCTCCTTGGTATTCCGAGGAGCATGCCTGTTTGAGTGTCATT
AAATTCTCAACTCTCTTATAC-TTTTTTGTAAAAGAGAGCTTGGACTGTGGAGGCTTGCT
GGCCACTTTTTGGGGTCAGCTCCTCTGAAATGCATTAGCGGAACCGTTTGCGATCTGCCA
CAAGTGTGATAAGTTATCTACACTGGCGAGGGGATTGCTCTCTGTAATGTTCAGCTTCTA
ATTGTCTCTACTTTGTGAGACTACTTTTGAATGCTTGACCTCAAATCAGGTAGGACTACC
CGCTGAACTTAA
>ABC2-40
TTTCCGTAGGTGAACCTGCGGAAGGATCATTATTGAATTATGTTTCTAGATAGGTTGTAG
CTGGCTCTTTTAGAGCATGTGCACGCCTGTTTGGACTTCATTTTCATCCACCTGTGCACC
TATTGTAGTCTTTGGTTGGGTTAGGAGGAAGTGATCATTGTATCAGCATCTGCTGGGAGT
GAGGACTTGCATTGTGAAAGCTTTGCTGTCCTTGATGTGATCATGGAATCTTTTTCTCAC
TAGAGTCTATGTCACTCATTATACTCTGTCGAATGTCATTGAATGTCTTTACATGGGCTT
GTATGCCTATGAAAATTGTAATACAACTTTCAGCAACGGATCTCTTGGCTCTCGCATCGA
TGAAGAACGCAGCGAAATGCGATAAGTAATGTGAATTGCAGAATTCAGTGAATCATCGAA
TCTTTGAACGCATCTTGCGCTCCTTGGTATTCCGAGGAGCATGCCTGTTTGAGTGTCATT
AAATTCTCAACTCTCTTATAC-TTTTTTGTAAAAGAGAGCTTGGACTGTGGAGGCTTGCT
GGCCACTTTTTGGGGTCAGCTCCTCTGAAATGCATTAGCGGAACCGTTTGCGATCTGCCA
CAAGTGTGATAAGTTATCTACACTGGCGAGGGGATTGCTCTCTGTAATGTTCAGCTTCTA
ATTGTCTCTACTTTGTGAGACTACTTTTGAATGCTTGACCTCAAATCAGGTAGGACTACC
CGCTGAACTTAA
>ABC2-45
TTTCCGTAGGTGAACCTGCGGAAGGATCATTATTGAATTATGTTTCTAGATAGGTTGTAG
CTGGCTCTTTTAGAGCATGTGCACGCCTGTTTGGACTTCATTTTCATCCACCTGTGCACC
TATTGTAGTCTTTGGTTGGGTTAGGAGGAAGTGATCATTGTATCAGCATCTGCTGGGAGT

GAGGACTTGCATTGTGAAAGCTTTGCTGTCCTTGATGTGATCATGGAATCTTTTTCTCAC
TAGAGTCTATGTCACTCATTATACTCTGTCGAATGTCATTGAATGTCTTTACATGGGCTT
GTATGCCTATGAAAATTGTAATACAACTTTCAGCAACGGATCTCTTGGCTCTCGCATCGA
TGAAGAACGCAGCGAAATGCGATAAGTAATGTGAATTGCAGAATTCAGTGAATCATCGAA
TCTTTGAACGCATCTTGCGCTCCTTGGTATTCCGAGGAGCATGCCTGTTTGAGTGTCATT
AAATTCTCAACTCTCTTATAC-TTTTTTGTAAAAGAGAGCTTGGACTGTGGAGGCTTGCT
GGCCACTTTTTGGGGTCAGCTCCTCTGAAATGCATTAGCGGAACCGTTTGCGATCTGCCA
CAAGTGTGATAAGTTATCTACACTGGCGAGGGGATTGCTCTCTGTAATGTTCAGCTTCTA
ATTGTCTCTACTTTGTGAGACTACTTTTGAATGCTTGACCTCAAATCAGGTAGGACTACC
CGCTGAACTTAA
>ABC2-48
TTTCCGTAGGTGAACCTGCGGAAGGATCATTATTGAATTATGTTTCTAGATAGGTTGTAG
CTGGCTCTTTTAGAGCATGTGCACGCCTGTTTGGACTTCATTTTCATCCACCTGTGCACC
TATTGTAGTCTTTGGTTGGGTTAGGAGGAAGTGATCATTGTATCAGCATCTGCTGGGAGT
GAGGACTTGCATTGTGAAAGCTTTGCTGTCCTTGATGTGATCATGGAATCTTTTTCTCAC
TAGAGTCTATGTCACTCATTATACTCTGTCGAATGTCATTGAATGTCTTTACATGGGCTT
GTATGCCTATGAAAATTGTAATACAACTTTCAGCAACGGATCTCTTGGCTCTCGCATCGA
TGAAGAACGCAGCGAAATGCGATAAGTAATGTGAATTGCAGAATTCAGTGAATCATCGAA
TCTTTGAACGCATCTTGCGCTCCTTGGTATTCCGAGGAGCATGCCTGTTTGAGTGTCATT
AAATTCTCAACTCTCTTATAC-TTTTTTGTAAAAGAGAGCTTGGACTGTGGAGGCTTGCT
GGCCACTTTTTGGGGTCAGCTCCTCTGAAATGCATTAGCGGAACCGTTTGCGATCTGCCA
CAAGTGTGATAAGTTATCTACACTGGCGAGGGGATTGCTCTCTGTAATGTTCAGCTTCTA
ATTGTCTCTACTTTGTGAGACTACTTTTGAATGCTTGACCTCAAATCAGGTAGGACTACC
CGCTGAACTTAA
>ABC2-50
TTTCCGTAGGTGAACCTGCGGAAGGATCATTATTGAATTATGTTTCTAGATAGGTTGTAG
CTGGCTCTTTTAGAGCATGTGCACGCCTGTTTGGACTTCATTTTCATCCACCTGTGCACC
TATTGTAGTCTTTGGTTGGGTTAGGAGGAAGTGATCATTGTATCAGCATCTGCTGGGAGT
GAGGACTTGCATTGTGAAAGCTTTGCTGTCCTTGATGTGATCATGGAATCTTTTTCTCAC
TAGAGTCTATGTCACTCATTATACTCTGTCGAATGTCATTGAATGTCTTTACATGGGCTT
GTATGCCTATGAAAATTGTAATACAACTTTCAGCAACGGATCTCTTGGCTCTCGCATCGA
TGAAGAACGCAGCGAAATGCGATAAGTAATGTGAATTGCAGAATTCAGTGAATCATCGAA
TCTTTGAACGCATCTTGCGCTCCTTGGTATTCCGAGGAGCATGCCTGTTTGAGTGTCATT
AAATTCTCAACTCTCTTATAC-TTTTTTGTAAAAGAGAGCTTGGACTGTGGAGGCTTGCT
GGCCACTTTTTGGGGTCAGCTCCTCTGAAATGCATTAGCGGAACCGTTTGCGATCTGCCA
CAAGTGTGATAAGTTATCTACACTGGCGAGGGGATTGCTCTCTGTAATGTTCAGCTTCTA
ATTGTCTCTACTTTGTGAGACTACTTTTGAATGCTTGACCTCAAATCAGGTAGGACTACC
CGCTGAACTTAA
>ABC2-69
TTTCCGTAGGTGAACCTGCGGAAGGATCATTATTGAATTATGTTTCTAGATAGGTTGTAG
CTGGCTCTTTTAGAGCATGTGCACGCCTGTTTGGACTTCATTTTCATCCACCTGTGCACC
TATTGTAGTCTTTGGTTGGGTTAGGAGGAAGTGATCATTGTATCAGCATCTGCTGGGAGT
GAGGACTTGCATTGTGAAAGCTTTGCTGTCCTTGATGTGATCATGGAATCTTTTTCTCAC
TAGAGTCTATGTCACTCATTATACTCTGTCGAATGTCATTGAATGTCTTTACATGGGCTT
GTATGCCTATGAAAATTGTAATACAACTTTCAGCAACGGATCTCTTGGCTCTCGCATCGA
TGAAGAACGCAGCGAAATGCGATAAGTAATGTGAATTGCAGAATTCAGTGAATCATCGAA
TCTTTGAACGCATCTTGCGCTCCTTGGTATTCCGAGGAGCATGCCTGTTTGAGTGTCATT
AAATTCTCAACTCTCTTATAC-TTTTTTGTAAAAGAGAGCTTGGACTGTGGAGGCTTGCT
GGCCACTTTTTGGGGTCAGCTCCTCTGAAATGCATTAGCGGAACCGTTTGCGATCTGCCA
CAAGTGTGATAAGTTATCTACACTGGCGAGGGGATTGCTCTCTGTAATGTTCAGCTTCTA

ATTGTCTCTACTTTGTGAGACTACTTTTGAATGCTTGACCTCAAATCAGGTAGGACTACC
CGCTGAACTTAA
>ABC2-71
TTTCCGTAGGTGAACCTGCGGAAGGATCATTATTGAATTATGTTTCTAGATAGGTTGTAG
CTGGCTCTTTTAGAGCATGTGCACGCCTGTTTGGACTTCATTTTCATCCACCTGTGCACC
TATTGTAGTCTTTGGTTGGGTTAGGAGGAAGTGATCATTGTATCAGCATCTGCTGGGAGT
GAGGACTTGCATTGTGAAAGCTTTGCTGTCCTTGATGTGATCATGGAATCTTTTTCTCAC
TAGAGTCTATGTCACTCATTATACTCTGTCGAATGTCATTGAATGTCTTTACATGGGCTT
GTATGCCTATGAAAATTGTAATACAACTTTCAGCAACGGATCTCTTGGCTCTCGCATCGA
TGAAGAACGCAGCGAAATGCGATAAGTAATGTGAATTGCAGAATTCAGTGAATCATCGAA
TCTTTGAACGCATCTTGCGCTCCTTGGTATTCCGAGGAGCATGCCTGTTTGAGTGTCATT
AAATTCTCAACTCTCTTATAC-TTTTTTGTAAAAGAGAGCTTGGACTGTGGAGGCTTGCT
GGCCACTTTTTGGGGTCAGCTCCTCTGAAATGCATTAGCGGAACCGTTTGCGATCTGCCA
CAAGTGTGATAAGTTATCTACACTGGCGAGGGGATTGCTCTCTGTAATGTTCAGCTTCTA
ATTGTCTCTACTTTGTGAGACTACTTTTGAATGCTTGACCTCAAATCAGGTAGGACTACC
CGCTGAACTTAA
>ABC3-3
TTTCCGTAGGTGAACCTGCGGAAGGATCATTATTGAATTATGTTTCTAGATAGGTTGTAG
CTGGCTCTTTTAGAGCATGTGCACGCCTGTTTGGACTTCATTTTCATCCACCTGTGCACC
TATTGTAGTCTTTGGTTGGGTTAGGAGGAAGTGATCATTGTATCAGCATCTGCTGGGAGT
GAGGACTTGCATTGTGAAAGCTTTGCTGTCCTTGATGTGATCATGGAATCTTTTTCTCAC
TAGAGTCTATGTCACTCATTATACTCTGTCGAATGTCATTGAATGTCTTTACATGGGCTT
GTATGCCTATGAAAATTGTAATACAACTTTCAGCAACGGATCTCTTGGCTCTCGCATCGA
TGAAGAACGCAGCGAAATGCGATAAGTAATGTGAATTGCAGAATTCAGTGAATCATCGAA
TCTTTGAACGCATCTTGCGCTCCTTGGTATTCCGAGGAGCATGCCTGTTTGAGTGTCATT
AAATTCTCAACTCTCTTATAC-TTTTTTGTAAAAGAGAGCTTGGACTGTGGAGGCTTGCT
GGCCACTTTTTGGGGTCAGCTCCTCTGAAATGCATTAGCGGAACCGTTTGCGATCTGCCA
CAAGTGTGATAAGTTATCTACACTGGCGAGGGGATTGCTCTCTGTAATGTTCAGCTTCTA
ATTGTCTCTACTTTGTGAGACTACTTTTGAATGCTTGACCTCAAATCAGGTAGGACTACC
CGCTGAACTTAA
>ABC3-10
TTTCCGTAGGTGAACCTGCGGAAGGATCATTATTGAATTATGTTTCTAGATAGGTTGTAG
CTGGCTCTTTTAGAGCATGTGCACGCCTGTTTGGACTTCATTTTCATCCACCTGTGCACC
TATTGTAGTCTTTGGTTGGGTTAGGAGGAAGTGATCATTGTATCAGCATCTGCTGGGAGT
GAGGACTTGCATTGTGAAAGCTTTGCTGTCCTTGATGTGATCATGGAATCTTTTTCTCAC
TAGAGTCTATGTCACTCATTATACTCTGTCGAATGTCATTGAATGTCTTTACATGGGCTT
GTATGCCTATGAAAATTGTAATACAACTTTCAGCAACGGATCTCTTGGCTCTCGCATCGA
TGAAGAACGCAGCGAAATGCGATAAGTAATGTGAATTGCAGAATTCAGTGAATCATCGAA
TCTTTGAACGCATCTTGCGCTCCTTGGTATTCCGAGGAGCATGCCTGTTTGAGTGTCATT
AAATTCTCAACTCTCTTATAC-TTTTTTGTAAAAGAGAGCTTGGACTGTGGAGGCTTGCT
GGCCACTTTTTGGGGTCAGCTCCTCTGAAATGCATTAGCGGAACCGTTTGCGATCTGCCA
CAAGTGTGATAAGTTATCTACACTGGCGAGGGGATTGCTCTCTGTAATGTTCAGCTTCTA
ATTGTCTCTACTTTGTGAGACTACTTTTGAATGCTTGACCTCAAATCAGGTAGGACTACC
CGCTGAACTTAA
>ABC3-11
TTTCCGTAGGTGAACCTGCGGAAGGATCATTATTGAATTATGTTTCTAGATAGGTTGTAG
CTGGCTCTTTTAGAGCATGTGCACGCCTGTTTGGACTTCATTTTCATCCACCTGTGCACC
TATTGTAGTCTTTGGTTGGGTTAGGAGGAAGTGATCATTGTATCAGCATCTGCTGGGAGT
GAGGACTTGCATTGTGAAAGCTTTGCTGTCCTTGATGTGATCATGGAATCTTTTTCTCAC
TAGAGTCTATGTCACTCATTATACTCTGTCGAATGTCATTGAATGTCTTTACATGGGCTT

GTATGCCTATGAAAATTGTAATACAACTTTCAGCAACGGATCTCTTGGCTCTCGCATCGA
TGAAGAACGCAGCGAAATGCGATAAGTAATGTGAATTGCAGAATTCAGTGAATCATCGAA
TCTTTGAACGCATCTTGCGCTCCTTGGTATTCCGAGGAGCATGCCTGTTTGAGTGTCATT
AAATTCTCAACTCTCTTATAC-TTTTTTGTAAAAGAGAGCTTGGACTGTGGAGGCTTGCT
GGCCACTTTTTGGGGTCAGCTCCTCTGAAATGCATTAGCGGAACCGTTTGCGATCTGCCA
CAAGTGTGATAAGTTATCTACACTGGCGAGGGGATTGCTCTCTGTAATGTTCAGCTTCTA
ATTGTCTCTACTTTGTGAGACTACTTTTGAATGCTTGACCTCAAATCAGGTAGGACTACC
CGCTGAACTTAA
>ABC3-12
TTTCCGTAGGTGAACCTGCGGAAGGATCATTATTGAATTATGTTTCTAGATAGGTTGTAG
CTGGCTCTTTTAGAGCATGTGCACGCCTGTTTGGACTTCATTTTCATCCACCTGTGCACC
TATTGTAGTCTTTGGTTGGGTTAGGAGGAAGTGATCATTGTATCAGCATCTGCTGGGAGT
GAGGACTTGCATTGTGAAAGCTTTGCTGTCCTTGATGTGATCATGGAATCTTTTTCTCAC
TAGAGTCTATGTCACTCATTATACTCTGTCGAATGTCATTGAATGTCTTTACATGGGCTT
GTATGCCTATGAAAATTGTAATACAACTTTCAGCAACGGATCTCTTGGCTCTCGCATCGA
TGAAGAACGCAGCGAAATGCGATAAGTAATGTGAATTGCAGAATTCAGTGAATCATCGAA
TCTTTGAACGCATCTTGCGCTCCTTGGTATTCCGAGGAGCATGCCTGTTTGAGTGTCATT
AAATTCTCAACTCTCTTATAC-TTTTTTGTAAAAGAGAGCTTGGACTGTGGAGGCTTGCT
GGCCACTTTTTGGGGTCAGCTCCTCTGAAATGCATTAGCGGAACCGTTTGCGATCTGCCA
CAAGTGTGATAAGTTATCTACACTGGCGAGGGGATTGCTCTCTGTAATGTTCAGCTTCTA
ATTGTCTCTACTTTGTGAGACTACTTTTGAATGCTTGACCTCAAATCAGGTAGGACTACC
CGCTGAACTTAA
>ABC3-17
TTTCCGTAGGTGAACCTGCGGAAGGATCATTATTGAATTATGTTTCTAGATAGGTTGTAG
CTGGCTCTTTTAGAGCATGTGCACGCCTGTTTGGACTTCATTTTCATCCACCTGTGCACC
TATTGTAGTCTTTGGTTGGGTTAGGAGGAAGTGATCATTGTATCAGCATCTGCTGGGAGT
GAGGACTTGCATTGTGAAAGCTTTGCTGTCCTTGATGTGATCATGGAATCTTTTTCTCAC
TAGAGTCTATGTCACTCATTATACTCTGTCGAATGTCATTGAATGTCTTTACATGGGCTT
GTATGCCTATGAAAATTGTAATACAACTTTCAGCAACGGATCTCTTGGCTCTCGCATCGA
TGAAGAACGCAGCGAAATGCGATAAGTAATGTGAATTGCAGAATTCAGTGAATCATCGAA
TCTTTGAACGCATCTTGCGCTCCTTGGTATTCCGAGGAGCATGCCTGTTTGAGTGTCATT
AAATTCTCAACTCTCTTATAC-TTTTTTGTAAAAGAGAGCTTGGACTGTGGAGGCTTGCT
GGCCACTTTTTGGGGTCAGCTCCTCTGAAATGCATTAGCGGAACCGTTTGCGATCTGCCA
CAAGTGTGATAAGTTATCTACACTGGCGAGGGGATTGCTCTCTGTAATGTTCAGCTTCTA
ATTGTCTCTACTTTGTGAGACTACTTTTGAATGCTTGACCTCAAATCAGGTAGGACTACC
CGCTGAACTTAA
>ABC3-21
TTTCCGTAGGTGAACCTGCGGAAGGATCATTATTGAATTATGTTTCTAGATAGGTTGTAG
CTGGCTCTTTTAGAGCATGTGCACGCCTGTTTGGACTTCATTTTCATCCACCTGTGCACC
TATTGTAGTCTTTGGTTGGGTTAGGAGGAAGTGATCATTGTATCAGCATCTGCTGGGAGT
GAGGACTTGCATTGTGAAAGCTTTGCTGTCCTTGATGTGATCATGGAATCTTTTTCTCAC
TAGAGTCTATGTCACTCATTATACTCTGTCGAATGTCATTGAATGTCTTTACATGGGCTT
GTATGCCTATGAAAATTGTAATACAACTTTCAGCAACGGATCTCTTGGCTCTCGCATCGA
TGAAGAACGCAGCGAAATGCGATAAGTAATGTGAATTGCAGAATTCAGTGAATCATCGAA
TCTTTGAACGCATCTTGCGCTCCTTGGTATTCCGAGGAGCATGCCTGTTTGAGTGTCATT
AAATTCTCAACTCTCTTATAC-TTTTTTGTAAAAGAGAGCTTGGACTGTGGAGGCTTGCT
GGCCACTTTTTGGGGTCAGCTCCTCTGAAATGCATTAGCGGAACCGTTTGCGATCTGCCA
CAAGTGTGATAAGTTATCTACACTGGCGAGGGGATTGCTCTCTGTAATGTTCAGCTTCTA
ATTGTCTCTACTTTGTGAGACTACTTTTGAATGCTTGACCTCAAATCAGGTAGGACTACC
CGCTGAACTTAA

>ABC3-26
TTTCCGTAGGTGAACCTGCGGAAGGATCATTATTGAATTATGTTTCTAGATAGGTTGTAG
CTGGCTCTTTTAGAGCATGTGCACGCCTGTTTGGACTTCATTTTCATCCACCTGTGCACC
TATTGTAGTCTTTGGTTGGGTTAGGAGGAAGTGATCATTGTATCAGCATCTGCTGGGAGT
GAGGACTTGCATTGTGAAAGCTTTGCTGTCCTTGATGTGATCATGGAATCTTTTTCTCAC
TAGAGTCTATGTCACTCATTATACTCTGTCGAATGTCATTGAATGTCTTTACATGGGCTT
GTATGCCTATGAAAATTGTAATACAACTTTCAGCAACGGATCTCTTGGCTCTCGCATCGA
TGAAGAACGCAGCGAAATGCGATAAGTAATGTGAATTGCAGAATTCAGTGAATCATCGAA
TCTTTGAACGCATCTTGCGCTCCTTGGTATTCCGAGGAGCATGCCTGTTTGAGTGTCATT
AAATTCTCAACTCTCTTATAC-TTTTTTGTAAAAGAGAGCTTGGACTGTGGAGGCTTGCT
GGCCACTTTTTGGGGTCAGCTCCTCTGAAATGCATTAGCGGAACCGTTTGCGATCTGCCA
CAAGTGTGATAAGTTATCTACACTGGCGAGGGGATTGCTCTCTGTAATGTTCAGCTTCTA
ATTGTCTCTACTTTGTGAGACTACTTTTGAATGCTTGACCTCAAATCAGGTAGGACTACC
CGCTGAACTTAA
>ABC3-28
TTTCCGTAGGTGAACCTGCGGAAGGATCATTATTGAATTATGTTTCTAGATAGGTTGTAG
CTGGCTCTTTTAGAGCATGTGCACGCCTGTTTGGACTTCATTTTCATCCACCTGTGCACC
TATTGTAGTCTTTGGTTGGGTTAGGAGGAAGTGATCATTGTATCAGCATCTGCTGGGAGT
GAGGACTTGCATTGTGAAAGCTTTGCTGTCCTTGATGTGATCATGGAATCTTTTTCTCAC
TAGAGTCTATGTCACTCATTATACTCTGTCGAATGTCATTGAATGTCTTTACATGGGCTT
GTATGCCTATGAAAATTGTAATACAACTTTCAGCAACGGATCTCTTGGCTCTCGCATCGA
TGAAGAACGCAGCGAAATGCGATAAGTAATGTGAATTGCAGAATTCAGTGAATCATCGAA
TCTTTGAACGCATCTTGCGCTCCTTGGTATTCCGAGGAGCATGCCTGTTTGAGTGTCATT
AAATTCTCAACTCTCTTATAC-TTTTTTGTAAAAGAGAGCTTGGACTGTGGAGGCTTGCT
GGCCACTTTTTGGGGTCAGCTCCTCTGAAATGCATTAGCGGAACCGTTTGCGATCTGCCA
CAAGTGTGATAAGTTATCTACACTGGCGAGGGGATTGCTCTCTGTAATGTTCAGCTTCTA
ATTGTCTCTACTTTGTGAGACTACTTTTGAATGCTTGACCTCAAATCAGGTAGGACTACC
CGCTGAACTTAA
>ABC3-29
TTTCCGTAGGTGAACCTGCGGAAGGATCATTATTGAATTATGTTTCTAGATAGGTTGTAG
CTGGCTCTTTTAGAGCATGTGCACGCCTGTTTGGACTTCATTTTCATCCACCTGTGCACC
TATTGTAGTCTTTGGTTGGGTTAGGAGGAAGTGATCATTGTATCAGCATCTGCTGGGAGT
GAGGACTTGCATTGTGAAAGCTTTGCTGTCCTTGATGTGATCATGGAATCTTTTTCTCAC
TAGAGTCTATGTCACTCATTATACTCTGTCGAATGTCATTGAATGTCTTTACATGGGCTT
GTATGCCTATGAAAATTGTAATACAACTTTCAGCAACGGATCTCTTGGCTCTCGCATCGA
TGAAGAACGCAGCGAAATGCGATAAGTAATGTGAATTGCAGAATTCAGTGAATCATCGAA
TCTTTGAACGCATCTTGCGCTCCTTGGTATTCCGAGGAGCATGCCTGTTTGAGTGTCATT
AAATTCTCAACTCTCTTATAC-TTTTTTGTAAAAGAGAGCTTGGACTGTGGAGGCTTGCT
GGCCACTTTTTGGGGTCAGCTCCTCTGAAATGCATTAGCGGAACCGTTTGCGATCTGCCA
CAAGTGTGATAAGTTATCTACACTGGCGAGGGGATTGCTCTCTGTAATGTTCAGCTTCTA
ATTGTCTCTACTTTGTGAGACTACTTTTGAATGCTTGACCTCAAATCAGGTAGGACTACC
CGCTGAACTTAA
>ABC3-36
TTTCCGTAGGTGAACCTGCGGAAGGATCATTATTGAATTATGTTTCTAGATAGGTTGTAG
CTGGCTCTTTTAGAGCATGTGCACGCCTGTTTGGACTTCATTTTCATCCACCTGTGCACC
TATTGTAGTCTTTGGTTGGGTTAGGAGGAAGTGATCATTGTATCAGCATCTGCTGGGAGT
GAGGACTTGCATTGTGAAAGCTTTGCTGTCCTTGATGTGATCATGGAATCTTTTTCTCAC
TAGAGTCTATGTCACTCATTATACTCTGTCGAATGTCATTGAATGTCTTTACATGGGCTT
GTATGCCTATGAAAATTGTAATACAACTTTCAGCAACGGATCTCTTGGCTCTCGCATCGA
TGAAGAACGCAGCGAAATGCGATAAGTAATGTGAATTGCAGAATTCAGTGAATCATCGAA

TCTTTGAACGCATCTTGCGCTCCTTGGTATTCCGAGGAGCATGCCTGTTTGAGTGTCATT
AAATTCTCAACTCTCTTATAC-TTTTTTGTAAAAGAGAGCTTGGACTGTGGAGGCTTGCT
GGCCACTTTTTGGGGTCAGCTCCTCTGAAATGCATTAGCGGAACCGTTTGCGATCTGCCA
CAAGTGTGATAAGTTATCTACACTGGCGAGGGGATTGCTCTCTGTAATGTTCAGCTTCTA
ATTGTCTCTACTTTGTGAGACTACTTTTGAATGCTTGACCTCAAATCAGGTAGGACTACC
CGCTGAACTTAA
>ABC3-44
TTTCCGTAGGTGAACCTGCGGAAGGATCATTATTGAATTATGTTTCTAGATAGGTTGTAG
CTGGCTCTTTTAGAGCATGTGCACGCCTGTTTGGACTTCATTTTCATCCACCTGTGCACC
TATTGTAGTCTTTGGTTGGGTTAGGAGGAAGTGATCATTGTATCAGCATCTGCTGGGAGT
GAGGACTTGCATTGTGAAAGCTTTGCTGTCCTTGATGTGATCATGGAATCTTTTTCTCAC
TAGAGTCTATGTCACTCATTATACTCTGTCGAATGTCATTGAATGTCTTTACATGGGCTT
GTATGCCTATGAAAATTGTAATACAACTTTCAGCAACGGATCTCTTGGCTCTCGCATCGA
TGAAGAACGCAGCGAAATGCGATAAGTAATGTGAATTGCAGAATTCAGTGAATCATCGAA
TCTTTGAACGCATCTTGCGCTCCTTGGTATTCCGAGGAGCATGCCTGTTTGAGTGTCATT
AAATTCTCAACTCTCTTATAC-TTTTTTGTAAAAGAGAGCTTGGACTGTGGAGGCTTGCT
GGCCACTTTTTGGGGTCAGCTCCTCTGAAATGCATTAGCGGAACCGTTTGCGATCTGCCA
CAAGTGTGATAAGTTATCTACACTGGCGAGGGGATTGCTCTCTGTAATGTTCAGCTTCTA
ATTGTCTCTACTTTGTGAGACTACTTTTGAATGCTTGACCTCAAATCAGGTAGGACTACC
CGCTGAACTTAA
>ABC3-52
TTTCCGTAGGTGAACCTGCGGAAGGATCATTATTGAATTATGTTTCTAGATAGGTTGTAG
CTGGCTCTTTTAGAGCATGTGCACGCCTGTTTGGACTTCATTTTCATCCACCTGTGCACC
TATTGTAGTCTTTGGTTGGGTTAGGAGGAAGTGATCATTGTATCAGCATCTGCTGGGAGT
GAGGACTTGCATTGTGAAAGCTTTGCTGTCCTTGATGTGATCATGGAATCTTTTTCTCAC
TAGAGTCTATGTCACTCATTATACTCTGTCGAATGTCATTGAATGTCTTTACATGGGCTT
GTATGCCTATGAAAATTGTAATACAACTTTCAGCAACGGATCTCTTGGCTCTCGCATCGA
TGAAGAACGCAGCGAAATGCGATAAGTAATGTGAATTGCAGAATTCAGTGAATCATCGAA
TCTTTGAACGCATCTTGCGCTCCTTGGTATTCCGAGGAGCATGCCTGTTTGAGTGTCATT
AAATTCTCAACTCTCTTATAC-TTTTTTGTAAAAGAGAGCTTGGACTGTGGAGGCTTGCT
GGCCACTTTTTGGGGTCAGCTCCTCTGAAATGCATTAGCGGAACCGTTTGCGATCTGCCA
CAAGTGTGATAAGTTATCTACACTGGCGAGGGGATTGCTCTCTGTAATGTTCAGCTTCTA
ATTGTCTCTACTTTGTGAGACTACTTTTGAATGCTTGACCTCAAATCAGGTAGGACTACC
CGCTGAACTTAA
>ABC4-6
TTTCCGTAGGTGAACCTGCGGAAGGATCATTATTGAATTATGTTTCTAGATAGGTTGTAG
CTGGCTCTTTTAGAGCATGTGCACGCCTGTTTGGACTTCATTTTCATCCACCTGTGCACC
TATTGTAGTCTTTGGTTGGGTTAGGAGGAAGTGATCATTGTATCAGCATCTGCTGGGAGT
GAGGACTTGCATTGTGAAAGCTTTGCTGTCCTTGATGTGATCATGGAATCTTTTTCTCAC
TAGAGTCTATGTCACTCATTATACTCTGTCGAATGTCATTGAATGTCTTTACATGGGCTT
GTATGCCTATGAAAATTGTAATACAACTTTCAGCAACGGATCTCTTGGCTCTCGCATCGA
TGAAGAACGCAGCGAAATGCGATAAGTAATGTGAATTGCAGAATTCAGTGAATCATCGAA
TCTTTGAACGCATCTTGCGCTCCTTGGTATTCCGAGGAGCATGCCTGTTTGAGTGTCATT
AAATTCTCAACTCTCTTATAC-TTTTTTGTAAAAGAGAGCTTGGACTGTGGAGGCTTGCT
GGCCACTTTTTGGGGTCAGCTCCTCTGAAATGCATTAGCGGAACCGTTTGCGATCTGCCA
CAAGTGTGATAAGTTATCTACACTGGCGAGGGGATTGCTCTCTGTAATGTTCAGCTTCTA
ATTGTCTCTACTTTGTGAGACTACTTTTGAATGCTTGACCTCAAATCAGGTAGGACTACC
CGCTGAACTTAA
>ABC4-7
TTTCCGTAGGTGAACCTGCGGAAGGATCATTATTGAATTATGTTTCTAGATAGGTTGTAG

CTGGCTCTTTTAGAGCATGTGCACGCCTGTTTGGACTTCATTTTCATCCACCTGTGCACC
TATTGTAGTCTTTGGTTGGGTTAGGAGGAAGTGATCATTGTATCAGCATCTGCTGGGAGT
GAGGACTTGCATTGTGAAAGCTTTGCTGTCCTTGATGTGATCATGGAATCTTTTTCTCAC
TAGAGTCTATGTCACTCATTATACTCTGTCGAATGTCATTGAATGTCTTTACATGGGCTT
GTATGCCTATGAAAATTGTAATACAACTTTCAGCAACGGATCTCTTGGCTCTCGCATCGA
TGAAGAACGCAGCGAAATGCGATAAGTAATGTGAATTGCAGAATTCAGTGAATCATCGAA
TCTTTGAACGCATCTTGCGCTCCTTGGTATTCCGAGGAGCATGCCTGTTTGAGTGTCATT
AAATTCTCAACTCTCTTATAC-TTTTTTGTAAAAGAGAGCTTGGACTGTGGAGGCTTGCT
GGCCACTTTTTGGGGTCAGCTCCTCTGAAATGCATTAGCGGAACCGTTTGCGATCTGCCA
CAAGTGTGATAAGTTATCTACACTGGCGAGGGGATTGCTCTCTGTAATGTTCAGCTTCTA
ATTGTCTCTACTTTGTGAGACTACTTTTGAATGCTTGACCTCAAATCAGGTAGGACTACC
CGCTGAACTTAA
>ABC4-8
TTTCCGTAGGTGAACCTGCGGAAGGATCATTATTGAATTATGTTTCTAGATAGGTTGTAG
CTGGCTCTTTTAGAGCATGTGCACGCCTGTTTGGACTTCATTTTCATCCACCTGTGCACC
TATTGTAGTCTTTGGTTGGGTTAGGAGGAAGTGATCATTGTATCAGCATCTGCTGGGAGT
GAGGACTTGCATTGTGAAAGCTTTGCTGTCCTTGATGTGATCATGGAATCTTTTTCTCAC
TAGAGTCTATGTCACTCATTATACTCTGTCGAATGTCATTGAATGTCTTTACATGGGCTT
GTATGCCTATGAAAATTGTAATACAACTTTCAGCAACGGATCTCTTGGCTCTCGCATCGA
TGAAGAACGCAGCGAAATGCGATAAGTAATGTGAATTGCAGAATTCAGTGAATCATCGAA
TCTTTGAACGCATCTTGCGCTCCTTGGTATTCCGAGGAGCATGCCTGTTTGAGTGTCATT
AAATTCTCAACTCTCTTATAC-TTTTTTGTAAAAGAGAGCTTGGACTGTGGAGGCTTGCT
GGCCACTTTTTGGGGTCAGCTCCTCTGAAATGCATTAGCGGAACCGTTTGCGATCTGCCA
CAAGTGTGATAAGTTATCTACACTGGCGAGGGGATTGCTCTCTGTAATGTTCAGCTTCTA
ATTGTCTCTACTTTGTGAGACTACTTTTGAATGCTTGACCTCAAATCAGGTAGGACTACC
CGCTGAACTTAA
>ABC4-11
TTTCCGTAGGTGAACCTGCGGAAGGATCATTATTGAATTATGTTTCTAGATAGGTTGTAG
CTGGCTCTTTTAGAGCATGTGCACGCCTGTTTGGACTTCATTTTCATCCACCTGTGCACC
TATTGTAGTCTTTGGTTGGGTTAGGAGGAAGTGATCATTGTATCAGCATCTGCTGGGAGT
GAGGACTTGCATTGTGAAAGCTTTGCTGTCCTTGATGTGATCATGGAATCTTTTTCTCAC
TAGAGTCTATGTCACTCATTATACTCTGTCGAATGTCATTGAATGTCTTTACATGGGCTT
GTATGCCTATGAAAATTGTAATACAACTTTCAGCAACGGATCTCTTGGCTCTCGCATCGA
TGAAGAACGCAGCGAAATGCGATAAGTAATGTGAATTGCAGAATTCAGTGAATCATCGAA
TCTTTGAACGCATCTTGCGCTCCTTGGTATTCCGAGGAGCATGCCTGTTTGAGTGTCATT
AAATTCTCAACTCTCTTATAC-TTTTTTGTAAAAGAGAGCTTGGACTGTGGAGGCTTGCT
GGCCACTTTTTGGGGTCAGCTCCTCTGAAATGCATTAGCGGAACCGTTTGCGATCTGCCA
CAAGTGTGATAAGTTATCTACACTGGCGAGGGGATTGCTCTCTGTAATGTTCAGCTTCTA
ATTGTCTCTACTTTGTGAGACTACTTTTGAATGCTTGACCTCAAATCAGGTAGGACTACC
CGCTGAACTTAA
>ABC4-24
TTTCCGTAGGTGAACCTGCGGAAGGATCATTATTGAATTATGTTTCTAGATAGGTTGTAG
CTGGCTCTTTTAGAGCATGTGCACGCCTGTTTGGACTTCATTTTCATCCACCTGTGCACC
TATTGTAGTCTTTGGTTGGGTTAGGAGGAAGTGATCATTGTATCAGCATCTGCTGGGAGT
GAGGACTTGCATTGTGAAAGCTTTGCTGTCCTTGATGTGATCATGGAATCTTTTTCTCAC
TAGAGTCTATGTCACTCATTATACTCTGTCGAATGTCATTGAATGTCTTTACATGGGCTT
GTATGCCTATGAAAATTGTAATACAACTTTCAGCAACGGATCTCTTGGCTCTCGCATCGA
TGAAGAACGCAGCGAAATGCGATAAGTAATGTGAATTGCAGAATTCAGTGAATCATCGAA
TCTTTGAACGCATCTTGCGCTCCTTGGTATTCCGAGGAGCATGCCTGTTTGAGTGTCATT
AAATTCTCAACTCTCTTATAC-TTTTTTGTAAAAGAGAGCTTGGACTGTGGAGGCTTGCT

GGCCACTTTTTGGGGTCAGCTCCTCTGAAATGCATTAGCGGAACCGTTTGCGATCTGCCA
CAAGTGTGATAAGTTATCTACACTGGCGAGGGGATTGCTCTCTGTAATGTTCAGCTTCTA
ATTGTCTCTACTTTGTGAGACTACTTTTGAATGCTTGACCTCAAATCAGGTAGGACTACC
CGCTGAACTTAA
>ABC4-37
TTTCCGTAGGTGAACCTGCGGAAGGATCATTATTGAATTATGTTTCTAGATAGGTTGTAG
CTGGCTCTTTTAGAGCATGTGCACGCCTGTTTGGACTTCATTTTCATCCACCTGTGCACC
TATTGTAGTCTTTGGTTGGGTTAGGAGGAAGTGATCATTGTATCAGCATCTGCTGGGAGT
GAGGACTTGCATTGTGAAAGCTTTGCTGTCCTTGATGTGATCATGGAATCTTTTTCTCAC
TAGAGTCTATGTCACTCATTATACTCTGTCGAATGTCATTGAATGTCTTTACATGGGCTT
GTATGCCTATGAAAATTGTAATACAACTTTCAGCAACGGATCTCTTGGCTCTCGCATCGA
TGAAGAACGCAGCGAAATGCGATAAGTAATGTGAATTGCAGAATTCAGTGAATCATCGAA
TCTTTGAACGCATCTTGCGCTCCTTGGTATTCCGAGGAGCATGCCTGTTTGAGTGTCATT
AAATTCTCAACTCTCTTATAC-TTTTTTGTAAAAGAGAGCTTGGACTGTGGAGGCTTGCT
GGCCACTTTTTGGGGTCAGCTCCTCTGAAATGCATTAGCGGAACCGTTTGCGATCTGCCA
CAAGTGTGATAAGTTATCTACACTGGCGAGGGGATTGCTCTCTGTAATGTTCAGCTTCTA
ATTGTCTCTACTTTGTGAGACTACTTTTGAATGCTTGACCTCAAATCAGGTAGGACTACC
CGCTGAACTTAA
>ABC4-41
TTTCCGTAGGTGAACCTGCGGAAGGATCATTATTGAATTATGTTTCTAGATAGGTTGTAG
CTGGCTCTTTTAGAGCATGTGCACGCCTGTTTGGACTTCATTTTCATCCACCTGTGCACC
TATTGTAGTCTTTGGTTGGGTTAGGAGGAAGTGATCATTGTATCAGCATCTGCTGGGAGT
GAGGACTTGCATTGTGAAAGCTTTGCTGTCCTTGATGTGATCATGGAATCTTTTTCTCAC
TAGAGTCTATGTCACTCATTATACTCTGTCGAATGTCATTGAATGTCTTTACATGGGCTT
GTATGCCTATGAAAATTGTAATACAACTTTCAGCAACGGATCTCTTGGCTCTCGCATCGA
TGAAGAACGCAGCGAAATGCGATAAGTAATGTGAATTGCAGAATTCAGTGAATCATCGAA
TCTTTGAACGCATCTTGCGCTCCTTGGTATTCCGAGGAGCATGCCTGTTTGAGTGTCATT
AAATTCTCAACTCTCTTATAC-TTTTTTGTAAAAGAGAGCTTGGACTGTGGAGGCTTGCT
GGCCACTTTTTGGGGTCAGCTCCTCTGAAATGCATTAGCGGAACCGTTTGCGATCTGCCA
CAAGTGTGATAAGTTATCTACACTGGCGAGGGGATTGCTCTCTGTAATGTTCAGCTTCTA
ATTGTCTCTACTTTGTGAGACTACTTTTGAATGCTTGACCTCAAATCAGGTAGGACTACC
CGCTGAACTTAA
>ABC4-45
TTTCCGTAGGTGAACCTGCGGAAGGATCATTATTGAATTATGTTTCTAGATAGGTTGTAG
CTGGCTCTTTTAGAGCATGTGCACGCCTGTTTGGACTTCATTTTCATCCACCTGTGCACC
TATTGTAGTCTTTGGTTGGGTTAGGAGGAAGTGATCATTGTATCAGCATCTGCTGGGAGT
GAGGACTTGCATTGTGAAAGCTTTGCTGTCCTTGATGTGATCATGGAATCTTTTTCTCAC
TAGAGTCTATGTCACTCATTATACTCTGTCGAATGTCATTGAATGTCTTTACATGGGCTT
GTATGCCTATGAAAATTGTAATACAACTTTCAGCAACGGATCTCTTGGCTCTCGCATCGA
TGAAGAACGCAGCGAAATGCGATAAGTAATGTGAATTGCAGAATTCAGTGAATCATCGAA
TCTTTGAACGCATCTTGCGCTCCTTGGTATTCCGAGGAGCATGCCTGTTTGAGTGTCATT
AAATTCTCAACTCTCTTATAC-TTTTTTGTAAAAGAGAGCTTGGACTGTGGAGGCTTGCT
GGCCACTTTTTGGGGTCAGCTCCTCTGAAATGCATTAGCGGAACCGTTTGCGATCTGCCA
CAAGTGTGATAAGTTATCTACACTGGCGAGGGGATTGCTCTCTGTAATGTTCAGCTTCTA
ATTGTCTCTACTTTGTGAGACTACTTTTGAATGCTTGACCTCAAATCAGGTAGGACTACC
CGCTGAACTTAA
>ABC4-48
TTTCCGTAGGTGAACCTGCGGAAGGATCATTATTGAATTATGTTTCTAGATAGGTTGTAG
CTGGCTCTTTTAGAGCATGTGCACGCCTGTTTGGACTTCATTTTCATCCACCTGTGCACC
TATTGTAGTCTTTGGTTGGGTTAGGAGGAAGTGATCATTGTATCAGCATCTGCTGGGAGT

GAGGACTTGCATTGTGAAAGCTTTGCTGTCCTTGATGTGATCATGGAATCTTTTTCTCAC
TAGAGTCTATGTCACTCATTATACTCTGTCGAATGTCATTGAATGTCTTTACATGGGCTT
GTATGCCTATGAAAATTGTAATACAACTTTCAGCAACGGATCTCTTGGCTCTCGCATCGA
TGAAGAACGCAGCGAAATGCGATAAGTAATGTGAATTGCAGAATTCAGTGAATCATCGAA
TCTTTGAACGCATCTTGCGCTCCTTGGTATTCCGAGGAGCATGCCTGTTTGAGTGTCATT
AAATTCTCAACTCTCTTATAC-TTTTTTGTAAAAGAGAGCTTGGACTGTGGAGGCTTGCT
GGCCACTTTTTGGGGTCAGCTCCTCTGAAATGCATTAGCGGAACCGTTTGCGATCTGCCA
CAAGTGTGATAAGTTATCTACACTGGCGAGGGGATTGCTCTCTGTAATGTTCAGCTTCTA
ATTGTCTCTACTTTGTGAGACTACTTTTGAATGCTTGACCTCAAATCAGGTAGGACTACC
CGCTGAACTTAA
>ABC4-49
TTTCCGTAGGTGAACCTGCGGAAGGATCATTATTGAATTATGTTTCTAGATAGGTTGTAG
CTGGCTCTTTTAGAGCATGTGCACGCCTGTTTGGACTTCATTTTCATCCACCTGTGCACC
TATTGTAGTCTTTGGTTGGGTTAGGAGGAAGTGATCATTGTATCAGCATCTGCTGGGAGT
GAGGACTTGCATTGTGAAAGCTTTGCTGTCCTTGATGTGATCATGGAATCTTTTTCTCAC
TAGAGTCTATGTCACTCATTATACTCTGTCGAATGTCATTGAATGTCTTTACATGGGCTT
GTATGCCTATGAAAATTGTAATACAACTTTCAGCAACGGATCTCTTGGCTCTCGCATCGA
TGAAGAACGCAGCGAAATGCGATAAGTAATGTGAATTGCAGAATTCAGTGAATCATCGAA
TCTTTGAACGCATCTTGCGCTCCTTGGTATTCCGAGGAGCATGCCTGTTTGAGTGTCATT
AAATTCTCAACTCTCTTATAC-TTTTTTGTAAAAGAGAGCTTGGACTGTGGAGGCTTGCT
GGCCACTTTTTGGGGTCAGCTCCTCTGAAATGCATTAGCGGAACCGTTTGCGATCTGCCA
CAAGTGTGATAAGTTATCTACACTGGCGAGGGGATTGCTCTCTGTAATGTTCAGCTTCTA
ATTGTCTCTACTTTGTGAGACTACTTTTGAATGCTTGACCTCAAATCAGGTAGGACTACC
CGCTGAACTTAA
>ABC4-63
TTTCCGTAGGTGAACCTGCGGAAGGATCATTATTGAATTATGTTTCTAGATAGGTTGTAG
CTGGCTCTTTTAGAGCATGTGCACGCCTGTTTGGACTTCATTTTCATCCACCTGTGCACC
TATTGTAGTCTTTGGTTGGGTTAGGAGGAAGTGATCATTGTATCAGCATCTGCTGGGAGT
GAGGACTTGCATTGTGAAAGCTTTGCTGTCCTTGATGTGATCATGGAATCTTTTTCTCAC
TAGAGTCTATGTCACTCATTATACTCTGTCGAATGTCATTGAATGTCTTTACATGGGCTT
GTATGCCTATGAAAATTGTAATACAACTTTCAGCAACGGATCTCTTGGCTCTCGCATCGA
TGAAGAACGCAGCGAAATGCGATAAGTAATGTGAATTGCAGAATTCAGTGAATCATCGAA
TCTTTGAACGCATCTTGCGCTCCTTGGTATTCCGAGGAGCATGCCTGTTTGAGTGTCATT
AAATTCTCAACTCTCTTATAC-TTTTTTGTAAAAGAGAGCTTGGACTGTGGAGGCTTGCT
GGCCACTTTTTGGGGTCAGCTCCTCTGAAATGCATTAGCGGAACCGTTTGCGATCTGCCA
CAAGTGTGATAAGTTATCTACACTGGCGAGGGGATTGCTCTCTGTAATGTTCAGCTTCTA
ATTGTCTCTACTTTGTGAGACTACTTTTGAATGCTTGACCTCAAATCAGGTAGGACTACC
CGCTGAACTTAA
>ABC4-64
TTTCCGTAGGTGAACCTGCGGAAGGATCATTATTGAATTATGTTTCTAGATAGGTTGTAG
CTGGCTCTTTTAGAGCATGTGCACGCCTGTTTGGACTTCATTTTCATCCACCTGTGCACC
TATTGTAGTCTTTGGTTGGGTTAGGAGGAAGTGATCATTGTATCAGCATCTGCTGGGAGT
GAGGACTTGCATTGTGAAAGCTTTGCTGTCCTTGATGTGATCATGGAATCTTTTTCTCAC
TAGAGTCTATGTCACTCATTATACTCTGTCGAATGTCATTGAATGTCTTTACATGGGCTT
GTATGCCTATGAAAATTGTAATACAACTTTCAGCAACGGATCTCTTGGCTCTCGCATCGA
TGAAGAACGCAGCGAAATGCGATAAGTAATGTGAATTGCAGAATTCAGTGAATCATCGAA
TCTTTGAACGCATCTTGCGCTCCTTGGTATTCCGAGGAGCATGCCTGTTTGAGTGTCATT
AAATTCTCAACTCTCTTATAC-TTTTTTGTAAAAGAGAGCTTGGACTGTGGAGGCTTGCT
GGCCACTTTTTGGGGTCAGCTCCTCTGAAATGCATTAGCGGAACCGTTTGCGATCTGCCA
CAAGTGTGATAAGTTATCTACACTGGCGAGGGGATTGCTCTCTGTAATGTTCAGCTTCTA

ATTGTCTCTACTTTGTGAGACTACTTTTGAATGCTTGACCTCAAATCAGGTAGGACTACC
CGCTGAACTTAA
>ABC4-73
TTTCCGTAGGTGAACCTGCGGAAGGATCATTATTGAATTATGTTTCTAGATAGGTTGTAG
CTGGCTCTTTTAGAGCATGTGCACGCCTGTTTGGACTTCATTTTCATCCACCTGTGCACC
TATTGTAGTCTTTGGTTGGGTTAGGAGGAAGTGATCATTGTATCAGCATCTGCTGGGAGT
GAGGACTTGCATTGTGAAAGCTTTGCTGTCCTTGATGTGATCATGGAATCTTTTTCTCAC
TAGAGTCTATGTCACTCATTATACTCTGTCGAATGTCATTGAATGTCTTTACATGGGCTT
GTATGCCTATGAAAATTGTAATACAACTTTCAGCAACGGATCTCTTGGCTCTCGCATCGA
TGAAGAACGCAGCGAAATGCGATAAGTAATGTGAATTGCAGAATTCAGTGAATCATCGAA
TCTTTGAACGCATCTTGCGCTCCTTGGTATTCCGAGGAGCATGCCTGTTTGAGTGTCATT
AAATTCTCAACTCTCTTATAC-TTTTTTGTAAAAGAGAGCTTGGACTGTGGAGGCTTGCT
GGCCACTTTTTGGGGTCAGCTCCTCTGAAATGCATTAGCGGAACCGTTTGCGATCTGCCA
CAAGTGTGATAAGTTATCTACACTGGCGAGGGGATTGCTCTCTGTAATGTTCAGCTTCTA
ATTGTCTCTACTTTGTGAGACTACTTTTGAATGCTTGACCTCAAATCAGGTAGGACTACC
CGCTGAACTTAA
>ABC4-74
TTTCCGTAGGTGAACCTGCGGAAGGATCATTATTGAATTATGTTTCTAGATAGGTTGTAG
CTGGCTCTTTTAGAGCATGTGCACGCCTGTTTGGACTTCATTTTCATCCACCTGTGCACC
TATTGTAGTCTTTGGTTGGGTTAGGAGGAAGTGATCATTGTATCAGCATCTGCTGGGAGT
GAGGACTTGCATTGTGAAAGCTTTGCTGTCCTTGATGTGATCATGGAATCTTTTTCTCAC
TAGAGTCTATGTCACTCATTATACTCTGTCGAATGTCATTGAATGTCTTTACATGGGCTT
GTATGCCTATGAAAATTGTAATACAACTTTCAGCAACGGATCTCTTGGCTCTCGCATCGA
TGAAGAACGCAGCGAAATGCGATAAGTAATGTGAATTGCAGAATTCAGTGAATCATCGAA
TCTTTGAACGCATCTTGCGCTCCTTGGTATTCCGAGGAGCATGCCTGTTTGAGTGTCATT
AAATTCTCAACTCTCTTATAC-TTTTTTGTAAAAGAGAGCTTGGACTGTGGAGGCTTGCT
GGCCACTTTTTGGGGTCAGCTCCTCTGAAATGCATTAGCGGAACCGTTTGCGATCTGCCA
CAAGTGTGATAAGTTATCTACACTGGCGAGGGGATTGCTCTCTGTAATGTTCAGCTTCTA
ATTGTCTCTACTTTGTGAGACTACTTTTGAATGCTTGACCTCAAATCAGGTAGGACTACC
CGCTGAACTTAA
>ABC4-76
TTTCCGTAGGTGAACCTGCGGAAGGATCATTATTGAATTATGTTTCTAGATAGGTTGTAG
CTGGCTCTTTTAGAGCATGTGCACGCCTGTTTGGACTTCATTTTCATCCACCTGTGCACC
TATTGTAGTCTTTGGTTGGGTTAGGAGGAAGTGATCATTGTATCAGCATCTGCTGGGAGT
GAGGACTTGCATTGTGAAAGCTTTGCTGTCCTTGATGTGATCATGGAATCTTTTTCTCAC
TAGAGTCTATGTCACTCATTATACTCTGTCGAATGTCATTGAATGTCTTTACATGGGCTT
GTATGCCTATGAAAATTGTAATACAACTTTCAGCAACGGATCTCTTGGCTCTCGCATCGA
TGAAGAACGCAGCGAAATGCGATAAGTAATGTGAATTGCAGAATTCAGTGAATCATCGAA
TCTTTGAACGCATCTTGCGCTCCTTGGTATTCCGAGGAGCATGCCTGTTTGAGTGTCATT
AAATTCTCAACTCTCTTATAC-TTTTTTGTAAAAGAGAGCTTGGACTGTGGAGGCTTGCT
GGCCACTTTTTGGGGTCAGCTCCTCTGAAATGCATTAGCGGAACCGTTTGCGATCTGCCA
CAAGTGTGATAAGTTATCTACACTGGCGAGGGGATTGCTCTCTGTAATGTTCAGCTTCTA
ATTGTCTCTACTTTGTGAGACTACTTTTGAATGCTTGACCTCAAATCAGGTAGGACTACC
CGCTGAACTTAA
>ABC4-78
TTTCCGTAGGTGAACCTGCGGAAGGATCATTATTGAATTATGTTTCTAGATAGGTTGTAG
CTGGCTCTTTTAGAGCATGTGCACGCCTGTTTGGACTTCATTTTCATCCACCTGTGCACC
TATTGTAGTCTTTGGTTGGGTTAGGAGGAAGTGATCATTGTATCAGCATCTGCTGGGAGT
GAGGACTTGCATTGTGAAAGCTTTGCTGTCCTTGATGTGATCATGGAATCTTTTTCTCAC
TAGAGTCTATGTCACTCATTATACTCTGTCGAATGTCATTGAATGTCTTTACATGGGCTT

GTATGCCTATGAAAATTGTAATACAACTTTCAGCAACGGATCTCTTGGCTCTCGCATCGA
TGAAGAACGCAGCGAAATGCGATAAGTAATGTGAATTGCAGAATTCAGTGAATCATCGAA
TCTTTGAACGCATCTTGCGCTCCTTGGTATTCCGAGGAGCATGCCTGTTTGAGTGTCATT
AAATTCTCAACTCTCTTATAC-TTTTTTGTAAAAGAGAGCTTGGACTGTGGAGGCTTGCT
GGCCACTTTTTGGGGTCAGCTCCTCTGAAATGCATTAGCGGAACCGTTTGCGATCTGCCA
CAAGTGTGATAAGTTATCTACACTGGCGAGGGGATTGCTCTCTGTAATGTTCAGCTTCTA
ATTGTCTCTACTTTGTGAGACTACTTTTGAATGCTTGACCTCAAATCAGGTAGGACTACC
CGCTGAACTTAA
>ABC5-19
TTTCCGTAGGTGAACCTGCGGAAGGATCATTATTGAATTATGTTTCTAGATAGGTTGTAG
CTGGCTCTTTTAGAGCATGTGCACGCCTGTTTGGACTTCATTTTCATCCACCTGTGCACC
TATTGTAGTCTTTGGTTGGGTTAGGAGGAAGTGATCATTGTATCAGCATCTGCTGGGAGT
GAGGACTTGCATTGTGAAAGCTTTGCTGTCCTTGATGTGATCATGGAATCTTTTTCTCAC
TAGAGTCTATGTCACTCATTATACTCTGTCGAATGTCATTGAATGTCTTTACATGGGCTT
GTATGCCTATGAAAATTGTAATACAACTTTCAGCAACGGATCTCTTGGCTCTCGCATCGA
TGAAGAACGCAGCGAAATGCGATAAGTAATGTGAATTGCAGAATTCAGTGAATCATCGAA
TCTTTGAACGCATCTTGCGCTCCTTGGTATTCCGAGGAGCATGCCTGTTTGAGTGTCATT
AAATTCTCAACTCTCTTATAC-TTTTTTGTAAAAGAGAGCTTGGACTGTGGAGGCTTGCT
GGCCACTTTTTGGGGTCAGCTCCTCTGAAATGCATTAGCGGAACCGTTTGCGATCTGCCA
CAAGTGTGATAAGTTATCTACACTGGCGAGGGGATTGCTCTCTGTAATGTTCAGCTTCTA
ATTGTCTCTACTTTGTGAGACTACTTTTGAATGCTTGACCTCAAATCAGGTAGGACTACC
CGCTGAACTTAA
>ABC5-22
TTTCCGTAGGTGAACCTGCGGAAGGATCATTATTGAATTATGTTTCTAGATAGGTTGTAG
CTGGCTCTTTTAGAGCATGTGCACGCCTGTTTGGACTTCATTTTCATCCACCTGTGCACC
TATTGTAGTCTTTGGTTGGGTTAGGAGGAAGTGATCATTGTATCAGCATCTGCTGGGAGT
GAGGACTTGCATTGTGAAAGCTTTGCTGTCCTTGATGTGATCATGGAATCTTTTTCTCAC
TAGAGTCTATGTCACTCATTATACTCTGTCGAATGTCATTGAATGTCTTTACATGGGCTT
GTATGCCTATGAAAATTGTAATACAACTTTCAGCAACGGATCTCTTGGCTCTCGCATCGA
TGAAGAACGCAGCGAAATGCGATAAGTAATGTGAATTGCAGAATTCAGTGAATCATCGAA
TCTTTGAACGCATCTTGCGCTCCTTGGTATTCCGAGGAGCATGCCTGTTTGAGTGTCATT
AAATTCTCAACTCTCTTATAC-TTTTTTGTAAAAGAGAGCTTGGACTGTGGAGGCTTGCT
GGCCACTTTTTGGGGTCAGCTCCTCTGAAATGCATTAGCGGAACCGTTTGCGATCTGCCA
CAAGTGTGATAAGTTATCTACACTGGCGAGGGGATTGCTCTCTGTAATGTTCAGCTTCTA
ATTGTCTCTACTTTGTGAGACTACTTTTGAATGCTTGACCTCAAATCAGGTAGGACTACC
CGCTGAACTTAA
>ABC5-28
TTTCCGTAGGTGAACCTGCGGAAGGATCATTATTGAATTATGTTTCTAGATAGGTTGTAG
CTGGCTCTTTTAGAGCATGTGCACGCCTGTTTGGACTTCATTTTCATCCACCTGTGCACC
TATTGTAGTCTTTGGTTGGGTTAGGAGGAAGTGATCATTGTATCAGCATCTGCTGGGAGT
GAGGACTTGCATTGTGAAAGCTTTGCTGTCCTTGATGTGATCATGGAATCTTTTTCTCAC
TAGAGTCTATGTCACTCATTATACTCTGTCGAATGTCATTGAATGTCTTTACATGGGCTT
GTATGCCTATGAAAATTGTAATACAACTTTCAGCAACGGATCTCTTGGCTCTCGCATCGA
TGAAGAACGCAGCGAAATGCGATAAGTAATGTGAATTGCAGAATTCAGTGAATCATCGAA
TCTTTGAACGCATCTTGCGCTCCTTGGTATTCCGAGGAGCATGCCTGTTTGAGTGTCATT
AAATTCTCAACTCTCTTATAC-TTTTTTGTAAAAGAGAGCTTGGACTGTGGAGGCTTGCT
GGCCACTTTTTGGGGTCAGCTCCTCTGAAATGCATTAGCGGAACCGTTTGCGATCTGCCA
CAAGTGTGATAAGTTATCTACACTGGCGAGGGGATTGCTCTCTGTAATGTTCAGCTTCTA
ATTGTCTCTACTTTGTGAGACTACTTTTGAATGCTTGACCTCAAATCAGGTAGGACTACC
CGCTGAACTTAA

>ABC5-32
TTTCCGTAGGTGAACCTGCGGAAGGATCATTATTGAATTATGTTTCTAGATAGGTTGTAG
CTGGCTCTTTTAGAGCATGTGCACGCCTGTTTGGACTTCATTTTCATCCACCTGTGCACC
TATTGTAGTCTTTGGTTGGGTTAGGAGGAAGTGATCATTGTATCAGCATCTGCTGGGAGT
GAGGACTTGCATTGTGAAAGCTTTGCTGTCCTTGATGTGATCATGGAATCTTTTTCTCAC
TAGAGTCTATGTCACTCATTATACTCTGTCGAATGTCATTGAATGTCTTTACATGGGCTT
GTATGCCTATGAAAATTGTAATACAACTTTCAGCAACGGATCTCTTGGCTCTCGCATCGA
TGAAGAACGCAGCGAAATGCGATAAGTAATGTGAATTGCAGAATTCAGTGAATCATCGAA
TCTTTGAACGCATCTTGCGCTCCTTGGTATTCCGAGGAGCATGCCTGTTTGAGTGTCATT
AAATTCTCAACTCTCTTATAC-TTTTTTGTAAAAGAGAGCTTGGACTGTGGAGGCTTGCT
GGCCACTTTTTGGGGTCAGCTCCTCTGAAATGCATTAGCGGAACCGTTTGCGATCTGCCA
CAAGTGTGATAAGTTATCTACACTGGCGAGGGGATTGCTCTCTGTAATGTTCAGCTTCTA
ATTGTCTCTACTTTGTGAGACTACTTTTGAATGCTTGACCTCAAATCAGGTAGGACTACC
CGCTGAACTTAA
>ABC5-35
TTTCCGTAGGTGAACCTGCGGAAGGATCATTATTGAATTATGTTTCTAGATAGGTTGTAG
CTGGCTCTTTTAGAGCATGTGCACGCCTGTTTGGACTTCATTTTCATCCACCTGTGCACC
TATTGTAGTCTTTGGTTGGGTTAGGAGGAAGTGATCATTGTATCAGCATCTGCTGGGAGT
GAGGACTTGCATTGTGAAAGCTTTGCTGTCCTTGATGTGATCATGGAATCTTTTTCTCAC
TAGAGTCTATGTCACTCATTATACTCTGTCGAATGTCATTGAATGTCTTTACATGGGCTT
GTATGCCTATGAAAATTGTAATACAACTTTCAGCAACGGATCTCTTGGCTCTCGCATCGA
TGAAGAACGCAGCGAAATGCGATAAGTAATGTGAATTGCAGAATTCAGTGAATCATCGAA
TCTTTGAACGCATCTTGCGCTCCTTGGTATTCCGAGGAGCATGCCTGTTTGAGTGTCATT
AAATTCTCAACTCTCTTATAC-TTTTTTGTAAAAGAGAGCTTGGACTGTGGAGGCTTGCT
GGCCACTTTTTGGGGTCAGCTCCTCTGAAATGCATTAGCGGAACCGTTTGCGATCTGCCA
CAAGTGTGATAAGTTATCTACACTGGCGAGGGGATTGCTCTCTGTAATGTTCAGCTTCTA
ATTGTCTCTACTTTGTGAGACTACTTTTGAATGCTTGACCTCAAATCAGGTAGGACTACC
CGCTGAACTTAA
>ABC5-43
TTTCCGTAGGTGAACCTGCGGAAGGATCATTATTGAATTATGTTTCTAGATAGGTTGTAG
CTGGCTCTTTTAGAGCATGTGCACGCCTGTTTGGACTTCATTTTCATCCACCTGTGCACC
TATTGTAGTCTTTGGTTGGGTTAGGAGGAAGTGATCATTGTATCAGCATCTGCTGGGAGT
GAGGACTTGCATTGTGAAAGCTTTGCTGTCCTTGATGTGATCATGGAATCTTTTTCTCAC
TAGAGTCTATGTCACTCATTATACTCTGTCGAATGTCATTGAATGTCTTTACATGGGCTT
GTATGCCTATGAAAATTGTAATACAACTTTCAGCAACGGATCTCTTGGCTCTCGCATCGA
TGAAGAACGCAGCGAAATGCGATAAGTAATGTGAATTGCAGAATTCAGTGAATCATCGAA
TCTTTGAACGCATCTTGCGCTCCTTGGTATTCCGAGGAGCATGCCTGTTTGAGTGTCATT
AAATTCTCAACTCTCTTATAC-TTTTTTGTAAAAGAGAGCTTGGACTGTGGAGGCTTGCT
GGCCACTTTTTGGGGTCAGCTCCTCTGAAATGCATTAGCGGAACCGTTTGCGATCTGCCA
CAAGTGTGATAAGTTATCTACACTGGCGAGGGGATTGCTCTCTGTAATGTTCAGCTTCTA
ATTGTCTCTACTTTGTGAGACTACTTTTGAATGCTTGACCTCAAATCAGGTAGGACTACC
CGCTGAACTTAA
>ABC5-62
TTTCCGTAGGTGAACCTGCGGAAGGATCATTATTGAATTATGTTTCTAGATAGGTTGTAG
CTGGCTCTTTTAGAGCATGTGCACGCCTGTTTGGACTTCATTTTCATCCACCTGTGCACC
TATTGTAGTCTTTGGTTGGGTTAGGAGGAAGTGATCATTGTATCAGCATCTGCTGGGAGT
GAGGACTTGCATTGTGAAAGCTTTGCTGTCCTTGATGTGATCATGGAATCTTTTTCTCAC
TAGAGTCTATGTCACTCATTATACTCTGTCGAATGTCATTGAATGTCTTTACATGGGCTT
GTATGCCTATGAAAATTGTAATACAACTTTCAGCAACGGATCTCTTGGCTCTCGCATCGA
TGAAGAACGCAGCGAAATGCGATAAGTAATGTGAATTGCAGAATTCAGTGAATCATCGAA

TCTTTGAACGCATCTTGCGCTCCTTGGTATTCCGAGGAGCATGCCTGTTTGAGTGTCATT
AAATTCTCAACTCTCTTATAC-TTTTTTGTAAAAGAGAGCTTGGACTGTGGAGGCTTGCT
GGCCACTTTTTGGGGTCAGCTCCTCTGAAATGCATTAGCGGAACCGTTTGCGATCTGCCA
CAAGTGTGATAAGTTATCTACACTGGCGAGGGGATTGCTCTCTGTAATGTTCAGCTTCTA
ATTGTCTCTACTTTGTGAGACTACTTTTGAATGCTTGACCTCAAATCAGGTAGGACTACC
CGCTGAACTTAA
>ABC5-65
TTTCCGTAGGTGAACCTGCGGAAGGATCATTATTGAATTATGTTTCTAGATAGGTTGTAG
CTGGCTCTTTTAGAGCATGTGCACGCCTGTTTGGACTTCATTTTCATCCACCTGTGCACC
TATTGTAGTCTTTGGTTGGGTTAGGAGGAAGTGATCATTGTATCAGCATCTGCTGGGAGT
GAGGACTTGCATTGTGAAAGCTTTGCTGTCCTTGATGTGATCATGGAATCTTTTTCTCAC
TAGAGTCTATGTCACTCATTATACTCTGTCGAATGTCATTGAATGTCTTTACATGGGCTT
GTATGCCTATGAAAATTGTAATACAACTTTCAGCAACGGATCTCTTGGCTCTCGCATCGA
TGAAGAACGCAGCGAAATGCGATAAGTAATGTGAATTGCAGAATTCAGTGAATCATCGAA
TCTTTGAACGCATCTTGCGCTCCTTGGTATTCCGAGGAGCATGCCTGTTTGAGTGTCATT
AAATTCTCAACTCTCTTATAC-TTTTTTGTAAAAGAGAGCTTGGACTGTGGAGGCTTGCT
GGCCACTTTTTGGGGTCAGCTCCTCTGAAATGCATTAGCGGAACCGTTTGCGATCTGCCA
CAAGTGTGATAAGTTATCTACACTGGCGAGGGGATTGCTCTCTGTAATGTTCAGCTTCTA
ATTGTCTCTACTTTGTGAGACTACTTTTGAATGCTTGACCTCAAATCAGGTAGGACTACC
CGCTGAACTTAA
>ABC5-69
TTTCCGTAGGTGAACCTGCGGAAGGATCATTATTGAATTATGTTTCTAGATAGGTTGTAG
CTGGCTCTTTTAGAGCATGTGCACGCCTGTTTGGACTTCATTTTCATCCACCTGTGCACC
TATTGTAGTCTTTGGTTGGGTTAGGAGGAAGTGATCATTGTATCAGCATCTGCTGGGAGT
GAGGACTTGCATTGTGAAAGCTTTGCTGTCCTTGATGTGATCATGGAATCTTTTTCTCAC
TAGAGTCTATGTCACTCATTATACTCTGTCGAATGTCATTGAATGTCTTTACATGGGCTT
GTATGCCTATGAAAATTGTAATACAACTTTCAGCAACGGATCTCTTGGCTCTCGCATCGA
TGAAGAACGCAGCGAAATGCGATAAGTAATGTGAATTGCAGAATTCAGTGAATCATCGAA
TCTTTGAACGCATCTTGCGCTCCTTGGTATTCCGAGGAGCATGCCTGTTTGAGTGTCATT
AAATTCTCAACTCTCTTATAC-TTTTTTGTAAAAGAGAGCTTGGACTGTGGAGGCTTGCT
GGCCACTTTTTGGGGTCAGCTCCTCTGAAATGCATTAGCGGAACCGTTTGCGATCTGCCA
CAAGTGTGATAAGTTATCTACACTGGCGAGGGGATTGCTCTCTGTAATGTTCAGCTTCTA
ATTGTCTCTACTTTGTGAGACTACTTTTGAATGCTTGACCTCAAATCAGGTAGGACTACC
CGCTGAACTTAA
>ABC5-76
TTTCCGTAGGTGAACCTGCGGAAGGATCATTATTGAATTATGTTTCTAGATAGGTTGTAG
CTGGCTCTTTTAGAGCATGTGCACGCCTGTTTGGACTTCATTTTCATCCACCTGTGCACC
TATTGTAGTCTTTGGTTGGGTTAGGAGGAAGTGATCATTGTATCAGCATCTGCTGGGAGT
GAGGACTTGCATTGTGAAAGCTTTGCTGTCCTTGATGTGATCATGGAATCTTTTTCTCAC
TAGAGTCTATGTCACTCATTATACTCTGTCGAATGTCATTGAATGTCTTTACATGGGCTT
GTATGCCTATGAAAATTGTAATACAACTTTCAGCAACGGATCTCTTGGCTCTCGCATCGA
TGAAGAACGCAGCGAAATGCGATAAGTAATGTGAATTGCAGAATTCAGTGAATCATCGAA
TCTTTGAACGCATCTTGCGCTCCTTGGTATTCCGAGGAGCATGCCTGTTTGAGTGTCATT
AAATTCTCAACTCTCTTATAC-TTTTTTGTAAAAGAGAGCTTGGACTGTGGAGGCTTGCT
GGCCACTTTTTGGGGTCAGCTCCTCTGAAATGCATTAGCGGAACCGTTTGCGATCTGCCA
CAAGTGTGATAAGTTATCTACACTGGCGAGGGGATTGCTCTCTGTAATGTTCAGCTTCTA
ATTGTCTCTACTTTGTGAGACTACTTTTGAATGCTTGACCTCAAATCAGGTAGGACTACC
CGCTGAACTTAA
>ABC5-77
TTTCCGTAGGTGAACCTGCGGAAGGATCATTATTGAATTATGTTTCTAGATAGGTTGTAG

CTGGCTCTTTTAGAGCATGTGCACGCCTGTTTGGACTTCATTTTCATCCACCTGTGCACC
TATTGTAGTCTTTGGTTGGGTTAGGAGGAAGTGATCATTGTATCAGCATCTGCTGGGAGT
GAGGACTTGCATTGTGAAAGCTTTGCTGTCCTTGATGTGATCATGGAATCTTTTTCTCAC
TAGAGTCTATGTCACTCATTATACTCTGTCGAATGTCATTGAATGTCTTTACATGGGCTT
GTATGCCTATGAAAATTGTAATACAACTTTCAGCAACGGATCTCTTGGCTCTCGCATCGA
TGAAGAACGCAGCGAAATGCGATAAGTAATGTGAATTGCAGAATTCAGTGAATCATCGAA
TCTTTGAACGCATCTTGCGCTCCTTGGTATTCCGAGGAGCATGCCTGTTTGAGTGTCATT
AAATTCTCAACTCTCTTATAC-TTTTTTGTAAAAGAGAGCTTGGACTGTGGAGGCTTGCT
GGCCACTTTTTGGGGTCAGCTCCTCTGAAATGCATTAGCGGAACCGTTTGCGATCTGCCA
CAAGTGTGATAAGTTATCTACACTGGCGAGGGGATTGCTCTCTGTAATGTTCAGCTTCTA
ATTGTCTCTACTTTGTGAGACTACTTTTGAATGCTTGACCTCAAATCAGGTAGGACTACC
CGCTGAACTTAA
>ABC5-84
TTTCCGTAGGTGAACCTGCGGAAGGATCATTATTGAATTATGTTTCTAGATAGGTTGTAG
CTGGCTCTTTTAGAGCATGTGCACGCCTGTTTGGACTTCATTTTCATCCACCTGTGCACC
TATTGTAGTCTTTGGTTGGGTTAGGAGGAAGTGATCATTGTATCAGCATCTGCTGGGAGT
GAGGACTTGCATTGTGAAAGCTTTGCTGTCCTTGATGTGATCATGGAATCTTTTTCTCAC
TAGAGTCTATGTCACTCATTATACTCTGTCGAATGTCATTGAATGTCTTTACATGGGCTT
GTATGCCTATGAAAATTGTAATACAACTTTCAGCAACGGATCTCTTGGCTCTCGCATCGA
TGAAGAACGCAGCGAAATGCGATAAGTAATGTGAATTGCAGAATTCAGTGAATCATCGAA
TCTTTGAACGCATCTTGCGCTCCTTGGTATTCCGAGGAGCATGCCTGTTTGAGTGTCATT
AAATTCTCAACTCTCTTATAC-TTTTTTGTAAAAGAGAGCTTGGACTGTGGAGGCTTGCT
GGCCACTTTTTGGGGTCAGCTCCTCTGAAATGCATTAGCGGAACCGTTTGCGATCTGCCA
CAAGTGTGATAAGTTATCTACACTGGCGAGGGGATTGCTCTCTGTAATGTTCAGCTTCTA
ATTGTCTCTACTTTGTGAGACTACTTTTGAATGCTTGACCTCAAATCAGGTAGGACTACC
CGCTGAACTTAA
>ABC6-8
TTTCCGTAGGTGAACCTGCGGAAGGATCATTATTGAATTATGTTTCTAGATAGGTTGTAG
CTGGCTCTTTTAGAGCATGTGCACGCCTGTTTGGACTTCATTTTCATCCACCTGTGCACC
TATTGTAGTCTTTGGTTGGGTTAGGAGGAAGTGATCATTGTATCAGCATCTGCTGGGAGT
GAGGACTTGCATTGTGAAAGCTTTGCTGTCCTTGATGTGATCATGGAATCTTTTTCTCAC
TAGAGTCTATGTCACTCATTATACTCTGTCGAATGTCATTGAATGTCTTTACATGGGCTT
GTATGCCTATGAAAATTGTAATACAACTTTCAGCAACGGATCTCTTGGCTCTCGCATCGA
TGAAGAACGCAGCGAAATGCGATAAGTAATGTGAATTGCAGAATTCAGTGAATCATCGAA
TCTTTGAACGCATCTTGCGCTCCTTGGTATTCCGAGGAGCATGCCTGTTTGAGTGTCATT
AAATTCTCAACTCTCTTATAC-TTTTTTGTAAAAGAGAGCTTGGACTGTGGAGGCTTGCT
GGCCACTTTTTGGGGTCAGCTCCTCTGAAATGCATTAGCGGAACCGTTTGCGATCTGCCA
CAAGTGTGATAAGTTATCTACACTGGCGAGGGGATTGCTCTCTGTAATGTTCAGCTTCTA
ATTGTCTCTACTTTGTGAGACTACTTTTGAATGCTTGACCTCAAATCAGGTAGGACTACC
CGCTGAACTTAA
>ABC6-11
TTTCCGTAGGTGAACCTGCGGAAGGATCATTATTGAATTATGTTTCTAGATAGGTTGTAG
CTGGCTCTTTTAGAGCATGTGCACGCCTGTTTGGACTTCATTTTCATCCACCTGTGCACC
TATTGTAGTCTTTGGTTGGGTTAGGAGGAAGTGATCATTGTATCAGCATCTGCTGGGAGT
GAGGACTTGCATTGTGAAAGCTTTGCTGTCCTTGATGTGATCATGGAATCTTTTTCTCAC
TAGAGTCTATGTCACTCATTATACTCTGTCGAATGTCATTGAATGTCTTTACATGGGCTT
GTATGCCTATGAAAATTGTAATACAACTTTCAGCAACGGATCTCTTGGCTCTCGCATCGA
TGAAGAACGCAGCGAAATGCGATAAGTAATGTGAATTGCAGAATTCAGTGAATCATCGAA
TCTTTGAACGCATCTTGCGCTCCTTGGTATTCCGAGGAGCATGCCTGTTTGAGTGTCATT
AAATTCTCAACTCTCTTATAC-TTTTTTGTAAAAGAGAGCTTGGACTGTGGAGGCTTGCT

GGCCACTTTTTGGGGTCAGCTCCTCTGAAATGCATTAGCGGAACCGTTTGCGATCTGCCA
CAAGTGTGATAAGTTATCTACACTGGCGAGGGGATTGCTCTCTGTAATGTTCAGCTTCTA
ATTGTCTCTACTTTGTGAGACTACTTTTGAATGCTTGACCTCAAATCAGGTAGGACTACC
CGCTGAACTTAA
>ABC6-20
TTTCCGTAGGTGAACCTGCGGAAGGATCATTATTGAATTATGTTTCTAGATAGGTTGTAG
CTGGCTCTTTTAGAGCATGTGCACGCCTGTTTGGACTTCATTTTCATCCACCTGTGCACC
TATTGTAGTCTTTGGTTGGGTTAGGAGGAAGTGATCATTGTATCAGCATCTGCTGGGAGT
GAGGACTTGCATTGTGAAAGCTTTGCTGTCCTTGATGTGATCATGGAATCTTTTTCTCAC
TAGAGTCTATGTCACTCATTATACTCTGTCGAATGTCATTGAATGTCTTTACATGGGCTT
GTATGCCTATGAAAATTGTAATACAACTTTCAGCAACGGATCTCTTGGCTCTCGCATCGA
TGAAGAACGCAGCGAAATGCGATAAGTAATGTGAATTGCAGAATTCAGTGAATCATCGAA
TCTTTGAACGCATCTTGCGCTCCTTGGTATTCCGAGGAGCATGCCTGTTTGAGTGTCATT
AAATTCTCAACTCTCTTATAC-TTTTTTGTAAAAGAGAGCTTGGACTGTGGAGGCTTGCT
GGCCACTTTTTGGGGTCAGCTCCTCTGAAATGCATTAGCGGAACCGTTTGCGATCTGCCA
CAAGTGTGATAAGTTATCTACACTGGCGAGGGGATTGCTCTCTGTAATGTTCAGCTTCTA
ATTGTCTCTACTTTGTGAGACTACTTTTGAATGCTTGACCTCAAATCAGGTAGGACTACC
CGCTGAACTTAA
>ABC6-24
TTTCCGTAGGTGAACCTGCGGAAGGATCATTATTGAATTATGTTTCTAGATAGGTTGTAG
CTGGCTCTTTTAGAGCATGTGCACGCCTGTTTGGACTTCATTTTCATCCACCTGTGCACC
TATTGTAGTCTTTGGTTGGGTTAGGAGGAAGTGATCATTGTATCAGCATCTGCTGGGAGT
GAGGACTTGCATTGTGAAAGCTTTGCTGTCCTTGATGTGATCATGGAATCTTTTTCTCAC
TAGAGTCTATGTCACTCATTATACTCTGTCGAATGTCATTGAATGTCTTTACATGGGCTT
GTATGCCTATGAAAATTGTAATACAACTTTCAGCAACGGATCTCTTGGCTCTCGCATCGA
TGAAGAACGCAGCGAAATGCGATAAGTAATGTGAATTGCAGAATTCAGTGAATCATCGAA
TCTTTGAACGCATCTTGCGCTCCTTGGTATTCCGAGGAGCATGCCTGTTTGAGTGTCATT
AAATTCTCAACTCTCTTATAC-TTTTTTGTAAAAGAGAGCTTGGACTGTGGAGGCTTGCT
GGCCACTTTTTGGGGTCAGCTCCTCTGAAATGCATTAGCGGAACCGTTTGCGATCTGCCA
CAAGTGTGATAAGTTATCTACACTGGCGAGGGGATTGCTCTCTGTAATGTTCAGCTTCTA
ATTGTCTCTACTTTGTGAGACTACTTTTGAATGCTTGACCTCAAATCAGGTAGGACTACC
CGCTGAACTTAA
>ABC6-27
TTTCCGTAGGTGAACCTGCGGAAGGATCATTATTGAATTATGTTTCTAGATAGGTTGTAG
CTGGCTCTTTTAGAGCATGTGCACGCCTGTTTGGACTTCATTTTCATCCACCTGTGCACC
TATTGTAGTCTTTGGTTGGGTTAGGAGGAAGTGATCATTGTATCAGCATCTGCTGGGAGT
GAGGACTTGCATTGTGAAAGCTTTGCTGTCCTTGATGTGATCATGGAATCTTTTTCTCAC
TAGAGTCTATGTCACTCATTATACTCTGTCGAATGTCATTGAATGTCTTTACATGGGCTT
GTATGCCTATGAAAATTGTAATACAACTTTCAGCAACGGATCTCTTGGCTCTCGCATCGA
TGAAGAACGCAGCGAAATGCGATAAGTAATGTGAATTGCAGAATTCAGTGAATCATCGAA
TCTTTGAACGCATCTTGCGCTCCTTGGTATTCCGAGGAGCATGCCTGTTTGAGTGTCATT
AAATTCTCAACTCTCTTATAC-TTTTTTGTAAAAGAGAGCTTGGACTGTGGAGGCTTGCT
GGCCACTTTTTGGGGTCAGCTCCTCTGAAATGCATTAGCGGAACCGTTTGCGATCTGCCA
CAAGTGTGATAAGTTATCTACACTGGCGAGGGGATTGCTCTCTGTAATGTTCAGCTTCTA
ATTGTCTCTACTTTGTGAGACTACTTTTGAATGCTTGACCTCAAATCAGGTAGGACTACC
CGCTGAACTTAA
>ABC6-30
TTTCCGTAGGTGAACCTGCGGAAGGATCATTATTGAATTATGTTTCTAGATAGGTTGTAG
CTGGCTCTTTTAGAGCATGTGCACGCCTGTTTGGACTTCATTTTCATCCACCTGTGCACC
TATTGTAGTCTTTGGTTGGGTTAGGAGGAAGTGATCATTGTATCAGCATCTGCTGGGAGT

GAGGACTTGCATTGTGAAAGCTTTGCTGTCCTTGATGTGATCATGGAATCTTTTTCTCAC
TAGAGTCTATGTCACTCATTATACTCTGTCGAATGTCATTGAATGTCTTTACATGGGCTT
GTATGCCTATGAAAATTGTAATACAACTTTCAGCAACGGATCTCTTGGCTCTCGCATCGA
TGAAGAACGCAGCGAAATGCGATAAGTAATGTGAATTGCAGAATTCAGTGAATCATCGAA
TCTTTGAACGCATCTTGCGCTCCTTGGTATTCCGAGGAGCATGCCTGTTTGAGTGTCATT
AAATTCTCAACTCTCTTATAC-TTTTTTGTAAAAGAGAGCTTGGACTGTGGAGGCTTGCT
GGCCACTTTTTGGGGTCAGCTCCTCTGAAATGCATTAGCGGAACCGTTTGCGATCTGCCA
CAAGTGTGATAAGTTATCTACACTGGCGAGGGGATTGCTCTCTGTAATGTTCAGCTTCTA
ATTGTCTCTACTTTGTGAGACTACTTTTGAATGCTTGACCTCAAATCAGGTAGGACTACC
CGCTGAACTTAA
>ABC6-43
TTTCCGTAGGTGAACCTGCGGAAGGATCATTATTGAATTATGTTTCTAGATAGGTTGTAG
CTGGCTCTTTTAGAGCATGTGCACGCCTGTTTGGACTTCATTTTCATCCACCTGTGCACC
TATTGTAGTCTTTGGTTGGGTTAGGAGGAAGTGATCATTGTATCAGCATCTGCTGGGAGT
GAGGACTTGCATTGTGAAAGCTTTGCTGTCCTTGATGTGATCATGGAATCTTTTTCTCAC
TAGAGTCTATGTCACTCATTATACTCTGTCGAATGTCATTGAATGTCTTTACATGGGCTT
GTATGCCTATGAAAATTGTAATACAACTTTCAGCAACGGATCTCTTGGCTCTCGCATCGA
TGAAGAACGCAGCGAAATGCGATAAGTAATGTGAATTGCAGAATTCAGTGAATCATCGAA
TCTTTGAACGCATCTTGCGCTCCTTGGTATTCCGAGGAGCATGCCTGTTTGAGTGTCATT
AAATTCTCAACTCTCTTATAC-TTTTTTGTAAAAGAGAGCTTGGACTGTGGAGGCTTGCT
GGCCACTTTTTGGGGTCAGCTCCTCTGAAATGCATTAGCGGAACCGTTTGCGATCTGCCA
CAAGTGTGATAAGTTATCTACACTGGCGAGGGGATTGCTCTCTGTAATGTTCAGCTTCTA
ATTGTCTCTACTTTGTGAGACTACTTTTGAATGCTTGACCTCAAATCAGGTAGGACTACC
CGCTGAACTTAA
>ABC6-46
TTTCCGTAGGTGAACCTGCGGAAGGATCATTATTGAATTATGTTTCTAGATAGGTTGTAG
CTGGCTCTTTTAGAGCATGTGCACGCCTGTTTGGACTTCATTTTCATCCACCTGTGCACC
TATTGTAGTCTTTGGTTGGGTTAGGAGGAAGTGATCATTGTATCAGCATCTGCTGGGAGT
GAGGACTTGCATTGTGAAAGCTTTGCTGTCCTTGATGTGATCATGGAATCTTTTTCTCAC
TAGAGTCTATGTCACTCATTATACTCTGTCGAATGTCATTGAATGTCTTTACATGGGCTT
GTATGCCTATGAAAATTGTAATACAACTTTCAGCAACGGATCTCTTGGCTCTCGCATCGA
TGAAGAACGCAGCGAAATGCGATAAGTAATGTGAATTGCAGAATTCAGTGAATCATCGAA
TCTTTGAACGCATCTTGCGCTCCTTGGTATTCCGAGGAGCATGCCTGTTTGAGTGTCATT
AAATTCTCAACTCTCTTATAC-TTTTTTGTAAAAGAGAGCTTGGACTGTGGAGGCTTGCT
GGCCACTTTTTGGGGTCAGCTCCTCTGAAATGCATTAGCGGAACCGTTTGCGATCTGCCA
CAAGTGTGATAAGTTATCTACACTGGCGAGGGGATTGCTCTCTGTAATGTTCAGCTTCTA
ATTGTCTCTACTTTGTGAGACTACTTTTGAATGCTTGACCTCAAATCAGGTAGGACTACC
CGCTGAACTTAA
>ABC6-56
TTTCCGTAGGTGAACCTGCGGAAGGATCATTATTGAATTATGTTTCTAGATAGGTTGTAG
CTGGCTCTTTTAGAGCATGTGCACGCCTGTTTGGACTTCATTTTCATCCACCTGTGCACC
TATTGTAGTCTTTGGTTGGGTTAGGAGGAAGTGATCATTGTATCAGCATCTGCTGGGAGT
GAGGACTTGCATTGTGAAAGCTTTGCTGTCCTTGATGTGATCATGGAATCTTTTTCTCAC
TAGAGTCTATGTCACTCATTATACTCTGTCGAATGTCATTGAATGTCTTTACATGGGCTT
GTATGCCTATGAAAATTGTAATACAACTTTCAGCAACGGATCTCTTGGCTCTCGCATCGA
TGAAGAACGCAGCGAAATGCGATAAGTAATGTGAATTGCAGAATTCAGTGAATCATCGAA
TCTTTGAACGCATCTTGCGCTCCTTGGTATTCCGAGGAGCATGCCTGTTTGAGTGTCATT
AAATTCTCAACTCTCTTATAC-TTTTTTGTAAAAGAGAGCTTGGACTGTGGAGGCTTGCT
GGCCACTTTTTGGGGTCAGCTCCTCTGAAATGCATTAGCGGAACCGTTTGCGATCTGCCA
CAAGTGTGATAAGTTATCTACACTGGCGAGGGGATTGCTCTCTGTAATGTTCAGCTTCTA

ATTGTCTCTACTTTGTGAGACTACTTTTGAATGCTTGACCTCAAATCAGGTAGGACTACC
CGCTGAACTTAA
>ABC7-34
TTTCCGTAGGTGAACCTGCGGAAGGATCATTATTGAATTATGTTTCTAGATAGGTTGTAG
CTGGCTCTTTTAGAGCATGTGCACGCCTGTTTGGACTTCATTTTCATCCACCTGTGCACC
TATTGTAGTCTTTGGTTGGGTTAGGAGGAAGTGATCATTGTATCAGCATCTGCTGGGAGT
GAGGACTTGCATTGTGAAAGCTTTGCTGTCCTTGATGTGATCATGGAATCTTTTTCTCAC
TAGAGTCTATGTCACTCATTATACTCTGTCGAATGTCATTGAATGTCTTTACATGGGCTT
GTATGCCTATGAAAATTGTAATACAACTTTCAGCAACGGATCTCTTGGCTCTCGCATCGA
TGAAGAACGCAGCGAAATGCGATAAGTAATGTGAATTGCAGAATTCAGTGAATCATCGAA
TCTTTGAACGCATCTTGCGCTCCTTGGTATTCCGAGGAGCATGCCTGTTTGAGTGTCATT
AAATTCTCAACTCTCTTATAC-TTTTTTGTAAAAGAGAGCTTGGACTGTGGAGGCTTGCT
GGCCACTTTTTGGGGTCAGCTCCTCTGAAATGCATTAGCGGAACCGTTTGCGATCTGCCA
CAAGTGTGATAAGTTATCTACACTGGCGAGGGGATTGCTCTCTGTAATGTTCAGCTTCTA
ATTGTCTCTACTTTGTGAGACTACTTTTGAATGCTTGACCTCAAATCAGGTAGGACTACC
CGCTGAACTTAA
>ABC7-37
TTTCCGTAGGTGAACCTGCGGAAGGATCATTATTGAATTATGTTTCTAGATAGGTTGTAG
CTGGCTCTTTTAGAGCATGTGCACGCCTGTTTGGACTTCATTTTCATCCACCTGTGCACC
TATTGTAGTCTTTGGTTGGGTTAGGAGGAAGTGATCATTGTATCAGCATCTGCTGGGAGT
GAGGACTTGCATTGTGAAAGCTTTGCTGTCCTTGATGTGATCATGGAATCTTTTTCTCAC
TAGAGTCTATGTCACTCATTATACTCTGTCGAATGTCATTGAATGTCTTTACATGGGCTT
GTATGCCTATGAAAATTGTAATACAACTTTCAGCAACGGATCTCTTGGCTCTCGCATCGA
TGAAGAACGCAGCGAAATGCGATAAGTAATGTGAATTGCAGAATTCAGTGAATCATCGAA
TCTTTGAACGCATCTTGCGCTCCTTGGTATTCCGAGGAGCATGCCTGTTTGAGTGTCATT
AAATTCTCAACTCTCTTATAC-TTTTTTGTAAAAGAGAGCTTGGACTGTGGAGGCTTGCT
GGCCACTTTTTGGGGTCAGCTCCTCTGAAATGCATTAGCGGAACCGTTTGCGATCTGCCA
CAAGTGTGATAAGTTATCTACACTGGCGAGGGGATTGCTCTCTGTAATGTTCAGCTTCTA
ATTGTCTCTACTTTGTGAGACTACTTTTGAATGCTTGACCTCAAATCAGGTAGGACTACC
CGCTGAACTTAA
>ABC7-42
TTTCCGTAGGTGAACCTGCGGAAGGATCATTATTGAATTATGTTTCTAGATAGGTTGTAG
CTGGCTCTTTTAGAGCATGTGCACGCCTGTTTGGACTTCATTTTCATCCACCTGTGCACC
TATTGTAGTCTTTGGTTGGGTTAGGAGGAAGTGATCATTGTATCAGCATCTGCTGGGAGT
GAGGACTTGCATTGTGAAAGCTTTGCTGTCCTTGATGTGATCATGGAATCTTTTTCTCAC
TAGAGTCTATGTCACTCATTATACTCTGTCGAATGTCATTGAATGTCTTTACATGGGCTT
GTATGCCTATGAAAATTGTAATACAACTTTCAGCAACGGATCTCTTGGCTCTCGCATCGA
TGAAGAACGCAGCGAAATGCGATAAGTAATGTGAATTGCAGAATTCAGTGAATCATCGAA
TCTTTGAACGCATCTTGCGCTCCTTGGTATTCCGAGGAGCATGCCTGTTTGAGTGTCATT
AAATTCTCAACTCTCTTATAC-TTTTTTGTAAAAGAGAGCTTGGACTGTGGAGGCTTGCT
GGCCACTTTTTGGGGTCAGCTCCTCTGAAATGCATTAGCGGAACCGTTTGCGATCTGCCA
CAAGTGTGATAAGTTATCTACACTGGCGAGGGGATTGCTCTCTGTAATGTTCAGCTTCTA
ATTGTCTCTACTTTGTGAGACTACTTTTGAATGCTTGACCTCAAATCAGGTAGGACTACC
CGCTGAACTTAA
>ABC7-47
TTTCCGTAGGTGAACCTGCGGAAGGATCATTATTGAATTATGTTTCTAGATAGGTTGTAG
CTGGCTCTTTTAGAGCATGTGCACGCCTGTTTGGACTTCATTTTCATCCACCTGTGCACC
TATTGTAGTCTTTGGTTGGGTTAGGAGGAAGTGATCATTGTATCAGCATCTGCTGGGAGT
GAGGACTTGCATTGTGAAAGCTTTGCTGTCCTTGATGTGATCATGGAATCTTTTTCTCAC
TAGAGTCTATGTCACTCATTATACTCTGTCGAATGTCATTGAATGTCTTTACATGGGCTT

GTATGCCTATGAAAATTGTAATACAACTTTCAGCAACGGATCTCTTGGCTCTCGCATCGA
TGAAGAACGCAGCGAAATGCGATAAGTAATGTGAATTGCAGAATTCAGTGAATCATCGAA
TCTTTGAACGCATCTTGCGCTCCTTGGTATTCCGAGGAGCATGCCTGTTTGAGTGTCATT
AAATTCTCAACTCTCTTATAC-TTTTTTGTAAAAGAGAGCTTGGACTGTGGAGGCTTGCT
GGCCACTTTTTGGGGTCAGCTCCTCTGAAATGCATTAGCGGAACCGTTTGCGATCTGCCA
CAAGTGTGATAAGTTATCTACACTGGCGAGGGGATTGCTCTCTGTAATGTTCAGCTTCTA
ATTGTCTCTACTTTGTGAGACTACTTTTGAATGCTTGACCTCAAATCAGGTAGGACTACC
CGCTGAACTTAA
>ABC7-49
TTTCCGTAGGTGAACCTGCGGAAGGATCATTATTGAATTATGTTTCTAGATAGGTTGTAG
CTGGCTCTTTTAGAGCATGTGCACGCCTGTTTGGACTTCATTTTCATCCACCTGTGCACC
TATTGTAGTCTTTGGTTGGGTTAGGAGGAAGTGATCATTGTATCAGCATCTGCTGGGAGT
GAGGACTTGCATTGTGAAAGCTTTGCTGTCCTTGATGTGATCATGGAATCTTTTTCTCAC
TAGAGTCTATGTCACTCATTATACTCTGTCGAATGTCATTGAATGTCTTTACATGGGCTT
GTATGCCTATGAAAATTGTAATACAACTTTCAGCAACGGATCTCTTGGCTCTCGCATCGA
TGAAGAACGCAGCGAAATGCGATAAGTAATGTGAATTGCAGAATTCAGTGAATCATCGAA
TCTTTGAACGCATCTTGCGCTCCTTGGTATTCCGAGGAGCATGCCTGTTTGAGTGTCATT
AAATTCTCAACTCTCTTATAC-TTTTTTGTAAAAGAGAGCTTGGACTGTGGAGGCTTGCT
GGCCACTTTTTGGGGTCAGCTCCTCTGAAATGCATTAGCGGAACCGTTTGCGATCTGCCA
CAAGTGTGATAAGTTATCTACACTGGCGAGGGGATTGCTCTCTGTAATGTTCAGCTTCTA
ATTGTCTCTACTTTGTGAGACTACTTTTGAATGCTTGACCTCAAATCAGGTAGGACTACC
CGCTGAACTTAA
>ABC7-54
TTTCCGTAGGTGAACCTGCGGAAGGATCATTATTGAATTATGTTTCTAGATAGGTTGTAG
CTGGCTCTTTTAGAGCATGTGCACGCCTGTTTGGACTTCATTTTCATCCACCTGTGCACC
TATTGTAGTCTTTGGTTGGGTTAGGAGGAAGTGATCATTGTATCAGCATCTGCTGGGAGT
GAGGACTTGCATTGTGAAAGCTTTGCTGTCCTTGATGTGATCATGGAATCTTTTTCTCAC
TAGAGTCTATGTCACTCATTATACTCTGTCGAATGTCATTGAATGTCTTTACATGGGCTT
GTATGCCTATGAAAATTGTAATACAACTTTCAGCAACGGATCTCTTGGCTCTCGCATCGA
TGAAGAACGCAGCGAAATGCGATAAGTAATGTGAATTGCAGAATTCAGTGAATCATCGAA
TCTTTGAACGCATCTTGCGCTCCTTGGTATTCCGAGGAGCATGCCTGTTTGAGTGTCATT
AAATTCTCAACTCTCTTATAC-TTTTTTGTAAAAGAGAGCTTGGACTGTGGAGGCTTGCT
GGCCACTTTTTGGGGTCAGCTCCTCTGAAATGCATTAGCGGAACCGTTTGCGATCTGCCA
CAAGTGTGATAAGTTATCTACACTGGCGAGGGGATTGCTCTCTGTAATGTTCAGCTTCTA
ATTGTCTCTACTTTGTGAGACTACTTTTGAATGCTTGACCTCAAATCAGGTAGGACTACC
CGCTGAACTTAA
>ABC8-4
TTTCCGTAGGTGAACCTGCGGAAGGATCATTATTGAATTATGTTTCTAGATAGGTTGTAG
CTGGCTCTTTTAGAGCATGTGCACGCCTGTTTGGACTTCATTTTCATCCACCTGTGCACC
TATTGTAGTCTTTGGTTGGGTTAGGAGGAAGTGATCATTGTATCAGCATCTGCTGGGAGT
GAGGACTTGCATTGTGAAAGCTTTGCTGTCCTTGATGTGATCATGGAATCTTTTTCTCAC
TAGAGTCTATGTCACTCATTATACTCTGTCGAATGTCATTGAATGTCTTTACATGGGCTT
GTATGCCTATGAAAATTGTAATACAACTTTCAGCAACGGATCTCTTGGCTCTCGCATCGA
TGAAGAACGCAGCGAAATGCGATAAGTAATGTGAATTGCAGAATTCAGTGAATCATCGAA
TCTTTGAACGCATCTTGCGCTCCTTGGTATTCCGAGGAGCATGCCTGTTTGAGTGTCATT
AAATTCTCAACTCTCTTATAC-TTTTTTGTAAAAGAGAGCTTGGACTGTGGAGGCTTGCT
GGCCACTTTTTGGGGTCAGCTCCTCTGAAATGCATTAGCGGAACCGTTTGCGATCTGCCA
CAAGTGTGATAAGTTATCTACACTGGCGAGGGGATTGCTCTCTGTAATGTTCAGCTTCTA
ATTGTCTCTACTTTGTGAGACTACTTTTGAATGCTTGACCTCAAATCAGGTAGGACTACC
CGCTGAACTTAA

>ABC8-17
TTTCCGTAGGTGAACCTGCGGAAGGATCATTATTGAATTATGTTTCTAGATAGGTTGTAG
CTGGCTCTTTTAGAGCATGTGCACGCCTGTTTGGACTTCATTTTCATCCACCTGTGCACC
TATTGTAGTCTTTGGTTGGGTTAGGAGGAAGTGATCATTGTATCAGCATCTGCTGGGAGT
GAGGACTTGCATTGTGAAAGCTTTGCTGTCCTTGATGTGATCATGGAATCTTTTTCTCAC
TAGAGTCTATGTCACTCATTATACTCTGTCGAATGTCATTGAATGTCTTTACATGGGCTT
GTATGCCTATGAAAATTGTAATACAACTTTCAGCAACGGATCTCTTGGCTCTCGCATCGA
TGAAGAACGCAGCGAAATGCGATAAGTAATGTGAATTGCAGAATTCAGTGAATCATCGAA
TCTTTGAACGCATCTTGCGCTCCTTGGTATTCCGAGGAGCATGCCTGTTTGAGTGTCATT
AAATTCTCAACTCTCTTATAC-TTTTTTGTAAAAGAGAGCTTGGACTGTGGAGGCTTGCT
GGCCACTTTTTGGGGTCAGCTCCTCTGAAATGCATTAGCGGAACCGTTTGCGATCTGCCA
CAAGTGTGATAAGTTATCTACACTGGCGAGGGGATTGCTCTCTGTAATGTTCAGCTTCTA
ATTGTCTCTACTTTGTGAGACTACTTTTGAATGCTTGACCTCAAATCAGGTAGGACTACC
CGCTGAACTTAA
>ABC8-23
TTTCCGTAGGTGAACCTGCGGAAGGATCATTATTGAATTATGTTTCTAGATAGGTTGTAG
CTGGCTCTTTTAGAGCATGTGCACGCCTGTTTGGACTTCATTTTCATCCACCTGTGCACC
TATTGTAGTCTTTGGTTGGGTTAGGAGGAAGTGATCATTGTATCAGCATCTGCTGGGAGT
GAGGACTTGCATTGTGAAAGCTTTGCTGTCCTTGATGTGATCATGGAATCTTTTTCTCAC
TAGAGTCTATGTCACTCATTATACTCTGTCGAATGTCATTGAATGTCTTTACATGGGCTT
GTATGCCTATGAAAATTGTAATACAACTTTCAGCAACGGATCTCTTGGCTCTCGCATCGA
TGAAGAACGCAGCGAAATGCGATAAGTAATGTGAATTGCAGAATTCAGTGAATCATCGAA
TCTTTGAACGCATCTTGCGCTCCTTGGTATTCCGAGGAGCATGCCTGTTTGAGTGTCATT
AAATTCTCAACTCTCTTATAC-TTTTTTGTAAAAGAGAGCTTGGACTGTGGAGGCTTGCT
GGCCACTTTTTGGGGTCAGCTCCTCTGAAATGCATTAGCGGAACCGTTTGCGATCTGCCA
CAAGTGTGATAAGTTATCTACACTGGCGAGGGGATTGCTCTCTGTAATGTTCAGCTTCTA
ATTGTCTCTACTTTGTGAGACTACTTTTGAATGCTTGACCTCAAATCAGGTAGGACTACC
CGCTGAACTTAA
>ABC8-30
TTTCCGTAGGTGAACCTGCGGAAGGATCATTATTGAATTATGTTTCTAGATAGGTTGTAG
CTGGCTCTTTTAGAGCATGTGCACGCCTGTTTGGACTTCATTTTCATCCACCTGTGCACC
TATTGTAGTCTTTGGTTGGGTTAGGAGGAAGTGATCATTGTATCAGCATCTGCTGGGAGT
GAGGACTTGCATTGTGAAAGCTTTGCTGTCCTTGATGTGATCATGGAATCTTTTTCTCAC
TAGAGTCTATGTCACTCATTATACTCTGTCGAATGTCATTGAATGTCTTTACATGGGCTT
GTATGCCTATGAAAATTGTAATACAACTTTCAGCAACGGATCTCTTGGCTCTCGCATCGA
TGAAGAACGCAGCGAAATGCGATAAGTAATGTGAATTGCAGAATTCAGTGAATCATCGAA
TCTTTGAACGCATCTTGCGCTCCTTGGTATTCCGAGGAGCATGCCTGTTTGAGTGTCATT
AAATTCTCAACTCTCTTATAC-TTTTTTGTAAAAGAGAGCTTGGACTGTGGAGGCTTGCT
GGCCACTTTTTGGGGTCAGCTCCTCTGAAATGCATTAGCGGAACCGTTTGCGATCTGCCA
CAAGTGTGATAAGTTATCTACACTGGCGAGGGGATTGCTCTCTGTAATGTTCAGCTTCTA
ATTGTCTCTACTTTGTGAGACTACTTTTGAATGCTTGACCTCAAATCAGGTAGGACTACC
CGCTGAACTTAA
>ABC8-38
TTTCCGTAGGTGAACCTGCGGAAGGATCATTATTGAATTATGTTTCTAGATAGGTTGTAG
CTGGCTCTTTTAGAGCATGTGCACGCCTGTTTGGACTTCATTTTCATCCACCTGTGCACC
TATTGTAGTCTTTGGTTGGGTTAGGAGGAAGTGATCATTGTATCAGCATCTGCTGGGAGT
GAGGACTTGCATTGTGAAAGCTTTGCTGTCCTTGATGTGATCATGGAATCTTTTTCTCAC
TAGAGTCTATGTCACTCATTATACTCTGTCGAATGTCATTGAATGTCTTTACATGGGCTT
GTATGCCTATGAAAATTGTAATACAACTTTCAGCAACGGATCTCTTGGCTCTCGCATCGA
TGAAGAACGCAGCGAAATGCGATAAGTAATGTGAATTGCAGAATTCAGTGAATCATCGAA

TCTTTGAACGCATCTTGCGCTCCTTGGTATTCCGAGGAGCATGCCTGTTTGAGTGTCATT
AAATTCTCAACTCTCTTATAC-TTTTTTGTAAAAGAGAGCTTGGACTGTGGAGGCTTGCT
GGCCACTTTTTGGGGTCAGCTCCTCTGAAATGCATTAGCGGAACCGTTTGCGATCTGCCA
CAAGTGTGATAAGTTATCTACACTGGCGAGGGGATTGCTCTCTGTAATGTTCAGCTTCTA
ATTGTCTCTACTTTGTGAGACTACTTTTGAATGCTTGACCTCAAATCAGGTAGGACTACC
CGCTGAACTTAA
>ABC9-1
TTTCCGTAGGTGAACCTGCGGAAGGATCATTATTGAATTATGTTTCTAGATAGGTTGTAG
CTGGCTCTTTTAGAGCATGTGCACGCCTGTTTGGACTTCATTTTCATCCACCTGTGCACC
TATTGTAGTCTTTGGTTGGGTTAGGAGGAAGTGATCATTGTATCAGCATCTGCTGGGAGT
GAGGACTTGCATTGTGAAAGCTTTGCTGTCCTTGATGTGATCATGGAATCTTTTTCTCAC
TAGAGTCTATGTCACTCATTATACTCTGTCGAATGTCATTGAATGTCTTTACATGGGCTT
GTATGCCTATGAAAATTGTAATACAACTTTCAGCAACGGATCTCTTGGCTCTCGCATCGA
TGAAGAACGCAGCGAAATGCGATAAGTAATGTGAATTGCAGAATTCAGTGAATCATCGAA
TCTTTGAACGCATCTTGCGCTCCTTGGTATTCCGAGGAGCATGCCTGTTTGAGTGTCATT
AAATTCTCAACTCTCTTATAC-TTTTTTGTAAAAGAGAGCTTGGACTGTGGAGGCTTGCT
GGCCACTTTTTGGGGTCAGCTCCTCTGAAATGCATTAGCGGAACCGTTTGCGATCTGCCA
CAAGTGTGATAAGTTATCTACACTGGCGAGGGGATTGCTCTCTGTAATGTTCAGCTTCTA
ATTGTCTCTACTTTGTGAGACTACTTTTGAATGCTTGACCTCAAATCAGGTAGGACTACC
CGCTGAACTTAA
>ABC9-4
TTTCCGTAGGTGAACCTGCGGAAGGATCATTATTGAATTATGTTTCTAGATAGGTTGTAG
CTGGCTCTTTTAGAGCATGTGCACGCCTGTTTGGACTTCATTTTCATCCACCTGTGCACC
TATTGTAGTCTTTGGTTGGGTTAGGAGGAAGTGATCATTGTATCAGCATCTGCTGGGAGT
GAGGACTTGCATTGTGAAAGCTTTGCTGTCCTTGATGTGATCATGGAATCTTTTTCTCAC
TAGAGTCTATGTCACTCATTATACTCTGTCGAATGTCATTGAATGTCTTTACATGGGCTT
GTATGCCTATGAAAATTGTAATACAACTTTCAGCAACGGATCTCTTGGCTCTCGCATCGA
TGAAGAACGCAGCGAAATGCGATAAGTAATGTGAATTGCAGAATTCAGTGAATCATCGAA
TCTTTGAACGCATCTTGCGCTCCTTGGTATTCCGAGGAGCATGCCTGTTTGAGTGTCATT
AAATTCTCAACTCTCTTATAC-TTTTTTGTAAAAGAGAGCTTGGACTGTGGAGGCTTGCT
GGCCACTTTTTGGGGTCAGCTCCTCTGAAATGCATTAGCGGAACCGTTTGCGATCTGCCA
CAAGTGTGATAAGTTATCTACACTGGCGAGGGGATTGCTCTCTGTAATGTTCAGCTTCTA
ATTGTCTCTACTTTGTGAGACTACTTTTGAATGCTTGACCTCAAATCAGGTAGGACTACC
CGCTGAACTTAA
>ABC9-16
TTTCCGTAGGTGAACCTGCGGAAGGATCATTATTGAATTATGTTTCTAGATAGGTTGTAG
CTGGCTCTTTTAGAGCATGTGCACGCCTGTTTGGACTTCATTTTCATCCACCTGTGCACC
TATTGTAGTCTTTGGTTGGGTTAGGAGGAAGTGATCATTGTATCAGCATCTGCTGGGAGT
GAGGACTTGCATTGTGAAAGCTTTGCTGTCCTTGATGTGATCATGGAATCTTTTTCTCAC
TAGAGTCTATGTCACTCATTATACTCTGTCGAATGTCATTGAATGTCTTTACATGGGCTT
GTATGCCTATGAAAATTGTAATACAACTTTCAGCAACGGATCTCTTGGCTCTCGCATCGA
TGAAGAACGCAGCGAAATGCGATAAGTAATGTGAATTGCAGAATTCAGTGAATCATCGAA
TCTTTGAACGCATCTTGCGCTCCTTGGTATTCCGAGGAGCATGCCTGTTTGAGTGTCATT
AAATTCTCAACTCTCTTATAC-TTTTTTGTAAAAGAGAGCTTGGACTGTGGAGGCTTGCT
GGCCACTTTTTGGGGTCAGCTCCTCTGAAATGCATTAGCGGAACCGTTTGCGATCTGCCA
CAAGTGTGATAAGTTATCTACACTGGCGAGGGGATTGCTCTCTGTAATGTTCAGCTTCTA
ATTGTCTCTACTTTGTGAGACTACTTTTGAATGCTTGACCTCAAATCAGGTAGGACTACC
CGCTGAACTTAA
>ABC9-29
TTTCCGTAGGTGAACCTGCGGAAGGATCATTATTGAATTATGTTTCTAGATAGGTTGTAG

CTGGCTCTTTTAGAGCATGTGCACGCCTGTTTGGACTTCATTTTCATCCACCTGTGCACC
TATTGTAGTCTTTGGTTGGGTTAGGAGGAAGTGATCATTGTATCAGCATCTGCTGGGAGT
GAGGACTTGCATTGTGAAAGCTTTGCTGTCCTTGATGTGATCATGGAATCTTTTTCTCAC
TAGAGTCTATGTCACTCATTATACTCTGTCGAATGTCATTGAATGTCTTTACATGGGCTT
GTATGCCTATGAAAATTGTAATACAACTTTCAGCAACGGATCTCTTGGCTCTCGCATCGA
TGAAGAACGCAGCGAAATGCGATAAGTAATGTGAATTGCAGAATTCAGTGAATCATCGAA
TCTTTGAACGCATCTTGCGCTCCTTGGTATTCCGAGGAGCATGCCTGTTTGAGTGTCATT
AAATTCTCAACTCTCTTATAC-TTTTTTGTAAAAGAGAGCTTGGACTGTGGAGGCTTGCT
GGCCACTTTTTGGGGTCAGCTCCTCTGAAATGCATTAGCGGAACCGTTTGCGATCTGCCA
CAAGTGTGATAAGTTATCTACACTGGCGAGGGGATTGCTCTCTGTAATGTTCAGCTTCTA
ATTGTCTCTACTTTGTGAGACTACTTTTGAATGCTTGACCTCAAATCAGGTAGGACTACC
CGCTGAACTTAA
>ABC9-35
TTTCCGTAGGTGAACCTGCGGAAGGATCATTATTGAATTATGTTTCTAGATAGGTTGTAG
CTGGCTCTTTTAGAGCATGTGCACGCCTGTTTGGACTTCATTTTCATCCACCTGTGCACC
TATTGTAGTCTTTGGTTGGGTTAGGAGGAAGTGATCATTGTATCAGCATCTGCTGGGAGT
GAGGACTTGCATTGTGAAAGCTTTGCTGTCCTTGATGTGATCATGGAATCTTTTTCTCAC
TAGAGTCTATGTCACTCATTATACTCTGTCGAATGTCATTGAATGTCTTTACATGGGCTT
GTATGCCTATGAAAATTGTAATACAACTTTCAGCAACGGATCTCTTGGCTCTCGCATCGA
TGAAGAACGCAGCGAAATGCGATAAGTAATGTGAATTGCAGAATTCAGTGAATCATCGAA
TCTTTGAACGCATCTTGCGCTCCTTGGTATTCCGAGGAGCATGCCTGTTTGAGTGTCATT
AAATTCTCAACTCTCTTATAC-TTTTTTGTAAAAGAGAGCTTGGACTGTGGAGGCTTGCT
GGCCACTTTTTGGGGTCAGCTCCTCTGAAATGCATTAGCGGAACCGTTTGCGATCTGCCA
CAAGTGTGATAAGTTATCTACACTGGCGAGGGGATTGCTCTCTGTAATGTTCAGCTTCTA
ATTGTCTCTACTTTGTGAGACTACTTTTGAATGCTTGACCTCAAATCAGGTAGGACTACC
CGCTGAACTTAA
>ABC9-37
TTTCCGTAGGTGAACCTGCGGAAGGATCATTATTGAATTATGTTTCTAGATAGGTTGTAG
CTGGCTCTTTTAGAGCATGTGCACGCCTGTTTGGACTTCATTTTCATCCACCTGTGCACC
TATTGTAGTCTTTGGTTGGGTTAGGAGGAAGTGATCATTGTATCAGCATCTGCTGGGAGT
GAGGACTTGCATTGTGAAAGCTTTGCTGTCCTTGATGTGATCATGGAATCTTTTTCTCAC
TAGAGTCTATGTCACTCATTATACTCTGTCGAATGTCATTGAATGTCTTTACATGGGCTT
GTATGCCTATGAAAATTGTAATACAACTTTCAGCAACGGATCTCTTGGCTCTCGCATCGA
TGAAGAACGCAGCGAAATGCGATAAGTAATGTGAATTGCAGAATTCAGTGAATCATCGAA
TCTTTGAACGCATCTTGCGCTCCTTGGTATTCCGAGGAGCATGCCTGTTTGAGTGTCATT
AAATTCTCAACTCTCTTATAC-TTTTTTGTAAAAGAGAGCTTGGACTGTGGAGGCTTGCT
GGCCACTTTTTGGGGTCAGCTCCTCTGAAATGCATTAGCGGAACCGTTTGCGATCTGCCA
CAAGTGTGATAAGTTATCTACACTGGCGAGGGGATTGCTCTCTGTAATGTTCAGCTTCTA
ATTGTCTCTACTTTGTGAGACTACTTTTGAATGCTTGACCTCAAATCAGGTAGGACTACC
CGCTGAACTTAA
>ABC9-50
TTTCCGTAGGTGAACCTGCGGAAGGATCATTATTGAATTATGTTTCTAGATAGGTTGTAG
CTGGCTCTTTTAGAGCATGTGCACGCCTGTTTGGACTTCATTTTCATCCACCTGTGCACC
TATTGTAGTCTTTGGTTGGGTTAGGAGGAAGTGATCATTGTATCAGCATCTGCTGGGAGT
GAGGACTTGCATTGTGAAAGCTTTGCTGTCCTTGATGTGATCATGGAATCTTTTTCTCAC
TAGAGTCTATGTCACTCATTATACTCTGTCGAATGTCATTGAATGTCTTTACATGGGCTT
GTATGCCTATGAAAATTGTAATACAACTTTCAGCAACGGATCTCTTGGCTCTCGCATCGA
TGAAGAACGCAGCGAAATGCGATAAGTAATGTGAATTGCAGAATTCAGTGAATCATCGAA
TCTTTGAACGCATCTTGCGCTCCTTGGTATTCCGAGGAGCATGCCTGTTTGAGTGTCATT
AAATTCTCAACTCTCTTATAC-TTTTTTGTAAAAGAGAGCTTGGACTGTGGAGGCTTGCT

GGCCACTTTTTGGGGTCAGCTCCTCTGAAATGCATTAGCGGAACCGTTTGCGATCTGCCA
CAAGTGTGATAAGTTATCTACACTGGCGAGGGGATTGCTCTCTGTAATGTTCAGCTTCTA
ATTGTCTCTACTTTGTGAGACTACTTTTGAATGCTTGACCTCAAATCAGGTAGGACTACC
CGCTGAACTTAA
>ABC9-56
TTTCCGTAGGTGAACCTGCGGAAGGATCATTATTGAATTATGTTTCTAGATAGGTTGTAG
CTGGCTCTTTTAGAGCATGTGCACGCCTGTTTGGACTTCATTTTCATCCACCTGTGCACC
TATTGTAGTCTTTGGTTGGGTTAGGAGGAAGTGATCATTGTATCAGCATCTGCTGGGAGT
GAGGACTTGCATTGTGAAAGCTTTGCTGTCCTTGATGTGATCATGGAATCTTTTTCTCAC
TAGAGTCTATGTCACTCATTATACTCTGTCGAATGTCATTGAATGTCTTTACATGGGCTT
GTATGCCTATGAAAATTGTAATACAACTTTCAGCAACGGATCTCTTGGCTCTCGCATCGA
TGAAGAACGCAGCGAAATGCGATAAGTAATGTGAATTGCAGAATTCAGTGAATCATCGAA
TCTTTGAACGCATCTTGCGCTCCTTGGTATTCCGAGGAGCATGCCTGTTTGAGTGTCATT
AAATTCTCAACTCTCTTATAC-TTTTTTGTAAAAGAGAGCTTGGACTGTGGAGGCTTGCT
GGCCACTTTTTGGGGTCAGCTCCTCTGAAATGCATTAGCGGAACCGTTTGCGATCTGCCA
CAAGTGTGATAAGTTATCTACACTGGCGAGGGGATTGCTCTCTGTAATGTTCAGCTTCTA
ATTGTCTCTACTTTGTGAGACTACTTTTGAATGCTTGACCTCAAATCAGGTAGGACTACC
CGCTGAACTTAA
>ABC10-2
TTTCCGTAGGTGAACCTGCGGAAGGATCATTATTGAATTATGTTTCTAGATAGGTTGTAG
CTGGCTCTTTTAGAGCATGTGCACGCCTGTTTGGACTTCATTTTCATCCACCTGTGCACC
TATTGTAGTCTTTGGTTGGGTTAGGAGGAAGTGATCATTGTATCAGCATCTGCTGGGAGT
GAGGACTTGCATTGTGAAAGCTTTGCTGTCCTTGATGTGATCATGGAATCTTTTTCTCAC
TAGAGTCTATGTCACTCATTATACTCTGTCGAATGTCATTGAATGTCTTTACATGGGCTT
GTATGCCTATGAAAATTGTAATACAACTTTCAGCAACGGATCTCTTGGCTCTCGCATCGA
TGAAGAACGCAGCGAAATGCGATAAGTAATGTGAATTGCAGAATTCAGTGAATCATCGAA
TCTTTGAACGCATCTTGCGCTCCTTGGTATTCCGAGGAGCATGCCTGTTTGAGTGTCATT
AAATTCTCAACTCTCTTATAC-TTTTTTGTAAAAGAGAGCTTGGACTGTGGAGGCTTGCT
GGCCACTTTTTGGGGTCAGCTCCTCTGAAATGCATTAGCGGAACCGTTTGCGATCTGCCA
CAAGTGTGATAAGTTATCTACACTGGCGAGGGGATTGCTCTCTGTAATGTTCAGCTTCTA
ATTGTCTCTACTTTGTGAGACTACTTTTGAATGCTTGACCTCAAATCAGGTAGGACTACC
CGCTGAACTTAA
>ABC10-3
TTTCCGTAGGTGAACCTGCGGAAGGATCATTATTGAATTATGTTTCTAGATAGGTTGTAG
CTGGCTCTTTTAGAGCATGTGCACGCCTGTTTGGACTTCATTTTCATCCACCTGTGCACC
TATTGTAGTCTTTGGTTGGGTTAGGAGGAAGTGATCATTGTATCAGCATCTGCTGGGAGT
GAGGACTTGCATTGTGAAAGCTTTGCTGTCCTTGATGTGATCATGGAATCTTTTTCTCAC
TAGAGTCTATGTCACTCATTATACTCTGTCGAATGTCATTGAATGTCTTTACATGGGCTT
GTATGCCTATGAAAATTGTAATACAACTTTCAGCAACGGATCTCTTGGCTCTCGCATCGA
TGAAGAACGCAGCGAAATGCGATAAGTAATGTGAATTGCAGAATTCAGTGAATCATCGAA
TCTTTGAACGCATCTTGCGCTCCTTGGTATTCCGAGGAGCATGCCTGTTTGAGTGTCATT
AAATTCTCAACTCTCTTATAC-TTTTTTGTAAAAGAGAGCTTGGACTGTGGAGGCTTGCT
GGCCACTTTTTGGGGTCAGCTCCTCTGAAATGCATTAGCGGAACCGTTTGCGATCTGCCA
CAAGTGTGATAAGTTATCTACACTGGCGAGGGGATTGCTCTCTGTAATGTTCAGCTTCTA
ATTGTCTCTACTTTGTGAGACTACTTTTGAATGCTTGACCTCAAATCAGGTAGGACTACC
CGCTGAACTTAA
>ABC10-4
TTTCCGTAGGTGAACCTGCGGAAGGATCATTATTGAATTATGTTTCTAGATAGGTTGTAG
CTGGCTCTTTTAGAGCATGTGCACGCCTGTTTGGACTTCATTTTCATCCACCTGTGCACC
TATTGTAGTCTTTGGTTGGGTTAGGAGGAAGTGATCATTGTATCAGCATCTGCTGGGAGT

GAGGACTTGCATTGTGAAAGCTTTGCTGTCCTTGATGTGATCATGGAATCTTTTTCTCAC
TAGAGTCTATGTCACTCATTATACTCTGTCGAATGTCATTGAATGTCTTTACATGGGCTT
GTATGCCTATGAAAATTGTAATACAACTTTCAGCAACGGATCTCTTGGCTCTCGCATCGA
TGAAGAACGCAGCGAAATGCGATAAGTAATGTGAATTGCAGAATTCAGTGAATCATCGAA
TCTTTGAACGCATCTTGCGCTCCTTGGTATTCCGAGGAGCATGCCTGTTTGAGTGTCATT
AAATTCTCAACTCTCTTATAC-TTTTTTGTAAAAGAGAGCTTGGACTGTGGAGGCTTGCT
GGCCACTTTTTGGGGTCAGCTCCTCTGAAATGCATTAGCGGAACCGTTTGCGATCTGCCA
CAAGTGTGATAAGTTATCTACACTGGCGAGGGGATTGCTCTCTGTAATGTTCAGCTTCTA
ATTGTCTCTACTTTGTGAGACTACTTTTGAATGCTTGACCTCAAATCAGGTAGGACTACC
CGCTGAACTTAA
>ABC10-5
TTTCCGTAGGTGAACCTGCGGAAGGATCATTATTGAATTATGTTTCTAGATAGGTTGTAG
CTGGCTCTTTTAGAGCATGTGCACGCCTGTTTGGACTTCATTTTCATCCACCTGTGCACC
TATTGTAGTCTTTGGTTGGGTTAGGAGGAAGTGATCATTGTATCAGCATCTGCTGGGAGT
GAGGACTTGCATTGTGAAAGCTTTGCTGTCCTTGATGTGATCATGGAATCTTTTTCTCAC
TAGAGTCTATGTCACTCATTATACTCTGTCGAATGTCATTGAATGTCTTTACATGGGCTT
GTATGCCTATGAAAATTGTAATACAACTTTCAGCAACGGATCTCTTGGCTCTCGCATCGA
TGAAGAACGCAGCGAAATGCGATAAGTAATGTGAATTGCAGAATTCAGTGAATCATCGAA
TCTTTGAACGCATCTTGCGCTCCTTGGTATTCCGAGGAGCATGCCTGTTTGAGTGTCATT
AAATTCTCAACTCTCTTATAC-TTTTTTGTAAAAGAGAGCTTGGACTGTGGAGGCTTGCT
GGCCACTTTTTGGGGTCAGCTCCTCTGAAATGCATTAGCGGAACCGTTTGCGATCTGCCA
CAAGTGTGATAAGTTATCTACACTGGCGAGGGGATTGCTCTCTGTAATGTTCAGCTTCTA
ATTGTCTCTACTTTGTGAGACTACTTTTGAATGCTTGACCTCAAATCAGGTAGGACTACC
CGCTGAACTTAA
>ABC10-10
TTTCCGTAGGTGAACCTGCGGAAGGATCATTATTGAATTATGTTTCTAGATAGGTTGTAG
CTGGCTCTTTTAGAGCATGTGCACGCCTGTTTGGACTTCATTTTCATCCACCTGTGCACC
TATTGTAGTCTTTGGTTGGGTTAGGAGGAAGTGATCATTGTATCAGCATCTGCTGGGAGT
GAGGACTTGCATTGTGAAAGCTTTGCTGTCCTTGATGTGATCATGGAATCTTTTTCTCAC
TAGAGTCTATGTCACTCATTATACTCTGTCGAATGTCATTGAATGTCTTTACATGGGCTT
GTATGCCTATGAAAATTGTAATACAACTTTCAGCAACGGATCTCTTGGCTCTCGCATCGA
TGAAGAACGCAGCGAAATGCGATAAGTAATGTGAATTGCAGAATTCAGTGAATCATCGAA
TCTTTGAACGCATCTTGCGCTCCTTGGTATTCCGAGGAGCATGCCTGTTTGAGTGTCATT
AAATTCTCAACTCTCTTATAC-TTTTTTGTAAAAGAGAGCTTGGACTGTGGAGGCTTGCT
GGCCACTTTTTGGGGTCAGCTCCTCTGAAATGCATTAGCGGAACCGTTTGCGATCTGCCA
CAAGTGTGATAAGTTATCTACACTGGCGAGGGGATTGCTCTCTGTAATGTTCAGCTTCTA
ATTGTCTCTACTTTGTGAGACTACTTTTGAATGCTTGACCTCAAATCAGGTAGGACTACC
CGCTGAACTTAA
>ABC10-12
TTTCCGTAGGTGAACCTGCGGAAGGATCATTATTGAATTATGTTTCTAGATAGGTTGTAG
CTGGCTCTTTTAGAGCATGTGCACGCCTGTTTGGACTTCATTTTCATCCACCTGTGCACC
TATTGTAGTCTTTGGTTGGGTTAGGAGGAAGTGATCATTGTATCAGCATCTGCTGGGAGT
GAGGACTTGCATTGTGAAAGCTTTGCTGTCCTTGATGTGATCATGGAATCTTTTTCTCAC
TAGAGTCTATGTCACTCATTATACTCTGTCGAATGTCATTGAATGTCTTTACATGGGCTT
GTATGCCTATGAAAATTGTAATACAACTTTCAGCAACGGATCTCTTGGCTCTCGCATCGA
TGAAGAACGCAGCGAAATGCGATAAGTAATGTGAATTGCAGAATTCAGTGAATCATCGAA
TCTTTGAACGCATCTTGCGCTCCTTGGTATTCCGAGGAGCATGCCTGTTTGAGTGTCATT
AAATTCTCAACTCTCTTATAC-TTTTTTGTAAAAGAGAGCTTGGACTGTGGAGGCTTGCT
GGCCACTTTTTGGGGTCAGCTCCTCTGAAATGCATTAGCGGAACCGTTTGCGATCTGCCA
CAAGTGTGATAAGTTATCTACACTGGCGAGGGGATTGCTCTCTGTAATGTTCAGCTTCTA

ATTGTCTCTACTTTGTGAGACTACTTTTGAATGCTTGACCTCAAATCAGGTAGGACTACC
CGCTGAACTTAA
>ABC10-18
TTTCCGTAGGTGAACCTGCGGAAGGATCATTATTGAATTATGTTTCTAGATAGGTTGTAG
CTGGCTCTTTTAGAGCATGTGCACGCCTGTTTGGACTTCATTTTCATCCACCTGTGCACC
TATTGTAGTCTTTGGTTGGGTTAGGAGGAAGTGATCATTGTATCAGCATCTGCTGGGAGT
GAGGACTTGCATTGTGAAAGCTTTGCTGTCCTTGATGTGATCATGGAATCTTTTTCTCAC
TAGAGTCTATGTCACTCATTATACTCTGTCGAATGTCATTGAATGTCTTTACATGGGCTT
GTATGCCTATGAAAATTGTAATACAACTTTCAGCAACGGATCTCTTGGCTCTCGCATCGA
TGAAGAACGCAGCGAAATGCGATAAGTAATGTGAATTGCAGAATTCAGTGAATCATCGAA
TCTTTGAACGCATCTTGCGCTCCTTGGTATTCCGAGGAGCATGCCTGTTTGAGTGTCATT
AAATTCTCAACTCTCTTATAC-TTTTTTGTAAAAGAGAGCTTGGACTGTGGAGGCTTGCT
GGCCACTTTTTGGGGTCAGCTCCTCTGAAATGCATTAGCGGAACCGTTTGCGATCTGCCA
CAAGTGTGATAAGTTATCTACACTGGCGAGGGGATTGCTCTCTGTAATGTTCAGCTTCTA
ATTGTCTCTACTTTGTGAGACTACTTTTGAATGCTTGACCTCAAATCAGGTAGGACTACC
CGCTGAACTTAA
>ABC10-36
TTTCCGTAGGTGAACCTGCGGAAGGATCATTATTGAATTATGTTTCTAGATAGGTTGTAG
CTGGCTCTTTTAGAGCATGTGCACGCCTGTTTGGACTTCATTTTCATCCACCTGTGCACC
TATTGTAGTCTTTGGTTGGGTTAGGAGGAAGTGATCATTGTATCAGCATCTGCTGGGAGT
GAGGACTTGCATTGTGAAAGCTTTGCTGTCCTTGATGTGATCATGGAATCTTTTTCTCAC
TAGAGTCTATGTCACTCATTATACTCTGTCGAATGTCATTGAATGTCTTTACATGGGCTT
GTATGCCTATGAAAATTGTAATACAACTTTCAGCAACGGATCTCTTGGCTCTCGCATCGA
TGAAGAACGCAGCGAAATGCGATAAGTAATGTGAATTGCAGAATTCAGTGAATCATCGAA
TCTTTGAACGCATCTTGCGCTCCTTGGTATTCCGAGGAGCATGCCTGTTTGAGTGTCATT
AAATTCTCAACTCTCTTATAC-TTTTTTGTAAAAGAGAGCTTGGACTGTGGAGGCTTGCT
GGCCACTTTTTGGGGTCAGCTCCTCTGAAATGCATTAGCGGAACCGTTTGCGATCTGCCA
CAAGTGTGATAAGTTATCTACACTGGCGAGGGGATTGCTCTCTGTAATGTTCAGCTTCTA
ATTGTCTCTACTTTGTGAGACTACTTTTGAATGCTTGACCTCAAATCAGGTAGGACTACC
CGCTGAACTTAA
>ABC10-38
TTTCCGTAGGTGAACCTGCGGAAGGATCATTATTGAATTATGTTTCTAGATAGGTTGTAG
CTGGCTCTTTTAGAGCATGTGCACGCCTGTTTGGACTTCATTTTCATCCACCTGTGCACC
TATTGTAGTCTTTGGTTGGGTTAGGAGGAAGTGATCATTGTATCAGCATCTGCTGGGAGT
GAGGACTTGCATTGTGAAAGCTTTGCTGTCCTTGATGTGATCATGGAATCTTTTTCTCAC
TAGAGTCTATGTCACTCATTATACTCTGTCGAATGTCATTGAATGTCTTTACATGGGCTT
GTATGCCTATGAAAATTGTAATACAACTTTCAGCAACGGATCTCTTGGCTCTCGCATCGA
TGAAGAACGCAGCGAAATGCGATAAGTAATGTGAATTGCAGAATTCAGTGAATCATCGAA
TCTTTGAACGCATCTTGCGCTCCTTGGTATTCCGAGGAGCATGCCTGTTTGAGTGTCATT
AAATTCTCAACTCTCTTATAC-TTTTTTGTAAAAGAGAGCTTGGACTGTGGAGGCTTGCT
GGCCACTTTTTGGGGTCAGCTCCTCTGAAATGCATTAGCGGAACCGTTTGCGATCTGCCA
CAAGTGTGATAAGTTATCTACACTGGCGAGGGGATTGCTCTCTGTAATGTTCAGCTTCTA
ATTGTCTCTACTTTGTGAGACTACTTTTGAATGCTTGACCTCAAATCAGGTAGGACTACC
CGCTGAACTTAA
>ABC10-44
TTTCCGTAGGTGAACCTGCGGAAGGATCATTATTGAATTATGTTTCTAGATAGGTTGTAG
CTGGCTCTTTTAGAGCATGTGCACGCCTGTTTGGACTTCATTTTCATCCACCTGTGCACC
TATTGTAGTCTTTGGTTGGGTTAGGAGGAAGTGATCATTGTATCAGCATCTGCTGGGAGT
GAGGACTTGCATTGTGAAAGCTTTGCTGTCCTTGATGTGATCATGGAATCTTTTTCTCAC
TAGAGTCTATGTCACTCATTATACTCTGTCGAATGTCATTGAATGTCTTTACATGGGCTT

GTATGCCTATGAAAATTGTAATACAACTTTCAGCAACGGATCTCTTGGCTCTCGCATCGA
TGAAGAACGCAGCGAAATGCGATAAGTAATGTGAATTGCAGAATTCAGTGAATCATCGAA
TCTTTGAACGCATCTTGCGCTCCTTGGTATTCCGAGGAGCATGCCTGTTTGAGTGTCATT
AAATTCTCAACTCTCTTATAC-TTTTTTGTAAAAGAGAGCTTGGACTGTGGAGGCTTGCT
GGCCACTTTTTGGGGTCAGCTCCTCTGAAATGCATTAGCGGAACCGTTTGCGATCTGCCA
CAAGTGTGATAAGTTATCTACACTGGCGAGGGGATTGCTCTCTGTAATGTTCAGCTTCTA
ATTGTCTCTACTTTGTGAGACTACTTTTGAATGCTTGACCTCAAATCAGGTAGGACTACC
CGCTGAACTTAA
>ABC10-46
TTTCCGTAGGTGAACCTGCGGAAGGATCATTATTGAATTATGTTTCTAGATAGGTTGTAG
CTGGCTCTTTTAGAGCATGTGCACGCCTGTTTGGACTTCATTTTCATCCACCTGTGCACC
TATTGTAGTCTTTGGTTGGGTTAGGAGGAAGTGATCATTGTATCAGCATCTGCTGGGAGT
GAGGACTTGCATTGTGAAAGCTTTGCTGTCCTTGATGTGATCATGGAATCTTTTTCTCAC
TAGAGTCTATGTCACTCATTATACTCTGTCGAATGTCATTGAATGTCTTTACATGGGCTT
GTATGCCTATGAAAATTGTAATACAACTTTCAGCAACGGATCTCTTGGCTCTCGCATCGA
TGAAGAACGCAGCGAAATGCGATAAGTAATGTGAATTGCAGAATTCAGTGAATCATCGAA
TCTTTGAACGCATCTTGCGCTCCTTGGTATTCCGAGGAGCATGCCTGTTTGAGTGTCATT
AAATTCTCAACTCTCTTATAC-TTTTTTGTAAAAGAGAGCTTGGACTGTGGAGGCTTGCT
GGCCACTTTTTGGGGTCAGCTCCTCTGAAATGCATTAGCGGAACCGTTTGCGATCTGCCA
CAAGTGTGATAAGTTATCTACACTGGCGAGGGGATTGCTCTCTGTAATGTTCAGCTTCTA
ATTGTCTCTACTTTGTGAGACTACTTTTGAATGCTTGACCTCAAATCAGGTAGGACTACC
CGCTGAACTTAA
>ABC11-1
TTTCCGTAGGTGAACCTGCGGAAGGATCATTATTGAATTATGTTTCTAGATAGGTTGTAG
CTGGCTCTTTTAGAGCATGTGCACGCCTGTTTGGACTTCATTTTCATCCACCTGTGCACC
TATTGTAGTCTTTGGTTGGGTTAGGAGGAAGTGATCATTGTATCAGCATCTGCTGGGAGT
GAGGACTTGCATTGTGAAAGCTTTGCTGTCCTTGATGTGATCATGGAATCTTTTTCTCAC
TAGAGTCTATGTCACTCATTATACTCTGTCGAATGTCATTGAATGTCTTTACATGGGCTT
GTATGCCTATGAAAATTGTAATACAACTTTCAGCAACGGATCTCTTGGCTCTCGCATCGA
TGAAGAACGCAGCGAAATGCGATAAGTAATGTGAATTGCAGAATTCAGTGAATCATCGAA
TCTTTGAACGCATCTTGCGCTCCTTGGTATTCCGAGGAGCATGCCTGTTTGAGTGTCATT
AAATTCTCAACTCTCTTATAC-TTTTTTGTAAAAGAGAGCTTGGACTGTGGAGGCTTGCT
GGCCACTTTTTGGGGTCAGCTCCTCTGAAATGCATTAGCGGAACCGTTTGCGATCTGCCA
CAAGTGTGATAAGTTATCTACACTGGCGAGGGGATTGCTCTCTGTAATGTTCAGCTTCTA
ATTGTCTCTACTTTGTGAGACTACTTTTGAATGCTTGACCTCAAATCAGGTAGGACTACC
CGCTGAACTTAA
>ABC11-2
TTTCCGTAGGTGAACCTGCGGAAGGATCATTATTGAATTATGTTTCTAGATAGGTTGTAG
CTGGCTCTTTTAGAGCATGTGCACGCCTGTTTGGACTTCATTTTCATCCACCTGTGCACC
TATTGTAGTCTTTGGTTGGGTTAGGAGGAAGTGATCATTGTATCAGCATCTGCTGGGAGT
GAGGACTTGCATTGTGAAAGCTTTGCTGTCCTTGATGTGATCATGGAATCTTTTTCTCAC
TAGAGTCTATGTCACTCATTATACTCTGTCGAATGTCATTGAATGTCTTTACATGGGCTT
GTATGCCTATGAAAATTGTAATACAACTTTCAGCAACGGATCTCTTGGCTCTCGCATCGA
TGAAGAACGCAGCGAAATGCGATAAGTAATGTGAATTGCAGAATTCAGTGAATCATCGAA
TCTTTGAACGCATCTTGCGCTCCTTGGTATTCCGAGGAGCATGCCTGTTTGAGTGTCATT
AAATTCTCAACTCTCTTATAC-TTTTTTGTAAAAGAGAGCTTGGACTGTGGAGGCTTGCT
GGCCACTTTTTGGGGTCAGCTCCTCTGAAATGCATTAGCGGAACCGTTTGCGATCTGCCA
CAAGTGTGATAAGTTATCTACACTGGCGAGGGGATTGCTCTCTGTAATGTTCAGCTTCTA
ATTGTCTCTACTTTGTGAGACTACTTTTGAATGCTTGACCTCAAATCAGGTAGGACTACC
CGCTGAACTTAA

>ABC11-4
TTTCCGTAGGTGAACCTGCGGAAGGATCATTATTGAATTATGTTTCTAGATAGGTTGTAG
CTGGCTCTTTTAGAGCATGTGCACGCCTGTTTGGACTTCATTTTCATCCACCTGTGCACC
TATTGTAGTCTTTGGTTGGGTTAGGAGGAAGTGATCATTGTATCAGCATCTGCTGGGAGT
GAGGACTTGCATTGTGAAAGCTTTGCTGTCCTTGATGTGATCATGGAATCTTTTTCTCAC
TAGAGTCTATGTCACTCATTATACTCTGTCGAATGTCATTGAATGTCTTTACATGGGCTT
GTATGCCTATGAAAATTGTAATACAACTTTCAGCAACGGATCTCTTGGCTCTCGCATCGA
TGAAGAACGCAGCGAAATGCGATAAGTAATGTGAATTGCAGAATTCAGTGAATCATCGAA
TCTTTGAACGCATCTTGCGCTCCTTGGTATTCCGAGGAGCATGCCTGTTTGAGTGTCATT
AAATTCTCAACTCTCTTATAC-TTTTTTGTAAAAGAGAGCTTGGACTGTGGAGGCTTGCT
GGCCACTTTTTGGGGTCAGCTCCTCTGAAATGCATTAGCGGAACCGTTTGCGATCTGCCA
CAAGTGTGATAAGTTATCTACACTGGCGAGGGGATTGCTCTCTGTAATGTTCAGCTTCTA
ATTGTCTCTACTTTGTGAGACTACTTTTGAATGCTTGACCTCAAATCAGGTAGGACTACC
CGCTGAACTTAA
>ABC11-6
TTTCCGTAGGTGAACCTGCGGAAGGATCATTATTGAATTATGTTTCTAGATAGGTTGTAG
CTGGCTCTTTTAGAGCATGTGCACGCCTGTTTGGACTTCATTTTCATCCACCTGTGCACC
TATTGTAGTCTTTGGTTGGGTTAGGAGGAAGTGATCATTGTATCAGCATCTGCTGGGAGT
GAGGACTTGCATTGTGAAAGCTTTGCTGTCCTTGATGTGATCATGGAATCTTTTTCTCAC
TAGAGTCTATGTCACTCATTATACTCTGTCGAATGTCATTGAATGTCTTTACATGGGCTT
GTATGCCTATGAAAATTGTAATACAACTTTCAGCAACGGATCTCTTGGCTCTCGCATCGA
TGAAGAACGCAGCGAAATGCGATAAGTAATGTGAATTGCAGAATTCAGTGAATCATCGAA
TCTTTGAACGCATCTTGCGCTCCTTGGTATTCCGAGGAGCATGCCTGTTTGAGTGTCATT
AAATTCTCAACTCTCTTATAC-TTTTTTGTAAAAGAGAGCTTGGACTGTGGAGGCTTGCT
GGCCACTTTTTGGGGTCAGCTCCTCTGAAATGCATTAGCGGAACCGTTTGCGATCTGCCA
CAAGTGTGATAAGTTATCTACACTGGCGAGGGGATTGCTCTCTGTAATGTTCAGCTTCTA
ATTGTCTCTACTTTGTGAGACTACTTTTGAATGCTTGACCTCAAATCAGGTAGGACTACC
CGCTGAACTTAA
>ABC11-26
TTTCCGTAGGTGAACCTGCGGAAGGATCATTATTGAATTATGTTTCTAGATAGGTTGTAG
CTGGCTCTTTTAGAGCATGTGCACGCCTGTTTGGACTTCATTTTCATCCACCTGTGCACC
TATTGTAGTCTTTGGTTGGGTTAGGAGGAAGTGATCATTGTATCAGCATCTGCTGGGAGT
GAGGACTTGCATTGTGAAAGCTTTGCTGTCCTTGATGTGATCATGGAATCTTTTTCTCAC
TAGAGTCTATGTCACTCATTATACTCTGTCGAATGTCATTGAATGTCTTTACATGGGCTT
GTATGCCTATGAAAATTGTAATACAACTTTCAGCAACGGATCTCTTGGCTCTCGCATCGA
TGAAGAACGCAGCGAAATGCGATAAGTAATGTGAATTGCAGAATTCAGTGAATCATCGAA
TCTTTGAACGCATCTTGCGCTCCTTGGTATTCCGAGGAGCATGCCTGTTTGAGTGTCATT
AAATTCTCAACTCTCTTATAC-TTTTTTGTAAAAGAGAGCTTGGACTGTGGAGGCTTGCT
GGCCACTTTTTGGGGTCAGCTCCTCTGAAATGCATTAGCGGAACCGTTTGCGATCTGCCA
CAAGTGTGATAAGTTATCTACACTGGCGAGGGGATTGCTCTCTGTAATGTTCAGCTTCTA
ATTGTCTCTACTTTGTGAGACTACTTTTGAATGCTTGACCTCAAATCAGGTAGGACTACC
CGCTGAACTTAA
>ABC11-27
TTTCCGTAGGTGAACCTGCGGAAGGATCATTATTGAATTATGTTTCTAGATAGGTTGTAG
CTGGCTCTTTTAGAGCATGTGCACGCCTGTTTGGACTTCATTTTCATCCACCTGTGCACC
TATTGTAGTCTTTGGTTGGGTTAGGAGGAAGTGATCATTGTATCAGCATCTGCTGGGAGT
GAGGACTTGCATTGTGAAAGCTTTGCTGTCCTTGATGTGATCATGGAATCTTTTTCTCAC
TAGAGTCTATGTCACTCATTATACTCTGTCGAATGTCATTGAATGTCTTTACATGGGCTT
GTATGCCTATGAAAATTGTAATACAACTTTCAGCAACGGATCTCTTGGCTCTCGCATCGA
TGAAGAACGCAGCGAAATGCGATAAGTAATGTGAATTGCAGAATTCAGTGAATCATCGAA

TCTTTGAACGCATCTTGCGCTCCTTGGTATTCCGAGGAGCATGCCTGTTTGAGTGTCATT
AAATTCTCAACTCTCTTATAC-TTTTTTGTAAAAGAGAGCTTGGACTGTGGAGGCTTGCT
GGCCACTTTTTGGGGTCAGCTCCTCTGAAATGCATTAGCGGAACCGTTTGCGATCTGCCA
CAAGTGTGATAAGTTATCTACACTGGCGAGGGGATTGCTCTCTGTAATGTTCAGCTTCTA
ATTGTCTCTACTTTGTGAGACTACTTTTGAATGCTTGACCTCAAATCAGGTAGGACTACC
CGCTGAACTTAA
>ABC11-30
TTTCCGTAGGTGAACCTGCGGAAGGATCATTATTGAATTATGTTTCTAGATAGGTTGTAG
CTGGCTCTTTTAGAGCATGTGCACGCCTGTTTGGACTTCATTTTCATCCACCTGTGCACC
TATTGTAGTCTTTGGTTGGGTTAGGAGGAAGTGATCATTGTATCAGCATCTGCTGGGAGT
GAGGACTTGCATTGTGAAAGCTTTGCTGTCCTTGATGTGATCATGGAATCTTTTTCTCAC
TAGAGTCTATGTCACTCATTATACTCTGTCGAATGTCATTGAATGTCTTTACATGGGCTT
GTATGCCTATGAAAATTGTAATACAACTTTCAGCAACGGATCTCTTGGCTCTCGCATCGA
TGAAGAACGCAGCGAAATGCGATAAGTAATGTGAATTGCAGAATTCAGTGAATCATCGAA
TCTTTGAACGCATCTTGCGCTCCTTGGTATTCCGAGGAGCATGCCTGTTTGAGTGTCATT
AAATTCTCAACTCTCTTATAC-TTTTTTGTAAAAGAGAGCTTGGACTGTGGAGGCTTGCT
GGCCACTTTTTGGGGTCAGCTCCTCTGAAATGCATTAGCGGAACCGTTTGCGATCTGCCA
CAAGTGTGATAAGTTATCTACACTGGCGAGGGGATTGCTCTCTGTAATGTTCAGCTTCTA
ATTGTCTCTACTTTGTGAGACTACTTTTGAATGCTTGACCTCAAATCAGGTAGGACTACC
CGCTGAACTTAA
>ABC11-31
TTTCCGTAGGTGAACCTGCGGAAGGATCATTATTGAATTATGTTTCTAGATAGGTTGTAG
CTGGCTCTTTTAGAGCATGTGCACGCCTGTTTGGACTTCATTTTCATCCACCTGTGCACC
TATTGTAGTCTTTGGTTGGGTTAGGAGGAAGTGATCATTGTATCAGCATCTGCTGGGAGT
GAGGACTTGCATTGTGAAAGCTTTGCTGTCCTTGATGTGATCATGGAATCTTTTTCTCAC
TAGAGTCTATGTCACTCATTATACTCTGTCGAATGTCATTGAATGTCTTTACATGGGCTT
GTATGCCTATGAAAATTGTAATACAACTTTCAGCAACGGATCTCTTGGCTCTCGCATCGA
TGAAGAACGCAGCGAAATGCGATAAGTAATGTGAATTGCAGAATTCAGTGAATCATCGAA
TCTTTGAACGCATCTTGCGCTCCTTGGTATTCCGAGGAGCATGCCTGTTTGAGTGTCATT
AAATTCTCAACTCTCTTATAC-TTTTTTGTAAAAGAGAGCTTGGACTGTGGAGGCTTGCT
GGCCACTTTTTGGGGTCAGCTCCTCTGAAATGCATTAGCGGAACCGTTTGCGATCTGCCA
CAAGTGTGATAAGTTATCTACACTGGCGAGGGGATTGCTCTCTGTAATGTTCAGCTTCTA
ATTGTCTCTACTTTGTGAGACTACTTTTGAATGCTTGACCTCAAATCAGGTAGGACTACC
CGCTGAACTTAA
>ABC11-33
TTTCCGTAGGTGAACCTGCGGAAGGATCATTATTGAATTATGTTTCTAGATAGGTTGTAG
CTGGCTCTTTTAGAGCATGTGCACGCCTGTTTGGACTTCATTTTCATCCACCTGTGCACC
TATTGTAGTCTTTGGTTGGGTTAGGAGGAAGTGATCATTGTATCAGCATCTGCTGGGAGT
GAGGACTTGCATTGTGAAAGCTTTGCTGTCCTTGATGTGATCATGGAATCTTTTTCTCAC
TAGAGTCTATGTCACTCATTATACTCTGTCGAATGTCATTGAATGTCTTTACATGGGCTT
GTATGCCTATGAAAATTGTAATACAACTTTCAGCAACGGATCTCTTGGCTCTCGCATCGA
TGAAGAACGCAGCGAAATGCGATAAGTAATGTGAATTGCAGAATTCAGTGAATCATCGAA
TCTTTGAACGCATCTTGCGCTCCTTGGTATTCCGAGGAGCATGCCTGTTTGAGTGTCATT
AAATTCTCAACTCTCTTATAC-TTTTTTGTAAAAGAGAGCTTGGACTGTGGAGGCTTGCT
GGCCACTTTTTGGGGTCAGCTCCTCTGAAATGCATTAGCGGAACCGTTTGCGATCTGCCA
CAAGTGTGATAAGTTATCTACACTGGCGAGGGGATTGCTCTCTGTAATGTTCAGCTTCTA
ATTGTCTCTACTTTGTGAGACTACTTTTGAATGCTTGACCTCAAATCAGGTAGGACTACC
CGCTGAACTTAA
>ABC11-37
TTTCCGTAGGTGAACCTGCGGAAGGATCATTATTGAATTATGTTTCTAGATAGGTTGTAG

CTGGCTCTTTTAGAGCATGTGCACGCCTGTTTGGACTTCATTTTCATCCACCTGTGCACC
TATTGTAGTCTTTGGTTGGGTTAGGAGGAAGTGATCATTGTATCAGCATCTGCTGGGAGT
GAGGACTTGCATTGTGAAAGCTTTGCTGTCCTTGATGTGATCATGGAATCTTTTTCTCAC
TAGAGTCTATGTCACTCATTATACTCTGTCGAATGTCATTGAATGTCTTTACATGGGCTT
GTATGCCTATGAAAATTGTAATACAACTTTCAGCAACGGATCTCTTGGCTCTCGCATCGA
TGAAGAACGCAGCGAAATGCGATAAGTAATGTGAATTGCAGAATTCAGTGAATCATCGAA
TCTTTGAACGCATCTTGCGCTCCTTGGTATTCCGAGGAGCATGCCTGTTTGAGTGTCATT
AAATTCTCAACTCTCTTATAC-TTTTTTGTAAAAGAGAGCTTGGACTGTGGAGGCTTGCT
GGCCACTTTTTGGGGTCAGCTCCTCTGAAATGCATTAGCGGAACCGTTTGCGATCTGCCA
CAAGTGTGATAAGTTATCTACACTGGCGAGGGGATTGCTCTCTGTAATGTTCAGCTTCTA
ATTGTCTCTACTTTGTGAGACTACTTTTGAATGCTTGACCTCAAATCAGGTAGGACTACC
CGCTGAACTTAA
>ABC11-49
TTTCCGTAGGTGAACCTGCGGAAGGATCATTATTGAATTATGTTTCTAGATAGGTTGTAG
CTGGCTCTTTTAGAGCATGTGCACGCCTGTTTGGACTTCATTTTCATCCACCTGTGCACC
TATTGTAGTCTTTGGTTGGGTTAGGAGGAAGTGATCATTGTATCAGCATCTGCTGGGAGT
GAGGACTTGCATTGTGAAAGCTTTGCTGTCCTTGATGTGATCATGGAATCTTTTTCTCAC
TAGAGTCTATGTCACTCATTATACTCTGTCGAATGTCATTGAATGTCTTTACATGGGCTT
GTATGCCTATGAAAATTGTAATACAACTTTCAGCAACGGATCTCTTGGCTCTCGCATCGA
TGAAGAACGCAGCGAAATGCGATAAGTAATGTGAATTGCAGAATTCAGTGAATCATCGAA
TCTTTGAACGCATCTTGCGCTCCTTGGTATTCCGAGGAGCATGCCTGTTTGAGTGTCATT
AAATTCTCAACTCTCTTATAC-TTTTTTGTAAAAGAGAGCTTGGACTGTGGAGGCTTGCT
GGCCACTTTTTGGGGTCAGCTCCTCTGAAATGCATTAGCGGAACCGTTTGCGATCTGCCA
CAAGTGTGATAAGTTATCTACACTGGCGAGGGGATTGCTCTCTGTAATGTTCAGCTTCTA
ATTGTCTCTACTTTGTGAGACTACTTTTGAATGCTTGACCTCAAATCAGGTAGGACTACC
CGCTGAACTTAA
>ABC12-7
TTTCCGTAGGTGAACCTGCGGAAGGATCATTATTGAATTATGTTTCTAGATAGGTTGTAG
CTGGCTCTTTTAGAGCATGTGCACGCCTGTTTGGACTTCATTTTCATCCACCTGTGCACC
TATTGTAGTCTTTGGTTGGGTTAGGAGGAAGTGATCATTGTATCAGCATCTGCTGGGAGT
GAGGACTTGCATTGTGAAAGCTTTGCTGTCCTTGATGTGATCATGGAATCTTTTTCTCAC
TAGAGTCTATGTCACTCATTATACTCTGTCGAATGTCATTGAATGTCTTTACATGGGCTT
GTATGCCTATGAAAATTGTAATACAACTTTCAGCAACGGATCTCTTGGCTCTCGCATCGA
TGAAGAACGCAGCGAAATGCGATAAGTAATGTGAATTGCAGAATTCAGTGAATCATCGAA
TCTTTGAACGCATCTTGCGCTCCTTGGTATTCCGAGGAGCATGCCTGTTTGAGTGTCATT
AAATTCTCAACTCTCTTATAC-TTTTTTGTAAAAGAGAGCTTGGACTGTGGAGGCTTGCT
GGCCACTTTTTGGGGTCAGCTCCTCTGAAATGCATTAGCGGAACCGTTTGCGATCTGCCA
CAAGTGTGATAAGTTATCTACACTGGCGAGGGGATTGCTCTCTGTAATGTTCAGCTTCTA
ATTGTCTCTACTTTGTGAGACTACTTTTGAATGCTTGACCTCAAATCAGGTAGGACTACC
CGCTGAACTTAA
>ABC12-8
TTTCCGTAGGTGAACCTGCGGAAGGATCATTATTGAATTATGTTTCTAGATAGGTTGTAG
CTGGCTCTTTTAGAGCATGTGCACGCCTGTTTGGACTTCATTTTCATCCACCTGTGCACC
TATTGTAGTCTTTGGTTGGGTTAGGAGGAAGTGATCATTGTATCAGCATCTGCTGGGAGT
GAGGACTTGCATTGTGAAAGCTTTGCTGTCCTTGATGTGATCATGGAATCTTTTTCTCAC
TAGAGTCTATGTCACTCATTATACTCTGTCGAATGTCATTGAATGTCTTTACATGGGCTT
GTATGCCTATGAAAATTGTAATACAACTTTCAGCAACGGATCTCTTGGCTCTCGCATCGA
TGAAGAACGCAGCGAAATGCGATAAGTAATGTGAATTGCAGAATTCAGTGAATCATCGAA
TCTTTGAACGCATCTTGCGCTCCTTGGTATTCCGAGGAGCATGCCTGTTTGAGTGTCATT
AAATTCTCAACTCTCTTATAC-TTTTTTGTAAAAGAGAGCTTGGACTGTGGAGGCTTGCT

GGCCACTTTTTGGGGTCAGCTCCTCTGAAATGCATTAGCGGAACCGTTTGCGATCTGCCA
CAAGTGTGATAAGTTATCTACACTGGCGAGGGGATTGCTCTCTGTAATGTTCAGCTTCTA
ATTGTCTCTACTTTGTGAGACTACTTTTGAATGCTTGACCTCAAATCAGGTAGGACTACC
CGCTGAACTTAA
>ABC12-10
TTTCCGTAGGTGAACCTGCGGAAGGATCATTATTGAATTATGTTTCTAGATAGGTTGTAG
CTGGCTCTTTTAGAGCATGTGCACGCCTGTTTGGACTTCATTTTCATCCACCTGTGCACC
TATTGTAGTCTTTGGTTGGGTTAGGAGGAAGTGATCATTGTATCAGCATCTGCTGGGAGT
GAGGACTTGCATTGTGAAAGCTTTGCTGTCCTTGATGTGATCATGGAATCTTTTTCTCAC
TAGAGTCTATGTCACTCATTATACTCTGTCGAATGTCATTGAATGTCTTTACATGGGCTT
GTATGCCTATGAAAATTGTAATACAACTTTCAGCAACGGATCTCTTGGCTCTCGCATCGA
TGAAGAACGCAGCGAAATGCGATAAGTAATGTGAATTGCAGAATTCAGTGAATCATCGAA
TCTTTGAACGCATCTTGCGCTCCTTGGTATTCCGAGGAGCATGCCTGTTTGAGTGTCATT
AAATTCTCAACTCTCTTATAC-TTTTTTGTAAAAGAGAGCTTGGACTGTGGAGGCTTGCT
GGCCACTTTTTGGGGTCAGCTCCTCTGAAATGCATTAGCGGAACCGTTTGCGATCTGCCA
CAAGTGTGATAAGTTATCTACACTGGCGAGGGGATTGCTCTCTGTAATGTTCAGCTTCTA
ATTGTCTCTACTTTGTGAGACTACTTTTGAATGCTTGACCTCAAATCAGGTAGGACTACC
CGCTGAACTTAA
>ABC12-11
TTTCCGTAGGTGAACCTGCGGAAGGATCATTATTGAATTATGTTTCTAGATAGGTTGTAG
CTGGCTCTTTTAGAGCATGTGCACGCCTGTTTGGACTTCATTTTCATCCACCTGTGCACC
TATTGTAGTCTTTGGTTGGGTTAGGAGGAAGTGATCATTGTATCAGCATCTGCTGGGAGT
GAGGACTTGCATTGTGAAAGCTTTGCTGTCCTTGATGTGATCATGGAATCTTTTTCTCAC
TAGAGTCTATGTCACTCATTATACTCTGTCGAATGTCATTGAATGTCTTTACATGGGCTT
GTATGCCTATGAAAATTGTAATACAACTTTCAGCAACGGATCTCTTGGCTCTCGCATCGA
TGAAGAACGCAGCGAAATGCGATAAGTAATGTGAATTGCAGAATTCAGTGAATCATCGAA
TCTTTGAACGCATCTTGCGCTCCTTGGTATTCCGAGGAGCATGCCTGTTTGAGTGTCATT
AAATTCTCAACTCTCTTATAC-TTTTTTGTAAAAGAGAGCTTGGACTGTGGAGGCTTGCT
GGCCACTTTTTGGGGTCAGCTCCTCTGAAATGCATTAGCGGAACCGTTTGCGATCTGCCA
CAAGTGTGATAAGTTATCTACACTGGCGAGGGGATTGCTCTCTGTAATGTTCAGCTTCTA
ATTGTCTCTACTTTGTGAGACTACTTTTGAATGCTTGACCTCAAATCAGGTAGGACTACC
CGCTGAACTTAA
>ABC12-12
TTTCCGTAGGTGAACCTGCGGAAGGATCATTATTGAATTATGTTTCTAGATAGGTTGTAG
CTGGCTCTTTTAGAGCATGTGCACGCCTGTTTGGACTTCATTTTCATCCACCTGTGCACC
TATTGTAGTCTTTGGTTGGGTTAGGAGGAAGTGATCATTGTATCAGCATCTGCTGGGAGT
GAGGACTTGCATTGTGAAAGCTTTGCTGTCCTTGATGTGATCATGGAATCTTTTTCTCAC
TAGAGTCTATGTCACTCATTATACTCTGTCGAATGTCATTGAATGTCTTTACATGGGCTT
GTATGCCTATGAAAATTGTAATACAACTTTCAGCAACGGATCTCTTGGCTCTCGCATCGA
TGAAGAACGCAGCGAAATGCGATAAGTAATGTGAATTGCAGAATTCAGTGAATCATCGAA
TCTTTGAACGCATCTTGCGCTCCTTGGTATTCCGAGGAGCATGCCTGTTTGAGTGTCATT
AAATTCTCAACTCTCTTATAC-TTTTTTGTAAAAGAGAGCTTGGACTGTGGAGGCTTGCT
GGCCACTTTTTGGGGTCAGCTCCTCTGAAATGCATTAGCGGAACCGTTTGCGATCTGCCA
CAAGTGTGATAAGTTATCTACACTGGCGAGGGGATTGCTCTCTGTAATGTTCAGCTTCTA
ATTGTCTCTACTTTGTGAGACTACTTTTGAATGCTTGACCTCAAATCAGGTAGGACTACC
CGCTGAACTTAA
>ABC12-14
TTTCCGTAGGTGAACCTGCGGAAGGATCATTATTGAATTATGTTTCTAGATAGGTTGTAG
CTGGCTCTTTTAGAGCATGTGCACGCCTGTTTGGACTTCATTTTCATCCACCTGTGCACC
TATTGTAGTCTTTGGTTGGGTTAGGAGGAAGTGATCATTGTATCAGCATCTGCTGGGAGT

GAGGACTTGCATTGTGAAAGCTTTGCTGTCCTTGATGTGATCATGGAATCTTTTTCTCAC
TAGAGTCTATGTCACTCATTATACTCTGTCGAATGTCATTGAATGTCTTTACATGGGCTT
GTATGCCTATGAAAATTGTAATACAACTTTCAGCAACGGATCTCTTGGCTCTCGCATCGA
TGAAGAACGCAGCGAAATGCGATAAGTAATGTGAATTGCAGAATTCAGTGAATCATCGAA
TCTTTGAACGCATCTTGCGCTCCTTGGTATTCCGAGGAGCATGCCTGTTTGAGTGTCATT
AAATTCTCAACTCTCTTATAC-TTTTTTGTAAAAGAGAGCTTGGACTGTGGAGGCTTGCT
GGCCACTTTTTGGGGTCAGCTCCTCTGAAATGCATTAGCGGAACCGTTTGCGATCTGCCA
CAAGTGTGATAAGTTATCTACACTGGCGAGGGGATTGCTCTCTGTAATGTTCAGCTTCTA
ATTGTCTCTACTTTGTGAGACTACTTTTGAATGCTTGACCTCAAATCAGGTAGGACTACC
CGCTGAACTTAA
>ABC12-16
TTTCCGTAGGTGAACCTGCGGAAGGATCATTATTGAATTATGTTTCTAGATAGGTTGTAG
CTGGCTCTTTTAGAGCATGTGCACGCCTGTTTGGACTTCATTTTCATCCACCTGTGCACC
TATTGTAGTCTTTGGTTGGGTTAGGAGGAAGTGATCATTGTATCAGCATCTGCTGGGAGT
GAGGACTTGCATTGTGAAAGCTTTGCTGTCCTTGATGTGATCATGGAATCTTTTTCTCAC
TAGAGTCTATGTCACTCATTATACTCTGTCGAATGTCATTGAATGTCTTTACATGGGCTT
GTATGCCTATGAAAATTGTAATACAACTTTCAGCAACGGATCTCTTGGCTCTCGCATCGA
TGAAGAACGCAGCGAAATGCGATAAGTAATGTGAATTGCAGAATTCAGTGAATCATCGAA
TCTTTGAACGCATCTTGCGCTCCTTGGTATTCCGAGGAGCATGCCTGTTTGAGTGTCATT
AAATTCTCAACTCTCTTATAC-TTTTTTGTAAAAGAGAGCTTGGACTGTGGAGGCTTGCT
GGCCACTTTTTGGGGTCAGCTCCTCTGAAATGCATTAGCGGAACCGTTTGCGATCTGCCA
CAAGTGTGATAAGTTATCTACACTGGCGAGGGGATTGCTCTCTGTAATGTTCAGCTTCTA
ATTGTCTCTACTTTGTGAGACTACTTTTGAATGCTTGACCTCAAATCAGGTAGGACTACC
CGCTGAACTTAA
>ABC12-21
TTTCCGTAGGTGAACCTGCGGAAGGATCATTATTGAATTATGTTTCTAGATAGGTTGTAG
CTGGCTCTTTTAGAGCATGTGCACGCCTGTTTGGACTTCATTTTCATCCACCTGTGCACC
TATTGTAGTCTTTGGTTGGGTTAGGAGGAAGTGATCATTGTATCAGCATCTGCTGGGAGT
GAGGACTTGCATTGTGAAAGCTTTGCTGTCCTTGATGTGATCATGGAATCTTTTTCTCAC
TAGAGTCTATGTCACTCATTATACTCTGTCGAATGTCATTGAATGTCTTTACATGGGCTT
GTATGCCTATGAAAATTGTAATACAACTTTCAGCAACGGATCTCTTGGCTCTCGCATCGA
TGAAGAACGCAGCGAAATGCGATAAGTAATGTGAATTGCAGAATTCAGTGAATCATCGAA
TCTTTGAACGCATCTTGCGCTCCTTGGTATTCCGAGGAGCATGCCTGTTTGAGTGTCATT
AAATTCTCAACTCTCTTATAC-TTTTTTGTAAAAGAGAGCTTGGACTGTGGAGGCTTGCT
GGCCACTTTTTGGGGTCAGCTCCTCTGAAATGCATTAGCGGAACCGTTTGCGATCTGCCA
CAAGTGTGATAAGTTATCTACACTGGCGAGGGGATTGCTCTCTGTAATGTTCAGCTTCTA
ATTGTCTCTACTTTGTGAGACTACTTTTGAATGCTTGACCTCAAATCAGGTAGGACTACC
CGCTGAACTTAA
>ABC12-23
TTTCCGTAGGTGAACCTGCGGAAGGATCATTATTGAATTATGTTTCTAGATAGGTTGTAG
CTGGCTCTTTTAGAGCATGTGCACGCCTGTTTGGACTTCATTTTCATCCACCTGTGCACC
TATTGTAGTCTTTGGTTGGGTTAGGAGGAAGTGATCATTGTATCAGCATCTGCTGGGAGT
GAGGACTTGCATTGTGAAAGCTTTGCTGTCCTTGATGTGATCATGGAATCTTTTTCTCAC
TAGAGTCTATGTCACTCATTATACTCTGTCGAATGTCATTGAATGTCTTTACATGGGCTT
GTATGCCTATGAAAATTGTAATACAACTTTCAGCAACGGATCTCTTGGCTCTCGCATCGA
TGAAGAACGCAGCGAAATGCGATAAGTAATGTGAATTGCAGAATTCAGTGAATCATCGAA
TCTTTGAACGCATCTTGCGCTCCTTGGTATTCCGAGGAGCATGCCTGTTTGAGTGTCATT
AAATTCTCAACTCTCTTATAC-TTTTTTGTAAAAGAGAGCTTGGACTGTGGAGGCTTGCT
GGCCACTTTTTGGGGTCAGCTCCTCTGAAATGCATTAGCGGAACCGTTTGCGATCTGCCA
CAAGTGTGATAAGTTATCTACACTGGCGAGGGGATTGCTCTCTGTAATGTTCAGCTTCTA

ATTGTCTCTACTTTGTGAGACTACTTTTGAATGCTTGACCTCAAATCAGGTAGGACTACC
CGCTGAACTTAA
>ABC12-26
TTTCCGTAGGTGAACCTGCGGAAGGATCATTATTGAATTATGTTTCTAGATAGGTTGTAG
CTGGCTCTTTTAGAGCATGTGCACGCCTGTTTGGACTTCATTTTCATCCACCTGTGCACC
TATTGTAGTCTTTGGTTGGGTTAGGAGGAAGTGATCATTGTATCAGCATCTGCTGGGAGT
GAGGACTTGCATTGTGAAAGCTTTGCTGTCCTTGATGTGATCATGGAATCTTTTTCTCAC
TAGAGTCTATGTCACTCATTATACTCTGTCGAATGTCATTGAATGTCTTTACATGGGCTT
GTATGCCTATGAAAATTGTAATACAACTTTCAGCAACGGATCTCTTGGCTCTCGCATCGA
TGAAGAACGCAGCGAAATGCGATAAGTAATGTGAATTGCAGAATTCAGTGAATCATCGAA
TCTTTGAACGCATCTTGCGCTCCTTGGTATTCCGAGGAGCATGCCTGTTTGAGTGTCATT
AAATTCTCAACTCTCTTATAC-TTTTTTGTAAAAGAGAGCTTGGACTGTGGAGGCTTGCT
GGCCACTTTTTGGGGTCAGCTCCTCTGAAATGCATTAGCGGAACCGTTTGCGATCTGCCA
CAAGTGTGATAAGTTATCTACACTGGCGAGGGGATTGCTCTCTGTAATGTTCAGCTTCTA
ATTGTCTCTACTTTGTGAGACTACTTTTGAATGCTTGACCTCAAATCAGGTAGGACTACC
CGCTGAACTTAA
>ABC12-30
TTTCCGTAGGTGAACCTGCGGAAGGATCATTATTGAATTATGTTTCTAGATAGGTTGTAG
CTGGCTCTTTTAGAGCATGTGCACGCCTGTTTGGACTTCATTTTCATCCACCTGTGCACC
TATTGTAGTCTTTGGTTGGGTTAGGAGGAAGTGATCATTGTATCAGCATCTGCTGGGAGT
GAGGACTTGCATTGTGAAAGCTTTGCTGTCCTTGATGTGATCATGGAATCTTTTTCTCAC
TAGAGTCTATGTCACTCATTATACTCTGTCGAATGTCATTGAATGTCTTTACATGGGCTT
GTATGCCTATGAAAATTGTAATACAACTTTCAGCAACGGATCTCTTGGCTCTCGCATCGA
TGAAGAACGCAGCGAAATGCGATAAGTAATGTGAATTGCAGAATTCAGTGAATCATCGAA
TCTTTGAACGCATCTTGCGCTCCTTGGTATTCCGAGGAGCATGCCTGTTTGAGTGTCATT
AAATTCTCAACTCTCTTATAC-TTTTTTGTAAAAGAGAGCTTGGACTGTGGAGGCTTGCT
GGCCACTTTTTGGGGTCAGCTCCTCTGAAATGCATTAGCGGAACCGTTTGCGATCTGCCA
CAAGTGTGATAAGTTATCTACACTGGCGAGGGGATTGCTCTCTGTAATGTTCAGCTTCTA
ATTGTCTCTACTTTGTGAGACTACTTTTGAATGCTTGACCTCAAATCAGGTAGGACTACC
CGCTGAACTTAA
>ABC12-41
TTTCCGTAGGTGAACCTGCGGAAGGATCATTATTGAATTATGTTTCTAGATAGGTTGTAG
CTGGCTCTTTTAGAGCATGTGCACGCCTGTTTGGACTTCATTTTCATCCACCTGTGCACC
TATTGTAGTCTTTGGTTGGGTTAGGAGGAAGTGATCATTGTATCAGCATCTGCTGGGAGT
GAGGACTTGCATTGTGAAAGCTTTGCTGTCCTTGATGTGATCATGGAATCTTTTTCTCAC
TAGAGTCTATGTCACTCATTATACTCTGTCGAATGTCATTGAATGTCTTTACATGGGCTT
GTATGCCTATGAAAATTGTAATACAACTTTCAGCAACGGATCTCTTGGCTCTCGCATCGA
TGAAGAACGCAGCGAAATGCGATAAGTAATGTGAATTGCAGAATTCAGTGAATCATCGAA
TCTTTGAACGCATCTTGCGCTCCTTGGTATTCCGAGGAGCATGCCTGTTTGAGTGTCATT
AAATTCTCAACTCTCTTATAC-TTTTTTGTAAAAGAGAGCTTGGACTGTGGAGGCTTGCT
GGCCACTTTTTGGGGTCAGCTCCTCTGAAATGCATTAGCGGAACCGTTTGCGATCTGCCA
CAAGTGTGATAAGTTATCTACACTGGCGAGGGGATTGCTCTCTGTAATGTTCAGCTTCTA
ATTGTCTCTACTTTGTGAGACTACTTTTGAATGCTTGACCTCAAATCAGGTAGGACTACC
CGCTGAACTTAA
>ABC12-44
TTTCCGTAGGTGAACCTGCGGAAGGATCATTATTGAATTATGTTTCTAGATAGGTTGTAG
CTGGCTCTTTTAGAGCATGTGCACGCCTGTTTGGACTTCATTTTCATCCACCTGTGCACC
TATTGTAGTCTTTGGTTGGGTTAGGAGGAAGTGATCATTGTATCAGCATCTGCTGGGAGT
GAGGACTTGCATTGTGAAAGCTTTGCTGTCCTTGATGTGATCATGGAATCTTTTTCTCAC
TAGAGTCTATGTCACTCATTATACTCTGTCGAATGTCATTGAATGTCTTTACATGGGCTT

GTATGCCTATGAAAATTGTAATACAACTTTCAGCAACGGATCTCTTGGCTCTCGCATCGA
TGAAGAACGCAGCGAAATGCGATAAGTAATGTGAATTGCAGAATTCAGTGAATCATCGAA
TCTTTGAACGCATCTTGCGCTCCTTGGTATTCCGAGGAGCATGCCTGTTTGAGTGTCATT
AAATTCTCAACTCTCTTATAC-TTTTTTGTAAAAGAGAGCTTGGACTGTGGAGGCTTGCT
GGCCACTTTTTGGGGTCAGCTCCTCTGAAATGCATTAGCGGAACCGTTTGCGATCTGCCA
CAAGTGTGATAAGTTATCTACACTGGCGAGGGGATTGCTCTCTGTAATGTTCAGCTTCTA
ATTGTCTCTACTTTGTGAGACTACTTTTGAATGCTTGACCTCAAATCAGGTAGGACTACC
CGCTGAACTTAA
>ABC12-46
TTTCCGTAGGTGAACCTGCGGAAGGATCATTATTGAATTATGTTTCTAGATAGGTTGTAG
CTGGCTCTTTTAGAGCATGTGCACGCCTGTTTGGACTTCATTTTCATCCACCTGTGCACC
TATTGTAGTCTTTGGTTGGGTTAGGAGGAAGTGATCATTGTATCAGCATCTGCTGGGAGT
GAGGACTTGCATTGTGAAAGCTTTGCTGTCCTTGATGTGATCATGGAATCTTTTTCTCAC
TAGAGTCTATGTCACTCATTATACTCTGTCGAATGTCATTGAATGTCTTTACATGGGCTT
GTATGCCTATGAAAATTGTAATACAACTTTCAGCAACGGATCTCTTGGCTCTCGCATCGA
TGAAGAACGCAGCGAAATGCGATAAGTAATGTGAATTGCAGAATTCAGTGAATCATCGAA
TCTTTGAACGCATCTTGCGCTCCTTGGTATTCCGAGGAGCATGCCTGTTTGAGTGTCATT
AAATTCTCAACTCTCTTATAC-TTTTTTGTAAAAGAGAGCTTGGACTGTGGAGGCTTGCT
GGCCACTTTTTGGGGTCAGCTCCTCTGAAATGCATTAGCGGAACCGTTTGCGATCTGCCA
CAAGTGTGATAAGTTATCTACACTGGCGAGGGGATTGCTCTCTGTAATGTTCAGCTTCTA
ATTGTCTCTACTTTGTGAGACTACTTTTGAATGCTTGACCTCAAATCAGGTAGGACTACC
CGCTGAACTTAA
>ABC11-16
TTTCCGTAGGTGAACCTGCGGAAGGATCATTATTGAATTATGTTTCTAGATAGGTTGTAG
CTGGCTCTTTTAGAGCATGTGCACGCCTGTTTGGACTTCATTTTCATCCACCTGTGCACC
TATTGTAGTCTTTGGTTGGGTTAGGAGGAAGTGATCATTGTATCAGCATCTGCTGGGAGT
GAGGACTTGCATTGTGAAAGCTTTGCTGTCCTTGATGTGATCATGGAATCTTTTTCTCAC
TAGAGTCTATGTCACTCATTATACTCTGTCGAATGTCATTGAATGTCTTTACATGGGCTT
GTATGCCTATGAAAATTGTAATACAACTTTCAGCAACGGATCTCTTGGCTCTCGCATCGA
TGAAGAACGCAGCGAAATGCGATAAGTAATGTGAATTGCAGAATTCAGTGAATCATCGAA
TCTTTGAACGCATCTTGCGCTCCTTGGTATTCCGAGGAGCATGCCTGTTTGAGTGTCATT
AAATTCTCAACTCTCTTATAC-TTTTTTGTAAAAGAGAGCTTGGACTGTGGAGGCTTGCT
GGCCACTTTTTGGGGTCAGCTCCTCTGAAATGCATTAGCGGAACCGTTTGCGATCTGCCA
CAAGTGTGATAAGTTATCTACACTGGCGAGGGGATTGCTCTCTGTAATGTTCAGCTTCTA
ATTGTCTCTACTTTGTGAGACTACTTTTGAATGCTTGACCTCAAATCAGGTAGGACTACC
CGCTGAACTTAA
>ABC5-38
TTTCCGTAGGTGAACCTGCGGAAGGATCATTATTGAATTATGTTTCTAGATAGGTTGTAG
CTGGCTCTTTTAGAGCATGTGCACGCCTGTTTGGACTTCATTTTCATCCACCTGTGCACC
TATTGTAGTCTTTGGTTGGGTTAGGAGGAAGTGATCATTGTATCAGCATCTGCTGGGAGT
GAGGACTTGCATTGTGAAAGCTTTGCTGTCCTTGATGTGATCATGGAATCTTTTTCTCAC
TAGAGTCTATGTCACTCATTATACTCTGTCGAATGTCATTGAATGTCTTTACATGGGCTT
GTATGCCTATGAAAATTGTAATACAACTTTCAGCAACGGATCTCTTGGCTCTCGCATCGA
TGAAGAACGCAGCGAAATGCGATAAGTAATGTGAATTGCAGAATTCAGTGAATCATCGAA
TCTTTGAACGCATCTTGCGCTCCTTGGTATTCCGAGGAGCATGCCTGTTTGAGTGTCATT
AAATTCTCAACTCTCTTATAC-TTTTTTGTAAAAGAGAGCTTGGACTGTGGAGGCTTGCT
GGCCACTTTTTGGGGTCAGCTCCTCTGAAATGCATTAGCGGAACCGTTTGCGATCTGCCA
CAAGTGTGATAAGTTATCTACACTGGCGAGGGGATTGCTCTCTGTAATGTTCAGCTTCTA
ATTGTCTCTACTTTGTGAGACTACTTTTGAATGCTTGACCTCAAATCAGGTAGGACTACC
CGCTGAACTTAA

>ABC2-28
TTTCCGTAGGTGAACCTGCGGAAGGATCATTATTGAATTATGTTTCTAGATAGGTTGTAG
CTGGCTCTTTTAGAGCATGTGCACGCCTGTTTGGACTTCATTTTCATCCACCTGTGCACC
TATTGTAGTCTTTGGTTGGGTTAGGAGGAAGTGATCATTGTATCAGCATCTGCTGGGAGT
GAGGACTTGCATTGTGAAAGCTTTGCTGTCCTTGATGTGATCATGGAATCTTTTTCTCAC
TAGAGTCTATGTCACTCATTATACTCTGTCGAATGTCATTGAATGTCTTTACATGGGCTT
GTATGCCTATGAAAATTGTAATACAACTTTCAGCAACGGATCTCTTGGCTCTCGCATCGA
TGAAGGACGCAGCGAAATGCGATAAGTAATGTGAATTGCAGAATTCAGTGAATCATCGAA
TCTTTGAACGCATCTTGCGCTCCTTGGTATTCCGAGGAGCATGCCTGTTTGAGTGTCATT
AAATTCTCAACTCTCTTATAC-TTTTTTGTAAAAGAGAGCTTGGACTGTGGAGGCTTGCT
GGCCACTTTTTGGGGTCAGCTCCTCTGAAATGCATTAGCGGAACCGTTTGCGATCTGCCA
CAAGTGTGATAAGTTATCTACACTGGCGAGGGGATTGCTCTCTGTAATGTTCAGCTTCTA
ATTGTCTCTACTTTGTGAGACTACTTTTGAATGCTTGACCTCAAATCAGGTAGGACTACC
CGCTGAACTTAA
>ABC7-36
TTTCCGTAGGTGAACCTGCGGAAGGATCATTATTGAATTATGTTTCTAGATAGGTTGTAG
CTGGCTCTTTTAGAGCATGTGCACGCCTGTTTGGACTTCATTTTCATCCACCTGTGCACC
TATTGTAGTCTTTGGTTGGGTTAGGAGGAAGTGATCATTGTATCAGCATCTGCTGGGAGT
GAGGACTTGCATTGTGAAAGCTTTGCTGTCCTTGATGTGATCATGGAATCTTTTTCTCAC
TAGAGTCTATGTCACTCATTATACTCTGTCGAATGTCATTGAATGTCTTTACATGGGCTT
GTATGCCTATGAAAATTGTAATACAACTTTCAGCAACGGATCTCTTGGCTCTCGCATCGA
TGAAGGACGCAGCGAAATGCGATAAGTAATGTGAATTGCAGAATTCAGTGAATCATCGAA
TCTTTGAACGCATCTTGCGCTCCTTGGTATTCCGAGGAGCATGCCTGTTTGAGTGTCATT
AAATTCTCAACTCTCTTATAC-TTTTTTGTAAAAGAGAGCTTGGACTGTGGAGGCTTGCT
GGCCACTTTTTGGGGTCAGCTCCTCTGAAATGCATTAGCGGAACCGTTTGCGATCTGCCA
CAAGTGTGATAAGTTATCTACACTGGCGAGGGGATTGCTCTCTGTAATGTTCAGCTTCTA
ATTGTCTCTACTTTGTGAGACTACTTTTGAATGCTTGACCTCAAATCAGGTAGGACTACC
CGCTGAACTTAA
>ABC1-7
TTTCCGTAGGTGAACCTGCGGAAGGATCATTATTGAATTATGTTTCTAGATAGGTTGTAG
CTGGCTCTTTTAGAGCATGTGCACGCCTGTTTGGACTTCATTTTCATCCACCTGTGCACC
TATTGTAGTCTTTGGTTGGGTTAGGAGGAAGTGATCATTGTATCAGCATCTGCTGGGAGT
GAGGACTTGCATTGTGAAAGCTTTGCTGTCCTTGATGTGATCATGGAATCTTTTTCTCAC
TAGAGTCTATGTCACTCATTATACTCTGTCGAATGTCATTGAATGTCTTTACATGGGCTT
GTATGCCTATGAAAATTGTAATACAACTTTCAGCAACGGATCTCTTGGCTCTCGCATCGA
TGAAGGACGCAGCGAAATGCGATAAGTAATGTGAATTGCAGAATTCAGTGAATCATCGAA
TCTTTGAACGCATCTTGCGCTCCTTGGTATTCCGAGGAGCATGCCTGTTTGAGTGTCATT
AAATTCTCAACTCTCTTATAC-TTTTTTGTAAAAGAGAGCTTGGACTGTGGAGGCTTGCT
GGCCACTTTTTGGGGTCAGCTCCTCTGAAATGCATTAGCGGAACCGTTTGCAATCTGCCA
CAAGTGTGATAAGTTATCTACACTGGCGAGGGGATTGCTCTCTGTAATGTTCAGCTTCTA
ATTGTCTCTACTTTGTGAGACTACTTTTGAATGCTTGACCTCAAATCAGGTAGGACTACC
CGCTGAACTTAA
>ABC4-77
TTTCCGTAGGTGAACCTGCGGAAGGATCATTATTGAATTATGTTTCTAGATAGGTTGTAG
CTGGCTCTTTTAGAGCATGTGCACGCCTGTTTGGACTTCATTTTCATCCACCTGTGCACC
TATTGTAGTCTTTGGTTGGGTTAGGAGGAAGTGATCATTGTATCAGCATCTGCTGGGAGT
GAGGACTTGCATTGTGAAAGCTTTGCTGTCCTTGATGTGATCATGGAATCTTTTTCTCAC
TAGAGTCTATGTCACTCATTATACTCTGTCGAATGTCATTGAATGTCTTTACATGGGCTT
GTATGCCTATGAAAATTGTAATACAACTTTCAGCAACGGATCTCTTGGCTCTCGCATCGA
TGAAGAACGCAGCGAAATGCGATAAGTAATGTGAATTGCAGAATTCAGTGAATCATCGAA

TCTTTGAACGCATCTTGCGCTCCTTGGTATTCCGAGGAGCATGCCTGTTTGAGTGTCATT
AAATTCTCAACTCTCTTATAC-TTTTTTGTAAAAGAGAGCTTGGACTGTGGAGGCTTGCT
GGCCACTTTTTGGGGTCAGCTCCTCTGAAATGCATTAGCGGAACCGTTTGCAATCTGCCA
CAAGTGTGATAAGTTATCTACACTGGCGAGGGGATTGCTCTCTGTAATGTTCAGCTTCTA
ATTGTCTCTACTTTGTGAGACTACTTTTGAATGCTTGACCTCAAATCAGGTAGGACTACC
CGCTGAACTTAA
>ABC10-22
TTTCCGTAGGTGAACCTGCGGAAGGATCATTATTGAATTATGTTTCTAGATAGGTTGTAG
CTGGCTCTTTTAGAGCATGTGCACGCCTGTTTGGACTTCATTTTCATCCACCTGTGCACC
TATTGTAGTCTTTGGTTGGGTTAGGAGGAAGTGATCATTGTATCAGCATCTGCTGGGAGT
GAGGACTTGCATTGTGAAAGCTTTGCTGTCCTTGATGTGATCATGGAATCTTTTTCTCAC
TAGAGTCTATGTCACTCATTATACTCTGTCGAATGTCATTGAATGTCTTTACATGGGCTT
GTATGCCTATGAAAATTGTAATACAACTTTCAGCAACGGATCTCTTGGCTCTCGCATCGA
TGAAGAACGCAGCGAAATGCGATAAGTAATGTGAATTGCAGAATTCAGTGAATCATCGAA
TCTTTGAACGCATCTTGCGCTCCTTGGTATTCCGAGGAGCATGCCTGTTTGAGTGTCATT
AAATTCTCAACTCTCTTATAC-TTTTTTGTAAAAGAGAGCTTGGACTGTGGAGGCTTGCT
GGCCACTTTTTGGGGTCAGCTCCTCTGAAATGCATTAGCGGAACCGTTTGCAATCTGCCA
CAAGTGTGATAAGTTATCTACACTGGCGAGGGGATTGCTCTCTGTAATGTTCAGCTTCTA
ATTGTCTCTACTTTGTGAGACTACTTTTGAATGCTTGACCTCAAATCAGGTAGGACTACC
CGCTGAACTTAA
>ABC3-45
TTTCCGTAGGTGAACCTGCGGAAGGATCATTATTGAATTATGTTTCTAGATAGGTTGTAG
CTGGCTCTTTTAGAGCATGTGCACGCCTGTTTGGACTTCATTTTCATCCACCTGTGCACC
TATTGTAGTCTTTGGTTGGGTTAGGAGGAAGTGATCATTGTATCAGCATCTGCTGGGAGT
GAGGACTTGCATTGTGAAAGCTTTGCTGTCCTTGATGTGATCATGGAATCTTTTTCTCAC
TAGAGTCTATGTCACTCATTATACTCTGTCGAATGTCATTGAATGTCTTTACATGGGCTT
ATATGCCTATGAAAATTGTAATACAACTTTCAGCAACGGATCTCTTGGCTCTCGCATCGA
TGAAGAACGCAGCGAAATGCGATAAGTAATGTGAATTGCAGAATTCAGTGAATCATCGAA
TCTTTGAACGCATCTTGCGCTCCTTGGTATTCCGAGGAGCATGCCTGTTTGAGTGTCATT
AAATTCTCAACTCTCTTATAC-TTTTTTGTAAAAGAGAGCTTGGACTGTGGAGGCTTGCT
GGCCACTTTTTGGGGTCAGCTCCTCTGAAATGCATTAGCGGAACCGTTTGCGATCTGCCA
CAAGTGTGATAAGTTATCTACACTGGCGAGGGGATTGCTCTCTGTAATGTTCAGCTTCTA
ATTGTCTCTACTTTGTGAGACTACTTTTGAATGCTTGACCTCAAATCAGGTAGGACTACC
CGCTGAACTTAA
>ABC12-32
TTTCCGTAGGTGAACCTGCGGAAGGATCATTATTGAATTATGTTTCTAGATAGGTTGTAG
CTGGCTCTTTTAGAGCATGTGCACGCCTGTTTGGACTTCATTTTCATCCACCTGTGCACC
TATTGTAGTCTTTGGTTGGGTTAGGAGGAAGTGATCATTGTATCAGCATCTGCTGGGAGT
GAGGACTTGCATTGTGAAAGCTTTGCTGTCCTTGATGTGATCATGGAATCTTTTTCTCAC
TAGAGTCTATGTCACTCATTATACTCTGTCGAATGTCATTGAATGTCTTTACATGGGCTT
ATATGCCTATGAAAATTGTAATACAACTTTCAGCAACGGATCTCTTGGCTCTCGCATCGA
TGAAGAACGCAGCGAAATGCGATAAGTAATGTGAATTGCAGAATTCAGTGAATCATCGAA
TCTTTGAACGCATCTTGCGCTCCTTGGTATTCCGAGGAGCATGCCTGTTTGAGTGTCATT
AAATTCTCAACTCTCTTATAC-TTTTTTGTAAAAGAGAGCTTGGACTGTGGAGGCTTGCT
GGCCACTTTTTGGGGTCAGCTCCTCTGAAATGCATTAGCGGAACCGTTTGCGATCTGCCA
CAAGTGTGATAAGTTATCTACACTGGCGAGGGGATTGCTCTCTGTAATGTTCAGCTTCTA
ATTGTCTCTACTTTGTGAGACTACTTTTGAATGCTTGACCTCAAATCAGGTAGGACTACC
CGCTGAACTTAA
>ABC3-35
TTTCCGTAGGTGAACCTGCGGAAGGATCATTATTGAATTATGTTTCTAGATAGGTTGTAG

CTGGCTCTTTTAGAGCATGTGCACGCCTGTTTGGACTTCATTTTCATCCACCTGTGCACC
TATTGTAGTCTTTGGTTGGGTTAGGAGGAAGTGATCATTGTATCAGCATCTGCTGGGAGT
GAGGACTTGCATTGTGAAAGCTTTGCTGTCCTTGATGTGATCATGGAATCTTTTTCTCAC
TAGAGTCTATGTCACTCATTATACTCTGTCGAATGTCATTGAATGTCTTTACATGGGCTT
ATATGCCTATGAAAATTGTAATACAACTTTCAGCAACGGATCTCTTGGCTCTCGCATCGA
TGAAGAACGCAGCGAAATGCGATAAGTAATGTGAATTGCAGAATTCAGTGAATCATCGAA
TCTTTGAACGCATCTTGCGCTCCTTGGTATTCCGAGGAGCATGCCTGTTTGAGTGTCATT
AAATTCTCAACTCTCTTATAC-TTTTTTGTAAAAGAGAGCTTGGACTGTGGAGGCTTGCT
GGCCACTTTTTGGGGTCAGCTCCTCTGAAATGCATTAGCGGAACCGTTTGCAATCTGCCA
CAAGTGTGATAAGTTATCTACACTGGCGAGGGGATTGCTCTCTGTAATGTTCAGCTTCTA
ATTGTCTCTACTTTGTGAGACTACTTTTGAATGCTTGACCTCAAATCAGGTAGGACTACC
CGCTGAACTTAA
>ABC3-25
TTTCCGTAGGTGAACCTGCGGAAGGATCATTATTGAATTATGTTTCTAGATAGGTTGTAG
CTGGCTCTTTTAGAGCATGTGCACGCCTGTTTGGACTTCATTTTCATCCACCTGTGCACC
TATTGTAGTCTTTGGTTGGGTTAGGAGGAAGTGATCATTGTATCAGCATCTGCTGGGAGT
GAGGACTTGCATTGTGAAAGCTTTGCTGTCCTTGATGTGATCATGGAATCTCTTTCTCAC
TAGAGTCTATGTCACTCATTATACTCTGTCGAATGTCATTGAATGTCTTTACATGGGCTT
ATATGCCTATGAAAATTGTAATACAACTTTCAGCAACGGATCTCTTGGCTCTCGCATCGA
TGAAGAACGCAGCGAAATGCGATAAGTAATGTGAATTGCAGAATTCAGTGAATCATCGAA
TCTTTGAACGCATCTTGCGCTCCTTGGTATTCCGAGGAGCATGCCTGTTTGAGTGTCATT
AAATTCTCAACTCTCTTATAC-TTTTTTGTAAAAGAGAGCTTGGACTGTGGAGGCTTGCT
GGCCACTTTTTGGGGTCAGCTCCTCTGAAATGCATTAGCGGAACCGTTTGCAATCTGCCA
CAAGTGTGATAAGTTATCTACACTGGCGAGGGGATTGCTCTCTGTAATGTTCAGCTTCTA
ATTGTCTCTACTTTGTGAGACTACTTTTGAATGCTTGACCTCAAATCAGGTAGGACTACC
CGCTGAACTTAA
>ABC12-43
TTTCCGTAGGTGAACCTGCGGAAGGATCATTATTGAATTATGTTTCTAGATAGGTTGTAG
CTGGCTCTTTTAGAGCATGTGCACGCCTGTTTGGACTTCATTTTCATCCACCTGTGCACC
TATTGTAGTCTTTGGTTGGGTTAGGAGGAAGTGATCATTGTATCAGCATCTGCTGGGAGT
GAGGACTTGCATTGTGAAAGCTTTGCTGTCCTTGATGTGATCATGGAATCTCTTTCTCAC
TAGAGTCTATGTCACTCATTATACTCTGTCGAATGTCATTGAATGTCTTTACATGGGCTT
ATATGCCTATGAAAATTGTAATACAACTTTCAGCAACGGATCTCTTGGCTCTCGCATCGA
TGAAGAACGCAGCGAAATGCGATAAGTAATGTGAATTGCAGAATTCAGTGAATCATCGAA
TCTTTGAACGCATCTTGCGCTCCTTGGTATTCCGAGGAGCATGCCTGTTTGAGTGTCATT
AAATTCTCAACTCTCTTATAC-TTTTTTGTAAAAGAGAGCTTGGACTGTGGAGGCTTGCT
GGCCACTTTTTGGGGTCAGCTCCTCTGAAATGCATTAGCGGAACCGTTTGCAATCTGCCA
CAAGTGTGATAAGTTATCTACACTGGCGAGGGGATTGCTCTCTGTAATGTTCAGCTTCTA
ATTGTCTCTACTTTGTGAGACTACTTTTGAATGCTTGACCTCAAATCAGGTAGGACTACC
CGCTGAACTTAA
>ABC11-5
TTTCCGTAGGTGAACCTGCGGAAGGATCATTATTGAATTATGTTTCTAGATAGGTTGTAG
CTGGCTCTTTTAGAGCATGTGCACGCCTGTTTGGACTTCATTTTCATCCACCTGTGCACC
TATTGTAGTCTTTGGTTGGGTTAGGAGGAAGTGATCATTGTATCAGCATCTGCTGGGAGT
GAGGACTTGCATTGTGAAAGCTTTGCTGTCCTTGATGTGATCATGGAATCTCTTTCTCAC
TAGAGTCTATGTCACTCATTATACTCTGTCGAATGTCATTGAATGTCTTTACATGGGCTT
GTATGCCTATGAAAATTGTAATACAACTTTCAGCAACGGATCTCTTGGCTCTCGCATCGA
TGAAGGACGCAGCGAAATGCGATAAGTAATGTGAATTGCAGAATTCAGTGAATCATCGAA
TCTTTGAACGCATCTTGCGCTCCTTGGTATTCCGAGGAGCATGCCTGTTTGAGTGTCATT
AAATTCTCAACTCTCTTATAC-TTTTTTGTAAAAGAGAGCTTGGACTGTGGAGGCTTGCT

GGCCACTTTTTGGGGTCAGCTCCTCTGAAATGCATTAGCGGAACCGTTTGCGATCTGCCA
CAAGTGTGATAAGTTATCTACACTGGCGAGGGGATTGCTCTCTGTAATGTTCAGCTTCTA
ATTGTCTCTACTTTGTGAGACTACTTTTGAATGCTTGACCTCAAATCAGGTAGGACTACC
CGCTGAACTTAA
>ABC9–26
TTTCCGTAGGTGAACCTGCGGAAGGATCATTATTGAATTATGTTTCTAGATAGGTTGTAG
CTGGCTCTTTTAGAGCATGTGCACGCCTGTTTGGACTTCATTTTCATCCACCTGTGCACC
TATTGTAGTCTTTGGTTGGGTTAGGAGGAAGTGATCATTGTATCAGCATCTGCTGGGAGT
GAGGACTTGCATTGTGAAAGCTTTGCTGTCCTTGATGTGATCATGGAATCTCTTTCTCAC
TAGAGTCTATGTCACTCATTATACTCTGTCGAATGTCATTGAATGTCTTTACATGGGCTT
GTATGCCTATGAAAATTGTAATACAACTTTCAGCAACGGATCTCTTGGCTCTCGCATCGA
TGAAGAACGCAGCGAAATGCGATAAGTAATGTGAATTGCAGAATTCAGTGAATCATCGAA
TCTTTGAACGCATCTTGCGCTCCTTGGTATTCCGAGGAGCATGCCTGTTTGAGTGTCATT
AAATTCTCAACTCTCTTATAC–TTTTTTGTAAAAGAGAGCTTGGACTGTGGAGGCTTGCT
GGCCACTTTTTGGGGTCAGCTCCTCTGAAATGCATTAGCGGAACCGTTTGCGATCTGCCA
CAAGTGTGATAAGTTATCTACACTGGCGAGGGGATTGCTCTCTGTAATGTTCAGCTTCTA
ATTGTCTCTACTTTGTGAGACTACTTTTGAATGCTTGACCTCAAATCAGGTAGGACTACC
CGCTGAACTTAA
>ABC10–51
TTTCCGTAGGTGAACCTGCGGAAGGATCATTATTGAATTATGTTTCTAGATAGGTTGTAG
CTGGCTCTTTTAGAGCATGTGCACGCCTGTTTGGACTTCATTTTCATCCACCTGTGCACC
TATTGTAGTCTTTGGTTGGGTTAGGAGGAAGTGATCATTGTATCAGCATCTGCTGGGAGT
GAGGACTTGCATTGTGAAAGCTTTGCTGTCCTTGATGTGATCATGGAATCTCTTTCTCAC
TAGAGTCTATGTCACTCATTATACTCTGTCGAATGTCATTGAATGTCTTTACATGGGCTT
GTATGCCTATGAAAATTGTAATACAACTTTCAGCAACGGATCTCTTGGCTCTCGCATCGA
TGAAGAACGCAGCGAAATGCGATAAGTAATGTGAATTGCAGAATTCAGTGAATCATCGAA
TCTTTGAACGCATCTTGCGCTCCTTGGTATTCCGAGGAGCATGCCTGTTTGAGTGTCATT
AAATTCTCAACTCTCTTATAC–TTTTTTGTAAAAGAGAGCTTGGACTGTGGAGGCTTGCT
GGCCACTTTTTGGGGTCAGCTCCTCTGAAATGCATTAGCGGAACCGTTTGCGATCTGCCA
CAAGTGTGATAAGTTATCTACACTGGCGAGGGGATTGCTCTCTGTAATGTTCAGCTTCTA
ATTGTCTCTACTTTGTGAGACTACTTTTGAATGCTTGACCTCAAATCAGGTAGGACTACC
CGCTGAACTTAA
>ABC5–17
TTTCCGTAGGTGAACCTGCGGAAGGATCATTATTGAATTATGTTTCTAGATAGGTTGTAG
CTGGCTCTTTTAGAGCATGTGCACGCCTGTTTGGACTTCATTTTCATCCACCTGTGCACC
TATTGTAGTCTTTGGTTGGGTTAGGAGGAAGTGATCATTGTATCAGCATCTGCTGGGAGT
GAGGACTTGCATTGTGAAAGCTTTGCTGTCCTTGATGTGATCATGGAATCTCTTTCTCAC
TAGAGTCTATGTCACTCATTATACTCTGTCGAATGTCATTGAATGTCTTTACATGGGCTT
GTATGCCTATGAAAATTGTAATACAACTTTCAGCAACGGATCTCTTGGCTCTCGCATCGA
TGAAGGACGCAGCGAAATGCGATAAGTAATGTGAATTGCAGAATTCAGTGAATCATCGAA
TCTTTGAACGCATCTTGCGCTCCTTGGTATTCCGAGGAGCATGCCTGTTTGAGTGTCATT
AAATTCTCAACTCTCTTATAC–TTTTTTGTAAAAGAGAGCTTGGACTGTGGAGGCTTGCT
GGCCACTTTTTGGGGTCAGCTCCTCTGAAATGCATTAGCGGAACCGTTTGTGATCTGCCA
CAAGTGTGATAAGTTATCTACACTGGCGAGGGGATTGCTCTCTGTAATGTTCAGCTTCTA
ATTGTCTCTACTTTGTGAGACTACTTTTGAATGCTTGACCTCAAATCAGGTAGGACTACC
CGCTGAACTTAA
>ABC5–41
TTTCCGTAGGTGAACCTGCGGAAGGATCATTATTGAATTATGTTTCTAGATAGGTTGTAG
CTGGCTCTTTTAGAGCATGTGCACGCCTGTTTGGACTTCATTTTCATCCACCTGTGCACC
TATTGTAGTCTTTGGTTGGGTTAGGAGGAAGTGATCATTGTATCAGCATCTGCTGGGAGT

GAGGACTTGCATTGTGAAAGCTTTGCTGTCCTTGATGTGATCATGGAATCTTTTTCTCAC
TAGAGTCTATGTCACTCATTATACTCTGTCGAATGTCATTGAATGTCTTTACATGGGCTT
GTATGCCTATGAAAATTGTAATACAACTTTCAGCAACGGATCTCTTGGCTCTCGCATCGA
TGAAGAACGCAGCGAAATGCGATAAGTAATGTGAATTGCAGAATTCAGTGAATCATCGAA
TCTTTGAACGCATCTTGCGCTCCTTGGTATTCCGAGGTGCATGCCTGTTTGAGTGTCATT
AAATTCTCAACTCTCTTATAC-TTTTTTGTAAAAGAGAGCTTGGACTGTGGAGGCTTGCT
GGCCACTTTTTGGGGTCAGCTCCTCTGAAATGCATTAGCGGAACCGTTTGCGATCTGCCA
CAAGTGTGATAAGTTATCTACACTGGCGAGGGGATTGCTCTCTGTAATGTTCAGCTTCTA
ATTGTCTCTACTTTGTGAGACTACTTTTGAATGCTTGACCTCAAATCAGGTAGGACTACC
CGCTGAACTTAA
>ABC1-23
TTTCCGTAGGTGAACCTGCGGAAGGATCATTATTGAATTATGTTTCTAGATAGGTTGTAG
CTGGCTCTTTTAGAGCATGTGCACGCCTGTTTGGACTTCATTTTCATCCACCTGTGCACC
TATTGTAGTCTTTGGTTGGGTTAGGAGGAAGTGATCATTGTATCAGCATCTGCTGGGAGT
GAGGACTTGCATTGTGAAAGCTTTGCTGTCCTTGATGTGATCATGGAATCTTTTTCTCAC
TAGAGTCTATGTCACTCATTATACTCTGTCGAATGTCATTGAATGTCTTTACATGGGCTT
GTATGCCTATGAAAATTGTAATACAACTTTCAGCAACGGATCTCTTGGCTCTCGCATCGA
TGAAGAACGCAGCGAAATGCGATAAGTAATGTGAATTGCAGAATTCAGTGAATCATCGAA
TCTTTGAACGCATCTTGCGCTCCTTGGTATTCCGAGGAGCATGCCTGTTTGAGTGTCATT
AAATTCTCAACTCTCTTATAC-TTTTTTGTAAAAGAGAGCTTGGACTGTGGAGGCTTGCT
GGCCACTTTTTGGGGTCAGCTCCTCTGAAATGCATTAGCGGAACCGTTTGCGATCTGCCA
CAAGTGTGATAAGTTATCTACACTGGCGAGGGGATTGCTCTCTGTAATGTTCAGCTTCTA
ATTGTCTCTACTTTGTGAGACAACTTTTGAATGCTTGACCTCAAATCAGGTAGGACTACC
CGCTGAACTTAA
>ABC2-32
TTTCCGTAGGTGAACCTGCGGAAGGATCATTATTGAATTATGTTTCTAGATAGGTTGTAG
CTGGCTCTTTTAGAGCATGTGCACGCCTGTTTGGACTTCATTTTCATCCACCTGTGCACC
TATTGTAGTCTTTGGTTGGGTTAGGAGGAAGTGATCATTGTATCAGCATCTGCTGGGAGT
GAGGACTTGCATTGTGAAAGCTTTGCTGTCCTTGATGTGATCATGGAATCTTTTTCTCAC
TAGAGTCTATGTCACTCATTATACTCTGTCGAATGTCATTGAATGTCTTTACATGGGCTT
GTATGCCTATGAAAATTGTAATACAACTTTCAGCAACGGATCTCTTGGCTCTCGCATCGA
TGAAGAACGCAGCGAAATGCGATAAGTAATGTGAATTGCAGAATTCAGTGAATCATCGAA
TCTTTGAACGCATCTTGCGCTCCTTGGTATTCCGAGGAGCATGCCTGTTTGAGTGTCATT
AAATTCTCAACTCTCTTATAC-TTTTTTGTAAAAGAGAGCTTGGACTGTGGAGGCTTGCT
GGCCACTTTTTGGGGTCAGCTCCTCTGAAATGCATTAGCGGAACCGTTTGCGATCTGCCA
CAAGTGTGATAAGTTATCTACACTGGCGAGGGGATTGCTCTCTGTAATGTTCAGCTTCTA
ATTGTCTCTACTTTGTGAGACAACTTTTGAATGCTTGACCTCAAATCAGGTAGGACTACC
CGCTGAACTTAA
>ABC3-18
TTTCCGTAGGTGAACCTGCGGAAGGATCATTATTGAATTATGTTTCTAGATAGGTTGTAG
CTGGCTCTTTTAGAGCATGTGCACGCCTGTTTGGACTTCATTTTCATCCACCTGTGCACC
TATTGTAGTCTTTGGTTGGGTTAGGAGGAAGTGATCATTGTATCAGCATCTGCTGGGAGT
GAGGACTTGCATTGTGAAAGCTTTGCTGTCCTTGATGTGATCATGGAATCTTTTTCTCAC
TAGAGTCTATGTCACTCATTATACTCTGTCGAATGTCATTGAATGTCTTTACATGGGCTT
GTATGCCTATGAAAATTGTAATACAACTTTCAGCAACGGATCTCTTGGCTCTCGCATCGA
TGAAGAACGCAGCGAAATGCGATAAGTAATGTGAATTGCAGAATTCAGTGAATCATCGAA
TCTTTGAACGCATCTTGCGCTCCTTGGTATTCCGAGGAGCATGCCTGTTTGAGTGTCATT
AAATTCTCAACTCTCTTATAC-TTTTTTGTAAAAGAGAGCTTGGACTGTGGAGGCTTGCT
GGCCACTTTTTGGGGTCAGCTCCTCTGAAATGCATTAGCGGAACCGTTTGCGATCTGCCA
CAAGTGTGATAAGTTATCTACACTGGCGAGGGGATTGCTCTCTGTAATGTTCAGCTTCTA

ATTGTCTCTACTTTGTGAGACAACTTTTGAATGCTTGACCTCAAATCAGGTAGGACTACC
CGCTGAACTTAA
>ABC5-73
TTTCCGTAGGTGAACCTGCGGAAGGATCATTATTGAATTATGTTTCTAGATAGGTTGTAG
CTGGCTCTTTTAGAGCATGTGCACGCCTGTTTGGACTTCATTTTCATCCACCTGTGCACC
TATTGTAGTCTTTGGTTGGGTTAGGAGGAAGTGATCATTGTATCAGCATCTGCTGGGAGT
GAGGACTTGCATTGTGAAAGCTTTGCTGTCCTTGATGTGATCATGGAATCTTTTTCTCAC
TAGAGTCTATGTCACTCATTATACTCTGTCGAATGTCATTGAATGTCTTTACATGGGCTT
GTATGCCTATGAAAATTGTAATACAACTTTCAGCAACGGATCTCTTGGCTCTCGCATCGA
TGAAGAACGCAGCGAAATGCGATAAGTAATGTGAATTGCAGAATTCAGTGAATCATCGAA
TCTTTGAACGCATCTTGCGCTCCTTGGTATTCCGAGGAGCATGCCTGTTTGAGTGTCATT
AAATTCTCAACTCTCTTATAC-TTTTTTGTAAAAGAGAGCTTGGACTGTGGAGGCTTGCT
GGCCACTTTTTGGGGTCAGCTCCTCTGAAATGCATTAGCGGAACCGTTTGCGATCTGCCA
CAAGTGTGATAAGTTATCTACACTGGCGAGGGGATTGCTCTCTGTAATGTTCAGCTTCTA
ATTGTCTCTACTTTGTGAGACAACTTTTGAATGCTTGACCTCAAATCAGGTAGGACTACC
CGCTGAACTTAA
>ABC2-46
TTTCCGTAGGTGAACCTGCGGAAGGATCATTATTGAATTATGTTTCTAGATAGGTTGTAG
CTGGCTCTTTTAGAGCATGTGCACGCCTGTTTGGACTTCATTTTCATCCACCTGTGCACC
TATTGTAGTCTTTGGTTGGGTTAGGAGGAAGTGATCATTGTATCAGCATCTGCTGGGAGT
GAGGACTTGCATTGTGAAAGCTTTGCTGTCCTTGATGTGATCATGGAATCTTTTTCTCAC
TAGAGTCTATGTCACTCATTATACTCTGTCGAATGTCATTGAATGTCTTTACATGGGCTT
GTATGCCTATGAAAATTGTAATACAACTTTCAGCAACGGATCTCTTGGCTCTCGCATCGA
TGAAGAACGCAGCGAAATGCGATAAGTAATGTGAATTGCAGAATTCAGTGAATCATCGAA
TCTTTGAACGCATCTTGCGCTCCTTGGTATTCCGAGGAGCATGCCTGTTTGAGTGTCATT
AAATTCTCAACTCTCTTATAC-TTTTTTGTAAAAGAGAGCTTGGACTGTGGAGGCTTGCT
GGCCACTTTTTGGGGTCAGCTCCTCTGAAATGCATTAGCGGAACCGTTTGCAATCTGCCA
CAAGTGTGATAAGTTATCTACACTGGCGAGGGGATTGCTCTCTGTAATGTTCAGCTTCTA
ATTGTCTCTACTTTGTGAGACAACTTTTGAATGCTTGACCTCAAATCAGGTAGGACTACC
CGCTGAACTTAA
>ABC8-52
TTTCCGTAGGTGAACCTGCGGAAGGATCATTATTGAATTATGTTTCTAGATAGGTTGTAG
CTGGCTCTTTTAGAGCATGTGCACGCCTGTTTGGACTTCATTTTCATCCACCTGTGCACC
TATTGTAGTCTTTGGTTGGGTTAGGAGGAAGTGATCATTGTATCAGCATCTGCTGGGAGT
GAGGACTTGCATTGTGAAAGCTTTGCTGTCCTTGATGTGATCATGGAATCTTTTTCTCAC
TAGAGTCTATGTCACTCATTATACTCTGTCGAATGTCATTGAATGTCTTTACATGGGCTT
GTATGCCTATGAAAATTGTAATACAACTTTCAGCAACGGATCTCTTGGCTCTCGCATCGA
TGAAGAACGCAGCGAAATGCGATAAGTAATGTGAATTGCAGAATTCAGTGAATCATCGAA
TCTTTGAACGCATCTTGCGCTCCTTGGTATTCCGAGGAGCATGCCTGTTTGAGTGTCATT
AAATTCTCAACTCTCTTATAC-TTTTTTGTAAAAGAGAGCTTGGACTGTGGAGGCTTGCT
GGCCACTTTTTGGGGTCAGCTCCTCTGAAATGCATTAGCGGAACCGTTTGCAATCTGCCA
CAAGTGTGATAAGTTATCTACACTGGCGAGGGGATTGCTCTCTGTAATGTTCAGCTTCTA
ATTGTCTCTACTTTGTGAGACAACTTTTGAATGCTTGACCTCAAATCAGGTAGGACTACC
CGCTGAACTTAA
>ABC9-39
TTTCCGTAGGTGAACCTGCGGAAGGATCATTATTGAATTATGTTTCTAGATAGGTTGTAG
CTGGCTCTTTTAGAGCATGTGCACGCCTGTTTGGACTTCATTTTCATCCACCTGTGCACC
TATTGTAGTCTTTGGTTGGGTTAGGAGGAAGTGATCATTGTATCAGCATCTGCTGGGAGT
GAGGACTTGCATTGTGAAAGCTTTGCTGTCCTTGATGTGATCATGGAATCTTTTTCTCAC
TAGAGTCTATGTCACTCATTATACTCTGTCGAATGTCATTGAATGTCTTTACATGGGCTT

GTATGCCTATGAAAATTGTAATACAACTTTCAGCAACGGATCTCTTGGCTCTCGCATCGA
TGAAGAACGCAGCGAAATGCGATAAGTAATGTGAATTGCAGAATTCAGTGAATCATCGAA
TCTTTGAACGCATCTTGCGCTCCTTGGTATTCCGAGGAGCATGCCTGTTTGAGTGTCATT
AAATTCTCAACTCTCTTATAC-TTTTTTGTAAAAGAGAGCTTGGACTGTGGAGGCTTGCT
GGCCACTTTTTGGGGTCAGCTCCTCTGAAATGCATTAGCGGAACCGTTTGCAATCTGCCA
CAAGTGTGATAAGTTATCTACACTGGCGAGGGGATTGCTCTCTGTAATGTTCAGCTTCTA
ATTGTCTCTACTTTGTGAGACAACTTTTGAATGCTTGACCTCAAATCAGGTAGGACTACC
CGCTGAACTTAA
>ABC9-51
TTTCCGTAGGTGAACCTGCGGAAGGATCATTATTGAATTATGTTTCTAGATAGGTTGTAG
CTGGCTCTTTTAGAGCATGTGCACGCCTGTTTGGACTTCATTTTCATCCACCTGTGCACC
TATTGTAGTCTTTGGTTGGGTTAGGAGGAAGTGATCATTGTATCAGCATCTGCTGGGAGT
GAGGACTTGCATTGTGAAAGCTTTGCTGTCCTTGATGTGATCATGGAATCTTTTTCTCAC
TAGAGTCTATGTCACTCATTATACTCTGTCGAATGTCATTGAATGTCTTTACATGGGCTT
GTATGCCTATGAAAATTGTAATACAACTTTCAGCAACGGATCTCTTGGCTCTCGCATCGA
TGAAGAACGCAGCGAAATGCGATAAGTAATGTGAATTGCAGAATTCAGTGAATCATCGAA
TCTTTGAACGCATCTTGCGCTCCTTGGTATTCCGAGGAGCATGCCTGTTTGAGTGTCATT
AAATTCTCAACTCTCTTATAC-TTTTTTGTAAAAGAGAGCTTGGACTGTGGAGGCTTGCT
GGCCACTTTTTGGGGTCAGCTCCTCTGAAATGCATTAGCGGAACCGTTTGCAATCTGCCA
CAAGTGTGATAAGTTATCTACACTGGCGAGGGGATTGCTCTCTGTAATGTTCAGCTTCTA
ATTGTCTCTACTTTGTGAGACAACTTTTGAATGCTTGACCTCAAATCAGGTAGGACTACC
CGCTGAACTTAA
>ABC10-37
TTTCCGTAGGTGAACCTGCGGAAGGATCATTATTGAATTATGTTTCTAGATAGGTTGTAG
CTGGCTCTTTTAGAGCATGTGCACGCCTGTTTGGACTTCATTTTCATCCACCTGTGCACC
TATTGTAGTCTTTGGTTGGGTTAGGAGGAAGTGATCATTGTATCAGCATCTGCTGGGAGT
GAGGACTTGCATTGTGAAAGCTTTGCTGTCCTTGATGTGATCATGGAATCTTTTTCTCAC
TAGAGTCTATGTCACTCATTATACTCTGTCGAATGTCATTGAATGTCTTTACATGGGCTT
GTATGCCTATGAAAATTGTAATACAACTTTCAGCAACGGATCTCTTGGCTCTCGCATCGA
TGAAGAACGCAGCGAAATGCGATAAGTAATGTGAATTGCAGAATTCAGTGAATCATCGAA
TCTTTGAACGCATCTTGCGCTCCTTGGTATTCCGAGGAGCATGCCTGTTTGAGTGTCATT
AAATTCTCAACTCTCTTATAC-TTTTTTGTAAAAGAGAGCTTGGACTGTGGAGGCTTGCT
GGCCACTTTTTGGGGTCAGCTCCTCTGAAATGCATTAGCGGAACCGTTTGCAATCTGCCA
CAAGTGTGATAAGTTATCTACACTGGCGAGGGGATTGCTCTCTGTAATGTTCAGCTTCTA
ATTGTCTCTACTTTGTGAGACAACTTTTGAATGCTTGACCTCAAATCAGGTAGGACTACC
CGCTGAACTTAA
>ABC11-22
TTTCCGTAGGTGAACCTGCGGAAGGATCATTATTGAATTATGTTTCTAGATAGGTTGTAG
CTGGCTCTTTTAGAGCATGTGCACGCCTGTTTGGACTTCATTTTCATCCACCTGTGCACC
TATTGTAGTCTTTGGTTGGGTTAGGAGGAAGTGATCATTGTATCAGCATCTGCTGGGAGT
GAGGACTTGCATTGTGAAAGCTTTGCTGTCCTTGATGTGATCATGGAATCTTTTTCTCAC
TAGAGTCTATGTCACTCATTATACTCTGTCGAATGTCATTGAATGTCTTTACATGGGCTT
GTATGCCTATGAAAATTGTAATACAACTTTCAGCAACGGATCTCTTGGCTCTCGCATCGA
TGAAGAACGCAGCGAAATGCGATAAGTAATGTGAATTGCAGAATTCAGTGAATCATCGAA
TCTTTGAACGCATCTTGCGCTCCTTGGTATTCCGAGGAGCATGCCTGTTTGAGTGTCATT
AAATTCTCAACTCTCTTATAC-TTTTTTGTAAAAGAGAGCTTGGACTGTGGAGGCTTGCT
GGCCACTTTTTGGGGTCAGCTCCTCTGAAATGCATTAGCGGAACCGTTTGCAATCTGCCA
CAAGTGTGATAAGTTATCTACACTGGCGAGGGGATTGCTCTCTGTAATGTTCAGCTTCTA
ATTGTCTCTACTTTGTGAGACAACTTTTGAATGCTTGACCTCAAATCAGGTAGGACTACC
CGCTGAACTTAA

>ABC12-35
TTTCCGTAGGTGAACCTGCGGAAGGATCATTATTGAATTATGTTTCTAGATAGGTTGTAG
CTGGCTCTTTTAGAGCATGTGCACGCCTGTTTGGACTTCATTTTCATCCACCTGTGCACC
TATTGTAGTCTTTGGTTGGGTTAGGAGGAAGTGATCATTGTATCAGCATCTGCTGGGAGT
GAGGACTTGCATTGTGAAAGCTTTGCTGTCCTTGATGTGATCATGGAATCTTTTTCTCAC
TAGAGTCTATGTCACTCATTATACTCTGTCGAATGTCATTGAATGTCTTTACATGGGCTT
GTATGCCTATGAAAATTGTAATACAACTTTCAGCAACGGATCTCTTGGCTCTCGCATCGA
TGAAGAACGCAGCGAAATGCGATAAGTAATGTGAATTGCAGAATTCAGTGAATCATCGAA
TCTTTGAACGCATCTTGCGCTCCTTGGTATTCCGAGGAGCATGCCTGTTTGAGTGTCATT
AAATTCTCAACTCTCTTATAC-TTTTTTGTAAAAGAGAGCTTGGACTGTGGAGGCTTGCT
GGCCACTTTTTGGGGTCAGCTCCTCTGAAATGCATTAGCGGAACCGTTTGCAATCTGCCA
CAAGTGTGATAAGTTATCTACACTGGCGAGGGGATTGCTCTCTGTAATGTTCAGCTTCTA
ATTGTCTCTACTTTGTGAGACAACTTTTGAATGCTTGACCTCAAATCAGGTAGGACTACC
CGCTGAACTTAA
>ABC7-29
TTTCCGTAGGTGAACCTGCGGAAGGATCATTATTGAATTATGTTTCTAGATAGGTTGTAG
CTGGCTCTTTTAGAGCATGTGCACGCCTGTTTGGACTTCATTTTCATCCACCTGTGCACC
TATTGTAGTCTTTGGTTGGGTTAGGAGGAAGTGATCATTGTATCAGCATCTGCTGGGAGT
GAGGACTTGCATTGTGAAAGCTTTGCTGTCCTTGATGTGATCATGGAATCTTTTTCTCAC
TAGAGTCTATGTCACTCATTATACTCTGTCGAATGTCATTGAATGTCTTTACATGGGCTT
GTATGCCTATGAAAATTGTAATACAACTTTCAGCAACGGATCTCTTGGCTCTCGCATCGA
TGAAGGACGCAGCGAAATGCGATAAGTAATGTGAATTGCAGAATTCAGTGAATCATCGAA
TCTTTGAACGCATCTTGCGCTCCTTGGTATTCCGAGGAGCATGCCTGTTTGAGTGTCATT
AAATTCTCAACTCTCTTATAC-TTTTTTGTAAAAGAGAGCTTGGACTGTGGAGGCTTGCT
GGCCACTTTTTGGGGTCAGCTCCTCTGAAATGCATTAGCGGAACCGTTTGCAATCTGCCA
CAAGTGTGATAAGTTATCTACACTGGCGAGGGGATTGCTCTCTGTAATGTTCAGCTTCTA
ATTGTCTCTACTTTGTGAGACAACTTTTGAATGCTTGACCTCAAATCAGGTAGGACTACC
CGCTGAACTTAA
>ABC10-15
TTTCCGTAGGTGAACCTGCGGAAGGATCATTATTGAATTATGTTTCTAGATAGGTTGTAG
CTGGCTCTTTTAGAGCATGTGCACGCCTGTTTGGACTTCATTTTCATCCACCTGTGCACC
TATTGTAGTCTTTGGTTGGGTTAGGAGGAAGTGATCATTGTATCAGCATCTGCTGGGAGT
GAGGACTTGCATTGTGAAAGCTTTGCTGTCCTTGATGTGATCATGGAATCTTTTTCTCAC
TAGAGTCTATGTCACTCATTATACTCTGTCGAATGTCATTGAATGTCTTTACATGGGCTT
GTATGCCTATGAAAATTGTAATACAACTTTCAGCAACGGATCTCTTGGCTCTCGCATCGA
TGAAGGACGCAGCGAAATGCGATAAGTAATGTGAATTGCAGAATTCAGTGAATCATCGAA
TCTTTGAACGCATCTTGCGCTCCTTGGTATTCCGAGGAGCATGCCTGTTTGAGTGTCATT
AAATTCTCAACTCTCTTATAC-TTTTTTGTAAAAGAGAGCTTGGACTGTGGAGGCTTGCT
GGCCACTTTTTGGGGTCAGCTCCTCTGAAATGCATTAGCGGAACCGTTTGCAATCTGCCA
CAAGTGTGATAAGTTATCTACACTGGCGAGGGGATTGCTCTCTGTAATGTTCAGCTTCTA
ATTGTCTCTACTTTGTGAGACAACTTTTGAATGCTTGACCTCAAATCAGGTAGGACTACC
CGCTGAACTTAA
>ABC6-52
TTTCCGTAGGTGAACCTGCGGAAGGATCATTATTGAATTATGTTTCTAGATAGGTTGTAG
CTGGCTCTTTTAGAGCATGTGCACGCCTGTTTGGACTTCATTTTCATCCACCTGTGCACC
TATTGTAGTCTTTGGTTGGGTTAGGAGGAAGTGATCATTGTATCAGCATCTGCTGGGAGT
GAGGACTTGCATTGTGAAAGCTTTGCTGTCCTTGATGTGATCATGGAATCTTTTTCTCAC
TAGAGTCTATGTCACTCATTATACTCTGTCGAATGTCATTGAATGTCTTTACATGGGCTT
GTATGCCTATGAAAATTGTAATACAACTTTCAGCAACGGATCTCTTGGCTCTCGCATCGA
TGAAGGACGCAGCGAAATGCGATAAGTAATGTGAATTGCAGAATTCAGTGAATCATCGAA

TCTTTGAACGCATCTTGCGCTCCTTGGTATTCCGAGGAGCATGCCTGTTTGAGTGTCATT
AAATTCTCAACTCTCTTATAC-TTTTTTGTAAAAGAGAGCTTGGACTGTGGAGGCTTGCT
GGCCACTTTTTGGGGTCAGCTCCTCTGAAATGCATTAGCGGAACCGTTTGCAATCTGCCA
CAAGTGTGATAAGTTATCTACACTGGCGAGGGGATTGCTCTCTGTAATGTTCAGCTTCTA
ATTGTCTCTACTTTGTGAGACAACTTTTGAATGCTTGACCTCAAATCAGGTAGGACTACC
CGCTGAACTTAA
>ABC8-20
TTTCCGTAGGTGAACCTGCGGAAGGATCATTATTGAATTATGTTTCTAGATAGGTTGTAG
CTGGCTCTTTTAGAGCATGTGCACGCCTGTTTGGACTTCATTTTCATCCACCTGTGCACC
TATTGTAGTCTTTGGTTGGGTTAGGAGGAAGTGATCATTGTATCAGCATCTGCTGGGAGT
GAGGACTTGCATTGTGAAAGCTTTGCTGTCCTTGATGTGATCATGGAATCTCTTTCTCAC
TAGAGTCTATGTCACTCATTATACTCTGTCGAATGTCATTGAATGTCTTTACATGGGCTT
GTATGCCTATGAAAATTGTAATACAACTTTCAGCAACGGATCTCTTGGCTCTCGCATCGA
TGAAGGACGCAGCGAAATGCGATAAGTAATGTGAATTGCAGAATTCAGTGAATCATCGAA
TCTTTGAACGCATCTTGCGCTCCTTGGTATTCCGAGGAGCATGCCTGTTTGAGTGTCATT
AAATTCTCAACTCTCTTATAC-TTTTTTGTAAAAGAGAGCTTGGACTGTGGAGGCTTGCT
GGCCACTTTTTGGGGTCAGCTCCTCTGAAATGCATTAGCGGAACCGTTTGCAATCTGCCA
CAAGTGTGATAAGTTATCTACACTGGCGAGGGGATTGCTCTCTGTAATGTTCAGCTTCTA
ATTGTCTCTACTTTGTGAGACAACTTTTGAATGCTTGACCTCAAATCAGGTAGGACTACC
CGCTGAACTTAA
>ABC3-20
TTTCCGTAGGTGAACCTGCGGAAGGATCATTATTGAATTATGTTTCTAGATAGGTTGTAG
CTGGCTCTTTTAGAGCATGTGCACGCCTGTTTGGACTTCATTTTCATCCACCTGTGCACC
TATTGTAGTCTTTGGTTGGGTTAGGAGGAAGTGATCATTGTATCAGCATCTGCTGGGAGT
GAGGACTTGCATTGTGAAAGCTTTGCTGTCCTTGATGTGATCATGGAATCTCTTTCTCAC
TAGAGTCTATGTCACTCATTATACTCTGTCGAATGTCATTGAATGTCTTTACATGGGCTT
ATATGCCTATGAAAATTGTAATACAACTTTCAGCAACGGATCTCTTGGCTCTCGCATCGA
TGAAGAACGCAGCGAAATGCGATAAGTAATGTGAATTGCAGAATTCAGTGAATCATCGAA
TCTTTGAACGCATCTTGCGCTCCTTGGTATTCCGAGGAGCATGCCTGTTTGAGTGTCATT
AAATTCTCAACTCTCTTATAC-TTTTTTGTAAAAGAGAGCTTGGACTGTGGAGGCTTGCT
GGCCACTTTTTGGGGTCAGCTCCTCTGAAATGCATTAGCGGAACCGTTTGCAATCTGCCA
CAAGTGTGATAAGTTATCTACACTGGCGAGGGGATTGCTCTCTGTAATGTTCAGCTTCTA
ATTGTCTCTACTTTGTGAGACAACTTTTGAATGCTTGACCTCAAATCAGGTAGGACTACC
CGCTGAACTTAA
>ABC11-43
TTTCCGTAGGTGAACCTGCGGAAGGATCATTATTGAATTATGTTTCTAGATAGGTTGTAG
CTGGCTCTTTTAGAGCATGTGCACGCCTGTTTGGACTTCATTTTCATCCACCTGTGCACC
TATTGTAGTCTTTGGTTGGGTTAGGAGGAAGTGATCATTGTATCAGCATCTGCTGGGAGT
GAGGACTTGCATTGTGAAAGCTTTGCTGTCCTTGATGTGATCATGGAATCTCTTTCTCAC
TAGAGTCTATGTCACTCATTATACTCTGTCGAATGTCATTGAATGTCTTTACATGGGCTT
GTATGCCTATGAAAATTGTAATACAACTTTCAGCAACGGATCTCTTGGCTCTCGCATCGA
TGAAGAACGCAGCGAAATGCGATAAGTAATGTGAATTGCAGAATTCAGTGAATCATCGAA
TCTTTGAACGCATCTTGCGCTCCTTGGTATTCCGAGGAGCATGCCTGTTTGAGTGTCATT
AAATTCTCAACTCTCTTATAC-TTTTTTGTAAAAGAGAGCTTGGACTGTGGAGGCTTGCT
GGCCACTTTTTGGGGTCAGCTCCTCTGAAATGCATTAGCGGAACCGTTTGCAATCTGCCA
CAAGTGTGATAAGTTATCTACACTGGCGAGGGGATTGCTCTCTGTAATGTTCAGCTTCTA
ATTGTCTCTACTTTGTGAGACAACTTTTGAATGCTTGACCTCAAATCAGGTAGGACTACC
CGCTGAACTTAA
>ABC8-2
TTTCCGTAGGTGAACCTGCGGAAGGATCATTATTGAATTATGTTTCTAGATAGGTTGTAG

CTGGCTCTTTTAGAGCATGTGCACGCCTGTTTGGACTTCATTTTCATCCACCTGTGCACC
TATTGTAGTCTTTGGTTGGGTTAGGAGGAAGTGGTCATTGTGTCAGCATCTGCTGGATGT
GAGGACTTGCATTGTGAAAGCTTTGCTGTCCTTGATGTGATCATGGAATCTCTTTCTCAC
TAGAGTCTATGTCACTCATTATACTCTGTCGAATGTCATTGAATGTCTTTACATGGGCTT
ATATGCCTATGAAAATTGTAATACAACTTTCAGCAACGGATCTCTTGGCTCTCGCATCGA
TGAAGAACGCAGCGAAATGCGATAAGTAATGTGAATTGCAGAATTCAGTGAATCATCGAA
TCTTTGAACGCATCTTGCGCTCCTTGGTATTCCGAGGAGCATGCCTGTTTGAGTGTCATT
AAATTCTCAACTCTCTTATAC-TTTTTTGTAAAAGAGAGCTTGGACTGTGGAGGCTTGCT
GGCCACTTTTTGGGGTCAGCTCCTCTGAAATGCATTAGCGGAACCGTTTGCGATCTGCCA
CAAGTGTGATAAGTTATCTACACTGGCGAGGGGATTGCTCTCTGTAATGTTCAGCTTCTA
ATTGTCTCTACTTTGTGAGACTACTTTTGAATGCTTGACCTCAAATCAGGTAGGACTACC
CGCTGAACTTAA
>ABC9-41
TTTCCGTAGGTGAACCTGCGGAAGGATCATTATTGAATTATGTTTCTAGATAGGTTGTAG
CTGGCTCTTTTAGAGCATGTGCACGCCTGTTTGGACTTCATTTTCATCCACCTGTGCACC
TATTGTAGTCTTTGGTTGGGTTAGGAGGAAGTGGTCATTGTGTCAGCATCTGCTGGATGT
GAGGACTTGCATTGTGAAAGCTTTGCTGTCCTTGATGTGATCATGGAATCTCTTTCTCAC
TAGAGTCTATGTCACTCATTATACTCTGTCGAATGTCATTGAATGTCTTTACATGGGCTT
GTATGCCTATGAAAATTGTAATACAACTTTCAGCAACGGATCTCTTGGCTCTCGCATCGA
TGAAGAACGCAGCGAAATGCGATAAGTAATGTGAATTGCAGAATTCAGTGAATCATCGAA
TCTTTGAACGCATCTTGCGCTCCTTGGTATTCCGAGGAGCATGCCTGTTTGAGTGTCATT
AAATTCTCAACTCTCTTATAC-TTTTTTGTAAAAGAGAGCTTGGACTGTGGAGGCTTGCT
GGCCACTTTTTGGGGTCAGCTCCTCTGAAATGCATTAGCGGAACCGTTTGCGATCTGCCA
CAAGTGTGATAAGTTATCTACACTGGCGAGGGGATTGCTCTCTGTAATGTTCAGCTTCTA
ATTGTCTCTACTTTGTGAGACTACTTTTGAATGCTTGACCTCAAATCAGGTAGGACTACC
CGCTGAACTTAA
>ABC5-44
TTTCCGTAGGTGAACCTGCGGAAGGATCATTATTGAATTATGTTTCTAGATAGGTTGTAG
CTGGCTCTTTTAGAGCATGTGCACGCCTGTTTGGACTTCATTTTCATCCACCTGTGCACC
TATTGTAGTCTTTGGTTGGGTTAGGGGGAAGTGGTCATTGTGTCAGCATCTGCTGGATGT
GAGGACTTGCATTGTGAAAGCTTTGCTGTCCTTGATGTGATCATGGAATCTCTTTCTCAC
TAGAGTCTATGTCACTCATTATACTCTGTCGAATGTCATTGAATGTCTTTACATGGGCTT
ATATGCCTATGAAAATTGTAATACAACTTTCAGCAACGGATCTCTTGGCTCTCGCATCGA
TGAAGAACGCAGCGAAATGCGATAAGTAATGTGAATTGCAGAATTCAGTGAATCATCGAA
TCTTTGAACGCATCTTGCGCTCCTTGGTATTCCGAGGAGCATGCCTGTTTGAGTGTCATT
AAATTCTCAACTCTCTTATAC-TTTTTTGTAAAAGAGAGCTTGGACTGTGGAGGCTTGCT
GGCCACTTTTTGGGGTCAGCTCCTCTGAAATGCATTAGCGGAACCGTTTGCGATCTGCCA
CAAGTGTGATAAGTTATCTACACTGGCGAGGGGATTGCTCTCTGTAATGTTCAGCTTCTA
ATTGTCTCTACTTTGTGAGACTACTTTTGAATGCTTGACCTCAAATCAGGTAGGACTACC
CGCTGAACTTAA
>ABC12-3
TTTCCGTAGGTGAACCTGCGGAAGGATCATTATTGAATTATGTTTCTAGATAGGTTGTAG
CTGGCTCTTTTAGAGCATGTGCACGCCTGTTTGGACTTCATTTTCATCCACCTGTGCACC
TATTGTAGTCTTTGGTTGGGTTAGGGGGAAGTGGTCATTGTGTCAGCATCTGCTGGATGT
GAGGACTTGCATTGTGAAAGCTTTGCTGTCCTTGATGTGATCATGGAATCTTTTTCTCAC
TAGAGTCTATGTCACTCATTATACTCTGTCGAATGTCATTGAATGTCTTTACATGGGCTT
GTATGCCTATGAAAATTGTAATACAACTTTCAGCAACGGATCTCTTGGCTCTCGCATCGA
TGAAGAACGCAGCGAAATGCGATAAGTAATGTGAATTGCAGAATTCAGTGAATCATCGAA
TCTTTGAACGCATCTTGCGCTCCTTGGTATTCCGAGGAGCATGCCTGTTTGAGTGTCATT
AAATTCTCAACTCTCTTATAC-TTTTTTGTAAAAGAGAGCTTGGACTGTGGAGGCTTGCT

GGCCACTTTTTGGGGTCAGCTCCTCTGAAATGCATTAGCGGAACCGTTTGCGATCTGCCA
CAAGTGTGATAAGTTATCTACACTGGCGAGGGGATTGCTCTCTGTAATGTTCAGCTTCTA
ATTGTCTCTACTTTGTGAGACTACTTTTGAATGCTTGACCTCAAATCAGGTAGGACTACC
CGCTGAACTTAA
>ABC7−50
TTTCCGTAGGTGAACCTGCGGAAGGATCATTATTGAATTATGTTTCTAGATAGGTTGTAG
CTGGCTCTTTTAGAGCATGTGCACGCCTGTTTGGACTTCATTTTCATCCACCTGTGCACC
TATTGTAGTCTTTGGTTGGGTTAGGAGGAAGTGGTCATTGTGTCAGCATCTGCTGGATGT
GAGGACTTGCATTGTGAAAGCTTTGCTGTCCTTGATGTGATCATGGAATCTCTTTCTCAC
TAGAGTCTATGTCACTCATTATACTCTGTCGAATGTCATTGAATGTCTTTACATGGGCTT
ATATGCCTATGAAAATTGTAATACAACTTTCAGCAACGGATCTCTTGGCTCTCGCATCGA
TGAAGAACGCAGCGAAATGCGATAAGTAATGTGAATTGCAGAATTCAGTGAATCATCGAA
TCTTTGAACGCATCTTGCGCTCCTTGGTATTCCGAGGAGCATGCCTGTTTGAGTGTCATT
AAATTCTCAACTCTCTTATAC−TTTTTTGTAAAAGAGAGCTTGGACTGTGGAGGCTTGCT
GGCCACTTTTTGGGGTCAGCTCCTCTGAAATGCATTAGCGGAACCGTTTGCAATCTGCCA
CAAGTGTGATAAGTTATCTACACTGACGAGGGGATTGCTCTCTGTAATGTTCAGCTTCTA
ATTGTCTCTACTTTGTGAGACTACTTTTGAATGCTTGACCTCAAATCAGGTAGGACTACC
CGCTGAACTTAA
>ABC6−14
TTTCCGTAGGTGAACCTGCGGAAGGATCATTATTGAATTATGTTTCTAGATAGGTTGTAG
CTGGCTCTTTTAGAGCATGTGCACGCCTGTTTGGACTTCATTTTCATCCACCTGTGCACC
TATTGTAGTCTTTGGTTGGGTTAGGAGGAAGTGGTCATTGTGTCAGCATCTGCTGGATGT
GAGGACTTGCATTGTGAAAGCTTTGCTGTCCTTGATGTGATCATGGAATCTCTTTCTCAC
TAGAGTCTATGTCACTCATTATACTCTGTCGAATGTCATTGAATGTCTTTACATGGGCTT
GTATGCCTATGAAAATTGTAATACAACTTTCAGCAACGGATCTCTTGGCTCTCGCATCGA
TGAAGGACGCAGCGAAATGCGATAAGTAATGTGAATTGCAGAATTCAGTGAATCATCGAA
TCTTTGAACGCATCTTGCGCTCCTTGGTATTCCGAGGAGCATGCCTGTTTGAGTGTCATT
AAATTCTCAACTCTCTTATAC−TTTTTTGTAAAAGAGAGCTTGGACTGTGGAGGCTTGCT
GGCCACTTTTTGGGGTCAGCTCCTCTGAAATGCATTAGCGGAACCGTTTGCAATCTGCCA
CAAGTGTGATAAGTTATCTACACTGGCGAGGGGATTGCTCTCTGTAATGTTCAGCTTCTA
ATTGTCTCTACTTTGTGAGACAACTTTTGAATGCTTGACCTCAAATCAGGTAGGACTACC
CGCTGAACTTAA
>ABC12−24
TTTCCGTAGGTGAACCTGCGGAAGGATCATTATTGAATTATGTTTCTAGATAGGTTGTAG
CTGGCTCTTTTAGAGCATGTGCACGCCTGTTTGGACTTCATTTTCATCCACCTGTGCACC
TATTGTAGTCTTTGGTTGGGTTAGGAGGAAGTGGTCATTGTGTCAGCATCTGCTGGATGT
GAGGACTTGCATTGTGAAAGCTTTGCTGTCCTTGATGTGATCATGGAATCTCTTTCTCAC
TAGAGTCTATGTCACTCATTATACTCTGTCGAATGTCATTGAATGTCTTTACATGGGCTT
GTATGCCTATGAAAATTGTAATACAACTTTCAGCAACGGATCTCTTGGCTCTCGCATCGA
TGAAGGACGCAGCGAAATGCGATAAGTAATGTGAATTGCAGAATTCAGTGAATCATCGAA
TCTTTGAACGCATCTTGCGCTCCTTGGTATTCCGAGGAGCATGCCTGTTTGAGTGTCATT
AAATTCTCAACTCTCTTATAC−TTTTTTGTAAAAGAGAGCTTGGACTGTGGAGGCTTGCT
GGCCACTTTTTGGGGTCAGCTCCTCTGAAATGCATTAGCGGAACCGTTTGCAATCTGCCA
CAAGTGTGATAAGTTATCTACACTGGCGAGGGGATTGCTCTCTGTAATGTTCAGCTTCTA
ATTGTCTCTACTTTGTGAGACAACTTTTGAATGCTTGACCTCAAATCAGGTAGGACTACC
CGCTGAACTTAA
>ABC8−33
TTTCCGTAGGTGAACCTGCGGAAGGATCATTATTGAATTATGTTTCTAGATAGGTTGTAG
CTGGCTCTTTTAGAGCATGTGCACGCCTGTTTGGACTTCATTTTCATCCACCTGTGCACC
TATTGTAGTCTTTGGTTGGGTTAGGAGGAAGTGATCATTGTATCAGCATCTGCTGGATGT

GAGGACTTGCATTGTGAAAGCTTTGCTGTCCTTGATGTGATCATGGAATCTCTTTCTCAC
TAGAGTCTATGTCACTCATTATACTCTGTCGAATGTCATTGAATGTCTTTACATGGGCTT
GTATGCCTATGAAAATTGTAATACAACTTTCAGCAACGGATCTCTTGGCTCTCGCATCGA
TGAAGGACGCAGCGAAATGCGATAAGTAATGTGAATTGCAGAATTCAGTGAATCATCGAA
TCTTTGAACGCATCTTGCGCTCCTTGGTATTCCGAGGAGCATGCCTGTTTGAGTGTCATT
AAATTCTCAACTCTCTTATAC-TTTTTTGTAAAAGAGAGCTTGGACTGTGGAGGCTTGCT
GGCCACTTTTTGGGGTCAGCTCCTCTGAAATGCATTAGCGGAACCGTTTGCAATCTGCCA
CAAGTGTGATAAGTTATCTACACTGGCGAGGGGATTGCTCTCTGTAATGTTCAGCTTCTA
ATTGTCTCTACTTTGTGAGACAACTTTTGAATGCTTGACCTCAAATCAGGTAGGACTACC
CGCTGAACTTAA
>ABC9-10
TTTCCGTAGGTGAACCTGCGGAAGGATCATTATTGAATTATGTTTCTAGATAGGTTGTAG
CTGGCTCTTTTAGAGCATGTGCACGCCTGTTTGGACTTCATTTTCATCCACCTGTGCACC
TATTGTAGTCTTTGGTTGGGTTAGGAGGAAGTGGTCATTGTGTCAGCATCTGCTGGATGT
GAGGACTTGCATTGTGAAAGCTTTGCTGTCCTTGATGTGATCATGGAATCTCTTTCTCAC
TAGAGTCTATGTCACTCATTATACTCTGTCGAATGTCATTGAATGTCTTTACATGGGCTT
ATATGCCTATGAAAATTGTAATACAACTTTCAGCAACGGATCTCTTGGCTCTCGCATCGA
TGAAGGACGCAGCGAAATGCGATAAGTAATGTGAATTGCAGAATTCAGTGAATCATCGAA
TCTTTGAACGCATCTTGCGCTCCTTGGTATTCCGAGGAGCATGCCTGTTTGAGTGTCATT
AAATTCTCAACTCTCTTATAC-TTTTTTGTAAAAGAGAGCTTGGACTGTGGAGGCTTGCT
GGCCACTTTTTGGGGTCAGCTCCTCTGAAATGCATTAGCGGAACCGTTTGCGATCTGCCA
CAAGTGTGATAAGTTATCTACACTGGCGAGGGGATTGCTCTCTGTAATGTTCAGCTTCTA
ATTGTCTCTACTTTGTGAGACAACTTTTGAATGCTTGACCTCAAATCAGGTAGGACTACC
CGCTGAACTTAA
>ABC7-35
TTTCCGTAGGTGAACCTGCGGAAGGATCATTATTGAATTATGTTTCTAGATAGGTTGTAG
CTGGCTCTTTTAGAGCATGTGCACGCCTGTTTGGACTTCATTTTCATCCACCTGTGCACC
TATTGTAGTCTTTGGTTGGGTTAGGAGGAAGTGATCATTGTATCAGCATCTGCTGGGAGT
GAGGACTTGCATTGTGAAAGCTTTGCTGTCCTTGATGTGATCATGGAATCTTTTTCTCAC
TAGAGTCTATGTCACTCATTATACTCTGTCGAATGTCATTGAATGTCTTTACATGGGCTT
GTATGCCTATGAAAATTGTAATACAACTTTCAGCAACGGATCTCTTGGCTCTCGCATCGA
TGAAGAACGCAGCGAAATGCGATAAGTAATGTGAATTGCAGAATTCAGTGAATCATCGAA
TCTTTGAACGCATCTTGCGCTCCTTGGTATTCCGAGGAGCATGCCTGTTTGAGTGTCATT
AAATTCTCAACTCTCTTATACTTTTTTTGTAAAAGAGAGCTTGGACTGTGGAGGCTTGCT
GGCCACTTTTTGGGGTCAGCTCCTCTGAAATGCATTAGCGGAACCGTTTGCGATCTGCCA
CAAGTGTGATAAGTTATCTACACTGGCGAGGGGATTGCTCTCTGTAATGTTCAGCTTCTA
ATTGTCTCTACTTTGTGAGACAACTTTTGAATGCTTGACCTCAAATCAGGTAGGACTACC
CGCTGAACTTAA
>ABC1-3
TTTCCGTAGGTGAACCTGCGGAAGGATCATTATTGAATTATGTTTCTAGATAGGTTGTAG
CTGGCTC-TTTAGAGCATGTGCACGCCTGTTTGGACTTCATTTTCATCCACCTGTGCACC
TATTGTAGTCTTTGGTTGGGTTAGGGGGAAGTGGTCATTGTGTCAGCATCTGCTGGATGT
GAGGACTTGCATTGTGAAAGCTTTGCTGTCCTTGATGTGATCATGGAATCTCTTTCTCAC
TAGAGTCTATGTCACTCATTATACTCTGTCGAATGTCATTGAATGTCTTTACATGGGCTT
GTATGCCTATGAAAATTGTAATACAACTTTCAGCAACGGATCTCTTGGCTCTCGCATCGA
TGAAGGACGCAGCGAAATGCGATAAGTAATGTGAATTGCAGAATTCAGTGAATCATCGAA
TCTTTGAACGCATCTTGCGCTCCTTGGTATTCCGAGGAGCATGCCTGTTTGAGTGTCATT
AAATTCTCAACTCTCTTATAC-TTTTTTGTAAAAGAGAGCTTGGACTGTGGAGGCTTGCT
GGCCACTTTTTGGGGTCAGCTCCTCTGAAATGCATTAGCGGAACCGTTTGCAATCTGCCA
CAAGTGTGATAAGTTATCTACACTGGCGAGGGGATTGCTCTCTGTAATGTTCAGCTTCTA

ATTGTCTCTACTTTGTGAGACAACTTTTGAATGCTTGACCTCAAATCAGGTAGGACTACC
CGCTGAACTTAA
>ABC5-47
TTTCCGTAGGTGAACCTGCGGAAGGATCATTATTGAATTATGTTTCTAGATAGGTTGTAG
CTGGCTC-TTTAGAGCATGTGCACGCCTGTTTGGACTTCATTTTCATCCACCTGTGCACC
TATTGTAGTCTTTGGTTGGGTTAGGGGGAAGTGGTCATTGTGTCAGCATCTGCTGGATGT
GAGGACTTGCATTGTGAAAGCTTTGCTGTCCTTGATGTGATCATGGAATCTCTTTCTCAC
TAGAGTCTATGTCACTCATTATACTCTGTCGAATGTCATTGAATGTCTTTACATGGGCTT
GTATGCCTATGAAAATTGTAATACAACTTTCAGCAACGGATCTCTTGGCTCTCGCATCGA
TGAAGGACGCAGCGAAATGCGATAAGTAATGTGAATTGCAGAATTCAGTGAATCATCGAA
TCTTTGAACGCATCTTGCGCTCCTTGGTATTCCGAGGAGCATGCCTGTTTGAGTGTCATT
AAATTCTCAACTCTCTTATAC-TTTTTTGTAAAAGAGAGCTTGGACTGTGGAGGCTTGCT
GGCCACTTTTTGGGGTCAGCTCCTCTGAAATGCATTAGCGGAACCGTTTGCAATCTGCCA
CAAGTGTGATAAGTTATCTACACTGGCGAGGGGATTGCTCTCTGTAATGTTCAGCTTCTA
ATTGTCTCTACTTTGTGAGACAACTTTTGAATGCTTGACCTCAAATCAGGTAGGACTACC
CGCTGAACTTAA
>ABC5-61
TTTCCGTAGGTGAACCTGCGGAAGGATCATTATTGAATTATGTTTCTAGATAGGTTGTAG
CTGGCTC-TTTAGAGCATGTGCACGCCTGTTTGGACTTCATTTTCATCCACCTGTGCACC
TATTGTAGTCTTTGGTTGGGTTAGGGGGAAGTGGTCATTGTGTCAGCATCTGCTGGATGT
GAGGACTTGCATTGTGAAAGCTTTGCTGTCCTTGATGTGATCATGGAATCTCTTTCTCAC
TAGAGTCTATGTCACTCATTATACTCTGTCGAATGTCATTGAATGTCTTTACATGGGCTT
GTATGCCTATGAAAATTGTAATACAACTTTCAGCAACGGATCTCTTGGCTCTCGCATCGA
TGAAGGACGCAGCGAAATGCGATAAGTAATGTGAATTGCAGAATTCAGTGAATCATCGAA
TCTTTGAACGCATCTTGCGCTCCTTGGTATTCCGAGGAGCATGCCTGTTTGAGTGTCATT
AAATTCTCAACTCTCTTATAC-TTTTTTGTAAAAGAGAGCTTGGACTGTGGAGGCTTGCT
GGCCACTTTTTGGGGTCAGCTCCTCTGAAATGCATTAGCGGAACCGTTTGCAATCTGCCA
CAAGTGTGATAAGTTATCTACACTGGCGAGGGGATTGCTCTCTGTAATGTTCAGCTTCTA
ATTGTCTCTACTTTGTGAGACAACTTTTGAATGCTTGACCTCAAATCAGGTAGGACTACC
CGCTGAACTTAA
>ABC9-3
TTTCCGTAGGTGAACCTGCGGAAGGATCATTATTGAATTATGTTTCTAGATAGGTTGTAG
CTGGCTC-TTTAGAGCATGTGCACGCCTGTTTGGACTTCATTTTCATCCACCTGTGCACC
TATTGTAGTCTTTGGTTGGGTTAGGGGGAAGTGGTCATTGTGTCAGCATCTGCTGGATGT
GAGGACTTGCATTGTGAAAGCTTTGCTGTCCTTGATGTGATCATGGAATCTCTTTCTCAC
TAGAGTCTATGTCACTCATTATACTCTGTCGAATGTCATTGAATGTCTTTACATGGGCTT
GTATGCCTATGAAAATTGTAATACAACTTTCAGCAACGGATCTCTTGGCTCTCGCATCGA
TGAAGGACGCAGCGAAATGCGATAAGTAATGTGAATTGCAGAATTCAGTGAATCATCGAA
TCTTTGAACGCATCTTGCGCTCCTTGGTATTCCGAGGAGCATGCCTGTTTGAGTGTCATT
AAATTCTCAACTCTCTTATAC-TTTTTTGTAAAAGAGAGCTTGGACTGTGGAGGCTTGCT
GGCCACTTTTTGGGGTCAGCTCCTCTGAAATGCATTAGCGGAACCGTTTGCAATCTGCCA
CAAGTGTGATAAGTTATCTACACTGGCGAGGGGATTGCTCTCTGTAATGTTCAGCTTCTA
ATTGTCTCTACTTTGTGAGACAACTTTTGAATGCTTGACCTCAAATCAGGTAGGACTACC
CGCTGAACTTAA
>ABC12-2
TTTCCGTAGGTGAACCTGCGGAAGGATCATTATTGAATTATGTTTCTAGATAGGTTGTAG
CTGGCTC-TTTAGAGCATGTGCACGCCTGTTTGGACTTCATTTTCATCCACCTGTGCACC
TATTGTAGTCTTTGGTTGGGTTAGGGGGAAGTGGTCATTGTGTCAGCATCTGCTGGATGT
GAGGACTTGCATTGTGAAAGCTTTGCTGTCCTTGATGTGATCATGGAATCTCTTTCTCAC
TAGAGTCTATGTCACTCATTATACTCTGTCGAATGTCATTGAATGTCTTTACATGGGCTT

GTATGCCTATGAAAATTGTAATACAACTTTCAGCAACGGATCTCTTGGCTCTCGCATCGA
TGAAGGACGCAGCGAAATGCGATAAGTAATGTGAATTGCAGAATTCAGTGAATCATCGAA
TCTTTGAACGCATCTTGCGCTCCTTGGTATTCCGAGGAGCATGCCTGTTTGAGTGTCATT
AAATTCTCAACTCTCTTATAC-TTTTTTGTAAAAGAGAGCTTGGACTGTGGAGGCTTGCT
GGCCACTTTTTGGGGTCAGCTCCTCTGAAATGCATTAGCGGAACCGTTTGCAATCTGCCA
CAAGTGTGATAAGTTATCTACACTGGCGAGGGGATTGCTCTCTGTAATGTTCAGCTTCTA
ATTGTCTCTACTTTGTGAGACAACTTTTGAATGCTTGACCTCAAATCAGGTAGGACTACC
CGCTGAACTTAA
>ABC7-25
TTTCCGTAGGTGAACCTGCGGAAGGATCATTATTGAATTATGTTTCTAGATAGGTTGTAG
CTGGCTC-TTTAGAGCATGTGCACGCCTGTTTGGACTTCATTTTCATCCACCTGTGCACC
TATTGTAGTCTTTGGTTGGGTTAGGGGGAAGTGGTCATTGTGTCAGCATCTGCTGGATGT
GAGGACTTGCATTGTGAAAGCTTTGCTGTCCTTGATGTGATCATGGAATCTCTTTCTCAC
TAGAGTCTATGTCACTCATTATACTCTGTCGAATGTCATTGAATGTCTTTACATGGGCTT
GTATGCCTATGAAAATTGTAATACAACTTTCAGCAACGGATCTCTTGGCTCTCGCATCGA
TGAAGGACGCAGCGAAATGCGATAAGTAATGTGAATTGCAGAATTCAGTGAATCATCGAA
TCTTTGAACGCATCTTGCGCTCCTTGGTATTCCGAGGAGCATGCCTGTTTGAGTGTCATT
AAATTCTCAACTCTCTTATAC-TTTTTTGTAAAAGAGAGCTTGGACTGTGGAGGCTTGCT
GGCCACTTTTTGGGGTCAGCTCCTCTGAAATGCATTAGCGGAACCGTTTGCAATCTGCCA
CAAGTGTGATAAGTTATCTACACTGGCGAGGGGATTGCTCTCTGTAATGTTCAGCTTCTA
ATTGTCTCTACTTTGTGAGACAACTTTTGAATGCTTGACCTCAAATCAGGTAGGACTACC
CGCTGAACTTAA
>ABC7-7
TTTCCGTAGGTGAACCTGCGGAAGGATCATTATTGAATTATGTTTCTAGATAGGTTGTAG
CTGGCTC-TTTAGAGCATGTGCACGCCTGTTTGGACTTCATTTTCATCCACCTGTGCACC
TATTGTAGTCTTTGGTTGGGTTAGGGGGAAGTGGTCATTGTGTCAGCATCTGCTGGATGT
GAGGACTTGCATTGTGAAAGCTTTGCTGTCCTTGATGTGATCATGGAATCTCTTTCTCAC
TAGAGTCTATGTCACTCATTATACTCTGTCGAATGTCATTGAATGTCTTTACATGGGCTT
GTATGCCTATGAAAATTGTAATACAACTTTCAGCAACGGATCTCTTGGCTCTCGCATCGA
TGAAGGACGCAGCGAAATGCGATAAGTAATGTGAATTGCAGAATTCAGTGAATCATCGAA
TCTTTGAACGCATCTTGCGCTCCTTGGTATTCCGAGGAGCATGCCTGTTTGAGTGTCATT
AAATTCTCAACTCTCTTATAC-TTTTTTGTAAAAGAGAGCTTGGACTGTGGAGGCTTGCT
GGCCACTTTTTGGGGTCAGCTCCTCTGAAATGCATTAGCGGAACCGTTTGCAATCTGCCA
CAAGTGTGATAAGTTATCTACACTGGCGAGGGGATTGCTCTCTGTAATGTTCAGCTTCTA
ATTGTCTCTACTTTGTGAGACAACTTTTGAATGCTTGACCTCAAATCAGGTAGGACTACC
CGCTGAACTTAA
>ABC5-34
TTTCCGTAGGTGAACCTGCGGAAGGATCATTATTGAATTATGTTTCTAGATAGGTTGTAG
CTGGCTC-TTTAGAGCATGTGCACGCCTGTTTGGACTTCATTTTCATCCACCTGTGCACC
TATTGTAGTCTTTGGTTGGGTTAGGGGGAAGTGGTCATTGTGTCAGCATCTGCTGGATGT
GAGGACTTGCATTGTGAAAGCTTTGCTGTCCTTGATGTGATCATGGAATCTCTTTCTCAC
TAGAGTCTATGTCACTCATTATACTCTGTCGAATGTCATTGAATGTCTTTACATGGGCTT
GTATGCCTATGAAAATTGTAATACAACTTTCAGCAACGGATCTCTTGGCTCTCGCATCGA
TGAAGGACGCAGCGAAATGCGATAAGTAATGTGAATTGCAGAATTCAGTGAATCATCGAA
TCTTTGAACGCATCTTGCGCTCCTTGGTATTCCGAGGAGCATGCCTGTTTGAGTGTCATT
AAATTCTCAACTCTCTTATAC-TTTTTTGTAAAAGAGAGCTTGGACTGTGGAGGCTTGCT
GGCCACTTTTTGGGGTCAGCTCCTCTGAAATGCATTAGCGGAACCGTTTGCAATCTGCCA
CAAGTGTGATAAGTTATCTACACTGGCGAGGGGATTGCTCTCTGTAATGTTCAGCTTCTA
ATTGTCTCTACTTTGTGAGACAACTTTTGAATGCTTGACCTCAAATCAGGTAGGACTACC
CGCTGAACTTAA

>ABC12-29
TTTCCGTAGGTGAACCTGCGGAAGGATCATTATTGAATTATGTTTCTAGATAGGTTGTAG
CTGGCTC-TTTAGAGCATGTGCACGCCTGTTTGGACTTCATTTTCATCCACCTGTGCACC
TATTGTAGTCTTTGGTTGGGTTAGGGGGAAGTGGTCATTGTGTCAGCATCTGCTGGATGT
GAGGACTTGCATTGTGAAAGCTTTGCTGTCCTTGATGTGATCATGGAATCTCTTTCTCAC
TAGAGTCTATGTCACTCATTATACTCTGTCGAATGTCATTGAATGTCTTTACATGGGCTT
GTATGCCTATGAAAATTGTAATACAACTTTCAGCAACGGATCTCTTGGCTCTCGCATCGA
TGAAGGACGCAGCGAAATGCGATAAGTAATGTGAATTGCAGAATTCAGTGAATCATCGAA
TCTTTGAACGCATCTTGCGCTCCTTGGTATTCCGAGGAGCATGCCTGTTTGAGTGTCATT
AAATTCTCAACTCTCTTATAC-TTTTTTGTAAAAGAGAGCTTGGACTGTGGAGGCTTGCT
GGCCACTTTTTGGGGTCAGCTCCTCTGAAATGCATTAGCGGAACCGTTTGCAATCTGCCA
CAAGTGTGATAAGTTATCTACACTGGCGAGGGGATTGCTCTCTGTAATGTTCAGCTTCTA
ATTGTCTCTACTTTGTGAGACAACTTTTGAATGCTTGACCTCAAATCAGGTAGGACTACC
CGCTGAACTTAA
>ABC7-21
TTTCCGTAGGTGAACCTGCGGAAGGATCATTATTGAATTATGTTTCTAGATAGGTTGTAG
CTGGCTC-TTTAGAGCATGTGCACGCCTGTTTGGACTTCATTTTCATCCACCTGTGCACC
TATTGTAGTCTTTGGTTGGGTTAGGGGGAAGTGGTCATTGTGTCAGCATCTGCTGGATGT
GAGGACTTGCATTGTGAAAGCTTTGCTGTCCTTGATGTGATCATGGAATCTCTTTCTCAC
TAGAGTCTATGTCACTCATTATACTCTGTCGAATGTCATTGAATGTCTTTACATGGGCTT
GTATGCCTATGAAAATTGTAATACAACTTTCAGCAACGGATCTCTTGGCTCTCGCATCGA
TGAAGGACGCAGCGAAATGCGATAAGTAATGTGAATTGCAGAATTCAGTGAATCATCGAA
TCTTTGAACGCATCTTGCGCTCCTTGGTATTCCGAGGAGCATGCCTGTTTGAGTGTCATT
AAATTCTCAACTCTCTTATAC-TTTTTTGTAAAAGAGAGCTTGGACTGTGGAGGCTTGCT
GGCCACTTTTTGGGGTCAGCTCCTCTGAAATGCATTAGCGGAACCGTTTGCAATCTGCCA
CAAGTGTGATAAGTTATCTACACTGGCGAGGGGATTGCTCTCTGTAATGTTCAGCTTCTA
ATTGTCTCTACTTTGTGAGACAACTTTTGAATGCTTGACCTCAAATCAGGTAGGACTACC
CGCTGAACTTAA
>ABC5-36
TTTCCGTAGGTGAACCTGCGGAAGGATCATTATTGAATTATGTTTCTAGATAGGTTGTAG
CTGGCTC-TTTAGAGCATGTGCACGCCTGTTTGGACTTCATTTTCATCCACCTGTGCACC
TATTGTAGTCTTTGGTTGGGTTAGGGGGAAGTGGTCATTGTGTCAGCATCTGCTGGATGT
GAGGACTTGCATTGTGAAAGCTTTGCTGTCCTTGATGTGATCATGGAATCTCTTTCTCAC
TAGAGTCTATGTCACTCATTATACTCTGTCGAATGTCATTGAATGTCTTTACATGGGCTT
GTATGCCTATGAAAATTGTAATACAACTTTCAGCAACGGATCTCTTGGCTCTCGCATCGA
TGAAGGACGCAGCGAAATGCGATAAGTAATGTGAATTGCAGAATTCAGTGAATCATCGAA
TCTTTGAACGCATCTTGCGCTCCTTGGTATTCCGAGGAGCATGCCTGTTTGAGTGTCATT
AAATTCTCAACTCTCTTATAC-TTTTTTGTAAAAGAGAGCTTGGACTGTGGAGGCTTGCT
GGCCACTTTTTGGGGTCAGCTCCTCTGAAATGCATTAGCGGAACCGTTTGCAATCTGCCA
CAAGTGTGATAAGTTATCTACACTGGCGAGGGGATTGCTCTCTGTAATGTTCAGCTTCTA
ATTGTCTCTACTTTGTGAGACAACTTTTGAATGCTTGACCTCAAATCAGGTAGGACTACC
CGCTGAACTTAA
>ABC8-45
TTTCCGTAGGTGAACCTGCGGAAGGATCATTATTGAATTATGTTTCTAGATAGGTTGTAG
CTGGCTC-TTTAGAGCATGTGCACGCCTGTTTGGACTTCATTTTCATCCACCTGTGCACC
TATTGTAGTCTTTGGTTGGGTTAGGGGGAAGTGGTCATTGTGTCAGCATCTGCTGGATGT
GAGGACTTGCATTGTGAAAGCTTTGCTGTCCTTGATGTGATCATGGAATCTCTTTCTCAC
TAGAGTCTATGTCACTCATTATACTCTGTCGAATGTCATTGAATGTCTTTACATGGGCTT
GTATGCCTATGAAAATTGTAATACAACTTTCAGCAACGGATCTCTTGGCTCTCGCATCGA
TGAAGGACGCAGCGAAATGCGATAAGTAATGTGAATTGCAGAATTCAGTGAATCATCGAA

TCTTTGAACGCATCTTGCGCTCCTTGGTATTCCGAGGAGCATGCCTGTTTGAGTGTCATT
AAATTCTCAACTCTCTTATAC-TTTTTTGTAAAAGAGAGCTTGGACTGTGGAGGCTTGCT
GGCCACTTTTTGGGGTCAGCTCCTCTGAAATGCATTAGCGGAACCGTTTGCAATCTGCCA
CAAGTGTGATAAGTTATCTACACTGGCGAGGGGATTGCTCTCTGTAATGTTCAGCTTCTA
ATTGTCTCTACTTTGTGAGACAACTTTTGAATGCTTGACCTCAAATCAGGTAGGACTACC
CGCTGAACTTAA
>ABC7-46
TTTCCGTAGGTGAACCTGCGGAAGGATCATTATTGAATTATGTTTCTAGATAGGTTGTAG
CTGGCTC-TTTAGAGCATGTGCACGCCTGTTTGGACTTCATTTTCATCCACCTGTGCACC
TATTGTAGTCTTTGGTTGGGTTAGGGGGAAGTGGTCATTGTGTCAGCATCTGCTGGATGT
GAGGACTTGCATTGTGAAAGCTTTGCTGTCCTTGATGTGATCATGGAATCTCTTTCTCAC
TAGAGTCTATGTCACTCATTATACTCTGTCGAATGTCATTGAATGTCTTTACATGGGCTT
GTATGCCTATGAAAATTGTAATACAACTTTCAGCAACGGATCTCTTGGCTCTCGCATCGA
TGAAGGACGCAGCGAAATGCGATAAGTAATGTGAATTGCAGAATTCAGTGAATCATCGAA
TCTTTGAACGCATCTTGCGCTCCTTGGTATTCCGAGGAGCATGCCTGTTTGAGTGTCATT
AAATTCTCAACTCTCTTATAC-TTTTTTGTAAAAGAGAGCTTGGACTGTGGAGGCTTGCT
GGCCACTTTTTGGGGTCAGCTCCTCTGAAATGCATTAGCGGAACCGTTTGCAATCTGCCA
CAAGTGTGATAAGTTATCTACACTGGCGAGGGGATTGCTCTCTGTAATGTTCAGCTTCTA
ATTGTCTCTACTTTGTGAGACAACTTTTGAATGCTTGACCTCAAATCAGGTAGGACTACC
CGCTGAACTTAA
>ABC11-36
TTTCCGTAGGTGAACCTGCGGAAGGATCATTATTGAATTATGTTTCTAGATAGGTTGTAG
CTGGCTC-TTTAGAGCATGTGCACGCCTGTTTGGACTTCATTTTCATCCACCTGTGCACC
TATTGTAGTCTTTGGTTGGGTTAGGGGGAAGTGGTCATTGTGTCAGCATCTGCTGGATGT
GAGGACTTGCATTGTGAAAGCTTTGCTGTCCTTGATGTGATCATGGAATCTCTTTCTCAC
TAGAGTCTATGTCACTCATTATACTCTGTCGAATGTCATTGAATGTCTTTACATGGGCTT
GTATGCCTATGAAAATTGTAATACAACTTTCAGCAACGGATCTCTTGGCTCTCGCATCGA
TGAAGGACGCAGCGAAATGCGATAAGTAATGTGAATTGCAGAATTCAGTGAATCATCGAA
TCTTTGAACGCATCTTGCGCTCCTTGGTATTCCGAGGAGCATGCCTGTTTGAGTGTCATT
AAATTCTCAACTCTCTTATAC-TTTTTTGTAAAAGAGAGCTTGGACTGTGGAGGCTTGCT
GGCCACTTTTTGGGGTCAGCTCCTCTGAAATGCATTAGCGGAACCGTTTGCAATCTGCCA
CAAGTGTGATAAGTTATCTACACTGGCGAGGGGATTGCTCTCTGTAATGTTCAGCTTCTA
ATTGTCTCTACTTTGTGAGACAACTTTTGAATGCTTGACCTCAAATCAGGTAGGACTACC
CGCTGAACTTAA
>ABC12-47
TTTCCGTAGGTGAACCTGCGGAAGGATCATTATTGAATTATGTTTCTAGATAGGTTGTAG
CTGGCTC-TTTAGAGCATGTGCACGCCTGTTTGGACTTCATTTTCATCCACCTGTGCACC
TATTGTAGTCTTTGGTTGGGTTAGGGGGAAGTGGTCATTGTGTCAGCATCTGCTGGATGT
GAGGACTTGCATTGTGAAAGCTTTGCTGTCCTTGATGTGATCATGGAATCTCTTTCTCAC
TAGAGTCTATGTCACTCATTATACTCTGTCGAATGTCATTGAATGTCTTTACATGGGCTT
GTATGCCTATGAAAATTGTAATACAACTTTCAGCAACGGATCTCTTGGCTCTCGCATCGA
TGAAGGACGCAGCGAAATGCGATAAGTAATGTGAATTGCAGAATTCAGTGAATCATCGAA
TCTTTGAACGCATCTTGCGCTCCTTGGTATTCCGAGGAGCATGCCTGTTTGAGTGTCATT
AAATTCTCAACTCTCTTATAC-TTTTTTGTAAAAGAGAGCTTGGACTGTGGAGGCTTGCT
GGCCACTTTTTGGGGTCAGCTCCTCTGAAATGCATTAGCGGAACCGTTTGCAATCTGCCA
CAAGTGTGATAAGTTATCTACACTGGCGAGGGGATTGCTCTCTGTAATGTTCAGCTTCTA
ATTGTCTCTACTTTGTGAGACAACTTTTGAATGCTTGACCTCAAATCAGGTAGGACTACC
CGCTGAACTTAA
>ABC5-18
TTTCCGTAGGTGAACCTGCGGAAGGATCATTATTGAATTATGTTTCTAGATAGGTTGTAG

CTGGCTC-TTTAGAGCATGTGCACGCCTGTTTGGACTTCATTTTCATCCACCTGTGCACC
TATTGTAGTCTTTGGTTGGGTTAGGGGGAAGTGGTCATTGTGTCAGCATCTGCTGGATGT
GAGGACTTGCATTGTGAAAGCTTTGCTGTCCTTGATGTGATCATGGAATCTCTTTCTCAC
TAGAGTCTATGTCACTCATTATACTCTGTCGAATGTCATTGAATGTCTTTACATGGGCTT
GTATGCCTATGAAAATTGTAATACAACTTTCAGCAACGGATCTCTTGGCTCTCGCATCGA
TGAAGGACGCAGCGAAATGCGATAAGTAATGTGAATTGCAGAATTCAGTGAATCATCGAA
TCTTTGAACGCATCTTGCGCTCCTTGGTATTCCGAGGAGCATGCCTGTTTGAGTGTCATT
AAATTCTCAACTCTCTTATAC-TTTTTTGTAAAAGAGAGCTTGGACTGTGGAGGCTTGCT
GGCCACTTTTTGGGGTCAGCTCCTCTGAAATGCATTAGCGGAACCGTTTGCAATCTGCCA
CAAGTGTGATAAGTTATCTACACTGGCGAGGGGATTGCTCTCTGTAATGTTCAGCTTCTA
ATTGTCTCTACTTTGTGAGACAACTTTTGAATGCTTGACCTCAAATCAGGTAGGACTACC
CGCTGAACTTAA
>ABC5-50
TTTCCGTAGGTGAACCTGCGGAAGGATCATTATTGAATTATGTTTCTAGATAGGTTGTAG
CTGGCTC-TTTAGAGCATGTGCACGCCTGTTTGGACTTCATTTTCATCCACCTGTGCACC
TATTGTAGTCTTTGGTTGGGTTAGGGGGAAGTGGTCATTGTGTCAGCATCTGCTGGATGT
GAGGACTTGCATTGTGAAAGCTTTGCTGTCCTTGATGTGATCATGGAATCTCTTTCTCAC
TAGAGTCTATGTCACTCATTATACTCTGTCGAATGTCATTGAATGTCTTTACATGGGCTT
GTATGCCTATGAAAATTGTAATACAACTTTCAGCAACGGATCTCTTGGCTCTCGCATCGA
TGAAGGACGCAGCGAAATGCGATAAGTAATGTGAATTGCAGAATTCAGTGAATCATCGAA
TCTTTGAACGCATCTTGCGCTCCTTGGTATTCCGAGGAGCATGCCTGTTTGAGTGTCATT
AAATTCTCAACTCTCTTATAC-TTTTTTGTAAAAGAGAGCTTGGACTGTGGAGGCTTGCT
GGCCACTTTTTGGGGTCAGCTCCTCTGAAATGCATTAGCGGAACCGTTTGCAATCTGCCA
CAAGTGTGATAAGTTATCTACACTGGCGAGGGGATTGCTCTCTGTAATGTTCAGCTTCTA
ATTGTCTCTACTTTGTGAGACAACTTTTGAATGCTTGACCTCAAATCAGGTAGGACTACC
CGCTGAACTTAA
>ABC11-28
TTTCCGTAGGTGAACCTGCGGAAGGATCATTATTGAATTATGTTTCTAGATAGGTTGTAG
CTGGCTC-TTTAGAGCATGTGCACGCCTGTTTGGACTTCATTTTCATCCACCTGTGCACC
TATTGTAGTCTTTGGTTGGGTTAGGGGGAAGTGGTCATTGTGTCAGCATCTGCTGGATGT
GAGGACTTGCATTGTGAAAGCTTTGCTGTCCTTGATGTGATCATGGAATCTCTTTCTCAC
TAGAGTCTATGTCACTCATTATACTCTGTCGAATGTCATTGAATGTCTTTACATGGGCTT
GTATGCCTATGAAAATTGTAATACAACTTTCAGCAACGGATCTCTTGGCTCTCGCATCGA
TGAAGGACGCAGCGAAATGCGATAAGTAATGTGAATTGCAGAATTCAGTGAATCATCGAA
TCTTTGAACGCATCTTGCGCTCCTTGGTATTCCGAGGAGCATGCCTGTTTGAGTGTCATT
AAATTCTCAACTCTCTTATAC-TTTTTTGTAAAAGAGAGCTTGGACTGTGGAGGCTTGCT
GGCCACTTTTTGGGGTCAGCTCCTCTGAAATGCATTAGCGGAACCGTTTGCAATCTGCCA
CAAGTGTGATAAGTTATCTACACTGGCGAGGGGATTGCTCTCTGTAATGTTCAGCTTCTA
ATTGTCTCTACTTTGTGAGACAACTTTTGAATGCTTGACCTCAAATCAGGTAGGACTACC
CGCTGAACTTAA
>ABC1-6
TTTCCGTAGGTGAACCTGCGGAAGGATCATTATTGAATTATGTTTCTAGATAGGTTGTAG
CTGGCTC-TTTAGAGCATGTGCACGCCTGTTTGGACTTCATTTTCATCCACCTGTGCACC
TATTGTAGTCTTTGGTTGGGTTAGGGGGAAGTGGTCATTGTGTCAGCATCTGCTGGATGT
GAGGACTTGCATTGTGAAAGCTTTGCTGTCCTTGATGTGATCATGGAATCTCTTTCTCAC
TAGAGTCTATGTCACTCATTATACTCTGTCGAATGTCATTGAATGTCTTTACATGGGCTT
GTATGCCTATGAAAATTGTAATACAACTTTCAGCAACGGATCTCTTGGCTCTCGCATCGA
TGAAGGACGCAGCGAAATGCGATAAGTAATGTGAATTGCAGAATTCAGTGAATCATCGAA
TCTTTGAACGCATCTTGCGCTCCTTGGTATTCCGAGGAGCATGCCTGTTTGAGTGTCATT
AAATTCTCAACTCTCTTATAC-TTTTTTGTAAAAGAGAGCTTGGACTGTGGAGGCTTGCT

GGCCACTTTTTGGGGTCAGCTCCTCTGAAATGCATTAGCGGAACCGTTTGCAATCTGCCA
CAAGTGTGATAAGTTATCTACACTGGCGAGGGGATTGCTCTCTGTAATGTTCAGCTTCTA
ATTGTCTCTACTTTGTGAGACAACTTTTGAATGCTTGACCTCAAATCAGGTAGGACTACC
CGCTGAACTTAA
>ABC1-10
TTTCCGTAGGTGAACCTGCGGAAGGATCATTATTGAATTATGTTTCTAGATAGGTTGTAG
CTGGCTC-TTTAGAGCATGTGCACGCCTGTTTGGACTTCATTTTCATCCACCTGTGCACC
TATTGTAGTCTTTGGTTGGGTTAGGGGGAAGTGGTCATTGTGTCAGCATCTGCTGGATGT
GAGGACTTGCATTGTGAAAGCTTTGCTGTCCTTGATGTGATCATGGAATCTCTTTCTCAC
TAGAGTCTATGTCACTCATTATACTCTGTCGAATGTCATTGAATGTCTTTACATGGGCTT
GTATGCCTATGAAAATTGTAATACAACTTTCAGCAACGGATCTCTTGGCTCTCGCATCGA
TGAAGGACGCAGCGAAATGCGATAAGTAATGTGAATTGCAGAATTCAGTGAATCATCGAA
TCTTTGAACGCATCTTGCGCTCCTTGGTATTCCGAGGAGCATGCCTGTTTGAGTGTCATT
AAATTCTCAACTCTCTTATAC-TTTTTTGTAAAAGAGAGCTTGGACTGTGGAGGCTTGCT
GGCCACTTTTTGGGGTCAGCTCCTCTGAAATGCATTAGCGGAACCGTTTGCAATCTGCCA
CAAGTGTGATAAGTTATCTACACTGGCGAGGGGATTGCTCTCTGTAATGTTCAGCTTCTA
ATTGTCTCTACTTTGTGAGACAACTTTTGAATGCTTGACCTCAAATCAGGTAGGACTACC
CGCTGAACTTAA
>ABC1-17
TTTCCGTAGGTGAACCTGCGGAAGGATCATTATTGAATTATGTTTCTAGATAGGTTGTAG
CTGGCTC-TTTAGAGCATGTGCACGCCTGTTTGGACTTCATTTTCATCCACCTGTGCACC
TATTGTAGTCTTTGGTTGGGTTAGGGGGAAGTGGTCATTGTGTCAGCATCTGCTGGATGT
GAGGACTTGCATTGTGAAAGCTTTGCTGTCCTTGATGTGATCATGGAATCTCTTTCTCAC
TAGAGTCTATGTCACTCATTATACTCTGTCGAATGTCATTGAATGTCTTTACATGGGCTT
GTATGCCTATGAAAATTGTAATACAACTTTCAGCAACGGATCTCTTGGCTCTCGCATCGA
TGAAGGACGCAGCGAAATGCGATAAGTAATGTGAATTGCAGAATTCAGTGAATCATCGAA
TCTTTGAACGCATCTTGCGCTCCTTGGTATTCCGAGGAGCATGCCTGTTTGAGTGTCATT
AAATTCTCAACTCTCTTATAC-TTTTTTGTAAAAGAGAGCTTGGACTGTGGAGGCTTGCT
GGCCACTTTTTGGGGTCAGCTCCTCTGAAATGCATTAGCGGAACCGTTTGCAATCTGCCA
CAAGTGTGATAAGTTATCTACACTGGCGAGGGGATTGCTCTCTGTAATGTTCAGCTTCTA
ATTGTCTCTACTTTGTGAGACAACTTTTGAATGCTTGACCTCAAATCAGGTAGGACTACC
CGCTGAACTTAA
>ABC1-1
TTTCCGTAGGTGAACCTGCGGAAGGATCATTATTGAATTATGTTTCTAGATAGGTTGTAG
CTGGCTC-TTTAGAGCATGTGCACGCCTGTTTGGACTTCATTTTCATCCACCTGTGCACC
TATTGTAGTCTTTGGTTGGGTTAGGGGGAAGTGGTCATTGTGTCAGCATCTGCTGGATGT
GAGGACTTGCATTGTGAAAGCTTTGCTGTCCTTGATGTGATCATGGAATCTCTTTCTCAC
TAGAGTCTATGTCACTCATTATACTCTGTCGAATGTCATTGAATGTCTTTACATGGGCTT
GTATGCCTATGAAAATTGTAATACAACTTTCAGCAACGGATCTCTTGGCTCTCGCATCGA
TGAAGGACGCAGCGAAATGCGATAAGTAATGTGAATTGCAGAATTCAGTGAATCATCGAA
TCTTTGAACGCATCTTGCGCTCCTTGGTATTCCGAGGAGCATGCCTGTTTGAGTGTCATT
AAATTCTCAACTCTCTTATAC-TTTTTTGTAAAAGAGAGCTTGGACTGTGGAGGCTTGCT
GGCCACTTTTTGGGGTCAGCTCCTCTGAAATGCATTAGCGGAACCGTTTGCAATCTGCCA
CAAGTGTGATAAGTTATCTACACTGGCGAGGGGATTGCTCTCTGTAATGTTCAGCTTCTA
ATTGTCTCTACTTTGTGAGACAACTTTTGAATGCTTGACCTCAAATCAGGTAGGACTACC
CGCTGAACTTAA
>ABC1-59
TTTCCGTAGGTGAACCTGCGGAAGGATCATTATTGAATTATGTTTCTAGATAGGTTGTAG
CTGGCTC-TTTAGAGCATGTGCACGCCTGTTTGGACTTCATTTTCATCCACCTGTGCACC
TATTGTAGTCTTTGGTTGGGTTAGGGGGAAGTGGTCATTGTGTCAGCATCTGCTGGATGT

GAGGACTTGCATTGTGAAAGCTTTGCTGTCCTTGATGTGATCATGGAATCTCTTTCTCAC
TAGAGTCTATGTCACTCATTATACTCTGTCGAATGTCATTGAATGTCTTTACATGGGCTT
GTATGCCTATGAAAATTGTAATACAACTTTCAGCAACGGATCTCTTGGCTCTCGCATCGA
TGAAGGACGCAGCGAAATGCGATAAGTAATGTGAATTGCAGAATTCAGTGAATCATCGAA
TCTTTGAACGCATCTTGCGCTCCTTGGTATTCCGAGGAGCATGCCTGTTTGAGTGTCATT
AAATTCTCAACTCTCTTATAC-TTTTTTGTAAAAGAGAGCTTGGACTGTGGAGGCTTGCT
GGCCACTTTTTGGGGTCAGCTCCTCTGAAATGCATTAGCGGAACCGTTTGCAATCTGCCA
CAAGTGTGATAAGTTATCTACACTGGCGAGGGGATTGCTCTCTGTAATGTTCAGCTTCTA
ATTGTCTCTACTTTGTGAGACAACTTTTGAATGCTTGACCTCAAATCAGGTAGGACTACC
CGCTGAACTTAA
>ABC4-58
TTTCCGTAGGTGAACCTGCGGAAGGATCATTATTGAATTATGTTTCTAGATAGGTTGTAG
CTGGCTC-TTTAGAGCATGTGCACGCCTGTTTGGACTTCATTTTCATCCACCTGTGCACC
TATTGTAGTCTTTGGTTGGGTTAGGGGGAAGTGGTCATTGTGTCAGCATCTGCTGGATGT
GAGGACTTGCATTGTGAAAGCTTTGCTGTCCTTGATGTGATCATGGAATCTCTTTCTCAC
TAGAGTCTATGTCACTCATTATACTCTGTCGAATGTCATTGAATGTCTTTACATGGGCTT
GTATGCCTATGAAAATTGTAATACAACTTTCAGCAACGGATCTCTTGGCTCTCGCATCGA
TGAAGGACGCAGCGAAATGCGATAAGTAATGTGAATTGCAGAATTCAGTGAATCATCGAA
TCTTTGAACGCATCTTGCGCTCCTTGGTATTCCGAGGAGCATGCCTGTTTGAGTGTCATT
AAATTCTCAACTCTCTTATAC-TTTTTTGTAAAAGAGAGCTTGGACTGTGGAGGCTTGCT
GGCCACTTTTTGGGGTCAGCTCCTCTGAAATGCATTAGCGGAACCGTTTGCAATCTGCCA
CAAGTGTGATAAGTTATCTACACTGGCGAGGGGATTGCTCTCTGTAATGTTCAGCTTCTA
ATTGTCTCTACTTTGTGAGACAACTTTTGAATGCTTGACCTCAAATCAGGTAGGACTACC
CGCTGAACTTAA
>ABC6-15
TTTCCGTAGGTGAACCTGCGGAAGGATCATTATTGAATTATGTTTCTAGATAGGTTGTAG
CTGGCTC-TTTAGAGCATGTGCACGCCTGTTTGGACTTCATTTTCATCCACCTGTGCACC
TATTGTAGTCTTTGGTTGGGTTAGGGGGAAGTGGTCATTGTGTCAGCATCTGCTGGATGT
GAGGACTTGCATTGTGAAAGCTTTGCTGTCCTTGATGTGATCATGGAATCTCTTTCTCAC
TAGAGTCTATGTCACTCATTATACTCTGTCGAATGTCATTGAATGTCTTTACATGGGCTT
GTATGCCTATGAAAATTGTAATACAACTTTCAGCAACGGATCTCTTGGCTCTCGCATCGA
TGAAGGACGCAGCGAAATGCGATAAGTAATGTGAATTGCAGAATTCAGTGAATCATCGAA
TCTTTGAACGCATCTTGCGCTCCTTGGTATTCCGAGGAGCATGCCTGTTTGAGTGTCATT
AAATTCTCAACTCTCTTATAC-TTTTTTGTAAAAGAGAGCTTGGACTGTGGAGGCTTGCT
GGCCACTTTTTGGGGTCAGCTCCTCTGAAATGCATTAGCGGAACCGTTTGCAATCTGCCA
CAAGTGTGATAAGTTATCTACACTGGCGAGGGGATTGCTCTCTGTAATGTTCAGCTTCTA
ATTGTCTCTACTTTGTGAGACAACTTTTGAATGCTTGACCTCAAATCAGGTAGGACTACC
CGCTGAACTTAA
>ABC6-42
TTTCCGTAGGTGAACCTGCGGAAGGATCATTATTGAATTATGTTTCTAGATAGGTTGTAG
CTGGCTC-TTTAGAGCATGTGCACGCCTGTTTGGACTTCATTTTCATCCACCTGTGCACC
TATTGTAGTCTTTGGTTGGGTTAGGGGGAAGTGGTCATTGTGTCAGCATCTGCTGGATGT
GAGGACTTGCATTGTGAAAGCTTTGCTGTCCTTGATGTGATCATGGAATCTCTTTCTCAC
TAGAGTCTATGTCACTCATTATACTCTGTCGAATGTCATTGAATGTCTTTACATGGGCTT
GTATGCCTATGAAAATTGTAATACAACTTTCAGCAACGGATCTCTTGGCTCTCGCATCGA
TGAAGGACGCAGCGAAATGCGATAAGTAATGTGAATTGCAGAATTCAGTGAATCATCGAA
TCTTTGAACGCATCTTGCGCTCCTTGGTATTCCGAGGAGCATGCCTGTTTGAGTGTCATT
AAATTCTCAACTCTCTTATAC-TTTTTTGTAAAAGAGAGCTTGGACTGTGGAGGCTTGCT
GGCCACTTTTTGGGGTCAGCTCCTCTGAAATGCATTAGCGGAACCGTTTGCAATCTGCCA
CAAGTGTGATAAGTTATCTACACTGGCGAGGGGATTGCTCTCTGTAATGTTCAGCTTCTA

ATTGTCTCTACTTTGTGAGACAACTTTTGAATGCTTGACCTCAAATCAGGTAGGACTACC
CGCTGAACTTAA
>ABC9-20
TTTCCGTAGGTGAACCTGCGGAAGGATCATTATTGAATTATGTTTCTAGATAGGTTGTAG
CTGGCTC-TTTAGAGCATGTGCACGCCTGTTTGGACTTCATTTTCATCCACCTGTGCACC
TATTGTAGTCTTTGGTTGGGTTAGGGGGAAGTGGTCATTGTGTCAGCATCTGCTGGATGT
GAGGACTTGCATTGTGAAAGCTTTGCTGTCCTTGATGTGATCATGGAATCTCTTTCTCAC
TAGAGTCTATGTCACTCATTATACTCTGTCGAATGTCATTGAATGTCTTTACATGGGCTT
GTATGCCTATGAAAATTGTAATACAACTTTCAGCAACGGATCTCTTGGCTCTCGCATCGA
TGAAGGACGCAGCGAAATGCGATAAGTAATGTGAATTGCAGAATTCAGTGAATCATCGAA
TCTTTGAACGCATCTTGCGCTCCTTGGTATTCCGAGGAGCATGCCTGTTTGAGTGTCATT
AAATTCTCAACTCTCTTATAC-TTTTTTGTAAAAGAGAGCTTGGACTGTGGAGGCTTGCT
GGCCACTTTTTGGGGTCAGCTCCTCTGAAATGCATTAGCGGAACCGTTTGCAATCTGCCA
CAAGTGTGATAAGTTATCTACACTGGCGAGGGGATTGCTCTCTGTAATGTTCAGCTTCTA
ATTGTCTCTACTTTGTGAGACAACTTTTGAATGCTTGACCTCAAATCAGGTAGGACTACC
CGCTGAACTTAA
>ABC8-29
TTTCCGTAGGTGAACCTGCGGAAGGATCATTATTGAATTATGTTTCTAGATAGGTTGTAG
CTGGCTC-TTTAGAGCATGTGCACGCCTGTTTGGACTTCATTTTCATCCACCTGTGCACC
TATTGTAGTCTTTGGTTGGGTTAGGGGGAAGTGGTCATTGTGTCAGCATCTGCTGGATGT
GAGGACTTGCATTGTGAAAGCTTTGCTGTCCTTGATGTGATCATGGAATCTCTTTCTCAC
TAGAGTCTATGTCACTCATTATACTCTGTCGAATGTCATTGAATGTCTTTACATGGGCTT
GTATGCCTATGAAAATTGTAATACAACTTTCAGCAACGGATCTCTTGGCTCTCGCATCGA
TGAAGGACGCAGCGAAATGCGATAAGTAATGTGAATTGCAGAATTCAGTGAATCATCGAA
TCTTTGAACGCATCTTGCGCTCCTTGGTATTCCGAGGAGCATGCCTGTTTGAGTGTCATT
AAATTCTCAACTCTCTTATAC-TTTTTTGTAAAAGAGAGCTTGGACTGTGGAGGCTTGCT
GGCCACTTTTTGGGGTCAGCTCCTCTGAAATGCATTAGCGGAACCGTTTGCAATCTGCCA
CAAGTGTGATAAGTTATCTACACTGGCGAGGGGATTGCTCTCTGTAATGTTCAGCTTCTA
ATTGTCTCTACTTTGTGAGACAACTTTTGAATGCTTGACCTCAAATCAGGTAGGACTACC
CGCTGAACTTAA
>ABC2-1
TTTCCGTAGGTGAACCTGCGGAAGGATCATTATTGAATTATGTTTCTAGATAGGTTGTAG
CTGGCTC-TTTAGAGCATGTGCACGCCTGTTTGGACTTCATTTTCATCCACCTGTGCACC
TATTGTAGTCTTTGGTTGGGTTAGGGGGAAGTGGTCATTGTGTCAGCATCTGCTGGATGT
GAGGACTTGCATTGTGAAAGCTTTGCTGTCCTTGATGTGATCATGGAATCTCTTTCTCAC
TAGAGTCTATGTCACTCATTATACTCTGTCGAATGTCATTGAATGTCTTTACATGGGCTT
GTATGCCTATGAAAATTGTAATACAACTTTCAGCAACGGATCTCTTGGCTCTCGCATCGA
TGAAGGACGCAGCGAAATGCGATAAGTAATGTGAATTGCAGAATTCAGTGAATCATCGAA
TCTTTGAACGCATCTTGCGCTCCTTGGTATTCCGAGGAGCATGCCTGTTTGAGTGTCATT
AAATTCTCAACTCTCTTATAC-TTTTTTGTAAAAGAGAGCTTGGACTGTGGAGGCTTGCT
GGCCACTTTTTGGGGTCAGCTCCTCTGAAATGCATTAGCGGAACCGTTTGCAATCTGCCA
CAAGTGTGATAAGTTATCTACACTGGCGAGGGGATTGCTCTCTGTAATGTTCAGCTTCTA
ATTGTCTCTACTTTGTGAGACAACTTTTGAATGCTTGACCTCAAATCAGGTAGGACTACC
CGCTGAACTTAA
>ABC6-13
TTTCCGTAGGTGAACCTGCGGAAGGATCATTATTGAATTATGTTTCTAGATAGGTTGTAG
CTGGCTC-TTTAGAGCATGTGCACGCCTGTTTGGACTTCATTTTCATCCACCTGTGCACC
TATTGTAGTCTTTGGTTGGGTTAGGGGGAAGTGGTCATTGTGTCAGCATCTGCTGGATGT
GAGGACTTGCATTGTGAAAGCTTTGCTGTCCTTGATGTGATCATGGAATCTCTTTCTCAC
TAGAGTCTATGTCACTCATTATACTCTGTCGAATGTCATTGAATGTCTTTACATGGGCTT

GTATGCCTATGAAAATTGTAATACAACTTTCAGCAACGGATCTCTTGGCTCTCGCATCGA
TGAAGGACGCAGCGAAATGCGATAAGTAATGTGAATTGCAGAATTCAGTGAATCATCGAA
TCTTTGAACGCATCTTGCGCTCCTTGGTATTCCGAGGAGCATGCCTGTTTGAGTGTCATT
AAATTCTCAACTCTCTTATAC-TTTTTTGTAAAAGAGAGCTTGGACTGTGGAGGCTTGCT
GGCCACTTTTTGGGGTCAGCTCCTCTGAAATGCATTAGCGGAACCGTTTGCAATCTGCCA
CAAGTGTGATAAGTTATCTACACTGGCGAGGGGATTGCTCTCTGTAATGTTCAGCTTCTA
ATTGTCTCTACTTTGTGAGACAACTTTTGAATGCTTGACCTCAAATCAGGTAGGACTACC
CGCTGAACTTAA
>ABC4-25
TTTCCGTAGGTGAACCTGCGGAAGGATCATTATTGAATTATGTTTCTAGATAGGTTGTAG
CTGGCTC-TTTAGAGCATGTGCACGCCTGTTTGGACTTCATTTTCATCCACCTGTGCACC
TATTGTAGTCTTTGGTTGGGTTAGGGGGAAGTGGTCATTGTGTCAGCATCTGCTGGATGT
GAGGACTTGCATTGTGAAAGCTTTGCTGTCCTTGATGTGATCATGGAATCTCTTTCTCAC
TAGAGTCTATGTCACTCATTATACTCTGTCGAATGTCATTGAATGTCTTTACATGGGCTT
GTATGCCTATGAAAATTGTAATACAACTTTCAGCAACGGATCTCTTGGCTCTCGCATCGA
TGAAGGACGCAGCGAAATGCGATAAGTAATGTGAATTGCAGAATTCAGTGAATCATCGAA
TCTTTGAACGCATCTTGCGCTCCTTGGTATTCCGAGGAGCATGCCTGTTTGAGTGTCATT
AAATTCTCAACTCTCTTATAC-TTTTTTGTAAAAGAGAGCTTGGACTGTGGAGGCTTGCT
GGCCACTTTTTGGGGTCAGCTCCTCTGAAATGCATTAGCGGAACCGTTTGCAATCTGCCA
CAAGTGTGATAAGTTATCTACACTGGCGAGGGGATTGCTCTCTGTAATGTTCAGCTTCTA
ATTGTCTCTACTTTGTGAGACAACTTTTGAATGCTTGACCTCAAATCAGGTAGGACTACC
CGCTGAACTTAA
>ABC7-28
TTTCCGTAGGTGAACCTGCGGAAGGATCATTATTGAATTATGTTTCTAGATAGGTTGTAG
CTGGCTC-TTTAGAGCATGTGCACGCCTGTTTGGACTTCATTTTCATCCACCTGTGCACC
TATTGTAGTCTTTGGTTGGGTTAGGGGGAAGTGGTCATTGTGTCAGCATCTGCTGGATGT
GAGGACTTGCATTGTGAAAGCTTTGCTGTCCTTGATGTGATCATGGAATCTCTTTCTCAC
TAGAGTCTATGTCACTCATTATACTCTGTCGAATGTCATTGAATGTCTTTACATGGGCTT
GTATGCCTATGAAAATTGTAATACAACTTTCAGCAACGGATCTCTTGGCTCTCGCATCGA
TGAAGGACGCAGCGAAATGCGATAAGTAATGTGAATTGCAGAATTCAGTGAATCATCGAA
TCTTTGAACGCATCTTGCGCTCCTTGGTATTCCGAGGAGCATGCCTGTTTGAGTGTCATT
AAATTCTCAACTCTCTTATAC-TTTTTTGTAAAAGAGAGCTTGGACTGTGGAGGCTTGCT
GGCCACTTTTTGGGGTCAGCTCCTCTGAAATGCATTAGCGGAACCGTTTGCAATCTGCCA
CAAGTGTGATAAGTTATCTACACTGGCGAGGGGATTGCTCTCTGTAATGTTCAGCTTCTA
ATTGTCTCTACTTTGTGAGACAACTTTTGAATGCTTGACCTCAAATCAGGTAGGACTACC
CGCTGAACTTAA
>ABC9-21
TTTCCGTAGGTGAACCTGCGGAAGGATCATTATTGAATTATGTTTCTAGATAGGTTGTAG
CTGGCTC-TTTAGAGCATGTGCACGCCTGTTTGGACTTCATTTTCATCCACCTGTGCACC
TATTGTAGTCTTTGGTTGGGTTAGGGGGAAGTGGTCATTGTGTCAGCATCTGCTGGATGT
GAGGACTTGCATTGTGAAAGCTTTGCTGTCCTTGATGTGATCATGGAATCTCTTTCTCAC
TAGAGTCTATGTCACTCATTATACTCTGTCGAATGTCATTGAATGTCTTTACATGGGCTT
GTATGCCTATGAAAATTGTAATACAACTTTCAGCAACGGATCTCTTGGCTCTCGCATCGA
TGAAGGACGCAGCGAAATGCGATAAGTAATGTGAATTGCAGAATTCAGTGAATCATCGAA
TCTTTGAACGCATCTTGCGCTCCTTGGTATTCCGAGGAGCATGCCTGTTTGAGTGTCATT
AAATTCTCAACTCTCTTATAC-TTTTTTGTAAAAGAGAGCTTGGACTGTGGAGGCTTGCT
GGCCACTTTTTGGGGTCAGCTCCTCTGAAATGCATTAGCGGAACCGTTTGCAATCTGCCA
CAAGTGTGATAAGTTATCTACACTGGCGAGGGGATTGCTCTCTGTAATGTTCAGCTTCTA
ATTGTCTCTACTTTGTGAGACAACTTTTGAATGCTTGACCTCAAATCAGGTAGGACTACC
CGCTGAACTTAA

>ABC10-14
TTTCCGTAGGTGAACCTGCGGAAGGATCATTATTGAATTATGTTTCTAGATAGGTTGTAG
CTGGCTC-TTTAGAGCATGTGCACGCCTGTTTGGACTTCATTTTCATCCACCTGTGCACC
TATTGTAGTCTTTGGTTGGGTTAGGGGGAAGTGGTCATTGTGTCAGCATCTGCTGGATGT
GAGGACTTGCATTGTGAAAGCTTTGCTGTCCTTGATGTGATCATGGAATCTCTTTCTCAC
TAGAGTCTATGTCACTCATTATACTCTGTCGAATGTCATTGAATGTCTTTACATGGGCTT
GTATGCCTATGAAAATTGTAATACAACTTTCAGCAACGGATCTCTTGGCTCTCGCATCGA
TGAAGGACGCAGCGAAATGCGATAAGTAATGTGAATTGCAGAATTCAGTGAATCATCGAA
TCTTTGAACGCATCTTGCGCTCCTTGGTATTCCGAGGAGCATGCCTGTTTGAGTGTCATT
AAATTCTCAACTCTCTTATAC-TTTTTTGTAAAAGAGAGCTTGGACTGTGGAGGCTTGCT
GGCCACTTTTTGGGGTCAGCTCCTCTGAAATGCATTAGCGGAACCGTTTGCAATCTGCCA
CAAGTGTGATAAGTTATCTACACTGGCGAGGGGATTGCTCTCTGTAATGTTCAGCTTCTA
ATTGTCTCTACTTTGTGAGACAACTTTTGAATGCTTGACCTCAAATCAGGTAGGACTACC
CGCTGAACTTAA
>ABC10-30
TTTCCGTAGGTGAACCTGCGGAAGGATCATTATTGAATTATGTTTCTAGATAGGTTGTAG
CTGGCTC-TTTAGAGCATGTGCACGCCTGTTTGGACTTCATTTTCATCCACCTGTGCACC
TATTGTAGTCTTTGGTTGGGTTAGGGGGAAGTGGTCATTGTGTCAGCATCTGCTGGATGT
GAGGACTTGCATTGTGAAAGCTTTGCTGTCCTTGATGTGATCATGGAATCTCTTTCTCAC
TAGAGTCTATGTCACTCATTATACTCTGTCGAATGTCATTGAATGTCTTTACATGGGCTT
GTATGCCTATGAAAATTGTAATACAACTTTCAGCAACGGATCTCTTGGCTCTCGCATCGA
TGAAGGACGCAGCGAAATGCGATAAGTAATGTGAATTGCAGAATTCAGTGAATCATCGAA
TCTTTGAACGCATCTTGCGCTCCTTGGTATTCCGAGGAGCATGCCTGTTTGAGTGTCATT
AAATTCTCAACTCTCTTATAC-TTTTTTGTAAAAGAGAGCTTGGACTGTGGAGGCTTGCT
GGCCACTTTTTGGGGTCAGCTCCTCTGAAATGCATTAGCGGAACCGTTTGCAATCTGCCA
CAAGTGTGATAAGTTATCTACACTGGCGAGGGGATTGCTCTCTGTAATGTTCAGCTTCTA
ATTGTCTCTACTTTGTGAGACAACTTTTGAATGCTTGACCTCAAATCAGGTAGGACTACC
CGCTGAACTTAA
>ABC11-32
TTTCCGTAGGTGAACCTGCGGAAGGATCATTATTGAATTATGTTTCTAGATAGGTTGTAG
CTGGCTC-TTTAGAGCATGTGCACGCCTGTTTGGACTTCATTTTCATCCACCTGTGCACC
TATTGTAGTCTTTGGTTGGGTTAGGGGGAAGTGGTCATTGTGTCAGCATCTGCTGGATGT
GAGGACTTGCATTGTGAAAGCTTTGCTGTCCTTGATGTGATCATGGAATCTCTTTCTCAC
TAGAGTCTATGTCACTCATTATACTCTGTCGAATGTCATTGAATGTCTTTACATGGGCTT
GTATGCCTATGAAAATTGTAATACAACTTTCAGCAACGGATCTCTTGGCTCTCGCATCGA
TGAAGGACGCAGCGAAATGCGATAAGTAATGTGAATTGCAGAATTCAGTGAATCATCGAA
TCTTTGAACGCATCTTGCGCTCCTTGGTATTCCGAGGAGCATGCCTGTTTGAGTGTCATT
AAATTCTCAACTCTCTTATAC-TTTTTTGTAAAAGAGAGCTTGGACTGTGGAGGCTTGCT
GGCCACTTTTTGGGGTCAGCTCCTCTGAAATGCATTAGCGGAACCGTTTGCAATCTGCCA
CAAGTGTGATAAGTTATCTACACTGGCGAGGGGATTGCTCTCTGTAATGTTCAGCTTCTA
ATTGTCTCTACTTTGTGAGACAACTTTTGAATGCTTGACCTCAAATCAGGTAGGACTACC
CGCTGAACTTAA
>ABC10-13
TTTCCGTAGGTGAACCTGCGGAAGGATCATTATTGAATTATGTTTCTAGATAGGTTGTAG
CTGGCTC-TTTAGAGCATGTGCACGCCTGTTTGGACTTCATTTTCATCCACCTGTGCACC
TATTGTAGTCTTTGGTTGGGTTAGGGGGAAGTGGTCATTGTGTCAGCATCTGCTGGATGT
GAGGACTTGCATTGTGAAAGCTTTGCTGTCCTTGATGTGATCATGGAATCTCTTTCTCAC
TAGAGTCTATGTCACTCATTATACTCTGTCGAATGTCATTGAATGTCTTTACATGGGCTT
GTATGCCTATGAAAATTGTAATACAACTTTCAGCAACGGATCTCTTGGCTCTCGCATCGA
TGAAGGACGCAGCGAAATGCGATAAGTAATGTGAATTGCAGAATTCAGTGAATCATCGAA

TCTTTGAACGCATCTTGCGCTCCTTGGTATTCCGAGGAGCATGCCTGTTTGAGTGTCATT
AAATTCTCAACTCTCTTATAC–TTTTTTGTAAAAGAGAGCTTGGACTGTGGAGGCTTGCT
GGCCACTTTTTGGGGTCAGCTCCTCTGAAATGCATTAGCGGAACCGTTTGCAATCTGCCA
CAAGTGTGATAAGTTATCTACACTGGCGAGGGGATTGCTCTCTGTAATGTTCAGCTTCTA
ATTGTCTCTACTTTGTGAGACAACTTTTGAATGCTTGACCTCAAATCAGGTAGGACTACC
CGCTGAACTTAA
>ABC6–31
TTTCCGTAGGTGAACCTGCGGAAGGATCATTATTGAATTATGTTTCTAGATAGGTTGTAG
CTGGCTC–TTTAGAGCATGTGCACGCCTGTTTGGACTTCATTTTCATCCACCTGTGCACC
TATTGTAGTCTTTGGTTGGGTTAGGGGGAAGTGGTCATTGTGTCAGCATCTGCTGGATGT
GAGGACTTGCATTGTGAAAGCTTTGCTGTCCTTGATGTGATCATGGAATCTCTTTCTCAC
TAGAGTCTATGTCACTCATTATACTCTGTCGAATGTCATTGAATGTCTTTACATGGGCTT
GTATGCCTATGAAAATTGTAATACAACTTTCAGCAACGGATCTCTTGGCTCTCGCATCGA
TGAAGGACGCAGCGAAATGCGATAAGTAATGTGAATTGCAGAATTCAGTGAATCATCGAA
TCTTTGAACGCATCTTGCGCTCCTTGGTATTCCGAGGAGCATGCCTGTTTGAGTGTCATT
AAATTCTCAACTCTCTTATAC–TTTTTTGTAAAAGAGAGCTTGGACTGTGGAGGCTTGCT
GGCCACTTTTTGGGGTCAGCTCCTCTGAAATGCATTAGCGGAACCGTTTGCAATCTGCCA
CAAGTGTGATAAGTTATCTACACTGGCGAGGGGATTGCTCTCTGTAATGTTCAGCTTCTA
ATTGTCTCTACTTTGTGAGACAACTTTTGAATGCTTGACCTCAAATCAGGTAGGACTACC
CGCTGAACTTAA
>ABC3–7
TTTCCGTAGGTGAACCTGCGGAAGGATCATTATTGAATTATGTTTCTAGATAGGTTGTAG
CTGGCTC–TTTAGAGCATGTGCACGCCTGTTTGGACTTCATTTTCATCCACCTGTGCACC
TATTGTAGTCTTTGGTTGGGTTAGGGGGAAGTGGTCATTGTGTCAGCATCTGCTGGATGT
GAGGACTTGCATTGTGAAAGCTTTGCTGTCCTTGATGTGATCATGGAATCTCTTTCTCAC
TAGAGTCTATGTCACTCATTATACTCTGTCGAATGTCATTGAATGTCTTTACATGGGCTT
GTATGCCTATGAAAATTGTAATACAACTTTCAGCAACGGATCTCTTGGCTCTCGCATCGA
TGAAGGACGCAGCGAAATGCGATAAGTAATGTGAATTGCAGAATTCAGTGAATCATCGAA
TCTTTGAACGCATCTTGCGCTCCTTGGTATTCCGAGGAGCATGCCTGTTTGAGTGTCATT
AAATTCTCAACTCTCTTATAC–TTTTTTGTAAAAGAGAGCTTGGACTGTGGAGGCTTGCT
GGCCACTTTTTGGGGTCAGCTCCTCTGAAATGCATTAGCGGAACCGTTTGCAATCTGCCA
CAAGTGTGATAAGTTATCTACACTGGCGAGGGGATTGCTCTCTGTAATGTTCAGCTTCTA
ATTGTCTCTACTTTGTGAGACAACTTTTGAATGCTTGACCTCAAATCAGGTAGGACTACC
CGCTGAACTTAA
>ABC3–23
TTTCCGTAGGTGAACCTGCGGAAGGATCATTATTGAATTATGTTTCTAGATAGGTTGTAG
CTGGCTC–TTTAGAGCATGTGCACGCCTGTTTGGACTTCATTTTCATCCACCTGTGCACC
TATTGTAGTCTTTGGTTGGGTTAGGGGGAAGTGGTCATTGTGTCAGCATCTGCTGGATGT
GAGGACTTGCATTGTGAAAGCTTTGCTGTCCTTGATGTGATCATGGAATCTCTTTCTCAC
TAGAGTCTATGTCACTCATTATACTCTGTCGAATGTCATTGAATGTCTTTACATGGGCTT
GTATGCCTATGAAAATTGTAATACAACTTTCAGCAACGGATCTCTTGGCTCTCGCATCGA
TGAAGGACGCAGCGAAATGCGATAAGTAATGTGAATTGCAGAATTCAGTGAATCATCGAA
TCTTTGAACGCATCTTGCGCTCCTTGGTATTCCGAGGAGCATGCCTGTTTGAGTGTCATT
AAATTCTCAACTCTCTTATAC–TTTTTTGTAAAAGAGAGCTTGGACTGTGGAGGCTTGCT
GGCCACTTTTTGGGGTCAGCTCCTCTGAAATGCATTAGCGGAACCGTTTGCAATCTGCCA
CAAGTGTGATAAGTTATCTACACTGGCGAGGGGATTGCTCTCTGTAATGTTCAGCTTCTA
ATTGTCTCTACTTTGTGAGACAACTTTTGAATGCTTGACCTCAAATCAGGTAGGACTACC
CGCTGAACTTAA
>ABC4–1
TTTCCGTAGGTGAACCTGCGGAAGGATCATTATTGAATTATGTTTCTAGATAGGTTGTAG

CTGGCTC-TTTAGAGCATGTGCACGCCTGTTTGGACTTCATTTTCATCCACCTGTGCACC
TATTGTAGTCTTTGGTTGGGTTAGGGGGAAGTGGTCATTGTGTCAGCATCTGCTGGATGT
GAGGACTTGCATTGTGAAAGCTTTGCTGTCCTTGATGTGATCATGGAATCTCTTTCTCAC
TAGAGTCTATGTCACTCATTATACTCTGTCGAATGTCATTGAATGTCTTTACATGGGCTT
GTATGCCTATGAAAATTGTAATACAACTTTCAGCAACGGATCTCTTGGCTCTCGCATCGA
TGAAGGACGCAGCGAAATGCGATAAGTAATGTGAATTGCAGAATTCAGTGAATCATCGAA
TCTTTGAACGCATCTTGCGCTCCTTGGTATTCCGAGGAGCATGCCTGTTTGAGTGTCATT
AAATTCTCAACTCTCTTATAC-TTTTTTGTAAAAGAGAGCTTGGACTGTGGAGGCTTGCT
GGCCACTTTTTGGGGTCAGCTCCTCTGAAATGCATTAGCGGAACCGTTTGCAATCTGCCA
CAAGTGTGATAAGTTATCTACACTGGCGAGGGGATTGCTCTCTGTAATGTTCAGCTTCTA
ATTGTCTCTACTTTGTGAGACAACTTTTGAATGCTTGACCTCAAATCAGGTAGGACTACC
CGCTGAACTTAA
>ABC4-35
TTTCCGTAGGTGAACCTGCGGAAGGATCATTATTGAATTATGTTTCTAGATAGGTTGTAG
CTGGCTC-TTTAGAGCATGTGCACGCCTGTTTGGACTTCATTTTCATCCACCTGTGCACC
TATTGTAGTCTTTGGTTGGGTTAGGGGGAAGTGGTCATTGTGTCAGCATCTGCTGGATGT
GAGGACTTGCATTGTGAAAGCTTTGCTGTCCTTGATGTGATCATGGAATCTCTTTCTCAC
TAGAGTCTATGTCACTCATTATACTCTGTCGAATGTCATTGAATGTCTTTACATGGGCTT
GTATGCCTATGAAAATTGTAATACAACTTTCAGCAACGGATCTCTTGGCTCTCGCATCGA
TGAAGGACGCAGCGAAATGCGATAAGTAATGTGAATTGCAGAATTCAGTGAATCATCGAA
TCTTTGAACGCATCTTGCGCTCCTTGGTATTCCGAGGAGCATGCCTGTTTGAGTGTCATT
AAATTCTCAACTCTCTTATAC-TTTTTTGTAAAAGAGAGCTTGGACTGTGGAGGCTTGCT
GGCCACTTTTTGGGGTCAGCTCCTCTGAAATGCATTAGCGGAACCGTTTGCAATCTGCCA
CAAGTGTGATAAGTTATCTACACTGGCGAGGGGATTGCTCTCTGTAATGTTCAGCTTCTA
ATTGTCTCTACTTTGTGAGACAACTTTTGAATGCTTGACCTCAAATCAGGTAGGACTACC
CGCTGAACTTAA
>ABC5-51
TTTCCGTAGGTGAACCTGCGGAAGGATCATTATTGAATTATGTTTCTAGATAGGTTGTAG
CTGGCTC-TTTAGAGCATGTGCACGCCTGTTTGGACTTCATTTTCATCCACCTGTGCACC
TATTGTAGTCTTTGGTTGGGTTAGGGGGAAGTGGTCATTGTGTCAGCATCTGCTGGATGT
GAGGACTTGCATTGTGAAAGCTTTGCTGTCCTTGATGTGATCATGGAATCTCTTTCTCAC
TAGAGTCTATGTCACTCATTATACTCTGTCGAATGTCATTGAATGTCTTTACATGGGCTT
GTATGCCTATGAAAATTGTAATACAACTTTCAGCAACGGATCTCTTGGCTCTCGCATCGA
TGAAGGACGCAGCGAAATGCGATAAGTAATGTGAATTGCAGAATTCAGTGAATCATCGAA
TCTTTGAACGCATCTTGCGCTCCTTGGTATTCCGAGGAGCATGCCTGTTTGAGTGTCATT
AAATTCTCAACTCTCTTATAC-TTTTTTGTAAAAGAGAGCTTGGACTGTGGAGGCTTGCT
GGCCACTTTTTGGGGTCAGCTCCTCTGAAATGCATTAGCGGAACCGTTTGCAATCTGCCA
CAAGTGTGATAAGTTATCTACACTGGCGAGGGGATTGCTCTCTGTAATGTTCAGCTTCTA
ATTGTCTCTACTTTGTGAGACAACTTTTGAATGCTTGACCTCAAATCAGGTAGGACTACC
CGCTGAACTTAA
>ABC5-66
TTTCCGTAGGTGAACCTGCGGAAGGATCATTATTGAATTATGTTTCTAGATAGGTTGTAG
CTGGCTC-TTTAGAGCATGTGCACGCCTGTTTGGACTTCATTTTCATCCACCTGTGCACC
TATTGTAGTCTTTGGTTGGGTTAGGGGGAAGTGGTCATTGTGTCAGCATCTGCTGGATGT
GAGGACTTGCATTGTGAAAGCTTTGCTGTCCTTGATGTGATCATGGAATCTCTTTCTCAC
TAGAGTCTATGTCACTCATTATACTCTGTCGAATGTCATTGAATGTCTTTACATGGGCTT
GTATGCCTATGAAAATTGTAATACAACTTTCAGCAACGGATCTCTTGGCTCTCGCATCGA
TGAAGGACGCAGCGAAATGCGATAAGTAATGTGAATTGCAGAATTCAGTGAATCATCGAA
TCTTTGAACGCATCTTGCGCTCCTTGGTATTCCGAGGAGCATGCCTGTTTGAGTGTCATT
AAATTCTCAACTCTCTTATAC-TTTTTTGTAAAAGAGAGCTTGGACTGTGGAGGCTTGCT

GGCCACTTTTTGGGGTCAGCTCCTCTGAAATGCATTAGCGGAACCGTTTGCAATCTGCCA
CAAGTGTGATAAGTTATCTACACTGGCGAGGGGATTGCTCTCTGTAATGTTCAGCTTCTA
ATTGTCTCTACTTTGTGAGACAACTTTTGAATGCTTGACCTCAAATCAGGTAGGACTACC
CGCTGAACTTAA
>ABC7-15
TTTCCGTAGGTGAACCTGCGGAAGGATCATTATTGAATTATGTTTCTAGATAGGTTGTAG
CTGGCTC-TTTAGAGCATGTGCACGCCTGTTTGGACTTCATTTTCATCCACCTGTGCACC
TATTGTAGTCTTTGGTTGGGTTAGGGGGAAGTGGTCATTGTGTCAGCATCTGCTGGATGT
GAGGACTTGCATTGTGAAAGCTTTGCTGTCCTTGATGTGATCATGGAATCTCTTTCTCAC
TAGAGTCTATGTCACTCATTATACTCTGTCGAATGTCATTGAATGTCTTTACATGGGCTT
GTATGCCTATGAAAATTGTAATACAACTTTCAGCAACGGATCTCTTGGCTCTCGCATCGA
TGAAGGACGCAGCGAAATGCGATAAGTAATGTGAATTGCAGAATTCAGTGAATCATCGAA
TCTTTGAACGCATCTTGCGCTCCTTGGTATTCCGAGGAGCATGCCTGTTTGAGTGTCATT
AAATTCTCAACTCTCTTATAC-TTTTTTGTAAAAGAGAGCTTGGACTGTGGAGGCTTGCT
GGCCACTTTTTGGGGTCAGCTCCTCTGAAATGCATTAGCGGAACCGTTTGCAATCTGCCA
CAAGTGTGATAAGTTATCTACACTGGCGAGGGGATTGCTCTCTGTAATGTTCAGCTTCTA
ATTGTCTCTACTTTGTGAGACAACTTTTGAATGCTTGACCTCAAATCAGGTAGGACTACC
CGCTGAACTTAA
>ABC7-31
TTTCCGTAGGTGAACCTGCGGAAGGATCATTATTGAATTATGTTTCTAGATAGGTTGTAG
CTGGCTC-TTTAGAGCATGTGCACGCCTGTTTGGACTTCATTTTCATCCACCTGTGCACC
TATTGTAGTCTTTGGTTGGGTTAGGGGGAAGTGGTCATTGTGTCAGCATCTGCTGGATGT
GAGGACTTGCATTGTGAAAGCTTTGCTGTCCTTGATGTGATCATGGAATCTCTTTCTCAC
TAGAGTCTATGTCACTCATTATACTCTGTCGAATGTCATTGAATGTCTTTACATGGGCTT
GTATGCCTATGAAAATTGTAATACAACTTTCAGCAACGGATCTCTTGGCTCTCGCATCGA
TGAAGGACGCAGCGAAATGCGATAAGTAATGTGAATTGCAGAATTCAGTGAATCATCGAA
TCTTTGAACGCATCTTGCGCTCCTTGGTATTCCGAGGAGCATGCCTGTTTGAGTGTCATT
AAATTCTCAACTCTCTTATAC-TTTTTTGTAAAAGAGAGCTTGGACTGTGGAGGCTTGCT
GGCCACTTTTTGGGGTCAGCTCCTCTGAAATGCATTAGCGGAACCGTTTGCAATCTGCCA
CAAGTGTGATAAGTTATCTACACTGGCGAGGGGATTGCTCTCTGTAATGTTCAGCTTCTA
ATTGTCTCTACTTTGTGAGACAACTTTTGAATGCTTGACCTCAAATCAGGTAGGACTACC
CGCTGAACTTAA
>ABC7-53
TTTCCGTAGGTGAACCTGCGGAAGGATCATTATTGAATTATGTTTCTAGATAGGTTGTAG
CTGGCTC-TTTAGAGCATGTGCACGCCTGTTTGGACTTCATTTTCATCCACCTGTGCACC
TATTGTAGTCTTTGGTTGGGTTAGGGGGAAGTGGTCATTGTGTCAGCATCTGCTGGATGT
GAGGACTTGCATTGTGAAAGCTTTGCTGTCCTTGATGTGATCATGGAATCTCTTTCTCAC
TAGAGTCTATGTCACTCATTATACTCTGTCGAATGTCATTGAATGTCTTTACATGGGCTT
GTATGCCTATGAAAATTGTAATACAACTTTCAGCAACGGATCTCTTGGCTCTCGCATCGA
TGAAGGACGCAGCGAAATGCGATAAGTAATGTGAATTGCAGAATTCAGTGAATCATCGAA
TCTTTGAACGCATCTTGCGCTCCTTGGTATTCCGAGGAGCATGCCTGTTTGAGTGTCATT
AAATTCTCAACTCTCTTATAC-TTTTTTGTAAAAGAGAGCTTGGACTGTGGAGGCTTGCT
GGCCACTTTTTGGGGTCAGCTCCTCTGAAATGCATTAGCGGAACCGTTTGCAATCTGCCA
CAAGTGTGATAAGTTATCTACACTGGCGAGGGGATTGCTCTCTGTAATGTTCAGCTTCTA
ATTGTCTCTACTTTGTGAGACAACTTTTGAATGCTTGACCTCAAATCAGGTAGGACTACC
CGCTGAACTTAA
>ABC8-3
TTTCCGTAGGTGAACCTGCGGAAGGATCATTATTGAATTATGTTTCTAGATAGGTTGTAG
CTGGCTC-TTTAGAGCATGTGCACGCCTGTTTGGACTTCATTTTCATCCACCTGTGCACC
TATTGTAGTCTTTGGTTGGGTTAGGGGGAAGTGGTCATTGTGTCAGCATCTGCTGGATGT

GAGGACTTGCATTGTGAAAGCTTTGCTGTCCTTGATGTGATCATGGAATCTCTTTCTCAC
TAGAGTCTATGTCACTCATTATACTCTGTCGAATGTCATTGAATGTCTTTACATGGGCTT
GTATGCCTATGAAAATTGTAATACAACTTTCAGCAACGGATCTCTTGGCTCTCGCATCGA
TGAAGGACGCAGCGAAATGCGATAAGTAATGTGAATTGCAGAATTCAGTGAATCATCGAA
TCTTTGAACGCATCTTGCGCTCCTTGGTATTCCGAGGAGCATGCCTGTTTGAGTGTCATT
AAATTCTCAACTCTCTTATAC-TTTTTTGTAAAAGAGAGCTTGGACTGTGGAGGCTTGCT
GGCCACTTTTTGGGGTCAGCTCCTCTGAAATGCATTAGCGGAACCGTTTGCAATCTGCCA
CAAGTGTGATAAGTTATCTACACTGGCGAGGGGATTGCTCTCTGTAATGTTCAGCTTCTA
ATTGTCTCTACTTTGTGAGACAACTTTTGAATGCTTGACCTCAAATCAGGTAGGACTACC
CGCTGAACTTAA
>ABC8-28
TTTCCGTAGGTGAACCTGCGGAAGGATCATTATTGAATTATGTTTCTAGATAGGTTGTAG
CTGGCTC-TTTAGAGCATGTGCACGCCTGTTTGGACTTCATTTTCATCCACCTGTGCACC
TATTGTAGTCTTTGGTTGGGTTAGGGGGAAGTGGTCATTGTGTCAGCATCTGCTGGATGT
GAGGACTTGCATTGTGAAAGCTTTGCTGTCCTTGATGTGATCATGGAATCTCTTTCTCAC
TAGAGTCTATGTCACTCATTATACTCTGTCGAATGTCATTGAATGTCTTTACATGGGCTT
GTATGCCTATGAAAATTGTAATACAACTTTCAGCAACGGATCTCTTGGCTCTCGCATCGA
TGAAGGACGCAGCGAAATGCGATAAGTAATGTGAATTGCAGAATTCAGTGAATCATCGAA
TCTTTGAACGCATCTTGCGCTCCTTGGTATTCCGAGGAGCATGCCTGTTTGAGTGTCATT
AAATTCTCAACTCTCTTATAC-TTTTTTGTAAAAGAGAGCTTGGACTGTGGAGGCTTGCT
GGCCACTTTTTGGGGTCAGCTCCTCTGAAATGCATTAGCGGAACCGTTTGCAATCTGCCA
CAAGTGTGATAAGTTATCTACACTGGCGAGGGGATTGCTCTCTGTAATGTTCAGCTTCTA
ATTGTCTCTACTTTGTGAGACAACTTTTGAATGCTTGACCTCAAATCAGGTAGGACTACC
CGCTGAACTTAA
>ABC9-25
TTTCCGTAGGTGAACCTGCGGAAGGATCATTATTGAATTATGTTTCTAGATAGGTTGTAG
CTGGCTC-TTTAGAGCATGTGCACGCCTGTTTGGACTTCATTTTCATCCACCTGTGCACC
TATTGTAGTCTTTGGTTGGGTTAGGGGGAAGTGGTCATTGTGTCAGCATCTGCTGGATGT
GAGGACTTGCATTGTGAAAGCTTTGCTGTCCTTGATGTGATCATGGAATCTCTTTCTCAC
TAGAGTCTATGTCACTCATTATACTCTGTCGAATGTCATTGAATGTCTTTACATGGGCTT
GTATGCCTATGAAAATTGTAATACAACTTTCAGCAACGGATCTCTTGGCTCTCGCATCGA
TGAAGGACGCAGCGAAATGCGATAAGTAATGTGAATTGCAGAATTCAGTGAATCATCGAA
TCTTTGAACGCATCTTGCGCTCCTTGGTATTCCGAGGAGCATGCCTGTTTGAGTGTCATT
AAATTCTCAACTCTCTTATAC-TTTTTTGTAAAAGAGAGCTTGGACTGTGGAGGCTTGCT
GGCCACTTTTTGGGGTCAGCTCCTCTGAAATGCATTAGCGGAACCGTTTGCAATCTGCCA
CAAGTGTGATAAGTTATCTACACTGGCGAGGGGATTGCTCTCTGTAATGTTCAGCTTCTA
ATTGTCTCTACTTTGTGAGACAACTTTTGAATGCTTGACCTCAAATCAGGTAGGACTACC
CGCTGAACTTAA
>ABC9-42
TTTCCGTAGGTGAACCTGCGGAAGGATCATTATTGAATTATGTTTCTAGATAGGTTGTAG
CTGGCTC-TTTAGAGCATGTGCACGCCTGTTTGGACTTCATTTTCATCCACCTGTGCACC
TATTGTAGTCTTTGGTTGGGTTAGGGGGAAGTGGTCATTGTGTCAGCATCTGCTGGATGT
GAGGACTTGCATTGTGAAAGCTTTGCTGTCCTTGATGTGATCATGGAATCTCTTTCTCAC
TAGAGTCTATGTCACTCATTATACTCTGTCGAATGTCATTGAATGTCTTTACATGGGCTT
GTATGCCTATGAAAATTGTAATACAACTTTCAGCAACGGATCTCTTGGCTCTCGCATCGA
TGAAGGACGCAGCGAAATGCGATAAGTAATGTGAATTGCAGAATTCAGTGAATCATCGAA
TCTTTGAACGCATCTTGCGCTCCTTGGTATTCCGAGGAGCATGCCTGTTTGAGTGTCATT
AAATTCTCAACTCTCTTATAC-TTTTTTGTAAAAGAGAGCTTGGACTGTGGAGGCTTGCT
GGCCACTTTTTGGGGTCAGCTCCTCTGAAATGCATTAGCGGAACCGTTTGCAATCTGCCA
CAAGTGTGATAAGTTATCTACACTGGCGAGGGGATTGCTCTCTGTAATGTTCAGCTTCTA

ATTGTCTCTACTTTGTGAGACAACTTTTGAATGCTTGACCTCAAATCAGGTAGGACTACC
CGCTGAACTTAA
>ABC9-54
TTTCCGTAGGTGAACCTGCGGAAGGATCATTATTGAATTATGTTTCTAGATAGGTTGTAG
CTGGCTC-TTTAGAGCATGTGCACGCCTGTTTGGACTTCATTTTCATCCACCTGTGCACC
TATTGTAGTCTTTGGTTGGGTTAGGGGGAAGTGGTCATTGTGTCAGCATCTGCTGGATGT
GAGGACTTGCATTGTGAAAGCTTTGCTGTCCTTGATGTGATCATGGAATCTCTTTCTCAC
TAGAGTCTATGTCACTCATTATACTCTGTCGAATGTCATTGAATGTCTTTACATGGGCTT
GTATGCCTATGAAAATTGTAATACAACTTTCAGCAACGGATCTCTTGGCTCTCGCATCGA
TGAAGGACGCAGCGAAATGCGATAAGTAATGTGAATTGCAGAATTCAGTGAATCATCGAA
TCTTTGAACGCATCTTGCGCTCCTTGGTATTCCGAGGAGCATGCCTGTTTGAGTGTCATT
AAATTCTCAACTCTCTTATAC-TTTTTTGTAAAAGAGAGCTTGGACTGTGGAGGCTTGCT
GGCCACTTTTTGGGGTCAGCTCCTCTGAAATGCATTAGCGGAACCGTTTGCAATCTGCCA
CAAGTGTGATAAGTTATCTACACTGGCGAGGGGATTGCTCTCTGTAATGTTCAGCTTCTA
ATTGTCTCTACTTTGTGAGACAACTTTTGAATGCTTGACCTCAAATCAGGTAGGACTACC
CGCTGAACTTAA
>ABC9-59
TTTCCGTAGGTGAACCTGCGGAAGGATCATTATTGAATTATGTTTCTAGATAGGTTGTAG
CTGGCTC-TTTAGAGCATGTGCACGCCTGTTTGGACTTCATTTTCATCCACCTGTGCACC
TATTGTAGTCTTTGGTTGGGTTAGGGGGAAGTGGTCATTGTGTCAGCATCTGCTGGATGT
GAGGACTTGCATTGTGAAAGCTTTGCTGTCCTTGATGTGATCATGGAATCTCTTTCTCAC
TAGAGTCTATGTCACTCATTATACTCTGTCGAATGTCATTGAATGTCTTTACATGGGCTT
GTATGCCTATGAAAATTGTAATACAACTTTCAGCAACGGATCTCTTGGCTCTCGCATCGA
TGAAGGACGCAGCGAAATGCGATAAGTAATGTGAATTGCAGAATTCAGTGAATCATCGAA
TCTTTGAACGCATCTTGCGCTCCTTGGTATTCCGAGGAGCATGCCTGTTTGAGTGTCATT
AAATTCTCAACTCTCTTATAC-TTTTTTGTAAAAGAGAGCTTGGACTGTGGAGGCTTGCT
GGCCACTTTTTGGGGTCAGCTCCTCTGAAATGCATTAGCGGAACCGTTTGCAATCTGCCA
CAAGTGTGATAAGTTATCTACACTGGCGAGGGGATTGCTCTCTGTAATGTTCAGCTTCTA
ATTGTCTCTACTTTGTGAGACAACTTTTGAATGCTTGACCTCAAATCAGGTAGGACTACC
CGCTGAACTTAA
>ABC11-45
TTTCCGTAGGTGAACCTGCGGAAGGATCATTATTGAATTATGTTTCTAGATAGGTTGTAG
CTGGCTC-TTTAGAGCATGTGCACGCCTGTTTGGACTTCATTTTCATCCACCTGTGCACC
TATTGTAGTCTTTGGTTGGGTTAGGGGGAAGTGGTCATTGTGTCAGCATCTGCTGGATGT
GAGGACTTGCATTGTGAAAGCTTTGCTGTCCTTGATGTGATCATGGAATCTCTTTCTCAC
TAGAGTCTATGTCACTCATTATACTCTGTCGAATGTCATTGAATGTCTTTACATGGGCTT
GTATGCCTATGAAAATTGTAATACAACTTTCAGCAACGGATCTCTTGGCTCTCGCATCGA
TGAAGGACGCAGCGAAATGCGATAAGTAATGTGAATTGCAGAATTCAGTGAATCATCGAA
TCTTTGAACGCATCTTGCGCTCCTTGGTATTCCGAGGAGCATGCCTGTTTGAGTGTCATT
AAATTCTCAACTCTCTTATAC-TTTTTTGTAAAAGAGAGCTTGGACTGTGGAGGCTTGCT
GGCCACTTTTTGGGGTCAGCTCCTCTGAAATGCATTAGCGGAACCGTTTGCAATCTGCCA
CAAGTGTGATAAGTTATCTACACTGGCGAGGGGATTGCTCTCTGTAATGTTCAGCTTCTA
ATTGTCTCTACTTTGTGAGACAACTTTTGAATGCTTGACCTCAAATCAGGTAGGACTACC
CGCTGAACTTAA
>ABC3-13
TTTCCGTAGGTGAACCTGCGGAAGGATCATTATTGAATTATGTTTCTAGATAGGTTGTAG
CTGGCTC-TTTAGAGCATGTGCACGCCTGTTTGGACTTCATTTTCATCCACCTGTGCACC
TATTGTAGTCTTTGGTTGGGTTAGGGGGAAGTGGTCATTGTGTCAGCATCTGCTGGATGT
GAGGACTTGCATTGTGAAAGCTTTGCTGTCCTTGATGTGATCATGGAATCTCTTTCTCAC
TAGAGTCTATGTCACTCATTATACTCTGTCGAATGTCATTGAATGTCTTTACATGGGCTT

GTATGCCTATGAAAATTGTAATACAACTTTCAGCAACGGATCTCTTGGCTCTCGCATCGA
TGAAGGACGCAGCGAAATGCGATAAGTAATGTGAATTGCAGAATTCAGTGAATCATCGAA
TCTTTGAACGCATCTTGCGCTCCTTGGTATTCCGAGGAGCATGCCTGTTTGAGTGTCATT
AAATTCTCAACTCTCTTATAC-TTTTTTGTAAAAGAGAGCTTGGACTGTGGAGGCTTGCT
GGCCACTTTTTGGGGTCAGCTCCTCTGAAATGCATTAGCGGAACCGTTTGCAATCTGCCA
CAAGTGTGATAAGTTATCTACACTGGCGAGGGGATTGCTCTCTGTAATGTTCAGCTTCTA
ATTGTCTCTACTTTGTGAGACAACTTTTGAATGCTTGACCTCAAATCAGGTAGGACTACC
CGCTGAACTTAA
>ABC8-11
TTTCCGTAGGTGAACCTGCGGAAGGATCATTATTGAATTATGTTTCTAGATAGGTTGTAG
CTGGCTC-TTTAGAGCATGTGCACGCCTGTTTGGACTTCATTTTCATCCACCTGTGCACC
TATTGTAGTCTTTGGTTGGGTTAGGGGGAAGTGGTCATTGTGTCAGCATCTGCTGGATGT
GAGGACTTGCATTGTGAAAGCTTTGCTGTCCTTGATGTGATCATGGAATCTCTTTCTCAC
TAGAGTCTATGTCACTCATTATACTCTGTCGAATGTCATTGAATGTCTTTACATGGGCTT
GTATGCCTATGAAAATTGTAATACAACTTTCAGCAACGGATCTCTTGGCTCTCGCATCGA
TGAAGGACGCAGCGAAATGCGATAAGTAATGTGAATTGCAGAATTCAGTGAATCATCGAA
TCTTTGAACGCATCTTGCGCTCCTTGGTATTCCGAGGAGCATGCCTGTTTGAGTGTCATT
AAATTCTCAACTCTCTTATAC-TTTTTTGTAAAAGAGAGCTTGGACTGTGGAGGCTTGCT
GGCCACTTTTTGGGGTCAGCTCCTCTGAAATGCATTAGCGGAACCGTTTGCAATCTGCCA
CAAGTGTGATAAGTTATCTACACTGGCGAGGGGATTGCTCTCTGTAATGTTCAGCTTCTA
ATTGTCTCTACTTTGTGAGACAACTTTTGAATGCTTGACCTCAAATCAGGTAGGACTACC
CGCTGAACTTAA
>ABC10-39
TTTCCGTAGGTGAACCTGCGGAAGGATCATTATTGAATTATGTTTCTAGATAGGTTGTAG
CTGGCTC-TTTAGAGCATGTGCACGCCTGTTTGGACTTCATTTTCATCCACCTGTGCACC
TATTGTAGTCTTTGGTTGGGTTAGGGGGAAGTGGTCATTGTGTCAGCATCTGCTGGATGT
GAGGACTTGCATTGTGAAAGCTTTGCTGTCCTTGATGTGATCATGGAATCTCTTTCTCAC
TAGAGTCTATGTCACTCATTATACTCTGTCGAATGTCATTGAATGTCTTTACATGGGCTT
GTATGCCTATGAAAATTGTAATACAACTTTCAGCAACGGATCTCTTGGCTCTCGCATCGA
TGAAGGACGCAGCGAAATGCGATAAGTAATGTGAATTGCAGAATTCAGTGAATCATCGAA
TCTTTGAACGCATCTTGCGCTCCTTGGTATTCCGAGGAGCATGCCTGTTTGAGTGTCATT
AAATTCTCAACTCTCTTATAC-TTTTTTGTAAAAGAGAGCTTGGACTGTGGAGGCTTGCT
GGCCACTTTTTGGGGTCAGCTCCTCTGAAATGCATTAGCGGAACCGTTTGCAATCTGCCA
CAAGTGTGATAAGTTATCTACACTGGCGAGGGGATTGCTCTCTGTAATGTTCAGCTTCTA
ATTGTCTCTACTTTGTGAGACAACTTTTGAATGCTTGACCTCAAATCAGGTAGGACTACC
CGCTGAACTTAA
>ABC11-34
TTTCCGTAGGTGAACCTGCGGAAGGATCATTATTGAATTATGTTTCTAGATAGGTTGTAG
CTGGCTC-TTTAGAGCATGTGCACGCCTGTTTGGACTTCATTTTCATCCACCTGTGCACC
TATTGTAGTCTTTGGTTGGGTTAGGGGGAAGTGGTCATTGTGTCAGCATCTGCTGGATGT
GAGGACTTGCATTGTGAAAGCTTTGCTGTCCTTGATGTGATCATGGAATCTCTTTCTCAC
TAGAGTCTATGTCACTCATTATACTCTGTCGAATGTCATTGAATGTCTTTACATGGGCTT
GTATGCCTATGAAAATTGTAATACAACTTTCAGCAACGGATCTCTTGGCTCTCGCATCGA
TGAAGGACGCAGCGAAATGCGATAAGTAATGTGAATTGCAGAATTCAGTGAATCATCGAA
TCTTTGAACGCATCTTGCGCTCCTTGGTATTCCGAGGAGCATGCCTGTTTGAGTGTCATT
AAATTCTCAACTCTCTTATAC-TTTTTTGTAAAAGAGAGCTTGGACTGTGGAGGCTTGCT
GGCCACTTTTTGGGGTCAGCTCCTCTGAAATGCATTAGCGGAACCGTTTGCAATCTGCCA
CAAGTGTGATAAGTTATCTACACTGGCGAGGGGATTGCTCTCTGTAATGTTCAGCTTCTA
ATTGTCTCTACTTTGTGAGACAACTTTTGAATGCTTGACCTCAAATCAGGTAGGACTACC
CGCTGAACTTAA

>ABC8-40
TTTCCGTAGGTGAACCTGCGGAAGGATCATTATTGAATTATGTTTCTAGATAGGTTGTAG
CTGGCTC-TTTAGAGCATGTGCACGCCTGTTTGGACTTCATTTTCATCCACCTGTGCACC
TATTGTAGTCTTTGGTTGGGTTAGGGGGAAGTGGTCATTGTGTCAGCATCTGCTGGATGT
GAGGACTTGCATTGTGAAAGCTTTGCTGTCCTTGATGTGATCATGGAATCTCTTTCTCAC
TAGAGTCTATGTCACTCATTATACTCTGTCGAATGTCATTGAATGTCTTTACATGGGCTT
GTATGCCTATGAAAATTGTAATACAACTTTCAGCAACGGATCTCTTGGCTCTCGCATCGA
TGAAGGACGCAGCGAAATGCGATAAGTAATGTGAATTGCAGAATTCAGTGAATCATCGAA
TCTTTGAACGCATCTTGCGCTCCTTGGTATTCCGAGGAGCATGCCTGTTTGAGTGTCATT
AAATTCTCAACTCTCTTATAC-TTTTTTGTAAAAGAGAGCTTGGACTGTGGAGGCTTGCT
GGCCACTTTTTGGGGTCAGCTCCTCTGAAATGCATTAGCGGAACCGTTTGCAATCTGCCA
CAAGTGTGATAAGTTATCTACACTGGCGAGGGGATTGCTCTCTGTAATGTTCAGCTTCTA
ATTGTCTCTACTTTGTGAGACAACTTTTGAATGCTTGACCTCAAATCAGGTAGGACTACC
CGCTGAACTTAA
>ABC10-23
TTTCCGTAGGTGAACCTGCGGAAGGATCATTATTGAATTATGTTTCTAGATAGGTTGTAG
CTGGCTC-TTTAGAGCATGTGCACGCCTGTTTGGACTTCATTTTCATCCACCTGTGCACC
TATTGTAGTCTTTGGTTGGGTTAGGGGGAAGTGGTCATTGTGTCAGCATCTGCTGGATGT
GAGGACTTGCATTGTGAAAGCTTTGCTGTCCTTGATGTGATCATGGAATCTCTTTCTCAC
TAGAGTCTATGTCACTCATTATACTCTGTCGAATGTCATTGAATGTCTTTACATGGGCTT
GTATGCCTATGAAAATTGTAATACAACTTTCAGCAACGGATCTCTTGGCTCTCGCATCGA
TGAAGGACGCAGCGAAATGCGATAAGTAATGTGAATTGCAGAATTCAGTGAATCATCGAA
TCTTTGAACGCATCTTGCGCTCCTTGGTATTCCGAGGAGCATGCCTGTTTGAGTGTCATT
AAATTCTCAACTCTCTTATAC-TTTTTTGTAAAAGAGAGCTTGGACTGTGGAGGCTTGCT
GGCCACTTTTTGGGGTCAGCTCCTCTGAAATGCATTAGCGGAACCGTTTGCAATCTGCCA
CAAGTGTGATAAGTTATCTACACTGGCGAGGGGATTGCTCTCTGTAATGTTCAGCTTCTA
ATTGTCTCTACTTTGTGAGACAACTTTTGAATGCTTGACCTCAAATCAGGTAGGACTACC
CGCTGAACTTAA
>ABC1-9
TTTCCGTAGGTGAACCTGCGGAAGGATCATTATTGAATTATGTTTCTAGATAGGTTGTAG
CTGGCTC-TTTAGAGCATGTGCACGCCTGTTTGGACTTCATTTTCATCCACCTGTGCACC
TATTGTAGTCTTTGGTTGGGTTAGGGGGAAGTGGTCATTGTGTCAGCATCTGCTGGATGT
GAGGACTTGCATTGTGAAAGCTTTGCTGTCCTTGATGTGATCATGGAATCTCTTTCTCAC
TAGAGTCTATGTCACTCATTATACTCTGTCGAATGTCATTGAATGTCTTTACATGGGCTT
GTATGCCTATGAAAATTGTAATACAACTTTCAGCAACGGATCTCTTGGCTCTCGCATCGA
TGAAGGACGCAGCGAAATGCGATAAGTAATGTGAATTGCAGAATTCAGTGAATCATCGAA
TCTTTGAACGCATCTTGCGCTCCTTGGTATTCCGAGGAGCATGCCTGTTTGAGTGTCATT
AAATTCTCAACTCTCTTATAC-TTTTTTGTAAAAGAGAGCTTGGACTGTGGAGGCTTGCT
GGCCACTTTTTGGGGTCAGCTCCTCTGAAATGCATTAGCGGAACCGTTTGCAATCTGCCA
CAAGTGTGATAAGTTATCTACACTGGCGAGGGGATTGCTCTCTGTAATGTTCAGCTTCTA
ATTGTCTCTACTTTGTGAGACAACTTTTGAATGCTTGACCTCAAATCAGGTAGGACTACC
CGCTGAACTTAA
>ABC1-28
TTTCCGTAGGTGAACCTGCGGAAGGATCATTATTGAATTATGTTTCTAGATAGGTTGTAG
CTGGCTC-TTTAGAGCATGTGCACGCCTGTTTGGACTTCATTTTCATCCACCTGTGCACC
TATTGTAGTCTTTGGTTGGGTTAGGGGGAAGTGGTCATTGTGTCAGCATCTGCTGGATGT
GAGGACTTGCATTGTGAAAGCTTTGCTGTCCTTGATGTGATCATGGAATCTCTTTCTCAC
TAGAGTCTATGTCACTCATTATACTCTGTCGAATGTCATTGAATGTCTTTACATGGGCTT
GTATGCCTATGAAAATTGTAATACAACTTTCAGCAACGGATCTCTTGGCTCTCGCATCGA
TGAAGGACGCAGCGAAATGCGATAAGTAATGTGAATTGCAGAATTCAGTGAATCATCGAA

TCTTTGAACGCATCTTGCGCTCCTTGGTATTCCGAGGAGCATGCCTGTTTGAGTGTCATT
AAATTCTCAACTCTCTTATAC-TTTTTTGTAAAAGAGAGCTTGGACTGTGGAGGCTTGCT
GGCCACTTTTTGGGGTCAGCTCCTCTGAAATGCATTAGCGGAACCGTTTGCAATCTGCCA
CAAGTGTGATAAGTTATCTACACTGGCGAGGGGATTGCTCTCTGTAATGTTCAGCTTCTA
ATTGTCTCTACTTTGTGAGACAACTTTTGAATGCTTGACCTCAAATCAGGTAGGACTACC
CGCTGAACTTAA
>ABC1-12
TTTCCGTAGGTGAACCTGCGGAAGGATCATTATTGAATTATGTTTCTAGATAGGTTGTAG
CTGGCTC-TTTAGAGCATGTGCACGCCTGTTTGGACTTCATTTTCATCCACCTGTGCACC
TATTGTAGTCTTTGGTTGGGTTAGGGGGAAGTGGTCATTGTGTCAGCATCTGCTGGATGT
GAGGACTTGCATTGTGAAAGCTTTGCTGTCCTTGATGTGATCATGGAATCTCTTTCTCAC
TAGAGTCTATGTCACTCATTATACTCTGTCGAATGTCATTGAATGTCTTTACATGGGCTT
GTATGCCTATGAAAATTGTAATACAACTTTCAGCAACGGATCTCTTGGCTCTCGCATCGA
TGAAGGACGCAGCGAAATGCGATAAGTAATGTGAATTGCAGAATTCAGTGAATCATCGAA
TCTTTGAACGCATCTTGCGCTCCTTGGTATTCCGAGGAGCATGCCTGTTTGAGTGTCATT
AAATTCTCAACTCTCTTATAC-TTTTTTGTAAAAGAGAGCTTGGACTGTGGAGGCTTGCT
GGCCACTTTTTGGGGTCAGCTCCTCTGAAATGCATTAGCGGAACCGTTTGCAATCTGCCA
CAAGTGTGATAAGTTATCTACACTGGCGAGGGGATTGCTCTCTGTAATGTTCAGCTTCTA
ATTGTCTCTACTTTGTGAGACAACTTTTGAATGCTTGACCTCAAATCAGGTAGGACTACC
CGCTGAACTTAA
>ABC1-19
TTTCCGTAGGTGAACCTGCGGAAGGATCATTATTGAATTATGTTTCTAGATAGGTTGTAG
CTGGCTC-TTTAGAGCATGTGCACGCCTGTTTGGACTTCATTTTCATCCACCTGTGCACC
TATTGTAGTCTTTGGTTGGGTTAGGGGGAAGTGGTCATTGTGTCAGCATCTGCTGGATGT
GAGGACTTGCATTGTGAAAGCTTTGCTGTCCTTGATGTGATCATGGAATCTCTTTCTCAC
TAGAGTCTATGTCACTCATTATACTCTGTCGAATGTCATTGAATGTCTTTACATGGGCTT
GTATGCCTATGAAAATTGTAATACAACTTTCAGCAACGGATCTCTTGGCTCTCGCATCGA
TGAAGGACGCAGCGAAATGCGATAAGTAATGTGAATTGCAGAATTCAGTGAATCATCGAA
TCTTTGAACGCATCTTGCGCTCCTTGGTATTCCGAGGAGCATGCCTGTTTGAGTGTCATT
AAATTCTCAACTCTCTTATAC-TTTTTTGTAAAAGAGAGCTTGGACTGTGGAGGCTTGCT
GGCCACTTTTTGGGGTCAGCTCCTCTGAAATGCATTAGCGGAACCGTTTGCAATCTGCCA
CAAGTGTGATAAGTTATCTACACTGGCGAGGGGATTGCTCTCTGTAATGTTCAGCTTCTA
ATTGTCTCTACTTTGTGAGACAACTTTTGAATGCTTGACCTCAAATCAGGTAGGACTACC
CGCTGAACTTAA
>ABC1-32
TTTCCGTAGGTGAACCTGCGGAAGGATCATTATTGAATTATGTTTCTAGATAGGTTGTAG
CTGGCTC-TTTAGAGCATGTGCACGCCTGTTTGGACTTCATTTTCATCCACCTGTGCACC
TATTGTAGTCTTTGGTTGGGTTAGGGGGAAGTGGTCATTGTGTCAGCATCTGCTGGATGT
GAGGACTTGCATTGTGAAAGCTTTGCTGTCCTTGATGTGATCATGGAATCTCTTTCTCAC
TAGAGTCTATGTCACTCATTATACTCTGTCGAATGTCATTGAATGTCTTTACATGGGCTT
GTATGCCTATGAAAATTGTAATACAACTTTCAGCAACGGATCTCTTGGCTCTCGCATCGA
TGAAGGACGCAGCGAAATGCGATAAGTAATGTGAATTGCAGAATTCAGTGAATCATCGAA
TCTTTGAACGCATCTTGCGCTCCTTGGTATTCCGAGGAGCATGCCTGTTTGAGTGTCATT
AAATTCTCAACTCTCTTATAC-TTTTTTGTAAAAGAGAGCTTGGACTGTGGAGGCTTGCT
GGCCACTTTTTGGGGTCAGCTCCTCTGAAATGCATTAGCGGAACCGTTTGCAATCTGCCA
CAAGTGTGATAAGTTATCTACACTGGCGAGGGGATTGCTCTCTGTAATGTTCAGCTTCTA
ATTGTCTCTACTTTGTGAGACAACTTTTGAATGCTTGACCTCAAATCAGGTAGGACTACC
CGCTGAACTTAA
>ABC1-11
TTTCCGTAGGTGAACCTGCGGAAGGATCATTATTGAATTATGTTTCTAGATAGGTTGTAG

CTGGCTC-TTTAGAGCATGTGCACGCCTGTTTGGACTTCATTTTCATCCACCTGTGCACC
TATTGTAGTCTTTGGTTGGGTTAGGGGGAAGTGGTCATTGTGTCAGCATCTGCTGGATGT
GAGGACTTGCATTGTGAAAGCTTTGCTGTCCTTGATGTGATCATGGAATCTCTTTCTCAC
TAGAGTCTATGTCACTCATTATACTCTGTCGAATGTCATTGAATGTCTTTACATGGGCTT
GTATGCCTATGAAAATTGTAATACAACTTTCAGCAACGGATCTCTTGGCTCTCGCATCGA
TGAAGGACGCAGCGAAATGCGATAAGTAATGTGAATTGCAGAATTCAGTGAATCATCGAA
TCTTTGAACGCATCTTGCGCTCCTTGGTATTCCGAGGAGCATGCCTGTTTGAGTGTCATT
AAATTCTCAACTCTCTTATAC-TTTTTTGTAAAAGAGAGCTTGGACTGTGGAGGCTTGCT
GGCCACTTTTTGGGGTCAGCTCCTCTGAAATGCATTAGCGGAACCGTTTGCAATCTGCCA
CAAGTGTGATAAGTTATCTACACTGGCGAGGGGATTGCTCTCTGTAATGTTCAGCTTCTA
ATTGTCTCTACTTTGTGAGACAACTTTTGAATGCTTGACCTCAAATCAGGTAGGACTACC
CGCTGAACTTAA
>ABC1-14
TTTCCGTAGGTGAACCTGCGGAAGGATCATTATTGAATTATGTTTCTAGATAGGTTGTAG
CTGGCTC-TTTAGAGCATGTGCACGCCTGTTTGGACTTCATTTTCATCCACCTGTGCACC
TATTGTAGTCTTTGGTTGGGTTAGGGGGAAGTGGTCATTGTGTCAGCATCTGCTGGATGT
GAGGACTTGCATTGTGAAAGCTTTGCTGTCCTTGATGTGATCATGGAATCTCTTTCTCAC
TAGAGTCTATGTCACTCATTATACTCTGTCGAATGTCATTGAATGTCTTTACATGGGCTT
GTATGCCTATGAAAATTGTAATACAACTTTCAGCAACGGATCTCTTGGCTCTCGCATCGA
TGAAGGACGCAGCGAAATGCGATAAGTAATGTGAATTGCAGAATTCAGTGAATCATCGAA
TCTTTGAACGCATCTTGCGCTCCTTGGTATTCCGAGGAGCATGCCTGTTTGAGTGTCATT
AAATTCTCAACTCTCTTATAC-TTTTTTGTAAAAGAGAGCTTGGACTGTGGAGGCTTGCT
GGCCACTTTTTGGGGTCAGCTCCTCTGAAATGCATTAGCGGAACCGTTTGCAATCTGCCA
CAAGTGTGATAAGTTATCTACACTGGCGAGGGGATTGCTCTCTGTAATGTTCAGCTTCTA
ATTGTCTCTACTTTGTGAGACAACTTTTGAATGCTTGACCTCAAATCAGGTAGGACTACC
CGCTGAACTTAA
>ABC1-18
TTTCCGTAGGTGAACCTGCGGAAGGATCATTATTGAATTATGTTTCTAGATAGGTTGTAG
CTGGCTC-TTTAGAGCATGTGCACGCCTGTTTGGACTTCATTTTCATCCACCTGTGCACC
TATTGTAGTCTTTGGTTGGGTTAGGGGGAAGTGGTCATTGTGTCAGCATCTGCTGGATGT
GAGGACTTGCATTGTGAAAGCTTTGCTGTCCTTGATGTGATCATGGAATCTCTTTCTCAC
TAGAGTCTATGTCACTCATTATACTCTGTCGAATGTCATTGAATGTCTTTACATGGGCTT
GTATGCCTATGAAAATTGTAATACAACTTTCAGCAACGGATCTCTTGGCTCTCGCATCGA
TGAAGGACGCAGCGAAATGCGATAAGTAATGTGAATTGCAGAATTCAGTGAATCATCGAA
TCTTTGAACGCATCTTGCGCTCCTTGGTATTCCGAGGAGCATGCCTGTTTGAGTGTCATT
AAATTCTCAACTCTCTTATAC-TTTTTTGTAAAAGAGAGCTTGGACTGTGGAGGCTTGCT
GGCCACTTTTTGGGGTCAGCTCCTCTGAAATGCATTAGCGGAACCGTTTGCAATCTGCCA
CAAGTGTGATAAGTTATCTACACTGGCGAGGGGATTGCTCTCTGTAATGTTCAGCTTCTA
ATTGTCTCTACTTTGTGAGACAACTTTTGAATGCTTGACCTCAAATCAGGTAGGACTACC
CGCTGAACTTAA
>ABC1-20
TTTCCGTAGGTGAACCTGCGGAAGGATCATTATTGAATTATGTTTCTAGATAGGTTGTAG
CTGGCTC-TTTAGAGCATGTGCACGCCTGTTTGGACTTCATTTTCATCCACCTGTGCACC
TATTGTAGTCTTTGGTTGGGTTAGGGGGAAGTGGTCATTGTGTCAGCATCTGCTGGATGT
GAGGACTTGCATTGTGAAAGCTTTGCTGTCCTTGATGTGATCATGGAATCTCTTTCTCAC
TAGAGTCTATGTCACTCATTATACTCTGTCGAATGTCATTGAATGTCTTTACATGGGCTT
GTATGCCTATGAAAATTGTAATACAACTTTCAGCAACGGATCTCTTGGCTCTCGCATCGA
TGAAGGACGCAGCGAAATGCGATAAGTAATGTGAATTGCAGAATTCAGTGAATCATCGAA
TCTTTGAACGCATCTTGCGCTCCTTGGTATTCCGAGGAGCATGCCTGTTTGAGTGTCATT
AAATTCTCAACTCTCTTATAC-TTTTTTGTAAAAGAGAGCTTGGACTGTGGAGGCTTGCT

GGCCACTTTTTGGGGTCAGCTCCTCTGAAATGCATTAGCGGAACCGTTTGCAATCTGCCA
CAAGTGTGATAAGTTATCTACACTGGCGAGGGGATTGCTCTCTGTAATGTTCAGCTTCTA
ATTGTCTCTACTTTGTGAGACAACTTTTGAATGCTTGACCTCAAATCAGGTAGGACTACC
CGCTGAACTTAA
>ABC1–21
TTTCCGTAGGTGAACCTGCGGAAGGATCATTATTGAATTATGTTTCTAGATAGGTTGTAG
CTGGCTC–TTTAGAGCATGTGCACGCCTGTTTGGACTTCATTTTCATCCACCTGTGCACC
TATTGTAGTCTTTGGTTGGGTTAGGGGGAAGTGGTCATTGTGTCAGCATCTGCTGGATGT
GAGGACTTGCATTGTGAAAGCTTTGCTGTCCTTGATGTGATCATGGAATCTCTTTCTCAC
TAGAGTCTATGTCACTCATTATACTCTGTCGAATGTCATTGAATGTCTTTACATGGGCTT
GTATGCCTATGAAAATTGTAATACAACTTTCAGCAACGGATCTCTTGGCTCTCGCATCGA
TGAAGGACGCAGCGAAATGCGATAAGTAATGTGAATTGCAGAATTCAGTGAATCATCGAA
TCTTTGAACGCATCTTGCGCTCCTTGGTATTCCGAGGAGCATGCCTGTTTGAGTGTCATT
AAATTCTCAACTCTCTTATAC–TTTTTTGTAAAAGAGAGCTTGGACTGTGGAGGCTTGCT
GGCCACTTTTTGGGGTCAGCTCCTCTGAAATGCATTAGCGGAACCGTTTGCAATCTGCCA
CAAGTGTGATAAGTTATCTACACTGGCGAGGGGATTGCTCTCTGTAATGTTCAGCTTCTA
ATTGTCTCTACTTTGTGAGACAACTTTTGAATGCTTGACCTCAAATCAGGTAGGACTACC
CGCTGAACTTAA
>ABC1–26
TTTCCGTAGGTGAACCTGCGGAAGGATCATTATTGAATTATGTTTCTAGATAGGTTGTAG
CTGGCTC–TTTAGAGCATGTGCACGCCTGTTTGGACTTCATTTTCATCCACCTGTGCACC
TATTGTAGTCTTTGGTTGGGTTAGGGGGAAGTGGTCATTGTGTCAGCATCTGCTGGATGT
GAGGACTTGCATTGTGAAAGCTTTGCTGTCCTTGATGTGATCATGGAATCTCTTTCTCAC
TAGAGTCTATGTCACTCATTATACTCTGTCGAATGTCATTGAATGTCTTTACATGGGCTT
GTATGCCTATGAAAATTGTAATACAACTTTCAGCAACGGATCTCTTGGCTCTCGCATCGA
TGAAGGACGCAGCGAAATGCGATAAGTAATGTGAATTGCAGAATTCAGTGAATCATCGAA
TCTTTGAACGCATCTTGCGCTCCTTGGTATTCCGAGGAGCATGCCTGTTTGAGTGTCATT
AAATTCTCAACTCTCTTATAC–TTTTTTGTAAAAGAGAGCTTGGACTGTGGAGGCTTGCT
GGCCACTTTTTGGGGTCAGCTCCTCTGAAATGCATTAGCGGAACCGTTTGCAATCTGCCA
CAAGTGTGATAAGTTATCTACACTGGCGAGGGGATTGCTCTCTGTAATGTTCAGCTTCTA
ATTGTCTCTACTTTGTGAGACAACTTTTGAATGCTTGACCTCAAATCAGGTAGGACTACC
CGCTGAACTTAA
>ABC1–34
TTTCCGTAGGTGAACCTGCGGAAGGATCATTATTGAATTATGTTTCTAGATAGGTTGTAG
CTGGCTC–TTTAGAGCATGTGCACGCCTGTTTGGACTTCATTTTCATCCACCTGTGCACC
TATTGTAGTCTTTGGTTGGGTTAGGGGGAAGTGGTCATTGTGTCAGCATCTGCTGGATGT
GAGGACTTGCATTGTGAAAGCTTTGCTGTCCTTGATGTGATCATGGAATCTCTTTCTCAC
TAGAGTCTATGTCACTCATTATACTCTGTCGAATGTCATTGAATGTCTTTACATGGGCTT
GTATGCCTATGAAAATTGTAATACAACTTTCAGCAACGGATCTCTTGGCTCTCGCATCGA
TGAAGGACGCAGCGAAATGCGATAAGTAATGTGAATTGCAGAATTCAGTGAATCATCGAA
TCTTTGAACGCATCTTGCGCTCCTTGGTATTCCGAGGAGCATGCCTGTTTGAGTGTCATT
AAATTCTCAACTCTCTTATAC–TTTTTTGTAAAAGAGAGCTTGGACTGTGGAGGCTTGCT
GGCCACTTTTTGGGGTCAGCTCCTCTGAAATGCATTAGCGGAACCGTTTGCAATCTGCCA
CAAGTGTGATAAGTTATCTACACTGGCGAGGGGATTGCTCTCTGTAATGTTCAGCTTCTA
ATTGTCTCTACTTTGTGAGACAACTTTTGAATGCTTGACCTCAAATCAGGTAGGACTACC
CGCTGAACTTAA
>ABC1–37
TTTCCGTAGGTGAACCTGCGGAAGGATCATTATTGAATTATGTTTCTAGATAGGTTGTAG
CTGGCTC–TTTAGAGCATGTGCACGCCTGTTTGGACTTCATTTTCATCCACCTGTGCACC
TATTGTAGTCTTTGGTTGGGTTAGGGGGAAGTGGTCATTGTGTCAGCATCTGCTGGATGT

GAGGACTTGCATTGTGAAAGCTTTGCTGTCCTTGATGTGATCATGGAATCTCTTTCTCAC
TAGAGTCTATGTCACTCATTATACTCTGTCGAATGTCATTGAATGTCTTTACATGGGCTT
GTATGCCTATGAAAATTGTAATACAACTTTCAGCAACGGATCTCTTGGCTCTCGCATCGA
TGAAGGACGCAGCGAAATGCGATAAGTAATGTGAATTGCAGAATTCAGTGAATCATCGAA
TCTTTGAACGCATCTTGCGCTCCTTGGTATTCCGAGGAGCATGCCTGTTTGAGTGTCATT
AAATTCTCAACTCTCTTATAC-TTTTTTGTAAAAGAGAGCTTGGACTGTGGAGGCTTGCT
GGCCACTTTTTGGGGTCAGCTCCTCTGAAATGCATTAGCGGAACCGTTTGCAATCTGCCA
CAAGTGTGATAAGTTATCTACACTGGCGAGGGGATTGCTCTCTGTAATGTTCAGCTTCTA
ATTGTCTCTACTTTGTGAGACAACTTTTGAATGCTTGACCTCAAATCAGGTAGGACTACC
CGCTGAACTTAA
>ABC1-39
TTTCCGTAGGTGAACCTGCGGAAGGATCATTATTGAATTATGTTTCTAGATAGGTTGTAG
CTGGCTC-TTTAGAGCATGTGCACGCCTGTTTGGACTTCATTTTCATCCACCTGTGCACC
TATTGTAGTCTTTGGTTGGGTTAGGGGGAAGTGGTCATTGTGTCAGCATCTGCTGGATGT
GAGGACTTGCATTGTGAAAGCTTTGCTGTCCTTGATGTGATCATGGAATCTCTTTCTCAC
TAGAGTCTATGTCACTCATTATACTCTGTCGAATGTCATTGAATGTCTTTACATGGGCTT
GTATGCCTATGAAAATTGTAATACAACTTTCAGCAACGGATCTCTTGGCTCTCGCATCGA
TGAAGGACGCAGCGAAATGCGATAAGTAATGTGAATTGCAGAATTCAGTGAATCATCGAA
TCTTTGAACGCATCTTGCGCTCCTTGGTATTCCGAGGAGCATGCCTGTTTGAGTGTCATT
AAATTCTCAACTCTCTTATAC-TTTTTTGTAAAAGAGAGCTTGGACTGTGGAGGCTTGCT
GGCCACTTTTTGGGGTCAGCTCCTCTGAAATGCATTAGCGGAACCGTTTGCAATCTGCCA
CAAGTGTGATAAGTTATCTACACTGGCGAGGGGATTGCTCTCTGTAATGTTCAGCTTCTA
ATTGTCTCTACTTTGTGAGACAACTTTTGAATGCTTGACCTCAAATCAGGTAGGACTACC
CGCTGAACTTAA
>ABC1-40
TTTCCGTAGGTGAACCTGCGGAAGGATCATTATTGAATTATGTTTCTAGATAGGTTGTAG
CTGGCTC-TTTAGAGCATGTGCACGCCTGTTTGGACTTCATTTTCATCCACCTGTGCACC
TATTGTAGTCTTTGGTTGGGTTAGGGGGAAGTGGTCATTGTGTCAGCATCTGCTGGATGT
GAGGACTTGCATTGTGAAAGCTTTGCTGTCCTTGATGTGATCATGGAATCTCTTTCTCAC
TAGAGTCTATGTCACTCATTATACTCTGTCGAATGTCATTGAATGTCTTTACATGGGCTT
GTATGCCTATGAAAATTGTAATACAACTTTCAGCAACGGATCTCTTGGCTCTCGCATCGA
TGAAGGACGCAGCGAAATGCGATAAGTAATGTGAATTGCAGAATTCAGTGAATCATCGAA
TCTTTGAACGCATCTTGCGCTCCTTGGTATTCCGAGGAGCATGCCTGTTTGAGTGTCATT
AAATTCTCAACTCTCTTATAC-TTTTTTGTAAAAGAGAGCTTGGACTGTGGAGGCTTGCT
GGCCACTTTTTGGGGTCAGCTCCTCTGAAATGCATTAGCGGAACCGTTTGCAATCTGCCA
CAAGTGTGATAAGTTATCTACACTGGCGAGGGGATTGCTCTCTGTAATGTTCAGCTTCTA
ATTGTCTCTACTTTGTGAGACAACTTTTGAATGCTTGACCTCAAATCAGGTAGGACTACC
CGCTGAACTTAA
>ABC1-41
TTTCCGTAGGTGAACCTGCGGAAGGATCATTATTGAATTATGTTTCTAGATAGGTTGTAG
CTGGCTC-TTTAGAGCATGTGCACGCCTGTTTGGACTTCATTTTCATCCACCTGTGCACC
TATTGTAGTCTTTGGTTGGGTTAGGGGGAAGTGGTCATTGTGTCAGCATCTGCTGGATGT
GAGGACTTGCATTGTGAAAGCTTTGCTGTCCTTGATGTGATCATGGAATCTCTTTCTCAC
TAGAGTCTATGTCACTCATTATACTCTGTCGAATGTCATTGAATGTCTTTACATGGGCTT
GTATGCCTATGAAAATTGTAATACAACTTTCAGCAACGGATCTCTTGGCTCTCGCATCGA
TGAAGGACGCAGCGAAATGCGATAAGTAATGTGAATTGCAGAATTCAGTGAATCATCGAA
TCTTTGAACGCATCTTGCGCTCCTTGGTATTCCGAGGAGCATGCCTGTTTGAGTGTCATT
AAATTCTCAACTCTCTTATAC-TTTTTTGTAAAAGAGAGCTTGGACTGTGGAGGCTTGCT
GGCCACTTTTTGGGGTCAGCTCCTCTGAAATGCATTAGCGGAACCGTTTGCAATCTGCCA
CAAGTGTGATAAGTTATCTACACTGGCGAGGGGATTGCTCTCTGTAATGTTCAGCTTCTA

ATTGTCTCTACTTTGTGAGACAACTTTTGAATGCTTGACCTCAAATCAGGTAGGACTACC
CGCTGAACTTAA
>ABC1-42
TTTCCGTAGGTGAACCTGCGGAAGGATCATTATTGAATTATGTTTCTAGATAGGTTGTAG
CTGGCTC-TTTAGAGCATGTGCACGCCTGTTTGGACTTCATTTTCATCCACCTGTGCACC
TATTGTAGTCTTTGGTTGGGTTAGGGGGAAGTGGTCATTGTGTCAGCATCTGCTGGATGT
GAGGACTTGCATTGTGAAAGCTTTGCTGTCCTTGATGTGATCATGGAATCTCTTTCTCAC
TAGAGTCTATGTCACTCATTATACTCTGTCGAATGTCATTGAATGTCTTTACATGGGCTT
GTATGCCTATGAAAATTGTAATACAACTTTCAGCAACGGATCTCTTGGCTCTCGCATCGA
TGAAGGACGCAGCGAAATGCGATAAGTAATGTGAATTGCAGAATTCAGTGAATCATCGAA
TCTTTGAACGCATCTTGCGCTCCTTGGTATTCCGAGGAGCATGCCTGTTTGAGTGTCATT
AAATTCTCAACTCTCTTATAC-TTTTTTGTAAAAGAGAGCTTGGACTGTGGAGGCTTGCT
GGCCACTTTTTGGGGTCAGCTCCTCTGAAATGCATTAGCGGAACCGTTTGCAATCTGCCA
CAAGTGTGATAAGTTATCTACACTGGCGAGGGGATTGCTCTCTGTAATGTTCAGCTTCTA
ATTGTCTCTACTTTGTGAGACAACTTTTGAATGCTTGACCTCAAATCAGGTAGGACTACC
CGCTGAACTTAA
>ABC1-44
TTTCCGTAGGTGAACCTGCGGAAGGATCATTATTGAATTATGTTTCTAGATAGGTTGTAG
CTGGCTC-TTTAGAGCATGTGCACGCCTGTTTGGACTTCATTTTCATCCACCTGTGCACC
TATTGTAGTCTTTGGTTGGGTTAGGGGGAAGTGGTCATTGTGTCAGCATCTGCTGGATGT
GAGGACTTGCATTGTGAAAGCTTTGCTGTCCTTGATGTGATCATGGAATCTCTTTCTCAC
TAGAGTCTATGTCACTCATTATACTCTGTCGAATGTCATTGAATGTCTTTACATGGGCTT
GTATGCCTATGAAAATTGTAATACAACTTTCAGCAACGGATCTCTTGGCTCTCGCATCGA
TGAAGGACGCAGCGAAATGCGATAAGTAATGTGAATTGCAGAATTCAGTGAATCATCGAA
TCTTTGAACGCATCTTGCGCTCCTTGGTATTCCGAGGAGCATGCCTGTTTGAGTGTCATT
AAATTCTCAACTCTCTTATAC-TTTTTTGTAAAAGAGAGCTTGGACTGTGGAGGCTTGCT
GGCCACTTTTTGGGGTCAGCTCCTCTGAAATGCATTAGCGGAACCGTTTGCAATCTGCCA
CAAGTGTGATAAGTTATCTACACTGGCGAGGGGATTGCTCTCTGTAATGTTCAGCTTCTA
ATTGTCTCTACTTTGTGAGACAACTTTTGAATGCTTGACCTCAAATCAGGTAGGACTACC
CGCTGAACTTAA
>ABC1-49
TTTCCGTAGGTGAACCTGCGGAAGGATCATTATTGAATTATGTTTCTAGATAGGTTGTAG
CTGGCTC-TTTAGAGCATGTGCACGCCTGTTTGGACTTCATTTTCATCCACCTGTGCACC
TATTGTAGTCTTTGGTTGGGTTAGGGGGAAGTGGTCATTGTGTCAGCATCTGCTGGATGT
GAGGACTTGCATTGTGAAAGCTTTGCTGTCCTTGATGTGATCATGGAATCTCTTTCTCAC
TAGAGTCTATGTCACTCATTATACTCTGTCGAATGTCATTGAATGTCTTTACATGGGCTT
GTATGCCTATGAAAATTGTAATACAACTTTCAGCAACGGATCTCTTGGCTCTCGCATCGA
TGAAGGACGCAGCGAAATGCGATAAGTAATGTGAATTGCAGAATTCAGTGAATCATCGAA
TCTTTGAACGCATCTTGCGCTCCTTGGTATTCCGAGGAGCATGCCTGTTTGAGTGTCATT
AAATTCTCAACTCTCTTATAC-TTTTTTGTAAAAGAGAGCTTGGACTGTGGAGGCTTGCT
GGCCACTTTTTGGGGTCAGCTCCTCTGAAATGCATTAGCGGAACCGTTTGCAATCTGCCA
CAAGTGTGATAAGTTATCTACACTGGCGAGGGGATTGCTCTCTGTAATGTTCAGCTTCTA
ATTGTCTCTACTTTGTGAGACAACTTTTGAATGCTTGACCTCAAATCAGGTAGGACTACC
CGCTGAACTTAA
>ABC1-55
TTTCCGTAGGTGAACCTGCGGAAGGATCATTATTGAATTATGTTTCTAGATAGGTTGTAG
CTGGCTC-TTTAGAGCATGTGCACGCCTGTTTGGACTTCATTTTCATCCACCTGTGCACC
TATTGTAGTCTTTGGTTGGGTTAGGGGGAAGTGGTCATTGTGTCAGCATCTGCTGGATGT
GAGGACTTGCATTGTGAAAGCTTTGCTGTCCTTGATGTGATCATGGAATCTCTTTCTCAC
TAGAGTCTATGTCACTCATTATACTCTGTCGAATGTCATTGAATGTCTTTACATGGGCTT

GTATGCCTATGAAAATTGTAATACAACTTTCAGCAACGGATCTCTTGGCTCTCGCATCGA
TGAAGGACGCAGCGAAATGCGATAAGTAATGTGAATTGCAGAATTCAGTGAATCATCGAA
TCTTTGAACGCATCTTGCGCTCCTTGGTATTCCGAGGAGCATGCCTGTTTGAGTGTCATT
AAATTCTCAACTCTCTTATAC-TTTTTTGTAAAAGAGAGCTTGGACTGTGGAGGCTTGCT
GGCCACTTTTTGGGGTCAGCTCCTCTGAAATGCATTAGCGGAACCGTTTGCAATCTGCCA
CAAGTGTGATAAGTTATCTACACTGGCGAGGGGATTGCTCTCTGTAATGTTCAGCTTCTA
ATTGTCTCTACTTTGTGAGACAACTTTTGAATGCTTGACCTCAAATCAGGTAGGACTACC
CGCTGAACTTAA
>ABC1-57
TTTCCGTAGGTGAACCTGCGGAAGGATCATTATTGAATTATGTTTCTAGATAGGTTGTAG
CTGGCTC-TTTAGAGCATGTGCACGCCTGTTTGGACTTCATTTTCATCCACCTGTGCACC
TATTGTAGTCTTTGGTTGGGTTAGGGGGAAGTGGTCATTGTGTCAGCATCTGCTGGATGT
GAGGACTTGCATTGTGAAAGCTTTGCTGTCCTTGATGTGATCATGGAATCTCTTTCTCAC
TAGAGTCTATGTCACTCATTATACTCTGTCGAATGTCATTGAATGTCTTTACATGGGCTT
GTATGCCTATGAAAATTGTAATACAACTTTCAGCAACGGATCTCTTGGCTCTCGCATCGA
TGAAGGACGCAGCGAAATGCGATAAGTAATGTGAATTGCAGAATTCAGTGAATCATCGAA
TCTTTGAACGCATCTTGCGCTCCTTGGTATTCCGAGGAGCATGCCTGTTTGAGTGTCATT
AAATTCTCAACTCTCTTATAC-TTTTTTGTAAAAGAGAGCTTGGACTGTGGAGGCTTGCT
GGCCACTTTTTGGGGTCAGCTCCTCTGAAATGCATTAGCGGAACCGTTTGCAATCTGCCA
CAAGTGTGATAAGTTATCTACACTGGCGAGGGGATTGCTCTCTGTAATGTTCAGCTTCTA
ATTGTCTCTACTTTGTGAGACAACTTTTGAATGCTTGACCTCAAATCAGGTAGGACTACC
CGCTGAACTTAA
>ABC1-63
TTTCCGTAGGTGAACCTGCGGAAGGATCATTATTGAATTATGTTTCTAGATAGGTTGTAG
CTGGCTC-TTTAGAGCATGTGCACGCCTGTTTGGACTTCATTTTCATCCACCTGTGCACC
TATTGTAGTCTTTGGTTGGGTTAGGGGGAAGTGGTCATTGTGTCAGCATCTGCTGGATGT
GAGGACTTGCATTGTGAAAGCTTTGCTGTCCTTGATGTGATCATGGAATCTCTTTCTCAC
TAGAGTCTATGTCACTCATTATACTCTGTCGAATGTCATTGAATGTCTTTACATGGGCTT
GTATGCCTATGAAAATTGTAATACAACTTTCAGCAACGGATCTCTTGGCTCTCGCATCGA
TGAAGGACGCAGCGAAATGCGATAAGTAATGTGAATTGCAGAATTCAGTGAATCATCGAA
TCTTTGAACGCATCTTGCGCTCCTTGGTATTCCGAGGAGCATGCCTGTTTGAGTGTCATT
AAATTCTCAACTCTCTTATAC-TTTTTTGTAAAAGAGAGCTTGGACTGTGGAGGCTTGCT
GGCCACTTTTTGGGGTCAGCTCCTCTGAAATGCATTAGCGGAACCGTTTGCAATCTGCCA
CAAGTGTGATAAGTTATCTACACTGGCGAGGGGATTGCTCTCTGTAATGTTCAGCTTCTA
ATTGTCTCTACTTTGTGAGACAACTTTTGAATGCTTGACCTCAAATCAGGTAGGACTACC
CGCTGAACTTAA
>ABC1-65
TTTCCGTAGGTGAACCTGCGGAAGGATCATTATTGAATTATGTTTCTAGATAGGTTGTAG
CTGGCTC-TTTAGAGCATGTGCACGCCTGTTTGGACTTCATTTTCATCCACCTGTGCACC
TATTGTAGTCTTTGGTTGGGTTAGGGGGAAGTGGTCATTGTGTCAGCATCTGCTGGATGT
GAGGACTTGCATTGTGAAAGCTTTGCTGTCCTTGATGTGATCATGGAATCTCTTTCTCAC
TAGAGTCTATGTCACTCATTATACTCTGTCGAATGTCATTGAATGTCTTTACATGGGCTT
GTATGCCTATGAAAATTGTAATACAACTTTCAGCAACGGATCTCTTGGCTCTCGCATCGA
TGAAGGACGCAGCGAAATGCGATAAGTAATGTGAATTGCAGAATTCAGTGAATCATCGAA
TCTTTGAACGCATCTTGCGCTCCTTGGTATTCCGAGGAGCATGCCTGTTTGAGTGTCATT
AAATTCTCAACTCTCTTATAC-TTTTTTGTAAAAGAGAGCTTGGACTGTGGAGGCTTGCT
GGCCACTTTTTGGGGTCAGCTCCTCTGAAATGCATTAGCGGAACCGTTTGCAATCTGCCA
CAAGTGTGATAAGTTATCTACACTGGCGAGGGGATTGCTCTCTGTAATGTTCAGCTTCTA
ATTGTCTCTACTTTGTGAGACAACTTTTGAATGCTTGACCTCAAATCAGGTAGGACTACC
CGCTGAACTTAA

>ABC1-67
TTTCCGTAGGTGAACCTGCGGAAGGATCATTATTGAATTATGTTTCTAGATAGGTTGTAG
CTGGCTC-TTTAGAGCATGTGCACGCCTGTTTGGACTTCATTTTCATCCACCTGTGCACC
TATTGTAGTCTTTGGTTGGGTTAGGGGGAAGTGGTCATTGTGTCAGCATCTGCTGGATGT
GAGGACTTGCATTGTGAAAGCTTTGCTGTCCTTGATGTGATCATGGAATCTCTTTCTCAC
TAGAGTCTATGTCACTCATTATACTCTGTCGAATGTCATTGAATGTCTTTACATGGGCTT
GTATGCCTATGAAAATTGTAATACAACTTTCAGCAACGGATCTCTTGGCTCTCGCATCGA
TGAAGGACGCAGCGAAATGCGATAAGTAATGTGAATTGCAGAATTCAGTGAATCATCGAA
TCTTTGAACGCATCTTGCGCTCCTTGGTATTCCGAGGAGCATGCCTGTTTGAGTGTCATT
AAATTCTCAACTCTCTTATAC-TTTTTTGTAAAAGAGAGCTTGGACTGTGGAGGCTTGCT
GGCCACTTTTTGGGGTCAGCTCCTCTGAAATGCATTAGCGGAACCGTTTGCAATCTGCCA
CAAGTGTGATAAGTTATCTACACTGGCGAGGGGATTGCTCTCTGTAATGTTCAGCTTCTA
ATTGTCTCTACTTTGTGAGACAACTTTTGAATGCTTGACCTCAAATCAGGTAGGACTACC
CGCTGAACTTAA
>ABC1-68
TTTCCGTAGGTGAACCTGCGGAAGGATCATTATTGAATTATGTTTCTAGATAGGTTGTAG
CTGGCTC-TTTAGAGCATGTGCACGCCTGTTTGGACTTCATTTTCATCCACCTGTGCACC
TATTGTAGTCTTTGGTTGGGTTAGGGGGAAGTGGTCATTGTGTCAGCATCTGCTGGATGT
GAGGACTTGCATTGTGAAAGCTTTGCTGTCCTTGATGTGATCATGGAATCTCTTTCTCAC
TAGAGTCTATGTCACTCATTATACTCTGTCGAATGTCATTGAATGTCTTTACATGGGCTT
GTATGCCTATGAAAATTGTAATACAACTTTCAGCAACGGATCTCTTGGCTCTCGCATCGA
TGAAGGACGCAGCGAAATGCGATAAGTAATGTGAATTGCAGAATTCAGTGAATCATCGAA
TCTTTGAACGCATCTTGCGCTCCTTGGTATTCCGAGGAGCATGCCTGTTTGAGTGTCATT
AAATTCTCAACTCTCTTATAC-TTTTTTGTAAAAGAGAGCTTGGACTGTGGAGGCTTGCT
GGCCACTTTTTGGGGTCAGCTCCTCTGAAATGCATTAGCGGAACCGTTTGCAATCTGCCA
CAAGTGTGATAAGTTATCTACACTGGCGAGGGGATTGCTCTCTGTAATGTTCAGCTTCTA
ATTGTCTCTACTTTGTGAGACAACTTTTGAATGCTTGACCTCAAATCAGGTAGGACTACC
CGCTGAACTTAA
>ABC1-70
TTTCCGTAGGTGAACCTGCGGAAGGATCATTATTGAATTATGTTTCTAGATAGGTTGTAG
CTGGCTC-TTTAGAGCATGTGCACGCCTGTTTGGACTTCATTTTCATCCACCTGTGCACC
TATTGTAGTCTTTGGTTGGGTTAGGGGGAAGTGGTCATTGTGTCAGCATCTGCTGGATGT
GAGGACTTGCATTGTGAAAGCTTTGCTGTCCTTGATGTGATCATGGAATCTCTTTCTCAC
TAGAGTCTATGTCACTCATTATACTCTGTCGAATGTCATTGAATGTCTTTACATGGGCTT
GTATGCCTATGAAAATTGTAATACAACTTTCAGCAACGGATCTCTTGGCTCTCGCATCGA
TGAAGGACGCAGCGAAATGCGATAAGTAATGTGAATTGCAGAATTCAGTGAATCATCGAA
TCTTTGAACGCATCTTGCGCTCCTTGGTATTCCGAGGAGCATGCCTGTTTGAGTGTCATT
AAATTCTCAACTCTCTTATAC-TTTTTTGTAAAAGAGAGCTTGGACTGTGGAGGCTTGCT
GGCCACTTTTTGGGGTCAGCTCCTCTGAAATGCATTAGCGGAACCGTTTGCAATCTGCCA
CAAGTGTGATAAGTTATCTACACTGGCGAGGGGATTGCTCTCTGTAATGTTCAGCTTCTA
ATTGTCTCTACTTTGTGAGACAACTTTTGAATGCTTGACCTCAAATCAGGTAGGACTACC
CGCTGAACTTAA
>ABC2-2
TTTCCGTAGGTGAACCTGCGGAAGGATCATTATTGAATTATGTTTCTAGATAGGTTGTAG
CTGGCTC-TTTAGAGCATGTGCACGCCTGTTTGGACTTCATTTTCATCCACCTGTGCACC
TATTGTAGTCTTTGGTTGGGTTAGGGGGAAGTGGTCATTGTGTCAGCATCTGCTGGATGT
GAGGACTTGCATTGTGAAAGCTTTGCTGTCCTTGATGTGATCATGGAATCTCTTTCTCAC
TAGAGTCTATGTCACTCATTATACTCTGTCGAATGTCATTGAATGTCTTTACATGGGCTT
GTATGCCTATGAAAATTGTAATACAACTTTCAGCAACGGATCTCTTGGCTCTCGCATCGA
TGAAGGACGCAGCGAAATGCGATAAGTAATGTGAATTGCAGAATTCAGTGAATCATCGAA

TCTTTGAACGCATCTTGCGCTCCTTGGTATTCCGAGGAGCATGCCTGTTTGAGTGTCATT
AAATTCTCAACTCTCTTATAC-TTTTTTGTAAAAGAGAGCTTGGACTGTGGAGGCTTGCT
GGCCACTTTTTGGGGTCAGCTCCTCTGAAATGCATTAGCGGAACCGTTTGCAATCTGCCA
CAAGTGTGATAAGTTATCTACACTGGCGAGGGGATTGCTCTCTGTAATGTTCAGCTTCTA
ATTGTCTCTACTTTGTGAGACAACTTTTGAATGCTTGACCTCAAATCAGGTAGGACTACC
CGCTGAACTTAA
>ABC2-7
TTTCCGTAGGTGAACCTGCGGAAGGATCATTATTGAATTATGTTTCTAGATAGGTTGTAG
CTGGCTC-TTTAGAGCATGTGCACGCCTGTTTGGACTTCATTTTCATCCACCTGTGCACC
TATTGTAGTCTTTGGTTGGGTTAGGGGGAAGTGGTCATTGTGTCAGCATCTGCTGGATGT
GAGGACTTGCATTGTGAAAGCTTTGCTGTCCTTGATGTGATCATGGAATCTCTTTCTCAC
TAGAGTCTATGTCACTCATTATACTCTGTCGAATGTCATTGAATGTCTTTACATGGGCTT
GTATGCCTATGAAAATTGTAATACAACTTTCAGCAACGGATCTCTTGGCTCTCGCATCGA
TGAAGGACGCAGCGAAATGCGATAAGTAATGTGAATTGCAGAATTCAGTGAATCATCGAA
TCTTTGAACGCATCTTGCGCTCCTTGGTATTCCGAGGAGCATGCCTGTTTGAGTGTCATT
AAATTCTCAACTCTCTTATAC-TTTTTTGTAAAAGAGAGCTTGGACTGTGGAGGCTTGCT
GGCCACTTTTTGGGGTCAGCTCCTCTGAAATGCATTAGCGGAACCGTTTGCAATCTGCCA
CAAGTGTGATAAGTTATCTACACTGGCGAGGGGATTGCTCTCTGTAATGTTCAGCTTCTA
ATTGTCTCTACTTTGTGAGACAACTTTTGAATGCTTGACCTCAAATCAGGTAGGACTACC
CGCTGAACTTAA
>ABC2-10
TTTCCGTAGGTGAACCTGCGGAAGGATCATTATTGAATTATGTTTCTAGATAGGTTGTAG
CTGGCTC-TTTAGAGCATGTGCACGCCTGTTTGGACTTCATTTTCATCCACCTGTGCACC
TATTGTAGTCTTTGGTTGGGTTAGGGGGAAGTGGTCATTGTGTCAGCATCTGCTGGATGT
GAGGACTTGCATTGTGAAAGCTTTGCTGTCCTTGATGTGATCATGGAATCTCTTTCTCAC
TAGAGTCTATGTCACTCATTATACTCTGTCGAATGTCATTGAATGTCTTTACATGGGCTT
GTATGCCTATGAAAATTGTAATACAACTTTCAGCAACGGATCTCTTGGCTCTCGCATCGA
TGAAGGACGCAGCGAAATGCGATAAGTAATGTGAATTGCAGAATTCAGTGAATCATCGAA
TCTTTGAACGCATCTTGCGCTCCTTGGTATTCCGAGGAGCATGCCTGTTTGAGTGTCATT
AAATTCTCAACTCTCTTATAC-TTTTTTGTAAAAGAGAGCTTGGACTGTGGAGGCTTGCT
GGCCACTTTTTGGGGTCAGCTCCTCTGAAATGCATTAGCGGAACCGTTTGCAATCTGCCA
CAAGTGTGATAAGTTATCTACACTGGCGAGGGGATTGCTCTCTGTAATGTTCAGCTTCTA
ATTGTCTCTACTTTGTGAGACAACTTTTGAATGCTTGACCTCAAATCAGGTAGGACTACC
CGCTGAACTTAA
>ABC2-12
TTTCCGTAGGTGAACCTGCGGAAGGATCATTATTGAATTATGTTTCTAGATAGGTTGTAG
CTGGCTC-TTTAGAGCATGTGCACGCCTGTTTGGACTTCATTTTCATCCACCTGTGCACC
TATTGTAGTCTTTGGTTGGGTTAGGGGGAAGTGGTCATTGTGTCAGCATCTGCTGGATGT
GAGGACTTGCATTGTGAAAGCTTTGCTGTCCTTGATGTGATCATGGAATCTCTTTCTCAC
TAGAGTCTATGTCACTCATTATACTCTGTCGAATGTCATTGAATGTCTTTACATGGGCTT
GTATGCCTATGAAAATTGTAATACAACTTTCAGCAACGGATCTCTTGGCTCTCGCATCGA
TGAAGGACGCAGCGAAATGCGATAAGTAATGTGAATTGCAGAATTCAGTGAATCATCGAA
TCTTTGAACGCATCTTGCGCTCCTTGGTATTCCGAGGAGCATGCCTGTTTGAGTGTCATT
AAATTCTCAACTCTCTTATAC-TTTTTTGTAAAAGAGAGCTTGGACTGTGGAGGCTTGCT
GGCCACTTTTTGGGGTCAGCTCCTCTGAAATGCATTAGCGGAACCGTTTGCAATCTGCCA
CAAGTGTGATAAGTTATCTACACTGGCGAGGGGATTGCTCTCTGTAATGTTCAGCTTCTA
ATTGTCTCTACTTTGTGAGACAACTTTTGAATGCTTGACCTCAAATCAGGTAGGACTACC
CGCTGAACTTAA
>ABC2-23
TTTCCGTAGGTGAACCTGCGGAAGGATCATTATTGAATTATGTTTCTAGATAGGTTGTAG

CTGGCTC-TTTAGAGCATGTGCACGCCTGTTTGGACTTCATTTTCATCCACCTGTGCACC
TATTGTAGTCTTTGGTTGGGTTAGGGGGAAGTGGTCATTGTGTCAGCATCTGCTGGATGT
GAGGACTTGCATTGTGAAAGCTTTGCTGTCCTTGATGTGATCATGGAATCTCTTTCTCAC
TAGAGTCTATGTCACTCATTATACTCTGTCGAATGTCATTGAATGTCTTTACATGGGCTT
GTATGCCTATGAAAATTGTAATACAACTTTCAGCAACGGATCTCTTGGCTCTCGCATCGA
TGAAGGACGCAGCGAAATGCGATAAGTAATGTGAATTGCAGAATTCAGTGAATCATCGAA
TCTTTGAACGCATCTTGCGCTCCTTGGTATTCCGAGGAGCATGCCTGTTTGAGTGTCATT
AAATTCTCAACTCTCTTATAC-TTTTTTGTAAAAGAGAGCTTGGACTGTGGAGGCTTGCT
GGCCACTTTTTGGGGTCAGCTCCTCTGAAATGCATTAGCGGAACCGTTTGCAATCTGCCA
CAAGTGTGATAAGTTATCTACACTGGCGAGGGGATTGCTCTCTGTAATGTTCAGCTTCTA
ATTGTCTCTACTTTGTGAGACAACTTTTGAATGCTTGACCTCAAATCAGGTAGGACTACC
CGCTGAACTTAA
>ABC2-24
TTTCCGTAGGTGAACCTGCGGAAGGATCATTATTGAATTATGTTTCTAGATAGGTTGTAG
CTGGCTC-TTTAGAGCATGTGCACGCCTGTTTGGACTTCATTTTCATCCACCTGTGCACC
TATTGTAGTCTTTGGTTGGGTTAGGGGGAAGTGGTCATTGTGTCAGCATCTGCTGGATGT
GAGGACTTGCATTGTGAAAGCTTTGCTGTCCTTGATGTGATCATGGAATCTCTTTCTCAC
TAGAGTCTATGTCACTCATTATACTCTGTCGAATGTCATTGAATGTCTTTACATGGGCTT
GTATGCCTATGAAAATTGTAATACAACTTTCAGCAACGGATCTCTTGGCTCTCGCATCGA
TGAAGGACGCAGCGAAATGCGATAAGTAATGTGAATTGCAGAATTCAGTGAATCATCGAA
TCTTTGAACGCATCTTGCGCTCCTTGGTATTCCGAGGAGCATGCCTGTTTGAGTGTCATT
AAATTCTCAACTCTCTTATAC-TTTTTTGTAAAAGAGAGCTTGGACTGTGGAGGCTTGCT
GGCCACTTTTTGGGGTCAGCTCCTCTGAAATGCATTAGCGGAACCGTTTGCAATCTGCCA
CAAGTGTGATAAGTTATCTACACTGGCGAGGGGATTGCTCTCTGTAATGTTCAGCTTCTA
ATTGTCTCTACTTTGTGAGACAACTTTTGAATGCTTGACCTCAAATCAGGTAGGACTACC
CGCTGAACTTAA
>ABC2-67
TTTCCGTAGGTGAACCTGCGGAAGGATCATTATTGAATTATGTTTCTAGATAGGTTGTAG
CTGGCTC-TTTAGAGCATGTGCACGCCTGTTTGGACTTCATTTTCATCCACCTGTGCACC
TATTGTAGTCTTTGGTTGGGTTAGGGGGAAGTGGTCATTGTGTCAGCATCTGCTGGATGT
GAGGACTTGCATTGTGAAAGCTTTGCTGTCCTTGATGTGATCATGGAATCTCTTTCTCAC
TAGAGTCTATGTCACTCATTATACTCTGTCGAATGTCATTGAATGTCTTTACATGGGCTT
GTATGCCTATGAAAATTGTAATACAACTTTCAGCAACGGATCTCTTGGCTCTCGCATCGA
TGAAGGACGCAGCGAAATGCGATAAGTAATGTGAATTGCAGAATTCAGTGAATCATCGAA
TCTTTGAACGCATCTTGCGCTCCTTGGTATTCCGAGGAGCATGCCTGTTTGAGTGTCATT
AAATTCTCAACTCTCTTATAC-TTTTTTGTAAAAGAGAGCTTGGACTGTGGAGGCTTGCT
GGCCACTTTTTGGGGTCAGCTCCTCTGAAATGCATTAGCGGAACCGTTTGCAATCTGCCA
CAAGTGTGATAAGTTATCTACACTGGCGAGGGGATTGCTCTCTGTAATGTTCAGCTTCTA
ATTGTCTCTACTTTGTGAGACAACTTTTGAATGCTTGACCTCAAATCAGGTAGGACTACC
CGCTGAACTTAA
>ABC2-77
TTTCCGTAGGTGAACCTGCGGAAGGATCATTATTGAATTATGTTTCTAGATAGGTTGTAG
CTGGCTC-TTTAGAGCATGTGCACGCCTGTTTGGACTTCATTTTCATCCACCTGTGCACC
TATTGTAGTCTTTGGTTGGGTTAGGGGGAAGTGGTCATTGTGTCAGCATCTGCTGGATGT
GAGGACTTGCATTGTGAAAGCTTTGCTGTCCTTGATGTGATCATGGAATCTCTTTCTCAC
TAGAGTCTATGTCACTCATTATACTCTGTCGAATGTCATTGAATGTCTTTACATGGGCTT
GTATGCCTATGAAAATTGTAATACAACTTTCAGCAACGGATCTCTTGGCTCTCGCATCGA
TGAAGGACGCAGCGAAATGCGATAAGTAATGTGAATTGCAGAATTCAGTGAATCATCGAA
TCTTTGAACGCATCTTGCGCTCCTTGGTATTCCGAGGAGCATGCCTGTTTGAGTGTCATT
AAATTCTCAACTCTCTTATAC-TTTTTTGTAAAAGAGAGCTTGGACTGTGGAGGCTTGCT

GGCCACTTTTTGGGGTCAGCTCCTCTGAAATGCATTAGCGGAACCGTTTGCAATCTGCCA
CAAGTGTGATAAGTTATCTACACTGGCGAGGGGATTGCTCTCTGTAATGTTCAGCTTCTA
ATTGTCTCTACTTTGTGAGACAACTTTTGAATGCTTGACCTCAAATCAGGTAGGACTACC
CGCTGAACTTAA
>ABC4-3
TTTCCGTAGGTGAACCTGCGGAAGGATCATTATTGAATTATGTTTCTAGATAGGTTGTAG
CTGGCTC-TTTAGAGCATGTGCACGCCTGTTTGGACTTCATTTTCATCCACCTGTGCACC
TATTGTAGTCTTTGGTTGGGTTAGGGGGAAGTGGTCATTGTGTCAGCATCTGCTGGATGT
GAGGACTTGCATTGTGAAAGCTTTGCTGTCCTTGATGTGATCATGGAATCTCTTTCTCAC
TAGAGTCTATGTCACTCATTATACTCTGTCGAATGTCATTGAATGTCTTTACATGGGCTT
GTATGCCTATGAAAATTGTAATACAACTTTCAGCAACGGATCTCTTGGCTCTCGCATCGA
TGAAGGACGCAGCGAAATGCGATAAGTAATGTGAATTGCAGAATTCAGTGAATCATCGAA
TCTTTGAACGCATCTTGCGCTCCTTGGTATTCCGAGGAGCATGCCTGTTTGAGTGTCATT
AAATTCTCAACTCTCTTATAC-TTTTTTGTAAAAGAGAGCTTGGACTGTGGAGGCTTGCT
GGCCACTTTTTGGGGTCAGCTCCTCTGAAATGCATTAGCGGAACCGTTTGCAATCTGCCA
CAAGTGTGATAAGTTATCTACACTGGCGAGGGGATTGCTCTCTGTAATGTTCAGCTTCTA
ATTGTCTCTACTTTGTGAGACAACTTTTGAATGCTTGACCTCAAATCAGGTAGGACTACC
CGCTGAACTTAA
>ABC4-21
TTTCCGTAGGTGAACCTGCGGAAGGATCATTATTGAATTATGTTTCTAGATAGGTTGTAG
CTGGCTC-TTTAGAGCATGTGCACGCCTGTTTGGACTTCATTTTCATCCACCTGTGCACC
TATTGTAGTCTTTGGTTGGGTTAGGGGGAAGTGGTCATTGTGTCAGCATCTGCTGGATGT
GAGGACTTGCATTGTGAAAGCTTTGCTGTCCTTGATGTGATCATGGAATCTCTTTCTCAC
TAGAGTCTATGTCACTCATTATACTCTGTCGAATGTCATTGAATGTCTTTACATGGGCTT
GTATGCCTATGAAAATTGTAATACAACTTTCAGCAACGGATCTCTTGGCTCTCGCATCGA
TGAAGGACGCAGCGAAATGCGATAAGTAATGTGAATTGCAGAATTCAGTGAATCATCGAA
TCTTTGAACGCATCTTGCGCTCCTTGGTATTCCGAGGAGCATGCCTGTTTGAGTGTCATT
AAATTCTCAACTCTCTTATAC-TTTTTTGTAAAAGAGAGCTTGGACTGTGGAGGCTTGCT
GGCCACTTTTTGGGGTCAGCTCCTCTGAAATGCATTAGCGGAACCGTTTGCAATCTGCCA
CAAGTGTGATAAGTTATCTACACTGGCGAGGGGATTGCTCTCTGTAATGTTCAGCTTCTA
ATTGTCTCTACTTTGTGAGACAACTTTTGAATGCTTGACCTCAAATCAGGTAGGACTACC
CGCTGAACTTAA
>ABC5-63
TTTCCGTAGGTGAACCTGCGGAAGGATCATTATTGAATTATGTTTCTAGATAGGTTGTAG
CTGGCTC-TTTAGAGCATGTGCACGCCTGTTTGGACTTCATTTTCATCCACCTGTGCACC
TATTGTAGTCTTTGGTTGGGTTAGGGGGAAGTGGTCATTGTGTCAGCATCTGCTGGATGT
GAGGACTTGCATTGTGAAAGCTTTGCTGTCCTTGATGTGATCATGGAATCTCTTTCTCAC
TAGAGTCTATGTCACTCATTATACTCTGTCGAATGTCATTGAATGTCTTTACATGGGCTT
GTATGCCTATGAAAATTGTAATACAACTTTCAGCAACGGATCTCTTGGCTCTCGCATCGA
TGAAGGACGCAGCGAAATGCGATAAGTAATGTGAATTGCAGAATTCAGTGAATCATCGAA
TCTTTGAACGCATCTTGCGCTCCTTGGTATTCCGAGGAGCATGCCTGTTTGAGTGTCATT
AAATTCTCAACTCTCTTATAC-TTTTTTGTAAAAGAGAGCTTGGACTGTGGAGGCTTGCT
GGCCACTTTTTGGGGTCAGCTCCTCTGAAATGCATTAGCGGAACCGTTTGCAATCTGCCA
CAAGTGTGATAAGTTATCTACACTGGCGAGGGGATTGCTCTCTGTAATGTTCAGCTTCTA
ATTGTCTCTACTTTGTGAGACAACTTTTGAATGCTTGACCTCAAATCAGGTAGGACTACC
CGCTGAACTTAA
>ABC6-39
TTTCCGTAGGTGAACCTGCGGAAGGATCATTATTGAATTATGTTTCTAGATAGGTTGTAG
CTGGCTC-TTTAGAGCATGTGCACGCCTGTTTGGACTTCATTTTCATCCACCTGTGCACC
TATTGTAGTCTTTGGTTGGGTTAGGGGGAAGTGGTCATTGTGTCAGCATCTGCTGGATGT

GAGGACTTGCATTGTGAAAGCTTTGCTGTCCTTGATGTGATCATGGAATCTCTTTCTCAC
TAGAGTCTATGTCACTCATTATACTCTGTCGAATGTCATTGAATGTCTTTACATGGGCTT
GTATGCCTATGAAAATTGTAATACAACTTTCAGCAACGGATCTCTTGGCTCTCGCATCGA
TGAAGGACGCAGCGAAATGCGATAAGTAATGTGAATTGCAGAATTCAGTGAATCATCGAA
TCTTTGAACGCATCTTGCGCTCCTTGGTATTCCGAGGAGCATGCCTGTTTGAGTGTCATT
AAATTCTCAACTCTCTTATAC–TTTTTTGTAAAAGAGAGCTTGGACTGTGGAGGCTTGCT
GGCCACTTTTTGGGGTCAGCTCCTCTGAAATGCATTAGCGGAACCGTTTGCAATCTGCCA
CAAGTGTGATAAGTTATCTACACTGGCGAGGGGATTGCTCTCTGTAATGTTCAGCTTCTA
ATTGTCTCTACTTTGTGAGACAACTTTTGAATGCTTGACCTCAAATCAGGTAGGACTACC
CGCTGAACTTAA
>ABC6–49
TTTCCGTAGGTGAACCTGCGGAAGGATCATTATTGAATTATGTTTCTAGATAGGTTGTAG
CTGGCTC–TTTAGAGCATGTGCACGCCTGTTTGGACTTCATTTTCATCCACCTGTGCACC
TATTGTAGTCTTTGGTTGGGTTAGGGGGAAGTGGTCATTGTGTCAGCATCTGCTGGATGT
GAGGACTTGCATTGTGAAAGCTTTGCTGTCCTTGATGTGATCATGGAATCTCTTTCTCAC
TAGAGTCTATGTCACTCATTATACTCTGTCGAATGTCATTGAATGTCTTTACATGGGCTT
GTATGCCTATGAAAATTGTAATACAACTTTCAGCAACGGATCTCTTGGCTCTCGCATCGA
TGAAGGACGCAGCGAAATGCGATAAGTAATGTGAATTGCAGAATTCAGTGAATCATCGAA
TCTTTGAACGCATCTTGCGCTCCTTGGTATTCCGAGGAGCATGCCTGTTTGAGTGTCATT
AAATTCTCAACTCTCTTATAC–TTTTTTGTAAAAGAGAGCTTGGACTGTGGAGGCTTGCT
GGCCACTTTTTGGGGTCAGCTCCTCTGAAATGCATTAGCGGAACCGTTTGCAATCTGCCA
CAAGTGTGATAAGTTATCTACACTGGCGAGGGGATTGCTCTCTGTAATGTTCAGCTTCTA
ATTGTCTCTACTTTGTGAGACAACTTTTGAATGCTTGACCTCAAATCAGGTAGGACTACC
CGCTGAACTTAA
>ABC7–19
TTTCCGTAGGTGAACCTGCGGAAGGATCATTATTGAATTATGTTTCTAGATAGGTTGTAG
CTGGCTC–TTTAGAGCATGTGCACGCCTGTTTGGACTTCATTTTCATCCACCTGTGCACC
TATTGTAGTCTTTGGTTGGGTTAGGGGGAAGTGGTCATTGTGTCAGCATCTGCTGGATGT
GAGGACTTGCATTGTGAAAGCTTTGCTGTCCTTGATGTGATCATGGAATCTCTTTCTCAC
TAGAGTCTATGTCACTCATTATACTCTGTCGAATGTCATTGAATGTCTTTACATGGGCTT
GTATGCCTATGAAAATTGTAATACAACTTTCAGCAACGGATCTCTTGGCTCTCGCATCGA
TGAAGGACGCAGCGAAATGCGATAAGTAATGTGAATTGCAGAATTCAGTGAATCATCGAA
TCTTTGAACGCATCTTGCGCTCCTTGGTATTCCGAGGAGCATGCCTGTTTGAGTGTCATT
AAATTCTCAACTCTCTTATAC–TTTTTTGTAAAAGAGAGCTTGGACTGTGGAGGCTTGCT
GGCCACTTTTTGGGGTCAGCTCCTCTGAAATGCATTAGCGGAACCGTTTGCAATCTGCCA
CAAGTGTGATAAGTTATCTACACTGGCGAGGGGATTGCTCTCTGTAATGTTCAGCTTCTA
ATTGTCTCTACTTTGTGAGACAACTTTTGAATGCTTGACCTCAAATCAGGTAGGACTACC
CGCTGAACTTAA
>ABC7–51
TTTCCGTAGGTGAACCTGCGGAAGGATCATTATTGAATTATGTTTCTAGATAGGTTGTAG
CTGGCTC–TTTAGAGCATGTGCACGCCTGTTTGGACTTCATTTTCATCCACCTGTGCACC
TATTGTAGTCTTTGGTTGGGTTAGGGGGAAGTGGTCATTGTGTCAGCATCTGCTGGATGT
GAGGACTTGCATTGTGAAAGCTTTGCTGTCCTTGATGTGATCATGGAATCTCTTTCTCAC
TAGAGTCTATGTCACTCATTATACTCTGTCGAATGTCATTGAATGTCTTTACATGGGCTT
GTATGCCTATGAAAATTGTAATACAACTTTCAGCAACGGATCTCTTGGCTCTCGCATCGA
TGAAGGACGCAGCGAAATGCGATAAGTAATGTGAATTGCAGAATTCAGTGAATCATCGAA
TCTTTGAACGCATCTTGCGCTCCTTGGTATTCCGAGGAGCATGCCTGTTTGAGTGTCATT
AAATTCTCAACTCTCTTATAC–TTTTTTGTAAAAGAGAGCTTGGACTGTGGAGGCTTGCT
GGCCACTTTTTGGGGTCAGCTCCTCTGAAATGCATTAGCGGAACCGTTTGCAATCTGCCA
CAAGTGTGATAAGTTATCTACACTGGCGAGGGGATTGCTCTCTGTAATGTTCAGCTTCTA

ATTGTCTCTACTTTGTGAGACAACTTTTGAATGCTTGACCTCAAATCAGGTAGGACTACC
CGCTGAACTTAA
>ABC7-52
TTTCCGTAGGTGAACCTGCGGAAGGATCATTATTGAATTATGTTTCTAGATAGGTTGTAG
CTGGCTC-TTTAGAGCATGTGCACGCCTGTTTGGACTTCATTTTCATCCACCTGTGCACC
TATTGTAGTCTTTGGTTGGGTTAGGGGGAAGTGGTCATTGTGTCAGCATCTGCTGGATGT
GAGGACTTGCATTGTGAAAGCTTTGCTGTCCTTGATGTGATCATGGAATCTCTTTCTCAC
TAGAGTCTATGTCACTCATTATACTCTGTCGAATGTCATTGAATGTCTTTACATGGGCTT
GTATGCCTATGAAAATTGTAATACAACTTTCAGCAACGGATCTCTTGGCTCTCGCATCGA
TGAAGGACGCAGCGAAATGCGATAAGTAATGTGAATTGCAGAATTCAGTGAATCATCGAA
TCTTTGAACGCATCTTGCGCTCCTTGGTATTCCGAGGAGCATGCCTGTTTGAGTGTCATT
AAATTCTCAACTCTCTTATAC-TTTTTTGTAAAAGAGAGCTTGGACTGTGGAGGCTTGCT
GGCCACTTTTTGGGGTCAGCTCCTCTGAAATGCATTAGCGGAACCGTTTGCAATCTGCCA
CAAGTGTGATAAGTTATCTACACTGGCGAGGGGATTGCTCTCTGTAATGTTCAGCTTCTA
ATTGTCTCTACTTTGTGAGACAACTTTTGAATGCTTGACCTCAAATCAGGTAGGACTACC
CGCTGAACTTAA
>ABC8-9
TTTCCGTAGGTGAACCTGCGGAAGGATCATTATTGAATTATGTTTCTAGATAGGTTGTAG
CTGGCTC-TTTAGAGCATGTGCACGCCTGTTTGGACTTCATTTTCATCCACCTGTGCACC
TATTGTAGTCTTTGGTTGGGTTAGGGGGAAGTGGTCATTGTGTCAGCATCTGCTGGATGT
GAGGACTTGCATTGTGAAAGCTTTGCTGTCCTTGATGTGATCATGGAATCTCTTTCTCAC
TAGAGTCTATGTCACTCATTATACTCTGTCGAATGTCATTGAATGTCTTTACATGGGCTT
GTATGCCTATGAAAATTGTAATACAACTTTCAGCAACGGATCTCTTGGCTCTCGCATCGA
TGAAGGACGCAGCGAAATGCGATAAGTAATGTGAATTGCAGAATTCAGTGAATCATCGAA
TCTTTGAACGCATCTTGCGCTCCTTGGTATTCCGAGGAGCATGCCTGTTTGAGTGTCATT
AAATTCTCAACTCTCTTATAC-TTTTTTGTAAAAGAGAGCTTGGACTGTGGAGGCTTGCT
GGCCACTTTTTGGGGTCAGCTCCTCTGAAATGCATTAGCGGAACCGTTTGCAATCTGCCA
CAAGTGTGATAAGTTATCTACACTGGCGAGGGGATTGCTCTCTGTAATGTTCAGCTTCTA
ATTGTCTCTACTTTGTGAGACAACTTTTGAATGCTTGACCTCAAATCAGGTAGGACTACC
CGCTGAACTTAA
>ABC8-14
TTTCCGTAGGTGAACCTGCGGAAGGATCATTATTGAATTATGTTTCTAGATAGGTTGTAG
CTGGCTC-TTTAGAGCATGTGCACGCCTGTTTGGACTTCATTTTCATCCACCTGTGCACC
TATTGTAGTCTTTGGTTGGGTTAGGGGGAAGTGGTCATTGTGTCAGCATCTGCTGGATGT
GAGGACTTGCATTGTGAAAGCTTTGCTGTCCTTGATGTGATCATGGAATCTCTTTCTCAC
TAGAGTCTATGTCACTCATTATACTCTGTCGAATGTCATTGAATGTCTTTACATGGGCTT
GTATGCCTATGAAAATTGTAATACAACTTTCAGCAACGGATCTCTTGGCTCTCGCATCGA
TGAAGGACGCAGCGAAATGCGATAAGTAATGTGAATTGCAGAATTCAGTGAATCATCGAA
TCTTTGAACGCATCTTGCGCTCCTTGGTATTCCGAGGAGCATGCCTGTTTGAGTGTCATT
AAATTCTCAACTCTCTTATAC-TTTTTTGTAAAAGAGAGCTTGGACTGTGGAGGCTTGCT
GGCCACTTTTTGGGGTCAGCTCCTCTGAAATGCATTAGCGGAACCGTTTGCAATCTGCCA
CAAGTGTGATAAGTTATCTACACTGGCGAGGGGATTGCTCTCTGTAATGTTCAGCTTCTA
ATTGTCTCTACTTTGTGAGACAACTTTTGAATGCTTGACCTCAAATCAGGTAGGACTACC
CGCTGAACTTAA
>ABC6-54
TTTCCGTAGGTGAACCTGCGGAAGGATCATTATTGAATTATGTTTCTAGATAGGTTGTAG
CTGGCTC-TTTAGAGCATGTGCACGCCTGTTTGGACTTCATTTTCATCCACCTGTGCACC
TATTGTAGTCTTTGGTTGGGTTAGGGGGAAGTGGTCATTGTGTCAGCATCTGCTGGATGT
GAGGACTTGCATTGTGAAAGCTTTGCTGTCCTTGATGTGATCATGGAATCTCTTTCTCAC
TAGAGTCTATGTCACTCATTATACTCTGTCGAATGTCATTGAATGTCTTTACATGGGCTT

GTATGCCTATGAAAATTGTAATACAACTTTCAGCAACGGATCTCTTGGCTCTCGCATCGA
TGAAGGACGCAGCGAAATGCGATAAGTAATGTGAATTGCAGAATTCAGTGAATCATCGAA
TCTTTGAACGCATCTTGCGCTCCTTGGTATTCCGAGGAGCATGCCTGTTTGAGTGTCATT
AAATTCTCAACTCTCTTATAC-TTTTTTGTAAAAGAGAGCTTGGACTGTGGAGGCTTGCT
GGCCACTTTTTGGGGTCAGCTCCTCTGAAATGCATTAGCGGAACCGTTTGCAATCTGCCA
CAAGTGTGATAAGTTATCTACACTGGCGAGGGGATTGCTCTCTGTAATGTTCAGCTTCTA
ATTGTCTCTACTTTGTGAGACAACTTTTGAATGCTTGACCTCAAATCAGGTAGGACTACC
CGCTGAACTTAA
>ABC8-56
TTTCCGTAGGTGAACCTGCGGAAGGATCATTATTGAATTATGTTTCTAGATAGGTTGTAG
CTGGCTC-TTTAGAGCATGTGCACGCCTGTTTGGACTTCATTTTCATCCACCTGTGCACC
TATTGTAGTCTTTGGTTGGGTTAGGGGGAAGTGGTCATTGTGTCAGCATCTGCTGGATGT
GAGGACTTGCATTGTGAAAGCTTTGCTGTCCTTGATGTGATCATGGAATCTCTTTCTCAC
TAGAGTCTATGTCACTCATTATACTCTGTCGAATGTCATTGAATGTCTTTACATGGGCTT
GTATGCCTATGAAAATTGTAATACAACTTTCAGCAACGGATCTCTTGGCTCTCGCATCGA
TGAAGGACGCAGCGAAATGCGATAAGTAATGTGAATTGCAGAATTCAGTGAATCATCGAA
TCTTTGAACGCATCTTGCGCTCCTTGGTATTCCGAGGAGCATGCCTGTTTGAGTGTCATT
AAATTCTCAACTCTCTTATAC-TTTTTTGTAAAAGAGAGCTTGGACTGTGGAGGCTTGCT
GGCCACTTTTTGGGGTCAGCTCCTCTGAAATGCATTAGCGGAACCGTTTGCAATCTGCCA
CAAGTGTGATAAGTTATCTACACTGGCGAGGGGATTGCTCTCTGTAATGTTCAGCTTCTA
ATTGTCTCTACTTTGTGAGACAACTTTTGAATGCTTGACCTCAAATCAGGTAGGACTACC
CGCTGAACTTAA
>ABC9-19
TTTCCGTAGGTGAACCTGCGGAAGGATCATTATTGAATTATGTTTCTAGATAGGTTGTAG
CTGGCTC-TTTAGAGCATGTGCACGCCTGTTTGGACTTCATTTTCATCCACCTGTGCACC
TATTGTAGTCTTTGGTTGGGTTAGGGGGAAGTGGTCATTGTGTCAGCATCTGCTGGATGT
GAGGACTTGCATTGTGAAAGCTTTGCTGTCCTTGATGTGATCATGGAATCTCTTTCTCAC
TAGAGTCTATGTCACTCATTATACTCTGTCGAATGTCATTGAATGTCTTTACATGGGCTT
GTATGCCTATGAAAATTGTAATACAACTTTCAGCAACGGATCTCTTGGCTCTCGCATCGA
TGAAGGACGCAGCGAAATGCGATAAGTAATGTGAATTGCAGAATTCAGTGAATCATCGAA
TCTTTGAACGCATCTTGCGCTCCTTGGTATTCCGAGGAGCATGCCTGTTTGAGTGTCATT
AAATTCTCAACTCTCTTATAC-TTTTTTGTAAAAGAGAGCTTGGACTGTGGAGGCTTGCT
GGCCACTTTTTGGGGTCAGCTCCTCTGAAATGCATTAGCGGAACCGTTTGCAATCTGCCA
CAAGTGTGATAAGTTATCTACACTGGCGAGGGGATTGCTCTCTGTAATGTTCAGCTTCTA
ATTGTCTCTACTTTGTGAGACAACTTTTGAATGCTTGACCTCAAATCAGGTAGGACTACC
CGCTGAACTTAA
>ABC9-24
TTTCCGTAGGTGAACCTGCGGAAGGATCATTATTGAATTATGTTTCTAGATAGGTTGTAG
CTGGCTC-TTTAGAGCATGTGCACGCCTGTTTGGACTTCATTTTCATCCACCTGTGCACC
TATTGTAGTCTTTGGTTGGGTTAGGGGGAAGTGGTCATTGTGTCAGCATCTGCTGGATGT
GAGGACTTGCATTGTGAAAGCTTTGCTGTCCTTGATGTGATCATGGAATCTCTTTCTCAC
TAGAGTCTATGTCACTCATTATACTCTGTCGAATGTCATTGAATGTCTTTACATGGGCTT
GTATGCCTATGAAAATTGTAATACAACTTTCAGCAACGGATCTCTTGGCTCTCGCATCGA
TGAAGGACGCAGCGAAATGCGATAAGTAATGTGAATTGCAGAATTCAGTGAATCATCGAA
TCTTTGAACGCATCTTGCGCTCCTTGGTATTCCGAGGAGCATGCCTGTTTGAGTGTCATT
AAATTCTCAACTCTCTTATAC-TTTTTTGTAAAAGAGAGCTTGGACTGTGGAGGCTTGCT
GGCCACTTTTTGGGGTCAGCTCCTCTGAAATGCATTAGCGGAACCGTTTGCAATCTGCCA
CAAGTGTGATAAGTTATCTACACTGGCGAGGGGATTGCTCTCTGTAATGTTCAGCTTCTA
ATTGTCTCTACTTTGTGAGACAACTTTTGAATGCTTGACCTCAAATCAGGTAGGACTACC
CGCTGAACTTAA

>ABC10-26
TTTCCGTAGGTGAACCTGCGGAAGGATCATTATTGAATTATGTTTCTAGATAGGTTGTAG
CTGGCTC-TTTAGAGCATGTGCACGCCTGTTTGGACTTCATTTTCATCCACCTGTGCACC
TATTGTAGTCTTTGGTTGGGTTAGGGGGAAGTGGTCATTGTGTCAGCATCTGCTGGATGT
GAGGACTTGCATTGTGAAAGCTTTGCTGTCCTTGATGTGATCATGGAATCTCTTTCTCAC
TAGAGTCTATGTCACTCATTATACTCTGTCGAATGTCATTGAATGTCTTTACATGGGCTT
GTATGCCTATGAAAATTGTAATACAACTTTCAGCAACGGATCTCTTGGCTCTCGCATCGA
TGAAGGACGCAGCGAAATGCGATAAGTAATGTGAATTGCAGAATTCAGTGAATCATCGAA
TCTTTGAACGCATCTTGCGCTCCTTGGTATTCCGAGGAGCATGCCTGTTTGAGTGTCATT
AAATTCTCAACTCTCTTATAC-TTTTTTGTAAAAGAGAGCTTGGACTGTGGAGGCTTGCT
GGCCACTTTTTGGGGTCAGCTCCTCTGAAATGCATTAGCGGAACCGTTTGCAATCTGCCA
CAAGTGTGATAAGTTATCTACACTGGCGAGGGGATTGCTCTCTGTAATGTTCAGCTTCTA
ATTGTCTCTACTTTGTGAGACAACTTTTGAATGCTTGACCTCAAATCAGGTAGGACTACC
CGCTGAACTTAA
>ABC10-28
TTTCCGTAGGTGAACCTGCGGAAGGATCATTATTGAATTATGTTTCTAGATAGGTTGTAG
CTGGCTC-TTTAGAGCATGTGCACGCCTGTTTGGACTTCATTTTCATCCACCTGTGCACC
TATTGTAGTCTTTGGTTGGGTTAGGGGGAAGTGGTCATTGTGTCAGCATCTGCTGGATGT
GAGGACTTGCATTGTGAAAGCTTTGCTGTCCTTGATGTGATCATGGAATCTCTTTCTCAC
TAGAGTCTATGTCACTCATTATACTCTGTCGAATGTCATTGAATGTCTTTACATGGGCTT
GTATGCCTATGAAAATTGTAATACAACTTTCAGCAACGGATCTCTTGGCTCTCGCATCGA
TGAAGGACGCAGCGAAATGCGATAAGTAATGTGAATTGCAGAATTCAGTGAATCATCGAA
TCTTTGAACGCATCTTGCGCTCCTTGGTATTCCGAGGAGCATGCCTGTTTGAGTGTCATT
AAATTCTCAACTCTCTTATAC-TTTTTTGTAAAAGAGAGCTTGGACTGTGGAGGCTTGCT
GGCCACTTTTTGGGGTCAGCTCCTCTGAAATGCATTAGCGGAACCGTTTGCAATCTGCCA
CAAGTGTGATAAGTTATCTACACTGGCGAGGGGATTGCTCTCTGTAATGTTCAGCTTCTA
ATTGTCTCTACTTTGTGAGACAACTTTTGAATGCTTGACCTCAAATCAGGTAGGACTACC
CGCTGAACTTAA
>ABC10-41
TTTCCGTAGGTGAACCTGCGGAAGGATCATTATTGAATTATGTTTCTAGATAGGTTGTAG
CTGGCTC-TTTAGAGCATGTGCACGCCTGTTTGGACTTCATTTTCATCCACCTGTGCACC
TATTGTAGTCTTTGGTTGGGTTAGGGGGAAGTGGTCATTGTGTCAGCATCTGCTGGATGT
GAGGACTTGCATTGTGAAAGCTTTGCTGTCCTTGATGTGATCATGGAATCTCTTTCTCAC
TAGAGTCTATGTCACTCATTATACTCTGTCGAATGTCATTGAATGTCTTTACATGGGCTT
GTATGCCTATGAAAATTGTAATACAACTTTCAGCAACGGATCTCTTGGCTCTCGCATCGA
TGAAGGACGCAGCGAAATGCGATAAGTAATGTGAATTGCAGAATTCAGTGAATCATCGAA
TCTTTGAACGCATCTTGCGCTCCTTGGTATTCCGAGGAGCATGCCTGTTTGAGTGTCATT
AAATTCTCAACTCTCTTATAC-TTTTTTGTAAAAGAGAGCTTGGACTGTGGAGGCTTGCT
GGCCACTTTTTGGGGTCAGCTCCTCTGAAATGCATTAGCGGAACCGTTTGCAATCTGCCA
CAAGTGTGATAAGTTATCTACACTGGCGAGGGGATTGCTCTCTGTAATGTTCAGCTTCTA
ATTGTCTCTACTTTGTGAGACAACTTTTGAATGCTTGACCTCAAATCAGGTAGGACTACC
CGCTGAACTTAA
>ABC11-7
TTTCCGTAGGTGAACCTGCGGAAGGATCATTATTGAATTATGTTTCTAGATAGGTTGTAG
CTGGCTC-TTTAGAGCATGTGCACGCCTGTTTGGACTTCATTTTCATCCACCTGTGCACC
TATTGTAGTCTTTGGTTGGGTTAGGGGGAAGTGGTCATTGTGTCAGCATCTGCTGGATGT
GAGGACTTGCATTGTGAAAGCTTTGCTGTCCTTGATGTGATCATGGAATCTCTTTCTCAC
TAGAGTCTATGTCACTCATTATACTCTGTCGAATGTCATTGAATGTCTTTACATGGGCTT
GTATGCCTATGAAAATTGTAATACAACTTTCAGCAACGGATCTCTTGGCTCTCGCATCGA
TGAAGGACGCAGCGAAATGCGATAAGTAATGTGAATTGCAGAATTCAGTGAATCATCGAA

TCTTTGAACGCATCTTGCGCTCCTTGGTATTCCGAGGAGCATGCCTGTTTGAGTGTCATT
AAATTCTCAACTCTCTTATAC-TTTTTTGTAAAAGAGAGCTTGGACTGTGGAGGCTTGCT
GGCCACTTTTTGGGGTCAGCTCCTCTGAAATGCATTAGCGGAACCGTTTGCAATCTGCCA
CAAGTGTGATAAGTTATCTACACTGGCGAGGGGATTGCTCTCTGTAATGTTCAGCTTCTA
ATTGTCTCTACTTTGTGAGACAACTTTTGAATGCTTGACCTCAAATCAGGTAGGACTACC
CGCTGAACTTAA
>ABC11-11
TTTCCGTAGGTGAACCTGCGGAAGGATCATTATTGAATTATGTTTCTAGATAGGTTGTAG
CTGGCTC-TTTAGAGCATGTGCACGCCTGTTTGGACTTCATTTTCATCCACCTGTGCACC
TATTGTAGTCTTTGGTTGGGTTAGGGGGAAGTGGTCATTGTGTCAGCATCTGCTGGATGT
GAGGACTTGCATTGTGAAAGCTTTGCTGTCCTTGATGTGATCATGGAATCTCTTTCTCAC
TAGAGTCTATGTCACTCATTATACTCTGTCGAATGTCATTGAATGTCTTTACATGGGCTT
GTATGCCTATGAAAATTGTAATACAACTTTCAGCAACGGATCTCTTGGCTCTCGCATCGA
TGAAGGACGCAGCGAAATGCGATAAGTAATGTGAATTGCAGAATTCAGTGAATCATCGAA
TCTTTGAACGCATCTTGCGCTCCTTGGTATTCCGAGGAGCATGCCTGTTTGAGTGTCATT
AAATTCTCAACTCTCTTATAC-TTTTTTGTAAAAGAGAGCTTGGACTGTGGAGGCTTGCT
GGCCACTTTTTGGGGTCAGCTCCTCTGAAATGCATTAGCGGAACCGTTTGCAATCTGCCA
CAAGTGTGATAAGTTATCTACACTGGCGAGGGGATTGCTCTCTGTAATGTTCAGCTTCTA
ATTGTCTCTACTTTGTGAGACAACTTTTGAATGCTTGACCTCAAATCAGGTAGGACTACC
CGCTGAACTTAA
>ABC11-23
TTTCCGTAGGTGAACCTGCGGAAGGATCATTATTGAATTATGTTTCTAGATAGGTTGTAG
CTGGCTC-TTTAGAGCATGTGCACGCCTGTTTGGACTTCATTTTCATCCACCTGTGCACC
TATTGTAGTCTTTGGTTGGGTTAGGGGGAAGTGGTCATTGTGTCAGCATCTGCTGGATGT
GAGGACTTGCATTGTGAAAGCTTTGCTGTCCTTGATGTGATCATGGAATCTCTTTCTCAC
TAGAGTCTATGTCACTCATTATACTCTGTCGAATGTCATTGAATGTCTTTACATGGGCTT
GTATGCCTATGAAAATTGTAATACAACTTTCAGCAACGGATCTCTTGGCTCTCGCATCGA
TGAAGGACGCAGCGAAATGCGATAAGTAATGTGAATTGCAGAATTCAGTGAATCATCGAA
TCTTTGAACGCATCTTGCGCTCCTTGGTATTCCGAGGAGCATGCCTGTTTGAGTGTCATT
AAATTCTCAACTCTCTTATAC-TTTTTTGTAAAAGAGAGCTTGGACTGTGGAGGCTTGCT
GGCCACTTTTTGGGGTCAGCTCCTCTGAAATGCATTAGCGGAACCGTTTGCAATCTGCCA
CAAGTGTGATAAGTTATCTACACTGGCGAGGGGATTGCTCTCTGTAATGTTCAGCTTCTA
ATTGTCTCTACTTTGTGAGACAACTTTTGAATGCTTGACCTCAAATCAGGTAGGACTACC
CGCTGAACTTAA
>ABC12-4
TTTCCGTAGGTGAACCTGCGGAAGGATCATTATTGAATTATGTTTCTAGATAGGTTGTAG
CTGGCTC-TTTAGAGCATGTGCACGCCTGTTTGGACTTCATTTTCATCCACCTGTGCACC
TATTGTAGTCTTTGGTTGGGTTAGGGGGAAGTGGTCATTGTGTCAGCATCTGCTGGATGT
GAGGACTTGCATTGTGAAAGCTTTGCTGTCCTTGATGTGATCATGGAATCTCTTTCTCAC
TAGAGTCTATGTCACTCATTATACTCTGTCGAATGTCATTGAATGTCTTTACATGGGCTT
GTATGCCTATGAAAATTGTAATACAACTTTCAGCAACGGATCTCTTGGCTCTCGCATCGA
TGAAGGACGCAGCGAAATGCGATAAGTAATGTGAATTGCAGAATTCAGTGAATCATCGAA
TCTTTGAACGCATCTTGCGCTCCTTGGTATTCCGAGGAGCATGCCTGTTTGAGTGTCATT
AAATTCTCAACTCTCTTATAC-TTTTTTGTAAAAGAGAGCTTGGACTGTGGAGGCTTGCT
GGCCACTTTTTGGGGTCAGCTCCTCTGAAATGCATTAGCGGAACCGTTTGCAATCTGCCA
CAAGTGTGATAAGTTATCTACACTGGCGAGGGGATTGCTCTCTGTAATGTTCAGCTTCTA
ATTGTCTCTACTTTGTGAGACAACTTTTGAATGCTTGACCTCAAATCAGGTAGGACTACC
CGCTGAACTTAA
>ABC12-9
TTTCCGTAGGTGAACCTGCGGAAGGATCATTATTGAATTATGTTTCTAGATAGGTTGTAG

CTGGCTC-TTTAGAGCATGTGCACGCCTGTTTGGACTTCATTTTCATCCACCTGTGCACC
TATTGTAGTCTTTGGTTGGGTTAGGGGGAAGTGGTCATTGTGTCAGCATCTGCTGGATGT
GAGGACTTGCATTGTGAAAGCTTTGCTGTCCTTGATGTGATCATGGAATCTCTTTCTCAC
TAGAGTCTATGTCACTCATTATACTCTGTCGAATGTCATTGAATGTCTTTACATGGGCTT
GTATGCCTATGAAAATTGTAATACAACTTTCAGCAACGGATCTCTTGGCTCTCGCATCGA
TGAAGGACGCAGCGAAATGCGATAAGTAATGTGAATTGCAGAATTCAGTGAATCATCGAA
TCTTTGAACGCATCTTGCGCTCCTTGGTATTCCGAGGAGCATGCCTGTTTGAGTGTCATT
AAATTCTCAACTCTCTTATAC-TTTTTTGTAAAAGAGAGCTTGGACTGTGGAGGCTTGCT
GGCCACTTTTTGGGGTCAGCTCCTCTGAAATGCATTAGCGGAACCGTTTGCAATCTGCCA
CAAGTGTGATAAGTTATCTACACTGGCGAGGGGATTGCTCTCTGTAATGTTCAGCTTCTA
ATTGTCTCTACTTTGTGAGACAACTTTTGAATGCTTGACCTCAAATCAGGTAGGACTACC
CGCTGAACTTAA
>ABC12-18
TTTCCGTAGGTGAACCTGCGGAAGGATCATTATTGAATTATGTTTCTAGATAGGTTGTAG
CTGGCTC-TTTAGAGCATGTGCACGCCTGTTTGGACTTCATTTTCATCCACCTGTGCACC
TATTGTAGTCTTTGGTTGGGTTAGGGGGAAGTGGTCATTGTGTCAGCATCTGCTGGATGT
GAGGACTTGCATTGTGAAAGCTTTGCTGTCCTTGATGTGATCATGGAATCTCTTTCTCAC
TAGAGTCTATGTCACTCATTATACTCTGTCGAATGTCATTGAATGTCTTTACATGGGCTT
GTATGCCTATGAAAATTGTAATACAACTTTCAGCAACGGATCTCTTGGCTCTCGCATCGA
TGAAGGACGCAGCGAAATGCGATAAGTAATGTGAATTGCAGAATTCAGTGAATCATCGAA
TCTTTGAACGCATCTTGCGCTCCTTGGTATTCCGAGGAGCATGCCTGTTTGAGTGTCATT
AAATTCTCAACTCTCTTATAC-TTTTTTGTAAAAGAGAGCTTGGACTGTGGAGGCTTGCT
GGCCACTTTTTGGGGTCAGCTCCTCTGAAATGCATTAGCGGAACCGTTTGCAATCTGCCA
CAAGTGTGATAAGTTATCTACACTGGCGAGGGGATTGCTCTCTGTAATGTTCAGCTTCTA
ATTGTCTCTACTTTGTGAGACAACTTTTGAATGCTTGACCTCAAATCAGGTAGGACTACC
CGCTGAACTTAA
>ABC12-51
TTTCCGTAGGTGAACCTGCGGAAGGATCATTATTGAATTATGTTTCTAGATAGGTTGTAG
CTGGCTC-TTTAGAGCATGTGCACGCCTGTTTGGACTTCATTTTCATCCACCTGTGCACC
TATTGTAGTCTTTGGTTGGGTTAGGGGGAAGTGGTCATTGTGTCAGCATCTGCTGGATGT
GAGGACTTGCATTGTGAAAGCTTTGCTGTCCTTGATGTGATCATGGAATCTCTTTCTCAC
TAGAGTCTATGTCACTCATTATACTCTGTCGAATGTCATTGAATGTCTTTACATGGGCTT
GTATGCCTATGAAAATTGTAATACAACTTTCAGCAACGGATCTCTTGGCTCTCGCATCGA
TGAAGGACGCAGCGAAATGCGATAAGTAATGTGAATTGCAGAATTCAGTGAATCATCGAA
TCTTTGAACGCATCTTGCGCTCCTTGGTATTCCGAGGAGCATGCCTGTTTGAGTGTCATT
AAATTCTCAACTCTCTTATAC-TTTTTTGTAAAAGAGAGCTTGGACTGTGGAGGCTTGCT
GGCCACTTTTTGGGGTCAGCTCCTCTGAAATGCATTAGCGGAACCGTTTGCAATCTGCCA
CAAGTGTGATAAGTTATCTACACTGGCGAGGGGATTGCTCTCTGTAATGTTCAGCTTCTA
ATTGTCTCTACTTTGTGAGACAACTTTTGAATGCTTGACCTCAAATCAGGTAGGACTACC
CGCTGAACTTAA
>ABC7-39
TTTCCGTAGGTGAACCTGCGGAAGGATCATTATTGAATTATGTTTCTAGATAGGTTGTAG
CTGGCTC-TTTAGAGCATGTGCACGCCTGTTTGGACTTCATTTTCATCCACCTGTGCACC
TATTGTAGTCTTTGGTTGGGTTAGGGGGAAGTGGTCATTGTGTCAGCATCTGCTGGATGT
GAGGACTTGCATTGTGAAAGCTTTGCTGTCCTTGATGTGATCATGGAATCTCTTTCTCAC
TAGAGTCTATGTCACTCATTATACTCTGTCGAATGTCATTGAATGTCTTTACATGGGCTT
GTATGCCTATGAAAATTGTAATACAACTTTCAGCAACGGATCTCTTGGCTCTCGCATCGA
TGAAGGACGCAGCGAAATGCGATAAGTAATGTGAATTGCAGAATTCAGTGAATCATCGAA
TCTTTGAACGCATCTTGCGCTCCTTGGTATTCCGAGGAGCATGCCTGTTTGAGTGTCATT
AAATTCTCAACTCTCTTATAC-TTTTTTGTAAAAGAGAGCTTGGACTGTGGAGGCTTGCT

GGCCACTTTTTGGGGTCAGCTCCTCTGAAATGCATTAGCGGAACCGTTTGCAATCTGCCA
CAAGTGTGATAAGTTATCTACACTGGCGAGGGGATTGCTCTCTGTAATGTTCAGCTTCTA
ATTGTCTCTACTTTGTGAGACAACTTTTGAATGCTTGACCTCAAATCAGGTAGGACTACC
CGCTGAACTTAA
>ABC5-86
TTTCCGTAGGTGAACCTGCGGAAGGATCATTATTGAATTATGTTTCTAGATAGGTTGTAG
CTGGCTC-TTTAGAGCATGTGCACGCCTGTTTGGACTTCATTTTCATCCACCTGTGCACC
TATTGTAGTCTTTGGTTGGGTTAGGGGGAAGTGGTCATTGTGTCAGCATCTGCTGGATGT
GAGGACTTGCATTGTGAAAGCTTTGCTGTCCTTGATGTGATCATGGAATCTCTTTCTCAC
TAGAGTCTATGTCACTCATTATACTCTGTCGAATGTCATTGAATGTCTTTACATGGGCTT
GTATGCCTATGAAAATTGTAATACAACTTTCAGCAACGGATCTCTTGGCTCTCGCATCGA
TGAAGGACGCAGCGAAATGCGATAAGTAATGTGAATTGCAGAATTCAGTGAATCATCGAA
TCTTTGAACGCATCTTGCGCTCCTTGGTATTCCGAGGAGCATGCCTGTTTGAGTGTCATT
AAATTCTCAACTCTCTTATAC-TTTTTTGTAAAAGAGAGCTTGGACTGTGGAGGCTTGCT
GGCCACTTTTTGGGGTCAGCTCCTCTGAAATGCATTAGCGGAACCGTTTGCAATCTGCCA
CAAGTGTGATAAGTTATCTACACTGGCGAGGGGATTGCTCTCTGTAATGTTCAGCTTCTA
ATTGTCTCTACTTTGTGAGACAACTTTTGAATGCTTGACCTCAAATCAGGTAGGACTACC
CGCTGAACTTAA
>ABC2-49
TTTCCGTAGGTGAACCTGCGGAAGGATCATTATTGAATTATGTTTCTAGATAGGTTGTAG
CTGGCTC-TTTAGAGCATGTGCACGCCTGTTTGGACTTCATTTTCATCCACCTGTGCACC
TATTGTAGTCTTTGGTTGGGTTAGGGGGAAGTGGTCATTGTGTCAGCATCTGCTGGATGT
GAGGACTTGCATTGTGAAAGCTTTGCTGTCCTTGATGTGATCATGGAATCTCTTTCTCAC
TAGAGTCTATGTCACTCATTATACTCTGTCGAATGTCATTGAATGTCTTTACATGGGCTT
GTATGCCTATGAAAATTGTAATACAACTTTCAGCAACGGATCTCTTGGCTCTCGCATCGA
TGAAGGACGCAGCGAAATGCGATAAGTAATGTGAATTGCAGAATTCAGTGAATCATCGAA
TCTTTGAACGCATCTTGCGCTCCTTGGTATTCCGAGGAGCATGCCTGTTTGAGTGTCATT
AAATTCTCAACTCTCTTATAC-TTTTTTGTAAAAGAGAGCTTGGACTGTGGAGGCTTGCT
GGCCACTTTTTGGGGTCAGCTCCTCTGAAATGCATTAGCGGAACCGTTTGCAATCTGCCA
CAAGTGTGATAAGTTATCTACACTGGCGAGGGGATTGCTCTCTGTAATGTTCAGCTTCTA
ATTGTCTCTACTTTGTGAGACAACTTTTGAATGCTTGACCTCAAATCAGGTAGGACTACC
CGCTGAACTTAA
>ABC1-13
TTTCCGTAGGTGAACCTGCGGAAGGATCATTATTGAATTATGTTTCTAGATAGGTTGTAG
CTGGCTC-TTTAGAGCATGTGCACGCCTGTTTGGACTTCATTTTCATCCACCTGTGCACC
TATTGTAGTCTTTGGTTGGGTTAGGGGGAAGTGGTCATTGTGTCAGCATCTGCTGGATGT
GAGGACTTGCATTGTGAAAGCTTTGCTGTCCTTGATGTGATCATGGAATCTCTTTCTCAC
TAGAGTCTATGTCACTCATTATACTCTGTCGAATGTCATTGAATGTCTTTACATGGGCTT
GTATGCCTATGAAAATTGTAATACAACTTTCAGCAACGGATCTCTTGGCTCTCGCATCGA
TGAAGGACGCAGCGAAATGCGATAAGTAATGTGAATTGCAGAATTCAGTGAATCATCGAA
TCTTTGAACGCATCTTGCGCTCCTTGGTATTCCGAGGAGCATGCCTGTTTGAGTGTCATT
AAATTCTCAACTCTCTTATAC-TTTTTTGTAAAAGAGAGCTTGGACTGTGGAGGCTTGCT
GGCCACTTTTTGGGGTCAGCTCCTCTGAAATGCATTAGCGGAACCGTTTGCAATCTGCCA
CAAGTGTGATAAGTTATCTACACTGGCGAGGGGATTGCTCTCTGTAATGTTCAGCTTCTA
ATTGTCTCTACTTTGTGAGACAACTTTTGAATGCTTGACCTCAAATCAGGTAGGACTACC
CGCTGAACTTAA
>ABC1-8
TTTCCGTAGGTGAACCTGCGGAAGGATCATTATTGAATTATGTTTCTAGATAGGTTGTAG
CTGGCTC-TTTAGAGCATGTGCACGCCTGTTTGGACTTCATTTTCATCCACCTGTGCACC
TATTGTAGTCTTTGGTTGGGTTAGGGGGAAGTGGTCATTGTGTCAGCATCTGCTGGATGT

GAGGACTTGCATTGTGAAAGCTTTGCTGTCCTTGATGTGATCATGGAATCTCTTTCTCAC
TAGAGTCTATGTCACTCATTATACTCTGTCGAATGTCATTGAATGTCTTTACATGGGCTT
GTATGCCTATGAAAATTGTAATACAACTTTCAGCAACGGATCTCTTGGCTCTCGCATCGA
TGAAGGACGCAGCGAAATGCGATAAGTAATGTGAATTGCAGAATTCAGTGAATCATCGAA
TCTTTGAACGCATCTTGCGCTCCTTGGTATTCCGAGGAGCATGCCTGTTTGAGTGTCATT
AAATTCTCAACTCTCTTATAC-TTTTTTGTAAAAGAGAGCTTGGACTGTGGAGGCTTGCT
GGCCACTTTTTGGGGTCAGCTCCTCTGAAATGCATTAGCGGAACCGTTTGCAATCTGCCA
CAAGTGTGATAAGTTATCTACACTGGCGAGGGGATTGCTCTCTGTAATGTTCAGCTTCTA
ATTGTCTCTACTTTGTGAGACAACTTTTGAATGCTTGACCTCAAATCAGGTAGGACTACC
CGCTGAACTTAA
>ABC1-35
TTTCCGTAGGTGAACCTGCGGAAGGATCATTATTGAATTATGTTTCTAGATAGGTTGTAG
CTGGCTC-TTTAGAGCATGTGCACGCCTGTTTGGACTTCATTTTCATCCACCTGTGCACC
TATTGTAGTCTTTGGTTGGGTTAGGGGGAAGTGGTCATTGTGTCAGCATCTGCTGGATGT
GAGGACTTGCATTGTGAAAGCTTTGCTGTCCTTGATGTGATCATGGAATCTCTTTCTCAC
TAGAGTCTATGTCACTCATTATACTCTGTCGAATGTCATTGAATGTCTTTACATGGGCTT
GTATGCCTATGAAAATTGTAATACAACTTTCAGCAACGGATCTCTTGGCTCTCGCATCGA
TGAAGGACGCAGCGAAATGCGATAAGTAATGTGAATTGCAGAATTCAGTGAATCATCGAA
TCTTTGAACGCATCTTGCGCTCCTTGGTATTCCGAGGAGCATGCCTGTTTGAGTGTCATT
AAATTCTCAACTCTCTTATAC-TTTTTTGTAAAAGAGAGCTTGGACTGTGGAGGCTTGCT
GGCCACTTTTTGGGGTCAGCTCCTCTGAAATGCATTAGCGGAACCGTTTGCAATCTGCCA
CAAGTGTGATAAGTTATCTACACTGGCGAGGGGATTGCTCTCTGTAATGTTCAGCTTCTA
ATTGTCTCTACTTTGTGAGACAACTTTTGAATGCTTGACCTCAAATCAGGTAGGACTACC
CGCTGAACTTAA
>ABC3-5
TTTCCGTAGGTGAACCTGCGGAAGGATCATTATTGAATTATGTTTCTAGATAGGTTGTAG
CTGGCTC-TTTAGAGCATGTGCACGCCTGTTTGGACTTCATTTTCATCCACCTGTGCACC
TATTGTAGTCTTTGGTTGGGTTAGGAGGAAGTGGTCATTGTGTCAGCATCTGCTGGATGT
GAGGACTTGCATTGTGAAAGCTTTGCTGTCCTTGATGTGATCATGGAATCTCTTTCTCAC
TAGAGTCTATGTCACTCATTATACTCTGTCGAATGTCATTGAATGTCTTTACATGGGCTT
GTATGCCTATGAAAATTGTAATACAACTTTCAGCAACGGATCTCTTGGCTCTCGCATCGA
TGAAGGACGCAGCGAAATGCGATAAGTAATGTGAATTGCAGAATTCAGTGAATCATCGAA
TCTTTGAACGCATCTTGCGCTCCTTGGTATTCCGAGGAGCATGCCTGTTTGAGTGTCATT
AAATTCTCAACTCTCTTATAC-TTTTTTGTAAAAGAGAGCTTGGACTGTGGAGGCTTGCT
GGCCACTTTTTGGGGTCAGCTCCTCTGAAATGCATTAGCGGAACCGTTTGCAATCTGCCA
CAAGTGTGATAAGTTATCTACACTGGCGAGGGGATTGCTCTCTGTAATGTTCAGCTTCTA
ATTGTCTCTACTTTGTGAGACAACTTTTGAATGCTTGACCTCAAATCAGGTAGGACTACC
CGCTGAACTTAA
>ABC5-68
TTTCCGTAGGTGAACCTGCGGAAGGATCATTATTGAATTATGTTTCTAGATAGGTTGTAG
CTGGCTC-TTTAGAGCATGTGCACGCCTGTTTGGACTTCATTTTCATCCACCTGTGCACC
TATTGTAGTCTTTGGTTGGGTTAGGAGGAAGTGGTCATTGTGTCAGCATCTGCTGGATGT
GAGGACTTGCATTGTGAAAGCTTTGCTGTCCTTGATGTGATCATGGAATCTCTTTCTCAC
TAGAGTCTATGTCACTCATTATACTCTGTCGAATGTCATTGAATGTCTTTACATGGGCTT
GTATGCCTATGAAAATTGTAATACAACTTTCAGCAACGGATCTCTTGGCTCTCGCATCGA
TGAAGGACGCAGCGAAATGCGATAAGTAATGTGAATTGCAGAATTCAGTGAATCATCGAA
TCTTTGAACGCATCTTGCGCTCCTTGGTATTCCGAGGAGCATGCCTGTTTGAGTGTCATT
AAATTCTCAACTCTCTTATAC-TTTTTTGTAAAAGAGAGCTTGGACTGTGGAGGCTTGCT
GGCCACTTTTTGGGGTCAGCTCCTCTGAAATGCATTAGCGGAACCGTTTGCAATCTGCCA
CAAGTGTGATAAGTTATCTACACTGGCGAGGGGATTGCTCTCTGTAATGTTCAGCTTCTA

ATTGTCTCTACTTTGTGAGACAACTTTTGAATGCTTGACCTCAAATCAGGTAGGACTACC
CGCTGAACTTAA
>ABC7-40
TTTCCGTAGGTGAACCTGCGGAAGGATCATTATTGAATTATGTTTCTAGATAGGTTGTAG
CTGGCTC-TTTAGAGCATGTGCACGCCTGTTTGGACTTCATTTTCATCCACCTGTGCACC
TATTGTAGTCTTTGGTTGGGTTAGGAGGAAGTGGTCATTGTGTCAGCATCTGCTGGATGT
GAGGACTTGCATTGTGAAAGCTTTGCTGTCCTTGATGTGATCATGGAATCTCTTTCTCAC
TAGAGTCTATGTCACTCATTATACTCTGTCGAATGTCATTGAATGTCTTTACATGGGCTT
GTATGCCTATGAAAATTGTAATACAACTTTCAGCAACGGATCTCTTGGCTCTCGCATCGA
TGAAGGACGCAGCGAAATGCGATAAGTAATGTGAATTGCAGAATTCAGTGAATCATCGAA
TCTTTGAACGCATCTTGCGCTCCTTGGTATTCCGAGGAGCATGCCTGTTTGAGTGTCATT
AAATTCTCAACTCTCTTATAC-TTTTTTGTAAAAGAGAGCTTGGACTGTGGAGGCTTGCT
GGCCACTTTTTGGGGTCAGCTCCTCTGAAATGCATTAGCGGAACCGTTTGCAATCTGCCA
CAAGTGTGATAAGTTATCTACACTGGCGAGGGGATTGCTCTCTGTAATGTTCAGCTTCTA
ATTGTCTCTACTTTGTGAGACAACTTTTGAATGCTTGACCTCAAATCAGGTAGGACTACC
CGCTGAACTTAA
>ABC8-53
TTTCCGTAGGTGAACCTGCGGAAGGATCATTATTGAATTATGTTTCTAGATAGGTTGTAG
CTGGCTC-TTTAGAGCATGTGCACGCCTGTTTGGACTTCATTTTCATCCACCTGTGCACC
TATTGTAGTCTTTGGTTGGGTTAGGAGGAAGTGGTCATTGTGTCAGCATCTGCTGGATGT
GAGGACTTGCATTGTGAAAGCTTTGCTGTCCTTGATGTGATCATGGAATCTCTTTCTCAC
TAGAGTCTATGTCACTCATTATACTCTGTCGAATGTCATTGAATGTCTTTACATGGGCTT
GTATGCCTATGAAAATTGTAATACAACTTTCAGCAACGGATCTCTTGGCTCTCGCATCGA
TGAAGGACGCAGCGAAATGCGATAAGTAATGTGAATTGCAGAATTCAGTGAATCATCGAA
TCTTTGAACGCATCTTGCGCTCCTTGGTATTCCGAGGAGCATGCCTGTTTGAGTGTCATT
AAATTCTCAACTCTCTTATAC-TTTTTTGTAAAAGAGAGCTTGGACTGTGGAGGCTTGCT
GGCCACTTTTTGGGGTCAGCTCCTCTGAAATGCATTAGCGGAACCGTTTGCAATCTGCCA
CAAGTGTGATAAGTTATCTACACTGGCGAGGGGATTGCTCTCTGTAATGTTCAGCTTCTA
ATTGTCTCTACTTTGTGAGACAACTTTTGAATGCTTGACCTCAAATCAGGTAGGACTACC
CGCTGAACTTAA
>ABC7-24
TTTCCGTAGGTGAACCTGCGGAAGGATCATTATTGAATTATGTTTCTAGATAGGTTGTAG
CTGGCTC-TTTAGAGCATGTGCACGCCTGTTTGGACTTCATTTTCATCCACCTGTGCACC
TATTGTAGTCTTTGGTTGGGTTAGGGGGAAGTGGTCATTGTGTCAGCATCTGCTGGATGT
GAGGACTTGCATTGTGAAAGCTTTGCTGTCCTTGATGTGATCATGGAATCTTTTTCTCAC
TAGAGTCTATGTCACTCATTATACTCTGTCGAATGTCATTGAATGTCTTTACATGGGCTT
GTATGCCTATGAAAATTGTAATACAACTTTCAGCAACGGATCTCTTGGCTCTCGCATCGA
TGAAGGACGCAGCGAAATGCGATAAGTAATGTGAATTGCAGAATTCAGTGAATCATCGAA
TCTTTGAACGCATCTTGCGCTCCTTGGTATTCCGAGGAGCATGCCTGTTTGAGTGTCATT
AAATTCTCAACTCTCTTATAC-TTTTTTGTAAAAGAGAGCTTGGACTGTGGAGGCTTGCT
GGCCACTTTTTGGGGTCAGCTCCTCTGAAATGCATTAGCGGAACCGTTTGCAATCTGCCA
CAAGTGTGATAAGTTATCTACACTGGCGAGGGGATTGCTCTCTGTAATGTTCAGCTTCTA
ATTGTCTCTACTTTGTGAGACAACTTTTGAATGCTTGACCTCAAATCAGGTAGGACTACC
CGCTGAACTTAA
>ABC12-38
TTTCCGTAGGTGAACCTGCGGAAGGATCATTATTGAATTATGTTTCTAGATAGGTTGTAG
CTGGCTC-TTTAGAGCATGTGCACGCCTGTTTGGACTTCATTTTCATCCACCTGTGCACC
TATTGTAGTCTTTGGTTGGGTTAGGGGGAAGTGGTCATTGTGTCAACATCTGCTGGATGT
GAGGACTTGCATTGTGAAAGCTTTGCTGTCCTTGATGTGATCATGGAATCTCTTTCTCAC
TAGAGTCTATGTCACTCATTATACTCTGTCGAATGTCATTGAATGTCTTTACATGGGCTT

GTATGCCTATGAAAATTGTAATACAACTTTCAGCAACGGATCTCTTGGCTCTCGCATCGA
TGAAGGACGCAGCGAAATGCGATAAGTAATGTGAATTGCAGAATTCAGTGAATCATCGAA
TCTTTGAACGCATCTTGCGCTCCTTGGTATTCCGAGGAGCATGCCTGTTTGAGTGTCATT
AAATTCTCAACTCTCTTATAC-TTTTTTGTAAAAGAGAGCTTGGACTGTGGAGGCTTGCT
GGCCACTTTTTGGGGTCAGCTCCTCTGAAATGCATTAGCGGAACCGTTTGCAATCTGCCA
CAAGTGTGATAAGTTATCTACACTGGCGAGGGGATTGCTCTCTGTAATGTTCAGCTTCTA
ATTGTCTCTACTTTGTGAGACAACTTTTGAATGCTTGACCTCAAATCAGGTAGGACTACC
CGCTGAACTTAA
>ABC6-12
TTTCCGTAGGTGAACCTGCGGAAGGATCATTATTGAATTATGTTTCTAGATAGGTTGTAG
CTGGCTC-TTTAGAGCATGTGCACGCCTGTTTGGACTTCATTTTCATCCACCTGTGCACC
TATTGTAGTCTTTGGTTGGGTTAGGAGGAAGTGGTCATTGTGTCAGCATCTGCTGGATGT
GAGGACTTGCATTGTGAAAGCTTTGCTGTCCTTGATGTGATCATGGAATCTCTTTCTCAC
TAGAGTCTATGTCACTCATTATACTCTGTCGAATGTCATTGAATGTCTTTACATGGGCTT
ATATGCCTATGAAAATTGTAATACAACTTTCAGCAACGGATCTCTTGGCTCTCGCATCGA
TGAAGGACGCAGCGAAATGCGATAAGTAATGTGAATTGCAGAATTCAGTGAATCATCGAA
TCTTTGAACGCATCTTGCGCTCCTTGGTATTCCGAGGAGCATGCCTGTTTGAGTGTCATT
AAATTCTCAACTCTCTTATAC-TTTTTTGTAAAAGAGAGCTTGGACTGTGGAGGCTTGCT
GGCCACTTTTTGGGGTCAGCTCCTCTGAAATGCATTAGCGGAACCGTTTGCAATCTGCCA
CAAGTGTGATAAGTTATCTACACTGGCGAGGGGATTGCTCTCTGTAATGTTCAGCTTCTA
ATTGTCTCTACTTTGTGAGACAACTTTTGAATGCTTGACCTCAAATCAGGTAGGACTACC
CGCTGAACTTAA
>ABC3-49
TTTCCGTAGGTGAACCTGCGGAAGGATCATTATTGAATTATGTTTCTAGATAGGTTGTAG
CTGGCTC-TTTAGAGCATGTGCACGCCTGTTTGGACTTCATTTTCATCCACCTGTGCACC
TATTGTAGTCTTTGGTTGGGTTAGGAGGAAGTGGTCATTGTGTCAGCATCTGCTGGATGT
GAGGACTTGCATTGTGAAAGCTTTGCTGTCCTTGATGTGATCATGGAATCTCTTTCTCAC
TAGAGTCTATGTCACTCATTATACTCTGTCGAATGTCATTGAATGTCTTTACATGGGCTT
ATATGCCTATGAAAATTGTAATACAACTTTCAGCAACGGATCTCTTGGCTCTCGCATCGA
TGAAGAACGCAGCGAAATGCGATAAGTAATGTGAATTGCAGAATTCAGTGAATCATCGAA
TCTTTGAACGCATCTTGCGCTCCTTGGTATTCCGAGGAGCATGCCTGTTTGAGTGTCATT
AAATTCTCAACTCTCTTATAC-TTTTTTGTAAAAGAGAGCTTGGACTGTGGAGGCTTGCT
GGCCACTTTTTGGGGTCAGCTCCTCTGAAATGCATTAGCGGAACCGTTTGCAATCTGCCA
CAAGTGTGATAAGTTATCTACACTGGCGAGGGGATTGCTCTCTGTAATGTTCAGCTTCTA
ATTGTCTCTACTTTGTGAGACAACTTTTGAATGCTTGACCTCAAATCAGGTAGGACTACC
CGCTGAACTTAA
>ABC4-5
TTTCCGTAGGTGAACCTGCGGAAGGATCATTATTGAATTATGTTTCTAGATAGGTTGTAG
CTGGCTC-TTTAGAGCATGTGCACGCCTGTTTGGACTTCATTTTCATCCACCTGTGCACC
TATTGTAGTCTTTGGTTGGGTTAGGAGGAAGTGGTCATTGTGTCAGCATCTGCTGGATGT
GAGGACTTGCATTGTGAAAGCTTTGCTGTCCTTGATGTGATCATGGAATCTCTTTCTCAC
TAGAGTCTATGTCACTCATTATACTCTGTCGAATGTCATTGAATGTCTTTACATGGGCTT
ATATGCCTATGAAAATTGTAATACAACTTTCAGCAACGGATCTCTTGGCTCTCGCATCGA
TGAAGAACGCAGCGAAATGCGATAAGTAATGTGAATTGCAGAATTCAGTGAATCATCGAA
TCTTTGAACGCATCTTGCGCTCCTTGGTATTCCGAGGAGCATGCCTGTTTGAGTGTCATT
AAATTCTCAACTCTCTTATAC-TTTTTTGTAAAAGAGAGCTTGGACTGTGGAGGCTTGCT
GGCCACTTTTTGGGGTCAGCTCCTCTGAAATGCATTAGCGGAACCGTTTGCAATCTGCCA
CAAGTGTGATAAGTTATCTACACTGGCGAGGGGATTGCTCTCTGTAATGTTCAGCTTCTA
ATTGTCTCTACTTTGTGAGACAACTTTTGAATGCTTGACCTCAAATCAGGTAGGACTACC
CGCTGAACTTAA

>ABC8-24
TTTCCGTAGGTGAACCTGCGGAAGGATCATTATTGAATTATGTTTCTAGATAGGTTGTAG
CTGGCTC-TTTAGAGCATGTGCACGCCTGTTTGGACTTCATTTTCATCCACCTGTGCACC
TATTGTAGTCTTTGGTTGGGTTAGGAGGAAGTGGTCATTGTGTCAGCATCTGCTGGATGT
GAGGACTTGCATTGTGAAAGCTTTGCTGTCCTTGATGTGATCATGGAATCTCTTTCTCAC
TAGAGTCTATGTCACTCATTATACTCTGTCGAATGTCATTGAATGTCTTTACATGGGCTT
ATATGCCTATGAAAATTGTAATACAACTTTCAGCAACGGATCTCTTGGCTCTCGCATCGA
TGAAGAACGCAGCGAAATGCGATAAGTAATGTGAATTGCAGAATTCAGTGAATCATCGAA
TCTTTGAACGCATCTTGCGCTCCTTGGTATTCCGAGGAGCATGCCTGTTTGAGTGTCATT
AAATTCTCAACTCTCTTATAC-TTTTTTGTAAAAGAGAGCTTGGACTGTGGAGGCTTGCT
GGCCACTTTTTGGGGTCAGCTCCTCTGAAATGCATTAGCGGAACCGTTTGCAATCTGCCA
CAAGTGTGATAAGTTATCTACACTGGCGAGGGGATTGCTCTCTGTAATGTTCAGCTTCTA
ATTGTCTCTACTTTGTGAGACAACTTTTGAATGCTTGACCTCAAATCAGGTAGGACTACC
CGCTGAACTTAA
>ABC9-15
TTTCCGTAGGTGAACCTGCGGAAGGATCATTATTGAATTATGTTTCTAGATAGGTTGTAG
CTGGCTC-TTTAGAGCATGTGCACGCCTGTTTGGACTTCATTTTCATCCACCTGTGCACC
TATTGTAGTCTTTGGTTGGGTTAGGAGGAAGTGGTCATTGTGTCAGCATCTGCTGGATGT
GAGGACTTGCATTGTGAAAGCTTTGCTGTCCTTGATGTGATCATGGAATCTCTTTCTCAC
TAGAGTCTATGTCACTCATTATACTCTGTCGAATGTCATTGAATGTCTTTACATGGGCTT
ATATGCCTATGAAAATTGTAATACAACTTTCAGCAACGGATCTCTTGGCTCTCGCATCGA
TGAAGAACGCAGCGAAATGCGATAAGTAATGTGAATTGCAGAATTCAGTGAATCATCGAA
TCTTTGAACGCATCTTGCGCTCCTTGGTATTCCGAGGAGCATGCCTGTTTGAGTGTCATT
AAATTCTCAACTCTCTTATAC-TTTTTTGTAAAAGAGAGCTTGGACTGTGGAGGCTTGCT
GGCCACTTTTTGGGGTCAGCTCCTCTGAAATGCATTAGCGGAACCGTTTGCAATCTGCCA
CAAGTGTGATAAGTTATCTACACTGGCGAGGGGATTGCTCTCTGTAATGTTCAGCTTCTA
ATTGTCTCTACTTTGTGAGACAACTTTTGAATGCTTGACCTCAAATCAGGTAGGACTACC
CGCTGAACTTAA
>ABC10-24
TTTCCGTAGGTGAACCTGCGGAAGGATCATTATTGAATTATGTTTCTAGATAGGTTGTAG
CTGGCTC-TTTAGAGCATGTGCACGCCTGTTTGGACTTCATTTTCATCCACCTGTGCACC
TATTGTAGTCTTTGGTTGGGTTAGGAGGAAGTGGTCATTGTGTCAGCATCTGCTGGATGT
GAGGACTTGCATTGTGAAAGCTTTGCTGTCCTTGATGTGATCATGGAATCTCTTTCTCAC
TAGAGTCTATGTCACTCATTATACTCTGTCGAATGTCATTGAATGTCTTTACATGGGCTT
ATATGCCTATGAAAATTGTAATACAACTTTCAGCAACGGATCTCTTGGCTCTCGCATCGA
TGAAGAACGCAGCGAAATGCGATAAGTAATGTGAATTGCAGAATTCAGTGAATCATCGAA
TCTTTGAACGCATCTTGCGCTCCTTGGTATTCCGAGGAGCATGCCTGTTTGAGTGTCATT
AAATTCTCAACTCTCTTATAC-TTTTTTGTAAAAGAGAGCTTGGACTGTGGAGGCTTGCT
GGCCACTTTTTGGGGTCAGCTCCTCTGAAATGCATTAGCGGAACCGTTTGCAATCTGCCA
CAAGTGTGATAAGTTATCTACACTGGCGAGGGGATTGCTCTCTGTAATGTTCAGCTTCTA
ATTGTCTCTACTTTGTGAGACAACTTTTGAATGCTTGACCTCAAATCAGGTAGGACTACC
CGCTGAACTTAA
>ABC11-55
TTTCCGTAGGTGAACCTGCGGAAGGATCATTATTGAATTATGTTTCTAGATAGGTTGTAG
CTGGCTC-TTTAGAGCATGTGCACGCCTGTTTGGACTTCATTTTCATCCACCTGTGCACC
TATTGTAGTCTTTGGTTGGGTTAGGAGGAAGTGGTCATTGTGTCAGCATCTGCTGGATGT
GAGGACTTGCATTGTGAAAGCTTTGCTGTCCTTGATGTGATCATGGAATCTCTTTCTCAC
TAGAGTCTATGTCACTCATTATACTCTGTCGAATGTCATTGAATGTCTTTACATGGGCTT
ATATGCCTATGAAAATTGTAATACAACTTTCAGCAACGGATCTCTTGGCTCTCGCATCGA
TGAAGAACGCAGCGAAATGCGATAAGTAATGTGAATTGCAGAATTCAGTGAATCATCGAA

TCTTTGAACGCATCTTGCGCTCCTTGGTATTCCGAGGAGCATGCCTGTTTGAGTGTCATT
AAATTCTCAACTCTCTTATAC-TTTTTTGTAAAAGAGAGCTTGGACTGTGGAGGCTTGCT
GGCCACTTTTTGGGGTCAGCTCCTCTGAAATGCATTAGCGGAACCGTTTGCAATCTGCCA
CAAGTGTGATAAGTTATCTACACTGGCGAGGGGATTGCTCTCTGTAATGTTCAGCTTCTA
ATTGTCTCTACTTTGTGAGACAACTTTTGAATGCTTGACCTCAAATCAGGTAGGACTACC
CGCTGAACTTAA
>ABC12-31
TTTCCGTAGGTGAACCTGCGGAAGGATCATTATTGAATTATGTTTCTAGATAGGTTGTAG
CTGGCTC-TTTAGAGCATGTGCACGCCTGTTTGGACTTCATTTTCATCCACCTGTGCACC
TATTGTAGTCTTTGGTTGGGTTAGGAGGAAGTGGTCATTGTGTCAGCATCTGCTGGATGT
GAGGACTTGCATTGTGAAAGCTTTGCTGTCCTTGATGTGATCATGGAATCTCTTTCTCAC
TAGAGTCTATGTCACTCATTATACTCTGTCGAATGTCATTGAATGTCTTTACATGGGCTT
ATATGCCTATGAAAATTGTAATACAACTTTCAGCAACGGATCTCTTGGCTCTCGCATCGA
TGAAGAACGCAGCGAAATGCGATAAGTAATGTGAATTGCAGAATTCAGTGAATCATCGAA
TCTTTGAACGCATCTTGCGCTCCTTGGTATTCCGAGGAGCATGCCTGTTTGAGTGTCATT
AAATTCTCAACTCTCTTATAC-TTTTTTGTAAAAGAGAGCTTGGACTGTGGAGGCTTGCT
GGCCACTTTTTGGGGTCAGCTCCTCTGAAATGCATTAGCGGAACCGTTTGCAATCTGCCA
CAAGTGTGATAAGTTATCTACACTGGCGAGGGGATTGCTCTCTGTAATGTTCAGCTTCTA
ATTGTCTCTACTTTGTGAGACAACTTTTGAATGCTTGACCTCAAATCAGGTAGGACTACC
CGCTGAACTTAA
>ABC12-53
TTTCCGTAGGTGAACCTGCGGAAGGATCATTATTGAATTATGTTTCTAGATAGGTTGTAG
CTGGCTC-TTTAGAGCATGTGCACGCCTGTTTGGACTTCATTTTCATCCACCTGTGCACC
TATTGTAGTCTTTGGTTGGGTTAGGAGGAAGTGGTCATTGTGTCAGCATCTGCTGGATGT
GAGGACTTGCATTGTGAAAGCTTTGCTGTCCTTGATGTGATCATGGAATCTCTTTCTCAC
TAGAGTCTATGTCACTCATTATACTCTGTCGAATGTCATTGAATGTCTTTACATGGGCTT
ATATGCCTATGAAAATTGTAATACAACTTTCAGCAACGGATCTCTTGGCTCTCGCATCGA
TGAAGAACGCAGCGAAATGCGATAAGTAATGTGAATTGCAGAATTCAGTGAATCATCGAA
TCTTTGAACGCATCTTGCGCTCCTTGGTATTCCGAGGAGCATGCCTGTTTGAGTGTCATT
AAATTCTCAACTCTCTTATAC-TTTTTTGTAAAAGAGAGCTTGGACTGTGGAGGCTTGCT
GGCCACTTTTTGGGGTCAGCTCCTCTGAAATGCATTAGCGGAACCGTTTGCAATCTGCCA
CAAGTGTGATAAGTTATCTACACTGGCGAGGGGATTGCTCTCTGTAATGTTCAGCTTCTA
ATTGTCTCTACTTTGTGAGACAACTTTTGAATGCTTGACCTCAAATCAGGTAGGACTACC
CGCTGAACTTAA
>ABC3-61
TTTCCGTAGGTGAACCTGCGGAAGGATCATTATTGAATTATGTTTCTAGATAGGTTGTAG
CTGGCTC-TTTAGAGCATGTGCACGCCTGTTTGGACTTCATTTTCATCCACCTGTGCACC
TATTGTAGTCTTTGGTTGGGTTAGGAGGAAGTGGTCATTGTGTCAGCATCTGCTGGATGT
GAGGACTTGCATTGTGAAAGCTTTGCTGTCCTTGATGTGATCATGGAATCTCTTTCTCAC
TAGAGTCTATGTCACTCATTATACTCTGTCGAATGTCATTGAATGTCTTTACATGGGCTT
ATATGCCTATGAAAATTGTAATACAACTTTCAGCAACGGATCTCTTGGCTCTCGCATCGA
TGAAGAACGCAGCGAAATGCGATAAGTAATGTGAATTGCAGAATTCAGTGAATCATCGAA
TCTTTGAACGCATCTTGCGCTCCTTGGTATTCCGAGGAGCATGCCTGTTTGAGTGTCATT
AAATTCTCAACTCTCTTATAC-TTTTTTGTAAAAGAGAGCTTGGACTGTGGAGGCTTGCT
GGCCACTTTTTGGGGTCAGCTCCTCTGAAATGCATTAGCGGAACCGTTTGCAATCTGCCA
CAAGTGTGATAAGTTATCTACACTGGCGAGGGGATTGCTCTCTGTAATGTTCAGCTTCTA
ATTGTCTCTACTTTGTGAGACAACTTTTGAATGCTTGACCTCAAATCAGGTAGGACTACC
CGCTGAACTTAA
>ABC6-23
TTTCCGTAGGTGAACCTGCGGAAGGATCATTATTGAATTATGTTTCTAGATAGGTTGTAG

CTGGCTC-TTTAGAGCATGTGCACGCCTGTTTGGACTTCATTTTCATCCACCTGTGCACC
TATTGTAGTCTTTGGTTGGGTTAGGGGGAAGTGGTCATTGTGTCAGCATCTGCTGGATGT
GAGGACTTGCATTGTGAAAGCTTTGCTGTCCTTGATGTGATCATGGAATCTCTTTCTCAC
TAGAGTCTATGTCACTCATTATACTCTGTCGAATGTCATTGAATGTCTTTACATGGGCTT
ATATGCCTATGAAAATTGTAATACAACTTTCAGCAACGGATCTCTTGGCTCTCGCATCGA
TGAAGAACGCAGCGAAATGCGATAAGTAATGTGAATTGCAGAATTCAGTGAATCATCGAA
TCTTTGAACGCATCTTGCGCTCCTTGGTATTCCGAGGAGCATGCCTGTTTGAGTGTCATT
AAATTCTCAACTCTCTTATAC-TTTTTTGTAAAAGAGAGCTTGGACTGTGGAGGCTTGCT
GGCCACTTTTTGGGGTCAGCTCCTCTGAAATGCATTAGCGGAACCGTTTGCAATCTGCCA
CAAGTGTGATAAGTTATCTACACTGGCGAGGGGATTGCTCTCTGTAATGTTCAGCTTCTA
ATTGTCTCTACTTTGTGAGACAACTTTTGAATGCTTGACCTCAAATCAGGTAGGACTACC
CGCTGAACTTAA
>ABC1-74
TTTCCGTAGGTGAACCTGCGGAAGGATCATTATTGAATTATGTTTCTAGATAGGTTGTAG
CTGGCTC-TTTAGAGCATGTGCACGCCTGTTTGGACTTCATTTTCATCCACCTGTGCACC
TATTGTAGTCTTTGGTTGGGTTAGGGGGAAGTGGTCATTGTGTCAGCATCTGCTGGATGT
GAGGACTTGCATTGTGAAAGCTTTGCTGTCCTTGATGTGATCATGGAATCTCTTTCTCAC
TAGAGTCTATGTCACTCATTATACTCTGTCGAATGTCATTGAATGTCTTTACATGGGCTT
GTATGCCTATGAAAATTGTAATACAACTTTCAGCAACGGATCTCTTGGCTCTCGCATCGA
TGAAGGACGCAGCGAAATGCGATAAGTAATGTGAATTGCAGAATTCAGTGAATCATCGAA
TCTTTGAACGCATCTTGCGCTCCTTGGTATTCCGAGGAGCATGCCTGTTTGAGTGTCATT
AAATTCTCAACTCTCTTATAC-TTTTTTGTAAAAGAGAGCTTGGACTGTGGAGGCTTGCT
GGCCACTTTTTGGGGTCAGCTCCTCTGAAATGCATTAGCGGAACCGTTTGCGATCTGCCA
CAAGTGTGATAAGTTATCTACACTGGCGAGGGGATTGCTCTCTGTAATGTTCAGCTTCTA
ATTGTCTCTACTTTGTGAGACAACTTTTGAATGCTTGACCTCAAATCAGGTAGGACTACC
CGCTGAACTTAA
>ABC8-26
TTTCCGTAGGTGAACCTGCGGAAGGATCATTATTGAATTATGTTTCTAGATAGGTTGTAG
CTGGCTC-TTTAGAGCATGTGCACGCCTGTTTGGACTTCATTTTCATCCACCTGTGCACC
TATTGTAGTCTTTGGTTGGGTTAGGGGGAAGTGGTCATTGTGTCAGCATCTGCTGGATGT
GAGGACTTGCATTGTGAAAGCTTTGCTGTCCTTGATGTGATCATGGAATCTCTTTCTCAC
TAGAGTCTATGTCACTCATTATACTCTGTCGAATGTCATTGAATGTCTTTACATGGGCTT
GTATGCCTATGAAAATTGTAATACAACTTTCAGCAACGGATCTCTTGGCTCTCGCATCGA
TGAAGAACGCAGCGAAATGCGATAAGTAATGTGAATTGCAGAATTCAGTGAATCATCGAA
TCTTTGAACGCATCTTGCGCTCCTTGGTATTCCGAGGAGCATGCCTGTTTGAGTGTCATT
AAATTCTCAACTCTCTTATAC-TTTTTTGTAAAAGAGAGCTTGGACTGTGGAGGCTTGCT
GGCCACTTTTTGGGGTCAGCTCCTCTGAAATGCATTAGCGGAACCGTTTGCGATCTGCCA
CAAGTGTGATAAGTTATCTACACTGGCGAGGGGATTGCTCTCTGTAATGTTCAGCTTCTA
ATTGTCTCTACTTTGTGAGACAACTTTTGAATGCTTGACCTCAAATCAGGTAGGACTACC
CGCTGAACTTAA
>ABC5-64
TTTCCGTAGGTGAACCTGCGGAAGGATCATTATTGAATTATGTTTCTAGATAGGTTGTAG
CTGGCTC-TTTAGAGCATGTGCACGCCTGTTTGGACTTCATTTTCATCCACCTGTGCACC
TATTGTAGTCTTTGGTTGGGTTAGGAGGAAGTGGTCATTGTGTCAGCATCTGCTGGATGT
GAGGACTTGCATTGTGAAAGCTTTGCTGTCCTTGATGTGATCATGGAATCTCTTTCTCAC
TAGAGTCTATGTCACTCATTATACTCTGTCGAATGTCATTGAATGTCTTTACATGGGCTT
GTATGCCTATGAAAATTGTAATACAACTTTCAGCAACGGATCTCTTGGCTCTCGCATCGA
TGAAGAACGCAGCGAAATGCGATAAGTAATGTGAATTGCAGAATTCAGTGAATCATCGAA
TCTTTGAACGCATCTTGCGCTCCTTGGTATTCCGAGGAGCATGCCTGTTTGAGTGTCATT
AAATTCTCAACTCTCTTATAC-TTTTTTGTAAAAGAGAGCTTGGACTGTGGAGGCTTGCT

GGCCACTTTTTGGGGTCAGCTCCTCTGAAATGCATTAGCGGAACCGTTTGCGATCTGCCA
CAAGTGTGATAAGTTATCTACACTGGCGAGGGGATTGCTCTCTGTAATGTTCAGCTTCTA
ATTGTCTCTACTTTGTGAGACAACTTTTGAATGCTTGACCTCAAATCAGGTAGGACTACC
CGCTGAACTTAA
>ABC10–11
TTTCCGTAGGTGAACCTGCGGAAGGATCATTATTGAATTATGTTTCTAGATAGGTTGTAG
CTGGCTC–TTTAGAGCATGTGCACGCCTGTTTGGACTTCATTTTCATCCACCTGTGCACC
TATTGTAGTCTTTGGTTGGGTTAGGAGGAAGTGGTCATTGTGTCAGCATCTGCTGGATGT
GAGGACTTGCATTGTGAAAGCTTTGCTGTCCTTGATGTGATCATGGAATCTCTTTCTCAC
TAGAGTCTATGTCACTCATTATACTCTGTCGAATGTCATTGAATGTCTTTACATGGGCTT
GTATGCCTATGAAAATTGTAATACAACTTTCAGCAACGGATCTCTTGGCTCTCGCATCGA
TGAAGAACGCAGCGAAATGCGATAAGTAATGTGAATTGCAGAATTCAGTGAATCATCGAA
TCTTTGAACGCATCTTGCGCTCCTTGGTATTCCGAGGAGCATGCCTGTTTGAGTGTCATT
AAATTCTCAACTCTCTTATAC–TTTTTTGTAAAAGAGAGCTTGGACTGTGGAGGCTTGCT
GGCCACTTTTTGGGGTCAGCTCCTCTGAAATGCATTAGCGGAACCGTTTGCAATCTGCCA
CAAGTGTGATAAGTTATCTACACTGGCGAGGGGATTGCTCTCTGTAATGTTCAGCTTCTA
ATTGTCTCTACTTTGTGAGACAACTTTTGAATGCTTGACCTCAAATCAGGTAGGACTACC
CGCTGAACTTAA
>ABC2–20
TTTCCGTAGGTGAACCTGCGGAAGGATCATTATTGAATTATGTTTCTAGATAGGTTGTAG
CTGGCTC–TTTAGAGCATGTGCACGCCTGTTTGGACTTCATTTTCATCCACCTGTGCACC
TATTGTAGTCTTTGGTTGGGTTAGGGGGAAGTGGTCATTGTGTCAGCATCTGCTGGATGT
GAGGACTTGCATTGTGAAAGCTTTGCTGTCCTTGATGTGATCATGGAATCTCTTTCTCAC
TAGAGTCTATGTCACTCATTATACTCTGTCGAATGTCATTGAATGTCTTTACATGGGCTT
GTATGCCTATGAAAATTGTAATACAACTTTCAGCAACGGATCTCTTGGCTCTCGCATCGA
TGAAGGACGCAGCGAAATGCGATAAGTAATGTGAATTGCAGAATTCAGTGAATCATCGAA
TCTTTGAACGCATCTTGCGCTCCTTGGTATTCCGAGGAGCATGCCTGTTTGAGTGTCATT
AAATTCTCAACTCTCTTATAC–TTTTTTGTAAAAGAGAGCTTGGACTGTGGAGGCTTGCT
GGCCACTTTTTGGGGTCAGCTCCTCTGAAATGCATTAGCGGAACCGTTTGCAATCTGCCA
CAAGTGTGATAAGTTATCTACACTGGGGAGGGGATTGCTCTCTGTAATGTTCAGCTTCTA
ATTGTCTCTACTTTGTGAGACAACTTTTGAATGCTTGACCTCAAATCAGGTAGGACTACC
CGCTGAACTTAA
>ABC1–58
TTTCCGTAGGTGAACCTGCGGAAGGATCATTATTGAATTATGTTTCTAGATAGGTTGTAG
CTGGCTC–TTTAGAGCATGTGCACGCCTGTTTGGACTTCATTTTCATCCACCTGTGCACC
TATTGTAGTCTTTGGTTGGGTTAGGGGGAAGTGGTCATTGTGTCAGCATCTGCTGGATGT
GAGGACTTGCATTGTGAAAGCTTTGCTGTCCTTGATGTGATCATGGAATCTCTTTCTCAC
TAGAGTCTATGTCACTCATTATACTCTGTCGAATGTCATTGAATGTATTTACATGGGCTT
GTATGCCTATGAAAATTGTAATACAACTTTCAGCAACGGATCTCTTGGCTCTCGCATCGA
TGAAGGACGCAGCGAAATGCGATAAGTAATGTGAATTGCAGAATTCAGTGAATCATCGAA
TCTTTGAACGCATCTTGCGCTCCTTGGTATTCCGAGGAGCATGCCTGTTTGAGTGTCATT
AAATTCTCAACTCTCTTATAC–TTTTTTGTAAAAGAGAGCTTGGACTGTGGAGGCTTGCT
GGCCACTTTTTGGGGTCAGCTCCTCTGAAATGCATTAGCGGAACCGTTTGCAATCTGCCA
CAAGTGTGATAAGTTATCTACACTGGCGAGGGGATTGCTCTCTGTAATGTTCAGCTTCTA
ATTGTCTCTACTTTGTGAGACAACTTTTGAATGCTTGACCTCAAATCAGGTAGGACTACC
CGCTGAACTTAA
>ABC9–36
TTTCCGTAGGTGAACCTGCGGAAGGATCATTATTGAATTATGTTTCTAGATAGGTTGTAG
CTGGCTC–TTTAGAGCATGTGCACGCCTGTTTGGACTTCATTTTCATCCACCTGTGCACC
TATTGTAGTCTTTGGTTGGGTTAGGGGGAAGTGGTCATTGTGTCAGCATCTGCTGGATGT

GAGGACTTGCATTGTGAAAGCTTTGCTGTCCTTGATGTGATCATGGAATCTCTTTCTCAC
TAGAGTCTATGTCACTCATTATACTCTGTCGAATGTCATTGAATGTCTTTACATGGGCTT
GTATGCTTATGAAAATTGTAATACAACTTTCAGCAACGGATCTCTTGGCTCTCGCATCGA
TGAAGAACGCAGCGAAATGCGATAAGTAATGTGAATTGCAGAATTCAGTGAATCATCGAA
TCTTTGAACGCATCTTGCGCTCCTTGGTATTCCGAGGAGCATGCCTGTTTGAGTGTCATT
AAATTCTCAACTCTCTTATAC-TTTTTTGTAAAAGAGAGCTTGGACTGTGGAGGCTTGCT
GGCCACTTTTTGGGGTCAGCTCCTCTGAAATGCATTAGCGGAACCGTTTGCGATCTGCCA
CAAGTGTGATAAGTTATCTACACTGGCGAGGGGATTGCTCTCTGCAATGTTCAGCTTCTA
ATTGTCTCTACTTTGTGAGACAACTTTTGAATGCTTGACCTCAAATCAGGTAGGACTACC
CGCTGAACTTAA
>ABC1-77
TTTCCGTAGGTGAACCTGCGGAAGGATCATTATTGAATTATGTTTCTAGATAGGTTGTAG
CTGGCTC-TTTAGAGCATGTGCACGCCTGTTTGGACTTCATTTTCATCCACCTGTGCACC
TATTGTAGTCTTTGGTTGGGTTAGGGGGAAGTGGTCATTGTGTCAGCATCTGCTGGATGT
GAGGACTTGCATTGTGAAAGCTTTGCTGTCCTTGATGTGATCATGGAATCTCTTTCTCAC
TAGAGTCTATGTCACTCATTATACTCTGTCGAATGTCATTGAATGTCTTTACATGGGCTT
GTATGCCTATGAAAATTGTAATACAACTTTCAGCAACGGATCTCTTGGCTCTCGCATCGA
TGAAGGACGCAGCGAAATGCGATAAGTAATGTGAATTGCAGAATTCAGTGAATCATCGAA
TCTTTGAACGCATCTTGCGCTCCTTGGTATTCCGAGGAGCATGCCTGTTTGAGTGTCATT
AAATTCTCAACTCTCTTATAC-TTTTTTGTAAAAGAGAGCTTGGACTGTGGAGGCTTGCT
GGCCACTTTTTGGGGTCAGCTCCTCTGAAATGCATTAGCGGAACCGTTTGCGATCTGCCA
CAAGTGTGATAAGTTATCTACACTGGCGAGGGGATTGCTCTCTGTAATGTTCAGCTTCTA
ATTGTCTCTACTTTGTGAGACTACTTTTGAATGCTTGACCTCAAATCAGGTAGGACTACC
CGCTGAACTTAA
>ABC5-46
TTTCCGTAGGTGAACCTGCGGAAGGATCATTATTGAATTATGTTTCTAGATAGGTTGTAG
CTGGCTC-TTTAGAGCATGTGCACGCCTGTTTGGACTTCATTTTCATCCACCTGTGCACC
TATTGTAGTCTTTGGTTGGGTTAGGGGGAAGTGGTCATTGTGTCAGCATCTGCTGGATGT
GAGGACTTGCATTGTGAAAGCTTTGCTGTCCTTGATGTGATCATGGAATCTCTTTCTCAC
TAGAGTCTATGTCACTCATTATACTCTGTCGAATGTCATTGAATGTCTTTACATGGGCTT
GTATGCCTATGAAAATTGTAATACAACTTTCAGCAACGGATCTCTTGGCTCTCGCATCGA
TGAAGGACGCAGCGAAATGCGATAAGTAATGTGAATTGCAGAATTCAGTGAATCATCGAA
TCTTTGAACGCATCTTGCGCTCCTTGGTATTCCGAGGAGCATGCCTGTTTGAGTGTCATT
AAATTCTCAACTCTCTTATAC-TTTTTTGTAAAAGAGAGCTTGGACTGTGGAGGCTTGCT
GGCCACTTTTTGGGGTCAGCTCCTCTGAAATGCATTAGCGGAACCGTTTGCGATCTGCCA
CAAGTGTGATAAGTTATCTACACTGGCGAGGGGATTGCTCTCTGTAATGTTCAGCTTCTA
ATTGTCTCTACTTTGTGAGACTACTTTTGAATGCTTGACCTCAAATCAGGTAGGACTACC
CGCTGAACTTAA
>ABC6-16
TTTCCGTAGGTGAACCTGCGGAAGGATCATTATTGAATTATGTTTCTAGATAGGTTGTAG
CTGGCTC-TTTAGAGCATGTGCACGCCTGTTTGGACTTCATTTTCATCCACCTGTGCACC
TATTGTAGTCTTTGGTTGGGTTAGGGGGAAGTGGTCATTGTGTCAGCATCTGCTGGATGT
GAGGACTTGCATTGTGAAAGCTTTGCTGTCCTTGATGTGATCATGGAATCTCTTTCTCAC
TAGAGTCTATGTCACTCATTATACTCTGTCGAATGTCATTGAATGTCTTTACATGGGCTT
GTATGCCTATGAAAATTGTAATACAACTTTCAGCAACGGATCTCTTGGCTCTCGCATCGA
TGAAGGACGCAGCGAAATGCGATAAGTAATGTGAATTGCAGAATTCAGTGAATCATCGAA
TCTTTGAACGCATCTTGCGCTCCTTGGTATTCCGAGGAGCATGCCTGTTTGAGTGTCATT
AAATTCTCAACTCTCTTATAC-TTTTTTGTAAAAGAGAGCTTGGACTGTGGAGGCTTGCT
GGCCACTTTTTGGGGTCAGCTCCTCTGAAATGCATTAGCGGAACCGTTTGCGATCTGCCA
CAAGTGTGATAAGTTATCTACACTGGCGAGGGGATTGCTCTCTGTAATGTTCAGCTTCTA

ATTGTCTCTACTTTGTGAGACTACTTTTGAATGCTTGACCTCAAATCAGGTAGGACTACC
CGCTGAACTTAA
>ABC9-40
TTTCCGTAGGTGAACCTGCGGAAGGATCATTATTGAATTATGTTTCTAGATAGGTTGTAG
CTGGCTC-TTTAGAGCATGTGCACGCCTGTTTGGACTTCATTTTCATCCACCTGTGCACC
TATTGTAGTCTTTGGTTGGGTTAGGGGGAAGTGGTCATTGTGTCAGCATCTGCTGGATGT
GAGGACTTGCATTGTGAAAGCTTTGCTGTCCTTGATGTGATCATGGAATCTCTTTCTCAC
TAGAGTCTATGTCACTCATTATACTCTGTCGAATGTCATTGAATGTCTTTACATGGGCTT
GTATGCCTATGAAAATTGTAATACAACTTTCAGCAACGGATCTCTTGGCTCTCGCATCGA
TGAAGGACGCAGCGAAATGCGATAAGTAATGTGAATTGCAGAATTCAGTGAATCATCGAA
TCTTTGAACGCATCTTGCGCTCCTTGGTATTCCGAGGAGCATGCCTGTTTGAGTGTCATT
AAATTCTCAACTCTCTTATAC-TTTTTTGTAAAAGAGAGCTTGGACTGTGGAGGCTTGCT
GGCCACTTTTTGGGGTCAGCTCCTCTGAAATGCATTAGCGGAACCGTTTGCGATCTGCCA
CAAGTGTGATAAGTTATCTACACTGGCGAGGGGATTGCTCTCTGTAATGTTCAGCTTCTA
ATTGTCTCTACTTTGTGAGACTACTTTTGAATGCTTGACCTCAAATCAGGTAGGACTACC
CGCTGAACTTAA
>ABC8-47
TTTCCGTAGGTGAACCTGCGGAAGGATCATTATTGAATTATGTTTCTAGATAGGTTGTAG
CTGGCTC-TTTAGAGCATGTGCACGCCTGTTTGGACTTCATTTTCATCCACCTGTGCACC
TATTGTAGTCTTTGGTTGGGTTAGGGGGAAGTGGTCATTGTGTCAGCATCTGCTGGATGT
GAGGACTTGCATTGTGAAAGCTTTGCTGTCCTTGATGTGATCATGGAATCTCTTTCTCAC
TAGAGTCTATGTCACTCATTATACTCTGTCGAATGTCATTGAATGTCTTTACATGGGCTT
GTATGCCTATGAAAATTGTAATACAACTTTCAGCAACGGATCTCTTGGCTCTCGCATCGA
TGAAGGACGCAGCGAAATGCGATAAGTAATGTGAATTGCAGAATTCAGTGAATCATCGAA
TCTTTGAACGCATCTTGCGCTCCTTGGTATTCCGAGGAGCATGCCTGTTTGAGTGTCATT
AAATTCTCAACTCTCTTATAC-TTTTTTGTAAAAGAGAGCTTGGACTGTGGAGGCTTGCT
GGCCACTTTTTGGGGTCAGCTCCTCTGAAATGCATTAGCGGAACCGTTTGCGATCTGCCA
CAAGTGTGATAAGTTATCTACACTGGCGAGGGGATTGCTCTCTGTAATGTTCAGCTTCTA
ATTGTCTCTACTTTGTGAGACTACTTTTGAATGCTTGACCTCAAATCAGGTAGGACTACC
CGCTGAACTTAA
>ABC8-1
TTTCCGTAGGTGAACCTGCGGAAGGATCATTATTGAATTATGTTTCTAGATAGGTTGTAG
CTGGCTC-TTTAGAGCATGTGCACGCCTGTTTGGACTTCATTTTCATCCACCTGTGCACC
TATTGTAGTCTTTGGTTGGGTTAGGGGGAAGTGGTCATTGTGTCAGCATCTGCTGGATGT
GAGGACTTGCATTGTGAAAGCTTTGCTGTCCTTGATGTGATCATGGAATCTCTTTCTCAC
TAGAGTCTATGTCACTCATTATACTCTGTCGAATGTCATTGAATGTCTTTACATGGGCTT
GTATGCCTATGAAAATTGTAATACAACTTTCAGCAACGGATCTCTTGGCTCTCGCATCGA
TGAAGGACGCAGCGAAATGCGATAAGTAATGTGAATTGCAGAATTCAGTGAATCATCGAA
TCTTTGAACGCATCTTGCGCTCCTTGGTATTCCGAGGAGCATGCCTGTTTGAGTGTCATT
AAATTCTCAACTCTCTTATAC-TTTTTTGTAAAAGAGAGCTTGGACTGTGGAGGCTTGCT
GGCCACTTTTTGGGGTCAGCTCCTCTGAAATGCATTAGCGGAACCGTTTGCGATCTGCCA
CAAGTGTGATAAGTTATCTACACTGGCGAGGGGATTGCTCTCTGTAATGTTCAGCTTCTA
ATTGTCTCTACTTTGTGAGACTACTTTTGAATGCTTGACCTCAAATCAGGTAGGACTACC
CGCTGAACTTAA
>ABC1-29
TTTCCGTAGGTGAACCTGCGGAAGGATCATTATTGAATTATGTTTCTAGATAGGTTGTAG
CTGGCTC-TTTAGAGCATGTGCACGCCTGTTTGGACTTCATTTTCATCCACCTGTGCACC
TATTGTAGTCTTTGGTTGGGTTAGGGGGAAGTGGTCATTGTGTCAGCATCTGCTGGATGT
GAGGACTTGCATTGTGAAAGCTTTGCTGTCCTTGATGTGATCATGGAATCTCTTTCTCAC
TAGAGTCTATGTCACTCATTATACTCTGTCGAATGTCATTGAATGTCTTTACATGGGCTT

GTATGCCTATGAAAATTGTAATACAACTTTCAGCAACGGATCTCTTGGCTCTCGCATCGA
TGAAGGACGCAGCGAAATGCGATAAGTAATGTGAATTGCAGAATTCAGTGAATCATCGAA
TCTTTGAACGCATCTTGCGCTCCTTGGTATTCCGAGGAGCATGCCTGTTTGAGTGTCATT
AAATTCTCAACTCTCTTATAC-TTTTTTGTAAAAGAGAGCTTGGACTGTGGAGGCTTGCT
GGCCACTTTTTGGGGTCAGCTCCTCTGAAATGCATTAGCGGAACCGTTTGCAATCTGCCA
CAAGTGTGATAAGTTATCTACACTGGCGAGGGGATTGCTCTCTGTAATGTTCAGCTTCTA
ATTGTCTCTACTTTGTGAGACTACTTTTGAATGCTTGACCTCAAATCAGGTAGGACTACC
CGCTGAACTTAA
>ABC5-48
TTTCCGTAGGTGAACCTGCGGAAGGATCATTATTGAATTATGTTTCTAGATAGGTTGTAG
CTGGCTC-TTTAGAGCATGTGCACGCCTGTTTGGACTTCATTTTCATCCACCTGTGCACC
TATTGTAGTCTTTGGTTGGGTTAGGGGGAAGTGGTCATTGTGTCAGCATCTGCTGGATGT
GAGGACTTGCATTGTGAAAGCTTTGCTGTCCTTGATGTGATCATGGAATCTCTTTCTCAC
TAGAGTCTATGTCACTCATTATACTCTGTCGAATGTCATTGAATGTCTTTACATGGGCTT
GTATGCCTATGAAAATTGTAATACAACTTTCAGCAACGGATCTCTTGGCTCTCGCATCGA
TGAAGGACGCAGCGAAATGCGATAAGTAATGTGAATTGCAGAATTCAGTGAATCATCGAA
TCTTTGAACGCATCTTGCGCTCCTTGGTATTCCGAGGAGCATGCCTGTTTGAGTGTCATT
AAATTCTCAACTCTCTTATAC-TTTTTTGTAAAAGAGAGCTTGGACTGTGGAGGCTTGCT
GGCCACTTTTTGGGGTCAGCTCCTCTGAAATGCATTAGCGGAACCGTTTGCAATCTGCCA
CAAGTGTGATAAGTTATCTACACTGGCGAGGGGATTGCTCTCTGTAATGTTCAGCTTCTA
ATTGTCTCTACTTTGTGAGACTACTTTTGAATGCTTGACCTCAAATCAGGTAGGACTACC
CGCTGAACTTAA
>ABC8-55
TTTCCGTAGGTGAACCTGCGGAAGGATCATTATTGAATTATGTTTCTAGATAGGTTGTAG
CTGGCTC-TTTAGAGCATGTGCACGCCTGTTTGGACTTCATTTTCATCCACCTGTGCACC
TATTGTAGTCTTTGGTTGGGTTAGGGGGAAGTGGTCATTGTGTCAGCATCTGCTGGATGT
GAGGACTTGCATTGTGAAAGCTTTGCTGTCCTTGATGTGATCATGGAATCTCTTTCTCAC
TAGAGTCTATGTCACTCATTATACTCTGTCGAATGTCATTGAATGTCTTTACATGGGCTT
GTATGCCTATGAAAATTGTAATACAACTTTCAGCAACGGATCTCTTGGCTCTCGCATCGA
TGAAGGACGCAGCGAAATGCGATAAGTAATGTGAATTGCAGAATTCAGTGAATCATCGAA
TCTTTGAACGCATCTTGCGCTCCTTGGTATTCCGAGGAGCATGCCTGTTTGAGTGTCATT
AAATTCTCAACTCTCTTATAC-TTTTTTGTAAAAGAGAGCTTGGACTGTGGAGGCTTGCT
GGCCACTTTTTGGGGTCAGCTCCTCTGAAATGCATTAGCGGAACCGTTTGCAATCTGCCA
CAAGTGTGATAAGTTATCTACACTGGCGAGGGGATTGCTCTCTGTAATGTTCAGCTTCTA
ATTGTCTCTACTTTGTGAGACTACTTTTGAATGCTTGACCTCAAATCAGGTAGGACTACC
CGCTGAACTTAA
>ABC4-61
TTTCCGTAGGTGAACCTGCGGAAGGATCATTATTGAATTATGTTTCTAGATAGGTTGTAG
CTGGCTC-TTTAGAGCATGTGCACGCCTGTTTGGACTTCATTTTCATCCACCTGTGCACC
TATTGTAGTCTTTGGTTGGGTTAGGGGGAAGTGGTCATTGTGTCAGCATCTGCTGGATGT
GAGGACTTGCATTGTGAAAGCTTTGCTGTCCTTGATGTGATCATGGAATCTCTTTCTCAC
TAGAGTCTATGTCACTCATTATACTCTGTCGAATGTCATTGAATGTCTTTACATGGGCTT
GTATGCCTATGAAAATTGTAATACAACTTTCAGCAACGGATCTCTTGGCTCTCGCATCGA
TGAAGGACGCAGCGAAATGCGATAAGTAATGTGAATTGCAGAATTCAGTGAATCATCGAA
TCTTTGAACGCATCTTGCGCTCCTTGGTATTCCGAGGAGCATGCCTGTTTGAGTGTCATT
AAATTCTCAACTCTCTTATAC-TTTTTTGTAAAAGAGAGCTTGGACTGTGGAGGCTTGCT
GGCCACTTTTTGGGGTCAGCTCCTCTGAAATGCATTAGCGGAACCGTTTGCAATCTGCCA
CAAGTGTGATAAGTTATCTACACTGGCGAGGGGATTGCTCTCTGTAATGTTCAGCTTCTA
ATTGTCTCTACTTTGTGAGACTACTTTTGAATGCTTGACCTCAAATCAGGTAGGACTACC
CGCTGAACTTAA

>ABC6-26
TTTCCGTAGGTGAACCTGCGGAAGGATCATTATTGAATTATGTTTCTAGATAGGTTGTAG
CTGGCTC-TTTAGAGCATGTGCACGCCTGTTTGGACTTCATTTTCATCCACCTGTGCACC
TATTGTAGTCTTTGGTTGGGTTAGGGGGAAGTGGTCATTGTGTCAGCATCTGCTGGATGT
GAGGACTTGCATTGTGAAAGCTTTGCTGTCCTTGATGTGATCATGGAATCTCTTTCTCAC
TAGAGTCTATGTCACTCATTATACTCTGTCGAATGTCATTGAATGTCTTTACATGGGCTT
GTATGCCTATGAAAATTGTAATACAACTTTCAGCAACGGATCTCTTGGCTCTCGCATCGA
TGAAGGACGCAGCGAAATGCGATAAGTAATGTGAATTGCAGAATTCAGTGAATCATCGAA
TCTTTGAACGCATCTTGCGCTCCTTGGTATTCCGAGGAGCATGCCTGTTTGAGTGTCATT
AAATTCTCAACTCTCTTATAC-TTTTTTGTAAAAGAGAGCTTGGACTGTGGAGGCTTGCT
GGCCACTTTTTGGGGTCAGCTCCTCTGAAATGCATTAGCGGAACCGTTTGCAATCTGCCA
CAAGTGTGATAAGTTATCTACACTGGCGAGGGGATTGCTCTCTGTAATGTTCAGCTTCTA
ATTGTCTCTACTTTGTGAGACTACTTTTGAATGCTTGACCTCAAATCAGGTAGGACTACC
CGCTGAACTTAA
>ABC6-25
TTTCCGTAGGTGAACCTGCGGAAGGATCATTATTGAATTATGTTTCTAGATAGGTTGTAG
CTGGCTC-TTTAGAGCATGTGCACGCCTGTTTGGACTTCATTTTCATCCACCTGTGCACC
TATTGTAGTCTTTGGTTGGGTTAGGGGGAAGTGGTCATTGTGTCAGCATCTGCTGGATGT
GAGGACTTGCATTGTGAAAGCTTTGCTGTCCTTGATGTGATCATGGAATCTCTTTCTCAC
TAGAGTCTATGTCACTCATTATACTCTGTCGAATGTCATTGAATGTCTTTACATGGGCTT
GTATGCCTATGAAAATTGTAATACAACTTTCAGCAACGGATCTCTTGGCTCTCGCATCGA
TGAAGAACGCAGCGAAATGCGATAAGTAATGTGAATTGCAGAATTCAGTGAATCATCGAA
TCTTTGAACGCATCTTGCGCTCCTTGGTATTCCGAGGAGCATGCCTGTTTGAGTGTCATT
AAATTCTCAACTCTCTTATAC-TTTTTTGTAAAAGAGAGCTTGGACTGTGGAGGCTTGCT
GGCCACTTTTTGGGGTCAGCTCCTCTGAAATGCATTAGCGGAACCGTTTGCAATCTGCCA
CAAGTGTGATAAGTTATCTACACTGGCGAGGGGATTGCTCTCTGTAATGTTCAGCTTCTA
ATTGTCTCTACTTTGTGAGACTACTTTTGAATGCTTGACCTCAAATCAGGTAGGACTACC
CGCTGAACTTAA
>ABC8-19
TTTCCGTAGGTGAACCTGCGGAAGGATCATTATTGAATTATGTTTCTAGATAGGTTGTAG
CTGGCTC-TTTAGAGCATGTGCACGCCTGTTTGGACTTCATTTTCATCCACCTGTGCACC
TATTGTAGTCTTTGGTTGGGTTAGGGGGAAGTGGTCATTGTGTCAGCATCTGCTGGATGT
GAGGACTTGCATTGTGAAAGCTTTGCTGTCCTTGATGTGATCATGGAATCTCTTTCTCAC
TAGAGTCTATGTCACTCATTATACTCTGTCGAATGTCATTGAATGTCTTTACATGGGCTT
GTATGCCTATGAAAATTGTAATACAACTTTCAGCAACGGATCTCTTGGCTCTCGCATCGA
TGAAGAACGCAGCGAAATGCGATAAGTAATGTGAATTGCAGAATTCAGTGAATCATCGAA
TCTTTGAACGCATCTTGCGCTCCTTGGTATTCCGAGGAGCATGCCTGTTTGAGTGTCATT
AAATTCTCAACTCTCTTATAC-TTTTTTGTAAAAGAGAGCTTGGACTGTGGAGGCTTGCT
GGCCACTTTTTGGGGTCAGCTCCTCTGAAATGCATTAGCGGAACCGTTTGCAATCTGCCA
CAAGTGTGATAAGTTATCTACACTGGCGAGGGGATTGCTCTCTGTAATGTTCAGCTTCTA
ATTGTCTCTACTTTGTGAGACTACTTTTGAATGCTTGACCTCAAATCAGGTAGGACTACC
CGCTGAACTTAA
>ABC6-33
TTTCCGTAGGTGAACCTGCGGAAGGATCATTATTGAATTATGTTTCTAGATAGGTTGTAG
CTGGCTC-TTTAGAGCATGTGCACGCCTGTTTGGACTTCATTTTCATCCACCTGTGCACC
TATTGTAGTCTTTGGTTGGGTTAGGGGGAAGTGGTCATTGTGTCAGCATCTGCTGGATGT
GAGGACTTGCATTGTGAAAGCTTTGCTGTCCTTGATGTGATCATGGAATCTCTTTCTCAC
TAGAGTCTATGTCACTCATTATACTCTGTCGAATGTCATTGAATGTCTTTACATGGGCTT
ATATGCCTATGAAAATTGTAATACAACTTTCAGCAACGGATCTCTTGGCTCTCGCATCGA
TGAAGAACGCAGCGAAATGCGATAAGTAATGTGAATTGCAGAATTCAGTGAATCATCGAA

TCTTTGAACGCATCTTGCGCTCCTTGGTATTCCGAGGAGCATGCCTGTTTGAGTGTCATT
AAATTCTCAACTCTCTTATAC-TTTTTTGTAAAAGAGAGCTTGGACTGTGGAGGCTTGCT
GGCCACTTTTTGGGGTCAGCTCCTCTGAAATGCATTAGCGGAACCGTTTGCGATCTGCCA
CAAGTGTGATAAGTTATCTACACTGGCGAGGGGATTGCTCTCTGTAATGTTCAGCTTCTA
ATTGTCTCTACTTTGTGAGACTACTTTTGAATGCTTGACCTCAAATCAGGTAGGACTACC
CGCTGAACTTAA
>ABC11-10
TTTCCGTAGGTGAACCTGCGGAAGGATCATTATTGAATTATGTTTCTAGATAGGTTGTAG
CTGGCTC-TTTAGAGCATGTGCACGCCTGTTTGGACTTCATTTTCATCCACCTGTGCACC
TATTGTAGTCTTTGGTTGGGTTAGGGGGAAGTGGTCATTGTGTCAGCATCTGCTGGATGT
GAGGACTTGCATTGTGAAAGCTTTGCTGTCCTTGATGTGATCATGGAATCTCTTTCTCAC
TAGAGTCTATGTCACTCATTATACTCTGTCGAATGTCATTGAATGTCTTTACATGGGCTT
ATATGCCTATGAAAATTGTAATACAACTTTCAGCAACGGATCTCTTGGCTCTCGCATCGA
TGAAGAACGCAGCGAAATGCGATAAGTAATGTGAATTGCAGAATTCAGTGAATCATCGAA
TCTTTGAACGCATCTTGCGCTCCTTGGTATTCCGAGGAGCATGCCTGTTTGAGTGTCATT
AAATTCTCAACTCTCTTATAC-TTTTTTGTAAAAGAGAGCTTGGACTGTGGAGGCTTGCT
GGCCACTTTTTGGGGTCAGCTCCTCTGAAATGCATTAGCGGAACCGTTTGCGATCTGCCA
CAAGTGTGATAAGTTATCTACACTGGCGAGGGGATTGCTCTCTGTAATGTTCAGCTTCTA
ATTGTCTCTACTTTGTGAGACTACTTTTGAATGCTTGACCTCAAATCAGGTAGGACTACC
CGCTGAACTTAA
>ABC6-44
TTTCCGTAGGTGAACCTGCGGAAGGATCATTATTGAATTATGTTTCTAGATAGGTTGTAG
CTGGCTC-TTTAGAGCATGTGCACGCCTGTTTGGACTTCATTTTCATCCACCTGTGCACC
TATTGTAGTCTTTGGTTGGGTTAGGGGGAAGTGGTCATTGTGTCAGCATCTGCTGGATGT
GAGGACTTGCATTGTGAAAGCTTTGCTGTCCTTGATGTGATCATGGAATCTCTTTCTCAC
TAGAGTCTATGTCACTCATTATACTCTGTCGAATGTCATTGAATGTCTTTACATGGGCTT
ATATGCCTATGAAAATTGTAATACAACTTTCAGCAACGGATCTCTTGGCTCTCGCATCGA
TGAAGGACGCAGCGAAATGCGATAAGTAATGTGAATTGCAGAATTCAGTGAATCATCGAA
TCTTTGAACGCATCTTGCGCTCCTTGGTATTCCGAGGAGCATGCCTGTTTGAGTGTCATT
AAATTCTCAACTCTCTTATAC-TTTTTTGTAAAAGAGAGCTTGGACTGTGGAGGCTTGCT
GGCCACTTTTTGGGGTCAGCTCCTCTGAAATGCATTAGCGGAACCGTTTGCGATCTGCCA
CAAGTGTGATAAGTTATCTACACTGGCGAGGGGATTGCTCTCTGTAATGTTCAGCTTCTA
ATTGTCTCTACTTTGTGAGACTACTTTTGAATGCTTGACCTCAAATCAGGTAGGACTACC
CGCTGAACTTAA
>ABC7-4
TTTCCGTAGGTGAACCTGCGGAAGGATCATTATTGAATTATGTTTCTAGATAGGTTGTAG
CTGGCTC-TTTAGAGCATGTGCACGCCTGTTTGGACTTCATTTTCATCCACCTGTGCACC
TATTGTAGTCTTTGGTTGGGTTAGGGGGAAGTGGTCATTGTGTCAGCATCTGCTGGATGT
GAGGACTTGCATTGTGAAAGCTTTGCTGTCCTTGATGTGATCATGGAATCTCTTTCTCAC
TAGAGTCTATGTCACTCATTATACTCTGTCGAATGTCATTGAATGTCTTTACATGGGCTT
ATATGCCTATGAAAATTGTAATACAACTTTCAGCAACGGATCTCTTGGCTCTCGCATCGA
TGAAGGACGCAGCGAAATGCGATAAGTAATGTGAATTGCAGAATTCAGTGAATCATCGAA
TCTTTGAACGCATCTTGCGCTCCTTGGTATTCCGAGGAGCATGCCTGTTTGAGTGTCATT
AAATTCTCAACTCTCTTATAC-TTTTTTGTAAAAGAGAGCTTGGACTGTGGAGGCTTGCT
GGCCACTTTTTGGGGTCAGCTCCTCTGAAATGCATTAGCGGAACCGTTTGCAATCTGCCA
CAAGTGTGATAAGTTATCTACACTGGCGAGGGGATTGCTCTCTGTAATGTTCAGCTTCTA
ATTGTCTCTACTTTGTGAGACTACTTTTGAATGCTTGACCTCAAATCAGGTAGGACTACC
CGCTGAACTTAA
>ABC2-72
TTTCCGTAGGTGAACCTGCGGAAGGATCATTATTGAATTATGTTTCTAGATAGGTTGTAG

CTGGCTC–TTTAGAGCATGTGCACGCCTGTTTGGACTTCATTTTCATCCACCTGTGCACC
TATTGTAGTCTTTGGTTGGGTTAGGAGGAAGTGGTCATTGTGTCAGCATCTGCTGGATGT
GAGGACTTGCATTGTGAAAGCTTTGCTGTCCTTGATGTGATCATGGAATCTCTTTCTCAC
TAGAGTCTATGTCACTCATTATACTCTGTCGAATGTCATTGAATGTCTTTACATGGGCTT
GTATGCCTATGAAAATTGTAATACAACTTTCAGCAACGGATCTCTTGGCTCTCGCATCGA
TGAAGAACGCAGCGAAATGCGATAAGTAATGTGAATTGCAGAATTCAGTGAATCATCGAA
TCTTTGAACGCATCTTGCGCTCCTTGGTATTCCGAGGAGCATGCCTGTTTGAGTGTCATT
AAATTCTCAACTCTCTTATAC–TTTTTTGTAAAAGAGAGCTTGGACTGTGGAGGCTTGCT
GGCCACTTTTTGGGGTCAGCTCCTCTGAAATGCATTAGCGGAACCGTTTGCGATCTGCCA
CAAGTGTGATAAGTTATCTACACTGGCGAGGGGATTGCTCTCTGTAATGTTCAGCTTCTA
ATTGTCTCTACTTTGTGAGACTACTTTTGAATGCTTGACCTCAAATCAGGTAGGACTACC
CGCTGAACTTAA
>ABC10–50
TTTCCGTAGGTGAACCTGCGGAAGGATCATTATTGAATTATGTTTCTAGATAGGTTGTAG
CTGGCTC–TTTAGAGCATGTGCACGCCTGTTTGGACTTCATTTTCATCCACCTGTGCACC
TATTGTAGTCTTTGGTTGGGTTAGGAGGAAGTGGTCATTGTGTCAGCATCTGCTGGATGT
GAGGACTTGCATTGTGAAAGCTTTGCTGTCCTTGATGTGATCATGGAATCTCTTTCTCAC
TAGAGTCTATGTCACTCATTATACTCTGTCGAATGTCATTGAATGTCTTTACATGGGCTT
GTATGCCTATGAAAATTGTAATACAACTTTCAGCAACGGATCTCTTGGCTCTCGCATCGA
TGAAGAACGCAGCGAAATGCGATAAGTAATGTGAATTGCAGAATTCAGTGAATCATCGAA
TCTTTGAACGCATCTTGCGCTCCTTGGTATTCCGAGGAGCATGCCTGTTTGAGTGTCATT
AAATTCTCAACTCTCTTATAC–TTTTTTGTAAAAGAGAGCTTGGACTGTGGAGGCTTGCT
GGCCACTTTTTGGGGTCAGCTCCTCTGAAATGCATTAGCGGAACCGTTTGCGATCTGCCA
CAAGTGTGATAAGTTATCTACACTGGCGAGGGGATTGCTCTCTGTAATGTTCAGCTTCTA
ATTGTCTCTACTTTGTGAGACTACTTTTGAATGCTTGACCTCAAATCAGGTAGGACTACC
CGCTGAACTTAA
>ABC3–67
TTTCCGTAGGTGAACCTGCGGAAGGATCATTATTGAATTATGTTTCTAGATAGGTTGTAG
CTGGCTC–TTTAGAGCATGTGCACGCCTGTTTGGACTTCATTTTCATCCACCTGTGCACC
TATTGTAGTCTTTGGTTGGGTTAGGAGGAAGTGGTCATTGTGTCAGCATCTGCTGGATGT
GAGGACTTGCATTGTGAAAGCTTTGCTGTCCTTGATGTGATCATGGAATCTCTTTCTCAC
TAGAGTCTATGTCACTCATTATACTCTGTCGAATGTCATTGAATGTCTTTACATGGGCTT
GTATGCCTATGAAAATTGTAATACAACTTTCAGCAACGGATCTCTTGGCTCTCGCATCGA
TGAAGAACGCAGCGAAATGCGATAAGTAATGTGAATTGCAGAATTCAGTGAATCATCGAA
TCTTTGAACGCATCTTGCGCTCCTTGGTATTCCGAGGAGCATGCCTGTTTGAGTGTCATT
AAATTCTCAACTCTCTTATAC–TTTTTTGTAAAAGAGAGCTTGGACTGTGGAGGCTTGCT
GGCCACTTTTTGGGGTCAGCTCCTCTGAAATGCATTAGCGGAACCGTTTGCGATCTGCCA
CAAGTGTGATAAGTTATCTACACTGGCGAGGGGATTGCTCTCTGTAATGTTCAGCTTCTA
ATTGTCTCTACTTTGTGAGACTACTTTTGAATGCTTGACCTCAAATCAGGTAGGACTACC
CGCTGAACTTAA
>ABC2–74
TTTCCGTAGGTGAACCTGCGGAAGGATCATTATTGAATTATGTTTCTAGATAGGTTGTAG
CTGGCTC–TTTAGAGCATGTGCACGCCTGTTTGGACTTCATTTTCATCCACCTGTGCACC
TATTGTAGTCTTTGGTTGGGTTAGGGGGAAGTGGTCATTGTGTCAGCATCTGCTGGATGT
GAGGACTTGCATTGTGAAAGCTTTGCTGTCCTTGATGTGATCATGGAATCTCTTTCTCAC
TAGAGTCTATGTCACTCATTATACTCTGTCGAATGTCATTGAATGTCTTTACATGGGCTT
GTATGCCTATGAAAATTGTAATACAACTTTCAGCAACGGATCTCTTGGCTCTCGCATCGA
TGAAGAACGCAGCGAAATGCGATAAGTAATGTGAATTGCAGAATTCAGTGAATCATCGAA
TCTTTGAACGCATCTTGCGCTCCTTGGTATTCCGAGGAGCATGCCTGTTTGAGTGTCATT
AAATTCTCAACTCTCTTATAC–TTTTTTGTAAAAGAGAGCTTGGACTGTGGAGGCTTGCT

GGCCACTTTTTGGGGTCAGCTCCTCTGAAATGCATTAGCGGAACCGTTTGCGATCTGCCA
CAAGTGTGATAAGTTATCTACACTGGCGAGGGGATTGCTCTCTGTAATGTTCAGCTTCTA
ATTGTCTCTACTTTGTGAGACTACTTTTGAATGCTTGACCTCAAATCAGGTAGGACTACC
CGCTGAACTTAA
>ABC3-16
TTTCCGTAGGTGAACCTGCGGAAGGATCATTATTGAATTATGTTTCTAGATAGGTTGTAG
CTGGCTC-TTTAGAGCATGTGCACGCCTGTTTGGACTTCATTTTCATCCACCTGTGCACC
TATTGTAGTCTTTGGTTGGGTTAGGGGGAAGTGGTCATTGTGTCAGCATCTGCTGGATGT
GAGGACTTGCATTGTGAAAGCTTTGCTGTCCTTGATGTGATCATGGAATCTCTTTCTCAC
TAGAGTCTATGTCACTCATTATACTCTGTCGAATGTCATTGAATGTCTTTACATGGGCTT
GTATGCCTATGAAAATTGTAATACAACTTTCAGCAACGGATCTCTTGGCTCTCGCATCGA
TGAAGAACGCAGCGAAATGCGATAAGTAATGTGAATTGCAGAATTCAGTGAATCATCGAA
TCTTTGAACGCATCTTGCGCTCCTTGGTATTCCGAGGAGCATGCCTGTTTGAGTGTCATT
AAATTCTCAACTCTCTTATAC-TTTTTTGTAAAAGAGAGCTTGGACTGTGGAGGCTTGCT
GGCCACTTTTTGGGGTCAGCTCCTCTGAAATGCATTAGCGGAACCGTTTGCGATCTGCCA
CAAGTGTGATAAGTTATCTACACTGGCGAGGGGATTGCTCTCTGTAATGTTCAGCTTCTA
ATTGTCTCTACTTTGTGAGACTACTTTTGAATGCTTGACCTCAAATCAGGTAGGACTACC
CGCTGAACTTAA
>ABC4-39
TTTCCGTAGGTGAACCTGCGGAAGGATCATTATTGAATTATGTTTCTAGATAGGTTGTAG
CTGGCTC-TTTAGAGCATGTGCACGCCTGTTTGGACTTCATTTTCATCCACCTGTGCACC
TATTGTAGTCTTTGGTTGGGTTAGGGGGAAGTGGTCATTGTGTCAGCATCTGCTGGATGT
GAGGACTTGCATTGTGAAAGCTTTGCTGTCCTTGATGTGATCATGGAATCTCTTTCTCAC
TAGAGTCTATGTCACTCATTATACTCTGTCGAATGTCATTGAATGTCTTTACATGGGCTT
GTATGCCTATGAAAATTGTAATACAACTTTCAGCAACGGATCTCTTGGCTCTCGCATCGA
TGAAGAACGCAGCGAAATGCGATAAGTAATGTGAATTGCAGAATTCAGTGAATCATCGAA
TCTTTGAACGCATCTTGCGCTCCTTGGTATTCCGAGGAGCATGCCTGTTTGAGTGTCATT
AAATTCTCAACTCTCTTATAC-TTTTTTGTAAAAGAGAGCTTGGACTGTGGAGGCTTGCT
GGCCACTTTTTGGGGTCAGCTCCTCTGAAATGCATTAGCGGAACCGTTTGCGATCTGCCA
CAAGTGTGATAAGTTATCTACACTGGCGAGGGGATTGCTCTCTGTAATGTTCAGCTTCTA
ATTGTCTCTACTTTGTGAGACTACTTTTGAATGCTTGACCTCAAATCAGGTAGGACTACC
CGCTGAACTTAA
>ABC8-37
TTTCCGTAGGTGAACCTGCGGAAGGATCATTATTGAATTATGTTTCTAGATAGGTTGTAG
CTGGCTC-TTTAGAGCATGTGCACGCCTGTTTGGACTTCATTTTCATCCACCTGTGCACC
TATTGTAGTCTTTGGTTGGGTTAGGGGGAAGTGGTCATTGTGTCAGCATCTGCTGGATGT
GAGGACTTGCATTGTGAAAGCTTTGCTGTCCTTGATGTGATCATGGAATCTCTTTCTCAC
TAGAGTCTATGTCACTCATTATACTCTGTCGAATGTCATTGAATGTCTTTACATGGGCTT
GTATGCCTATGAAAATTGTAATACAACTTTCAGCAACGGATCTCTTGGCTCTCGCATCGA
TGAAGAACGCAGCGAAATGCGATAAGTAATGTGAATTGCAGAATTCAGTGAATCATCGAA
TCTTTGAACGCATCTTGCGCTCCTTGGTATTCCGAGGAGCATGCCTGTTTGAGTGTCATT
AAATTCTCAACTCTCTTATAC-TTTTTTGTAAAAGAGAGCTTGGACTGTGGAGGCTTGCT
GGCCACTTTTTGGGGTCAGCTCCTCTGAAATGCATTAGCGGAACCGTTTGCGATCTGCCA
CAAGTGTGATAAGTTATCTACACTGGCGAGGGGATTGCTCTCTGTAATGTTCAGCTTCTA
ATTGTCTCTACTTTGTGAGACTACTTTTGAATGCTTGACCTCAAATCAGGTAGGACTACC
CGCTGAACTTAA
>ABC9-23
TTTCCGTAGGTGAACCTGCGGAAGGATCATTATTGAATTATGTTTCTAGATAGGTTGTAG
CTGGCTC-TTTAGAGCATGTGCACGCCTGTTTGGACTTCATTTTCATCCACCTGTGCACC
TATTGTAGTCTTTGGTTGGGTTAGGGGGAAGTGGTCATTGTGTCAGCATCTGCTGGATGT

GAGGACTTGCATTGTGAAAGCTTTGCTGTCCTTGATGTGATCATGGAATCTCTTTCTCAC
TAGAGTCTATGTCACTCATTATACTCTGTCGAATGTCATTGAATGTCTTTACATGGGCTT
GTATGCCTATGAAAATTGTAATACAACTTTCAGCAACGGATCTCTTGGCTCTCGCATCGA
TGAAGAACGCAGCGAAATGCGATAAGTAATGTGAATTGCAGAATTCAGTGAATCATCGAA
TCTTTGAACGCATCTTGCGCTCCTTGGTATTCCGAGGAGCATGCCTGTTTGAGTGTCATT
AAATTCTCAACTCTCTTATAC-TTTTTTGTAAAAGAGAGCTTGGACTGTGGAGGCTTGCT
GGCCACTTTTTGGGGTCAGCTCCTCTGAAATGCATTAGCGGAACCGTTTGCGATCTGCCA
CAAGTGTGATAAGTTATCTACACTGGCGAGGGGATTGCTCTCTGTAATGTTCAGCTTCTA
ATTGTCTCTACTTTGTGAGACTACTTTTGAATGCTTGACCTCAAATCAGGTAGGACTACC
CGCTGAACTTAA
>ABC11-15
TTTCCGTAGGTGAACCTGCGGAAGGATCATTATTGAATTATGTTTCTAGATAGGTTGTAG
CTGGCTC-TTTAGAGCATGTGCACGCCTGTTTGGACTTCATTTTCATCCACCTGTGCACC
TATTGTAGTCTTTGGTTGGGTTAGGGGGAAGTGGTCATTGTGTCAGCATCTGCTGGATGT
GAGGACTTGCATTGTGAAAGCTTTGCTGTCCTTGATGTGATCATGGAATCTCTTTCTCAC
TAGAGTCTATGTCACTCATTATACTCTGTCGAATGTCATTGAATGTCTTTACATGGGCTT
GTATGCCTATGAAAATTGTAATACAACTTTCAGCAACGGATCTCTTGGCTCTCGCATCGA
TGAAGAACGCAGCGAAATGCGATAAGTAATGTGAATTGCAGAATTCAGTGAATCATCGAA
TCTTTGAACGCATCTTGCGCTCCTTGGTATTCCGAGGAGCATGCCTGTTTGAGTGTCATT
AAATTCTCAACTCTCTTATAC-TTTTTTGTAAAAGAGAGCTTGGACTGTGGAGGCTTGCT
GGCCACTTTTTGGGGTCAGCTCCTCTGAAATGCATTAGCGGAACCGTTTGCGATCTGCCA
CAAGTGTGATAAGTTATCTACACTGGCGAGGGGATTGCTCTCTGTAATGTTCAGCTTCTA
ATTGTCTCTACTTTGTGAGACTACTTTTGAATGCTTGACCTCAAATCAGGTAGGACTACC
CGCTGAACTTAA
>ABC7-56
TTTCCGTAGGTGAACCTGCGGAAGGATCATTATTGAATTATGTTTCTAGATAGGTTGTAG
CTGGCTC-TTTAGAGCATGTGCACGCCTGTTTGGACTTCATTTTCATCCACCTGTGCACC
TATTGTAGTCTTTGGTTGGGTTAGGGGGAAGTGGTCATTGTGTCAGCATCTGCTGGATGT
GAGGACTTGCATTGTGAAAGCTTTGCTGTCCTTGATGTGATCATGGAATCTCTTTCTCAC
TAGAGTCTATGTCACTCATTATACTCTGTCGAATGTCATTGAATGTCTTTACATGGGCTT
GTATGCCTATGAAAATTGTAATACAACTTTCAGCAACGGATCTCTTGGCTCTCGCATCGA
TGAAGAACGCAGCGAAATGCGATAAGTAATGTGAATTGCAGAATTCAGTGAATCATCGAA
TCTTTGAACGCATCTTGCGCTCCTTGGTATTCCGAGGAGCATGCCTGTTTGAGTGTCATT
AAATTCTCAACTCTCTTATAC-TTTTTTGTAAAAGAGAGCTTGGACTGTGGAGGCTTGCT
GGCCACTTTTTGGGGTCAGCTCCTCTGAAATGCATTAGCGGAACCGTTTGCGATCTGCCA
CAAGTGTGATAAGTTATCTACACTGGCGAGGGGATTGCTCTCTGTAATGTTCAGCTTCTA
ATTGTCTCTACTTTGTGAGACTACTTTTGAATGCTTGACCTCAAATCAGGTAGGACTACC
CGCTGAACTTAA
>ABC6-6
TTTCCGTAGGTGAACCTGCGGAAGGATCATTATTGAATTATGTTTCTAGATAGGTTGTAG
CTGGCTC-TTTAGAGCATGTGCACGCCTGTTTGGACTTCATTTTCATCCACCTGTGCACC
TATTGTAGTCTTTGGTTGGGTTAGGGGGAAGTGGTCATTGTGTCAGCATCTGCTGGATGT
GAGGACTTGCATTGTGAAAGCTTTGCTGTCCTTGATGTGATCATGGAATCTTTTTCTCAC
TAGAGTCTATGTCACTCATTATACTCTGTCGAATGTCATTGAATGTCTTTACATGGGCTT
GTATGCCTATGAAAATTGTAATACAACTTTCAGCAACGGATCTCTTGGCTCTCGCATCGA
TGAAGAACGCAGCGAAATGCGATAAGTAATGTGAATTGCAGAATTCAGTGAATCATCGAA
TCTTTGAACGCATCTTGCGCTCCTTGGTATTCCGAGGAGCATGCCTGTTTGAGTGTCATT
AAATTCTCAACTCTCTTATAC-TTTTTTGTAAAAGAGAGCTTGGACTGTGGAGGCTTGCT
GGCCACTTTTTGGGGTCAGCTCCTCTGAAATGCATTAGCGGAACCGTTTGCGATCTGCCA
CAAGTGTGATAAGTTATCTACACTGGCGAGGGGATTGCTCTCTGTAATGTTCAGCTTCTA

ATTGTCTCTACTTTGTGAGACTACTTTTGAATGCTTGACCTCAAATCAGGTAGGACTACC
CGCTGAACTTAA
>ABC8-60
TTTCCGTAGGTGAACCTGCGGAAGGATCATTATTGAATTATGTTTCTAGATAGGTTGTAG
CTGGCTC-TTTAGAGCATGTGCACGCCTGTTTGGACTTCATTTTCATCCACCTGTGCACC
TATTGTAGTCTTTGGTTGGGTTAGGGGGAAGTGGTCATTGTGTCAGCATCTGCTGGATGT
GAGGACTTGCATTGTGAAAGCTTTGCTGTCCTTGATGTGATCATGGAATCTTTTTCTCAC
TAGAGTCTATGTCACTCATTATACTCTGTCGAATGTCATTGAATGTCTTTACATGGGCTT
GTATGCCTATGAAAATTGTAATACAACTTTCAGCAACGGATCTCTTGGCTCTCGCATCGA
TGAAGAACGCAGCGAAATGCGATAAGTAATGTGAATTGCAGAATTCAGTGAATCATCGAA
TCTTTGAACGCATCTTGCGCTCCTTGGTATTCCGAGGAGCATGCCTGTTTGAGTGTCATT
AAATTCTCAACTCTCTTATAC-TTTTTTGTAAAAGAGAGCTTGGACTGTGGAGGCTTGCT
GGCCACTTTTTGGGGTCAGCTCCTCTGAAATGCATTAGCGGAACCGTTTGCGATCTGCCA
CAAGTGTGATAAGTTATCTACACTGGCGAGGGGATTGCTCTCTGTAATGTTCAGCTTCTA
ATTGTCTCTACTTTGTGAGACTACTTTTGAATGCTTGACCTCAAATCAGGTAGGACTACC
CGCTGAACTTAA
>ABC6-28
TTTCCGTAGGTGAACCTGCGGAAGGATCATTATTGAATTATGTTTCTAGATAGGTTGTAG
CTGGCTC-TTTAGAGCATGTGCACGCCTGTTTGGACTTCATTTTCATCCACCTGTGCACC
TATTGTAGTCTTTGGTTGGGTTAGGAGGAAGTGGTCATTGTGTCAGCATCTGCTGGATGT
GAGGACTTGCATTGTGAAAGCTTTGCTGTCCTTGATGTGATCATGGAATCTTTTTCTCAC
TAGAGTCTATGTCACTCATTATACTCTGTCGAATGTCATTGAATGTCTTTACATGGGCTT
GTATGCCTATGAAAATTGTAATACAACTTTCAGCAACGGATCTCTTGGCTCTCGCATCGA
TGAAGAACGCAGCGAAATGCGATAAGTAATGTGAATTGCAGAATTCAGTGAATCATCGAA
TCTTTGAACGCATCTTGCGCTCCTTGGTATTCCGAGGAGCATGCCTGTTTGAGTGTCATT
AAATTCTCAACTCTCTTATAC-TTTTTTGTAAAAGAGAGCTTGGACTGTGGAGGCTTGCT
GGCCACTTTTTGGGGTCAGCTCCTCTGAAATGCATTAGCGGAACCGTTTGCGATCTGCCA
CAAGTGTGATAAGTTATCTACACTGGCGAGGGGATTGCTCTCTGTAATGTTCAGCTTCTA
ATTGTCTCTACTTTGTGAGACTACTTTTGAATGCTTGACCTCAAATCAGGTAGGACTACC
CGCTGAACTTAA
>ABC7-13
TTTCCGTAGGTGAACCTGCGGAAGGATCATTATTGAATTATGTTTCTAGATAGGTTGTAG
CTGGCTC-TTTAGAGCATGTGCACGCCTGTTTGGACTTCATTTTCATCCACCTGTGCACC
TATTGTAGTCTTTGGTTGGGTTAGGAGGAAGTGGTCATTGTGTCAGCATCTGCTGGATGT
GAGGACTTGCATTGTGAAAGCTTTGCTGTCCTTGATGTGATCATGGAATCTTTTTCTCAC
TAGAGTCTATGTCACTCATTATACTCTGTCGAATGTCATTGAATGTCTTTACATGGGCTT
GTATGCCTATGAAAATTGTAATACAACTTTCAGCAACGGATCTCTTGGCTCTCGCATCGA
TGAAGAACGCAGCGAAATGCGATAAGTAATGTGAATTGCAGAATTCAGTGAATCATCGAA
TCTTTGAACGCATCTTGCGCTCCTTGGTATTCCGAGGAGCATGCCTGTTTGAGTGTCATT
AAATTCTCAACTCTCTTATAC-TTTTTTGTAAAAGAGAGCTTGGACTGTGGAGGCTTGCT
GGCCACTTTTTGGGGTCAGCTCCTCTGAAATGCATTAGCGGAACCGTTTGCGATCTGCCA
CAAGTGTGATAAGTTATCTACACTGGCGAGGGGATTGCTCTCTGTAATGTTCAGCTTCTA
ATTGTCTCTACTTTGTGAGACTACTTTTGAATGCTTGACCTCAAATCAGGTAGGACTACC
CGCTGAACTTAA
>ABC2-41
TTTCCGTAGGTGAACCTGCGGAAGGATCATTATTGAATTATGTTTCTAGATAGGTTGTAG
CTGGCTC-TTTAGAGCATGTGCACGCCTGTTTGGACTTCATTTTCATCCACCTGTGCACC
TATTGTAGTCTTTGGTTGGGTTAGGGGGAAGTGGTCATTGTGTCAGCATCTGCTGGATGT
GAGGACTTGCATTGTGAAAGCTTTGCTGTCCTTGATGTGATCATGGAATCTCTTTCTCAC
TAGAGTCTATGTCACTCATTATACTCTGTCGAATGTCATTGAATGTCTTTACATGGGCTT

GTATGCCTATGAAAATTGTAATACAACTTTCAGCAACGGATCTCTTGGCTCTCGCATCGA
TGAAGAACGCAGCGAAATGCGATAAGTAATGTGAATTGCAGAATTCAGTGAATCATCGAA
TCTTTGAACGCATCTTGCGCTCCTTGGTATTCCGAGGAGCATGCCTGTTTGAGTGTCATT
AAATTCTCAACTCTCTTATAC-TTTTTTGTAAAAGAGAGCTTGGACTGTGGAGGCTTGCT
GGCCACTTTTTGGGGTCAGCTCCTCTGAAATACATTAGCGGAACCGTTTGCGATCTGCCA
CAAGTGTGATAAGTTATCTACACTGGCGAGGGGATTGCTCTCTGTAATGTTCAGCTTCTA
ATTGTCTCTACTTTGTGAGACTACTTTTGAATGCTTGACCTCAAATCAGGTAGGACTACC
CGCTGAACTTAA
>ABC10-43
TTTCCGTAGGTGAACCTGCGGAAGGATCATTATTGAATTATGTTTCTAGATAGGTTGTAG
CTGGCTC-TTTAGAGCATGTGCACGCCTGTTTGGACTTCATTTTCATCCACCTGTGCACC
TATTGTAGTCTTTGGTTGGGTTAGGAGGAAGTGGTCATTGTGTCAGCATCTGCTGGATGT
GAGGACTTGCATTGTGAAAGCTTTGCTGTCCTTGATGTGATCATGGAATCTTTTTCTCAC
TAGAGTCTATGTCACTCATTATACTCTGTCGAATGTCATTGAATGTCTTTACATGGGCTT
ATATGCCTATGAAAATTGTAATACAACTTTCAGCAACGGATCTCTTGGCTCTCGCATCGA
TGAAGAACGCAGCGAAATGCGATAAGTAATGTGAATTGCAGAATTCAGTGAATCATCGAA
TCTTTGAACGCATCTTGCGCTCCTTGGTATTCCGAGGAGCATGCCTGTTTGAGTGTCATT
AAATTCTCAACTCTCTTATAC-TTTTTTGTAAAAGAGAGCTTGGACTGTGGAGGCTTGCT
GGCCACTTTTTGGGGTCAGCTCCTCTGAAATGCATTAGCGGAACCGTTTGCGATCTGCCA
CAAGTGTGATAAGTTATCTACACTGGCGAGGGGATTGCTCTCTGTAATGTTCAGCTTCTA
ATTGTCTCTACTTTGTGAGACTACTTTTGAATGCTTGACCTCAAATCAGGTAGGACTACC
CGCTGAACTTAA
>ABC3-38
TTTCCGTAGGTGAACCTGCGGAAGGATCATTATTGAATTATGTTTCTAGATAGGTTGTAG
CTGGCTC-TTTAGAGCATGTGCACGCCTGTTTGGACTTCATTTTCATCCACCTGTGCACC
TATTGTAGTCTTTGGTTGGGTTAGGAGGAAGTGGTCATTGTGTCAGCATCTGCTGGATGT
GAGGACTTGCATTGTGAAAGCTTTGCTGTCCTTGATGTGATCATGGAATCTCTTTCTCAC
TAGAGTCTATGTCACTCATTATACTCTGTCGAATGTCATTGAATGTCTTTACATGGGCTT
ATATGCCTATGAAAATTGTAATACAACTTTCAGCAACGGATCTCTTGGCTCTCGCATCGA
TGAAGAACGCAGCGAAATGCGATAAGTAATGTGAATTGCAGAATTCAGTGAATCATCGAA
TCTTTGAACGCATCTTGCGCTCCTTGGTATTCCGAGGAGCATGCCTGTTTGAGTGTCATT
AAATTCTCAACTCTCTTATAC-TTTTTTGTAAAAGAGAGCTTGGACTGTGGAGGCTTGCT
GGCCACTTTTTGGGGTCAGCTCCTCTGAAATGCATTAGCGGAACCGTTTGCGATCTGCCA
CAAGTGTGATAAGTTATCTACACTGGCGAGGGGATTGCTCTCTGTAATGTTCAGCTTCTA
ATTGTCTCTACTTTGTGAGACTACTTTTGAATGCTTGACCTCAAATCAGGTAGGACTACC
CGCTGAACTTAA
>ABC4-15
TTTCCGTAGGTGAACCTGCGGAAGGATCATTATTGAATTATGTTTCTAGATAGGTTGTAG
CTGGCTC-TTTAGAGCATGTGCACGCCTGTTTGGACTTCATTTTCATCCACCTGTGCACC
TATTGTAGTCTTTGGTTGGGTTAGGAGGAAGTGGTCATTGTGTCAGCATCTGCTGGATGT
GAGGACTTGCATTGTGAAAGCTTTGCTGTCCTTGATGTGATCATGGAATCTCTTTCTCAC
TAGAGTCTATGTCACTCATTATACTCTGTCGAATGTCATTGAATGTCTTTACATGGGCTT
ATATGCCTATGAAAATTGTAATACAACTTTCAGCAACGGATCTCTTGGCTCTCGCATCGA
TGAAGAACGCAGCGAAATGCGATAAGTAATGTGAATTGCAGAATTCAGTGAATCATCGAA
TCTTTGAACGCATCTTGCGCTCCTTGGTATTCCGAGGAGCATGCCTGTTTGAGTGTCATT
AAATTCTCAACTCTCTTATAC-TTTTTTGTAAAAGAGAGCTTGGACTGTGGAGGCTTGCT
GGCCACTTTTTGGGGTCAGCTCCTCTGAAATGCATTAGCGGAACCGTTTGCGATCTGCCA
CAAGTGTGATAAGTTATCTACACTGGCGAGGGGATTGCTCTCTGTAATGTTCAGCTTCTA
ATTGTCTCTACTTTGTGAGACTACTTTTGAATGCTTGACCTCAAATCAGGTAGGACTACC
CGCTGAACTTAA

>ABC5-83
TTTCCGTAGGTGAACCTGCGGAAGGATCATTATTGAATTATGTTTCTAGATAGGTTGTAG
CTGGCTC-TTTAGAGCATGTGCACGCCTGTTTGGACTTCATTTTCATCCACCTGTGCACC
TATTGTAGTCTTTGGTTGGGTTAGGAGGAAGTGGTCATTGTGTCAGCATCTGCTGGATGT
GAGGACTTGCATTGTGAAAGCTTTGCTGTCCTTGATGTGATCATGGAATCTCTTTCTCAC
TAGAGTCTATGTCACTCATTATACTCTGTCGAATGTCATTGAATGTCTTTACATGGGCTT
ATATGCCTATGAAAATTGTAATACAACTTTCAGCAACGGATCTCTTGGCTCTCGCATCGA
TGAAGAACGCAGCGAAATGCGATAAGTAATGTGAATTGCAGAATTCAGTGAATCATCGAA
TCTTTGAACGCATCTTGCGCTCCTTGGTATTCCGAGGAGCATGCCTGTTTGAGTGTCATT
AAATTCTCAACTCTCTTATAC-TTTTTTGTAAAAGAGAGCTTGGACTGTGGAGGCTTGCT
GGCCACTTTTTGGGGTCAGCTCCTCTGAAATGCATTAGCGGAACCGTTTGCGATCTGCCA
CAAGTGTGATAAGTTATCTACACTGGCGAGGGGATTGCTCTCTGTAATGTTCAGCTTCTA
ATTGTCTCTACTTTGTGAGACTACTTTTGAATGCTTGACCTCAAATCAGGTAGGACTACC
CGCTGAACTTAA
>ABC6-7
TTTCCGTAGGTGAACCTGCGGAAGGATCATTATTGAATTATGTTTCTAGATAGGTTGTAG
CTGGCTC-TTTAGAGCATGTGCACGCCTGTTTGGACTTCATTTTCATCCACCTGTGCACC
TATTGTAGTCTTTGGTTGGGTTAGGAGGAAGTGGTCATTGTGTCAGCATCTGCTGGATGT
GAGGACTTGCATTGTGAAAGCTTTGCTGTCCTTGATGTGATCATGGAATCTCTTTCTCAC
TAGAGTCTATGTCACTCATTATACTCTGTCGAATGTCATTGAATGTCTTTACATGGGCTT
ATATGCCTATGAAAATTGTAATACAACTTTCAGCAACGGATCTCTTGGCTCTCGCATCGA
TGAAGAACGCAGCGAAATGCGATAAGTAATGTGAATTGCAGAATTCAGTGAATCATCGAA
TCTTTGAACGCATCTTGCGCTCCTTGGTATTCCGAGGAGCATGCCTGTTTGAGTGTCATT
AAATTCTCAACTCTCTTATAC-TTTTTTGTAAAAGAGAGCTTGGACTGTGGAGGCTTGCT
GGCCACTTTTTGGGGTCAGCTCCTCTGAAATGCATTAGCGGAACCGTTTGCGATCTGCCA
CAAGTGTGATAAGTTATCTACACTGGCGAGGGGATTGCTCTCTGTAATGTTCAGCTTCTA
ATTGTCTCTACTTTGTGAGACTACTTTTGAATGCTTGACCTCAAATCAGGTAGGACTACC
CGCTGAACTTAA
>ABC7-22
TTTCCGTAGGTGAACCTGCGGAAGGATCATTATTGAATTATGTTTCTAGATAGGTTGTAG
CTGGCTC-TTTAGAGCATGTGCACGCCTGTTTGGACTTCATTTTCATCCACCTGTGCACC
TATTGTAGTCTTTGGTTGGGTTAGGAGGAAGTGGTCATTGTGTCAGCATCTGCTGGATGT
GAGGACTTGCATTGTGAAAGCTTTGCTGTCCTTGATGTGATCATGGAATCTCTTTCTCAC
TAGAGTCTATGTCACTCATTATACTCTGTCGAATGTCATTGAATGTCTTTACATGGGCTT
ATATGCCTATGAAAATTGTAATACAACTTTCAGCAACGGATCTCTTGGCTCTCGCATCGA
TGAAGAACGCAGCGAAATGCGATAAGTAATGTGAATTGCAGAATTCAGTGAATCATCGAA
TCTTTGAACGCATCTTGCGCTCCTTGGTATTCCGAGGAGCATGCCTGTTTGAGTGTCATT
AAATTCTCAACTCTCTTATAC-TTTTTTGTAAAAGAGAGCTTGGACTGTGGAGGCTTGCT
GGCCACTTTTTGGGGTCAGCTCCTCTGAAATGCATTAGCGGAACCGTTTGCGATCTGCCA
CAAGTGTGATAAGTTATCTACACTGGCGAGGGGATTGCTCTCTGTAATGTTCAGCTTCTA
ATTGTCTCTACTTTGTGAGACTACTTTTGAATGCTTGACCTCAAATCAGGTAGGACTACC
CGCTGAACTTAA
>ABC8-35
TTTCCGTAGGTGAACCTGCGGAAGGATCATTATTGAATTATGTTTCTAGATAGGTTGTAG
CTGGCTC-TTTAGAGCATGTGCACGCCTGTTTGGACTTCATTTTCATCCACCTGTGCACC
TATTGTAGTCTTTGGTTGGGTTAGGAGGAAGTGGTCATTGTGTCAGCATCTGCTGGATGT
GAGGACTTGCATTGTGAAAGCTTTGCTGTCCTTGATGTGATCATGGAATCTCTTTCTCAC
TAGAGTCTATGTCACTCATTATACTCTGTCGAATGTCATTGAATGTCTTTACATGGGCTT
ATATGCCTATGAAAATTGTAATACAACTTTCAGCAACGGATCTCTTGGCTCTCGCATCGA
TGAAGAACGCAGCGAAATGCGATAAGTAATGTGAATTGCAGAATTCAGTGAATCATCGAA

TCTTTGAACGCATCTTGCGCTCCTTGGTATTCCGAGGAGCATGCCTGTTTGAGTGTCATT
AAATTCTCAACTCTCTTATAC-TTTTTTGTAAAAGAGAGCTTGGACTGTGGAGGCTTGCT
GGCCACTTTTTGGGGTCAGCTCCTCTGAAATGCATTAGCGGAACCGTTTGCGATCTGCCA
CAAGTGTGATAAGTTATCTACACTGGCGAGGGGATTGCTCTCTGTAATGTTCAGCTTCTA
ATTGTCTCTACTTTGTGAGACTACTTTTGAATGCTTGACCTCAAATCAGGTAGGACTACC
CGCTGAACTTAA
>ABC9-28
TTTCCGTAGGTGAACCTGCGGAAGGATCATTATTGAATTATGTTTCTAGATAGGTTGTAG
CTGGCTC-TTTAGAGCATGTGCACGCCTGTTTGGACTTCATTTTCATCCACCTGTGCACC
TATTGTAGTCTTTGGTTGGGTTAGGAGGAAGTGGTCATTGTGTCAGCATCTGCTGGATGT
GAGGACTTGCATTGTGAAAGCTTTGCTGTCCTTGATGTGATCATGGAATCTCTTTCTCAC
TAGAGTCTATGTCACTCATTATACTCTGTCGAATGTCATTGAATGTCTTTACATGGGCTT
ATATGCCTATGAAAATTGTAATACAACTTTCAGCAACGGATCTCTTGGCTCTCGCATCGA
TGAAGAACGCAGCGAAATGCGATAAGTAATGTGAATTGCAGAATTCAGTGAATCATCGAA
TCTTTGAACGCATCTTGCGCTCCTTGGTATTCCGAGGAGCATGCCTGTTTGAGTGTCATT
AAATTCTCAACTCTCTTATAC-TTTTTTGTAAAAGAGAGCTTGGACTGTGGAGGCTTGCT
GGCCACTTTTTGGGGTCAGCTCCTCTGAAATGCATTAGCGGAACCGTTTGCGATCTGCCA
CAAGTGTGATAAGTTATCTACACTGGCGAGGGGATTGCTCTCTGTAATGTTCAGCTTCTA
ATTGTCTCTACTTTGTGAGACTACTTTTGAATGCTTGACCTCAAATCAGGTAGGACTACC
CGCTGAACTTAA
>ABC9-53
TTTCCGTAGGTGAACCTGCGGAAGGATCATTATTGAATTATGTTTCTAGATAGGTTGTAG
CTGGCTC-TTTAGAGCATGTGCACGCCTGTTTGGACTTCATTTTCATCCACCTGTGCACC
TATTGTAGTCTTTGGTTGGGTTAGGAGGAAGTGGTCATTGTGTCAGCATCTGCTGGATGT
GAGGACTTGCATTGTGAAAGCTTTGCTGTCCTTGATGTGATCATGGAATCTCTTTCTCAC
TAGAGTCTATGTCACTCATTATACTCTGTCGAATGTCATTGAATGTCTTTACATGGGCTT
ATATGCCTATGAAAATTGTAATACAACTTTCAGCAACGGATCTCTTGGCTCTCGCATCGA
TGAAGAACGCAGCGAAATGCGATAAGTAATGTGAATTGCAGAATTCAGTGAATCATCGAA
TCTTTGAACGCATCTTGCGCTCCTTGGTATTCCGAGGAGCATGCCTGTTTGAGTGTCATT
AAATTCTCAACTCTCTTATAC-TTTTTTGTAAAAGAGAGCTTGGACTGTGGAGGCTTGCT
GGCCACTTTTTGGGGTCAGCTCCTCTGAAATGCATTAGCGGAACCGTTTGCGATCTGCCA
CAAGTGTGATAAGTTATCTACACTGGCGAGGGGATTGCTCTCTGTAATGTTCAGCTTCTA
ATTGTCTCTACTTTGTGAGACTACTTTTGAATGCTTGACCTCAAATCAGGTAGGACTACC
CGCTGAACTTAA
>ABC11-41
TTTCCGTAGGTGAACCTGCGGAAGGATCATTATTGAATTATGTTTCTAGATAGGTTGTAG
CTGGCTC-TTTAGAGCATGTGCACGCCTGTTTGGACTTCATTTTCATCCACCTGTGCACC
TATTGTAGTCTTTGGTTGGGTTAGGAGGAAGTGGTCATTGTGTCAGCATCTGCTGGATGT
GAGGACTTGCATTGTGAAAGCTTTGCTGTCCTTGATGTGATCATGGAATCTCTTTCTCAC
TAGAGTCTATGTCACTCATTATACTCTGTCGAATGTCATTGAATGTCTTTACATGGGCTT
ATATGCCTATGAAAATTGTAATACAACTTTCAGCAACGGATCTCTTGGCTCTCGCATCGA
TGAAGAACGCAGCGAAATGCGATAAGTAATGTGAATTGCAGAATTCAGTGAATCATCGAA
TCTTTGAACGCATCTTGCGCTCCTTGGTATTCCGAGGAGCATGCCTGTTTGAGTGTCATT
AAATTCTCAACTCTCTTATAC-TTTTTTGTAAAAGAGAGCTTGGACTGTGGAGGCTTGCT
GGCCACTTTTTGGGGTCAGCTCCTCTGAAATGCATTAGCGGAACCGTTTGCGATCTGCCA
CAAGTGTGATAAGTTATCTACACTGGCGAGGGGATTGCTCTCTGTAATGTTCAGCTTCTA
ATTGTCTCTACTTTGTGAGACTACTTTTGAATGCTTGACCTCAAATCAGGTAGGACTACC
CGCTGAACTTAA
>ABC11-54
TTTCCGTAGGTGAACCTGCGGAAGGATCATTATTGAATTATGTTTCTAGATAGGTTGTAG

CTGGCTC-TTTAGAGCATGTGCACGCCTGTTTGGACTTCATTTTCATCCACCTGTGCACC
TATTGTAGTCTTTGGTTGGGTTAGGAGGAAGTGGTCATTGTGTCAGCATCTGCTGGATGT
GAGGACTTGCATTGTGAAAGCTTTGCTGTCCTTGATGTGATCATGGAATCTCTTTCTCAC
TAGAGTCTATGTCACTCATTATACTCTGTCGAATGTCATTGAATGTCTTTACATGGGCTT
ATATGCCTATGAAAATTGTAATACAACTTTCAGCAACGGATCTCTTGGCTCTCGCATCGA
TGAAGAACGCAGCGAAATGCGATAAGTAATGTGAATTGCAGAATTCAGTGAATCATCGAA
TCTTTGAACGCATCTTGCGCTCCTTGGTATTCCGAGGAGCATGCCTGTTTGAGTGTCATT
AAATTCTCAACTCTCTTATAC-TTTTTTGTAAAAGAGAGCTTGGACTGTGGAGGCTTGCT
GGCCACTTTTTGGGGTCAGCTCCTCTGAAATGCATTAGCGGAACCGTTTGCGATCTGCCA
CAAGTGTGATAAGTTATCTACACTGGCGAGGGGATTGCTCTCTGTAATGTTCAGCTTCTA
ATTGTCTCTACTTTGTGAGACTACTTTTGAATGCTTGACCTCAAATCAGGTAGGACTACC
CGCTGAACTTAA
>ABC8-32
TTTCCGTAGGTGAACCTGCGGAAGGATCATTATTGAATTATGTTTCTAGATAGGTTGTAG
CTGGCTC-TTTAGAGCATGTGCACGCCTGTTTGGACTTCATTTTCATCCACCTGTGCACC
TATTGTAGTCTTTGGTTGGGTTAGGAGGAAGTGGTCATTGTGTCAGCATCTGCTGGATGT
GAGGACTTGCATTGTGAAAGCTTTGCTGTCCTTGATGTGATCATGGAATCTCTTTCTCAC
TAGAGTCTATGTCACTCATTATACTCTGTCGAATGTCATTGAATGTCTTTACATGGGCTT
GTATGCCTATGAAAATTGTAATACAACTTTCAGCAACGGATCTCTTGGCTCTCGCATCGA
TGAAGAACGCAGCGAAATGCGATAAGTAATGTGAATTGCAGAATTCAGTGAATCATCGAA
TCTTTGAACGCATCTTGCGCTCCTTGGTATTCCGAGGAGCATGCCTGTTTGAGTGTCATT
AAATTCTCAACTCTCTTATAC-TTTTTTGTAAAAGAGAGCTTGGACTGTGGAGGCTTGCT
GGCCACTTTTTGGGGTCAGCTCCTCTGAAATGCATTAGCGGAACCGTTTGCAATCTGCCA
CAAGTGTGATAAGTTATCTACACTGGCGAGGGGATTGCTCTCTGTAATGTTCAGCTTCTA
ATTGTCTCTACTTTGTGAGACTACTTTTGAATGCTTGACCTCAAATCAGGTAGGACTACC
CGCTGAACTTAA
>ABC9-18
TTTCCGTAGGTGAACCTGCGGAAGGATCATTATTGAATTATGTTTCTAGATAGGTTGTAG
CTGGCTC-TTTAGAGCATGTGCACGCCTGTTTGGACTTCATTTTCATCCACCTGTGCACC
TATTGTAGTCTTTGGTTGGGTTAGGAGGAAGTGGTCATTGTGTCAGCATCTGCTGGATGT
GAGGACTTGCATTGTGAAAGCTTTGCTGTCCTTGATGTGATCATGGAATCTCTTTCTCAC
TAGAGTCTATGTCACTCATTATACTCTGTCGAATGTCATTGAATGTCTTTACATGGGCTT
ATATGCCTATGAAAATTGTAATACAACTTTCAGCAACGGATCTCTTGGCTCTCGCATCGA
TGAAGAACGCAGCGAAATGCGATAAGTAATGTGAATTGCAGAATTCAGTGAATCATCGAA
TCTTTGAACGCATCTTGCGCTCCTTGGTATTCCGAGGAGCATGCCTGTTTGAGTGTCATT
AAATTCTCAACTCTCTTATAC-TTTTTTGTAAAAGAGAGCTTGGACTGTGGAGGCTTGCT
GGCCACTTTTTGGGGTCAGCTCCTCTGAAATGCATTAGCGGAACCGTTTGCAATCTGCCA
CAAGTGTGATAAGTTATCTACACTGGCGAGGGGATTGCTCTCTGTAATGTTCAGCTTCTA
ATTGTCTCTACTTTGTGAGACTACTTTTGAATGCTTGACCTCAAATCAGGTAGGACTACC
CGCTGAACTTAA
>ABC4-2
TTTCCGTAGGTGAACCTGCGGAAGGATCATTATTGAATTATGTTTCTAGATAGGTTGTAG
CTGGCTC-TTTAGAGCATGTGCACGCCTGTTTGGACTTCATTTTCATCCACCTGTGCACC
TATTGTAGTCTTTGGTTGGGTTAGGAGGAAGTGGTCATTGTGTCAGCATCTGCTGGATGT
GAGGACTTGCATTGTGAAAGCTTTGCTGTCCTTGATGTGATCATGGAATCTCTTTCTCAC
TAGAGTCTATGTCACTCATTATACTCTGTCGAATGTCATTGAATGTCTTTACATGGGCTT
GTATGCCTATGAAAATTGTAATACAACTTTCAGCAACGGATCTCTTGGCTCTCGCATCGA
TGAAGGACGCAGCGAAATGCGATAAGTAATGTGAATTGCAGAATTCAGTGAATCATCGAA
TCTTTGAACGCATCTTGCGCTCCTTGGTATTCCGAGGAGCATGCCTGTTTGAGTGTCATT
AAATTCTCAACTCTCTTATAC-TTTTTTGTAAAAGAGAGCTTGGACTGTGGAGGCTTGCT

GGCCACTTTTTGGGGTCAGCTCCTCTGAAATGCATTAGCGGAACCGTTTGCGATCTGCCA
CAAGTGTGATAAGTTATCTACACTGGCGAGGGGATTGCTCTCTGTAATGTTCAGCTTCTA
ATTGTCTCTACTTTGTGAGACTACTTTTGAATGCTTGACCTCAAATCAGGTAGGACTACC
CGCTGAACTTAA
>ABC11-38
TTTCCGTAGGTGAACCTGCGGAAGGATCATTATTGAATTATGTTTCTAGATAGGTTGTAG
CTGGCTC-TTTAGAGCATGTGCACGCCTGTTTGGACTTCATTTTCATCCACCTGTGCACC
TATTGTAGTCTTTGGTTGGGTTAGGAGGAAGTGGTCATTGTGTCAGCATCTGCTGGATGT
GAGGACTTGCATTGTGAAAGCTTTGCTGTCCTTGATGTGATCATGGAATCTCTTTCTCAC
TAGAGTCTATGTCACTCATTATACTCTGTCGAATGTCATTGAATGTCTTTACATGGGCTT
GTATGCCTATGAAAATTGTAATACAACTTTCAGCAACGGATCTCTTGGCTCTCGCATCGA
TGAAGGACGCAGCGAAATGCGATAAGTAATGTGAATTGCAGAATTCAGTGAATCATCGAA
TCTTTGAACGCATCTTGCGCTCCTTGGTATTCCGAGGAGCATGCCTGTTTGAGTGTCATT
AAATTCTCAACTCTCTTATAC-TTTTTTGTAAAAGAGAGCTTGGACTGTGGAGGCTTGCT
GGCCACTTTTTGGGGTCAGCTCCTCTGAAATGCATTAGCGGAACCGTTTGCGATCTGCCA
CAAGTGTGATAAGTTATCTACACTGGCGAGGGGATTGCTCTCTGTAATGTTCAGCTTCTA
ATTGTCTCTACTTTGTGAGACTACTTTTGAATGCTTGACCTCAAATCAGGTAGGACTACC
CGCTGAACTTAA
>ABC8-12
TTTCCGTAGGTGAACCTGCGGAAGGATCATTATTGAATTATGTTTCTAGATAGGTTGTAG
CTGGCTC-TTTAGAGCATGTGCACGCCTGTTTGGACTTCATTTTCATCCACCTGTGCACC
TATTGTAGTCTTTGGTTGGGTTAGGAGGAAGTGGTCATTGTGTCAGCATCTGCTGGATGT
GAGGACTTGCATTGTGAAAGCTTTGCTGTCCTTGATGTGATCATGGAATCTCTTTCTCAC
TAGAGTCTATGTCACTCATTATACTCTGTCGAATGTCATTGAATGTCTTTACATGGGCTT
ATATGCCTATGAAAATTGTAATACAACTTTCAGCAACGGATCTCTTGGCTCTCGCATCGA
TGAAGGACGCAGCGAAATGCGATAAGTAATGTGAATTGCAGAATTCAGTGAATCATCGAA
TCTTTGAACGCATCTTGCGCTCCTTGGTATTCCGAGGAGCATGCCTGTTTGAGTGTCATT
AAATTCTCAACTCTCTTATAC-TTTTTTGTAAAAGAGAGCTTGGACTGTGGAGGCTTGCT
GGCCACTTTTTGGGGTCAGCTCCTCTGAAATGCATTAGCGGAACCGTTTGCGATCTGCCA
CAAGTGTGATAAGTTATCTACACTGGCGAGGGGATTGCTCTCTGTAATGTTCAGCTTCTA
ATTGTCTCTACTTTGTGAGACTACTTTTGAATGCTTGACCTCAAATCAGGTAGGACTACC
CGCTGAACTTAA
>ABC4-22
TTTCCGTAGGTGAACCTGCGGAAGGATCATTATTGAATTATGTTTCTAGATAGGTTGTAG
CTGGCTC-TTTAGAGCATGTGCACGCCTGTTTGGACTTCATTTTCATCCACCTGTGCACC
TATTGTAGTCTTTGGTTGGGTTAGGAGGAAGTGGTCATTGTGTCAGCATCTGCTGGATGT
GAGGACTTGCATTGTGAAAGCTTTGCTGTCCTTGATGTGATCATGGAATCTCTTTCTCAC
TAGAGTCTATGTCACTCATTATACTCTGTCGAATGTCATTGAATGTCTTTACATGGGCTT
ATATGCCTATGAAAATTGTAATACAACTTTCAGCAACGGATCTCTTGGCTCTCGCATCGA
TGAAGGACGCAGCGAAATGCGATAAGTAATGTGAATTGCAGAATTCAGTGAATCATCGAA
TCTTTGAACGCATCTTGCGCTCCTTGGTATTCCGAGGAGCATGCCTGTTTGAGTGTCATT
AAATTCTCAACTCTCTTATAC-TTTTTTGTAAAAGAGAGCTTGGACTGTGGAGGCTTGCT
GGCCACTTTTTGGGGTCAGCTCCTCTGAAATGCATTAGCGGAACCGTTTGCGATCTGCCA
CAAGTGTGATAAGTTATCTACACTGGCGAGGGGATTGCTCTCTGTAATGTTCAGCTTCTA
ATTGTCTCTACTTTGTGAGACTACTTTTGAATGCTTGACCTCAAATCAGGTAGGACTACC
CGCTGAACTTAA
>ABC11-21
TTTCCGTAGGTGAACCTGCGGAAGGATCATTATTGAATTATGTTTCTAGATAGGTTGTAG
CTGGCTC-TTTAGAGCATGTGCACGCCTGTTTGGACTTCATTTTCATCCACCTGTGCACC
TATTGTAGTCTTTGGTTGGGTTAGGAGGAAGTGGTCATTGTGTCAGCATCTGCTGGATGT

GAGGACTTGCATTGTGAAAGCTTTGCTGTCCTTGATGTGATCATGGAATCTTTTTCTCAC
TAGAGTCTATGTCACTCATTATACTCTGTCGAATGTCATTGAATGTCTTTACATGGGCTT
GTATGCCTATGAAAATTGTAATACAACTTTCAGCAACGGATCTCTTGGCTCTCGCATCGA
TGAAGGACGCAGCGAAATGCGATAAGTAATGTGAATTGCAGAATTCAGTGAATCATCGAA
TCTTTGAACGCATCTTGCGCTCCTTGGTATTCCGAGGAGCATGCCTGTTTGAGTGTCATT
AAATTCTCAACTCTCTTATAC-TTTTTTGTAAAAGAGAGCTTGGACTGTGGAGGCTTGCT
GGCCACTTTTTGGGGTCAGCTCCTCTGAAATGCATTAGCGGAACCGTTTGCGATCTGCCA
CAAGTGTGATAAGTTATCTACACTGGCGAGGGGATTGCTCTCTGTAATGTTCAGCTTCTA
ATTGTCTCTACTTTGTGAGACTACTTTTGAATGCTTGACCTCAAATCAGGTAGGACTACC
CGCTGAACTTAA
>ABC6-19
TTTCCGTAGGTGAACCTGCGGAAGGATCATTATTGAATTATGTTTCTAGATAGGTTGTAG
CTGGCTC-TTTAGAGCATGTGCACGCCTGTTTGGACTTCATTTTCATCCACCTGTGCACC
TATTGTAGTCTTTGGTTGGGTTAGGGGGAAGTGGTCATTGTGTCAGCATCTGCTGGATGT
GAGGACTTGCATTGTGAAAGCTTTGCTGTCCTTGATGTGATCATGGAATCTTTTTCTCAC
TAGAGTCTATGTCACTCATTATACTCTGTCGAATGTCATTGAATGTCTTTACATGGGCTT
GTATGCCTATGAAAATTGTAATACAACTTTCAGCAACGGATCTCTTGGCTCTCGCATCGA
TGAAGAACGCAGCGAAATGCGATAAGTAATGTGAATTGCAGAATTCAGTGAATCATCGAA
TCTTTGAACGCATCTTGCGCTCCTTGGTATTCCGAGGAGCATGCCTGTTTGAGTGTCATT
AAATTCTCAACTCTCTTATAC-TTTTTTGTAAAAGAGAGCTTGGACTGTGGAGGCTTGCT
GACCACTTTTTGGGGTCAGCTCCTCTGAAATGCATTAGCGGAACCGTTTGCGATCTGCCA
CAAGTGTGATAAGTTATCTACACTGGCGAGGGGATTGCTCTCTGTAATGTTCAGCTTCTA
ATTGTCTCTACTTTGTGAGACTACTTTTGAATGCTTGACCTCAAATCAGGTAGGACTACC
CGCTGAACTTAA
>ABC2-70
TTTCCGTAGGTGAACCTGCGGAAGGATCATTATTGAATTATGTTTCTAGATAGGTTGTAG
CTGGCTC-TTTAGAGCATGTGCACGCCTGTTTGGACTTCATTTTCATCCACCTGTGCACC
TATTGTAGTCTTTGGTTGGGTTAGGAGGAAGTGATCATTGTATCAGCATCTGCTGGATGT
GAGGACTTGCATTGTGAAAGCTTTGCTGTCCTTGATGTGATCATGGAATCTCTTTCTCAC
TAGAGTCTATGTCACTCATTATACTCTGTCGAATGTCATTGAATGTCTTTACATGGGCTT
GTATGCCTATGAAAATTGTAATACAACTTTCAGCAACGGATCTCTTGGCTCTCGCATCGA
TGAAGAACGCAGCGAAATGCGATAAGTAATGTGAATTGCAGAATTCAGTGAATCATCGAA
TCTTTGAACGCATCTTGCGCTCCTTGGTATTCCGAGGAGCATGCCTGTTTGAGTGTCATT
AAATTCTCAACTCTCTTATAC-TTTTTTGTAAAAGAGAGCTTGGACTGTGGAGGCTTGCT
GGCCACTTTTTGGGGTCAGCTCCTCTGAAATGCATTAGCGGAACCGTTTGCGATCTGCCA
CAAGTGTGATAAGTTATCTACACTGGCGAGGGGATTGCTCTCTGTAATGTTCAGCTTCTA
ATTGTCTCTACTTTGTGAGACTACTTTTGAATGCTTGACCTCAAATCAGGTAGGACTACC
CGCTGAACTTAA
>ABC2-22
TTTCCGTAGGTGAACCTGCGGAAGGATCATTATTGAATTATGTTTCTAGATAGGTTGTAG
CTGGCTC-TTTAGAGCATGTGCACGCCTGTTTGGACTTCATTTTCATCCACCTGTGCACC
TATTGTAGTCTTTGGTTGGGTTAGGAGGAAGTGATCATTGTATCAGCATCTGCTGGGAGT
GAGGACTTGCATTGTGAAAGCTTTGCTGTCCTTGATGTGATCATGGAATCTTTTTCTCAC
TAGAGTCTATGTCACTCATTATACTCTGTCGAATGTCATTGAATGTCTTTACATGGGCTT
GTATGCCTATGAAAATTGTAATACAACTTTCAGCAACGGATCTCTTGGCTCTCGCATCGA
TGAAGAACGCAGCGAAATGCGATAAGTAATGTGAATTGCAGAATTCAGTGAATCATCGAA
TCTTTGAACGCATCTTGCGCTCCTTGGTATTCCGAGGAGCATGCCTGTTTGAGTGTCATT
AAATTCTCAACTCTCTTATAC-TTTTTTGTAAAAGAGAGCTTGGACTGTGGAGGCTTGCT
GGCCACTTTTTGGGGTCAGCTCCTCTGAAATGCATTAGCGGAACCGTTTGCGATCTGCCA
CAAGTGTGATAAGTTATCTACACTGGCGAGGGGATTGCTCTCTGTAATGTTCAGCTTCTA

ATTGTCTCTACTTTGTGAGACTACTTTTGAATGCTTGACCTCAAATCAGGTAGGACTACC
CGCTGAACTTAA
>ABC3-24
TTTCCGTAGGTGAACCTGCGGAAGGATCATTATTGAATTATGTTTCTAGATAGGTTGTAG
CTGGCTC-TTTAGAGCATGTGCACGCCTGTTTGGACTTCATTTTCATCCACCTGTGCACC
TATTGTAGTCTTTGGTTGGGTTAGGAGGAAGTGATCATTGTATCAGCATCTGCTGGGAGT
GAGGACTTGCATTGTGAAAGCTTTGCTGTCCTTGATGTGATCATGGAATCTTTTTCTCAC
TAGAGTCTATGTCACTCATTATACTCTGTCGAATGTCATTGAATGTCTTTACATGGGCTT
GTATGCCTATGAAAATTGTAATACAACTTTCAGCAACGGATCTCTTGGCTCTCGCATCGA
TGAAGAACGCAGCGAAATGCGATAAGTAATGTGAATTGCAGAATTCAGTGAATCATCGAA
TCTTTGAACGCATCTTGCGCTCCTTGGTATTCCGAGGAGCATGCCTGTTTGAGTGTCATT
AAATTCTCAACTCTCTTATAC-TTTTTTGTAAAAGAGAGCTTGGACTGTGGAGGCTTGCT
GGCCACTTTTTGGGGTCAGCTCCTCTGAAATGCATTAGCGGAACCGTTTGCGATCTGCCA
CAAGTGTGATAAGTTATCTACACTGGCGAGGGGATTGCTCTCTGTAATGTTCAGCTTCTA
ATTGTCTCTACTTTGTGAGACTACTTTTGAATGCTTGACCTCAAATCAGGTAGGACTACC
CGCTGAACTTAA
>ABC7-57
TTTCCGTAGGTGAACCTGCGGAAGGATCATTATTGAATTATGTTTCTAGATAGGTTGTAG
CTGGCTC-TTTAGAGCATGTGCACGCCTGTTTGGACTTCATTTTCATCCACCTGTGCACC
TATTGTAGTCTTTGGTTGGGTTAGGAGGAAGTGATCATTGTATCAGCATCTGCTGGGAGT
GAGGACTTGCATTGTGAAAGCTTTGCTGTCCTTGATGTGATCATGGAATCTTTTTCTCAC
TAGAGTCTATGTCACTCATTATACTCTGTCGAATGTCATTGAATGTCTTTACATGGGCTT
GTATGCCTATGAAAATTGTAATACAACTTTCAGCAACGGATCTCTTGGCTCTCGCATCGA
TGAAGAACGCAGCGAAATGCGATAAGTAATGTGAATTGCAGAATTCAGTGAATCATCGAA
TCTTTGAACGCATCTTGCGCTCCTTGGTATTCCGAGGAGCATGCCTGTTTGAGTGTCATT
AAATTCTCAACTCTCTTATAC-TTTTTTGTAAAAGAGAGCTTGGACTGTGGAGGCTTGCT
GGCCACTTTTTGGGGTCAGCTCCTCTGAAATGCATTAGCGGAACCGTTTGCGATCTGCCA
CAAGTGTGATAAGTTATCTACACTGGCGAGGGGATTGCTCTCTGTAATGTTCAGCTTCTA
ATTGTCTCTACTTTGTGAGACTACTTTTGAATGCTTGACCTCAAATCAGGTAGGACTACC
CGCTGAACTTAA
>ABC9-52
TTTCCGTAGGTGAACCTGCGGAAGGATCATTATTGAATTATGTTTCTAGATAGGTTGTAG
CTGGCTC-TTTAGAGCATGTGCACGCCTGTTTGGACTTCATTTTCATCCACCTGTGCACC
TATTGTAGTCTTTGGTTGGGTTAGGAGGAAGTGATCATTGTATCAGCATCTGCTGGGAGT
GAGGACTTGCATTGTGAAAGCTTTGCTGTCCTTGATGTGATCATGGAATCTTTTTCTCAC
TAGAGTCTATGTCACTCATTATACTCTGTCGAATGTCATTGAATGTCTTTACATGGGCTT
GTATGCCTATGAAAATTGTAATACAACTTTCAGCAACGGATCTCTTGGCTCTCGCATCGA
TGAAGAACGCAGCGAAATGCGATAAGTAATGTGAATTGCAGAATTCAGTGAATCATCGAA
TCTTTGAACGCATCTTGCGCTCCTTGGTATTCCGAGGAGCATGCCTGTTTGAGTGTCATT
AAATTCTCAACTCTCTTATAC-TTTTTTGTAAAAGAGAGCTTGGACTGTGGAGGCTTGCT
GGCCACTTTTTGGGGTCAGCTCCTCTGAAATGCATTAGCGGAACCGTTTGCGATCTGCCA
CAAGTGTGATAAGTTATCTACACTGGCGAGGGGATTGCTCTCTGTAATGTTCAGCTTCTA
ATTGTCTCTACTTTGTGAGACTACTTTTGAATGCTTGACCTCAAATCAGGTAGGACTACC
CGCTGAACTTAA
>ABC12-19
TTTCCGTAGGTGAACCTGCGGAAGGATCATTATTGAATTATGTTTCTAGATAGGTTGTAG
CTGGCTC-TTTAGAGCATGTGCACGCCTGTTTGGACTTCATTTTCATCCACCTGTGCACC
TATTGTAGTCTTTGGTTGGGTTAGGAGGAAGTGATCATTGTATCAGCATCTGCTGGGAGT
GAGGACTTGCATTGTGAAAGCTTTGCTGTCCTTGATGTGATCATGGAATCTTTTTCTCAC
TAGAGTCTATGTCACTCATTATACTCTGTCGAATGTCATTGAATGTCTTTACATGGGCTT

GTATGCCTATGAAAATTGTAATACAACTTTCAGCAACGGATCTCTTGGCTCTCGCATCGA
TGAAGAACGCAGCGAAATGCGATAAGTAATGTGAATTGCAGAATTCAGTGAATCATCGAA
TCTTTGAACGCATCTTGCGCTCCTTGGTATTCCGAGGAGCATGCCTGTTTGAGTGTCATT
AAATTCTCAACTCTCTTATAC-TTTTTTGTAAAAGAGAGCTTGGACTGTGGAGGCTTGCT
GGCCACTTTTTGGGGTCAGCTCCTCTGAAATGCATTAGCGGAACCGTTTGCGATCTGCCA
CAAGTGTGATAAGTTATCTACACTGGCGAGGGGATTGCTCTCTGTAATGTTCAGCTTCTA
ATTGTCTCTACTTTGTGAGACTACTTTTGAATGCTTGACCTCAAATCAGGTAGGACTACC
CGCTGAACTTAA
>ABC2-29
TTTCCGTAGGTGAACCTGCGGAAGGATCATTATTGAATTATGTTTCTAGATAGGTTGTAG
CTGGCTC-TTTAGAGCATGTGCACGCCTGTTTGGACTTCATTTTCATCCACCTGTGCACC
TATTGTAGTCTTTGGTTGGGTTAGGAGGAAGTGATCATTGTATCAGCATCTGCTGGGAGT
GAGGACTTGCATTGTGAAAGCTTTGCTGTCCTTGATGTGATCATGGAATCTTTTTCTCAC
TAGAGTCTATGTCACTCATTATACTCTGTCGAATGTCATTGAATGTCTTTACATGGGCTT
ATATGCCTATGAAAATTGTAATACAACTTTCAGCAACGGATCTCTTGGCTCTCGCATCGA
TGAAGAACGCAGCGAAATGCGATAAGTAATGTGAATTGCAGAATTCAGTGAATCATCGAA
TCTTTGAACGCATCTTGCGCTCCTTGGTATTCCGAGGAGCATGCCTGTTTGAGTGTCATT
AAATTCTCAACTCTCTTATAC-TTTTTTGTAAAAGAGAGCTTGGACTGTGGAGGCTTGCT
GGCCACTTTTTGGGGTCAGCTCCTCTGAAATGCATTAGCGGAACCGTTTGCGATCTGCCA
CAAGTGTGATAAGTTATCTACACTGGCGAGGGGATTGCTCTCTGTAATGTTCAGCTTCTA
ATTGTCTCTACTTTGTGAGACTACTTTTGAATGCTTGACCTCAAATCAGGTAGGACTACC
CGCTGAACTTAA
>ABC12-42
TTTCCGTAGGTGAACCTGCGGAAGGATCATTATTGAATTATGTTTCTAGATAGGTTGTAG
CTGGCTC-TTTAGAGCATGTGCACGCCTGTTTGGACTTCATTTTCATCCACCTGTGCACC
TATTGTAGTCTTTGGTTGGGTTAGGAGGAAGTGATCATTGTATCAGCATCTGCTGGGAGT
GAGGACTTGCATTGTGAAAGCTTTGCTGTCCTTGATGTGATCATGGAATCTTTTTCTCAC
TAGAGTCTATGTCACTCATTATACTCTGTCGAATGTCATTGAATGTCTTTACATGGGCTT
ATATGCCTATGAAAATTGTAATACAACTTTCAGCAACGGATCTCTTGGCTCTCGCATCGA
TGAAGAACGCAGCGAAATGCGATAAGTAATGTGAATTGCAGAATTCAGTGAATCATCGAA
TCTTTGAACGCATCTTGCGCTCCTTGGTATTCCGAGGAGCATGCCTGTTTGAGTGTCATT
AAATTCTCAACTCTCTTATAC-TTTTTTGTAAAAGAGAGCTTGGACTGTGGAGGCTTGCT
GGCCACTTTTTGGGGTCAGCTCCTCTGAAATGCATTAGCGGAACCGTTTGCGATCTGCCA
CAAGTGTGATAAGTTATCTACACTGGCGAGGGGATTGCTCTCTGTAATGTTCAGCTTCTA
ATTGTCTCTACTTTGTGAGACTACTTTTGAATGCTTGACCTCAAATCAGGTAGGACTACC
CGCTGAACTTAA
>ABC2-27
TTTCCGTAGGTGAACCTGCGGAAGGATCATTATTGAATTATGTTTCTAGATAGGTTGTAG
CTGGCTC-TTTAGAGCATGTGCACGCCTGTTTGGACTTCATTTTCATCCACCTGTGCACC
TATTGTAGTCTTTGGTTGGGTTAGGAGGAAGTGATCATTGTATCAGCATCTGCTGGGAGT
GAGGACTTGCATTGTGAAAGCTTTGCTGTCCTTGATGTGATCATGGAATCTTTTTCTCAC
TAGAGTCTATGTCACTCATTATACTCTGTCGAATGTCATTGAATGTCTTTACATGGGCTT
ATATGCCTATGAAAATTGTAATACAACTTTCAGCAACGGATCTCTTGGCTCTCGCATCGA
TGAAGAACGCAGCGAAATGCGATAAGTAATGTGAATTGCAGAATTCAGTGAATCATCGAA
TCTTTGAACGCATCTTGCGCTCCTTGGTATTCCGAGGAGCATGCCTGTTTGAGTGTCATT
AAATTCTCAACTCTCTTATAC-TTTTTTGTAAAAGAGAGCTTGGACTGTGGAGGCTTGCT
GGCCACTTTTTGGGGTCAGCTCCTCTGAAATGCATTAGCGGAACCGTTTGCGATCTGCCA
CAAGTGTGATAAGTTATCTACACTGGCGAGGGGATTGCTCTCTGTAATGTTCAGCTTCTA
ATTGTCTCTACTTTGTGAGACTACTTTTGAATGCTTGACCTCAAATCAGGTAGGACTACC
CGCTGAACTTAA

>ABC5-67
TTTCCGTAGGTGAACCTGCGGAAGGATCATTATTGAATTATGTTTCTAGATAGGTTGTAG
CTGGCTC-TTTAGAGCATGTGCACGCCTGTTTGGACTTCATTTTCATCCACCTGTGCACC
TATTGTAGTCTTTGGTTGGGTTAGGAGGAAGTGATCATTGTATCAGCATCTGCTGGGAGT
GAGGACTTGCATTGTGAAAGCTTTGCTGTCCTTGATGTGATCATGGAATCTCTTTCTCAC
TAGAGTCTATGTCACTCATTATACTCTGTCGAATGTCATTGAATGTCTTTACATGGGCTT
GTATGCCTATGAAAATTGTAATACAACTTTCAGCAACGGATCTCTTGGCTCTCGCATCGA
TGAAGAACGCAGCGAAATGCGATAAGTAATGTGAATTGCAGAATTCAGTGAATCATCGAA
TCTTTGAACGCATCTTGCGCTCCTTGGTATTCCGAGGAGCATGCCTGTTTGAGTGTCATT
AAATTCTCAACTCTCTTATAC-TTTTTTGTAAAAGAGAGCTTGGACTGTGGAGGCTTGCT
GGCCACTTTTTGGGGTCAGCTCCTCTGAAATGCATTAGCGGAACCGTTTGCGATCTGCCA
CAAGTGTGATAAGTTATCTACACTGGCGAGGGGATTGCTCTCTGTAATGTTCAGCTTCTA
ATTGTCTCTACTTTGTGAGACTACTTTTGAATGCTTGACCTCAAATCAGGTAGGACTACC
CGCTGAACTTAA
>ABC8-13
TTTCCGTAGGTGAACCTGCGGAAGGATCATTATTGAATTATGTTTCTAGATAGGTTGTAG
CTGGCTC-TTTAGAGCATGTGCACGCCTGTTTGGACTTCATTTTCATCCACCTGTGCACC
TATTGTAGTCTTTGGTTGGGTTAGGAGGAAGTGATCATTGTATCAGCATCTGCTGGGAGT
GAGGACTTGCATTGTGAAAGCTTTGCTGTCCTTGATGTGATCATGGAATCTTTTTCTCAC
TAGAGTCTATGTCACTCATTATACTCTGTCGAATGTCATTGAATGTCTTTACATGGGCTT
GTATGCCTATGAAAATTGTAATACAACTTTCAGCAACGGATCTCTTGGCTCTCGCATCGA
TGAAGGACGCAGCGAAATGCGATAAGTAATGTGAATTGCAGAATTCAGTGAATCATCGAA
TCTTTGAACGCATCTTGCGCTCCTTGGTATTCCGAGGAGCATGCCTGTTTGAGTGTCATT
AAATTCTCAACTCTCTTATAC-TTTTTTGTAAAAGAGAGCTTGGACTGTGGAGGCTTGCT
GGCCACTTTTTGGGGTCAGCTCCTCTGAAATGCATTAGCGGAACCGTTTGCGATCTGCCA
CAAGTGTGATAAGTTATCTACACTGGCGAGGGGATTGCTCTCTGTAATGTTCAGCTTCTA
ATTGTCTCTACTTTGTGAGACTACTTTTGAATGCTTGACCTCAAATCAGGTAGGACTACC
CGCTGAACTTAA
>ABC10-19
TTTCCGTAGGTGAACCTGCGGAAGGATCATTATTGAATTATGTTTCTAGATAGGTTGTAG
CTGGCTC-TTTAGAGCATGTGCACGCCTGTTTGGACTTCATTTTCATCCACCTGTGCACC
TATTGTAGTCTTTGGTTGGGTTAGGAGGAAGTGATCATTGTATCAGCATCTGCTGGGAGT
GAGGACTTGCATTGTGAAAGCTTTGCTGTCCTTGATGTGATCATGGAATCTTTTTCTCAC
TAGAGTCTATGTCACTCATTATACTCTGTCGAATGTCATTGAATGTCTTTACATGGGCTT
GTATGCCTATGAAAATTGTAATACAACTTTCAGCAACGGATCTCTTGGCTCTCGCATCGA
TGAAGAACGCAGCGAAATGCGATAAGTAATGTGAATTGCAGAATTCAGTGAATCATCGAA
TCTTTGAACGCATCTTGCGCTCCTTGGTATTCCGAGGAGCATGCCTGTTTGAGTGTCATT
AAATTCTCAACTCTCTTATAC-TTTTTTGTAAAAGAGAGCTTGGACTGTGGAGGCTTGCT
GGCCACTTTTTGGGGTCAGCTCCTCTGAAATGCATTAGCGGAACCGTTTGCAATCTGCCA
CAAGTGTGATAAGTTATCTACACTGGCGAGGGGATTGCTCTCTGTAATGTTCAGCTTCTA
ATTGTCTCTACTTTGTGAGACTACTTTTGAATGCTTGACCTCAAATCAGGTAGGACTACC
CGCTGAACTTAA
>ABC3-65
TTTCCGTAGGTGAACCTGCGGAAGGATCATTATTGAATTATGTTTCTAGATAGGTTGTAG
CTGGCTC-TTTAGAGCATGTGCACGCCTGTTTGGACTTCATTTTCATCCACCTGTGCACC
TATTGTAGTCTTTGGTTGGGTTAGGAGGAAGTGATCATTGTATCAGCATCTGCTGGGAGT
GAGGACTTGCATTGTGAAAGCTTTGCTGTCCTTGATGTGATCATGGAATCTCTTTCTCAC
TAGAGTCTATGTCACTCATTATACTCTGTCGAATGTCATTGAATGTCTTTACATGGGCTT
ATATGCCTATGAAAATTGTAATACAACTTTCAGCAACGGATCTCTTGGCTCTCGCATCGA
TGAAGAACGCAGCGAAATGCGATAAGTAATGTGAATTGCAGAATTCAGTGAATCATCGAA

TCTTTGAACGCATCTTGCGCTCCTTGGTATTCCGAGGAGCATGCCTGTTTGAGTGTCATT
AAATTCTCAACTCTCTTATAC-TTTTTTGTAAAAGAGAGCTTGGACTGTGGAGGCTTGCT
GGCCACTTTTTGGGGTCAGCTCCTCTGAAATGCATTAGCGGAACCGTTTGCAATCTGCCA
CAAGTGTGATAAGTTATCTACACTGGCGAGGGGATTGCTCTCTGTAATGTTCAGCTTCTA
ATTGTCTCTACTTTGTGAGACTACTTTTGAATGCTTGACCTCAAATCAGGTAGGACTACC
CGCTGAACTTAA
>ABC2-36
TTTCCGTAGGTGAACCTGCGGAAGGATCATTATTGAATTATGTTTCTAGATAGGTTGTAG
CTGGCTC-TTTAGAGCATGTGCACGCCTGTTTGGACTTCATTTTCATCCACCTGTGCACC
TATTGTAGTCTTTGGTTGGGTTAGGAGGAAGTGATCATTGTATCAGCATCTGCTGGGAGT
GAGGACTTGCATTGTGAAAGCTTTGCTGTCCTTGATGTGATCATGGAATCTTTTTCTCAC
TAGAGTCTATGTCACTCATTATACTCTGTCGAATGTCATTGAATGTCTTTACATGGGCTT
GTATGCCTATGAAAATTGTAATACAACTTTCAGCAACGGATCTCTTGGCTCTCGCATCGA
TGAAGAACGCAGCGAAATGCGATAAGTAATGTGAATTGCAGAATTCAGTGAATCATCGAA
TCTTTGAACGCATCTTGCGCTCCTTGGTATTCCGAGGAGCATGCCTGTTTGAGTGTCATT
AAATTCTCAACTCTCTTATAC-TTTTTTGTAAAAGAGAGCTTGGACTGTGGAGGCTTGCT
GGCCACTTTTTGGGGTCAGCTCCTCTGAAATGCATTAGCGGAACCGTTTGCGATCTGCCA
CAAGTGTGATAAGTTATCTACACTGGCGAGGGGATTGCTCTCTGTAATGTTCAGCTTCTA
ATTGTCTCTACTTTGTGAGACAACTTTTGAATGCTTGACCTCAAATCAGGTAGGACTACC
CGCTGAACTTAA
>ABC6-22
TTTCCGTAGGTGAACCTGCGGAAGGATCATTATTGAATTATGTTTCTAGATAGGTTGTAG
CTGGCTC-TTTAGAGCATGTGCACGCCTGTTTGGACTTCATTTTCATCCACCTGTGCACC
TATTGTAGTCTTTGGTTGGGTTAGGAGGAAGTGATCATTGTATCAGCATCTGCTGGGAGT
GAGGACTTGCATTGTGAAAGCTTTGCTGTCCTTGATGTGATCATGGAATCTTTTTCTCAC
TAGAGTCTATGTCACTCATTATACTCTGTCGAATGTCATTGAATGTCTTTACATGGGCTT
ATATGCCTATGAAAATTGTAATACAACTTTCAGCAACGGATCTCTTGGCTCTCGCATCGA
TGAAGAACGCAGCGAAATGCGATAAGTAATGTGAATTGCAGAATTCAGTGAATCATCGAA
TCTTTGAACGCATCTTGCGCTCCTTGGTATTCCGAGGAGCATGCCTGTTTGAGTGTCATT
AAATTCTCAACTCTCTTATAC-TTTTTTGTAAAAGAGAGCTTGGACTGTGGAGGCTTGCT
GGCCACTTTTTGGGGTCAGCTCCTCTGAAATGCATTAGCGGAACCGTTTGCGATCTGCCA
CAAGTGTGATAAGTTATCTACACTGGCGAGGGGATTGCTCTCTGTAATGTTCAGCTTCTA
ATTGTCTCTACTTTGTGAGACAACTTTTGAATGCTTGACCTCAAATCAGGTAGGACTACC
CGCTGAACTTAA
>ABC7-26
TTTCCGTAGGTGAACCTGCGGAAGGATCATTATTGAATTATGTTTCTAGATAGGTTGTAG
CTGGCTC-TTTAGAGCATGTGCACGCCTGTTTGGACTTCATTTTCATCCACCTGTGCACC
TATTGTAGTCTTTGGTTGGGTTAGGAGGAAGTGATCATTGTATCAGCATCTGCTGGGAGT
GAGGACTTGCATTGTGAAAGCTTTGCTGTCCTTGATGTGATCATGGAATCTTTTTCTCAC
TAGAGTCTATGTCACTCATTATACTCTGTCGAATGTCATTGAATGTCTTTACATGGGCTT
GTATGCCTATGAAAATTGTAATACAACTTTCAGCAACGGATCTCTTGGCTCTCGCATCGA
TGAAGGACGCAGCGAAATGCGATAAGTAATGTGAATTGCAGAATTCAGTGAATCATCGAA
TCTTTGAACGCATCTTGCGCTCCTTGGTATTCCGAGGAGCATGCCTGTTTGAGTGTCATT
AAATTCTCAACTCTCTTATAC-TTTTTTGTAAAAGAGAGCTTGGACTGTGGAGGCTTGCT
GGCCACTTTTTGGGGTCAGCTCCTCTGAAATGCATTAGCGGAACCGTTTGCAATCTGCCA
CAAGTGTGATAAGTTATCTACACTGGCGAGGGGATTGCTCTCTGTAATGTTCAGCTTCTA
ATTGTCTCTACTTTGTGAGACAACTTTTGAATGCTTGACCTCAAATCAGGTAGGACTACC
CGCTGAACTTAA
>ABC6-32
TTTCCGTAGGTGAACCTGCGGAAGGATCATTATTGAATTATGTTTCTAGATAGGTTGTAG

CTGGCTC-TTTAGAGCATGTGCACGCCTGTTTGGACTTCATTTTCATCCACCTGTGCACC
TATTGTAGTCTTTGGTTGGGTTAGGAGGAAGTGATCATTGTATCAGCATCTGCTGGGAGT
GAGGACTTGCATTGTGAAAGCTTTGCTGTCCTTGATGTGATCATGGAATCTTTTTCTCAC
TAGAGTCTATGTCACTCATTATACTCTGTCGAATGTCATTGAATGTCTTTACATGGGCTT
GTATGCCTATGAAAATTGTAATACAACTTTCAGCAACGGATCTCTTGGCTCTCGCATCGA
TGAAGGACGCAGCGAAATGCGATAAGTAATGTGAATTGCAGAATTCAGTGAATCATCGAA
TCTTTGAACGCATCTTGCGCTCCTTGGTATTCCGAGGAGCATGCCTGTTTGAGTGTCATT
AAATTCTCAACTCTCTTATAC-TTTTTTGTAAAAGAGAGCTTGGACTGTGGAGGCTTGCT
GGCCACTTTTTGGGGTCAGCTCCTCTGAAATGCATTAGCGGAACCGTTTGCAATCTGCCA
CAAGTGTGATAAGTTATCTACACTGGCGAGGGGATTGCTCTCTGTAATGTTCAGCTTCTA
ATTGTCTCTACTTTGTGAGACAACTTTTGAATGCTTGACCTCAAATCAGGTAGGACTACC
CGCTGAACTTAA
>ABC9-22
TTTCCGTAGGTGAACCTGCGGAAGGATCATTATTGAATTATGTTTCTAGATAGGTTGTAG
CTGGCTC-TTTAGAGCATGTGCACGCCTGTTTGGACTTCATTTTCATCCACCTGTGCACC
TATTGTAGTCTTTGGTTGGGTTAGGAGGAAGTGATCATTGTATCAGCATCTGCTGGGAGT
GAGGACTTGCATTGTGAAAGCTTTGCTGTCCTTGATGTGATCATGGAATCTTTTTCTCAC
TAGAGTCTATGTCACTCATTATACTCTGTCGAATGTCATTGAATGTCTTTACATGGGCTT
GTATGCCTATGAAAATTGTAATACAACTTTCAGCAACGGATCTCTTGGCTCTCGCATCGA
TGAAGGACGCAGCGAAATGCGATAAGTAATGTGAATTGCAGAATTCAGTGAATCATCGAA
TCTTTGAACGCATCTTGCGCTCCTTGGTATTCCGAGGAGCATGCCTGTTTGAGTGTCATT
AAATTCTCAACTCTCTTATAC-TTTTTTGTAAAAGAGAGCTTGGACTGTGGAGGCTTGCT
GGCCACTTTTTGGGGTCAGCTCCTCTGAAATGCATTAGCGGAACCGTTTGCGATCTGCCA
CAAGTGTGATAAGTTATCTACACTGGCGAGGGGATTGCTCTCTGTAATGTTCAGCTTCTA
ATTGTCTCTACTTTGTGAGACAACTTTTGAATGCTTGACCTCAAATCAGGTAGGACTACC
CGCTGAACTTAA
>ABC6-34
TTTCCGTAGGTGAACCTGCGGAAGGATCATTATTGAATTATGTTTCTAGATAGGTTGTAG
CTGGCTC-TTTAGAGCATGTGCACGCCTGTTTGGACTTCATTTTCATCCACCTGTGCACC
TATTGTAGTCTTTGGTTGGGTTAGGAGGAAGTGGTCATTGTGTCAGCATCTGCTGGGAGT
GAGGACTTGCATTGTGAAAGCTTTGCTGTCCTTGATGTGATCATGGAATCTTTTTCTCAC
TAGAGTCTATGTCACTCATTATACTCTGTCGAATGTCATTGAATGTCTTTACATGGGCTT
GTATGCCTATGAAAATTGTAATACAACTTTCAGCAACGGATCTCTTGGCTCTCGCATCGA
TGAAGAACGCAGCGAAATGCGATAAGTAATGTGAATTGCAGAATTCAGTGAATCATCGAA
TCTTTGAACGCATCTTGCGCTCCTTGGTATTCCGAGGAGCATGCCTGTTTGAGTGTCATT
AAATTCTCAACTCTCTTATAC-TTTTTTGTAAAAGAGAGCTTGGACTGTGGAGGCTTGCT
GGCCACTTTTTGGGGTCAGCTCCTCTGAAATGCATTAGCGGAACCGTTTGCGATCTGCCA
CAAGTGTGATAAGTTATCTACACTGGCGAGGGGATTGCTCTCTGTAATGTTCAGCTTCTA
ATTGTCTCTACTTTGTGAGACTACTTTTGAATGCTTGACCTCAAATCAGGTAGGACTACC
CGCTGAACTTAA
>ABC7-38
TTTCCGTAGGTGAACCTGCGGAAGGATCATTATTGAATTATGTTTCTAGATAGGTTGTAG
CTGGCTC-TTTAGAGCATGTGCACGCCTGTTTGGACTTCATTTTCATCCACCTGTGCACC
TATTGTAGTCTTTGGTTGGGTTAGGGGGAAGTGGTCATTGTGTCAGCATCTGCTGGGAGT
GAGGACTTGCATTGTGAAAGCTTTGCTGTCCTTGATGTGATCATGGAATCTTTTTCTCAC
TAGAGTCTATGTCACTCATTATACTCTGTCGAATGTCATTGAATGTCTTTACATGGGCTT
GTATGCCTATGAAAATTGTAATACAACTTTCAGCAACGGATCTCTTGGCTCTCGCATCGA
TGAAGGACGCAGCGAAATGCGATAAGTAATGTGAATTGCAGAATTCAGTGAATCATCGAA
TCTTTGAACGCATCTTGCGCTCCTTGGTATTCCGAGGAGCATGCCTGTTTGAGTGTCATT
AAATTCTCAACTCTCTTATAC-TTTTTTGTAAAAGAGAGCTTGGACTGTGGAGGCTTGCT

GGCCACTTTTTGGGGTCAGCTCCTCTGAAATGCATTAGCGGAACCGTTTGCAATCTGCCA
CAAGTGTGATAAGTTATCTACACTGGCGAGGGGATTGCTCTCTGTAATGTTCAGCTTCTA
ATTGTCTCTACTTTGTGAGACAACTTTTGAATGCTTGACCTCAAATCAGGTAGGACTACC
CGCTGAACTTAA
>ABC10-20
TTTCCGTAGGTGAACCTGCGGAAGGATCATTATTGAATTATGTTTCTAGATAGGTTGTAG
CTGGCTC-TTTAGAGCATGTGCACGCCTGTTTGGACTTCATTTTCATCCACCTGTGCACC
TATTGTAGTCTTTGGTTGGGTTAGGGGGAAGTGGTCATTGTGTCAGCATCTGCTGGGAGT
GAGGACTTGCATTGTGAAAGCTTTGCTGTCCTTGATGTGATCATGGAATCTTTTTCTCAC
TAGAGTCTATGTCACTCATTATACTCTGTCGAATGTCATTGAATGTCTTTACATGGGCTT
GTATGCCTATGAAAATTGTAATACAACTTTCAGCAACGGATCTCTTGGCTCTCGCATCGA
TGAAGGACGCAGCGAAATGCGATAAGTAATGTGAATTGCAGAATTCAGTGAATCATCGAA
TCTTTGAACGCATCTTGCGCTCCTTGGTATTCCGAGGAGCATGCCTGTTTGAGTGTCATT
AAATTCTCAACTCTCTTATAC-TTTTTTGTAAAAGAGAGCTTGGACTGTGGAGGCTTGCT
GGCCACTTTTTGGGGTCAGCTCCTCTGAAATGCATTAGCGGAACCGTTTGCAATCTGCCA
CAAGTGTGATAAGTTATCTACACTGGCGAGGGGATTGCTCTCTGTAATGTTCAGCTTCTA
ATTGTCTCTACTTTGTGAGACTACTTTTGAATGCTTGACCTCAAATCAGGTAGGACTACC
CGCTGAACTTAA
>ABC1-2
TTTCCGTAGGTGAACCTGCGGAAGGATCATTATTGAATTATGTTTCTAGATAGGTTGTAG
CTGGCTCTTTTAGAGCATGTGCACGCCTGTTTGGACTTCATTTTCATCCACCTGTGCACC
TATTGTAGTCTTTGGTTGGGTTAGGAGGAAGTGATCATTGTATCAGCATCTGCTGGGAGT
GAGGACTTGCATTGTGAAAGCTTTGCTGTCCTTGATGTGATCATGGAATCTTTTTCTCAC
TAGAGTCTATGTCACTCATTATACTCTGTCGAATGTCATTGAATGTCTTTACATGGGCTT
GTATGCCTATGAAAATTGTAATACAACTTTCAGCAACGGATCTCTTGGCTCTCGCATCGA
TGAAGAACGCAGCGAAATGCGATAAGTAATGTGAATTGCAGAATTCAGTGAATCATCGAA
TCTTTGAACGCATCTTGCGCTCCTTGGTATTCCGAGGAGCATGCCTGTTTGAGTGTCATT
AAATTCTCAACTCTCTTCTAC--TTTTTGTAAAAGAGAGCTTGGACTGTGGAGGCTTGCT
GGCCACTTTTTGGGGTCAGCTCCTCTGAAATGCATTAGCGGAACCGTTTGCGATCTGCCA
CAAGTGTGATAAGTTATCTACACTGGCGAGGGGATTGCTCTCTGTAATGTTCAGCTTCTA
ATTGTCTCTACTTTGTGAGACTACTTTTGAATGCTTGACCTCAAATCAGGTAGGACTACC
CGCTGAACTTAA
>ABC2-78
TTTCCGTAGGTGAACCTGCGGAAGGATCATTATTGAATTATGTTTCTAGATAGGTTGTAG
CTGGCTCTTTTAGAGCATGTGCACGCCTGTTTGGACTTCATTTTCATCCACCTGTGCACC
TATTGTAGTCTTTGGTTGGGTTAGGAGGAAGTGATCATTGTATCAGCATCTGCTGGGAGT
GAGGACTTGCATTGTGAAAGCTTTGCTGTCCTTGATGTGATCATGGAATCTTTTTCTCAC
TAGAGTCTATGTCACTCATTATACTCTGTCGAATGTCATTGAATGTCTTTACATGGGCTT
GTATGCCTATGAAAATTGTAATACAACTTTCAGCAACGGATCTCTTGGCTCTCGCATCGA
TGAAGAACGCAGCGAAATGCGATAAGTAATGTGAATTGCAGAATTCAGTGAATCATCGAA
TCTTTGAACGCATCTTGCGCTCCTTGGTATTCCGAGGAGCATGCCTGTTTGAGTGTCATT
AAATTCTCAACTCTCTTCTAC--TTTTTGTAAAAGAGAGCTTGGACTGTGGAGGCTTGCT
GGCCACTTTTTGGGGTCAGCTCCTCTGAAATGCATTAGCGGAACCGTTTGCGATCTGCCA
CAAGTGTGATAAGTTATCTACACTGGCGAGGGGATTGCTCTCTGTAATGTTCAGCTTCTA
ATTGTCTCTACTTTGTGAGACTACTTTTGAATGCTTGACCTCAAATCAGGTAGGACTACC
CGCTGAACTTAA
>ABC3-32
TTTCCGTAGGTGAACCTGCGGAAGGATCATTATTGAATTATGTTTCTAGATAGGTTGTAG
CTGGCTCTTTTAGAGCATGTGCACGCCTGTTTGGACTTCATTTTCATCCACCTGTGCACC
TATTGTAGTCTTTGGTTGGGTTAGGAGGAAGTGATCATTGTATCAGCATCTGCTGGGAGT

GAGGACTTGCATTGTGAAAGCTTTGCTGTCCTTGATGTGATCATGGAATCTTTTTCTCAC
TAGAGTCTATGTCACTCATTATACTCTGTCGAATGTCATTGAATGTCTTTACATGGGCTT
GTATGCCTATGAAAATTGTAATACAACTTTCAGCAACGGATCTCTTGGCTCTCGCATCGA
TGAAGAACGCAGCGAAATGCGATAAGTAATGTGAATTGCAGAATTCAGTGAATCATCGAA
TCTTTGAACGCATCTTGCGCTCCTTGGTATTCCGAGGAGCATGCCTGTTTGAGTGTCATT
AAATTCTCAACTCTCTTCTAC--TTTTTGTAAAAGAGAGCTTGGACTGTGGAGGCTTGCT
GGCCACTTTTTGGGGTCAGCTCCTCTGAAATGCATTAGCGGAACCGTTTGCGATCTGCCA
CAAGTGTGATAAGTTATCTACACTGGCGAGGGGATTGCTCTCTGTAATGTTCAGCTTCTA
ATTGTCTCTACTTTGTGAGACTACTTTTGAATGCTTGACCTCAAATCAGGTAGGACTACC
CGCTGAACTTAA
>ABC4-65
TTTCCGTAGGTGAACCTGCGGAAGGATCATTATTGAATTATGTTTCTAGATAGGTTGTAG
CTGGCTCTTTTAGAGCATGTGCACGCCTGTTTGGACTTCATTTTCATCCACCTGTGCACC
TATTGTAGTCTTTGGTTGGGTTAGGAGGAAGTGATCATTGTATCAGCATCTGCTGGGAGT
GAGGACTTGCATTGTGAAAGCTTTGCTGTCCTTGATGTGATCATGGAATCTTTTTCTCAC
TAGAGTCTATGTCACTCATTATACTCTGTCGAATGTCATTGAATGTCTTTACATGGGCTT
GTATGCCTATGAAAATTGTAATACAACTTTCAGCAACGGATCTCTTGGCTCTCGCATCGA
TGAAGAACGCAGCGAAATGCGATAAGTAATGTGAATTGCAGAATTCAGTGAATCATCGAA
TCTTTGAACGCATCTTGCGCTCCTTGGTATTCCGAGGAGCATGCCTGTTTGAGTGTCATT
AAATTCTCAACTCTCTTCTAC--TTTTTGTAAAAGAGAGCTTGGACTGTGGAGGCTTGCT
GGCCACTTTTTGGGGTCAGCTCCTCTGAAATGCATTAGCGGAACCGTTTGCGATCTGCCA
CAAGTGTGATAAGTTATCTACACTGGCGAGGGGATTGCTCTCTGTAATGTTCAGCTTCTA
ATTGTCTCTACTTTGTGAGACTACTTTTGAATGCTTGACCTCAAATCAGGTAGGACTACC
CGCTGAACTTAA
>ABC5-30
TTTCCGTAGGTGAACCTGCGGAAGGATCATTATTGAATTATGTTTCTAGATAGGTTGTAG
CTGGCTCTTTTAGAGCATGTGCACGCCTGTTTGGACTTCATTTTCATCCACCTGTGCACC
TATTGTAGTCTTTGGTTGGGTTAGGAGGAAGTGATCATTGTATCAGCATCTGCTGGGAGT
GAGGACTTGCATTGTGAAAGCTTTGCTGTCCTTGATGTGATCATGGAATCTTTTTCTCAC
TAGAGTCTATGTCACTCATTATACTCTGTCGAATGTCATTGAATGTCTTTACATGGGCTT
GTATGCCTATGAAAATTGTAATACAACTTTCAGCAACGGATCTCTTGGCTCTCGCATCGA
TGAAGAACGCAGCGAAATGCGATAAGTAATGTGAATTGCAGAATTCAGTGAATCATCGAA
TCTTTGAACGCATCTTGCGCTCCTTGGTATTCCGAGGAGCATGCCTGTTTGAGTGTCATT
AAATTCTCAACTCTCTTCTAC--TTTTTGTAAAAGAGAGCTTGGACTGTGGAGGCTTGCT
GGCCACTTTTTGGGGTCAGCTCCTCTGAAATGCATTAGCGGAACCGTTTGCGATCTGCCA
CAAGTGTGATAAGTTATCTACACTGGCGAGGGGATTGCTCTCTGTAATGTTCAGCTTCTA
ATTGTCTCTACTTTGTGAGACTACTTTTGAATGCTTGACCTCAAATCAGGTAGGACTACC
CGCTGAACTTAA
>ABC6-41
TTTCCGTAGGTGAACCTGCGGAAGGATCATTATTGAATTATGTTTCTAGATAGGTTGTAG
CTGGCTCTTTTAGAGCATGTGCACGCCTGTTTGGACTTCATTTTCATCCACCTGTGCACC
TATTGTAGTCTTTGGTTGGGTTAGGAGGAAGTGATCATTGTATCAGCATCTGCTGGGAGT
GAGGACTTGCATTGTGAAAGCTTTGCTGTCCTTGATGTGATCATGGAATCTTTTTCTCAC
TAGAGTCTATGTCACTCATTATACTCTGTCGAATGTCATTGAATGTCTTTACATGGGCTT
GTATGCCTATGAAAATTGTAATACAACTTTCAGCAACGGATCTCTTGGCTCTCGCATCGA
TGAAGAACGCAGCGAAATGCGATAAGTAATGTGAATTGCAGAATTCAGTGAATCATCGAA
TCTTTGAACGCATCTTGCGCTCCTTGGTATTCCGAGGAGCATGCCTGTTTGAGTGTCATT
AAATTCTCAACTCTCTTCTAC--TTTTTGTAAAAGAGAGCTTGGACTGTGGAGGCTTGCT
GGCCACTTTTTGGGGTCAGCTCCTCTGAAATGCATTAGCGGAACCGTTTGCGATCTGCCA
CAAGTGTGATAAGTTATCTACACTGGCGAGGGGATTGCTCTCTGTAATGTTCAGCTTCTA

ATTGTCTCTACTTTGTGAGACTACTTTTGAATGCTTGACCTCAAATCAGGTAGGACTACC
CGCTGAACTTAA
>ABC7-33
TTTCCGTAGGTGAACCTGCGGAAGGATCATTATTGAATTATGTTTCTAGATAGGTTGTAG
CTGGCTCTTTTAGAGCATGTGCACGCCTGTTTGGACTTCATTTTCATCCACCTGTGCACC
TATTGTAGTCTTTGGTTGGGTTAGGAGGAAGTGATCATTGTATCAGCATCTGCTGGGAGT
GAGGACTTGCATTGTGAAAGCTTTGCTGTCCTTGATGTGATCATGGAATCTTTTTCTCAC
TAGAGTCTATGTCACTCATTATACTCTGTCGAATGTCATTGAATGTCTTTACATGGGCTT
GTATGCCTATGAAAATTGTAATACAACTTTCAGCAACGGATCTCTTGGCTCTCGCATCGA
TGAAGAACGCAGCGAAATGCGATAAGTAATGTGAATTGCAGAATTCAGTGAATCATCGAA
TCTTTGAACGCATCTTGCGCTCCTTGGTATTCCGAGGAGCATGCCTGTTTGAGTGTCATT
AAATTCTCAACTCTCTTCTAC--TTTTTGTAAAAGAGAGCTTGGACTGTGGAGGCTTGCT
GGCCACTTTTTGGGGTCAGCTCCTCTGAAATGCATTAGCGGAACCGTTTGCGATCTGCCA
CAAGTGTGATAAGTTATCTACACTGGCGAGGGGATTGCTCTCTGTAATGTTCAGCTTCTA
ATTGTCTCTACTTTGTGAGACTACTTTTGAATGCTTGACCTCAAATCAGGTAGGACTACC
CGCTGAACTTAA
>ABC8-22
TTTCCGTAGGTGAACCTGCGGAAGGATCATTATTGAATTATGTTTCTAGATAGGTTGTAG
CTGGCTCTTTTAGAGCATGTGCACGCCTGTTTGGACTTCATTTTCATCCACCTGTGCACC
TATTGTAGTCTTTGGTTGGGTTAGGAGGAAGTGATCATTGTATCAGCATCTGCTGGGAGT
GAGGACTTGCATTGTGAAAGCTTTGCTGTCCTTGATGTGATCATGGAATCTTTTTCTCAC
TAGAGTCTATGTCACTCATTATACTCTGTCGAATGTCATTGAATGTCTTTACATGGGCTT
GTATGCCTATGAAAATTGTAATACAACTTTCAGCAACGGATCTCTTGGCTCTCGCATCGA
TGAAGAACGCAGCGAAATGCGATAAGTAATGTGAATTGCAGAATTCAGTGAATCATCGAA
TCTTTGAACGCATCTTGCGCTCCTTGGTATTCCGAGGAGCATGCCTGTTTGAGTGTCATT
AAATTCTCAACTCTCTTCTAC--TTTTTGTAAAAGAGAGCTTGGACTGTGGAGGCTTGCT
GGCCACTTTTTGGGGTCAGCTCCTCTGAAATGCATTAGCGGAACCGTTTGCGATCTGCCA
CAAGTGTGATAAGTTATCTACACTGGCGAGGGGATTGCTCTCTGTAATGTTCAGCTTCTA
ATTGTCTCTACTTTGTGAGACTACTTTTGAATGCTTGACCTCAAATCAGGTAGGACTACC
CGCTGAACTTAA
>ABC9-32
TTTCCGTAGGTGAACCTGCGGAAGGATCATTATTGAATTATGTTTCTAGATAGGTTGTAG
CTGGCTCTTTTAGAGCATGTGCACGCCTGTTTGGACTTCATTTTCATCCACCTGTGCACC
TATTGTAGTCTTTGGTTGGGTTAGGAGGAAGTGATCATTGTATCAGCATCTGCTGGGAGT
GAGGACTTGCATTGTGAAAGCTTTGCTGTCCTTGATGTGATCATGGAATCTTTTTCTCAC
TAGAGTCTATGTCACTCATTATACTCTGTCGAATGTCATTGAATGTCTTTACATGGGCTT
GTATGCCTATGAAAATTGTAATACAACTTTCAGCAACGGATCTCTTGGCTCTCGCATCGA
TGAAGAACGCAGCGAAATGCGATAAGTAATGTGAATTGCAGAATTCAGTGAATCATCGAA
TCTTTGAACGCATCTTGCGCTCCTTGGTATTCCGAGGAGCATGCCTGTTTGAGTGTCATT
AAATTCTCAACTCTCTTCTAC--TTTTTGTAAAAGAGAGCTTGGACTGTGGAGGCTTGCT
GGCCACTTTTTGGGGTCAGCTCCTCTGAAATGCATTAGCGGAACCGTTTGCGATCTGCCA
CAAGTGTGATAAGTTATCTACACTGGCGAGGGGATTGCTCTCTGTAATGTTCAGCTTCTA
ATTGTCTCTACTTTGTGAGACTACTTTTGAATGCTTGACCTCAAATCAGGTAGGACTACC
CGCTGAACTTAA
>ABC12-45
TTTCCGTAGGTGAACCTGCGGAAGGATCATTATTGAATTATGTTTCTAGATAGGTTGTAG
CTGGCTCTTTTAGAGCATGTGCACGCCTGTTTGGACTTCATTTTCATCCACCTGTGCACC
TATTGTAGTCTTTGGTTGGGTTAGGAGGAAGTGATCATTGTATCAGCATCTGCTGGGAGT
GAGGACTTGCATTGTGAAAGCTTTGCTGTCCTTGATGTGATCATGGAATCTTTTTCTCAC
TAGAGTCTATGTCACTCATTATACTCTGTCGAATGTCATTGAATGTCTTTACATGGGCTT

GTATGCCTATGAAAATTGTAATACAACTTTCAGCAACGGATCTCTTGGCTCTCGCATCGA
TGAAGAACGCAGCGAAATGCGATAAGTAATGTGAATTGCAGAATTCAGTGAATCATCGAA
TCTTTGAACGCATCTTGCGCTCCTTGGTATTCCGAGGAGCATGCCTGTTTGAGTGTCATT
AAATTCTCAACTCTCTTCTAC--TTTTTGTAAAAGAGAGCTTGGACTGTGGAGGCTTGCT
GGCCACTTTTTGGGGTCAGCTCCTCTGAAATGCATTAGCGGAACCGTTTGCGATCTGCCA
CAAGTGTGATAAGTTATCTACACTGGCGAGGGGATTGCTCTCTGTAATGTTCAGCTTCTA
ATTGTCTCTACTTTGTGAGACTACTTTTGAATGCTTGACCTCAAATCAGGTAGGACTACC
CGCTGAACTTAA
>ABC6-1
TTTCCGTAGGTGAACCTGCGGAAGGATCATTATTGAATTATGTTTCTAGATAGGTTGTAG
CTGGCTCTTTTAGAGCATGTGCACGCCTGTTTGGACTTCATTTTCATCCACCTGTGCACC
TATTGTAGTCTTTGGTTGGGTTAGGAGGAAGTGATCATTGTATCAGCATCTGCTGGGAGT
GAGGACTTGCATTGTGAAAGCTTTGCTGTCCTTGATGTGATCATGGAATCTTTTTCTCAC
TAGAGTCTATGTCACTCATTATACTCTGTCGAATGTCATTGAATGTCTTTACATGGGCTT
GTATGCCTATGAAAATTGTAATACAACTTTCAGCAACGGATCTCTTGGCTCTCGCATCGA
TGAAGAACGCAGCGAAATGCGATAAGTAATGTGAATTGCAGAATTCAGTGAATCATCGAA
TCTTTGAACGCATCTTGCGCTCCTTGGTATTCCGAGGAGCATGCCTGTTTGAGTGTCATT
AAATTCTCAACTCTCTTCTAC--TTTTTGTAAAAGAGAGCTTGGACTGTGGAGGCTTGCT
GGCCACTTTTTGGGGTCAGCTCCTCTGAAATGCATTAGCGGAACCGTTTGCGATCTGCCA
CAAGTGTGATAAGTTATCTACACTGGCGAGGGGATTGCTCTCTGTAATGTTCAGCTTCTA
ATTGTCTCTACTTTGTGAGACTACTTTTGAATGCTTGACCTCAAATCAGGTAGGACTACC
CGCTGAACTTAA
>ABC7-2
TTTCCGTAGGTGAACCTGCGGAAGGATCATTATTGAATTATGTTTCTAGATAGGTTGTAG
CTGGCTCTTTTAGAGCATGTGCACGCCTGTTTGGACTTCATTTTCATCCACCTGTGCACC
TATTGTAGTCTTTGGTTGGGTTAGGAGGAAGTGATCATTGTATCAGCATCTGCTGGGAGT
GAGGACTTGCATTGTGAAAGCTTTGCTGTCCTTGATGTGATCATGGAATCTTTTTCTCAC
TAGAGTCTATGTCACTCATTATACTCTGTCGAATGTCATTGAATGTCTTTACATGGGCTT
ATATGCCTATGAAAATTGTAATACAACTTTCAGCAACGGATCTCTTGGCTCTCGCATCGA
TGAAGAACGCAGCGAAATGCGATAAGTAATGTGAATTGCAGAATTCAGTGAATCATCGAA
TCTTTGAACGCATCTTGCGCTCCTTGGTATTCCGAGGAGCATGCCTGTTTGAGTGTCATT
AAATTCTCAACTCTCTTCTAC--TTTTTGTAAAAGAGAGCTTGGACTGTGGAGGCTTGCT
GGCCACTTTTTGGGGTCAGCTCCTCTGAAATGCATTAGCGGAACCGTTTGCGATCTGCCA
CAAGTGTGATAAGTTATCTACACTGGCGAGGGGATTGCTCTCTGTAATGTTCAGCTTCTA
ATTGTCTCTACTTTGTGAGACTACTTTTGAATGCTTGACCTCAAATCAGGTAGGACTACC
CGCTGAACTTAA
>ABC8-57
TTTCCGTAGGTGAACCTGCGGAAGGATCATTATTGAATTATGTTTCTAGATAGGTTGTAG
CTGGCTCTTTTAGAGCATGTGCACGCCTGTTTGGACTTCATTTTCATCCACCTGTGCACC
TATTGTAGTCTTTGGTTGGGTTAGGAGGAAGTGATCATTGTATCAGCATCTGCTGGGAGT
GAGGACTTGCATTGTGAAAGCTTTGCTGTCCTTGATGTGATCATGGAATCTTTTTCTCAC
TAGAGTCTATGTCACTCATTATACTCTGTCGAATGTCATTGAATGTCTTTACATGGGCTT
ATATGCCTATGAAAATTGTAATACAACTTTCAGCAACGGATCTCTTGGCTCTCGCATCGA
TGAAGAACGCAGCGAAATGCGATAAGTAATGTGAATTGCAGAATTCAGTGAATCATCGAA
TCTTTGAACGCATCTTGCGCTCCTTGGTATTCCGAGGAGCATGCCTGTTTGAGTGTCATT
AAATTCTCAACTCTCTTCTAC--TTTTTGTAAAAGAGAGCTTGGACTGTGGAGGCTTGCT
GGCCACTTTTTGGGGTCAGCTCCTCTGAAATGCATTAGCGGAACCGTTTGCGATCTGCCA
CAAGTGTGATAAGTTATCTACACTGGCGAGGGGATTGCTCTCTGTAATGTTCAGCTTCTA
ATTGTCTCTACTTTGTGAGACTACTTTTGAATGCTTGACCTCAAATCAGGTAGGACTACC
CGCTGAACTTAA

>ABC6-45
TTTCCGTAGGTGAACCTGCGGAAGGATCATTATTGAATTATGTTTCTAGATAGGTTGTAG
CTGGCTCTTTTAGAGCATGTGCACGCCTGTTTGGACTTCATTTTCATCCACCTGTGCACC
TATTGTAGTCTTTGGTTGGGTTAGGAGGAAGTGATCATTGTATCAGCATCTGCTGGGAGT
GAGGACTTGCATTGTGAAAGCTTTGCTGTCCTTGATGTGATCATGGAATCTCTTTCTCAC
TAGAGTCTATGTCACTCATTATACTCTGTCGAATGTCATTGAATGTCTTTACATGGGCTT
ATATGCCTATGAAAATTGTAATACAACTTTCAGCAACGGATCTCTTGGCTCTCGCATCGA
TGAAGAACGCAGCGAAATGCGATAAGTAATGTGAATTGCAGAATTCAGTGAATCATCGAA
TCTTTGAACGCATCTTGCGCTCCTTGGTATTCCGAGGAGCATGCCTGTTTGAGTGTCATT
AAATTCTCAACTCTCTTCTAC--TTTTTGTAAAAGAGAGCTTGGACTGTGGAGGCTTGCT
GGCCACTTTTTGGGGTCAGCTCCTCTGAAATGCATTAGCGGAACCGTTTGCGATCTGCCA
CAAGTGTGATAAGTTATCTACACTGGCGAGGGGATTGCTCTCTGTAATGTTCAGCTTCTA
ATTGTCTCTACTTTGTGAGACTACTTTTGAATGCTTGACCTCAAATCAGGTAGGACTACC
CGCTGAACTTAA
>ABC10-49
TTTCCGTAGGTGAACCTGCGGAAGGATCATTATTGAATTATGTTTCTAGATAGGTTGTAG
CTGGCTCTTTTAGAGCATGTGCACGCCTGTTTGGACTTCATTTTCATCCACCTGTGCACC
TATTGTAGTCTTTGGTTGGGTTAGGAGGAAGTGATCATTGTATCAGCATCTGCTGGGAGT
GAGGACTTGCATTGTGAAAGCTTTGCTGTCCTTGATGTGATCATGGAATCTCTTTCTCAC
TAGAGTCTATGTCACTCATTATACTCTGTCGAATGTCATTGAATGTCTTTACATGGGCTT
GTATGCCTATGAAAATTGTAATACAACTTTCAGCAACGGATCTCTTGGCTCTCGCATCGA
TGAAGAACGCAGCGAAATGCGATAAGTAATGTGAATTGCAGAATTCAGTGAATCATCGAA
TCTTTGAACGCATCTTGCGCTCCTTGGTATTCCGAGGAGCATGCCTGTTTGAGTGTCATT
AAATTCTCAACTCTCTTCTAC--TTTTTGTAAAAGAGAGCTTGGACTGTGGAGGCTTGCT
GGCCACTTTTTGGGGTCAGCTCCTCTGAAATGCATTAGCGGAACCGTTTGCGATCTGCCA
CAAGTGTGATAAGTTATCTACACTGGCGAGGGGATTGCTCTCTGTAATGTTCAGCTTCTA
ATTGTCTCTACTTTGTGAGACTACTTTTGAATGCTTGACCTCAAATCAGGTAGGACTACC
CGCTGAACTTAA
>ABC5-20
TTTCCGTAGGTGAACCTGCGGAAGGATCATTATTGAATTATGTTTCTAGATAGGTTGTAG
CTGGCTCTTTTAGAGCATGTGCACGCCTGTTTGGACTTCATTTTCATCCACCTGTGCACC
TATTGTAGTCTTTGGTTGGGTTAGGAGGAAGTGATCATTGTATCAGCATCTGCTGGGAGT
GAGGACTTGCATTGTGAAAGCTTTGCTGTCCTTGATGTGATCATGGAATCTCTTTCTCAC
TAGAGTCTATGTCACTCATTATACTCTGTCGAATGTCATTGAATGTCTTTACATGGGCTT
GTATGCCTATGAAAATTGTAATACAACTTTCAGCAACGGATCTCTTGGCTCTCGCATCGA
TGAAGAACGCAGCGAAATGCGATAAGTAATGTGAATTGCAGAATTCAGTGAATCATCGAA
TCTTTGAACGCATCTTGCGCTCCTTGGTATTCCGAGGAGCATGCCTGTTTGAGTGTCATT
AAATTCTCAACTCTCTTCTAC--TTTTTGTAAAAGAGAGCTTGGACTGTGGAGGCTTGCT
GGCCACTTTTTGGGGTCAGCTCCTCTGAAATGCATTAGCGGAACCGTTTGCAATCTGCCA
CAAGTGTGATAAGTTATCTACACTGGCGAGGGGATTGCTCTCTGTAATGTTCAGCTTCTA
ATTGTCTCTACTTTGTGAGACTACTTTTGAATGCTTGACCTCAAATCAGGTAGGACTACC
CGCTGAACTTAA
>ABC10-7
TTTCCGTAGGTGAACCTGCGGAAGGATCATTATTGAATTATGTTTCTAGATAGGTTGTAG
CTGGCTCTTTTAGAGCATGTGCACGCCTGTTTGGACTTCATTTTCATCCACCTGTGCACC
TATTGTAGTCTTTGGTTGGGTTAGGAGGAAGTGATCATTGTATCAGCATCTGCTGGGAGT
GAGGACTTGCATTGTGAAAGCTTTGCTGTCCTTGATGTGATCATGGAATCTTTTTCTCAC
TAGAGTCTATGTCACTCATTATACTCTGTCGAATGTCATTGAATGTCTTTACATGGGCTT
GTATGCCTATGAAAATTGTAATACAACTTTCAGCAACGGATCTCTTGGCTCTCGCATCGA
TGAAGGACGCAGCGAAATGCGATAAGTAATGTGAATTGCAGAATTCAGTGAATCATCGAA

TCTTTGAACGCATCTTGCGCTCCTTGGTATTCCGAGGAGCATGCCTGTTTGAGTGTCATT
AAATTCTCAACTCTCTTCTAC--TTTTTGTAAAAGAGAGCTTGGACTGTGGAGGCTTGCT
GGCCACTTTTTGGGGTCAGCTCCTCTGAAATGCATTAGCGGAACCGTTTGCGATCTGCCA
CAAGTGTGATAAGTTATCTACACTGGCGAGGGGATTGCTCTCTGTAATGTTCAGCTTCTA
ATTGTCTCTACTTTGTGAGACTACTTTTGAATGCTTGACCTCAAATCAGGTAGGACTACC
CGCTGAACTTAA
>ABC12-54
TTTCCGTAGGTGAACCTGCGGAAGGATCATTATTGAATTATGTTTCTAGATAGGTTGTAG
CTGGCTCTTTTAGAGCATGTGCACGCCTGTTTGGACTTCATTTTCATCCACCTGTGCACC
TATTGTAGTCTTTGGTTGGGTTAGGAGGAAGTGATCATTGTATCAGCATCTGCTGGGAGT
GAGGACTTGCATTGTGAAAGCTTTGCTGTCCTTGATGTGATCATGGAATCTCTTTCTCAC
TAGAGTCTATGTCACTCATTATACTCTGTCGAATGTCATTGAATGTCTTTACATGGGCTT
GTATGCCTATGAAAATTGTAATACAACTTTCAGCAACGGATCTCTTGGCTCTCGCATCGA
TGAAGGACGCAGCGAAATGCGATAAGTAATGTGAATTGCAGAATTCAGTGAATCATCGAA
TCTTTGAACGCATCTTGCGCTCCTTGGTATTCCGAGGAGCATGCCTGTTTGAGTGTCATT
AAATTCTCAACTCTCTTCTAC--TTTTTGTAAAAGAGAGCTTGGACTGTGGAGGCTTGCT
GGCCACTTTTTGGGGTCAGCTCCTCTGAAATGCATTAGCGGAACCGTTTGCGATCTGCCA
CAAGTGTGATAAGTTATCTACACTGGCGAGGGGATTGCTCTCTGTAATGTTCAGCTTCTA
ATTGTCTCTACTTTGTGAGACTACTTTTGAATGCTTGACCTCAAATCAGGTAGGACTACC
CGCTGAACTTAA
>ABC6-36
TTTCCGTAGGTGAACCTGCGGAAGGATCATTATTGAATTATGTTTCTAGATAGGTTGTAG
CTGGCTCTTTTAGAGCATGTGCACGCCTGTTTGGACTTCATTTTCATCCACCTGTGCACC
TATTGTAGTCTTTGGTTGGGTTAGGAGGAAGTGATCATTGTATCAGCATCTGCTGGGAGT
GAGGACTTGCATTGTGAAAGCTTTGCTGTCCTTGATGTGATCATGGAATCTTTTTCTCAC
TAGAGTCTATGTCACTCATTATACTCTGTCGAATGTCATTGAATGTCTTTACATGGGCTT
GTATGCCTATGAAAATTGTAATACAACTTTCAGCAACGGATCTCTTGGCTCTCGCATCGA
TGAAGAACGCAGCGAAATGCGATAAGTAATGTGAATTGCAGAATTCAGTGAATCATCGAA
TCTTTGAACGCATCTTGCGCTCCTTGGTATTCCGAGGAGCATGCCTGTTTGAGTGTCATT
AAATTCTCAACTCTCTTCTAC--TTTTTGTAAAAGAGAGCTTGGACTGTGGAGGCTTGCT
GGCCACTTTTTGGGGTCAGCTCCTCTGAAATGCATTAGCGGAACCGTTTGCGATCTGCCA
CAAGTGTGATAAGTTATCTACACTGGCGAGGGGATTGTTCTCTGTAATGTTCAGCTTCTA
ATTGTCTCTACTTTGTGAGACTACTTTTGAATGCTTGACCTCAAATCAGGTAGGACTACC
CGCTGAACTTAA
>ABC6-58
TTTCCGTAGGTGAACCTGCGGAAGGATCATTATTGAATTATGTTTCTAGATAGGTTGTAG
CTGGCTCTTTTAGAGCATGTGCACGCCTGTTTGGACTTCATTTTCATCCACCTGTGCACC
TATTGTAGTCTTTGGTTGGGTTAGGAGGAAGTGATCATTGTATCAGCATCTGCTGGGAGT
GAGGACTTGCATTGTGAAAGCTTTGCTGTCCTTGATGTGATCATGGAATCTTTTTCTCAC
TAGAGTCTATGTCACTCATTATACTCTGTCGAATGTCATTGAATGTCTTTACATGGGCTT
GTATGCCTATGAAAATTGTAATACAACTTTCAGCAACGGATCTCTTGGCTCTCGCATCGA
TGAAGAACGCAGCGAAATGCGATAAGTAATGTGAATTGCAGAATTCAGTGAATCATCGAA
TCTTTGAACGCATCTTGCGCTCCTTGGTATTCCGAGGAGCATGCCTGTTTGAGTGTCATT
AAATTCTCAACTCTCTTCTAC--TTTTTGTAAAAGAGAGCTTGGACTGTGGAGGCTTGCT
GGCCACTTTTTGGGATCAGCTCCTCTGAAATGCATTAGCGGAACCGTTTGCGATCTGCCA
CAAGTGTGATAAGTTATCTACACTGGCGAGGGGATTGCTCTCTGTAATGTTCAGCTTCTA
ATTGTCTCTACTTTGTGAGACTACTTTTGAATGCTTGACCTCAAATCAGGTAGGACTACC
CGCTGAACTTAA
>ABC10-8
TTTCCGTAGGTGAACCTGCGGAAGGATCATTATTGAATTATGTTTCTAGATAGGTTGTAG

CTGGCTCTTTTAGAGCATGTGCACGCCTGTTTGGACTTCATTTTCATCCACCTGTGCACC
TATTGTAGTCTTTGGTTGGGTTAGGAGGAAGTGATCATTGTATCAGCATCTGCTGGGAGT
GAGGACTTGCATTGTGAAAGCTTTGCTGTCCTTGATGTGATCATGGAATCTCTTTCTCAC
TAGAGTCTATGTCACTCATTATACTCTGTCGAATGTCATTGAATGTCTTTACATGGGCTT
ATATGCCTATGAAAATTGTAATACAACTTTCAGCAACGGATCTCTTGGCTCTCGCATCGA
TGAAGAACGCAGCGAAATGCGATAAGTAATGTGAATTGCAGAATTCAGTGAATCATCGAA
TCTTTGAACGCATCTTGCGCTCCTTGGTATTCCGAGGAGCATGCCTGTTTGAGTGTCATT
AAATTCTCAACTCTCTTCTAC--TTTTTGTAAAAGAGAGCTTGGACTGTGGAGGCTTGCT
GGCCACTTTTTGGGGTCAGCTCCTCTGAAATGCATTAGCGGAACCGTTTGCGATCTGCCA
CAAGTGTGATAAGTTATCTACACTGGCGAGGGGATTGCTCTCTGTAATGTTCAGCTTCTA
ATTGTCTCTACTTTGTGAGACAACTTTTGAATGCTTGACCTCAAATCAGGTAGGACTACC
CGCTGAACTTAA
>ABC9-2
TTTCCGTAGGTGAACCTGCGGAAGGATCATTATTGAATTATGTTTCTAGATAGGTTGTAG
CTGGCTCTTTTAGAGCATGTGCACGCCTGTTTGGACTTCATTTTCATCCACCTGTGCACC
TATTGTAGTCTTTGGTTGGGTTAGGAGGAAGTGATCATTGTATCAGCATCTGCTGGGAGT
GAGGACTTGCATTGTGAAAGCTTTGCTGTCCTTGATGTGATCATGGAATCTTTTTCTCAC
TAGAGTCTATGTCACTCATTATACTCTGTCGAATGTCATTGAATGTCTTTACATGGGCTT
GTATGCCTATGAAAATTGTAATACAACTTTCAGCAACGGATCTCTTGGCTCTCGCATCGA
TGAAGAACGCAGCGAAATGCGATAAGTAATGTGAATTGCAGAATTCAGTGAATCATCGAA
TCTTTGAACGCATCTTTCGCTCCTTGGTATTCCGAGGAGCATGCCTGTTTGAGTGTCATT
AAATTCTCAACTCTCTTCTAC--TTTTTGTAAAAGAGAGCTTGGACTGTGGAGGCTTGCT
GACCACTTTTTGGGGTCAGCTCCTCTGAAATGCATTAGCGGAACCGTTTGCGATCTGCCA
CAAGTGTGATAAGTTATCTACACTGGCGAGGGGATTGCTCTCTGTAATGTTCAGCTTCTA
ATTGTCTCTACTTTGTGAGACTACTTTTGAATGCTTGACCTCAAATCAGGTAGGACTACC
CGCTGAACTTAA
>ABC1-71
TTTCCGTAGGTGAACCTGCGGAAGGATCATTATTGAATTATGTTTCTAGATAGGTTGTAG
CTGGCTCTTTTAGAGCATGTGCACGCCTGTTTGGACTTCATTTTCATCCACCTGTGCACC
TATTGTAGTCTTTGGTTGGGTTAGGAGGAAGTGGTCATTGTGTCAGCATCTGCTGGATGT
GAGGACTTGCATTGTGAAAGCTTTGCTGTCCTTGATGTGATCATGGAATCTCTTTCTCAC
TAGAGTCTATGTCACTCATTATACTCTGTCGAATGTCATTGAATGTCTTTACATGGGCTT
ATATGCCTATGAAAATTGTAATACAACTTTCAGCAACGGATCTCTTGGCTCTCGCATCGA
TGAAGAACGCAGCGAAATGCGATAAGTAATGTGAATTGCAGAATTCAGTGAATCATCGAA
TCTTTGAACGCATCTTGCGCTCCTTGGTATTCCGAGGAGCATGCCTGTTTGAGTGTCATT
AAATTCTCAACTCTCTTCTAC--TTTTTGTAAAAGAGAGCTTGGACTGTGGAGGCTTGCT
GGCCACTTTTTGGGGTCAGCTCCTCTGAAATGCATTAGCGGAACCGTTTGCGATCTGCCA
CAAGTGTGATAAGTTATCTACACTGGCGAGGGGATTGCTCTCTGTAATGTTCAGCTTCTA
ATTGTCTCTACTTTGTGAGACTACTTTTGAATGCTTGACCTCAAATCAGGTAGGACTACC
CGCTGAACTTAA
>ABC10-27
TTTCCGTAGGTGAACCTGCGGAAGGATCATTATTGAATTATGTTTCTAGATAGGTTGTAG
CTGGCTCTTTTAGAGCATGTGCACGCCTGTTTGGACTTCATTTTCATCCACCTGTGCACC
TATTGTAGTCTTTGGTTGGGTTAGGAGGAAGTGGTCATTGTGTCAGCATCTGCTGGATGT
GAGGACTTGCATTGTGAAAGCTTTGCTGTCCTTGATGTGATCATGGAATCTCTTTCTCAC
TAGAGTCTATGTCACTCATTATACTCTGTCGAATGTCATTGAATGTCTTTACATGGGCTT
ATATGCCTATGAAAATTGTAATACAACTTTCAGCAACGGATCTCTTGGCTCTCGCATCGA
TGAAGAACGCAGCGAAATGCGATAAGTAATGTGAATTGCAGAATTCAGTGAATCATCGAA
TCTTTGAACGCATCTTGCGCTCCTTGGTATTCCGAGGAGCATGCCTGTTTGAGTGTCATT
AAATTCTCAACTCTCTTCTAC--TTTTTGTAAAAGAGAGCTTGGACTGTGGAGGCTTGCT

GGCCACTTTTTGGGGTCAGCTCCTCTGAAATGCATTAGCGGAACCGTTTGCGATCTGCCA
CAAGTGTGATAAGTTATCTACACTGGCGAGGGGATTGCTCTCTGTAATGTTCAGCTTCTA
ATTGTCTCTACTTTGTGAGACTACTTTTGAATGCTTGACCTCAAATCAGGTAGGACTACC
CGCTGAACTTAA
>ABC4-57
TTTCCGTAGGTGAACCTGCGGAAGGATCATTATTGAATTATGTTTCTAGATAGGTTGTAG
CTGGCTCTTTTAGAGCATGTGCACGCCTGTTTGGACTTCATTTTCATCCACCTGTGCACC
TATTGTAGTCTTTGGTTGGGTTAGGAGGAAGTGATCATTGTGTCAGCATCTGCTGGATGT
GAGGACTTGCATTGTGAAAGCTTTGCTGTCCTTGATGTGATCATGGAATCTCTTTCTCAC
TAGAGTCTATGTCACTCATTATACTCTGTCGAATGTCATTGAATGTCTTTACATGGGCTT
ATATGCCTATGAAAATTGTAATACAACTTTCAGCAACGGATCTCTTGGCTCTCGCATCGA
TGAAGAACGCAGCGAAATGCGATAAGTAATGTGAATTGCAGAATTCAGTGAATCATCGAA
TCTTTGAACGCATCTTGCGCTCCTTGGTATTCCGAGGAGCATGCCTGTTTGAGTGTCATT
AAATTCTCAACTCTCTTCTAC--TTTTTGTAAAAGAGAGCTTGGACTGTGGAGGCTTGCT
GGCCACTTTTTGGGGTCAGCTCCTCTGAAATGCATTAGCGGAACCGTTTGCGATCTGCCA
CAAGTGTGATAAGTTATCTACACTGGCGAGGGGATTGCTCTCTGTAATGTTCAGCTTCTA
ATTGTCTCTACTTTGTGAGACTACTTTTGAATGCTTGACCTCAAATCAGGTAGGACTACC
CGCTGAACTTAA
>ABC9-55
TTTCCGTAGGTGAACCTGCGGAAGGATCATTATTGAATTATGTTTCTAGATAGGTTGTAG
CTGGCTCTTTTAGAGCATGTGCACGCCTGTTTGGACTTCATTTTCATCCACCTGTGCACC
TATTGTAGTCTTTGGTTGGGTTAGGAGGAAGTGATCATTGTGTCAGCATCTGCTGGATGT
GAGGACTTGCATTGTGAAAGCTTTGCTGTCCTTGATGTGATCATGGAATCTCTTTCTCAC
TAGAGTCTATGTCACTCATTATACTCTGTCGAATGTCATTGAATGTCTTTACATGGGCTT
ATATGCCTATGAAAATTGTAATACAACTTTCAGCAACGGATCTCTTGGCTCTCGCATCGA
TGAAGGACGCAGCGAAATGCGATAAGTAATGTGAATTGCAGAATTCAGTGAATCATCGAA
TCTTTGAACGCATCTTGCGCTCCTTGGTATTCCGAGGAGCATGCCTGTTTGAGTGTCATT
AAATTCTCAACTCTCTTCTAC--TTTTTGTAAAAGAGAGCTTGGACTGTGGAGGCTTGCT
GGCCACTTTTTGGGGTCAGCTCCTCTGAAATGCATTAGCGGAACCGTTTGCGATCTGCCA
CAAGTGTGATAAGTTATCTACACTGGCGAGGGGATTGCTCTCTGTAATGTTCAGCTTCTA
ATTGTCTCTACTTTGTGAGACTACTTTTGAATGCTTGACCTCAAATCAGGTAGGACTACC
CGCTGAACTTAA
>ABC2-73
TTTCCGTAGGTGAACCTGCGGAAGGATCATTATTGAATTATGTTTCTAGATAGGTTGTAG
CTGGCTCTTTTAGAGCATGTGCACGCCTGTTTGGACTTCATTTTCATCCACCTGTGCACC
TATTGTAGTCTTTGGTTGGGTTAGGGGGAAGTGGTCATTGTGTCAGCATCTGCTGGATGT
GAGGACTTGCATTGTGAAAGCTTTGCTGTCCTTGATGTGATCATGGAATCTCTTTCTCAC
TAGAGTCTATGTCACTCATTATACTCTGTCGAATGTCATTGAATGTCTTTACATGGGCTT
GTATGCCTATGAAAATTGTAATACAACTTTCAGCAACGGATCTCTTGGCTCTCGCATCGA
TGAAGAACGCAGCGAAATGCGATAAGTAATGTGAATTGCAGAATTCAGTGAATCATCGAA
TCTTTGAACGCATCTTGCGCTCCTTGGTATTCCGAGGAGCATGCCTGTTTGAGTGTCATT
AAATTCTCAACTCTCTTCTAC--TTTTTGTAAAAGAGAGCTTGGACTGTGGAGGCTTGCT
GGCCACTTTTTGGGGTCAGCTCCTCTGAAATGCATTAGCGGAACCGTTTGCGATCTGCCA
CAAGTGTGATAAGTTATCTACACTGGCGAGGGGATTGCTCTCTGTAATGTTCAGCTTCTA
ATTGTCTCTACTTTGTGAGACTACTTTTGAATGCTTGACCTCAAATCAGGTAGGACTACC
CGCTGAACTTAA
>ABC4-71
TTTCCGTAGGTGAACCTGCGGAAGGATCATTATTGAATTATGTTTCTAGATAGGTTGTAG
CTGGCTCTTTTAGAGCATGTGCACGCCTGTTTGGACTTCATTTTCATCCACCTGTGCACC
TATTGTAGTCTTTGGTTGGGTTAGGAGGAAGTGGTCATTGTGTCAGCATCTGCTGGATGT

GAGGACTTGCATTGTGAAAGCTTTGCTGTCCTTGATGTGATCATGGAATCTCTTTCTCAC
TAGAGTCTATGTCACTCATTATACTCTGTCGAATGTCATTGAATGTCTTTACATGGGCTT
GTATGCCTATGAAAATTGTAATACAACTTTCAGCAACGGATCTCTTGGCTCTCGCATCGA
TGAAGAACGCAGCGAAATGCGATAAGTAATGTGAATTGCAGAATTCAGTGAATCATCGAA
TCTTTGAACGCATCTTGCGCTCCTTGGTATTCCGAGGAGCATGCCTGTTTGAGTGTCATT
AAATTCTCAACTCTCTTCTAC--TTTTTGTAAAAGAGAGCTTGGACTGTGGAGGCTTGCT
GGCCACTTTTTGGGGTCAGCTCCTCTGAAATGCATTAGCGGAACCGTTTGCGATCTGCCA
CAAGTGTGATAAGTTATCTACACTGGCGAGGGGATTGCTCTCTGTAATGTTCAGCTTCTA
ATTGTCTCTACTTTGTGAGACTACTTTTGAATGCTTGACCTCAAATCAGGTAGGACTACC
CGCTGAACTTAA
>ABC3-34
TTTCCGTAGGTGAACCTGCGGAAGGATCATTATTGAATTATGTTTCTAGATAGGTTGTAG
CTGGCTCTTTTAGAGCATGTGCACGCCTGTTTGGACTTCATTTTCATCCACCTGTGCACC
TATTGTAGTCTTTGGTTGGGTTAGGAGGAAGTGGTCATTGTGTCAGCATCTGCTGGATGT
GAGGACTTGCATTGTGAAAGCTTTGCTGTCCTTGATGTGATCATGGAATCTCTTTCTCAC
TAGAGTCTATGTCACTCATTATACTCTGTCGAATGTCATTGAATGTCTTTACATGGGCTT
GTATGCCTATGAAAATTGTAATACAACTTTCAGCAACGGATCTCTTGGCTCTCGCATCGA
TGAAGAACGCAGCGAAATGCGATAAGTAATGTGAATTGCAGAATTCAGTGAATCATCGAA
TCTTTGAACGCATCTTGCGCTCCTTGGTATTCCGAGGAGCATGCCTGTTTGAGTGTCATT
AAATTCTCAACTCTCTTCTAC--TTTTTGTAAAAGAGAGCTTGGACTGTGGAGGCTTGCT
GGCCACTTTTTGGGGTCAGCTCCTCTGAAATGCATTAGCGGAACCGTTTGCAATCTGCCA
CAAGTGTGATAAGTTATCTACACTGGCGAGGGGATTGCTCTCTGTAATGTTCAGCTTCTA
ATTGTCTCTACTTTGTGAGACTACTTTTGAATGCTTGACCTCAAATCAGGTAGGACTACC
CGCTGAACTTAA
>ABC3-9
TTTCCGTAGGTGAACCTGCGGAAGGATCATTATTGAATTATGTTTCTAGATAGGTTGTAG
CTGGCTCTTTTAGAGCATGTGCACGCCTGTTTGGACTTCATTTTCATCCACCTGTGCACC
TATTGTAGTCTTTGGTTGGGTTAGGGGGAAGTGGTCATTGTGTCAGCATCTGCTGGATGT
GAGGACTTGCATTGTGAAAGCTTTGCTGTCCTTGATGTGATCATGGAATCTCTTTCTCAC
TAGAGTCTATGTCACTCATTATACTCTGTCGAATGTCATTGAATGTCTTTACATGGGCTT
GTATGCCTATGAAAATTGTAATACAACTTTCAGCAACGGATCTCTTGGCTCTCGCATCGA
TGAAGAACGCAGCGAAATGCGATAAGTAATGTGAATTGCAGAATTCAGTGAATCATCGAA
TCTTTGAACGCATCTTGCGCTCCTTGGTATTCCGAGGAGCATGCCTGTTTGAGTGTCATT
AAATTCTCAACTCTCTTCTAC--TTTTTGTAAAAGAGAGCTTGGACTGTGGAGGCTTGCT
GGCCACTTTTTGGGGTCAGCTCCTCTGAAATGCATTAGCGGAACCGTTTGCAATCTGCCA
CAAGTGTGATAAGTTATCTACACTGGCGAGGGGATTGCTCTCTGTAATGTTCAGCTTCTA
ATTGTCTCTACTTTGTGAGACTACTTTTGAATGCTTGACCTCAAATCAGGTAGGACTACC
CGCTGAACTTAA
>ABC3-40
TTTCCGTAGGTGAACCTGCGGAAGGATCATTATTGAATTATGTTTCTAGATAGGTTGTAG
CTGGCTCTTTTAGAGCATGTGCACGCCTGTTTGGACTTCATTTTCATCCACCTGTGCACC
TATTGTAGTCTTTGGTTGGGTTAGGAGGAAGTGATCATTGTATCAGCATCTGCTGGATGT
GAGGACTTGCATTGTGAAAGCTTTGCTGTCCTTGATGTGATCATGGAATCTCTTTCTCAC
TAGAGTCTATGTCACTCATTATACTCTGTCGAATGTCATTGAATGTCTTTACATGGGCTT
GTATGCCTATGAAAATTGTAATACAACTTTCAGCAACGGATCTCTTGGCTCTCGCATCGA
TGAAGGACGCAGCGAAATGCGATAAGTAATGTGAATTGCAGAATTCAGTGAATCATCGAA
TCTTTGAACGCATCTTGCGCTCCTTGGTATTCCGAGGAGCATGCCTGTTTGAGTGTCATT
AAATTCTCAACTCTCTTCTAC--TTTTTGTAAAAGAGAGCTTGGACTGTGGAGGCTTGCT
GGCCACTTTTTGGGGTCAGCTCCTCTGAAATGCATTAGCGGAACCGTTTGCGATCTGCCA
CAAGTGTGATAAGTTATCTACACTGGCGAGGGGATTGCTCTCTGTAATGTTCAGCTTCTA

ATTGTCTCTACTTTGTGAGACTACTTTTGAATGCTTGACCTCAAATCAGGTAGGACTACC
CGCTGAACTTAA
>ABC9-44
TTTCCGTAGGTGAACCTGCGGAAGGATCATTATTGAATTATGTTTCTAGATAGGTTGTAG
CTGGCTCTTTTAGAGCATGTGCACGCCTGTTTGGACTTCATTTTCATCCACCTGTGCACC
TATTGTAGTCTTTGGTTGGGTTAGGAGGAAGTGATCATTGTATCAGCATCTGCTGGGAGT
GAGGACTTGCATTGTGAAAGCTTTGCTGTCCTTGATGTGATCATGGAATCTTTTTCTCAC
TAGAGTCTATGTCACTCATTATACTCTGTCGAATGTCATTGAATGTCTTTACATGGGCTT
GTATGCCTATGAAAATTGTAATACAACTTTCAGCAACGGATCTCTTGGCTCTCGCATCGA
TGAAGGACGCAGCGAAATGCGATAAGTAATGTGAATTGCAGAATTCAGTGAATCATCGAA
TCTTTGAACGCATCTTGCGCTCCTTGGTATTCCGAGGAGCATGCCTGTTTGAGTGTCATT
AAATTCTCAACTCTCTTATAC--TTTTTGTAAAAGAGAGCTTGGACTGTGGAGGCTTGCT
GGCCACTTTTTGGGGTCAGCTCCTCTGAAATGCATTAGCGGAACCGTTTGCGATCTGCCA
CAAGTGTGATAAGTTATCTACACTGGCGAGGGGATTGCTCTCTGTAATGTTCAGCTTCTA
ATTGTCTCTACTTTGTGAGACAACTTTTGAATGCTTGACCTCAAATCAGGTAGGACTACC
CGCTGAACTTAA
>ABC6-3
TTTCCGTAGGTGAACCTGCGGAAGGATCATTATTGAATTATGTTTCTAGATAGGTTGTAG
CTGGCTC-TTTAGAGCATGTGCACGCCTGTTTGGACTTCATTTTCATCCACCTGTGCACC
TATTGTAGTCTTTGGTTGGGTTAGGGGGAAGTGGTCATTGTGTCAGCATCTGCTGGATGT
GAGGACTTGCATTGTGAAAGCTTTGCTGTCCTTGATGTGATCATGGAATCTCTTTCTCAC
TAGAGTCTATGTCACTCATTATACTCTGTCGAATGTCATTGAATGTCTTTACATGGGCTT
GTATGCCTATGAAAATTGTAATACAACTTTCAGCAACGGATCTCTTGGCTCTCGCATCGA
TGAAGGACGCAGCGAAATGCGATAAGTAATGTGAATTGCAGAATTCAGTGAATCATCGAA
TCTTTGAACGCATCTTGCGCTCCTTGGTATTCCGAGGAGCATGCCTGTTTGAGTGTCATT
AAATTCTCAACTCTCTTATAC--TTTTTGTAAAAGAGAGCTTGGACTGTGGAGGCTTGCT
GGCCACTTTTTGGGGTCAGCTCCTCTGAAATGCATTAGCGGAACCGTTTGCAATCTGCCA
CAAGTGTGATAAGTTATCTACACTGGCGAGGGGATTGCTCTCTGTAATGTTCAGCTTCTA
ATTGTCTCTACTTTGTGAGACAACTTTTGAATGCTTGACCTCAAATCAGGTAGGACTACC
CGCTGAACTTAA
>ABC1-54
TTTCCGTAGGTGAACCTGCGGAAGGATCATTATTGAATTATGTTTCTAGATAGGTTGTAG
CTGGCTC-TTTAGAGCATGTGTACGCCTGTTTGGACTTCATTTTCATCCACCTGTGCACC
TATTGTAGTCTTTGGTTGGGTTAGGGGGAAGTGGTCATTGTGTCAGCATCTGCTGGATGT
GAGGACTTGCATTGTGAAAGCTTTGCTGTCCTTGATGTGATCATGGAATCTCTTTCTCAC
TAGAGTCTATGTCACTCATTATACTCTGTCGAATGTCATTGAATGTCTTTACATGGGCTT
GTATGCCTATGAAAATTGTAATACAACTTTCAGCAACGGATCTCTTGGCTCTCGCATCGA
TGAAGGACGCAGCGAAATGCGATAAGTAATGTGAATTGCAGAATTCAGTGAATCATCGAA
TCTTTGAACGCATCTTGCGCTCCTTGGTATTCCGAGGAGCATGCCTGTTTGAGTGTCATT
AAATTCTCAACTCTCTTATAC--TTTTTGTAAAAGAGAGCTTGGACTGTGGAGGCTTGCT
GGCCACTTTTTGGGGTCAGCTCCTCTGAAATGCATTAGCGGAACCGTTTGCAATCTGCCA
CAAGTGTGATAAGTTATCTACACTGGCGAGGGGATTGCTCTCTGTAATGTTCAGCTTCTA
ATTGTCTCTACTTTGTGAGACAACTTTTGAATGCTTGACCTCAAATCAGGTAGGACTACC
CGCTGAACTTAA
>ABC3-6
TTTCCGTAGGTGAACCTGCGGAAGGATCATTATTGAATTATGTTTCTAGATAGGTTGTAG
CTGGCTC-TTTAGAGCATGTGCACGCCTGTTTGGACTTCATTTTCATCCACCTGTGCACC
TATTGTAGTCTTTGGTTGGGTTAGGGGGAAGTGGTCATTGTGTCAGCATCTGCTGGATGT
GAGGACTTGCATTGTGAAAGCTTTGCTGTCCTTGATGTGATCATGGAATCTCTTTCTCAC
TAGAGTCTATGTCACTCATTATACTCTGTCGAATGTCATTGAATGTCTTTACATGGGCTT

GTATGCCTATGAAAATTGTAATACAACTTTCAGCAACGGATCTCTTGGCTCTCGCATCGA
TGAAGGACGCAGCGAAATGCGATAAGTAATGTGAATTGCAGAATTCAGTGAATCATCGAA
TCTTTGAACGCATCTTGCGCTCCTTGGTATTCCGAGGAGCATGCCTGTTTGAGTGTCATT
AAATTCTCAACTCTCTTATAC--TTTTTGTAAAAGAGAGCTTGGACTGTGGAGGCTTGCT
GGCCACTTTTTGGGGTCAGCTCCTCTGAAATGCATTAGCGGAACCGTTTGCAATCTGCCA
CAAGTGTGATAAGTTATCTACACTGGCGAGGGGATTACTCTCTGTAATGTTCAGCTTCTA
ATTGTCTCTACTTTGTGAGACAACTTTTGAATGCTTGACCTCAAATCAGGTAGGACTACC
CGCTGAACTTAA
>ABC9-45
TTTCCGTAGGTGAACCTGCGGAAGGATCATTATTGAATTATGTTTCTAGATAGGTTGTAG
CTGGCTC-TTTAGAGCATGTGCACGCCTGTTTGGACTTCATTTTCATCCACCTGTGCACC
TATTGTAGTCTTTGGTTGGGTTAGGGGGAAGTGGTCATTGTGTCAGCATCTGCTGGATGT
GAGGACTTGCATTGTGAAAGCTTTGCTGTCCTTGATGTGATCATGGAATCTCTTTCTCAC
TAGAGTCTATGTCACTCATTATACTCTGTCGAATGTCATTGAATGTCTTTACATGGGCTT
GTATGCCTATGAAAATTGTAATACAACTTTCAGCAACGGATCTCTTGGCTCTCGCATCGA
TGAAGGACGCAACGAAATGCGATAAGTAATGTGAATTGCAGAATTCAGTGAATCATCGAA
TCTTTGAACGCATCTTGCGCTCCTTGGTATTCCGAGGAGCATGCCTGTTTGAGTGTCATT
AAATTCTCAACTCTCTTATAC--TTTTTGTAAAAGAGAGCTTGGACTGTGGAGGCTTGCT
GGCCACTTTTTGGGGTCAGCTCCTCTGAAATGCATTAGCGGAACCGTTTGCAATCTGCCA
CAAGTGTGATAAGTTATCTACACTGGCGAGGGGATTGCTCTCTGTAATGTTCAGCTTCTA
ATTGTCTCTACTTTGTGAGACAACTTTTGAATGCTTGACCTCAAATCAGGTAGGACTACC
CGCTGAACTTAA
>ABC3-63
TTTCCGTAGGTGAACCTGCGGAAGGATCATTATTGAATTATGTTTCTAGATAGGTTGTAG
CTGGCTC-TTTAGAGCATGTGCACGCCTGTTTGGACTTCATTTTCATCCACCTGTGCACC
TATTGTAGTCTTTGGTTGGGTTAGGAGGAAGTGGTCATTGTGTCAGCATCTGCTGGATGT
GAGGACTTGCATTGTGAAAGCTTTGCTGTCCTTGATGTGATCATGGAATCTCTTTCTCAC
TAGAGTCTATGTCACTCATTATACTCTGTCGAATGTCATTGAATGTCTTTACATGGGCTT
ATATGCCTATGAAAATTGTAATACAACTTTCAGCAACGGATCTCTTGGCTCTCGCATCGA
TGAAGAACGCAGCGAAATGCGATAAGTAATGTGAATTGCAGAATTCAGTGAATCATCGAA
TCTTTGAACGCATCTTGCGCTCCTTGGTATTCCGAGGAGCATGCCTGTTTGAGTGTCATT
AAATTCTCAACTCTCTTCTAC--TTTTTGTAAAAGAGAGCTTGGACTGTGGAGGCTTGCT
GGCCACTTTTTGGGGTCAGCTCCTCTGAAATGCATTAGCGGAACCGTTTGCGATCTGCCA
CAAGTGTGATAAGTTATCTACACTGGCGAGGGGATTGCTCTCTGTAATGTTCAGCTTCTA
ATTGTCTCTACTTTGTGAGACAACTTTTGAATGCTTGACCTCAAATCAGGTAGGACTACC
CGCTGAACTTAA
>ABC9-48
TTTCCGTAGGTGAACCTGCGGAAGGATCATTATTGAATTATGTTTCTAGATAGGTTGTAG
CTGGCTC-TTTAGAGCATGTGCACGCCTGTTTGGACTTCATTTTCATCCACCTGTGCACC
TATTGTAGTCTTTGGTTGGGTTAGGAGGAAGTGGTCATTGTGTCAGCATCTGCTGGATGT
GAGGACTTGCATTGTGAAAGCTTTGCTGTCCTTGATGTGATCATGGAATCTCTTTCTCAC
TAGAGTCTATGTCACTCATTATACTCTGTCGAATGTCATTGAATGTCTTTACATGGGCTT
ATATGCCTATGAAAATTGTAATACAACTTTCAGCAACGGATCTCTTGGCTCTCGCATCGA
TGAAGAACGCAGCGAAATGCGATAAGTAATGTGAATTGCAGAATTCAGTGAATCATCGAA
TCTTTGAACGCATCTTGCGCTCCTTGGTATTCCGAGGAGCATGCCTGTTTGAGTGTCATT
AAATTCTCAACTCTCTTCTAC--TTTTTGTAAAAGAGAGCTTGGACTGTGGAGGCTTGCT
GGCCACTTTTTGGGGTCAGCTCCTCTGAAATGCATTAGCGGAACCGTTTGCGATCTGCCA
CAAGTGTGATAAGTTATCTACACTGGCGAGGGGATTGCTCTCTGTAATGTTCAGCTTCTA
ATTGTCTCTACTTTGTGAGACAACTTTTGAATGCTTGACCTCAAATCAGGTAGGACTACC
CGCTGAACTTAA

>ABC11-3
TTTCCGTAGGTGAACCTGCGGAAGGATCATTATTGAATTATGTTTCTAGATAGGTTGTAG
CTGGCTC-TTTAGAGCATGTGCACGCCTGTTTGGACTTCATTTTCATCCACCTGTGCACC
TATTGTAGTCTTTGGTTGGGTTAGGAGGAAGTGGTCATTGTGTCAGCATCTGCTGGATGT
GAGGACTTGCATTGTGAAAGCTTTGCTGTCCTTGATGTGATCATGGAATCTCTTTCTCAC
TAGAGTCTATGTCACTCATTATACTCTGTCGAATGTCATTGAATGTCTTTACATGGGCTT
ATATGCCTATGAAAATTGTAATACAACTTTCAGCAACGGATCTCTTGGCTCTCGCATCGA
TGAAGAACGCAGCGAAATGCGATAAGTAATGTGAATTGCAGAATTCAGTGAATCATCGAA
TCTTTGAACGCATCTTGCGCTCCTTGGTATTCCGAGGAGCATGCCTGTTTGAGTGTCATT
AAATTCTCAACTCTCTTCTAC--TTTTTGTAAAAGAGAGCTTGGACTGTGGAGGCTTGCT
GGCCACTTTTTGGGGTCAGCTCCTCTGAAATGCATTAGCGGAACCGTTTGCGATCTGCCA
CAAGTGTGATAAGTTATCTACACTGGCGAGGGGATTGCTCTCTGTAATGTTCAGCTTCTA
ATTGTCTCTACTTTGTGAGACAACTTTTGAATGCTTGACCTCAAATCAGGTAGGACTACC
CGCTGAACTTAA
>ABC5-75
TTTCCGTAGGTGAACCTGCGGAAGGATCATTATTGAATTATGTTTCTAGATAGGTTGTAG
CTGGCTC-TTTAGAGCATGTGCACGCCTGTTTGGACTTCATTTTCATCCACCTGTGCACC
TATTGTAGTCTTTGGTTGGGTTAGGGGGAAGTGGTCATTGTGTCAGCATCTGCTGGATGT
GAGGACTTGCATTGTGAAAGCTTTGCTGTCCTTGATGTGATCATGGAATCTCTTTCTCAC
TAGAGTCTATGTCACTCATTATACTCTGTCGAATGTCATTGAATGTCTTTACATGGGCTT
ATATGCCTATGAAAATTGTAATACAACTTTCAGCAACGGATCTCTTGGCTCTCGCATCGA
TGAAGAACGCAGCGAAATGCGATAAGTAATGTGAATTGCAGAATTCAGTGAATCATCGAA
TCTTTGAACGCATCTTGCGCTCCTTGGTATTCCGAGGAGCATGCCTGTTTGAGTGTCATT
AAATTCTCAACTCTCTTCTAC--TTTTTGTAAAAGAGAGCTTGGACTGTGGAGGCTTGCT
GGCCACTTTTTGGGGTCAGCTCCTCTGAAATGCATTAGCGGAACCGTTTGCGATCTGCCA
CAAGTGTGATAAGTTATCTACACTGGCGAGGGGATTGCTCTCTGTAATGTTCAGCTTCTA
ATTGTCTCTACTTTGTGAGACAACTTTTGAATGCTTGACCTCAAATCAGGTAGGACTACC
CGCTGAACTTAA
>ABC5-39
TTTCCGTAGGTGAACCTGCGGAAGGATCATTATTGAATTATGTTTCTAGATAGGTTGTAG
CTGGCTC-TTTAGAGCATGTGCACGCCTGTTTGGACTTCATTTTCATCCACCTGTGCACC
TATTGTAGTCTTTGGTTGGGTTAGGGGGAAGTGGTCATTGTGTCAGCATCTGCTGGATGT
GAGGACTTGCATTGTGAAAGCTTTGCTGTCCTTGATGTGATCATGGAATCTCTTTCTCAC
TAGAGTCTATGTCACTCATTATACTCTGTCGAATGTCATTGAATGTCTTTACATGGGCTT
GTATGCCTATGAAAATTGTAATACAACTTTCAGCAACGGATCTCTTGGCTCTCGCATCGA
TGAAGAACGCAGCGAAATGCGATAAGTAATGTGAATTGCAGAATTCAGTGAATCATCGAA
TCTTTGAACGCATCTTGCGCTCCTTGGTATTCCGAGGAGCATGCCTGTTTGAGTGTCATT
AAATTCTCAACTCTCTTCTAC--TTTTTGTAAAAGAGAGCTTGGACTGTGGAGGCTTGCT
GGCCACTTTTTGGGGTCAGCTCCTCTGAAATGCATTAGCGGAACCGTTTGCGATCTGCCA
CAAGTGTGATAAGTTATCTACACTGGCGAGGGGATTGCTCTCTGTAATGTTCAGCTTCTA
ATTGTCTCTACTTTGTGAGACAACTTTTGAATGCTTGACCTCAAATCAGGTAGGACTACC
CGCTGAACTTAA
>ABC9-57
TTTCCGTAGGTGAACCTGCGGAAGGATCATTATTGAATTATGTTTCTAGATAGGTTGTAG
CTGGCTC-TTTAGAGCATGTGCACGCCTGTTTGGACTTCATTTTCATCCACCTGTGCACC
TATTGTAGTCTTTGGTTGGGTTAGGGGGAAGTGGTCATTGTGTCAGCATCTGCTGGATGT
GAGGACTTGCATTGTGAAAGCTTTGCTGTCCTTGATGTGATCATGGAATCTCTTTCTCAC
TAGAGTCTATGTCACTCATTATACTCTGTCGAATGTCATTGAATGTCTTTACATGGGCTT
GTATGCCTATGAAAATTGTAATACAACTTTCAGCAACGGATCTCTTGGCTCTCGCATCGA
TGAAGAACGCAGCGAAATGCGATAAGTAATGTGAATTGCAGAATTCAGTGAATCATCGAA

TCTTTGAACGCATCTTGCGCTCCTTGGTATTCCGAGGAGCATGCCTGTTTGAGTGTCATT
AAATTCTCAACTCTCTTCTAC--TTTTTGTAAAAGAGAGCTTGGACTGTGGAGGCTTGCT
GGCCACTTTTTGGGGTCAGCTCCTCTGAAATGCATTAGCGGAACCGTTTGCGATCTGCCA
CAAGTGTGATAAGTTATCTACACTGGCGAGGGGATTGCTCTCTGTAATGTTCAGCTTCTA
ATTGTCTCTACTTTGTGAGACAACTTTTGAATGCTTGACCTCAAATCAGGTAGGACTACC
CGCTGAACTTAA
>ABC8-61
TTTCCGTAGGTGAACCTGCGGAAGGATCATTATTGAATTATGTTTCTAGATAGGTTGTAG
CTGGCTC-TTTAGAGCATGTGCACGCCTGTTTGGACTTCATTTTCATCCACCTGTGCACC
TATTGTAGTCTTTGGTTGGGTTAGGGGGAAGTGGTCATTGTGTCAGCATCTGCTGGATGT
GAGGACTTGCATTGTGAAAGCTTTGCTGTCCTTGATGTGATCATGGAATCTCTTTCTCAC
TAGAGTCTATGTCACTCATTATACTCTGTCGAATGTCATTGAATGTCTTTACATGGGCTT
GTATGCCTATGAAAATTGTAATACAACTTTCAGCAACGGATCTCTTGGCTCTCGCATCGA
TGAAGGACGCAGCGAAATGCGATAAGTAATGTGAATTGCAGAATTCAGTGAATCATCGAA
TCTTTGAACGCATCTTGCGCTCCTTGGTATTCCGAGGAGCATGCCTGTTTGAGTGTCATT
AAATTCTCAACTCTCTTCTAC--TTTTTGTAAAAGAGAGCTTGGACTGTGGAGGCTTGCT
GGCCACTTTTTGGGGTCAGCTCCTCTGAAATGCATTAGCGGAACCGTTTGCGATCTGCCA
CAAGTGTGATAAGTTATCTACACTGGCGAGGGGATTGCTCTCTGTAATGTTCAGCTTCTA
ATTGTCTCTACTTTGTGAGACAACTTTTGAATGCTTGACCTCAAATCAGGTAGGACTACC
CGCTGAACTTAA
>ABC3-33
TTTCCGTAGGTGAACCTGCGGAAGGATCATTATTGAATTATGTTTCTAGATAGGTTGTAG
CTGGCTC-TTTAGAGCATGTGCACGCCTGTTTGGACTTCATTTTCATCCACCTGTGCACC
TATTGTAGTCTTTGGTTGGGTTAGGGGGAAGTGGTCATTGTGTCAGCATCTGCTGGATGT
GAGGACTTGCATTGTGAAAGCTTTGCTGTCCTTGATGTGATCATGGAATCTCTTTCTCAC
TAGAGTCTATGTCACTCATTATACTCTGTCGAATGTCATTGAATGTCTTTACATGGGCTT
GTATGCCTATGAAAATTGTAATACAACTTTCAGCAACGGATCTCTTGGCTCTCGCATCGA
TGAAGGACGCAGCGAAATGCGATAAGTAATGTGAATTGCAGAATTCAGTGAATCATCGAA
TCTTTGAACGCATCTTGCGCTCCTTGGTATTCCGAGGAGCATGCCTGTTTGAGTGTCATT
AAATTCTCAACTCTCTTCTAC--TTTTTGTAAAAGAGAGCTTGGACTGTGGAGGCTTGCT
GGCCACTTTTTGGGGTCAGCTCCTCTGAAATGCATTAGCGGAACCGTTTGCAATCTGCCA
CAAGTGTGATAAGTTATCTACACTGGCGAGGGGATTGCTCTCTGTAATGTTCAGCTTCTA
ATTGTCTCTACTTTGTGAGACAACTTTTGAATGCTTGACCTCAAATCAGGTAGGACTACC
CGCTGAACTTAA
>ABC3-53
TTTCCGTAGGTGAACCTGCGGAAGGATCATTATTGAATTATGTTTCTAGATAGGTTGTAG
CTGGCTC-TTTAGAGCATGTGCACGCCTGTTTGGACTTCATTTTCATCCACCTGTGCACC
TATTGTAGTCTTTGGTTGGGTTAGGGGGAAGTGGTCATTGTGTCAGCATCTGCTGGATGT
GAGGACTTGCATTGTGAAAGCTTTGCTGTCCTTGATGTGATCATGGAATCTCTTTCTCAC
TAGAGTCTATGTCACTCATTATACTCTGTCGAATGTCATTGAATGTCTTTACATGGGCTT
GTATGCCTATGAAAATTGTAATACAACTTTCAGCAACGGATCTCTTGGCTCTCGCATCGA
TGAAGGACGCAGCGAAATGCGATAAGTAATGTGAATTGCAGAATTCAGTGAATCATCGAA
TCTTTGAACGCATCTTGCGCTCCTTGGTATTCCGAGGAGCATGCCTGTTTGAGTGTCATT
AAATTCTCAACTCTCTTCTAC--TTTTTGTAAAAGAGAGCTTGGACTGTGGAGGCTTGCT
GGCCACTTTTTGGGGTCAGCTCCTCTGAAATGCATTAGCGGAACCGTTTGCAATCTGCCA
CAAGTGTGATCAGTTATCTACACTGGCGAGGGGATTGCTCTCTGTAATGTTCAGCTTCTA
ATTGTCTCTACTTTGTGAGACAACTTTTGAATGCTTGACCTCAAATCAGGTAGGACTACC
CGCTGAACTTAA
>ABC1-5
TTTCCGTAGGTGAACCTGCGGAAGGATCATTATTGAATTATGTTTCTAGATAGGTTGTAG

CTGGCTC-TTTAGAGCATGTGCACGCCTGTTTGGACTTCATTTTCATCCACCTGTGCACC
TATTGTAGTCTTTGGTTGGGTTAGGAGGAAGTGGTCATTGTGTCAGCATCTGCTGGATGT
GAGGACTTGCATTGTGAAAGCTTTGCTGTCCTTGATGTGATCATGGAATCTCTTTCTCAC
TAGAGTCTATGTCACTCATTATACTCTGTCGAATGTCATTGAATGTCTTTACATGGGCTT
ATATGCCTATGAAAATTGTAATACAACTTTCAGCAACGGATCTCTTGGCTCTCGCATCGA
TGAAGAACGCAGCGAAATGCGATAAGTAATGTGAATTGCAGAATTCAGTGAATCATCGAA
TCTTTGAACGCATCTTGCGCTCCTTGGTATTCCGAGGAGCATGCCTGTTTGAGTGTCATT
AAATTCTCAACTCTCTTCTAC--TTTTTGTAAAAGAGAGCTTGGACTGTGGAGGCTTGCT
GGCCACTTTTTGGGGTCAGCTCCTCTGAAATGCATTAGCGGAACCGTTTGCGATCTGCCA
CAAGTGTGATAAGTTATCTACACTGGCGAGGGGATTGCTCTCTGTAATGTTCAGCTTCTA
ATTGTCTCTACTTTGTGAGACTACTTTTGAATGCTTGACCTCAAATCAGGTAGGACTACC
CGCTGAACTTAA
>ABC5-49
TTTCCGTAGGTGAACCTGCGGAAGGATCATTATTGAATTATGTTTCTAGATAGGTTGTAG
CTGGCTC-TTTAGAGCATGTGCACGCCTGTTTGGACTTCATTTTCATCCACCTGTGCACC
TATTGTAGTCTTTGGTTGGGTTAGGAGGAAGTGGTCATTGTGTCAGCATCTGCTGGATGT
GAGGACTTGCATTGTGAAAGCTTTGCTGTCCTTGATGTGATCATGGAATCTCTTTCTCAC
TAGAGTCTATGTCACTCATTATACTCTGTCGAATGTCATTGAATGTCTTTACATGGGCTT
ATATGCCTATGAAAATTGTAATACAACTTTCAGCAACGGATCTCTTGGCTCTCGCATCGA
TGAAGAACGCAGCGAAATGCGATAAGTAATGTGAATTGCAGAATTCAGTGAATCATCGAA
TCTTTGAACGCATCTTGCGCTCCTTGGTATTCCGAGGAGCATGCCTGTTTGAGTGTCATT
AAATTCTCAACTCTCTTCTAC--TTTTTGTAAAAGAGAGCTTGGACTGTGGAGGCTTGCT
GGCCACTTTTTGGGGTCAGCTCCTCTGAAATGCATTAGCGGAACCGTTTGCGATCTGCCA
CAAGTGTGATAAGTTATCTACACTGGCGAGGGGATTGCTCTCTGTAATGTTCAGCTTCTA
ATTGTCTCTACTTTGTGAGACTACTTTTGAATGCTTGACCTCAAATCAGGTAGGACTACC
CGCTGAACTTAA
>ABC3-48
TTTCCGTAGGTGAACCTGCGGAAGGATCATTATTGAATTATGTTTCTAGATAGGTTGTAG
CTGGCTC-TTTAGAGCATGTGCACGCCTGTTTGGACTTCATTTTCATCCACCTGTGCACC
TATTGTAGTCTTTGGTTGGGTTAGGAGGAAGTGGTCATTGTGTCAGCATCTGCTGGATGT
GAGGACTTGCATTGTGAAAGCTTTGCTGTCCTTGATGTGATCATGGAATCTCTTTCTCAC
TAGAGTCTATGTCACTCATTATACTCTGTCGAATGTCATTGAATGTCTTTACATGGGCTT
ATATGCCTATGAAAATTGTAATACAACTTTCAGCAACGGATCTCTTGGCTCTCGCATCGA
TGAAGAACGCAGCGAAATGCGATAAGTAATGTGAATTGCAGAATTCAGTGAATCATCGAA
TCTTTGAACGCATCTTGCGCTCCTTGGTATTCCGAGGAGCATGCCTGTTTGAGTGTCATT
AAATTCTCAACTCTCTTCTAC--TTTTTGTAAAAGAGAGCTTGGACTGTGGAGGCTTGCT
GGCCACTTTTTGGGGTCAGCTCCTCTGAAATGCATTAGCGGAACCGTTTGCGATCTGCCA
CAAGTGTGATAAGTTATCTACACTGGCGAGGGGATTGCTCTCTGTAATGTTCAGCTTCTA
ATTGTCTCTACTTTGTGAGACTACTTTTGAATGCTTGACCTCAAATCAGGTAGGACTACC
CGCTGAACTTAA
>ABC1-60
TTTCCGTAGGTGAACCTGCGGAAGGATCATTATTGAATTATGTTTCTAGATAGGTTGTAG
CTGGCTC-TTTAGAGCATGTGCACGCCTGTTTGGACTTCATTTTCATCCACCTGTGCACC
TATTGTAGTCTTTGGTTGGGTTAGGAGGAAGTGGTCATTGTGTCAGCATCTGCTGGATGT
GAGGACTTGCATTGTGAAAGCTTTGCTGTCCTTGATGTGATCATGGAATCTCTTTCTCAC
TAGAGTCTATGTCACTCATTATACTCTGTCGAATGTCATTGAATGTCTTTACATGGGCTT
ATATGCCTATGAAAATTGTAATACAACTTTCAGCAACGGATCTCTTGGCTCTCGCATCGA
TGAAGAACGCAGCGAAATGCGATAAGTAATGTGAATTGCAGAATTCAGTGAATCATCGAA
TCTTTGAACGCATCTTGCGCTCCTTGGTATTCCGAGGAGCATGCCTGTTTGAGTGTCATT
AAATTCTCAACTCTCTTCTAC--TTTTTGTAAAAGAGAGCTTGGACTGTGGAGGCTTGCT

GGCCACTTTTTGGGGTCAGCTCCTCTGAAATGCATTAGCGGAACCGTTTGCGATCTGCCA
CAAGTGTGATAAGTTATCTACACTGGCGAGGGGATTGCTCTCTGTAATGTTCAGCTTCTA
ATTGTCTCTACTTTGTGAGACTACTTTTGAATGCTTGACCTCAAATCAGGTAGGACTACC
CGCTGAACTTAA
>ABC1-47
TTTCCGTAGGTGAACCTGCGGAAGGATCATTATTGAATTATGTTTCTAGATAGGTTGTAG
CTGGCTC-TTTAGAGCATGTGCACGCCTGTTTGGACTTCATTTTCATCCACCTGTGCACC
TATTGTAGTCTTTGGTTGGGTTAGGAGGAAGTGGTCATTGTGTCAGCATCTGCTGGATGT
GAGGACTTGCATTGTGAAAGCTTTGCTGTCCTTGATGTGATCATGGAATCTCTTTCTCAC
TAGAGTCTATGTCACTCATTATACTCTGTCGAATGTCATTGAATGTCTTTACATGGGCTT
ATATGCCTATGAAAATTGTAATACAACTTTCAGCAACGGATCTCTTGGCTCTCGCATCGA
TGAAGAACGCAGCGAAATGCGATAAGTAATGTGAATTGCAGAATTCAGTGAATCATCGAA
TCTTTGAACGCATCTTGCGCTCCTTGGTATTCCGAGGAGCATGCCTGTTTGAGTGTCATT
AAATTCTCAACTCTCTTCTAC--TTTTTGTAAAAGAGAGCTTGGACTGTGGAGGCTTGCT
GGCCACTTTTTGGGGTCAGCTCCTCTGAAATGCATTAGCGGAACCGTTTGCGATCTGCCA
CAAGTGTGATAAGTTATCTACACTGGCGAGGGGATTGCTCTCTGTAATGTTCAGCTTCTA
ATTGTCTCTACTTTGTGAGACTACTTTTGAATGCTTGACCTCAAATCAGGTAGGACTACC
CGCTGAACTTAA
>ABC12-48
TTTCCGTAGGTGAACCTGCGGAAGGATCATTATTGAATTATGTTTCTAGATAGGTTGTAG
CTGGCTC-TTTAGAGCATGTGCACGCCTGTTTGGACTTCATTTTCATCCACCTGTGCACC
TATTGTAGTCTTTGGTTGGGTTAGGAGGAAGTGGTCATTGTGTCAGCATCTGCTGGATGT
GAGGACTTGCATTGTGAAAGCTTTGCTGTCCTTGATGTGATCATGGAATCTCTTTCTCAC
TAGAGTCTATGTCACTCATTATACTCTGTCGAATGTCATTGAATGTCTTTACATGGGCTT
ATATGCCTATGAAAATTGTAATACAACTTTCAGCAACGGATCTCTTGGCTCTCGCATCGA
TGAAGAACGCAGCGAAATGCGATAAGTAATGTGAATTGCAGAATTCAGTGAATCATCGAA
TCTTTGAACGCATCTTGCGCTCCTTGGTATTCCGAGGAGCATGCCTGTTTGAGTGTCATT
AAATTCTCAACTCTCTTCTAC--TTTTTGTAAAAGAGAGCTTGGACTGTGGAGGCTTGCT
GGCCACTTTTTGGGGTCAGCTCCTCTGAAATGCATTAGCGGAACCGTTTGCGATCTGCCA
CAAGTGTGATAAGTTATCTACACTGGCGAGGGGATTGCTCTCTGTAATGTTCAGCTTCTA
ATTGTCTCTACTTTGTGAGACTACTTTTGAATGCTTGACCTCAAATCAGGTAGGACTACC
CGCTGAACTTAA
>ABC7-32
TTTCCGTAGGTGAACCTGCGGAAGGATCATTATTGAATTATGTTTCTAGATAGGTTGTAG
CTGGCTC-TTTAGAGCATGTGCACGCCTGTTTGGACTTCATTTTCATCCACCTGTGCACC
TATTGTAGTCTTTGGTTGGGTTAGGAGGAAGTGGTCATTGTGTCAGCATCTGCTGGATGT
GAGGACTTGCATTGTGAAAGCTTTGCTGTCCTTGATGTGATCATGGAATCTCTTTCTCAC
TAGAGTCTATGTCACTCATTATACTCTGTCGAATGTCATTGAATGTCTTTACATGGGCTT
ATATGCCTATGAAAATTGTAATACAACTTTCAGCAACGGATCTCTTGGCTCTCGCATCGA
TGAAGAACGCAGCGAAATGCGATAAGTAATGTGAATTGCAGAATTCAGTGAATCATCGAA
TCTTTGAACGCATCTTGCGCTCCTTGGTATTCCGAGGAGCATGCCTGTTTGAGTGTCATT
AAATTCTCAACTCTCTTCTAC--TTTTTGTAAAAGAGAGCTTGGACTGTGGAGGCTTGCT
GGCCACTTTTTGGGGTCAGCTCCTCTGAAATGCATTAGCGGAACCGTTTGCGATCTGCCA
CAAGTGTGATAAGTTATCTACACTGGCGAGGGGATTGCTCTCTGTAATGTTCAGCTTCTA
ATTGTCTCTACTTTGTGAGACTACTTTTGAATGCTTGACCTCAAATCAGGTAGGACTACC
CGCTGAACTTAA
>ABC8-10
TTTCCGTAGGTGAACCTGCGGAAGGATCATTATTGAATTATGTTTCTAGATAGGTTGTAG
CTGGCTC-TTTAGAGCATGTGCACGCCTGTTTGGACTTCATTTTCATCCACCTGTGCACC
TATTGTAGTCTTTGGTTGGGTTAGGAGGAAGTGGTCATTGTGTCAGCATCTGCTGGATGT

GAGGACTTGCATTGTGAAAGCTTTGCTGTCCTTGATGTGATCATGGAATCTCTTTCTCAC
TAGAGTCTATGTCACTCATTATACTCTGTCGAATGTCATTGAATGTCTTTACATGGGCTT
ATATGCCTATGAAAATTGTAATACAACTTTCAGCAACGGATCTCTTGGCTCTCGCATCGA
TGAAGAACGCAGCGAAATGCGATAAGTAATGTGAATTGCAGAATTCAGTGAATCATCGAA
TCTTTGAACGCATCTTGCGCTCCTTGGTATTCCGAGGAGCATGCCTGTTTGAGTGTCATT
AAATTCTCAACTCTCTTCTAC--TTTTTGTAAAAGAGAGCTTGGACTGTGGAGGCTTGCT
GGCCACTTTTTGGGGTCAGCTCCTCTGAAATGCATTAGCGGAACCGTTTGCGATCTGCCA
CAAGTGTGATAAGTTATCTACACTGGCGAGGGGATTGCTCTCTGTAATGTTCAGCTTCTA
ATTGTCTCTACTTTGTGAGACTACTTTTGAATGCTTGACCTCAAATCAGGTAGGACTACC
CGCTGAACTTAA
>ABC12-5
TTTCCGTAGGTGAACCTGCGGAAGGATCATTATTGAATTATGTTTCTAGATAGGTTGTAG
CTGGCTC-TTTAGAGCATGTGCACGCCTGTTTGGACTTCATTTTCATCCACCTGTGCACC
TATTGTAGTCTTTGGTTGGGTTAGGAGGAAGTGGTCATTGTGTCAGCATCTGCTGGATGT
GAGGACTTGCATTGTGAAAGCTTTGCTGTCCTTGATGTGATCATGGAATCTCTTTCTCAC
TAGAGTCTATGTCACTCATTATACTCTGTCGAATGTCATTGAATGTCTTTACATGGGCTT
ATATGCCTATGAAAATTGTAATACAACTTTCAGCAACGGATCTCTTGGCTCTCGCATCGA
TGAAGAACGCAGCGAAATGCGATAAGTAATGTGAATTGCAGAATTCAGTGAATCATCGAA
TCTTTGAACGCATCTTGCGCTCCTTGGTATTCCGAGGAGCATGCCTGTTTGAGTGTCATT
AAATTCTCAACTCTCTTCTAC--TTTTTGTAAAAGAGAGCTTGGACTGTGGAGGCTTGCT
GGCCACTTTTTGGGGTCAGCTCCTCTGAAATGCATTAGCGGAACCGTTTGCGATCTGCCA
CAAGTGTGATAAGTTATCTACACTGGCGAGGGGATTGCTCTCTGTAATGTTCAGCTTCTA
ATTGTCTCTACTTTGTGAGACTACTTTTGAATGCTTGACCTCAAATCAGGTAGGACTACC
CGCTGAACTTAA
>ABC11-46
TTTCCGTAGGTGAACCTGCGGAAGGATCATTATTGAATTATGTTTCTAGATAGGTTGTAG
CTGGCTC-TTTAGAGCATGTGCACGCCTGTTTGGACTTCATTTTCATCCACCTGTGCACC
TATTGTAGTCTTTGGTTGGGTTAGGAGGAAGTGGTCATTGTGTCAGCATCTGCTGGATGT
GAGGACTTGCATTGTGAAAGCTTTGCTGTCCTTGATGTGATCATGGAATCTCTTTCTCAC
TAGAGTCTATGTCACTCATTATACTCTGTCGAATGTCATTGAATGTCTTTACATGGGCTT
ATATGCCTATGAAAATTGTAATACAACTTTCAGCAACGGATCTCTTGGCTCTCGCATCGA
TGAAGAACGCAGCGAAATGCGATAAGTAATGTGAATTGCAGAATTCAGTGAATCATCGAA
TCTTTGAACGCATCTTGCGCTCCTTGGTATTCCGAGGAGCATGCCTGTTTGAGTGTCATT
AAATTCTCAACTCTCTTCTAC--TTTTTGTAAAAGAGAGCTTGGACTGTGGAGGCTTGCT
GGCCACTTTTTGGGGTCAGCTCCTCTGAAATGCATTAGCGGAACCGTTTGCGATCTGCCA
CAAGTGTGATAAGTTATCTACACTGGCGAGGGGATTGCTCTCTGTAATGTTCAGCTTCTA
ATTGTCTCTACTTTGTGAGACTACTTTTGAATGCTTGACCTCAAATCAGGTAGGACTACC
CGCTGAACTTAA
>ABC10-25
TTTCCGTAGGTGAACCTGCGGAAGGATCATTATTGAATTATGTTTCTAGATAGGTTGTAG
CTGGCTC-TTTAGAGCATGTGCACGCCTGTTTGGACTTCATTTTCATCCACCTGTGCACC
TATTGTAGTCTTTGGTTGGGTTAGGAGGAAGTGGTCATTGTGTCAGCATCTGCTGGATGT
GAGGACTTGCATTGTGAAAGCTTTGCTGTCCTTGATGTGATCATGGAATCTCTTTCTCAC
TAGAGTCTATGTCACTCATTATACTCTGTCGAATGTCATTGAATGTCTTTACATGGGCTT
ATATGCCTATGAAAATTGTAATACAACTTTCAGCAACGGATCTCTTGGCTCTCGCATCGA
TGAAGAACGCAGCGAAATGCGATAAGTAATGTGAATTGCAGAATTCAGTGAATCATCGAA
TCTTTGAACGCATCTTGCGCTCCTTGGTATTCCGAGGAGCATGCCTGTTTGAGTGTCATT
AAATTCTCAACTCTCTTCTAC--TTTTTGTAAAAGAGAGCTTGGACTGTGGAGGCTTGCT
GGCCACTTTTTGGGGTCAGCTCCTCTGAAATGCATTAGCGGAACCGTTTGCGATCTGCCA
CAAGTGTGATAAGTTATCTACACTGGCGAGGGGATTGCTCTCTGTAATGTTCAGCTTCTA

ATTGTCTCTACTTTGTGAGACTACTTTTGAATGCTTGACCTCAAATCAGGTAGGACTACC
CGCTGAACTTAA
>ABC11-25
TTTCCGTAGGTGAACCTGCGGAAGGATCATTATTGAATTATGTTTCTAGATAGGTTGTAG
CTGGCTC-TTTAGAGCATGTGCACGCCTGTTTGGACTTCATTTTCATCCACCTGTGCACC
TATTGTAGTCTTTGGTTGGGTTAGGAGGAAGTGGTCATTGTGTCAGCATCTGCTGGATGT
GAGGACTTGCATTGTGAAAGCTTTGCTGTCCTTGATGTGATCATGGAATCTCTTTCTCAC
TAGAGTCTATGTCACTCATTATACTCTGTCGAATGTCATTGAATGTCTTTACATGGGCTT
ATATGCCTATGAAAATTGTAATACAACTTTCAGCAACGGATCTCTTGGCTCTCGCATCGA
TGAAGAACGCAGCGAAATGCGATAAGTAATGTGAATTGCAGAATTCAGTGAATCATCGAA
TCTTTGAACGCATCTTGCGCTCCTTGGTATTCCGAGGAGCATGCCTGTTTGAGTGTCATT
AAATTCTCAACTCTCTTCTAC--TTTTTGTAAAAGAGAGCTTGGACTGTGGAGGCTTGCT
GGCCACTTTTTGGGGTCAGCTCCTCTGAAATGCATTAGCGGAACCGTTTGCGATCTGCCA
CAAGTGTGATAAGTTATCTACACTGGCGAGGGGATTGCTCTCTGTAATGTTCAGCTTCTA
ATTGTCTCTACTTTGTGAGACTACTTTTGAATGCTTGACCTCAAATCAGGTAGGACTACC
CGCTGAACTTAA
>ABC8-18
TTTCCGTAGGTGAACCTGCGGAAGGATCATTATTGAATTATGTTTCTAGATAGGTTGTAG
CTGGCTC-TTTAGAGCATGTGCACGCCTGTTTGGACTTCATTTTCATCCACCTGTGCACC
TATTGTAGTCTTTGGTTGGGTTAGGAGGAAGTGGTCATTGTGTCAGCATCTGCTGGATGT
GAGGACTTGCATTGTGAAAGCTTTGCTGTCCTTGATGTGATCATGGAATCTCTTTCTCAC
TAGAGTCTATGTCACTCATTATACTCTGTCGAATGTCATTGAATGTCTTTACATGGGCTT
ATATGCCTATGAAAATTGTAATACAACTTTCAGCAACGGATCTCTTGGCTCTCGCATCGA
TGAAGAACGCAGCGAAATGCGATAAGTAATGTGAATTGCAGAATTCAGTGAATCATCGAA
TCTTTGAACGCATCTTGCGCTCCTTGGTATTCCGAGGAGCATGCCTGTTTGAGTGTCATT
AAATTCTCAACTCTCTTCTAC--TTTTTGTAAAAGAGAGCTTGGACTGTGGAGGCTTGCT
GGCCACTTTTTGGGGTCAGCTCCTCTGAAATGCATTAGCGGAACCGTTTGCGATCTGCCA
CAAGTGTGATAAGTTATCTACACTGGCGAGGGGATTGCTCTCTGTAATGTTCAGCTTCTA
ATTGTCTCTACTTTGTGAGACTACTTTTGAATGCTTGACCTCAAATCAGGTAGGACTACC
CGCTGAACTTAA
>ABC8-58
TTTCCGTAGGTGAACCTGCGGAAGGATCATTATTGAATTATGTTTCTAGATAGGTTGTAG
CTGGCTC-TTTAGAGCATGTGCACGCCTGTTTGGACTTCATTTTCATCCACCTGTGCACC
TATTGTAGTCTTTGGTTGGGTTAGGAGGAAGTGGTCATTGTGTCAGCATCTGCTGGATGT
GAGGACTTGCATTGTGAAAGCTTTGCTGTCCTTGATGTGATCATGGAATCTCTTTCTCAC
TAGAGTCTATGTCACTCATTATACTCTGTCGAATGTCATTGAATGTCTTTACATGGGCTT
ATATGCCTATGAAAATTGTAATACAACTTTCAGCAACGGATCTCTTGGCTCTCGCATCGA
TGAAGAACGCAGCGAAATGCGATAAGTAATGTGAATTGCAGAATTCAGTGAATCATCGAA
TCTTTGAACGCATCTTGCGCTCCTTGGTATTCCGAGGAGCATGCCTGTTTGAGTGTCATT
AAATTCTCAACTCTCTTCTAC--TTTTTGTAAAAGAGAGCTTGGACTGTGGAGGCTTGCT
GGCCACTTTTTGGGGTCAGCTCCTCTGAAATGCATTAGCGGAACCGTTTGCGATCTGCCA
CAAGTGTGATAAGTTATCTACACTGGCGAGGGGATTGCTCTCTGTAATGTTCAGCTTCTA
ATTGTCTCTACTTTGTGAGACTACTTTTGAATGCTTGACCTCAAATCAGGTAGGACTACC
CGCTGAACTTAA
>ABC3-14
TTTCCGTAGGTGAACCTGCGGAAGGATCATTATTGAATTATGTTTCTAGATAGGTTGTAG
CTGGCTC-TTTAGAGCATGTGCACGCCTGTTTGGACTTCATTTTCATCCACCTGTGCACC
TATTGTAGTCTTTGGTTGGGTTAGGAGGAAGTGGTCATTGTGTCAGCATCTGCTGGATGT
GAGGACTTGCATTGTGAAAGCTTTGCTGTCCTTGATGTGATCATGGAATCTCTTTCTCAC
TAGAGTCTATGTCACTCATTATACTCTGTCGAATGTCATTGAATGTCTTTACATGGGCTT

ATATGCCTATGAAAATTGTAATACAACTTTCAGCAACGGATCTCTTGGCTCTCGCATCGA
TGAAGAACGCAGCGAAATGCGATAAGTAATGTGAATTGCAGAATTCAGTGAATCATCGAA
TCTTTGAACGCATCTTGCGCTCCTTGGTATTCCGAGGAGCATGCCTGTTTGAGTGTCATT
AAATTCTCAACTCTCTTCTAC--TTTTTGTAAAAGAGAGCTTGGACTGTGGAGGCTTGCT
GGCCACTTTTTGGGGTCAGCTCCTCTGAAATGCATTAGCGGAACCGTTTGCGATCTGCCA
CAAGTGTGATAAGTTATCTACACTGGCGAGGGGATTGCTCTCTGTAATGTTCAGCTTCTA
ATTGTCTCTACTTTGTGAGACTACTTTTGAATGCTTGACCTCAAATCAGGTAGGACTACC
CGCTGAACTTAA
>ABC3-1
TTTCCGTAGGTGAACCTGCGGAAGGATCATTATTGAATTATGTTTCTAGATAGGTTGTAG
CTGGCTC-TTTAGAGCATGTGCACGCCTGTTTGGACTTCATTTTCATCCACCTGTGCACC
TATTGTAGTCTTTGGTTGGGTTAGGAGGAAGTGGTCATTGTGTCAGCATCTGCTGGATGT
GAGGACTTGCATTGTGAAAGCTTTGCTGTCCTTGATGTGATCATGGAATCTCTTTCTCAC
TAGAGTCTATGTCACTCATTATACTCTGTCGAATGTCATTGAATGTCTTTACATGGGCTT
ATATGCCTATGAAAATTGTAATACAACTTTCAGCAACGGATCTCTTGGCTCTCGCATCGA
TGAAGAACGCAGCGAAATGCGATAAGTAATGTGAATTGCAGAATTCAGTGAATCATCGAA
TCTTTGAACGCATCTTGCGCTCCTTGGTATTCCGAGGAGCATGCCTGTTTGAGTGTCATT
AAATTCTCAACTCTCTTCTAC--TTTTTGTAAAAGAGAGCTTGGACTGTGGAGGCTTGCT
GGCCACTTTTTGGGGTCAGCTCCTCTGAAATGCATTAGCGGAACCGTTTGCGATCTGCCA
CAAGTGTGATAAGTTATCTACACTGGCGAGGGGATTGCTCTCTGTAATGTTCAGCTTCTA
ATTGTCTCTACTTTGTGAGACTACTTTTGAATGCTTGACCTCAAATCAGGTAGGACTACC
CGCTGAACTTAA
>ABC3-46
TTTCCGTAGGTGAACCTGCGGAAGGATCATTATTGAATTATGTTTCTAGATAGGTTGTAG
CTGGCTC-TTTAGAGCATGTGCACGCCTGTTTGGACTTCATTTTCATCCACCTGTGCACC
TATTGTAGTCTTTGGTTGGGTTAGGAGGAAGTGGTCATTGTGTCAGCATCTGCTGGATGT
GAGGACTTGCATTGTGAAAGCTTTGCTGTCCTTGATGTGATCATGGAATCTCTTTCTCAC
TAGAGTCTATGTCACTCATTATACTCTGTCGAATGTCATTGAATGTCTTTACATGGGCTT
ATATGCCTATGAAAATTGTAATACAACTTTCAGCAACGGATCTCTTGGCTCTCGCATCGA
TGAAGAACGCAGCGAAATGCGATAAGTAATGTGAATTGCAGAATTCAGTGAATCATCGAA
TCTTTGAACGCATCTTGCGCTCCTTGGTATTCCGAGGAGCATGCCTGTTTGAGTGTCATT
AAATTCTCAACTCTCTTCTAC--TTTTTGTAAAAGAGAGCTTGGACTGTGGAGGCTTGCT
GGCCACTTTTTGGGGTCAGCTCCTCTGAAATGCATTAGCGGAACCGTTTGCGATCTGCCA
CAAGTGTGATAAGTTATCTACACTGGCGAGGGGATTGCTCTCTGTAATGTTCAGCTTCTA
ATTGTCTCTACTTTGTGAGACTACTTTTGAATGCTTGACCTCAAATCAGGTAGGACTACC
CGCTGAACTTAA
>ABC7-5
TTTCCGTAGGTGAACCTGCGGAAGGATCATTATTGAATTATGTTTCTAGATAGGTTGTAG
CTGGCTC-TTTAGAGCATGTGCACGCCTGTTTGGACTTCATTTTCATCCACCTGTGCACC
TATTGTAGTCTTTGGTTGGGTTAGGAGGAAGTGGTCATTGTGTCAGCATCTGCTGGATGT
GAGGACTTGCATTGTGAAAGCTTTGCTGTCCTTGATGTGATCATGGAATCTCTTTCTCAC
TAGAGTCTATGTCACTCATTATACTCTGTCGAATGTCATTGAATGTCTTTACATGGGCTT
ATATGCCTATGAAAATTGTAATACAACTTTCAGCAACGGATCTCTTGGCTCTCGCATCGA
TGAAGAACGCAGCGAAATGCGATAAGTAATGTGAATTGCAGAATTCAGTGAATCATCGAA
TCTTTGAACGCATCTTGCGCTCCTTGGTATTCCGAGGAGCATGCCTGTTTGAGTGTCATT
AAATTCTCAACTCTCTTCTAC--TTTTTGTAAAAGAGAGCTTGGACTGTGGAGGCTTGCT
GGCCACTTTTTGGGGTCAGCTCCTCTGAAATGCATTAGCGGAACCGTTTGCGATCTGCCA
CAAGTGTGATAAGTTATCTACACTGGCGAGGGGATTGCTCTCTGTAATGTTCAGCTTCTA
ATTGTCTCTACTTTGTGAGACTACTTTTGAATGCTTGACCTCAAATCAGGTAGGACTACC
CGCTGAACTTAA

>ABC8-62
TTTCCGTAGGTGAACCTGCGGAAGGATCATTATTGAATTATGTTTCTAGATAGGTTGTAG
CTGGCTC-TTTAGAGCATGTGCACGCCTGTTTGGACTTCATTTTCATCCACCTGTGCACC
TATTGTAGTCTTTGGTTGGGTTAGGAGGAAGTGGTCATTGTGTCAGCATCTGCTGGATGT
GAGGACTTGCATTGTGAAAGCTTTGCTGTCCTTGATGTGATCATGGAATCTCTTTCTCAC
TAGAGTCTATGTCACTCATTATACTCTGTCGAATGTCATTGAATGTCTTTACATGGGCTT
ATATGCCTATGAAAATTGTAATACAACTTTCAGCAACGGATCTCTTGGCTCTCGCATCGA
TGAAGAACGCAGCGAAATGCGATAAGTAATGTGAATTGCAGAATTCAGTGAATCATCGAA
TCTTTGAACGCATCTTGCGCTCCTTGGTATTCCGAGGAGCATGCCTGTTTGAGTGTCATT
AAATTCTCAACTCTCTTCTAC--TTTTTGTAAAAGAGAGCTTGGACTGTGGAGGCTTGCT
GGCCACTTTTTGGGGTCAGCTCCTCTGAAATGCATTAGCGGAACCGTTTGCGATCTGCCA
CAAGTGTGATAAGTTATCTACACTGGCGAGGGGATTGCTCTCTGTAATGTTCAGCTTCTA
ATTGTCTCTACTTTGTGAGACTACTTTTGAATGCTTGACCTCAAATCAGGTAGGACTACC
CGCTGAACTTAA
>ABC8-63
TTTCCGTAGGTGAACCTGCGGAAGGATCATTATTGAATTATGTTTCTAGATAGGTTGTAG
CTGGCTC-TTTAGAGCATGTGCACGCCTGTTTGGACTTCATTTTCATCCACCTGTGCACC
TATTGTAGTCTTTGGTTGGGTTAGGAGGAAGTGGTCATTGTGTCAGCATCTGCTGGATGT
GAGGACTTGCATTGTGAAAGCTTTGCTGTCCTTGATGTGATCATGGAATCTCTTTCTCAC
TAGAGTCTATGTCACTCATTATACTCTGTCGAATGTCATTGAATGTCTTTACATGGGCTT
ATATGCCTATGAAAATTGTAATACAACTTTCAGCAACGGATCTCTTGGCTCTCGCATCGA
TGAAGAACGCAGCGAAATGCGATAAGTAATGTGAATTGCAGAATTCAGTGAATCATCGAA
TCTTTGAACGCATCTTGCGCTCCTTGGTATTCCGAGGAGCATGCCTGTTTGAGTGTCATT
AAATTCTCAACTCTCTTCTAC--TTTTTGTAAAAGAGAGCTTGGACTGTGGAGGCTTGCT
GGCCACTTTTTGGGGTCAGCTCCTCTGAAATGCATTAGCGGAACCGTTTGCGATCTGCCA
CAAGTGTGATAAGTTATCTACACTGGCGAGGGGATTGCTCTCTGTAATGTTCAGCTTCTA
ATTGTCTCTACTTTGTGAGACTACTTTTGAATGCTTGACCTCAAATCAGGTAGGACTACC
CGCTGAACTTAA
>ABC4-13
TTTCCGTAGGTGAACCTGCGGAAGGATCATTATTGAATTATGTTTCTAGATAGGTTGTAG
CTGGCTC-TTTAGAGCATGTGCACGCCTGTTTGGACTTCATTTTCATCCACCTGTGCACC
TATTGTAGTCTTTGGTTGGGTTAGGAGGAAGTGGTCATTGTGTCAGCATCTGCTGGATGT
GAGGACTTGCATTGTGAAAGCTTTGCTGTCCTTGATGTGATCATGGAATCTCTTTCTCAC
TAGAGTCTATGTCACTCATTATACTCTGTCGAATGTCATTGAATGTCTTTACATGGGCTT
ATATGCCTATGAAAATTGTAATACAACTTTCAGCAACGGATCTCTTGGCTCTCGCATCGA
TGAAGAACGCAGCGAAATGCGATAAGTAATGTGAATTGCAGAATTCAGTGAATCATCGAA
TCTTTGAACGCATCTTGCGCTCCTTGGTATTCCGAGGAGCATGCCTGTTTGAGTGTCATT
AAATTCTCAACTCTCTTCTAC--TTTTTGTAAAAGAGAGCTTGGACTGTGGAGGCTTGCT
GGCCACTTTTTGGGGTCAGCTCCTCTGAAATGCATTAGCGGAACCGTTTGCGATCTGCCA
CAAGTGTGATAAGTTATCTACACTGGCGAGGGGATTGCTCTCTGTAATGTTCAGCTTCTA
ATTGTCTCTACTTTGTGAGACTACTTTTGAATGCTTGACCTCAAATCAGGTAGGACTACC
CGCTGAACTTAA
>ABC4-33
TTTCCGTAGGTGAACCTGCGGAAGGATCATTATTGAATTATGTTTCTAGATAGGTTGTAG
CTGGCTC-TTTAGAGCATGTGCACGCCTGTTTGGACTTCATTTTCATCCACCTGTGCACC
TATTGTAGTCTTTGGTTGGGTTAGGAGGAAGTGGTCATTGTGTCAGCATCTGCTGGATGT
GAGGACTTGCATTGTGAAAGCTTTGCTGTCCTTGATGTGATCATGGAATCTCTTTCTCAC
TAGAGTCTATGTCACTCATTATACTCTGTCGAATGTCATTGAATGTCTTTACATGGGCTT
ATATGCCTATGAAAATTGTAATACAACTTTCAGCAACGGATCTCTTGGCTCTCGCATCGA
TGAAGAACGCAGCGAAATGCGATAAGTAATGTGAATTGCAGAATTCAGTGAATCATCGAA

TCTTTGAACGCATCTTGCGCTCCTTGGTATTCCGAGGAGCATGCCTGTTTGAGTGTCATT
AAATTCTCAACTCTCTTCTAC--TTTTTGTAAAAGAGAGCTTGGACTGTGGAGGCTTGCT
GGCCACTTTTTGGGGTCAGCTCCTCTGAAATGCATTAGCGGAACCGTTTGCGATCTGCCA
CAAGTGTGATAAGTTATCTACACTGGCGAGGGGATTGCTCTCTGTAATGTTCAGCTTCTA
ATTGTCTCTACTTTGTGAGACTACTTTTGAATGCTTGACCTCAAATCAGGTAGGACTACC
CGCTGAACTTAA
>ABC8-44
TTTCCGTAGGTGAACCTGCGGAAGGATCATTATTGAATTATGTTTCTAGATAGGTTGTAG
CTGGCTC-TTTAGAGCATGTGCACGCCTGTTTGGACTTCATTTTCATCCACCTGTGCACC
TATTGTAGTCTTTGGTTGGGTTAGGAGGAAGTGGTCATTGTGTCAGCATCTGCTGGATGT
GAGGACTTGCATTGTGAAAGCTTTGCTGTCCTTGATGTGATCATGGAATCTCTTTCTCAC
TAGAGTCTATGTCACTCATTATACTCTGTCGAATGTCATTGAATGTCTTTACATGGGCTT
ATATGCCTATGAAAATTGTAATACAACTTTCAGCAACGGATCTCTTGGCTCTCGCATCGA
TGAAGAACGCAGCGAAATGCGATAAGTAATGTGAATTGCAGAATTCAGTGAATCATCGAA
TCTTTGAACGCATCTTGCGCTCCTTGGTATTCCGAGGAGCATGCCTGTTTGAGTGTCATT
AAATTCTCAACTCTCTTCTAC--TTTTTGTAAAAGAGAGCTTGGACTGTGGAGGCTTGCT
GGCCACTTTTTGGGGTCAGCTCCTCTGAAATGCATTAGCGGAACCGTTTGCGATCTGCCA
CAAGTGTGATAAGTTATCTACACTGGCGAGGGGATTGCTCTCTGTAATGTTCAGCTTCTA
ATTGTCTCTACTTTGTGAGACTACTTTTGAATGCTTGACCTCAAATCAGGTAGGACTACC
CGCTGAACTTAA
>ABC12-49
TTTCCGTAGGTGAACCTGCGGAAGGATCATTATTGAATTATGTTTCTAGATAGGTTGTAG
CTGGCTC-TTTAGAGCATGTGCACGCCTGTTTGGACTTCATTTTCATCCACCTGTGCACC
TATTGTAGTCTTTGGTTGGGTTAGGAGGAAGTGGTCATTGTGTCAGCATCTGCTGGATGT
GAGGACTTGCATTGTGAAAGCTTTGCTGTCCTTGATGTGATCATGGAATCTCTTTCTCAC
TAGAGTCTATGTCACTCATTATACTCTGTCGAATGTCATTGAATGTCTTTACATGGGCTT
ATATGCCTATGAAAATTGTAATACAACTTTCAGCAACGGATCTCTTGGCTCTCGCATCGA
TGAAGAACGCAGCGAAATGCGATAAGTAATGTGAATTGCAGAATTCAGTGAATCATCGAA
TCTTTGAACGCATCTTGCGCTCCTTGGTATTCCGAGGAGCATGCCTGTTTGAGTGTCATT
AAATTCTCAACTCTCTTCTAC--TTTTTGTAAAAGAGAGCTTGGACTGTGGAGGCTTGCT
GGCCACTTTTTGGGGTCAGCTCCTCTGAAATGCATTAGCGGAACCGTTTGCGATCTGCCA
CAAGTGTGATAAGTTATCTACACTGGCGAGGGGATTGCTCTCTGTAATGTTCAGCTTCTA
ATTGTCTCTACTTTGTGAGACTACTTTTGAATGCTTGACCTCAAATCAGGTAGGACTACC
CGCTGAACTTAA
>ABC11-17
TTTCCGTAGGTGAACCTGCGGAAGGATCATTATTGAATTATGTTTCTAGATAGGTTGTAG
CTGGCTC-TTTAGAGCATGTGCACGCCTGTTTGGACTTCATTTTCATCCACCTGTGCACC
TATTGTAGTCTTTGGTTGGGTTAGGAGGAAGTGGTCATTGTGTCAGCATCTGCTGGATGT
GAGGACTTGCATTGTGAAAGCTTTGCTGTCCTTGATGTGATCATGGAATCTCTTTCTCAC
TAGAGTCTATGTCACTCATTATACTCTGTCGAATGTCATTGAATGTCTTTACATGGGCTT
ATATGCCTATGAAAATTGTAATACAACTTTCAGCAACGGATCTCTTGGCTCTCGCATCGA
TGAAGAACGCAGCGAAATGCGATAAGTAATGTGAATTGCAGAATTCAGTGAATCATCGAA
TCTTTGAACGCATCTTGCGCTCCTTGGTATTCCGAGGAGCATGCCTGTTTGAGTGTCATT
AAATTCTCAACTCTCTTCTAC--TTTTTGTAAAAGAGAGCTTGGACTGTGGAGGCTTGCT
GGCCACTTTTTGGGGTCAGCTCCTCTGAAATGCATTAGCGGAACCGTTTGCGATCTGCCA
CAAGTGTGATAAGTTATCTACACTGGCGAGGGGATTGCTCTCTGTAATGTTCAGCTTCTA
ATTGTCTCTACTTTGTGAGACTACTTTTGAATGCTTGACCTCAAATCAGGTAGGACTACC
CGCTGAACTTAA
>ABC3-68
TTTCCGTAGGTGAACCTGCGGAAGGATCATTATTGAATTATGTTTCTAGATAGGTTGTAG

CTGGCTC-TTTAGAGCATGTGCACGCCTGTTTGGACTTCATTTTCATCCACCTGTGCACC
TATTGTAGTCTTTGGTTGGGTTAGGAGGAAGTGGTCATTGTGTCAGCATCTGCTGGATGT
GAGGACTTGCATTGTGAAAGCTTTGCTGTCCTTGATGTGATCATGGAATCTCTTTCTCAC
TAGAGTCTATGTCACTCATTATACTCTGTCGAATGTCATTGAATGTCTTTACATGGGCTT
ATATGCCTATGAAAATTGTAATACAACTTTCAGCAACGGATCTCTTGGCTCTCGCATCGA
TGAAGAACGCAGCGAAATGCGATAAGTAATGTGAATTGCAGAATTCAGTGAATCATCGAA
TCTTTGAACGCATCTTGCGCTCCTTGGTATTCCGAGGAGCATGCCTGTTTGAGTGTCATT
AAATTCTCAACTCTCTTCTAC--TTTTTGTAAAAGAGAGCTTGGACTGTGGAGGCTTGCT
GGCCACTTTTTGGGGTCAGCTCCTCTGAAATGCATTAGCGGAACCGTTTGCGATCTGCCA
CAAGTGTGATAAGTTATCTACACTGGCGAGGGGATTGCTCTCTGTAATGTTCAGCTTCTA
ATTGTCTCTACTTTGTGAGACTACTTTTGAATGCTTGACCTCAAATCAGGTAGGACTACC
CGCTGAACTTAA
>ABC3-30
TTTCCGTAGGTGAACCTGCGGAAGGATCATTATTGAATTATGTTTCTAGATAGGTTGTAG
CTGGCTC-TTTAGAGCATGTGCACGCCTGTTTGGACTTCATTTTCATCCACCTGTGCACC
TATTGTAGTCTTTGGTTGGGTTAGGAGGAAGTGGTCATTGTGTCAGCATCTGCTGGATGT
GAGGACTTGCATTGTGAAAGCTTTGCTGTCCTTGATGTGATCATGGAATCTCTTTCTCAC
TAGAGTCTATGTCACTCATTATACTCTGTCGAATGTCATTGAATGTCTTTACATGGGCTT
ATATGCCTATGAAAATTGTAATACAACTTTCAGCAACGGATCTCTTGGCTCTCGCATCGA
TGAAGAACGCAGCGAAATGCGATAAGTAATGTGAATTGCAGAATTCAGTGAATCATCGAA
TCTTTGAACGCATCTTGCGCTCCTTGGTATTCCGAGGAGCATGCCTGTTTGAGTGTCATT
AAATTCTCAACTCTCTTCTAC--TTTTTGTAAAAGAGAGCTTGGACTGTGGAGGCTTGCT
GGCCACTTTTTGGGGTCAGCTCCTCTGAAATGCATTAGCGGAACCGTTTGCGATCTGCCA
CAAGTGTGATAAGTTATCTACACTGGCGAGGGGATTGCTCTCTGTAATGTTCAGCTTCTA
ATTGTCTCTACTTTGTGAGACTACTTTTGAATGCTTGACCTCAAATCAGGTAGGACTACC
CGCTGAACTTAA
>ABC11-14
TTTCCGTAGGTGAACCTGCGGAAGGATCATTATTGAATTATGTTTCTAGATAGGTTGTAG
CTGGCTC-TTTAGAGCATGTGCACGCCTGTTTGGACTTCATTTTCATCCACCTGTGCACC
TATTGTAGTCTTTGGTTGGGTTAGGAGGAAGTGGTCATTGTGTCAGCATCTGCTGGATGT
GAGGACTTGCATTGTGAAAGCTTTGCTGTCCTTGATGTGATCATGGAATCTCTTTCTCAC
TAGAGTCTATGTCACTCATTATACTCTGTCGAATGTCATTGAATGTCTTTACATGGGCTT
ATATGCCTATGAAAATTGTAATACAACTTTCAGCAACGGATCTCTTGGCTCTCGCATCGA
TGAAGAACGCAGCGAAATGCGATAAGTAATGTGAATTGCAGAATTCAGTGAATCATCGAA
TCTTTGAACGCATCTTGCGCTCCTTGGTATTCCGAGGAGCATGCCTGTTTGAGTGTCATT
AAATTCTCAACTCTCTTCTAC--TTTTTGTAAAAGAGAGCTTGGACTGTGGAGGCTTGCT
GGCCACTTTTTGGGGTCAGCTCCTCTGAAATGCATTAGCGGAACCGTTTGCGATCTGCCA
CAAGTGTGATAAGTTATCTACACTGGCGAGGGGATTGCTCTCTGTAATGTTCAGCTTCTA
ATTGTCTCTACTTTGTGAGACTACTTTTGAATGCTTGACCTCAAATCAGGTAGGACTACC
CGCTGAACTTAA
>ABC10-29
TTTCCGTAGGTGAACCTGCGGAAGGATCATTATTGAATTATGTTTCTAGATAGGTTGTAG
CTGGCTC-TTTAGAGCATGTGCACGCCTGTTTGGACTTCATTTTCATCCACCTGTGCACC
TATTGTAGTCTTTGGTTGGGTTAGGAGGAAGTGGTCATTGTGTCAGCATCTGCTGGATGT
GAGGACTTGCATTGTGAAAGCTTTGCTGTCCTTGATGTGATCATGGAATCTCTTTCTCAC
TAGAGTCTATGTCACTCATTATACTCTGTCGAATGTCATTGAATGTCTTTACATGGGCTT
ATATGCCTATGAAAATTGTAATACAACTTTCAGCAACGGATCTCTTGGCTCTCGCATCGA
TGAAGAACGCAGCGAAATGCGATAAGTAATGTGAATTGCAGAATTCAGTGAATCATCGAA
TCTTTGAACGCATCTTGCGCTCCTTGGTATTCCGAGGAGCATGCCTGTTTGAGTGTCATT
AAATTCTCAACTCTCTTCTAC--TTTTTGTAAAAGAGAGCTTGGACTGTGGAGGCTTGCT

GGCCACTTTTTGGGGTCAGCTCCTCTGAAATGCATTAGCGGAACCGTTTGCGATCTGCCA
CAAGTGTGATAAGTTATCTACACTGGCGAGGGGATTGCTCTCTGTAATGTTCAGCTTCTA
ATTGTCTCTACTTTGTGAGACTACTTTTGAATGCTTGACCTCAAATCAGGTAGGACTACC
CGCTGAACTTAA
>ABC4-72
TTTCCGTAGGTGAACCTGCGGAAGGATCATTATTGAATTATGTTTCTAGATAGGTTGTAG
CTGGCTC-TTTAGAGCATGTGCACGCCTGTTTGGACTTCATTTTCATCCACCTGTGCACC
TATTGTAGTCTTTGGTTGGGTTAGGAGGAAGTGGTCATTGTGTCAGCATCTGCTGGATGT
GAGGACTTGCATTGTGAAAGCTTTGCTGTCCTTGATGTGATCATGGAATCTCTTTCTCAC
TAGAGTCTATGTCACTCATTATACTCTGTCGAATGTCATTGAATGTCTTTACATGGGCTT
ATATGCCTATGAAAATTGTAATACAACTTTCAGCAACGGATCTCTTGGCTCTCGCATCGA
TGAAGAACGCAGCGAAATGCGATAAGTAATGTGAATTGCAGAATTCAGTGAATCATCGAA
TCTTTGAACGCATCTTGCGCTCCTTGGTATTCCGAGGAGCATGCCTGTTTGAGTGTCATT
AAATTCTCAACTCTCTTCTAC--TTTTTGTAAAAGAGAGCTTGGACTGTGGAGGCTTGCT
GGCCACTTTTTGGGGTCAGCTCCTCTGAAATGCATTAGCGGAACCGTTTGCGATCTGCCA
CAAGTGTGATAAGTTATCTACACTGGCGAGGGGATTGCTCTCTGTAATGTTCAGCTTCTA
ATTGTCTCTACTTTGTGAGACTACTTTTGAATGCTTGACCTCAAATCAGGTAGGACTACC
CGCTGAACTTAA
>ABC6-57
TTTCCGTAGGTGAACCTGCGGAAGGATCATTATTGAATTATGTTTCTAGATAGGTTGTAG
CTGGCTC-TTTAGAGCATGTGCACGCCTGTTTGGACTTCATTTTCATCCACCTGTGCACC
TATTGTAGTCTTTGGTTGGGTTAGGAGGAAGTGGTCATTGTGTCAGCATCTGCTGGATGT
GAGGACTTGCATTGTGAAAGCTTTGCTGTCCTTGATGTGATCATGGAATCTCTTTCTCAC
TAGAGTCTATGTCACTCATTATACTCTGTCGAATGTCATTGAATGTCTTTACATGGGCTT
ATATGCCTATGAAAATTGTAATACAACTTTCAGCAACGGATCTCTTGGCTCTCGCATCGA
TGAAGAACGCAGCGAAATGCGATAAGTAATGTGAATTGCAGAATTCAGTGAATCATCGAA
TCTTTGAACGCATCTTGCGCTCCTTGGTATTCCGAGGAGCATGCCTGTTTGAGTGTCATT
AAATTCTCAACTCTCTTCTAC--TTTTTGTAAAAGAGAGCTTGGACTGTGGAGGCTTGCT
GGCCACTTTTTGGGGTCAGCTCCTCTGAAATGCATTAGCGGAACCGTTTGCGATCTGCCA
CAAGTGTGATAAGTTATCTACACTGGCGAGGGGATTGCTCTCTGTAATGTTCAGCTTCTA
ATTGTCTCTACTTTGTGAGACTACTTTTGAATGCTTGACCTCAAATCAGGTAGGACTACC
CGCTGAACTTAA
>ABC3-41
TTTCCGTAGGTGAACCTGCGGAAGGATCATTATTGAATTATGTTTCTAGATAGGTTGTAG
CTGGCTC-TTTAGAGCATGTGCACGCCTGTTTGGACTTCATTTTCATCCACCTGTGCACC
TATTGTAGTCTTTGGTTGGGTTAGGAGGAAGTGGTCATTGTGTCAGCATCTGCTGGATGT
GAGGACTTGCATTGTGAAAGCTTTGCTGTCCTTGATGTGATCATGGAATCTCTTTCTCAC
TAGAGTCTATGTCACTCATTATACTCTGTCGAATGTCATTGAATGTCTTTACATGGGCTT
ATATGCCTATGAAAATTGTAATACAACTTTCAGCAACGGATCTCTTGGCTCTCGCATCGA
TGAAGAACGCAGCGAAATGCGATAAGTAATGTGAATTGCAGAATTCAGTGAATCATCGAA
TCTTTGAACGCATCTTGCGCTCCTTGGTATTCCGAGGAGCATGCCTGTTTGAGTGTCATT
AAATTCTCAACTCTCTTCTAC--TTTTTGTAAAAGAGAGCTTGGACTGTGGAGGCTTGCT
GGCCACTTTTTGGGGTCAGCTCCTCTGAAATGCATTAGCGGAACCGTTTGCGATCTGCCA
CAAGTGTGATAAGTTATCTACACTGGCGAGGGGATTGCTCTCTGTAATGTTCAGCTTCTA
ATTGTCTCTACTTTGTGAGACTACTTTTGAATGCTTGACCTCAAATCAGGTAGGACTACC
CGCTGAACTTAA
>ABC9-30
TTTCCGTAGGTGAACCTGCGGAAGGATCATTATTGAATTATGTTTCTAGATAGGTTGTAG
CTGGCTC-TTTAGAGCATGTGCACGCCTGTTTGGACTTCATTTTCATCCACCTGTGCACC
TATTGTAGTCTTTGGTTGGGTTAGGAGGAAGTGGTCATTGTGTCAGCATCTGCTGGATGT

GAGGACTTGCATTGTGAAAGCTTTGCTGTCCTTGATGTGATCATGGAATCTCTTTCTCAC
TAGAGTCTATGTCACTCATTATACTCTGTCGAATGTCATTGAATGTCTTTACATGGGCTT
ATATGCCTATGAAAATTGTAATACAACTTTCAGCAACGGATCTCTTGGCTCTCGCATCGA
TGAAGAACGCAGCGAAATGCGATAAGTAATGTGAATTGCAGAATTCAGTGAATCATCGAA
TCTTTGAACGCATCTTGCGCTCCTTGGTATTCCGAGGAGCATGCCTGTTTGAGTGTCATT
AAATTCTCAACTCTCTTCTAC--TTTTTGTAAAAGAGAGCTTGGACTGTGGAGGCTTGCT
GGCCACTTTTTGGGGTCAGCTCCTCTGAAATGCATTAGCGGAACCGTTTGCGATCTGCCA
CAAGTGTGATAAGTTATCTACACTGGCGAGGGGATTGCTCTCTGTAATGTTCAGCTTCTA
ATTGTCTCTACTTTGTGAGACTACTTTTGAATGCTTGACCTCAAATCAGGTAGGACTACC
CGCTGAACTTAA
>ABC11-47
TTTCCGTAGGTGAACCTGCGGAAGGATCATTATTGAATTATGTTTCTAGATAGGTTGTAG
CTGGCTC-TTTAGAGCATGTGCACGCCTGTTTGGACTTCATTTTCATCCACCTGTGCACC
TATTGTAGTCTTTGGTTGGGTTAGGAGGAAGTGGTCATTGTGTCAGCATCTGCTGGATGT
GAGGACTTGCATTGTGAAAGCTTTGCTGTCCTTGATGTGATCATGGAATCTCTTTCTCAC
TAGAGTCTATGTCACTCATTATACTCTGTCGAATGTCATTGAATGTCTTTACATGGGCTT
ATATGCCTATGAAAATTGTAATACAACTTTCAGCAACGGATCTCTTGGCTCTCGCATCGA
TGAAGAACGCAGCGAAATGCGATAAGTAATGTGAATTGCAGAATTCAGTGAATCATCGAA
TCTTTGAACGCATCTTGCGCTCCTTGGTATTCCGAGGAGCATGCCTGTTTGAGTGTCATT
AAATTCTCAACTCTCTTCTAC--TTTTTGTAAAAGAGAGCTTGGACTGTGGAGGCTTGCT
GGCCACTTTTTGGGGTCAGCTCCTCTGAAATGCATTAGCGGAACCGTTTGCGATCTGCCA
CAAGTGTGATAAGTTATCTACACTGGCGAGGGGATTGCTCTCTGTAATGTTCAGCTTCTA
ATTGTCTCTACTTTGTGAGACTACTTTTGAATGCTTGACCTCAAATCAGGTAGGACTACC
CGCTGAACTTAA
>ABC1-38
TTTCCGTAGGTGAACCTGCGGAAGGATCATTATTGAATTATGTTTCTAGATAGGTTGTAG
CTGGCTC-TTTAGAGCATGTGCACGCCTGTTTGGACTTCATTTTCATCCACCTGTGCACC
TATTGTAGTCTTTGGTTGGGTTAGGAGGAAGTGGTCATTGTGTCAGCATCTGCTGGATGT
GAGGACTTGCATTGTGAAAGCTTTGCTGTCCTTGATGTGATCATGGAATCTCTTTCTCAC
TAGAGTCTATGTCACTCATTATACTCTGTCGAATGTCATTGAATGTCTTTACATGGGCTT
ATATGCCTATGAAAATTGTAATACAACTTTCAGCAACGGATCTCTTGGCTCTCGCATCGA
TGAAGAACGCAGCGAAATGCGATAAGTAATGTGAATTGCAGAATTCAGTGAATCATCGAA
TCTTTGAACGCATCTTGCGCTCCTTGGTATTCCGAGGAGCATGCCTGTTTGAGTGTCATT
AAATTCTCAACTCTCTTCTAC--TTTTTGTAAAAGAGAGCTTGGACTGTGGAGGCTTGCT
GGCCACTTTTTGGGGTCAGCTCCTCTGAAATGCATTAGCGGAACCGTTTGCGATCTGCCA
CAAGTGTGATAAGTTATCTACACTGGCGAGGGGATTGCTCTCTGTAATGTTCAGCTTCTA
ATTGTCTCTACTTTGTGAGACTACTTTTGAATGCTTGACCTCAAATCAGGTAGGACTACC
CGCTGAACTTAA
>ABC11-13
TTTCCGTAGGTGAACCTGCGGAAGGATCATTATTGAATTATGTTTCTAGATAGGTTGTAG
CTGGCTC-TTTAGAGCATGTGCACGCCTGTTTGGACTTCATTTTCATCCACCTGTGCACC
TATTGTAGTCTTTGGTTGGGTTAGGAGGAAGTGGTCATTGTGTCAGCATCTGCTGGATGT
GAGGACTTGCATTGTGAAAGCTTTGCTGTCCTTGATGTGATCATGGAATCTCTTTCTCAC
TAGAGTCTATGTCACTCATTATACTCTGTCGAATGTCATTGAATGTCTTTACATGGGCTT
ATATGCCTATGAAAATTGTAATACAACTTTCAGCAACGGATCTCTTGGCTCTCGCATCGA
TGAAGAACGCAGCGAAATGCGATAAGTAATGTGAATTGCAGAATTCAGTGAATCATCGAA
TCTTTGAACGCATCTTGCGCTCCTTGGTATTCCGAGGAGCATGCCTGTTTGAGTGTCATT
AAATTCTCAACTCTCTTCTAC--TTTTTGTAAAAGAGAGCTTGGACTGTGGAGGCTTGCT
GGCCACTTTTTGGGGTCAGCTCCTCTGAAATGCATTAGCGGAACCGTTTGCGATCTGCCA
CAAGTGTGATAAGTTATCTACACTGGCGAGGGGATTGCTCTCTGTAATGTTCAGCTTCTA

ATTGTCTCTACTTTGTGAGACTACTTTTGAATGCTTGACCTCAAATCAGGTAGGACTACC
CGCTGAACTTAA
>ABC11-44
TTTCCGTAGGTGAACCTGCGGAAGGATCATTATTGAATTATGTTTCTAGATAGGTTGTAG
CTGGCTC-TTTAGAGCATGTGCACGCCTGTTTGGACTTCATTTTCATCCACCTGTGCACC
TATTGTAGTCTTTGGTTGGGTTAGGAGGAAGTGGTCATTGTGTCAGCATCTGCTGGATGT
GAGGACTTGCATTGTGAAAGCTTTGCTGTCCTTGATGTGATCATGGAATCTCTTTCTCAC
TAGAGTCTATGTCACTCATTATACTCTGTCGAATGTCATTGAATGTCTTTACATGGGCTT
ATATGCCTATGAAAATTGTAATACAACTTTCAGCAACGGATCTCTTGGCTCTCGCATCGA
TGAAGAACGCAGCGAAATGCGATAAGTAATGTGAATTGCAGAATTCAGTGAATCATCGAA
TCTTTGAACGCATCTTGCGCTCCTTGGTATTCCGAGGAGCATGCCTGTTTGAGTGTCATT
AAATTCTCAACTCTCTTCTAC--TTTTTGTAAAAGAGAGCTTGGACTGTGGAGGCTTGCT
GGCCACTTTTTGGGGTCAGCTCCTCTGAAATGCATTAGCGGAACCGTTTGCGATCTGCCA
CAAGTGTGATAAGTTATCTACACTGGCGAGGGGATTGCTCTCTGTAATGTTCAGCTTCTA
ATTGTCTCTACTTTGTGAGACTACTTTTGAATGCTTGACCTCAAATCAGGTAGGACTACC
CGCTGAACTTAA
>ABC10-6
TTTCCGTAGGTGAACCTGCGGAAGGATCATTATTGAATTATGTTTCTAGATAGGTTGTAG
CTGGCTC-TTTAGAGCATGTGCACGCCTGTTTGGACTTCATTTTCATCCACCTGTGCACC
TATTGTAGTCTTTGGTTGGGTTAGGAGGAAGTGGTCATTGTGTCAGCATCTGCTGGATGT
GAGGACTTGCATTGTGAAAGCTTTGCTGTCCTTGATGTGATCATGGAATCTCTTTCTCAC
TAGAGTCTATGTCACTCATTATACTCTGTCGAATGTCATTGAATGTCTTTACATGGGCTT
ATATGCCTATGAAAATTGTAATACAACTTTCAGCAACGGATCTCTTGGCTCTCGCATCGA
TGAAGAACGCAGCGAAATGCGATAAGTAATGTGAATTGCAGAATTCAGTGAATCATCGAA
TCTTTGAACGCATCTTGCGCTCCTTGGTATTCCGAGGAGCATGCCTGTTTGAGTGTCATT
AAATTCTCAACTCTCTTCTAC--TTTTTGTAAAAGAGAGCTTGGACTGTGGAGGCTTGCT
GGCCACTTTTTGGGGTCAGCTCCTCTGAAATGCATTAGCGGAACCGTTTGCGATCTGCCA
CAAGTGTGATAAGTTATCTACACTGGCGAGGGGATTGCTCTCTGTAATGTTCAGCTTCTA
ATTGTCTCTACTTTGTGAGACTACTTTTGAATGCTTGACCTCAAATCAGGTAGGACTACC
CGCTGAACTTAA
>ABC4-18
TTTCCGTAGGTGAACCTGCGGAAGGATCATTATTGAATTATGTTTCTAGATAGGTTGTAG
CTGGCTC-TTTAGAGCATGTGCACGCCTGTTTGGACTTCATTTTCATCCACCTGTGCACC
TATTGTAGTCTTTGGTTGGGTTAGGAGGAAGTGGTCATTGTGTCAGCATCTGCTGGATGT
GAGGACTTGCATTGTGAAAGCTTTGCTGTCCTTGATGTGATCATGGAATCTCTTTCTCAC
TAGAGTCTATGTCACTCATTATACTCTGTCGAATGTCATTGAATGTCTTTACATGGGCTT
ATATGCCTATGAAAATTGTAATACAACTTTCAGCAACGGATCTCTTGGCTCTCGCATCGA
TGAAGAACGCAGCGAAATGCGATAAGTAATGTGAATTGCAGAATTCAGTGAATCATCGAA
TCTTTGAACGCATCTTGCGCTCCTTGGTATTCCGAGGAGCATGCCTGTTTGAGTGTCATT
AAATTCTCAACTCTCTTCTAC--TTTTTGTAAAAGAGAGCTTGGACTGTGGAGGCTTGCT
GGCCACTTTTTGGGGTCAGCTCCTCTGAAATGCATTAGCGGAACCGTTTGCGATCTGCCA
CAAGTGTGATAAGTTATCTACACTGGCGAGGGGATTGCTCTCTGTAATGTTCAGCTTCTA
ATTGTCTCTACTTTGTGAGACTACTTTTGAATGCTTGACCTCAAATCAGGTAGGACTACC
CGCTGAACTTAA
>ABC5-40
TTTCCGTAGGTGAACCTGCGGAAGGATCATTATTGAATTATGTTTCTAGATAGGTTGTAG
CTGGCTC-TTTAGAGCATGTGCACGCCTGTTTGGACTTCATTTTCATCCACCTGTGCACC
TATTGTAGTCTTTGGTTGGGTTAGGAGGAAGTGGTCATTGTGTCAGCATCTGCTGGATGT
GAGGACTTGCATTGTGAAAGCTTTGCTGTCCTTGATGTGATCATGGAATCTCTTTCTCAC
TAGAGTCTATGTCACTCATTATACTCTGTCGAATGTCATTGAATGTCTTTACATGGGCTT

ATATGCCTATGAAAATTGTAATACAACTTTCAGCAACGGATCTCTTGGCTCTCGCATCGA
TGAAGAACGCAGCGAAATGCGATAAGTAATGTGAATTGCAGAATTCAGTGAATCATCGAA
TCTTTGAACGCATCTTGCGCTCCTTGGTATTCCGAGGAGCATGCCTGTTTGAGTGTCATT
AAATTCTCAACTCTCTTCTAC--TTTTTGTAAAAGAGAGCTTGGACTGTGGAGGCTTGCT
GGCCACTTTTTGGGGTCAGCTCCTCTGAAATGCATTAGCGGAACCGTTTGCGATCTGCCA
CAAGTGTGATAAGTTATCTACACTGGCGAGGGGATTGCTCTCTGTAATGTTCAGCTTCTA
ATTGTCTCTACTTTGTGAGACTACTTTTGAATGCTTGACCTCAAATCAGGTAGGACTACC
CGCTGAACTTAA
>ABC4-70
TTTCCGTAGGTGAACCTGCGGAAGGATCATTATTGAATTATGTTTCTAGATAGGTTGTAG
CTGGCTC-TTTAGAGCATGTGCACGCCTGTTTGGACTTCATTTTCATCCACCTGTGCACC
TATTGTAGTCTTTGGTTGGGTTAGGAGGAAGTGGTCATTGTGTCAGCATCTGCTGGATGT
GAGGACTTGCATTGTGAAAGCTTTGCTGTCCTTGATGTGATCATGGAATCTCTTTCTCAC
TAGAGTCTATGTCACTCATTATACTCTGTCGAATGTCATTGAATGTCTTTACATGGGCTT
ATATGCCTATGAAAATTGTAATACAACTTTCAGCAACGGATCTCTTGGCTCTCGCATCGA
TGAAGAACGCAGCGAAATGCGATAAGTAATGTGAATTGCAGAATTCAGTGAATCATCGAA
TCTTTGAACGCATCTTGCGCTCCTTGGTATTCCGAGGAGCATGCCTGTTTGAGTGTCATT
AAATTCTCAACTCTCTTCTAC--TTTTTGTAAAAGAGAGCTTGGACTGTGGAGGCTTGCT
GGCCACTTTTTGGGGTCAGCTCCTCTGAAATGCATTAGCGGAACCGTTTGCGATCTGCCA
CAAGTGTGATAAGTTATCTACACTGGCGAGGGGATTGCTCTCTGTAATGTTCAGCTTCTA
ATTGTCTCTACTTTGTGAGACTACTTTTGAATGCTTGACCTCAAATCAGGTAGGACTACC
CGCTGAACTTAA
>ABC8-15
TTTCCGTAGGTGAACCTGCGGAAGGATCATTATTGAATTATGTTTCTAGATAGGTTGTAG
CTGGCTC-TTTAGAGCATGTGCACGCCTGTTTGGACTTCATTTTCATCCACCTGTGCACC
TATTGTAGTCTTTGGTTGGGTTAGGAGGAAGTGGTCATTGTGTCAGCATCTGCTGGATGT
GAGGACTTGCATTGTGAAAGCTTTGCTGTCCTTGATGTGATCATGGAATCTCTTTCTCAC
TAGAGTCTATGTCACTCATTATACTCTGTCGAATGTCATTGAATGTCTTTACATGGGCTT
ATATGCCTATGAAAATTGTAATACAACTTTCAGCAACGGATCTCTTGGCTCTCGCATCGA
TGAAGAACGCAGCGAAATGCGATAAGTAATGTGAATTGCAGAATTCAGTGAATCATCGAA
TCTTTGAACGCATCTTGCGCTCCTTGGTATTCCGAGGAGCATGCCTGTTTGAGTGTCATT
AAATTCTCAACTCTCTTCTAC--TTTTTGTAAAAGAGAGCTTGGACTGTGGAGGCTTGCT
GGCCACTTTTTGGGGTCAGCTCCTCTGAAATGCATTAGCGGAACCGTTTGCGATCTGCCA
CAAGTGTGATAAGTTATCTACACTGGCGAGGGGATTGCTCTCTGTAATGTTCAGCTTCTA
ATTGTCTCTACTTTGTGAGACTACTTTTGAATGCTTGACCTCAAATCAGGTAGGACTACC
CGCTGAACTTAA
>ABC8-31
TTTCCGTAGGTGAACCTGCGGAAGGATCATTATTGAATTATGTTTCTAGATAGGTTGTAG
CTGGCTC-TTTAGAGCATGTGCACGCCTGTTTGGACTTCATTTTCATCCACCTGTGCACC
TATTGTAGTCTTTGGTTGGGTTAGGAGGAAGTGGTCATTGTGTCAGCATCTGCTGGATGT
GAGGACTTGCATTGTGAAAGCTTTGCTGTCCTTGATGTGATCATGGAATCTCTTTCTCAC
TAGAGTCTATGTCACTCATTATACTCTGTCGAATGTCATTGAATGTCTTTACATGGGCTT
ATATGCCTATGAAAATTGTAATACAACTTTCAGCAACGGATCTCTTGGCTCTCGCATCGA
TGAAGAACGCAGCGAAATGCGATAAGTAATGTGAATTGCAGAATTCAGTGAATCATCGAA
TCTTTGAACGCATCTTGCGCTCCTTGGTATTCCGAGGAGCATGCCTGTTTGAGTGTCATT
AAATTCTCAACTCTCTTCTAC--TTTTTGTAAAAGAGAGCTTGGACTGTGGAGGCTTGCT
GGCCACTTTTTGGGGTCAGCTCCTCTGAAATGCATTAGCGGAACCGTTTGCGATCTGCCA
CAAGTGTGATAAGTTATCTACACTGGCGAGGGGATTGCTCTCTGTAATGTTCAGCTTCTA
ATTGTCTCTACTTTGTGAGACTACTTTTGAATGCTTGACCTCAAATCAGGTAGGACTACC
CGCTGAACTTAA

>ABC9-27
TTTCCGTAGGTGAACCTGCGGAAGGATCATTATTGAATTATGTTTCTAGATAGGTTGTAG
CTGGCTC-TTTAGAGCATGTGCACGCCTGTTTGGACTTCATTTTCATCCACCTGTGCACC
TATTGTAGTCTTTGGTTGGGTTAGGAGGAAGTGGTCATTGTGTCAGCATCTGCTGGATGT
GAGGACTTGCATTGTGAAAGCTTTGCTGTCCTTGATGTGATCATGGAATCTCTTTCTCAC
TAGAGTCTATGTCACTCATTATACTCTGTCGAATGTCATTGAATGTCTTTACATGGGCTT
ATATGCCTATGAAAATTGTAATACAACTTTCAGCAACGGATCTCTTGGCTCTCGCATCGA
TGAAGAACGCAGCGAAATGCGATAAGTAATGTGAATTGCAGAATTCAGTGAATCATCGAA
TCTTTGAACGCATCTTGCGCTCCTTGGTATTCCGAGGAGCATGCCTGTTTGAGTGTCATT
AAATTCTCAACTCTCTTCTAC--TTTTTGTAAAAGAGAGCTTGGACTGTGGAGGCTTGCT
GGCCACTTTTTGGGGTCAGCTCCTCTGAAATGCATTAGCGGAACCGTTTGCGATCTGCCA
CAAGTGTGATAAGTTATCTACACTGGCGAGGGGATTGCTCTCTGTAATGTTCAGCTTCTA
ATTGTCTCTACTTTGTGAGACTACTTTTGAATGCTTGACCTCAAATCAGGTAGGACTACC
CGCTGAACTTAA
>ABC11-51
TTTCCGTAGGTGAACCTGCGGAAGGATCATTATTGAATTATGTTTCTAGATAGGTTGTAG
CTGGCTC-TTTAGAGCATGTGCACGCCTGTTTGGACTTCATTTTCATCCACCTGTGCACC
TATTGTAGTCTTTGGTTGGGTTAGGAGGAAGTGGTCATTGTGTCAGCATCTGCTGGATGT
GAGGACTTGCATTGTGAAAGCTTTGCTGTCCTTGATGTGATCATGGAATCTCTTTCTCAC
TAGAGTCTATGTCACTCATTATACTCTGTCGAATGTCATTGAATGTCTTTACATGGGCTT
ATATGCCTATGAAAATTGTAATACAACTTTCAGCAACGGATCTCTTGGCTCTCGCATCGA
TGAAGAACGCAGCGAAATGCGATAAGTAATGTGAATTGCAGAATTCAGTGAATCATCGAA
TCTTTGAACGCATCTTGCGCTCCTTGGTATTCCGAGGAGCATGCCTGTTTGAGTGTCATT
AAATTCTCAACTCTCTTCTAC--TTTTTGTAAAAGAGAGCTTGGACTGTGGAGGCTTGCT
GGCCACTTTTTGGGGTCAGCTCCTCTGAAATGCATTAGCGGAACCGTTTGCGATCTGCCA
CAAGTGTGATAAGTTATCTACACTGGCGAGGGGATTGCTCTCTGTAATGTTCAGCTTCTA
ATTGTCTCTACTTTGTGAGACTACTTTTGAATGCTTGACCTCAAATCAGGTAGGACTACC
CGCTGAACTTAA
>ABC12-20
TTTCCGTAGGTGAACCTGCGGAAGGATCATTATTGAATTATGTTTCTAGATAGGTTGTAG
CTGGCTC-TTTAGAGCATGTGCACGCCTGTTTGGACTTCATTTTCATCCACCTGTGCACC
TATTGTAGTCTTTGGTTGGGTTAGGAGGAAGTGGTCATTGTGTCAGCATCTGCTGGATGT
GAGGACTTGCATTGTGAAAGCTTTGCTGTCCTTGATGTGATCATGGAATCTCTTTCTCAC
TAGAGTCTATGTCACTCATTATACTCTGTCGAATGTCATTGAATGTCTTTACATGGGCTT
ATATGCCTATGAAAATTGTAATACAACTTTCAGCAACGGATCTCTTGGCTCTCGCATCGA
TGAAGAACGCAGCGAAATGCGATAAGTAATGTGAATTGCAGAATTCAGTGAATCATCGAA
TCTTTGAACGCATCTTGCGCTCCTTGGTATTCCGAGGAGCATGCCTGTTTGAGTGTCATT
AAATTCTCAACTCTCTTCTAC--TTTTTGTAAAAGAGAGCTTGGACTGTGGAGGCTTGCT
GGCCACTTTTTGGGGTCAGCTCCTCTGAAATGCATTAGCGGAACCGTTTGCGATCTGCCA
CAAGTGTGATAAGTTATCTACACTGGCGAGGGGATTGCTCTCTGTAATGTTCAGCTTCTA
ATTGTCTCTACTTTGTGAGACTACTTTTGAATGCTTGACCTCAAATCAGGTAGGACTACC
CGCTGAACTTAA
>ABC1-31
TTTCCGTAGGTGAACCTGCGGAAGGATCATTATTGAATTATGTTTCTAGATAGGTTGTAG
CTGGCTC-TTTAGAGCATGTGCACGCCTGTTTGGACTTCATTTTCATCCACCTGTGCACC
TATTGTAGTCTTTGGTTGGGTTAGGAGGAAGTGGTCATTGTGTCAGCATCTGCTGGATGT
GAGGACTTGCATTGTGAAAGCTTTGCTGTCCTTGATGTGATCATGGAATCTCTTTCTCAC
TAGAGTCTATGTCACTCATTATACTCTGTCGAATGTCATTGAATGTCTTTACATGGGCTT
ATATGCCTATGAAAATTGTAATACAACTTTCAGCAACGGATCTCTTGGCTCTCGCATCGA
TGAAGAACGCAGCGAAATGCGATAAGTAATGTGAATTGCAGAATTCAGTGAATCATCGAA

TCTTTGAACGCATCTTGCGCTCCTTGGTATTCCGAGGAGCATGCCTGTTTGAGTGTCATT
AAATTCTCAACTCTCTTCTAC--TTTTTGTAAAAGAGAGCTTGGACTGTGGAGGCTTGCT
GGCCACTTTTTGGGGTCAGCTCCTCTGAAATGCATTAGCGGAACCGTTTGCGATCTGCCA
CAAGTGTGATAAGTTATCTACACTGGCGAGGGGATTGCTCTCTGTAATGTTCAGCTTCTA
ATTGTCTCTACTTTGTGAGACTACTTTTGAATGCTTGACCTCAAATCAGGTAGGACTACC
CGCTGAACTTAA
>ABC1-56
TTTCCGTAGGTGAACCTGCGGAAGGATCATTATTGAATTATGTTTCTAGATAGGTTGTAG
CTGGCTC-TTTAGAGCATGTGCACGCCTGTTTGGACTTCATTTTCATCCACCTGTGCACC
TATTGTAGTCTTTGGTTGGGTTAGGAGGAAGTGGTCATTGTGTCAGCATCTGCTGGATGT
GAGGACTTGCATTGTGAAAGCTTTGCTGTCCTTGATGTGATCATGGAATCTCTTTCTCAC
TAGAGTCTATGTCACTCATTATACTCTGTCGAATGTCATTGAATGTCTTTACATGGGCTT
ATATGCCTATGAAAATTGTAATACAACTTTCAGCAACGGATCTCTTGGCTCTCGCATCGA
TGAAGAACGCAGCGAAATGCGATAAGTAATGTGAATTGCAGAATTCAGTGAATCATCGAA
TCTTTGAACGCATCTTGCGCTCCTTGGTATTCCGAGGAGCATGCCTGTTTGAGTGTCATT
AAATTCTCAACTCTCTTCTAC--TTTTTGTAAAAGAGAGCTTGGACTGTGGAGGCTTGCT
GGCCACTTTTTGGGGTCAGCTCCTCTGAAATGCATTAGCGGAACCGTTTGCGATCTGCCA
CAAGTGTGATAAGTTATCTACACTGGCGAGGGGATTGCTCTCTGTAATGTTCAGCTTCTA
ATTGTCTCTACTTTGTGAGACTACTTTTGAATGCTTGACCTCAAATCAGGTAGGACTACC
CGCTGAACTTAA
>ABC2-9
TTTCCGTAGGTGAACCTGCGGAAGGATCATTATTGAATTATGTTTCTAGATAGGTTGTAG
CTGGCTC-TTTAGAGCATGTGCACGCCTGTTTGGACTTCATTTTCATCCACCTGTGCACC
TATTGTAGTCTTTGGTTGGGTTAGGAGGAAGTGGTCATTGTGTCAGCATCTGCTGGATGT
GAGGACTTGCATTGTGAAAGCTTTGCTGTCCTTGATGTGATCATGGAATCTCTTTCTCAC
TAGAGTCTATGTCACTCATTATACTCTGTCGAATGTCATTGAATGTCTTTACATGGGCTT
ATATGCCTATGAAAATTGTAATACAACTTTCAGCAACGGATCTCTTGGCTCTCGCATCGA
TGAAGAACGCAGCGAAATGCGATAAGTAATGTGAATTGCAGAATTCAGTGAATCATCGAA
TCTTTGAACGCATCTTGCGCTCCTTGGTATTCCGAGGAGCATGCCTGTTTGAGTGTCATT
AAATTCTCAACTCTCTTCTAC--TTTTTGTAAAAGAGAGCTTGGACTGTGGAGGCTTGCT
GGCCACTTTTTGGGGTCAGCTCCTCTGAAATGCATTAGCGGAACCGTTTGCGATCTGCCA
CAAGTGTGATAAGTTATCTACACTGGCGAGGGGATTGCTCTCTGTAATGTTCAGCTTCTA
ATTGTCTCTACTTTGTGAGACTACTTTTGAATGCTTGACCTCAAATCAGGTAGGACTACC
CGCTGAACTTAA
>ABC2-33
TTTCCGTAGGTGAACCTGCGGAAGGATCATTATTGAATTATGTTTCTAGATAGGTTGTAG
CTGGCTC-TTTAGAGCATGTGCACGCCTGTTTGGACTTCATTTTCATCCACCTGTGCACC
TATTGTAGTCTTTGGTTGGGTTAGGAGGAAGTGGTCATTGTGTCAGCATCTGCTGGATGT
GAGGACTTGCATTGTGAAAGCTTTGCTGTCCTTGATGTGATCATGGAATCTCTTTCTCAC
TAGAGTCTATGTCACTCATTATACTCTGTCGAATGTCATTGAATGTCTTTACATGGGCTT
ATATGCCTATGAAAATTGTAATACAACTTTCAGCAACGGATCTCTTGGCTCTCGCATCGA
TGAAGAACGCAGCGAAATGCGATAAGTAATGTGAATTGCAGAATTCAGTGAATCATCGAA
TCTTTGAACGCATCTTGCGCTCCTTGGTATTCCGAGGAGCATGCCTGTTTGAGTGTCATT
AAATTCTCAACTCTCTTCTAC--TTTTTGTAAAAGAGAGCTTGGACTGTGGAGGCTTGCT
GGCCACTTTTTGGGGTCAGCTCCTCTGAAATGCATTAGCGGAACCGTTTGCGATCTGCCA
CAAGTGTGATAAGTTATCTACACTGGCGAGGGGATTGCTCTCTGTAATGTTCAGCTTCTA
ATTGTCTCTACTTTGTGAGACTACTTTTGAATGCTTGACCTCAAATCAGGTAGGACTACC
CGCTGAACTTAA
>ABC2-34
TTTCCGTAGGTGAACCTGCGGAAGGATCATTATTGAATTATGTTTCTAGATAGGTTGTAG

CTGGCTC-TTTAGAGCATGTGCACGCCTGTTTGGACTTCATTTTCATCCACCTGTGCACC
TATTGTAGTCTTTGGTTGGGTTAGGAGGAAGTGGTCATTGTGTCAGCATCTGCTGGATGT
GAGGACTTGCATTGTGAAAGCTTTGCTGTCCTTGATGTGATCATGGAATCTCTTTCTCAC
TAGAGTCTATGTCACTCATTATACTCTGTCGAATGTCATTGAATGTCTTTACATGGGCTT
ATATGCCTATGAAAATTGTAATACAACTTTCAGCAACGGATCTCTTGGCTCTCGCATCGA
TGAAGAACGCAGCGAAATGCGATAAGTAATGTGAATTGCAGAATTCAGTGAATCATCGAA
TCTTTGAACGCATCTTGCGCTCCTTGGTATTCCGAGGAGCATGCCTGTTTGAGTGTCATT
AAATTCTCAACTCTCTTCTAC--TTTTTGTAAAAGAGAGCTTGGACTGTGGAGGCTTGCT
GGCCACTTTTTGGGGTCAGCTCCTCTGAAATGCATTAGCGGAACCGTTTGCGATCTGCCA
CAAGTGTGATAAGTTATCTACACTGGCGAGGGGATTGCTCTCTGTAATGTTCAGCTTCTA
ATTGTCTCTACTTTGTGAGACTACTTTTGAATGCTTGACCTCAAATCAGGTAGGACTACC
CGCTGAACTTAA
>ABC2-39
TTTCCGTAGGTGAACCTGCGGAAGGATCATTATTGAATTATGTTTCTAGATAGGTTGTAG
CTGGCTC-TTTAGAGCATGTGCACGCCTGTTTGGACTTCATTTTCATCCACCTGTGCACC
TATTGTAGTCTTTGGTTGGGTTAGGAGGAAGTGGTCATTGTGTCAGCATCTGCTGGATGT
GAGGACTTGCATTGTGAAAGCTTTGCTGTCCTTGATGTGATCATGGAATCTCTTTCTCAC
TAGAGTCTATGTCACTCATTATACTCTGTCGAATGTCATTGAATGTCTTTACATGGGCTT
ATATGCCTATGAAAATTGTAATACAACTTTCAGCAACGGATCTCTTGGCTCTCGCATCGA
TGAAGAACGCAGCGAAATGCGATAAGTAATGTGAATTGCAGAATTCAGTGAATCATCGAA
TCTTTGAACGCATCTTGCGCTCCTTGGTATTCCGAGGAGCATGCCTGTTTGAGTGTCATT
AAATTCTCAACTCTCTTCTAC--TTTTTGTAAAAGAGAGCTTGGACTGTGGAGGCTTGCT
GGCCACTTTTTGGGGTCAGCTCCTCTGAAATGCATTAGCGGAACCGTTTGCGATCTGCCA
CAAGTGTGATAAGTTATCTACACTGGCGAGGGGATTGCTCTCTGTAATGTTCAGCTTCTA
ATTGTCTCTACTTTGTGAGACTACTTTTGAATGCTTGACCTCAAATCAGGTAGGACTACC
CGCTGAACTTAA
>ABC2-44
TTTCCGTAGGTGAACCTGCGGAAGGATCATTATTGAATTATGTTTCTAGATAGGTTGTAG
CTGGCTC-TTTAGAGCATGTGCACGCCTGTTTGGACTTCATTTTCATCCACCTGTGCACC
TATTGTAGTCTTTGGTTGGGTTAGGAGGAAGTGGTCATTGTGTCAGCATCTGCTGGATGT
GAGGACTTGCATTGTGAAAGCTTTGCTGTCCTTGATGTGATCATGGAATCTCTTTCTCAC
TAGAGTCTATGTCACTCATTATACTCTGTCGAATGTCATTGAATGTCTTTACATGGGCTT
ATATGCCTATGAAAATTGTAATACAACTTTCAGCAACGGATCTCTTGGCTCTCGCATCGA
TGAAGAACGCAGCGAAATGCGATAAGTAATGTGAATTGCAGAATTCAGTGAATCATCGAA
TCTTTGAACGCATCTTGCGCTCCTTGGTATTCCGAGGAGCATGCCTGTTTGAGTGTCATT
AAATTCTCAACTCTCTTCTAC--TTTTTGTAAAAGAGAGCTTGGACTGTGGAGGCTTGCT
GGCCACTTTTTGGGGTCAGCTCCTCTGAAATGCATTAGCGGAACCGTTTGCGATCTGCCA
CAAGTGTGATAAGTTATCTACACTGGCGAGGGGATTGCTCTCTGTAATGTTCAGCTTCTA
ATTGTCTCTACTTTGTGAGACTACTTTTGAATGCTTGACCTCAAATCAGGTAGGACTACC
CGCTGAACTTAA
>ABC2-47
TTTCCGTAGGTGAACCTGCGGAAGGATCATTATTGAATTATGTTTCTAGATAGGTTGTAG
CTGGCTC-TTTAGAGCATGTGCACGCCTGTTTGGACTTCATTTTCATCCACCTGTGCACC
TATTGTAGTCTTTGGTTGGGTTAGGAGGAAGTGGTCATTGTGTCAGCATCTGCTGGATGT
GAGGACTTGCATTGTGAAAGCTTTGCTGTCCTTGATGTGATCATGGAATCTCTTTCTCAC
TAGAGTCTATGTCACTCATTATACTCTGTCGAATGTCATTGAATGTCTTTACATGGGCTT
ATATGCCTATGAAAATTGTAATACAACTTTCAGCAACGGATCTCTTGGCTCTCGCATCGA
TGAAGAACGCAGCGAAATGCGATAAGTAATGTGAATTGCAGAATTCAGTGAATCATCGAA
TCTTTGAACGCATCTTGCGCTCCTTGGTATTCCGAGGAGCATGCCTGTTTGAGTGTCATT
AAATTCTCAACTCTCTTCTAC--TTTTTGTAAAAGAGAGCTTGGACTGTGGAGGCTTGCT

GGCCACTTTTTGGGGTCAGCTCCTCTGAAATGCATTAGCGGAACCGTTTGCGATCTGCCA
CAAGTGTGATAAGTTATCTACACTGGCGAGGGGATTGCTCTCTGTAATGTTCAGCTTCTA
ATTGTCTCTACTTTGTGAGACTACTTTTGAATGCTTGACCTCAAATCAGGTAGGACTACC
CGCTGAACTTAA
>ABC2-68
TTTCCGTAGGTGAACCTGCGGAAGGATCATTATTGAATTATGTTTCTAGATAGGTTGTAG
CTGGCTC-TTTAGAGCATGTGCACGCCTGTTTGGACTTCATTTTCATCCACCTGTGCACC
TATTGTAGTCTTTGGTTGGGTTAGGAGGAAGTGGTCATTGTGTCAGCATCTGCTGGATGT
GAGGACTTGCATTGTGAAAGCTTTGCTGTCCTTGATGTGATCATGGAATCTCTTTCTCAC
TAGAGTCTATGTCACTCATTATACTCTGTCGAATGTCATTGAATGTCTTTACATGGGCTT
ATATGCCTATGAAAATTGTAATACAACTTTCAGCAACGGATCTCTTGGCTCTCGCATCGA
TGAAGAACGCAGCGAAATGCGATAAGTAATGTGAATTGCAGAATTCAGTGAATCATCGAA
TCTTTGAACGCATCTTGCGCTCCTTGGTATTCCGAGGAGCATGCCTGTTTGAGTGTCATT
AAATTCTCAACTCTCTTCTAC--TTTTTGTAAAAGAGAGCTTGGACTGTGGAGGCTTGCT
GGCCACTTTTTGGGGTCAGCTCCTCTGAAATGCATTAGCGGAACCGTTTGCGATCTGCCA
CAAGTGTGATAAGTTATCTACACTGGCGAGGGGATTGCTCTCTGTAATGTTCAGCTTCTA
ATTGTCTCTACTTTGTGAGACTACTTTTGAATGCTTGACCTCAAATCAGGTAGGACTACC
CGCTGAACTTAA
>ABC2-75
TTTCCGTAGGTGAACCTGCGGAAGGATCATTATTGAATTATGTTTCTAGATAGGTTGTAG
CTGGCTC-TTTAGAGCATGTGCACGCCTGTTTGGACTTCATTTTCATCCACCTGTGCACC
TATTGTAGTCTTTGGTTGGGTTAGGAGGAAGTGGTCATTGTGTCAGCATCTGCTGGATGT
GAGGACTTGCATTGTGAAAGCTTTGCTGTCCTTGATGTGATCATGGAATCTCTTTCTCAC
TAGAGTCTATGTCACTCATTATACTCTGTCGAATGTCATTGAATGTCTTTACATGGGCTT
ATATGCCTATGAAAATTGTAATACAACTTTCAGCAACGGATCTCTTGGCTCTCGCATCGA
TGAAGAACGCAGCGAAATGCGATAAGTAATGTGAATTGCAGAATTCAGTGAATCATCGAA
TCTTTGAACGCATCTTGCGCTCCTTGGTATTCCGAGGAGCATGCCTGTTTGAGTGTCATT
AAATTCTCAACTCTCTTCTAC--TTTTTGTAAAAGAGAGCTTGGACTGTGGAGGCTTGCT
GGCCACTTTTTGGGGTCAGCTCCTCTGAAATGCATTAGCGGAACCGTTTGCGATCTGCCA
CAAGTGTGATAAGTTATCTACACTGGCGAGGGGATTGCTCTCTGTAATGTTCAGCTTCTA
ATTGTCTCTACTTTGTGAGACTACTTTTGAATGCTTGACCTCAAATCAGGTAGGACTACC
CGCTGAACTTAA
>ABC2-79
TTTCCGTAGGTGAACCTGCGGAAGGATCATTATTGAATTATGTTTCTAGATAGGTTGTAG
CTGGCTC-TTTAGAGCATGTGCACGCCTGTTTGGACTTCATTTTCATCCACCTGTGCACC
TATTGTAGTCTTTGGTTGGGTTAGGAGGAAGTGGTCATTGTGTCAGCATCTGCTGGATGT
GAGGACTTGCATTGTGAAAGCTTTGCTGTCCTTGATGTGATCATGGAATCTCTTTCTCAC
TAGAGTCTATGTCACTCATTATACTCTGTCGAATGTCATTGAATGTCTTTACATGGGCTT
ATATGCCTATGAAAATTGTAATACAACTTTCAGCAACGGATCTCTTGGCTCTCGCATCGA
TGAAGAACGCAGCGAAATGCGATAAGTAATGTGAATTGCAGAATTCAGTGAATCATCGAA
TCTTTGAACGCATCTTGCGCTCCTTGGTATTCCGAGGAGCATGCCTGTTTGAGTGTCATT
AAATTCTCAACTCTCTTCTAC--TTTTTGTAAAAGAGAGCTTGGACTGTGGAGGCTTGCT
GGCCACTTTTTGGGGTCAGCTCCTCTGAAATGCATTAGCGGAACCGTTTGCGATCTGCCA
CAAGTGTGATAAGTTATCTACACTGGCGAGGGGATTGCTCTCTGTAATGTTCAGCTTCTA
ATTGTCTCTACTTTGTGAGACTACTTTTGAATGCTTGACCTCAAATCAGGTAGGACTACC
CGCTGAACTTAA
>ABC3-2
TTTCCGTAGGTGAACCTGCGGAAGGATCATTATTGAATTATGTTTCTAGATAGGTTGTAG
CTGGCTC-TTTAGAGCATGTGCACGCCTGTTTGGACTTCATTTTCATCCACCTGTGCACC
TATTGTAGTCTTTGGTTGGGTTAGGAGGAAGTGGTCATTGTGTCAGCATCTGCTGGATGT

GAGGACTTGCATTGTGAAAGCTTTGCTGTCCTTGATGTGATCATGGAATCTCTTTCTCAC
TAGAGTCTATGTCACTCATTATACTCTGTCGAATGTCATTGAATGTCTTTACATGGGCTT
ATATGCCTATGAAAATTGTAATACAACTTTCAGCAACGGATCTCTTGGCTCTCGCATCGA
TGAAGAACGCAGCGAAATGCGATAAGTAATGTGAATTGCAGAATTCAGTGAATCATCGAA
TCTTTGAACGCATCTTGCGCTCCTTGGTATTCCGAGGAGCATGCCTGTTTGAGTGTCATT
AAATTCTCAACTCTCTTCTAC--TTTTTGTAAAAGAGAGCTTGGACTGTGGAGGCTTGCT
GGCCACTTTTTGGGGTCAGCTCCTCTGAAATGCATTAGCGGAACCGTTTGCGATCTGCCA
CAAGTGTGATAAGTTATCTACACTGGCGAGGGGATTGCTCTCTGTAATGTTCAGCTTCTA
ATTGTCTCTACTTTGTGAGACTACTTTTGAATGCTTGACCTCAAATCAGGTAGGACTACC
CGCTGAACTTAA
>ABC3-4
TTTCCGTAGGTGAACCTGCGGAAGGATCATTATTGAATTATGTTTCTAGATAGGTTGTAG
CTGGCTC-TTTAGAGCATGTGCACGCCTGTTTGGACTTCATTTTCATCCACCTGTGCACC
TATTGTAGTCTTTGGTTGGGTTAGGAGGAAGTGGTCATTGTGTCAGCATCTGCTGGATGT
GAGGACTTGCATTGTGAAAGCTTTGCTGTCCTTGATGTGATCATGGAATCTCTTTCTCAC
TAGAGTCTATGTCACTCATTATACTCTGTCGAATGTCATTGAATGTCTTTACATGGGCTT
ATATGCCTATGAAAATTGTAATACAACTTTCAGCAACGGATCTCTTGGCTCTCGCATCGA
TGAAGAACGCAGCGAAATGCGATAAGTAATGTGAATTGCAGAATTCAGTGAATCATCGAA
TCTTTGAACGCATCTTGCGCTCCTTGGTATTCCGAGGAGCATGCCTGTTTGAGTGTCATT
AAATTCTCAACTCTCTTCTAC--TTTTTGTAAAAGAGAGCTTGGACTGTGGAGGCTTGCT
GGCCACTTTTTGGGGTCAGCTCCTCTGAAATGCATTAGCGGAACCGTTTGCGATCTGCCA
CAAGTGTGATAAGTTATCTACACTGGCGAGGGGATTGCTCTCTGTAATGTTCAGCTTCTA
ATTGTCTCTACTTTGTGAGACTACTTTTGAATGCTTGACCTCAAATCAGGTAGGACTACC
CGCTGAACTTAA
>ABC3-8
TTTCCGTAGGTGAACCTGCGGAAGGATCATTATTGAATTATGTTTCTAGATAGGTTGTAG
CTGGCTC-TTTAGAGCATGTGCACGCCTGTTTGGACTTCATTTTCATCCACCTGTGCACC
TATTGTAGTCTTTGGTTGGGTTAGGAGGAAGTGGTCATTGTGTCAGCATCTGCTGGATGT
GAGGACTTGCATTGTGAAAGCTTTGCTGTCCTTGATGTGATCATGGAATCTCTTTCTCAC
TAGAGTCTATGTCACTCATTATACTCTGTCGAATGTCATTGAATGTCTTTACATGGGCTT
ATATGCCTATGAAAATTGTAATACAACTTTCAGCAACGGATCTCTTGGCTCTCGCATCGA
TGAAGAACGCAGCGAAATGCGATAAGTAATGTGAATTGCAGAATTCAGTGAATCATCGAA
TCTTTGAACGCATCTTGCGCTCCTTGGTATTCCGAGGAGCATGCCTGTTTGAGTGTCATT
AAATTCTCAACTCTCTTCTAC--TTTTTGTAAAAGAGAGCTTGGACTGTGGAGGCTTGCT
GGCCACTTTTTGGGGTCAGCTCCTCTGAAATGCATTAGCGGAACCGTTTGCGATCTGCCA
CAAGTGTGATAAGTTATCTACACTGGCGAGGGGATTGCTCTCTGTAATGTTCAGCTTCTA
ATTGTCTCTACTTTGTGAGACTACTTTTGAATGCTTGACCTCAAATCAGGTAGGACTACC
CGCTGAACTTAA
>ABC3-27
TTTCCGTAGGTGAACCTGCGGAAGGATCATTATTGAATTATGTTTCTAGATAGGTTGTAG
CTGGCTC-TTTAGAGCATGTGCACGCCTGTTTGGACTTCATTTTCATCCACCTGTGCACC
TATTGTAGTCTTTGGTTGGGTTAGGAGGAAGTGGTCATTGTGTCAGCATCTGCTGGATGT
GAGGACTTGCATTGTGAAAGCTTTGCTGTCCTTGATGTGATCATGGAATCTCTTTCTCAC
TAGAGTCTATGTCACTCATTATACTCTGTCGAATGTCATTGAATGTCTTTACATGGGCTT
ATATGCCTATGAAAATTGTAATACAACTTTCAGCAACGGATCTCTTGGCTCTCGCATCGA
TGAAGAACGCAGCGAAATGCGATAAGTAATGTGAATTGCAGAATTCAGTGAATCATCGAA
TCTTTGAACGCATCTTGCGCTCCTTGGTATTCCGAGGAGCATGCCTGTTTGAGTGTCATT
AAATTCTCAACTCTCTTCTAC--TTTTTGTAAAAGAGAGCTTGGACTGTGGAGGCTTGCT
GGCCACTTTTTGGGGTCAGCTCCTCTGAAATGCATTAGCGGAACCGTTTGCGATCTGCCA
CAAGTGTGATAAGTTATCTACACTGGCGAGGGGATTGCTCTCTGTAATGTTCAGCTTCTA

ATTGTCTCTACTTTGTGAGACTACTTTTGAATGCTTGACCTCAAATCAGGTAGGACTACC
CGCTGAACTTAA
>ABC3-31
TTTCCGTAGGTGAACCTGCGGAAGGATCATTATTGAATTATGTTTCTAGATAGGTTGTAG
CTGGCTC-TTTAGAGCATGTGCACGCCTGTTTGGACTTCATTTTCATCCACCTGTGCACC
TATTGTAGTCTTTGGTTGGGTTAGGAGGAAGTGGTCATTGTGTCAGCATCTGCTGGATGT
GAGGACTTGCATTGTGAAAGCTTTGCTGTCCTTGATGTGATCATGGAATCTCTTTCTCAC
TAGAGTCTATGTCACTCATTATACTCTGTCGAATGTCATTGAATGTCTTTACATGGGCTT
ATATGCCTATGAAAATTGTAATACAACTTTCAGCAACGGATCTCTTGGCTCTCGCATCGA
TGAAGAACGCAGCGAAATGCGATAAGTAATGTGAATTGCAGAATTCAGTGAATCATCGAA
TCTTTGAACGCATCTTGCGCTCCTTGGTATTCCGAGGAGCATGCCTGTTTGAGTGTCATT
AAATTCTCAACTCTCTTCTAC--TTTTTGTAAAAGAGAGCTTGGACTGTGGAGGCTTGCT
GGCCACTTTTTGGGGTCAGCTCCTCTGAAATGCATTAGCGGAACCGTTTGCGATCTGCCA
CAAGTGTGATAAGTTATCTACACTGGCGAGGGGATTGCTCTCTGTAATGTTCAGCTTCTA
ATTGTCTCTACTTTGTGAGACTACTTTTGAATGCTTGACCTCAAATCAGGTAGGACTACC
CGCTGAACTTAA
>ABC3-50
TTTCCGTAGGTGAACCTGCGGAAGGATCATTATTGAATTATGTTTCTAGATAGGTTGTAG
CTGGCTC-TTTAGAGCATGTGCACGCCTGTTTGGACTTCATTTTCATCCACCTGTGCACC
TATTGTAGTCTTTGGTTGGGTTAGGAGGAAGTGGTCATTGTGTCAGCATCTGCTGGATGT
GAGGACTTGCATTGTGAAAGCTTTGCTGTCCTTGATGTGATCATGGAATCTCTTTCTCAC
TAGAGTCTATGTCACTCATTATACTCTGTCGAATGTCATTGAATGTCTTTACATGGGCTT
ATATGCCTATGAAAATTGTAATACAACTTTCAGCAACGGATCTCTTGGCTCTCGCATCGA
TGAAGAACGCAGCGAAATGCGATAAGTAATGTGAATTGCAGAATTCAGTGAATCATCGAA
TCTTTGAACGCATCTTGCGCTCCTTGGTATTCCGAGGAGCATGCCTGTTTGAGTGTCATT
AAATTCTCAACTCTCTTCTAC--TTTTTGTAAAAGAGAGCTTGGACTGTGGAGGCTTGCT
GGCCACTTTTTGGGGTCAGCTCCTCTGAAATGCATTAGCGGAACCGTTTGCGATCTGCCA
CAAGTGTGATAAGTTATCTACACTGGCGAGGGGATTGCTCTCTGTAATGTTCAGCTTCTA
ATTGTCTCTACTTTGTGAGACTACTTTTGAATGCTTGACCTCAAATCAGGTAGGACTACC
CGCTGAACTTAA
>ABC3-69
TTTCCGTAGGTGAACCTGCGGAAGGATCATTATTGAATTATGTTTCTAGATAGGTTGTAG
CTGGCTC-TTTAGAGCATGTGCACGCCTGTTTGGACTTCATTTTCATCCACCTGTGCACC
TATTGTAGTCTTTGGTTGGGTTAGGAGGAAGTGGTCATTGTGTCAGCATCTGCTGGATGT
GAGGACTTGCATTGTGAAAGCTTTGCTGTCCTTGATGTGATCATGGAATCTCTTTCTCAC
TAGAGTCTATGTCACTCATTATACTCTGTCGAATGTCATTGAATGTCTTTACATGGGCTT
ATATGCCTATGAAAATTGTAATACAACTTTCAGCAACGGATCTCTTGGCTCTCGCATCGA
TGAAGAACGCAGCGAAATGCGATAAGTAATGTGAATTGCAGAATTCAGTGAATCATCGAA
TCTTTGAACGCATCTTGCGCTCCTTGGTATTCCGAGGAGCATGCCTGTTTGAGTGTCATT
AAATTCTCAACTCTCTTCTAC--TTTTTGTAAAAGAGAGCTTGGACTGTGGAGGCTTGCT
GGCCACTTTTTGGGGTCAGCTCCTCTGAAATGCATTAGCGGAACCGTTTGCGATCTGCCA
CAAGTGTGATAAGTTATCTACACTGGCGAGGGGATTGCTCTCTGTAATGTTCAGCTTCTA
ATTGTCTCTACTTTGTGAGACTACTTTTGAATGCTTGACCTCAAATCAGGTAGGACTACC
CGCTGAACTTAA
>ABC4-4
TTTCCGTAGGTGAACCTGCGGAAGGATCATTATTGAATTATGTTTCTAGATAGGTTGTAG
CTGGCTC-TTTAGAGCATGTGCACGCCTGTTTGGACTTCATTTTCATCCACCTGTGCACC
TATTGTAGTCTTTGGTTGGGTTAGGAGGAAGTGGTCATTGTGTCAGCATCTGCTGGATGT
GAGGACTTGCATTGTGAAAGCTTTGCTGTCCTTGATGTGATCATGGAATCTCTTTCTCAC
TAGAGTCTATGTCACTCATTATACTCTGTCGAATGTCATTGAATGTCTTTACATGGGCTT

ATATGCCTATGAAAATTGTAATACAACTTTCAGCAACGGATCTCTTGGCTCTCGCATCGA
TGAAGAACGCAGCGAAATGCGATAAGTAATGTGAATTGCAGAATTCAGTGAATCATCGAA
TCTTTGAACGCATCTTGCGCTCCTTGGTATTCCGAGGAGCATGCCTGTTTGAGTGTCATT
AAATTCTCAACTCTCTTCTAC--TTTTTGTAAAAGAGAGCTTGGACTGTGGAGGCTTGCT
GGCCACTTTTTGGGGTCAGCTCCTCTGAAATGCATTAGCGGAACCGTTTGCGATCTGCCA
CAAGTGTGATAAGTTATCTACACTGGCGAGGGGATTGCTCTCTGTAATGTTCAGCTTCTA
ATTGTCTCTACTTTGTGAGACTACTTTTGAATGCTTGACCTCAAATCAGGTAGGACTACC
CGCTGAACTTAA
>ABC4-9
TTTCCGTAGGTGAACCTGCGGAAGGATCATTATTGAATTATGTTTCTAGATAGGTTGTAG
CTGGCTC-TTTAGAGCATGTGCACGCCTGTTTGGACTTCATTTTCATCCACCTGTGCACC
TATTGTAGTCTTTGGTTGGGTTAGGAGGAAGTGGTCATTGTGTCAGCATCTGCTGGATGT
GAGGACTTGCATTGTGAAAGCTTTGCTGTCCTTGATGTGATCATGGAATCTCTTTCTCAC
TAGAGTCTATGTCACTCATTATACTCTGTCGAATGTCATTGAATGTCTTTACATGGGCTT
ATATGCCTATGAAAATTGTAATACAACTTTCAGCAACGGATCTCTTGGCTCTCGCATCGA
TGAAGAACGCAGCGAAATGCGATAAGTAATGTGAATTGCAGAATTCAGTGAATCATCGAA
TCTTTGAACGCATCTTGCGCTCCTTGGTATTCCGAGGAGCATGCCTGTTTGAGTGTCATT
AAATTCTCAACTCTCTTCTAC--TTTTTGTAAAAGAGAGCTTGGACTGTGGAGGCTTGCT
GGCCACTTTTTGGGGTCAGCTCCTCTGAAATGCATTAGCGGAACCGTTTGCGATCTGCCA
CAAGTGTGATAAGTTATCTACACTGGCGAGGGGATTGCTCTCTGTAATGTTCAGCTTCTA
ATTGTCTCTACTTTGTGAGACTACTTTTGAATGCTTGACCTCAAATCAGGTAGGACTACC
CGCTGAACTTAA
>ABC4-12
TTTCCGTAGGTGAACCTGCGGAAGGATCATTATTGAATTATGTTTCTAGATAGGTTGTAG
CTGGCTC-TTTAGAGCATGTGCACGCCTGTTTGGACTTCATTTTCATCCACCTGTGCACC
TATTGTAGTCTTTGGTTGGGTTAGGAGGAAGTGGTCATTGTGTCAGCATCTGCTGGATGT
GAGGACTTGCATTGTGAAAGCTTTGCTGTCCTTGATGTGATCATGGAATCTCTTTCTCAC
TAGAGTCTATGTCACTCATTATACTCTGTCGAATGTCATTGAATGTCTTTACATGGGCTT
ATATGCCTATGAAAATTGTAATACAACTTTCAGCAACGGATCTCTTGGCTCTCGCATCGA
TGAAGAACGCAGCGAAATGCGATAAGTAATGTGAATTGCAGAATTCAGTGAATCATCGAA
TCTTTGAACGCATCTTGCGCTCCTTGGTATTCCGAGGAGCATGCCTGTTTGAGTGTCATT
AAATTCTCAACTCTCTTCTAC--TTTTTGTAAAAGAGAGCTTGGACTGTGGAGGCTTGCT
GGCCACTTTTTGGGGTCAGCTCCTCTGAAATGCATTAGCGGAACCGTTTGCGATCTGCCA
CAAGTGTGATAAGTTATCTACACTGGCGAGGGGATTGCTCTCTGTAATGTTCAGCTTCTA
ATTGTCTCTACTTTGTGAGACTACTTTTGAATGCTTGACCTCAAATCAGGTAGGACTACC
CGCTGAACTTAA
>ABC4-16
TTTCCGTAGGTGAACCTGCGGAAGGATCATTATTGAATTATGTTTCTAGATAGGTTGTAG
CTGGCTC-TTTAGAGCATGTGCACGCCTGTTTGGACTTCATTTTCATCCACCTGTGCACC
TATTGTAGTCTTTGGTTGGGTTAGGAGGAAGTGGTCATTGTGTCAGCATCTGCTGGATGT
GAGGACTTGCATTGTGAAAGCTTTGCTGTCCTTGATGTGATCATGGAATCTCTTTCTCAC
TAGAGTCTATGTCACTCATTATACTCTGTCGAATGTCATTGAATGTCTTTACATGGGCTT
ATATGCCTATGAAAATTGTAATACAACTTTCAGCAACGGATCTCTTGGCTCTCGCATCGA
TGAAGAACGCAGCGAAATGCGATAAGTAATGTGAATTGCAGAATTCAGTGAATCATCGAA
TCTTTGAACGCATCTTGCGCTCCTTGGTATTCCGAGGAGCATGCCTGTTTGAGTGTCATT
AAATTCTCAACTCTCTTCTAC--TTTTTGTAAAAGAGAGCTTGGACTGTGGAGGCTTGCT
GGCCACTTTTTGGGGTCAGCTCCTCTGAAATGCATTAGCGGAACCGTTTGCGATCTGCCA
CAAGTGTGATAAGTTATCTACACTGGCGAGGGGATTGCTCTCTGTAATGTTCAGCTTCTA
ATTGTCTCTACTTTGTGAGACTACTTTTGAATGCTTGACCTCAAATCAGGTAGGACTACC
CGCTGAACTTAA

>ABC4-17
TTTCCGTAGGTGAACCTGCGGAAGGATCATTATTGAATTATGTTTCTAGATAGGTTGTAG
CTGGCTC-TTTAGAGCATGTGCACGCCTGTTTGGACTTCATTTTCATCCACCTGTGCACC
TATTGTAGTCTTTGGTTGGGTTAGGAGGAAGTGGTCATTGTGTCAGCATCTGCTGGATGT
GAGGACTTGCATTGTGAAAGCTTTGCTGTCCTTGATGTGATCATGGAATCTCTTTCTCAC
TAGAGTCTATGTCACTCATTATACTCTGTCGAATGTCATTGAATGTCTTTACATGGGCTT
ATATGCCTATGAAAATTGTAATACAACTTTCAGCAACGGATCTCTTGGCTCTCGCATCGA
TGAAGAACGCAGCGAAATGCGATAAGTAATGTGAATTGCAGAATTCAGTGAATCATCGAA
TCTTTGAACGCATCTTGCGCTCCTTGGTATTCCGAGGAGCATGCCTGTTTGAGTGTCATT
AAATTCTCAACTCTCTTCTAC--TTTTTGTAAAAGAGAGCTTGGACTGTGGAGGCTTGCT
GGCCACTTTTTGGGGTCAGCTCCTCTGAAATGCATTAGCGGAACCGTTTGCGATCTGCCA
CAAGTGTGATAAGTTATCTACACTGGCGAGGGGATTGCTCTCTGTAATGTTCAGCTTCTA
ATTGTCTCTACTTTGTGAGACTACTTTTGAATGCTTGACCTCAAATCAGGTAGGACTACC
CGCTGAACTTAA
>ABC4-36
TTTCCGTAGGTGAACCTGCGGAAGGATCATTATTGAATTATGTTTCTAGATAGGTTGTAG
CTGGCTC-TTTAGAGCATGTGCACGCCTGTTTGGACTTCATTTTCATCCACCTGTGCACC
TATTGTAGTCTTTGGTTGGGTTAGGAGGAAGTGGTCATTGTGTCAGCATCTGCTGGATGT
GAGGACTTGCATTGTGAAAGCTTTGCTGTCCTTGATGTGATCATGGAATCTCTTTCTCAC
TAGAGTCTATGTCACTCATTATACTCTGTCGAATGTCATTGAATGTCTTTACATGGGCTT
ATATGCCTATGAAAATTGTAATACAACTTTCAGCAACGGATCTCTTGGCTCTCGCATCGA
TGAAGAACGCAGCGAAATGCGATAAGTAATGTGAATTGCAGAATTCAGTGAATCATCGAA
TCTTTGAACGCATCTTGCGCTCCTTGGTATTCCGAGGAGCATGCCTGTTTGAGTGTCATT
AAATTCTCAACTCTCTTCTAC--TTTTTGTAAAAGAGAGCTTGGACTGTGGAGGCTTGCT
GGCCACTTTTTGGGGTCAGCTCCTCTGAAATGCATTAGCGGAACCGTTTGCGATCTGCCA
CAAGTGTGATAAGTTATCTACACTGGCGAGGGGATTGCTCTCTGTAATGTTCAGCTTCTA
ATTGTCTCTACTTTGTGAGACTACTTTTGAATGCTTGACCTCAAATCAGGTAGGACTACC
CGCTGAACTTAA
>ABC4-46
TTTCCGTAGGTGAACCTGCGGAAGGATCATTATTGAATTATGTTTCTAGATAGGTTGTAG
CTGGCTC-TTTAGAGCATGTGCACGCCTGTTTGGACTTCATTTTCATCCACCTGTGCACC
TATTGTAGTCTTTGGTTGGGTTAGGAGGAAGTGGTCATTGTGTCAGCATCTGCTGGATGT
GAGGACTTGCATTGTGAAAGCTTTGCTGTCCTTGATGTGATCATGGAATCTCTTTCTCAC
TAGAGTCTATGTCACTCATTATACTCTGTCGAATGTCATTGAATGTCTTTACATGGGCTT
ATATGCCTATGAAAATTGTAATACAACTTTCAGCAACGGATCTCTTGGCTCTCGCATCGA
TGAAGAACGCAGCGAAATGCGATAAGTAATGTGAATTGCAGAATTCAGTGAATCATCGAA
TCTTTGAACGCATCTTGCGCTCCTTGGTATTCCGAGGAGCATGCCTGTTTGAGTGTCATT
AAATTCTCAACTCTCTTCTAC--TTTTTGTAAAAGAGAGCTTGGACTGTGGAGGCTTGCT
GGCCACTTTTTGGGGTCAGCTCCTCTGAAATGCATTAGCGGAACCGTTTGCGATCTGCCA
CAAGTGTGATAAGTTATCTACACTGGCGAGGGGATTGCTCTCTGTAATGTTCAGCTTCTA
ATTGTCTCTACTTTGTGAGACTACTTTTGAATGCTTGACCTCAAATCAGGTAGGACTACC
CGCTGAACTTAA
>ABC4-50
TTTCCGTAGGTGAACCTGCGGAAGGATCATTATTGAATTATGTTTCTAGATAGGTTGTAG
CTGGCTC-TTTAGAGCATGTGCACGCCTGTTTGGACTTCATTTTCATCCACCTGTGCACC
TATTGTAGTCTTTGGTTGGGTTAGGAGGAAGTGGTCATTGTGTCAGCATCTGCTGGATGT
GAGGACTTGCATTGTGAAAGCTTTGCTGTCCTTGATGTGATCATGGAATCTCTTTCTCAC
TAGAGTCTATGTCACTCATTATACTCTGTCGAATGTCATTGAATGTCTTTACATGGGCTT
ATATGCCTATGAAAATTGTAATACAACTTTCAGCAACGGATCTCTTGGCTCTCGCATCGA
TGAAGAACGCAGCGAAATGCGATAAGTAATGTGAATTGCAGAATTCAGTGAATCATCGAA

TCTTTGAACGCATCTTGCGCTCCTTGGTATTCCGAGGAGCATGCCTGTTTGAGTGTCATT
AAATTCTCAACTCTCTTCTAC--TTTTTGTAAAAGAGAGCTTGGACTGTGGAGGCTTGCT
GGCCACTTTTTGGGGTCAGCTCCTCTGAAATGCATTAGCGGAACCGTTTGCGATCTGCCA
CAAGTGTGATAAGTTATCTACACTGGCGAGGGGATTGCTCTCTGTAATGTTCAGCTTCTA
ATTGTCTCTACTTTGTGAGACTACTTTTGAATGCTTGACCTCAAATCAGGTAGGACTACC
CGCTGAACTTAA
>ABC4-62
TTTCCGTAGGTGAACCTGCGGAAGGATCATTATTGAATTATGTTTCTAGATAGGTTGTAG
CTGGCTC-TTTAGAGCATGTGCACGCCTGTTTGGACTTCATTTTCATCCACCTGTGCACC
TATTGTAGTCTTTGGTTGGGTTAGGAGGAAGTGGTCATTGTGTCAGCATCTGCTGGATGT
GAGGACTTGCATTGTGAAAGCTTTGCTGTCCTTGATGTGATCATGGAATCTCTTTCTCAC
TAGAGTCTATGTCACTCATTATACTCTGTCGAATGTCATTGAATGTCTTTACATGGGCTT
ATATGCCTATGAAAATTGTAATACAACTTTCAGCAACGGATCTCTTGGCTCTCGCATCGA
TGAAGAACGCAGCGAAATGCGATAAGTAATGTGAATTGCAGAATTCAGTGAATCATCGAA
TCTTTGAACGCATCTTGCGCTCCTTGGTATTCCGAGGAGCATGCCTGTTTGAGTGTCATT
AAATTCTCAACTCTCTTCTAC--TTTTTGTAAAAGAGAGCTTGGACTGTGGAGGCTTGCT
GGCCACTTTTTGGGGTCAGCTCCTCTGAAATGCATTAGCGGAACCGTTTGCGATCTGCCA
CAAGTGTGATAAGTTATCTACACTGGCGAGGGGATTGCTCTCTGTAATGTTCAGCTTCTA
ATTGTCTCTACTTTGTGAGACTACTTTTGAATGCTTGACCTCAAATCAGGTAGGACTACC
CGCTGAACTTAA
>ABC4-75
TTTCCGTAGGTGAACCTGCGGAAGGATCATTATTGAATTATGTTTCTAGATAGGTTGTAG
CTGGCTC-TTTAGAGCATGTGCACGCCTGTTTGGACTTCATTTTCATCCACCTGTGCACC
TATTGTAGTCTTTGGTTGGGTTAGGAGGAAGTGGTCATTGTGTCAGCATCTGCTGGATGT
GAGGACTTGCATTGTGAAAGCTTTGCTGTCCTTGATGTGATCATGGAATCTCTTTCTCAC
TAGAGTCTATGTCACTCATTATACTCTGTCGAATGTCATTGAATGTCTTTACATGGGCTT
ATATGCCTATGAAAATTGTAATACAACTTTCAGCAACGGATCTCTTGGCTCTCGCATCGA
TGAAGAACGCAGCGAAATGCGATAAGTAATGTGAATTGCAGAATTCAGTGAATCATCGAA
TCTTTGAACGCATCTTGCGCTCCTTGGTATTCCGAGGAGCATGCCTGTTTGAGTGTCATT
AAATTCTCAACTCTCTTCTAC--TTTTTGTAAAAGAGAGCTTGGACTGTGGAGGCTTGCT
GGCCACTTTTTGGGGTCAGCTCCTCTGAAATGCATTAGCGGAACCGTTTGCGATCTGCCA
CAAGTGTGATAAGTTATCTACACTGGCGAGGGGATTGCTCTCTGTAATGTTCAGCTTCTA
ATTGTCTCTACTTTGTGAGACTACTTTTGAATGCTTGACCTCAAATCAGGTAGGACTACC
CGCTGAACTTAA
>ABC5-21
TTTCCGTAGGTGAACCTGCGGAAGGATCATTATTGAATTATGTTTCTAGATAGGTTGTAG
CTGGCTC-TTTAGAGCATGTGCACGCCTGTTTGGACTTCATTTTCATCCACCTGTGCACC
TATTGTAGTCTTTGGTTGGGTTAGGAGGAAGTGGTCATTGTGTCAGCATCTGCTGGATGT
GAGGACTTGCATTGTGAAAGCTTTGCTGTCCTTGATGTGATCATGGAATCTCTTTCTCAC
TAGAGTCTATGTCACTCATTATACTCTGTCGAATGTCATTGAATGTCTTTACATGGGCTT
ATATGCCTATGAAAATTGTAATACAACTTTCAGCAACGGATCTCTTGGCTCTCGCATCGA
TGAAGAACGCAGCGAAATGCGATAAGTAATGTGAATTGCAGAATTCAGTGAATCATCGAA
TCTTTGAACGCATCTTGCGCTCCTTGGTATTCCGAGGAGCATGCCTGTTTGAGTGTCATT
AAATTCTCAACTCTCTTCTAC--TTTTTGTAAAAGAGAGCTTGGACTGTGGAGGCTTGCT
GGCCACTTTTTGGGGTCAGCTCCTCTGAAATGCATTAGCGGAACCGTTTGCGATCTGCCA
CAAGTGTGATAAGTTATCTACACTGGCGAGGGGATTGCTCTCTGTAATGTTCAGCTTCTA
ATTGTCTCTACTTTGTGAGACTACTTTTGAATGCTTGACCTCAAATCAGGTAGGACTACC
CGCTGAACTTAA
>ABC5-25
TTTCCGTAGGTGAACCTGCGGAAGGATCATTATTGAATTATGTTTCTAGATAGGTTGTAG

CTGGCTC-TTTAGAGCATGTGCACGCCTGTTTGGACTTCATTTTCATCCACCTGTGCACC
TATTGTAGTCTTTGGTTGGGTTAGGAGGAAGTGGTCATTGTGTCAGCATCTGCTGGATGT
GAGGACTTGCATTGTGAAAGCTTTGCTGTCCTTGATGTGATCATGGAATCTCTTTCTCAC
TAGAGTCTATGTCACTCATTATACTCTGTCGAATGTCATTGAATGTCTTTACATGGGCTT
ATATGCCTATGAAAATTGTAATACAACTTTCAGCAACGGATCTCTTGGCTCTCGCATCGA
TGAAGAACGCAGCGAAATGCGATAAGTAATGTGAATTGCAGAATTCAGTGAATCATCGAA
TCTTTGAACGCATCTTGCGCTCCTTGGTATTCCGAGGAGCATGCCTGTTTGAGTGTCATT
AAATTCTCAACTCTCTTCTAC--TTTTTGTAAAAGAGAGCTTGGACTGTGGAGGCTTGCT
GGCCACTTTTTGGGGTCAGCTCCTCTGAAATGCATTAGCGGAACCGTTTGCGATCTGCCA
CAAGTGTGATAAGTTATCTACACTGGCGAGGGGATTGCTCTCTGTAATGTTCAGCTTCTA
ATTGTCTCTACTTTGTGAGACTACTTTTGAATGCTTGACCTCAAATCAGGTAGGACTACC
CGCTGAACTTAA
>ABC2-51
TTTCCGTAGGTGAACCTGCGGAAGGATCATTATTGAATTATGTTTCTAGATAGGTTGTAG
CTGGCTC-TTTAGAGCATGTGCACGCCTGTTTGGACTTCATTTTCATCCACCTGTGCACC
TATTGTAGTCTTTGGTTGGGTTAGGAGGAAGTGGTCATTGTGTCAGCATCTGCTGGATGT
GAGGACTTGCATTGTGAAAGCTTTGCTGTCCTTGATGTGATCATGGAATCTCTTTCTCAC
TAGAGTCTATGTCACTCATTATACTCTGTCGAATGTCATTGAATGTCTTTACATGGGCTT
ATATGCCTATGAAAATTGTAATACAACTTTCAGCAACGGATCTCTTGGCTCTCGCATCGA
TGAAGAACGCAGCGAAATGCGATAAGTAATGTGAATTGCAGAATTCAGTGAATCATCGAA
TCTTTGAACGCATCTTGCGCTCCTTGGTATTCCGAGGAGCATGCCTGTTTGAGTGTCATT
AAATTCTCAACTCTCTTCTAC--TTTTTGTAAAAGAGAGCTTGGACTGTGGAGGCTTGCT
GGCCACTTTTTGGGGTCAGCTCCTCTGAAATGCATTAGCGGAACCGTTTGCGATCTGCCA
CAAGTGTGATAAGTTATCTACACTGGCGAGGGGATTGCTCTCTGTAATGTTCAGCTTCTA
ATTGTCTCTACTTTGTGAGACTACTTTTGAATGCTTGACCTCAAATCAGGTAGGACTACC
CGCTGAACTTAA
>ABC5-27
TTTCCGTAGGTGAACCTGCGGAAGGATCATTATTGAATTATGTTTCTAGATAGGTTGTAG
CTGGCTC-TTTAGAGCATGTGCACGCCTGTTTGGACTTCATTTTCATCCACCTGTGCACC
TATTGTAGTCTTTGGTTGGGTTAGGAGGAAGTGGTCATTGTGTCAGCATCTGCTGGATGT
GAGGACTTGCATTGTGAAAGCTTTGCTGTCCTTGATGTGATCATGGAATCTCTTTCTCAC
TAGAGTCTATGTCACTCATTATACTCTGTCGAATGTCATTGAATGTCTTTACATGGGCTT
ATATGCCTATGAAAATTGTAATACAACTTTCAGCAACGGATCTCTTGGCTCTCGCATCGA
TGAAGAACGCAGCGAAATGCGATAAGTAATGTGAATTGCAGAATTCAGTGAATCATCGAA
TCTTTGAACGCATCTTGCGCTCCTTGGTATTCCGAGGAGCATGCCTGTTTGAGTGTCATT
AAATTCTCAACTCTCTTCTAC--TTTTTGTAAAAGAGAGCTTGGACTGTGGAGGCTTGCT
GGCCACTTTTTGGGGTCAGCTCCTCTGAAATGCATTAGCGGAACCGTTTGCGATCTGCCA
CAAGTGTGATAAGTTATCTACACTGGCGAGGGGATTGCTCTCTGTAATGTTCAGCTTCTA
ATTGTCTCTACTTTGTGAGACTACTTTTGAATGCTTGACCTCAAATCAGGTAGGACTACC
CGCTGAACTTAA
>ABC5-29
TTTCCGTAGGTGAACCTGCGGAAGGATCATTATTGAATTATGTTTCTAGATAGGTTGTAG
CTGGCTC-TTTAGAGCATGTGCACGCCTGTTTGGACTTCATTTTCATCCACCTGTGCACC
TATTGTAGTCTTTGGTTGGGTTAGGAGGAAGTGGTCATTGTGTCAGCATCTGCTGGATGT
GAGGACTTGCATTGTGAAAGCTTTGCTGTCCTTGATGTGATCATGGAATCTCTTTCTCAC
TAGAGTCTATGTCACTCATTATACTCTGTCGAATGTCATTGAATGTCTTTACATGGGCTT
ATATGCCTATGAAAATTGTAATACAACTTTCAGCAACGGATCTCTTGGCTCTCGCATCGA
TGAAGAACGCAGCGAAATGCGATAAGTAATGTGAATTGCAGAATTCAGTGAATCATCGAA
TCTTTGAACGCATCTTGCGCTCCTTGGTATTCCGAGGAGCATGCCTGTTTGAGTGTCATT
AAATTCTCAACTCTCTTCTAC--TTTTTGTAAAAGAGAGCTTGGACTGTGGAGGCTTGCT

GGCCACTTTTTGGGGTCAGCTCCTCTGAAATGCATTAGCGGAACCGTTTGCGATCTGCCA
CAAGTGTGATAAGTTATCTACACTGGCGAGGGGATTGCTCTCTGTAATGTTCAGCTTCTA
ATTGTCTCTACTTTGTGAGACTACTTTTGAATGCTTGACCTCAAATCAGGTAGGACTACC
CGCTGAACTTAA
>ABC5-31
TTTCCGTAGGTGAACCTGCGGAAGGATCATTATTGAATTATGTTTCTAGATAGGTTGTAG
CTGGCTC-TTTAGAGCATGTGCACGCCTGTTTGGACTTCATTTTCATCCACCTGTGCACC
TATTGTAGTCTTTGGTTGGGTTAGGAGGAAGTGGTCATTGTGTCAGCATCTGCTGGATGT
GAGGACTTGCATTGTGAAAGCTTTGCTGTCCTTGATGTGATCATGGAATCTCTTTCTCAC
TAGAGTCTATGTCACTCATTATACTCTGTCGAATGTCATTGAATGTCTTTACATGGGCTT
ATATGCCTATGAAAATTGTAATACAACTTTCAGCAACGGATCTCTTGGCTCTCGCATCGA
TGAAGAACGCAGCGAAATGCGATAAGTAATGTGAATTGCAGAATTCAGTGAATCATCGAA
TCTTTGAACGCATCTTGCGCTCCTTGGTATTCCGAGGAGCATGCCTGTTTGAGTGTCATT
AAATTCTCAACTCTCTTCTAC--TTTTTGTAAAAGAGAGCTTGGACTGTGGAGGCTTGCT
GGCCACTTTTTGGGGTCAGCTCCTCTGAAATGCATTAGCGGAACCGTTTGCGATCTGCCA
CAAGTGTGATAAGTTATCTACACTGGCGAGGGGATTGCTCTCTGTAATGTTCAGCTTCTA
ATTGTCTCTACTTTGTGAGACTACTTTTGAATGCTTGACCTCAAATCAGGTAGGACTACC
CGCTGAACTTAA
>ABC5-33
TTTCCGTAGGTGAACCTGCGGAAGGATCATTATTGAATTATGTTTCTAGATAGGTTGTAG
CTGGCTC-TTTAGAGCATGTGCACGCCTGTTTGGACTTCATTTTCATCCACCTGTGCACC
TATTGTAGTCTTTGGTTGGGTTAGGAGGAAGTGGTCATTGTGTCAGCATCTGCTGGATGT
GAGGACTTGCATTGTGAAAGCTTTGCTGTCCTTGATGTGATCATGGAATCTCTTTCTCAC
TAGAGTCTATGTCACTCATTATACTCTGTCGAATGTCATTGAATGTCTTTACATGGGCTT
ATATGCCTATGAAAATTGTAATACAACTTTCAGCAACGGATCTCTTGGCTCTCGCATCGA
TGAAGAACGCAGCGAAATGCGATAAGTAATGTGAATTGCAGAATTCAGTGAATCATCGAA
TCTTTGAACGCATCTTGCGCTCCTTGGTATTCCGAGGAGCATGCCTGTTTGAGTGTCATT
AAATTCTCAACTCTCTTCTAC--TTTTTGTAAAAGAGAGCTTGGACTGTGGAGGCTTGCT
GGCCACTTTTTGGGGTCAGCTCCTCTGAAATGCATTAGCGGAACCGTTTGCGATCTGCCA
CAAGTGTGATAAGTTATCTACACTGGCGAGGGGATTGCTCTCTGTAATGTTCAGCTTCTA
ATTGTCTCTACTTTGTGAGACTACTTTTGAATGCTTGACCTCAAATCAGGTAGGACTACC
CGCTGAACTTAA
>ABC5-42
TTTCCGTAGGTGAACCTGCGGAAGGATCATTATTGAATTATGTTTCTAGATAGGTTGTAG
CTGGCTC-TTTAGAGCATGTGCACGCCTGTTTGGACTTCATTTTCATCCACCTGTGCACC
TATTGTAGTCTTTGGTTGGGTTAGGAGGAAGTGGTCATTGTGTCAGCATCTGCTGGATGT
GAGGACTTGCATTGTGAAAGCTTTGCTGTCCTTGATGTGATCATGGAATCTCTTTCTCAC
TAGAGTCTATGTCACTCATTATACTCTGTCGAATGTCATTGAATGTCTTTACATGGGCTT
ATATGCCTATGAAAATTGTAATACAACTTTCAGCAACGGATCTCTTGGCTCTCGCATCGA
TGAAGAACGCAGCGAAATGCGATAAGTAATGTGAATTGCAGAATTCAGTGAATCATCGAA
TCTTTGAACGCATCTTGCGCTCCTTGGTATTCCGAGGAGCATGCCTGTTTGAGTGTCATT
AAATTCTCAACTCTCTTCTAC--TTTTTGTAAAAGAGAGCTTGGACTGTGGAGGCTTGCT
GGCCACTTTTTGGGGTCAGCTCCTCTGAAATGCATTAGCGGAACCGTTTGCGATCTGCCA
CAAGTGTGATAAGTTATCTACACTGGCGAGGGGATTGCTCTCTGTAATGTTCAGCTTCTA
ATTGTCTCTACTTTGTGAGACTACTTTTGAATGCTTGACCTCAAATCAGGTAGGACTACC
CGCTGAACTTAA
>ABC5-70
TTTCCGTAGGTGAACCTGCGGAAGGATCATTATTGAATTATGTTTCTAGATAGGTTGTAG
CTGGCTC-TTTAGAGCATGTGCACGCCTGTTTGGACTTCATTTTCATCCACCTGTGCACC
TATTGTAGTCTTTGGTTGGGTTAGGAGGAAGTGGTCATTGTGTCAGCATCTGCTGGATGT

GAGGACTTGCATTGTGAAAGCTTTGCTGTCCTTGATGTGATCATGGAATCTCTTTCTCAC
TAGAGTCTATGTCACTCATTATACTCTGTCGAATGTCATTGAATGTCTTTACATGGGCTT
ATATGCCTATGAAAATTGTAATACAACTTTCAGCAACGGATCTCTTGGCTCTCGCATCGA
TGAAGAACGCAGCGAAATGCGATAAGTAATGTGAATTGCAGAATTCAGTGAATCATCGAA
TCTTTGAACGCATCTTGCGCTCCTTGGTATTCCGAGGAGCATGCCTGTTTGAGTGTCATT
AAATTCTCAACTCTCTTCTAC--TTTTTGTAAAAGAGAGCTTGGACTGTGGAGGCTTGCT
GGCCACTTTTTGGGGTCAGCTCCTCTGAAATGCATTAGCGGAACCGTTTGCGATCTGCCA
CAAGTGTGATAAGTTATCTACACTGGCGAGGGGATTGCTCTCTGTAATGTTCAGCTTCTA
ATTGTCTCTACTTTGTGAGACTACTTTTGAATGCTTGACCTCAAATCAGGTAGGACTACC
CGCTGAACTTAA
>ABC5-74
TTTCCGTAGGTGAACCTGCGGAAGGATCATTATTGAATTATGTTTCTAGATAGGTTGTAG
CTGGCTC-TTTAGAGCATGTGCACGCCTGTTTGGACTTCATTTTCATCCACCTGTGCACC
TATTGTAGTCTTTGGTTGGGTTAGGAGGAAGTGGTCATTGTGTCAGCATCTGCTGGATGT
GAGGACTTGCATTGTGAAAGCTTTGCTGTCCTTGATGTGATCATGGAATCTCTTTCTCAC
TAGAGTCTATGTCACTCATTATACTCTGTCGAATGTCATTGAATGTCTTTACATGGGCTT
ATATGCCTATGAAAATTGTAATACAACTTTCAGCAACGGATCTCTTGGCTCTCGCATCGA
TGAAGAACGCAGCGAAATGCGATAAGTAATGTGAATTGCAGAATTCAGTGAATCATCGAA
TCTTTGAACGCATCTTGCGCTCCTTGGTATTCCGAGGAGCATGCCTGTTTGAGTGTCATT
AAATTCTCAACTCTCTTCTAC--TTTTTGTAAAAGAGAGCTTGGACTGTGGAGGCTTGCT
GGCCACTTTTTGGGGTCAGCTCCTCTGAAATGCATTAGCGGAACCGTTTGCGATCTGCCA
CAAGTGTGATAAGTTATCTACACTGGCGAGGGGATTGCTCTCTGTAATGTTCAGCTTCTA
ATTGTCTCTACTTTGTGAGACTACTTTTGAATGCTTGACCTCAAATCAGGTAGGACTACC
CGCTGAACTTAA
>ABC5-80
TTTCCGTAGGTGAACCTGCGGAAGGATCATTATTGAATTATGTTTCTAGATAGGTTGTAG
CTGGCTC-TTTAGAGCATGTGCACGCCTGTTTGGACTTCATTTTCATCCACCTGTGCACC
TATTGTAGTCTTTGGTTGGGTTAGGAGGAAGTGGTCATTGTGTCAGCATCTGCTGGATGT
GAGGACTTGCATTGTGAAAGCTTTGCTGTCCTTGATGTGATCATGGAATCTCTTTCTCAC
TAGAGTCTATGTCACTCATTATACTCTGTCGAATGTCATTGAATGTCTTTACATGGGCTT
ATATGCCTATGAAAATTGTAATACAACTTTCAGCAACGGATCTCTTGGCTCTCGCATCGA
TGAAGAACGCAGCGAAATGCGATAAGTAATGTGAATTGCAGAATTCAGTGAATCATCGAA
TCTTTGAACGCATCTTGCGCTCCTTGGTATTCCGAGGAGCATGCCTGTTTGAGTGTCATT
AAATTCTCAACTCTCTTCTAC--TTTTTGTAAAAGAGAGCTTGGACTGTGGAGGCTTGCT
GGCCACTTTTTGGGGTCAGCTCCTCTGAAATGCATTAGCGGAACCGTTTGCGATCTGCCA
CAAGTGTGATAAGTTATCTACACTGGCGAGGGGATTGCTCTCTGTAATGTTCAGCTTCTA
ATTGTCTCTACTTTGTGAGACTACTTTTGAATGCTTGACCTCAAATCAGGTAGGACTACC
CGCTGAACTTAA
>ABC5-85
TTTCCGTAGGTGAACCTGCGGAAGGATCATTATTGAATTATGTTTCTAGATAGGTTGTAG
CTGGCTC-TTTAGAGCATGTGCACGCCTGTTTGGACTTCATTTTCATCCACCTGTGCACC
TATTGTAGTCTTTGGTTGGGTTAGGAGGAAGTGGTCATTGTGTCAGCATCTGCTGGATGT
GAGGACTTGCATTGTGAAAGCTTTGCTGTCCTTGATGTGATCATGGAATCTCTTTCTCAC
TAGAGTCTATGTCACTCATTATACTCTGTCGAATGTCATTGAATGTCTTTACATGGGCTT
ATATGCCTATGAAAATTGTAATACAACTTTCAGCAACGGATCTCTTGGCTCTCGCATCGA
TGAAGAACGCAGCGAAATGCGATAAGTAATGTGAATTGCAGAATTCAGTGAATCATCGAA
TCTTTGAACGCATCTTGCGCTCCTTGGTATTCCGAGGAGCATGCCTGTTTGAGTGTCATT
AAATTCTCAACTCTCTTCTAC--TTTTTGTAAAAGAGAGCTTGGACTGTGGAGGCTTGCT
GGCCACTTTTTGGGGTCAGCTCCTCTGAAATGCATTAGCGGAACCGTTTGCGATCTGCCA
CAAGTGTGATAAGTTATCTACACTGGCGAGGGGATTGCTCTCTGTAATGTTCAGCTTCTA

ATTGTCTCTACTTTGTGAGACTACTTTTGAATGCTTGACCTCAAATCAGGTAGGACTACC
CGCTGAACTTAA
>ABC6-9
TTTCCGTAGGTGAACCTGCGGAAGGATCATTATTGAATTATGTTTCTAGATAGGTTGTAG
CTGGCTC-TTTAGAGCATGTGCACGCCTGTTTGGACTTCATTTTCATCCACCTGTGCACC
TATTGTAGTCTTTGGTTGGGTTAGGAGGAAGTGGTCATTGTGTCAGCATCTGCTGGATGT
GAGGACTTGCATTGTGAAAGCTTTGCTGTCCTTGATGTGATCATGGAATCTCTTTCTCAC
TAGAGTCTATGTCACTCATTATACTCTGTCGAATGTCATTGAATGTCTTTACATGGGCTT
ATATGCCTATGAAAATTGTAATACAACTTTCAGCAACGGATCTCTTGGCTCTCGCATCGA
TGAAGAACGCAGCGAAATGCGATAAGTAATGTGAATTGCAGAATTCAGTGAATCATCGAA
TCTTTGAACGCATCTTGCGCTCCTTGGTATTCCGAGGAGCATGCCTGTTTGAGTGTCATT
AAATTCTCAACTCTCTTCTAC--TTTTTGTAAAAGAGAGCTTGGACTGTGGAGGCTTGCT
GGCCACTTTTTGGGGTCAGCTCCTCTGAAATGCATTAGCGGAACCGTTTGCGATCTGCCA
CAAGTGTGATAAGTTATCTACACTGGCGAGGGGATTGCTCTCTGTAATGTTCAGCTTCTA
ATTGTCTCTACTTTGTGAGACTACTTTTGAATGCTTGACCTCAAATCAGGTAGGACTACC
CGCTGAACTTAA
>ABC6-17
TTTCCGTAGGTGAACCTGCGGAAGGATCATTATTGAATTATGTTTCTAGATAGGTTGTAG
CTGGCTC-TTTAGAGCATGTGCACGCCTGTTTGGACTTCATTTTCATCCACCTGTGCACC
TATTGTAGTCTTTGGTTGGGTTAGGAGGAAGTGGTCATTGTGTCAGCATCTGCTGGATGT
GAGGACTTGCATTGTGAAAGCTTTGCTGTCCTTGATGTGATCATGGAATCTCTTTCTCAC
TAGAGTCTATGTCACTCATTATACTCTGTCGAATGTCATTGAATGTCTTTACATGGGCTT
ATATGCCTATGAAAATTGTAATACAACTTTCAGCAACGGATCTCTTGGCTCTCGCATCGA
TGAAGAACGCAGCGAAATGCGATAAGTAATGTGAATTGCAGAATTCAGTGAATCATCGAA
TCTTTGAACGCATCTTGCGCTCCTTGGTATTCCGAGGAGCATGCCTGTTTGAGTGTCATT
AAATTCTCAACTCTCTTCTAC--TTTTTGTAAAAGAGAGCTTGGACTGTGGAGGCTTGCT
GGCCACTTTTTGGGGTCAGCTCCTCTGAAATGCATTAGCGGAACCGTTTGCGATCTGCCA
CAAGTGTGATAAGTTATCTACACTGGCGAGGGGATTGCTCTCTGTAATGTTCAGCTTCTA
ATTGTCTCTACTTTGTGAGACTACTTTTGAATGCTTGACCTCAAATCAGGTAGGACTACC
CGCTGAACTTAA
>ABC6-18
TTTCCGTAGGTGAACCTGCGGAAGGATCATTATTGAATTATGTTTCTAGATAGGTTGTAG
CTGGCTC-TTTAGAGCATGTGCACGCCTGTTTGGACTTCATTTTCATCCACCTGTGCACC
TATTGTAGTCTTTGGTTGGGTTAGGAGGAAGTGGTCATTGTGTCAGCATCTGCTGGATGT
GAGGACTTGCATTGTGAAAGCTTTGCTGTCCTTGATGTGATCATGGAATCTCTTTCTCAC
TAGAGTCTATGTCACTCATTATACTCTGTCGAATGTCATTGAATGTCTTTACATGGGCTT
ATATGCCTATGAAAATTGTAATACAACTTTCAGCAACGGATCTCTTGGCTCTCGCATCGA
TGAAGAACGCAGCGAAATGCGATAAGTAATGTGAATTGCAGAATTCAGTGAATCATCGAA
TCTTTGAACGCATCTTGCGCTCCTTGGTATTCCGAGGAGCATGCCTGTTTGAGTGTCATT
AAATTCTCAACTCTCTTCTAC--TTTTTGTAAAAGAGAGCTTGGACTGTGGAGGCTTGCT
GGCCACTTTTTGGGGTCAGCTCCTCTGAAATGCATTAGCGGAACCGTTTGCGATCTGCCA
CAAGTGTGATAAGTTATCTACACTGGCGAGGGGATTGCTCTCTGTAATGTTCAGCTTCTA
ATTGTCTCTACTTTGTGAGACTACTTTTGAATGCTTGACCTCAAATCAGGTAGGACTACC
CGCTGAACTTAA
>ABC6-35
TTTCCGTAGGTGAACCTGCGGAAGGATCATTATTGAATTATGTTTCTAGATAGGTTGTAG
CTGGCTC-TTTAGAGCATGTGCACGCCTGTTTGGACTTCATTTTCATCCACCTGTGCACC
TATTGTAGTCTTTGGTTGGGTTAGGAGGAAGTGGTCATTGTGTCAGCATCTGCTGGATGT
GAGGACTTGCATTGTGAAAGCTTTGCTGTCCTTGATGTGATCATGGAATCTCTTTCTCAC
TAGAGTCTATGTCACTCATTATACTCTGTCGAATGTCATTGAATGTCTTTACATGGGCTT

ATATGCCTATGAAAATTGTAATACAACTTTCAGCAACGGATCTCTTGGCTCTCGCATCGA
TGAAGAACGCAGCGAAATGCGATAAGTAATGTGAATTGCAGAATTCAGTGAATCATCGAA
TCTTTGAACGCATCTTGCGCTCCTTGGTATTCCGAGGAGCATGCCTGTTTGAGTGTCATT
AAATTCTCAACTCTCTTCTAC--TTTTTGTAAAAGAGAGCTTGGACTGTGGAGGCTTGCT
GGCCACTTTTTGGGGTCAGCTCCTCTGAAATGCATTAGCGGAACCGTTTGCGATCTGCCA
CAAGTGTGATAAGTTATCTACACTGGCGAGGGGATTGCTCTCTGTAATGTTCAGCTTCTA
ATTGTCTCTACTTTGTGAGACTACTTTTGAATGCTTGACCTCAAATCAGGTAGGACTACC
CGCTGAACTTAA
>ABC6-47
TTTCCGTAGGTGAACCTGCGGAAGGATCATTATTGAATTATGTTTCTAGATAGGTTGTAG
CTGGCTC-TTTAGAGCATGTGCACGCCTGTTTGGACTTCATTTTCATCCACCTGTGCACC
TATTGTAGTCTTTGGTTGGGTTAGGAGGAAGTGGTCATTGTGTCAGCATCTGCTGGATGT
GAGGACTTGCATTGTGAAAGCTTTGCTGTCCTTGATGTGATCATGGAATCTCTTTCTCAC
TAGAGTCTATGTCACTCATTATACTCTGTCGAATGTCATTGAATGTCTTTACATGGGCTT
ATATGCCTATGAAAATTGTAATACAACTTTCAGCAACGGATCTCTTGGCTCTCGCATCGA
TGAAGAACGCAGCGAAATGCGATAAGTAATGTGAATTGCAGAATTCAGTGAATCATCGAA
TCTTTGAACGCATCTTGCGCTCCTTGGTATTCCGAGGAGCATGCCTGTTTGAGTGTCATT
AAATTCTCAACTCTCTTCTAC--TTTTTGTAAAAGAGAGCTTGGACTGTGGAGGCTTGCT
GGCCACTTTTTGGGGTCAGCTCCTCTGAAATGCATTAGCGGAACCGTTTGCGATCTGCCA
CAAGTGTGATAAGTTATCTACACTGGCGAGGGGATTGCTCTCTGTAATGTTCAGCTTCTA
ATTGTCTCTACTTTGTGAGACTACTTTTGAATGCTTGACCTCAAATCAGGTAGGACTACC
CGCTGAACTTAA
>ABC6-51
TTTCCGTAGGTGAACCTGCGGAAGGATCATTATTGAATTATGTTTCTAGATAGGTTGTAG
CTGGCTC-TTTAGAGCATGTGCACGCCTGTTTGGACTTCATTTTCATCCACCTGTGCACC
TATTGTAGTCTTTGGTTGGGTTAGGAGGAAGTGGTCATTGTGTCAGCATCTGCTGGATGT
GAGGACTTGCATTGTGAAAGCTTTGCTGTCCTTGATGTGATCATGGAATCTCTTTCTCAC
TAGAGTCTATGTCACTCATTATACTCTGTCGAATGTCATTGAATGTCTTTACATGGGCTT
ATATGCCTATGAAAATTGTAATACAACTTTCAGCAACGGATCTCTTGGCTCTCGCATCGA
TGAAGAACGCAGCGAAATGCGATAAGTAATGTGAATTGCAGAATTCAGTGAATCATCGAA
TCTTTGAACGCATCTTGCGCTCCTTGGTATTCCGAGGAGCATGCCTGTTTGAGTGTCATT
AAATTCTCAACTCTCTTCTAC--TTTTTGTAAAAGAGAGCTTGGACTGTGGAGGCTTGCT
GGCCACTTTTTGGGGTCAGCTCCTCTGAAATGCATTAGCGGAACCGTTTGCGATCTGCCA
CAAGTGTGATAAGTTATCTACACTGGCGAGGGGATTGCTCTCTGTAATGTTCAGCTTCTA
ATTGTCTCTACTTTGTGAGACTACTTTTGAATGCTTGACCTCAAATCAGGTAGGACTACC
CGCTGAACTTAA
>ABC7-1
TTTCCGTAGGTGAACCTGCGGAAGGATCATTATTGAATTATGTTTCTAGATAGGTTGTAG
CTGGCTC-TTTAGAGCATGTGCACGCCTGTTTGGACTTCATTTTCATCCACCTGTGCACC
TATTGTAGTCTTTGGTTGGGTTAGGAGGAAGTGGTCATTGTGTCAGCATCTGCTGGATGT
GAGGACTTGCATTGTGAAAGCTTTGCTGTCCTTGATGTGATCATGGAATCTCTTTCTCAC
TAGAGTCTATGTCACTCATTATACTCTGTCGAATGTCATTGAATGTCTTTACATGGGCTT
ATATGCCTATGAAAATTGTAATACAACTTTCAGCAACGGATCTCTTGGCTCTCGCATCGA
TGAAGAACGCAGCGAAATGCGATAAGTAATGTGAATTGCAGAATTCAGTGAATCATCGAA
TCTTTGAACGCATCTTGCGCTCCTTGGTATTCCGAGGAGCATGCCTGTTTGAGTGTCATT
AAATTCTCAACTCTCTTCTAC--TTTTTGTAAAAGAGAGCTTGGACTGTGGAGGCTTGCT
GGCCACTTTTTGGGGTCAGCTCCTCTGAAATGCATTAGCGGAACCGTTTGCGATCTGCCA
CAAGTGTGATAAGTTATCTACACTGGCGAGGGGATTGCTCTCTGTAATGTTCAGCTTCTA
ATTGTCTCTACTTTGTGAGACTACTTTTGAATGCTTGACCTCAAATCAGGTAGGACTACC
CGCTGAACTTAA

>ABC7-3
TTTCCGTAGGTGAACCTGCGGAAGGATCATTATTGAATTATGTTTCTAGATAGGTTGTAG
CTGGCTC-TTTAGAGCATGTGCACGCCTGTTTGGACTTCATTTTCATCCACCTGTGCACC
TATTGTAGTCTTTGGTTGGGTTAGGAGGAAGTGGTCATTGTGTCAGCATCTGCTGGATGT
GAGGACTTGCATTGTGAAAGCTTTGCTGTCCTTGATGTGATCATGGAATCTCTTTCTCAC
TAGAGTCTATGTCACTCATTATACTCTGTCGAATGTCATTGAATGTCTTTACATGGGCTT
ATATGCCTATGAAAATTGTAATACAACTTTCAGCAACGGATCTCTTGGCTCTCGCATCGA
TGAAGAACGCAGCGAAATGCGATAAGTAATGTGAATTGCAGAATTCAGTGAATCATCGAA
TCTTTGAACGCATCTTGCGCTCCTTGGTATTCCGAGGAGCATGCCTGTTTGAGTGTCATT
AAATTCTCAACTCTCTTCTAC--TTTTTGTAAAAGAGAGCTTGGACTGTGGAGGCTTGCT
GGCCACTTTTTGGGGTCAGCTCCTCTGAAATGCATTAGCGGAACCGTTTGCGATCTGCCA
CAAGTGTGATAAGTTATCTACACTGGCGAGGGGATTGCTCTCTGTAATGTTCAGCTTCTA
ATTGTCTCTACTTTGTGAGACTACTTTTGAATGCTTGACCTCAAATCAGGTAGGACTACC
CGCTGAACTTAA
>ABC7-9
TTTCCGTAGGTGAACCTGCGGAAGGATCATTATTGAATTATGTTTCTAGATAGGTTGTAG
CTGGCTC-TTTAGAGCATGTGCACGCCTGTTTGGACTTCATTTTCATCCACCTGTGCACC
TATTGTAGTCTTTGGTTGGGTTAGGAGGAAGTGGTCATTGTGTCAGCATCTGCTGGATGT
GAGGACTTGCATTGTGAAAGCTTTGCTGTCCTTGATGTGATCATGGAATCTCTTTCTCAC
TAGAGTCTATGTCACTCATTATACTCTGTCGAATGTCATTGAATGTCTTTACATGGGCTT
ATATGCCTATGAAAATTGTAATACAACTTTCAGCAACGGATCTCTTGGCTCTCGCATCGA
TGAAGAACGCAGCGAAATGCGATAAGTAATGTGAATTGCAGAATTCAGTGAATCATCGAA
TCTTTGAACGCATCTTGCGCTCCTTGGTATTCCGAGGAGCATGCCTGTTTGAGTGTCATT
AAATTCTCAACTCTCTTCTAC--TTTTTGTAAAAGAGAGCTTGGACTGTGGAGGCTTGCT
GGCCACTTTTTGGGGTCAGCTCCTCTGAAATGCATTAGCGGAACCGTTTGCGATCTGCCA
CAAGTGTGATAAGTTATCTACACTGGCGAGGGGATTGCTCTCTGTAATGTTCAGCTTCTA
ATTGTCTCTACTTTGTGAGACTACTTTTGAATGCTTGACCTCAAATCAGGTAGGACTACC
CGCTGAACTTAA
>ABC7-11
TTTCCGTAGGTGAACCTGCGGAAGGATCATTATTGAATTATGTTTCTAGATAGGTTGTAG
CTGGCTC-TTTAGAGCATGTGCACGCCTGTTTGGACTTCATTTTCATCCACCTGTGCACC
TATTGTAGTCTTTGGTTGGGTTAGGAGGAAGTGGTCATTGTGTCAGCATCTGCTGGATGT
GAGGACTTGCATTGTGAAAGCTTTGCTGTCCTTGATGTGATCATGGAATCTCTTTCTCAC
TAGAGTCTATGTCACTCATTATACTCTGTCGAATGTCATTGAATGTCTTTACATGGGCTT
ATATGCCTATGAAAATTGTAATACAACTTTCAGCAACGGATCTCTTGGCTCTCGCATCGA
TGAAGAACGCAGCGAAATGCGATAAGTAATGTGAATTGCAGAATTCAGTGAATCATCGAA
TCTTTGAACGCATCTTGCGCTCCTTGGTATTCCGAGGAGCATGCCTGTTTGAGTGTCATT
AAATTCTCAACTCTCTTCTAC--TTTTTGTAAAAGAGAGCTTGGACTGTGGAGGCTTGCT
GGCCACTTTTTGGGGTCAGCTCCTCTGAAATGCATTAGCGGAACCGTTTGCGATCTGCCA
CAAGTGTGATAAGTTATCTACACTGGCGAGGGGATTGCTCTCTGTAATGTTCAGCTTCTA
ATTGTCTCTACTTTGTGAGACTACTTTTGAATGCTTGACCTCAAATCAGGTAGGACTACC
CGCTGAACTTAA
>ABC7-14
TTTCCGTAGGTGAACCTGCGGAAGGATCATTATTGAATTATGTTTCTAGATAGGTTGTAG
CTGGCTC-TTTAGAGCATGTGCACGCCTGTTTGGACTTCATTTTCATCCACCTGTGCACC
TATTGTAGTCTTTGGTTGGGTTAGGAGGAAGTGGTCATTGTGTCAGCATCTGCTGGATGT
GAGGACTTGCATTGTGAAAGCTTTGCTGTCCTTGATGTGATCATGGAATCTCTTTCTCAC
TAGAGTCTATGTCACTCATTATACTCTGTCGAATGTCATTGAATGTCTTTACATGGGCTT
ATATGCCTATGAAAATTGTAATACAACTTTCAGCAACGGATCTCTTGGCTCTCGCATCGA
TGAAGAACGCAGCGAAATGCGATAAGTAATGTGAATTGCAGAATTCAGTGAATCATCGAA

TCTTTGAACGCATCTTGCGCTCCTTGGTATTCCGAGGAGCATGCCTGTTTGAGTGTCATT
AAATTCTCAACTCTCTTCTAC--TTTTTGTAAAAGAGAGCTTGGACTGTGGAGGCTTGCT
GGCCACTTTTTGGGGTCAGCTCCTCTGAAATGCATTAGCGGAACCGTTTGCGATCTGCCA
CAAGTGTGATAAGTTATCTACACTGGCGAGGGGATTGCTCTCTGTAATGTTCAGCTTCTA
ATTGTCTCTACTTTGTGAGACTACTTTTGAATGCTTGACCTCAAATCAGGTAGGACTACC
CGCTGAACTTAA
>ABC8-7
TTTCCGTAGGTGAACCTGCGGAAGGATCATTATTGAATTATGTTTCTAGATAGGTTGTAG
CTGGCTC-TTTAGAGCATGTGCACGCCTGTTTGGACTTCATTTTCATCCACCTGTGCACC
TATTGTAGTCTTTGGTTGGGTTAGGAGGAAGTGGTCATTGTGTCAGCATCTGCTGGATGT
GAGGACTTGCATTGTGAAAGCTTTGCTGTCCTTGATGTGATCATGGAATCTCTTTCTCAC
TAGAGTCTATGTCACTCATTATACTCTGTCGAATGTCATTGAATGTCTTTACATGGGCTT
ATATGCCTATGAAAATTGTAATACAACTTTCAGCAACGGATCTCTTGGCTCTCGCATCGA
TGAAGAACGCAGCGAAATGCGATAAGTAATGTGAATTGCAGAATTCAGTGAATCATCGAA
TCTTTGAACGCATCTTGCGCTCCTTGGTATTCCGAGGAGCATGCCTGTTTGAGTGTCATT
AAATTCTCAACTCTCTTCTAC--TTTTTGTAAAAGAGAGCTTGGACTGTGGAGGCTTGCT
GGCCACTTTTTGGGGTCAGCTCCTCTGAAATGCATTAGCGGAACCGTTTGCGATCTGCCA
CAAGTGTGATAAGTTATCTACACTGGCGAGGGGATTGCTCTCTGTAATGTTCAGCTTCTA
ATTGTCTCTACTTTGTGAGACTACTTTTGAATGCTTGACCTCAAATCAGGTAGGACTACC
CGCTGAACTTAA
>ABC8-27
TTTCCGTAGGTGAACCTGCGGAAGGATCATTATTGAATTATGTTTCTAGATAGGTTGTAG
CTGGCTC-TTTAGAGCATGTGCACGCCTGTTTGGACTTCATTTTCATCCACCTGTGCACC
TATTGTAGTCTTTGGTTGGGTTAGGAGGAAGTGGTCATTGTGTCAGCATCTGCTGGATGT
GAGGACTTGCATTGTGAAAGCTTTGCTGTCCTTGATGTGATCATGGAATCTCTTTCTCAC
TAGAGTCTATGTCACTCATTATACTCTGTCGAATGTCATTGAATGTCTTTACATGGGCTT
ATATGCCTATGAAAATTGTAATACAACTTTCAGCAACGGATCTCTTGGCTCTCGCATCGA
TGAAGAACGCAGCGAAATGCGATAAGTAATGTGAATTGCAGAATTCAGTGAATCATCGAA
TCTTTGAACGCATCTTGCGCTCCTTGGTATTCCGAGGAGCATGCCTGTTTGAGTGTCATT
AAATTCTCAACTCTCTTCTAC--TTTTTGTAAAAGAGAGCTTGGACTGTGGAGGCTTGCT
GGCCACTTTTTGGGGTCAGCTCCTCTGAAATGCATTAGCGGAACCGTTTGCGATCTGCCA
CAAGTGTGATAAGTTATCTACACTGGCGAGGGGATTGCTCTCTGTAATGTTCAGCTTCTA
ATTGTCTCTACTTTGTGAGACTACTTTTGAATGCTTGACCTCAAATCAGGTAGGACTACC
CGCTGAACTTAA
>ABC8-42
TTTCCGTAGGTGAACCTGCGGAAGGATCATTATTGAATTATGTTTCTAGATAGGTTGTAG
CTGGCTC-TTTAGAGCATGTGCACGCCTGTTTGGACTTCATTTTCATCCACCTGTGCACC
TATTGTAGTCTTTGGTTGGGTTAGGAGGAAGTGGTCATTGTGTCAGCATCTGCTGGATGT
GAGGACTTGCATTGTGAAAGCTTTGCTGTCCTTGATGTGATCATGGAATCTCTTTCTCAC
TAGAGTCTATGTCACTCATTATACTCTGTCGAATGTCATTGAATGTCTTTACATGGGCTT
ATATGCCTATGAAAATTGTAATACAACTTTCAGCAACGGATCTCTTGGCTCTCGCATCGA
TGAAGAACGCAGCGAAATGCGATAAGTAATGTGAATTGCAGAATTCAGTGAATCATCGAA
TCTTTGAACGCATCTTGCGCTCCTTGGTATTCCGAGGAGCATGCCTGTTTGAGTGTCATT
AAATTCTCAACTCTCTTCTAC--TTTTTGTAAAAGAGAGCTTGGACTGTGGAGGCTTGCT
GGCCACTTTTTGGGGTCAGCTCCTCTGAAATGCATTAGCGGAACCGTTTGCGATCTGCCA
CAAGTGTGATAAGTTATCTACACTGGCGAGGGGATTGCTCTCTGTAATGTTCAGCTTCTA
ATTGTCTCTACTTTGTGAGACTACTTTTGAATGCTTGACCTCAAATCAGGTAGGACTACC
CGCTGAACTTAA
>ABC9-5
TTTCCGTAGGTGAACCTGCGGAAGGATCATTATTGAATTATGTTTCTAGATAGGTTGTAG

CTGGCTC-TTTAGAGCATGTGCACGCCTGTTTGGACTTCATTTTCATCCACCTGTGCACC
TATTGTAGTCTTTGGTTGGGTTAGGAGGAAGTGGTCATTGTGTCAGCATCTGCTGGATGT
GAGGACTTGCATTGTGAAAGCTTTGCTGTCCTTGATGTGATCATGGAATCTCTTTCTCAC
TAGAGTCTATGTCACTCATTATACTCTGTCGAATGTCATTGAATGTCTTTACATGGGCTT
ATATGCCTATGAAAATTGTAATACAACTTTCAGCAACGGATCTCTTGGCTCTCGCATCGA
TGAAGAACGCAGCGAAATGCGATAAGTAATGTGAATTGCAGAATTCAGTGAATCATCGAA
TCTTTGAACGCATCTTGCGCTCCTTGGTATTCCGAGGAGCATGCCTGTTTGAGTGTCATT
AAATTCTCAACTCTCTTCTAC--TTTTTGTAAAAGAGAGCTTGGACTGTGGAGGCTTGCT
GGCCACTTTTTGGGGTCAGCTCCTCTGAAATGCATTAGCGGAACCGTTTGCGATCTGCCA
CAAGTGTGATAAGTTATCTACACTGGCGAGGGGATTGCTCTCTGTAATGTTCAGCTTCTA
ATTGTCTCTACTTTGTGAGACTACTTTTGAATGCTTGACCTCAAATCAGGTAGGACTACC
CGCTGAACTTAA
>ABC9-9
TTTCCGTAGGTGAACCTGCGGAAGGATCATTATTGAATTATGTTTCTAGATAGGTTGTAG
CTGGCTC-TTTAGAGCATGTGCACGCCTGTTTGGACTTCATTTTCATCCACCTGTGCACC
TATTGTAGTCTTTGGTTGGGTTAGGAGGAAGTGGTCATTGTGTCAGCATCTGCTGGATGT
GAGGACTTGCATTGTGAAAGCTTTGCTGTCCTTGATGTGATCATGGAATCTCTTTCTCAC
TAGAGTCTATGTCACTCATTATACTCTGTCGAATGTCATTGAATGTCTTTACATGGGCTT
ATATGCCTATGAAAATTGTAATACAACTTTCAGCAACGGATCTCTTGGCTCTCGCATCGA
TGAAGAACGCAGCGAAATGCGATAAGTAATGTGAATTGCAGAATTCAGTGAATCATCGAA
TCTTTGAACGCATCTTGCGCTCCTTGGTATTCCGAGGAGCATGCCTGTTTGAGTGTCATT
AAATTCTCAACTCTCTTCTAC--TTTTTGTAAAAGAGAGCTTGGACTGTGGAGGCTTGCT
GGCCACTTTTTGGGGTCAGCTCCTCTGAAATGCATTAGCGGAACCGTTTGCGATCTGCCA
CAAGTGTGATAAGTTATCTACACTGGCGAGGGGATTGCTCTCTGTAATGTTCAGCTTCTA
ATTGTCTCTACTTTGTGAGACTACTTTTGAATGCTTGACCTCAAATCAGGTAGGACTACC
CGCTGAACTTAA
>ABC9-34
TTTCCGTAGGTGAACCTGCGGAAGGATCATTATTGAATTATGTTTCTAGATAGGTTGTAG
CTGGCTC-TTTAGAGCATGTGCACGCCTGTTTGGACTTCATTTTCATCCACCTGTGCACC
TATTGTAGTCTTTGGTTGGGTTAGGAGGAAGTGGTCATTGTGTCAGCATCTGCTGGATGT
GAGGACTTGCATTGTGAAAGCTTTGCTGTCCTTGATGTGATCATGGAATCTCTTTCTCAC
TAGAGTCTATGTCACTCATTATACTCTGTCGAATGTCATTGAATGTCTTTACATGGGCTT
ATATGCCTATGAAAATTGTAATACAACTTTCAGCAACGGATCTCTTGGCTCTCGCATCGA
TGAAGAACGCAGCGAAATGCGATAAGTAATGTGAATTGCAGAATTCAGTGAATCATCGAA
TCTTTGAACGCATCTTGCGCTCCTTGGTATTCCGAGGAGCATGCCTGTTTGAGTGTCATT
AAATTCTCAACTCTCTTCTAC--TTTTTGTAAAAGAGAGCTTGGACTGTGGAGGCTTGCT
GGCCACTTTTTGGGGTCAGCTCCTCTGAAATGCATTAGCGGAACCGTTTGCGATCTGCCA
CAAGTGTGATAAGTTATCTACACTGGCGAGGGGATTGCTCTCTGTAATGTTCAGCTTCTA
ATTGTCTCTACTTTGTGAGACTACTTTTGAATGCTTGACCTCAAATCAGGTAGGACTACC
CGCTGAACTTAA
>ABC9-58
TTTCCGTAGGTGAACCTGCGGAAGGATCATTATTGAATTATGTTTCTAGATAGGTTGTAG
CTGGCTC-TTTAGAGCATGTGCACGCCTGTTTGGACTTCATTTTCATCCACCTGTGCACC
TATTGTAGTCTTTGGTTGGGTTAGGAGGAAGTGGTCATTGTGTCAGCATCTGCTGGATGT
GAGGACTTGCATTGTGAAAGCTTTGCTGTCCTTGATGTGATCATGGAATCTCTTTCTCAC
TAGAGTCTATGTCACTCATTATACTCTGTCGAATGTCATTGAATGTCTTTACATGGGCTT
ATATGCCTATGAAAATTGTAATACAACTTTCAGCAACGGATCTCTTGGCTCTCGCATCGA
TGAAGAACGCAGCGAAATGCGATAAGTAATGTGAATTGCAGAATTCAGTGAATCATCGAA
TCTTTGAACGCATCTTGCGCTCCTTGGTATTCCGAGGAGCATGCCTGTTTGAGTGTCATT
AAATTCTCAACTCTCTTCTAC--TTTTTGTAAAAGAGAGCTTGGACTGTGGAGGCTTGCT

GGCCACTTTTTGGGGTCAGCTCCTCTGAAATGCATTAGCGGAACCGTTTGCGATCTGCCA
CAAGTGTGATAAGTTATCTACACTGGCGAGGGGATTGCTCTCTGTAATGTTCAGCTTCTA
ATTGTCTCTACTTTGTGAGACTACTTTTGAATGCTTGACCTCAAATCAGGTAGGACTACC
CGCTGAACTTAA
>ABC10-1
TTTCCGTAGGTGAACCTGCGGAAGGATCATTATTGAATTATGTTTCTAGATAGGTTGTAG
CTGGCTC-TTTAGAGCATGTGCACGCCTGTTTGGACTTCATTTTCATCCACCTGTGCACC
TATTGTAGTCTTTGGTTGGGTTAGGAGGAAGTGGTCATTGTGTCAGCATCTGCTGGATGT
GAGGACTTGCATTGTGAAAGCTTTGCTGTCCTTGATGTGATCATGGAATCTCTTTCTCAC
TAGAGTCTATGTCACTCATTATACTCTGTCGAATGTCATTGAATGTCTTTACATGGGCTT
ATATGCCTATGAAAATTGTAATACAACTTTCAGCAACGGATCTCTTGGCTCTCGCATCGA
TGAAGAACGCAGCGAAATGCGATAAGTAATGTGAATTGCAGAATTCAGTGAATCATCGAA
TCTTTGAACGCATCTTGCGCTCCTTGGTATTCCGAGGAGCATGCCTGTTTGAGTGTCATT
AAATTCTCAACTCTCTTCTAC--TTTTTGTAAAAGAGAGCTTGGACTGTGGAGGCTTGCT
GGCCACTTTTTGGGGTCAGCTCCTCTGAAATGCATTAGCGGAACCGTTTGCGATCTGCCA
CAAGTGTGATAAGTTATCTACACTGGCGAGGGGATTGCTCTCTGTAATGTTCAGCTTCTA
ATTGTCTCTACTTTGTGAGACTACTTTTGAATGCTTGACCTCAAATCAGGTAGGACTACC
CGCTGAACTTAA
>ABC10-9
TTTCCGTAGGTGAACCTGCGGAAGGATCATTATTGAATTATGTTTCTAGATAGGTTGTAG
CTGGCTC-TTTAGAGCATGTGCACGCCTGTTTGGACTTCATTTTCATCCACCTGTGCACC
TATTGTAGTCTTTGGTTGGGTTAGGAGGAAGTGGTCATTGTGTCAGCATCTGCTGGATGT
GAGGACTTGCATTGTGAAAGCTTTGCTGTCCTTGATGTGATCATGGAATCTCTTTCTCAC
TAGAGTCTATGTCACTCATTATACTCTGTCGAATGTCATTGAATGTCTTTACATGGGCTT
ATATGCCTATGAAAATTGTAATACAACTTTCAGCAACGGATCTCTTGGCTCTCGCATCGA
TGAAGAACGCAGCGAAATGCGATAAGTAATGTGAATTGCAGAATTCAGTGAATCATCGAA
TCTTTGAACGCATCTTGCGCTCCTTGGTATTCCGAGGAGCATGCCTGTTTGAGTGTCATT
AAATTCTCAACTCTCTTCTAC--TTTTTGTAAAAGAGAGCTTGGACTGTGGAGGCTTGCT
GGCCACTTTTTGGGGTCAGCTCCTCTGAAATGCATTAGCGGAACCGTTTGCGATCTGCCA
CAAGTGTGATAAGTTATCTACACTGGCGAGGGGATTGCTCTCTGTAATGTTCAGCTTCTA
ATTGTCTCTACTTTGTGAGACTACTTTTGAATGCTTGACCTCAAATCAGGTAGGACTACC
CGCTGAACTTAA
>ABC10-16
TTTCCGTAGGTGAACCTGCGGAAGGATCATTATTGAATTATGTTTCTAGATAGGTTGTAG
CTGGCTC-TTTAGAGCATGTGCACGCCTGTTTGGACTTCATTTTCATCCACCTGTGCACC
TATTGTAGTCTTTGGTTGGGTTAGGAGGAAGTGGTCATTGTGTCAGCATCTGCTGGATGT
GAGGACTTGCATTGTGAAAGCTTTGCTGTCCTTGATGTGATCATGGAATCTCTTTCTCAC
TAGAGTCTATGTCACTCATTATACTCTGTCGAATGTCATTGAATGTCTTTACATGGGCTT
ATATGCCTATGAAAATTGTAATACAACTTTCAGCAACGGATCTCTTGGCTCTCGCATCGA
TGAAGAACGCAGCGAAATGCGATAAGTAATGTGAATTGCAGAATTCAGTGAATCATCGAA
TCTTTGAACGCATCTTGCGCTCCTTGGTATTCCGAGGAGCATGCCTGTTTGAGTGTCATT
AAATTCTCAACTCTCTTCTAC--TTTTTGTAAAAGAGAGCTTGGACTGTGGAGGCTTGCT
GGCCACTTTTTGGGGTCAGCTCCTCTGAAATGCATTAGCGGAACCGTTTGCGATCTGCCA
CAAGTGTGATAAGTTATCTACACTGGCGAGGGGATTGCTCTCTGTAATGTTCAGCTTCTA
ATTGTCTCTACTTTGTGAGACTACTTTTGAATGCTTGACCTCAAATCAGGTAGGACTACC
CGCTGAACTTAA
>ABC10-21
TTTCCGTAGGTGAACCTGCGGAAGGATCATTATTGAATTATGTTTCTAGATAGGTTGTAG
CTGGCTC-TTTAGAGCATGTGCACGCCTGTTTGGACTTCATTTTCATCCACCTGTGCACC
TATTGTAGTCTTTGGTTGGGTTAGGAGGAAGTGGTCATTGTGTCAGCATCTGCTGGATGT

GAGGACTTGCATTGTGAAAGCTTTGCTGTCCTTGATGTGATCATGGAATCTCTTTCTCAC
TAGAGTCTATGTCACTCATTATACTCTGTCGAATGTCATTGAATGTCTTTACATGGGCTT
ATATGCCTATGAAAATTGTAATACAACTTTCAGCAACGGATCTCTTGGCTCTCGCATCGA
TGAAGAACGCAGCGAAATGCGATAAGTAATGTGAATTGCAGAATTCAGTGAATCATCGAA
TCTTTGAACGCATCTTGCGCTCCTTGGTATTCCGAGGAGCATGCCTGTTTGAGTGTCATT
AAATTCTCAACTCTCTTCTAC--TTTTTGTAAAAGAGAGCTTGGACTGTGGAGGCTTGCT
GGCCACTTTTTGGGGTCAGCTCCTCTGAAATGCATTAGCGGAACCGTTTGCGATCTGCCA
CAAGTGTGATAAGTTATCTACACTGGCGAGGGGATTGCTCTCTGTAATGTTCAGCTTCTA
ATTGTCTCTACTTTGTGAGACTACTTTTGAATGCTTGACCTCAAATCAGGTAGGACTACC
CGCTGAACTTAA
>ABC10-31
TTTCCGTAGGTGAACCTGCGGAAGGATCATTATTGAATTATGTTTCTAGATAGGTTGTAG
CTGGCTC-TTTAGAGCATGTGCACGCCTGTTTGGACTTCATTTTCATCCACCTGTGCACC
TATTGTAGTCTTTGGTTGGGTTAGGAGGAAGTGGTCATTGTGTCAGCATCTGCTGGATGT
GAGGACTTGCATTGTGAAAGCTTTGCTGTCCTTGATGTGATCATGGAATCTCTTTCTCAC
TAGAGTCTATGTCACTCATTATACTCTGTCGAATGTCATTGAATGTCTTTACATGGGCTT
ATATGCCTATGAAAATTGTAATACAACTTTCAGCAACGGATCTCTTGGCTCTCGCATCGA
TGAAGAACGCAGCGAAATGCGATAAGTAATGTGAATTGCAGAATTCAGTGAATCATCGAA
TCTTTGAACGCATCTTGCGCTCCTTGGTATTCCGAGGAGCATGCCTGTTTGAGTGTCATT
AAATTCTCAACTCTCTTCTAC--TTTTTGTAAAAGAGAGCTTGGACTGTGGAGGCTTGCT
GGCCACTTTTTGGGGTCAGCTCCTCTGAAATGCATTAGCGGAACCGTTTGCGATCTGCCA
CAAGTGTGATAAGTTATCTACACTGGCGAGGGGATTGCTCTCTGTAATGTTCAGCTTCTA
ATTGTCTCTACTTTGTGAGACTACTTTTGAATGCTTGACCTCAAATCAGGTAGGACTACC
CGCTGAACTTAA
>ABC10-32
TTTCCGTAGGTGAACCTGCGGAAGGATCATTATTGAATTATGTTTCTAGATAGGTTGTAG
CTGGCTC-TTTAGAGCATGTGCACGCCTGTTTGGACTTCATTTTCATCCACCTGTGCACC
TATTGTAGTCTTTGGTTGGGTTAGGAGGAAGTGGTCATTGTGTCAGCATCTGCTGGATGT
GAGGACTTGCATTGTGAAAGCTTTGCTGTCCTTGATGTGATCATGGAATCTCTTTCTCAC
TAGAGTCTATGTCACTCATTATACTCTGTCGAATGTCATTGAATGTCTTTACATGGGCTT
ATATGCCTATGAAAATTGTAATACAACTTTCAGCAACGGATCTCTTGGCTCTCGCATCGA
TGAAGAACGCAGCGAAATGCGATAAGTAATGTGAATTGCAGAATTCAGTGAATCATCGAA
TCTTTGAACGCATCTTGCGCTCCTTGGTATTCCGAGGAGCATGCCTGTTTGAGTGTCATT
AAATTCTCAACTCTCTTCTAC--TTTTTGTAAAAGAGAGCTTGGACTGTGGAGGCTTGCT
GGCCACTTTTTGGGGTCAGCTCCTCTGAAATGCATTAGCGGAACCGTTTGCGATCTGCCA
CAAGTGTGATAAGTTATCTACACTGGCGAGGGGATTGCTCTCTGTAATGTTCAGCTTCTA
ATTGTCTCTACTTTGTGAGACTACTTTTGAATGCTTGACCTCAAATCAGGTAGGACTACC
CGCTGAACTTAA
>ABC10-33
TTTCCGTAGGTGAACCTGCGGAAGGATCATTATTGAATTATGTTTCTAGATAGGTTGTAG
CTGGCTC-TTTAGAGCATGTGCACGCCTGTTTGGACTTCATTTTCATCCACCTGTGCACC
TATTGTAGTCTTTGGTTGGGTTAGGAGGAAGTGGTCATTGTGTCAGCATCTGCTGGATGT
GAGGACTTGCATTGTGAAAGCTTTGCTGTCCTTGATGTGATCATGGAATCTCTTTCTCAC
TAGAGTCTATGTCACTCATTATACTCTGTCGAATGTCATTGAATGTCTTTACATGGGCTT
ATATGCCTATGAAAATTGTAATACAACTTTCAGCAACGGATCTCTTGGCTCTCGCATCGA
TGAAGAACGCAGCGAAATGCGATAAGTAATGTGAATTGCAGAATTCAGTGAATCATCGAA
TCTTTGAACGCATCTTGCGCTCCTTGGTATTCCGAGGAGCATGCCTGTTTGAGTGTCATT
AAATTCTCAACTCTCTTCTAC--TTTTTGTAAAAGAGAGCTTGGACTGTGGAGGCTTGCT
GGCCACTTTTTGGGGTCAGCTCCTCTGAAATGCATTAGCGGAACCGTTTGCGATCTGCCA
CAAGTGTGATAAGTTATCTACACTGGCGAGGGGATTGCTCTCTGTAATGTTCAGCTTCTA

ATTGTCTCTACTTTGTGAGACTACTTTTGAATGCTTGACCTCAAATCAGGTAGGACTACC
CGCTGAACTTAA
>ABC10-34
TTTCCGTAGGTGAACCTGCGGAAGGATCATTATTGAATTATGTTTCTAGATAGGTTGTAG
CTGGCTC-TTTAGAGCATGTGCACGCCTGTTTGGACTTCATTTTCATCCACCTGTGCACC
TATTGTAGTCTTTGGTTGGGTTAGGAGGAAGTGGTCATTGTGTCAGCATCTGCTGGATGT
GAGGACTTGCATTGTGAAAGCTTTGCTGTCCTTGATGTGATCATGGAATCTCTTTCTCAC
TAGAGTCTATGTCACTCATTATACTCTGTCGAATGTCATTGAATGTCTTTACATGGGCTT
ATATGCCTATGAAAATTGTAATACAACTTTCAGCAACGGATCTCTTGGCTCTCGCATCGA
TGAAGAACGCAGCGAAATGCGATAAGTAATGTGAATTGCAGAATTCAGTGAATCATCGAA
TCTTTGAACGCATCTTGCGCTCCTTGGTATTCCGAGGAGCATGCCTGTTTGAGTGTCATT
AAATTCTCAACTCTCTTCTAC--TTTTTGTAAAAGAGAGCTTGGACTGTGGAGGCTTGCT
GGCCACTTTTTGGGGTCAGCTCCTCTGAAATGCATTAGCGGAACCGTTTGCGATCTGCCA
CAAGTGTGATAAGTTATCTACACTGGCGAGGGGATTGCTCTCTGTAATGTTCAGCTTCTA
ATTGTCTCTACTTTGTGAGACTACTTTTGAATGCTTGACCTCAAATCAGGTAGGACTACC
CGCTGAACTTAA
>ABC10-35
TTTCCGTAGGTGAACCTGCGGAAGGATCATTATTGAATTATGTTTCTAGATAGGTTGTAG
CTGGCTC-TTTAGAGCATGTGCACGCCTGTTTGGACTTCATTTTCATCCACCTGTGCACC
TATTGTAGTCTTTGGTTGGGTTAGGAGGAAGTGGTCATTGTGTCAGCATCTGCTGGATGT
GAGGACTTGCATTGTGAAAGCTTTGCTGTCCTTGATGTGATCATGGAATCTCTTTCTCAC
TAGAGTCTATGTCACTCATTATACTCTGTCGAATGTCATTGAATGTCTTTACATGGGCTT
ATATGCCTATGAAAATTGTAATACAACTTTCAGCAACGGATCTCTTGGCTCTCGCATCGA
TGAAGAACGCAGCGAAATGCGATAAGTAATGTGAATTGCAGAATTCAGTGAATCATCGAA
TCTTTGAACGCATCTTGCGCTCCTTGGTATTCCGAGGAGCATGCCTGTTTGAGTGTCATT
AAATTCTCAACTCTCTTCTAC--TTTTTGTAAAAGAGAGCTTGGACTGTGGAGGCTTGCT
GGCCACTTTTTGGGGTCAGCTCCTCTGAAATGCATTAGCGGAACCGTTTGCGATCTGCCA
CAAGTGTGATAAGTTATCTACACTGGCGAGGGGATTGCTCTCTGTAATGTTCAGCTTCTA
ATTGTCTCTACTTTGTGAGACTACTTTTGAATGCTTGACCTCAAATCAGGTAGGACTACC
CGCTGAACTTAA
>ABC10-40
TTTCCGTAGGTGAACCTGCGGAAGGATCATTATTGAATTATGTTTCTAGATAGGTTGTAG
CTGGCTC-TTTAGAGCATGTGCACGCCTGTTTGGACTTCATTTTCATCCACCTGTGCACC
TATTGTAGTCTTTGGTTGGGTTAGGAGGAAGTGGTCATTGTGTCAGCATCTGCTGGATGT
GAGGACTTGCATTGTGAAAGCTTTGCTGTCCTTGATGTGATCATGGAATCTCTTTCTCAC
TAGAGTCTATGTCACTCATTATACTCTGTCGAATGTCATTGAATGTCTTTACATGGGCTT
ATATGCCTATGAAAATTGTAATACAACTTTCAGCAACGGATCTCTTGGCTCTCGCATCGA
TGAAGAACGCAGCGAAATGCGATAAGTAATGTGAATTGCAGAATTCAGTGAATCATCGAA
TCTTTGAACGCATCTTGCGCTCCTTGGTATTCCGAGGAGCATGCCTGTTTGAGTGTCATT
AAATTCTCAACTCTCTTCTAC--TTTTTGTAAAAGAGAGCTTGGACTGTGGAGGCTTGCT
GGCCACTTTTTGGGGTCAGCTCCTCTGAAATGCATTAGCGGAACCGTTTGCGATCTGCCA
CAAGTGTGATAAGTTATCTACACTGGCGAGGGGATTGCTCTCTGTAATGTTCAGCTTCTA
ATTGTCTCTACTTTGTGAGACTACTTTTGAATGCTTGACCTCAAATCAGGTAGGACTACC
CGCTGAACTTAA
>ABC10-45
TTTCCGTAGGTGAACCTGCGGAAGGATCATTATTGAATTATGTTTCTAGATAGGTTGTAG
CTGGCTC-TTTAGAGCATGTGCACGCCTGTTTGGACTTCATTTTCATCCACCTGTGCACC
TATTGTAGTCTTTGGTTGGGTTAGGAGGAAGTGGTCATTGTGTCAGCATCTGCTGGATGT
GAGGACTTGCATTGTGAAAGCTTTGCTGTCCTTGATGTGATCATGGAATCTCTTTCTCAC
TAGAGTCTATGTCACTCATTATACTCTGTCGAATGTCATTGAATGTCTTTACATGGGCTT

ATATGCCTATGAAAATTGTAATACAACTTTCAGCAACGGATCTCTTGGCTCTCGCATCGA
TGAAGAACGCAGCGAAATGCGATAAGTAATGTGAATTGCAGAATTCAGTGAATCATCGAA
TCTTTGAACGCATCTTGCGCTCCTTGGTATTCCGAGGAGCATGCCTGTTTGAGTGTCATT
AAATTCTCAACTCTCTTCTAC--TTTTTGTAAAAGAGAGCTTGGACTGTGGAGGCTTGCT
GGCCACTTTTTGGGGTCAGCTCCTCTGAAATGCATTAGCGGAACCGTTTGCGATCTGCCA
CAAGTGTGATAAGTTATCTACACTGGCGAGGGGATTGCTCTCTGTAATGTTCAGCTTCTA
ATTGTCTCTACTTTGTGAGACTACTTTTGAATGCTTGACCTCAAATCAGGTAGGACTACC
CGCTGAACTTAA
>ABC10-48
TTTCCGTAGGTGAACCTGCGGAAGGATCATTATTGAATTATGTTTCTAGATAGGTTGTAG
CTGGCTC-TTTAGAGCATGTGCACGCCTGTTTGGACTTCATTTTCATCCACCTGTGCACC
TATTGTAGTCTTTGGTTGGGTTAGGAGGAAGTGGTCATTGTGTCAGCATCTGCTGGATGT
GAGGACTTGCATTGTGAAAGCTTTGCTGTCCTTGATGTGATCATGGAATCTCTTTCTCAC
TAGAGTCTATGTCACTCATTATACTCTGTCGAATGTCATTGAATGTCTTTACATGGGCTT
ATATGCCTATGAAAATTGTAATACAACTTTCAGCAACGGATCTCTTGGCTCTCGCATCGA
TGAAGAACGCAGCGAAATGCGATAAGTAATGTGAATTGCAGAATTCAGTGAATCATCGAA
TCTTTGAACGCATCTTGCGCTCCTTGGTATTCCGAGGAGCATGCCTGTTTGAGTGTCATT
AAATTCTCAACTCTCTTCTAC--TTTTTGTAAAAGAGAGCTTGGACTGTGGAGGCTTGCT
GGCCACTTTTTGGGGTCAGCTCCTCTGAAATGCATTAGCGGAACCGTTTGCGATCTGCCA
CAAGTGTGATAAGTTATCTACACTGGCGAGGGGATTGCTCTCTGTAATGTTCAGCTTCTA
ATTGTCTCTACTTTGTGAGACTACTTTTGAATGCTTGACCTCAAATCAGGTAGGACTACC
CGCTGAACTTAA
>ABC11-8
TTTCCGTAGGTGAACCTGCGGAAGGATCATTATTGAATTATGTTTCTAGATAGGTTGTAG
CTGGCTC-TTTAGAGCATGTGCACGCCTGTTTGGACTTCATTTTCATCCACCTGTGCACC
TATTGTAGTCTTTGGTTGGGTTAGGAGGAAGTGGTCATTGTGTCAGCATCTGCTGGATGT
GAGGACTTGCATTGTGAAAGCTTTGCTGTCCTTGATGTGATCATGGAATCTCTTTCTCAC
TAGAGTCTATGTCACTCATTATACTCTGTCGAATGTCATTGAATGTCTTTACATGGGCTT
ATATGCCTATGAAAATTGTAATACAACTTTCAGCAACGGATCTCTTGGCTCTCGCATCGA
TGAAGAACGCAGCGAAATGCGATAAGTAATGTGAATTGCAGAATTCAGTGAATCATCGAA
TCTTTGAACGCATCTTGCGCTCCTTGGTATTCCGAGGAGCATGCCTGTTTGAGTGTCATT
AAATTCTCAACTCTCTTCTAC--TTTTTGTAAAAGAGAGCTTGGACTGTGGAGGCTTGCT
GGCCACTTTTTGGGGTCAGCTCCTCTGAAATGCATTAGCGGAACCGTTTGCGATCTGCCA
CAAGTGTGATAAGTTATCTACACTGGCGAGGGGATTGCTCTCTGTAATGTTCAGCTTCTA
ATTGTCTCTACTTTGTGAGACTACTTTTGAATGCTTGACCTCAAATCAGGTAGGACTACC
CGCTGAACTTAA
>ABC11-12
TTTCCGTAGGTGAACCTGCGGAAGGATCATTATTGAATTATGTTTCTAGATAGGTTGTAG
CTGGCTC-TTTAGAGCATGTGCACGCCTGTTTGGACTTCATTTTCATCCACCTGTGCACC
TATTGTAGTCTTTGGTTGGGTTAGGAGGAAGTGGTCATTGTGTCAGCATCTGCTGGATGT
GAGGACTTGCATTGTGAAAGCTTTGCTGTCCTTGATGTGATCATGGAATCTCTTTCTCAC
TAGAGTCTATGTCACTCATTATACTCTGTCGAATGTCATTGAATGTCTTTACATGGGCTT
ATATGCCTATGAAAATTGTAATACAACTTTCAGCAACGGATCTCTTGGCTCTCGCATCGA
TGAAGAACGCAGCGAAATGCGATAAGTAATGTGAATTGCAGAATTCAGTGAATCATCGAA
TCTTTGAACGCATCTTGCGCTCCTTGGTATTCCGAGGAGCATGCCTGTTTGAGTGTCATT
AAATTCTCAACTCTCTTCTAC--TTTTTGTAAAAGAGAGCTTGGACTGTGGAGGCTTGCT
GGCCACTTTTTGGGGTCAGCTCCTCTGAAATGCATTAGCGGAACCGTTTGCGATCTGCCA
CAAGTGTGATAAGTTATCTACACTGGCGAGGGGATTGCTCTCTGTAATGTTCAGCTTCTA
ATTGTCTCTACTTTGTGAGACTACTTTTGAATGCTTGACCTCAAATCAGGTAGGACTACC
CGCTGAACTTAA

>ABC11-19
TTTCCGTAGGTGAACCTGCGGAAGGATCATTATTGAATTATGTTTCTAGATAGGTTGTAG
CTGGCTC-TTTAGAGCATGTGCACGCCTGTTTGGACTTCATTTTCATCCACCTGTGCACC
TATTGTAGTCTTTGGTTGGGTTAGGAGGAAGTGGTCATTGTGTCAGCATCTGCTGGATGT
GAGGACTTGCATTGTGAAAGCTTTGCTGTCCTTGATGTGATCATGGAATCTCTTTCTCAC
TAGAGTCTATGTCACTCATTATACTCTGTCGAATGTCATTGAATGTCTTTACATGGGCTT
ATATGCCTATGAAAATTGTAATACAACTTTCAGCAACGGATCTCTTGGCTCTCGCATCGA
TGAAGAACGCAGCGAAATGCGATAAGTAATGTGAATTGCAGAATTCAGTGAATCATCGAA
TCTTTGAACGCATCTTGCGCTCCTTGGTATTCCGAGGAGCATGCCTGTTTGAGTGTCATT
AAATTCTCAACTCTCTTCTAC--TTTTTGTAAAAGAGAGCTTGGACTGTGGAGGCTTGCT
GGCCACTTTTTGGGGTCAGCTCCTCTGAAATGCATTAGCGGAACCGTTTGCGATCTGCCA
CAAGTGTGATAAGTTATCTACACTGGCGAGGGGATTGCTCTCTGTAATGTTCAGCTTCTA
ATTGTCTCTACTTTGTGAGACTACTTTTGAATGCTTGACCTCAAATCAGGTAGGACTACC
CGCTGAACTTAA
>ABC11-20
TTTCCGTAGGTGAACCTGCGGAAGGATCATTATTGAATTATGTTTCTAGATAGGTTGTAG
CTGGCTC-TTTAGAGCATGTGCACGCCTGTTTGGACTTCATTTTCATCCACCTGTGCACC
TATTGTAGTCTTTGGTTGGGTTAGGAGGAAGTGGTCATTGTGTCAGCATCTGCTGGATGT
GAGGACTTGCATTGTGAAAGCTTTGCTGTCCTTGATGTGATCATGGAATCTCTTTCTCAC
TAGAGTCTATGTCACTCATTATACTCTGTCGAATGTCATTGAATGTCTTTACATGGGCTT
ATATGCCTATGAAAATTGTAATACAACTTTCAGCAACGGATCTCTTGGCTCTCGCATCGA
TGAAGAACGCAGCGAAATGCGATAAGTAATGTGAATTGCAGAATTCAGTGAATCATCGAA
TCTTTGAACGCATCTTGCGCTCCTTGGTATTCCGAGGAGCATGCCTGTTTGAGTGTCATT
AAATTCTCAACTCTCTTCTAC--TTTTTGTAAAAGAGAGCTTGGACTGTGGAGGCTTGCT
GGCCACTTTTTGGGGTCAGCTCCTCTGAAATGCATTAGCGGAACCGTTTGCGATCTGCCA
CAAGTGTGATAAGTTATCTACACTGGCGAGGGGATTGCTCTCTGTAATGTTCAGCTTCTA
ATTGTCTCTACTTTGTGAGACTACTTTTGAATGCTTGACCTCAAATCAGGTAGGACTACC
CGCTGAACTTAA
>ABC11-24
TTTCCGTAGGTGAACCTGCGGAAGGATCATTATTGAATTATGTTTCTAGATAGGTTGTAG
CTGGCTC-TTTAGAGCATGTGCACGCCTGTTTGGACTTCATTTTCATCCACCTGTGCACC
TATTGTAGTCTTTGGTTGGGTTAGGAGGAAGTGGTCATTGTGTCAGCATCTGCTGGATGT
GAGGACTTGCATTGTGAAAGCTTTGCTGTCCTTGATGTGATCATGGAATCTCTTTCTCAC
TAGAGTCTATGTCACTCATTATACTCTGTCGAATGTCATTGAATGTCTTTACATGGGCTT
ATATGCCTATGAAAATTGTAATACAACTTTCAGCAACGGATCTCTTGGCTCTCGCATCGA
TGAAGAACGCAGCGAAATGCGATAAGTAATGTGAATTGCAGAATTCAGTGAATCATCGAA
TCTTTGAACGCATCTTGCGCTCCTTGGTATTCCGAGGAGCATGCCTGTTTGAGTGTCATT
AAATTCTCAACTCTCTTCTAC--TTTTTGTAAAAGAGAGCTTGGACTGTGGAGGCTTGCT
GGCCACTTTTTGGGGTCAGCTCCTCTGAAATGCATTAGCGGAACCGTTTGCGATCTGCCA
CAAGTGTGATAAGTTATCTACACTGGCGAGGGGATTGCTCTCTGTAATGTTCAGCTTCTA
ATTGTCTCTACTTTGTGAGACTACTTTTGAATGCTTGACCTCAAATCAGGTAGGACTACC
CGCTGAACTTAA
>ABC11-39
TTTCCGTAGGTGAACCTGCGGAAGGATCATTATTGAATTATGTTTCTAGATAGGTTGTAG
CTGGCTC-TTTAGAGCATGTGCACGCCTGTTTGGACTTCATTTTCATCCACCTGTGCACC
TATTGTAGTCTTTGGTTGGGTTAGGAGGAAGTGGTCATTGTGTCAGCATCTGCTGGATGT
GAGGACTTGCATTGTGAAAGCTTTGCTGTCCTTGATGTGATCATGGAATCTCTTTCTCAC
TAGAGTCTATGTCACTCATTATACTCTGTCGAATGTCATTGAATGTCTTTACATGGGCTT
ATATGCCTATGAAAATTGTAATACAACTTTCAGCAACGGATCTCTTGGCTCTCGCATCGA
TGAAGAACGCAGCGAAATGCGATAAGTAATGTGAATTGCAGAATTCAGTGAATCATCGAA

TCTTTGAACGCATCTTGCGCTCCTTGGTATTCCGAGGAGCATGCCTGTTTGAGTGTCATT
AAATTCTCAACTCTCTTCTAC--TTTTTGTAAAAGAGAGCTTGGACTGTGGAGGCTTGCT
GGCCACTTTTTGGGGTCAGCTCCTCTGAAATGCATTAGCGGAACCGTTTGCGATCTGCCA
CAAGTGTGATAAGTTATCTACACTGGCGAGGGGATTGCTCTCTGTAATGTTCAGCTTCTA
ATTGTCTCTACTTTGTGAGACTACTTTTGAATGCTTGACCTCAAATCAGGTAGGACTACC
CGCTGAACTTAA
>ABC11-48
TTTCCGTAGGTGAACCTGCGGAAGGATCATTATTGAATTATGTTTCTAGATAGGTTGTAG
CTGGCTC-TTTAGAGCATGTGCACGCCTGTTTGGACTTCATTTTCATCCACCTGTGCACC
TATTGTAGTCTTTGGTTGGGTTAGGAGGAAGTGGTCATTGTGTCAGCATCTGCTGGATGT
GAGGACTTGCATTGTGAAAGCTTTGCTGTCCTTGATGTGATCATGGAATCTCTTTCTCAC
TAGAGTCTATGTCACTCATTATACTCTGTCGAATGTCATTGAATGTCTTTACATGGGCTT
ATATGCCTATGAAAATTGTAATACAACTTTCAGCAACGGATCTCTTGGCTCTCGCATCGA
TGAAGAACGCAGCGAAATGCGATAAGTAATGTGAATTGCAGAATTCAGTGAATCATCGAA
TCTTTGAACGCATCTTGCGCTCCTTGGTATTCCGAGGAGCATGCCTGTTTGAGTGTCATT
AAATTCTCAACTCTCTTCTAC--TTTTTGTAAAAGAGAGCTTGGACTGTGGAGGCTTGCT
GGCCACTTTTTGGGGTCAGCTCCTCTGAAATGCATTAGCGGAACCGTTTGCGATCTGCCA
CAAGTGTGATAAGTTATCTACACTGGCGAGGGGATTGCTCTCTGTAATGTTCAGCTTCTA
ATTGTCTCTACTTTGTGAGACTACTTTTGAATGCTTGACCTCAAATCAGGTAGGACTACC
CGCTGAACTTAA
>ABC12-1
TTTCCGTAGGTGAACCTGCGGAAGGATCATTATTGAATTATGTTTCTAGATAGGTTGTAG
CTGGCTC-TTTAGAGCATGTGCACGCCTGTTTGGACTTCATTTTCATCCACCTGTGCACC
TATTGTAGTCTTTGGTTGGGTTAGGAGGAAGTGGTCATTGTGTCAGCATCTGCTGGATGT
GAGGACTTGCATTGTGAAAGCTTTGCTGTCCTTGATGTGATCATGGAATCTCTTTCTCAC
TAGAGTCTATGTCACTCATTATACTCTGTCGAATGTCATTGAATGTCTTTACATGGGCTT
ATATGCCTATGAAAATTGTAATACAACTTTCAGCAACGGATCTCTTGGCTCTCGCATCGA
TGAAGAACGCAGCGAAATGCGATAAGTAATGTGAATTGCAGAATTCAGTGAATCATCGAA
TCTTTGAACGCATCTTGCGCTCCTTGGTATTCCGAGGAGCATGCCTGTTTGAGTGTCATT
AAATTCTCAACTCTCTTCTAC--TTTTTGTAAAAGAGAGCTTGGACTGTGGAGGCTTGCT
GGCCACTTTTTGGGGTCAGCTCCTCTGAAATGCATTAGCGGAACCGTTTGCGATCTGCCA
CAAGTGTGATAAGTTATCTACACTGGCGAGGGGATTGCTCTCTGTAATGTTCAGCTTCTA
ATTGTCTCTACTTTGTGAGACTACTTTTGAATGCTTGACCTCAAATCAGGTAGGACTACC
CGCTGAACTTAA
>ABC12-13
TTTCCGTAGGTGAACCTGCGGAAGGATCATTATTGAATTATGTTTCTAGATAGGTTGTAG
CTGGCTC-TTTAGAGCATGTGCACGCCTGTTTGGACTTCATTTTCATCCACCTGTGCACC
TATTGTAGTCTTTGGTTGGGTTAGGAGGAAGTGGTCATTGTGTCAGCATCTGCTGGATGT
GAGGACTTGCATTGTGAAAGCTTTGCTGTCCTTGATGTGATCATGGAATCTCTTTCTCAC
TAGAGTCTATGTCACTCATTATACTCTGTCGAATGTCATTGAATGTCTTTACATGGGCTT
ATATGCCTATGAAAATTGTAATACAACTTTCAGCAACGGATCTCTTGGCTCTCGCATCGA
TGAAGAACGCAGCGAAATGCGATAAGTAATGTGAATTGCAGAATTCAGTGAATCATCGAA
TCTTTGAACGCATCTTGCGCTCCTTGGTATTCCGAGGAGCATGCCTGTTTGAGTGTCATT
AAATTCTCAACTCTCTTCTAC--TTTTTGTAAAAGAGAGCTTGGACTGTGGAGGCTTGCT
GGCCACTTTTTGGGGTCAGCTCCTCTGAAATGCATTAGCGGAACCGTTTGCGATCTGCCA
CAAGTGTGATAAGTTATCTACACTGGCGAGGGGATTGCTCTCTGTAATGTTCAGCTTCTA
ATTGTCTCTACTTTGTGAGACTACTTTTGAATGCTTGACCTCAAATCAGGTAGGACTACC
CGCTGAACTTAA
>ABC12-22
TTTCCGTAGGTGAACCTGCGGAAGGATCATTATTGAATTATGTTTCTAGATAGGTTGTAG

CTGGCTC-TTTAGAGCATGTGCACGCCTGTTTGGACTTCATTTTCATCCACCTGTGCACC
TATTGTAGTCTTTGGTTGGGTTAGGAGGAAGTGGTCATTGTGTCAGCATCTGCTGGATGT
GAGGACTTGCATTGTGAAAGCTTTGCTGTCCTTGATGTGATCATGGAATCTCTTTCTCAC
TAGAGTCTATGTCACTCATTATACTCTGTCGAATGTCATTGAATGTCTTTACATGGGCTT
ATATGCCTATGAAAATTGTAATACAACTTTCAGCAACGGATCTCTTGGCTCTCGCATCGA
TGAAGAACGCAGCGAAATGCGATAAGTAATGTGAATTGCAGAATTCAGTGAATCATCGAA
TCTTTGAACGCATCTTGCGCTCCTTGGTATTCCGAGGAGCATGCCTGTTTGAGTGTCATT
AAATTCTCAACTCTCTTCTAC--TTTTTGTAAAAGAGAGCTTGGACTGTGGAGGCTTGCT
GGCCACTTTTTGGGGTCAGCTCCTCTGAAATGCATTAGCGGAACCGTTTGCGATCTGCCA
CAAGTGTGATAAGTTATCTACACTGGCGAGGGGATTGCTCTCTGTAATGTTCAGCTTCTA
ATTGTCTCTACTTTGTGAGACTACTTTTGAATGCTTGACCTCAAATCAGGTAGGACTACC
CGCTGAACTTAA
>ABC12-27
TTTCCGTAGGTGAACCTGCGGAAGGATCATTATTGAATTATGTTTCTAGATAGGTTGTAG
CTGGCTC-TTTAGAGCATGTGCACGCCTGTTTGGACTTCATTTTCATCCACCTGTGCACC
TATTGTAGTCTTTGGTTGGGTTAGGAGGAAGTGGTCATTGTGTCAGCATCTGCTGGATGT
GAGGACTTGCATTGTGAAAGCTTTGCTGTCCTTGATGTGATCATGGAATCTCTTTCTCAC
TAGAGTCTATGTCACTCATTATACTCTGTCGAATGTCATTGAATGTCTTTACATGGGCTT
ATATGCCTATGAAAATTGTAATACAACTTTCAGCAACGGATCTCTTGGCTCTCGCATCGA
TGAAGAACGCAGCGAAATGCGATAAGTAATGTGAATTGCAGAATTCAGTGAATCATCGAA
TCTTTGAACGCATCTTGCGCTCCTTGGTATTCCGAGGAGCATGCCTGTTTGAGTGTCATT
AAATTCTCAACTCTCTTCTAC--TTTTTGTAAAAGAGAGCTTGGACTGTGGAGGCTTGCT
GGCCACTTTTTGGGGTCAGCTCCTCTGAAATGCATTAGCGGAACCGTTTGCGATCTGCCA
CAAGTGTGATAAGTTATCTACACTGGCGAGGGGATTGCTCTCTGTAATGTTCAGCTTCTA
ATTGTCTCTACTTTGTGAGACTACTTTTGAATGCTTGACCTCAAATCAGGTAGGACTACC
CGCTGAACTTAA
>ABC12-33
TTTCCGTAGGTGAACCTGCGGAAGGATCATTATTGAATTATGTTTCTAGATAGGTTGTAG
CTGGCTC-TTTAGAGCATGTGCACGCCTGTTTGGACTTCATTTTCATCCACCTGTGCACC
TATTGTAGTCTTTGGTTGGGTTAGGAGGAAGTGGTCATTGTGTCAGCATCTGCTGGATGT
GAGGACTTGCATTGTGAAAGCTTTGCTGTCCTTGATGTGATCATGGAATCTCTTTCTCAC
TAGAGTCTATGTCACTCATTATACTCTGTCGAATGTCATTGAATGTCTTTACATGGGCTT
ATATGCCTATGAAAATTGTAATACAACTTTCAGCAACGGATCTCTTGGCTCTCGCATCGA
TGAAGAACGCAGCGAAATGCGATAAGTAATGTGAATTGCAGAATTCAGTGAATCATCGAA
TCTTTGAACGCATCTTGCGCTCCTTGGTATTCCGAGGAGCATGCCTGTTTGAGTGTCATT
AAATTCTCAACTCTCTTCTAC--TTTTTGTAAAAGAGAGCTTGGACTGTGGAGGCTTGCT
GGCCACTTTTTGGGGTCAGCTCCTCTGAAATGCATTAGCGGAACCGTTTGCGATCTGCCA
CAAGTGTGATAAGTTATCTACACTGGCGAGGGGATTGCTCTCTGTAATGTTCAGCTTCTA
ATTGTCTCTACTTTGTGAGACTACTTTTGAATGCTTGACCTCAAATCAGGTAGGACTACC
CGCTGAACTTAA
>ABC12-36
TTTCCGTAGGTGAACCTGCGGAAGGATCATTATTGAATTATGTTTCTAGATAGGTTGTAG
CTGGCTC-TTTAGAGCATGTGCACGCCTGTTTGGACTTCATTTTCATCCACCTGTGCACC
TATTGTAGTCTTTGGTTGGGTTAGGAGGAAGTGGTCATTGTGTCAGCATCTGCTGGATGT
GAGGACTTGCATTGTGAAAGCTTTGCTGTCCTTGATGTGATCATGGAATCTCTTTCTCAC
TAGAGTCTATGTCACTCATTATACTCTGTCGAATGTCATTGAATGTCTTTACATGGGCTT
ATATGCCTATGAAAATTGTAATACAACTTTCAGCAACGGATCTCTTGGCTCTCGCATCGA
TGAAGAACGCAGCGAAATGCGATAAGTAATGTGAATTGCAGAATTCAGTGAATCATCGAA
TCTTTGAACGCATCTTGCGCTCCTTGGTATTCCGAGGAGCATGCCTGTTTGAGTGTCATT
AAATTCTCAACTCTCTTCTAC--TTTTTGTAAAAGAGAGCTTGGACTGTGGAGGCTTGCT

GGCCACTTTTTGGGGTCAGCTCCTCTGAAATGCATTAGCGGAACCGTTTGCGATCTGCCA
CAAGTGTGATAAGTTATCTACACTGGCGAGGGGATTGCTCTCTGTAATGTTCAGCTTCTA
ATTGTCTCTACTTTGTGAGACTACTTTTGAATGCTTGACCTCAAATCAGGTAGGACTACC
CGCTGAACTTAA
>ABC12-37
TTTCCGTAGGTGAACCTGCGGAAGGATCATTATTGAATTATGTTTCTAGATAGGTTGTAG
CTGGCTC-TTTAGAGCATGTGCACGCCTGTTTGGACTTCATTTTCATCCACCTGTGCACC
TATTGTAGTCTTTGGTTGGGTTAGGAGGAAGTGGTCATTGTGTCAGCATCTGCTGGATGT
GAGGACTTGCATTGTGAAAGCTTTGCTGTCCTTGATGTGATCATGGAATCTCTTTCTCAC
TAGAGTCTATGTCACTCATTATACTCTGTCGAATGTCATTGAATGTCTTTACATGGGCTT
ATATGCCTATGAAAATTGTAATACAACTTTCAGCAACGGATCTCTTGGCTCTCGCATCGA
TGAAGAACGCAGCGAAATGCGATAAGTAATGTGAATTGCAGAATTCAGTGAATCATCGAA
TCTTTGAACGCATCTTGCGCTCCTTGGTATTCCGAGGAGCATGCCTGTTTGAGTGTCATT
AAATTCTCAACTCTCTTCTAC--TTTTTGTAAAAGAGAGCTTGGACTGTGGAGGCTTGCT
GGCCACTTTTTGGGGTCAGCTCCTCTGAAATGCATTAGCGGAACCGTTTGCGATCTGCCA
CAAGTGTGATAAGTTATCTACACTGGCGAGGGGATTGCTCTCTGTAATGTTCAGCTTCTA
ATTGTCTCTACTTTGTGAGACTACTTTTGAATGCTTGACCTCAAATCAGGTAGGACTACC
CGCTGAACTTAA
>ABC12-39
TTTCCGTAGGTGAACCTGCGGAAGGATCATTATTGAATTATGTTTCTAGATAGGTTGTAG
CTGGCTC-TTTAGAGCATGTGCACGCCTGTTTGGACTTCATTTTCATCCACCTGTGCACC
TATTGTAGTCTTTGGTTGGGTTAGGAGGAAGTGGTCATTGTGTCAGCATCTGCTGGATGT
GAGGACTTGCATTGTGAAAGCTTTGCTGTCCTTGATGTGATCATGGAATCTCTTTCTCAC
TAGAGTCTATGTCACTCATTATACTCTGTCGAATGTCATTGAATGTCTTTACATGGGCTT
ATATGCCTATGAAAATTGTAATACAACTTTCAGCAACGGATCTCTTGGCTCTCGCATCGA
TGAAGAACGCAGCGAAATGCGATAAGTAATGTGAATTGCAGAATTCAGTGAATCATCGAA
TCTTTGAACGCATCTTGCGCTCCTTGGTATTCCGAGGAGCATGCCTGTTTGAGTGTCATT
AAATTCTCAACTCTCTTCTAC--TTTTTGTAAAAGAGAGCTTGGACTGTGGAGGCTTGCT
GGCCACTTTTTGGGGTCAGCTCCTCTGAAATGCATTAGCGGAACCGTTTGCGATCTGCCA
CAAGTGTGATAAGTTATCTACACTGGCGAGGGGATTGCTCTCTGTAATGTTCAGCTTCTA
ATTGTCTCTACTTTGTGAGACTACTTTTGAATGCTTGACCTCAAATCAGGTAGGACTACC
CGCTGAACTTAA
>ABC12-40
TTTCCGTAGGTGAACCTGCGGAAGGATCATTATTGAATTATGTTTCTAGATAGGTTGTAG
CTGGCTC-TTTAGAGCATGTGCACGCCTGTTTGGACTTCATTTTCATCCACCTGTGCACC
TATTGTAGTCTTTGGTTGGGTTAGGAGGAAGTGGTCATTGTGTCAGCATCTGCTGGATGT
GAGGACTTGCATTGTGAAAGCTTTGCTGTCCTTGATGTGATCATGGAATCTCTTTCTCAC
TAGAGTCTATGTCACTCATTATACTCTGTCGAATGTCATTGAATGTCTTTACATGGGCTT
ATATGCCTATGAAAATTGTAATACAACTTTCAGCAACGGATCTCTTGGCTCTCGCATCGA
TGAAGAACGCAGCGAAATGCGATAAGTAATGTGAATTGCAGAATTCAGTGAATCATCGAA
TCTTTGAACGCATCTTGCGCTCCTTGGTATTCCGAGGAGCATGCCTGTTTGAGTGTCATT
AAATTCTCAACTCTCTTCTAC--TTTTTGTAAAAGAGAGCTTGGACTGTGGAGGCTTGCT
GGCCACTTTTTGGGGTCAGCTCCTCTGAAATGCATTAGCGGAACCGTTTGCGATCTGCCA
CAAGTGTGATAAGTTATCTACACTGGCGAGGGGATTGCTCTCTGTAATGTTCAGCTTCTA
ATTGTCTCTACTTTGTGAGACTACTTTTGAATGCTTGACCTCAAATCAGGTAGGACTACC
CGCTGAACTTAA
>ABC5-82
TTTCCGTAGGTGAACCTGCGGAAGGATCATTATTGAATTATGTTTCTAGATAGGTTGTAG
CTGGCTC-TTTAGAGCATGTGCACGCCTGTTTGGACTTCATTTTCATCCACCTGTGCACC
TATTGTAGTCTTTGGTTGGGTTAGGAGGAAGTGGTCATTGTGTCAGCATCTGCTGGATGT

GAGGACTTGCATTGTGAAAGCTTTGCTGTCCTTGATGTGATCATGGAATCTCTTTCTCAC
TAGAGTCTATGTCACTCATTATACTCTGTCGAATGTCATTGAATGTCTTTACATGGGCTT
ATATGCCTATGAAAATTGTAATACAACTTTCAGCAACGGATCTCTTGGCTCTCGCATCGA
TGAAGAACGCAGCGAAATGCGATAAGTAATGTGAATTGCAGAATTCAGTGAATCATCGAA
TCTTTGAACGCATCTTGCGCTCCTTGGTATTCCGAGGAGCATGCCTGTTTGAGTGTCATT
AAATTCTCAACTCTCTTCTAC--TTTTTGTAAAAGAGAGCTTGGACTGTGGAGGCTTGCT
GGCCACTTTTTGGGGTCAGCTCCTCTGAAATGCATTAGCGGAACCGTTTGCGATCTGCCA
CAAGTGTGATAAGTTATCTACACTGGCGAGGGGATTGCTCTCTGTAATGTTCAGCTTCTA
ATTGTCTCTACTTTGTGAGACTACTTTTGAATGCTTGACCTCAAATCAGGTAGGACTACC
CGCTGAACTTAA
>ABC3-47
TTTCCGTAGGTGAACCTGCGGAAGGATCATTATTGAATTATGTTTCTAGATAGGTTGTAG
CTGGCTC-TTTAGAGCATGTGCACGCCTGTTTGGACTTCATTTTCATCCACCTGTGCACC
TATTGTAGTCTTTGGTTGGGTTAGGAGGAAGTGGTCATTGTGTCAGCATCTGCTGGATGT
GAGGACTTGCATTGTGAAAGCTTTGCTGTCCTTGATGTGATCATGGAATCTCTTTCTCAC
TAGAGTCTATGTCACTCATTATACTCTGTCGAATGTCATTGAATGTCTTTACATGGGCTT
ATATGCCTATGAAAATTGTAATACAACTTTCAGCAACGGATCTCTTGGCTCTCGCATCGA
TGAAGAACGCAGCGAAATGCGATAAGTAATGTGAATTGCAGAATTCAGTGAATCATCGAA
TCTTTGAACGCATCTTGCGCTCCTTGGTATTCCGAGGAGCATGCCTGTTTGAGTGTCATT
AAATTCTCAACTCTCTTCTAC--TTTTTGTAAAAGAGAGCTTGGACTGTGGAGGCTTGCT
GGCCACTTTTTGGGGTCAGCTCCTCTGAAATGCATTAGCGGAACCGTTTGCGATCTGCCA
CAAGTGTGATAAGTTATCTACACTGGCGAGGGGATTGCTCTCTGTAATGTTCAGCTTCTA
ATTGTCTCTACTTTGTGAGACTACTTTTGAATGCTTGACCTCAAATCAGGTAGGACTACC
CGCTGAACTTAA
>ABC10-47
TTTCCGTAGGTGAACCTGCGGAAGGATCATTATTGAATTATGTTTCTAGATAGGTTGTAG
CTGGCTC-TTTAGAGCATGTGCACGCCTGTTTGGACTTCATTTTCATCCACCTGTGCACC
TATTGTAGTCTTTGGTTGGGTTAGGAGGAAGTGGTCATTGTGTCAGCATCTGCTGGATGT
GAGGACTTGCATTGTGAAAGCTTTGCTGTCCTTGATGTGATCATGGAATCTCTTTCTCAC
TAGAGTCTATGTCACTCATTATACTCTGTCGAATGTCATTGAATGTCTTTACATGGGCTT
ATATGCCTATGAAAATTGTAATACAACTTTCAGCAACGGATCTCTTGGCTCTCGCATCGA
TGAAGAACGCAGCGAAATGCGATAAGTAATGTGAATTGCAGAATTCAGTGAATCATCGAA
TCTTTGAACGCATCTTGCGCTCCTTGGTATTCCGAGGAGCATGCCTGTTTGAGTGTCATT
AAATTCTCAACTCTCTTCTAC--TTTTTGTAAAAGAGAGCTTGGACTGTGGAGGCTTGCT
GGCCACTTTTTGGGGTCAGCTCCTCTGAAATGCATTAGCGGAACCGTTTGCGATCTGCCA
CAAGTGTGATAAGTTATCTACACTGGCGAGGGGATTGCTCTCTGTAATGTTCAGCTTCTA
ATTGTCTCTACTTTGTGAGACTACTTTTGAATGCTTGACCTCAAATCAGGTAGGACTACC
CGCTGAACTTAA
>ABC5-81
TTTCCGTAGGTGAACCTGCGGAAGGATCATTATTGAATTATGTTTCTAGATAGGTTGTAG
CTGGCTC-TTTAGAGCATGTGCACGCCTGTTTGGACTTCATTTTCATCCACCTGTGCACC
TATTGTAGTCTTTGGTTGGGTTAGGGGGAAGTGGTCATTGTGTCAGCATCTGCTGGATGT
GAGGACTTGCATTGTGAAAGCTTTGCTGTCCTTGATGTGATCATGGAATCTCTTTCTCAC
TAGAGTCTATGTCACTCATTATACTCTGTCGAATGTCATTGAATGTCTTTACATGGGCTT
ATATGCCTATGAAAATTGTAATACAACTTTCAGCAACGGATCTCTTGGCTCTCGCATCGA
TGAAGAACGCAGCGAAATGCGATAAGTAATGTGAATTGCAGAATTCAGTGAATCATCGAA
TCTTTGAACGCATCTTGCGCTCCTTGGTATTCCGAGGAGCATGCCTGTTTGAGTGTCATT
AAATTCTCAACTCTCTTCTAC--TTTTTGTAAAAGAGAGCTTGGACTGTGGAGGCTTGCT
GGCCACTTTTTGGGGTCAGCTCCTCTGAAATGCATTAGCGGAACCGTTTGCGATCTGCCA
CAAGTGTGATAAGTTATCTACACTGGCGAGGGGATTGCTCTCTGTAATGTTCAGCTTCTA

ATTGTCTCTACTTTGTGAGACTACTTTTGAATGCTTGACCTCAAATCAGGTAGGACTACC
CGCTGAACTTAA
>ABC8-49
TTTCCGTAGGTGAACCTGCGGAAGGATCATTATTGAATTATGTTTCTAGATAGGTTGTAG
CTGGCTC-TTTAGAGCATGTGCACGCCTGTTTGGACTTCATTTTCATCCACCTGTGCACC
TATTGTAGTCTTTGGTTGGGTTAGGGGGAAGTGGTCATTGTGTCAGCATCTGCTGGATGT
GAGGACTTGCATTGTGAAAGCTTTGCTGTCCTTGATGTGATCATGGAATCTCTTTCTCAC
TAGAGTCTATGTCACTCATTATACTCTGTCGAATGTCATTGAATGTCTTTACATGGGCTT
ATATGCCTATGAAAATTGTAATACAACTTTCAGCAACGGATCTCTTGGCTCTCGCATCGA
TGAAGAACGCAGCGAAATGCGATAAGTAATGTGAATTGCAGAATTCAGTGAATCATCGAA
TCTTTGAACGCATCTTGCGCTCCTTGGTATTCCGAGGAGCATGCCTGTTTGAGTGTCATT
AAATTCTCAACTCTCTTCTAC--TTTTTGTAAAAGAGAGCTTGGACTGTGGAGGCTTGCT
GGCCACTTTTTGGGGTCAGCTCCTCTGAAATGCATTAGCGGAACCGTTTGCGATCTGCCA
CAAGTGTGATAAGTTATCTACACTGGCGAGGGGATTGCTCTCTGTAATGTTCAGCTTCTA
ATTGTCTCTACTTTGTGAGACTACTTTTGAATGCTTGACCTCAAATCAGGTAGGACTACC
CGCTGAACTTAA
>ABC9-38
TTTCCGTAGGTGAACCTGCGGAAGGATCATTATTGAATTATGTTTCTAGATAGGTTGTAG
CTGGCTC-TTTAGAGCATGTGCACGCCTGTTTGGACTTCATTTTCATCCACCTGTGCACC
TATTGTAGTCTTTGGTTGGGTTAGGGGGAAGTGGTCATTGTGTCAGCATCTGCTGGATGT
GAGGACTTGCATTGTGAAAGCTTTGCTGTCCTTGATGTGATCATGGAATCTCTTTCTCAC
TAGAGTCTATGTCACTCATTATACTCTGTCGAATGTCATTGAATGTCTTTACATGGGCTT
ATATGCCTATGAAAATTGTAATACAACTTTCAGCAACGGATCTCTTGGCTCTCGCATCGA
TGAAGAACGCAGCGAAATGCGATAAGTAATGTGAATTGCAGAATTCAGTGAATCATCGAA
TCTTTGAACGCATCTTGCGCTCCTTGGTATTCCGAGGAGCATGCCTGTTTGAGTGTCATT
AAATTCTCAACTCTCTTCTAC--TTTTTGTAAAAGAGAGCTTGGACTGTGGAGGCTTGCT
GGCCACTTTTTGGGGTCAGCTCCTCTGAAATGCATTAGCGGAACCGTTTGCGATCTGCCA
CAAGTGTGATAAGTTATCTACACTGGCGAGGGGATTGCTCTCTGTAATGTTCAGCTTCTA
ATTGTCTCTACTTTGTGAGACTACTTTTGAATGCTTGACCTCAAATCAGGTAGGACTACC
CGCTGAACTTAA
>ABC11-53
TTTCCGTAGGTGAACCTGCGGAAGGATCATTATTGAATTATGTTTCTAGATAGGTTGTAG
CTGGCTC-TTTAGAGCATGTGCACGCCTGTTTGGACTTCATTTTCATCCACCTGTGCACC
TATTGTAGTCTTTGGTTGGGTTAGGGGGAAGTGGTCATTGTGTCAGCATCTGCTGGATGT
GAGGACTTGCATTGTGAAAGCTTTGCTGTCCTTGATGTGATCATGGAATCTCTTTCTCAC
TAGAGTCTATGTCACTCATTATACTCTGTCGAATGTCATTGAATGTCTTTACATGGGCTT
ATATGCCTATGAAAATTGTAATACAACTTTCAGCAACGGATCTCTTGGCTCTCGCATCGA
TGAAGAACGCAGCGAAATGCGATAAGTAATGTGAATTGCAGAATTCAGTGAATCATCGAA
TCTTTGAACGCATCTTGCGCTCCTTGGTATTCCGAGGAGCATGCCTGTTTGAGTGTCATT
AAATTCTCAACTCTCTTCTAC--TTTTTGTAAAAGAGAGCTTGGACTGTGGAGGCTTGCT
GGCCACTTTTTGGGGTCAGCTCCTCTGAAATGCATTAGCGGAACCGTTTGCGATCTGCCA
CAAGTGTGATAAGTTATCTACACTGGCGAGGGGATTGCTCTCTGTAATGTTCAGCTTCTA
ATTGTCTCTACTTTGTGAGACTACTTTTGAATGCTTGACCTCAAATCAGGTAGGACTACC
CGCTGAACTTAA
>ABC12-34
TTTCCGTAGGTGAACCTGCGGAAGGATCATTATTGAATTATGTTTCTAGATAGGTTGTAG
CTGGCTC-TTTAGAGCATGTGCACGCCTGTTTGGACTTCATTTTCATCCACCTGTGCACC
TATTGTAGTCTTTGGTTGGGTTAGGGGGAAGTGGTCATTGTGTCAGCATCTGCTGGATGT
GAGGACTTGCATTGTGAAAGCTTTGCTGTCCTTGATGTGATCATGGAATCTCTTTCTCAC
TAGAGTCTATGTCACTCATTATACTCTGTCGAATGTCATTGAATGTCTTTACATGGGCTT

ATATGCCTATGAAAATTGTAATACAACTTTCAGCAACGGATCTCTTGGCTCTCGCATCGA
TGAAGAACGCAGCGAAATGCGATAAGTAATGTGAATTGCAGAATTCAGTGAATCATCGAA
TCTTTGAACGCATCTTGCGCTCCTTGGTATTCCGAGGAGCATGCCTGTTTGAGTGTCATT
AAATTCTCAACTCTCTTCTAC--TTTTTGTAAAAGAGAGCTTGGACTGTGGAGGCTTGCT
GGCCACTTTTTGGGGTCAGCTCCTCTGAAATGCATTAGCGGAACCGTTTGCGATCTGCCA
CAAGTGTGATAAGTTATCTACACTGGCGAGGGGATTGCTCTCTGTAATGTTCAGCTTCTA
ATTGTCTCTACTTTGTGAGACTACTTTTGAATGCTTGACCTCAAATCAGGTAGGACTACC
CGCTGAACTTAA
>ABC7-55
TTTCCGTAGGTGAACCTGCGGAAGGATCATTATTGAATTATGTTTCTAGATAGGTTGTAG
CTGGCTC-TTTAGAGCATGTGCACGCCTGTTTGGACTTCATTTTCATCCACCTGTGCACC
TATTGTAGTCTTTGGTTGGGTTAGGAGGAAGTGATCATTGTGTCAGCATCTGCTGGATGT
GAGGACTTGCATTGTGAAAGCTTTGCTGTCCTTGATGTGATCATGGAATCTCTTTCTCAC
TAGAGTCTATGTCACTCATTATACTCTGTCGAATGTCATTGAATGTCTTTACATGGGCTT
ATATGCCTATGAAAATTGTAATACAACTTTCAGCAACGGATCTCTTGGCTCTCGCATCGA
TGAAGAACGCAGCGAAATGCGATAAGTAATGTGAATTGCAGAATTCAGTGAATCATCGAA
TCTTTGAACGCATCTTGCGCTCCTTGGTATTCCGAGGAGCATGCCTGTTTGAGTGTCATT
AAATTCTCAACTCTCTTCTAC--TTTTTGTAAAAGAGAGCTTGGACTGTGGAGGCTTGCT
GGCCACTTTTTGGGGTCAGCTCCTCTGAAATGCATTAGCGGAACCGTTTGCGATCTGCCA
CAAGTGTGATAAGTTATCTACACTGGCGAGGGGATTGCTCTCTGTAATGTTCAGCTTCTA
ATTGTCTCTACTTTGTGAGACTACTTTTGAATGCTTGACCTCAAATCAGGTAGGACTACC
CGCTGAACTTAA
>ABC12-50
TTTCCGTAGGTGAACCTGCGGAAGGATCATTATTGAATTATGTTTCTAGATAGGTTGTAG
CTGGCTC-TTTAGAGCATGTGCACGCCTGTTTGGACTTCATTTTCATCCACCTGTGCACC
TATTGTAGTCTTTGGTTGGGTTAGGAGGAAGTGGTCATTGTGTCAGCATCTGCTGGATGT
GAGGACTTGCATTGTGAAAGCTTTGCTGTCCTTGATGTGATCATGGAATCTCTTTCTCAC
TAGAGTCTATGTCACTCATTATACTCTGTCGAATGTCATTGAATGTCTTTACATGGGCTT
ATATGCCTATGAAAATTGTAATACAACTTTCAGCAACGGATCTCTTGGCTCTCGCATCGA
TGAAGAACGCAGCGAAATGCGATAAGTAATGTGAATTGCAGAATTCAGTGAATCATCGAA
TCTTTGAACGCATCTTGCGCTCCTTGGTATTCCGAGGAGCATGCCTGTTTGAGTGTCATT
AAATTCTCAACTCTCTTCTAC--TTTTTGTAAAAGAGAGCTTGGACTGTGGAGGCTTGCT
GGCCACTTTTTGGGGTCAGCTCCTCTGAAATGCATTAGCGGAACCGTTTGCGATCTGCCA
CAAGTGTGATAAGTTATCTGCACTGGCGAGGGGATTGCTCTCTGTAATGTTCAGCTTCTA
ATTGTCTCTACTTTGTGAGACTACTTTTGAATGCTTGACCTCAAATCAGGTAGGACTACC
CGCTGAACTTAA
>ABC9-13
TTTCCGTAGGTGAACCTGCGGAAGGATCATTATTGAATTATGTTTCTAGATAGGTTGTAG
CTGGCTC-TTTAGAGCATGTGCACGCCTGTTTGGACTTCATTTTCATCCACCTGTGCACC
TATTGTAGTCTTTGGTTGGGTTAGGAGGAAGTGGTCATTGTGTCAGCATCTGCTGGATGT
GAGGACTTGCATTGTGAAAGCTTTGCTGTCCTTGATGTGATCATGGAATCTCTTTCTCAC
TAGAGTCTATGTCACTCATTATACTCTGTCGAATGTCATTGAATGTCTTTACATGGGCTT
ATATGCCTATGAAAATTGTAATACAACTTTCAGCAACGGATCTCTTGGCTCTCGCATCGA
TGAAGAACGCAGCGAAATGCGATAAGTAATGTGAATTGCAGAATTCAGTGAATCATCGAA
TCTTTGAACGCATCTTGCGCTCCTTGGTATTCCGAGGAGCATGCCTGTTTGAGTGTCATT
AAATTCTCAACTCTCTTCTAC--TTTTTGTAAAAGAGAGCTTGGACTGTGGAGGCTTGCT
GGCCACTTTTTGGGGTCAGCTCCTCTGAAATGCATTAGCGGAACCGTTTGCGATCTGCCA
CAAGTGTGATAAGTTATCTACACTGGCGAGGGGATTGTTCTCTGTAATGTTCAGCTTCTA
ATTGTCTCTACTTTGTGAGACTACTTTTGAATGCTTGACCTCAAATCAGGTAGGACTACC
CGCTGAACTTAA

>ABC4-38
TTTCCGTAGGTGAACCTGCGGAAGGATCATTATTGAATTATGTTTCTAGATAGGTTGTAG
CTGGCTC-TTTAGAGCATGTGCACGCCTGTTTGGACTTCATTTTCATCCACCTGTGCACC
TATTGTAGTCTTTGGTTGGGTTAGGAGGAAGTGGTCATTGTGTCAGCATCTGCTGGATGT
GAGGACTTGCATTGTGAAAGCTTTGCTGTCCTTGATGTGATCATGGAATCTCTTTCTCAC
TAGAGTCTATGTCACTCATTATACTCTGTCGAATGTCATTGAATGTCTTTACATGGGCTT
ATATGCCTATGAAAATTGTAATACAACTTTCAGCAACGGATCTCTTGGCTCTCGCATCGA
TGAAGAACGCAGCGAAATGCGATAAGTAATGTGAATTGCAGAATTCAGTGAATCATCGAA
TCTTTGAACGCATCTTGCGCTCCTTGGTATTCCGAGGAGCATGCCTGTTTGAGTGTCATT
AAATTCTCAACTCTCTTCTAC--TTTTTGTAAAAGAGAGCTTGGACTGTGGAGGCTTGCT
GGCCACTTTTTGGGGTCAGCTCCTCTGAAATGCATTAGTGGAACCGTTTGCGATCTGCCA
CAAGTGTGATAAGTTATCTACACTGGCGAGGGGATTGCTCTCTGTAATGTTCAGCTTCTA
ATTGTCTCTACTTTGTGAGACTACTTTTGAATGCTTGACCTCAAATCAGGTAGGACTACC
CGCTGAACTTAA
>ABC11-9
TTTCCGTAGGTGAACCTGCGGAAGGATCATTATTGAATTATGTTTCTAGATAGGTTGTAG
CTGGCTC-TTTAGAGCATGTGCACGCCTGTTTGGACTTCATTTTCATCCACCTGTGCACC
TATTGTAGTCTTTGGTTGGGTTAGGAGGAAGTGGTCATTGTGTCAGCATCTGCTGGATGT
GAGGACTTGCATTGTGAAAGCTTTGTTGTCCTTGATGTGATCATGGAATCTCTTTCTCAC
TAGAGTCTATGTCACTCATTATACTCTGTCGAATGTCATTGAATGTCTTTACATGGGCTT
ATATGCCTATGAAAATTGTAATACAACTTTCAGCAACGGATCTCTTGGCTCTCGCATCGA
TGAAGAACGCAGCGAAATGCGATAAGTAATGTGAATTGCAGAATTCAGTGAATCATCGAA
TCTTTGAACGCATCTTGCGCTCCTTGGTATTCCGAGGAGCATGCCTGTTTGAGTGTCATT
AAATTCTCAACTCTCTTCTAC--TTTTTGTAAAAGAGAGCTTGGACTGTGGAGGCTTGCT
GGCCACTTTTTGGGGTCAGCTCCTCTGAAATGCATTAGCGGAACCGTTTGCGATCTGCCA
CAAGTGTGATAAGTTATCTACACTGGCGAGGGGATTGCTCTCTGTAATGTTCAGCTTCTA
ATTGTCTCTACTTTGTGAGACTACTTTTGAATGCTTGACCTCAAATCAGGTAGGACTACC
CGCTGAACTTAA
>ABC8-43
TTTCCGTAGGTGAACCTGCGGAAGGATCATTATTGAATTATGTTTCTAGATAGGTTGTAG
CTGGCTC-TTTAGAGCATGTGCACGCCTGTTTGGACTTCATTTTCATCCACCTGTGCACC
TATTGTAGTCTTTGGTTGGGTTAGGAGGAAGTGGTCATTGTGTCAGCATCTGCTGGATGT
GAGGACTTGCATTGTGAAAGCTTTGCTGTCCTTGATGTGATCATGGAATCTCTTTCTCAC
TAGAGTCTATGTCACTCATTATACTCTGTCGAATGTCATTGAATGTCTTTACATGGGCTT
ATATGCCTATGAAAATTGTAATACAACTTTCAGCAACGGATCTCTTGGCTCTCGCATCGA
TGAAGAACGCAGCGAAATGCGATAAGTAATGTGAATTGCAGAATTCAGTGAATCATCGAA
TCTTTGAACACATCTTGCGCTCCTTGGTATTCCGAGGAGCATGCCTGTTTGAGTGTCATT
AAATTCTCAACTCTCTTCTAC--TTTTTGTAAAAGAGAGCTTGGACTGTGGAGGCTTGCT
GGCCACTTTTTGGGGTCAGCTCCTCTGAAATGCATTAGCGGAACCGTTTGCGATCTGCCA
CAAGTGTGATAAGTTATCTACACTGGCGAGGGGATTGCTCTCTGTAATGTTCAGCTTCTA
ATTGTCTCTACTTTGTGAGACTACTTTTGAATGCTTGACCTCAAATCAGGTAGGACTACC
CGCTGAACTTAA
>ABC7-30
TTTCCGTAGGTGAACCTGCGGAAGGATCATTATTGAATTATGTTTCTAGATAGGTTGTAG
CTGGCTC-TTTAGAGCATGTGCACGCCTGTTTGGACTTCATTTTCATCCACCTGTGCACC
TATTGTAGTCTTTGGTTGGGTTAGGAGGAAGTGGTCATTGTGTCAGCATCTGCTGGATGT
GAGGACTTACATTGTGAAAGCTTTGCTGTCCTTGATGTGATCATGGAATCTCTTTCTCAC
TAGAGTCTATGTCACTCATTATACTCTGTCGAATGTCATTGAATGTCTTTACATGGGCTT
ATATGCCTATGAAAATTGTAATACAACTTTCAGCAACGGATCTCTTGGCTCTCGCATCGA
TGAAGAACGCAGCGAAATGCGATAAGTAATGTGAATTGCAGAATTCAGTGAATCATCGAA

TCTTTGAACGCATCTTGCGCTCCTTGGTATTCCGAGGAGCATGCCTGTTTGAGTGTCATT
AAATTCTCAACTCTCTTCTAC--TTTTTGTAAAAGAGAGCTTGGACTGTGGAGGCTTGCT
GGCCACTTTTTGGGGTCAGCTCCTCTGAAATGCATTAGCGGAACCGTTTGCGATCTGCCA
CAAGTGTGATAAGTTATCTACACTGGCGAGGGGATTGCTCTCTGTAATGTTCAGCTTCTA
ATTGTCTCTACTTTGTGAGACTACTTTTGAATGCTTGACCTCAAATCAGGTAGGACTACC
CGCTGAACTTAA
>ABC1-51
TTTCCGTAGGTGAACCTGCGGAAGGATCATTATTGAATTATGTTTCTAGATAGGTTGTAG
CTGGCTC-TTTAGAGCATGTGCACGCCTGTTTGGACTTCATTTTCATCCACCTGTGCACC
TATTGTAGTCTTTGGTTGGGTTAGGGGGAAGTGGTCATTGTGTCAGCATCTGCTGGATGT
GAGGACTTGCATTGTGAAAGCTTTGCTGTCCTTGATGTGATCATGGAATCTCTTTCTCAC
TAGAGTCTATGTCACTCATTATACTCTGTCGAATGTCATTGAATGTCTTTACATGGGCTT
GTATGCCTATGAAAATTGTAATACAACTTTCAGCAACGGATCTCTTGGCTCTCGCATCGA
TGAAGGACGCAGCGAAATGCGATAAGTAATGTGAATTGCAGAATTCAGTGAATCATCGAA
TCTTTGAACGCATCTTGCGCTCCTTGGTATTCCGAGGAGCATGCCTGTTTGAGTGTCATT
AAATTCTCAACTCTCTTCTAC--TTTTTGTAAAAGAGAGCTTGGACTGTGGAGGCTTGCT
GGCCACTTTTTGGGGTCAGCTCCTCTGAAATGCATTAGCGGAACCGTTTGCGATCTGCCA
CAAGTGTGATAAGTTATCTACACTGGCGAGGGGATTGCTCTCTGTAATGTTCAGCTTCTA
ATTGTCTCTACTTTGTGAGACTACTTTTGAATGCTTGACCTCAAATCAGGTAGGACTACC
CGCTGAACTTAA
>ABC7-12
TTTCCGTAGGTGAACCTGCGGAAGGATCATTATTGAATTATGTTTCTAGATAGGTTGTAG
CTGGCTC-TTTAGAGCATGTGCACGCCTGTTTGGACTTCATTTTCATCCACCTGTGCACC
TATTGTAGTCTTTGGTTGGGTTAGGGGGAAGTGGTCATTGTGTCAGCATCTGCTGGATGT
GAGGACTTGCATTGTGAAAGCTTTGCTGTCCTTGATGTGATCATGGAATCTCTTTCTCAC
TAGAGTCTATGTCACTCATTATACTCTGTCGAATGTCATTGAATGTCTTTACATGGGCTT
GTATGCCTATGAAAATTGTAATACAACTTTCAGCAACGGATCTCTTGGCTCTCGCATCGA
TGAAGGACGCAGCGAAATGCGATAAGTAATGTGAATTGCAGAATTCAGTGAATCATCGAA
TCTTTGAACGCATCTTGCGCTCCTTGGTATTCCGAGGAGCATGCCTGTTTGAGTGTCATT
AAATTCTCAACTCTCTTCTAC--TTTTTGTAAAAGAGAGCTTGGACTGTGGAGGCTTGCT
GGCCACTTTTTGGGGTCAGCTCCTCTGAAATGCATTAGCGGAACCGTTTGCGATCTGCCA
CAAGTGTGATAAGTTATCTACACTGGCGAGGGGATTGCTCTCTGTAATGTTCAGCTTCTA
ATTGTCTCTACTTTGTGAGACTACTTTTGAATGCTTGACCTCAAATCAGGTAGGACTACC
CGCTGAACTTAA
>ABC8-5
TTTCCGTAGGTGAACCTGCGGAAGGATCATTATTGAATTATGTTTCTAGATAGGTTGTAG
CTGGCTC-TTTAGAGCATGTGCACGCCTGTTTGGACTTCATTTTCATCCACCTGTGCACC
TATTGTAGTCTTTGGTTGGGTTAGGGGGAAGTGGTCATTGTGTCAGCATCTGCTGGATGT
GAGGACTTGCATTGTGAAAGCTTTGCTGTCCTTGATGTGATCATGGAATCTCTTTCTCAC
TAGAGTCTATGTCACTCATTATACTCTGTCGAATGTCATTGAATGTCTTTACATGGGCTT
GTATGCCTATGAAAATTGTAATACAACTTTCAGCAACGGATCTCTTGGCTCTCGCATCGA
TGAAGGACGCAGCGAAATGCGATAAGTAATGTGAATTGCAGAATTCAGTGAATCATCGAA
TCTTTGAACGCATCTTGCGCTCCTTGGTATTCCGAGGAGCATGCCTGTTTGAGTGTCATT
AAATTCTCAACTCTCTTCTAC--TTTTTGTAAAAGAGAGCTTGGACTGTGGAGGCTTGCT
GGCCACTTTTTGGGGTCAGCTCCTCTGAAATGCATTAGCGGAACCGTTTGCGATCTGCCA
CAAGTGTGATAAGTTATCTACACTGGCGAGGGGATTGCTCTCTGTAATGTTCAGCTTCTA
ATTGTCTCTACTTTGTGAGACTACTTTTGAATGCTTGACCTCAAATCAGGTAGGACTACC
CGCTGAACTTAA
>ABC5-24
TTTCCGTAGGTGAACCTGCGGAAGGATCATTATTGAATTATGTTTCTAGATAGGTTGTAG

CTGGCTC-TTTAGAGCATGTGCACGCCTGTTTGGACTTCATTTTCATCCACCTGTGCACC
TATTGTAGTCTTTGGTTGGGTTAGGGGGAAGTGGTCATTGTGTCAGCATCTGCTGGATGT
GAGGACTTGCATTGTGAAAGCTTTGCTGTCCTTGATGTGATCATGGAATCTCTTTCTCAC
TAGAGTCTATGTCACTCATTATACTCTGTCGAATGTCATTGAATGTCTTTACATGGGCTT
GTATGCCTATGAAAATTGTAATACAACTTTCAGCAACGGATCTCTTGGCTCTCGCATCGA
TGAAGAACGCAGCGAAATGCGATAAGTAATGTGAATTGCAGAATTCAGTGAATCATCGAA
TCTTTGAACGCATCTTGCGCTCCTTGGTATTCCGAGGAGCATGCCTGTTTGAGTGTCATT
AAATTCTCAACTCTCTTCTAC--TTTTTGTAAAAGAGAGCTTGGACTGTGGAGGCTTGCT
GGCCACTTTTTGGGGTCAGCTCCTCTGAAATGCATTAGCGGAACCGTTTGCGATCTGCCA
CAAGTGTGATAAGTTATCTACACTGGCGAGGGGATTGCTCTCTGTAATGTTCAGCTTCTA
ATTGTCTCTACTTTGTGAGACTACTTTTGAATGCTTGACCTCAAATCAGGTAGGACTACC
CGCTGAACTTAA
>ABC8-6
TTTCCGTAGGTGAACCTGCGGAAGGATCATTATTGAATTATGTTTCTAGATAGGTTGTAG
CTGGCTC-TTTAGAGCATGTGCACGCCTGTTTGGACTTCATTTTCATCCACCTGTGCACC
TATTGTAGTCTTTGGTTGGGTTAGGGGGAAGTGGTCATTGTGTCAGCATCTGCTGGATGT
GAGGACTTGCATTGTGAAAGCTTTGCTGTCCTTGATGTGATCATGGAATCTCTTTCTCAC
TAGAGTCTATGTCACTCATTATACTCTGTCGAATGTCATTGAATGTCTTTACATGGGCTT
GTATGCCTATGAAAATTGTAATACAACTTTCAGCAACGGATCTCTTGGCTCTCGCATCGA
TGAAGAACGCAGCGAAATGCGATAAGTAATGTGAATTGCAGAATTCAGTGAATCATCGAA
TCTTTGAACGCATCTTGCGCTCCTTGGTATTCCGAGGAGCATGCCTGTTTGAGTGTCATT
AAATTCTCAACTCTCTTCTAC--TTTTTGTAAAAGAGAGCTTGGACTGTGGAGGCTTGCT
GGCCACTTTTTGGGGTCAGCTCCTCTGAAATGCATTAGCGGAACCGTTTGCGATCTGCCA
CAAGTGTGATAAGTTATCTACACTGGCGAGGGGATTGCTCTCTGTAATGTTCAGCTTCTA
ATTGTCTCTACTTTGTGAGACTACTTTTGAATGCTTGACCTCAAATCAGGTAGGACTACC
CGCTGAACTTAA
>ABC11-42
TTTCCGTAGGTGAACCTGCGGAAGGATCATTATTGAATTATGTTTCTAGATAGGTTGTAG
CTGGCTC-TTTAGAGCATGTGCACGCCTGTTTGGACTTCATTTTCATCCACCTGTGCACC
TATTGTAGTCTTTGGTTGGGTTAGGGGGAAGTGGTCATTGTGTCAGCATCTGCTGGATGT
GAGGACTTGCATTGTGAAAGCTTTGCTGTCCTTGATGTGATCATGGAATCTCTTTCTCAC
TAGAGTCTATGTCACTCATTATACTCTGTCGAATGTCATTGAATGTCTTTACATGGGCTT
GTATGCCTATGAAAATTGTAATACAACTTTCAGCAACGGATCTCTTGGCTCTCGCATCGA
TGAAGAACGCAGCGAAATGCGATAAGTAATGTGAATTGCAGAATTCAGTGAATCATCGAA
TCTTTGAACGCATCTTGCGCTCCTTGGTATTCCGAGGAGCATGCCTGTTTGAGTGTCATT
AAATTCTCAACTCTCTTCTAC--TTTTTGTAAAAGAGAGCTTGGACTGTGGAGGCTTGCT
GGCCACTTTTTGGGGTCAGCTCCTCTGAAATGCATTAGCGGAACCGTTTGCGATCTGCCA
CAAGTGTGATAAGTTATCTACACTGGCGAGGGGATTGCTCTCTGTAATGTTCAGCTTCTA
ATTGTCTCTACTTTGTGAGACTACTTTTGAATGCTTGACCTCAAATCAGGTAGGACTACC
CGCTGAACTTAA
>ABC12-28
TTTCCGTAGGTGAACCTGCGGAAGGATCATTATTGAATTATGTTTCTAGATAGGTTGTAG
CTGGCTC-TTTAGAGCATGTGCACGCCTGTTTGGACTTCATTTTCATCCACCTGTGCACC
TATTGTAGTCTTTGGTTGGGTTAGGGGGAAGTGGTCATTGTGTCAGCATCTGCTGGATGT
GAGGACTTGCATTGTGAAAGCTTTGCTGTCCTTGATGTGATCATGGAATCTCTTTCTCAC
TAGAGTCTATGTCACTCATTATACTCTGTCGAATGTCATTGAATGTCTTTACATGGGCTT
GTATGCCTATGAAAATTGTAATACAACTTTCAGCAACGGATCTCTTGGCTCTCGCATCGA
TGAAGAACGCAGCGAAATGCGATAAGTAATGTGAATTGCAGAATTCAGTGAATCATCGAA
TCTTTGAACGCATCTTGCGCTCCTTGGTATTCCGAGGAGCATGCCTGTTTGAGTGTCATT
AAATTCTCAACTCTCTTCTAC--TTTTTGTAAAAGAGAGCTTGGACTGTGGAGGCTTGCT

GGCCACTTTTTGGGGTCAGCTCCTCTGAAATGCATTAGCGGAACCGTTTGCGATCTGCCA
CAAGTGTGATAAGTTATCTACACTGGCGAGGGGATTGCTCTCTGTAATGTTCAGCTTCTA
ATTGTCTCTACTTTGTGAGACTACTTTTGAATGCTTGACCTCAAATCAGGTAGGACTACC
CGCTGAACTTAA
>ABC3-22
TTTCCGTAGGTGAACCTGCGGAAGGATCATTATTGAATTATGTTTCTAGATAGGTTGTAG
CTGGCTC-TTTAGAGCATGTGCACGCCTGTTTGGACTTCATTTTCATCCACCTGTGCACC
TATTGTAGTCTTTGGTTGGGTTAGGAGGAAGTGGTCATTGTGTCAGCATCTGCTGGATGT
GAGGACTTGCATTGTGAAAGCTTTGCTGTCCTTGATGTGATCATGGAATCTCTTTCTCAC
TAGAGTCTATGTCACTCATTATACTCTGTCGAATGTCATTGAATGTCTTTACATGGGCTT
ATATGCCTATGAAAATTGTAATACAACTTTCAGCAACGGATCTCTTGGCTCTCGCATCGA
TGAAGGACGCAGCGAAATGCGATAAGTAATGTGAATTGCAGAATTCAGTGAATCATCGAA
TCTTTGAACGCATCTTGCGCTCCTTGGTATTCCGAGGAGCATGCCTGTTTGAGTGTCATT
AAATTCTCAACTCTCTTCTAC--TTTTTGTAAAAGAGAGCTTGGACTGTGGAGGCTTGCT
GGCCACTTTTTGGGGTCAGCTCCTCTGAAATGCATTAGCGGAACCGTTTGCGATCTGCCA
CAAGTGTGATAAGTTATCTACACTGGCGAGGGGATTGCTCTCTGTAATGTTCAGCTTCTA
ATTGTCTCTACTTTGTGAGACTACTTTTGAATGCTTGACCTCAAATCAGGTAGGACTACC
CGCTGAACTTAA
>ABC3-39
TTTCCGTAGGTGAACCTGCGGAAGGATCATTATTGAATTATGTTTCTAGATAGGTTGTAG
CTGGCTC-TTTAGAGCATGTGCACGCCTGTTTGGACTTCATTTTCATCCACCTGTGCACC
TATTGTAGTCTTTGGTTGGGTTAGGAGGAAGTGGTCATTGTGTCAGCATCTGCTGGATGT
GAGGACTTGCATTGTGAAAGCTTTGCTGTCCTTGATGTGATCATGGAATCTCTTTCTCAC
TAGAGTCTATGTCACTCATTATACTCTGTCGAATGTCATTGAATGTCTTTACATGGGCTT
GTATGCCTATGAAAATTGTAATACAACTTTCAGCAACGGATCTCTTGGCTCTCGCATCGA
TGAAGGACGCAGCGAAATGCGATAAGTAATGTGAATTGCAGAATTCAGTGAATCATCGAA
TCTTTGAACGCATCTTGCGCTCCTTGGTATTCCGAGGAGCATGCCTGTTTGAGTGTCATT
AAATTCTCAACTCTCTTCTAC--TTTTTGTAAAAGAGAGCTTGGACTGTGGAGGCTTGCT
GGCCACTTTTTGGGGTCAGCTCCTCTGAAATGCATTAGCGGAACCGTTTGCGATCTGCCA
CAAGTGTGATAAGTTATCTACACTGGCGAGGGGATTGCTCTCTGTAATGTTCAGCTTCTA
ATTGTCTCTACTTTGTGAGACTACTTTTGAATGCTTGACCTCAAATCAGGTAGGACTACC
CGCTGAACTTAA
>ABC4-59
TTTCCGTAGGTGAACCTGCGGAAGGATCATTATTGAATTATGTTTCTAGATAGGTTGTAG
CTGGCTC-TTTAGAGCATGTGCACGCCTGTTTGGACTTCATTTTCATCCACCTGTGCACC
TATTGTAGTCTTTGGTTGGGTTAGGAGGAAGTGGTCATTGTGTCAGCATCTGCTGGATGT
GAGGACTTGCATTGTGAAAGCTTTGCTGTCCTTGATGTGATCATGGAATCTCTTTCTCAC
TAGAGTCTATGTCACTCATTATACTCTGTCGAATGTCATTGAATGTCTTTACATGGGCTT
GTATGCCTATGAAAATTGTAATACAACTTTCAGCAACGGATCTCTTGGCTCTCGCATCGA
TGAAGAACGCAGCGAAATGCGATAAGTAATGTGAATTGCAGAATTCAGTGAATCATCGAA
TCTTTGAACGCATCTTGCGCTCCTTGGTATTCCGAGGAGCATGCCTGTTTGAGTGTCATT
AAATTCTCAACTCTCTTCTAC--TTTTTGTAAAAGAGAGCTTGGACTGTGGAGGCTTGCT
GGCCACTTTTTGGGGTCAGCTCCTCTGAAATGCATTAGCGGAACCGTTTGCGATCTGCCA
CAAGTGTGATAAGTTATCTACACTGGCGAGGGGATTGCTCTCTGTAATGTTCAGCTTCTA
ATTGTCTCTACTTTGTGAGACTACTTTTGAATGCTTGACCTCAAATCAGGTAGGACTACC
CGCTGAACTTAA
>ABC6-5
TTTCCGTAGGTGAACCTGCGGAAGGATCATTATTGAATTATGTTTCTAGATAGGTTGTAG
CTGGCTC-TTTAGAGCATGTGCACGCCTGTTTGGACTTCATTTTCATCCACCTGTGCACC
TATTGTAGTCTTTGGTTGGGTTAGGAGGAAGTGGTCATTGTGTCAGCATCTGCTGGATGT

GAGGACTTGCATTGTGAAAGCTTTGCTGTCCTTGATGTGATCATGGAATCTCTTTCTCAC
TAGAGTCTATGTCACTCATTATACTCTGTCGAATGTCATTGAATGTCTTTACATGGGCTT
GTATGCCTATGAAAATTGTAATACAACTTTCAGCAACGGATCTCTTGGCTCTCGCATCGA
TGAAGAACGCAGCGAAATGCGATAAGTAATGTGAATTGCAGAATTCAGTGAATCATCGAA
TCTTTGAACGCATCTTGCGCTCCTTGGTATTCCGAGGAGCATGCCTGTTTGAGTGTCATT
AAATTCTCAACTCTCTTCTAC--TTTTTGTAAAAGAGAGCTTGGACTGTGGAGGCTTGCT
GGCCACTTTTTGGGGTCAGCTCCTCTGAAATGCATTAGCGGAACCGTTTGCGATCTGCCA
CAAGTGTGATAAGTTATCTACACTGGCGAGGGGATTGCTCTCTGTAATGTTCAGCTTCTA
ATTGTCTCTACTTTGTGAGACTACTTTTGAATGCTTGACCTCAAATCAGGTAGGACTACC
CGCTGAACTTAA
>ABC7-48
TTTCCGTAGGTGAACCTGCGGAAGGATCATTATTGAATTATGTTTCTAGATAGGTTGTAG
CTGGCTC-TTTAGAGCATGTGCACGCCTGTTTGGACTTCATTTTCATCCACCTGTGCACC
TATTGTAGTCTTTGGTTGGGTTAGGAGGAAGTGGTCATTGTGTCAGCATCTGCTGGATGT
GAGGACTTGCATTGTGAAAGCTTTGCTGTCCTTGATGTGATCATGGAATCTCTTTCTCAC
TAGAGTCTATGTCACTCATTATACTCTGTCGAATGTCATTGAATGTCTTTACATGGGCTT
GTATGCCTATGAAAATTGTAATACAACTTTCAGCAACGGATCTCTTGGCTCTCGCATCGA
TGAAGAACGCAGCGAAATGCGATAAGTAATGTGAATTGCAGAATTCAGTGAATCATCGAA
TCTTTGAACGCATCTTGCGCTCCTTGGTATTCCGAGGAGCATGCCTGTTTGAGTGTCATT
AAATTCTCAACTCTCTTCTAC--TTTTTGTAAAAGAGAGCTTGGACTGTGGAGGCTTGCT
GGCCACTTTTTGGGGTCAGCTCCTCTGAAATGCATTAGCGGAACCGTTTGCGATCTGCCA
CAAGTGTGATAAGTTATCTACACTGGCGAGGGGATTGCTCTCTGTAATGTTCAGCTTCTA
ATTGTCTCTACTTTGTGAGACTACTTTTGAATGCTTGACCTCAAATCAGGTAGGACTACC
CGCTGAACTTAA
>ABC11-29
TTTCCGTAGGTGAACCTGCGGAAGGATCATTATTGAATTATGTTTCTAGATAGGTTGTAG
CTGGCTC-TTTAGAGCATGTGCACGCCTGTTTGGACTTCATTTTCATCCACCTGTGCACC
TATTGTAGTCTTTGGTTGGGTTAGGAGGAAGTGGTCATTGTGTCAGCATCTGCTGGATGT
GAGGACTTGCATTGTGAAAGCTTTGCTGTCCTTGATGTGATCATGGAATCTCTTTCTCAC
TAGAGTCTATGTCACTCATTATACTCTGTCGAATGTCATTGAATGTCTTTACATGGGCTT
GTATGCCTATGAAAATTGTAATACAACTTTCAGCAACGGATCTCTTGGCTCTCGCATCGA
TGAAGAACGCAGCGAAATGCGATAAGTAATGTGAATTGCAGAATTCAGTGAATCATCGAA
TCTTTGAACGCATCTTGCGCTCCTTGGTATTCCGAGGAGCATGCCTGTTTGAGTGTCATT
AAATTCTCAACTCTCTTCTAC--TTTTTGTAAAAGAGAGCTTGGACTGTGGAGGCTTGCT
GGCCACTTTTTGGGGTCAGCTCCTCTGAAATGCATTAGCGGAACCGTTTGCGATCTGCCA
CAAGTGTGATAAGTTATCTACACTGGCGAGGGGATTGCTCTCTGTAATGTTCAGCTTCTA
ATTGTCTCTACTTTGTGAGACTACTTTTGAATGCTTGACCTCAAATCAGGTAGGACTACC
CGCTGAACTTAA
>ABC6-53
TTTCCGTAGGTGAACCTGCGGAAGGATCATTATTGAATTATGTTTCTAGATAGGTTGTAG
CTGGCTC-TTTAGAGCATGTGCACGCCTGTTTGGACTTCATTTTCATCCACCTGTGCACC
TATTGTAGTCTTTGGTTGGGTTAGGAGGAAGTGGTCATTGTGTCAGCATCTGCTGGATGT
GAGGACTTGCATTGTGAAAGCTTTGCTGTCCTTGATGTGATCATGGAATCTTTTTCTCAC
TAGAGTCTATGTCACTCATTATACTCTGTCGAATGTCATTGAATGTCTTTACATGGGCTT
GTATGCCTATGAAAATTGTAATACAACTTTCAGCAACGGATCTCTTGGCTCTCGCATCGA
TGAAGAACGCAGCGAAATGCGATAAGTAATGTGAATTGCAGAATTCAGTGAATCATCGAA
TCTTTGAACGCATCTTGCGCTCCTTGGTATTCCGAGGAGCATGCCTGTTTGAGTGTCATT
AAATTCTCAACTCTCTTCTAC--TTTTTGTAAAAGAGAGCTTGGACTGTGGAGGCTTGCT
GGCCACTTTTTGGGGTCAGCTCCTCTGAAATGCATTAGCGGAACCGTTTGCGATCTGCCA
CAAGTGTGATAAGTTATCTACACTGGCGAGGGGATTGCTCTCTGTAATGTTCAGCTTCTA

ATTGTCTCTACTTTGTGAGACTACTTTTGAATGCTTGACCTCAAATCAGGTAGGACTACC
CGCTGAACTTAA
>ABC9-8
TTTCCGTAGGTGAACCTGCGGAAGGATCATTATTGAATTATGTTTCTAGATAGGTTGTAG
CTGGCTC-TTTAGAGCATGTGCACGCCTGTTTGGACTTCATTTTCATCCACCTGTGCACC
TATTGTAGTCTTTGGTTGGGTTAGGAGGAAGTGGTCATTGTGTCAGCATCTGCTGGATGT
GAGGACTTGCATTGTGAAAGCTTTGCTGTCCTTGATGTGATCATGGAATCTTTTTCTCAC
TAGAGTCTATGTCACTCATTATACTCTGTCGAATGTCATTGAATGTCTTTACATGGGCTT
GTATGCCTATGAAAATTGTAATACAACTTTCAGCAACGGATCTCTTGGCTCTCGCATCGA
TGAAGAACGCAGCGAAATGCGATAAGTAATGTGAATTGCAGAATTCAGTGAATCATCGAA
TCTTTGAACGCATCTTGCGCTCCTTGGTATTCCGAGGAGCATGCCTGTTTGAGTGTCATT
AAATTCTCAACTCTCTTCTAC--TTTTTGTAAAAGAGAGCTTGGACTGTGGAGGCTTGCT
GGCCACTTTTTGGGGTCAGCTCCTCTGAAATGCATTAGCGGAACCGTTTGCGATCTGCCA
CAAGTGTGATAAGTTATCTACACTGGCGAGGGGATTGCTCTCTGTAATGTTCAGCTTCTA
ATTGTCTCTACTTTGTGAGACTACTTTTGAATGCTTGACCTCAAATCAGGTAGGACTACC
CGCTGAACTTAA
>ABC7-6
TTTCCGTAGGTGAACCTGCGGAAGGATCATTATTGAATTATGTTTCTAGATAGGTTGTAG
CTGGCTC-TTTAGAGCATGTGCACGCCTGTTTGGACTTCATTTTCATCCACCTGTGCACC
TATTGTAGTCTTTGGTTGGGTTAGGAGGAAGTGGTCATTGTGTCAGCATCTGCTGGATGT
GAGGACTTGCATTGTGAAAGCTTTGCTGTCCTTGATGTGATCATGGAATCTTTTTCTCAC
TAGAGTCTATGTCACTCATTATACTCTGTCGAATGTCATTGAATGTCTTTACATGGGCTT
ATATGCCTATGAAAATTGTAATACAACTTTCAGCAACGGATCTCTTGGCTCTCGCATCGA
TGAAGAACGCAGCGAAATGCGATAAGTAATGTGAATTGCAGAATTCAGTGAATCATCGAA
TCTTTGAACGCATCTTGCGCTCCTTGGTATTCCGAGGAGCATGCCTGTTTGAGTGTCATT
AAATTCTCAACTCTCTTCTAC--TTTTTGTAAAAGAGAGCTTGGACTGTGGAGGCTTGCT
GGCCACTTTTTGGGGTCAGCTCCTCTGAAATGCATTAGCGGAACCGTTTGCGATCTGCCA
CAAGTGTGATAAGTTATCTACACTGGCGAGGGGATTGCTCTCTGTAATGTTCAGCTTCTA
ATTGTCTCTACTTTGTGAGACTACTTTTGAATGCTTGACCTCAAATCAGGTAGGACTACC
CGCTGAACTTAA
>ABC6-55
TTTCCGTAGGTGAACCTGCGGAAGGATCATTATTGAATTATGTTTCTAGATAGGTTGTAG
CTGGCTC-TTTAGAGCATGTGCACGCCTGTTTGGACTTCATTTTCATCCACCTGTGCACC
TATTGTAGTCTTTGGTTGGGTTAGGAGGAAGTGGTCATTGTGTCAGCATCTGCTGGATGT
GAGGACTTGCATTGTGAAAGCTTTGCTGTCCTTGATGTGATCATGGAATCTTTTTCTCAC
TAGAGTCTATGTCACTCATTATACTCTGTCGAATGTCATTGAATGTCTTTACATGGGCTT
ATATGCCTATGAAAATTGTAATACAACTTTCAGCAACGGATCTCTTGGCTCTCGCATCGA
TGAAGAACGCAGCGAAATGCGATAAGTAATGTGAATTGCAGAATTCAGTGAATCATCGAA
TCTTTGAACGCATCTTGCGCTCCTTGGTATTCCGAGGAGCATGCCTGTTTGAGTGTCATT
AAATTCTCAACTCTCTTCTAC--TTTTTGTAAAAGAGAGCTTGGACTGTGGAGGCTTGCT
GGCCACTTTTTGGGGTCAGCTCCTCTGAAATGCATTAGCGGAACCGTTTGCGATCTGCCA
CAAGTGTGATAAGTTATCTACACTGGCGAGGGGATTGCTCTCTGTAATGTTCAGCTTCTA
ATTGTCTCTACTTTGTGAGACTACTTTTGAATGCTTGACCTCAAATCAGGTAGGACTACC
CGCTGAACTTAA
>ABC9-43
TTTCCGTAGGTGAACCTGCGGAAGGATCATTATTGAATTATGTTTCTAGATAGGTTGTAG
CTGGCTC-TTTAGAGCATGTGCACGCCTGTTTGGACTTCATTTTCATCCACCTGTGCACC
TATTGTAGTCTTTGGTTGGGTTAGGGGGAAGTGGTCATTGTGTCAGCATCTGCTGGATGT
GAGGACTTGCATTGTGAAAGCTTTGCTGTCCTTGATGTGATCATGGAATCTTTTTCTCAC
TAGAGTCTATGTCACTCATTATACTCTGTCGAATGTCATTGAATGTCTTTACATGGGCTT

GTATGCCTATGAAAATTGTAATACAACTTTCAGCAACGGATCTCTTGGCTCTCGCATCGA
TGAAGAACGCAGCGAAATGCGATAAGTAATGTGAATTGCAGAATTCAGTGAATCATCGAA
TCTTTGAACGCATCTTGCGCTCCTTGGTATTCCGAGGAGCATGCCTGTTTGAGTGTCATT
AAATTCTCAACTCTCTTCTAC--TTTTTGTAAAAGAGAGCTTGGACTGTGGAGGCTTGCT
GGCCACTTTTTGGGGTCAGCTCCTCTGAAATGCATTAGCGGAACCGTTTGCGATCTGCCA
CAAGTGTGATAAGTTATCTACACTGGCGAGGGGATTGCTCTCTGTAATGTTCAGCTTCTA
ATTGTCTCTACTTTGTGAGACTACTTTTGAATGCTTGACCTCAAATCAGGTAGGACTACC
CGCTGAACTTAA
>ABC4-43
TTTCCGTAGGTGAACCTGCGGAAGGATCATTATTGAATTATGTTTCTAGATAGGTTGTAG
CTGGCTC-TTTAGAGCATGTGCACGCCTGTTTGGACTTCATTTTCATCCACCTGTGCACC
TATTGTAGTCTTTGGTTGGGTTAGGGGGAAGTGGTCATTGTGTCAGCATCTGCTGGATGT
GAGGACTTGCATTGTGAAAGCTTTGCTGTCCTTGATGTGATCATGGAATCTTTTTCTCAC
TAGAGTCTATGTCACTCATTATACTCTGTCGAATGTCATTGAATGTCTTTACATGGGCTT
ATATGCCTATGAAAATTGTAATACAACTTTCAGCAACGGATCTCTTGGCTCTCGCATCGA
TGAAGAACGCAGCGAAATGCGATAAGTAATGTGAATTGCAGAATTCAGTGAATCATCGAA
TCTTTGAACGCATCTTGCGCTCCTTGGTATTCCGAGGAGCATGCCTGTTTGAGTGTCATT
AAATTCTCAACTCTCTTCTAC--TTTTTGTAAAAGAGAGCTTGGACTGTGGAGGCTTGCT
GGCCACTTTTTGGGGTCAGCTCCTCTGAAATGCATTAGCGGAACCGTTTGCGATCTGCCA
CAAGTGTGATAAGTTATCTACACTGGCGAGGGGATTGCTCTCTGTAATGTTCAGCTTCTA
ATTGTCTCTACTTTGTGAGACTACTTTTGAATGCTTGACCTCAAATCAGGTAGGACTACC
CGCTGAACTTAA
>ABC4-10
TTTCCGTAGGTGAACCTGCGGAAGGATCATTATTGAATTATGTTTCTAGATAGGTTGTAG
CTGGCTC-TTTAGAGCATGTGCACGCCTGTTTGGACTTCATTTTCATCCACCTGTGCACC
TATTGTAGTCTTTGGTTGGGTTAGGGGGAAGTGGTCATTGTGTCAGCATCTGCTGGATGT
GAGGACTTGCATTGTGAAAGCTTTGCTGTCCTTGATGTGATCATGGAATCTCTTTCTCAC
TAGAGTCTATGTCACTCATTATACTCTGTCGAATGTCATTGAATGTCTTTACATGGGCTT
GTATGCCTATGAAAATTGTAATACAACTTTCAGCAACGGATCTCTTGGCTCTCGCATCGA
TGAAGAACGCAGCGAAATGCGATAAGTAATGTGAATTGCAGAATTCAGTGAATCATCGAA
TCTTTGAACGCATCTTGCGCTCCTTGGTATTCCGAGGAGCATGCCTGTTTGAGTGTCATT
AAATTCTCAACTCTCTTCTAC--TTTTTGTAAAAGAGAGCTTGGACTGTGGAGGCTTGCT
GGCCACTTTTTGGGGTCAGCTCCTCTGAAATGCATTAGCGGAACCGTTTGCAATCTGCCA
CAAGTGTGATAAGTTATCTACACTGGCGAGGGGATTGCTCTCTGTAATGTTCAGCTTCTA
ATTGTCTCTACTTTGTGAGACTACTTTTGAATGCTTGACCTCAAATCAGGTAGGACTACC
CGCTGAACTTAA
>ABC7-10
TTTCCGTAGGTGAACCTGCGGAAGGATCATTATTGAATTATGTTTCTAGATAGGTTGTAG
CTGGCTC-TTTAGAGCATGTGCACGCCTGTTTGGACTTCATTTTCATCCACCTGTGCACC
TATTGTAGTCTTTGGTTGGGTTAGGGGGAAGTGGTCATTGTGTCAGCATCTGCTGGATGT
GAGGACTTGCATTGTGAAAGCTTTGCTGTCCTTGATGTGATCATGGAATCTCTTTCTCAC
TAGAGTCTATGTCACTCATTATACTCTGTCGAATGTCATTGAATGTCTTTACATGGGCTT
ATATGCCTATGAAAATTGTAATACAACTTTCAGCAACGGATCTCTTGGCTCTCGCATCGA
TGAAGAACGCAGCGAAATGCGATAAGTAATGTGAATTGCAGAATTCAGTGAATCATCGAA
TCTTTGAACGCATCTTGCGCTCCTTGGTATTCCGAGGAGCATGCCTGTTTGAGTGTCATT
AAATTCTCAACTCTCTTCTAC--TTTTTGTAAAAGAGAGCTTGGACTGTGGAGGCTTGCT
GGCCACTTTTTGGGGTCAGCTCCTCTGAAATGCATTAGCGGAACCGTTTGCAATCTGCCA
CAAGTGTGATAAGTTATCTACACTGGCGAGGGGATTGCTCTCTGTAATGTTCAGCTTCTA
ATTGTCTCTACTTTGTGAGACTACTTTTGAATGCTTGACCTCAAATCAGGTAGGACTACC
CGCTGAACTTAA

```
>ABC10-42
TTTCCGTAGGTGAACCTGCGGAAGGATCATTATTGAATTATGTTTCTAGATAGGTTGTAG
CTGGCTC-TTTAGAGCATGTGCACGCCTGTTTGGACTTCATTTTCATCCACCTGTGCACC
TATTGTAGTCTTTGGTTGGGTTAGGGGGAAGTGGTCATTGTGTCAGCATCTGCTGGATGT
GAGGACTTGCATTGTGAAAGCTTTGCTGTCCTTGATGTGATCATGGAATCTCTTTCTCAC
TAGAGTCTATGTCACTCATTATACTCTGTCGAATGTCATTGAATGTCTTTACATGGGCTT
ATATGCCTATGAAAATTGTAATACAACTTTCAGCAACGGATCTCTTGGCTCTCGCATCGA
TGAAGAACGCAGCGAAATGCGATAAGTAATGTGAATTGCAGAATTCAGTGAATCATCGAA
TCTTTGAACGCATCTTGCGCTCCTTGGTATTCCGAGGAGCATGTCTGTTTGAGTGTCATT
AAATTCTCAACTCTCTTCTAC--TTTTTGTAAAAGAGAGCTTGGACTGTGGAGGCTTGCT
GGCCACTTTTTGGGGTCAGCTCCTCTGAAATGCATTAGCGGAACCGTTTGCGATCTGCCA
CAAGTGTGATAAGTTATCTACACTGGCGAGGGGATTGCTCTCTGTAATGTTCAGCTTCTA
ATTGTCTCTACTTTGTGAGACTACTTTTGAATGCTTGACCTCAAATCAGGTAGGACTACC
CGCTGAACTTAA
>ABC12-17
TTTCCGTAGGTGAACCTGCGGAAGGATCATTATTGAATTATGTTTCTAGATAGGTTGTAG
CTGGCTC-TTTAGAGCATGTGCACGCCTGTTTGGACTTCATTTTCATCCACCTGTGCACC
TATTGTAGTCTTTGGTTGGGTTAGGAGGAAGTGGTCATTGTGTCAGCATCTGCTGGATGT
GAGGACTTGCATTGTGAAAGCTTTGCTGTCCTTGATGTGATCATGGAATCTCTTTCTCAC
TAGAGTCTATGTCACTCATTATACTCAGTCGAATGTCATTGAATGTCTTTACATGGGCTT
ATATGCCTATGAAAATTGTAATACAACTTTCAGCAACGGATCTCTTGGCTCTCGCATCGA
TGAAGAACGCAGCGAAATGCGATAAGTAATGTGAATTGCAGAATTCAGTGAATCATCGAA
TCTTTGAACGCATCTTGCGCTCCTTGGTATTCCGAGGAGCATGCCTGTTTGAGTGTCATT
AAATTCTCAACTCTCTTCTAC--TTTTTGTAAAAGAGAGCTTGGACTGTGGAGGCTTGCT
GGCCACTTTTTGGGGTCAGCTCCTCTGAAATGCATTAGCGGAACCGTTTGCGATCTGCCA
CAAGTGTGATAAGTTATCTACACTGGCGAGGGGATTGCTCTCTGTAATGTTCAGCTTCTA
ATTGTCTCTACTTTGTGAGACTACTTTTGAATGCTTGACCTCAAATCAGGTAGGACTACC
CGCTGAACTTAA
>ABC2-15
TTTCCGTAGGTGAACCTGCGGAAGGATCATTATTGAATTATGTTTCTAGATAGGTTGTAG
CTGGCTC-TTTAGAGCATGTGCACGCCTGTTTGGACTTCATTTTCATCCACCTGTGCACC
TATTGTAGTCTTTGGTTGGGTTAGGAGGAAGTGGTCATTGTGTCAGCATCTGCTGGATGT
GAGGACTTGCATTGTGAAAGCTTTGCTGTCCTTGATGTGATCATGGAATCTCTTTCTCAC
TAGAGTCTATGTCACTCATTATACTCTGTCGAATGTCATTGAATGTCTTTACATGGGCTT
ATATGCCTATGAAAATTGTAATACAACTTTCAGCAACGGATCTCTTGGCTCTCGCATCGA
TGAAGAACGCAGCGAAATGCGATAAGTAATGTGAATTGCAGAATTCAGTGAATCATCGAA
TCTTTGAACGCATCTTGCGCTCCTTGGTATTCCGAGGAGCATGCATGTTTGAGTGTCATT
AAATTCTCAACTCTCTTCTAC--TTTTTGTAAAAGAGAGCTTGGACTGTGGAGGCTTGCT
GGCCACTTTTTGGGGTCAGCTCCTCTGAAATGCATTAGCGGAACCGTTTGCGATCTGCCA
CAAGTGTGATAAGTTATCTACACTGGCGAGGGGATTGCTCTCTGTAATGTTCAGCTTCTA
ATTGTCTCTACTTTGTGAGACTACTTTTGAATGCTTGACCTCAAATCAGGTAGGACTACC
CGCTGAACTTAA
>ABC6-29
TTTCCGTAGGTGAACCTGCGGAAGGATCATTATTGAATTATGTTTCTAGATAGGTTGTAG
CTGGCTC-TTTAGAGCATGTGCACGCCTGTTTGGACTTCATTTTCATCCACCTGTGCACC
TATTGTAGTCTTTGGTTGGGTTAGGGGGAAGTGGTCATTGTGTCAGCATCTGCTGGATGT
GAGGACTTGCATTGTGAAAGCTTTGCTGTCCTTGATGTGATCATGGAATCTCTTTCTCAC
TAGAGTCTATGTCACTCATTATACTCTGTCGAATGTCATTGAATGTCTTTACATGGGCGT
GTATGCCTATGAAAATTGTAATACAACTTTCAGCAACGGATCTCTTGGCTCTCGCATCGA
TGGAGGACGCAGCGAAATGCGATAAGTAATGTGAATTGCAGAATTCAGTGAATCATCGAA
```

TCTTTGAACGCATCTTGCGCTCCTTGGTATTCCGAGGAGCATGCCTGTTTGAGTGTCATT
AAATTCTCAACTCTCTTCTAC--TTTTTGTAAAAGAGAGCTTGGACTGTGGAGGCTTGCT
GGCCACTTTTTGGGGTCAGCTCCTCTGAAATGCATTAGCGGAACCGTTTGCGATCTGCCA
CAAGTGTGATAAGTTATCTACACTGGCGAGGGGATTGCTCTCTGTAATGTTCAGCTTCTA
ATTGTCTCTACTTTGTGAGACTACTTTTGAATGCTTGACCTCAAATCAGGTAGGACTACC
CGCTGAACTTAA
>ABC7-18
TTTCCGTAGGTGAACCTGCGGAAGGATCATTATTGAATTATGTTTCTAGATAGGTTGTAG
CTGGCTC-TTTAGAGCATGTGCACGCCTGTTTGGACTTCATTTTCATCCACCTGTGCACC
TATTGTAGTCTTTGGTTGGGTTAGGAGGAAGTGATCATTGTATCAGCATCTGCTGGGAGT
GAGGACTTGCATTGTGAAAGCTTTGCTGTCCTTGATGTGATCATGGAATCTTTTTCTCAC
TAGAGTCTATGTCACTCATTATACTCTGTCGAATGTCATTGAATGTCTTTACATGGGCTT
ATATGCCTATGAAAATTGTAATACAACTTTCAGCAACGGATCTCTTGGCTCTCGCATCGA
TGAAGAACGCAGCGAAATGCGATAAGTAATGTGAATTGCAGAATTCAGTGAATCATCGAA
TCTTTGAACGCATCTTGCGCTCCTTGGTATTCCGAGGAGCATGCCTGTTTGAGTGTCATT
AAATTCTCAACTCTCTTCTAC--TTTTTGTAAAAGAGAGCTTGGACTGTGGAGGCTTGCT
GGCCACTTTTTGGGGTCAGCTCCTCTGAAATGCATTAGCGGAACCGTTTGCGATCTGCCA
CAAGTGTGATAAGTTATCTACACTGGCGAGGGGATTGCTCTCTGTAATGTTCAGCTTCTA
ATTGTCTCTACTTTGTGAGACTACTTTTGAATGCTTGACCTCAAATCAGGTAGGACTACC
CGCTGAACTTAA
>ABC9-6
TTTCCGTAGGTGAACCTGCGGAAGGATCATTATTGAATTATGTTTCTAGATAGGTTGTAG
CTGGCTC-TTTAGAGCATGTGCACGCCTGTTTGGACTTCATTTTCATCCACCTGTGCACC
TATTGTAGTCTTTGGTTGGGTTAGGGGGAAGTGATCATTGTATCAGCATCTGCTGGGAGT
GAGGACTTGCATTGTGAAAGCTTTGCTGTCCTTGATGTGATCATGGAATCTTTTTCTCAC
TAGAGTCTATGTCACTCATTATACTCTGTCGAATGTCATTGAATGTCTTTACATGGGCTT
GTATGCCTATGAAAATTGTAATACAACTTTCAGCAACGGATCTCTTGGCTCTCGCATCGA
TGAAGAACGCAGCGAAATGCGATAAGTAATGTGAATTGCAGAATTCAGTGAATCATCGAA
TCTTTGAACGCATCTTGCGCTCCTTGGTATTCCGAGGAGCATGCCTGTTTGAGTGTCATT
AAATTCTCAACTCTCTTCTAC--TTTTTGTAAAAGAGAGCTTGGACTGTGGAGGCTTGCT
GGCCACTTTTTGGGGTCAGCTCCTCTGAAATGCATTAGCGGAACCGTTTGCGATCTGCCA
CAAGTGTGATAAGTTATCTACACTGGCGAGGGGATTGCTCTCTGTAATGTTCAGCTTCTA
ATTGTCTCTACTTTGTGAGACTACTTTTGAATGCTTGACCTCAAATCAGGTAGGACTACC
CGCTGAACTTAA
>ABC7-45
TTTCCGTAGGTGAACCTGCGGAAGGATCATTATTGAATTATGTTTCTAGATAGGTTGTAG
CTGGCTC-TTTAGAGCATGTGCACGCCTGTTTGGACTTCATTTTCATCCACCTGTGCACC
TATTGTAGTCTTTGGTTGGGTTAGGGGGAAGTGGTCATTGTGTCAGCATCTGCTGGGAGT
GAGGACTTGCATTGTGAAAGCTTTGCTGTCCTTGATGTGATCATGGAATCTTTTTCTCAC
TAGAGTCTATGTCACTCATTATACTCTGTCGAATGTCATTGAATGTCTTTACATGGGCTT
GTATGCCTATGAAAATTGTAATACAACTTTCAGCAACGGATCTCTTGGCTCTCGCATCGA
TGAAGAACGCAGCGAAATGCGATAAGTAATGTGAATTGCAGAATTCAGTGAATCATCGAA
TCTTTGAACGCATCTTGCGCTCCTTGGTATTCCGAGGAGCATGCCTGTTTGAGTGTCATT
AAATTCTCAACTCTCTTCTAC--TTTTTGTAAAAGAGAGCTTGGACTGTGGAGGCTTGCT
GGCCACTTTTTGGGGTCAGCTCCTCTGAAATGCATTAGCGGAACCGTTTGCAATCTGCCA
CAAGTGTGATAAGTTATCTACACTGGCGAGGGGATTGCTCTCTGTAATGTTCAGCTTCTA
ATTGTCTCTACTTTGTGAGACAACTTTTGAATGCTTGACCTCAAATCAGGTAGGACTACC
CGCTGAACTTAA
>ABC7-27
TTTCCGTAGGTGAACCTGCGGAAGGATCATTATTGAATTATGTTTCTAGATAGGTTGTAG

CTGGCTC-TTTAGAGCATGTGCACGCCTGTTTGGACTTCATTTTCATCCACCTGTGCACC
TATTGTAGTCTTTGGTTGGGTTAGGAGGAAGTGATCATTGTATCAGCATCTGCTGGGAGT
GAGGACTTGCATTGTGAAAGCTTTGCTGTCCTTGATGTGATCATGGAATCTTTTTCTCAC
TAGAGTCTATGTCACTCATTATACTCTGTCGAATGTCATTGAATGTCTTTACATGGGCTT
GTATGCCTATGAAAATTGTAATACAACTTTCAGCAACGGATCTCTTGGCTCTCGCATCGA
TGAAGAACGCAGCGAAATGCGATAAGTAATGTGAATTGCAGAATTCAGTGAATCATCGAA
TCTTTGAACGCATCTTGCGCTCCTTGGTATTCCGAGGAGCATGCCTGTTTGAGTGTCATT
AAATTCTCAACTCTCTTCTAC--TTTTTGTAAAAGAGAGCTTGGACTGTGGAGGCTTGCT
GGCCACTTTTTGGGGTCAGCTCCTCTGAAATGCATTAGCGGAACCGTTTGCGATCTGCCA
CAAGTGTGATAAGTTATCTACACTGGCGAGGGGATTGCTCTCTGTAATGTTCAGCTTCTA
ATTGTCTCTACTTTGTGAGACAACTTTTGAATGCTTGACCTCAAATCAGGTAGGACTACC
CGCTGAACTTAA
>ABC9-11
TTTCCGTAGGTGAACCTGCGGAAGGATCATTATTGAATTATGTTTCTAGATAGGTTGTAG
CTGGCTC-TTTAGAGCATGTGCACGCCTGTTTGGACTTCATTTTCATCCACCTGTGCACC
TATTGTAGTCTTTGGTTGGGTTAGGAGGAAGTGGTCATTGTATCAGCATCTGCTGGGAGT
GAGGACTTGCATTGTGAAAGCTTTGCTGTCCTTGATGTGATCATGGAATCTTTTTCTCAC
TAGAGTCTATGTCACTCATTATACTCTGTCGAATGTCATTGAATGTCTTTACATGGGCTT
GTATGCCTATGAAAATTGTAATACAACTTTCAGCAACGGATCTCTTGGCTCTCGCATCGA
TGAAGAACGCAGCGAAATGCGATAAGTAATGTGAATTGCAGAATTCAGTGAATCATCGAA
TCTTTGAACGCATCTTGCGCTCCTTGGTATTCCGAGGAGCATGCCTGTTTGAGTGTCATT
AAATTCTCAACTCTCTTCTAC--TTTTTGTAAAAGAGAGCTTGGACTGTGGAGGCTTGCT
GGCCACTTTTTGGGGTCAGCTCCTCTGAAATGCATTAGCGGAACCGTTTGCGATCTGCCA
CAAGTGTGATAAGTTATCTACACTGGCGAGGGGATTGCTCTCTGTAATGTTCAGCTTCTA
ATTGTCTCTACTTTGTGAGACAACTTTTGAATGCTTGACCTCAAATCAGGTAGGACTACC
CGCTGAACTTAA
>ABC3-37
TTTCCGTAGGTGAACCTGCGGAAGGATCATTATTGAATTATGTTTCTAGATAGGTTGTAG
CTGGCTC-TTTAGAGCATGTGCACGCCTGTTTGGACTTCATTTTCATCCACCTGTGCACC
TATTGTAGTCTTTGGTTGGGTTAGGAGGAAGTGGTCATTGTGTCAGCATCTGCTGGATGT
GAGGACTTGCATTGTGAAAGCTTTGCTGTCCTTGATGTGATCATGGAATCTCTTTCTCAC
TAGAGTCTATGTCACTCATTATACTCTGTCGAATGTCATTGAATGTCTTTACATGGGCTT
ATATGCCTATGAAAATTGTAATACAACTTTCAGCAACGGATCTCTTGGCTCTCGCATCGA
TGAAGAACGCAGCGAAATGCGATAAGTAATGTGAATTGCAGAATTCAGTGAATCATCGAA
TCTTTGAACGCATCTTGCGCTCCTTGGTATTCCGAGGAGCATGCCTGTTTGAGTGTCATT
AAATTCTCAACTCTCTTCTAC--TTTTTGTAAAAGAGAGCTTGGACTGTGGAGGCTTGCT
GGCCAC-TTTTGGGGTCAGCTCCTCTGAAATGCATTAGCGGAACCGTTTGCGATCTGCCA
CAAGTGTGATAAGTTATCTACACTGGCGAGGGGATTGCTCTCTGTAATGTTCAGCTTCTA
ATTGTCTCTACTTTGTGAGACTACTTTTGAATGCTTGACCTCAAATCAGGTAGGACTACC
CGCTGAACTTAA
>ABC3-19
TTTCCGTAGGTGAACCTGCGGAAGGATCATTATTGAATTATGTTTCTAGATAGGTTGTAG
CTGGCTC-TTTAGAGCATGTGCACGCCTGTTTGGACTTCATTTTCATCCACCTGTGCACC
TATTGTAGTCTTTGGTTGGGTTAGGAGGAAGTGGTCATTGTGTCAGCATCTGCTGGATGT
GAGGACTTGCATTGTGAAAGCTTTGCTGTCCTTGATGTGATCATGGAATCTCTTTCTCAC
TAGAGTCTATGTCACTCATTATACTCTGTCGAATGTCATTGAATGTCTTTACATGGGCTT
ATATGCCTATGAAAATTGTAATACAACTTTCAGCAACGGATCTCTTGGCTCTCGCATCGA
TGAAGAACGCAGCGAAATGCGATAAGTAATGTGAATTGCAGAATTCAGTGAATCATCGAA
TCTTTGAACGCATCTTGCGCTCCTTGGTATTCCGAGGAGCATGCCTGTTTGAGTGTCATT
AAATTCTCAACTCTCTTCTAC--TTTTTGTAAAAGAGAGCTTGGACTGTGGAGGCTTGCT

GGCCAC-TTTTGGGGTCAGCTCCTCTGAAATGCATTAGCGGAACCGTTTGCGATCTGCCA
CAAGTGTGATAAGTTATCTACACTGGCGAGGGGATTGCTCTCTGTAATGTTCAGCTTCTA
ATTGTCTCTACTTTGTGAGACTACTTTTGAATGCTTGACCTCAAATCAGGTAGGACTACC
CGCTGAACTTAA
