# Supplementary material for: Patterns of PCR Amplification Artifacts of the Fungal Barcode Marker in a Hybrid Mushroom
Source: Front Microbiol. 2019 Nov 19;10:2686. doi: 10.3389/fmicb.2019.02686 (PMC6877668; doi:10.3389/fmicb.2019.02686)
Supplement: Supplementary file 11 [file Data_Sheet_11.PDF]

>AB1–2
TTTCCGTAGGTGAACCTGCGGAAGGATCATTATTGAATTATGTTTCTAGATAGGTTGTAG
CTGGCTCTTTTAGAGCATGTGCACGCCTGTTTGGACTTCATTTTCATCCACCTGTGCACC
TATTGTAGTCTTTGGTTGGGTTAGGAGGAAGTGATCATTGTATCAGCATCTGCTGGGAGT
GAGGACTTGCATTGTGAAAGCTTTGCTGTCCTTGATGTGATCATGGAATCTTTTTCTCAC
TAGAGTCTATGTCACTCATTATACTCTGTCGAATGTCATTGAATGTCTTTACATGGGCTT
GTATGCCTATGAAAATTGTAATACAACTTTCAGCAACGGATCTCTTGGCTCTCGCATCGA
TGAAGAACGCAGCGAAATGCGATAAGTAATGTGAATTGCAGAATTCAGTGAATCATCGAA
TCTTTGAACGCATCTTGCGCTCCTTGGTATTCCGAGGAGCATGCCTGTTTGAGTGTCATT
AAATTCTCAACTCTCTTATACTTTTTTGTAAAAGAGAGCTTGGACTGTGGAGGCTTGCTG
GCCACTTTTTGGGGTCAGCTCCTCTGAAATGCATTAGCGGAACCGTTTGCGATCTGCCAC
AAGTGTGATAAGTTATCTACACTGGCGAGGGGATTGCTCTCTGTAATGTTCAGCTTCTAA
TTGTCTCTACTTTGTGAGACTACTTTTGAATGCTTGACCTCAAATCAGGTAGGACTACCC
GCTGAACTTAA
>AB1–3
TTTCCGTAGGTGAACCTGCGGAAGGATCATTATTGAATTATGTTTCTAGATAGGTTGTAG
CTGGCTCTTTTAGAGCATGTGCACGCCTGTTTGGACTTCATTTTCATCCACCTGTGCACC
TATTGTAGTCTTTGGTTGGGTTAGGAGGAAGTGATCATTGTATCAGCATCTGCTGGGAGT
GAGGACTTGCATTGTGAAAGCTTTGCTGTCCTTGATGTGATCATGGAATCTTTTTCTCAC
TAGAGTCTATGTCACTCATTATACTCTGTCGAATGTCATTGAATGTCTTTACATGGGCTT
GTATGCCTATGAAAATTGTAATACAACTTTCAGCAACGGATCTCTTGGCTCTCGCATCGA
TGAAGAACGCAGCGAAATGCGATAAGTAATGTGAATTGCAGAATTCAGTGAATCATCGAA
TCTTTGAACGCATCTTGCGCTCCTTGGTATTCCGAGGAGCATGCCTGTTTGAGTGTCATT
AAATTCTCAACTCTCTTATACTTTTTTGTAAAAGAGAGCTTGGACTGTGGAGGCTTGCTG
GCCACTTTTTGGGGTCAGCTCCTCTGAAATGCATTAGCGGAACCGTTTGCGATCTGCCAC
AAGTGTGATAAGTTATCTACACTGGCGAGGGGATTGCTCTCTGTAATGTTCAGCTTCTAA
TTGTCTCTACTTTGTGAGACTACTTTTGAATGCTTGACCTCAAATCAGGTAGGACTACCC
GCTGAACTTAA
>AB1–5
TTTCCGTAGGTGAACCTGCGGAAGGATCATTATTGAATTATGTTTCTAGATAGGTTGTAG
CTGGCTCTTTTAGAGCATGTGCACGCCTGTTTGGACTTCATTTTCATCCACCTGTGCACC
TATTGTAGTCTTTGGTTGGGTTAGGAGGAAGTGATCATTGTATCAGCATCTGCTGGGAGT
GAGGACTTGCATTGTGAAAGCTTTGCTGTCCTTGATGTGATCATGGAATCTTTTTCTCAC
TAGAGTCTATGTCACTCATTATACTCTGTCGAATGTCATTGAATGTCTTTACATGGGCTT
GTATGCCTATGAAAATTGTAATACAACTTTCAGCAACGGATCTCTTGGCTCTCGCATCGA
TGAAGAACGCAGCGAAATGCGATAAGTAATGTGAATTGCAGAATTCAGTGAATCATCGAA
TCTTTGAACGCATCTTGCGCTCCTTGGTATTCCGAGGAGCATGCCTGTTTGAGTGTCATT
AAATTCTCAACTCTCTTATACTTTTTTGTAAAAGAGAGCTTGGACTGTGGAGGCTTGCTG
GCCACTTTTTGGGGTCAGCTCCTCTGAAATGCATTAGCGGAACCGTTTGCGATCTGCCAC
AAGTGTGATAAGTTATCTACACTGGCGAGGGGATTGCTCTCTGTAATGTTCAGCTTCTAA
TTGTCTCTACTTTGTGAGACTACTTTTGAATGCTTGACCTCAAATCAGGTAGGACTACCC
GCTGAACTTAA
>AB1–9
TTTCCGTAGGTGAACCTGCGGAAGGATCATTATTGAATTATGTTTCTAGATAGGTTGTAG
CTGGCTCTTTTAGAGCATGTGCACGCCTGTTTGGACTTCATTTTCATCCACCTGTGCACC
TATTGTAGTCTTTGGTTGGGTTAGGAGGAAGTGATCATTGTATCAGCATCTGCTGGGAGT
GAGGACTTGCATTGTGAAAGCTTTGCTGTCCTTGATGTGATCATGGAATCTTTTTCTCAC
TAGAGTCTATGTCACTCATTATACTCTGTCGAATGTCATTGAATGTCTTTACATGGGCTT
GTATGCCTATGAAAATTGTAATACAACTTTCAGCAACGGATCTCTTGGCTCTCGCATCGA
TGAAGAACGCAGCGAAATGCGATAAGTAATGTGAATTGCAGAATTCAGTGAATCATCGAA

TCTTTGAACGCATCTTGCGCTCCTTGGTATTCCGAGGAGCATGCCTGTTTGAGTGTCATT
AAATTCTCAACTCTCTTATACTTTTTTGTAAAAGAGAGCTTGGACTGTGGAGGCTTGCTG
GCCACTTTTTGGGGTCAGCTCCTCTGAAATGCATTAGCGGAACCGTTTGCGATCTGCCAC
AAGTGTGATAAGTTATCTACACTGGCGAGGGGATTGCTCTCTGTAATGTTCAGCTTCTAA
TTGTCTCTACTTTGTGAGACTACTTTTGAATGCTTGACCTCAAATCAGGTAGGACTACCC
GCTGAACTTAA
>AB1-11
TTTCCGTAGGTGAACCTGCGGAAGGATCATTATTGAATTATGTTTCTAGATAGGTTGTAG
CTGGCTCTTTTAGAGCATGTGCACGCCTGTTTGGACTTCATTTTCATCCACCTGTGCACC
TATTGTAGTCTTTGGTTGGGTTAGGAGGAAGTGATCATTGTATCAGCATCTGCTGGGAGT
GAGGACTTGCATTGTGAAAGCTTTGCTGTCCTTGATGTGATCATGGAATCTTTTTCTCAC
TAGAGTCTATGTCACTCATTATACTCTGTCGAATGTCATTGAATGTCTTTACATGGGCTT
GTATGCCTATGAAAATTGTAATACAACTTTCAGCAACGGATCTCTTGGCTCTCGCATCGA
TGAAGAACGCAGCGAAATGCGATAAGTAATGTGAATTGCAGAATTCAGTGAATCATCGAA
TCTTTGAACGCATCTTGCGCTCCTTGGTATTCCGAGGAGCATGCCTGTTTGAGTGTCATT
AAATTCTCAACTCTCTTATACTTTTTTGTAAAAGAGAGCTTGGACTGTGGAGGCTTGCTG
GCCACTTTTTGGGGTCAGCTCCTCTGAAATGCATTAGCGGAACCGTTTGCGATCTGCCAC
AAGTGTGATAAGTTATCTACACTGGCGAGGGGATTGCTCTCTGTAATGTTCAGCTTCTAA
TTGTCTCTACTTTGTGAGACTACTTTTGAATGCTTGACCTCAAATCAGGTAGGACTACCC
GCTGAACTTAA
>AB1-14
TTTCCGTAGGTGAACCTGCGGAAGGATCATTATTGAATTATGTTTCTAGATAGGTTGTAG
CTGGCTCTTTTAGAGCATGTGCACGCCTGTTTGGACTTCATTTTCATCCACCTGTGCACC
TATTGTAGTCTTTGGTTGGGTTAGGAGGAAGTGATCATTGTATCAGCATCTGCTGGGAGT
GAGGACTTGCATTGTGAAAGCTTTGCTGTCCTTGATGTGATCATGGAATCTTTTTCTCAC
TAGAGTCTATGTCACTCATTATACTCTGTCGAATGTCATTGAATGTCTTTACATGGGCTT
GTATGCCTATGAAAATTGTAATACAACTTTCAGCAACGGATCTCTTGGCTCTCGCATCGA
TGAAGAACGCAGCGAAATGCGATAAGTAATGTGAATTGCAGAATTCAGTGAATCATCGAA
TCTTTGAACGCATCTTGCGCTCCTTGGTATTCCGAGGAGCATGCCTGTTTGAGTGTCATT
AAATTCTCAACTCTCTTATACTTTTTTGTAAAAGAGAGCTTGGACTGTGGAGGCTTGCTG
GCCACTTTTTGGGGTCAGCTCCTCTGAAATGCATTAGCGGAACCGTTTGCGATCTGCCAC
AAGTGTGATAAGTTATCTACACTGGCGAGGGGATTGCTCTCTGTAATGTTCAGCTTCTAA
TTGTCTCTACTTTGTGAGACTACTTTTGAATGCTTGACCTCAAATCAGGTAGGACTACCC
GCTGAACTTAA
>AB1-20
TTTCCGTAGGTGAACCTGCGGAAGGATCATTATTGAATTATGTTTCTAGATAGGTTGTAG
CTGGCTCTTTTAGAGCATGTGCACGCCTGTTTGGACTTCATTTTCATCCACCTGTGCACC
TATTGTAGTCTTTGGTTGGGTTAGGAGGAAGTGATCATTGTATCAGCATCTGCTGGGAGT
GAGGACTTGCATTGTGAAAGCTTTGCTGTCCTTGATGTGATCATGGAATCTTTTTCTCAC
TAGAGTCTATGTCACTCATTATACTCTGTCGAATGTCATTGAATGTCTTTACATGGGCTT
GTATGCCTATGAAAATTGTAATACAACTTTCAGCAACGGATCTCTTGGCTCTCGCATCGA
TGAAGAACGCAGCGAAATGCGATAAGTAATGTGAATTGCAGAATTCAGTGAATCATCGAA
TCTTTGAACGCATCTTGCGCTCCTTGGTATTCCGAGGAGCATGCCTGTTTGAGTGTCATT
AAATTCTCAACTCTCTTATACTTTTTTGTAAAAGAGAGCTTGGACTGTGGAGGCTTGCTG
GCCACTTTTTGGGGTCAGCTCCTCTGAAATGCATTAGCGGAACCGTTTGCGATCTGCCAC
AAGTGTGATAAGTTATCTACACTGGCGAGGGGATTGCTCTCTGTAATGTTCAGCTTCTAA
TTGTCTCTACTTTGTGAGACTACTTTTGAATGCTTGACCTCAAATCAGGTAGGACTACCC
GCTGAACTTAA
>AB1-23
TTTCCGTAGGTGAACCTGCGGAAGGATCATTATTGAATTATGTTTCTAGATAGGTTGTAG

CTGGCTCTTTTAGAGCATGTGCACGCCTGTTTGGACTTCATTTTCATCCACCTGTGCACC
TATTGTAGTCTTTGGTTGGGTTAGGAGGAAGTGATCATTGTATCAGCATCTGCTGGGAGT
GAGGACTTGCATTGTGAAAGCTTTGCTGTCCTTGATGTGATCATGGAATCTTTTTCTCAC
TAGAGTCTATGTCACTCATTATACTCTGTCGAATGTCATTGAATGTCTTTACATGGGCTT
GTATGCCTATGAAAATTGTAATACAACTTTCAGCAACGGATCTCTTGGCTCTCGCATCGA
TGAAGAACGCAGCGAAATGCGATAAGTAATGTGAATTGCAGAATTCAGTGAATCATCGAA
TCTTTGAACGCATCTTGCGCTCCTTGGTATTCCGAGGAGCATGCCTGTTTGAGTGTCATT
AAATTCTCAACTCTCTTATACTTTTTTGTAAAAGAGAGCTTGGACTGTGGAGGCTTGCTG
GCCACTTTTTGGGGTCAGCTCCTCTGAAATGCATTAGCGGAACCGTTTGCGATCTGCCAC
AAGTGTGATAAGTTATCTACACTGGCGAGGGGATTGCTCTCTGTAATGTTCAGCTTCTAA
TTGTCTCTACTTTGTGAGACTACTTTTGAATGCTTGACCTCAAATCAGGTAGGACTACCC
GCTGAACTTAA
>AB1-26
TTTCCGTAGGTGAACCTGCGGAAGGATCATTATTGAATTATGTTTCTAGATAGGTTGTAG
CTGGCTCTTTTAGAGCATGTGCACGCCTGTTTGGACTTCATTTTCATCCACCTGTGCACC
TATTGTAGTCTTTGGTTGGGTTAGGAGGAAGTGATCATTGTATCAGCATCTGCTGGGAGT
GAGGACTTGCATTGTGAAAGCTTTGCTGTCCTTGATGTGATCATGGAATCTTTTTCTCAC
TAGAGTCTATGTCACTCATTATACTCTGTCGAATGTCATTGAATGTCTTTACATGGGCTT
GTATGCCTATGAAAATTGTAATACAACTTTCAGCAACGGATCTCTTGGCTCTCGCATCGA
TGAAGAACGCAGCGAAATGCGATAAGTAATGTGAATTGCAGAATTCAGTGAATCATCGAA
TCTTTGAACGCATCTTGCGCTCCTTGGTATTCCGAGGAGCATGCCTGTTTGAGTGTCATT
AAATTCTCAACTCTCTTATACTTTTTTGTAAAAGAGAGCTTGGACTGTGGAGGCTTGCTG
GCCACTTTTTGGGGTCAGCTCCTCTGAAATGCATTAGCGGAACCGTTTGCGATCTGCCAC
AAGTGTGATAAGTTATCTACACTGGCGAGGGGATTGCTCTCTGTAATGTTCAGCTTCTAA
TTGTCTCTACTTTGTGAGACTACTTTTGAATGCTTGACCTCAAATCAGGTAGGACTACCC
GCTGAACTTAA
>AB1-28
TTTCCGTAGGTGAACCTGCGGAAGGATCATTATTGAATTATGTTTCTAGATAGGTTGTAG
CTGGCTCTTTTAGAGCATGTGCACGCCTGTTTGGACTTCATTTTCATCCACCTGTGCACC
TATTGTAGTCTTTGGTTGGGTTAGGAGGAAGTGATCATTGTATCAGCATCTGCTGGGAGT
GAGGACTTGCATTGTGAAAGCTTTGCTGTCCTTGATGTGATCATGGAATCTTTTTCTCAC
TAGAGTCTATGTCACTCATTATACTCTGTCGAATGTCATTGAATGTCTTTACATGGGCTT
GTATGCCTATGAAAATTGTAATACAACTTTCAGCAACGGATCTCTTGGCTCTCGCATCGA
TGAAGAACGCAGCGAAATGCGATAAGTAATGTGAATTGCAGAATTCAGTGAATCATCGAA
TCTTTGAACGCATCTTGCGCTCCTTGGTATTCCGAGGAGCATGCCTGTTTGAGTGTCATT
AAATTCTCAACTCTCTTATACTTTTTTGTAAAAGAGAGCTTGGACTGTGGAGGCTTGCTG
GCCACTTTTTGGGGTCAGCTCCTCTGAAATGCATTAGCGGAACCGTTTGCGATCTGCCAC
AAGTGTGATAAGTTATCTACACTGGCGAGGGGATTGCTCTCTGTAATGTTCAGCTTCTAA
TTGTCTCTACTTTGTGAGACTACTTTTGAATGCTTGACCTCAAATCAGGTAGGACTACCC
GCTGAACTTAA
>AB1-38
TTTCCGTAGGTGAACCTGCGGAAGGATCATTATTGAATTATGTTTCTAGATAGGTTGTAG
CTGGCTCTTTTAGAGCATGTGCACGCCTGTTTGGACTTCATTTTCATCCACCTGTGCACC
TATTGTAGTCTTTGGTTGGGTTAGGAGGAAGTGATCATTGTATCAGCATCTGCTGGGAGT
GAGGACTTGCATTGTGAAAGCTTTGCTGTCCTTGATGTGATCATGGAATCTTTTTCTCAC
TAGAGTCTATGTCACTCATTATACTCTGTCGAATGTCATTGAATGTCTTTACATGGGCTT
GTATGCCTATGAAAATTGTAATACAACTTTCAGCAACGGATCTCTTGGCTCTCGCATCGA
TGAAGAACGCAGCGAAATGCGATAAGTAATGTGAATTGCAGAATTCAGTGAATCATCGAA
TCTTTGAACGCATCTTGCGCTCCTTGGTATTCCGAGGAGCATGCCTGTTTGAGTGTCATT
AAATTCTCAACTCTCTTATACTTTTTTGTAAAAGAGAGCTTGGACTGTGGAGGCTTGCTG

GCCACTTTTTGGGGTCAGCTCCTCTGAAATGCATTAGCGGAACCGTTTGCGATCTGCCAC
AAGTGTGATAAGTTATCTACACTGGCGAGGGGATTGCTCTCTGTAATGTTCAGCTTCTAA
TTGTCTCTACTTTGTGAGACTACTTTTGAATGCTTGACCTCAAATCAGGTAGGACTACCC
GCTGAACTTAA
>AB1–39
TTTCCGTAGGTGAACCTGCGGAAGGATCATTATTGAATTATGTTTCTAGATAGGTTGTAG
CTGGCTCTTTTAGAGCATGTGCACGCCTGTTTGGACTTCATTTTCATCCACCTGTGCACC
TATTGTAGTCTTTGGTTGGGTTAGGAGGAAGTGATCATTGTATCAGCATCTGCTGGGAGT
GAGGACTTGCATTGTGAAAGCTTTGCTGTCCTTGATGTGATCATGGAATCTTTTTCTCAC
TAGAGTCTATGTCACTCATTATACTCTGTCGAATGTCATTGAATGTCTTTACATGGGCTT
GTATGCCTATGAAAATTGTAATACAACTTTCAGCAACGGATCTCTTGGCTCTCGCATCGA
TGAAGAACGCAGCGAAATGCGATAAGTAATGTGAATTGCAGAATTCAGTGAATCATCGAA
TCTTTGAACGCATCTTGCGCTCCTTGGTATTCCGAGGAGCATGCCTGTTTGAGTGTCATT
AAATTCTCAACTCTCTTATACTTTTTTGTAAAAGAGAGCTTGGACTGTGGAGGCTTGCTG
GCCACTTTTTGGGGTCAGCTCCTCTGAAATGCATTAGCGGAACCGTTTGCGATCTGCCAC
AAGTGTGATAAGTTATCTACACTGGCGAGGGGATTGCTCTCTGTAATGTTCAGCTTCTAA
TTGTCTCTACTTTGTGAGACTACTTTTGAATGCTTGACCTCAAATCAGGTAGGACTACCC
GCTGAACTTAA
>AB1–43
TTTCCGTAGGTGAACCTGCGGAAGGATCATTATTGAATTATGTTTCTAGATAGGTTGTAG
CTGGCTCTTTTAGAGCATGTGCACGCCTGTTTGGACTTCATTTTCATCCACCTGTGCACC
TATTGTAGTCTTTGGTTGGGTTAGGAGGAAGTGATCATTGTATCAGCATCTGCTGGGAGT
GAGGACTTGCATTGTGAAAGCTTTGCTGTCCTTGATGTGATCATGGAATCTTTTTCTCAC
TAGAGTCTATGTCACTCATTATACTCTGTCGAATGTCATTGAATGTCTTTACATGGGCTT
GTATGCCTATGAAAATTGTAATACAACTTTCAGCAACGGATCTCTTGGCTCTCGCATCGA
TGAAGAACGCAGCGAAATGCGATAAGTAATGTGAATTGCAGAATTCAGTGAATCATCGAA
TCTTTGAACGCATCTTGCGCTCCTTGGTATTCCGAGGAGCATGCCTGTTTGAGTGTCATT
AAATTCTCAACTCTCTTATACTTTTTTGTAAAAGAGAGCTTGGACTGTGGAGGCTTGCTG
GCCACTTTTTGGGGTCAGCTCCTCTGAAATGCATTAGCGGAACCGTTTGCGATCTGCCAC
AAGTGTGATAAGTTATCTACACTGGCGAGGGGATTGCTCTCTGTAATGTTCAGCTTCTAA
TTGTCTCTACTTTGTGAGACTACTTTTGAATGCTTGACCTCAAATCAGGTAGGACTACCC
GCTGAACTTAA
>AB1–48
TTTCCGTAGGTGAACCTGCGGAAGGATCATTATTGAATTATGTTTCTAGATAGGTTGTAG
CTGGCTCTTTTAGAGCATGTGCACGCCTGTTTGGACTTCATTTTCATCCACCTGTGCACC
TATTGTAGTCTTTGGTTGGGTTAGGAGGAAGTGATCATTGTATCAGCATCTGCTGGGAGT
GAGGACTTGCATTGTGAAAGCTTTGCTGTCCTTGATGTGATCATGGAATCTTTTTCTCAC
TAGAGTCTATGTCACTCATTATACTCTGTCGAATGTCATTGAATGTCTTTACATGGGCTT
GTATGCCTATGAAAATTGTAATACAACTTTCAGCAACGGATCTCTTGGCTCTCGCATCGA
TGAAGAACGCAGCGAAATGCGATAAGTAATGTGAATTGCAGAATTCAGTGAATCATCGAA
TCTTTGAACGCATCTTGCGCTCCTTGGTATTCCGAGGAGCATGCCTGTTTGAGTGTCATT
AAATTCTCAACTCTCTTATACTTTTTTGTAAAAGAGAGCTTGGACTGTGGAGGCTTGCTG
GCCACTTTTTGGGGTCAGCTCCTCTGAAATGCATTAGCGGAACCGTTTGCGATCTGCCAC
AAGTGTGATAAGTTATCTACACTGGCGAGGGGATTGCTCTCTGTAATGTTCAGCTTCTAA
TTGTCTCTACTTTGTGAGACTACTTTTGAATGCTTGACCTCAAATCAGGTAGGACTACCC
GCTGAACTTAA
>AB2–1
TTTCCGTAGGTGAACCTGCGGAAGGATCATTATTGAATTATGTTTCTAGATAGGTTGTAG
CTGGCTCTTTTAGAGCATGTGCACGCCTGTTTGGACTTCATTTTCATCCACCTGTGCACC
TATTGTAGTCTTTGGTTGGGTTAGGAGGAAGTGATCATTGTATCAGCATCTGCTGGGAGT

GAGGACTTGCATTGTGAAAGCTTTGCTGTCCTTGATGTGATCATGGAATCTTTTTCTCAC
TAGAGTCTATGTCACTCATTATACTCTGTCGAATGTCATTGAATGTCTTTACATGGGCTT
GTATGCCTATGAAAATTGTAATACAACTTTCAGCAACGGATCTCTTGGCTCTCGCATCGA
TGAAGAACGCAGCGAAATGCGATAAGTAATGTGAATTGCAGAATTCAGTGAATCATCGAA
TCTTTGAACGCATCTTGCGCTCCTTGGTATTCCGAGGAGCATGCCTGTTTGAGTGTCATT
AAATTCTCAACTCTCTTATACTTTTTTGTAAAAGAGAGCTTGGACTGTGGAGGCTTGCTG
GCCACTTTTTGGGGTCAGCTCCTCTGAAATGCATTAGCGGAACCGTTTGCGATCTGCCAC
AAGTGTGATAAGTTATCTACACTGGCGAGGGGATTGCTCTCTGTAATGTTCAGCTTCTAA
TTGTCTCTACTTTGTGAGACTACTTTTGAATGCTTGACCTCAAATCAGGTAGGACTACCC
GCTGAACTTAA
>AB2-4
TTTCCGTAGGTGAACCTGCGGAAGGATCATTATTGAATTATGTTTCTAGATAGGTTGTAG
CTGGCTCTTTTAGAGCATGTGCACGCCTGTTTGGACTTCATTTTCATCCACCTGTGCACC
TATTGTAGTCTTTGGTTGGGTTAGGAGGAAGTGATCATTGTATCAGCATCTGCTGGGAGT
GAGGACTTGCATTGTGAAAGCTTTGCTGTCCTTGATGTGATCATGGAATCTTTTTCTCAC
TAGAGTCTATGTCACTCATTATACTCTGTCGAATGTCATTGAATGTCTTTACATGGGCTT
GTATGCCTATGAAAATTGTAATACAACTTTCAGCAACGGATCTCTTGGCTCTCGCATCGA
TGAAGAACGCAGCGAAATGCGATAAGTAATGTGAATTGCAGAATTCAGTGAATCATCGAA
TCTTTGAACGCATCTTGCGCTCCTTGGTATTCCGAGGAGCATGCCTGTTTGAGTGTCATT
AAATTCTCAACTCTCTTATACTTTTTTGTAAAAGAGAGCTTGGACTGTGGAGGCTTGCTG
GCCACTTTTTGGGGTCAGCTCCTCTGAAATGCATTAGCGGAACCGTTTGCGATCTGCCAC
AAGTGTGATAAGTTATCTACACTGGCGAGGGGATTGCTCTCTGTAATGTTCAGCTTCTAA
TTGTCTCTACTTTGTGAGACTACTTTTGAATGCTTGACCTCAAATCAGGTAGGACTACCC
GCTGAACTTAA
>AB2-5
TTTCCGTAGGTGAACCTGCGGAAGGATCATTATTGAATTATGTTTCTAGATAGGTTGTAG
CTGGCTCTTTTAGAGCATGTGCACGCCTGTTTGGACTTCATTTTCATCCACCTGTGCACC
TATTGTAGTCTTTGGTTGGGTTAGGAGGAAGTGATCATTGTATCAGCATCTGCTGGGAGT
GAGGACTTGCATTGTGAAAGCTTTGCTGTCCTTGATGTGATCATGGAATCTTTTTCTCAC
TAGAGTCTATGTCACTCATTATACTCTGTCGAATGTCATTGAATGTCTTTACATGGGCTT
GTATGCCTATGAAAATTGTAATACAACTTTCAGCAACGGATCTCTTGGCTCTCGCATCGA
TGAAGAACGCAGCGAAATGCGATAAGTAATGTGAATTGCAGAATTCAGTGAATCATCGAA
TCTTTGAACGCATCTTGCGCTCCTTGGTATTCCGAGGAGCATGCCTGTTTGAGTGTCATT
AAATTCTCAACTCTCTTATACTTTTTTGTAAAAGAGAGCTTGGACTGTGGAGGCTTGCTG
GCCACTTTTTGGGGTCAGCTCCTCTGAAATGCATTAGCGGAACCGTTTGCGATCTGCCAC
AAGTGTGATAAGTTATCTACACTGGCGAGGGGATTGCTCTCTGTAATGTTCAGCTTCTAA
TTGTCTCTACTTTGTGAGACTACTTTTGAATGCTTGACCTCAAATCAGGTAGGACTACCC
GCTGAACTTAA
>AB2-6
TTTCCGTAGGTGAACCTGCGGAAGGATCATTATTGAATTATGTTTCTAGATAGGTTGTAG
CTGGCTCTTTTAGAGCATGTGCACGCCTGTTTGGACTTCATTTTCATCCACCTGTGCACC
TATTGTAGTCTTTGGTTGGGTTAGGAGGAAGTGATCATTGTATCAGCATCTGCTGGGAGT
GAGGACTTGCATTGTGAAAGCTTTGCTGTCCTTGATGTGATCATGGAATCTTTTTCTCAC
TAGAGTCTATGTCACTCATTATACTCTGTCGAATGTCATTGAATGTCTTTACATGGGCTT
GTATGCCTATGAAAATTGTAATACAACTTTCAGCAACGGATCTCTTGGCTCTCGCATCGA
TGAAGAACGCAGCGAAATGCGATAAGTAATGTGAATTGCAGAATTCAGTGAATCATCGAA
TCTTTGAACGCATCTTGCGCTCCTTGGTATTCCGAGGAGCATGCCTGTTTGAGTGTCATT
AAATTCTCAACTCTCTTATACTTTTTTGTAAAAGAGAGCTTGGACTGTGGAGGCTTGCTG
GCCACTTTTTGGGGTCAGCTCCTCTGAAATGCATTAGCGGAACCGTTTGCGATCTGCCAC
AAGTGTGATAAGTTATCTACACTGGCGAGGGGATTGCTCTCTGTAATGTTCAGCTTCTAA

TTGTCTCTACTTTGTGAGACTACTTTTGAATGCTTGACCTCAAATCAGGTAGGACTACCC
GCTGAACTTAA
>AB2–8
TTTCCGTAGGTGAACCTGCGGAAGGATCATTATTGAATTATGTTTCTAGATAGGTTGTAG
CTGGCTCTTTTAGAGCATGTGCACGCCTGTTTGGACTTCATTTTCATCCACCTGTGCACC
TATTGTAGTCTTTGGTTGGGTTAGGAGGAAGTGATCATTGTATCAGCATCTGCTGGGAGT
GAGGACTTGCATTGTGAAAGCTTTGCTGTCCTTGATGTGATCATGGAATCTTTTTCTCAC
TAGAGTCTATGTCACTCATTATACTCTGTCGAATGTCATTGAATGTCTTTACATGGGCTT
GTATGCCTATGAAAATTGTAATACAACTTTCAGCAACGGATCTCTTGGCTCTCGCATCGA
TGAAGAACGCAGCGAAATGCGATAAGTAATGTGAATTGCAGAATTCAGTGAATCATCGAA
TCTTTGAACGCATCTTGCGCTCCTTGGTATTCCGAGGAGCATGCCTGTTTGAGTGTCATT
AAATTCTCAACTCTCTTATACTTTTTTGTAAAAGAGAGCTTGGACTGTGGAGGCTTGCTG
GCCACTTTTTGGGGTCAGCTCCTCTGAAATGCATTAGCGGAACCGTTTGCGATCTGCCAC
AAGTGTGATAAGTTATCTACACTGGCGAGGGGATTGCTCTCTGTAATGTTCAGCTTCTAA
TTGTCTCTACTTTGTGAGACTACTTTTGAATGCTTGACCTCAAATCAGGTAGGACTACCC
GCTGAACTTAA
>AB2–9
TTTCCGTAGGTGAACCTGCGGAAGGATCATTATTGAATTATGTTTCTAGATAGGTTGTAG
CTGGCTCTTTTAGAGCATGTGCACGCCTGTTTGGACTTCATTTTCATCCACCTGTGCACC
TATTGTAGTCTTTGGTTGGGTTAGGAGGAAGTGATCATTGTATCAGCATCTGCTGGGAGT
GAGGACTTGCATTGTGAAAGCTTTGCTGTCCTTGATGTGATCATGGAATCTTTTTCTCAC
TAGAGTCTATGTCACTCATTATACTCTGTCGAATGTCATTGAATGTCTTTACATGGGCTT
GTATGCCTATGAAAATTGTAATACAACTTTCAGCAACGGATCTCTTGGCTCTCGCATCGA
TGAAGAACGCAGCGAAATGCGATAAGTAATGTGAATTGCAGAATTCAGTGAATCATCGAA
TCTTTGAACGCATCTTGCGCTCCTTGGTATTCCGAGGAGCATGCCTGTTTGAGTGTCATT
AAATTCTCAACTCTCTTATACTTTTTTGTAAAAGAGAGCTTGGACTGTGGAGGCTTGCTG
GCCACTTTTTGGGGTCAGCTCCTCTGAAATGCATTAGCGGAACCGTTTGCGATCTGCCAC
AAGTGTGATAAGTTATCTACACTGGCGAGGGGATTGCTCTCTGTAATGTTCAGCTTCTAA
TTGTCTCTACTTTGTGAGACTACTTTTGAATGCTTGACCTCAAATCAGGTAGGACTACCC
GCTGAACTTAA
>AB2–13
TTTCCGTAGGTGAACCTGCGGAAGGATCATTATTGAATTATGTTTCTAGATAGGTTGTAG
CTGGCTCTTTTAGAGCATGTGCACGCCTGTTTGGACTTCATTTTCATCCACCTGTGCACC
TATTGTAGTCTTTGGTTGGGTTAGGAGGAAGTGATCATTGTATCAGCATCTGCTGGGAGT
GAGGACTTGCATTGTGAAAGCTTTGCTGTCCTTGATGTGATCATGGAATCTTTTTCTCAC
TAGAGTCTATGTCACTCATTATACTCTGTCGAATGTCATTGAATGTCTTTACATGGGCTT
GTATGCCTATGAAAATTGTAATACAACTTTCAGCAACGGATCTCTTGGCTCTCGCATCGA
TGAAGAACGCAGCGAAATGCGATAAGTAATGTGAATTGCAGAATTCAGTGAATCATCGAA
TCTTTGAACGCATCTTGCGCTCCTTGGTATTCCGAGGAGCATGCCTGTTTGAGTGTCATT
AAATTCTCAACTCTCTTATACTTTTTTGTAAAAGAGAGCTTGGACTGTGGAGGCTTGCTG
GCCACTTTTTGGGGTCAGCTCCTCTGAAATGCATTAGCGGAACCGTTTGCGATCTGCCAC
AAGTGTGATAAGTTATCTACACTGGCGAGGGGATTGCTCTCTGTAATGTTCAGCTTCTAA
TTGTCTCTACTTTGTGAGACTACTTTTGAATGCTTGACCTCAAATCAGGTAGGACTACCC
GCTGAACTTAA
>AB2–17
TTTCCGTAGGTGAACCTGCGGAAGGATCATTATTGAATTATGTTTCTAGATAGGTTGTAG
CTGGCTCTTTTAGAGCATGTGCACGCCTGTTTGGACTTCATTTTCATCCACCTGTGCACC
TATTGTAGTCTTTGGTTGGGTTAGGAGGAAGTGATCATTGTATCAGCATCTGCTGGGAGT
GAGGACTTGCATTGTGAAAGCTTTGCTGTCCTTGATGTGATCATGGAATCTTTTTCTCAC
TAGAGTCTATGTCACTCATTATACTCTGTCGAATGTCATTGAATGTCTTTACATGGGCTT

GTATGCCTATGAAAATTGTAATACAACTTTCAGCAACGGATCTCTTGGCTCTCGCATCGA
TGAAGAACGCAGCGAAATGCGATAAGTAATGTGAATTGCAGAATTCAGTGAATCATCGAA
TCTTTGAACGCATCTTGCGCTCCTTGGTATTCCGAGGAGCATGCCTGTTTGAGTGTCATT
AAATTCTCAACTCTCTTATACTTTTTTGTAAAAGAGAGCTTGGACTGTGGAGGCTTGCTG
GCCACTTTTTGGGGTCAGCTCCTCTGAAATGCATTAGCGGAACCGTTTGCGATCTGCCAC
AAGTGTGATAAGTTATCTACACTGGCGAGGGGATTGCTCTCTGTAATGTTCAGCTTCTAA
TTGTCTCTACTTTGTGAGACTACTTTTGAATGCTTGACCTCAAATCAGGTAGGACTACCC
GCTGAACTTAA
>AB2-18
TTTCCGTAGGTGAACCTGCGGAAGGATCATTATTGAATTATGTTTCTAGATAGGTTGTAG
CTGGCTCTTTTAGAGCATGTGCACGCCTGTTTGGACTTCATTTTCATCCACCTGTGCACC
TATTGTAGTCTTTGGTTGGGTTAGGAGGAAGTGATCATTGTATCAGCATCTGCTGGGAGT
GAGGACTTGCATTGTGAAAGCTTTGCTGTCCTTGATGTGATCATGGAATCTTTTTCTCAC
TAGAGTCTATGTCACTCATTATACTCTGTCGAATGTCATTGAATGTCTTTACATGGGCTT
GTATGCCTATGAAAATTGTAATACAACTTTCAGCAACGGATCTCTTGGCTCTCGCATCGA
TGAAGAACGCAGCGAAATGCGATAAGTAATGTGAATTGCAGAATTCAGTGAATCATCGAA
TCTTTGAACGCATCTTGCGCTCCTTGGTATTCCGAGGAGCATGCCTGTTTGAGTGTCATT
AAATTCTCAACTCTCTTATACTTTTTTGTAAAAGAGAGCTTGGACTGTGGAGGCTTGCTG
GCCACTTTTTGGGGTCAGCTCCTCTGAAATGCATTAGCGGAACCGTTTGCGATCTGCCAC
AAGTGTGATAAGTTATCTACACTGGCGAGGGGATTGCTCTCTGTAATGTTCAGCTTCTAA
TTGTCTCTACTTTGTGAGACTACTTTTGAATGCTTGACCTCAAATCAGGTAGGACTACCC
GCTGAACTTAA
>AB2-21
TTTCCGTAGGTGAACCTGCGGAAGGATCATTATTGAATTATGTTTCTAGATAGGTTGTAG
CTGGCTCTTTTAGAGCATGTGCACGCCTGTTTGGACTTCATTTTCATCCACCTGTGCACC
TATTGTAGTCTTTGGTTGGGTTAGGAGGAAGTGATCATTGTATCAGCATCTGCTGGGAGT
GAGGACTTGCATTGTGAAAGCTTTGCTGTCCTTGATGTGATCATGGAATCTTTTTCTCAC
TAGAGTCTATGTCACTCATTATACTCTGTCGAATGTCATTGAATGTCTTTACATGGGCTT
GTATGCCTATGAAAATTGTAATACAACTTTCAGCAACGGATCTCTTGGCTCTCGCATCGA
TGAAGAACGCAGCGAAATGCGATAAGTAATGTGAATTGCAGAATTCAGTGAATCATCGAA
TCTTTGAACGCATCTTGCGCTCCTTGGTATTCCGAGGAGCATGCCTGTTTGAGTGTCATT
AAATTCTCAACTCTCTTATACTTTTTTGTAAAAGAGAGCTTGGACTGTGGAGGCTTGCTG
GCCACTTTTTGGGGTCAGCTCCTCTGAAATGCATTAGCGGAACCGTTTGCGATCTGCCAC
AAGTGTGATAAGTTATCTACACTGGCGAGGGGATTGCTCTCTGTAATGTTCAGCTTCTAA
TTGTCTCTACTTTGTGAGACTACTTTTGAATGCTTGACCTCAAATCAGGTAGGACTACCC
GCTGAACTTAA
>AB2-22
TTTCCGTAGGTGAACCTGCGGAAGGATCATTATTGAATTATGTTTCTAGATAGGTTGTAG
CTGGCTCTTTTAGAGCATGTGCACGCCTGTTTGGACTTCATTTTCATCCACCTGTGCACC
TATTGTAGTCTTTGGTTGGGTTAGGAGGAAGTGATCATTGTATCAGCATCTGCTGGGAGT
GAGGACTTGCATTGTGAAAGCTTTGCTGTCCTTGATGTGATCATGGAATCTTTTTCTCAC
TAGAGTCTATGTCACTCATTATACTCTGTCGAATGTCATTGAATGTCTTTACATGGGCTT
GTATGCCTATGAAAATTGTAATACAACTTTCAGCAACGGATCTCTTGGCTCTCGCATCGA
TGAAGAACGCAGCGAAATGCGATAAGTAATGTGAATTGCAGAATTCAGTGAATCATCGAA
TCTTTGAACGCATCTTGCGCTCCTTGGTATTCCGAGGAGCATGCCTGTTTGAGTGTCATT
AAATTCTCAACTCTCTTATACTTTTTTGTAAAAGAGAGCTTGGACTGTGGAGGCTTGCTG
GCCACTTTTTGGGGTCAGCTCCTCTGAAATGCATTAGCGGAACCGTTTGCGATCTGCCAC
AAGTGTGATAAGTTATCTACACTGGCGAGGGGATTGCTCTCTGTAATGTTCAGCTTCTAA
TTGTCTCTACTTTGTGAGACTACTTTTGAATGCTTGACCTCAAATCAGGTAGGACTACCC
GCTGAACTTAA

>AB2-24
TTTCCGTAGGTGAACCTGCGGAAGGATCATTATTGAATTATGTTTCTAGATAGGTTGTAG
CTGGCTCTTTTAGAGCATGTGCACGCCTGTTTGGACTTCATTTTCATCCACCTGTGCACC
TATTGTAGTCTTTGGTTGGGTTAGGAGGAAGTGATCATTGTATCAGCATCTGCTGGGAGT
GAGGACTTGCATTGTGAAAGCTTTGCTGTCCTTGATGTGATCATGGAATCTTTTTCTCAC
TAGAGTCTATGTCACTCATTATACTCTGTCGAATGTCATTGAATGTCTTTACATGGGCTT
GTATGCCTATGAAAATTGTAATACAACTTTCAGCAACGGATCTCTTGGCTCTCGCATCGA
TGAAGAACGCAGCGAAATGCGATAAGTAATGTGAATTGCAGAATTCAGTGAATCATCGAA
TCTTTGAACGCATCTTGCGCTCCTTGGTATTCCGAGGAGCATGCCTGTTTGAGTGTCATT
AAATTCTCAACTCTCTTATACTTTTTTGTAAAAGAGAGCTTGGACTGTGGAGGCTTGCTG
GCCACTTTTTGGGGTCAGCTCCTCTGAAATGCATTAGCGGAACCGTTTGCGATCTGCCAC
AAGTGTGATAAGTTATCTACACTGGCGAGGGGATTGCTCTCTGTAATGTTCAGCTTCTAA
TTGTCTCTACTTTGTGAGACTACTTTTGAATGCTTGACCTCAAATCAGGTAGGACTACCC
GCTGAACTTAA
>AB2-25
TTTCCGTAGGTGAACCTGCGGAAGGATCATTATTGAATTATGTTTCTAGATAGGTTGTAG
CTGGCTCTTTTAGAGCATGTGCACGCCTGTTTGGACTTCATTTTCATCCACCTGTGCACC
TATTGTAGTCTTTGGTTGGGTTAGGAGGAAGTGATCATTGTATCAGCATCTGCTGGGAGT
GAGGACTTGCATTGTGAAAGCTTTGCTGTCCTTGATGTGATCATGGAATCTTTTTCTCAC
TAGAGTCTATGTCACTCATTATACTCTGTCGAATGTCATTGAATGTCTTTACATGGGCTT
GTATGCCTATGAAAATTGTAATACAACTTTCAGCAACGGATCTCTTGGCTCTCGCATCGA
TGAAGAACGCAGCGAAATGCGATAAGTAATGTGAATTGCAGAATTCAGTGAATCATCGAA
TCTTTGAACGCATCTTGCGCTCCTTGGTATTCCGAGGAGCATGCCTGTTTGAGTGTCATT
AAATTCTCAACTCTCTTATACTTTTTTGTAAAAGAGAGCTTGGACTGTGGAGGCTTGCTG
GCCACTTTTTGGGGTCAGCTCCTCTGAAATGCATTAGCGGAACCGTTTGCGATCTGCCAC
AAGTGTGATAAGTTATCTACACTGGCGAGGGGATTGCTCTCTGTAATGTTCAGCTTCTAA
TTGTCTCTACTTTGTGAGACTACTTTTGAATGCTTGACCTCAAATCAGGTAGGACTACCC
GCTGAACTTAA
>AB2-33
TTTCCGTAGGTGAACCTGCGGAAGGATCATTATTGAATTATGTTTCTAGATAGGTTGTAG
CTGGCTCTTTTAGAGCATGTGCACGCCTGTTTGGACTTCATTTTCATCCACCTGTGCACC
TATTGTAGTCTTTGGTTGGGTTAGGAGGAAGTGATCATTGTATCAGCATCTGCTGGGAGT
GAGGACTTGCATTGTGAAAGCTTTGCTGTCCTTGATGTGATCATGGAATCTTTTTCTCAC
TAGAGTCTATGTCACTCATTATACTCTGTCGAATGTCATTGAATGTCTTTACATGGGCTT
GTATGCCTATGAAAATTGTAATACAACTTTCAGCAACGGATCTCTTGGCTCTCGCATCGA
TGAAGAACGCAGCGAAATGCGATAAGTAATGTGAATTGCAGAATTCAGTGAATCATCGAA
TCTTTGAACGCATCTTGCGCTCCTTGGTATTCCGAGGAGCATGCCTGTTTGAGTGTCATT
AAATTCTCAACTCTCTTATACTTTTTTGTAAAAGAGAGCTTGGACTGTGGAGGCTTGCTG
GCCACTTTTTGGGGTCAGCTCCTCTGAAATGCATTAGCGGAACCGTTTGCGATCTGCCAC
AAGTGTGATAAGTTATCTACACTGGCGAGGGGATTGCTCTCTGTAATGTTCAGCTTCTAA
TTGTCTCTACTTTGTGAGACTACTTTTGAATGCTTGACCTCAAATCAGGTAGGACTACCC
GCTGAACTTAA
>AB2-34
TTTCCGTAGGTGAACCTGCGGAAGGATCATTATTGAATTATGTTTCTAGATAGGTTGTAG
CTGGCTCTTTTAGAGCATGTGCACGCCTGTTTGGACTTCATTTTCATCCACCTGTGCACC
TATTGTAGTCTTTGGTTGGGTTAGGAGGAAGTGATCATTGTATCAGCATCTGCTGGGAGT
GAGGACTTGCATTGTGAAAGCTTTGCTGTCCTTGATGTGATCATGGAATCTTTTTCTCAC
TAGAGTCTATGTCACTCATTATACTCTGTCGAATGTCATTGAATGTCTTTACATGGGCTT
GTATGCCTATGAAAATTGTAATACAACTTTCAGCAACGGATCTCTTGGCTCTCGCATCGA
TGAAGAACGCAGCGAAATGCGATAAGTAATGTGAATTGCAGAATTCAGTGAATCATCGAA

TCTTTGAACGCATCTTGCGCTCCTTGGTATTCCGAGGAGCATGCCTGTTTGAGTGTCATT
AAATTCTCAACTCTCTTATACTTTTTTGTAAAAGAGAGCTTGGACTGTGGAGGCTTGCTG
GCCACTTTTTGGGGTCAGCTCCTCTGAAATGCATTAGCGGAACCGTTTGCGATCTGCCAC
AAGTGTGATAAGTTATCTACACTGGCGAGGGGATTGCTCTCTGTAATGTTCAGCTTCTAA
TTGTCTCTACTTTGTGAGACTACTTTTGAATGCTTGACCTCAAATCAGGTAGGACTACCC
GCTGAACTTAA
>AB2-35
TTTCCGTAGGTGAACCTGCGGAAGGATCATTATTGAATTATGTTTCTAGATAGGTTGTAG
CTGGCTCTTTTAGAGCATGTGCACGCCTGTTTGGACTTCATTTTCATCCACCTGTGCACC
TATTGTAGTCTTTGGTTGGGTTAGGAGGAAGTGATCATTGTATCAGCATCTGCTGGGAGT
GAGGACTTGCATTGTGAAAGCTTTGCTGTCCTTGATGTGATCATGGAATCTTTTTCTCAC
TAGAGTCTATGTCACTCATTATACTCTGTCGAATGTCATTGAATGTCTTTACATGGGCTT
GTATGCCTATGAAAATTGTAATACAACTTTCAGCAACGGATCTCTTGGCTCTCGCATCGA
TGAAGAACGCAGCGAAATGCGATAAGTAATGTGAATTGCAGAATTCAGTGAATCATCGAA
TCTTTGAACGCATCTTGCGCTCCTTGGTATTCCGAGGAGCATGCCTGTTTGAGTGTCATT
AAATTCTCAACTCTCTTATACTTTTTTGTAAAAGAGAGCTTGGACTGTGGAGGCTTGCTG
GCCACTTTTTGGGGTCAGCTCCTCTGAAATGCATTAGCGGAACCGTTTGCGATCTGCCAC
AAGTGTGATAAGTTATCTACACTGGCGAGGGGATTGCTCTCTGTAATGTTCAGCTTCTAA
TTGTCTCTACTTTGTGAGACTACTTTTGAATGCTTGACCTCAAATCAGGTAGGACTACCC
GCTGAACTTAA
>AB2-40
TTTCCGTAGGTGAACCTGCGGAAGGATCATTATTGAATTATGTTTCTAGATAGGTTGTAG
CTGGCTCTTTTAGAGCATGTGCACGCCTGTTTGGACTTCATTTTCATCCACCTGTGCACC
TATTGTAGTCTTTGGTTGGGTTAGGAGGAAGTGATCATTGTATCAGCATCTGCTGGGAGT
GAGGACTTGCATTGTGAAAGCTTTGCTGTCCTTGATGTGATCATGGAATCTTTTTCTCAC
TAGAGTCTATGTCACTCATTATACTCTGTCGAATGTCATTGAATGTCTTTACATGGGCTT
GTATGCCTATGAAAATTGTAATACAACTTTCAGCAACGGATCTCTTGGCTCTCGCATCGA
TGAAGAACGCAGCGAAATGCGATAAGTAATGTGAATTGCAGAATTCAGTGAATCATCGAA
TCTTTGAACGCATCTTGCGCTCCTTGGTATTCCGAGGAGCATGCCTGTTTGAGTGTCATT
AAATTCTCAACTCTCTTATACTTTTTTGTAAAAGAGAGCTTGGACTGTGGAGGCTTGCTG
GCCACTTTTTGGGGTCAGCTCCTCTGAAATGCATTAGCGGAACCGTTTGCGATCTGCCAC
AAGTGTGATAAGTTATCTACACTGGCGAGGGGATTGCTCTCTGTAATGTTCAGCTTCTAA
TTGTCTCTACTTTGTGAGACTACTTTTGAATGCTTGACCTCAAATCAGGTAGGACTACCC
GCTGAACTTAA
>AB2-44
TTTCCGTAGGTGAACCTGCGGAAGGATCATTATTGAATTATGTTTCTAGATAGGTTGTAG
CTGGCTCTTTTAGAGCATGTGCACGCCTGTTTGGACTTCATTTTCATCCACCTGTGCACC
TATTGTAGTCTTTGGTTGGGTTAGGAGGAAGTGATCATTGTATCAGCATCTGCTGGGAGT
GAGGACTTGCATTGTGAAAGCTTTGCTGTCCTTGATGTGATCATGGAATCTTTTTCTCAC
TAGAGTCTATGTCACTCATTATACTCTGTCGAATGTCATTGAATGTCTTTACATGGGCTT
GTATGCCTATGAAAATTGTAATACAACTTTCAGCAACGGATCTCTTGGCTCTCGCATCGA
TGAAGAACGCAGCGAAATGCGATAAGTAATGTGAATTGCAGAATTCAGTGAATCATCGAA
TCTTTGAACGCATCTTGCGCTCCTTGGTATTCCGAGGAGCATGCCTGTTTGAGTGTCATT
AAATTCTCAACTCTCTTATACTTTTTTGTAAAAGAGAGCTTGGACTGTGGAGGCTTGCTG
GCCACTTTTTGGGGTCAGCTCCTCTGAAATGCATTAGCGGAACCGTTTGCGATCTGCCAC
AAGTGTGATAAGTTATCTACACTGGCGAGGGGATTGCTCTCTGTAATGTTCAGCTTCTAA
TTGTCTCTACTTTGTGAGACTACTTTTGAATGCTTGACCTCAAATCAGGTAGGACTACCC
GCTGAACTTAA
>AB2-45
TTTCCGTAGGTGAACCTGCGGAAGGATCATTATTGAATTATGTTTCTAGATAGGTTGTAG

CTGGCTCTTTTAGAGCATGTGCACGCCTGTTTGGACTTCATTTTCATCCACCTGTGCACC
TATTGTAGTCTTTGGTTGGGTTAGGAGGAAGTGATCATTGTATCAGCATCTGCTGGGAGT
GAGGACTTGCATTGTGAAAGCTTTGCTGTCCTTGATGTGATCATGGAATCTTTTTCTCAC
TAGAGTCTATGTCACTCATTATACTCTGTCGAATGTCATTGAATGTCTTTACATGGGCTT
GTATGCCTATGAAAATTGTAATACAACTTTCAGCAACGGATCTCTTGGCTCTCGCATCGA
TGAAGAACGCAGCGAAATGCGATAAGTAATGTGAATTGCAGAATTCAGTGAATCATCGAA
TCTTTGAACGCATCTTGCGCTCCTTGGTATTCCGAGGAGCATGCCTGTTTGAGTGTCATT
AAATTCTCAACTCTCTTATACTTTTTTGTAAAAGAGAGCTTGGACTGTGGAGGCTTGCTG
GCCACTTTTTGGGGTCAGCTCCTCTGAAATGCATTAGCGGAACCGTTTGCGATCTGCCAC
AAGTGTGATAAGTTATCTACACTGGCGAGGGGATTGCTCTCTGTAATGTTCAGCTTCTAA
TTGTCTCTACTTTGTGAGACTACTTTTGAATGCTTGACCTCAAATCAGGTAGGACTACCC
GCTGAACTTAA
>AB2-47
TTTCCGTAGGTGAACCTGCGGAAGGATCATTATTGAATTATGTTTCTAGATAGGTTGTAG
CTGGCTCTTTTAGAGCATGTGCACGCCTGTTTGGACTTCATTTTCATCCACCTGTGCACC
TATTGTAGTCTTTGGTTGGGTTAGGAGGAAGTGATCATTGTATCAGCATCTGCTGGGAGT
GAGGACTTGCATTGTGAAAGCTTTGCTGTCCTTGATGTGATCATGGAATCTTTTTCTCAC
TAGAGTCTATGTCACTCATTATACTCTGTCGAATGTCATTGAATGTCTTTACATGGGCTT
GTATGCCTATGAAAATTGTAATACAACTTTCAGCAACGGATCTCTTGGCTCTCGCATCGA
TGAAGAACGCAGCGAAATGCGATAAGTAATGTGAATTGCAGAATTCAGTGAATCATCGAA
TCTTTGAACGCATCTTGCGCTCCTTGGTATTCCGAGGAGCATGCCTGTTTGAGTGTCATT
AAATTCTCAACTCTCTTATACTTTTTTGTAAAAGAGAGCTTGGACTGTGGAGGCTTGCTG
GCCACTTTTTGGGGTCAGCTCCTCTGAAATGCATTAGCGGAACCGTTTGCGATCTGCCAC
AAGTGTGATAAGTTATCTACACTGGCGAGGGGATTGCTCTCTGTAATGTTCAGCTTCTAA
TTGTCTCTACTTTGTGAGACTACTTTTGAATGCTTGACCTCAAATCAGGTAGGACTACCC
GCTGAACTTAA
>AB2-49
TTTCCGTAGGTGAACCTGCGGAAGGATCATTATTGAATTATGTTTCTAGATAGGTTGTAG
CTGGCTCTTTTAGAGCATGTGCACGCCTGTTTGGACTTCATTTTCATCCACCTGTGCACC
TATTGTAGTCTTTGGTTGGGTTAGGAGGAAGTGATCATTGTATCAGCATCTGCTGGGAGT
GAGGACTTGCATTGTGAAAGCTTTGCTGTCCTTGATGTGATCATGGAATCTTTTTCTCAC
TAGAGTCTATGTCACTCATTATACTCTGTCGAATGTCATTGAATGTCTTTACATGGGCTT
GTATGCCTATGAAAATTGTAATACAACTTTCAGCAACGGATCTCTTGGCTCTCGCATCGA
TGAAGAACGCAGCGAAATGCGATAAGTAATGTGAATTGCAGAATTCAGTGAATCATCGAA
TCTTTGAACGCATCTTGCGCTCCTTGGTATTCCGAGGAGCATGCCTGTTTGAGTGTCATT
AAATTCTCAACTCTCTTATACTTTTTTGTAAAAGAGAGCTTGGACTGTGGAGGCTTGCTG
GCCACTTTTTGGGGTCAGCTCCTCTGAAATGCATTAGCGGAACCGTTTGCGATCTGCCAC
AAGTGTGATAAGTTATCTACACTGGCGAGGGGATTGCTCTCTGTAATGTTCAGCTTCTAA
TTGTCTCTACTTTGTGAGACTACTTTTGAATGCTTGACCTCAAATCAGGTAGGACTACCC
GCTGAACTTAA
>AB2-51
TTTCCGTAGGTGAACCTGCGGAAGGATCATTATTGAATTATGTTTCTAGATAGGTTGTAG
CTGGCTCTTTTAGAGCATGTGCACGCCTGTTTGGACTTCATTTTCATCCACCTGTGCACC
TATTGTAGTCTTTGGTTGGGTTAGGAGGAAGTGATCATTGTATCAGCATCTGCTGGGAGT
GAGGACTTGCATTGTGAAAGCTTTGCTGTCCTTGATGTGATCATGGAATCTTTTTCTCAC
TAGAGTCTATGTCACTCATTATACTCTGTCGAATGTCATTGAATGTCTTTACATGGGCTT
GTATGCCTATGAAAATTGTAATACAACTTTCAGCAACGGATCTCTTGGCTCTCGCATCGA
TGAAGAACGCAGCGAAATGCGATAAGTAATGTGAATTGCAGAATTCAGTGAATCATCGAA
TCTTTGAACGCATCTTGCGCTCCTTGGTATTCCGAGGAGCATGCCTGTTTGAGTGTCATT
AAATTCTCAACTCTCTTATACTTTTTTGTAAAAGAGAGCTTGGACTGTGGAGGCTTGCTG

GCCACTTTTTGGGGTCAGCTCCTCTGAAATGCATTAGCGGAACCGTTTGCGATCTGCCAC
AAGTGTGATAAGTTATCTACACTGGCGAGGGGATTGCTCTCTGTAATGTTCAGCTTCTAA
TTGTCTCTACTTTGTGAGACTACTTTTGAATGCTTGACCTCAAATCAGGTAGGACTACCC
GCTGAACTTAA
>AB2-52
TTTCCGTAGGTGAACCTGCGGAAGGATCATTATTGAATTATGTTTCTAGATAGGTTGTAG
CTGGCTCTTTTAGAGCATGTGCACGCCTGTTTGGACTTCATTTTCATCCACCTGTGCACC
TATTGTAGTCTTTGGTTGGGTTAGGAGGAAGTGATCATTGTATCAGCATCTGCTGGGAGT
GAGGACTTGCATTGTGAAAGCTTTGCTGTCCTTGATGTGATCATGGAATCTTTTTCTCAC
TAGAGTCTATGTCACTCATTATACTCTGTCGAATGTCATTGAATGTCTTTACATGGGCTT
GTATGCCTATGAAAATTGTAATACAACTTTCAGCAACGGATCTCTTGGCTCTCGCATCGA
TGAAGAACGCAGCGAAATGCGATAAGTAATGTGAATTGCAGAATTCAGTGAATCATCGAA
TCTTTGAACGCATCTTGCGCTCCTTGGTATTCCGAGGAGCATGCCTGTTTGAGTGTCATT
AAATTCTCAACTCTCTTATACTTTTTTGTAAAAGAGAGCTTGGACTGTGGAGGCTTGCTG
GCCACTTTTTGGGGTCAGCTCCTCTGAAATGCATTAGCGGAACCGTTTGCGATCTGCCAC
AAGTGTGATAAGTTATCTACACTGGCGAGGGGATTGCTCTCTGTAATGTTCAGCTTCTAA
TTGTCTCTACTTTGTGAGACTACTTTTGAATGCTTGACCTCAAATCAGGTAGGACTACCC
GCTGAACTTAA
>AB2-53
TTTCCGTAGGTGAACCTGCGGAAGGATCATTATTGAATTATGTTTCTAGATAGGTTGTAG
CTGGCTCTTTTAGAGCATGTGCACGCCTGTTTGGACTTCATTTTCATCCACCTGTGCACC
TATTGTAGTCTTTGGTTGGGTTAGGAGGAAGTGATCATTGTATCAGCATCTGCTGGGAGT
GAGGACTTGCATTGTGAAAGCTTTGCTGTCCTTGATGTGATCATGGAATCTTTTTCTCAC
TAGAGTCTATGTCACTCATTATACTCTGTCGAATGTCATTGAATGTCTTTACATGGGCTT
GTATGCCTATGAAAATTGTAATACAACTTTCAGCAACGGATCTCTTGGCTCTCGCATCGA
TGAAGAACGCAGCGAAATGCGATAAGTAATGTGAATTGCAGAATTCAGTGAATCATCGAA
TCTTTGAACGCATCTTGCGCTCCTTGGTATTCCGAGGAGCATGCCTGTTTGAGTGTCATT
AAATTCTCAACTCTCTTATACTTTTTTGTAAAAGAGAGCTTGGACTGTGGAGGCTTGCTG
GCCACTTTTTGGGGTCAGCTCCTCTGAAATGCATTAGCGGAACCGTTTGCGATCTGCCAC
AAGTGTGATAAGTTATCTACACTGGCGAGGGGATTGCTCTCTGTAATGTTCAGCTTCTAA
TTGTCTCTACTTTGTGAGACTACTTTTGAATGCTTGACCTCAAATCAGGTAGGACTACCC
GCTGAACTTAA
>AB2-57
TTTCCGTAGGTGAACCTGCGGAAGGATCATTATTGAATTATGTTTCTAGATAGGTTGTAG
CTGGCTCTTTTAGAGCATGTGCACGCCTGTTTGGACTTCATTTTCATCCACCTGTGCACC
TATTGTAGTCTTTGGTTGGGTTAGGAGGAAGTGATCATTGTATCAGCATCTGCTGGGAGT
GAGGACTTGCATTGTGAAAGCTTTGCTGTCCTTGATGTGATCATGGAATCTTTTTCTCAC
TAGAGTCTATGTCACTCATTATACTCTGTCGAATGTCATTGAATGTCTTTACATGGGCTT
GTATGCCTATGAAAATTGTAATACAACTTTCAGCAACGGATCTCTTGGCTCTCGCATCGA
TGAAGAACGCAGCGAAATGCGATAAGTAATGTGAATTGCAGAATTCAGTGAATCATCGAA
TCTTTGAACGCATCTTGCGCTCCTTGGTATTCCGAGGAGCATGCCTGTTTGAGTGTCATT
AAATTCTCAACTCTCTTATACTTTTTTGTAAAAGAGAGCTTGGACTGTGGAGGCTTGCTG
GCCACTTTTTGGGGTCAGCTCCTCTGAAATGCATTAGCGGAACCGTTTGCGATCTGCCAC
AAGTGTGATAAGTTATCTACACTGGCGAGGGGATTGCTCTCTGTAATGTTCAGCTTCTAA
TTGTCTCTACTTTGTGAGACTACTTTTGAATGCTTGACCTCAAATCAGGTAGGACTACCC
GCTGAACTTAA
>AB2-60
TTTCCGTAGGTGAACCTGCGGAAGGATCATTATTGAATTATGTTTCTAGATAGGTTGTAG
CTGGCTCTTTTAGAGCATGTGCACGCCTGTTTGGACTTCATTTTCATCCACCTGTGCACC
TATTGTAGTCTTTGGTTGGGTTAGGAGGAAGTGATCATTGTATCAGCATCTGCTGGGAGT

GAGGACTTGCATTGTGAAAGCTTTGCTGTCCTTGATGTGATCATGGAATCTTTTTCTCAC
TAGAGTCTATGTCACTCATTATACTCTGTCGAATGTCATTGAATGTCTTTACATGGGCTT
GTATGCCTATGAAAATTGTAATACAACTTTCAGCAACGGATCTCTTGGCTCTCGCATCGA
TGAAGAACGCAGCGAAATGCGATAAGTAATGTGAATTGCAGAATTCAGTGAATCATCGAA
TCTTTGAACGCATCTTGCGCTCCTTGGTATTCCGAGGAGCATGCCTGTTTGAGTGTCATT
AAATTCTCAACTCTCTTATACTTTTTTGTAAAAGAGAGCTTGGACTGTGGAGGCTTGCTG
GCCACTTTTTGGGGTCAGCTCCTCTGAAATGCATTAGCGGAACCGTTTGCGATCTGCCAC
AAGTGTGATAAGTTATCTACACTGGCGAGGGGATTGCTCTCTGTAATGTTCAGCTTCTAA
TTGTCTCTACTTTGTGAGACTACTTTTGAATGCTTGACCTCAAATCAGGTAGGACTACCC
GCTGAACTTAA
>AB3-2
TTTCCGTAGGTGAACCTGCGGAAGGATCATTATTGAATTATGTTTCTAGATAGGTTGTAG
CTGGCTCTTTTAGAGCATGTGCACGCCTGTTTGGACTTCATTTTCATCCACCTGTGCACC
TATTGTAGTCTTTGGTTGGGTTAGGAGGAAGTGATCATTGTATCAGCATCTGCTGGGAGT
GAGGACTTGCATTGTGAAAGCTTTGCTGTCCTTGATGTGATCATGGAATCTTTTTCTCAC
TAGAGTCTATGTCACTCATTATACTCTGTCGAATGTCATTGAATGTCTTTACATGGGCTT
GTATGCCTATGAAAATTGTAATACAACTTTCAGCAACGGATCTCTTGGCTCTCGCATCGA
TGAAGAACGCAGCGAAATGCGATAAGTAATGTGAATTGCAGAATTCAGTGAATCATCGAA
TCTTTGAACGCATCTTGCGCTCCTTGGTATTCCGAGGAGCATGCCTGTTTGAGTGTCATT
AAATTCTCAACTCTCTTATACTTTTTTGTAAAAGAGAGCTTGGACTGTGGAGGCTTGCTG
GCCACTTTTTGGGGTCAGCTCCTCTGAAATGCATTAGCGGAACCGTTTGCGATCTGCCAC
AAGTGTGATAAGTTATCTACACTGGCGAGGGGATTGCTCTCTGTAATGTTCAGCTTCTAA
TTGTCTCTACTTTGTGAGACTACTTTTGAATGCTTGACCTCAAATCAGGTAGGACTACCC
GCTGAACTTAA
>AB3-6
TTTCCGTAGGTGAACCTGCGGAAGGATCATTATTGAATTATGTTTCTAGATAGGTTGTAG
CTGGCTCTTTTAGAGCATGTGCACGCCTGTTTGGACTTCATTTTCATCCACCTGTGCACC
TATTGTAGTCTTTGGTTGGGTTAGGAGGAAGTGATCATTGTATCAGCATCTGCTGGGAGT
GAGGACTTGCATTGTGAAAGCTTTGCTGTCCTTGATGTGATCATGGAATCTTTTTCTCAC
TAGAGTCTATGTCACTCATTATACTCTGTCGAATGTCATTGAATGTCTTTACATGGGCTT
GTATGCCTATGAAAATTGTAATACAACTTTCAGCAACGGATCTCTTGGCTCTCGCATCGA
TGAAGAACGCAGCGAAATGCGATAAGTAATGTGAATTGCAGAATTCAGTGAATCATCGAA
TCTTTGAACGCATCTTGCGCTCCTTGGTATTCCGAGGAGCATGCCTGTTTGAGTGTCATT
AAATTCTCAACTCTCTTATACTTTTTTGTAAAAGAGAGCTTGGACTGTGGAGGCTTGCTG
GCCACTTTTTGGGGTCAGCTCCTCTGAAATGCATTAGCGGAACCGTTTGCGATCTGCCAC
AAGTGTGATAAGTTATCTACACTGGCGAGGGGATTGCTCTCTGTAATGTTCAGCTTCTAA
TTGTCTCTACTTTGTGAGACTACTTTTGAATGCTTGACCTCAAATCAGGTAGGACTACCC
GCTGAACTTAA
>AB3-8
TTTCCGTAGGTGAACCTGCGGAAGGATCATTATTGAATTATGTTTCTAGATAGGTTGTAG
CTGGCTCTTTTAGAGCATGTGCACGCCTGTTTGGACTTCATTTTCATCCACCTGTGCACC
TATTGTAGTCTTTGGTTGGGTTAGGAGGAAGTGATCATTGTATCAGCATCTGCTGGGAGT
GAGGACTTGCATTGTGAAAGCTTTGCTGTCCTTGATGTGATCATGGAATCTTTTTCTCAC
TAGAGTCTATGTCACTCATTATACTCTGTCGAATGTCATTGAATGTCTTTACATGGGCTT
GTATGCCTATGAAAATTGTAATACAACTTTCAGCAACGGATCTCTTGGCTCTCGCATCGA
TGAAGAACGCAGCGAAATGCGATAAGTAATGTGAATTGCAGAATTCAGTGAATCATCGAA
TCTTTGAACGCATCTTGCGCTCCTTGGTATTCCGAGGAGCATGCCTGTTTGAGTGTCATT
AAATTCTCAACTCTCTTATACTTTTTTGTAAAAGAGAGCTTGGACTGTGGAGGCTTGCTG
GCCACTTTTTGGGGTCAGCTCCTCTGAAATGCATTAGCGGAACCGTTTGCGATCTGCCAC
AAGTGTGATAAGTTATCTACACTGGCGAGGGGATTGCTCTCTGTAATGTTCAGCTTCTAA

TTGTCTCTACTTTGTGAGACTACTTTTGAATGCTTGACCTCAAATCAGGTAGGACTACCC
GCTGAACTTAA
>AB3-9
TTTCCGTAGGTGAACCTGCGGAAGGATCATTATTGAATTATGTTTCTAGATAGGTTGTAG
CTGGCTCTTTTAGAGCATGTGCACGCCTGTTTGGACTTCATTTTCATCCACCTGTGCACC
TATTGTAGTCTTTGGTTGGGTTAGGAGGAAGTGATCATTGTATCAGCATCTGCTGGGAGT
GAGGACTTGCATTGTGAAAGCTTTGCTGTCCTTGATGTGATCATGGAATCTTTTTCTCAC
TAGAGTCTATGTCACTCATTATACTCTGTCGAATGTCATTGAATGTCTTTACATGGGCTT
GTATGCCTATGAAAATTGTAATACAACTTTCAGCAACGGATCTCTTGGCTCTCGCATCGA
TGAAGAACGCAGCGAAATGCGATAAGTAATGTGAATTGCAGAATTCAGTGAATCATCGAA
TCTTTGAACGCATCTTGCGCTCCTTGGTATTCCGAGGAGCATGCCTGTTTGAGTGTCATT
AAATTCTCAACTCTCTTATACTTTTTTGTAAAAGAGAGCTTGGACTGTGGAGGCTTGCTG
GCCACTTTTTGGGGTCAGCTCCTCTGAAATGCATTAGCGGAACCGTTTGCGATCTGCCAC
AAGTGTGATAAGTTATCTACACTGGCGAGGGGATTGCTCTCTGTAATGTTCAGCTTCTAA
TTGTCTCTACTTTGTGAGACTACTTTTGAATGCTTGACCTCAAATCAGGTAGGACTACCC
GCTGAACTTAA
>AB3-10
TTTCCGTAGGTGAACCTGCGGAAGGATCATTATTGAATTATGTTTCTAGATAGGTTGTAG
CTGGCTCTTTTAGAGCATGTGCACGCCTGTTTGGACTTCATTTTCATCCACCTGTGCACC
TATTGTAGTCTTTGGTTGGGTTAGGAGGAAGTGATCATTGTATCAGCATCTGCTGGGAGT
GAGGACTTGCATTGTGAAAGCTTTGCTGTCCTTGATGTGATCATGGAATCTTTTTCTCAC
TAGAGTCTATGTCACTCATTATACTCTGTCGAATGTCATTGAATGTCTTTACATGGGCTT
GTATGCCTATGAAAATTGTAATACAACTTTCAGCAACGGATCTCTTGGCTCTCGCATCGA
TGAAGAACGCAGCGAAATGCGATAAGTAATGTGAATTGCAGAATTCAGTGAATCATCGAA
TCTTTGAACGCATCTTGCGCTCCTTGGTATTCCGAGGAGCATGCCTGTTTGAGTGTCATT
AAATTCTCAACTCTCTTATACTTTTTTGTAAAAGAGAGCTTGGACTGTGGAGGCTTGCTG
GCCACTTTTTGGGGTCAGCTCCTCTGAAATGCATTAGCGGAACCGTTTGCGATCTGCCAC
AAGTGTGATAAGTTATCTACACTGGCGAGGGGATTGCTCTCTGTAATGTTCAGCTTCTAA
TTGTCTCTACTTTGTGAGACTACTTTTGAATGCTTGACCTCAAATCAGGTAGGACTACCC
GCTGAACTTAA
>AB3-11
TTTCCGTAGGTGAACCTGCGGAAGGATCATTATTGAATTATGTTTCTAGATAGGTTGTAG
CTGGCTCTTTTAGAGCATGTGCACGCCTGTTTGGACTTCATTTTCATCCACCTGTGCACC
TATTGTAGTCTTTGGTTGGGTTAGGAGGAAGTGATCATTGTATCAGCATCTGCTGGGAGT
GAGGACTTGCATTGTGAAAGCTTTGCTGTCCTTGATGTGATCATGGAATCTTTTTCTCAC
TAGAGTCTATGTCACTCATTATACTCTGTCGAATGTCATTGAATGTCTTTACATGGGCTT
GTATGCCTATGAAAATTGTAATACAACTTTCAGCAACGGATCTCTTGGCTCTCGCATCGA
TGAAGAACGCAGCGAAATGCGATAAGTAATGTGAATTGCAGAATTCAGTGAATCATCGAA
TCTTTGAACGCATCTTGCGCTCCTTGGTATTCCGAGGAGCATGCCTGTTTGAGTGTCATT
AAATTCTCAACTCTCTTATACTTTTTTGTAAAAGAGAGCTTGGACTGTGGAGGCTTGCTG
GCCACTTTTTGGGGTCAGCTCCTCTGAAATGCATTAGCGGAACCGTTTGCGATCTGCCAC
AAGTGTGATAAGTTATCTACACTGGCGAGGGGATTGCTCTCTGTAATGTTCAGCTTCTAA
TTGTCTCTACTTTGTGAGACTACTTTTGAATGCTTGACCTCAAATCAGGTAGGACTACCC
GCTGAACTTAA
>AB3-12
TTTCCGTAGGTGAACCTGCGGAAGGATCATTATTGAATTATGTTTCTAGATAGGTTGTAG
CTGGCTCTTTTAGAGCATGTGCACGCCTGTTTGGACTTCATTTTCATCCACCTGTGCACC
TATTGTAGTCTTTGGTTGGGTTAGGAGGAAGTGATCATTGTATCAGCATCTGCTGGGAGT
GAGGACTTGCATTGTGAAAGCTTTGCTGTCCTTGATGTGATCATGGAATCTTTTTCTCAC
TAGAGTCTATGTCACTCATTATACTCTGTCGAATGTCATTGAATGTCTTTACATGGGCTT

GTATGCCTATGAAAATTGTAATACAACTTTCAGCAACGGATCTCTTGGCTCTCGCATCGA
TGAAGAACGCAGCGAAATGCGATAAGTAATGTGAATTGCAGAATTCAGTGAATCATCGAA
TCTTTGAACGCATCTTGCGCTCCTTGGTATTCCGAGGAGCATGCCTGTTTGAGTGTCATT
AAATTCTCAACTCTCTTATACTTTTTTGTAAAAGAGAGCTTGGACTGTGGAGGCTTGCTG
GCCACTTTTTGGGGTCAGCTCCTCTGAAATGCATTAGCGGAACCGTTTGCGATCTGCCAC
AAGTGTGATAAGTTATCTACACTGGCGAGGGGATTGCTCTCTGTAATGTTCAGCTTCTAA
TTGTCTCTACTTTGTGAGACTACTTTTGAATGCTTGACCTCAAATCAGGTAGGACTACCC
GCTGAACTTAA
>AB3-13
TTTCCGTAGGTGAACCTGCGGAAGGATCATTATTGAATTATGTTTCTAGATAGGTTGTAG
CTGGCTCTTTTAGAGCATGTGCACGCCTGTTTGGACTTCATTTTCATCCACCTGTGCACC
TATTGTAGTCTTTGGTTGGGTTAGGAGGAAGTGATCATTGTATCAGCATCTGCTGGGAGT
GAGGACTTGCATTGTGAAAGCTTTGCTGTCCTTGATGTGATCATGGAATCTTTTTCTCAC
TAGAGTCTATGTCACTCATTATACTCTGTCGAATGTCATTGAATGTCTTTACATGGGCTT
GTATGCCTATGAAAATTGTAATACAACTTTCAGCAACGGATCTCTTGGCTCTCGCATCGA
TGAAGAACGCAGCGAAATGCGATAAGTAATGTGAATTGCAGAATTCAGTGAATCATCGAA
TCTTTGAACGCATCTTGCGCTCCTTGGTATTCCGAGGAGCATGCCTGTTTGAGTGTCATT
AAATTCTCAACTCTCTTATACTTTTTTGTAAAAGAGAGCTTGGACTGTGGAGGCTTGCTG
GCCACTTTTTGGGGTCAGCTCCTCTGAAATGCATTAGCGGAACCGTTTGCGATCTGCCAC
AAGTGTGATAAGTTATCTACACTGGCGAGGGGATTGCTCTCTGTAATGTTCAGCTTCTAA
TTGTCTCTACTTTGTGAGACTACTTTTGAATGCTTGACCTCAAATCAGGTAGGACTACCC
GCTGAACTTAA
>AB3-15
TTTCCGTAGGTGAACCTGCGGAAGGATCATTATTGAATTATGTTTCTAGATAGGTTGTAG
CTGGCTCTTTTAGAGCATGTGCACGCCTGTTTGGACTTCATTTTCATCCACCTGTGCACC
TATTGTAGTCTTTGGTTGGGTTAGGAGGAAGTGATCATTGTATCAGCATCTGCTGGGAGT
GAGGACTTGCATTGTGAAAGCTTTGCTGTCCTTGATGTGATCATGGAATCTTTTTCTCAC
TAGAGTCTATGTCACTCATTATACTCTGTCGAATGTCATTGAATGTCTTTACATGGGCTT
GTATGCCTATGAAAATTGTAATACAACTTTCAGCAACGGATCTCTTGGCTCTCGCATCGA
TGAAGAACGCAGCGAAATGCGATAAGTAATGTGAATTGCAGAATTCAGTGAATCATCGAA
TCTTTGAACGCATCTTGCGCTCCTTGGTATTCCGAGGAGCATGCCTGTTTGAGTGTCATT
AAATTCTCAACTCTCTTATACTTTTTTGTAAAAGAGAGCTTGGACTGTGGAGGCTTGCTG
GCCACTTTTTGGGGTCAGCTCCTCTGAAATGCATTAGCGGAACCGTTTGCGATCTGCCAC
AAGTGTGATAAGTTATCTACACTGGCGAGGGGATTGCTCTCTGTAATGTTCAGCTTCTAA
TTGTCTCTACTTTGTGAGACTACTTTTGAATGCTTGACCTCAAATCAGGTAGGACTACCC
GCTGAACTTAA
>AB3-16
TTTCCGTAGGTGAACCTGCGGAAGGATCATTATTGAATTATGTTTCTAGATAGGTTGTAG
CTGGCTCTTTTAGAGCATGTGCACGCCTGTTTGGACTTCATTTTCATCCACCTGTGCACC
TATTGTAGTCTTTGGTTGGGTTAGGAGGAAGTGATCATTGTATCAGCATCTGCTGGGAGT
GAGGACTTGCATTGTGAAAGCTTTGCTGTCCTTGATGTGATCATGGAATCTTTTTCTCAC
TAGAGTCTATGTCACTCATTATACTCTGTCGAATGTCATTGAATGTCTTTACATGGGCTT
GTATGCCTATGAAAATTGTAATACAACTTTCAGCAACGGATCTCTTGGCTCTCGCATCGA
TGAAGAACGCAGCGAAATGCGATAAGTAATGTGAATTGCAGAATTCAGTGAATCATCGAA
TCTTTGAACGCATCTTGCGCTCCTTGGTATTCCGAGGAGCATGCCTGTTTGAGTGTCATT
AAATTCTCAACTCTCTTATACTTTTTTGTAAAAGAGAGCTTGGACTGTGGAGGCTTGCTG
GCCACTTTTTGGGGTCAGCTCCTCTGAAATGCATTAGCGGAACCGTTTGCGATCTGCCAC
AAGTGTGATAAGTTATCTACACTGGCGAGGGGATTGCTCTCTGTAATGTTCAGCTTCTAA
TTGTCTCTACTTTGTGAGACTACTTTTGAATGCTTGACCTCAAATCAGGTAGGACTACCC
GCTGAACTTAA

>AB3-20
TTTCCGTAGGTGAACCTGCGGAAGGATCATTATTGAATTATGTTTCTAGATAGGTTGTAG
CTGGCTCTTTTAGAGCATGTGCACGCCTGTTTGGACTTCATTTTCATCCACCTGTGCACC
TATTGTAGTCTTTGGTTGGGTTAGGAGGAAGTGATCATTGTATCAGCATCTGCTGGGAGT
GAGGACTTGCATTGTGAAAGCTTTGCTGTCCTTGATGTGATCATGGAATCTTTTTCTCAC
TAGAGTCTATGTCACTCATTATACTCTGTCGAATGTCATTGAATGTCTTTACATGGGCTT
GTATGCCTATGAAAATTGTAATACAACTTTCAGCAACGGATCTCTTGGCTCTCGCATCGA
TGAAGAACGCAGCGAAATGCGATAAGTAATGTGAATTGCAGAATTCAGTGAATCATCGAA
TCTTTGAACGCATCTTGCGCTCCTTGGTATTCCGAGGAGCATGCCTGTTTGAGTGTCATT
AAATTCTCAACTCTCTTATACTTTTTTGTAAAAGAGAGCTTGGACTGTGGAGGCTTGCTG
GCCACTTTTTGGGGTCAGCTCCTCTGAAATGCATTAGCGGAACCGTTTGCGATCTGCCAC
AAGTGTGATAAGTTATCTACACTGGCGAGGGGATTGCTCTCTGTAATGTTCAGCTTCTAA
TTGTCTCTACTTTGTGAGACTACTTTTGAATGCTTGACCTCAAATCAGGTAGGACTACCC
GCTGAACTTAA
>AB3-23
TTTCCGTAGGTGAACCTGCGGAAGGATCATTATTGAATTATGTTTCTAGATAGGTTGTAG
CTGGCTCTTTTAGAGCATGTGCACGCCTGTTTGGACTTCATTTTCATCCACCTGTGCACC
TATTGTAGTCTTTGGTTGGGTTAGGAGGAAGTGATCATTGTATCAGCATCTGCTGGGAGT
GAGGACTTGCATTGTGAAAGCTTTGCTGTCCTTGATGTGATCATGGAATCTTTTTCTCAC
TAGAGTCTATGTCACTCATTATACTCTGTCGAATGTCATTGAATGTCTTTACATGGGCTT
GTATGCCTATGAAAATTGTAATACAACTTTCAGCAACGGATCTCTTGGCTCTCGCATCGA
TGAAGAACGCAGCGAAATGCGATAAGTAATGTGAATTGCAGAATTCAGTGAATCATCGAA
TCTTTGAACGCATCTTGCGCTCCTTGGTATTCCGAGGAGCATGCCTGTTTGAGTGTCATT
AAATTCTCAACTCTCTTATACTTTTTTGTAAAAGAGAGCTTGGACTGTGGAGGCTTGCTG
GCCACTTTTTGGGGTCAGCTCCTCTGAAATGCATTAGCGGAACCGTTTGCGATCTGCCAC
AAGTGTGATAAGTTATCTACACTGGCGAGGGGATTGCTCTCTGTAATGTTCAGCTTCTAA
TTGTCTCTACTTTGTGAGACTACTTTTGAATGCTTGACCTCAAATCAGGTAGGACTACCC
GCTGAACTTAA
>AB3-28
TTTCCGTAGGTGAACCTGCGGAAGGATCATTATTGAATTATGTTTCTAGATAGGTTGTAG
CTGGCTCTTTTAGAGCATGTGCACGCCTGTTTGGACTTCATTTTCATCCACCTGTGCACC
TATTGTAGTCTTTGGTTGGGTTAGGAGGAAGTGATCATTGTATCAGCATCTGCTGGGAGT
GAGGACTTGCATTGTGAAAGCTTTGCTGTCCTTGATGTGATCATGGAATCTTTTTCTCAC
TAGAGTCTATGTCACTCATTATACTCTGTCGAATGTCATTGAATGTCTTTACATGGGCTT
GTATGCCTATGAAAATTGTAATACAACTTTCAGCAACGGATCTCTTGGCTCTCGCATCGA
TGAAGAACGCAGCGAAATGCGATAAGTAATGTGAATTGCAGAATTCAGTGAATCATCGAA
TCTTTGAACGCATCTTGCGCTCCTTGGTATTCCGAGGAGCATGCCTGTTTGAGTGTCATT
AAATTCTCAACTCTCTTATACTTTTTTGTAAAAGAGAGCTTGGACTGTGGAGGCTTGCTG
GCCACTTTTTGGGGTCAGCTCCTCTGAAATGCATTAGCGGAACCGTTTGCGATCTGCCAC
AAGTGTGATAAGTTATCTACACTGGCGAGGGGATTGCTCTCTGTAATGTTCAGCTTCTAA
TTGTCTCTACTTTGTGAGACTACTTTTGAATGCTTGACCTCAAATCAGGTAGGACTACCC
GCTGAACTTAA
>AB3-32
TTTCCGTAGGTGAACCTGCGGAAGGATCATTATTGAATTATGTTTCTAGATAGGTTGTAG
CTGGCTCTTTTAGAGCATGTGCACGCCTGTTTGGACTTCATTTTCATCCACCTGTGCACC
TATTGTAGTCTTTGGTTGGGTTAGGAGGAAGTGATCATTGTATCAGCATCTGCTGGGAGT
GAGGACTTGCATTGTGAAAGCTTTGCTGTCCTTGATGTGATCATGGAATCTTTTTCTCAC
TAGAGTCTATGTCACTCATTATACTCTGTCGAATGTCATTGAATGTCTTTACATGGGCTT
GTATGCCTATGAAAATTGTAATACAACTTTCAGCAACGGATCTCTTGGCTCTCGCATCGA
TGAAGAACGCAGCGAAATGCGATAAGTAATGTGAATTGCAGAATTCAGTGAATCATCGAA

TCTTTGAACGCATCTTGCGCTCCTTGGTATTCCGAGGAGCATGCCTGTTTGAGTGTCATT
AAATTCTCAACTCTCTTATACTTTTTTGTAAAAGAGAGCTTGGACTGTGGAGGCTTGCTG
GCCACTTTTTGGGGTCAGCTCCTCTGAAATGCATTAGCGGAACCGTTTGCGATCTGCCAC
AAGTGTGATAAGTTATCTACACTGGCGAGGGGATTGCTCTCTGTAATGTTCAGCTTCTAA
TTGTCTCTACTTTGTGAGACTACTTTTGAATGCTTGACCTCAAATCAGGTAGGACTACCC
GCTGAACTTAA
>AB3−36
TTTCCGTAGGTGAACCTGCGGAAGGATCATTATTGAATTATGTTTCTAGATAGGTTGTAG
CTGGCTCTTTTAGAGCATGTGCACGCCTGTTTGGACTTCATTTTCATCCACCTGTGCACC
TATTGTAGTCTTTGGTTGGGTTAGGAGGAAGTGATCATTGTATCAGCATCTGCTGGGAGT
GAGGACTTGCATTGTGAAAGCTTTGCTGTCCTTGATGTGATCATGGAATCTTTTTCTCAC
TAGAGTCTATGTCACTCATTATACTCTGTCGAATGTCATTGAATGTCTTTACATGGGCTT
GTATGCCTATGAAAATTGTAATACAACTTTCAGCAACGGATCTCTTGGCTCTCGCATCGA
TGAAGAACGCAGCGAAATGCGATAAGTAATGTGAATTGCAGAATTCAGTGAATCATCGAA
TCTTTGAACGCATCTTGCGCTCCTTGGTATTCCGAGGAGCATGCCTGTTTGAGTGTCATT
AAATTCTCAACTCTCTTATACTTTTTTGTAAAAGAGAGCTTGGACTGTGGAGGCTTGCTG
GCCACTTTTTGGGGTCAGCTCCTCTGAAATGCATTAGCGGAACCGTTTGCGATCTGCCAC
AAGTGTGATAAGTTATCTACACTGGCGAGGGGATTGCTCTCTGTAATGTTCAGCTTCTAA
TTGTCTCTACTTTGTGAGACTACTTTTGAATGCTTGACCTCAAATCAGGTAGGACTACCC
GCTGAACTTAA
>AB3−41
TTTCCGTAGGTGAACCTGCGGAAGGATCATTATTGAATTATGTTTCTAGATAGGTTGTAG
CTGGCTCTTTTAGAGCATGTGCACGCCTGTTTGGACTTCATTTTCATCCACCTGTGCACC
TATTGTAGTCTTTGGTTGGGTTAGGAGGAAGTGATCATTGTATCAGCATCTGCTGGGAGT
GAGGACTTGCATTGTGAAAGCTTTGCTGTCCTTGATGTGATCATGGAATCTTTTTCTCAC
TAGAGTCTATGTCACTCATTATACTCTGTCGAATGTCATTGAATGTCTTTACATGGGCTT
GTATGCCTATGAAAATTGTAATACAACTTTCAGCAACGGATCTCTTGGCTCTCGCATCGA
TGAAGAACGCAGCGAAATGCGATAAGTAATGTGAATTGCAGAATTCAGTGAATCATCGAA
TCTTTGAACGCATCTTGCGCTCCTTGGTATTCCGAGGAGCATGCCTGTTTGAGTGTCATT
AAATTCTCAACTCTCTTATACTTTTTTGTAAAAGAGAGCTTGGACTGTGGAGGCTTGCTG
GCCACTTTTTGGGGTCAGCTCCTCTGAAATGCATTAGCGGAACCGTTTGCGATCTGCCAC
AAGTGTGATAAGTTATCTACACTGGCGAGGGGATTGCTCTCTGTAATGTTCAGCTTCTAA
TTGTCTCTACTTTGTGAGACTACTTTTGAATGCTTGACCTCAAATCAGGTAGGACTACCC
GCTGAACTTAA
>AB3−42
TTTCCGTAGGTGAACCTGCGGAAGGATCATTATTGAATTATGTTTCTAGATAGGTTGTAG
CTGGCTCTTTTAGAGCATGTGCACGCCTGTTTGGACTTCATTTTCATCCACCTGTGCACC
TATTGTAGTCTTTGGTTGGGTTAGGAGGAAGTGATCATTGTATCAGCATCTGCTGGGAGT
GAGGACTTGCATTGTGAAAGCTTTGCTGTCCTTGATGTGATCATGGAATCTTTTTCTCAC
TAGAGTCTATGTCACTCATTATACTCTGTCGAATGTCATTGAATGTCTTTACATGGGCTT
GTATGCCTATGAAAATTGTAATACAACTTTCAGCAACGGATCTCTTGGCTCTCGCATCGA
TGAAGAACGCAGCGAAATGCGATAAGTAATGTGAATTGCAGAATTCAGTGAATCATCGAA
TCTTTGAACGCATCTTGCGCTCCTTGGTATTCCGAGGAGCATGCCTGTTTGAGTGTCATT
AAATTCTCAACTCTCTTATACTTTTTTGTAAAAGAGAGCTTGGACTGTGGAGGCTTGCTG
GCCACTTTTTGGGGTCAGCTCCTCTGAAATGCATTAGCGGAACCGTTTGCGATCTGCCAC
AAGTGTGATAAGTTATCTACACTGGCGAGGGGATTGCTCTCTGTAATGTTCAGCTTCTAA
TTGTCTCTACTTTGTGAGACTACTTTTGAATGCTTGACCTCAAATCAGGTAGGACTACCC
GCTGAACTTAA
>AB3−43
TTTCCGTAGGTGAACCTGCGGAAGGATCATTATTGAATTATGTTTCTAGATAGGTTGTAG

CTGGCTCTTTTAGAGCATGTGCACGCCTGTTTGGACTTCATTTTCATCCACCTGTGCACC
TATTGTAGTCTTTGGTTGGGTTAGGAGGAAGTGATCATTGTATCAGCATCTGCTGGGAGT
GAGGACTTGCATTGTGAAAGCTTTGCTGTCCTTGATGTGATCATGGAATCTTTTTCTCAC
TAGAGTCTATGTCACTCATTATACTCTGTCGAATGTCATTGAATGTCTTTACATGGGCTT
GTATGCCTATGAAAATTGTAATACAACTTTCAGCAACGGATCTCTTGGCTCTCGCATCGA
TGAAGAACGCAGCGAAATGCGATAAGTAATGTGAATTGCAGAATTCAGTGAATCATCGAA
TCTTTGAACGCATCTTGCGCTCCTTGGTATTCCGAGGAGCATGCCTGTTTGAGTGTCATT
AAATTCTCAACTCTCTTATACTTTTTTGTAAAAGAGAGCTTGGACTGTGGAGGCTTGCTG
GCCACTTTTTGGGGTCAGCTCCTCTGAAATGCATTAGCGGAACCGTTTGCGATCTGCCAC
AAGTGTGATAAGTTATCTACACTGGCGAGGGGATTGCTCTCTGTAATGTTCAGCTTCTAA
TTGTCTCTACTTTGTGAGACTACTTTTGAATGCTTGACCTCAAATCAGGTAGGACTACCC
GCTGAACTTAA
>AB3-44
TTTCCGTAGGTGAACCTGCGGAAGGATCATTATTGAATTATGTTTCTAGATAGGTTGTAG
CTGGCTCTTTTAGAGCATGTGCACGCCTGTTTGGACTTCATTTTCATCCACCTGTGCACC
TATTGTAGTCTTTGGTTGGGTTAGGAGGAAGTGATCATTGTATCAGCATCTGCTGGGAGT
GAGGACTTGCATTGTGAAAGCTTTGCTGTCCTTGATGTGATCATGGAATCTTTTTCTCAC
TAGAGTCTATGTCACTCATTATACTCTGTCGAATGTCATTGAATGTCTTTACATGGGCTT
GTATGCCTATGAAAATTGTAATACAACTTTCAGCAACGGATCTCTTGGCTCTCGCATCGA
TGAAGAACGCAGCGAAATGCGATAAGTAATGTGAATTGCAGAATTCAGTGAATCATCGAA
TCTTTGAACGCATCTTGCGCTCCTTGGTATTCCGAGGAGCATGCCTGTTTGAGTGTCATT
AAATTCTCAACTCTCTTATACTTTTTTGTAAAAGAGAGCTTGGACTGTGGAGGCTTGCTG
GCCACTTTTTGGGGTCAGCTCCTCTGAAATGCATTAGCGGAACCGTTTGCGATCTGCCAC
AAGTGTGATAAGTTATCTACACTGGCGAGGGGATTGCTCTCTGTAATGTTCAGCTTCTAA
TTGTCTCTACTTTGTGAGACTACTTTTGAATGCTTGACCTCAAATCAGGTAGGACTACCC
GCTGAACTTAA
>AB3-45
TTTCCGTAGGTGAACCTGCGGAAGGATCATTATTGAATTATGTTTCTAGATAGGTTGTAG
CTGGCTCTTTTAGAGCATGTGCACGCCTGTTTGGACTTCATTTTCATCCACCTGTGCACC
TATTGTAGTCTTTGGTTGGGTTAGGAGGAAGTGATCATTGTATCAGCATCTGCTGGGAGT
GAGGACTTGCATTGTGAAAGCTTTGCTGTCCTTGATGTGATCATGGAATCTTTTTCTCAC
TAGAGTCTATGTCACTCATTATACTCTGTCGAATGTCATTGAATGTCTTTACATGGGCTT
GTATGCCTATGAAAATTGTAATACAACTTTCAGCAACGGATCTCTTGGCTCTCGCATCGA
TGAAGAACGCAGCGAAATGCGATAAGTAATGTGAATTGCAGAATTCAGTGAATCATCGAA
TCTTTGAACGCATCTTGCGCTCCTTGGTATTCCGAGGAGCATGCCTGTTTGAGTGTCATT
AAATTCTCAACTCTCTTATACTTTTTTGTAAAAGAGAGCTTGGACTGTGGAGGCTTGCTG
GCCACTTTTTGGGGTCAGCTCCTCTGAAATGCATTAGCGGAACCGTTTGCGATCTGCCAC
AAGTGTGATAAGTTATCTACACTGGCGAGGGGATTGCTCTCTGTAATGTTCAGCTTCTAA
TTGTCTCTACTTTGTGAGACTACTTTTGAATGCTTGACCTCAAATCAGGTAGGACTACCC
GCTGAACTTAA
>AB3-50
TTTCCGTAGGTGAACCTGCGGAAGGATCATTATTGAATTATGTTTCTAGATAGGTTGTAG
CTGGCTCTTTTAGAGCATGTGCACGCCTGTTTGGACTTCATTTTCATCCACCTGTGCACC
TATTGTAGTCTTTGGTTGGGTTAGGAGGAAGTGATCATTGTATCAGCATCTGCTGGGAGT
GAGGACTTGCATTGTGAAAGCTTTGCTGTCCTTGATGTGATCATGGAATCTTTTTCTCAC
TAGAGTCTATGTCACTCATTATACTCTGTCGAATGTCATTGAATGTCTTTACATGGGCTT
GTATGCCTATGAAAATTGTAATACAACTTTCAGCAACGGATCTCTTGGCTCTCGCATCGA
TGAAGAACGCAGCGAAATGCGATAAGTAATGTGAATTGCAGAATTCAGTGAATCATCGAA
TCTTTGAACGCATCTTGCGCTCCTTGGTATTCCGAGGAGCATGCCTGTTTGAGTGTCATT
AAATTCTCAACTCTCTTATACTTTTTTGTAAAAGAGAGCTTGGACTGTGGAGGCTTGCTG

GCCACTTTTTGGGGTCAGCTCCTCTGAAATGCATTAGCGGAACCGTTTGCGATCTGCCAC
AAGTGTGATAAGTTATCTACACTGGCGAGGGGATTGCTCTCTGTAATGTTCAGCTTCTAA
TTGTCTCTACTTTGTGAGACTACTTTTGAATGCTTGACCTCAAATCAGGTAGGACTACCC
GCTGAACTTAA
>AB3-55
TTTCCGTAGGTGAACCTGCGGAAGGATCATTATTGAATTATGTTTCTAGATAGGTTGTAG
CTGGCTCTTTTAGAGCATGTGCACGCCTGTTTGGACTTCATTTTCATCCACCTGTGCACC
TATTGTAGTCTTTGGTTGGGTTAGGAGGAAGTGATCATTGTATCAGCATCTGCTGGGAGT
GAGGACTTGCATTGTGAAAGCTTTGCTGTCCTTGATGTGATCATGGAATCTTTTTCTCAC
TAGAGTCTATGTCACTCATTATACTCTGTCGAATGTCATTGAATGTCTTTACATGGGCTT
GTATGCCTATGAAAATTGTAATACAACTTTCAGCAACGGATCTCTTGGCTCTCGCATCGA
TGAAGAACGCAGCGAAATGCGATAAGTAATGTGAATTGCAGAATTCAGTGAATCATCGAA
TCTTTGAACGCATCTTGCGCTCCTTGGTATTCCGAGGAGCATGCCTGTTTGAGTGTCATT
AAATTCTCAACTCTCTTATACTTTTTTGTAAAAGAGAGCTTGGACTGTGGAGGCTTGCTG
GCCACTTTTTGGGGTCAGCTCCTCTGAAATGCATTAGCGGAACCGTTTGCGATCTGCCAC
AAGTGTGATAAGTTATCTACACTGGCGAGGGGATTGCTCTCTGTAATGTTCAGCTTCTAA
TTGTCTCTACTTTGTGAGACTACTTTTGAATGCTTGACCTCAAATCAGGTAGGACTACCC
GCTGAACTTAA
>AB3-57
TTTCCGTAGGTGAACCTGCGGAAGGATCATTATTGAATTATGTTTCTAGATAGGTTGTAG
CTGGCTCTTTTAGAGCATGTGCACGCCTGTTTGGACTTCATTTTCATCCACCTGTGCACC
TATTGTAGTCTTTGGTTGGGTTAGGAGGAAGTGATCATTGTATCAGCATCTGCTGGGAGT
GAGGACTTGCATTGTGAAAGCTTTGCTGTCCTTGATGTGATCATGGAATCTTTTTCTCAC
TAGAGTCTATGTCACTCATTATACTCTGTCGAATGTCATTGAATGTCTTTACATGGGCTT
GTATGCCTATGAAAATTGTAATACAACTTTCAGCAACGGATCTCTTGGCTCTCGCATCGA
TGAAGAACGCAGCGAAATGCGATAAGTAATGTGAATTGCAGAATTCAGTGAATCATCGAA
TCTTTGAACGCATCTTGCGCTCCTTGGTATTCCGAGGAGCATGCCTGTTTGAGTGTCATT
AAATTCTCAACTCTCTTATACTTTTTTGTAAAAGAGAGCTTGGACTGTGGAGGCTTGCTG
GCCACTTTTTGGGGTCAGCTCCTCTGAAATGCATTAGCGGAACCGTTTGCGATCTGCCAC
AAGTGTGATAAGTTATCTACACTGGCGAGGGGATTGCTCTCTGTAATGTTCAGCTTCTAA
TTGTCTCTACTTTGTGAGACTACTTTTGAATGCTTGACCTCAAATCAGGTAGGACTACCC
GCTGAACTTAA
>AB3-58
TTTCCGTAGGTGAACCTGCGGAAGGATCATTATTGAATTATGTTTCTAGATAGGTTGTAG
CTGGCTCTTTTAGAGCATGTGCACGCCTGTTTGGACTTCATTTTCATCCACCTGTGCACC
TATTGTAGTCTTTGGTTGGGTTAGGAGGAAGTGATCATTGTATCAGCATCTGCTGGGAGT
GAGGACTTGCATTGTGAAAGCTTTGCTGTCCTTGATGTGATCATGGAATCTTTTTCTCAC
TAGAGTCTATGTCACTCATTATACTCTGTCGAATGTCATTGAATGTCTTTACATGGGCTT
GTATGCCTATGAAAATTGTAATACAACTTTCAGCAACGGATCTCTTGGCTCTCGCATCGA
TGAAGAACGCAGCGAAATGCGATAAGTAATGTGAATTGCAGAATTCAGTGAATCATCGAA
TCTTTGAACGCATCTTGCGCTCCTTGGTATTCCGAGGAGCATGCCTGTTTGAGTGTCATT
AAATTCTCAACTCTCTTATACTTTTTTGTAAAAGAGAGCTTGGACTGTGGAGGCTTGCTG
GCCACTTTTTGGGGTCAGCTCCTCTGAAATGCATTAGCGGAACCGTTTGCGATCTGCCAC
AAGTGTGATAAGTTATCTACACTGGCGAGGGGATTGCTCTCTGTAATGTTCAGCTTCTAA
TTGTCTCTACTTTGTGAGACTACTTTTGAATGCTTGACCTCAAATCAGGTAGGACTACCC
GCTGAACTTAA
>AB4-3
TTTCCGTAGGTGAACCTGCGGAAGGATCATTATTGAATTATGTTTCTAGATAGGTTGTAG
CTGGCTCTTTTAGAGCATGTGCACGCCTGTTTGGACTTCATTTTCATCCACCTGTGCACC
TATTGTAGTCTTTGGTTGGGTTAGGAGGAAGTGATCATTGTATCAGCATCTGCTGGGAGT

GAGGACTTGCATTGTGAAAGCTTTGCTGTCCTTGATGTGATCATGGAATCTTTTTCTCAC
TAGAGTCTATGTCACTCATTATACTCTGTCGAATGTCATTGAATGTCTTTACATGGGCTT
GTATGCCTATGAAAATTGTAATACAACTTTCAGCAACGGATCTCTTGGCTCTCGCATCGA
TGAAGAACGCAGCGAAATGCGATAAGTAATGTGAATTGCAGAATTCAGTGAATCATCGAA
TCTTTGAACGCATCTTGCGCTCCTTGGTATTCCGAGGAGCATGCCTGTTTGAGTGTCATT
AAATTCTCAACTCTCTTATACTTTTTTGTAAAAGAGAGCTTGGACTGTGGAGGCTTGCTG
GCCACTTTTTGGGGTCAGCTCCTCTGAAATGCATTAGCGGAACCGTTTGCGATCTGCCAC
AAGTGTGATAAGTTATCTACACTGGCGAGGGGATTGCTCTCTGTAATGTTCAGCTTCTAA
TTGTCTCTACTTTGTGAGACTACTTTTGAATGCTTGACCTCAAATCAGGTAGGACTACCC
GCTGAACTTAA
>AB4-5
TTTCCGTAGGTGAACCTGCGGAAGGATCATTATTGAATTATGTTTCTAGATAGGTTGTAG
CTGGCTCTTTTAGAGCATGTGCACGCCTGTTTGGACTTCATTTTCATCCACCTGTGCACC
TATTGTAGTCTTTGGTTGGGTTAGGAGGAAGTGATCATTGTATCAGCATCTGCTGGGAGT
GAGGACTTGCATTGTGAAAGCTTTGCTGTCCTTGATGTGATCATGGAATCTTTTTCTCAC
TAGAGTCTATGTCACTCATTATACTCTGTCGAATGTCATTGAATGTCTTTACATGGGCTT
GTATGCCTATGAAAATTGTAATACAACTTTCAGCAACGGATCTCTTGGCTCTCGCATCGA
TGAAGAACGCAGCGAAATGCGATAAGTAATGTGAATTGCAGAATTCAGTGAATCATCGAA
TCTTTGAACGCATCTTGCGCTCCTTGGTATTCCGAGGAGCATGCCTGTTTGAGTGTCATT
AAATTCTCAACTCTCTTATACTTTTTTGTAAAAGAGAGCTTGGACTGTGGAGGCTTGCTG
GCCACTTTTTGGGGTCAGCTCCTCTGAAATGCATTAGCGGAACCGTTTGCGATCTGCCAC
AAGTGTGATAAGTTATCTACACTGGCGAGGGGATTGCTCTCTGTAATGTTCAGCTTCTAA
TTGTCTCTACTTTGTGAGACTACTTTTGAATGCTTGACCTCAAATCAGGTAGGACTACCC
GCTGAACTTAA
>AB4-6
TTTCCGTAGGTGAACCTGCGGAAGGATCATTATTGAATTATGTTTCTAGATAGGTTGTAG
CTGGCTCTTTTAGAGCATGTGCACGCCTGTTTGGACTTCATTTTCATCCACCTGTGCACC
TATTGTAGTCTTTGGTTGGGTTAGGAGGAAGTGATCATTGTATCAGCATCTGCTGGGAGT
GAGGACTTGCATTGTGAAAGCTTTGCTGTCCTTGATGTGATCATGGAATCTTTTTCTCAC
TAGAGTCTATGTCACTCATTATACTCTGTCGAATGTCATTGAATGTCTTTACATGGGCTT
GTATGCCTATGAAAATTGTAATACAACTTTCAGCAACGGATCTCTTGGCTCTCGCATCGA
TGAAGAACGCAGCGAAATGCGATAAGTAATGTGAATTGCAGAATTCAGTGAATCATCGAA
TCTTTGAACGCATCTTGCGCTCCTTGGTATTCCGAGGAGCATGCCTGTTTGAGTGTCATT
AAATTCTCAACTCTCTTATACTTTTTTGTAAAAGAGAGCTTGGACTGTGGAGGCTTGCTG
GCCACTTTTTGGGGTCAGCTCCTCTGAAATGCATTAGCGGAACCGTTTGCGATCTGCCAC
AAGTGTGATAAGTTATCTACACTGGCGAGGGGATTGCTCTCTGTAATGTTCAGCTTCTAA
TTGTCTCTACTTTGTGAGACTACTTTTGAATGCTTGACCTCAAATCAGGTAGGACTACCC
GCTGAACTTAA
>AB4-7
TTTCCGTAGGTGAACCTGCGGAAGGATCATTATTGAATTATGTTTCTAGATAGGTTGTAG
CTGGCTCTTTTAGAGCATGTGCACGCCTGTTTGGACTTCATTTTCATCCACCTGTGCACC
TATTGTAGTCTTTGGTTGGGTTAGGAGGAAGTGATCATTGTATCAGCATCTGCTGGGAGT
GAGGACTTGCATTGTGAAAGCTTTGCTGTCCTTGATGTGATCATGGAATCTTTTTCTCAC
TAGAGTCTATGTCACTCATTATACTCTGTCGAATGTCATTGAATGTCTTTACATGGGCTT
GTATGCCTATGAAAATTGTAATACAACTTTCAGCAACGGATCTCTTGGCTCTCGCATCGA
TGAAGAACGCAGCGAAATGCGATAAGTAATGTGAATTGCAGAATTCAGTGAATCATCGAA
TCTTTGAACGCATCTTGCGCTCCTTGGTATTCCGAGGAGCATGCCTGTTTGAGTGTCATT
AAATTCTCAACTCTCTTATACTTTTTTGTAAAAGAGAGCTTGGACTGTGGAGGCTTGCTG
GCCACTTTTTGGGGTCAGCTCCTCTGAAATGCATTAGCGGAACCGTTTGCGATCTGCCAC
AAGTGTGATAAGTTATCTACACTGGCGAGGGGATTGCTCTCTGTAATGTTCAGCTTCTAA

TTGTCTCTACTTTGTGAGACTACTTTTGAATGCTTGACCTCAAATCAGGTAGGACTACCC
GCTGAACTTAA
>AB4-9
TTTCCGTAGGTGAACCTGCGGAAGGATCATTATTGAATTATGTTTCTAGATAGGTTGTAG
CTGGCTCTTTTAGAGCATGTGCACGCCTGTTTGGACTTCATTTTCATCCACCTGTGCACC
TATTGTAGTCTTTGGTTGGGTTAGGAGGAAGTGATCATTGTATCAGCATCTGCTGGGAGT
GAGGACTTGCATTGTGAAAGCTTTGCTGTCCTTGATGTGATCATGGAATCTTTTTCTCAC
TAGAGTCTATGTCACTCATTATACTCTGTCGAATGTCATTGAATGTCTTTACATGGGCTT
GTATGCCTATGAAAATTGTAATACAACTTTCAGCAACGGATCTCTTGGCTCTCGCATCGA
TGAAGAACGCAGCGAAATGCGATAAGTAATGTGAATTGCAGAATTCAGTGAATCATCGAA
TCTTTGAACGCATCTTGCGCTCCTTGGTATTCCGAGGAGCATGCCTGTTTGAGTGTCATT
AAATTCTCAACTCTCTTATACTTTTTTGTAAAAGAGAGCTTGGACTGTGGAGGCTTGCTG
GCCACTTTTTGGGGTCAGCTCCTCTGAAATGCATTAGCGGAACCGTTTGCGATCTGCCAC
AAGTGTGATAAGTTATCTACACTGGCGAGGGGATTGCTCTCTGTAATGTTCAGCTTCTAA
TTGTCTCTACTTTGTGAGACTACTTTTGAATGCTTGACCTCAAATCAGGTAGGACTACCC
GCTGAACTTAA
>AB4-11
TTTCCGTAGGTGAACCTGCGGAAGGATCATTATTGAATTATGTTTCTAGATAGGTTGTAG
CTGGCTCTTTTAGAGCATGTGCACGCCTGTTTGGACTTCATTTTCATCCACCTGTGCACC
TATTGTAGTCTTTGGTTGGGTTAGGAGGAAGTGATCATTGTATCAGCATCTGCTGGGAGT
GAGGACTTGCATTGTGAAAGCTTTGCTGTCCTTGATGTGATCATGGAATCTTTTTCTCAC
TAGAGTCTATGTCACTCATTATACTCTGTCGAATGTCATTGAATGTCTTTACATGGGCTT
GTATGCCTATGAAAATTGTAATACAACTTTCAGCAACGGATCTCTTGGCTCTCGCATCGA
TGAAGAACGCAGCGAAATGCGATAAGTAATGTGAATTGCAGAATTCAGTGAATCATCGAA
TCTTTGAACGCATCTTGCGCTCCTTGGTATTCCGAGGAGCATGCCTGTTTGAGTGTCATT
AAATTCTCAACTCTCTTATACTTTTTTGTAAAAGAGAGCTTGGACTGTGGAGGCTTGCTG
GCCACTTTTTGGGGTCAGCTCCTCTGAAATGCATTAGCGGAACCGTTTGCGATCTGCCAC
AAGTGTGATAAGTTATCTACACTGGCGAGGGGATTGCTCTCTGTAATGTTCAGCTTCTAA
TTGTCTCTACTTTGTGAGACTACTTTTGAATGCTTGACCTCAAATCAGGTAGGACTACCC
GCTGAACTTAA
>AB4-16
TTTCCGTAGGTGAACCTGCGGAAGGATCATTATTGAATTATGTTTCTAGATAGGTTGTAG
CTGGCTCTTTTAGAGCATGTGCACGCCTGTTTGGACTTCATTTTCATCCACCTGTGCACC
TATTGTAGTCTTTGGTTGGGTTAGGAGGAAGTGATCATTGTATCAGCATCTGCTGGGAGT
GAGGACTTGCATTGTGAAAGCTTTGCTGTCCTTGATGTGATCATGGAATCTTTTTCTCAC
TAGAGTCTATGTCACTCATTATACTCTGTCGAATGTCATTGAATGTCTTTACATGGGCTT
GTATGCCTATGAAAATTGTAATACAACTTTCAGCAACGGATCTCTTGGCTCTCGCATCGA
TGAAGAACGCAGCGAAATGCGATAAGTAATGTGAATTGCAGAATTCAGTGAATCATCGAA
TCTTTGAACGCATCTTGCGCTCCTTGGTATTCCGAGGAGCATGCCTGTTTGAGTGTCATT
AAATTCTCAACTCTCTTATACTTTTTTGTAAAAGAGAGCTTGGACTGTGGAGGCTTGCTG
GCCACTTTTTGGGGTCAGCTCCTCTGAAATGCATTAGCGGAACCGTTTGCGATCTGCCAC
AAGTGTGATAAGTTATCTACACTGGCGAGGGGATTGCTCTCTGTAATGTTCAGCTTCTAA
TTGTCTCTACTTTGTGAGACTACTTTTGAATGCTTGACCTCAAATCAGGTAGGACTACCC
GCTGAACTTAA
>AB4-20
TTTCCGTAGGTGAACCTGCGGAAGGATCATTATTGAATTATGTTTCTAGATAGGTTGTAG
CTGGCTCTTTTAGAGCATGTGCACGCCTGTTTGGACTTCATTTTCATCCACCTGTGCACC
TATTGTAGTCTTTGGTTGGGTTAGGAGGAAGTGATCATTGTATCAGCATCTGCTGGGAGT
GAGGACTTGCATTGTGAAAGCTTTGCTGTCCTTGATGTGATCATGGAATCTTTTTCTCAC
TAGAGTCTATGTCACTCATTATACTCTGTCGAATGTCATTGAATGTCTTTACATGGGCTT

GTATGCCTATGAAAATTGTAATACAACTTTCAGCAACGGATCTCTTGGCTCTCGCATCGA
TGAAGAACGCAGCGAAATGCGATAAGTAATGTGAATTGCAGAATTCAGTGAATCATCGAA
TCTTTGAACGCATCTTGCGCTCCTTGGTATTCCGAGGAGCATGCCTGTTTGAGTGTCATT
AAATTCTCAACTCTCTTATACTTTTTTGTAAAAGAGAGCTTGGACTGTGGAGGCTTGCTG
GCCACTTTTTGGGGTCAGCTCCTCTGAAATGCATTAGCGGAACCGTTTGCGATCTGCCAC
AAGTGTGATAAGTTATCTACACTGGCGAGGGGATTGCTCTCTGTAATGTTCAGCTTCTAA
TTGTCTCTACTTTGTGAGACTACTTTTGAATGCTTGACCTCAAATCAGGTAGGACTACCC
GCTGAACTTAA
>AB4−23
TTTCCGTAGGTGAACCTGCGGAAGGATCATTATTGAATTATGTTTCTAGATAGGTTGTAG
CTGGCTCTTTTAGAGCATGTGCACGCCTGTTTGGACTTCATTTTCATCCACCTGTGCACC
TATTGTAGTCTTTGGTTGGGTTAGGAGGAAGTGATCATTGTATCAGCATCTGCTGGGAGT
GAGGACTTGCATTGTGAAAGCTTTGCTGTCCTTGATGTGATCATGGAATCTTTTTCTCAC
TAGAGTCTATGTCACTCATTATACTCTGTCGAATGTCATTGAATGTCTTTACATGGGCTT
GTATGCCTATGAAAATTGTAATACAACTTTCAGCAACGGATCTCTTGGCTCTCGCATCGA
TGAAGAACGCAGCGAAATGCGATAAGTAATGTGAATTGCAGAATTCAGTGAATCATCGAA
TCTTTGAACGCATCTTGCGCTCCTTGGTATTCCGAGGAGCATGCCTGTTTGAGTGTCATT
AAATTCTCAACTCTCTTATACTTTTTTGTAAAAGAGAGCTTGGACTGTGGAGGCTTGCTG
GCCACTTTTTGGGGTCAGCTCCTCTGAAATGCATTAGCGGAACCGTTTGCGATCTGCCAC
AAGTGTGATAAGTTATCTACACTGGCGAGGGGATTGCTCTCTGTAATGTTCAGCTTCTAA
TTGTCTCTACTTTGTGAGACTACTTTTGAATGCTTGACCTCAAATCAGGTAGGACTACCC
GCTGAACTTAA
>AB4−24
TTTCCGTAGGTGAACCTGCGGAAGGATCATTATTGAATTATGTTTCTAGATAGGTTGTAG
CTGGCTCTTTTAGAGCATGTGCACGCCTGTTTGGACTTCATTTTCATCCACCTGTGCACC
TATTGTAGTCTTTGGTTGGGTTAGGAGGAAGTGATCATTGTATCAGCATCTGCTGGGAGT
GAGGACTTGCATTGTGAAAGCTTTGCTGTCCTTGATGTGATCATGGAATCTTTTTCTCAC
TAGAGTCTATGTCACTCATTATACTCTGTCGAATGTCATTGAATGTCTTTACATGGGCTT
GTATGCCTATGAAAATTGTAATACAACTTTCAGCAACGGATCTCTTGGCTCTCGCATCGA
TGAAGAACGCAGCGAAATGCGATAAGTAATGTGAATTGCAGAATTCAGTGAATCATCGAA
TCTTTGAACGCATCTTGCGCTCCTTGGTATTCCGAGGAGCATGCCTGTTTGAGTGTCATT
AAATTCTCAACTCTCTTATACTTTTTTGTAAAAGAGAGCTTGGACTGTGGAGGCTTGCTG
GCCACTTTTTGGGGTCAGCTCCTCTGAAATGCATTAGCGGAACCGTTTGCGATCTGCCAC
AAGTGTGATAAGTTATCTACACTGGCGAGGGGATTGCTCTCTGTAATGTTCAGCTTCTAA
TTGTCTCTACTTTGTGAGACTACTTTTGAATGCTTGACCTCAAATCAGGTAGGACTACCC
GCTGAACTTAA
>AB4−25
TTTCCGTAGGTGAACCTGCGGAAGGATCATTATTGAATTATGTTTCTAGATAGGTTGTAG
CTGGCTCTTTTAGAGCATGTGCACGCCTGTTTGGACTTCATTTTCATCCACCTGTGCACC
TATTGTAGTCTTTGGTTGGGTTAGGAGGAAGTGATCATTGTATCAGCATCTGCTGGGAGT
GAGGACTTGCATTGTGAAAGCTTTGCTGTCCTTGATGTGATCATGGAATCTTTTTCTCAC
TAGAGTCTATGTCACTCATTATACTCTGTCGAATGTCATTGAATGTCTTTACATGGGCTT
GTATGCCTATGAAAATTGTAATACAACTTTCAGCAACGGATCTCTTGGCTCTCGCATCGA
TGAAGAACGCAGCGAAATGCGATAAGTAATGTGAATTGCAGAATTCAGTGAATCATCGAA
TCTTTGAACGCATCTTGCGCTCCTTGGTATTCCGAGGAGCATGCCTGTTTGAGTGTCATT
AAATTCTCAACTCTCTTATACTTTTTTGTAAAAGAGAGCTTGGACTGTGGAGGCTTGCTG
GCCACTTTTTGGGGTCAGCTCCTCTGAAATGCATTAGCGGAACCGTTTGCGATCTGCCAC
AAGTGTGATAAGTTATCTACACTGGCGAGGGGATTGCTCTCTGTAATGTTCAGCTTCTAA
TTGTCTCTACTTTGTGAGACTACTTTTGAATGCTTGACCTCAAATCAGGTAGGACTACCC
GCTGAACTTAA

>AB4–26
TTTCCGTAGGTGAACCTGCGGAAGGATCATTATTGAATTATGTTTCTAGATAGGTTGTAG
CTGGCTCTTTTAGAGCATGTGCACGCCTGTTTGGACTTCATTTTCATCCACCTGTGCACC
TATTGTAGTCTTTGGTTGGGTTAGGAGGAAGTGATCATTGTATCAGCATCTGCTGGGAGT
GAGGACTTGCATTGTGAAAGCTTTGCTGTCCTTGATGTGATCATGGAATCTTTTTCTCAC
TAGAGTCTATGTCACTCATTATACTCTGTCGAATGTCATTGAATGTCTTTACATGGGCTT
GTATGCCTATGAAAATTGTAATACAACTTTCAGCAACGGATCTCTTGGCTCTCGCATCGA
TGAAGAACGCAGCGAAATGCGATAAGTAATGTGAATTGCAGAATTCAGTGAATCATCGAA
TCTTTGAACGCATCTTGCGCTCCTTGGTATTCCGAGGAGCATGCCTGTTTGAGTGTCATT
AAATTCTCAACTCTCTTATACTTTTTTGTAAAAGAGAGCTTGGACTGTGGAGGCTTGCTG
GCCACTTTTTGGGGTCAGCTCCTCTGAAATGCATTAGCGGAACCGTTTGCGATCTGCCAC
AAGTGTGATAAGTTATCTACACTGGCGAGGGGATTGCTCTCTGTAATGTTCAGCTTCTAA
TTGTCTCTACTTTGTGAGACTACTTTTGAATGCTTGACCTCAAATCAGGTAGGACTACCC
GCTGAACTTAA
>AB4–28
TTTCCGTAGGTGAACCTGCGGAAGGATCATTATTGAATTATGTTTCTAGATAGGTTGTAG
CTGGCTCTTTTAGAGCATGTGCACGCCTGTTTGGACTTCATTTTCATCCACCTGTGCACC
TATTGTAGTCTTTGGTTGGGTTAGGAGGAAGTGATCATTGTATCAGCATCTGCTGGGAGT
GAGGACTTGCATTGTGAAAGCTTTGCTGTCCTTGATGTGATCATGGAATCTTTTTCTCAC
TAGAGTCTATGTCACTCATTATACTCTGTCGAATGTCATTGAATGTCTTTACATGGGCTT
GTATGCCTATGAAAATTGTAATACAACTTTCAGCAACGGATCTCTTGGCTCTCGCATCGA
TGAAGAACGCAGCGAAATGCGATAAGTAATGTGAATTGCAGAATTCAGTGAATCATCGAA
TCTTTGAACGCATCTTGCGCTCCTTGGTATTCCGAGGAGCATGCCTGTTTGAGTGTCATT
AAATTCTCAACTCTCTTATACTTTTTTGTAAAAGAGAGCTTGGACTGTGGAGGCTTGCTG
GCCACTTTTTGGGGTCAGCTCCTCTGAAATGCATTAGCGGAACCGTTTGCGATCTGCCAC
AAGTGTGATAAGTTATCTACACTGGCGAGGGGATTGCTCTCTGTAATGTTCAGCTTCTAA
TTGTCTCTACTTTGTGAGACTACTTTTGAATGCTTGACCTCAAATCAGGTAGGACTACCC
GCTGAACTTAA
>AB4–29
TTTCCGTAGGTGAACCTGCGGAAGGATCATTATTGAATTATGTTTCTAGATAGGTTGTAG
CTGGCTCTTTTAGAGCATGTGCACGCCTGTTTGGACTTCATTTTCATCCACCTGTGCACC
TATTGTAGTCTTTGGTTGGGTTAGGAGGAAGTGATCATTGTATCAGCATCTGCTGGGAGT
GAGGACTTGCATTGTGAAAGCTTTGCTGTCCTTGATGTGATCATGGAATCTTTTTCTCAC
TAGAGTCTATGTCACTCATTATACTCTGTCGAATGTCATTGAATGTCTTTACATGGGCTT
GTATGCCTATGAAAATTGTAATACAACTTTCAGCAACGGATCTCTTGGCTCTCGCATCGA
TGAAGAACGCAGCGAAATGCGATAAGTAATGTGAATTGCAGAATTCAGTGAATCATCGAA
TCTTTGAACGCATCTTGCGCTCCTTGGTATTCCGAGGAGCATGCCTGTTTGAGTGTCATT
AAATTCTCAACTCTCTTATACTTTTTTGTAAAAGAGAGCTTGGACTGTGGAGGCTTGCTG
GCCACTTTTTGGGGTCAGCTCCTCTGAAATGCATTAGCGGAACCGTTTGCGATCTGCCAC
AAGTGTGATAAGTTATCTACACTGGCGAGGGGATTGCTCTCTGTAATGTTCAGCTTCTAA
TTGTCTCTACTTTGTGAGACTACTTTTGAATGCTTGACCTCAAATCAGGTAGGACTACCC
GCTGAACTTAA
>AB4–30
TTTCCGTAGGTGAACCTGCGGAAGGATCATTATTGAATTATGTTTCTAGATAGGTTGTAG
CTGGCTCTTTTAGAGCATGTGCACGCCTGTTTGGACTTCATTTTCATCCACCTGTGCACC
TATTGTAGTCTTTGGTTGGGTTAGGAGGAAGTGATCATTGTATCAGCATCTGCTGGGAGT
GAGGACTTGCATTGTGAAAGCTTTGCTGTCCTTGATGTGATCATGGAATCTTTTTCTCAC
TAGAGTCTATGTCACTCATTATACTCTGTCGAATGTCATTGAATGTCTTTACATGGGCTT
GTATGCCTATGAAAATTGTAATACAACTTTCAGCAACGGATCTCTTGGCTCTCGCATCGA
TGAAGAACGCAGCGAAATGCGATAAGTAATGTGAATTGCAGAATTCAGTGAATCATCGAA

TCTTTGAACGCATCTTGCGCTCCTTGGTATTCCGAGGAGCATGCCTGTTTGAGTGTCATT
AAATTCTCAACTCTCTTATACTTTTTTGTAAAAGAGAGCTTGGACTGTGGAGGCTTGCTG
GCCACTTTTTGGGGTCAGCTCCTCTGAAATGCATTAGCGGAACCGTTTGCGATCTGCCAC
AAGTGTGATAAGTTATCTACACTGGCGAGGGGATTGCTCTCTGTAATGTTCAGCTTCTAA
TTGTCTCTACTTTGTGAGACTACTTTTGAATGCTTGACCTCAAATCAGGTAGGACTACCC
GCTGAACTTAA
>AB4−33
TTTCCGTAGGTGAACCTGCGGAAGGATCATTATTGAATTATGTTTCTAGATAGGTTGTAG
CTGGCTCTTTTAGAGCATGTGCACGCCTGTTTGGACTTCATTTTCATCCACCTGTGCACC
TATTGTAGTCTTTGGTTGGGTTAGGAGGAAGTGATCATTGTATCAGCATCTGCTGGGAGT
GAGGACTTGCATTGTGAAAGCTTTGCTGTCCTTGATGTGATCATGGAATCTTTTTCTCAC
TAGAGTCTATGTCACTCATTATACTCTGTCGAATGTCATTGAATGTCTTTACATGGGCTT
GTATGCCTATGAAAATTGTAATACAACTTTCAGCAACGGATCTCTTGGCTCTCGCATCGA
TGAAGAACGCAGCGAAATGCGATAAGTAATGTGAATTGCAGAATTCAGTGAATCATCGAA
TCTTTGAACGCATCTTGCGCTCCTTGGTATTCCGAGGAGCATGCCTGTTTGAGTGTCATT
AAATTCTCAACTCTCTTATACTTTTTTGTAAAAGAGAGCTTGGACTGTGGAGGCTTGCTG
GCCACTTTTTGGGGTCAGCTCCTCTGAAATGCATTAGCGGAACCGTTTGCGATCTGCCAC
AAGTGTGATAAGTTATCTACACTGGCGAGGGGATTGCTCTCTGTAATGTTCAGCTTCTAA
TTGTCTCTACTTTGTGAGACTACTTTTGAATGCTTGACCTCAAATCAGGTAGGACTACCC
GCTGAACTTAA
>AB4−35
TTTCCGTAGGTGAACCTGCGGAAGGATCATTATTGAATTATGTTTCTAGATAGGTTGTAG
CTGGCTCTTTTAGAGCATGTGCACGCCTGTTTGGACTTCATTTTCATCCACCTGTGCACC
TATTGTAGTCTTTGGTTGGGTTAGGAGGAAGTGATCATTGTATCAGCATCTGCTGGGAGT
GAGGACTTGCATTGTGAAAGCTTTGCTGTCCTTGATGTGATCATGGAATCTTTTTCTCAC
TAGAGTCTATGTCACTCATTATACTCTGTCGAATGTCATTGAATGTCTTTACATGGGCTT
GTATGCCTATGAAAATTGTAATACAACTTTCAGCAACGGATCTCTTGGCTCTCGCATCGA
TGAAGAACGCAGCGAAATGCGATAAGTAATGTGAATTGCAGAATTCAGTGAATCATCGAA
TCTTTGAACGCATCTTGCGCTCCTTGGTATTCCGAGGAGCATGCCTGTTTGAGTGTCATT
AAATTCTCAACTCTCTTATACTTTTTTGTAAAAGAGAGCTTGGACTGTGGAGGCTTGCTG
GCCACTTTTTGGGGTCAGCTCCTCTGAAATGCATTAGCGGAACCGTTTGCGATCTGCCAC
AAGTGTGATAAGTTATCTACACTGGCGAGGGGATTGCTCTCTGTAATGTTCAGCTTCTAA
TTGTCTCTACTTTGTGAGACTACTTTTGAATGCTTGACCTCAAATCAGGTAGGACTACCC
GCTGAACTTAA
>AB4−38
TTTCCGTAGGTGAACCTGCGGAAGGATCATTATTGAATTATGTTTCTAGATAGGTTGTAG
CTGGCTCTTTTAGAGCATGTGCACGCCTGTTTGGACTTCATTTTCATCCACCTGTGCACC
TATTGTAGTCTTTGGTTGGGTTAGGAGGAAGTGATCATTGTATCAGCATCTGCTGGGAGT
GAGGACTTGCATTGTGAAAGCTTTGCTGTCCTTGATGTGATCATGGAATCTTTTTCTCAC
TAGAGTCTATGTCACTCATTATACTCTGTCGAATGTCATTGAATGTCTTTACATGGGCTT
GTATGCCTATGAAAATTGTAATACAACTTTCAGCAACGGATCTCTTGGCTCTCGCATCGA
TGAAGAACGCAGCGAAATGCGATAAGTAATGTGAATTGCAGAATTCAGTGAATCATCGAA
TCTTTGAACGCATCTTGCGCTCCTTGGTATTCCGAGGAGCATGCCTGTTTGAGTGTCATT
AAATTCTCAACTCTCTTATACTTTTTTGTAAAAGAGAGCTTGGACTGTGGAGGCTTGCTG
GCCACTTTTTGGGGTCAGCTCCTCTGAAATGCATTAGCGGAACCGTTTGCGATCTGCCAC
AAGTGTGATAAGTTATCTACACTGGCGAGGGGATTGCTCTCTGTAATGTTCAGCTTCTAA
TTGTCTCTACTTTGTGAGACTACTTTTGAATGCTTGACCTCAAATCAGGTAGGACTACCC
GCTGAACTTAA
>AB4−39
TTTCCGTAGGTGAACCTGCGGAAGGATCATTATTGAATTATGTTTCTAGATAGGTTGTAG

CTGGCTCTTTTAGAGCATGTGCACGCCTGTTTGGACTTCATTTTCATCCACCTGTGCACC
TATTGTAGTCTTTGGTTGGGTTAGGAGGAAGTGATCATTGTATCAGCATCTGCTGGGAGT
GAGGACTTGCATTGTGAAAGCTTTGCTGTCCTTGATGTGATCATGGAATCTTTTTCTCAC
TAGAGTCTATGTCACTCATTATACTCTGTCGAATGTCATTGAATGTCTTTACATGGGCTT
GTATGCCTATGAAAATTGTAATACAACTTTCAGCAACGGATCTCTTGGCTCTCGCATCGA
TGAAGAACGCAGCGAAATGCGATAAGTAATGTGAATTGCAGAATTCAGTGAATCATCGAA
TCTTTGAACGCATCTTGCGCTCCTTGGTATTCCGAGGAGCATGCCTGTTTGAGTGTCATT
AAATTCTCAACTCTCTTATACTTTTTTGTAAAAGAGAGCTTGGACTGTGGAGGCTTGCTG
GCCACTTTTTGGGGTCAGCTCCTCTGAAATGCATTAGCGGAACCGTTTGCGATCTGCCAC
AAGTGTGATAAGTTATCTACACTGGCGAGGGGATTGCTCTCTGTAATGTTCAGCTTCTAA
TTGTCTCTACTTTGTGAGACTACTTTTGAATGCTTGACCTCAAATCAGGTAGGACTACCC
GCTGAACTTAA
>AB4-40
TTTCCGTAGGTGAACCTGCGGAAGGATCATTATTGAATTATGTTTCTAGATAGGTTGTAG
CTGGCTCTTTTAGAGCATGTGCACGCCTGTTTGGACTTCATTTTCATCCACCTGTGCACC
TATTGTAGTCTTTGGTTGGGTTAGGAGGAAGTGATCATTGTATCAGCATCTGCTGGGAGT
GAGGACTTGCATTGTGAAAGCTTTGCTGTCCTTGATGTGATCATGGAATCTTTTTCTCAC
TAGAGTCTATGTCACTCATTATACTCTGTCGAATGTCATTGAATGTCTTTACATGGGCTT
GTATGCCTATGAAAATTGTAATACAACTTTCAGCAACGGATCTCTTGGCTCTCGCATCGA
TGAAGAACGCAGCGAAATGCGATAAGTAATGTGAATTGCAGAATTCAGTGAATCATCGAA
TCTTTGAACGCATCTTGCGCTCCTTGGTATTCCGAGGAGCATGCCTGTTTGAGTGTCATT
AAATTCTCAACTCTCTTATACTTTTTTGTAAAAGAGAGCTTGGACTGTGGAGGCTTGCTG
GCCACTTTTTGGGGTCAGCTCCTCTGAAATGCATTAGCGGAACCGTTTGCGATCTGCCAC
AAGTGTGATAAGTTATCTACACTGGCGAGGGGATTGCTCTCTGTAATGTTCAGCTTCTAA
TTGTCTCTACTTTGTGAGACTACTTTTGAATGCTTGACCTCAAATCAGGTAGGACTACCC
GCTGAACTTAA
>AB4-46
TTTCCGTAGGTGAACCTGCGGAAGGATCATTATTGAATTATGTTTCTAGATAGGTTGTAG
CTGGCTCTTTTAGAGCATGTGCACGCCTGTTTGGACTTCATTTTCATCCACCTGTGCACC
TATTGTAGTCTTTGGTTGGGTTAGGAGGAAGTGATCATTGTATCAGCATCTGCTGGGAGT
GAGGACTTGCATTGTGAAAGCTTTGCTGTCCTTGATGTGATCATGGAATCTTTTTCTCAC
TAGAGTCTATGTCACTCATTATACTCTGTCGAATGTCATTGAATGTCTTTACATGGGCTT
GTATGCCTATGAAAATTGTAATACAACTTTCAGCAACGGATCTCTTGGCTCTCGCATCGA
TGAAGAACGCAGCGAAATGCGATAAGTAATGTGAATTGCAGAATTCAGTGAATCATCGAA
TCTTTGAACGCATCTTGCGCTCCTTGGTATTCCGAGGAGCATGCCTGTTTGAGTGTCATT
AAATTCTCAACTCTCTTATACTTTTTTGTAAAAGAGAGCTTGGACTGTGGAGGCTTGCTG
GCCACTTTTTGGGGTCAGCTCCTCTGAAATGCATTAGCGGAACCGTTTGCGATCTGCCAC
AAGTGTGATAAGTTATCTACACTGGCGAGGGGATTGCTCTCTGTAATGTTCAGCTTCTAA
TTGTCTCTACTTTGTGAGACTACTTTTGAATGCTTGACCTCAAATCAGGTAGGACTACCC
GCTGAACTTAA
>AB4-47
TTTCCGTAGGTGAACCTGCGGAAGGATCATTATTGAATTATGTTTCTAGATAGGTTGTAG
CTGGCTCTTTTAGAGCATGTGCACGCCTGTTTGGACTTCATTTTCATCCACCTGTGCACC
TATTGTAGTCTTTGGTTGGGTTAGGAGGAAGTGATCATTGTATCAGCATCTGCTGGGAGT
GAGGACTTGCATTGTGAAAGCTTTGCTGTCCTTGATGTGATCATGGAATCTTTTTCTCAC
TAGAGTCTATGTCACTCATTATACTCTGTCGAATGTCATTGAATGTCTTTACATGGGCTT
GTATGCCTATGAAAATTGTAATACAACTTTCAGCAACGGATCTCTTGGCTCTCGCATCGA
TGAAGAACGCAGCGAAATGCGATAAGTAATGTGAATTGCAGAATTCAGTGAATCATCGAA
TCTTTGAACGCATCTTGCGCTCCTTGGTATTCCGAGGAGCATGCCTGTTTGAGTGTCATT
AAATTCTCAACTCTCTTATACTTTTTTGTAAAAGAGAGCTTGGACTGTGGAGGCTTGCTG

GCCACTTTTTGGGGTCAGCTCCTCTGAAATGCATTAGCGGAACCGTTTGCGATCTGCCAC
AAGTGTGATAAGTTATCTACACTGGCGAGGGGATTGCTCTCTGTAATGTTCAGCTTCTAA
TTGTCTCTACTTTGTGAGACTACTTTTGAATGCTTGACCTCAAATCAGGTAGGACTACCC
GCTGAACTTAA
>AB4−48
TTTCCGTAGGTGAACCTGCGGAAGGATCATTATTGAATTATGTTTCTAGATAGGTTGTAG
CTGGCTCTTTTAGAGCATGTGCACGCCTGTTTGGACTTCATTTTCATCCACCTGTGCACC
TATTGTAGTCTTTGGTTGGGTTAGGAGGAAGTGATCATTGTATCAGCATCTGCTGGGAGT
GAGGACTTGCATTGTGAAAGCTTTGCTGTCCTTGATGTGATCATGGAATCTTTTTCTCAC
TAGAGTCTATGTCACTCATTATACTCTGTCGAATGTCATTGAATGTCTTTACATGGGCTT
GTATGCCTATGAAAATTGTAATACAACTTTCAGCAACGGATCTCTTGGCTCTCGCATCGA
TGAAGAACGCAGCGAAATGCGATAAGTAATGTGAATTGCAGAATTCAGTGAATCATCGAA
TCTTTGAACGCATCTTGCGCTCCTTGGTATTCCGAGGAGCATGCCTGTTTGAGTGTCATT
AAATTCTCAACTCTCTTATACTTTTTTGTAAAAGAGAGCTTGGACTGTGGAGGCTTGCTG
GCCACTTTTTGGGGTCAGCTCCTCTGAAATGCATTAGCGGAACCGTTTGCGATCTGCCAC
AAGTGTGATAAGTTATCTACACTGGCGAGGGGATTGCTCTCTGTAATGTTCAGCTTCTAA
TTGTCTCTACTTTGTGAGACTACTTTTGAATGCTTGACCTCAAATCAGGTAGGACTACCC
GCTGAACTTAA
>AB4−49
TTTCCGTAGGTGAACCTGCGGAAGGATCATTATTGAATTATGTTTCTAGATAGGTTGTAG
CTGGCTCTTTTAGAGCATGTGCACGCCTGTTTGGACTTCATTTTCATCCACCTGTGCACC
TATTGTAGTCTTTGGTTGGGTTAGGAGGAAGTGATCATTGTATCAGCATCTGCTGGGAGT
GAGGACTTGCATTGTGAAAGCTTTGCTGTCCTTGATGTGATCATGGAATCTTTTTCTCAC
TAGAGTCTATGTCACTCATTATACTCTGTCGAATGTCATTGAATGTCTTTACATGGGCTT
GTATGCCTATGAAAATTGTAATACAACTTTCAGCAACGGATCTCTTGGCTCTCGCATCGA
TGAAGAACGCAGCGAAATGCGATAAGTAATGTGAATTGCAGAATTCAGTGAATCATCGAA
TCTTTGAACGCATCTTGCGCTCCTTGGTATTCCGAGGAGCATGCCTGTTTGAGTGTCATT
AAATTCTCAACTCTCTTATACTTTTTTGTAAAAGAGAGCTTGGACTGTGGAGGCTTGCTG
GCCACTTTTTGGGGTCAGCTCCTCTGAAATGCATTAGCGGAACCGTTTGCGATCTGCCAC
AAGTGTGATAAGTTATCTACACTGGCGAGGGGATTGCTCTCTGTAATGTTCAGCTTCTAA
TTGTCTCTACTTTGTGAGACTACTTTTGAATGCTTGACCTCAAATCAGGTAGGACTACCC
GCTGAACTTAA
>AB4−53
TTTCCGTAGGTGAACCTGCGGAAGGATCATTATTGAATTATGTTTCTAGATAGGTTGTAG
CTGGCTCTTTTAGAGCATGTGCACGCCTGTTTGGACTTCATTTTCATCCACCTGTGCACC
TATTGTAGTCTTTGGTTGGGTTAGGAGGAAGTGATCATTGTATCAGCATCTGCTGGGAGT
GAGGACTTGCATTGTGAAAGCTTTGCTGTCCTTGATGTGATCATGGAATCTTTTTCTCAC
TAGAGTCTATGTCACTCATTATACTCTGTCGAATGTCATTGAATGTCTTTACATGGGCTT
GTATGCCTATGAAAATTGTAATACAACTTTCAGCAACGGATCTCTTGGCTCTCGCATCGA
TGAAGAACGCAGCGAAATGCGATAAGTAATGTGAATTGCAGAATTCAGTGAATCATCGAA
TCTTTGAACGCATCTTGCGCTCCTTGGTATTCCGAGGAGCATGCCTGTTTGAGTGTCATT
AAATTCTCAACTCTCTTATACTTTTTTGTAAAAGAGAGCTTGGACTGTGGAGGCTTGCTG
GCCACTTTTTGGGGTCAGCTCCTCTGAAATGCATTAGCGGAACCGTTTGCGATCTGCCAC
AAGTGTGATAAGTTATCTACACTGGCGAGGGGATTGCTCTCTGTAATGTTCAGCTTCTAA
TTGTCTCTACTTTGTGAGACTACTTTTGAATGCTTGACCTCAAATCAGGTAGGACTACCC
GCTGAACTTAA
>AB4−54
TTTCCGTAGGTGAACCTGCGGAAGGATCATTATTGAATTATGTTTCTAGATAGGTTGTAG
CTGGCTCTTTTAGAGCATGTGCACGCCTGTTTGGACTTCATTTTCATCCACCTGTGCACC
TATTGTAGTCTTTGGTTGGGTTAGGAGGAAGTGATCATTGTATCAGCATCTGCTGGGAGT

GAGGACTTGCATTGTGAAAGCTTTGCTGTCCTTGATGTGATCATGGAATCTTTTTCTCAC
TAGAGTCTATGTCACTCATTATACTCTGTCGAATGTCATTGAATGTCTTTACATGGGCTT
GTATGCCTATGAAAATTGTAATACAACTTTCAGCAACGGATCTCTTGGCTCTCGCATCGA
TGAAGAACGCAGCGAAATGCGATAAGTAATGTGAATTGCAGAATTCAGTGAATCATCGAA
TCTTTGAACGCATCTTGCGCTCCTTGGTATTCCGAGGAGCATGCCTGTTTGAGTGTCATT
AAATTCTCAACTCTCTTATACTTTTTTGTAAAAGAGAGCTTGGACTGTGGAGGCTTGCTG
GCCACTTTTTGGGGTCAGCTCCTCTGAAATGCATTAGCGGAACCGTTTGCGATCTGCCAC
AAGTGTGATAAGTTATCTACACTGGCGAGGGGATTGCTCTCTGTAATGTTCAGCTTCTAA
TTGTCTCTACTTTGTGAGACTACTTTTGAATGCTTGACCTCAAATCAGGTAGGACTACCC
GCTGAACTTAA
>AB4-59
TTTCCGTAGGTGAACCTGCGGAAGGATCATTATTGAATTATGTTTCTAGATAGGTTGTAG
CTGGCTCTTTTAGAGCATGTGCACGCCTGTTTGGACTTCATTTTCATCCACCTGTGCACC
TATTGTAGTCTTTGGTTGGGTTAGGAGGAAGTGATCATTGTATCAGCATCTGCTGGGAGT
GAGGACTTGCATTGTGAAAGCTTTGCTGTCCTTGATGTGATCATGGAATCTTTTTCTCAC
TAGAGTCTATGTCACTCATTATACTCTGTCGAATGTCATTGAATGTCTTTACATGGGCTT
GTATGCCTATGAAAATTGTAATACAACTTTCAGCAACGGATCTCTTGGCTCTCGCATCGA
TGAAGAACGCAGCGAAATGCGATAAGTAATGTGAATTGCAGAATTCAGTGAATCATCGAA
TCTTTGAACGCATCTTGCGCTCCTTGGTATTCCGAGGAGCATGCCTGTTTGAGTGTCATT
AAATTCTCAACTCTCTTATACTTTTTTGTAAAAGAGAGCTTGGACTGTGGAGGCTTGCTG
GCCACTTTTTGGGGTCAGCTCCTCTGAAATGCATTAGCGGAACCGTTTGCGATCTGCCAC
AAGTGTGATAAGTTATCTACACTGGCGAGGGGATTGCTCTCTGTAATGTTCAGCTTCTAA
TTGTCTCTACTTTGTGAGACTACTTTTGAATGCTTGACCTCAAATCAGGTAGGACTACCC
GCTGAACTTAA
>AB5-1
TTTCCGTAGGTGAACCTGCGGAAGGATCATTATTGAATTATGTTTCTAGATAGGTTGTAG
CTGGCTCTTTTAGAGCATGTGCACGCCTGTTTGGACTTCATTTTCATCCACCTGTGCACC
TATTGTAGTCTTTGGTTGGGTTAGGAGGAAGTGATCATTGTATCAGCATCTGCTGGGAGT
GAGGACTTGCATTGTGAAAGCTTTGCTGTCCTTGATGTGATCATGGAATCTTTTTCTCAC
TAGAGTCTATGTCACTCATTATACTCTGTCGAATGTCATTGAATGTCTTTACATGGGCTT
GTATGCCTATGAAAATTGTAATACAACTTTCAGCAACGGATCTCTTGGCTCTCGCATCGA
TGAAGAACGCAGCGAAATGCGATAAGTAATGTGAATTGCAGAATTCAGTGAATCATCGAA
TCTTTGAACGCATCTTGCGCTCCTTGGTATTCCGAGGAGCATGCCTGTTTGAGTGTCATT
AAATTCTCAACTCTCTTATACTTTTTTGTAAAAGAGAGCTTGGACTGTGGAGGCTTGCTG
GCCACTTTTTGGGGTCAGCTCCTCTGAAATGCATTAGCGGAACCGTTTGCGATCTGCCAC
AAGTGTGATAAGTTATCTACACTGGCGAGGGGATTGCTCTCTGTAATGTTCAGCTTCTAA
TTGTCTCTACTTTGTGAGACTACTTTTGAATGCTTGACCTCAAATCAGGTAGGACTACCC
GCTGAACTTAA
>AB5-3
TTTCCGTAGGTGAACCTGCGGAAGGATCATTATTGAATTATGTTTCTAGATAGGTTGTAG
CTGGCTCTTTTAGAGCATGTGCACGCCTGTTTGGACTTCATTTTCATCCACCTGTGCACC
TATTGTAGTCTTTGGTTGGGTTAGGAGGAAGTGATCATTGTATCAGCATCTGCTGGGAGT
GAGGACTTGCATTGTGAAAGCTTTGCTGTCCTTGATGTGATCATGGAATCTTTTTCTCAC
TAGAGTCTATGTCACTCATTATACTCTGTCGAATGTCATTGAATGTCTTTACATGGGCTT
GTATGCCTATGAAAATTGTAATACAACTTTCAGCAACGGATCTCTTGGCTCTCGCATCGA
TGAAGAACGCAGCGAAATGCGATAAGTAATGTGAATTGCAGAATTCAGTGAATCATCGAA
TCTTTGAACGCATCTTGCGCTCCTTGGTATTCCGAGGAGCATGCCTGTTTGAGTGTCATT
AAATTCTCAACTCTCTTATACTTTTTTGTAAAAGAGAGCTTGGACTGTGGAGGCTTGCTG
GCCACTTTTTGGGGTCAGCTCCTCTGAAATGCATTAGCGGAACCGTTTGCGATCTGCCAC
AAGTGTGATAAGTTATCTACACTGGCGAGGGGATTGCTCTCTGTAATGTTCAGCTTCTAA

TTGTCTCTACTTTGTGAGACTACTTTTGAATGCTTGACCTCAAATCAGGTAGGACTACCC
GCTGAACTTAA
>AB5-4
TTTCCGTAGGTGAACCTGCGGAAGGATCATTATTGAATTATGTTTCTAGATAGGTTGTAG
CTGGCTCTTTTAGAGCATGTGCACGCCTGTTTGGACTTCATTTTCATCCACCTGTGCACC
TATTGTAGTCTTTGGTTGGGTTAGGAGGAAGTGATCATTGTATCAGCATCTGCTGGGAGT
GAGGACTTGCATTGTGAAAGCTTTGCTGTCCTTGATGTGATCATGGAATCTTTTTCTCAC
TAGAGTCTATGTCACTCATTATACTCTGTCGAATGTCATTGAATGTCTTTACATGGGCTT
GTATGCCTATGAAAATTGTAATACAACTTTCAGCAACGGATCTCTTGGCTCTCGCATCGA
TGAAGAACGCAGCGAAATGCGATAAGTAATGTGAATTGCAGAATTCAGTGAATCATCGAA
TCTTTGAACGCATCTTGCGCTCCTTGGTATTCCGAGGAGCATGCCTGTTTGAGTGTCATT
AAATTCTCAACTCTCTTATACTTTTTTGTAAAAGAGAGCTTGGACTGTGGAGGCTTGCTG
GCCACTTTTTGGGGTCAGCTCCTCTGAAATGCATTAGCGGAACCGTTTGCGATCTGCCAC
AAGTGTGATAAGTTATCTACACTGGCGAGGGGATTGCTCTCTGTAATGTTCAGCTTCTAA
TTGTCTCTACTTTGTGAGACTACTTTTGAATGCTTGACCTCAAATCAGGTAGGACTACCC
GCTGAACTTAA
>AB5-10
TTTCCGTAGGTGAACCTGCGGAAGGATCATTATTGAATTATGTTTCTAGATAGGTTGTAG
CTGGCTCTTTTAGAGCATGTGCACGCCTGTTTGGACTTCATTTTCATCCACCTGTGCACC
TATTGTAGTCTTTGGTTGGGTTAGGAGGAAGTGATCATTGTATCAGCATCTGCTGGGAGT
GAGGACTTGCATTGTGAAAGCTTTGCTGTCCTTGATGTGATCATGGAATCTTTTTCTCAC
TAGAGTCTATGTCACTCATTATACTCTGTCGAATGTCATTGAATGTCTTTACATGGGCTT
GTATGCCTATGAAAATTGTAATACAACTTTCAGCAACGGATCTCTTGGCTCTCGCATCGA
TGAAGAACGCAGCGAAATGCGATAAGTAATGTGAATTGCAGAATTCAGTGAATCATCGAA
TCTTTGAACGCATCTTGCGCTCCTTGGTATTCCGAGGAGCATGCCTGTTTGAGTGTCATT
AAATTCTCAACTCTCTTATACTTTTTTGTAAAAGAGAGCTTGGACTGTGGAGGCTTGCTG
GCCACTTTTTGGGGTCAGCTCCTCTGAAATGCATTAGCGGAACCGTTTGCGATCTGCCAC
AAGTGTGATAAGTTATCTACACTGGCGAGGGGATTGCTCTCTGTAATGTTCAGCTTCTAA
TTGTCTCTACTTTGTGAGACTACTTTTGAATGCTTGACCTCAAATCAGGTAGGACTACCC
GCTGAACTTAA
>AB5-11
TTTCCGTAGGTGAACCTGCGGAAGGATCATTATTGAATTATGTTTCTAGATAGGTTGTAG
CTGGCTCTTTTAGAGCATGTGCACGCCTGTTTGGACTTCATTTTCATCCACCTGTGCACC
TATTGTAGTCTTTGGTTGGGTTAGGAGGAAGTGATCATTGTATCAGCATCTGCTGGGAGT
GAGGACTTGCATTGTGAAAGCTTTGCTGTCCTTGATGTGATCATGGAATCTTTTTCTCAC
TAGAGTCTATGTCACTCATTATACTCTGTCGAATGTCATTGAATGTCTTTACATGGGCTT
GTATGCCTATGAAAATTGTAATACAACTTTCAGCAACGGATCTCTTGGCTCTCGCATCGA
TGAAGAACGCAGCGAAATGCGATAAGTAATGTGAATTGCAGAATTCAGTGAATCATCGAA
TCTTTGAACGCATCTTGCGCTCCTTGGTATTCCGAGGAGCATGCCTGTTTGAGTGTCATT
AAATTCTCAACTCTCTTATACTTTTTTGTAAAAGAGAGCTTGGACTGTGGAGGCTTGCTG
GCCACTTTTTGGGGTCAGCTCCTCTGAAATGCATTAGCGGAACCGTTTGCGATCTGCCAC
AAGTGTGATAAGTTATCTACACTGGCGAGGGGATTGCTCTCTGTAATGTTCAGCTTCTAA
TTGTCTCTACTTTGTGAGACTACTTTTGAATGCTTGACCTCAAATCAGGTAGGACTACCC
GCTGAACTTAA
>AB5-14
TTTCCGTAGGTGAACCTGCGGAAGGATCATTATTGAATTATGTTTCTAGATAGGTTGTAG
CTGGCTCTTTTAGAGCATGTGCACGCCTGTTTGGACTTCATTTTCATCCACCTGTGCACC
TATTGTAGTCTTTGGTTGGGTTAGGAGGAAGTGATCATTGTATCAGCATCTGCTGGGAGT
GAGGACTTGCATTGTGAAAGCTTTGCTGTCCTTGATGTGATCATGGAATCTTTTTCTCAC
TAGAGTCTATGTCACTCATTATACTCTGTCGAATGTCATTGAATGTCTTTACATGGGCTT

GTATGCCTATGAAAATTGTAATACAACTTTCAGCAACGGATCTCTTGGCTCTCGCATCGA
TGAAGAACGCAGCGAAATGCGATAAGTAATGTGAATTGCAGAATTCAGTGAATCATCGAA
TCTTTGAACGCATCTTGCGCTCCTTGGTATTCCGAGGAGCATGCCTGTTTGAGTGTCATT
AAATTCTCAACTCTCTTATACTTTTTTGTAAAAGAGAGCTTGGACTGTGGAGGCTTGCTG
GCCACTTTTTGGGGTCAGCTCCTCTGAAATGCATTAGCGGAACCGTTTGCGATCTGCCAC
AAGTGTGATAAGTTATCTACACTGGCGAGGGGATTGCTCTCTGTAATGTTCAGCTTCTAA
TTGTCTCTACTTTGTGAGACTACTTTTGAATGCTTGACCTCAAATCAGGTAGGACTACCC
GCTGAACTTAA
>AB5-15
TTTCCGTAGGTGAACCTGCGGAAGGATCATTATTGAATTATGTTTCTAGATAGGTTGTAG
CTGGCTCTTTTAGAGCATGTGCACGCCTGTTTGGACTTCATTTTCATCCACCTGTGCACC
TATTGTAGTCTTTGGTTGGGTTAGGAGGAAGTGATCATTGTATCAGCATCTGCTGGGAGT
GAGGACTTGCATTGTGAAAGCTTTGCTGTCCTTGATGTGATCATGGAATCTTTTTCTCAC
TAGAGTCTATGTCACTCATTATACTCTGTCGAATGTCATTGAATGTCTTTACATGGGCTT
GTATGCCTATGAAAATTGTAATACAACTTTCAGCAACGGATCTCTTGGCTCTCGCATCGA
TGAAGAACGCAGCGAAATGCGATAAGTAATGTGAATTGCAGAATTCAGTGAATCATCGAA
TCTTTGAACGCATCTTGCGCTCCTTGGTATTCCGAGGAGCATGCCTGTTTGAGTGTCATT
AAATTCTCAACTCTCTTATACTTTTTTGTAAAAGAGAGCTTGGACTGTGGAGGCTTGCTG
GCCACTTTTTGGGGTCAGCTCCTCTGAAATGCATTAGCGGAACCGTTTGCGATCTGCCAC
AAGTGTGATAAGTTATCTACACTGGCGAGGGGATTGCTCTCTGTAATGTTCAGCTTCTAA
TTGTCTCTACTTTGTGAGACTACTTTTGAATGCTTGACCTCAAATCAGGTAGGACTACCC
GCTGAACTTAA
>AB5-20
TTTCCGTAGGTGAACCTGCGGAAGGATCATTATTGAATTATGTTTCTAGATAGGTTGTAG
CTGGCTCTTTTAGAGCATGTGCACGCCTGTTTGGACTTCATTTTCATCCACCTGTGCACC
TATTGTAGTCTTTGGTTGGGTTAGGAGGAAGTGATCATTGTATCAGCATCTGCTGGGAGT
GAGGACTTGCATTGTGAAAGCTTTGCTGTCCTTGATGTGATCATGGAATCTTTTTCTCAC
TAGAGTCTATGTCACTCATTATACTCTGTCGAATGTCATTGAATGTCTTTACATGGGCTT
GTATGCCTATGAAAATTGTAATACAACTTTCAGCAACGGATCTCTTGGCTCTCGCATCGA
TGAAGAACGCAGCGAAATGCGATAAGTAATGTGAATTGCAGAATTCAGTGAATCATCGAA
TCTTTGAACGCATCTTGCGCTCCTTGGTATTCCGAGGAGCATGCCTGTTTGAGTGTCATT
AAATTCTCAACTCTCTTATACTTTTTTGTAAAAGAGAGCTTGGACTGTGGAGGCTTGCTG
GCCACTTTTTGGGGTCAGCTCCTCTGAAATGCATTAGCGGAACCGTTTGCGATCTGCCAC
AAGTGTGATAAGTTATCTACACTGGCGAGGGGATTGCTCTCTGTAATGTTCAGCTTCTAA
TTGTCTCTACTTTGTGAGACTACTTTTGAATGCTTGACCTCAAATCAGGTAGGACTACCC
GCTGAACTTAA
>AB5-21
TTTCCGTAGGTGAACCTGCGGAAGGATCATTATTGAATTATGTTTCTAGATAGGTTGTAG
CTGGCTCTTTTAGAGCATGTGCACGCCTGTTTGGACTTCATTTTCATCCACCTGTGCACC
TATTGTAGTCTTTGGTTGGGTTAGGAGGAAGTGATCATTGTATCAGCATCTGCTGGGAGT
GAGGACTTGCATTGTGAAAGCTTTGCTGTCCTTGATGTGATCATGGAATCTTTTTCTCAC
TAGAGTCTATGTCACTCATTATACTCTGTCGAATGTCATTGAATGTCTTTACATGGGCTT
GTATGCCTATGAAAATTGTAATACAACTTTCAGCAACGGATCTCTTGGCTCTCGCATCGA
TGAAGAACGCAGCGAAATGCGATAAGTAATGTGAATTGCAGAATTCAGTGAATCATCGAA
TCTTTGAACGCATCTTGCGCTCCTTGGTATTCCGAGGAGCATGCCTGTTTGAGTGTCATT
AAATTCTCAACTCTCTTATACTTTTTTGTAAAAGAGAGCTTGGACTGTGGAGGCTTGCTG
GCCACTTTTTGGGGTCAGCTCCTCTGAAATGCATTAGCGGAACCGTTTGCGATCTGCCAC
AAGTGTGATAAGTTATCTACACTGGCGAGGGGATTGCTCTCTGTAATGTTCAGCTTCTAA
TTGTCTCTACTTTGTGAGACTACTTTTGAATGCTTGACCTCAAATCAGGTAGGACTACCC
GCTGAACTTAA

>AB5-22
TTTCCGTAGGTGAACCTGCGGAAGGATCATTATTGAATTATGTTTCTAGATAGGTTGTAG
CTGGCTCTTTTAGAGCATGTGCACGCCTGTTTGGACTTCATTTTCATCCACCTGTGCACC
TATTGTAGTCTTTGGTTGGGTTAGGAGGAAGTGATCATTGTATCAGCATCTGCTGGGAGT
GAGGACTTGCATTGTGAAAGCTTTGCTGTCCTTGATGTGATCATGGAATCTTTTTCTCAC
TAGAGTCTATGTCACTCATTATACTCTGTCGAATGTCATTGAATGTCTTTACATGGGCTT
GTATGCCTATGAAAATTGTAATACAACTTTCAGCAACGGATCTCTTGGCTCTCGCATCGA
TGAAGAACGCAGCGAAATGCGATAAGTAATGTGAATTGCAGAATTCAGTGAATCATCGAA
TCTTTGAACGCATCTTGCGCTCCTTGGTATTCCGAGGAGCATGCCTGTTTGAGTGTCATT
AAATTCTCAACTCTCTTATACTTTTTTGTAAAAGAGAGCTTGGACTGTGGAGGCTTGCTG
GCCACTTTTTGGGGTCAGCTCCTCTGAAATGCATTAGCGGAACCGTTTGCGATCTGCCAC
AAGTGTGATAAGTTATCTACACTGGCGAGGGGATTGCTCTCTGTAATGTTCAGCTTCTAA
TTGTCTCTACTTTGTGAGACTACTTTTGAATGCTTGACCTCAAATCAGGTAGGACTACCC
GCTGAACTTAA
>AB5-24
TTTCCGTAGGTGAACCTGCGGAAGGATCATTATTGAATTATGTTTCTAGATAGGTTGTAG
CTGGCTCTTTTAGAGCATGTGCACGCCTGTTTGGACTTCATTTTCATCCACCTGTGCACC
TATTGTAGTCTTTGGTTGGGTTAGGAGGAAGTGATCATTGTATCAGCATCTGCTGGGAGT
GAGGACTTGCATTGTGAAAGCTTTGCTGTCCTTGATGTGATCATGGAATCTTTTTCTCAC
TAGAGTCTATGTCACTCATTATACTCTGTCGAATGTCATTGAATGTCTTTACATGGGCTT
GTATGCCTATGAAAATTGTAATACAACTTTCAGCAACGGATCTCTTGGCTCTCGCATCGA
TGAAGAACGCAGCGAAATGCGATAAGTAATGTGAATTGCAGAATTCAGTGAATCATCGAA
TCTTTGAACGCATCTTGCGCTCCTTGGTATTCCGAGGAGCATGCCTGTTTGAGTGTCATT
AAATTCTCAACTCTCTTATACTTTTTTGTAAAAGAGAGCTTGGACTGTGGAGGCTTGCTG
GCCACTTTTTGGGGTCAGCTCCTCTGAAATGCATTAGCGGAACCGTTTGCGATCTGCCAC
AAGTGTGATAAGTTATCTACACTGGCGAGGGGATTGCTCTCTGTAATGTTCAGCTTCTAA
TTGTCTCTACTTTGTGAGACTACTTTTGAATGCTTGACCTCAAATCAGGTAGGACTACCC
GCTGAACTTAA
>AB5-25
TTTCCGTAGGTGAACCTGCGGAAGGATCATTATTGAATTATGTTTCTAGATAGGTTGTAG
CTGGCTCTTTTAGAGCATGTGCACGCCTGTTTGGACTTCATTTTCATCCACCTGTGCACC
TATTGTAGTCTTTGGTTGGGTTAGGAGGAAGTGATCATTGTATCAGCATCTGCTGGGAGT
GAGGACTTGCATTGTGAAAGCTTTGCTGTCCTTGATGTGATCATGGAATCTTTTTCTCAC
TAGAGTCTATGTCACTCATTATACTCTGTCGAATGTCATTGAATGTCTTTACATGGGCTT
GTATGCCTATGAAAATTGTAATACAACTTTCAGCAACGGATCTCTTGGCTCTCGCATCGA
TGAAGAACGCAGCGAAATGCGATAAGTAATGTGAATTGCAGAATTCAGTGAATCATCGAA
TCTTTGAACGCATCTTGCGCTCCTTGGTATTCCGAGGAGCATGCCTGTTTGAGTGTCATT
AAATTCTCAACTCTCTTATACTTTTTTGTAAAAGAGAGCTTGGACTGTGGAGGCTTGCTG
GCCACTTTTTGGGGTCAGCTCCTCTGAAATGCATTAGCGGAACCGTTTGCGATCTGCCAC
AAGTGTGATAAGTTATCTACACTGGCGAGGGGATTGCTCTCTGTAATGTTCAGCTTCTAA
TTGTCTCTACTTTGTGAGACTACTTTTGAATGCTTGACCTCAAATCAGGTAGGACTACCC
GCTGAACTTAA
>AB5-26
TTTCCGTAGGTGAACCTGCGGAAGGATCATTATTGAATTATGTTTCTAGATAGGTTGTAG
CTGGCTCTTTTAGAGCATGTGCACGCCTGTTTGGACTTCATTTTCATCCACCTGTGCACC
TATTGTAGTCTTTGGTTGGGTTAGGAGGAAGTGATCATTGTATCAGCATCTGCTGGGAGT
GAGGACTTGCATTGTGAAAGCTTTGCTGTCCTTGATGTGATCATGGAATCTTTTTCTCAC
TAGAGTCTATGTCACTCATTATACTCTGTCGAATGTCATTGAATGTCTTTACATGGGCTT
GTATGCCTATGAAAATTGTAATACAACTTTCAGCAACGGATCTCTTGGCTCTCGCATCGA
TGAAGAACGCAGCGAAATGCGATAAGTAATGTGAATTGCAGAATTCAGTGAATCATCGAA

TCTTTGAACGCATCTTGCGCTCCTTGGTATTCCGAGGAGCATGCCTGTTTGAGTGTCATT
AAATTCTCAACTCTCTTATACTTTTTTGTAAAAGAGAGCTTGGACTGTGGAGGCTTGCTG
GCCACTTTTTGGGGTCAGCTCCTCTGAAATGCATTAGCGGAACCGTTTGCGATCTGCCAC
AAGTGTGATAAGTTATCTACACTGGCGAGGGGATTGCTCTCTGTAATGTTCAGCTTCTAA
TTGTCTCTACTTTGTGAGACTACTTTTGAATGCTTGACCTCAAATCAGGTAGGACTACCC
GCTGAACTTAA
>AB5-29
TTTCCGTAGGTGAACCTGCGGAAGGATCATTATTGAATTATGTTTCTAGATAGGTTGTAG
CTGGCTCTTTTAGAGCATGTGCACGCCTGTTTGGACTTCATTTTCATCCACCTGTGCACC
TATTGTAGTCTTTGGTTGGGTTAGGAGGAAGTGATCATTGTATCAGCATCTGCTGGGAGT
GAGGACTTGCATTGTGAAAGCTTTGCTGTCCTTGATGTGATCATGGAATCTTTTTCTCAC
TAGAGTCTATGTCACTCATTATACTCTGTCGAATGTCATTGAATGTCTTTACATGGGCTT
GTATGCCTATGAAAATTGTAATACAACTTTCAGCAACGGATCTCTTGGCTCTCGCATCGA
TGAAGAACGCAGCGAAATGCGATAAGTAATGTGAATTGCAGAATTCAGTGAATCATCGAA
TCTTTGAACGCATCTTGCGCTCCTTGGTATTCCGAGGAGCATGCCTGTTTGAGTGTCATT
AAATTCTCAACTCTCTTATACTTTTTTGTAAAAGAGAGCTTGGACTGTGGAGGCTTGCTG
GCCACTTTTTGGGGTCAGCTCCTCTGAAATGCATTAGCGGAACCGTTTGCGATCTGCCAC
AAGTGTGATAAGTTATCTACACTGGCGAGGGGATTGCTCTCTGTAATGTTCAGCTTCTAA
TTGTCTCTACTTTGTGAGACTACTTTTGAATGCTTGACCTCAAATCAGGTAGGACTACCC
GCTGAACTTAA
>AB5-36
TTTCCGTAGGTGAACCTGCGGAAGGATCATTATTGAATTATGTTTCTAGATAGGTTGTAG
CTGGCTCTTTTAGAGCATGTGCACGCCTGTTTGGACTTCATTTTCATCCACCTGTGCACC
TATTGTAGTCTTTGGTTGGGTTAGGAGGAAGTGATCATTGTATCAGCATCTGCTGGGAGT
GAGGACTTGCATTGTGAAAGCTTTGCTGTCCTTGATGTGATCATGGAATCTTTTTCTCAC
TAGAGTCTATGTCACTCATTATACTCTGTCGAATGTCATTGAATGTCTTTACATGGGCTT
GTATGCCTATGAAAATTGTAATACAACTTTCAGCAACGGATCTCTTGGCTCTCGCATCGA
TGAAGAACGCAGCGAAATGCGATAAGTAATGTGAATTGCAGAATTCAGTGAATCATCGAA
TCTTTGAACGCATCTTGCGCTCCTTGGTATTCCGAGGAGCATGCCTGTTTGAGTGTCATT
AAATTCTCAACTCTCTTATACTTTTTTGTAAAAGAGAGCTTGGACTGTGGAGGCTTGCTG
GCCACTTTTTGGGGTCAGCTCCTCTGAAATGCATTAGCGGAACCGTTTGCGATCTGCCAC
AAGTGTGATAAGTTATCTACACTGGCGAGGGGATTGCTCTCTGTAATGTTCAGCTTCTAA
TTGTCTCTACTTTGTGAGACTACTTTTGAATGCTTGACCTCAAATCAGGTAGGACTACCC
GCTGAACTTAA
>AB5-38
TTTCCGTAGGTGAACCTGCGGAAGGATCATTATTGAATTATGTTTCTAGATAGGTTGTAG
CTGGCTCTTTTAGAGCATGTGCACGCCTGTTTGGACTTCATTTTCATCCACCTGTGCACC
TATTGTAGTCTTTGGTTGGGTTAGGAGGAAGTGATCATTGTATCAGCATCTGCTGGGAGT
GAGGACTTGCATTGTGAAAGCTTTGCTGTCCTTGATGTGATCATGGAATCTTTTTCTCAC
TAGAGTCTATGTCACTCATTATACTCTGTCGAATGTCATTGAATGTCTTTACATGGGCTT
GTATGCCTATGAAAATTGTAATACAACTTTCAGCAACGGATCTCTTGGCTCTCGCATCGA
TGAAGAACGCAGCGAAATGCGATAAGTAATGTGAATTGCAGAATTCAGTGAATCATCGAA
TCTTTGAACGCATCTTGCGCTCCTTGGTATTCCGAGGAGCATGCCTGTTTGAGTGTCATT
AAATTCTCAACTCTCTTATACTTTTTTGTAAAAGAGAGCTTGGACTGTGGAGGCTTGCTG
GCCACTTTTTGGGGTCAGCTCCTCTGAAATGCATTAGCGGAACCGTTTGCGATCTGCCAC
AAGTGTGATAAGTTATCTACACTGGCGAGGGGATTGCTCTCTGTAATGTTCAGCTTCTAA
TTGTCTCTACTTTGTGAGACTACTTTTGAATGCTTGACCTCAAATCAGGTAGGACTACCC
GCTGAACTTAA
>AB5-39
TTTCCGTAGGTGAACCTGCGGAAGGATCATTATTGAATTATGTTTCTAGATAGGTTGTAG

CTGGCTCTTTTAGAGCATGTGCACGCCTGTTTGGACTTCATTTTCATCCACCTGTGCACC
TATTGTAGTCTTTGGTTGGGTTAGGAGGAAGTGATCATTGTATCAGCATCTGCTGGGAGT
GAGGACTTGCATTGTGAAAGCTTTGCTGTCCTTGATGTGATCATGGAATCTTTTTCTCAC
TAGAGTCTATGTCACTCATTATACTCTGTCGAATGTCATTGAATGTCTTTACATGGGCTT
GTATGCCTATGAAAATTGTAATACAACTTTCAGCAACGGATCTCTTGGCTCTCGCATCGA
TGAAGAACGCAGCGAAATGCGATAAGTAATGTGAATTGCAGAATTCAGTGAATCATCGAA
TCTTTGAACGCATCTTGCGCTCCTTGGTATTCCGAGGAGCATGCCTGTTTGAGTGTCATT
AAATTCTCAACTCTCTTATACTTTTTTGTAAAAGAGAGCTTGGACTGTGGAGGCTTGCTG
GCCACTTTTTGGGGTCAGCTCCTCTGAAATGCATTAGCGGAACCGTTTGCGATCTGCCAC
AAGTGTGATAAGTTATCTACACTGGCGAGGGGATTGCTCTCTGTAATGTTCAGCTTCTAA
TTGTCTCTACTTTGTGAGACTACTTTTGAATGCTTGACCTCAAATCAGGTAGGACTACCC
GCTGAACTTAA
>AB5-43
TTTCCGTAGGTGAACCTGCGGAAGGATCATTATTGAATTATGTTTCTAGATAGGTTGTAG
CTGGCTCTTTTAGAGCATGTGCACGCCTGTTTGGACTTCATTTTCATCCACCTGTGCACC
TATTGTAGTCTTTGGTTGGGTTAGGAGGAAGTGATCATTGTATCAGCATCTGCTGGGAGT
GAGGACTTGCATTGTGAAAGCTTTGCTGTCCTTGATGTGATCATGGAATCTTTTTCTCAC
TAGAGTCTATGTCACTCATTATACTCTGTCGAATGTCATTGAATGTCTTTACATGGGCTT
GTATGCCTATGAAAATTGTAATACAACTTTCAGCAACGGATCTCTTGGCTCTCGCATCGA
TGAAGAACGCAGCGAAATGCGATAAGTAATGTGAATTGCAGAATTCAGTGAATCATCGAA
TCTTTGAACGCATCTTGCGCTCCTTGGTATTCCGAGGAGCATGCCTGTTTGAGTGTCATT
AAATTCTCAACTCTCTTATACTTTTTTGTAAAAGAGAGCTTGGACTGTGGAGGCTTGCTG
GCCACTTTTTGGGGTCAGCTCCTCTGAAATGCATTAGCGGAACCGTTTGCGATCTGCCAC
AAGTGTGATAAGTTATCTACACTGGCGAGGGGATTGCTCTCTGTAATGTTCAGCTTCTAA
TTGTCTCTACTTTGTGAGACTACTTTTGAATGCTTGACCTCAAATCAGGTAGGACTACCC
GCTGAACTTAA
>AB5-44
TTTCCGTAGGTGAACCTGCGGAAGGATCATTATTGAATTATGTTTCTAGATAGGTTGTAG
CTGGCTCTTTTAGAGCATGTGCACGCCTGTTTGGACTTCATTTTCATCCACCTGTGCACC
TATTGTAGTCTTTGGTTGGGTTAGGAGGAAGTGATCATTGTATCAGCATCTGCTGGGAGT
GAGGACTTGCATTGTGAAAGCTTTGCTGTCCTTGATGTGATCATGGAATCTTTTTCTCAC
TAGAGTCTATGTCACTCATTATACTCTGTCGAATGTCATTGAATGTCTTTACATGGGCTT
GTATGCCTATGAAAATTGTAATACAACTTTCAGCAACGGATCTCTTGGCTCTCGCATCGA
TGAAGAACGCAGCGAAATGCGATAAGTAATGTGAATTGCAGAATTCAGTGAATCATCGAA
TCTTTGAACGCATCTTGCGCTCCTTGGTATTCCGAGGAGCATGCCTGTTTGAGTGTCATT
AAATTCTCAACTCTCTTATACTTTTTTGTAAAAGAGAGCTTGGACTGTGGAGGCTTGCTG
GCCACTTTTTGGGGTCAGCTCCTCTGAAATGCATTAGCGGAACCGTTTGCGATCTGCCAC
AAGTGTGATAAGTTATCTACACTGGCGAGGGGATTGCTCTCTGTAATGTTCAGCTTCTAA
TTGTCTCTACTTTGTGAGACTACTTTTGAATGCTTGACCTCAAATCAGGTAGGACTACCC
GCTGAACTTAA
>AB5-45
TTTCCGTAGGTGAACCTGCGGAAGGATCATTATTGAATTATGTTTCTAGATAGGTTGTAG
CTGGCTCTTTTAGAGCATGTGCACGCCTGTTTGGACTTCATTTTCATCCACCTGTGCACC
TATTGTAGTCTTTGGTTGGGTTAGGAGGAAGTGATCATTGTATCAGCATCTGCTGGGAGT
GAGGACTTGCATTGTGAAAGCTTTGCTGTCCTTGATGTGATCATGGAATCTTTTTCTCAC
TAGAGTCTATGTCACTCATTATACTCTGTCGAATGTCATTGAATGTCTTTACATGGGCTT
GTATGCCTATGAAAATTGTAATACAACTTTCAGCAACGGATCTCTTGGCTCTCGCATCGA
TGAAGAACGCAGCGAAATGCGATAAGTAATGTGAATTGCAGAATTCAGTGAATCATCGAA
TCTTTGAACGCATCTTGCGCTCCTTGGTATTCCGAGGAGCATGCCTGTTTGAGTGTCATT
AAATTCTCAACTCTCTTATACTTTTTTGTAAAAGAGAGCTTGGACTGTGGAGGCTTGCTG

GCCACTTTTTGGGGTCAGCTCCTCTGAAATGCATTAGCGGAACCGTTTGCGATCTGCCAC
AAGTGTGATAAGTTATCTACACTGGCGAGGGGATTGCTCTCTGTAATGTTCAGCTTCTAA
TTGTCTCTACTTTGTGAGACTACTTTTGAATGCTTGACCTCAAATCAGGTAGGACTACCC
GCTGAACTTAA
>AB5-49
TTTCCGTAGGTGAACCTGCGGAAGGATCATTATTGAATTATGTTTCTAGATAGGTTGTAG
CTGGCTCTTTTAGAGCATGTGCACGCCTGTTTGGACTTCATTTTCATCCACCTGTGCACC
TATTGTAGTCTTTGGTTGGGTTAGGAGGAAGTGATCATTGTATCAGCATCTGCTGGGAGT
GAGGACTTGCATTGTGAAAGCTTTGCTGTCCTTGATGTGATCATGGAATCTTTTTCTCAC
TAGAGTCTATGTCACTCATTATACTCTGTCGAATGTCATTGAATGTCTTTACATGGGCTT
GTATGCCTATGAAAATTGTAATACAACTTTCAGCAACGGATCTCTTGGCTCTCGCATCGA
TGAAGAACGCAGCGAAATGCGATAAGTAATGTGAATTGCAGAATTCAGTGAATCATCGAA
TCTTTGAACGCATCTTGCGCTCCTTGGTATTCCGAGGAGCATGCCTGTTTGAGTGTCATT
AAATTCTCAACTCTCTTATACTTTTTTGTAAAAGAGAGCTTGGACTGTGGAGGCTTGCTG
GCCACTTTTTGGGGTCAGCTCCTCTGAAATGCATTAGCGGAACCGTTTGCGATCTGCCAC
AAGTGTGATAAGTTATCTACACTGGCGAGGGGATTGCTCTCTGTAATGTTCAGCTTCTAA
TTGTCTCTACTTTGTGAGACTACTTTTGAATGCTTGACCTCAAATCAGGTAGGACTACCC
GCTGAACTTAA
>AB5-50
TTTCCGTAGGTGAACCTGCGGAAGGATCATTATTGAATTATGTTTCTAGATAGGTTGTAG
CTGGCTCTTTTAGAGCATGTGCACGCCTGTTTGGACTTCATTTTCATCCACCTGTGCACC
TATTGTAGTCTTTGGTTGGGTTAGGAGGAAGTGATCATTGTATCAGCATCTGCTGGGAGT
GAGGACTTGCATTGTGAAAGCTTTGCTGTCCTTGATGTGATCATGGAATCTTTTTCTCAC
TAGAGTCTATGTCACTCATTATACTCTGTCGAATGTCATTGAATGTCTTTACATGGGCTT
GTATGCCTATGAAAATTGTAATACAACTTTCAGCAACGGATCTCTTGGCTCTCGCATCGA
TGAAGAACGCAGCGAAATGCGATAAGTAATGTGAATTGCAGAATTCAGTGAATCATCGAA
TCTTTGAACGCATCTTGCGCTCCTTGGTATTCCGAGGAGCATGCCTGTTTGAGTGTCATT
AAATTCTCAACTCTCTTATACTTTTTTGTAAAAGAGAGCTTGGACTGTGGAGGCTTGCTG
GCCACTTTTTGGGGTCAGCTCCTCTGAAATGCATTAGCGGAACCGTTTGCGATCTGCCAC
AAGTGTGATAAGTTATCTACACTGGCGAGGGGATTGCTCTCTGTAATGTTCAGCTTCTAA
TTGTCTCTACTTTGTGAGACTACTTTTGAATGCTTGACCTCAAATCAGGTAGGACTACCC
GCTGAACTTAA
>AB5-52
TTTCCGTAGGTGAACCTGCGGAAGGATCATTATTGAATTATGTTTCTAGATAGGTTGTAG
CTGGCTCTTTTAGAGCATGTGCACGCCTGTTTGGACTTCATTTTCATCCACCTGTGCACC
TATTGTAGTCTTTGGTTGGGTTAGGAGGAAGTGATCATTGTATCAGCATCTGCTGGGAGT
GAGGACTTGCATTGTGAAAGCTTTGCTGTCCTTGATGTGATCATGGAATCTTTTTCTCAC
TAGAGTCTATGTCACTCATTATACTCTGTCGAATGTCATTGAATGTCTTTACATGGGCTT
GTATGCCTATGAAAATTGTAATACAACTTTCAGCAACGGATCTCTTGGCTCTCGCATCGA
TGAAGAACGCAGCGAAATGCGATAAGTAATGTGAATTGCAGAATTCAGTGAATCATCGAA
TCTTTGAACGCATCTTGCGCTCCTTGGTATTCCGAGGAGCATGCCTGTTTGAGTGTCATT
AAATTCTCAACTCTCTTATACTTTTTTGTAAAAGAGAGCTTGGACTGTGGAGGCTTGCTG
GCCACTTTTTGGGGTCAGCTCCTCTGAAATGCATTAGCGGAACCGTTTGCGATCTGCCAC
AAGTGTGATAAGTTATCTACACTGGCGAGGGGATTGCTCTCTGTAATGTTCAGCTTCTAA
TTGTCTCTACTTTGTGAGACTACTTTTGAATGCTTGACCTCAAATCAGGTAGGACTACCC
GCTGAACTTAA
>AB5-53
TTTCCGTAGGTGAACCTGCGGAAGGATCATTATTGAATTATGTTTCTAGATAGGTTGTAG
CTGGCTCTTTTAGAGCATGTGCACGCCTGTTTGGACTTCATTTTCATCCACCTGTGCACC
TATTGTAGTCTTTGGTTGGGTTAGGAGGAAGTGATCATTGTATCAGCATCTGCTGGGAGT

GAGGACTTGCATTGTGAAAGCTTTGCTGTCCTTGATGTGATCATGGAATCTTTTTCTCAC
TAGAGTCTATGTCACTCATTATACTCTGTCGAATGTCATTGAATGTCTTTACATGGGCTT
GTATGCCTATGAAAATTGTAATACAACTTTCAGCAACGGATCTCTTGGCTCTCGCATCGA
TGAAGAACGCAGCGAAATGCGATAAGTAATGTGAATTGCAGAATTCAGTGAATCATCGAA
TCTTTGAACGCATCTTGCGCTCCTTGGTATTCCGAGGAGCATGCCTGTTTGAGTGTCATT
AAATTCTCAACTCTCTTATACTTTTTTGTAAAAGAGAGCTTGGACTGTGGAGGCTTGCTG
GCCACTTTTTGGGGTCAGCTCCTCTGAAATGCATTAGCGGAACCGTTTGCGATCTGCCAC
AAGTGTGATAAGTTATCTACACTGGCGAGGGGATTGCTCTCTGTAATGTTCAGCTTCTAA
TTGTCTCTACTTTGTGAGACTACTTTTGAATGCTTGACCTCAAATCAGGTAGGACTACCC
GCTGAACTTAA
>AB5-54
TTTCCGTAGGTGAACCTGCGGAAGGATCATTATTGAATTATGTTTCTAGATAGGTTGTAG
CTGGCTCTTTTAGAGCATGTGCACGCCTGTTTGGACTTCATTTTCATCCACCTGTGCACC
TATTGTAGTCTTTGGTTGGGTTAGGAGGAAGTGATCATTGTATCAGCATCTGCTGGGAGT
GAGGACTTGCATTGTGAAAGCTTTGCTGTCCTTGATGTGATCATGGAATCTTTTTCTCAC
TAGAGTCTATGTCACTCATTATACTCTGTCGAATGTCATTGAATGTCTTTACATGGGCTT
GTATGCCTATGAAAATTGTAATACAACTTTCAGCAACGGATCTCTTGGCTCTCGCATCGA
TGAAGAACGCAGCGAAATGCGATAAGTAATGTGAATTGCAGAATTCAGTGAATCATCGAA
TCTTTGAACGCATCTTGCGCTCCTTGGTATTCCGAGGAGCATGCCTGTTTGAGTGTCATT
AAATTCTCAACTCTCTTATACTTTTTTGTAAAAGAGAGCTTGGACTGTGGAGGCTTGCTG
GCCACTTTTTGGGGTCAGCTCCTCTGAAATGCATTAGCGGAACCGTTTGCGATCTGCCAC
AAGTGTGATAAGTTATCTACACTGGCGAGGGGATTGCTCTCTGTAATGTTCAGCTTCTAA
TTGTCTCTACTTTGTGAGACTACTTTTGAATGCTTGACCTCAAATCAGGTAGGACTACCC
GCTGAACTTAA
>AB5-60
TTTCCGTAGGTGAACCTGCGGAAGGATCATTATTGAATTATGTTTCTAGATAGGTTGTAG
CTGGCTCTTTTAGAGCATGTGCACGCCTGTTTGGACTTCATTTTCATCCACCTGTGCACC
TATTGTAGTCTTTGGTTGGGTTAGGAGGAAGTGATCATTGTATCAGCATCTGCTGGGAGT
GAGGACTTGCATTGTGAAAGCTTTGCTGTCCTTGATGTGATCATGGAATCTTTTTCTCAC
TAGAGTCTATGTCACTCATTATACTCTGTCGAATGTCATTGAATGTCTTTACATGGGCTT
GTATGCCTATGAAAATTGTAATACAACTTTCAGCAACGGATCTCTTGGCTCTCGCATCGA
TGAAGAACGCAGCGAAATGCGATAAGTAATGTGAATTGCAGAATTCAGTGAATCATCGAA
TCTTTGAACGCATCTTGCGCTCCTTGGTATTCCGAGGAGCATGCCTGTTTGAGTGTCATT
AAATTCTCAACTCTCTTATACTTTTTTGTAAAAGAGAGCTTGGACTGTGGAGGCTTGCTG
GCCACTTTTTGGGGTCAGCTCCTCTGAAATGCATTAGCGGAACCGTTTGCGATCTGCCAC
AAGTGTGATAAGTTATCTACACTGGCGAGGGGATTGCTCTCTGTAATGTTCAGCTTCTAA
TTGTCTCTACTTTGTGAGACTACTTTTGAATGCTTGACCTCAAATCAGGTAGGACTACCC
GCTGAACTTAA
>AB5-63
TTTCCGTAGGTGAACCTGCGGAAGGATCATTATTGAATTATGTTTCTAGATAGGTTGTAG
CTGGCTCTTTTAGAGCATGTGCACGCCTGTTTGGACTTCATTTTCATCCACCTGTGCACC
TATTGTAGTCTTTGGTTGGGTTAGGAGGAAGTGATCATTGTATCAGCATCTGCTGGGAGT
GAGGACTTGCATTGTGAAAGCTTTGCTGTCCTTGATGTGATCATGGAATCTTTTTCTCAC
TAGAGTCTATGTCACTCATTATACTCTGTCGAATGTCATTGAATGTCTTTACATGGGCTT
GTATGCCTATGAAAATTGTAATACAACTTTCAGCAACGGATCTCTTGGCTCTCGCATCGA
TGAAGAACGCAGCGAAATGCGATAAGTAATGTGAATTGCAGAATTCAGTGAATCATCGAA
TCTTTGAACGCATCTTGCGCTCCTTGGTATTCCGAGGAGCATGCCTGTTTGAGTGTCATT
AAATTCTCAACTCTCTTATACTTTTTTGTAAAAGAGAGCTTGGACTGTGGAGGCTTGCTG
GCCACTTTTTGGGGTCAGCTCCTCTGAAATGCATTAGCGGAACCGTTTGCGATCTGCCAC
AAGTGTGATAAGTTATCTACACTGGCGAGGGGATTGCTCTCTGTAATGTTCAGCTTCTAA

TTGTCTCTACTTTGTGAGACTACTTTTGAATGCTTGACCTCAAATCAGGTAGGACTACCC
GCTGAACTTAA
>AB5-66
TTTCCGTAGGTGAACCTGCGGAAGGATCATTATTGAATTATGTTTCTAGATAGGTTGTAG
CTGGCTCTTTTAGAGCATGTGCACGCCTGTTTGGACTTCATTTTCATCCACCTGTGCACC
TATTGTAGTCTTTGGTTGGGTTAGGAGGAAGTGATCATTGTATCAGCATCTGCTGGGAGT
GAGGACTTGCATTGTGAAAGCTTTGCTGTCCTTGATGTGATCATGGAATCTTTTTCTCAC
TAGAGTCTATGTCACTCATTATACTCTGTCGAATGTCATTGAATGTCTTTACATGGGCTT
GTATGCCTATGAAAATTGTAATACAACTTTCAGCAACGGATCTCTTGGCTCTCGCATCGA
TGAAGAACGCAGCGAAATGCGATAAGTAATGTGAATTGCAGAATTCAGTGAATCATCGAA
TCTTTGAACGCATCTTGCGCTCCTTGGTATTCCGAGGAGCATGCCTGTTTGAGTGTCATT
AAATTCTCAACTCTCTTATACTTTTTTGTAAAAGAGAGCTTGGACTGTGGAGGCTTGCTG
GCCACTTTTTGGGGTCAGCTCCTCTGAAATGCATTAGCGGAACCGTTTGCGATCTGCCAC
AAGTGTGATAAGTTATCTACACTGGCGAGGGGATTGCTCTCTGTAATGTTCAGCTTCTAA
TTGTCTCTACTTTGTGAGACTACTTTTGAATGCTTGACCTCAAATCAGGTAGGACTACCC
GCTGAACTTAA
>AB5-71
TTTCCGTAGGTGAACCTGCGGAAGGATCATTATTGAATTATGTTTCTAGATAGGTTGTAG
CTGGCTCTTTTAGAGCATGTGCACGCCTGTTTGGACTTCATTTTCATCCACCTGTGCACC
TATTGTAGTCTTTGGTTGGGTTAGGAGGAAGTGATCATTGTATCAGCATCTGCTGGGAGT
GAGGACTTGCATTGTGAAAGCTTTGCTGTCCTTGATGTGATCATGGAATCTTTTTCTCAC
TAGAGTCTATGTCACTCATTATACTCTGTCGAATGTCATTGAATGTCTTTACATGGGCTT
GTATGCCTATGAAAATTGTAATACAACTTTCAGCAACGGATCTCTTGGCTCTCGCATCGA
TGAAGAACGCAGCGAAATGCGATAAGTAATGTGAATTGCAGAATTCAGTGAATCATCGAA
TCTTTGAACGCATCTTGCGCTCCTTGGTATTCCGAGGAGCATGCCTGTTTGAGTGTCATT
AAATTCTCAACTCTCTTATACTTTTTTGTAAAAGAGAGCTTGGACTGTGGAGGCTTGCTG
GCCACTTTTTGGGGTCAGCTCCTCTGAAATGCATTAGCGGAACCGTTTGCGATCTGCCAC
AAGTGTGATAAGTTATCTACACTGGCGAGGGGATTGCTCTCTGTAATGTTCAGCTTCTAA
TTGTCTCTACTTTGTGAGACTACTTTTGAATGCTTGACCTCAAATCAGGTAGGACTACCC
GCTGAACTTAA
>AB5-77
TTTCCGTAGGTGAACCTGCGGAAGGATCATTATTGAATTATGTTTCTAGATAGGTTGTAG
CTGGCTCTTTTAGAGCATGTGCACGCCTGTTTGGACTTCATTTTCATCCACCTGTGCACC
TATTGTAGTCTTTGGTTGGGTTAGGAGGAAGTGATCATTGTATCAGCATCTGCTGGGAGT
GAGGACTTGCATTGTGAAAGCTTTGCTGTCCTTGATGTGATCATGGAATCTTTTTCTCAC
TAGAGTCTATGTCACTCATTATACTCTGTCGAATGTCATTGAATGTCTTTACATGGGCTT
GTATGCCTATGAAAATTGTAATACAACTTTCAGCAACGGATCTCTTGGCTCTCGCATCGA
TGAAGAACGCAGCGAAATGCGATAAGTAATGTGAATTGCAGAATTCAGTGAATCATCGAA
TCTTTGAACGCATCTTGCGCTCCTTGGTATTCCGAGGAGCATGCCTGTTTGAGTGTCATT
AAATTCTCAACTCTCTTATACTTTTTTGTAAAAGAGAGCTTGGACTGTGGAGGCTTGCTG
GCCACTTTTTGGGGTCAGCTCCTCTGAAATGCATTAGCGGAACCGTTTGCGATCTGCCAC
AAGTGTGATAAGTTATCTACACTGGCGAGGGGATTGCTCTCTGTAATGTTCAGCTTCTAA
TTGTCTCTACTTTGTGAGACTACTTTTGAATGCTTGACCTCAAATCAGGTAGGACTACCC
GCTGAACTTAA
>AB5-78
TTTCCGTAGGTGAACCTGCGGAAGGATCATTATTGAATTATGTTTCTAGATAGGTTGTAG
CTGGCTCTTTTAGAGCATGTGCACGCCTGTTTGGACTTCATTTTCATCCACCTGTGCACC
TATTGTAGTCTTTGGTTGGGTTAGGAGGAAGTGATCATTGTATCAGCATCTGCTGGGAGT
GAGGACTTGCATTGTGAAAGCTTTGCTGTCCTTGATGTGATCATGGAATCTTTTTCTCAC
TAGAGTCTATGTCACTCATTATACTCTGTCGAATGTCATTGAATGTCTTTACATGGGCTT

GTATGCCTATGAAAATTGTAATACAACTTTCAGCAACGGATCTCTTGGCTCTCGCATCGA
TGAAGAACGCAGCGAAATGCGATAAGTAATGTGAATTGCAGAATTCAGTGAATCATCGAA
TCTTTGAACGCATCTTGCGCTCCTTGGTATTCCGAGGAGCATGCCTGTTTGAGTGTCATT
AAATTCTCAACTCTCTTATACTTTTTTGTAAAAGAGAGCTTGGACTGTGGAGGCTTGCTG
GCCACTTTTTGGGGTCAGCTCCTCTGAAATGCATTAGCGGAACCGTTTGCGATCTGCCAC
AAGTGTGATAAGTTATCTACACTGGCGAGGGGATTGCTCTCTGTAATGTTCAGCTTCTAA
TTGTCTCTACTTTGTGAGACTACTTTTGAATGCTTGACCTCAAATCAGGTAGGACTACCC
GCTGAACTTAA
>AB5-80
TTTCCGTAGGTGAACCTGCGGAAGGATCATTATTGAATTATGTTTCTAGATAGGTTGTAG
CTGGCTCTTTTAGAGCATGTGCACGCCTGTTTGGACTTCATTTTCATCCACCTGTGCACC
TATTGTAGTCTTTGGTTGGGTTAGGAGGAAGTGATCATTGTATCAGCATCTGCTGGGAGT
GAGGACTTGCATTGTGAAAGCTTTGCTGTCCTTGATGTGATCATGGAATCTTTTTCTCAC
TAGAGTCTATGTCACTCATTATACTCTGTCGAATGTCATTGAATGTCTTTACATGGGCTT
GTATGCCTATGAAAATTGTAATACAACTTTCAGCAACGGATCTCTTGGCTCTCGCATCGA
TGAAGAACGCAGCGAAATGCGATAAGTAATGTGAATTGCAGAATTCAGTGAATCATCGAA
TCTTTGAACGCATCTTGCGCTCCTTGGTATTCCGAGGAGCATGCCTGTTTGAGTGTCATT
AAATTCTCAACTCTCTTATACTTTTTTGTAAAAGAGAGCTTGGACTGTGGAGGCTTGCTG
GCCACTTTTTGGGGTCAGCTCCTCTGAAATGCATTAGCGGAACCGTTTGCGATCTGCCAC
AAGTGTGATAAGTTATCTACACTGGCGAGGGGATTGCTCTCTGTAATGTTCAGCTTCTAA
TTGTCTCTACTTTGTGAGACTACTTTTGAATGCTTGACCTCAAATCAGGTAGGACTACCC
GCTGAACTTAA
>AB5-82
TTTCCGTAGGTGAACCTGCGGAAGGATCATTATTGAATTATGTTTCTAGATAGGTTGTAG
CTGGCTCTTTTAGAGCATGTGCACGCCTGTTTGGACTTCATTTTCATCCACCTGTGCACC
TATTGTAGTCTTTGGTTGGGTTAGGAGGAAGTGATCATTGTATCAGCATCTGCTGGGAGT
GAGGACTTGCATTGTGAAAGCTTTGCTGTCCTTGATGTGATCATGGAATCTTTTTCTCAC
TAGAGTCTATGTCACTCATTATACTCTGTCGAATGTCATTGAATGTCTTTACATGGGCTT
GTATGCCTATGAAAATTGTAATACAACTTTCAGCAACGGATCTCTTGGCTCTCGCATCGA
TGAAGAACGCAGCGAAATGCGATAAGTAATGTGAATTGCAGAATTCAGTGAATCATCGAA
TCTTTGAACGCATCTTGCGCTCCTTGGTATTCCGAGGAGCATGCCTGTTTGAGTGTCATT
AAATTCTCAACTCTCTTATACTTTTTTGTAAAAGAGAGCTTGGACTGTGGAGGCTTGCTG
GCCACTTTTTGGGGTCAGCTCCTCTGAAATGCATTAGCGGAACCGTTTGCGATCTGCCAC
AAGTGTGATAAGTTATCTACACTGGCGAGGGGATTGCTCTCTGTAATGTTCAGCTTCTAA
TTGTCTCTACTTTGTGAGACTACTTTTGAATGCTTGACCTCAAATCAGGTAGGACTACCC
GCTGAACTTAA
>AB5-98
TTTCCGTAGGTGAACCTGCGGAAGGATCATTATTGAATTATGTTTCTAGATAGGTTGTAG
CTGGCTCTTTTAGAGCATGTGCACGCCTGTTTGGACTTCATTTTCATCCACCTGTGCACC
TATTGTAGTCTTTGGTTGGGTTAGGAGGAAGTGATCATTGTATCAGCATCTGCTGGGAGT
GAGGACTTGCATTGTGAAAGCTTTGCTGTCCTTGATGTGATCATGGAATCTTTTTCTCAC
TAGAGTCTATGTCACTCATTATACTCTGTCGAATGTCATTGAATGTCTTTACATGGGCTT
GTATGCCTATGAAAATTGTAATACAACTTTCAGCAACGGATCTCTTGGCTCTCGCATCGA
TGAAGAACGCAGCGAAATGCGATAAGTAATGTGAATTGCAGAATTCAGTGAATCATCGAA
TCTTTGAACGCATCTTGCGCTCCTTGGTATTCCGAGGAGCATGCCTGTTTGAGTGTCATT
AAATTCTCAACTCTCTTATACTTTTTTGTAAAAGAGAGCTTGGACTGTGGAGGCTTGCTG
GCCACTTTTTGGGGTCAGCTCCTCTGAAATGCATTAGCGGAACCGTTTGCGATCTGCCAC
AAGTGTGATAAGTTATCTACACTGGCGAGGGGATTGCTCTCTGTAATGTTCAGCTTCTAA
TTGTCTCTACTTTGTGAGACTACTTTTGAATGCTTGACCTCAAATCAGGTAGGACTACCC
GCTGAACTTAA

>AB5-101
TTTCCGTAGGTGAACCTGCGGAAGGATCATTATTGAATTATGTTTCTAGATAGGTTGTAG
CTGGCTCTTTTAGAGCATGTGCACGCCTGTTTGGACTTCATTTTCATCCACCTGTGCACC
TATTGTAGTCTTTGGTTGGGTTAGGAGGAAGTGATCATTGTATCAGCATCTGCTGGGAGT
GAGGACTTGCATTGTGAAAGCTTTGCTGTCCTTGATGTGATCATGGAATCTTTTTCTCAC
TAGAGTCTATGTCACTCATTATACTCTGTCGAATGTCATTGAATGTCTTTACATGGGCTT
GTATGCCTATGAAAATTGTAATACAACTTTCAGCAACGGATCTCTTGGCTCTCGCATCGA
TGAAGAACGCAGCGAAATGCGATAAGTAATGTGAATTGCAGAATTCAGTGAATCATCGAA
TCTTTGAACGCATCTTGCGCTCCTTGGTATTCCGAGGAGCATGCCTGTTTGAGTGTCATT
AAATTCTCAACTCTCTTATACTTTTTTGTAAAAGAGAGCTTGGACTGTGGAGGCTTGCTG
GCCACTTTTTGGGGTCAGCTCCTCTGAAATGCATTAGCGGAACCGTTTGCGATCTGCCAC
AAGTGTGATAAGTTATCTACACTGGCGAGGGGATTGCTCTCTGTAATGTTCAGCTTCTAA
TTGTCTCTACTTTGTGAGACTACTTTTGAATGCTTGACCTCAAATCAGGTAGGACTACCC
GCTGAACTTAA
>AB6-5
TTTCCGTAGGTGAACCTGCGGAAGGATCATTATTGAATTATGTTTCTAGATAGGTTGTAG
CTGGCTCTTTTAGAGCATGTGCACGCCTGTTTGGACTTCATTTTCATCCACCTGTGCACC
TATTGTAGTCTTTGGTTGGGTTAGGAGGAAGTGATCATTGTATCAGCATCTGCTGGGAGT
GAGGACTTGCATTGTGAAAGCTTTGCTGTCCTTGATGTGATCATGGAATCTTTTTCTCAC
TAGAGTCTATGTCACTCATTATACTCTGTCGAATGTCATTGAATGTCTTTACATGGGCTT
GTATGCCTATGAAAATTGTAATACAACTTTCAGCAACGGATCTCTTGGCTCTCGCATCGA
TGAAGAACGCAGCGAAATGCGATAAGTAATGTGAATTGCAGAATTCAGTGAATCATCGAA
TCTTTGAACGCATCTTGCGCTCCTTGGTATTCCGAGGAGCATGCCTGTTTGAGTGTCATT
AAATTCTCAACTCTCTTATACTTTTTTGTAAAAGAGAGCTTGGACTGTGGAGGCTTGCTG
GCCACTTTTTGGGGTCAGCTCCTCTGAAATGCATTAGCGGAACCGTTTGCGATCTGCCAC
AAGTGTGATAAGTTATCTACACTGGCGAGGGGATTGCTCTCTGTAATGTTCAGCTTCTAA
TTGTCTCTACTTTGTGAGACTACTTTTGAATGCTTGACCTCAAATCAGGTAGGACTACCC
GCTGAACTTAA
>AB6-17
TTTCCGTAGGTGAACCTGCGGAAGGATCATTATTGAATTATGTTTCTAGATAGGTTGTAG
CTGGCTCTTTTAGAGCATGTGCACGCCTGTTTGGACTTCATTTTCATCCACCTGTGCACC
TATTGTAGTCTTTGGTTGGGTTAGGAGGAAGTGATCATTGTATCAGCATCTGCTGGGAGT
GAGGACTTGCATTGTGAAAGCTTTGCTGTCCTTGATGTGATCATGGAATCTTTTTCTCAC
TAGAGTCTATGTCACTCATTATACTCTGTCGAATGTCATTGAATGTCTTTACATGGGCTT
GTATGCCTATGAAAATTGTAATACAACTTTCAGCAACGGATCTCTTGGCTCTCGCATCGA
TGAAGAACGCAGCGAAATGCGATAAGTAATGTGAATTGCAGAATTCAGTGAATCATCGAA
TCTTTGAACGCATCTTGCGCTCCTTGGTATTCCGAGGAGCATGCCTGTTTGAGTGTCATT
AAATTCTCAACTCTCTTATACTTTTTTGTAAAAGAGAGCTTGGACTGTGGAGGCTTGCTG
GCCACTTTTTGGGGTCAGCTCCTCTGAAATGCATTAGCGGAACCGTTTGCGATCTGCCAC
AAGTGTGATAAGTTATCTACACTGGCGAGGGGATTGCTCTCTGTAATGTTCAGCTTCTAA
TTGTCTCTACTTTGTGAGACTACTTTTGAATGCTTGACCTCAAATCAGGTAGGACTACCC
GCTGAACTTAA
>AB6-22
TTTCCGTAGGTGAACCTGCGGAAGGATCATTATTGAATTATGTTTCTAGATAGGTTGTAG
CTGGCTCTTTTAGAGCATGTGCACGCCTGTTTGGACTTCATTTTCATCCACCTGTGCACC
TATTGTAGTCTTTGGTTGGGTTAGGAGGAAGTGATCATTGTATCAGCATCTGCTGGGAGT
GAGGACTTGCATTGTGAAAGCTTTGCTGTCCTTGATGTGATCATGGAATCTTTTTCTCAC
TAGAGTCTATGTCACTCATTATACTCTGTCGAATGTCATTGAATGTCTTTACATGGGCTT
GTATGCCTATGAAAATTGTAATACAACTTTCAGCAACGGATCTCTTGGCTCTCGCATCGA
TGAAGAACGCAGCGAAATGCGATAAGTAATGTGAATTGCAGAATTCAGTGAATCATCGAA

TCTTTGAACGCATCTTGCGCTCCTTGGTATTCCGAGGAGCATGCCTGTTTGAGTGTCATT
AAATTCTCAACTCTCTTATACTTTTTTGTAAAAGAGAGCTTGGACTGTGGAGGCTTGCTG
GCCACTTTTTGGGGTCAGCTCCTCTGAAATGCATTAGCGGAACCGTTTGCGATCTGCCAC
AAGTGTGATAAGTTATCTACACTGGCGAGGGGATTGCTCTCTGTAATGTTCAGCTTCTAA
TTGTCTCTACTTTGTGAGACTACTTTTGAATGCTTGACCTCAAATCAGGTAGGACTACCC
GCTGAACTTAA
>AB6-23
TTTCCGTAGGTGAACCTGCGGAAGGATCATTATTGAATTATGTTTCTAGATAGGTTGTAG
CTGGCTCTTTTAGAGCATGTGCACGCCTGTTTGGACTTCATTTTCATCCACCTGTGCACC
TATTGTAGTCTTTGGTTGGGTTAGGAGGAAGTGATCATTGTATCAGCATCTGCTGGGAGT
GAGGACTTGCATTGTGAAAGCTTTGCTGTCCTTGATGTGATCATGGAATCTTTTTCTCAC
TAGAGTCTATGTCACTCATTATACTCTGTCGAATGTCATTGAATGTCTTTACATGGGCTT
GTATGCCTATGAAAATTGTAATACAACTTTCAGCAACGGATCTCTTGGCTCTCGCATCGA
TGAAGAACGCAGCGAAATGCGATAAGTAATGTGAATTGCAGAATTCAGTGAATCATCGAA
TCTTTGAACGCATCTTGCGCTCCTTGGTATTCCGAGGAGCATGCCTGTTTGAGTGTCATT
AAATTCTCAACTCTCTTATACTTTTTTGTAAAAGAGAGCTTGGACTGTGGAGGCTTGCTG
GCCACTTTTTGGGGTCAGCTCCTCTGAAATGCATTAGCGGAACCGTTTGCGATCTGCCAC
AAGTGTGATAAGTTATCTACACTGGCGAGGGGATTGCTCTCTGTAATGTTCAGCTTCTAA
TTGTCTCTACTTTGTGAGACTACTTTTGAATGCTTGACCTCAAATCAGGTAGGACTACCC
GCTGAACTTAA
>AB6-25
TTTCCGTAGGTGAACCTGCGGAAGGATCATTATTGAATTATGTTTCTAGATAGGTTGTAG
CTGGCTCTTTTAGAGCATGTGCACGCCTGTTTGGACTTCATTTTCATCCACCTGTGCACC
TATTGTAGTCTTTGGTTGGGTTAGGAGGAAGTGATCATTGTATCAGCATCTGCTGGGAGT
GAGGACTTGCATTGTGAAAGCTTTGCTGTCCTTGATGTGATCATGGAATCTTTTTCTCAC
TAGAGTCTATGTCACTCATTATACTCTGTCGAATGTCATTGAATGTCTTTACATGGGCTT
GTATGCCTATGAAAATTGTAATACAACTTTCAGCAACGGATCTCTTGGCTCTCGCATCGA
TGAAGAACGCAGCGAAATGCGATAAGTAATGTGAATTGCAGAATTCAGTGAATCATCGAA
TCTTTGAACGCATCTTGCGCTCCTTGGTATTCCGAGGAGCATGCCTGTTTGAGTGTCATT
AAATTCTCAACTCTCTTATACTTTTTTGTAAAAGAGAGCTTGGACTGTGGAGGCTTGCTG
GCCACTTTTTGGGGTCAGCTCCTCTGAAATGCATTAGCGGAACCGTTTGCGATCTGCCAC
AAGTGTGATAAGTTATCTACACTGGCGAGGGGATTGCTCTCTGTAATGTTCAGCTTCTAA
TTGTCTCTACTTTGTGAGACTACTTTTGAATGCTTGACCTCAAATCAGGTAGGACTACCC
GCTGAACTTAA
>AB6-28
TTTCCGTAGGTGAACCTGCGGAAGGATCATTATTGAATTATGTTTCTAGATAGGTTGTAG
CTGGCTCTTTTAGAGCATGTGCACGCCTGTTTGGACTTCATTTTCATCCACCTGTGCACC
TATTGTAGTCTTTGGTTGGGTTAGGAGGAAGTGATCATTGTATCAGCATCTGCTGGGAGT
GAGGACTTGCATTGTGAAAGCTTTGCTGTCCTTGATGTGATCATGGAATCTTTTTCTCAC
TAGAGTCTATGTCACTCATTATACTCTGTCGAATGTCATTGAATGTCTTTACATGGGCTT
GTATGCCTATGAAAATTGTAATACAACTTTCAGCAACGGATCTCTTGGCTCTCGCATCGA
TGAAGAACGCAGCGAAATGCGATAAGTAATGTGAATTGCAGAATTCAGTGAATCATCGAA
TCTTTGAACGCATCTTGCGCTCCTTGGTATTCCGAGGAGCATGCCTGTTTGAGTGTCATT
AAATTCTCAACTCTCTTATACTTTTTTGTAAAAGAGAGCTTGGACTGTGGAGGCTTGCTG
GCCACTTTTTGGGGTCAGCTCCTCTGAAATGCATTAGCGGAACCGTTTGCGATCTGCCAC
AAGTGTGATAAGTTATCTACACTGGCGAGGGGATTGCTCTCTGTAATGTTCAGCTTCTAA
TTGTCTCTACTTTGTGAGACTACTTTTGAATGCTTGACCTCAAATCAGGTAGGACTACCC
GCTGAACTTAA
>AB6-30
TTTCCGTAGGTGAACCTGCGGAAGGATCATTATTGAATTATGTTTCTAGATAGGTTGTAG

CTGGCTCTTTTAGAGCATGTGCACGCCTGTTTGGACTTCATTTTCATCCACCTGTGCACC
TATTGTAGTCTTTGGTTGGGTTAGGAGGAAGTGATCATTGTATCAGCATCTGCTGGGAGT
GAGGACTTGCATTGTGAAAGCTTTGCTGTCCTTGATGTGATCATGGAATCTTTTTCTCAC
TAGAGTCTATGTCACTCATTATACTCTGTCGAATGTCATTGAATGTCTTTACATGGGCTT
GTATGCCTATGAAAATTGTAATACAACTTTCAGCAACGGATCTCTTGGCTCTCGCATCGA
TGAAGAACGCAGCGAAATGCGATAAGTAATGTGAATTGCAGAATTCAGTGAATCATCGAA
TCTTTGAACGCATCTTGCGCTCCTTGGTATTCCGAGGAGCATGCCTGTTTGAGTGTCATT
AAATTCTCAACTCTCTTATACTTTTTTGTAAAAGAGAGCTTGGACTGTGGAGGCTTGCTG
GCCACTTTTTGGGGTCAGCTCCTCTGAAATGCATTAGCGGAACCGTTTGCGATCTGCCAC
AAGTGTGATAAGTTATCTACACTGGCGAGGGGATTGCTCTCTGTAATGTTCAGCTTCTAA
TTGTCTCTACTTTGTGAGACTACTTTTGAATGCTTGACCTCAAATCAGGTAGGACTACCC
GCTGAACTTAA
>AB6-31
TTTCCGTAGGTGAACCTGCGGAAGGATCATTATTGAATTATGTTTCTAGATAGGTTGTAG
CTGGCTCTTTTAGAGCATGTGCACGCCTGTTTGGACTTCATTTTCATCCACCTGTGCACC
TATTGTAGTCTTTGGTTGGGTTAGGAGGAAGTGATCATTGTATCAGCATCTGCTGGGAGT
GAGGACTTGCATTGTGAAAGCTTTGCTGTCCTTGATGTGATCATGGAATCTTTTTCTCAC
TAGAGTCTATGTCACTCATTATACTCTGTCGAATGTCATTGAATGTCTTTACATGGGCTT
GTATGCCTATGAAAATTGTAATACAACTTTCAGCAACGGATCTCTTGGCTCTCGCATCGA
TGAAGAACGCAGCGAAATGCGATAAGTAATGTGAATTGCAGAATTCAGTGAATCATCGAA
TCTTTGAACGCATCTTGCGCTCCTTGGTATTCCGAGGAGCATGCCTGTTTGAGTGTCATT
AAATTCTCAACTCTCTTATACTTTTTTGTAAAAGAGAGCTTGGACTGTGGAGGCTTGCTG
GCCACTTTTTGGGGTCAGCTCCTCTGAAATGCATTAGCGGAACCGTTTGCGATCTGCCAC
AAGTGTGATAAGTTATCTACACTGGCGAGGGGATTGCTCTCTGTAATGTTCAGCTTCTAA
TTGTCTCTACTTTGTGAGACTACTTTTGAATGCTTGACCTCAAATCAGGTAGGACTACCC
GCTGAACTTAA
>AB6-33
TTTCCGTAGGTGAACCTGCGGAAGGATCATTATTGAATTATGTTTCTAGATAGGTTGTAG
CTGGCTCTTTTAGAGCATGTGCACGCCTGTTTGGACTTCATTTTCATCCACCTGTGCACC
TATTGTAGTCTTTGGTTGGGTTAGGAGGAAGTGATCATTGTATCAGCATCTGCTGGGAGT
GAGGACTTGCATTGTGAAAGCTTTGCTGTCCTTGATGTGATCATGGAATCTTTTTCTCAC
TAGAGTCTATGTCACTCATTATACTCTGTCGAATGTCATTGAATGTCTTTACATGGGCTT
GTATGCCTATGAAAATTGTAATACAACTTTCAGCAACGGATCTCTTGGCTCTCGCATCGA
TGAAGAACGCAGCGAAATGCGATAAGTAATGTGAATTGCAGAATTCAGTGAATCATCGAA
TCTTTGAACGCATCTTGCGCTCCTTGGTATTCCGAGGAGCATGCCTGTTTGAGTGTCATT
AAATTCTCAACTCTCTTATACTTTTTTGTAAAAGAGAGCTTGGACTGTGGAGGCTTGCTG
GCCACTTTTTGGGGTCAGCTCCTCTGAAATGCATTAGCGGAACCGTTTGCGATCTGCCAC
AAGTGTGATAAGTTATCTACACTGGCGAGGGGATTGCTCTCTGTAATGTTCAGCTTCTAA
TTGTCTCTACTTTGTGAGACTACTTTTGAATGCTTGACCTCAAATCAGGTAGGACTACCC
GCTGAACTTAA
>AB6-35
TTTCCGTAGGTGAACCTGCGGAAGGATCATTATTGAATTATGTTTCTAGATAGGTTGTAG
CTGGCTCTTTTAGAGCATGTGCACGCCTGTTTGGACTTCATTTTCATCCACCTGTGCACC
TATTGTAGTCTTTGGTTGGGTTAGGAGGAAGTGATCATTGTATCAGCATCTGCTGGGAGT
GAGGACTTGCATTGTGAAAGCTTTGCTGTCCTTGATGTGATCATGGAATCTTTTTCTCAC
TAGAGTCTATGTCACTCATTATACTCTGTCGAATGTCATTGAATGTCTTTACATGGGCTT
GTATGCCTATGAAAATTGTAATACAACTTTCAGCAACGGATCTCTTGGCTCTCGCATCGA
TGAAGAACGCAGCGAAATGCGATAAGTAATGTGAATTGCAGAATTCAGTGAATCATCGAA
TCTTTGAACGCATCTTGCGCTCCTTGGTATTCCGAGGAGCATGCCTGTTTGAGTGTCATT
AAATTCTCAACTCTCTTATACTTTTTTGTAAAAGAGAGCTTGGACTGTGGAGGCTTGCTG

GCCACTTTTTGGGGTCAGCTCCTCTGAAATGCATTAGCGGAACCGTTTGCGATCTGCCAC
AAGTGTGATAAGTTATCTACACTGGCGAGGGGATTGCTCTCTGTAATGTTCAGCTTCTAA
TTGTCTCTACTTTGTGAGACTACTTTTGAATGCTTGACCTCAAATCAGGTAGGACTACCC
GCTGAACTTAA
>AB6-36
TTTCCGTAGGTGAACCTGCGGAAGGATCATTATTGAATTATGTTTCTAGATAGGTTGTAG
CTGGCTCTTTTAGAGCATGTGCACGCCTGTTTGGACTTCATTTTCATCCACCTGTGCACC
TATTGTAGTCTTTGGTTGGGTTAGGAGGAAGTGATCATTGTATCAGCATCTGCTGGGAGT
GAGGACTTGCATTGTGAAAGCTTTGCTGTCCTTGATGTGATCATGGAATCTTTTTCTCAC
TAGAGTCTATGTCACTCATTATACTCTGTCGAATGTCATTGAATGTCTTTACATGGGCTT
GTATGCCTATGAAAATTGTAATACAACTTTCAGCAACGGATCTCTTGGCTCTCGCATCGA
TGAAGAACGCAGCGAAATGCGATAAGTAATGTGAATTGCAGAATTCAGTGAATCATCGAA
TCTTTGAACGCATCTTGCGCTCCTTGGTATTCCGAGGAGCATGCCTGTTTGAGTGTCATT
AAATTCTCAACTCTCTTATACTTTTTTGTAAAAGAGAGCTTGGACTGTGGAGGCTTGCTG
GCCACTTTTTGGGGTCAGCTCCTCTGAAATGCATTAGCGGAACCGTTTGCGATCTGCCAC
AAGTGTGATAAGTTATCTACACTGGCGAGGGGATTGCTCTCTGTAATGTTCAGCTTCTAA
TTGTCTCTACTTTGTGAGACTACTTTTGAATGCTTGACCTCAAATCAGGTAGGACTACCC
GCTGAACTTAA
>AB6-38
TTTCCGTAGGTGAACCTGCGGAAGGATCATTATTGAATTATGTTTCTAGATAGGTTGTAG
CTGGCTCTTTTAGAGCATGTGCACGCCTGTTTGGACTTCATTTTCATCCACCTGTGCACC
TATTGTAGTCTTTGGTTGGGTTAGGAGGAAGTGATCATTGTATCAGCATCTGCTGGGAGT
GAGGACTTGCATTGTGAAAGCTTTGCTGTCCTTGATGTGATCATGGAATCTTTTTCTCAC
TAGAGTCTATGTCACTCATTATACTCTGTCGAATGTCATTGAATGTCTTTACATGGGCTT
GTATGCCTATGAAAATTGTAATACAACTTTCAGCAACGGATCTCTTGGCTCTCGCATCGA
TGAAGAACGCAGCGAAATGCGATAAGTAATGTGAATTGCAGAATTCAGTGAATCATCGAA
TCTTTGAACGCATCTTGCGCTCCTTGGTATTCCGAGGAGCATGCCTGTTTGAGTGTCATT
AAATTCTCAACTCTCTTATACTTTTTTGTAAAAGAGAGCTTGGACTGTGGAGGCTTGCTG
GCCACTTTTTGGGGTCAGCTCCTCTGAAATGCATTAGCGGAACCGTTTGCGATCTGCCAC
AAGTGTGATAAGTTATCTACACTGGCGAGGGGATTGCTCTCTGTAATGTTCAGCTTCTAA
TTGTCTCTACTTTGTGAGACTACTTTTGAATGCTTGACCTCAAATCAGGTAGGACTACCC
GCTGAACTTAA
>AB6-40
TTTCCGTAGGTGAACCTGCGGAAGGATCATTATTGAATTATGTTTCTAGATAGGTTGTAG
CTGGCTCTTTTAGAGCATGTGCACGCCTGTTTGGACTTCATTTTCATCCACCTGTGCACC
TATTGTAGTCTTTGGTTGGGTTAGGAGGAAGTGATCATTGTATCAGCATCTGCTGGGAGT
GAGGACTTGCATTGTGAAAGCTTTGCTGTCCTTGATGTGATCATGGAATCTTTTTCTCAC
TAGAGTCTATGTCACTCATTATACTCTGTCGAATGTCATTGAATGTCTTTACATGGGCTT
GTATGCCTATGAAAATTGTAATACAACTTTCAGCAACGGATCTCTTGGCTCTCGCATCGA
TGAAGAACGCAGCGAAATGCGATAAGTAATGTGAATTGCAGAATTCAGTGAATCATCGAA
TCTTTGAACGCATCTTGCGCTCCTTGGTATTCCGAGGAGCATGCCTGTTTGAGTGTCATT
AAATTCTCAACTCTCTTATACTTTTTTGTAAAAGAGAGCTTGGACTGTGGAGGCTTGCTG
GCCACTTTTTGGGGTCAGCTCCTCTGAAATGCATTAGCGGAACCGTTTGCGATCTGCCAC
AAGTGTGATAAGTTATCTACACTGGCGAGGGGATTGCTCTCTGTAATGTTCAGCTTCTAA
TTGTCTCTACTTTGTGAGACTACTTTTGAATGCTTGACCTCAAATCAGGTAGGACTACCC
GCTGAACTTAA
>AB6-49
TTTCCGTAGGTGAACCTGCGGAAGGATCATTATTGAATTATGTTTCTAGATAGGTTGTAG
CTGGCTCTTTTAGAGCATGTGCACGCCTGTTTGGACTTCATTTTCATCCACCTGTGCACC
TATTGTAGTCTTTGGTTGGGTTAGGAGGAAGTGATCATTGTATCAGCATCTGCTGGGAGT

GAGGACTTGCATTGTGAAAGCTTTGCTGTCCTTGATGTGATCATGGAATCTTTTTCTCAC
TAGAGTCTATGTCACTCATTATACTCTGTCGAATGTCATTGAATGTCTTTACATGGGCTT
GTATGCCTATGAAAATTGTAATACAACTTTCAGCAACGGATCTCTTGGCTCTCGCATCGA
TGAAGAACGCAGCGAAATGCGATAAGTAATGTGAATTGCAGAATTCAGTGAATCATCGAA
TCTTTGAACGCATCTTGCGCTCCTTGGTATTCCGAGGAGCATGCCTGTTTGAGTGTCATT
AAATTCTCAACTCTCTTATACTTTTTTGTAAAAGAGAGCTTGGACTGTGGAGGCTTGCTG
GCCACTTTTTGGGGTCAGCTCCTCTGAAATGCATTAGCGGAACCGTTTGCGATCTGCCAC
AAGTGTGATAAGTTATCTACACTGGCGAGGGGATTGCTCTCTGTAATGTTCAGCTTCTAA
TTGTCTCTACTTTGTGAGACTACTTTTGAATGCTTGACCTCAAATCAGGTAGGACTACCC
GCTGAACTTAA
>AB7-1
TTTCCGTAGGTGAACCTGCGGAAGGATCATTATTGAATTATGTTTCTAGATAGGTTGTAG
CTGGCTCTTTTAGAGCATGTGCACGCCTGTTTGGACTTCATTTTCATCCACCTGTGCACC
TATTGTAGTCTTTGGTTGGGTTAGGAGGAAGTGATCATTGTATCAGCATCTGCTGGGAGT
GAGGACTTGCATTGTGAAAGCTTTGCTGTCCTTGATGTGATCATGGAATCTTTTTCTCAC
TAGAGTCTATGTCACTCATTATACTCTGTCGAATGTCATTGAATGTCTTTACATGGGCTT
GTATGCCTATGAAAATTGTAATACAACTTTCAGCAACGGATCTCTTGGCTCTCGCATCGA
TGAAGAACGCAGCGAAATGCGATAAGTAATGTGAATTGCAGAATTCAGTGAATCATCGAA
TCTTTGAACGCATCTTGCGCTCCTTGGTATTCCGAGGAGCATGCCTGTTTGAGTGTCATT
AAATTCTCAACTCTCTTATACTTTTTTGTAAAAGAGAGCTTGGACTGTGGAGGCTTGCTG
GCCACTTTTTGGGGTCAGCTCCTCTGAAATGCATTAGCGGAACCGTTTGCGATCTGCCAC
AAGTGTGATAAGTTATCTACACTGGCGAGGGGATTGCTCTCTGTAATGTTCAGCTTCTAA
TTGTCTCTACTTTGTGAGACTACTTTTGAATGCTTGACCTCAAATCAGGTAGGACTACCC
GCTGAACTTAA
>AB7-4
TTTCCGTAGGTGAACCTGCGGAAGGATCATTATTGAATTATGTTTCTAGATAGGTTGTAG
CTGGCTCTTTTAGAGCATGTGCACGCCTGTTTGGACTTCATTTTCATCCACCTGTGCACC
TATTGTAGTCTTTGGTTGGGTTAGGAGGAAGTGATCATTGTATCAGCATCTGCTGGGAGT
GAGGACTTGCATTGTGAAAGCTTTGCTGTCCTTGATGTGATCATGGAATCTTTTTCTCAC
TAGAGTCTATGTCACTCATTATACTCTGTCGAATGTCATTGAATGTCTTTACATGGGCTT
GTATGCCTATGAAAATTGTAATACAACTTTCAGCAACGGATCTCTTGGCTCTCGCATCGA
TGAAGAACGCAGCGAAATGCGATAAGTAATGTGAATTGCAGAATTCAGTGAATCATCGAA
TCTTTGAACGCATCTTGCGCTCCTTGGTATTCCGAGGAGCATGCCTGTTTGAGTGTCATT
AAATTCTCAACTCTCTTATACTTTTTTGTAAAAGAGAGCTTGGACTGTGGAGGCTTGCTG
GCCACTTTTTGGGGTCAGCTCCTCTGAAATGCATTAGCGGAACCGTTTGCGATCTGCCAC
AAGTGTGATAAGTTATCTACACTGGCGAGGGGATTGCTCTCTGTAATGTTCAGCTTCTAA
TTGTCTCTACTTTGTGAGACTACTTTTGAATGCTTGACCTCAAATCAGGTAGGACTACCC
GCTGAACTTAA
>AB7-6
TTTCCGTAGGTGAACCTGCGGAAGGATCATTATTGAATTATGTTTCTAGATAGGTTGTAG
CTGGCTCTTTTAGAGCATGTGCACGCCTGTTTGGACTTCATTTTCATCCACCTGTGCACC
TATTGTAGTCTTTGGTTGGGTTAGGAGGAAGTGATCATTGTATCAGCATCTGCTGGGAGT
GAGGACTTGCATTGTGAAAGCTTTGCTGTCCTTGATGTGATCATGGAATCTTTTTCTCAC
TAGAGTCTATGTCACTCATTATACTCTGTCGAATGTCATTGAATGTCTTTACATGGGCTT
GTATGCCTATGAAAATTGTAATACAACTTTCAGCAACGGATCTCTTGGCTCTCGCATCGA
TGAAGAACGCAGCGAAATGCGATAAGTAATGTGAATTGCAGAATTCAGTGAATCATCGAA
TCTTTGAACGCATCTTGCGCTCCTTGGTATTCCGAGGAGCATGCCTGTTTGAGTGTCATT
AAATTCTCAACTCTCTTATACTTTTTTGTAAAAGAGAGCTTGGACTGTGGAGGCTTGCTG
GCCACTTTTTGGGGTCAGCTCCTCTGAAATGCATTAGCGGAACCGTTTGCGATCTGCCAC
AAGTGTGATAAGTTATCTACACTGGCGAGGGGATTGCTCTCTGTAATGTTCAGCTTCTAA

TTGTCTCTACTTTGTGAGACTACTTTTGAATGCTTGACCTCAAATCAGGTAGGACTACCC
GCTGAACTTAA
>AB7-11
TTTCCGTAGGTGAACCTGCGGAAGGATCATTATTGAATTATGTTTCTAGATAGGTTGTAG
CTGGCTCTTTTAGAGCATGTGCACGCCTGTTTGGACTTCATTTTCATCCACCTGTGCACC
TATTGTAGTCTTTGGTTGGGTTAGGAGGAAGTGATCATTGTATCAGCATCTGCTGGGAGT
GAGGACTTGCATTGTGAAAGCTTTGCTGTCCTTGATGTGATCATGGAATCTTTTTCTCAC
TAGAGTCTATGTCACTCATTATACTCTGTCGAATGTCATTGAATGTCTTTACATGGGCTT
GTATGCCTATGAAAATTGTAATACAACTTTCAGCAACGGATCTCTTGGCTCTCGCATCGA
TGAAGAACGCAGCGAAATGCGATAAGTAATGTGAATTGCAGAATTCAGTGAATCATCGAA
TCTTTGAACGCATCTTGCGCTCCTTGGTATTCCGAGGAGCATGCCTGTTTGAGTGTCATT
AAATTCTCAACTCTCTTATACTTTTTTGTAAAAGAGAGCTTGGACTGTGGAGGCTTGCTG
GCCACTTTTTGGGGTCAGCTCCTCTGAAATGCATTAGCGGAACCGTTTGCGATCTGCCAC
AAGTGTGATAAGTTATCTACACTGGCGAGGGGATTGCTCTCTGTAATGTTCAGCTTCTAA
TTGTCTCTACTTTGTGAGACTACTTTTGAATGCTTGACCTCAAATCAGGTAGGACTACCC
GCTGAACTTAA
>AB7-19
TTTCCGTAGGTGAACCTGCGGAAGGATCATTATTGAATTATGTTTCTAGATAGGTTGTAG
CTGGCTCTTTTAGAGCATGTGCACGCCTGTTTGGACTTCATTTTCATCCACCTGTGCACC
TATTGTAGTCTTTGGTTGGGTTAGGAGGAAGTGATCATTGTATCAGCATCTGCTGGGAGT
GAGGACTTGCATTGTGAAAGCTTTGCTGTCCTTGATGTGATCATGGAATCTTTTTCTCAC
TAGAGTCTATGTCACTCATTATACTCTGTCGAATGTCATTGAATGTCTTTACATGGGCTT
GTATGCCTATGAAAATTGTAATACAACTTTCAGCAACGGATCTCTTGGCTCTCGCATCGA
TGAAGAACGCAGCGAAATGCGATAAGTAATGTGAATTGCAGAATTCAGTGAATCATCGAA
TCTTTGAACGCATCTTGCGCTCCTTGGTATTCCGAGGAGCATGCCTGTTTGAGTGTCATT
AAATTCTCAACTCTCTTATACTTTTTTGTAAAAGAGAGCTTGGACTGTGGAGGCTTGCTG
GCCACTTTTTGGGGTCAGCTCCTCTGAAATGCATTAGCGGAACCGTTTGCGATCTGCCAC
AAGTGTGATAAGTTATCTACACTGGCGAGGGGATTGCTCTCTGTAATGTTCAGCTTCTAA
TTGTCTCTACTTTGTGAGACTACTTTTGAATGCTTGACCTCAAATCAGGTAGGACTACCC
GCTGAACTTAA
>AB7-25
TTTCCGTAGGTGAACCTGCGGAAGGATCATTATTGAATTATGTTTCTAGATAGGTTGTAG
CTGGCTCTTTTAGAGCATGTGCACGCCTGTTTGGACTTCATTTTCATCCACCTGTGCACC
TATTGTAGTCTTTGGTTGGGTTAGGAGGAAGTGATCATTGTATCAGCATCTGCTGGGAGT
GAGGACTTGCATTGTGAAAGCTTTGCTGTCCTTGATGTGATCATGGAATCTTTTTCTCAC
TAGAGTCTATGTCACTCATTATACTCTGTCGAATGTCATTGAATGTCTTTACATGGGCTT
GTATGCCTATGAAAATTGTAATACAACTTTCAGCAACGGATCTCTTGGCTCTCGCATCGA
TGAAGAACGCAGCGAAATGCGATAAGTAATGTGAATTGCAGAATTCAGTGAATCATCGAA
TCTTTGAACGCATCTTGCGCTCCTTGGTATTCCGAGGAGCATGCCTGTTTGAGTGTCATT
AAATTCTCAACTCTCTTATACTTTTTTGTAAAAGAGAGCTTGGACTGTGGAGGCTTGCTG
GCCACTTTTTGGGGTCAGCTCCTCTGAAATGCATTAGCGGAACCGTTTGCGATCTGCCAC
AAGTGTGATAAGTTATCTACACTGGCGAGGGGATTGCTCTCTGTAATGTTCAGCTTCTAA
TTGTCTCTACTTTGTGAGACTACTTTTGAATGCTTGACCTCAAATCAGGTAGGACTACCC
GCTGAACTTAA
>AB7-27
TTTCCGTAGGTGAACCTGCGGAAGGATCATTATTGAATTATGTTTCTAGATAGGTTGTAG
CTGGCTCTTTTAGAGCATGTGCACGCCTGTTTGGACTTCATTTTCATCCACCTGTGCACC
TATTGTAGTCTTTGGTTGGGTTAGGAGGAAGTGATCATTGTATCAGCATCTGCTGGGAGT
GAGGACTTGCATTGTGAAAGCTTTGCTGTCCTTGATGTGATCATGGAATCTTTTTCTCAC
TAGAGTCTATGTCACTCATTATACTCTGTCGAATGTCATTGAATGTCTTTACATGGGCTT

GTATGCCTATGAAAATTGTAATACAACTTTCAGCAACGGATCTCTTGGCTCTCGCATCGA
TGAAGAACGCAGCGAAATGCGATAAGTAATGTGAATTGCAGAATTCAGTGAATCATCGAA
TCTTTGAACGCATCTTGCGCTCCTTGGTATTCCGAGGAGCATGCCTGTTTGAGTGTCATT
AAATTCTCAACTCTCTTATACTTTTTTGTAAAAGAGAGCTTGGACTGTGGAGGCTTGCTG
GCCACTTTTTGGGGTCAGCTCCTCTGAAATGCATTAGCGGAACCGTTTGCGATCTGCCAC
AAGTGTGATAAGTTATCTACACTGGCGAGGGGATTGCTCTCTGTAATGTTCAGCTTCTAA
TTGTCTCTACTTTGTGAGACTACTTTTGAATGCTTGACCTCAAATCAGGTAGGACTACCC
GCTGAACTTAA
>AB7-29
TTTCCGTAGGTGAACCTGCGGAAGGATCATTATTGAATTATGTTTCTAGATAGGTTGTAG
CTGGCTCTTTTAGAGCATGTGCACGCCTGTTTGGACTTCATTTTCATCCACCTGTGCACC
TATTGTAGTCTTTGGTTGGGTTAGGAGGAAGTGATCATTGTATCAGCATCTGCTGGGAGT
GAGGACTTGCATTGTGAAAGCTTTGCTGTCCTTGATGTGATCATGGAATCTTTTTCTCAC
TAGAGTCTATGTCACTCATTATACTCTGTCGAATGTCATTGAATGTCTTTACATGGGCTT
GTATGCCTATGAAAATTGTAATACAACTTTCAGCAACGGATCTCTTGGCTCTCGCATCGA
TGAAGAACGCAGCGAAATGCGATAAGTAATGTGAATTGCAGAATTCAGTGAATCATCGAA
TCTTTGAACGCATCTTGCGCTCCTTGGTATTCCGAGGAGCATGCCTGTTTGAGTGTCATT
AAATTCTCAACTCTCTTATACTTTTTTGTAAAAGAGAGCTTGGACTGTGGAGGCTTGCTG
GCCACTTTTTGGGGTCAGCTCCTCTGAAATGCATTAGCGGAACCGTTTGCGATCTGCCAC
AAGTGTGATAAGTTATCTACACTGGCGAGGGGATTGCTCTCTGTAATGTTCAGCTTCTAA
TTGTCTCTACTTTGTGAGACTACTTTTGAATGCTTGACCTCAAATCAGGTAGGACTACCC
GCTGAACTTAA
>AB7-32
TTTCCGTAGGTGAACCTGCGGAAGGATCATTATTGAATTATGTTTCTAGATAGGTTGTAG
CTGGCTCTTTTAGAGCATGTGCACGCCTGTTTGGACTTCATTTTCATCCACCTGTGCACC
TATTGTAGTCTTTGGTTGGGTTAGGAGGAAGTGATCATTGTATCAGCATCTGCTGGGAGT
GAGGACTTGCATTGTGAAAGCTTTGCTGTCCTTGATGTGATCATGGAATCTTTTTCTCAC
TAGAGTCTATGTCACTCATTATACTCTGTCGAATGTCATTGAATGTCTTTACATGGGCTT
GTATGCCTATGAAAATTGTAATACAACTTTCAGCAACGGATCTCTTGGCTCTCGCATCGA
TGAAGAACGCAGCGAAATGCGATAAGTAATGTGAATTGCAGAATTCAGTGAATCATCGAA
TCTTTGAACGCATCTTGCGCTCCTTGGTATTCCGAGGAGCATGCCTGTTTGAGTGTCATT
AAATTCTCAACTCTCTTATACTTTTTTGTAAAAGAGAGCTTGGACTGTGGAGGCTTGCTG
GCCACTTTTTGGGGTCAGCTCCTCTGAAATGCATTAGCGGAACCGTTTGCGATCTGCCAC
AAGTGTGATAAGTTATCTACACTGGCGAGGGGATTGCTCTCTGTAATGTTCAGCTTCTAA
TTGTCTCTACTTTGTGAGACTACTTTTGAATGCTTGACCTCAAATCAGGTAGGACTACCC
GCTGAACTTAA
>AB7-33
TTTCCGTAGGTGAACCTGCGGAAGGATCATTATTGAATTATGTTTCTAGATAGGTTGTAG
CTGGCTCTTTTAGAGCATGTGCACGCCTGTTTGGACTTCATTTTCATCCACCTGTGCACC
TATTGTAGTCTTTGGTTGGGTTAGGAGGAAGTGATCATTGTATCAGCATCTGCTGGGAGT
GAGGACTTGCATTGTGAAAGCTTTGCTGTCCTTGATGTGATCATGGAATCTTTTTCTCAC
TAGAGTCTATGTCACTCATTATACTCTGTCGAATGTCATTGAATGTCTTTACATGGGCTT
GTATGCCTATGAAAATTGTAATACAACTTTCAGCAACGGATCTCTTGGCTCTCGCATCGA
TGAAGAACGCAGCGAAATGCGATAAGTAATGTGAATTGCAGAATTCAGTGAATCATCGAA
TCTTTGAACGCATCTTGCGCTCCTTGGTATTCCGAGGAGCATGCCTGTTTGAGTGTCATT
AAATTCTCAACTCTCTTATACTTTTTTGTAAAAGAGAGCTTGGACTGTGGAGGCTTGCTG
GCCACTTTTTGGGGTCAGCTCCTCTGAAATGCATTAGCGGAACCGTTTGCGATCTGCCAC
AAGTGTGATAAGTTATCTACACTGGCGAGGGGATTGCTCTCTGTAATGTTCAGCTTCTAA
TTGTCTCTACTTTGTGAGACTACTTTTGAATGCTTGACCTCAAATCAGGTAGGACTACCC
GCTGAACTTAA

>AB7-34
TTTCCGTAGGTGAACCTGCGGAAGGATCATTATTGAATTATGTTTCTAGATAGGTTGTAG
CTGGCTCTTTTAGAGCATGTGCACGCCTGTTTGGACTTCATTTTCATCCACCTGTGCACC
TATTGTAGTCTTTGGTTGGGTTAGGAGGAAGTGATCATTGTATCAGCATCTGCTGGGAGT
GAGGACTTGCATTGTGAAAGCTTTGCTGTCCTTGATGTGATCATGGAATCTTTTTCTCAC
TAGAGTCTATGTCACTCATTATACTCTGTCGAATGTCATTGAATGTCTTTACATGGGCTT
GTATGCCTATGAAAATTGTAATACAACTTTCAGCAACGGATCTCTTGGCTCTCGCATCGA
TGAAGAACGCAGCGAAATGCGATAAGTAATGTGAATTGCAGAATTCAGTGAATCATCGAA
TCTTTGAACGCATCTTGCGCTCCTTGGTATTCCGAGGAGCATGCCTGTTTGAGTGTCATT
AAATTCTCAACTCTCTTATACTTTTTTGTAAAAGAGAGCTTGGACTGTGGAGGCTTGCTG
GCCACTTTTTGGGGTCAGCTCCTCTGAAATGCATTAGCGGAACCGTTTGCGATCTGCCAC
AAGTGTGATAAGTTATCTACACTGGCGAGGGGATTGCTCTCTGTAATGTTCAGCTTCTAA
TTGTCTCTACTTTGTGAGACTACTTTTGAATGCTTGACCTCAAATCAGGTAGGACTACCC
GCTGAACTTAA
>AB7-37
TTTCCGTAGGTGAACCTGCGGAAGGATCATTATTGAATTATGTTTCTAGATAGGTTGTAG
CTGGCTCTTTTAGAGCATGTGCACGCCTGTTTGGACTTCATTTTCATCCACCTGTGCACC
TATTGTAGTCTTTGGTTGGGTTAGGAGGAAGTGATCATTGTATCAGCATCTGCTGGGAGT
GAGGACTTGCATTGTGAAAGCTTTGCTGTCCTTGATGTGATCATGGAATCTTTTTCTCAC
TAGAGTCTATGTCACTCATTATACTCTGTCGAATGTCATTGAATGTCTTTACATGGGCTT
GTATGCCTATGAAAATTGTAATACAACTTTCAGCAACGGATCTCTTGGCTCTCGCATCGA
TGAAGAACGCAGCGAAATGCGATAAGTAATGTGAATTGCAGAATTCAGTGAATCATCGAA
TCTTTGAACGCATCTTGCGCTCCTTGGTATTCCGAGGAGCATGCCTGTTTGAGTGTCATT
AAATTCTCAACTCTCTTATACTTTTTTGTAAAAGAGAGCTTGGACTGTGGAGGCTTGCTG
GCCACTTTTTGGGGTCAGCTCCTCTGAAATGCATTAGCGGAACCGTTTGCGATCTGCCAC
AAGTGTGATAAGTTATCTACACTGGCGAGGGGATTGCTCTCTGTAATGTTCAGCTTCTAA
TTGTCTCTACTTTGTGAGACTACTTTTGAATGCTTGACCTCAAATCAGGTAGGACTACCC
GCTGAACTTAA
>AB7-38
TTTCCGTAGGTGAACCTGCGGAAGGATCATTATTGAATTATGTTTCTAGATAGGTTGTAG
CTGGCTCTTTTAGAGCATGTGCACGCCTGTTTGGACTTCATTTTCATCCACCTGTGCACC
TATTGTAGTCTTTGGTTGGGTTAGGAGGAAGTGATCATTGTATCAGCATCTGCTGGGAGT
GAGGACTTGCATTGTGAAAGCTTTGCTGTCCTTGATGTGATCATGGAATCTTTTTCTCAC
TAGAGTCTATGTCACTCATTATACTCTGTCGAATGTCATTGAATGTCTTTACATGGGCTT
GTATGCCTATGAAAATTGTAATACAACTTTCAGCAACGGATCTCTTGGCTCTCGCATCGA
TGAAGAACGCAGCGAAATGCGATAAGTAATGTGAATTGCAGAATTCAGTGAATCATCGAA
TCTTTGAACGCATCTTGCGCTCCTTGGTATTCCGAGGAGCATGCCTGTTTGAGTGTCATT
AAATTCTCAACTCTCTTATACTTTTTTGTAAAAGAGAGCTTGGACTGTGGAGGCTTGCTG
GCCACTTTTTGGGGTCAGCTCCTCTGAAATGCATTAGCGGAACCGTTTGCGATCTGCCAC
AAGTGTGATAAGTTATCTACACTGGCGAGGGGATTGCTCTCTGTAATGTTCAGCTTCTAA
TTGTCTCTACTTTGTGAGACTACTTTTGAATGCTTGACCTCAAATCAGGTAGGACTACCC
GCTGAACTTAA
>AB7-39
TTTCCGTAGGTGAACCTGCGGAAGGATCATTATTGAATTATGTTTCTAGATAGGTTGTAG
CTGGCTCTTTTAGAGCATGTGCACGCCTGTTTGGACTTCATTTTCATCCACCTGTGCACC
TATTGTAGTCTTTGGTTGGGTTAGGAGGAAGTGATCATTGTATCAGCATCTGCTGGGAGT
GAGGACTTGCATTGTGAAAGCTTTGCTGTCCTTGATGTGATCATGGAATCTTTTTCTCAC
TAGAGTCTATGTCACTCATTATACTCTGTCGAATGTCATTGAATGTCTTTACATGGGCTT
GTATGCCTATGAAAATTGTAATACAACTTTCAGCAACGGATCTCTTGGCTCTCGCATCGA
TGAAGAACGCAGCGAAATGCGATAAGTAATGTGAATTGCAGAATTCAGTGAATCATCGAA

TCTTTGAACGCATCTTGCGCTCCTTGGTATTCCGAGGAGCATGCCTGTTTGAGTGTCATT
AAATTCTCAACTCTCTTATACTTTTTTGTAAAAGAGAGCTTGGACTGTGGAGGCTTGCTG
GCCACTTTTTGGGGTCAGCTCCTCTGAAATGCATTAGCGGAACCGTTTGCGATCTGCCAC
AAGTGTGATAAGTTATCTACACTGGCGAGGGGATTGCTCTCTGTAATGTTCAGCTTCTAA
TTGTCTCTACTTTGTGAGACTACTTTTGAATGCTTGACCTCAAATCAGGTAGGACTACCC
GCTGAACTTAA
>AB7–42
TTTCCGTAGGTGAACCTGCGGAAGGATCATTATTGAATTATGTTTCTAGATAGGTTGTAG
CTGGCTCTTTTAGAGCATGTGCACGCCTGTTTGGACTTCATTTTCATCCACCTGTGCACC
TATTGTAGTCTTTGGTTGGGTTAGGAGGAAGTGATCATTGTATCAGCATCTGCTGGGAGT
GAGGACTTGCATTGTGAAAGCTTTGCTGTCCTTGATGTGATCATGGAATCTTTTTCTCAC
TAGAGTCTATGTCACTCATTATACTCTGTCGAATGTCATTGAATGTCTTTACATGGGCTT
GTATGCCTATGAAAATTGTAATACAACTTTCAGCAACGGATCTCTTGGCTCTCGCATCGA
TGAAGAACGCAGCGAAATGCGATAAGTAATGTGAATTGCAGAATTCAGTGAATCATCGAA
TCTTTGAACGCATCTTGCGCTCCTTGGTATTCCGAGGAGCATGCCTGTTTGAGTGTCATT
AAATTCTCAACTCTCTTATACTTTTTTGTAAAAGAGAGCTTGGACTGTGGAGGCTTGCTG
GCCACTTTTTGGGGTCAGCTCCTCTGAAATGCATTAGCGGAACCGTTTGCGATCTGCCAC
AAGTGTGATAAGTTATCTACACTGGCGAGGGGATTGCTCTCTGTAATGTTCAGCTTCTAA
TTGTCTCTACTTTGTGAGACTACTTTTGAATGCTTGACCTCAAATCAGGTAGGACTACCC
GCTGAACTTAA
>AB7–43
TTTCCGTAGGTGAACCTGCGGAAGGATCATTATTGAATTATGTTTCTAGATAGGTTGTAG
CTGGCTCTTTTAGAGCATGTGCACGCCTGTTTGGACTTCATTTTCATCCACCTGTGCACC
TATTGTAGTCTTTGGTTGGGTTAGGAGGAAGTGATCATTGTATCAGCATCTGCTGGGAGT
GAGGACTTGCATTGTGAAAGCTTTGCTGTCCTTGATGTGATCATGGAATCTTTTTCTCAC
TAGAGTCTATGTCACTCATTATACTCTGTCGAATGTCATTGAATGTCTTTACATGGGCTT
GTATGCCTATGAAAATTGTAATACAACTTTCAGCAACGGATCTCTTGGCTCTCGCATCGA
TGAAGAACGCAGCGAAATGCGATAAGTAATGTGAATTGCAGAATTCAGTGAATCATCGAA
TCTTTGAACGCATCTTGCGCTCCTTGGTATTCCGAGGAGCATGCCTGTTTGAGTGTCATT
AAATTCTCAACTCTCTTATACTTTTTTGTAAAAGAGAGCTTGGACTGTGGAGGCTTGCTG
GCCACTTTTTGGGGTCAGCTCCTCTGAAATGCATTAGCGGAACCGTTTGCGATCTGCCAC
AAGTGTGATAAGTTATCTACACTGGCGAGGGGATTGCTCTCTGTAATGTTCAGCTTCTAA
TTGTCTCTACTTTGTGAGACTACTTTTGAATGCTTGACCTCAAATCAGGTAGGACTACCC
GCTGAACTTAA
>AB7–44
TTTCCGTAGGTGAACCTGCGGAAGGATCATTATTGAATTATGTTTCTAGATAGGTTGTAG
CTGGCTCTTTTAGAGCATGTGCACGCCTGTTTGGACTTCATTTTCATCCACCTGTGCACC
TATTGTAGTCTTTGGTTGGGTTAGGAGGAAGTGATCATTGTATCAGCATCTGCTGGGAGT
GAGGACTTGCATTGTGAAAGCTTTGCTGTCCTTGATGTGATCATGGAATCTTTTTCTCAC
TAGAGTCTATGTCACTCATTATACTCTGTCGAATGTCATTGAATGTCTTTACATGGGCTT
GTATGCCTATGAAAATTGTAATACAACTTTCAGCAACGGATCTCTTGGCTCTCGCATCGA
TGAAGAACGCAGCGAAATGCGATAAGTAATGTGAATTGCAGAATTCAGTGAATCATCGAA
TCTTTGAACGCATCTTGCGCTCCTTGGTATTCCGAGGAGCATGCCTGTTTGAGTGTCATT
AAATTCTCAACTCTCTTATACTTTTTTGTAAAAGAGAGCTTGGACTGTGGAGGCTTGCTG
GCCACTTTTTGGGGTCAGCTCCTCTGAAATGCATTAGCGGAACCGTTTGCGATCTGCCAC
AAGTGTGATAAGTTATCTACACTGGCGAGGGGATTGCTCTCTGTAATGTTCAGCTTCTAA
TTGTCTCTACTTTGTGAGACTACTTTTGAATGCTTGACCTCAAATCAGGTAGGACTACCC
GCTGAACTTAA
>AB7–46
TTTCCGTAGGTGAACCTGCGGAAGGATCATTATTGAATTATGTTTCTAGATAGGTTGTAG

CTGGCTCTTTTAGAGCATGTGCACGCCTGTTTGGACTTCATTTTCATCCACCTGTGCACC
TATTGTAGTCTTTGGTTGGGTTAGGAGGAAGTGATCATTGTATCAGCATCTGCTGGGAGT
GAGGACTTGCATTGTGAAAGCTTTGCTGTCCTTGATGTGATCATGGAATCTTTTTCTCAC
TAGAGTCTATGTCACTCATTATACTCTGTCGAATGTCATTGAATGTCTTTACATGGGCTT
GTATGCCTATGAAAATTGTAATACAACTTTCAGCAACGGATCTCTTGGCTCTCGCATCGA
TGAAGAACGCAGCGAAATGCGATAAGTAATGTGAATTGCAGAATTCAGTGAATCATCGAA
TCTTTGAACGCATCTTGCGCTCCTTGGTATTCCGAGGAGCATGCCTGTTTGAGTGTCATT
AAATTCTCAACTCTCTTATACTTTTTTGTAAAAGAGAGCTTGGACTGTGGAGGCTTGCTG
GCCACTTTTTGGGGTCAGCTCCTCTGAAATGCATTAGCGGAACCGTTTGCGATCTGCCAC
AAGTGTGATAAGTTATCTACACTGGCGAGGGGATTGCTCTCTGTAATGTTCAGCTTCTAA
TTGTCTCTACTTTGTGAGACTACTTTTGAATGCTTGACCTCAAATCAGGTAGGACTACCC
GCTGAACTTAA
>AB7−52
TTTCCGTAGGTGAACCTGCGGAAGGATCATTATTGAATTATGTTTCTAGATAGGTTGTAG
CTGGCTCTTTTAGAGCATGTGCACGCCTGTTTGGACTTCATTTTCATCCACCTGTGCACC
TATTGTAGTCTTTGGTTGGGTTAGGAGGAAGTGATCATTGTATCAGCATCTGCTGGGAGT
GAGGACTTGCATTGTGAAAGCTTTGCTGTCCTTGATGTGATCATGGAATCTTTTTCTCAC
TAGAGTCTATGTCACTCATTATACTCTGTCGAATGTCATTGAATGTCTTTACATGGGCTT
GTATGCCTATGAAAATTGTAATACAACTTTCAGCAACGGATCTCTTGGCTCTCGCATCGA
TGAAGAACGCAGCGAAATGCGATAAGTAATGTGAATTGCAGAATTCAGTGAATCATCGAA
TCTTTGAACGCATCTTGCGCTCCTTGGTATTCCGAGGAGCATGCCTGTTTGAGTGTCATT
AAATTCTCAACTCTCTTATACTTTTTTGTAAAAGAGAGCTTGGACTGTGGAGGCTTGCTG
GCCACTTTTTGGGGTCAGCTCCTCTGAAATGCATTAGCGGAACCGTTTGCGATCTGCCAC
AAGTGTGATAAGTTATCTACACTGGCGAGGGGATTGCTCTCTGTAATGTTCAGCTTCTAA
TTGTCTCTACTTTGTGAGACTACTTTTGAATGCTTGACCTCAAATCAGGTAGGACTACCC
GCTGAACTTAA
>AB7−53
TTTCCGTAGGTGAACCTGCGGAAGGATCATTATTGAATTATGTTTCTAGATAGGTTGTAG
CTGGCTCTTTTAGAGCATGTGCACGCCTGTTTGGACTTCATTTTCATCCACCTGTGCACC
TATTGTAGTCTTTGGTTGGGTTAGGAGGAAGTGATCATTGTATCAGCATCTGCTGGGAGT
GAGGACTTGCATTGTGAAAGCTTTGCTGTCCTTGATGTGATCATGGAATCTTTTTCTCAC
TAGAGTCTATGTCACTCATTATACTCTGTCGAATGTCATTGAATGTCTTTACATGGGCTT
GTATGCCTATGAAAATTGTAATACAACTTTCAGCAACGGATCTCTTGGCTCTCGCATCGA
TGAAGAACGCAGCGAAATGCGATAAGTAATGTGAATTGCAGAATTCAGTGAATCATCGAA
TCTTTGAACGCATCTTGCGCTCCTTGGTATTCCGAGGAGCATGCCTGTTTGAGTGTCATT
AAATTCTCAACTCTCTTATACTTTTTTGTAAAAGAGAGCTTGGACTGTGGAGGCTTGCTG
GCCACTTTTTGGGGTCAGCTCCTCTGAAATGCATTAGCGGAACCGTTTGCGATCTGCCAC
AAGTGTGATAAGTTATCTACACTGGCGAGGGGATTGCTCTCTGTAATGTTCAGCTTCTAA
TTGTCTCTACTTTGTGAGACTACTTTTGAATGCTTGACCTCAAATCAGGTAGGACTACCC
GCTGAACTTAA
>AB8−3
TTTCCGTAGGTGAACCTGCGGAAGGATCATTATTGAATTATGTTTCTAGATAGGTTGTAG
CTGGCTCTTTTAGAGCATGTGCACGCCTGTTTGGACTTCATTTTCATCCACCTGTGCACC
TATTGTAGTCTTTGGTTGGGTTAGGAGGAAGTGATCATTGTATCAGCATCTGCTGGGAGT
GAGGACTTGCATTGTGAAAGCTTTGCTGTCCTTGATGTGATCATGGAATCTTTTTCTCAC
TAGAGTCTATGTCACTCATTATACTCTGTCGAATGTCATTGAATGTCTTTACATGGGCTT
GTATGCCTATGAAAATTGTAATACAACTTTCAGCAACGGATCTCTTGGCTCTCGCATCGA
TGAAGAACGCAGCGAAATGCGATAAGTAATGTGAATTGCAGAATTCAGTGAATCATCGAA
TCTTTGAACGCATCTTGCGCTCCTTGGTATTCCGAGGAGCATGCCTGTTTGAGTGTCATT
AAATTCTCAACTCTCTTATACTTTTTTGTAAAAGAGAGCTTGGACTGTGGAGGCTTGCTG

GCCACTTTTTGGGGTCAGCTCCTCTGAAATGCATTAGCGGAACCGTTTGCGATCTGCCAC
AAGTGTGATAAGTTATCTACACTGGCGAGGGGATTGCTCTCTGTAATGTTCAGCTTCTAA
TTGTCTCTACTTTGTGAGACTACTTTTGAATGCTTGACCTCAAATCAGGTAGGACTACCC
GCTGAACTTAA
>AB8-4
TTTCCGTAGGTGAACCTGCGGAAGGATCATTATTGAATTATGTTTCTAGATAGGTTGTAG
CTGGCTCTTTTAGAGCATGTGCACGCCTGTTTGGACTTCATTTTCATCCACCTGTGCACC
TATTGTAGTCTTTGGTTGGGTTAGGAGGAAGTGATCATTGTATCAGCATCTGCTGGGAGT
GAGGACTTGCATTGTGAAAGCTTTGCTGTCCTTGATGTGATCATGGAATCTTTTTCTCAC
TAGAGTCTATGTCACTCATTATACTCTGTCGAATGTCATTGAATGTCTTTACATGGGCTT
GTATGCCTATGAAAATTGTAATACAACTTTCAGCAACGGATCTCTTGGCTCTCGCATCGA
TGAAGAACGCAGCGAAATGCGATAAGTAATGTGAATTGCAGAATTCAGTGAATCATCGAA
TCTTTGAACGCATCTTGCGCTCCTTGGTATTCCGAGGAGCATGCCTGTTTGAGTGTCATT
AAATTCTCAACTCTCTTATACTTTTTGTAAAAGAGAGCTTGGACTGTGGAGGCTTGCTG
GCCACTTTTTGGGGTCAGCTCCTCTGAAATGCATTAGCGGAACCGTTTGCGATCTGCCAC
AAGTGTGATAAGTTATCTACACTGGCGAGGGGATTGCTCTCTGTAATGTTCAGCTTCTAA
TTGTCTCTACTTTGTGAGACTACTTTTGAATGCTTGACCTCAAATCAGGTAGGACTACCC
GCTGAACTTAA
>AB8-6
TTTCCGTAGGTGAACCTGCGGAAGGATCATTATTGAATTATGTTTCTAGATAGGTTGTAG
CTGGCTCTTTTAGAGCATGTGCACGCCTGTTTGGACTTCATTTTCATCCACCTGTGCACC
TATTGTAGTCTTTGGTTGGGTTAGGAGGAAGTGATCATTGTATCAGCATCTGCTGGGAGT
GAGGACTTGCATTGTGAAAGCTTTGCTGTCCTTGATGTGATCATGGAATCTTTTTCTCAC
TAGAGTCTATGTCACTCATTATACTCTGTCGAATGTCATTGAATGTCTTTACATGGGCTT
GTATGCCTATGAAAATTGTAATACAACTTTCAGCAACGGATCTCTTGGCTCTCGCATCGA
TGAAGAACGCAGCGAAATGCGATAAGTAATGTGAATTGCAGAATTCAGTGAATCATCGAA
TCTTTGAACGCATCTTGCGCTCCTTGGTATTCCGAGGAGCATGCCTGTTTGAGTGTCATT
AAATTCTCAACTCTCTTATACTTTTTGTAAAAGAGAGCTTGGACTGTGGAGGCTTGCTG
GCCACTTTTTGGGGTCAGCTCCTCTGAAATGCATTAGCGGAACCGTTTGCGATCTGCCAC
AAGTGTGATAAGTTATCTACACTGGCGAGGGGATTGCTCTCTGTAATGTTCAGCTTCTAA
TTGTCTCTACTTTGTGAGACTACTTTTGAATGCTTGACCTCAAATCAGGTAGGACTACCC
GCTGAACTTAA
>AB8-7
TTTCCGTAGGTGAACCTGCGGAAGGATCATTATTGAATTATGTTTCTAGATAGGTTGTAG
CTGGCTCTTTTAGAGCATGTGCACGCCTGTTTGGACTTCATTTTCATCCACCTGTGCACC
TATTGTAGTCTTTGGTTGGGTTAGGAGGAAGTGATCATTGTATCAGCATCTGCTGGGAGT
GAGGACTTGCATTGTGAAAGCTTTGCTGTCCTTGATGTGATCATGGAATCTTTTTCTCAC
TAGAGTCTATGTCACTCATTATACTCTGTCGAATGTCATTGAATGTCTTTACATGGGCTT
GTATGCCTATGAAAATTGTAATACAACTTTCAGCAACGGATCTCTTGGCTCTCGCATCGA
TGAAGAACGCAGCGAAATGCGATAAGTAATGTGAATTGCAGAATTCAGTGAATCATCGAA
TCTTTGAACGCATCTTGCGCTCCTTGGTATTCCGAGGAGCATGCCTGTTTGAGTGTCATT
AAATTCTCAACTCTCTTATACTTTTTGTAAAAGAGAGCTTGGACTGTGGAGGCTTGCTG
GCCACTTTTTGGGGTCAGCTCCTCTGAAATGCATTAGCGGAACCGTTTGCGATCTGCCAC
AAGTGTGATAAGTTATCTACACTGGCGAGGGGATTGCTCTCTGTAATGTTCAGCTTCTAA
TTGTCTCTACTTTGTGAGACTACTTTTGAATGCTTGACCTCAAATCAGGTAGGACTACCC
GCTGAACTTAA
>AB8-8
TTTCCGTAGGTGAACCTGCGGAAGGATCATTATTGAATTATGTTTCTAGATAGGTTGTAG
CTGGCTCTTTTAGAGCATGTGCACGCCTGTTTGGACTTCATTTTCATCCACCTGTGCACC
TATTGTAGTCTTTGGTTGGGTTAGGAGGAAGTGATCATTGTATCAGCATCTGCTGGGAGT

GAGGACTTGCATTGTGAAAGCTTTGCTGTCCTTGATGTGATCATGGAATCTTTTTCTCAC
TAGAGTCTATGTCACTCATTATACTCTGTCGAATGTCATTGAATGTCTTTACATGGGCTT
GTATGCCTATGAAAATTGTAATACAACTTTCAGCAACGGATCTCTTGGCTCTCGCATCGA
TGAAGAACGCAGCGAAATGCGATAAGTAATGTGAATTGCAGAATTCAGTGAATCATCGAA
TCTTTGAACGCATCTTGCGCTCCTTGGTATTCCGAGGAGCATGCCTGTTTGAGTGTCATT
AAATTCTCAACTCTCTTATACTTTTTTGTAAAAGAGAGCTTGGACTGTGGAGGCTTGCTG
GCCACTTTTTGGGGTCAGCTCCTCTGAAATGCATTAGCGGAACCGTTTGCGATCTGCCAC
AAGTGTGATAAGTTATCTACACTGGCGAGGGGATTGCTCTCTGTAATGTTCAGCTTCTAA
TTGTCTCTACTTTGTGAGACTACTTTTGAATGCTTGACCTCAAATCAGGTAGGACTACCC
GCTGAACTTAA
>AB8-10
TTTCCGTAGGTGAACCTGCGGAAGGATCATTATTGAATTATGTTTCTAGATAGGTTGTAG
CTGGCTCTTTTAGAGCATGTGCACGCCTGTTTGGACTTCATTTTCATCCACCTGTGCACC
TATTGTAGTCTTTGGTTGGGTTAGGAGGAAGTGATCATTGTATCAGCATCTGCTGGGAGT
GAGGACTTGCATTGTGAAAGCTTTGCTGTCCTTGATGTGATCATGGAATCTTTTTCTCAC
TAGAGTCTATGTCACTCATTATACTCTGTCGAATGTCATTGAATGTCTTTACATGGGCTT
GTATGCCTATGAAAATTGTAATACAACTTTCAGCAACGGATCTCTTGGCTCTCGCATCGA
TGAAGAACGCAGCGAAATGCGATAAGTAATGTGAATTGCAGAATTCAGTGAATCATCGAA
TCTTTGAACGCATCTTGCGCTCCTTGGTATTCCGAGGAGCATGCCTGTTTGAGTGTCATT
AAATTCTCAACTCTCTTATACTTTTTTGTAAAAGAGAGCTTGGACTGTGGAGGCTTGCTG
GCCACTTTTTGGGGTCAGCTCCTCTGAAATGCATTAGCGGAACCGTTTGCGATCTGCCAC
AAGTGTGATAAGTTATCTACACTGGCGAGGGGATTGCTCTCTGTAATGTTCAGCTTCTAA
TTGTCTCTACTTTGTGAGACTACTTTTGAATGCTTGACCTCAAATCAGGTAGGACTACCC
GCTGAACTTAA
>AB8-11
TTTCCGTAGGTGAACCTGCGGAAGGATCATTATTGAATTATGTTTCTAGATAGGTTGTAG
CTGGCTCTTTTAGAGCATGTGCACGCCTGTTTGGACTTCATTTTCATCCACCTGTGCACC
TATTGTAGTCTTTGGTTGGGTTAGGAGGAAGTGATCATTGTATCAGCATCTGCTGGGAGT
GAGGACTTGCATTGTGAAAGCTTTGCTGTCCTTGATGTGATCATGGAATCTTTTTCTCAC
TAGAGTCTATGTCACTCATTATACTCTGTCGAATGTCATTGAATGTCTTTACATGGGCTT
GTATGCCTATGAAAATTGTAATACAACTTTCAGCAACGGATCTCTTGGCTCTCGCATCGA
TGAAGAACGCAGCGAAATGCGATAAGTAATGTGAATTGCAGAATTCAGTGAATCATCGAA
TCTTTGAACGCATCTTGCGCTCCTTGGTATTCCGAGGAGCATGCCTGTTTGAGTGTCATT
AAATTCTCAACTCTCTTATACTTTTTTGTAAAAGAGAGCTTGGACTGTGGAGGCTTGCTG
GCCACTTTTTGGGGTCAGCTCCTCTGAAATGCATTAGCGGAACCGTTTGCGATCTGCCAC
AAGTGTGATAAGTTATCTACACTGGCGAGGGGATTGCTCTCTGTAATGTTCAGCTTCTAA
TTGTCTCTACTTTGTGAGACTACTTTTGAATGCTTGACCTCAAATCAGGTAGGACTACCC
GCTGAACTTAA
>AB8-14
TTTCCGTAGGTGAACCTGCGGAAGGATCATTATTGAATTATGTTTCTAGATAGGTTGTAG
CTGGCTCTTTTAGAGCATGTGCACGCCTGTTTGGACTTCATTTTCATCCACCTGTGCACC
TATTGTAGTCTTTGGTTGGGTTAGGAGGAAGTGATCATTGTATCAGCATCTGCTGGGAGT
GAGGACTTGCATTGTGAAAGCTTTGCTGTCCTTGATGTGATCATGGAATCTTTTTCTCAC
TAGAGTCTATGTCACTCATTATACTCTGTCGAATGTCATTGAATGTCTTTACATGGGCTT
GTATGCCTATGAAAATTGTAATACAACTTTCAGCAACGGATCTCTTGGCTCTCGCATCGA
TGAAGAACGCAGCGAAATGCGATAAGTAATGTGAATTGCAGAATTCAGTGAATCATCGAA
TCTTTGAACGCATCTTGCGCTCCTTGGTATTCCGAGGAGCATGCCTGTTTGAGTGTCATT
AAATTCTCAACTCTCTTATACTTTTTTGTAAAAGAGAGCTTGGACTGTGGAGGCTTGCTG
GCCACTTTTTGGGGTCAGCTCCTCTGAAATGCATTAGCGGAACCGTTTGCGATCTGCCAC
AAGTGTGATAAGTTATCTACACTGGCGAGGGGATTGCTCTCTGTAATGTTCAGCTTCTAA

TTGTCTCTACTTTGTGAGACTACTTTTGAATGCTTGACCTCAAATCAGGTAGGACTACCC
GCTGAACTTAA
>AB8−18
TTTCCGTAGGTGAACCTGCGGAAGGATCATTATTGAATTATGTTTCTAGATAGGTTGTAG
CTGGCTCTTTTAGAGCATGTGCACGCCTGTTTGGACTTCATTTTCATCCACCTGTGCACC
TATTGTAGTCTTTGGTTGGGTTAGGAGGAAGTGATCATTGTATCAGCATCTGCTGGGAGT
GAGGACTTGCATTGTGAAAGCTTTGCTGTCCTTGATGTGATCATGGAATCTTTTTCTCAC
TAGAGTCTATGTCACTCATTATACTCTGTCGAATGTCATTGAATGTCTTTACATGGGCTT
GTATGCCTATGAAAATTGTAATACAACTTTCAGCAACGGATCTCTTGGCTCTCGCATCGA
TGAAGAACGCAGCGAAATGCGATAAGTAATGTGAATTGCAGAATTCAGTGAATCATCGAA
TCTTTGAACGCATCTTGCGCTCCTTGGTATTCCGAGGAGCATGCCTGTTTGAGTGTCATT
AAATTCTCAACTCTCTTATACTTTTTTGTAAAAGAGAGCTTGGACTGTGGAGGCTTGCTG
GCCACTTTTTGGGGTCAGCTCCTCTGAAATGCATTAGCGGAACCGTTTGCGATCTGCCAC
AAGTGTGATAAGTTATCTACACTGGCGAGGGGATTGCTCTCTGTAATGTTCAGCTTCTAA
TTGTCTCTACTTTGTGAGACTACTTTTGAATGCTTGACCTCAAATCAGGTAGGACTACCC
GCTGAACTTAA
>AB8−22
TTTCCGTAGGTGAACCTGCGGAAGGATCATTATTGAATTATGTTTCTAGATAGGTTGTAG
CTGGCTCTTTTAGAGCATGTGCACGCCTGTTTGGACTTCATTTTCATCCACCTGTGCACC
TATTGTAGTCTTTGGTTGGGTTAGGAGGAAGTGATCATTGTATCAGCATCTGCTGGGAGT
GAGGACTTGCATTGTGAAAGCTTTGCTGTCCTTGATGTGATCATGGAATCTTTTTCTCAC
TAGAGTCTATGTCACTCATTATACTCTGTCGAATGTCATTGAATGTCTTTACATGGGCTT
GTATGCCTATGAAAATTGTAATACAACTTTCAGCAACGGATCTCTTGGCTCTCGCATCGA
TGAAGAACGCAGCGAAATGCGATAAGTAATGTGAATTGCAGAATTCAGTGAATCATCGAA
TCTTTGAACGCATCTTGCGCTCCTTGGTATTCCGAGGAGCATGCCTGTTTGAGTGTCATT
AAATTCTCAACTCTCTTATACTTTTTTGTAAAAGAGAGCTTGGACTGTGGAGGCTTGCTG
GCCACTTTTTGGGGTCAGCTCCTCTGAAATGCATTAGCGGAACCGTTTGCGATCTGCCAC
AAGTGTGATAAGTTATCTACACTGGCGAGGGGATTGCTCTCTGTAATGTTCAGCTTCTAA
TTGTCTCTACTTTGTGAGACTACTTTTGAATGCTTGACCTCAAATCAGGTAGGACTACCC
GCTGAACTTAA
>AB8−23
TTTCCGTAGGTGAACCTGCGGAAGGATCATTATTGAATTATGTTTCTAGATAGGTTGTAG
CTGGCTCTTTTAGAGCATGTGCACGCCTGTTTGGACTTCATTTTCATCCACCTGTGCACC
TATTGTAGTCTTTGGTTGGGTTAGGAGGAAGTGATCATTGTATCAGCATCTGCTGGGAGT
GAGGACTTGCATTGTGAAAGCTTTGCTGTCCTTGATGTGATCATGGAATCTTTTTCTCAC
TAGAGTCTATGTCACTCATTATACTCTGTCGAATGTCATTGAATGTCTTTACATGGGCTT
GTATGCCTATGAAAATTGTAATACAACTTTCAGCAACGGATCTCTTGGCTCTCGCATCGA
TGAAGAACGCAGCGAAATGCGATAAGTAATGTGAATTGCAGAATTCAGTGAATCATCGAA
TCTTTGAACGCATCTTGCGCTCCTTGGTATTCCGAGGAGCATGCCTGTTTGAGTGTCATT
AAATTCTCAACTCTCTTATACTTTTTTGTAAAAGAGAGCTTGGACTGTGGAGGCTTGCTG
GCCACTTTTTGGGGTCAGCTCCTCTGAAATGCATTAGCGGAACCGTTTGCGATCTGCCAC
AAGTGTGATAAGTTATCTACACTGGCGAGGGGATTGCTCTCTGTAATGTTCAGCTTCTAA
TTGTCTCTACTTTGTGAGACTACTTTTGAATGCTTGACCTCAAATCAGGTAGGACTACCC
GCTGAACTTAA
>AB8−24
TTTCCGTAGGTGAACCTGCGGAAGGATCATTATTGAATTATGTTTCTAGATAGGTTGTAG
CTGGCTCTTTTAGAGCATGTGCACGCCTGTTTGGACTTCATTTTCATCCACCTGTGCACC
TATTGTAGTCTTTGGTTGGGTTAGGAGGAAGTGATCATTGTATCAGCATCTGCTGGGAGT
GAGGACTTGCATTGTGAAAGCTTTGCTGTCCTTGATGTGATCATGGAATCTTTTTCTCAC
TAGAGTCTATGTCACTCATTATACTCTGTCGAATGTCATTGAATGTCTTTACATGGGCTT

GTATGCCTATGAAAATTGTAATACAACTTTCAGCAACGGATCTCTTGGCTCTCGCATCGA
TGAAGAACGCAGCGAAATGCGATAAGTAATGTGAATTGCAGAATTCAGTGAATCATCGAA
TCTTTGAACGCATCTTGCGCTCCTTGGTATTCCGAGGAGCATGCCTGTTTGAGTGTCATT
AAATTCTCAACTCTCTTATACTTTTTTGTAAAAGAGAGCTTGGACTGTGGAGGCTTGCTG
GCCACTTTTTGGGGTCAGCTCCTCTGAAATGCATTAGCGGAACCGTTTGCGATCTGCCAC
AAGTGTGATAAGTTATCTACACTGGCGAGGGGATTGCTCTCTGTAATGTTCAGCTTCTAA
TTGTCTCTACTTTGTGAGACTACTTTTGAATGCTTGACCTCAAATCAGGTAGGACTACCC
GCTGAACTTAA
>AB8−26
TTTCCGTAGGTGAACCTGCGGAAGGATCATTATTGAATTATGTTTCTAGATAGGTTGTAG
CTGGCTCTTTTAGAGCATGTGCACGCCTGTTTGGACTTCATTTTCATCCACCTGTGCACC
TATTGTAGTCTTTGGTTGGGTTAGGAGGAAGTGATCATTGTATCAGCATCTGCTGGGAGT
GAGGACTTGCATTGTGAAAGCTTTGCTGTCCTTGATGTGATCATGGAATCTTTTTCTCAC
TAGAGTCTATGTCACTCATTATACTCTGTCGAATGTCATTGAATGTCTTTACATGGGCTT
GTATGCCTATGAAAATTGTAATACAACTTTCAGCAACGGATCTCTTGGCTCTCGCATCGA
TGAAGAACGCAGCGAAATGCGATAAGTAATGTGAATTGCAGAATTCAGTGAATCATCGAA
TCTTTGAACGCATCTTGCGCTCCTTGGTATTCCGAGGAGCATGCCTGTTTGAGTGTCATT
AAATTCTCAACTCTCTTATACTTTTTTGTAAAAGAGAGCTTGGACTGTGGAGGCTTGCTG
GCCACTTTTTGGGGTCAGCTCCTCTGAAATGCATTAGCGGAACCGTTTGCGATCTGCCAC
AAGTGTGATAAGTTATCTACACTGGCGAGGGGATTGCTCTCTGTAATGTTCAGCTTCTAA
TTGTCTCTACTTTGTGAGACTACTTTTGAATGCTTGACCTCAAATCAGGTAGGACTACCC
GCTGAACTTAA
>AB8−29
TTTCCGTAGGTGAACCTGCGGAAGGATCATTATTGAATTATGTTTCTAGATAGGTTGTAG
CTGGCTCTTTTAGAGCATGTGCACGCCTGTTTGGACTTCATTTTCATCCACCTGTGCACC
TATTGTAGTCTTTGGTTGGGTTAGGAGGAAGTGATCATTGTATCAGCATCTGCTGGGAGT
GAGGACTTGCATTGTGAAAGCTTTGCTGTCCTTGATGTGATCATGGAATCTTTTTCTCAC
TAGAGTCTATGTCACTCATTATACTCTGTCGAATGTCATTGAATGTCTTTACATGGGCTT
GTATGCCTATGAAAATTGTAATACAACTTTCAGCAACGGATCTCTTGGCTCTCGCATCGA
TGAAGAACGCAGCGAAATGCGATAAGTAATGTGAATTGCAGAATTCAGTGAATCATCGAA
TCTTTGAACGCATCTTGCGCTCCTTGGTATTCCGAGGAGCATGCCTGTTTGAGTGTCATT
AAATTCTCAACTCTCTTATACTTTTTTGTAAAAGAGAGCTTGGACTGTGGAGGCTTGCTG
GCCACTTTTTGGGGTCAGCTCCTCTGAAATGCATTAGCGGAACCGTTTGCGATCTGCCAC
AAGTGTGATAAGTTATCTACACTGGCGAGGGGATTGCTCTCTGTAATGTTCAGCTTCTAA
TTGTCTCTACTTTGTGAGACTACTTTTGAATGCTTGACCTCAAATCAGGTAGGACTACCC
GCTGAACTTAA
>AB8−31
TTTCCGTAGGTGAACCTGCGGAAGGATCATTATTGAATTATGTTTCTAGATAGGTTGTAG
CTGGCTCTTTTAGAGCATGTGCACGCCTGTTTGGACTTCATTTTCATCCACCTGTGCACC
TATTGTAGTCTTTGGTTGGGTTAGGAGGAAGTGATCATTGTATCAGCATCTGCTGGGAGT
GAGGACTTGCATTGTGAAAGCTTTGCTGTCCTTGATGTGATCATGGAATCTTTTTCTCAC
TAGAGTCTATGTCACTCATTATACTCTGTCGAATGTCATTGAATGTCTTTACATGGGCTT
GTATGCCTATGAAAATTGTAATACAACTTTCAGCAACGGATCTCTTGGCTCTCGCATCGA
TGAAGAACGCAGCGAAATGCGATAAGTAATGTGAATTGCAGAATTCAGTGAATCATCGAA
TCTTTGAACGCATCTTGCGCTCCTTGGTATTCCGAGGAGCATGCCTGTTTGAGTGTCATT
AAATTCTCAACTCTCTTATACTTTTTTGTAAAAGAGAGCTTGGACTGTGGAGGCTTGCTG
GCCACTTTTTGGGGTCAGCTCCTCTGAAATGCATTAGCGGAACCGTTTGCGATCTGCCAC
AAGTGTGATAAGTTATCTACACTGGCGAGGGGATTGCTCTCTGTAATGTTCAGCTTCTAA
TTGTCTCTACTTTGTGAGACTACTTTTGAATGCTTGACCTCAAATCAGGTAGGACTACCC
GCTGAACTTAA

>AB8-33
TTTCCGTAGGTGAACCTGCGGAAGGATCATTATTGAATTATGTTTCTAGATAGGTTGTAG
CTGGCTCTTTTAGAGCATGTGCACGCCTGTTTGGACTTCATTTTCATCCACCTGTGCACC
TATTGTAGTCTTTGGTTGGGTTAGGAGGAAGTGATCATTGTATCAGCATCTGCTGGGAGT
GAGGACTTGCATTGTGAAAGCTTTGCTGTCCTTGATGTGATCATGGAATCTTTTTCTCAC
TAGAGTCTATGTCACTCATTATACTCTGTCGAATGTCATTGAATGTCTTTACATGGGCTT
GTATGCCTATGAAAATTGTAATACAACTTTCAGCAACGGATCTCTTGGCTCTCGCATCGA
TGAAGAACGCAGCGAAATGCGATAAGTAATGTGAATTGCAGAATTCAGTGAATCATCGAA
TCTTTGAACGCATCTTGCGCTCCTTGGTATTCCGAGGAGCATGCCTGTTTGAGTGTCATT
AAATTCTCAACTCTCTTATACTTTTTTGTAAAAGAGAGCTTGGACTGTGGAGGCTTGCTG
GCCACTTTTTGGGGTCAGCTCCTCTGAAATGCATTAGCGGAACCGTTTGCGATCTGCCAC
AAGTGTGATAAGTTATCTACACTGGCGAGGGGATTGCTCTCTGTAATGTTCAGCTTCTAA
TTGTCTCTACTTTGTGAGACTACTTTTGAATGCTTGACCTCAAATCAGGTAGGACTACCC
GCTGAACTTAA
>AB8-34
TTTCCGTAGGTGAACCTGCGGAAGGATCATTATTGAATTATGTTTCTAGATAGGTTGTAG
CTGGCTCTTTTAGAGCATGTGCACGCCTGTTTGGACTTCATTTTCATCCACCTGTGCACC
TATTGTAGTCTTTGGTTGGGTTAGGAGGAAGTGATCATTGTATCAGCATCTGCTGGGAGT
GAGGACTTGCATTGTGAAAGCTTTGCTGTCCTTGATGTGATCATGGAATCTTTTTCTCAC
TAGAGTCTATGTCACTCATTATACTCTGTCGAATGTCATTGAATGTCTTTACATGGGCTT
GTATGCCTATGAAAATTGTAATACAACTTTCAGCAACGGATCTCTTGGCTCTCGCATCGA
TGAAGAACGCAGCGAAATGCGATAAGTAATGTGAATTGCAGAATTCAGTGAATCATCGAA
TCTTTGAACGCATCTTGCGCTCCTTGGTATTCCGAGGAGCATGCCTGTTTGAGTGTCATT
AAATTCTCAACTCTCTTATACTTTTTTGTAAAAGAGAGCTTGGACTGTGGAGGCTTGCTG
GCCACTTTTTGGGGTCAGCTCCTCTGAAATGCATTAGCGGAACCGTTTGCGATCTGCCAC
AAGTGTGATAAGTTATCTACACTGGCGAGGGGATTGCTCTCTGTAATGTTCAGCTTCTAA
TTGTCTCTACTTTGTGAGACTACTTTTGAATGCTTGACCTCAAATCAGGTAGGACTACCC
GCTGAACTTAA
>AB8-35
TTTCCGTAGGTGAACCTGCGGAAGGATCATTATTGAATTATGTTTCTAGATAGGTTGTAG
CTGGCTCTTTTAGAGCATGTGCACGCCTGTTTGGACTTCATTTTCATCCACCTGTGCACC
TATTGTAGTCTTTGGTTGGGTTAGGAGGAAGTGATCATTGTATCAGCATCTGCTGGGAGT
GAGGACTTGCATTGTGAAAGCTTTGCTGTCCTTGATGTGATCATGGAATCTTTTTCTCAC
TAGAGTCTATGTCACTCATTATACTCTGTCGAATGTCATTGAATGTCTTTACATGGGCTT
GTATGCCTATGAAAATTGTAATACAACTTTCAGCAACGGATCTCTTGGCTCTCGCATCGA
TGAAGAACGCAGCGAAATGCGATAAGTAATGTGAATTGCAGAATTCAGTGAATCATCGAA
TCTTTGAACGCATCTTGCGCTCCTTGGTATTCCGAGGAGCATGCCTGTTTGAGTGTCATT
AAATTCTCAACTCTCTTATACTTTTTTGTAAAAGAGAGCTTGGACTGTGGAGGCTTGCTG
GCCACTTTTTGGGGTCAGCTCCTCTGAAATGCATTAGCGGAACCGTTTGCGATCTGCCAC
AAGTGTGATAAGTTATCTACACTGGCGAGGGGATTGCTCTCTGTAATGTTCAGCTTCTAA
TTGTCTCTACTTTGTGAGACTACTTTTGAATGCTTGACCTCAAATCAGGTAGGACTACCC
GCTGAACTTAA
>AB8-36
TTTCCGTAGGTGAACCTGCGGAAGGATCATTATTGAATTATGTTTCTAGATAGGTTGTAG
CTGGCTCTTTTAGAGCATGTGCACGCCTGTTTGGACTTCATTTTCATCCACCTGTGCACC
TATTGTAGTCTTTGGTTGGGTTAGGAGGAAGTGATCATTGTATCAGCATCTGCTGGGAGT
GAGGACTTGCATTGTGAAAGCTTTGCTGTCCTTGATGTGATCATGGAATCTTTTTCTCAC
TAGAGTCTATGTCACTCATTATACTCTGTCGAATGTCATTGAATGTCTTTACATGGGCTT
GTATGCCTATGAAAATTGTAATACAACTTTCAGCAACGGATCTCTTGGCTCTCGCATCGA
TGAAGAACGCAGCGAAATGCGATAAGTAATGTGAATTGCAGAATTCAGTGAATCATCGAA

TCTTTGAACGCATCTTGCGCTCCTTGGTATTCCGAGGAGCATGCCTGTTTGAGTGTCATT
AAATTCTCAACTCTCTTATACTTTTTTGTAAAAGAGAGCTTGGACTGTGGAGGCTTGCTG
GCCACTTTTTGGGGTCAGCTCCTCTGAAATGCATTAGCGGAACCGTTTGCGATCTGCCAC
AAGTGTGATAAGTTATCTACACTGGCGAGGGGATTGCTCTCTGTAATGTTCAGCTTCTAA
TTGTCTCTACTTTGTGAGACTACTTTTGAATGCTTGACCTCAAATCAGGTAGGACTACCC
GCTGAACTTAA
>AB8-39
TTTCCGTAGGTGAACCTGCGGAAGGATCATTATTGAATTATGTTTCTAGATAGGTTGTAG
CTGGCTCTTTTAGAGCATGTGCACGCCTGTTTGGACTTCATTTTCATCCACCTGTGCACC
TATTGTAGTCTTTGGTTGGGTTAGGAGGAAGTGATCATTGTATCAGCATCTGCTGGGAGT
GAGGACTTGCATTGTGAAAGCTTTGCTGTCCTTGATGTGATCATGGAATCTTTTTCTCAC
TAGAGTCTATGTCACTCATTATACTCTGTCGAATGTCATTGAATGTCTTTACATGGGCTT
GTATGCCTATGAAAATTGTAATACAACTTTCAGCAACGGATCTCTTGGCTCTCGCATCGA
TGAAGAACGCAGCGAAATGCGATAAGTAATGTGAATTGCAGAATTCAGTGAATCATCGAA
TCTTTGAACGCATCTTGCGCTCCTTGGTATTCCGAGGAGCATGCCTGTTTGAGTGTCATT
AAATTCTCAACTCTCTTATACTTTTTTGTAAAAGAGAGCTTGGACTGTGGAGGCTTGCTG
GCCACTTTTTGGGGTCAGCTCCTCTGAAATGCATTAGCGGAACCGTTTGCGATCTGCCAC
AAGTGTGATAAGTTATCTACACTGGCGAGGGGATTGCTCTCTGTAATGTTCAGCTTCTAA
TTGTCTCTACTTTGTGAGACTACTTTTGAATGCTTGACCTCAAATCAGGTAGGACTACCC
GCTGAACTTAA
>AB8-45
TTTCCGTAGGTGAACCTGCGGAAGGATCATTATTGAATTATGTTTCTAGATAGGTTGTAG
CTGGCTCTTTTAGAGCATGTGCACGCCTGTTTGGACTTCATTTTCATCCACCTGTGCACC
TATTGTAGTCTTTGGTTGGGTTAGGAGGAAGTGATCATTGTATCAGCATCTGCTGGGAGT
GAGGACTTGCATTGTGAAAGCTTTGCTGTCCTTGATGTGATCATGGAATCTTTTTCTCAC
TAGAGTCTATGTCACTCATTATACTCTGTCGAATGTCATTGAATGTCTTTACATGGGCTT
GTATGCCTATGAAAATTGTAATACAACTTTCAGCAACGGATCTCTTGGCTCTCGCATCGA
TGAAGAACGCAGCGAAATGCGATAAGTAATGTGAATTGCAGAATTCAGTGAATCATCGAA
TCTTTGAACGCATCTTGCGCTCCTTGGTATTCCGAGGAGCATGCCTGTTTGAGTGTCATT
AAATTCTCAACTCTCTTATACTTTTTTGTAAAAGAGAGCTTGGACTGTGGAGGCTTGCTG
GCCACTTTTTGGGGTCAGCTCCTCTGAAATGCATTAGCGGAACCGTTTGCGATCTGCCAC
AAGTGTGATAAGTTATCTACACTGGCGAGGGGATTGCTCTCTGTAATGTTCAGCTTCTAA
TTGTCTCTACTTTGTGAGACTACTTTTGAATGCTTGACCTCAAATCAGGTAGGACTACCC
GCTGAACTTAA
>AB8-51
TTTCCGTAGGTGAACCTGCGGAAGGATCATTATTGAATTATGTTTCTAGATAGGTTGTAG
CTGGCTCTTTTAGAGCATGTGCACGCCTGTTTGGACTTCATTTTCATCCACCTGTGCACC
TATTGTAGTCTTTGGTTGGGTTAGGAGGAAGTGATCATTGTATCAGCATCTGCTGGGAGT
GAGGACTTGCATTGTGAAAGCTTTGCTGTCCTTGATGTGATCATGGAATCTTTTTCTCAC
TAGAGTCTATGTCACTCATTATACTCTGTCGAATGTCATTGAATGTCTTTACATGGGCTT
GTATGCCTATGAAAATTGTAATACAACTTTCAGCAACGGATCTCTTGGCTCTCGCATCGA
TGAAGAACGCAGCGAAATGCGATAAGTAATGTGAATTGCAGAATTCAGTGAATCATCGAA
TCTTTGAACGCATCTTGCGCTCCTTGGTATTCCGAGGAGCATGCCTGTTTGAGTGTCATT
AAATTCTCAACTCTCTTATACTTTTTTGTAAAAGAGAGCTTGGACTGTGGAGGCTTGCTG
GCCACTTTTTGGGGTCAGCTCCTCTGAAATGCATTAGCGGAACCGTTTGCGATCTGCCAC
AAGTGTGATAAGTTATCTACACTGGCGAGGGGATTGCTCTCTGTAATGTTCAGCTTCTAA
TTGTCTCTACTTTGTGAGACTACTTTTGAATGCTTGACCTCAAATCAGGTAGGACTACCC
GCTGAACTTAA
>AB8-53
TTTCCGTAGGTGAACCTGCGGAAGGATCATTATTGAATTATGTTTCTAGATAGGTTGTAG

CTGGCTCTTTTAGAGCATGTGCACGCCTGTTTGGACTTCATTTTCATCCACCTGTGCACC
TATTGTAGTCTTTGGTTGGGTTAGGAGGAAGTGATCATTGTATCAGCATCTGCTGGGAGT
GAGGACTTGCATTGTGAAAGCTTTGCTGTCCTTGATGTGATCATGGAATCTTTTTCTCAC
TAGAGTCTATGTCACTCATTATACTCTGTCGAATGTCATTGAATGTCTTTACATGGGCTT
GTATGCCTATGAAAATTGTAATACAACTTTCAGCAACGGATCTCTTGGCTCTCGCATCGA
TGAAGAACGCAGCGAAATGCGATAAGTAATGTGAATTGCAGAATTCAGTGAATCATCGAA
TCTTTGAACGCATCTTGCGCTCCTTGGTATTCCGAGGAGCATGCCTGTTTGAGTGTCATT
AAATTCTCAACTCTCTTATACTTTTTTGTAAAAGAGAGCTTGGACTGTGGAGGCTTGCTG
GCCACTTTTTGGGGTCAGCTCCTCTGAAATGCATTAGCGGAACCGTTTGCGATCTGCCAC
AAGTGTGATAAGTTATCTACACTGGCGAGGGGATTGCTCTCTGTAATGTTCAGCTTCTAA
TTGTCTCTACTTTGTGAGACTACTTTTGAATGCTTGACCTCAAATCAGGTAGGACTACCC
GCTGAACTTAA
>AB9-3
TTTCCGTAGGTGAACCTGCGGAAGGATCATTATTGAATTATGTTTCTAGATAGGTTGTAG
CTGGCTCTTTTAGAGCATGTGCACGCCTGTTTGGACTTCATTTTCATCCACCTGTGCACC
TATTGTAGTCTTTGGTTGGGTTAGGAGGAAGTGATCATTGTATCAGCATCTGCTGGGAGT
GAGGACTTGCATTGTGAAAGCTTTGCTGTCCTTGATGTGATCATGGAATCTTTTTCTCAC
TAGAGTCTATGTCACTCATTATACTCTGTCGAATGTCATTGAATGTCTTTACATGGGCTT
GTATGCCTATGAAAATTGTAATACAACTTTCAGCAACGGATCTCTTGGCTCTCGCATCGA
TGAAGAACGCAGCGAAATGCGATAAGTAATGTGAATTGCAGAATTCAGTGAATCATCGAA
TCTTTGAACGCATCTTGCGCTCCTTGGTATTCCGAGGAGCATGCCTGTTTGAGTGTCATT
AAATTCTCAACTCTCTTATACTTTTTTGTAAAAGAGAGCTTGGACTGTGGAGGCTTGCTG
GCCACTTTTTGGGGTCAGCTCCTCTGAAATGCATTAGCGGAACCGTTTGCGATCTGCCAC
AAGTGTGATAAGTTATCTACACTGGCGAGGGGATTGCTCTCTGTAATGTTCAGCTTCTAA
TTGTCTCTACTTTGTGAGACTACTTTTGAATGCTTGACCTCAAATCAGGTAGGACTACCC
GCTGAACTTAA
>AB9-6
TTTCCGTAGGTGAACCTGCGGAAGGATCATTATTGAATTATGTTTCTAGATAGGTTGTAG
CTGGCTCTTTTAGAGCATGTGCACGCCTGTTTGGACTTCATTTTCATCCACCTGTGCACC
TATTGTAGTCTTTGGTTGGGTTAGGAGGAAGTGATCATTGTATCAGCATCTGCTGGGAGT
GAGGACTTGCATTGTGAAAGCTTTGCTGTCCTTGATGTGATCATGGAATCTTTTTCTCAC
TAGAGTCTATGTCACTCATTATACTCTGTCGAATGTCATTGAATGTCTTTACATGGGCTT
GTATGCCTATGAAAATTGTAATACAACTTTCAGCAACGGATCTCTTGGCTCTCGCATCGA
TGAAGAACGCAGCGAAATGCGATAAGTAATGTGAATTGCAGAATTCAGTGAATCATCGAA
TCTTTGAACGCATCTTGCGCTCCTTGGTATTCCGAGGAGCATGCCTGTTTGAGTGTCATT
AAATTCTCAACTCTCTTATACTTTTTTGTAAAAGAGAGCTTGGACTGTGGAGGCTTGCTG
GCCACTTTTTGGGGTCAGCTCCTCTGAAATGCATTAGCGGAACCGTTTGCGATCTGCCAC
AAGTGTGATAAGTTATCTACACTGGCGAGGGGATTGCTCTCTGTAATGTTCAGCTTCTAA
TTGTCTCTACTTTGTGAGACTACTTTTGAATGCTTGACCTCAAATCAGGTAGGACTACCC
GCTGAACTTAA
>AB9-7
TTTCCGTAGGTGAACCTGCGGAAGGATCATTATTGAATTATGTTTCTAGATAGGTTGTAG
CTGGCTCTTTTAGAGCATGTGCACGCCTGTTTGGACTTCATTTTCATCCACCTGTGCACC
TATTGTAGTCTTTGGTTGGGTTAGGAGGAAGTGATCATTGTATCAGCATCTGCTGGGAGT
GAGGACTTGCATTGTGAAAGCTTTGCTGTCCTTGATGTGATCATGGAATCTTTTTCTCAC
TAGAGTCTATGTCACTCATTATACTCTGTCGAATGTCATTGAATGTCTTTACATGGGCTT
GTATGCCTATGAAAATTGTAATACAACTTTCAGCAACGGATCTCTTGGCTCTCGCATCGA
TGAAGAACGCAGCGAAATGCGATAAGTAATGTGAATTGCAGAATTCAGTGAATCATCGAA
TCTTTGAACGCATCTTGCGCTCCTTGGTATTCCGAGGAGCATGCCTGTTTGAGTGTCATT
AAATTCTCAACTCTCTTATACTTTTTTGTAAAAGAGAGCTTGGACTGTGGAGGCTTGCTG

GCCACTTTTTGGGGTCAGCTCCTCTGAAATGCATTAGCGGAACCGTTTGCGATCTGCCAC
AAGTGTGATAAGTTATCTACACTGGCGAGGGGATTGCTCTCTGTAATGTTCAGCTTCTAA
TTGTCTCTACTTTGTGAGACTACTTTTGAATGCTTGACCTCAAATCAGGTAGGACTACCC
GCTGAACTTAA
>AB9−16
TTTCCGTAGGTGAACCTGCGGAAGGATCATTATTGAATTATGTTTCTAGATAGGTTGTAG
CTGGCTCTTTTAGAGCATGTGCACGCCTGTTTGGACTTCATTTTCATCCACCTGTGCACC
TATTGTAGTCTTTGGTTGGGTTAGGAGGAAGTGATCATTGTATCAGCATCTGCTGGGAGT
GAGGACTTGCATTGTGAAAGCTTTGCTGTCCTTGATGTGATCATGGAATCTTTTTCTCAC
TAGAGTCTATGTCACTCATTATACTCTGTCGAATGTCATTGAATGTCTTTACATGGGCTT
GTATGCCTATGAAAATTGTAATACAACTTTCAGCAACGGATCTCTTGGCTCTCGCATCGA
TGAAGAACGCAGCGAAATGCGATAAGTAATGTGAATTGCAGAATTCAGTGAATCATCGAA
TCTTTGAACGCATCTTGCGCTCCTTGGTATTCCGAGGAGCATGCCTGTTTGAGTGTCATT
AAATTCTCAACTCTCTTATACTTTTTTGTAAAAGAGAGCTTGGACTGTGGAGGCTTGCTG
GCCACTTTTTGGGGTCAGCTCCTCTGAAATGCATTAGCGGAACCGTTTGCGATCTGCCAC
AAGTGTGATAAGTTATCTACACTGGCGAGGGGATTGCTCTCTGTAATGTTCAGCTTCTAA
TTGTCTCTACTTTGTGAGACTACTTTTGAATGCTTGACCTCAAATCAGGTAGGACTACCC
GCTGAACTTAA
>AB9−19
TTTCCGTAGGTGAACCTGCGGAAGGATCATTATTGAATTATGTTTCTAGATAGGTTGTAG
CTGGCTCTTTTAGAGCATGTGCACGCCTGTTTGGACTTCATTTTCATCCACCTGTGCACC
TATTGTAGTCTTTGGTTGGGTTAGGAGGAAGTGATCATTGTATCAGCATCTGCTGGGAGT
GAGGACTTGCATTGTGAAAGCTTTGCTGTCCTTGATGTGATCATGGAATCTTTTTCTCAC
TAGAGTCTATGTCACTCATTATACTCTGTCGAATGTCATTGAATGTCTTTACATGGGCTT
GTATGCCTATGAAAATTGTAATACAACTTTCAGCAACGGATCTCTTGGCTCTCGCATCGA
TGAAGAACGCAGCGAAATGCGATAAGTAATGTGAATTGCAGAATTCAGTGAATCATCGAA
TCTTTGAACGCATCTTGCGCTCCTTGGTATTCCGAGGAGCATGCCTGTTTGAGTGTCATT
AAATTCTCAACTCTCTTATACTTTTTTGTAAAAGAGAGCTTGGACTGTGGAGGCTTGCTG
GCCACTTTTTGGGGTCAGCTCCTCTGAAATGCATTAGCGGAACCGTTTGCGATCTGCCAC
AAGTGTGATAAGTTATCTACACTGGCGAGGGGATTGCTCTCTGTAATGTTCAGCTTCTAA
TTGTCTCTACTTTGTGAGACTACTTTTGAATGCTTGACCTCAAATCAGGTAGGACTACCC
GCTGAACTTAA
>AB9−22
TTTCCGTAGGTGAACCTGCGGAAGGATCATTATTGAATTATGTTTCTAGATAGGTTGTAG
CTGGCTCTTTTAGAGCATGTGCACGCCTGTTTGGACTTCATTTTCATCCACCTGTGCACC
TATTGTAGTCTTTGGTTGGGTTAGGAGGAAGTGATCATTGTATCAGCATCTGCTGGGAGT
GAGGACTTGCATTGTGAAAGCTTTGCTGTCCTTGATGTGATCATGGAATCTTTTTCTCAC
TAGAGTCTATGTCACTCATTATACTCTGTCGAATGTCATTGAATGTCTTTACATGGGCTT
GTATGCCTATGAAAATTGTAATACAACTTTCAGCAACGGATCTCTTGGCTCTCGCATCGA
TGAAGAACGCAGCGAAATGCGATAAGTAATGTGAATTGCAGAATTCAGTGAATCATCGAA
TCTTTGAACGCATCTTGCGCTCCTTGGTATTCCGAGGAGCATGCCTGTTTGAGTGTCATT
AAATTCTCAACTCTCTTATACTTTTTTGTAAAAGAGAGCTTGGACTGTGGAGGCTTGCTG
GCCACTTTTTGGGGTCAGCTCCTCTGAAATGCATTAGCGGAACCGTTTGCGATCTGCCAC
AAGTGTGATAAGTTATCTACACTGGCGAGGGGATTGCTCTCTGTAATGTTCAGCTTCTAA
TTGTCTCTACTTTGTGAGACTACTTTTGAATGCTTGACCTCAAATCAGGTAGGACTACCC
GCTGAACTTAA
>AB9−23
TTTCCGTAGGTGAACCTGCGGAAGGATCATTATTGAATTATGTTTCTAGATAGGTTGTAG
CTGGCTCTTTTAGAGCATGTGCACGCCTGTTTGGACTTCATTTTCATCCACCTGTGCACC
TATTGTAGTCTTTGGTTGGGTTAGGAGGAAGTGATCATTGTATCAGCATCTGCTGGGAGT

GAGGACTTGCATTGTGAAAGCTTTGCTGTCCTTGATGTGATCATGGAATCTTTTTCTCAC
TAGAGTCTATGTCACTCATTATACTCTGTCGAATGTCATTGAATGTCTTTACATGGGCTT
GTATGCCTATGAAAATTGTAATACAACTTTCAGCAACGGATCTCTTGGCTCTCGCATCGA
TGAAGAACGCAGCGAAATGCGATAAGTAATGTGAATTGCAGAATTCAGTGAATCATCGAA
TCTTTGAACGCATCTTGCGCTCCTTGGTATTCCGAGGAGCATGCCTGTTTGAGTGTCATT
AAATTCTCAACTCTCTTATACTTTTTTGTAAAAGAGAGCTTGGACTGTGGAGGCTTGCTG
GCCACTTTTTGGGGTCAGCTCCTCTGAAATGCATTAGCGGAACCGTTTGCGATCTGCCAC
AAGTGTGATAAGTTATCTACACTGGCGAGGGGATTGCTCTCTGTAATGTTCAGCTTCTAA
TTGTCTCTACTTTGTGAGACTACTTTTGAATGCTTGACCTCAAATCAGGTAGGACTACCC
GCTGAACTTAA
>AB9–28
TTTCCGTAGGTGAACCTGCGGAAGGATCATTATTGAATTATGTTTCTAGATAGGTTGTAG
CTGGCTCTTTTAGAGCATGTGCACGCCTGTTTGGACTTCATTTTCATCCACCTGTGCACC
TATTGTAGTCTTTGGTTGGGTTAGGAGGAAGTGATCATTGTATCAGCATCTGCTGGGAGT
GAGGACTTGCATTGTGAAAGCTTTGCTGTCCTTGATGTGATCATGGAATCTTTTTCTCAC
TAGAGTCTATGTCACTCATTATACTCTGTCGAATGTCATTGAATGTCTTTACATGGGCTT
GTATGCCTATGAAAATTGTAATACAACTTTCAGCAACGGATCTCTTGGCTCTCGCATCGA
TGAAGAACGCAGCGAAATGCGATAAGTAATGTGAATTGCAGAATTCAGTGAATCATCGAA
TCTTTGAACGCATCTTGCGCTCCTTGGTATTCCGAGGAGCATGCCTGTTTGAGTGTCATT
AAATTCTCAACTCTCTTATACTTTTTTGTAAAAGAGAGCTTGGACTGTGGAGGCTTGCTG
GCCACTTTTTGGGGTCAGCTCCTCTGAAATGCATTAGCGGAACCGTTTGCGATCTGCCAC
AAGTGTGATAAGTTATCTACACTGGCGAGGGGATTGCTCTCTGTAATGTTCAGCTTCTAA
TTGTCTCTACTTTGTGAGACTACTTTTGAATGCTTGACCTCAAATCAGGTAGGACTACCC
GCTGAACTTAA
>AB9–29
TTTCCGTAGGTGAACCTGCGGAAGGATCATTATTGAATTATGTTTCTAGATAGGTTGTAG
CTGGCTCTTTTAGAGCATGTGCACGCCTGTTTGGACTTCATTTTCATCCACCTGTGCACC
TATTGTAGTCTTTGGTTGGGTTAGGAGGAAGTGATCATTGTATCAGCATCTGCTGGGAGT
GAGGACTTGCATTGTGAAAGCTTTGCTGTCCTTGATGTGATCATGGAATCTTTTTCTCAC
TAGAGTCTATGTCACTCATTATACTCTGTCGAATGTCATTGAATGTCTTTACATGGGCTT
GTATGCCTATGAAAATTGTAATACAACTTTCAGCAACGGATCTCTTGGCTCTCGCATCGA
TGAAGAACGCAGCGAAATGCGATAAGTAATGTGAATTGCAGAATTCAGTGAATCATCGAA
TCTTTGAACGCATCTTGCGCTCCTTGGTATTCCGAGGAGCATGCCTGTTTGAGTGTCATT
AAATTCTCAACTCTCTTATACTTTTTTGTAAAAGAGAGCTTGGACTGTGGAGGCTTGCTG
GCCACTTTTTGGGGTCAGCTCCTCTGAAATGCATTAGCGGAACCGTTTGCGATCTGCCAC
AAGTGTGATAAGTTATCTACACTGGCGAGGGGATTGCTCTCTGTAATGTTCAGCTTCTAA
TTGTCTCTACTTTGTGAGACTACTTTTGAATGCTTGACCTCAAATCAGGTAGGACTACCC
GCTGAACTTAA
>AB9–30
TTTCCGTAGGTGAACCTGCGGAAGGATCATTATTGAATTATGTTTCTAGATAGGTTGTAG
CTGGCTCTTTTAGAGCATGTGCACGCCTGTTTGGACTTCATTTTCATCCACCTGTGCACC
TATTGTAGTCTTTGGTTGGGTTAGGAGGAAGTGATCATTGTATCAGCATCTGCTGGGAGT
GAGGACTTGCATTGTGAAAGCTTTGCTGTCCTTGATGTGATCATGGAATCTTTTTCTCAC
TAGAGTCTATGTCACTCATTATACTCTGTCGAATGTCATTGAATGTCTTTACATGGGCTT
GTATGCCTATGAAAATTGTAATACAACTTTCAGCAACGGATCTCTTGGCTCTCGCATCGA
TGAAGAACGCAGCGAAATGCGATAAGTAATGTGAATTGCAGAATTCAGTGAATCATCGAA
TCTTTGAACGCATCTTGCGCTCCTTGGTATTCCGAGGAGCATGCCTGTTTGAGTGTCATT
AAATTCTCAACTCTCTTATACTTTTTTGTAAAAGAGAGCTTGGACTGTGGAGGCTTGCTG
GCCACTTTTTGGGGTCAGCTCCTCTGAAATGCATTAGCGGAACCGTTTGCGATCTGCCAC
AAGTGTGATAAGTTATCTACACTGGCGAGGGGATTGCTCTCTGTAATGTTCAGCTTCTAA

TTGTCTCTACTTTGTGAGACTACTTTTGAATGCTTGACCTCAAATCAGGTAGGACTACCC
GCTGAACTTAA
>AB9-32
TTTCCGTAGGTGAACCTGCGGAAGGATCATTATTGAATTATGTTTCTAGATAGGTTGTAG
CTGGCTCTTTTAGAGCATGTGCACGCCTGTTTGGACTTCATTTTCATCCACCTGTGCACC
TATTGTAGTCTTTGGTTGGGTTAGGAGGAAGTGATCATTGTATCAGCATCTGCTGGGAGT
GAGGACTTGCATTGTGAAAGCTTTGCTGTCCTTGATGTGATCATGGAATCTTTTTCTCAC
TAGAGTCTATGTCACTCATTATACTCTGTCGAATGTCATTGAATGTCTTTACATGGGCTT
GTATGCCTATGAAAATTGTAATACAACTTTCAGCAACGGATCTCTTGGCTCTCGCATCGA
TGAAGAACGCAGCGAAATGCGATAAGTAATGTGAATTGCAGAATTCAGTGAATCATCGAA
TCTTTGAACGCATCTTGCGCTCCTTGGTATTCCGAGGAGCATGCCTGTTTGAGTGTCATT
AAATTCTCAACTCTCTTATACTTTTTTGTAAAAGAGAGCTTGGACTGTGGAGGCTTGCTG
GCCACTTTTTGGGGTCAGCTCCTCTGAAATGCATTAGCGGAACCGTTTGCGATCTGCCAC
AAGTGTGATAAGTTATCTACACTGGCGAGGGGATTGCTCTCTGTAATGTTCAGCTTCTAA
TTGTCTCTACTTTGTGAGACTACTTTTGAATGCTTGACCTCAAATCAGGTAGGACTACCC
GCTGAACTTAA
>AB9-33
TTTCCGTAGGTGAACCTGCGGAAGGATCATTATTGAATTATGTTTCTAGATAGGTTGTAG
CTGGCTCTTTTAGAGCATGTGCACGCCTGTTTGGACTTCATTTTCATCCACCTGTGCACC
TATTGTAGTCTTTGGTTGGGTTAGGAGGAAGTGATCATTGTATCAGCATCTGCTGGGAGT
GAGGACTTGCATTGTGAAAGCTTTGCTGTCCTTGATGTGATCATGGAATCTTTTTCTCAC
TAGAGTCTATGTCACTCATTATACTCTGTCGAATGTCATTGAATGTCTTTACATGGGCTT
GTATGCCTATGAAAATTGTAATACAACTTTCAGCAACGGATCTCTTGGCTCTCGCATCGA
TGAAGAACGCAGCGAAATGCGATAAGTAATGTGAATTGCAGAATTCAGTGAATCATCGAA
TCTTTGAACGCATCTTGCGCTCCTTGGTATTCCGAGGAGCATGCCTGTTTGAGTGTCATT
AAATTCTCAACTCTCTTATACTTTTTTGTAAAAGAGAGCTTGGACTGTGGAGGCTTGCTG
GCCACTTTTTGGGGTCAGCTCCTCTGAAATGCATTAGCGGAACCGTTTGCGATCTGCCAC
AAGTGTGATAAGTTATCTACACTGGCGAGGGGATTGCTCTCTGTAATGTTCAGCTTCTAA
TTGTCTCTACTTTGTGAGACTACTTTTGAATGCTTGACCTCAAATCAGGTAGGACTACCC
GCTGAACTTAA
>AB9-34
TTTCCGTAGGTGAACCTGCGGAAGGATCATTATTGAATTATGTTTCTAGATAGGTTGTAG
CTGGCTCTTTTAGAGCATGTGCACGCCTGTTTGGACTTCATTTTCATCCACCTGTGCACC
TATTGTAGTCTTTGGTTGGGTTAGGAGGAAGTGATCATTGTATCAGCATCTGCTGGGAGT
GAGGACTTGCATTGTGAAAGCTTTGCTGTCCTTGATGTGATCATGGAATCTTTTTCTCAC
TAGAGTCTATGTCACTCATTATACTCTGTCGAATGTCATTGAATGTCTTTACATGGGCTT
GTATGCCTATGAAAATTGTAATACAACTTTCAGCAACGGATCTCTTGGCTCTCGCATCGA
TGAAGAACGCAGCGAAATGCGATAAGTAATGTGAATTGCAGAATTCAGTGAATCATCGAA
TCTTTGAACGCATCTTGCGCTCCTTGGTATTCCGAGGAGCATGCCTGTTTGAGTGTCATT
AAATTCTCAACTCTCTTATACTTTTTTGTAAAAGAGAGCTTGGACTGTGGAGGCTTGCTG
GCCACTTTTTGGGGTCAGCTCCTCTGAAATGCATTAGCGGAACCGTTTGCGATCTGCCAC
AAGTGTGATAAGTTATCTACACTGGCGAGGGGATTGCTCTCTGTAATGTTCAGCTTCTAA
TTGTCTCTACTTTGTGAGACTACTTTTGAATGCTTGACCTCAAATCAGGTAGGACTACCC
GCTGAACTTAA
>AB9-40
TTTCCGTAGGTGAACCTGCGGAAGGATCATTATTGAATTATGTTTCTAGATAGGTTGTAG
CTGGCTCTTTTAGAGCATGTGCACGCCTGTTTGGACTTCATTTTCATCCACCTGTGCACC
TATTGTAGTCTTTGGTTGGGTTAGGAGGAAGTGATCATTGTATCAGCATCTGCTGGGAGT
GAGGACTTGCATTGTGAAAGCTTTGCTGTCCTTGATGTGATCATGGAATCTTTTTCTCAC
TAGAGTCTATGTCACTCATTATACTCTGTCGAATGTCATTGAATGTCTTTACATGGGCTT

GTATGCCTATGAAAATTGTAATACAACTTTCAGCAACGGATCTCTTGGCTCTCGCATCGA
TGAAGAACGCAGCGAAATGCGATAAGTAATGTGAATTGCAGAATTCAGTGAATCATCGAA
TCTTTGAACGCATCTTGCGCTCCTTGGTATTCCGAGGAGCATGCCTGTTTGAGTGTCATT
AAATTCTCAACTCTCTTATACTTTTTTGTAAAAGAGAGCTTGGACTGTGGAGGCTTGCTG
GCCACTTTTTGGGGTCAGCTCCTCTGAAATGCATTAGCGGAACCGTTTGCGATCTGCCAC
AAGTGTGATAAGTTATCTACACTGGCGAGGGGATTGCTCTCTGTAATGTTCAGCTTCTAA
TTGTCTCTACTTTGTGAGACTACTTTTGAATGCTTGACCTCAAATCAGGTAGGACTACCC
GCTGAACTTAA
>AB9-45
TTTCCGTAGGTGAACCTGCGGAAGGATCATTATTGAATTATGTTTCTAGATAGGTTGTAG
CTGGCTCTTTTAGAGCATGTGCACGCCTGTTTGGACTTCATTTTCATCCACCTGTGCACC
TATTGTAGTCTTTGGTTGGGTTAGGAGGAAGTGATCATTGTATCAGCATCTGCTGGGAGT
GAGGACTTGCATTGTGAAAGCTTTGCTGTCCTTGATGTGATCATGGAATCTTTTTCTCAC
TAGAGTCTATGTCACTCATTATACTCTGTCGAATGTCATTGAATGTCTTTACATGGGCTT
GTATGCCTATGAAAATTGTAATACAACTTTCAGCAACGGATCTCTTGGCTCTCGCATCGA
TGAAGAACGCAGCGAAATGCGATAAGTAATGTGAATTGCAGAATTCAGTGAATCATCGAA
TCTTTGAACGCATCTTGCGCTCCTTGGTATTCCGAGGAGCATGCCTGTTTGAGTGTCATT
AAATTCTCAACTCTCTTATACTTTTTTGTAAAAGAGAGCTTGGACTGTGGAGGCTTGCTG
GCCACTTTTTGGGGTCAGCTCCTCTGAAATGCATTAGCGGAACCGTTTGCGATCTGCCAC
AAGTGTGATAAGTTATCTACACTGGCGAGGGGATTGCTCTCTGTAATGTTCAGCTTCTAA
TTGTCTCTACTTTGTGAGACTACTTTTGAATGCTTGACCTCAAATCAGGTAGGACTACCC
GCTGAACTTAA
>AB9-48
TTTCCGTAGGTGAACCTGCGGAAGGATCATTATTGAATTATGTTTCTAGATAGGTTGTAG
CTGGCTCTTTTAGAGCATGTGCACGCCTGTTTGGACTTCATTTTCATCCACCTGTGCACC
TATTGTAGTCTTTGGTTGGGTTAGGAGGAAGTGATCATTGTATCAGCATCTGCTGGGAGT
GAGGACTTGCATTGTGAAAGCTTTGCTGTCCTTGATGTGATCATGGAATCTTTTTCTCAC
TAGAGTCTATGTCACTCATTATACTCTGTCGAATGTCATTGAATGTCTTTACATGGGCTT
GTATGCCTATGAAAATTGTAATACAACTTTCAGCAACGGATCTCTTGGCTCTCGCATCGA
TGAAGAACGCAGCGAAATGCGATAAGTAATGTGAATTGCAGAATTCAGTGAATCATCGAA
TCTTTGAACGCATCTTGCGCTCCTTGGTATTCCGAGGAGCATGCCTGTTTGAGTGTCATT
AAATTCTCAACTCTCTTATACTTTTTTGTAAAAGAGAGCTTGGACTGTGGAGGCTTGCTG
GCCACTTTTTGGGGTCAGCTCCTCTGAAATGCATTAGCGGAACCGTTTGCGATCTGCCAC
AAGTGTGATAAGTTATCTACACTGGCGAGGGGATTGCTCTCTGTAATGTTCAGCTTCTAA
TTGTCTCTACTTTGTGAGACTACTTTTGAATGCTTGACCTCAAATCAGGTAGGACTACCC
GCTGAACTTAA
>AB9-50
TTTCCGTAGGTGAACCTGCGGAAGGATCATTATTGAATTATGTTTCTAGATAGGTTGTAG
CTGGCTCTTTTAGAGCATGTGCACGCCTGTTTGGACTTCATTTTCATCCACCTGTGCACC
TATTGTAGTCTTTGGTTGGGTTAGGAGGAAGTGATCATTGTATCAGCATCTGCTGGGAGT
GAGGACTTGCATTGTGAAAGCTTTGCTGTCCTTGATGTGATCATGGAATCTTTTTCTCAC
TAGAGTCTATGTCACTCATTATACTCTGTCGAATGTCATTGAATGTCTTTACATGGGCTT
GTATGCCTATGAAAATTGTAATACAACTTTCAGCAACGGATCTCTTGGCTCTCGCATCGA
TGAAGAACGCAGCGAAATGCGATAAGTAATGTGAATTGCAGAATTCAGTGAATCATCGAA
TCTTTGAACGCATCTTGCGCTCCTTGGTATTCCGAGGAGCATGCCTGTTTGAGTGTCATT
AAATTCTCAACTCTCTTATACTTTTTTGTAAAAGAGAGCTTGGACTGTGGAGGCTTGCTG
GCCACTTTTTGGGGTCAGCTCCTCTGAAATGCATTAGCGGAACCGTTTGCGATCTGCCAC
AAGTGTGATAAGTTATCTACACTGGCGAGGGGATTGCTCTCTGTAATGTTCAGCTTCTAA
TTGTCTCTACTTTGTGAGACTACTTTTGAATGCTTGACCTCAAATCAGGTAGGACTACCC
GCTGAACTTAA

>AB9-52
TTTCCGTAGGTGAACCTGCGGAAGGATCATTATTGAATTATGTTTCTAGATAGGTTGTAG
CTGGCTCTTTTAGAGCATGTGCACGCCTGTTTGGACTTCATTTTCATCCACCTGTGCACC
TATTGTAGTCTTTGGTTGGGTTAGGAGGAAGTGATCATTGTATCAGCATCTGCTGGGAGT
GAGGACTTGCATTGTGAAAGCTTTGCTGTCCTTGATGTGATCATGGAATCTTTTTCTCAC
TAGAGTCTATGTCACTCATTATACTCTGTCGAATGTCATTGAATGTCTTTACATGGGCTT
GTATGCCTATGAAAATTGTAATACAACTTTCAGCAACGGATCTCTTGGCTCTCGCATCGA
TGAAGAACGCAGCGAAATGCGATAAGTAATGTGAATTGCAGAATTCAGTGAATCATCGAA
TCTTTGAACGCATCTTGCGCTCCTTGGTATTCCGAGGAGCATGCCTGTTTGAGTGTCATT
AAATTCTCAACTCTCTTATACTTTTTTGTAAAAGAGAGCTTGGACTGTGGAGGCTTGCTG
GCCACTTTTTGGGGTCAGCTCCTCTGAAATGCATTAGCGGAACCGTTTGCGATCTGCCAC
AAGTGTGATAAGTTATCTACACTGGCGAGGGGATTGCTCTCTGTAATGTTCAGCTTCTAA
TTGTCTCTACTTTGTGAGACTACTTTTGAATGCTTGACCTCAAATCAGGTAGGACTACCC
GCTGAACTTAA
>AB9-54
TTTCCGTAGGTGAACCTGCGGAAGGATCATTATTGAATTATGTTTCTAGATAGGTTGTAG
CTGGCTCTTTTAGAGCATGTGCACGCCTGTTTGGACTTCATTTTCATCCACCTGTGCACC
TATTGTAGTCTTTGGTTGGGTTAGGAGGAAGTGATCATTGTATCAGCATCTGCTGGGAGT
GAGGACTTGCATTGTGAAAGCTTTGCTGTCCTTGATGTGATCATGGAATCTTTTTCTCAC
TAGAGTCTATGTCACTCATTATACTCTGTCGAATGTCATTGAATGTCTTTACATGGGCTT
GTATGCCTATGAAAATTGTAATACAACTTTCAGCAACGGATCTCTTGGCTCTCGCATCGA
TGAAGAACGCAGCGAAATGCGATAAGTAATGTGAATTGCAGAATTCAGTGAATCATCGAA
TCTTTGAACGCATCTTGCGCTCCTTGGTATTCCGAGGAGCATGCCTGTTTGAGTGTCATT
AAATTCTCAACTCTCTTATACTTTTTTGTAAAAGAGAGCTTGGACTGTGGAGGCTTGCTG
GCCACTTTTTGGGGTCAGCTCCTCTGAAATGCATTAGCGGAACCGTTTGCGATCTGCCAC
AAGTGTGATAAGTTATCTACACTGGCGAGGGGATTGCTCTCTGTAATGTTCAGCTTCTAA
TTGTCTCTACTTTGTGAGACTACTTTTGAATGCTTGACCTCAAATCAGGTAGGACTACCC
GCTGAACTTAA
>AB10-1
TTTCCGTAGGTGAACCTGCGGAAGGATCATTATTGAATTATGTTTCTAGATAGGTTGTAG
CTGGCTCTTTTAGAGCATGTGCACGCCTGTTTGGACTTCATTTTCATCCACCTGTGCACC
TATTGTAGTCTTTGGTTGGGTTAGGAGGAAGTGATCATTGTATCAGCATCTGCTGGGAGT
GAGGACTTGCATTGTGAAAGCTTTGCTGTCCTTGATGTGATCATGGAATCTTTTTCTCAC
TAGAGTCTATGTCACTCATTATACTCTGTCGAATGTCATTGAATGTCTTTACATGGGCTT
GTATGCCTATGAAAATTGTAATACAACTTTCAGCAACGGATCTCTTGGCTCTCGCATCGA
TGAAGAACGCAGCGAAATGCGATAAGTAATGTGAATTGCAGAATTCAGTGAATCATCGAA
TCTTTGAACGCATCTTGCGCTCCTTGGTATTCCGAGGAGCATGCCTGTTTGAGTGTCATT
AAATTCTCAACTCTCTTATACTTTTTTGTAAAAGAGAGCTTGGACTGTGGAGGCTTGCTG
GCCACTTTTTGGGGTCAGCTCCTCTGAAATGCATTAGCGGAACCGTTTGCGATCTGCCAC
AAGTGTGATAAGTTATCTACACTGGCGAGGGGATTGCTCTCTGTAATGTTCAGCTTCTAA
TTGTCTCTACTTTGTGAGACTACTTTTGAATGCTTGACCTCAAATCAGGTAGGACTACCC
GCTGAACTTAA
>AB10-4
TTTCCGTAGGTGAACCTGCGGAAGGATCATTATTGAATTATGTTTCTAGATAGGTTGTAG
CTGGCTCTTTTAGAGCATGTGCACGCCTGTTTGGACTTCATTTTCATCCACCTGTGCACC
TATTGTAGTCTTTGGTTGGGTTAGGAGGAAGTGATCATTGTATCAGCATCTGCTGGGAGT
GAGGACTTGCATTGTGAAAGCTTTGCTGTCCTTGATGTGATCATGGAATCTTTTTCTCAC
TAGAGTCTATGTCACTCATTATACTCTGTCGAATGTCATTGAATGTCTTTACATGGGCTT
GTATGCCTATGAAAATTGTAATACAACTTTCAGCAACGGATCTCTTGGCTCTCGCATCGA
TGAAGAACGCAGCGAAATGCGATAAGTAATGTGAATTGCAGAATTCAGTGAATCATCGAA

TCTTTGAACGCATCTTGCGCTCCTTGGTATTCCGAGGAGCATGCCTGTTTGAGTGTCATT
AAATTCTCAACTCTCTTATACTTTTTTGTAAAAGAGAGCTTGGACTGTGGAGGCTTGCTG
GCCACTTTTTGGGGTCAGCTCCTCTGAAATGCATTAGCGGAACCGTTTGCGATCTGCCAC
AAGTGTGATAAGTTATCTACACTGGCGAGGGGATTGCTCTCTGTAATGTTCAGCTTCTAA
TTGTCTCTACTTTGTGAGACTACTTTTGAATGCTTGACCTCAAATCAGGTAGGACTACCC
GCTGAACTTAA
>AB10-12
TTTCCGTAGGTGAACCTGCGGAAGGATCATTATTGAATTATGTTTCTAGATAGGTTGTAG
CTGGCTCTTTTAGAGCATGTGCACGCCTGTTTGGACTTCATTTTCATCCACCTGTGCACC
TATTGTAGTCTTTGGTTGGGTTAGGAGGAAGTGATCATTGTATCAGCATCTGCTGGGAGT
GAGGACTTGCATTGTGAAAGCTTTGCTGTCCTTGATGTGATCATGGAATCTTTTTCTCAC
TAGAGTCTATGTCACTCATTATACTCTGTCGAATGTCATTGAATGTCTTTACATGGGCTT
GTATGCCTATGAAAATTGTAATACAACTTTCAGCAACGGATCTCTTGGCTCTCGCATCGA
TGAAGAACGCAGCGAAATGCGATAAGTAATGTGAATTGCAGAATTCAGTGAATCATCGAA
TCTTTGAACGCATCTTGCGCTCCTTGGTATTCCGAGGAGCATGCCTGTTTGAGTGTCATT
AAATTCTCAACTCTCTTATACTTTTTTGTAAAAGAGAGCTTGGACTGTGGAGGCTTGCTG
GCCACTTTTTGGGGTCAGCTCCTCTGAAATGCATTAGCGGAACCGTTTGCGATCTGCCAC
AAGTGTGATAAGTTATCTACACTGGCGAGGGGATTGCTCTCTGTAATGTTCAGCTTCTAA
TTGTCTCTACTTTGTGAGACTACTTTTGAATGCTTGACCTCAAATCAGGTAGGACTACCC
GCTGAACTTAA
>AB10-18
TTTCCGTAGGTGAACCTGCGGAAGGATCATTATTGAATTATGTTTCTAGATAGGTTGTAG
CTGGCTCTTTTAGAGCATGTGCACGCCTGTTTGGACTTCATTTTCATCCACCTGTGCACC
TATTGTAGTCTTTGGTTGGGTTAGGAGGAAGTGATCATTGTATCAGCATCTGCTGGGAGT
GAGGACTTGCATTGTGAAAGCTTTGCTGTCCTTGATGTGATCATGGAATCTTTTTCTCAC
TAGAGTCTATGTCACTCATTATACTCTGTCGAATGTCATTGAATGTCTTTACATGGGCTT
GTATGCCTATGAAAATTGTAATACAACTTTCAGCAACGGATCTCTTGGCTCTCGCATCGA
TGAAGAACGCAGCGAAATGCGATAAGTAATGTGAATTGCAGAATTCAGTGAATCATCGAA
TCTTTGAACGCATCTTGCGCTCCTTGGTATTCCGAGGAGCATGCCTGTTTGAGTGTCATT
AAATTCTCAACTCTCTTATACTTTTTTGTAAAAGAGAGCTTGGACTGTGGAGGCTTGCTG
GCCACTTTTTGGGGTCAGCTCCTCTGAAATGCATTAGCGGAACCGTTTGCGATCTGCCAC
AAGTGTGATAAGTTATCTACACTGGCGAGGGGATTGCTCTCTGTAATGTTCAGCTTCTAA
TTGTCTCTACTTTGTGAGACTACTTTTGAATGCTTGACCTCAAATCAGGTAGGACTACCC
GCTGAACTTAA
>AB10-20
TTTCCGTAGGTGAACCTGCGGAAGGATCATTATTGAATTATGTTTCTAGATAGGTTGTAG
CTGGCTCTTTTAGAGCATGTGCACGCCTGTTTGGACTTCATTTTCATCCACCTGTGCACC
TATTGTAGTCTTTGGTTGGGTTAGGAGGAAGTGATCATTGTATCAGCATCTGCTGGGAGT
GAGGACTTGCATTGTGAAAGCTTTGCTGTCCTTGATGTGATCATGGAATCTTTTTCTCAC
TAGAGTCTATGTCACTCATTATACTCTGTCGAATGTCATTGAATGTCTTTACATGGGCTT
GTATGCCTATGAAAATTGTAATACAACTTTCAGCAACGGATCTCTTGGCTCTCGCATCGA
TGAAGAACGCAGCGAAATGCGATAAGTAATGTGAATTGCAGAATTCAGTGAATCATCGAA
TCTTTGAACGCATCTTGCGCTCCTTGGTATTCCGAGGAGCATGCCTGTTTGAGTGTCATT
AAATTCTCAACTCTCTTATACTTTTTTGTAAAAGAGAGCTTGGACTGTGGAGGCTTGCTG
GCCACTTTTTGGGGTCAGCTCCTCTGAAATGCATTAGCGGAACCGTTTGCGATCTGCCAC
AAGTGTGATAAGTTATCTACACTGGCGAGGGGATTGCTCTCTGTAATGTTCAGCTTCTAA
TTGTCTCTACTTTGTGAGACTACTTTTGAATGCTTGACCTCAAATCAGGTAGGACTACCC
GCTGAACTTAA
>AB10-24
TTTCCGTAGGTGAACCTGCGGAAGGATCATTATTGAATTATGTTTCTAGATAGGTTGTAG

CTGGCTCTTTTAGAGCATGTGCACGCCTGTTTGGACTTCATTTTCATCCACCTGTGCACC
TATTGTAGTCTTTGGTTGGGTTAGGAGGAAGTGATCATTGTATCAGCATCTGCTGGGAGT
GAGGACTTGCATTGTGAAAGCTTTGCTGTCCTTGATGTGATCATGGAATCTTTTTCTCAC
TAGAGTCTATGTCACTCATTATACTCTGTCGAATGTCATTGAATGTCTTTACATGGGCTT
GTATGCCTATGAAAATTGTAATACAACTTTCAGCAACGGATCTCTTGGCTCTCGCATCGA
TGAAGAACGCAGCGAAATGCGATAAGTAATGTGAATTGCAGAATTCAGTGAATCATCGAA
TCTTTGAACGCATCTTGCGCTCCTTGGTATTCCGAGGAGCATGCCTGTTTGAGTGTCATT
AAATTCTCAACTCTCTTATACTTTTTTGTAAAAGAGAGCTTGGACTGTGGAGGCTTGCTG
GCCACTTTTTGGGGTCAGCTCCTCTGAAATGCATTAGCGGAACCGTTTGCGATCTGCCAC
AAGTGTGATAAGTTATCTACACTGGCGAGGGGATTGCTCTCTGTAATGTTCAGCTTCTAA
TTGTCTCTACTTTGTGAGACTACTTTTGAATGCTTGACCTCAAATCAGGTAGGACTACCC
GCTGAACTTAA
>AB10-25
TTTCCGTAGGTGAACCTGCGGAAGGATCATTATTGAATTATGTTTCTAGATAGGTTGTAG
CTGGCTCTTTTAGAGCATGTGCACGCCTGTTTGGACTTCATTTTCATCCACCTGTGCACC
TATTGTAGTCTTTGGTTGGGTTAGGAGGAAGTGATCATTGTATCAGCATCTGCTGGGAGT
GAGGACTTGCATTGTGAAAGCTTTGCTGTCCTTGATGTGATCATGGAATCTTTTTCTCAC
TAGAGTCTATGTCACTCATTATACTCTGTCGAATGTCATTGAATGTCTTTACATGGGCTT
GTATGCCTATGAAAATTGTAATACAACTTTCAGCAACGGATCTCTTGGCTCTCGCATCGA
TGAAGAACGCAGCGAAATGCGATAAGTAATGTGAATTGCAGAATTCAGTGAATCATCGAA
TCTTTGAACGCATCTTGCGCTCCTTGGTATTCCGAGGAGCATGCCTGTTTGAGTGTCATT
AAATTCTCAACTCTCTTATACTTTTTTGTAAAAGAGAGCTTGGACTGTGGAGGCTTGCTG
GCCACTTTTTGGGGTCAGCTCCTCTGAAATGCATTAGCGGAACCGTTTGCGATCTGCCAC
AAGTGTGATAAGTTATCTACACTGGCGAGGGGATTGCTCTCTGTAATGTTCAGCTTCTAA
TTGTCTCTACTTTGTGAGACTACTTTTGAATGCTTGACCTCAAATCAGGTAGGACTACCC
GCTGAACTTAA
>AB10-27
TTTCCGTAGGTGAACCTGCGGAAGGATCATTATTGAATTATGTTTCTAGATAGGTTGTAG
CTGGCTCTTTTAGAGCATGTGCACGCCTGTTTGGACTTCATTTTCATCCACCTGTGCACC
TATTGTAGTCTTTGGTTGGGTTAGGAGGAAGTGATCATTGTATCAGCATCTGCTGGGAGT
GAGGACTTGCATTGTGAAAGCTTTGCTGTCCTTGATGTGATCATGGAATCTTTTTCTCAC
TAGAGTCTATGTCACTCATTATACTCTGTCGAATGTCATTGAATGTCTTTACATGGGCTT
GTATGCCTATGAAAATTGTAATACAACTTTCAGCAACGGATCTCTTGGCTCTCGCATCGA
TGAAGAACGCAGCGAAATGCGATAAGTAATGTGAATTGCAGAATTCAGTGAATCATCGAA
TCTTTGAACGCATCTTGCGCTCCTTGGTATTCCGAGGAGCATGCCTGTTTGAGTGTCATT
AAATTCTCAACTCTCTTATACTTTTTTGTAAAAGAGAGCTTGGACTGTGGAGGCTTGCTG
GCCACTTTTTGGGGTCAGCTCCTCTGAAATGCATTAGCGGAACCGTTTGCGATCTGCCAC
AAGTGTGATAAGTTATCTACACTGGCGAGGGGATTGCTCTCTGTAATGTTCAGCTTCTAA
TTGTCTCTACTTTGTGAGACTACTTTTGAATGCTTGACCTCAAATCAGGTAGGACTACCC
GCTGAACTTAA
>AB10-31
TTTCCGTAGGTGAACCTGCGGAAGGATCATTATTGAATTATGTTTCTAGATAGGTTGTAG
CTGGCTCTTTTAGAGCATGTGCACGCCTGTTTGGACTTCATTTTCATCCACCTGTGCACC
TATTGTAGTCTTTGGTTGGGTTAGGAGGAAGTGATCATTGTATCAGCATCTGCTGGGAGT
GAGGACTTGCATTGTGAAAGCTTTGCTGTCCTTGATGTGATCATGGAATCTTTTTCTCAC
TAGAGTCTATGTCACTCATTATACTCTGTCGAATGTCATTGAATGTCTTTACATGGGCTT
GTATGCCTATGAAAATTGTAATACAACTTTCAGCAACGGATCTCTTGGCTCTCGCATCGA
TGAAGAACGCAGCGAAATGCGATAAGTAATGTGAATTGCAGAATTCAGTGAATCATCGAA
TCTTTGAACGCATCTTGCGCTCCTTGGTATTCCGAGGAGCATGCCTGTTTGAGTGTCATT
AAATTCTCAACTCTCTTATACTTTTTTGTAAAAGAGAGCTTGGACTGTGGAGGCTTGCTG

GCCACTTTTTGGGGTCAGCTCCTCTGAAATGCATTAGCGGAACCGTTTGCGATCTGCCAC
AAGTGTGATAAGTTATCTACACTGGCGAGGGGATTGCTCTCTGTAATGTTCAGCTTCTAA
TTGTCTCTACTTTGTGAGACTACTTTTGAATGCTTGACCTCAAATCAGGTAGGACTACCC
GCTGAACTTAA
>AB10-35
TTTCCGTAGGTGAACCTGCGGAAGGATCATTATTGAATTATGTTTCTAGATAGGTTGTAG
CTGGCTCTTTTAGAGCATGTGCACGCCTGTTTGGACTTCATTTTCATCCACCTGTGCACC
TATTGTAGTCTTTGGTTGGGTTAGGAGGAAGTGATCATTGTATCAGCATCTGCTGGGAGT
GAGGACTTGCATTGTGAAAGCTTTGCTGTCCTTGATGTGATCATGGAATCTTTTTCTCAC
TAGAGTCTATGTCACTCATTATACTCTGTCGAATGTCATTGAATGTCTTTACATGGGCTT
GTATGCCTATGAAAATTGTAATACAACTTTCAGCAACGGATCTCTTGGCTCTCGCATCGA
TGAAGAACGCAGCGAAATGCGATAAGTAATGTGAATTGCAGAATTCAGTGAATCATCGAA
TCTTTGAACGCATCTTGCGCTCCTTGGTATTCCGAGGAGCATGCCTGTTTGAGTGTCATT
AAATTCTCAACTCTCTTATACTTTTTTGTAAAAGAGAGCTTGGACTGTGGAGGCTTGCTG
GCCACTTTTTGGGGTCAGCTCCTCTGAAATGCATTAGCGGAACCGTTTGCGATCTGCCAC
AAGTGTGATAAGTTATCTACACTGGCGAGGGGATTGCTCTCTGTAATGTTCAGCTTCTAA
TTGTCTCTACTTTGTGAGACTACTTTTGAATGCTTGACCTCAAATCAGGTAGGACTACCC
GCTGAACTTAA
>AB10-43
TTTCCGTAGGTGAACCTGCGGAAGGATCATTATTGAATTATGTTTCTAGATAGGTTGTAG
CTGGCTCTTTTAGAGCATGTGCACGCCTGTTTGGACTTCATTTTCATCCACCTGTGCACC
TATTGTAGTCTTTGGTTGGGTTAGGAGGAAGTGATCATTGTATCAGCATCTGCTGGGAGT
GAGGACTTGCATTGTGAAAGCTTTGCTGTCCTTGATGTGATCATGGAATCTTTTTCTCAC
TAGAGTCTATGTCACTCATTATACTCTGTCGAATGTCATTGAATGTCTTTACATGGGCTT
GTATGCCTATGAAAATTGTAATACAACTTTCAGCAACGGATCTCTTGGCTCTCGCATCGA
TGAAGAACGCAGCGAAATGCGATAAGTAATGTGAATTGCAGAATTCAGTGAATCATCGAA
TCTTTGAACGCATCTTGCGCTCCTTGGTATTCCGAGGAGCATGCCTGTTTGAGTGTCATT
AAATTCTCAACTCTCTTATACTTTTTTGTAAAAGAGAGCTTGGACTGTGGAGGCTTGCTG
GCCACTTTTTGGGGTCAGCTCCTCTGAAATGCATTAGCGGAACCGTTTGCGATCTGCCAC
AAGTGTGATAAGTTATCTACACTGGCGAGGGGATTGCTCTCTGTAATGTTCAGCTTCTAA
TTGTCTCTACTTTGTGAGACTACTTTTGAATGCTTGACCTCAAATCAGGTAGGACTACCC
GCTGAACTTAA
>AB10-45
TTTCCGTAGGTGAACCTGCGGAAGGATCATTATTGAATTATGTTTCTAGATAGGTTGTAG
CTGGCTCTTTTAGAGCATGTGCACGCCTGTTTGGACTTCATTTTCATCCACCTGTGCACC
TATTGTAGTCTTTGGTTGGGTTAGGAGGAAGTGATCATTGTATCAGCATCTGCTGGGAGT
GAGGACTTGCATTGTGAAAGCTTTGCTGTCCTTGATGTGATCATGGAATCTTTTTCTCAC
TAGAGTCTATGTCACTCATTATACTCTGTCGAATGTCATTGAATGTCTTTACATGGGCTT
GTATGCCTATGAAAATTGTAATACAACTTTCAGCAACGGATCTCTTGGCTCTCGCATCGA
TGAAGAACGCAGCGAAATGCGATAAGTAATGTGAATTGCAGAATTCAGTGAATCATCGAA
TCTTTGAACGCATCTTGCGCTCCTTGGTATTCCGAGGAGCATGCCTGTTTGAGTGTCATT
AAATTCTCAACTCTCTTATACTTTTTTGTAAAAGAGAGCTTGGACTGTGGAGGCTTGCTG
GCCACTTTTTGGGGTCAGCTCCTCTGAAATGCATTAGCGGAACCGTTTGCGATCTGCCAC
AAGTGTGATAAGTTATCTACACTGGCGAGGGGATTGCTCTCTGTAATGTTCAGCTTCTAA
TTGTCTCTACTTTGTGAGACTACTTTTGAATGCTTGACCTCAAATCAGGTAGGACTACCC
GCTGAACTTAA
>AB10-51
TTTCCGTAGGTGAACCTGCGGAAGGATCATTATTGAATTATGTTTCTAGATAGGTTGTAG
CTGGCTCTTTTAGAGCATGTGCACGCCTGTTTGGACTTCATTTTCATCCACCTGTGCACC
TATTGTAGTCTTTGGTTGGGTTAGGAGGAAGTGATCATTGTATCAGCATCTGCTGGGAGT

GAGGACTTGCATTGTGAAAGCTTTGCTGTCCTTGATGTGATCATGGAATCTTTTTCTCAC
TAGAGTCTATGTCACTCATTATACTCTGTCGAATGTCATTGAATGTCTTTACATGGGCTT
GTATGCCTATGAAAATTGTAATACAACTTTCAGCAACGGATCTCTTGGCTCTCGCATCGA
TGAAGAACGCAGCGAAATGCGATAAGTAATGTGAATTGCAGAATTCAGTGAATCATCGAA
TCTTTGAACGCATCTTGCGCTCCTTGGTATTCCGAGGAGCATGCCTGTTTGAGTGTCATT
AAATTCTCAACTCTCTTATACTTTTTTGTAAAAGAGAGCTTGGACTGTGGAGGCTTGCTG
GCCACTTTTTGGGGTCAGCTCCTCTGAAATGCATTAGCGGAACCGTTTGCGATCTGCCAC
AAGTGTGATAAGTTATCTACACTGGCGAGGGGATTGCTCTCTGTAATGTTCAGCTTCTAA
TTGTCTCTACTTTGTGAGACTACTTTTGAATGCTTGACCTCAAATCAGGTAGGACTACCC
GCTGAACTTAA
>AB10-52
TTTCCGTAGGTGAACCTGCGGAAGGATCATTATTGAATTATGTTTCTAGATAGGTTGTAG
CTGGCTCTTTTAGAGCATGTGCACGCCTGTTTGGACTTCATTTTCATCCACCTGTGCACC
TATTGTAGTCTTTGGTTGGGTTAGGAGGAAGTGATCATTGTATCAGCATCTGCTGGGAGT
GAGGACTTGCATTGTGAAAGCTTTGCTGTCCTTGATGTGATCATGGAATCTTTTTCTCAC
TAGAGTCTATGTCACTCATTATACTCTGTCGAATGTCATTGAATGTCTTTACATGGGCTT
GTATGCCTATGAAAATTGTAATACAACTTTCAGCAACGGATCTCTTGGCTCTCGCATCGA
TGAAGAACGCAGCGAAATGCGATAAGTAATGTGAATTGCAGAATTCAGTGAATCATCGAA
TCTTTGAACGCATCTTGCGCTCCTTGGTATTCCGAGGAGCATGCCTGTTTGAGTGTCATT
AAATTCTCAACTCTCTTATACTTTTTTGTAAAAGAGAGCTTGGACTGTGGAGGCTTGCTG
GCCACTTTTTGGGGTCAGCTCCTCTGAAATGCATTAGCGGAACCGTTTGCGATCTGCCAC
AAGTGTGATAAGTTATCTACACTGGCGAGGGGATTGCTCTCTGTAATGTTCAGCTTCTAA
TTGTCTCTACTTTGTGAGACTACTTTTGAATGCTTGACCTCAAATCAGGTAGGACTACCC
GCTGAACTTAA
>AB10-53
TTTCCGTAGGTGAACCTGCGGAAGGATCATTATTGAATTATGTTTCTAGATAGGTTGTAG
CTGGCTCTTTTAGAGCATGTGCACGCCTGTTTGGACTTCATTTTCATCCACCTGTGCACC
TATTGTAGTCTTTGGTTGGGTTAGGAGGAAGTGATCATTGTATCAGCATCTGCTGGGAGT
GAGGACTTGCATTGTGAAAGCTTTGCTGTCCTTGATGTGATCATGGAATCTTTTTCTCAC
TAGAGTCTATGTCACTCATTATACTCTGTCGAATGTCATTGAATGTCTTTACATGGGCTT
GTATGCCTATGAAAATTGTAATACAACTTTCAGCAACGGATCTCTTGGCTCTCGCATCGA
TGAAGAACGCAGCGAAATGCGATAAGTAATGTGAATTGCAGAATTCAGTGAATCATCGAA
TCTTTGAACGCATCTTGCGCTCCTTGGTATTCCGAGGAGCATGCCTGTTTGAGTGTCATT
AAATTCTCAACTCTCTTATACTTTTTTGTAAAAGAGAGCTTGGACTGTGGAGGCTTGCTG
GCCACTTTTTGGGGTCAGCTCCTCTGAAATGCATTAGCGGAACCGTTTGCGATCTGCCAC
AAGTGTGATAAGTTATCTACACTGGCGAGGGGATTGCTCTCTGTAATGTTCAGCTTCTAA
TTGTCTCTACTTTGTGAGACTACTTTTGAATGCTTGACCTCAAATCAGGTAGGACTACCC
GCTGAACTTAA
>AB11-1
TTTCCGTAGGTGAACCTGCGGAAGGATCATTATTGAATTATGTTTCTAGATAGGTTGTAG
CTGGCTCTTTTAGAGCATGTGCACGCCTGTTTGGACTTCATTTTCATCCACCTGTGCACC
TATTGTAGTCTTTGGTTGGGTTAGGAGGAAGTGATCATTGTATCAGCATCTGCTGGGAGT
GAGGACTTGCATTGTGAAAGCTTTGCTGTCCTTGATGTGATCATGGAATCTTTTTCTCAC
TAGAGTCTATGTCACTCATTATACTCTGTCGAATGTCATTGAATGTCTTTACATGGGCTT
GTATGCCTATGAAAATTGTAATACAACTTTCAGCAACGGATCTCTTGGCTCTCGCATCGA
TGAAGAACGCAGCGAAATGCGATAAGTAATGTGAATTGCAGAATTCAGTGAATCATCGAA
TCTTTGAACGCATCTTGCGCTCCTTGGTATTCCGAGGAGCATGCCTGTTTGAGTGTCATT
AAATTCTCAACTCTCTTATACTTTTTTGTAAAAGAGAGCTTGGACTGTGGAGGCTTGCTG
GCCACTTTTTGGGGTCAGCTCCTCTGAAATGCATTAGCGGAACCGTTTGCGATCTGCCAC
AAGTGTGATAAGTTATCTACACTGGCGAGGGGATTGCTCTCTGTAATGTTCAGCTTCTAA

TTGTCTCTACTTTGTGAGACTACTTTTGAATGCTTGACCTCAAATCAGGTAGGACTACCC
GCTGAACTTAA
>AB11-2
TTTCCGTAGGTGAACCTGCGGAAGGATCATTATTGAATTATGTTTCTAGATAGGTTGTAG
CTGGCTCTTTTAGAGCATGTGCACGCCTGTTTGGACTTCATTTTCATCCACCTGTGCACC
TATTGTAGTCTTTGGTTGGGTTAGGAGGAAGTGATCATTGTATCAGCATCTGCTGGGAGT
GAGGACTTGCATTGTGAAAGCTTTGCTGTCCTTGATGTGATCATGGAATCTTTTTCTCAC
TAGAGTCTATGTCACTCATTATACTCTGTCGAATGTCATTGAATGTCTTTACATGGGCTT
GTATGCCTATGAAAATTGTAATACAACTTTCAGCAACGGATCTCTTGGCTCTCGCATCGA
TGAAGAACGCAGCGAAATGCGATAAGTAATGTGAATTGCAGAATTCAGTGAATCATCGAA
TCTTTGAACGCATCTTGCGCTCCTTGGTATTCCGAGGAGCATGCCTGTTTGAGTGTCATT
AAATTCTCAACTCTCTTATACTTTTTTGTAAAAGAGAGCTTGGACTGTGGAGGCTTGCTG
GCCACTTTTTGGGGTCAGCTCCTCTGAAATGCATTAGCGGAACCGTTTGCGATCTGCCAC
AAGTGTGATAAGTTATCTACACTGGCGAGGGGATTGCTCTCTGTAATGTTCAGCTTCTAA
TTGTCTCTACTTTGTGAGACTACTTTTGAATGCTTGACCTCAAATCAGGTAGGACTACCC
GCTGAACTTAA
>AB11-16
TTTCCGTAGGTGAACCTGCGGAAGGATCATTATTGAATTATGTTTCTAGATAGGTTGTAG
CTGGCTCTTTTAGAGCATGTGCACGCCTGTTTGGACTTCATTTTCATCCACCTGTGCACC
TATTGTAGTCTTTGGTTGGGTTAGGAGGAAGTGATCATTGTATCAGCATCTGCTGGGAGT
GAGGACTTGCATTGTGAAAGCTTTGCTGTCCTTGATGTGATCATGGAATCTTTTTCTCAC
TAGAGTCTATGTCACTCATTATACTCTGTCGAATGTCATTGAATGTCTTTACATGGGCTT
GTATGCCTATGAAAATTGTAATACAACTTTCAGCAACGGATCTCTTGGCTCTCGCATCGA
TGAAGAACGCAGCGAAATGCGATAAGTAATGTGAATTGCAGAATTCAGTGAATCATCGAA
TCTTTGAACGCATCTTGCGCTCCTTGGTATTCCGAGGAGCATGCCTGTTTGAGTGTCATT
AAATTCTCAACTCTCTTATACTTTTTTGTAAAAGAGAGCTTGGACTGTGGAGGCTTGCTG
GCCACTTTTTGGGGTCAGCTCCTCTGAAATGCATTAGCGGAACCGTTTGCGATCTGCCAC
AAGTGTGATAAGTTATCTACACTGGCGAGGGGATTGCTCTCTGTAATGTTCAGCTTCTAA
TTGTCTCTACTTTGTGAGACTACTTTTGAATGCTTGACCTCAAATCAGGTAGGACTACCC
GCTGAACTTAA
>AB11-20
TTTCCGTAGGTGAACCTGCGGAAGGATCATTATTGAATTATGTTTCTAGATAGGTTGTAG
CTGGCTCTTTTAGAGCATGTGCACGCCTGTTTGGACTTCATTTTCATCCACCTGTGCACC
TATTGTAGTCTTTGGTTGGGTTAGGAGGAAGTGATCATTGTATCAGCATCTGCTGGGAGT
GAGGACTTGCATTGTGAAAGCTTTGCTGTCCTTGATGTGATCATGGAATCTTTTTCTCAC
TAGAGTCTATGTCACTCATTATACTCTGTCGAATGTCATTGAATGTCTTTACATGGGCTT
GTATGCCTATGAAAATTGTAATACAACTTTCAGCAACGGATCTCTTGGCTCTCGCATCGA
TGAAGAACGCAGCGAAATGCGATAAGTAATGTGAATTGCAGAATTCAGTGAATCATCGAA
TCTTTGAACGCATCTTGCGCTCCTTGGTATTCCGAGGAGCATGCCTGTTTGAGTGTCATT
AAATTCTCAACTCTCTTATACTTTTTTGTAAAAGAGAGCTTGGACTGTGGAGGCTTGCTG
GCCACTTTTTGGGGTCAGCTCCTCTGAAATGCATTAGCGGAACCGTTTGCGATCTGCCAC
AAGTGTGATAAGTTATCTACACTGGCGAGGGGATTGCTCTCTGTAATGTTCAGCTTCTAA
TTGTCTCTACTTTGTGAGACTACTTTTGAATGCTTGACCTCAAATCAGGTAGGACTACCC
GCTGAACTTAA
>AB11-23
TTTCCGTAGGTGAACCTGCGGAAGGATCATTATTGAATTATGTTTCTAGATAGGTTGTAG
CTGGCTCTTTTAGAGCATGTGCACGCCTGTTTGGACTTCATTTTCATCCACCTGTGCACC
TATTGTAGTCTTTGGTTGGGTTAGGAGGAAGTGATCATTGTATCAGCATCTGCTGGGAGT
GAGGACTTGCATTGTGAAAGCTTTGCTGTCCTTGATGTGATCATGGAATCTTTTTCTCAC
TAGAGTCTATGTCACTCATTATACTCTGTCGAATGTCATTGAATGTCTTTACATGGGCTT

GTATGCCTATGAAAATTGTAATACAACTTTCAGCAACGGATCTCTTGGCTCTCGCATCGA
TGAAGAACGCAGCGAAATGCGATAAGTAATGTGAATTGCAGAATTCAGTGAATCATCGAA
TCTTTGAACGCATCTTGCGCTCCTTGGTATTCCGAGGAGCATGCCTGTTTGAGTGTCATT
AAATTCTCAACTCTCTTATACTTTTTTGTAAAAGAGAGCTTGGACTGTGGAGGCTTGCTG
GCCACTTTTTGGGGTCAGCTCCTCTGAAATGCATTAGCGGAACCGTTTGCGATCTGCCAC
AAGTGTGATAAGTTATCTACACTGGCGAGGGGATTGCTCTCTGTAATGTTCAGCTTCTAA
TTGTCTCTACTTTGTGAGACTACTTTTGAATGCTTGACCTCAAATCAGGTAGGACTACCC
GCTGAACTTAA
>AB11-37
TTTCCGTAGGTGAACCTGCGGAAGGATCATTATTGAATTATGTTTCTAGATAGGTTGTAG
CTGGCTCTTTTAGAGCATGTGCACGCCTGTTTGGACTTCATTTTCATCCACCTGTGCACC
TATTGTAGTCTTTGGTTGGGTTAGGAGGAAGTGATCATTGTATCAGCATCTGCTGGGAGT
GAGGACTTGCATTGTGAAAGCTTTGCTGTCCTTGATGTGATCATGGAATCTTTTTCTCAC
TAGAGTCTATGTCACTCATTATACTCTGTCGAATGTCATTGAATGTCTTTACATGGGCTT
GTATGCCTATGAAAATTGTAATACAACTTTCAGCAACGGATCTCTTGGCTCTCGCATCGA
TGAAGAACGCAGCGAAATGCGATAAGTAATGTGAATTGCAGAATTCAGTGAATCATCGAA
TCTTTGAACGCATCTTGCGCTCCTTGGTATTCCGAGGAGCATGCCTGTTTGAGTGTCATT
AAATTCTCAACTCTCTTATACTTTTTTGTAAAAGAGAGCTTGGACTGTGGAGGCTTGCTG
GCCACTTTTTGGGGTCAGCTCCTCTGAAATGCATTAGCGGAACCGTTTGCGATCTGCCAC
AAGTGTGATAAGTTATCTACACTGGCGAGGGGATTGCTCTCTGTAATGTTCAGCTTCTAA
TTGTCTCTACTTTGTGAGACTACTTTTGAATGCTTGACCTCAAATCAGGTAGGACTACCC
GCTGAACTTAA
>AB11-41
TTTCCGTAGGTGAACCTGCGGAAGGATCATTATTGAATTATGTTTCTAGATAGGTTGTAG
CTGGCTCTTTTAGAGCATGTGCACGCCTGTTTGGACTTCATTTTCATCCACCTGTGCACC
TATTGTAGTCTTTGGTTGGGTTAGGAGGAAGTGATCATTGTATCAGCATCTGCTGGGAGT
GAGGACTTGCATTGTGAAAGCTTTGCTGTCCTTGATGTGATCATGGAATCTTTTTCTCAC
TAGAGTCTATGTCACTCATTATACTCTGTCGAATGTCATTGAATGTCTTTACATGGGCTT
GTATGCCTATGAAAATTGTAATACAACTTTCAGCAACGGATCTCTTGGCTCTCGCATCGA
TGAAGAACGCAGCGAAATGCGATAAGTAATGTGAATTGCAGAATTCAGTGAATCATCGAA
TCTTTGAACGCATCTTGCGCTCCTTGGTATTCCGAGGAGCATGCCTGTTTGAGTGTCATT
AAATTCTCAACTCTCTTATACTTTTTTGTAAAAGAGAGCTTGGACTGTGGAGGCTTGCTG
GCCACTTTTTGGGGTCAGCTCCTCTGAAATGCATTAGCGGAACCGTTTGCGATCTGCCAC
AAGTGTGATAAGTTATCTACACTGGCGAGGGGATTGCTCTCTGTAATGTTCAGCTTCTAA
TTGTCTCTACTTTGTGAGACTACTTTTGAATGCTTGACCTCAAATCAGGTAGGACTACCC
GCTGAACTTAA
>AB11-43
TTTCCGTAGGTGAACCTGCGGAAGGATCATTATTGAATTATGTTTCTAGATAGGTTGTAG
CTGGCTCTTTTAGAGCATGTGCACGCCTGTTTGGACTTCATTTTCATCCACCTGTGCACC
TATTGTAGTCTTTGGTTGGGTTAGGAGGAAGTGATCATTGTATCAGCATCTGCTGGGAGT
GAGGACTTGCATTGTGAAAGCTTTGCTGTCCTTGATGTGATCATGGAATCTTTTTCTCAC
TAGAGTCTATGTCACTCATTATACTCTGTCGAATGTCATTGAATGTCTTTACATGGGCTT
GTATGCCTATGAAAATTGTAATACAACTTTCAGCAACGGATCTCTTGGCTCTCGCATCGA
TGAAGAACGCAGCGAAATGCGATAAGTAATGTGAATTGCAGAATTCAGTGAATCATCGAA
TCTTTGAACGCATCTTGCGCTCCTTGGTATTCCGAGGAGCATGCCTGTTTGAGTGTCATT
AAATTCTCAACTCTCTTATACTTTTTTGTAAAAGAGAGCTTGGACTGTGGAGGCTTGCTG
GCCACTTTTTGGGGTCAGCTCCTCTGAAATGCATTAGCGGAACCGTTTGCGATCTGCCAC
AAGTGTGATAAGTTATCTACACTGGCGAGGGGATTGCTCTCTGTAATGTTCAGCTTCTAA
TTGTCTCTACTTTGTGAGACTACTTTTGAATGCTTGACCTCAAATCAGGTAGGACTACCC
GCTGAACTTAA

>AB11-48
TTTCCGTAGGTGAACCTGCGGAAGGATCATTATTGAATTATGTTTCTAGATAGGTTGTAG
CTGGCTCTTTTAGAGCATGTGCACGCCTGTTTGGACTTCATTTTCATCCACCTGTGCACC
TATTGTAGTCTTTGGTTGGGTTAGGAGGAAGTGATCATTGTATCAGCATCTGCTGGGAGT
GAGGACTTGCATTGTGAAAGCTTTGCTGTCCTTGATGTGATCATGGAATCTTTTTCTCAC
TAGAGTCTATGTCACTCATTATACTCTGTCGAATGTCATTGAATGTCTTTACATGGGCTT
GTATGCCTATGAAAATTGTAATACAACTTTCAGCAACGGATCTCTTGGCTCTCGCATCGA
TGAAGAACGCAGCGAAATGCGATAAGTAATGTGAATTGCAGAATTCAGTGAATCATCGAA
TCTTTGAACGCATCTTGCGCTCCTTGGTATTCCGAGGAGCATGCCTGTTTGAGTGTCATT
AAATTCTCAACTCTCTTATACTTTTTTGTAAAAGAGAGCTTGGACTGTGGAGGCTTGCTG
GCCACTTTTTGGGGTCAGCTCCTCTGAAATGCATTAGCGGAACCGTTTGCGATCTGCCAC
AAGTGTGATAAGTTATCTACACTGGCGAGGGGATTGCTCTCTGTAATGTTCAGCTTCTAA
TTGTCTCTACTTTGTGAGACTACTTTTGAATGCTTGACCTCAAATCAGGTAGGACTACCC
GCTGAACTTAA
>AB11-57
TTTCCGTAGGTGAACCTGCGGAAGGATCATTATTGAATTATGTTTCTAGATAGGTTGTAG
CTGGCTCTTTTAGAGCATGTGCACGCCTGTTTGGACTTCATTTTCATCCACCTGTGCACC
TATTGTAGTCTTTGGTTGGGTTAGGAGGAAGTGATCATTGTATCAGCATCTGCTGGGAGT
GAGGACTTGCATTGTGAAAGCTTTGCTGTCCTTGATGTGATCATGGAATCTTTTTCTCAC
TAGAGTCTATGTCACTCATTATACTCTGTCGAATGTCATTGAATGTCTTTACATGGGCTT
GTATGCCTATGAAAATTGTAATACAACTTTCAGCAACGGATCTCTTGGCTCTCGCATCGA
TGAAGAACGCAGCGAAATGCGATAAGTAATGTGAATTGCAGAATTCAGTGAATCATCGAA
TCTTTGAACGCATCTTGCGCTCCTTGGTATTCCGAGGAGCATGCCTGTTTGAGTGTCATT
AAATTCTCAACTCTCTTATACTTTTTTGTAAAAGAGAGCTTGGACTGTGGAGGCTTGCTG
GCCACTTTTTGGGGTCAGCTCCTCTGAAATGCATTAGCGGAACCGTTTGCGATCTGCCAC
AAGTGTGATAAGTTATCTACACTGGCGAGGGGATTGCTCTCTGTAATGTTCAGCTTCTAA
TTGTCTCTACTTTGTGAGACTACTTTTGAATGCTTGACCTCAAATCAGGTAGGACTACCC
GCTGAACTTAA
>AB12-1
TTTCCGTAGGTGAACCTGCGGAAGGATCATTATTGAATTATGTTTCTAGATAGGTTGTAG
CTGGCTCTTTTAGAGCATGTGCACGCCTGTTTGGACTTCATTTTCATCCACCTGTGCACC
TATTGTAGTCTTTGGTTGGGTTAGGAGGAAGTGATCATTGTATCAGCATCTGCTGGGAGT
GAGGACTTGCATTGTGAAAGCTTTGCTGTCCTTGATGTGATCATGGAATCTTTTTCTCAC
TAGAGTCTATGTCACTCATTATACTCTGTCGAATGTCATTGAATGTCTTTACATGGGCTT
GTATGCCTATGAAAATTGTAATACAACTTTCAGCAACGGATCTCTTGGCTCTCGCATCGA
TGAAGAACGCAGCGAAATGCGATAAGTAATGTGAATTGCAGAATTCAGTGAATCATCGAA
TCTTTGAACGCATCTTGCGCTCCTTGGTATTCCGAGGAGCATGCCTGTTTGAGTGTCATT
AAATTCTCAACTCTCTTATACTTTTTTGTAAAAGAGAGCTTGGACTGTGGAGGCTTGCTG
GCCACTTTTTGGGGTCAGCTCCTCTGAAATGCATTAGCGGAACCGTTTGCGATCTGCCAC
AAGTGTGATAAGTTATCTACACTGGCGAGGGGATTGCTCTCTGTAATGTTCAGCTTCTAA
TTGTCTCTACTTTGTGAGACTACTTTTGAATGCTTGACCTCAAATCAGGTAGGACTACCC
GCTGAACTTAA
>AB12-5
TTTCCGTAGGTGAACCTGCGGAAGGATCATTATTGAATTATGTTTCTAGATAGGTTGTAG
CTGGCTCTTTTAGAGCATGTGCACGCCTGTTTGGACTTCATTTTCATCCACCTGTGCACC
TATTGTAGTCTTTGGTTGGGTTAGGAGGAAGTGATCATTGTATCAGCATCTGCTGGGAGT
GAGGACTTGCATTGTGAAAGCTTTGCTGTCCTTGATGTGATCATGGAATCTTTTTCTCAC
TAGAGTCTATGTCACTCATTATACTCTGTCGAATGTCATTGAATGTCTTTACATGGGCTT
GTATGCCTATGAAAATTGTAATACAACTTTCAGCAACGGATCTCTTGGCTCTCGCATCGA
TGAAGAACGCAGCGAAATGCGATAAGTAATGTGAATTGCAGAATTCAGTGAATCATCGAA

TCTTTGAACGCATCTTGCGCTCCTTGGTATTCCGAGGAGCATGCCTGTTTGAGTGTCATT
AAATTCTCAACTCTCTTATACTTTTTTGTAAAAGAGAGCTTGGACTGTGGAGGCTTGCTG
GCCACTTTTTGGGGTCAGCTCCTCTGAAATGCATTAGCGGAACCGTTTGCGATCTGCCAC
AAGTGTGATAAGTTATCTACACTGGCGAGGGGATTGCTCTCTGTAATGTTCAGCTTCTAA
TTGTCTCTACTTTGTGAGACTACTTTTGAATGCTTGACCTCAAATCAGGTAGGACTACCC
GCTGAACTTAA
>AB12-16
TTTCCGTAGGTGAACCTGCGGAAGGATCATTATTGAATTATGTTTCTAGATAGGTTGTAG
CTGGCTCTTTTAGAGCATGTGCACGCCTGTTTGGACTTCATTTTCATCCACCTGTGCACC
TATTGTAGTCTTTGGTTGGGTTAGGAGGAAGTGATCATTGTATCAGCATCTGCTGGGAGT
GAGGACTTGCATTGTGAAAGCTTTGCTGTCCTTGATGTGATCATGGAATCTTTTTCTCAC
TAGAGTCTATGTCACTCATTATACTCTGTCGAATGTCATTGAATGTCTTTACATGGGCTT
GTATGCCTATGAAAATTGTAATACAACTTTCAGCAACGGATCTCTTGGCTCTCGCATCGA
TGAAGAACGCAGCGAAATGCGATAAGTAATGTGAATTGCAGAATTCAGTGAATCATCGAA
TCTTTGAACGCATCTTGCGCTCCTTGGTATTCCGAGGAGCATGCCTGTTTGAGTGTCATT
AAATTCTCAACTCTCTTATACTTTTTTGTAAAAGAGAGCTTGGACTGTGGAGGCTTGCTG
GCCACTTTTTGGGGTCAGCTCCTCTGAAATGCATTAGCGGAACCGTTTGCGATCTGCCAC
AAGTGTGATAAGTTATCTACACTGGCGAGGGGATTGCTCTCTGTAATGTTCAGCTTCTAA
TTGTCTCTACTTTGTGAGACTACTTTTGAATGCTTGACCTCAAATCAGGTAGGACTACCC
GCTGAACTTAA
>AB12-17
TTTCCGTAGGTGAACCTGCGGAAGGATCATTATTGAATTATGTTTCTAGATAGGTTGTAG
CTGGCTCTTTTAGAGCATGTGCACGCCTGTTTGGACTTCATTTTCATCCACCTGTGCACC
TATTGTAGTCTTTGGTTGGGTTAGGAGGAAGTGATCATTGTATCAGCATCTGCTGGGAGT
GAGGACTTGCATTGTGAAAGCTTTGCTGTCCTTGATGTGATCATGGAATCTTTTTCTCAC
TAGAGTCTATGTCACTCATTATACTCTGTCGAATGTCATTGAATGTCTTTACATGGGCTT
GTATGCCTATGAAAATTGTAATACAACTTTCAGCAACGGATCTCTTGGCTCTCGCATCGA
TGAAGAACGCAGCGAAATGCGATAAGTAATGTGAATTGCAGAATTCAGTGAATCATCGAA
TCTTTGAACGCATCTTGCGCTCCTTGGTATTCCGAGGAGCATGCCTGTTTGAGTGTCATT
AAATTCTCAACTCTCTTATACTTTTTTGTAAAAGAGAGCTTGGACTGTGGAGGCTTGCTG
GCCACTTTTTGGGGTCAGCTCCTCTGAAATGCATTAGCGGAACCGTTTGCGATCTGCCAC
AAGTGTGATAAGTTATCTACACTGGCGAGGGGATTGCTCTCTGTAATGTTCAGCTTCTAA
TTGTCTCTACTTTGTGAGACTACTTTTGAATGCTTGACCTCAAATCAGGTAGGACTACCC
GCTGAACTTAA
>AB12-24
TTTCCGTAGGTGAACCTGCGGAAGGATCATTATTGAATTATGTTTCTAGATAGGTTGTAG
CTGGCTCTTTTAGAGCATGTGCACGCCTGTTTGGACTTCATTTTCATCCACCTGTGCACC
TATTGTAGTCTTTGGTTGGGTTAGGAGGAAGTGATCATTGTATCAGCATCTGCTGGGAGT
GAGGACTTGCATTGTGAAAGCTTTGCTGTCCTTGATGTGATCATGGAATCTTTTTCTCAC
TAGAGTCTATGTCACTCATTATACTCTGTCGAATGTCATTGAATGTCTTTACATGGGCTT
GTATGCCTATGAAAATTGTAATACAACTTTCAGCAACGGATCTCTTGGCTCTCGCATCGA
TGAAGAACGCAGCGAAATGCGATAAGTAATGTGAATTGCAGAATTCAGTGAATCATCGAA
TCTTTGAACGCATCTTGCGCTCCTTGGTATTCCGAGGAGCATGCCTGTTTGAGTGTCATT
AAATTCTCAACTCTCTTATACTTTTTTGTAAAAGAGAGCTTGGACTGTGGAGGCTTGCTG
GCCACTTTTTGGGGTCAGCTCCTCTGAAATGCATTAGCGGAACCGTTTGCGATCTGCCAC
AAGTGTGATAAGTTATCTACACTGGCGAGGGGATTGCTCTCTGTAATGTTCAGCTTCTAA
TTGTCTCTACTTTGTGAGACTACTTTTGAATGCTTGACCTCAAATCAGGTAGGACTACCC
GCTGAACTTAA
>AB12-25
TTTCCGTAGGTGAACCTGCGGAAGGATCATTATTGAATTATGTTTCTAGATAGGTTGTAG

CTGGCTCTTTTAGAGCATGTGCACGCCTGTTTGGACTTCATTTTCATCCACCTGTGCACC
TATTGTAGTCTTTGGTTGGGTTAGGAGGAAGTGATCATTGTATCAGCATCTGCTGGGAGT
GAGGACTTGCATTGTGAAAGCTTTGCTGTCCTTGATGTGATCATGGAATCTTTTTCTCAC
TAGAGTCTATGTCACTCATTATACTCTGTCGAATGTCATTGAATGTCTTTACATGGGCTT
GTATGCCTATGAAAATTGTAATACAACTTTCAGCAACGGATCTCTTGGCTCTCGCATCGA
TGAAGAACGCAGCGAAATGCGATAAGTAATGTGAATTGCAGAATTCAGTGAATCATCGAA
TCTTTGAACGCATCTTGCGCTCCTTGGTATTCCGAGGAGCATGCCTGTTTGAGTGTCATT
AAATTCTCAACTCTCTTATACTTTTTTGTAAAAGAGAGCTTGGACTGTGGAGGCTTGCTG
GCCACTTTTTGGGGTCAGCTCCTCTGAAATGCATTAGCGGAACCGTTTGCGATCTGCCAC
AAGTGTGATAAGTTATCTACACTGGCGAGGGGATTGCTCTCTGTAATGTTCAGCTTCTAA
TTGTCTCTACTTTGTGAGACTACTTTTGAATGCTTGACCTCAAATCAGGTAGGACTACCC
GCTGAACTTAA
>AB12-30
TTTCCGTAGGTGAACCTGCGGAAGGATCATTATTGAATTATGTTTCTAGATAGGTTGTAG
CTGGCTCTTTTAGAGCATGTGCACGCCTGTTTGGACTTCATTTTCATCCACCTGTGCACC
TATTGTAGTCTTTGGTTGGGTTAGGAGGAAGTGATCATTGTATCAGCATCTGCTGGGAGT
GAGGACTTGCATTGTGAAAGCTTTGCTGTCCTTGATGTGATCATGGAATCTTTTTCTCAC
TAGAGTCTATGTCACTCATTATACTCTGTCGAATGTCATTGAATGTCTTTACATGGGCTT
GTATGCCTATGAAAATTGTAATACAACTTTCAGCAACGGATCTCTTGGCTCTCGCATCGA
TGAAGAACGCAGCGAAATGCGATAAGTAATGTGAATTGCAGAATTCAGTGAATCATCGAA
TCTTTGAACGCATCTTGCGCTCCTTGGTATTCCGAGGAGCATGCCTGTTTGAGTGTCATT
AAATTCTCAACTCTCTTATACTTTTTTGTAAAAGAGAGCTTGGACTGTGGAGGCTTGCTG
GCCACTTTTTGGGGTCAGCTCCTCTGAAATGCATTAGCGGAACCGTTTGCGATCTGCCAC
AAGTGTGATAAGTTATCTACACTGGCGAGGGGATTGCTCTCTGTAATGTTCAGCTTCTAA
TTGTCTCTACTTTGTGAGACTACTTTTGAATGCTTGACCTCAAATCAGGTAGGACTACCC
GCTGAACTTAA
>AB12-32
TTTCCGTAGGTGAACCTGCGGAAGGATCATTATTGAATTATGTTTCTAGATAGGTTGTAG
CTGGCTCTTTTAGAGCATGTGCACGCCTGTTTGGACTTCATTTTCATCCACCTGTGCACC
TATTGTAGTCTTTGGTTGGGTTAGGAGGAAGTGATCATTGTATCAGCATCTGCTGGGAGT
GAGGACTTGCATTGTGAAAGCTTTGCTGTCCTTGATGTGATCATGGAATCTTTTTCTCAC
TAGAGTCTATGTCACTCATTATACTCTGTCGAATGTCATTGAATGTCTTTACATGGGCTT
GTATGCCTATGAAAATTGTAATACAACTTTCAGCAACGGATCTCTTGGCTCTCGCATCGA
TGAAGAACGCAGCGAAATGCGATAAGTAATGTGAATTGCAGAATTCAGTGAATCATCGAA
TCTTTGAACGCATCTTGCGCTCCTTGGTATTCCGAGGAGCATGCCTGTTTGAGTGTCATT
AAATTCTCAACTCTCTTATACTTTTTTGTAAAAGAGAGCTTGGACTGTGGAGGCTTGCTG
GCCACTTTTTGGGGTCAGCTCCTCTGAAATGCATTAGCGGAACCGTTTGCGATCTGCCAC
AAGTGTGATAAGTTATCTACACTGGCGAGGGGATTGCTCTCTGTAATGTTCAGCTTCTAA
TTGTCTCTACTTTGTGAGACTACTTTTGAATGCTTGACCTCAAATCAGGTAGGACTACCC
GCTGAACTTAA
>AB12-34
TTTCCGTAGGTGAACCTGCGGAAGGATCATTATTGAATTATGTTTCTAGATAGGTTGTAG
CTGGCTCTTTTAGAGCATGTGCACGCCTGTTTGGACTTCATTTTCATCCACCTGTGCACC
TATTGTAGTCTTTGGTTGGGTTAGGAGGAAGTGATCATTGTATCAGCATCTGCTGGGAGT
GAGGACTTGCATTGTGAAAGCTTTGCTGTCCTTGATGTGATCATGGAATCTTTTTCTCAC
TAGAGTCTATGTCACTCATTATACTCTGTCGAATGTCATTGAATGTCTTTACATGGGCTT
GTATGCCTATGAAAATTGTAATACAACTTTCAGCAACGGATCTCTTGGCTCTCGCATCGA
TGAAGAACGCAGCGAAATGCGATAAGTAATGTGAATTGCAGAATTCAGTGAATCATCGAA
TCTTTGAACGCATCTTGCGCTCCTTGGTATTCCGAGGAGCATGCCTGTTTGAGTGTCATT
AAATTCTCAACTCTCTTATACTTTTTTGTAAAAGAGAGCTTGGACTGTGGAGGCTTGCTG

GCCACTTTTTGGGGTCAGCTCCTCTGAAATGCATTAGCGGAACCGTTTGCGATCTGCCAC
AAGTGTGATAAGTTATCTACACTGGCGAGGGGATTGCTCTCTGTAATGTTCAGCTTCTAA
TTGTCTCTACTTTGTGAGACTACTTTTGAATGCTTGACCTCAAATCAGGTAGGACTACCC
GCTGAACTTAA
>AB12-41
TTTCCGTAGGTGAACCTGCGGAAGGATCATTATTGAATTATGTTTCTAGATAGGTTGTAG
CTGGCTCTTTTAGAGCATGTGCACGCCTGTTTGGACTTCATTTTCATCCACCTGTGCACC
TATTGTAGTCTTTGGTTGGGTTAGGAGGAAGTGATCATTGTATCAGCATCTGCTGGGAGT
GAGGACTTGCATTGTGAAAGCTTTGCTGTCCTTGATGTGATCATGGAATCTTTTTCTCAC
TAGAGTCTATGTCACTCATTATACTCTGTCGAATGTCATTGAATGTCTTTACATGGGCTT
GTATGCCTATGAAAATTGTAATACAACTTTCAGCAACGGATCTCTTGGCTCTCGCATCGA
TGAAGAACGCAGCGAAATGCGATAAGTAATGTGAATTGCAGAATTCAGTGAATCATCGAA
TCTTTGAACGCATCTTGCGCTCCTTGGTATTCCGAGGAGCATGCCTGTTTGAGTGTCATT
AAATTCTCAACTCTCTTATACTTTTTTGTAAAAGAGAGCTTGGACTGTGGAGGCTTGCTG
GCCACTTTTTGGGGTCAGCTCCTCTGAAATGCATTAGCGGAACCGTTTGCGATCTGCCAC
AAGTGTGATAAGTTATCTACACTGGCGAGGGGATTGCTCTCTGTAATGTTCAGCTTCTAA
TTGTCTCTACTTTGTGAGACTACTTTTGAATGCTTGACCTCAAATCAGGTAGGACTACCC
GCTGAACTTAA
>AB12-45
TTTCCGTAGGTGAACCTGCGGAAGGATCATTATTGAATTATGTTTCTAGATAGGTTGTAG
CTGGCTCTTTTAGAGCATGTGCACGCCTGTTTGGACTTCATTTTCATCCACCTGTGCACC
TATTGTAGTCTTTGGTTGGGTTAGGAGGAAGTGATCATTGTATCAGCATCTGCTGGGAGT
GAGGACTTGCATTGTGAAAGCTTTGCTGTCCTTGATGTGATCATGGAATCTTTTTCTCAC
TAGAGTCTATGTCACTCATTATACTCTGTCGAATGTCATTGAATGTCTTTACATGGGCTT
GTATGCCTATGAAAATTGTAATACAACTTTCAGCAACGGATCTCTTGGCTCTCGCATCGA
TGAAGAACGCAGCGAAATGCGATAAGTAATGTGAATTGCAGAATTCAGTGAATCATCGAA
TCTTTGAACGCATCTTGCGCTCCTTGGTATTCCGAGGAGCATGCCTGTTTGAGTGTCATT
AAATTCTCAACTCTCTTATACTTTTTTGTAAAAGAGAGCTTGGACTGTGGAGGCTTGCTG
GCCACTTTTTGGGGTCAGCTCCTCTGAAATGCATTAGCGGAACCGTTTGCGATCTGCCAC
AAGTGTGATAAGTTATCTACACTGGCGAGGGGATTGCTCTCTGTAATGTTCAGCTTCTAA
TTGTCTCTACTTTGTGAGACTACTTTTGAATGCTTGACCTCAAATCAGGTAGGACTACCC
GCTGAACTTAA
>AB12-46
TTTCCGTAGGTGAACCTGCGGAAGGATCATTATTGAATTATGTTTCTAGATAGGTTGTAG
CTGGCTCTTTTAGAGCATGTGCACGCCTGTTTGGACTTCATTTTCATCCACCTGTGCACC
TATTGTAGTCTTTGGTTGGGTTAGGAGGAAGTGATCATTGTATCAGCATCTGCTGGGAGT
GAGGACTTGCATTGTGAAAGCTTTGCTGTCCTTGATGTGATCATGGAATCTTTTTCTCAC
TAGAGTCTATGTCACTCATTATACTCTGTCGAATGTCATTGAATGTCTTTACATGGGCTT
GTATGCCTATGAAAATTGTAATACAACTTTCAGCAACGGATCTCTTGGCTCTCGCATCGA
TGAAGAACGCAGCGAAATGCGATAAGTAATGTGAATTGCAGAATTCAGTGAATCATCGAA
TCTTTGAACGCATCTTGCGCTCCTTGGTATTCCGAGGAGCATGCCTGTTTGAGTGTCATT
AAATTCTCAACTCTCTTATACTTTTTTGTAAAAGAGAGCTTGGACTGTGGAGGCTTGCTG
GCCACTTTTTGGGGTCAGCTCCTCTGAAATGCATTAGCGGAACCGTTTGCGATCTGCCAC
AAGTGTGATAAGTTATCTACACTGGCGAGGGGATTGCTCTCTGTAATGTTCAGCTTCTAA
TTGTCTCTACTTTGTGAGACTACTTTTGAATGCTTGACCTCAAATCAGGTAGGACTACCC
GCTGAACTTAA
>AB12-48
TTTCCGTAGGTGAACCTGCGGAAGGATCATTATTGAATTATGTTTCTAGATAGGTTGTAG
CTGGCTCTTTTAGAGCATGTGCACGCCTGTTTGGACTTCATTTTCATCCACCTGTGCACC
TATTGTAGTCTTTGGTTGGGTTAGGAGGAAGTGATCATTGTATCAGCATCTGCTGGGAGT

GAGGACTTGCATTGTGAAAGCTTTGCTGTCCTTGATGTGATCATGGAATCTTTTTCTCAC
TAGAGTCTATGTCACTCATTATACTCTGTCGAATGTCATTGAATGTCTTTACATGGGCTT
GTATGCCTATGAAAATTGTAATACAACTTTCAGCAACGGATCTCTTGGCTCTCGCATCGA
TGAAGAACGCAGCGAAATGCGATAAGTAATGTGAATTGCAGAATTCAGTGAATCATCGAA
TCTTTGAACGCATCTTGCGCTCCTTGGTATTCCGAGGAGCATGCCTGTTTGAGTGTCATT
AAATTCTCAACTCTCTTATACTTTTTTGTAAAAGAGAGCTTGGACTGTGGAGGCTTGCTG
GCCACTTTTTGGGGTCAGCTCCTCTGAAATGCATTAGCGGAACCGTTTGCGATCTGCCAC
AAGTGTGATAAGTTATCTACACTGGCGAGGGGATTGCTCTCTGTAATGTTCAGCTTCTAA
TTGTCTCTACTTTGTGAGACTACTTTTGAATGCTTGACCTCAAATCAGGTAGGACTACCC
GCTGAACTTAA
>AB12-56
TTTCCGTAGGTGAACCTGCGGAAGGATCATTATTGAATTATGTTTCTAGATAGGTTGTAG
CTGGCTCTTTTAGAGCATGTGCACGCCTGTTTGGACTTCATTTTCATCCACCTGTGCACC
TATTGTAGTCTTTGGTTGGGTTAGGAGGAAGTGATCATTGTATCAGCATCTGCTGGGAGT
GAGGACTTGCATTGTGAAAGCTTTGCTGTCCTTGATGTGATCATGGAATCTTTTTCTCAC
TAGAGTCTATGTCACTCATTATACTCTGTCGAATGTCATTGAATGTCTTTACATGGGCTT
GTATGCCTATGAAAATTGTAATACAACTTTCAGCAACGGATCTCTTGGCTCTCGCATCGA
TGAAGAACGCAGCGAAATGCGATAAGTAATGTGAATTGCAGAATTCAGTGAATCATCGAA
TCTTTGAACGCATCTTGCGCTCCTTGGTATTCCGAGGAGCATGCCTGTTTGAGTGTCATT
AAATTCTCAACTCTCTTATACTTTTTTGTAAAAGAGAGCTTGGACTGTGGAGGCTTGCTG
GCCACTTTTTGGGGTCAGCTCCTCTGAAATGCATTAGCGGAACCGTTTGCGATCTGCCAC
AAGTGTGATAAGTTATCTACACTGGCGAGGGGATTGCTCTCTGTAATGTTCAGCTTCTAA
TTGTCTCTACTTTGTGAGACTACTTTTGAATGCTTGACCTCAAATCAGGTAGGACTACCC
GCTGAACTTAA
>AB12-59
TTTCCGTAGGTGAACCTGCGGAAGGATCATTATTGAATTATGTTTCTAGATAGGTTGTAG
CTGGCTCTTTTAGAGCATGTGCACGCCTGTTTGGACTTCATTTTCATCCACCTGTGCACC
TATTGTAGTCTTTGGTTGGGTTAGGAGGAAGTGATCATTGTATCAGCATCTGCTGGGAGT
GAGGACTTGCATTGTGAAAGCTTTGCTGTCCTTGATGTGATCATGGAATCTTTTTCTCAC
TAGAGTCTATGTCACTCATTATACTCTGTCGAATGTCATTGAATGTCTTTACATGGGCTT
GTATGCCTATGAAAATTGTAATACAACTTTCAGCAACGGATCTCTTGGCTCTCGCATCGA
TGAAGAACGCAGCGAAATGCGATAAGTAATGTGAATTGCAGAATTCAGTGAATCATCGAA
TCTTTGAACGCATCTTGCGCTCCTTGGTATTCCGAGGAGCATGCCTGTTTGAGTGTCATT
AAATTCTCAACTCTCTTATACTTTTTTGTAAAAGAGAGCTTGGACTGTGGAGGCTTGCTG
GCCACTTTTTGGGGTCAGCTCCTCTGAAATGCATTAGCGGAACCGTTTGCGATCTGCCAC
AAGTGTGATAAGTTATCTACACTGGCGAGGGGATTGCTCTCTGTAATGTTCAGCTTCTAA
TTGTCTCTACTTTGTGAGACTACTTTTGAATGCTTGACCTCAAATCAGGTAGGACTACCC
GCTGAACTTAA
>AB12-62
TTTCCGTAGGTGAACCTGCGGAAGGATCATTATTGAATTATGTTTCTAGATAGGTTGTAG
CTGGCTCTTTTAGAGCATGTGCACGCCTGTTTGGACTTCATTTTCATCCACCTGTGCACC
TATTGTAGTCTTTGGTTGGGTTAGGAGGAAGTGATCATTGTATCAGCATCTGCTGGGAGT
GAGGACTTGCATTGTGAAAGCTTTGCTGTCCTTGATGTGATCATGGAATCTTTTTCTCAC
TAGAGTCTATGTCACTCATTATACTCTGTCGAATGTCATTGAATGTCTTTACATGGGCTT
GTATGCCTATGAAAATTGTAATACAACTTTCAGCAACGGATCTCTTGGCTCTCGCATCGA
TGAAGAACGCAGCGAAATGCGATAAGTAATGTGAATTGCAGAATTCAGTGAATCATCGAA
TCTTTGAACGCATCTTGCGCTCCTTGGTATTCCGAGGAGCATGCCTGTTTGAGTGTCATT
AAATTCTCAACTCTCTTATACTTTTTTGTAAAAGAGAGCTTGGACTGTGGAGGCTTGCTG
GCCACTTTTTGGGGTCAGCTCCTCTGAAATGCATTAGCGGAACCGTTTGCGATCTGCCAC
AAGTGTGATAAGTTATCTACACTGGCGAGGGGATTGCTCTCTGTAATGTTCAGCTTCTAA

TTGTCTCTACTTTGTGAGACTACTTTTGAATGCTTGACCTCAAATCAGGTAGGACTACCC
GCTGAACTTAA
>AB8-12
TTTCCGTAGGTGAACCTGCGGAAGGATCATTATTGAATTATGTTTCTAGATAGGTTGTAG
CTGGCTCTTTTAGAGCATGTGCACGCCTGTTTGGACTTCATTTTCATCCACCTGTGCACC
TATTGTAGTCTTTGGTTGGGTTAGGGGGAAGTGATCATTGTATCAGCATCTGCTGGGAGT
GAGGACTTGCATTGTGAAAGCTTTGCTGTCCTTGATGTGATCATGGAATCTTTTTCTCAC
TAGAGTCTATGTCACTCATTATACTCTGTCGAATGTCATTGAATGTCTTTACATGGGCTT
GTATGCCTATGAAAATTGTAATACAACTTTCAGCAACGGATCTCTTGGCTCTCGCATCGA
TGAAGAACGCAGCGAAATGCGATAAGTAATGTGAATTGCAGAATTCAGTGAATCATCGAA
TCTTTGAACGCATCTTGCGCTCCTTGGTATTCCGAGGAGCATGCCTGTTTGAGTGTCATT
AAATTCTCAACTCTCTTATACTTTTTTGTAAAAGAGAGCTTGGACTGTGGAGGCTTGCTG
GCCACTTTTTGGGGTCAGCTCCTCTGAAATGCATTAGCGGAACCGTTTGCGATCTGCCAC
AAGTGTGATAAGTTATCTACACTGGCGAGGGGATTGCTCTCTGTAATGTTCAGCTTCTAA
TTGTCTCTACTTTGTGAGACTACTTTTGAATGCTTGACCTCAAATCAGGTAGGACTACCC
GCTGAACTTAA
>AB3-1
TTTCCGTAGGTGAACCTGCGGAAGGATCATTATTGAATTATGTTTCTAGATAGGTTGTAG
CTGGCTCTTTTAGAGCATGTGCACGCCTGTTTGGACTTCATTTTCATCCACCTGTGCACC
TATTGTAGTCTTTGGTTGGGTTAGGAGGAAGTGATCATTGTATCAGCATCTGCTGGGAGT
GAGGACTTGCATTGTGAAAGCTTTGCTGTCCTTGATGTGATCATGGAATCTTTTTCTCAC
TAGAGTCTATGTCACTCATTATACTCTGTCGAATGTCATTGAATGTCTTTACATGGGCTT
GTATGCCTATGAAAATTGTAATACAACTTTCAGCAACGGATCTCTTGGCTCTCGCATCGA
TGAAGAACGCAGCGAAATGCGATAAGTAATGTGAATTGCAGAATTCAGTGAATCATCGAA
TCTTTGAACGCATCTTGCGCTCCTTGGTATTCCGAGGAGCATGCCTGTTTGAGTGTCATT
AAATTCTCAACTCTCTTATACTTTTTTGTAAAAGAGAGCTTGGACTGTGGAGGCTTGCTG
GCCACTTTTTGGGGTCAGCTCCTCTGAAATGCATTAGCGGAACCGTTTGCAATCTGCCAC
AAGTGTGATAAGTTATCTACACTGGCGAGGGGATTGCTCTCTGTAATGTTCAGCTTCTAA
TTGTCTCTACTTTGTGAGACTACTTTTGAATGCTTGACCTCAAATCAGGTAGGACTACCC
GCTGAACTTAA
>AB12-18
TTTCCGTAGGTGAACCTGCGGAAGGATCATTATTGAATTATGTTTCTAGATAGGTTGTAG
CTGGCTCTTTTAGAGCATGTGCACGCCTGTTTGGACTTCATTTTCATCCACCTGTGCACC
TATTGTAGTCTTTGGTTGGGTTAGGAGGAAGTGATCATTGTATCAGCATCTGCTGGGAGT
GAGGACTTGCATTGTGAAAGCTTTGCTGTCCTTGATGTGATCATGGAATCTTTTTCTCAC
TAGAGTCTATGTCACTCATTATACTCTGTCGAATGTCATTGAATGTCTTTACATGGGCTT
GTATGCCTATGAAAATTGTAATACAACTTTCAGCAACGGATCTCTTGGCTCTCGCATCGA
TGAAGAACGCAGCGAAATGCGATAAGTAATGTGAATTGCAGAATTCAGTGAATCATCGAA
TCTTTGAACGCATCTTGCGCTCCTTGGTATTCCGAGGAGCATGCCTGTTTGAGTGTCATT
AAATTCTCAACTCTCTTATACTTTTTTGTAAAAGAGAGCTTGGACTGTGGAGGCTTGCTG
GCCACTTTTTGGGGTCAGCTCCTCTGAAATGCATTAGCGGAACCGTTTGCAATCTGCCAC
AAGTGTGATAAGTTATCTACACTGGCGAGGGGATTGCTCTCTGTAATGTTCAGCTTCTAA
TTGTCTCTACTTTGTGAGACTACTTTTGAATGCTTGACCTCAAATCAGGTAGGACTACCC
GCTGAACTTAA
>AB3-47
TTTCCGTAGGTGAACCTGCGGAAGGATCATTATTGAATTATGTTTCTAGATAGGTTGTAG
CTGGCTCTTTTAGAGCATGTGCACGCCTGTTTGGACTTCATTTTCATCCACCTGTGCACC
TATTGTAGTCTTTGGTTGGGTTAGGAGGAAGTGATCATTGTATCAGCATCTGCTGGGAGT
GAGGACTTGCATTGTGAAAGCTTTGCTGTCCTTGATGTGATCATGGAATCTTTTTCTCAC
TAGAGTCTATGTCACTCATTATACTCTGTCGAATGTCATTGAATGTCTTTACATGGGCTT

GTATGCCTATGAAAATTGTAATACAACTTTCAGCAACGGATCTCTTGGCTCTCGCATCGA
TGAAGAACGCAGCGAAATGCGATAAGTAATGTGAATTGCAGAATTCAGTGAATCATCGAA
TCTTTGAACGCATCTTGCGCTCCTTGGTATTCCGAGGAGCATGCCTGTTTGAGTGTCATT
AAATTCTCAACTCTCTTATACTTTTTTGTAAAAGAGAGCTTGGACTGTGGAGGCTTGCTG
GCCACTTTTTGGGGTCAGCTCCTCTGAAATGCATTAGCGGAACCGTTTGCAATCTGCCAC
AAGTGTGATAAGTTATCTACACTGGCGAGGGGATTGCTCTCTGTAATGTTCAGCTTCTAA
TTGTCTCTACTTTGTGAGACTACTTTTGAATGCTTGACCTCAAATCAGGTAGGACTACCC
GCTGAACTTAA
>AB6–6
TTTCCGTAGGTGAACCTGCGGAAGGATCATTATTGAATTATGTTTCTAGATAGGTTGTAG
CTGGCTCTTTTAGAGCATGTGCACGCCTGTTTGGACTTCATTTTCATCCACCTGTGCACC
TATTGTAGTCTTTGGTTGGGTTAGGAGGAAGTGATCATTGTATCAGCATCTGCTGGGAGT
GAGGACTTGCATTGTGAAAGCTTTGCTGTCCTTGATGTGATCATGGAATCTTTTTCTCAC
TAGAGTCTATGTCACTCATTATACTCTGTCGAATGTCATTGAATGTCTTTACATGGGCTT
GTATGCCTATGAAAATTGTAATACAACTTTCAGCAACGGATCTCTTGGCTCTCGCATCGA
TGAAGAACGCAGCGAAATGCGATAAGTAATGTGAATTGCAGAATTCAGTGAATCATCGAA
TCTTTGAACGCATCTTGCGCTCCTTGGTATTCCGAGGAGCATGCCTGTTTGAGTGTCATT
AAATTCTCAACTCTCTTATACTTTTTTGTAAAAGAGAGCTTGGACTGTGGAGGCTTGCTG
GCCACTTTTTGGGGTCAGCTCCTCTGAAATGCATTAGCGGAACCGTTTGCAATCTGCCAC
AAGTGTGATAAGTTATCTACACTGGCGAGGGGATTGCTCTCTGTAATGTTCAGCTTCTAA
TTGTCTCTACTTTGTGAGACTACTTTTGAATGCTTGACCTCAAATCAGGTAGGACTACCC
GCTGAACTTAA
>AB7–10
TTTCCGTAGGTGAACCTGCGGAAGGATCATTATTGAATTATGTTTCTAGATAGGTTGTAG
CTGGCTCTTTTAGAGCATGTGCACGCCTGTTTGGACTTCATTTTCATCCACCTGTGCACC
TATTGTAGTCTTTGGTTGGGTTAGGAGGAAGTGATCATTGTATCAGCATCTGCTGGGAGT
GAGGACTTGCATTGTGAAAGCTTTGCTGTCCTTGATGTGATCATGGAATCTTTTTCTCAC
TAGAGTCTATGTCACTCATTATACTCTGTCGAATGTCATTGAATGTCTTTACATGGGCTT
GTATGCCTATGAAAATTGTAATACAACTTTCAGCAACGGATCTCTTGGCTCTCGCATCGA
TGAAGAACGCAGCGAAATGCGATAAGTAATGTGAATTGCAGAATTCAGTGAATCATCGAA
TCTTTGAACGCATCTTGCGCTCCTTGGTATTCCGAGGAGCATGCCTGTTTGAGTGTCATT
AAATTCTCAACTCTCTTATACTTTTTTGTAAAAGAGAGCTTGGACTGTGGAGGCTTGCTG
GCCACTTTTTGGGGTCAGCTCCTCTGAAATGCATTAGCGGAACCGTTTGCAATCTGCCAC
AAGTGTGATAAGTTATCTACACTGGCGAGGGGATTGCTCTCTGTAATGTTCAGCTTCTAA
TTGTCTCTACTTTGTGAGACTACTTTTGAATGCTTGACCTCAAATCAGGTAGGACTACCC
GCTGAACTTAA
>AB7–49
TTTCCGTAGGTGAACCTGCGGAAGGATCATTATTGAATTATGTTTCTAGATAGGTTGTAG
CTGGCTCTTTTAGAGCATGTGCACGCCTGTTTGGACTTCATTTTCATCCACCTGTGCACC
TATTGTAGTCTTTGGTTGGGTTAGGAGGAAGTGATCATTGTATCAGCATCTGCTGGGAGT
GAGGACTTGCATTGTGAAAGCTTTGCTGTCCTTGATGTGATCATGGAATCTTTTTCTCAC
TAGAGTCTATGTCACTCATTATACTCTGTCGAATGTCATTGAATGTCTTTACATGGGCTT
GTATGCCTATGAAAATTGTAATACAACTTTCAGCAACGGATCTCTTGGCTCTCGCATCGA
TGAAGAACGCAGCGAAATGCGATAAGTAATGTGAATTGCAGAATTCAGTGAATCATCGAA
TCTTTGAACGCATCTTGCGCTCCTTGGTATTCCGAGGAGCATGCCTGTTTGAGTGTCATT
AAATTCTCAACTCTCTTATACTTTTTTGTAAAAGAGAGCTTGGACTGTGGAGGCTTGCTG
GCCACTTTTTGGGGTCAGCTCCTCTGAAATGCATTAGCGGAACCGTTTGCAATCTGCCAC
AAGTGTGATAAGTTATCTACACTGGCGAGGGGATTGCTCTCTGTAATGTTCAGCTTCTAA
TTGTCTCTACTTTGTGAGACTACTTTTGAATGCTTGACCTCAAATCAGGTAGGACTACCC
GCTGAACTTAA

>AB8-47
TTTCCGTAGGTGAACCTGCGGAAGGATCATTATTGAATTATGTTTCTAGATAGGTTGTAG
CTGGCTCTTTTAGAGCATGTGCACGCCTGTTTGGACTTCATTTTCATCCACCTGTGCACC
TATTGTAGTCTTTGGTTGGGTTAGGAGGAAGTGATCATTGTATCAGCATCTGCTGGGAGT
GAGGACTTGCATTGTGAAAGCTTTGCTGTCCTTGATGTGATCATGGAATCTTTTTCTCAC
TAGAGTCTATGTCACTCATTATACTCTGTCGAATGTCATTGAATGTCTTTACATGGGCTT
GTATGCCTATGAAAATTGTAATACAACTTTCAGCAACGGATCTCTTGGCTCTCGCATCGA
TGAAGAACGCAGCGAAATGCGATAAGTAATGTGAATTGCAGAATTCAGTGAATCATCGAA
TCTTTGAACGCATCTTGCGCTCCTTGGTATTCCGAGGAGCATGCCTGTTTGAGTGTCATT
AAATTCTCAACTCTCTTATACTTTTTTGTAAAAGAGAGCTTGGACTGTGGAGGCTTGCTG
GCCACTTTTTGGGGTCAGCTCCTCTGAAATGCATTAGCGGAACCGTTTGCAATCTGCCAC
AAGTGTGATAAGTTATCTACACTGGCGAGGGGATTGCTCTCTGTAATGTTCAGCTTCTAA
TTGTCTCTACTTTGTGAGACTACTTTTGAATGCTTGACCTCAAATCAGGTAGGACTACCC
GCTGAACTTAA
>AB9-36
TTTCCGTAGGTGAACCTGCGGAAGGATCATTATTGAATTATGTTTCTAGATAGGTTGTAG
CTGGCTCTTTTAGAGCATGTGCACGCCTGTTTGGACTTCATTTTCATCCACCTGTGCACC
TATTGTAGTCTTTGGTTGGGTTAGGAGGAAGTGATCATTGTATCAGCATCTGCTGGGAGT
GAGGACTTGCATTGTGAAAGCTTTGCTGTCCTTGATGTGATCATGGAATCTTTTTCTCAC
TAGAGTCTATGTCACTCATTATACTCTGTCGAATGTCATTGAATGTCTTTACATGGGCTT
GTATGCCTATGAAAATTGTAATACAACTTTCAGCAACGGATCTCTTGGCTCTCGCATCGA
TGAAGAACGCAGCGAAATGCGATAAGTAATGTGAATTGCAGAATTCAGTGAATCATCGAA
TCTTTGAACGCATCTTGCGCTCCTTGGTATTCCGAGGAGCATGCCTGTTTGAGTGTCATT
AAATTCTCAACTCTCTTATACTTTTTTGTAAAAGAGAGCTTGGACTGTGGAGGCTTGCTG
GCCACTTTTTGGGGTCAGCTCCTCTGAAATGCATTAGCGGAACCGTTTGCAATCTGCCAC
AAGTGTGATAAGTTATCTACACTGGCGAGGGGATTGCTCTCTGTAATGTTCAGCTTCTAA
TTGTCTCTACTTTGTGAGACTACTTTTGAATGCTTGACCTCAAATCAGGTAGGACTACCC
GCTGAACTTAA
>AB11-45
TTTCCGTAGGTGAACCTGCGGAAGGATCATTATTGAATTATGTTTCTAGATAGGTTGTAG
CTGGCTCTTTTAGAGCATGTGCACGCCTGTTTGGACTTCATTTTCATCCACCTGTGCACC
TATTGTAGTCTTTGGTTGGGTTAGGAGGAAGTGATCATTGTATCAGCATCTGCTGGGAGT
GAGGACTTGCATTGTGAAAGCTTTGCTGTCCTTGATGTGATCATGGAATCTTTTTCTCAC
TAGAGTCTATGTCACTCATTATACTCTGTCGAATGTCATTGAATGTCTTTACATGGGCTT
GTATGCCTATGAAAATTGTAATACAACTTTCAGCAACGGATCTCTTGGCTCTCGCATCGA
TGAAGAACGCAGCGAAATGCGATAAGTAATGTGAATTGCAGAATTCAGTGAATCATCGAA
TCTTTGAACGCATCTTGCGCTCCTTGGTATTCCGAGGAGCATGCCTGTTTGAGTGTCATT
AAATTCTCAACTCTCTTATACTTTTTTGTAAAAGAGAGCTTGGACTGTGGAGGCTTGCTG
GCCACTTTTTGGGGTCAGCTCCTCTGAAATGCATTAGCGGAACCGTTTGCAATCTGCCAC
AAGTGTGATAAGTTATCTACACTGGCGAGGGGATTGCTCTCTGTAATGTTCAGCTTCTAA
TTGTCTCTACTTTGTGAGACTACTTTTGAATGCTTGACCTCAAATCAGGTAGGACTACCC
GCTGAACTTAA
>AB3-18
TTTCCGTAGGTGAACCTGCGGAAGGATCATTATTGAATTATGTTTCTAGATAGGTTGTAG
CTGGCTCTTTTAGAGCATGTGCACGCCTGTTTGGACTTCATTTTCATCCACCTGTGCACC
TATTGTAGTCTTTGGTTGGGTTAGGAGGAAGTGATCATTGTATCAGCATCTGCTGGGAGT
GAGGACTTGCATTGTGAAAGCTTTGCTGTCCTTGATGTGATCATGGAATCTTTTTCTCAC
TAGAGTCTATGTCACTCATTATACTCTGTCGAATGTCATTGAATGTCTTTACATGGGCTT
GTATGCCTATGAAAATTGTAATACAACTTTCAGCAACGGATCTCTTGGCTCTCGCATCGA
TGAAGAACGCAGCGAAATGCGATAAGTAATGTGAATTGCAGAATTCAGTGAATCATCGAA

TCTTTGAACGCATCTTGCGCTCCTTGGTATTCCGAGGAGCATGCCTGTTTGAGTGTCATT
AAATTCTCAACTCTCTTATACTTTTTTGTAAAAGAGAGCTTGGACTGTGGAGGCTTGCTG
GCCACTTTTTGGGGTCAGCTCCTCTGAAATGCATTAGCGGAACCGTTTGCAATCTGCCAC
AAGTGTGATAAGTTATCTACACTGGCGAGGGGATTGCTCTCTGTAATGTTCAGCTTCTAA
TTGTCTCTACTTTGTGAGACTACTTTTGAATGCTTGACCTCAAATCAGGTAGGACTACCC
GCTGAACTTAA
>AB9–44
TTTCCGTAGGTGAACCTGCGGAAGGATCATTATTGAATTATGTTTCTAGATAGGTTGTAG
CTGGCTCTTTTAGAGCATGTGCACGCCTGTTTGGACTTCATTTTCATCCACCTGTGCACC
TATTGTAGTCTTTGGTTGGGTTAGGAGGAAGTGATCATTGTATCAGCATCTGCTGGGAGT
GAGGACTTGCATTGTGAAAGCTTTGCTGTCCTTGATGTGATCATGGAATCTTTTTCTCAC
TAGAGTCTATGTCACTCATTATACTCTGTCGAATGTCATTGAATGTCTTTACATGGGCTT
GTATGCCTATGAAAATTGTAATACAACTTTCAGCAACGGATCTCTTGGCTCTCGCATCGA
TGAAGAACGCAGCGAAATGCGATAAGTAATGTGAATTGCAGAATTCAGTGAATCATCGAA
TCTTTGAACGCATCTTGCGCTCCTTGGTATTCCGAGGAGCATGCCTGTTTGAGTGTCATT
AAATTCTCAACTCTCTTATACTTTTTTGTAAAAGAGAGCTTGGACTGTGGAGGCTTGCTG
GCCACTTTTTGGGGTCAGCTCCTCTGAAATGCATTAGCGGAACCGTTTGCGATCTACCAC
AAGTGTGATAAGTTATCTACACTGGCGAGGGGATTGCTCTCTGTAATGTTCAGCTTCTAA
TTGTCTCTACTTTGTGAGACTACTTTTGAATGCTTGACCTCAAATCAGGTAGGACTACCC
GCTGAACTTAA
>AB10–41
TTTCCGTAGGTGAACCTGCGGAAGGATCATTATTGAATTATGTTTCTAGATAGGTTGTAG
CTGGCTCTTTTAGAGCATGTGCACGCCTGTTTGGACTTCATTTTCATCCACCTGTGCACC
TATTGTAGTCTTTGGTTGGGTTAGGAGGAAGTGATCATTGTATCAGCATCTGCTGGGAGT
GAGGACTTGCATTGTGAAAGCTTTGCTGTCCTTGATGTGATCATGGAATCTTTTTCTCAC
TAGAGTCTATGTCACTCATTATACTCTGTCGAATGTCATTGAATGTCTTTACATGGGCTT
GTATGCCTATGAAAATTGTAATACAACTTTCAGCAACGGATCTCTTGGCTCTCGCATCGA
TGAAGAACGCAGCGAAATGCGATAAGTAATGTGAATTGCAGAATTCAGTGAATCATCGAA
TCTTTGAACGCATCTTGCGCTCCTTGGTATTCCGAGGAGCATGCCTATTTGAGTGTCATT
AAATTCTCAACTCTCTTATACTTTTTTGTAAAAGAGAGCTTGGACTGTGGAGGCTTGCTG
GCCACTTTTTGGGGTCAGCTCCTCTGAAATGCATTAGCGGAACCGTTTGCGATCTGCCAC
AAGTGTGATAAGTTATCTACACTGGCGAGGGGATTGCTCTCTGTAATGTTCAGCTTCTAA
TTGTCTCTACTTTGTGAGACTACTTTTGAATGCTTGACCTCAAATCAGGTAGGACTACCC
GCTGAACTTAA
>AB3–38
TTTCCGTAGGTGAACCTGCGGAAGGATCATTATTGAATTATGTTTCTAGATAGGTTGTAG
CTGGCTCTTTTAGAGCATGTGCACGCCTGTTTGGACTTCATTTTCATCCACCTGTGCACC
TATTGTAGTCTTTGGTTGGGTTAGGAGGAAGTGATCATTGTATCAGCATCTGCTGGGAGT
GAGGACTTGCATTGTGAAAGCTTTGCTGTCCTTGATGTGATCATGGAATCTTTTTCTCAC
TAGAGTCTATGTCACTCATTATACTCTGTCGAATGTCATTGAATGTCTTTACATGGGCTT
GTATGCCTATGAAAATTGTAATACAACTTTCAGCAACGGATCTCTTGGCTCTCGCATCGA
TGAAGAACGCAGCGAAATGCGATAAGTAATGTGAATTGCAGAATTCAGTGAATCATCGAA
TCTTTGAACGCATCTTGCGCTCCTTGGTATTCCGAGGAGCATGCCTGTTTGAGTGTCATT
AAATTCTCAACTCTCTTATACTTTTTTGTAAAAGAGAGCTTGGACTGTGGAGGCTTGCTG
GTCACTTTTTGGGGTCAGCTCCTCTGAAATGCATTAGCGGAACCGTTTGCGATCTGCCAC
AAGTGTGATAAGTTATCTACACTGGCGAGGGGATTGCTCTCTGTAATGTTCAGCTTCTAA
TTGTCTCTACTTTGTGAGACTACTTTTGAATGCTTGACCTCAAATCAGGTAGGACTACCC
GCTGAACTTAA
>AB3–52
TTTCCGTAGGTGAACCTGCGGAAGGATCATTATTGAATTATGTTTCTAGATAGGTTGTAG

CTGGCTCTTTTAGAGCATGTGCACGCCTGTTTGGACTTCATTTTCATCCACCTGTGCACC
TATTGTAGTCTTTGGTTGGGTTAGGAGGAAGTGATCATTGTATCAGCATCTGCTGGGAGT
GAGGACTTGCATTGTGAAAGCTTTGCTGTCCTTGATGTGATCATGGAATCTTTTTCTCAC
TAGAGTCTATGTCACTCATTATACTCTGTCGAATGTCATTGAATGTCTTTACATGGGCTT
GTATGCCTATGAAAATTGTAATACAACTTTCAGCAACGGATCTCTTGGCTCTCGCATCGA
TGAAGAACGCAGCGAAATGCGATAAGTAATGTGAATTGCAGAATTCAGTGAATCATCGAA
TCTTTGAACGCATCTTGCGCTCCTTGGTATTCCGAGGAGCATGCCTGTTTGAGTGTCATT
AAATTCTCAACTCTCTTATACTTTTTTGTAAAAGAGAGCTTGGACTGTGGAGGCTTGCTG
GTCACTTTTTGGGGTCAGCTCCTCTGAAATGCATTAGCGGAACCGTTTGCGATCTGCCAC
AAGTGTGATAAGTTATCTACACTGGCGAGGGGATTGCTCTCTGTAATGTTCAGCTTCTAA
TTGTCTCTACTTTGTGAGACTACTTTTGAATGCTTGACCTCAAATCAGGTAGGACTACCC
GCTGAACTTAA
>AB6–46
TTTCCGTAGGTGAACCTGCGGAAGGATCATTATTGAATTATGTTTCTAGATAGGTTGTAG
CTGGCTCTTTTAGAGCATGTGCACGCCTGTTTGGACTTCATTTTCATCCACCTGTGCACC
TATTGTAGTCTTTGGTTGGGTTAGGAGGAAGTGATCATTGTATCAGCATCTGCTGGGAGT
GAGGACTTGCATTGTGAAAGCTTTGCTGTCCTTGATGTGATCATGGAATCTTTTTCTCAC
TAGAGTCTATGTCACTCATTATACTCTGTCGAATGTCATTGAATGTCTTTACATGGGCTT
GTATGCCTATGAAAATTGTAATACAACTTTCAGCAACGGATCTCTTGGCTCTCGCATCGA
TGAAGAACGCAGCGAAATGCGATAAGTAATGTGAATTGCAGAATTCAGTGAATCATCGAA
TCTTTGAACGCATCTTGCGCTCCTTGGTATTCCGAGGAGCATGCCTGTTTGAGTGTCATT
AAATTCTCAACTCTCTTATACTTTTTTGTAAAAGAGAGCTTGGACTGTGGAGGCTTGCTG
GCCACTTTTTGGGGTCAGCTCCTCTGAAATGCATTAGCGGAACCGTTTGTGATCTGCCAC
AAGTGTGATAAGTTATCTACACTGGCGAGGGGATTGCTCTCTGTAATGTTCAGCTTCTAA
TTGTCTCTACTTTGTGAGACTACTTTTGAATGCTTGACCTCAAATCAGGTAGGACTACCC
GCTGAACTTAA
>AB10–8
TTTCCGTAGGTGAACCTGCGGAAGGATCATTATTGAATTATGTTTCTAGATAGGTTGTAG
CTGGCTCTTTTAGAGCATGTGCACGCCTGTTTGGACTTCATTTTCATCCACCTGTGCACC
TATTGTAGTCTTTGGTTGGGTTAGGAGGAAGTGATCATTGTATCAGCATCTGCTGGGAGT
GAGGACTTGCATTGTGAAAGCTTTGCTGTCCTTGATGTGATCATGGAATCTCTTTCTCAC
TAGAGTCTATGTCACTCATTATACTCTGTCGAATGTCATTGAATGTCTTTACATGGGCTT
GTATGCCTATGAAAATTGTAATACAACTTTCAGCAACGGATCTCTTGGCTCTCGCATCGA
TGAAGAACGCAGCGAAATGCGATAAGTAATGTGAATTGCAGAATTCAGTGAATCATCGAA
TCTTTGAACGCATCTTGCGCTCCTTGGTATTCCGAGGAGCATGCCTGTTTGAGTGTCATT
AAATTCTCAACTCTCTTATACTTTTTTGTAAAAGAGAGCTTGGACTGTGGAGGCTTGCTG
GCCACTTTTTGGGGTCAGCTCCTCTGAAATGCATTAGCGGAACCGTTTGCGATCTGCCAC
AAGTGTGATAAGTTATCTACACTGGCGAGGGGATTGCTCTCTGTAATGTTCAGCTTCTAA
TTGTCTCTACTTTGTGAGACTACTTTTGAATGCTTGACCTCAAATCAGGTAGGACTACCC
GCTGAACTTAA
>AB5–31
TTTCCGTAGGTGAACCTGCGGAAGGATCATTATTGAATTATGTTTCTAGATAGGTTGTAG
CTGGCTCTTTTAGAGCATGTGCACGCCTGTTTGGACTTCATTTTCATCCACCTGTGCACC
TATTGTAGTCTTTGGTTGGGTTAGGAGGAAGTGATCATTGTATCAGCATCTGCTGGGAGT
GAGGACTTGCATTGTGAAAGCTTTGCTGTCCTTGATGTGATCATGGAATCTCTTTCTCAC
TAGAGTCTATGTCACTCATTATACTCTGTCGAATGTCATTGAATGTCTTTACATGGGCTT
GTATGCCTATGAAAATTGTAATACAACTTTCAGCAACGGATCTCTTGGCTCTCGCATCGA
TGAAGAACGCAGCGAAATGCGATAAGTAATGTGAATTGCAGAATTCAGTGAATCATCGAA
TCTTTGAACGCATCTTGCGCTCCTTGGTATTCCGAGGAGCATGCCTGTTTGAGTGTCATT
AAATTCTCAACTCTCTTATACTTTTTTGTAAAAGAGAGCTTGGACTGTGGAGGCTTGCTG

GCCACTTTTTGGGGTCAGCTCCTCTGAAATGCATTAGCGGAACCGTTTGCGATCTGCCAC
AAGTGTGATAAGTTATCTACACTGGCGAGGGGATTGCTCTCTGTAATGTTCAGCTTCTAA
TTGTCTCTACTTTGTGAGACTACTTTTGAATGCTTGACCTCAAATCAGGTAGGACTACCC
GCTGAACTTAA
>AB1–15
TTTCCGTAGGTGAACCTGCGGAAGGATCATTATTGAATTATGTTTCTAGATAGGTTGTAG
CTGGCTCTTTTAGAGCATGTGCACGCCTGTTTGGACTTCATTTTCATCCACCTGTGCACC
TATTGTAGTCTTTGGTTGGGTTAGGAGGAAGTGATCATTGTATCAGCATCTGCTGGGAGT
GAGGACTTGCATTGTGAAAGCTTTGCTGTCCTTGATGTGATCATGGAATCTCTTTCTCAC
TAGAGTCTATGTCACTCATTATACTCTGTCGAATGTCATTGAATGTCTTTACATGGGCTT
GTATGCCTATGAAAATTGTAATACAACTTTCAGCAACGGATCTCTTGGCTCTCGCATCGA
TGAAGAACGCAGCGAAATGCGATAAGTAATGTGAATTGCAGAATTCAGTGAATCATCGAA
TCTTTGAACGCATCTTGCGCTCCTTGGTATTCCGAGGAGCATGCCTGTTTGAGTGTCATT
AAATTCTCAACTCTCTTATACTTTTTTGTAAAAGAGAGCTTGGACTGTGGAGGCTTGCTG
GCCACTTTTTGGGGTCAGCTCCTCTGAAATGCATTAGCGGAACCGTTTGCGATCTGCCAC
AAGTGTGATAAGTTATCTACACTGGCGAGGGGATTGCTCTCTGTAATGTTCAGCTTCTAA
TTGTCTCTACTTTGTGAGACTACTTTTGAATGCTTGACCTCAAATCAGGTAGGACTACCC
GCTGAACTTAA
>AB6–45
TTTCCGTAGGTGAACCTGCGGAAGGATCATTATTGAATTATGTTTCTAGATAGGTTGTAG
CTGGCTCTTTTAGAGCATGTGCACGCCTGTTTGGACTTCATTTTCATCCACCTGTGCACC
TATTGTAGTCTTTGGTTGGGTTAGGAGGAAGTGATCATTGTATCAGCATCTGCTGGGAGT
GAGGACTTGCATTGTGAAAGCTTTGCTGTCCTTGATGTGATCATGGAATCTCTTTCTCAC
TAGAGTCTATGTCACTCATTATACTCTGTCGAATGTCATTGAATGTCTTTACATGGGCTT
GTATGCCTATGAAAATTGTAATACAACTTTCAGCAACGGATCTCTTGGCTCTCGCATCGA
TGAAGGACGCAGCGAAATGCGATAAGTAATGTGAATTGCAGAATTCAGTGAATCATCGAA
TCTTTGAACGCATCTTGCGCTCCTTGGTATTCCGAGGAGCATGCCTGTTTGAGTGTCATT
AAATTCTCAACTCTCTTATACTTTTTTGTAAAAGAGAGCTTGGACTGTGGAGGCTTGCTG
GCCACTTTTTGGGGTCAGCTCCTCTGAAATGCATTAGCGGAACCGTTTGCGATCTGCCAC
AAGTGTGATAAGTTATCTACACTGGCGAGGGGATTGCTCTCTGTAATGTTCAGCTTCTAA
TTGTCTCTACTTTGTGAGACTACTTTTGAATGCTTGACCTCAAATCAGGTAGGACTACCC
GCTGAACTTAA
>AB10–2
TTTCCGTAGGTGAACCTGCGGAAGGATCATTATTGAATTATGTTTCTAGATAGGTTGTAG
CTGGCTCTTTTAGAGCATGTGCACGCCTGTTTGGACTTCATTTTCATCCACCTGTGCACC
TATTGTAGTCTTTGGTTGGGTTAGGAGGAAGTGATCATTGTATCAGCATCTGCTGGGAGT
GAGGACTTGCATTGTGAAAGCTTTGCTGTCCTTGATGTGATCATGGAATCTCTTTCTCAC
TAGAGTCTATGTCACTCATTATACTCTGTCGAATGTCATTGAATGTCTTTACATGGGCTT
GTATGCCTATGAAAATTGTAATACAACTTTCAGCAACGGATCTCTTGGCTCTCGCATCGA
TGAAGGACGCAGCGAAATGCGATAAGTAATGTGAATTGCAGAATTCAGTGAATCATCGAA
TCTTTGAACGCATCTTGCGCTCCTTGGTATTCCGAGGAGCATGCCTGTTTGAGTGTCATT
AAATTCTCAACTCTCTTATACTTTTTTGTAAAAGAGAGCTTGGACTGTGGAGGCTTGCTG
GCCACTTTTTGGGGTCAGCTCCTCTGAAATGCATTAGCGGAACCGTTTGCGATCTGCCAC
AAGTGTGATAAGTTATCTACACTGGCGAGGGGATTGCTCTCTGTAATGTTCAGCTTCTAA
TTGTCTCTACTTTGTGAGACTACTTTTGAATGCTTGACCTCAAATCAGGTAGGACTACCC
GCTGAACTTAA
>AB9–20
TTTCCGTAGGTGAACCTGCGGAAGGATCATTATTGAATTATGTTTCTAGATAGGTTGTAG
CTGGCTCTTTTAGAGCATGTGCACGCCTGTTTGGACTTCATTTTCATCCACCTGTGCACC
TATTGTAGTCTTTGGTTGGGTTAGGAGGAAGTGATCATTGTATCAGCATCTGCTGGGAGT

GAGGACTTGCATTGTGAAAGCTTTGCTGTCCTTGATGTGATCATGGAATCTCTTTCTCAC
TAGAGTCTATGTCACTCATTATACTCTGTCGAATGTCATTGAATGTCTTTACATGGGCTT
GTATGCCTATGAAAATTGTAATACAACTTTCAGCAACGGATCTCTTGGCTCTCGCATCGA
TGAAGGACGCAGCGAAATGCGATAAGTAATGTGAATTGCAGAATTCAGTGAATCATCGAA
TCTTTGAACGCATCTTGCGCTCCTTGGTATTCCGAGGAGCATGCCTGTTTGAGTGTCATT
AAATTCTCAACTCTCTTATACTTTTTTGTAAAAGAGAGCTTGGACTGTGGAGGCTTGCTG
GCCACTTTTTGGGGTCAGCTCCTCTGAAATGCATTAGCGGAACCGTTTGCGATCTGCCAC
AAGTGTGATAAGTTATCTACACTGGCGAGGGGATTGCTCTCTGTAATGTTCAGCTTCTAA
TTGTCTCTACTTTGTGAGACTACTTTTGAATGCTTGACCTCAAATCAGGTAGGACTACCC
GCTGAACTTAA
>AB5-30
TTTCCGTAGGTGAACCTGCGGAAGGATCATTATTGAATTATGTTTCTAGATAGGTTGTAG
CTGGCTCTTTTAGAGCATGTGCACGCCTGTTTGGACTTCATTTTCATCCACCTGTGCACC
TATTGTAGTCTTTGGTTGGGTTAGGAGGAAGTGATCATTGTATCAGCATCTGCTGGGAGT
GAGGACTTGCATTGTGAAAGCTTTGCTGTCCTTGATGTGATCATGGAATCTCTTTCTCAC
TAGAGTCTATGTCACTCATTATACTCTGTCGAATGTCATTGAATGTCTTTACATGGGCTT
GTATGCCTATGAAAATTGTAATACAACTTTCAGCAACGGATCTCTTGGCTCTCGCATCGA
TGAAGGACGCAGCGAAATGCGATAAGTAATGTGAATTGCAGAATTCAGTGAATCATCGAA
TCTTTGAACGCATCTTGCGCTCCTTGGTATTCCGAGGAGCATGCCTGTTTGAGTGTCATT
AAATTCTCAACTCTCTTATACTTTTTTGTAAAAGAGAGCTTGGACTGTGGAGGCTTGCTG
GCCACTTTTTGGGGTCAGCTCCTCTGAAATGCATTAGCGGAACCGTTTGCGATCTGCCAC
AAGTGTGATAAGTTATCTACACTGGCGAGGGGATTGCTCTCTGTAATGTTCAGCTTCTAA
TTGTCTCTACTTTGTGAGACTACTTTTGAATGCTTGACCTCAAATCAGGTAGGACTACCC
GCTGAACTTAA
>AB12-31
TTTCCGTAGGTGAACCTGCGGAAGGATCATTATTGAATTATGTTTCTAGATAGGTTGTAG
CTGGCTCTTTTAGAGCATGTGCACGCCTGTTTGGACTTCATTTTCATCCACCTGTGCACC
TATTGTAGTCTTTGGTTGGGTTAGGAGGAAGTGATCATTGTATCAGCATCTGCTGGGAGT
GAGGACTTGCATTGTGAAAGCTTTGCTGTCCTTGATGTGATCATGGAATCTCTTTCTCAC
TAGAGTCTATGTCACTCATTATACTCTGTCGAATGTCATTGAATGTCTTTACATGGGCTT
GTATGCCTATGAAAATTGTAATACAACTTTCAGCAACGGATCTCTTGGCTCTCGCATCGA
TGAAGGACGCAGCGAAATGCGATAAGTAATGTGAATTGCAGAATTCAGTGAATCATCGAA
TCTTTGAACGCATCTTGCGCTCCTTGGTATTCCGAGGAGCATGCCTGTTTGAGTGTCATT
AAATTCTCAACTCTCTTATACTTTTTTGTAAAAGAGAGCTTGGACTGTGGAGGCTTGCTG
GCCACTTTTTGGGGTCAGCTCCTCTGAAATGCATTAGCGGAACCGTTTGCAATCTGCCAC
AAGTGTGATAAGTTATCTACACTGGCGAGGGGATTGCTCTCTGTAATGTTCAGCTTCTAA
TTGTCTCTACTTTGTGAGACTACTTTTGAATGCTTGACCTCAAATCAGGTAGGACTACCC
GCTGAACTTAA
>AB11-55
TTTCCGTAGGTGAACCTGCGGAAGGATCATTATTGAATTATGTTTCTAGATAGGTTGTAG
CTGGCTCTTTTAGAGCATGTGCACGCCTGTTTGGACTTCATTTTCATCCACCTGTGCACC
TATTGTAGTCTTTGGTTGGGTTAGGAGGAAGTGATCATTGTATCAGCATCTGCTGGGAGT
GAGGACTTGCATTGTGAAAGCTTTGCTGTCCTTGATGTGATCATGGAATCTCTTTCTCAC
TAGAGTCTATGTCACTCATTATACTCTGTCGAATGTCATTGAATGTCTTTACATGGGCTT
GTATGCCTATGAAAATTGTAATACAACTTTCAGCAACGGATCTCTTGGCTCTCGCATCGA
TGAAGGACGCAGCGAAATGCGATAAGTAATGTGAATTGCAGAATTCAGTGAATCATCGAA
TCTTTGAACGCATCTTGCGCTCCTTGGTATTCCGAGGAGCATGCCTGTTTGAGTGTCATT
AAATTCTCAACTCTCTTATACTTTTTTGTAAAAGAGAGCTTGGACTGTGGAGGCTTGCTG
ACCACTTTTTGGGGTCAGCTCCTCTGAAATGCATTAGCGGAACCGTTTGCGATCTGCCAC
AAGTGTGATAAGTTATCTACACTGGCGAGGGGATTGCTCTCTGTAATGTTCAGCTTCTAA

TTGTCTCTACTTTGTGAGACTACTTTTGAATGCTTGACCTCAAATCAGGTAGGACTACCC
GCTGAACTTAA
>AB3-49
TTTCCGTAGGTGAACCTGCGGAAGGATCATTATTGAATTATGTTTCTAGATAGGTTGTAG
CTGGCTCTTTTAGAGCATGTGCACGCCTGTTTGGACTTCATTTTCATCCACCTGTGCACC
TATTGTAGTCTTTGGTTGGGTTAGGAGGAAGTGATCATTGTATCAGCATCTGCTGGGAGT
GAGGACTTGCATTGTGAAAGCTTTGCTGTCCTTGATGTGATCATGGAATCTTTTTCTCAC
TAGAGTCTATGTCACTCATTATACTCTGTCGAATGTCATTGAATGTCTTTACATGGGCTT
GTATGCCTATGAAAATTGTAATACAACTTTCAGCAACGGATCTCTTGGCTCTCGCATCGA
TGAAGGACGCAGCGAAATGCGATAAGTAATGTGAATTGCAGAATTCAGTGAATCATCGAA
TCTTTGAACGCATCTTGCGCTCCTTGGTATTCCGAGGAGCATGCCTGTTTGAGTGTCATT
AAATTCTCAACTCTCTTATACTTTTTTGTAAAAGAGAGCTTGGACTGTGGAGGCTTGCTG
GCCACTTTTTGGGGTCAGCTCCTCTGAAATGCATTAGCGGAACCGTTTGCGATCTGCCAC
AAGTGTGATAAGTTATCTACACTGGCGAGGGGATTGCTCTCTGTAATGTTCAGCTTCTAA
TTGTCTCTACTTTGTGAGACTACTTTTGAATGCTTGACCTCAAATCAGGTAGGACTACCC
GCTGAACTTAA
>AB8-13
TTTCCGTAGGTGAACCTGCGGAAGGATCATTATTGAATTATGTTTCTAGATAGGTTGTAG
CTGGCTCTTTTAGAGCATGTGCACGCCTGTTTGGACTTCATTTTCATCCACCTGTGCACC
TATTGTAGTCTTTGGTTGGGTTAGGAGGAAGTGATCATTGTATCAGCATCTGCTGGGAGT
GAGGACTTGCATTGTGAAAGCTTTGCTGTCCTTGATGTGATCATGGAATCTTTTTCTCAC
TAGAGTCTATGTCACTCATTATACTCTGTCGAATGTCATTGAATGTCTTTACATGGGCTT
GTATGCCTATGAAAATTGTAATACAACTTTCAGCAACGGATCTCTTGGCTCTCGCATCGA
TGAAGGACGCAGCGAAATGCGATAAGTAATGTGAATTGCAGAATTCAGTGAATCATCGAA
TCTTTGAACGCATCTTGCGCTCCTTGGTATTCCGAGGAGCATGCCTGTTTGAGTGTCATT
AAATTCTCAACTCTCTTATACTTTTTTGTAAAAGAGAGCTTGGACTGTGGAGGCTTGCTG
GCCACTTTTTGGGGTCAGCTCCTCTGAAATGCATTAGCGGAACCGTTTGCGATCTGCCAC
AAGTGTGATAAGTTATCTACACTGGCGAGGGGATTGCTCTCTGTAATGTTCAGCTTCTAA
TTGTCTCTACTTTGTGAGACTACTTTTGAATGCTTGACCTCAAATCAGGTAGGACTACCC
GCTGAACTTAA
>AB10-40
TTTCCGTAGGTGAACCTGCGGAAGGATCATTATTGAATTATGTTTCTAGATAGGTTGTAG
CTGGCTCTTTTAGAGCATGTGCACGCCTGTTTGGACTTCATTTTCATCCACCTGTGCACC
TATTGTAGTCTTTGGTTGGGTTAGGAGGAAGTGATCATTGTATCAGCATCTGCTGGGAGT
GAGGACTTGCATTGTGAAAGCTTTGCTGTCCTTGATGTGATCATGGAATCTTTTTCTCAC
TAGAGTCTATGTCACTCATTATACTCTGTCGAATGTCATTGAATGTCTTTACATGGGCTT
GTATGCCTATGAAAATTGTAATACAACTTTCAGCAACGGATCTCTTGGCTCTCGCATCGA
TGAAGGACGCAGCGAAATGCGATAAGTAATGTGAATTGCAGAATTCAGTGAATCATCGAA
TCTTTGAACGCATCTTGCGCTCCTTGGTATTCCGAGGAGCATGCCTGTTTGAGTGTCATT
AAATTCTCAACTCTCTTATACTTTTTTGTAAAAGAGAGCTTGGACTGTGGAGGCTTGCTG
GCCACTTTTTGGGGTCAGCTCCTCTGAAATGCATTAGCGGAACCGTTTGCGATCTGCCAC
AAGTGTGATAAGTTATCTACACTGGCGAGGGGATTGCTCTCTGTAATGTTCAGCTTCTAA
TTGTCTCTACTTTGTGAGACTACTTTTGAATGCTTGACCTCAAATCAGGTAGGACTACCC
GCTGAACTTAA
>AB11-12
TTTCCGTAGGTGAACCTGCGGAAGGATCATTATTGAATTATGTTTCTAGATAGGTTGTAG
CTGGCTCTTTTAGAGCATGTGCACGCCTGTTTGGACTTCATTTTCATCCACCTGTGCACC
TATTGTAGTCTTTGGTTGGGTTAGGAGGAAGTGATCATTGTATCAGCATCTGCTGGGAGT
GAGGACTTGCATTGTGAAAGCTTTGCTGTCCTTGATGTGATCATGGAATCTTTTTCTCAC
TAGAGTCTATGTCACTCATTATACTCTGTCGAATGTCATTGAATGTCTTTACATGGGCTT

GTATGCCTATGAAAATTGTAATACAACTTTCAGCAACGGATCTCTTGGCTCTCGCATCGA
TGAAGGACGCAGCGAAATGCGATAAGTAATGTGAATTGCAGAATTCAGTGAATCATCGAA
TCTTTGAACGCATCTTGCGCTCCTTGGTATTCCGAGGAGCATGCCTGTTTGAGTGTCATT
AAATTCTCAACTCTCTTATACTTTTTTGTAAAAGAGAGCTTGGACTGTGGAGGCTTGCTG
GCCACTTTTTGGGGTCAGCTCCTCTGAAATGCATTAGCGGAACCGTTTGCGATCTGCCAC
AAGTGTGATAAGTTATCTACACTGGCGAGGGGATTGCTCTCTGTAATGTTCAGCTTCTAA
TTGTCTCTACTTTGTGAGACTACTTTTGAATGCTTGACCTCAAATCAGGTAGGACTACCC
GCTGAACTTAA
>AB12-14
TTTCCGTAGGTGAACCTGCGGAAGGATCATTATTGAATTATGTTTCTAGATAGGTTGTAG
CTGGCTCTTTTAGAGCATGTGCACGCCTGTTTGGACTTCATTTTCATCCACCTGTGCACC
TATTGTAGTCTTTGGTTGGGTTAGGAGGAAGTGATCATTGTATCAGCATCTGCTGGGAGT
GAGGACTTGCATTGTGAAAGCTTTGCTGTCCTTGATGTGATCATGGAATCTTTTTCTCAC
TAGAGTCTATGTCACTCATTATACTCTGTCGAATGTCATTGAATGTCTTTACATGGGCTT
GTATGCCTATGAAAATTGTAATACAACTTTCAGCAACGGATCTCTTGGCTCTCGCATCGA
TGAAGGACGCAGCGAAATGCGATAAGTAATGTGAATTGCAGAATTCAGTGAATCATCGAA
TCTTTGAACGCATCTTGCGCTCCTTGGTATTCCGAGGAGCATGCCTGTTTGAGTGTCATT
AAATTCTCAACTCTCTTATACTTTTTTGTAAAAGAGAGCTTGGACTGTGGAGGCTTGCTG
GCCACTTTTTGGGGTCAGCTCCTCTGAAATGCATTAGCGGAACCGTTTGCGATCTGCCAC
AAGTGTGATAAGTTATCTACACTGGCGAGGGGATTGCTCTCTGTAATGTTCAGCTTCTAA
TTGTCTCTACTTTGTGAGACTACTTTTGAATGCTTGACCTCAAATCAGGTAGGACTACCC
GCTGAACTTAA
>AB10-16
TTTCCGTAGGTGAACCTGCGGAAGGATCATTATTGAATTATGTTTCTAGATAGGTTGTAG
CTGGCTCTTTTAGAGCATGTGCACGCCTGTTTGGACTTCATTTTCATCCACCTGTGCACC
TATTGTAGTCTTTGGTTGGGTTAGGAGGAAGTGATCATTGTATCAGCATCTGCTGGGAGT
GAGGACTTGCATTGTGAAAGCTTTGCTGTCCTTGATGTGATCATGGAATCTTTTTCTCAC
TAGAGTCTATGTCACTCATTATACTCTGTCGAATGTCATTGAATGTCTTTACATGGGCTT
GTATGCCTATGAAAATTGTAATACAACTTTCAGCAACGGATCTCTTGGCTCTCGCATCGA
TGAAGGACGCAGCGAAATGCGATAAGTAATGTGAATTGCAGAATTCAGTGAATCATCGAA
TCTTTGAACGCATCTTGCGCTCCTTGGTATTCCGAGGAGCATGCCTGTTTGAGTGTCATT
AAATTCTCAACTCTCTTATACTTTTTTGTAAAAGAGAGCTTGGACTGTGGAGGCTTGCTG
GCCACTTTTTGGGGTCAGCTCCTCTGAAATGCATTAGCGGAACCGTTTGCGATCTGCCAC
AAGTGTGATAAGTTATCTACACTGGCGAGGGGATTACTCTCTGTAATGTTCAGCTTCTAA
TTGTCTCTACTTTGTGAGACTACTTTTGAATGCTTGACCTCAAATCAGGTAGGACTACCC
GCTGAACTTAA
>AB12-27
TTTCCGTAGGTGAACCTGCGGAAGGATCATTATTGAATTATGTTTCTAGATAGGTTGTAG
CTGGCTCTTTTAGAGCATGTGCACGCCTGTTTGGACTTCATTTTCATCCACCTGTGCACC
TATTGTAGTCTTTGGTTGGGTTAGGAGGAAGTGATCATTGTATCAGCATCTGCTGGGAGT
GAGGACTTGCATTGTGAAAGCTTTGCTGTCCTTGATGTGATCATGGAATCTTTTTCTCAC
TAGAGTCTATGTCACTCATTATATTCTGTCGAATGTCATTGAATGTCTTTACATGGGCTT
GTATGCCTATGAAAATTGTAATACAACTTTCAGCAACGGATCTCTTGGCTCTCGCATCGA
TGAAGGACGCAGCGAAATGCGATAAGTAATGTGAATTGCAGAATTCAGTGAATCATCGAA
TCTTTGAACGCATCTTGCGCTCCTTGGTATTCCGAGGAGCATGCCTGTTTGAGTGTCATT
AAATTCTCAACTCTCTTATACTTTTTTGTAAAAGAGAGCTTGGACTGTGGAGGCTTGCTG
GCCACTTTTTGGGGTCAGCTCCTCTGAAATGCATTAGCGGAACCGTTTGCGATCTGCCAC
AAGTGTGATAAGTTATCTACACTGGCGAGGGGATTGCTCTCTGTAATGTTCAGCTTCTAA
TTGTCTCTACTTTGTGAGACTACTTTTGAATGCTTGACCTCAAATCAGGTAGGACTACCC
GCTGAACTTAA

>AB6-24
TTTCCGTAGGTGAACCTGCGGAAGGATCATTATTGAATTATGTTTCTAGATAGGTTGTAG
CTGGCTCTTTTAGAGCATGTGCACGCCTGTTTGGACTTCATTTTCATCCACCTGTGCACC
TATTGTAGTCTTTGGTTGGGTTAGGAGGAAGTGATCATTGTATCAGCATCTGCTGGGAGT
GAGGACTTGCATTGTGAAAGCTTTGCTGTCCTTGATGTGATCATGGAATCTTTTTCTCAC
TAGAGTCTATGTCACTCATTATACTCTGTCGAATGTCATTGAATGTCTTTACATGGGCTT
GTATGCCTATGAAAATTGTAATACAACTTTCAGCAACGGATCTCTTGGCTCTCGCATCGA
TGAAGAACGCAGCGAAATGCGATAAGTAATGTGAATTGTAGAATTCAGTGAATCATCGAA
TCTTTGAACGCATCTTGCGCTCCTTGGTATTCCGAGGAGCATGCCTGTTTGAGTGTCATT
AAATTCTCAACTCTCTTATACTTTTTTGTAAAAGAGAGCTTGGACTGTGGAGGCTTGCTG
ACCACTTTTTGGGGTCAGCTCCTCTGAAATGCATTAGCGGAACCGTTTGCGATCTGCCAC
AAGTGTGATAAGTTATCTACACTGGCGAGGGGATTGCTCTCTGTAATGTTCAGCTTCTAA
TTGTCTCTACTTTGTGAGACTACTTTTGAATGCTTGACCTCAAATCAGGTAGGACTACCC
GCTGAACTTAA
>AB3-29
TTTCCGTAGGTGAACCTGCGGAAGGATCATTATTGAATTATGTTTCTAGATAGGTTGTAG
CTGGCTCTTTTAGAGCATGTGCACGCCTGTTTGGACTTCATTTTCATCCACCTGTGCACC
TATTGTAGTCTTTGGTTGGGTTAGGAGGAAGTGATCATTGTATCAGCATCTGCTGGGAGT
GAGGACTTGCATTGTGAAAGCTTTGCTGTCCTTGATGTGATCATGGAATCTTTTTCTCAC
TAGAGTCTATGTCACTCATTATACTCTGTCGAATGTCATTGAATGTCTTTACATGGGCTT
GTATGCCTATGAAAATTGTAATACAACTTTCAGCAACGGATCTCTTGGCTCTCGCATCGA
TGAAGGACGCAGCGAAATGCGATAAGTAATGTGAATTGCAGAATTCAGTGAATCATCGAA
TCTTTGAACGCATCTTGCGCTCCTTGGTATTCCGAGGAGCATGCCTGTTTGAGTGTCATT
AAATTCTCAACTCTCTTATACTTTTTTGTAAAAGAGAGCTTGGACTGTGGAGGCTTGCTG
GCCACTTTTTGGGGTCAGCTCCTCTGAAATGCATTAGCGGAACCGTTTGCGATCTGCCAC
AAGTGTGATAAGTTATCTACACTGGCGAGGGGATTGCTCTCTGTAATGTTCAGCTTCTAA
TTGTCTCTACTTTGTGAGACAACTTTTGAATGCTTGACCTCAAATCAGGTAGGACTACCC
GCTGAACTTAA
>AB7-48
TTTCCGTAGGTGAACCTGCGGAAGGATCATTATTGAATTATGTTTCTAGATAGGTTGTAG
CTGGCTCTTTTAGAGCATGTGCACGCCTGTTTGGACTTCATTTTCATCCACCTGTGCACC
TATTGTAGTCTTTGGTTGGGTTAGGAGGAAGTGATCATTGTATCAGCATCTGCTGGGAGT
GAGGACTTGCATTGTGAAAGCTTTGCTGTCCTTGATGTGATCATGGAATCTTTTTCTCAC
TAGAGTCTATGTCACTCATTATACTCTGTCGAATGTCATTGAATGTCTTTACATGGGCTT
GTATGCCTATGAAAATTGTAATACAACTTTCAGCAACGGATCTCTTGGCTCTCGCATCGA
TGAAGGACGCAGCGAAATGCGATAAGTAATGTGAATTGCAGAATTCAGTGAATCATCGAA
TCTTTGAACGCATCTTGCGCTCCTTGGTATTCCGAGGAGCATGCCTGTTTGAGTGTCATT
AAATTCTCAACTCTCTTATACTTTTTTGTAAAAGAGAGCTTGGACTGTGGAGGCTTGCTG
GCCACTTTTTGGGGTCAGCTCCTCTGAAATGCATTAGCGGAACCGTTTGCGATCTGCCAC
AAGTGTGATAAGTTATCTACACTGGCGAGGGGATTGCTCTCTGTAATGTTCAGCTTCTAA
TTGTCTCTACTTTGTGAGACAACTTTTGAATGCTTGACCTCAAATCAGGTAGGACTACCC
GCTGAACTTAA
>AB8-44
TTTCCGTAGGTGAACCTGCGGAAGGATCATTATTGAATTATGTTTCTAGATAGGTTGTAG
CTGGCTCTTTTAGAGCATGTGCACGCCTGTTTGGACTTCATTTTCATCCACCTGTGCACC
TATTGTAGTCTTTGGTTGGGTTAGGAGGAAGTGATCATTGTATCAGCATCTGCTGGGAGT
GAGGACTTGCATTGTGAAAGCTTTGCTGTCCTTGATGTGATCATGGAATCTCTTTCTCAC
TAGAGTCTATGTCACTCATTATACTCTGTCGAATGTCATTGAATGTCTTTACATGGGCTT
GTATGCCTATGAAAATTGTAATACAACTTTCAGCAACGGATCTCTTGGCTCTCGCATCGA
TGAAGGACGCAGCGAAATGCGATAAGTAATGTGAATTGCAGAATTCAGTGAATCATCGAA

TCTTTGAACGCATCTTGCGCTCCTTGGTATTCCGAGGAGCATGCCTGTTTGAGTGTCATT
AAATTCTCAACTCTCTTATACTTTTTTGTAAAAGAGAGCTTGGACTGTGGAGGCTTGCTG
GCCACTTTTTGGGGTCAGCTCCTCTGAAATGCATTAGCGGAACCGTTTGCGATCTGCCAC
AAGTGTGATAAGTTATCTACACTGGCGAGGGGATTGCTCTCTGTAATGTTCAGCTTCTAA
TTGTCTCTACTTTGTGAGACAACTTTTGAATGCTTGACCTCAAATCAGGTAGGACTACCC
GCTGAACTTAA
>AB2−27
TTTCCGTAGGTGAACCTGCGGAAGGATCATTATTGAATTATGTTTCTAGATAGGTTGTAG
CTGGCTCTTTTAGAGCATGTGCACGCCTGTTTGGACTTCATTTTCATCCACCTGTGCACC
TATTGTAGTCTTTGGTTGGGTTAGGAGGAAGTGATCATTGTATCAGCATCTGCTGGGAGT
GAGGACTTGCATTGTGAAAGCTTTGCTGTCCTTGATGTGATCATGGAATCTTTTTCTCAC
TAGAGTCTATGTCACTCATTATACTCTGTCGAATGTCATTGAATGTCTTTACATGGGCTT
GTATGCCTATGAAAATTGTAATACAACTTTCAGCAACGGATCTCTTGGCTCTCGCATCGA
TGAAGGACGCAGCGAAATGCGATAAGTAATGTGAATTGCAGAATTCAGTGAATCATCGAA
TCTTTGAACGCATCTTGCGCTCCTTGGTATTCCGAGGAGCATGCCTGTTTGAGTGTCATT
AAATTCTCAACTCTCTTATACTTTTTTGTAAAAGAGAGCTTGGACTGTGGAGGCTTGCTG
GCCACTTTTTGGGGTCAGCTCCTCTGAAATGCATTAGCGGAACCGTTTGCAATCTGCCAC
AAGTGTGATAAGTTATCTACACTGGCGAGGGGATTGCTCTCTGTAATGTTCAGCTTCTAA
TTGTCTCTACTTTGTGAGACAACTTTTGAATGCTTGACCTCAAATCAGGTAGGACTACCC
GCTGAACTTAA
>AB3−19
TTTCCGTAGGTGAACCTGCGGAAGGATCATTATTGAATTATGTTTCTAGATAGGTTGTAG
CTGGCTCTTTTAGAGCATGTGCACGCCTGTTTGGACTTCATTTTCATCCACCTGTGCACC
TATTGTAGTCTTTGGTTGGGTTAGGAGGAAGTGATCATTGTATCAGCATCTGCTGGGAGT
GAGGACTTGCATTGTGAAAGCTTTGCTGTCCTTGATGTGATCATGGAATCTTTTTCTCAC
TAGAGTCTATGTCACTCATTATACTCTGTCGAATGTCATTGAATGTCTTTACATGGGCTT
GTATGCCTATGAAAATTGTAATACAACTTTCAGCAACGGATCTCTTGGCTCTCGCATCGA
TGAAGGACGCAGCGAAATGCGATAAGTAATGTGAATTGCAGAATTCAGTGAATCATCGAA
TCTTTGAACGCATCTTGCGCTCCTTGGTATTCCGAGGAGCATGCCTGTTTGAGTGTCATT
AAATTCTCAACTCTCTTATACTTTTTTGTAAAAGAGAGCTTGGACTGTGGAGGCTTGCTG
GCCACTTTTTGGGGTCAGCTCCTCTGAAATGCATTAGCGGAACCGTTTGCAATCTGCCAC
AAGTGTGATAAGTTATCTACACTGGCGAGGGGATTGCTCTCTGTAATGTTCAGCTTCTAA
TTGTCTCTACTTTGTGAGACAACTTTTGAATGCTTGACCTCAAATCAGGTAGGACTACCC
GCTGAACTTAA
>AB6−47
TTTCCGTAGGTGAACCTGCGGAAGGATCATTATTGAATTATGTTTCTAGATAGGTTGTAG
CTGGCTCTTTTAGAGCATGTGCACGCCTGTTTGGACTTCATTTTCATCCACCTGTGCACC
TATTGTAGTCTTTGGTTGGGTTAGGAGGAAGTGATCATTGTATCAGCATCTGCTGGGAGT
GAGGACTTGCATTGTGAAAGCTTTGCTGTCCTTGATGTGATCATGGAATCTTTTTCTCAC
TAGAGTCTATGTCACTCATTATACTCTGTCGAATGTCATTGAATGTCTTTACATGGGCTT
GTATGCCTATGAAAATTGTAATACAACTTTCAGCAACGGATCTCTTGGCTCTCGCATCGA
TGAAGGACGCAGCGAAATGCGATAAGTAATGTGAATTGCAGAATTCAGTGAATCATCGAA
TCTTTGAACGCATCTTGCGCTCCTTGGTATTCCGAGGAGCATGCCTGTTTGAGTGTCATT
AAATTCTCAACTCTCTTATACTTTTTTGTAAAAGAGAGCTTGGACTGTGGAGGCTTGCTG
GCCACTTTTTGGGGTCAGCTCCTCTGAAATGCATTAGCGGAACCGTTTGCAATCTGCCAC
AAGTGTGATAAGTTATCTACACTGGCGAGGGGATTGCTCTCTGTAATGTTCAGCTTCTAA
TTGTCTCTACTTTGTGAGACAACTTTTGAATGCTTGACCTCAAATCAGGTAGGACTACCC
GCTGAACTTAA
>AB7−21
TTTCCGTAGGTGAACCTGCGGAAGGATCATTATTGAATTATGTTTCTAGATAGGTTGTAG

CTGGCTCTTTTAGAGCATGTGCACGCCTGTTTGGACTTCATTTTCATCCACCTGTGCACC
TATTGTAGTCTTTGGTTGGGTTAGGAGGAAGTGATCATTGTATCAGCATCTGCTGGGAGT
GAGGACTTGCATTGTGAAAGCTTTGCTGTCCTTGATGTGATCATGGAATCTTTTTCTCAC
TAGAGTCTATGTCACTCATTATACTCTGTCGAATGTCATTGAATGTCTTTACATGGGCTT
GTATGCCTATGAAAATTGTAATACAACTTTCAGCAACGGATCTCTTGGCTCTCGCATCGA
TGAAGGACGCAGCGAAATGCGATAAGTAATGTGAATTGCAGAATTCAGTGAATCATCGAA
TCTTTGAACGCATCTTGCGCTCCTTGGTATTCCGAGGAGCATGCCTGTTTGAGTGTCATT
AAATTCTCAACTCTCTTATACTTTTTTGTAAAAGAGAGCTTGGACTGTGGAGGCTTGCTG
GCCACTTTTTGGGGTCAGCTCCTCTGAAATGCATTAGCGGAACCGTTTGCAATCTGCCAC
AAGTGTGATAAGTTATCTACACTGGCGAGGGGATTGCTCTCTGTAATGTTCAGCTTCTAA
TTGTCTCTACTTTGTGAGACAACTTTTGAATGCTTGACCTCAAATCAGGTAGGACTACCC
GCTGAACTTAA
>AB7-45
TTTCCGTAGGTGAACCTGCGGAAGGATCATTATTGAATTATGTTTCTAGATAGGTTGTAG
CTGGCTCTTTTAGAGCATGTGCACGCCTGTTTGGACTTCATTTTCATCCACCTGTGCACC
TATTGTAGTCTTTGGTTGGGTTAGGAGGAAGTGATCATTGTATCAGCATCTGCTGGGAGT
GAGGACTTGCATTGTGAAAGCTTTGCTGTCCTTGATGTGATCATGGAATCTTTTTCTCAC
TAGAGTCTATGTCACTCATTATACTCTGTCGAATGTCATTGAATGTCTTTACATGGGCTT
GTATGCCTATGAAAATTGTAATACAACTTTCAGCAACGGATCTCTTGGCTCTCGCATCGA
TGAAGGACGCAGCGAAATGCGATAAGTAATGTGAATTGCAGAATTCAGTGAATCATCGAA
TCTTTGAACGCATCTTGCGCTCCTTGGTATTCCGAGGAGCATGCCTGTTTGAGTGTCATT
AAATTCTCAACTCTCTTATACTTTTTTGTAAAAGAGAGCTTGGACTGTGGAGGCTTGCTG
GCCACTTTTTGGGGTCAGCTCCTCTGAAATGCATTAGCGGAACCGTTTGCAATCTGCCAC
AAGTGTGATAAGTTATCTACACTGGCGAGGGGATTGCTCTCTGTAATGTTCAGCTTCTAA
TTGTCTCTACTTTGTGAGACAACTTTTGAATGCTTGACCTCAAATCAGGTAGGACTACCC
GCTGAACTTAA
>AB11-51
TTTCCGTAGGTGAACCTGCGGAAGGATCATTATTGAATTATGTTTCTAGATAGGTTGTAG
CTGGCTCTTTTAGAGCATGTGCACGCCTGTTTGGACTTCATTTTCATCCACCTGTGCACC
TATTGTAGTCTTTGGTTGGGTTAGGAGGAAGTGATCATTGTATCAGCATCTGCTGGGAGT
GAGGACTTGCATTGTGAAAGCTTTGCTGTCCTTGATGTGATCATGGAATCTTTTTCTCAC
TAGAGTCTATGTCACTCATTATACTCTGTCGAATGTCATTGAATGTCTTTACATGGGCTT
GTATGCCTATGAAAATTGTAATACAACTTTCAGCAACGGATCTCTTGGCTCTCGCATCGA
TGAAGGACGCAGCGAAATGCGATAAGTAATGTGAATTGCAGAATTCAGTGAATCATCGAA
TCTTTGAACGCATCTTGCGCTCCTTGGTATTCCGAGGAGCATGCCTGTTTGAGTGTCATT
AAATTCTCAACTCTCTTATACTTTTTTGTAAAAGAGAGCTTGGACTGTGGAGGCTTGCTG
GCCACTTTTTGGGGTCAGCTCCTCTGAAATGCATTAGCGGAACCGTTTGCAATCTGCCAC
AAGTGTGATAAGTTATCTACACTGGCGAGGGGATTGCTCTCTGTAATGTTCAGCTTCTAA
TTGTCTCTACTTTGTGAGACAACTTTTGAATGCTTGACCTCAAATCAGGTAGGACTACCC
GCTGAACTTAA
>AB12-40
TTTCCGTAGGTGAACCTGCGGAAGGATCATTATTGAATTATGTTTCTAGATAGGTTGTAG
CTGGCTCTTTTAGAGCATGTGCACGCCTGTTTGGACTTCATTTTCATCCACCTGTGCACC
TATTGTAGTCTTTGGTTGGGTTAGGAGGAAGTGATCATTGTATCAGCATCTGCTGGGAGT
GAGGACTTGCATTGTGAAAGCTTTGCTGTCCTTGATGTGATCATGGAATCTTTTTCTCAC
TAGAGTCTATGTCACTCATTATACTCTGTCGAATGTCATTGAATGTCTTTACATGGGCTT
GTATGCCTATGAAAATTGTAATACAACTTTCAGCAACGGATCTCTTGGCTCTCGCATCGA
TGAAGGACGCAGCGAAATGCGATAAGTAATGTGAATTGCAGAATTCAGTGAATCATCGAA
TCTTTGAACGCATCTTGCGCTCCTTGGTATTCCGAGGAGCATGCCTGTTTGAGTGTCATT
AAATTCTCAACTCTCTTATACTTTTTTGTAAAAGAGAGCTTGGACTGTGGAGGCTTGCTG

GCCACTTTTTGGGGTCAGCTCCTCTGAAATGCATTAGCGGAACCGTTTGCAATCTGCCAC
AAGTGTGATAAGTTATCTACACTGGCGAGGGGATTGCTCTCTGTAATGTTCAGCTTCTAA
TTGTCTCTACTTTGTGAGACAACTTTTGAATGCTTGACCTCAAATCAGGTAGGACTACCC
GCTGAACTTAA
>AB12–63
TTTCCGTAGGTGAACCTGCGGAAGGATCATTATTGAATTATGTTTCTAGATAGGTTGTAG
CTGGCTCTTTTAGAGCATGTGCACGCCTGTTTGGACTTCATTTTCATCCACCTGTGCACC
TATTGTAGTCTTTGGTTGGGTTAGGAGGAAGTGATCATTGTATCAGCATCTGCTGGGAGT
GAGGACTTGCATTGTGAAAGCTTTGCTGTCCTTGATGTGATCATGGAATCTTTTTCTCAC
TAGAGTCTATGTCACTCATTATACTCTGTCGAATGTCATTGAATGTCTTTACATGGGCTT
GTATGCCTATGAAAATTGTAATACAACTTTCAGCAACGGATCTCTTGGCTCTCGCATCGA
TGAAGGACGCAGCGAAATGCGATAAGTAATGTGAATTGCAGAATTCAGTGAATCATCGAA
TCTTTGAACGCATCTTGCGCTCCTTGGTATTCCGAGGAGCATGCCTGTTTGAGTGTCATT
AAATTCTCAACTCTCTTATACTTTTTTGTAAAAGAGAGCTTGGACTGTGGAGGCTTGCTG
GCCACTTTTTGGGGTCAGCTCCTCTGAAATGCATTAGCGGAACCGTTTGCAATCTGCCAC
AAGTGTGATAAGTTATCTACACTGGCGAGGGGATTGCTCTCTGTAATGTTCAGCTTCTAA
TTGTCTCTACTTTGTGAGACAACTTTTGAATGCTTGACCTCAAATCAGGTAGGACTACCC
GCTGAACTTAA
>AB6–50
TTTCCGTAGGTGAACCTGCGGAAGGATCATTATTGAATTATGTTTCTAGATAGGTTGTAG
CTGGCTCTTTTAGAGCATGTGCACGCCTGTTTGGACTTCATTTTCATCCACCTGTGCACC
TATTGTAGTCTTTGGTTGGGTTAGGAGGAAGTGATCATTGTATCAGCATCTGCTGGGAGT
GAGGACTTGCATTGTGAAAGCTTTGCTGTCCTTGATGTGATCATGGAATCTTTTTCTCAC
TAGAGTCTATGTCACTCATTATACTCTGTCGAATGTCATTGAATGTCTTTACATGGGCTT
GTATGCCTATGAAAATTGTAATACAACTTTCAGCAACGGATCTCTTGGCTCTCGCATCGA
TGAAGGACGCAGCGAAATGCGATAAGTAATGTGAATTGCAGAATTCAGTGAATCATCGAA
TCTTTGAACGCATCTTGCGCTCCTTGGTATTCCGAGGAGCATGCCTGTTTGAGTGTCATT
AAATTCTCAACTCTCTTATACTTTTTTGTAAAAGAGAGCTTGGACTGTGGAGGCTTGCTG
GCCACTTTTTGGGGTCAGCTCCTCTGAAATGCATTAGCGGAACCGTTTGCAATCTGCCAC
AAGTGTGATAAGTTATCTACACTGGCGAGGGGATTGCTCTCTGTAATGTTCAGCTTCTAA
TTGTCTCTACTTTGTGAGACAACTTTTGAATGCTTGACCTCAAATCAGGTAGGACTACCC
GCTGAACTTAA
>AB3–7
TTTCCGTAGGTGAACCTGCGGAAGGATCATTATTGAATTATGTTTCTAGATAGGTTGTAG
CTGGCTCTTTTAGAGCATGTGCACGCCTGTTTGGACTTCATTTTCATCCACCTGTGCACC
TATTGTAGTCTTTGGTTGGGTTAGGAGGAAGTGATCATTGTATCAGCATCTGCTGGGAGT
GAGGACTTGCATTGTGAAAGCTTTGCTGTCCTTGATGTGATCATGGAATCTCTTTCTCAC
TAGAGTCTATGTCACTCATTATACTCTGTCGAATGTCATTGAATGTCTTTACATGGGCTT
GTATGCCTATGAAAATTGTAATACAACTTTCAGCAACGGATCTCTTGGCTCTCGCATCGA
TGAAGGACGCAGCGAAATGCGATAAGTAATGTGAATTGCAGAATTCAGTGAATCATCGAA
TCTTTGAACGCATCTTGCGCTCCTTGGTATTCCGAGGAGCATGCCTGTTTGAGTGTCATT
AAATTCTCAACTCTCTTATACTTTTTTGTAAAAGAGAGCTTGGACTGTGGAGGCTTGCTG
GCCACTTTTTGGGGTCAGCTCCTCTGAAATGCATTAGCGGAACCGTTTGCAATCTGCCAC
AAGTGTGATAAGTTATCTACACTGGCGAGGGGATTGCTCTCTGTAATGTTCAGCTTCTAA
TTGTCTCTACTTTGTGAGACAACTTTTGAATGCTTGACCTCAAATCAGGTAGGACTACCC
GCTGAACTTAA
>AB8–27
TTTCCGTAGGTGAACCTGCGGAAGGATCATTATTGAATTATGTTTCTAGATAGGTTGTAG
CTGGCTCTTTTAGAGCATGTGCACGCCTGTTTGGACTTCATTTTCATCCACCTGTGCACC
TATTGTAGTCTTTGGTTGGGTTAGGAGGAAGTGATCATTGTATCAGCATCTGCTGGGAGT

GAGGACTTGCATTGTGAAAGCTTTGCTGTCCTTGATGTGATCATGGAATCTCTTTCTCAC
TAGAGTCTATGTCACTCATTATACTCTGTCGAATGTCATTGAATGTCTTTACATGGGCTT
GTATGCCTATGAAAATTGTAATACAACTTTCAGCAACGGATCTCTTGGCTCTCGCATCGA
TGAAGGACGCAGCGAAATGCGATAAGTAATGTGAATTGCAGAATTCAGTGAATCATCGAA
TCTTTGAACGCATCTTGCGCTCCTTGGTATTCCGAGGAGCATGCCTGTTTGAGTGTCATT
AAATTCTCAACTCTCTTATACTTTTTTGTAAAAGAGAGCTTGGACTGTGGAGGCTTGCTG
GCCACTTTTTGGGGTCAGCTCCTCTGAAATGCATTAGCGGAACCGTTTGCAATCTGCCAC
AAGTGTGATAAGTTATCTACACTGGCGAGGGGATTGCTCTCTGTAATGTTCAGCTTCTAA
TTGTCTCTACTTTGTGAGACAACTTTTGAATGCTTGACCTCAAATCAGGTAGGACTACCC
GCTGAACTTAA
>AB12–36
TTTCCGTAGGTGAACCTGCGGAAGGATCATTATTGAATTATGTTTCTAGATAGGTTGTAG
CTGGCTCTTTTAGAGCATGTGCACGCCTGTTTGGACTTCATTTTCATCCACCTGTGCACC
TATTGTAGTCTTTGGTTGGGTTAGGAGGAAGTGATCATTGTATCAGCATCTGCTGGGAGT
GAGGACTTGCATTGTGAAAGCTTTGCTGTCCTTGATGTGATCATGGAATCTCTTTCTCAC
TAGAGTCTATGTCACTCATTATACTCTGTCGAATGTCATTGAATGTCTTTACATGGGCTT
GTATGCCTATGAAAATTGTAATACAACTTTCAGCAACGGATCTCTTGGCTCTCGCATCGA
TGAAGGACGCAGCGAAATGCGATAAGTAATGTGAATTGCAGAATTCAGTGAATCATCGAA
TCTTTGAACGCATCTTGCGCTCCTTGGTATTCCGAGGAGCATGCCTGTTTGAGTGTCATT
AAATTCTCAACTCTCTTATACTTTTTTGTAAAAGAGAGCTTGGACTGTGGAGGCTTGCTG
GCCACTTTTTGGGGTCAGCTCCTCTGAAATGCATTAGCGGAACCGTTTGCAATCTGCCAC
AAGTGTGATAAGTTATCTACACTGGCGAGGGGATTGCTCTCTGTAATGTTCAGCTTCTAA
TTGTCTCTACTTTGTGAGACAACTTTTGAATGCTTGACCTCAAATCAGGTAGGACTACCC
GCTGAACTTAA
>AB1–44
TTTCCGTAGGTGAACCTGCGGAAGGATCATTATTGAATTATGTTTCTAGATAGGTTGTAG
CTGGCTCTTTTAGAGCATGTGCACGCCTGTTTGGACTTCATTTTCATCCACCTGTGCACC
TATTGTAGTCTTTGGTTGGGTTAGGAGGAAGTGATCATTGTATCAGCATCTGCTGGGAGT
GAGGACTTGCATTGTGAAAGCTTTGCTGTCCTTGATGTGATCATGGAATCTTTTTCTCAC
TAGAGTCTATGTCACTCATTATACTCTGTCGAATGTCATTGAATGTCTTTACATGGGCTT
GTATGCCTATGAAAATTGTAATACAACTTTCAGCAACGGATCTCTTGGCTCTCGCATCGA
TGAAGAACGCAGCGAAATGCGATAAGTAATGTGAATTGCAGAATTCAGTGAATCATCGAA
TCTTTGAACGCATCTTGCGCTCCTTGGTATTCCGAGGAGCATGCCTGTTTGAGTGTCATT
AAATTCTCAACTCTCTTATACTTTTTTGTAAAAGAGAGCTTGGACTGTGGAGGCTTGCTG
GCCACTTTTTGGGGTCAGCTCCTCTGAAATGCATTAGCGGAACCGTTTGCGATCTGCCAC
AAGTGTGATAAGTTATCTACACTGGCGAGGGGATTGCTCTCTGTAATGTTCAGCTTCTAA
TTGTCTCTACTTTGTGAGACAACTTTTGAATGCTTGACCTCAAATCAGGTAGGACTACCC
GCTGAACTTAA
>AB2–16
TTTCCGTAGGTGAACCTGCGGAAGGATCATTATTGAATTATGTTTCTAGATAGGTTGTAG
CTGGCTCTTTTAGAGCATGTGCACGCCTGTTTGGACTTCATTTTCATCCACCTGTGCACC
TATTGTAGTCTTTGGTTGGGTTAGGAGGAAGTGATCATTGTATCAGCATCTGCTGGGAGT
GAGGACTTGCATTGTGAAAGCTTTGCTGTCCTTGATGTGATCATGGAATCTTTTTCTCAC
TAGAGTCTATGTCACTCATTATACTCTGTCGAATGTCATTGAATGTCTTTACATGGGCTT
GTATGCCTATGAAAATTGTAATACAACTTTCAGCAACGGATCTCTTGGCTCTCGCATCGA
TGAAGAACGCAGCGAAATGCGATAAGTAATGTGAATTGCAGAATTCAGTGAATCATCGAA
TCTTTGAACGCATCTTGCGCTCCTTGGTATTCCGAGGAGCATGCCTGTTTGAGTGTCATT
AAATTCTCAACTCTCTTATACTTTTTTGTAAAAGAGAGCTTGGACTGTGGAGGCTTGCTG
GCCACTTTTTGGGGTCAGCTCCTCTGAAATGCATTAGCGGAACCGTTTGCGATCTGCCAC
AAGTGTGATAAGTTATCTACACTGGCGAGGGGATTGCTCTCTGTAATGTTCAGCTTCTAA

TTGTCTCTACTTTGTGAGACAACTTTTGAATGCTTGACCTCAAATCAGGTAGGACTACCC
GCTGAACTTAA
>AB3-53
TTTCCGTAGGTGAACCTGCGGAAGGATCATTATTGAATTATGTTTCTAGATAGGTTGTAG
CTGGCTCTTTTAGAGCATGTGCACGCCTGTTTGGACTTCATTTTCATCCACCTGTGCACC
TATTGTAGTCTTTGGTTGGGTTAGGAGGAAGTGATCATTGTATCAGCATCTGCTGGGAGT
GAGGACTTGCATTGTGAAAGCTTTGCTGTCCTTGATGTGATCATGGAATCTTTTTCTCAC
TAGAGTCTATGTCACTCATTATACTCTGTCGAATGTCATTGAATGTCTTTACATGGGCTT
GTATGCCTATGAAAATTGTAATACAACTTTCAGCAACGGATCTCTTGGCTCTCGCATCGA
TGAAGAACGCAGCGAAATGCGATAAGTAATGTGAATTGCAGAATTCAGTGAATCATCGAA
TCTTTGAACGCATCTTGCGCTCCTTGGTATTCCGAGGAGCATGCCTGTTTGAGTGTCATT
AAATTCTCAACTCTCTTATACTTTTTTGTAAAAGAGAGCTTGGACTGTGGAGGCTTGCTG
GCCACTTTTTGGGGTCAGCTCCTCTGAAATGCATTAGCGGAACCGTTTGCGATCTGCCAC
AAGTGTGATAAGTTATCTACACTGGCGAGGGGATTGCTCTCTGTAATGTTCAGCTTCTAA
TTGTCTCTACTTTGTGAGACAACTTTTGAATGCTTGACCTCAAATCAGGTAGGACTACCC
GCTGAACTTAA
>AB3-59
TTTCCGTAGGTGAACCTGCGGAAGGATCATTATTGAATTATGTTTCTAGATAGGTTGTAG
CTGGCTCTTTTAGAGCATGTGCACGCCTGTTTGGACTTCATTTTCATCCACCTGTGCACC
TATTGTAGTCTTTGGTTGGGTTAGGAGGAAGTGATCATTGTATCAGCATCTGCTGGGAGT
GAGGACTTGCATTGTGAAAGCTTTGCTGTCCTTGATGTGATCATGGAATCTTTTTCTCAC
TAGAGTCTATGTCACTCATTATACTCTGTCGAATGTCATTGAATGTCTTTACATGGGCTT
GTATGCCTATGAAAATTGTAATACAACTTTCAGCAACGGATCTCTTGGCTCTCGCATCGA
TGAAGAACGCAGCGAAATGCGATAAGTAATGTGAATTGCAGAATTCAGTGAATCATCGAA
TCTTTGAACGCATCTTGCGCTCCTTGGTATTCCGAGGAGCATGCCTGTTTGAGTGTCATT
AAATTCTCAACTCTCTTATACTTTTTTGTAAAAGAGAGCTTGGACTGTGGAGGCTTGCTG
GCCACTTTTTGGGGTCAGCTCCTCTGAAATGCATTAGCGGAACCGTTTGCGATCTGCCAC
AAGTGTGATAAGTTATCTACACTGGCGAGGGGATTGCTCTCTGTAATGTTCAGCTTCTAA
TTGTCTCTACTTTGTGAGACAACTTTTGAATGCTTGACCTCAAATCAGGTAGGACTACCC
GCTGAACTTAA
>AB6-7
TTTCCGTAGGTGAACCTGCGGAAGGATCATTATTGAATTATGTTTCTAGATAGGTTGTAG
CTGGCTCTTTTAGAGCATGTGCACGCCTGTTTGGACTTCATTTTCATCCACCTGTGCACC
TATTGTAGTCTTTGGTTGGGTTAGGAGGAAGTGATCATTGTATCAGCATCTGCTGGGAGT
GAGGACTTGCATTGTGAAAGCTTTGCTGTCCTTGATGTGATCATGGAATCTTTTTCTCAC
TAGAGTCTATGTCACTCATTATACTCTGTCGAATGTCATTGAATGTCTTTACATGGGCTT
GTATGCCTATGAAAATTGTAATACAACTTTCAGCAACGGATCTCTTGGCTCTCGCATCGA
TGAAGAACGCAGCGAAATGCGATAAGTAATGTGAATTGCAGAATTCAGTGAATCATCGAA
TCTTTGAACGCATCTTGCGCTCCTTGGTATTCCGAGGAGCATGCCTGTTTGAGTGTCATT
AAATTCTCAACTCTCTTATACTTTTTTGTAAAAGAGAGCTTGGACTGTGGAGGCTTGCTG
GCCACTTTTTGGGGTCAGCTCCTCTGAAATGCATTAGCGGAACCGTTTGCGATCTGCCAC
AAGTGTGATAAGTTATCTACACTGGCGAGGGGATTGCTCTCTGTAATGTTCAGCTTCTAA
TTGTCTCTACTTTGTGAGACAACTTTTGAATGCTTGACCTCAAATCAGGTAGGACTACCC
GCTGAACTTAA
>AB6-43
TTTCCGTAGGTGAACCTGCGGAAGGATCATTATTGAATTATGTTTCTAGATAGGTTGTAG
CTGGCTCTTTTAGAGCATGTGCACGCCTGTTTGGACTTCATTTTCATCCACCTGTGCACC
TATTGTAGTCTTTGGTTGGGTTAGGAGGAAGTGATCATTGTATCAGCATCTGCTGGGAGT
GAGGACTTGCATTGTGAAAGCTTTGCTGTCCTTGATGTGATCATGGAATCTTTTTCTCAC
TAGAGTCTATGTCACTCATTATACTCTGTCGAATGTCATTGAATGTCTTTACATGGGCTT

GTATGCCTATGAAAATTGTAATACAACTTTCAGCAACGGATCTCTTGGCTCTCGCATCGA
TGAAGAACGCAGCGAAATGCGATAAGTAATGTGAATTGCAGAATTCAGTGAATCATCGAA
TCTTTGAACGCATCTTGCGCTCCTTGGTATTCCGAGGAGCATGCCTGTTTGAGTGTCATT
AAATTCTCAACTCTCTTATACTTTTTTGTAAAAGAGAGCTTGGACTGTGGAGGCTTGCTG
GCCACTTTTTGGGGTCAGCTCCTCTGAAATGCATTAGCGGAACCGTTTGCGATCTGCCAC
AAGTGTGATAAGTTATCTACACTGGCGAGGGGATTGCTCTCTGTAATGTTCAGCTTCTAA
TTGTCTCTACTTTGTGAGACAACTTTTGAATGCTTGACCTCAAATCAGGTAGGACTACCC
GCTGAACTTAA
>AB7-31
TTTCCGTAGGTGAACCTGCGGAAGGATCATTATTGAATTATGTTTCTAGATAGGTTGTAG
CTGGCTCTTTTAGAGCATGTGCACGCCTGTTTGGACTTCATTTTCATCCACCTGTGCACC
TATTGTAGTCTTTGGTTGGGTTAGGAGGAAGTGATCATTGTATCAGCATCTGCTGGGAGT
GAGGACTTGCATTGTGAAAGCTTTGCTGTCCTTGATGTGATCATGGAATCTTTTTCTCAC
TAGAGTCTATGTCACTCATTATACTCTGTCGAATGTCATTGAATGTCTTTACATGGGCTT
GTATGCCTATGAAAATTGTAATACAACTTTCAGCAACGGATCTCTTGGCTCTCGCATCGA
TGAAGAACGCAGCGAAATGCGATAAGTAATGTGAATTGCAGAATTCAGTGAATCATCGAA
TCTTTGAACGCATCTTGCGCTCCTTGGTATTCCGAGGAGCATGCCTGTTTGAGTGTCATT
AAATTCTCAACTCTCTTATACTTTTTTGTAAAAGAGAGCTTGGACTGTGGAGGCTTGCTG
GCCACTTTTTGGGGTCAGCTCCTCTGAAATGCATTAGCGGAACCGTTTGCGATCTGCCAC
AAGTGTGATAAGTTATCTACACTGGCGAGGGGATTGCTCTCTGTAATGTTCAGCTTCTAA
TTGTCTCTACTTTGTGAGACAACTTTTGAATGCTTGACCTCAAATCAGGTAGGACTACCC
GCTGAACTTAA
>AB8-50
TTTCCGTAGGTGAACCTGCGGAAGGATCATTATTGAATTATGTTTCTAGATAGGTTGTAG
CTGGCTCTTTTAGAGCATGTGCACGCCTGTTTGGACTTCATTTTCATCCACCTGTGCACC
TATTGTAGTCTTTGGTTGGGTTAGGAGGAAGTGATCATTGTATCAGCATCTGCTGGGAGT
GAGGACTTGCATTGTGAAAGCTTTGCTGTCCTTGATGTGATCATGGAATCTTTTTCTCAC
TAGAGTCTATGTCACTCATTATACTCTGTCGAATGTCATTGAATGTCTTTACATGGGCTT
GTATGCCTATGAAAATTGTAATACAACTTTCAGCAACGGATCTCTTGGCTCTCGCATCGA
TGAAGAACGCAGCGAAATGCGATAAGTAATGTGAATTGCAGAATTCAGTGAATCATCGAA
TCTTTGAACGCATCTTGCGCTCCTTGGTATTCCGAGGAGCATGCCTGTTTGAGTGTCATT
AAATTCTCAACTCTCTTATACTTTTTTGTAAAAGAGAGCTTGGACTGTGGAGGCTTGCTG
GCCACTTTTTGGGGTCAGCTCCTCTGAAATGCATTAGCGGAACCGTTTGCGATCTGCCAC
AAGTGTGATAAGTTATCTACACTGGCGAGGGGATTGCTCTCTGTAATGTTCAGCTTCTAA
TTGTCTCTACTTTGTGAGACAACTTTTGAATGCTTGACCTCAAATCAGGTAGGACTACCC
GCTGAACTTAA
>AB9-25
TTTCCGTAGGTGAACCTGCGGAAGGATCATTATTGAATTATGTTTCTAGATAGGTTGTAG
CTGGCTCTTTTAGAGCATGTGCACGCCTGTTTGGACTTCATTTTCATCCACCTGTGCACC
TATTGTAGTCTTTGGTTGGGTTAGGAGGAAGTGATCATTGTATCAGCATCTGCTGGGAGT
GAGGACTTGCATTGTGAAAGCTTTGCTGTCCTTGATGTGATCATGGAATCTTTTTCTCAC
TAGAGTCTATGTCACTCATTATACTCTGTCGAATGTCATTGAATGTCTTTACATGGGCTT
GTATGCCTATGAAAATTGTAATACAACTTTCAGCAACGGATCTCTTGGCTCTCGCATCGA
TGAAGAACGCAGCGAAATGCGATAAGTAATGTGAATTGCAGAATTCAGTGAATCATCGAA
TCTTTGAACGCATCTTGCGCTCCTTGGTATTCCGAGGAGCATGCCTGTTTGAGTGTCATT
AAATTCTCAACTCTCTTATACTTTTTTGTAAAAGAGAGCTTGGACTGTGGAGGCTTGCTG
GCCACTTTTTGGGGTCAGCTCCTCTGAAATGCATTAGCGGAACCGTTTGCGATCTGCCAC
AAGTGTGATAAGTTATCTACACTGGCGAGGGGATTGCTCTCTGTAATGTTCAGCTTCTAA
TTGTCTCTACTTTGTGAGACAACTTTTGAATGCTTGACCTCAAATCAGGTAGGACTACCC
GCTGAACTTAA

>AB9-31
TTTCCGTAGGTGAACCTGCGGAAGGATCATTATTGAATTATGTTTCTAGATAGGTTGTAG
CTGGCTCTTTTAGAGCATGTGCACGCCTGTTTGGACTTCATTTTCATCCACCTGTGCACC
TATTGTAGTCTTTGGTTGGGTTAGGAGGAAGTGATCATTGTATCAGCATCTGCTGGGAGT
GAGGACTTGCATTGTGAAAGCTTTGCTGTCCTTGATGTGATCATGGAATCTTTTTCTCAC
TAGAGTCTATGTCACTCATTATACTCTGTCGAATGTCATTGAATGTCTTTACATGGGCTT
GTATGCCTATGAAAATTGTAATACAACTTTCAGCAACGGATCTCTTGGCTCTCGCATCGA
TGAAGAACGCAGCGAAATGCGATAAGTAATGTGAATTGCAGAATTCAGTGAATCATCGAA
TCTTTGAACGCATCTTGCGCTCCTTGGTATTCCGAGGAGCATGCCTGTTTGAGTGTCATT
AAATTCTCAACTCTCTTATACTTTTTTGTAAAAGAGAGCTTGGACTGTGGAGGCTTGCTG
GCCACTTTTTGGGGTCAGCTCCTCTGAAATGCATTAGCGGAACCGTTTGCGATCTGCCAC
AAGTGTGATAAGTTATCTACACTGGCGAGGGGATTGCTCTCTGTAATGTTCAGCTTCTAA
TTGTCTCTACTTTGTGAGACAACTTTTGAATGCTTGACCTCAAATCAGGTAGGACTACCC
GCTGAACTTAA
>AB10-14
TTTCCGTAGGTGAACCTGCGGAAGGATCATTATTGAATTATGTTTCTAGATAGGTTGTAG
CTGGCTCTTTTAGAGCATGTGCACGCCTGTTTGGACTTCATTTTCATCCACCTGTGCACC
TATTGTAGTCTTTGGTTGGGTTAGGAGGAAGTGATCATTGTATCAGCATCTGCTGGGAGT
GAGGACTTGCATTGTGAAAGCTTTGCTGTCCTTGATGTGATCATGGAATCTTTTTCTCAC
TAGAGTCTATGTCACTCATTATACTCTGTCGAATGTCATTGAATGTCTTTACATGGGCTT
GTATGCCTATGAAAATTGTAATACAACTTTCAGCAACGGATCTCTTGGCTCTCGCATCGA
TGAAGAACGCAGCGAAATGCGATAAGTAATGTGAATTGCAGAATTCAGTGAATCATCGAA
TCTTTGAACGCATCTTGCGCTCCTTGGTATTCCGAGGAGCATGCCTGTTTGAGTGTCATT
AAATTCTCAACTCTCTTATACTTTTTTGTAAAAGAGAGCTTGGACTGTGGAGGCTTGCTG
GCCACTTTTTGGGGTCAGCTCCTCTGAAATGCATTAGCGGAACCGTTTGCGATCTGCCAC
AAGTGTGATAAGTTATCTACACTGGCGAGGGGATTGCTCTCTGTAATGTTCAGCTTCTAA
TTGTCTCTACTTTGTGAGACAACTTTTGAATGCTTGACCTCAAATCAGGTAGGACTACCC
GCTGAACTTAA
>AB10-33
TTTCCGTAGGTGAACCTGCGGAAGGATCATTATTGAATTATGTTTCTAGATAGGTTGTAG
CTGGCTCTTTTAGAGCATGTGCACGCCTGTTTGGACTTCATTTTCATCCACCTGTGCACC
TATTGTAGTCTTTGGTTGGGTTAGGAGGAAGTGATCATTGTATCAGCATCTGCTGGGAGT
GAGGACTTGCATTGTGAAAGCTTTGCTGTCCTTGATGTGATCATGGAATCTTTTTCTCAC
TAGAGTCTATGTCACTCATTATACTCTGTCGAATGTCATTGAATGTCTTTACATGGGCTT
GTATGCCTATGAAAATTGTAATACAACTTTCAGCAACGGATCTCTTGGCTCTCGCATCGA
TGAAGAACGCAGCGAAATGCGATAAGTAATGTGAATTGCAGAATTCAGTGAATCATCGAA
TCTTTGAACGCATCTTGCGCTCCTTGGTATTCCGAGGAGCATGCCTGTTTGAGTGTCATT
AAATTCTCAACTCTCTTATACTTTTTTGTAAAAGAGAGCTTGGACTGTGGAGGCTTGCTG
GCCACTTTTTGGGGTCAGCTCCTCTGAAATGCATTAGCGGAACCGTTTGCGATCTGCCAC
AAGTGTGATAAGTTATCTACACTGGCGAGGGGATTGCTCTCTGTAATGTTCAGCTTCTAA
TTGTCTCTACTTTGTGAGACAACTTTTGAATGCTTGACCTCAAATCAGGTAGGACTACCC
GCTGAACTTAA
>AB11-5
TTTCCGTAGGTGAACCTGCGGAAGGATCATTATTGAATTATGTTTCTAGATAGGTTGTAG
CTGGCTCTTTTAGAGCATGTGCACGCCTGTTTGGACTTCATTTTCATCCACCTGTGCACC
TATTGTAGTCTTTGGTTGGGTTAGGAGGAAGTGATCATTGTATCAGCATCTGCTGGGAGT
GAGGACTTGCATTGTGAAAGCTTTGCTGTCCTTGATGTGATCATGGAATCTTTTTCTCAC
TAGAGTCTATGTCACTCATTATACTCTGTCGAATGTCATTGAATGTCTTTACATGGGCTT
GTATGCCTATGAAAATTGTAATACAACTTTCAGCAACGGATCTCTTGGCTCTCGCATCGA
TGAAGAACGCAGCGAAATGCGATAAGTAATGTGAATTGCAGAATTCAGTGAATCATCGAA

TCTTTGAACGCATCTTGCGCTCCTTGGTATTCCGAGGAGCATGCCTGTTTGAGTGTCATT
AAATTCTCAACTCTCTTATACTTTTTTGTAAAAGAGAGCTTGGACTGTGGAGGCTTGCTG
GCCACTTTTTGGGGTCAGCTCCTCTGAAATGCATTAGCGGAACCGTTTGCGATCTGCCAC
AAGTGTGATAAGTTATCTACACTGGCGAGGGGATTGCTCTCTGTAATGTTCAGCTTCTAA
TTGTCTCTACTTTGTGAGACAACTTTTGAATGCTTGACCTCAAATCAGGTAGGACTACCC
GCTGAACTTAA
>AB11-21
TTTCCGTAGGTGAACCTGCGGAAGGATCATTATTGAATTATGTTTCTAGATAGGTTGTAG
CTGGCTCTTTTAGAGCATGTGCACGCCTGTTTGGACTTCATTTTCATCCACCTGTGCACC
TATTGTAGTCTTTGGTTGGGTTAGGAGGAAGTGATCATTGTATCAGCATCTGCTGGGAGT
GAGGACTTGCATTGTGAAAGCTTTGCTGTCCTTGATGTGATCATGGAATCTTTTTCTCAC
TAGAGTCTATGTCACTCATTATACTCTGTCGAATGTCATTGAATGTCTTTACATGGGCTT
GTATGCCTATGAAAATTGTAATACAACTTTCAGCAACGGATCTCTTGGCTCTCGCATCGA
TGAAGAACGCAGCGAAATGCGATAAGTAATGTGAATTGCAGAATTCAGTGAATCATCGAA
TCTTTGAACGCATCTTGCGCTCCTTGGTATTCCGAGGAGCATGCCTGTTTGAGTGTCATT
AAATTCTCAACTCTCTTATACTTTTTTGTAAAAGAGAGCTTGGACTGTGGAGGCTTGCTG
GCCACTTTTTGGGGTCAGCTCCTCTGAAATGCATTAGCGGAACCGTTTGCGATCTGCCAC
AAGTGTGATAAGTTATCTACACTGGCGAGGGGATTGCTCTCTGTAATGTTCAGCTTCTAA
TTGTCTCTACTTTGTGAGACAACTTTTGAATGCTTGACCTCAAATCAGGTAGGACTACCC
GCTGAACTTAA
>AB11-39
TTTCCGTAGGTGAACCTGCGGAAGGATCATTATTGAATTATGTTTCTAGATAGGTTGTAG
CTGGCTCTTTTAGAGCATGTGCACGCCTGTTTGGACTTCATTTTCATCCACCTGTGCACC
TATTGTAGTCTTTGGTTGGGTTAGGAGGAAGTGATCATTGTATCAGCATCTGCTGGGAGT
GAGGACTTGCATTGTGAAAGCTTTGCTGTCCTTGATGTGATCATGGAATCTTTTTCTCAC
TAGAGTCTATGTCACTCATTATACTCTGTCGAATGTCATTGAATGTCTTTACATGGGCTT
GTATGCCTATGAAAATTGTAATACAACTTTCAGCAACGGATCTCTTGGCTCTCGCATCGA
TGAAGAACGCAGCGAAATGCGATAAGTAATGTGAATTGCAGAATTCAGTGAATCATCGAA
TCTTTGAACGCATCTTGCGCTCCTTGGTATTCCGAGGAGCATGCCTGTTTGAGTGTCATT
AAATTCTCAACTCTCTTATACTTTTTTGTAAAAGAGAGCTTGGACTGTGGAGGCTTGCTG
GCCACTTTTTGGGGTCAGCTCCTCTGAAATGCATTAGCGGAACCGTTTGCGATCTGCCAC
AAGTGTGATAAGTTATCTACACTGGCGAGGGGATTGCTCTCTGTAATGTTCAGCTTCTAA
TTGTCTCTACTTTGTGAGACAACTTTTGAATGCTTGACCTCAAATCAGGTAGGACTACCC
GCTGAACTTAA
>AB12-10
TTTCCGTAGGTGAACCTGCGGAAGGATCATTATTGAATTATGTTTCTAGATAGGTTGTAG
CTGGCTCTTTTAGAGCATGTGCACGCCTGTTTGGACTTCATTTTCATCCACCTGTGCACC
TATTGTAGTCTTTGGTTGGGTTAGGAGGAAGTGATCATTGTATCAGCATCTGCTGGGAGT
GAGGACTTGCATTGTGAAAGCTTTGCTGTCCTTGATGTGATCATGGAATCTTTTTCTCAC
TAGAGTCTATGTCACTCATTATACTCTGTCGAATGTCATTGAATGTCTTTACATGGGCTT
GTATGCCTATGAAAATTGTAATACAACTTTCAGCAACGGATCTCTTGGCTCTCGCATCGA
TGAAGAACGCAGCGAAATGCGATAAGTAATGTGAATTGCAGAATTCAGTGAATCATCGAA
TCTTTGAACGCATCTTGCGCTCCTTGGTATTCCGAGGAGCATGCCTGTTTGAGTGTCATT
AAATTCTCAACTCTCTTATACTTTTTTGTAAAAGAGAGCTTGGACTGTGGAGGCTTGCTG
GCCACTTTTTGGGGTCAGCTCCTCTGAAATGCATTAGCGGAACCGTTTGCGATCTGCCAC
AAGTGTGATAAGTTATCTACACTGGCGAGGGGATTGCTCTCTGTAATGTTCAGCTTCTAA
TTGTCTCTACTTTGTGAGACAACTTTTGAATGCTTGACCTCAAATCAGGTAGGACTACCC
GCTGAACTTAA
>AB12-37
TTTCCGTAGGTGAACCTGCGGAAGGATCATTATTGAATTATGTTTCTAGATAGGTTGTAG

CTGGCTCTTTTAGAGCATGTGCACGCCTGTTTGGACTTCATTTTCATCCACCTGTGCACC
TATTGTAGTCTTTGGTTGGGTTAGGAGGAAGTGATCATTGTATCAGCATCTGCTGGGAGT
GAGGACTTGCATTGTGAAAGCTTTGCTGTCCTTGATGTGATCATGGAATCTTTTTCTCAC
TAGAGTCTATGTCACTCATTATACTCTGTCGAATGTCATTGAATGTCTTTACATGGGCTT
GTATGCCTATGAAAATTGTAATACAACTTTCAGCAACGGATCTCTTGGCTCTCGCATCGA
TGAAGAACGCAGCGAAATGCGATAAGTAATGTGAATTGCAGAATTCAGTGAATCATCGAA
TCTTTGAACGCATCTTGCGCTCCTTGGTATTCCGAGGAGCATGCCTGTTTGAGTGTCATT
AAATTCTCAACTCTCTTATACTTTTTTGTAAAAGAGAGCTTGGACTGTGGAGGCTTGCTG
GCCACTTTTTGGGGTCAGCTCCTCTGAAATGCATTAGCGGAACCGTTTGCGATCTGCCAC
AAGTGTGATAAGTTATCTACACTGGCGAGGGGATTGCTCTCTGTAATGTTCAGCTTCTAA
TTGTCTCTACTTTGTGAGACAACTTTTGAATGCTTGACCTCAAATCAGGTAGGACTACCC
GCTGAACTTAA
>AB1–51
TTTCCGTAGGTGAACCTGCGGAAGGATCATTATTGAATTATGTTTCTAGATAGGTTGTAG
CTGGCTCTTTTAGAGCATGTGCACGCCTGTTTGGACTTCATTTTCATCCACCTGTGCACC
TATTGTAGTCTTTGGTTGGGTTAGGAGGAAGTGATCATTGTATCAGCATCTGCTGGGAGT
GAGGACTTGCATTGTGAAAGCTTTGCTGTCCTTGATGTGATCATGGAATCTTTTTCTCAC
TAGAGTCTATGTCACTCATTATACTCTGTCGAATGTCATTGAATGTCTTTACATGGGCTT
GTATGCCTATGAAAATTGTAATACAACTTTCAGCAACGGATCTCTTGGCTCTCGCATCGA
TGAAGAACGCAGCGAAATGCGATAAGTAATGTGAATTGCAGAATTCAGTGAATCATCGAA
TCTTTGAACGCATCTTGCGCTCCTTGGTATTCCGAGGAGCATGCCTGTTTGAGTGTCATT
AAATTCTCAACTCTCTTATACTTTTTTGTAAAAGAGAGCTTGGACTGTGGAGGCTTGCTG
GCCACTTTTTGGGGTCAGCTCCTCTGAAATGCATTAGCGGAACCGTTTGCAATCTGCCAC
AAGTGTGATAAGTTATCTACACTGGCGAGGGGATTGCTCTCTGTAATGTTCAGCTTCTAA
TTGTCTCTACTTTGTGAGACAACTTTTGAATGCTTGACCTCAAATCAGGTAGGACTACCC
GCTGAACTTAA
>AB1–29
TTTCCGTAGGTGAACCTGCGGAAGGATCATTATTGAATTATGTTTCTAGATAGGTTGTAG
CTGGCTCTTTTAGAGCATGTGCACGCCTGTTTGGACTTCATTTTCATCCACCTGTGCACC
TATTGTAGTCTTTGGTTGGGTTAGGAGGAAGTGATCATTGTATCAGCATCTGCTGGGAGT
GAGGACTTGCATTGTGAAAGCTTTGCTGTCCTTGATGTGATCATGGAATCTTTTTCTCAC
TAGAGTCTATGTCACTCATTATACTCTGTCGAATGTCATTGAATGTCTTTACATGGGCTT
GTATGCCTATGAAAATTGTAATACAACTTTCAGCAACGGATCTCTTGGCTCTCGCATCGA
TGAAGAACGCAGCGAAATGCGATAAGTAATGTGAATTGCAGAATTCAGTGAATCATCGAA
TCTTTGAACGCATCTTGCGCTCCTTGGTATTCCGAGGAGCATGCCTGTTTGAGTGTCATT
AAATTCTCAACTCTCTTATACTTTTTTGTAAAAGAGAGCTTGGACTGTGGAGGCTTGCTG
GCCACTTTTTGGGGTCAGCTCCTCTGAAATGCATTAGCGGAACCGTTTGCAATCTGCCAC
AAGTGTGATAAGTTATCTACACTGGCGAGGGGATTGCTCTCTGTAATGTTCAGCTTCTAA
TTGTCTCTACTTTGTGAGACAACTTTTGAATGCTTGACCTCAAATCAGGTAGGACTACCC
GCTGAACTTAA
>AB1–31
TTTCCGTAGGTGAACCTGCGGAAGGATCATTATTGAATTATGTTTCTAGATAGGTTGTAG
CTGGCTCTTTTAGAGCATGTGCACGCCTGTTTGGACTTCATTTTCATCCACCTGTGCACC
TATTGTAGTCTTTGGTTGGGTTAGGAGGAAGTGATCATTGTATCAGCATCTGCTGGGAGT
GAGGACTTGCATTGTGAAAGCTTTGCTGTCCTTGATGTGATCATGGAATCTTTTTCTCAC
TAGAGTCTATGTCACTCATTATACTCTGTCGAATGTCATTGAATGTCTTTACATGGGCTT
GTATGCCTATGAAAATTGTAATACAACTTTCAGCAACGGATCTCTTGGCTCTCGCATCGA
TGAAGAACGCAGCGAAATGCGATAAGTAATGTGAATTGCAGAATTCAGTGAATCATCGAA
TCTTTGAACGCATCTTGCGCTCCTTGGTATTCCGAGGAGCATGCCTGTTTGAGTGTCATT
AAATTCTCAACTCTCTTATACTTTTTTGTAAAAGAGAGCTTGGACTGTGGAGGCTTGCTG

GCCACTTTTTGGGGTCAGCTCCTCTGAAATGCATTAGCGGAACCGTTTGCAATCTGCCAC
AAGTGTGATAAGTTATCTACACTGGCGAGGGGATTGCTCTCTGTAATGTTCAGCTTCTAA
TTGTCTCTACTTTGTGAGACAACTTTTGAATGCTTGACCTCAAATCAGGTAGGACTACCC
GCTGAACTTAA
>AB2−3
TTTCCGTAGGTGAACCTGCGGAAGGATCATTATTGAATTATGTTTCTAGATAGGTTGTAG
CTGGCTCTTTTAGAGCATGTGCACGCCTGTTTGGACTTCATTTTCATCCACCTGTGCACC
TATTGTAGTCTTTGGTTGGGTTAGGAGGAAGTGATCATTGTATCAGCATCTGCTGGGAGT
GAGGACTTGCATTGTGAAAGCTTTGCTGTCCTTGATGTGATCATGGAATCTTTTTCTCAC
TAGAGTCTATGTCACTCATTATACTCTGTCGAATGTCATTGAATGTCTTTACATGGGCTT
GTATGCCTATGAAAATTGTAATACAACTTTCAGCAACGGATCTCTTGGCTCTCGCATCGA
TGAAGAACGCAGCGAAATGCGATAAGTAATGTGAATTGCAGAATTCAGTGAATCATCGAA
TCTTTGAACGCATCTTGCGCTCCTTGGTATTCCGAGGAGCATGCCTGTTTGAGTGTCATT
AAATTCTCAACTCTCTTATACTTTTTTGTAAAAGAGAGCTTGGACTGTGGAGGCTTGCTG
GCCACTTTTTGGGGTCAGCTCCTCTGAAATGCATTAGCGGAACCGTTTGCAATCTGCCAC
AAGTGTGATAAGTTATCTACACTGGCGAGGGGATTGCTCTCTGTAATGTTCAGCTTCTAA
TTGTCTCTACTTTGTGAGACAACTTTTGAATGCTTGACCTCAAATCAGGTAGGACTACCC
GCTGAACTTAA
>AB3−25
TTTCCGTAGGTGAACCTGCGGAAGGATCATTATTGAATTATGTTTCTAGATAGGTTGTAG
CTGGCTCTTTTAGAGCATGTGCACGCCTGTTTGGACTTCATTTTCATCCACCTGTGCACC
TATTGTAGTCTTTGGTTGGGTTAGGAGGAAGTGATCATTGTATCAGCATCTGCTGGGAGT
GAGGACTTGCATTGTGAAAGCTTTGCTGTCCTTGATGTGATCATGGAATCTTTTTCTCAC
TAGAGTCTATGTCACTCATTATACTCTGTCGAATGTCATTGAATGTCTTTACATGGGCTT
GTATGCCTATGAAAATTGTAATACAACTTTCAGCAACGGATCTCTTGGCTCTCGCATCGA
TGAAGAACGCAGCGAAATGCGATAAGTAATGTGAATTGCAGAATTCAGTGAATCATCGAA
TCTTTGAACGCATCTTGCGCTCCTTGGTATTCCGAGGAGCATGCCTGTTTGAGTGTCATT
AAATTCTCAACTCTCTTATACTTTTTTGTAAAAGAGAGCTTGGACTGTGGAGGCTTGCTG
GCCACTTTTTGGGGTCAGCTCCTCTGAAATGCATTAGCGGAACCGTTTGCAATCTGCCAC
AAGTGTGATAAGTTATCTACACTGGCGAGGGGATTGCTCTCTGTAATGTTCAGCTTCTAA
TTGTCTCTACTTTGTGAGACAACTTTTGAATGCTTGACCTCAAATCAGGTAGGACTACCC
GCTGAACTTAA
>AB3−51
TTTCCGTAGGTGAACCTGCGGAAGGATCATTATTGAATTATGTTTCTAGATAGGTTGTAG
CTGGCTCTTTTAGAGCATGTGCACGCCTGTTTGGACTTCATTTTCATCCACCTGTGCACC
TATTGTAGTCTTTGGTTGGGTTAGGAGGAAGTGATCATTGTATCAGCATCTGCTGGGAGT
GAGGACTTGCATTGTGAAAGCTTTGCTGTCCTTGATGTGATCATGGAATCTTTTTCTCAC
TAGAGTCTATGTCACTCATTATACTCTGTCGAATGTCATTGAATGTCTTTACATGGGCTT
GTATGCCTATGAAAATTGTAATACAACTTTCAGCAACGGATCTCTTGGCTCTCGCATCGA
TGAAGAACGCAGCGAAATGCGATAAGTAATGTGAATTGCAGAATTCAGTGAATCATCGAA
TCTTTGAACGCATCTTGCGCTCCTTGGTATTCCGAGGAGCATGCCTGTTTGAGTGTCATT
AAATTCTCAACTCTCTTATACTTTTTTGTAAAAGAGAGCTTGGACTGTGGAGGCTTGCTG
GCCACTTTTTGGGGTCAGCTCCTCTGAAATGCATTAGCGGAACCGTTTGCAATCTGCCAC
AAGTGTGATAAGTTATCTACACTGGCGAGGGGATTGCTCTCTGTAATGTTCAGCTTCTAA
TTGTCTCTACTTTGTGAGACAACTTTTGAATGCTTGACCTCAAATCAGGTAGGACTACCC
GCTGAACTTAA
>AB4−8
TTTCCGTAGGTGAACCTGCGGAAGGATCATTATTGAATTATGTTTCTAGATAGGTTGTAG
CTGGCTCTTTTAGAGCATGTGCACGCCTGTTTGGACTTCATTTTCATCCACCTGTGCACC
TATTGTAGTCTTTGGTTGGGTTAGGAGGAAGTGATCATTGTATCAGCATCTGCTGGGAGT

GAGGACTTGCATTGTGAAAGCTTTGCTGTCCTTGATGTGATCATGGAATCTTTTTCTCAC
TAGAGTCTATGTCACTCATTATACTCTGTCGAATGTCATTGAATGTCTTTACATGGGCTT
GTATGCCTATGAAAATTGTAATACAACTTTCAGCAACGGATCTCTTGGCTCTCGCATCGA
TGAAGAACGCAGCGAAATGCGATAAGTAATGTGAATTGCAGAATTCAGTGAATCATCGAA
TCTTTGAACGCATCTTGCGCTCCTTGGTATTCCGAGGAGCATGCCTGTTTGAGTGTCATT
AAATTCTCAACTCTCTTATACTTTTTTGTAAAAGAGAGCTTGGACTGTGGAGGCTTGCTG
GCCACTTTTTGGGGTCAGCTCCTCTGAAATGCATTAGCGGAACCGTTTGCAATCTGCCAC
AAGTGTGATAAGTTATCTACACTGGCGAGGGGATTGCTCTCTGTAATGTTCAGCTTCTAA
TTGTCTCTACTTTGTGAGACAACTTTTGAATGCTTGACCTCAAATCAGGTAGGACTACCC
GCTGAACTTAA
>AB4-41
TTTCCGTAGGTGAACCTGCGGAAGGATCATTATTGAATTATGTTTCTAGATAGGTTGTAG
CTGGCTCTTTTAGAGCATGTGCACGCCTGTTTGGACTTCATTTTCATCCACCTGTGCACC
TATTGTAGTCTTTGGTTGGGTTAGGAGGAAGTGATCATTGTATCAGCATCTGCTGGGAGT
GAGGACTTGCATTGTGAAAGCTTTGCTGTCCTTGATGTGATCATGGAATCTTTTTCTCAC
TAGAGTCTATGTCACTCATTATACTCTGTCGAATGTCATTGAATGTCTTTACATGGGCTT
GTATGCCTATGAAAATTGTAATACAACTTTCAGCAACGGATCTCTTGGCTCTCGCATCGA
TGAAGAACGCAGCGAAATGCGATAAGTAATGTGAATTGCAGAATTCAGTGAATCATCGAA
TCTTTGAACGCATCTTGCGCTCCTTGGTATTCCGAGGAGCATGCCTGTTTGAGTGTCATT
AAATTCTCAACTCTCTTATACTTTTTTGTAAAAGAGAGCTTGGACTGTGGAGGCTTGCTG
GCCACTTTTTGGGGTCAGCTCCTCTGAAATGCATTAGCGGAACCGTTTGCAATCTGCCAC
AAGTGTGATAAGTTATCTACACTGGCGAGGGGATTGCTCTCTGTAATGTTCAGCTTCTAA
TTGTCTCTACTTTGTGAGACAACTTTTGAATGCTTGACCTCAAATCAGGTAGGACTACCC
GCTGAACTTAA
>AB6-18
TTTCCGTAGGTGAACCTGCGGAAGGATCATTATTGAATTATGTTTCTAGATAGGTTGTAG
CTGGCTCTTTTAGAGCATGTGCACGCCTGTTTGGACTTCATTTTCATCCACCTGTGCACC
TATTGTAGTCTTTGGTTGGGTTAGGAGGAAGTGATCATTGTATCAGCATCTGCTGGGAGT
GAGGACTTGCATTGTGAAAGCTTTGCTGTCCTTGATGTGATCATGGAATCTTTTTCTCAC
TAGAGTCTATGTCACTCATTATACTCTGTCGAATGTCATTGAATGTCTTTACATGGGCTT
GTATGCCTATGAAAATTGTAATACAACTTTCAGCAACGGATCTCTTGGCTCTCGCATCGA
TGAAGAACGCAGCGAAATGCGATAAGTAATGTGAATTGCAGAATTCAGTGAATCATCGAA
TCTTTGAACGCATCTTGCGCTCCTTGGTATTCCGAGGAGCATGCCTGTTTGAGTGTCATT
AAATTCTCAACTCTCTTATACTTTTTTGTAAAAGAGAGCTTGGACTGTGGAGGCTTGCTG
GCCACTTTTTGGGGTCAGCTCCTCTGAAATGCATTAGCGGAACCGTTTGCAATCTGCCAC
AAGTGTGATAAGTTATCTACACTGGCGAGGGGATTGCTCTCTGTAATGTTCAGCTTCTAA
TTGTCTCTACTTTGTGAGACAACTTTTGAATGCTTGACCTCAAATCAGGTAGGACTACCC
GCTGAACTTAA
>AB8-9
TTTCCGTAGGTGAACCTGCGGAAGGATCATTATTGAATTATGTTTCTAGATAGGTTGTAG
CTGGCTCTTTTAGAGCATGTGCACGCCTGTTTGGACTTCATTTTCATCCACCTGTGCACC
TATTGTAGTCTTTGGTTGGGTTAGGAGGAAGTGATCATTGTATCAGCATCTGCTGGGAGT
GAGGACTTGCATTGTGAAAGCTTTGCTGTCCTTGATGTGATCATGGAATCTTTTTCTCAC
TAGAGTCTATGTCACTCATTATACTCTGTCGAATGTCATTGAATGTCTTTACATGGGCTT
GTATGCCTATGAAAATTGTAATACAACTTTCAGCAACGGATCTCTTGGCTCTCGCATCGA
TGAAGAACGCAGCGAAATGCGATAAGTAATGTGAATTGCAGAATTCAGTGAATCATCGAA
TCTTTGAACGCATCTTGCGCTCCTTGGTATTCCGAGGAGCATGCCTGTTTGAGTGTCATT
AAATTCTCAACTCTCTTATACTTTTTTGTAAAAGAGAGCTTGGACTGTGGAGGCTTGCTG
GCCACTTTTTGGGGTCAGCTCCTCTGAAATGCATTAGCGGAACCGTTTGCAATCTGCCAC
AAGTGTGATAAGTTATCTACACTGGCGAGGGGATTGCTCTCTGTAATGTTCAGCTTCTAA

TTGTCTCTACTTTGTGAGACAACTTTTGAATGCTTGACCTCAAATCAGGTAGGACTACCC
GCTGAACTTAA
>AB9-26
TTTCCGTAGGTGAACCTGCGGAAGGATCATTATTGAATTATGTTTCTAGATAGGTTGTAG
CTGGCTCTTTTAGAGCATGTGCACGCCTGTTTGGACTTCATTTTCATCCACCTGTGCACC
TATTGTAGTCTTTGGTTGGGTTAGGAGGAAGTGATCATTGTATCAGCATCTGCTGGGAGT
GAGGACTTGCATTGTGAAAGCTTTGCTGTCCTTGATGTGATCATGGAATCTTTTTCTCAC
TAGAGTCTATGTCACTCATTATACTCTGTCGAATGTCATTGAATGTCTTTACATGGGCTT
GTATGCCTATGAAAATTGTAATACAACTTTCAGCAACGGATCTCTTGGCTCTCGCATCGA
TGAAGAACGCAGCGAAATGCGATAAGTAATGTGAATTGCAGAATTCAGTGAATCATCGAA
TCTTTGAACGCATCTTGCGCTCCTTGGTATTCCGAGGAGCATGCCTGTTTGAGTGTCATT
AAATTCTCAACTCTCTTATACTTTTTTGTAAAAGAGAGCTTGGACTGTGGAGGCTTGCTG
GCCACTTTTTGGGGTCAGCTCCTCTGAAATGCATTAGCGGAACCGTTTGCAATCTGCCAC
AAGTGTGATAAGTTATCTACACTGGCGAGGGGATTGCTCTCTGTAATGTTCAGCTTCTAA
TTGTCTCTACTTTGTGAGACAACTTTTGAATGCTTGACCTCAAATCAGGTAGGACTACCC
GCTGAACTTAA
>AB9-35
TTTCCGTAGGTGAACCTGCGGAAGGATCATTATTGAATTATGTTTCTAGATAGGTTGTAG
CTGGCTCTTTTAGAGCATGTGCACGCCTGTTTGGACTTCATTTTCATCCACCTGTGCACC
TATTGTAGTCTTTGGTTGGGTTAGGAGGAAGTGATCATTGTATCAGCATCTGCTGGGAGT
GAGGACTTGCATTGTGAAAGCTTTGCTGTCCTTGATGTGATCATGGAATCTTTTTCTCAC
TAGAGTCTATGTCACTCATTATACTCTGTCGAATGTCATTGAATGTCTTTACATGGGCTT
GTATGCCTATGAAAATTGTAATACAACTTTCAGCAACGGATCTCTTGGCTCTCGCATCGA
TGAAGAACGCAGCGAAATGCGATAAGTAATGTGAATTGCAGAATTCAGTGAATCATCGAA
TCTTTGAACGCATCTTGCGCTCCTTGGTATTCCGAGGAGCATGCCTGTTTGAGTGTCATT
AAATTCTCAACTCTCTTATACTTTTTTGTAAAAGAGAGCTTGGACTGTGGAGGCTTGCTG
GCCACTTTTTGGGGTCAGCTCCTCTGAAATGCATTAGCGGAACCGTTTGCAATCTGCCAC
AAGTGTGATAAGTTATCTACACTGGCGAGGGGATTGCTCTCTGTAATGTTCAGCTTCTAA
TTGTCTCTACTTTGTGAGACAACTTTTGAATGCTTGACCTCAAATCAGGTAGGACTACCC
GCTGAACTTAA
>AB10-11
TTTCCGTAGGTGAACCTGCGGAAGGATCATTATTGAATTATGTTTCTAGATAGGTTGTAG
CTGGCTCTTTTAGAGCATGTGCACGCCTGTTTGGACTTCATTTTCATCCACCTGTGCACC
TATTGTAGTCTTTGGTTGGGTTAGGAGGAAGTGATCATTGTATCAGCATCTGCTGGGAGT
GAGGACTTGCATTGTGAAAGCTTTGCTGTCCTTGATGTGATCATGGAATCTTTTTCTCAC
TAGAGTCTATGTCACTCATTATACTCTGTCGAATGTCATTGAATGTCTTTACATGGGCTT
GTATGCCTATGAAAATTGTAATACAACTTTCAGCAACGGATCTCTTGGCTCTCGCATCGA
TGAAGAACGCAGCGAAATGCGATAAGTAATGTGAATTGCAGAATTCAGTGAATCATCGAA
TCTTTGAACGCATCTTGCGCTCCTTGGTATTCCGAGGAGCATGCCTGTTTGAGTGTCATT
AAATTCTCAACTCTCTTATACTTTTTTGTAAAAGAGAGCTTGGACTGTGGAGGCTTGCTG
GCCACTTTTTGGGGTCAGCTCCTCTGAAATGCATTAGCGGAACCGTTTGCAATCTGCCAC
AAGTGTGATAAGTTATCTACACTGGCGAGGGGATTGCTCTCTGTAATGTTCAGCTTCTAA
TTGTCTCTACTTTGTGAGACAACTTTTGAATGCTTGACCTCAAATCAGGTAGGACTACCC
GCTGAACTTAA
>AB11-24
TTTCCGTAGGTGAACCTGCGGAAGGATCATTATTGAATTATGTTTCTAGATAGGTTGTAG
CTGGCTCTTTTAGAGCATGTGCACGCCTGTTTGGACTTCATTTTCATCCACCTGTGCACC
TATTGTAGTCTTTGGTTGGGTTAGGAGGAAGTGATCATTGTATCAGCATCTGCTGGGAGT
GAGGACTTGCATTGTGAAAGCTTTGCTGTCCTTGATGTGATCATGGAATCTTTTTCTCAC
TAGAGTCTATGTCACTCATTATACTCTGTCGAATGTCATTGAATGTCTTTACATGGGCTT

GTATGCCTATGAAAATTGTAATACAACTTTCAGCAACGGATCTCTTGGCTCTCGCATCGA
TGAAGAACGCAGCGAAATGCGATAAGTAATGTGAATTGCAGAATTCAGTGAATCATCGAA
TCTTTGAACGCATCTTGCGCTCCTTGGTATTCCGAGGAGCATGCCTGTTTGAGTGTCATT
AAATTCTCAACTCTCTTATACTTTTTTGTAAAAGAGAGCTTGGACTGTGGAGGCTTGCTG
GCCACTTTTTGGGGTCAGCTCCTCTGAAATGCATTAGCGGAACCGTTTGCAATCTGCCAC
AAGTGTGATAAGTTATCTACACTGGCGAGGGGATTGCTCTCTGTAATGTTCAGCTTCTAA
TTGTCTCTACTTTGTGAGACAACTTTTGAATGCTTGACCTCAAATCAGGTAGGACTACCC
GCTGAACTTAA
>AB11−30
TTTCCGTAGGTGAACCTGCGGAAGGATCATTATTGAATTATGTTTCTAGATAGGTTGTAG
CTGGCTCTTTTAGAGCATGTGCACGCCTGTTTGGACTTCATTTTCATCCACCTGTGCACC
TATTGTAGTCTTTGGTTGGGTTAGGAGGAAGTGATCATTGTATCAGCATCTGCTGGGAGT
GAGGACTTGCATTGTGAAAGCTTTGCTGTCCTTGATGTGATCATGGAATCTTTTTCTCAC
TAGAGTCTATGTCACTCATTATACTCTGTCGAATGTCATTGAATGTCTTTACATGGGCTT
GTATGCCTATGAAAATTGTAATACAACTTTCAGCAACGGATCTCTTGGCTCTCGCATCGA
TGAAGAACGCAGCGAAATGCGATAAGTAATGTGAATTGCAGAATTCAGTGAATCATCGAA
TCTTTGAACGCATCTTGCGCTCCTTGGTATTCCGAGGAGCATGCCTGTTTGAGTGTCATT
AAATTCTCAACTCTCTTATACTTTTTTGTAAAAGAGAGCTTGGACTGTGGAGGCTTGCTG
GCCACTTTTTGGGGTCAGCTCCTCTGAAATGCATTAGCGGAACCGTTTGCAATCTGCCAC
AAGTGTGATAAGTTATCTACACTGGCGAGGGGATTGCTCTCTGTAATGTTCAGCTTCTAA
TTGTCTCTACTTTGTGAGACAACTTTTGAATGCTTGACCTCAAATCAGGTAGGACTACCC
GCTGAACTTAA
>AB12−35
TTTCCGTAGGTGAACCTGCGGAAGGATCATTATTGAATTATGTTTCTAGATAGGTTGTAG
CTGGCTCTTTTAGAGCATGTGCACGCCTGTTTGGACTTCATTTTCATCCACCTGTGCACC
TATTGTAGTCTTTGGTTGGGTTAGGAGGAAGTGATCATTGTATCAGCATCTGCTGGGAGT
GAGGACTTGCATTGTGAAAGCTTTGCTGTCCTTGATGTGATCATGGAATCTTTTTCTCAC
TAGAGTCTATGTCACTCATTATACTCTGTCGAATGTCATTGAATGTCTTTACATGGGCTT
GTATGCCTATGAAAATTGTAATACAACTTTCAGCAACGGATCTCTTGGCTCTCGCATCGA
TGAAGAACGCAGCGAAATGCGATAAGTAATGTGAATTGCAGAATTCAGTGAATCATCGAA
TCTTTGAACGCATCTTGCGCTCCTTGGTATTCCGAGGAGCATGCCTGTTTGAGTGTCATT
AAATTCTCAACTCTCTTATACTTTTTTGTAAAAGAGAGCTTGGACTGTGGAGGCTTGCTG
GCCACTTTTTGGGGTCAGCTCCTCTGAAATGCATTAGCGGAACCGTTTGCAATCTGCCAC
AAGTGTGATAAGTTATCTACACTGGCGAGGGGATTGCTCTCTGTAATGTTCAGCTTCTAA
TTGTCTCTACTTTGTGAGACAACTTTTGAATGCTTGACCTCAAATCAGGTAGGACTACCC
GCTGAACTTAA
>AB10−36
TTTCCGTAGGTGAACCTGCGGAAGGATCATTATTGAATTATGTTTCTAGATAGGTTGTAG
CTGGCTCTTTTAGAGCATGTGCACGCCTGTTTGGACTTCATTTTCATCCACCTGTGCACC
TATTGTAGTCTTTGGTTGGGTTAGGAGGAAGTGATCATTGTATCAGCATCTGCTGGGAGT
GAGGACTTGCATTGTGAAAGCTTTGCTGTCCTTGATGTGATCATGGAATCTTTTTCTCAC
TAGAGTCTATGTCACTCATTATACTCTGTCGAATGTCATTGAATGTCTTTACATGGGCTT
GTATGCCTATGAAAATTGTAATACAACTTTCAGCAACGGATCTCTTGGCTCTCGCATCGA
TGAAGAACGCAGCGAAATGCGATAAGTAATGTGAATTGCAGAATTCAGTGAATCATCGAA
TCTTTGAACGCATCTTGCGCTCCTTGGTATTCCGAGGAGCATGCCTGTTTGAGTGTCATT
AAATTCTCAACTCTCTTATACTTTTTTGTAAAAGAGAGCTTGGACTGTGGAGGCTTGCTG
GCCACTTTTTGGGGTCAGCTCCTCTGAAATGCATTAGCGGAACCGTTTGCAATCTGCCAC
AAGTGTGATAAGTTATCTACACTGGCGAGGGGATTGCTCTCTGTAATGTTCAGCTTCTAA
TTGTCTCTACTTTGTGAGACAACTTTTGAATGCTTGACCTCAAATCAGGTAGGACTACCC
GCTGAACTTAA

>AB7–23
TTTCCGTAGGTGAACCTGCGGAAGGATCATTATTGAATTATGTTTCTAGATAGGTTGTAG
CTGGCTCTTTTAGAGCATGTGCACGCCTGTTTGGACTTCATTTTCATCCACCTGTGCACC
TATTGTAGTCTTTGGTTGGGTTAGGAGGAAGTGATCATTGTATCAGCATCTGCTGGGAGT
GAGGACTTGCATTGTGAAAGCTTTGCTGTCCTTGATGTGATCATGGAATCTCTTTCTCAC
TAGAGTCTATGTCACTCATTATACTCTGTCGAATGTCATTGAATGTCTTTACATGGGCTT
GTATGCCTATGAAAATTGTAATACAACTTTCAGCAACGGATCTCTTGGCTCTCGCATCGA
TGAAGAACGCAGCGAAATGCGATAAGTAATGTGAATTGCAGAATTCAGTGAATCATCGAA
TCTTTGAACGCATCTTGCGCTCCTTGGTATTCCGAGGAGCATGCCTGTTTGAGTGTCATT
AAATTCTCAACTCTCTTATACTTTTTTGTAAAAGAGAGCTTGGACTGTGGAGGCTTGCTG
GCCACTTTTTGGGGTCAGCTCCTCTGAAATGCATTAGCGGAACCGTTTGCAATCTGCCAC
AAGTGTGATAAGTTATCTACACTGGCGAGGGGATTGCTCTCTGTAATGTTCAGCTTCTAA
TTGTCTCTACTTTGTGAGACAACTTTTGAATGCTTGACCTCAAATCAGGTAGGACTACCC
GCTGAACTTAA
>AB11–36
TTTCCGTAGGTGAACCTGCGGAAGGATCATTATTGAATTATGTTTCTAGATAGGTTGTAG
CTGGCTCTTTTAGAGCATGTGCACGCCTGTTTGGACTTCATTTTCATCCACCTGTGCACC
TATTGTAGTCTTTGGTTGGGTTAGGAGGAAGTGATCATTGTATCAGCATCTGCTGGGAGT
GAGGACTTGCATTGTGAAAGCTTTGCTGTCCTTGATGTGATCATGGAATCTTTTTCTCAC
TAGAGTCTATGTCACTCATTATACTCTGTCGAATGTCATTGAATGTCTTTACATGGGCTT
GTATGTCTATGAAAATTGTAATACAACTTTCAGCAACGGATCTCTTGGCTCTCGCATCGA
TGAAGAACGCAGCGAAATGCGATAAGTAATGTGAATTGCAGAATTCAGTGAATCATCGAA
TCTTTGAACGCATCTTGCGCTCCTTGGTATTCCGAGGAGCATGCCTGTTTGAGTGTCATT
AAATTCTCAACTCTCTTATACTTTTTTGTAAAAGAGAGCTTGGACTGTGGAGGCTTGCTG
GCCACTTTTTGGGGTCAGCTCCTCTGAAATGCATTAGCGGAACCGTTTGCAATCTGCCAC
AAGTGTGATAAGTTATCTACACTGGCGAGGGGATTGCTCTCTGTAATGTTCAGCTTCTAA
TTGTCTCTACTTTGTGAGACAACTTTTGAATGCTTGACCTCAAATCAGGTAGGACTACCC
GCTGAACTTAA
>AB9–38
TTTCCGTAGGTGAACCTGCGGAAGGATCATTATTGAATTATGTTTCTAGATAGGTTGTAG
CTGGCTCTTTTAGAGCATGTGCACGCCTGTTTGGACTTCATTTTCATCCACCTGTGCACC
TATTGTAGTCTTTGGTTGGGTTAGGAGGAAGTGATCATTGTATCAGCATCTGCTGGGAGT
GAGGACTTGCATTGTGAAAGCTTTGCTGTCCTTGATGTGATCATGGAATCTTTTTCTCAC
TAGAGTCTATGTCACTCATTATACTCTGTCGAATGTCATTGAATGTCTTTACATGGGCTT
GTATGCCTATGAAAATTGTAATACAACTTTCAGCAACGGATCTCTTGGCTCTCGCATCGA
TGAAGAACGCAGCGAAATGCGATAAGTAATGTGAATTGCAGAATTCAGTGAATCATCGAA
TCTTTGAACGCATCTTGCGCTCCTTGGTATTCCGAGGAGCATGTCTGTTTGAGTGTCATT
AAATTCTCAACTCTCTTATACTTTTTTGTAAAAGAGAGCTTGGACTGTGGAGGCTTGCTG
ACCACTTTTTGGGGTCAGCTCCTCTGAAATGCATTAGCGGAACCGTTTGCAATCTGCCAC
AAGTGTGATAAGTTATCTACACTGGCGAGGGGATTGCTCTCTGTAATGTTCAGCTTCTAA
TTGTCTCTACTTTGTGAGACAACTTTTGAATGCTTGACCTCAAATCAGGTAGGACTACCC
GCTGAACTTAA
>AB11–52
TTTCCGTAGGTGAACCTGCGGAAGGATCATTATTGAATTATGTTTCTAGATAGGTTGTAG
CTGGCTCTTTTAGAGCATGTGCACGCCTGTTTGGACTTCATTTTCATCCACCTGTGCACC
TATTGAAGTCTTTGGTTGGGTTAGGAGGAAGTGATCATTGTATCAGCATCTGCTGGGAGT
GAGGACTTGCATTGTGAAAGCTTTGCTGTCCTTGATGTGATCATGGAATCTTTTTCTCAC
TAGAGTCTATGTCACTCATTATACTCTGTCGAATGTCATTGAATGTCTTTACATGGGCTT
GTATGCCTATGAAAATTGTAATACAACTTTCAGCAACGGATCTCTTGGCTCTCGCATCGA
TGAAGGACGCAGCGAAATGCGATAAGTAATGTGAATTGCAGAATTCAGTGAATCATCGAA

TCTTTGAACGCATCTTGCGCTCCTTGGTATTCCGAGGAGCATGCCTGTTTGAGTGTCATT
AAATTCTCAACTCTCTTATACTTTTTTGTAAAAGAGAGCTTGGACTGTGGAGGCTTGCTG
GCCACTTTTTGGGGTCAGCTCCTCTGAAATGCATTAGCGGAACCGTTTGCAATCTGCCAC
AAGTGTGATAAGTTATCTACACTGGCGAGGGGATTGCTCTCTGTAATGTTCAGCTTCTAA
TTGTCTCTACTTTGTGAGACAACTTTTGAATGCTTGACCTCAAATCAGGTAGGACTACCC
GCTGAACTTAA
>AB12-61
TTTCCGTAGGTGAACCTGCGGAAGGATCATTATTGAATTATGTTTCTAGATAGGTTGTAG
CTGGCTCTTTTAGAGCATGTGCACGCCTGTTTGGACTTCATTTTCATCCACCTGTGCACC
TATTGTAGTCTTTGGTTGGGTTAGGAGGAAGTGGTCATTGTGTCAGCATCTGCTGGGTGT
GAGGACTTGCATTGTGAAAGCTTTGCTGTCCTTGATGTGATCATGGAATCTCTTTCTCAC
TAGAGTCTATGTCACTCATTATACTCTGTCGAATGTCATTGAATGTCTTTACATGGGCTT
GTATGCCTATGAAAATTGTAATACAACTTTCAGCAACGGATCTCTTGGCTCTCGCATCGA
TGAAGAACGCAGCGAAATGCGATAAGTAATGTGAATTGCAGAATTCAGTGAATCATCGAA
TCTTTGAACGCATCTTGCGCTCCTTGGTATTCCGAGGAGCATGCCTGTTTGAGTGTCATT
AAATTCTCAACTCTCTTATACTTTTTTGTAAAAGAGAGCTTGGACTGTGGAGGCTTGCTG
GCCACTTTTTGGGGTCAGCTCCTCTGAAATGCATTAGCGGAACCGTTTGCGATCTGCCAC
AAGTGTGATAAGTTATCTACACTGGCGAGGGGATTGCTCTCTGTAATGTTCAGCTTCTAA
TTGTCTCTACTTTGTGAGACTACTTTTGAATGCTTGACCTCAAATCAGGTAGGACTACCC
GCTGAACTTAA
>AB6-48
TTTCCGTAGGTGAACCTGCGGAAGGATCATTATTGAATTATGTTTCTAGATAGGTTGTAG
CTGGCTCTTTTAGAGCATGTGCACGCCTGTTTGGACTTCATTTTCATCCACCTGTGCACC
TATTGTAGTCTTTGGTTGGGTTAGGAGGAAGTGATCATTGTGTCAGCATCTGCTGGATGT
GAGGACTTGCATTGTGAAAGCTTTGCTGTCCTTGATGTGATCATGGAATCTCTTTCTCAC
TAGAGTCTATGTCACTCATTATACTCTGTCGAATGTCATTGAATGTCTTTACATGGGCTT
GTATGCCTATGAAAATTGTAATACAACTTTCAGCAACGGATCTCTTGGCTCTCGCATCGA
TGAAGAACGCAGCGAAATGCGATAAGTAATGTGAATTGCAGAATTCAGTGAATCATCGAA
TCTTTGAACGCATCTTGCGCTCCTTGGTATTCCGAGGAGCATGCCTGTTTGAGTGTCATT
AAATTCTCAACTCTCTTATACTTTTTTGTAAAAGAGAGCTTGGACTGTGGAGGCTTGCTG
GCCACTTTTTGGGGTCAGCTCCTCTGAAATGCATTAGCGGAACCGTTTGCGATCTGCCAC
AAGTGTGATAAGTTATCTACACTGGCGAGGGGATTGCTCTCTGTAATGTTCAGCTTCTAA
TTGTCTCTACTTTGTGAGACTACTTTTGAATGCTTGACCTCAAATCAGGTAGGACTACCC
GCTGAACTTAA
>AB6-8
TTTCCGTAGGTGAACCTGCGGAAGGATCATTATTGAATTATGTTTCTAGATAGGTTGTAG
CTGGCTCTTTTAGAGCATGTGCACGCCTGTTTGGACTTCATTTTCATCCACCTGTGCACC
TATTGTAGTCTTTGGTTGGGTTAGGGGGAAGTGGTCATTGTGTCAGCATCTGCTGGATGT
GAGGACTTGCATTGTGAAAGCTTTGCTGTCCTTGATGTGATCATGGAATCTCTTTCTCAC
TAGAGTCTATGTCACTCATTATACTCTGTCGAATGTCATTGAATGTCTTTACATGGGCTT
GTATGCCTATGAAAATTGTAATACAACTTTCAGCAACGGATCTCTTGGCTCTCGCATCGA
TGAAGAACGCAGCGAAATGCGATAAGTAATGTGAATTGCAGAATTCAGTGAATCATCGAA
TCTTTGAACGCATCTTGCGCTCCTTGGTATTCCGAGGAGCATGCCTGTTTGAGTGTCATT
AAATTCTCAACTCTCTTATACTTTTTTGTAAAAGAGAGCTTGGACTGTGGAGGCTTGCTG
GCCACTTTTTGGGGTCAGCTCCTCTGAAATGCATTAGCGGAACCGTTTGCGATCTGCCAC
AAGTGTGATAAGTTATCTACACTGGCGAGGGGATTGCTCTCTGTAATGTTCAGCTTCTAA
TTGTCTCTACTTTGTGAGACTACTTTTGAATGCTTGACCTCAAATCAGGTAGGACTACCC
GCTGAACTTAA
>AB6-15
TTTCCGTAGGTGAACCTGCGGAAGGATCATTATTGAATTATGTTTCTAGATAGGTTGTAG

CTGGCTCTTTTAGAGCATGTGCACGCCTGTTTGGACTTCATTTTCATCCACCTGTGCACC
TATTGTAGTCTTTGGTTGGGTTAGGGGGAAGTGGTCATTGTGTCAGCATCTGCTGGATGT
GAGGACTTGCATTGTGAAAGCTTTGCTGTCCTTGATGTGATCATGGAATCTTTTTCTCAC
TAGAGTCTATGTCACTCATTATACTCTGTCGAATGTCATTGAATGTCTTTACATGGGCTT
GTATGCCTATGAAAATTGTAATACAACTTTCAGCAACGGATCTCTTGGCTCTCGCATCGA
TGAAGAACGCAGCGAAATGCGATAAGTAATGTGAATTGCAGAATTCAGTGAATCATCGAA
TCTTTGAACGCATCTTGCGCTCCTTGGTATTCCGAGGAGCATGCCTGTTTGAGTGTCATT
AAATTCTCAACTCTCTTATACTTTTTTGTAAAAGAGAGCTTGGACTGTGGAGGCTTGCTG
GCCACTTTTTGGGGTCAGCTCCTCTGAAATGCATTAGCGGAACCGTTTGCGATCTGCCAC
AAGTGTGATAAGTTATCTACACTGGCGAGGGGATTGCTCTCTGTAATGTTCAGCTTCTAA
TTGTCTCTACTTTGTGAGACTACTTTTGAATGCTTGACCTCAAATCAGGTAGGACTACCC
GCTGAACTTAA
>AB7-24
TTTCCGTAGGTGAACCTGCGGAAGGATCATTATTGAATTATGTTTCTAGATAGGTTGTAG
CTGGCTCTTTTAGAGCATGTGCACGCCTGTTTGGACTTCATTTTCATCCACCTGTGCACC
TATTGTAGTCTTTGGTTGGGTTAGGGGGAAGTGGTCATTGTGTCAGCATCTGCTGGATGT
GAGGACTTGCATTGTGAAAGCTTTGCTGTCCTTGATGTGATCATGGAATCTTTTTCTCAC
TAGAGTCTATGTCACTCATTATACTCTGTCGAATGTCATTGAATGTCTTTACATGGGCTT
GTATGCCTATGAAAATTGTAATACAACTTTCAGCAACGGATCTCTTGGCTCTCGCATCGA
TGAAGAACGCAGCGAAATGCGATAAGTAATGTGAATTGCAGAATTCAGTGAATCATCGAA
TCTTTGAACGCATCTTGCGCTCCTTGGTATTCCGAGGAGCATGCCTGTTTGAGTGTCATT
AAATTCTCAACTCTCTTATACTTTTTTGTAAAAGAGAGCTTGGACTGTGGAGGCTTGCTG
GCCACTTTTTGGGGTCAGCTCCTCTGAAATGCATTAGCGGAACCGTTTGCGATCTGCCAC
AAGTGTGATAAGTTATCTACACTGGCGAGGGGATTGCTCTCTGTAATGTTCAGCTTCTAA
TTGTCTCTACTTTGTGAGACTACTTTTGAATGCTTGACCTCAAATCAGGTAGGACTACCC
GCTGAACTTAA
>AB9-5
TTTCCGTAGGTGAACCTGCGGAAGGATCATTATTGAATTATGTTTCTAGATAGGTTGTAG
CTGGCTCTTTTAGAGCATGTGCACGCCTGTTTGGACTTCATTTTCATCCACCTGTGCACC
TATTGTAGTCTTTGGTTGGGTTAGGGGGAAGTGGTCATTGTGTCAGCATCTGCTGGATGT
GAGGACTTGCATTGTGAAAGCTTTGCTGTCCTTGATGTGATCATGGAATCTTTTTCTCAC
TAGAGTCTATGTCACTCATTATACTCTGTCGAATGTCATTGAATGTCTTTACATGGGCTT
GTATGCCTATGAAAATTGTAATACAACTTTCAGCAACGGATCTCTTGGCTCTCGCATCGA
TGAAGAACGCAGCGAAATGCGATAAGTAATGTGAATTGCAGAATTCAGTGAATCATCGAA
TCTTTGAACGCATCTTGCGCTCCTTGGTATTCCGAGGAGCATGCCTGTTTGAGTGTCATT
AAATTCTCAACTCTCTTATACTTTTTTGTAAAAGAGAGCTTGGACTGTGGAGGCTTGCTG
GCCACTTTTTGGGGTCAGCTCCTCTGAAATGCATTAGCGGAACCGTTTGCGATCTGCCAC
AAGTGTGATAAGTTATCTACACTGGCGAGGGGATTGCTCTCTGTAATGTTCAGCTTCTAA
TTGTCTCTACTTTGTGAGACTACTTTTGAATGCTTGACCTCAAATCAGGTAGGACTACCC
GCTGAACTTAA
>AB12-58
TTTCCGTAGGTGAACCTGCGGAAGGATCATTATTGAATTATGTTTCTAGATAGGTTGTAG
CTGGCTCTTTTAGAGCATGTGCACGCCTGTTTGGACTTCATTTTCATCCACCTGTGCACC
TATTGTAGTCTTTGGTTGGGTTAGGAGGAAGTGATCATTGTATCAGCATCTGCTGGATGT
GAGGACTTGCATTGTGAAAGCTTTGCTGTCCTTGATGTGATCATGGAATCTTTTTCTCAC
TAGAGTCTATGTCACTCATTATACTCTGTCGAATGTCATTGAATGTCTTTACATGGGCTT
GTATGCCTATGAAAATTGTAATACAACTTTCAGCAACGGATCTCTTGGCTCTCGCATCGA
TGAAGAACGCAGCGAAATGCGATAAGTAATGTGAATTGCAGAATTCAGTGAATCATCGAA
TCTTTGAACGCATCTTGCGCTCCTTGGTATTCCGAGGAGCATGCCTGTTTGAGTGTCATT
AAATTCTCAACTCTCTTATACTTTTTTGTAAAAGAGAGCTTGGACTGTGGAGGCTTGCTG

GCCACTTTTTGGGGTCAGCTCCTCTGAAATGCATTAGCGGAACCGTTTGCGATCTGCCAC
AAGTGTGATAAGTTATCTACACTGGCGAGGGGATTGCTCTCTGTAATGTTCAGCTTCTAA
TTGTCTCTACTTTGTGAGACTACTTTTGAATGCTTGACCTCAAATCAGGTAGGACTACCC
GCTGAACTTAA
>AB12-57
TTTCCGTAGGTGAACCTGCGGAAGGATCATTATTGAATTATGTTTCTAGATAGGTTGTAG
CTGGCTCTTTTAGAGCATGTGCACGCCTGTTTGGACTTCATTTTCATCCACCTGTGCACC
TATTGTAGTCTTTGGTTGGGTTAGGGGGAAGTGGTCATTGTGTCAGCATCTGCTGGATGT
GAGGACTTGCATTGTGAAAGCTTTGCTGTCCTTGATGTGATCATGGAATCTCTTTCTCAC
TAGAGTCTATGTCACTCATTATACTCTGTCGAATGTCATTGAATGTCTTTACATGGGCTT
GTATGCCTATGAAAATTGTAATACAACTTTCAGCAACGGATCTCTTGGCTCTCGCATCGA
TGAAGAACGCAGCGAAATGCGATAAGTAATGTGAATTGCAGAATTCAGTGAATCATCGAA
TCTTTGAACGCATCTTGCGCTCCTTGGTATTCCGAGGAGCATGCCTGTTTGAGTGTCATT
AAATTCTCAACTCTCTTATACTTTTTTGTAAAAGAGAGCTTGGACTGTGGAGGCTTGCTG
GCCACTTTTTGGGGTCAGCTCCTCTGAAATGCATTAGCGGAACCGTTTGCAATCTGCCAC
AAGTGTGATAAGTTATCTACACTGGCGAGGGGATTGCTCTCTGTAATGTTCAGCTTCTAA
TTGTCTCTACTTTGTGAGACAACTTTTGAATGCTTGACCTCAAATCAGGTAGGACTACCC
GCTGAACTTAA
>AB8-15
TTTCCGTAGGTGAACCTGCGGAAGGATCATTATTGAATTATGTTTCTAGATAGGTTGTAG
CTGGCTCTTTTAGAGCATGTGCACGCCTGTTTGGACTTCATTTTCATCCACCTGTGCACC
TATTGTAGTCTTTGGTTGGGTTAGGGGGAAGTGGTCATTGTGTCAGCATCTGCTGGATGT
GAGGACTTGCATTGTGAAAGCTTTGCTGTCCTTGATGTGATCATGGAATCTCTTTCTCAC
TAGAGTCTATGTCACTCATTATACTCTGTCGAATGTCATTGAATGTCTTTACATGGGCTT
GTATGCCTATGAAAATTGTAATACAACTTTCAGCAACGGATCTCTTGGCTCTCGCATCGA
TGAAGAACGCAGCGAAATGCGATAAGTAATGTGAATTGCAGAATTCAGTGAATCATCGAA
TCTTTGAACGCATCTTGCGCTCCTTGGTATTCCGAGGAGCATGCCTGTTTGAGTGTCATT
AAATTCTCAACTCTCTTATACTTTTTTGTAAAAGAGAGCTTGGACTGTGGAGGCTTGCTG
GCCACTTTTTGGGGTCAGCTCCTCTGAAATGCATTAGCGGAACCGTTTGCGATCTGCCAC
AAGTGTGATAAGTTATCTACACTGGCGAGGGGATTGCTCTCTGTAATGTTCAGCTTCTAA
TTGTCTCTACTTTGTGAGACAACTTTTGAATGCTTGACCTCAAATCAGGTAGGACTACCC
GCTGAACTTAA
>AB10-5
TTTCCGTAGGTGAACCTGCGGAAGGATCATTATTGAATTATGTTTCTAGATAGGTTGTAG
CTGGCTCTTTTAGAGCATGTGCACGCCTGTTTGGACTTCATTTTCATCCACCTGTGCACC
TATTGTAGTCTTTGGTTGGGTTAGGGGGAAGTGGTCATTGTGTCAGCATCTGCTGGATGT
GAGGACTTGCATTGTGAAAGCTTTGCTGTCCTTGATGTGATCATGGAATCTTTTTCTCAC
TAGAGTCTATGTCACTCATTATACTCTGTCGAATGTCATTGAATGTCTTTACATGGGCTT
GTATGCCTATGAAAATTGTAATACAACTTTCAGCAACGGATCTCTTGGCTCTCGCATCGA
TGAAGAACGCAGCGAAATGCGATAAGTAATGTGAATTGCAGAATTCAGTGAATCATCGAA
TCTTTGAACGCATCTTGCGCTCCTTGGTATTCCGAGGAGCATGCCTGTTTGAGTGTCATT
AAATTCTCAACTCTCTTATACTTTTTTGTAAAAGAGAGCTTGGACTGTGGAGGCTTGCTG
GCCACTTTTTGGGGTCAGCTCCTCTGAAATGCATTAGCGGAACCGTTTGCAATCTGCCAC
AAGTGTGATAAGTTATCTACACTGGCGAGGGGATTGCTCTCTGTAATGTTCAGCTTCTAA
TTGTCTCTACTTTGTGAGACAACTTTTGAATGCTTGACCTCAAATCAGGTAGGACTACCC
GCTGAACTTAA
>AB10-29
TTTCCGTAGGTGAACCTGCGGAAGGATCATTATTGAATTATGTTTCTAGATAGGTTGTAG
CTGGCTCTTTTAGAGCATGTGCACGCCTGTTTGGACTTCATTTTCATCCACCTGTGCACC
TATTGTAGTCTTTGGTTGGGTTAGGGGGAAGTGGTCATTGTGTCAGCATCTGCTGGATGT

GAGGACTTGCATTGTGAAAGCTTTGCTGTCCTTGATGTGATCATGGAATCTCTTTCTCAC
TAGAGTCTATGTCACTCATTATACTCTGTCGAATGTCATTGAATGTCTTTACATGGGCTT
GTATGCCTATGAAAATTGTAATACAACTTTCAGCAACGGATCTCTTGGCTCTCGCATCGA
TGAAGGACGCAGCGAAATGCGATAAGTAATGTGAATTGCAGAATTCAGTGAATCATCGAA
TCTTTGAACGCATCTTGCGCTCCTTGGTATTCCGAGGAGCATGCCTGTTTGAGTGTCATT
AAATTCTCAACTCTCTTATACTTTTTTGTAAAAGAGAGCTTGGACTGTGGAGGCTTGCTG
GCCACTTTTTGGGGTCAGCTCCTCTGAAATGCATTAGCGGAACCGTTTGCGATCTGCCAC
AAGTGTGATAAGTTATCTACACTGGCGAGGGGATTGCTCTCTGTAATGTTCAGCTTCTAA
TTGTCTCTACTTTGTGAGACAACTTTTGAATGCTTGACCTCAAATCAGGTAGGACTACCC
GCTGAACTTAA
>AB3-60
TTTCCGTAGGTGAACCTGCGGAAGGATCATTATTGAATTATGTTTCTAGATAGGTTGTAG
CTGGCTCTTTTAGAGCATGTGCACGCCTGTTTGGACTTCATTTTCATCCACCTGTGCACC
TATTGTAGTCTTTGGTTGGGTTAGGGGGAAGTGGTCATTGTGTCAGCATCTGCTGGATGT
GAGGACTTGCATTGTGAAAGCTTTGCTGTCCTTGATGTGATCATGGAATCTCTTTCTCAC
TAGAGTCTATGTCACTCATTATACTCTGTCGAATGTCATTGAATGTCTTTACATGGGCTT
GTATGCCTATGAAAATTGTAATACAACTTTCAGCAACGGATCTCTTGGCTCTCGCATCGA
TGAAGGACGCAGCGAAATGCGATAAGTAATGTGAATTGCAGAATTCAGTGAATCATCGAA
TCTTTGAACGCATCTTGCGCTCCTTGGTATTCCGAGGAGCATGCCTGTTTGAGTGTCATT
AAATTCTCAACTCTCTTATACTTTTTTGTAAAAGAGAGCTTGGACTGTGGAGGCTTGCTG
GCCACTTTTTGGGGTCAGCTCCTCTGAAATGCATTAGCGGAACCGTTTGCAATCTGCCAC
AAGTGTGATAAGTTATCTACACTGGCGAGGGGATTGCTCTCTGTAATGTTCAGCTTCTAA
TTGTCTCTACTTTGTGAGACAACTTTTGAATGCTTGACCTCAAATCAGGTAGGACTACCC
GCTGAACTTAA
>AB11-44
TTTCCGTAGGTGAACCTGCGGAAGGATCATTATTGAATTATGTTTCTAGATAGGTTGTAG
CTGGCTCTTTTAGAGCATGTGCACGCCTGTTTGGACTTCATTTTCATCCACCTGTGCACC
TATTGTAGTCTTTGGTTGGGTTAGGGGGAAGTGGTCATTGTGTCAGCATCTGCTGGATGT
GAGGACTTGCATTGTGAAAGCTTTGCTGTCCTTGATGTGATCATGGAATCTCTTTCTCAC
TAGAGTCTATGTCACTCATTATACTCTGTCGAATGTCATTGAATGTCTTTACATGGGCTT
GTATGCCTATGAAAATTGTAATACAACTTTCAGCAACGGATCTCTTGGCTCTCGCATCGA
TGAAGGACGCAGCGAAATGCGATAAGTAATGTGAATTGCAGAATTCAGTGAATCATCGAA
TCTTTGAACGCATCTTGCGCTCCTTGGTATTCCGAGGAGCATGCCTGTTTGAGTGTCATT
AAATTCTCAACTCTCTTATACTTTTTTGTAAAAGAGAGCTTGGACTGTGGAGGCTTGCTG
GCCACTTTTTGGGGTCAGCTCCTCTGAAATGCATTAGCGGAACCGTTTGCAATCTGCCAC
AAGTGTGATAAGTTATCTACACTGGCGAGGGGATTGCTCTCTGTAATGTTCAGCTTCTAA
TTGTCTCTACTTTGTGAGACAACTTTTGAATGCTTGACCTCAAATCAGGTAGGACTACCC
GCTGAACTTAA
>AB9-14
TTTCCGTAGGTGAACCTGCGGAAGGATCATTATTGAATTATGTTTCTAGATAGGTTGTAG
CTGGCTCTTTTAGAGCATGTGCACGCCTGTTTGGACTTCATTTTCATCCACCTGTGCACC
TATTGTAGTCTTTGGTTGGGTTAGGAGGAAGTGGTCATTGTGTCAGCATCTGCTGGATGT
GAGGACTTGCATTGTGAAAGCTTTGCTGTCCTTGATGTGATCATGGAATCTCTTTCTCAC
TAGAGTCTATGTCACTCATTATACTCTGTCGAATGTCATTGAATGTCTTTACATGGGCTT
GTATGCCTATGAAAATTGTAATACAACTTTCAGCAACGGATCTCTTGGCTCTCGCATCGA
TGAAGGACGCAGCGAAATGCGATAAGTAATGTGAATTGCAGAATTCAGTGAATCATCGAA
TCTTTGAACGCATCTTGCGCTCCTTGGTATTCCGAGGAGCATGCCTGTTTGAGTGTCATT
AAATTCTCAACTCTCTTATACTTTTTTGTAAAAGAGAGCTTGGACTGTGGAGGCTTGCTG
GCCACTTTTTGGGGTCAGCTCCTCTGAAATGCATTAGCGGAACCGTTTGCAATCTGCCAC
AAGTGTGATAAGTTATCTACACTGGCGAGGGGATTGCTCTCTGTAATGTTCAGCTTCTAA

TTGTCTCTACTTTGTGAGACAACTTTTGAATGCTTGACCTCAAATCAGGTAGGACTACCC
GCTGAACTTAA
>AB11-32
TTTCCGTAGGTGAACCTGCGGAAGGATCATTATTGAATTATGTTTCTAGATAGGTTGTAG
CTGGCTCTTTTAGAGCATGTGCACGCCTGTTTGGACTTCATTTTCATCCACCTGTGCACC
TATTGTAGTCTTTGGTTGGGTTAGGGGGAAGTGGTCATTGTGTCAGCATCTGCTGGATGT
GAGGACTTGCATTGTGAAAGCTTTGCTGTCCTTGATGTGATCATGGAATCTCTTTCTCAC
TAGAGTCTATGTCACTCATTATACTCTGTCGAATGTCATTGAATGTCTTTACATGGGCTT
GTATGCCTATGAAAATTGTAATACAACTTTCAGCAACGGATCTCTTGGCTCTCGCATCGA
TGAAGGACGCAGCGAAATGCGATAAGTAATGTGAATTGCAGAATTCAGTGAATCATCGAA
TCTTTGAACGCATCTTGCGCTCCTTGGTATTCCGAGGAGCATGCCTGTTTGAGTGTCATT
AAATTCTCAACTCTCTTATACTTTTTTGTAAAAGAGAGCTTGGACTGTGGAGGCTCGCTG
GCCACTTTTTGGGGTCAGCTCCTCTGAAATGCATTAGCGGAACCGTTTGCAATCTGCCAC
AAGTGTGATAAGTTATCTACACTGGCGAGGGGATTGCTCTCTGTAATGTTCAGCTTCTAA
TTGTCTCTACTTTGTGAGACAACTTTTGAATGCTTGACCTCAAATCAGGTAGGACTACCC
GCTGAACTTAA
>AB11-11
TTTCCGTAGGTGAACCTGCGGAAGGATCATTATTGAATTATGTTTCTAGATAGGTTGTAG
CTGGCTCTTTTAGAGCATGTGCACGCCTGTTTGGACTTCATTTTCATCCACCTGTGCACC
TATTGTAGTCTTTGGTTGGGTTAGGGGGAAGTGGTCATTGTGTCAGCATCTGCTGGGAGT
GAGGACTTGCATTGTGAAAGCTTTGCTGTCCTTGATGTGATCATGGAATCTTTTTCTCAC
TAGAGTCTATGTCACTCATTATACTCTGTCGAATGTCATTGAATGTCTTTACATGGGCTT
GTATGCCTATGAAAATTGTAATACAACTTTCAGCAACGGATCTCTTGGCTCTCGCATCGA
TGAAGAACGCAGCGAAATGCGATAAGTAATGTGAATTGCAGAATTCAGTGAATCATCGAA
TCTTTGAACGCATCTTGCGCTCCTTGGTATTCCGAGGAGCATGCCTGTTTGAGTGTCATT
AAATTCTCAACTCTCTTATACTTTTTTGTAAAAGAGAGCTTGGACTGTGGAGGCTTGCTG
GCCACTTTTTGGGGTCAGCTCCTCTGAAATGCATTAGCGGAACCGTTTGCAATCTGCCAC
AAGTGTGATAAGTTATCTACACTGGCGAGGGGATTGCTCTCTGTAATGTTCAGCTTCTAA
TTGTCTCTACTTTGTGAGACAACTTTTGAATGCTTGACCTCAAATCAGGTAGGACTACCC
GCTGAACTTAA
>AB1-46
TTTCCGTAGGTGAACCTGCGGAAGGATCATTATTGAATTATGTTTCTAGATAGGTTGTAG
CTGGCTC-TTTAGAGCATGTGCACGCCTGTTTGGACTTCATTTTCATCCACCTGTGCACC
TATTGTAGTCTTTGGTTGGGTTAGGGGGAAGTGGTCATTGTGTCAGCATCTGCTGGATGT
GAGGACTTGCATTGTGAAAGCTTTGCTGTCCTTGATGTGATCATGGAATCTCTTTCTCAC
TAGAGTCTATGTCACTCATTATACTCTGTCGAATGTCATTGAATGTCTTTACATGGGCTT
GTATGCCTATGAAAATTGTAATACAACTTTCAGCAACGGATCTCTTGGCTCTCGCATCGA
TGAAGGACGCAGCGAAATGCGATAAGTAATGTGAATTGCAGAATTCAGTGAATCATCGAA
TCTTTGAACGCATCTTGCGCTCCTTGGTATTCCGAGGAGCATGCCTGTTTGAGTGTCATT
AAATTCTCAACTCTCTTATACTTTTTTGTAAAAGAGAGCTTGGACTGTGGAGGCTTGCTG
GCCACTTTTTGGGGTCAGCTCCTCTGAAATGCATTAGCGGAACCGTTTGCAATCTGCCAC
AAGTGTGATAAGTTATCTACACTGGCGAGGGGATTGCTCTCTGTAATGTTCAGCTTCTAA
TTGTCTCTACTTTGTGAGACAACTTTTGAATGCTTGACCTCAAATCAGGTAGGACTACCC
GCTGAACTTAA
>AB3-37
TTTCCGTAGGTGAACCTGCGGAAGGATCATTATTGAATTATGTTTCTAGATAGGTTGTAG
CTGGCTC-TTTAGAGCATGTGCACGCCTGTTTGGACTTCATTTTCATCCACCTGTGCACC
TATTGTAGTCTTTGGTTGGGTTAGGGGGAAGTGGTCATTGTGTCAGCATCTGCTGGATGT
GAGGACTTGCATTGTGAAAGCTTTGCTGTCCTTGATGTGATCATGGAATCTCTTTCTCAC
TAGAGTCTATGTCACTCATTATACTCTGTCGAATGTCATTGAATGTCTTTACATGGGCTT

GTATGCCTATGAAAATTGTAATACAACTTTCAGCAACGGATCTCTTGGCTCTCGCATCGA
TGAAGGACGCAGCGAAATGCGATAAGTAATGTGAATTGCAGAATTCAGTGAATCATCGAA
TCTTTGAACGCATCTTGCGCTCCTTGGTATTCCGAGGAGCATGCCTGTTTGAGTGTCATT
AAATTCTCAACTCTCTTATACTTTTTTGTAAAAGAGAGCTTGGACTGTGGAGGCTTGCTG
GCCACTTTTTGGGGTCAGCTCCTCTGAAATGCATTAGCGGAACCGTTTGCAATCTGCCAC
AAGTGTGATAAGTTATCTACACTGGCGAGGGGATTGCTCTCTGTAATGTTCAGCTTCTAA
TTGTCTCTACTTTGTGAGACAACTTTTGAATGCTTGACCTCAAATCAGGTAGGACTACCC
GCTGAACTTAA
>AB8-2
TTTCCGTAGGTGAACCTGCGGAAGGATCATTATTGAATTATGTTTCTAGATAGGTTGTAG
CTGGCTC-TTTAGAGCATGTGCACGCCTGTTTGGACTTCATTTTCATCCACCTGTGCACC
TATTGTAGTCTTTGGTTGGGTTAGGGGGAAGTGGTCATTGTGTCAGCATCTGCTGGATGT
GAGGACTTGCATTGTGAAAGCTTTGCTGTCCTTGATGTGATCATGGAATCTCTTTCTCAC
TAGAGTCTATGTCACTCATTATACTCTGTCGAATGTCATTGAATGTCTTTACATGGGCTT
GTATGCCTATGAAAATTGTAATACAACTTTCAGCAACGGATCTCTTGGCTCTCGCATCGA
TGAAGGACGCAGCGAAATGCGATAAGTAATGTGAATTGCAGAATTCAGTGAATCATCGAA
TCTTTGAACGCATCTTGCGCTCCTTGGTATTCCGAGGAGCATGCCTGTTTGAGTGTCATT
AAATTCTCAACTCTCTTATACTTTTTTGTAAAAGAGAGCTTGGACTGTGGAGGCTTGCTG
GCCACTTTTTGGGGTCAGCTCCTCTGAAATGCATTAGCGGAACCGTTTGCAATCTGCCAC
AAGTGTGATAAGTTATCTACACTGGCGAGGGGATTGCTCTCTGTAATGTTCAGCTTCTAA
TTGTCTCTACTTTGTGAGACAACTTTTGAATGCTTGACCTCAAATCAGGTAGGACTACCC
GCTGAACTTAA
>AB8-54
TTTCCGTAGGTGAACCTGCGGAAGGATCATTATTGAATTATGTTTCTAGATAGGTTGTAG
CTGGCTC-TTTAGAGCATGTGCACGCCTGTTTGGACTTCATTTTCATCCACCTGTGCACC
TATTGTAGTCTTTGGTTGGGTTAGGGGGAAGTGGTCATTGTGTCAGCATCTGCTGGATGT
GAGGACTTGCATTGTGAAAGCTTTGCTGTCCTTGATGTGATCATGGAATCTCTTTCTCAC
TAGAGTCTATGTCACTCATTATACTCTGTCGAATGTCATTGAATGTCTTTACATGGGCTT
GTATGCCTATGAAAATTGTAATACAACTTTCAGCAACGGATCTCTTGGCTCTCGCATCGA
TGAAGGACGCAGCGAAATGCGATAAGTAATGTGAATTGCAGAATTCAGTGAATCATCGAA
TCTTTGAACGCATCTTGCGCTCCTTGGTATTCCGAGGAGCATGCCTGTTTGAGTGTCATT
AAATTCTCAACTCTCTTATACTTTTTTGTAAAAGAGAGCTTGGACTGTGGAGGCTTGCTG
GCCACTTTTTGGGGTCAGCTCCTCTGAAATGCATTAGCGGAACCGTTTGCAATCTGCCAC
AAGTGTGATAAGTTATCTACACTGGCGAGGGGATTGCTCTCTGTAATGTTCAGCTTCTAA
TTGTCTCTACTTTGTGAGACAACTTTTGAATGCTTGACCTCAAATCAGGTAGGACTACCC
GCTGAACTTAA
>AB11-19
TTTCCGTAGGTGAACCTGCGGAAGGATCATTATTGAATTATGTTTCTAGATAGGTTGTAG
CTGGCTC-TTTAGAGCATGTGCACGCCTGTTTGGACTTCATTTTCATCCACCTGTGCACC
TATTGTAGTCTTTGGTTGGGTTAGGGGGAAGTGGTCATTGTGTCAGCATCTGCTGGATGT
GAGGACTTGCATTGTGAAAGCTTTGCTGTCCTTGATGTGATCATGGAATCTCTTTCTCAC
TAGAGTCTATGTCACTCATTATACTCTGTCGAATGTCATTGAATGTCTTTACATGGGCTT
GTATGCCTATGAAAATTGTAATACAACTTTCAGCAACGGATCTCTTGGCTCTCGCATCGA
TGAAGGACGCAGCGAAATGCGATAAGTAATGTGAATTGCAGAATTCAGTGAATCATCGAA
TCTTTGAACGCATCTTGCGCTCCTTGGTATTCCGAGGAGCATGCCTGTTTGAGTGTCATT
AAATTCTCAACTCTCTTATACTTTTTTGTAAAAGAGAGCTTGGACTGTGGAGGCTTGCTG
GCCACTTTTTGGGGTCAGCTCCTCTGAAATGCATTAGCGGAACCGTTTGCAATCTGCCAC
AAGTGTGATAAGTTATCTACACTGGCGAGGGGATTGCTCTCTGTAATGTTCAGCTTCTAA
TTGTCTCTACTTTGTGAGACAACTTTTGAATGCTTGACCTCAAATCAGGTAGGACTACCC
GCTGAACTTAA

>AB11-26
TTTCCGTAGGTGAACCTGCGGAAGGATCATTATTGAATTATGTTTCTAGATAGGTTGTAG
CTGGCTC-TTTAGAGCATGTGCACGCCTGTTTGGACTTCATTTTCATCCACCTGTGCACC
TATTGTAGTCTTTGGTTGGGTTAGGGGGAAGTGGTCATTGTGTCAGCATCTGCTGGATGT
GAGGACTTGCATTGTGAAAGCTTTGCTGTCCTTGATGTGATCATGGAATCTCTTTCTCAC
TAGAGTCTATGTCACTCATTATACTCTGTCGAATGTCATTGAATGTCTTTACATGGGCTT
GTATGCCTATGAAAATTGTAATACAACTTTCAGCAACGGATCTCTTGGCTCTCGCATCGA
TGAAGGACGCAGCGAAATGCGATAAGTAATGTGAATTGCAGAATTCAGTGAATCATCGAA
TCTTTGAACGCATCTTGCGCTCCTTGGTATTCCGAGGAGCATGCCTGTTTGAGTGTCATT
AAATTCTCAACTCTCTTATACTTTTTTGTAAAAGAGAGCTTGGACTGTGGAGGCTTGCTG
GCCACTTTTTGGGGTCAGCTCCTCTGAAATGCATTAGCGGAACCGTTTGCAATCTGCCAC
AAGTGTGATAAGTTATCTACACTGGCGAGGGGATTGCTCTCTGTAATGTTCAGCTTCTAA
TTGTCTCTACTTTGTGAGACAACTTTTGAATGCTTGACCTCAAATCAGGTAGGACTACCC
GCTGAACTTAA
>AB11-53
TTTCCGTAGGTGAACCTGCGGAAGGATCATTATTGAATTATGTTTCTAGATAGGTTGTAG
CTGGCTC-TTTAGAGCATGTGCACGCCTGTTTGGACTTCATTTTCATCCACCTGTGCACC
TATTGTAGTCTTTGGTTGGGTTAGGGGGAAGTGGTCATTGTGTCAGCATCTGCTGGATGT
GAGGACTTGCATTGTGAAAGCTTTGCTGTCCTTGATGTGATCATGGAATCTCTTTCTCAC
TAGAGTCTATGTCACTCATTATACTCTGTCGAATGTCATTGAATGTCTTTACATGGGCTT
GTATGCCTATGAAAATTGTAATACAACTTTCAGCAACGGATCTCTTGGCTCTCGCATCGA
TGAAGGACGCAGCGAAATGCGATAAGTAATGTGAATTGCAGAATTCAGTGAATCATCGAA
TCTTTGAACGCATCTTGCGCTCCTTGGTATTCCGAGGAGCATGCCTGTTTGAGTGTCATT
AAATTCTCAACTCTCTTATACTTTTTTGTAAAAGAGAGCTTGGACTGTGGAGGCTTGCTG
GCCACTTTTTGGGGTCAGCTCCTCTGAAATGCATTAGCGGAACCGTTTGCAATCTGCCAC
AAGTGTGATAAGTTATCTACACTGGCGAGGGGATTGCTCTCTGTAATGTTCAGCTTCTAA
TTGTCTCTACTTTGTGAGACAACTTTTGAATGCTTGACCTCAAATCAGGTAGGACTACCC
GCTGAACTTAA
>AB6-2
TTTCCGTAGGTGAACCTGCGGAAGGATCATTATTGAATTATGTTTCTAGATAGGTTGTAG
CTGGCTC-TTTAGAGCATGTGCACGCCTGTTTGGACTTCATTTTCATCCACCTGTGCACC
TATTGTAGTCTTTGGTTGGGTTAGGGGGAAGTGGTCATTGTGTCAGCATCTGCTGGATGT
GAGGACTTGCATTGTGAAAGCTTTGCTGTCCTTGATGTGATCATGGAATCTCTTTCTCAC
TAGAGTCTATGTCACTCATTATACTCTGTCGAATGTCATTGAATGTCTTTACATGGGCTT
GTATGCCTATGAAAATTGTAATACAACTTTCAGCAACGGATCTCTTGGCTCTCGCATCGA
TGAAGGACGCAGCGAAATGCGATAAGTAATGTGAATTGCAGAATTCAGTGAATCATCGAA
TCTTTGAACGCATCTTGCGCTCCTTGGTATTCCGAGGAGCATGCCTGTTTGAGTGTCATT
AAATTCTCAACTCTCTTATACTTTTTTGTAAAAGAGAGCTTGGACTGTGGAGGCTTGCTG
GCCACTTTTTGGGGTCAGCTCCTCTGAAATGCATTAGCGGAACCGTTTGCAATCTGCCAC
AAGTGTGATAAGTTATCTACACTGGCGAGGGGATTGCTCTCTGTAATGTTCAGCTTCTAA
TTGTCTCTACTTTGTGAGACAACTTTTGAATGCTTGACCTCAAATCAGGTAGGACTACCC
GCTGAACTTAA
>AB6-26
TTTCCGTAGGTGAACCTGCGGAAGGATCATTATTGAATTATGTTTCTAGATAGGTTGTAG
CTGGCTC-TTTAGAGCATGTGCACGCCTGTTTGGACTTCATTTTCATCCACCTGTGCACC
TATTGTAGTCTTTGGTTGGGTTAGGGGGAAGTGGTCATTGTGTCAGCATCTGCTGGATGT
GAGGACTTGCATTGTGAAAGCTTTGCTGTCCTTGATGTGATCATGGAATCTCTTTCTCAC
TAGAGTCTATGTCACTCATTATACTCTGTCGAATGTCATTGAATGTCTTTACATGGGCTT
GTATGCCTATGAAAATTGTAATACAACTTTCAGCAACGGATCTCTTGGCTCTCGCATCGA
TGAAGGACGCAGCGAAATGCGATAAGTAATGTGAATTGCAGAATTCAGTGAATCATCGAA

TCTTTGAACGCATCTTGCGCTCCTTGGTATTCCGAGGAGCATGCCTGTTTGAGTGTCATT
AAATTCTCAACTCTCTTATACTTTTTTGTAAAAGAGAGCTTGGACTGTGGAGGCTTGCTG
GCCACTTTTTGGGGTCAGCTCCTCTGAAATGCATTAGCGGAACCGTTTGCAATCTGCCAC
AAGTGTGATAAGTTATCTACACTGGCGAGGGGATTGCTCTCTGTAATGTTCAGCTTCTAA
TTGTCTCTACTTTGTGAGACAACTTTTGAATGCTTGACCTCAAATCAGGTAGGACTACCC
GCTGAACTTAA
>AB7-41
TTTCCGTAGGTGAACCTGCGGAAGGATCATTATTGAATTATGTTTCTAGATAGGTTGTAG
CTGGCTC-TTTAGAGCATGTGCACGCCTGTTTGGACTTCATTTTCATCCACCTGTGCACC
TATTGTAGTCTTTGGTTGGGTTAGGGGGAAGTGGTCATTGTGTCAGCATCTGCTGGATGT
GAGGACTTGCATTGTGAAAGCTTTGCTGTCCTTGATGTGATCATGGAATCTCTTTCTCAC
TAGAGTCTATGTCACTCATTATACTCTGTCGAATGTCATTGAATGTCTTTACATGGGCTT
GTATGCCTATGAAAATTGTAATACAACTTTCAGCAACGGATCTCTTGGCTCTCGCATCGA
TGAAGGACGCAGCGAAATGCGATAAGTAATGTGAATTGCAGAATTCAGTGAATCATCGAA
TCTTTGAACGCATCTTGCGCTCCTTGGTATTCCGAGGAGCATGCCTGTTTGAGTGTCATT
AAATTCTCAACTCTCTTATACTTTTTTGTAAAAGAGAGCTTGGACTGTGGAGGCTTGCTG
GCCACTTTTTGGGGTCAGCTCCTCTGAAATGCATTAGCGGAACCGTTTGCAATCTGCCAC
AAGTGTGATAAGTTATCTACACTGGCGAGGGGATTGCTCTCTGTAATGTTCAGCTTCTAA
TTGTCTCTACTTTGTGAGACAACTTTTGAATGCTTGACCTCAAATCAGGTAGGACTACCC
GCTGAACTTAA
>AB9-21
TTTCCGTAGGTGAACCTGCGGAAGGATCATTATTGAATTATGTTTCTAGATAGGTTGTAG
CTGGCTC-TTTAGAGCATGTGCACGCCTGTTTGGACTTCATTTTCATCCACCTGTGCACC
TATTGTAGTCTTTGGTTGGGTTAGGGGGAAGTGGTCATTGTGTCAGCATCTGCTGGATGT
GAGGACTTGCATTGTGAAAGCTTTGCTGTCCTTGATGTGATCATGGAATCTCTTTCTCAC
TAGAGTCTATGTCACTCATTATACTCTGTCGAATGTCATTGAATGTCTTTACATGGGCTT
GTATGCCTATGAAAATTGTAATACAACTTTCAGCAACGGATCTCTTGGCTCTCGCATCGA
TGAAGGACGCAGCGAAATGCGATAAGTAATGTGAATTGCAGAATTCAGTGAATCATCGAA
TCTTTGAACGCATCTTGCGCTCCTTGGTATTCCGAGGAGCATGCCTGTTTGAGTGTCATT
AAATTCTCAACTCTCTTATACTTTTTTGTAAAAGAGAGCTTGGACTGTGGAGGCTTGCTG
GCCACTTTTTGGGGTCAGCTCCTCTGAAATGCATTAGCGGAACCGTTTGCAATCTGCCAC
AAGTGTGATAAGTTATCTACACTGGCGAGGGGATTGCTCTCTGTAATGTTCAGCTTCTAA
TTGTCTCTACTTTGTGAGACAACTTTTGAATGCTTGACCTCAAATCAGGTAGGACTACCC
GCTGAACTTAA
>AB10-28
TTTCCGTAGGTGAACCTGCGGAAGGATCATTATTGAATTATGTTTCTAGATAGGTTGTAG
CTGGCTC-TTTAGAGCATGTGCACGCCTGTTTGGACTTCATTTTCATCCACCTGTGCACC
TATTGTAGTCTTTGGTTGGGTTAGGGGGAAGTGGTCATTGTGTCAGCATCTGCTGGATGT
GAGGACTTGCATTGTGAAAGCTTTGCTGTCCTTGATGTGATCATGGAATCTCTTTCTCAC
TAGAGTCTATGTCACTCATTATACTCTGTCGAATGTCATTGAATGTCTTTACATGGGCTT
GTATGCCTATGAAAATTGTAATACAACTTTCAGCAACGGATCTCTTGGCTCTCGCATCGA
TGAAGGACGCAGCGAAATGCGATAAGTAATGTGAATTGCAGAATTCAGTGAATCATCGAA
TCTTTGAACGCATCTTGCGCTCCTTGGTATTCCGAGGAGCATGCCTGTTTGAGTGTCATT
AAATTCTCAACTCTCTTATACTTTTTTGTAAAAGAGAGCTTGGACTGTGGAGGCTTGCTG
GCCACTTTTTGGGGTCAGCTCCTCTGAAATGCATTAGCGGAACCGTTTGCAATCTGCCAC
AAGTGTGATAAGTTATCTACACTGGCGAGGGGATTGCTCTCTGTAATGTTCAGCTTCTAA
TTGTCTCTACTTTGTGAGACAACTTTTGAATGCTTGACCTCAAATCAGGTAGGACTACCC
GCTGAACTTAA
>AB10-39
TTTCCGTAGGTGAACCTGCGGAAGGATCATTATTGAATTATGTTTCTAGATAGGTTGTAG

CTGGCTC-TTTAGAGCATGTGCACGCCTGTTTGGACTTCATTTTCATCCACCTGTGCACC
TATTGTAGTCTTTGGTTGGGTTAGGGGGAAGTGGTCATTGTGTCAGCATCTGCTGGATGT
GAGGACTTGCATTGTGAAAGCTTTGCTGTCCTTGATGTGATCATGGAATCTCTTTCTCAC
TAGAGTCTATGTCACTCATTATACTCTGTCGAATGTCATTGAATGTCTTTACATGGGCTT
GTATGCCTATGAAAATTGTAATACAACTTTCAGCAACGGATCTCTTGGCTCTCGCATCGA
TGAAGGACGCAGCGAAATGCGATAAGTAATGTGAATTGCAGAATTCAGTGAATCATCGAA
TCTTTGAACGCATCTTGCGCTCCTTGGTATTCCGAGGAGCATGCCTGTTTGAGTGTCATT
AAATTCTCAACTCTCTTATACTTTTTTGTAAAAGAGAGCTTGGACTGTGGAGGCTTGCTG
GCCACTTTTTGGGGTCAGCTCCTCTGAAATGCATTAGCGGAACCGTTTGCAATCTGCCAC
AAGTGTGATAAGTTATCTACACTGGCGAGGGGATTGCTCTCTGTAATGTTCAGCTTCTAA
TTGTCTCTACTTTGTGAGACAACTTTTGAATGCTTGACCTCAAATCAGGTAGGACTACCC
GCTGAACTTAA
>AB11-10
TTTCCGTAGGTGAACCTGCGGAAGGATCATTATTGAATTATGTTTCTAGATAGGTTGTAG
CTGGCTC-TTTAGAGCATGTGCACGCCTGTTTGGACTTCATTTTCATCCACCTGTGCACC
TATTGTAGTCTTTGGTTGGGTTAGGGGGAAGTGGTCATTGTGTCAGCATCTGCTGGATGT
GAGGACTTGCATTGTGAAAGCTTTGCTGTCCTTGATGTGATCATGGAATCTCTTTCTCAC
TAGAGTCTATGTCACTCATTATACTCTGTCGAATGTCATTGAATGTCTTTACATGGGCTT
GTATGCCTATGAAAATTGTAATACAACTTTCAGCAACGGATCTCTTGGCTCTCGCATCGA
TGAAGGACGCAGCGAAATGCGATAAGTAATGTGAATTGCAGAATTCAGTGAATCATCGAA
TCTTTGAACGCATCTTGCGCTCCTTGGTATTCCGAGGAGCATGCCTGTTTGAGTGTCATT
AAATTCTCAACTCTCTTATACTTTTTTGTAAAAGAGAGCTTGGACTGTGGAGGCTTGCTG
GCCACTTTTTGGGGTCAGCTCCTCTGAAATGCATTAGCGGAACCGTTTGCAATCTGCCAC
AAGTGTGATAAGTTATCTACACTGGCGAGGGGATTGCTCTCTGTAATGTTCAGCTTCTAA
TTGTCTCTACTTTGTGAGACAACTTTTGAATGCTTGACCTCAAATCAGGTAGGACTACCC
GCTGAACTTAA
>AB12-22
TTTCCGTAGGTGAACCTGCGGAAGGATCATTATTGAATTATGTTTCTAGATAGGTTGTAG
CTGGCTC-TTTAGAGCATGTGCACGCCTGTTTGGACTTCATTTTCATCCACCTGTGCACC
TATTGTAGTCTTTGGTTGGGTTAGGGGGAAGTGGTCATTGTGTCAGCATCTGCTGGATGT
GAGGACTTGCATTGTGAAAGCTTTGCTGTCCTTGATGTGATCATGGAATCTCTTTCTCAC
TAGAGTCTATGTCACTCATTATACTCTGTCGAATGTCATTGAATGTCTTTACATGGGCTT
GTATGCCTATGAAAATTGTAATACAACTTTCAGCAACGGATCTCTTGGCTCTCGCATCGA
TGAAGGACGCAGCGAAATGCGATAAGTAATGTGAATTGCAGAATTCAGTGAATCATCGAA
TCTTTGAACGCATCTTGCGCTCCTTGGTATTCCGAGGAGCATGCCTGTTTGAGTGTCATT
AAATTCTCAACTCTCTTATACTTTTTTGTAAAAGAGAGCTTGGACTGTGGAGGCTTGCTG
GCCACTTTTTGGGGTCAGCTCCTCTGAAATGCATTAGCGGAACCGTTTGCAATCTGCCAC
AAGTGTGATAAGTTATCTACACTGGCGAGGGGATTGCTCTCTGTAATGTTCAGCTTCTAA
TTGTCTCTACTTTGTGAGACAACTTTTGAATGCTTGACCTCAAATCAGGTAGGACTACCC
GCTGAACTTAA
>AB12-54
TTTCCGTAGGTGAACCTGCGGAAGGATCATTATTGAATTATGTTTCTAGATAGGTTGTAG
CTGGCTC-TTTAGAGCATGTGCACGCCTGTTTGGACTTCATTTTCATCCACCTGTGCACC
TATTGTAGTCTTTGGTTGGGTTAGGGGGAAGTGGTCATTGTGTCAGCATCTGCTGGATGT
GAGGACTTGCATTGTGAAAGCTTTGCTGTCCTTGATGTGATCATGGAATCTCTTTCTCAC
TAGAGTCTATGTCACTCATTATACTCTGTCGAATGTCATTGAATGTCTTTACATGGGCTT
GTATGCCTATGAAAATTGTAATACAACTTTCAGCAACGGATCTCTTGGCTCTCGCATCGA
TGAAGGACGCAGCGAAATGCGATAAGTAATGTGAATTGCAGAATTCAGTGAATCATCGAA
TCTTTGAACGCATCTTGCGCTCCTTGGTATTCCGAGGAGCATGCCTGTTTGAGTGTCATT
AAATTCTCAACTCTCTTATACTTTTTTGTAAAAGAGAGCTTGGACTGTGGAGGCTTGCTG

GCCACTTTTTGGGGTCAGCTCCTCTGAAATGCATTAGCGGAACCGTTTGCAATCTGCCAC
AAGTGTGATAAGTTATCTACACTGGCGAGGGGATTGCTCTCTGTAATGTTCAGCTTCTAA
TTGTCTCTACTTTGTGAGACAACTTTTGAATGCTTGACCTCAAATCAGGTAGGACTACCC
GCTGAACTTAA
>AB6-11
TTTCCGTAGGTGAACCTGCGGAAGGATCATTATTGAATTATGTTTCTAGATAGGTTGTAG
CTGGCTC-TTTAGAGCATGTGCACGCCTGTTTGGACTTCATTTTCATCCACCTGTGCACC
TATTGTAGTCTTTGGTTGGGTTAGGGGGAAGTGGTCATTGTGTCAGCATCTGCTGGATGT
GAGGACTTGCATTGTGAAAGCTTTGCTGTCCTTGATGTGATCATGGAATCTCTTTCTCAC
TAGAGTCTATGTCACTCATTATACTCTGTCGAATGTCATTGAATGTCTTTACATGGGCTT
GTATGCCTATGAAAATTGTAATACAACTTTCAGCAACGGATCTCTTGGCTCTCGCATCGA
TGAAGGACGCAGCGAAATGCGATAAGTAATGTGAATTGCAGAATTCAGTGAATCATCGAA
TCTTTGAACGCATCTTGCGCTCCTTGGTATTCCGAGGAGCATGCCTGTTTGAGTGTCATT
AAATTCTCAACTCTCTTATACTTTTTTGTAAAAGAGAGCTTGGACTGTGGAGGCTTGCTG
GCCACTTTTTGGGGTCAGCTCCTCTGAAATGCATTAGCGGAACCGTTTGCAATCTGCCAC
AAGTGTGATAAGTTATCTACACTGGCGAGGGGATTGCTCTCTGTAATGTTCAGCTTCTAA
TTGTCTCTACTTTGTGAGACAACTTTTGAATGCTTGACCTCAAATCAGGTAGGACTACCC
GCTGAACTTAA
>AB12-7
TTTCCGTAGGTGAACCTGCGGAAGGATCATTATTGAATTATGTTTCTAGATAGGTTGTAG
CTGGCTC-TTTAGAGCATGTGCACGCCTGTTTGGACTTCATTTTCATCCACCTGTGCACC
TATTGTAGTCTTTGGTTGGGTTAGGGGGAAGTGGTCATTGTGTCAGCATCTGCTGGATGT
GAGGACTTGCATTGTGAAAGCTTTGCTGTCCTTGATGTGATCATGGAATCTCTTTCTCAC
TAGAGTCTATGTCACTCATTATACTCTGTCGAATGTCATTGAATGTCTTTACATGGGCTT
GTATGCCTATGAAAATTGTAATACAACTTTCAGCAACGGATCTCTTGGCTCTCGCATCGA
TGAAGGACGCAGCGAAATGCGATAAGTAATGTGAATTGCAGAATTCAGTGAATCATCGAA
TCTTTGAACGCATCTTGCGCTCCTTGGTATTCCGAGGAGCATGCCTGTTTGAGTGTCATT
AAATTCTCAACTCTCTTATACTTTTTTGTAAAAGAGAGCTTGGACTGTGGAGGCTTGCTG
GCCACTTTTTGGGGTCAGCTCCTCTGAAATGCATTAGCGGAACCGTTTGCAATCTGCCAC
AAGTGTGATAAGTTATCTACACTGGCGAGGGGATTGCTCTCTGTAATGTTCAGCTTCTAA
TTGTCTCTACTTTGTGAGACAACTTTTGAATGCTTGACCTCAAATCAGGTAGGACTACCC
GCTGAACTTAA
>AB12-50
TTTCCGTAGGTGAACCTGCGGAAGGATCATTATTGAATTATGTTTCTAGATAGGTTGTAG
CTGGCTC-TTTAGAGCATGTGCACGCCTGTTTGGACTTCATTTTCATCCACCTGTGCACC
TATTGTAGTCTTTGGTTGGGTTAGGGGGAAGTGGTCATTGTGTCAGCATCTGCTGGATGT
GAGGACTTGCATTGTGAAAGCTTTGCTGTCCTTGATGTGATCATGGAATCTCTTTCTCAC
TAGAGTCTATGTCACTCATTATACTCTGTCGAATGTCATTGAATGTCTTTACATGGGCTT
GTATGCCTATGAAAATTGTAATACAACTTTCAGCAACGGATCTCTTGGCTCTCGCATCGA
TGAAGGACGCAGCGAAATGCGATAAGTAATGTGAATTGCAGAATTCAGTGAATCATCGAA
TCTTTGAACGCATCTTGCGCTCCTTGGTATTCCGAGGAGCATGCCTGTTTGAGTGTCATT
AAATTCTCAACTCTCTTATACTTTTTTGTAAAAGAGAGCTTGGACTGTGGAGGCTTGCTG
GCCACTTTTTGGGGTCAGCTCCTCTGAAATGCATTAGCGGAACCGTTTGCAATCTGCCAC
AAGTGTGATAAGTTATCTACACTGGCGAGGGGATTGCTCTCTGTAATGTTCAGCTTCTAA
TTGTCTCTACTTTGTGAGACAACTTTTGAATGCTTGACCTCAAATCAGGTAGGACTACCC
GCTGAACTTAA
>AB11-7
TTTCCGTAGGTGAACCTGCGGAAGGATCATTATTGAATTATGTTTCTAGATAGGTTGTAG
CTGGCTC-TTTAGAGCATGTGCACGCCTGTTTGGACTTCATTTTCATCCACCTGTGCACC
TATTGTAGTCTTTGGTTGGGTTAGGGGGAAGTGGTCATTGTGTCAGCATCTGCTGGATGT

GAGGACTTGCATTGTGAAAGCTTTGCTGTCCTTGATGTGATCATGGAATCTCTTTCTCAC
TAGAGTCTATGTCACTCATTATACTCTGTCGAATGTCATTGAATGTCTTTACATGGGCTT
GTATGCCTATGAAAATTGTAATACAACTTTCAGCAACGGATCTCTTGGCTCTCGCATCGA
TGAAGGACGCAGCGAAATGCGATAAGTAATGTGAATTGCAGAATTCAGTGAATCATCGAA
TCTTTGAACGCATCTTGCGCTCCTTGGTATTCCGAGGAGCATGCCTGTTTGAGTGTCATT
AAATTCTCAACTCTCTTATACTTTTTTGTAAAAGAGAGCTTGGACTGTGGAGGCTTGCTG
GCCACTTTTTGGGGTCAGCTCCTCTGAAATGCATTAGCGGAACCGTTTGCAATCTGCCAC
AAGTGTGATAAGTTATCTACACTGGCGAGGGGATTGCTCTCTGTAATGTTCAGCTTCTAA
TTGTCTCTACTTTGTGAGACAACTTTTGAATGCTTGACCTCAAATCAGGTAGGACTACCC
GCTGAACTTAA
>AB3–3
TTTCCGTAGGTGAACCTGCGGAAGGATCATTATTGAATTATGTTTCTAGATAGGTTGTAG
CTGGCTC–TTTAGAGCATGTGCACGCCTGTTTGGACTTCATTTTCATCCACCTGTGCACC
TATTGTAGTCTTTGGTTGGGTTAGGGGGAAGTGGTCATTGTGTCAGCATCTGCTGGATGT
GAGGACTTGCATTGTGAAAGCTTTGCTGTCCTTGATGTGATCATGGAATCTCTTTCTCAC
TAGAGTCTATGTCACTCATTATACTCTGTCGAATGTCATTGAATGTCTTTACATGGGCTT
GTATGCCTATGAAAATTGTAATACAACTTTCAGCAACGGATCTCTTGGCTCTCGCATCGA
TGAAGGACGCAGCGAAATGCGATAAGTAATGTGAATTGCAGAATTCAGTGAATCATCGAA
TCTTTGAACGCATCTTGCGCTCCTTGGTATTCCGAGGAGCATGCCTGTTTGAGTGTCATT
AAATTCTCAACTCTCTTATACTTTTTTGTAAAAGAGAGCTTGGACTGTGGAGGCTTGCTG
GCCACTTTTTGGGGTCAGCTCCTCTGAAATGCATTAGCGGAACCGTTTGCAATCTGCCAC
AAGTGTGATAAGTTATCTACACTGGCGAGGGGATTGCTCTCTGTAATGTTCAGCTTCTAA
TTGTCTCTACTTTGTGAGACAACTTTTGAATGCTTGACCTCAAATCAGGTAGGACTACCC
GCTGAACTTAA
>AB12–19
TTTCCGTAGGTGAACCTGCGGAAGGATCATTATTGAATTATGTTTCTAGATAGGTTGTAG
CTGGCTC–TTTAGAGCATGTGCACGCCTGTTTGGACTTCATTTTCATCCACCTGTGCACC
TATTGTAGTCTTTGGTTGGGTTAGGGGGAAGTGGTCATTGTGTCAGCATCTGCTGGATGT
GAGGACTTGCATTGTGAAAGCTTTGCTGTCCTTGATGTGATCATGGAATCTCTTTCTCAC
TAGAGTCTATGTCACTCATTATACTCTGTCGAATGTCATTGAATGTCTTTACATGGGCTT
GTATGCCTATGAAAATTGTAATACAACTTTCAGCAACGGATCTCTTGGCTCTCGCATCGA
TGAAGGACGCAGCGAAATGCGATAAGTAATGTGAATTGCAGAATTCAGTGAATCATCGAA
TCTTTGAACGCATCTTGCGCTCCTTGGTATTCCGAGGAGCATGCCTGTTTGAGTGTCATT
AAATTCTCAACTCTCTTATACTTTTTTGTAAAAGAGAGCTTGGACTGTGGAGGCTTGCTG
GCCACTTTTTGGGGTCAGCTCCTCTGAAATGCATTAGCGGAACCGTTTGCAATCTGCCAC
AAGTGTGATAAGTTATCTACACTGGCGAGGGGATTGCTCTCTGTAATGTTCAGCTTCTAA
TTGTCTCTACTTTGTGAGACAACTTTTGAATGCTTGACCTCAAATCAGGTAGGACTACCC
GCTGAACTTAA
>AB6–13
TTTCCGTAGGTGAACCTGCGGAAGGATCATTATTGAATTATGTTTCTAGATAGGTTGTAG
CTGGCTC–TTTAGAGCATGTGCACGCCTGTTTGGACTTCATTTTCATCCACCTGTGCACC
TATTGTAGTCTTTGGTTGGGTTAGGGGGAAGTGGTCATTGTGTCAGCATCTGCTGGATGT
GAGGACTTGCATTGTGAAAGCTTTGCTGTCCTTGATGTGATCATGGAATCTCTTTCTCAC
TAGAGTCTATGTCACTCATTATACTCTGTCGAATGTCATTGAATGTCTTTACATGGGCTT
GTATGCCTATGAAAATTGTAATACAACTTTCAGCAACGGATCTCTTGGCTCTCGCATCGA
TGAAGGACGCAGCGAAATGCGATAAGTAATGTGAATTGCAGAATTCAGTGAATCATCGAA
TCTTTGAACGCATCTTGCGCTCCTTGGTATTCCGAGGAGCATGCCTGTTTGAGTGTCATT
AAATTCTCAACTCTCTTATACTTTTTTGTAAAAGAGAGCTTGGACTGTGGAGGCTTGCTG
GCCACTTTTTGGGGTCAGCTCCTCTGAAATGCATTAGCGGAACCGTTTGCAATCTGCCAC
AAGTGTGATAAGTTATCTACACTGGCGAGGGGATTGCTCTCTGTAATGTTCAGCTTCTAA

TTGTCTCTACTTTGTGAGACAACTTTTGAATGCTTGACCTCAAATCAGGTAGGACTACCC
GCTGAACTTAA
>AB8-17
TTTCCGTAGGTGAACCTGCGGAAGGATCATTATTGAATTATGTTTCTAGATAGGTTGTAG
CTGGCTC-TTTAGAGCATGTGCACGCCTGTTTGGACTTCATTTTCATCCACCTGTGCACC
TATTGTAGTCTTTGGTTGGGTTAGGGGGAAGTGGTCATTGTGTCAGCATCTGCTGGATGT
GAGGACTTGCATTGTGAAAGCTTTGCTGTCCTTGATGTGATCATGGAATCTCTTTCTCAC
TAGAGTCTATGTCACTCATTATACTCTGTCGAATGTCATTGAATGTCTTTACATGGGCTT
GTATGCCTATGAAAATTGTAATACAACTTTCAGCAACGGATCTCTTGGCTCTCGCATCGA
TGAAGGACGCAGCGAAATGCGATAAGTAATGTGAATTGCAGAATTCAGTGAATCATCGAA
TCTTTGAACGCATCTTGCGCTCCTTGGTATTCCGAGGAGCATGCCTGTTTGAGTGTCATT
AAATTCTCAACTCTCTTATACTTTTTTGTAAAAGAGAGCTTGGACTGTGGAGGCTTGCTG
GCCACTTTTTGGGGTCAGCTCCTCTGAAATGCATTAGCGGAACCGTTTGCAATCTGCCAC
AAGTGTGATAAGTTATCTACACTGGCGAGGGGATTGCTCTCTGTAATGTTCAGCTTCTAA
TTGTCTCTACTTTGTGAGACAACTTTTGAATGCTTGACCTCAAATCAGGTAGGACTACCC
GCTGAACTTAA
>AB11-14
TTTCCGTAGGTGAACCTGCGGAAGGATCATTATTGAATTATGTTTCTAGATAGGTTGTAG
CTGGCTC-TTTAGAGCATGTGCACGCCTGTTTGGACTTCATTTTCATCCACCTGTGCACC
TATTGTAGTCTTTGGTTGGGTTAGGGGGAAGTGGTCATTGTGTCAGCATCTGCTGGATGT
GAGGACTTGCATTGTGAAAGCTTTGCTGTCCTTGATGTGATCATGGAATCTCTTTCTCAC
TAGAGTCTATGTCACTCATTATACTCTGTCGAATGTCATTGAATGTCTTTACATGGGCTT
GTATGCCTATGAAAATTGTAATACAACTTTCAGCAACGGATCTCTTGGCTCTCGCATCGA
TGAAGGACGCAGCGAAATGCGATAAGTAATGTGAATTGCAGAATTCAGTGAATCATCGAA
TCTTTGAACGCATCTTGCGCTCCTTGGTATTCCGAGGAGCATGCCTGTTTGAGTGTCATT
AAATTCTCAACTCTCTTATACTTTTTTGTAAAAGAGAGCTTGGACTGTGGAGGCTTGCTG
GCCACTTTTTGGGGTCAGCTCCTCTGAAATGCATTAGCGGAACCGTTTGCAATCTGCCAC
AAGTGTGATAAGTTATCTACACTGGCGAGGGGATTGCTCTCTGTAATGTTCAGCTTCTAA
TTGTCTCTACTTTGTGAGACAACTTTTGAATGCTTGACCTCAAATCAGGTAGGACTACCC
GCTGAACTTAA
>AB1-10
TTTCCGTAGGTGAACCTGCGGAAGGATCATTATTGAATTATGTTTCTAGATAGGTTGTAG
CTGGCTC-TTTAGAGCATGTGCACGCCTGTTTGGACTTCATTTTCATCCACCTGTGCACC
TATTGTAGTCTTTGGTTGGGTTAGGGGGAAGTGGTCATTGTGTCAGCATCTGCTGGATGT
GAGGACTTGCATTGTGAAAGCTTTGCTGTCCTTGATGTGATCATGGAATCTCTTTCTCAC
TAGAGTCTATGTCACTCATTATACTCTGTCGAATGTCATTGAATGTCTTTACATGGGCTT
GTATGCCTATGAAAATTGTAATACAACTTTCAGCAACGGATCTCTTGGCTCTCGCATCGA
TGAAGGACGCAGCGAAATGCGATAAGTAATGTGAATTGCAGAATTCAGTGAATCATCGAA
TCTTTGAACGCATCTTGCGCTCCTTGGTATTCCGAGGAGCATGCCTGTTTGAGTGTCATT
AAATTCTCAACTCTCTTATACTTTTTTGTAAAAGAGAGCTTGGACTGTGGAGGCTTGCTG
GCCACTTTTTGGGGTCAGCTCCTCTGAAATGCATTAGCGGAACCGTTTGCAATCTGCCAC
AAGTGTGATAAGTTATCTACACTGGCGAGGGGATTGCTCTCTGTAATGTTCAGCTTCTAA
TTGTCTCTACTTTGTGAGACAACTTTTGAATGCTTGACCTCAAATCAGGTAGGACTACCC
GCTGAACTTAA
>AB7-8
TTTCCGTAGGTGAACCTGCGGAAGGATCATTATTGAATTATGTTTCTAGATAGGTTGTAG
CTGGCTC-TTTAGAGCATGTGCACGCCTGTTTGGACTTCATTTTCATCCACCTGTGCACC
TATTGTAGTCTTTGGTTGGGTTAGGGGGAAGTGGTCATTGTGTCAGCATCTGCTGGATGT
GAGGACTTGCATTGTGAAAGCTTTGCTGTCCTTGATGTGATCATGGAATCTCTTTCTCAC
TAGAGTCTATGTCACTCATTATACTCTGTCGAATGTCATTGAATGTCTTTACATGGGCTT

GTATGCCTATGAAAATTGTAATACAACTTTCAGCAACGGATCTCTTGGCTCTCGCATCGA
TGAAGGACGCAGCGAAATGCGATAAGTAATGTGAATTGCAGAATTCAGTGAATCATCGAA
TCTTTGAACGCATCTTGCGCTCCTTGGTATTCCGAGGAGCATGCCTGTTTGAGTGTCATT
AAATTCTCAACTCTCTTATACTTTTTTGTAAAAGAGAGCTTGGACTGTGGAGGCTTGCTG
GCCACTTTTTGGGGTCAGCTCCTCTGAAATGCATTAGCGGAACCGTTTGCAATCTGCCAC
AAGTGTGATAAGTTATCTACACTGGCGAGGGGATTGCTCTCTGTAATGTTCAGCTTCTAA
TTGTCTCTACTTTGTGAGACAACTTTTGAATGCTTGACCTCAAATCAGGTAGGACTACCC
GCTGAACTTAA
>AB2–20
TTTCCGTAGGTGAACCTGCGGAAGGATCATTATTGAATTATGTTTCTAGATAGGTTGTAG
CTGGCTC–TTTAGAGCATGTGCACGCCTGTTTGGACTTCATTTTCATCCACCTGTGCACC
TATTGTAGTCTTTGGTTGGGTTAGGGGGAAGTGGTCATTGTGTCAGCATCTGCTGGATGT
GAGGACTTGCATTGTGAAAGCTTTGCTGTCCTTGATGTGATCATGGAATCTCTTTCTCAC
TAGAGTCTATGTCACTCATTATACTCTGTCGAATGTCATTGAATGTCTTTACATGGGCTT
GTATGCCTATGAAAATTGTAATACAACTTTCAGCAACGGATCTCTTGGCTCTCGCATCGA
TGAAGGACGCAGCGAAATGCGATAAGTAATGTGAATTGCAGAATTCAGTGAATCATCGAA
TCTTTGAACGCATCTTGCGCTCCTTGGTATTCCGAGGAGCATGCCTGTTTGAGTGTCATT
AAATTCTCAACTCTCTTATACTTTTTTGTAAAAGAGAGCTTGGACTGTGGAGGCTTGCTG
GCCACTTTTTGGGGTCAGCTCCTCTGAAATGCATTAGCGGAACCGTTTGCAATCTGCCAC
AAGTGTGATAAGTTATCTACACTGGCGAGGGGATTGCTCTCTGTAATGTTCAGCTTCTAA
TTGTCTCTACTTTGTGAGACAACTTTTGAATGCTTGACCTCAAATCAGGTAGGACTACCC
GCTGAACTTAA
>AB6–27
TTTCCGTAGGTGAACCTGCGGAAGGATCATTATTGAATTATGTTTCTAGATAGGTTGTAG
CTGGCTC–TTTAGAGCATGTGCACGCCTGTTTGGACTTCATTTTCATCCACCTGTGCACC
TATTGTAGTCTTTGGTTGGGTTAGGGGGAAGTGGTCATTGTGTCAGCATCTGCTGGATGT
GAGGACTTGCATTGTGAAAGCTTTGCTGTCCTTGATGTGATCATGGAATCTCTTTCTCAC
TAGAGTCTATGTCACTCATTATACTCTGTCGAATGTCATTGAATGTCTTTACATGGGCTT
GTATGCCTATGAAAATTGTAATACAACTTTCAGCAACGGATCTCTTGGCTCTCGCATCGA
TGAAGGACGCAGCGAAATGCGATAAGTAATGTGAATTGCAGAATTCAGTGAATCATCGAA
TCTTTGAACGCATCTTGCGCTCCTTGGTATTCCGAGGAGCATGCCTGTTTGAGTGTCATT
AAATTCTCAACTCTCTTATACTTTTTTGTAAAAGAGAGCTTGGACTGTGGAGGCTTGCTG
GCCACTTTTTGGGGTCAGCTCCTCTGAAATGCATTAGCGGAACCGTTTGCAATCTGCCAC
AAGTGTGATAAGTTATCTACACTGGCGAGGGGATTGCTCTCTGTAATGTTCAGCTTCTAA
TTGTCTCTACTTTGTGAGACAACTTTTGAATGCTTGACCTCAAATCAGGTAGGACTACCC
GCTGAACTTAA
>AB10–19
TTTCCGTAGGTGAACCTGCGGAAGGATCATTATTGAATTATGTTTCTAGATAGGTTGTAG
CTGGCTC–TTTAGAGCATGTGCACGCCTGTTTGGACTTCATTTTCATCCACCTGTGCACC
TATTGTAGTCTTTGGTTGGGTTAGGGGGAAGTGGTCATTGTGTCAGCATCTGCTGGATGT
GAGGACTTGCATTGTGAAAGCTTTGCTGTCCTTGATGTGATCATGGAATCTCTTTCTCAC
TAGAGTCTATGTCACTCATTATACTCTGTCGAATGTCATTGAATGTCTTTACATGGGCTT
GTATGCCTATGAAAATTGTAATACAACTTTCAGCAACGGATCTCTTGGCTCTCGCATCGA
TGAAGGACGCAGCGAAATGCGATAAGTAATGTGAATTGCAGAATTCAGTGAATCATCGAA
TCTTTGAACGCATCTTGCGCTCCTTGGTATTCCGAGGAGCATGCCTGTTTGAGTGTCATT
AAATTCTCAACTCTCTTATACTTTTTTGTAAAAGAGAGCTTGGACTGTGGAGGCTTGCTG
GCCACTTTTTGGGGTCAGCTCCTCTGAAATGCATTAGCGGAACCGTTTGCAATCTGCCAC
AAGTGTGATAAGTTATCTACACTGGCGAGGGGATTGCTCTCTGTAATGTTCAGCTTCTAA
TTGTCTCTACTTTGTGAGACAACTTTTGAATGCTTGACCTCAAATCAGGTAGGACTACCC
GCTGAACTTAA

>AB11-9
TTTCCGTAGGTGAACCTGCGGAAGGATCATTATTGAATTATGTTTCTAGATAGGTTGTAG
CTGGCTC-TTTAGAGCATGTGCACGCCTGTTTGGACTTCATTTTCATCCACCTGTGCACC
TATTGTAGTCTTTGGTTGGGTTAGGGGGAAGTGGTCATTGTGTCAGCATCTGCTGGATGT
GAGGACTTGCATTGTGAAAGCTTTGCTGTCCTTGATGTGATCATGGAATCTCTTTCTCAC
TAGAGTCTATGTCACTCATTATACTCTGTCGAATGTCATTGAATGTCTTTACATGGGCTT
GTATGCCTATGAAAATTGTAATACAACTTTCAGCAACGGATCTCTTGGCTCTCGCATCGA
TGAAGGACGCAGCGAAATGCGATAAGTAATGTGAATTGCAGAATTCAGTGAATCATCGAA
TCTTTGAACGCATCTTGCGCTCCTTGGTATTCCGAGGAGCATGCCTGTTTGAGTGTCATT
AAATTCTCAACTCTCTTATACTTTTTTGTAAAAGAGAGCTTGGACTGTGGAGGCTTGCTG
GCCACTTTTTGGGGTCAGCTCCTCTGAAATGCATTAGCGGAACCGTTTGCAATCTGCCAC
AAGTGTGATAAGTTATCTACACTGGCGAGGGGATTGCTCTCTGTAATGTTCAGCTTCTAA
TTGTCTCTACTTTGTGAGACAACTTTTGAATGCTTGACCTCAAATCAGGTAGGACTACCC
GCTGAACTTAA
>AB2-12
TTTCCGTAGGTGAACCTGCGGAAGGATCATTATTGAATTATGTTTCTAGATAGGTTGTAG
CTGGCTC-TTTAGAGCATGTGCACGCCTGTTTGGACTTCATTTTCATCCACCTGTGCACC
TATTGTAGTCTTTGGTTGGGTTAGGGGGAAGTGGTCATTGTGTCAGCATCTGCTGGATGT
GAGGACTTGCATTGTGAAAGCTTTGCTGTCCTTGATGTGATCATGGAATCTCTTTCTCAC
TAGAGTCTATGTCACTCATTATACTCTGTCGAATGTCATTGAATGTCTTTACATGGGCTT
GTATGCCTATGAAAATTGTAATACAACTTTCAGCAACGGATCTCTTGGCTCTCGCATCGA
TGAAGGACGCAGCGAAATGCGATAAGTAATGTGAATTGCAGAATTCAGTGAATCATCGAA
TCTTTGAACGCATCTTGCGCTCCTTGGTATTCCGAGGAGCATGCCTGTTTGAGTGTCATT
AAATTCTCAACTCTCTTATACTTTTTTGTAAAAGAGAGCTTGGACTGTGGAGGCTTGCTG
GCCACTTTTTGGGGTCAGCTCCTCTGAAATGCATTAGCGGAACCGTTTGCAATCTGCCAC
AAGTGTGATAAGTTATCTACACTGGCGAGGGGATTGCTCTCTGTAATGTTCAGCTTCTAA
TTGTCTCTACTTTGTGAGACAACTTTTGAATGCTTGACCTCAAATCAGGTAGGACTACCC
GCTGAACTTAA
>AB5-37
TTTCCGTAGGTGAACCTGCGGAAGGATCATTATTGAATTATGTTTCTAGATAGGTTGTAG
CTGGCTC-TTTAGAGCATGTGCACGCCTGTTTGGACTTCATTTTCATCCACCTGTGCACC
TATTGTAGTCTTTGGTTGGGTTAGGGGGAAGTGGTCATTGTGTCAGCATCTGCTGGATGT
GAGGACTTGCATTGTGAAAGCTTTGCTGTCCTTGATGTGATCATGGAATCTCTTTCTCAC
TAGAGTCTATGTCACTCATTATACTCTGTCGAATGTCATTGAATGTCTTTACATGGGCTT
GTATGCCTATGAAAATTGTAATACAACTTTCAGCAACGGATCTCTTGGCTCTCGCATCGA
TGAAGGACGCAGCGAAATGCGATAAGTAATGTGAATTGCAGAATTCAGTGAATCATCGAA
TCTTTGAACGCATCTTGCGCTCCTTGGTATTCCGAGGAGCATGCCTGTTTGAGTGTCATT
AAATTCTCAACTCTCTTATACTTTTTTGTAAAAGAGAGCTTGGACTGTGGAGGCTTGCTG
GCCACTTTTTGGGGTCAGCTCCTCTGAAATGCATTAGCGGAACCGTTTGCAATCTGCCAC
AAGTGTGATAAGTTATCTACACTGGCGAGGGGATTGCTCTCTGTAATGTTCAGCTTCTAA
TTGTCTCTACTTTGTGAGACAACTTTTGAATGCTTGACCTCAAATCAGGTAGGACTACCC
GCTGAACTTAA
>AB5-32
TTTCCGTAGGTGAACCTGCGGAAGGATCATTATTGAATTATGTTTCTAGATAGGTTGTAG
CTGGCTC-TTTAGAGCATGTGCACGCCTGTTTGGACTTCATTTTCATCCACCTGTGCACC
TATTGTAGTCTTTGGTTGGGTTAGGGGGAAGTGGTCATTGTGTCAGCATCTGCTGGATGT
GAGGACTTGCATTGTGAAAGCTTTGCTGTCCTTGATGTGATCATGGAATCTCTTTCTCAC
TAGAGTCTATGTCACTCATTATACTCTGTCGAATGTCATTGAATGTCTTTACATGGGCTT
GTATGCCTATGAAAATTGTAATACAACTTTCAGCAACGGATCTCTTGGCTCTCGCATCGA
TGAAGGACGCAGCGAAATGCGATAAGTAATGTGAATTGCAGAATTCAGTGAATCATCGAA

TCTTTGAACGCATCTTGCGCTCCTTGGTATTCCGAGGAGCATGCCTGTTTGAGTGTCATT
AAATTCTCAACTCTCTTATACTTTTTTGTAAAAGAGAGCTTGGACTGTGGAGGCTTGCTG
GCCACTTTTTGGGGTCAGCTCCTCTGAAATGCATTAGCGGAACCGTTTGCAATCTGCCAC
AAGTGTGATAAGTTATCTACACTGGCGAGGGGATTGCTCTCTGTAATGTTCAGCTTCTAA
TTGTCTCTACTTTGTGAGACAACTTTTGAATGCTTGACCTCAAATCAGGTAGGACTACCC
GCTGAACTTAA
>AB10-15
TTTCCGTAGGTGAACCTGCGGAAGGATCATTATTGAATTATGTTTCTAGATAGGTTGTAG
CTGGCTC-TTTAGAGCATGTGCACGCCTGTTTGGACTTCATTTTCATCCACCTGTGCACC
TATTGTAGTCTTTGGTTGGGTTAGGGGGAAGTGGTCATTGTGTCAGCATCTGCTGGATGT
GAGGACTTGCATTGTGAAAGCTTTGCTGTCCTTGATGTGATCATGGAATCTCTTTCTCAC
TAGAGTCTATGTCACTCATTATACTCTGTCGAATGTCATTGAATGTCTTTACATGGGCTT
GTATGCCTATGAAAATTGTAATACAACTTTCAGCAACGGATCTCTTGGCTCTCGCATCGA
TGAAGGACGCAGCGAAATGCGATAAGTAATGTGAATTGCAGAATTCAGTGAATCATCGAA
TCTTTGAACGCATCTTGCGCTCCTTGGTATTCCGAGGAGCATGCCTGTTTGAGTGTCATT
AAATTCTCAACTCTCTTATACTTTTTTGTAAAAGAGAGCTTGGACTGTGGAGGCTTGCTG
GCCACTTTTTGGGGTCAGCTCCTCTGAAATGCATTAGCGGAACCGTTTGCAATCTGCCAC
AAGTGTGATAAGTTATCTACACTGGCGAGGGGATTGCTCTCTGTAATGTTCAGCTTCTAA
TTGTCTCTACTTTGTGAGACAACTTTTGAATGCTTGACCTCAAATCAGGTAGGACTACCC
GCTGAACTTAA
>AB1-25
TTTCCGTAGGTGAACCTGCGGAAGGATCATTATTGAATTATGTTTCTAGATAGGTTGTAG
CTGGCTC-TTTAGAGCATGTGCACGCCTGTTTGGACTTCATTTTCATCCACCTGTGCACC
TATTGTAGTCTTTGGTTGGGTTAGGGGGAAGTGGTCATTGTGTCAGCATCTGCTGGATGT
GAGGACTTGCATTGTGAAAGCTTTGCTGTCCTTGATGTGATCATGGAATCTCTTTCTCAC
TAGAGTCTATGTCACTCATTATACTCTGTCGAATGTCATTGAATGTCTTTACATGGGCTT
GTATGCCTATGAAAATTGTAATACAACTTTCAGCAACGGATCTCTTGGCTCTCGCATCGA
TGAAGGACGCAGCGAAATGCGATAAGTAATGTGAATTGCAGAATTCAGTGAATCATCGAA
TCTTTGAACGCATCTTGCGCTCCTTGGTATTCCGAGGAGCATGCCTGTTTGAGTGTCATT
AAATTCTCAACTCTCTTATACTTTTTTGTAAAAGAGAGCTTGGACTGTGGAGGCTTGCTG
GCCACTTTTTGGGGTCAGCTCCTCTGAAATGCATTAGCGGAACCGTTTGCAATCTGCCAC
AAGTGTGATAAGTTATCTACACTGGCGAGGGGATTGCTCTCTGTAATGTTCAGCTTCTAA
TTGTCTCTACTTTGTGAGACAACTTTTGAATGCTTGACCTCAAATCAGGTAGGACTACCC
GCTGAACTTAA
>AB5-59
TTTCCGTAGGTGAACCTGCGGAAGGATCATTATTGAATTATGTTTCTAGATAGGTTGTAG
CTGGCTC-TTTAGAGCATGTGCACGCCTGTTTGGACTTCATTTTCATCCACCTGTGCACC
TATTGTAGTCTTTGGTTGGGTTAGGGGGAAGTGGTCATTGTGTCAGCATCTGCTGGATGT
GAGGACTTGCATTGTGAAAGCTTTGCTGTCCTTGATGTGATCATGGAATCTCTTTCTCAC
TAGAGTCTATGTCACTCATTATACTCTGTCGAATGTCATTGAATGTCTTTACATGGGCTT
GTATGCCTATGAAAATTGTAATACAACTTTCAGCAACGGATCTCTTGGCTCTCGCATCGA
TGAAGGACGCAGCGAAATGCGATAAGTAATGTGAATTGCAGAATTCAGTGAATCATCGAA
TCTTTGAACGCATCTTGCGCTCCTTGGTATTCCGAGGAGCATGCCTGTTTGAGTGTCATT
AAATTCTCAACTCTCTTATACTTTTTTGTAAAAGAGAGCTTGGACTGTGGAGGCTTGCTG
GCCACTTTTTGGGGTCAGCTCCTCTGAAATGCATTAGCGGAACCGTTTGCAATCTGCCAC
AAGTGTGATAAGTTATCTACACTGGCGAGGGGATTGCTCTCTGTAATGTTCAGCTTCTAA
TTGTCTCTACTTTGTGAGACAACTTTTGAATGCTTGACCTCAAATCAGGTAGGACTACCC
GCTGAACTTAA
>AB7-14
TTTCCGTAGGTGAACCTGCGGAAGGATCATTATTGAATTATGTTTCTAGATAGGTTGTAG

CTGGCTC-TTTAGAGCATGTGCACGCCTGTTTGGACTTCATTTTCATCCACCTGTGCACC
TATTGTAGTCTTTGGTTGGGTTAGGGGGAAGTGGTCATTGTGTCAGCATCTGCTGGATGT
GAGGACTTGCATTGTGAAAGCTTTGCTGTCCTTGATGTGATCATGGAATCTCTTTCTCAC
TAGAGTCTATGTCACTCATTATACTCTGTCGAATGTCATTGAATGTCTTTACATGGGCTT
GTATGCCTATGAAAATTGTAATACAACTTTCAGCAACGGATCTCTTGGCTCTCGCATCGA
TGAAGGACGCAGCGAAATGCGATAAGTAATGTGAATTGCAGAATTCAGTGAATCATCGAA
TCTTTGAACGCATCTTGCGCTCCTTGGTATTCCGAGGAGCATGCCTGTTTGAGTGTCATT
AAATTCTCAACTCTCTTATACTTTTTTGTAAAAGAGAGCTTGGACTGTGGAGGCTTGCTG
GCCACTTTTTGGGGTCAGCTCCTCTGAAATGCATTAGCGGAACCGTTTGCAATCTGCCAC
AAGTGTGATAAGTTATCTACACTGGCGAGGGGATTGCTCTCTGTAATGTTCAGCTTCTAA
TTGTCTCTACTTTGTGAGACAACTTTTGAATGCTTGACCTCAAATCAGGTAGGACTACCC
GCTGAACTTAA
>AB11-13
TTTCCGTAGGTGAACCTGCGGAAGGATCATTATTGAATTATGTTTCTAGATAGGTTGTAG
CTGGCTC-TTTAGAGCATGTGCACGCCTGTTTGGACTTCATTTTCATCCACCTGTGCACC
TATTGTAGTCTTTGGTTGGGTTAGGGGGAAGTGGTCATTGTGTCAGCATCTGCTGGATGT
GAGGACTTGCATTGTGAAAGCTTTGCTGTCCTTGATGTGATCATGGAATCTCTTTCTCAC
TAGAGTCTATGTCACTCATTATACTCTGTCGAATGTCATTGAATGTCTTTACATGGGCTT
GTATGCCTATGAAAATTGTAATACAACTTTCAGCAACGGATCTCTTGGCTCTCGCATCGA
TGAAGGACGCAGCGAAATGCGATAAGTAATGTGAATTGCAGAATTCAGTGAATCATCGAA
TCTTTGAACGCATCTTGCGCTCCTTGGTATTCCGAGGAGCATGCCTGTTTGAGTGTCATT
AAATTCTCAACTCTCTTATACTTTTTTGTAAAAGAGAGCTTGGACTGTGGAGGCTTGCTG
GCCACTTTTTGGGGTCAGCTCCTCTGAAATGCATTAGCGGAACCGTTTGCAATCTGCCAC
AAGTGTGATAAGTTATCTACACTGGCGAGGGGATTGCTCTCTGTAATGTTCAGCTTCTAA
TTGTCTCTACTTTGTGAGACAACTTTTGAATGCTTGACCTCAAATCAGGTAGGACTACCC
GCTGAACTTAA
>AB10-30
TTTCCGTAGGTGAACCTGCGGAAGGATCATTATTGAATTATGTTTCTAGATAGGTTGTAG
CTGGCTC-TTTAGAGCATGTGCACGCCTGTTTGGACTTCATTTTCATCCACCTGTGCACC
TATTGTAGTCTTTGGTTGGGTTAGGGGGAAGTGGTCATTGTGTCAGCATCTGCTGGATGT
GAGGACTTGCATTGTGAAAGCTTTGCTGTCCTTGATGTGATCATGGAATCTCTTTCTCAC
TAGAGTCTATGTCACTCATTATACTCTGTCGAATGTCATTGAATGTCTTTACATGGGCTT
GTATGCCTATGAAAATTGTAATACAACTTTCAGCAACGGATCTCTTGGCTCTCGCATCGA
TGAAGGACGCAGCGAAATGCGATAAGTAATGTGAATTGCAGAATTCAGTGAATCATCGAA
TCTTTGAACGCATCTTGCGCTCCTTGGTATTCCGAGGAGCATGCCTGTTTGAGTGTCATT
AAATTCTCAACTCTCTTATACTTTTTTGTAAAAGAGAGCTTGGACTGTGGAGGCTTGCTG
GCCACTTTTTGGGGTCAGCTCCTCTGAAATGCATTAGCGGAACCGTTTGCAATCTGCCAC
AAGTGTGATAAGTTATCTACACTGGCGAGGGGATTGCTCTCTGTAATGTTCAGCTTCTAA
TTGTCTCTACTTTGTGAGACAACTTTTGAATGCTTGACCTCAAATCAGGTAGGACTACCC
GCTGAACTTAA
>AB2-37
TTTCCGTAGGTGAACCTGCGGAAGGATCATTATTGAATTATGTTTCTAGATAGGTTGTAG
CTGGCTC-TTTAGAGCATGTGCACGCCTGTTTGGACTTCATTTTCATCCACCTGTGCACC
TATTGTAGTCTTTGGTTGGGTTAGGGGGAAGTGGTCATTGTGTCAGCATCTGCTGGATGT
GAGGACTTGCATTGTGAAAGCTTTGCTGTCCTTGATGTGATCATGGAATCTCTTTCTCAC
TAGAGTCTATGTCACTCATTATACTCTGTCGAATGTCATTGAATGTCTTTACATGGGCTT
GTATGCCTATGAAAATTGTAATACAACTTTCAGCAACGGATCTCTTGGCTCTCGCATCGA
TGAAGGACGCAGCGAAATGCGATAAGTAATGTGAATTGCAGAATTCAGTGAATCATCGAA
TCTTTGAACGCATCTTGCGCTCCTTGGTATTCCGAGGAGCATGCCTGTTTGAGTGTCATT
AAATTCTCAACTCTCTTATACTTTTTTGTAAAAGAGAGCTTGGACTGTGGAGGCTTGCTG

GCCACTTTTTGGGGTCAGCTCCTCTGAAATGCATTAGCGGAACCGTTTGCAATCTGCCAC
AAGTGTGATAAGTTATCTACACTGGCGAGGGGATTGCTCTCTGTAATGTTCAGCTTCTAA
TTGTCTCTACTTTGTGAGACAACTTTTGAATGCTTGACCTCAAATCAGGTAGGACTACCC
GCTGAACTTAA
>AB4-1
TTTCCGTAGGTGAACCTGCGGAAGGATCATTATTGAATTATGTTTCTAGATAGGTTGTAG
CTGGCTC-TTTAGAGCATGTGCACGCCTGTTTGGACTTCATTTTCATCCACCTGTGCACC
TATTGTAGTCTTTGGTTGGGTTAGGGGGAAGTGGTCATTGTGTCAGCATCTGCTGGATGT
GAGGACTTGCATTGTGAAAGCTTTGCTGTCCTTGATGTGATCATGGAATCTCTTTCTCAC
TAGAGTCTATGTCACTCATTATACTCTGTCGAATGTCATTGAATGTCTTTACATGGGCTT
GTATGCCTATGAAAATTGTAATACAACTTTCAGCAACGGATCTCTTGGCTCTCGCATCGA
TGAAGGACGCAGCGAAATGCGATAAGTAATGTGAATTGCAGAATTCAGTGAATCATCGAA
TCTTTGAACGCATCTTGCGCTCCTTGGTATTCCGAGGAGCATGCCTGTTTGAGTGTCATT
AAATTCTCAACTCTCTTATACTTTTTTGTAAAAGAGAGCTTGGACTGTGGAGGCTTGCTG
GCCACTTTTTGGGGTCAGCTCCTCTGAAATGCATTAGCGGAACCGTTTGCAATCTGCCAC
AAGTGTGATAAGTTATCTACACTGGCGAGGGGATTGCTCTCTGTAATGTTCAGCTTCTAA
TTGTCTCTACTTTGTGAGACAACTTTTGAATGCTTGACCTCAAATCAGGTAGGACTACCC
GCTGAACTTAA
>AB7-7
TTTCCGTAGGTGAACCTGCGGAAGGATCATTATTGAATTATGTTTCTAGATAGGTTGTAG
CTGGCTC-TTTAGAGCATGTGCACGCCTGTTTGGACTTCATTTTCATCCACCTGTGCACC
TATTGTAGTCTTTGGTTGGGTTAGGGGGAAGTGGTCATTGTGTCAGCATCTGCTGGATGT
GAGGACTTGCATTGTGAAAGCTTTGCTGTCCTTGATGTGATCATGGAATCTCTTTCTCAC
TAGAGTCTATGTCACTCATTATACTCTGTCGAATGTCATTGAATGTCTTTACATGGGCTT
GTATGCCTATGAAAATTGTAATACAACTTTCAGCAACGGATCTCTTGGCTCTCGCATCGA
TGAAGGACGCAGCGAAATGCGATAAGTAATGTGAATTGCAGAATTCAGTGAATCATCGAA
TCTTTGAACGCATCTTGCGCTCCTTGGTATTCCGAGGAGCATGCCTGTTTGAGTGTCATT
AAATTCTCAACTCTCTTATACTTTTTTGTAAAAGAGAGCTTGGACTGTGGAGGCTTGCTG
GCCACTTTTTGGGGTCAGCTCCTCTGAAATGCATTAGCGGAACCGTTTGCAATCTGCCAC
AAGTGTGATAAGTTATCTACACTGGCGAGGGGATTGCTCTCTGTAATGTTCAGCTTCTAA
TTGTCTCTACTTTGTGAGACAACTTTTGAATGCTTGACCTCAAATCAGGTAGGACTACCC
GCTGAACTTAA
>AB8-28
TTTCCGTAGGTGAACCTGCGGAAGGATCATTATTGAATTATGTTTCTAGATAGGTTGTAG
CTGGCTC-TTTAGAGCATGTGCACGCCTGTTTGGACTTCATTTTCATCCACCTGTGCACC
TATTGTAGTCTTTGGTTGGGTTAGGGGGAAGTGGTCATTGTGTCAGCATCTGCTGGATGT
GAGGACTTGCATTGTGAAAGCTTTGCTGTCCTTGATGTGATCATGGAATCTCTTTCTCAC
TAGAGTCTATGTCACTCATTATACTCTGTCGAATGTCATTGAATGTCTTTACATGGGCTT
GTATGCCTATGAAAATTGTAATACAACTTTCAGCAACGGATCTCTTGGCTCTCGCATCGA
TGAAGGACGCAGCGAAATGCGATAAGTAATGTGAATTGCAGAATTCAGTGAATCATCGAA
TCTTTGAACGCATCTTGCGCTCCTTGGTATTCCGAGGAGCATGCCTGTTTGAGTGTCATT
AAATTCTCAACTCTCTTATACTTTTTTGTAAAAGAGAGCTTGGACTGTGGAGGCTTGCTG
GCCACTTTTTGGGGTCAGCTCCTCTGAAATGCATTAGCGGAACCGTTTGCAATCTGCCAC
AAGTGTGATAAGTTATCTACACTGGCGAGGGGATTGCTCTCTGTAATGTTCAGCTTCTAA
TTGTCTCTACTTTGTGAGACAACTTTTGAATGCTTGACCTCAAATCAGGTAGGACTACCC
GCTGAACTTAA
>AB9-8
TTTCCGTAGGTGAACCTGCGGAAGGATCATTATTGAATTATGTTTCTAGATAGGTTGTAG
CTGGCTC-TTTAGAGCATGTGCACGCCTGTTTGGACTTCATTTTCATCCACCTGTGCACC
TATTGTAGTCTTTGGTTGGGTTAGGGGGAAGTGGTCATTGTGTCAGCATCTGCTGGATGT

GAGGACTTGCATTGTGAAAGCTTTGCTGTCCTTGATGTGATCATGGAATCTCTTTCTCAC
TAGAGTCTATGTCACTCATTATACTCTGTCGAATGTCATTGAATGTCTTTACATGGGCTT
GTATGCCTATGAAAATTGTAATACAACTTTCAGCAACGGATCTCTTGGCTCTCGCATCGA
TGAAGGACGCAGCGAAATGCGATAAGTAATGTGAATTGCAGAATTCAGTGAATCATCGAA
TCTTTGAACGCATCTTGCGCTCCTTGGTATTCCGAGGAGCATGCCTGTTTGAGTGTCATT
AAATTCTCAACTCTCTTATACTTTTTTGTAAAAGAGAGCTTGGACTGTGGAGGCTTGCTG
GCCACTTTTTGGGGTCAGCTCCTCTGAAATGCATTAGCGGAACCGTTTGCAATCTGCCAC
AAGTGTGATAAGTTATCTACACTGGCGAGGGGATTGCTCTCTGTAATGTTCAGCTTCTAA
TTGTCTCTACTTTGTGAGACAACTTTTGAATGCTTGACCTCAAATCAGGTAGGACTACCC
GCTGAACTTAA
>AB10-47
TTTCCGTAGGTGAACCTGCGGAAGGATCATTATTGAATTATGTTTCTAGATAGGTTGTAG
CTGGCTC-TTTAGAGCATGTGCACGCCTGTTTGGACTTCATTTTCATCCACCTGTGCACC
TATTGTAGTCTTTGGTTGGGTTAGGGGGAAGTGGTCATTGTGTCAGCATCTGCTGGATGT
GAGGACTTGCATTGTGAAAGCTTTGCTGTCCTTGATGTGATCATGGAATCTCTTTCTCAC
TAGAGTCTATGTCACTCATTATACTCTGTCGAATGTCATTGAATGTCTTTACATGGGCTT
GTATGCCTATGAAAATTGTAATACAACTTTCAGCAACGGATCTCTTGGCTCTCGCATCGA
TGAAGGACGCAGCGAAATGCGATAAGTAATGTGAATTGCAGAATTCAGTGAATCATCGAA
TCTTTGAACGCATCTTGCGCTCCTTGGTATTCCGAGGAGCATGCCTGTTTGAGTGTCATT
AAATTCTCAACTCTCTTATACTTTTTTGTAAAAGAGAGCTTGGACTGTGGAGGCTTGCTG
GCCACTTTTTGGGGTCAGCTCCTCTGAAATGCATTAGCGGAACCGTTTGCAATCTGCCAC
AAGTGTGATAAGTTATCTACACTGGCGAGGGGATTGCTCTCTGTAATGTTCAGCTTCTAA
TTGTCTCTACTTTGTGAGACAACTTTTGAATGCTTGACCTCAAATCAGGTAGGACTACCC
GCTGAACTTAA
>AB2-58
TTTCCGTAGGTGAACCTGCGGAAGGATCATTATTGAATTATGTTTCTAGATAGGTTGTAG
CTGGCTC-TTTAGAGCATGTGCACGCCTGTTTGGACTTCATTTTCATCCACCTGTGCACC
TATTGTAGTCTTTGGTTGGGTTAGGGGGAAGTGGTCATTGTGTCAGCATCTGCTGGATGT
GAGGACTTGCATTGTGAAAGCTTTGCTGTCCTTGATGTGATCATGGAATCTCTTTCTCAC
TAGAGTCTATGTCACTCATTATACTCTGTCGAATGTCATTGAATGTCTTTACATGGGCTT
GTATGCCTATGAAAATTGTAATACAACTTTCAGCAACGGATCTCTTGGCTCTCGCATCGA
TGAAGGACGCAGCGAAATGCGATAAGTAATGTGAATTGCAGAATTCAGTGAATCATCGAA
TCTTTGAACGCATCTTGCGCTCCTTGGTATTCCGAGGAGCATGCCTGTTTGAGTGTCATT
AAATTCTCAACTCTCTTATACTTTTTTGTAAAAGAGAGCTTGGACTGTGGAGGCTTGCTG
GCCACTTTTTGGGGTCAGCTCCTCTGAAATGCATTAGCGGAACCGTTTGCAATCTGCCAC
AAGTGTGATAAGTTATCTACACTGGCGAGGGGATTGCTCTCTGTAATGTTCAGCTTCTAA
TTGTCTCTACTTTGTGAGACAACTTTTGAATGCTTGACCTCAAATCAGGTAGGACTACCC
GCTGAACTTAA
>AB11-27
TTTCCGTAGGTGAACCTGCGGAAGGATCATTATTGAATTATGTTTCTAGATAGGTTGTAG
CTGGCTC-TTTAGAGCATGTGCACGCCTGTTTGGACTTCATTTTCATCCACCTGTGCACC
TATTGTAGTCTTTGGTTGGGTTAGGGGGAAGTGGTCATTGTGTCAGCATCTGCTGGATGT
GAGGACTTGCATTGTGAAAGCTTTGCTGTCCTTGATGTGATCATGGAATCTCTTTCTCAC
TAGAGTCTATGTCACTCATTATACTCTGTCGAATGTCATTGAATGTCTTTACATGGGCTT
GTATGCCTATGAAAATTGTAATACAACTTTCAGCAACGGATCTCTTGGCTCTCGCATCGA
TGAAGGACGCAGCGAAATGCGATAAGTAATGTGAATTGCAGAATTCAGTGAATCATCGAA
TCTTTGAACGCATCTTGCGCTCCTTGGTATTCCGAGGAGCATGCCTGTTTGAGTGTCATT
AAATTCTCAACTCTCTTATACTTTTTTGTAAAAGAGAGCTTGGACTGTGGAGGCTTGCTG
GCCACTTTTTGGGGTCAGCTCCTCTGAAATGCATTAGCGGAACCGTTTGCAATCTGCCAC
AAGTGTGATAAGTTATCTACACTGGCGAGGGGATTGCTCTCTGTAATGTTCAGCTTCTAA

TTGTCTCTACTTTGTGAGACAACTTTTGAATGCTTGACCTCAAATCAGGTAGGACTACCC
GCTGAACTTAA
>AB4-12
TTTCCGTAGGTGAACCTGCGGAAGGATCATTATTGAATTATGTTTCTAGATAGGTTGTAG
CTGGCTC-TTTAGAGCATGTGCACGCCTGTTTGGACTTCATTTTCATCCACCTGTGCACC
TATTGTAGTCTTTGGTTGGGTTAGGGGGAAGTGGTCATTGTGTCAGCATCTGCTGGATGT
GAGGACTTGCATTGTGAAAGCTTTGCTGTCCTTGATGTGATCATGGAATCTCTTTCTCAC
TAGAGTCTATGTCACTCATTATACTCTGTCGAATGTCATTGAATGTCTTTACATGGGCTT
GTATGCCTATGAAAATTGTAATACAACTTTCAGCAACGGATCTCTTGGCTCTCGCATCGA
TGAAGGACGCAGCGAAATGCGATAAGTAATGTGAATTGCAGAATTCAGTGAATCATCGAA
TCTTTGAACGCATCTTGCGCTCCTTGGTATTCCGAGGAGCATGCCTGTTTGAGTGTCATT
AAATTCTCAACTCTCTTATACTTTTTTGTAAAAGAGAGCTTGGACTGTGGAGGCTTGCTG
GCCACTTTTTGGGGTCAGCTCCTCTGAAATGCATTAGCGGAACCGTTTGCAATCTGCCAC
AAGTGTGATAAGTTATCTACACTGGCGAGGGGATTGCTCTCTGTAATGTTCAGCTTCTAA
TTGTCTCTACTTTGTGAGACAACTTTTGAATGCTTGACCTCAAATCAGGTAGGACTACCC
GCTGAACTTAA
>AB6-29
TTTCCGTAGGTGAACCTGCGGAAGGATCATTATTGAATTATGTTTCTAGATAGGTTGTAG
CTGGCTC-TTTAGAGCATGTGCACGCCTGTTTGGACTTCATTTTCATCCACCTGTGCACC
TATTGTAGTCTTTGGTTGGGTTAGGGGGAAGTGGTCATTGTGTCAGCATCTGCTGGATGT
GAGGACTTGCATTGTGAAAGCTTTGCTGTCCTTGATGTGATCATGGAATCTCTTTCTCAC
TAGAGTCTATGTCACTCATTATACTCTGTCGAATGTCATTGAATGTCTTTACATGGGCTT
GTATGCCTATGAAAATTGTAATACAACTTTCAGCAACGGATCTCTTGGCTCTCGCATCGA
TGAAGGACGCAGCGAAATGCGATAAGTAATGTGAATTGCAGAATTCAGTGAATCATCGAA
TCTTTGAACGCATCTTGCGCTCCTTGGTATTCCGAGGAGCATGCCTGTTTGAGTGTCATT
AAATTCTCAACTCTCTTATACTTTTTTGTAAAAGAGAGCTTGGACTGTGGAGGCTTGCTG
GCCACTTTTTGGGGTCAGCTCCTCTGAAATGCATTAGCGGAACCGTTTGCAATCTGCCAC
AAGTGTGATAAGTTATCTACACTGGCGAGGGGATTGCTCTCTGTAATGTTCAGCTTCTAA
TTGTCTCTACTTTGTGAGACAACTTTTGAATGCTTGACCTCAAATCAGGTAGGACTACCC
GCTGAACTTAA
>AB3-5
TTTCCGTAGGTGAACCTGCGGAAGGATCATTATTGAATTATGTTTCTAGATAGGTTGTAG
CTGGCTC-TTTAGAGCATGTGCACGCCTGTTTGGACTTCATTTTCATCCACCTGTGCACC
TATTGTAGTCTTTGGTTGGGTTAGGGGGAAGTGGTCATTGTGTCAGCATCTGCTGGATGT
GAGGACTTGCATTGTGAAAGCTTTGCTGTCCTTGATGTGATCATGGAATCTCTTTCTCAC
TAGAGTCTATGTCACTCATTATACTCTGTCGAATGTCATTGAATGTCTTTACATGGGCTT
GTATGCCTATGAAAATTGTAATACAACTTTCAGCAACGGATCTCTTGGCTCTCGCATCGA
TGAAGGACGCAGCGAAATGCGATAAGTAATGTGAATTGCAGAATTCAGTGAATCATCGAA
TCTTTGAACGCATCTTGCGCTCCTTGGTATTCCGAGGAGCATGCCTGTTTGAGTGTCATT
AAATTCTCAACTCTCTTATACTTTTTTGTAAAAGAGAGCTTGGACTGTGGAGGCTTGCTG
GCCACTTTTTGGGGTCAGCTCCTCTGAAATGCATTAGCGGAACCGTTTGCAATCTGCCAC
AAGTGTGATAAGTTATCTACACTGGCGAGGGGATTGCTCTCTGTAATGTTCAGCTTCTAA
TTGTCTCTACTTTGTGAGACAACTTTTGAATGCTTGACCTCAAATCAGGTAGGACTACCC
GCTGAACTTAA
>AB5-34
TTTCCGTAGGTGAACCTGCGGAAGGATCATTATTGAATTATGTTTCTAGATAGGTTGTAG
CTGGCTC-TTTAGAGCATGTGCACGCCTGTTTGGACTTCATTTTCATCCACCTGTGCACC
TATTGTAGTCTTTGGTTGGGTTAGGGGGAAGTGGTCATTGTGTCAGCATCTGCTGGATGT
GAGGACTTGCATTGTGAAAGCTTTGCTGTCCTTGATGTGATCATGGAATCTCTTTCTCAC
TAGAGTCTATGTCACTCATTATACTCTGTCGAATGTCATTGAATGTCTTTACATGGGCTT

GTATGCCTATGAAAATTGTAATACAACTTTCAGCAACGGATCTCTTGGCTCTCGCATCGA
TGAAGGACGCAGCGAAATGCGATAAGTAATGTGAATTGCAGAATTCAGTGAATCATCGAA
TCTTTGAACGCATCTTGCGCTCCTTGGTATTCCGAGGAGCATGCCTGTTTGAGTGTCATT
AAATTCTCAACTCTCTTATACTTTTTTGTAAAAGAGAGCTTGGACTGTGGAGGCTTGCTG
GCCACTTTTTGGGGTCAGCTCCTCTGAAATGCATTAGCGGAACCGTTTGCAATCTGCCAC
AAGTGTGATAAGTTATCTACACTGGCGAGGGGATTGCTCTCTGTAATGTTCAGCTTCTAA
TTGTCTCTACTTTGTGAGACAACTTTTGAATGCTTGACCTCAAATCAGGTAGGACTACCC
GCTGAACTTAA
>AB5-46
TTTCCGTAGGTGAACCTGCGGAAGGATCATTATTGAATTATGTTTCTAGATAGGTTGTAG
CTGGCTC-TTTAGAGCATGTGCACGCCTGTTTGGACTTCATTTTCATCCACCTGTGCACC
TATTGTAGTCTTTGGTTGGGTTAGGGGGAAGTGGTCATTGTGTCAGCATCTGCTGGATGT
GAGGACTTGCATTGTGAAAGCTTTGCTGTCCTTGATGTGATCATGGAATCTCTTTCTCAC
TAGAGTCTATGTCACTCATTATACTCTGTCGAATGTCATTGAATGTCTTTACATGGGCTT
GTATGCCTATGAAAATTGTAATACAACTTTCAGCAACGGATCTCTTGGCTCTCGCATCGA
TGAAGGACGCAGCGAAATGCGATAAGTAATGTGAATTGCAGAATTCAGTGAATCATCGAA
TCTTTGAACGCATCTTGCGCTCCTTGGTATTCCGAGGAGCATGCCTGTTTGAGTGTCATT
AAATTCTCAACTCTCTTATACTTTTTTGTAAAAGAGAGCTTGGACTGTGGAGGCTTGCTG
GCCACTTTTTGGGGTCAGCTCCTCTGAAATGCATTAGCGGAACCGTTTGCAATCTGCCAC
AAGTGTGATAAGTTATCTACACTGGCGAGGGGATTGCTCTCTGTAATGTTCAGCTTCTAA
TTGTCTCTACTTTGTGAGACAACTTTTGAATGCTTGACCTCAAATCAGGTAGGACTACCC
GCTGAACTTAA
>AB10-22
TTTCCGTAGGTGAACCTGCGGAAGGATCATTATTGAATTATGTTTCTAGATAGGTTGTAG
CTGGCTC-TTTAGAGCATGTGCACGCCTGTTTGGACTTCATTTTCATCCACCTGTGCACC
TATTGTAGTCTTTGGTTGGGTTAGGGGGAAGTGGTCATTGTGTCAGCATCTGCTGGATGT
GAGGACTTGCATTGTGAAAGCTTTGCTGTCCTTGATGTGATCATGGAATCTCTTTCTCAC
TAGAGTCTATGTCACTCATTATACTCTGTCGAATGTCATTGAATGTCTTTACATGGGCTT
GTATGCCTATGAAAATTGTAATACAACTTTCAGCAACGGATCTCTTGGCTCTCGCATCGA
TGAAGGACGCAGCGAAATGCGATAAGTAATGTGAATTGCAGAATTCAGTGAATCATCGAA
TCTTTGAACGCATCTTGCGCTCCTTGGTATTCCGAGGAGCATGCCTGTTTGAGTGTCATT
AAATTCTCAACTCTCTTATACTTTTTTGTAAAAGAGAGCTTGGACTGTGGAGGCTTGCTG
GCCACTTTTTGGGGTCAGCTCCTCTGAAATGCATTAGCGGAACCGTTTGCAATCTGCCAC
AAGTGTGATAAGTTATCTACACTGGCGAGGGGATTGCTCTCTGTAATGTTCAGCTTCTAA
TTGTCTCTACTTTGTGAGACAACTTTTGAATGCTTGACCTCAAATCAGGTAGGACTACCC
GCTGAACTTAA
>AB5-48
TTTCCGTAGGTGAACCTGCGGAAGGATCATTATTGAATTATGTTTCTAGATAGGTTGTAG
CTGGCTC-TTTAGAGCATGTGCACGCCTGTTTGGACTTCATTTTCATCCACCTGTGCACC
TATTGTAGTCTTTGGTTGGGTTAGGGGGAAGTGGTCATTGTGTCAGCATCTGCTGGATGT
GAGGACTTGCATTGTGAAAGCTTTGCTGTCCTTGATGTGATCATGGAATCTCTTTCTCAC
TAGAGTCTATGTCACTCATTATACTCTGTCGAATGTCATTGAATGTCTTTACATGGGCTT
GTATGCCTATGAAAATTGTAATACAACTTTCAGCAACGGATCTCTTGGCTCTCGCATCGA
TGAAGGACGCAGCGAAATGCGATAAGTAATGTGAATTGCAGAATTCAGTGAATCATCGAA
TCTTTGAACGCATCTTGCGCTCCTTGGTATTCCGAGGAGCATGCCTGTTTGAGTGTCATT
AAATTCTCAACTCTCTTATACTTTTTTGTAAAAGAGAGCTTGGACTGTGGAGGCTTGCTG
GCCACTTTTTGGGGTCAGCTCCTCTGAAATGCATTAGCGGAACCGTTTGCAATCTGCCAC
AAGTGTGATAAGTTATCTACACTGGCGAGGGGATTGCTCTCTGTAATGTTCAGCTTCTAA
TTGTCTCTACTTTGTGAGACAACTTTTGAATGCTTGACCTCAAATCAGGTAGGACTACCC
GCTGAACTTAA

>AB3-21
TTTCCGTAGGTGAACCTGCGGAAGGATCATTATTGAATTATGTTTCTAGATAGGTTGTAG
CTGGCTC-TTTAGAGCATGTGCACGCCTGTTTGGACTTCATTTTCATCCACCTGTGCACC
TATTGTAGTCTTTGGTTGGGTTAGGGGGAAGTGGTCATTGTGTCAGCATCTGCTGGATGT
GAGGACTTGCATTGTGAAAGCTTTGCTGTCCTTGATGTGATCATGGAATCTCTTTCTCAC
TAGAGTCTATGTCACTCATTATACTCTGTCGAATGTCATTGAATGTCTTTACATGGGCTT
GTATGCCTATGAAAATTGTAATACAACTTTCAGCAACGGATCTCTTGGCTCTCGCATCGA
TGAAGGACGCAGCGAAATGCGATAAGTAATGTGAATTGCAGAATTCAGTGAATCATCGAA
TCTTTGAACGCATCTTGCGCTCCTTGGTATTCCGAGGAGCATGCCTGTTTGAGTGTCATT
AAATTCTCAACTCTCTTATACTTTTTTGTAAAAGAGAGCTTGGACTGTGGAGGCTTGCTG
GCCACTTTTTGGGGTCAGCTCCTCTGAAATGCATTAGCGGAACCGTTTGCAATCTGCCAC
AAGTGTGATAAGTTATCTACACTGGCGAGGGGATTGCTCTCTGTAATGTTCAGCTTCTAA
TTGTCTCTACTTTGTGAGACAACTTTTGAATGCTTGACCTCAAATCAGGTAGGACTACCC
GCTGAACTTAA
>AB6-34
TTTCCGTAGGTGAACCTGCGGAAGGATCATTATTGAATTATGTTTCTAGATAGGTTGTAG
CTGGCTC-TTTAGAGCATGTGCACGCCTGTTTGGACTTCATTTTCATCCACCTGTGCACC
TATTGTAGTCTTTGGTTGGGTTAGGGGGAAGTGGTCATTGTGTCAGCATCTGCTGGATGT
GAGGACTTGCATTGTGAAAGCTTTGCTGTCCTTGATGTGATCATGGAATCTCTTTCTCAC
TAGAGTCTATGTCACTCATTATACTCTGTCGAATGTCATTGAATGTCTTTACATGGGCTT
GTATGCCTATGAAAATTGTAATACAACTTTCAGCAACGGATCTCTTGGCTCTCGCATCGA
TGAAGGACGCAGCGAAATGCGATAAGTAATGTGAATTGCAGAATTCAGTGAATCATCGAA
TCTTTGAACGCATCTTGCGCTCCTTGGTATTCCGAGGAGCATGCCTGTTTGAGTGTCATT
AAATTCTCAACTCTCTTATACTTTTTTGTAAAAGAGAGCTTGGACTGTGGAGGCTTGCTG
GCCACTTTTTGGGGTCAGCTCCTCTGAAATGCATTAGCGGAACCGTTTGCAATCTGCCAC
AAGTGTGATAAGTTATCTACACTGGCGAGGGGATTGCTCTCTGTAATGTTCAGCTTCTAA
TTGTCTCTACTTTGTGAGACAACTTTTGAATGCTTGACCTCAAATCAGGTAGGACTACCC
GCTGAACTTAA
>AB10-38
TTTCCGTAGGTGAACCTGCGGAAGGATCATTATTGAATTATGTTTCTAGATAGGTTGTAG
CTGGCTC-TTTAGAGCATGTGCACGCCTGTTTGGACTTCATTTTCATCCACCTGTGCACC
TATTGTAGTCTTTGGTTGGGTTAGGGGGAAGTGGTCATTGTGTCAGCATCTGCTGGATGT
GAGGACTTGCATTGTGAAAGCTTTGCTGTCCTTGATGTGATCATGGAATCTCTTTCTCAC
TAGAGTCTATGTCACTCATTATACTCTGTCGAATGTCATTGAATGTCTTTACATGGGCTT
GTATGCCTATGAAAATTGTAATACAACTTTCAGCAACGGATCTCTTGGCTCTCGCATCGA
TGAAGGACGCAGCGAAATGCGATAAGTAATGTGAATTGCAGAATTCAGTGAATCATCGAA
TCTTTGAACGCATCTTGCGCTCCTTGGTATTCCGAGGAGCATGCCTGTTTGAGTGTCATT
AAATTCTCAACTCTCTTATACTTTTTTGTAAAAGAGAGCTTGGACTGTGGAGGCTTGCTG
GCCACTTTTTGGGGTCAGCTCCTCTGAAATGCATTAGCGGAACCGTTTGCAATCTGCCAC
AAGTGTGATAAGTTATCTACACTGGCGAGGGGATTGCTCTCTGTAATGTTCAGCTTCTAA
TTGTCTCTACTTTGTGAGACAACTTTTGAATGCTTGACCTCAAATCAGGTAGGACTACCC
GCTGAACTTAA
>AB2-61
TTTCCGTAGGTGAACCTGCGGAAGGATCATTATTGAATTATGTTTCTAGATAGGTTGTAG
CTGGCTC-TTTAGAGCATGTGCACGCCTGTTTGGACTTCATTTTCATCCACCTGTGCACC
TATTGTAGTCTTTGGTTGGGTTAGGGGGAAGTGGTCATTGTGTCAGCATCTGCTGGATGT
GAGGACTTGCATTGTGAAAGCTTTGCTGTCCTTGATGTGATCATGGAATCTCTTTCTCAC
TAGAGTCTATGTCACTCATTATACTCTGTCGAATGTCATTGAATGTCTTTACATGGGCTT
GTATGCCTATGAAAATTGTAATACAACTTTCAGCAACGGATCTCTTGGCTCTCGCATCGA
TGAAGGACGCAGCGAAATGCGATAAGTAATGTGAATTGCAGAATTCAGTGAATCATCGAA

TCTTTGAACGCATCTTGCGCTCCTTGGTATTCCGAGGAGCATGCCTGTTTGAGTGTCATT
AAATTCTCAACTCTCTTATACTTTTTTGTAAAAGAGAGCTTGGACTGTGGAGGCTTGCTG
GCCACTTTTTGGGGTCAGCTCCTCTGAAATGCATTAGCGGAACCGTTTGCAATCTGCCAC
AAGTGTGATAAGTTATCTACACTGGCGAGGGGATTGCTCTCTGTAATGTTCAGCTTCTAA
TTGTCTCTACTTTGTGAGACAACTTTTGAATGCTTGACCTCAAATCAGGTAGGACTACCC
GCTGAACTTAA
>AB5-9
TTTCCGTAGGTGAACCTGCGGAAGGATCATTATTGAATTATGTTTCTAGATAGGTTGTAG
CTGGCTC-TTTAGAGCATGTGCACGCCTGTTTGGACTTCATTTTCATCCACCTGTGCACC
TATTGTAGTCTTTGGTTGGGTTAGGGGGAAGTGGTCATTGTGTCAGCATCTGCTGGATGT
GAGGACTTGCATTGTGAAAGCTTTGCTGTCCTTGATGTGATCATGGAATCTCTTTCTCAC
TAGAGTCTATGTCACTCATTATACTCTGTCGAATGTCATTGAATGTCTTTACATGGGCTT
GTATGCCTATGAAAATTGTAATACAACTTTCAGCAACGGATCTCTTGGCTCTCGCATCGA
TGAAGGACGCAGCGAAATGCGATAAGTAATGTGAATTGCAGAATTCAGTGAATCATCGAA
TCTTTGAACGCATCTTGCGCTCCTTGGTATTCCGAGGAGCATGCCTGTTTGAGTGTCATT
AAATTCTCAACTCTCTTATACTTTTTTGTAAAAGAGAGCTTGGACTGTGGAGGCTTGCTG
GCCACTTTTTGGGGTCAGCTCCTCTGAAATGCATTAGCGGAACCGTTTGCAATCTGCCAC
AAGTGTGATAAGTTATCTACACTGGCGAGGGGATTGCTCTCTGTAATGTTCAGCTTCTAA
TTGTCTCTACTTTGTGAGACAACTTTTGAATGCTTGACCTCAAATCAGGTAGGACTACCC
GCTGAACTTAA
>AB5-33
TTTCCGTAGGTGAACCTGCGGAAGGATCATTATTGAATTATGTTTCTAGATAGGTTGTAG
CTGGCTC-TTTAGAGCATGTGCACGCCTGTTTGGACTTCATTTTCATCCACCTGTGCACC
TATTGTAGTCTTTGGTTGGGTTAGGGGGAAGTGGTCATTGTGTCAGCATCTGCTGGATGT
GAGGACTTGCATTGTGAAAGCTTTGCTGTCCTTGATGTGATCATGGAATCTCTTTCTCAC
TAGAGTCTATGTCACTCATTATACTCTGTCGAATGTCATTGAATGTCTTTACATGGGCTT
GTATGCCTATGAAAATTGTAATACAACTTTCAGCAACGGATCTCTTGGCTCTCGCATCGA
TGAAGGACGCAGCGAAATGCGATAAGTAATGTGAATTGCAGAATTCAGTGAATCATCGAA
TCTTTGAACGCATCTTGCGCTCCTTGGTATTCCGAGGAGCATGCCTGTTTGAGTGTCATT
AAATTCTCAACTCTCTTATACTTTTTTGTAAAAGAGAGCTTGGACTGTGGAGGCTTGCTG
GCCACTTTTTGGGGTCAGCTCCTCTGAAATGCATTAGCGGAACCGTTTGCAATCTGCCAC
AAGTGTGATAAGTTATCTACACTGGCGAGGGGATTGCTCTCTGTAATGTTCAGCTTCTAA
TTGTCTCTACTTTGTGAGACAACTTTTGAATGCTTGACCTCAAATCAGGTAGGACTACCC
GCTGAACTTAA
>AB1-19
TTTCCGTAGGTGAACCTGCGGAAGGATCATTATTGAATTATGTTTCTAGATAGGTTGTAG
CTGGCTC-TTTAGAGCATGTGCACGCCTGTTTGGACTTCATTTTCATCCACCTGTGCACC
TATTGTAGTCTTTGGTTGGGTTAGGGGGAAGTGGTCATTGTGTCAGCATCTGCTGGATGT
GAGGACTTGCATTGTGAAAGCTTTGCTGTCCTTGATGTGATCATGGAATCTCTTTCTCAC
TAGAGTCTATGTCACTCATTATACTCTGTCGAATGTCATTGAATGTCTTTACATGGGCTT
GTATGCCTATGAAAATTGTAATACAACTTTCAGCAACGGATCTCTTGGCTCTCGCATCGA
TGAAGGACGCAGCGAAATGCGATAAGTAATGTGAATTGCAGAATTCAGTGAATCATCGAA
TCTTTGAACGCATCTTGCGCTCCTTGGTATTCCGAGGAGCATGCCTGTTTGAGTGTCATT
AAATTCTCAACTCTCTTATACTTTTTTGTAAAAGAGAGCTTGGACTGTGGAGGCTTGCTG
GCCACTTTTTGGGGTCAGCTCCTCTGAAATGCATTAGCGGAACCGTTTGCAATCTGCCAC
AAGTGTGATAAGTTATCTACACTGGCGAGGGGATTGCTCTCTGTAATGTTCAGCTTCTAA
TTGTCTCTACTTTGTGAGACAACTTTTGAATGCTTGACCTCAAATCAGGTAGGACTACCC
GCTGAACTTAA
>AB9-47
TTTCCGTAGGTGAACCTGCGGAAGGATCATTATTGAATTATGTTTCTAGATAGGTTGTAG

CTGGCTC-TTTAGAGCATGTGCACGCCTGTTTGGACTTCATTTTCATCCACCTGTGCACC
TATTGTAGTCTTTGGTTGGGTTAGGGGGAAGTGGTCATTGTGTCAGCATCTGCTGGATGT
GAGGACTTGCATTGTGAAAGCTTTGCTGTCCTTGATGTGATCATGGAATCTCTTTCTCAC
TAGAGTCTATGTCACTCATTATACTCTGTCGAATGTCATTGAATGTCTTTACATGGGCTT
GTATGCCTATGAAAATTGTAATACAACTTTCAGCAACGGATCTCTTGGCTCTCGCATCGA
TGAAGGACGCAGCGAAATGCGATAAGTAATGTGAATTGCAGAATTCAGTGAATCATCGAA
TCTTTGAACGCATCTTGCGCTCCTTGGTATTCCGAGGAGCATGCCTGTTTGAGTGTCATT
AAATTCTCAACTCTCTTATACTTTTTTGTAAAAGAGAGCTTGGACTGTGGAGGCTTGCTG
GCCACTTTTTGGGGTCAGCTCCTCTGAAATGCATTAGCGGAACCGTTTGCAATCTGCCAC
AAGTGTGATAAGTTATCTACACTGGCGAGGGGATTGCTCTCTGTAATGTTCAGCTTCTAA
TTGTCTCTACTTTGTGAGACAACTTTTGAATGCTTGACCTCAAATCAGGTAGGACTACCC
GCTGAACTTAA
>AB6-20
TTTCCGTAGGTGAACCTGCGGAAGGATCATTATTGAATTATGTTTCTAGATAGGTTGTAG
CTGGCTC-TTTAGAGCATGTGCACGCCTGTTTGGACTTCATTTTCATCCACCTGTGCACC
TATTGTAGTCTTTGGTTGGGTTAGGGGGAAGTGGTCATTGTGTCAGCATCTGCTGGATGT
GAGGACTTGCATTGTGAAAGCTTTGCTGTCCTTGATGTGATCATGGAATCTCTTTCTCAC
TAGAGTCTATGTCACTCATTATACTCTGTCGAATGTCATTGAATGTCTTTACATGGGCTT
GTATGCCTATGAAAATTGTAATACAACTTTCAGCAACGGATCTCTTGGCTCTCGCATCGA
TGAAGGACGCAGCGAAATGCGATAAGTAATGTGAATTGCAGAATTCAGTGAATCATCGAA
TCTTTGAACGCATCTTGCGCTCCTTGGTATTCCGAGGAGCATGCCTGTTTGAGTGTCATT
AAATTCTCAACTCTCTTATACTTTTTTGTAAAAGAGAGCTTGGACTGTGGAGGCTTGCTG
GCCACTTTTTGGGGTCAGCTCCTCTGAAATGCATTAGCGGAACCGTTTGCAATCTGCCAC
AAGTGTGATAAGTTATCTACACTGGCGAGGGGATTGCTCTCTGTAATGTTCAGCTTCTAA
TTGTCTCTACTTTGTGAGACAACTTTTGAATGCTTGACCTCAAATCAGGTAGGACTACCC
GCTGAACTTAA
>AB12-11
TTTCCGTAGGTGAACCTGCGGAAGGATCATTATTGAATTATGTTTCTAGATAGGTTGTAG
CTGGCTC-TTTAGAGCATGTGCACGCCTGTTTGGACTTCATTTTCATCCACCTGTGCACC
TATTGTAGTCTTTGGTTGGGTTAGGGGGAAGTGGTCATTGTGTCAGCATCTGCTGGATGT
GAGGACTTGCATTGTGAAAGCTTTGCTGTCCTTGATGTGATCATGGAATCTCTTTCTCAC
TAGAGTCTATGTCACTCATTATACTCTGTCGAATGTCATTGAATGTCTTTACATGGGCTT
GTATGCCTATGAAAATTGTAATACAACTTTCAGCAACGGATCTCTTGGCTCTCGCATCGA
TGAAGGACGCAGCGAAATGCGATAAGTAATGTGAATTGCAGAATTCAGTGAATCATCGAA
TCTTTGAACGCATCTTGCGCTCCTTGGTATTCCGAGGAGCATGCCTGTTTGAGTGTCATT
AAATTCTCAACTCTCTTATACTTTTTTGTAAAAGAGAGCTTGGACTGTGGAGGCTTGCTG
GCCACTTTTTGGGGTCAGCTCCTCTGAAATGCATTAGCGGAACCGTTTGCAATCTGCCAC
AAGTGTGATAAGTTATCTACACTGGCGAGGGGATTGCTCTCTGTAATGTTCAGCTTCTAA
TTGTCTCTACTTTGTGAGACAACTTTTGAATGCTTGACCTCAAATCAGGTAGGACTACCC
GCTGAACTTAA
>AB1-52
TTTCCGTAGGTGAACCTGCGGAAGGATCATTATTGAATTATGTTTCTAGATAGGTTGTAG
CTGGCTC-TTTAGAGCATGTGCACGCCTGTTTGGACTTCATTTTCATCCACCTGTGCACC
TATTGTAGTCTTTGGTTGGGTTAGGGGGAAGTGGTCATTGTGTCAGCATCTGCTGGATGT
GAGGACTTGCATTGTGAAAGCTTTGCTGTCCTTGATGTGATCATGGAATCTCTTTCTCAC
TAGAGTCTATGTCACTCATTATACTCTGTCGAATGTCATTGAATGTCTTTACATGGGCTT
GTATGCCTATGAAAATTGTAATACAACTTTCAGCAACGGATCTCTTGGCTCTCGCATCGA
TGAAGGACGCAGCGAAATGCGATAAGTAATGTGAATTGCAGAATTCAGTGAATCATCGAA
TCTTTGAACGCATCTTGCGCTCCTTGGTATTCCGAGGAGCATGCCTGTTTGAGTGTCATT
AAATTCTCAACTCTCTTATACTTTTTTGTAAAAGAGAGCTTGGACTGTGGAGGCTTGCTG

GCCACTTTTTGGGGTCAGCTCCTCTGAAATGCATTAGCGGAACCGTTTGCAATCTGCCAC
AAGTGTGATAAGTTATCTACACTGGCGAGGGGATTGCTCTCTGTAATGTTCAGCTTCTAA
TTGTCTCTACTTTGTGAGACAACTTTTGAATGCTTGACCTCAAATCAGGTAGGACTACCC
GCTGAACTTAA
>AB3-40
TTTCCGTAGGTGAACCTGCGGAAGGATCATTATTGAATTATGTTTCTAGATAGGTTGTAG
CTGGCTC-TTTAGAGCATGTGCACGCCTGTTTGGACTTCATTTTCATCCACCTGTGCACC
TATTGTAGTCTTTGGTTGGGTTAGGGGGAAGTGGTCATTGTGTCAGCATCTGCTGGATGT
GAGGACTTGCATTGTGAAAGCTTTGCTGTCCTTGATGTGATCATGGAATCTCTTTCTCAC
TAGAGTCTATGTCACTCATTATACTCTGTCGAATGTCATTGAATGTCTTTACATGGGCTT
GTATGCCTATGAAAATTGTAATACAACTTTCAGCAACGGATCTCTTGGCTCTCGCATCGA
TGAAGGACGCAGCGAAATGCGATAAGTAATGTGAATTGCAGAATTCAGTGAATCATCGAA
TCTTTGAACGCATCTTGCGCTCCTTGGTATTCCGAGGAGCATGCCTGTTTGAGTGTCATT
AAATTCTCAACTCTCTTATACTTTTTTGTAAAAGAGAGCTTGGACTGTGGAGGCTTGCTG
GCCACTTTTTGGGGTCAGCTCCTCTGAAATGCATTAGCGGAACCGTTTGCAATCTGCCAC
AAGTGTGATAAGTTATCTACACTGGCGAGGGGATTGCTCTCTGTAATGTTCAGCTTCTAA
TTGTCTCTACTTTGTGAGACAACTTTTGAATGCTTGACCTCAAATCAGGTAGGACTACCC
GCTGAACTTAA
>AB8-55
TTTCCGTAGGTGAACCTGCGGAAGGATCATTATTGAATTATGTTTCTAGATAGGTTGTAG
CTGGCTC-TTTAGAGCATGTGCACGCCTGTTTGGACTTCATTTTCATCCACCTGTGCACC
TATTGTAGTCTTTGGTTGGGTTAGGGGGAAGTGGTCATTGTGTCAGCATCTGCTGGATGT
GAGGACTTGCATTGTGAAAGCTTTGCTGTCCTTGATGTGATCATGGAATCTCTTTCTCAC
TAGAGTCTATGTCACTCATTATACTCTGTCGAATGTCATTGAATGTCTTTACATGGGCTT
GTATGCCTATGAAAATTGTAATACAACTTTCAGCAACGGATCTCTTGGCTCTCGCATCGA
TGAAGGACGCAGCGAAATGCGATAAGTAATGTGAATTGCAGAATTCAGTGAATCATCGAA
TCTTTGAACGCATCTTGCGCTCCTTGGTATTCCGAGGAGCATGCCTGTTTGAGTGTCATT
AAATTCTCAACTCTCTTATACTTTTTTGTAAAAGAGAGCTTGGACTGTGGAGGCTTGCTG
GCCACTTTTTGGGGTCAGCTCCTCTGAAATGCATTAGCGGAACCGTTTGCAATCTGCCAC
AAGTGTGATAAGTTATCTACACTGGCGAGGGGATTGCTCTCTGTAATGTTCAGCTTCTAA
TTGTCTCTACTTTGTGAGACAACTTTTGAATGCTTGACCTCAAATCAGGTAGGACTACCC
GCTGAACTTAA
>AB8-43
TTTCCGTAGGTGAACCTGCGGAAGGATCATTATTGAATTATGTTTCTAGATAGGTTGTAG
CTGGCTC-TTTAGAGCATGTGCACGCCTGTTTGGACTTCATTTTCATCCACCTGTGCACC
TATTGTAGTCTTTGGTTGGGTTAGGGGGAAGTGGTCATTGTGTCAGCATCTGCTGGATGT
GAGGACTTGCATTGTGAAAGCTTTGCTGTCCTTGATGTGATCATGGAATCTCTTTCTCAC
TAGAGTCTATGTCACTCATTATACTCTGTCGAATGTCATTGAATGTCTTTACATGGGCTT
GTATGCCTATGAAAATTGTAATACAACTTTCAGCAACGGATCTCTTGGCTCTCGCATCGA
TGAAGGACGCAGCGAAATGCGATAAGTAATGTGAATTGCAGAATTCAGTGAATCATCGAA
TCTTTGAACGCATCTTGCGCTCCTTGGTATTCCGAGGAGCATGCCTGTTTGAGTGTCATT
AAATTCTCAACTCTCTTATACTTTTTTGTAAAAGAGAGCTTGGACTGTGGAGGCTTGCTG
GCCACTTTTTGGGGTCAGCTCCTCTGAAATGCATTAGCGGAACCGTTTGCAATCTGCCAC
AAGTGTGATAAGTTATCTACACTGGCGAGGGGATTGCTCTCTGTAATGTTCAGCTTCTAA
TTGTCTCTACTTTGTGAGACAACTTTTGAATGCTTGACCTCAAATCAGGTAGGACTACCC
GCTGAACTTAA
>AB11-49
TTTCCGTAGGTGAACCTGCGGAAGGATCATTATTGAATTATGTTTCTAGATAGGTTGTAG
CTGGCTC-TTTAGAGCATGTGCACGCCTGTTTGGACTTCATTTTCATCCACCTGTGCACC
TATTGTAGTCTTTGGTTGGGTTAGGGGGAAGTGGTCATTGTGTCAGCATCTGCTGGATGT

GAGGACTTGCATTGTGAAAGCTTTGCTGTCCTTGATGTGATCATGGAATCTCTTTCTCAC
TAGAGTCTATGTCACTCATTATACTCTGTCGAATGTCATTGAATGTCTTTACATGGGCTT
GTATGCCTATGAAAATTGTAATACAACTTTCAGCAACGGATCTCTTGGCTCTCGCATCGA
TGAAGGACGCAGCGAAATGCGATAAGTAATGTGAATTGCAGAATTCAGTGAATCATCGAA
TCTTTGAACGCATCTTGCGCTCCTTGGTATTCCGAGGAGCATGCCTGTTTGAGTGTCATT
AAATTCTCAACTCTCTTATACTTTTTTGTAAAAGAGAGCTTGGACTGTGGAGGCTTGCTG
GCCACTTTTTGGGGTCAGCTCCTCTGAAATGCATTAGCGGAACCGTTTGCAATCTGCCAC
AAGTGTGATAAGTTATCTACACTGGCGAGGGGATTGCTCTCTGTAATGTTCAGCTTCTAA
TTGTCTCTACTTTGTGAGACAACTTTTGAATGCTTGACCTCAAATCAGGTAGGACTACCC
GCTGAACTTAA
>AB11-56
TTTCCGTAGGTGAACCTGCGGAAGGATCATTATTGAATTATGTTTCTAGATAGGTTGTAG
CTGGCTC-TTTAGAGCATGTGCACGCCTGTTTGGACTTCATTTTCATCCACCTGTGCACC
TATTGTAGTCTTTGGTTGGGTTAGGGGGAAGTGGTCATTGTGTCAGCATCTGCTGGATGT
GAGGACTTGCATTGTGAAAGCTTTGCTGTCCTTGATGTGATCATGGAATCTCTTTCTCAC
TAGAGTCTATGTCACTCATTATACTCTGTCGAATGTCATTGAATGTCTTTACATGGGCTT
GTATGCCTATGAAAATTGTAATACAACTTTCAGCAACGGATCTCTTGGCTCTCGCATCGA
TGAAGGACGCAGCGAAATGCGATAAGTAATGTGAATTGCAGAATTCAGTGAATCATCGAA
TCTTTGAACGCATCTTGCGCTCCTTGGTATTCCGAGGAGCATGCCTGTTTGAGTGTCATT
AAATTCTCAACTCTCTTATACTTTTTTGTAAAAGAGAGCTTGGACTGTGGAGGCTTGCTG
GCCACTTTTTGGGGTCAGCTCCTCTGAAATGCATTAGCGGAACCGTTTGCAATCTGCCAC
AAGTGTGATAAGTTATCTACACTGGCGAGGGGATTGCTCTCTGTAATGTTCAGCTTCTAA
TTGTCTCTACTTTGTGAGACAACTTTTGAATGCTTGACCTCAAATCAGGTAGGACTACCC
GCTGAACTTAA
>AB11-58
TTTCCGTAGGTGAACCTGCGGAAGGATCATTATTGAATTATGTTTCTAGATAGGTTGTAG
CTGGCTC-TTTAGAGCATGTGCACGCCTGTTTGGACTTCATTTTCATCCACCTGTGCACC
TATTGTAGTCTTTGGTTGGGTTAGGGGGAAGTGGTCATTGTGTCAGCATCTGCTGGATGT
GAGGACTTGCATTGTGAAAGCTTTGCTGTCCTTGATGTGATCATGGAATCTCTTTCTCAC
TAGAGTCTATGTCACTCATTATACTCTGTCGAATGTCATTGAATGTCTTTACATGGGCTT
GTATGCCTATGAAAATTGTAATACAACTTTCAGCAACGGATCTCTTGGCTCTCGCATCGA
TGAAGGACGCAGCGAAATGCGATAAGTAATGTGAATTGCAGAATTCAGTGAATCATCGAA
TCTTTGAACGCATCTTGCGCTCCTTGGTATTCCGAGGAGCATGCCTGTTTGAGTGTCATT
AAATTCTCAACTCTCTTATACTTTTTTGTAAAAGAGAGCTTGGACTGTGGAGGCTTGCTG
GCCACTTTTTGGGGTCAGCTCCTCTGAAATGCATTAGCGGAACCGTTTGCAATCTGCCAC
AAGTGTGATAAGTTATCTACACTGGCGAGGGGATTGCTCTCTGTAATGTTCAGCTTCTAA
TTGTCTCTACTTTGTGAGACAACTTTTGAATGCTTGACCTCAAATCAGGTAGGACTACCC
GCTGAACTTAA
>AB3-26
TTTCCGTAGGTGAACCTGCGGAAGGATCATTATTGAATTATGTTTCTAGATAGGTTGTAG
CTGGCTC-TTTAGAGCATGTGCACGCCTGTTTGGACTTCATTTTCATCCACCTGTGCACC
TATTGTAGTCTTTGGTTGGGTTAGGGGGAAGTGGTCATTGTGTCAGCATCTGCTGGATGT
GAGGACTTGCATTGTGAAAGCTTTGCTGTCCTTGATGTGATCATGGAATCTCTTTCTCAC
TAGAGTCTATGTCACTCATTATACTCTGTCGAATGTCATTGAATGTCTTTACATGGGCTT
GTATGCCTATGAAAATTGTAATACAACTTTCAGCAACGGATCTCTTGGCTCTCGCATCGA
TGAAGGACGCAGCGAAATGCGATAAGTAATGTGAATTGCAGAATTCAGTGAATCATCGAA
TCTTTGAACGCATCTTGCGCTCCTTGGTATTCCGAGGAGCATGCCTGTTTGAGTGTCATT
AAATTCTCAACTCTCTTATACTTTTTTGTAAAAGAGAGCTTGGACTGTGGAGGCTTGCTG
GCCACTTTTTGGGGTCAGCTCCTCTGAAATGCATTAGCGGAACCGTTTGCAATCTGCCAC
AAGTGTGATAAGTTATCTACACTGGCGAGGGGATTGCTCTCTGTAATGTTCAGCTTCTAA

TTGTCTCTACTTTGTGAGACAACTTTTGAATGCTTGACCTCAAATCAGGTAGGACTACCC
GCTGAACTTAA
>AB6-51
TTTCCGTAGGTGAACCTGCGGAAGGATCATTATTGAATTATGTTTCTAGATAGGTTGTAG
CTGGCTC-TTTAGAGCATGTGCACGCCTGTTTGGACTTCATTTTCATCCACCTGTGCACC
TATTGTAGTCTTTGGTTGGGTTAGGGGGAAGTGGTCATTGTGTCAGCATCTGCTGGATGT
GAGGACTTGCATTGTGAAAGCTTTGCTGTCCTTGATGTGATCATGGAATCTCTTTCTCAC
TAGAGTCTATGTCACTCATTATACTCTGTCGAATGTCATTGAATGTCTTTACATGGGCTT
GTATGCCTATGAAAATTGTAATACAACTTTCAGCAACGGATCTCTTGGCTCTCGCATCGA
TGAAGGACGCAGCGAAATGCGATAAGTAATGTGAATTGCAGAATTCAGTGAATCATCGAA
TCTTTGAACGCATCTTGCGCTCCTTGGTATTCCGAGGAGCATGCCTGTTTGAGTGTCATT
AAATTCTCAACTCTCTTATACTTTTTTGTAAAAGAGAGCTTGGACTGTGGAGGCTTGCTG
GCCACTTTTTGGGGTCAGCTCCTCTGAAATGCATTAGCGGAACCGTTTGCAATCTGCCAC
AAGTGTGATAAGTTATCTACACTGGCGAGGGGATTGCTCTCTGTAATGTTCAGCTTCTAA
TTGTCTCTACTTTGTGAGACAACTTTTGAATGCTTGACCTCAAATCAGGTAGGACTACCC
GCTGAACTTAA
>AB6-52
TTTCCGTAGGTGAACCTGCGGAAGGATCATTATTGAATTATGTTTCTAGATAGGTTGTAG
CTGGCTC-TTTAGAGCATGTGCACGCCTGTTTGGACTTCATTTTCATCCACCTGTGCACC
TATTGTAGTCTTTGGTTGGGTTAGGGGGAAGTGGTCATTGTGTCAGCATCTGCTGGATGT
GAGGACTTGCATTGTGAAAGCTTTGCTGTCCTTGATGTGATCATGGAATCTCTTTCTCAC
TAGAGTCTATGTCACTCATTATACTCTGTCGAATGTCATTGAATGTCTTTACATGGGCTT
GTATGCCTATGAAAATTGTAATACAACTTTCAGCAACGGATCTCTTGGCTCTCGCATCGA
TGAAGGACGCAGCGAAATGCGATAAGTAATGTGAATTGCAGAATTCAGTGAATCATCGAA
TCTTTGAACGCATCTTGCGCTCCTTGGTATTCCGAGGAGCATGCCTGTTTGAGTGTCATT
AAATTCTCAACTCTCTTATACTTTTTTGTAAAAGAGAGCTTGGACTGTGGAGGCTTGCTG
GCCACTTTTTGGGGTCAGCTCCTCTGAAATGCATTAGCGGAACCGTTTGCAATCTGCCAC
AAGTGTGATAAGTTATCTACACTGGCGAGGGGATTGCTCTCTGTAATGTTCAGCTTCTAA
TTGTCTCTACTTTGTGAGACAACTTTTGAATGCTTGACCTCAAATCAGGTAGGACTACCC
GCTGAACTTAA
>AB7-2
TTTCCGTAGGTGAACCTGCGGAAGGATCATTATTGAATTATGTTTCTAGATAGGTTGTAG
CTGGCTC-TTTAGAGCATGTGCACGCCTGTTTGGACTTCATTTTCATCCACCTGTGCACC
TATTGTAGTCTTTGGTTGGGTTAGGGGGAAGTGGTCATTGTGTCAGCATCTGCTGGATGT
GAGGACTTGCATTGTGAAAGCTTTGCTGTCCTTGATGTGATCATGGAATCTCTTTCTCAC
TAGAGTCTATGTCACTCATTATACTCTGTCGAATGTCATTGAATGTCTTTACATGGGCTT
GTATGCCTATGAAAATTGTAATACAACTTTCAGCAACGGATCTCTTGGCTCTCGCATCGA
TGAAGGACGCAGCGAAATGCGATAAGTAATGTGAATTGCAGAATTCAGTGAATCATCGAA
TCTTTGAACGCATCTTGCGCTCCTTGGTATTCCGAGGAGCATGCCTGTTTGAGTGTCATT
AAATTCTCAACTCTCTTATACTTTTTTGTAAAAGAGAGCTTGGACTGTGGAGGCTTGCTG
GCCACTTTTTGGGGTCAGCTCCTCTGAAATGCATTAGCGGAACCGTTTGCAATCTGCCAC
AAGTGTGATAAGTTATCTACACTGGCGAGGGGATTGCTCTCTGTAATGTTCAGCTTCTAA
TTGTCTCTACTTTGTGAGACAACTTTTGAATGCTTGACCTCAAATCAGGTAGGACTACCC
GCTGAACTTAA
>AB9-10
TTTCCGTAGGTGAACCTGCGGAAGGATCATTATTGAATTATGTTTCTAGATAGGTTGTAG
CTGGCTC-TTTAGAGCATGTGCACGCCTGTTTGGACTTCATTTTCATCCACCTGTGCACC
TATTGTAGTCTTTGGTTGGGTTAGGGGGAAGTGGTCATTGTGTCAGCATCTGCTGGATGT
GAGGACTTGCATTGTGAAAGCTTTGCTGTCCTTGATGTGATCATGGAATCTCTTTCTCAC
TAGAGTCTATGTCACTCATTATACTCTGTCGAATGTCATTGAATGTCTTTACATGGGCTT

GTATGCCTATGAAAATTGTAATACAACTTTCAGCAACGGATCTCTTGGCTCTCGCATCGA
TGAAGGACGCAGCGAAATGCGATAAGTAATGTGAATTGCAGAATTCAGTGAATCATCGAA
TCTTTGAACGCATCTTGCGCTCCTTGGTATTCCGAGGAGCATGCCTGTTTGAGTGTCATT
AAATTCTCAACTCTCTTATACTTTTTTGTAAAAGAGAGCTTGGACTGTGGAGGCTTGCTG
GCCACTTTTTGGGGTCAGCTCCTCTGAAATGCATTAGCGGAACCGTTTGCAATCTGCCAC
AAGTGTGATAAGTTATCTACACTGGCGAGGGGATTGCTCTCTGTAATGTTCAGCTTCTAA
TTGTCTCTACTTTGTGAGACAACTTTTGAATGCTTGACCTCAAATCAGGTAGGACTACCC
GCTGAACTTAA
>AB9-12
TTTCCGTAGGTGAACCTGCGGAAGGATCATTATTGAATTATGTTTCTAGATAGGTTGTAG
CTGGCTC-TTTAGAGCATGTGCACGCCTGTTTGGACTTCATTTTCATCCACCTGTGCACC
TATTGTAGTCTTTGGTTGGGTTAGGGGGAAGTGGTCATTGTGTCAGCATCTGCTGGATGT
GAGGACTTGCATTGTGAAAGCTTTGCTGTCCTTGATGTGATCATGGAATCTCTTTCTCAC
TAGAGTCTATGTCACTCATTATACTCTGTCGAATGTCATTGAATGTCTTTACATGGGCTT
GTATGCCTATGAAAATTGTAATACAACTTTCAGCAACGGATCTCTTGGCTCTCGCATCGA
TGAAGGACGCAGCGAAATGCGATAAGTAATGTGAATTGCAGAATTCAGTGAATCATCGAA
TCTTTGAACGCATCTTGCGCTCCTTGGTATTCCGAGGAGCATGCCTGTTTGAGTGTCATT
AAATTCTCAACTCTCTTATACTTTTTTGTAAAAGAGAGCTTGGACTGTGGAGGCTTGCTG
GCCACTTTTTGGGGTCAGCTCCTCTGAAATGCATTAGCGGAACCGTTTGCAATCTGCCAC
AAGTGTGATAAGTTATCTACACTGGCGAGGGGATTGCTCTCTGTAATGTTCAGCTTCTAA
TTGTCTCTACTTTGTGAGACAACTTTTGAATGCTTGACCTCAAATCAGGTAGGACTACCC
GCTGAACTTAA
>AB1-34
TTTCCGTAGGTGAACCTGCGGAAGGATCATTATTGAATTATGTTTCTAGATAGGTTGTAG
CTGGCTC-TTTAGAGCATGTGCACGCCTGTTTGGACTTCATTTTCATCCACCTGTGCACC
TATTGTAGTCTTTGGTTGGGTTAGGGGGAAGTGGTCATTGTGTCAGCATCTGCTGGATGT
GAGGACTTGCATTGTGAAAGCTTTGCTGTCCTTGATGTGATCATGGAATCTCTTTCTCAC
TAGAGTCTATGTCACTCATTATACTCTGTCGAATGTCATTGAATGTCTTTACATGGGCTT
GTATGCCTATGAAAATTGTAATACAACTTTCAGCAACGGATCTCTTGGCTCTCGCATCGA
TGAAGGACGCAGCGAAATGCGATAAGTAATGTGAATTGCAGAATTCAGTGAATCATCGAA
TCTTTGAACGCATCTTGCGCTCCTTGGTATTCCGAGGAGCATGCCTGTTTGAGTGTCATT
AAATTCTCAACTCTCTTATACTTTTTTGTAAAAGAGAGCTTGGACTGTGGAGGCTTGCTG
GCCACTTTTTGGGGTCAGCTCCTCTGAAATGCATTAGCGGAACCGTTTGCAATCTGCCAC
AAGTGTGATAAGTTATCTACACTGGCGAGGGGATTGCTCTCTGTAATGTTCAGCTTCTAA
TTGTCTCTACTTTGTGAGACAACTTTTGAATGCTTGACCTCAAATCAGGTAGGACTACCC
GCTGAACTTAA
>AB10-55
TTTCCGTAGGTGAACCTGCGGAAGGATCATTATTGAATTATGTTTCTAGATAGGTTGTAG
CTGGCTC-TTTAGAGCATGTGCACGCCTGTTTGGACTTCATTTTCATCCACCTGTGCACC
TATTGTAGTCTTTGGTTGGGTTAGGGGGAAGTGGTCATTGTGTCAGCATCTGCTGGATGT
GAGGACTTGCATTGTGAAAGCTTTGCTGTCCTTGATGTGATCATGGAATCTCTTTCTCAC
TAGAGTCTATGTCACTCATTATACTCTGTCGAATGTCATTGAATGTCTTTACATGGGCTT
GTATGCCTATGAAAATTGTAATACAACTTTCAGCAACGGATCTCTTGGCTCTCGCATCGA
TGAAGGACGCAGCGAAATGCGATAAGTAATGTGAATTGCAGAATTCAGTGAATCATCGAA
TCTTTGAACGCATCTTGCGCTCCTTGGTATTCCGAGGAGCATGCCTGTTTGAGTGTCATT
AAATTCTCAACTCTCTTATACTTTTTTGTAAAAGAGAGCTTGGACTGTGGAGGCTTGCTG
GCCACTTTTTGGGGTCAGCTCCTCTGAAATGCATTAGCGGAACCGTTTGCAATCTGCCAC
AAGTGTGATAAGTTATCTACACTGGCGAGGGGATTGCTCTCTGTAATGTTCAGCTTCTAA
TTGTCTCTACTTTGTGAGACAACTTTTGAATGCTTGACCTCAAATCAGGTAGGACTACCC
GCTGAACTTAA

>AB11-6
TTTCCGTAGGTGAACCTGCGGAAGGATCATTATTGAATTATGTTTCTAGATAGGTTGTAG
CTGGCTC-TTTAGAGCATGTGCACGCCTGTTTGGACTTCATTTTCATCCACCTGTGCACC
TATTGTAGTCTTTGGTTGGGTTAGGGGGAAGTGGTCATTGTGTCAGCATCTGCTGGATGT
GAGGACTTGCATTGTGAAAGCTTTGCTGTCCTTGATGTGATCATGGAATCTCTTTCTCAC
TAGAGTCTATGTCACTCATTATACTCTGTCGAATGTCATTGAATGTCTTTACATGGGCTT
GTATGCCTATGAAAATTGTAATACAACTTTCAGCAACGGATCTCTTGGCTCTCGCATCGA
TGAAGGACGCAGCGAAATGCGATAAGTAATGTGAATTGCAGAATTCAGTGAATCATCGAA
TCTTTGAACGCATCTTGCGCTCCTTGGTATTCCGAGGAGCATGCCTGTTTGAGTGTCATT
AAATTCTCAACTCTCTTATACTTTTTTGTAAAAGAGAGCTTGGACTGTGGAGGCTTGCTG
GCCACTTTTTGGGGTCAGCTCCTCTGAAATGCATTAGCGGAACCGTTTGCAATCTGCCAC
AAGTGTGATAAGTTATCTACACTGGCGAGGGGATTGCTCTCTGTAATGTTCAGCTTCTAA
TTGTCTCTACTTTGTGAGACAACTTTTGAATGCTTGACCTCAAATCAGGTAGGACTACCC
GCTGAACTTAA
>AB11-31
TTTCCGTAGGTGAACCTGCGGAAGGATCATTATTGAATTATGTTTCTAGATAGGTTGTAG
CTGGCTC-TTTAGAGCATGTGCACGCCTGTTTGGACTTCATTTTCATCCACCTGTGCACC
TATTGTAGTCTTTGGTTGGGTTAGGGGGAAGTGGTCATTGTGTCAGCATCTGCTGGATGT
GAGGACTTGCATTGTGAAAGCTTTGCTGTCCTTGATGTGATCATGGAATCTCTTTCTCAC
TAGAGTCTATGTCACTCATTATACTCTGTCGAATGTCATTGAATGTCTTTACATGGGCTT
GTATGCCTATGAAAATTGTAATACAACTTTCAGCAACGGATCTCTTGGCTCTCGCATCGA
TGAAGGACGCAGCGAAATGCGATAAGTAATGTGAATTGCAGAATTCAGTGAATCATCGAA
TCTTTGAACGCATCTTGCGCTCCTTGGTATTCCGAGGAGCATGCCTGTTTGAGTGTCATT
AAATTCTCAACTCTCTTATACTTTTTTGTAAAAGAGAGCTTGGACTGTGGAGGCTTGCTG
GCCACTTTTTGGGGTCAGCTCCTCTGAAATGCATTAGCGGAACCGTTTGCAATCTGCCAC
AAGTGTGATAAGTTATCTACACTGGCGAGGGGATTGCTCTCTGTAATGTTCAGCTTCTAA
TTGTCTCTACTTTGTGAGACAACTTTTGAATGCTTGACCTCAAATCAGGTAGGACTACCC
GCTGAACTTAA
>AB12-2
TTTCCGTAGGTGAACCTGCGGAAGGATCATTATTGAATTATGTTTCTAGATAGGTTGTAG
CTGGCTC-TTTAGAGCATGTGCACGCCTGTTTGGACTTCATTTTCATCCACCTGTGCACC
TATTGTAGTCTTTGGTTGGGTTAGGGGGAAGTGGTCATTGTGTCAGCATCTGCTGGATGT
GAGGACTTGCATTGTGAAAGCTTTGCTGTCCTTGATGTGATCATGGAATCTCTTTCTCAC
TAGAGTCTATGTCACTCATTATACTCTGTCGAATGTCATTGAATGTCTTTACATGGGCTT
GTATGCCTATGAAAATTGTAATACAACTTTCAGCAACGGATCTCTTGGCTCTCGCATCGA
TGAAGGACGCAGCGAAATGCGATAAGTAATGTGAATTGCAGAATTCAGTGAATCATCGAA
TCTTTGAACGCATCTTGCGCTCCTTGGTATTCCGAGGAGCATGCCTGTTTGAGTGTCATT
AAATTCTCAACTCTCTTATACTTTTTTGTAAAAGAGAGCTTGGACTGTGGAGGCTTGCTG
GCCACTTTTTGGGGTCAGCTCCTCTGAAATGCATTAGCGGAACCGTTTGCAATCTGCCAC
AAGTGTGATAAGTTATCTACACTGGCGAGGGGATTGCTCTCTGTAATGTTCAGCTTCTAA
TTGTCTCTACTTTGTGAGACAACTTTTGAATGCTTGACCTCAAATCAGGTAGGACTACCC
GCTGAACTTAA
>AB12-3
TTTCCGTAGGTGAACCTGCGGAAGGATCATTATTGAATTATGTTTCTAGATAGGTTGTAG
CTGGCTC-TTTAGAGCATGTGCACGCCTGTTTGGACTTCATTTTCATCCACCTGTGCACC
TATTGTAGTCTTTGGTTGGGTTAGGGGGAAGTGGTCATTGTGTCAGCATCTGCTGGATGT
GAGGACTTGCATTGTGAAAGCTTTGCTGTCCTTGATGTGATCATGGAATCTCTTTCTCAC
TAGAGTCTATGTCACTCATTATACTCTGTCGAATGTCATTGAATGTCTTTACATGGGCTT
GTATGCCTATGAAAATTGTAATACAACTTTCAGCAACGGATCTCTTGGCTCTCGCATCGA
TGAAGGACGCAGCGAAATGCGATAAGTAATGTGAATTGCAGAATTCAGTGAATCATCGAA

TCTTTGAACGCATCTTGCGCTCCTTGGTATTCCGAGGAGCATGCCTGTTTGAGTGTCATT
AAATTCTCAACTCTCTTATACTTTTTTGTAAAAGAGAGCTTGGACTGTGGAGGCTTGCTG
GCCACTTTTTGGGGTCAGCTCCTCTGAAATGCATTAGCGGAACCGTTTGCAATCTGCCAC
AAGTGTGATAAGTTATCTACACTGGCGAGGGGATTGCTCTCTGTAATGTTCAGCTTCTAA
TTGTCTCTACTTTGTGAGACAACTTTTGAATGCTTGACCTCAAATCAGGTAGGACTACCC
GCTGAACTTAA
>AB11-59
TTTCCGTAGGTGAACCTGCGGAAGGATCATTATTGAATTATGTTTCTAGATAGGTTGTAG
CTGGCTC-TTTAGAGCATGTGCACGCCTGTTTGGACTTCATTTTCATCCACCTGTGCACC
TATTGTAGTCTTTGGTTGGGTTAGGGGGAAGTGGTCATTGTGTCAGCATCTGCTGGATGT
GAGGACTTGCATTGTGAAAGCTTTGCTGTCCTTGATGTGATCATGGAATCTCTTTCTCAC
TAGAGTCTATGTCACTCATTATACTCTGTCGAATGTCATTGAATGTCTTTACATGGGCTT
GTATGCCTATGAAAATTGTAATACAACTTTCAGCAACGGATCTCTTGGCTCTCGCATCGA
TGAAGGACGCAGCGAAATGCGATAAGTAATGTGAATTGCAGAATTCAGTGAATCATCGAA
TCTTTGAACGCATCTTGCGCTCCTTGGTATTCCGAGGAGCATGCCTGTTTGAGTGTCATT
AAATTCTCAACTCTCTTATACTTTTTTGTAAAAGAGAGCTTGGACTGTGGAGGCTTGCTG
GCCACTTTTTGGGGTCAGCTCCTCTGAAATGCATTAGCGGAACCGTTTGCAATCTGCCAC
AAGTGTGATAAGTTATCTACACTGGCGAGGGGATTGCTCTCTGTAATGTTCAGCTTCTAA
TTGTCTCTACTTTGTGAGACAACTTTTGAATGCTTGACCTCAAATCAGGTAGGACTACCC
GCTGAACTTAA
>AB12-4
TTTCCGTAGGTGAACCTGCGGAAGGATCATTATTGAATTATGTTTCTAGATAGGTTGTAG
CTGGCTC-TTTAGAGCATGTGCACGCCTGTTTGGACTTCATTTTCATCCACCTGTGCACC
TATTGTAGTCTTTGGTTGGGTTAGGGGGAAGTGGTCATTGTGTCAGCATCTGCTGGATGT
GAGGACTTGCATTGTGAAAGCTTTGCTGTCCTTGATGTGATCATGGAATCTCTTTCTCAC
TAGAGTCTATGTCACTCATTATACTCTGTCGAATGTCATTGAATGTCTTTACATGGGCTT
GTATGCCTATGAAAATTGTAATACAACTTTCAGCAACGGATCTCTTGGCTCTCGCATCGA
TGAAGGACGCAGCGAAATGCGATAAGTAATGTGAATTGCAGAATTCAGTGAATCATCGAA
TCTTTGAACGCATCTTGCGCTCCTTGGTATTCCGAGGAGCATGCCTGTTTGAGTGTCATT
AAATTCTCAACTCTCTTATACTTTTTTGTAAAAGAGAGCTTGGACTGTGGAGGCTTGCTG
GCCACTTTTTGGGGTCAGCTCCTCTGAAATGCATTAGCGGAACCGTTTGCAATCTGCCAC
AAGTGTGATAAGTTATCTACACTGGCGAGGGGATTGCTCTCTGTAATGTTCAGCTTCTAA
TTGTCTCTACTTTGTGAGACAACTTTTGAATGCTTGACCTCAAATCAGGTAGGACTACCC
GCTGAACTTAA
>AB10-54
TTTCCGTAGGTGAACCTGCGGAAGGATCATTATTGAATTATGTTTCTAGATAGGTTGTAG
CTGGCTC-TTTAGAGCATGTGCACGCCTGTTTGGACTTCATTTTCATCCACCTGTGCACC
TATTGTAGTCTTTGGTTGGGTTAGGGGGAAGTGGTCATTGTGTCAGCATCTGCTGGATGT
GAGGACTTGCATTGTGAAAGCTTTGCTGTCCTTGATGTGATCATGGAATCTCTTTCTCAC
TAGAGTCTATGTCACTCATTATACTCTGTCGAATGTCATTGAATGTCTTTACATGGGCTT
GTATGCCTATGAAAATTGTAATACAACTTTCAGCAACGGATCTCTTGGCTCTCGCATCGA
TGAAGGACGCAGCGAAATGCGATAAGTAATGTGAATTGCAGAATTCAGTGAATCATCGAA
TCTTTGAACGCATCTTGCGCTCCTTGGTATTCCGAGGAGCATGCCTGTTTGAGTGTCATT
AAATTCTCAACTCTCTTATACTTTTTTGTAAAAGAGAGCTTGGACTGTGGAGGCTTGCTG
GCCACTTTTTGGGGTCAGCTCCTCTGAAATGCATTAGCGGAACCGTTTGCAATCTGCCAC
AAGTGTGATAAGTTATCTACACTGGCGAGGGGATTGCTCTCTGTAATGTTCAGCTTCTAA
TTGTCTCTACTTTGTGAGACAACTTTTGAATGCTTGACCTCAAATCAGGTAGGACTACCC
GCTGAACTTAA
>AB2-64
TTTCCGTAGGTGAACCTGCGGAAGGATCATTATTGAATTATGTTTCTAGATAGGTTGTAG

CTGGCTC-TTTAGAGCATGTGCACGCCTGTTTGGACTTCATTTTCATCCACCTGTGCACC
TATTGTAGTCTTTGGTTGGGTTAGGGGGAAGTGGTCATTGTGTCAGCATCTGCTGGATGT
GAGGACTTGCATTGTGAAAGCTTTGCTGTCCTTGATGTGATCATGGAATCTCTTTCTCAC
TAGAGTCTATGTCACTCATTATACTCTGTCGAATGTCATTGAATGTCTTTACATGGGCTT
GTATGCCTATGAAAATTGTAATACAACTTTCAGCAACGGATCTCTTGGCTCTCGCATCGA
TGAAGGACGCAGCGAAATGCGATAAGTAATGTGAATTGCAGAATTCAGTGAATCATCGAA
TCTTTGAACGCATCTTGCGCTCCTTGGTATTCCGAGGAGCATGCCTGTTTGAGTGTCATT
AAATTCTCAACTCTCTTATACTTTTTTGTAAAAGAGAGCTTGGACTGTGGAGGCTTGCTG
GCCACTTTTTGGGGTCAGCTCCTCTGAAATGCATTAGCGGAACCGTTTGCAATCTGCCAC
AAGTGTGATAAGTTATCTACACTGGCGAGGGGATTGCTCTCTGTAATGTTCAGCTTCTAA
TTGTCTCTACTTTGTGAGACAACTTTTGAATGCTTGACCTCAAATCAGGTAGGACTACCC
GCTGAACTTAA
>AB3-63
TTTCCGTAGGTGAACCTGCGGAAGGATCATTATTGAATTATGTTTCTAGATAGGTTGTAG
CTGGCTC-TTTAGAGCATGTGCACGCCTGTTTGGACTTCATTTTCATCCACCTGTGCACC
TATTGTAGTCTTTGGTTGGGTTAGGGGGAAGTGGTCATTGTGTCAGCATCTGCTGGATGT
GAGGACTTGCATTGTGAAAGCTTTGCTGTCCTTGATGTGATCATGGAATCTCTTTCTCAC
TAGAGTCTATGTCACTCATTATACTCTGTCGAATGTCATTGAATGTCTTTACATGGGCTT
GTATGCCTATGAAAATTGTAATACAACTTTCAGCAACGGATCTCTTGGCTCTCGCATCGA
TGAAGGACGCAGCGAAATGCGATAAGTAATGTGAATTGCAGAATTCAGTGAATCATCGAA
TCTTTGAACGCATCTTGCGCTCCTTGGTATTCCGAGGAGCATGCCTGTTTGAGTGTCATT
AAATTCTCAACTCTCTTATACTTTTTTGTAAAAGAGAGCTTGGACTGTGGAGGCTTGCTG
GCCACTTTTTGGGGTCAGCTCCTCTGAAATGCATTAGCGGAACCGTTTGCAATCTGCCAC
AAGTGTGATAAGTTATCTACACTGGCGAGGGGATTGCTCTCTGTAATGTTCAGCTTCTAA
TTGTCTCTACTTTGTGAGACAACTTTTGAATGCTTGACCTCAAATCAGGTAGGACTACCC
GCTGAACTTAA
>AB4-52
TTTCCGTAGGTGAACCTGCGGAAGGATCATTATTGAATTATGTTTCTAGATAGGTTGTAG
CTGGCTC-TTTAGAGCATGTGCACGCCTGTTTGGACTTCATTTTCATCCACCTGTGCACC
TATTGTAGTCTTTGGTTGGGTTAGGGGGAAGTGGTCATTGTGTCAGCATCTGCTGGATGT
GAGGACTTGCATTGTGAAAGCTTTGCTGTCCTTGATGTGATCATGGAATCTCTTTCTCAC
TAGAGTCTATGTCACTCATTATACTCTGTCGAATGTCATTGAATGTCTTTACATGGGCTT
GTATGCCTATGAAAATTGTAATACAACTTTCAGCAACGGATCTCTTGGCTCTCGCATCGA
TGAAGGACGCAGCGAAATGCGATAAGTAATGTGAATTGCAGAATTCAGTGAATCATCGAA
TCTTTGAACGCATCTTGCGCTCCTTGGTATTCCGAGGAGCATGCCTGTTTGAGTGTCATT
AAATTCTCAACTCTCTTATACTTTTTTGTAAAAGAGAGCTTGGACTGTGGAGGCTTGCTG
GCCACTTTTTGGGGTCAGCTCCTCTGAAATGCATTAGCGGAACCGTTTGCAATCTGCCAC
AAGTGTGATAAGTTATCTACACTGGCGAGGGGATTGCTCTCTGTAATGTTCAGCTTCTAA
TTGTCTCTACTTTGTGAGACAACTTTTGAATGCTTGACCTCAAATCAGGTAGGACTACCC
GCTGAACTTAA
>AB6-53
TTTCCGTAGGTGAACCTGCGGAAGGATCATTATTGAATTATGTTTCTAGATAGGTTGTAG
CTGGCTC-TTTAGAGCATGTGCACGCCTGTTTGGACTTCATTTTCATCCACCTGTGCACC
TATTGTAGTCTTTGGTTGGGTTAGGGGGAAGTGGTCATTGTGTCAGCATCTGCTGGATGT
GAGGACTTGCATTGTGAAAGCTTTGCTGTCCTTGATGTGATCATGGAATCTCTTTCTCAC
TAGAGTCTATGTCACTCATTATACTCTGTCGAATGTCATTGAATGTCTTTACATGGGCTT
GTATGCCTATGAAAATTGTAATACAACTTTCAGCAACGGATCTCTTGGCTCTCGCATCGA
TGAAGGACGCAGCGAAATGCGATAAGTAATGTGAATTGCAGAATTCAGTGAATCATCGAA
TCTTTGAACGCATCTTGCGCTCCTTGGTATTCCGAGGAGCATGCCTGTTTGAGTGTCATT
AAATTCTCAACTCTCTTATACTTTTTTGTAAAAGAGAGCTTGGACTGTGGAGGCTTGCTG

GCCACTTTTTGGGGTCAGCTCCTCTGAAATGCATTAGCGGAACCGTTTGCAATCTGCCAC
AAGTGTGATAAGTTATCTACACTGGCGAGGGGATTGCTCTCTGTAATGTTCAGCTTCTAA
TTGTCTCTACTTTGTGAGACAACTTTTGAATGCTTGACCTCAAATCAGGTAGGACTACCC
GCTGAACTTAA
>AB7–55
TTTCCGTAGGTGAACCTGCGGAAGGATCATTATTGAATTATGTTTCTAGATAGGTTGTAG
CTGGCTC–TTTAGAGCATGTGCACGCCTGTTTGGACTTCATTTTCATCCACCTGTGCACC
TATTGTAGTCTTTGGTTGGGTTAGGGGGAAGTGGTCATTGTGTCAGCATCTGCTGGATGT
GAGGACTTGCATTGTGAAAGCTTTGCTGTCCTTGATGTGATCATGGAATCTCTTTCTCAC
TAGAGTCTATGTCACTCATTATACTCTGTCGAATGTCATTGAATGTCTTTACATGGGCTT
GTATGCCTATGAAAATTGTAATACAACTTTCAGCAACGGATCTCTTGGCTCTCGCATCGA
TGAAGGACGCAGCGAAATGCGATAAGTAATGTGAATTGCAGAATTCAGTGAATCATCGAA
TCTTTGAACGCATCTTGCGCTCCTTGGTATTCCGAGGAGCATGCCTGTTTGAGTGTCATT
AAATTCTCAACTCTCTTATACTTTTTTGTAAAAGAGAGCTTGGACTGTGGAGGCTTGCTG
GCCACTTTTTGGGGTCAGCTCCTCTGAAATGCATTAGCGGAACCGTTTGCAATCTGCCAC
AAGTGTGATAAGTTATCTACACTGGCGAGGGGATTGCTCTCTGTAATGTTCAGCTTCTAA
TTGTCTCTACTTTGTGAGACAACTTTTGAATGCTTGACCTCAAATCAGGTAGGACTACCC
GCTGAACTTAA
>AB7–40
TTTCCGTAGGTGAACCTGCGGAAGGATCATTATTGAATTATGTTTCTAGATAGGTTGTAG
CTGGCTC–TTTAGAGCATGTGCACGCCTGTTTGGACTTCATTTTCATCCACCTGTGCACC
TATTGTAGTCTTTGGTTGGGTTAGGGGGAAGTGGTCATTGTGTCAGCATCTGCTGGATGT
GAGGACTTGCATTGTGAAAGCTTTGCTGTCCTTGATGTGATCATGGAATCTCTTTCTCAC
TAGAGTCTATGTCACTCATTATACTCTGTCGAATGTCATTGAATGTCTTTACATGGGCTT
GTATGCCTATGAAAATTGTAATACAACTTTCAGCAACGGATCTCTTGGCTCTCGCATCGA
TGAAGGACGCAGCGAAATGCGATAAGTAATGTGAATTGCAGAATTCAGTGAATCATCGAA
TCTTTGAACGCATCTTGCGCTCCTTGGTATTCCGAGGAGCATGCCTGTTTGAGTGTCATT
AAATTCTCAACTCTCTTATACTTTTTTGTAAAAGAGAGCTTGGACTGTGGAGGCTTGCTG
GCCACTTTTTGGGGTCAGCTCCTCTGAAATGCATTAGCGGAACCGTTTGCAATCTGCCAC
AAGTGTGATAAGTTATCTACACTGGCGAGGGGATTGCTCTCTGTAATGTTCAGCTTCTAA
TTGTCTCTACTTTGTGAGACAACTTTTGAATGCTTGACCTCAAATCAGGTAGGACTACCC
GCTGAACTTAA
>AB7–54
TTTCCGTAGGTGAACCTGCGGAAGGATCATTATTGAATTATGTTTCTAGATAGGTTGTAG
CTGGCTC–TTTAGAGCATGTGCACGCCTGTTTGGACTTCATTTTCATCCACCTGTGCACC
TATTGTAGTCTTTGGTTGGGTTAGGGGGAAGTGGTCATTGTGTCAGCATCTGCTGGATGT
GAGGACTTGCATTGTGAAAGCTTTGCTGTCCTTGATGTGATCATGGAATCTCTTTCTCAC
TAGAGTCTATGTCACTCATTATACTCTGTCGAATGTCATTGAATGTCTTTACATGGGCTT
GTATGCCTATGAAAATTGTAATACAACTTTCAGCAACGGATCTCTTGGCTCTCGCATCGA
TGAAGGACGCAGCGAAATGCGATAAGTAATGTGAATTGCAGAATTCAGTGAATCATCGAA
TCTTTGAACGCATCTTGCGCTCCTTGGTATTCCGAGGAGCATGCCTGTTTGAGTGTCATT
AAATTCTCAACTCTCTTATACTTTTTTGTAAAAGAGAGCTTGGACTGTGGAGGCTTGCTG
GCCACTTTTTGGGGTCAGCTCCTCTGAAATGCATTAGCGGAACCGTTTGCAATCTGCCAC
AAGTGTGATAAGTTATCTACACTGGCGAGGGGATTGCTCTCTGTAATGTTCAGCTTCTAA
TTGTCTCTACTTTGTGAGACAACTTTTGAATGCTTGACCTCAAATCAGGTAGGACTACCC
GCTGAACTTAA
>AB8–57
TTTCCGTAGGTGAACCTGCGGAAGGATCATTATTGAATTATGTTTCTAGATAGGTTGTAG
CTGGCTC–TTTAGAGCATGTGCACGCCTGTTTGGACTTCATTTTCATCCACCTGTGCACC
TATTGTAGTCTTTGGTTGGGTTAGGGGGAAGTGGTCATTGTGTCAGCATCTGCTGGATGT

GAGGACTTGCATTGTGAAAGCTTTGCTGTCCTTGATGTGATCATGGAATCTCTTTCTCAC
TAGAGTCTATGTCACTCATTATACTCTGTCGAATGTCATTGAATGTCTTTACATGGGCTT
GTATGCCTATGAAAATTGTAATACAACTTTCAGCAACGGATCTCTTGGCTCTCGCATCGA
TGAAGGACGCAGCGAAATGCGATAAGTAATGTGAATTGCAGAATTCAGTGAATCATCGAA
TCTTTGAACGCATCTTGCGCTCCTTGGTATTCCGAGGAGCATGCCTGTTTGAGTGTCATT
AAATTCTCAACTCTCTTATACTTTTTTGTAAAAGAGAGCTTGGACTGTGGAGGCTTGCTG
GCCACTTTTTGGGGTCAGCTCCTCTGAAATGCATTAGCGGAACCGTTTGCAATCTGCCAC
AAGTGTGATAAGTTATCTACACTGGCGAGGGGATTGCTCTCTGTAATGTTCAGCTTCTAA
TTGTCTCTACTTTGTGAGACAACTTTTGAATGCTTGACCTCAAATCAGGTAGGACTACCC
GCTGAACTTAA
>AB8-56
TTTCCGTAGGTGAACCTGCGGAAGGATCATTATTGAATTATGTTTCTAGATAGGTTGTAG
CTGGCTC-TTTAGAGCATGTGCACGCCTGTTTGGACTTCATTTTCATCCACCTGTGCACC
TATTGTAGTCTTTGGTTGGGTTAGGGGGAAGTGGTCATTGTGTCAGCATCTGCTGGATGT
GAGGACTTGCATTGTGAAAGCTTTGCTGTCCTTGATGTGATCATGGAATCTCTTTCTCAC
TAGAGTCTATGTCACTCATTATACTCTGTCGAATGTCATTGAATGTCTTTACATGGGCTT
GTATGCCTATGAAAATTGTAATACAACTTTCAGCAACGGATCTCTTGGCTCTCGCATCGA
TGAAGGACGCAGCGAAATGCGATAAGTAATGTGAATTGCAGAATTCAGTGAATCATCGAA
TCTTTGAACGCATCTTGCGCTCCTTGGTATTCCGAGGAGCATGCCTGTTTGAGTGTCATT
AAATTCTCAACTCTCTTATACTTTTTTGTAAAAGAGAGCTTGGACTGTGGAGGCTTGCTG
GCCACTTTTTGGGGTCAGCTCCTCTGAAATGCATTAGCGGAACCGTTTGCAATCTGCCAC
AAGTGTGATAAGTTATCTACACTGGCGAGGGGATTGCTCTCTGTAATGTTCAGCTTCTAA
TTGTCTCTACTTTGTGAGACAACTTTTGAATGCTTGACCTCAAATCAGGTAGGACTACCC
GCTGAACTTAA
>AB9-27
TTTCCGTAGGTGAACCTGCGGAAGGATCATTATTGAATTATGTTTCTAGATAGGTTGTAG
CTGGCTC-TTTAGAGCATGTGCACGCCTGTTTGGACTTCATTTTCATCCACCTGTGCACC
TATTGTAGTCTTTGGTTGGGTTAGGGGGAAGTGGTCATTGTGTCAGCATCTGCTGGATGT
GAGGACTTGCATTGTGAAAGCTTTGCTGTCCTTGATGTGATCATGGAATCTCTTTCTCAC
TAGAGTCTATGTCACTCATTATACTCTGTCGAATGTCATTGAATGTCTTTACATGGGCTT
GTATGCCTATGAAAATTGTAATACAACTTTCAGCAACGGATCTCTTGGCTCTCGCATCGA
TGAAGGACGCAGCGAAATGCGATAAGTAATGTGAATTGCAGAATTCAGTGAATCATCGAA
TCTTTGAACGCATCTTGCGCTCCTTGGTATTCCGAGGAGCATGCCTGTTTGAGTGTCATT
AAATTCTCAACTCTCTTATACTTTTTTGTAAAAGAGAGCTTGGACTGTGGAGGCTTGCTG
GCCACTTTTTGGGGTCAGCTCCTCTGAAATGCATTAGCGGAACCGTTTGCAATCTGCCAC
AAGTGTGATAAGTTATCTACACTGGCGAGGGGATTGCTCTCTGTAATGTTCAGCTTCTAA
TTGTCTCTACTTTGTGAGACAACTTTTGAATGCTTGACCTCAAATCAGGTAGGACTACCC
GCTGAACTTAA
>AB9-49
TTTCCGTAGGTGAACCTGCGGAAGGATCATTATTGAATTATGTTTCTAGATAGGTTGTAG
CTGGCTC-TTTAGAGCATGTGCACGCCTGTTTGGACTTCATTTTCATCCACCTGTGCACC
TATTGTAGTCTTTGGTTGGGTTAGGGGGAAGTGGTCATTGTGTCAGCATCTGCTGGATGT
GAGGACTTGCATTGTGAAAGCTTTGCTGTCCTTGATGTGATCATGGAATCTCTTTCTCAC
TAGAGTCTATGTCACTCATTATACTCTGTCGAATGTCATTGAATGTCTTTACATGGGCTT
GTATGCCTATGAAAATTGTAATACAACTTTCAGCAACGGATCTCTTGGCTCTCGCATCGA
TGAAGGACGCAGCGAAATGCGATAAGTAATGTGAATTGCAGAATTCAGTGAATCATCGAA
TCTTTGAACGCATCTTGCGCTCCTTGGTATTCCGAGGAGCATGCCTGTTTGAGTGTCATT
AAATTCTCAACTCTCTTATACTTTTTTGTAAAAGAGAGCTTGGACTGTGGAGGCTTGCTG
GCCACTTTTTGGGGTCAGCTCCTCTGAAATGCATTAGCGGAACCGTTTGCAATCTGCCAC
AAGTGTGATAAGTTATCTACACTGGCGAGGGGATTGCTCTCTGTAATGTTCAGCTTCTAA

TTGTCTCTACTTTGTGAGACAACTTTTGAATGCTTGACCTCAAATCAGGTAGGACTACCC
GCTGAACTTAA
>AB9-13
TTTCCGTAGGTGAACCTGCGGAAGGATCATTATTGAATTATGTTTCTAGATAGGTTGTAG
CTGGCTC-TTTAGAGCATGTGCACGCCTGTTTGGACTTCATTTTCATCCACCTGTGCACC
TATTGTAGTCTTTGGTTGGGTTAGGGGGAAGTGGTCATTGTGTCAGCATCTGCTGGATGT
GAGGACTTGCATTGTGAAAGCTTTGCTGTCCTTGATGTGATCATGGAATCTCTTTCTCAC
TAGAGTCTATGTCACTCATTATACTCTGTCGAATGTCATTGAATGTCTTTACATGGGCTT
GTATGCCTATGAAAATTGTAATACAACTTTCAGCAACGGATCTCTTGGCTCTCGCATCGA
TGAAGGACGCAGCGAAATGCGATAAGTAATGTGAATTGCAGAATTCAGTGAATCATCGAA
TCTTTGAACGCATCTTGCGCTCCTTGGTATTCCGAGGAGCATGCCTGTTTGAGTGTCATT
AAATTCTCAACTCTCTTATACTTTTTTGTAAAAGAGAGCTTGGACTGTGGAGGCTTGCTG
GCCACTTTTTGGGGTCAGCTCCTCTGAAATGCATTAGCGGAACCGTTTGCAATCTGCCAC
AAGTGTGATAAGTTATCTACACTGGCGAGGGGATTGCTCTCTGTAATGTTCAGCTTCTAA
TTGTCTCTACTTTGTGAGACAACTTTTGAATGCTTGACCTCAAATCAGGTAGGACTACCC
GCTGAACTTAA
>AB5-27
TTTCCGTAGGTGAACCTGCGGAAGGATCATTATTGAATTATGTTTCTAGATAGGTTGTAG
CTGGCTC-TTTAGAGCATGTGCACGCCTGTTTGGACTTCATTTTCATCCACCTGTGCACC
TATTGTAGTCTTTGGTTGGGTTAGGGGGAAGTGGTCATTGTGTCAGCATCTGCTGGATGT
GAGGACTTGCATTGTGAAAGCTTTGCTGTCCTTGATGTGATCATGGAATCTCTTTCTCAC
TAGAGTCTATGTCACTCATTATACTCTGTCGAATGTCATTGAATGTCTTTACATGGGCTT
GTATGCCTATGAAAATTGTAATACAACTTTCAGCAACGGATCTCTTGGCTCTCGCATCGA
TGAAGGACGCAGCGAAATGCGATAAGTAATGTGAATTGCAGAATTCAGTGAATCATCGAA
TCTTTGAACGCATCTTGCGCTCCTTGGTATTCCGAGGAGCATGCCTGTTTGAGTGTCATT
AAATTCTCAACTCTCTTATACTTTTTTGTAAAAGAGAGCTTGGACTGTGGAGGCTTGCTG
GCCACTTTTTGGGGTCAGCTCCTCTGAAATGCATTAGCGGAACCGTTTGCAATCTGCCAC
AAGTGTGATAAGTTATCTACACTGGCGAGGGGATTGCTCTCTGTAATGTTCAGCTTCTAA
TTGTCTCTACTTTGTGAGACAACTTTTGAATGCTTGACCTCAAATCAGGTAGGACTACCC
GCTGAACTTAA
>AB1-13
TTTCCGTAGGTGAACCTGCGGAAGGATCATTATTGAATTATGTTTCTAGATAGGTTGTAG
CTGGCTC-TTTAGAGCATGTGCACGCCTGTTTGGACTTCATTTTCATCCACCTGTGCACC
TATTGTAGTCTTTGGTTGGGTTAGGGGGAAGTGGTCATTGTGTCAGCATCTGCTGGATGT
GAGGACTTGCATTGTGAAAGCTTTGCTGTCCTTGATGTGATCATGGAATCTCTTTCTCAC
TAGAGTCTATGTCACTCATTATACTCTGTCGAATGTCATTGAATGTCTTTACATGGGCTT
GTATGCCTATGAAAATTGTAATACAACTTTCAGCAACGGATCTCTTGGCTCTCGCATCGA
TGAAGGACGCAGCGAAATGCGATAAGTAATGTGAATTGCAGAATTCAGTGAATCATCGAA
TCTTTGAACGCATCTTGCGCTCCTTGGTATTCCGAGGAGCATGCCTGTTTGAGTGTCATT
AAATTCTCAACTCTCTTATACTTTTTTGTAAAAGAGAGCTTGGACTGTGGAGGCTTGCTG
GCCACTTTTTGGGGTCAGCTCCTCTGAAATGCATTAGCGGAACCGTTTGCAATCTGCCAC
AAGTGTGATAAGTTATCTACACTGGCGAGGGGATTGCTCTCTGTAATGTTCAGCTTCTAA
TTGTCTCTACTTTGTGAGACAACTTTTGAATGCTTGACCTCAAATCAGGTAGGACTACCC
GCTGAACTTAA
>AB2-66
TTTCCGTAGGTGAACCTGCGGAAGGATCATTATTGAATTATGTTTCTAGATAGGTTGTAG
CTGGCTC-TTTAGAGCATGTGCACGCCTGTTTGGACTTCATTTTCATCCACCTGTGCACC
TATTGTAGTCTTTGGTTGGGTTAGGGGGAAGTGGTCATTGTGTCAGCATCTGCTGGATGT
GAGGACTTGCATTGTGAAAGCTTTGCTGTCCTTGATGTGATCATGGAATCTCTTTCTCAC
TAGAGTCTATGTCACTCATTATACTCTGTCGAATGTCATTGAATGTCTTTACATGGGCTT

GTATGCCTATGAAAATTGTAATACAACTTTCAGCAACGGATCTCTTGGCTCTCGCATCGA
TGAAGGACGCAGCGAAATGCGATAAGTAATGTGAATTGCAGAATTCAGTGAATCATCGAA
TCTTTGAACGCATCTTGCGCTCCTTGGTATTCCGAGGAGCATGCCTGTTTGAGTGTCATT
AAATTCTCAACTCTCTTATACTTTTTTGTAAAAGAGAGCTTGGACTGTGGAGGCTTGCTG
GCCACTTTTTGGGGTCAGCTCCTCTGAAATGCATTAGCGGAACCGTTTGCAATCTGCCAC
AAGTGTGATAAGTTATCTACACTGGCGAGGGGATTGCTCTCTGTAATGTTCAGCTTCTAA
TTGTCTCTACTTTGTGAGACAACTTTTGAATGCTTGACCTCAAATCAGGTAGGACTACCC
GCTGAACTTAA
>AB1–27
TTTCCGTAGGTGAACCTGCGGAAGGATCATTATTGAATTATGTTTCTAGATAGGTTGTAG
CTGGCTC–TTTAGAGCATGTGCACGCCTGTTTGGACTTCATTTTCATCCACCTGTGCACC
TATTGTAGTCTTTGGTTGGGTTAGGGGGAAGTGGTCATTGTGTCAGCATCTGCTGGATGT
GAGGACTTGCATTGTGAAAGCTTTGCTGTCCTTGATGTGATCATGGAATCTCTTTCTCAC
TAGAGTCTATGTCACTCATTATACTCTGTCGAATGTCATTGAATGTCTTTACATGGGCTT
GTATGCCTATGAAAATTGTAATACAACTTTCAGCAACGGATCTCTTGGCTCTCGCATCGA
TGAAGGACGCAGCGAAATGCGATAAGTAATGTGAATTGCAGAATTCAGTGAATCATCGAA
TCTTTGAACGCATCTTGCGCTCCTTGGTATTCCGAGGAGCATGCCTGTTTGAGTGTCATT
AAATTCTCAACTCTCTTATACTTTTTTGTAAAAGAGAGCTTGGACTGTGGAGGCTTGCTG
GCCACTTTTTGGGGTCAGCTCCTCTGAAATGCATTAGCGGAACCGTTTGCAATCTGCCAC
AAGTGTGATAAGTTATCTACACTGGCGAGGGGATTGCTCTCTGTAATGTTCAGCTTCTAA
TTGTCTCTACTTTGTGAGACAACTTTTGAATGCTTGACCTCAAATCAGGTAGGACTACCC
GCTGAACTTAA
>AB1–32
TTTCCGTAGGTGAACCTGCGGAAGGATCATTATTGAATTATGTTTCTAGATAGGTTGTAG
CTGGCTC–TTTAGAGCATGTGCACGCCTGTTTGGACTTCATTTTCATCCACCTGTGCACC
TATTGTAGTCTTTGGTTGGGTTAGGGGGAAGTGGTCATTGTGTCAGCATCTGCTGGATGT
GAGGACTTGCATTGTGAAAGCTTTGCTGTCCTTGATGTGATCATGGAATCTCTTTCTCAC
TAGAGTCTATGTCACTCATTATACTCTGTCGAATGTCATTGAATGTCTTTACATGGGCTT
GTATGCCTATGAAAATTGTAATACAACTTTCAGCAACGGATCTCTTGGCTCTCGCATCGA
TGAAGGACGCAGCGAAATGCGATAAGTAATGTGAATTGCAGAATTCAGTGAATCATCGAA
TCTTTGAACGCATCTTGCGCTCCTTGGTATTCCGAGGAGCATGCCTGTTTGAGTGTCATT
AAATTCTCAACTCTCTTATACTTTTTTGTAAAAGAGAGCTTGGACTGTGGAGGCTTGCTG
GCCACTTTTTGGGGTCAGCTCCTCTGAAATGCATTAGCGGAACCGTTTGCAATCTGCCAC
AAGTGTGATAAGTTATCTACACTGGCGAGGGGATTGCTCTCTGTAATGTTCAGCTTCTAA
TTGTCTCTACTTTGTGAGACAACTTTTGAATGCTTGACCTCAAATCAGGTAGGACTACCC
GCTGAACTTAA
>AB4–19
TTTCCGTAGGTGAACCTGCGGAAGGATCATTATTGAATTATGTTTCTAGATAGGTTGTAG
CTGGCTC–TTTAGAGCATGTGCACGCCTGTTTGGACTTCATTTTCATCCACCTGTGCACC
TATTGTAGTCTTTGGTTGGGTTAGGGGGAAGTGGTCATTGTGTCAGCATCTGCTGGATGT
GAGGACTTGCATTGTGAAAGCTTTGCTGTCCTTGATGTGATCATGGAATCTCTTTCTCAC
TAGAGTCTATGTCACTCATTATACTCTGTCGAATGTCATTGAATGTCTTTACATGGGCTT
GTATGCCTATGAAAATTGTAATACAACTTTCAGCAACGGATCTCTTGGCTCTCGCATCGA
TGAAGGACGCAGCGAAATGCGATAAGTAATGTGAATTGCAGAATTCAGTGAATCATCGAA
TCTTTGAACGCATCTTGCGCTCCTTGGTATTCCGAGGAGCATGCCTGTTTGAGTGTCATT
AAATTCTCAACTCTCTTATACTTTTTTGTAAAAGAGAGCTTGGACTGTGGAGGCTTGCTG
GCCACTTTTTGGGGTCAGCTCCTCTGAAATGCATTAGCGGAACCGTTTGCAATCTGCCAC
AAGTGTGATAAGTTATCTACACTGGCGAGGGGATTGCTCTCTGTAATGTTCAGCTTCTAA
TTGTCTCTACTTTGTGAGACAACTTTTGAATGCTTGACCTCAAATCAGGTAGGACTACCC
GCTGAACTTAA

>AB1–42
TTTCCGTAGGTGAACCTGCGGAAGGATCATTATTGAATTATGTTTCTAGATAGGTTGTAG
CTGGCTC–TTTAGAGCATGTGCACGCCTGTTTGGACTTCATTTTCATCCACCTGTGCACC
TATTGTAGTCTTTGGTTGGGTTAGGGGGAAGTGGTCATTGTGTCAGCATCTGCTGGATGT
GAGGACTTGCATTGTGAAAGCTTTGCTGTCCTTGATGTGATCATGGAATCTCTTTCTCAC
TAGAGTCTATGTCACTCATTATACTCTGTCGAATGTCATTGAATGTCTTTACATGGGCTT
GTATGCCTATGAAAATTGTAATACAACTTTCAGCAACGGATCTCTTGGCTCTCGCATCGA
TGAAGGACGCAGCGAAATGCGATAAGTAATGTGAATTGCAGAATTCAGTGAATCATCGAA
TCTTTGAACGCATCTTGCGCTCCTTGGTATTCCGAGGAGCATGCCTGTTTGAGTGTCATT
AAATTCTCAACTCTCTTATACTTTTTTGTAAAAGAGAGCTTGGACTGTGGAGGCTTGCTG
GCCACTTTTTGGGGTCAGCTCCTCTGAAATGCATTAGCGGAACCGTTTGCAATCTGCCAC
AAGTGTGATAAGTTATCTACACTGGCGAGGGGATTGCTCTCTGTAATGTTCAGCTTCTAA
TTGTCTCTACTTTGTGAGACAACTTTTGAATGCTTGACCTCAAATCAGGTAGGACTACCC
GCTGAACTTAA
>AB2–67
TTTCCGTAGGTGAACCTGCGGAAGGATCATTATTGAATTATGTTTCTAGATAGGTTGTAG
CTGGCTC–TTTAGAGCATGTGCACGCCTGTTTGGACTTCATTTTCATCCACCTGTGCACC
TATTGTAGTCTTTGGTTGGGTTAGGGGGAAGTGGTCATTGTGTCAGCATCTGCTGGATGT
GAGGACTTGCATTGTGAAAGCTTTGCTGTCCTTGATGTGATCATGGAATCTCTTTCTCAC
TAGAGTCTATGTCACTCATTATACTCTGTCGAATGTCATTGAATGTCTTTACATGGGCTT
GTATGCCTATGAAAATTGTAATACAACTTTCAGCAACGGATCTCTTGGCTCTCGCATCGA
TGAAGGACGCAGCGAAATGCGATAAGTAATGTGAATTGCAGAATTCAGTGAATCATCGAA
TCTTTGAACGCATCTTGCGCTCCTTGGTATTCCGAGGAGCATGCCTGTTTGAGTGTCATT
AAATTCTCAACTCTCTTATACTTTTTTGTAAAAGAGAGCTTGGACTGTGGAGGCTTGCTG
GCCACTTTTTGGGGTCAGCTCCTCTGAAATGCATTAGCGGAACCGTTTGCAATCTGCCAC
AAGTGTGATAAGTTATCTACACTGGCGAGGGGATTGCTCTCTGTAATGTTCAGCTTCTAA
TTGTCTCTACTTTGTGAGACAACTTTTGAATGCTTGACCTCAAATCAGGTAGGACTACCC
GCTGAACTTAA
>AB5–56
TTTCCGTAGGTGAACCTGCGGAAGGATCATTATTGAATTATGTTTCTAGATAGGTTGTAG
CTGGCTC–TTTAGAGCATGTGCACGCCTGTTTGGACTTCATTTTCATCCACCTGTGCACC
TATTGTAGTCTTTGGTTGGGTTAGGGGGAAGTGGTCATTGTGTCAGCATCTGCTGGATGT
GAGGACTTGCATTGTGAAAGCTTTGCTGTCCTTGATGTGATCATGGAATCTCTTTCTCAC
TAGAGTCTATGTCACTCATTATACTCTGTCGAATGTCATTGAATGTCTTTACATGGGCTT
GTATGCCTATGAAAATTGTAATACAACTTTCAGCAACGGATCTCTTGGCTCTCGCATCGA
TGAAGGACGCAGCGAAATGCGATAAGTAATGTGAATTGCAGAATTCAGTGAATCATCGAA
TCTTTGAACGCATCTTGCGCTCCTTGGTATTCCGAGGAGCATGCCTGTTTGAGTGTCATT
AAATTCTCAACTCTCTTATACTTTTTTGTAAAAGAGAGCTTGGACTGTGGAGGCTTGCTG
GCCACTTTTTGGGGTCAGCTCCTCTGAAATGCATTAGCGGAACCGTTTGCAATCTGCCAC
AAGTGTGATAAGTTATCTACACTGGCGAGGGGATTGCTCTCTGTAATGTTCAGCTTCTAA
TTGTCTCTACTTTGTGAGACAACTTTTGAATGCTTGACCTCAAATCAGGTAGGACTACCC
GCTGAACTTAA
>AB7–15
TTTCCGTAGGTGAACCTGCGGAAGGATCATTATTGAATTATGTTTCTAGATAGGTTGTAG
CTGGCTC–TTTAGAGCATGTGCACGCCTGTTTGGACTTCATTTTCATCCACCTGTGCACC
TATTGTAGTCTTTGGTTGGGTTAGGGGGAAGTGGTCATTGTGTCAGCATCTGCTGGATGT
GAGGACTTGCATTGTGAAAGCTTTGCTGTCCTTGATGTGATCATGGAATCTCTTTCTCAC
TAGAGTCTATGTCACTCATTATACTCTGTCGAATGTCATTGAATGTCTTTACATGGGCTT
GTATGCCTATGAAAATTGTAATACAACTTTCAGCAACGGATCTCTTGGCTCTCGCATCGA
TGAAGGACGCAGCGAAATGCGATAAGTAATGTGAATTGCAGAATTCAGTGAATCATCGAA

TCTTTGAACGCATCTTGCGCTCCTTGGTATTCCGAGGAGCATGCCTGTTTGAGTGTCATT
AAATTCTCAACTCTCTTATACTTTTTTGTAAAAGAGAGCTTGGACTGTGGAGGCTTGCTG
GCCACTTTTTGGGGTCAGCTCCTCTGAAATGCATTAGCGGAACCGTTTGCAATCTGCCAC
AAGTGTGATAAGTTATCTACACTGGCGAGGGGATTGCTCTCTGTAATGTTCAGCTTCTAA
TTGTCTCTACTTTGTGAGACAACTTTTGAATGCTTGACCTCAAATCAGGTAGGACTACCC
GCTGAACTTAA
>AB8-46
TTTCCGTAGGTGAACCTGCGGAAGGATCATTATTGAATTATGTTTCTAGATAGGTTGTAG
CTGGCTC-TTTAGAGCATGTGCACGCCTGTTTGGACTTCATTTTCATCCACCTGTGCACC
TATTGTAGTCTTTGGTTGGGTTAGGGGGAAGTGGTCATTGTGTCAGCATCTGCTGGATGT
GAGGACTTGCATTGTGAAAGCTTTGCTGTCCTTGATGTGATCATGGAATCTCTTTCTCAC
TAGAGTCTATGTCACTCATTATACTCTGTCGAATGTCATTGAATGTCTTTACATGGGCTT
GTATGCCTATGAAAATTGTAATACAACTTTCAGCAACGGATCTCTTGGCTCTCGCATCGA
TGAAGGACGCAGCGAAATGCGATAAGTAATGTGAATTGCAGAATTCAGTGAATCATCGAA
TCTTTGAACGCATCTTGCGCTCCTTGGTATTCCGAGGAGCATGCCTGTTTGAGTGTCATT
AAATTCTCAACTCTCTTATACTTTTTTGTAAAAGAGAGCTTGGACTGTGGAGGCTTGCTG
GCCACTTTTTGGGGTCAGCTCCTCTGAAATGCATTAGCGGAACCGTTTGCAATCTGCCAC
AAGTGTGATAAGTTATCTACACTGGCGAGGGGATTGCTCTCTGTAATGTTCAGCTTCTAA
TTGTCTCTACTTTGTGAGACAACTTTTGAATGCTTGACCTCAAATCAGGTAGGACTACCC
GCTGAACTTAA
>AB9-9
TTTCCGTAGGTGAACCTGCGGAAGGATCATTATTGAATTATGTTTCTAGATAGGTTGTAG
CTGGCTC-TTTAGAGCATGTGCACGCCTGTTTGGACTTCATTTTCATCCACCTGTGCACC
TATTGTAGTCTTTGGTTGGGTTAGGGGGAAGTGGTCATTGTGTCAGCATCTGCTGGATGT
GAGGACTTGCATTGTGAAAGCTTTGCTGTCCTTGATGTGATCATGGAATCTCTTTCTCAC
TAGAGTCTATGTCACTCATTATACTCTGTCGAATGTCATTGAATGTCTTTACATGGGCTT
GTATGCCTATGAAAATTGTAATACAACTTTCAGCAACGGATCTCTTGGCTCTCGCATCGA
TGAAGGACGCAGCGAAATGCGATAAGTAATGTGAATTGCAGAATTCAGTGAATCATCGAA
TCTTTGAACGCATCTTGCGCTCCTTGGTATTCCGAGGAGCATGCCTGTTTGAGTGTCATT
AAATTCTCAACTCTCTTATACTTTTTTGTAAAAGAGAGCTTGGACTGTGGAGGCTTGCTG
GCCACTTTTTGGGGTCAGCTCCTCTGAAATGCATTAGCGGAACCGTTTGCAATCTGCCAC
AAGTGTGATAAGTTATCTACACTGGCGAGGGGATTGCTCTCTGTAATGTTCAGCTTCTAA
TTGTCTCTACTTTGTGAGACAACTTTTGAATGCTTGACCTCAAATCAGGTAGGACTACCC
GCTGAACTTAA
>AB10-34
TTTCCGTAGGTGAACCTGCGGAAGGATCATTATTGAATTATGTTTCTAGATAGGTTGTAG
CTGGCTC-TTTAGAGCATGTGCACGCCTGTTTGGACTTCATTTTCATCCACCTGTGCACC
TATTGTAGTCTTTGGTTGGGTTAGGGGGAAGTGGTCATTGTGTCAGCATCTGCTGGATGT
GAGGACTTGCATTGTGAAAGCTTTGCTGTCCTTGATGTGATCATGGAATCTCTTTCTCAC
TAGAGTCTATGTCACTCATTATACTCTGTCGAATGTCATTGAATGTCTTTACATGGGCTT
GTATGCCTATGAAAATTGTAATACAACTTTCAGCAACGGATCTCTTGGCTCTCGCATCGA
TGAAGGACGCAGCGAAATGCGATAAGTAATGTGAATTGCAGAATTCAGTGAATCATCGAA
TCTTTGAACGCATCTTGCGCTCCTTGGTATTCCGAGGAGCATGCCTGTTTGAGTGTCATT
AAATTCTCAACTCTCTTATACTTTTTTGTAAAAGAGAGCTTGGACTGTGGAGGCTTGCTG
GCCACTTTTTGGGGTCAGCTCCTCTGAAATGCATTAGCGGAACCGTTTGCAATCTGCCAC
AAGTGTGATAAGTTATCTACACTGGCGAGGGGATTGCTCTCTGTAATGTTCAGCTTCTAA
TTGTCTCTACTTTGTGAGACAACTTTTGAATGCTTGACCTCAAATCAGGTAGGACTACCC
GCTGAACTTAA
>AB11-25
TTTCCGTAGGTGAACCTGCGGAAGGATCATTATTGAATTATGTTTCTAGATAGGTTGTAG

CTGGCTC-TTTAGAGCATGTGCACGCCTGTTTGGACTTCATTTTCATCCACCTGTGCACC
TATTGTAGTCTTTGGTTGGGTTAGGGGGAAGTGGTCATTGTGTCAGCATCTGCTGGATGT
GAGGACTTGCATTGTGAAAGCTTTGCTGTCCTTGATGTGATCATGGAATCTCTTTCTCAC
TAGAGTCTATGTCACTCATTATACTCTGTCGAATGTCATTGAATGTCTTTACATGGGCTT
GTATGCCTATGAAAATTGTAATACAACTTTCAGCAACGGATCTCTTGGCTCTCGCATCGA
TGAAGGACGCAGCGAAATGCGATAAGTAATGTGAATTGCAGAATTCAGTGAATCATCGAA
TCTTTGAACGCATCTTGCGCTCCTTGGTATTCCGAGGAGCATGCCTGTTTGAGTGTCATT
AAATTCTCAACTCTCTTATACTTTTTTGTAAAAGAGAGCTTGGACTGTGGAGGCTTGCTG
GCCACTTTTTGGGGTCAGCTCCTCTGAAATGCATTAGCGGAACCGTTTGCAATCTGCCAC
AAGTGTGATAAGTTATCTACACTGGCGAGGGGATTGCTCTCTGTAATGTTCAGCTTCTAA
TTGTCTCTACTTTGTGAGACAACTTTTGAATGCTTGACCTCAAATCAGGTAGGACTACCC
GCTGAACTTAA
>AB12-53
TTTCCGTAGGTGAACCTGCGGAAGGATCATTATTGAATTATGTTTCTAGATAGGTTGTAG
CTGGCTC-TTTAGAGCATGTGCACGCCTGTTTGGACTTCATTTTCATCCACCTGTGCACC
TATTGTAGTCTTTGGTTGGGTTAGGGGGAAGTGGTCATTGTGTCAGCATCTGCTGGATGT
GAGGACTTGCATTGTGAAAGCTTTGCTGTCCTTGATGTGATCATGGAATCTCTTTCTCAC
TAGAGTCTATGTCACTCATTATACTCTGTCGAATGTCATTGAATGTCTTTACATGGGCTT
GTATGCCTATGAAAATTGTAATACAACTTTCAGCAACGGATCTCTTGGCTCTCGCATCGA
TGAAGGACGCAGCGAAATGCGATAAGTAATGTGAATTGCAGAATTCAGTGAATCATCGAA
TCTTTGAACGCATCTTGCGCTCCTTGGTATTCCGAGGAGCATGCCTGTTTGAGTGTCATT
AAATTCTCAACTCTCTTATACTTTTTTGTAAAAGAGAGCTTGGACTGTGGAGGCTTGCTG
GCCACTTTTTGGGGTCAGCTCCTCTGAAATGCATTAGCGGAACCGTTTGCAATCTGCCAC
AAGTGTGATAAGTTATCTACACTGGCGAGGGGATTGCTCTCTGTAATGTTCAGCTTCTAA
TTGTCTCTACTTTGTGAGACAACTTTTGAATGCTTGACCTCAAATCAGGTAGGACTACCC
GCTGAACTTAA
>AB2-68
TTTCCGTAGGTGAACCTGCGGAAGGATCATTATTGAATTATGTTTCTAGATAGGTTGTAG
CTGGCTC-TTTAGAGCATGTGCACGCCTGTTTGGACTTCATTTTCATCCACCTGTGCACC
TATTGTAGTCTTTGGTTGGGTTAGGGGGAAGTGGTCATTGTGTCAGCATCTGCTGGATGT
GAGGACTTGCATTGTGAAAGCTTTGCTGTCCTTGATGTGATCATGGAATCTCTTTCTCAC
TAGAGTCTATGTCACTCATTATACTCTGTCGAATGTCATTGAATGTCTTTACATGGGCTT
GTATGCCTATGAAAATTGTAATACAACTTTCAGCAACGGATCTCTTGGCTCTCGCATCGA
TGAAGGACGCAGCGAAATGCGATAAGTAATGTGAATTGCAGAATTCAGTGAATCATCGAA
TCTTTGAACGCATCTTGCGCTCCTTGGTATTCCGAGGAGCATGCCTGTTTGAGTGTCATT
AAATTCTCAACTCTCTTATACTTTTTTGTAAAAGAGAGCTTGGACTGTGGAGGCTTGCTG
GCCACTTTTTGGGGTCAGCTCCTCTGAAATGCATTAGCGGAACCGTTTGCAATCTGCCAC
AAGTGTGATAAGTTATCTACACTGGCGAGGGGATTGCTCTCTGTAATGTTCAGCTTCTAA
TTGTCTCTACTTTGTGAGACAACTTTTGAATGCTTGACCTCAAATCAGGTAGGACTACCC
GCTGAACTTAA
>AB2-7
TTTCCGTAGGTGAACCTGCGGAAGGATCATTATTGAATTATGTTTCTAGATAGGTTGTAG
CTGGCTC-TTTAGAGCATGTGCACGCCTGTTTGGACTTCATTTTCATCCACCTGTGCACC
TATTGTAGTCTTTGGTTGGGTTAGGGGGAAGTGGTCATTGTGTCAGCATCTGCTGGATGT
GAGGACTTGCATTGTGAAAGCTTTGCTGTCCTTGATGTGATCATGGAATCTCTTTCTCAC
TAGAGTCTATGTCACTCATTATACTCTGTCGAATGTCATTGAATGTCTTTACATGGGCTT
GTATGCCTATGAAAATTGTAATACAACTTTCAGCAACGGATCTCTTGGCTCTCGCATCGA
TGAAGGACGCAGCGAAATGCGATAAGTAATGTGAATTGCAGAATTCAGTGAATCATCGAA
TCTTTGAACGCATCTTGCGCTCCTTGGTATTCCGAGGAGCATGCCTGTTTGAGTGTCATT
AAATTCTCAACTCTCTTATACTTTTTTGTAAAAGAGAGCTTGGACTGTGGAGGCTTGCTG

GCCACTTTTTGGGGTCAGCTCCTCTGAAATGCATTAGCGGAACCGTTTGCAATCTGCCAC
AAGTGTGATAAGTTATCTACACTGGCGAGGGGATTGCTCTCTGTAATGTTCAGCTTCTAA
TTGTCTCTACTTTGTGAGACAACTTTTGAATGCTTGACCTCAAATCAGGTAGGACTACCC
GCTGAACTTAA
>AB2-39
TTTCCGTAGGTGAACCTGCGGAAGGATCATTATTGAATTATGTTTCTAGATAGGTTGTAG
CTGGCTC-TTTAGAGCATGTGCACGCCTGTTTGGACTTCATTTTCATCCACCTGTGCACC
TATTGTAGTCTTTGGTTGGGTTAGGGGGAAGTGGTCATTGTGTCAGCATCTGCTGGATGT
GAGGACTTGCATTGTGAAAGCTTTGCTGTCCTTGATGTGATCATGGAATCTCTTTCTCAC
TAGAGTCTATGTCACTCATTATACTCTGTCGAATGTCATTGAATGTCTTTACATGGGCTT
GTATGCCTATGAAAATTGTAATACAACTTTCAGCAACGGATCTCTTGGCTCTCGCATCGA
TGAAGGACGCAGCGAAATGCGATAAGTAATGTGAATTGCAGAATTCAGTGAATCATCGAA
TCTTTGAACGCATCTTGCGCTCCTTGGTATTCCGAGGAGCATGCCTGTTTGAGTGTCATT
AAATTCTCAACTCTCTTATACTTTTTTGTAAAAGAGAGCTTGGACTGTGGAGGCTTGCTG
GCCACTTTTTGGGGTCAGCTCCTCTGAAATGCATTAGCGGAACCGTTTGCAATCTGCCAC
AAGTGTGATAAGTTATCTACACTGGCGAGGGGATTGCTCTCTGTAATGTTCAGCTTCTAA
TTGTCTCTACTTTGTGAGACAACTTTTGAATGCTTGACCTCAAATCAGGTAGGACTACCC
GCTGAACTTAA
>AB4
TTTCCGTAGGTGAACCTGCGGAAGGATCATTATTGAATTATGTTTCTAGATAGGTTGTAG
CTGGCTC-TTTAGAGCATGTGCACGCCTGTTTGGACTTCATTTTCATCCACCTGTGCACC
TATTGTAGTCTTTGGTTGGGTTAGGGGGAAGTGGTCATTGTGTCAGCATCTGCTGGATGT
GAGGACTTGCATTGTGAAAGCTTTGCTGTCCTTGATGTGATCATGGAATCTCTTTCTCAC
TAGAGTCTATGTCACTCATTATACTCTGTCGAATGTCATTGAATGTCTTTACATGGGCTT
GTATGCCTATGAAAATTGTAATACAACTTTCAGCAACGGATCTCTTGGCTCTCGCATCGA
TGAAGGACGCAGCGAAATGCGATAAGTAATGTGAATTGCAGAATTCAGTGAATCATCGAA
TCTTTGAACGCATCTTGCGCTCCTTGGTATTCCGAGGAGCATGCCTGTTTGAGTGTCATT
AAATTCTCAACTCTCTTATACTTTTTTGTAAAAGAGAGCTTGGACTGTGGAGGCTTGCTG
GCCACTTTTTGGGGTCAGCTCCTCTGAAATGCATTAGCGGAACCGTTTGCAATCTGCCAC
AAGTGTGATAAGTTATCTACACTGGCGAGGGGATTGCTCTCTGTAATGTTCAGCTTCTAA
TTGTCTCTACTTTGTGAGACAACTTTTGAATGCTTGACCTCAAATCAGGTAGGACTACCC
GCTGAACTTAA
>AB5
TTTCCGTAGGTGAACCTGCGGAAGGATCATTATTGAATTATGTTTCTAGATAGGTTGTAG
CTGGCTC-TTTAGAGCATGTGCACGCCTGTTTGGACTTCATTTTCATCCACCTGTGCACC
TATTGTAGTCTTTGGTTGGGTTAGGGGGAAGTGGTCATTGTGTCAGCATCTGCTGGATGT
GAGGACTTGCATTGTGAAAGCTTTGCTGTCCTTGATGTGATCATGGAATCTCTTTCTCAC
TAGAGTCTATGTCACTCATTATACTCTGTCGAATGTCATTGAATGTCTTTACATGGGCTT
GTATGCCTATGAAAATTGTAATACAACTTTCAGCAACGGATCTCTTGGCTCTCGCATCGA
TGAAGGACGCAGCGAAATGCGATAAGTAATGTGAATTGCAGAATTCAGTGAATCATCGAA
TCTTTGAACGCATCTTGCGCTCCTTGGTATTCCGAGGAGCATGCCTGTTTGAGTGTCATT
AAATTCTCAACTCTCTTATACTTTTTTGTAAAAGAGAGCTTGGACTGTGGAGGCTTGCTG
GCCACTTTTTGGGGTCAGCTCCTCTGAAATGCATTAGCGGAACCGTTTGCAATCTGCCAC
AAGTGTGATAAGTTATCTACACTGGCGAGGGGATTGCTCTCTGTAATGTTCAGCTTCTAA
TTGTCTCTACTTTGTGAGACAACTTTTGAATGCTTGACCTCAAATCAGGTAGGACTACCC
GCTGAACTTAA
>AB6
TTTCCGTAGGTGAACCTGCGGAAGGATCATTATTGAATTATGTTTCTAGATAGGTTGTAG
CTGGCTC-TTTAGAGCATGTGCACGCCTGTTTGGACTTCATTTTCATCCACCTGTGCACC
TATTGTAGTCTTTGGTTGGGTTAGGGGGAAGTGGTCATTGTGTCAGCATCTGCTGGATGT

GAGGACTTGCATTGTGAAAGCTTTGCTGTCCTTGATGTGATCATGGAATCTCTTTCTCAC
TAGAGTCTATGTCACTCATTATACTCTGTCGAATGTCATTGAATGTCTTTACATGGGCTT
GTATGCCTATGAAAATTGTAATACAACTTTCAGCAACGGATCTCTTGGCTCTCGCATCGA
TGAAGGACGCAGCGAAATGCGATAAGTAATGTGAATTGCAGAATTCAGTGAATCATCGAA
TCTTTGAACGCATCTTGCGCTCCTTGGTATTCCGAGGAGCATGCCTGTTTGAGTGTCATT
AAATTCTCAACTCTCTTATACTTTTTTGTAAAAGAGAGCTTGGACTGTGGAGGCTTGCTG
GCCACTTTTTGGGGTCAGCTCCTCTGAAATGCATTAGCGGAACCGTTTGCAATCTGCCAC
AAGTGTGATAAGTTATCTACACTGGCGAGGGGATTGCTCTCTGTAATGTTCAGCTTCTAA
TTGTCTCTACTTTGTGAGACAACTTTTGAATGCTTGACCTCAAATCAGGTAGGACTACCC
GCTGAACTTAA
>AB37-21
TTTCCGTAGGTGAACCTGCGGAAGGATCATTATTGAATTATGTTTCTAGATAGGTTGTAG
CTGGCTC-TTTAGAGCATGTGCACGCCTGTTTGGACTTCATTTTCATCCACCTGTGCACC
TATTGTAGTCTTTGGTTGGGTTAGGGGGAAGTGGTCATTGTGTCAGCATCTGCTGGATGT
GAGGACTTGCATTGTGAAAGCTTTGCTGTCCTTGATGTGATCATGGAATCTCTTTCTCAC
TAGAGTCTATGTCACTCATTATACTCTGTCGAATGTCATTGAATGTCTTTACATGGGCTT
GTATGCCTATGAAAATTGTAATACAACTTTCAGCAACGGATCTCTTGGCTCTCGCATCGA
TGAAGGACGCAGCGAAATGCGATAAGTAATGTGAATTGCAGAATTCAGTGAATCATCGAA
TCTTTGAACGCATCTTGCGCTCCTTGGTATTCCGAGGAGCATGCCTGTTTGAGTGTCATT
AAATTCTCAACTCTCTTATACTTTTTTGTAAAAGAGAGCTTGGACTGTGGAGGCTTGCTG
GCCACTTTTTGGGGTCAGCTCCTCTGAAATGCATTAGCGGAACCGTTTGCAATCTGCCAC
AAGTGTGATAAGTTATCTACACTGGCGAGGGGATTGCTCTCTGTAATGTTCAGCTTCTAA
TTGTCTCTACTTTGTGAGACAACTTTTGAATGCTTGACCTCAAATCAGGTAGGACTACCC
GCTGAACTTAA
>AB37-22
TTTCCGTAGGTGAACCTGCGGAAGGATCATTATTGAATTATGTTTCTAGATAGGTTGTAG
CTGGCTC-TTTAGAGCATGTGCACGCCTGTTTGGACTTCATTTTCATCCACCTGTGCACC
TATTGTAGTCTTTGGTTGGGTTAGGGGGAAGTGGTCATTGTGTCAGCATCTGCTGGATGT
GAGGACTTGCATTGTGAAAGCTTTGCTGTCCTTGATGTGATCATGGAATCTCTTTCTCAC
TAGAGTCTATGTCACTCATTATACTCTGTCGAATGTCATTGAATGTCTTTACATGGGCTT
GTATGCCTATGAAAATTGTAATACAACTTTCAGCAACGGATCTCTTGGCTCTCGCATCGA
TGAAGGACGCAGCGAAATGCGATAAGTAATGTGAATTGCAGAATTCAGTGAATCATCGAA
TCTTTGAACGCATCTTGCGCTCCTTGGTATTCCGAGGAGCATGCCTGTTTGAGTGTCATT
AAATTCTCAACTCTCTTATACTTTTTTGTAAAAGAGAGCTTGGACTGTGGAGGCTTGCTG
GCCACTTTTTGGGGTCAGCTCCTCTGAAATGCATTAGCGGAACCGTTTGCAATCTGCCAC
AAGTGTGATAAGTTATCTACACTGGCGAGGGGATTGCTCTCTGTAATGTTCAGCTTCTAA
TTGTCTCTACTTTGTGAGACAACTTTTGAATGCTTGACCTCAAATCAGGTAGGACTACCC
GCTGAACTTAA
>AB1-1
TTTCCGTAGGTGAACCTGCGGAAGGATCATTATTGAATTATGTTTCTAGATAGGTTGTAG
CTGGCTC-TTTAGAGCATGTGCACGCCTGTTTGGACTTCATTTTCATCCACCTGTGCACC
TATTGTAGTCTTTGGTTGGGTTAGGGGGAAGTGGTCATTGTGTCAGCATCTGCTGGATGT
GAGGACTTGCATTGTGAAAGCTTTGCTGTCCTTGATGTGATCATGGAATCTCTTTCTCAC
TAGAGTCTATGTCACTCATTATACTCTGTCGAATGTCATTGAATGTCTTTACATGGGCTT
GTATGCCTATGAAAATTGTAATACAACTTTCAGCAACGGATCTCTTGGCTCTCGCATCGA
TGAAGGACGCAGCGAAATGCGATAAGTAATGTGAATTGCAGAATTCAGTGAATCATCGAA
TCTTTGAACGCATCTTGCGCTCCTTGGTATTCCGAGGAGCATGCCTGTTTGAGTGTCATT
AAATTCTCAACTCTCTTATACTTTTTTGTAAAAGAGAGCTTGGACTGTGGAGGCTTGCTG
GCCACTTTTTGGGGTCAGCTCCTCTGAAATGCATTAGCGGAACCGTTTGCAATCTGCCAC
AAGTGTGATAAGTTATCTACACTGGCGAGGGGATTGCTCTCTGTAATGTTCAGCTTCTAA

TTGTCTCTACTTTGTGAGACAACTTTTGAATGCTTGACCTCAAATCAGGTAGGACTACCC
GCTGAACTTAA
>AB1-8
TTTCCGTAGGTGAACCTGCGGAAGGATCATTATTGAATTATGTTTCTAGATAGGTTGTAG
CTGGCTC-TTTAGAGCATGTGCACGCCTGTTTGGACTTCATTTTCATCCACCTGTGCACC
TATTGTAGTCTTTGGTTGGGTTAGGGGGAAGTGGTCATTGTGTCAGCATCTGCTGGATGT
GAGGACTTGCATTGTGAAAGCTTTGCTGTCCTTGATGTGATCATGGAATCTCTTTCTCAC
TAGAGTCTATGTCACTCATTATACTCTGTCGAATGTCATTGAATGTCTTTACATGGGCTT
GTATGCCTATGAAAATTGTAATACAACTTTCAGCAACGGATCTCTTGGCTCTCGCATCGA
TGAAGGACGCAGCGAAATGCGATAAGTAATGTGAATTGCAGAATTCAGTGAATCATCGAA
TCTTTGAACGCATCTTGCGCTCCTTGGTATTCCGAGGAGCATGCCTGTTTGAGTGTCATT
AAATTCTCAACTCTCTTATACTTTTTTGTAAAAGAGAGCTTGGACTGTGGAGGCTTGCTG
GCCACTTTTTGGGGTCAGCTCCTCTGAAATGCATTAGCGGAACCGTTTGCAATCTGCCAC
AAGTGTGATAAGTTATCTACACTGGCGAGGGGATTGCTCTCTGTAATGTTCAGCTTCTAA
TTGTCTCTACTTTGTGAGACAACTTTTGAATGCTTGACCTCAAATCAGGTAGGACTACCC
GCTGAACTTAA
>AB1-53
TTTCCGTAGGTGAACCTGCGGAAGGATCATTATTGAATTATGTTTCTAGATAGGTTGTAG
CTGGCTC-TTTAGAGCATGTGCACGCCTGTTTGGACTTCATTTTCATCCACCTGTGCACC
TATTGTAGTCTTTGGTTGGGTTAGGGGGAAGTGGTCATTGTGTCAGCATCTGCTGGATGT
GAGGACTTGCATTGTGAAAGCTTTGCTGTCCTTGATGTGATCATGGAATCTCTTTCTCAC
TAGAGTCTATGTCACTCATTATACTCTGTCGAATGTCATTGAATGTCTTTACATGGGCTT
GTATGCCTATGAAAATTGTAATACAACTTTCAGCAACGGATCTCTTGGCTCTCGCATCGA
TGAAGGACGCAGCGAAATGCGATAAGTAATGTGAATTGCAGAATTCAGTGAATCATCGAA
TCTTTGAACGCATCTTGCGCTCCTTGGTATTCCGAGGAGCATGCCTGTTTGAGTGTCATT
AAATTCTCAACTCTCTTATACTTTTTTGTAAAAGAGAGCTTGGACTGTGGAGGCTTGCTG
GCCACTTTTTGGGGTCAGCTCCTCTGAAATGCATTAGCGGAACCGTTTGCAATCTGCCAC
AAGTGTGATAAGTTATCTACACTGGCGAGGGGATTGCTCTCTGTAATGTTCAGCTTCTAA
TTGTCTCTACTTTGTGAGACAACTTTTGAATGCTTGACCTCAAATCAGGTAGGACTACCC
GCTGAACTTAA
>AB1-21
TTTCCGTAGGTGAACCTGCGGAAGGATCATTATTGAATTATGTTTCTAGATAGGTTGTAG
CTGGCTC-TTTAGAGCATGTGCACGCCTGTTTGGACTTCATTTTCATCCACCTGTGCACC
TATTGTAGTCTTTGGTTGGGTTAGGGGGAAGTGGTCATTGTGTCAGCATCTGCTGGATGT
GAGGACTTGCATTGTGAAAGCTTTGCTGTCCTTGATGTGATCATGGAATCTCTTTCTCAC
TAGAGTCTATGTCACTCATTATACTCTGTCGAATGTCATTGAATGTCTTTACATGGGCTT
GTATGCCTATGAAAATTGTAATACAACTTTCAGCAACGGATCTCTTGGCTCTCGCATCGA
TGAAGGACGCAGCGAAATGCGATAAGTAATGTGAATTGCAGAATTCAGTGAATCATCGAA
TCTTTGAACGCATCTTGCGCTCCTTGGTATTCCGAGGAGCATGCCTGTTTGAGTGTCATT
AAATTCTCAACTCTCTTATACTTTTTTGTAAAAGAGAGCTTGGACTGTGGAGGCTTGCTG
GCCACTTTTTGGGGTCAGCTCCTCTGAAATGCATTAGCGGAACCGTTTGCAATCTGCCAC
AAGTGTGATAAGTTATCTACACTGGCGAGGGGATTGCTCTCTGTAATGTTCAGCTTCTAA
TTGTCTCTACTTTGTGAGACAACTTTTGAATGCTTGACCTCAAATCAGGTAGGACTACCC
GCTGAACTTAA
>AB1-54
TTTCCGTAGGTGAACCTGCGGAAGGATCATTATTGAATTATGTTTCTAGATAGGTTGTAG
CTGGCTC-TTTAGAGCATGTGCACGCCTGTTTGGACTTCATTTTCATCCACCTGTGCACC
TATTGTAGTCTTTGGTTGGGTTAGGGGGAAGTGGTCATTGTGTCAGCATCTGCTGGATGT
GAGGACTTGCATTGTGAAAGCTTTGCTGTCCTTGATGTGATCATGGAATCTCTTTCTCAC
TAGAGTCTATGTCACTCATTATACTCTGTCGAATGTCATTGAATGTCTTTACATGGGCTT

GTATGCCTATGAAAATTGTAATACAACTTTCAGCAACGGATCTCTTGGCTCTCGCATCGA
TGAAGGACGCAGCGAAATGCGATAAGTAATGTGAATTGCAGAATTCAGTGAATCATCGAA
TCTTTGAACGCATCTTGCGCTCCTTGGTATTCCGAGGAGCATGCCTGTTTGAGTGTCATT
AAATTCTCAACTCTCTTATACTTTTTTGTAAAAGAGAGCTTGGACTGTGGAGGCTTGCTG
GCCACTTTTTGGGGTCAGCTCCTCTGAAATGCATTAGCGGAACCGTTTGCAATCTGCCAC
AAGTGTGATAAGTTATCTACACTGGCGAGGGGATTGCTCTCTGTAATGTTCAGCTTCTAA
TTGTCTCTACTTTGTGAGACAACTTTTGAATGCTTGACCTCAAATCAGGTAGGACTACCC
GCTGAACTTAA
>AB1-30
TTTCCGTAGGTGAACCTGCGGAAGGATCATTATTGAATTATGTTTCTAGATAGGTTGTAG
CTGGCTC-TTTAGAGCATGTGCACGCCTGTTTGGACTTCATTTTCATCCACCTGTGCACC
TATTGTAGTCTTTGGTTGGGTTAGGGGGAAGTGGTCATTGTGTCAGCATCTGCTGGATGT
GAGGACTTGCATTGTGAAAGCTTTGCTGTCCTTGATGTGATCATGGAATCTCTTTCTCAC
TAGAGTCTATGTCACTCATTATACTCTGTCGAATGTCATTGAATGTCTTTACATGGGCTT
GTATGCCTATGAAAATTGTAATACAACTTTCAGCAACGGATCTCTTGGCTCTCGCATCGA
TGAAGGACGCAGCGAAATGCGATAAGTAATGTGAATTGCAGAATTCAGTGAATCATCGAA
TCTTTGAACGCATCTTGCGCTCCTTGGTATTCCGAGGAGCATGCCTGTTTGAGTGTCATT
AAATTCTCAACTCTCTTATACTTTTTTGTAAAAGAGAGCTTGGACTGTGGAGGCTTGCTG
GCCACTTTTTGGGGTCAGCTCCTCTGAAATGCATTAGCGGAACCGTTTGCAATCTGCCAC
AAGTGTGATAAGTTATCTACACTGGCGAGGGGATTGCTCTCTGTAATGTTCAGCTTCTAA
TTGTCTCTACTTTGTGAGACAACTTTTGAATGCTTGACCTCAAATCAGGTAGGACTACCC
GCTGAACTTAA
>AB1-40
TTTCCGTAGGTGAACCTGCGGAAGGATCATTATTGAATTATGTTTCTAGATAGGTTGTAG
CTGGCTC-TTTAGAGCATGTGCACGCCTGTTTGGACTTCATTTTCATCCACCTGTGCACC
TATTGTAGTCTTTGGTTGGGTTAGGGGGAAGTGGTCATTGTGTCAGCATCTGCTGGATGT
GAGGACTTGCATTGTGAAAGCTTTGCTGTCCTTGATGTGATCATGGAATCTCTTTCTCAC
TAGAGTCTATGTCACTCATTATACTCTGTCGAATGTCATTGAATGTCTTTACATGGGCTT
GTATGCCTATGAAAATTGTAATACAACTTTCAGCAACGGATCTCTTGGCTCTCGCATCGA
TGAAGGACGCAGCGAAATGCGATAAGTAATGTGAATTGCAGAATTCAGTGAATCATCGAA
TCTTTGAACGCATCTTGCGCTCCTTGGTATTCCGAGGAGCATGCCTGTTTGAGTGTCATT
AAATTCTCAACTCTCTTATACTTTTTTGTAAAAGAGAGCTTGGACTGTGGAGGCTTGCTG
GCCACTTTTTGGGGTCAGCTCCTCTGAAATGCATTAGCGGAACCGTTTGCAATCTGCCAC
AAGTGTGATAAGTTATCTACACTGGCGAGGGGATTGCTCTCTGTAATGTTCAGCTTCTAA
TTGTCTCTACTTTGTGAGACAACTTTTGAATGCTTGACCTCAAATCAGGTAGGACTACCC
GCTGAACTTAA
>AB1-58
TTTCCGTAGGTGAACCTGCGGAAGGATCATTATTGAATTATGTTTCTAGATAGGTTGTAG
CTGGCTC-TTTAGAGCATGTGCACGCCTGTTTGGACTTCATTTTCATCCACCTGTGCACC
TATTGTAGTCTTTGGTTGGGTTAGGGGGAAGTGGTCATTGTGTCAGCATCTGCTGGATGT
GAGGACTTGCATTGTGAAAGCTTTGCTGTCCTTGATGTGATCATGGAATCTCTTTCTCAC
TAGAGTCTATGTCACTCATTATACTCTGTCGAATGTCATTGAATGTCTTTACATGGGCTT
GTATGCCTATGAAAATTGTAATACAACTTTCAGCAACGGATCTCTTGGCTCTCGCATCGA
TGAAGGACGCAGCGAAATGCGATAAGTAATGTGAATTGCAGAATTCAGTGAATCATCGAA
TCTTTGAACGCATCTTGCGCTCCTTGGTATTCCGAGGAGCATGCCTGTTTGAGTGTCATT
AAATTCTCAACTCTCTTATACTTTTTTGTAAAAGAGAGCTTGGACTGTGGAGGCTTGCTG
GCCACTTTTTGGGGTCAGCTCCTCTGAAATGCATTAGCGGAACCGTTTGCAATCTGCCAC
AAGTGTGATAAGTTATCTACACTGGCGAGGGGATTGCTCTCTGTAATGTTCAGCTTCTAA
TTGTCTCTACTTTGTGAGACAACTTTTGAATGCTTGACCTCAAATCAGGTAGGACTACCC
GCTGAACTTAA

>AB1-45
TTTCCGTAGGTGAACCTGCGGAAGGATCATTATTGAATTATGTTTCTAGATAGGTTGTAG
CTGGCTC-TTTAGAGCATGTGCACGCCTGTTTGGACTTCATTTTCATCCACCTGTGCACC
TATTGTAGTCTTTGGTTGGGTTAGGGGGAAGTGGTCATTGTGTCAGCATCTGCTGGATGT
GAGGACTTGCATTGTGAAAGCTTTGCTGTCCTTGATGTGATCATGGAATCTCTTTCTCAC
TAGAGTCTATGTCACTCATTATACTCTGTCGAATGTCATTGAATGTCTTTACATGGGCTT
GTATGCCTATGAAAATTGTAATACAACTTTCAGCAACGGATCTCTTGGCTCTCGCATCGA
TGAAGGACGCAGCGAAATGCGATAAGTAATGTGAATTGCAGAATTCAGTGAATCATCGAA
TCTTTGAACGCATCTTGCGCTCCTTGGTATTCCGAGGAGCATGCCTGTTTGAGTGTCATT
AAATTCTCAACTCTCTTATACTTTTTTGTAAAAGAGAGCTTGGACTGTGGAGGCTTGCTG
GCCACTTTTTGGGGTCAGCTCCTCTGAAATGCATTAGCGGAACCGTTTGCAATCTGCCAC
AAGTGTGATAAGTTATCTACACTGGCGAGGGGATTGCTCTCTGTAATGTTCAGCTTCTAA
TTGTCTCTACTTTGTGAGACAACTTTTGAATGCTTGACCTCAAATCAGGTAGGACTACCC
GCTGAACTTAA
>AB1-50
TTTCCGTAGGTGAACCTGCGGAAGGATCATTATTGAATTATGTTTCTAGATAGGTTGTAG
CTGGCTC-TTTAGAGCATGTGCACGCCTGTTTGGACTTCATTTTCATCCACCTGTGCACC
TATTGTAGTCTTTGGTTGGGTTAGGGGGAAGTGGTCATTGTGTCAGCATCTGCTGGATGT
GAGGACTTGCATTGTGAAAGCTTTGCTGTCCTTGATGTGATCATGGAATCTCTTTCTCAC
TAGAGTCTATGTCACTCATTATACTCTGTCGAATGTCATTGAATGTCTTTACATGGGCTT
GTATGCCTATGAAAATTGTAATACAACTTTCAGCAACGGATCTCTTGGCTCTCGCATCGA
TGAAGGACGCAGCGAAATGCGATAAGTAATGTGAATTGCAGAATTCAGTGAATCATCGAA
TCTTTGAACGCATCTTGCGCTCCTTGGTATTCCGAGGAGCATGCCTGTTTGAGTGTCATT
AAATTCTCAACTCTCTTATACTTTTTTGTAAAAGAGAGCTTGGACTGTGGAGGCTTGCTG
GCCACTTTTTGGGGTCAGCTCCTCTGAAATGCATTAGCGGAACCGTTTGCAATCTGCCAC
AAGTGTGATAAGTTATCTACACTGGCGAGGGGATTGCTCTCTGTAATGTTCAGCTTCTAA
TTGTCTCTACTTTGTGAGACAACTTTTGAATGCTTGACCTCAAATCAGGTAGGACTACCC
GCTGAACTTAA
>AB2-2
TTTCCGTAGGTGAACCTGCGGAAGGATCATTATTGAATTATGTTTCTAGATAGGTTGTAG
CTGGCTC-TTTAGAGCATGTGCACGCCTGTTTGGACTTCATTTTCATCCACCTGTGCACC
TATTGTAGTCTTTGGTTGGGTTAGGGGGAAGTGGTCATTGTGTCAGCATCTGCTGGATGT
GAGGACTTGCATTGTGAAAGCTTTGCTGTCCTTGATGTGATCATGGAATCTCTTTCTCAC
TAGAGTCTATGTCACTCATTATACTCTGTCGAATGTCATTGAATGTCTTTACATGGGCTT
GTATGCCTATGAAAATTGTAATACAACTTTCAGCAACGGATCTCTTGGCTCTCGCATCGA
TGAAGGACGCAGCGAAATGCGATAAGTAATGTGAATTGCAGAATTCAGTGAATCATCGAA
TCTTTGAACGCATCTTGCGCTCCTTGGTATTCCGAGGAGCATGCCTGTTTGAGTGTCATT
AAATTCTCAACTCTCTTATACTTTTTTGTAAAAGAGAGCTTGGACTGTGGAGGCTTGCTG
GCCACTTTTTGGGGTCAGCTCCTCTGAAATGCATTAGCGGAACCGTTTGCAATCTGCCAC
AAGTGTGATAAGTTATCTACACTGGCGAGGGGATTGCTCTCTGTAATGTTCAGCTTCTAA
TTGTCTCTACTTTGTGAGACAACTTTTGAATGCTTGACCTCAAATCAGGTAGGACTACCC
GCTGAACTTAA
>AB2-10
TTTCCGTAGGTGAACCTGCGGAAGGATCATTATTGAATTATGTTTCTAGATAGGTTGTAG
CTGGCTC-TTTAGAGCATGTGCACGCCTGTTTGGACTTCATTTTCATCCACCTGTGCACC
TATTGTAGTCTTTGGTTGGGTTAGGGGGAAGTGGTCATTGTGTCAGCATCTGCTGGATGT
GAGGACTTGCATTGTGAAAGCTTTGCTGTCCTTGATGTGATCATGGAATCTCTTTCTCAC
TAGAGTCTATGTCACTCATTATACTCTGTCGAATGTCATTGAATGTCTTTACATGGGCTT
GTATGCCTATGAAAATTGTAATACAACTTTCAGCAACGGATCTCTTGGCTCTCGCATCGA
TGAAGGACGCAGCGAAATGCGATAAGTAATGTGAATTGCAGAATTCAGTGAATCATCGAA

TCTTTGAACGCATCTTGCGCTCCTTGGTATTCCGAGGAGCATGCCTGTTTGAGTGTCATT
AAATTCTCAACTCTCTTATACTTTTTTGTAAAAGAGAGCTTGGACTGTGGAGGCTTGCTG
GCCACTTTTTGGGGTCAGCTCCTCTGAAATGCATTAGCGGAACCGTTTGCAATCTGCCAC
AAGTGTGATAAGTTATCTACACTGGCGAGGGGATTGCTCTCTGTAATGTTCAGCTTCTAA
TTGTCTCTACTTTGTGAGACAACTTTTGAATGCTTGACCTCAAATCAGGTAGGACTACCC
GCTGAACTTAA
>AB2−11
TTTCCGTAGGTGAACCTGCGGAAGGATCATTATTGAATTATGTTTCTAGATAGGTTGTAG
CTGGCTC−TTTAGAGCATGTGCACGCCTGTTTGGACTTCATTTTCATCCACCTGTGCACC
TATTGTAGTCTTTGGTTGGGTTAGGGGGAAGTGGTCATTGTGTCAGCATCTGCTGGATGT
GAGGACTTGCATTGTGAAAGCTTTGCTGTCCTTGATGTGATCATGGAATCTCTTTCTCAC
TAGAGTCTATGTCACTCATTATACTCTGTCGAATGTCATTGAATGTCTTTACATGGGCTT
GTATGCCTATGAAAATTGTAATACAACTTTCAGCAACGGATCTCTTGGCTCTCGCATCGA
TGAAGGACGCAGCGAAATGCGATAAGTAATGTGAATTGCAGAATTCAGTGAATCATCGAA
TCTTTGAACGCATCTTGCGCTCCTTGGTATTCCGAGGAGCATGCCTGTTTGAGTGTCATT
AAATTCTCAACTCTCTTATACTTTTTTGTAAAAGAGAGCTTGGACTGTGGAGGCTTGCTG
GCCACTTTTTGGGGTCAGCTCCTCTGAAATGCATTAGCGGAACCGTTTGCAATCTGCCAC
AAGTGTGATAAGTTATCTACACTGGCGAGGGGATTGCTCTCTGTAATGTTCAGCTTCTAA
TTGTCTCTACTTTGTGAGACAACTTTTGAATGCTTGACCTCAAATCAGGTAGGACTACCC
GCTGAACTTAA
>AB2−38
TTTCCGTAGGTGAACCTGCGGAAGGATCATTATTGAATTATGTTTCTAGATAGGTTGTAG
CTGGCTC−TTTAGAGCATGTGCACGCCTGTTTGGACTTCATTTTCATCCACCTGTGCACC
TATTGTAGTCTTTGGTTGGGTTAGGGGGAAGTGGTCATTGTGTCAGCATCTGCTGGATGT
GAGGACTTGCATTGTGAAAGCTTTGCTGTCCTTGATGTGATCATGGAATCTCTTTCTCAC
TAGAGTCTATGTCACTCATTATACTCTGTCGAATGTCATTGAATGTCTTTACATGGGCTT
GTATGCCTATGAAAATTGTAATACAACTTTCAGCAACGGATCTCTTGGCTCTCGCATCGA
TGAAGGACGCAGCGAAATGCGATAAGTAATGTGAATTGCAGAATTCAGTGAATCATCGAA
TCTTTGAACGCATCTTGCGCTCCTTGGTATTCCGAGGAGCATGCCTGTTTGAGTGTCATT
AAATTCTCAACTCTCTTATACTTTTTTGTAAAAGAGAGCTTGGACTGTGGAGGCTTGCTG
GCCACTTTTTGGGGTCAGCTCCTCTGAAATGCATTAGCGGAACCGTTTGCAATCTGCCAC
AAGTGTGATAAGTTATCTACACTGGCGAGGGGATTGCTCTCTGTAATGTTCAGCTTCTAA
TTGTCTCTACTTTGTGAGACAACTTTTGAATGCTTGACCTCAAATCAGGTAGGACTACCC
GCTGAACTTAA
>AB2−41
TTTCCGTAGGTGAACCTGCGGAAGGATCATTATTGAATTATGTTTCTAGATAGGTTGTAG
CTGGCTC−TTTAGAGCATGTGCACGCCTGTTTGGACTTCATTTTCATCCACCTGTGCACC
TATTGTAGTCTTTGGTTGGGTTAGGGGGAAGTGGTCATTGTGTCAGCATCTGCTGGATGT
GAGGACTTGCATTGTGAAAGCTTTGCTGTCCTTGATGTGATCATGGAATCTCTTTCTCAC
TAGAGTCTATGTCACTCATTATACTCTGTCGAATGTCATTGAATGTCTTTACATGGGCTT
GTATGCCTATGAAAATTGTAATACAACTTTCAGCAACGGATCTCTTGGCTCTCGCATCGA
TGAAGGACGCAGCGAAATGCGATAAGTAATGTGAATTGCAGAATTCAGTGAATCATCGAA
TCTTTGAACGCATCTTGCGCTCCTTGGTATTCCGAGGAGCATGCCTGTTTGAGTGTCATT
AAATTCTCAACTCTCTTATACTTTTTTGTAAAAGAGAGCTTGGACTGTGGAGGCTTGCTG
GCCACTTTTTGGGGTCAGCTCCTCTGAAATGCATTAGCGGAACCGTTTGCAATCTGCCAC
AAGTGTGATAAGTTATCTACACTGGCGAGGGGATTGCTCTCTGTAATGTTCAGCTTCTAA
TTGTCTCTACTTTGTGAGACAACTTTTGAATGCTTGACCTCAAATCAGGTAGGACTACCC
GCTGAACTTAA
>AB2−42
TTTCCGTAGGTGAACCTGCGGAAGGATCATTATTGAATTATGTTTCTAGATAGGTTGTAG

CTGGCTC-TTTAGAGCATGTGCACGCCTGTTTGGACTTCATTTTCATCCACCTGTGCACC
TATTGTAGTCTTTGGTTGGGTTAGGGGGAAGTGGTCATTGTGTCAGCATCTGCTGGATGT
GAGGACTTGCATTGTGAAAGCTTTGCTGTCCTTGATGTGATCATGGAATCTCTTTCTCAC
TAGAGTCTATGTCACTCATTATACTCTGTCGAATGTCATTGAATGTCTTTACATGGGCTT
GTATGCCTATGAAAATTGTAATACAACTTTCAGCAACGGATCTCTTGGCTCTCGCATCGA
TGAAGGACGCAGCGAAATGCGATAAGTAATGTGAATTGCAGAATTCAGTGAATCATCGAA
TCTTTGAACGCATCTTGCGCTCCTTGGTATTCCGAGGAGCATGCCTGTTTGAGTGTCATT
AAATTCTCAACTCTCTTATACTTTTTTGTAAAAGAGAGCTTGGACTGTGGAGGCTTGCTG
GCCACTTTTTGGGGTCAGCTCCTCTGAAATGCATTAGCGGAACCGTTTGCAATCTGCCAC
AAGTGTGATAAGTTATCTACACTGGCGAGGGGATTGCTCTCTGTAATGTTCAGCTTCTAA
TTGTCTCTACTTTGTGAGACAACTTTTGAATGCTTGACCTCAAATCAGGTAGGACTACCC
GCTGAACTTAA
>AB2-50
TTTCCGTAGGTGAACCTGCGGAAGGATCATTATTGAATTATGTTTCTAGATAGGTTGTAG
CTGGCTC-TTTAGAGCATGTGCACGCCTGTTTGGACTTCATTTTCATCCACCTGTGCACC
TATTGTAGTCTTTGGTTGGGTTAGGGGGAAGTGGTCATTGTGTCAGCATCTGCTGGATGT
GAGGACTTGCATTGTGAAAGCTTTGCTGTCCTTGATGTGATCATGGAATCTCTTTCTCAC
TAGAGTCTATGTCACTCATTATACTCTGTCGAATGTCATTGAATGTCTTTACATGGGCTT
GTATGCCTATGAAAATTGTAATACAACTTTCAGCAACGGATCTCTTGGCTCTCGCATCGA
TGAAGGACGCAGCGAAATGCGATAAGTAATGTGAATTGCAGAATTCAGTGAATCATCGAA
TCTTTGAACGCATCTTGCGCTCCTTGGTATTCCGAGGAGCATGCCTGTTTGAGTGTCATT
AAATTCTCAACTCTCTTATACTTTTTTGTAAAAGAGAGCTTGGACTGTGGAGGCTTGCTG
GCCACTTTTTGGGGTCAGCTCCTCTGAAATGCATTAGCGGAACCGTTTGCAATCTGCCAC
AAGTGTGATAAGTTATCTACACTGGCGAGGGGATTGCTCTCTGTAATGTTCAGCTTCTAA
TTGTCTCTACTTTGTGAGACAACTTTTGAATGCTTGACCTCAAATCAGGTAGGACTACCC
GCTGAACTTAA
>AB2-55
TTTCCGTAGGTGAACCTGCGGAAGGATCATTATTGAATTATGTTTCTAGATAGGTTGTAG
CTGGCTC-TTTAGAGCATGTGCACGCCTGTTTGGACTTCATTTTCATCCACCTGTGCACC
TATTGTAGTCTTTGGTTGGGTTAGGGGGAAGTGGTCATTGTGTCAGCATCTGCTGGATGT
GAGGACTTGCATTGTGAAAGCTTTGCTGTCCTTGATGTGATCATGGAATCTCTTTCTCAC
TAGAGTCTATGTCACTCATTATACTCTGTCGAATGTCATTGAATGTCTTTACATGGGCTT
GTATGCCTATGAAAATTGTAATACAACTTTCAGCAACGGATCTCTTGGCTCTCGCATCGA
TGAAGGACGCAGCGAAATGCGATAAGTAATGTGAATTGCAGAATTCAGTGAATCATCGAA
TCTTTGAACGCATCTTGCGCTCCTTGGTATTCCGAGGAGCATGCCTGTTTGAGTGTCATT
AAATTCTCAACTCTCTTATACTTTTTTGTAAAAGAGAGCTTGGACTGTGGAGGCTTGCTG
GCCACTTTTTGGGGTCAGCTCCTCTGAAATGCATTAGCGGAACCGTTTGCAATCTGCCAC
AAGTGTGATAAGTTATCTACACTGGCGAGGGGATTGCTCTCTGTAATGTTCAGCTTCTAA
TTGTCTCTACTTTGTGAGACAACTTTTGAATGCTTGACCTCAAATCAGGTAGGACTACCC
GCTGAACTTAA
>AB2-62
TTTCCGTAGGTGAACCTGCGGAAGGATCATTATTGAATTATGTTTCTAGATAGGTTGTAG
CTGGCTC-TTTAGAGCATGTGCACGCCTGTTTGGACTTCATTTTCATCCACCTGTGCACC
TATTGTAGTCTTTGGTTGGGTTAGGGGGAAGTGGTCATTGTGTCAGCATCTGCTGGATGT
GAGGACTTGCATTGTGAAAGCTTTGCTGTCCTTGATGTGATCATGGAATCTCTTTCTCAC
TAGAGTCTATGTCACTCATTATACTCTGTCGAATGTCATTGAATGTCTTTACATGGGCTT
GTATGCCTATGAAAATTGTAATACAACTTTCAGCAACGGATCTCTTGGCTCTCGCATCGA
TGAAGGACGCAGCGAAATGCGATAAGTAATGTGAATTGCAGAATTCAGTGAATCATCGAA
TCTTTGAACGCATCTTGCGCTCCTTGGTATTCCGAGGAGCATGCCTGTTTGAGTGTCATT
AAATTCTCAACTCTCTTATACTTTTTTGTAAAAGAGAGCTTGGACTGTGGAGGCTTGCTG

GCCACTTTTTGGGGTCAGCTCCTCTGAAATGCATTAGCGGAACCGTTTGCAATCTGCCAC
AAGTGTGATAAGTTATCTACACTGGCGAGGGGATTGCTCTCTGTAATGTTCAGCTTCTAA
TTGTCTCTACTTTGTGAGACAACTTTTGAATGCTTGACCTCAAATCAGGTAGGACTACCC
GCTGAACTTAA
>AB2-63
TTTCCGTAGGTGAACCTGCGGAAGGATCATTATTGAATTATGTTTCTAGATAGGTTGTAG
CTGGCTC-TTTAGAGCATGTGCACGCCTGTTTGGACTTCATTTTCATCCACCTGTGCACC
TATTGTAGTCTTTGGTTGGGTTAGGGGGAAGTGGTCATTGTGTCAGCATCTGCTGGATGT
GAGGACTTGCATTGTGAAAGCTTTGCTGTCCTTGATGTGATCATGGAATCTCTTTCTCAC
TAGAGTCTATGTCACTCATTATACTCTGTCGAATGTCATTGAATGTCTTTACATGGGCTT
GTATGCCTATGAAAATTGTAATACAACTTTCAGCAACGGATCTCTTGGCTCTCGCATCGA
TGAAGGACGCAGCGAAATGCGATAAGTAATGTGAATTGCAGAATTCAGTGAATCATCGAA
TCTTTGAACGCATCTTGCGCTCCTTGGTATTCCGAGGAGCATGCCTGTTTGAGTGTCATT
AAATTCTCAACTCTCTTATACTTTTTTGTAAAAGAGAGCTTGGACTGTGGAGGCTTGCTG
GCCACTTTTTGGGGTCAGCTCCTCTGAAATGCATTAGCGGAACCGTTTGCAATCTGCCAC
AAGTGTGATAAGTTATCTACACTGGCGAGGGGATTGCTCTCTGTAATGTTCAGCTTCTAA
TTGTCTCTACTTTGTGAGACAACTTTTGAATGCTTGACCTCAAATCAGGTAGGACTACCC
GCTGAACTTAA
>AB3-22
TTTCCGTAGGTGAACCTGCGGAAGGATCATTATTGAATTATGTTTCTAGATAGGTTGTAG
CTGGCTC-TTTAGAGCATGTGCACGCCTGTTTGGACTTCATTTTCATCCACCTGTGCACC
TATTGTAGTCTTTGGTTGGGTTAGGGGGAAGTGGTCATTGTGTCAGCATCTGCTGGATGT
GAGGACTTGCATTGTGAAAGCTTTGCTGTCCTTGATGTGATCATGGAATCTCTTTCTCAC
TAGAGTCTATGTCACTCATTATACTCTGTCGAATGTCATTGAATGTCTTTACATGGGCTT
GTATGCCTATGAAAATTGTAATACAACTTTCAGCAACGGATCTCTTGGCTCTCGCATCGA
TGAAGGACGCAGCGAAATGCGATAAGTAATGTGAATTGCAGAATTCAGTGAATCATCGAA
TCTTTGAACGCATCTTGCGCTCCTTGGTATTCCGAGGAGCATGCCTGTTTGAGTGTCATT
AAATTCTCAACTCTCTTATACTTTTTTGTAAAAGAGAGCTTGGACTGTGGAGGCTTGCTG
GCCACTTTTTGGGGTCAGCTCCTCTGAAATGCATTAGCGGAACCGTTTGCAATCTGCCAC
AAGTGTGATAAGTTATCTACACTGGCGAGGGGATTGCTCTCTGTAATGTTCAGCTTCTAA
TTGTCTCTACTTTGTGAGACAACTTTTGAATGCTTGACCTCAAATCAGGTAGGACTACCC
GCTGAACTTAA
>AB3-48
TTTCCGTAGGTGAACCTGCGGAAGGATCATTATTGAATTATGTTTCTAGATAGGTTGTAG
CTGGCTC-TTTAGAGCATGTGCACGCCTGTTTGGACTTCATTTTCATCCACCTGTGCACC
TATTGTAGTCTTTGGTTGGGTTAGGGGGAAGTGGTCATTGTGTCAGCATCTGCTGGATGT
GAGGACTTGCATTGTGAAAGCTTTGCTGTCCTTGATGTGATCATGGAATCTCTTTCTCAC
TAGAGTCTATGTCACTCATTATACTCTGTCGAATGTCATTGAATGTCTTTACATGGGCTT
GTATGCCTATGAAAATTGTAATACAACTTTCAGCAACGGATCTCTTGGCTCTCGCATCGA
TGAAGGACGCAGCGAAATGCGATAAGTAATGTGAATTGCAGAATTCAGTGAATCATCGAA
TCTTTGAACGCATCTTGCGCTCCTTGGTATTCCGAGGAGCATGCCTGTTTGAGTGTCATT
AAATTCTCAACTCTCTTATACTTTTTTGTAAAAGAGAGCTTGGACTGTGGAGGCTTGCTG
GCCACTTTTTGGGGTCAGCTCCTCTGAAATGCATTAGCGGAACCGTTTGCAATCTGCCAC
AAGTGTGATAAGTTATCTACACTGGCGAGGGGATTGCTCTCTGTAATGTTCAGCTTCTAA
TTGTCTCTACTTTGTGAGACAACTTTTGAATGCTTGACCTCAAATCAGGTAGGACTACCC
GCTGAACTTAA
>AB4-10
TTTCCGTAGGTGAACCTGCGGAAGGATCATTATTGAATTATGTTTCTAGATAGGTTGTAG
CTGGCTC-TTTAGAGCATGTGCACGCCTGTTTGGACTTCATTTTCATCCACCTGTGCACC
TATTGTAGTCTTTGGTTGGGTTAGGGGGAAGTGGTCATTGTGTCAGCATCTGCTGGATGT

GAGGACTTGCATTGTGAAAGCTTTGCTGTCCTTGATGTGATCATGGAATCTCTTTCTCAC
TAGAGTCTATGTCACTCATTATACTCTGTCGAATGTCATTGAATGTCTTTACATGGGCTT
GTATGCCTATGAAAATTGTAATACAACTTTCAGCAACGGATCTCTTGGCTCTCGCATCGA
TGAAGGACGCAGCGAAATGCGATAAGTAATGTGAATTGCAGAATTCAGTGAATCATCGAA
TCTTTGAACGCATCTTGCGCTCCTTGGTATTCCGAGGAGCATGCCTGTTTGAGTGTCATT
AAATTCTCAACTCTCTTATACTTTTTTGTAAAAGAGAGCTTGGACTGTGGAGGCTTGCTG
GCCACTTTTTGGGGTCAGCTCCTCTGAAATGCATTAGCGGAACCGTTTGCAATCTGCCAC
AAGTGTGATAAGTTATCTACACTGGCGAGGGGATTGCTCTCTGTAATGTTCAGCTTCTAA
TTGTCTCTACTTTGTGAGACAACTTTTGAATGCTTGACCTCAAATCAGGTAGGACTACCC
GCTGAACTTAA
>AB4-21
TTTCCGTAGGTGAACCTGCGGAAGGATCATTATTGAATTATGTTTCTAGATAGGTTGTAG
CTGGCTC-TTTAGAGCATGTGCACGCCTGTTTGGACTTCATTTTCATCCACCTGTGCACC
TATTGTAGTCTTTGGTTGGGTTAGGGGGAAGTGGTCATTGTGTCAGCATCTGCTGGATGT
GAGGACTTGCATTGTGAAAGCTTTGCTGTCCTTGATGTGATCATGGAATCTCTTTCTCAC
TAGAGTCTATGTCACTCATTATACTCTGTCGAATGTCATTGAATGTCTTTACATGGGCTT
GTATGCCTATGAAAATTGTAATACAACTTTCAGCAACGGATCTCTTGGCTCTCGCATCGA
TGAAGGACGCAGCGAAATGCGATAAGTAATGTGAATTGCAGAATTCAGTGAATCATCGAA
TCTTTGAACGCATCTTGCGCTCCTTGGTATTCCGAGGAGCATGCCTGTTTGAGTGTCATT
AAATTCTCAACTCTCTTATACTTTTTTGTAAAAGAGAGCTTGGACTGTGGAGGCTTGCTG
GCCACTTTTTGGGGTCAGCTCCTCTGAAATGCATTAGCGGAACCGTTTGCAATCTGCCAC
AAGTGTGATAAGTTATCTACACTGGCGAGGGGATTGCTCTCTGTAATGTTCAGCTTCTAA
TTGTCTCTACTTTGTGAGACAACTTTTGAATGCTTGACCTCAAATCAGGTAGGACTACCC
GCTGAACTTAA
>AB4-22
TTTCCGTAGGTGAACCTGCGGAAGGATCATTATTGAATTATGTTTCTAGATAGGTTGTAG
CTGGCTC-TTTAGAGCATGTGCACGCCTGTTTGGACTTCATTTTCATCCACCTGTGCACC
TATTGTAGTCTTTGGTTGGGTTAGGGGGAAGTGGTCATTGTGTCAGCATCTGCTGGATGT
GAGGACTTGCATTGTGAAAGCTTTGCTGTCCTTGATGTGATCATGGAATCTCTTTCTCAC
TAGAGTCTATGTCACTCATTATACTCTGTCGAATGTCATTGAATGTCTTTACATGGGCTT
GTATGCCTATGAAAATTGTAATACAACTTTCAGCAACGGATCTCTTGGCTCTCGCATCGA
TGAAGGACGCAGCGAAATGCGATAAGTAATGTGAATTGCAGAATTCAGTGAATCATCGAA
TCTTTGAACGCATCTTGCGCTCCTTGGTATTCCGAGGAGCATGCCTGTTTGAGTGTCATT
AAATTCTCAACTCTCTTATACTTTTTTGTAAAAGAGAGCTTGGACTGTGGAGGCTTGCTG
GCCACTTTTTGGGGTCAGCTCCTCTGAAATGCATTAGCGGAACCGTTTGCAATCTGCCAC
AAGTGTGATAAGTTATCTACACTGGCGAGGGGATTGCTCTCTGTAATGTTCAGCTTCTAA
TTGTCTCTACTTTGTGAGACAACTTTTGAATGCTTGACCTCAAATCAGGTAGGACTACCC
GCTGAACTTAA
>AB4-45
TTTCCGTAGGTGAACCTGCGGAAGGATCATTATTGAATTATGTTTCTAGATAGGTTGTAG
CTGGCTC-TTTAGAGCATGTGCACGCCTGTTTGGACTTCATTTTCATCCACCTGTGCACC
TATTGTAGTCTTTGGTTGGGTTAGGGGGAAGTGGTCATTGTGTCAGCATCTGCTGGATGT
GAGGACTTGCATTGTGAAAGCTTTGCTGTCCTTGATGTGATCATGGAATCTCTTTCTCAC
TAGAGTCTATGTCACTCATTATACTCTGTCGAATGTCATTGAATGTCTTTACATGGGCTT
GTATGCCTATGAAAATTGTAATACAACTTTCAGCAACGGATCTCTTGGCTCTCGCATCGA
TGAAGGACGCAGCGAAATGCGATAAGTAATGTGAATTGCAGAATTCAGTGAATCATCGAA
TCTTTGAACGCATCTTGCGCTCCTTGGTATTCCGAGGAGCATGCCTGTTTGAGTGTCATT
AAATTCTCAACTCTCTTATACTTTTTTGTAAAAGAGAGCTTGGACTGTGGAGGCTTGCTG
GCCACTTTTTGGGGTCAGCTCCTCTGAAATGCATTAGCGGAACCGTTTGCAATCTGCCAC
AAGTGTGATAAGTTATCTACACTGGCGAGGGGATTGCTCTCTGTAATGTTCAGCTTCTAA

TTGTCTCTACTTTGTGAGACAACTTTTGAATGCTTGACCTCAAATCAGGTAGGACTACCC
GCTGAACTTAA
>AB4-51
TTTCCGTAGGTGAACCTGCGGAAGGATCATTATTGAATTATGTTTCTAGATAGGTTGTAG
CTGGCTC-TTTAGAGCATGTGCACGCCTGTTTGGACTTCATTTTCATCCACCTGTGCACC
TATTGTAGTCTTTGGTTGGGTTAGGGGGAAGTGGTCATTGTGTCAGCATCTGCTGGATGT
GAGGACTTGCATTGTGAAAGCTTTGCTGTCCTTGATGTGATCATGGAATCTCTTTCTCAC
TAGAGTCTATGTCACTCATTATACTCTGTCGAATGTCATTGAATGTCTTTACATGGGCTT
GTATGCCTATGAAAATTGTAATACAACTTTCAGCAACGGATCTCTTGGCTCTCGCATCGA
TGAAGGACGCAGCGAAATGCGATAAGTAATGTGAATTGCAGAATTCAGTGAATCATCGAA
TCTTTGAACGCATCTTGCGCTCCTTGGTATTCCGAGGAGCATGCCTGTTTGAGTGTCATT
AAATTCTCAACTCTCTTATACTTTTTTGTAAAAGAGAGCTTGGACTGTGGAGGCTTGCTG
GCCACTTTTTGGGGTCAGCTCCTCTGAAATGCATTAGCGGAACCGTTTGCAATCTGCCAC
AAGTGTGATAAGTTATCTACACTGGCGAGGGGATTGCTCTCTGTAATGTTCAGCTTCTAA
TTGTCTCTACTTTGTGAGACAACTTTTGAATGCTTGACCTCAAATCAGGTAGGACTACCC
GCTGAACTTAA
>AB5-2
TTTCCGTAGGTGAACCTGCGGAAGGATCATTATTGAATTATGTTTCTAGATAGGTTGTAG
CTGGCTC-TTTAGAGCATGTGCACGCCTGTTTGGACTTCATTTTCATCCACCTGTGCACC
TATTGTAGTCTTTGGTTGGGTTAGGGGGAAGTGGTCATTGTGTCAGCATCTGCTGGATGT
GAGGACTTGCATTGTGAAAGCTTTGCTGTCCTTGATGTGATCATGGAATCTCTTTCTCAC
TAGAGTCTATGTCACTCATTATACTCTGTCGAATGTCATTGAATGTCTTTACATGGGCTT
GTATGCCTATGAAAATTGTAATACAACTTTCAGCAACGGATCTCTTGGCTCTCGCATCGA
TGAAGGACGCAGCGAAATGCGATAAGTAATGTGAATTGCAGAATTCAGTGAATCATCGAA
TCTTTGAACGCATCTTGCGCTCCTTGGTATTCCGAGGAGCATGCCTGTTTGAGTGTCATT
AAATTCTCAACTCTCTTATACTTTTTTGTAAAAGAGAGCTTGGACTGTGGAGGCTTGCTG
GCCACTTTTTGGGGTCAGCTCCTCTGAAATGCATTAGCGGAACCGTTTGCAATCTGCCAC
AAGTGTGATAAGTTATCTACACTGGCGAGGGGATTGCTCTCTGTAATGTTCAGCTTCTAA
TTGTCTCTACTTTGTGAGACAACTTTTGAATGCTTGACCTCAAATCAGGTAGGACTACCC
GCTGAACTTAA
>AB5-12
TTTCCGTAGGTGAACCTGCGGAAGGATCATTATTGAATTATGTTTCTAGATAGGTTGTAG
CTGGCTC-TTTAGAGCATGTGCACGCCTGTTTGGACTTCATTTTCATCCACCTGTGCACC
TATTGTAGTCTTTGGTTGGGTTAGGGGGAAGTGGTCATTGTGTCAGCATCTGCTGGATGT
GAGGACTTGCATTGTGAAAGCTTTGCTGTCCTTGATGTGATCATGGAATCTCTTTCTCAC
TAGAGTCTATGTCACTCATTATACTCTGTCGAATGTCATTGAATGTCTTTACATGGGCTT
GTATGCCTATGAAAATTGTAATACAACTTTCAGCAACGGATCTCTTGGCTCTCGCATCGA
TGAAGGACGCAGCGAAATGCGATAAGTAATGTGAATTGCAGAATTCAGTGAATCATCGAA
TCTTTGAACGCATCTTGCGCTCCTTGGTATTCCGAGGAGCATGCCTGTTTGAGTGTCATT
AAATTCTCAACTCTCTTATACTTTTTTGTAAAAGAGAGCTTGGACTGTGGAGGCTTGCTG
GCCACTTTTTGGGGTCAGCTCCTCTGAAATGCATTAGCGGAACCGTTTGCAATCTGCCAC
AAGTGTGATAAGTTATCTACACTGGCGAGGGGATTGCTCTCTGTAATGTTCAGCTTCTAA
TTGTCTCTACTTTGTGAGACAACTTTTGAATGCTTGACCTCAAATCAGGTAGGACTACCC
GCTGAACTTAA
>AB5-42
TTTCCGTAGGTGAACCTGCGGAAGGATCATTATTGAATTATGTTTCTAGATAGGTTGTAG
CTGGCTC-TTTAGAGCATGTGCACGCCTGTTTGGACTTCATTTTCATCCACCTGTGCACC
TATTGTAGTCTTTGGTTGGGTTAGGGGGAAGTGGTCATTGTGTCAGCATCTGCTGGATGT
GAGGACTTGCATTGTGAAAGCTTTGCTGTCCTTGATGTGATCATGGAATCTCTTTCTCAC
TAGAGTCTATGTCACTCATTATACTCTGTCGAATGTCATTGAATGTCTTTACATGGGCTT

GTATGCCTATGAAAATTGTAATACAACTTTCAGCAACGGATCTCTTGGCTCTCGCATCGA
TGAAGGACGCAGCGAAATGCGATAAGTAATGTGAATTGCAGAATTCAGTGAATCATCGAA
TCTTTGAACGCATCTTGCGCTCCTTGGTATTCCGAGGAGCATGCCTGTTTGAGTGTCATT
AAATTCTCAACTCTCTTATACTTTTTTGTAAAAGAGAGCTTGGACTGTGGAGGCTTGCTG
GCCACTTTTTGGGGTCAGCTCCTCTGAAATGCATTAGCGGAACCGTTTGCAATCTGCCAC
AAGTGTGATAAGTTATCTACACTGGCGAGGGGATTGCTCTCTGTAATGTTCAGCTTCTAA
TTGTCTCTACTTTGTGAGACAACTTTTGAATGCTTGACCTCAAATCAGGTAGGACTACCC
GCTGAACTTAA
>AB5-57
TTTCCGTAGGTGAACCTGCGGAAGGATCATTATTGAATTATGTTTCTAGATAGGTTGTAG
CTGGCTC-TTTAGAGCATGTGCACGCCTGTTTGGACTTCATTTTCATCCACCTGTGCACC
TATTGTAGTCTTTGGTTGGGTTAGGGGGAAGTGGTCATTGTGTCAGCATCTGCTGGATGT
GAGGACTTGCATTGTGAAAGCTTTGCTGTCCTTGATGTGATCATGGAATCTCTTTCTCAC
TAGAGTCTATGTCACTCATTATACTCTGTCGAATGTCATTGAATGTCTTTACATGGGCTT
GTATGCCTATGAAAATTGTAATACAACTTTCAGCAACGGATCTCTTGGCTCTCGCATCGA
TGAAGGACGCAGCGAAATGCGATAAGTAATGTGAATTGCAGAATTCAGTGAATCATCGAA
TCTTTGAACGCATCTTGCGCTCCTTGGTATTCCGAGGAGCATGCCTGTTTGAGTGTCATT
AAATTCTCAACTCTCTTATACTTTTTTGTAAAAGAGAGCTTGGACTGTGGAGGCTTGCTG
GCCACTTTTTGGGGTCAGCTCCTCTGAAATGCATTAGCGGAACCGTTTGCAATCTGCCAC
AAGTGTGATAAGTTATCTACACTGGCGAGGGGATTGCTCTCTGTAATGTTCAGCTTCTAA
TTGTCTCTACTTTGTGAGACAACTTTTGAATGCTTGACCTCAAATCAGGTAGGACTACCC
GCTGAACTTAA
>AB5-58
TTTCCGTAGGTGAACCTGCGGAAGGATCATTATTGAATTATGTTTCTAGATAGGTTGTAG
CTGGCTC-TTTAGAGCATGTGCACGCCTGTTTGGACTTCATTTTCATCCACCTGTGCACC
TATTGTAGTCTTTGGTTGGGTTAGGGGGAAGTGGTCATTGTGTCAGCATCTGCTGGATGT
GAGGACTTGCATTGTGAAAGCTTTGCTGTCCTTGATGTGATCATGGAATCTCTTTCTCAC
TAGAGTCTATGTCACTCATTATACTCTGTCGAATGTCATTGAATGTCTTTACATGGGCTT
GTATGCCTATGAAAATTGTAATACAACTTTCAGCAACGGATCTCTTGGCTCTCGCATCGA
TGAAGGACGCAGCGAAATGCGATAAGTAATGTGAATTGCAGAATTCAGTGAATCATCGAA
TCTTTGAACGCATCTTGCGCTCCTTGGTATTCCGAGGAGCATGCCTGTTTGAGTGTCATT
AAATTCTCAACTCTCTTATACTTTTTTGTAAAAGAGAGCTTGGACTGTGGAGGCTTGCTG
GCCACTTTTTGGGGTCAGCTCCTCTGAAATGCATTAGCGGAACCGTTTGCAATCTGCCAC
AAGTGTGATAAGTTATCTACACTGGCGAGGGGATTGCTCTCTGTAATGTTCAGCTTCTAA
TTGTCTCTACTTTGTGAGACAACTTTTGAATGCTTGACCTCAAATCAGGTAGGACTACCC
GCTGAACTTAA
>AB5-67
TTTCCGTAGGTGAACCTGCGGAAGGATCATTATTGAATTATGTTTCTAGATAGGTTGTAG
CTGGCTC-TTTAGAGCATGTGCACGCCTGTTTGGACTTCATTTTCATCCACCTGTGCACC
TATTGTAGTCTTTGGTTGGGTTAGGGGGAAGTGGTCATTGTGTCAGCATCTGCTGGATGT
GAGGACTTGCATTGTGAAAGCTTTGCTGTCCTTGATGTGATCATGGAATCTCTTTCTCAC
TAGAGTCTATGTCACTCATTATACTCTGTCGAATGTCATTGAATGTCTTTACATGGGCTT
GTATGCCTATGAAAATTGTAATACAACTTTCAGCAACGGATCTCTTGGCTCTCGCATCGA
TGAAGGACGCAGCGAAATGCGATAAGTAATGTGAATTGCAGAATTCAGTGAATCATCGAA
TCTTTGAACGCATCTTGCGCTCCTTGGTATTCCGAGGAGCATGCCTGTTTGAGTGTCATT
AAATTCTCAACTCTCTTATACTTTTTTGTAAAAGAGAGCTTGGACTGTGGAGGCTTGCTG
GCCACTTTTTGGGGTCAGCTCCTCTGAAATGCATTAGCGGAACCGTTTGCAATCTGCCAC
AAGTGTGATAAGTTATCTACACTGGCGAGGGGATTGCTCTCTGTAATGTTCAGCTTCTAA
TTGTCTCTACTTTGTGAGACAACTTTTGAATGCTTGACCTCAAATCAGGTAGGACTACCC
GCTGAACTTAA

>AB5-68
TTTCCGTAGGTGAACCTGCGGAAGGATCATTATTGAATTATGTTTCTAGATAGGTTGTAG
CTGGCTC-TTTAGAGCATGTGCACGCCTGTTTGGACTTCATTTTCATCCACCTGTGCACC
TATTGTAGTCTTTGGTTGGGTTAGGGGGAAGTGGTCATTGTGTCAGCATCTGCTGGATGT
GAGGACTTGCATTGTGAAAGCTTTGCTGTCCTTGATGTGATCATGGAATCTCTTTCTCAC
TAGAGTCTATGTCACTCATTATACTCTGTCGAATGTCATTGAATGTCTTTACATGGGCTT
GTATGCCTATGAAAATTGTAATACAACTTTCAGCAACGGATCTCTTGGCTCTCGCATCGA
TGAAGGACGCAGCGAAATGCGATAAGTAATGTGAATTGCAGAATTCAGTGAATCATCGAA
TCTTTGAACGCATCTTGCGCTCCTTGGTATTCCGAGGAGCATGCCTGTTTGAGTGTCATT
AAATTCTCAACTCTCTTATACTTTTTTGTAAAAGAGAGCTTGGACTGTGGAGGCTTGCTG
GCCACTTTTTGGGGTCAGCTCCTCTGAAATGCATTAGCGGAACCGTTTGCAATCTGCCAC
AAGTGTGATAAGTTATCTACACTGGCGAGGGGATTGCTCTCTGTAATGTTCAGCTTCTAA
TTGTCTCTACTTTGTGAGACAACTTTTGAATGCTTGACCTCAAATCAGGTAGGACTACCC
GCTGAACTTAA
>AB6-3
TTTCCGTAGGTGAACCTGCGGAAGGATCATTATTGAATTATGTTTCTAGATAGGTTGTAG
CTGGCTC-TTTAGAGCATGTGCACGCCTGTTTGGACTTCATTTTCATCCACCTGTGCACC
TATTGTAGTCTTTGGTTGGGTTAGGGGGAAGTGGTCATTGTGTCAGCATCTGCTGGATGT
GAGGACTTGCATTGTGAAAGCTTTGCTGTCCTTGATGTGATCATGGAATCTCTTTCTCAC
TAGAGTCTATGTCACTCATTATACTCTGTCGAATGTCATTGAATGTCTTTACATGGGCTT
GTATGCCTATGAAAATTGTAATACAACTTTCAGCAACGGATCTCTTGGCTCTCGCATCGA
TGAAGGACGCAGCGAAATGCGATAAGTAATGTGAATTGCAGAATTCAGTGAATCATCGAA
TCTTTGAACGCATCTTGCGCTCCTTGGTATTCCGAGGAGCATGCCTGTTTGAGTGTCATT
AAATTCTCAACTCTCTTATACTTTTTTGTAAAAGAGAGCTTGGACTGTGGAGGCTTGCTG
GCCACTTTTTGGGGTCAGCTCCTCTGAAATGCATTAGCGGAACCGTTTGCAATCTGCCAC
AAGTGTGATAAGTTATCTACACTGGCGAGGGGATTGCTCTCTGTAATGTTCAGCTTCTAA
TTGTCTCTACTTTGTGAGACAACTTTTGAATGCTTGACCTCAAATCAGGTAGGACTACCC
GCTGAACTTAA
>AB6-9
TTTCCGTAGGTGAACCTGCGGAAGGATCATTATTGAATTATGTTTCTAGATAGGTTGTAG
CTGGCTC-TTTAGAGCATGTGCACGCCTGTTTGGACTTCATTTTCATCCACCTGTGCACC
TATTGTAGTCTTTGGTTGGGTTAGGGGGAAGTGGTCATTGTGTCAGCATCTGCTGGATGT
GAGGACTTGCATTGTGAAAGCTTTGCTGTCCTTGATGTGATCATGGAATCTCTTTCTCAC
TAGAGTCTATGTCACTCATTATACTCTGTCGAATGTCATTGAATGTCTTTACATGGGCTT
GTATGCCTATGAAAATTGTAATACAACTTTCAGCAACGGATCTCTTGGCTCTCGCATCGA
TGAAGGACGCAGCGAAATGCGATAAGTAATGTGAATTGCAGAATTCAGTGAATCATCGAA
TCTTTGAACGCATCTTGCGCTCCTTGGTATTCCGAGGAGCATGCCTGTTTGAGTGTCATT
AAATTCTCAACTCTCTTATACTTTTTTGTAAAAGAGAGCTTGGACTGTGGAGGCTTGCTG
GCCACTTTTTGGGGTCAGCTCCTCTGAAATGCATTAGCGGAACCGTTTGCAATCTGCCAC
AAGTGTGATAAGTTATCTACACTGGCGAGGGGATTGCTCTCTGTAATGTTCAGCTTCTAA
TTGTCTCTACTTTGTGAGACAACTTTTGAATGCTTGACCTCAAATCAGGTAGGACTACCC
GCTGAACTTAA
>AB6-16
TTTCCGTAGGTGAACCTGCGGAAGGATCATTATTGAATTATGTTTCTAGATAGGTTGTAG
CTGGCTC-TTTAGAGCATGTGCACGCCTGTTTGGACTTCATTTTCATCCACCTGTGCACC
TATTGTAGTCTTTGGTTGGGTTAGGGGGAAGTGGTCATTGTGTCAGCATCTGCTGGATGT
GAGGACTTGCATTGTGAAAGCTTTGCTGTCCTTGATGTGATCATGGAATCTCTTTCTCAC
TAGAGTCTATGTCACTCATTATACTCTGTCGAATGTCATTGAATGTCTTTACATGGGCTT
GTATGCCTATGAAAATTGTAATACAACTTTCAGCAACGGATCTCTTGGCTCTCGCATCGA
TGAAGGACGCAGCGAAATGCGATAAGTAATGTGAATTGCAGAATTCAGTGAATCATCGAA

TCTTTGAACGCATCTTGCGCTCCTTGGTATTCCGAGGAGCATGCCTGTTTGAGTGTCATT
AAATTCTCAACTCTCTTATACTTTTTTGTAAAAGAGAGCTTGGACTGTGGAGGCTTGCTG
GCCACTTTTTGGGGTCAGCTCCTCTGAAATGCATTAGCGGAACCGTTTGCAATCTGCCAC
AAGTGTGATAAGTTATCTACACTGGCGAGGGGATTGCTCTCTGTAATGTTCAGCTTCTAA
TTGTCTCTACTTTGTGAGACAACTTTTGAATGCTTGACCTCAAATCAGGTAGGACTACCC
GCTGAACTTAA
>AB6−19
TTTCCGTAGGTGAACCTGCGGAAGGATCATTATTGAATTATGTTTCTAGATAGGTTGTAG
CTGGCTC−TTTAGAGCATGTGCACGCCTGTTTGGACTTCATTTTCATCCACCTGTGCACC
TATTGTAGTCTTTGGTTGGGTTAGGGGGAAGTGGTCATTGTGTCAGCATCTGCTGGATGT
GAGGACTTGCATTGTGAAAGCTTTGCTGTCCTTGATGTGATCATGGAATCTCTTTCTCAC
TAGAGTCTATGTCACTCATTATACTCTGTCGAATGTCATTGAATGTCTTTACATGGGCTT
GTATGCCTATGAAAATTGTAATACAACTTTCAGCAACGGATCTCTTGGCTCTCGCATCGA
TGAAGGACGCAGCGAAATGCGATAAGTAATGTGAATTGCAGAATTCAGTGAATCATCGAA
TCTTTGAACGCATCTTGCGCTCCTTGGTATTCCGAGGAGCATGCCTGTTTGAGTGTCATT
AAATTCTCAACTCTCTTATACTTTTTTGTAAAAGAGAGCTTGGACTGTGGAGGCTTGCTG
GCCACTTTTTGGGGTCAGCTCCTCTGAAATGCATTAGCGGAACCGTTTGCAATCTGCCAC
AAGTGTGATAAGTTATCTACACTGGCGAGGGGATTGCTCTCTGTAATGTTCAGCTTCTAA
TTGTCTCTACTTTGTGAGACAACTTTTGAATGCTTGACCTCAAATCAGGTAGGACTACCC
GCTGAACTTAA
>AB6−32
TTTCCGTAGGTGAACCTGCGGAAGGATCATTATTGAATTATGTTTCTAGATAGGTTGTAG
CTGGCTC−TTTAGAGCATGTGCACGCCTGTTTGGACTTCATTTTCATCCACCTGTGCACC
TATTGTAGTCTTTGGTTGGGTTAGGGGGAAGTGGTCATTGTGTCAGCATCTGCTGGATGT
GAGGACTTGCATTGTGAAAGCTTTGCTGTCCTTGATGTGATCATGGAATCTCTTTCTCAC
TAGAGTCTATGTCACTCATTATACTCTGTCGAATGTCATTGAATGTCTTTACATGGGCTT
GTATGCCTATGAAAATTGTAATACAACTTTCAGCAACGGATCTCTTGGCTCTCGCATCGA
TGAAGGACGCAGCGAAATGCGATAAGTAATGTGAATTGCAGAATTCAGTGAATCATCGAA
TCTTTGAACGCATCTTGCGCTCCTTGGTATTCCGAGGAGCATGCCTGTTTGAGTGTCATT
AAATTCTCAACTCTCTTATACTTTTTTGTAAAAGAGAGCTTGGACTGTGGAGGCTTGCTG
GCCACTTTTTGGGGTCAGCTCCTCTGAAATGCATTAGCGGAACCGTTTGCAATCTGCCAC
AAGTGTGATAAGTTATCTACACTGGCGAGGGGATTGCTCTCTGTAATGTTCAGCTTCTAA
TTGTCTCTACTTTGTGAGACAACTTTTGAATGCTTGACCTCAAATCAGGTAGGACTACCC
GCTGAACTTAA
>AB6−39
TTTCCGTAGGTGAACCTGCGGAAGGATCATTATTGAATTATGTTTCTAGATAGGTTGTAG
CTGGCTC−TTTAGAGCATGTGCACGCCTGTTTGGACTTCATTTTCATCCACCTGTGCACC
TATTGTAGTCTTTGGTTGGGTTAGGGGGAAGTGGTCATTGTGTCAGCATCTGCTGGATGT
GAGGACTTGCATTGTGAAAGCTTTGCTGTCCTTGATGTGATCATGGAATCTCTTTCTCAC
TAGAGTCTATGTCACTCATTATACTCTGTCGAATGTCATTGAATGTCTTTACATGGGCTT
GTATGCCTATGAAAATTGTAATACAACTTTCAGCAACGGATCTCTTGGCTCTCGCATCGA
TGAAGGACGCAGCGAAATGCGATAAGTAATGTGAATTGCAGAATTCAGTGAATCATCGAA
TCTTTGAACGCATCTTGCGCTCCTTGGTATTCCGAGGAGCATGCCTGTTTGAGTGTCATT
AAATTCTCAACTCTCTTATACTTTTTTGTAAAAGAGAGCTTGGACTGTGGAGGCTTGCTG
GCCACTTTTTGGGGTCAGCTCCTCTGAAATGCATTAGCGGAACCGTTTGCAATCTGCCAC
AAGTGTGATAAGTTATCTACACTGGCGAGGGGATTGCTCTCTGTAATGTTCAGCTTCTAA
TTGTCTCTACTTTGTGAGACAACTTTTGAATGCTTGACCTCAAATCAGGTAGGACTACCC
GCTGAACTTAA
>AB6−42
TTTCCGTAGGTGAACCTGCGGAAGGATCATTATTGAATTATGTTTCTAGATAGGTTGTAG

CTGGCTC-TTTAGAGCATGTGCACGCCTGTTTGGACTTCATTTTCATCCACCTGTGCACC
TATTGTAGTCTTTGGTTGGGTTAGGGGGAAGTGGTCATTGTGTCAGCATCTGCTGGATGT
GAGGACTTGCATTGTGAAAGCTTTGCTGTCCTTGATGTGATCATGGAATCTCTTTCTCAC
TAGAGTCTATGTCACTCATTATACTCTGTCGAATGTCATTGAATGTCTTTACATGGGCTT
GTATGCCTATGAAAATTGTAATACAACTTTCAGCAACGGATCTCTTGGCTCTCGCATCGA
TGAAGGACGCAGCGAAATGCGATAAGTAATGTGAATTGCAGAATTCAGTGAATCATCGAA
TCTTTGAACGCATCTTGCGCTCCTTGGTATTCCGAGGAGCATGCCTGTTTGAGTGTCATT
AAATTCTCAACTCTCTTATACTTTTTTGTAAAAGAGAGCTTGGACTGTGGAGGCTTGCTG
GCCACTTTTTGGGGTCAGCTCCTCTGAAATGCATTAGCGGAACCGTTTGCAATCTGCCAC
AAGTGTGATAAGTTATCTACACTGGCGAGGGGATTGCTCTCTGTAATGTTCAGCTTCTAA
TTGTCTCTACTTTGTGAGACAACTTTTGAATGCTTGACCTCAAATCAGGTAGGACTACCC
GCTGAACTTAA
>AB7-9
TTTCCGTAGGTGAACCTGCGGAAGGATCATTATTGAATTATGTTTCTAGATAGGTTGTAG
CTGGCTC-TTTAGAGCATGTGCACGCCTGTTTGGACTTCATTTTCATCCACCTGTGCACC
TATTGTAGTCTTTGGTTGGGTTAGGGGGAAGTGGTCATTGTGTCAGCATCTGCTGGATGT
GAGGACTTGCATTGTGAAAGCTTTGCTGTCCTTGATGTGATCATGGAATCTCTTTCTCAC
TAGAGTCTATGTCACTCATTATACTCTGTCGAATGTCATTGAATGTCTTTACATGGGCTT
GTATGCCTATGAAAATTGTAATACAACTTTCAGCAACGGATCTCTTGGCTCTCGCATCGA
TGAAGGACGCAGCGAAATGCGATAAGTAATGTGAATTGCAGAATTCAGTGAATCATCGAA
TCTTTGAACGCATCTTGCGCTCCTTGGTATTCCGAGGAGCATGCCTGTTTGAGTGTCATT
AAATTCTCAACTCTCTTATACTTTTTTGTAAAAGAGAGCTTGGACTGTGGAGGCTTGCTG
GCCACTTTTTGGGGTCAGCTCCTCTGAAATGCATTAGCGGAACCGTTTGCAATCTGCCAC
AAGTGTGATAAGTTATCTACACTGGCGAGGGGATTGCTCTCTGTAATGTTCAGCTTCTAA
TTGTCTCTACTTTGTGAGACAACTTTTGAATGCTTGACCTCAAATCAGGTAGGACTACCC
GCTGAACTTAA
>AB7-13
TTTCCGTAGGTGAACCTGCGGAAGGATCATTATTGAATTATGTTTCTAGATAGGTTGTAG
CTGGCTC-TTTAGAGCATGTGCACGCCTGTTTGGACTTCATTTTCATCCACCTGTGCACC
TATTGTAGTCTTTGGTTGGGTTAGGGGGAAGTGGTCATTGTGTCAGCATCTGCTGGATGT
GAGGACTTGCATTGTGAAAGCTTTGCTGTCCTTGATGTGATCATGGAATCTCTTTCTCAC
TAGAGTCTATGTCACTCATTATACTCTGTCGAATGTCATTGAATGTCTTTACATGGGCTT
GTATGCCTATGAAAATTGTAATACAACTTTCAGCAACGGATCTCTTGGCTCTCGCATCGA
TGAAGGACGCAGCGAAATGCGATAAGTAATGTGAATTGCAGAATTCAGTGAATCATCGAA
TCTTTGAACGCATCTTGCGCTCCTTGGTATTCCGAGGAGCATGCCTGTTTGAGTGTCATT
AAATTCTCAACTCTCTTATACTTTTTTGTAAAAGAGAGCTTGGACTGTGGAGGCTTGCTG
GCCACTTTTTGGGGTCAGCTCCTCTGAAATGCATTAGCGGAACCGTTTGCAATCTGCCAC
AAGTGTGATAAGTTATCTACACTGGCGAGGGGATTGCTCTCTGTAATGTTCAGCTTCTAA
TTGTCTCTACTTTGTGAGACAACTTTTGAATGCTTGACCTCAAATCAGGTAGGACTACCC
GCTGAACTTAA
>AB7-18
TTTCCGTAGGTGAACCTGCGGAAGGATCATTATTGAATTATGTTTCTAGATAGGTTGTAG
CTGGCTC-TTTAGAGCATGTGCACGCCTGTTTGGACTTCATTTTCATCCACCTGTGCACC
TATTGTAGTCTTTGGTTGGGTTAGGGGGAAGTGGTCATTGTGTCAGCATCTGCTGGATGT
GAGGACTTGCATTGTGAAAGCTTTGCTGTCCTTGATGTGATCATGGAATCTCTTTCTCAC
TAGAGTCTATGTCACTCATTATACTCTGTCGAATGTCATTGAATGTCTTTACATGGGCTT
GTATGCCTATGAAAATTGTAATACAACTTTCAGCAACGGATCTCTTGGCTCTCGCATCGA
TGAAGGACGCAGCGAAATGCGATAAGTAATGTGAATTGCAGAATTCAGTGAATCATCGAA
TCTTTGAACGCATCTTGCGCTCCTTGGTATTCCGAGGAGCATGCCTGTTTGAGTGTCATT
AAATTCTCAACTCTCTTATACTTTTTTGTAAAAGAGAGCTTGGACTGTGGAGGCTTGCTG

GCCACTTTTTGGGGTCAGCTCCTCTGAAATGCATTAGCGGAACCGTTTGCAATCTGCCAC
AAGTGTGATAAGTTATCTACACTGGCGAGGGGATTGCTCTCTGTAATGTTCAGCTTCTAA
TTGTCTCTACTTTGTGAGACAACTTTTGAATGCTTGACCTCAAATCAGGTAGGACTACCC
GCTGAACTTAA
>AB7-20
TTTCCGTAGGTGAACCTGCGGAAGGATCATTATTGAATTATGTTTCTAGATAGGTTGTAG
CTGGCTC-TTTAGAGCATGTGCACGCCTGTTTGGACTTCATTTTCATCCACCTGTGCACC
TATTGTAGTCTTTGGTTGGGTTAGGGGGAAGTGGTCATTGTGTCAGCATCTGCTGGATGT
GAGGACTTGCATTGTGAAAGCTTTGCTGTCCTTGATGTGATCATGGAATCTCTTTCTCAC
TAGAGTCTATGTCACTCATTATACTCTGTCGAATGTCATTGAATGTCTTTACATGGGCTT
GTATGCCTATGAAAATTGTAATACAACTTTCAGCAACGGATCTCTTGGCTCTCGCATCGA
TGAAGGACGCAGCGAAATGCGATAAGTAATGTGAATTGCAGAATTCAGTGAATCATCGAA
TCTTTGAACGCATCTTGCGCTCCTTGGTATTCCGAGGAGCATGCCTGTTTGAGTGTCATT
AAATTCTCAACTCTCTTATACTTTTTTGTAAAAGAGAGCTTGGACTGTGGAGGCTTGCTG
GCCACTTTTTGGGGTCAGCTCCTCTGAAATGCATTAGCGGAACCGTTTGCAATCTGCCAC
AAGTGTGATAAGTTATCTACACTGGCGAGGGGATTGCTCTCTGTAATGTTCAGCTTCTAA
TTGTCTCTACTTTGTGAGACAACTTTTGAATGCTTGACCTCAAATCAGGTAGGACTACCC
GCTGAACTTAA
>AB7-50
TTTCCGTAGGTGAACCTGCGGAAGGATCATTATTGAATTATGTTTCTAGATAGGTTGTAG
CTGGCTC-TTTAGAGCATGTGCACGCCTGTTTGGACTTCATTTTCATCCACCTGTGCACC
TATTGTAGTCTTTGGTTGGGTTAGGGGGAAGTGGTCATTGTGTCAGCATCTGCTGGATGT
GAGGACTTGCATTGTGAAAGCTTTGCTGTCCTTGATGTGATCATGGAATCTCTTTCTCAC
TAGAGTCTATGTCACTCATTATACTCTGTCGAATGTCATTGAATGTCTTTACATGGGCTT
GTATGCCTATGAAAATTGTAATACAACTTTCAGCAACGGATCTCTTGGCTCTCGCATCGA
TGAAGGACGCAGCGAAATGCGATAAGTAATGTGAATTGCAGAATTCAGTGAATCATCGAA
TCTTTGAACGCATCTTGCGCTCCTTGGTATTCCGAGGAGCATGCCTGTTTGAGTGTCATT
AAATTCTCAACTCTCTTATACTTTTTTGTAAAAGAGAGCTTGGACTGTGGAGGCTTGCTG
GCCACTTTTTGGGGTCAGCTCCTCTGAAATGCATTAGCGGAACCGTTTGCAATCTGCCAC
AAGTGTGATAAGTTATCTACACTGGCGAGGGGATTGCTCTCTGTAATGTTCAGCTTCTAA
TTGTCTCTACTTTGTGAGACAACTTTTGAATGCTTGACCTCAAATCAGGTAGGACTACCC
GCTGAACTTAA
>AB8-1
TTTCCGTAGGTGAACCTGCGGAAGGATCATTATTGAATTATGTTTCTAGATAGGTTGTAG
CTGGCTC-TTTAGAGCATGTGCACGCCTGTTTGGACTTCATTTTCATCCACCTGTGCACC
TATTGTAGTCTTTGGTTGGGTTAGGGGGAAGTGGTCATTGTGTCAGCATCTGCTGGATGT
GAGGACTTGCATTGTGAAAGCTTTGCTGTCCTTGATGTGATCATGGAATCTCTTTCTCAC
TAGAGTCTATGTCACTCATTATACTCTGTCGAATGTCATTGAATGTCTTTACATGGGCTT
GTATGCCTATGAAAATTGTAATACAACTTTCAGCAACGGATCTCTTGGCTCTCGCATCGA
TGAAGGACGCAGCGAAATGCGATAAGTAATGTGAATTGCAGAATTCAGTGAATCATCGAA
TCTTTGAACGCATCTTGCGCTCCTTGGTATTCCGAGGAGCATGCCTGTTTGAGTGTCATT
AAATTCTCAACTCTCTTATACTTTTTTGTAAAAGAGAGCTTGGACTGTGGAGGCTTGCTG
GCCACTTTTTGGGGTCAGCTCCTCTGAAATGCATTAGCGGAACCGTTTGCAATCTGCCAC
AAGTGTGATAAGTTATCTACACTGGCGAGGGGATTGCTCTCTGTAATGTTCAGCTTCTAA
TTGTCTCTACTTTGTGAGACAACTTTTGAATGCTTGACCTCAAATCAGGTAGGACTACCC
GCTGAACTTAA
>AB8-30
TTTCCGTAGGTGAACCTGCGGAAGGATCATTATTGAATTATGTTTCTAGATAGGTTGTAG
CTGGCTC-TTTAGAGCATGTGCACGCCTGTTTGGACTTCATTTTCATCCACCTGTGCACC
TATTGTAGTCTTTGGTTGGGTTAGGGGGAAGTGGTCATTGTGTCAGCATCTGCTGGATGT

GAGGACTTGCATTGTGAAAGCTTTGCTGTCCTTGATGTGATCATGGAATCTCTTTCTCAC
TAGAGTCTATGTCACTCATTATACTCTGTCGAATGTCATTGAATGTCTTTACATGGGCTT
GTATGCCTATGAAAATTGTAATACAACTTTCAGCAACGGATCTCTTGGCTCTCGCATCGA
TGAAGGACGCAGCGAAATGCGATAAGTAATGTGAATTGCAGAATTCAGTGAATCATCGAA
TCTTTGAACGCATCTTGCGCTCCTTGGTATTCCGAGGAGCATGCCTGTTTGAGTGTCATT
AAATTCTCAACTCTCTTATACTTTTTTGTAAAAGAGAGCTTGGACTGTGGAGGCTTGCTG
GCCACTTTTTGGGGTCAGCTCCTCTGAAATGCATTAGCGGAACCGTTTGCAATCTGCCAC
AAGTGTGATAAGTTATCTACACTGGCGAGGGGATTGCTCTCTGTAATGTTCAGCTTCTAA
TTGTCTCTACTTTGTGAGACAACTTTTGAATGCTTGACCTCAAATCAGGTAGGACTACCC
GCTGAACTTAA
>AB8-42
TTTCCGTAGGTGAACCTGCGGAAGGATCATTATTGAATTATGTTTCTAGATAGGTTGTAG
CTGGCTC-TTTAGAGCATGTGCACGCCTGTTTGGACTTCATTTTCATCCACCTGTGCACC
TATTGTAGTCTTTGGTTGGGTTAGGGGGAAGTGGTCATTGTGTCAGCATCTGCTGGATGT
GAGGACTTGCATTGTGAAAGCTTTGCTGTCCTTGATGTGATCATGGAATCTCTTTCTCAC
TAGAGTCTATGTCACTCATTATACTCTGTCGAATGTCATTGAATGTCTTTACATGGGCTT
GTATGCCTATGAAAATTGTAATACAACTTTCAGCAACGGATCTCTTGGCTCTCGCATCGA
TGAAGGACGCAGCGAAATGCGATAAGTAATGTGAATTGCAGAATTCAGTGAATCATCGAA
TCTTTGAACGCATCTTGCGCTCCTTGGTATTCCGAGGAGCATGCCTGTTTGAGTGTCATT
AAATTCTCAACTCTCTTATACTTTTTTGTAAAAGAGAGCTTGGACTGTGGAGGCTTGCTG
GCCACTTTTTGGGGTCAGCTCCTCTGAAATGCATTAGCGGAACCGTTTGCAATCTGCCAC
AAGTGTGATAAGTTATCTACACTGGCGAGGGGATTGCTCTCTGTAATGTTCAGCTTCTAA
TTGTCTCTACTTTGTGAGACAACTTTTGAATGCTTGACCTCAAATCAGGTAGGACTACCC
GCTGAACTTAA
>AB9-24
TTTCCGTAGGTGAACCTGCGGAAGGATCATTATTGAATTATGTTTCTAGATAGGTTGTAG
CTGGCTC-TTTAGAGCATGTGCACGCCTGTTTGGACTTCATTTTCATCCACCTGTGCACC
TATTGTAGTCTTTGGTTGGGTTAGGGGGAAGTGGTCATTGTGTCAGCATCTGCTGGATGT
GAGGACTTGCATTGTGAAAGCTTTGCTGTCCTTGATGTGATCATGGAATCTCTTTCTCAC
TAGAGTCTATGTCACTCATTATACTCTGTCGAATGTCATTGAATGTCTTTACATGGGCTT
GTATGCCTATGAAAATTGTAATACAACTTTCAGCAACGGATCTCTTGGCTCTCGCATCGA
TGAAGGACGCAGCGAAATGCGATAAGTAATGTGAATTGCAGAATTCAGTGAATCATCGAA
TCTTTGAACGCATCTTGCGCTCCTTGGTATTCCGAGGAGCATGCCTGTTTGAGTGTCATT
AAATTCTCAACTCTCTTATACTTTTTTGTAAAAGAGAGCTTGGACTGTGGAGGCTTGCTG
GCCACTTTTTGGGGTCAGCTCCTCTGAAATGCATTAGCGGAACCGTTTGCAATCTGCCAC
AAGTGTGATAAGTTATCTACACTGGCGAGGGGATTGCTCTCTGTAATGTTCAGCTTCTAA
TTGTCTCTACTTTGTGAGACAACTTTTGAATGCTTGACCTCAAATCAGGTAGGACTACCC
GCTGAACTTAA
>AB9-43
TTTCCGTAGGTGAACCTGCGGAAGGATCATTATTGAATTATGTTTCTAGATAGGTTGTAG
CTGGCTC-TTTAGAGCATGTGCACGCCTGTTTGGACTTCATTTTCATCCACCTGTGCACC
TATTGTAGTCTTTGGTTGGGTTAGGGGGAAGTGGTCATTGTGTCAGCATCTGCTGGATGT
GAGGACTTGCATTGTGAAAGCTTTGCTGTCCTTGATGTGATCATGGAATCTCTTTCTCAC
TAGAGTCTATGTCACTCATTATACTCTGTCGAATGTCATTGAATGTCTTTACATGGGCTT
GTATGCCTATGAAAATTGTAATACAACTTTCAGCAACGGATCTCTTGGCTCTCGCATCGA
TGAAGGACGCAGCGAAATGCGATAAGTAATGTGAATTGCAGAATTCAGTGAATCATCGAA
TCTTTGAACGCATCTTGCGCTCCTTGGTATTCCGAGGAGCATGCCTGTTTGAGTGTCATT
AAATTCTCAACTCTCTTATACTTTTTTGTAAAAGAGAGCTTGGACTGTGGAGGCTTGCTG
GCCACTTTTTGGGGTCAGCTCCTCTGAAATGCATTAGCGGAACCGTTTGCAATCTGCCAC
AAGTGTGATAAGTTATCTACACTGGCGAGGGGATTGCTCTCTGTAATGTTCAGCTTCTAA

TTGTCTCTACTTTGTGAGACAACTTTTGAATGCTTGACCTCAAATCAGGTAGGACTACCC
GCTGAACTTAA
>AB9-46
TTTCCGTAGGTGAACCTGCGGAAGGATCATTATTGAATTATGTTTCTAGATAGGTTGTAG
CTGGCTC-TTTAGAGCATGTGCACGCCTGTTTGGACTTCATTTTCATCCACCTGTGCACC
TATTGTAGTCTTTGGTTGGGTTAGGGGGAAGTGGTCATTGTGTCAGCATCTGCTGGATGT
GAGGACTTGCATTGTGAAAGCTTTGCTGTCCTTGATGTGATCATGGAATCTCTTTCTCAC
TAGAGTCTATGTCACTCATTATACTCTGTCGAATGTCATTGAATGTCTTTACATGGGCTT
GTATGCCTATGAAAATTGTAATACAACTTTCAGCAACGGATCTCTTGGCTCTCGCATCGA
TGAAGGACGCAGCGAAATGCGATAAGTAATGTGAATTGCAGAATTCAGTGAATCATCGAA
TCTTTGAACGCATCTTGCGCTCCTTGGTATTCCGAGGAGCATGCCTGTTTGAGTGTCATT
AAATTCTCAACTCTCTTATACTTTTTTGTAAAAGAGAGCTTGGACTGTGGAGGCTTGCTG
GCCACTTTTTGGGGTCAGCTCCTCTGAAATGCATTAGCGGAACCGTTTGCAATCTGCCAC
AAGTGTGATAAGTTATCTACACTGGCGAGGGGATTGCTCTCTGTAATGTTCAGCTTCTAA
TTGTCTCTACTTTGTGAGACAACTTTTGAATGCTTGACCTCAAATCAGGTAGGACTACCC
GCTGAACTTAA
>AB10-7
TTTCCGTAGGTGAACCTGCGGAAGGATCATTATTGAATTATGTTTCTAGATAGGTTGTAG
CTGGCTC-TTTAGAGCATGTGCACGCCTGTTTGGACTTCATTTTCATCCACCTGTGCACC
TATTGTAGTCTTTGGTTGGGTTAGGGGGAAGTGGTCATTGTGTCAGCATCTGCTGGATGT
GAGGACTTGCATTGTGAAAGCTTTGCTGTCCTTGATGTGATCATGGAATCTCTTTCTCAC
TAGAGTCTATGTCACTCATTATACTCTGTCGAATGTCATTGAATGTCTTTACATGGGCTT
GTATGCCTATGAAAATTGTAATACAACTTTCAGCAACGGATCTCTTGGCTCTCGCATCGA
TGAAGGACGCAGCGAAATGCGATAAGTAATGTGAATTGCAGAATTCAGTGAATCATCGAA
TCTTTGAACGCATCTTGCGCTCCTTGGTATTCCGAGGAGCATGCCTGTTTGAGTGTCATT
AAATTCTCAACTCTCTTATACTTTTTTGTAAAAGAGAGCTTGGACTGTGGAGGCTTGCTG
GCCACTTTTTGGGGTCAGCTCCTCTGAAATGCATTAGCGGAACCGTTTGCAATCTGCCAC
AAGTGTGATAAGTTATCTACACTGGCGAGGGGATTGCTCTCTGTAATGTTCAGCTTCTAA
TTGTCTCTACTTTGTGAGACAACTTTTGAATGCTTGACCTCAAATCAGGTAGGACTACCC
GCTGAACTTAA
>AB10-9
TTTCCGTAGGTGAACCTGCGGAAGGATCATTATTGAATTATGTTTCTAGATAGGTTGTAG
CTGGCTC-TTTAGAGCATGTGCACGCCTGTTTGGACTTCATTTTCATCCACCTGTGCACC
TATTGTAGTCTTTGGTTGGGTTAGGGGGAAGTGGTCATTGTGTCAGCATCTGCTGGATGT
GAGGACTTGCATTGTGAAAGCTTTGCTGTCCTTGATGTGATCATGGAATCTCTTTCTCAC
TAGAGTCTATGTCACTCATTATACTCTGTCGAATGTCATTGAATGTCTTTACATGGGCTT
GTATGCCTATGAAAATTGTAATACAACTTTCAGCAACGGATCTCTTGGCTCTCGCATCGA
TGAAGGACGCAGCGAAATGCGATAAGTAATGTGAATTGCAGAATTCAGTGAATCATCGAA
TCTTTGAACGCATCTTGCGCTCCTTGGTATTCCGAGGAGCATGCCTGTTTGAGTGTCATT
AAATTCTCAACTCTCTTATACTTTTTTGTAAAAGAGAGCTTGGACTGTGGAGGCTTGCTG
GCCACTTTTTGGGGTCAGCTCCTCTGAAATGCATTAGCGGAACCGTTTGCAATCTGCCAC
AAGTGTGATAAGTTATCTACACTGGCGAGGGGATTGCTCTCTGTAATGTTCAGCTTCTAA
TTGTCTCTACTTTGTGAGACAACTTTTGAATGCTTGACCTCAAATCAGGTAGGACTACCC
GCTGAACTTAA
>AB10-10
TTTCCGTAGGTGAACCTGCGGAAGGATCATTATTGAATTATGTTTCTAGATAGGTTGTAG
CTGGCTC-TTTAGAGCATGTGCACGCCTGTTTGGACTTCATTTTCATCCACCTGTGCACC
TATTGTAGTCTTTGGTTGGGTTAGGGGGAAGTGGTCATTGTGTCAGCATCTGCTGGATGT
GAGGACTTGCATTGTGAAAGCTTTGCTGTCCTTGATGTGATCATGGAATCTCTTTCTCAC
TAGAGTCTATGTCACTCATTATACTCTGTCGAATGTCATTGAATGTCTTTACATGGGCTT

GTATGCCTATGAAAATTGTAATACAACTTTCAGCAACGGATCTCTTGGCTCTCGCATCGA
TGAAGGACGCAGCGAAATGCGATAAGTAATGTGAATTGCAGAATTCAGTGAATCATCGAA
TCTTTGAACGCATCTTGCGCTCCTTGGTATTCCGAGGAGCATGCCTGTTTGAGTGTCATT
AAATTCTCAACTCTCTTATACTTTTTTGTAAAAGAGAGCTTGGACTGTGGAGGCTTGCTG
GCCACTTTTTGGGGTCAGCTCCTCTGAAATGCATTAGCGGAACCGTTTGCAATCTGCCAC
AAGTGTGATAAGTTATCTACACTGGCGAGGGGATTGCTCTCTGTAATGTTCAGCTTCTAA
TTGTCTCTACTTTGTGAGACAACTTTTGAATGCTTGACCTCAAATCAGGTAGGACTACCC
GCTGAACTTAA
>AB10-21
TTTCCGTAGGTGAACCTGCGGAAGGATCATTATTGAATTATGTTTCTAGATAGGTTGTAG
CTGGCTC-TTTAGAGCATGTGCACGCCTGTTTGGACTTCATTTTCATCCACCTGTGCACC
TATTGTAGTCTTTGGTTGGGTTAGGGGGAAGTGGTCATTGTGTCAGCATCTGCTGGATGT
GAGGACTTGCATTGTGAAAGCTTTGCTGTCCTTGATGTGATCATGGAATCTCTTTCTCAC
TAGAGTCTATGTCACTCATTATACTCTGTCGAATGTCATTGAATGTCTTTACATGGGCTT
GTATGCCTATGAAAATTGTAATACAACTTTCAGCAACGGATCTCTTGGCTCTCGCATCGA
TGAAGGACGCAGCGAAATGCGATAAGTAATGTGAATTGCAGAATTCAGTGAATCATCGAA
TCTTTGAACGCATCTTGCGCTCCTTGGTATTCCGAGGAGCATGCCTGTTTGAGTGTCATT
AAATTCTCAACTCTCTTATACTTTTTTGTAAAAGAGAGCTTGGACTGTGGAGGCTTGCTG
GCCACTTTTTGGGGTCAGCTCCTCTGAAATGCATTAGCGGAACCGTTTGCAATCTGCCAC
AAGTGTGATAAGTTATCTACACTGGCGAGGGGATTGCTCTCTGTAATGTTCAGCTTCTAA
TTGTCTCTACTTTGTGAGACAACTTTTGAATGCTTGACCTCAAATCAGGTAGGACTACCC
GCTGAACTTAA
>AB11-3
TTTCCGTAGGTGAACCTGCGGAAGGATCATTATTGAATTATGTTTCTAGATAGGTTGTAG
CTGGCTC-TTTAGAGCATGTGCACGCCTGTTTGGACTTCATTTTCATCCACCTGTGCACC
TATTGTAGTCTTTGGTTGGGTTAGGGGGAAGTGGTCATTGTGTCAGCATCTGCTGGATGT
GAGGACTTGCATTGTGAAAGCTTTGCTGTCCTTGATGTGATCATGGAATCTCTTTCTCAC
TAGAGTCTATGTCACTCATTATACTCTGTCGAATGTCATTGAATGTCTTTACATGGGCTT
GTATGCCTATGAAAATTGTAATACAACTTTCAGCAACGGATCTCTTGGCTCTCGCATCGA
TGAAGGACGCAGCGAAATGCGATAAGTAATGTGAATTGCAGAATTCAGTGAATCATCGAA
TCTTTGAACGCATCTTGCGCTCCTTGGTATTCCGAGGAGCATGCCTGTTTGAGTGTCATT
AAATTCTCAACTCTCTTATACTTTTTTGTAAAAGAGAGCTTGGACTGTGGAGGCTTGCTG
GCCACTTTTTGGGGTCAGCTCCTCTGAAATGCATTAGCGGAACCGTTTGCAATCTGCCAC
AAGTGTGATAAGTTATCTACACTGGCGAGGGGATTGCTCTCTGTAATGTTCAGCTTCTAA
TTGTCTCTACTTTGTGAGACAACTTTTGAATGCTTGACCTCAAATCAGGTAGGACTACCC
GCTGAACTTAA
>AB11-4
TTTCCGTAGGTGAACCTGCGGAAGGATCATTATTGAATTATGTTTCTAGATAGGTTGTAG
CTGGCTC-TTTAGAGCATGTGCACGCCTGTTTGGACTTCATTTTCATCCACCTGTGCACC
TATTGTAGTCTTTGGTTGGGTTAGGGGGAAGTGGTCATTGTGTCAGCATCTGCTGGATGT
GAGGACTTGCATTGTGAAAGCTTTGCTGTCCTTGATGTGATCATGGAATCTCTTTCTCAC
TAGAGTCTATGTCACTCATTATACTCTGTCGAATGTCATTGAATGTCTTTACATGGGCTT
GTATGCCTATGAAAATTGTAATACAACTTTCAGCAACGGATCTCTTGGCTCTCGCATCGA
TGAAGGACGCAGCGAAATGCGATAAGTAATGTGAATTGCAGAATTCAGTGAATCATCGAA
TCTTTGAACGCATCTTGCGCTCCTTGGTATTCCGAGGAGCATGCCTGTTTGAGTGTCATT
AAATTCTCAACTCTCTTATACTTTTTTGTAAAAGAGAGCTTGGACTGTGGAGGCTTGCTG
GCCACTTTTTGGGGTCAGCTCCTCTGAAATGCATTAGCGGAACCGTTTGCAATCTGCCAC
AAGTGTGATAAGTTATCTACACTGGCGAGGGGATTGCTCTCTGTAATGTTCAGCTTCTAA
TTGTCTCTACTTTGTGAGACAACTTTTGAATGCTTGACCTCAAATCAGGTAGGACTACCC
GCTGAACTTAA

>AB11-15
TTTCCGTAGGTGAACCTGCGGAAGGATCATTATTGAATTATGTTTCTAGATAGGTTGTAG
CTGGCTC-TTTAGAGCATGTGCACGCCTGTTTGGACTTCATTTTCATCCACCTGTGCACC
TATTGTAGTCTTTGGTTGGGTTAGGGGGAAGTGGTCATTGTGTCAGCATCTGCTGGATGT
GAGGACTTGCATTGTGAAAGCTTTGCTGTCCTTGATGTGATCATGGAATCTCTTTCTCAC
TAGAGTCTATGTCACTCATTATACTCTGTCGAATGTCATTGAATGTCTTTACATGGGCTT
GTATGCCTATGAAAATTGTAATACAACTTTCAGCAACGGATCTCTTGGCTCTCGCATCGA
TGAAGGACGCAGCGAAATGCGATAAGTAATGTGAATTGCAGAATTCAGTGAATCATCGAA
TCTTTGAACGCATCTTGCGCTCCTTGGTATTCCGAGGAGCATGCCTGTTTGAGTGTCATT
AAATTCTCAACTCTCTTATACTTTTTTGTAAAAGAGAGCTTGGACTGTGGAGGCTTGCTG
GCCACTTTTTGGGGTCAGCTCCTCTGAAATGCATTAGCGGAACCGTTTGCAATCTGCCAC
AAGTGTGATAAGTTATCTACACTGGCGAGGGGATTGCTCTCTGTAATGTTCAGCTTCTAA
TTGTCTCTACTTTGTGAGACAACTTTTGAATGCTTGACCTCAAATCAGGTAGGACTACCC
GCTGAACTTAA
>AB11-17
TTTCCGTAGGTGAACCTGCGGAAGGATCATTATTGAATTATGTTTCTAGATAGGTTGTAG
CTGGCTC-TTTAGAGCATGTGCACGCCTGTTTGGACTTCATTTTCATCCACCTGTGCACC
TATTGTAGTCTTTGGTTGGGTTAGGGGGAAGTGGTCATTGTGTCAGCATCTGCTGGATGT
GAGGACTTGCATTGTGAAAGCTTTGCTGTCCTTGATGTGATCATGGAATCTCTTTCTCAC
TAGAGTCTATGTCACTCATTATACTCTGTCGAATGTCATTGAATGTCTTTACATGGGCTT
GTATGCCTATGAAAATTGTAATACAACTTTCAGCAACGGATCTCTTGGCTCTCGCATCGA
TGAAGGACGCAGCGAAATGCGATAAGTAATGTGAATTGCAGAATTCAGTGAATCATCGAA
TCTTTGAACGCATCTTGCGCTCCTTGGTATTCCGAGGAGCATGCCTGTTTGAGTGTCATT
AAATTCTCAACTCTCTTATACTTTTTTGTAAAAGAGAGCTTGGACTGTGGAGGCTTGCTG
GCCACTTTTTGGGGTCAGCTCCTCTGAAATGCATTAGCGGAACCGTTTGCAATCTGCCAC
AAGTGTGATAAGTTATCTACACTGGCGAGGGGATTGCTCTCTGTAATGTTCAGCTTCTAA
TTGTCTCTACTTTGTGAGACAACTTTTGAATGCTTGACCTCAAATCAGGTAGGACTACCC
GCTGAACTTAA
>AB11-22
TTTCCGTAGGTGAACCTGCGGAAGGATCATTATTGAATTATGTTTCTAGATAGGTTGTAG
CTGGCTC-TTTAGAGCATGTGCACGCCTGTTTGGACTTCATTTTCATCCACCTGTGCACC
TATTGTAGTCTTTGGTTGGGTTAGGGGGAAGTGGTCATTGTGTCAGCATCTGCTGGATGT
GAGGACTTGCATTGTGAAAGCTTTGCTGTCCTTGATGTGATCATGGAATCTCTTTCTCAC
TAGAGTCTATGTCACTCATTATACTCTGTCGAATGTCATTGAATGTCTTTACATGGGCTT
GTATGCCTATGAAAATTGTAATACAACTTTCAGCAACGGATCTCTTGGCTCTCGCATCGA
TGAAGGACGCAGCGAAATGCGATAAGTAATGTGAATTGCAGAATTCAGTGAATCATCGAA
TCTTTGAACGCATCTTGCGCTCCTTGGTATTCCGAGGAGCATGCCTGTTTGAGTGTCATT
AAATTCTCAACTCTCTTATACTTTTTTGTAAAAGAGAGCTTGGACTGTGGAGGCTTGCTG
GCCACTTTTTGGGGTCAGCTCCTCTGAAATGCATTAGCGGAACCGTTTGCAATCTGCCAC
AAGTGTGATAAGTTATCTACACTGGCGAGGGGATTGCTCTCTGTAATGTTCAGCTTCTAA
TTGTCTCTACTTTGTGAGACAACTTTTGAATGCTTGACCTCAAATCAGGTAGGACTACCC
GCTGAACTTAA
>AB11-29
TTTCCGTAGGTGAACCTGCGGAAGGATCATTATTGAATTATGTTTCTAGATAGGTTGTAG
CTGGCTC-TTTAGAGCATGTGCACGCCTGTTTGGACTTCATTTTCATCCACCTGTGCACC
TATTGTAGTCTTTGGTTGGGTTAGGGGGAAGTGGTCATTGTGTCAGCATCTGCTGGATGT
GAGGACTTGCATTGTGAAAGCTTTGCTGTCCTTGATGTGATCATGGAATCTCTTTCTCAC
TAGAGTCTATGTCACTCATTATACTCTGTCGAATGTCATTGAATGTCTTTACATGGGCTT
GTATGCCTATGAAAATTGTAATACAACTTTCAGCAACGGATCTCTTGGCTCTCGCATCGA
TGAAGGACGCAGCGAAATGCGATAAGTAATGTGAATTGCAGAATTCAGTGAATCATCGAA

TCTTTGAACGCATCTTGCGCTCCTTGGTATTCCGAGGAGCATGCCTGTTTGAGTGTCATT
AAATTCTCAACTCTCTTATACTTTTTTGTAAAAGAGAGCTTGGACTGTGGAGGCTTGCTG
GCCACTTTTTGGGGTCAGCTCCTCTGAAATGCATTAGCGGAACCGTTTGCAATCTGCCAC
AAGTGTGATAAGTTATCTACACTGGCGAGGGGATTGCTCTCTGTAATGTTCAGCTTCTAA
TTGTCTCTACTTTGTGAGACAACTTTTGAATGCTTGACCTCAAATCAGGTAGGACTACCC
GCTGAACTTAA
>AB11-35
TTTCCGTAGGTGAACCTGCGGAAGGATCATTATTGAATTATGTTTCTAGATAGGTTGTAG
CTGGCTC-TTTAGAGCATGTGCACGCCTGTTTGGACTTCATTTTCATCCACCTGTGCACC
TATTGTAGTCTTTGGTTGGGTTAGGGGGAAGTGGTCATTGTGTCAGCATCTGCTGGATGT
GAGGACTTGCATTGTGAAAGCTTTGCTGTCCTTGATGTGATCATGGAATCTCTTTCTCAC
TAGAGTCTATGTCACTCATTATACTCTGTCGAATGTCATTGAATGTCTTTACATGGGCTT
GTATGCCTATGAAAATTGTAATACAACTTTCAGCAACGGATCTCTTGGCTCTCGCATCGA
TGAAGGACGCAGCGAAATGCGATAAGTAATGTGAATTGCAGAATTCAGTGAATCATCGAA
TCTTTGAACGCATCTTGCGCTCCTTGGTATTCCGAGGAGCATGCCTGTTTGAGTGTCATT
AAATTCTCAACTCTCTTATACTTTTTTGTAAAAGAGAGCTTGGACTGTGGAGGCTTGCTG
GCCACTTTTTGGGGTCAGCTCCTCTGAAATGCATTAGCGGAACCGTTTGCAATCTGCCAC
AAGTGTGATAAGTTATCTACACTGGCGAGGGGATTGCTCTCTGTAATGTTCAGCTTCTAA
TTGTCTCTACTTTGTGAGACAACTTTTGAATGCTTGACCTCAAATCAGGTAGGACTACCC
GCTGAACTTAA
>AB12-8
TTTCCGTAGGTGAACCTGCGGAAGGATCATTATTGAATTATGTTTCTAGATAGGTTGTAG
CTGGCTC-TTTAGAGCATGTGCACGCCTGTTTGGACTTCATTTTCATCCACCTGTGCACC
TATTGTAGTCTTTGGTTGGGTTAGGGGGAAGTGGTCATTGTGTCAGCATCTGCTGGATGT
GAGGACTTGCATTGTGAAAGCTTTGCTGTCCTTGATGTGATCATGGAATCTCTTTCTCAC
TAGAGTCTATGTCACTCATTATACTCTGTCGAATGTCATTGAATGTCTTTACATGGGCTT
GTATGCCTATGAAAATTGTAATACAACTTTCAGCAACGGATCTCTTGGCTCTCGCATCGA
TGAAGGACGCAGCGAAATGCGATAAGTAATGTGAATTGCAGAATTCAGTGAATCATCGAA
TCTTTGAACGCATCTTGCGCTCCTTGGTATTCCGAGGAGCATGCCTGTTTGAGTGTCATT
AAATTCTCAACTCTCTTATACTTTTTTGTAAAAGAGAGCTTGGACTGTGGAGGCTTGCTG
GCCACTTTTTGGGGTCAGCTCCTCTGAAATGCATTAGCGGAACCGTTTGCAATCTGCCAC
AAGTGTGATAAGTTATCTACACTGGCGAGGGGATTGCTCTCTGTAATGTTCAGCTTCTAA
TTGTCTCTACTTTGTGAGACAACTTTTGAATGCTTGACCTCAAATCAGGTAGGACTACCC
GCTGAACTTAA
>AB12-13
TTTCCGTAGGTGAACCTGCGGAAGGATCATTATTGAATTATGTTTCTAGATAGGTTGTAG
CTGGCTC-TTTAGAGCATGTGCACGCCTGTTTGGACTTCATTTTCATCCACCTGTGCACC
TATTGTAGTCTTTGGTTGGGTTAGGGGGAAGTGGTCATTGTGTCAGCATCTGCTGGATGT
GAGGACTTGCATTGTGAAAGCTTTGCTGTCCTTGATGTGATCATGGAATCTCTTTCTCAC
TAGAGTCTATGTCACTCATTATACTCTGTCGAATGTCATTGAATGTCTTTACATGGGCTT
GTATGCCTATGAAAATTGTAATACAACTTTCAGCAACGGATCTCTTGGCTCTCGCATCGA
TGAAGGACGCAGCGAAATGCGATAAGTAATGTGAATTGCAGAATTCAGTGAATCATCGAA
TCTTTGAACGCATCTTGCGCTCCTTGGTATTCCGAGGAGCATGCCTGTTTGAGTGTCATT
AAATTCTCAACTCTCTTATACTTTTTTGTAAAAGAGAGCTTGGACTGTGGAGGCTTGCTG
GCCACTTTTTGGGGTCAGCTCCTCTGAAATGCATTAGCGGAACCGTTTGCAATCTGCCAC
AAGTGTGATAAGTTATCTACACTGGCGAGGGGATTGCTCTCTGTAATGTTCAGCTTCTAA
TTGTCTCTACTTTGTGAGACAACTTTTGAATGCTTGACCTCAAATCAGGTAGGACTACCC
GCTGAACTTAA
>AB12-21
TTTCCGTAGGTGAACCTGCGGAAGGATCATTATTGAATTATGTTTCTAGATAGGTTGTAG

CTGGCTC-TTTAGAGCATGTGCACGCCTGTTTGGACTTCATTTTCATCCACCTGTGCACC
TATTGTAGTCTTTGGTTGGGTTAGGGGGAAGTGGTCATTGTGTCAGCATCTGCTGGATGT
GAGGACTTGCATTGTGAAAGCTTTGCTGTCCTTGATGTGATCATGGAATCTCTTTCTCAC
TAGAGTCTATGTCACTCATTATACTCTGTCGAATGTCATTGAATGTCTTTACATGGGCTT
GTATGCCTATGAAAATTGTAATACAACTTTCAGCAACGGATCTCTTGGCTCTCGCATCGA
TGAAGGACGCAGCGAAATGCGATAAGTAATGTGAATTGCAGAATTCAGTGAATCATCGAA
TCTTTGAACGCATCTTGCGCTCCTTGGTATTCCGAGGAGCATGCCTGTTTGAGTGTCATT
AAATTCTCAACTCTCTTATACTTTTTTGTAAAAGAGAGCTTGGACTGTGGAGGCTTGCTG
GCCACTTTTTGGGGTCAGCTCCTCTGAAATGCATTAGCGGAACCGTTTGCAATCTGCCAC
AAGTGTGATAAGTTATCTACACTGGCGAGGGGATTGCTCTCTGTAATGTTCAGCTTCTAA
TTGTCTCTACTTTGTGAGACAACTTTTGAATGCTTGACCTCAAATCAGGTAGGACTACCC
GCTGAACTTAA
>AB12-33
TTTCCGTAGGTGAACCTGCGGAAGGATCATTATTGAATTATGTTTCTAGATAGGTTGTAG
CTGGCTC-TTTAGAGCATGTGCACGCCTGTTTGGACTTCATTTTCATCCACCTGTGCACC
TATTGTAGTCTTTGGTTGGGTTAGGGGGAAGTGGTCATTGTGTCAGCATCTGCTGGATGT
GAGGACTTGCATTGTGAAAGCTTTGCTGTCCTTGATGTGATCATGGAATCTCTTTCTCAC
TAGAGTCTATGTCACTCATTATACTCTGTCGAATGTCATTGAATGTCTTTACATGGGCTT
GTATGCCTATGAAAATTGTAATACAACTTTCAGCAACGGATCTCTTGGCTCTCGCATCGA
TGAAGGACGCAGCGAAATGCGATAAGTAATGTGAATTGCAGAATTCAGTGAATCATCGAA
TCTTTGAACGCATCTTGCGCTCCTTGGTATTCCGAGGAGCATGCCTGTTTGAGTGTCATT
AAATTCTCAACTCTCTTATACTTTTTTGTAAAAGAGAGCTTGGACTGTGGAGGCTTGCTG
GCCACTTTTTGGGGTCAGCTCCTCTGAAATGCATTAGCGGAACCGTTTGCAATCTGCCAC
AAGTGTGATAAGTTATCTACACTGGCGAGGGGATTGCTCTCTGTAATGTTCAGCTTCTAA
TTGTCTCTACTTTGTGAGACAACTTTTGAATGCTTGACCTCAAATCAGGTAGGACTACCC
GCTGAACTTAA
>AB12-42
TTTCCGTAGGTGAACCTGCGGAAGGATCATTATTGAATTATGTTTCTAGATAGGTTGTAG
CTGGCTC-TTTAGAGCATGTGCACGCCTGTTTGGACTTCATTTTCATCCACCTGTGCACC
TATTGTAGTCTTTGGTTGGGTTAGGGGGAAGTGGTCATTGTGTCAGCATCTGCTGGATGT
GAGGACTTGCATTGTGAAAGCTTTGCTGTCCTTGATGTGATCATGGAATCTCTTTCTCAC
TAGAGTCTATGTCACTCATTATACTCTGTCGAATGTCATTGAATGTCTTTACATGGGCTT
GTATGCCTATGAAAATTGTAATACAACTTTCAGCAACGGATCTCTTGGCTCTCGCATCGA
TGAAGGACGCAGCGAAATGCGATAAGTAATGTGAATTGCAGAATTCAGTGAATCATCGAA
TCTTTGAACGCATCTTGCGCTCCTTGGTATTCCGAGGAGCATGCCTGTTTGAGTGTCATT
AAATTCTCAACTCTCTTATACTTTTTTGTAAAAGAGAGCTTGGACTGTGGAGGCTTGCTG
GCCACTTTTTGGGGTCAGCTCCTCTGAAATGCATTAGCGGAACCGTTTGCAATCTGCCAC
AAGTGTGATAAGTTATCTACACTGGCGAGGGGATTGCTCTCTGTAATGTTCAGCTTCTAA
TTGTCTCTACTTTGTGAGACAACTTTTGAATGCTTGACCTCAAATCAGGTAGGACTACCC
GCTGAACTTAA
>AB7-17
TTTCCGTAGGTGAACCTGCGGAAGGATCATTATTGAATTATGTTTCTAGATAGGTTGTAG
CTGGCTC-TTTAGAGCATGTGCACGCCTGTTTGGACTTCATTTTCATCCACCTGTGCACC
TATTGTAGTCTTTGGTTGGGTTAGGGGGAAGTGGTCATTGTGTCAGCATCTGCTGGATGT
GAGGACTTGCATTGTGAAAGCTTTGCTGTCCTTGATGTGATCATGGAATCTCTTTCTCAC
TAGAGTCTATGTCACTCATTATACTCTGTCGAATGTCATTGAATGTCTTTACATGGGCTT
GTATGCCTATGAAAATTGTAATACAACTTTCAGCAACGGATCTCTTGGCTCTCGCATCGA
TGAAGAACGCAGCGAAATGCGATAAGTAATGTGAATTGCAGAATTCAGTGAATCATCGAA
TCTTTGAACGCATCTTGCGCTCCTTGGTATTCCGAGGAGCATGCCTGTTTGAGTGTCATT
AAATTCTCAACTCTCTTATACTTTTTTGTAAAAGAGAGCTTGGACTGTGGAGGCTTGCTG

GCCACTTTTTGGGGTCAGCTCCTCTGAAATGCATTAGCGGAACCGTTTGCAATCTGCCAC
AAGTGTGATAAGTTATCTACACTGGCGAGGGGATTGCTCTCTGTAATGTTCAGCTTCTAA
TTGTCTCTACTTTGTGAGACAACTTTTGAATGCTTGACCTCAAATCAGGTAGGACTACCC
GCTGAACTTAA
>AB10-42
TTTCCGTAGGTGAACCTGCGGAAGGATCATTATTGAATTATGTTTCTAGATAGGTTGTAG
CTGGCTC-TTTAGAGCATGTGCACGCCTGTTTGGACTTCATTTTCATCCACCTGTGCACC
TATTGTAGTCTTTGGTTGGGTTAGGGGGAAGTGGTCATTGTGTCAGCATCTGCTGGATGT
GAGGACTTGCATTGTGAAAGCTTTGCTGTCCTTGATGTGATCATGGAATCTCTTTCTCAC
TAGAGTCTATGTCACTCATTATACTCTGTCGAATGTCATTGAATGTCTTTACATGGGCTT
GTATGCCTATGAAAATTGTAATACAACTTTCAGCAACGGATCTCTTGGCTCTCGCATCGA
TGAAGAACGCAGCGAAATGCGATAAGTAATGTGAATTGCAGAATTCAGTGAATCATCGAA
TCTTTGAACGCATCTTGCGCTCCTTGGTATTCCGAGGAGCATGCCTGTTTGAGTGTCATT
AAATTCTCAACTCTCTTATACTTTTTTGTAAAAGAGAGCTTGGACTGTGGAGGCTTGCTG
GCCACTTTTTGGGGTCAGCTCCTCTGAAATGCATTAGCGGAACCGTTTGCAATCTGCCAC
AAGTGTGATAAGTTATCTACACTGGCGAGGGGATTGCTCTCTGTAATGTTCAGCTTCTAA
TTGTCTCTACTTTGTGAGACAACTTTTGAATGCTTGACCTCAAATCAGGTAGGACTACCC
GCTGAACTTAA
>AB11-18
TTTCCGTAGGTGAACCTGCGGAAGGATCATTATTGAATTATGTTTCTAGATAGGTTGTAG
CTGGCTC-TTTAGAGCATGTGCACGCCTGTTTGGACTTCATTTTCATCCACCTGTGCACC
TATTGTAGTCTTTGGTTGGGTTAGGGGGAAGTGGTCATTGTGTCAGCATCTGCTGGATGT
GAGGACTTGCATTGTGAAAGCTTTGCTGTCCTTGATGTGATCATGGAATCTCTTTCTCAC
TAGAGTCTATGTCACTCATTATACTCTGTCGAATGTCATTGAATGTCTTTACATGGGCTT
GTATGCCTATGAAAATTGTAATACAACTTTCAGCAACGGATCTCTTGGCTCTCGCATCGA
TGAAGAACGCAGCGAAATGCGATAAGTAATGTGAATTGCAGAATTCAGTGAATCATCGAA
TCTTTGAACGCATCTTGCGCTCCTTGGTATTCCGAGGAGCATGCCTGTTTGAGTGTCATT
AAATTCTCAACTCTCTTATACTTTTTTGTAAAAGAGAGCTTGGACTGTGGAGGCTTGCTG
GCCACTTTTTGGGGTCAGCTCCTCTGAAATGCATTAGCGGAACCGTTTGCAATCTGCCAC
AAGTGTGATAAGTTATCTACACTGGCGAGGGGATTGCTCTCTGTAATGTTCAGCTTCTAA
TTGTCTCTACTTTGTGAGACAACTTTTGAATGCTTGACCTCAAATCAGGTAGGACTACCC
GCTGAACTTAA
>AB12-29
TTTCCGTAGGTGAACCTGCGGAAGGATCATTATTGAATTATGTTTCTAGATAGGTTGTAG
CTGGCTC-TTTAGAGCATGTGCACGCCTGTTTGGACTTCATTTTCATCCACCTGTGCACC
TATTGTAGTCTTTGGTTGGGTTAGGGGGAAGTGGTCATTGTGTCAGCATCTGCTGGATGT
GAGGACTTGCATTGTGAAAGCTTTGCTGTCCTTGATGTGATCATGGAATCTCTTTCTCAC
TAGAGTCTATGTCACTCATTATACTCTGTCGAATGTCATTGAATGTCTTTACATGGGCTT
GTATGCCTATGAAAATTGTAATACAACTTTCAGCAACGGATCTCTTGGCTCTCGCATCGA
TGAAGAACGCAGCGAAATGCGATAAGTAATGTGAATTGCAGAATTCAGTGAATCATCGAA
TCTTTGAACGCATCTTGCGCTCCTTGGTATTCCGAGGAGCATGCCTGTTTGAGTGTCATT
AAATTCTCAACTCTCTTATACTTTTTTGTAAAAGAGAGCTTGGACTGTGGAGGCTTGCTG
GCCACTTTTTGGGGTCAGCTCCTCTGAAATGCATTAGCGGAACCGTTTGCAATCTGCCAC
AAGTGTGATAAGTTATCTACACTGGCGAGGGGATTGCTCTCTGTAATGTTCAGCTTCTAA
TTGTCTCTACTTTGTGAGACAACTTTTGAATGCTTGACCTCAAATCAGGTAGGACTACCC
GCTGAACTTAA
>AB2-26
TTTCCGTAGGTGAACCTGCGGAAGGATCATTATTGAATTATGTTTCTAGATAGGTTGTAG
CTGGCTC-TTTAGAGCATGTGCACGCCTGTTTGGACTTCATTTTCATCCACCTGTGCACC
TATTGTAGTCTTTGGTTGGGTTAGGGGGAAGTGGTCATTGTGTCAGCATCTGCTGGATGT

GAGGACTTGCATTGTGAAAGCTTTGCTGTCCTTGATGTGATCATGGAATCTCTTTCTCAC
TAGAGTCTATGTCACTCATTATACTCTGTCGAATGTCATTGAATGTCTTTACATGGGCTT
GTATGCCTATGAAAATTGTAATACAACTTTCAGCAACGGATCTCTTGGCTCTCGCATCGA
TGAAGAACGCAGCGAAATGCGATAAGTAATGTGAATTGCAGAATTCAGTGAATCATCGAA
TCTTTGAACGCATCTTGCGCTCCTTGGTATTCCGAGGAGCATGCCTGTTTGAGTGTCATT
AAATTCTCAACTCTCTTATACTTTTTTGTAAAAGAGAGCTTGGACTGTGGAGGCTTGCTG
GCCACTTTTTGGGGTCAGCTCCTCTGAAATGCATTAGCGGAACCGTTTGCGATCTGCCAC
AAGTGTGATAAGTTATCTACACTGGCGAGGGGATTGCTCTCTGTAATGTTCAGCTTCTAA
TTGTCTCTACTTTGTGAGACAACTTTTGAATGCTTGACCTCAAATCAGGTAGGACTACCC
GCTGAACTTAA
>AB9-37
TTTCCGTAGGTGAACCTGCGGAAGGATCATTATTGAATTATGTTTCTAGATAGGTTGTAG
CTGGCTC-TTTAGAGCATGTGCACGCCTGTTTGGACTTCATTTTCATCCACCTGTGCACC
TATTGTAGTCTTTGGTTGGGTTAGGGGGAAGTGGTCATTGTGTCAGCATCTGCTGGATGT
GAGGACTTGCATTGTGAAAGCTTTGCTGTCCTTGATGTGATCATGGAATCTTTTTCTCAC
TAGAGTCTATGTCACTCATTATACTCTGTCGAATGTCATTGAATGTCTTTACATGGGCTT
GTATGCCTATGAAAATTGTAATACAACTTTCAGCAACGGATCTCTTGGCTCTCGCATCGA
TGAAGAACGCAGCGAAATGCGATAAGTAATGTGAATTGCAGAATTCAGTGAATCATCGAA
TCTTTGAACGCATCTTGCGCTCCTTGGTATTCCGAGGAGCATGCCTGTTTGAGTGTCATT
AAATTCTCAACTCTCTTATACTTTTTTGTAAAAGAGAGCTTGGACTGTGGAGGCTTGCTG
GCCACTTTTTGGGGTCAGCTCCTCTGAAATGCATTAGCGGAACCGTTTGCAATCTGCCAC
AAGTGTGATAAGTTATCTACACTGGCGAGGGGATTGCTCTCTGTAATGTTCAGCTTCTAA
TTGTCTCTACTTTGTGAGACAACTTTTGAATGCTTGACCTCAAATCAGGTAGGACTACCC
GCTGAACTTAA
>AB4-18
TTTCCGTAGGTGAACCTGCGGAAGGATCATTATTGAATTATGTTTCTAGATAGGTTGTAG
CTGGCTC-TTTAGAGCATGTGCACGCCTGTTTGGACTTCATTTTCATCCACCTGTGCACC
TATTGTAGTCTTTGGTTGGGTTAGGGGGAAGTGGTCATTGTGTCAGCATCTGCTGGATGT
GAGGACTTGCATTGTGAAAGCTTTGCTGTCCTTGATGTGATCATGGAATCTTTTTCTCAC
TAGAGTCTATGTCACTCATTATACTCTGTCGAATGTCATTGAATGTCTTTACATGGGCTT
GTATGCCTATGAAAATTGTAATACAACTTTCAGCAACGGATCTCTTGGCTCTCGCATCGA
TGAAGAACGCAGCGAAATGCGATAAGTAATGTGAATTGCAGAATTCAGTGAATCATCGAA
TCTTTGAACGCATCTTGCGCTCCTTGGTATTCCGAGGAGCATGCCTGTTTGAGTGTCATT
AAATTCTCAACTCTCTTATACTTTTTTGTAAAAGAGAGCTTGGACTGTGGAGGCTTGCTG
GCCACTTTTTGGGGTCAGCTCCTCTGAAATGCATTAGCGGAACCGTTTGCGATCTGCCAC
AAGTGTGATAAGTTATCTACACTGGCGAGGGGATTGCTCTCTGTAATGTTCAGCTTCTAA
TTGTCTCTACTTTGTGAGACAACTTTTGAATGCTTGACCTCAAATCAGGTAGGACTACCC
GCTGAACTTAA
>AB12-38
TTTCCGTAGGTGAACCTGCGGAAGGATCATTATTGAATTATGTTTCTAGATAGGTTGTAG
CTGGCTC-TTTAGAGCATGTGCACGCCTGTTTGGACTTCATTTTCATCCACCTGTGCACC
TATTGTAGTCTTTGGTTGGGTTAGGGGGAAGTGGTCATTGTGTCAGCATCTGCTGGATGT
GAGGACTTGCATTGTGAAAGCTTTGCTGTCCTTGATGTGATCATGGAATCTTTTTCTCAC
TAGAGTCTATGTCACTCATTATACTCTGTCGAATGTCATTGAATGTCTTTACATGGGCTT
GTATGCCTATGAAAATTGTAATACAACTTTCAGCAACGGATCTCTTGGCTCTCGCATCGA
TGAAGAACGCAGCGAAATGCGATAAGTAATGTGAATTGCAGAATTCAGTGAATCATCGAA
TCTTTGAACGCATCTTGCGCTCCTTGGTATTCCGAGGAGCATGCCTGTTTGAGTGTCATT
AAATTCTCAACTCTCTTATACTTTTTTGTAAAAGAGAGCTTGGACTGTGGAGGCTTGCTG
GCCACTTTTTGGGGTCAGCTCCTCTGAAATGCATTAGCGGAACCGTTTGCGATCTGCCAC
AAGTGTGATAAGTTATCTACACTGGCGAGGGGATTGCTCTCTGTAATGTTCAGCTTCTAA

TTGTCTCTACTTTGTGAGACAACTTTTGAATGCTTGACCTCAAATCAGGTAGGACTACCC
GCTGAACTTAA
>AB5-19
TTTCCGTAGGTGAACCTGCGGAAGGATCATTATTGAATTATGTTTCTAGATAGGTTGTAG
CTGGCTC-TTTAGAGCATGTGCACGCCTGTTTGGACTTCATTTTCATCCACCTGTGCACC
TATTGTAGTCTTTGGTTGGGTTAGGAGGAAGTGGTCATTGTGTCAGCATCTGCTGGATGT
GAGGACTTGCATTGTGAAAGCTTTGCTGTCCTTGATGTGATCATGGAATCTCTTTCTCAC
TAGAGTCTATGTCACTCATTATACTCTGTCGAATGTCATTGAATGTCTTTACATGGGCTT
GTATGCCTATGAAAATTGTAATACAACTTTCAGCAACGGATCTCTTGGCTCTCGCATCGA
TGAAGGACGCAGCGAAATGCGATAAGTAATGTGAATTGCAGAATTCAGTGAATCATCGAA
TCTTTGAACGCATCTTGCGCTCCTTGGTATTCCGAGGAGCATGCCTGTTTGAGTGTCATT
AAATTCTCAACTCTCTTATACTTTTTTGTAAAAGAGAGCTTGGACTGTGGAGGCTTGCTG
GCCACTTTTTGGGGTCAGCTCCTCTGAAATGCATTAGCGGAACCGTTTGCAATCTGCCAC
AAGTGTGATAAGTTATCTACACTGGCGAGGGGATTGCTCTCTGTAATGTTCAGCTTCTAA
TTGTCTCTACTTTGTGAGACAACTTTTGAATGCTTGACCTCAAATCAGGTAGGACTACCC
GCTGAACTTAA
>AB2-54
TTTCCGTAGGTGAACCTGCGGAAGGATCATTATTGAATTATGTTTCTAGATAGGTTGTAG
CTGGCTC-TTTAGAGCATGTGCACGCCTGTTTGGACTTCATTTTCATCCACCTGTGCACC
TATTGTAGTCTTTGGTTGGGTTAGGGGGAAGTGGTCATTGTGTCAGCATCTGCTGGATGT
GAGGACTTGCATTGTGAAAGCTTTGCTGTCCTTGATGTGATCATGGAATCTCTTTCTCAC
TAGAGTCTATGTCACTCATTATACTCTGTCGAATGTCATTGAATGTCTTTACATGGACTT
GTATGCCTATGAAAATTGTAATACAACTTTCAGCAACGGATCTCTTGGCTCTCGCATCGA
TGAAGGACGCAGCGAAATGCGATAAGTAATGTGAATTGCAGAATTCAGTGAATCATCGAA
TCTTTGAACGCATCTTGCGCTCCTTGGTATTCCGAGGAGCATGCCTGTTTGAGTGTCATT
AAATTCTCAACTCTCTTATACTTTTTTGTAAAAGAGAGCTTGGACTGTGGAGGCTTGCTG
GCCACTTTTTGGGGTCAGCTCCTCTGAAATGCATTAGCGGAACCGTTTGCAATCTGCCAC
AAGTGTGATAAGTTATCTACACTGGCGAGGGGATTGCTCTCTGTAATGTTCAGCTTCTAA
TTGTCTCTACTTTGTGAGACAACTTTTGAATGCTTGACCTCAAATCAGGTAGGACTACCC
GCTGAACTTAA
>AB5-41
TTTCCGTAGGTGAACCTGCGGAAGGATCATTATTGAATTATGTTTCTAGATAGGTTGTAG
CTGGCTC-TTTAGAGCATGTGCACGCCTGTTTGGACTTCATTTTCATCCACCTGTGCACC
TATTGTAGTCTTTGGTTGGGTTAGGGGGAAGTGGTCATTGTGTCAGCATCTGCTGGATGT
GAGGACTTGCATTGTGAAAGCTTTGCTGTCCTTGATGTGATCATGGAATCTCTTTCTCAC
TAGAGTCTATGTCACTCATTATACTCTGTCGAATGTCATTGAATGTCTTTACATGGGCTT
GTATGCCTATGAAAATTGTAATACAACTTTCAGCAACGGATCTCTTGGCTCTCGCATCGA
TGAAGGACGCAGCGAAATGCGATAAGTAATGTGAATTGCAGAATTCAGTGAATCATCGAA
TCTTTGAACGCATCTTGCGCTCCTTGGTATTCCGAGGAGCATGCCTGTTTGAGTGTCATT
AAATTCTCAACTCTCTTATACTTTTTTGTAAAAGAGAGCTTGGACTGTGGAGGTTTGCTG
GCCACTTTTTGGGGTCAGCTCCTCTGAAATGCATTAGCGGAACCGTTTGCAATCTGCCAC
AAGTGTGATAAGTTATCTACACTGGCGAGGGGATTGCTCTCTGTAATGTTCAGCTTCTAA
TTGTCTCTACTTTGTGAGACAACTTTTGAATGCTTGACCTCAAATCAGGTAGGACTACCC
GCTGAACTTAA
>AB9-17
TTTCCGTAGGTGAACCTGCGGAAGGATCATTATTGAATTATGTTTCTAGATAGGTTGTAG
CTGGCTC-TTTAGAGCATGTGCACGCCTGTTTGGACTTCATTTTCATCCACCTGTGCATC
TATTGTAGTCTTTGGTTGGGTTAGGGGGAAGTGGTCATTGTGTCAGCATCTGCTGGATGT
GAGGACTTGCATTGTGAAAGCTTTGCTGTCCTTGATGTGATCATGGAATCTCTTTCTCAC
TAGAGTCTATGTCACTCATTATACTCTGTCGAATGTCATTGAATGTCTTTACATGGGCTT

GTATGCCTATGAAAATTGTAATACAACTTTCAGCAACGGATCTcTTGGCTCTCGCATCGA
TGAAGGACGCAGCGAAATGCGATAAGTAATGTGAATTGCAGAATTCAGTGAATCATCGAA
TCTTTGAACGCATCTTGCGCTCCTTGGTATTCCGAGGAGCATGCCTGTTTGAGTGTCATT
AAATTCTCAACTCTCTTATACTTTTTTGTAAAAGAGAGCTTGGACTGTGGAGGCTTGCTG
GCCACTTTTTGGGGTCAGCTCCTCTGAAATGCATTAGCGGAACCGTTTGCAATCTGCCAC
AAGTGTGATAAGTTATCTACACTGGCGAGGGGATTGCTCTCTGTAATGTTCAGCTTCTAA
TTGTCTCTACTTTGTGAGACAACTTTTGAATGCTTGACCTCAAATCAGGTAGGACTACCC
ACTGAACTTAA
>AB6–14
TTTCCGTAGGTGAACCTGCGGAAGGATCATTATTGAATTATGTTTCTAGATAGGTTGTAG
CTGGCTC–TTTAGAGCATGTGCACGCCTGTTTGGACTTCATTTTCATCCACCTGTGCACC
TATTGTAGTCTTTGGTTGGGTTAGGGGGAAGTGGTCATTGTGTCAGCATCTGCTGGATGT
GTGGACTTGCATTGTGAAAGCTTTGCTGTCCTTGATGTGATCATGGAATCTCTTTCTCAC
TAGAGTCTATGTCACTCATTATACTCTGTCGAATGTCATTGAATGTCTTTACATGGGCTT
GTATGCCTATGAAAATTGTAATACAACTTTCAGCAACGGATCTCTTGGCTCTCGCATCGA
TGAAGGACGCAGCGAAATGCGATAAGTAATGTGAATTGCAGAATTCAGTGAATCATCGAA
TCTTTGAACGCATCTTGCGCTCCTTGGTATTCCGAGGAGCATGCCTGTTTGAGTGTCATT
AAATTCTCAACTCTCTTATACTTTTTTGTAAAAGAGAGCTTGGACTGTGGAGGCTTGCTG
GCCACTTTTTGGGGTCAGCTCCTCTGAAATGCATTAGCGGAACCGTTTGCAATCTGCCAC
AAGTGTGATAAGTTATCTACACTGGCGAGGGGATTGCTCTCTGTAATGTTCAGCTTCTAA
TTGTCTCTACTTTGTGAGACAACTTTTGAATGCTTGACCTCAAATCAGGTAGGACTACCC
GCTGAACTTAA
>AB7–12
TTTCCGTAGGTGAACCTGCGGAAGGATCATTATTGAATTATGTTTCTAGATAGGTTGTAG
CTGGCTC–TTTAGAGCATGTGCACGCCTGTTTGGACTTCATTTTCATCCACCTGTGCACC
TATTGTAGTCTTTGGTTGGGTTAGGGGGAAGTGGTCATTGTGTCAGCATCTGCTGGATGT
GAGGACTTGCATTGTGAAAGCTTTGCTGTCCTTGATGTGATCATGGAATCTCTTTCTCAC
TAGAGTCTATGTCACTCATTATACTCTGTCGAATGTCATTGAATGTCTTTACATGGGCTT
GTATGCCTATGAAAATTGTAATACAACTTTCAGCAACGGATCTCTTGGCTCTCGCATCGA
TGAAGGACGCAGCGAAATGCGATAAGTAATGTGAATTGCAGAATTCAGTGAATCATCGAA
TCTTTGAACGCATCTTGCGCTCATTGGTATTCCGAGGAGCATGCCTGTTTGAGTGTCATT
AAATTCTCAACTCTCTTATACTTTTTTGTAAAAGAGAGCTTGGACTGTGGAGGCTTGCTG
GCCACTTTTTGGGGTCAGCTCCTCTGAAATGCATTAGCGGAACCGTTTGCAATCTGCCAC
AAGTGTGATAAGTTATCTACACTGGCGAGGGGATTGCTCTCTGTAATGTTCAGCTTCTAA
TTGTCTCTACTTTGTGAGACAACTTTTGAATGCTTGACCTCAAATCAGGTAGGACTACCC
GCTGAACTTAA
>AB5–18
TTTCCGTAGGTGAACCTGCGGAAGGATCATTATTGAATTATGTTTCTAGATAGGTTGTAG
CTGGCTC–TTTAGAGCATGTGCACGCCTGTTTGGACTTCATTTTCATCCACCTGTGCACC
TATTGTAGTCTTTGGTTGGGTTAGGGGGAAGTGGTCATTGTGTCAGCATCTGCTGGATGT
GAGGACTTGCATTGTGAAAGCTTTGCTGTCCTTGATGTGATCATGGAATCTTTTTCTCAC
TAGAGTCTATGTCACTCATTATACTCTGTCGAATGTCATTGAATGTCTTTACATGGGCTT
GTATGCCTATGAAAATTGTAATACAACTTTCAGCAACGGATCTCTTGGCTCTCGCATCGA
TGAAGGACGCAGCGAAATGCGATAAGTAATGTGAATTGCAGAATTCAGTGAATCATCGAA
TCTTTGAACGCATCTTGCGCTCCTTGGTATTCCGAGGAGCATGCCTGTTTGAGTGTCATT
AAATTCTCAACTCTCTTATACTTTTTTGTAAAAGAGAGCTTGGACTGTGGAGGCTTGCTG
GCCACTTTTTGGGGTCAGCTCCTCTGAAATGCATTAGCGGAACCGTTTGCGATCTGCCAC
AAGTGTGATAAGTTATCTACACTGGCGAGGGGATTGCTCTCTGTAATGTTCAGCTTCTAA
TTGTCTCTACTTTGTGAGACTACTTTTGAATGCTTGACCTCAAATCAGGTAGGACTACCC
GCTGAACTTAA

>AB10-17
TTTCCGTAGGTGAACCTGCGGAAGGATCATTATTGAATTATGTTTCTAGATAGGTTGTAG
CTGGCTC-TTTAGAGCATGTGCACGCCTGTTTGGACTTCATTTTCATCCACCTGTGCACC
TATTGTAGTCTTTGGTTGGGTTAGGGGGAAGTGGTCATTGTGTCAGCATCTGCTGGATGT
GAGGACTTGCATTGTGAAAGCTTTGCTGTCCTTGATGTGATCATGGAATCTTTTTCTCAC
TAGAGTCTATGTCACTCATTATACTCTGTCGAATGTCATTGAATGTCTTTACATGGGCTT
GTATGCCTATGAAAATTGTAATACAACTTTCAGCAACGGATCTCTTGGCTCTCGCATCGA
TGAAGGACGCAGCGAAATGCGATAAGTAATGTGAATTGCAGAATTCAGTGAATCATCGAA
TCTTTGAACGCATCTTGCGCTCCTTGGTATTCCGAGGAGCATGCCTGTTTGAGTGTCATT
AAATTCTCAACTCTCTTATACTTTTTTGTAAAAGAGAGCTTGGACTGTGGAGGCTTGCTG
GCCACTTTTTGGGGTCAGCTCCTCTGAAATGCATTAGCGGAACCGTTTGCGATCTGCCAC
AAGTGTGATAAGTTATCTACACTGGCGAGGGGATTGCTCTCTGTAATGTTCAGCTTCTAA
TTGTCTCTACTTTGTGAGACTACTTTTGAATGCTTGACCTCAAATCAGGTAGGACTACCC
GCTGAACTTAA
>AB7-30
TTTCCGTAGGTGAACCTGCGGAAGGATCATTATTGAATTATGTTTCTAGATAGGTTGTAG
CTGGCTC-TTTAGAGCATGTGCACGCCTGTTTGGACTTCATTTTCATCCACCTGTGCACC
TATTGTAGTCTTTGGTTGGGTTAGGGGGAAGTGGTCATTGTGTCAGCATCTGCTGGATGT
GAGGACTTGCATTGTGAAAGCTTTGCTGTCCTTGATGTGATCATGGAATCTCTTTCTCAC
TAGAGTCTATGTCACTCATTATACTCTGTCGAATGTCATTGAATGTCTTTACATGGGCTT
GTATGCCTATGAAAATTGTAATACAACTTTCAGCAACGGATCTCTTGGCTCTCGCATCGA
TGAAGGACGCAGCGAAATGCGATAAGTAATGTGAATTGCAGAATTCAGTGAATCATCGAA
TCTTTGAACGCATCTTGCGCTCCTTGGTATTCCGAGGAGCATGCCTGTTTGAGTGTCATT
AAATTCTCAACTCTCTTATACTTTTTTGTAAAAGAGAGCTTGGACTGTGGAGGCTTGCTG
GCCACTTTTTGGGGTCAGCTCCTCTGAAATGCATTAGCGGAACCGTTTGCGATCTGCCAC
AAGTGTGATAAGTTATCTACACTGGCGAGGGGATTGCTCTCTGTAATGTTCAGCTTCTAA
TTGTCTCTACTTTGTGAGACTACTTTTGAATGCTTGACCTCAAATCAGGTAGGACTACCC
GCTGAACTTAA
>AB9-11
TTTCCGTAGGTGAACCTGCGGAAGGATCATTATTGAATTATGTTTCTAGATAGGTTGTAG
CTGGCTC-TTTAGAGCATGTGCACGCCTGTTTGGACTTCATTTTCATCCACCTGTGCACC
TATTGTAGTCTTTGGTTGGGTTAGGGGGAAGTGGTCATTGTGTCAGCATCTGCTGGATGT
GAGGACTTGCATTGTGAAAGCTTTGCTGTCCTTGATGTGATCATGGAATCTCTTTCTCAC
TAGAGTCTATGTCACTCATTATACTCTGTCGAATGTCATTGAATGTCTTTACATGGGCTT
GTATGCCTATGAAAATTGTAATACAACTTTCAGCAACGGATCTCTTGGCTCTCGCATCGA
TGAAGGACGCAGCGAAATGCGATAAGTAATGTGAATTGCAGAATTCAGTGAATCATCGAA
TCTTTGAACGCATCTTGCGCTCCTTGGTATTCCGAGGAGCATGCCTGTTTGAGTGTCATT
AAATTCTCAACTCTCTTATACTTTTTTGTAAAAGAGAGCTTGGACTGTGGAGGCTTGCTG
GCCACTTTTTGGGGTCAGCTCCTCTGAAATGCATTAGCGGAACCGTTTGCGATCTGCCAC
AAGTGTGATAAGTTATCTACACTGGCGAGGGGATTGCTCTCTGTAATGTTCAGCTTCTAA
TTGTCTCTACTTTGTGAGACTACTTTTGAATGCTTGACCTCAAATCAGGTAGGACTACCC
GCTGAACTTAA
>AB7-3
TTTCCGTAGGTGAACCTGCGGAAGGATCATTATTGAATTATGTTTCTAGATAGGTTGTAG
CTGGCTC-TTTAGAGCATGTGCACGCCTGTTTGGACTTCATTTTCATCCACCTGTGCACC
TATTGTAGTCTTTGGTTGGGTTAGGGGGAAGTGGTCATTGTGTCAGCATCTGCTGGATGT
GAGGACTTGCATTGTGAAAGCTTTGCTGTCCTTGATGTGATCATGGAATCTCTTTCTCAC
TAGAGTCTATGTCACTCATTATACTCTGTCGAATGTCATTGAATGTCTTTACATGGGCTT
GTATGCCTATGAAAATTGTAATACAACTTTCAGCAACGGATCTCTTGGCTCTCGCATCGA
TGAAGGACGCAGCGAAATGCGATAAGTAATGTGAATTGCAGAATTCAGTGAATCATCGAA

TCTTTGAACGCATCTTGCGCTCCTTGGTATTCCGAGGAGCATGCCTGTTTGAGTGTCATT
AAATTCTCAACTCTCTTATACTTTTTTGTAAAAGAGAGCTTGGACTGTGGAGGCTTGCTG
GCCACTTTTTGGGGTCAGCTCCTCTGAAATGCATTAGCGGAACCGTTTGCGATCTGCCAC
AAGTGTGATAAGTTATCTACACTGGCGAGGGGATTGCTCTCTGTAATGTTCAGCTTCTAA
TTGTCTCTACTTTGTGAGACTACTTTTGAATGCTTGACCTCAAATCAGGTAGGACTACCC
GCTGAACTTAA
>AB2-29
TTTCCGTAGGTGAACCTGCGGAAGGATCATTATTGAATTATGTTTCTAGATAGGTTGTAG
CTGGCTC-TTTAGAGCATGTGCACGCCTGTTTGGACTTCATTTTCATCCACCTGTGCACC
TATTGTAGTCTTTGGTTGGGTTAGGGGGAAGTGGTCATTGTGTCAGCATCTGCTGGATGT
GAGGACTTGCATTGTGAAAGCTTTGCTGTCCTTGATGTGATCATGGAATCTCTTTCTCAC
TAGAGTCTATGTCACTCATTATACTCTGTCGAATGTCATTGAATGTCTTTACATGGGCTT
GTATGCCTATGAAAATTGTAATACAACTTTCAGCAACGGATCTCTTGGCTCTCGCATCGA
TGAAGGACGCAGCGAAATGCGATAAGTAATGTGAATTGCAGAATTCAGTGAATCATCGAA
TCTTTGAACGCATCTTGCGCTCCTTGGTATTCCGAGGAGCATGCCTGTTTGAGTGTCATT
AAATTCTCAACTCTCTTATACTTTTTTGTAAAAGAGAGCTTGGACTGTGGAGGCTTGCTG
GCCACTTTTTGGGGTCAGCTCCTCTGAAATGCATTAGCGGAACCGTTTGCGATCTGCCAC
AAGTGTGATAAGTTATCTACACTGGCGAGGGGATTGCTCTCTGTAATGTTCAGCTTCTAA
TTGTCTCTACTTTGTGAGACTACTTTTGAATGCTTGACCTCAAATCAGGTAGGACTACCC
GCTGAACTTAA
>AB3-14
TTTCCGTAGGTGAACCTGCGGAAGGATCATTATTGAATTATGTTTCTAGATAGGTTGTAG
CTGGCTC-TTTAGAGCATGTGCACGCCTGTTTGGACTTCATTTTCATCCACCTGTGCACC
TATTGTAGTCTTTGGTTGGGTTAGGGGGAAGTGGTCATTGTGTCAGCATCTGCTGGATGT
GAGGACTTGCATTGTGAAAGCTTTGCTGTCCTTGATGTGATCATGGAATCTCTTTCTCAC
TAGAGTCTATGTCACTCATTATACTCTGTCGAATGTCATTGAATGTCTTTACATGGGCTT
GTATGCCTATGAAAATTGTAATACAACTTTCAGCAACGGATCTCTTGGCTCTCGCATCGA
TGAAGGACGCAGCGAAATGCGATAAGTAATGTGAATTGCAGAATTCAGTGAATCATCGAA
TCTTTGAACGCATCTTGCGCTCCTTGGTATTCCGAGGAGCATGCCTGTTTGAGTGTCATT
AAATTCTCAACTCTCTTATACTTTTTTGTAAAAGAGAGCTTGGACTGTGGAGGCTTGCTG
GCCACTTTTTGGGGTCAGCTCCTCTGAAATGCATTAGCGGAACCGTTTGCGATCTGCCAC
AAGTGTGATAAGTTATCTACACTGGCGAGGGGATTGCTCTCTGTAATGTTCAGCTTCTAA
TTGTCTCTACTTTGTGAGACTACTTTTGAATGCTTGACCTCAAATCAGGTAGGACTACCC
GCTGAACTTAA
>AB4-13
TTTCCGTAGGTGAACCTGCGGAAGGATCATTATTGAATTATGTTTCTAGATAGGTTGTAG
CTGGCTC-TTTAGAGCATGTGCACGCCTGTTTGGACTTCATTTTCATCCACCTGTGCACC
TATTGTAGTCTTTGGTTGGGTTAGGGGGAAGTGGTCATTGTGTCAGCATCTGCTGGATGT
GAGGACTTGCATTGTGAAAGCTTTGCTGTCCTTGATGTGATCATGGAATCTCTTTCTCAC
TAGAGTCTATGTCACTCATTATACTCTGTCGAATGTCATTGAATGTCTTTACATGGGCTT
GTATGCCTATGAAAATTGTAATACAACTTTCAGCAACGGATCTCTTGGCTCTCGCATCGA
TGAAGGACGCAGCGAAATGCGATAAGTAATGTGAATTGCAGAATTCAGTGAATCATCGAA
TCTTTGAACGCATCTTGCGCTCCTTGGTATTCCGAGGAGCATGCCTGTTTGAGTGTCATT
AAATTCTCAACTCTCTTATACTTTTTTGTAAAAGAGAGCTTGGACTGTGGAGGCTTGCTG
GCCACTTTTTGGGGTCAGCTCCTCTGAAATGCATTAGCGGAACCGTTTGCGATCTGCCAC
AAGTGTGATAAGTTATCTACACTGGCGAGGGGATTGCTCTCTGTAATGTTCAGCTTCTAA
TTGTCTCTACTTTGTGAGACTACTTTTGAATGCTTGACCTCAAATCAGGTAGGACTACCC
GCTGAACTTAA
>AB5-40
TTTCCGTAGGTGAACCTGCGGAAGGATCATTATTGAATTATGTTTCTAGATAGGTTGTAG

CTGGCTC-TTTAGAGCATGTGCACGCCTGTTTGGACTTCATTTTCATCCACCTGTGCACC
TATTGTAGTCTTTGGTTGGGTTAGGGGGAAGTGGTCATTGTGTCAGCATCTGCTGGATGT
GAGGACTTGCATTGTGAAAGCTTTGCTGTCCTTGATGTGATCATGGAATCTCTTTCTCAC
TAGAGTCTATGTCACTCATTATACTCTGTCGAATGTCATTGAATGTCTTTACATGGGCTT
GTATGCCTATGAAAATTGTAATACAACTTTCAGCAACGGATCTCTTGGCTCTCGCATCGA
TGAAGGACGCAGCGAAATGCGATAAGTAATGTGAATTGCAGAATTCAGTGAATCATCGAA
TCTTTGAACGCATCTTGCGCTCCTTGGTATTCCGAGGAGCATGCCTGTTTGAGTGTCATT
AAATTCTCAACTCTCTTATACTTTTTTGTAAAAGAGAGCTTGGACTGTGGAGGCTTGCTG
GCCACTTTTTGGGGTCAGCTCCTCTGAAATGCATTAGCGGAACCGTTTGCGATCTGCCAC
AAGTGTGATAAGTTATCTACACTGGCGAGGGGATTGCTCTCTGTAATGTTCAGCTTCTAA
TTGTCTCTACTTTGTGAGACTACTTTTGAATGCTTGACCTCAAATCAGGTAGGACTACCC
GCTGAACTTAA
>AB7-26
TTTCCGTAGGTGAACCTGCGGAAGGATCATTATTGAATTATGTTTCTAGATAGGTTGTAG
CTGGCTC-TTTAGAGCATGTGCACGCCTGTTTGGACTTCATTTTCATCCACCTGTGCACC
TATTGTAGTCTTTGGTTGGGTTAGGGGGAAGTGGTCATTGTGTCAGCATCTGCTGGATGT
GAGGACTTGCATTGTGAAAGCTTTGCTGTCCTTGATGTGATCATGGAATCTCTTTCTCAC
TAGAGTCTATGTCACTCATTATACTCTGTCGAATGTCATTGAATGTCTTTACATGGGCTT
GTATGCCTATGAAAATTGTAATACAACTTTCAGCAACGGATCTCTTGGCTCTCGCATCGA
TGAAGGACGCAGCGAAATGCGATAAGTAATGTGAATTGCAGAATTCAGTGAATCATCGAA
TCTTTGAACGCATCTTGCGCTCCTTGGTATTCCGAGGAGCATGCCTGTTTGAGTGTCATT
AAATTCTCAACTCTCTTATACTTTTTTGTAAAAGAGAGCTTGGACTGTGGAGGCTTGCTG
GCCACTTTTTGGGGTCAGCTCCTCTGAAATGCATTAGCGGAACCGTTTGCGATCTGCCAC
AAGTGTGATAAGTTATCTACACTGGCGAGGGGATTGCTCTCTGTAATGTTCAGCTTCTAA
TTGTCTCTACTTTGTGAGACTACTTTTGAATGCTTGACCTCAAATCAGGTAGGACTACCC
GCTGAACTTAA
>AB8-16
TTTCCGTAGGTGAACCTGCGGAAGGATCATTATTGAATTATGTTTCTAGATAGGTTGTAG
CTGGCTC-TTTAGAGCATGTGCACGCCTGTTTGGACTTCATTTTCATCCACCTGTGCACC
TATTGTAGTCTTTGGTTGGGTTAGGGGGAAGTGGTCATTGTGTCAGCATCTGCTGGATGT
GAGGACTTGCATTGTGAAAGCTTTGCTGTCCTTGATGTGATCATGGAATCTCTTTCTCAC
TAGAGTCTATGTCACTCATTATACTCTGTCGAATGTCATTGAATGTCTTTACATGGGCTT
GTATGCCTATGAAAATTGTAATACAACTTTCAGCAACGGATCTCTTGGCTCTCGCATCGA
TGAAGGACGCAGCGAAATGCGATAAGTAATGTGAATTGCAGAATTCAGTGAATCATCGAA
TCTTTGAACGCATCTTGCGCTCCTTGGTATTCCGAGGAGCATGCCTGTTTGAGTGTCATT
AAATTCTCAACTCTCTTATACTTTTTTGTAAAAGAGAGCTTGGACTGTGGAGGCTTGCTG
GCCACTTTTTGGGGTCAGCTCCTCTGAAATGCATTAGCGGAACCGTTTGCGATCTGCCAC
AAGTGTGATAAGTTATCTACACTGGCGAGGGGATTGCTCTCTGTAATGTTCAGCTTCTAA
TTGTCTCTACTTTGTGAGACTACTTTTGAATGCTTGACCTCAAATCAGGTAGGACTACCC
GCTGAACTTAA
>AB8-25
TTTCCGTAGGTGAACCTGCGGAAGGATCATTATTGAATTATGTTTCTAGATAGGTTGTAG
CTGGCTC-TTTAGAGCATGTGCACGCCTGTTTGGACTTCATTTTCATCCACCTGTGCACC
TATTGTAGTCTTTGGTTGGGTTAGGGGGAAGTGGTCATTGTGTCAGCATCTGCTGGATGT
GAGGACTTGCATTGTGAAAGCTTTGCTGTCCTTGATGTGATCATGGAATCTCTTTCTCAC
TAGAGTCTATGTCACTCATTATACTCTGTCGAATGTCATTGAATGTCTTTACATGGGCTT
GTATGCCTATGAAAATTGTAATACAACTTTCAGCAACGGATCTCTTGGCTCTCGCATCGA
TGAAGGACGCAGCGAAATGCGATAAGTAATGTGAATTGCAGAATTCAGTGAATCATCGAA
TCTTTGAACGCATCTTGCGCTCCTTGGTATTCCGAGGAGCATGCCTGTTTGAGTGTCATT
AAATTCTCAACTCTCTTATACTTTTTTGTAAAAGAGAGCTTGGACTGTGGAGGCTTGCTG

GCCACTTTTTGGGGTCAGCTCCTCTGAAATGCATTAGCGGAACCGTTTGCGATCTGCCAC
AAGTGTGATAAGTTATCTACACTGGCGAGGGGATTGCTCTCTGTAATGTTCAGCTTCTAA
TTGTCTCTACTTTGTGAGACTACTTTTGAATGCTTGACCTCAAATCAGGTAGGACTACCC
GCTGAACTTAA
>AB9-4
TTTCCGTAGGTGAACCTGCGGAAGGATCATTATTGAATTATGTTTCTAGATAGGTTGTAG
CTGGCTC-TTTAGAGCATGTGCACGCCTGTTTGGACTTCATTTTCATCCACCTGTGCACC
TATTGTAGTCTTTGGTTGGGTTAGGGGGAAGTGGTCATTGTGTCAGCATCTGCTGGATGT
GAGGACTTGCATTGTGAAAGCTTTGCTGTCCTTGATGTGATCATGGAATCTCTTTCTCAC
TAGAGTCTATGTCACTCATTATACTCTGTCGAATGTCATTGAATGTCTTTACATGGGCTT
GTATGCCTATGAAAATTGTAATACAACTTTCAGCAACGGATCTCTTGGCTCTCGCATCGA
TGAAGGACGCAGCGAAATGCGATAAGTAATGTGAATTGCAGAATTCAGTGAATCATCGAA
TCTTTGAACGCATCTTGCGCTCCTTGGTATTCCGAGGAGCATGCCTGTTTGAGTGTCATT
AAATTCTCAACTCTCTTATACTTTTTTGTAAAAGAGAGCTTGGACTGTGGAGGCTTGCTG
GCCACTTTTTGGGGTCAGCTCCTCTGAAATGCATTAGCGGAACCGTTTGCGATCTGCCAC
AAGTGTGATAAGTTATCTACACTGGCGAGGGGATTGCTCTCTGTAATGTTCAGCTTCTAA
TTGTCTCTACTTTGTGAGACTACTTTTGAATGCTTGACCTCAAATCAGGTAGGACTACCC
GCTGAACTTAA
>AB9-39
TTTCCGTAGGTGAACCTGCGGAAGGATCATTATTGAATTATGTTTCTAGATAGGTTGTAG
CTGGCTC-TTTAGAGCATGTGCACGCCTGTTTGGACTTCATTTTCATCCACCTGTGCACC
TATTGTAGTCTTTGGTTGGGTTAGGGGGAAGTGGTCATTGTGTCAGCATCTGCTGGATGT
GAGGACTTGCATTGTGAAAGCTTTGCTGTCCTTGATGTGATCATGGAATCTCTTTCTCAC
TAGAGTCTATGTCACTCATTATACTCTGTCGAATGTCATTGAATGTCTTTACATGGGCTT
GTATGCCTATGAAAATTGTAATACAACTTTCAGCAACGGATCTCTTGGCTCTCGCATCGA
TGAAGGACGCAGCGAAATGCGATAAGTAATGTGAATTGCAGAATTCAGTGAATCATCGAA
TCTTTGAACGCATCTTGCGCTCCTTGGTATTCCGAGGAGCATGCCTGTTTGAGTGTCATT
AAATTCTCAACTCTCTTATACTTTTTTGTAAAAGAGAGCTTGGACTGTGGAGGCTTGCTG
GCCACTTTTTGGGGTCAGCTCCTCTGAAATGCATTAGCGGAACCGTTTGCGATCTGCCAC
AAGTGTGATAAGTTATCTACACTGGCGAGGGGATTGCTCTCTGTAATGTTCAGCTTCTAA
TTGTCTCTACTTTGTGAGACTACTTTTGAATGCTTGACCTCAAATCAGGTAGGACTACCC
GCTGAACTTAA
>AB12-9
TTTCCGTAGGTGAACCTGCGGAAGGATCATTATTGAATTATGTTTCTAGATAGGTTGTAG
CTGGCTC-TTTAGAGCATGTGCACGCCTGTTTGGACTTCATTTTCATCCACCTGTGCACC
TATTGTAGTCTTTGGTTGGGTTAGGGGGAAGTGGTCATTGTGTCAGCATCTGCTGGATGT
GAGGACTTGCATTGTGAAAGCTTTGCTGTCCTTGATGTGATCATGGAATCTCTTTCTCAC
TAGAGTCTATGTCACTCATTATACTCTGTCGAATGTCATTGAATGTCTTTACATGGGCTT
GTATGCCTATGAAAATTGTAATACAACTTTCAGCAACGGATCTCTTGGCTCTCGCATCGA
TGAAGGACGCAGCGAAATGCGATAAGTAATGTGAATTGCAGAATTCAGTGAATCATCGAA
TCTTTGAACGCATCTTGCGCTCCTTGGTATTCCGAGGAGCATGCCTGTTTGAGTGTCATT
AAATTCTCAACTCTCTTATACTTTTTTGTAAAAGAGAGCTTGGACTGTGGAGGCTTGCTG
GCCACTTTTTGGGGTCAGCTCCTCTGAAATGCATTAGCGGAACCGTTTGCGATCTGCCAC
AAGTGTGATAAGTTATCTACACTGGCGAGGGGATTGCTCTCTGTAATGTTCAGCTTCTAA
TTGTCTCTACTTTGTGAGACTACTTTTGAATGCTTGACCTCAAATCAGGTAGGACTACCC
GCTGAACTTAA
>AB1-7
TTTCCGTAGGTGAACCTGCGGAAGGATCATTATTGAATTATGTTTCTAGATAGGTTGTAG
CTGGCTC-TTTAGAGCATGTGCACGCCTGTTTGGACTTCATTTTCATCCACCTGTGCACC
TATTGTAGTCTTTGGTTGGGTTAGGGGGAAGTGGTCATTGTGTCAGCATCTGCTGGATGT

GAGGACTTGCATTGTGAAAGCTTTGCTGTCCTTGATGTGATCATGGAATCTTTTTCTCAC
TAGAGTCTATGTCACTCATTATACTCTGTCGAATGTCATTGAATGTCTTTACATGGGCTT
GTATGCCTATGAAAATTGTAATACAACTTTCAGCAACGGATCTCTTGGCTCTCGCATCGA
TGAAGAACGCAGCGAAATGCGATAAGTAATGTGAATTGCAGAATTCAGTGAATCATCGAA
TCTTTGAACGCATCTTGCGCTCCTTGGTATTCCGAGGAGCATGCCTGTTTGAGTGTCATT
AAATTCTCAACTCTCTTATACTTTTTTGTAAAAGAGAGCTTGGACTGTGGAGGCTTGCTG
GCCACTTTTTGGGGTCAGCTCCTCTGAAATGCATTAGCGGAACCGTTTGCGATCTGCCAC
AAGTGTGATAAGTTATCTACACTGGCGAGGGGATTGCTCTCTGTAATGTTCAGCTTCTAA
TTGTCTCTACTTTGTGAGACTACTTTTGAATGCTTGACCTCAAATCAGGTAGGACTACCC
GCTGAACTTAA
>AB2-30
TTTCCGTAGGTGAACCTGCGGAAGGATCATTATTGAATTATGTTTCTAGATAGGTTGTAG
CTGGCTC-TTTAGAGCATGTGCACGCCTGTTTGGACTTCATTTTCATCCACCTGTGCACC
TATTGTAGTCTTTGGTTGGGTTAGGGGGAAGTGGTCATTGTGTCAGCATCTGCTGGATGT
GAGGACTTGCATTGTGAAAGCTTTGCTGTCCTTGATGTGATCATGGAATCTTTTTCTCAC
TAGAGTCTATGTCACTCATTATACTCTGTCGAATGTCATTGAATGTCTTTACATGGGCTT
GTATGCCTATGAAAATTGTAATACAACTTTCAGCAACGGATCTCTTGGCTCTCGCATCGA
TGAAGAACGCAGCGAAATGCGATAAGTAATGTGAATTGCAGAATTCAGTGAATCATCGAA
TCTTTGAACGCATCTTGCGCTCCTTGGTATTCCGAGGAGCATGCCTGTTTGAGTGTCATT
AAATTCTCAACTCTCTTATACTTTTTTGTAAAAGAGAGCTTGGACTGTGGAGGCTTGCTG
GCCACTTTTTGGGGTCAGCTCCTCTGAAATGCATTAGCGGAACCGTTTGCGATCTGCCAC
AAGTGTGATAAGTTATCTACACTGGCGAGGGGATTGCTCTCTGTAATGTTCAGCTTCTAA
TTGTCTCTACTTTGTGAGACTACTTTTGAATGCTTGACCTCAAATCAGGTAGGACTACCC
GCTGAACTTAA
>AB6-1
TTTCCGTAGGTGAACCTGCGGAAGGATCATTATTGAATTATGTTTCTAGATAGGTTGTAG
CTGGCTC-TTTAGAGCATGTGCACGCCTGTTTGGACTTCATTTTCATCCACCTGTGCACC
TATTGTAGTCTTTGGTTGGGTTAGGGGGAAGTGGTCATTGTGTCAGCATCTGCTGGATGT
GAGGACTTGCATTGTGAAAGCTTTGCTGTCCTTGATGTGATCATGGAATCTTTTTCTCAC
TAGAGTCTATGTCACTCATTATACTCTGTCGAATGTCATTGAATGTCTTTACATGGGCTT
GTATGCCTATGAAAATTGTAATACAACTTTCAGCAACGGATCTCTTGGCTCTCGCATCGA
TGAAGAACGCAGCGAAATGCGATAAGTAATGTGAATTGCAGAATTCAGTGAATCATCGAA
TCTTTGAACGCATCTTGCGCTCCTTGGTATTCCGAGGAGCATGCCTGTTTGAGTGTCATT
AAATTCTCAACTCTCTTATACTTTTTTGTAAAAGAGAGCTTGGACTGTGGAGGCTTGCTG
GCCACTTTTTGGGGTCAGCTCCTCTGAAATGCATTAGCGGAACCGTTTGCGATCTGCCAC
AAGTGTGATAAGTTATCTACACTGGCGAGGGGATTGCTCTCTGTAATGTTCAGCTTCTAA
TTGTCTCTACTTTGTGAGACTACTTTTGAATGCTTGACCTCAAATCAGGTAGGACTACCC
GCTGAACTTAA
>AB11-8
TTTCCGTAGGTGAACCTGCGGAAGGATCATTATTGAATTATGTTTCTAGATAGGTTGTAG
CTGGCTC-TTTAGAGCATGTGCACGCCTGTTTGGACTTCATTTTCATCCACCTGTGCACC
TATTGTAGTCTTTGGTTGGGTTAGGGGGAAGTGGTCATTGTGTCAGCATCTGCTGGATGT
GAGGACTTGCATTGTGAAAGCTTTGCTGTCCTTGATGTGATCATGGAATCTTTTTCTCAC
TAGAGTCTATGTCACTCATTATACTCTGTCGAATGTCATTGAATGTCTTTACATGGGCTT
GTATGCCTATGAAAATTGTAATACAACTTTCAGCAACGGATCTCTTGGCTCTCGCATCGA
TGAAGAACGCAGCGAAATGCGATAAGTAATGTGAATTGCAGAATTCAGTGAATCATCGAA
TCTTTGAACGCATCTTGCGCTCCTTGGTATTCCGAGGAGCATGCCTGTTTGAGTGTCATT
AAATTCTCAACTCTCTTATACTTTTTTGTAAAAGAGAGCTTGGACTGTGGAGGCTTGCTG
GCCACTTTTTGGGGTCAGCTCCTCTGAAATGCATTAGCGGAACCGTTTGCGATCTGCCAC
AAGTGTGATAAGTTATCTACACTGGCGAGGGGATTGCTCTCTGTAATGTTCAGCTTCTAA

TTGTCTCTACTTTGTGAGACTACTTTTGAATGCTTGACCTCAAATCAGGTAGGACTACCC
GCTGAACTTAA
>AB12-6
TTTCCGTAGGTGAACCTGCGGAAGGATCATTATTGAATTATGTTTCTAGATAGGTTGTAG
CTGGCTC-TTTAGAGCATGTGCACGCCTGTTTGGACTTCATTTTCATCCACCTGTGCACC
TATTGTAGTCTTTGGTTGGGTTAGGGGGAAGTGGTCATTGTGTCAGCATCTGCTGGATGT
GAGGACTTGCATTGTGAAAGCTTTGCTGTCCTTGATGTGATCATGGAATCTTTTTCTCAC
TAGAGTCTATGTCACTCATTATACTCTGTCGAATGTCATTGAATGTCTTTACATGGGCTT
GTATGCCTATGAAAATTGTAATACAACTTTCAGCAACGGATCTCTTGGCTCTCGCATCGA
TGAAGAACGCAGCGAAATGCGATAAGTAATGTGAATTGCAGAATTCAGTGAATCATCGAA
TCTTTGAACGCATCTTGCGCTCCTTGGTATTCCGAGGAGCATGCCTGTTTGAGTGTCATT
AAATTCTCAACTCTCTTATACTTTTTTGTAAAAGAGAGCTTGGACTGTGGAGGCTTGCTG
GCCACTTTTTGGGGTCAGCTCCTCTGAAATGCATTAGCGGAACCGTTTGCGATCTGCCAC
AAGTGTGATAAGTTATCTACACTGGCGAGGGGATTGCTCTCTGTAATGTTCAGCTTCTAA
TTGTCTCTACTTTGTGAGACTACTTTTGAATGCTTGACCTCAAATCAGGTAGGACTACCC
GCTGAACTTAA
>AB1-37
TTTCCGTAGGTGAACCTGCGGAAGGATCATTATTGAATTATGTTTCTAGATAGGTTGTAG
CTGGCTC-TTTAGAGCATGTGCACGCCTGTTTGGACTTCATTTTCATCCACCTGTGCACC
TATTGTAGTCTTTGGTTGGGTTAGGGGGAAGTGGTCATTGTGTCAGCATCTGCTGGATGT
GAGGACTTGCATTGTGAAAGCTTTGCTGTCCTTGATGTGATCATGGAATCTCTTTCTCAC
TAGAGTCTATGTCACTCATTATACTCTGTCGAATGTCATTGAATGTCTTTACATGGGCTT
GTATGCCTATGAAAATTGTAATACAACTTTCAGCAACGGATCTCTTGGCTCTCGCATCGA
TGAAGAACGCAGCGAAATGCGATAAGTAATGTGAATTGCAGAATTCAGTGAATCATCGAA
TCTTTGAACGCATCTTGCGCTCCTTGGTATTCCGAGGAGCATGCCTGTTTGAGTGTCATT
AAATTCTCAACTCTCTTATACTTTTTTGTAAAAGAGAGCTTGGACTGTGGAGGCTTGCTG
GCCACTTTTTGGGGTCAGCTCCTCTGAAATGCATTAGCGGAACCGTTTGCGATCTGCCAC
AAGTGTGATAAGTTATCTACACTGGCGAGGGGATTGCTCTCTGTAATGTTCAGCTTCTAA
TTGTCTCTACTTTGTGAGACTACTTTTGAATGCTTGACCTCAAATCAGGTAGGACTACCC
GCTGAACTTAA
>AB2-14
TTTCCGTAGGTGAACCTGCGGAAGGATCATTATTGAATTATGTTTCTAGATAGGTTGTAG
CTGGCTC-TTTAGAGCATGTGCACGCCTGTTTGGACTTCATTTTCATCCACCTGTGCACC
TATTGTAGTCTTTGGTTGGGTTAGGGGGAAGTGGTCATTGTGTCAGCATCTGCTGGATGT
GAGGACTTGCATTGTGAAAGCTTTGCTGTCCTTGATGTGATCATGGAATCTCTTTCTCAC
TAGAGTCTATGTCACTCATTATACTCTGTCGAATGTCATTGAATGTCTTTACATGGGCTT
GTATGCCTATGAAAATTGTAATACAACTTTCAGCAACGGATCTCTTGGCTCTCGCATCGA
TGAAGAACGCAGCGAAATGCGATAAGTAATGTGAATTGCAGAATTCAGTGAATCATCGAA
TCTTTGAACGCATCTTGCGCTCCTTGGTATTCCGAGGAGCATGCCTGTTTGAGTGTCATT
AAATTCTCAACTCTCTTATACTTTTTTGTAAAAGAGAGCTTGGACTGTGGAGGCTTGCTG
GCCACTTTTTGGGGTCAGCTCCTCTGAAATGCATTAGCGGAACCGTTTGCGATCTGCCAC
AAGTGTGATAAGTTATCTACACTGGCGAGGGGATTGCTCTCTGTAATGTTCAGCTTCTAA
TTGTCTCTACTTTGTGAGACTACTTTTGAATGCTTGACCTCAAATCAGGTAGGACTACCC
GCTGAACTTAA
>AB2-23
TTTCCGTAGGTGAACCTGCGGAAGGATCATTATTGAATTATGTTTCTAGATAGGTTGTAG
CTGGCTC-TTTAGAGCATGTGCACGCCTGTTTGGACTTCATTTTCATCCACCTGTGCACC
TATTGTAGTCTTTGGTTGGGTTAGGGGGAAGTGGTCATTGTGTCAGCATCTGCTGGATGT
GAGGACTTGCATTGTGAAAGCTTTGCTGTCCTTGATGTGATCATGGAATCTCTTTCTCAC
TAGAGTCTATGTCACTCATTATACTCTGTCGAATGTCATTGAATGTCTTTACATGGGCTT

GTATGCCTATGAAAATTGTAATACAACTTTCAGCAACGGATCTCTTGGCTCTCGCATCGA
TGAAGAACGCAGCGAAATGCGATAAGTAATGTGAATTGCAGAATTCAGTGAATCATCGAA
TCTTTGAACGCATCTTGCGCTCCTTGGTATTCCGAGGAGCATGCCTGTTTGAGTGTCATT
AAATTCTCAACTCTCTTATACTTTTTTGTAAAAGAGAGCTTGGACTGTGGAGGCTTGCTG
GCCACTTTTTGGGGTCAGCTCCTCTGAAATGCATTAGCGGAACCGTTTGCGATCTGCCAC
AAGTGTGATAAGTTATCTACACTGGCGAGGGGATTGCTCTCTGTAATGTTCAGCTTCTAA
TTGTCTCTACTTTGTGAGACTACTTTTGAATGCTTGACCTCAAATCAGGTAGGACTACCC
GCTGAACTTAA
>AB3-4
TTTCCGTAGGTGAACCTGCGGAAGGATCATTATTGAATTATGTTTCTAGATAGGTTGTAG
CTGGCTC-TTTAGAGCATGTGCACGCCTGTTTGGACTTCATTTTCATCCACCTGTGCACC
TATTGTAGTCTTTGGTTGGGTTAGGGGGAAGTGGTCATTGTGTCAGCATCTGCTGGATGT
GAGGACTTGCATTGTGAAAGCTTTGCTGTCCTTGATGTGATCATGGAATCTCTTTCTCAC
TAGAGTCTATGTCACTCATTATACTCTGTCGAATGTCATTGAATGTCTTTACATGGGCTT
GTATGCCTATGAAAATTGTAATACAACTTTCAGCAACGGATCTCTTGGCTCTCGCATCGA
TGAAGAACGCAGCGAAATGCGATAAGTAATGTGAATTGCAGAATTCAGTGAATCATCGAA
TCTTTGAACGCATCTTGCGCTCCTTGGTATTCCGAGGAGCATGCCTGTTTGAGTGTCATT
AAATTCTCAACTCTCTTATACTTTTTTGTAAAAGAGAGCTTGGACTGTGGAGGCTTGCTG
GCCACTTTTTGGGGTCAGCTCCTCTGAAATGCATTAGCGGAACCGTTTGCGATCTGCCAC
AAGTGTGATAAGTTATCTACACTGGCGAGGGGATTGCTCTCTGTAATGTTCAGCTTCTAA
TTGTCTCTACTTTGTGAGACTACTTTTGAATGCTTGACCTCAAATCAGGTAGGACTACCC
GCTGAACTTAA
>AB6-12
TTTCCGTAGGTGAACCTGCGGAAGGATCATTATTGAATTATGTTTCTAGATAGGTTGTAG
CTGGCTC-TTTAGAGCATGTGCACGCCTGTTTGGACTTCATTTTCATCCACCTGTGCACC
TATTGTAGTCTTTGGTTGGGTTAGGGGGAAGTGGTCATTGTGTCAGCATCTGCTGGATGT
GAGGACTTGCATTGTGAAAGCTTTGCTGTCCTTGATGTGATCATGGAATCTCTTTCTCAC
TAGAGTCTATGTCACTCATTATACTCTGTCGAATGTCATTGAATGTCTTTACATGGGCTT
GTATGCCTATGAAAATTGTAATACAACTTTCAGCAACGGATCTCTTGGCTCTCGCATCGA
TGAAGAACGCAGCGAAATGCGATAAGTAATGTGAATTGCAGAATTCAGTGAATCATCGAA
TCTTTGAACGCATCTTGCGCTCCTTGGTATTCCGAGGAGCATGCCTGTTTGAGTGTCATT
AAATTCTCAACTCTCTTATACTTTTTTGTAAAAGAGAGCTTGGACTGTGGAGGCTTGCTG
GCCACTTTTTGGGGTCAGCTCCTCTGAAATGCATTAGCGGAACCGTTTGCGATCTGCCAC
AAGTGTGATAAGTTATCTACACTGGCGAGGGGATTGCTCTCTGTAATGTTCAGCTTCTAA
TTGTCTCTACTTTGTGAGACTACTTTTGAATGCTTGACCTCAAATCAGGTAGGACTACCC
GCTGAACTTAA
>AB6-41
TTTCCGTAGGTGAACCTGCGGAAGGATCATTATTGAATTATGTTTCTAGATAGGTTGTAG
CTGGCTC-TTTAGAGCATGTGCACGCCTGTTTGGACTTCATTTTCATCCACCTGTGCACC
TATTGTAGTCTTTGGTTGGGTTAGGGGGAAGTGGTCATTGTGTCAGCATCTGCTGGATGT
GAGGACTTGCATTGTGAAAGCTTTGCTGTCCTTGATGTGATCATGGAATCTCTTTCTCAC
TAGAGTCTATGTCACTCATTATACTCTGTCGAATGTCATTGAATGTCTTTACATGGGCTT
GTATGCCTATGAAAATTGTAATACAACTTTCAGCAACGGATCTCTTGGCTCTCGCATCGA
TGAAGAACGCAGCGAAATGCGATAAGTAATGTGAATTGCAGAATTCAGTGAATCATCGAA
TCTTTGAACGCATCTTGCGCTCCTTGGTATTCCGAGGAGCATGCCTGTTTGAGTGTCATT
AAATTCTCAACTCTCTTATACTTTTTTGTAAAAGAGAGCTTGGACTGTGGAGGCTTGCTG
GCCACTTTTTGGGGTCAGCTCCTCTGAAATGCATTAGCGGAACCGTTTGCGATCTGCCAC
AAGTGTGATAAGTTATCTACACTGGCGAGGGGATTGCTCTCTGTAATGTTCAGCTTCTAA
TTGTCTCTACTTTGTGAGACTACTTTTGAATGCTTGACCTCAAATCAGGTAGGACTACCC
GCTGAACTTAA

>AB7–5
TTTCCGTAGGTGAACCTGCGGAAGGATCATTATTGAATTATGTTTCTAGATAGGTTGTAG
CTGGCTC–TTTAGAGCATGTGCACGCCTGTTTGGACTTCATTTTCATCCACCTGTGCACC
TATTGTAGTCTTTGGTTGGGTTAGGGGGAAGTGGTCATTGTGTCAGCATCTGCTGGATGT
GAGGACTTGCATTGTGAAAGCTTTGCTGTCCTTGATGTGATCATGGAATCTCTTTCTCAC
TAGAGTCTATGTCACTCATTATACTCTGTCGAATGTCATTGAATGTCTTTACATGGGCTT
GTATGCCTATGAAAATTGTAATACAACTTTCAGCAACGGATCTCTTGGCTCTCGCATCGA
TGAAGAACGCAGCGAAATGCGATAAGTAATGTGAATTGCAGAATTCAGTGAATCATCGAA
TCTTTGAACGCATCTTGCGCTCCTTGGTATTCCGAGGAGCATGCCTGTTTGAGTGTCATT
AAATTCTCAACTCTCTTATACTTTTTTGTAAAAGAGAGCTTGGACTGTGGAGGCTTGCTG
GCCACTTTTTGGGGTCAGCTCCTCTGAAATGCATTAGCGGAACCGTTTGCGATCTGCCAC
AAGTGTGATAAGTTATCTACACTGGCGAGGGGATTGCTCTCTGTAATGTTCAGCTTCTAA
TTGTCTCTACTTTGTGAGACTACTTTTGAATGCTTGACCTCAAATCAGGTAGGACTACCC
GCTGAACTTAA
>AB7–36
TTTCCGTAGGTGAACCTGCGGAAGGATCATTATTGAATTATGTTTCTAGATAGGTTGTAG
CTGGCTC–TTTAGAGCATGTGCACGCCTGTTTGGACTTCATTTTCATCCACCTGTGCACC
TATTGTAGTCTTTGGTTGGGTTAGGGGGAAGTGGTCATTGTGTCAGCATCTGCTGGATGT
GAGGACTTGCATTGTGAAAGCTTTGCTGTCCTTGATGTGATCATGGAATCTCTTTCTCAC
TAGAGTCTATGTCACTCATTATACTCTGTCGAATGTCATTGAATGTCTTTACATGGGCTT
GTATGCCTATGAAAATTGTAATACAACTTTCAGCAACGGATCTCTTGGCTCTCGCATCGA
TGAAGAACGCAGCGAAATGCGATAAGTAATGTGAATTGCAGAATTCAGTGAATCATCGAA
TCTTTGAACGCATCTTGCGCTCCTTGGTATTCCGAGGAGCATGCCTGTTTGAGTGTCATT
AAATTCTCAACTCTCTTATACTTTTTTGTAAAAGAGAGCTTGGACTGTGGAGGCTTGCTG
GCCACTTTTTGGGGTCAGCTCCTCTGAAATGCATTAGCGGAACCGTTTGCGATCTGCCAC
AAGTGTGATAAGTTATCTACACTGGCGAGGGGATTGCTCTCTGTAATGTTCAGCTTCTAA
TTGTCTCTACTTTGTGAGACTACTTTTGAATGCTTGACCTCAAATCAGGTAGGACTACCC
GCTGAACTTAA
>AB7–51
TTTCCGTAGGTGAACCTGCGGAAGGATCATTATTGAATTATGTTTCTAGATAGGTTGTAG
CTGGCTC–TTTAGAGCATGTGCACGCCTGTTTGGACTTCATTTTCATCCACCTGTGCACC
TATTGTAGTCTTTGGTTGGGTTAGGGGGAAGTGGTCATTGTGTCAGCATCTGCTGGATGT
GAGGACTTGCATTGTGAAAGCTTTGCTGTCCTTGATGTGATCATGGAATCTCTTTCTCAC
TAGAGTCTATGTCACTCATTATACTCTGTCGAATGTCATTGAATGTCTTTACATGGGCTT
GTATGCCTATGAAAATTGTAATACAACTTTCAGCAACGGATCTCTTGGCTCTCGCATCGA
TGAAGAACGCAGCGAAATGCGATAAGTAATGTGAATTGCAGAATTCAGTGAATCATCGAA
TCTTTGAACGCATCTTGCGCTCCTTGGTATTCCGAGGAGCATGCCTGTTTGAGTGTCATT
AAATTCTCAACTCTCTTATACTTTTTTGTAAAAGAGAGCTTGGACTGTGGAGGCTTGCTG
GCCACTTTTTGGGGTCAGCTCCTCTGAAATGCATTAGCGGAACCGTTTGCGATCTGCCAC
AAGTGTGATAAGTTATCTACACTGGCGAGGGGATTGCTCTCTGTAATGTTCAGCTTCTAA
TTGTCTCTACTTTGTGAGACTACTTTTGAATGCTTGACCTCAAATCAGGTAGGACTACCC
GCTGAACTTAA
>AB8–5
TTTCCGTAGGTGAACCTGCGGAAGGATCATTATTGAATTATGTTTCTAGATAGGTTGTAG
CTGGCTC–TTTAGAGCATGTGCACGCCTGTTTGGACTTCATTTTCATCCACCTGTGCACC
TATTGTAGTCTTTGGTTGGGTTAGGGGGAAGTGGTCATTGTGTCAGCATCTGCTGGATGT
GAGGACTTGCATTGTGAAAGCTTTGCTGTCCTTGATGTGATCATGGAATCTCTTTCTCAC
TAGAGTCTATGTCACTCATTATACTCTGTCGAATGTCATTGAATGTCTTTACATGGGCTT
GTATGCCTATGAAAATTGTAATACAACTTTCAGCAACGGATCTCTTGGCTCTCGCATCGA
TGAAGAACGCAGCGAAATGCGATAAGTAATGTGAATTGCAGAATTCAGTGAATCATCGAA

TCTTTGAACGCATCTTGCGCTCCTTGGTATTCCGAGGAGCATGCCTGTTTGAGTGTCATT
AAATTCTCAACTCTCTTATACTTTTTTGTAAAAGAGAGCTTGGACTGTGGAGGCTTGCTG
GCCACTTTTTGGGGTCAGCTCCTCTGAAATGCATTAGCGGAACCGTTTGCGATCTGCCAC
AAGTGTGATAAGTTATCTACACTGGCGAGGGGATTGCTCTCTGTAATGTTCAGCTTCTAA
TTGTCTCTACTTTGTGAGACTACTTTTGAATGCTTGACCTCAAATCAGGTAGGACTACCC
GCTGAACTTAA
>AB8−38
TTTCCGTAGGTGAACCTGCGGAAGGATCATTATTGAATTATGTTTCTAGATAGGTTGTAG
CTGGCTC−TTTAGAGCATGTGCACGCCTGTTTGGACTTCATTTTCATCCACCTGTGCACC
TATTGTAGTCTTTGGTTGGGTTAGGGGGAAGTGGTCATTGTGTCAGCATCTGCTGGATGT
GAGGACTTGCATTGTGAAAGCTTTGCTGTCCTTGATGTGATCATGGAATCTCTTTCTCAC
TAGAGTCTATGTCACTCATTATACTCTGTCGAATGTCATTGAATGTCTTTACATGGGCTT
GTATGCCTATGAAAATTGTAATACAACTTTCAGCAACGGATCTCTTGGCTCTCGCATCGA
TGAAGAACGCAGCGAAATGCGATAAGTAATGTGAATTGCAGAATTCAGTGAATCATCGAA
TCTTTGAACGCATCTTGCGCTCCTTGGTATTCCGAGGAGCATGCCTGTTTGAGTGTCATT
AAATTCTCAACTCTCTTATACTTTTTTGTAAAAGAGAGCTTGGACTGTGGAGGCTTGCTG
GCCACTTTTTGGGGTCAGCTCCTCTGAAATGCATTAGCGGAACCGTTTGCGATCTGCCAC
AAGTGTGATAAGTTATCTACACTGGCGAGGGGATTGCTCTCTGTAATGTTCAGCTTCTAA
TTGTCTCTACTTTGTGAGACTACTTTTGAATGCTTGACCTCAAATCAGGTAGGACTACCC
GCTGAACTTAA
>AB9−2
TTTCCGTAGGTGAACCTGCGGAAGGATCATTATTGAATTATGTTTCTAGATAGGTTGTAG
CTGGCTC−TTTAGAGCATGTGCACGCCTGTTTGGACTTCATTTTCATCCACCTGTGCACC
TATTGTAGTCTTTGGTTGGGTTAGGGGGAAGTGGTCATTGTGTCAGCATCTGCTGGATGT
GAGGACTTGCATTGTGAAAGCTTTGCTGTCCTTGATGTGATCATGGAATCTCTTTCTCAC
TAGAGTCTATGTCACTCATTATACTCTGTCGAATGTCATTGAATGTCTTTACATGGGCTT
GTATGCCTATGAAAATTGTAATACAACTTTCAGCAACGGATCTCTTGGCTCTCGCATCGA
TGAAGAACGCAGCGAAATGCGATAAGTAATGTGAATTGCAGAATTCAGTGAATCATCGAA
TCTTTGAACGCATCTTGCGCTCCTTGGTATTCCGAGGAGCATGCCTGTTTGAGTGTCATT
AAATTCTCAACTCTCTTATACTTTTTTGTAAAAGAGAGCTTGGACTGTGGAGGCTTGCTG
GCCACTTTTTGGGGTCAGCTCCTCTGAAATGCATTAGCGGAACCGTTTGCGATCTGCCAC
AAGTGTGATAAGTTATCTACACTGGCGAGGGGATTGCTCTCTGTAATGTTCAGCTTCTAA
TTGTCTCTACTTTGTGAGACTACTTTTGAATGCTTGACCTCAAATCAGGTAGGACTACCC
GCTGAACTTAA
>AB9−51
TTTCCGTAGGTGAACCTGCGGAAGGATCATTATTGAATTATGTTTCTAGATAGGTTGTAG
CTGGCTC−TTTAGAGCATGTGCACGCCTGTTTGGACTTCATTTTCATCCACCTGTGCACC
TATTGTAGTCTTTGGTTGGGTTAGGGGGAAGTGGTCATTGTGTCAGCATCTGCTGGATGT
GAGGACTTGCATTGTGAAAGCTTTGCTGTCCTTGATGTGATCATGGAATCTCTTTCTCAC
TAGAGTCTATGTCACTCATTATACTCTGTCGAATGTCATTGAATGTCTTTACATGGGCTT
GTATGCCTATGAAAATTGTAATACAACTTTCAGCAACGGATCTCTTGGCTCTCGCATCGA
TGAAGAACGCAGCGAAATGCGATAAGTAATGTGAATTGCAGAATTCAGTGAATCATCGAA
TCTTTGAACGCATCTTGCGCTCCTTGGTATTCCGAGGAGCATGCCTGTTTGAGTGTCATT
AAATTCTCAACTCTCTTATACTTTTTTGTAAAAGAGAGCTTGGACTGTGGAGGCTTGCTG
GCCACTTTTTGGGGTCAGCTCCTCTGAAATGCATTAGCGGAACCGTTTGCGATCTGCCAC
AAGTGTGATAAGTTATCTACACTGGCGAGGGGATTGCTCTCTGTAATGTTCAGCTTCTAA
TTGTCTCTACTTTGTGAGACTACTTTTGAATGCTTGACCTCAAATCAGGTAGGACTACCC
GCTGAACTTAA
>AB10−3
TTTCCGTAGGTGAACCTGCGGAAGGATCATTATTGAATTATGTTTCTAGATAGGTTGTAG

CTGGCTC-TTTAGAGCATGTGCACGCCTGTTTGGACTTCATTTTCATCCACCTGTGCACC
TATTGTAGTCTTTGGTTGGGTTAGGGGGAAGTGGTCATTGTGTCAGCATCTGCTGGATGT
GAGGACTTGCATTGTGAAAGCTTTGCTGTCCTTGATGTGATCATGGAATCTCTTTCTCAC
TAGAGTCTATGTCACTCATTATACTCTGTCGAATGTCATTGAATGTCTTTACATGGGCTT
GTATGCCTATGAAAATTGTAATACAACTTTCAGCAACGGATCTCTTGGCTCTCGCATCGA
TGAAGAACGCAGCGAAATGCGATAAGTAATGTGAATTGCAGAATTCAGTGAATCATCGAA
TCTTTGAACGCATCTTGCGCTCCTTGGTATTCCGAGGAGCATGCCTGTTTGAGTGTCATT
AAATTCTCAACTCTCTTATACTTTTTTGTAAAAGAGAGCTTGGACTGTGGAGGCTTGCTG
GCCACTTTTTGGGGTCAGCTCCTCTGAAATGCATTAGCGGAACCGTTTGCGATCTGCCAC
AAGTGTGATAAGTTATCTACACTGGCGAGGGGATTGCTCTCTGTAATGTTCAGCTTCTAA
TTGTCTCTACTTTGTGAGACTACTTTTGAATGCTTGACCTCAAATCAGGTAGGACTACCC
GCTGAACTTAA
>AB11-50
TTTCCGTAGGTGAACCTGCGGAAGGATCATTATTGAATTATGTTTCTAGATAGGTTGTAG
CTGGCTC-TTTAGAGCATGTGCACGCCTGTTTGGACTTCATTTTCATCCACCTGTGCACC
TATTGTAGTCTTTGGTTGGGTTAGGGGGAAGTGGTCATTGTGTCAGCATCTGCTGGATGT
GAGGACTTGCATTGTGAAAGCTTTGCTGTCCTTGATGTGATCATGGAATCTCTTTCTCAC
TAGAGTCTATGTCACTCATTATACTCTGTCGAATGTCATTGAATGTCTTTACATGGGCTT
GTATGCCTATGAAAATTGTAATACAACTTTCAGCAACGGATCTCTTGGCTCTCGCATCGA
TGAAGAACGCAGCGAAATGCGATAAGTAATGTGAATTGCAGAATTCAGTGAATCATCGAA
TCTTTGAACGCATCTTGCGCTCCTTGGTATTCCGAGGAGCATGCCTGTTTGAGTGTCATT
AAATTCTCAACTCTCTTATACTTTTTTGTAAAAGAGAGCTTGGACTGTGGAGGCTTGCTG
GCCACTTTTTGGGGTCAGCTCCTCTGAAATGCATTAGCGGAACCGTTTGCGATCTGCCAC
AAGTGTGATAAGTTATCTACACTGGCGAGGGGATTGCTCTCTGTAATGTTCAGCTTCTAA
TTGTCTCTACTTTGTGAGACTACTTTTGAATGCTTGACCTCAAATCAGGTAGGACTACCC
GCTGAACTTAA
>AB12-44
TTTCCGTAGGTGAACCTGCGGAAGGATCATTATTGAATTATGTTTCTAGATAGGTTGTAG
CTGGCTC-TTTAGAGCATGTGCACGCCTGTTTGGACTTCATTTTCATCCACCTGTGCACC
TATTGTAGTCTTTGGTTGGGTTAGGGGGAAGTGGTCATTGTGTCAGCATCTGCTGGATGT
GAGGACTTGCATTGTGAAAGCTTTGCTGTCCTTGATGTGATCATGGAATCTCTTTCTCAC
TAGAGTCTATGTCACTCATTATACTCTGTCGAATGTCATTGAATGTCTTTACATGGGCTT
GTATGCCTATGAAAATTGTAATACAACTTTCAGCAACGGATCTCTTGGCTCTCGCATCGA
TGAAGAACGCAGCGAAATGCGATAAGTAATGTGAATTGCAGAATTCAGTGAATCATCGAA
TCTTTGAACGCATCTTGCGCTCCTTGGTATTCCGAGGAGCATGCCTGTTTGAGTGTCATT
AAATTCTCAACTCTCTTATACTTTTTTGTAAAAGAGAGCTTGGACTGTGGAGGCTTGCTG
GCCACTTTTTGGGGTCAGCTCCTCTGAAATGCATTAGCGGAACCGTTTGCGATCTGCCAC
AAGTGTGATAAGTTATCTACACTGGCGAGGGGATTGCTCTCTGTAATGTTCAGCTTCTAA
TTGTCTCTACTTTGTGAGACTACTTTTGAATGCTTGACCTCAAATCAGGTAGGACTACCC
GCTGAACTTAA
>AB11-38
TTTCCGTAGGTGAACCTGCGGAAGGATCATTATTGAATTATGTTTCTAGATAGGTTGTAG
CTGGCTC-TTTAGAGCATGTGCACGCCTGTTTGGACTTCATTTTCATCCACCTGTGCACC
TATTGTAGTCTTTGGTTGGGTTAGGAGGAAGTGGTCATTGTGTCAGCATCTGCTGGATGT
GAGGACTTGCATTGTGAAAGCTTTGCTGTCCTTGATGTGATCATGGAATCTCTTTCTCAC
TAGAGTCTATGTCACTCATTATACTCTGTCGAATGTCATTGAATGTCTTTACATGGGCTT
GTATGCCTATGAAAATTGTAATACAACTTTCAGCAACGGATCTCTTGGCTCTCGCATCGA
TGAAGAACGCAGCGAAATGCGATAAGTAATGTGAATTGCAGAATTCAGTGAATCATCGAA
TCTTTGAACGCATCTTGCGCTCCTTGGTATTCCGAGGAGCATGCCTGTTTGAGTGTCATT
AAATTCTCAACTCTCTTATACTTTTTTGTAAAAGAGAGCTTGGACTGTGGAGGCTTGCTG

GCCACTTTTTGGGGTCAGCTCCTCTGAAATGCATTAGCGGAACCGTTTGCGATCTGCCAC
AAGTGTGATAAGTTATCTACACTGGCGAGGGGATTGCTCTCTGTAATGTTCAGCTTCTAA
TTGTCTCTACTTTGTGAGACTACTTTTGAATGCTTGACCTCAAATCAGGTAGGACTACCC
GCTGAACTTAA
>AB2-28
TTTCCGTAGGTGAACCTGCGGAAGGATCATTATTGAATTATGTTTCTAGATAGGTTGTAG
CTGGCTC-TTTAGAGCATGTGCACGCCTGTTTGGACTTCATTTTCATCCACCTGTGCACC
TATTGTAGTCTTTGGTTGGGTTAGGGGGAAGTGGTCATTGTGTCAGCATCTGCTGGATGT
GAGGACTTGCATTGTGAAAGCTTTGCTGTCCTTGATGTGATCATGGAATCTCTTTCTCAC
TAGAGTCTATGTCACTCATTATACTCTGTCGAATGTCATTGAATGTCTTTACATGGGCTT
GTATGCCTATGAAAATTGTAATACAACTTTCAGCAACGGATCTCTTGGCTCTCGCATCGA
TGAAGGACGCAGCGAAATGCGATAAGTAATGTGAATTGTAGAATTCAGTGAATCATCGAA
TCTTTGAACGCATCTTGCGCTCCTTGGTATTCCGAGGAGCATGCCTGTTTGAGTGTCATT
AAATTCTCAACTCTCTTATACTTTTTTGTAAAAGAGAGCTTGGACTGTGGAGGCTTGCTG
GCCACTTTTTGGGGTCAGCTCCTCTGAAATGCATTAGCGGAACCGTTTGCGATCTGCCAC
AAGTGTGATAAGTTATCTACACTGGCGAGGGGATTGCTCTCTGTAATGTTCAGCTTCTAA
TTGTCTCTACTTTGTGAGACTACTTTTGAATGCTTGACCTCAAATCAGGTAGGACTACCC
GCTGAACTTAA
>AB5-5
TTTCCGTAGGTGAACCTGCGGAAGGATCATTATTGAATTATGTTTCTAGATAGGTTGTAG
CTGGCTC-TTTAGAGCATGTGCACGCCTGTTTGGACTTCATTTTCATCCACCTGTGCACC
TATTGTAGTCTTTGGTTGGGTTAGGGGGAAGTGGTCATTGTGTCAGCATCTGCTGGATGT
GAGGACTTGCATTGTGAAAGCTTTGCTGTCCTTGATGTGATCATGGAATCTCTTTCTCAC
TAGAGTCTATGTCACTCATTATACTCTGTCGAATGTCATTGAATGTCTTTACATGGGCTT
GTATGCCTATGAAAATTGTAATACAACTTTCAGCAACGGATCTCTTGGCTCTCGCATCGA
TGAAGAACGCAGCGAAATGCGATAAGTAATGTGAATTGCAGAATTCAGTGAATCATCGAA
TCTTTGAACGCATCTTGCGCTCCTTGGTATTCCGAGGAGCATGCCTGTTTGAGTGTCATT
AAATTCTCAACTCTCTTATACTTTTTTGTAAAAGAGAGCTTGGACTGTGGAGGCTTGCTG
GCCACTTTTTGGGGTCAGCTCCTCTGAAATGCATTAGCGGAACCGTTTGCAATCTGCCAC
AAGTGTGATAAGTTATCTACACTGGCGAGGGGATTGCTCTCTGTAATGTTCAGCTTCTAA
TTGTCTCTACTTTGTGAGACTACTTTTGAATGCTTGACCTCAAATCAGGTAGGACTACCC
GCTGAACTTAA
>AB8-41
TTTCCGTAGGTGAACCTGCGGAAGGATCATTATTGAATTATGTTTCTAGATAGGTTGTAG
CTGGCTC-TTTAGAGCATGTGCACGCCTGTTTGGACTTCATTTTCATCCACCTGTGCACC
TATTGTAGTCTTTGGTTGGGTTAGGGGGAAGTGGTCATTGTGTCAGCATCTGCTGGATGT
GAGGACTTGCATTGTGAAAGCTTTGCTGTCCTTGATGTGATCATGGAATCTCTTTCTCAC
TAGAGTCTATGTCACTCATTATACTCTGTCGAATGTCATTGAATGTCTTTACATGGGCTT
GTATGCCTATGAAAATTGTAATACAACTTTCAGCAACGGATCTCTTGGCTCTCGCATCGA
TGAAGAACGCAGCGAAATGCGATAAGTAATGTGAATTGCAGAATTCAGTGAATCATCGAA
TCTTTGAACGCATCTTGCGCTCCTTGGTATTCCGAGGAGCATGCCTGTTTGAGTGTCATT
AAATTCTCAACTCTCTTATACTTTTTTGTAAAAGAGAGCTTGGACTGTGGAGGCTTGCTG
GCCACTTTTTGGGGTCAGCTCCTCTGAAATGCATTAGCGGAACCGTTTGCAATCTGCCAC
AAGTGTGATAAGTTATCTACACTGGCGAGGGGATTGCTCTCTGTAATGTTCAGCTTCTAA
TTGTCTCTACTTTGTGAGACTACTTTTGAATGCTTGACCTCAAATCAGGTAGGACTACCC
GCTGAACTTAA
>AB9-1
TTTCCGTAGGTGAACCTGCGGAAGGATCATTATTGAATTATGTTTCTAGATAGGTTGTAG
CTGGCTC-TTTAGAGCATGTGCACGCCTGTTTGGACTTCATTTTCATCCACCTGTGCACC
TATTGTAGTCTTTGGTTGGGTTAGGGGGAAGTGGTCATTGTGTCAGCATCTGCTGGATGT

GAGGACTTGCATTGTGAAAGCTTTGCTGTCCTTGATGTGATCATGGAATCTCTTTCTCAC
TAGAGTCTATGTCACTCATTATACTCTGTCGAATGTCATTGAATGTCTTTACATGGGCTT
GTATGCCTATGAAAATTGTAATACAACTTTCAGCAACGGATCTCTTGGCTCTCGCATCGA
TGAAGAACGCAGCGAAATGCGATAAGTAATGTGAATTGCAGAATTCAGTGAATCATCGAA
TCTTTGAACGCATCTTGCGCTCCTTGGTATTCCGAGGAGCATGCCTGTTTGAGTGTCATT
AAATTCTCAACTCTCTTATACTTTTTTGTAAAAGAGAGCTTGGACTGTGGAGGCTTGCTG
GCCACTTTTTGGGGTCAGCTCCTCTGAAATGCATTAGCGGAACCGTTTGCAATCTGCCAC
AAGTGTGATAAGTTATCTACACTGGCGAGGGGATTGCTCTCTGTAATGTTCAGCTTCTAA
TTGTCTCTACTTTGTGAGACTACTTTTGAATGCTTGACCTCAAATCAGGTAGGACTACCC
GCTGAACTTAA
>AB12-47
TTTCCGTAGGTGAACCTGCGGAAGGATCATTATTGAATTATGTTTCTAGATAGGTTGTAG
CTGGCTC-TTTAGAGCATGTGCACGCCTGTTTGGACTTCATTTTCATCCACCTGTGCACC
TATTGTAGTCTTTGGTTGGGTTAGGGGGAAGTGGTCATTGTGTCAGCATCTGCTGGATGT
GAGGACTTGCATTGTGAAAGCTTTGCTGTCCTTGATGTGATCATGGAATCTCTTTCTCAC
TAGAGTCTATGTCACTCATTATACTCTGTCGAATGTCATTGAATGTCTTTACATGGGCTT
GTATGCCTATGAAAATTGTAATACAACTTTCAGCAACGGATCTCTTGGCTCTCGCATCGA
TGAAGAACGCAGCGAAATGCGATAAGTAATGTGAATTGCAGAATTCAGTGAATCATCGAA
TCTTTGAACGCATCTTGCGCTCCTTGGTATTCCGAGGAGCATGCCTGTTTGAGTGTCATT
AAATTCTCAACTCTCTTATACTTTTTTGTAAAAGAGAGCTTGGACTGTGGAGGCTTGCTG
GCCACTTTTTGGGGTCAGCTCCTCTGAAATGCATTAGCGGAACCGTTTGCAATCTGCCAC
AAGTGTGATAAGTTATCTACACTGGCGAGGGGATTGCTCTCTGTAATGTTCAGCTTCTAA
TTGTCTCTACTTTGTGAGACTACTTTTGAATGCTTGACCTCAAATCAGGTAGGACTACCC
GCTGAACTTAA
>AB1-22
TTTCCGTAGGTGAACCTGCGGAAGGATCATTATTGAATTATGTTTCTAGATAGGTTGTAG
CTGGCTC-TTTAGAGCATGTGCACGCCTGTTTGGACTTCATTTTCATCCACCTGTGCACC
TATTGTAGTCTTTGGTTGGGTTAGGGGGAAGTGGTCATTGTGTCAGCATCTGCTGGATGT
GAGGACTTGCATTGTGAAAGCTTTGCTGTCCTTGATGTGATCATGGAATCTCTTTCTCAC
TAGAGTCTATGTCACTCATTATACTCTGTCGAATGTCATTGAATGTCTTTACATGGGCTT
GTATGCCTATGAAAATTGTAATACAACTTTCAGCAACGGATCTCTTGGCTCTCGCATCGA
TGAAGGACGCAGCGAAATGCGATAAGTAATGTGAATTGCAGAATTCAGTGAATCATCGAA
TCTTTGAACGCATCTTGCGCTCCTTGGTATTCCGAGGAGCATGCCTGTTTGAGTGTCATT
AAATTCTCAACTCTCTTATACTTTTTTGTAAAAGAGAGCTTGGACTGTGGAGGCTTGCTG
GCCACTTTTTGGGGTCAGCTCCTCTGAAATGCATTAGCGGAACCGTTTGCAATCTGCCAC
AAGTGTGATAAGTTATCTACACTGGCGAGGGGATTGCTCTCTGTAATGTTCAGCTTCTAA
TTGTCTCTACTTTGTGAGACTACTTTTGAATGCTTGACCTCAAATCAGGTAGGACTACCC
GCTGAACTTAA
>AB4-17
TTTCCGTAGGTGAACCTGCGGAAGGATCATTATTGAATTATGTTTCTAGATAGGTTGTAG
CTGGCTC-TTTAGAGCATGTGCACGCCTGTTTGGACTTCATTTTCATCCACCTGTGCACC
TATTGTAGTCTTTGGTTGGGTTAGGGGGAAGTGGTCATTGTGTCAGCATCTGCTGGATGT
GAGGACTTGCATTGTGAAAGCTTTGCTGTCCTTGATGTGATCATGGAATCTCTTTCTCAC
TAGAGTCTATGTCACTCATTATACTCTGTCGAATGTCATTGAATGTCTTTACATGGGCTT
GTATGCCTATGAAAATTGTAATACAACTTTCAGCAACGGATCTCTTGGCTCTCGCATCGA
TGAAGGACGCAGCGAAATGCGATAAGTAATGTGAATTGCAGAATTCAGTGAATCATCGAA
TCTTTGAACGCATCTTGCGCTCCTTGGTATTCCGAGGAGCATGCCTGTTTGAGTGTCATT
AAATTCTCAACTCTCTTATACTTTTTTGTAAAAGAGAGCTTGGACTGTGGAGGCTTGCTG
GCCACTTTTTGGGGTCAGCTCCTCTGAAATGCATTAGCGGAACCGTTTGCAATCTGCCAC
AAGTGTGATAAGTTATCTACACTGGCGAGGGGATTGCTCTCTGTAATGTTCAGCTTCTAA

TTGTCTCTACTTTGTGAGACTACTTTTGAATGCTTGACCTCAAATCAGGTAGGACTACCC
GCTGAACTTAA
>AB5-7
TTTCCGTAGGTGAACCTGCGGAAGGATCATTATTGAATTATGTTTCTAGATAGGTTGTAG
CTGGCTC-TTTAGAGCATGTGCACGCCTGTTTGGACTTCATTTTCATCCACCTGTGCACC
TATTGTAGTCTTTGGTTGGGTTAGGGGGAAGTGGTCATTGTGTCAGCATCTGCTGGATGT
GAGGACTTGCATTGTGAAAGCTTTGCTGTCCTTGATGTGATCATGGAATCTCTTTCTCAC
TAGAGTCTATGTCACTCATTATACTCTGTCGAATGTCATTGAATGTCTTTACATGGGCTT
GTATGCCTATGAAAATTGTAATACAACTTTCAGCAACGGATCTCTTGGCTCTCGCATCGA
TGAAGGACGCAGCGAAATGCGATAAGTAATGTGAATTGCAGAATTCAGTGAATCATCGAA
TCTTTGAACGCATCTTGCGCTCCTTGGTATTCCGAGGAGCATGCCTGTTTGAGTGTCATT
AAATTCTCAACTCTCTTATACTTTTTTGTAAAAGAGAGCTTGGACTGTGGAGGCTTGCTG
GCCACTTTTTGGGGTCAGCTCCTCTGAAATGCATTAGCGGAACCGTTTGCAATCTGCCAC
AAGTGTGATAAGTTATCTACACTGGCGAGGGGATTGCTCTCTGTAATGTTCAGCTTCTAA
TTGTCTCTACTTTGTGAGACTACTTTTGAATGCTTGACCTCAAATCAGGTAGGACTACCC
GCTGAACTTAA
>AB5-55
TTTCCGTAGGTGAACCTGCGGAAGGATCATTATTGAATTATGTTTCTAGATAGGTTGTAG
CTGGCTC-TTTAGAGCATGTGCACGCCTGTTTGGACTTCATTTTCATCCACCTGTGCACC
TATTGTAGTCTTTGGTTGGGTTAGGGGGAAGTGGTCATTGTGTCAGCATCTGCTGGATGT
GAGGACTTGCATTGTGAAAGCTTTGCTGTCCTTGATGTGATCATGGAATCTCTTTCTCAC
TAGAGTCTATGTCACTCATTATACTCTGTCGAATGTCATTGAATGTCTTTACATGGGCTT
GTATGCCTATGAAAATTGTAATACAACTTTCAGCAACGGATCTCTTGGCTCTCGCATCGA
TGAAGGACGCAGCGAAATGCGATAAGTAATGTGAATTGCAGAATTCAGTGAATCATCGAA
TCTTTGAACGCATCTTGCGCTCCTTGGTATTCCGAGGAGCATGCCTGTTTGAGTGTCATT
AAATTCTCAACTCTCTTATACTTTTTTGTAAAAGAGAGCTTGGACTGTGGAGGCTTGCTG
GCCACTTTTTGGGGTCAGCTCCTCTGAAATGCATTAGCGGAACCGTTTGCAATCTGCCAC
AAGTGTGATAAGTTATCTACACTGGCGAGGGGATTGCTCTCTGTAATGTTCAGCTTCTAA
TTGTCTCTACTTTGTGAGACTACTTTTGAATGCTTGACCTCAAATCAGGTAGGACTACCC
GCTGAACTTAA
>AB7-35
TTTCCGTAGGTGAACCTGCGGAAGGATCATTATTGAATTATGTTTCTAGATAGGTTGTAG
CTGGCTC-TTTAGAGCATGTGCACGCCTGTTTGGACTTCATTTTCATCCACCTGTGCACC
TATTGTAGTCTTTGGTTGGGTTAGGGGGAAGTGGTCATTGTGTCAGCATCTGCTGGATGT
GAGGACTTGCATTGTGAAAGCTTTGCTGTCCTTGATGTGATCATGGAATCTCTTTCTCAC
TAGAGTCTATGTCACTCATTATACTCTGTCGAATGTCATTGAATGTCTTTACATGGGCTT
GTATGCCTATGAAAATTGTAATACAACTTTCAGCAACGGATCTCTTGGCTCTCGCATCGA
TGAAGGACGCAGCGAAATGCGATAAGTAATGTGAATTGCAGAATTCAGTGAATCATCGAA
TCTTTGAACGCATCTTGCGCTCCTTGGTATTCCGAGGAGCATGCCTGTTTGAGTGTCATT
AAATTCTCAACTCTCTTATACTTTTTTGTAAAAGAGAGCTTGGACTGTGGAGGCTTGCTG
GCCACTTTTTGGGGTCAGCTCCTCTGAAATGCATTAGCGGAACCGTTTGCAATCTGCCAC
AAGTGTGATAAGTTATCTACACTGGCGAGGGGATTGCTCTCTGTAATGTTCAGCTTCTAA
TTGTCTCTACTTTGTGAGACTACTTTTGAATGCTTGACCTCAAATCAGGTAGGACTACCC
GCTGAACTTAA
>AB9-41
TTTCCGTAGGTGAACCTGCGGAAGGATCATTATTGAATTATGTTTCTAGATAGGTTGTAG
CTGGCTC-TTTAGAGCATGTGCACGCCTGTTTGGACTTCATTTTCATCCACCTGTGCACC
TATTGTAGTCTTTGGTTGGGTTAGGGGGAAGTGGTCATTGTGTCAGCATCTGCTGGATGT
GAGGACTTGCATTGTGAAAGCTTTGCTGTCCTTGATGTGATCATGGAATCTCTTTCTCAC
TAGAGTCTATGTCACTCATTATACTCTGTCGAATGTCATTGAATGTCTTTACATGGGCTT

GTATGCCTATGAAAATTGTAATACAACTTTCAGCAACGGATCTCTTGGCTCTCGCATCGA
TGAAGGACGCAGCGAAATGCGATAAGTAATGTGAATTGCAGAATTCAGTGAATCATCGAA
TCTTTGAACGCATCTTGCGCTCCTTGGTATTCCGAGGAGCATGCCTGTTTGAGTGTCATT
AAATTCTCAACTCTCTTATACTTTTTTGTAAAAGAGAGCTTGGACTGTGGAGGCTTGCTG
GCCACTTTTTGGGGTCAGCTCCTCTGAAATGCATTAGCGGAACCGTTTGCAATCTGCCAC
AAGTGTGATAAGTTATCTACACTGGCGAGGGGATTGCTCTCTGTAATGTTCAGCTTCTAA
TTGTCTCTACTTTGTGAGACTACTTTTGAATGCTTGACCTCAAATCAGGTAGGACTACCC
GCTGAACTTAA
>AB10-46
TTTCCGTAGGTGAACCTGCGGAAGGATCATTATTGAATTATGTTTCTAGATAGGTTGTAG
CTGGCTC-TTTAGAGCATGTGCACGCCTGTTTGGACTTCATTTTCATCCACCTGTGCACC
TATTGTAGTCTTTGGTTGGGTTAGGGGGAAGTGGTCATTGTGTCAGCATCTGCTGGATGT
GAGGACTTGCATTGTGAAAGCTTTGCTGTCCTTGATGTGATCATGGAATCTCTTTCTCAC
TAGAGTCTATGTCACTCATTATACTCTGTCGAATGTCATTGAATGTCTTTACATGGGCTT
GTATGCCTATGAAAATTGTAATACAACTTTCAGCAACGGATCTCTTGGCTCTCGCATCGA
TGAAGGACGCAGCGAAATGCGATAAGTAATGTGAATTGCAGAATTCAGTGAATCATCGAA
TCTTTGAACGCATCTTGCGCTCCTTGGTATTCCGAGGAGCATGCCTGTTTGAGTGTCATT
AAATTCTCAACTCTCTTATACTTTTTTGTAAAAGAGAGCTTGGACTGTGGAGGCTTGCTG
GCCACTTTTTGGGGTCAGCTCCTCTGAAATGCATTAGCGGAACCGTTTGCAATCTGCCAC
AAGTGTGATAAGTTATCTACACTGGCGAGGGGATTGCTCTCTGTAATGTTCAGCTTCTAA
TTGTCTCTACTTTGTGAGACTACTTTTGAATGCTTGACCTCAAATCAGGTAGGACTACCC
GCTGAACTTAA
>AB10-48
TTTCCGTAGGTGAACCTGCGGAAGGATCATTATTGAATTATGTTTCTAGATAGGTTGTAG
CTGGCTC-TTTAGAGCATGTGCACGCCTGTTTGGACTTCATTTTCATCCACCTGTGCACC
TATTGTAGTCTTTGGTTGGGTTAGGGGGAAGTGGTCATTGTGTCAGCATCTGCTGGATGT
GAGGACTTGCATTGTGAAAGCTTTGCTGTCCTTGATGTGATCATGGAATCTCTTTCTCAC
TAGAGTCTATGTCACTCATTATACTCTGTCGAATGTCATTGAATGTCTTTACATGGGCTT
GTATGCCTATGAAAATTGTAATACAACTTTCAGCAACGGATCTCTTGGCTCTCGCATCGA
TGAAGGACGCAGCGAAATGCGATAAGTAATGTGAATTGCAGAATTCAGTGAATCATCGAA
TCTTTGAACGCATCTTGCGCTCCTTGGTATTCCGAGGAGCATGCCTGTTTGAGTGTCATT
AAATTCTCAACTCTCTTATACTTTTTTGTAAAAGAGAGCTTGGACTGTGGAGGCTTGCTG
GCCACTTTTTGGGGTCAGCTCCTCTGAAATGCATTAGCGGAACCGTTTGCAATCTGCCAC
AAGTGTGATAAGTTATCTACACTGGCGAGGGGATTGCTCTCTGTAATGTTCAGCTTCTAA
TTGTCTCTACTTTGTGAGACTACTTTTGAATGCTTGACCTCAAATCAGGTAGGACTACCC
GCTGAACTTAA
>AB10-50
TTTCCGTAGGTGAACCTGCGGAAGGATCATTATTGAATTATGTTTCTAGATAGGTTGTAG
CTGGCTC-TTTAGAGCATGTGCACGCCTGTTTGGACTTCATTTTCATCCACCTGTGCACC
TATTGTAGTCTTTGGTTGGGTTAGGGGGAAGTGGTCATTGTGTCAGCATCTGCTGGATGT
GAGGACTTGCATTGTGAAAGCTTTGCTGTCCTTGATGTGATCATGGAATCTCTTTCTCAC
TAGAGTCTATGTCACTCATTATACTCTGTCGAATGTCATTGAATGTCTTTACATGGGCTT
GTATGCCTATGAAAATTGTAATACAACTTTCAGCAACGGATCTCTTGGCTCTCGCATCGA
TGAAGGACGCAGCGAAATGCGATAAGTAATGTGAATTGCAGAATTCAGTGAATCATCGAA
TCTTTGAACGCATCTTGCGCTCCTTGGTATTCCGAGGAGCATGCCTGTTTGAGTGTCATT
AAATTCTCAACTCTCTTATACTTTTTTGTAAAAGAGAGCTTGGACTGTGGAGGCTTGCTG
GCCACTTTTTGGGGTCAGCTCCTCTGAAATGCATTAGCGGAACCGTTTGCAATCTGCCAC
AAGTGTGATAAGTTATCTACACTGGCGAGGGGATTGCTCTCTGTAATGTTCAGCTTCTAA
TTGTCTCTACTTTGTGAGACTACTTTTGAATGCTTGACCTCAAATCAGGTAGGACTACCC
GCTGAACTTAA

>AB12-23
TTTCCGTAGGTGAACCTGCGGAAGGATCATTATTGAATTATGTTTCTAGATAGGTTGTAG
CTGGCTC-TTTAGAGCATGTGCACGCCTGTTTGGACTTCATTTTCATCCACCTGTGCACC
TATTGTAGTCTTTGGTTGGGTTAGGGGGAAGTGGTCATTGTGTCAGCATCTGCTGGATGT
GAGGACTTGCATTGTGAAAGCTTTGCTGTCCTTGATGTGATCATGGAATCTCTTTCTCAC
TAGAGTCTATGTCACTCATTATACTCTGTCGAATGTCATTGAATGTCTTTACATGGGCTT
GTATGCCTATGAAAATTGTAATACAACTTTCAGCAACGGATCTCTTGGCTCTCGCATCGA
TGAAGGACGCAGCGAAATGCGATAAGTAATGTGAATTGCAGAATTCAGTGAATCATCGAA
TCTTTGAACGCATCTTGCGCTCCTTGGTATTCCGAGGAGCATGCCTGTTTGAGTGTCATT
AAATTCTCAACTCTCTTATACTTTTTTGTAAAAGAGAGCTTGGACTGTGGAGGCTTGCTG
GCCACTTTTTGGGGTCAGCTCCTCTGAAATGCATTAGCGGAACCGTTTGCAATCTGCCAC
AAGTGTGATAAGTTATCTACACTGGCGAGGGGATTGCTCTCTGTAATGTTCAGCTTCTAA
TTGTCTCTACTTTGTGAGACTACTTTTGAATGCTTGACCTCAAATCAGGTAGGACTACCC
GCTGAACTTAA
>AB12-28
TTTCCGTAGGTGAACCTGCGGAAGGATCATTATTGAATTATGTTTCTAGATAGGTTGTAG
CTGGCTC-TTTAGAGCATGTGCACGCCTGTTTGGACTTCATTTTCATCCACCTGTGCACC
TATTGTAGTCTTTGGTTGGGTTAGGGGGAAGTGGTCATTGTGTCAGCATCTGCTGGATGT
GAGGACTTGCATTGTGAAAGCTTTGCTGTCCTTGATGTGATCATGGAATCTCTTTCTCAC
TAGAGTCTATGTCACTCATTATACTCTGTCGAATGTCATTGAATGTCTTTACATGGGCTT
GTATGCCTATGAAAATTGTAATACAACTTTCAGCAACGGATCTCTTGGCTCTCGCATCGA
TGAAGGACGCAGCGAAATGCGATAAGTAATGTGAATTGCAGAATTCAGTGAATCATCGAA
TCTTTGAACGCATCTTGCGCTCCTTGGTATTCCGAGGAGCATGCCTGTTTGAGTGTCATT
AAATTCTCAACTCTCTTATACTTTTTTGTAAAAGAGAGCTTGGACTGTGGAGGCTTGCTG
GCCACTTTTTGGGGTCAGCTCCTCTGAAATGCATTAGCGGAACCGTTTGCAATCTGCCAC
AAGTGTGATAAGTTATCTACACTGGCGAGGGGATTGCTCTCTGTAATGTTCAGCTTCTAA
TTGTCTCTACTTTGTGAGACTACTTTTGAATGCTTGACCTCAAATCAGGTAGGACTACCC
GCTGAACTTAA
>AB9-42
TTTCCGTAGGTGAACCTGCGGAAGGATCATTATTGAATTATGTTTCTAGATAGGTTGTAG
CTGGCTC-TTTAGAGCATGTGCACGCCTGTTTGGACTTCATTTTCATCCACCTGTGCACC
TATTGTAGTCTTTGGTTGGGTTAGGGGGAAGTGGTCATTGTGTCAGCATCTGCTGGATGT
GAGGACTTGCATTGTGAAAGCTTTGCTGTCCTTGATGTGATCATGGAATCTCTTTCTCAC
TAGAGTCTATGTCACTCATTATACTCTGTCGAATGTCATTGAATGTCTTTACATGGGCTT
GTATGCCTATGAAAATTGTAATACAACTTTCAGCAACGGATCTCTTGGCTCTCGCATCGA
TGAAGGACGCAGCGAAATGCGATAAGTAATGTGAATTGCAGAATTCAGTGAATCATCGAA
TCTTTGAACGCATCTTGCGCTCCTTGGTATTCCGAGGAGCATGCCTGTTTGAGTGTCATT
AAATTCTCAACTCTCTTATACTTTTTTGTAAAAGAGAGCTTGGACTGTGGAGGCTTGCTG
GCCACTTTTTGGGGTCAGCTCCTCTGAAATGCATTAGCGGAACCGTTTGCAATCTGCCAC
AAGTGTGATAAGTTATCTACACTGGCGAGGGGATTGCTCTCTGTAATGTTCAGTTTCTAA
TTGTCTCTACTTTGTGAGACTACTTTTGAATGCTTGACCTCAAATCAGGTAGGACTACCC
GCTGAACTTAA
>AB6-21
TTTCCGTAGGTGAACCTGCGGAAGGATCATTATTGAATTATGTTTCTAGATAGGTTGTAG
CTGGCTC-TTTAGAGCATGTGCACGCCTGTTTGGACTTCATTTTCATCCACCTGTGCACC
TATTGTAGTCTTTGGTTGGGTTAGGAGGAAGTGATCATTGTATCAGCATCTGCTGGGAGT
GAGGACTTGCATTGTGAAAGCTTTGCTGTCCTTGATGTGATCATGGAATCTCTTTCTCAC
TAGAGTCTATGTCACTCATTATACTCTGTCGAATGTCATTGAATGTCTTTACATGGGCTT
GTATGCCTATGAAAATTGTAATACAACTTTCAGCAACGGATCTCTTGGCTCTCGCATCGA
TGAAGAACGCAGCGAAATGCGATAAGTAATGTGAATTGCAGAATTCAGTGAATCATCGAA

TCTTTGAACGCATCTTGCGCTCCTTGGTATTCCGAGGAGCATGCCTGTTTGAGTGTCATT
AAATTCTCAACTCTCTTATACTTTTTTGTAAAAGAGAGCTTGGACTGTGGAGGCTTGCTG
GCCACTTTTTGGGGTCAGCTCCTCTGAAATGCATTAGCGGAACCGTTTGCAATCTGCCAC
AAGTGTGATAAGTTATCTACACTGGCGAGGGGATTGCTCTCTGTAATGTTCAGCTTCTAA
TTGTCTCTACTTTGTGAGACAACTTTTGAATGCTTGACCTCAAATCAGGTAGGACTACCC
GCTGAACTTAA
>AB6-4
TTTCCGTAGGTGAACCTGCGGAAGGATCATTATTGAATTATGTTTCTAGATAGGTTGTAG
CTGGCTC-TTTAGAGCATGTGCACGCCTGTTTGGACTTCATTTTCATCCACCTGTGCACC
TATTGTAGTCTTTGGTTGGGTTAGGAGGAAGTGATCATTGTATCAGCATCTGCTGGGAGT
GAGGACTTGCATTGTGAAAGCTTTGCTGTCCTTGATGTGATCATGGAATCTTTTTCTCAC
TAGAGTCTATGTCACTCATTATACTCTGTCGAATGTCATTGAATGTCTTTACATGGGCTT
GTATGCCTATGAAAATTGTAATACAACTTTCAGCAACGGATCTCTTGGCTCTCGCATCGA
TGAAGAACGCAGCGAAATGCGATAAGTAATGTGAATTGCAGAATTCAGTGAATCATCGAA
TCTTTGAACGCATCTTGCGCTCCTTGGTATTCCGAGGAGCATGCCTGTTTGAGTGTCATT
AAATTCTCAACTCTCTTATACTTTTTTGTAAAAGAGAGCTTGGACTGTGGAGGCTTGCTG
GCCACTTTTTGGGGTCAGCTCCTCTGAAATGCATTAGCGGAACCGTTTGCAATCTGCCAC
AAGTGTGATAAGTTATCTACACTGGCGAGGGGATTGCTCTCTGTAATGTTCAGCTTCTAA
TTGTCTCTACTTTGTGAGACAACTTTTGAATGCTTGACCTCAAATCAGGTAGGACTACCC
GCTGAACTTAA
>AB10-26
TTTCCGTAGGTGAACCTGCGGAAGGATCATTATTGAATTATGTTTCTAGATAGGTTGTAG
CTGGCTC-TTTAGAGCATGTGCACGCCTGTTTGGACTTCATTTTCATCCACCTGTGCACC
TATTGTAGTCTTTGGTTGGGTTAGGAGGAAGTGATCATTGTATCAGCATCTGCTGGGAGT
GAGGACTTGCATTGTGAAAGCTTTGCTGTCCTTGATGTGATCATGGAATCTTTTTCTCAC
TAGAGTCTATGTCACTCATTATACTCTGTCGAATGTCATTGAATGTCTTTACATGGGCTT
GTATGCCTATGAAAATTGTAATACAACTTTCAGCAACGGATCTCTTGGCTCTCGCATCGA
TGAAGAACGCAGCGAAATGCGATAAGTAATGTGAATTGCAGAATTCAGTGAATCATCGAA
TCTTTGAACGCATCTTGCGCTCCTTGGTATTCCGAGGAGCATGCCTGTTTGAGTGTCATT
AAATTCTCAACTCTCTTATACTTTTTTGTAAAAGAGAGCTTGGACTGTGGAGGCTTGCTG
GCCACTTTTTGGGGTCAGCTCCTCTGAAATGCATTAGCGGAACCGTTTGCAATCTGCCAC
AAGTGTGATAAGTTATCTACACTGGCGAGGGGATTGCTCTCTGTAATGTTCAGCTTCTAA
TTGTCTCTACTTTGTGAGACAACTTTTGAATGCTTGACCTCAAATCAGGTAGGACTACCC
GCTGAACTTAA
>AB9-15
TTTCCGTAGGTGAACCTGCGGAAGGATCATTATTGAATTATGTTTCTAGATAGGTTGTAG
CTGGCTC-TTTAGAGCATGTGCACGCCTGTTTGGACTTCATTTTCATCCACCTGTGCACC
TATTGTAGTCTTTGGTTGGGTTAGGAGGAAGTGATCATTGTATCAGCATCTGCTGGGAGT
GAGGACTTGCATTGTGAAAGCTTTGCTGTCCTTGATGTGATCATGGAATCTTTTTCTCAC
TAGAGTCTATGTCACTCATTATACTCTGTCGAATGTCATTGAATGTCTTTACATGGGCTT
GTATGCCTATGAAAATTGTAATACAACTTTCAGCAACGGATCTCTTGGCTCTCGCATCGA
TGAAGAACGCAGCGAAATGCGATAAGTAATGTGAATTGCAGAATTCAGTGAATCATCGAA
TCTTTGAACGCATCTTGCGCTCCTTGGTATTCCGAGGAGCATGCCTGTTTGAGTGTCATT
AAATTCTCAACTCTCTTATACTTTTTTGTAAAAGAGAGCTTGGACTGTGGAGGCTTGCTG
GCCACTTTTTGGGGTCAGCTCCTCTGAAATGCATTAGCGGAACCGTTTGCGATCTGCCAC
AAGTGTGATAAGTTATCTACACTGGCGAGGGGATTGCTCTCTGTAATGTTCAGCTTCTAA
TTGTCTCTACTTTGTGAGACAACTTTTGAATGCTTGACCTCAAATCAGGTAGGACTACCC
GCTGAACTTAA
>AB10-44
TTTCCGTAGGTGAACCTGCGGAAGGATCATTATTGAATTATGTTTCTAGATAGGTTGTAG

CTGGCTC-TTTAGAGCATGTGCACGCCTGTTTGGACTTCATTTTCATCCACCTGTGCACC
TATTGTAGTCTTTGGTTGGGTTAGGAGGAAGTGATCATTGTATCAGCATCTGCTGGGAGT
GAGGACTTGCATTGTGAAAGCTTTGCTGTCCTTGATGTGATCATGGAATCTTTTTCTCAC
TAGAGTCTATGTCACTCATTATACTCTGTCGAATGTCATTGAATGTCTTTACATGGGCTT
GTATGCCTATGAAAATTGTAATACAACTTTCAGCAACGGATCTCTTGGCTCTCGCATCGA
TGAAGGACGCAGCGAAATGCGATAAGTAATGTGAATTGCAGAATTCAGTGAATCATCGAA
TCTTTGAACGCATCTTGCGCTCCTTGGTATTCCGAGGAGCATGCCTGTTTGAGTGTCATT
AAATTCTCAACTCTCTTATACTTTTTTGTAAAAGAGAGCTTGGACTGTGGAGGCTTGCTG
GCCACTTTTTGGGGTCAGCTCCTCTGAAATGCATTAGCGGAACCGTTTGCAATCTGCCAC
AAGTGTGATAAGTTATCTACACTGGCGAGGGGATTGCTCTCTGTAATGTTCAGCTTCTAA
TTGTCTCTACTTTGTGAGACAACTTTTGAATGCTTGACCTCAAATCAGGTAGGACTACCC
GCTGAACTTAA
>AB8-40
TTTCCGTAGGTGAACCTGCGGAAGGATCATTATTGAATTATGTTTCTAGATAGGTTGTAG
CTGGCTC-TTTAGAGCATGTGCACGCCTGTTTGGACTTCATTTTCATCCACCTGTGCACC
TATTGTAGTCTTTGGTTGGGTTAGGAGGAAGTGATCATTGTATCAGCATCTGCTGGGAGT
GAGGACTTGCATTGTGAAAGCTTTGCTGTCCTTGATGTGATCATGGAATCTTTTTCTCAC
TAGAGTCTATGTCACTCATTATACTCTGTCGAATGTCATTGAATGTCTTTACATGGGCTT
GTATGCCTATGAAAATTGTAATACAACTTTCAGCAACGGATCTCTTGGCTCTCGCATCGA
TGAAGGACGCAGCGAAATGCGATAAGTAATGTGAATTGCAGAATTCAGTGAATCATCGAA
TCTTTGAACGCATCTTGCGCTCCTTGGTATTCCGAGGAGCATGCCTGTTTGAGTGTCATT
AAATTCTCAACTCTCTTATACTTTTTTGTAAAAGAGAGCTTGGACTGTGGAGGCTTGCTG
GCCACTTTTTGGGGTCAGCTCCTCTGAAATGCATTAGCGGAACCGTTTGCGATCTGCCAC
AAGTGTGATAAGTTATCTACACTGGCGAGGGGATTGCTCTCTGTAATGTTCAGCTTCTAA
TTGTCTCTACTTTGTGAGACAACTTTTGAATGCTTGACCTCAAATCAGGTAGGACTACCC
GCTGAACTTAA
>AB8-32
TTTCCGTAGGTGAACCTGCGGAAGGATCATTATTGAATTATGTTTCTAGATAGGTTGTAG
CTGGCTC-TTTAGAGCATGTGCACGCCTGTTTGGACTTCATTTTCATCCACCTGTGCACC
TATTGTAGTCTTTGGTTGGGTTAGGAGGAAGTGATCATTGTATCAGCATCTGCTGGGAGT
GAGGACTTGCATTGTGAAAGCTTTGCTGTCCTTGATGTGATCATGGAATCTTTTTCTCAC
TAGAGTCTATGTCACTCATTATACTCTGTCGAATGTCATTGAATGTCTTTACATGGGCTT
GTATGCCTATGAAAATTGTAATACAACTTTCAGCAACGGATCTCTTGGCTCTCGCATCGA
TGAAGGACGCAGCGAAATGCGATAAGTAATGTGAATTGCAGAATTCAGTGAATCATCGAA
TCTTTGAACGCATCTTGCGCTCCTTGGTATTCCGAGGAGCATGCCTGTTTGAGTGTCATT
AAATTCTCAACTCTCTTATACTTTTTTGTAAAAGAGAGCTTGGACTGTGGAGGCTTGCTG
GCCACTTTTTGGGGTCAGCTCCTCTGAAATGCATTAGCGGAACCGTTTGCAATCTGCCAC
AAGTGTGATAAGTTATCTACACTGGCGAGGGGATTGCTCTCTGTAATGTTCAGCTTCTAA
TTGTCTCTACTTTGTGAGACTACTTTTGAATGCTTGACCTCAAATCAGGTAGGACTACCC
GCTGAACTTAA
>AB6-37
TTTCCGTAGGTGAACCTGCGGAAGGATCATTATTGAATTATGTTTCTAGATAGGTTGTAG
CTGGCTC-TTTAGAGCATGTGCACGCCTGTTTGGACTTCATTTTCATCCACCTGTGCACC
TATTGTAGTCTTTGGTTGGGTTAGGAGGAAGTGATCATTGTATCAGCATCTGCTGGGAGT
GAGGACTTGCATTGTGAAAGCTTTGCTGTCCTTGATGTGATCATGGAATCTTTTTCTCAC
TAGAGTCTATGTCACTCATTATACTCTGTCGAATGTCATTGAATGTCTTTACATGGGCTT
GTATGCCTATGAAAATTGTAATACAACTTTCAGCAACGGATCTCTTGGCTCTCGCATCGA
TGAAGGACGCAGCGAAATGCGATAAGTAATGTGAATTGCAGAATTCAGTGAATCATCGAA
TCTTTGAACGCATCTTGCGCTCCTTGGTATTCCGAGGAGCATGCCTGTTTGAGTGTCATT
AAATTCTCAACTCTCTTATACTTTTTTGTAAAAGAGAGCTTGGACTGTGGAGGCTTGCTG

GCCACTTTTTGGGGTCAGCTCCTCTGAAATGCATTAGCGGAACCGTTTGCGATCTGCCAC
AAGTGTGATAAGTTATCTACACTGGCGAGGGGATTGCTCTCTGTAATGTTCAGCTTCTAA
TTGTCTCTACTTTGTGAGACTACTTTTGAATGCTTGACCTCAAATCAGGTAGGACTACCC
GCTGAACTTAA
>AB1-47
TTTCCGTAGGTGAACCTGCGGAAGGATCATTATTGAATTATGTTTCTAGATAGGTTGTAG
CTGGCTC-TTTAGAGCATGTGCACGCCTGTTTGGACTTCATTTTCATCCACCTGTGCACC
TATTGTAGTCTTTGGTTGGGTTAGGAGGAAGTGATCATTGTATCAGCATCTGCTGGGAGT
GAGGACTTGCATTGTGAAAGCTTTGCTGTCCTTGATGTGATCATGGAATCTTTTTCTCAC
TAGAGTCTATGTCACTCATTATACTCTGTCGAATGTCATTGAATGTCTTTACATGGGCTT
GTATGCCTATGAAAATTGTAATACAACTTTCAGCAACGGATCTCTTGGCTCTCGCATCGA
TGAAGAACGCAGCGAAATGCGATAAGTAATGTGAATTGCAGAATTCAGTGAATCATCGAA
TCTTTGAACGCATCTTGCGCTCCTTGGTATTCCGAGGAGCATGCCTGTTTGAGTGTCATT
AAATTCTCAACTCTCTTATACTTTTTTGTAAAAGAGAGCTTGGACTGTGGAGGCTTGCTG
GCCACTTTTTGGGGTCAGCTCCTCTGAAATGCATTAGCGGAACCGTTTGCAATCTGCCAC
AAGTGTGATAAGTTATCTACACTGGCGAGGGGATTGCTCTCTGTAATGTTCAGCTTCTAA
TTGTCTCTACTTTGTGAGACTACTTTTGAATGCTTGACCTCAAATCAGGTAGGACTACCC
GCTGAACTTAA
>AB9-18
TTTCCGTAGGTGAACCTGCGGAAGGATCATTATTGAATTATGTTTCTAGATAGGTTGTAG
CTGGCTC-TTTAGAGCATGTGCACGCCTGTTTGGACTTCATTTTCATCCACCTGTGCACC
TATTGTAGTCTTTGGTTGGGTTAGGAGGAAGTGATCATTGTATCAGCATCTGCTGGGAGT
GAGGACTTGCATTGTGAAAGCTTTGCTGTCCTTGATGTGATCATGGAATCTTTTTCTCAC
TAGAGTCTATGTCACTCATTATACTCTGTCGAATGTCATTGAATGTCTTTACATGGGCTT
GTATGCCTATGAAAATTGTAATACAACTTTCAGCAACGGATCTCTTGGCTCTCGCATCGA
TGAAGAACGCAGCGAAATGCGATAAGTAATGTGAATTGCAGAATTCAGTGAATCATCGAA
TCTTTGAACGCATCTTGCGCTCCTTGGTATTCCGAGGAGCATGCCTGTTTGAGTGTCATT
AAATTCTCAACTCTCTTATACTTTTTTGTAAAAGAGAGCTTGGACTGTGGAGGCTTGCTG
GCCACTTTTTGGGGTCAGCTCCTCTGAAATGCATTAGCGGAACCGTTTGCAATCTGCCAC
AAGTGTGATAAGTTATCTACACTGGCGAGGGGATTGCTCTCTGTAATGTTCAGCTTCTAA
TTGTCTCTACTTTGTGAGACTACTTTTGAATGCTTGACCTCAAATCAGGTAGGACTACCC
GCTGAACTTAA
>AB11-54
TTTCCGTAGGTGAACCTGCGGAAGGATCATTATTGAATTATGTTTCTAGATAGGTTGTAG
CTGGCTC-TTTAGAGCATGTGCACGCCTGTTTGGACTTCATTTTCATCCACCTGTGCACC
TATTGTAGTCTTTGGTTGGGTTAGGAGGAAGTGATCATTGTATCAGCATCTGCTGGGAGT
GAGGACTTGCATTGTGAAAGCTTTGCTGTCCTTGATGTGATCATGGAATCTTTTTCTCAC
TAGAGTCTATGTCACTCATTATACTCTGTCGAATGTCATTGAATGTCTTTACATGGGCTT
GTATGCCTATGAAAATTGTAATACAACTTTCAGCAACGGATCTCTTGGCTCTCGCATCGA
TGAAGAACGCAGCGAAATGCGATAAGTAATGTGAATTGCAGAATTCAGTGAATCATCGAA
TCTTTGAACGCATCTTGCGCTCCTTGGTATTCCGAGGAGCATGCCTGTTTGAGTGTCATT
AAATTCTCAACTCTCTTATACTTTTTTGTAAAAGAGAGCTTGGACTGTGGAGGCTTGCTG
GCCACTTTTTGGGGTCAGCTCCTCTGAAATGCATTAGCGGAACCGTTTGCAATCTGCCAC
AAGTGTGATAAGTTATCTACACTGGCGAGGGGATTGCTCTCTGTAATGTTCAGCTTCTAA
TTGTCTCTACTTTGTGAGACTACTTTTGAATGCTTGACCTCAAATCAGGTAGGACTACCC
GCTGAACTTAA
>AB2-32
TTTCCGTAGGTGAACCTGCGGAAGGATCATTATTGAATTATGTTTCTAGATAGGTTGTAG
CTGGCTC-TTTAGAGCATGTGCACGCCTGTTTGGACTTCATTTTCATCCACCTGTGCACC
TATTGTAGTCTTTGGTTGGGTTAGGAGGAAGTGATCATTGTATCAGCATCTGCTGGGAGT

GAGGACTTGCATTGTGAAAGCTTTGCTGTCCTTGATGTGATCATGGAATCTTTTTCTCAC
TAGAGTCTATGTCACTCATTATACTCTGTCGAATGTCATTGAATGTCTTTACATGGGCTT
GTATGCCTATGAAAATTGTAATACAACTTTCAGCAACGGATCTCTTGGCTCTCGCATCGA
TGAAGAACGCAGCGAAATGCGATAAGTAATGTGAATTGCAGAATTCAGTGAATCATCGAA
TCTTTGAACGCATCTTGCGCTCCTTGGTATTCCGAGGAGCATGCCTGTTTGAGTGTCATT
AAATTCTCAACTCTCTTATACTTTTTTGTAAAAGAGAGCTTGGACTGTGGAGGCTTGCTG
GCCACTTTTTGGGGTCAGCTCCTCTGAAATGCATTAGCGGAACCGTTTGCGATCTGCCAC
AAGTGTGATAAGTTATCTACACTGGCGAGGGGATTGCTCTCTGTAATGTTCAGCTTCTAA
TTGTCTCTACTTTGTGAGACTACTTTTGAATGCTTGACCTCAAATCAGGTAGGACTACCC
GCTGAACTTAA
>AB6-10
TTTCCGTAGGTGAACCTGCGGAAGGATCATTATTGAATTATGTTTCTAGATAGGTTGTAG
CTGGCTC-TTTAGAGCATGTGCACGCCTGTTTGGACTTCATTTTCATCCACCTGTGCACC
TATTGTAGTCTTTGGTTGGGTTAGGAGGAAGTGATCATTGTATCAGCATCTGCTGGGAGT
GAGGACTTGCATTGTGAAAGCTTTGCTGTCCTTGATGTGATCATGGAATCTTTTTCTCAC
TAGAGTCTATGTCACTCATTATACTCTGTCGAATGTCATTGAATGTCTTTACATGGGCTT
GTATGCCTATGAAAATTGTAATACAACTTTCAGCAACGGATCTCTTGGCTCTCGCATCGA
TGAAGAACGCAGCGAAATGCGATAAGTAATGTGAATTGCAGAATTCAGTGAATCATCGAA
TCTTTGAACGCATCTTGCGCTCCTTGGTATTCCGAGGAGCATGCCTGTTTGAGTGTCATT
AAATTCTCAACTCTCTTATACTTTTTTGTAAAAGAGAGCTTGGACTGTGGAGGCTTGCTG
GCCACTTTTTGGGGTCAGCTCCTCTGAAATGCATTAGCGGAACCGTTTGCGATCTGCCAC
AAGTGTGATAAGTTATCTACACTGGCGAGGGGATTGCTCTCTGTAATGTTCAGCTTCTAA
TTGTCTCTACTTTGTGAGACTACTTTTGAATGCTTGACCTCAAATCAGGTAGGACTACCC
GCTGAACTTAA
>AB6-44
TTTCCGTAGGTGAACCTGCGGAAGGATCATTATTGAATTATGTTTCTAGATAGGTTGTAG
CTGGCTC-TTTAGAGCATGTGCACGCCTGTTTGGACTTCATTTTCATCCACCTGTGCACC
TATTGTAGTCTTTGGTTGGGTTAGGAGGAAGTGATCATTGTATCAGCATCTGCTGGGAGT
GAGGACTTGCATTGTGAAAGCTTTGCTGTCCTTGATGTGATCATGGAATCTTTTTCTCAC
TAGAGTCTATGTCACTCATTATACTCTGTCGAATGTCATTGAATGTCTTTACATGGGCTT
GTATGCCTATGAAAATTGTAATACAACTTTCAGCAACGGATCTCTTGGCTCTCGCATCGA
TGAAGAACGCAGCGAAATGCGATAAGTAATGTGAATTGCAGAATTCAGTGAATCATCGAA
TCTTTGAACGCATCTTGCGCTCCTTGGTATTCCGAGGAGCATGCCTGTTTGAGTGTCATT
AAATTCTCAACTCTCTTATACTTTTTTGTAAAAGAGAGCTTGGACTGTGGAGGCTTGCTG
GCCACTTTTTGGGGTCAGCTCCTCTGAAATGCATTAGCGGAACCGTTTGCGATCTGCCAC
AAGTGTGATAAGTTATCTACACTGGCGAGGGGATTGCTCTCTGTAATGTTCAGCTTCTAA
TTGTCTCTACTTTGTGAGACTACTTTTGAATGCTTGACCTCAAATCAGGTAGGACTACCC
GCTGAACTTAA
>AB11-28
TTTCCGTAGGTGAACCTGCGGAAGGATCATTATTGAATTATGTTTCTAGATAGGTTGTAG
CTGGCTC-TTTAGAGCATGTGCACGCCTGTTTGGACTTCATTTTCATCCACCTGTGCACC
TATTGTAGTCTTTGGTTGGGTTAGGAGGAAGTGATCATTGTATCAGCATCTGCTGGGAGT
GAGGACTTGCATTGTGAAAGCTTTGCTGTCCTTGATGTGATCATGGAATCTTTTTCTCAC
TAGAGTCTATGTCACTCATTATACTCTGTCGAATGTCATTGAATGTCTTTACATGGGCTT
GTATGCCTATGAAAATTGTAATACAACTTTCAGCAACGGATCTCTTGGCTCTCGCATCGA
TGAAGAACGCAGCGAAATGCGATAAGTAATGTGAATTGCAGAATTCAGTGAATCATCGAA
TCTTTGAACGCATCTTGCGCTCCTTGGTATTCCGAGGAGCATGCCTGTTTGAGTGTCATT
AAATTCTCAACTCTCTTATACTTTTTTGTAAAAGAGAGCTTGGACTGTGGAGGCTTGCTG
GCCACTTTTTGGGGTCAGCTCCTCTGAAATGCATTAGCGGAACCGTTTGCGATCTGCCAC
AAGTGTGATAAGTTATCTACACTGGCGAGGGGATTGCTCTCTGTAATGTTCAGCTTCTAA

TTGTCTCTACTTTGTGAGACTACTTTTGAATGCTTGACCTCAAATCAGGTAGGACTACCC
GCTGAACTTAA
>AB1-35
TTTCCGTAGGTGAACCTGCGGAAGGATCATTATTGAATTATGTTTCTAGATAGGTTGTAG
CTGGCTC-TTTAGAGCATGTGCACGCCTGTTTGGACTTCATTTTCATCCACCTGTGCACC
TATTGTAGTCTTTGGTTGGGTTAGGAGGAAGTGATCATTGTATCAGCATCTGCTGGGAGT
GAGGACTTGCATTGTGAAAGCTTTGCTGTCCTTGATGTGATCATGGAATCTTTTTCTCAC
TAGAGTCTATGTCACTCATTATACTCTGTCGAATGTCATTGAATGTCTTTACATGGGCTT
GTATGCCTATGAAAATTGTAATACAACTTTCAGCAACGGATCTCTTGGCTCTCGCATCGA
TGAAGAACGCAGCGAAATGCGATAAGTAATGTGAATTGCAGAATTCAGTGAATCATCGAA
TCTTTGAACGCATCTTGCGCTCCTTGGTATTCCGAGGAGCATGCCTGTTTGAGTGTCATT
AAATTCTCAACTCTCTTATACTTTTTTGTAAAAGAGAGCTTGGACTGTGGAGGCTTGCTG
GCCACTTTTTGGGGTCAGCTCCTCTGAAATGCATTAGCGGAACCGTTTGCGATCTGCCAC
AAGTGTGATAAGTTATCTACACTGGCGAGGGGATTGCTCTCTGTAATGTTCAGCTTCTAA
TTGTCTCTACTTTGTGAGACTACTTTTGAATGCTTGACCTCAAATCAGGTAGGACTACCC
GCTGAACTTAA
>AB4-32
TTTCCGTAGGTGAACCTGCGGAAGGATCATTATTGAATTATGTTTCTAGATAGGTTGTAG
CTGGCTC-TTTAGAGCATGTGCACGCCTGTTTGGACTTCATTTTCATCCACCTGTGCACC
AATTGTAGTCTTTGGTTGGGTTAGGAGGAAGTGATCATTGTATCAGCATCTGCTGGGAGT
GAGGACTTGCATTGTGAAAGCTTTGCTGTCCTTGATGTGATCATGGAATCTTTTTCTCAC
TAGAGTCTATGTCACTCATTATACTCTGTCGAATGTCATTGAATGTCTTTACATGGGCTT
GTATGCCTATGAAAATTGTAATACAACTTTCAGCAACGGATCTCTTGGCTCTCGCATCGA
TGAAGAACGCAGCGAAATGCGATAAGTAATGTGAATTGCAGAATTCAGTGAATCATCGAA
TCTTTGAACGCATCTTGCGCTCCTTGGTATTCCGAGGAGCATGCCTGTTTGAGTGTCATT
AAATTCTCAACTCTCTTATACTTTTTTGTAAAAGAGAGCTTGGACTGTGGAGGCTTGCTG
GCCACTTTTTGGGGTCAGCTCCTCTGAAATGCATTAGCGGAACCGTTTGCGATCTGCCAC
AAGTGTGATAAGTTATCTACACTGGCGAGGGGATTGCTCTCTGTAATGTTCAGCTTCTAA
TTGTCTCTACTTTGTGAGACTACTTTTGAATGCTTGACCTCAAATCAGGTAGGACTACCC
GCTGAACTTAA
>AB10-23
TTTCCGTAGGTGAACCTGCGGAAGGATCATTATTGAATTATGTTTCTAGATAGGTTGTAG
CTGGCTC-TTTAGAGCATGTGCACGCCTGTTTGGACTTCATTTTCATCCACCTGTGCACC
TATTGTAGTCTTTGGTTGGGTTAGGGGGAAGTGGTCATTGTGTCAGCATCTGCTGGGAGT
GAGGACTTGCATTGTGAAAGCTTTGCTGTCCTTGATGTGATCATGGAATCTTTTTCTCAC
TAGAGTCTATGTCACTCATTATACTCTGTCGAATGTCATTGAATGTCTTTACATGGGCTT
GTATGCCTATGAAAATTGTAATACAACTTTCAGCAACGGATCTCTTGGCTCTCGCATCGA
TGAAGAACGCAGCGAAATGCGATAAGTAATGTGAATTGCAGAATTCAGTGAATCATCGAA
TCTTTGAACGCATCTTGCGCTCCTTGGTATTCCGAGGAGCATGCCTGTTTGAGTGTCATT
AAATTCTCAACTCTCTTATACTTTTTTGTAAAAGAGAGCTTGGACTGTGGAGGCTTGCTG
GCCACTTTTTGGGGTCAGCTCCTCTGAAATGCATTAGCGGAACCGTTTGCGATCTGCCAC
AAGTGTGATAAGTTATCTACACTGGCGAGGGGATTGCTCTCTGTAATGTTCAGCTTCTAA
TTGTCTCTACTTTGTGAGACTACTTTTGAATGCTTGACCTCAAATCAGGTAGGACTACCC
GCTGAACTTAA
